# Assembly of *Schizosaccharomyces cryophilus* chromosomes and their comparative genomic analyses revealed principles of genome evolution of the haploid fission yeasts

**DOI:** 10.1038/s41598-018-32525-9

**Published:** 2018-10-02

**Authors:** Lajos Ács-Szabó, László Attila Papp, Zsuzsa Antunovics, Matthias Sipiczki, Ida Miklós

**Affiliations:** Department of Genetics and Applied Microbiology, Faculty of Science and Technology, University of Debrecen, Debrecen, Hungary

## Abstract

The fission yeast clade, which has a distinct life history from other yeasts, can provide important clues about evolutionary changes. To reveal these changes the large *S. cryophilus* supercontigs were assembled into chromosomes using synteny relationships and the conserved pericentromeric, subtelomeric genes. Togetherness of the supercontigs was confirmed by PCR. Investigation of the gene order revealed localisation of the rDNA arrays, more than 300 new conserved orthologues and proved that *S. cryophilus* supercontigs were mosaics of collinear blocks. PFGE analysis showed that size of the *S. cryophilus* chromosomes differ from the *S. pombe* chromosomes. Comparative genomic analyses of the newly assembled chromosomes confirmed that the closest relative of *S. cryophilus* was *S. octosporus* not just in sequence similarity but also in a structural way, and revealed that preservation of the conserved regions did not arise from the lower number of chromosomal rearrangements. Translocations were more typical in the closely related species, while the number of inversions increased with the phylogenetic distances. Our data suggested that sites of the chromosomal rearrangements were not random and often associated with repetitive sequences, structural- and nucleotide evolution might correlate. Chromosomal rearrangements of the fission yeasts compared to other lineages were also discussed.

## Introduction

Although sequencing processes are becoming more and more accurate and fast, assembly of draft genomic sequences remains a serious challenge in many cases, in turn they are necessary to perform extensive and thorough comparative evolutionary studies. These large-scale comparative studies of the ever-increasing numbers of sequences enable us to discover similarities and differences between the genomes, gain insight into genome structures and learn how genomes function and evolve.

Thus, investigation of *Haemophilus influenzae* and *Escherichia coli* sequences revealed an important role of the gene shuffling in bacterial evolution^1^, while comparison of *Saccharomyces cerevisiae* and *Candida albicans* genome sequences shed light on the fact that small inversions could be common forms of the chromosomal rearrangements^2^. Other analyses helped us to identify rapidly evolving genes^3,4^, while comparison of *Hemiascomycetes* whole genome sequences drew our attention to numerous interesting features, like mechanisms of a single gene-, segmental- and whole genome duplications or showed that nucleotide and structural evolution depend on two different molecular clocks reviewed in^5^. Moreover, a novel form of evolution (mesosynteny) was also identified by studying filamentous fungi genomes^6^. A genome and proteome sequence comparison of the *Schizosaccharomyces pombe* and *S. cereviaise* provided insight into the functional similarities and differences between the budding and fission yeasts^7^, while a study of *Schizosaccharomyces* species showed that the fission yeasts could have unusually stable genome structures^8^.

As *Schizosaccharomyces* species (*Schizosaccharomyces pombe, S. japonicus, S. octosporus* and *S. cryophilus*) have haploid genomes and distinct life history from other yeasts, this clade can provide an attractive model for the genome evolution studies, which was hindered by lack of the assembled *S. cryophilus* chromosomes. In order to expand our knowledge obtained from previous analyses, the aim of this project was to assemble the *S. cryophilus* large supercontigs into chromosomes based on the data available and use these chromosomes for comparative genomic studies. Accordingly, we suggest here a hypothetical genome assembly based on synteny relationships and validated by molecular experiments. Finally, the newly assembled *S. cryophilus* genome was used for comparative analyses, which revealed important features of the *Schizosaccharomyces* genomes.

## Results

### Assembly of the *S. cryophilus* supercontigs based on synteny and the conserved pericentromeric- and subtelomeric genes revealed that *S. cryophilus* supercontigs were mosaics of collinear blocks belonging to the different chromosomes of its related species

Since the *S. cryophilus* database (Broad) contained only supercontigs (Scs) and no chromosomes, which would be necessary to perform extensive comparative evolutionary studies with the *S. cryophilus* genome, we decided to assemble those Scs. Earlier results showed that gene order and gene content were remarkably conserved in genomes of the related fission yeast species^8^, thus we supposed that identification of locally collinear blocks (LCBs)(conserved regions of the chromosomes) could allow us to set order of the *S. cryophilus* largest Scs. To identify the LCBs, sequence alignments were carried out by Mauve program using the closely related *S. octosporus* and *S. pombe* DNA sequences as reference genomes. In the first alignments, the *S. cryophilus* Scs were in default order (1–9) and orientation (Supplementary Fig. S1a,b), while later we reordered the Scs with Mauve by using the synteny relationships (Supplementary Fig. S1c,d). These alignments revealed that the *S. cryophilus* Scs are mosaics of LCBs belonging to different chromosomes of the related species (Fig. S1). At the same time these alignments showed that “automatic” reordering of the Scs based on global synteny could not provide a reliable order. Namely, completely different Sc orders were obtained depending on the reference genomes (Sc order: Sc5,3,2,6,8,9,1,7,4 - with *S. pombe* reference genome and Sc4,9,5,8,7,2,6,3,1-with *S. octosporus* reference genome) (Supplementary Fig. S1c,d). Consequently, we tried to reveal the true order and orientation of the Scs by manual identification of the pericentromeric and subtelomeric genes based on earlier results that higher degree of conservation were typical in these regions^8–12^. Thus, the translated sequences of 70 *S. pombe* and *S. octosporus* pericentromeric- and 180 subtelomeric genes were collected and their putative orthologues in *S. cryophilus* genome were identified by BLASTp program. Our results showed that pericentromeric- and subtelomeric gene orders were highly conserved also in the *S. cryophilus* genome (Supplementary Tables S1 and S2).

Although we failed to find the corresponding orthologues of every subtelomeric genes (Supplementary Table S2), the successfully identified *S. cryophilus* genes enabled us to find those Scs which contained subtelomeric genes. Based on these findings, we concluded that Sc3, Sc4, Sc6 and Sc7 could have subtelomeric ends, while the pericentromeric genes of *S. cryophilus* could be found on the Sc2, Sc3, Sc4, Sc5, Sc7, Sc9 (Supplementary Table S1). tRNA genes, which tend to be located close to the centromeres^13^^,^^8^ were also found on these supercontigs.

**Figure 1:**
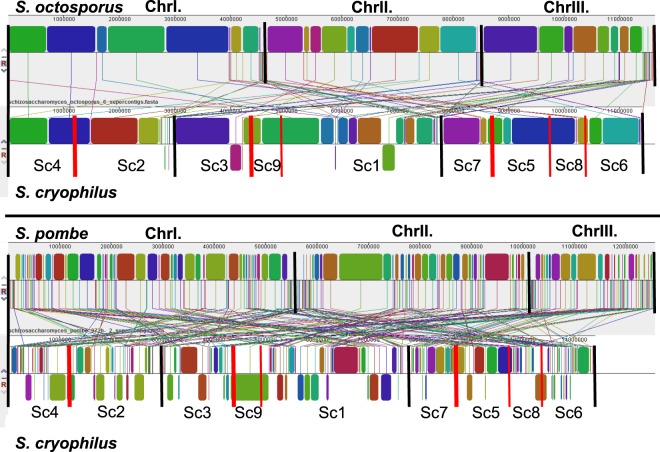
Pairwise whole genome alignments of the species created by Mauve^49^ aligner, where the newly established supercontig (Sc) order of *S. cryophilus* was used. The large overlapping locally collinear blocks (LCBs) between the adjacent Scs provide support for the current Sc order. The alignments also indicate that *S. cryophilus* closest relative is *S. octosporus*. Colourful rectangles represent LCBs. Sizes of the rectangles are proportional to the genomic extensions of LCBs. LCBs below a genome’s centre line are in inverted orientation relative to the reference genome. Vertical black lines indicate the chromosome boundaries, vertical red lines show the inner supercontig boundaries of *S. cryophilus*. Wider red lines indicate positions of the centromeric regions in the case of *S. cryophilus*.

Considering these data, additional Mauve alignments were carried out which suggested the following Sc order: Sc4-Sc2; Sc3-Sc9-Sc1 and Sc7-Sc5-Sc8-Sc6 (Fig. 1). This state of the hypothetic assembly seemed to be reliable and also shed light on further neighbouring contigs, such as Sc9-Sc1; Sc5-Sc8, Sc8-Sc6 (Fig. 1).

### Validation of the neighbouring supercontigs by PCR

**Figure 2:**
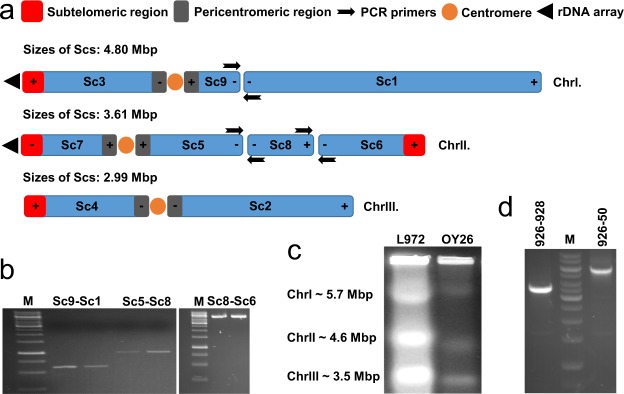
(**a**) Hypothetic chromosomes of *S. cryophilus* and positions of the primers (black arrows). “+” and “−” indicate the original orientation of the supercontigs available in the corresponding databases. In the case of (+−) the orientation coincides with the default orientation, (−+) refers to inverted orientations. (**b**) PCR validation of togetherness of Sc9-Sc1 (lane: 1., 2.; primers: 666–667), Sc5-Sc8 (lane: 3.,4.; primers: 668–669) and Sc8-Sc6 (lane: 5.,6.; primers: 794–795) supercontig pairs. M: 1 Kb DNA ladder. (**c**) Pulsed-field gel electrophoresis of the chromosomal DNA of *S. pombe* (L972) and *S. cryophilus* (OY26). (**d**) PCR validation of localisation of the rDNA arrays on the *S. cryophilus* chromosomes. M: 1 Kb DNA ladder; 926, 928, 50: PCR primers. Gel photos of (**b**–**d**) are cropped, full-length gels are presented in Supplementary Figs S7–S10.

To validate adjacency of the neighbouring Scs (Sc9-Sc1; Sc5-Sc8, Sc8-Sc6) suggested by the last Mauve alignment (Fig. 1), PCR amplifications were carried out with sequence specific primers (Table 1). Primers were designed to hybridize to the corresponding contig ends of the concerning Scs (Fig. 2a). After optimisation of the PCR parameters, we managed to amplify those PCR products which confirmed togetherness of the Sc9-Sc1, Sc5-Sc8 and Sc8-Sc6 (Fig. 2b). PCR fragments were also approved by sequencing (Genbank accessions: MH605091- MH605096, Supplementary File S1). Concatenated chromosome sequences of *S. cryophilus* in fasta format are available in Supplementary Files S2–S4.

**Table 1:** List of strains and primers used in this study. Sc: supercontig.

Species	Collection number	Description
*S. pombe*	0–1	Wild-type strain L972h-
*S. cryophilus*	6–21; CBS11777	Wild-type strain
**Primers**	**Sequence (5′-3′)**	**Description**
		For proving relation of supercontigs
Sc9 Forw_B	tagtttatggccgccacagt	Sc9 and Sc1
Sc1 Rev_B	ccgtctgctttctcagtttg	Sc9 and Sc1
Sc5 Forw_C	gcttcaagctgccacatttt	Sc5 and Sc8
Sc8 Rew_C	gcgatctctttagcatttcca	Sc5 and Sc8
Sc6endF	ggaaataccttttggcgact	Sc6 and Sc8
Sc8startR	ggtctaagggggcagattta	Sc6 and Sc8
		For detecting localization of
NL4^55^	ggtccgtgtttcaagacgg	ribosomal DNA
18S rDNA1	tcattacggcggtcctagaa	ribosomal DNA
SPOG_04999	tgttggtgttgatgagcagc	ribosomal DNA

### Determination of the sizes of *S. cryophilus* chromosomes and localisation of the rDNA arrays

To investigate total sizes of the *S. cryophilus* chromosomes, we carried out a karyotypic analysis which confimed that *S. cryophilus* had three chromosomes similarly to the related *S. pombe* (Fig. 2c) (https://www.pombase.org/)^8^, and revealed that *S. cryophilus* chromosomes differ in size from the *S. pombe* chromosomes (Fig. 2c). Since *S. cryophilus* Scs were mosaics of collinear blocks belonging to the different chromosomes of its closely related species, we classified its chromosomes depending on their sizes (Figs 1 and S1). Thus hereinafter the largest chromosome was designated as ChrI, while the smallest one as ChrIII, similarly to *S. pombe* chromosomes (Fig. 2c). Consequently, *S. cryophilus* ChrI seemed to be larger in the karyotypic analysis than *S. pombe* ChrI (5.7Mbp). *S. cryophilus* ChrII and ChrIII were smaller than 4.6 Mbp and 3.5 Mbp, respectively (Fig. 2c). At the same time, we have to mention that differences between calculated length of the coherent Scs (Fig. 2a) and real sizes of the chromosomes found in the karyotypic analysis (Fig. 2c) could arise from the lack of certain chromosomal regions, such as unplaced small contigs (~0,2Mbp overall), centromeres, telomeres, or the unknown localisation of rDNA arrays.

Since extensions of rDNA arrays can significantly influence sizes of the chromosomes, we wanted to establish the possible locations of 18S-5,8S-28S rDNA genes on the *S. cryophilus* chromosomes. Based on synteny between *S. octosporus* and *S. cryophilus* we managed to find orthologues of the *S. octosporus* rDNA genes in the subtelomeric region of Sc7. This data could also be confirmed by PCR (Fig. 2d), as PCR fragments were successfully amplified with primers 928–926 and 926–50 (928 binds to the 18 S subunit, 50 binds to the 28 S subunit (D1/D2 domain), while 926 binds to the gene SPOG04999 which is the closest one to the rDNA array according to the sequence file). A further rDNA array was supposed to be on the Sc3 by synteny investigations of the *S. octosporus* rDNA close genes (Supplementary Table S2, Supplementary Fig. S2), which might correlate with the large size of *S. cryophilus* ChrI observed in the PFGE analysis (Fig. 2c).

### Nucleotide sequence similarity and gene order comparisons confirmed that *S. cryophilus* closest relative was *S. octosporus*

**Figure 3:**
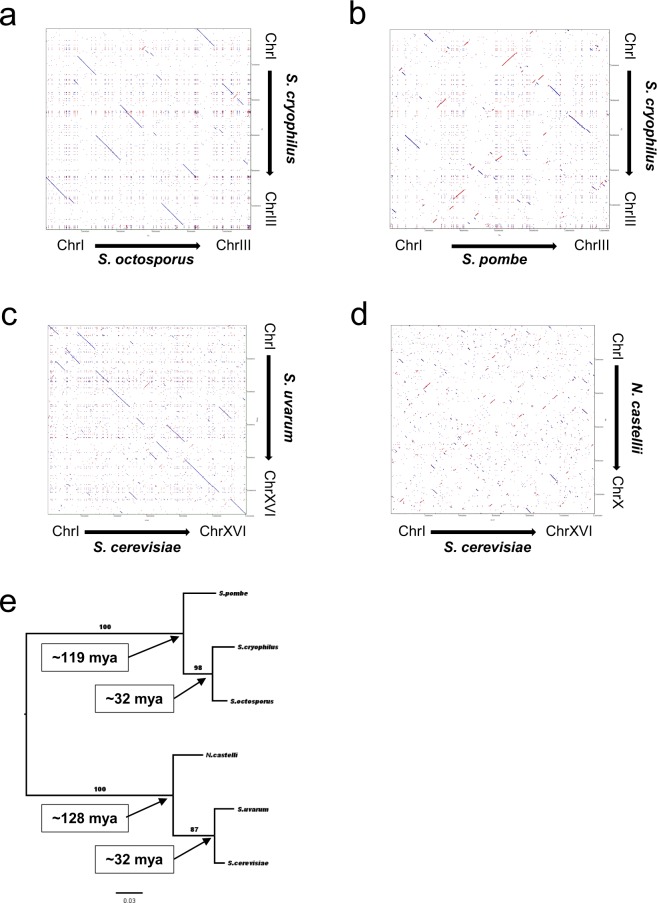
Dot-plot alignments created with YASS^50^ (E < 1.0E-30) using the concatenated whole genome sequences of the species. The alignments clearly show that interchromosomal translocations were more frequent than inversions in short evolutionary terms. Blue lines represent homologous segments in the same orientation, while red lines represent inverted segments. (**a**) *S. octosporus* and *S. cryophilus* alignment; (**b**) *S. pombe* and *S. cryophilus* alignment; (**c**) *S. cerevisiae* and *S. uvarum* alignment; (**d**) *S. cerevisiae* and *N. castelli* alignment. First species located to the horizontal axis and the latter located to the vertical axis. High resolution pictures of these alignments are available in Supplementary Fig. 4(a–d). (**e**) Phylogenetic relations of the concerning species. Values at the branches are statistical supports come from 100 bootstrap replicates. Numbers in the boxes indicate the dates of divergences^8^. Mya: million years ago.

**Figure 4:**
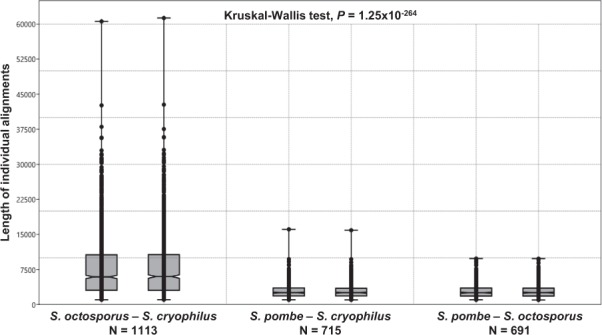
Length distributions of individual DNA alignments created by YASS^50^ (E = 0). Box plots indicate the 25–75 percent quartiles. Horizontal lines within the boxes show the medians of the samples, notches indicate the 95 percent confidence intervals for the medians. Minimal and maximal values are depicted by the whiskers, plots on the whiskers show individual values. N: sample size. Alignments of *S. octosporus-S. cryophilus* are significantly different from the other alignments (Kruskal-Wallis test, *P* = 1.25 × 10^−264^). Pairwise statistics (Mann-Whitney U tests) are presented in Supplementary Table 3.

To gain more information on the relation of the species, the common putative orthologues shared by *S. pombe, S. octosporus* and *S. cryophilus* were manually identified (Supplementary Table S3). As a result, we managed to find 4580 1:1:1 putative orthologous genes (89–92% of the whole gene content of the three species), in contrast to 4218 genes shared by the four fission yeast species^8^. This was in a good agreement with the phylogenetic distances of the species. Synteny analyses of these common orthologous proteins confirmed the close relation of *S. cryophilus* and *S. octosporus*^8,14^ (Supplementary Fig. S3), which was also supported by additional DNA level studies. Namely, we created whole genome dot-plots using the concatenated DNA sequences of the species and the newly established Sc order of the *S. cryophilus* genome with different parameters (E = 0; E < 1.0E-30; alignment size >1000 nt) (Supplementary Table S4). These dot-plot alignments revealed more consecutive homologous DNA sequences between *S. cryophilus* and *S. octosporus* (Fig. 3a, Supplementary Fig. S4a) than between *S. cryophilus* and *S. pombe* (Fig. 3b, Supplementary Fig. S4b) regardless of the level of strictness. Statistical analyses of the pairwise alignments also proved that *S. cryophilus* closest relative was *S. octosporus* (Fig. 4, Supplementary Table S4).

### Study of chromosomal rearrangements showed that interchromosomal translocations were more frequent than inversions in the closely related species, while the number of inverted segments became higher with the increasing phylogenetic distances

Hereinafter, we were interested in the chromosomal rearrangements of *S. cryophilus*. To obtain information about their number and types, we examined the created whole genome dot-plots and compared them with certain *Saccharomycotina* genomes. These dot-plot alignments shed light on that the frequency of translocations (that are mainly interchromosomal translocations) was higher compared to the frequency of inversions in the closest relatives than in the distantly related species. While number of the inverted sequences became higher with increasing phylogenetic distances (Fig. 3a,b). We observed the same tendency in the alignments of *S. pombe* and *S. octosporus* (Supplementary Fig. S6a,b). This latter observation was also supported by GRIMM analysis^15^, which could estimate the minimal number of changes in the lineages. As we were interested only in gross chromosomal changes, we used manually selected LCBs larger than 20.000 nucleotides extracted from Mauve alignments. According to optimal rearrangement scenarios provided by GRIMM, 7 translocations and 4 inversions could occur between the closely related *S. octosporus* and *S. cryophilus*, 9 and 54 between the distantly related *S. pombe* and *S. cryophilus*, and 14 and 53 between *S. pombe-S. octosporus*, respectively. Interestingly, the same tendency was emerged in those *Saccharomycotina* species, whose dates of divergences approximately matched with the *Schizosaccharomyces* species investigated^8^ (Fig. 4c–e).

### Mauve alignments and GRIMM analyses suggested that a higher number of gross chromosomal rearrangements occurred in the fission yeast genomes than in the budding yeast genomes

As reported by Rhind^8^, conservation of the gene content is significantly higher in *Schizosaccharomyces* than within *Saccharomyces* or *Kluyveromyces*, both of which have much lower amino acid divergence. We assumed that the observed conservation of gene content/order might be in relation to the fact that lower number of rearrangements could occur in the genomes of fission yeast. Thus, to compare dynamics of the genome evolutionary changes of the fission yeasts to the sampled *Saccharomycotina* species we created Mauve alignments (Supplementary Fig. S4e,f) and performed GRIMM rearrangement analyses with all of the extracted LCBs regardless of their size (Table 2).

**Table 2:** Comparisons of multi chromosomal distances (MCDs) among the species.

	Number of Chrs	Multi Chromosomal Distance (MCD)						Gross changes/all changes percentages
		per whole genomes		per chromosomes		per Megabases		
		all changes	gross changes	all changes	gross changes	all changes	gross changes	
*So - Scry*	3 -3	46	11	15.33	3.67	3.97	0.95	24%
*Sp - Scry*	3-3	150	63	50.00	21.00	12.50	5.25	42%
*Su - Scer*	16-16	72	5	4.50	0.31	6.10	0.42	7%
*Nc - Scer*	10–16	607	102	37.94	6.38	51.88	8.72	17%

The data indicate that the chromosomes of fission yeasts bore more gross rearrangements than the chromosomes of budding yeasts. Values were estimated by GRIMM^15^ using the data of LCBs extracted from the pairwise whole genome alignments created by Mauve aligner^49^. Since GRIMM estimates optimal rearrangement scenarios by transforming one genome to another via rearrangement events, the given values in the table correspond to one genome. For example MCD values per chromosomes in the case of *So – Scry* correspond to 3 chromosomes not 6. *So: S. octosporus; Scry: S. cryophilus; Sp: S. pombe; Su: S. uvarum; Scer: S. cerevisiae; Nc: N. castelli*. Chrs: chromosomes. All changes mean that we considered every rearrangement events regardless of the sizes and positions of the concerning LCBs. While in the case of gross changes we excluded the subtelomeric regions because these are inclined to undergo rearrangements and just the LCBs > 20 000 nucleotides were considered.

Based on optimal rearrangement scenarios, the multichromosomal distances (MCDs) (by means of the number of changes that possibly occurred) were proved to be higher in the Saccharomycotina species than in the fission yeasts when we considered all the changes possibly occurred (Table 2). These findings might suggest that fewer chromosomal rearrangements occurred in the genomes of the fission yeasts than in the budding yeast. However, according to an alternative analysis, where we excluded the subtelomeric regions, because these segments are inclined to undergo rearrangements and used only LCBs larger than 20000 nucleotides showed different result. That is, considering only the gross changes we could find less chromosomal rearrangements in all pairs of species and our data coincided with the findings of Fischer in the case of *S. cerevisiae* and *S. uvarum*^16,17^ (Table 2). Later, numbers of the gross changes were compared to the chromosome numbers and sizes of the genomes, and these ratios clearly suggest that more gross rearrangements happened per chromosome (or per megabase) in the fission yeasts genomes (Table 2). That is, individual chromosomes of the fission yeasts bore many more large scale translocations and inversions than chromosomes of the budding yeasts (Table 2).

### Breakpoint analyses suggested that sites of chromosomal rearrangements could not be random

In order to obtain information on the sites of chromosomal rearrangements, we identified the chromosomal breakpoints between large LCBs (>20 000 nts) in the YASS and Mauve alignments. To ensure that breakpoints were correctly revealed, we examined both *S. octosporus – S. cryophilus* and *S. cryophilus – S. octosporus* alignments. The analyses revealed 19 breakpoints. In the next step, the genes located to the edges of the LCBs were identified and mainly 5S rDNA genes were found (5S rDNAs were associated with 12 breakage sites from the 19) (Supplementary Fig. S5). Thus, we assumed that rearrangements could happen along these repeated sequences. A similar data was found earlier, where inversion endpoints were correlated with repeated sequences^18,19^. In other cases, the rearrangements occurred in large intergenic regions (>1000 nts).

**Figure 5:**
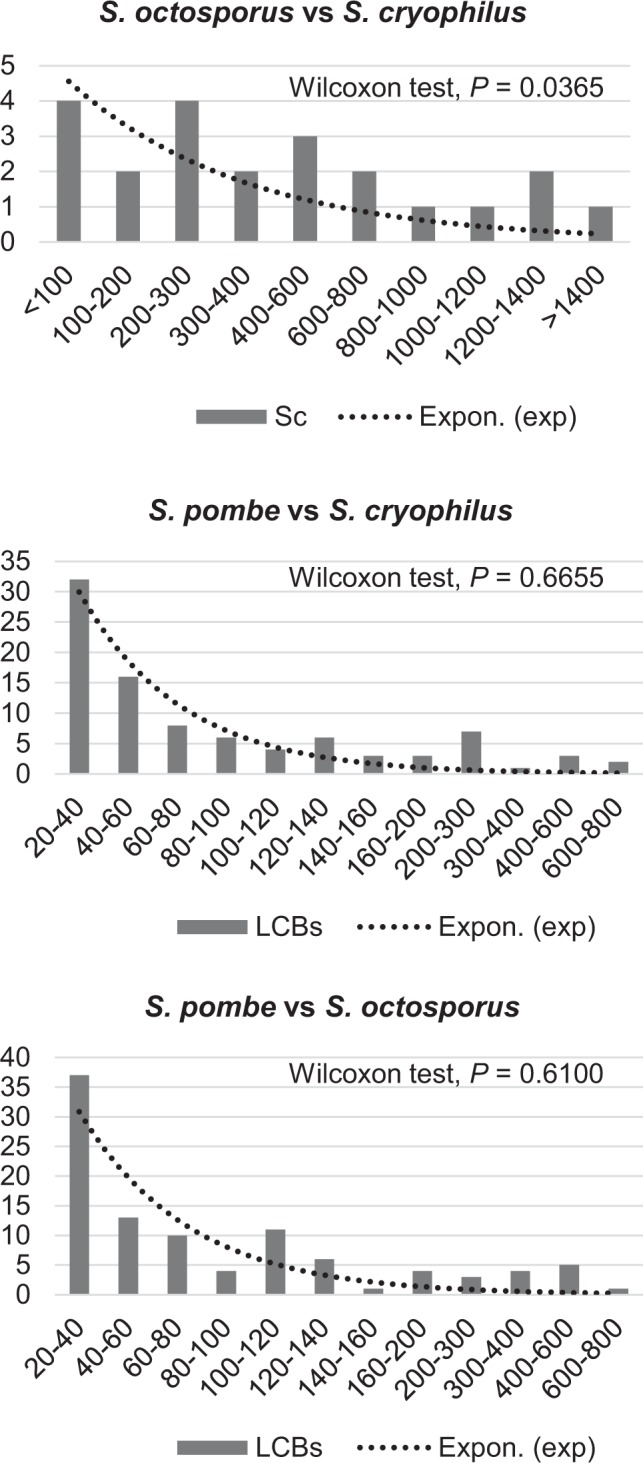
Comparison of histograms of LCBs’ lengths to random breakage model of chromosomal evolution (dashed lines). According to random breakage model distances between breakpoints should follow an exponential distribution of the form f(x) = 1/L e − x/L, where L is the average size of all syntenic segments. Numbers on the horizontal axes indicate the distribution of the lengths of LCBs in Kb. Values of the vertical axes show the frequency of LCBs at a given length. Analysis of *S. octosporus – S. cryophilus* proved that the concerning values did not show agreement with the random breakage model. However, values of *S. pombe – S. cryophilus* and of *S. pombe – S. octosporus* did not diverge largely from the model prediction.

The random breakage model of chromosomal evolution considers distribution of lengths between breakpoints and supposes that lengths of LCBs between rearrangements should be exponentially distributed^20–22^. To reveal trends in chromosomal evolution of the fission yeasts we also computed distribution of the lengths of LCBs between rearrangements and observed in the *S. octosporus – S. cryophilus* analysis that the concerning values did not show agreement with the random breakage model (Fig. 5), in contrast to some *Aspergillus* species^23^. However, values of the more distantly related *S. pombe – S. cryophilus* and of *S. pombe – S. octosporus* did not diverge largely from the model prediction (Fig. 5).

### Structural and sequence evolution in the fission yeast genomes might be correlated

Later, structural and sequence evolution in the fission yeasts was investigated in two considered scenarios. In the first case - depending on the Mauve alignments - we found 224 LCBs in *S. cryophilus* (Fig. 1) and 226 LCBs in *S. octosporus* using *S. pombe* as reference genome (Supplementary Fig. S6), while estimation of MCDs established by GRIMM showed 150 for *S. cryophilus* and 156 for *S. octosporus* (Table 3). Based on these findings, the overall rate of genome reorganization seemed to be almost the same in the two different lineages.

**Table 3:** Comparison of sequence and structural evolution of the fission yeasts.

	Amino acid identity^8^	Mauve analysis		Manual analysis	
		Mauve LCBs	MCD	Manual LCBs	MCD
*S. cryophilus - S. octosporus*	85.00%	59	46	26	11
*S. pombe - S. cryophilus*	66.40%	224	150	111	63
*S. pombe - S. octosporus*	65.60%	226	156	112	67

The results suggested that structural differences might correlate (Pearson’s *r* = 0.99; *P* = 0.0176) with the established amino acid divergence (1-identity). Values of amino acid identity originated from^8^. Mauve LCBs: locally collinear blocks (conserved regions of the chromosomes) established by Mauve^49^. Manual LCBs: manually selected LCBs larger than 20 000 nucleotides. MCD: multi-chromosomal distance (by means of number of changes occurred) estimated by GRIMM^15^.

Later we examined the previous results of GRIMM analysis (submission of the LCBs larger than 20 000 nucleotides). The overall MCDs were 63 in the *S. cryophilus* and 67 in the *S. octosporus* lineage (Table 3). These results suggested that structural differences might correlate (Pearson’s *r* = 0.99; *P* = 0.0176) with the established amino acid divergence^8^ (Table 3), similarly to certain vertebrates, nematodes and arthropods^24–26^ and differently from the *Aspergillus* species^23^. However, genome evolution (either sequence or structural) of *S. octosporus* seems to be somewhat faster than genome evolution of *S. cryophilus* (Table 3).

## Discussion

As genome sequencing has become less expensive, thousands of genome projects have been launched recently. However, performing a genome assembly with good quality is still a serious challenge^27^. Consequently, many genomes remained in the state of draft genomes, which could be sufficient for certain experimental studies, but not for the analysis of large scale genomic changes^28,29^.

Since fission yeasts have a distinct life history from other yeasts^30,31^, share important biological features, such as chromosome structure and metabolism, G2/M cell cycle control, cytokinesis, or the spliceosome components with metazoans^32,33^, reviewed in^34^, and they have haploid chromosome sets, these species (*S. pombe, S. octosporus, S. japonicus, S. cryophilus*) can provide an attractive model for genome evolution studies.

In order to introduce the genome of the recently described species *S. cryophilus*^14^ into comparative genomic analyses of the fission yeasts and study of the chromosomal changes, we decided to assemble the *S. cryophilus* large Scs into chromosomes. Thus, genome sequence alignments, BLAST searches and investigation of the synteny relationships were carried out with the *S. cryophilus* Scs. Their results revealed that *S. octosporus* is the closest relative of *S. cryophilus* (Figs 1, 3 and 4, Supplementary Figs S1, S3 and S4), which was in good agreement with earlier data obtained from protein sequences alignments and investigation of rRNA genes^8,14^.

These results also revealed that *S. cryophilus* Scs were mosaics of collinear blocks belonging to the different chromosomes of its related species (Supplementary Fig. S1, Fig. 1) and the subtelomeric- and pericentomeric genes were conserved between *S. cryophilus* and *S. octosporus* (Supplementary Tables S1 and S2, Supplementary Fig. S2), similarly to its relatives and several *Saccharomyces* species^8,9^. These conserved genes and the following Mauve alignments allowed us to determine order of the Scs. They suggested the following order: Sc3-9-1 (ChrI), Sc7-5-8-6 (ChrII) and Sc4-2 (ChrIII) (Fig. 1). Togetherness of Sc9-Sc1, Sc5-Sc8-Sc6 was also proved by PCR reactions (Fig. 2a,b) and confirmed by the re-sequencing of the *S. cryophilus* genome^35^. At the same time, sequencing of the *S. cryophilus* centromers suggested an exchange in the localisation of Sc7 and Sc4^35^.

The Scs investigated belonged to three chromosomes, as the Pulsed Field Gel Electrophoresis proved it. However the *S. cryophilus* chromosomes had different sizes compared to *S. pombe* chromosomes (Fig. 2c). Since calculated length of the coherent Scs differed from the real size of the chromosomes (Fig. 2a,c), we assumed that there were unassembled and unidentified regions of the *S. cryophilus* chromosomes. To reduce the missing regions, we tried to find positions of the rDNA arrays, which often located in subtelomeric regions and their extensions could exceed 1Mbp^32,36,37^. According to synteny analysis of the sequences deposited on Broad, one rDNA array could be found on Sc7 (ChrIII^35^)(Fig. 2a), which was confirmed also by PCR reactions (Fig. 2d). While study of the gene content and order in the regions located next to the *S. octosporus* rDNA arrays shed light on a further *S. cryophilus* rDNA array on the Sc3 (ChrI) (Supplementary Table S2, Supplementary Fig. S2, Fig. 2a). These localisations are in good agreement with the re-sequencing data^35^, which also has revealed a third array on the ChrII and further atypical centromere-proximal rDNA repeats^35^.

Later, the genome conservation and chromosomal rearrangements were investigated using the newly established *S. cryophilus* chromosomes. Since the reordering of genetic elements could occur by different mechanisms, we primarily wanted to learn what kind of rearrangements formed the current *S. cryophilus* chromosome structure. YASS and Mauve analyses revealed a high number of chromosomal rearrangements, which were mainly interchromosomal translocations in the closely related species (Figs 1 and 3a, Supplementary Fig. S1). At the same time, the whole genome alignments also showed that the number of inversions increased with phylogenetic distance, which was also supported by GRIMM analysis (Fig. 3b). Interestingly, these data were not fission yeast-specific, since similar tendency was obtained from those *Saccharomycotina* species, whose dates of divergences approximately matched the *Schizosaccharomyces* species investigated (Fig. 3c,d,e). These data arose the question, whether the interchromosomal translocations could be more sustainable than inversions in short evolutionary terms? Since effects of both rearrangements types can be extensive, as they can change the gene expression pattern of a genome^38^, consequently they can lead to elevation of the fitness in certain environments^38,39^ or even reproductive isolation^40,41^, this possibility does not seem probable. Instead, we suppose that the underlying mechanisms generating rearrangements were responsible for the greater frequency of translocations. Accordingly, a decreasing number of translocations between the distantly related species do not necessarily originate from less translocation events; rather we suppose that the greater number of inversions tend to blur the traces of interchromosomal translocation events^2^.

Study of genomes of the related species^8^ and our earlier sequence alignments shed light on the highly conserved gene orders of the fission yeasts (Fig. 1, Supplementary Figs S2 and S3). Thus, we could suppose that number of chromosomal rearrangements were low, which could preserve the large LCBs. In contrast, our analyses suggested that more gross chromosomal changes could occur in the genomes of *S. cryophilus* and *S. octosporus* than in *S. cerevisiae* and *S. uvarum* (Table 2). Moreover, the breakpoint analysis pointed out that 5S rDNAs were often associated with breakage sites (Supplementary Fig. S5). These latter results resembled those data where rearrangement endpoints were correlated with repeated sequences^18,19,42^. The random breakage model prediction also supported that the chromosomal rearrangements in these two fission yeasts species probably did not occur randomly (Fig. 5). At the same time we have to take note that this trend seemed to be less obvious with increasing phylogenetic distances (Fig. 5). Consequently, we assume that inner regions of the large LCBs could contain a lower number of those sequences which predisposing to DNA breakage or recombination. This idea might be supported by data of other studies, where these large structural variations mainly occurred in positions of the low gene density regions^43^. Furthermore, repetitive sequences, which are inclined to attract insertions of the transposons that can also cause changes in the genome, seemed to be situated rather in the centromere or telomere regions^35^. That is, LCBs can be under stronger selection pressure. Moreover, the 3D architecture of the genomes could also contribute to the highly conserved gene order reviewed in^44^.

Besides this, we proved that the structural and sequence evolution in the fission yeast genomes might be correlated with the previously established amino acid divergences^8^ (Table 3), similarly to certain vertebrates, nematodes and arthropods^24–26^ and differently from the *Aspergillus* species^23^. However, a slight difference was discernible between *S. octosporus* and *S. cryophilus* compared to *S. pombe*, since genome evolution of the former might be faster (Table 3).

Taken together, we propose here a hypothetical assembly of the *S. cryophilus* Scs, whose comparative genomic analyses provided insights into genome evolution of the haploid *Schizosaccharomyces* species.

## Materials and Methods

### Yeast strains and media

The strains used in this study are listed in Table 1. Compositions of the rich culture media were the following: YPL: 2% glucose, 1% Scharlau casein tryptic peptone, 1% yeast extract, pH 6.7–6.9. YPA: YPL + 2% agar. YEL: 1% yeast extract (Scharlau, 07-079-500), 3% glucose (VWR). YEA: YEL + 2% agar. *S. pombe* cells were cultured at 30 °C, while *S. cryophilus* was incubated at 25 °C.

### DNA isolation and PCR amplification

Genomic DNA was isolated from exponential-phase yeast cultures grown either in YEL or in YPL with the glass bead method^45^. These genomic DNA and the primers listed in Table 1 were used in the PCR reactions. Since certain Scs contained overlapping sequences at their ends, the PCR primers were designed to hybridize outside these overlapping regions. Parameters for PCR reactions were optimised individually for each reaction. Parameters of Sc adjacency validation and rDNA amplification: 95 °C-3 min; 95 °C-30 sec; 54 °C-30 sec; 72 °C-3-5 min (steps 2–4 were repeated 30X); 72 °C-10 min. Gel electrophoresis was carried out in 1% agarose gel in 1xTBE buffer. Gels were stained with ethidium bromide and photos were taken by UV-Transilluminator (UVP Bio-Doc-It Imaging System). Gel photos were cropped in Microsoft Office PowerPoint 2013.

### Pulsed-field electrophoresis of the chromosomal DNA

Chromosomal preparations were obtained as described previously^46^. The samples (chromosomes in 1.5% LM agarose) were placed into the wells of 1% agarose gel. 0.5 × TBE cooled to 14 °C was used as a buffer. Electrophoresis was carried out on the CHEF-DR III apparatus (Bio-Rad) at 50 V in the following mode: (1) 48 h 2400 sec; (2) 70 h 3000 sec; and (3) 24 h 3300 sec^41^. After electrophoresis, the gel was stained with ethidium bromide, washed in distilled water and photographed with Olympus C-4000 Zoom digital camera under UV light. Background and contrast of gel photo was adjusted in and was cropped in Microsoft Office PowerPoint 2013.

### Bioinformatics

#### Genome sequence data

The nucleotid sequences of *Schizosaccharomyces pombe* (L972 h^−^)*, S. octosporus* (yFS286) and *S. cryophilus* (OY26) were downloaded from the database of Broad Institute (http://www.broadinstitute.org/annotation/genome/schizosaccharomyces_group/MultiDownloads.html), whose data were relocated in the meantime to the FungiDB (http://fungidb.org/fungidb/). Individual chromosome sequences with annotations were downloaded from NCBI with the following accession numbers: CU329670, CU329671 and CU329672 for *S. pombe*, KE503206, KE503207 and KE503208 for *S. octosporus*, KE546988, KE546989, KE546990, KE546991, KE546992, KE546993, KE546994, KE546995 and KE546996 for the contigs of *S. cryophilus*. The annotated files were imported to the SnapGene Viewer software (http://www.snapgene.com/products/snapgene_viewer). Chromosome sequences of *Saccharomyces cerevisiae* (S288C) were downloaded from *Saccharomyces* Genome Database (http://www.yeastgenome.org/download-data/sequence), *S. bayanus var. uvarum* (CBS7001) sequences were obtained from (http://www.saccharomycessensustricto.org)^47^. Individual chromosome sequences of *Naumovozyma castelli* (CBS 4309) were downloaded from GenBank with the following accessions: HE576752-HE576761.

#### BLAST analyses and sequence comparison

BLASTp search was performed in the website of Broad Institute (http://www.broadinstitute.org/annotation/genome/schizosaccharomyces_group/MultiHome.html) with the following parameters: E value: 1e-3, matrix: BLOSUM62 and BLOSUM45 and default parameters were used for the others. After the retirement of the *Schizosaccharomyces* website at Broad Institute, NCBI BLASTp search (http://blast.ncbi.nlm.nih.gov/Blast.cgi?PAGE=Proteins) and BLASTp search at PomBase (http://genomebrowser.pombase.org/Multi/Tools/Blast?db=core) were performed with standard parameters. We used the sequences of *S. pombe* as reference to identify the putative orthologues of *S. octosporus* and *S. cryophilus*. To make sure the results are reliable, reciprocal BLAST analyses were also carried out. Beside the sequence similarities, genes in the neighbourhood and predicted protein domains were also considered in the orthology inference. We ignored the single genes, only orthologues within synteny blocks were considered. To perform pairwise alignment a Needleman-Wunsch algorithm was used at the website of EMBL-EBI (http://www.ebi.ac.uk/Tools/psa/emboss_needle/nucleotide.html)^48^.

#### Whole genome alignments and rearrangement analyses

Whole genome alignments were generated with Mauve aligner using the progressive Mauve algorithm with standard parameters except minimum LCB weight, which was adjusted to 40^49^. Whole genome dot-plots were created with YASS (http://bioinfo.lifl.fr/yass/yass.php)^50^ with the following parameters: E value: 1.0E-30; X-drop: 50; window range: 100–200000; window incr.: 2X; hit criterion: double and default parameters were used for the others. For the nucleotide comparison we extracted the individual alignments in tabular form from all three pairwise alignments (*S. octosporus – S. cryophilus, S. pombe – S. cryophilus, S. pombe – S. octosporus*), but we considered only the statistically most significant (E value: 0) alignments and filtered out the non-syntenic repetitive regions like 5S RNAs, tRNAs and high copy number genes. The number of large scale inversions and translocations between the compared genomes were estimated with GRIMM v2.01 (http://grimm.ucsd.edu/cgi-bin/grimm.cgi)^15^.

#### Synteny analyses

Shared synteny of the subtelomeric genes were presented with the online tool Genome Synteny Viewer GSV (http://cas-bioinfo.cas.unt.edu/gsv/homepage.php)^51^ using the manually curated list of putative orthologues. Visualizations of whole genome syntenic relationships were displayed using the OrthoClusterDB online platform with the following parameters: order and strandedness: -r -s, synteny block size lower bound: 2, upper bound: 2000 and default parameters were used for the others (http://genome.sfu.ca/cgi-bin/orthoclusterdb/runortho.cgi)^52^.

#### Breakpoint analyses and breakage model

Chromosomal breakpoints were determined in the whole genome alignments (either in Mauve or in YASS) and inspected manually. Breakpoint associated sequences were extracted from the generated dot-plots and were identified in the corresponding annotated sequence files using SnapGene Viewer. To determine whether conserved LCBs in fission yeasts follow random breakage, the distribution of lengths of syntenic regions between large rearrangements were analysed. According to the random breakage model distances between breakpoints should follow an exponential distribution of the form f(x) = 1/L e−x/L, where L is the average size of all syntenic segments^20,23^.

#### Phylogenetic tree construction

Phylogenetic tree was created at the website of Phylogeny.fr (http://www.phylogeny.fr/)^53^ using the concatenation of 3 evolutionarily conserved protein sequences of the concerning species (Supplementary Table S5). The sequences were submitted to a manually adjusted workflow consisting of MUSCLE for alignment, GBLOCKS for curation of the alignment and PhyML with WAG substitution model for phylogeny. The number of substitution rate category was adjusted to 4, gamma distribution parameter and proportions of invariable sites were both estimated. Branch support was estimated from bootstrap analysis (100 replicates). The tree was displayed with FigTree v1.4.2 (http://tree.bio.ed.ac.uk/software/figtree/).

#### Statistical analyses

Normal distributions of the data were tested by Shapiro-Wilk and Anderson-Darling tests. Since most of our datasets proved not to be normally distributed, Kruskal-Wallis test was used for multiple comparison followed by pairwise Mann-Whitney U as post-hoc test. Wilcoxon signed rank test was used in the case of related pairwise datasets. Correlation of the data was tested by linear Pearson correlation test. *P* values were considered significant below the alpha level 0.05. All statistical analyses were performed in PAST v.3.20 software (https://folk.uio.no/ohammer/past/)^54^ and in Microsoft Office Excel 2013.

## Electronic supplementary material

Supplementary file 2

Supplementary file 3

Supplementary file 4

Supplementary tables

Supplementary figures

Supplementary file 1

## Data Availability

The sequences generated during the current study are available in the GenBank repository with the following accession numbers: MH605091- MH605096. Other data generated or analysed during this study are included in this published article (and its Supplementary Information files).
