# Supplementary material for: Assembly of Schizosaccharomyces cryophilus chromosomes and their comparative genomic analyses revealed principles of genome evolution of the haploid fission yeasts
Source: Sci Rep. 2018 Oct 2;8:14629. doi: 10.1038/s41598-018-32525-9 (PMC6168568; doi:10.1038/s41598-018-32525-9)
Supplement: Supplementary file 1 — Supplementary file 2 [file 41598_2018_32525_MOESM1_ESM.pdf]

Supplementary information

Assembly of *Schizosaccharomyces cryophilus* chromosomes and their comparative genomic analyses revealed principles of genome evolution of the haploid fission yeasts

Lajos Ács-Szabó, László Attila Papp, Zsuzsa Antunovics, Matthias Sipiczki and Ida Miklós

Department of Genetics and Applied Microbiology, Faculty of Science and Technology, University of Debrecen, Hungary

Correspondence: Ida Miklós, Department of Genetics and Applied Microbiology, Faculty of Science and Technology, University of Debrecen, 4032 Debrecen, Egyetem tér 1., Hungary

E-mail: miklos.ida@science.unideb.hu

Tel: +36-52-518-600/62056

>Chromosome I of *Schizosaccharomyces cryophilus*

caaaaactcaattgattattactgtaaaatcaaagactaagtatctttttactttaaaatattgataataaaagaatcagatcttcttaagtatga
gcattattgtatctactatttttgataatgcatttgtctttaagtaaatacactttgaggtttatagaaacataaaattaaatgattattagagagaag
tctaaatagaatgatgacttaatgaatcctttcaaggaaaagactgattataaaaaatttcagtaaagtacagtgttatatatactttgacttcttg
atagattggagctctgtagggctagagtaaactataataaatgcctcgattgtgaataatgaattgagaagaaaacagccataaattacaattag
ccaagtttcgtaatctttattagatccttcaaataataaaaatacggacaggataatcaatgagaggaattccgagagtaaatcttcataaaaaa
attttacgatcggccactaacaccgtgagaaatacattagaccttacagctataacttggtgaagtaaattgattaatcacgtaaatccaaaaaa
aaaatatgcgttttgattcagggactatatatatattcattacttcttgccatgacaggtttttttcaataccccttgacaactaaaaagtcctaaaa
tacttaagggagttgaagaatttcattgggaagctgataacttcttcaagtatttaattatgacaatagagcattgggattcctaagtgtaactcag
aatcgtaattgtagaaaatctcgtttcatacagagaaatcctaatttatcataagggttttgagtcgattgtttttattactatctgtttatatatatta
aatgtcataaattaaaaaaataaatcataacttattctattatgcgtaattaagatgagaacggaaattgctgcgcaagaaaaaacatatgatta
tttcctcaaaaacttttcccaaacaattagtacatggcccatccaccgaagctgagattacctgcccagtgatccatcgacttctatcttcagcaag
aaaagcgacgatttcagcaatgtcagaaggtaatccaatgcgattatcaaatggagtcatttttttttgcaaactgaattacttcatccgaaagtac
cccctttaacatatcggtatcaacagctcctggattaacttgatttacggtatggctaaataagatcgtcttcctactttcaatttagttaagcagatc
cctggacattactagcgtgtatatacgtagtacaactaagtccaattaacgtatacaattctacaatcgtagatattaattccacttgatttttattct
catagaccgtgagaattattaacgtgacacattgacttaacaccgcgcaaacataagtgacataaatttacttgttactcgagaaaatacagata
ggactaacccatggaatgattactaacgaaaaaattatgatgactaatacgatgatattacaaatctttgtctatataagacagaatgacaagcc
tttcagagcttaaaccactttcccatttaagatgtcagaaaatagcttgcccctccaaaacaaagtagcaatcgttacaggagcttcacgaggaat
tggagcagcaatagctgaaactctggcacgccgtggtgctaaagtagccattaccttcacatctgacagcagtattgatgcgactgaacagttggt
gaaaaaaataaagggttttggcacctcagcggatgcgatcaaaattcaggcagatttgcgtgatgtttcaagtgctaaacatattgttaatgccac
agttcaagcttttggtcctaccatccatatccttgttaacaatgcagggtacgcaggtccattaaaacctataggaacaataactatggaagattat
aacagtatgtttgatgtaaatgttcgtggtgtctttttttatgacagaagcgatagtgccacaccttcccaaaaaagaaaaagaaaatggaggaag
gatcataaattttggaagtgttgctggaaggttgggatttcttcagcagcctgtttattgtgcaagcaaagcagcaatcgaaggttttactagatgt
tgggccgccgaattaggccctcgagggcataccgttaatcaagttaatccaggagctgttgataccgatatgttaaagggggtactttcggatgaa
gtaattcagtttgcaaagaaaacgactccatttgaaaatcgcattggattaccttctgacattgctgaaatcgtcgcttttcttgctgaagatagaa
gtcgatggatcactgggcaggtaatctcagcttctggtggatgggccatgtattagatggtttagaggaaattaagtagaccatcacataagcagt
attcaatcttttgaactaattgccgttctttcacgtgttggaatcttttgaaagagtttttaataacgatcgattcaagcccttactttagtaaaggat
gtcgtaaatgaatatatttttggacatggagagatattcatttgcgattctgacgttgtcgttgcagacgtttcatacttatactcaactggattatct
aatattcattataaagaaggaatcaactacacgatagatcttgtcaagtgtatatatttttcactattctcttcgtacttaatttagtgcaatgtttaa
gctcttttctgaagtaatcctctaatcaatttaatagctaaaatattttaaaagtagttactaaattctgtttcacattaatagaaagttccgttatcgt
tcctgtgacagtattattttcacgagaagattccttattatgctataaaaaatcgagaactacatatacatttgctgtctgttgacatattgcctaag
agaaatttccattgttccacatttaattttcattttaggagaaagcatagaaattgtactaaataaagtacgaaaaaatagtgaagaaaatatcct
ttacaagatccatcataaacttgatcccttttttacacatcagttaataaccctattaaattagaacatcaagcttcaatttttttaaagctacattatt
tctatttctaaacatgataaactttatctttcttaaatcgattttcctttacattatgctattattcatttgctttacctttaaagagccaaaatgtgtaa
gaagaaatagccaagcgacttttttaatttgattatcttgaagtttaagtaaattaaaaggtgtctaaaatttacagtatagaaatactacgtattta
aaagtagtgaaaaaggttggatgactattaatttgtttattgagggttaaacaaacgttagataatgaaactagttgcataccttctctgactccaa
atctgaccattcttaaaggaaccgaatttccagctgaaaccagattctttcaattttacttattacgattggaattttggatgtgtttaacaagtaaat
tgctttcgactttacctttagttctcttgaacttcgcnnnnnnnnnnnnnnnnnnnnnnnnnnnnnnnnnnnnnnnnnnnnnnnnn
nnnnnnnnnnnnnnnnnnnnnnnnnnnnnnnnnnnnnnnnnnnnnnnnnnnnnnncaatttttaaagctacattatttctatttctaaaca
tgataaactttatctttcttaaatcgattttcctttacattatgctattattcatttgctttacctttaaagagccaaaatgtgtaagaagaaatagcca
agcgacttttttaatttgattatcttgaagttaagtaaattaaaaggtgtctaaaatttacagtatagaaatactacgtatttaaaagtagtgaaaaa
ggttggatgactattaatttgtttattgagggttaaacaaacgttagataatgaaactagttgcataccttctctgactccaaatctgaccattctta
aaggaaccgaatttccagctgaaaccagattctttcaattttacttattacgattggaattttggatgtgtttaacaagtaaattgctttcgactttac
ctttagttctcttgaacttcgcatttagtggaactgagaatatttacattcaaaaaggaaacaaaaagcttccgtcgaataaactcataggagaaa
aaatccgataaaagctgaatccgatgtcttttatactccctgcatcaatatatggaagctaacagattttcatttccggaaaattcaattggttcaa
gtacaatacgttgttaatcagcagctttagctttcgtatgtgttgtgtaacattgtaccatttctataatttatattctatcagttagcaaatgggcga
acagtgtttctttaatttagctaacactattgattggaatcttcttatgtaagtagattaaaaatatttatcagaaaatgttattgatagtgcatttcta

gtaagaacttgcatttatgtggttttcacttggatagaatgagttgtcgcgannnnnnnnnnnnnnnnnnnnnnnnnnnnnnnnnn
nnnnnnnnnnnnnnnnnnnnnnnnnnnnnnnnnnnnnnnnnnnnnnnnnnnnnnnnnnnnnnnnnnnnnnttcttaaaggaaccgaatt
tccagctgaaaccagattctttcaattttacttattacgattggaattttggatgtgtttaacaagtaaattgctttcgactttacctttagttctcttga
acttcgcatttagtggaactgagaatatttacattcaaaaaggaaacaaaaagcttccgtcgaataaactcataggagaaaaaatccgataaaa
gctgaatccgatgtcttttatactccctgcatcaatatatggaagctaacagattttcatttccggaaaattcaattggttcaagtacaatacgttgtt
aatcagcagctttagctttcgtatgtgttgtgtaacattgtaccatttctataatttatattctatcagttagcaaatgggcgaacagtgcttctttaat
ttagctaacaatacggtcgctattgattggaatcttcttatgtaagtagattaaaaatatttatcagaaaatgttattgatagtgcatttctagtaag
aacttgcatttatgtggttttcacttggatagaatgagttgtcgcgagattactagtggtatggttttgcttctttgtaaactattattatagtattttcc
ttggattaaaatttaaatgctcaactgaaacacaacatcgcccatgcttttgagaaatcaacaaaaggattatgtaccattaccactgcgaacgac
tacgagtaaataattggggcttcagagcaatcctactagctcaacttcgtgaagtgcagaatatgccgatatcattggctcacgacaaatgcaaa
tatttggcattcaaaagcagtatgctcaaaacaacaatacttctacggatgtcattcttcaatactcataatcatagatcctaagggaatccagaa
aactgataagctaaatcataagcactcattaaaccgacaacatcgcggagcctttctttcaatttaatagtttcattagggttaaatcttattccata
cccgtttaaataagaagtgcactgatcttttgttaacagatcgatatcttcaactgatctaatttgaggtaatttttcagtaggttcctctccatttaa
aaaaggtacaacgtcgataggaaaccctaaggatcttcgctgaacgtttgctattctgtcaattcttgcttccattcgacctattctgttaaatagtc
ctgaaaatccattttcatcattcacccacatctgaaaccatcttggaggtgtatcctcaagagtacgctctctttgtctgaattgggaagctacattc
agaatctgttgcatttggagacgaacattaacctcattctcaatactccttcccttggtagcaatttcaaagtcttttaaaaacctcataacatcctc
agatgtttcagtcgtgggcggaggaatattgaattcatttacaaaaactcttgcatccatcttaaattagtatccacttatttgtcactatgctagaa
aaaggttggtaaaacttgtaagcttaggataaacagaattttgtagtcggatgaaaataaatagtagatgtctgcgcttctatctgtcagagcaat
ccttaataaaattacaaaccatttggtaatgattatactatggagtatatgacttactattgtcacgttaataattctcacggtctatgagaataaaa
atcaagtggaattaatatctacgattgtagaattgtatacgttaattcgacttagttgtactacgtatctagatggtagtaatgtccagggatctggt
taactaaattgaaagtaggaaaacgatcttatttagccagaccagtcgaattttccaatctgaagagttcgactgtggattcatactttatatatctt
cgctgaaacaactaaagtaatctatctctttgtataacaaactttctctgtatataagacttccgttccgaaaggctttccgatttgttgaaatcgtat
tttctttgttttgtttaccttttgtaatatcttatccttatcctttagtcttattgcatttgctaatacttagaaatatgctcttatgatagtgtattgttata
tttataatataaacaagacaactatcaacgttaataaacaaacaaacaaaacagtttgataggttgaacgatcgtcactgattagttaaagtttat
ggtttgtttacttcccagttttaggttactatataaacgcccgttcttctcagacgaattcttcagttaggaagattttgctggcagaggataatagac
gtgtctaagtaacataatcattagtataaccaggacgttactaaagatatatcactccagatatttagtaacttattttagagtaatcaacttgaca
tttaaagcaattacaattgtattccactcacattcttataactatacggagtattttcccatgacagtatccaaataattatcttcctaaccgaagtg
gaatcgttagatccgaatacgcaaatcaataagccaaaatgtctcagaacgcattgagtccaaatattctcagcgaatattttcaacaatacgag
gagcatatcaatgaattaacgactagaatgagtctagtgacgcaaggaatgattgatattaaggaagagaaaaagaaagaactaacgtcccaa
atggaaattgatgaagactacatatccgtcgaatacactgacatcatcagaatgaccatcagagcttatggagaaaacgctcacgtatacatttc
acgattaaatcagaaagaacccgacttatatgactctgagaaagaatcgacaaaagtctttttaaagaaattagaaaatgttttcgaattgttacc
attagtattcgtttcagatcgagagaaaataggatatctttataacagatctcctgaaaatcttaaggaaaagataaaagaatccgttaagaaac
cgggaccacatagaacattcaaaaatatttataatcaagtttattcgaaagaacaattttcgacttggttgactcaattacatacagtcatggatca
agcattgaaacttgaagtaaatacgcaagagtacagacattggttcgaagaattagataacgaaacaagattcaaaatgcatcttttcatggtac
atccttttgctataagaaaagaacttcttagaaacaattatgaaactggtgattatgacaagtcaataaatatcacgaaagaaaaatttcgttacg
gcaatttccgaagatatgagaaacataatgggcgaacattttcaaatacgaaacctttaaacaaaaggtttcctagtaaagacgaatttaagaa
aagcgagaacgataaaccaaaattcaagaaattggaatgactagatgattgttgtccagcactacagaccaaaacaaatcacacaagaatttg
ataaagaccgaacatttgtaggaataactaaacacattctaaaagttactgggaagttaacattaccagtggaaatccgaggaagaaaatacca
cgaaacataaactatatcgccaaggattgaagaagacgccattatcggttggtcattaattgaaaagatgaacaaattagaaggtttaagcaaa
attgaaataaaagaaagccacgatgagaattttaagaataatgaggaaattaaaaaaattatgaaacctttttcagaaatttttgaggaacctac
taggactccaatacctgaacactcagagagtgatttgaaaatagaattaacagaagaattaccaacgtatgcgccaaaattaagaccttataca
cagaaggaaagagaatgcataaaggaaaagactgacgaattattaaaacaaggcaggattcgtcgaagctcttcaaagtttttatgtgtgccat
tgattgttccaaaaaagggaacagatagatatagaatgttagtaaattacaagcctttaaatgcgataacaaaaagagataaatatccgatacc
taacttcgaagaaattataaattggttaaacggagccaaagttttctcaacattagacttgaaggacgcatatcatttacttaggatcagggaagg
agatgagtataaaactgcttttagaacacctttcggatcatttgaatatacagttatgccatttggaataacaaatgcgccagcgaattttcaaag
atatattaacaaagtgttacttccttttattggcatatttgttatcgtttatttagacgatattctcgtattttcatcatcagaaaaggaccatgaaaag
catttgcaacaggttttcgaaagattgcagaaagctaacctatacgcaaaccgagaaaaaatgtcatttttttcacaaacaaagtaaagtttctag
gccacatattctccgataaaggtattgaaattgatccagagaggattcaagcagtcgcaaattggtcaatacccaatactcttagaagattacaa

ccatttttagaattcgtaaattacagccgacgatttatttatccaaattagcgagacctttatatgatctaaccaagaaaaacactaaagttaaatg
gactgatcgtcaacaacaagcattcttacaagttaagaaagagctcgaaaatgtttctatgctcagacattatgagccacatcgagaaactagat
tagaaacggatgcttctgattttgctataggagccatactctcccaaaaacaagacgacgaaatttatcatcccatcgcgtttcattcagagacatt
tacagatcggcaacgaatttggccaacatgggtaaaagaagcatatgcaattatagcaactttacgtaaatggtatcatttttggcaggtactga
caaaccatttcaaattcttactgatcataagaatttaacatcaatgttcactaaggttcacacagacactcactcaaaaatcatgcgatgggcaac
tgaactagatttatacaattttgaaatcgaataccgccctggtcgaaaaaatgaacctgcagacgcactttctcgtcgtccagatcacaaaactga
aatagttaatcgagtcgtggaaacagacattctcacaaacatattcgactgcgtgagacaggagtatttccatcacaagaatttaaagaatattct
taatggaataaatcgtaacccacaatacactgtgaaaaatggtctcatcgtcaatgatcaaggacaaattgtcattcctaaactaccacaacttgt
acaactcatatgcaatcagtttcatgatcagccagtctcaggaggacatcgtggaaaaatgtccacaacaaatcgcatccgcgaaaagttttact
ggaaaggtatgaccgcagacatccaaaaatatgtcaaaaattgtgacatatgtcaacgacatataactcgaaatcatcaacgatatggtcaatt
acaaccgttacccattgcagaaaagccattcagacattattctatggattttattaacaggattaccacctnnnnnnnnnnnnnnnnnnn
nnnnnnnnnnnnnnnnnnnnnnnnnnnnnnnnnnnnnnnnnnnnnnnnnnnnnnnnnnnnnnnnnn
nnnnnnnnnnnnnnnnnnnnnnnnnnnnnnnnnnnnnnnnnnnnnnnnacccattgcagaaaaccattcagacat
tattctatggattttataacaggattaccaccttctcaaggtaaagatgcaattctaaccattgtagatcgttttaccaaaatggcttggtttatcgct
tgtaacacaacaacaaccacggatcagttaattgaaatattcgagaacaaaatcatatccagttttggatatccaattgatattgtttcagaccga
ggacctgtttttatttcagaaaagtggcgacaatatgccaaagaccataacatacaccttatgttatcttcaggatatcatccacaaacaaacgga
caaacagagcgcgtcaatcaaatcttggagtcatatcttcgttgctacgtgaacaaacatttaacaaattgggtcaattatcttctcaaagctcagt
tatgttataactcttcacagcactctgctacaaaaatagctccgattaatgctgcttttggtattcaagctatagttaccgcttgggatacgcaaatc
ctggatgaactagatattccttatttaccggacagaacagaatccatcgatgaaatacgtgaacgtatcagaagagcaaatgataaatacgcaa
agtattacaaccaaaagacaattccacctcgatttttcaacctaacgatcaggtattagtcaaaaatggcaaagtgggatttttccatcaatcaaa
taaacttgaaccaacattgttggtccattttcagtaaaaaggaaaataggagaacataactatgagttaaattttcctgaaacgatacgaagacg
ctatcctgctagttttcatgtatccgaactcgaaccttacaacgcggacttaagctcaaacaattttatgcaaaacatcattattgatgaaattttag
aagctgataacctagaaaagccttctcactacaattgtttatatagatcgcaactttcagattatgactaacgctcagtcacctgggttcctagaga
catcgtccgaaaaaaggcatactcaaagctcattgaatttactcgtcgcgcttctccaacagaagaaccgcaatgaaaataagagtcttccaatt
caaggaagtgaaagactcttaggcagggaggtgtcataattgggtccctcgttagcattcataatataacaatccgttataagaatggaatatta
gctaatatcagttaatattcataagctaaaagaattgtgcttgataataagtaaacaaaaatttgaaaatgcaaatttctccttgaagcagaacat
acgattaaagatttactaacgtatgataattagtactatttataaatcagtaacatttgaagttttatagcgaaatctttagatcggaagatttgact
gatcaggctaaataagattgacacttagtcgtttcatttagttaatcagatccctggacatattactattactctaagtagcactaacacgtccaatt
ccaatagacaactttgtaactgtatttattacttccactcgatttacattctcataacgtgtgaaatttctcccgtgacaataaagcactttgcaataa
ttatgctatggagtatattactgtacatcaatgttaaacaaacaaactgagaaaacagactgaaaaattaaataataattaagtttattgtttcttt
atttcctagttttagattactatataaacgcccgttcttatcacacaaaatcttcagttacgaagattttgctggcagacctaataaacttgtttaata
acatattcattagtataaccaggacgttactaaagatatatcactccagatatttagtaacttatttttagagtattcaacttgacatttaaagcaat
tacaactgttttccacttacactcatctaactatacggaatatttcctgtgacagtatcgaggtaactaaggattgttatgttctatgccctctagccc
tgacggcagcgatcactcagatacctgacataagattagtagcagtcagccaaggctatgaatacagggtttgccaatagggtaacccggaaaa
tttgcgatcagtacatatcttaaactgattaactgcctgctatatgccagtataggctgctttgacatggcagagataaccaaagaccgtagtacg
acatagtgcgatgttggaatcttaagcatcataatccatatttgttttactgtataagatccctcgaacattggttctagtttgttgttctgttaaaag
gatccgattacccttattaactaagacacaatagcatagttgaattattttgaggatgagcgtccctgttatagtgtgcagcgtatttttttgttcttttt
cctcagtcgctggcttgcgtattgtctcacatcaaaacgaaggattaggtacataataaaaccgcacttgatgtagatagggaaaatcccagtctg
tctgttatcgaaggaaggccacattttgttgttatatattgtgagatgtcagaataaaatcgagctttgactgaaacaagggtctaattactcaaat
gttcttgagtagtaagatgattttcgagggtttaatcgattctcagtattgccatttctttgaagataggctacgttgtattttgtggttttgtggtcttg
catgttgtatttccatttatcggaagtaaaaacactttttctttgtctgacacaatgtcttctcagaaaccgttatttcctacaattctttcgtttaatat
ttgataatttttaaattcgagtccctttttccttacgatgtgtgcaatcctgtaatcaagtccataaaaagatgttataagggctttttttgttattggt
aatggttgtaactttccatatttttagtgatttctagataatgtcattgtgatatgtagcagtttctcatatatacctgcacgtctcttagtaactttcct
tcgactgcttcctgttgtaggttgatcatgatacttatcaaacatagcttgtatatgttgtagcagctctatcccaattaagatctgccttcgagtaaa
accaattttgactgagaagtcagagtcagaatcaatacccgtaaaactattgtgaaattctctgtgaggagggtgttcatctctcatgtaatcgaa
atgtttgtcaaatgatcagcttctatcacataatagatcgttgaagtctttagatcttcttggcgagatacggcatcagatgggtcactctttcgttct
gatcaataacgaatgtcaaagctacttagatttaatttcgcggaccatctttgcatttttttcgtcaatatctgtacgaatttcgtaaacattgtcgtta
gaattttatgatatgtttaaagttcggaaggttcatctggatctgtaagcaagctggaacattttctaatggttgatattataaaatatgcttcttttc

cctacgttaaacttttgctgatctgttaacgatgttggaataaaatgcggctggatgaagatttccatcgtctttgttgttagaatagcatagctccg
ggagcaaagttagaagcatcagtttccaacctagtttgtctttttgggtcataatgccttagcataagaataatttcgattccttttcagaaaatat
gtggccaaaggatttagctttgtcattgcaaaattgacatctctatgatttctcctttatttttctggaatacttcttgcaaatgtactttgcgttactct
tccgatcaagattgatagtctaagtataatggaaatgctgagcaaaggaaaaatatcttgtttatatatccttgagatttttctggtgcgtttgttaa
tccaatcttcgaatcagtagtaacggagtacggaggcaacgatatcttggtattctctgaatacatataaaaaatggcttttatttattcttgaactt
tcgcatcaatttgctatgtttttgatcttcctccgctatcgtgccggcgtataaaaaatccaatttcccaatttttcagggagggcccatcctatgaga
actcatcttcagaattgtagtacatatggactctcataaacctttgacatcaagtttatcttgcgttgaatcccgaaagttcgattttctttgttggat
ttacgcttatttcgttttgcaaaacctgattatgtattgttgttacgaccgcaacgtgatttccatgctcgtaattttgtttgaggggctcgtttccaaa
ataatttgaatggatgcgaatgccacgactttcttgattgtatccatatctaatccgtcgagccacttgtgaattacatcaacgtctaattcaccacg
agcttccctttgctaaatatataggaacgggacgaaatcaacttttccctcgttcctttcagaaacgtctgttcagagacaatcttaaaggtcctctg
tgggcctggtacctttacaaattctcatatctgtctttggtaaagttctggggatttggaatataaaaaagcaattctctcgtggtcagatgaaaata
ctttaggtatgtattcaaaaaattttacagattgttcagctggctcaattattccaggactgagcgggtggatcatgttcaaatacgagtttgttcttg
gctcatatgcttcatgtacgaacgctcataatatcccaaggtttttctgggatatagtcttcatttaattccaattgatttgattgtctcgtttgctgatt
ttttgtgaaaatattcatatcgtcatggcctgtatttttgcagtaattgatttggttgtgcgagtatgacttccacatttatttcagttcaaaatttgcct
gtccatcctcattaaatttcaagttgcttattttaatttcaatgattcctctcagcaaaactggaagataaattgtcacggcagaaatcaaaaaccg
taaaattagtgttaagtcaagtggaataatcaacaaaagttgtaacttcatgtaccgaattggactctgctgtactacgtgtatagacgctagtaat
gtccagggtctggttaactaaattgaaaatgtgaaaggcgatcttatataccagtcgaatactccaatctgaagattttgactgaagactcgtgtc
actaataaagcaagaagtttccaacaatggaatggttacctaaaaagtttaagaagcagtacctttcagttttgaacgaataaagaaatgatggt
atcattaacatctttttatattaattttcgatgttaactaatacatagccaatccaccagaagccgaaatcacctgtccagtgatccatcgactgct
accatcgacaagaaaactagcaacctctgctatctcttgaggttgcccatagcgattctcaaatggagtatttttgcgagctctacgcctccacttc
caaatcccttcagcatgtcggtgtcaacggctcctggattgatttgatttacggtatggccttgagggcctaattcggcggcccaacatcttgtgaa
accttcgactgctgctttgcttgcacagtatacagaatgttgaggaaatcctatccttccagcaacacttccgatattaatgattcttcctccattttct
ggaagatatggcactatcgcttctgtcatgaaaaagacgccacgaacatttacatcaaaaatactcgaataatcttctatggtgattgttcctatag
gttttggttgacctgcgtaccctgcattgttaacaaggatatggatggtaggaccaaaagcttgaactgtggcatcaacaatatgtttagcacttga
aacatcacgcaaatctgcctgaattttgatcgcatccgctgaggtgccaaaaccctttattttttcaccaactgttcagtcgcatcaatgctgctgt
cagatgtgaaggtaatggctactttagcaccacggcgtgccagagtttcagctattgctgctccaattcctcgtgaagctcctgtaacgattgctac
tttgttttggaggggcaagctattttctgacatcttaaatgggaaagtggtttaagctctgaaaggcttgtcattctgtcttatatagacaaagatttg
taatatcatcgtattagtcatcataatttttcgttagtaatcattccatgggttagtcctatttgtattttctcgagtaacaaggaaatttatgtcactt
atgtttgcgtggtgttaagtcaatgtcaccataggtgttttgtaaacatttgaaatattacagtaggcttgtctgtcacaagcaatctcacgccgtat
attttcaggtggaaactgaacgctgctgtacaacatacatagattctagtaatgtccagggatctagttaaattcaatggatgacagtaaacgcga
ttttatacacatatcggtggaatctttcgatctcgagattctttggaggacctttaatttaattataattacaatagaataattattgagaagcattac
ggaagaaatttccatttcagaaaagagcttaaacacggcactaaattaagtacgaagagaatagtgaaaaatatatacacttgacaagatctat
cgtgtagttgattccttctttataatgaatattagataatccagttgagtataagtataaaacgtctgcaacgacaacgtcaaaatcgcaaatgaat
atctctccatgtccaaaaatatattcatttacgacatcctttatttactaaagtaagggcttgaatcgatcgttattaaaaactctttcaaaagattcc
aacacgtgaaagaacggcaattagttcaaaagattgaatactgcttatgtgatggtctacttaatttcctctaaaccatctaatacatggcccatcc
accagaagctgaaattacctgcccagtgatccatcgacttctatcttcagcaacaaaagcagcaacctcagcaatgtcagaaggtaatccaatgc
gattttcaaatggagtcatttttttgcaaactgaattatttcatctggaactcccttaaacatatctgtttcaacagctcctggattaacttgatttac
ggtatggccttgagggcctaattcggcggcccaacatcttgtaaaaccttcgattgctgctttgcttgcacagtatacagaattttgaggaaatccc
atccttccacctaagcttccgatattaatgattcttcctccatttttgggaagatatggcacaattgcttctgtcatgaaaaagacgccacgaacatt
tacatcaaaaatactcgaataatcttctatggtgattgttcctataggttttggttgacctgcgcatcccgcattgttgacaaggatatggatggtgg
aaccaaaagcttgtactgtggtatcaacaatatgtttagcacttgaaacatcacgtaaatctgcctgaattttgattgcatctgctgaggtgccaaa
acccttaattttttcaccaatagttctgccgcatcaatgctgctgtcagaggtaaaggtaatggctactttagcaccacggcgtgccaaagtttca
gctattgctgctccaattcctcgtgaagctcctgtaacgattgctactttgttttgaagagaaagattgtattctgacattttacatggttgtggttgcg
tcctccgatcttcggaacgcagatagattaaaggttacacaggtttgtcacttctttgtaaatgtctgtcattgtcattatcatgataggtgttttgtta
aaatcaaagactacagtacgcttactattcggattgacgaatattagtgaatgccataaggctaaaggatgagatattacaaatagtaaacgaa
gcatggaaactttttcaaaatgtttataaaccacggaattattaaaataaagaaacagattacgctagcaatttcaaaaagctatatgaagaggt
aactgtcttaaccaaaaaaacttttgtaggatagcttctctgaaatataatatggacaatttgaggtgtttcaacaattcatcatcatttatcgttctt
ttatttttgtcacatatcgtcttttagtcatcaattgttgtcgcaatacaaccgtagttccaaataccaaaaggccagattcgatgagtcgagtgac

aagttatttacatggagtccttcttatataaatccttttttgcgtctgctattgcatgcttaatgttttattctctacgtgttttgagtaaaaagcattttac
taagttggattaaaaaacggaataaaaacgacttcactgagaacgattgtcatgtctgatattcgaatagtttacattgtatattgaaaaaaggta
tgatgcaacaacccgtcccatacgtaatatttagtactttttaagaacatcagcgaaggaaataccgaaattcagacgggtaaaaatatatgaca
cgatgtgtggtatataaagacgatggacaacgcgtaaaatgatctgccaattgtcaaataccgaagaaaatatgcatctgtcaactcatacgtgg
atgaggcctgagcctctagaaacgaccttgaaacgcgctagtgaattaggttatgaaagtatcgaattggctggtattcctgaacagtatccaatt
gagcagacgaaaaagctactcgataagtatggactaaaatgctggggaacagtgactttacaaatgggtgatcatgatcttgcggcttctaaccc
tgagcaacgaaaaaagacagtacaatacgtcaaagatgttgtagatatggctgctggtttgggtgggagcatcacaagtgtcgtgccggtaacg
gtaggtaagcttgcaccaacctcctccccagagaatgaatggaattgggtgattgaaggtttgaaggaagttcataagcatgccgtatctcgcgg
gataaaaataggtttggagccgttgaaccgtttcgaaacatatttcttgaatcgaatcgatcaagcgcttgctttagccgatgcggtcggccctga
gattggtattgcgttagatccatttcatttaaacattgaagaagccaatttgttttcagcaattaaatcggctggtacaagaattgttgacgtacatg
tttcagacaataatagacttccagttggagaaggaagcttggactggaagaaaataattgatatgttgaatgaattaggatataaaggaggatta
gctgtcgaatttatgccccctattgacagaacaccagttagtccatatcctcaacaacttgaaacaaagcctgttgaggttcctccagcattacgaa
aatttattgaagatcatgcttcaagcattttaaatgatgattactacactagtctaatgagaaaatcagccgaaagtgttcaagctcttctcgggaa
ataatcttaatacaaatattagcaacacctatttgtatgagataatatacgaataataatattaatgacgttgttaatttataacttgatacttttgct
ttgtcgaagagtgtttagggtaaacaattcagaggtaactcttatattggcatatttagcatataccacagtcgttttcaggggaaaaggaaaaaa
atctgcgattaagagaattattagctgaactatttcttacactaacattaaaagttcaaagacaagcaggagaataggttcaagagactgaattg
aatcaataagtatgttacatgtagaatttaagcttcttaggttctcacttcgctttaagtgtaacaaattcgtggttttaggtttgaagctctataatt
attaataccttttgagacatagacagccagccaagttaggtttatttaaataactaaaaaaggtgtaatataagatgagtcacgaaagacgaaag
caaagcgtttttttgggtaatacatctttaattaagtttgaatatccaatccaagaatatcggaggccttattctaacgatcacactttactatttttcc
tacgcatgacgctcagaaacgtttcagcatgcttttaagcactataagactacgattactaagatttaactaacaggcctcttatattattacagta
cgtgaaccattcttttgtttattccattttgaacataacagtagaaactacattgatacttttacagtcgaacaaacatacaaaatagtcaaaatca
taaggttaagcatagtaattcattattgacataactatattttcgtaatttatctcgtcgttccattcagagataatttgagttaaaagatcaattttttc
acaggacttcatcctcaatgttcgtaattatgaagcctttcattatgctcaagttcttcctttgcgtgttccttagcttcttcagacacgttcggattgt
gcgtggtagccttatatcctctttcaacatttcttgggtttttacctcctgatccggactcgctgtattccgcaccatgactttctaggtattctttttcat
gttgtttagtctcttcagagacatttggattatgtaaagcagctttatgtcctgcgttaactctttttgaatctgccattatcgattgtttttatttgata
agccatagatggtacttaagttatttataactattttaagtggcaagaatagtctgagaaacgtcaatctttatttcgagaacatcactttcattgac
atttgcatatttaattttaacgttagatgacgcgtatatgtcacacaataacttatataacaaagtagtgctacatattttttttttttttcttttcttgg
aagaaattacactgacgtaaatattcctattatttcgaaagaatctgaatgactttgcttatgtttagtctgagaagccttagatatgctatatgtca
caatcccctactcctaagcaattatcctttgcaatataattagaaaataattattatttcttaaatggtttacttatatctacttaaacaaaaaaatca
ataaaataaatattcataagaaaaatggttcaaaaaggtatttcttcaggataaatttgcctaattgctttggaacatatgaggttcggctttgttc
ataggcggagacacgttttcagccgtctcttttgcaaccttttcagtagcttcgcgaatgcgttctcccaaattatggtgcacccgaccaaatacctt
atattggcgctcacgaatttcgggaagagcaccggaaagatggccagcaacgtttttgacaaaattgtcttgttggccaggtttcttaccgagaac
attcaaccaaaaggctctaggctgctcaaaatcgatgtcggtaatttcatccatgtggtaagtaacttcaccaacccatttttcatgcttctcatttg
gctcatgatactcctttgctaagggctgtaatgaagaaggatagtttggcatgctgccttggttgtcagtgacattcatgggaccatcacgagaata
attaaaaacggggcatttagggctgtttacaggaatttgctcaaagttggcacctaagcgatggcgatgagtatcgggataactaaaagtacgaa
cttgcaagacaggatcagcggaaatctcgataccaggaaccatatgagagggagagaaagcagcttgctcaatttcagcgaaaaagttttgag
ggttttgatttaaagtgaaacgaccgacttcttgcataggaacatctttgtggggccaaaccttggtaaggtcaaagatgttgtatctgaatttctc
agcctcctcaggagtcatggtttggacataaagagtccagctgggatagtcaccgcgctcaatggaatcaaagaggtctttgcgagcatggtcag
gattagatccatccaaggccttagcttcctcatcagttaagcctttggtaccttgatcagaaataaaatgccatttgcagtagtaaaattcgccttta
tcattgacaaacttgtacgtatggctagagaatccattcatgaaacggtaaccatagggcgttccaccgagatcggaaaagagaatcatgacctg
atgaatagattcttcgttttgagatagataatcccaaaacatggtcgcatccttcatgtcagtttgcgggtttctcttttgagtatgaatgaacaaag
ggaatttggcggggtcacgaatgaaaaaagtgggagtattgttaccaaccatatcaaagattccgtcttcggtatagaacttgagagcgaaacca
cgagggtcacgagcactgtcgggagtgccgcgttcaccacccacggtagagaaacgagccaacatgggagtcttctttccaacctcagagaaca
ttgcatgcttagtgtacttggtgatatccttggtgcactcaaaaacaccatgggcaccagagcctttagcatgaacaacacgttctggaatacgctc
acgatcaaagtgttggaaaacgtcaatcaagtggaaatcttgaagcagaatagggccactttttccaacacgggcgctggccattgggttgaaa
atggggcagcccgtgttagttctataaacgttttgtgacataattctgggggatcttgaaatgttacgcttgctaatgtaaggagtagttactttagg
aaaacaaactcgaaaaccgcgagacgatgacactttggaaaatgtcatgtaagctcagaataaaagagagtagaataaaagagaatgaaata
tacagaaattcactcttgtagaatcctgagtgaaaaggtttaaattgcagtggatatgaacggcgacattttgaaaagaaaataaagctcctttta

tattgtattcctgcactgtctagaattatgtaattcaagatcaatgaatcatattcttacatatagaattggccgaggcacgattggcttgtcatcaa
gtacgtaattgtggtagactgtttaacgatccaataaaacagcttacaatagttctataacattaagtattgcttatgcgagagaggttggtagtga
tcggctgttaatccttatgtaagtcattttcctagaacaaaaattggcttgtctagaacatgctaaaaattgaagcgacacttataaagaaataaa
aataaatagataaaagatgctgtaagaaagaaaaaagtgtttacagaatgtttgcttataaattcgttttatttgttggatgaacggttaaagatg
aagatgtgtacaaatctattctgttgtggattctactacatacataacaatgaattttagttttcttccttggtaaaggactgcaataatattctttgtt
attattatttatgtttatgtataattctgtatgactactgcaagttaatgctcttgataatctttcaataagattagtaaagtataagtattaatattgg
taaagacgtatgaaaagtgtaagaaacaataatttcatgtaaaaagatatatagcatgtggatggaaggtatgaacgaaactcgtcttcctgtttt
cacaataatttatcgatgttattatgaatatcaaaatatagttatccttgtctcatccttgcgatgcaacttgattaaaccaaagaaaaaaagtttta
agtttctaagaaaataaagcataatacctgaaaaatagtagaaaagaaagcgaagaggagaaaaagtatgaaacggggattttgcgaataaa
gcaggtaagggacaaaaaccatgaaacgcagttgtttacttcaagtccaattggttaaactcatcttcagcaaacttttcatcttgcaaattctaac
actggttagattgaatactttcagcaaacatgttgtgacaaaagagattgcataccttttgatgctccgccaaagcagccaaacggcgctttctatg
agcaaaataagattgaccaagcataacagtaccaccaacagttccaaatagcaaaagggaaaccgatcctttctggatgaagtcgtaaatacg
gccacgattcattttgaaggctagaattaattagcattcattctaatacaaacattaagatgatctcttaaatactactcaaaatatcccatcatcat
agacaatcaccgtcatacggatggcgaccgttgaatcacttgcttacctatcaattcaaaaagtagtatgaaaacaaaattgccctaggatatctg
taaagtaaagaaaaaaagattaaaaagatgttatacgatgacttctggaaggcacccaagaatgtgatctagctaccgtttccggtctgcattga
gttggcatgaaaccagaatagagagcagtacaatatttttctacgcaacacagcgtatatttaaattttaagcaacctacacacttgtattatcaa
aaaaatgtttttaaaaaaaaaaaataatttttggctttttctattgaatgtatctattataagcttatgtttacacgtggtgtgcgactcacttattct
atatccgttgcttttcctaaaaaaaaaagttcgctatttctgttaacgcacttttaaccagtacgaacacagctgattaagaacgctactgtgctcgc
tattttaacactcagcaatgctgaaacagaaacaacagacgagccgacctaagaaatcgaaagcaaggcttgtttttttataggcttttagaaa
aataaattttggggaaaaacaagcaggaagcctcgctgagaatttgaaaatcgcatagggaagaaatagcaattcgaagtagcccaaaattttc
tatcgaatttgttggcatgtcgaagagtcttgagccttcagcaaataataagggctttattgtcaatgctttaaaaaaagaactgagattagatggt
cgatccttgactgactttcgagatcttaaaatcgaatttggtgaggactatggtcaggtagacatttctttgggttctactaggtaagttagactttttt
ttgttatattatttctttcttttaatacatattgctaacacacgcttaccttatagggttatggcacgtatctctgctgaaattacaaagccgtattcgg
atcgaccttttgatggaatatttgcaattacaaccgagcttacgcctctcgctagtcctgcttttgagacaggaaggtatgagaaattttgcactctt
tgatgtagagattactaacatgtatagggtatctgagcaagaagttatcatatcccgcctaattgaacaagctattcgacgttcaaacgctttagat
acagaatctctttgtatcatttccgggcaaaagtgctggagcgttcgagctagtgttcattttataaatcatgatggaaatttagtggatgctgcttg
tattgcggttataacgggactctgtcattttcgaaggcctgaaattaccgttcttggtgacgaagttacggtgcattctattgaagaacgtgtccctg
ttcctctttccgttctccataccccaatttgtgtaacttttagctttttttgaagatggaagtttgtcagctatagatgcatctcttgaagaagaagagct
gcgtactggtagtatgaccgtcactcttaacaagaaccgcgaaatttgtcaaatctttaaggctggtggtgttacaatagaggcttctagtgtggtt
gcatgtgctcatactgctttccaaaaaaacgacgtctatcatttccgaaattcaacgagctttagatgaggatttgtccaaaaaaagagacgcaatttt
ttggaggctctgctgaaaatcaacggtcttaaaaaaggatgtattttgatactgcttactggttatagattgaatcctaatgattttgttaatatcat
gaaattcttcataagaaaaatcatagttcccttttcatacacaaaatattacaagccaatgcttacttatctaagacaaaatgaaagaaaatacgt
ataaaaccacaagtaaattcagcaacggcaacaaaaggggaactattgtattactagttaaaagaaaaaaccaacaacaagcaagcggtaag
gaaacagtggatacaagtttaagtaaagggcatttagctaatacagaaaaatttgatttaggggaaggtaaaaaacacatgtgtgacatagcac
agctgcatggattggagaggacgaacgttgttcaagagagcagacaactgccagattggccttatctatttcgcagagatatgtattgttttgtaa
gcataaaattattttccacaggaaaaggataatgtttattgtttaacaaggacaaaacagaccgcagcaaaaccatgtttatatattttaaacaac
acaaagactctgaaaatatataagcaaaagtaaaaacgaagtaaacagctaacaaaaaacgaaaccaataagtaatataacaaccctattttt
tttaacatttggtgataatgatagccatgcattgcgcatttagataacgaaacgagcatgtatagaaaatcaggatggttgaaaacttacataaa
acaagaacataaagaccgaaccaaaaatttatattcagaatatacataatcatatatgaatgaaagtattaggtagaatacccattaatgagag
cgacgatccttttcaagttcaagttgctggtaaagcaagaaaagagctttctcattttcctcatgggtacccgtaatagtaaacatccgttcaccag
tttcgtcatgaggctctttggcaatggaaatcttgctaccgctggtacggcgaatttcagaaatcttggaacctcctcgaccaattatacagccgac
catgtctgcgggaatactaatgttctgagtgactttaggttgttgtaagaaaggcagttgagtggtggggaatacttgagcaccataggaaacga
agttttcctggactccttcattggcagcgggtggtgtggcagatgcgactggctgtggaggaggagctccctgagcaagggatggtaggggttgg
gtgatacgagaaacaggattgtaaaatacagtgccagccccgcgttcccaatcatcaataaggcattttccgatttcccagattgctgcgtgtaag
ttttctacggttccatgaacttcaacagtacgctcagtagactgaggaagcatatctttggaagcaatcatgcgacagctacatttgtcttgaatgt
gctttatacgcaagccattcttgccgatgatactacccatgagggaatgagcaattaggagacgaagcttccgaggagcagcagggtcaatagg
agtgccatcagcattcgtgctgttacgagcaaagatatcaacaatgaatctataagcacttacgacattttctaagctgccactgacagtaaggac
acggtcatggacgttcgggacagctttagtgacaccggcttttacattggtagaggtacggagttcggcaacgtttttgcctgctttgccaatgaca

ataccagcctcacgggtggacaacagtgcacgcaaagtgagttgttgattagcataatttgtatcatccatggggggcttggagctagtggaagg
agcggtggtcgtgataggaagttcttgttgagaagcggggatgggttgtgattgttgcatggattgttgaagtccgggagtaacgctgttctggat
agtagaaagaggagttgaagtagttgaagtagctaaagaagaagcacttgcaaatgtaggaggtactttgggtaaggaagcagcaacggcgg
gtgcagaggcgtcttcgggttcgtcttggaaagtcacgtcgaaaaacgaattttcttttcttcaattgactttggaattgcaaaatggttatatgcc
atagatgatacaggtacagtattggaaagttggataggacacatatgcgtttttaattaggaagaatgcaattgaaaaaaccgtatatgtgttaa
accttggaatatttagcagctttcggccaagatatgaattgatggataaagcgataggatactgcgtgaaagggatttcaagggccggtcaatag
aaaacgattgaaacaaaactttttcaagtgtttttatttagataaaaacacctatttaaaaagctttgaaactttacaaacgtataggaaaaacg
gggataggagcacggttatggtgaaggaagcaaaatgagtgaagaggagggaaaaaaatttgctgtgggaaggatctggattgttcggaacta
gcgttgtagcagatccgctattttcgtataacgaatggtaaagtacgttttcgtagtcagaataggaaaattcgtaacgttaacctggtaaagttgc
agtaataaacaaaggagattgttataaaggatggatgttgtttttttgaattatggaataagcaatttttccgatcactttttaaaaatggtaagg
aaaagattgattaataatatattttggctcccatttaaatggtttatatcgtagtattttggttgatagagaactaggtggatcaaagggtaaaggg
cacatgatacggttgtttgtaaggtagcaagtttttaaggttgtaaaagaaagacgaagaaaaccatgttgacagatacttcttagcaatcattcg
aagctgacggtcgctacgagtcatgaaatcttcatatttgatggtctaatcgaatataggattcttcttcattgcagcaggatgactttttcatttccc
catgtaaagtgttaaatcaagacttagttaaagaacaaagagacaatcaagcacaatcataatcggattccttcaattagttccaggggtttgccc
tgatcaaaatagctgtggaaaagcaaagaagatagcagcaggaacttcttttagtttgacagcaatatttaatgaaaagaaaaagaaaattaca
atgtattcgttacgaaaagaatctttctcggtacatattaaataaccttcaggattagaatcagggattttccaaataatcaaaactccacctaaag
aacacaatggtacaacttgaatatgaagaaatggaaatttgttatatacttgcattaaaacccgacaataagcattgaaataaagaatgtggact
gtaaccaaagcatttttcgtaggaccttcgtaactgttgctgtgtactagaaatcatttttcaaccagttattgtttggcaacgttaatcagtttcct
tctaggataccgtccgggtaagttagataagcttaccgatcctcgtattttcttttcctgcatccctgaatgaaagaaagcgaaaataagttgattg
gtgataaatgtcaaccttacgaataaaacagcttactaagtatttagagtatgggagtttgaaactaaaacttaatcttctttaaaaatggcggtat
aagtatataaacattactggtgggtgcgcatactcacttaagcttttctttttagcatttctataaaggtgaggggtttttcacattaaagagagacc
ggtatattatattagtagctacttaatcactgtaaacacaaaagggattcaagtgtatttcgtctttgttgccttaaatacaagtaacaaaaatgac
attggtccatattagataacttgaggttagccttttctgtgtagtcaaagattaaataatcactatatgtgcattgaaaagtaatgctaacaaagaa
agtagaaatagtagtaatcatttccaattagcattacttaatataggactttcaagtatgttgaaagttaacagctgcatggtgtgtgcacttgacc
gaaatgtaaatcaatatactgtaagaaatatttgatacaaatgttatctaaaaaatttacatccaagccaaataatcttatgtgcacaatgaatac
tttatgtaaacaaaccagatataaaacaaaatgcccacaaatacgacgagaaacccttattatattaaagttacctagagggaagactaatatgt
aagcactaatcttagtagtaaacaaagtacatgcagaagacaaaaaaggggaaaggaagagagatggtcaaaagttgcataggtgatatagc
taaccaatttaacgttgaaaggtatagccgtttcctctccatgagttggctgaataggtcatgagattcattaggaatagagcagtacattgtgtac
acagccaatacgcacaccacaagtcatatttgagattgaaaacaaaaaaagactaaggaaagctcgaataatgtctgtagaagataaataaat
tcaatgattaacaaaaaatgcaaacataacgaaccaatccacttttaccaacgataccgaaacttgatttgctcgcggtcggtaagatccataaa
ctcttcattcttaacagaatatgctatagaaggatcattagccatcaaagcatctcgccttttgttttcataaagataataaccaattaatataaagg
cgatcgccataattaaacaaagagcaacaaccatagcctgaatcccctcaatatattctggggtttggccagatttgaacaactggggagcgata
atgtttcctaaggaataaaaaacaaaaacagttgcattagtaacgctcttcttcgtgtaaccagcaacgttagaagaaatgagcgacaacaaca
atgcactagctgcaggtacccacattgtgaaccatacacccaccaaacggccataaggattggattgagggattttccaaatcatgaggcatcca
accaaatttaagcaactgcacaaagcaccaattaacagacgtccgttcttgcaaatcttacagagaataccactgacgatcatggcagcaacttc
aatagcacctaagggcatgttaaggatggctgatttcaattcaccatagcccaacccattgataattaaagtgctgaaaacaccgataccattggt
aatcatacatataccagtaaacagagttatcaaaattacttggggatcacgaaacgcttcccaagcttgagagagtttgaaagttttgttttctaa
accagttctattatctctcaccctttccaaagcgattcgttgctcacgaggactaaggaaacgtgcttttgcgggattactggggacatatacaaaa
acaaggccccaaaccagcgacattaagcctatgacaataaaaatgtatctccatgaagcaattttatggtgaatatgacctaaaccataagata
aaatagacccgatgatctgagcgacgccattaaatgcataccaacacagagttctcatcggttgttcgtcacgtttataccacatggctgttatatt
gataaatgctggtgtaatagaagcttcaaacacaccggcgagaaaacgaagagctaatagagaggagccattacgacaagcagccatcaagc
aaaccatggcgctccaaagtatcacatcaatgagaataaataaggaaaggggaactttttgcataatataagcggcagggtattgagccacaag
ataaccaatatagaataacgtagtcatactactataaatgtgaccgtcgatatgggtatcactcttcataccgtatagagcagcataagacaagg
ccgtcttgtccaaatattgaatcatgtatgtaatacacatgactggcatcattacgaaatcgatcttccaaagaacctttctggcttctttgtctgtga
atgtaaactcatcatcaacgagcaattcatctgccacgacatccacagctcctacaccttctgttgttggtttttcagttgcaaaatccttgatgcta
gcctcggcttttagctcggaagacgatgcgcttttctcaattgaactacgactatgagcagtttctgaagaaggcatcttgaagtcgattttaagtgt
aagtgaaagagaaacaatcaataatttttaaatggtacagttactcctgatagattataaatagagatactttaagaaaataacaagaacaacc
ggattaatcatcaagtagaatcttcaacttaacggagaaaaacaagcaagacttttagctagtatccccaatttacctaaattactcatgtttttag

taattctcattcttcaaatatagtagattctatcattctttatacccgcataatcttggataaagcagccacttgcttatcgttctttaacaatcattga
taaaaaatcatttgatcatagggtatataaaacaatgacaaagttgagctcatgatcgataataataataaacaacctttatcttcaggaag
ccattattatatttcctccctttacctttacaaccattttgtattaatgagtttcctatcaatacgagtggtaaaagaacaagacttttgatacatttaa
aatgattccagtgtgatcaattcgggagctttttttataacacagaggacttatctgtcatctctttatcatcaagaggagaatctaaacgccaaggg
ttgaatctaaaaggttaaagtttctataaaagttaccaataaatgagcttatttcaatatttccaaaattagttctgtcactgagaattcgacattttc
gcatattgtaaaatacttttatatctattatcgtaacgagttttcctttgtttattaaatcaagaattaaagcattatttgcgtagcaacatgagaatt
ctacgcactggaggggtacgataatggattactatgagatgaattctcaaagacgaaatacagatggtcttctcttacagaggatgaatatcata
gtaaagtgaggactaaaataccccagtttctggagtcgaggtcggtttactattatattcttcttactgaaggtgacaatacaaaatacagaaaat
gtacaacacttaagaatcaactacaagttcttatatctatcttcatcaagattaatattaacatctgagacccataggatacggtataatataactt
cgtttatgctaatagaaatccaaaataaattagatcaacaaaacaacggctaacaagcaggtgaaggccatcatgcaagacaaaggtttagtac
tagaacccttagactcattggatccactatctttgcttgtagaataagcagtgcttgctacatcgctagtataggtcgaagagccgctcgtataggt
gctgtgatgcgtattaacagaagaaaaagtacttggaggagcagaagaggaactggaagaaccagtaaatgaaagtgacccgggatatgaag
acgtcggatatgatgcagaagaagaatagtagctcgtaggagtccctataaagttttcaaatgaactcaagctagcctctggataataaacttga
ggcatgccagtggttactgtaacgtgtaatgtacccgaagaacctacggtagttgttgtattgctcaaaacttcgcgaaccttggttccttccttgaa
tttagaagcgacatcgtaagcaactttgtcagtattcgactcgtaatttgtatagacaccgagaacatcacccttttgaacaaatgcataacgtaa
gtcgtaatcaagaaattcgtggttcgtagtcgtccatttatcgttgtcgtgcatagcttgcttacgcagagcgatggttgaacgaattaattgataaa
taggtgagttcttattgtaaccgtaattccacaaagcttcacgattatccgaggtaccaggaccaccagtcaggtgttgctcttgtccataatatatt
ataggaatgccagtatgaagtaagagaaacacaatggctcctttattttgcgaggcatcatctgtaatataagggtaacggggcaaatcatggtt
ttccaaaaagttggctactatggacatatcttgaccggaacaagcctcctgaaactgagtatcaatttcatgaagatactggaatgcagcaccagt
attgttgaaagcaaattcgatgccttgtcgaattggataactagtaatgctatcaatttgattacgaacagagcaggaaatattagggttgtacga
aaacatctcaccaacggaaaaaacaccagcagccttgagataatccggaaagaagttggggttcatttgcttgatagcgtccactcggataccat
cgataccataacgttgaacgacatcgtgaacatgctcgttcaaatatttagaaactacatcggactcgagatctaagtctgcaagtatgatttcatt
atcaccactccagcaatcttggatatcagaatcatttgtgctagcataatcaatccagcaaaaggggtgaaagtattcagatgaattgaaaggat
gaagatgagagtaagtaatattatcagggggtccgggaatggccattgagttggcaagggcatcgaacataacccacatatcacgattatgcat
atcgttaataagatcaattaaatcgtcttcggaaccccaatgctcatttatacgagtaaaatcactgttccagtatccgtgataagcctcaccgtctt
ctccatatgttccttcaacgttttcatcaataggagacatccaaatagcgttaaagcccatttcctgaatataatctagcttagattggatgcctttcc
aagtaccaccgcagtacttgctagaacccttttcacactcagggtttgaagtatcgttgacggcaaaccgatccgtcaaaacttgatagatgacgc
ggtctcgccattcttcactggattttgcccatccttggtgtagcttactgagcacacctaaagcaagtaatgaaatctgcaataatcccattataaa
aatgattataaacacagacggaaatgcacaagagaatgaatgatttcaaaatgaatggatatacaataaacgagaatcaaaactatgcaatgc
tataataaaaaaaggaatgcgaatgcaaaaacaaaaaaaaaaaaagaagaaacgatgcaaagaaggaaaagaaatgcaaaatactaaaa
agaactgttcgctgaaaatatgagagtatcaaaaaaaatcaacaaaaagaacccatgtatctttaaaaaggaaaataacaaaaagaaagtag
gacaattttttagcgtcccaaggacaaaatcagccggtgttccgtacagtgcagtaaaatgtgccggttgagtgaagaacaaagtcacgctccaa
agagaaaaagctttcttttataggcttcgggaataatttaatctcgttgacagtgggtaaaaagaggcttgtatgtttacataagcgtaaacaaga
tgacacaatccagaactttaaagcaagcaaaggaagaactacactcgcccggatcattgggttcgtataaaccggactattgccgggtctcattg
attattagtacccggaatgatcttcgtaagtgtagtttaggtaaaggtaggaagatattagaaagaaatgattgagtgagaaacaagcgtaatct
cttctgataattcaaaaatttgcatgtttaacatgttgagtaagagaaaatgatgaacaagaataacagtcggaaaaaactcagtactacgcaac
ggcattttccctatttccatagcgacgttatatcctcggaaaattgatactaaccgaatagcctgccatatcagaaagaagaacaacgggtcatag
aaatccaataatattgagcatatcgttcgttgaatgcataaagttaattagaatgtaaacaagaacctcacgttacgcctagcagctattgaacat
tcccgaagtagattctccttaggctagtaaaacatatccatagtgtattacggtttcttttgagatacaaaaggttttaggtgatgatgctgaagctt
aatcttgattccatatggtcaaacttgcgttgacagtaatgttttgtttcaccgagtacttaacttgactagtacatataatataatacagaagaagc
aagaaacattcggacttaaaacccttttggagaacaaagaaacgaaatagcaaaggccactaaaaattattacgcaattcctaaaagtttcgct
aaaaggttaggaccgaggcttgtaatgcagtatttgacttgcagacaagtatcctagaagcgttttgtatccctattttaactggaagacttaataa
acaacagaaatcaattcgtgaaaatgaataccaaattagactaccaaaggttttaaaaaaaaataaagtatgactggtttttttctttcaattagag
gtttctgttcaaggtggtgttctaatatgctcctcgatttttttcgaacaagaaatttaaccttagagagtttgttcgtcgattgctctggattgtgtag
ctgctttattagactttagtagttttagtagcttattttattagtgtttcagccatagatctatgcaaaatgggttcatttttgtttttatttgatgcagac
aaacataacattcggtccaccaaaatttaaactgttgaaaactagcgaaacagaaagtaaattatacttatatatatatataatgttcgtatgtatt
atacccacctcaaaatctcgtaaaccatttttttttgaataccgacgttacacgaaagcttggatagcaaaattacagtcgttgtgctttttaaataa
aaaacaaagaaaaaaaaaaacaacattgtccttcgtttatgctgatgaaaatgtttggtgacctggaattacacgtgtgacagaggttctctctgcc

acaatagataatcaatatcccaaagttttccttttgtttgaacattgatgaatttttgttttaattattggaaagtcaattgaaacgaattgatttaat
acactacagacagtcaacgactggaatagtcaatcctatggtgacatataagcttactgaccagcaaaagggagaattacaagcgcaagattcg
gtcattgaggggaacgagctaaaagaatgccttttaaaacttaaaaaagtaaaaaaagatattgattttaagccatttctacgagggatacaatt
gtcgtcaattagtacgcctcttccaaatgattccgttgctcgacagaaggaattgatgaaaaaggaattagatgatctaagagaaaagaatctat
atgacggaattcagctattgccatctatggcgggtgtatccttcgatagtcagcttcaggaaaaagcggaaaacaagaaaataaagaatcaatt
aagtgccataatcaacattcttttcacagtgataggaagtgcttttgcagtttggtgctgtacctcttcttttgaagtcgaaaaggtaatatgcgtgg
tttctttgtgcaatgttcaacttgaatactaactgtttcatagagagtcgctatgtgcggcatttcttcgattttagttttaatcgctgatacatttcttt
acactcggtttctctctttaccaccagtgcaaccgtcaccgaataccaaaggtgaagtcgtctattcctggagtaccaacgatccactctctcaaga
tgatcagattttgtcttttacgaagagtcctgattctattaaagaaaaaaagaataattaattcttatccccattttcctacgttaaaaacgaatgcg
aagtttacctcgtgccagaatttaaacaatcagactttctagagcacaaaccccgtcaaacaagattatggattatggttaaattacaggctttcta
ttgaaagatgaaatggtcttttgattttcttataaatacatatatgattgaacttccagtaagccggttgttttgaattcgctttccaaaaacgctgtt
ctttttttatggtgatcaatggtaattcttattaacgtttactatgctctgaaagatttggcatttagcgttctggttgaaaaggacctttgtgattctc
aagtgttttaagtcaaatagatgatcaagtatagaactatgctaaaaacatatatccaacggacatcgatttgagtaaaaacactgggtgaaagc
aatttaggattgttcaaagtgttacgttctgtttcttatgcttttttggaaacagcttaacctttgttgggaaaattaatagcttgatattttcgaaac
gagtgaggctgaagttatttttggaagttatattgtcatttttaaaatcgcatttctcgctaaattaattgtaaccacagcttgggtcatttcatcgac
taatgtgatttgaataagaaacaatacgaatgcaatgtcatcaaaggtttctacaagacgtgaaggtttataatgaattaagctcattgattaata
actagattcgttatttgtcattaatatcgtaactgttcgtatcatatttaatttgctggttcgaacatgtgtttattgccttcatttataactttattttcg
gttgcagcaagcttttcggcagctttttcagtagactccttgattcgagctcccaattcaggatctactttttcaaataccttatactgtctttgacgta
ctacgggaattgctttggacaaatgcaaagcaacgtttttgacaaaattgtcttgttgtcccggttgctttcctagtaccttctcccagaatgctctag
gttgctcaaaatcgacgtcggtaatttcatccatgtggtaagtgacttcaccaacccatttttcatgcttctcattgggctcatgatactcttttgctaa
gggctgtaatgaagaaggatagtttggcatgctgccttggttgtcagtgacattcatgggaccatcacgagaataattaaaaacggggcatctag
cactgttcacaggaatttgctcaaagttggcacctaagcgatggcgatgtgtatcgggataactgaaagtacgaacttgcaagacagggtcagg
agaaacttcaacacctggtagcatatgagagggagagaaagcagcttgctcaatttcagcgaaaaagttttgagggttttgattcaaagtgaaac
gaccgacttcttgcataggaacatctttgtggggccaaaccttggtaaggtcaaaggagttatatctgaatttctcggcttcttcgggtgttaatacc
tgcacgtaaagagtccagctaggatagtcgccgcgatcgattgcttcgaagagatcctgacgtgcaaattcttgatttaatccaatcatatcagta
gcctcctcatgagtcataccttttgttccctggtcggaaaggaagtgccatttgcagtagtgaaactcacctttatcgttgacaaatttgtaagtatg
gctagaaaagccatccataaatcgatagccatagggtgtaccaccgagatcggaaaaaagaatcatgacctgatgaatagattcttcgttttgag
atagataatcccaataggcggtgttgtctcgtagggacgtttgaggattgcgtttttgtgagtggataaacacaggaaacttggcaggatctcgca
agaagaaaacaggggtattatttccaaccatgtcgaaaataccatcttccgcatagaatttcagagcgaatcctcgcggatcacggaaagtgtcg
gggctaccttgttcaccactaacttgggaaaatcttgctagcataggagttttctttcctattttattaaacatcttcaatttggtgtattttgttatatc
cttggtgacctcaaaaaggccaaaagcaccggaaccttttgcgtgcacgacgcgctctggaattctttcacgatcaaatttctggtatacatcgatc
aaatgaaagtcttgtaataagacaggacctcccttgccaatacgagcactagctagtggattgtagatgggacatcctgtactagttgtgtacact
ggcgggccttcgcctcctactaaattcttaacttcttcaagcatctccgaattggattgatactaaaaatcttgaataatttatcatgcgtgttaaca
gcccatatataaagcacataacctcgtaaattggagacgtaactctgaactattgtttacataatgtaaacaattggaattgtaagcgtcacgatt
ggaagatatgaaatcacagactataaaaacggtggcgagcgcatctcaatttataatgaaatttttgcattttattctacatagagatatataaatt
aatacttttaactagataaatttaattctatcaatacatattggtcagaaataaagagttatacaggaaaagacatggcgaatgatttgcatagaa
cgatactatgattatttaccaacattacaaagtcgcaatgctacctgtttacactatttggccatccagtctatagtctatttacacgaaatatgttca
ttatagccattgattcctgtgttgtatattaagagcgctaaggcaggtcactgctgaagcaagctttcttgccttacgaatgaagcccgagaatata
agcaattcataatgtttatgttatgagtttcaaatttttatacagcatacaatataaggaaagtatatacttgtaataacaataataatgatgatgat
gaataaaatcagccctcactacattccaatttcaaagtacatttttgttattacgaaactgtatgagaatacttttatgtttgtaaaatatcaaact
gataccatttcaggaatctttccacgatacaaactatctcttcttttttttttaattttttaatttttaattttttttttttaaatctgtgttcatgattcaa
ttgaatttacaaatttgaaattttgaaattttttataacgacaaagaagggaaaataaataaacgcgggtaacattcatcaagtaacgatgttat
gatcagaaattttcttgctcattttcacttatggaaagccaaaatattagcatagacgaatagataagatgtctttaccagtggaaaatccaaacc
aaaaagatgggacgtttgggatcagtcgtcgaagatccagaattgagcatcgccctaccgaattcctgaatgaattgaggcccgagatagagtct
atcagagaagaatatgaatcgaatcatgatataaaagagaaaaacgaatattatatttcttcaaatgagttggacaatagtggggctcctagtttt
gctgatgtatccaatgactcaaaaattgacaacgcaaatatgattcctcctaagcggtctatcgctttaattttggttaatagtatcatgtcagatat
gtcttttggcgttgctttaccaacatctccgtcttatacagaaaaacttgggggtacggatgcattctctggtttagtggttgggattccgactcttat
ttcgttattgatgctgtatccaatgctaaaacttgctacaccaaagtctgcaaagggatatacattgtattatcgaccaatgttggtctctgtcttcgc

tcatagcatcggacacttattgtattgtatggcagatcaagcaaattggctttatttgatccttattggccgtatgatgaatggtataggtttcaccat
gtttttatatcacaaaaagtacacaacagataagttacttgttggacccaatcggcgaacgtttcttgctactttaaacatcctagctcaaacattg
ggttttatggcaggtccatttttgggtggtattttagcaaaaggtacaaaaaaatttggaaaatcgagtttggaaccaatacactggtggctcatggt
tcatgatgtatatatggattatttttggtgtttttttggtcatttttttacaaagaagtatacccgcaagatgatcgtacagttttggaaaagcctgttga
cgttgaggaaaacactggtccattgtcactgaaacatatttatttactgtctttttgggactagctgctttcgtgtcttattttaacatttctggctatc
aaaccagtatacccatttacgcgaataatctctacaattataatgcttatcaatctggtaattttatttcattggcagcattggtaatttccccgtttgt
cttcttgagcacttttctctctcgatggttagaagaccgacatattatgctaattgggtttatgattggaattatagctcttgttctacatcttgttttgg
atgctctccacagaatacctgttcaagcatactttgtgttcttttcaattatgatgtttggatttagcataggttcagcaccgctagtttcattagcttc
caaacagatgaagcctaaatatcatgttttgagcagtgtactttttcaagtgggattgtcgctttccaacaccgtaggcgcaatctgtggtggtgct
atatacagtatgacgacggtcggtttcataggtctttgtatcgggttatctgttgttgcatttttacaatctctaacgttgtggaattctatgaagctg
aagttgcattaagtatgactcgtcatctacgtgtttgacagaatattgtgattatcctgtcctttataagcctttggctaatgttgagatatggcttag
gaaaaagtttttggaagtaaaaaaatatttacaaacatttacttgatgcatacatcatagcactatggctgtcgggatatgacgcgattgaaatga
aagggttgtttgtgatttcgggtttgtacaattttccaagctgaagcagttaacgaaggctctcttgcctaagcgttttctatttctaaacatgtgtcg
ttgacgaagtagaattattatcatatccataggaggccgctaattaaaatcatgttacaataagggacgtgagcataacacacgtatcatctatcc
ttcttttacatgtgaagtctttaatcaggcgattcatggttgatgatttataggttcatggagccttaaaaaataatgcagaggccgctaaacttagc
aaaatttatatacatggggttcattaggatttggttatggtttacaggttatatataaaaaaaaataaataaaagatacactgtttttagttttatat
attacatcttcgtgcttaaaaaatatgtaagttgacggtttttgtagtttagtagagacaaataatactgaactatcattgaaattagtctgcgtgag
gtaagtatattagcaaaattattaattatgtgtaaatttggaaaaaagaaggattgtgatgatcaaaagcaaaatatcgctggttcacttcaaagt
tccacgctttctcggtaaacaacatactcattttggaaggggttccaaatataacaaggaatgatgacttcaataaaatagcgaatcagccactgc
actagactgcctttgctaacatattcataaaacggaagcatatcaatgattaaaccattaaaatcaaaaaggagagggacaatggagaagatac
taatgcagtaagtcagtgaacggttacaaactttcgcagagtattcagacttaaaattcgtttggatcctttctttatacagcaaaagaatgacga
ataaccagattattctggaagcagactcatttaaccattgatcaactaatagcagaggttcataatgataataatcacgaccttctaacatatata
gtctaagaaatatgcgaaagagaaatgggaaaaccaatccagtaccgacactgataatgaatttgggttgaacaggtcgccaaaccccaaaac
caaaagaggcaagaaaaaggagcaaacgatgcacaaacatgcgaaagctgatttgaagaaaacgataaagctctaatctccaactttccgaa
gaaaacaatgagggattaatagccgtgctaataatgagcagcaataatccatattgaataggtagtactttctcaggttgggacgagagtgcgta
gatccagtaaccagctgatagcagagaaataacagtagtaaccagtggggtatagttcttgttcgtctttgggtcttctattctttgaaattcatatt
tatacttcgaaaaatgaatatcaatgtttgaatccaagcttgtcaatgacctcgttgccacacaataaaagcccgaatcgtttattctgtaagaact
aacgttacccgtcgacgtcgacactatatcgttaataacagaacttctgttgcactcgaatggacccgtccactcttctcgcttatccgatgggaag
taaaccgaagttataagttcatctgtctcagagtgtaaataataagaaaaaagattttccatgctttctaagggacttaaagtacattcccggttct
ttacattaaataaacttatggcgacagtttatcatcaccccaataaaaaaaattggcatacaaagggtgcatttgcacgttcaccttttcacagatt
actctcgtatgaaactctttttgttagggtccttgacaatgatagataattgggcattgctcactgtaagcaagaaacagagtgaaaacaaccaaa
atagccagttcctaactgacactagataacttttcatttttctacgtcccttgtttcagtagtaggtaggaaacaggaaaataatagaaacttataa
gtggggttcctaatgtcaaattgtaaagttttctaatttgaattcaatgtctgtacaacaaattttagagcttggagtctttcaaccatccatcttcat
ctacttttcattgcttcgtctgcttcgttgatataatcataaaatgacatattcgcttggacgcaaaaaaattcagcgaattggaaaagtcttttcctt
tacaattttatcctttaccatctagatttttgctctagttgacatgtttccaagggagtggaatccctctgtcctcatcagtaaacctttgtttatttacg
ttatgcaagggttcggtctttgtttttctttcaacgatataagaaaatatatttgttccttgaatgtaatctcaacgcgagtggaactaacgtcgtgat
tccttttaccgtcctcaaaaacggatgcgttatttccttgaaaccaccgaatctccgtcaccctatcagatcaatacttggtgaaaaattttgaatga
atctagtggaaaagcagcccatgcatcatcgattggtgaaaggcatgagcaatggaggaagaagagcagagaacaagcacggcattttcctaa
ctcgttcgttgatgcgattttacgtcacagaaaagaaacgtcaagaatatcttttgaagtcttaaaaggtatataagcagacgactagctatgttta
atcaacacaataaacatatttgaactttttttgaattataaataatggtcgctgtcggattttctttacctaaagttactctctgggaagacaagccca
accaaaacgttgaattgcctagccaaggcaagattatcgttgtcggtgtccccggcgctttcactcctccttgctcttctcatgtccctggctacgtc
gccaacgaaaaggcctttgaagctaagggcgtggccggtatttacattgttgctgtcaatgacgttttctgcactgctgcctggaagaagtcttttg
aaggcggtgaacaaagcaaggttcacttcctcgctgactggaacggtcaattcaccaaggctttcgatgctacctttgacgccagcggccttttag
gacctcttcgctctaagcgttatgccgccgttgttgaggatggcaaggttaccaaatcttttgttgaagatgaggtcactaacgttggtgtcacttct
gctgacaatgtcctcaagtctctctaaatcaaaacgtaaacaacagaatttacaaagaaaaagagcatgaacaatacattcaggaacgaaact
agatgctctcgttgatgcatggtaacaccgtgtcatgtcatgatgtattgatgacgaatagatgtaatgaatgaaaattgtcttagtgtttccgatta
atatgatagatcgtcgtgtaaggtaatcgtaatatgatactgtttagaaatccattttcactgttagtggtgttggtttattgctttgtctttgatttcgt
aagaggagatggggttatttagcagaaaagatccatttgggtttaatcaagttgggttacagcatagtttgtatccacgagagccgtttcttgtaa

aaggaccaaaccatcagatcctgcttatttctctttgcttttaagttattttttttaatttatgccattcaattgaacaagatgttgaaaaacgttgaa
agtgttatacttgtccttcacacaagcctttcgcctttcttttgtcactccccattaaaggcgacacacacacattactcattcacacagagcatgtat
gtttccatgtaacgagtttaattgaagtcaaataggatagccactaattccatatttttatttcttttatatgtgatttccttttgtaagctaatatcatc
caactaccaaaagaaaacggattgttttggaatttctgattttctctagagtccttcctgttcttgtcatttctttgttgtttatggatcttttactctgca
tcatgaaagtttccaaggcattgacgcttacctttgctggtatcatagccgtgtcttctcttgtcgttgctttttcccctccacaagaccacctcattt
aaaatcctggtccaagcgtcaattgattttgtattgtgaaagaaatgggttagaaacttccggttcttatttggatttactttcccgtgttgagcagc
accaaaaatcgcataccgttgctgattaaacaaatataaaaacaaaagacgctttattaaatcttcctctatatggcttccttcttgtacgacacat
ggattgaggagacaaacgcccagttcttgctgagacaaaatgattgtcgtcccatccacccttgtcgaaaagcgaaaggaaactcgacataaca
tcgtatccgctttcttgtcggaagcatgtgtgtataatctgctttttacaagacagctgggttgcttggcatgtcgaatgttttctatctttattcagcat
ctcccttccttcatccgtgggaggatggatttaaacgtaaagatacatgccttatttttcgtttttttactgtagatggaaaatttgtccttccatagaa
tagtagtatgttaaaatgagattttctattgaacagtaacccagtctaccaaatcttcagtagacgtttcctttttttgtatttttcaccttaaagatttg
attttttttcctccttttctatgaattcattttcttcctttgcttccaatcttgtgtacgatttgataattccagctaccccaaagagaacacatttttgaa
gacgtatgcaaaatggacgctagactcaatcataagttttcacaaagagcaaatgggttaaagaacccgcttgcaaaggcattacttgctttaat
ggaggaaaaacagacgaatttgtccgttgccgtcgacttgacaaataaggctgacatccttgctctcgtagataaaattggtccttttgtatgtgtc
atcaagacccatgtggatgtcattgaagattttgaccaagattttattcaacaattaactgctctttctgagaaacatcgattctatatctttgaaga
ccgtaaatttgccgatattggaaatacagtcaaacttcagtactcttctggtgtttacaaaattgcttcttgggctcacattaccaactgtcataccg
ttcctggagaaggcattgttcagggtttgaaagaagttggcttacctcttggtcgcggtttgctcctcttggcagaaatgtcttccaaggggagcttg
gccactggatcctacactcaaaccactttagaatgggcagaaaagcataatgatttttgcatcggcttcattgctggtcgtcgtttccccaaccttc
agcatgactttattacaatgtctcctggtattggattggatgtaaagggcgatggtttgggtcaacaatatcgtacacctcatgaagttatagtgaa
ttgtggcagcgatattatcattgttggccgtggaatttatggagctggtcgggaccccgtggtcgaagcccaacgttacaaacaagcaggatgga
atgcctatcaagaacgtcttagtggaaagtaatgaatttggttagcttcaacttgattctatacttggattgagtctcaaacatggatgaaattggg
tatttttctatggaaccatgagatgtataatagtaatcttttttttgttaaatgtctatagctttatttcatatcacgattgaaagtttggaaatttcaat
gtaaagaacttacatgtacctattcgatgaaactagacagaggaagtaatcctcgttccttcaccttctcaaccaatagagggtataacctcgcaa
agtatgttgagagctcttcccatcttgttgctaatccatatcaggtagaatttcaaggaaagtcaaggtattcaagaaacaagcggagacgaata
gaatcaagaatttataaatgggaatctattttcattgcctagaaaatagtacatgatgagtgcataaaacgaacgtaaactttaagtctttagaaa
aaaattcgtaagagcgtcaagaaggcaccgccgcacataagaactaaaaagaaaactaaaatgaattggttactagatgaagcttgagcaaac
tagcggaaacacatacgggctatattacggggaaccatgctgggggatgcttagcaacgctgtggtcttttgagtttgtttttacgcgtcgagtaa
cattttttgaaattgagcatttgcacgacgctgagatggagtttgaacggcctacgtttataattagtgagaattcatttatgaaaaagaaggtct
aacaaaggataaggaaaggcacttacaggcattatagcaaataaaaatatattcagattcagattcttcctttagaagactcttacagatgttctc
ttcaaaaaggtcgttggtaccaatgggcgtatttttaaaacaaggcgtcgcgtaaatgtagaataacgcaacagaagaaacggtggaagtata
ctatgatatcactctatagataaaatgtaaacaaaaaagaatggaatgctgaatttcttctgaaaatactttgtttctagctagttcgtttctatcaa
atgtctaaacagcaaataaacttaaaatatgaacgactcaaaacagctatgctgccagctttgatttgatttcctttgctcttcacaagttttcaaa
gtaaacagatgaagtattggcaatacagccgcgctatacattcaaaccatcaattcggataataaaaatgtatgctatatttcttactaaagaagt
taaaagaattgtaagagaagcgcattctcaagccttttgctcgataacacagaacttcgtgggttattttaaactgatgatctcttcacccttttcta
gttgtttctatctcgtttacttatactctacttgtcaaaaattcattacttgtattcacttcaagtctgtcgaaaaataacactacagctttataaattta
attttatagactctttgcttgccctaactttcacctagaattcattttggtagcgttgcaattagaagggaaaacctatattataaatgtcataatttt
aagatttatattttaacggaaacctcttcagttttttttttttttcgtgttctaagtaaaccacaatctaactgtatagtattatcaagaaataaactg
gtctcttgtgtacacttccttttcgcactatacattaccgctatacgcttagaccaaacagacatatatgttccttaacaaacagttaattagcacaa
aagaaattaagccggagaggcgcaaacaaagaaagcaaaaaaagatagatagaaacaaaaaacgaaagcacttggtccgaagaaaggatc
atggccaagtggttaagacagttaaaagtataaataattattcattattaatgagtgtcgcatatccaaaacaatcaaaagatcatcggaaagctt
atacacagaagaaaaggcataaagcccattaggattagataagagagcaagatttcaaaacggctttaaattatagatagtgtaaaacgtatac
atgccccaaagaaaaaacaactgaagcttaatcttttgcataaacctctcgtaaaagcttagcaaatacgtcaccgaaatcacgcataaacacat
gttctccaaatcgagcacaagcagatcgtgcattaactcgcatctcctgttgttcttgaggactaagggtcaaagctttatggtaagcttctgcatat
tcaccaatggtagtggcatgaaatcctgtgggttttcctatccaagggatcacaatgtcaaactttggtccacccgagttgttaacaacagggatc
agaccagctgccatgtattcaacaacaccaataccaaaatgttcgttccacatatagttaactcctatcgagcaggtcccgagatactctacaact
ttaggccatggagcatcgacaataaattcaaccttgtccaatatcttcaattctccagcaagttgtttcaaagattctactagcttcatatctatttcg
ttacggacactgccaacgagtaatagctttgcaggagaatcagggtgttttcctatataaagggcaaaactgtgaagtaattctttatgattcttct
caggtcgatactgagcaagataaagtatgttaggttcacgcgaacgaataatatcaaccttttcaagttctgaagtattgcagggtggaaacacc

acagataagttgatgtccttaccccataaggaagcaatatgattttgcgtccaagaagaattagtcattacgaaatccgcatgacgacctgcttga
ctatatattttagcaaaccatctccaataagcacccttgactttactaacaatggaaaccttcttcagagacttcaacatatcactagaaattgtcg
gataatgaacgtaagcaccaacaggaatattaagaaatcctttaacgattacaaaagtaaaagcgtaacccatcgtatcaatgaaaatatctgg
agcaaaccggcaaattgcttcaaatcccaaaatcatactacccacggattgtccaagcaaagtaaaacgaggccaaacggttggagaaacaag
aaaccgaagctttaagggaacaaagatcacttttgaagaatccaaatcgatttcgaatgttgtttttaccctccgaagcgcctcttctgaagaaac
agggtctccagtgtagataagggaaatgacattaggaaaatctgtttggacagactttacagccgtccataatacacgctctccgcctcctccggc
attgcaataaggatggaaaaatccaacagtacgagcaaatttaggcacaacacccaatttttcatatacgtcggctttacgttttgctacacgctg
cagaagatgtcgccctagggcatgacatacaaaacgacgaagttctcgatgaactgccaacagcgcaattacaccagctatgcaaagcgtgga
caacatatgtatagctactaaaagagtttataaaatttcaacaggaaagaaacaataaacaaagtaaacgataaataagagggaaaacaagg
aagagaaggaagcccaatctaggagttaccaagctgttagtaagactcgcgggagaatgacaacgaaatctaccggaagagctattgatatac
aaagccttttcagaatttctagaagttcaaattaaaccagctccgatccctttaaatgaatgatatatgaataatctatgaaaacggtagttgctag
aagaaaacaacacaatactaatattacttctcacataatattaaagagggacctaaagaaataataagggttgaatattcattgaaaaaaaacc
ttgacaattgtaactacggtatattttgggtgtataaaacgcgcttctcaatatcgttcttcaaactagccagaagctgagtttgccagaatataac
accaaagaaagcaacccatgtgtaacgttaagttgtcgagagcctgagttttctcaggaattcgatgactgacttccacctttgttaatactgaagc
ttatttaatgaattctacagatttaccatgaagacaataggcaggatgccaacaaaaccattttcattcattctgtattaagtttattccaactcggt
aatgttgccgcgctcagagacatagataccatccaagaacttacgaatatccttgttacggacattgcagatttgcttgatgttggcagcagattgt
gagacattttccaaagagttgccctcgatgatcaattcatccttgacgttagcggacatggaaacggtaacaccgggcaaacacttgatgacacg
agtaatacgttcacccaagaagttacggatctcaacaacaccaccgttttcggacaagttgatgttgatgggaaagtgtgcgtaaacaagacgca
tcttgtagcggaagccttgtgtaacaccaacgatcatgttattgatgatggaatgaacggtacggaggcaagcattgtgcaaacgagaaccgtgc
cagacaataaatttgataacattaccttgcttcttgatctctaagtcaatatggcgcaagctctgtttcaaaacaccgcgaggaccagtcacagta
actgcgcgagctttaatatcgacgctaactcccttaggaatagctaaagtttcgtccttgtaaaggtcgcggcccatcctgtattcttttggatttttt
tcgtatgcacggcactcagcaagttctagttttgttgtggattacttactcatgaaaagcagtcacgggtgactcaaccctcggtctgtttagggta
agaccacgtgactgctttttccctgaaaaagtgggtgcacagttcagcgcacaactagtttactcttgaccaacgacgaacgacgagatggcatt
aaagacctttgaacttcgtaagcaatctcaagagaaattgggtgagcaattgcaagagctccgtcaagagctcgcctctctccgcgtgcaaaaga
ttgccggtggttctggttccaagttatccaagatgtaagtttgaaaaagcccgaagaaattaggattgggaatgattgaaagcaatttgtatacat
aaacaaattgcttttgtgtcttgtacattgaagtcctcttgaaaatgaaaccgagaagatagaaagaaatcttcattcggaagtgagtacgggcac
tcaatcgtggctactttttcgtttcgggcttataagtctccagaacatatttgataactaacctctttgtagcaagaccactcgtaaggacattgctcg
tatcttgactgttatcaaccaatccaaccgtcttgccgttcgtgaggcctacaagaacaagaagtaccttcctcttgatcttcgtcaaaagaagact
cgtgctatccgtcgcgctcttactccttatgagaagagccgtaagacccttaagcaagtcaagaaggatcgctacttccctctccgcaagtacgcc
ctcaaggcttaaattggttaggctgattaaaataaagtaattgctcctgcatccattaaattgtatacctctcttttaatagtattttgtacagtatta
gtagtcttttcactatctttgtagagcgatgctgactggcaagtacctttcaattagatcagttttccttagttcatttgtatataaatataacaagta
acgctaaaacataggcatcgttttcgttcaccgtttccattcctcaggttgttgattcttccatacaaggtggtttctatttggtttgaatggcaactca
aagacaaattcggcttttgaaacagctacaaaatattgattcttcccgtttaacaagtactactgcacaatgtctcattgttaagctgatcctagatt
cacctcttgcaacgacaattgcattgttaaatccagaacaaacaagctatgatgaatggagatatctctgttccttgactgactgcttttcatgtgct
ttatgctcacaaaagtctattccttctgaattacatcctatagcacaaaaagtttttggctccacacttcttccaaagctgacaatatgggtccaata
ctatagctctcaatttgtatcgtattgcaattctacaaccattaatttacacgatttccctgttgtatttttcttccaatctcctcttggtgttcagctgtt
ttttcgaacctgggaaacaattcaatattcttccaaagaattactttttccaagtttctatcagtatgctcattctctgctgcatttgagcttgcccgg
ctttgccaaaactatgacaatcgttctcagttagtacagttactttgtcattcttcctcattgaaaagattattcctcctttcagaattttatgaccttttt
aagcgccattatacttcaagaaaacccagaagatcttgtgtacgatcttacgaaagaatgtttattgttggctagaacacacgagtttgtccgtctt
tcgctcaagagaatgaactcactgttccttttatgtcttccgaggcatccagaaattattaataaagatttttgtgttgaatggactgaactagctga
gagacatagttgtctagaagagtggctttatcttctaaatacaatttgcaacttcaagtcatgcagagcagtcattcataaagactgtgctgatcttt
tagaggaacaagttcattcaagaacggctaagatggttcgcatcaaactggctattcaatatgatcctaaaagtatagtacctcgagttcgagatt
ttctcgaatgtggtgtctatgactcgcttcttttagaagtgctgcgacttacgtctactttgggctccgtgaaaaaattggttatggactattcgaacg
tcttaaaacaactgatagatggcttaaacataaactcatccttaactgaggttaactcttcagctgttatactttacaatctttgcagatttaaagtc
caaaaagcagaaaatgagcaagagttgcaacgtttgcgtacaatggctgaagcagattcatcttacactgcttcccccgaagatgagcaaggac
caactgaaaatagaatacaagtaatactccgtcttcaggttttacaaaaattatattcattatctcatacccatccctctttgaatgagtttatttcaa
aaattctagtacatatcgcgagtgtccaaaaaaatcgatccgaacttgtgcaatatggtgctataaagtttttaactaagccttattttacagaaag
gtcaaattttaatgcaggtcatgctttagcaaagattctcatcagtacagcgcctcaaagtgtatttactggctctttaccgtctaatcgagcaattg

atcctatatcaaaagtacttcttgttagtgaagaaacggacaacgaatatcctattttattagcaaaatttgaatctttattggcactaacaaattta
gcatccatggacgaaaatagtcgccaagctattactttgaaatcatgggctgccatcgacaacttattaattagtaaccatgaattaatccaaaga
gcaactgttgaacttgtcaacaatctgtcactttctccatattgcttaattaagttcattggcgaaaacagttcaggatccgaagcgacacgtctga
agatcctcatggcattaagtgatgctgacgatgaagcgacaaagttggcagcatgtgggactttagtgcaaattagtagttgcgaagctggttgta
aagctataatggacatttattcgaactttgaatttgttattcgtatgctgaaggatgaaaacgttggtgtccaacatcgtgcattagtttgtatttgca
attttgttttctctgatgatgaagcaatctttgcagcatttcaaaagactcctcgagccttactcgcagtgaaaacatataagtccgctcaaagaga
gctaatgatgttgaagaaagaagctcttgcgaggattgaaagcttagcaaaaacgtgaatgaagggaacaatagaaaaaaaatctaaagaca
acagtaatgtaaagttaatgaatttacatatattagattcttaatataaataataggacccaaaacttatgttatccgcatggtgcaatgacttgta
agttgcagaatcaggatatgcaaaagaataaaaaaaaaaaaaaaaaaaaaaaaaagaaattgaactggtaccttctatgtcggaaatcgaatc
aatgcttggttcttttacatttgaatggtcactcggcggaacttggttggaatgttgtttggctttaaataatgtttacgtctctcttcgggacgcttct
ccccgtgaaatagctcatcatgtttttgaaaatgttcaatatcttcaggatggttccagttttcgtcaggttcgtcaagtccggcaggatgatactttt
ccacatgatgcatttcgaattcttcttcttcatctccatgatgtcctgggaaaccaaaatcagtaaattctcctccttgtttacgaaagttcaaaaatt
cgtccaaagtgacaattcgattttgtgaagcatcaacttttttttaaaatatctaccaaaatttcatttttcttggagaaaggaacatcatcattttcaa
aaacaccgtaaatattaagaatatcccgatcagtccagtatcctttgtttgctaaatcatgcagacgaaaaaaggattcgtctgtaaatttatcgat
atgatgctcttctttcatatgtttttcaatccatccagcaagcactgtttttaaaaaaagaataaaaacgaaacattttgataactttgcaatcatgtt
gttcgtcttcaaatcaattttaatatatgcgtagtgggtagtaggttacttgatatttcttacgttcgaaacaatatgttgagtagttacagcctttta
agaagtatccagaagaatgaaacaaaaaaaaaagatcagaaatgaaaatgaacaaaagaaaagacaacaaaattggtatatcatttcaaagg
gtttattcaggatcagccatagtacagaacagagcctaactttgcttttaatttaagacctctattcttcttgtttttattttttttattgtgcaactttcg
gctcactacttcatttaataagcttcatttcagcgtttgcttacattgtttgaacgctaggatgaagtaaataaacaaatcaaagagtaaaaaaac
gaagtttctggtttatgaaaaggaagtaaaggaagttttcaggaatttttttattcttttttttttcttttgtctcattgcaaagtcaacaacttaattt
tctttcatcgttcgtatcctttgtaaatatgcaatagaaaccccatttcaggctcagttttatatatcagtaacatgtcttattacaattgatatgataa
tatacatatcaatttcaaaaaaccatgtcttcttagttgtttcaagtagaaaatacctgtttctttgtaggatcacttcaaagtatgcgagcgtgtctt
taacgctaacattcaattttataatttcgtttatttcatgattctttgatatgtttcttttcagctcatcaagagtccttgtggtaaacacttgcctgtac
ccatttagtcctttctacctttgagcatgtcagaagggcgatccaaaatttgctctgtctgcagagtaagtattagcaagtatagatgtcctggatgt
gaatcttgctattgctctttaaattgcaataaaatacataaagagcagacaaattgttcaggcaaaagagaccccacaaagtttattcctaaaaa
ccaacttgtcagccacttaaattctgattataattttctttccggtttggaaaaggttatagaaaagaaggagaaagatgaaactacctctattcac
cgtgcagatcgaaatcgggcacagctaaaacggacgatcgaaaagacaggaattaacattcatctagcacctgcttcaacaaaaaaaagaaa
ggataataaaacgcattttgatagaaaaagaaggcatgtagtttggaccatcgaatggcaactccatgatacttccattcagggatctccaattgc
tgtttgcttgacgcatcataatagtgagatgaactctattgataaactatgggatagtttccttcaagaacaaaaagataaattatctgtggatctc
tcaaagtttcagctggaaaaccatccgcaatgggtcatgaaggcgaggcgatcggtttctcgcggtgatgtttacaaaagaataccacctttcata
ccattatcatctgtattgcaacaaatagagatttttgagtttccaacggttcatgttcttcctagtgatgcttctgtcatcacactttctgattacgaaa
catcttctgaagaagaagaagaagaagaggaggaaggtagtgaaagctcagatagtgagtcttcttccgataacaacgacaaagaaagtaatg
gtgaaacatcagattcagcttcttcagatggagaatcttcaagaagccccaagcaaacgccaattcccactttggatgattcatcaaataaagag
aaaaaaaaagactcacaaagtaatgcaccagatgctcgttctgctgatgtacttgattcaaataaagcggaattaaatataattgaagactcgtt
gagtcttcctcctctattcggttcatttttccaaaagaaagattgaatgtttattcaaaaagcataaagcataacttatatctttgaatttttagggtt
ttttgacaataagggtttcgattaatagtttctgtaaaatacggcggtctttttaataatattcatgatattgaattcatttttagtttattttatttcatt
atttgcaacgctcgtttcttcctttagcaaacaaggtccactgaagaactccgtttacgtttacttctacagaaccactcatcatttctcaattatgcc
tcatctgcatgctagcgacatagaaccgtattgaatgagactgaacctgtgtcactcccagctctatagcctggaacaccgacttacatagtgcta
caacaatcagaattctggtctaatgatgatatactgccaagactacaggatgttacacggtacttggtgagtaatggtgacattaaagctgccctg
ttactgaatgggtagatatactccaagctaacgagtatgatcgtgctattactgaagaggatatatcaaatatgagaggggttgattaccaaacg
atagacccgcttgatctacataaggtccgccactcgaaatcgttcactctcccagagaatcttcacaactacattacttaggtattggacgatactg
tgaagtactatagttctaattgtagtgattaagatgctttattaagcaggttgctaacatctgattatttatgttgttttaaacaaagggtgtaggtga
tctatcatgcctttcgtagtagtattagcctatgttgatacaactaagaaagctactatgctagtgacacatacaattaaccaccttgtaaacatgt
ataaataacattgctaataggcgaacgacagcttttcatttatgtatatttgctcaagtcctagtgtcaacaatttaatggaaatcgttctagttgtct
gccttttgttttcttctgccattgtaaaactacgaactggtgtgagctgtttaattagaatccaatttaaatagaaaaagtatctatactacaccttttt
ttgtttttttctttcttcaaaaccaagcttgagaaatctgaaccgcttaccttgtattacaaaatcgaaataaatttcttcttttatatttcttacaattc
atcctttacttattttatttttatttttattgaattctttctcataaacatcattttctcttctcaaaacgtttgtacttctctgcaaacctctatcctactgc
ccatttacagcaccatggcaaaacgatataatgatgagtcggaagacactcaaatgaatgagattatgaaagctccagatatggatttcataaat

attggtaggaagtttcttggagggtagaacacaacgctgactttgaactgtagattttgaatttttgaccctcaacctattgactttcatgcctttaa
aaatttgttgaaacagttgctaggctatgatcacacaaacgttaacattagtgctctctctgatttaattttaagccaacctttactaggatctactgt
caaggtcgatggaaataattctgacccttatgctatgctttctgtcattaatcttaacagcagaagagtaagtctataccagcggcttactgcaata
agcctgaaaattacatgaatcaatgaagatgatggcaatgctaatatataattaggaagagccggtgataaaggaacttacctcctatgtactat
ctagaatttcagcttctaaccctaacatggagaagcaagttcgtgagttattgtctccagaatcgaaagcacaggttggtttaattgtcaacgaac
ggttgatcaacatgcccgttcaggttatccctcctatgtacaacatgcttttagaagaaatgcaatgggctatcgatgaaaatgaaccttacaactt
tactcattatctctcgttgtcaaggacgtacgtcgaggtcgcttccaagctcgatgacgaagatgaacgacccaaaaagaaaggtaaaaaggca
ggagaacaagaaatttgttacttccatccagaggatgaacaatttcgtcaagctgcattgtccgttgccgattatccttttgccaaccaggacttca
accctgatgccaaccgagtgttccaggatgctggtatcaaacctcaaggagaattgattttgctgtcacatgacgctttcaagtccttagtacctca
actgttaaatgtgtacagtgcttgatacatacgactacgcctcctaaaaattcaaaaacatcttttacttgatttcgttactttggttttgctgggaat
ttggatctttaggtttttctggataaataaatagtatattcatattcaaaataatttgtatcaaaagtcgacggtcgtaacatatttcaatctccatg
tatcgccctacgctcaattaatttacacttgtccattgtaatcaaaaatttcttatagcacacagaacatgttcgaatctgcatcaagagagcctgat
taaaatatacttttcttataccttttttatttttagatcttgttttctctattcagatcatttaccaagtgccttcggggatttctctttattaacctcgc
taaaaaattcagcgaagtagaattctttaaaggcaattgaatctatcgaaaaaatatttttcatgaatgctcaagagggcgaaaatcctttcacca
attttgaagatgctgtagacgttactgaagatgttcaagattggcgctttttgtcgaatgctaaagtgtatgcaatgactgtctaaaccgtcaacag
agttctgcaatttttttggttttagagacatattctaatattaatttagagagcaaggtaactacacaattcccaagaggggagaaaaagattttgag
ccagatggaacgcagaaacaagagggaattttagagctcagccgtaaggctatgtacgatgcattatcagtagaaagacttatatctttgtaagt
ttgcaatggctggccccttaatcttatagagaagcgtttgctaactggtcgttcaagtaagcatgttattattggggtatggaattgggaaaagaaa
atggcgtgtgttgaaaaagtgcatggccctttttttaaaacaatgggacaaggtgattcaaaaaatcgtatgtggcttctccctgaagaaacacta
tatttggtagaacgaggatccatggaatgctgggcaccagatggtgtccctatgagcctacaatctctctacgcagcaactataccttttttgtggct
ctttggaaaactacattgtatatgctcatttgcgaagatgcgggttttcagttgtacgatcaagacaaatacaaagaaactacgataacttatcta
aatctatggataatgcaaggataggcttatggagtgtcgggatgaaaaaggttttgtctgcttttcattatatgagctatatgaaactctggttgcc
aaaatgcaaaaaagctcacggcctcttaccggacaaaacgtacagagactatagacaagtatacgaggaaattcaagttgtccccggatactct
ccaaaatcatttagctgtgagaaagagcctgaagctccctttcaacttacctttgacgtctataaaccatcccaaatttttaaaaaatcttccttgaa
agaaccagattttagaatctgtgtcgttgatgctcaaagtacaatcgtgccaacgtgtacgcaacttaatcgtctcttcggcatagtccctatccaa
gaaaatacgtctaagcatatgatgcaaagactaaaagaaggagttaggaatgtcatttttgctgttgttgactgtggtgtcgttagttatgttcgat
tttctgatgtatgtatgaatgaaaacttatttcttgagcaaccaagaaagcgcggaaaaggagatggaaacagaaagggactaataaacaataa
ttacaatagaggtaattgatatattttcctttttgtttacctttcagcctctaatgtatgattggtaaagtccaaaaaaagaattacatcgactgtcgg
ttcatcagacacttcttttcagaagagtcaatgacttttgtctatgattacctattatatactaagtgaagcgagactcatttcaaatagaagcattc
aatttgaaatctatatgctcaactaattcaatgataaagtaccctatagtccatgaggagagtttcagttctactcatcatgttcaatatagaaaca
gcattttcaatgatcaaaggaaatgatttctttcagttgagggagttattaaaactttttgcattttattattgaaaaggtcgaatattcaattcaata
ggcatagtccgtttcaaatatcacttttaaaagagatgaatgcatgctttctttgggtaaacgtgcaaatctaaatattattgtacttttattaattga
actgttcatttggttttctcaaaatttgtgaaaattaattgccgacgctattagcagtaataaagcaaatctttaaataatttgaaagtaacaactta
aatttttaatttttaattatataaatgattaagaaacgtgtttttcagaacagcagttctttgagtagcagtagtaagccagcccatacggatatcaa
agtcaccaactcccaactctgtcattcacagcaaaccctcacaaaagaataagaaagttgaagttttaggatgccaatcagtaatatgagatcat
ttacaagaaacttgaacaagatctctcaaaatcgaggcattcatggagtctcgtattccccacaaaaattacaaggtatttcgttcgctggtagag
aaacagctagtcctactggatccttatcggttgtgattcgtgctggtagtcgttttcaacctgatgctggtgtcgctcacctgttggagaagtttgcct
ttaaaaacactgaggatcgctccgctttgcgcatcactcgtgaatcagaaattttaggaggtcagctttcgactcaattgactcgcgaacacattat
attgactgcacgtttcttaaatgagtatttggaatattatgctcgtttacttgccgaagttgtcgatgcttctcaatatcttggcttccagctaaaaga
agaagttctaccgaccgcaaaattggaaagctctttatggaggagtaacctcttgaacgtggccatggccaaactccacgagaaggctttccatc
gaggtcttggcgatttgccctatctcccagtgggcgctaacgcttctatctcgcagattactgattacgctaaagctgcctacgtgaagtccaatttt
accgttgtttcttccggccctgatgtccaaaaggctgctgattcagtcgcaaagtatttctcggttatccgtgacggaaaccctcttcccgcttctcct
agtaaggttgcatctggagaatctcgtatctactctagcaagcctgtgaaccatttcttgcttggcttccctaccaactctgctccttctgctgagctt
tacgttttgtcctctatcctcggtggacactcctctatcaagtggtctcacggtaacactttgcttgctaaagcctctgaaaaggcttctgaattcaaa
gccaccgctgccgctaacttgcatccttactccgatgctagccttttgtccattgttgtttccggtacttgcactaaggctatcagggctactgcttcc
gaatccgttcaagcaatcaagtccttaacttcttctattcctgctgaaactattaaggctgctgttgccgatgctaagaccaagtatcttacctcttac
gaacccagtgtcttaaaccctgtctctaccacttctctctttgccacaaattatcaacctgagtccttcatttctgctttagatgctgtcacacctgaat
ccgtcgccaggactgcttctactttattatctcgtcctgctagcaccgtcgcagttggtaagcttaaccacttgccttactacgatgagctttaaagtc

atcctcaatctttttgttcttttagtatttaaattgaacagcattctgatatagctcaaacagtacctgataattttttcaaattccaagtcttattattt
gaaccagaatattcgacgagtctcggaattactttcttttacagcattgctgtgttatggtttttccatcaaaggtattcaataaaacacgattgttt
cattaggaaatttctatatcagcttgactacgaatccatgaaataaatctatttaccattaagcccaaattcagcgtaaaaagaaagaaaagcaa
aaagtgattcgctttatatcaattagttctctagctccattaaaagaaagatcatgtgtaccatctttagacgtagaatctgtatcattactgaatat
cattattaggtttgtgctctttgaatgtaaaccctccaaattcgttttgattatcgtttcctatattatacatatatacatcgcctccgttacagcagac
tgaaagttttgttttatcagaactaaagactccatgagtgatcgcaaagtccagctctgcaaaattttgtacgaatggctcattcaatcggaggtcc
caaactttcacgaggccatcactacctccagtaacaaataggtttccactcggaacccattgggccatagtaacgcctgcatctacttcttcctggt
tagcatcatcaaaatgaatcatttttgttcttccatgttttaaaacattcaaaaccttatttggtttacgagaatcccaaacaaaagtctgattgtctg
tcccgctagatgaaacaagaaacccggagttgctgattgtagcatgattgatatctctttgtgaggtattaaacacaatggcaggttggtaaatcc
gtgtatcgatgtaataaacctttcctctctgagagctgttactgctggctacaaatccaatgtgcgatggatgcatataaagggaagtaaatgcttc
gtcactcgtattaaacctttgaataaaattcccgttgttagcgtcaaataaagcaagacttccgcgcgagtcagatagatgaaaactatcataccc
tattacaacacgaggagaggaattatcaaacgaagaagagaattcaattaatgatgcagagtaatttttttcaatgctttaggatatataagtttt
tgagtagaaagaatactgcctgaggttttgtcggttgtaagcatataaccaagaccgtctttgcatgccacagcaagtacattatttttagacaaa
gaaagtttatgaatacgatcattaggctcatcaaatattccacaaagggttccctccatgtcccaaacctttaccgtattatcgaatgcaccagtat
acaaaaagtgtccattttgggaaaagcaaagatcagaaatggttgaaaactcgttctctccgtagatatttactttgtagtgaccttccaacgcctg
aacggtatccaattggaaatcacccagccataaattgccaagtctattgtatgcaccatcctgagcggtacttccaaaagcaaattttccgaataa
aggattaaaatcgacgcatactgtatcaccacttgtttcgttaaagtatttccaaggtgtcataaatagaatggactcacttagcatcaggttgata
caatttcctcgatgaaaataagatgctttagcattgtaaagcttttcaaaatactttgatagatagtgagttcgttcttgtttcttcaactgcaaggct
ccataatctgcaacaggtgttagatttgggcagtcgacctcgaatttctgacaatcctcataggttcgtcctagtaatcgtgaagaaatataaaatg
gaaaggcatcgtggaaccgaacactgttaacaggtccatataccccttttggcctctttaaggatttgacattcttgaggagtgaaatcaacgtgaa
ggatagaaggctttaatacatctttcggcgtgttatataaaagatccaaatgttctctggctttttttacttaagtatggacgagatttaggatagtag
acgccagaacttaaacgcaacatcaactcctcatcttcattaatatcttttgtttcttttttttcttaaattcgctttaataggttgggagacacgacca
cgaggaatttgtacttgatcgaaaggatcatataatgcaaacccatcctctaaatcttcatcactccattgattctcaaatcctgtattctgtaaaaa
atcattgttccagtcagttgaagcttttagtcgtttcgccgatggcaaatcctcattcatgccattaaacaagaattttgaagtcgtattctatattac
cgtagatttatcctagtaatgtttttattcacccaaacgattagcgctgactttcaaaacaagcaacttgagaaatcttttaatatgaataaatactt
ttgtgtgctatttacaggggactttactttactattgaatatatgtaacaacagtaggaagaggtggtagtaatttcttatataaaacacaatttaaa
tatatcttctcttatacccttagtgttttatgttgataatagactagtccaacgtttataaatataaaaaaagcttttattatattacatctaaataaat
aaattaataaatagcaaaatctaaaattctttaaccaaattcaatctttagaactgcatgcactgctttctaaaaaaaaataatagcaaataaag
atggtataaaagataaataaacttttccaaagattcaaaacttgtatgtaaagaaagggctaacaaaggtaaaacaagaacggacatgtcatta
agacaaattcatcattagatgaataaataatatgcagcaagatctaatattttgataataattttagttggaaaacaaaaattgcatgtcatctacc
gagagttggttgagagccttttcatcttgatctattgtagcatggatcatctgtgctttcttctcttgaagttctataattttactctcaatggaattctc
gatgcagagagtagtaacacgaattggtcgcttttgcccaattcgatgaattcgatccatggcttgccattgcacagctccattccaccatgggtcc
atcataaacacctgagaagcttcagtaaggttcaaggcaacgccaccagcttttagagaaacaaggaaaatggtgatgtttatatctttactaaa
ggcctcaattgttgctgctcttgcttttggagtcataccaccatccaacttaacgcagttaaacccagcttttctaagtcgccaatgaatcagatcaa
gcattgaagtaaactgagaaaatacaattgattttatggtcctatcttttcttctcaaaagatataattcttcaactaaggcttcgattttggtagaa
gaacgccaattactcatgtcaatacgattaagaattgacgcattcttaaatttctcttctgaaaattcttccatgggaggtgcactgaggtctatgct
tagcggaacaaaacacgcaggacatgtaacatcttctgattcaccagcagcgtttaaaaattcagaaatacatagccgacaaaatgtatgatga
catctagactcgatggcatcttgtgcaacttcatcacatattctacaaactatattttcttgggtgtcgatgtccacagttttcgtcttacccgccaag
acaagatcaggatgatctgccatttgacgcattctagtaatcaattggaaaatgtttgcatagttatttaagacgacaccttgtgcaagataagtgt
taaacttccttttggagtccatgtaaagtgactgataaacatcttcttcttcttcattaaataaatctcgtcgcacttctacgacacgaggaggaagt
cccaaatcatcggccctctctaattttgttcgtcgaagcataatgttgttcaaaagtcgatgaacttttgcaaagccagctcttccaggtccttcgctt
ccaaacttctgtatcggcttcaacatctccgcgttaaaatagcaagtgtgactcatcggtttatgaccacaatgatcacaattacttctatctgaaa
actgccactgaagagacttgcaatcgcattgcaaacagtaataaaaagcaaatggatcagcgcgtagaaatcgaagtagggagaataactcgc
caattctgttttgcaaaggtgtacccgaaaggcaaatccgcctcttcgttttaagagtgcaaacagctcttgccgtattacaagtcctggattttata
ccatgtgcttcgtctaaaatgattctgtaaaattctaaattatgtaacaaggatttctccttgacaagcccctctttacggcgaaatcctgaacgttc
ttttcgataagccgattcaatcacgttgtaacttgtcaaaactacatcaaattttgacaaattctccatagaaatgttcctggattggccataataca
gatatgtgcttaatgctttattagtatgaacatcaatttcctccttccattgcattatagcaactacgggagctacaaccaacgtcggttcccctcga
ggtgtagaaagtaaaagaccaattgtttggatggttttacccatacccatttcatctgccaaaattcctcctccaaaggaagattcttcctgatgac

ggagccaatatacaccttctcgctggaatggtaaaagatttaatactaactcctttggttgttctatctgctgtacctgcgttggagcttcccgctcg
agagcttcccatacattgtccagctcagggtgatttctaactaatcggtgatgggtcctttcgtacggtggtattcgagttgaaggagttctagaag
gagacgaagatacttgagacatgggtcgtccttttccttttcctttccgcttggcctgagcgagaggaattatgtcctcatccgtttcagtagaagag
tcatcttctttcacagaatttaaggaagtattcacaggaacattatcctcattatcactttcgtttagttcaattacactagcaacctcatcactttcat
cagcgtattcagaaccatcagattcttgagatcctcgaggttcatctgaagggccagacgagaaggtctccacagatgaagacagactagattg
atattttcttgaccttaatacacgtccctcattcattttaacattagttggatctaattcaagtttccttttcctctcttgaccctccaaacgttgcaaac
ctttcttctttgtatcaactttgggtatattctctttcgtttctataaaggttttattatttgacagtaagaaatcatcatttatcatgttctcattgcacg
atccgctctgatcctcagtatccaattgtctggtatcaggctccttggtatcactaactttaaaaagaggagactgttcgtcaaaagaaatattcttt
ctaccggcattcagtgaattaattagactttcatcctctgtatcacttgcggcaattatatcagcaatgggcgacgatgaatcaaccaggtttatgtc
ttcattgctatgttcgtcttcctttttacctaataaaacagaaacaaaggtctggcttccctttcgcggcaaatcttgttgagttgagggtgtgtcatt
aatgacattaggcccgttgaaaggacacaaattatgggcgttttgattttctgaagaaccaattggtttccttaaaccattttcatcaacaatttccc
ttgatatatccggtttttcagtcagaacgtccatttgtagttcgctctgggtaagtttgccacgcgccctcaaaatttaattaagagccatagttatcc
tctcccaaataaaaaaaagacgcgaatgtcaagaagtcgcgtaagaggacccaattcagcgttgactgaatttttaagggtatgttagtgtctat
aataaaaattaacgaatttatagtctcaagggatcaatacatctgccttgggtagagcacgccctccaagatcagaatccacgtctcaacaaact
gatcaagaatctattgcatcaatcactgaagctgaagaaacaccattatcggaagaatcgcctctcatcgagacaacaactgttcaacttcagaa
tccacccaggtctcgaggtagaagtgtgaagaagaagaaaaaacccatagaagataataatgatgatgatagtgagtataacacaaatac
tggttttggtcgtgctggattttcatacaagtcgcgtgagaacgcaggtaaattagacttttgtgttcactgccagtgtcgctttacaattacgcctta
cagtaaatatagtgagaaagaaaatggatggctttgctacccttgtagtcgtggtgttgaagaacagaaagttcctgaagttcacactcgaaagc
gacgtgccatgacaaggaagaaagctgcggcggctaccatggatgaagaactgaatgttccgaagcttcaagacctttgtattcgcatcattgcg
gaatatattcacgatatcgaagcgttgggagacattggacaaataaacatgaacaaaataagccagatcatttctaaaaacagatctttagatg
atacaacagtgcaattatttttgactggagaccaaacggaactcaagttgtacgactgctccaaattaacagttgaagctctgttgcaaattgttca
atactgtcctcatttacataccctacatttgacttattgtggacaaatgaaagatgaagtgcttgaactatattctgaaaagttgactgaactaaag
gacttttctatcaaaggtgctttccttgtatccactgatacgtggatttccttcttaaagaaacgtggtccacaactgacaagacttgaaattacaga
tacagcgaaagttcgccctcctgttataaatacgattgtggattattgtcccaatcttacgagtctgactttatcaagaatcttttacatggatgatg
aatgtgtccgattactttcgggctgtaaaaatctcaaaaccctacggattgagtcacctggcgatgtcgttactgatggaagcatattggatgtttt
aaatcaaattggcagtggtcttcaaactcttagtttggctggctgctgcaaattaacggacgaactattacgacaaggcattggcccctgttgtggt
cgtcttcgtaacctggatttaacagctttatctgaattaacggatgacgaagcagcttcgatgtttggcaattggaatatacagtctggtttagaaa
acctatctctacgaagatgtttagggctcggcgatcgaactgttcgtgctgttttggtaaactcggggcattctctttctagtttggatttaaatggttt
atcatatgtcacgcataattctcttcaatatttagcaacgttccctcttcccaaattacaaacattggacgtaagctggatccgtgatatgaatgaca
aacttgtttgtgagtttgaggaaaataaacccaccctggagaaactgttagtatggggcaataatcatgtactaatgccgacgaaacgacttttgc
taattggaagagaagtgcaataatttgaattatagaaaactaaaactatgccattactatggtttttctagattatttatttttgtttatttaaatgaa
catgaaggttaataaggctgtaatactagtagttggaatagattcatatgcttggtaaaaaaattgcttcggccgggaatcgaacccgggtcgctt
cgatggcaacgaagcattataccactaaaccaccaaagcatagttagcactagacaagatatgtagcatattatctgccaaagcaaatgaatcc
ttgtatagtctcagagttgcaagcttttatgaacgcactcgaacagtgagtgatcagaaaaaaagagcagcaaaaagaagcttacagcacccgg
attcccatgttgtctccaaccatagtatgaacgagaccctcagacgttttcacttaactgcagtgatcggacgggaactggtgttcacctaggtatg
ggcgtagacagttcactgtctttttataatatatatctttataatttaatccaacattcttgcacgtggaggttgtagagcaggcttggttgagccat
ttgaaagatagatgttcaatcagcgtcaaacttcattgtttttgttcatgaatgaaaaggatatttcttttgaaatagggatcgattctataactagc
gacttcttggatatatcgagtatattctactcttgtttatttacattagacgaaatgtaagtgacattgaactttttgtgaactatgtaggattaaatg
ctactgaaagaagtttgtttatataggaataagaatatgaaaaaaatgtcattgttatttccatgaaataccaacttaagccatagatcggatcag
acctatgtaataaaaaaaaagtactggcttcaaatcatagcaactattagcgtgtccctaagaaagaaggaataaatttcagcataagtaaatg
aatctatcttgatacatatttttctacactttttcaaatatcctcattctttttccccacgaataagtctttaatatcggtacacaaacacttgtctgatt
tgttataagcttccttgaatgaatattaacgaagataaaatacaaaagaagaacgcatctagtattagctaggtcagtctctcaactgtcctagctt
gcatacgctttcgacatacaccaacactcattatattatcattgatatcgaaaaatcgatagcaatattcatcttccaaaagcaatcaacttaacca
gaagctgaatattgttagaaatattactagatgaaagcaaatttttcttaaaaaccacctagcactagcatcaacatttcacacttttacggaggtt
caattcagcaactctcttcctgatacttcataaaaactaaataaaaaaatgggaagagaaatcataacgattcaagccggacaatgcggtaatca
aagtaagtggctttcgtaatttgaagcttgttgctaattcaccttttctttagttggctctcagttttggcaacaactgtgtcttgaacatggtattagt
cctgatggcacattagaaagttatgctacggatggtgtagatcgaaaagatgtctttttttatcaagtatgaacaataaggatcgacttgaagact
aactaaccttttcacccagtcagatgataatagatatattccaagagcacttttaattgatcttgaaccaagggtaagagaaattacatttattaat

tcgaagcacgctaaccacaaatacctctaggtggtatccaacatccttggagatacatacgggaatttatataacccggaaaatattttagtaaca
aaaaacggtggtggtgctggaaataattgggctcatggatactcttatgcagaccggatcttcgaagatatcatggacatgatagaccgtgaagc
tgacggaagcgattctttggaaggattttctcttttacattctattgctggtggtacaggatcaggattgggatcatatttgttagaacggttaaatg
atagattcccgaagaaaattgtccaaacctactctgtatttcctaataatcagtcagtttcggatgttgttgttcaaccatataacagtcttcttacac
tcaaaaggttaactctcaacgctgatgcagttgttgttttggacaatgctgcactggctcatattgctgccgaccgtctccacatccaaaatcccact
tttcatcaacaaaatcaattggtaagtttaatttgaatttatttgttctgacagcacgtaggtttctactgtgatgtctgctagtaccaatacccttcg
atatcctggttacatgaacaacgatttggtcagtattatagcttctttgatcccaactcctcgctgtcactttctctctacatcttataccccctttacg
aaccaacaagtggaagaagctaaaaccattcgaaaaactacagttttagaagtcatgagaaggttactccaaccaaagaaccgaatggtctctg
ttaaccctagtaaacaaagttgctttatcagtcttttgaacatcattcaaggagaagctgatcccactgatgtacacaagtctcttctccgtattcgt
gaaaggaagcttgcaacctttataccatggggacccgcaagtatccaagttgcactttccaaaaaagtctccgtatataaaaacaaatcatcgtgtt
agcggactgatgctggcaaatcatacaagcatcgcatcggtaagtattatgggttgcagttgaaaccatcaattatcagaaactcagaatctaatt
ttaatttagctgttcaagaggactttagatcaatacgatcgtttacgaaagcggaatgcttttcttgaccaatacaaaaaggagtcaatttttgaag
ataatttagacgaatttgattcttcaaggtatgtcatttatccttttattgttcattgttcaagaagcttacagcatttgcaaacagagaggttgtctc
caacttgattcaagagtacgaggcttgtgaaagccccaactatctatccatgtaatgatattttaggatctagactttatttttaccaaaaaaaaat
acatacccaaggcaaacagtatttagcgaatgaactcgatgaatatataagaccaaacatttcctatttatatagacagacattgattacaccaa
cctatgaagtaaagcatttttacgtctctccagcaaaatccctttctacctcttcacgtgcaggaatgtgctcgattacgatgggcttccaattctcca
acgaatgggttctttcttgagataatcgttccattctttgatcagatattgtttttcgtagtcctgtaacgaggcttcttcttccaaaggcgtataacc
ataataccaagcatcgagctgtcgctgttgcatctcttgttttcgcaagcgttcaggtacaaaagtcatatcagcttcaaataactcttttacgccag
gtaattctctagcacgcccataatagcgataatcatcgatctcaccgatcttctcaccatcatccgcaaagagtttcgctttgctgtatatataattt
attcctccaagatcgcgaatttgaatttcccaagcatgcttctcacgcatcaattgattaatagcatcattcaaatctcgaacttgatagtccggca
attgagcttatgttgtggttagtatcgataaaattgatttcttttgttgtacctaccactctgaatcttattaatttttttgaaaggtctttaatgacact
ttgtcggtgtttttcacaggtagctactgaatttatgtttttatattcctaggtctttttccacaggcttgcttccgaccacatactaagtattaagtt
gttagccagcaagcgcttcaaatacatgcgactcccaaacatacctgatttgaggacatgatgataacaactttttccaaacgtttacttcactacc
aaccacagacttagacttcgacttaacgtgaaacacataaaaattaatcaaacaaataatttctacaaggcaattgttttgctacgcatatgcaaa
ctctgcttcccctattttcccaaggcagtcgtttacctttatgatctgcttccacttacaagatgttctcgttatagttattatatacaatcgagggaat
aagtataatagtaacagtgcttttcaatagaagatttcacacggcttcttccatactataccaatttctggtggtttcgtattgatctttagacacgca
aagtcaatatagtgaagtacactacactgcgtcttcattgctaaacggctgcgatgaaagtttaaacaccatttagcgaatttaaagcaaacgac
attaaacagaaagttcacggtctgcaagggaatctgtgaaacacggaaattcagagggaaaagaggattttatttattatttcttgtagaatacc
agagttctattttggtcgtttggaattttgaaaagtacagtgaatttgatccacatgcgcttggttctcctatgaaggaaacatttagtactccggaa
gtcgattggcaaacgtcctctcaatggataataaaaaaagaggatttggactatacgccctctgctctcgacggaattcctttggtacaagaagaa
attcagaggtccaaaggctgtaactttatcatcaacgtgggcttgagattaaaattgtaagttttttttcttttttttcttaaataaaggttgtttaatt
aacctagactaggcctcaacttacattagcgacagcaaatgcttactttcatcggttttaccttcgattttccctgaaggaatatcactattatgata
ttgcagcgacttgtgtttttttagcaagcaaagtagaagattcgaacaggaagctgagagatatcatcatcaattgcgcaaaggttgctcaaaag
aataacaacatccttatagatgagcagaccaaagaatactggcgctggagggatgttattctttacacagaagaaattttactggaagcaatatg
cttcgattttaaagtagaccaccccttcaacgatttgttgcgtttaatccaacattatgtccccgatcacaaaaacctcacgaaaatttcctggacct
atatcaatgacagtacgagaagtatttcttgtttgctttttccaaggaaagtgattgcagcagcagcttttcagtacggccttgaaaaaaccaaaa
catcacttcctctcaatgaagcaggcactccttcatggttaatcgaaaataacatttcaatggatgatatacacggtaagtgttaaattttaaagtt
atgatactaatcgtctcaatagctgtcatgaacttaatccattctttgtacagacaaatcaacagtggaaagcccgttccacaaggtgaagttaaa
tcagattcgcagatgccgtctcaatccagtactccttcaacaatggctgcaagctctgcccaatcaacaccgcaagttgaacctgctattacaaag
ctctcctttgaaacagggcaaaaagcccctccttctcaggctgactcccttaacactcctcaaagtgggcagatagaacaagaacagcgtgctgc
tgaggcggacaaaaaagaagaaacccagggagtgatggaaactagctagaaaaagatacaatgaaccttgtattcctcttaatttttcaatatt
acatctttatgtaagatttaacaataatagtattatacaattgggtttgcaacaggcgaagcaaagcgaataagcagttttcgagtttagtgcttgc
tctctatttggttatccaggatccgtatttttgggatcttttggctctccaactcagaaaactatatctacatatgtcttttatacatagggcaaaaac
cgcacctaaaatactttttgtacctttgttaatctatttgggaagagtcgtcccaaaaattttcctgctaaaggaagtaagacaattggtcgtagaa
tccgaaggatttcagttgtaaacccatctaaaaatgtatggaaaacagaaaaaaaagtccataattcagaatgttttacttctgcatcctcattagt
cactaggttttgctgttcaatttgatcatctaattttcggagccagttttctcttaattcttcgtcgtcttcctgtgtatagactgaaatgtttttttgat
atagtttgttagctctttgtgaatagaacgtatccatggtaaagcacataataatatctgcgtagtggaaaaccgatctacacagtaatagacaga
gcagtgtgtaaataaactaaaaactatgtcataagaaggagcttcacaaaatcgaacgagcatacatggaagaatcggaaggatggccgacga

aaacaaaggtcgttgcatcacaaataacatggagctttcatcagccacttgccggcacacccagagacctgtctctctgaagatcgaatacacag
acttgcctataccgagacaaacacctgtcccaagtgcaatctgcactaatccagattcgatgttttggcaaagaccaacagcatgtaagacccga
caattggctttcatgggtcgagcaattatatattcattgaggcattgaggacatctagtgtttgttgagcttgtaacgtaagagactaagcagctttc
gtgggcaattaatgagcatttgcaaggccgtctccagggatctttaggattgcgcacgccgtcaaactcatcatagcaaatccagcatcgtaaag
ggtatcttttatcaacgcaggagttttccattactaccttacaagtttgaattcagtcgtccaataattaatagaattcagtgaatatcctgggcaaa
atgttgcatttttttgttcaatatgggataaaaatgtttcctttgattaaaattatcaaaaaaatcgaattttacagggttttgctatccttcgaaattt
gaattggaagagtaagaagttgttccaatggaccaaaaattttagcaataaataaattaaagttgaaaataaaaactaaaagaaacaaaagaa
attataaaacgctgataagtagtcaattggggtgattgcgctgggtatttgttgaatgtgtggacgagcgtaatatggagtttggccttttgggtaa
ggcggaaggccgtcgcgtaccagtcatggatgctgatgaagttcttaccggaggacgaagacgtgaagaacttttaggaaataaactacgtcttg
aagcgctcgatgtcctttgagggacctgaggatcatgaggagtgggtgccgaggatgaagtgggatatgaagaagaaatggctcgatttggcac
ggaagacctagttttgggaacagaagagtaagagctaactcttctattcgcagaagggttcaaaacggaaggacgacctgtaccaccagacga
agaagttcgaggagggggatggaggatgggtttcgagaagaaaatgaaggaactcttgaatttcttccacgaaaaatacttgagcgtttctttt
gagctgtcaaagcttcctctttttctctgcgagcctgctcttctaattctcgttggtgctgtaattgaagttgctgctgcttttgcaactcctgctcttgc
tgtagtttttccttctgtaatacctcaatctcctcaagatcttgcataggccccttccagtccgtatttgatagtaactgttgcgtgtgatctgtttggcc
ctgtaccgaagaccatatgtttactaactgattagcagactgaatggatttttcaaaagaatgaagtttttctgatgatatcttcagacttcccgtaa
gtttttctatcacttggttaaatgtttttagtagctgaagttcctgccgcaagtcgcttgacattcttaaatataactcctaaaagtatcttgaatcaat
ttttttcatagaatgatgttagattcagacagatttttagtatgaaatcttcagtaaggaaacttgaatgctggaaataaccgtgaagtttcaggaa
taaatttataaacagaattgaatgaaagaaggaaaaaggttttaataaatataaataccgactattgtgctgatatttcctttttgtagaagtgttt
cgttcctttgaaagtattccacgaaagcaagtgaaagtatgcctgttgtgcgatactttttctttctataaaatatgtaaatttatccatagccaaca
gtaattagaaaaagaacattgaaaataaaattgatcttgatctttatattgaattttgaaggatcgctttgaaatagcaaagtacaaatccaccaa
aaactgaaatcaaactaccgtaacgattcaaaaccatcgtacttgaaaaccttttgctgtctgtctttcagcccacctagggtccgcgatgattaca
ttttgttttgtttacaagttgcatactaaggaaaccagcgttctatgctttaataaaatgccaacaagggttttttctcaaatgaaaacgataacaag
aagaaaaaaggaaaatatttacaaacaacatgggatttttgaaatcatccttgtttgtttacactgttactccgtttgtttagattgctacttatgaa
agcaatgagtgttcagagtagcgcgtcgcgttttaacaacaacacccatccattccattgtaagtaaaacataaaaaacgcgagtttttaagaatt
gcaggcttcctgaactttattcctttttataagaagtttttctttgctcaattcatcgttccttggataaaaagaagccctacaattccctaggccaga
tattcatatttcatcttattctctaatcaatatactcttattatctaccttcagctcaaaacgaaaggttcatgagattgtagaggagttcatctagttg
cttacaataacaattgttattgatacaacaggtttaactacctctattatattatcttcattattatagtacagcaaaagtttcgtgcaccagaaaaa
ttatacagtctgtggttttatcctcatcatttcgatttttttattttgaattactttttataaatccccttttgtcattttggatttttttgatttcattttcatttt
gtgattcgcaaatcttggttttctttgttattattcaccgttaaggaaggaaccgtacctaaggctctttcttcctggaccactttctatatcacataca
atctcaaaattacccagtttctatccatttcgtttatttgggttactgtatattagagattcactctactgttgctccatttatcttcaatcactgttctac
ttaacccaagaaaaatggagcctactactccgcccttgcggcccgtatgtactcccaccactccagaatcaccatcgaactctaagctcgacctcg
aaagctattttgattcagaagcgaatgactcatctgccagctatactgggttaccgaatgctgaagcttcttgcaatgggctaccacccaccccctgc
aaaaactcctaaaaagcgactgtttccttctttactccgagaacggaacaatcaaacaaatgaaccgatcgaggaagatgctggtaataatccct
ttttgggtccgcctgcgccagctgaagtgattgcgaaacggaaacgtgaaatggaagtaccaccttctgacggtttattttatattttccgtggaaa
gaaggtcaaaaaagtgttttcagaaggaaccgacttgtcatcattaaaacccaggcgattgtttccggaagccgcaagcagttcctcggcaacac
ctttatctgataggattgattcttcacaacatcctcatatcggtgttcttcctccagagtataatcgtgaacaaaagtccttatatggatcgtcttctct
ttttggaccaaccacggctactgctacgtctttaccattccgaaatcgtggttcccatccgccttcctcccatacttcttcatttagcagtggaaggac
tcgacagctccccaggtaataggaaagctttggaagacataagctaccttttatgattgaggctttgatcgttcggttccctcgtttttttattatata
atatccgtatgctttaactaagcaagataattttctttctctctttttttttttatattttctttcagtgctggtcgctggtaatgcttgtctttggcaaccgt
aaggaaatttctatgaatatttttacggttgatgtatgcatgatccattcattcaacgagatttcccttagtattgtattctttatggcaggttccaag
aaaagacatactatacccttcccttttcacatactgatgattttctttcccgattcctataataattccctaatctaattgcatttctcgaagcaatcga
tttgacctcggcttcctaaaaagtctaaggtttacgagatttattattccatcactgagttagacacgactggcttgttcaaacccccatttttttattg
ttttcatgtttttttatttgtgttgaaaacatagttactccaataggaatagataaagaattaagtagatatttctaataatatgatttaatttttataat
acgtttccatgctttgtttactttcttcactttatttagaccttactatatcacccatctgaggttgagagactttaatcataattgttccgtctagaata
ctgttgcactattggttaaaccttgttttgtgtcagcaacatcatctggtctaggtactttgccaacctaaaactccaaacaaaaaagtgtctgaatg
atctgtttgcacagactaaatttctcataattggaaaaaaaaggttcagggatagttctttagctttttgctccgaataatttgaacctatggaataa
atctataaaggtataagatgcttgtttactaaatggttgtttgttttcatgtgacgttaaattggttgaaaggtagcaaccgagattattagagtcac
aaaatgtgtggggctattgcgctaatatacgttataccgcgggttcttggtttacatacaagcctagggcttccactttgacctctgcatatactcag

ctcagacttggcaagattagttagaaagtcccactttcttgaaacgtgttatatataagttaaattattgttaacgaagtttctaatttacattcctca
taagcatagaaatccttgcttaaattagttacgtttacgaattacttgtttgacgatctctttaaagcatttacataatgattgaaacgattgctac
gaagcccttcgaaggccaacgccctggtacctccggtctgcgtaaaaaggtgaccgttttccaacaacctggctatttggagaactttgtccaagc
catcatggatattgtggaaccctcctcgaaaggctctcatttggtattgggtggtgatggtagatatttgaatgaccatgccattcaagtcatctgct
ctattgctgccggaaacggtgtagaaaagctagtaattggtgttaatggctatttgtccactcctgccgctagtcatgtcattcgcaaatacaagtt
acacggtggtattattttaactgcctctcataatgccggtggtcccaacaacgattttggtatcaaatacaaccttggcaatggtgctcctgctcccg
agggagtaactgaaaagatgtattcgattaccaaaactatttcagaatacaaggcagctaagattccccaagttgacttgagtactgttggtgaa
cgcaaatatgggcccttgactgtggaaattattgaccctgtcaaagattacgtggaatttatgaagcaaatttttgattttgatatgattcgcagctt
tatttccaagaaccccgactttacatttgtgtttgacgcactccacggtgtaactggtccttatggattggcaattttccacagggagctcggcttac
ccgaaagctcttgtgacaactgcaagcctttgcctgattttggtggtggtcatcctgatcccaatcttacctatgccaaatcgttggtcgaacgcgtt
aaccgcgaaaagattgtttttggcgctgcgtctgatggtgacggtgatcgtaacatgatttatggatacgaatcgttcgttacccctagtgatagtg
ttgcgttaatcgcacaccacgcagacttaattccctatttccgcgagggaggtattcacggttatgcccgttcgatgccaacatcaggagccattga
tttggttggtaaatacaagggtaaagacgtctatgaagtacctaccggatggaagttcttttgcaatttgttcgatgccaaacgtttgtctgtctgtg
gtgaagaatcctttggtacaggcagtgaccacatccgtgagaaggatggtatgtggggtattttgagttggttgaacattttagccgctttgaatgc
tcaaaaccccaagatcaagaccattaaggatgttatgaaagatttctacagcatctatggacgaactttcttcacgcgctatgattatgaagaatt
ggacagcgaagctgcaggcaaggtaattcaaaagttcagagatgttgtagaagccaaagatatcgttggcaaggaagtcttaccaggttttgtta
ttgctaaggcaggagatttcgcctaccgtgatccagttgacggaagtgtcagtgagcaccaaggattgtatgcaaaatttgataatggcagtcgta
ttgtgactcgcctttcaggaacaggatccagtggtgctactttgcgattgtacattgagaagcatgaatcggattctagaaagtttgagttgaatgc
acaagaggccttgaagccggtgatagacgtggcattgaacttgttgtcattagaagcattgactggacgaaaggagcctacagtaattacttaaa
gagaagggatagattaagcataaattaatatttatattacttgaacaaagaatataaaactgtcaaataagtatgtaatatgtaagaaaaaagg
acagagcataagacatttattttctaggaaatttcatagctgtcatagaaatagagtgtgtaataagaaaatatatggagggaaagtttttcagga
taaaggaacgagtaacgaaagatggtggtgaagattacgaaccaattagaaaaaaaaaaacatcgttcaccatgaggtaaattcaaatttccg
taagggtcggttaagctgattttccgaaagagcaaaattagggtgatatcacataggcgaaaagcgaaaaagccgggataggtaaacaaacgg
gagattctaacaaaagagagagaaaaagacaatatctatggtgaaaagaagataaaatatggaattgaaacgattgatatggaatcgagagg
gtattgagagttgagaaggggagcaattgagaattatagaaaaaaaaacaagaggtttgagggaatgcagggtacgggaagaaaagggaaa
tgttagaaacgggacagaacattgttagataacaacgaagaagtatcaagaagagagtggaacaacatattcaacggatgatgggattacgct
tctctgctaagcgcctgacggtacgcgatatgcgatcatggactgtgagactaaagattctgcgaattcgatgaaggaggaacgtcatggatttca
catcgttcaatggaagatgacgtaaacttgaggcttccgagtcatgaaacggggtaccgtgatggaagaaagaaagatcggagaaagttaaag
aacagaacaaggcatcattggggaagtaataggccaatcaaaagcagaaggggtgactccagtaacaacggggatgaagtatgcgataaggt
gtgaatcgaaagaacccttacccaatggataccacgaaatcattatgatgcttgatcaagggggtcgaaaagtataaatacaggcaggtaaatg
gaattgtggtactacagctcattcaaatcctgaatcgatctattagtaattattaaaaatggcaattcccaaggttggtattaacggtttcggtcgta
ttggacgtattgtcctccgtaacgctattcaagcaaagtctgttcaagtcgttgctgtcaatgaccccttcattgaactcgagtatatggcttatatgt
tcaagtatgattccactcacggccaatacaagggtgaagttgacattaagaatggaaagttggtgattgatggtaatcccatcactgtcttcaacg
agaaggagcctgccgacatcaagtggggctctgtcggtgctgaatatgtcattgaatccactggtgtcttcaccacccaagaagctgcttctgctc
acttgaagggtggtgctaagcgtgtcgtcatctctgctccttctaaggatgctcctatgtacgtcgttggtgtcaaccatgagactttcaaacctgac
caaaaggtcatctctaacgcctcttgtaccaccaactgcttggccccttggctaaagttattaacgatgccttcggtattgaggagggtttgatga
ccaccgtccactctgctactgctacccaaaagactgttgatggtccctccaagaaggactggcgtggtggtcgtaccgccagtgacaacatcatcc
cctcttccaccggtgctgccaaggctgtcggtaaggttattccttctctcgacggcaagctcactggtatggccttccgtgttcctaccactgatgtct
ccgttgttgacttgaccgctaagctctctaagcctactacttatgaagacatcaagaaggccgtcaaggccgctgctgatggcccccctcaagggta
tcatcacctacactgaagatgctgttgtctcccaagacttcgttggagacatcagttcttctatcttcgatgccaccgctggtattcaactttctcccc
agtttgttaagctcgttgcctggtacgacaacgaatacggctactctcgccgtgttgttgacttggttgcctacactgcctccaaggacaagtaact
agctagcacgccggatgtcaagaagcgacgagtgaacgtttaggagttttcatttttatttttaacgaagaaatatttttcacctacgaatttttgat
gccttcactcagttctttcacgctttttaacgtacatataatctaaataacgatttttgactattctcttgaaaaaaaaacaattcagagcaagtaa
tgtaatattgcgagaatataaaacgttctcctaataatcccttattcatttggtgtagtctttaactaatttttcaaagttggatgagtaattgtagttt
tgcgtagtgttttgaagtggtattttgtagtttattatggacgacgatacattcggaccggaagtatttgcattcgtttggggtacttttaattttacac
tttaccaaccacgttgttacatccatgccgagagcacagaaaggattgttgttggtggagtgcgacccaactgttaagcaactcattcttaacattg
atgaaaagtcaccaggcattgttattgaagaaattgacgatgagcgtctgctggtgaacgagcatcgtcttgaacaaatcaaggccgaactaga
gcgtcgccttgaagaaaatacttatcaaattgaagattaattttttggtttttaaatgagtgatttaatcttttcattatattatctattctataaggc

aaactttctgcgaccgaccatttgtagtcgttaaaaaagttagcaaaacctcttgttactgtccgaatttctcaaaagatggtttagtatactgttct
ccacctaagaaaggatgtggatgcatcaggcatcaggccatgtcaaagtcaaaccaagtcatgggaacgccaactttcttacactgcgtcagcg
ggggcatcgttgtttccaccggtcccacccaatgtagagctcgaagtaaatgcctggcccttgattcctgcaaacgtgtaacaaagacctgtcaga
ctcaaaactacgccagcggcgattaaactggtagctgagaaaatgcaccccaaaaaggtcaccagtgcaaaaatagaatatataagctataatt
cctcatgttagtcattggcactttgaacagtacagtacagaaggaagcttaccccacgataatagttgctttcaaagatactcgtaaatgcttgaa
atttctctgaaaccggacatatgcgagtcaagaagggaagttcaataaaaatcaacatcacaccttccacgatggttaaggccgagaacaagac
tactagcgtaacatgaaacaagttgacaattcccaatataatacacacttcatccatatgttagaaagtaaaatcagagaaaagcaaaacagtt
gcgcattgcaaagaagtatgagctatagcttagtcttttgcacattgagttcaccaggacacatttcaaaaccacaagacggcagttctcaaaca
agaaaacactttttcatactttcctttacaatctccatttggtttccttcctaaatttccttttacttacagagaatcgaaagaatgccgagccattga
gcgtaaattctgcaagttttgttagcatacgtctttccagccatccaagtcccacatacgagaaattccaactcttaagctctccgaaaaacgtgct
tttcattcgatctaaaattatagactgaaacatattgaattaaggattatgctaagcgcaaacactttgctccaaataccacaacattgtctgttcgt
tccatttaaggaggtcgaacaatcattcgtatctcattttccgcatcactactgatcgcttaagctatgagcttatctgatcagataaatagaaaca
gagaatttcatttgatgtgttcacaaagttactatgtatgtttcaattatcatgaattgagaatcatctaaacttcagattgtagtctcaccgattctc
tttgaagatgatatcgggtgttggtgaacaagttcatgaaaacaaaacatcccgaaacaccccaccaggacaaacgatcctgattagaaggaga
aaagtgtagcgtatcgtcaagttttctatttcaattttctatgaatcaggttcgtgcttgttcacgaccgtcaacaaaacctaagtggtaccctctgtt
tctcatacgccaataattgttatttttgattcaaccttcatattctagaatatctctagtacaatcaaaagtctcagtgctcacaaagtaattgtctat
tcatcgctggttttcaatagtatcgttcctgcagatcaggcatcaagctattcagaaatagagtacttctcccttgctttttagaaaattcggtcatt
ctttgtattcatgtaatttcaaccattcattctttttccgcccacaagatactacttacttttccctccgtccgttgttgccttatgaataactttactctg
cttccattttccttttgtatttcgtgaagcaatatcagctactcttgatttcggaggctgttcagaggttttctggttgaatggtcgagctgatcttcgc
cattcgggagttgagtttccgccgggtttcttagaagatttcggtacaaaactttgattttcatccttgtttgttgttgctgattctcttggaggaattc
cttctttaggtgcgttcgtatcgtcttttcctgcaattctctcaatttcggctagtttttccgtgcttttatttgtttttcatctctttttcatccaaggatt
tactaaacttcgtttctttcttctggtggtttcctttgtcagctttgctttcattttccaacttcaatttttgcatctgcttttccagaaaagaaattctacc
gccgccaggtctataccgaggaatatcttctggagcaacatatcctggtctcacttgtatttgttgcctcacacttccatctttccttctgctttctggg
ataacgaatttgccttctaccaaacgcgctctgtcatcgattagtaataaattctgcaattccgcacaaacaaacccagaaaaagactttttcccct
ccattagggtgtatattttacctttactcaatctctaaaggctaaattcggttcttggtttgtcgtttaagtgttgcagtgccaaaatcttctgtccccc
aaaaagaccattatttgctttcgcatatcgttaaagctctactttgttatcttttatttttatgtatttttaaatataaagcattgatcaattctcttgtc
gattagcatgccatggtaccgagcgagacgaaccgacctttatctcgatgtgctctaatacgcagacggttgctgctgtttcttcaatcccatgcgt
tgatgtactcttttatgtggtctgaatcagctaaaaagtctctacttaacgaagtattttctgcacttctgggttacgatcatactctatggcaagctc
tcttaccagaaaagcctgttatcgactatgattttaagctacgttatgctcagggacatagagaaggtgatgaatatcgtcctggtgtatgcggtct
ttcttgtggtcgtatattcaagaaaggtgaggtcttttatcggtgcaaaacttgtagtgttgacagtaattctgcactttgtgtgaaatgtttccgcgc
tacagaccatcatggacatgagacctcctttacgattagcgctggaagcggaggctgctgcgattgtggaaattccgctgcttggattcgtgacat
gccatgcaaaatccacaatcgtttacaaaattcagataaaatccaaagtgacagtcaaacagaaatgaatgaaaaagtggcatcgagtattcaa
acaacagtagaatgcgtactggattttattctcgacgtattccgctgtagccctgaaaacctgaaaaagatgccaactcatgaatctatcattgaa
gacgaacgctctagtcggttgagcgaagacaaatacggagatgttgatgattcttgtaatctatattctttaatactttggaacgacgaaaaacac
tccttcaagcaattttatgaacaaataactatcgcatttgatgcacctaacgactcatttggacagcgactatcaaatataattaatgataccggaa
gagcttgtgtggtgacagactcaaatattaaagaattattaagaattggtcacagactcgcacaaattaatttgaccgtaactatacgatctatga
gggatacttttcgcgaggaatgttgcggtgttttattagaatggcttgctgatatcgctggaagcaaagtatgcggcgatggaagttatttcagaac
tgtaatctgtaaagaacttttaaggccttgggattgcggcttacaatcaaattttcatttgaaatcttcatcctatacaggatccgaaatagtcgcaa
tagataatccagatgcattccttagtgaaactcttgatcgctccgtacaaagtttattacaatccagtaacgagaattctgaaaacgaaatagata
tggatccctttcagaatggtacctcgcattggacaccctcttatgtactgaataacaaacatgaagggttatcaaaaacccgatttgactactttttc
ttatatgacctgaagctttggaaaagcttaagatacaaattgcaagaactttacttgggttattttatcaatgtgccagggttcaaagaaatcatgg
gtgcccgtatcgctattttgtatcggaggctggccgagctattcctgcttttagacagagaacctgagcattcagttatatttttttcaatgcaaatat
tcactgtttttggaatagcaagagtgttgattagcgattttgatttttttgacaactgtaaatgcaactctttatactttttttacgtataaagtcttgact
gccccaaactttgttgatgaccgggctatcatacgacttgattccgctgcatttcattctcgtaggtatattcatatttttcatcacatacagtttatgt
tggggattgattgcgttgccgaaacagtccgggaggacgatcgctttttgaagcaatatgttgactttttttctcttttccaaggtatgagtccttata
ctcgtgcaatttcacaacatgttgaatgggaaagtgattcatggatgtacgttttgaacgttagtctgcaagtagcaaagctttgtagacacgtagg
aaatgttttcgctgatttagattctaataaacttaagatcgcaattgattatttaattcaggcgataatacagccaagaaatcgtcctgctgactctt
ggagtaacactgaaccgtcaacgaccggaacgtggaaggatacgactggggttactcacttgattgaatatgatatttctttccaacctatttcatt

tcaccatccattacattggttactaacttatctattatcgttccatatgcaaaaagttgactatgagaaagtctggtctgaaagtgatcttttacgagt
tgtagatcacccattaagggtttgcgcttggcttagccaaatgcgttcgaaactctggataaggaatggaacgacactgcgtgatcaagcacatc
actacaggaatctaagtttccatgaatatacatttgatttggatcttatcttgctgcaactcgtcttggtatatggtaatcctgatgttgttcttcctat
atacatagaaagatttcaactttgtgatcaatttatcggtaaatattttgttccacataagcattatgatctgcctcaattatcaagcatgcttgaag
agtttatgcttttgcttatatctttagtgagtcatacattcgtgttggacaagtggagtatgtgttattgcataaaatatgaaatcgcacatctacttt
gctttcagtctttatcgtattcggaaataaccaaacgtgtttgtgaacactttttaaatcacaaagattttgagaagactttacatgaaataacagat
tttaagccagccgagggtataaacgatactggaacttttgctttgaaggatgagtactataacttggtagacccctttagtattcattactcaagaa
atcaacgtgaagaagcggaaacaatacttagaaaaaaatacgcaaaaagacatgatgaacaattggatgatgttgtgtatgaagcccattacc
ctataattacatctacaacaactcttcacgttcttcagagtgatgctttcctgtcgattttatggcattctataaattacgtttacttatacccatctgat
caaggtaaatgtgaaggtcttttaatgacaactttgcatgcttgcttagttgcattaattgctgaaagagatgacgaaccaaggttcgccttgaagc
tttgtgaaacaaagtttcctgtggttacagggctcaagcagtattgtaactctccggatgtttccctatttacagtgctgtatcagttaaaagatcat
actgactttgatttcgcaagaaaaaagcttgaatacttattgtctatcttgaaagagcggatcccttcatgctacgagaagttatatcgggagaca
ataactgtttcttcttccaaagtcgttcagtcattatcggaggccgaacaatcagagcaacgtcaagcaaagattcgtttagcgaaagaacgcca
gcggcgtattatggaacaatttcgtttacaacaaactaagttcttagaaaacaatacagactttgaagtatctgattcagaaatggatgacgctga
tacctcctcaattaccagttcacttagtacaaggttgtttttggaacctccagtcgaatcttgcttgctgtgtcaagaggaacttaaggataaacgtc
cttatggcacacttgcattcattcaaaggagttctgtaattcgatttcttccctctatcgatactgattattcacatgagatctacgatttacctgtcaa
cttggaccgcaaagtctcatctcgtccgtatggtatggctgcttacaataaacataatctagatagtgttgaaaattatcattcacacacttcaggg
aattcctactcagaatctgaaggcctcgacgatttagaatcaaaagatgttctaaaaggatttccttcggactctttggatcgagggttgtatgcta
atagttgtggacatttaatgcatattgattgtttcaaaaatttaatgacttctgtaacattaactacccgatcaaacccttatcgaaatcaccctcat
aatttaagcatgaaagagtttttgtgtccattatgcaagggactttgtaatacaattttacccattctttggaggccaaaggaagaactggatttctc
aaaagtcaaagtcaatgactgcgctttaacatcatggttattttctacttctgtgaaaaacaagttggtgccgaaaaatcaatcgtatccttcaact
acaacctttgttcaaaatactggttcatataaccccaactttttggatgttttgcagaacacatttaaagattcattgaaagacgtgtatacccttaa
cgctggaaataactctacagaaagcctaacaccgcattcagatagttatctatcaagcgcagcagagtcttcaccttttaaatcacttccgcatga
aggaaggtccttagaagatcgcttcattgtttctgaagacatatttgaattatatcgtcgactaaatgaagtggtggacttaaattcttcactttact
caagcgattatcttcctttaaatggtaagataaccaatatcttgaagctttttcttatacattgactcaagtcgaattagctgctcgagggaacgta
aaagggagcaacagtacattgtcaaacgtttggatttacgatatcagtaagaaacaacaaatttttttacgcgtcctatcagaaacaatgaaaac
gtacacgcttttatgcgcccatgactccgaaaaaagcatagatggcttgatgcaagaatatgaatttattagctattgccaacaaaaaagggtatt
cggaaaagtttttcattcattggattctcctcttacaaatgcaataagtgatgatagagttgaacctctattacagaaggatccatttcgcgaatttg
cagaagcaagtgtttctggtcttataatctgtgaagagtcatttcatgctttgactcaactttattatttgtccgacattattaggaacttatgggtttt
acttagaagcaaagtggcattggtacaactcatgcataatcggtctagtggaatagtagattcgacgaggcttaaagggtttcaattactggtatc
tcgtgtttgggaatggagtggattggctagatatcctaataatcctgggtatctaaatatcaatgatttaaatgattatcgacttctatctcttcttta
ccactttgtggaaaagtttgagcttgtttttctaagaaagtctgcattgctatggtactgtcgttatggagtatcttttgaggagcaatcaatagggg
acgatgcggcttgtgagcagaagcgcctagaaaatcgacttcacttggatgatgtagaaaacttatcaaataagttattgaacacgaatgattcc
caggaatggtctatcgtggaatattggtgtaaatcatttgcgttaaatgacagcctgtgcgaatcccctcgtttatatagcccttctattccagagtt
agttcccttaccctttgagcacgataagttattcgaacttcttttagggaaaaaatgctccaggtgcctttctgagccattagagccggccatctgtt
tattctgtggacagtttttgtgtttccaaagccattgttgtgcattaaatggtattggtgagtgcaatttacatatgcagcaatgtgctggagatgttg
gaatctttctcattgccaagagatgtgcaatattatatctaaatccgccactcggcagctttgcagttgctcctttttggacatctacggtgaacca
gatctcggtttacgacgaggtcgttctcagtacttgtcccaaaagcgatatgatgaaaccgtcagaaacatatggctaaatggttttgtaccttcttt
cattgcaaggaagctggatggtgttaatgacacaggaggttgggaaactctttagcaattctatcaaaatttgctttaaaaatatttattggcaact
gtactataattctgctaggagcttacagttaggttgaatttcatttactggacttcaaacacattttcattgtacgttatccttaatcttttcatgcatta
aacacgataaacatagtaaaaaataaatctggtagtttgtggacgttgtcgttaaaataaattaaaaaaaaaaaaatgtagagtttgcagctgat
attggattgatgggttaatgagagatactttcttcattcagttttcaaccatatatgaaacttcggcaggcatcgagttgcttttctctttggcaaaag
cacacaagactgaattgtttatattttcaagtaacagctaggaagtaacattagcacttgctctgcaaaagttcaggcacaaaattgtggtgaggt
aaacgatcgattttttttttaaattcgaatagacgtctagggtattttttttgattgttgaatgagacaataaaggagaaacagggagaacggctag
gcgaaaagaagaacaaaaaaaaacaagaaacgatataatacacgcgattcatagaagcgaaaagggttaattcggggaatcaaacggatag
aataataaagagaagaaaaaagcataattcgagatgtttttgctgagtcaagtttcggacattataagcatccatccatcaaatttctggaaaccc
acaaaagaagcattggcggaggagatacataaaaaatacgcaaataaggtgattcaagaagttgggcttgcaatttgcgtgtatgactttacga
aagtaggagaaggaatcatcaagtatggtaatggatcgtcatttatgaatggtatggatggacagaagtccagagaaacagaagcagtgttcga

gatctttagagctaattttaggaaagtcacgtttcggctgattgtatttcggcccttccgtggagaagtgatgctaggtagaatcaaatcttgcagc
gaagatggtatacgaggtatggtgcatgctaactagggaaggataagcaggtgtgcaacaactgacaatgtatagtaacgcttggattttttgat
gatctatttattccaaaagacatgttgtttgacccatgtgtgtatcgaccagaagagcgtgcatgggtatggaaactagaaggcgaggacggaca
gcctgggccagagttgtactttgatgtagacgaagaaattagatttcaaattgaatctgaagagtttgtcgacatttcacccaagagaaataagg
gtgcaacagctgttactggttcagaagcactcgaatcaatggctccctataccatcattgtaagtttttcaagtatatcagtctataatattcgaaat
atggaagaaagctaactatatataggcttcctgtgcacgagatggtctcggtattcccgtctggtggaagtagacaggctagctagcatatcgctt
gaatgaaattcacaagtaagtattggctgatgtcttaaatgtttccttcaacttatcgaagctggtcggcggttcataagcggacactccattcaaa
aggagagggcttagatcatgtttaaaaagtaaaattcaaaaggaaaatttgtttccaaatcctttgacatgcattcttgttttcgatcttattcattgt
ccaattcatttcttctgtttttttgtttatcaacagcatagcttcaacaagcccttataccaaaataagccatcgtttttttccattcgtagacaatccttt
gtgatgccttcaccttccttacaacttctttcactttgtaatacatttcgaaagcttattcatcattagcgtaatcgtacgatatactgtggacccactt
aacaaaattcggtatttgcaaaccacatcgataatgaccaattaaaatttccaatcgctgtagaggaaggctcgcaacatattggtaaaattgcg
caacacggcatctcatttgtgcgatatccgtgctactgacgtatctgccttccccatataaacaaatggtcttctcgtctttctgatttgtccttgcata
gcttagaaagtttcgggtgtctcggagactttgtttcaagttgcgtattgcaaaactggaattgccagcatttattacttagaactttccgtaactgtg
gttttctctttctccttcttccaactacttcattttgttgtcagcggcagttttttgtttttactggttttagcactgaacagtattaatttttgtacttttgt
atctgttgtagaagaagcttgataggatccagtatttgtgtttaacttgattttataagccgcatatttttctcgtttttcttttctttgtcaactttggtt
aaactttttgctcatgctttcgaatcggatttctccagcagtaaggacgtccaagtcgtctgcttctactttgggcgcttttgttttggcccgtctggct
catactcaccacaaagtgttaatcgtcggtggtggttcggccggtatctctgcagcccatcagatctataacaaattctccaagtatcgttttgctaa
tggcgagggtaaagatacttctatcaaatctggtgaaataggtattgtcgacggcgccaaagaccatcattatcaaccaggatggacattggctg
gcgccggacttagctctgttgctcgtaatcagcgtgatctcagtagcttggtaccgtctgacaaattcaaattgcaccccgaatttgtgcagtcttta
gaccctaaggacaacaaaatcgttacccagggtggccatgaaatctcttacgactaccttgttatgactgctggtatccactctgacttcaatcgca
tcaagggtttgagcgaagctcttgacgacccttctgttcctgttactaccatctactcggctaagcatgctgattctgtctttcccttaatccagaaaa
ctacatccggcactgctatctttactcaacccagcggtcccttgaaatgtgctggtgcccctcaaaagattatgtggatggctgaggattactggcg
ccgtcaaaatgtacgcaacaatattcaagtctctttttactctggttgtcccactctcttctccgtgaagcgttactcggatgccttagaacgtcaaa
accaaacccttcaccgtaacgttcatctcaactacaacaacaatctcatcgaagtaaatggtaaggaacgcaaggccatcttccaaaacaccgct
gataataccacctttgaacgtccttttgatcttttgcacgccgttcctttcatgcgttctcatgatttcattgctaagagtgaacttgccgacaaaact
ggtttcgtcgctgttgatcccgctaccactcaatctaccaaattccctaacgtctttgcaattggtgactgctctggccttcctactagcaagacgttc
gctgctattacttctcaaacacctgtaatggttcacaacctctggagcttagttaatggcaaaaacctgactgcggcttacgacggatattcttcttg
ccccttgttgactagttacggcaaacttattttggctgaattcaagtataatcaagtacccagtgaatctttcggcagatactctcgcttcctcgacc
aatctgttcctcgtcgtccttattattacctcaaaaaggatgttttcccttttgtttactggaattttgccatgcgtaatggtcaatggtttggtaagcg
tggtctcttccctcctcacttccccatcaactaaattaatcatgacttaaaataagttgagagtaaacacttgtttttagccctgtctcgtgagtattt
acagtcgatctctcgctcttcccacctttggtaaacattgaggttacttaaaatttttctgctgcaaatttgcagtacttttctttccccttgtgataaa
agaacattatttatctgaataaccgtttactttcttagatatttggttttgtttatatgtttctatgtatgttatgatattgctcaacattttctgatttcca
tccaacatagatttttttattgtacgttttcatatataaatacgcatctttacaagcaaatgagatgtattttattaaaaattcttcaattatatcgtttgt
aggattgtacgtgatatatacgtgaaaatacgacacattttacagtactcagtccgttcatagtccaccaatttctttcccaaaaatattcaccaatc
acttagaaacacacattattcattcgtaaactaccttcttacatgttggcaacttcaaaattctgaatatacaatatgtaaggaaactatctaacat
ccttcagcttaaaaagcacggcgaacaggggaacggatgaaaggagtatccttgtaagcttcatgggtcttttgagcgcgttgagcaacaccttg
gggagtgaactggtacttctcgtacaattgcttagcagtaccagactcaccaaaggaagtcataccgaaagcttcgtggacataacgagtccagc
ccaaagcgctataaacctcaacagacatagcagggatgccatcggggatgacagaaagacggtagctttcagtttgctcttcgaaaacttccca
gcagggcaaagagacaacacgagcctccaaattgtattcactcttcaaaaccttgacggcttcaaggcaaagagagacttcagaaccagtacca
acgagagtaacgttagcattcttgttctcgtagagtacataaccacccttactagcattctcaatggtagagttttcaagttgagggaggttttgac
gagtcaaagccaagatggagggactgctatcatgagtcaaagcactgaggtaagcggcagaagtctcgttaccgtcagcagggcgccagcagt
agatattgggcatagcgcggaagtgagcgaaggtctcaatgggttggtgagtaggaccatcttcacccaaaccaatagaatcgtgagtagcgac
gtagataacacggctgttgttcaaagcagccatacggacggcgccagcaccgtaagaaacgaagttcaagaaagtaccaccataaggaatgat
gggaccatagacagccaaaccgttcatgataccagccataccgtgttcacgaataccgtagcggatgtaacggccagcataagatccgatattg
gtgctgggatgttggaagtcggcagcgccttgccagcgagtcaagttggaaggagtcaagtcagcagagccaccaacaagctcaggaatgacg
gggcaaagagcatccaagacaatttcagagagcttacgagtggcaacggcagcatcgccaggcttgtagagagggaggttcttttcccagcctt
cagggagctttctgttaagaatacgctccaattgattgtataggtcaggatgagccttggcatattcagcgtacatagcatcataagccttttgagc
agcacggttttcatcaacgcgcttttgatagaaatcatagacctcagcaggaacttggaaggtttgttcggggttgaaaccgaagagctttttgact

tgaacagcatcgtcaggcttgagagggctgccgtgaacactgtgagtaccttgttgttgagagccgaaaccaatggtggtcttaaggttaatcaa
agtaggcttgtcttggcaggcgacagcttcacggaaagccttctcgatgctttcaaggtcagagtcaccattttcgacacgcaaaatttgccagcc
gtaagcctcataacgcttttcgacatcctcgtcgaaggaaagttcggtgttaccatcgatggtaaccttgttgttatcccagatagcaaccaagtta
ccaagcttcaagtgaccagccaaagaggaagcttccgaagagataccctcttgaagacagccatcacccaagaagcagaaggtgctattgtcg
aagatatcgaaaccaggtctgttataaacggcggcagaatgagccttaccaatggcgagaccaacggcggcggcgataccttgacccaaaggg
ccagcaccagtctcaacgttcaaagtggggtcatgagtctcagggtgaccaggggtcttgctaccaacttgacggaacttcttcaagtcgtcgatg
gacatttggtagccgagcaaatggcacataacgtattgcaaaacacaagcatggccattagaaagaatgaaacgatcacgattcaaccacttgg
gggaggaagggttgaacttcatgatgcggctgaaaagaacatggctagcaggggccaaacccataggagcaccaggatgtccggacttagcat
tggaagtggtgtcgacggacaaagcacgaatggtgttaatggcaagttgatcaatctctgagtaaggcatgatgaatgcttatatgtcgtgaatgt
ctatagtaataaactttggatataaaaaaaataagaataaaatgaacaggtcgtctataatgaagtaccaaataaatctcgcgatccctactttg
gtattttcttcaatgtgtcgggtgcgttgatcgaaagtggggactctgagaatatatagtccgggtccggcaaaaagaatttcgaagaaggagttg
aacttgtgatcatccgaagctttgcgtcaacccctgatgttacgcgaaaatccacctcgcacggacagatccgaagaaattctcgacgtccgtgct
gacacacgaaattcattgaaaatttaattaaaacaaacaaaaaacaacagaattgagtcgaatttttgtaaaaaggagtaacttttatttttttt
ctttgttctaaggctcctcaatgcccgcatggaggcaatttaacccaatttcgtcgcagtttctgaacgtttttacgttgacgcgtcgcttctgtcgtc
tgaatgcttcttcggagaaactatctgaaccctaaattttatcagcaatctactatcggaaaacagatttgtgtaactcattatagttatttgaggcc
tttaatgcggtacgctatatatggtataccgatatattcccgttcgctattctataaacaatcaaagtgtcaacagttgacacttaattaaaacaag
gggcctactatagcagcaaaatttgctgtttttcgatagtggaataaggatttaattcaattaaaacttggactgatctctgtactaggtacgacttg
attcttccaacaaaggaaaagtctcatgtgacgcgttgatctctcacactgatttttggatagggactttttaagctaaataaaaaaaaaaaactg
aatacggaagttaagcacatatagactcttaaagtgtagaacacaaaaaacactcacagcggttgctgctacgtcttctctcctacacgcaactcc
actccactcccagccgtaatgctgcgcaaaaagcttccaaagcgtttggaagcttttgatgcatttccaaaagtagtgaatgagtatagaaaacc
aggatcttcccgaggaggagccttttcgttatctgtaggtttttcattctcttgctcttccttcttgagtgtttttcttacgtgaaaggtgttcgagagc
aaaggattgtggtctccgacaaagtttctgaactaatggatctcaatgttgatgttacgattgcaatgccttgtacccatgttcgcgttgacgcagct
gacaaaaccaacgacattgttatggctaccgatatgctgacccttgaagaaacggatttaagtgccatgaaacattcatccactgtgcatcgccct
ggaagaaactccgaataccgttgggtgcgatctggtaaatttcgcacgaagaaaaagccccttacaggagctggcagcgcatgtagaatttatg
gaaagattaccgtgaatcgtgtccacggccaattgcacattaccgcccccggatggggctacggccgttctaacataccctttcatgcgctcaattt
tactcattattttgaagaattgtcctttggtgactattatccagctattttaaattctctagatggtcattatggatttacagataaacaagcctttggt
tttcagtattacttgtcgattttgcctacaagctacaaatcttcatttcgctctctcgaaacgaatcaatattccctaaccgaaaattctgtgccaaga
caactcggattccgttccattccacctggaatctttattgaatatgatatagagcctctaggtgtggaaattgttgataaatatcctagtatcctaaa
atcatttttacgagtcttatccatttcgggcgggctttacattgtcatggattggctggataagtattatagtagtcggccgactgcaaaggctgagg
acgatatgcttggactacttggtaaagaggtgaactaaaaaaaatttgcatcagtcgattgtttatgataccgtatgactacattattccttctatat
acactaaatgtgcatagctcaaatagacttcgctacgattgttgcgaggctttttgaaattgtcgcaaaataaagaaaacaaaaaaaaaaaaaa
aaaaacaatggaagaaattaaatgacaaattaaacctaaactaaaactaaacccaaataagtactttttaaatcccaaaacccaatcatcattt
ctagatataagcttacaactagaaacttttcacgtgaatttaccctttagcacattccggtgtttgggattgcaacttacgagtctttcggtcgcttcc
taaacccaacccaaggatacctgtgttcaatccttccaaaagcatgcaggccttacaaattcgattactggatataaagccacatcgttcacactg
agtttgctgaggaagcttatcctgaactgaatgagaaagttgcatagcttctccagaatagataatatctaaaatcgaactcggtcgaatgctttc
caattgcttgatcatggctcgagcagttccccggaatgcctcgggagaataggtacattcggtagagaaataatccagtcttttataatgagcgta
caaaacaatttccttttcgtatgtgtatttgaaaggcttggagcgctttgtaggcgaagtttctgactgagtagtgatttcggtgcttctgggaagac
gagctacatcacctcgtagtaagttcatcaaaacagtttcagcaatatcgtcagcattgtggccggtaaccaggtgatggatgtctaaactaagcg
cagctcgatccaaagcttgacggcgaaatacaccgcagtaggtgcaattgtttttcattccaatacgagccaccacattgtccatggtccatccgtc
atataaatcggcatagctaaccacagtcattggaagttcgtactgctgttgattccgtttcacagtggccagagagtcatctctgtatccgcgaatt
ccttcatccacgctaattaaatgcaattcaatgccgtagttgtatcgttcgttcaaaagtttcattacataagccaaaactgtactgtcttttcctcca
ctagcaccgataccaaccttttcccctcgatgaaagagtttgttttcggtaatgacattgtgaatttctgtttcaaacacatagtagaagcaatcttt
gcaaatcctttgacctgtcttgggacgcactagcgcaggtcgattggtatcacataattcacataacttacttcgcattgttatagacagcttgctgg
ttttttgttaaaaatttctgctttctcgccgtatggtgggagcacaggtataaagagaataaggataatcctaacagtgcgttattatggtttaccga
acttagagaagttaccatcgacttctataacaaataaacaaaccatattcggtgaagttgtgggttatatggaactaaacagaaaactgtagtaa
tacacctatagagcttgacctttgaaatgatacacaaaaggaattccccttaaaccatgacgactgctgtttttccaaggagccatggggcataaa
tacaaaaagactgaacgggaagaaatagtcgcagatttcaaagaaaacgttcacaagttgtcttctttacacgtaggcgggtagactactagac
atcaacaacgctaaatcatcggagcttaatagatcagattgtggggaagtgaaagaatgcgacgaaacgctaaacataaataaggacagtaca

atcgaaccgacttgagatcacagaagggtcgaaaacacaaacattacaaatgaaaaataaaactcgaatgtttaatttttcttatttcatataac
aagagctttcattttccttcggggtatatgagtataggcatcggatgttccaaaccggcgataggtattttatatatatataaaggagcggtagtat
gctttcttctttctccaagtataaggtatcctttttggaaagagcatgtttcggtttctttgtttattttcatagacaaaacaaacaatgataatcacca
actttgttttccttttcattgaaaaatctattcgtacttacttactataattaaaacttctttttttaggtacagaatccaaaaaggtcagatgtagctt
actgtgacttatgacgcgtctttcgacgctcttcccttcttttcgttccttgctcccttatcgtttcgactttaattacacgtcaaggggagatttgggg
aaacgcctcgtgtatgagattgttagggacggctaaccgtatcttaaatttgaaatcactggcttcgaagtctacgtattctgtctaattttgctgttg
tttatcacattctacgactcacaaattttttttgtgacatagttgctttcgtctgcaacaacatcataaatggttaccttctaaactgttagtgttaac
atacacatagaaacttcggtacatttcccttgacaatccagcgccttgcatacgagccatcgcatcaatacgtcatacaaagcaacatttagggaa
ccgctaggggtacattgaactttttagcacgtaaccctcgccgactccttattttatttcatatataaaaaaagggtaaccacaacagctaccaaa
agacgatttgtttctagcattctcaaaacttgagttgctatcgagtagtgctttgctacgagaaagcatcgtacttattttaaaattggctatcaatc
attttcatatccttttcttaacgtttgtttcgacagttggaaatttttgcaggttagagacttgcttgccttcactgtgaggctatcctcggattctactg
gacggaaactaggcatttgacattaacgaatatttgttcctgttcgattcgggtttttatttaaacaaaaaaaaaaaggagcaactgaattttccct
tgaaaagttaagtcttttcacttttattgctcaacttccaagccacaaagtctgattttagagagtgccaataatcaacggaaaccttatattttgat
ccttttagcttttggtttcaatcataatagcacgactttctttcttggtaattcagaagaaaagtttgctcagcatatctttttatcctactctttgcaat
ctcaaattaatatttgctgactgcattttacatcctttatactaagttggatttcttttcctgaatattggattacccaggagtcttttggatttgaaga
atttcttgctttcttggaatcatatagaatctggtatttttccttgctagtcgaatactcatcggacacttcttcgtttacttcccatttcattcttatttga
ctccaatcttacaaaaaagaaaaaaagagctctggtttgaacttgcccatcatatctaagccttttatttatttaagcttatttcaaacccattttata
ccattgaatgttttatttttattaagtattaatccgttgtttacgcttgacatttccatacttctctgttcttatttgtttcttttcctttcaagttcttaactg
cattttagttttcttgccgattgcttcatcgtttatttccctatttcaggtgtttatattattttggttcaaaaatattcttggattcaatctttcagattttt
aacatcatgtcagcaagaaacatcgtgactctaagtcataatccagctattacatatagctctaaagacgacgattttattgaagacccaactcca
tcgccagcgaatgagaacgttagctggatctcttggttctgttctcgaccaggccgagaatattttgttgaagttcgagaagagtttattgaagattt
gtttaatttaactggcttaaatctcgctgtacccttttataatgaagcgctggaccttatccttgataggaataatcctgaaactttaaaaaagttgg
atatgggtgtcgttgaaactagtgcgcaaattctttacggtctgatacatcaacgttatattattacccggatgggcttacatcaaatggccgaaaa
atatagcacaggaatctttggctgctgtcctcgggtgaattgttgttatacgcacgttcttcctgctggccggtccgatgtcgtcggtaggatgcctg
tcgtactgttttgtccgaactgcttggatttatatgcgccatctagctctaaatataaaaatattgatggatctttttttggtacgacattccctcatct
gttctttgaaacctaccctgaacttaatcctaaaagggccattccatgcggtaaaatctaccagcccagaatttacggattcaaggttagtgaattc
agtaaaaccggccctcgaatgcactggttacgcatgtatttgaccgatagtagtgatagtgattctgaaagtgaaagtgaaagtgattagttctta
tgatgataatgttattataaaaaacgctcttcatttctcctttttcgtctgaccgcgcctacgttacctttctatccgctattataccttattacctttctc
tataaaaccataatggctactttgttttaattagttttcagtagcttttcatgtgtcgtaataatgacttatggaaagtttagtacggtcgtaaaagga
cttttggggttatttatgtttatcttttgctttttaccaaaatgaagtttagggtttatattgagttggtttatttaacatatccttaaaggtgttgtttaa
aaactgtggataataataatccatcaaaagttaattacaaaatgtttttaggatggaaaggctttatactataacctaatcctcgcgatccggaaa
actacgtgattgttgcaggtaattaataaaatgagcctttggatatatagtgtgtattattgtatatcgtaaaatatgtagaattagctttattactaa
aaaagagaatcaatggtcaggactgtaatcatttcgtctagacgcgcttgccgcgcttcttaattttgcgttgacgttctcgatccatacgcttctttt
gttcagcttcaggaaggttgttaatatctgaagtagtggacttcttactttttgcagctttcttagcagcggaagcatgaacaacttcttcaagggcc
agtttacgagaggaagtcaattttctggttatctcgggtcgccattggaatttagacgtgttatcagcgatcgacaataaagcagtaaataaatca
ctagcctgaagagtttgaagctcactaattgaagccacattatcgacacgcatagaagcatcgatacgaggttgttcaacatattctttttcatttg
caggggggagcagaaggttgatcagtaatgataagatactcgagattcttcaagcaagactgaagagaagggagaaattgagggaggttaagta
cgcgttcagtacattcaagattttcggacataagaacatgagtttctggaagcgatggatgagaagatattctagtaaaactaagatcgaaccta
gtttcacgaagaatacgcatacaatctttacgaacaatggcaaatacgaatggttgccaaggcttgctatgattgccaaggttgagtaacacttgg
aagcggtctgcaacaggtaaaactggagacatgacatttcctaagactatttcgacaagttgatttataccataggtaaacagatcttgacgagg
aaagagctgcaaagtagtgtaaacattctttacattcactctacctgtgagatacgaagagtactcggtaggagagacatcaataatgggcgaca
tatttcttccaggaccatattgcgcaaactgactacgaaacatggattgcaaagagttataccagcgagaagcaaagtcggcatttttacgcttttt
tgtaacaaaagagattacatataaagcaaacaaagacaaaatgactaactcaattttgaaatcttgccagctaacctttctaagcattccaggctt
ctcagcgtcgacaaattcctcctctgtatatggctcttccgtgctttcatcttctgccaaagcaccataaatgaatgctaaaacagagagtaaccac
ctttggttcatttttctaaggttcaccgaggaaagaatgttaacaccagaaaggaggtgaacgctctttgaaagtggagttttcagttttagatgctt
agtggtcaatgcataaatagttctttatatatcttatctttattaaagaattcaacgaaggtcgcggtggtgtagttgggagcatgacagactgaag
atctgttggtcaccggttcgatcccggttcgcgacactttttcctgtttttccactttgatattaatttaataattgttggatggtatttaatttactat
taataacgtaaacatatttgaagggaatgatattacattcgaaaaaaaaggagttgctttttattattcaatacaagtcaatgtgaaagaccagg

aagctgaagaagattgaaaaaacaaaagaaaatttattttggaagatagtttatgaggttgaaacggaagtttaatcagaagcccgttcgtaat
gctttcatactgtgttctaaaaataagcaatatattaacgtggtacgaaatagtacaatacctatctcacggggattaatcattgacgagtgtattg
taattgagtttagaactagtgatggataattgtaccaggaacttgaaattcaattaggttttgtgtactaaatttttgataaacattccaacacatgt
ggttgactaatgaagctactcaacgcttactaacaagttcaatggctcgttcagaggtaaaaagttacgaagattttatatgtaatttcttagaagg
acaaaagaatttttattttattcattatcgcatttcttttgttcttgcattcatgtttatttactatagtgttgtatatgttaggtaacatctatagtattg
ggtcctctctcgacagtatcacagcgtggaagaataactcattcatatttaaaagtctataaaaataatatcaaaagttcttatgtactatgtatga
accactttatgttgtgtgaaacaaattcactgacttgactcgcaaagtattctcagacgaagcgctataccgttcaaaatggaaacattaactattt
aatcaagaccaatttatttacttagcttcattgcatttcatctttgtctttattgtgtgtttctttattttattttattttattttattttattttttcctctgtt
tctcttgtctgtttttgttattttataacggcagtttttgaggtaacattcgtgtagtacaatatctggaaacaaatatgcgatcgctaaatgtcatttt
gacatgctttttatatgaggaataatgctgtagcagctgcttagttcgctttataaagttaatacgaaaagacatgctacttttgctttatgagttga
aggaaactattaacgttatgctagattacagacgagctcaattgatgaagcgtgcctttacattataaagtttggctcttttaatttagacatggaa
aaaattctacaggaatctctttagaaagttatggtcgattcagcaatatagcttttcaccttttgcatgcttcctagtcaaccagggtattattgaatt
acatatttctattgtaacaggaatcttcatccatagtcaaaaatcattacacctagtcatagtagtgcttgttcattcagcagatgagtttacagaat
accaaatttatttatctttgaatacttcctaaacgcttatataattgcttattaactgcatctatcaataaagtcgcggtggtgtagttgggagcatga
cagactgaagatctgttggtcactggttcgatcccggttcgtgacactttttctgatttttcgacttcgcaaattgaacgtcttgttgtaatcaaaca
caaaaataaagtgcactatctagtgatgaacgtataacaaagttagttgctgttgcatcataagtcttctgaggccatagaaatcgtttctatctgt
ctttttggactgaaaaaccaagcgagtgaaacactttgacatctcccagtacatcatcttacaaaacaatactgaacccattgtatgagtgcggga
tttggtctctatagcaagatgcaattatatgagttactgaatgtcttagggagaagaaagacaagacctccaattgttgctttaaacaagtactttt
tgtagaattatatgcatccctcaattgcaacagctctcttcaacatatttatcttactccttcattaataaataaaagaacaacatgaatacaggttt
tgtttaccgttaagccatacttctccataataccaccattcccaagagatggaaagtatattactaatattttattagaattatcagatgaaaatatt
ttattgaagatccatttcatgactcttataaatcataaaacctgaactatggtaaaatcagtaaggaaatgtatatagaaataaacacatacaaat
atagtatctcttattcaagaatccaatgactatcgtaatgtttgtttattcatagtaatgggaccagaccgataatactaaaatggagattatctaa
gcgagaactatggtatccaatagcttgcagtccagctcagctcaaaagttaaaaccccattgactcttgaacttgctctgaaaatttcccgttttcc
attaccctcctttttttcattccatataagtccaaacttggcaaatcccatctcataagaaacatgtcgaacagaattttcgaggccgaagaagtcgt
agaacacaatagtcctaaggacctctatatggtttttgagggaaaagtgtacgatgtgacccaattcgccgaggatcatcctggtggtttagacat
tttgttggattacggtggtaagtgcctcttatttctttcgtgataggatagaaaagcaatgctgctggacgaatccgttcaaagtatatgagaaacc
ctttctatgtttagtagccgtatatgtgtctgcttatttgaatttggccaggaataatttaccgcttttcttatgtgggcatatgttctctatatgagctg
caaatctcgcatggaatatggtcattcagtttgcaagcagtcggtgcaatgattgccagatttaattaccgattcattttgtttatattcatttttttatg
ctaatttccttttcctgtttaggccaagactgtaccaaggcatatcgcgatgttggccattcagccgctgccgatgagttacttgaagaaatgtacat
tggtgatttaaaacccggggcagaagaaaaattgttggaaatgaggcaacctcgtaccgaggaaaacgacgctccctcgtatgtgttataacatc
tggcatcataaggaagctttcttgtgtgtactaaaaggaaagcttctttgtatgcctcgttttatttttttcttttactattctttttttctaacaaagttgta
gtatcttcatgatccttgctttgcttatcatccctgcgttagctgtaattggttatgtaaaattcaagggttagacaaggatttactattccaggattat
gaatatccttatattattaatgattcagaaaatcagatgcatactgtaatcaaggcatcaagcaacaatgactttgctttctttttttactaagatcttt
tataacaattaacgacaaagtatgagttcatgtagcattggacatcacatattaaattaatatgctcattagtattaaattatattccaaaaactgt
atagagtcccaaagtttaagtttttctaatctggaaacattgcattccttgaggttaatccatatgaatgtaatatgttcaagacgggctttcaccca
tcgttggctttactttggacaaattcacaatcatttttccttgaatcatgtctgtgttgaacttcatagcctttatctcccgaagaaaaatgtccaactt
cttttggatatactcttgatcctgttctgtcggtgtactcctttcatattctggcgttagatccagtgagttcatttgatccatcagctcccttaattaga
cagttaataagtagaattcatacaaattaaagcttactcataagtgttttcatcttgatttctttcaggagcctccgtccttagtcgcatagaaaata
cgtctatgaatttgttccttgagaggttaaaaatccgattctggcttttttttcttcaaattcctcagtcttactttgcattttcatatactttagtttccaa
ttcttcttcttcttgacgtttccttaggctcatatctgcctcagcagtaggagcagtcctttgaattcttaaggtattagcgattgcagaactcaaaag
gtcattctattatttacccttcattaggaagaagtggagtaacttcatcaatctacgtccttgttaggctccatctaataatgttgaattctcaaacct
catcagaactgccaaagcgtgagcagcaatttcccattattaactttaacaataactaaaaagcaaagaagattaattgaaaggcttttattcgct
ctcttttaaacaattgtgctttttcagtatgctggtgctttccaggttctcgatggtagtgtgtaagtaatgtttgtaataaaccaattgcggacttttc
atagatgtttatattcaaaggattctttcactttagattaaaactattataccatgttaatcgtaagcttcctattctagctcagtatcagttatgtaca
aacagtcatttttctgattcattcatatacttcatgtaaataccttaaacctctctctctaatatacaaataaaagtatgcatggaatcttatgctata
cctctaagttcagtgtctccgcggaaagcaagttttatctcatcatcagctatggcaaggcgaacacgagcgtttctactgcttttattctcaaggta
cagaacaccagtttcactcagactactaagaatataatgaaactctggacgacgttgtaaaatttggccaagctcagttgcatctatagtggtcat
agattttatcaaacggtccacttcgtcaactacttcgtagacataggattcggcgaatccagaacgacgcatacgattgacgatagcgcataaga

acaccttctgcatgaaagataaatctcgcaagacacgctgaagtggagaagctgtcatttccgttatagcttgatgaattaagataggagtaacct
ttccgtccttgctttctgctaattcagaagatctccggcaaatatccaaagctcgacgagcatctccactgactgcagccacttttcttgcagcgaat
cgaatagcgtcagaagaaaacaactcttcatctttaacttcttccagacgtgaagctataattgtttctagttgattatgggtataaggttcaaagg
gcactcttgaaagacctaaacgggaggatatgcgattcgacaaaattcgttcaggaagatccatcgtattagctacagcaataacaataagtcgt
gagtgcggaagggatggccagttaaaaaagttatataacaccttttggttattcgttacaagttgatctaactcatccattaaaacaacgcatgaa
catcgattcggtgaaggatggttgaaacgattatccagtaattccatagcatgaagaggggtcacacgttcaccagttaaggattcccatagaga
cgaatatgcttggttcgcattcgttaccttcattccatttatttcacaaaacgtaaattctgctagctgcccttctctggccatttcttgcaaattccat
accacttcatggacagtagcggtttttccagtgcctggtgttccagatatgtataggcatccaccggtttcttcctcaattgcactttccaagttagtg
aagatggtagtaaactcattatctctgcattgaagagtatttggaatcgctgacacatgaagcatagctctcgcttttctatgaggcgaattttgaa
attctttcattgataacattcgtattggaagggggggtgatttgcagaggttgcttgtagccactacccatttgttagtaaggtaataaaaacaaaat
agtaatcatacgttcttctaggtgtagacattgcgctgcctgatgcattccttgaccgtttgatttttcgtctgggagttctaggtgtcccatactcttc
atcagacgaggagactacaggggaagcatcatcggaagataaactttcaacctcctttattagctcgtattcatcattatctccttcaacattctttg
aaaccgatcttttctttggccggcctctgcctctagaaccagccttctttttggtagtctaagggtttcttccatctcaactaataaatcttctattttg
tctaaattatgggaatagtcggaccaattgaggggctctgtatacttgttgactttcaaatggactcctctgtatacaaaaaaatttcgagcaaaat
ggtctttcttctttgatggtatgccttgaggaaatcttttgaagaattcttctttagaatagatattgcaatgctctataatgctagacgtcgggttttc
atctcgcccgcaagacaggtaaacttcatttacatgacgatctggcaataaatactctggcttaatttccatgcgttttgcaactgctctactgtacc
agatagcttcataatacttctctcccgaatgtttagtgattgccttgtcgtataactttgtaataacaccaacatagaagctagaatccccatcattc
acagtaatatcatcacctagacgatatataatgccatccttctgtagctttcgataataagatcgtcctgcttcagtctgaaacggtttctcaatcca
tataccatgcacctgttcttcatcgatgtcgctgctatctgattcatcgtttgagtcaagttcttcttgaggtaaatttcgaattaacatttggcttctta
atgattttctacggcccatttttaactaattttgctttttgcatgaacgtgtttataggcagaattgggaacttacttaaatataattcgtatagccagt
agtgaatatacaacagatgttcataagtaaaatttttagtaaaggcatttacgcttctatttgttgtaatttttataaaaatacaataaatactttaa
aattctaaatcttttatcaaaccaaaaatccaacatacaggttcagacctgctagctatttacaaaattagtaatttattcattgtggttaaaataac
aacgaatttaaactttagtatatatttagccagattccaaagcaaatgcagatataaatgttagagataatttcatcaggtttaatggttttcatatt
taacaaagacgtgttttataattaaagggcatttaaagagagaacaatttcatcatacggggcttcttgtttcactgagacagcagattttgtggtt
aaagctaagtaggtaagtaagaaatacgtatgataaatcgtacctaaaagatgactgaaagcttgtgcggccacagagcgttgtaatgtacagg
gtaagagctctgagaaggtcatctctccaccgttttcaaatattgctgtttttgtgtattcatagaaattagcacactctcgttccatatgtaagaaat
ttccaatattttcttcttgagtatttttgtagagagaaaagtcatcttcaagttgcaaagataattcagagtttaagtgatcatgatactgactagatt
ccaaagcaggcaagaactgcaatcggttattcattgaaggactaggtgtaagtctttttccatagcgagtgtcaagcaatggagttgaatgttcac
taccattttgagattgacttttagaagagtttaatagactgtttgaacgagaggaagtattccaaggcatatttaactctaaagacccctgagcatc
acgacccatttcaatatctgaattttccaagttaggcattggcaactcaacatcatcagcttctgtttggttgtgaactggttctggttgaagtttacc
aacccattcttgaataataggatggaatgattcccaatgatacaatttttttagctgtttttttcttttcaagtgaggttccttttcgtcgcatatgcttgg
cagtttcaagggctttcatcctttccacatacgtggacctccaatgggaaagggtttttgtactcagctcgatagattcgtcaggttcaatttttttgaa
ttcttctacgtttaacaggctcttcctgaggttgaatttcaagatttgggtctactacagattccaagggaaccggaaggggtaaaacatcatcgtc
taattcatatatccgaatccgagattcttgttcttcgtcgtgcgcttcaatgggggattgagcatcgggatgatcctcttcgtcttgagaattagcag
gcgtaagttgaggcatcttgtcctgtgtcgaaatatcatgcacctcaccattttcgtcaaattcaaagtccaaaacctcatctggaaaaagattatt
tgacgcttcataatcaaatctcgacataggggcgtggttttgaaggctaaaggattgagtcgcgttgccaaaggtatttgaagcttctggaagtgtt
tgtaacgacgtcaagtttgtatgatcaaatgaaattgaatcattgttcaaggtaaaattgaggtccaaagagggaagactgagaccgttcgataa
tgtcaattcgggtacaaaggctgggtcatcagcaaggattagattatcagtactatatatcattagtaacttaactttatgttttaaacaataaagt
cgcgtactttgatttttctacgcgcgcgtcgatactcttagaaagcttgccatttatattatcaagctctttgcgtagtcttgaatgcagtgaagaaac
ttgtgctaaaaaatgtaagtaaaccggacttttgggatttgttgacgtcgaaaaacgcgtatcgtacattggaagaaagaatattgatgggcata
aacgcgtgcaacaccaatcattaagttactactaaggcgtaaagctagcggttcgggagaatacgcgacaaattcacaagcttcatcgatattaa
cagacattatatctttttttatgaagtttccggagagagtgctttgaccccagagtagctgcgagcctgaaatcattagtattagcatcaattaaagc
aattatttaccaaactacacccattcccccttcttttttgtcaggacgtattgatcataaaacatggttttggttaaacgcgtccgcccagtacaaat
tctataaaacgaatcgaatataacgtatttgagtgcattcctataagtcaagcttgggcagttcattgagaatcagaaataaacattagtcttgca
aataatattgtaaatgtgagttgagttacctttgaaatttgctgtaagactttcgaactagttttctacaggaagattcgctaaccttgaagcatcgt
acattcttgtgcaatgccaattgtagttttaatatatttcattgactttggaattagcttaacgtgctcacagctgaatcaaactataacatagagat
gtaaaaggagcgattcacgtgctaatttacgaatcaaagagaaatggttccctaaatgtttagctaaagtatgtcttgatcgacataaaccaacta
aatgaggagtatagtgggtttacataatgctcaaatcgccaaacttctcgcggtaaccagggacaacccatttaccctttgtaaccatagtctcaa

caatcttttcgacctcagggacagcagtcttcatatcttcaatagaaatttcattggtaggacgagcttgttcaatgtttttcaaagtagaactcaag
cttcccaattcagtcttgaccatttcaacgttcttcttagcactttcgacagccttgccttcaaaggcgtcaattgccttcagttgagaagccaaattg
atcttaacaggcttaaaagatttcatagaagactcaatacggttcacgatttcttggttcctaagcacctgacggtaaaatgcaaaatcaaccgtt
gtagcttgttccttcagggttccaagtcttccagcggcttgggcatgacgtgaacggaagttggcaacagcagaagcagtgctagcgtccaatttc
aacttgctagggatggttgcccaatctatagctttaacagcagaactaaccgatttagacatcgcgaatttgtaaagctaaaacaattaagcagc
aaacaatgtttttttataaggtcgacgggccgaaaaacacaaattacagactacttatgagagagggggagagggaaagttaccgttgcgtgaa
gcaggtcccttcttaattaggctcgaactagatttagcgaatacaattagtatatgcaacaagtatacgtaaaatatgatacctattaaatttatat
atattatacgattagttttaatccaaataaccaaaaagcagtttccttctcgacttcacttttgtaatattataggatactatctgattttatgattata
atgaggaagtcaactattcaaaatatttacaaaccaatgaaacgttactttcatcttttgacattaataaatatattttcaaacaaaaagaaaatat
ttggtcctttttaattgaggtaaccaccttatagcctttcccttcgtccatttccatagatttactactatagcaaatacatttttttaaatatcaaaatc
acacaaacaaattttctgtagccagaaaagtattgcaagcgaaataaagtaatgtgtagcttaatcaaataaccttccacgagcaaaccaattcc
tttctcgactcacttctcaacttgatagcttattgtttaaaatggacatatgtatatgactattcagtaacgagaaacatttcgaaagatcggcatct
ttcgacaaattcatgaatcataagtctgtaagacatcagatttgaggatgaactatctgaatccactttcagtcatctacgggctgaacaataaaa
caaatatagaatttatgagaagagttcatatttcataacatattaaatacgagtaacatgtaggaattccgtccaactcaattcaccaaaaaagg
aaaacgtcatatacaaaaaataaaaacaaaaagaactaaaaaacttattctcgaaggcgagcttccaattcagccttacgagcagcacgaaga
cttttaatgtcgtcttgtttggtgttgtaacttacgttgagctaaaaataggcgttagagtacaccgatgattgtgctaaacttaccttgttaaaagg
gttgttggggtgcttctggaactctctatacataacatcttcgtactgattaagacgaagaccaggactttccttacgaagttcgggaagtcgtgatt
ctttgtattcattaaacgccggtttaaaacggcgttcaggatggcgatcaatttttttccttggacccagaatccaaactaagtaggttcaaagcatc
gtcaatgttgcttgcgctatatgaggctggttgagcaggttcctccagaaaagcatccaaagataaatctttacgagcaccagcctttttaccctttt
ccaccttttgatggaatgctagccatttcttcttgttctaaccgagtacgctctgctttcttttgagctgctaaacgtcgcttttcctcttcttgctccttc
ttattgttactcttgattcctttcgaccatttttcactttcctcagcttccttctttttttgcgtcaacttcgtccttcctgccttttgcagcttctgccttttct
gctctcttcttaggatttcccattgtttgtggtatgtaaaaaagttcaaatagtacgtacgattcgttagatggaagtctttcatagacaaatgatctg
attagtaacaaataaattttaaactaagattttgaataattatataaatgaaataaaataataaaactatttttttatttttttgttttctctggtgaaa
gtgtatttttctctttgccccagtaagaccttactcaagcaaagactacctgtgaaacaatcgtcagagagaaacaagtctactgagaatgtttttta
atgtttttcctatgctaaatgaaacgaatgctatgaaaaaaggcataagtacttacatagaaatttagctaagcaatgggattgggctgaaatctc
aggaaaccacatagagtcttttcattttttttttttgatcaaaagccctttttgagctcatattcccaagcttaaattgaacaatccaaaaacgacagg
taacatatacagggtagcaatatcgatgtaactgcaacttacaacgatacacttcataataaaatggaagacatttaaataaatatgatattcga
gtataaaaatgatgtttccttcttttcttgtgaaaacaaatgaaaaataaaaaagatagattttaaagaaggcatttcgtagagactagctaaaac
cgtcgagttgactgaggaagcaaaatacaacactgattcttttgacgctcagtcttacctttctatttaccaaattatcgctagcttaaaactccaac
gaatgccatatactctaccttcaatgaagaatgatcgctttctttcttgatacctttcttgcctttgtttacatcaacttaataatgtaaaatagcgata
ctgcgttgtggattccttgaatcttctttggtcatatctcaaacatcgaaaacataaagaatcaagttttgttttcatttttgtgaaaatatttctctac
ggtgtagtaaatttcgaaggattccaacggtatttatcaggtttggcttgccattcttaaatttgtgcacttcatctcagttccctgtattttgagttttc
ataatcattataacggaagcttccaacagttaaggatttatctcctataactttcttttcgtccaggtttatcctttataagttgaatcgaacggctta
cttatcaactatatatataaccttgattctattagttccttcttaccttactttcagctttcagtcctttttttttttctatacatacagatatatatgttaaga
aacctaaaatttaggtcagtaatttcatctgcattatgagtttttttaaactcctggagagcgtcaaaatatacagttaagcaaaaactagacgaca
ttgagcttcccgagagtgcttccgtacttcctgtaactgtacagttttgcattaaggaatttatttcatctcttgtaagccccgttcatccccaactatt
atctcctgatgaaataacgaaaacctttcatgacttttacagaaaaacagatgaatttatgctttcaactctaattcccaatcctgaatctccaagt
caagatgtgcctttactttcccctgaggaaatggaaacccagaagatggcaaagcaacaccttgtaaaacaaaaagatgaatggatgaatatta
ttgaaggtatcgtatgcgagtatttatatgacaggtgagttttctattcttaatactactttgctattgattctaatgcatacctagacttttctgtttga
gcactagcaccgatgaaaataaagatgaccttcttaaaaagttcgcctcttctgaagaaaaaaacggtttagtggagtgtctttccattgaagcag
agataaagaagcgccttgaaaatgtcgcagagactttctttgctctcagtagactaaaaacgcctttgctgaaactgaatctgtttatgaatgttag
tgaaaaaatcgttgaggcatcaaatttgccgaaacaagaattaaatgctgataatttattaaatctaactatcttctgcatattatcttttcagggtt
tacaattagtgtctcatttgaattttgttttgcgttttcgaaacccagattacttaactgggcaacaaaagtattgcttgacaacgtttgaagctgca
atttctttcatcctccgtgcgtgtccaaatctactgacccattgtcccatgcaggcaacggacgatcctctatcattagaggcctctgctaaggatga
atttccgtcttccgattctaatcaagcagaccttgaaacaaataatgaacaacgatcttcgctttcaaacttaagaggtttgggcgtagctcttgaa
aaatcttattcctcttttgtcaataaagtagctgggcatgctcttcgtaactctactttgtcgacagacaacgttgtctccgttaatgatccacctcta
gaacggtttcttaccgtgacagacgcctccgagctgaaggtcggcgaaataaacattttgcttgcagattacaaacgcttagctcgtctaatttttg
acaaacaagttgaaagttcgacctgatttgttgactcatcgtgttaaaaaatgctgctagcccattctataatgattttaatgtcccatttttcttata

attttccatttagaatctaataattgcttcttgtcatttatattcctgtaattcattgttcgtttgtgatattgtttattaaattgaacatctaccatactct
agcgtaattcattataccgtgtcgcgtcttattataatatgctttcgttttatttacagccaaactttttcaaacaataaataatgcccaagtagcact
gcggctatggaggattcgaaactcttccaggaactagaacaatcgattcaggataatcaaaattcaccttcatttcagggttctaataaagttcttc
atttggctctatcgtatttaaatgccaaccgccaagatgctcattgggtttgtaaatcgcacttgaagattgctgtacgggaatgctttttttctattttc
ttttcaggatgaaaatgatttttttgacgtggtttaaaaaacacttggacgaacgactccaagtttgcccttcctgcatattaagctatcatcaattaa
ttgaagaattcaagactttatgccttaatattttccattttgatccaaacactctcgctatagtgcttgagaagatatttcaatgggatgtttccagac
taatgaaggttttacaaagtattcctaagattgactctagtgcctcttccaaacctattttgtgtgctttttatgaaattctatataatgtagaattttt
aaagaatgaaacccttttcacgttgtttcgaaagaggttcttggaggctggatttcttcgactatctaaaaagttggttccaggtgttgtgtacctttt
attttctgatgatgacgccttacggaaatgggcgttcggtattctcagcggttgtgaagtaatccccgcgaatgaatttgtgaacctccaagcacca
tttcttgaggaaattcattctttaaggaaaagtacatccgacgacgtatatgtaaagcgctttttgaatgctttttcggctcttttgaataatgtctcat
ttgattattttgaatccttaggaaaacatggatttgaaccaatttcctttatcttacgcgggttggagaaagcaccgttgagtcaatatccctctttct
tagattgtctgcattctttattaaaatgttataaacttaggttttggaaagcttctaaacttgaaccctctcaagttgttactttgatttttgaaagcga
agctcatcaacattgggtagaacaaatgataaagcaaaataaagattgcaatagcaagattttggattggaccataccctttctcaactcgatttc
tttagaaaaacacctacctgctgtacaaatgttgcttgaaaagtgcaatgtactttttacaatatctgaaggaatcacgtatatgaataatcccatt
ttcaaatgtatttgtgaaattcattctttgctttttaacgaatactttcagattatatctcatagcgaagattcaccaaaagcatatgtggattccttgc
atctaactgtatgctcgaatttcgatctctttttttctaatctcaaagattatatcaattcttttgaacaaccatcgtttaagaaagctttcgaatatcg
cttgaacatcgatgtttatgctttaagacatagttatcgaaagtacgctcagaagcgtaatgatgtaaacataaacttgcatcttactgaaaagtct
gtatcaactttttggagaaaattactttctctcctcgtcgaacaaagtcaaaccttggcttgttctattttccgatgcatttcatctcttcatcttataga
caaacttgatccaccgaagactgatgatgcctttttgtctgcttataataaagcactagactcgttttgccagtttttgtctgacatgttgatgataat
tcaacgttggagtctttcatctttaaaatctttttttggaaaatcctcacgtttttttctgcgctaataggattcctttttttctccaatttctgaattttcaaa
taatgcatggactataattaatctgtcaactgatactgaaagcataggtgaagccatagacaagttgctaaaatatcggttttcggattcacttag
agcgctctcaggtatttatttacaatgggttaaatatagggcgtttagcagtgttaagcggctggtttatcaaggaaaaacgatcatcacacatctt
ttccacccattagatggaatgttacatggtactagcatatcgttcaaagatgaagataactgtgatgccttgaaaacatattgggataatttatgga
agtttcttgctcatttttttattgctttaccaagttggccagtaaaacatgaaaaagaagttctcgttgacataatgcatgtttctttagactgttgtca
aatgcttttaaaaagctttcatcaggtctccgaatttatagctggcatcaatatttctgtctccaattccaaaccagaagatagcgacgtttctaaga
aagcattagctaattcagttcttaactccctaataacactttcctattggcttaaattgaacgattcgagtttattatcttcagttgtcagaattatttg
tgctatgctgaaattattcaataacttgtctttgccaattaatcaaaatactgttgatatgattcaaaagtcttctgcagcacgagatgggtctacga
tccttactttatccgaacgggaagatttgtttcttgcaatttcaccttatttacctgagcatttacaaacaaagcctttcagttcaactgcaggcgaa
acatcaaattcagaacaagtggctttgacgagtccggatgtctatgtgatagatgaaggaccgaagcaacaaaggaaactctcttctaaatctca
agaaagtgatactaaggttgtttccgccaatgaagataaagggacagcaaaccattttactctttcatcaaaaacattgtccctgcttgataaaga
gaaggacgcaattgaaaagccacaacccgctcgtcgaaattatattcagaaggtcggcccaaaatctgctaacttgactatcggagaaacttca
gagttacagtcacgcagaactttgaataagttggattctgtagctccaaaaaagaagcctatagaacctaaacggaacttacaattgacttcaaa
tgatgcagaatcttccgaatcagagtcagagtcagagtcagaatccgattcaaataataaggaaaatgaaaataatgggcttttctctttaatgcg
tgatgtctctaatcagaataaatcaaatgctccacgtcgtcaagtacaacttttggatataaattccttgaagactaataatgtcgtacatcctgca
cagcgtcacagggaagctcatcagagtatttatacctctcggcttcgtttatttccagatgttcaaagtttttataaaattcttttaagctggaatcca
attctccaatctgatacaactgtcgatgaaaaaaatttgagatgtcgaagtattgaaattgcttactcttctccagatacttacgagaaaacctttat
gccgtttttattcaatgaatgttgggctcaaattcgcggagctattgaggaagatcaatatactccgttagaattgacaatgaatactcgtaccact
gttgataactttgttgagctaggtttttcctccagaacaatcactgaattacagtatctcagcgatactgacatatgcgtattatccaagtcaaaaa
aacctagctcaccagaaaaaaaagagccgtcaactcttgctaaaattcagtctctaactaggaagaaagaatcaatcgatatttttgttcgtgtta
attctgatgcaaaaacgctacaagaaatgattcctggtgccaagttttacttacagaaactttttagtgcgaccaccagtttgcgcgaatactccgc
attgaagtcacttccatttgttaatctttccagcgatattttacatgccagaattagcaaacactcggagtatgctaatagtgaaggcctaaaaaaa
attgtgaaatcatatggagttaatgagcctcaagctaaagcaattaacgcagcttctagtaatgatggatttacacttgtgcagggtcctcctggta
caggaaaaacaaaaactattttaggtatgattggtgcgatattgacttccaaaggtcagggtttacggtttgctgcccctgggcagtcaaatccca
ttcaacaaaatcagaaaaatagggttttgatctgtgctcctagtaatgctgcaatagacgaaattttattgcgtttaaaagatggcgtatacgacc
atgaaggaataaagtttatgcctaaagtaattcgaataggtttcacagagtcaattaatgttcatgctaaggaatttacactagaagaacagatg
gttaagcaaatggaaattacaaatttaaagaagcatgaaaattcttctgatgccggcgaagtcagaaaaaaacatgacgctattcttttggaacg
taacaagttaagagaaaatattgatttaactcgccaagctggtaaggatagctcctctttggaattaaggctcagagaaattacacgtcaaaaaa
attcccttgaacaaagtttagacgaaatgcgcagcacgcaacaaaatgccaacaggagtatggatttagcgaaaaagcaaatacaaaatcaac

ttcttcaggatgctgatgtagtttgtgcaactttgtctgctagcggtcatgagacgttgctaaatgccaatataagtttcccgactgtaatcatagat
gaagctgctcaagcagtcgagcttagctctcttatccctttgaaatatgattgcaaacgttgtattatggtcggtgatccgaaccagttaccaccaa
ctgtgctgtcgaagtctgcttcaaaaaacggttatgatcagtcactttatgtccggatgtttaaaaagaatccaagtaatgcctatcttttaagcatc
caataccgaatgcacccagaaattagcaaatttcccagtcattacttttaccaatcgaagctaattgatggaccaaatatggtttctgttgcagatc
gaccatggcatcatgatgctctatttggtgtttatcgtttttttaacgtgcaaggtaaagaatctgtttcaaaatctaaatcattgtataatactgatg
aagcatcatttgttcttttattatatgaaaaactttcacaaaactttatcaatattgactttgaaggaaagattggtattgttactccttatcgaagcc
aagtccaagaacttcgctaccaatttcagaaaaaatttggatcaattatttttaagtacttggacatacatacagttgatggttttcaaggacaaga
aaaggacattatcatcttctcttgcgtcaggagttccatgggaaatggtataggatttttacaagacgtacgtcgtttgaacgttgctttaactaga
gcaaggtcttcattgtacattgtgggcagtactgaacctttactgcaaaatgaagtctttcgtaacttgattgaggacgccaaagcgaggaaatgt
tggatgggtgttagttttgaacgactattgcattggaaatctgatacgaaaaaactaaagtcaattaaggaacagcataaattggatgataactta
gtaaaggaagaatcggatactttaccacgtaatgagcgctcaccatcgttaactcctttctcagaaccgaaacacgaagttctaaatgcgcaggc
ctctccagatatgaatgatcataataaagatacaaaagaaattagaccttccggcgacatagaaaattcagatatgatcaattcaaaaaagctta
agaagccttttaaggaaaagaaggacgaggaaaaacagaaagagactgctggctcgagtattttacagaaaaaacgtgaaaacagttttagtg
attcgactgatagtaagaaaataaaaaaatcgaaggaagaaaaaaagcaacacgaagataaaagatcttctaaaaagtcaaagaaaccgaa
atctaagttggctctggcggctttggagcaaggttttcgcccgccaagttgattcgaagaatataaattttaaatatattcgtggttcatctgccagc
tacggcttagctctttaccccttggctatcttgctaatccactttatcttcttatgtttcaattattcctaatcgtttaacagagttatgtatacagtgat
ataatctttggggacgcgctattgggtttggagttacccatgttttattagttttctgatgcttatattaaagcggtttttacagttctctcctttttgtgt
gtgtgtgtttcgtatcttacttccggtctgttgtaatttatgtgacagaagaagccttcaaagagcaataaaagaatgaaaggcgatggtaacaac
attatagcagatctaaattatttaaaaagtttgatggaaaaatcagcttatatctagtatcatctagtatcgattgtgaatgttcggggctattaggc
ataactaacttggaaccgaagaccttagttcgtttataaaattatttgagtcttctcgagaaagcaccttaaaacacgcgatttctttcattgccatg
gatggatcagcttcatgcattccatgcacaattaacccagaaatgcactcctgagtattcaaagagtttatttgatggagacatcaagtttgaattt
tctcgtacgaatggttttcctttttttttaagaaaacttgactggagtgtaccttatctaagactgaagaatcatttctcgtgaaaaaggtttggaaat
caattgcatcaaaatcaaaattgatatctcttgatattactgcattttggttagagcaactagagctcgtattagaaaatggttatcttattcatcgtc
catggatggcgctaatttttttgttacgacagaaacctcacgcgttgtgggaatgtttttaaagtggaaataacaggtacagttgtaggattgtacat
tgaaatgaacgatgtgatattacatgaaaggatactaaatccgtccaagaatttggtaaaacaaatcgataaatggaaaaacaatcttattcagc
cagaccagttgaatcctacaatctcaagatttcgactagactaatgctacatatatttatttcaatgtagtaaacatgatctattctttgtctcataag
tttctatttgagttgagtaatcgctgattccacattgtttttacatatagaatagatagtaattctttatagttattaccgtgattatagcgaaaattat
aaaaacaggatcctgatatgacacctcccgcaataaatggacgctgtgaaaagatagagatgctatattgagcgatgaagatgactagtgatca
cttacatttttagcatgtggattaacatctctccatctttcaattgagttctgtcaaaatcattaccgaaacgccacaattctaagtcacgggcactgt
ctttatatgcttcattcttgataaagtttccacttcttattaaccccaactttgtggtataatccagccagcagttgcagattagagaaacatcatcag
aaatagcggtaaggggccgcccgttttgacttgtttcttcatcagggccattttctatattatatagctctaatatagagtacccatgtattccaaga
ttacgtttagcagcatcctccaattcggatatgatacctttgttaatgcttgcttcagtaggccaaacgctaacatggttgttttccccttgaatttgat
aattccaacagtttgcctggtcacctaagatgtcaatcaccgtactaataatagcccaagtaacggctggtgtatctatctgagcatcaactgcatt
ctgtaaattctcatttggcctgataaattggtcataatccctcttgtaattcgaaaggatattataatcttcaatattcatagtgggaactggtaagc
aaagaatggtgaaattcactgatctatccttgtctatcgcatgcagttatatatctacatggatgattcactaaccgttatgacacaaaggctatttg
aaactcgatagctaggtcttcaaagcttatcgaattcaaattcgcctgtctgatgtttacaacttatagcccgtcaaattttatattcctgaagcagg
ttgagttaatctattaaaaggactgttaaaagaaatggctcagttactcaatgagaacggttggtccacaagttttggattacttgctttaagaaga
gagcccataatacgccataacggtttagtttagtcttcttttttttgtgtttgaaggtggttttccacaaaacgtataatgattataaagagatgctt
atcctttcgtttgaatctatatgtatgacactaaagggctctactccaagtctccataccttcagcatctcatagatcctataattcacaaatattagc
tgaaaagctcagtaatgagtagtactgctaaaaaacttccgtcaggtgtatctttatcaaagtccttgttggacaatgatgaatagacaatgttcac
gtgaagcagtacgttactaccgtaccatttttctactatctctactatctcaattctacatcatattcagacatcaatgaatggtggtacagttttattt
cttggtgggcttatattgtctaagagtagtttcgcaatgtagactagaaaagaaggattaaaatattaaaagaactctatataaattttaaaatca
attaatcaattccttttaaaataaatataatttcatggctagaattgatctaagataggatgccgataccaccccaactacaagcatcatctccatg
atgcttggtgtaggcgtccaaagcaagactaagttgttgtttaccagtgcatccactaaactttccataacgaccattataggggtcactggtaata
tcagcacatgcctcttggtcataaaagcacaattcaccaaaaacttgacctatcttggctccttctgggtcgccattcacagagcaacgaaggtttt
tgacagcatcgtcgcataaagaggcattaggggtgaccgggaaggcctcgaaagtgacagcccagctagtttcgtttctggcagggcattcggg
agcattacctaggggctgataagaatgcttcgacaccttttcgggattgacagtagcccattgttttttaagttgtcaaaatcgacacttttaacac
gctcaccggtagaagttatattgactaacccatagccatttacctcctcggagaactcgtaaacaatacctccgctccaaacgtcagtcatgttttt

ggagtaaatcgcctggacttcagtgaagatacgggggtgaatgatgttgcatccaaattcagaaaagaagatgggaacagtgatgttttggagc
tcatttgtacgagcagcataaccggaagcatagaagttagagtcaccacaccattcatagttattgtaccccaaaaagtcagcctctgtagcattg
tcttgaggggtattggtagcgttacaggttaagtaagcagcgagttcattacgaataacgggaatatcggcgcctgcataaccaacaggaatgct
acgataatttttggacttgatgtaggactttaagtcacggacaacagaacggacgtaggcagcagaagcagcattggtagcattattaacaacct
cattaccagcaaaaaagccagcagtgttgttatacttggaaaaaacgtcaatgacctgtttgtagctatcgtactgagtcaaggtccaagtcgggt
tggctcggtcgatgccggtagaggggttggcgacgtctaaaaagacatagatgctttcattcttgaaagcttccatacaataatcatgatccaagc
tagcgttcacggaatatacacggatagagtttccaccaagagagcggatcaacttggcgtcgttgctacaattcttgttattggcatcggccaaag
gatcaatgacggtgctggtcacttcagcatctacttggggttgataggcgacacctttaacataaaactgggtatcattttggaagaactttcgtcc
tttgattgtcaaaggttcgattgcaaagacagtggtcgatgccgtcaagagtgaaagagcaagatgaagaaattgcataacgattatgatggtaa
taaaatgaaaataacgaaatcaaacgaaatgaagcagagaaaataacttctactgttgttcaaatacctgaagaatgctgaacgaaaaacaga
gatgactttaaaggacttttctaacaagaaatgtaatagaaaatgataaataaaacgcaagagagagaaacgaaaaacaaagaaagacaaa
aggacaccgaatttttatatttattttggtttagtttggtttgtttatattttgttggaggatccaaatcaaccagttcccaacactagtcttaggtaca
attcctttccggtaagaattttctaactgtcagcatcgctccatgcttccatctatatatatagacggaaaaacttcggaactccgcaaaggcctcg
atagaccataatacggcatactttcgggatgacaaagaattcatattccgtaagaacaatgacgccggaatcccaggaacagacgtatgaattg
gagctaaataaggtcacataacgagtggagcttgcataaacccgaggctttatttctcactaccgaagagggaagagggaaagcagtgatattc
gtacaaatgagggaacacttctgtgatgtttttgtttatgttttaatgttccgcggagatcaatttttttcggttggaacggatttagcaaggaatcca
cgtttgcgcttgatcggattggcatgtctccttggtttatacggtaaggaacaaaaagaaataatcgtcggtttcattgtgtcgccatctacatttcct
gaatatggaaatatcaagtacaaggtggacaataaccgacttgacactcctttctgtatgattgtgtttaagaagtattccttgttgagaattggttt
catgactatccttgacgatttaataaacgttttaagcaatttttagtttttttttggtttcatttcaaaccttttatttttcattgtttgtattttatatttct
ctttcctgtttctctctgaaaaccgaatagtttcagtaggcatcaattcagtaatatagctaggtggttacataggagtaagatgagaaaaaggttc
attaagagacaccgggaaaatataaaaagaccttttttttttatttcatttctatttcatagaatattaccatatcccaaaccaaagcttgcttataa
agcaagtttgttttcatttatgtactcaagggagatgaatcacgaggatagcgacaatacttatcttggacgcttaacacagtatatacaccttccg
ttgaacttaataccttatattcataatcataaattccagtgattgtgtgcatttccaaaatcttaccaggtcctttccttccaatggctcttcgacaata
cgtaaaggaaaagggagggatacctgtaaagtgaaacgagatttccgcttgatcaccttcgtgaatattttcaatcacctctccaccgtcactaac
ccttactgttggaatgtcatacacttggttttgaatcggaggatcaagttcagcgcggcaaggacttccttggtcggatatcgatttcatagtcacc
aaaccagggaactcagctattcttgtgaaaatatgctggttgctttttgcatgtttttttattcccattaaattcataatctatttcaaagggcggcact
ccttgcaaagcataagaaagtcgatctccgacacagaaatattcatgagattcaattggaaccatggaagccttttctgcaacacttaccgttaca
aatggatcttctttgggaatactagattgacatccattggcatcttgcacatgcagcaagcgcactgtatgctttccaagcgttaattgatctttggg
aaatataaatttgtaaatgggcccaaacacttcattcactgttattttattaacagctcccgtcatctcgtttttgatttcaaatcctaaaataaacgg
tcctttgccgttcaatttcacaccaatctgttgttcctttgagtaattactgtcagaatgtatacagaatgtgtaaagcttattggaatccaaaaacg
aagctacaggtaacttatttaccttctggtgaataatcactctgtgcaagcctgattcatccactattcgcgcctctactttgtcatacaaggcatcc
gacaaacgaagaaattcatatgtatatgtaccaaacagtgaagtttctgttttcagtagaatggaactttgcatagtagatacttctgatcgttgct
tcgaatgatgcttgccattttcttctactagagacttttcatactccaaaagatatggactggcacctttaagctcaatatcaaatgcactactgtca
ccagcacaaacgggttcttgttcgtagtggtaggcattcgaacttttccaggaaggctccgaaggcatagacaaagagggcttaggaagccattc
aacttcataagtttgtaaggaattctgaatggatcccgggcaagagctatcccttacggaaactaatgagtacgttcctgctttgttaacgtttaga
tggctatttggatcatagaaaacggcttgattgcgagtagttttattgtccggagacacatgatcgtactcaatataccaaggcttttcaccagcaa
gtctcaaaggaatagatagcaaagagttttccacagactctaaacgatagctttcgtcagaggaataaaatgtagcagtgggtctttgacgacgt
actttaatgatcgtatcagcggtattcaaagcacgcttacagccattcgcatcctcaacgcttactaaagtgatagtatattgagatccgtgtataa
gagctggagtctctattatgtagcgttgactcttagactcaacaacttgaatgctttcacgtttgttgtttctaagaatttcgtactcgagcttccatg
gagatgagccaactaatgaaacaggaattttcatagtatcgccaatacagcagcttttaactattgaactcatttctccgttcatattttcttcaaaa
gcagcatcagccaaagggaaaacagtttgctctttactgtaagcagcgccagcaagttctttgtatccgtagttagcatcttctactcctaaaataa
cgtagcggtattttcctgactttttaggagtaaaatttagctgatagcgtgtacgatcagtcgtgacatattgtactcgagaagctttattgtctttga
tcatccgataagcaaccttaaagggaggtgtgccttcaaactcaagatcggcacgtgcacctatagaaccagcacactgatccgagatttcctca
aatccaacatgcagcttggtttcaggaggtgtcacaaccaaacaagtatttggtactaaaacatctcctgaacaatatttcgaagaaacaccaga
aagcacatatataccagtttctttaacttttataaatgacgaatgaggctctagcttaaatacattttgagagttaggggcaactgtgaggttttgtg
gatctaaacctaaatcatatcgattaagtgaaacttgcaagtcatccgggaacttataggatgtagcaattgacaaagaaacctcttttccgggga
aaaggttggcaggattttcaagtgaacattcacggaatttgacttcaggtctctgatggacgagtactttgtaagtgtgactagacaattggtcag
gctgatcttcgttagtggaatattttgcgaaaaactcttcaacagaagggaagacgtaagtgtttccaaagacatcagtgacattttcaattgcata

cagccattccccatatgtttctaaaggagcagtgataggaatttgaactttagaagaatgtgcatactgtacatctaatcctttataaaatgcaata
tcttgaggattgttagagagcataagaggatgcaaacggtaatcctttggaacgacggaatcgataaagtgactatatgatcgctttccgtcccat
gttttgtaagcaacctttaagggaggtacaccgaatacgtctaattcgatggaatcttcgtcgcccacacatttctcatgagctcgatcactaggttt
agaggaagcaaaaaccgctgaagggcaatcaacgaccacagcttcggagcgaacaacccgaacgcccaaattcgatttatcgacgactttttc
agtttataaaggccatgtttgttggctttaatataaatagtctgcaaacgagcatctttgggattccgcataccagttgagataagctttttcaaaga
acggccagaaatctcaatttcattttctatatcagaggaaaagtctctgtaggaatacacaattgatttcggagtggtcgaattaaactgaacggc
gaattcgacgaaaccacttttgtcattttcgggcaaacagaaatgttggttattaggatttaattttgcagtgccctctgggaggatttggacagtat
attgacctaaaatccgggaagaattatgaagtacagacctaggatttattttatgttctgcgatcgcgtactccttttgcttccaatatggaaaaatt
atacttccaagagttacaacccgtaaactagtaatggaaaccaatagcacgttaaccgcagcaaaagcgacaatctgcaaaatcgttgtggtag
gttgaaacgaaagccagggtatacgaaaacgaggtaatatttgcaaatataatgcgtctattagtatccattttaccaaaaaactcgtttttgaac
cctctataaaactactagttcctaacctaaaaagatcgtaaattttgtaagcttgaagcgctacaagcacgccaatggcgtaaagtcgctgactag
gagcatcaataattgattctgatactagaggccttggtcgttcacgagaactgacgcgggagaccatagcataccttgggtgtagaaaggtggta
ctggtcagaaggaaggaccctccaagaacaccttcacattcttccaccgcttcattcaggtgaagttgattttcattctttaattgatttacaggatg
gaattagatgaaacccagttcgaaaaatttataggattgttcagcattagacctgaggattttccatacaaccaagatgttaggttgaacattaag
tcattgcgacaaaatcaaatagacaagcagctctttttgatgttttattctctttcattgatattattaaggagccagaggcgtattatcctccatcc
tccatgaacgatttgaaaaatctctttttaatcattgactcttctaatatcgatgaacttaaacagcagtgcttttattactacttattaaaagattgg
ggaaaaagtgaaacatatgccaatcaaatattattacctttcaatttccgtcaattaatcgatggttattacgctttagatcatttgaactttgaggt
atgctagacgagaatgtgaatttattagtttcctatggcttttcattctatcgcttgggcttattgagctaacaaaatataggaagcgcttgatcactt
tttacagccggatgtgttaccaaatttcccagacaaaattttggaaacgttttatcgacacgacaagttcttccaaatgattcgttttgtacagtttgt
gaaccctcctttggatacatttagaaaacaagagtgctatactctcgctcttgcaagacatagttttctgagcgcctttaactttatggtattgttata
attacttataaagctgctaacaaatctcagaaaaaatgcatgcaaccgcttgtgttgtgggaaggacttcttcatgtagttttagactcgaatgata
aagactcttgcaagttaatagtttcacttcctttaaccgaagaagaggtcgatattatggtttccgttttaagctcgaaaaaagaacctttatacctc
gatactctcttagctttcctcatacagtcaggcaggatacatgaagcctgtcagttagcatcttcgaatctctaccttgacaagtctccgatttttgcg
gctttacaaaggctaggcgtctcataataaatttattttttctcttacttgtacaataattatgatttatccggatgctactcttcatgaagttatttta
attccttaaactaactagtaccgttcgaaaaaatgatatataaatattttagtacccaaagtaaaccattgaaaagaatgacaaagaattacaag
atggtttggttacacacgaaaggtataaatgaacgtagatagcaagacatgccaaatttaacgaaacttataatgttgcaatgacaaaacccctt
tagggcgttcaggtaatgaggtggatgatggctttgaagacacagtagatggtgattgtaaagagaatgaacgtttagcttttcgatttagagcat
tctggccaattttgcttaagggtgttgagggacgtggtgtcgttgaagtctcgtaagaaaacgaagaaaaccaggatttcggagctggtgaagga
gaagcgatggaaatattttctttttcatcttttaaagatctttcattaatgacattcttttcatcaggctgcttttgagcaaagtacttgctacatccac
ctggtgctgtaaatggaggaatgttttcgctcttcctcaccaaatggctaacgtctgacaaagatttgtcaaacctgcgtggttctgaagcggtgttt
accgatgacaatggttcccgttgaggtgttttcatctgttcatttccaccttcacctttcgtcagggaatccacagattcttgcttttgagacggtaga
aacttatccatcggtctttcttgactgggaatgcaagtcatactagcaattttctttggacgcttatttggcggtcgcgttcccataaaaaaggtaga
aatatctgtcttagtgatacccttcttttagtagattcagtataattttcttttctgggagaattgaagtttgtcatagtattcttttcggtaaaagcag
acttggtcactggatcaaatctaccctctgcaattgcaattgctatttggtcttcgatgaagggaccgatataagcatcctcttcaggggaaagctc
tctagatattggcgacatatgcacaatacgtttaagcttagggcaataaactcgctgatgctgaaaagcaaggtctgctaataaaaattctttctc
aaaagcaatcggtactttcagacttttgtcaaggcgcatcgcacggatagcagcacgaggatctggatatcgaataatatagcgtaaagcagttt
ttaggcccattccagcgattccatcagtataatcacacccagaaaaaatggacatatggcgaaatttttcatcagaaagcattcttaaatctacct
cttgcttttaaagaggtcctcacgttgtatttgaacgcagtttccaaacccatccattttgaagaggactctttttgcaccaaaaacaagcatgtca
gaatcctcagttataatgccatcaatgaccttttctttttccaagtaaacaagctgggaatctgcttcatatggtgctacaattgatgaaattccatg
ttccttcagtgcagctattaattttgcggccatttccggcgtgacatcgacacagtgggagaattgcgtaaaagcttggcttctctttccttcatccca
tagcttctttgcaaatttgaaagcttcttcccggcgctcttttcgtttttgctcagtaggtgctttgcaaggtaaaggccctccatcgaagacaacaat
tggcttcacaccataatattgaagcattaaagcttgatggattgcatatttaatgtatttatctgtgtctttctgaaatgccaattcatgagcacatgt
aaacacacctttgtgtagccaaacatacccatcaacccctaatgtcttacctgaaaactcctctacgtgggagggagtttgagaggactttaagaa
tcctaatagtccctttattcccatagtatgaggagcatctgccgtagcttaaaagagtgacaagttggttatgaagctttctctcgaacccaaagaa
gacgactttcaacgaatacggatacattaggatacgttaaatagacttttatttattatgaaaaaacgagagtctatatttaaaaatatcttattaa
taaaacgttgcttttcgtatggctattatgtaaataggcatgtattaaagaaccaattaacctcaagtattaaataaacaaggtaaagaacgcaac
attttcaatcatagcgtcaaatattctgatcttcagttgttttaagtagtttaacattatgtttcaccaataagtccgataaataatcatcctgtaaag
ctgtcataaacatataatatatggaaagatttagaaacccatttaaaaaattattgggcataagcttccaactgataaaatgttagtaaaagcgtt

cacaatagtttcaagaaaaaaaaaataaaaacttacattaatggaaagcgtttttgaagaattttgctcttggtcgcggtaaatccctaccaaggct
tcagatttactgaataattggtttttaagggatataacaacgaactgaaagccatttgacgcatgttggcgaatgtaattcgcaatttttgaaacat
tggtctgatccaaggctgcatcaatttcgtcaagtacgaaaaacggagaaggctgatatgaatgaattgcaaagagtaaagctaaagctgccat
tgtcttttcgccccccgataattgatccatatctcgaaatcgtttcatgggaggcatagcatgaaatctgatacctcctaaatatggctcatctgaat
cgtcaagtgtcaaatatgcagtaccgccaagaggaaaagctggacttttcgttagttctttatatatagggtcaatttgttcggaaatatgattaaa
cgcctcctgaaactttttcaggcgctgttctttcacactgttaaatcgttctttggcggttttagccgactttctgacttctccaaattcatcatccaaa
cgcgaaagtcgttgctcaactacttccagcctttccattgctttcatgtttggcgacattctttcgagttcctcgtcgtattcacgtaacttctcttgaa
gaactgtacccatactctcggagccatcacccttcaattcgtcatccaactcctcatagtccacctcaattccaaacttaccaaaagcagcttgcac
ggttttggggtcgctatcgatgtccattgtgtcaacattttgtccaaactcatctatagggatgttagataatgatccttcggataacggaagagga
atgtcttcaagtttacatttcctcacaatggcatgccaatctgaagcgaagcgttcaatctcagattctgcaagagtcacatcatttcgaagctttgt
aatttcagctaaaaagcgcttattttctaacttatgttgagatatcgttcgtaatacttcctcatttttggatttcgtttcggcagactcttcttttaata
ggtctaattctgcttcggcagtagcaacttctgtttccaaaacttcgtgcttttgttcatattctttaatagataattcatctttgcgaataaattccttc
agtctatttaatcgaagttcaatatccgaagttctttgtcgctcaaaacctaatcgattttccagcaaagctttttgatttgcaaactcaagttgttttt
gtgagtattgctgagtaaaagacctttgtagttcctcataggatcgaatattcgtaatctttgttcttttgcagaagttaaaaaatattttatcttcaa
tttcatgagtctgttgaagaagttcgctttgatattcctccatttccgacaactctcgttcagaagactctaattctggtttcttttgtctgtaaagttcg
ttaaaatgatctgactcttttcgtttgtcttctaatgatcttagcttagcattgaggctttctttcattagggataattcagattcaagattatgtatatt
gatcgtgtcctgttccacaactaaagatgccgccttttcagtttgtatcttatcaatccgcgatatcaaatcattctggttttgtttaagctgttctattt
cacgatcatcccaatgctttgcggatctagtactagaagacccaccagtaattagaccagtcttgtgaatcacagtcccctccaaggtaacagtct
tagcattggcacgtttgttgtaagataaatctcgggcaacatttaagttatcgcaaatgagtgtatttccaagggcgctcacgacaacacgttcata
ctgaggctcaaaatttacaacgtcaattgctaagcgagcgcctgcatgcgtaccacggaagctttgattgataggctttacagctattgtgtccatc
ggaaggaaagtcattacaccaatgcgctgctctctaatatattcaatacattccttagcagtagcctgtgactcaacgacaatggcgtcaaagttt
ctacctaaagcggcaaccaaagcactttcgtatttcttttgtgtgggactgcataaatcaataactctccctttaattccaggataaatacgttttatt
gcgtaaagcgcttctcgtttctttatatcttgattcgactcatttctatctgcattggcttcaagtagtttttgtaaacaaacatgtaattcatcattaa
gatcacgttcctgttgacataagtgggaataatttgcacgcttttgttgtatggattgacgagcggattccaattcatcaattcgacgattaacctca
gttataagagcgtctttctcctctgttaaattatgaatcttttctttagattgagtctttctcattttcaaatcttcaactacttccgctaatgcatttctt
gcttgagaagcaactttgattttgcgatttagtgattgtagctctgtaagggttttggaattttgtaagtcagcttgggtccgtaagtcatcatactct
ttttgatcttcaggagaaagtttttgaatacgttgtttttcccgttcatgaatattccgaatcagctgctcttcagcagcgtttaacgaagcaagctgg
ttttctagcatctgtaaggtactagattgagaagtataatctttttcaacttctccaactttcctttgaatcttttgtaaattcacggtcgattctgaag
ccttctcagcaagagatattaattctggtcgcttggaagcaataaatttctcttgcttacgaacgttcctatcaaacaaaagtaattgccgatgaat
agcaccttcttgctctcttaaagactcaactttagcagaagacgcctcttggcgtttggctaattctcgagtggagttttgaatttttttgatatccttg
gtattgaagtgaacgtttttttcgatgtgatacaatttccataacaagaagattagcgaggcaatatctctctgctctttcttgctctcataaagttct
gcctcggctttctgctcttggtattgtttgatttcagcgtttatgccccttttcttgttaaatgaatgtgctgaaagattaacggcataatcttgttcttg
cttgtacttttcatagtcacctttgaactccaaagaaccacatatttgttcaataagacgagaaagatcgacgggagattgagaagctatcgcttc
aacatctccttggaaaactaaaaagtttcttgcacgtacaagaatattctgatcttctaaagacttgcaatattccgaaaattgagcaggttggtca
ttgattttgtattcggcactaccattaggggtaataacgcgtttatactcgcgttcttcaccgtcgtccaattcataaaccagttttacatatgcactg
cgtggagattgagagtcatcgaaagaagtagacccttgactactcataacccttcctctataaataagctcgcgaatgtttgacgatcgcagatga
gaagacttcacacctagtacaaaagagatggcatccatcaaattcgactttcctgaaccattcggcccaatgaccgatgtgaaatcttcaaaggg
tcctatcgtttggcgtcctcgataggacttaaagttctctacttctaatcgtaacagtcgtcccatcaaggttgtggttaaaggaaggctgtttattgc
gacgcgctttcacctatacgaggagacgcgtgagcagtcgtcgcggtaatattcgttaattcattcatgagttttattagcaacaaacaagataaa
tacaattcattttttcttttaaaattcaaattactaaaatcctgtcaaatccaaatcttcttttctggaaaacctcgttaggcttctttagtaaacaagc
attccatttgagatttttaataatagaatgattcatattttattatttatattcatatatttaaagaaacagtaaatccttttctgtgtcatcatttggct
ttatgttaactgtatgaacctgtgattagcagaatgctgcgatttaaaacactaccacttctccttgcgaaggcataaagcattgaaaggggtcgtc
agaggcagaatacaaaacctatctatattatatcatctttatttttaatcgttgcgaaaagcaatagttcatggatgttgacaataatatatcgagt
gaatctaaaactgctagtcaggaatcaaagaatggcccaattactatggaagtggacggctctgaaggcacctatgtaaacgaaaacggtgttg
ataagactagtatggaagttgatgactcacatctttcagaagacacagctttgtcaaagtccagtaaacggaagcgtacgaatgcagaatccac
agaagcaggcaatgaacgaaagttggagcggcaacgacttcaagaggaaaaggcaagaaagaaagaagaagatcgtcttcgtcgtgaacga
gaacgagagcaacgagagatagagaaaaaacagcgagaagaagaacgactagctgcgaagaagaagcgtgaactggaaaaaatggagaa
agacctcgaacaacaaaggaaaaaagaggagcgtgatcaaaagcaaagagaaaaggaagaggcgctgcgtgttagacaagagcaaaccgc

taataaagaaaagcagcaacttcgactaaatagttttttttctaaaggaaaagcttctaacaaagagtctcctgttagtgagcaagaacaaccag
caacggattttgataagacgtttcgtcccttttttcttaaaagccaagcagcatttgcaaaatttccccttgcctatactctttcgatcaactggaca
agctgttgacttctacctccgacgactcattaaccttaaaaaaattacttttatcgcatcgctcttctcaggctttatcagaaccacttcatgcaaaa
ctccctacaggcttcaaatctcagtgggtgaaacactttatggataaattccaagatcccaacgttagtaacccggaagaaatactacaacaggt
ctctaaagcccaagttaaacttatatactttgctcaagacattcgtcctccttattttggaacgtttaccaaacagcataccgaagaaagcatatat
gtcaatccatggcttgaagacaaaactatagactacggttatgactccgaagctgaatgggttgctgatgatgaagaagaaggagaagatttgg
atagcgaagatgatgagttggataactctgacgataatctggacgaagtggatgctggattcgttgatgatgacaatgacaaagaaatcattact
tctggcaaaccaaatcgtttggttggtccgttacagccgattatcgaaggaccgtcttgggatttaaattttctctctaattttgcttgcatctctttat
tagaccctattaccccttctccacctcctctagttttcgaattgatcctcacagagattattgggttcctcaagaaagatcatctgatgtgtcagtttc
ttccattgttcaagatacctctccatctagcttacaagtgaaattgccatctgaagatctacctagttttctgggttatgtcaccagatctcacgaaa
acaaaatcctcttaattgagcatctacgacaaatttttcctcatgtcactaagaatgttataagcgatacgcttaacaaagtcgctgttcgccgtgg
taaagcagtttctgatggttgggatataaaacaagaatttcaatctttcctttcttcttgatgtcaattcaagaataccttagtctttctactatatggt
caaggatccaaagcatgtaacattatatttataggtcattacttactataatatgaaatgcatatccactaaacattgtatattattaatttaatcaa
taccactatataaggctcattatatatacctgtagtttataagacttaatatataataaatttgaaatcagataacactcattaataaatttcaaact
tcattcggtttcagattcatgtttcagtttcatacattcattaatcaggtgaacgttaagataactaacgtttctttccgtatggtaatcaattcacacc
tgcagtagctatgtttttctgcgtcttatatcccgagtatgcagccttaggtattgaagtttcagcgttcaccggatatgctgaggctggaggataca
tattttgccaattaaaattatcataaactggactggtggcatagacattattgtctacaggcatatacaaattcattttaggatacatagacacagg
tggtgtttccgtccggtctaagttcgaatatgcatcgtttaaagcggtaatgctcttggtaagaacattatactcacgggcacgccacatacgcctct
tttgagtttgacctttaaatttcttttgctcggcagaatctgcaaaacgaatttgcaaaggaggtaaggttgggtcaatgcaataatcattgaaact
atcaatgatttcctcagctacgaaacgatcctgcatgcgagcaaaaccaacgcctcgagattgattcttagaatcacgaagcacacgatttgaaa
caattctgtgaggtttgaaaagattactaatgtcgtcctcattccaatggagaggaagattggagatatacagattggtgctatctttatcttgaag
attttgcaatcttgcgctgaaactctctttggcaaatgagcattgataaccacacaaggtcatagcagatatgcataagactgcagatttgagctc
ctcaaagcaagcaaatccgaatcctttgcataagttattattaacatcaatgatggctttactactcacaaccttaccaaagcggttgcaatacag
cagtaaactttcatcggttgtagtaggaggaaggccacgaatgtaaacgtttttttgtttttcctggattttccgtctttttctcttcctgagatatggga
aacatactaggaatagctggagggatagctggatgcatggacacaagacgagtgagatacgcattttgttgctttaattccggtttagacgcatta
tccgatctgagagcatcctcggattcagaacgcacatcttctcttggttttgtatgtgtataaggagtcatatttgtagagggtgcgggaaatccgg
aagagttgttaccgacagcgggattaggaggcacagacgggagatatgctggttgatttaaatatgggacattcggctgtcctacgggcgttact
ggaggatacattgaagtattctgaggggcatgcactgccattaactgctgagtaactccattggaaacataaggtttcatataaggatgagttcca
ttcattggataatgttgctgctgagcctgccactgtgctccgtaacaaggtgtagcagcagggacaccaaatggaggacgaaaagcagggacac
caggacgtccacctgctgtttgttgggaggcagcataataaggaggataatatgatccaggtaaataaggattaggctgtgggacggtagtaac
attattgttacttagataagcattagattcttggaagcgagtttgataattagggtgttcgttaatatatgcttctgtttgagtattcatggaatagcc
attcgtttgatttgtatattgggaatgtgctccctggtaatgtctacgttgagagctaggagtatgatcacttgacaacaccatatcgctggatccac
gagtcgtgtgtgggcttgtgccaggagtcagtaaaacaacacctgaatccatactttcggttttcttgatatcattacttagaagctcgaccgttgtt
tgactagttggagttggtaactcagtagttgagaggggatcgtggattgaagaagacataagcctttgatctttcgttggagtgcttaattcctgca
tactattaagatattctattgaaataaaagtaggtatactacagttcttgggttaagatggaattggttgttggtaagtttgtttagcttgggcttgct
aattaatgccatcaaaacaaacaacctgtacgcatatactacgattgcatacatgttttactctataggatgattctttcaaaatattatttaatgaa
ttgtgtattaaatttctctgcaaatcttctttttcagaagtatcaacttttctaaaggggactcttcagagatactccttacttggtcaagatctcacag
tcttagaataaaaggaaaaagtaatagaagaagtacattaaaaactaattttgtcagtttatttttattcatctatacggataaaagcaatgtcaa
ttgatgtgtatgtcttaatgaatttactgtggatcaaactagcaaagtaacaaataacttcgattagatctattacaatcacttatccctgaacgttg
ttttgagctaaaatgagcttgtaaacaaaagccagtttccataggaaatatatgactttcattacgagtaagtgtaattccaaatcactagagttat
gcttattagtgaaagtttctttagctttattttgcgctcacttattataaagctgagagggaaatgtatagagtaatatttaaaaactatactataac
atttattattcataagcgatctccgttcgtattcttgcttttatcagtgtatgagtatgaaagcttacagtaggaagctttattcaacaaaaattctta
atgtaaatttctattcctttggagccagttcattttactttgaggagagttttccataaaatcatttgatgaactttttcatttttcatcccactctctgg
aatcttcgcttatatatcaaccgtgaaatggtgacaaaggaaaatagtttaaaaccatttattcactgtttgctgaaacaagtattgctgtgatcga
tgaaacatttgcatttgcacgtatttcataaaagttcgcattactctatttttctttcaggatttagcatatagtattgcgctttaagtgatagtaaata
ctctcagctacatagattgattaaagtatatggagttccttttgcgtataggagttaattattttctcgaaaagtaaaaaagtgagacgataaacac
atcgggtatttacagtaaacattgccaactactatttgaacaactctcagtatcatttatggcttagtttcgaaatattaattctttatacctttaatt
cttaaatgtcattgtcaatttgaaggtatctgctgagtttattgaattacttaaactatgagtcactatatttttgagcggtgttttagctgagaatatt

gtatctgatgaataaaattgtctaccctccaatttctatgaggttggtatacttataagtttccgaacacattagatttttattacatctacattgatta
aacaatcattgaacaactgtttgcctttacagattttgttctatagccattctttcgaaacgaacattctaacattgacaatgacataaatacattac
caggtatcttcaagaaaccgattttcatcacgaaggctttttatacgcttagccccgtcctcttctaccgtggtaagtatcgtagccaaagcatttgt
aacgtccttggccatgcgctcagcatcaccacaaatgtaaaaccaagcgccagcacttattaattcattaacaagggcagattgctgaagaagcc
gatgctgcacataaagcttctgatcctgctcacgggaaaatgcggtgtataattcaaacgagtctttcatcaatttttgtatgtatcccattcttctt
tgtacaagaaatcagtatccgatcgtcgacaaccgtaaaataaaattgtctttgcaaccttcactccctgagaagccaaatgagcacgttccataa
caaaaccacggaagggggcgatgcccgtgcctggccctaccatgatgatagggacttcaggaggtgctaagtgaaatgtggacctctttatgaa
catcgggattttaccagttaatttttgacgaggaccttccaaagtatattgcaaattattaggatgaggagttaattctccattttgatgaaggcaat
gagcaagcaggtaattagtagtcaaaccataaaaaatatgattttgatcaacccattcttttctgtcaacaacagctgttacatggaccttatcagg
gcaagacaagctactcgacgaaatggaataatacctgggttttaaatgaccaaggttttccaaaagcaaagagaatggaagcttcgtataagga
ataccggaggaaacgcgacgaagtacctgtgctaaattcaaatgcatgtcagcaacgtttgctttgaagtaatccttatcgcttccaagtttagtca
gctcattcttggcttcatctgtaggagcaaaaggagcaattaaaaagatcaattgacgggaaattataccacagatttcaaagtaatagcgaaat
acggcatcataagttgtcggactcaaaataggtagcttatctagcggattaaaaggtttcacaataactaccatgttcctcttatccttcaaaccaa
gaatatccaaaagttcatcgacagcttgagatgggttcataggacaaatcgaaacgtagtcacctgtttggtatttcatgtttgaaccggagatat
ccaattccagatgaagacagttacgggtatcgtttttgaataactcgcgagaaacaacaggagtggaattaaaaggatttgccggagaaggagg
tgcttttgaaacgattcccttgagctgctgacgactagactcaccgaggaaaacggttgggctttcactggatatatcaggtttttcgataacatcg
tacaagggtttatactcttcacgtacttctttgagattgtaaaccttagcaatttcagggaatacttcgtctttccattgtaaatagtcctcttctaata
agccggcggcatcgtcacccaaacctataggtcctatacgttgagcccccaaagcgttcatagcagaatcaactcgcttagccatggcattgtaat
gttcataggtatgatttcctaaaccaaaaatagcataccgtacgtttgctaaggggcggtcttcaaccccatttccgctggagaaaatcgtatcttc
gctctcaattagttctatgaaactttcagcattgtcggttggttcaccttcaccataggtagctaagaaaaaaaccaaaagcttggaggggtcaag
cttatccatgtcagtaaagtcaaagtcatccaagtcttcggtagagttggcaaacttgtacttggacttggcttccgttgaaaaacggtaagcgaa
atcttcagcggtaccagtttgggaaccgaaaagtaccatagcgttcaagttttcctcttctatttttcaaaaacaccattattggaagaaaccactt
ctttttcaacgtgcttggtccgactggagaaaaaattgtatgcgaagtatccaaaagtgataattaaagcgataccgagtccaataatatctgttgt
cttcataatttacagcctgtatatttttctatgctttctaggctcaatcgatatcggagaaatagcaagggaatgaaaggaaaaggttttctaaaa
caagaaaagaaaaagaaaaagaaacgaatgttaagactgtaatctatgaaggtatgcaatgttgaaaacaagacaagtcaactgtattacaa
atcaaacgatccagcgttcgaattggtatctcttagctttctaaaaacaagtaaacaacgtgattttcaatagttatgaaaccttcgaaatggttgc
tactatactatgactttgctcaatacaaaacaataaatctttgagagtaaatggagccttacaaagattaaagattcaacgcaaaagaagatgag
actcagcacctcgaacgcggtagagccaaaaacgggatattaaaggaactgcaaaaaaaaaagagagaaaaaaggctctagggtattttcaa
gaatgtttcttttcgaataaattgcttaaataattccagcctcaaatcaagaacggctactgttcccgtgtcaatcaattaacaagtgcacggtgga
ggagaactgagagagttttatgagatgaaagaaataaatagaggaattaatggaaaaagataaaaactaaagagaaggcgatgaaaagggt
atgtgatcggtgctatgaaaactcgccaaacactcatatataggaatgtgaatttttagaatagttcgatttttttagaaccaaaatctgatacagg
tgaataacattcaaaaaataatgctctttttgaatgtttttttatgtgttggatgctcataaaaagtttgatttctgctattcaaggagttgtgatact
aacgagggtaaaattaggtaaatgaaactctaaaaataaaaatagaagaaaggaaaagaaattgcctatggctggataccttttgtaatgcatc
cagtcataagcaaccaaactcaattttgtacatatgtatgcttttgtgagacctattagttacgtcgcaaatgattcaacataaacatcaagaaaa
cagaaggtagaaaacgttggaaaaggacgcaaccctatttttagtctggtccttgtacttcaaaacttttaaatctacatcaaatagtaaacacc
aatagcaagttaagctggacattatattcatttattactatacaaagataaagaagaaaccagtctgaaagagttcagcgctgatacagcaaga
atgtaaggagatccttacctgtaggcttgatcctcaatggaacctggccttacgttcaagtaaacaatgtttaaattataagcaacatctgataaa
acgcgttaaaggaaaagatagagaaagaaagaattaaagcgcagaacaagcgggaaagaggtgtaaagaggaaatggaggtgaataggaa
gtatacgtaacaaatgcataaaattatattttcagaaagaaaattgccatttaggcaacagcaaactgttgagatttataggtacgttgttgaaga
gcatccaaataagaagttcgaagtttgcgattggttacaccgccgataccaaaggcaggaagaattctaatgacacgggcaaatgtactcttcaa
caagtttttacgaatttggaccatgcggttcgcaactgttatgttgggaccgccgcggttgtatatgtcgagagccatcaagtggaggggagtgct
aacaaactgcacaatgcaaggggtagctagttgagcagtaacaagaccaccaggcaaaaatggagcaatcataggtggtatattgaaagaaa
atgcaatggttagagcatcacggagaccataaagtccgtagctttgccagcccagaggacgaggagagccagtaccccagaatttggtgaaaa
agctgtctttaagaatggatagaccactgttagcagcggtgacggaggagaacttcacaaagcccgtagtttcagccttataaacagggtttttgt
aacgaacattgtcaatggtgtcgatggtattggcggtcatataagtgccggaataaatggaaaacacaagggcaaagggaagactaaaaataa
atgcgtggggacgggaaaggatgcgcttaacggagtgggagacggaacgcaagagagaactacggccgctcgtgttttcgacgatcgaacgat
caatggtaagaatgaagggggaaatcaacgcactagcagtggtggctgccatggcgtctataccgatccgtttccataggttggaggagggagg
cttggcggtaggaagaggttgatgttgattcaaggccattttaaataaacaaagaaatgaagaagaaagagtgaaagacgaacgacgtgtaac

aacgacaaataaagacaacagaaaccgtgaatgtgtaaaatcgataattttaaaaacgaatgaatccaaagaatctccacgaaccaagaaaa
gagatctttatgtcacgtaaaaacaaatgacaagtgtattctatgatgtctggaaagtgagtttaaaaaagcaaaaataaactatgcaatcaaat
gatagagataggcaatgtaagaaaccccgagaatacgcagcaaacaaatgaaacaaatatgcctgaaagaaagtagctcctgtatagaaaaa
aaaaaagccaactaacaaaaaacaaaaaacgtatcaatgacaaaagtttcgtctaacgatcgcggcagggcaaaaggatcctcaaatttacta
aaaggagtagtttcaaggaaaaccaagaatttcagatagaagaagtcaatcaagttcgttgttttcagaaaaaaaagcaaccaaaacaaaacc
caacttcaaaaacgaatggatcggtataggaaaaaggattgtgaaggctatcagtcagaccaactttcacgtcagaacacaagccgcttggga
agtgagaaaaaaagcgaaggaatcttccctctttatattggacgaaaggcagattaaatttttcaaaaccggatggcattccttcacaatcttaac
atagcgtactgcaacgcttcctctttaagagaaatctgatgtttttactatgtatgaagaacagagcagtttggattgttgtagccaaccaagtcaa
gccatttagacaatgaatgtatagctcattgtccgctcgctctattttgctcatgcaattatgcgagattggtttgtatagtcagtaaataaaagcta
aattaaacaaaacaagaaaatttgtagcgttcagtttgggctccagagttcccttcatctttacattcgctttgtttatctccttcggtacttgtttttc
ttttctttggtttattcttgcgtgctgctgcgtcaatatcccgatactaaaaatttttctatccttctagcgaggatcctttcgttattacgataaacaat
ttgcagcaaaacaagacaaggtgtcgaaaaaaggaccagggaaaagtggatcgaatgcatgttaattcaaatcttccaaacaaaccattgtag
gcttttatcctggtaattgttgctggtgattcatccattcttactggtacccttccatccgtcacttacattccattgcaaatcccaatcccaagtccga
cttgaaaatttagttgcaaactgcgtgacctttgatttaagaccttgttgcatgcatgtcatcatacgagatatacgagaaataaggggtctttatct
cttctgctcttccttggcggctttattttattcgcttctcggttgataccatgcttgataacgttcttttgcgaagcaaaatttttagttttaaaatcggtt
tctgcaatgaagcgatgaaaccttgtttgttacttttgtagatcaagttgaaattgtggagttctgacacaacccttgaggctccatgttttgagtatt
cgtcttggaaagggtagtagatctcaccatgtgttcagacgacacattctctcagtctttttttttttattataggaaagtttttataggtaattagattt
ttaatatttgtatccttgtctcctgtccggttttccatttcttggacactcattcgtcgtgttgtgcctagagagagtaaggccttgtgctcacggtttga
gttgcaggtccataaattcatgttcatgtccagtattcataagctttgtaaagccgttctttcctttttcccttttcttttaaaaaccaaaattaggtag
acaatctcataaccagattcaataatgctgccgtctcagtactcgcgtaaaagttacaagcgtccatcaaaactcgcaaatgatggacaaaattct
cttcatatcgctttaaaatttcttccattttgtccatccttacgtcgtcgtatgtctcctcgcctgtctctgcaagttgcaaggatcttgtaaatgaactg
gtgtacgacgcaaacatgccgcaagtccctagaagctttgcattcacctttagcaattttgagtttgtcaacatacattcttttaaacaggtatccaa
aaaatcaacatgctcttgcattaaattgtctacagtacgcgcatgttcaatacttttcataaatacaggccagtgcgtctcgatgacttcgttcatac
tgtagtagcgaattttctgtaccagtgtgagcatacgggttcgcagaaaccatgccttctttttccattgctcaatccgaggaaatggcgatttgtttt
tccaagtgggtttcttgctatgatgcagccaactgttttccaattgcatttctacacgcttgaccattaaaaaaaaccgaaataataactgatatcg
aataatcgcttttcgcgataaaattaaggataagggaaacggaactttatatccaaactgcatcgcttcaaatccatcaatttctttttccattgctc
ctttcgatgagaagctttgactcttttggtcactcccactgtccatccgattcgttaaatggtccatgattcccttttcactttgtaccaacgccgaatc
ttccaatccactaatgctcacaatatgcatcagccagtcaattaaagacgtctgattcaattcaagtgtcaagtactccttaaacgggtcgactgct
gtaattgtccccggttgccggagggacaggtccagctgcgactgtagtttggttgcggaaataagctttacaggctttcgtagttcttgctgtgccgt
atctaaaaaggtagagaaaaaatcagactgatccaaaaagaaataatgtttcaaagcatgcaagtgagaataaagtgactgtgaagattgcag
caacagcaaaagtgactcgtttgcatagacatatgcatccataatgttttgggtaaaattttcatcttcaaatgaatctggccattcatactttttttc
ttcagggctgactgtaaaagtttgacaagcattctctccagaggcctgcttgcactcattaacaacattcaagtacttccctgcaagcaaaacctta
tctttaagctgggatagctgtgtaggaacctgatcttcacgaacaacatagcgcttttcccagtattcgtctgtatagtcatcgtcaagcctttctga
acttaaacttttgtgagatttgatcataaattcttcatatgggtcgtcgattttgccttcgtgaatccaagaattcaagaatctcatatagggacttga
ggactctcggagaagaaaggttaaaatgtttcgagctatagggtcaccagcataatttgttaaggcatctgttaagtaggttagaacacgaccac
ctttacagactttttcttcgatattgaactatgcataatttgtgatatgtcatttccctggtttagcttttccagtaagtcgtcaatattgtccaaaccc
atatcgtcctcctgctgatcatgctctgtcgacaatacaagatcgcgtaatataaaatacactagtctcatagaacgagaagtaggtagaatatat
aaacgaagagtttgcaaagaaaaaccaggatccacttggctcctgtgttcgcattgcatgagaagtacaaggtaatccgacataaatcttctcaa
agctgcacacaatgcatggtttatgtatccatattcaaattgacttctagattcaataaatgtaacaatactcaagtagtacgagcccatttccaac
gtttcagaaacgacatccttaagccccaaatcgaaaccaggacatatagaaaaagacggaggagaaacgattctttgtgaatctgttagattgg
aagcatcttgtaaatgaagatagttcccttccatccccataaatgcgtttaaaagctcatcaatcaaacacttttcttggtctcgcagagataaatg
ggaagggttcctggtattttttcaagatacaaataatttaattgcgatctggcattaagcggcgattgagcatcctgtattaaagaaacccgatac
acattttgctcatgagaagaactcttggaaagtggctctattccagcgtcttcattcgagagcatagaggcgtttgaccggaccataagttttttta
aatggataaatcgatagaaggaactataatttgtttgaagctcggtataagagatttactagtataaataaaaaatatgccttttaaccatgtaga
aaggtaggagaatgaatactttcttgtaatatcttattacttttggtttcacaaatggttgtaaacactgtcgagcttgatgatatgtcaggtggaa
agtatcgttttacaggtatatacaatgcaaaggaacatatagagaccttcttatttaaatggcagagaatgagtcagatgcagtggaagcatgga
aatgatatgcaattaattaatcagggaataaaaattacaaattcatcttttggatgtgataaaacacatacaaaatgaagccttttactggttttaa
ctactgaattgtaggatccagaacgacttgcattctactatttttttttttaatttttttttaataaataaaaaaaagaattgatcgacatgtacgctgg

tcatttttttcttcgttttcactatttgtttacactgtatacctgacatttagggaacttttatttgttctcaagcagctgacattttctaccatgaaaatt
caaatctaaggttaactttttttctttttttcttttcttttctcatgattcatcttttccttcttgtatttatgcgtatttttttttgtttactcgaatctgataactc
gattttttttttttttttcgtttgttgagggccactcaccctttggtaaaattgcttattttttttcaccctgaactataaaatttgcttcgtttgttatatgc
aaacagttgtaaagaatacaatacatttttgtttcttctagtgaaatttccaactcatatgcagcatggttttatttgcttttgtagtgatatatgttcg
ttagctatcttcgcacttttagaaagtataaaaaaaaaaaggatttttttttcttcttcatgtgaaactatagtatagtgtaaatgtttgctaattgtaac
gcttgagtagtcaattgaggaattaagcggtatttcgcttaaatacgttgtaatagtacacaacctcagcaaaacacaacttttacaaaaaaaattg
aatattgacggttttctacagatggtaagatgcgttataattagaaatcattattaaaataaagatggcgatattaacttacatctagactcgggaa
actggtaatactgtcttggtgaccggcggtgctggatatattggctctcatacggtaagacttagaaatagggagtttggattgatttagcgttgga
agactgttttgtttggtctcgtacataggagtacaggaatgaaaacttcaaatggtaacccctttactatatgcaatcgaggagtgttttccataca
caacattttggactttttgctgaaaaaaatacactcgaaactaaatgctaactgctctaggtaatcgtcttgttggagaagggatacaatgttgtta
ttgttgacaatttgtgtaattccagattcgaagctattaagcgaatccaaaagttgtcagggaaagaccttatattttatagggttgatttattgaat
gagaaagatttggacgaggtgttttcaaaacacccagtctcctctgtaatccattttgcaggattaaaggcagtgggtgagtcagtaaaaatcccc
cttacctattacaaaaacaacattactggcactgttcacttgctggaatgcatgaaaagacataatgttacagattttgtgttttcttcgtccgcaac
cgtttatggtgatcctactcgtcctggtggaaccattcccattcctgagtcatgtcctcgtgaggctgccaatccctatggaaaaaccaagcttttca
ttgaaaacctcattgaggacgagtgcaaagcttccggatcattaaacgccgcgcttcttcgctacttcaatccaggtggtgctcatccaactggtga
attgggtgaagatcctttgggcgtcccaaacaatctcattccttatattgcgcaagttgctgtcggccgtcgggaatatttgagtgtctttggtaatg
attatccgactcccgatggaactcctattcgtgattatattcacgtctgcgatcttgcagaagctcatgttgcttctttagcctatttacaacgaaatt
atgttggatgccgtgcttggaatttgggttctggtactggcagtacggttttgcaaatccatcgtgctttttccaaggctgtcggaacggaacttcctt
acaagattgaccctagacgtgatggcgacgttgttaacttgactgctgatccaaagcgcgctaatgaagaactcaaatggaagaccaatcgaag
tattgatgaaatatgtgctgacgtctggaattggcaacgtaagcatccttatggctttgacttttacaaggctaattctgatgccaatttaaaataat
tctagcctgtattactataacaaacaaaagaaaaggtcgtaattggaatttgctctcagcttgtttgttttaattttcgctttttcgttttttttttcgctc
ctgtttatactattaattatttaattcacgaattagtcttggggttcataagttttactgaaaatttcctgtctgattaaagtacgtgacctacactcgt
tttgacatgatgtacaaagtattgtgtcctttttttttctttctaactatccattgagctattttccttctttttcagaaaacttgtgcatgctgttaattta
cgatcctctcttttaataaattattggttgtggccatgtttcggaacgtaatttagttattcttttctattttctttttatttcctttattttcctaaatatta
tctttgtacttgcaataatgaaaggaaataaaagaaaggtcaaattaccctagttaccgacaaagcattatgtagaaaccgattaaaggcgtatt
aaattctttatttaaattatttcgaagaatttttttaccaagtggttttcttgtaatatacacggtaaaccctacataagaattgcgcacaaggtatcca
tccatgcacacagaataccaacactgttgacattacataaaagggtactattagctatatctgcctgtcatgccggaaaaagagaatatagaaga
agaaaacaaaaagcaggtggaagaaaaaagcattgaagcttccaaaaccgaggataatatggcaacgaaacgtaagcatgaatttaatgaag
aaaacgcggaaatacccgagaagaaaccaaaagaaacaactgtagaagaaaagaatgaagaaaatgttgaggaagtggacagccaaaaag
agaaaaaagatttggtaagtattagcggtacaattacttttggtgcattttgattatattatttaagggcgcggaacgaatgaaaactaatatgtcg
tttagaaaaagattgatgacacatctccaaccaaaaaaccgtctactcaaacagaagacgagtctggggctgaaacaagtacctcaaaatccga
aggaaaacctgtttcaggagaggaaccaagaaatgaaagtcaggaaaaagcagaaccatcatcaaaaccaagtgctaagcttcctagtttctc
caagtttgcgtccttttccagttcatcctcttctcctttcgcatctatagcgacttcacaatcaccagaaagaaaagaagatactactccttcgaaag
tgcccgcattcggtgctaagtatgcaaatctcagtatgaacgaaatattagcaagcacaaaacaggaaagcaatgacgccgaagatgacaagc
atagaaagcaagaacaagaaaagaaaagctttgatgagttattaacaaatgcaattcccaacgaatctgctgcaactcctgaatctagtgagga
tccatcagctgctaaggaagccgcaacagaagattcagtagaaaaaagtggtatacaaattagccagttaagtgaaaatgaaatgataactgg
cgaggaaaaggaaaagaccctttatagtatccgtgctcgtttatatgtcgttgacggagaaaagaggatttggaaagagcgaggccatggagttt
taaaggtcaacgttcctctggaaggatcagaaggagtccaccgcttagtaatgcgaaatgatgcggttcatcgagtcattatgaacgttcctttgtt
tcaaggaatgtctgagcaagctttccagattgcgtcggcatccagcggtggttctgcaaactatgtaaaggtttttgtcattgaaaatggaaagtct
gttttgtacgcagtacgtgtcaaagacaacgaatcggcgagccaactcaaggatcatatagtagccgcaatccctaaggaaggcgaaaacagta
atggagaacaggcgaagaagacaaaggagtcagtttaacgctctttgttttgtttcgaatatcagagttgagaatgaattaaagaaggttgttgtc
actcttttctccccctttttcttttgtcctcaagagaaaactattgcccctttttttgtttatcccatatcacattctcaatcctaaacagctactgtcatcg
atacgttcgtgtgtttatacagctatgtttatgttgacaagagtctttcttattcatcgtagaaatcatattaggaaccttgtatcctcagaatataaa
aatgaatgcaatgaagtttttttactttaactttttttttctttcttggaatgccaatcactaaacccttctctgatgaagctatatttttctaatgtaaa
atcctttttttgcaaatctaaacacagcagacctttagcatcctacttaaaccagttgtagcatttaaaaacgcctaagttatttaaattcattagtgtt
ctatattactactaatcctatatcgttgtctatacatctcaagagaataatatcgacttaagttattatattacttctatgcaattgttcaatgggagg
catatggcgattctcgctttaagtgtgtttttacatttagtttatgtcatgaatgcaattacacagctaaaacatatttgccttgtgaaattgcacgta
gagcaaagatgatgctttacgtcagttcacttacattgatgcaatgtttcaaaggcgtatatatatatgtagtataatccctatgatcttcttcttacc

aaagacaacatttaagaattggattcacttgttaacgtaaatttcccaggtcacgattatcaccttgaagatttcgagtttgagtctcctttcttgttg
cttgcataatatttccattactacttctctcataataagaattgattgaaattgtaagattgatccttagcatttgacttgtttacttgtcctaggtgct
gattcaagattggacaccttttacactttcatcaaaaagagtattgagctaattccctttacaattccataatttcattaaggttctccctattatcctt
tcattcttcatttaccaattttatttgtgttctcttattgcataacttcgcattatgcgggtccaacaattatttttgatcaacacccttgcggcagttgg
gctgtatttttgcttttatggattctccacatacaaaaatcaggatgtctctgcaatcaaaacctggctcgatgaacattccgtgccatacggtactc
gatcgagtcctgctgagtttagggactttctcgtcgagcattatgagtctttgattccaagctttaaaagtgtaaatgattttcaacgatccagctgg
ctcggtaaaaaaaccaacccttcgtattttggcacagccaaacacaagatcaatcaagctagccacaaaataggagacgcggcttctgatgcaa
aagataccacttctgatgtgtgggaggaccagaaatctcgctttttttgatgcttggaacgactctcaacttcgtgctttcttagctcgttacagtgac
gcatttgctcctaaagagaagaaaggcttgcttgagaagctctctcctgcttctcttcgtaagctcgccgttaaggagtataagcggttgggttcca
aactaggtgaaactggcgattgggtatacgatacctggtctgacaatgagcttcgtacctggcttcacaatatgggggttccgattcaggctcatg
agtcccgtcatcatattctcgataaggtcaaaaagacagtgtctgaaaagttagattctggcaagcccatcattgaatccaccaaaaaggatgcc
acaaagaaagccggcaaagctggtcattatgttgacaaagttagtgagaaagccgaagactctgtggattatgcctctggtaaaatcactgaaa
ccggcgagagggctagtgaaagcttacaagaagaggctgacaggcttcatgaagcttcaaagggcaaaggccgcatcgctacttggtggtctga
atctggattaaaagcatatttggacgctcgcggcattcctgcttatcaaccatctgctcttgataaactatgggcaatggctcgtcgccaatactac
cttaggacgaacggatacaaagctttgaaagaaaagacaaaggagcatgcatcttctgtcactgacactgcttcaggtgccgcttctgatgcttct
acggcagcgaccgatgcattccacgctgcttcttcaaacgctggccaactcaaagatgagattaaaggcgcaggaagctcaaatgcacatgttgt
ttcttcgaaaactggggatttaaagcataaggcttcgggtgtttcttcttacgcatctgataaatttgaacaaggttccgaaatagccaaagcccaa
aaggaagctgcttcttcgaaagctcgcgaattggttgatgaaaccgtgctagaaaagtggaaggactctaagctgcgcgaattcctctatttgcgt
ggtgtacccgtcccaaagaagagcaacaagcaacaattgatagacaaagttcgtaaacatttcaacaaaggaactattcctcactgggcggtgt
acttcaacacgcttagctcaaaggaattaaataattggatccaagattacaagaaatactaccatggcaagcttcatccttctaaggatcgcgag
catttggttcagaatgcttgcaatatttatcgttcccttacaaatcaaggcgataagtccgttcttaagaaggcccagaagaagttcccaaaagctt
ccctttccgaatatactgactggtcaaatgaggatttgaagtctgctttgtctgaatacggagagccaccagcaaagccttttaatcgccgtgatgc
tattgagaaattaaaacatcacgacttgttgttttatggacctgttctaagtgaaggacaaacccaaggtatatttggatttttgcaacgtgcagcc
agttatttagggtttggaaagcgtcctactcctgagaaaaagcttgaagaagacattgttaaagcaaaaaaagatttaccgaaaatggcttctaa
gtacgctacatccgcctcagactatgtggagtcaaatgccaaaaagttccaaacagcttaattggttttgtttaagtcccaccataatcttgcttttt
ttgttaagtttttgtatgatgtatagtgaattaatatatataatttctaacctgatcaaaactcagtttagaaggaaatattatgaatgaaattaatct
aaggaaaaaccaaagcagcgaagtaaagatagaattactatggatccaattcagcatcattaattgacacttttcaaaagaatctgctgatgaat
atcatctaaatccaaagcaattgctaagataggaagtatctggtcacggtgttcttgattctctaaaaattgaatcaaaacattccttgcatattcct
tgtcaataacctctttcgagagaccatttaagtgttctgcttttgattcacctgcactcaaaagtactttcggtgtaatctcattgcttttaacttccga
aagttgtacaagagtctcatcctttctatccaaaacttgtatttttttgtattaattctttaacttcgtcacgagaatcgactaattcatttcctacatttc
gtaacatctcctgtaagttttggttctcttcttgaatcctagaagtttcctccgatttagcatcattagtttctttcaagtatttaatttcttttctcaaac
tctccatttcttttgacaagagacctttcgattcatcagcatctacattatttaatttcgtttctgcaagcgttttccttagagcttcaatttcctcagag
aagtccttgttctttaactgtaattctgaaattaaagaatctttttcatcttctaagactttattttcttggtgttcaccgttggcttcggtagtcactgtt
ttcttcttcttcttgtttttttttcttcttcttcgatgaagagctgaccgaaggtgcaggcgagactgtagagttttcttttaagccattttcttggttttgc
ggctctaaatcatccttaactgctaaggacgtagccatcgtagcatatagtagagaccaggaagtttcacttccagaaatgtgtccagcaaaagc
ctcaaatattcacgaatttaaagccttactttctctctttttttgttaaaaattgataaaaattaaatacaaatacaaatacaaatagtgaaatgttta
taatgactggaattcttcttatgtacacttcacttcttatagtataaaagtattgttgaaagaaccccgtttatgttaaatatattagtaactttctatc
atattatatgtaatatatattttcattcatgagagaatttgaatcacatttaagggagaggattaccaaatgcatgctctttcaagtagtcaggtcct
ttttcttgatattcagctcgagatatagacacgttcttgaattcttctgtaacgctccattcagaggctcctttccatgcatctagcaggggatcggat
gcactatatacattcaatggggaattgactggtaggacactcgttaaatctctttcaatacgctcaacaattccaggaaattttgagagacctcca
gttacaacaatatttttgacgagtttattctgttcttccgagttatgtctttgtaaaatgccgcgaacaatctcaactattccagcttgatctaagcca
acaatgctaggagtgaaaagaacttctggaactcgcaggcgttctacgttaaaatgaagctgaaacgaatgagcaacgctctctggatcaaagg
gactgactcctcgtgtaaacgcatagagcaaagtacttcggggatcagtaagggcatcatatgtattttccggttgaaataaaggatcgtattcta
aaagttgtttttctaatgcataaatttcctccacgaaacgctttccctcttcttctaactgctcagcatttagaacgtcatggtatactttccagtcttc
atcactagcgccaaaggtatcttctgactgtccttttcgtcgcctttttttgaataggttcactggctaatgtagcgagacttttcattctcatttgtgaa
gtgtgtgatttgcgatcgttaagctcgaatttcagccttcgattttcctgaattttatccaaaagatgtttgtgtaattcacgcttttgcttgacccaga
ctgaaaaactttcctttcgtaaatgctcatcagcttcttgtttttcttgcaaaaccgcctcttcaactgccttatcggcttttgcacgcatcctggcatc
ataatttgccttcaaaagcttctgatttcgtttttgacgaagtccagcttcatctaattcagaatcagggacatttaccaaaggaatgcttgctcta

catcaatatctgcgagatcagcttcagaatcatcttgttgatgttgctgttgacgtaaagcatctctttctgccctgcgaattctagattgcacgttct
tcaattgggcattcaattgagcttcgtcagaaaacccagcttcttgtaaagctttttgatactctcgtttcgaaagaacaccactttgttcctgtaagc
gtcgaagtgttttgagctctttttcgcgctcagcagctttttcttttcttttaatagcagcttgagcctggagtcttcgcccactttctctctttctttcag
caatctgttcaagttcctcctgcgatttttcttgcgctactgcttcggcgtaaggaaattgcaaaacaacgtcctctcgggctaaaatatccttgtcta
gcgcgtgagcaacctcagtattataatcaggagaaattcgacaatagttatgcataagagattgagcctgagaaggaagcattttgatagggaa
aaatggatatttcatttggaaaagacggagcaaataagatgatgaggaagcaccaccccaggaaatacgtttggcttcgtgggcaagtttctggc
catccaagataggcagaatatgagaagtagaatatccaaggtttagagctataccactagaatccttacctgcattatggtaaaaggaaaaaag
accatctacgccatatgcaaccgaagggacctgatataactcaaacagtagttcagtcatcgtggagcggctataagaggggttcataaaggatt
cagtcatgcaaacaggatgatcttgatgatcaattccaagttttaagaaggtataatcaagtacactttccatcacatcccagttattgataacatt
ttgctcgaaaggagagcgtgcaatagaacgggcaccaacttcaaccaaagtatcatttcccacaagtgtggaagtcctggaaagctttctgtctct
gtatctagacattagattgtcgaatttcagccgaggttccttttctcctccccatccagcgcgaagttgccaagatcctagaaacagtcttagtatca
atcaaaaataagttaagtgagggcaaaatgaagctcaaatttgcttcgctaaagtgtttccctgaaagagctctttctgataaaccatcaaaaag
aattctcaatatttaacaccaaacgaaaatgttataacttaccattatcgattactatgggaatttcgttagatacgttgctgaacgctggcgtatgt
ccaggaaacgtaggatcacgaataacatgaaatatgccagtcattctgaaattaaggcaaaaaaagtaatgaaatcagaaaatgtatttaaaa
gattacaagcactgattcatccgataaacccttgatacattaccaaaattagtaagtgctcaaaggagagtttatcaaaaggcgtaaccagagat
tattgttcgatagacaaggtcaaatatcaataatcagcaaattgaacagtacacttttgccttagctattgggtacagtgagtgagaacaagatag
ttcgtaagtctataaaacaatctgtatgtgagatccaataccaaaattttgagataattaattaactggaaaacaaacgacaaaagaaagtcaga
caagcctttttgatggtgcaaattaaatgaatctggaaaaagtacaggcaattgggttgacgagctcagaaagtagaattaaaaaaatctaaat
ataaaaatgagttttagtagttagagatatttggaatcttgtaatgaaacgctgctttcacgttcatgcacttcgttggtgagaagaatcgacttctc
gaattgcatgcacagtagttgccgttagccattcttctgcactttgtgacgaaaagtgtgaattgacttgttaatactatttaaaggaattgaaaac
gcatttaacaagtcagagaggctttttttttcgataaaaactgacaaatagtagcaaactgagtctcataagattgaacgtctataacatctgtaat
caattaatcggagtttttataattacaatttgaatctttcaagtctcaataattattatgggacgagctgaagctggtaccccaaaggctatatcga
atgctcttaaaagcaaaggattgcagcgtttacgctggtattgttctgcttgccagaaacaaatgagagatgagaatggattcaaatgccatacg
caaagtgaaggtcatattcgaaggatgactttaattgctgagaatcccggcaaacacattcaagatttttccaaccaatttttgcgggatttcgtta
cgcttctccggaccgctcatggagagaaaaaaagttcaatttaataaattttatcaagagtatattcgagataagcaacatgtacatatgaacgcg
actcgctggcatactctaacagagttctgcaagtttttgggtcgacagagcatttgtcgtgtggaagaaacggaaaaagggtttttttatttcctatat
agataaaaaatccagaggctcttcgtcgagctgaagctaatcgaaagagagaacttcaagaaaagtctgatgaggagactcatttacgattatta
aacgaacaaattcaacgagcaaaggaagcttacactatgcaacatgcagacgaagagaacaaatctataagtcatgaactacaacgacctga
aaaccaaggcgttcaactacaattgtcttcatctaccccttctacgaatgaaaagcctttttcggaaaataatttgtcggatacttcctccctctctcc
aaaactagcagctcctacaagctcctcgtcaaaaccgctttttttcagtgccttctaaaccgagatcaaaaaaatgtgtttgcaacgttggacaagtct
cgaaaaaaagacattagagccttctgcaaatagaaaacaaaaagatgctgataccaaacgtccccggtcagcaatggaagaaatcattatacaa
gagactgcacgagagaagcgaagacatgtttctacttaaggatgtacaggacatttttttaaacgtctcttttgtttgcaactggtagcttagggttt
atatttattctttagattaatgtacagtagtagtggttgatataacgattatttatgtcaacaagttaataatgaaattcaaaaagatgaacagata
cttggatacgtataatgccttgtatgacaagcaatgtggaatgttaggaggaaacttgttgctgtgaaataaattaagcgaacttactttattataa
tcagagtattcacagttagttgtatccaccaactgtctatattccggacgcgttgtccaaattcatcgagcgctcaatctattcgtgaaccaacaatt
cagaaatggcatccgaattcataaatcaggttcagcagcttttggaaagaattcctccagtaacaagatatgcattaattagcatgattacattaa
cagtcctatgtttgtgtgagcttctttcactgagtcaatatgcattaagcttccattctgtttttttacaaacgtgagctctatagactcatcagtaactt
cctctttgtgggaacaaaattcgatttcgtttttaatttgtattcccgtacgttcggaagtgctttggacttattttcctgaaattgttgctaatggtaat
tgctttagtttatcagcatggatcgttccttgaaaaccatgtttttcaaagaaatacaaaaagctacataatttatttattaaaaataatcctcatca
tagacgtaagcaaatgactgagtcagtctgctggtttgctttatgctaatgtctttgaactagacttttagtttactctctggaataggttcattccttt
tctcttctctttcctgtgcaatggcttatacatggtcattgtacaatgcctatcagacggtacaagtgtttttcgtatttcagcttccaggaaagtatct
tccctattgcttacttggaatgagtcttatttccggtggcctaggtgccttaattgtatccggttttggcattttagctggccttattgtttctaagttgg
gttctcttcaaccaaactcggggttttctgttcatgcacctagtaatcatagtaccaccagtgagagatcttcgactgctttcaaaggccgtggaaa
aaggcttggtggctgaaaatgcaccatgtcgctggatacgcaaaatggtgcaaacgaagctatgttgcttttcatgccgagatgaataaatcactt
tattaaatcaagtatatttaaaggctttatactgccgtctataaatatctataaagaattccattgctttctaagggttctgagactatttatgaatca
aactaataaattcagtaaacaactgaagtatattatttcttgcaattaaaccaaagtgtaactgttttagtttttcattcccaatatgctctcgctgtt
gaccagggaccttccacactattttcctagagccaattgaggtcttccaagactaaattatattatggaaggctcaaaatggggagatttttttaaa
aagacgttgacaaacgttgaatcaagccttgacaaagttttagaggttcctagccatgaaggaggaagttctgaggcaccgaagtctaaagacg

aggttattcgtaaattggttgaagaaggacatgctttatccaaacatgaattaaagttgaataacacaattaagaatttaaggaagaatttgaatg
aagctgaatcaaaattgaagagactggaagttgcaaagtcgagagagacatccgatacaaatgatgattcagaagttaacgttcaagaacgac
tgggatctatcaaaaatgattatgagaatcgaattgttcttcttgaaaaagaaaacgagttattaaagcgcaaagttgaagaagctacggtagat
agcatggaagttgtccgtttaacacgacaagttgagacattatcgactcaacagtctattcaaaaaagccaatggcttcgctctgaagagaagct
taaagctgaagtagaatttttaaacgaaaataaccgcaaactggctcaagccgctcaagaagagtccaagactgccaacgaaaagctttctggg
tttacagaaaaagaagaagattacaaaaaacaattgaaatctcagaaggatgaaatcgagaatttattaaaatctaactctgatattcggcaag
agtatcaattaaagattcaactcttacaacaagaactagaatctactgcggtcaaagcacctactgagacattcccggatacatctgagactcttg
atactaactataccaatgaagatgacggattttacgctaatgatgatgacgataatgaacatactcctacaaatgtttctaaacctaatctggcaa
ataattggaacgacatagataaggagaagcaaaatatcgattcccgttcagatataaacacaacttcgtctaatttgaatcgaaatcgatcgccg
tctttggagcttaaggaggagaacttgaatgaagagagcgggtttttttccaaaagatgattttattcaggactcagaagttacaaagcaagaagt
tttggaaaactacttagatacgcttgcgatctctcctacagaaccaaaagcaccccgactgccgcgaactgtgtcagaaggtaaaaggatggga
agacagtcctcatcaaggttcgcttcaatgaatagcatggttatgtctcccatgtcagcctcacatgactttgacgctttgtcttctttgtcatatacg
ccaagtaatggcgaaaagccagcgaataacactggcccagatatgagtcttctggaacaactatcatcgacgattcgccgcttagaggcagaat
tacagactacaaaacagcagattgtacagctcattaatcaacgcgatcaagcacgccaagaaattgtaaattcgtatgtggatcgagaaacgaa
agaagcgtcagaacgagaacttattgagctacgtgagctatacacaaatcttcaaaagaaacacgtagaaacgcaaactaccttaaagcaacg
aagcgaccgagtctttgagcttgagctggatataaaagagatgcgacagctttaccaaagtcaaattgatctcttggccagaaattgagccaagg
tatataagtttgcaaaaggaattcatactgattggtttgcgaccatagaacatatagacatataatcttaaaatcgtcattttattaaacaattcttt
cctgagagcatctgtgcttcacataagaactagcgaaacccagataaatgtatcgtagcatgaaagaagccacatcacttcatcccaatattta
atttttatcataaccaagccagactaatgatcactactgctcggatttttgacttaattgctgccgcctagttgctcaagtacagcttcaatttggtca
ccatcctccatatcaagctattcaaagtaagattcatattcaatagaaagaatggttcatttatacatacctctgctggagtttggtcaggtctaata
cgctcaccgtcgaccaagaagcgcaaactattcaaagttttaccttgacgagcacagtaaattttcataagcttgctaaactcggttgttttttttat
tttaaaaaaaacctcattgttgtcctaaagtcattactgcgttagcctctattgttcttgttcgtttaactcaaagattttgaaagaaagcggaaatta
gaacaccatagacgaatattatgatctcccgaatgctcagaattctactctcaaaattaatcttacctgtcctacaactttgagattaatgtgttcag
tcgaaggtttgacatcttgttgagaagtctcgccgccagtgggagtcattgctccattatcaatattagatacattcgaagctggtgaatcagacat
cttgtgattcctttgtttttccaagtgaaaaggactgaggtgtatatcggtcaaaagttttatggtgtatacagtaatccatccgcggtgtctcctcg
cttcatgcgcggtccaccgactttgtaagacgtcgttattcatacataatccgtggctatttatttatgtttaacgaatacgtgcgataatagtttaaa
attaaagtcctggaacatttcctggattatagatttaggtcgatatacgtttcttatttattttctttattgtttttattatgtttcgaaggttattagaaa
ggtttcatatgtacatatcatattaaggaggaaccacagtgtaattattataatgctatgtgtatggtattaagcacttaacaaaaccgtacaccca
agtgaaaataggaaagttgagccattgagggaccaaagtcgctttatttcctcaatttattatttaaacagctctctctgattagtagagaatctgg
aagtgaaaggatatttaagcgcctatatgtttattgagcgcaggctactgtccggttaaatagactataaacgaatccttacgaagcagatattctt
atttattttatcaggcatccgtcatgtcttacatgaacaacacatttcaatccaattatgattactccaaagataataactttatgtcgaccgacatt
ggaccctccatgcgggagttgaaagtacaaaatcgacgcagtaggccaatggagaatccaggtgctgacaatccatatattcctaaattcatttc
gacagctccttggtacgcgcaagatgagcaagctgtggaaaggttagcgcatcaacgactaggaaagaaagaaccacccagtggtggccgctc
ttctattgaggaatcttggtacgctcgtggtaaaagagcaggacctgctgcaaccaaatacaggaaaggcgcttgtgagaactgcggtgccatga
gccataaattaaaagattgcatggagcgtccgcgaaagcgtggagcccgctggactggaaaggatatccaagcagatgaagttatacaagagg
ttgatgttagttgggacgccaaacgtgaccggtggaatggttttgatgctagtgactataaaaaggtgattgaacgatatgaaaagatggatgctt
tacagcagatgcataatagcacagagagacaaacggaaggtactgataacttcggaaaagatacgccgtcatctacaaataaagaagaatcta
acggtggtgcagactctggagtaacaacgcccagtttgcgtatgcgtgaagatgtagtcggttatttacgtactgataataaaggtcttcagtacg
aaccaaagtcaagatcgatgcgggatactacagggtctcatcttttgagcgattccacaaatggatctggtttcatcaaagcttccggaggagata
aggaagactttgagaaactacagacgtttgcgtgggaagctgaacgaagtggaaatcgtgtgcatgtagtggctaatccaacggctggtgagtt
ggagtatcggaagaatcgtcagtcgaaagctgttaatcaaaaacaaattgaacagagcatcttggaaaagtatggagatgggacagcaaagag
gaaacgacctgcaggaccatcttttccttccaacagtacattagattctgctcctagtcaacaggatgtctcgaaaaaggacgacagcccccagc
gggacttaaagacagagaagccaaccggtacagagaatgttactattcttgaaggtgatttgtgagaaaactcaaataaattaaaacaggaagc
ttttacttttacaaatctaattgtatgatttttggttttggttttgcacggttgatcgtcgttctcgttattatacttagtgtattttaacgaactgaaact
ggaccaggtggaactgagagcaatagaaaatgagtttttttactggaaccatgtcatctaaggtatctaaaaagaagttaaaatctttagagtatc
gatcgaaaaagtttgcaaagaaatcgaagacgctcgaagatcatgaaaaagaggtagaagatcgtaatgttgctcaggagcaaaaattgaaca
aaaacgctcaagatgaagcaactaaaaattctgaagccaagacaactgaaaaggataagcaagaaaagaagcggccattagaaggagagtc
agagccacccaaaggaaaacttaaaaaacaaaagactgataaaaaggaaaagcgagtcattttgtttgtgggtaatttgccaaaaaactcgga

cgtagaaacattgcaattgcattttaagcgagctggacaggcgcctcaagtacgaattcccacagagaaggggacaggtcgacaacgaggata
tgcatttgtagagtatattaatccccgctcagatgtcatatccaaggccctcaagtttcaccacacactttataaagatcgaaagattaacatcgag
ttaactgctggtggaggaggaaagaaggagacccgaatgaataaaattaaggagaagaacaagaagtggactgaagaacgtagggagcgtg
ttgccggcgaagagaaaaaggcaatggaagctaaacactcgaaagagtccgtcgttcaagaaggtgcgcagtccggtattcatcctgatcgtct
gcatatgctaaaataagagcatagtgctttctaaaactacggaaggtttaagagaaagcttctgtgagggtttaatcatgaaatctgataatttga
gtatataattttatttagaggatacggtactggttacaatttaatttgtttattttcttggaaaatcaagagtcatgttttaggtattgcaaatcagtct
ttattattcaaagcagaatagtgtttgacatttggatgcttttatgacgtgtagaattatattataacaaattactctactatagaggttttataaacc
agtgtgcatcattggaaaagaagaaattagaaattagcgagaaaaaaatatactatttggcaagtaaatgtgaacctcatatcggcatttttctgt
ttatttttcattctgaatttgcgcaaatgttgtttttgtgcataaaagtttggtttggttctgtgactgctttttcaacaccaaacattttcggtagaca
aggtggaaattaccaacccataattttcctccaacaggacgcaacatgcctcactcattcggtctcagagctcgtacccgttacactttccaacgtg
gcttcagagagcatggtcaaattcgtttgtccacttacttgaagacctacaaggttggtgacattgtcgacatcaaagccaatggtgccgtccaaa
agggtatgcctcacaagtactaccatggtaagaccggtattgtctacaacgttactcaatcttccgttggtgtcttgatctacaagattgttggcaac
cgctacatggagaagcgtgtcaacgtccgtattgaacacgttaagcactccaagtgccgtcaagatttcttgaaccgtgtcaaggtcaacgagga
aaagcgtaaggaggctaagactcaaggtaagactgtccaactccgtcgtcaacctgctcctcctgccaaggctcacgttgtcgctactgacaaca
acaagcccatcactcttcatcctgctccttatgatacttttatctaatctggacggcttcatttgttgtaggagaatggttaaaaaaatttttgcttaa
catatgctagaaagtgggtagtgacttttagaatacgtaggaaaagagtaaacatggtgtaaacagagaatgaaagtagtagatctgtaagag
gtaagtggtaagccaaggaatgcattgaccatttgtcttgatgcttcggtgaacttgtaggaagaaaaccaggtcgaatctatctgcaatgatgga
aaggaaagcgtgggtagcagagcatggaactatgggtgctcgtgggattgtgaattcgtaagtttgttttcaattgaatgaaaaggggttgttctg
tgaaacctctagagcacgatctctggcatttcgaataatattcttgttgaaaatatttaatagtggagttccaaaaagggaatgagacttgtgtctt
ttcaaccagatttatcggtcaattcctcaaatgcaatcgaattccatcctttctactacttgcgatatgttcaaggaaatactgtacggtgagcctaa
tcatccaaataaaagtcgaatcaagtgtagtttatggcattcgatgtctaagaaaaatatatagctatgtacaaatgatttatttcttgctttttca
ggaacccttccccttcataagcaggctagttttaagaaatagagatttgacaaatgggtttgtgaagcatttccctaaaaagttagcatgatctttc
catctttgaattctttttattttctaaaatatgaatcgttaattcccgctacagattgtcttcagtgagctaccaaatctcccatatttctccatctacta
aacacctttactttatatattctccaactgctatgaggtaggagacaaaccacataaactggaattgattaaaaagaaaattgcatctttttcgtat
ttatgttgaattcattcagccattggccgtacaaatcatgcttttctgcgcggtcgagctggagacggttctttagtcaaaatgtagtctctatcaga
cctttaagccgatcttttttttaaaaaagaatattaaatacaattctgtacctttttcatctccaagctcacctgatcctcaaattttgaactttttaca
gaagcgtattcaaacggctcctcgcttccccaagccaagcgctttgctggagcttggcaaaccaaggttaacggttttggttgtcttaacaactatg
tcatcttatgccttggctccatatcctggtctctcgtttggtactcttgtgtggttaaccatgggcactgccctttgcagtgtaagtgcgaacgcgttca
accaatgtatggaaccaacccttgattgtcagatggcacgtacccgagggcgtccgattccccgtggtgctgtccgtcctgaatatggttggttatt
tgctactaccaccggaattttgggcacttccatgtcttttctagtaaaccccgttgttggttggttaggtttgaccaatatagtactatatatgggattt
tataccccccataaagcgtatctcaattatcaatacatgggtcggatcaattgtaggtgcgatgcctcctttaatgggatgggctgcctgttctggag
gtgatctttcccattctggtgcattctttacagccgctattctctacgcttggcaatttccgcacttcaatgctttttcgactatggttgctgatgattat
aagcagtgtggctaccaaatggcatcatggaagaggcctgctttgaatgctcgagttgctctacgttattctttgctcttccttcctttatgctatggt
ttcgtctggtctggaattgtcaatccttcgtatatggttggtgcttcagccgccaatttttatttgatcgcaaaagcctggcaattttacagaaatcct
cagaaaacaaatgctcgttcattattctttgcaagtctactacatctacctttaatttttatcttgactttagcaagccacatgtttaaagtttggaaa
gacgctgaaaatttagaaccggagaacccatcatctggtgattaaatcaccactctttttcgtctatgaattaaaaagaaagctataatgttgtata
ttccccacttgcttcccactatacagtaatatatttacttaaattagcatgctcgctcatatgtggctagtgttttattttttttgtatacatgtaaagt
attttgtttcaaggtagacgaaatgaccgaaactggttgtacagaatgacaatatgcaaaattttcactttcatatttataggttatacatgcgtgg
aaattttgagtaggatgtcggaggcattcgacgttcgttggtgagggaatctataaagtttgtggattttaataggaacctggtagagctgaaaag
ttagatgagattaccctggtaataatagtcttaagcccttgaccagtcgactttacccttagaatcgataagaccaagtcgttgataaatgttagga
ggacgaagagagaggccttcacgccaaagctgtttgcattttgatttgacttccttgcgtttttgttctaaatctttctcgttgggcttccctgcggcgt
aaagccattctctagtaacactaatgaattctttagctgcttcaacatatttgtcatccaaagtcttaaattgctctttgctgccgacgccagcaggtt
ctggaggcgcattatcataaattttggattcattaaactggccttgcatagaaattgggatgtatttatcgtcaaggtaatctttgacttgataaggt
ttacagaaagtgatttttttacgcacttctgcatccaaaaagtatttaccaatactccaaacaccttcaaacaaggccattcgaaaaccagaaccg
acaaccaaggcctggcccagaatttctggataatagttttggaaggtctcggcaaaaaccttggcaaattccaagtcaatgtttttctagaaaaa
taggttaaatcgactaagcccaagacgccttttaaagagttttgttcggagtccaaaaataaacgagcattttccatggcataaagaataagcgc
cttaagttcttgcatattttgtgcgtccttagggggggatgaagttatgaaggttcaagaaaacgattggccttccagctctgtcttgaccccaaataa
agtacatagaagctttcacaaagtcttgatttaaacccttttcgccgcgcaacataatgccacgtaagtccacttgatgtctccagcgaatagcgtt

ggtaagcatactagaagcatcgttgacgttgaactttcgggcttttaagaaacgaaggagggtcaagtcaaaatcgtcaaatgcacattgttcaa
aaaaattaacatacatttgatgttcggaatcaaaagcagatttgaactcgggttgttcaatctcggcgaatattttggaccaagtagtttggagtac
ttgtcgtttgtcgcttggaacttcagtcatatttgctagcaaaggaggatgaattttttttcaacagatgaaagccacgaagagtatgataccctga
aaaaaaagcgaaataaaccgaaaacctagtagggaatcccgaaagtctttgatccctgtagaaaccgtttgggggggaggaaaggaaagaaat
caaagacagaaaatgaatgaaaacttacactgagtctattttttggaaccttgaatcagatgattctattcacaaaaacaattttaatatgatca
gacctaaccaacagtcttgagaatgcctatttatcacactgcaatgtacactttttgaaaaaaatgtacttgtttaggcactttttaataagcgaaa
gtatggtaaagggaaaatgttatgatcagaagaacactggtatgactttcgctttcccttgctagtagtagcttatgccccttggggttgaaaaac
tctgaagttcttcattccaaaaagaggtaacgtaaacaaagctacggttgagtaacaaggtcttcgctaagtttaatgtttgtttggtttgttaagta
aataaataaataacagagagggagagggagagggagagtttgtatgagctcaaatataggttatttgacgaagtatgagtcaaagatcggtag
attggagacatgcgccgaaacccgatttggtttaagaactcaagagaaagttaagaaactaaaattggtttcatcatgtctgaatggagaagaca
gacgtgttgatagattatgttaagcttggcttcctctagttaactcgcaataaaaacgaatggcatgaccattcgtttctattcattgctgcttgtttct
tgaaccaacaaaagagaaattccatcgtgtctacttcaacagtaatctgttccctttctgcttatatttattttatttattatttattcttttagtttttaa
cttgttcaatataacaaggtgaattttccatcgattttcctgcaatgacatgaaacacaaagtccacacataatggtacccattgtttcaagttgatt
gtttcgtccatactaagcttcgcatgaagctttctggaatattctttccgtttcctctaagtcgaccttctcgccatctacacgtaatccattaacgtttt
tgtcatcatctatataagtgctataacgtgcccgtacaacttgttttagaagcttaaacaatctacccattcttacttcattactttcatttttttattgt
ttggtttgtacctgtataaagaaaggtaaagtcattgatataataagatggatttcaatcctgctctcatcattgtagacatgcaaaacgattttgta
acaccagaatttgcaggccatcttcccggtgctacttctattattcctaacattaacgaactcttgagcgataatttcaaatgggactatactgttgc
aacaaaggatttgcatcctgctgaccacgcatcgtttcggacttctcatgggccttctgtgaagcccaaagggactgttattgaaattaatgcccat
ggaaaaacatataaacaacaactttggctaagccattgtgtgaatggtactgcaggtgccgagtttcatccagacctacgaatggataaagttga
tttcatccttcctaaaggaacggataccctcgtagagtcctattctggattttacgatgctattggtagagacaatggcctgaagaaaatgctagac
gaacgtaatattaccgacattttgttgtcggcgttgccggtgacgtttgtgttcgtgatacagctcttcatgcgaaggagcattatcgaacatatgt
tattgaagatggcatctgtatgtctacagaagaaagcggccaaaagaccttgcgtgaatttcacgatgctggaatctcacttataaagatgaatg
atccgttactggagtttgtgaaaaaccgcactaaatcatctaattagtttacgatgattattgtcctatattaaaatactgttgaacttttctaaaatt
cgctcctttttttaaccgagaaagacataactatttgcactcacatgctctgccgtcgggtgtgcctcataggacattcggtgaaatcccctcgagga
gtttggtgatagtacaatagaattatctcccgaatcgtataatgctgcgtccacaaagcttgcctctcgttccattggcaaagcgtacgcaggaag
aatttgaagcggcatctttacgcaattaaatgtcctaaaatcgttagtttcttctatttatttattcgcttttcttgttgattaccttaatctggatccaa
ttgaaatttgtaatatgtattctacctgaaagtcagatccaacgtttattgtcgcttctctgtccggaatccgaatttgacattccattgtatgtgtacc
caaagtatcgattcctttccagttatagtagttattcttccttctctcattaatcacttccttttgtacacaaatccaagtttctttacgaatagaattac
gtttttttgctcgcgacaatgcatctggttggtatcttttacctagcggtcctcgaaatatggtaatgcgtttgaagagggaaaggttcacatatttg
atttcgtaatcaccataattttccactttaatattcgcattaatccgttctcctccgagccattcagatcttggaagtctgactgttatcccattggcag
tattatgcttgcataatccaagttttgttcttggtttactgacacacattaatggcggtgataacaagaaagtagatgaaagaaccctaggaagga
cgttgatttcttgacattctcttacctttttttactttatccccatattggtaaatcgcattgcttctctggttagtcttttctcaattagcccagatcgaaa
agtacgtacgcaaaaatataatagtttatattgaactgtggtgtggattgtcttccaggtcccacttgcccgtaaagttccatttgaaaagctactg
gtataccttgagtatgctgaatatcaatcaaataatctcctgttgtgttccttgagtgttctgccaattcgtttggaacagtaaactttcgatccattat
gttctttcctgtacagtaaaatactgttggatgtccctgtttacactggctgatcccgagaatctgagtttcaattcgtattagtccaatcggtcgctc
ctttggcttaacagctaagtgtatcagtccattcaactcagattcttggaaaaatattggagaatcaaattcgaaagttaatacaacctctgacgac
ttgagcttggacgaagacgttaggtcgtagatgacttccttggccgcataattcgctgtagaacatgtgattattgtttcatttttctatgaaataat
tgattattccttttaatgattttcatcgcgtcaaacaggtagtttgttctgcctaaaacactgtttgtttacttattcacgagtcctcgagtttatattcc
tactgacctcggaaaaacaaacggtattagaacaacaaaaaacaactatcaaatgctattagctcaacttattaatgtttatttctatcggaaact
caaaaaaatatacccggataataatataagcattatttcccggttctttattctgtcatcatatgtagtagtcgagtatatacaagattccacaatat
caataaaacagatagcagcataaaatagtgatattacattttcaaaattgaatgtagagatttcataaatcagaagtcattgagaaaccttgatg
caagacagtagcaaaattcagatcattatagaagcgtacatcaaaatttaagctttgtgaaagcttctattcctcttggaaagaagtgttgaaatg
agagctatgctgtctggatagagaatcgtagttattaggttctgtttcattatcaaaaaaaattcgtaggaaccgtttgagatacatcattcgtagca
ctagctatacctggattcggatacgaagtggttttctgcagctcatcatcaaaaagattctttgctccttcaatctcttggatttgaggttgttcagag
ttagaaggatttaaaccatattcagcatgattatgtaccggattgtttgcatagctttcaggagaaagttgtttcaaatacggattcttggttgagag
cataggcggttgaaattcgtgtgaattccaatcactctgtatgctacttgtcctttcgagttggttaaaagaggggaagaaagacttgtcttcagtgt
cagacagatttggatttgacgacgaagtagggtttgaggcgacttttcctgtttctgcaaagaagttgttcggaattgttttctcgttagtttgcaatg
cttgaccagggtaattcaattcttcaggattgatatatggattcgtggaagggcggcttatgtacttctgattagcagcagttgatttccgacgacg

caaagaagaaaatacctcgtcatcaccttctaacttcaaattagagtgagaagaagagtaaggtaagccctgtgaaagcgattttgaatgccga
aatgctttatgcggtcccgctaatgcatcagtttcgaaattcacaggtgacttgtgattagtcgtttgaggatcttgaatttcgaactttagaaattc
attaggaaatgcactggatgatttaaaagaatgggaaggacctggtaattcatctgaagagccagtcttttctgacttctgagcatccatgacaat
agaagaagaaggtggatgattttgagactcatctttaacattcgcatcctgactattagactctgtaggtctcaaaaaactcgcaaaagcatcatc
tgccaactttgcaatttcatcttctcgctttgaaagttcataagctttttgagaagacatatcagtctgattcgttacttgtgcttccaattcactcaga
tctggatatcgtgaagcaatatcatcttctagatcgccagtagtaaattcaggtaccgacttcgaacctgtctttgcaaaatcagttggtgcatagt
gactagacgcacctgttgtagagtgggagacagggaatggattcttactattacttgaaacataaggagtagagggtacagaatcagtaacgac
tgaagcaggacgagatatcgcttgttgattatgataactcaagctccttgaagaccccaaatcaactggatgattcattgacatattctgttgaacg
ggaacgggaatacgtatttttgagatgcaccagacatatctgcttgcacatagaaagggttagacactggatgagaagggtaagtaggaggtg
tcataggaggcacaggaggtcgagaagtttgaatatgagaggcggacattggtgcagacgaggggatttgttggttaaaggctaatccttgaga
ttgtctaacaagggacattacttttctattactcatatcataaaaggagggatcgttgccggcataaaaatcaccgaacggcaatggttttccgcg
aagccgacaaacttcatacattaattggaagatgttagggcgtaaacaagggtttggctgaagcaaagcaataataatattctttagagaatgag
aataatgaggaaaaggaggaaaagcataagaagcagtaaggatcgcatttggtccttgagtttcaaagggagtagtgtaataacaaagcttat
aaagcaggacacccaaagcccacatatcactttttcatcaattgccagcccggcgtaaaaattaatcatctcgggggctcgatactgataggtg
gtaaaagtagccaggtttttttccaatgcatgaatttcagtagaattttctgcggcatgcataggttcggtcacactgccaaaatcgcaaagcttaa
acgaagagaaagaagtcataaggatattctctacttttaagtctcgatggattaagggaggccgtagacgatgcattgctgctacaccttgtgtga
catccgaaaggatctttaaaacttctgcttccgacaatcgtgtctgcaaacgctgattcatgaaatcaatgagtccgccaccagaacaatattcca
tcaaaagaagaacttcataatctttttttcttcactcagaggcaagacacaagaatcatagtaagtaacaatcttgggatttgatttgagcaattt
ctgtttcagaggaacattccgttagtaaatccggcagatacactttttgagccacttaccattgtgttgatttccgatttgatgtgattcaaagcttgt
tcgtctgcggcatacatgcgttttagaactgctgaaagaggagacccttctgcattggagaatgcttgcacagcataaacatgcgagaaaccacc
tgaaacaaagaaaaccagttaataattgtttccatcataacgtttcataaaatgcatcctttttaccttctgacaagtacttttcaatcgtgatattgt
actttccaaccacatatttcgatccaaccaaaagctcatctttaaaaagaaagctagcgggatcagacatcctagttgtgcctttctttctatctttc
agtaaggatgaattatcgctggacacaacccgggagtgcaaaacgatggaaattcgttgttcttttactcgttagtttcatttaatattcctatttatt
tttactcactctttttctttttcaaaaatattaaaaacccaacttgtagtactaatttgcagttggtgcgttttgtttgttcgtccttcgtacttggtatct
tcttttaggctagttcttccagtagacatcagcgatttgtatagtaggaacaagaagcaattcttgtttttatttaaagaagtcaattttattttcaaa
tatgattcctttatggttccttctgattctcgttttctttttgaaagacgcttttcactagccatgcatgatctgcgaggtcaactttcggaaccgtatta
ttactgtaagtagcgattagtattgtcttaaaaaagtctcctcgcgaagctagaaaacctagaattcttttcgaacagatacaagtcctcacatatc
gtttgctagatcgcattggttttggcgtttctttaaaaacatctttgagtttttctttccatgaaaaagaaagcaagaatgagtgccactaaaacgcg
agagcacgccaattgggattgccatgtttctactaccaacacaaaaatgtacaaaatgaaacgaaccttgaacaatattgctactcatctcattca
ctaccacgataattaacacgctctttctcctgcttcttagttgacaatccttgctactttttgttcatcctaatacgatattttggtttgtttacattttag
cgcatcgctaaaagtacgctatgtggattagtgagcggtgagctcctttgcatgatttcattatttgggacaaaacatttcgacctaaatcaaaacg
tgaaaaaaagcagaattttgtggattgctcctttttccttctttaagcgtatgtcattttatctttcctatgcagtctgtgtatttggagcacctctgtttg
aggcttgctttgtttacgctatcgggtgacaaattgacatgtttactaacattggaacacagaataaaaatctcttagagttcttgctggagcgaat
tttttcaaaatgtcacaaactagagttactagaaggtcacagagacaacaggaagccttggggactagtttgaataagcgtgaattggaagatca
tgaagctcctgttccaacaaaaccatcgaagaagcgtgttaaggtttctagtggtcctaaacctaaaaggaccactggaagaaaactaaacagc
attcccgctctccctaatgagctgttgaatgtttttgtatttggtaccggttccatgaatgaactcggcatgggcgacttggacgttgatgtcgcttat
cgacctcgtcttaacccgaatttacctagagataaaatcggcgtcgttgatttagcggttggtggcatgcactcagctgtgctttctcatgatggccg
cgttttcacttggggtgttaatgacgactttgccttgggtcgtcctactaaaaaccaaactgacaaagagggaaacgcaattgataacgatttatt
agaaggaacccccagtcaagtcactggcgctattcagcgtcttaaagttaccaaagtttgttgtagtgataaccttacgagtgttatcacagaaaa
cggcagttgcttcagttggggtacttttcgtgtaagtttttcataactgtgttttatagtgcctttctgtgagatttgtcgatattgttataaatatcctct
atcgtggcgtctcctacgatcggtttcattcctttattttgtatgatctttgctaacgaaatgtagtgttctgatggtattttgggattcagcgaatcta
gcaagcgtgcctcagagcctgtgcaaatgccgcttcccgaagttgcacagcttgctgctggcactgatcatatacttgctttgacaacaaccggaa
aagtttatacctggggtaatggtcagcaatgtcagctcggacgtcgcatgatggatcgtcgtcgactgcaaggtctaaaccctgagcctcttgcac
ttaaaaacatagtagctgttggtgctggttcttaccattcctttgccataaataataagggaaaggtttatgcatggggcttgaatatgacaaaaca
atgcgctattgctgatgaagaagacgaagaagaaggtatagttaccaaacctaccttagtagaagccttagagccttacaaaattaaagccatc
actggtggtgaacatcacagtcttgcattattagaagatggacgagtattggcttggggtcgtcaagatcgtcatcaacttggtatacccgatgat
gcattacctgaatctcttaccaaagatgagaagggatacaattacagtatatctactcccactattatccctggactctcgaatgttgtccaagtttg
ctgtggtactcaccatagtttagcagtaactaatgatggtaaagtatattcgtggggttcgtctgagaattatgaggttggacaaggtgacaccga

tgaagaaattgtagtacctacgcttgttcgctcaaaagctattacgaatgctagtatccgagttgctggtgccggtggtcaattttctgtgattgctg
gtgctcctaatgaaccagaaacaaaacctgccgaaaacggagtaaagactgacaatgaagtaaagtctgaaagtgagaataactctactcctc
cacagcaagaagaaaatgaagaaaagaagcctgtcacttctgagtctacagaaatgcatattgaccagcaacctcctactaccgaagatcaaa
caactacccagagtactgaagaaaaaaaggaacctgctgaggaagccgattctacctcttcttaataatatttcagctatatattacactaaaaat
aataattttggttgtattgtgcaaataaatgtacatttaacgagcaaactgccgtacgattaattgtagtttcgtgaatggatgaaacggttgccaa
gttattaaaggaacttgaaggttcttcaaaaccctcttcaacatcaaaggaagtgtcaaatgataccaattcatctcagactttcaattccgaacta
gatccttcattcaacaatcaaaaggcttctcaggaacttgctcttgtcgaaaatattcgatcatatccagcagctttacgctatgtcttttcattgcaa
gtaaaaaacccggagaagctgcgtaaagtaaaaaagttgaaaaccatgcaggaacgccaagaacgcgattggttcatggaacgccaaagact
aattcaccatcatcagtctaaaaataaattaaaaaatctattgacacctctttcgacttctctgggtcatgcatcttctcttgaaaggcattcttctac
aagcaacaagaactctctgagcgaacacgacagggaacttgaagaagtattatctgaatttgataaaaaggttgttttattaaccgaacgtatgt
attccgaccaaagaaaggcactttctgagctgcgtattccgctattcctcattccttcatcggaacctttaacagaagagcaaagcaacttaatga
ggctgttacaggatctatttagtgaatgaaacgagctaataggttatctttaaaattatattagcaaaaaagttctttcttatacttagtaaatagca
tatataggaatgatttctatgaaaacccaaaaatataagattaccaaaataatatttatttcattaccttgaatactaacagcttaacaaaattatt
gacaaggtacaattttccttacacatacagacattatatacaaaaatccaaaattccgcagtaaaaaaaaagaaacccattaaatctttgtacttc
aaaaacagacaaccatttgataaagcctgcaaacgatcatataaggtctgtaatcgccaacgaggaaagacaggcttccaaacaacatttcag
aaagttcaagaaattaagcgataaaactgaacttgtaaaaaaaggtaagtaaacgatcgttttgaaaatggtagtcttgcgcaaaatctttaacg
aaaagctaccaaatataaatcccaaaaaaaaccaaaaaaaacataacacttaaataagaaaccaatgtaaataaaagaacgataggtagaat
ctatacagaactatatacagaaaaccaataacatacttccacttataatatcgtcgtaaaaaatcaatgattcgttacctgtttaggtgtccctccg
acgatccaatgtatgaaaaaacttcatagacccactccgagataatgagttggatctaggtttacctttaatttgggaactatttacctttagatca
cccatattaggactacgatcaagaatgtcagctgtggaaaagttcttattcgattgacggctgaaagaacgccatcgtttaatggttccacttttag
agttataaagatgtactctagaatggccatcagattgtgttaccaacgtcgaggaagaggcattgggagaagtcggactgggaactaagggagg
taacgaagaaagaggaatagtctttatgaagcttccttgatgactgattaaaaataaaagtgcattctgtgaaattttgtagtctttagagtatact
tcgtgactaggatgggataggataccaggttgaaaaatagcggctaaattttcggcggacataagatttatgtgagaatttgaagcgaaaacag
caagcaaatctaatagatagagaagaagctgtcgatttggaataggaagcttatctatacgttggcgatacatttcaatacgatcattatccgtca
aattagaaagagtcatgggctcacggaaaagctcatagtaatctagaggaattacatgctcaggcatcaagttaataaatcttcgaaagacgttg
gcggcatcatgaacggtatagccattccattcgaagctacgaccgtaatctgggctcgtgctaaacgctttctgaagtaactgaattctttttgaag
agccattaactcggaatattccttttacttgtgtagcattttgttttaagaaaaatccacattttgcaactactgccggtatataaccataaaacactt
tttcaccatcttttcgggtgaggaaaatggctactttggaaacttccaaagcttcagaaaggcttaatccaaatagttcattctcagttgctttatga
attctcaattgcttccaccaggttattagtgatttttttcccatatcgtttgaacatatgggctgatacctcaatcattcacttgatattggaaggaata
tgttttcctttcctcctttttcttcgaatgaggcgtcacactgccaagtttttgaatcctaacgtcttttgggcaacacttgcacggatggccggtccttt
tacacaatgcaaatgaaaactttttttctttattataaacgtaaatcacttattttctaaggaataaataaacagaatatcgaccaattttgatatttt
ttgaaatctgttgtatgtaacgagagggacagacagttcctacctgtggatactttaatcaagatcggttgagcccggtatttccgtggcagatcgg
tagccttgataggtttatacgatttactacgggatcactttcatgcggaaagtaatgatgtaatataattgtacagagtatctgtttgtaaacaaac
aatgcttgtgcagaacagatgaaaccaaaacaaagctaataaattgcttaatagaataaatttcttttcctttttatatacggtaatgaaagcgta
aaagttctcaatggaatgaacagtttacagtttgtatttcagtgacatgaaagtgatgaaatactgtcattgaataagtactagaggaatttcatat
acaataccttacgagtatttgttcgtcattgaatacttatatttgtttatatttcaattaaaaaactgcgcttacgatcaaactaaacacctggttttt
acttgcaatacgaatagctgtggttgaattaaatgatttttctgtacagtccctctaacagtaacaaaaaaaaagtaaaatgaaacgaagtaattg
aactgaaacaaaattgccttcaagtattagggatatttttttcccattgtttgtttttattagagctaaaataatgcctgtaataatattcgtatatcg
caaaaataaatataaactcgataagcggataaccggactactttatatgtaataagctccgaacaagttttaagtcagaaccagatttccattatt
tacagtaaataattacagttaatgtagtaaaaataactggggttcctagttctgtctctgtttttctttgtttcctttttttttttacattttgatctatca
aagctatcagttgctatcttttcctatcttttgatttaaattgggtttttctcccggcataattttctatcgttctttatattactttttcatttttttttggtttt
catcagtatgcgactgtctagcttctctttatggtttttggctttcatcataggttctgtttcggcccggttttctgatttcgagtcttttaaaaatacatt
caaccttcaggaacatttaggcagtcttagcatttatcatgaaccacatttcactggattgagctctgcatttccggaatcttgtgaaattcgtcaag
tccatgttttgcagcgccatggttcacgaaaccctgctagcgacgacggtggttccagtgccaaatctcttgttcaaatgcaagaaaagcttacca
atggatcgcttccaattaattatacaaagcctgataacccctttcagtttatcaagtcctgggttccagtcattgaacttcgtaatgccgataagctt
tccagtagtggccgtgtggaattgtttaaattgggccgtgaagtctttactcggtataacatgctgtttgatgccgatgtttatgaaataaatacagc
ggctcaaggtcgtgtcgtcgagtctgcagaatggtacagttacggcatgtttggaaatgaaattgctaacaagacaaaattcgtctaccttgctga
ggatgagactcctggtgctaattctctttccaactataattcttgtcctgtgtactctgacaacaacatcgactacaagcaggtagaagaggctcat

tccatatgggaaaacattttcttagttcctatcagaaaccgtcttaacccttacttttcaaactataatctcacaaatgatgacgttaggagctttttc
tacttgtgtgcttacgagctcactttaaaggatgaaagtggcttttgttctcttttcactccttctgaaatcatcaattttgaatacgattacgacttgg
aattttccctttggggcggtccgtcttctaaatgggctaatgttttgggtggtccttatatccataacttggccaaatctcttcgttccgtttataatga
aagcaattaccaaaaaacttttttgctttcacccacgatggccaaatccttcctattgaagctgctcttggtttcttcccagatatcactcccgagca
ccctctccctttaacttacaatccttatacttattctctgaaaacttcgtcattcgttccttttgctggaaacttgatcaccgagctttttcaatgccaag
atcagaagtattatgtacgacacctggttaaccaacaagtcttccctttgaccgactgtggttatggcccttctaattcttcagatggtatgtgtgag
ctttctgcgtatcttaattctcccgttcgttctaattctacttacaatggtaccgacacctttaatgctgcttgccaagcaaaccctactaatgtcaca
gttcgttactagaatatctatctgactttaccactatctcatttatagattgtgaatttactttctggtaaatgaatcttaaccatatccgaaccttgat
ccattataaaataaaaagcgtatgccaatgctatagcctttaagtattagttgactaggccattcgacttttgactttcctgtcccaatgaatgttca
ggtgaagcccatgctcttaagcctcctcttattttttatgctttgcagagtaggagatccataaagtagataaaagggtttaaaaagataccattag
gaagagagcgtactaaacgcatcaaacaaaggggacacctgcttcatcatgaacgttcccaaagtacttttctgctgcttcaaagacataaatga
taaaagaaaagatattttctgaataaattgtaatatgatttaactcttaagcagttttatgtttaaggaagcactttttttgattcaaatagcaaaaat
ggaatcccatgaacgtactgcatattccacctaacagcattttcttttataaaagagctaaaatttcagcttaataaaccccctattacactgtcctat
tccaatttccctgagcaattcaagaggacaaatttcgctataccacaggtttccccaactaccatacaagactaggtgaacttacattcgcatcgtt
tcgttaatgagaacaagctaatagaaccttcatgtttgtttaataaaagctaagctgggattatggaacatcagacggcatcttgcgcctatacaa
aactagaacttgtaggccgtggttcatacggtgctgtatacaaagggtatggttatattctttactattctataattgcagaattgactgactcgtat
cagtcttcgaaatgctacaaaagagattgtagcaattaaagtcttgaatctggacacagcagaagacgaagtatcagatatccaaaaagaaatt
actgttttaagtgaattaaagcaacgtgaatctgaaaatatcgttaaataccatggatcatacttggtaggtacaaatttatggatcattatggact
attgccagggcggcagtgttcgtactttaatggaagctggtcaattaagtgagccttatatttctattatccttcgtgaaactttacaggccttaaaa
ttcattcatcatgctggtattattcaccgtgatataaaagcagctaacattcttgtttctgtgtctggtaatgtcaagctttgcgattttggtgtcgctg
cagaaatcaatattaatcgaaggaagcgaacaacttttattggcactccctattggatggctcctgaagttattcgtgatggacaagaatacaatg
tcatggtacgtattacagccttaagcctttgttttataagtacggcgtttgctaatacagttaggctgatatatggtctttaggaattacggcttacga
aatcgcgactggttctcctcctcatgccaaagaagacccatttcgtgcagttttccttattgcgcatactcccccaccaaagctcgaaggaaactat
agcagcttattaaaggaatttattgctttttgtttacatgttattccgaataagcgtttggatgcctctgaacttttaaaatcaaagtttatcaagcaa
tacgcacggctacctggaaatgagctgtacaatctgataaaacgttatgaaaattggcaagcggctggcggtgttccggatactttaatgaatcc
acacgagcctgaaaatggcagcgaccaagataatgatacttctgaaacaatgttggatgatgattgggagtttggaacagtcaaaaaaacacat
caacaacgttcctcctcgtcttcagggtctgaaaccccaacttccaagccgacctcgtctacagctgtatctgccacagaaaacgagtctcatcctc
ttatgcagctttttcaaaatagctctatgccgaatgaagatccctctaacattggccgaggtattgcaaaccaagaatcgaatattaaaccttcata
ctctcagattgaactgccaagttttgacaaggaagatgaatgtttgaaaaacaaaccaactgttaatccgaaacaatcgagatctccagaagaat
cagaattggttgtcaatcggagcagaggaaatgctatgaattctatagaccagaatgtgaagcgtccattaccacgcgtggtgcaaagacaaat
gaatatgggcaaaagaggcatatccatgtctcctatgaaaggccctcttcgatttccatcctcactagatccccagtctaggagtgtttccatgggt
gcctttgaacacttgaacacttcagcagataccagcgcccataaaacgcactccttttttacccccaatagatgttgctagatcatctaataaccaac
cttcaaaagctacttctccgacaattcccaaacaccctatcaattcacccactgtaaagccttttaactcgaaattacgagcacgtttacccccatt
atctattaatcctcttcaatcgaacacagctttaaatgaaaataatgaacccccagctgttaagccattaccattggaattatttgatgatagttctt
ataacatgcatactatgaagtcctctataaaattggaattagacaatctcttgacagaaatggacatctacttgaaaggattggaaatgagcctcg
ccaaagaaaaagatttttaatgacttattacgagtttgttttatcgttattgtttttctttattatttatttaagtctctgaagtataaataccatcttca
gtgagttgcgtgttgctcagagaatttttgatttaattttactaccgtcaaattaacaataaaaatcctaattgataatccataaaaatttcattaat
aaacgactttgaggtttgtatttagtcgtctaatagactaaaagaccgtgccagtaaattgtcagaacctctttttgttgattggtatatctatataa
atattttttcttttgcatcattcactttataagagtggattacttagccagaaataagcatctattttattggccaatttgaggttctttgatgatttttct
gtttttgtaaccttcatctcctgagcgacagcgtaatcaaacatctcaggtgtaccatacatttttaagacattaaggaacgtcttacaaggatagt
aaacagaaagggattcctctctgaaaatttgatatgctattccgaatcttcctgtttgtaaactccattccagaaacacagattttatcaaattatct
tctggtaaggccagattctttagttgccgtaataattgaaaagtcatttgcgggtttttccttacaatcaaatagtaatatatggcgagataaaactc
gttagtagtcaaaaaatgatcaaaaagagaactaaaaaaaattgcttcgaagcttctatcacggaaataatcgggtaacgttgcatcttgagtca
cggtgttcacgtaggctttaatttcatatgtagaggttagtaaaggatatagaagagtttgaaaccaaacgatggtcaacaaatcttcttttccttg
aaatatgtcgtataggtttcttgatctcctatacatatatttatcatacaacgaaaggattctagcggttttgttcggtggatcatgaggcctaaggt
atgctttatttcgatcagaagccactattaaaagttttctcgtgtgctgtaaattacaaaaactcaacgaattaaaaatgacttgacgcaaagaac
ggctttcgggattcttttccttaagcgcattactacgtttacttatgaatgcacggttcgactttttcgaaacttgatttgaacgggaagaagaatcg
gcaactgttgaagcattttgtccatgccttactttgtttgagaccaaagtttggttttgagtttgggaacgataattccaaggggtatatgcaaaaga

acgagaggagattgtggtataaggatgtgaatataatacacaatgaaagcgggaaaaatgcctatgcagtcgaatattactgtggtagaccctg
catctcgcatggaacatagcaaggatccaatggtgaaatttccatagaagcccgcagacgaaaggctatagtataatgcttgaaaccagaatttt
atagttaaaaaacttcttcaaagaaggaagacaatcaacagacgccttgccttgttttcgaactgttataaaacaattacgggaccaaaattctca
gtagtaaagctattccaaaaatcagtctctggaaaagaataacgaataatgaagatataataaagttattttatcctttctctatactgacaacga
aaatcgataaaccgatcctaaaacgaatgaaagcatttatttacctgcttttcattcctttttctttcttattctattagaattgcagtataatactaat
taattcatgaacctggcaaaatagaaaggcaatctcgcaagtggaagatggtagtagtagtctaccagttaagtctatagtctgcaactatagat
atacaagatcggtcacacgaatgcaattacctatttagtataaataattgaatttgaatagcttaaagaagtttatttactatcattttaatatatcc
tatgttaaatgaactaatagtaattatcaagattttacttactcttagttaaagaatatgatctaaggtgagtgaaactcttccaaataaactcaga
aataattaaaaataatgaacgttaaatgacaaataatttataaggacaatctgcagcgacttgcgaagtgaaattacgagaacaagaagacaa
aattagcaagtcaaatatcccaaaatatttttaagatacagtagaggaattagacgctgagaacctcggcggtaattcattcgtctcaaaagacg
ccaaaagagcggagtggggaaacagttcataaataaattgcaaaatctgaacacgataatgaaattggtctaaaatattgaagcaaaatttggc
taggcgctttgacaaaaagactcgaggggttttagatcgtatttttgttaactgacgagcggattcattcgccaatacatgattcgacaaattggaa
atatacttgacgacttctttctcaatatttgttaagttgtcagctaaaaatgactgagggtatgtgagtgttctatcactcaagtgttcagcctcttttc
ctggagtcgaaattacacggcatgcatgtactgggatacaaacaccttgcgaagtccccatcaatgacattaaactataccaatgcaaatttgag
agaatttgtacgccacgactagtccttgaaattaaccctaaaacaaaaaatgctgtcccacggacagtaggaatttttgattcttccgcgaatttg
acaataagaggcacagaatcatatttatccagaaagtgaacccctgtctctgcttttcctatatttccgatagcccataatgcacttttcagacgac
gaatctgtaatgagtctacttcttcaacatactcccttaatatattcatgaaggaaccaaagtgaccgcttgtctctaaaacagagcaaccttccgg
tgacttcaccaattctccgtagaaatgtaatggaagaatatcatctccattagttgaaatctctgcactagcaatatccatgttggaaataaatgtc
agaaagccatttagttcaattaaatcaacgtatgcttcatttcgatggtaaaaccaattttctagttcgtgttcaataaaattaatttctgacaaata
gtaaaagccgacagtggtagccaagaacctcaataacaaaggtgagcctatttcacctagatgggccaaagatggctgtagttgaacaacctga
gcaagcagattttcgtttttagcgcagacatcgtccaacgctttgacagcaacttgacatacgtctagagaaggatcatacaattgaaaaatcaa
aagcgatatagcccaatgattcaagttgtcatttcctgattctgaatttatcaatgttaatagatgtcgtgttgcatgtaatcgaacattttgttgcgc
agtacttaaagcctttgaaaatataattctgctgtggccctctaagcgataatccacattcgttaagagacaaatcaacaaatcatctctgtttcga
atttcggcaatgtgatatagagtagtaaatatcctccatcgttccataatcgcatgtccctctttctgactggaaaggactttaagcatgcaaaagt
aaccatgcacgagagttgtctcgaggcgctcgctggaaaagatcggattcgctacttgaccagaatattcgtcaagctgtgacaaagcttcagca
atttgctttatgagtttgctttccgataagtatttgacccccttcgggatttgccaaaagcgtcttaaaaaccaaacacccgacattgatatatttctga
ttagccgtcgtgttttctatagaagaaaacctatcagaaaagggcttataaaaagataacaatctcttcataaatttcgtcgtgcgaagggtttcat
ctattctcttatgactcagcagaggtccttccataatttgagctaatgtatcccatctccatttctgatagttttttgttgttaagacattagtttccgcc
agcatattcctaaagtgtgagtcatcaatctgcaatcccatcttcagtttaacatgttctacctgcctttgaccccgaaacgtgtttccagataaccc
cccttcatgaatactggatgcatgaaaaccggtttcagtctggcattttacacggttcaagctttcaatagattgaagaactgaagtggcgacaaa
tctatcttcagcagtaaatttggaagccatattgaataaagatggaagtgactgaattttagcgcctaactgaataggaagtagttgatctgaaag
acgtaaaacttcaccaagcagacacgttgcttttttgaaagaaattgatcatcattagcacggatcacacatacgattgattcaaccaaacccag
ttccaaaaaaataaacaatatgagagccataaaatggtggttaagagacaaaaaatttctataagacccgtcggacagattaagcgtgcttgttc
taatgtcttcatgaacattttgactttttgctggaaacggcttctttccagcaataatgcacgattcatgtattgttgaacaatcaaccttaaaaata
agcaaaaataaatctattatatcattacgggcagagaacgtcggaacacgaagggaatctacaagagatcgaagggcttggaaatcctcagta
gagaaagccaaaagtccaggccaggaatttagtagaacagagatgactttagcacttgagcgaatagctctagacgtttgctctggctgccctcc
ggaaggactatataagtcataaagcgtgggtgaagtaaatggggataataaaataccaatatcgtgaggtagacttaaataacggcaagttac
agggttatctagaagataaatcagagccaaagctgcggttgctgccaactcctctgaaatggaaccgtcaacaagggaggatattaacactctc
aaaccattagctcgcaataaaatctgtggttttaaaatcattaattcaatcaatgtttcgcttgcgataccccctacttgcatctgttgaatgttcaca
aatagcaattaatgtgtttgtaatgcctaaaagcatatgttcatgttcttctgtacttgagaacgcatatagtgtacgtattaatcgaaacgcctgag
ctctttcggtctcataagaaggtccaactgttataatggattttgaaagcaaccaagtcaaattaagtgactggcagacagcgaacgccgaaggg
tcagtgagtaggtttcttatgacgcgatatacacaagcagaaacgtctggtacgttgagcatgagcatgaagtgaagggtaggaagcatatagta
agccctgggaagacagtccttttcagggtgctcatgcaaatactgacaaatgtcattagaaagttggataacttctctagtggaaagtttcgagca
agcatttcgtactgctgttgtccaatcttggagaactaaattaggtgcttcattggattgaaagaattgaattctattatgcaaagatggataaaca
gataaaggtttgtctctcctctcttcatctttgtcgatagcagaatattgggtgactataggatccagttttttggaaggacctagatagtcatttttg
taagtgataaaccgacttgtctggagttaccccgctggtgtcttgtatgtgtttcaggatgactttgggctttctgaagtgatggatcccaagaactg
attccagactccgtagcttcattttgaatatccatctgaatgttttcaatggatttcttaagtttctctattgatctcctacaggactccaaatccgatt
cgagattatcctttaagctcgctcttcgttctattggttcatgtttaaatctcgcaccaaaatccattaataactgttcagtcttatgacatgctgcgta

ttcattgtcaagttttcttttaagctcatcaattaactcgtcattttcttctacgacaccaggtaatttttccataatggagctcgtttccttactgtttgc
actaaagtgttggctttccggtcccttttgcataggtctcatagctatatactaacaagggaaaaacatgaagaactttccataaggacttgcataa
gttaagaaacaacaatctttgcttagaaatgttgaatacgctaagttcaattccttagccaattcttctccaaagcaaatctatttttctttgtttattt
tcatataataagaaatccagtttttttcaaatataatttatattactaccatgaagactcagcagcgattctattgtgttttcgtatagttgtattttcc
ctaaggagtattaattataacgaaccagtacaaattgcgcaataataaataaagtctcattaaacgagcatttgattcaaaaacaagagttaaat
attgtaatataagcataagatttatacgattatatatgtgagaatacattctagtaacagcaaataggcggctctgttggagggtgccagaggaa
gtcctgactttatcaattgttactcatcgttcattactctaaggagtatataattagtaccatgtcaccttgagcaagacaaaaaatctatggtgaat
tgagtaattagactgggttatattgattcaaagagagtagaataagaaaacttgtgaataagtagactggttccaaacaaatacattctaaggga
gaatttgttggtatccgaagcaacttgcttattgatacatctgaattcgtcaaccaaggacattagaacaagattagttgtttacgtagcaacgaat
atagtaaggattatttaaaatacagttgcgatttttcagcacatatacgaatcctaactccattcatacttcttcgtatctttttcctaatatagacta
gaaccattggaagaatgaaagaattactttagtgctttgttcttatggtatctctgtaacggcttcatcaactttttccaagttgaatctagagttta
atcagactgataaacatgcctttcaaacttatagaacctttcgagagaaagcatatctacagcttacgaaaataataagcagtggataacatact
gaccggataatatatagacgggatgaacaccggttgtttggcagattacgtgagaaatcaaggacagtataatattaaaaacttttagatccaaa
gataccaaaggatcacgaaaacaaaactagaaattataggaacaaaagcaaaaatataagccactgaaatgaaaagaaggggattgaaacg
attaaacctgaaattcatgtacacattataatcataaattgacaggtaacaaaaaaacctttccttaaaacaagcatgttaccaaatttccttaata
tacttcaaggaaagaggattctgccaagtcgccaattggttagaagcttcactatgagacatactcccgaatttcatgcaaatgtcccgcaatgca
tcgtcgacacctcgtaataaaccttgtttaccaccacaaaggtatattttaccgttcgggtggttcaaaacattccatacaatatcaccttttttgacg
taattcgtcctgcacgtaaactcgtttgcatgaacgtgattcaacgactaagtgatgaggcccttccctgggatcattgtgaaactctaacgcatca
acttcggtattattttgattgctaacggaattagaagagtcagaacgagagtcgtcattccggtgttcccattcgccatggtacaattcatccaactc
ggagtcacggcaaccgtgaaaaatccagacttttccagcacaagatgcattcatgagacggcgctgaacaaatccgcggaaaggtgcgatacc
aacaccggtgccaattagacacatagggccatcggaagtgaagtgcgtaacaaaagcgttcctatgtaagccacgatacatggggaggattaa
gtctcttttaccgtccatccattgcttcgctaaacgatacaagaagccagaaccgataccgtaacgaataccgccaccagccttttcgatctctacg
agagcaaccgcaaattctaagattccattatgctgagcgggatcgttggataaagaataccatctaggcattagttggggtagaatacttaagag
gtgatccagaggaagcttgacactagaaaacgcttccaagagtgtaagcaaagtaacattgttattcgctctaagatcacagaaagcacgttga
cccaaacggctaaccaaaaacaaaagaatttgtttttctgcagggctttcggcatattcggccaataaacgaattaactgctttttaggggggaacg
gacatgaagtcagaagaccatttcaagagctcccgtcgagtagtaggaagctctctagccatgtcctcagcccaaatggtaggccagcgaccac
cattagttttcaaaaggacaggagcatcagcgacgtcgggaggaacatgcaaaagatggagcaaatgacttacagcgtcatcgctgttgggagc
catgagaccaaatgaaccacctaccatccactgttcaccttcaggaagaggataatcagaaacgtcaatttcaaaatggaaagcacgcttatag
gcaccgggctttgttagttcgcggatatccctaacacgagacggatagacatggaatggtggatgaggctggacaaatgaaggcggcatcttttc
aggaataagggagctttgatggaaggaatccgcggacttggcttcaggagtcaatggagcaaaggaaatattacagggaggaagtttgggaat
acgatcgatcgtaatgttatcgtgcaagagatgaaggtcgaggttgagagacttgaaatgcggatttaaggataggtttgacatagcatggacaa
gattttgagaaggatcatctacatgggtactttcgtttgcgtctttaccggaagagcagcgacagttcacacaacccaactcagccgccgctgaag
aggaatgaggaaggtttacggtagacgaagagggggagttgggagagagatgttgatgttttggagcaatcagggaaggagccattggatcta
aaaaagcacagagaaacaatccaaaaagagggaattataaaacaaaatgtaaaaatgacaaaaaaaaagattaggaaccaacgaaacaaa
taataaattcaaatgataaataggtaagtcggcaaactcggctatgtcgatatagaaaacaaatgtagaccgaacctgttcttttgtcaaattcca
aagcgatgcaacgaaattgcaaaacaagagatgagtcgtacaatcaatgaaccctatatgtgtataacaaaatcaaattaccgggaacgaagg
gtattcacgattcggcaagaagaaagtgactactaggaaccagcaagaaaggatccaaaaggaaagagtattttttgtacttatttggaataaat
tgaaaacccacaaggcaggtatgaagtaaaatccgtgaaagtatgggaaaggcaaagtgatggtagaagaagagtattgtgatcagataaaa
atttaaagcaatatatggtttagaaatatcatgagatttagaataccgagctggttcaacattggtaaagtacatgtaacgaaatgcaacaaaca
aagtaaaacgggaagcgagtttttgattggagcgaaagaaagccaagcagagccaaaaaaaagaaaaagaaagctttcaatttgctttctgta
aagtaaactgcgtttttaagagcaggataaaatggtttgaaaataggagttggaaagccatattcccattcaacagggacggatgagtgtaaaa
gcatcgtccgcttggatctgaaaggatctgcaagaagaatttgtgttctcacgtttgagtctcagaaccccaacttggacagaaagtgttaagaaa
aaaaaaagaaaagaaagttttgattttaaaaaaaatatcaagattaaaaagaaaaaaaagctcaaaaaatgctttctagccattgttgtcgttat
atcctcaacgccagcctgatgaaataaatgtagatcctttcgcaactccaacgaagcgagtcgggttttgcgtcatgctccaatcaatggaagcgg
ttatctgatcaaggccatcaatgcagatcatctattgctaagaagaagtcataggccacttgtggtggtgctcttgcaattctttagtcaagagcag
caaaggatccacaaaggattcacgaatcattcacagggtagatgttgcatcgagaatcaacgacatgcttttcttttgtgccctaagaaatttctg
gttccaatatgtaaaacaaaatacggatatttatatttatatttatttacggtgctttctaagtacaggattgaaagcttggaaataggaacatcag
attactataccaaagaagcatcacttactatagataagttttccgtttttttatttcatcacccaaggaacccatggattatttttcatgtttccgttcct

cttcggattccattgcactcccgtataaagcataagaaagaaaaagtcggatccgataaaccaagataatgacatcactcctttgcatttctctac
aaacgctgctgcctccattccacagttcaatgtctgtctttgtcttgttgccttgtcggtggtcccatccagcaatccatactcacgcttttgccacgta
agaggatggtgggaagtcagaggaagagcaacgtaaaagcgcattaatcgctaataaaataaagacgcggtccatgcttacgtttaccaatca
aaattcattaattttcatttttacgaacgtttccacgctaaagtttttcaaggtgtccgtgcttccaatgtgcctgtgcttggtgtttaccggaacccgt
aattggatcttgtcctgcctttagattcttgatcgagctccttaaccttcctcctcctaataacaaacgtacagaccatggagaaggtttcaggacta
agcataaaggttcatactactgcacaaaatgtttgaaataaggttgattctatgtttcatgaaacatgagtcgttgcggagaagtataacgcgtaa
acaaagcacaagcgatgaaagtaaacaaatagaaaaatcatttctttccctttttctttttggtggatttttcgacggtcttactggtaaatcctca
caaatcgtatcggtgcttctagttttcgcacgaatgctattttattttttccaagacctttaaagaacattagcatttttcaccgaaatcgttgcttttc
tttgctacttaattgtttcctgataacacgtaacatgacttacccttcccggtgaagtcataactggacgcatttttatttatagggataaagcgagc
tggactctctctatactcttctacaggcactacggactgcgaaggaactgtttgtcgattgcctttttcgagcaactttattgattccttctcttagcc
atatttacggacatctcgtttccccgttagaaacgttataggcttttgtttttgttttgaattgaattaattcgaagcattcacttgtttccttcttctttttt
gttttcatcaactttttctctacgcattctttggtgatttaacatagcttccaatatggtatcaacaaccactgttcctgcaaaccaggccgctgacaa
ggttttggcgaatgaatcgaaagattccatattcaaggtcgccattgttggtgctggcggtattaactttggttctcccgaaggtccttggaataatg
cccaacgaattgagcaaaacttaggccaacgtctccgagtcaacgctttgattaatcccattcctaatgaaaccaaccgtgttttggattcaaagc
gtgattctaacgtttcttacgcctacaacgaaactaagatgtatacctccatgaccgaatacttggactatttgtcaagacatcccgaagatgaac
ctaatgccttcgtcatcggtattcctcccgacttccacggttccaacaaggttggtgccgacatggaattgcaaatcgttcgccaattccccaatgc
agctctcttcatcgaaaagcccattacctccgctgctgtcgaggacgctttcgatgttgttcgtcaacttgagcaacaccaagctattatctccattg
gctacatgttccgttatttgaaggtcgtcgaagctgcgaagaagtacattcaggacaacaatcttaacatcgtctgtaccatcgcccgctacaact
cctcttacattcacaactccaaggtcttttggtggtacgtttccaagtctggtggtcctgtagttgagcaaggcactcacttctgtgacttgtctcgct
actttggaggtgatgtagacatttcaactgttaaagtcaaccgcgttctttctagcgatccatgtggtaagctttctgctcttcccatcgatgaaggc
tcgattcccgaacatgaacgtgttccccgctttaccgctgcttcttggaagtacaagtctggtgccgtcggtattctggctcacagtatcattctcca
aggtactcaatacgatacttgcttggagctgcaagctgatggtcactacattcgtatcgtcgacctctacggcactcccagattgtacattcgctca
cctgaatccgacaccgagcgtattgttaacttccccgatgatgatccttatttctcagagtttgaagctttcattggtgttgctgagggtaagcttcct
cgtgagcgtattttgtctgactttgaggatggcaccaagacttatgaacttacaaaggccattacaaataactagacaagatattcataacgttag
aagcacttgaatatttgatgatactatttaaggttgtttttgggttttaataaaaaattcgacctcctttttttccattcagataccttgtttttctgcctt
tatcatccttgtttaccttttgaagggaccggtaacagagggcgagttctggtcgcttgaccattgtaattttcgtcctttttttttgttagaaattttt
aatgaacgttttctctcttttcatgagttttgttgtaattgaatgctaaatagtctcgaacgtcgttaaactacagaataaactttcaatatcttctcat
ttcattagcgttgtattttgtgttacattaaaacaacttaaatatccataacatagctgaaaggttagcaatttcaaagaatctttaaactacaactt
acttcgcaggaaattgaccagtaataccgtttaaggtgccttcagaccacccattcccagcgtcgcgctgagtgacacgaataatgtctccctcgc
gaatgcttatttccaactcgccttgtgcttggtaatcaaaaacagcttgtacatagtgttcatcaacagattcggtagccgattttgttggataaact
gagtttatatcaaactttacataagacgtagggagtaaaccaacagaattgccaattttcacgtaaatccagccacttccatctaaatattacatta
gttcattatgtttagtaatttcaataacacttactgtccggttctaaaagagtaaatgtttggccttgtgaagcactcaagacaccttcttgggatcc
agtgaaatcataaatcagtgtcccatccacttgctccacatcctgaggatgttgggtttcatcattgttcaaagaaggtgtttggtgtaaagaaggt
gcataagcatgacgagaaccaacgctgttagcgtatccatctttatatgtgtcatatgggtttgaggacaaaagtgatccttctcgttgagcgctgc
cgaaatcccctgtacttgatgtggcatcttcatgatcatcttccagggattggccacagttagcaggaacatatagttgacatcgcacatggcattt
gtatttgcagtctataatttatgttaacgttattataactttttgctttttttgcagcttttaaaattcataccctcacaggacaaaccgcgtccccaaa
ctttagagcggcagaagtaacacttaacaggaattttaaagctagcatccttaaatgtgtggaacttcattccttcttcaatatttccagcatcttga
attattttgttaacgcgtacattaagatcgtggattgtgtttttaattaaaacctctttgctccctagtttcacggcagctattttagattcatatggctg
ttcaagtgtcgttgaatcatcaactcgtaaaccttctaactgacccgataggttttgcagttctgcctcagccttttctatttccgatttggagttaacc
aatgtgtttcgaagaaaatttgtactcgcaccatccacagctaacgcattggtgtcatgccaaattgggctaggttcaaatatcaaaagtgaaggc
tcattccaaggtttagcgttatgctgaccaaacattacagaatctaatgaaggctcgtttttgtcaatttctgagttaagtacatctgcttcctcgctt
aaggatttgtaacagcgtttttcacatgcgattccggatttccaggtatcgttcaagtttttcactctgtactcgttcaacttttgcatatgatctaaaa
ggtttggaagggtttcttgatgataacgctgcatatgagcattgtagacaagaatacgaagaatatacatattcttcttatctagcatatcattttgc
gaagttttaaatagtttttcggttttctcatcattgtgtttgtctctcttttgccttgctgattccaaactctcgcaagattcatggtaattcaatttagct
cttttagccaaactcaataagtattctctctgctccaaaaacttttcatataggttgtcataagacttctttaaactgtcaatgtttgtttctaatcgctt
tgcctgctcattagtttcataaacaaaatcatccgcaagtttctgatgaattttcgatgattgcacaagttcatccaatattttcgaccacgttgttaa
agaagcgctgtactcaaatgttagtatggctaaaacaatgaaagagagaaccattccccaaactttatcttcccataaatgcaacttaccattcca
aagagccagcagatttagtaggggtttcaccgaccgaaagaagtgatgatttttttgttctttttttcgacaaattttcttgaaagacttgaaagcttc

tgagagtattccctctctattgttacataggttagtttacaggctgttcgctggcgaggtgaaataattcttatgcaactcgtaccaattgagctcctt
tccttatagtattgttgaatgtcatctagccatttagttccgttatgtacctaatcattagaatcaactcaaaaccaagtttgaagcatgcttcctcct
acccaactatcaacaattttgaagtcgtcctaaaaatcattaagtcagtaagaaactcttaaatacattttcccttatcctatgagctaacttacgtg
caattcggagctaaatttataggaattaagttccatcgcgatgtacttggtctttaaaaaagagaagttatagacaatcgaaaaaggataaaatt
ggtgaatcaaagatagaagaaatagtagaatttaattcaagttaaaagtaagaccgaattgattgttggtacaaagatggtatgtgcttatatta
aaccattgggagggtgtaagacgtataactcaacttgactagagccaactacaccaaacaagaaaaggcaacgtacaaattgggtggtagttat
tcttcgattttctactttttgttttatatttgctttacaaaaagaaattgaacacagtgaccaggatgtcgacggcagtgagacaagaaaagaagag
tgactgggcaaatctcgattcagatgaagaagtcgagcgtatcagtgaaaatattaataacttacgaacttcagaaaacgggcaaaaggatgaa
gagcccaaagtggatctaagcagtcgtattggtcccaaagttagcgaggagcctttaaaacaggacgaaacaaaagtggaaaccaaagacga
aaaggagggcgctactaaggatgaagaagagaaggcaaacgcgaaagcgcaagaagaagaatccaacttaattcaaaacaagaatgaagta
cgcgtaaagcttgctgacttgcaagctgaccctaattcccctctcttttctgttaaaagttttgaagaattgaacttgaagcctgaactattgaaggg
tgtatattctatgaaattccaaaaaccatccaaaattcaagagaaagcattgcccctcttgctcgctgatcctcctcgtaacatgatcggtcaatcc
caatcgggtactggcaagacggcagcatttgctttaactatgctttcacgcgtcgataccactattgccaaacctcaagccatttgtttggccccctc
tcgagaactcgctcgtcaaatcatggatgttgtagaagaaatgggaaaatatactggcgtacaaactgcatttgctattaaggatgttgttcctaa
aggtcaagttataaatgctcaaattgtcataggtactcctggtactgtcatggatctagccaagagaagacaactcgatcttaaacaaattcatgc
ttttgtattggatgaggctgataacatgttagatcaacaaggtctggctgatcagtctatgcgaattaagcgtttattgcccaagaacactcaggta
gtcttattcagtgccactttctctgagcaagtaggagaattcgccaagcatttcgctcctaatgccaatgaaattcgtcttaaaactgaagaactta
gtgtcgagggaattcggcagttgtacatggattgccagagtgaacagcacaaatatgatgtgcttgtcgagctatatggtcttttgaccattggtca
gtctatcatcttttgcaggaagaaagatacggctgaggagattgctcgacgtatggttcaagacggacatacggtatcttgtttaactggtaatttg
gatggtgctcaacgagatcatattatggattctttccgtgctggtacctcaaaagtattgattactactaacgtaatcgctcgtggtattgatgttctt
caagtcaacatggttgtcaattacgacatgcctttagattctgccaacaagcctgatccccaaacttatcttcaccgtattggtcgtaccggtcggtt
tggccgtgtaggtgttagtatcaattttgttcatgatgaaaaaagctggcgagaaatgaaggcgattgaggactattttggtcgcgccataacacg
cgttcctacggatgattatgaagaacttgagaaagtcgtcaaggatgcacttaaaatgtagaatagcaatgtttagttagatggaatggtttcttg
gtctttagtcataataaaatactagaaaagacatggacgagtatatcatatacgtatttacgaaggtacctacgtggattgcacaaagaaaacgc
gaaagacacttacaaagatgtaaaagagacaaacacaaaataaagctatagaacatacactaagggaggaatagtaaaaagaagcacctg
acaaaattgtatacgagacttatcttaagcagaaagagatttttcaacaacctcacgagcaaagttaggaaggacgaaagaagcagaatggat
atcgctgttgtaatacttgcagagtttcttttcttgctcaggggtccatttgcgtacaggtttgctcaagtcagtagaggcattcttggaagcaacaa
caaagccaatttgaccagagggataagtagggatggtagtgtaggcataacggacgttggggaaagcagttttgacggactttaagacatcgcc
aataacgccaaggtgaagccagatgcactcagcctgggtagtgataacaccgccttcacggagagcatcagcaagaagttcgaagtaaggtttc
tggaagagagcttcggcaggaccatcaggatcagaagagtcggtgacgatggcatcaaaggtgttcttgtagtccttcaagaactggaaaccat
caccaatgtggaccttgaccttaggatgactgagaccctcagccatttcagggagatattgtttagaaaccttgaggacgtcctcatcaatgtcac
acaaaactgcctcttccacgcaatcatgcttaacaacttcacgaaggacaccaccgtcaccaccaccgatgacaagaactttcttaggattgggg
tgggaattgagagcgatatgagcaatcatttcttggtaagaaaattcatcacgctctgtgcattgaatagcaccgtctaaaaccaatacacgacc
ataagtttcgctttcaaaaaccaagacatcttggaacttggacttgccagcataaagaacctttttgacctgtaagctcatagcttgtccaggccac
atgtggttaatttcgcggaaccatccgtccttgatgagaggatgagaaatttcttgagcagacatcttttaactaatttaaaggagccggaaaagg
ttctgtttaacgtacaaactctgtttgagcaaggaatcgctgttgttaaaagaaaaacagtcaaacaatgtgagaaatctgcgttagatctcattg
gaatccattagtaaatatgaaatggaagtataggctagtcgtaggaaatggataaaaaaaagaggtcaatgaagctgaattaaaatagttaata
attcgcttttgttaagaaaactatattaaatatgttttcgcatcaactttccgatagtaacgtatatgcgaacagattccccaaagtgatgaaaaga
gcagaatttatttttccctttttcgtattctttagtaactaaatgaagagtcgaagtcttatcggaaccctcatgacaaggcgcataagtttccttgt
actggaattctcgacagagcaaaccgcttgtcactaccgtcgaaaattagatgcatacctaggcaacgtattctttcttttttttcttgttatcgtagg
aaactcaatagaatcataattattaaggatgttcctttttggtctgcgatttctcaagcatcatcaagggtacggtaaaaaacataaggatgtttga
gtatgtaagttagctaacgccaaaaggattcaggtttctaataaaaatgctcttcagaagctgaagacattacctgttcgaaagcaattgcctcta
agttacttacaaccaattcgaggtgtttcaacatctgttgcagacacagcggtagcagctacaactaataccgccaacgcagttggtattagtaat
tcttggtggccgtatgcttttcttcaaaatgcagcttacacaatcaatgtgtatgccggagctccctggtgggtgtctattattgctacaaccattggt
gttcggtttgccctttaccacttatgttgaagtcttttcgaacaagctcgaagctttcagtacttcagcctgaaatgaagaaagaactcgatgccat
caaatcagccaagttagaaaatgaccagatgcgccttacccagcacagtatggctctaagagctttgtacattaagcatcagatcaatccgttgtc
catcttcatgcttcctctcacccagagtgttgtcttctttagctttttctatgcaatccgtcaaatggctcgagtgcctgccgaaggtttcaaggaagg
cggttgtgcttggtttcaggacttgtcagcagcagatccttactacattttgcctatgataaacgcagcattcatgtttacgggaatgcaattaaacc

gatcaaacaccgcatctaccatcggcaattctcccaattggagtaccttttttctttctttgttgtcttctgagtcctcttttcaccataaaacttccttct
gtatgtatcgcgtccaacgactgtatcagaatcactaacgaaacttataggctatttttctgtactggattcctagcagtatctttaacatcgttcaa
ggtttcattttgaaaagacctgccgttcgtcagaaacttggattcgctcctcttccgccttctttagactctgccatatcaggcaaaaatcttttacga
aatcctttcaaggcagttcgtgacatgtataagggtattaaagactccattttgggaagatatgatcagcttaaaattgatatggaaaaacgggcc
ctagctaccaggtctgtcaatgttgttcgacctaatgatcattatagaaagctgaaagaattgaaccgaaaaatggctatcgaaaagaaaaaggt
ctctgagaaaaaatgatgagttacccactatccaattccccactctttttgaaaatgcttgttgcataaaacgtctttaaattcatttgacctcaaat
agccagttgtcccctttaccatacaatgttttaatttaggaaataatatagtctttcagatacgtttcataaaagtatatgcctaattaagtcaaaat
tatccattagttaatagtatcgatttaattctggtaaatacaggtaatcaattgcttttatgttctgtttatttctaataacgtcttttattataaaatctt
atttcgcttattttgttgggtgtatcgtattcaatggcttgactttcgatttctaacctctccattactgctaactccaatcattcctctgcgtacctttcg
tttaaatcttcgacaatgcctttcgaattgaaaagtgtgtgggctcctgctccttctacaaagctttcacaaccatgtaaattgtcaacggatcgtaa
aggcgaacgaattgcttatgctgtatgttggcatctagatgtaaagaaccgtcaaactaattgaatgtagacaaataaagctatcatacttagaa
gtatttccaatcctgaagaatgctatcaatataatgaacatactgctcctactagtgttgctaaattcagcccttccggattttacgtagcgtctggt
ggtatgaatagtcgaggagcatttctgcttctttcatataatattatatattcgtggatgattaaccagtctttctttagatgtacacggaaatgtacg
catttgggattgtgtcggtgaagacaaaatcctaaaaaacaatgttacagctataagtggtagaattaacgatttggattggggtaaggatgaat
taaaaaatttatttaaatactaatttatatctagacggagacaatcaaagaattattgccgttggagaaggaaaagaacggtatgaataccagct
ttaaattcaagagtaattaaagctaattcagtatatcagatacggccatgcttttacagcagattctggaaactcagtcggtgaggtgagtcttttt
cttctttaaatggaatgtctcattttatataactaaccattgtagatttttggtcattctggaactgtcaacagcgtcgctatgcgcaagcaacgccc
gcttcgcgctgctacggcctctgatgataattccatcaatttttccatggtgtaccttatcgattcaatagcagcattcgctgccacaataaattcgt
ttatgatgtacgttatgcccctaacgatgaaaagttcgcttctgttggtgcagatggaaaagtatttttatttgatggaaaaaccggtgaacaagtg
atggaagtcaatgctcacaaaggttctattttctctctctcctggtcgccagattccaagaagttcgtaacctcatctgctgaccgaactgttaaagt
ttggaatgctgacgatggctccttaattcgtgaatggccttgtacgaatgaacaacaagtcgggaacgtttggccttctgaaaatttgattatatca
gttgatgtgaaaggcgtattggtttatcttaatattgacaaagaagaacctgaacgattagtttacggacatcagcgttctatcacatcgtctttcct
ttcttcagataagaaggctttatataccgcttcttatgatggagtagctttagtctgggacgttgaatctcaaactgattcttctttacaagggccctc
tcacagtaatcaagtcatggatatgactaaacttggagataatgtcatttctgtaggaatggacgatacattgcgagctttggacaccaaatcgaa
ttcctttattccagaaagcgtatatgccaccggttatcagccagtgggtgtgtgtgctgttggcgattgtgttgtgttgtccactgtctcagatataca
agtacttcgtgatttaaaatgtgtttctacagctaaaacaatctatcaaccaagctgtatggcagctcacccttcgaaacaagaattcgcagttggt
ggagaggtaagtgtttatattgttataaaatatctaattcattaggattcctgtgtgtatgttcattctcttgaaaaagacgaactttgcgagatagc
acaactaaaaaatcatacatctccgatcacttcaatggcttactcgtttgatgggaaataccttgcttgcggtgatgcttctggaaaagtcgtccttt
acgatgctgaaactcgacaaatcatcacttctcgctgggcttttcatactggtcgaattctgggtatgtcttggaattctaactcaactcacttggca
actgcttctcttgatacgaatgttcatgtttactctgtcgagcgtcctatgaaatacgtcgctgtaaaaaatgctcattccttaggtgctacacaagtt
cattggatatcagaagatgagattgtgagtactggaagtgatgcagccgtcaaagtttggaaactttctttttaaagtaaacctttcaatttagattt
acgaattttggcagcatatttatgaaaattaaataatcctctgaatgaaatatatatcttatttaaagaaataaaaagatccaaaaacacaaaaa
tgaggatactatcagaaacattaaaaattcaataggtcgttggtatcaaaaaacgcaagcatatactaaattagataaatacattaattcaaaag
taaagagtgaagataatgaataaaaaggaagaattagtatgatcatcaaaacgatggttaaacgcctttccacgtggcataaaccacccatctt
cctatcttttcagctaaaattaaagcccggtattctccagtttcgttaatgactgatgatagtatggaacgcgtgatgcgaccgtttgaaaattcttc
atgcaatcgtaaaatgttaccggtcggcgtttggattgcgagcctagttgagagggcttcagtttcgttagaagaaaaagcggttacatagaagtc
gtatttgaccccagctcgcagtataccgcattggggagaagagatatataaatcaacattcgggcactggggtataggatatcgactaagaaact
cgaatgatttgtttttccatgatgagcaaaaggaatggatacagccatcggatgaggtattgactttgctggactagctactccagtgcgaccgg
catatatacgtagcacagcaaacttactattcaaaggcatcatagctttaatcctaaccactctcagattctttttttcccaaaaactttgaaccaaa
acatgtttgtgtgatttagaaaaatctggcgtagaagggggtgtactgtaattatctacttcagcgacacactcaatttcctttggtgcctccatatc
aatctgcaaaaattggtaatcagaaagatgaaggatgctagtttcataaccacgaagacgcagacccaagtcaaaatatacagacgaaaccca
cggcaaagccataacaatcggcattggtaaatcgggcataataaactgctgatctggattctctggaatatgcgtatataggcattcattgggctt
cattaggaaatagaagttattggcgactgaagattttaagacctgctgtgggtgcgtggggcttgcaaaactagcatccattaggcgaacttcatc
attgaatgtaataacattccaagcatggttcacttttatatctctagtatagtaaatgtcatctgacgatttaagatatccctcaatgatttcacacca
cattcctaaaccttgcagcatttcttttactagtaacgaaacttcaaacggagttcctttaccagacagcaagacgtcatgagccgaacgatgtgat
tttccatttggagggtgatttgtataggagacattttccgagacgtagatataaacagctctcaattgatgtaagggagactcaatcccatgcacta
gataatcttttgcgaactctttgggatccaggtgtatcctttgggaaatgaaagaacgagtcttcttatcaattgcaccgaagttcattttatgtaca
tccatgcccgccgagaattttacgggctccccgtcagccaatacgtttccaaaggtattttcaagttcttccaatggcttgtaaacaggcatagaaa

gatcattaaaagttttctcccgttttttattaaggtcaaattgagtgtaagttattgttcgatgtaagtcttcgtgaacagcggccattgtttgaggag
aggctggacgaacagttttctggtgagcagaaggcgacaaggtagctgagctgggttgagattgaaaagagctagatggaattcgtttcgtcgat
gaccctcggtttaggatttttttgaaacgttttctttaaaaatcctgaactttttttacggaatgattgagcggtagaaggccgctttatttccaagtga
gggttggttagttgagaagcactactctcagtttgagtaagagggagcagtgttgaaattggtctcaaaatgggtgctggagtactctgaaatcta
cgatgaagaggagcacgattgctgaaagacgatgtgtcaggagtcaaacggtctgctttttttctgatcgggaatagaggaagctacaggttggg
aaggctttgactttccatgaacgggtggtaatggtgattctttggaccttccattcaattctagatcagtatttgtgtccattcgatttgatgaaggcg
taccagaacggttcgtgcttttacctcccattgcgttaagtgactcaattacattctccatggcactccgaataggagaaggacttgttcgagaaac
atcctgtgtatttgcggaacgcttcgactgaatagattctgatccgcggctgtgtatcgagctagctcggcttcttcttgaaaagacagacgacggg
cgagagaatgactttaagaaagagccgtgatcttctttcttcggacttaagctactatccctttagcttctgaataatatttggactcgtcgctttcaa
attctttgccgataattcaggtccagatgaagtaagaggagaatcgggttcgtctaaagataaacgagagcttacgctgcttcttttcgaataaga
gctattgggtcttgatgtggatatattgaggacttgtacaaaattgctaggaaagattccttgcgtgtttgtattgatgtgccttccgatccaccactt
tccatcaccaaggctttgacattctatcaaatttccaccttcaaaagataaatctcctatcttaagttagtgctgccattttacttgcagataaggatc
cagaaaagatttcacgtatgctgctagttgaacaaaccttcgcgttccccaggccacgcataaagagcacgaaccatgcagggaagttgcttagg
aatagacatccctactgaactatataaacgcttccaatttcgattttgttgggtatacgttgatagcctgcggaagtacctaatttccaaggtgttgt
tatggtacttttaagttatattatactttccttaagcgaagggcaaggacgaacattggttttctttttttttgtgcaaaagagtgcgcagataaagt
acttggtgtatacttgtcaaataaataaggtaattaatgctgaatcattccaatttcgacaaagacagcttgcagttaactcttttccagaatgcgg
tttaggtcctcagacgtcttggttcattaagacaattcaacatagggtgtaagtaggagacgaaagactgatatatctgcattataagtacgatatt
agatattcatctttcgtttgctttattacttcgtttatcgtgataagcattctaaaagtactagtttctcttacaataaaattcgcgtagataacttaga
tacttacgtcggttcttactgcttgaactctagtagtgatccttcctttcccaactactgcttcttcttctatttctttaaacgcataatttaatctaataa
gtaattaacattaagtgaatattcataagtttttgatatttttgctcctttgttcgaccgttcatgaaggtcgaaagctaagtttcaatctttacgcatc
gcaacggtttacgacttgtgttggttttgcaagttcttttatttcgatatttaaatctggttatggactgtctatggatgatctgatagcttcttttgcgg
caggtaaccacagtcaaacagacaaagtttggtctgaaattactccgttttcaacaacttcacctacagaagatattatttcagaagttgcagcttc
tgagatttatagttggaaagacaatgcagttcagttagatcctttttaattttgatgaattcaataggcaattagacaaactctatgggcctcaaaag
ccaccaaaaagaatatcaatggcttctaagagctacgttgggataacccctacctctgattctgtttttcaaaagttactccggttggatgctcctg
agcatggcttgcctgagctaaggcatggtgccgttagtaaattactacgagagcacatgtcccttttggaaccaagaaagaacaaatgttcatcga
aaaactggggctgtattttgctggtcaacagcctacaaaaaacaaaagtctaaagaacgaattcagctcactagtcctgctacttcccctgcttca
aagtctcctggaaatactgtgttattgtcgccaaatatgaatcaatcttccgttagacaaggtgcagatatgtataagccctctttaaacaaaaacg
gcacgctttcaaataataaaatcaagcccatgaaccgtcaagttaatctattatcttcatctaaagaaagtgaggaagaggttctccctctttccga
tagttcgtctcccacctccgatgacaatacaaaacactcacaagcaaggcaaattaacaatacaaaagaaggactttcagcgataagcacacca
ggcgtgaaaaagctagatcttcagaatcatggttctgttcctgcgaatattgagtcattaccttcaaaaccagatcctgtacaattgattcaatata
ataccacaaaaaaaagctcagatataacggatgaaattaactccataccttcgacggaatggggtgattggatgagtgcaaaaacagattcatc
gtcaagaaactttaatgatcgacaaaccgatttattacttctttcatctcgtgatgaatcagaaaagctgagcaagcattctgaagaagcttcgga
gtatgcaccatcgttgccgtagcttttagtttctgacttcctgaagttgattgctatagctttccatatttattaatgagctttgagttacaatatgcta
gatataaaactgttgaatagctataattatttaattgtgagtaaatttccaaatataagttttcattttcatattctattacgatagttgaattgcatct
tcgtatagtctgttatgaacatatagtttcttgctgattgtttctataaaaacataccattctctgggggaagagggcatgtctgtttttttcatctctgt
tccaacatatcctttgtactgatcggaaagttctcttcaaccggctttcttcatagtcatcaaggctactatttttacgcttctcaaataaatacacag
aaatgggaaagggagacgagggcagtgtcatagctaaaggcagttttcctccaaaagaaaaaacaccaccgaactggtatgtcgaggctagtt
gcgacacgcaatataaagctgccataccaccctgtccaaaggaaaagaagaaaatgcgcgttgagagtattccccctgtcaacaatgcgcctaat
tagttctctaagaactgaaaggcatttggaaaaatctgcttcggattctagatccccttcatgtcaaaatgaacatcttctccccacatccagtttc
cttccggtgtaccaaagtcaagaggaagccgatatacacctcgtaaagatatataggatgtgttcggtaatgggatattccttgcagcttgtgcaa
agctttgttcagaatctcctaatccatgcatcatgataactacattatatgtctgttccttagcaggaataacttgaccgtgagcatttgaaggcaaa
caatctgtaaggaatgacatattccaagaaatgactatacgacgcttcgtaaaactcagaagaacaaggataaagcttcgttttttttaaaacttcc
aacaaatcaagaatgcagtctcaacacctacgtagtatcgctttcaggctcgtcttgatgagctaacacaatttcacgaatgatttgagcaattgct
tcaggatgtatattattttcgcatagacttacgcaaagagaaagagctgttttgtctaaccccacgcctagcaaagttcctatttcgtgtaagatatc
tatggtttccttggctgaatcggctgaggacatgaaaaggattaacaaaagaaaagaaagaacaagcttcttccttggcagtactataagtttaa
tctagcaactttttgctaatgaaaagctgtagtttccttagtcacaaggtgctttcagcatggtgtagtatagtgggtataggtctcaaatatatattt
actttctcatatagaagccagccttgtttcttataggaatgatcctttttgtttgaaagttatttagtataaaagatatttacgtaaatttagttgactc
attcgccttgttctagctttactctgtaaaaggatgccatgtttccactttcagctgaatcgtatattttgaaagagaaaattggatcaggatctttcg

gcattgtctggagggcaattgaaaagtcctctggtactattttggctgtcaaacaggtaagttgaaatttaagaatccacataaccgaccattgcg
agcaagctactatacgaagaatgaagttctcgttggtttttttgaatatataagttgaacttgaatgctaagccgtctttactcacccttcaccattt
ggttttatgttaattacgatcgtcataattctaactgaacatagattgatttggaagcgtctgtggatgatatagctgaaattgaacaagaggtttta
atgctttctaattgcagtcatcctaatgttatacggtattacgggtgcttcgtccaaggacatgtcctttggatactaatggaacatatggatggagg
ttctgtttctgggcttctaaaaaagcaaggtcttgaagaaggaatgatatctataatattacgagaaaccctgaaaggtttgacctatctgcacaa
ccaaaacaaaatacacagagacataaaagcggcaaatatacttttgtcttcttccacgggtgaagtcaaacttgcagattttggcgttgctgctca
actttcgaacgccgcttccagacgatatacctttgtaggaaccccttttggatggctccagaagttatacaacaaaccagctacggaacagctgc
agatatttggtccttaggaatcacggctatagagatggcagaggcagcgcctcctagagcaacaatgcatcctatgcgtgtcatatttgaaattcc
acaatcagaaccgccaaagctgtcttctgattatggtttggaatttcgagattttgtatctcgttgtttaaagctccatcctgcttctcgtcgttcctcg
tcttctctgctagagcacccctttattaaacgctcgggtaagattgaactcctaattcccttactattggaaagcaaaattttctctaatgaacaaca
aaattctgaagatgatttcgattcaaataccactgatacgttaaaaaagccaagctctacatcaagatcgaaaaatacgacctttgatgatgattg
gaattttgaagatactgttcgcattacttctccgaatgaaacaaaaaggaatatctccaccgaaacggcaaaaccacaaaagccccagtcttcttt
ttccaattcaggtttttatccttcgtccgtaaattcaactaatgctgaagctactttgaagaagtccataaaatactcatccaaagaaaactcttata
ctccatctcatttttctaaaccttctcgtccaaaaagtctagcaacttcgtgctttgactcaatagtttcttcaattatttcagatcctaatttgtccgct
gatcaaaagtcctctttaaaattattaagttccacattcaattcattagatgaagatacctcttctcgtttaatgaacagtattatgtcagacagcaa
gcttaatgccaagaaaaagaaaagaggacctattgctcaactattgttttctcgatggctagaagaaactgataaacgtagaagttcaagttagt
gacatttttccgaacgatgaaacgtattcaatgaaagaattgcataatttattcatattatatattagtaaatacatttataaagagcttatatagt
aacgaaagttcaaacattattgaaaatgttttgtatagcttcttgtacattccaatttgattctactagaatacgctcagccttttcacgaactatag
aggcgtataccattaagtagtgcaatctgacagtagttagcataatcatcatactaaaacaaaaaatacttacttagcttcccgtttgacggaatc
agcataatccaaccatttctttttgacgacgtcccaagtcgaagcttgttcttgagcattcacaagcttaacttttgcatgtatcaatgctgacacag
gacgatgatcagaagcacggacctcatgtcgtctgtaattgtcggcgtgtatgtagtccatatcacctcgaaagcagatacgatcgcaccaagct
ggaaccctcttcttttcacttgaatcgtaccgatcactattgacatcatatttgtatgtaggagcaaaagttatttcaggttcactgaatgttcgtaac
cggaaacctgcattgcgtttcctttcttgcgtcaattgatcactttgtaaaagcgtttttatgtcattctttttcaacaaatcattgactttgggccttaa
aagattgattctataattaaggtcgccatgcaaaatacaaacctcatgatccaatatttgagaaccatcaccacctgcttcaaaaaagttcaaccg
atccatttcattgctttctggggtcaaggctgcggagtctaatattgttgccaaatcattattccttgctgccttactcgtctgaccagcagctaaatg
gcagttcactatgcagaatgaagtatcatctataagaaaacgaacgacgatagcacctttgtttccatgtaatccacccaatccagttttgacagt
agtggtttgtagcattcgaaccttgctttgcaaagcacttttaacaaaaatgcaagagaaaagaccaactaaattttcacagaccaaaacttgat
agccatattttcgatcacaccgtgtcatttcagattctagtttttcacgccataaacgatactgcctggaaatgttgtcactatttgaagaactgtttt
tagtgctagatgaaagcaaacttttggcagttagtttttttattttcaaggtcaaccaattcttgaaagccgaatatcactatgtctggatatccatttt
cttcaagtaaagcctttatgaaatcaggaccagcgtcctgacttctgaattcagacggtttggaagcaccagcattccaggaacagactaacgca
gtaatatcttcaaaggagctatacttcgggaagagctttctcatttctgtagataaccaatcgcccatcattaatccatcccaaaactttacaattgg
agaacaaaggctaaccacttgtaaacgtttagctttccaaatactgtttacatctatacctaagatagtatttgcaccactttgactaactttgtgag
catgccatgtttttagtaatctccaagggctgacagaagcatcaaaaacataaatcattccagtagtgtaagctgcccatatagtgctgccaacac
caaccaaagaacttatacgataaaaggagctactcgtcacaagttccataaatcgatattgcgtttttgaatatattgaaattttaccatcaacatg
gccgtaaaagacaaggtcgggaaagctagccatagctgttccgcaagatatatcaccaataagtcctgggggagtcatcggtttttgaaggata
gatggacgttcagtattagcagggtcatatacataaatagacttgccgactacggcccataataaattatcgtctaataccatggcatgcgaagc
atggggaacaatacggacacttttcggatcagactgtacacttaatcccataatcctatcttcttgccaaacaagaagtcgaccagcttcatcaag
cgtccaaacttcatttttacatgtcaatatatgagagattgatgtatgaagagtggttcttttgtttactatatggtgatttacaacatcaatctccca
aagagcaccatcgcgagtaccaatccaaacgaatcttccatcatcggctactttattaccagagggtttaaaagacattgcggttactttaacatc
tcccaaaggcatatgccaattgcataaacccgtaatggaatcataaacccgtaatgcaccattaccagctaaaacaaggcgctcaccacttacaa
caaaggcacgtgcttcaaaatctaaattgatagaatcagcgccgttcataaaagtcggttgattcctacggacacatgatacatctggtaaagta
cctttcagaattgtatctgtctgagaggtagaaggggaaccagaagatatcgagatgtcctgggcgtcgctcatagcagaggaagaagttggcct
tatgcctgaagataacgatcttccaggttgaggagatacaaggaaaggaggaggcgatacggaatcgctttcattcgtcatattattcaatgatat
atctggtgccatttggctaggtcggggaggtaattttggtttttcagaagataacatcggctccgacgcagacagactaatatttgacatgggtga
agataccgaagaaattcgcctgcttggcaagctacccgaaaattcgtttgaagtagatgtataaatagtatgagaagattttcgagcaggaggtt
ttggcggtcgttgaggagtgcgacttggaacctgtttagccatttcaatacgcgactgatgcacagcttctgcagcgacagacttaggtggtctgg
gtgaaagaaccgggggttgaacatcaacatgctgactctgtaaacgcggagggcggggtatggaacttgaagtggaatccaaagtttcagaatc
atgtttaaacggattatcgtgttgtttagaaaactgaagggaactcttggctgggataccttcatcttcctgctcagacgatagtgtcgaggaggat

tcaggatctgattcagatattaagtcttcttccacttcatctttctcatcagtaggtttttcctcagtatagcttggacttgaatccaaatttggttttgc
tggtgatgagaatcctcttgtaccccctttggttttgttacataatcaggtctaggacgattcgttaagtcagtgtaatctcgtgaactgacagatc
gcgaaggtttattaggcaaaggagaaacagaaatgaaagaattggacgcagtaggtgatgatggcataaaagcgtccgtatcagaagatgact
cttgattttgagaaaatgcttttcgtaaaatttcaacgctaggtttatttttcacttcattgtttacgtcttcgcttttcacggattctggcaacgaattt
gacccactcgacttttctccctgggtggacgtatttccaactgaaggaaaagagggaggtatttgggttccttgatatttagaaactggtttttcagt
attatttgcattctgttcctgagaattcaaatgctttttaacggggtatttgtaccattgcttgtaacatcgtttgagctaagagattcaaattttgac
cgcaatgacagcaccgattgagggaattcgggttctttttggtcattactcgagtcctgccgagaggccataatccatgtttacacagttaaaagta
tgagtgcaataaaaactactaaattcccagaaatctccctatttatatatttatatcgtccttctagattcaaacctggataagcaattatatttgta
attattcttcgtaaaattcacttgagtgttggaagaaggcagagttcacgaacgaagtgtggcacgattatatgccttgctcattttaatcggttcat
ttaaacagtataaagtccattacaagcgaaaccttatgattgtagaaagatcaaaaaatgcaattcaatattgacagccgttcagaaatatttttg
acataacaaaaagtttttttcttttacttctttacgagggtgaaaggtgcactaaaagcttcgtatttagattaaaatatgtgaataactgcttggt
ttcttttatagactaagggtgcttacttagacgtctttaaataactaatgacaatttagcactttttttttacttactataatatcgtcaacatttgggg
gggttcaattatcaaatctttagttatcataaaatttatacgtaaaacaaggaactaatattgtgtttatataaattggtttttgacacgaccgttact
ggacatacgctaaaccttttttcccaactctgtactcaatggtaaagatgttgacgtacaattctattgcaaaaaagatcaataagtcctttggaac
atttctgacaagatatcgggttacatgacttgaaaaggaaagacagaatgactaaaaaagaacgttgggttcagtgaaaatcaagattagcata
gaattcaaaacataaatgtatgattgaaccatagaagagaaacagatagcatggaccctagaccgccactttcatgtctttacaaaatccgagac
taggaataatgaaacgaaaagagagtaggaattaagaaaataaaaaattttagattaaccatgggataacaaataaaaaagaaggaaaaac
gcggtcaaaacgatcatcttagtcgtcggttgaaagattgaaaaggcacagaaaagtcatggtacgtatgcaaaaatgtttgggatcatgaaaa
ttagcgcttacgagaagagctgtttaactttagtcgatcaatttcaagctcgagttgtttaatttggacatgctgagcttcaagatctttgttcaaag
agtcacgttcagcaattaatctttgttcccattgtcggaagcgagcctcttcttttcgaacctgttcggtaaatcttcttcttaggttttcttcctcctctt
taaacttcgaattgtcaatagtcctcaatttcggttcaccgtattggcgaacctgcatctgttcctgtcgatattgctcataaataatgtcttcagttct
ctgaataagatctaacatacagcttcgaaccaggagatttcgtaattgtttgaaatcacagtgttcttcattctcaacctcgacggtgccccaagga
tacttgcgacctttcacagtacggccatcagcggtttgtatatcatcttcgctaccaacaattgcgaaaggcattgcgttgatgataccttgaatttg
gcgttggaaaactgggtctccctcatccatacttggcttgtatatgttgacctgatgatagtcaagcacttgtgctatgcgagtcttatacatggcca
aatcatgtcgggtgtacatatcggacttggcgattacaggaatcaagttgacacgcttgcagagacgcttcatagcctccaaatccatagggcgt
aaaccatttttcaccggacgtaggaaatacaagcatgcatgaatacgcatgtcaataatctttgttctgtctggttgttgatcctgtcgcatatatga
ttcatgctgatcctctataaactctacgacagattcccagcaaccggtgttattaataaaatctccaaatccaggggtatcaactacagtaagtcgc
aagtggaaattcttttcctccaattctgctttggtaatttcaatttccacactcttctccatatgcttcgagcggcgcgtctcaatgtccatatgaggct
taatcgtagtggaaaacaatgtattacagaacgtcgttttaccgagccctgattcaccacaaagcatgagcgtcaaagcagcaccattacgcgac
acaattttgtgtctttgattaggtaagtcagcaattccaacagatagagtctcgtcttcattcattttcgcgtcaatctatctaatacttgaaaccttg
ctaagtcgcgaattgttctatggatagcggatttacgtgtgttctttgtgtttgaaaacctttctggcaataaaaaccttgcagattctcttctttctat
aaatattactaattgaactttctgatgaaggatgcaggccgcaacaatccaagttcaatattcgctttgttctaacagaatgtcggttcttactgga
accagatcctccccacagcaggactgttggctggtccttttcgctcaaaacctcggtaagcggcttttatgaaatcgaacaaggtgcttctgttcca
atcatgagttcgttgcagtgcactaatatacaaacacctatatatcatactagaaactgtatgatatttccttttctttctgtataaagagcaatcat
atttcctggggctgtttcaactcgttcgataccactatgaatttattttacttctagctttaaagtttgttataaaaaagccttctgatccaagagtttg
caattagtcgtaatgatgaaaaactgaatgttttatgagatttaataaaaaatcggttgtctgaattagtatctttttactacgttccaattgatcctt
gaaaaactatatcattcatcgtcttgggtgtaagtctttttaatttattctattttatgctcatgttcttaattttctgtttgtttacaccaatgtaaattc
acagtaagtagcatgctactttactcttgaggaaatttttaccaactttttctataggccagagcttgttagaatttaggcctttttaaggccaaac
gagaaggtataactcttttgttttcttttctatttgtatataatttgtcttgataacgccttaaaaagtgagtagcttgtggctttcgtatatattgcaat
atatcaagatttcaagtgttggatttggtgctaggttataaaccttcatttttttggcaaatatcatttttttgttttgttattgggctatcagcttgaaa
aggtaactaggttctcctattcaaacactccttttctgtttacagcattacttgactatcattatggtcttctctaagtataaagaatttgtagaggac
ggtgatcttgtggtggcttggattggaaggaataaactgattcctctgaaaattgaagctgaccaaactttccacaatcaatatggtgctttccccc
attcagaaatgattggcaaacggtatgggcaacaaattagctccactgctaaacaaggattcatttaccttcttcagcccactcctgaactttggac
attagccctacctcatcgtacccagattgtttatacacctgatatatctattattttccaaaagcttcgcattgtccatgggacaagggttattgaggc
cggaactggaagtgcaagcatgtcccacgcattatcaaggaccgttggcccatctggaaagctttatagttatgaataccatgcttccagatacca
gacggctctacaagagttccgcgaccacaacatgctagaaggaataaacggaaacactcatcttatgcacagggacgtttgtaaggatggttttg
aaattaaggaatcaccactagaagtggatgctatctttttggatcttccagctccttgggaggcaattcctcacttaattaaacacgttagtcgtaat
agtagttcgcgcatttgttgcttttctccttgtatggagcaaattcagcgttctgcatctgctttgcaagactctggttggtgtgacatcgagatgcta

gaggtctcttacaaacaatgggctgccaggaaaactagagtaatacatatcaacgatgcaatcaacagattgaaggaagtaaagcgtcatcga
acggaaggatttgagcgcagaaaagaaaggcaacgtttagaaaaacagcttgccgaatctgctgagaatggtgttgagcctactgattcacaac
cttctactcctagtgaaacaccatccaaaagccctgctatgacagaaaaggtcaacaaacgtattcgtgaaggtgatgaggattacgagtggag
caatgttgcacgtgttgattcaaatttaaaaagccatactagttatcttttgtttgctgtccatcttccttctcaacttgacaacgaaattgagtctac
agaataaatactttcacactaaccaaagtaagaataaaaacaagctgccttttatttttcggatatataggaaacaataaatatacttataagctc
aattttcctaagtcgtactgattatcattattacaaaacgtcattaatgtacacatcccttcaagaagtattacctaatcccgtattaagtttttttggtt
ggaccatcttggatagcatctggtgaggcagatcttttttgggatcattcaatcggccttgaatgtcggattcctgagaaaagcgatcaagctgtc
ttttcagttcaagaagaatgactatattattttgtaattccacgtatttttcagaaacttttgcagtcggcataattaagcgtgatgacacgcctaatt
cattcatcgtagcataaactttctgaactaatgaagctttgacagcaggaatcttctgcgaacgaacgaagcagccactgtgtaacttctcatgcc
atgaaatacctgattttgcatcgtttggagatggaccatatctgactttgggagcattcaacgtaagtggacgggcaggcgtgctgacagaactac
ttacaccagaagatggagccgaataagtggatgtaacatcttctgctttagtgacccggcgagcgtttaccatgtcttgaattaacccacttactcc
tgcgctggtgtggaacgattgaagatcctcagttgtcttttgttcatcaagtaattttagcacttcctctcgatcactcaacagcttggattgcaaag
actcaattctcttgagttctatgaacaaagcctcttcctcagccacctcttcaggtgttctcgaagccaaaccaacgagataatttttccgaattact
tcttgctccttattatactccatgctattaagcaaagaactctgagcggctgtcatgctgttgatgggatttcgagctgtcagaatcttccgagatac
agtgtaaaaccgatctttaagatcttctaatgtacgatttttagctggccattgataacgatcagctatcacaaaaaatcgaagatcaaactctcta
cacagcttaaataaataatccgtttcttcctttgaccagtcttcatctgttaagagttaatatgacttattgtacactcttaaattatcgtaccctccaa
taattcacgatattcctcttccgtatagtcctgaataaaaagggttgtattaaacttttcaaatttataagctgaattaagtcgttagcataatagctc
ataattctttaactgctaagcttatatagcacttaggagctatttcaacaaaacacatacgagcttccgattctgcttctgactttaaaacccaatgg
tataatgtaaggtcatcttctctagttgggtttttgaacggatgccagaccctatgtagtattagtatattttcccctatttcttgtagaaaaaaaaaa
aaacaatatagaaactacagacgtttcatttgtacaggaacactcactgatcacatttgaagtgcttaacgtgaataacacatccagtttctacat
accaattctttgctttctggttactcttcggcttttctttaaattttttttgataaatagctaatggtgcagaattttcaccaagcaacgaaaaaagttc
acgagagactccttctggacgtctttcgccatggcttttcggtttctgtttagtccctgctcctggtgggggaagctcaaaaacgtcccggatatctg
cactgctcattatcgagtgatacggactttgcagatatactactgaaattgtttcgtaccgccactttcttggtggttagtttgaaatttagcaatcgt
taaagaattaccttatgtagtaataacggattggagtggttctctgcaaaatgctcgctcgttccgcagaaaaccaatactttcttagatgtaaata
actccttttgtcttttgttgtttgttttttttttttttttttttctaaacatattactatatctatagttgtgccgcgctctatcctttttacaatcatccatc
tctattggagggagatataataggtgtatatcgcagtcttgaattagcattatatctatatagattttcgtataaaaattacgacgatgtgtttctatt
aacatctttggtaactaatgaaagcatagcgagcatcataaatcatgttcatcaggtctcatagaaaagcgttacaacttaaaaaagtataacac
tcaattagcacataaatggtatcgggttatcattaagtactagaaacaatttcactagcttgcaataaagattcaaccatctgttcgcattctttgcg
tcgctgaacggtaacttcactttcttccaaaagtttctcgtagctgttatttttgtaaatctgttcaagtagttcgcgctggatatgctcctttgagtta
cgaatcatcttcagagaaacggacttgggaaccatatcagctaaagtcctcttcacgatgttaaaataagaagtaatcagtaattctaacagttag
tttaattttccatttccaataaaaacttacttataacttcagtatcagtcttttcacggtcagaaagagtggtggaagcacgaagaacgggtggtgg
aggctccattgctgcgagtctcttcttgttctttgagccaaaaaagcttccaaagaaactatttgaactgcttgttggtgtctccacagtctgagcag
gattgtttgttaagggttttcctgtcttaggatcaattggaatcggtttattgctttggctttgtacaatagccattgcttgatgtccagataaaaagtc
agggtgaacagtatttatgtaagaaccttccatagcgaccaagtcacttactaaaacggaagttggcttcatgcatttcttaaagaagcccatagc
aactttgaaaaattcgtccttcaactgagggaagcgcttgaatataggcttttgcaatagctggttcagaattcgaactaattcatcaaagataagt
gaaacgcacttttgtgagggatcttccaggcgcttaatttgttgtttcacgataagctcaaatgcttgcgtccccataaacaaactaggagaagga
ccagccgagttgtaaagaattgtacgaatatctgtatctttgacttcatcaaaaggatccactgcttgtatgccatttgagaagatctcgtgaaaga
caaaagctatacgtgcaccgccagataattcggtaatagacaagtcctcactgttaccatcaagaaccgtacgaaattcattacaaaaatctgtt
ataagatttaaaacgatgctggaactgtcaccaaccaaagaatcaccaagtgactgtaattcagcctggtattttgctaaagcagcattgatacg
aaccttgatttcagggagagtattacgaatatgatgcatcagaatcttttttgttagtaatcaaagagaacagtccaacttaccatattcaatttcc
gagcaaggaaaggagtaccgcaatactgagcctttgaactgtaagaaggatgactttcaaaaaactccttttccgcttccagagccaaacgaat
ggatttttaccctcgatatctttttgaccacggttaatgactggcacatagcctaaacgcaaaggaataatgcgaccagcaagaatatcaacgac
atcagtacctttgtccattaaatcaacctttgtcagtacgccgatggtacgcaaaccctctggatcaacctccctcgcaagtttcaaaccgtctgaa
tttgcaaggtcggtattagcggcatttacggcaagaataatggcgttattcttggaaatgtatttcaaaaccatttcacgaatttgcttttcaatgtc
acgaggttggtcaccaacaggaacttttgtaagtcctggaagatccactaaagtcaatgtcaagacgtgaggagagtaaatgcgtaaataaatt
ggaacagaagaaattcctacattctttcctgtcttttcttcagtttctctaactatttcgctcctgattctttcaaagtcatacatcttttgaccgggaa
gatggaggaattcaccccattcggtattgttgttttcgtcggtcccctctttcgatgagctattagtgttctcattcttggaattctccgggtttggtcta
tttattaattgtaaaaccaatggtcgtctagttacaattcctgtgccacgaggaagaaagtctcttccgacgatgttttccaacacagaagattttcc

gcttgactgtgagccaagtacctaaaagaattggttattactatggaaaacaaacaaagcagcactaaagcaaacaaactttcataccgtaatct
gtggtaaatcaatcaggttttgaacaccaactgtggaaaaggcttcttgaagttgattgacaactttaatcaaggatggatccattcttgcttttcg
aatccacttaatcaggaaaatacttttctataattgggttattttttggtgtatgtttgtggtgaattgctgtttatgaatgtggtaacgctgcttcttcc
tcattaacgttcgatgttgagtgtcgttaaaaataaatagcgattgctttagtttgcattaatttacgataaggtaaaaggcaatcctttttactttac
aaaacaaacaaagcgttcgggggtttttttaaaaaaaataaaaaagtgtgtacttctattgaaagcctgttgtatatgcaaaaattttaaaaatg
gagacctttttgatagtactactcaattgatatccctaaaaaatccggcaaacttgaaaaattccgaatttaatccctttttgatggagtctccaattt
tcttgtagcttttaaattaaattaaatttaaatacaagactacttcattcatattattgtttataattgaaactcttatttgcaggtttatctgatcaatt
cttatatactaagggtggctcccttgtatatgcctgcctttcgcgaattaactgaattaaggcaaatgtcagtaaaggccctttcttagatcatttgtt
gatcattctttttataaaaaattatactcttctttttattttttttttttgatggttgcttgtgtgttatagtggtctttgagacgtaataatcaaaagcc
gaaatattgatacttcgtccgtttggtttgagtcataagcactagaggatttattaaacttgatcaattaacttattaaagaaatccgttcatgttcg
ataacacagaccgtcagacgatacttgcgacactactgttgaaaccgattcagaccgtattccaatttgtacatgagagcggcggtaaagcagat
gttaaagacattcaatctttgtttataccaaaacgaacaattccgttggcaaactatattgctttgacttcttgtggagggctaaaaacaatgctcc
aatccaagcagctcaagaatatttttcaatattcaaagagatttgtaactattactacaactaatgcacagcaagtatctacttcgaaagtttatcc
caaattggttacctttgtcaaagagcaaggacaaaataggtctccagagttctgggagaatatcaataagcaactatcaatatcttataaaaata
aaaagaaaaaagagaaactgttccctgaggatgcattgtctgaaaccaataacaacgcgaattctctgacctgcatccctcgtaaacataagga
aaatatggagcttgcaagctttattgatgagaaaaaaggaatagaggaagatattgagcactttacttccttactttatatcctatgcaggctttc
ttttggcatttaaaaaattcaatagacgggataaagcgtcgagatcttaccacagtattaaaaaaccaggaaacctgtcctattccattggaaga
aagagactatttgattttatcccaattgggaggcattgacagaattgctacttctgactatctaagtcctcttatattatatgataccacctcaggga
ctattaaattgaaaaatcctaatgaaaaaatttctcttaaacttctgcgacagtgtctatgtaaatctgtcaaggagcgtcgtgcatctttgaaacc
cgatgcatggaggagtataagtacttacctgaggaaggcaactgtcaagtatagctcttctcctatatttaatgaacaaaagcaagttactcttac
cggagactcaaatccgattataagcttggaatctaaatccaacctctttgaaggttctggtcttttgccccttggttacttggcagatcaatcacgtt
cttatttttccaattctatcaaatttttacttacagacttgctattggagcctcttcgagctctgcttgtttatttttatttttacaaatctcccatgtctat
tggacagatcgaacgtttttttccaagtcattttagaatcgcatttttttacaaaattctactaaaaccgcattactgctggtaggtggtattgtgaatat
tttgtcgtcggcgtcttcaaggcacctttttgaatgtatagacaataatggaataaaggtaagccatccattgagaacgatgttttctgaaagaatg
tttccggaattattcaaatctgttgaacagattgtgtccgagtccttttctgaacgtctgttgggaaatgataaaaaaactattcacaaaccttatgga
tgagctgtcctctgccaatttgggtagcctacaggattacatgcgaaactcaaacataaaatggaagatggtatatttgcttgttccgactttattg
agtttaagccgcgagtttgccagagaccctcacctttcatcggaaaagatttttttcagcttttcaccataaggaaaatatggagaattcttctttatt
ttcaacaccacttttgcagctggttgaacgaatgggtggcatcgctaaatttttatcttcacaagaatttgttcctttatttaagatagatgaagata
caatgcaacccatgattgagaatgaacagaaattacttataactgtgtttgtttctgtatataaattctcgaccaatatacagctacaaaacccagc
gctgatagacgatttacggaacagtttaaacagctactttcaggatgaaaaggatgctgagcataaaaaagtcattgaagctggatcgatcatctg
agtttcaagctcaaactgtaaaaaaatattgaaaaagcggagcaaaatacagaaggactacaagtatccaatgttgaagaaagccagttccgtg
ataaagcaaaagcaatttcatcaagcctttctgatttactaaatgcggaaaaaagagaaaatgaaatttctgatggactttcgaaatacgcaaca
aatctactatttggagaaaatcaaccatattcagtcgatcatatgaatctgcttattttgagaagatcagctaagggtattcgctccaaaaaagag
atccagtcatttttgaatagctatttcttggtcaagttggagcaatcatttgtcgacgggtacttgatgaatttagaaaagttgggtctgattaccga
ttcttatccacgtaagcttacgccaattggagaagaatttttaagccatcccaatgagtgggacccacttaaaatgtcggaaagcaactttatggtt
gtttcaagcaaacttcagaaaacgagagagagagatatttataaggtatatttagacaaaaagatcgataaaagacgggaggaattggtgctta
aattcataaaaaaaatacgaaagcttttaagtgaaggtcttaatatgcctgtccaagtttataccttaggttcgtataggactggtttaatgacaaa
tcattcagatatcgatttggttgctacttctaaaagtccccttttaaagaaatttaacactattactaagttaattttcaaaagatatagagaagtga
ttccgatacgacatgccaaggctcctgtaattaaactttctacaccagaaggtttccgttgtgacctaagttttgattccatgttcgccgctcaaaat
tcatgcttaattgagaaatatttaagtatagatgaaagagtcaaagtttttgtttcaactataaaaagctgggcatcacttcgcatgattgacaaat
cctattttagtgtgccttcgggttatacgtggtgcataatggctctattttaccttcaacaactttcgcctccattattaccaaatctccaagcgaaaa
attccgaattttcttacaagttgtctgactcgacgcatgaatcagtaaattgttggtttgaacaaaacatagaaccatataaaaaaacagcgaagc
ttaacaaaaactctgtcaatgatttgctgcttggttttttttattattacagctcctcaaaaagtggctcttttaattggaatcgagatgcagttgatat
atcttgtggtcgccctattatgaaaagtacagttaaagaagccagcgaatctcatatgatggttatagatccgtttcttaaaaccaagaatctaac
atcagtcgtcacttctgctacaagggatttagtacagtatgagatggaaagagcatatcgaatattgagtgatcccaatgctacgattaaacaatt
aatgagtccgtctgttcgatgaagaagaatttcctgaacagatattacaagatttgggaaattagacttgatttacgttccttgtcgttttgttatgct
tataaaaacaagaattgggatctacagcttggttacattctcttgaaatatctaaacgcgcaccgatagaagaaactggtttatttaatttagtcct
actaatatactaaaaatgtacatactataattactacccccctaatcgcaacaatatatttatccatccaatattaaaaatgtttttttatcaatatca

atataaagtaactaaattatctaaaataggagtacatataaccttgaaatatatcatattaaaataaagctcactgaagcgaagtactgtaagta
gagaatttgcaggttacctacctttgacaaaaaccgttattttgcacttcagcaacttgagtctttaaattttgatgatattgtatggataatgactta
gacatgactggagaagaggacaaacttgtttccctgggcacggatgtgttttctcgtgaacgatccgttggtcaaaatatggacgaaaataataa
cagacttaacgaaaatgatcaagcaagagcatcaactgatacatcagagctacaaaaagtagcagaaactaatgagaataattcgtatgctcc
ggaaaccgtttacaaccttcaatcagatttcaatgaagaaggaacacaagctggaatttttcaagatgaacgccatgacagaacttttgctttaca
caaggtggtcgtgcctcctgactacaagtacaatcccattgagcagcatgttccaaacgacccacctgcaaaatcatattcttttcagcttgatcca
ttccaagctactgcgatggcttgtatagaaagatgtgaatcagttttagtaagcgcacatacgtccgcaggtaagacagtaatcgctgaatacgct
gtcgctcaatccctcaaaaacaagcaacgagttgtctacactagtcctattaaatcgttgagtaaccaaaaatatcgtgaattgctctccgagtttg
gtgatgttggtcttctaaccggcgacgtatctatcaatccatcagctagctgtttagtaatgactactgaaatattgcgttctatgttatatagaaatt
ccgaacttatgcatgaagtagcctgggttattttcgacgaaattcactacatgcaagataaagatcgcggagtggtttgggaggaaacccttattc
ttctaccaaacgcggttcgctacgtttttctctccgcgacccttccaaacgctatgcagtttgctcgttggattgcttccatacattgccagccttgcc
atgttgtatatactgattatcgaccaacaccattacaacatttcatatatccccatggtgctgatggtatatacatggttgtcgatgaaaagaatcgt
ttcaaagatgaaagctttgcaaaggtattggaagtacttcaaagcactgttgagtcacacgaaaaccgccaatctagaaaaaagggcaagcga
acaacatcactgcaacgcataattagtatggttataactaatcattatgatcctttaattgtgttttgctttagtaaaaaagaatgtgaagctaatgt
ctttcagttaaacaaggttgatctcaatggtgacgaaaaaaaggatttaataaatgaaattatagattctgctttacttcaattaaataaggaaga
tcgagagattccgcaattcaatcatatgagatcgtttcttttgcgaggaattggctttcatcattccggactgctgccgattatgaaagagatagttg
aaatcctctttcaggagggattagtgagaatattatttgctactgagacatttgccataggtttgaatatgccagcaaggacggttttatttacaaat
gctcaaaagttctctggcactaactttcgttggctgacgtctggagagtatatgcagatgagcggtagagccggcagacgaggtattgacccaaa
gggtttatcaattgtgatggttgatcaatctatagatgagcaaactgctcgtgcattaatgaatggtcagcccgcatctctttattctgcttttcattt
gagttacaatatgattttgagtctaatgagaattgaaggaataagtccagaggaccttctacgaaagagtttttatcaatttcagaaaatggaatc
acttcctgtgcttgcgcacaagctcgacaaatacaaggtcaaagagaaatctattgaagtgaaagacgaacaatccttaagaagattccatgatc
tgaaattgcagttggagcgttatgggagagatattaagcatattattacggaaccagaaaattgcttaccatatttacagccaggaaggctagcg
caggtaaaaattcatgatacattatttccttggggattattgtgaattctactaagagaatggattcctcaactccaaaagtggatcctcaggata
tgtacataattgatatattactcccactatcgtcaaaatcaacctctaatagcaatttacgatctactcaactatttgcacctttacctaatgaaaag
ccagtatatgaagtcattgctgttttcctgtctgtcattgaaaacttttcttctcttagggttcttcttccaaaagatttgagttctcatgaaagcagaa
gaagtacttatcaaaacgtccttgaaattcaaaaaaagttgaacgacgaaatgcccttgctggatcctgtcgaacacatgaacataaaagaaac
tgcgttgaaacttcatgttagaaaagttgaaattttagaacccaagtttttagaaagcccctactataaagatccatcgtttagatcggagtatcac
aaatatacggaaaaattagacttaaaaaatcgtataaaagatttgagctcgaagattgggactactgaatccatcatccatctaaaggaattgaa
aagtcgacagcgaacacttcgacgcctcggattcattactgctgataacgtgattgatattaagggaagagttgcttgtgaaataagtacgggcg
atgagcttttaataactgaaatgatttttcaaggtatttttaattccctccctcccgatctttttgctgctactctaagctgttttgtatatcaggaaaa
atctgatgtttcaacgcttaaccttaaggaaccgtattctgcaacatataaaactatattagacattgcaaagcgaattgtgacagtatccgtgga
aagcagactagatattgacgaagatgaatatgttcatcaatttaagccggacatgatggaagccgtttctagatggataaatggaggcaccttctt
agagatttgtgaaatgacaaggttgtatgaaggaagtattgttcgaacttttagacgattatcggaactgttaaaacaatttcgaaacgctgcatc
cgttttaggtaattatgaactacaagaaaagtctgcccaaacagaacaattactttatcgcgatataatatcttcttcttctttgtatttttaattgga
ttctagaacttggagtttatacttttaactttttattttcagagcacaatagttcctaattttgattgaaagtgatgaaggatatcattcttattagatt
atagattaaactggaattatgtaccatgatcacctgatagataaatatatatatatttttttttttttttgtataaaagcataagaaagcattatgctt
gggtgacagtaaactatacctgaatgttaagcttgcatcggtataagtcaattgtcaatgactcatggacaaaagatcctttaacccataaaagta
gttgagtagttcccaaaagacttgttctgctttggttacagactaattggcatttattatgctttaaatcttcctgtaaaatgaatgaatgatccagac
agaacggccagctgctacccagggcaggcttgttaatacgaagctgtttgacataaacaagagaggtagatagataaacagcaatgttaaattt
tctagttagcatgcgaagttgtctccctaaagacatcatagatgcatgggcgtaagagatattagcttttgccatgaggagtccaagaggattgga
aaaagagtcaataaataaggcataagatacatcctcattctggcaaagatcactctctaactcatgaatggcgtcttgtataccttcagcgtcgaa
ggctcgaataatagctgtttttccaacgaaggtaacggtaaagctgtcggattaaaaattccaagtgtatctatccagataattttatcaaacttc
ccatgatattgtctcaaaatccaataaagcacaagatttgcagaatcaccactaaactgagtgacttggttcatattgaacaacagcttttaactat
ttcaatactaaatttttgttaattttcaaagaattaggattaatcgatattgctctagcagagcaatctgagcttcagtgaaagagggattgaataa
ccattaaatatactgtactactttttaaaagagaatgaagcaaatcagatagactctgttctatatggttacatttagttttcacctttataaagcca
gtaattgccgttctcttcgcgaaggtaaaaccaatcgctagtgaaagctattcctttagaaaggcgacatagattcttacaatactcctaaaattaa
taagtgaaacttaaatgtatattaaaaaaaaaagcaaacatatagaattaatatattatccgttaaaatctattacacgaaacccgaccaatctttg
tttccataaatcaaagatcaaacacaaatgaaaaaagtaaaatctcgaagtaggcttaagcatcaataacagcaattatattaagagaaaaatg

gtatgtaaacatagcttcatggatactgaatttgtgaatcgtaaacccaaattcgatgctagttgtttatgttttaacatttttttaccttccatatcatt
acaacaaagtgattcccaattctttaaacaaataagacggccacattttgaagagatgcaaaaaaattggcacatgaagtcctttttttacaacacg
ctcaacttgtcttcaacaaggaaagccaagatattagattttatatgtttcaaaaacagcagaaaacatcagcaaatataaagtttatcccaatta
ttaaaaatacatgacacccattcaatgaatgtagcagcagggctgcaattaatatggtgaactttaggcataagtcccgaaatctgcagggaaag
aggaaggaccttgtaagtttggatagtggggagcattacaacgtaaacaattacttcggcgacggaaattagtgaagccacattcacaaagcca
atcaccttgatcagtagcaaaagaagctttagggacatctttaaaccggttttgctcctcaggacgattggcattagggttcggatatgggtgatta
ttagcagctatagaaccaagagatagctgagaatatggatatacacatgcatttgtaggactagtaccaaagacaccagatgaagtgcttggat
aattaccgttggtattgttattagcgtttgacgacatcgacgtgtggtctgcgacgacagcggccgttgcacggctagcaccgcagcgtaggcaac
aaacgtttttggcaaaattatgatatccacagccctcagaaccacatttccagtcaccagcccggaaaggaacattaccaccaaacgtggaacg
ggaagagtgattgttaacaatgtttgttgcattgtgatgctgatcggcctggctattaacttcattagcatgatggataccaaagccatagttgtga
gggatatgcgaattaccgttgccataagactgaccataagggaactcgggaggaaatgacgaagttgcagcagttgctgaagcatgaccagga
ccagggaaagagcaacggaaacaagaaattctacgttgaaaattgctgaaaccgcacatggggcaattccagtcgccaggacgggggcggttt
ttgctggaagggaaaggaatcaaaatttccgatgccttatccaaaacctttgggcagctaggagtaattgcaagcatgcgatcgccaatacataa
cccattcaggccaagagcatctgatgcgtcttcatggctgccgaagaggacaaaaccggtaccagttgatttataaggctcgggtgtcttaagagt
ccagagggcaattggatgaatgccatgattcgtgaaccaaccttctaattcggattgagtaacgtcgttggttaggccaccgatgtggaccacctt
ggagcgctccaagtaaaacagtctcgcgtcagcttgacaatcgatcggctgagtgaggacattaggaaaagaaggtagtctggcttcgcgctca
atttttcgcagcagaagagtagtagcattagcaaggcgccagcattgcgtcaagcctttggtaatataagggacttctgtgggggaaagctgaat
tgttttacattcattaatgggaggcaatgaatcaacatctagctttccaaaaatatgtgacagtgtaattgtgtaaggtggaacggagcctgaacg
gatttgccatttcgcatattcactggagagatcaaatagacgagggtgacgcatataagaagggaggataacgccgtgatcttttgcctctttggg
taaaagaacccgtaattctcgcccattcatgacgacaaatgtgaaaggcttttcctgtaagttgagtcgttcttggatggaattgtccacatattgt
atcgcttcttgtaaagaagacaccgttccgcagattccatcttttgacgactcttcccgaatgctacaagactcgcaatgttgtttttctaatgtttta
ccatccaaaatgacccagcaaatttcatggatttcgcacggagcccgaccgatgcttattccatggtcatctaaattcgtttcaaaatgtaggaca
accaaatcgcctacagattccggcagaggcattgtatggaatcaaagggacttgagtggtgaccataaaacacaaacctcaagcagggtttctg
ttacctgtaaatacagtcacataagtaacattcaagctgtaacgaagcttaatgtatcgttatatatgtgtgttaaaggattttgttttgcagtaata
aacaaggaggaaacaaggaatcgtagtaagatggaagaaagaatcaggcaccgtgttgtgtttgctggatttttgtttacataggagtttcattgt
tggcaacgcaatcaaacaggaaatgattttgattctcctgatccatttatcttgctatgcatacttcattcgcattgcttttccaaccaggaaacgttt
agtacgtagcatctcacagaaacaaactaaaagccagcgaattgaacgaaaaatcatcgaattgaaaagagatttttttaatagtgaacccaact
ctcttttttttttttgttaaaaacttccttgttattatcattcaagaaggactacgatagtatgatagtatgatactatgagaatatgataccatccatg
gtgcaagcgttttctcgatactctcgctcccaagtgctttgcttcatagggaaggggttttcgactttacaaatgaaaaaatccttccttaaagtttgt
aacatcctttaccgcagcggtagcacaattgtatcttttttttaaaacactcgttcccctttggtaaatcattttaagcgagtgtaacaagaccctatc
ctctgcaaaggcagaatccaaaacttgctaagaacagcattttggagaccaacggattcattgaaatttttcattcgcttctttctaaacgactcat
ttttgttacatatctgtaaaattcaattagaaagaaaggatggctggagttgacgctaaaggaaggagagctcaaaaaaaagagcatgaaagcta
cccaaaagaagaagggagttgctgagcaaaagcaggaaccgatctcggatgtttcagcagaatcacatgaaaattcagattctgtttcccaaaa
aaattctagcaataatgaagaaaagtcggaagagcaaggtgaaactcgagttcaggagaaattgccagagaaagaaaaacctcaacatcaac
aagaaacaaaagtagacgatcaggatgaagacgaagacgaggaggaagaggatgaggatgaagttgaactttcagacctggaaggtgtcga
gttagaagaggacgctgatcttgttcgtaagaggaaattggctttaaataacacaattgctcttgaaagcatttatgaaaaactcaagtatcctga
tgacatttctatcgttgaaaaccaggctgtcactacaaaggaacctattgtaattgctgacgtagaagatgatcttacaagagaacttgcattctat
aagcagggtgttgagagtaccaaagctgcctttgctcaattggaaaagcacaaggttcctataactcgtccttccgattactttgccgaaatgctg
aaatcggatgcccacatggagaaggtccgccaagaattagttaaggaagctaccgctaagaagctctccgaacaagccaagaaacaacgtgg
gcttaaaaaattcggcaaacaggttcaagttgctaagcaagaagaacgtcagcgtacaaagagggaaactttggaaaagattaatttacttaag
cgcaagcatactggttctgatttgaccaccgaagatgattttgatgttacacttgcgaaagccgaaaaagaagaaaaggccaaggccaaggctc
ctgctaataaaaaaggcgaccgtcagcctaacttgaaacgacaaaagaaagacgaaaaattcggttttggaggtagaaaacgtggtacgaaga
gcaatgacgctgacagtcttgctgctacggaattcggcagaaaaggcttaaaggccttaaagggcaataaaagtcgccctggaaaagctcgccg
tcaaaaagctagaaagtaaatagggttttattttgggtaaatcaatatctatggttagagtatttaatatctaatttcttaaaagttgtaagtttatat
acaaccttcgtttatcttccttttcttgcagttttatgatctccatgttccatagaaagtataatctcttaattgacaaattgatgtatctgatagaatc
aactaaccgtgctttcgataacttgagcgccagtcatttaataacgatgaactagctaattttaagagtttacagtactcttgccaagaagattccg
gataattgttctcctctaaacattgatacatttggtctattgaaagcgcccgcgcttcataataaccagcaagtgagagctgagtcgttatctccttg
cttacctcgtattcccaatggaaaattctgtgcgcaagtcgcgatctctcgtaacaactacttccaagaagccaatctttttttggattcttcatgatac

tctagatcattttctaaaaccttgaaaagttagcgctttagtatttgaaattgtagcccataccttgtaagtgtcacgattgtataaatggcctttaaa
gcgcacaagctgtgtggataaacgtgaatgtatattacaagcatcacacccatatgtagcttcgcatctcctgttcgtcattatcgggcggtttttaa
gaatattaatgaaatttggtttccagatactagaaatcacattggattccactgaggaaaaaagtcgccttcgaatcgttttacgactaaaaagaa
aatgttcatctatgcttctatctatttgaagattcagtatttgcttaatgagatgtcgaaggaaatttgcaaaatgagctcggagaccctggtggctt
gtcattgaaaattctggaggaagtaatgcacgagcacctagcgtttcttcgtcttcctcttcatctactataaaagactcatcattcgaagaatcatc
atcgctagtatccaccttttcactcgccggttctgaaatgtcttcttcggtctctatattagaatcaccaagactacgctttcctaattattgttagtaa
gtaatcataaaaaaatattccttacttctgataagtgccaatttttcatgaaacgccttcttagctgggtttctaaaacgcttacgcttatcttctttct
catcatgtacaggctctaaattctccacttcttgctttagttccgtgtccaagtggtccctttcatcttcatttgaattttttcattggaagaccgttcc
ggagatgagtgattaaactcaacagattcatcctcagaagagcgaggaagatatttccttcttttcgatttttttgagactaaacctgatccattgct
tggtagggacgcagttgatgagtagtatgagtcggaattatcagattcagagtctgaaagtattaacttctgagaaggtcgtttacctttggacgg
agatataacttcagtatctgaaaggtcgaaatcagtttcttgctcctctctttcatcaaactccggcaaagttctctggtcattcgaatctttcatagg
agtagagtcaactgcagagttcatcgattgatcttccttattgatttcataaccttttaaaaaactctttttctcttgactatacgaaccatccgcggg
gtcgctgtttttaactatgcctggcagttcataatcattagtcgaaatgagatccatttgcttattctcagcaaatgcagtgtcttcttgagaggagg
atggaattggaagaatcagttcatctgtgatagtattttctttgcgattcttatgcttggattcttctgttgtttgttttgagcataatatgggccgctgt
tgtttaaccacgatctcaggtaaaaaatttttttgtttctttcttaaatccttgtgcttttgttctttctccagaatgttcaatgagtccaaatccgttcg
ggaataagagctttgtctttccttattcgtttcaggttcatttttgaacaacttgctaacatactttctatattttgggtccccttttccaatttccggtt
caacttcttacgattctttctaagctttcgtgtcattttctttgtactcattttaagtgagcagaagattaaatagatattcagatatcgttaagaacg
ataataataactctttttccaaatgaaatatttcaggtgaaattgtatacaatcgatatatattatactgggtcttaactgaacagcccaaaagctct
aaaattcagagacctgcttaaatctttttaaaactcttttctataaatacttttatatttaaatttgcatagcttactttgatttacggtcaaaaagca
gaccaagcgcaggttcgctactggtaaaggtgaatttgccaacagccagcataaaggatggtatatttgtccaccgttaatgactttttgaacaa
actaagctcttagtagaagcatatcctgatacagtaagaactgttttacttttgacttaaaatataccgtctaacagtggtatatagacaaaattaa
ctacgcgttatcgaattgatgaagatcaacagagttaccttgttgcaaaagcatatgaaagtacagcaggaatatgcttaaagtttagaacagac
aaagctgccgagttgagccggttaatgctcattgcaagtaaacttggttatgtttctaacaagttggaaatccctcctgaaccatctggtatgtatat
ctcttgtttttttaaaaagagagaaatgatgaatataaccttaacctgtgtatatgaacattttaaatcgtatagcttcctttgagatgtgtttcatatt
ccagacatcatttctatgaaaccaattcatataactaacagctttaaactagaaactgaacctgcagctcccaataagcctgtcgaagtgaagtca
gaacctcaggctgctcctagtaagagctccaagaaaaaaaagaaaggaaagaagaaataaaattcatcttaccgatacttgtgtttttaaaggtt
taagttactctgttccactgtctttattattttaatcatggctgcttataacgattacttactaaaggctcaatcttttgatcggttagaaatcttcctct
gagaaaatgaatcatatctcttcgatatattttacttttactatttgtgctgtatcatgtatcgttctttcttcctctactccttctggtcgttcgtctgcat
tgattgtattactcgtacttacattgtagtcattggagcacttgaaatatagatgttaaggtattttctgaaacggtttgaatattaaatattaatgat
tcaattcaattcttttattctattctagtccagttagtgggtacaagattcacaatatcttgcaaagcttccttaagtcaaagctcaaattataagtac
cttaggctgaaaatagaaaaaagctttttgaaatgaattaaattacttatttagtgcaatgaacacacacatgatctatttttaggagttgttttata
accattttcccaagtactagctgacatacctacctcctataagcatgacttttccggagatttcataaaatcaattgcttttctcttctatttataatttt
aagaaatatttacaaatttttaaagaaactaataaagatagtattgaagttgagtggatattacctacctagtttggaattcataatacaagagg
gcctgtgctttttctgcttatactgagcaacttaagaaattgagcaatctcagatgttataatttgaaagtattatcattatattgagcagtctaagc
tactcaagtactatttgaccacctaattggttttgatcaattttggtatcttcattatttggagtccatttttaattaagacatcaagctagcttcattgc
tgtcctccgactacttcgagaatacgatagtaggaagcggtattgtattctttaatctgttttcgttccttttttgttattcttcctttttttgctttactctta
gtctataattggtaacggtctactccatgaccaattcaattgagactttccccccgaaagattccagggaattcttgatcaagtccgggatagcag
gtggaatcgcgggctgcgttgcaaagactgtaattgcacctttggatagagtgaagattctctatcagactaatcatgcagcataccgaggttatg
ctttctctcgccatggtttaactaaatccataaagcgtatttaccatgtaagcggtgttcgaggtttgtatcaaggccacacagctactctatatcgt
atttttccctacgcaggtattaagtttgttgcttatgagcaagtacggcgactgcttatacactcagaagattatgaaaccaatggtcggagatttct
ttcgggttctttagctggtaagataactatactttgaaaatttgcattttaaccatataggtatgtgttctgtctttttttacttatccattggaattaatt
cgggttcgacttgcatatataacaggttcaagtaataagccaagacttttgaatgttacaagccaagtattgcacgaacgagattttggcaatctg
aaagaaacccctttacgtacctcaattagaaggatttcaaacttctatcgtggattttctgtaaccttgatggggattttcccctatgctggaatgtct
tttttagctcatgatcttgctaccgatttttttccataatcctgtaatcaaaagagctcttcgttctgagaaggatgaacggcatttaagtacttggtct
gaacttacagccggtgccctggctggtgtttgtggccaaacgatttcgtaccctttttgaagtatgtagaagaaaaaatgcaagtaggcggtgttgaa
agtcgttctcggttcctaaaattacaaaacgttataaaatctacatatagagacgctggattgagaggttttttttgttggtcttacgatcggatactt
aaaagtgatcccaatggttagtacgagtttttttgtttacactcgaagcaaatcctacttgggtattgattagaaatgagagaaatgagctttgtttt
attcaataaaatatagagacttttgtatattatgtccacaagtctttaagacatgtcttacttctatgagaaaaaaaaaggcttaacatattagatag

acagtaaaacagcaaaaggcagaagtgtaccagcataacattataattatcaactcgaaaataggttggaggttaggccccattcctttatagca
aacagcgaatgatccaaataaatccaaactccgaaggctaaacaacttttttttcaataatgagtgaaaaaactaaaagcattttgtataaaacc
cgtatacgtattttaatcccagggaaaaaagtcggccaaaaccaaaaatggaaatagaaaagaaaggaaatatctaacatatgacacaacatt
ttgttgaagaagaatcagggttctgagccgcagcaggacccttagaagctttagtagctggctgagattttgctacttgtgtagcttgtttagtatg
aagtcctctttcttcagatttattaaaatgacggattgttctgacaaggctgtaaaaggattcttcgacgtttagtcgaagttttgctgaagtttcaac
gtaaatgcatttcatacttctagcaagagcctctccttcagctcgagaaatgaccctttcgttctccaaatcgcacttattagcgactaaaacaacg
ggaaagctatctttgtctttgactcttaatatctgttgataaaacgtatttatctcatcaaaagatgaccgagaagttatgttgtatactagtagaaa
accttcaccagttcgcatatactgctcccgcatagcagagtattcttcttgaccagctgtatcaagaacgtctaaaagagccccttctccatcaatct
cacactttttttcgataggaatcctcaattgttggatcatactcatctacaaaatgggattgaatcagttgaatcgtaagagcacttttaccgacacc
gccgtcgcctacaacaactaatttatactctctcaaataggtagactaattgaaacattagtatttcatacagataactctcattcactgaaacgag
gtaaactggcataccttcatattacttgtaaaaaagaatatttctaagagtttaaacgtctaaaatatattcctcttttaagtaaactcagagatcca
aaacttgaaaggcgtatatggtggagttctagtagaagaattccccaagtccctcgtttgtctaggatactgaagtggaataatccctaaataaac
aaaataaaaaatacgaataccaaagatgtggaattctaaagagaacagaattaattatgaataaggaattatgaattgtgaaaattcaaacga
ataattgactctacatttaagtaaaatatttagcgatttctcctaaaacgagaaagtaatgaagaatcatacaaactatcgaaagaatagtaatgt
aaattattgtatttcccaaaaagatgtatatatttatatgttgcggttttcgcaattttttcattaagaagttcattagcgcaagaatttaggaatgta
atctagcaagactttccgataaatttaccaactcattcacagaaatgttcaagggtagtcctccgttctttccttgaacgatatcatcctgtatagac
agaggcatgtcaacgataccttctaataaattaccatctataagcttactcggaagctcttcagcaacttcagaatgacaccttctccagtcttcat
gacttagtccgcctgtcgaaggtaaaacgcgccttacgttatcctgaagctgcaagagtatgtcagcattatcattcgcatctccaataagcattat
agcaccatccactgtgacggagatgagtctcggttgtacaaagcttcctgcctgaagctttgttaatgtagcagttcgagttttgtttacgaattctc
ccaagtagtactgatccaagcagcgcaaccgtttacggtcggctatttgcggtgaatgcacgtcccgtagaaaaacaaatgcgttaccatcagca
tcacagacaaagtatttatgtttgtctagcttatcaacggatgatacccatattggcttaaaatccctggctaattcggtcagctggttttctgaaaa
atttatcacagaaatcgacttcatcaagtcgccgattactatatcatcatcgtcaatacagaggtctatggtataagtaggagttcgaagacaacc
ttcgacgtgaaaacttccttcacgaaaagcaaacgtactaactgtagcatttataccagctacaattagacctttgtaactttcgagtgtgtttacc
gatccttgcaccttataagttgaaattaaattgaggttgttcttattgacagtaaacataagcaatcgccccgaatcgggtgtatcctgatcaggat
agttatatcctgtaccaacgattatgctagagttagtctctagatacactatactctctaccatttcatttgcatcaaactcaaactgacatacttctt
ctaatgtcaatttatcaaagactcgtagagaagattttaggctctgttcttgacttcctaaagcatcctctagttgcatacaaaggacaaagtagtg
tttttctgaagagcaaatcctccgtgggaaaccttccactgggattgtttgtatagtcaacccttgaatggcaccaagagaagccataactaatttg
gaacccgtacaatatacaagatccgtatttaaggtagggtgacagaaccttgtaatactaacgatttcttttgagggaaaaggagtaaaaaccaa
tctttttgattccatatggataaattgctgagtttcagctatagaaacgagggtgcttttatctttaaaaattaggggttttatgctaacaggcgtgtt
tcctaggcgtattaccttaggttgaatgttgtaaataccatgttcaccgaaagaatattgcaaaaactgaccatcattcgtagttacatataggaaa
tcgttcacctgattactgaacgaaaaacaagtcaaacttcgaggtacatcttttaggctatattgacacgtctcttggatactaccttggctgatctt
taaacccaagactgaacaagaccatagtcctatgaagcaaatagagtcatccaaaaagccaaggcatgagatttccgaatcacactgatacctá
gcaatttcttccaacatacgaagaaaaattatagtgccacctgttaaggcaacacaaaccaaatcttcatatatagtgccattagttatgtttgctt
ggggagaccaggatattagtttgcttccattgaataagcggacattagaagtagtaatttgcaaaagttgctttccctcatccactacaccgacact
taacgtagaagaatcacaagaaaggtcagtacatattgaaaattcaccttcgggagagacataaagtacccgagtttcactaggaaagcttata
atgatatagttgtcataatcagccttaaacgagacagagaagacttccttaatactttccatatctattatgcctatatcctgaattgaaacagtatt
ttttaagacacgtatacttccatccttgtaagcaccgctgcaagtaagtatagaagatccagaactttcatgttcgactgtaaaatcacttatagga
gctatgttaggaaatatctgtaaagtctgtacagttaactcttggttagccaaaccaggattatctaactgtaaaaccgcggaatctccataatggc
ttcccaaaaaaactttattttggggaagggaaactagacaagaggcaatagaagactcgccaagcttttctaagtccattttcaaaccattatgtg
agaattcagaatagaaatggtatataaagccgctttcatcagttacaaagaaatggttcggatcatacttgacggaaaataaatcttcaagtgga
tcggaaaccaaaattcctgtgaatgctgatattggataagtgagtaaggactttgaagctttaagctccttgctgatgtagtatacatacatttcac
caaagatgaatacaccaccattgttgtgcgcaataagcttaccttcctctatatcgtgacaaactaaatcatcttcgtctatctcacattcgcgaatg
tttattgtgtaagtagacagatggacaacagatttgctgtcgcgatataaaacagctaaggtgggaactgaaggagtgtggagcatgcaaatatc
aataacatccaattctttgatgcggacattaaaattatcatgaaggaaagagtccatactgtttagacgtcgtttttttagagttgacaattggaata
attgtcagaagcccttgataaacatgcaaacataagacacgctggaaagggtctacgagtagtatagggccagattgactttcgcggagagaac
gcacactacaatcctgtattttgattccatttgcaattgattgattttcagaatcccagaaaagagtgaaataatggaaagagtcagtagcaacaa
taatatggtctgttgacgatagtgcagggcgaaatgaatttaaaccaacaatacgtccaaatatatttgtataagttaaaagctgaagattttttttc
atcgttgtactcgtaaatctcgatacagttgcttttggcaattattacattgcacgacgtcgaatcaacaaactgacaaacaacagcatttctcact

gacgaaggcttgtgtatataagacacataactcatttcttctataattcaattcaagtaaacgaacgaacttgtttttcactaaaggttcctatttc
aattggaccataaacaaaaatttaacagaacgatggtgagtgttaaatttacaacgtctaagtccaaacgatttcgaaaaagacgacagcagat
ttctagagtgttcgaaaaaaatattatgaaaataaataaataagggaatagtgaattttaccttttccgtacacgtctgtttagaaagggttaaca
ctcaaagagccaaacaagtatgatactatcccagagggtataacattactcaactgttactcaaaaatgttatagtaagtcataatactaaaggg
gatattacacagatgttacttcgtcgacattcgtaaataaaaaacgaaaagatgatgattcaacgatagaatagatggattaagctttcaaaaag
aattcggataagcagtgctgctctcataaaccctttgagaaaaagtttttttggtcgagctgttcaacttgatttacgatactgatacttgaagcta
agcagctagacagccatactggatgtatgaataataacaattttcttgtactgcatgatattttagaaagaaaatgatttctcatcattatttcatca
ataaaaaactgttgagcgatagttctatttattcatattgtactagaaaattgtttgaatacgatatcatattgggaaagaaataaagaccattcat
gtcctgaaagtcattatgacagtgagtggtatcaaagtagtctaaggaaaaaaaaaatgtaagatttcattcaagctacttcagtagaaaggtcg
agtggaagttcttcttgcatgagctttggcgtaaacaaataaatctactgttccatgttctactctttggaaagcgacttaatcgagttacaagctat
actgtcaaacattgtagcatataaattggttagacggttcagttacagatctccgtaggtaaacattctaagtcttggataattatggaaggttaag
ctaattcatgtttgcaatataccgttatattgaaacaaaacacaaagtaatagcagaagtggaagattctgttccttctatgagtcatttgtcacta
gtatagcatagatggaatggtcctcatattgacttggtttgtttctcattatgcagagtaaataattacactaacttttacacttgaaaaatatactta
ttatgatgggttggatctacctattgcttttacagaaagaggacataccattgcattcgcgattagcgatttccagtattttgaagtcattttgccggg
accaccattctctaagtaaacattgtattttgaaatcattaaaatctttattattaggtggaaggtgcctgtaatagtcacttattttgtttttactgcc
ttccaataccagagctatacatacttcatagcctaacaaccctcttgctttgccagcaactagaaacattcttatattgttgctcagtaccattcaat
atgtcgtctgaagctcgtatgtttagtatttctgatacaatcgtcaaggagatcgaccgatttcgcttgagactcaagaaatctctattgtttgcctac
atttttaagatagataaaagctctaaagaagtagtaccagatggagaaattatgcaaatagaaagtgtagaagaaatcgccgacgaattgtctg
aaacgcagcctcgttacattcttgtttcttaccctacgaaaacaactgatggtcgtttgagtacaccccttttatgatttattggagaccgagtagct
cgcctcacgatttatctatgatttatgcatcagccaaggtctggtttcaggataaatctcaagtgcacaaagtcatggaagtacgagaagttgagg
atattactacggatgcagtagaagagttccttcattaattcgttttttccttctagttcttttctctggaaggatggatgactatgtttcgcagcctta
cataatgttagataacgatttcagtaactgtctgtctttctttcctctatacgtttcctttacacaataatagttacttttgcttattcaatttcatgaga
atttatgttgacgaaagtcgatgacagatctttccttgatttactagtccatcacagtttttttatgttgtgtgaagtcaatttcattgaatatacttttg
aagtacaaactttttggaggtctcaaagagttacccaatatgaacaagatactctgagagtgcttcaactcaaagtttccaaactttgccaaaaga
agaattttccaaaagtccctttacaggaccttgcttggctccatattcggccttcggtccagcaaccgtagcaacaaatttgggatttccgcaaatg
atacaaagagtctcacttaaagggacgttaacattatcccatttcataatatcgtctacatctatcaagcgatcttggattgtgcggacacgaacat
tgggacattctttctctaattgctccttcaaagcaggctcttgacctggtcgttcgcaatagacgatatcaacaggaactttcgagttctgagctaac
tgatagataggagcaaaaccggtaccggcaacaataccaacaattctagagaacttagtgaaatctagctttgtagtcttgaagggaccacgaa
gttcgatggtgtctccttctttttggaatggatatgggaactcacaggtccatcttcatagttgcggatgtagaactttaaattatcctcattgatgt
ataaaggggtataaggacgctgaacttgcatatctgggttgcgaatagtgactttggtaataggatcccaattcttcatattttcagtagaaccctg
gggagtgagagtgaatatactggcatcaggagtaattttggtaatcttagagacagtaaagggagaataaacttccggattaaggccgataggc
ttattctgatttttataggcaaagtacccaccgacgcagccaactgaaaggccacctagcaaaaatttggaaaatcttttataattagcagacattt
caaaaaaaaggatcgtaaactgctataactaaggaatgagagaggaggggtattttaatctttgacgaagagagtgctgttggtgaagttagaa
aacttatcaatggcataaatatttattaagggaaggaggcagcttattgttgtttaaatgaaagcaaattcaaagataaaaaagcttataaattct
gagaattctttaaaaaatgatgaatgaaagaattttcgactttaaattttacaagacaagtctatcatggcattctcaaatttaaaattaaaatcgc
tctattattttgaacaaaaaagaatcagactagtactctaagttggtttggcctgtttttgagttaataagaaaaaagagcaagaggattccttgc
tcgaaaagaaagaatggaaacccaaaaccagagaaaaacaaagaatcattaaacgaaaaatgaaatcatctaaattacaacatgcttattttg
caagttcttgagtgccaaaatccttgataaccaaataatcacggtaagcgacatactcagtttccgtaaagcccaaaatgcttccaatttcattgct
cctttttctcttgatcaattcaatttcggtactgctatagtttaccaaggctctgccgattacaacgccattgaaaactatattcactgcttgatgagc
agcaaaatggccgtttaccttggtaacacctaccggtaataaaccagcgcggtttgttcttgttattgcttcgtgggcgcctccatcaatttccaagc
ttccctgtggcttcaggccatgcaaaagccaaaaatggcgatcccgcattcgattttgctttttggcaacaaaatgagtatataatggtaggccctc
aactttttctaaatcatcgacagacgtggatgcgtgtcgaatgatttcaaggatattggatggtttcgagccatgggaaatcacaacatttacgccc
gaagaagtacctaattcagcggcaataagttttgtcttcatccctccagtaccaacattacttcctggggaagaaacgtctgcgattaccgaagag
gagtcatgaaccacacgaataggttttgcatcagggtttgagcgaggattggccgtgtacatacactccacatctgtcaataaaaagagatagtc
tgcattaatcattcctgcagtaattgctgacagtgtatcattatcaccaaatcgaatttcctgcactgaaagcgtatcgttttcattaacgatgggga
caacacccaaatgcaaaagctcagaaattgtattagcagcgtttacatattgtgttcgctatatcaagtcattagtatgtcttcaaacaaaatgaa
aagggtaaacccatacctctgctatatcatttctagttattaaaatctgtgcaattggttgacggagttgagaaaacaagttatcccaaagagata
ttaatctaccctgaccaacggcagcaatagcttgtacagcagaaagcttagaagggcgcttggggagatccaatcgtctcagacccattgcgata

ccaccacttgacaccaagacgacattatggcccagttttcgaagtttgacaacagtctccacaatcaaagacaagttagaaataagtggttcatg
cgttctctcatcgcagatagaagaagtgcctgcataaagttaatgaaagttattcatacacaaatataaggagctactgcttcaccttttctaagat
ttacaaaagttcataggtcacacttctagtacttaccaagcttgattactacagtataagctttttgcgattgcttaacttttggagccattctttagtt
ggtttttagagatacaaaaatctaaaatctcgaacaaaattttgtagtacacccgaacttctaatgagtctatgaaatggacaagtttttttgttgat
agagcaacggtagtgatatgcttagtccaaggaagtacaagatgtaaaatcttacagtatgtcgaataactaatgttagtactgttgatatttctct
tttagttttattcatacatgacagtaaatacacttttttttctggttataatttactgtatttagtaaaaggaaatttccatcggcaaacagtaagttccc
taacaggattctatgaattgatggcatctcattccttacttttgtttactttttacaaaaataaaagatatacgatggatatctgtgttcattattgctt
catttctaatctactagctcattattgatccaacattagagttcatcatcatttctagaagatataaaagaacggagaatgttgagacgagccttaa
aatcgtctgcatttgcaagagcaatggatcttcggcgtaaaattttggataagcgctttgcttccttggtaacccagctttcatctttcaaagctttct
caaagacgttcctgtagtactgagtccaacgagttctattttccatggtaaattgttttaccttttcgaaaacaacttccttggctgctcgagaactat
ccacgaactctgaagcaaaatcatcgagaatggtaattcggcctgctgagggcaataagctgccatcggcagaacgctgggttccagttttctca
tttaaatagtcagtcaagctttccaaagagcgatcgccgttgtaagcatcaggcttgtctttacgacccttttggaaaagtttaatcgtagggaagc
tagcgacttgatgtttcatcccaatgtcggcataaatatcaccattgactttggctatttcaacgtttggttcattcttgaaaactgaggcaagctttt
cataagtaggagctaaacgcttacagtaaccacaccagtcggcatagaattccaccaagacgtctttggtgtcatccatgacaattttatcaaagc
tagcagaatctaattcaacaacgctactaggaaatgcatttttcttaagcttcttgacaccggtcttctcagagacaaattgtgtaaggctatccaa
atcacgggcgtgagaatattgttcaggatccgagccatcagctgggtaccatatcaacgtagggaatccacgaatttgatactttttcccaacttc
gccatatgtgtcagcatcaattttgccaattagtatatcatcgtcctcctcaaatgtttgtcccaattcttcatacactggaagaagattcttacagtg
accacaccagggagcataaaactcaatcaatgcgcctttttagcagagcgaattccattttcgagttgagaaaggccagtaaattcgaagacat
ttttggcagctaccaaagcaaaaagccaaaaagaaagtgtcactaacaaagggactttcatattcaaaatttggtactataaagattcaaaaatt
caatatgagaccggaaatccaaatttattgacaaccccaggtttggttcttgaagaaaacacaaattcgtagtgatgatgatattccgaggaacg
aacatgttctcttaataaactacaccatgtcgctaagcaatttaatgcactacacggtgtcgttataataaagattgcaccataaccgtaggtcagt
aaacgctataccgcaatacagtggataccacattaaagtattaagtcaaagacggaacaggacgctctgtggagtaagaccaaaacaccaact
ttttgggcctttgagacttgtaaagtttttgcaaattccatcatcataaaaaatttaaggctataagaatcaggatggttaagctcgaagaagtcgtt
gacgaaactgaattacagacccagggacaaaacgtcgccgaaaaggataaatacatctacgctgaggaagacgttgaagaatccgattccgat
gaatctgactttgagggactcgaagatgagacgatatttgaccgtatcgctgctttgaaggaagttgttcccgttacatggcgcgttaggattgcgg
acagtgcaaagtgtgctgtttccggtgtttctcgactttctagctttggaggaaagtctttgtgggttctctctacctctgctcttcttttaggtgttcctt
ttatgatgtctgtagaagacgaagctcagctttcagaatacgaaaagcaattgaaggaccaacgtggtgccaacgaggtaaggaattatgaatt
gtactttcattcacggcatagtgactaactatttactaggtgattgctcccggagctactagcaatgctcttccccaataatcgaatcgtaagggag
aagacaatttaggaattagcaggaagtttacttctttcgtatgtaatgatttggatgattattgcatctgtaaaatcaaaaataaaaattaaattttc
ctgtggactttttttatatcgatactactcagtaatttttctggcaagataagacctatattttcacagacgtaagcttcatagaagaaacaaaaaaa
gaacaaagaaagtgaaaagaaatattcctaatatatagtgaaaaactgttctgaatctttttttttctcttaacttgttttagtgatttgaccaggct
gaaggtgtaaaatcaatttgaatattctaaatgtattccaaaatataagggatcaatttttgttatgtgtgccgttgtacccaaggttaacagaaaa
ttccggatccgtgcctgccaaagcaatgagatttcacccgttgtggacaatacagatttcaggacagacgtacatgactgaatttttgatccatttc
tttcatctccagcgcgagaaaagaacttaccacgccaaagctcagctcctatctgaagtttactttcataagagtagatattgaaatcgtattgtgc
actaaacgcttttacatgcggctctgctgtcgaaaaggtggaagttatgtgaccaacaattggatttaaagtacaggttaaaataaacggtgaat
aaagatgagaaggtccactatggagtcgcatacctagacttgcaccagcacattttgtcaaaatgccatagtatgtttcaaaacccaaactccag
cgcatattcgaagtagttccgatggtttcgttcattttttgcatatggtcttcctctagatttagattccataaggctctgagtcctatcattgcttctgt
cgtctcatacatcaattccaggctcctcttaccagtatcgtgctgccattgaaagcacatctatacccgtaatcattagtaatggattccatttataa
taaaacttaccgttcctccatgtggatcgtcaatctcgttggttgccaacacagaaaatagactccatggagacaatctcctcgcaaatattgcatc
taattttttggaaggtaaaaagatgcatccataaagaagagtagacttcttaggaagattgaagtcttcgaattcccttgggtaatgaataaaagg
aacgtcaacatgcttgtatccttcatagaatttttgtaattccgtcaggggatcgatttttgcattctccaaattcacgttactatatatgtaggaaag
agatccattaaggatcggcatagctgaaatctcacattttgaaacaaaatttggggtcgttaatgaggaaagctgacaattgattccagaaggga
tgtcaaatcttaggatgtctagatttatgttaatttgactgtacagaaattcagaagacttactgtcggctgtctgtgtgagattgcagtataaatta
tgaacattccagttcgtttttttagatattcatagcaaatataatcagtgaagtttaacattctattagatcttcgtatttactccattatatgtgttga
tcttgtagtaactattttcgctgagtccacttgcaggaagtgtagactgccaatatactcgctttgtttgggtttatattaataaaggaggttaaaag
taaaatttgatatatgaactagaaaacgctgtaaactacttttgtaatatattcaaatcaaataatacacctcacctcctaaatatatcgtaaatca
tctacaaatttaaatttctctcaatcgtagaatcttaagcaatttatttctagcattactttagtaaaataataacgacataaaaaacatcaaatatc
ccaggagcattacgctcaaaattttcggtcataaacgaaggatgcgaaaagagtactcacttagaattaggtaattataaaagaaagaaagga

aattatggacagtggttctctttaaacaaaattttcatataaattcaacgctcgctgtatccccaataaaagatttctgagaaattttttctctcaga
ttagcagccgtaccgtgcaaagaaggattccaaacgcgaacgctaccatcatccgatatacttgccagctgattttcattcgaccaatctacagcg
gttacatcttttgcatgccctccaactaacatggcagatgaggaggaattgtctcgagtagtatcaaaaagtatcgctccgctgttttcatcctgtat
acctcctccgcaggccaacgtcgaaccatcgggactacaagcgactttcacatagaaagaagaaattcttaatctcgggtcattgtacaatttaca
aggcccagattctaaatgctgggaagaatattcataaactgtattgtcccttgaagccgcgtaaacacgatctcccataggcgaggtacaaacac
tggtgataccataatcgcgtctcgaagttttacttcctggggttgaagaaagaggaagtggacgaactgtgtgaatgttccgaagatcccataatt
taagtgtagagtttgcagaacaggaagacactacttgagaagtagtgttcggtaaccagacggcagacgtaactgaacaatctctattttgtcttt
catgagctttttttattttgaggaccggtttttgatgatgttcgccatcaacggttattccatgagttctcatatcccaaaaaatgagtgatccatccc
tagagcacgtcactatattgtaaggagaatcattgcaaaagttaacttgtttaacggaatgagaatgatatccttcaagacctctacgaccgagtc
tggtgatgcattgctgtgtggcaaggtcgaacactttggctgtttggtcaccagaggatgtagcgagcaaagagtcatcgcttgagaaattaacgt
taaagatagcattattatgagcgagccatccatgaagccgacgcgctacatacgcagttagtaacaagctaacaagaaatacgttttcaacatac
ctgacggctgactttcttcatcttgccggttaaagtacctggaatcaaacaattctaaagcacccgtctctgtgcatacagcaagtaatgattcatt
atttgcaaacccaaggcaaaatggaagttgattgagcatcatcaagcattctgatggacgagaataaaatgtttctaagtttagtctacttccaac
atgacctataattatcgttagcgaccaaacataaataattgattaagtacacttacgatttgaaggaatgtgatttatgcgagaacgtgctcccc c
aagctgtctagctaataactccttacaaaactgactaggtttttttcggggaagtgggaggcttgtgcaagttttcggaagtatcaaacagtacacg
tttcttcggctttgttggcttcccgttaaagtcgagaaaatcgtacagtttatgtttttttcgatgtgccagcaggggaggagtctggagtaggagga
agagagttatttacggccagagtaatatctcttaaaccaccaacatcttctggtaaacaaggtaagtctaaaaccatgttgttttgtttatcctggc
aacactatataacggatgtcgtgtgttccggtttaattaagttgggttggaaggagtaagtatattaaaaagacgcgacgcggatcacgcgacgc
gtactcagtccatgaggacatttgttcgttactcgtacactatttgttgggtaacgataagtaaaataaataaacaaacaacattggaagagttaa
ttgtcgatatctttgcttcggaaccttttttttttttatagagaatcttagataataacttacttatactttgcttttcctagcatgtcccatcttcaagcta
tagtgcgctatgaacacctatgagcggtttctccctctgtaacgtttattaaaatgactctagtgcttaaagtaaatgtactatttataatattattta
tgttactgctcttttcctttttattctttaagggtcccgcgaagtaggttaatctcttataggacgcgacgcgtctgatttctcattacctctacgaaatt
tgagtcagcattttcatgcattcaggccaatatagaactgcattatttgaacttacgagaatcatagactaaaatcaaataatgtctcgttcagtta
ccccatctagcgatgatactggagatggagcttcatctcctattagattgtctcaaacaatggatcctttaagagataattctgatccctcttccgaa
gaagaaaacagtgacgatgactacgagaaaactatcaattcaaagcggaagaggcctatttccggaagaagcagaacagccaagagatcgaa
agcgaacgcatttatcgaagacgaccatcaagaagagattcctaataaaatatacggtatgcgaaatttgctgaagattccttctatctaactgaa
tttgaagatgacctattacagaacactgcaagtgtaaaggacatagttatagaatggcttacaaagtatgaaaaagatcaacaaggttctctggt
tgagttggttaattttattttaaaagtatgttcggtttaatactttccaaatctaacagttagtgctgcgcatgtaatcgaacggtaaatgaattcgac
gtgcaagatcaagagtctgtctctgtcacgctttcccaaattcaactctcagtcgaaaaagtacgtgatgattacctacttcaatagctgacgtttat
tttcagacaacaacccgtgaatatcctttaaattctaaaagtcctaaatttcgcaactttcgaaaaaaactcgctcagttccttacctactttgtttca
cagctttcatctagaaaatatctttatgagagcagtatttttgaaaatatcatgtcctggattgtagcaatgtcttcctctaccatgaggccgattcg
acatactgccacagtttttttgcttgattattatgtctgctttatgtgaagaatcaagcgaatttttatctgagcacgccatagcatctaagcagttgac
gaaagaggaaaaaagggctcgccctaataataatcgcattcaagaattgagtgactcacttcaaacaattattaagcagcaagacactatatca
gagtatctaagtggctactttgacaggtaaatattcttttaagatttcagttgtctaacttttaagtgtttttgtacatcgatacagagatgttgaacc
aaagatacgaattgattgtctgaaagagcttggcaattggattaatatagtccctagtgtctttttttctggatcctacctaagatacctaggttgga
tgctatcagatacgaatacaactgttcgtctaacggtaatcaaagttttgcaaaagattttcgaaacagaatcctttttttggaggactgagacattt
cacttcaaggtttaaagagagaatcatcgaaataagctgcgtagatgccgaggctaccgttcgtatagcctcaattcgtttgtgcaatactatcag
gtcatgtggtttttttagaagatgacgaggttatgaaaatatcgcagcttattttggatgtaaacccaagaattcagcgtgaggcaatacaattttta
tgttccgtagtgcaagaagctacagacgaaaaattagattcttggggaggtgaggaagctatttctgaatccttttcctcggaaaagatcaattctt
ttaatatttcttggataaaatatgctcaactctcgaaaacagtggaagttttgcaggatttgttcaaaaacgatccgacggattcaaaactgtttca
tgtttttcaaaggaacatatcagattcgactcctataactcaggcatttttgaatgcgtgtagattggataatgattatcagtcatgggagaggatt
gctagttatatattatttgacgattacggttctaagtctgtagacacagtggatggcgttctacaattctgcaaactttctgagagccaagaatgtat
tttactgcaactgcttgcctcttcccttctgaatatgtcagattctgagtttgtggttcctaaaaaaaattggaaaacaacaaccagaggaaagaaac
gagcttgaaaaagatgatgaaaaggaagtaatatatttaaggcttcttcctctgttgaacagcatcatagaaagagaagcagcttcccctacctt
gctgcaccatagtctgcgattgctgtttacgcttgatttcaggcagtatacgcacatgcaactttcaaaggcttttgaaaatcttctcttaaatttatc
aaagttctttttgacaagcaattattcaaatatcattcaagattgcacggttgtgtttctacgcatacagtctgttccagtcttagaggagaggtcgt
ccttcaaatataccgagatgtgtgatcaaagtattcaagaatttcttgaactatttctctccttcaacctacgtgacggtattatcaccaaggatgat
catttacgttttgaagcatgtttgaagcgtatggaaggctgcacatccataagaaggataacggacaatacggaatttaagactatttatgataaa

ttatcagatttgttatctcgaaatcctaatgcccttgaagattcactcaagttccctgctttacattctcaacaaagtttgttttttggctccttttcca
agaagaatcactgaacaaagaagttgttcgatcttctggggaaacccttttaattgtttgatgtttctgattaacgaagacagctcgggcatacta
aaacttcaagccacccgaattttattggaagtaattattatggtgtcgagtttcggagctaccaaggggagcgaaattgaggaatttattcctggc
gttgagggatcaaaaaatagagatatggttcattcgttattctctgttttacaagtatggctgaatgcttatgcaaagatgatccaggtttcacctat
aaagctagtgaatgaaaaaataagttggattccgacaaatatgccggcaaatccattgaacaggcagttgttagagcaaatatgctgtgatttgg
caggaaaattgctacttacgggttctcttacaaatgtactcttaaacgaagactggaaaatattaaaaacaatcaaagggaattttggttcggcta
tagacatgttgtttgttgagtttaaagttgatgaaagagcttgagtttatatatatatttgcgagcaataagtaatgaacgttatggaatagtaata
caattttataccttaaaaccgattatcctaagtaaacaatgatttaataactactttactttttcccccgtgcatcaactcacgatacttttgaatta
gttcttgacgttcttgtccaagggcttgccgtctggctagagattcaggttggcctctgctattgaactttggaacgttttcaattgtaattttttcttc
aagttcagttgaggaagcttgggtggagctagtacagtttcaccaaaagcaggcttttcctgtatgccagcccaaggatcgggagagccttttcgt
tttttatgcgactctacatggatgcgttttctacaatagttagaaaaaataagacgtttagcgtctaatcatacccagagacatctgcctcccaactc
ttatcctccagatttttctcagcttcatcacgttcacgctgacgcttttcttggtgcttctcaatcttctttttatctttcttgtctgtaaattcatcaatcg
tcgcttttcctgttttaaatgctaccggaatagcttgattcactcgcagactgaattcacgcatgctttcaccaggttgtcgcttcaattgctcattatt
ttttggtttattcttttttttctctattttctcggttttctttggcttgttttcagaaaattcttttagtttcataagtctcttgaatgactttggcgtatcatc
gtctaattcagcttcattcgcagctggagcattgttgtagctagagaaaattggttagtaattgttgatttaagtaaactattacttaccctaatttct
ttcgctcagcatcacgagtcgcctttttttttcgttttcgtaggcatattggttttaggatggtatttttttgcaatgcaatcatggttaactacaggttaa
ataagcaaacgtaaagtagaaatacctttttaattcatttttctaaattattttgtagatattgtcagcttctcaagatcaaatgatggaaaaaaagt
gtaaacaaattgaaaccaaggcaaaatatggaagtaggaagaaaaatgaaagaaatacaaaaaaagaaaaaagagcgcgacaaaaagag
atgaaaagattgtactaaaattttactgctgttatttatttaccttttcactgaactatttgttttgtaatttcgttcgccaatttatattcatctgattt
aggggtatgtagcttctaatgttatttttacaaactttttttttcttcttttttttttgaatgcactgcttacagattgatctgacttgttatagtagagc
tataatgttgatctttcctaatttaacctgatggctctcttaagtaagtcttgacatgaaaacttagagaacattgtgagattataactgtcttctagt
aaattaaattcaataaaagtcaccccaatagaacgcattcacgtcagcgctcttgtaccaggttcttcctgacagcaacagcaaaagctgaataa
aaggagcaagaaagagataagtggtcatcaggaagagtcaaataaaaactaaggcttttacaaaacattccgtttagcattggttctgttagact
ataaattttattaacgagtacaactatgataaactcgatttgcaacaacataagagataaaactattacgagtgaattctagaggatcaattatat
aattaaaatgagcatgtaaaatagaacatgccactattactacaatttatttacctttacctttaaaattgttcctgacttaactaagtgacaccgtc
acttgaatatagctattgttattttctacagctaaagtaccattcaccgttcaggttgactttgaaggaaaactcttgatgaatagctggaatcaaat
cgtctaggtttttgttcgctctttttttgtaagttatttttttgatccagtaggttaatactcgtttgcattttcgactatttaaattactcccttccgcttctg
agttaatacatccttctatattttttttggttcatttaaaaaaaaaaaaaaaacactggtttttagctccaaaaatttgaattggctcttagaattgctgaa
tgcaggatccgattctgtttttgtgattttttttttacacgaaaagcctaaatcgaaaaaataattcagatatcccttcttttatatttgaattttctaat
tttcgggttgctatattctttcatacccatatttcaccttaaattttttgtgtctaatatttgcatgattttatgcttctaattccctctatttttatttctctt
tcttttaatcaagtctcatcttttgccgaatttatcgacctaatagtttattctcagatatgtttcctttgggaagtgttgcagaaacagccgctggag
aacgagggttcgttcgttatgtcggtgaagttgaaaaccgtaaaggcacctttgtaggattggaattatttcctgaatacgcggaatccggaaaaa
acagaggggtcgtagatggaaaagagtattttaagacaaggaatggagatagaacaggtatttttttacctgtcgataagtgtaaattagcttcct
ctatagctgcttcgccctcatcatctccaaaaggtagtgaaaattatatcgcatctccagctcccacttctcttcctatgtctcccaatttacaatcttc
tatcccaaggctaactgcttttcctccgaatagcaacttaaacgtaaacagcatttcgtcgacagctctaacccccacggaaaagatccttcaaaa
acgtatcgaagacctactcttcgaacgtcaaactcatcaacaacagttggaggaagtcctctcaagcgttgatcagcttcaggctttagtcacaaa
ctttacggatcagcaggatgagctagatgagctacgcgagcgaataaccatgaaagaggaacgtattcaacaaatgagagaagaagcttctca
aaggcgtcatgattttaaaacaactatcgagtatttagaagattctacatctcgtgtagttgaatcttacgaacatcgaatatccgaacttgaattt
caattggaagcatattccagcgataagcaaggagaaacaataataacattatgtcaagaacggaatgaagcactaagtcacattgaagtcttag
aggaacgccttgagaaattgcttaaatcacgaacgcctgcagaaaacggggttgtcaatgggaaacaccagagcataatggaaagcggctccc
caactttgaccaatatttcatatgaaagtcattcacgcggtaaagcttctcgagaattacccgaaaaccatccacaacgccgccagacattagagt
tttatgaaattgaaatcgaggtattacgaaacaaggctgaatcgttgcaggcggactgcgaagaaaagaattactacattggtgagttagaggct
cgtttgggtaaaactagttttactacatctggcacggaacctaagcatagctctacagccgagcgagaaaagctgcttgatcgaattacggagctt
gaatctacgatagaaagaatgacgctccatgataacttggaagctaaaaacctgtcttccgtaaatagtatcccagccaatgctgtgtcacaaca
taccgataataattccgtttggtgtgaagtttgtgaaaattcaagtcatactttagctgaatgccctaccgtttttggtacaaaagaagaaaactga
cttcgtaaaccatgattatacatgtatatttattgcatttttagctttgaatgaatgaataatgtttcagatacctacttcaaatgagctattgcttttct
tatccagaaataatttcttttaatactgaattatttactttgttacgctttatttttggctttgccaaaaacctataccatcaaaacagatgggatatcg
aatctaatcatacactgaaacctttatacattaaatagtcttaacaaatttcattaagctcgtgaaaaatcatggtcataaattaagcgtcatcatt

cgcaaatacatgaattactgtataagtttcagatgagactttaaatattgctcgctttctttaggaccattactacccgcaggataggtcaaaggct
gagtaggagaatctaaaatttcatcaaaaatgcgccaggcatactcgacttcgtcgtaccggacaaattttgtaggattgcccttaatagcatcct
cgaataagatttcatatgcatcccttttgtgtttcatctcttctgcaaactgctctttgaagtttaaattcaaagaggcattaacgcaactacgctccg
aactcatgggcttattgacaaaggcagtcaaagtaacttcctggtttggttgtactgtaaatgaaatatagtggtcttgattaacgtcagaaaaga
caccagagtccttccctttcaaataatatctgatttcaacctttcctttcgacatgctctttcctgaatcaaaaacaaaaggaacgcctttccaacgt
tcattgtctacgtataatgtggttgcaacataagtagtagctttgctttcctttggtacccccctctaaatccttataacctggcttatcctgaccagca
gtatactggcccaagatagagtccttcaaggaagcaggctttgtggcctttaatactttgaccttttcgtaacgaacatcatctggaccttcagattt
tggttcatccatagcagctaccgtgaaaatttgagccatgtggttttggagaacgtctcgaacaacaccgttgatgtcgtagtagcctacacggcct
tcacaaccaccactttcccacatagaaattcctacagatttgatgtggtgattattaagcaatgcatccaaactgtgtgggttggcgaaccgtagg
gtaggaatattgattaccatgtctttagctaagtaatggtcaatacggtagatttcgtcttccttccatttttttatgtagttcttctttgagtttgataga
agaatcataattatcgccaaatggtttctcaatgataattctggtcttcccatctggaagataggcatggtcatgaatgagatttcctacagaaacg
taggctgaagggggtaaagccacgtaaaaaatacgaagactctttgagggatcagtgcttttctctaatccctgaagatacttattaaactcttta
aatgagtctgcttggtcgtattgaccacagaagtaagaaactcgcttgataaattcatcatagatcttagtttcctctccctgtggttgaatgtaatc
ttttaaagagtttcgaaagtcattttcggatagtttacttctggcatagcctaccacataaaacgactgacggttcacagtgtttctcaaatacagat
tgtagagtgctggaaacgttttcttatgggctaaatgacccgatgcgccaaagacaataaaactcaccatttgtaaagtacaagtgtgatattttat
tcgattgaaaagtataaagaagaagaaagtagacacttgctcgtcaacgtaaaaaatcactcagaacacacaaacagaactggattatagtga
tattcagtcaaaaactaattgagaagaggattggattgccttttatgccagtcagacgacacgataccgaaaaatgaaaagaacggaaacacat
gaaatcatattcaagcaaagagtaggaatgacgaatgcgcgcaaaggtcccatgaaatcaacatattcatttgaggaaagagggcattctcgaa
ggaaacttcaagcaagacattttactttgtaccattctgttccaaattatatcgtttcgcattttccatacaaattcaaaactttttttgatttcatgaaa
cactcttcatgaaatgatatccatttgtttacagcatatgcgctgtagtcaacatcatgttcacgtgtgggtggcagaaattaatgtaatccatcatt
tttcacatatgggcttcatcatccatcatccaacatgcaaatatgaatgttttttacgtcaataaaacttgaatcgatttcctacagggagatgactg
tgttttcgagaataatgtgaaaatggcaattaaggtaggtaggtaggtagtctgttatcgacaggaactcatattgtgaagtagaaaaataacac
agggttgaagcagagtgagtacagttgcaagggaacgaaagaaagaaatacttatatatatacacaaaaacaataattttagaaatatacatg
gattatctacgttggaatcaaagctatagcttttatagtttcgaaagattagagattaataatgcaaatattgatttaggactatttaagtcaggta
atcacaaaagtagacgtagaacataaattcgttcgtatgctctgaaacaggaatgtaaagctgttttagatacggaaaacagtgcggtagaaag
tttcaccttgaatagttgcagccttcaattctatttcagaaggaaagcgactgccatcagcggcagcaaaggtaccagcaccataagaagagccg
ccatggggggaatcgagttcagaagcaatagcaaaggtgttcttgtaacccaaaggaacaaagatcataccatggtggataaagctagtcaag
gcagtgaagccagtggtttcttgaccacctccttgagtaccagttgagaagaactgaccaaagtattttccatgcaaggcacctttagcccaaaga
ccaccggtaccatcccagaaagcacggaattgagcgcaaggggtaccatagcgagtgggatagccaaagaggaaagcatcgtatttagtcaaa
gtgtctggtgtagcaacggggtaattgggtttcttaggggcatgcatcttgctcaaagcttcatcagacaaaatctcggggagtaggtaaatgtct
gccttaccaccagcagcttcaatacccttttctcagcctctgcgagtttgccaacatggccgtgagtagagtagtaaataatagcaacagtgttgt
tcatgataatgggcaatgctagtaattagaataacaaatcaacaatagtaagtgtttggaataagccaactacaattgatgatgaattctggacg
gggatactcacacctatttataacctttttttttatacatgatttaatcaaaatgattcaaaatcattctatggaaaaatctatttgtgagcaatcaga
aaacaataagacatagccctgaatgatttgatgactaatgaccatagattgactgaaaataacgagaaattttctttgctttgtcagcgagtatca
gttctcctcattcttcttactaaacaatgatttttattgtttccacccatttctgtgtcgtttcagccccattttgaggcccttttcatgccttgttctacc
atctcctaagcaagcggctgttttcatgaatcagtttttggtggtgatgtttagaaaaaccagtcagaatttttattggctcgtttaagaaataaact
tttttaaaaaaactcgcgttaccatattattacgtatttattacgtatatttattgaatcataaattgaatacggtagcgcgcaaaggtttcttggaa
ataataacgagctgaattcaaacttgttaatatcttaaagtgagagaattctttttttcttttattatcctataggggacaatctatgtttgttttgattc
tcaaaacaaagctatggttgctgttagaaaggtagaaataagcacaaaggcatttgaaattgcttaacacgaagagaaaaagactcaagagga
aagaactatttttgaaaacgaaatttgttgctttccatttccatctattcatggtagtcatcatcaaacgaacattggtttgtttaccgtgtgttaatat
aattggagactaaaacttcagtcagtcaacatttcgattctatttgttatttaattgtttatgaatgatgatctattagtaattgattataatttatagt
atatttgtacattttctatgcggaaactacctcttacttgataagcatctggaactagtcaaattattttgcaaagttgaaactaccgtttcgaaaga
ctttacttcttttctataaacaaaacacttctagaaaattctgcaaaccaattttaaaaggaaaatctaccgaaaccattgcttctccctttgtaataa
ctgaataacttctttttaacaaatattttaacaaaaattttaatataatatgctcattaaacactataccagacaaggcctcgtggcccaatggtta
aggcgcctgactacggatcaggagattccaggttcgactcctggcggggtcaaattttttttgatatttatatgaaacaatctcatacatggagatct
aaactcgtaaaaagaaaaaagacttgtaactttaagaacatattgtacaatcctttttatctttttattttattttaattttattttactttttttatttcg
ctttattttctaatatgttctgcttggttatctcaaactctaccatacacttatcccatgcttttgacttttctaggaaatattataattatattaatatttt
agctttactagacttattaaatctcccacctttgctcggtatatttccaaaaaaggtacaacaaaataataaaacactggttcatgaagtatacact

agaatgcacccgacgaattaaataaggtaaatgcaattacaaccattcttcttttgtattttttccgtttaatcggtttcttctgattctgcttttca
ttatccttttctgaaagcgtggcatccaaagcaatcttggcattcgccaaacgttgaatgtgttcttcgatcgtatttttaaccacgaatttaataact
gtcacctctttttctgtcctacacgatgagcccgatcttccgcttgcaaatcatcgaatggattaaatgacacatcgtaaagaatcaccatattggc
acaagccaagttgataccaaatccaccagcctttgttgacagtaggaaaacattgatactttcatcagcatagaattgatcaatcaagtcttgtcg
gaaatcgacttgtgttgatccatcaaatctcaaaaatttcaaattcaaacttttcataaccaattggagaatgtcaaggacctgtgtaaattgacta
aacaaaacaatacgatctcccctggcaatggcgttctccaacaaagttttcagttttctgactttagtagcattcatccaaggttcgtctttcaactg
gaagggttgaattgtagagaatttgccgcaaaggttatgtaactcaatatccgacatatattgcatatcttcgaatatatagttttcattcgccttctt
atactgtggctcattcataattgccttagccatttttcgaagtatttcatccttataaagaatacgaaacaacattgggtgatccgcaagcttccgaa
gttgaaccaaaacaaaaccagcagtactcttctttttgcgagattggcatttgtatctaaattcgttttcatgacattctcatctaaaagattattga
cgctttgcttgttcgcaaattcatcatatctctttcgctcttcttcagagaactcacaaaattctataatgcgtaccttcttgggtagagcatccagta
cttgactctttttacggcgtaaaacgaatggcgccatcatcatcttggctctggaaactcgctgttctgaaagtagtgctctttcaaaatcactctct
ggcgacttcttcatcgtaaaaataacatccaaactcttcaaaccgtagtcaaatacatgaggaagaataaatgctaacaacgaaattaattctttc
aaattgttttgaaggggagtaccagttaacagtacacgaaagtctgcaggaatactcatcaggtgacgataacgttcggaagcacgattcttaag
ataatgtccctcatcataaatgcaaacgttaaatttttgattacgcaaaaataaccgatcagctttactagttgcagccaaacggtatgttgttaac
atgacattgtaggaatctttgttggaattaattctttcccgaatctcttcacgttcgatttgagaaccatagtacagctcaattttcagttttggacag
aactttgcaaactcacgcaaccaattctccatggtggatgcaggtgcaataaccaaatgaggaccattaacattcttatccatcagcattgcgaaa
aaggcaatcgtttgacatgtttttccaagacccatttcatcagccaaaatacctgctaacttgagttcataaagcaaatacagccaattgatacct
taatctgataatcttgtaaactcacatcttgcgaaaaggaagctgggggacttgttatgaaattagcattggaaggagtgccaatgtttttcatttg
atcaaaactaacaatagaagtttctccttcttcaaaagtagaggagcttggtaaaccccagttttcaactcctcgctgaatttttccacccaactgtt
cacattgacggatcaaagcatcaacaacaaagtaaccacgcataatttccatacaaatgccaacgagtcgaggacctaagggagtttttcacgt
ctaccacgtctacccttgggaattacgggacgaggttgtttaacgaccaaagcagactcaaggtcggcaaagggtcgtttggcaaccataaattc
cgcttcggctagagtgcaaccggtcatatcagcaatgtcttgcgttgaacatcggttcatatacttgagaacttcctctttcaaagccgcagtgtca
attcgtgatgcccaacgatcagcattagattcatcgtcagagtacatatcgtcattactgtcatcatcggcttcaggttcatacgttttgcgagaaga
gatgggcaaagtaaattggagtccattcgtcacattgccgctagcagcgtatctttcacgcatggttgggcgatgagcagatgttctaggaggttg
cgtctgcgaaacgagagactgtgatgaaagcgaaagaggtaaggagtcactagcgtcttcctcttcatccgcataagagggcatcgtgccaacc
attcctggatatggtggctgcatattgaaattttgagtgtccaagaaattcggcactcctccatagggtatctgtggattggcaaaaggagtggcgt
agggatttacaggggggaacagaaggaatcgttccagggaaagattgaggaaggaagctttcaggagtcgtcatactaggggcggctaagccag
gagcaatgtttccagcttgtttgtaaacctcaaggttcgaaagaggtgttgtggattgaacgcgtttcgacatttccgacaaaatttgaagacgag
catttatgtcgccaccagattcattggctttttctgatgcttctgagagagagtctgcaatgaaaggggggaaatactggcttgcgcttggtgtcata
gggaagatcctcataatgaccattttcagcatgatccatttttcgtttgccgtccattttgtgcaaaattgatataagatgttggtaataagaaaggt
atacaagaaagagtacagcagagcgtgataagaacgaattcttcggttcttagcagcaaagaaaaaaaaaaaaactttttaaaagggaaaaca
agaaattgtcaacggatttaattggcaagacaaaaaactcgaccaattttagctgttacttgtgattttctaaaaatcaacgatgatcgtaaagta
aagcttaattgttgtcttatacaagcttttctagggaaaaagaaaataaaagtatgcttgcttcagtaattgctcgaacaagaaaaaggatgagct
gtaagaagcatacacgctgtttgcgtcgagtcaaggaaggggcaaaaaagtatttttataagaaaaaaaaaacaaactttaagaagacaaata
attaaacctagagtagcccaagttctttgcgattagcagacaccaacagctttgaaggagcaaacactttcttgatgatcgactgagaaagcaaa
aatttctaaccttcgatttcaaacttcttcaattttaaaaaatgtaattaaagaagaaacaactctgcaaaggagaaatagccagtcaaatttatgt
tacctgctacttcgcttcgcaatataaagcaattttggtactatagacaagaacacgctacgcctggtagttgttcgtggaggggtttgcttgaggt
atgtatgtgggtggtggtgaatttttttatttaaagtactgtaccacagaattaaaaaaagaaaaggaaaagttacgcagggcgcagtaaacataa
tacggaaacggaacgaaacctagcgcaagctgctccttttacgcatactcagtaagcaggaagaaaggagagagaaatagtcagaaaaaggtt
tttttaggaatttcatgaggatatatgccatggattggttcatgtgttacgaatttaatgagtcatgcaaattttggacgacggccttgtgatagaaa
gcgaccgatttttggcttctttcttttctcttttgattttattcatttatttactgagtaccatatcaatgcatggatttttttgacataaaagaattga
gcaccatcgtaatcaagattataggatgtaattatacatagcatttcgcatctaatgggtacgtaaacaaacactatcgctgtactatgcctaacg
aacgagggcagggtgggtggtattgttcggttgaattgagattcaacttaccacgaaccattaccttcattcaaggaaataaaaaagaatccgat
tgcaattggagatcagcttccctgtttaggattttctctaaatagatactttgacaagaaaaagtattgattctttacttacgtatagttcagaagta
catatatatctggaatcttgttaggggtgccttttgaatcatcgtacaggattcgataggtctggaattaataaatagaaagaaagaaatgcaagt
atataattcttagaatttaaacattacatacaaagattcgtgcatcctcactctttgtcgcccagattactcttcctagctttcttttcagattcttgta
aagatcctttgtttgataaaatcggtttctaagcaaagaccatcatcgcttccaatctaattgcgttgcgttgtttgatagttctcgacacaagatac
tgacattcacggcaatcccttttccctttgaaaaggatccatatagtttctttgcatgccgaaggctacgaaatgccaactttgcgagacaagacgt

ccttccatttgtgaacagtgcctgggaagagagctgacaagtttttatacatcaaaggaacagtatcagaaggagatattttctcgtttggaacat
ataagacaacaccaaggaaaacatgtcttatatcgagagcgaatttccgaaaaagaaatgcttttaaataagcttgacagattgtctcatatcca
atctgataagctgtctgctctatccattctcaacgaaaggatccataccctaaaacatgggatcactaggaggagagagtcccttgatctgaaga
gcaaagatttgaatgacagtttcatgaggaatcgagatctgtatgccgagagatatattcattctattcagcagtctgagaccctgcaagctagac
ttgctaagcttcaacaaacactgattcgaaaggttctcgatatgtatcagctccattggcgcagggacgtcctattgctgtcttttgacgttgttccta
aacaatctccagaacacgaacgtagtgctgattccgttgatgctagcttagcatatcagctttcaaagctgacaatgagtgtatccggatccggtt
ctggttatgaggaaaatgtacaacgccgaaaccagacgttcttggaacaagctaggcaattaaacgattctgttacgatttctggagtttatgtat
gtgtaaaaaataaagaaagagtgtttctgaatccagaatcttctctgactttatcattcatctgcatttttctagcgcagtacttaagcgtgactcttc
cttgcccaattcaactaccttccccagatcaaaaaatgctcccggtttattcgaatgatcagacgctatatctcatgtacaacataggatggcttgc
atcgatttgtggcgcattcactattcctaaaggattgtctcaaatacaacaaatggaacttctccaatgtacaggattctggttagttcaattacggt
ctaaaccagtagaagtaagaagctttgccagcactcctcagctggaaatggatttcgacatcttttatcgcttgtcgtgtgctcataatccctatgct
tttagttatggcaatgtgagatctaaaccttcttccaaagcattccagcttatttcttaaagcagcagtatctctatatgttgggatactattgcttca
gaacgatattcttttctctttttttttattcttcctgaatactgcttcagagaaggagcatttatgtatattttagtttatgattcatgccttctaatttgttt
aatttgtttacatatgtttattaatgttgatttttgtcgtgtttatactttcatcatatgacgattattttcatcaaataagacttagtcaactaattgtgt
aaaagtaagtgtaaaaagagctagcgaacggtcttatagaaagagagagagagagagcaaaaaaaggataaaagtaaattaaaagacaatt
aattggaaagattgttcaaaacagaacgaagagccgttttgtctgggttttcagaaatgggttggatagagcgagtttgattgatgatgtctctggt
tttatcaaaataaacctcttgaaggaagttacgcattcgagtttccatctcctcaactagacgtccaatgtttccgacttcgctgtcctgttcacttgc
aggcatacgctgagaagtttggcgagttaaatggccagaaagatttaatgctccagaaacaagggacgaacttctgtcagaagatccgaggtaa
agtattatagtgctagttaaacgatagtcaaaaacgtccgtttctgaagtgggtaagcactcaaagacatggatagaatcccaaccgcaggtacc
agagcttgaggctgaatgaacgttagactctgtaaaaacttaatggataagcaataaaaaacaaaccttttttaataagtacagcgccagcgtag
gaatcgtcttggtcccaaagatatacactgctaacaccaccttcatagtacaaatctaaatatacacgcatagcttcattgagtgccaactctaact
ttctcacacgatcactggaaacgaggccatcctctaaaggagggtcatatttattcgtccaaggagaacgataggaatcgccatcacgattgaag
tcgcaaagtagatattggttaccagactcagagcaagtacctatttgcaagggttgatccacagaagagagaaggacatcagccaaatcgggag
ctacagctagaatggcatctaaatttttagaaatgtccttgggatttaagcggcgaagtaagtccagggcggcgtcttcggactgattgttgttagt
aaagaatgttcagatatctatgtcccctataactagtgtaataaaacaagtaggactctcttcatatataaacattagctaagggctattgaagctc
aaaaaaaactttagcaaaatattttattagtctttctcacttttggtaaaacggcacactttgtcttagagcgcatgctttgccagaaaacgaaatg
aaaatagatcaagcaaataaggacatcagaaaacacttaccaacataatgaatcaaagaaaagtgaataaacttcgaaaatatctacagatac
taacgatcaatgaaccggtttcaatgacaaaaaaaagcaatatgcaatcgaattgctttgcacttatggccaatgaagtattatatcagtttttca
acagaaaatcttttgattaataacgactataatccaaaatgatggaatccactaaaataactgtaagagagaaggaagtgacagaaggacttgt
tggtgtatggagtgacaattcgtgtacacagtatcgaaatgaagtgaaggagacatatccagaacgaacaacttccaagatatttatttaagacc
agtcacgttcatataaccaatttagaaatgtctggaatctcttgaagcatccaactgaagcctacactagtttctatttgtggtggtacttttgtctct
gtgcttgctatgacaaccagcctagcaatgggcggcgaacacactctaacaaaccaaaacagaaccactatgccttatgtgatttgtttacccaa
gcataaaaaaagatgaaagataacttggttggtgtcatacaagatttgttagattttgatttctacataatcacaaacaatatgatggaaggaatt
ttttaactgttaagtcggatcaaaaatgaaagctgccagaccgaagaaatggaggaaattattataatatacggaaagactctgaaggagagga
ccttgttcttttctacataacaattgagactaacattttgagacctcatgcattcactgcaacaattggagctatagcatgttacaagcaaaagtcgt
cattttattcacctgctaaacgaactgtatgcccttgaagcaaaacaaccgatgggtctctacgtaatgaaatagttgagttggcttcctctgggta
ccgtataaaagcctctgtgttcatgactttcttgttgttaacagtgactgtattattaagcgtatgtcttttctcttggactgccattccttatcaatgcg
aaattgcacggtacgagtctcagttacggaaaacagttcttcggctttggagataaagtgtcagttgtatttaatgaagcatgaacttcattgctgc
tgccatcaccaattgactatgattttacctacttctcttccttttcctaaacttcatatttttatgattcggtataccgtaaaataacgtttgctcgttcat
tgagatactgttgtcgcagcctttgcgcaaaatgcagcttaccgagctctatgaacaatatgttttcatttctatagtactatactgaaaacggatgt
ggtttgttataattgccacctgagataatgatgttctgatgatctaattattggaatactaggtggtgcaatttgcatcaacgaagagaagtcgcttt
cgatcatgttcgataaagttcgattcacgctttggccttcttttccaagagaaggttgcatggagacgcttgcgtgctggagcgccttatttttttttt
tttttttttttttttttttttgtttttgtttagtacgtacgacgacgacgacgtacgtacgtacgtaacgtaacgttnnnnnnnnnnnnnnnnnn
nnnnnnnnnnnnnnnnnnnnnnnnnnnnnnnnnnnnnnnnnnnnnnnnnnnnnnnnnnnnnnnnnnnnnnnnnnnnnnn
nnnngaagagaagtcgctttcgatcatgttcgataaagttcgattcacgctttggccttcttttccaagagtaaggttgcatggagacgcgttcgtc
gtgtcgtggagcgtcttttattttttttttttttttttttttttttttgttttggacttgaaaggccaccgacagagattccaagaactcctacttctgtttg
atttgtctttgcctttctccttatgattttatcaactgtaggcaagtaggtgtaactttggcttattgcgcgtacctgcatatattttaacaaagccacg
tagacatgaatgaagacgcgtagaacgcgcttccgagtagaagcttataatggggttattcgtaaaacaataatgatcgcttcacgtcaaatcat

gcatgcgaccggaagcgctcaatcaagattgaaattcatacttggtgaactgctgtaaagatacgtgaatggtatggtggccgtttttagctacgc
ggtttgtttacaacaaataaactgcagtgtgtatgacatagtgtcaacgctgattggcccttcatctaacgaataaaagttccttgtaatcgatcttc
cgcctctattctaatctccatcgaaaccctaaccctgattaggtccgggacttatacctacatgttccctccttccctcaacaaacaacaattatttta
cttatttatcgattcttccaatactgaaaccttatttctcactattgcaaatagtgaattgaaggttgtcggatagtatctgaggaatttaatcgaagt
tagaaggcctgctgtattaatgcgggtactgcagatcgtgaatatttaatatttctgtgaactatattatttttttaaagtggctgtgagaacagtgaa
ctatacatgaccagtgagaatattatatcgtaagtaggttttgttgttgcgcaagggatttccatatgggagagattagcgagaagtttcttttctta
cgcttttaaacgttacactcgataagtgaatataattttaggaggaataaaaggttatatgcttccgagtacaaagtagacaacaaaaataacatt
tggatggtgaaagattttggttccctttggtttacaactttgcgaagatattgaccacgaatactttaaaatttctgttctctttgtctcagtttatttcg
atgtctagtttgttcactatctcgactcttctctcgcagtcttcattccagcgcagtttattgttctcaattactaacattcagcagtgtgaaatcctcg
cagggtaaagttaatatttcaaacgactctggtaaatatactttgggttcaatgaacgtctctaatatcgatattactgtgacgcacccttattctgc
cgaaaccgcctctataagcaaaagtggattgggtaaaagaaacgactcgacatttacacctagatcaccagagccttccatgaatggggaaggt
aagaaaacttcgtatttccccttaaatgagaatgaagatggaacgctagatttgtttggtgataatgaactcgaggaagaaaatttacaatatca
ggagaacgactattcttctcaatacttgcatcctacaccgccgtatacgaactatgacgatgagtctccgtcttcaccgtctcaagcgtccgaagga
gctagtaaaaagcacatactagaaagcaatacttctaccaaattggactctaatgaagctcctaacaagcgccaacgctatgacggaacggacg
ttgagcatgaacaacctcattctgtaaatgctatcaactccgttcaacctgaacgaaaaccttcacctatagaaatgcaattaaaatcagagcttt
ctgtagtccctccttcctctaaagtttcaaatgtcgctggcggcgccgctcaagttatctcagatctaccggatagcgcagttgaggaaaaagaatt
tctgtcttcgaaagagtatcccccaatgacaaaagagcaacacaagtacattcatgccatgcttcgtcaacttcgtcgtggtcgcgattcaattcct
ttccgtgctccggtcgattcagtaaagcaaaacattcctgattatccttcaatagttaagcacccaatggatttaggcaccattcaaaaaaaatttt
caagtggtcgttatgccagtgctgagaactttattgacgatatgagcttgatgtttgacaattgctttctatataatggtacagaatctccagtaggt
gtgatgggaaaaaatttacaggctacatttgaacgacaattaaagcaattgccttccccgtatgttactagctcctctagacctggacgacgtcctc
gtaatacgactacagctagtaggcccacgagtcggacgcgacgacagtctgctttatcatcaggtcgagagacgatgtatgatctaaaacctcat
cgcagaaaagatgcagctgagatgaaattctgtcagtctgttttgaaggagttgctgaaaaagcagcatgaatcctatgcttatcctttttacaag
ccagttaatcctatagcctgtggctgtcccgattattataaagtcatcaagcatcccatggatctttgtacgatgcagaataaattgaaccacaatg
agtatgcatctcttaaagctttcgaagctgatatgttgcttatgttcaagaactgctataaatttaatcctcctggcacgccagtacatattatggga
aaaaagcttgagtccgtgtttcagaaattgtgggccgagcagcctgattttgattctgaagcctatccttcactttcatctgtgaatgctgactacta
ttatggggacaacgatgtgttcgatagcgcggatgagttcttagatgatggagaagaatttgaggctgtcaatcgacagatatatagacttcaga
gcactttacgtgcgatgaaaacgagagctcgttcctcttcagtttctcgttctcgaagtttgagtattgataactttccaccaattacctatgaaatgc
agaatgaattagctgaacaatgcaattacctaacagctgaccaactgacgcatgttgcggaaattctacgttcagcccttcctcatcttcgaaaaa
cagacgaaatagaaattgatgtttcgtcgatgcctcctgatgtattctttaaagtttactactatgtatgcaaaggggatgaaacagctgccgatca
tattctaatgtctcctcaacctcagaaggagagaaagggtcgtgctttgtctgaacatgaacaggcagagaagattcgccagcttcgtgctcaact
tgatcgctttgctggaatagctagtcaaaacaaaggattgagtgattctacagctgcttatcagtcaaaggcgtttgctacggatgaatcgtcgtct
gaagatgatggcgaaagcagcgaaagcagtgattcagcgtgagctaatgcggtaggagctttttttctttaattttcaaggtttcggtttattatctc
attattataattcgtgttattcttaatttttcaggtttcttgcgctgtacatatgtgttttgcttatgttgaatttcttccttttaatactttcacattctttat
tgttgaacatttttcactcgcatgatcttctacgtaacggttacttcgtgatttctaaattgaaatctagttccaatttgttatgattcttttgatataat
gttgtttttggtgaactgtcttaatcagtctgtcggttttctctgtatttcattttaatttactttttttctgcgattttaaagcacagttttgttctatctat
actgaaaaccgcagttttgatttacaatgtctcatctcttgtacactagttagcgtttatatttgatacgtttaataaaactatttttaaaaagataaa
ttacgagtaaacaacgtgcgaatttagagcttctacgtaagaaagattttatagaagaatgtaagtactagccgctgctgctatacaatgaataat
tgaaaaaacatgccgagtaccttttttcttttgatttaagctttttatttgtgtgtcactgcacatgataaaattttaacgatgcgttatttcgtgcgct
atcctaagtgggttagcttaacatttgtcaaacagcaaaccgcttcaccaggatcctgacagcaatcaatcccaaatggcagctaggagtcggcc
caaatcgacattccggacggtacgggataacggaataaaccttggggcttttcaagagtcttctaagctttatgctagtataacagatgctttaga
ttctcagcgtctgcgtattttcgattcgcaagagggatcgctcaagaccgaatatgctttagaagggaatcagcgggtcagtagtatctgctggga
tcaagctgttgaatatgatgttgaacctatagatggttctataggtggctctggtcatgtcgtactccttggtacagaacaaggagagattatagta
tactcagaggctttgggcacagttacaaaaacatatggttttggccaattgggtaaaatcaccaaagttgctcttctgagctcatttgcatttgccat
tgacagttttggaaagttggttcaatttaatgttaataatggttctgctattcaaattatgaagattactactccttctcgtgaattttccggtgtatat
ccttccccttgcattccaaattttgctatcgtcacctcgcacagcgcgcatttgttggcacttccggattcgaatcctgtcgatactttaacaactcat
acaacgcttgttcagtctgttgtttactcttcagcctcaacgccgtcgcaaaatttcgtcctttttgcgacagctgctgaacaagaccgatttgtcaat
cttttcagcaagcaagtttccgataagaagctcactgatgcaccttccaaaaatattggagcacttgtttgtgaaaatgatgtacaaggactagca
agttgcgagaaaatcgaagaagccactgggaaaaagggtagtattttagcagcgctgtcagtagacggtactattgaaatatttgataatccatg

ggagtcgaaactttcatctggatcaggaggactagcctctctatcccgtaagcgtaaacaattaacaagtcattctaatctcagaatccgcttctct
cgttctcgtggcggagttcctattacactcgagtccattagtttcgtcaatgcagatactattgtggtagcatggaaagaggctactcgcaccatttt
tgagagcgtcccttggaaaattctgtcttctcaggctacggatggactattagaagtagttcgtcccaaatcccgggatgtcaataaagtaggaaa
acccgtcgtcacttatgatgaagcaaatgccaccgttacatcaggtgtaatgcaaaaacatgctgctggtattcctgatattcccactgaggaaga
agcgcaggcagaagagcaggaacctagtcttgctgaacgattgcagacacttgccaagttagaccaacaagctcaggctactcctacacaggta
tctgcttcttcttctttagcaactgtgttagctcaggctttgcgaacaaacgatcaagctcttcttgagtcatgctttaataatcaaagtttggaagta
attgaaaacactgttcgccgtttagactctacattggctcctgccttgttggataaagtagctgatcggttagctcgtcgcccgttaagagcagata
catttttgacttggattcgttgtactttaattttccacggtggtcatcttagcttaatccgtgatctgaaagatcagcttgctactcttcgttcagtgttg
gaaacaagagcttccaaatacacaagcatgcttgcgttgcaaggaagattggatatggtgttttctcagattgctttacgaaaagctggtggctca
aaggataatgctgagaacgaagagcccattactgtatattatgacggttttgaggaatacgaggacatggaagaagatgaagatgaagacgag
gaggacatggattctgaagaggatgatatttcagaagatttatctgaagatgaagaccatgtaacaaatgatcaggcgccttcggacgaagatt
acgatcaagaattacacgaggaggacgttcagcatgatagttcagatgaagaaaactaactcggtggttttctttgattttaaaatgtgattttctt
attaaatcatgggtgagtttttatattggttttggattgcgttacttaatctatatatatatgtgtgtgtgtgtcgataggtgaacataaaaactttttt
caggtagaaaaaaataaaggtttggttttagtgcgttaaactttggcagaagtaatataactaaaagaattgtattcacggtttgtatttgactata
actgtttaaatcgatctgaaaactataaagaaaattatattttcacaaaaggatacattgcttatcttggtgttgagcaaagtatgcttcgatcacc
actacgacttactactacagtaaaatcaattctctatatacctgcacagtagaatattaatgaaaactgccaatgacttcaaatgtagatggaaaa
ctggaaatcatagttcggtttgcttctgctatgcctgatctctcattagaagttcaaaatgcaggagaaacaacaatccgggaacttttcaagatta
ttcgaagtcgaacaccggaatgtaaggacaaacacctaaaactgatatttcaaggtcgtcttttatctccttcgttcacaatagaaagagcagtaa
gggggaactggcattatgaaccccatagacaaggtaacgaatcgcgaaaagcatttattcattgtattgtcggacctcagctatcaccaaacgaa
ttggccccagaagaccaagaattaagtcaacttcagcgctccagcgcttcacaagcggaagcgtcggcatcacatcctacagagatgctgagag
gttttgaccgacttcgtgaagctggatttacagaacctgaaatttccagtttaaggtcccaatttcaccaagcccgtggtacgaatttagaggctctt
acagaagatgcaatacgcgaagctgaggacgattggatagataacggggcacaaacttcatcagcagatgaatttgacatgtcttacgaaactc
ttttggctggcgtgctggtcggtttttttggtggtgctcttgcatgctattttctttggcagaggactatgttttccctgcgaatgcagctttcaatcttg
gtaggaattatttgcaattttgtttacggagttttgcattcttatcgatggtaaaaaattagaagtattgcttactgtagcatcaaattgatcggtact
ttctaatctgaactgaagtacctttcttatgttttcgcatttaagaaattcccgccaatacttggacgttttatcttatttaaaaaatatctgtcgggta
ctacgaatgattcatttgtctaccacttttatgactatggatcgctttttttttctctctctccttttgttgcttaatttaaacatgcaatttaacaacact
ggataaatatcattaatgatctttctatcctctgattgccaggttaactttattactgttgtttatgcacgttgcactctatggtttataatgactatgat
atgactctatttgttgaaataaatcggttttgtaattcacatttcacactgtataatacaccgagtagttgtttaccaagcgtatattactgggttttct
caggtgttttgtttacaaaatagtgattgacaacattcgttcgttaaagcatagtagtgaacaacagtggcaagtgaccttaacagcaatttacga
tacttttagacacaaaaaaatttaaataccattttatgcttgtgaaatttcatccttcaacagcaacctagtcaaccatcagatcttgcggaattgcca
tttcgcaacgttcaaatctttgggagacatgacatcctctgtgctaattcaaggtcgtgaagaagacaatgtcctcaaactgcttgaggacttgtta
aatgccgaaacttctaaggactgtgccaatattggcgaaaaaataggagaaatttttaaaaaggatgaaccactagttgctttgaaaacaactg
gatttttggatggattagagaaggctggtcgtaataaaaaatcaggcttccacagggagtctgcaatgatcggttttgcttctgttattcggaagat
cggtcgtccttgcgaggtcgatttccttccttatcttccagttatgctagatttgttctctgatagaggtgaagtcgttagagctgctgctaagatggc
tactgatgctttgctgaatgttctccccccctgttgcagttgaaactcgtttaattccctctttaattgactatttggatagctcagccgtaaagtggcca
agtaaggtagctgctcttgaattgctgggctcgttggcaaaatctttccgtgacgctgttgcaacagcaatgggctctttgattccttgcattcgaga
acgcatgcatgataccaaaggcgaggtttctcgagctgctatcaagtgcatgttagatctttgcactgttgtcgaaaataatgacattattcctcat
attcccaagttggtcgattgcatggctcgtccagagaccctggaagattgtatcaaagaattgagtgctacaacctttgttgctactgttgaatccg
ttgctttagcagttttggttcccatcttgaaacgtgcacttgctgaacgttctcaaattatgttgcgtatgactgttattatcactgacaacttatgtaa
gcttgttcccgatcctgctgaggcttcggactttctcccagagttgattccagaagtcgaaaggatcgctcaaggtgctgctatgcctgaagtccgt
gctcttgcgtctcatgcccttaccactttaaacaaggctgctgctgcccaagctgcaaaggccgccaatgatgctgagagacaagcgctttctggc
gttcgtaacagacttcaacaattcgtcactgaacatgcgacatgtcctcagaactttaatatggaggaaattttgagttatgtttgtgatgcctatgt
agccttctataatcacaagaattttaataaggagacttgggttaatgagatgggtgtcccgtatctgactcccttcgttggtgaaactgctgcttccg
attgtgtttccaaagcttatgatgatcttcactcgttttacaaatttattaatggtcctgatatttcaaacttacatattgaggacgaagagttagtta
acacagacttctcgctagcttatggtggccgtttgcttttgaaccacactaatttacgcctttacagaggccatcgttatggtgttgttggtcataacg
gttgtggtaaatctacattactgaaagcaatcgctgattataaggtcgaaaatttccctcctcctgagcaagtgaagacttgctttgtggctcatag
cttacagggtgaagataccagtattgctattcttgactttgttgctaacgacccttccctagctgctatgaaagtcactcgagaggaagccgcagct
gcgttaaagtctgttggcttcacagaagaaatgcaaaaggatccagttgcctctttgtccggtggatggaagatgaaattagaactggctcgtgcc

atgttgcagaaagctgatattcttttgttggacgaacctactaaccatttggacgctgcaaacattgcttgggtggaagcttatttgaacgctcaga
ctcaaattacttgccttatggtgtctcacgactcttcctttttggatcacgtttgtacagacattattcattatgaaggtgtcaagaataatgcaaaga
aattgggttattatcctggcaatctttctgctttcgttgagaagaagcctgaggccaaatcttattatactctttcagctacgaatgaaaagttcgttt
tccctccccctggtattcttgctggagtacgttccaacactagagtcatcctaaaaatgactaatgcaagctatacttatcctggttcttccaagaaa
tcattagatgatgttactgttggtctctctttatcttcccgtgttgcgattctcggtccaaatggtgctggtaaatctactcttattaaagtattgatcgg
tgaggttattcctcaagaaggccgtgttttcaagcatcctaaccttcgggttggttatgtagcacaacatgctttccatcatttagatcaacatttag
agaaaacacccagccagtatattcaatggagatatgctggtggtcaagaccgtgaggtcttagaaaaggagactcgcaaactaacagatgagg
accgtgctcaacttcaacgtgaaattactgtggatggtgaacgtcgtagggtagaagctctgattggtcgtcaaaagttcaagaaatctttccaat
atgaaattaagtggcttgggaagcctcataagtacaacaattggattcctcgtgaattattgcttgaaaacgggttccaaaagtttgttcaagcctt
cgatgatatggagtcatctcgtgaaggtcttggttttagagatcttatccctgaagaaatccgtaagcactttgaagatgttggacttcctggtgata
ttgctgattacagtcctttgagcagcttatctggtgggcagaaagtgaaggttgttatagctgcttgcttgtggaacaacccccaacttttagtcttg
gatgaacccacaaacttcttggatagagatgctcttggcggtctagcaactgctattcgtgattggggtggtggtgttgttatgatttctcacaacgc
tgaatttgtctctgcactttgtcctgagcaatggcatgtcgtcgccggaaaggtcacaaacaaagacaaggcagctgttgacaacgagaagtttg
aagatttatccgaaaaggatttgaagaagattgaatccaagactactaagaagaagaagctcactcgaaatgaaatgaaagccaaagaacgtc
gtgctcgtgatagagagctcgcatggttgtcctcacctaagggtactcccaagccaaagactttcttctctgacgacgaagaataaatgatttagt
gtacagtaaatgtctgtatgatccggtgcgtttatgtacttattttacttccttctttttgggtccagtaaaagtttggtggaacaggttaataattta
tgcatcatttaaaaattgatgattccaaaagaaataattgttatatatgtgggtttggtttggtgtttaatagatattttactagacagttatatccgt
ttttaggtttcaataatttagctttttgatttttgattttgaaaatttataaagtatatattaagtcgatgaaatatgaaaataaaaaacacgaagta
tgtagtacacactaaaaactcgtatatcaactctaataagcgcatcacaactcaagagttaagcagtagtaaattcatacatacgtattataagtt
cacattaccaaatgtcttcaaaaaggtaaattttatgtaataagacgaaatatagaaagctaaagagattcagataaaattaggtcaatgatgaa
tttgaataagttcatgtttactggatataatgtcagcagagattgcggatattgtcgaagtggagagcctactgaacacttcggacttttatcgaag
gaactaaaatgcgaggtatgtctagttaaattaatcttaatgaagcttttagctaacgtttgcatacggtcaggcgtatcaagaccttgttgacaga
ggctggaggtaatacaaaagagtttcattttcactgttactgatagttatagacgttcaggacattatttgtataagccaaacttgaggacctcttg
ctgtccattgtacacgattcggtatgtaaatgcaagtactacagggcaatggcttatgtaggatatatgtataaacatactaacggtgaagattga
atgctgatgagtttcaaattactaaggtgcaaaaaaaaactataaaaaaatgggccaaattggtaactggcaaaccactgaatcagaaggctac
gaagaatagtatcgaataccttaagaacgcatttgagcaaattgagggatatgaagatcaaacgcactcatacacggtcaaattagaatcggct
gagtacaccgatgaaaaatttgaactgttcaagaaatatcaaataagcgtccatcatgagtccgaagaggaagtaacgaaaaaaggatttgaa
agatttctatgctcctctcctcttgttactaacaataaatacggatcttttcatcaactgtatcgtataaatggtattttagttgctgttggtgtactcg
atttgttacctcatgctgtatcaagtgtttacttgttttatgatccggagtactcatctttctcgcttggtcgactaagcgcttgtcgtgaaattcttta
gcagctgaagaaaaatatcaattctattatatgggtatacttgatatcgtataaagtgagttagttgaattaaccgttaattaggatattacattca
caagtgtacgaagatgcgatacaaaggtgattacagtcccagttatctgcttaatccggtaagtaaagggttggagtgttactttttacttacaaa
gcattaaggaaacaaacgattggctatcaataaaacatttcacgcagttgtgggaaaagggagcatcagattatgtaacttttgaaaatgttgat
gagaataatgacgagttaaaatccaaaagagtcgagcctttaccaaaattatcagacattgacgaagaagagccccccctctgtttatgaacgga
gcctaccgggaattttggatgaagacgctgccaaatctctcttgcgcaaaaattatattaccattggagactcaactttaccactgaatgttagtaa
tgagattagtgtgatctgtgctaacgattaaaaggctgttttaggattattcccaagattggagaacctctatattgaagcgaacgcggcacttgga
agtaatgtcggtaaggattatactctcgtcttttcacaataatttcttttctattgtgtatgtgtttagcatttatcagtcgctacgtaagaaacgctga
atactggaaagctttgaaatcgcctaaattgagacgagacaaacaacctttatactggtctagctgtggatgactttaagaataccggatattatg
ggttgcaaaactacgaatgcaaggacaattcacaaaatagtatagaaaaaataaaacaaaatatctttattgttaaggtaattacttatttcatga
gatggtatccttttatatgatattagtctgaagtattgtcactttcaccgtcctcttggtagcttcgcctaacgactatccaccatttttctttcgtctgg
aacaaccggacttgtggaaataacaagaggtttgaatttttgttttattaatatacatatatatatatatatatatttggactctaaaatagggactg
atcttgtcaaacgttatagattactttgcaagtcataaaccaaaagttcgtcaggaaagcgtacattttcaaaatagaggcttcttctgcttaaagt
tggattacttgtttttatatgagaatactatctttataacttctggtgaagcatggattaaaaagatctaaactttgcattgcacgtttccgcctgttg
aaacgtcgttgataatttattgagtttttggtttttttgcttgttaattttataattgtttgtataccctcttttgattgaaaagcttgcgtttttgtttacaa
gtttttgatatcctaagcatcttttggcccttatttagcatctttctatcgcgctgattatatttatatctttattttttaagtatttattagcaagtgtttc
gtcggtaactgctgttccatccgtttttttatttttgatttccctttattactttaatctttatattcacatttcgtcagttaaaagatcactttctttttttgtt
agaagtttgcttcggtttccttttatacagataatatgaaaaaatacttgtggagaacaccgtctatggcaaatcatcatgggcggacgtctaagg
aagttatgaaatgtaatactgtcgatgatttttttttctataatgcgcaaagtgggtgatggaacttttggttctgtacatttggctacccgtaaaggtt
cgacaaaggtagtagccattaaatccatgaagagaaagctattgaaaatatcagacgctgctcggctacgtgaagtgcattctttgcttcatctgt

cagaacatgaaaatattgttaatattttcgacctttatattgatcgatctttttgtttacatatcgtaatggaataccttgaatgcaatctgtttcagtt
gattaattcccgacgagacaaaccttttttcttacaacactgttagagatatcatgagccagatatttagaggtttaggtcatatacacacgaacgg
cttctttcatcgagacatgaaacccgagaatattttggtatcaccaatagaaaattctagtttctacaaggtgaaaattgcagactttggtcttgctc
gggaaattaattccaatccaccctacacggaatatgtctctactcgctggtatcgggcccctgagttattacttcgggattcatattattcatatcct
gttgacatatacgctgcaggttgtatggcttttgaaattgcaacattgcagccaatatttcctggaatggatgacattgatcaggtgtataaaatat
gtgaaattttaggaagcccagacgaggaatctcaaaatatgggatttggaggtggcggtgcttggaagcaggcagagaagttaactagagagct
gggaataagtttgcccaagttagtacctttgaatctaaaagagttattttcgccgccatggaatgggccttttgcttccatgctcacccaacttttac
aatggaatccagagtcgagacccaccgctcaaaaatgtctagagcttgaattttttaactcagaagaagaaaagtttgaagaacccaaaacgaa
tcaagttgggaaagctggtgatactcaagtttctgtatcgatgtatccatttccgaatgcatttacaaaagttacgttaccttatgttgcggataagg
ctgatactacgtcttctaagctattgcaaaagttccacaaagggtttaccggctttaagcgtacgtttcaaaagtcaagtaagcagaagaagccct
ccaagaagacagctcagacgatttccacaccaatggatcgtccgccttctcctttttttcctatacttcctcaaattcggccatcaacgcctttgaac
ttgaaattgagaaactacttgctttcaactgcggaatataatgtagcaagtaagagtatgcctatgtttatggaaacctcgcaatctgaagtttcga
gccagtcaaacgacaaagagaacaaacaaatcggaaactcaacaaatcctagtcctcgaagtaatttcgagccagttatcccgagaaagtcgtt
accatcgtattctgagccagaaactacttctcctacaaccttgaacgacaagagtaccacctttcattcaaacaaattcagtggatattctacatgc
agctcaagatctacagaacaaacgtattcaattttgttggattctgctgattgggaacaaactgcactttggaagaaaagtagcttaaagggtgat
tccgatgcccaagtaacgaagcgacctttaaagaacttgaatagcgatattgacgatgatgatcgtgacgacagtggtaaagccccgaatatgg
atttaagaaagtcgtcgattttgtcttcaaatatccttgaacaggatttcccggattttggtgttacatcgcttgcagatacattgggtgttcctgggt
tgaattcaagaaccgactctcatgtgacatctcaaacaatatcctaattgcatgatggatagataattttggacctttctttgcttattttttttgtca
ttcttttattgtgtagactacgttgtttacacgcattctatttttctatcttcttgactttcttttgtttccttcttctattcaaaagtctaacagtgcttgct
gttgactcaccataggcaaccctctcacatgaaatcggttttctttttaacataggctgccttaattatttatatctaaatacccttattacttcacatt
cttctttcattgactgggggaaatttaagatgtcgcttatattcctaaccccggaaatgaacaataatctctttatcattatccctcctcgataacga
atcaattccttctttctataataaaattgacctttgcttttgaaccgacttagaaaaacggaaaaataatactcgtaccgtaacgaaaaatctacca
tatgaccttgaaagttgccagaatgaatgtttacatgatttacaaaattgtgtccctcattggaattttcgccgaaccttaaatggataaactacta
atagtgatttcatctgagtcaattatgctctcattgatatcgtttatttctgccacgacgtcattgagtttgaaatttcgtcagtcatttcttgcacttta
tatagacggggaatcaccatcttgtccacaacttaattataatcatagtcattaaggtttattatagtgagtatcagatttgttggctgtttctatcaa
ttcagttgttttaaagttccattgtttctcgtttgcgatttcctaaaggcaaatctacgatcggtaaaaatcatttcgtgattgtcgactccttgtgcttt
actttggttttacttaatttcgtttctcctgttgctttctcttttcataccgcttggtgaagactgtaaaacagatcctccaattatcttttgtctatttact
tacttacgtatacactcgcttaattttttgctttgcttcagaatgactggcgataacaaatacttggacggtgggttgtccacccttcagaaatgcttc
aaagccttctttgacaagcattcggacaaaatgggtagctctgataatgtctccggaacttataatgacagtggtagctacatttaccttgtttggtc
ggttgctgtttatgccgaggctatggcagataccttaaagtttacaaacaagtttgaaaaaaagtttgaaaacgtatttcaatgtttaaagaggta
ctggtctccagagtacaatgcttgttgtgctagttactattttcaaggaaacaatgatgtttattttgacgataacgctcaagtagccattgctctgg
ctaccgctggatactatacttctaatagctctcggaaaaagcagtacttagacagagccgaagcaattatttcttttcttatcaatcgtggatggaa
tcaacagaaaggcggtgtagcttggcatgtgaactctggaagtcctcctggaggtaacttgaatgcttgttccactgccatgtcagccctggctgct
ttgcgacttgctttcgctgtggatgacaataagaagaaacaacagctcgtatcgtttgcttggaattgtgttgagtggattcataataatttggttga
caactctcatctcgtttgcgatggtctaagtatgaaggacggcaaatggacccttgactctgtaaagtttacttacaataccggcgctactttggct
agtatgtctttgcttctcggatttgaggatattattaaggggcttcccaatattggcaaaatacattcttacatggaaaacatggccgaagctgctct
taacacgaaaagtgctatgtacgatcaatcctgcaagaccgatttcaaaacttggaaagataataccttttttcgctcagcatctggctgagggatt
aatgacttttttcttttgctatgcccacttcttcttacgcaaagcctgctcagcaaatggtctttgatcaagcagagtttataatgaagtacatccgcga
tcccaaggacggtctctatttccgtgatttgaatctctataaaatcagtcaagatttaacgaatacctttaacaaaatttttcatgctaaccagaatt
ttcagccagataaagatgaacgcatccaaggtgatggtccagttgaaaaacaaccactctgcaaatcattgattggaagctcaggtgccgctcgg
atgctatttcccgctgctgaaataaaaaattcgtagcgaaaatcctcaaaagggtcctattttaggaagacgaccctcgggcggcaaagatgacaa
aagttgttttatatgctaagttccttactttcccttcgcattctccactttttaaatacctctctacccactacctaattatggagattttatgaacggca
ttttatcacccaagacggttccttgttcaatgaagaaaaatatactataaacttctttacagagttatttaagcaataaaatattattttcctctatca
tatataaagataaccttctgcaagctttatttggttttataagtaagttgcatggcgacttcgattgggtttgaaatatgaacgtctattgtctgttcc
cctttgaaagtgatgatttcttgactctgcgtttgggttgttcattttcatgccatgaccgtgcgaattattgttcaccgaacttaaacctggacgacc
taaaaatatttcttttaaaacttcggaatcattgaaaccctttgacataatggatgcttcgtccatatcctataatgttagcatgcagtctactgctct
ctcatacccggtaacattgagctgcctgagttgctgctatttgcatagccgaacccttttttaatgtgatgtccaacgccgcggacaacaccatcaca
atgaacttcagaaatccagataaccttgttcgtttccctaatcgtaatatgtatatatagaagtgatatagagcgtggcgtgctttccttacaactga

ccgtagaagctggtaagccagacaaaggccctcttgagtccagcggttcaattctcctgtttgtaagctttcgataacgggtttcaactatttgcca
agttctttcatcttctctgaatgatgaaacagtagttaaataacgcagtatatatggctcaactttgctagtgcttcgaaaactagtgggtgtatcag
aaccggaactataagaacttgcaggacttagagaattttccccaatgtcgttctgtaatttataaaatcttctcctttttccgctaatattttatttt
gtttgcgatgcgctcttcgttttcagttctttcgcgtccaagtttcgctaattgatgacaatgatcaattaaactgttgcaaatactgcttagtttatgc
gcactcgaagatgatttttccgtgatttcatcaagcataacatctgttgaatccaacaactggttaatttgatggtgctttgcacatattgaatctcct
tgagacttcaattgatataaaactagattgatttcattaggtcgtaacttcgctgggagaagattttcattagaattttcatcattgctttgagtcgaa
atcgacgcatgtttgtcagttaaatttaagttcgttaagcgttctttgatttttatgtaggacatgtagattttactgtcatagtcattatatttcaggtt
ttgtttcaaaagattgagcctttccctttttcaattggttttgaagtttaagcaaaagccttgtaccgatatcatcacctaaacagagcgaacatatgt
ctcttagatgaaggcatgtttcctcctaaatgagtatgttaatcgaatactcaaaaataatggacttacatgttgagacactaaacccagtttttcct
tttctaggatagccgcgtttctttttttctttgcctgtgagctattagtattatcctgttaattcaaaaatacaaaccaaagttaactctagttctttgag
atgggtttcctcgttctctttctcggaaattaggtcttgatgctgaagcctaagggtttgctaagatgagttggtcaaaatgaaaaatcctttaaact
ttacttactaactccctttgaaacttccgtattcgttcctctgacacttcattaagacttaacctttctttttccagcgaatgctgtacattgtaagcaa
gatagtctctcattctattccttacaaaattatctttcaattgcctgtaaagtccatttttctgataagcgtgatttcctgtgattttagcaggcgcatta
ctaaatgaagagtcgagttgatcctaaagtggattagcttgatattgatgcttcccacttacgactggagtgtttttggatctaactggaatttcga
catctcttttgctgttaatgaaggtacgcccatggaagtcatgttatttcccaataaagcattaatagcagtgttatttatagttgtatcgtttcctctg
gaagtttgattcctccattgctcaaagcaatcccaaagtcgcttctgttgctctaataaagcattaagcataagcttgctttccttggatgtgccaga
gtcatccggtttattcgcagctagctgttctttggagtctgaacttcctcttttgaatcgtttactcgagttttcgtctccacttatcctatctgaagcat
tgatgtgctttcgatttggcgtcaaatgctcattgtcgagtacagtaccaagtcctgggatattctgaaaatcctcagaccaaggccatttcttaaa
agacatctcataaaattctaatccttgaataagacaaatgaattgattagcttatttgcattcaaatgggagttgtgctaaaggcttgttagtttgtt
tacatactgcccaacataaacaatacctttaacaaattttaacgagagcgcttaaaagttacttaatattataggcgttagtattatctgtcatagt
agaaatcttacaccgtataataatgtatttttaatggaataaacaattctagctgcctctaggttgaaattggctgtactatgtgtatagatgctagt
aatgtccaggaatttatctggtgaactaaatagaatgcaagagaaacgatctcatttagccataccagttgtatctccagcctataaaggctttga
ctgtagattaacgaatgtatatatccttgtttttcaaatcaattaatcgacttgtcaattgataattcattgcatacgtgctttcgtttttattgtttata
tacatcatgttaggccacaattccttatgctgtagcaatcatactaatgcaattgacatacggaatatttttgaggtgacagttctgtgcagtaataa
atatacaaggctccattcctatcgttacagttataagcggtttaattaaatcaatccatccaagtattttaatccttatttttttaagcatctatggct
tatatatatattaccgaaaatgaatgagaatagatattacaatataaaaaaacattaatcaaaacacatcacaaccaaaataaaaagaacgag
aattaaggacagacataatccaaccaagaaggatgcatatttcccagccagctaccctcaatatccattccttgatcggcattggcccagcttaac
aatgaagagtcattggcgagatcaggcgtagttttgttcgcatcccatgcagccccaactttttgaatatattcatccaagtcattagcatccaatg
aaaaagaattatcatcgggaagagaaaaccaactcatgtctgagggcatgggagtcggcccaggggtcaaagacgcactggaacgacctgttg
atacactgggtgtataattaccaacaaaattcgcttggttggctttattgtttttagaagcaacttcggctatccagcgttgccaagccataaatcct
gattgactagaattattatttcctgctgagcgtatgcctttcaagtgcgtgtcgaataaacgtataattctagcacaattctttgctggatcattgccc
tctgccatgtaatcgagagctgcgtatgcgtattccatagcaatgtcgtcttcttggcaaggctcagttacacaatgtaataaaacaacgagagca
ctcgaaaacgtataattataatcaaaaaaggaatagcaagatagctgagaatgctgttgaagtaaatgtacaagagaaattaaatgtcgagctg
caagaatgcaatcctcgttgatacgattcactctgggtgagttctttgcatttttcattttatgcaacaatacaggacgagacataataattattgctt
gatcatacgtcatgtgaagatgaacgttagcacggaataaagggtcttttggatccaaattttcaaggatctggaaagaaggtaaagattgcttc
caatcgtttagatcggctcgtaaagattcaataatagttatcggattctttttgtaatttacagttccatatagctgttccatagtctttcccatgattt
gagcaatttttgacatggatataatattatgaacgtttgaagccgaaccttcaatttcgagctctgggacaacagtaggtaaagcagcatcacatt
cgctatccgcaattgaaagaggcctcccagtggcaatgcttattaaacggtccattgtatatacagaccaccataaacgattgcggagttcttttgt
tttagggtcgatatcgggattttcacaactacgatgtaaaccgttggcgacggcagcggtaagagcaagaccaaaataagtataggagctctttt
gagaaagtgttgactgaaggtataagcccattaataaagaaacctgcacaaggctaggtttacattgcaggataacttgaggaagcaaaaggc
gagcagtatcaaaaaaactgttttccaggattgtcgagtaaggtgcttgcagattggacctcaaggtgagcgaattgacttcccaaagcaaagatc
atcatgactaaacaaagaaaaccaggatcaactgaaggattttgataattcataatgatattcagtcggtagcgtaaaagtgtttcatgatagaa
gaagaagtttgcttgaacatggcgataaaatgtttttattaggaaatcagcaacttcagagggaggtaaaagatttaggccttgattaactgcact
tacatcatgctcattaggagaataaacagggtgcgaatgtgaataagtgctcaagtcagaaaaggagggatcagcgagtatttcgctttgaaca
gaatcaacaaaagcttcaccagccgaagcaccaatgtaacgagactctccgttttctacttttatttttccaatgagtctcataatctcgtcgacatt
ctgatcgttagaatcaccgtttgatgcagtgttgtcatattcttcttgataatgattggtgttgttatcattccgttgtcgactttctggatacaagata
ggttccgtattgtattgtaggttattgatatcaggcgtggcggcagggggatgcaaaaaatcataatggatggagaaaggagatgtcgcagtaac
gtttgagaaaatttcatttggaaggaccggatctggtcgtttactaagagaagtggacaaatccactaatgagtccgttgaatactgggagatctt

tgtgccacaaagccgctctattatgtattcaagacactgatacctcttcgaaaggttcataaaatattctcgtttcatttcggcgcctctttcctcccg
cattggatatacacattgcaagtcgtttaaagcgcatgctttgcaagggttgccgccttcacaacgaattttttttcgttttacagtttaaacaggcta
ctccaatacgacgtcttcgttcattcggcacgcgccgacggcgattatgttcttcattttttattgcctcaaagaatgcagttgtagttttaggtgaaa
aggcattgttcatttttcctttttagaaatagtattcgtaataacctttagtttacaaaaatcataccaaacgttattaaatatggtattttatactgggt
tgagggagaatggattggtcttggtagtgattcactacaccgggcgtaggtgtagtacagaatgtagataacaataacaaaaatatctaaaatat
aaaattcatttgtttgaaaaattgaaaattgaaaaatgttgaattcaagccatagtcgtagggtggaaaaaatgtatatgcgtctttgcatagaaa
ctcaagtaggaaaagtctcacaaagcaggaataaaagacgaaggatgttgttacactttttcaaacattcttgcgatggaaaaacaaagcaagt
cgggttaaattagtgttgaatggatacgagctcatctcacctaaacagcacgtcgcgtaacgatttcttcagcaaaaatacagctttgaaagaact
gttttattggtttaaattaaaaggttcacgaattttgtacaatcaactcttcacgtagtgatcttgtcgcgtagtctcttttctaccaagtatcctttttc
atcgattgctttgttttcctatgaatgatatctagtcaacgtacgtgaactgtggaaaaaaagtttgtgaacgcttttctattcatttacgttctcttcttt
aaaataaaatgctaatgtaagatcagaccgtctaagtacgcaaccagcgattcgcgcgtacgaagcattttccgcgcccggttttagctttaattc
gaagtttctaaatgaagcatttggtggcaccggattgaagcgaggatttgtttctgaaatttgcggtgctcccggaatgggtaagacgagcctttct
ttagctctcacagcaaatgcactgctttcaggaaccagagtcctttggatagaaacgtgtcatccgatccctctatctaggttaagggctttgttac
aaagtcaagtcccatcatctcaggaggaagataatttaagtagcacgttggacgaatacttgcagctcttagatgtttattatgcgccttatttggc
aaatttactggtatatttgaaaagttttgacgatgattcgaatctggatgatgtcggtttaatcattattgataacttgtctatgccaattcaattggc
ctttcctacgtgtccagatgactatgcatatttgaggcaacgccgaaacaccagctcacgtaaaacctcgatggattccttaggaaaagaaaatat
gaattccggcatcgaaaaagaagttgcttccaaagagctggatgagccttttcccaattctcaaacgaatctaaacaaacgaaaacgtgttgttg
gagaaatcagttctattttgagtagaattgcagctacatatcaaatagctatacttgttactactcaaatgacctcgaaagtggtgactggaatgg
gtgcaaagcttatcccgtttcttagcaacaattggcttgacaaggtctcttcgcgcttagttttgtattgccggcatcctacggaaaatgataagttc
gttgacaattctgaatcgaatctcagttatcaatcactacgttacgcttacatggcaaaacaactgccaggcatctccatcgactctgaacttgtat
ttcaagttacagaaaatgggttaatggattgtcggagcttaccaataaacagctcacagcgcagaaaacgtagcttgatggagacagacttatg
atgattaaatgtcttcacaatgcgcagtaatgttaatccagcctttacgctgttataatattttaaaatgttatattatattaaattctgactcatgat
atgttttgatcttgacaaaacctgtcgaaatgttctaatcattgtttacacagctctgatttcatcatcttgtaatatctttatacaggaaaaatcacg
gattgtcatgtttatgaaataatgaataaaggatttgtaactagaagctggaagataatgaattaaggcgaattattagttctcattgccagtgatt
aaagcacgaactgactcaccgaaagatgacagcacatttcgaaattaattaagtgttgaaattatgacgtctgggaagcctttaagtaattaaa
agatcccaaggatcacccaaaagccaaccggccatcgtctattttcatcttcttttcaaaagaacgcggttgttgactataaaagaagtcagtgttt
cactagaacgaagtgaactttatttaactttcaacattgcaagtgagcatttcatttgaatcggttagcgcttaagtgatcaatcatttttaagatga
actcctctcgctcttactcgattcatactcttcgagctccacctacgtctcatcgccgtcaggaagttccccgctccacgacgaagagaaatttcaa
aatcccacgcatgggtacacaagtacgcaccaatcatgtaggaatgtttactcctgaaatggctaagcgtcttgctgctctcgttaagatggagaa
aggcatgcttaggacttacgaggtccttgctgatgagcgcaaagcttgtgccaaccaattgtcctattggggtgaggactgtgatgatgatattag
cgatataagtgataagatcggtgttctcctttaccaaattggcgaacaagaagatcatatgattgacaagcacgatcaatatcgtgtttctttgaag
actattcgtaatattgaagcgaatgttcaacctgcgcgtcagaggaaagaaaagcttctgaatgctatctatgagatgagacaacgcgaacccga
ctctcccaaactcatttcaatggaacaagaattggttcgtgaagaggctgcttgcttggttgccgatgctcagcttacaaacatcactcgcgaaaa
ctttaagcttgctatgaacatcaacattagtactcttcttgaacattccgaaaagattgctgttctatgtggctatgctaagaagatacttgacctcct
tgatgacaccccaattacgcctggtgaacctcgtcctatatatgatggatacgacatcaccaaagattatgttttagaagctgagaaagaggtttc
taactggcagtctccttttgttccttctgaacctttgaccgacattgatggacttccaatccagagccattaccagccagaataccaaagaaactac
actcgatacaacgaaggtgcagatcgtggaaatcgtcagcagaacaatgaacctggttatgtgactggtacaactcatacttacactggaagaa
gctatgagcgtccacagggtgaagaatttcagcctaatgttcttcaagacacccaatatgttgataacttcgaagtaggcgaagaggacgatctt
gatgttgaatcccaacctggacatgagcaaggttcttatcctcctcagtctcatgccgtcgcagcgtaaactgaagctagacgtcatgatggcatt
gaaacagcttttagcgttttacgattctaacgctctgtattggttaagttatatatctttggttaagtcattgcatcccttttttatcgtctatctatttcat
tggattttacagttttaataacgtgttcacattaattctccaatttaacttgcgtttctttctttagttattcattggtatcgatgatccaggaattttttttt
tgtttttcttcttaataatgataaatagtaataataactcaaatgttggaagtgtatatatacattatgaacggtttattgaatttttagttaccaac
cttagtgaattagatgctgtttttgtgaataatatagctaataaaaataaggtatgattcgtgtattcataatgaaaccaattgaccttagtaacata
ctgaagttgttttttttttatttctttttcattaccctctattcttttaactttcaaattattagtaaacaaactgctggctgtccaaataagaatggtaa
cttcagaaacactagatattcaaacaagaagaaatactaaagattattcttataggtcatgaatagttatgctttgtttacatcgatgtttatacaa
aagcgcgatattatttacttgatacttattaaagttacattgtcacttacttcatgatttagtctcttcatctcttcaaaaaaaatcgtttgatgagttgtt
cctatataagtattggttattcatttcatttttaaggaaacgaaaatagtaagtataattataggagcttctcatttgagcacagagttttctgctag
ctgagctagagaaaatcatgaatcaggatgtacgaattctcccagaagcgatatatattagttctttgaaaaaagcccaatgccttcgcattgaca

gatcaagtggcgctctcgatttaagtcaattagaggaattgcttgaaatacccccctcagcttagcaatattcaactgtacggaatcattcgcctcaa
gttgtttaaatacattatcttaattactaaatgtgaacataataacactttcttggggaacaatgtattccgtgcaaaaagttttgctattcttccgat
tactaaaactatctcagctcctttagaacctcaaacagtgcaggacgaagaagaacggtattatttgtatctcttgaagcgacatctttaccatgg
gcaaattctgtttagtccttcaatggatctcacaaattctttacagcgttctcgtaacgtttcttcaggttgtaacagaaagctgaaaccgaactttc
gctttttctggaataagtacaccaatgaagagctactatctttggcaatgaataatacccagtttttagagtatatccagcctgtgatccaaggaaa
tatatcttctaccaatttgtttgttaagacgcatcatgttcaactccatatcataactcgacatgcaacagatcattcaggaactagatacttttctag
aggagttgatcagaatggaaaagttgcaaactttaatgagacagagcaggttttattgatcgattctaccctagtgccggacgaacggattcttct
ttcgtatactcagatcagaggcagtataccgatgttttgggctgaaattaatgatttaagatacttacctcggctggtggttcaacctactacatata
gtgaagcagcctttgaacggcatttccaagatttagcctccgattatcaaagtgagattcttgttgtcagtttattagatcataaaggtcgtgaagc
gatgctacaaaagaactttaaaaagatttgtacggaatctccaaagccagtaaaatactatgagtttgattatcattctcaaggaagcaaagaca
ttcctttattacttgctagtttgaaaagtgccttatcacaagggagcttttattctgagtacggttctcgagttgtttctcagcagcaaaaaataatac
gcgcaaattgtatggattgcttggatcgtacaaatgtcgtgcaaacagaagtatccctcttaattttaaatttgcaattgcagacagtaggaatcct
ttcgtattctgagacgattgaagattttcaggaatttgttcaagggtttcgaaatgtttgggctagcacaggtgactacatcagccatttgtacacgg
gtacacctgcattgaaaggagatgtaactcgtaatgggaaaagaacgactaagggaatgtataaggattttaccaactgtttaaagcgatatgta
tataataatttctgcgatggtgttcttcaagacagctttgacttgggtcttggtgcttttcgcccaattacttctttttcttcctctataatgtttcatcga
acttcgtgggcccattacattccacccacttattcagtcgtatccatcatatggttgataatgcaaggactcatgggtacaggttctttgctatttcaa
ttgtcattgggcataccagctctttttcttttctttatgtaatactacatagaaaaagctatataaattggcctcggttgttgcctccaaaatacgcta
gtacaggatggttctcaattcgtcacttgttgagatcctggtctacaaaagtttttcgttttcttcgttttggttctttcaagaaaaaggtataggctaa
tggtatagttattctcaactagtctagataacccgaaaaaacttgcgcgtatttccaagatcacgtggacctaaggtaccctcccattaacatatat
cttcgctttttgatcttagattgcattgattaacattctcatgaacatgactaatgaatgaaactacaaattcacatccgtttcttctatgatatgaac
tttctggtactccccgttaagtataaccaaataagcttttggtttttgacaccgcataaaaataataaaatacagtaaatagaattttatgatgaa
gtgttacataaatcatacatatttttagtaaccattggcagagcatgccaatttagctgttgtatccattatgattctattatacatcttcagtactgtt
aaactatgatataacggaatcctgaattatctcaaacagaaatatgaaagaaataactttgctaatgactgtttattagagaggacttgacaacct
catttcttatttgtaggtaacacgatacctgtttataaccgtcattgggttgtgcaatttaggtttgtgtcattagaagcaaacacagtatttgagga
atttgtttacgtgttcctaccatcgtaccttgtcttttgcatatatcaagcaatttgggaaaattgaaaacattgggtcaatttcggtgttttctttctta
ttttcgccttaggaaaatatagggacaggattgcttgtattttggaccagaaagtataagcacccgagaggctcagtcgacatttgataaagatag
aattcaggacatctatggtgtcgacactgtaggctctatactagagagttcatgaagggagtatcctcctcatttctgttattgagagttggaaacc
taaagaaccgttaactgcgtgaattaagttgtttaattgagtttggggtaattataattaaatcatggaaaatattgaaggatatataggactgtgt
gtttgcttcctgggcttaccggcttcaggtaaaacattttctgctggcaagctttcacgctatttgagttggatgtctgtgaagacggaggtttacagt
tcagaaaagtatctagaaactgagtacgggagctcggaaataatccctcctaggtattcgatgacaaaggtgaacttgattttcaatgacttgga
agcattttacaaggagggcggacatgtaacaattctggattttaatgaatgcaatctggagcttcgccagaacattgtcaaagctgccgaaaagc
gaagtatattgattatgtttgttgagatagtatgcacagaccagaaatttatagaggagaatattaccgatatttgcaagaactctccttcctataa
acatttgcctctcgaaaatgccaaggacaaactccgggaagtaatcaaatgctatcaaaaagattatactcctctgtccgaagcagaagactgta
catttgtcagaatcatcgacttttgcaatgaagttaacattcataatataaagaattactggcagtcaaggattgtctattatttatctaaccttcgta
cccaacgacgaagcatctggttgagtagacacggcgaaagcgaattcaatgttcaaggtcggattggaggcaactcaggactaagctcccagg
gcgaaaagtacgcttctttacttcctgagtacgtttcaaagtttgtggtcgggtcgaagggtttaaccgtatggacatcctcaatgaagcgtacaat
tcaaactgccagtcatctggactatcaaaagctggagtggaaggctttggatgagctagatgcaggtatttttgatggcttcacctatgactatatt
gaagaaaagtatcccgatgaagctcgaaagcgaagctatgataaatttcactatcgctatcgtggtggagagtcttatatggatgttgtacgaag
gttagaaccgataattatggaattagagcgacaaggagacgtcttcatcatctgtcatcaagctatccttcgttgcatttatggttactatcacaatt
tttctctagaagaattacctttttgaacgttcctttacacacgatcttgaaacttacacctactacgtttgagaccttagaagagagattaacaata
ccagtctccgctgtttccacgcaacgtggaaagcattagactcatcattctatttttatactttttgcaatccatcattgctaaaattcgtttacttttct
attgtacaagaccggtatgctaagtaacaaatactccattcaagtaaatcagcgttatgaagtcttgtatgttaatgatacggtcagccccaataa
tgagaggtgaatttgtaattcaaatatcctttggaaatcaatgaattttgaaatcataacttgaagaagagcagctagatttttaatcaacattat
acattaataatctatacattatttaagtgagtcaagaatgttgggaaaaataaaattaaacgggtgacttgaataaaccaaatgaacatgaatttt
tgatagaatcatttaagggaaaggtcagaacctttaatagcagacgttggagaccttcgtttgcagaattaagccgacaataaggccataaagcc
ctaaaacctctgcgaaaattaaaattaaaaccatgctaacaaagattcggtcttgtctcataaaactttgcacacccttgtcgccgacaataccga
tggcatagcccgcagcgactccagtgagaccgacggccaatccggcagaaaggtgaacaaatccacttaaaaatgttagaaatcggttatgaa
gcaacatgcatagtcaacaatacttggattacagatatataagtgcatgaatttttcaaatgtgcacattctcttccattgtacttacctaaacaga

gagtattcactgtcgggagacatatcaccagcaattaagacagacatcactaaaccataaacctaccaagaggatcattagaatatatcaatcgt
gattgaaacagtttcacatacaccgataataccgctcattacgactggaatcaaagactacaaacatccaaacagttagcatactaatcatcgttc
gactatgaaagtcctcaccttcataacaatttcaggtcgaaaagctcctacagctgcaattcctctaccggctaaagccgtgccgtagcctgcccc
caggcaagaaaaaaccattgacgcacaaacacctagcaatgagtgcaattccaaaaatccgtacctgcaaagccaaaaaaagaagagtatat
agggctaatgttagagacagtatcaaacattttcttaccataattcgctagccatttttatctaatgtaacttttgttatgaatgtagttaccaggact
tacagaagatgccaaagcactagttcagtttagcggactctggcactactcgttatttcaaataagggttacataattattaccttagtaaacagta
gcacaaacaaattgattttacaatataaatggatggaaagctgttagctgtattacactacgataacggtctctttacaaagtattgctgggctctt
tacagcatcacgaatgtaatcattatggaggagaataatctcctacgattctaatctagttaggacatcattaggcaaaaaacaataaataaaga
aaaaatcaaaacgaatgataaaagagtaaaaggaaacaaaccgcaatccatagaaacaaaaagttttagcttgctttaatctgaagaagaaa
ggccgtcatagtacgaaaaacaagagctggctgatagaatattagcgggaaaaaagaacagtaaatacctttttacactattttaggaggcaca
ttaacgacatgtttcataaaaaagttgtaaatataatgtccatggtagagcaatcgctcatagtctgcacgcaaaaatctgtagcaagaaagaca
ctcatatctttgaagcaaagttgtaaagtcatccatcctcattatacacaacatgtcggtaacaatatagtatttcttgattttacgtagcaattggt
acaaagcttggatttcagatttatccaaaaatggaatccgctccacgtctctgtgtcgcatattccgaagaacgcgtaactcagccgctaccataa
aagaagtctctggcaagggttcgtatatgtcaaagttgatgttatcaggtttttgactgttcatggccgcatgttcgttagcaaagaatagttcgctg
catgataacttaagccagtaaggaaataaaacactgttctcgtctaccattggtaagcatgaaagatataagtctcttctccactgttcctgcttat
ggtattgcatcggtgtcaaagttttccccgatttgctatagttcagaaacagccggtcggggtaaaacgttcttaataattcgtattcaaattttgtt
acacttgtcgtcggttctaagattcgaagagagatgattcgctttagcaaattttgttctttccattcatccgcagaatagaagaacgactttttaat
acctccgtctggtgctcagcagctcgcgaatgcgtaccacacatttcatatatatattccgcggaatgactcggtgcgagttcattgtgaaactgct
ctaaggacttttcgtgctcttcgatttcttcctcggacggtgatggaacaggttcaagtcctaaatgaagaagtatgttgttgcgtgtattagcataa
tcaagaacagcaccataaaatactcgtcctaatccatcggaaacgacttcatatatagatccattcctcttcaaggacttcgtcataaaaattctat
ggtttgcgtacttactatagtagacggaacgcttttctaggcgttcaccacgccttggtaaagcagcatagtgatctcccaccatatgtttattgcga
aatgcttgttcaaacacgctgtcgggagaaaatcgaaagtcttctctctttttggcgcataaatcatgaaattggcttaagtaaaactgcacctcgt
cttctccattaaggaagctaggctcatcgagggaaattagttccgcaatagtaattagctcatgagcttcaaacaaatctcttgttggtttgtttgct
aaaatattataaaaaaccggctgatgaaccacttttggtgacttcttctcaacatgtgtaactttgtatcgggctgatttagctacttcgtccaagta
cttaaaacaaattttcggcgtgacaagcgatgttatttttaagttaacgtaccaccaatcgtccagttcatcttgaggcaaaatatccagaatggat
acagctttcagacctgactcttgcttataagcagcgtgatacagagctgcatacaagaagtcataatctcccttcggaatatacttcctaattaaaa
tttgattccttatgtctcgatcggactttgcttcggcggttgaaatagaagccgcaaccgccatgctttggaaagactctttaaacagctgaggaat
gttaacttgtttcgtataatcagattttgtgcggcgatcagctccttgaatcatgctaaacataaaagaattagaaatacatcttcgatcatattggt
ttagcttccgctcgatctcaggatctccaaattttcctaaccataagaagttatgaggcatgctcctcaaagactctagtttctctaaagatttttcac
ccttcactatcatcgtttcataatatctaagacgccattctatatagagagcaattacggtcctcgcattatgaaactgcaacgcagtaaaaaaatc
aataggctcgacttctgtgcaatttttttgatattctgaaggcgcgctttgttgactcgatattcattcggcttacatctgcgagattggtgatgtccc
agaaaaggagggaaacaagaggaagtttttattttagagtaattagcgcgtgaatttgcatcctccaattgggtttttaagggaattccgaattcg
tcaaaacaatctgaatagtcactgagattgtcgacaacacttgaaggatcggtatcatctaatagactgtatatctttgttttatcagagagcgcct
tcctatgttcggtatccacttctttattaaaaggtcccgcagaacttgcagataacagagagtcacttgttgacaacggactcatatcatctgtgaa
agggctaggggaattctcatctgggagttttcctgccgtttggaggttagtaataagaaaagcatcttcattgtcatctgcataagaataacttgag
ttgaaaccagctgatatagggaacggtttatcaatgtcctccatttcaacttgaaactttgaggggtctttcatagaaggatggtaacatttccattc
catgttcagattgttcagattttgatgcgtaagcttattgggtgcggatttacatgaagccttcttttcgtcgatatcatttctgtgctgctcgtctgag
caataagcacggcttggctttgctaacaaagggattgcaggacgaggaagtttttgatttggcttaattctaaaccttgaaaggtcattctctgatg
gacaagaagaagagcctgaatccaaaacggttgaaagttttgagtcaacagtagggtaacgtgtattaaagggagtggacggtcttttccatttt
tcaatatcgttatattttagattttcagatgaaatttttggtagagcttcgattgctttatttttactcaattctcctctttccatgcgattagcagtacg
gtctttttcggttgggaagcagcaaaacactttcatgaaaaaacaaaaaatcttttgaaagatgttgaagaatcccatgatggatgcaattgagg
gaagactaatgcaatataaccagggtaaacaaacaacaacaaaggtaaagatggagcaaataaataaaacaggcaaaatatagatatatctt
ccaaatggtttttaaggatacaaaatggaatttcccagtgcaggaaataaggatttgaagaaaaagggcacagggcggcgtaaacaataataa
tacaaattgagtatgaaactcaaggttatcgaattaacaagaaatcgaatcaatgaatttctacagccgttgataaatatataagtgaatagtaat
gcttttgatgaagtaaaagccgcgagcgatcaatcaattataaaaatgctttaggtataaaatgcttgagcgaaaaaaataatggaataatatag
gaaaaaggtttgtatcgttgaggataacaagccatttataatctaagcaaacaagctttttttgtcaacaaacaaacatctgccaagtgtaaacaa
ccaagtagctctggtttataggactaggacggcttgtagtctataaccgactttttttaactttattaattgttcattaattcaatagtatagatttctct
tttcgacagcttaatagaagtgaattagtttatttacaataaagcaaatatttcgcagatatgacatattcatgcaaattccgttacttgagtacctt

ataaaatcaatcctaattcattcccatgtaatgttctataagatagttcattttaatatttatgagtattgtccttggatgaaaagttttaccagcgac
gtccacatcagataggaagatcttggagaatgtttatactaagtttgtgattggggatgtacatcggtaagatttaagcttataatttcgctagcgc
tatatacgtaactgatccagccaaatacatacaaccaaaacaaacgtctaatgaaaaataaacactaataacccaaacacttcctttttaaatat
tactacattctgtaaatcattagatactagattgaatgatcttaaacgtattatatgtctaaacatatacaaccaatgtcgtctacctactttccgaa
aggaagcaaagttgggttttcatttttgatatttggcctcttaaacaatcttctatatgtggttattttgtctgctgcacttgatttagtaggtgctaac
atatccaaaggtgttgttttgctttctaatatcgtaccttcactttcttgcaagcttttcgctccaatattgcaagttcacaaatactcagcttccaaga
gactactgttttgtgctttcctatcttttttggaatgcaatggattacctggtcatcctccgtctcatctaggatgctcggcgtagcgttagcagccgt
ttcttcaagttttggagagattacttttcttcagctttctagcagatatcaatcgaccgccttaacctgctggagctcaggtactggcttggctggctt
gtttggtgcatccttctaccttgtgttcacaacatggtttaactttactgtgcgtacgacactcttaatttccagttttcttcctttatttttaatttttaat
tacttctttatcttaccaacggctgaatatcagcatatctctgtttctcaccaagttccgtttgatgctattcaccggggagaaactagttctcccaca
aacgttctttcttctatgagagtattttttaccttgccaaaccctttttcttatctcatatgctaccactgtttttggtctacctttctgaatatacaatc
aacgtgggagtagcacctactctactgttttcccaagaagattcgggcttgagctcatttcgagatttctatcctacctatcaaactatgtatcagct
cggggttttcatatcacgctcttccataggttgggtcaaggttccttacgttttagagcttgctataatccaatttggcattttgggttttacaatcctc
cagtctctcttttatttcattcatagctaccatttttttgttgttaatcatctttatggaaggacttattggtggaactgtttatgtcaatgtctatcacact
ttgcaaaaccttggtcatgtcgacagtgaaatggccataagcattgtcagtccatctgatagcactggtatatttgtcgcttctcttctttctttaatc
cttgaacccactctgtgcggctaccaagtacggcatggccgagattggtgtaaccgtatttagacctatttcattgagcatcatacaacgtcaagta
tttcgctaatacgtatatagctagtttttcgttcaagtttttcggattgtgaccacatattaatatttttattacctttttttttgtgtaaaaaaaaaaaa
aaacaatttaaataagtttattcgtcaaaagaaagtaatcagcgtcgtgaatcagatattcgtctatcctagtaagtgacgtttgtttgaaaattca
acaaatctatggtaactaaatgagctttacggactctcaatcttaacattgaatgtcaaacgttgacataatcttaccagttcttggaaatcccata
tcctctcagagaaactcaataacagcaatttaatggcagagaagcaactaatatctctgttcgacgatgaatattgtactaaatatactattctcat
cgcgagtacattgggccgtatgaaagagtccagagactctgctttttacgaaaatgaacatcaattgtcgggtgaagaaataaaagatttagaat
tgttccatgaaaggtttgatgagtgggctataacggttgtaaaaagaacaggaagtgctttagacccatccacgcctaaggatgcgacggaagtc
cggaaattcgactattttagcgaagcagaaaagggtgaatgttttgtaaaatgtttgcttcttcttttaatctctttaggagattattcatccaattcg
cgccaactactttatcttatttcaagagacttaagtcagcctgaagaagtcccccacaaggcagagtttatcacgtcgtctatgctactagaaactt
ttgaaaaactcgaatcaaacgaaaaaatgtatgaactatctcaaacccagcaactccggaagcgcattactatgggattagctggcgttgccgg
aggggcattgatcggtattacaggcggactggctgcaccttttgtaatcagcggattaggagtactattcgcaggactaggacttggtacagtgat
tggggcttcttatttgggggctgtgattacatctgctcctatgattaccacactttttggaagctttggggcaaaaatgagtattgatcaagttcatgc
ggtttcacgagggattcaggattttgaatttcttccattaaatgtttcttctaacctttccatgacaattggtgtggccggatggcttgaaagcggtca
agatgctgcagatgcatggtatcctttaaccattggtgataaatcatattattgggggatttgtatgctttaaaatttgaagttcaagccttgattg
atttagggaaagttatatctcgcattttatttgcaaccgggttgggttgggtccaagatgaagttattaaacgaaccgttttggctcctttagctgct
gctctttggcctttaagccttttaagaatcgggaatatattgggtaattcttggagagtggcatttaacctttccattaaagctggtgaagctcttgcc
aacgctctgtgtatgagagcgcaaggacaacgtccagttacgttgatcggattctctttgggatcaagaactataattgaatgtttgcttaaattag
ctgatcgaggcgaaattggcatcgtagagaatgtcatattgatgggaaccccatcatctacagacccaaagctttggaggagaatgcgatgtgtt
gtttcaggaagatttgtaaatgtttattctaagtatgactacgtcctgcaactggcttataaactgaacagcattcaagcccatgctgcaggcttgg
agcccattgctgtaaacgcatcatgcgtagaaaacgttgacgttggcgacttgattgaaggacatcttcagtatcgttggcttctcccaaaaatatt
aaaggagcgacttggatattctcacatatcagataaaatgattgtacagcttcaaatgaaagaaaaagactttgaacagtctcagcgtgaacatt
tcgataaaactacggaagaacaagaaattatatttgatgcttcagatcattccaaataaatcaatgaagcttggtaactcacaaaagtatcaaaa
tatatttattgttaagctttttggttatttatttttgcaacttgctaggattctattttcgaaatgttcttttctgcaatctggttcattccaaacgttatca
atgattttacatgtctaatttctttcctatactaattacttaattctacaaatcaaaccattctccttttcagttagtttatgttgattctctttaaaataa
tatcgtaaattgtttcacctgctaggaaggagtatctgtcctttacaaaaattttctcaacagtttctatatttgctttggcgtattcatcagttcctatt
caatttttcaaatatcttttttttgtaaataatctaaagtggtaattggaagcggtcaagaaatatgagtatctttggaatagtcgccgtgtcaata
atatgtgcaatcgcctttttattcgtctcatatggagctcttcgtctaataaatacggctagaagaagaagcattatgaataacgctgattcatcctc
tatgcgatcttggaaatttttgaagaacccctttgctcgttctggaaagtttgatgctttggatgccgatgatatgtgggacagtcgagttgaagaa
acagaattaagtaccattccgtcagctgctaactatttcgagaatgctactgaaaggtctccatttgttactacagaactgagctctcagcgagatt
cttcacccgtttcccatgaaaagaattcagagagcgaagctttcacgtctccatttcatccacagaatgcaccgttctcgcagaacaaagccattt
aaagtcttacgttttcattcttgcgtcaagttacttcaagtcgctgcattgttatttatgtttaattaagttttccatacatatagaaaaaaaaaacatt
gctgaaaagctcaccttacgcatctagctttggatggagattgtttattaaagctggcttcctatcatatttcaatcatttatccctacaatatactta
agcattccctggacattctttttgaaattgtttcaacatttttatttgtcttaattctctcctttttcctgcccttcattgttttcgaatttcgtttgttttgc

atattattgttcgcagatttacctacatttactaccctcgaaaatcgaactggaatctgaacaaagcagtttttcgtgtttgactgaatttatgttcca
aaatatcagttctgactacctgtttaatgatttttattataccagcgttcattaatatactgttattaatcatgagagcattttgtttcacttagacagtt
acttgttcaaaatgtatacttttcatattttatagtttcattaaagtaggatccttgtcgctaatgtgcgatagagatcattcctatgttgcttaacttc
ggtataaaattcatttctcatttcctttcaccatctttcttttactaggaacaaatgagtcaaatcattttagataatattcgtaacgatgagactgta
taccatgtaaggaaaataatttttgttatactatatagaatcttgcttatcgtttaaggtcgttcgcaatgctaatcaacatttttataacagagatt
tgtcattgttcatggtagggttaccaaaataaattctcacacttcccgaatcgtaaccgtgaagcagcacaatgatacattccccgaacaagcctg
ggtcgtcactggctcttacttcaaagctcttgttcacgtcgtgccaggcgtaaatgacatccgatttcgaacagacactgggtttgagctccctttac
gcttgaattaccaagtattaaatgataaccctttctatcggattggccttgttgtaggaagagattctgatttaagatttgacgctccttatggtatga
agaatgaccttgacgaagcgatccgtaaattgcgctgcgcagcttatctttggcaagcctttaccgcagaatgtatgtacagaaatggattcggta
ggcgtacatttcgtctggaagaagaaatccaacctgacacaatgtcttgtcaatcaaaatggggtactgaaaggttaactgcttcaatccatgtttt
gagatcagagaaaacagctgaggaaattcgttctactcctcctgatcagcttttccatgtcgctgggaacgccgttgataacctgaatctcccaga
gccatggcactacgtctgcatgctgttagatacccgctataatccttcttcgaaagaagtccgaggtcatgtcgcgttaggaggtggtactgatatg
cacaaattaggagtttttggttcacattctttgcattcttttcctagcgcagttgaatatgtggtacctgttttcagtgatgcccgaagggttcccgatt
atttagcaaatgatgccaatgaaagctctactgtttgggagtgtgctaatattggaattggagctatacttcatgaacttggccatactttagggtg
cccccaccaacctgacggcattatgctacgttcttatccaatctttaaccgtagttttacaacgagggagtttgaatgtgtccgaacaggaagtaaa
ggccttgctcccgtacttgcgaaggatgaatgctcctggcattacttggatctactgagattctattatcatccttgtttccgacttccttctgaccac
gtttatcctacagatgttgaaacaagctactatgtacgtgacgaacagatagttttcacgaataagactgggatattccttgcggaaatcgaatat
aatggtcagactaaggggtggaagattttcaatcctcctacggaagaggctggttttactgacgcagaaatacgatccctttcaaatgccaaccct
ggacaagattatcgtattcgcatcctatctaggaataataaagccatagatttaagtgatatccctggtatcatcaggaatgcaaaaatgtctacc
aactttggaaacgtatatcgctccgaaagatttggtcttcgtggttgcaatgggaacgaattgtctgatctgttgtttcaacctgataagaagatta
ctaaaatacgcgtgcattgtggtcttgctttagacggtattgaagcctttttggctcagattctgcccttcttggaaaccgtgggggaagtcctcac
gatttcgatacagaaggtagtcgcatttccgggtttcttattcgcagtggagcctgggtagatggaattagtattattttggaaaacggtaacacca
gccccttttttggtaatgcgaatggcggtggtcaaaaactatataaaattcctgaaggctccagattggttggcttttacggaacactagcggtaag
attggattttacttcatttaatcaacaatgtactaatactttatagggctttatggattctgttggatttttcattcaatagatttgacagctttggagat
catttattttcatatagcttatttagaatttttgtaactcttttcagtcctttaagaattttagagctaagaaaaccatcagattcatgttaactagtta
actttctatcaatgtcatgattagatcttttattgttcttccattttcatatgcataaacgcaaaacattaaacaaaaactcctttgccagaagcttca
acgatattactcaatgaatgaatgttttgattccattcgcgtaagcgtgtttccatactggcaacctgagcgtgattgaggattcttgattgaacaga
agagatatttacgatttgtgcaacttggtcaatcacgccggtgattaggccaacgctaagagcacgcataattaataactcaacttcatgggaag
gaatgcgagttgctttagcaattgtatggaaagttagcgtacgctgattaggaggtaactggaaaacaagctcaatcaaagccataagacgaac
tttttggccaagtaatcccacagaactttgtaagagaggaatcttgttaatgtgtgccatgagacgctcaaatttagccaagtcaccaacgttcata
gccacgaccaaatcatgtagccattcattgtgagtacccaccaaaacctggaagacggggtgtaacaaaagttcaccaaagttataaatgtcac
ccaggatagcagcgacactaaggtcaatagcgcgctcctctatttccatccgagacagttgttccaaatcaacacaggatagatagagaaggca
atgacgatagtattgagcataatccgctttagccttgtaataatctgcactgacgctatagtaagaagcatgaatgatattttccacgtaatcaatt
cgttcaatgagtttatcagacttaatcaaaatatctctggctgcatccagatcgttaaagacaagcttgattctaccgatagctaccaaagcataa
acgtaggcatctttgaatttttcttcatcaataccttcaatcagctgggttaaattgcgaaatgcctcctcatttgaacaagattcaaaggcctttaa
agcaatgtaaacagctttgagctgatttatatttgatcgaaacgcgctaacaaagtttgtgtaaagatcaagtcgtaatggaatagtttcgggggt
ctcaaaaaactgaataagtttatctgttaattgtttccataatctaaaaaagttaattttagcccgagatatcaagatttgtgtttttctatacactttt
cataaacaacatactttcgttcatatagatcttccatagcaaaataatattcttgaaattgctcaggagccctagctgcctgatcctgcaagaaatt
tgacatactaactgccatttcggtatccattttgtgaaaggatgttgttgaggtggtagtggttaaggtaactctgaggatttaaaggtcaccgtttt
gacacacctgaacaacattgagtagcttgttttagaataacccttcaaaagataagtatgatctgagttgtttttatttgctttctaaaaacaggga
aaacaacaataatgtagtaataattggaatttcaaattgtctattttcatatgttatgatgtacaagaaatgtaaaactatagcttgtaacagctttt
aaacattactcataagactatactataaagatatttatctgaaagcgctgatcaaaaatttactctgtaaacaaaagaaaaaacaacccatagtc
accgtctcagtatggatctatacaagaagacagggggggttttgcaaaacgaagcataattatagctatcattgagcctttagcttttgtttacttt
aagaaagcttttgaaaacattttctcaagctccgaactccaaatagtgtattttcgtctgcaccgccaagcgtccaagtggaatgtgccaagtcga
tcagtttcatgttatacaatgagctcccattttttcctggttcgtgcatacgatcctccataccctctagatcgtatgccaacaagagactagatgac
cgaaactccataggaagggatgaaatgtctttctcgaaatccgcaagtaaagtggaaaacctttccaccaaaaaaattcttaagatggtttgaaca
aggggaaaaaaaaaactgcgagcccttgtaagatcgtatccttcccaagagttttcccccatgctgtagaatacgttgcataagactgggactttttt
atcccacactaagattccggtaatccggaatccaaggcttccacttgtggtcaattgtgacacaacatctaatctagcacgtttctcaggagaagc

atccaatgcccagagctgtctacctagcttaatatccataacgcaaggatatttcatagaatgtgtaatattttctagcaccaaagcgcgacagcg
caacgcatgatttggtaaaccatccatatccaattgcagctctcttgcatgttccgtatcccatttcccgtaaaactgcggaagccacttccgaagt
gcattacattcatcacaagattcataaaagtaaatctcactgtctagacaaggctttaccaggtatcgttcatcctcactagcaaggacttctccat
ggccagcagcttgtaaattgaacggtttgagtttcattcagtcactcagcgtccgttcttttttcattcaaaaaaaaaagaaaatcaatacataca
ttctatattgaaaccaaatgccaataagtatggtggttttctgtagaacacaaagtaacagtgtaacttcgaaatcgtattatttttcaaaggctac
tacttctgttcatctatgtttatcaaagttcttctcttttgcttttaatttattttttactgaatcttcatgagatggctttccatgaaaactctgcaaaat
ccaccaaggtaacaatgttaccctcctagcaaataaaagtcaccgagcaaatttttcttaccttgggcgagcatatttcattataggtgtaataact
cacttatttaagcaaggaacccgtaaatcatgtctgtcaatcctaaggtaagttactataatcacgaatactgaagaaattattggaaagcgatgc
gtttggaacggaatttctcaaatcaaagttaatgtacaaattcatgataaagtggatggctgattgagttttctgctacatgaaaggcttatgggttt
tcttcagtattcttttatattttgttttctatgagactttatgctaactaatgttttcaaggcttttcctttagccgacactggcttaactcaacaaattct
cgacttggtgcaacaaggctctcattacaagcaacttcgtaagggtgctaacgaggctactaagactttgaaccgtggtataagcgaattcattgt
catggctgccgacactgagcccatcgagattttgttgcatcttcctcttctttgcgaagacaagaacgttccctacgtctttgttcccagtaaggctg
ctcttggacgtgcttgcggtgtcagccgtcctgtgatttcagctagtattactaccaacgaggctagtgatttggctcctcaaatccaagccatcag
attggctattgaaaagcttcttatttagaggtcctgaaataaaatggcggcatgtgaatgtcatctttttttgattcatagtatatgttggctgtaagtt
ttcgtacaatttcttgggaaccaatttctgaaacactaaaacgtaataggaactgtaacactattgtagaataaatttaagtgtaaatggtgtcttt
gaaggttcaggcagaattctggttaaatatattccccaaagtctcaaacagcagcaaatttcagttctttcattaatgctgcaaattttcataacttt
ttgttacaaattcaattctctcttacattttccatggattcttctgagcatctttagtaaattgctattggtaaaaaataccaattctgtaagttcctga
acataattgttgttcttaattaattactttttctagaacatatacggacattctaaaatcaattaagaaggtgattgcaggttcacagacttgttagc
gtaatattctttctattctattccttctagaaaccattaaattctctaagcctgagtccgacttgactaccaccaccacgctgcatgtacaagatgaa
gtaccaaaaggtatctttctttgcactattgtgctctttatatgtccaaaattgtgtggccgataatgaaattgaaaatgaacctttagtttttaaggt
aagttgttactgttctagtttgtccatattttagaagtttatcgtcaattggaacaaaaaatctattaaggacttataaaaaacaaatgatatggtca
ttttgctaggagtgttgtaaaaaaacttgatgcgctaactagtttcgatagcctactgaggtgaaaggtcctgtggtcgaacaattcttaggagact
ggactgaacgttggattccttctcatgctaaacgctttgtaaatggtgtagaagaaatgagttatgttggtgaatgggcagttgaggaaggaacat
cacccggtgctgttactggtgagcagggattggttatgaaggatgaggcagctcaccacgccatctcttatgaatttgacgagcctgtaaacgatt
atgaaaaagatttagttgtccaatacgaagttaacgctcaagatggattaaactgtggtggtgcctatctgaagcttttggctgaacgccctaatg
gtgaaatgtctaatgccgttccttacagagttatgtttggtcctgataaatgtggtatcaacgatcgtgtccacttcattttcaaacatgaaaacccc
atctctcatgtggtttctgaaaagcacttggatagtaagcctgcttctcatctcaaacctgggatcactaatctatacactcttgttgtccatcctgat
caaacttacgaagtccgtatcaatggtaatatacttcgttctggctcgttgttcaccgactttcttccccctgtactgcctcctttagagatctatgatc
ctgaagaccttaagcctcaggactgggtagatgaagctgaaattcctgaccccaatgctgttaagcctgatgactgggatgaagatgctcctcgta
tgattcacgaccctgatgcattgcaacccgaggattggcttgcggatgagcctctttacattccagatcctaaagccgagaagcctgaagattgga
atgacgaagaagatggtgattgggttcctcctgaggttcctaaccctaagtgtcttgaagtctctggttgtggagaatggaaggctcccatgatcc
gtaatcctaacttccgcggtccttggactcctcctatgattcctaaccctgattatattggtgaatggatcccacgcaagattccaaatcccgagtac
tttgacgacgaacatccatctcatttcggccccatttatggcattggctttgaactctggactatgcaacctaatattcgtttttctaatatttaccttg
gtcattccatcgaagatgctgaacgcttgggtaatgaaacctttattcccaaatcgaaggttgagcaatctttgttggtcaagcaaaaatcagaaa
ataagccatctatgcatgccgatgaagaagaaaataagtacttgacccttttccttgatgcctatgacactattaaagccaagcttcccccatcgat
tacggatcaactcgacccttatgttgctactattgctcaatctcctgaaattgcaatagctattttcgctgtcgcttcctcagttattgctgttgttatca
cttgcttttactacttttcatcctcatcctcttcttctcctgctcctctctctgcaggcgtcaccgaagctgaaaaggaacagcaagcaaaatttagac
aagaagatgagaatgataatattgacgtttcttatgctcctgaattagagtccactcccaagagtgaggacgtcaatgctgaataagatgtttatc
gacgaatctttttgaatgttcagtttacgcttgaataacgttacaaatatatagtatcttaagatttaataattgaaaaaatcgtttgcctaaattttg
tctatactaattacatatttagtttgataatgatggaataggttgtaattaatttaagccctaataatgttcaagtctataatgtgatcaatatcgtaa
tgtaaattctagtcgtacctataacctgattatccctatttaatttcagtactgactctgtctagttgctccttcatgggaatgacggtgcaatagtttg
aaggaaaccaccctctttcactattgcgttttcttccataccaccatccagtttctgttccatttgagtcagtcttactcaaaacagcaatgatgtctc
ctttcttcaatccgagttccacagttggttctcttggtacaaagtcaaattccgctttacaaaattcaagatcttgtaataactttgaatttttctctttt
gcaataatttttttcctttcgtaaatctttttgaacatttttattagcacatatggaaaaacaaatagagaagcgaataaaacaccagagagaagtt
tggatttttttagaggaaggcttggattcttcaacatcaaaggaattatagtcactcatatttggtggtttcgccaatttgattcccagtatgttataaa
tgaatcgctttagtcgtcttattaatgaaatgatgccgaacaaagacccaatagacacctttagtttagataaattttccatcactgaaacaaaagt
gttataactcatatgagctgccattaaagttgattccataacttgagcaatactaccaacagagctgacgattccttctataactcttaaagaagag
acgggttcagttgtaggtccgttggattctacagaagggatcccattaaaggagggtaaaaaagaattgaaattgggatagttcccatagtttaa

ccccatataccctggagcgttatagaacctatttcctaaggcatatgaagggtggttcaaatacgaacctgactggactgactgtacattcccatc
aagtccaccttgaggataagctggtaattctggcgcttgggttggagcaattttgtcttggtccatactgtaataaagaaaatgaggttcttccctta
gaaatctggaagtcaaattataaattaaccgtgtaagctacttcaaatggaaggccataaaaattacaacagtaatttgcttttccatcgaatttg
aaatttccagcaatatgatatagtcaccagtatttttctagtaaaattcgtaagatagcccgaaatatctttcaaatcttaagaaaaaaaaacattt
caaaaaatagagatagtagtgaaagtaaggcgcaaatagaactgtgttgaaaagaaaggcatacgatttatataaaaaaaataatttgattca
ctgttattctctattcatattttccgactgtaaagaaaagaaaattaattaagaacataaataattttgtaaaaagattctattaaagtactcattgc
tctgcatcctgggtagctaaataattactaacgacttctgacttggcataatcttctataaaacctttgagatatggtaatattttcgtaaataaattt
cgttcccttaataaaagattcgtagcaacacctttgcgcgatgatatgtattggtcactttctaaattatgtttgagggctgcttcggctaactgctca
acaaggcaaaatactctcaagaaatacaagtcttttaaagaagactttaatattaattttgggagttcctcaatagtggttctttgcccttcgaactc
ccattggggttctatatccccgtattcgttttgctccaaatgaaaaaaagtgaagtcttgaatattcaaaatcgaaaaatataaaaatggagcatg
ccaccataaaccagtaggatccgaagcaaactcgatcattggtggtcttttacaacaacttcgttttacaacatccaagggatcgttgggatctttg
ggaattaagatctttacaacgtcaattgtaccatctcctatgctttgtggaatgaaaccgtatgaaaatagaaattccccagcacctttggaactac
cataattaaaagtgatttcatctccaacagaaatagtcttctgagcatacacttgtatagcgtctgaagtaaaatcccatctggaattggcactatt
tgagtgattacacaaatcaatcacaggagacaaaactgtattacccgatggtctttcgaggcatcgactattgtataaggcatcagcatgtatcca
atcagacaaagtgatttttcctggccaaaggtttccatatcgggttcttaattcttcccattcttctctcaaaaaattttgtttggccaagacaggcat
gaacaatgaagttccttgaagaccctgtatatcactttcaggccactgaactggggtatcaatataagatggcaaagtagataagtagagattcc
attcactctccttttcgtttgacccatttaagtgacagaggatgaacagaacctgctttctggatggcataacatcccactcttcggactcggcgatc
gaagcaaaggatttattcttctttcgatattctgtaattgattcttcacaaataacttttactaatggtatctccaacagtagttgcccagagtcgcat
ttctctaaacatcgcagccctcttccttttccttcaatttcagcgatttcaagtttaatacatttcttcacaaaagcgtttatcaattcgtttgagcttgg
atgcatgggaggctgtccacctttgagaattcttaataaaccaggtttcaagaattttggcaacagtaggcagctataataagcttttcagtggtag
acgaaaatcggtgatattgtaattaaacgtgatacaacttggttaatagattacattaatagtagattataatggaataagtgtgaaaaatggttg
tttcgtaggatctttccaaattcgctaaagacagcataagactgatgcctttactgtccattctgtctctattgaatggttatgtctgtagagttctca
gttagaaataaaattgatacatcagtgaaaagaaagaaaaaagcttttcaatagttttaagaaaatggtcagatgtaattaagagatacctaata
gcaagcttgaatgaccgctaataattcagcatatctctcaagaaaagcacattaacaagggcagcattgcgcaaagaaactcaaaggtaaaatt
caaggtgactacatcgtgaaaataatgacaagagataagaaacaagcctgcataaagaacctatggctaagtagaattattctaattaacgcag
gtgaaatttactatttttatttaggaatttagttatcgtaccaagaaaattatgagaacttccaccgattaccacaaacgacacattcacaaaacgt
cgtcataggttcatctgcagatctggtttgcatttgataataggaaaccttcttttgcctacattttccgcacgtaaacagatcggtgatagccttttg
aggtttagcaccttgagcatgaaacaggttttcttgttcaagcttagcatcctctttacggcggtcttcggaagcaagttctgcagagttcatgttgc
ttaggcgttgtgcacttatatcttcagatataacagaagcacgaagctttgggttacttttatcttttaaattcatatatagcgaccgcattcgattac
gatactcagaccctgtatttccggaagcacgagctaaaacttgtgcatcaatagcaacggcacgtttcaaaatcacggaagaatcggcgtccgtg
tccattgccaaggcattgtatataagaccaacacaattgttgcggattttatcatctgtcaaattaacgtcaacattgtcacttttaaaagtacgttg
tccattagaggactggctttgagcttgtctttgagttggggttccaatgcctgctcctttagaagctggagaagcaccattggtaccaccagtggttg
tagcttttagtggtctgcctctgctcacatcggttttccatttttaacaatatcgcgtgcaagttcattaactttttcatttgggtaagatcgtagcttt
cctactactagaccgagcctggtttcctaattatcaagttagttatcctcttgtctgagagcaatttctgcgaaaaaaatcaaatttccttctcctccc
cctttaaaagttactggcttcctaaaatagttcaagtctcaaattacctttaataattcctcagtagcaacgacgcctgttctcaaacggctcatgat
gtcgagcattgtctatcaaaaattaatacaacatttcaatcgacctatatatagatatagatatatgaatttacctccgtattcttagattgaatggc
tttttccaaggaagccttggcagatcgaatttcttctgaatccatcgtgctatttactgattttagacggaccagcaatatatatgcatttaattatgc
ctttaccaaaccaaactcactttggctaacaactattctgtattttccaacctcttggactagagtagtggtaggtagaaacttatgcattcagaaat
atcgcttagtacttagttaatttttaccacttacctcaaagttgacttatggtcaatcaataagaaaaacaagtcgtgcattgaaaataataaaagg
aatcattcatcggaaggaatattaactggaaagctctggtatatcatcctgcattggtttctggtatacacaagagtttctttgagatttcacgcgta
ctagtagatacacaaagactattgtgaaatacaacgaagaagaaactgacgaaaagaaaagaatacgcgaatccggatcctctgaagcaata
cattgttgacgggagatttatactcagtattttgaagtttaaccatggcaagataattcactcggatatcttttgatatcaagacttttcatacgaaa
cgtttcagcgctcttttgcacctggtcaatctttttgctagcatacatcaagacatatttctagccatacattatcgttccttctcgcgtttttttaata
atttagatttaagtataaatgcctaccgaaagcagctctaaggatcaattgaactcaactgaagtattatcagatgtggatgcagacataagttgg
gaggaagcagcgacttccttactcacagaacttgaagaatcctctttttcttctccaagtccttctaaattatctaatactaaaccgtccgtgttgca
agagcattcaaacattaattctaaccaatgtatccaaccgacattggaaggcaagattgtgcgggatttgagctttaaaccaaggttggtgacag
aatatgagcaagaaaattattgggagaaagatatagaagctttaaattattgggtttatcctgaaatggatgaatttcgtgaataccaaaggtgct
tttgtgaattagctttttaccataaccttttattagcattgcctacaggcttagggaaaacctttatctctgctgtcgtaatgctaaatttttatcgttgg

tttcctaccggaaaagtgatatttattgctcccacaaaacctcttttacaacagcaacgaacagccgttatcgaaattacgggaatacatgcatat
aatacaactgaattaaacggaaatgtctcaatagaagctcgctgtaaagaataccgttcgaagagaatctttttttatgactccacagactctgaat
aatgatttgcgttcggggttcgtcgatgcaagaagcatagtatgtttagttattgatgaggcacataaggctacaggaagtcatgcttatgcgcaa
gtaattcgaactattcttaaacataacagtcactttcgagttttagctttaactgcaactcctggttctactacttcagctgttcaaaaagtcattgat
tgcttacatatatctaaagttattgtacgtaacgaggaaagtgtggacattcgaccgtatgttttccataagaacattcaggttcttaaagttgagct
tactcgtgacatgcaatacttaaaggctgactttgcagacatgtactttccatttttttgattttcttcgaaaaaagaaagttattcctggtacatgtga
tcttaaaaatctgaaatcttacgttcttttattggccttaaggaaatattcttttagacaaaaggaaaaccaagataaagaaaagctaaaaattttc
ggctattttaagttacttattacttgtgctcatgctatgtatcttttagattgtcatgggattattcagttttatcagaagcttcacgagatacagacag
aaacagaagaaagaaagagtggagaactatatcgattattttcaagtaatccatttcatttttatatgaatcatttgcaacatctaattgcaaatga
tcattttatgaaccatccaaaaattacgcaccttaccggtattttaaaagaacactttcaacaggcaactcaactaaatagtaattccagagttatg
atatttacagagtatcgtattacggctgaacaaatactgaaagctttttcgaacgaaaagcctctcattagaacttctctattcgttggccaagcaa
actctgtggactctgtggggatgagccaaaaccttcagaaagagattatatcccaatttaaagctggcatcattaacgttttggttgctacttgcatt
ggtgaagagggtttagatattggggatacagatatgattgtttgctacgatacttctagctcacctatccgtatgattcaaagaatgggaagaaca
ggccgtaaaaaggatggaaaggttttcatccttgttaccaaaaactctgaagatgtaaaatgggagagagcgaaggcttcatatagaagagttc
aaaaagtgattgaaaatgggcaaaaattgatacttaaaacggatgttcctcgtttacttccaccgacatgtagtccaacaatcaggtttcagaaat
taaactcaattcatcagcagtcatttgcgggtttaactgataatgataaacgagatgaatcacaagttgaaactcttgggtcacaaccaagacgc
cagttaccgtcgattgtcgatgatgaatttgaagatatgaaaagcaatcttagaatacctacgcctgaaatcaaactgcgaagatatttagcgga
ctatagaagtacagtctatcatacccatggtacttccataaacctaacaaagtcagccatagtctcgaaagtgagaacccccctacaaaattggta
ccaagtctttacttgactttcaaatttcagctcttcaaacgaaattcaagagttgtacccatgaagagatacaacggtataaaaagtatgaggga
aatggaagcggaagaaaagaaagctcatctgataatactttaatgtttaattgcataatttagaaatatcaaacccaaaaatgaaacagtcgtac
tttaatgactaagtagatttatttataataccaagaattagataatgaaaagaaaagtccgaacttagctggctatttaattggatttaatttttcgg
cttcttaagtatgcttgaaataagtattccacagcttctgtgttcatttttgtatcttgccaactgtcaccccagcaataaatgccatgatctcttaca
atgacggcgcatgcttctggataagctaggatagctgcttttaaactatcatgaaggtctctttcatgcgccgtgttgtctataactatttaaacagt
tagcaattcggtcatagttatattgatccatacagggaatttttacggtgtccgttatggaataaaaaccatttttcttcgttccacggggaatatgt
ctaagcacttcaaaaccagacgctgagaaataatctgaattaggaaatagttctgaaagtaagatggcttcctgagaatgagtatggatgcatgc
ataagcatcgcgacagcttcataggttaatcatttaccagaaaaaaaaaaagaataaaaaatttcacatacgtataagctgctaaaaataaagga
gtacactgagaaggcttgaggggttgtaaaggcttgtgaatgtattcacgagtcctacattctgttaacactcatcggtcgataatcggagacgta
ttcatacttcaaagacataacgaagagatgctctaatcgcatcctttctttttgaacaccagatggagcaatgacgattgcatctctgtaacagtta
aaaaacatttttttcaattaattaataccctgaacggatagtaattcctccactatttactgttagatttcaaactatatggaaccatttacccagtgc
cagttacccagccagaattatacaaatcctggcatattgtacaaataagctcaccgcaaatccgaagatcgccgccttctaaacaacctaattctc
cactattcatatgtgctagatattcggtgaaaaaacgaaacgtccaccaaagtaaatcgtcttacaaatatatggcagttgcttaatggagtaata
tagaggattcgcagattagtacacttagacaaacgtgattttgttaacgaatatatttttattaagtgtattattattataaactggaaaagaaaaa
aatagtaactgaattgcgtggctatatacattaaaaaactaagagatatgtgtgcttaaacggggtacactatagaacaattaatcatcaccaaa
aatagcattgaaaaggctctcaggagaaactttgggttgaggaggacgattggagagactttcatcgactgtctttggtaaggcttcatatgaaat
agagggtcgctcactggaaagtataggagaaatttcatgctttatctcaggggcacgaacatggaagcgtttacaaagcaagcgatttgggtaa
aaatctgatatttgtcgaggcatgctcaatatagaagtatcattagcttgatctgtggtttctgatattgctatagcattacgggggttatcaccgga
agaagaagtaaagcgcatggacaaattccgagacattggacgatataaagatgcggtttgggcaaactcggaaagatgtgtagttcgcatttct
gttaacagcttaggcatatcttgaatatgagctttcagaaaaatttcgtacatcccagctcgctcatcgttgtacggattgtccttcctggaaagagc
atgtttggctgtgtccaaatcaattatcctccattcttgctcgtcagaaggactagtatgcgatagcgttccttgattgtcctggttgtttttctcagag
tcgaaaagcgcttctgaacgttcacttgtgtttgtgcttttatgaaatgaggatgcatgtgaaacagggttagtggaagatgagaatcgtgatttcc
aaccttcaggaacctccggtgcaggaaaccattgatttgtaatagtctctggtgaataaataacgacgaaaccagcaagagctggtaaaccatcc
atacaattggtcgagggtgcaaaagaggattttagtcgactttttttagaaacaaaggtatgctttccgatagtaggtatagaaggtgtatttttctt
ccttgaattaatgcttctgtcaaatcgagatttgggtggcccaaggttatacgggtcttcgtcctcttcattatcgtcaaccgcaacacctaaaccaa
aagcatggttccctggtttctttttttaaatctttatttaaaattttctgttgattgatagtttgatcctgttgatatccaagcccatgaatgttgtacttg
gctgagtcttctgcctcgtaaaaatccttagctgtagagaccacgaaatccttcccgttccatccaaaatttcttaatatcttttcaccaattgttgttt
gttttggtccaagctctcttaatattgggtcatcaaggggaatagatttttctaatgtgtcttcctgaaaactgtaaactctggacatctcttgatcat
ttctatcttcttcatccataatgtcttccaaggaaagaccaatttttgaccccgatctgtcttggaacttgaccaagcttttggctgccatccttcctt
cgatcccaccgaattataatatcctgctgagaatccacctgtgaatgcaccatgaaaccgctttctatttctttcatccgttgctgcttgtttccacac

tggcaaattatggctgttttgatatgcctctaagtggactggtgtaccataaatacagtgtggatgtctgtcaagttctttatttgctctctttctgttg
ctttgatacattgaagaaattaggagacacttcccgttcttattgagacttcatcaaacgcttctacatatataagcgaggatactttcaaattgtat
gttgaattttgcttctaggagttttatgcagacaacgaagggatggacttacaaactgtgcaacaagcaatttaaaaaaaataaataaaagaaa
ggatagaaaaagctgagcttcaaactcctatactgaaattaagttgtcgattgctactttaccatcagaaggaacgttacttgagaatacgaagat
tgctacttcttaaggcatggtaaataaaggatgtttataagaagggaaggcggttatagatagcatcgttttataaaatataacttgttttagaata
aatattgatcgcataatcacaggactttggttttcgagcatggagcacagaaagtttggtctttcttttgtagcgataatccagtaatttcatatgca
ggtgaaattaaataaccatgtttctttgagactaataaattaaagaaaaaaaaatcctacagtcttcgttgatgagctaagattgttatgagttcat
ccttcttacgaacatgatcaatggttgcagtataatgcaatagaagtgttaggattagtttcctgttataagtctgcattcattctcttgataatttgct
gcaaaagccataaattttaatttgatttcttatttttatgcagcctatatttttcgttttcttttgtttactactccatcgtttcaccaccaaatttgaaa
agctagtgttgcacctgcaaacacttgtaaaaattcaattatttagtatcgtaaagccaagctctatatcctttattaaaataaaaaaaagagatat
aataatattggccttggctagtttttgttccattttcataactttgaaaagttaatgaacctttagtttgtaaagataaatggatggatatttcaaatt
atgttgcaaattttcattttgacgaaaaatatgctgcagcttttgaaaatggtatgttttaaagtaagttttcttgtcatgaaacaggaaagaatagc
taacgtatgtttctctgtctagcagatataagtacaattgatctcttaactcttgatatctctgatttggagaagcggacaaatcgtaataaagatga
gcttattcaaattgtacaacaggtttttcttttacttcgtcctaagagaaaagttgcgtcacgaataagccagagtttttttaacaactggagatcgaa
tactggatcagaagcttcacggaggcattcccgttggtcaattgactgaaatttgcggtgagagtggttctggaaaatctcaactatgtatgcagct
ttgtttaatggtccaacttcctcgtgcttttggtggcttaaataaagcggccgtgtttatatccaccgaatcggggcttgaaacaaagcgtttatttg
aactggcaaaatatttaccagatcgatttcctgaagctgaagaaagcgacgtatttattcggaatcctggtgacagggtatataccatcctttgtcc
agatttggaatctcaagaacatattattcaataccagcttcctatactatttgaacgcgatagtataggactagttgtactagacagcgttgctgct
aattatcgtgctgaattgaggtataatcggtcaaaaagccattacacggatttgaataatattgccaccagaggaaatcaattgggaaaacttgc
atctatacttcgtgatcttagtcgacggcatgaagcagctgtcgttattgcaaaccaagtgagtgatcgactcactcgggattacggcgcaatcgg
gctgtttagcttagactatcagtctcagtggttcaatggatgggatgatttggatcctaatcctaagataccttccttgggtcttgtttggacaaataa
tgttaacacgagattggctcttattaagcgtactgataatgcagctgtgaaagcaagaaggacactacgagttgtttactctccaaatgctgcacg
aatcgatgttccagtatttataggttctatgggtatttttgttcccaaggaagaaaaagacgctttgaaccccttattataaagtgcattaattctgtg
cacagaaagaacataatcaactgtattgcagtatctgtttttccaacggagatataactaggttaagcctttctttctattttaccccccttcaaata
gaggcctttttttggtgaatagtgcgtcttgagttattacagaagagaaattgattttattcaaaaaccctattgctttttgtttgatttaggaaggaac
cttaattgcatctgcatgcttgacgatccctcccttcagttcactttcacttacgaccctttataagatttttttaaaaaattaccgaatgctgcagatat
ttacttaagggatttttattttatcataataatgaagttttaattaagtgaatctacagaggaccttgcgatagagcataattgaaacaaactttgtg
gaacaaaatgtcactacccattgatcgaattaataccaaagatatcctttctaatgaactttcaaatcaagtaaactcatttcctctacatatctact
tcaatatacgaggaaagttacttatgattgaaagatcggaattattgtacttaccacattgtcttattggaatgttatttacgaaaggattctctccc
ttattacaaaatgaaggtggtagtcgcgagacttgtttaaagtgccatctaatcgatcctgctgtttgcagttggttgttggactcatatttacgatta
tttcttaagaatccgaattattctgctttaagagttagagaagatcctttgaaagaaacgacgttcgtctttttgctggtagaattctgtgacatatat
gtgatacccgaaaattcagggcaaggagtgagcctaagtgtaaaagagaaacttttggaacattactacagaagctggaacatttttgaaacct
gtccgaattccttttttattggcgataataaggaacggaaagctttcgttcatttgatgaatgaatggtaagaataaacaagaggttcaatcttata
cttaacagttgctaatgttttctattacccagtggtttcagtataagggataaatggagtcgaagagtcaaagagccaaaacgaatgtcattatctt
catatcatatttctacattacggtgtacgcctgaaaatgcgaatacatgtgaaaaactattaagattttggaaaagcccaggggtatgacacgtcg
gatcagcaattttatttttttatgtttatactaactaagatttttgacagcataaatgttggtggtttgaaaacccgcatacgttcaataacactgttct
aaatgtttggcgccgcaaattttggactttggaacttagcgttacaggataaaccaaaaacatgtcaaactttcggaactcagctattaccataat
gtgctagaattttactcttttccttttacgattattcttatgttgattattatataatttattcatagcattctctttcttcatatagttaaatatactttcc
taattcagtaaactaattgcagtcatatacgtcctttgtaagcatcttgttgcgaacatctgtgaaaaagtatggaggatgaatttgctattcagtca
ttcaaaccggctctcagtaccaatctaacacttcaaaaaaagttattaccaatatttaaatatcgtaagaagcctttactttaaagaatggacatca
agctaatggtgcttcaggagagcaacttttgtatgccattgagacgtttcaggttacgctcatacatggacacacaggatgcggaaagacgactc
gtaagggagaaatcttagaaatcaactgatgagtatctaactgaaaactagaactgcctcaatatttgtatgaaacaggatggactaaagacgg
aaagataattgggtgtacgcaggtattgtactctttttttcttatttattcaatattaattttattgattcactagcctagaagattggttgctagaagcg
tcgcagctcgtgtctcagaagaactgactacagaagttgggaaaacatgcgggtacaaaattcaatttgagcataatatatccgacgaaacgag
aattgagtacatgactgatggtacccttctaaaagaaatccttacagatcctctgttagagcgctattctgttattatcattgatgaagtacacgaa
cgaactataaatatagatcttcttttaggaattttgaaaaggtatgacttaagggaattctaggtaactaactggcaggattcttattaaacgccgt
gatttgagagtaattcttgctagtgcctctgccaatattcaaaagttaaatgactttttttacgaaaaggataagattgaaaccaaaagtatttcaa
ttgaaggaaagctattccctgtggatgttatgtaccttgaaaaccctactgaaaattatgtgaatacagcaattgagacggtcttgaatataaattc

atcttatccgtcgggtgatattttggtatttctcacagataggagagaaattgaggaatgcgtgaagcgtatcgaatcatcggctgtaacaactcct
gatgacttaccaatgcttgtaccattacctttgcattcgggtctaacattagacgaacagctacgggtttttgagatttatgagaacagatttagaa
aagtgattttctcgacgaatattgccgaagcaagtgtaaccattgatggaatcgtttacgttgtagactgtggtttctgcaagcaacgtattttcaat
ccttatagtagagtttcaaaattaattcgtattccaatatcaaaatctaatgcagttcaaagaatgggaagggcaggaagaactaattctggaaa
gtgttttcgactgttccctaaaactatctatgattctttgaaggatgaaagcgatgccgaaataagtcacagtgatttattacccgctgtgtttttct
gaaagcgattggtttaaggaacatataccagtttcccttttatattcctcctcctacagtacatattatcgcagctttagaggtatgcttgaattttttt
attacgatgttaataaattcaaaggatttgtatttttaggtgctattgatgattactcaaatttgacggaccctactggattgcaaatggtggagtt
acctctagatgcaagattatcgaaagccgtaagttttgctctgtttaatatgcttcttgaatcgtctctctgactttgaatagctgctctcctctgctag
ctatgattgtacttacgcaatgttaaatattgtttctgttatgatggcaggggatgtcttctaccgcccttctagtaatcaatacgaagctactgcttg
ccatgctacatttaccactgaagaaggcgatatcctaactggtttaaatggtaatgcgtgtttttatagttggatgttactgacaatttagttttcgaa
tcatttctgaatcgaagaacagatatgaaatggtgcaaggcgaactttttgaactataatacattgagtcatgctagtgaaattcgaaagcacatt
cttccctacttgcaacgttttcatatatcaccagagaagcggataagagatatagatccttcgaagatgatagaatgtcttttacaaggatttttcg
aaatgtagcgcatctacaggatgacggttcttacagaacgattttaggatctccagttttggttgatcataaaagtacattaattaacaaaggcat
accttggattatgtttacttcgagtaagtgacagatcatgaatttttactaaatagcttctagtggttgaatatgaaggtcgagcatttattaacggg
gtatcaaaaattgaaagtaaatggttagagaagttttatcatagaaaaaataaaaaataaatagtgatttttgggtaataattgagttttcagtgt
gttgtcacagtcatcgtacttattgccaagtagttcgtaccttcctccactgactttgataaagcaattatgagtgatttggaaaatgtatggataaa
tgtcgattatgtatactaattaagttatagatacgccttaaaagacttgcaaagttacaacgctctagttctggtgaaaatgtttcaaatagaagca
gagagacgagcgaggaacatgaatcgaaaaaaccaaatttaaaacaggacgaagacaagactactgttagtagccctcctacagagaaaaag
aaggaaacagtagtaccttctcgtccaagtaatcaacaactaaaggcattttcccgtgaagagtgggtacacgttgtaacatgtcaaacgctcaa
tataactttagactcggaagataaagggaaattttatttagaatcttttaaaaaggacttggaaagcgaagggcgaccttgcgcacttaacgaag
ataatgttgattcagcattgctaaccagattgtcgacgactggtgataatacattcaaatatttattaaattcctggagttctgtttataatgtcctaa
aaaagttgccgaaggatgaacaccttccgtttaaaacgaattatcttacgagcttgaaatacctattagtaagttatgctggcataactatattgtt
gcccgatactttcaattgctcttccattgactttgcagatttacttacaaagtctcctgggataccaccggaatttatcgttgattttgttgctcgttat
gaaaatgagggattagatgaatttttgttcctgtgttggaaagtttgtcggtaaagattagtcttatgaatgtggaaacgtttcaaatgaatgttat
tcaagtgttgtttcaattgatttctttgaaacccatcgctgtgctagttcctcaactaccatcttggtgtccttcaacaagccctggtgagcttgaatat
aaaaccttcttaggaagaatttctagtctttctgtttttactcaggaagttgcgaatcgctacttttcaaactccagggaacgctctatgcgggacat
tgcttcttctataagttccttgggattaattgtttctagttaccaagaacaactttaccaaatcattagtgttcttattcgaacctcttcagaaattcgt
gaaaaagtcttagatttttggcaatggttgtaaatgttaatcataagcggcagtcacttcatgttgatcagttcaacataacttcagatgcttgtat
gcttaatctggcttatgttatgaatagattatcagaaccgtttttggatatcaaatattcaaaaattgacagggttcaaccggagtatttacgcagg
catcctcgtatagacttcaaagaggaaacaaagcttaatgctgatcaaaaatcttcggaagaatttttttctcatggttttgaaggacccaataatt
ttatatctgatatttttttcattaacctttcttatcatcatttcggtatcaatgctgctgttaaagctcttgagcaaactattgtgagtatccgagattca
gagaaattgagagagcgacttgaagctgaacaaaccataaattccaatacgtttttggctagacgactttccgcgcaaattgatcgtcttcatcaa
cgaatcgatttagatcgatcctttgttcactgttatgagattatgcttgctcaaccatctgaaacctcgagatcctatagttttctaaatttcgtcatg
gcatggttgttgcgagtagctaacaataatgcatctgactatccaaaggcacccattgtactacctttctcagattccgctcctgaacttttcaaaaa
ccttccagaatactttattgaagcgattactgattatgtcctttctctcttcaaaacttcttcatctactttaaatactcacccattagaaacattatgt
gaattttgtgttactttgttgactaaatcatcgtatataaaaaatccatatttgcgtgcgaagcttgtggaaattctttttatggagtacagactcat
cctggtcaacctgatgtgatgaccgacgtcttgtcaacctcgaaagttgctacgaaatggctaattcctgctttaatgggcttttatattgagattga
gtctactggtcagtctactcagttttatgataaatttaatattcgattttacatctgtgaggttttccgtactatatggaaattgccagcttatttcgga
aaattggaacaggaacagaaaaccaatttgcctttttttgtgaaattcgtggctttgatgctaaatgatgctacatatcttttagacgaggctctcct
gaagttgaaggatatacataacttacaagaaagggttaatgaagctatcaacacaggtgaatccaatcaaaacgttcaagagtctcaacagaat
ttagtagcaacagagcgccaggcttcagtctattgtcagctgggtaacgaaactattatgatgctacgattgtttacatcatctattcccaaagcat
tttgtgctctagaaatagttgagagactggctgctatgttaaattataaccttcaagcactctgtggtccaagatgtagaaatcttaaagtgcgaga
tcccacaaagtataattttgaccctaaatctttattatccacgattctggatgtgtatttgaatctgtgcaatgaaggaaactttgttgaagcagttg
cccatgatggacgctcttacaacaaggaaatttttgaaagagcaattggaattgtcaaaaaacacaacttgaaatcttcgcttgacattgaaacc
atcctaaactttgtcaacgaagtggagaaatttaggttgaaagaggaaaatgaagaagaagacatgggcgaagcccctgatcatttccttgatc
ctctgatgttcacgattatgaaagatcctgttattttgcctagatcgggtgtctcaattgacaaaagtactattaaagcacaccttttgagtgacaac
acggatccattcaaccgcacccctttgactttggatgatgttacacctagtaagtttttacatgattgtcttagcttgcacatttactaacacaaactt
agatgatgcgttacgagaagaaattgaagcatatatgaaagctttgaaggaaaaaagagctaaaagaaacgagtaattttgacgaatgcaagt

gatagatgactgttgagttaaaataaagacgacaacacattgtttaatcagaaaatttgaagtaaatatttttagtacataaaagagaaatcttag
attaaatgatgtcgttaaacatttttataagaataaattattatgctaatgaaagcagataaaaatgtaatggtaaactattatcgtcgatggaccc
attaaaatacatgtttgagttcattaacattagataaagcagacataatagtagccgaattatacggcagttgatggaaaaacattgaaggttta
aagaagaacgcaacgcttcttctttttggtctttgttccgctgttagctttatatttcagcatagaggcacgggcagcggtttcaaatacttcttgcac
accctcattaagtttagcagagcactcaaggtatttataggcgccaatctttttagcgacagtttcgccgtcctcataaggaattggcttttgagag
gttttggaaagttcctcaattactttgggatcattgcgcaaatcatttttgcaaccgacaagtatgataggaagattggagcagaagtgcaaaact
tcagaaatccatttttcttgcacattgtctaaactttcaggagcatcaatggaaaagcagattaagacgacatgagaatctggataggaaagagg
acgaagacggtcatagtcttcttgaccagcagtatcccaaagagctaactcaatgtgacgaccgtcgacatccacgtctgcaacgtaattttcaaa
gactgtaggcacatacacctcaggaaatgtacccttggagaaaacgctaaggagaagggttagcatgttttcattataaaggtacgaatacgtac
ataagcaaacaagttttaccgcaagcaccatctccaacgatgaccagtttacgacgaagttctgtggacatgataacaagggatattaataagct
tcaagggaagaataatatgtccttttcagaatttaaatttaagtgagtttgaagaaaaaaaattaggaatgattaggcaaaaatattgaagaaac
ccctttcaaaaaatagaaagaataaagaatgctataagaaagagtagcaaattttgaaaaagagagaaaagaaatgcgaattagatgaaaac
ttagaagtaaaacagccaaagaaagtagtaagaagactttgaggaagcctttagaatatgaaattcttatcaattctaatgctttcttttgatgaa
ttaatgagtaaagtaaacaaatgaagacgagtagtggtaaagagtaattgcattggcaacgcttcttgccctcacttaaaggggaaaataacatc
ctacgacttgcgtcataaaaatcatttaaggtagattaaagtaatttaaaaaagtgttacaaatttggctatcttgcttttggtaatgcgtaaagctg
cgtatagctatcaaggtaacggctgctttatgtttacgaacagttgtttacatgaaatttcagctttttcggtggtacgtgtctgtgtgtttgctcgcat
ttctattattttaaattaattttcttaaagggaattacaaaggatctatagggagaaatttgctatttatgatattttgtattaaatgagcatgatgca
gtacttgtccagtcaacgaaaatccattcattctataaacaagacgaatttctggaaaacatcaaaaagaatactttgtcccgtgctttcaagtact
aagtatacaccacatagttaggtatgagtttgaaaatgcaaactgtgtcatgcaagcttttcaagaagcataatcagtacctattcattggaaaga
acactttttttctggatctcaattttcatagttacattcaataaaattgatcgtaaaccaaagtgaaatagaacgatcgatggagatcctttttcttatt
cttaaacgtaggcgaaaaataaacacgaaataaacaagagctataaattcaaatagtgcaaactaaaatcaatacattacatacatatctacat
atatactttttctttatttattttactttcaaacaggaattgtttacattcctcaatctccgtttgtttacaaaatactgatatacgctctattgcgctaat
gagcttaatggatattgacaaggcgatgctaaacgcagagcagagacatgcacttgcagccaaatcgttaccgtacgaatgccataaaaacgtt
ttgaaccgggtggaattggtgaaaatagtcgcttctttgtatttgtcgcgttgatattgattaaaagagactttagtttgttccaccttgtccaagtat
tcattctgtgacgctctcctctgagccatttctagaaacataactagaaaagcgaattttgcgataagtaatccctgctagtataagttttcgtgagc
ataaagcatgaaatcaaaagaagaagaggaagagtatgatctctctatgtctatggtagacgaaccaagctatacagaccttgcagacgattct
actttagaacccctaaccaagcaagtaagccctggaacgaagttgcggatgaatgtcggcaactggtttaagaatggcatggcgatgctgggga
atttttatctctcagctttttccttgttattatagtcttggtcggaattgcatttgatgttggtggaaagttttgtggactggtatgcatttcaataattt
agaaatatttattttacgataccatcattttcattttcatattttcagcagtttacggattgtgctaacttagatgttcactttaggttttgacactcgct
ctggaagcgtatttttgttctgttgctttgttaaagctttttggacttcgaaaaattgccgtcattcttcattttcttgaacccttactcgtacttcagcta
ttaattattgtccttaatatttcacccagtcttgaacaaaacaaaattgcaaactatgtcattcatgcttggaatatgatacttttacactgcactcct
cttttcaatttgttagaaggctttgcatctttgcttgccatacaagctcttggtcatttaggtcgctggcttgtacattacaagtccgaaaactggatg
gtatgttgatttactatttaagctatgttatcttttatctaactatcagtttttcattctccttaacgctagtagcgttatctcgatgagtttatatttact
gtaccgcgtttcctcattcacgatatcaaatcctaatgcagtcatgattggtttttctttggctgctgttacggtagtttccatttatggtgtctcttcgg
gaagagctagtctttctgaggcttctctaatggtacgtcttatttttcattcgttcattatttttaacaatttctagttttcgtacattgcttacactgtat
atatggtttgcactgattttggaaatccaactacaatttcaacgcaaaaaccaaactttgactacctaccaccggacgtgttacagaggtaagtttt
ttaatggtttcacacctcctattttcaagaaaatgccattgctaacatcaaatagtgttcatcatctactttcttctatctctgctatttttccaaagac
cctttcaaacattgcttcctttatggttgcagccattaggacagttgcacctagcgtgtttgctacgttcgcctatcgcataacagttatgtatgcagt
gactcgaatccttccagctatacagcagaatatagtctatcttgaatatagtcgaacaacaaagcagagcttatggacggttatctcaccttgtat
attgatcgctgtctatacaaatttactccttcagcatctgtatcctacgccttcttttggttcccttaccaaccagattctttgttcagcagagatatgg
cgatgggtcagtgcgatattgaccattattttatacgctgttgaattagcatacagcaaagagagtaatacgggacaagctcttgcaagtcatttta
agctggactaattatgctgcaaaagccattcaataccaatatttatcttctcacgctcgtttctctgttcttttcccctaatatagatactttagtttta
ccttagttctactttattaatttaattttagcagtcgaatttttatcattgatataagtttgtttaaattttaaacttgtaatcaaagtagggctacttc
aaactacatcttacactttagcaccgttcaaaatacatttccatagttttatcctttttcaacacatcatacacctcaaaataagcaaatcacctttat
atattaatgattttaagctctaggcttctccttcttttcgtggaagagggcagcaagaccgacaccagcgaccttgacgaccttgaaacggacacc
gggaatatcacccttagccttacccttacgaccgaaaccagaaagcaagacttcgtcgttctcgtcaacaaagttaagacaaccgtcgttgggaa
cgaaagcggtaaccttcttaccgttcttaatcaattggacacgaacgcacttacgaatggcagagttgggttgcttggcttcaacaccaatcttttc
gacgacgataccttggcgtgagaagaaccaccgaaaggagaagacttgaaggcagtacctaacaagcgcttcttgtagtgagcatcagccca

acgctcttcacgacggtgagtacggagcttacgagcggcgttcaatcctgcgggtttacccattttgtagtttagaaggttgcggctaatttggaaa
gagaattgtttccctttcctttatggtataggcagtcacaaaaccctacccttcatgttttagtgaaaataggtgccacatcgttataaaactttaaa
gacgtattggagcgaaggaaaagagccatgaaaattcaaataaacagaggaacgaaaccagaacactgaaaggtgtgagctgctgctttttga
tattttgaaagggtttcagggttaattgaggatattatatatataaagtcgattattaatataaacgaaggacttgtgagacgtcttcttagaaaca
ttcaattttaattgagccaactgaaaaagtatatcatctcatggcagaagaaaccgcggacattcttgagcctacttcatttttattcccaaatgag
caagcttcaaatcaattagttgaggaggccatcagatacttctctgctgtcaaagttccttcatctagtcctattcagaaagtcgttacgaagaatc
ttgatgcttggcaggtctataaccaactagccattgctgcaaagcatacactagatgatcttccagatttagatgttgaggaggatagaccgtcag
atgaagagagttatgcatctgaagactcgcaggaagaaaatcaattcgaagcattaccagccgagaatccagctgaggatgtggagaatgagg
aagaagaatttttagggttttctgataggggagatgaagagaacgatcatttgagcgatgaaaatattgagaacgaggatttggaaactggcaa
ttctaatgaggaggacgagaacgaacaagtgcaagaagatcaagagcccaaaaaagacgtgtttggtttaaacgacaaattctttgatatagac
gaattcaataagcagactttagctttggaagaacaggaggacgagcaaaatcacgagattggcgaagaagaagaagacgacattgatttgttt
gttgatcctgaagaagaagatgaggaggaagaaggtggccctcgtgctgattcaatcttgtatgaagactttttggtccaaaatctgcgaaagg
ccgtcgtgaagttagtaaaaagaagaagcgtgacgctgatgctagtaagaaaaaaaaggcgaagagagtaccaaaacccgaggaatctgacg
aagaagaagatcaagaggaaccccaaaacgatgataatacctttgaacgcgttagaaaagacctttttgccgatgaagaggaggaagagaat
gagaattctcaacaactcctctcttcttatgaacgtgacaaggctcgaattagtcaacaaattaaggagttagaatctgaaaacgtcgccaagaa
accctggacattgattggtgaagcaacatccaaaagccgtcccagtaactcacttcttgatgttgatattgattttgaaactggtgcaaaacccgta
cccgtgcaaactgaagagacaacatctgcacttgaagacgtaattaagaatcgcatcctagagaagagattcgacgatgtacctcgtaaagcac
ctagctcacttcccgaatttcgtccaagcgagctatttgagcttgatgaaaataagcctcagcaatctctggctgatatatatgaggagcagtacat
gaagaaagcgaaccccgatacttatcaaagtggagttgacaaaaagcgccaggacgattataatgagattaaaaccttgtttgaagatgtcagt
tacactttggatgctctctcttcttggcactatattccagctcctgctgaggccaatgtgcaagttgtatctaacgcccctacattagcgatggaaga
agctcaacctactgcttcatctgatgccatggctttggctcctcaagaagtttatcaaccctctatggataatcgccagaacgagactgtcgaacgc
tctggtatcgctgtatcaaaggctgaaatggatcactcagagaagcaagctcgtcgtagaagagttcgtactaagcacgctgaaaaacgcaaac
agttgggtgaaaagagacgtggtactgaaaaagagcaagtcgtgcgtcaactgtctcgtgctgatgttcaagtcattggtgctggcggagaacgt
aaaagaatctcttctaaatctaaagataaacctgctgcggcgctatcctctaatcagcttttactttaagattaatgttttaacggttcattttctgca
ttcattaaaaagaattgtttgggatataaaaatttttgttttggactttacaattgggtcgcggtgacatagtaattagaattttagggataaaagat
aataaattatagtattgaagataaattatagtattgaaggctttctcatttgttaattcgttcattcataccactgattccacttttcttctagttccgg
aaggcatctaaatggaacgctttctacgctttcaaatccctctttgacgcttggcttttgaaactttgagcggaacgaaacatacgccacttctggt
aggcctttaatctgtaaagcgttagtatttcacaataattggcacgcctgatgaataatagatacctcttgctgaatatatctaaaagtgttattgtg
tttagccaattgttcaggtacttctaaataaatacaacgcgcgggaacattaaattctttagctagcttaacccaaacttctctagcctcataagta
ggactggtattatctaaacgttcagtaaacaccatgcagttgcaaacgtaccaataacgactgatttattatttttaatgcttccactgctgcttta
cacaacgtgctttagttttaagagcatcttgattaactcgttgatatccaaaaggcactatttgcgattcacatagagtgcttttaccagaagctgg
ataaccaacaaggacaacaatttcttgatgttttgctggtacaaatttataaggctgggaggttttatcctcttgaaggaacatttttggatgaaat
gaagaaaaatctgggcgatcaaagggctcgcctgtaaaaaaatgctctggagtatcaaattttataccaatattctcggcaaacttgaggtcggt
agagttatgattacgaggacgtcctgcggcatcgccaacaaaaaaagcaagagatttgtctgagtgaattaataatcacgatccttttgcttgaa
aacatgatacttacctatcggatgagcccacctttctacaaagtgatcccacataccttgcaaaggcttacgatacttatctcttaacaatgcggca
tatagaattacaggaagatccagtgactctaatatagcacgaacttttatttgaaactgatgtcccgttgatgcgcgtctaggaattccattctgatt
actgaaaatgacaatagagaagccctcggaatccaactttttaaagttggaacgacattcggatgccaccagagccaatctcctgcgtctttggc
gaaaactcgacccgacttggtttttattaaggtcccatccagatcaaatgtagcaactttttcggatttcttaggttttccatacgttgctatgtacaa
agactctgtaatctcccaagtcaatttgccgtctgttactgtagggtctttattttttgtttttatactcagagtcatcaaaataagaatttatagaa
gattgaggtgctaatttacgtttccccatgtcgaaaagctgtggaataaaacggtaaaatcaaaattaagggttaatagtccaattaccaaattaa
acgattactagtttcgaacctgcttcacagtcttctaaggttacgtttataataaatagacattgattcgtcaaaccaaaccaaattaactgcttctt
tccaaaatttcagaaaaaaatatttataaaaacatgaaacaaaaaaatcagacagaagagtgctttggtggacaaattatgtaaatggaaagc
aagtctacctagtatcatattaagtgaaatcaactgccattgaacgatataccaaggtgtcacttaataagtgacggttttctgtatatcaagaaat
tctacttggcttatttatttccattttagaataatagtttatgtaaataataaataaagtagcaatcctccatacttctggcctggacactattttaaa
aaggacaaactggatatctcaagcaccaaaaaattcttcctgaagtgtaatctaaaagtattggccctaggcgtcctttttttgtagtattccaaa
tgtttattaactatctccagtgttcagatgctgtcattactcggactaaaatactaaagaatattgtttttacccagcggtataactatataatgtcaa
caaacggttaataaacaacacaattgactgaaaattcaaaagagcggatgtatttcttttatctatagctctattaaactatgagtttccaggtctc
tctgaaagtttctctggaaactcttttgtaactgaactgacaattatacttgaacattcaactcgtcacagttgggcatgttgaaatcatcaatatta

tgatttgataaagctatgctatattagttaacatctataagcttaaaagaatccccgcgttacataaagtaaacaaataacaataattgtgataca
gaaaatggaatggaacttgtggaacaaagaaacagattttgttagcgattttagaaaagctatatattcatcagtctactgtcagaatcttcagat
tggatgatttgactggttcggctaacttggaccacttttcctgcaaatcattaagttggtcaggtatatggacatgaatgattagtgtctatacacgt
aattcaaccaagtccaccaggagtgtatgcaacaacactattctagttacttattccactaaaaatacattcttttaccgggggggggggagaatt
ctgccatgatcgaagacaaacaattaaaataattgttcagtctggcggtttttctcaattttgacttagaaagatcttgtttatcgagctattttagtt
tgttttgctgcaagagacaatgtggttagtctacaaggcatcaggaacattaccaaggaactatttcaagccaatattaacaatcatttttttaaat
attctggacaatgaacaattgtattccacccaattagaattaatagtaaatgagtctgcattcccgtaacagggcatattttatataatatcttatta
acagaaaccatcacgaggaagaatatgtatttagtttaaaggaagattcaaacaaacattttattgctatatttatttttacacttgttgtttttacta
gtttggtatcctaaaggtgtgaagtacaaagaatttaattgacaataaataagcatgaaaacgatggtccgaagaaaaaaaaatatttgattctt
taaaatagaataatttttatttttcttctatataaaattatactgttcataatattccgaatcactttgaaagccttttatcacggaagcgaaagttga
cttccaccattcccaaagacaaaatgagtgaattcagtactcgtcaagtcggtgctttgaacaccttggattatcaactctttattgaaaagaacgg
ttcccctgtttctgcttggcacgatgtccctttgtatgccaacgctgagaagaccgttttgaacatgatcgtcgaaattcctcgctggagccaagcca
agcttgaaatcaccaaggaagctactttgaaccccatcaagcaagacaccaagaagggtaagcttcgttacgtccgtaattgcttccctcaccac
ggttacatttggaactatggtgccttccctcaaaccttcgaggatcccaacgttgttcaccccgagactaaggccaagggtgatagtgatcctttgg
atgtttgtgaaattggtgaggcccgtggttacaccggtcaaatcaagcaggtcaaggttttgggtgttatggccctccttgatgaaggtgaaactg
actggaaggttatcgtcattgatgtcaatgatcctttggcccccaagttgaatgacatcgaagatgtcgagcgtcacatgcctggcttgatccgtgc
taccaacgagtggttccgtatctacaagatccctgacggtaagcctgaaaactcctttgccttcagcggtgaatgcaagaaccgtaagtatgctga
ggaagttgtccgtgaatgcaatgaatcttgggaacgtttgatcactggtaaaactgatgctaagtctgaattcgccttggtcaacactactgtctct
ggctctgtcgccaatgatgtttctgttgccaacactatccctgctgctaatgaacttgctcctgctgatgttgacccctctgttcacaagtggttctac
atctctggctcccctctttaagcatattcattagaagcgagttgtagactgtacgttatagatacataccttccttgagaataaagttgtttcttccga
ttattttttagtgcttcttagtatccaccaatccatcgggtttccatcaaaacatttctactagtattaagttgaacgtcatattcgtttgcgacaagac
gatccgaaagcttataaatttaacaataaatttaacaaaccttcctatttctttgtttttataaaacatgcatcaatctctaatcaaatcaaaaaaat
ttactaaaaatcaatacttgttgaaacgttattgacttcagctaagctacttaaatgcagatatattatttcattaatattcattataaagacatatta
cattcatcacaaaatcgcatagaaaactgacaatcgaatgcttcattatcttcgagttatttatcttccattataaagttcagctaaacgcttgagta
taagcctagcgccttcgtttgtcttccagacttcatgtggagcatcattaactatatcccaaactgtattgtctgtaagcttgcttaattctctttgctct
tccgaccatttcttcacacttttcaaagtatttcccgaagtaataatactaactggtaacttcttgggaagcacagcttgtgcagattgtaattcgtat
tttgagaatgttggtcctgtgattgactcttcaagcttagcttttatccatcgatcagacgtccaagaataaagtccataaacacgatcttttctattt
cgtcccataaaaatagttccaagcaaacgatttatacccaaaggtgaaagccatcctcgaagaaacaagaaaaaacgactgacgaagctacca
gatcgctccaaagaatggactgatccggagtctatcagcaacatgcctttcagttctccgagatggcgagcagcaaaaatattagaataggtacc
tccaataccatgaccaactaaaatgtaagggccatgcacgtccgcttgcgcaagggcttccgtaagcagatcttcaaccatgccgatagaggag
ggtgatccaatgttatcagaccaacccataccaggacgatcccaaacgcagacacgggtgtcaggaggaataaaatcaagatcagcttgatcct
tgacgttgtcttgattccaatagtttttggttggaatgtctccagtatgatggagatttaggatccattccttaaaagggtgcacagacacttcacct
ccttcaattaggacagtggagcggttgggagaacttgtgcttccaaaacattccaagtggattctagctttactatcggttaccgtgtaaagtttcc
cgggcgcggtaagattaccgtcgtaagcacgaagagctaaggtcaatacggtatggatggtcagaatagtaaggataatggaaaagatcatag
aaattgttaatttgctaaatcttctccaaggccaagtagtaggggacaaagtccaacgttcactggaaccaacgacatattctggatgttcttcttt
gtactgacgaagaagcatcctgtcagttacgataatccagatactcattaagaaagcccaaaaaacgaaaaagattcctctccaaccttctctat
gtcgaagaatagggctgatgacaacaagtagtacatcaatggctaagaggaaagcgttaatgcgttgagtaatcctttcaggtgcattaggaga
atatacaaaggtaagttgcatcgccaggaaaagtgctgacaaggcggtaaatccaattggtgtaaagccggtggacaaatgactcaaaatcgg
aatggatatgaagacatttatgagtagtagcaaatgaacaattaacgtaaaaattagtaggcttaaagaggtacttcgaatccagtaaaagagt
ggtgttaaagcatataggtttgtctcttctggaatgactccctgatgagagcctaaaaatctagggagtaaggtagaccgttcattcatatcaccgg
cactggccggtctgggtgcgggagcattgggattgacatccatgttttctgatacagtattctttatattttccgcgaatgtttagtgaaataagggg
ttcgttgctataataagtcctctactgtatccggtaaaagaaattattggcaaactaaacaatatatttaaaaatcgtttttcttttcgttgattttaca
gaatagtgtggttcttcgtgtgcaagaagcgaaatgaatgtgaattcaacgattattatccaaaaaaaataaagctccggtcaagtaggttcttta
atatttgtaaaacaaatatataccgtgtttatacgtaaacgatgtaggaatacctactgcgtcgactcaacgtaatggattgatatgagcttgattt
tggccatcggcagattctaaacaaggcaaatgaagtataagataatgagtcagaaagagagaaaaaaaagccttcttttagctgggaaaacgc
acagcttgtgacgttcgatattagattcacatgaagtctctttctgacaacttccgcttgtcaagaaaatgtttgtgtaccaaaatagaacttctaac
tgaaagagacagcttgtattcccaaggatcttctagaactcctcacactttttcaaaatgaaaaaccatcaaaaaccataagagctcaatttgttt
accatggcgtcatcaataagtaacgcagcaaagtgttttcatagaggcaaatggcactgtctaagaaggcacgccaccacagaatgcaaagtgg

gggaacaaattctatagctagccaggaagggaaaggtaaaagaagagaaaggaagcaaacattcgcggtagctcattttgagaagataaaca
aattgagagtaaaggaaggattgtacggttgaaaacgtgtcatggataccgaaaaaggaagccaagatgaatacgggttattgtgatttggaaa
agttcgtaacagtattcaaatgagccgtaatttcttcaccttttactcgaattttcttattaaactgcctttcattattcttcatttatttccacattgta
acgaaaaaatggctcgttttctatattttaggtggatctacccttactgctaattaatcaacgtttgtttactttttgcgttccattacagtcatataa
taaaataacgaatgtaaacaattagaattatcaagtataacgcacacattaaatttcgttaaaagcttcaagaaagcattgcacggctttctaccc
gcatcaacacaagcgcgtttccatccgaaatacaatcaaaagttcaatttttcaatcattttccttttcgtacctggaattgtttattgtatacaatttt
ttgagctactgttttgtttgagcacggaaagtcattacttggctgtgttataatacagtttaaccacgttaccttactggtctaagttcctgataaatt
cgcttgaaaagcttgtcttatcttaaaaatgagctctgatggatttgattatacgaatctgttaccaattgcaaatgtggcgcgtataatgaaaagc
gctcttcctgaaaacgcaaaaatatcaaaggtatcaattgttctgcaaatgtgcttccaaatgtcaccacaaaggaatagataacatatgtccatt
tccaataatctttgatggctttcataagcccaaaattagggtcttggggtgttacatccttatatatttctatctactttgctttgacttgggcctgaag
ttaacaaatcataggaagccaaggattgcgttcaagattgtgtcagtgaatttatctcgtttattacgagtgaaggtatgctgtgccttattactttc
atcgttttgttaactcacgtatagcttctgaacaatgcacacaggaaaaacgtaaaacaatcaccggagaagatgttttgcttgccatgaataccc
ttgggtttgagaattacgctgaagtattgaaaatttctctagcaaagtaccgtgaggtatggattctatcaaaagagcttgcatagtcaagtgata
acactctttaaacaaatcactttaagcttaaactaacctaatatagcaacaagccaggtcggccagcatgaaagaatccaaacaaactcgcattg
aagaagattaataattgtaaaggaagttcatgatcttgcttaaaacttatgcatatgaaaagtccttaatgcgcttttcttttcttttctttttttgtttg
ttttttctcttgttccttttaagtattacatcctccaccaattgtgtaggtatctttgctaattctgcctcaatacccttgctgccctgagagtatttttat
gtttacgtaaaagcgtttggagtccatttttttctttcacaaagtaagttaccggaaaggctgaaaggctcaaaggctcaaagataaattgcaatcg
tagtctaattgactatcattccagttgctcctctgtatattaatagcgaccagcaatcacagctttttttttggtctattgttgtcatcgctaagtatca
catacacagttagctaatttttaaatgaagtgaattgataagcatcttattttttcttgtacgtgtatactactcaaatcttttctaactgccatctat
gaataagtatttaataaaatcgtttatattaagtatgtatcttattcaattgatcgaacatttgattttagtatttttatacatttaataccatgtcata
aaagaattccccttttattttcagaatccaccttccttccaccacggatgtttgagtatgcaaaccgctttgcaatctttcacataaaaggatttaaag
gattctacctacaaccattatataaaagaaaacttagcagttttaatctggatccctcactatctcagcttcataaaaaattttcgagccactaaaac
ttaccgtttaccactacatcaacgtacccttatgaccatcccaaccgagtcgattgagtggcctgctaataaggttaggagcactttcttaaaatttt
ttgaagagcgaggacataccgtcgttcctagctcctctgtagtcccttacgatgatcctacgcttctctttgccaatgctggtatgaaccagtttaag
cctatttttttgggaactgtagacccttctcacgaatattccaagcttaaacgtgctgttgactctcaaaagtgtattcgtgctggtggtaagcataat
gatcttgaagatgtcggcagagacaattatcatcacaccatgtttgaaatgttgggcaattggtcatttggtgattatttcaagaaggaggctattc
agatggcctgggtattgcttaccgaagtgtatggtctagaaagggatcagctttatgtcacctactttggtggtcatgaagaatctggcttagaacc
tgatttggaagctcgtcaactttggcttgacattggtatccacgagtctcgtgttattcctggagaccttaaagataatttctgggaaatgggtgatc
aaggcccttgcggtccctgttcagaaattcactatgatcgtattggcaatcgctctgtacctgaactcgtcaacatggatgatcctaatgtcctgga
aatttggaacattgtgttcatacaatacaaccgtgaaaaagatggctctcttcgccccctccctaatcgccatgttgacaccggtatgggttttgag
cgtctagtgtctgtcattcaaaataagacatctaattacgatactgatgtgttcactccgctctttaaaaaaaatacaagagcttaccggtgctcgtcc
atataccggaaaaatgggtgacgaagatgtggatggtatcgatactgcttatcgtgtagttgctgatcatgttcgtaccttgacttttgctattagtg
atggcggtgtccctaataacgaaggacgcggttacgttttgcgtcgtatcctgagacgtggtgctcgctatgtccgtaaaaagtttggtgttcctatc
ggaagctttttctcccagctctctcttactgtcgtcgaacagatgggtgaattctttccagagctttacagaaaggtcaacgatgtacgtgaattatt
agacgacgaagaagtgtcctttttctaaaactttggatcgcggtgaaaagatgtttgaacaatatgcagctgctgccaaaaagtcttccagcaatat
attgcaaggaaatgatgtttggcgtctatacgagacatatggttttccagtcgatcttactcacttaatggctgaagaagcaggtattaagattgac
gaacctggttttgaagctgctcaaactcattcgaaagaaatgagtaagcgtgctaccaagagtggctcttctggtggcgaactgttagttttggac
gttcacgccttgggtgatcttgcaaaaatggacgaagtacccaagactgatgatatttataagcacgatcatgctactttagaatctgccgttaaa
gctatctaccataaggatggcttccaaactgttactaaagattttgaatctggtgagcaatttggaatcttgcttgatcgtaccaacttttacgctga
acaaggtggtcaagaatacgacactggacatatcgtcattgatggcgttgctgatttccgtgtaactaacgttcaagtttatgctggttatgttcttc
atacaggtttttttagaatacggcaatctgaacatcggtgataaggttacctgtgaatatgatgagattcgtcgttggcacttaatgaacaatcatac
tgtcacccatgttctgaaccttgctcttcgtaatgttcttggggagggtattgagcaacgtggttctttggtttctcaagaaaagatgagatttgattt
ttcttacaaatcttcccttccaattgataagcttttggaagtcgaaaatttctgcaacgatcaaattagaaaggatttaccagtttattctaagaatg
tccccttgaaggatacaaaggaaattaatggtctacgtgccgtttttggagaagtctatcccgacccagttagacttgtttgtatcggtgtcgacatt
gataatcttttacagaatcctaagaatcctgaatggtataattacagtattgaattctgtggtggtactcatgttgctaaatctggtgaaattaaaga
ctttgtcattttggaggaaagcggtattgctaaaggaattcgtcgtatcgtatccgttacatccaccgaagctaatcgtgtttctcgtgttgctaatg
aatttgacgcccgcattaatgaattagaaaagatgccttttggtgctgctaaagaagccgaattgaaaaaggttggcgtagatctttctaagctta
cgatatctgccgtccgtaaacataacatgagagaacgtgttgccaaaatcacaaagcagttccaagaccaggtaaaggctgctaatgctgctga

gcaaaaagaagctgtaaatatggtagttgaatatttcaaggagaaccctgaagctcatttcgttgtcgctaaggtccctatctctgccaaccctaa
agccttgagctttgctctcgcccatacgaagaagaaccttaaaggcaagagtgtctaccttgtagcttctgacaatacaaaggttgctcatgcttgc
cttgtgtcaaatgaggctttgcaaaagatcactcctcaagaatggtctcaaaaggtttgtttacaaattggtggacgtagtggtggtaaaggtgat
acttgtcaaggtgttggtgagaaggctgagaatgttgatgctgccattgaagaagctatcgaattcttccagggtaaattgactttgtagttttata
atagtatggatttgtacattaattgtgcatgttaatagagatattgttttgaatgtaaaggatttacattttagttcgcattttaattgatgagtttgat
ttcagaaagttgtgggagtgtatgttgttatatagaacgattcacacaccaatctaatattaatgttatttattctcagtgaccttagtctttttcagtt
attcgaaaagtatatataaaataacgaattctgagttatgtctttggtactatatggcagctcagatacagaaagtagtgaggaatcagactttta
cgtgaaacaggttccaaaactccctgactttattaaagatctttatagaagtaagaaaaatcaactggaaattatttactaaacaaacgttgatta
gaaaagcgtaaaactttcgatagtcccaatttccacgaaggaagaaagaggacgaaggctcatgtcgaaggtttatgggcagttcaaatttactt
agatggtatgaatgctatacatcccaagcaaagcttttcgctaaccgtaaagttgattttgataattcctctaaacaaaagattcaggaaattata
gcttcgcaatcagagttcagaagtttgtttacttcggaatttggtgttgtgaggagtttgcacttgagtttgtcagaactatttagaattgcatctgat
gaaagaaaagctgttttaacaatttggagacgggagtgtcagcacttacaacacaggtaagtgatgaaattgacttaatatgaattgtaacttac
tcattgttttaaagagcaatcagatacaagcttcaggacccatgtcttctttttaatgatgatagaactcgaatgtttttgtactgaaagcaacattt
gaaagtgctgatcttttgaaaaaattgctagaaatttcaaataacttaatggaaaaatacgaagggaaagaattacgaaaggttcgttttatttta
ccataaaagttcgctcatagcaacaagtctaacctgaattcttacaggactttctaccccatgtttcgtttgcttgggcacatgccaaacaaggtga
ttttgaagcttgggagagttgcgacagggaaaatctacgatttatagaactagaaacactgttgaaacaattcgaatcggcgatgctaaaatccg
tctcattagaaaataacagcatgaaaatggtaatgggacataaaattcactctgtcactctgcaatagatttctacttgagactcgcgaagaatga
ctccttgttcaaatcaattcaaaaagcatccctgaaagttttgatctaggataagaaacttaataattacaatacatacttggaatgcctacgtaca
gaatttgtaaccaaactatgacctgtgtccatccaagtatggactactatgaatatgttctgctgaacattacattacctactagaatgcttcggtaa
aaattgcaataaaaatacaaaaataccttactattctcgtaacatatataaaaatagtttctcagcttttataaacacaagttttgattttgtgttttt
tttttgagttcgttacacttcgttactataaacaacaaactatagcgtacgtttctaaagttgaaccctacaatttgagaataaggtacacagctttt
gaaccgtcctttcaccagccaggagttagaaagttgttcaatacaccgagtccaagaatttacggttcagaaaaaggggttcttcgatagaaaac
aagaaaaaaaaagactgcaatcatgaatcagagtgaatgggctcctgtagtctcagtttcgcgggtttgtaagtggcagaagtcaaatgaaagaa
gtatgttaacaaaaaacagacgctcatgtaaatgaggagtttccccgtgagtattgggactatgaaaacatggaagaaacttttgggtatcagga
caattacgaaattttacggaaagtgggacgaggaaaatacagtgaggttttcgaaggaataaatgttttgaacaactctaaatgtgttataaagg
tgctgaagcctgttaaatataagaagatcaagcgtgaaatcaaaattttacaaaacttaacaggaggccctaatatcatagctttgcttgatgttg
ttcgtgatccagaggtaagctacaataaacacggtgacctaatattctgtatcagacgggttgtctgtcatggtgctgtacagcaccatgtttcacc
gttcgtttttaatacaggaaaagcagactaacgaatttttagagtaaaacaccttccttaatttttgaatatgtagataacattgattttcggacact
gtaccctacattatctgactatgatattcgatactattcatatgaattgctgaaggcattggatttctgccattcccgaggtattatgcatcgtgatgt
aaaacctcataacgttatgattgatcacaaaaatggaacactccgcctaatcgattggggtttggctgaattttatcatgcgggaatggagtataa
tgttcgtgtcgctagtcgatatttcaaagggccagaacttctggttgattttcgtgaatatggtatgtataaaagaattatgatgaaaggccttgtca
tgtatttttccgcgatatactaaccgtatagattactcgttggacatttggtcctttggtgttatgtttgctgcattgattttcaagaaagataccttctt
ccgtggtcgcgataattatgaccagttggttaagattgcgaaagtcttgggaacggatgaccttttttgcttacattcaaaaataccaaatcgtttta
gacagacaatatgacaacatccttgggcattaccccaagcgtgactggtactctttcgtgaatcgagaaaaccgacaattggcgaatgacgatgc
cattgattttctttcgagacttcttcgttacgatcatcaggagcggttaacctgtaaagaagcaatggcgcaccccttattttaatattttgaagcagtt
gtaaatttatttactgaattataatatccctaattgcatttataatggatatttttttacacaccaaaatcatggaaatatcgactcattgcttggaaaa
cgccaatttttcagctcagggcacgttcgatagtcgttggtttatactctagttgttttcagcatatgtagctgagtttctgagattctttttgatacag
gtggctcaaaaagttgtaattaccacttaatggcagccctcatttcctcccttcacttttgttcccgaatggaaaggtatgatacttccttttcaaatt
ataggcatttatcataataaaaattccttccataagctgcttgatttcttttttttttattttttttatttgcaatttcactttctatggatatcgcgattcca
atatgtttaaacatagacaaatgggagaactaaatttgtaataaaatcaacagagaagagaattcattaacagaataagatattttatgtataga
acagctgaaatatagaagttgcttttgttagtacgaatagacaaatcgacaatgccttgatatttgtctcttttttttttttttttttttattttattaaact
tttctgatttacaatcttgttaattagttgaatttattcaatcctttgtttgaaactatttgctgctcatccttggcagattttatttgtttttctttctactt
gtgtgttattctaaagaatccaccaccaagttcaatcagttttgtaagggaagacaagaaacggggaatttaagaaatcaaaagaaattgattaa
aggagaaaagtttgaagagtataaaggaactcaagctgttcaattggagttaatttttaacaaaagcgtgacaagtaagaaagaatatcttggg
aagtttgatatctttgcatattttcctagctggcagaacatttataagaaaagagatgctacgaaaagaaccaacggccattcaaattactgcaga
agatgtctttgagtatgatgatgaaaagctgaaagaaaaccacgatttcagctcaccgtctcatgatgtaccacatcgacccaaagatacgtata
cgtccatgcaagagaagaaaaatttatcaaaagagcgaagaataggaattgcacctggacaagacaattatgcactcccctaaacagaaggta
acgaatctgtgtacaactgaatgaatatgtcagtagttgtaagttgtatacaggagatgaaccatacacatattttattaatgctatgctaaggatt

gactgatagcgtgtcatcgggctaacaatctaaattgaaattaagatgctatgaatgaagctttcaaaaaaacgggttatccaaattatatatgta
gagaagtgacaaaataaaaaaaaagaaaaagaaaagcacttggaaaatcaacgaaaaggcgtaaaagtagaatagcaaatgttttattagat
caagtgcgtaatataccctatacatctcaaaataaggacgaatctaaattattaagagaaaataataggtgcgggacgaaattatagagaagag
aatgaggggatgaaagaagggtttaagcagacttgtcagaaatcttggctttctgtttagccacaagagcatcatgctcatcaatgtcaatagctt
cccaaggatacatgatccagtagtcgggggtgttgcagccggcaaagtagtagttgtcgagaagttctttatcgagctctgcattcttgggtttgac
cttgttgtggacgacaaaaataccaaacctagtcttctcatgttcacggttcagtaactttgcttgtttcgcaacatcgcgctcaagctcacggaca
ccgtagtggagagtagtacgggtatcgtcgacctcgtcaacaatcaaaatattttttccgaccaagctgaccatacccaaagtgctgaaatccaa
ccattgagtacgcttgacctcgacaccagggacttcttcaggagaatcggtggttagttcctcatacaagctaagaccaatggcctgaataggaat
gtttttgcttcccttctttttcaagaaagtacggagaatgcgagcaggaataaaaccaccaccgccaatggcaataattacatctgggcgaaaaa
cctgtagtatgttattggcttgttcagcacacattttatgaatatcgttgtagctgtagtaaagacgaactggctccatgtttgtagatagatcaaaa
acaaaaaaaatgcaaattacgaaagaaaaggaaaacaaaggataagatggaaatacaattcaggtcctaatggaggatttaggagaacaatg
aattaaattagatgaagtcaaaacaacaaaggaaaaaatacaaatcaaggttcttcgggacagattgttttataaacgtcgtttgcgctctgtca
aaatccctatttatactttttttcaaggaaacctatctgattatcacaatccgaaaattgggaatttacagtcgaacatttagcttagaagtcacgaa
ccctaccctaatatcagttaatggatgcttaagaaattcctagatgtaggtagtagggtcatgaaggtgatgggtagatcaaggggaaccaaaag
actgaactgaaactggagtggtttctacaagagtctaacgttacatcctcgtggccataactcaatgatgatcctggagccacccatggtcttgtcg
gttctacaccacacagtcccttacatcagaatcgacaggtctttggagtccatgcttcagccagttttatggttaggaacgcaacaatgtgttcaac
cagagtagtagatgtggtgattgtatctgaacgcacccattggttatttaaagataaacaaggaagaacttcataggaatgaaatagtagacgta
aaaattagaaaggaatcaagttggagaatgaaataaaaaaacggttgaagaaacgaatccttagaaaacgagtacgctctctcaactatggaa
caattgtaatagaacgtttggtttggcttaatttgattttccttttttttttttttttttttttcaattcattgtatagtactgttcctgtattgtttttgtta
aacatatctcaaaaatctaagcatactttttatatatgctttatagtatccttaaacttccgattaagcatatgcttagaatgatcctatcatgctgtt
attagagatgatctgatatcaatctctacaaaatcccaactaaacaccatttattgcaagacttttctttttctttctttctccctttatatttttttgttc
tcctaaaaaaaaacttaatttatcttttgtccgtctctttttggtgctcttctcaacgttggaccacctcatctcttcttgcttttgccatatctactgga
aataagttatataaagttataaactcaaaccaccaaatcgctaaccttgatctctctgagattaaaagctaaaactaccaatttttgtgaatactac
aatacaaaaaaaattaaccctcaacttttgattgccatcaaaaaaatagaatactggcttgttgaagttctctaaattgcaagcatcgcattgcta
cttttcggcgactttttccctttcatcactttacactaagcagtagaaagtttgcgtgagaggaattaattgcctttgacgtggcttaacttgtgttttt
ataaaatttttcggcttgttttgattttgttctgttcttatctttttctggtttttatttttcgtattggtttatttcattttgttgatttttcccttctttcttct
cgattcagtcttccatagtcttcatggatctttcacgtcaaacccgtattgtcttactattaagcattgacatcacctttttctttattgaaattattacc
ggctatgcgattgactcgctggctctcatagctgattccttccatatgcttaatgacattgttagcttgctcgttgctctttgggccaccaagcttgcc
cattctaccgactatgagtccaaatacacttatggctggcaaagagctgaaattttgggtgctttggccaatggcgtcttcttaattgccctttgcat
gttcattttcatggaagccatccaacgctttatcgaaccgccttccgttagcaatcctaaacttatgcttgtcgtcggctccatgggtcttctcagtaa
tatcattggtatgttccttttccatgaccatagtcatgctcatggccacgatcactcatctcatgctcgtaactacgaattcccagaagaaatcgatg
acatcgaatctgttttaccttccaccatcgtcaatcgctgtcattttcaagatgtccctcatgaccatcctgctgccgacatcgctgctacggaaaac
tccccccttgctaagctacattggcttaccctctggctccgcctctccccgaaaatcttctagcaatcatggtgctattcccacttcttcgtctcaaaaa
ggacaaaaatccaaacgtaatcttaacatgcatggcgttttccttcacgtattgggagatgcccttggcaatgttggcgtgatcagcgctgccttgg
tcattggatatacggattactcttggcgctatctttttgatccttgtgtaagtgttttgctcacttttattatccttctcagtgccattcccctttgcaagt
ctgccgccctgattttattgcaggttgctcctcagagcattcgcattgaagatgtctcacacctaattggtcaattaaacggcgttgaatcagttcat
gagcttcacatctggcaactgtcggatgtaaagttgattgctactgttcacgtttgtgttactcttcctgatgatcagggcgagtcttatatgaagttg
accaaggacatcaataaggttcttcaatcctttggtatccatgaatgtactattcaaccagaatttgctaatcacccgtccctttgccctactgctaa
ctgaaatatgtgccattcctgaacgcacattcaatgaccacataacaacaataagggatacttttacatcctttcgcttcctttttacgatcttatgc
ctattatcaatttaataattatggcctaaagtaataaaatcacgtttgatttggaccgcttacttatgcttccgtcagactaattgaaatttttctatct
tgtttagattttatatttatcgatttgtactattcattcactaaaactttttaaaaatctaagattattgaaaaattcatcaaattgtaactttgtatatg
tgaaatattttgagtaaaaattgtcttttaattggcttcatcagttttattgttattctttttttgagtcaattatattcttattcattctctattaatttattc
ttttgatctttatctattaagtctgtatttttttgtactttaattctaaagtcaacttcaaaattgaattattgctgcttttacaagacttggtttttttcttaa
ctcttgcttccattaaccaccagacatactttttttctagagtcaacgtcttggatgttctaacctttcctcagctaccctttacatatttttcacctccaa
cgtagacgggatcaattacccaaatattaatgatttaatagttggatacatggaacaagcaatctcggtttctagaaatggtatgttattttccttct
ctgaacctgtcactaaatttttcttgcagactttagatcgtcaataaacgagaactaccacagtgggactgtgcccatgaatgaaaactatcagaa
tggaagtgagaatgcccagatagaatccgttgcttttattcgctattttggtgaaaaactctttcattatccttccctctacaccgcatttgtggagat
tgtaaaagctttggatgtgcaaatgtatgaaaatcggtttttctaagattttcagctaactttcctttacagtattgactttcctgggtttattgaacga

gtgcacattcttttgaatgacttcccagagttgctgcttgagcttaatactctccttccttcttcctaccagtttttaccatcctccgcagaaaataagg
ggccgcagtttgctactcccactgggtcagtatcgcggccttactcttacgctgctagctatgctgacgttcccagttgctaccaccgagctattgct
tatattgcaaaggtaaaaaagtcattgaaaaatagcggtacatcttttcagcaatttcagctacttcttcaacaattttcgagctctcagataagttt
ggaagagcttcaaagtagattaacctctttactcagcgattttcctgcattaagtcaacagtttcaacagtttctaccaaattcggtttttttacagctc
aacacctcctttaggatcatatcctatacgaacggttcagagctccaactttcgactcagtaacctttcggatttctatcctccaccgtcctccgctca
tggtactgtctctacagtttttccgtccgtttctcagcctgatgctattgatttttttcaaacatgttaaacaccagttaccaagtctcgatatatatcat
gaatttttaaaattaattaatctttatgtgcaaaagattatttctcgggatacattgttagccagaagctttgcgtttttaaggtcccttcctgacttat
ggaaaagctttcaattgatgttgcattttaatcctcaagaattccctaatatttattatactgccacatctgatcactctgattacggaccaagttatc
gtcttcttcctttagaggaacgtatgattccgtgtagtggaagagacgagttagcctggtctattctaaacgatgaatgggtcagtcatcctacttg
ggcatcagaggaaagcggttttattgtccaacgtaaaacaccctatgaagatgccatgacaaagctggaagaagaacgttacgaatttgatcga
cacatcgaagcaattaactggactatcaaagcacttaaaaatctggcagatcgtctgggagagatgccagatgatgaacggaatagctatcaac
taccgcctggtttaggtttgcagtcgaaaagtatttacgagaaaacaatcaagttagtctacactgctgatcattataaggttgtattaaatgctct
agaaagtatgccttcagctactcttcctatagttctaaaacgattgatcgagaaaaaagaagaatggcaatcagcgaaagaaagtttgcaaccta
tctggagaaatacagagtttaaaaattatgataaaagcttggatccacaatgcatatactttaaggctaaggatagaaaagttgttactccaaag
cttttacttgccgaagcagagaccatacgcgctcagacgtctacaaagtttccttttctgagtgaaacttcttttgatttttcaaccatatacgataat
gaacacattctttttgacgtgtgttacatagtgtgtacatacgtggtatgtaattccccttccggtctcaagaaggtcgaaaactttttttaaaagtac
gctacctcttttttttggaattgataaagaaaaaattagccaagtttttagatcctgtttttcatggtcctaattatgattcttccgttgataatacgtctg
gaagcaaaatagtacgccgaaaacgatcaaattccattactcaattgactgagctttcaaaacatcaaaaaataagtgaccaacgacaaagcc
gatcggcggctgccgctaagaagaagggggatgtcactagtaacgctctgaattatgacgacgctccttctgacaatggcagttcagcttcatctt
caaaacagcaattatctcgtcctgctgcagctattgtggcaagcttgaaatatcctagtcatcctgaccattgcttgttggaaggaagaaatagcg
agaaagctgtcccggataaccaaacggcgcaagaagagaagtttccatttatgaaatccgacattgttaacgaaattgagcaatacggctattct
gcaatatatgtctttttccgtctattcaacatgttatacgaacggttactattattgaaaaatctggaaaactgcgtggctgatgctcagaaacaaa
taaaaccaaatccagtggcccaagagcacaagttatggaggaatagagaaagtgagttacctgagctccccgaagaaagtacatattacgata
aaaccttcgtcatgtcgttacgacttatatacggtgtcctcgatcagaatcaatttgaagattacatacgtttttattatggcaataaagcctacgag
ctgtacacggttgataagctggtgtggtcaatagctaaacaggtacaccacatcgtttcagacggaaaatttaaacacgttttaagtgtcattgaa
gaatacaatagccagtccacaccaaaacgatcctatgacgagtttttatatcgcatgaatttggaacgatatctaaatccagatgaaatgttattt
cggttctcgtggaattttgacgtaaagcgattcaccattaacttgttgcgacgtgctaatgtaactattgatgagacgttagaaagccagcggcaa
ggatggaaaaagtatttggaagattatattgatcagaaagagactgattgtgtctcttacagacattatcggtgtcctttcttatgcagaacaattc
cttttgggggaacatccggaatggcccgattgtcattacaaaaaagacttatgtgttcgcttaatttccagtccgacttgaaagccaggctttgtatt
aattcttttaagctactatatgttccttgtacccaagacgcttactcgagccgtgtgtatctgaaattgaataactctgaatctcagaatatacaatt
aactcgctgttcgcgctggaaggcacggctaagcactctacagaacaagttgtttgatggtcaacctgatgatgtttcatttgaatcactatttatat
agaagtgctattaatgtatttagaaaattacttccctaaagctgtttaaattttttttgatagttgacatttaatgttttaaaaattaaattgttttaata
tatttctttcttatatcctctttattgttgcgcttatgggattagtcacatgttgactggaatgtttgtttacatttgcagttgccctagtaacaactttgt
ttacgttgtaacggcaccttcaggaatttgtcaagagacaaaatctcaaatggctagcgtcgcaataaaagagccaaaagctgctataactccaa
aaaagaagacaaagccttcccgtttatgttacactcctcctaccaacattgcaaatagcaagaaaaaaaatcctgtatacccgttatgattgtatag
gagagggaggatttgctagatgctttcgagttaaagatgaaagcggaaatatatatgcagctaaagttatagccaaagcatcattacaaaatga
aaaaactcgacaaaagctttttggagaaattaaagtgcatcagtctatgagtcacccaaacgttgtaggactcattgactgttttgaagataaaac
aaatgtctatcttgttttggaactttgtgaacataagtcattgatggagttgttacgaaagagaaaactgctgacggaaccagaagtacgatttttg
atgcttcaaatattaggagctttaaaatatatgcataaaaaaagagtaattcatcgggacttaaagctcggaaatatcatgttagacgaaagcaa
taatgtaaaaatcggtgatttcggtttagctgccttgcttatggacgacgaagaacgaaagatgacaatttgtggtactcctaactacattgcgcct
gaaattttattcaattcaaaagaagggcattcatttgaggtagacctttggagtgctggcgtggtcatgtacgctcttctagtgggaaagcctccttt
ccaggacaaggaagtaaagactatatataaaaagatcaaagcaaactcttacagcttccctcctaatatccctatttcatcggaagctaaaaatc
taatcgcttccctgttgacccaggatccatctattcgcccgagcgttgatgacatagcaaaccatgatttttttcattcaggctacatggcttcctcct
tacctgatgatattctacacattcaaccagtatggccttctacacaatctagatcaagctttcaaagaaatttggattatgtagcgtcggctgctggc
gtcggttttggtaaatcagctggaactgaaaaaaataaaccctatgcattaaagacagatgagggtgatgatgatcatatgctccccagcgttttg
agccctcgagatcgagttaacccagtcatgaaaattggaccagaaacaaaacccagttctactaagctatcttctatctttgaagcagctcgcaa
aacgagtgaggaaacacttccttcaagagtaaaagccctgaaagaagaatctcggtccatcccgaaatccaaacttacacgttctgatgctccag
ataacgaaacccgcacaggttcagtttcaattgccaatttgtcggataacattgtatggattaacataaaaaaaactgctcttaaaattggtatgg

ggcttgaatcaagaccagtggcctctagtcaaggaccaattgcagccaaaaatatcctgttcatcaccaagtgggttgactattccaacaagtatg
gtctgggatatcagttatcaaatgagtcggtgggtgtgcatttcaacgatcaaacctccctcgttttctccgccgatgaaaatatgatcgaatacat
tactcatcaaaagaactcagaggcgaagcattcgatattttctacttccaaggcgcctgatgctctaaaaaacaaaatttatcttttaaaacatttt
aaaagctatatggtgcaaaatttgtcaagggctgttcaagatgaaacacttgacgatatcgatatcaagaacgctggttctatgatttttatgcata
attatctcaggactcgacaagcaattatgtttcgcttaagtaatggaacttaccaggtaagaaatacgattacaaatggatgcttgaatgaaatct
gttgctaacatactttagttcaactttgttgaccacgtcaaagttgttgtttcgcctagtgtacaaggcattatggttctagacaaagagaaagaaa
aaatggtttttacattggaagaagcatgctggttcactgatgatctgcggtcacgcttgaaatatattcgtgaagtactagaatcttgggcttcgaa
attagaatccactcattaatcgcgtattactttacaaacctattattacctcttttatgtgctagttcacaatttgctaattttgattccattttattcagt
ctttcatattgtctcctttccatcttgttctttcagtgttacgttctttttaacattactttatgaaccttttgctgtcatttttatgttttcattacttgatg
aagcctcacctggcatcatacttgtttgttttgaatatgtacatcattcatttgcctgctcatataatcttggtaaccggtattttcagactttttttgat
gagtgactaatgatacagtttaagaaataatactttttttcgtattaattaaaaattaaaaccggctcttatagagaataaaacgagctttaaagc
tcctgcatttcgctataatagcttagctagatgactctaaacaacaattggtagtatgcttaaaaacgtcacggcaatcgtggcgctactgtattga
tttagcagcctaagaatgggtaaacaagtcacctacaagcctcgccctagcctatctacttgagcaattaagtctacaaacctgactgacaattta
gatgtatctatctatcttggtgtctaccttgagaaccaactgattcaacaagaaaaatcttcccacgaaattttcttgaatacatacctatctagttg
ggatttatacgaaacatgggtctaacctaaccaatcaattgtatgaaattgaactccttttggaaaattttcataccgtggattcaccgtagtcgtc
gtagaggtagattcttagagtaatagtatcattcaatattatatcttaaaattaattattttgatgacatcttcccgcaatgattgattcattctctgat
actaagatggtttacgcttggaaacatataatcacaataaaattttagatcactatgcggtgtttagcgaagcgtactttttgctcttagtaacgtta
ctgattcgttatgctacgcaattaagtcataattaaaagatgctttaggcatcaaaacgatccgaaagcacatatcaaacagtgtaatatgctcaa
aaatcgagttttgacgagacctttgatcaaaggtacggctataattctttagcttacagacaagaacaaatgttaacagtgattaggttctcaattg
acaaacgtgccgaaatgtgcgtccttcatacagccgatgatacctattagagcgtatggacatcttccatttaggcccataagtaggtctgctgtat
cattttcggattccaggctgaatcgtgcgtattttggtatgaagtatgggttcccaatgagatattcatcaaatgcaccaaaagaaagctctgaaa
aggatgtcgcagaccctagcacgtttgaacaacagaagcccacagctccagtaaagaaggcttctatgtggcagcggctgaagggggtgtttt
gcatttttgggatggaactaagcttcttggagtggaattcaaaatttcctctaaacttgtatacaaaatggcagtaggttatgagttgacaagacgg
gagagtcgccaattgactagaacacttaaggatattggtcgtcttgttcccttttcaatgtttgttctcgtaccttttgctgaattgcttttgcctgtcgc
tgtgaagctgttcccaaatttgcttcctagcacttttgaggatcccaaagataagcaagcaaagaaagagaaacttcgtaaagtacgctcggacg
tttccgaagtgcttcggggtactatcaaatctggaaagtttaccttctctgatgaaacacgcaaatcaaaagaattccgtaatttctttttaaaagtt
cgcactagtggacaatctccctcccgtgaggaattaattaatgtctgtcaatatttcaaggatgacattaccctagataacttgtctcgtgcccaatt
aattgccctttgccgatacatgaatttgaatgcttttggtaccgaccccttgcttcgctataacatccgttcaagaatgcgtcaaattcgccgggatg
atagagccatctatatagagggcattaattccttatccgttcctgaattgttcaatgcttgcaactctcgtggtattcgttctcaaggtctttctcccgc
taagcttagggatgaattgagcgtatggttagatatgcgtattaagcatggaattccgtctgttattttgatgctttctaacgctttctcttatggttac
catgagggtacttatgaatctcgctgggatgctttacaagacacgcttgcatcaatccctgacgaactttaccatgagactgtggttgatatgccta
gtgaagaagtctctaataagcaacgtcttgaaattttgagagagcaagaagaactcattgaggaagaagctgaaaccctgatatcgcgaaca
aggaaaaagatagtgccaaagctgaacccactaattctgctcaacccaaagacaacaagaaagtataatgtttttggtaacttcataagcataa
acttacattcaaccaattagaaaatatcaggggcatcagttggagagctcctggtctcccatctctcctttttcttatattatttaaagttatgtctttc
actgttctttttgattacttactagttgttaatgcactttcatggttacctgctttagcgatttttgatttctatgtttttaaaaaaaaaagttggatga
ataggattttcgaatgttcaaaacgtattgaattttgctattaatagcagataggaagtgtaggagtgagaaaaatggctagaaatttggacattg
acgaacatttagggtataaagtatttttatattcgtaagtaaaaggaaatatataaactaaaacgaaaagagtaatttaaggactagtggtttggt
gaagaagttcgttggctgctaccaaaacttctttcggaagacctgtgttttgttagtttcataaccttcagtgtcaaaacttaccggctacttcagct
acccttagtccgtgggatctcatgttaacaccctctcgaatcttataatcgaaagtgaatgtatcagtattctaccaagttagctttcgggtgaataa
ttaaaagatacagataacttacctcgttaacaattatatcagtgcagtagcacttaatgtttttatatgctttaaatttctctgctaattcatgagcat
gagtagcgaatagtgtgcgagccctattaacatcagacaaatatttcaagcacccataagctatcgctaaaccatctctagcagtagtcccgcgtc
caacttcgtccataatgataaaagatcggtttgttgcatttttaaaatgaaagcagtttccatcatctccaccataaaggtggactgttgatgatat
aaattatcggcggacccaatcctgctaaatatctgatccacaatacctatttttgcataactcgccggtacgaagctacccatctgcgctaaaatag
atataactgcattttgtctcaagaatgtgcttttaccggccatgttcggaccagttaaaagccagatattatcgccggaatttccaaccaaacagtc
attcggtgtaaactgaagtaatttattcttaagaccttttcaactataggatggcgaccttgaattattgtatttgatagagaatcatccaatattgg
tcgagaaaactcgcattctgcagctaaatcagccaacgacgtagatatatcaagctcatctaatatattagcagtttcacgtatttttttatgctctt
caagcaccgtatcaataattatttgaagtactctagcttcttcttgatgaataaaagatttaacactctcaagctccaaaccaagagaagaccatt
caggaagttgaatgctagcggttgctttggtagtatggaatactgtagcctttgggaaaacggacatgaattcctttagcaaaaggctattggatg

atttcatgtgaacatagtataacttcgagggatttttccgtaaagttgctttgctttcgaatttttatccaaagtattctgtagcttgtcgtatgaaag
tagtaggctttctatacgtttatgcaaattttgtaaagtttcattgaaagtcggtgtgataatccaaatatcagaaaaattttgcttgaacccgttttc
aggaacggacgttcgcttcttagcaccgctgacgatcgactccgattcagacaattctgttgactgagcagcaatgtcctcggcttctttttcttcct
ctgtacgttttcttaccaaagcactttcatttaaagcttctgaaatgagagatgctaaagcatcatgctgatataaagcatttaacaaaggtttcaa
agttttagaacttgtcttggtaagaagactttctatatttttataatggaaaagttattggaaagtcccaataaatcgtaagcatttccacgacgca
tcaaaagccgttgaagtattctgtgggtatcgttcgatcgtttaagtaaatgccttatttctgttcgaagttgatcacgatggataaacacttcgact
aaatcaagccttctattgatttcagaaatgtcagtagaaggagagcataatcgctgtgtcaacaaacgagatccagatttagttaccgacctcttt
attgcatgcaatagactaccggtatagcgattttcctgtaaggatgatcttatttcaagtcctttcatagcggaagcgtcaatcagcatggagtcac
gtaaagatacgtgaataggagcccgaagattagtatgtgaatttagtaaacgatcggaaacatacgacaacaagacagaaccagctctcttctc
tataatagagcaattctcgatgtatgtaggctcaatttctttctctaaataagcagaccattgctggaagctccaatcttgcatgggagtgtaagat
acaaaataaatacttcgatcaatactctccaaaatagggtgagagccaacgccttgtaggcgattatccaaaacaatttccttcggttgaatacgt
gctaaatccccgaccaaacgttgatgatcactagtttgggaaaaaaattcaccggttgataaatccaaccacgataatccgatacgaagactag
aagacttttcgtttaatccttcagaaacgttatcattttgattagcattatttacagaatcttgcgaaataatagacatcacataattactctggaaa
tggtccataaagttctcatcaatgagagtacctggggttattacacgtgtaacaaagcgagtgtacatatttttgaagagaaatcatcagccgga
cgaagagcttcttcacttagagcaacacatctctttaggtcttctaccagaattttcagatacctgtcaagcttaaagaaaggaaatccagccata
gaaacttcataatgggaagtcttctttttgaaactcgtaaattcaatagaggacctaattcctctgcttggtcaaagtacaactcataaaagttac
caactctcgttagcagaatgcaatcggcgaatttttttgttgaaggttaacttcctttaataaaggaggcagaagagtttttctttgggttgaatc
aattccaatggaggccccaaagcaccttgaagcaaatgcacatctttcacttttggtgacttatttgcaacatgacaacaagtaaatcgccgacaa
ttttgaagaaaattcagagagcaataccgaatccatttacttctccatattagcatgctttcaggggattccagtactaacctgggttatagtagttc
agtaagagtcttttgtttcataagggagataaaagtaataaataactaagacaatagtatttaatgaaaattaaataaatcaataaaaagtttcat
aaataaatggatgaatgaaaaagaaaatatgcaaatttgtatttgtagtcgaaatgtttttcatatgtaaaatggtataactaagaccttggaaga
tgaggaaagtgaactctgtttatgagcgttttacaaaacacttcaaacatacgtaaagctagatgagagtgcctatggaattccttgatgacctctt
ttttttcttttttaccataggataaagtacatacggatatcatcaaaataacttcattaacatacatgatatgtacaaaacttttaggagaatagaa
ggagtatttgaaacaaaagcaagggttttaaagcaaaactactcattttggtcatcgttgtcaactaactgggtccgtttcaattctaattccgttat
catacgattgacatcggccaggaaacgattgcgtaaaatgggattcgtagcttcatctgcgtcttttctacgtttctggatggaagattccaattcg
gaaataaattcgcgcaccagtcttgtctgtcgtttgctttcgcgagtttcatcatcggctactaattagttagttaaggcaagctctaggaaaaaaa
ataaacataccttcattatcttcttcctcttcatcctcttcatcctcctcatcgctgactgattcttcaccagcaacttggggttctgcagtagactctct
cgtgtcttgactaagttcaagcattccttgttcgatttggccagccaaatcatcttcgtccattccatcaaacatgtcaccaggtatccgttgatcttg
gtcacccacaccaacaccggttgtctgcatactgtcaaatgaaatatcttctgacaacgcaacactggagtccctggctaacatagaatggtccac
aagttcgcttgtggtgctaatcgctctttcatccatggcaagcagtcgatctacttcgttttcgacagcttctatggtgcggttggaaacacgtttacg
aaatctttttgacgaacccaatgcataggaggcgtaagaccatgaggataaacataatcagattgttttaaggtggttttcagtacatcgttttca
tgctctatgcgaccagtaactaatagcatttgacagatatcagcagctttaaaaataactttttatcaaatgatttgttcgattcaataacgcacgg
tagatcaactaatttagctccatacaaatgtccattgacatgaacaacagcacgtcgttggtctgaacgaattagcatactttttattcagctagtat
tacaactcacgaacctttaaatttaacccacacatcagctcccctgcctatctcgcggttttcgattgccttacgaacgtactcgcagtcttcacctg
gcatcatccgaagaatgatttgttcttcaatgtacggatcttcttctcgatcagatgcttctgaatcatatcctaggcctggttcccgcacacgtttaa
gacgaattttcgtcaaccgcggacctttgggctctcgcgcttttattttgattttaggaattgccgacggtactccacttgctggaatctgtggtttcg
gttcttctggcggcggtggaggaggacccgttcgaatctttaattttaccatttctataccccaattccaatatctcgtgaaatgcagttggtagcat
ttgtttgcctacagaaaagtttcgcaagctcgcaagctttcgttatcctaatcggttttgtcacctacagacgataccttacgtattttataattataa
ttaagctttagtaataaagttaaagagttccattacttgactctattatctagcgttacaagaatcaagagcatttgatataataagaaaataaaat
atttattagttcttgtatgtgtatctaagtggggaaaaaccgccgataaaaggactatttctactccaatatctgcacgagtatttacagaaaagcg
gatacattcaagtggtttagactccattcgtatgggagagtctaggaaaaatccaaggaaaaacaaaataaataacaagttttatagcatcctta
gagaaggtaagtgatataaaaaacgatcagattcattaggtttagtattaaaaagcagtcgtggctattaattaaactcgaaacggaatatgaaa
ttgagcattcattgtaaagcaaaaagtaaaacttcccgaaacattgcaaattattagcgtaacaagttagtattagcagaacattacggggagtg
ggagggttcaaaaggttaggggttcatttttcaatttttttataaaataaataaatgaaagaaaaaaaaaaataattgaaaaaagaaagaatggaa
agctagtcatttatattttcattattagaggagggttgacaattaataaaaaaacatcattagctgaaaggtaaaggtaagcaaataaccattttc
cttaggaaaaaccataaaatacttgagactcactccatgatgtagatgcctcaaggatgaatatcagttacatgaggatggtgacgagtagccgg
caattcaaacttctcagtccacttattatcttcgcgccatttttggaataagagtcaacagcttttatagcatctgcgacgtcttggggtgtaaactt
tttcgacatgttgtgaatgctctcttcaggacgtaaggtagcacgaccaatcattaaaagctcttcgtctgtaacaaatggaatgccaagttcctcc

aaagttactgggagatggcattgggacatgaaagcaacgagcttgttaaattcttcaagaggccagttttccaaaattaactgagtcaacgttcc
ataggctaccttttcgccgtgcattaagcgatgaatgtttccgtgagcagtggtaagaccattatggatagcatgagctgcggccaaatttccgttc
tcaaaacctaagccagaaagcaaggtattagcctcaactgtatgttcgaaagcttctgtacaaacaccacgtaaattagaaagaataccagcaa
ccgcatatttttcaatagtctccttgcaagcgcgagcaatagcagctccagcaatggtagggacaccaccagcaaaggatagggagttagaacg
catgatgctctcagtttccacataagttgaaagggcatcaccgataccactaatgagaaaacgtactggcgcttttacaattacatcagtatcgac
cataataaggtttgggttagtggggtagaagctatacttttgaaactcgccttcagatgtgtaaatgacggaaagagaagcagtagcagcatctg
aagaggccgtcgtcggagcgataatgcaagggattttgatttcgttagccaagtacttggcagtgtcaattgtcttaccgccgccaaccccgatga
cgatttgagcgtcggaaggacattcagcttgtaaacgatgaatttctttaagactggactcaccaccaaagataagctttttgactttcatgccgtt
agattccaaagagttgacgatggtgggagcacatatattccaaatattgggatcagcaaggacaaccgccgaatcagtagcccagtttttcacaa
aagtaaaggaacggccaaagatattgcgtccctgggcatatttctggggtgaagtaaaaaggcgatctgttgttctttccttttcccaacccatttc
cttgggtcgttccgattcaagtggagaatagaatcgcttgcccaaaccagtgctgactctgctttgcttataagccaaaagggagagctgacgag
gaagatgtgcaggaatccccttggttatagaatgacggtgcatcgaaagcatgctaaacaagaaaaaagcaaaccaggagagaagcaaattc
aaacaattcaataaagaaaagtaaatgaaacggataatatttgggagtatttgaataaactaaaaacccaggggagacgttagtgatgaaagg
acgaaacacaaatataagacactaaagtcgtttgtttaccatacaagcccaagcagagacacaaacaaaacaacggaaaaattagttgatgaa
tcaaaaatataatagggaaaaaagcaaaagtattatttaaaaacttttgctttatatactgaatactggactggatttgaagagcaagtagactta
ggaatccaaggaaaacaagaaaacccaacaattctgcacaggtaaacaaaccaatgggtaaatgttccaaaaaatgggataagtactctttgtt
tagatataaacaaaatggtattcaagattgggaatgctaggatagagaaaaaatggtcctcaagcgcaacaaatatagtggaagactgtgtgat
gacacaagaatccctcttttgaaaaggactaaagttcaacaaattaaaccagataggcccgaaataaaatcttaaacattcgtgaatcgtagact
cttataacgtcatgttcaaaagtcaataaaaagaggcggcgagaccagaaaataagctctaagtttccttgcggagctaaacagtgaaaaggct
gacataatttgcgggacccgcacactttaattctctgaaactcgaaaggatcccattcgttcatagcagcgattgtttacaaaaagtggagacaat
gctatggacttcataattatgcgctcatcttcatggaaagcaaatatagaattgaaaaaatctcggatgtatccgaaaaaaaaaaaattaaagttt
aaactcaacactcccccattggggaaggaaatgggccagatatttgagtcgcgtgtctcacacccgcacttattgttcgagtaatcctagcgcact
ttttaagtggaaaatcgccgttgccgtgtcgtcgtagtccaacgcactgtaagcatatcgcgcatgacctcgtgccgcctcgacgcggtcaagatg
atcaaacccgctaaaaccgaaacgagccgcgggagttccagaggcagtccccggaagagtggaggaagccattgaggatcgaggagtcatttg
acttatggggaaaacaggtgagcctgagacagctgttgtcgcttcatcgaaaacctccgaatgatctgtatctgcggattccttttgtatatccgac
attttcttttggagttcggtatcgacaggaggtgatacttttgtgcgttgttcttcatgaccatgaaagctgcgtcttgttgttggttcactagggatta
cgtcctcatcgaatcttgtcggtgttaattttgacaacgaattttgcaggcgaggaaggtcaaaagattcaatagcgtccttcgagttggcagagtc
gttctgtaaagcttcttgttccaaaggagcaatagaaggaggacgacccgtcattgttgaggataaacctgaagtctcatccagatgggagctag
agggtttcacccctgtttcttttcctggtgatacatccgtttcatgtttagcaagtcgtccagaagtctgtcgtgctggcgtgggcttggggagaaag
gtcgaggaggattccatttgttttccttcatctctctttagaatttcttcatccttctccaaatttgtgggctctttttgcgtagaagactttgtcttcgaa
atccctactgcgcgtagcttacaaaacttgatgtatttttgtgcctataacgttagtattagtctagcgtatgtttttcttatgctaaagcacaaaaga
taattacctgttcgtccaaaggtccccaaacctttaagatttcaaaaaagtctttagctgctaaatatgccaggacatccggcatactgagcttact
cttttcttccgcttgtagcaacacctccatggcaaatgagtttacataagcctttgcgctttcttcatcgttaacgttttcgttttcacccaactctttcc
gtaactaaatatactgatcagtttcccgcacttatcaaaccacccgtctgaccctctttcgtctacatacctcttctaaacgttcaactaaacgcaat
aaaaagtcccgacaagcatttgttaatccagtagagctcgataagcccacctgagttccccaatacaagcctgaaaaattagctctgtgctccctt
gttaaacatagctgtaaaatgatgttcactcatttgttatatgcatccagtcgagaatgactttttttatgcgtcatttctcttctgcacaaacagactt
acaccagtaagcaatgacaggattatacctgtttagctcattgaatctttgaataaagctttgaatcgaagataattctttaggtattgttgctaact
caattatactcattcttttagtcaatacgtgaacaatggcaaactatgcgtttttgcgtactttttaaatggacaattcgtgaataataatatcaactg
ttttaaaaatttgaataaatactcttttacacctgtaaatcaccgtgacgatctatttttgttgaattgcaattgaatgtagcgtgttctgtaatagta
gtaatctaccatctataaccactacaccataccccttcagttacagaagactgagcctttcttgaggttgaacggtttatatacttcattttatttattt
acatcactttgtagaagttacttcatacagaacgttggacttggcattcttctctgcttcctgaatttttacgtgtttttgtttcaattctccttgatatcg
tcaatcacttttaatactttctcttttcatgcattttctctagatttttgaagtagttagttgttgctttgttttaccgaaaattcatttgaactttccaatc
tgaaatgcatatcaaaactcctatttggcttctcttgtttgctttttcttcgctaattcttttgcggcatcttctttattcggcccaaacactgtagaatt
gggtacgagaacactttcaaaatttgtcaaccaacatggaccaacgtttgttgtttttatgctccttggtgtggatactgtaaaaaactaggccctg
tttttcaaaactttccaataaactacacaatcttataccttttgccgccgtaaactgtgatactgaaagcaataagcccgtctgcgcacgatatga
agtgaaaggatttccgagcatcaaattccttttcccttccccattccaaccatctgcaatcttcagtgcaaattacgaaggggatcgcagttatggg
tcaatgtacaaattcgtttctcaaaactacccggtcacgattaccccaatcaaaagtgatgttcaaatgagaaaatatttaaattcaagaaaaaac
gttacaaaggcgatgttggttactgagaagtcaaaacccacaattatgtacaaaaccctggcaaatcatttctccaagctatcgttcagtatctgtt

caccagaaaaaatctcattagatcttctcggtatagatgcaaaggaaatcaataagtttcctatgcttttgttgcagcatcctggtcaaaaatatcc
aacaatatatcgcggttcaatggagcgtgaagctatggtacgttttctcgaacagcagttacaggaatttagtttcagtgaatatcttgattccttct
gtactcgccaaaactgtcttttatatttttacgagaatgaacatgaaaaagaaacgtttccagctgaggaaattgccaaaaaatatcccaagata
cgtttgctgtctcttcctgcatcctcagaatcttccgaattgattcgcaaccatctagatctggggtatagtgatgctcttttaattaatcttttaaaat
cgcatttccttgcaaaaccttttggtactcacgtgttacgctggcttgatgatataaaagtcggacaagcgggcagtttaacctccttaccgaaaaa
cttcattcaaaacgtctcctagaagttagaaaaaaaaaaagagttaaatgtttacgcattcaagaggatgatggtatccctacatagaatgaactt
aaagatatattttattatcattacgacatataaaataagaaataaatagtaaaagattaaaaaaggattagtaattactccaatagtttataaaac
cgtgaatggaaacctttcaaagcatacagaccgcaccaattttaagtactcttaggggatattaatatctcctctttacttgtctgtaaacgtagatt
ttctgggagatcacagcgtagtcgcagaacttcgtgtatattcatatcgctgaagccagcctttcgcttgtcatgcattaccgctggtaaagcatca
gttctgtgttgttagaaacatttgggaaagcataacttacttgaccattaataaacttagcaattgctgcaacgttctaaatttattgttgactacatc
aataccccagcttcgtaatgctgaataaaaaaagttcaaatcactaaaatacgttagtattatggaaagcatgtagaatcttggaaaaacttcaa
catactcttgtagaatcagggcaccagttggagtgacttttgagcgaaatgcgtatccaattaatacagcggcaatattaattgcagctttctttttc
agagcaaacaacgagggatcattaggaccaagttcttggatagacaacaacaactgatcaaggtatgtttgaaccaacttcgtcatagactgtc
gttgttttggtgatgtatcaatttgctcctctgacatagtgtatgatatgtgtttgtatggagatataagaaaatctaaatgggcactcaaaaccttt
acagaagcatagaacaaattgttgacgcgatattcaagatttgtaatagctaagctcaaagtattaatcgtttctttcctcgaagacgcagtaaaa
ccaatggagggtataataatagaagtaatatatgctgaataaaagcccattatctttttgcattcgcggatatcgattaaaaagctcaaattaaag
tcagaacgtttgtcatattgctggatatagttgttgatggacgataaagcttcatttatgtatcggtttctgactaactcgtccagcagcttcaccag
ggaaaaaagtatcgtagttagatcttgctgggacatcaaaagggatgctcgatttaaggcctcagcatgccaaccaataaaagaatatatattttt
tgaatccagatgaccttcgtcttcaggtattgacagtgtactttcggtcacggcggcacctgagttgattaaattctcattcacacctgcaattctta
gtagataaccttctgtttttgtattaaaacgagtctgtgattccgttgacgaaatttttgatggttgaatagggttcataagtgatcttaaaagaccg
ggcttggctcgttgtttgaaaggattcgcagctagaaacttatagacattcagccaaaacattgaagttaaagattgcctctctcggttgagatact
catttggctcgacatacggaactagtaaatcctccaaatattgatttaaagcaaggaacagggggggagttatcagaaacattaaacccgcgatg
agtcaaaattttttcaactcaactaaaagctctcttaaagaagaaactattgtttgtaaggatcttaaatataccaggtttgtcttagaacgggca
acttccataatctcttctatacgattttggatagactgtccaaaaattctctgaaaaagtgtttgtaaaacaagctctggttttggaaaaacacgttt
tatgacagcacagtctctgtcaacgacagagcatagttcgttaaaaagggaagaaaggctatttatggaagttggcacaggttggaaaggatttt
ggagggcgttccaaatactatcttcttccaatccttttctcgattcaagcttgtcggcagctataaaaaattcgtgttgattaacaaactcccgaatt
acagacgccccgccgttaaattcctgcaaaatagttccaaaagaagccattcctttccagttaggctttctatactcattattaaaaaactttagaa
aactggtttctaaaaactcggaaaacttttctatgcgatttaacgtttcagaagctccgggcaattcaatttcatttgcgaaacttgatagttgtcga
gttctttgagcgcataaaagcattttatcatgactgtttgttcgaaatagatccatgagagattgagagcttccagaacgaaattccaaataatact
gaatcaagtcggcagaagccagaatcctctttcgatgattttcagcgagttctaattcttttccaacatctacaagaccgcctctcatatcttctaca
ttgtcgtttaaatgccttgataggtccaacacctgggaaaacttgcctcgtaggtgattggatttcttttggtagtcatactgaatgtgtttggatgca
gagtcatattttttatcttctcttccaaatttgatcgtaatttcgtcaattcagagtttcctttttcaaataaggaaattgagttttctaaggagagca
ctgttcaagattgttaataccttcagcagcaatagcatagatcaatgagccattaatgaatactctaaactgattataacagatgattcattcgttt
aggcacgtaaagatggagttgacgcggcctttggctagtgatcaaccgggttaaagatgatatatataaaacatacgttggttaggagcgctttct
gaaggattgtaaagcggcttagagaattgctcaataaattcaatatcagacagttccgcctacaacatgatttgttagtcatatttgttgaaatgat
gcttcatacattgtcgccaagagattctagcaatttatcatcaaaatcattaatttcatcttttacagacatgtctactcaaaagacaatatagttgg
actagagagaactactaggaaagagctttgtgcgagcaagtggtgagaagtgtaagcgaagaataagctgtaggtcgaaaagcaccataacta
tgttggtatctatagtttaaatttttattctttttcaatcctttgtttcgtaaatttatattttgagaactctgtgcagttgtctcagatagaaaacgtta
cgttctctaacttgaaagcttttaatcaaagttggactatatcgtattcgatacatattcaaatgacgatagaattttcttatcctttcattctacatgt
tgatttttctactgttcctagttactttggatcttaagtaaaagggtgtgtaaagcttgacagattagctgaattatattcgcattgttgatctttttca
acatgttctggatataaagcaaaaaaagaaccagcaaatgagtcatgcatatatttcatgttcatgaaaaaaatggccaaaagttaaaatcaaa
acttcagttaatctgaatcatattcttcagaagaacccgtaaattctgcttcatcatcggaatcgcttaagacggacttggttgtgacatgatggcgt
tttgttttgcgtacaacctttggagggaccatggaaggtttttatcagaacgaatgctcgttgaggtgctagccttcattccattagctatctttttgc
tttctcgctcgtcttctgccgcaacgtgaccttcttgtacagctcggataattttgtcaatccattcatccgggagagccgtgattccatccaaagtgt
ttcttaaatcatcttcaccaaggcgatttttaacgtttttaacatacgcggtgtgacacacccccaatgtttccaagcccagctttgaaatctccca
gagtcaacaaatgtaccgaagcgtacagcacctgctggaatttttgaactttactaattcgttagctttatttagaaataaagtcagattctaaaaa
agcatattctctctcttgttaacagtaccagcccagtaccaattccaatatagaactggactgtgaatgaacagttccacaactggtttttcatactc
ttatatagaagcaataggtaatttttcaacaagtctagaaactcccttttcatgaactttttcagaatcttcccaagaaacgtaccgaccacataaa

gaacctttacatttggaacgactattggaagcaatctcaacccggtatccgcttccgccctgttccatgattgttggatagcaaacaatacttcgttt
caaatattaaatgactgcagtttgatacggttcaacaaacctttgttgttgttaacgaatccaaggcttgcaagttgaaatactgtctttttcgatc
gaaatcaggctaaacgaaatgaacggtgtcaactataagatacaagattggagagcataattctagcagggataaagaatacaattcagccag
ccaaaagtcacaacgtttctcatgcttttccattcatgatgtttaatatgtttaatcagagataggaatctcttgcgagctgaggataatacaacttc
cttgccttgaaggaaactgtgagatacgacagatgtttgagctaaaaaagaaaaacaaaagaaaaacaaaaaagaaacaccaaagtaaatct
aaagcgaaagtatgtacattcgaaatcaaaaaaaggatctgccaaaggtaacatttttttcgttagtataatatcattttgaatgttgctattcataa
aatgagagacatattaaaagaagctgttcagcattcaaacacttaaattattactattttcctgcgtagaaacaaccttgtctgattgccaaaggc
gcatttgcttttatatgcgatttcgtaacactttatctgatatcgtacaagtaacaatgcctggaggtagaagaattttttttctgtatgaatgagaaa
gtagagaaataagattctaaccatctaaaagaaaattattaaagtgttagagcattatgcgatcttttggttcttttatttacgttttgcatcttccttc
tgagtaccttttttctaatacgcgttgcattttgttattttatataacgaaacttgtcgcacaacgttgatgaatgagacatcagataacgggcttatc
agattttatacttgccttgaacaaaacaccataaattttcaaaagggaattacagaattttgtctgtttacaattcaatcctataaaactttaactat
ttctaggtgaaaggtagtttctgtgatccgtcacaaaaagctgtaagcttcttaccttattttccgttaacagatgtatctctgtataatcgtggtaaa
cgtctaagtttgatattatttttactatattgttttactcgttttccctttgtttatcttctagtttttttggtttcctgatccggtttgtttttgggtttttgttt
tcttctggtcttcgagaatgttgcaaagatcagttcgaactcctgtcggtacacgtccaacatttttcaaaaagctttttggaaatgaaccatattca
ccttctacacgaggccaatttctggctccggcaagtaaaggatgtttaaaaaagatagttgtaagaacctgctgtcaatattctagtcgacggagg
tattctactggaaaacggtttttgggtcatgtctcaaactacttatcccaaggaaaaagcccgtcaggttcatctcattctacctcctctgcgtccga
tttgctaccgtctttttctctgtaaacgaacaattgcagacactgttgcgaaccccttcatttcaaaatcttcagatcaagcttgatgaactaaaat
atgacgaatgtttttctaatcctacacccatcccccagaaaaaagtttcctttacacccactaccgttgattctgctcctcttgatcctctcctttccga
tagtcctacatctcctcacccaccttcttttgttcagcctcatcccccgtttcacgttttttctgcccctgttttagacattcgtactttaaccaatcctgg
tgctataaaacgtacatacaatgtcactttggatgtctcaaaataccctttacgggaaggcagcgattggaagatcggtggatcttttggtgttatg
cctcctaatcccgataatgaaattttccgtttagctcatctactgaatattcctttaaacgagctatatgcaaccaaaattcttcatacctctggtgga
aggtggccgactatttggggagaggaaaaaccacgtgcattgacaacttcaatttatcatatacttaagtggtgcacagattttctttctaagccgc
cgtctaaagatttctatcgtttgttagcaaaatattcaacaaattccgttgagcaccatatccttcttggactttcgaattgctctcaaaatatatgct
ctgaaaatttctccgtttccgacatattggatgcgtttccgaacgtacgcccaccagtggatgaagttatttcactgctacctcagttaatgcctagg
tggtactctatatccaatgatcctgatctcgggaaaggagtcttggaaatggcttttacagtacaagaatttacggctccagatggacagacgcgt
actggtatttgttctgggtttttggaaaatgtggctttagaatatttaaaatgtcaacaaaacggtaccttaaaaaacaaaaaatttacgttaccaa
tgttccgtggtgttcagcagaatccgtttgcaaaggaattccataatgatggaccaatgtgccttattggtgccggagttggtatcgctccattccgt
ggctttgtgcaaagacgccttgcaaacgcatcctgtacaggaaaagtttggattattcagggttgtcgggatcagactttggacgaactttatcatg
gtgaatggaatactatacccgggcatcacacaaatcccaaatgtcgagcaaagaaattggtagttgaatcgagaaatggaaagcgagagtatg
ttcaagatgctgttcgcagacacggaaaaattatatgggatgtcatcaatcataaaaatggacgcatttatctttgtggttctggaaaagcctttgc
tactgaaattgaaaaggctatcatggatattgccatgaaatatggtgggtataagaaagaggaggcagctcgccaattagaatattggcaaagc
ccaataaattacaaattcgttaaagaagtctggtaatgtattggtatttgctacagggaaaaggaaaggatcatttggtaaccatggctaacgatt
ttgatgattgacatattttctatgattcttatttattatatatatcatgaataataatccttaccaaaatactctagtaagagtcaagtaattctttaaa
atagctataccatggcatgaaacataggaaaacagcctgtggtattcatcaatcgtacttcgtatatttggcctccagaaaaatttgtttttcaacc
aaatcaagttttgtagcccgtgtacggcccaatttacaaagtttctctaagacgctttcggttacattgttacaatttctgggtacttcaggttccgct
gacattcgagcacgagtttcaaaagaaatggtcaataaaatggaactaataaatgttaacatggatggaacagaaagggtaatagctgcaggt
aaataacgaagatataagcagaaaatacgatcagtacttccagcggactcaaggcatttggttaattgacatagtcccctgccattagtggtagt
accctaagcaaatgaaacggttagctttatcccaccaaaaaaagataatcactatttaaatatcatacctctggaaatacaacgacgaccttgcc
agtcttgctggcatctttacagatttcggagagtggtcgaagatctttttttagagggagatagcataaagcttaatctagaaaagcatgaaaagaa
agaagaagtagatgaaacaggaaaaacatttgattgacaattcgtttttacgaatatacaatcaaacaaagacgaaacgattaagacatctaca
ggactcgtatggttgacagcaacaatatcaccaggctgtaaactatccccttgtacaaacgatctgaaaaattagcttgctgaaaatgaaattccg
ttgacttacccaacaagcgtataggaaagtcggaagcaacccaatgagaatagtaaaattcgtgaaagtgaacgttcaataaacccagctatag
acggctgaaagtatagtagcttcttgaagcacgcagaccataacccaaagtacaagagaccggagataatgcataacgggatgcgaactaaag
ctacaggtaagataatcaaccatttaaagcttgtaactgatggtttctctgcataaattggttgaaacggagcaatgcctgtgccagcgtctaaga
aagaaaccagttagtctttaaataatggaagatatacagtttgcagtttcaacaagttttatcagagaaaagaaaacttagagaagtttatgtagt
taattgaaatctccaaaaggatacttgtttgaaaaataaagctgaaagttcagttcttacctctccaacgggtgaatttctccattattcaaatatg
atgataaacttttattactttcgatacgttagttcttcagaacagttataaacaggaattgatagtcttaagacagtaaattgaaattccaattgata
cgttacagactgctgtacgctcggtaggcttagcttatgtaccttaaagaatgaaaacaattacgaacaatttctccaaccgaaacttcaaagtct

atggatattgtttattgtataattacaaaataaatgtatttttcggtcagatatcatagagagaccaggatgacaagtcgacgattcagccagatt
tctcaagaggataatagtagtagtgaagaagaaattcccattgaagaaaatgttggtcaagctaaacttcattctagttctataaacaagaaaat
tcggaacactgaaaacacgccgactgaggaaaaaagcgggttaaatccccttacttgggaaaagaaggagctgttagagaggaggaatcaatt
gtacagtcaaatatatttgttagagcaagatattgtacgattccaaaaacagactttggatccaaacttacagaagcaagaacatcaaatgctta
aggagctcgttgggttattcttcgagacttcaccggcttcggaagatagacagtctactaaaaacgacagtaatgatgtttttattccttctgcggc
acctaatattaatcgcccagttagggtctctttttcaggactacggattcttacccaccagtctacagatgtgagcgatgtacattgcatgcacgaa
tttaagggattatctgattttttccgtttttggattttcatatgtatatccatattcgtacaactgattatgctatccttgagatgtttagtacggtagct
tcatttgcaagaaacgagcttcaatctgtgttagagaaatcagcgaatgcaagggatgtagtgacagcattaagggcaatctcaatgtttggata
ttatcatgcaattcgctccgacgattggacatatttttaagtaaattcgatgggttagcgagagtcgatttaactacatctgagctacatgttcaaa
gtaaactatacaaggtttcgttacagcataacatctgttttgacgagtttggatttgccaaagcggcatataacatattatttagaccaaaaaaaat
accccaaaagagcgaagagtctatttcattacttacaaaaaaaattaatcatcgattccatgaattaatcagtcttttcggatttcggaaaggttct
gaaatcttactagagatattgtttaaaccagccaacggataagaatcagggtatattaatagggaaacgaattggatcgcatcggacaaaacgc
actaaaaaatagagtttatagaaatagaatgaaaaattcgatgtgaatatatacagtttaagaatgctttggaaactctgagtactccacctcacg
ctttacacctgcttccggtagttgtacagactccatcactcggttgatcgactcaagtggcgaccgctcctttttcacctttttagaaggagattgag
gctttgtaactttggtggaagcagtgagttttgcttgccctgaagtccgtttcttggcagctaaagaagtgtttgagtgagcctttggtctccacctgg
gagcttctaaataaggcttcatagtacgaatagcagcctgacaatctccttcaacgataatatcacacaagttcgaaaactcctttgtaggagaat
caaaattcagccaaattactgaccctttctgacggtgaacgcaattactcatttcctttatgattcttttaacgccgggaattttacaggaagtacct
gcaatgaccaaacagtcaggccttgatttcaaatcctgcgaacacacggctccaattgcgtctgaatctggatgcccttcattataaagcacaatt
cgtggacgtaaacatccttccatgacaagtcgctttccagcaatgcgacggacctcattttcagtcctgcattcggggcaagccgtcagaccatctt
tctcgaagagaccaggatggaagggtcggacaaagggacatctcgtacatctaacaacttctaatgtcccatggagtgggatagtggtaggcca
aggcgctgtttgaggcaagggagtgcacgtttggagaccttcaagccgggtttcaaggaaatctatattttgagtatacaaacgaagaagtttgg
aatcctgggctagttttgacaaaaaatgatgaaagtcggtgggggcagcattgtctgaaagttcatgaagctttttaatcatttcatgaaaaacgtt
tacacttttgagatcgcgatacactcctccatcaaatagttcttttcctgaacaatttaatttatactctgaccggagagaagagaagagaccaacg
gaggaccgaaaatcaggaatacctgctccacacgaaatgccagcacctgtacaaattgttagaataaatctgaaaatgcattaaatttcatctca
caggatctgtgagttccagatgacaaataacataccggtaacgatcactattcgttttgcttttcttaaagcggcaattgcaggattcaaatcaaca
ttgtctgaagaaagctgaatcttaatgggagaatctgcaatttgttcgtggcttttcagtcccgtctcggtggattgcaattttcttttctttgactcat
cattgatggggagcagctcatccggcttcatattttcaagacaaaatgaaaacgatattattcgttttttgtaaattttatgaattacgtccaagaa
tcgacatttagggtactttcccgttatttagagaagtttggggtaagtgatggtgatgtgtaatggacacacatatgggtcaggaattggaaagac
tcttccaagggagtgttgagttgacagtttataaacatatgtttcataaacaaaacataatggaagaaccttacagaggatgaaaccagcaagtt
tgaagacttttcgaagatatatatgtttattcgaataaaagaacataatttgaatcaattatttttttggggtttttatgtgtctataaagcggcaatc
cgagatttagaaaccggtctttgttgagcaatgaaaaagctagcctttttcgtccaaacctcctgggtaaacctaatcattcaattcaattgtaaag
ttcattctacctcaattgttagggacaatttcaacgatgagtgaacattttcaccaaaagtgcttcatattccaattgggatatgcactgttgcgaat
ctaaaggaataggtgtttcaagtgagtatataagttttgttaaaattggttggctttccttctttttcacttttgattttacaagtgactttcatgttgct
acgtgctggagccaaagaacagaagaagaatactaagtgcagcttttgtatttcattcttcattcctagttgaaaaagctatatacgctagtactc
acattagagtcggtatctactctatagatcaagttcactttgggctgactcgaataaaagcaaatgaaaagagaataaagtgaatttagaagtga
gtaattgaataaataaaacatagattataaatacacataattcgtcagcatcgtcacaggaaactcagaaaacgaaacacttctttctcgaggaa
agtgaaactttctcctggtgctttaaataaaagaaatggttaaaaaaagaaataaaaaacaatgtacggagagttttcttgcttgaccagatcat
gcacctacaaaagtaggtttagattgaaaggaaaaccagaaatagaagccataaaatagtcatatatcatctctaaaggtaatttcaagcaagt
agaacccctaaacgtaccaaaataacctgaaatgttgttaaggtgctttttttcatacaggcgaggattccgccaacaacgcaacaatacaagcaa
acaacataccactgcttgaattagcctacaccgaggtttcctctggttttcttggtgtatctgactaaaaaagaaaggattaaaccgttgaggttta
ttaaaattttgaattcctaaatttacaagaggagcagccagtcttctaggcaacccttaaatcaatcttgccagaagcttggtgcgagggaaatca
tgagctccaatcatactaatggcgcatttgaagatcctaatggtatagcaaggacggaagttgcaagtgtagcacaacccaatatgcaaacttcc
atagaattgcctgttctctctacaaatgaagggtccaactatctaagcaactcctcttcgtatccttctcagacttctttaacttcctatggtgattctg
aaaatgttaccagtgctgctaacagcacggtggtatcttcagctttatcgactcctcaaatcgagtactctgccagcttcgatgaactcagagtaga
tggtaatagacgttcaggccgtgtaaagcgtgccgtttatccagaccagtcattctttgatatggatgaccaagaaattcccacatctaaaaaaag
gaaaagcaaggtggcaccagtccaaagaaggtctcctgaaaaaagtgaagatgatgccgatgcttatgatcgcaatcataagacgagctatgt
acctactgaagttagaacttccactcggtcctcaaagggtggagtcaactataatgagcaagatctttatggagatttggataacgaagaagagg
aaatggaggaagaagttgaagaagaatacgaacctgccattgactttgttttgaaccatcggaaacgcgaagacgccgaagatgatgatccca

agaaaagttaccaataccttataaaatggcaagatatgtctcatttgcataatacgtgggaagattatgcttctgctacttctgttcgtggttttaag
aaagttgataattacattaagcaaaacattatctatgatcgagaaattcgggaagatcctagtactacttacgaggatttggaggctctagacatt
gaccgcgagcggaaaaatatggtatacgaggaatacaagatcgttgaacgtgtcgtagcctctgaacctaatgaggctggcaaaacagaatatt
ttgtcaaatggaaacagttgccttatgataactgcacttgggaagatggtaaccttatttacagtatggctcctaatgaagtatttcaatttcttcag
agagaaaacagcccgtatttaccttccaaaggtgtgttttataacacaaggcctccttatcgtaaattagaaaagcaaccgtcctatataaaagg
aggagaagtaagggatttccaattaacgggtattaattggatggcttatttgtggcacagaaatgaaaacggcatcttggctgatgaaatgggtc
tgggtaaaaccgtacaagctgtttgtttcctttcctatttagtccattccttaaggcagcatggtccttttctaattgttgttccgctttcaacagtacc
agcttggcaagaaacattggcaaattgggcaccagacttgaacagtatttgctatactggaaatacagaatctcgagcaaatatacgagagtac
gagttctacatgtcttctaataatcggaagctcaaatttaatgttcttttgacaacgtatgagtacatattgaaagataagcatgagctcaataatg
ttcgatggcaatatcttgcaattgatgaagctcatcgtttaaaaaatagtggatcttcactttacgaaaccctttcccaatttaaaacttccaataga
cttttaattaccggtaccccgttacaaaataatttaaaggagctggcatccttggttaatttcctaatgcctggtaaattttatatcaaagaagaact
taattttgacgagcctaatgaagaacaggaacgtgatatccgcgatcttcaacaaagattacaaccttttattctccgcagattaaaaaaagatgt
ggaaaagtcactaccatcaaaatcggagcgtattctgcgagtcgaattatcagacatgcaaactcagtggtacagaaatattcttaccaaaaact
accgcgctcttattggtagtactgatgggagaaatcagctctctctattaaatattgttgtggagcttaagaaagcctctaaccatccgtatctattc
cctggaacggaagagaaatggatgactggtagaaaaatgaccagagaagacacccttcgtggtatcattatgaatagtggaaaattggttttgtt
ggacaaattgttacaaagacttaaacgagacggtcatcgtgtattaatttttagccaaatggtgaaaatgctaaacatattaggagagtacatgtc
tcttcgaggctataattatcaacgtcttgatggaaccattcctgcgtccgtacgaagggtatcaattgatcatttcaatgctcctgaaagcccggatt
ttgtttttctattaagtactagagcaggcggtttaggtattaatttaagtactgctgatacggttattatttttgattcggattggaaccctcaagcgg
atcttcaggcaatggctcgtgctcatagaattggtcaaaaaaaccacgtgaacgtttatcgtttttttgtctagagatacaattgaagaagatatttt
ggagcgtgctaggaggaaaatgattttggaatatgctattatatctttgggtgtcactgagaagtcgaagacgtctaaaaacgataagtatagtg
ctgaagaacttagtgctatattgaaatttggtgcatcaaatatgtttaaagcgaatgaaaaccagaaaaaattagaaaacatgaatttagatgat
atcttgagacatgctgaagatcatgattcgtctaatgatgttggcggagctagtatgggaggagaagaatttttaaaacaatttgaagttaccgat
tataaagcggatgatcttaaatgggacgacattatacctgaagaagacttggaaagaattgaagaagaagaacgagtattagctgctcaacga
gctgaagaggaagaacgtgaacgaagagaagaagagcttcgagaagctgaagaagagcaaagttctcgtgcttcaaagcgaactactaagtc
cattactaagcgccaacaacgccgagaagaagcaatacgtgagaaagaagttcgtcagctttatcgtgctatgattaaatttggtttagtcgaag
aaaggttcagtgacatcgtgaaagaagcggaattagaagctactgatcccaaacgaatcttacaagtatcgtctgatatagtgaaagcatgtga
gaaggctgttgaacaagttgaagcagaggatattaaaagcaaacaacctcgtaaagcgattttgattgaatatcgaggagtgaaacacattaat
gcggaaacaattacccagagagtaaaagatttaacctatttgcatcgagcgtataaaggtttagacccactaaagcaacgtattggttatcccat
ccgaagtgtccacagttggaactgtacatggggtattaaggaagattctatgttgcttgcgggtattgatcggcacggattcggatcatggcaagc
aattaaaatggatccggatctaggcttgcaaaacaaaatattcttagaggaagctaagaatgataaagattcaagaaatgttccgagcgcagta
catttagtacgccgaggagaatatttattaactgttgtccgagagcatcctgacctcttcacaacaaagacagatcaagcaacgaagcgtaaata
cactcgtaaaagcagcaatacgaaaggttctggtcggcaaaccactttggatgattcagttgcatccaaaaagaagaatcgtggaaaaaagca
ggcacaagatgatgataacacagaagaagaagaaacttcacctgatggaacggttagggaagaggaggaagaggaggaagaggagaaatc
aaagcctgaagcccctaagcgagcattgcgctccaatactggtaataaaccacagagaacaagccgaaggactaccagaaattcttccaaagc
accgtcggcgatggacacacttacggctgctgctgctttagatgctgagttggatactgattctcctaagaaaccggaaaaaacagaagctgaag
atgattcgataaaaaaagaaaacaaggattcctcggaagctatctcctcttaagttaaccggaagagataaagtaaaatggattcaggatgcttc
ttgggtacattgtgatttgtctcaatgtaaacatggaagaatataacaacagatatctatttcagtgtatattgaactactatcaattattaaggttt
ctaattgcatataagttttgttatctttttgtaagtaatactaattttgtttaagattatcaaataaagcgtttggactatgctaataatgttgctcaag
ttttcatttaaaaaaatgaaatgaagaaaaaaagtagacattcttaaaaagtagagctacccaaaagcgacattcacacaataataaatgaggag
atatccatcaatactaaagttgtcatataaattaccaataacttcgtcaggagcaggagcaaaacaatagtttacatataataactaaagtcgta
agttagacccccctcgcaatcaaaatataactcaccaaggatgaatcgggttgtaaaccaagctcttttttttaagaatcgaattactttgtcaaagc
gatggcttgcattaatcgcgaatacgtttttacgcagcaaaggagttcttccaatggctttaaaccgtaaattcactgaatgatactagttaattca
actctgctcttgacgaatttatccctaacctttctatttcccttttcattgtaatcgcttatgatttcatttattttttcctgtagatttcctatttctctggt
atgaggggactctagcctcttattcaattgatcttcaacatccttcgaggaagaagacattgtgtttattgaaagcaatgaaaagttaaatcattct
atttggtcgttgttacgatgatagcctttgtacaacgctgcagtaattgttggttttgactatatgaaacataatgatacgtatatttcgtgcgctaat
agagatcagctcaatgtcagttactcaccagtgtatcgcctttgattccttatgtaaatacatttttattagacagtgtgaattacctgacaattaag
atttacagaaaatattctttataaaccattgcttgaagatcttagaacttctgttatttcagaatacatggcaattatcattagatatattatataact
attttcaactgctctagaaccacactgacacataatataaattagttggaaaaaatagaattgtatttccagggcatttacatctttgagttaacag

aaaggaaattctaataaattgacactcactcacttcggattcggaatctcataaaaaattataaaaggcgagcgagaatagataaataactaaa
gcttctttaatgtctgatccaattcttgcttatcaacaacaacaccagacgctgagcatttggccttctgtttcagtttagcacataaatcatcgatgt
ccaattgagaaaagttggggtgactcataattttagaccaaacttcgggacactttatataagtttcttccttatttggaacaacttcatcctcaaca
ttgaattgtggattctgcggtaatggactaacaccaggttgtttatcttgtaccttatttcgatcgaaggatggaccgttagaaatattggataaatc
aacactagcatagtccggtggcacgttaataatgtcataagggccgtccttctttagactagaagggtctgcgaatagtttattttcgttgaaaaag
tccaaagtagcaggagtatgctgtccaatgccgaacattgaaaattcgtcgccctgattctcatcttcagtatccctaaagtatgttctaaacatgt
cattggcatcgccctcatcattgaaataatctttatccaaattttcagtaggttctctccaagtagagaataaatctgctatggaattgctctcatgat
ttacattattaagaacgttagtgcttggttcgaatattgcatcgtaatcaggtaaattattatgagctagttgatccacagatttaggttcttgagatt
gaatcgtttcatcagtatacatagacttagaaaacggtttcgaggatagaatttctaaattgagaggctcaggattcgcatgattcacgaagcgat
ccaaagggtcattgtttatattctcctcggtaagaatatcaacactcgcacgatgaggagtactcttcgttaacatggaacaagcaatggaaccac
atgccgaagacagtttttggcaaaactgctttccatccatagaaggaatctgatggtcggacgagtcgtcttgatgaagagaaagagaggtaga
acttcgtttatgtccgttgaattgctgttgaggagagttagaggaaaccagactttctggggacaaggtccccctcactgaactattagaatctacc
aaaccaggaactccatattgagtaccgttcgctgacatcgagtccgggttcgatgaagaattagaattactttgcgttgaaggaagagtatcaaa
tgtagagtttttatatggttcgacagatggtgcatttatgttattggaaacagaaaaaagagaagggtctcgctgaggcaagctcatttcaaaggt
aaacgatcccttccgcaaagcagcaagttcatcttccagttgcttaactcgctttctcaattcatcattctccacattcgttgtcgaatgaagctgctg
aagactcacaacttgagtttccaaagtcttaaggtggtcttcttttcgtctacggaaagcacgctgtgcagcacgattctgtgcttttctctttgaaa
gcgattcattctcctcagtctttcgaccaaactttttgggcttttcttcaaaaaattcgtcatttaaatctggaaaagactgattctcatccgacgacg
atcgttcaacattcatgtgacctacaatgggacctcgtttttggacgcactcatcgctgtggacgagttttgatcaggattggtggcaacggaaga
aggattgacctcccttgattgtaaggaggaatatacaacaggcaaagtttccgcattagtagacatgataaatgattatagaaacgaattagata
tgcaaattaaaaaaccccccaaactttgcacactggtgaatataagaattaaaagaaaagaacagaaaagaaaagaaaagaatttaaggtgg
tataagataagaagaaaacaaatgcaacagaataacaaaagtctagtgcgatgcaacgaaaataaaaaaaaacgaaaaatagaaaaaaa
aacaacagaaaaaggaaaccaatggaaccgtaattaagcttgtaattctttaggaaagaaaaatttcaaaaaaccgaacgaaccaaataatca
cttgatttttccagactttattatctagaaaagaaaaatatgttgaaagggtttaagacgtaaattcacaaaatagaacagcaacccaattttaaa
aagtactgtaactatacacgtagggcaggggagcaaggatctttgtaagaattctagccgacgatataaatgcaaccacactcagattttctgaa
aagaatagaattcaatcacatttacacctaagcaacacctatttattaaagaaaaggctcaacagcaaggaaatcgtatagcagttaagttaag
gaaaaaagggcccgaaaatcctcaaagaaaggcgaaaactaccaattgatccagctttagcgaaatttttaggaaaaaaataagaattaacc
tgtttttcaaggaactgagctctttttactcaattttcttagctcaagtgaaaaactgggtaactgcgaaagacaaatttcgaggtccgctcaagttc
ctttacgcatccctggagtggagtgacgcaaatcctcaattatagaggattctaggcttccctttattcagtatataaggaggtctacagaagctttt
ttacgtccttaacaattttactgcgcgtttacgaaacgtaacaaagatgaaggtagtttccaattcgaagggacttgttactttccaggaattttgtt
tatttacctgtagtttgcttgaaaaagtcactttttagaacagttatgaaagggatcccggttcatttaccaaaaagaaaaaggaggagaaagaa
aaaaatgatcttttgcaaactatatagtttgtattgccagtaaaaatctctatgtaatattttatattcttactattgaaacctgatgagcatttgatct
catataatgaggtaggtaataaataagggaccgcttgttaatcatctagttttaacagtaactttttataaaatttaagggttgcaaaatctctaga
taaaataaacgaaacatctcttggctgaaaagaattgcagaacgaagcatgattggaacctcctttttagaagcattcattgatccagatgtttta
tttttttttaaaaggagcaatatatttataatactataaataatcaatcactttcaggatggaatatgtaaaaatatgctcaatgaaatggtaaata
aacagtggccaaatcaatgatcagaaagtgacaagtaacggagtcacaccttcgtactataacaactttaatataattttcagaataacagccaa
ggaagaattcatgggacttgaaatttcatttaaatatactagaaaataaaaaaaattcttagacttgagcaggaattgcatgacttagttgacgtgt
gttaagttatccgtccaactaactcggcccgaagaagctcctataaatgtttctctaaaaatctacctcttcaaattttgaaagcaataaggttgcc
attgccgttttaattgcttttcctgtgagccgatttaccgtcgagtctttatgagaagcttacacagaggagtatgtactaaggtttcaagtttagcac
ttccacagcaatataaaccttttcaatttctgagaattagaaatataaataaaaagaactctgcgactacttaaagacgtaacgttcattaaaggt
gcttccctcgtatcataaagttgattgtcataagctatcggcaggatacgcaatcctgaagttcaacctgattgaagtcttctatttcccaatcattgt
tgtgactgatttcaagtagaatgaaatccaggcgaaaaccaaaaagttggtaattgcagattagctggtagatttgctactttctcattaactcttg
cacgactatcgttaaccctgtcttgcacttctttagtcttttatttcagtgtattccggtaaagattggctttcttgatcatgagggtgagaatttaggg
agccgttggtttgcgcatttgataatttctgttccctatcgagcctttccttttcttttcttggtaaccgtaaacattccttgtttggaattcccactaga
atgaataacacgaaagatacaatagggatagcaaagaaaagcgaaatgactaagtgcgctttcccatggtattttaaagtcgacgccaaaaca
agatgtataaagcatatgaagaaaagaaagcaaaatagtaaacccagttttctattcgtgtttaataagtctctcttcagataggcgatatctttgt
acaactcgtggttgaaacatgttgaatccgctgctttaaaattgaggatggttctttgcggaggaatcgctgtgaagttgtaagaaaggaaagag
gatggtattcgacgtcagacggaagggagaagatggatgatattgaaaccagctattcccattcaaagtcaccaccatctttttttcagaatcttaa
gaaggggtcaacccgaagagaattttgaggaaactgctcctttaacgtctgaagttgctcttagtcatgaaggggattataacagacgtctttggc

aatgtgtactaaaaagatttaaggattgaaaaatttcttgattttgatatccaatacatatttaaaactttgtttttttcctttcataatatatattatat
ggagatactcggacaactattgggagagcgactatgagggttttgcatacttgactattattccttgctcaatttttgctcttgcctgcttaattgcca
ttcgtcattactttaacccattgttattgaatttcaagtagattcgattcctttgaatgcttttgaatcataattatttcttttcatgaacggaagatttg
tcaagaaaaaggtgacacaacgaaaaagtaactttaccaatgtttcaagcatggtgccaatcaaaaaattggttcttccaagaatcagagttttt
ctaaggatgcctccttagagtttccttccaggaattagatgtttacaagaagaggttctactgaaattggcgcaagctttatctgaaaagtcaagc
gccatgcctactgaacatgaatgattatgaaaatacaggagcttcttgtttaaactataaacagaagcattcaccacccttgtaaatggacgattt
aacaagacccatccttttatgaaaccggcaacttgggacgacttattactttgatatattttaattaacaattggtttgtcttataatctagtgtttctt
attggaaattgccagagtaaagaaagaaaaatctgacggaattctgtaccggataaagctacaagtcttggttacacatgataacttaaacctga
aattattccttttgtttttacatgttttataaaatattggcaagtaaactcgttccgcaggaagccgatgaagccgataactactgaatgttcagctct
actttcaatcaagaatgtctgagtatttttttgaaccaaataaccaagatgcactgtcagcatgtacattataattaccagataaaaatttacagcga
tttcaaccttactgatcctgtttttttgcccgttacggctaggtcaaagcgataatccttagtgcatctgttccgcttggaccaattcttctttccaataa
tcatatccgaatttcctttatctctttgggtttttctttttccagtatgtctgtctttgtatctaaacttatattttttatagagctctttacatgtaaactaa
gtttgattaaatgatttttttatgaaaagaaaagcaacgtatcactaaaaaatggcgatacatatcaaatactgcatcttactattaatactttaaat
tgttgcatatgaactacaattgaactggaagaaggtaatagcatctatgcttttcttctgttattactagtaaagcctcatgtccgattacgttttgca
atgagaaatatgtagtttggaatttcatttgggtccatacagcgctattaattcaacttgggaatagtagacaaatcaagtgtaaaagcaatgaag
caaggtttgcaaacagacataccgaagttatttgaactaatacttaactcattttattagtaatcaagcatttcttcttcgtctaacttcatttgttga
gctatcagaaagcataaatgacttttggtgcaacttaatactccagtaccatttttcaaaaatctattgtactgcattaccaaaaacccatccgata
gcttatagagtacttggctttgctatatcttttacgaaactctacgaattgaaccttatgctacgcaccagacttattcttctctactgtctcataaaa
cttttccaattgaacctgaaactttgaattgtaccatttctttgagccagaagataaaacgataaaataaccccaccttaacggtatctgcgaaga
gacaaggtgttgtgacggagccaagtggcatgcttgggagtgttgttgaaacggtgacccttggcaataccacgagacttcttaccaatagaggt
aagaccacgagcctcacggtgcttgtgaacagagttgcagatccagttgatacgaggatcacggcggacggccttatgagagggatcgacgcag
atgacttcgtagaacttataggtagcgtcttggttaacccagtaggagttaagaacacgcaagttagcacaacggcgaccgacacgctcttcagc
agtggaacggagagagcgttggtacttgaggtggttaacaccttcatggacgggcttaccgtagacttgacccttaggaacaggacgcttacgac
caccacgacgaacacggatgcggtagacgacgtaaccttgcttggccttgtaacccaaacgacgagccttgtcaggacgagaagggcgactag
cacggtgaataacattcatctgacggtattcccaagcacgaactcgagacaagaacaagttcacatcagattgcttcttcttagaaagctcttcca
aatacttataagcacccatttttaattttctggcttcctacgaaaaaggcaagatggaagttcgttgtcactgagaaaaagtttcgacctgcactac
ccactcgtgactgctcttttatgtttttctttcacccacacaaatctttccttcgccaaatcgcatacatggagtaataattataattatgtttactttt
caataaagaatatactttaagttcaagcttatattccgagatactaagtcgcattgggctaactacaatcataaacaactttttgaaaggtagttat
ttattaacattataacttgaatttataaacttttaaaccaaattcactgtccttttcactacccaaacctaggattatgtacatattaaatagtcaagg
catacaaaaccataatgtcaaccaaacaataataacaaaagtcaaaaaacaaatagacttaccatatatcaaaatatcgttaagtcaaaactta
aagagcgtataagttgttaggaattttttttttttttttactctatccttataatgtatgcagttttctcaagaaggacaacggatcaccaaaagttga
agcgtccctttattcaaggtagtaatattagaggtaacataatagaaagcaaaacatctttcttaaccagcaaaagagacattccaaccagggtt
tccggtagcatatcctaccagactatcaatagttgattcttggtaatttcgtaaatcatccgaaggccatagaccattccattgatgccaatccgcat
tcttcgacaatccaaagtgagacatcaaatctgaagaataagtttcttgaacgctatgattggcaataattggagattcagctatatacataaaat
gcttttccaaatccaacatttttttcttcatcaataggctcatttgcaattgtgtcattaacagcagaaccggaagatgtacgctgtacccagtccgcc
caaatttcagacatcctttcgcacaatgcaccaatagagccccagtaatttttcatttccaacattaatcttaaatctatttccaaattatgcctagc
agtggatgcagccagaacatttgagccaaaagctgcttcagtttgaatgcaaaaagctgtataaacacctgaagctataaacggggaagttaat
attacattatgctttctgcaatcatcgacaatcattgatattgcattagcacaccgtaggcacatttcacgtgcagctttatgtaaccgccgtgcagc
actattgacgtggttttctggcttctgttcagcttgaaactcattatgatgagagtcatgagagttatgccacacagatgaaaaagaaattgtatttc
ctgtcaacgataccgtacttgacgttcttgacaactgattattttcagactcaccaacggaacggaaaagccacattaacgtgtaatgataagcta
aatgaaggaacgcatactgagcaccttgctgtatagatagatgattttccaaacctaccactgagtatctcaatctttgaggaagcccatcagccc
attgataaagagtctcactaatttgggtaaaatcagaatttctatgccaatacggatgagaatcccttcgtttgccatcgtgcttaaaatacttcac
agctcttccccaaattgctattatcttcacaatgtatgctgcaacacccatgttttccgtggtatctgagggaattactatgctaggaacaccttcta
atattcttccgtccaaagtctgggtaactccagcgatttgtttggtgaacaataactcatgtacagggagttgtatcccaatatcgttttcgtccaaa
aattgcaagtgttcatgagaagaaacacatctatccatcagaaaacaggaccaaaaggttcgacgtctaagttcacgttcaaggaaagaaatcc
gaggaccagagtcaatattatcaagatccaatggattgcttccttgttcgcaatttagctgtaacgcagcggccatacgaagagccataccaccat
acatccaacttttaccattttccccagttcccgagtcatgcaacgacaataataggagggcagcaactaatgacagatccggcctatcaaaattat
ctaacgccagtctacgagcctctcttgcaaattgttcaccagccatataaggaggagaatactgatcatgcatacgagaagaaaagcgtgcgga

aacggcacagatagcataaaccagaaccaaaggaattgtattactattaagagattcaaaaaacgtgggacgatgaaataagttataagcctg
accatggcaacaatgaaaaaatacctcagctaaatgaattcgtaattccaaagggggcaattctgtcactggagtagatggcatggtaaggcta
gtcaatggttcaggaattttagcgttaatgagagcggcttctcgctcattctcatccttttcattaagggagttcgtgttaagagagtaattgttagtt
gtaataacaggttgcgtagcaatgttagacatatctggatacgtagaagaagcaaatgaattacctgctttattttcaaagaagggaaattgagg
ttttggagaataacctccataagtagaataaagagggtactcactcgaggaaagcggcgggatcgtactggaaggaaaaatcgagttgtcagcg
gcagtagggtgagaaggaacatacggtaaaggatccggaggaggaattgatgtagctgcgggagtgacagggactgtcgttgcttgctgtggtt
ggtattgaacggcaggacttagcggagtaggagcggcaacagcagcaggggttggattggaagtattagcggataccataggcaaaacggaa
gcgacgtctgcaataggcacacctgcggagaaactttggtttttcgggatagaaggattggcggtggaagggacatacatgacttcggaaggat
aaatattgttactagaagttggattagaagttgattcaggagaagcagaattcgaatcattgcggtcaagaacgagattttagaaagcgaagca
gacatcattgatacctgcggattaccaacaggaacgcgagaattcatcatgtaagaatatgaagaaggagcattcagagacgaagagctagac
tgttttgataagttcaaatttacagaagaagatgatgatggaggcgccgaatcacgagggctaccaacttgagttaacggcgtctgttcagataa
aggctcttgtggatgagagccagatgaatttgctgcggcagcagccgctgccgcagcacgattctgttgttgctgaaggtgtatttgttgatgatgg
tgaatctcttcatgccttgaatgggatcggcggggattagacttataaacgcaaggtatactatgcttcgagcattgatcacaggatggacgctct
cctgagcattttatcttctttttctacatgtctggcaagcaagaggtaaccgttttcgttccttgcccgcttttgtttttcgtgaacccagctaggaa
gcgaaggtgaattcggtttcatgtcttctatattcgtttcttcgcccatctgtccatcttgctggttcgtcatattcactctcgtctgcgttgcatttgaa
agtatcgatacaggactcgattgttgcatccttaatcgatcatccaacaatatcaagcctttcaattctatccaatctgacccccgatcttataactc
gactactttttcaatcctgtgctatttttatttctttagtggcgtgcaaaaggcaactttcctatctcatcaatgtatattaaggaagcaccaaagaa
tacggtgaagagttacatttcatcgctaaaacttatttactgtaacagagagaacaacggaggaagcatatgtaaacattatcatgtttttatttgc
ataagtctccagcgtttttacgagatacatgtgaaaattggcaaagtttccaataaaaagtctgctttattggggggttcaaaacaaaattcggttat
tttgagaacgaacaaacgtggtaacaaagattttgtctgagataatcagcatccttactttcacctacaaacctaactactatttttatcattttcttt
agttttcttataatattgtagttagttcttgatttacaacgttatcgattacttgactttttgctttgctccttcgcttcttacaactctaatcattatcgca
tattacgcaaaaacggacaatccatcaggtctagcacttttgctatgtttatgaaatagcggttcacagtttttttttttctaattctaatcctcttcat
ttttcttttattactatacgaaaaattaaagtatagtacttgagaaacctgttcatcttcataaatagtcattcgtcaaattttcaagctctcttatact
tttaaagaaccatccataattcgtcgtaaagttatacaaacagattgacgtgcttgttcgctgtttaccttgttacctaccaagtacgactgaagaa
acagattggaaaccttgagcccttcatccttcatgctgccaatctcatgaccctcgtgttcgtacgtcaatttgtccttatctgttagagtaatcgaga
gggtagtagatcgaagacaagttccggttccagggaatgtatctcggaagccatgaatcatacgctcttcattctcgtcgttgcgatccttgcgcaa
gagcattgcttttgtaaagccaccttttagatgtttaaagttggtatttcgtgcaggaaggatacgaactgcgtattgaattttacgtttcaaaagtt
cttcaataatacgacttccaacttcagcttccttcaaactcatttctactcccacattcgggtccacttgctcatttaaactttctgaatggtacgaa
gatcttgtttacgaatatagagctctatatcataaacgtaaatccccaaaaacgaaacaaaccttggtcctgatcctagtaaaaacatgcttttac
ggctgttatccttcttcaaattgtattcatcagcaagacctgtcagcttgtcaacgaagctttcgcattgtaatttctgagttttgattgctgttccaac
tgccgtgacagaagtcaaggccataagtgcaggccgcttccaaatccagttttgacaaaatttaaacgagaatgtttgaggtccaaacatcttaa
acgagtaattaccaaatgttccgatggtttgatgttctaatcagtcaggttaccctaaggaactaatagtaagtgaatagattgctttaaggtatga
ctactctttatagttataccacaaaactgaaaacatttagaataaaatagtaataaaaacagcatattttgaaaatgtaaaaacaaaataaaaat
aggttaatttaatacaatagaggaaaggaatccattcatctaactgctaaacaattaaattaattccacgtcatataaactcagaacacttattct
aaaaacctgaaaaacgttagaggctagcaaagttcctaatatctgctttgttgttcggctattaaacaatgaaaagatagcgttatccatcttcctc
ataagcttctcagattcaatataaagtccttcttgttacgactaataagtttgaaaaagactcctagtgtaagattgtcgattaaatgaattcaatat
gagattttcataggtttatatccttaaattcataaaaaggtatcacttatgcaccattggatttcttagatcatagtaaacaatccatatttctcttgtc
agtggcttttcttaaataccttgaagaatatttatattagcatcacttttacaaaaagacttccttttatctttgcacttgatatatgtccaggattttt
tgacgaagaaaagcaatccagcatttcaaccacaactagtaggttctgaagaatttgctttaaattcttcctaaacttcctttcagtttccatatttc
gtatgaaatcgatagaggcgacacagaaaagtcaacttaaataaagcaggtccctaattagaagctttatccatcaaaaaaaaagtattatatg
tatacaatctttccaatgaaaataatatgcattaattgctatacttccaggtctttatcaatggtactttatttggcaagttgcatctgtcttgggcatt
tcttgccatatgttattaatagtagtccacctacttccgagaacttttaattcattcttaggttttcttggagatgcacttttatattttgacggtttcaa
cgtcgacttcaaccttcctttagagactttgctagatgaaaacgtttctaaagatttttttatcttgcaagggatctgaaaatgtactgtagacatcat
cctttactaaagcaatacgccagcttggatgaccttttccgtaagcaattcgagcataatcggcagaatcaaatttgacataagctatatcatttttt
gaagaatcctgatctttacaaaaccgcacatccaacttagatggaataaaagcaaaatatgagaggatatcttcctcagccgtagtcttagcgat
ttcgctaataacaatcgtgtgtgagcttacagaaggatccgctttattaattcttgaaattgaggttggcgttggtgattgttccaagtaggaagaa
tagaagtgatcagagacagaagagtttgagcaagaatgattcaaggaaggtgatcgttgatctaaaatagaaagacatgttgaacttgatggtg
ggggtgtggattccttacaaagggctgctatagaagaagttgaagttggagatcctattggatgcccctgagagtaagcatttttgtcatatatccc

agaatttcccatatgggataatgaaggagaatccaaataggattcactttggaaagctgacggtaaatcactatcggagagtgaaacctggtcat
cacttttctttttttttcattagataactgacttagagtttgctcattacccgactcctccggttgttgatgactcagtctgcgtttcgttaccctcttttc
tgataagattattcgaggtgcttttggttttaagtcagactttgtcttggatttccgcctggatttcgcagcaggtttcgagagagatgaaaggggtt
cattagtgttcaaatttaaatctaaggggtattttcctccttcctcttgttttctttctttaagtaaaagattttcctcacgctgtactttttttgttcattt
tcatcaacattataaggttgcaaaagtccggcagctacgagtccctttcttttctgccaatctttcaaagccatagaggcgccaatgagattttcctc
tggctcccatgtattatcgtatgaatcataaccagtccactttatatagtattcattttcaccaaaattattaattcgatctgcaagaatgcgttcgac
ttcatacacgtctggctctacattcctttcgcttctagaatgcttcacggatttcctggaattagccatgacaatagaaaagcgtgtaacaatcttaa
atagaaatattaattgatattaaaattaacaaaatagaagaatatgcaaatgaattcaatgatcgttcgtaaggcatccagagtaatgagtgttg
agtatctttaaaagaaaactaattttcttacataaatgcttcttcatcggccaagtccgaaattcaaagaagtttgttaagaatttattcctcaacgc
gagattggctaaatgaggcaccaaaagaaagcaatgagtgctaagaagcacaagtttaaatctaaatgatacttataaatacataaactttactt
ggaacttgtttgtgatggacagaaacctgttttagggcggtgttgttgtaggaaatggtggtgacagagggatcttattaaataatgcagagtttta
ggtcgtttctgttatagcagttacttgagattatctaaaaccgttaggaaatctccatactaagatagcaatgaaattatttacgctgaaagagaat
caatgttatccaattccatgggtactcctgaccatatcacttagcttcttacaaagcgaaaatcaatcagtgagtattgaaatgtaagcaaaacctt
taatggtcttctttcgaagttaatatgcttggttttgcgacataagtatcaaagcctatgagcatcagaggaacatccaggacgatgcaacaccttg
tatgtgcaaacatggctaaagtgctcttccattttgctactttcacttgtcaagctcgtttgctttatgtgatttgaacccttttaggaaggtaaaccc
aaaagcaccgcgtgcgctaccgctttttggcatatgcctaattaaagataaaactattttgtacataaagttccatcttggatttcgcttgctaatg
actccctatatcagcatttttgttcaaattctttgtaatacgatacaagtttttgtatgattgttacctggttggagaatttaatgcgccaaaggtga
gacaataaaaaatatcaaaaataagactattcgaatcattttcaaagcatgcttgatatatatccattgaagaataattcagtggaatctatgtta
cgaattgttttgcattgtgttttcttgaatgcctcaaattatgtggagtaaaaaccaaggaagcgaaaatagcctaacaaaatttcaacacaaacc
tgacttttattctttatgaacagctaaacaaaatgtttctttcgtctcaatagtacgtacgccttgtcataataaggaatgtttgtttacctcataatat
cctcacaaatagcgtagcgtacctaaaaagtgctaaaaagaataacaaacatttaccgctactctctatagttgattttaggtaattataggatttt
ggcttcttttgctttattttgtttattgattcgttcctattcgttatggactaacctggaggatgaagttgggtaactgtcaataatgggtcctttggaa
cttgacaacaacaaactacgatttttttagaatcattcaacgctcatagagcaacggaaacgaagagaaaaaagggtaggtgaagaagagatttt
gaacggaaattggtttattgacacaaagaaccagataccaagtgaagaaaacgaacgatgtgccaagaagacgaatatgattacttgttcaaa
actgtattgatcggtgatagcggagtcggaaagtcgaatctgttaatgcgtttcacaagaaatgaatttaatatcgaaagcaagagtaccattggt
gtggagtttgctactagaaacattgtattagacagcaagaaaatcaaagctcaaatctgggatacagctggtcaggagcgctaccgggcaatta
catctgcttactatcgtggtgctgtcggcgctctcattgtttatgatattaccaagcagtcgtcctttgataacgttggccgctggctgaaggaattgc
gcgagcatgctgacagcaacatcgtcattatgcttgtcggcaacaagaccgatttgctgcatttgcgtgctgtatcaactgaagaagctcaggcat
tcgcaggtatgtatttttcatatctccccatttgctccctctgctccgaggaactcgctcaaatgggagttatcactgcttaatatgtttataccttttt
gtacgttgactaatctttctatagctgaaaacaacttgtcgtttatagagacttctgctatggatgccagcaacgtagaagaagcttttcaaactgt
cttaactggtatgtagatggtcgtcgtcttcgtattcagtactaacaaatatcttagaaatttttagaattgtctctaacagatctctcgaaagtggtg
atgaaggtgttcatcccatgagtggtcaaactctcagcatcactcctactatgaacgacgcgaacaagaagaaatcttcttctcaatgttgctaact
ttttacgggatttctcaatgcatataatctaatttgattccttgtctgcctttttcaagcttgcatcaacgtttgctcctttatatcgtccaagaattata
agttgattagtctttgataatcttcgcacaccctcttattttattgtcctgtcgcttgttcataggatgaagaggtttgtatatctagcctagtatgttct
aacacacggctatcgaagaattctcagaaatgtaccttctcgccttgtatacagcattttccgcattcccgggtttcttccctttatccagcattagta
tgaaatatttgattttgtttaaagaaagaaaaacattaactctgtggtagacgattactttaatggaaacgtatgtcataagataggaattcttcga
cgtctagctaaatctcaattaaaaagaatacacactgttagtaaactgtgaccatgattatgaattattatatcattatttatccctttacttataatt
tacgtgtccaaatatatttaaattcacataactctacaagcttaaatgggaaagtcgaaattgctagcccattgacgaacttgttctttcaacttaa
agatttccggaatttgacttccgtcaccaacaaagtttttaaagtctttaagtttggtctttccattctccttggcatgcttgttcaatttgatagttagc
tgaacaacttcgtcaataaggtcgacgatccgtacaaaatcttgttcgttgaagcctcgagtggtgcaagccggggtaccaatacgcaaaccatg
gggaataagtgcagacttgtcttcaggaacggtgttcttgttagccgagatattgatcaactccaaaatacgctcaaccctagctccgtcaatatct
ttactagtcaagtcgacaagaactaaatgggtgtcagtaccaccagaaaccaatttgtaatctttctttgcaaatgcatcggccattgctttggcgt
tcttgacaacattcttttgatattcaataaattcaggagtttttgcttggcctagagctacagccaaagcagtgattgtgtggttgtgagggccacct
tgatgaccaggaaagacagaaaagttaattttgtcttccaattcatagtaaacagggttgcccttcttgtcgtgattgcgaagaccacggcggtag
aagatcatagcgcctcgaggtccacgaagggatttatgggttgtagtggtgacaatatcggcatattcaaaaggagaaggaatgataccagcag
caacaagaccagaaatatgggccatatcacaaagcaagtaagcattacacttgtcagcgatttcacgcatgcgtttataatcaattaagcgggaa
taggcacttgcacctgcaacgatcaccttgggacggaattgcatagcagacttctccaaggcatcgtaatcaattataccggtttcggaattgatg
ccataaggcatggtacagaaatatgtagaaaccgcagaaattgacttctgaggtgtagaaaatccatgagataagtgtcctccgtgaggcaaat

ccaaacccattagacggtcatggggtttcataacagcttgataagcttgaaggttggcaggcgaacctgaatggggctgtacgttgacaccccaa
agctccgaattcaagcaaaaagcttcaagggcacgcttttgacacaaagattccgcttgatcgatgaattcattaccaccataatatctagctccc
gggtaaccttcgctatacttgttttgcatcacactaccaaggacatccataacagccttggaagtaaagttttcactagcaattaatcctatcgact
ccttttgtcgcttctcttcagattttaagattttgccgacctcagggtcatattggaagactgaacactgaagaagctatttccgttagtaattcattc
ttcaatatatgctgtttatttcttgtcaaactcgggtttggtaaagttgattttctcatgataaacatccaagtttgacagcaatcaattatttatgcag
ctactataccttttttgtgcactcggtaaagtgtaaaaccgcttgaaaacaaaactaatacctgttttcttaggaatctgtcgtgccaatgccatag
tggtaagtgataattaaggtcacgaaactgaaagtaaagtaggaacaagaaaaaagaattcttggctttgtcatttgtttgaacatgatgattctt
gattatcaatgtttttcgcggaaaatcatgtccgacatgatacacaggtataccataaatattttttgtcaacggtattgtaccggtatagaaacaa
ctagattccaagagcgtttcattccttcttagttccacttttatcgatcttaatcccctttagattcatacagcccacatttccttccttcaaatagtctg
gtacgtaaagcttactagtgtatctattacttcttcctttcctctcatcaaagacagttcctataatttaaattactaaagaacatttagtgatgttga
aaatcctaaaaccatataatttgaaatttacataagattctatatacaaatttatatagcttgtcatagatcaaaacaaatcaataatccatcaatt
ttttggtcaatattttcaattttttgacattatattttgctccttgttttgttccaaaaatcaatgaccaaaataaacgaaatgaataagttaatactc
cgacttccatcaataaaaatagctgtaaactttagctcaatgggttaattcctggttacgattaagtaacaccatttaccatgattcattggaaata
acctcattatctctaccaacagctgtaatttcagccaaatcttccttttcttccaattgaattctaccacttccagaggcgatacgatcacgttcaccg
gctgccagtttgaatgcaacacgacgagtgtaatcagtaaagcttctagcacgaggtccctccaaagtttccatatacttgtttatgatcgaatcat
catcaacaagtttgaacattgctaagaaggcggcctctgcagcaagtttgattggaataacaatagcacgaatacaactgaagacagcaggtgc
aagaatattgagatgaggtcggacgatgtcatatttttcttttgacaagattcgaatgataaccagagatagacgcttggaatcttgggatccaga
tgggctaacctccaggctaattctcaaagcctcgaacaaaacctttgactcgttgaagttttgaggaacgtcagcgagaagtgctttaccggcagc
caagacggcattctcggtaatataggtctctttttgctggcatgcatccgatattgcttgacagacactttcgctcaaacccatctcgataatcttctt
tgatccatatttaatgactgcattcaatatcaagattgagtattcatccaaagtttcaacgctgaaaatcttggtatcaagtagctgcctggcacga
gtgtcggtaagttgattaaacaaagcgccatacatcttggcacagataatcatatattcatttgtttcagatgatatttctcttaacaattgatcaac
agattcagctgaggcttcattcatgttttggccggatttagaaacgaccgcgaataatgcattcagcatagccttgcgcacaccagcatcaggagt
acgagcacctgctaccaattctgttacaataggagcgagacgagtttgtaaggtaataagagtacccaaagctgtagctgctctactgcgaatga
cttcactcgaaggatcacccaaacttttagcaaatgtacgttgtaactgaggcaaaaagggacgcaaaaatgtaggaattttagatagtatgatg
ttaagggtatacaaaatcgcactcttaacttcaactgggaatctttcgccacttatacgaatcaacggaccagtaatctgtgtgacatagggacga
agtttatcgggctcagcacgcataactatgtctgcaacacctaaagcagattgttcacgttgctcgtttgtaccgtgcatcaaaccatgcaggaag
ataggcaaaattgaactaataccctggggcagttcaaatgcaggcaatgtggtatcatgtacaccaacatcgtgtaatgacttgtatgtaatatta
atcactgattcaatcctgtctttacgcattgaagacgtgagggcatgctgagcagcaacagctgcagcgataacatcagtgctcctatcttcaaac
aatctaacaagatgactgatccaatcaggtacaaagcggaaatagtccagtttagagttttgaaagaatgtaagcatgtgcttagcagcaaaca
aacgcttcttgtaatcttcattctcagtaaatgagtaaagatgagccataagcaccgtgagtccatcttggtcttttacagatagaacgattgtgtc
aactgcttcttctaagctttgcaaattgtcatctttcgcaacaagtatggattccatgagggcattaaggattgacggtaatcgtctgtttaatgtag
gaccagctgcttgcgcaagagaagccaaagcccgagcattgaacgcagagattggttgtttaataagagtagggataaggacagggaaaattg
aggcagagcgacgagcaattatttctttaagtgcggacaatgcatattcggacttgtcatcagattcaagcaatttgagcaattcggggagaacct
catcaaccgcacgatcacctatcactgtttggagaatatcgaatgcttcagctgcagtttctcttacttccaaatctgaatccatgagtgcttcacgt
acagaataaacgaattggtcactataaatttcaagctgatcatgtgaagcactagaaatcaactcttgcaaagcaatacaaacaccaatcctatc
ttgtctatcagaagactctaaaccagccttcagtgatggaagaagttgggacaaaacatcaaaaccagctttcttcaataaatctcctaagctctt
aacacacatggcacgacggtcatttccagtgctgttcaagttgcccacaataagagctgttaacgtagacatgatatctctaacagtacgaggag
tatttacaacaatattcttccagatctgttgagcatgagtcctaacaacagcagcaatatcttgacgaaggatgtaaagtgaggataacacacggt
catgacgctcagaaccgatagtctccaataaggctttacgattaacatcagtcctggttccttcctcactttcatcagtttcttcttgcaatgtcttttt
gttaatacctgcaagcttatagatgagatcaccaacaagttgtactgaagaaagtctaatacgccaattatcatcaaataaacctttctcaagttc
agggagaagtaaatcgacagcacgggtagcataattattgacaatcatcttagcagctcttaatgaagcagtttgtacaagttcggactcatcag
ctaagccagaaagaataggtggaatagcacgagcaaggtatggctggaaacgagctccgaatgtagccggcaaataaataagaagagaaata
aagctttccctgatataaggagtaggactcgaagtattttgtaagatttcaggcagaacatcttcaagacgggtcaagcccaaaccagagagaac
ctcagacaaaccttgagcagcaccttgtcgatcaacttcactacactcacttctaagaatattgaacaattcaggaataagagttgggaacttagt
ttcacccaatttctccattaacgatcctaaagctttagcagcagttgcacgggtatcaggcacagggtcgataagtacttcccgtaatttgggcatt
aatgcatctagataaattgcgagattttcaggttcagtcaaacttgccattaatccaaaaatctttgcagattgacgctttgtagatgcattgcgttc
tctcataccatactgaataatgggtatgactaaagccaacgaaggagaatccaaataatgaacgaaagcagtcttaagaatggcagataatgca
tcatcagtgaaacgtgtacagtcacttaaggccttaaggagaataggtacaaggttctgtatttcaggattggaaatgacttcaccgaaacgcaa

caaacttttatttgccgtattgcgaacctgagaatgagagtcagtcaaaacctctgtcagcttgggaacaatagtaggtaaagagactgataact
gtttgggcgccatgaaggacattaaaccgagaatttctacagaagcacgcttagaccgccagttgtaatcgttcaagccatccaacaagctagga
agcaaaagtttaactccataagcgcttaactggctcattatatgtttcacagattccaaagtagcttctctaacttcagttgcactgtcaccaaagg
aagaaagcaataaaggaagtaaatcaggtaaataaggctcgaaataaattcctaacgtacgcgaaaaggcttccacacaaaacaaagcaccc
tgtctgattgatgaactttgcctgttcgacattgattcgcataattgtgccataatatcaaaatccttaaaggctttaatgcccaaacccttaaccaa
tccagccaaaccataagctgttccttttctctcggcaaaagatgaagcatttattaaaccggttgtcaaacggtcataatattcttttgctttaggaa
gtagctttttgatcaatggtgaaatacatgaagcaacagctaattgcacactttcactgggagtggacaaagcagaaattaagttttcgataacca
caactaatcgttggtcattctctcgcagatgctgtgcaacggtaccaaagagtactacagttgcctcacgtaatctatcattggcctcagttggcaa
agtatcaacgttaagtttagtttcgaacaattccatcaatgcctccacttgataggaaccaaaagcagtaatcgcttgttttccggctaccaacata
ttggaagctacagttactgaactatcagtaacgggaatctgagcatctgactgaacagaagacagcaaaaattccaaaaaaggtaccagtaaa
cttgaagaaatgtactgcgaaatatgcacaaagattatactgacaccttccctggtaacaatacctagatcccgaccgatagtctctttctttatga
taccgtattcatcatattccggaggtgaaggtaaggcgttcttactatatatttccattaatttcttggggagaacagatgagaaatcttcaaattca
tcgatcaagtcgacaaacgattttccgataagagtgtgaagataattagatttaacatcaagcaaaggtaatagttcggaatacgagtcttcattc
gcgtctaaaccgttttgcgtcgatatctgatgagcaacggcagcgttagattcattgtcatcataaagctccaagaaaatttccttaatgaagctat
attgggttaagtctaagacttgcaaagactgtaaaacggctgagcgaacagaactttcgttgtgacaagcatgttgtaaaagaatatttaattcgt
tatcagtgtaagatgtacggataccttgcacaagggaaagtaatgagttcttgatgtcctgataatgagcaggaacagtctcaagtaaatgcaaa
agtgctttcaaaaacatagcccgcatgcccctcaaacagaaagaatatggtgcttgaaatcctaacatttcaatcgctaataataagtgctcagtc
gatttttcgtcatctttagtagcgttttcataacaaagcttattaaccaatggaaaaatgcaagagaatgcttcaggctcaaaatatgcttgttcaat
ggcaaagcgtaccttataaaggaaagaatcaataatactctcatcaacgctaggattagcgttaagagcattgagaatggttatagctaaagaa
tccgaatatgcagattcacccaagcgagatggaagaccagaggcagctacaaccaacttgaagactactaaagccaggtcgccagtaaatggt
gtactcaaatgtagaactttaccaaaaaccagcacatcaatagcttcctcaacccacaatccaggacacaattggacagcatttcccatactttga
ataattccaagaccacgctcaagggatacaacaaggcgtctaacagactgtctaattttggattccttctcgagttgagcgtccaccaaagcctgc
tgatctttgttcaacttcataggcttcctctcagcttgcttagctcgaacttctgcttcccaacgctttgtttcataatccttcgtattcttgtccatttttt
gagtcttcttttctaatacgttctcccagagaattccatcaggagttttccaaatttcatattccaaatctgagacatctgctaatttgagattaacaa
gctgaccacggaaaaggtttacaaaaagaggagtggaagcctctggagcaacaaaaacaatcatcgaaatagaagaaatcattgctttctcctt
ttcagatctaacgtcactggacaatgcaacttcgatgtacttcacgatatcgtgaattcgttcagtgacaagtgttgcaggatctctatgcgaaagc
tggcataactgaatccaatcaaattggtcaacatatgcgaaggaaaggaataaaagttcaatcagataaaaatcatagtctgcaatctctttcgg
atttacgtcggaaatgtttgaggaaattgttaagagaattgattttaatagctgaaccaacttcttataagaattctcatcaaacttatcatccttttt
agaggcctcagactgataatgcaaattccaaaactgagagcagatgattttacgcaaaccaggattaactagtaccgcattctttaaagcttcaa
aagcaattttgctggtctggttgccagagaagttaaataaatttattattgactggaaccaggaagaagccaagctatcagttaagtctttggcat
agacactagcaaaattacaaagaccacgaagccaccataatttcgcggaatcagtagttaatttattaataattttgtttgaaaagagaatagtat
ctccatttacagagctgacaagcccatcgagtaagctttgcaaatcgtcgacagatttaagttcggtaagttccctatcacttcgttctttgttcatta
agcacgaatacacaacaggggccaaaatgcttccattctgtaaagcattggtaggattacttaaagcagccttgcttgatttattcaagtgctcca
agtaaaagtttaggaactccaaacgagaaggctcatcggctcgcaaatcccaagctaaatcggccaaagctaatatccaatgctgacgctcgta
tgcaactggcttatctaatgattttgaaaggaagtcgtaaatgcttctacttaaagaaatatttgacgatagaagcgatttgacattccttgtaacc
acagctgctaggtgataaaaactgctttcttgaaccttcatgaacaaaggcaggacttcattaagtaaagaagcagaatctacttctttaacagag
agatgagaaagacactcaatataaagattgcgatgttcattagaggtaactttgttcgttttcaaagcagaaaccaatttcaaagcaacatccata
agcattttggagtccttggcatgcttacaaaggaccgaaaatgtttgaaagcagttttgtcgaacagttgcattcgaactcttcaaatttgaagcaa
aggcctcgtaaagactagtcagcagtaaagaagaggcatcctttccagaatcggcgaaaccaatagccaatgatgatagaatacctgcaaaga
caagttccggagaacgtaataaagccttctcgatagaaggcaataggtcactggacaatttatctaaagataacaacaatccaatgctataaca
gaatccctcaccagcaaacctgtgtaaagccggttttgctgcaaggatgtgttgaggaatcatctttacaaaagggtctatattttctgatgccaaa
gcgcgaggctccgaagacaattgaaaatagtaacaagtgcccagtatgacaccgtataatggcaaagctttcggaagaacgagagctggtttgg
tttcgttcaaaagaaaagaaataaaagaggacaaatcattcagttgagcaaggaataaggaacggtaacaacgacgtgttgagtgcaaggcac
tacgaataatcgcgggttttccatcccgaaggacgacattcaagaattggctttgaagaggaagaatttccagcatccagtatttgaattcattgg
aagggagattagaaaggatgcagttcacccaaatcaaagcattaaaagatattgaaatagagctgctaggaatgcgctccaagcgaatagaag
tgcttcgaagacgatttacaagcgcacaaagagcaatcgaaggagataacttcgttatatgtgaaagtgtagcacatagttgtaagcgacattca
cgtgaaacatttctaggtaaaacttccataatattatctatgatagcaacgatactttttttcatcaatttctatatagtatcagtatcaaaatacctca
aagacctttgttaacacgtaccattggtttgattcacatgtcgtagaatatccaacataacctttttcgtaccaagaaaagcaagaatcgtttcact

gcttatcaaggtagaataaagcgttttcttttcattttgaaacgcttctattcctggtccctcgaccgccatggtgaaaaccgggaatatgaggtttg
gtggttatggaaaggtgaaaggttcttttgtgcagacgagaattggtcacttatatgaagagaagcgaagcgcgaattagaatgtcgtaatgcaa
aataaatgtacaaaagttttattaaaaaaatactagctaaatacataataaaagttagctataaatagaagcgaacttattttttcttctcagtga
atcgtatgcctaattacagaatagacaaaacaccaaaagttcgatttctaaaagtcctccaaaaaaaatgaacgttagatgaatagatagaaaa
acagtgtgacttatttaatcactaccatacatttcacatgtaccttatggttgaacaaatcacacagctttttatgaaagtgaaaaggagaaaaac
gatcatgacactttccgtttcatcatctttcatttttaaatcttaatgaccaaaatttgatgcctagaattgtgattctaaacaatcttcttaaacctga
aaagtctttttcttataatttatttactagagttcgttcaaaaaagtcctttaagtctgtttaataaggcatcgttattttacgttccattattataggaa
cgtgcacaacaaagttctgctgtgggttgcgactcactatatgcagcatttgaccttccattgtacaagatcagctagtaagaaccatctctcacag
attattccttgaaagaaaaagtcctaaataaaaagggagacgactttcgcaatatggtagcaacctttaaaaacgctggacagcgacaaaacca
tcgcgaacgctctcagcctaatgagcgtcgaaaattgggattgttggagaaacggaaagactatgtggctcgcgctcaagattataataataagc
agaaaaaagttaagcgtttgcgcgaaatagcgcaagaaagaaatccagatgagttttactatggaatgactcacaaaaagacgaagaacggc
gttcctcgtgaagaacgagaggattcttccattgatatggatactataaaaatcttaaagacacaagatattggttggattcgtcatcatcgagat
attgagcgctcgaaaattcaagaattagaacagaaaatgcacatggtcggagctcaacgttttaaatcggagcctcggaagcacacattgtttgt
cgatggaaaaaaagaagcagccaggtttgacccagctgaacactttcagaccagtgccgaattggtcaatcgagctgaaaatcgattacgcgtt
gatcaattaggagaaacgaatatgaaggtagacgagttttcaagtcgccttcacagtaagttgaaagagaaaacggctagagaattgcttttgc
ggaaaaagcgggatgctaagcttgcagcagccgaagaacgtgttcagttagaacggttattacaaggaaaaggaggccgccagaaaaagaaa
actgtaaatggaaaacccgtttataaatggagaaacgaacgtaaaaggtaagctcaaatcgctactggtcgctactggtttggtcactttaagaa
gactataagatgaaaagacggaaacgaaagaatccgtcctcttaattttgttggtttttcacatctattacttgggtttggtttttttgcataattggta
tctgataataaaatgaatcttttctacctaaaacatcgtatacatttcccgttaattagtattgattgatgagtatagcaatgcttatgtctaataaat
gacccaaaaaattaaccactctttcctttcgtttttctcacagagctcctaggacactttcagggaaaatgagagatgttcatttgaccaaaatcta
aaaaaatattaaaccgaattatactataagcacctagataatactagacgcactccttcacaattgtttacacaattcctaaaaagtcttccatag
aatgaacaaactttgaacatgcaatactcgcacttaaaggatagtcgctgatactaatcttctcgtccacccagccgtctgaaaagtcatccggac
catgagccatggcaccaatggcaatgcaaacagattgattaggttccaaagtttccaggtacttaggagtaggaacagtgggtgcatcaaaaga
taaagtagcctttcgacaattaggaggaaggtaatcggtaacagggtttttgataacctttagcaatttctcattaccattcacacttcgaatggag
agcttgtggaggagttgaaccatcaaaccagaaaaacgcttaaatgtacgagggatacgaacagaaggattcacttccacgagaaccttcttgg
ctgtatgaatataaacctgcagacgtccggccttgttaagaggggaatccaataatgttaacagacactggtgagtaatgtctggacgagcttga
gcaatgtttctgtttaatttcttcaagataccttgatggtcatcacaattcaaaagctggtattttgcatcctttgcctttccaactttgtaaatctcca
aacaagcttggtccaaaacaacgatcaatcgttgggtattggtattatgagagttgagaggacgagcgggcactggagctaaatgagtagtcgtt
ctgtgaacagatcctgcaggaagagggcgacttccagtatttttttgctccgttacttcttcctccttttctttttcatcttcttgtccatctacaattccc
gctggagatccttcatcattcgactcggactctttcgtcgtaaactgttccacagcactttgattctcgcgtaatcttcggagttcttcatcctctatttt
ttcagtattatctggaacaattgactcctctaccgctgtttcttgttcaggcgaattgtcaatgccatttttcccattctgagcggttctcttaacaattt
tcggctggttcgacttctctgaaacctccaaagaagacctcgactttcttttagagtatgctggcatgaattctaaatgcaacgactttgaatgaca
gttggcttttgtcaagttgcgccctggagaaaattgagtcgctctttcttgatacagcacactaaagtacatttagatatcacataacgattacgaa
cgaatatccttatttcacgtttatactgcttactagttccttattcttaattccggatagaagaaactatactagaaaagttggtattcataagatggt
acatacaacacttggcttttctacagaagtgtttcatatctcttcactaatgcaacaattactaacttataactttcacgcatataatgtatataaaa
atgctttcttattgactgctaaattactactttcttacactgaaagcagatcgaatcctttaaaaggtgagcagcaagagaaacacttaagctggat
ctcaaattgtctgtatttatgtcgacaccattgatgcctctacaccaatacatacattttaagaagaaaaattaaactattccaattgcaattaaca
actagaatcgcttaaaagaaaaatccaaagcggtgttggaatgcaagatgtatgcacaattcggataaataagaaaatctctgcgatagtaatc
tttgaaacgtagattatatttccaaaaaaattaccgcagaatgaaacctctactttcgttcacctccttttgccaactgttaaaaacagtcagcaatt
aggctccagtcgtcgtaatattgattacatgactgatgataatcatcataaaaaaacagctatcctttgataaacgttcagttaatcaaaaggccg
ctggtatccacaggaaaaaagaagcaaaaaggaattaaacacataaaaagaatattcttaatcatcaaactcaaattaaatatttagtttgacgt
gcttaaaaatgataaaagagaattgaaaaaaaagaacgaagagatgaggatagtaaaatatagacgagctagccctcatccaaggctcttaa
aatgaattaattaaaacacataaaaaccagtcagaataattaaaacagcaaagaatctcataaaccaaattcattggaaaaatattaaggcaat
ccaatcaatctaggaaaatacacgccaaaaacaaaacccctcacggatcaaaattgacataaggagacaaggataaaaagagtcgttgatttg
ttcataaaggtgtttgtagtcatatatggctgaccgagtaagaacccgaatatatagcacaacactaacaccgtacattgaacacaacgaaacca
taaataaataatgagtcccgtcgcaaagaaccaaatctattaattcgttaacaatcgtaaacgactccagcaaaaaattagaaaagacgcgaca
cagatatctcgtataatatcgcaaaccaaaagacttgaagaaaaaccactcttccgttccaaaaaaaaagaggaacataagtgacgagaataa
aatagacatagggagtcatttctgaagtgtctttctttggcggaggtgaggtcttgctgaattaccaacaatggcaacaactaaaaccaaaggag

cagatgtaacaaaaaaaggatgtaaaggaagcaaatagtaagtagaatggaaactgtaccagaaaagccggagcaaaacaggaaatgaact
atgaataaaagaaacataactatacacgcgagaagaagcaacttcttatgcacttccgtgatggagcatacggaaaagacgttgttcacggtca
cgatgaaatagcaaatctaaggctgaagtagaatggcaatgaaaaccatcagcaagggaattagattgaaagtgagaaggtgagacttgtcta
taagatgcatgatcgaagataggaacacctactccttcgcaggaaggtgtagaagaagagtgagtcatactgggtacagcatgattattaatata
attatcagacacagagcatccacttgagggaggagagtaccagcacatgttggctgcgggcacatgagctggggcaggaagagcctgggaagg
tgtgggaggtaaagaagcataaatttgttgactgggagaaggtgaggcaaatgaagactcgggcttctcgactgcatgggatgcattaaggaaa
gtagggatagggagattagaagcggcaggagaatgagtaaaagtgggaccagcataaagagacgaattaagttcggaagaagacatggtag
aagaggtagtggactttgcactaccaggaggattaggcgaggaagaattcgagttttcttcaacaccattagccacattcaaaaagtgaaactgc
tcgttctttccaggagattgattcttcacacgaccagaagagccgttgctgttcttacgagagccgccagaaacacctccatttggctttaactttcg
gctgtggccattactgccattgcgaggcttttcaacatgtaaagcgttttccgcttcgtttaagccaaatggaatactctttgccttcattgctttcgg
ggacgctgttcccttgtcgcggcgtgaagttatcgatggcgaatccaaactaaattctacatcgtggaaagaaaccattcctccaccagaactagc
tttgttgaactttctgttgcgctgaattctatgtccaattcgtttttccatttttttctcagttcaaactttacacaatcactgatactcatgaaattcgttg
aaatgatattggtaaaggataactcttaaagccgcaagttaacgcctccatatacgtcggcaatcttgaaattcgtagtaacctattctgcgttaag
tctctttaaatacaataacttcaccactacctaaaattttgccacctgcttgtttgtttacgaagtggactaaaaggcacttgaattgtttttttctttca
tcttacatcagtttaaattggctgactggtcttttttttttcaaggaaaccggttccatatgacctcagcttgagaagtgtttttacgaaaagtaaaca
aaccccgtaagcgaatgcgctaaccttgaacgccttcaagccatcagatttcgctttttttgcttgtttatagggagggtttctttttattttatttctac
aaggttccttgttacatttaggtgaaatataagaatcaaaaacaaggcaccattctgttcgttgattgactattatcgttgtgttggctacttggaag
ctcctttgaccgaatttttttattatggaccgatggtatgagcctgactttaaccttcgatcccttcgggtcgcagatctaaaaaggattttgcaaaat
gctaatgttgaatatcctgtttatggaagaaaagcggactacctacaacttgttgagcggctgcgtaaacgaagggcaggcggcaactcgatttc
tgtcaaaactactccaagtgatttttccagcacttcttctgcaaattccaacctcaactctgataataaaaatgcacttcctagttcccgtgccagtg
catcctccctaaaatccacgcaagcaaccggtaccagtcgtagctcctcaacccaatccaaaccttctctatcgcggtcttcctcgtcctcctcatct
tctcctcctacagaaacagaggaaactgctcacctgtcatcattccctactggcttccagtttccccatacctcaaaaaacaatacctttcagtcttc
cgtattagaaggcagccccaatgtcgagacttctataaccttgaccagtcccccaccattatcatctacaccaaagttccggcctcatgcctcaccc
cgcgattcttttcctatatcgtctatggatagttctaccttgcaaggttcacctaggggtatacggaaactcaagcccatttcttcaagtagcccgag
acaaactccagggaagcctatagtgagccctcgtcgcccttttccatcatcatcaaatggttccctctcttatcggaatcaacgttcccccgttaag
gaaactcagccaatttctaggaagctcacaaaagacaatccgctctcgtttttagatgatgatcttgattcttctgaagaagatgctctatttgaag
attatgtacataagcaagaccattctaactatacaaagcgctcattatgctcaaagttggctcgaaaagctttccgcctttattcggttatgagaga
tgcatcttccttgccgaactttttttcattcccagcatcgtattcatcccccgcaggtattgacaattgtacttggtatcttcgtagcgattttcttgactt
tatggagagaagaggtctatcgtgctggattttgtgatgtaccttcttctgggaattcaacaatccttcaaaaatttggacttggggcttcatgtact
ccttgtccatcccactccaaatgtttgcctaatttcgaacttcagtgtgattcaggttacattttaagcgagccttggtattctaggtttggcctgtggc
caaaaaaaaaatgtgttggtgatacaaatagggaagaaagtgtaacaattttctatgaagaatgtttggacgttttgcgaacatggaattcaatt
ctccaatgtggtcgtttgtctgatgatcttattgaaaaaaataaaacgctttccaaaacaaatccgtacgttttcgaggaattaaaaggatctaag
atgacacatcatcctctattttctatagccttattacgacatacactgttggaaaagaaatccccaaacctctctttaggtgattttaatgatttattt
aaagcagctttgtatcagcttgctaatgcagaagaaattgaaataaatgaagaaaatattggttcccatagctgggctaatgtacctttaagatgc
cgacttcgacgacaggtttatcgaacaatttggaagtataaatttactcttcttctttttatcctagtcaccatctgtctttataaggcttatacttattt
ttacttacgcaaaaaagttcagaagtatttaccttttgcttcacgtttctgtatcgattccttaaagcgacaaaaggctttatatcaaagttcacgta
cttcccagcctgttgttccgctaattgagatgcatgatttattgcttcattcacatggtgtattagagcagtcagatctcactgaaaaagaaggtcaa
ttgttatgggattcagtcgtcaaaaaagttgaacaagtgggaagtatacggacccgcgaagctgaagtagatggagaatggacccgtgtttggg
agtgggtgggtacgaatcctctcgatcctttaccttcgcagtaatctattcgcatatttactttaattgacgaacatgtaaattataaactttaaagat
aacaatggaaagaatgtcttttgatatattgttgaaatcttgatgctacatgaattcttaatgcaatggatggaaaggaaatttagcaacgtgtact
ccttttaagtttacgtaacgggcattatatgcgaatcgtgtaagtaatttcttttagtgccataaacagtaggcatgatctataatacatctaaacttt
ttgatcgcagtctgcctaaaaagtatacgcatctgatttttaattcgccattggtatcccaaattttattaaacttatttagattgacattattacgtta
aatagacactgaccggtaaattaaaactccacacaactgtttcttgtaaaccaagatgtcatttgaaaagattcggttcctgaggtcattttaatta
taacgacgatacgagaggaaaatcggtcactggttcatcttcagcttcctcatcttcgtccaatccaggaactggctgccattcattataaaatggt
tggacaatcgacgaagattttgatagacgataaggatcgaaggggaaaaacgaatcgattgtttctaatccatcccgaattgatccctttctgtttt
gctccaaaattgaataaatatacataaagttcaagtgatttgctatcctagcaaactgatgaacaacattaggtgagcagtatcgcaatggattta
agcgggaaagaacggatctatgaagaacttccaggcctggaagccattctgccggcttgggatccattggatctgtctcatccgagattcgcagc
tcttgccaacggaaacagaaaatgtataatacgctttggttaatggcataaaaaacagaatgtttggatatcaaatcatcggatagctcgttttcg

cattgatccaagtaggcctcgatccaacgagtcattaggccgacaataacttgaatcgtcgaggcttccaaagttttagcacgagacacataaga
cccgaggtacatggctccactaatccgaagcgtatagggttgagatggatccaagcaaacttccgtgaggactcccaagaaaatgtcggtaaatt
ctggatttagttgggaagcccagaagattaaaaactgtgtataccgacagcgaaaggttttaagaacagtattaacaaatgatcgaatcagacta
ttgtaaacaacgtaacgatccctactttggtttaaattctttgttaaataagaaaacaatgtatacaaaagctggtccagttgcagcaaggtttgttt
ggtatcgacaggagacaagggatcatcgttcaatagagtactagaagtaagatcctccgaagtaccagcgtggcgctcatacaaagcagatgc
agttaatacatcggagctagcatcatctgaaatatctgattcctcatcatcctcgatatcaagttctacttgaatctcaacatcaacttgaatgatttt
atcaataattgcatgcaatacaggacctttaagagatggtacatattcacaaatacttaaaacgttcgaaatataggtcatttgtgccagaatact
ttcatccttgtacggaaactcctcaagtatacaactatacaagtaagaatgcgcacgagggacaagatctaacacatattttaaagccatatgag
catgttcataatgaattgctaaactattactctggtagcacatgtgctggataagcatggtcatgaccatgggcaaataggtagattgagcagaaa
gcaaatgtcccaaaaaccttgtgtagcagcggacaaaaggttcatttctcgatacccacactgagcccagtatgctcataatcaaagatgagcaa
gaactatcaagtcgagaaacgttgcatgtaagggcagataataaattttgaatctgaatagatgaaggagcatcttttgcatcaggccttataga
gaattgtctcctcatatcttcatatgcagcaacatttcccgcggttttatcatcaagggctttactaacgaatgcgcgaaacatgtgctgcattaatt
gagagtcatgtttggatgcattaccttcagatgggaggacgctagaatcggtaggaggattcatcatgccattcgaaagggaagaatctattaac
gttgaagcttgaataattgacggcatccttaagcaagtttcataaactagtttatgttttgtgtaaaaaaaataaaaaaattgtccaaaattcactt
ctctattcagagggctatagagtaataattaaatttctaacaaaacatatgttagcataagaatgggagacctcttcgtttgtcatggaacttatttt
tacattagagcaattgcctctcaagtcagcgttgatagaaatgagagtgtccagcttcaaagcaattttctcagcaaaaacaccatctgagcactt
ctacttactttatgttttcaagaaatgtccgatgtacacaatatcctctaaagtaaaaaaaacttttccaatgcaacaaagatgtgaaagatgtgg
agtctgtcgtgaaaacttcacaattgacgataataagccgctcgaaaacagtgaaatattaataaattaaattaaattaaaccagtataatgcag
tttcccctaaattaccagcaattcctattattattaagttttgttgttggtaaaagaaaagtttctacttgctggtagtaaatgtgttcgtttactgaag
gttgctacacctcgttaattgatgctgcaaaaaaacaaaacaatatatcttggataaacgatttattaggcaaggatagaaaacttgatcagtga
gagcattatctatattcgtaaaaaactaaatcacttttgatgaaggtcgttggataatggtttgaacgctttttgtgtggacgcatgcttgacttttg
atcgcgaagcatttgtctataaagaccgtaaattccagtgcttagaggaagaatgtctctattgcatctcccagatatgaaataatgatgcatgtcc
tccattgtgaagacacttcgataattaggtccagcaaagtatttgtcagaaggtatcaaggacgctgcataaggataggaggagttctctggagg
aggcgagatccatgaatttgttctctccatccatactggtatagttgtcgatttcagaggcgtttcgggatcactcatgtaaataggtaaagtgcctt
gagcaatagtccccaaaatcttagaaggagccacactaggttgatttgtttccggatctattcgtttacacaaactcagatacggctttaactttga
gtgtaatgagggatctgtgagcatctgacgcattggttcgtttctacctccagtaatgagtaccacgtcaaagtctagaaaagaaaaaccttcatc
tgtataatgctttggttcgataaattcttcaagggtacatagtatgctgtagatatccatagcctctttggaaggtcccatgatgcttcctttccatcc
acatgtataagaagggtctggtctagctgtccttggaggatcaattggtcttggaaacacaaattcaaccagaaaaccacgatcggtaaagtaac
gccaaggaagacacacctgttctacgtcgaatccttcgtccgccaggacaattgccaaatgaggagataaatgagcattccggccaaatacagg
ggcatttgctgcagcaaaaggtaaaggatttggttgtaatccaacgtcaacgtcgttgattatgtccttgtttgatatctgaaaataaaatgccatg
acggaacgcgacgaagccacaatctaaacgaattggtctctcaataatgtttaatttttaaacaaattcaatccaaagtctttgtataagttgtggt
aaagactagtaggaaagtatacgaagaaggtttttgttttccaaaaagagagcaaaaataaataaataaataaataaacaagacttctactgat
ttctgtactttccaacgtttgtagtgctataaaaatattctgtgtaatctctatatatataacgatcattaaacgagaaaataggctgacgtttcgat
attacctcgaaccacccaactttgcatttattcaattacttcatcatttgtttaaaattgagcaagacgaagccggtaaacaagaaatattgggatg
tttttgcttttatagacgattgagtaattagaggtcttttgtagtaagcggattcagctgctttttaaagtccgaaaacttaagcaaattctgcttcta
gcacttctatttcatctcattttgttttctttttggtcattttactcatgaagcttagggtattttgtgaagaatttcctatccgacaatggattcacaca
gccagttttggattggttgacaccactggtaccgtagcggacctttgcaaaacaatctgggaatggaatgaaggtcccccattctggaaacttcag
cttttaaatgaaggatacgctgtccccatgacaggaatcttggcccgttacttaaaggaagatgcaaagatccaactaataggacaggatttacg
ttctatatggaaactagaaaacttttctacttctccttatgaattcatttctagcgttggtcaacttcctgaaaatactgaacaaatgcatcaggcaa
acttggagcatctcgtatcatggaaggatgtagagatgtgttcataaattacgggattataaatttagtactcgagttggtttagatagctgttaca
ctagcaagaagacatatgttaattttggacagcaaaaaaagaattaaaccagagaacggatctcttccaatattaccggttatgcaaaattcaac
agcaagttggcataattttattttgcgatgaaaacatctattcttgattgaccacggagtccaatttttcacattgttttattattaaaatgtataaag
actcaagcattacgatgacagcacttacaatgaattctttttttcaacttgggcaaagatttgttttaaaaaaaaacatagatagcatcaattagaat
actttgcagcaagagaaaacgactacctaaacttcaactctttaaaaacatcctttccctacaacaactcgcaagagcacgtttgttttcaacgta
aacaacctaatacatagacacaagggaagattacattttcaagtcaggtaatctaacgcaaattttgaagctatcgaatccaacccgaaccgatg
caaacgcaaaacgtaaattttattgcttacaagccttcaccaatggtgacggggaactcaaagttgacggtcttaccggtgagtttgtggtagacg
ctagagaaagaggacaacttgtagtcaacagtgctggcttctctgttgtcaaggaagaccttgatggacttgcggccgtcagtgccttggcgggta
cgcttaccgatgataacggtagggaaaacaatgtcttcaagaatggcactgtgaacagaggtcaaagtacgagagcgagggcgcttttgaccaa

cacgggacttacgaccgggcttgggcaaaatacgacgttgagcgatgaaaataacgtgacggtcagcaaacttcttctcgagctcgcgggtcaa
acgagcctggcacttgtggaaagcggcgagaagaggctggggcacgaaaacaacgattgccttctttccagctccaacttcgacttcacgagca
gaagtgatttggagaggacggagctccttggccatgtccttagaagagttctctaaatcaaagagacattgagcaacaaggaggtcggtttcact
gggctgggagctcgaacgcttgacaatcttgttgagagcagacattgctgttgtgtagaaaagaaaatgtttcgactctcccatattctcttttgtg
actgctctcttcggtttgggtaacactgtccatcccatttggtactgttacttggttgatggtagctgtattgggtaaataggacttccacataattttt
ttctagtaaattataacaatgtcatattaaaactgaaattatcaaattccaatagcgcaattcattttcccaatttctcctaggctttccaattcaaga
tgcgtttacctgtctaaaaaacagtcacatttcacaacacagacaaagtatacagtacgagaaaatgttagtttacgagtcgcttttgcgcgcgtt
agacgctttcagcgttctataaggagcctgttaatatgatagcagtatgattaggaaggaagtcgcactgaaaagcgtttacagaggtgctgcttg
aaaaatgctcatcagtaggaattttatagctttaaaatcatttttttttcttttcttttctttttcttaattcttaattctctgagtgttagggagcattata
agaggtggtagctttcgtcaaatctcaatgtttacaaatagtcgtcgctattggttgggaacatgtaaacaaaagaaacttataggtaggtaggtt
tcttttctgtggaatttcattttgtcgtttctatagttgttttataaataaatgaagaactttttgcatcttttcaccgcccatacttactgacttgatttg
attgtttcttactacagatttagtgaagtgtgtaaataagtaaatctaaaataaaataataggccttttgcgattgaataaagaaaatgagaaaac
gatttcctttcactttaagtgcacaaaacttgagagtcgcattggtctgtccatttcgaaatttaagttgctaccacatattgtttgatatatttgacg
gtaaacaaaccaattggactggcattgagaattcatttctagttgattttagaaccaagaacaaaagttacaaaaagggaagtttaaaatcaaca
gaaacgaagaccgcctcaataggattccacacagtagctttatgagaactttttcggtagaagaatgaaggatctgctttgcatttcaagtagtta
acttgtttttctttttttgtagagaaaaaaaatatatgctaaataaccaccattatgaaatcttaaatcaagaattgcagtttgtttacattgccaaa
atgtatcctttccaataagcgacttttagctatctttctataaattataaatatgtactttgtttgtatttaactttactataatagtcatttataaaata
tgccatgaaatcgtttatgagcgaccttggtaaagaacggtttgtaaataataaatatgggggttttctgttatttccaattatggttaatccataaa
ataatatttattatttatgaaccctctttaggcatgttgcttactgtttttttactatgcgttttagagaatatgatattctcataagtaagaccgacgc
gtctttctcttcccaagaaccacaatctttacaatgtctgaatctcggcaagagttgctggcttggatcaaccaagtaagtaaataatgaagaatgt
caaaaaaaaaagatttctaacatatttgtttcttaggtcacaaaacttggtctttcaaggattgaggattgtggaaaaggttacgccatgcttgta
attatggattctatttatcaagatgttcccctaaaaaaagtgaactttgaatgcaacaatgattatcagtacataaacaattggaaagttcttcagc
aagtatttttaaggaaaggaatcgataaagtggttgacccagaacgtctttcacgttgtaaaatgcaggataatttagagtttgttcaatgggcaa
agcgtttttgggaccaatattacccaggcggtgactatgatgctttggcgcgtcgtggtggacgcggcctggtacaacatggagcatctcccaatg
ctcctagaagaaggcaagtgtcatctggaagtaccggacagcccgctccacgatctagtttgaatacaacaggcgctgctacaaacaatactgcc
gctgtacttcgtgccaagcaagctcaacaacagattgccaatttagaaggccaattattagaagcaaatgaaacaatgtttgcattggaacgcga
gagagacttttatttcaataaattacgcgaaatcgaaattttagttcaaacacatttgagtgcagaaccaatgtcccatgaaagtatgttagagag
aatacaaacaatcctttattctactgaagatggctttgagcttcctccagaaacagaaagatcgacggatgtgtatccacttgtggatcataacat
gagtaatgtcgttgatgaacctcacatggaagaagccaagcccgtagatatgtcctctaatcccggtgctcctgactttgtgcgcgctcgtttacaa
agtttagaagttgacgatgatgacgaaaatcttactttttaaagtaatcatatttcgctcattctttttatttccttattctgtttcgatgcattctcttac
acattgtccttgtctttcttttcattttgtgcaaaaatcacttaccttttactagcggcgatctgggtatgaccattgttaaaatatgtgatgttttctaa
tttatcatcgtcttttattggcgttatttcttcatgtttgcaaaatcttaataaaacttatggttgtcatttataagaaatttcgttgtgccagataaccc
atactccaaacttccttgtcaaaatttattatatatatatatatttcaaagactgaatttgaaaaagtattcatactacctttgaaatgattctcttatc
gagatttcccggtttgctcattgaagctatcctatttgcaaatactattttagtagttgtcatggcagaaaaggtttaatctttttgcattaaaacaag
gtggaaagtaatgccagcaaataccatacaagcttagccaaatctgtaattgtaagtttaaccccagaaaagccttcgaaataaccaaaatcat
attcgttacctaaatataacataacaagctttttcattaataagaaccaagactacacaggaaagctatttattgaccatactcttgttttgtggtta
agtctttaaataatgcaaataagacttctcctaatttgactatccaattccgaaagctacctgttctttacatcacaaaacccgaacttctactcaat
gacttaaattagtcattaaatgttattattacttgctggaagtttagtttccatatgccgaaagaagtggagcagtatcttgatcttaatgaagagtt
cataaaaactctagcaggtataaggaaaagactgcgaagtcgatatttaaatcctcaaaccttagaagatttcattcacgcattagcccatgttcg
agctatgaaacggctaaactcattttgctctaactttgaaggagaatatacatcacagtccggtcaatcaagtcaatcagcacaggaagcatcatt
caccgttgtaaacaatgcagacgtgcgtcaatcgatggatccctctttctttcaaggaaacatactggagttaattaatgattacgctgagtactcc
acgcaattaatttctatgggaaccagaaagtcgtgcattacaccttcaaccgaacctaataggtcgaaaaagaaaacagcaggtattctagactg
cccagacgaaattcttatattaatattccaaaagtgctttgatgctacctacaaagaaagcatcccatttgcttttacttatcgaagggattggcac
acattacttgatgatttgccatatacttgtacccgctttcgtaaaattctgagcccaaagaacgatgggttttggaagcgtgtcttagacttatataa
aaaacctgaggttccgtcttctcagtttgatacttcatctaggagtcttccattcccttacgttcaaatgccaaatgttcttcctgtcttgttagatcca
agtatcgaaacatattcgaatagctcaccttcatctagcttggcctcgttaccctcgcagattcaaacacaaaacaccctttctactggaagtcctt
gtgaagagccttcttacattgagtttgcttgtaatgtagttggtcgttgtactgtatgcaaattgctgcctaagcgtacaaagagtcgggaatgcat
aaagtcgttttatagatcatctattgcaactaacatttgtgatcaatgcttagaggcaatcatagacttttatgagccagtaagtcggtacgattttt

atgtgatggtgaatcaatttggtgttggttatattaccaggccaggtcaacgatttccttcttggctttctgaacatgtccaacttgcacgagatgaa
gaaaaggcatacaaatacatctcttcctcacaaataaaatttgcaagtgacctgtttaggttattccaatcacaattctgaaactatctacgagaat
ttacttgcaagctgctagctacaggaacttccctatacagaagacttgattctttgaaggaatgattcaacgtatatcaaatatcatacggttactt
agttgaaagacaatgaacgaacaataaaaataagattactatgagaaatgaccaaaacccttttgtcaatattaatacaagtaccgtaaccaac
gatgaagtttaaaatataatatcctatataggtgaatcggaaaaggaaaaacgaggtaaaaagatacggcaagtaaaaagtatgccaatcaag
agctttatacccgattaagataacttttttttaaaaaaagaaacaaacgttataaaaggatgcacctttgttcctgagaaaggaactatcgcaatga
ccaaattaaagaccaacacattacgaaaaacaggccggaaaatagcataaaagattcaaaagcccttctaggaatttttaggaaaagatggaa
cttatcaattgaatgagtgggtagtaagaaattcaaagaaaaataagcaccagtttttttttctatatgcattgtcttgaaaaaaatagttgataag
tatttactagagcacatcctaggccaacgacgtcaattgaaaaccatttatttaagcctcaacaccagggagagtgcagttcatagcaatatggcc
aggttgattgcacttatagcataattgaccatcgttggattgttggcattcaaagctacgatgaccaatcttaccacaagaataacacttgacaccc
atggtacagtcacgagcctggtgaccaacagaaccacaagcataacagttcataggacgggatccaaaacggccgttagtgcggcagtctctag
caatgtgaccaacgcgtccacatttgtagcattcagcgccttggcggggattaggattaggattggggttggaagggcattcacgaacaagatgg
ccaccagtgccacaagcgtaacaggtcttttcttgctggggttcagtgcaatcactggccttgtgtccggtttgattgcagttatagcagatggcgc
ccttggtgcattcacgagcttgatgtccgttttcaccacaattgtaacagcggaatccggcacgggggaaagaaggggcggcttgtgactccatat
tttcttccatagtgattatgatttaaaatccgggttgtattaaaactttgggaattttctcgtaaaaattcgcttgaaaaaaatatacagagttgtga
ctgttcttgcggggcaatattggtacctgaagtaggtaacaatgcggcgagtttcacgaatataaacaagggaaaacgagaaattggcttgcttt
atttgttttataatcaataaagcttataatgttgctcttcatttcagcgagcttcattttcaattcgttcaagttgaacagaggatttagcaatatgcat
tgtataatcaaatagaacgaatgttttctacaatacgattttataaacaagagttgcttcaaagtcagtaagtttttattgcaattgtatggttcatgt
tggtcattgtttgttttatctaagaaaaacgaagaaaaatcgttaggaagaaggtaaaatctttcatgcatcaaaggtaaatttctttcggaaagt
gcgtaatgcgtggctttttttattttctctttttctcttttacccatgaaaatcttgcatttgggaattcttgctgggtaacatatcgtattattttcgctat
gatgatgagaacccaacgataacggacactcagtaataacttacagtacaaactggttatagcgctcggaattccttcagtgttgtaatatgattt
gttgtttttttagaatactatacgttcaaaaaatctaatgtgtgtttgatctatattggcaaatattcaaaccccggcaaaaagaattgcttcggccgg
gaatcgaacccgggtcgcttcgatggcaacgaagcattataccactaaaccaccaaagcagttgttataagactaatattctttatcatatataac
ttagtaaggtattggacactgaagctatgtaagtgtttggtttgcaaaagcaaaaaaatttcttagtaagtcatagacttgtaaacttgcattgaaa
ggaagagtccagaaataggaagactcagaaaaaaagtagtgcagtacccaatttccatgatgccttttacacatacaatgcaaacagggctatc
ttactcggacggttttgctgaactgcaatcaccggagcgaaattagaatgtgtgcagtcgcaataggtaatctatgaacaactttaaatttaaagg
atattatatctgatatattttgatctcctaagaattgtgaacttgaaaatcgctgtttggttatagaagaagaaaaaaaaaaaaaaaaaaaaaa
aaaaaaaaaaaaaaaaaaaggaatactaatactttaaagattagaagcatacctgcatcattgtgaaaactataactgttctgttgttttgtaatt
tatttaccaaaatttattcacggcatggaaataaaaaggcaattcatttagaacgagtaaaattcagcatagtaagaaaacaaaaatgtatatttt
tttcaaagaaaaaaaagtcatgctttgtaaataagaatggggaaaaacttcagaaatatagactatggctttcccggaatgtagtcgtcaaagat
agtagctaccatctatcataaacagccctttttttcattgctgtgtaaactgtgcaatgtcttaccatttccaaactttttcaactctctgattgagagat
aattggaagtttattggtgctttagccattcgttgtttgctacattctacagtaagtggagcatttttgttcgtcacaagttcaactctttgctcacaag
aagtaaatttaataaaataatcttcaacttaacaggagccttcatctactgtataatattcgtgtgtatttccaatactgctgttgggcaactagtaa
atggtattggacgagtagttggtgctataaattttgttgtttgtcaaaaattttataaagtttttatttattgctttgggttggtgttcgtaagctcaact
gaacacaccagttcgcctatgcttcccgggaatcaacaaagggatgatgcactagttcctccgccgacaacgacgaatgaagaaattgaacttc
agagcaatcctaagctaatgctaatttgtttacctttcttttgccttgagtcttaactcttccgattgaaagtcactaaatgaaaaagggaacattac
aagcggtttattttgaggaagatttatttctatgccttggttaagcgcttttacattttatttattaacagattttaactacgatataatccttgtatcaa
tatgaattggatgttgacgaaaacaattgtacaaaaggaaggcatgttgcgttttagcccgtcgacaagaaataaatgggttggttgatatcaatc
tagaatcttatatgcatacatctgctattatttataattgtaattgaatacaatcgtttcatctttttcttacaaaacgtgactccatcgaatttacattt
gggtagtgtttgggtttactgctactctggaggtaacaatctgcagcaattttagaacagtttgtacatttttttcgaataactcgagaaagtattgag
aaagtaaacataataatcttaaaattaataaaaaaatgtccttaggtttgctatatccttagtgtttaagaggtcctcagggttaaccaacgagaa
ttggtggctttgttcgatagtatgttcatatacttagagacaacttcactgggaatacgtgtgtcgtatggtttataataaaatttatttaaatatctg
ctaacaaacaatacggaagtaaaagtttctgtattctacttgagatccagaggataatatcctatatctattagacggctggaaattcattaacag
gaggaaaggttatcattcttctgacccttagcgacgattggactcatataaactaatctagtggtattccttatttcagtaaactctgataagatcag
tggcttactagaataagttatccctatacaaacactatatgttttatttttgtttctatctaagcgatactcattaaaaggtttttaggtcacctatgac
acgaacctttaaaccatctacatctaagtaaagaacctcttgtaatatcgacagacttatgcccatttaactttactaattagctatgcccacagctc
aattcttgaatgactacatttctttacactatatttagtctatctacgtacgacaagttatgctgctttggtggtttagtggtataatgcttcgttgccat
cgaagcgacccgggttcgattcccggccgaagcaatgtttttcagcagtttgcccaacacagatgaaagtacactgtagaaccaaatttttttggct

cgaatgaaatacgctgtttggcctttttccacaataaagtatgtttattactgtgctctaacgatgtattttagtaaatacccttcagtaagaaatca
atagttgcctgcatggtacgagttcatttcaatgtgtataatcgagtttcagatttcataaagtgcaaaagccatgtctattgacggttcaagtgca
ggtagttaatcgtgtcaaacgaatgacttgaaacagcatttatagaagcgaactatactatttgtctagttgtcgtaatctaggtgatccttgtgtgc
ttttacagtaactaacgggctcataagaaacggcaattactagatttcatagcttcatccttggtcagtctcgcttggaaaagttaaaaaataaac
gaaaagtcttgatccattcggatttagcaattctggattgcagattgatttaaaccgaatattggaagtcataatctgtcaatcttctataaaatttt
aaatataaaatattaaattgctcagcgtagcatgcatctagaatactatgaaagaaccccatacaatcgacaagaattacgcatgaagtcaaaa
agaaatcttcataagattacttaaaagatcaaaaatatgaagcttactttacaaatttttactatatcccttaaagagcctagtttttctgtgtaata
gacacacattgctttgttttctaaaatgtgcaccgagtacatcaattgttaagataatatttctaaaaataatactaagaagttgaaaaacctttgc
ttcggccgggaatcgaacccgggtcgcttcgatggcaacgaagcattataccactaaaccaccaaagcagtagatgatatgcaatatataattac
ggttatgtaactcgattatagtaactgaaaagttgatacttctatgcaagaatgaatatggacaggagagggaagtatttattgttcttctattttat
cacaataatacttagttcacaatttctctctcacatctacagatggatctactcacttcggcttaccattaaaggcggtagtacacgaaagcaaagc
aagtacaaaataatccgttaaatgtaaattgcattcctttctaggatagtctattttatcaagtaagcgtacgctatattatttttggaaattaccaa
atccattttcaattgccaaccgatatttatcactatagcttttcgacgatggcccaccaattatctcgatcgtaaccatcctctataagtttccctcca
gccttttgaactagctcgctgagctctcctttccggaacaaatggtagtatcgctggtacgcaacgttttcgcttggatggtgtccttttaaatcttct
ggttttgcattaggatgttcgtattgccgttttaaaacccaaggaacgtggacgtcttgaggtccattttcagaaaacccacgacgagaattatttt
gttcaagagcccaaacaaaaaataatgctcttcctcccttacgaagtactctcaaaacttcttggacagcttgcagacgtcgatcttcattgctgaa
atgatgaatgactgcaatgctcaatgcaaagtcagttcgattacttggatgaggaacatgcaagccgtctgccactatcataggtcctagttggct
tgcaattctaactagtttttcacaacgatcacttcctatcaaataaatgttcggattcactttttgatactttccatttccacatccaatgtctaaaccg
attgctcctacaggtaaagaattgaggaaatgctctacgactggccaaggctttttttctctgttaacgaaaggcgaatctcaacaaatttttctgt
agtttcacttaccttgtaacgagtgtctgaaaaatgagtagctattttgtcatataccctgtgaacatgctcattttcgtactctaattcgtttcgttca
ttcatagttgcttttcttttagaaaaaaaataccactttctccttaaattgaagaatgcttgaatatttggaaaagtcaagttttcattcgttactgtct
tctagtagctttattaataaaaagatatgatatatcacccataaaatgagatattgaatttagtaagaaataaatttaccggaatatgtatatataa
attaaggacccgtacttgtcacacagaacgtaagtacatatatacagtaaaatctactatcaaaaaatcgtaatgtatatcaaaattactagcttt
tattctatcataacttataccaaaacctttgcatagacaaaacatcaacataataaaaaaaaaatatctgaatgctcaagctcctaaattgagctgt
gtcaacagattatcaatcatatccattctgtgattataatctttttcatacggtgttaacgtatccacataaccgtccacacacaaatcttcatggta
aataccatagcgtttcgaaagaaaatagcgaatatcatcaataagttcaattgtgtcagtttcctgacgatgaaggatacgatcagcactttcgac
gacaagttcatgaaactctggatcatcaatgatttcttccaaatgatttgccatgtactcagaagcatattgctccaaccttgtcgttgatgtgtccc
atgcggttcgcaatatatcataaatactgtctttcggctctttttgctttgtgattgaaattgaagccaaagacttcaaactgcgttctttatcgagtg
aaagcatgtctgcaacgtaaatgacgtctaatgcaagaaaaggtgggatatcggctttgtctgaatataggaattcgagaacaatttcaacaatt
tcgctagaaaaaggaagagaaacaactggtaaactttggttaccctctaagaaagggccagtgaacatatccataaagtattcagatctgcaga
gaattgcccgatgaacggggtagcgatgagtctcagattgtaaataggcatcatggaaagagcactgaagaggtaaattttcaatcggttctgat
atcgtttgcctgtagtttcgaactatttctctcaaaaaacgacccatatcctcttgtgtatgtattaattggcgctttctccactctggggaacgcatct
gttcttggttactttgataggtatcgacaaaatctgtcaaagaaaatttcttggcaattgacaccaaagcggatgtatcatgcttggctaacaaagg
atgataatctaggtacaaaaaccgtaaaacagcctccaattctttttcgtttctcttgatagaaacttctttctgttcatcacctaatttcaagaatttt
gctttaaaatacgggctttttgaggccaaatagaatttatgaccaggtaaagatatactgttaccagaaaaagtaacatcagaggtaaaatgaaa
agcaggattggaaagcaaagatgtgatatgactagcatatggttgagactcatccacggccttcgtgatgttgtaagacaaaagcattttacgga
tgcgatcattcaatgcaccgtaaagacagcgctctccttgaaaggtatcacgctcgcaaattgctccgttctccaataaatatttaacaactgccag
ttagtttctagttaggagttgcgtgccatcatttatgacggttacataccctcttcatgaccacacaggctggcaagaacaagtggtgagtagtcaa
agatatcaactcgattaatagggacattgtagttttcaatcatactagagttactgtcagcgcttgtttccataaacgaaaagaaacaaatggagc
caaaaaattctgatgaaagagttcatctgtatgatccatttaacgtaccttcgaacaacctctaaatcgcctcggcggcaagcttcacacaattctc
gaaattcctcactttcatcgacagagaacagccctgaattggatgatttttctcgtccatagctttcgaccaaagagcaaatttgttgaaacctttc
tgtaaagattcagtacctgatgcttgctcagaagcattcatttctgttgaaggatgtctttcagcagtcattctgatcctcttgatgctaataaatatt
caaatcttataaaaaagaaatggggttctctcagtaatgttgtaaacgacacaattgatgaaatacgaaaaaaatcgttcaaacaggctcagttt
tccacaacctaaaaaatacctttgattgaataatctactaatttaatccatgaagattatttctgaaattctgaacaaccgacacttgcagtgcgat
attgtgaagaggcttcgttggtgcttttcgtacactgctagtaccctgcacaaatcccaattcaggtctatattagaaagtgattagctatatctaaa
aacaaaagaaaacaaaacacaaagtagaaatggatgatatctttgtgaatgtacgttcctctgggataagttctgatcgaaaaatcaatcgcca
atggactgtgagtcaattaaagacaaagctggttcctgttgtagggattccagaagtatatcaaatagtaaaatatgaacctgcttctgttgcttct
caaccacatacattttcgagcgaggaagaaaataccattttgtctcttcacaatattgaagaactaagtacgattatcatcgaagatacacgccct

cctcacttgcggcccaattatgaggatctttccgaagtagagaaatatgagatgtctcaagaagagtatgagcgccgcgatgattctgttttggca
tggaagaagcgtaatcgattgggtcgatttgatcctgcctacgaggagacaatgagctcaaagaaggattctttggagcgagagctaaaggact
tacaggtgaatatcaatgctcgctgctgtgcaagtggagaacggtatggaacgattcgttacattgggtttgtccctgaaatttccaagagctcact
ttggatcggcgtcgaatttgatgagcctgtaggaaagaatgacggttctgttcaaggaaagcgttatttcactacaaggaacaagcacggttcttt
tttgaaacctacggatgtagaggttggcgatttccctgaggaggatattttagccgacctttagttaggaactttagattatgcatatagatttcaga
agattttgaatgaatttatgatgatacaaaacattgttagaacaacatgaatgtttctgctacaattcaaagaccgatccccaatacattgctttta
caattagaaaaactgtttcctactatctggctttattttttactgaataaatatataaaaagtaactatatacgtttttcaaagtagctctccaatattt
catgattcctataggaaacttgctgtcagtagcatgcatacatggtaaaacatcctatcttaaaaaataccgttttcatgattatttttgggctatat
ctttatttttaaagaaatttaatcttttacattcatagactactcaaacacatctcattctaacctcctaagcactctcgtgtaaaatgaaaaaaa
cgatgaaaccactacgtttgcttcacccataactgaagaatctgacacgagaaagggattcaatgcagcctttgttcctccttctatagatactaca
actcccgaaaaaagcaactactttcatacaacgattgcacctgatgtccccaaagatcaacctttccgcttaggtgtagatgaagcaggtcgtggt
cctgttttgggtcccatggtttatgcagtcgcatattgtcccgtcgattttgacctaacacattttgggtttgcagacagtaaaacattgacttcggtc
aagcgctcccaactcttgcaagatatttgcaacacggaggatgagcttggtcgtcatgttggctggtccgttcaaagtatctctgcccaggagctg
gctgccgggatgctgcgcctgcacaacaagtacaatctcaattcccaggcacacgacaccaccatggatctaattcaaaaggtttatgattcagg
tgttcgagtaactgaaatttacgtggatacggtgggtcctccagacacatatcaacagaaacttcaagcacgttttcctcaagcaaaagtgaaggt
aacgaagaaagcagattctctattccctattgtgtctctagcatctatttgtgcgaaagtaactcgtgatattcaactcgaacttgtccgatatctttt
acagactcaagattggggttctggttattcggccgacgcgaggacaacgaaatggctacgctcctcagttcatcctgtatttggctggaaaggag
atattgtacgctactcatggcaaacggctcgtgacattcttgaggtggcttcttcgaaaacttcatctccatcttttgttgatgtcgactggcatgaa
gaaacctctgctcctcttaattttacaattcgtaaaccaagcaccaaaaaccgttcatggtttggaacagactttgttttctaataatcgactttcaa
tcgtttatatactcttcttttgaatcattccctgttttgttttccctcagatcttttcactactaatttatttctttttatgcttaccttccattgagaataaa
tttcattttgcatcctatagcactttcttttgtttacaaattaacgatgcgctttagttcgttgaacacttcaccaccacttttccgcggattataatctg
cagttaaccaagcactccgtctcatagaaattcacttttgtttcggtttttcttatagttcattatgactggtatgagaatcatcctcgttggtcccccc
ggtgccggtaagggtacccaagctcccaatatccaaaagaagtatggcattgctcatcttgctaccggtgacatgttgcgctcccaagttgctcgc
ggcactgatcttggtaaggaagctaagcaaatcatggatcaaggtggtcttgtcagcgatgatatcgttgctggtatgattcgcgatgagttcatg
aacaatcccgcctgtaagaacggattcatccttgatggtttccctcgtaccgttgtccaagctgagaagcttgatggcttgcttaaggatttgaagc
ttgaattaaatgctgttcttgaattacaagttgatgatgaattgctagtccgtcgtattaccggtcgtctcgttcatcctgcctctggtcgcagttacc
atactgaattcaaccctcctaaggtccccatgaaggacgatattaccggtgagtctctcgtacaacgttccgatgacaatgctgatgctttacgca
agcgtttggttacttaccatgatcaaaccgctcctgttgttgattactataagaaggaagacaagtggcatcctattcaagccgctcaaaagcctg
aagaagtctggcaacaaattgttgaaattcttgaaaagtcctcttaagtgtatagtttactcaagaattttcctatgaacaagttttcagttcttagg
gtttcaattttgcttcaaatagacattgtaagaaccattcatgaagtgttttcgttcatagacgtgttctttcaataaacttcttttgtttttacaatata
atctaaaagaacaaaactgtagaccaacaaacaagtatttagatttattaaaaaattatcacaaaaaaattagaaagtaaaaaagccatcttat
agattattccatttcctgctttaccgctttcggaggtgttgtgtcaaccgattcatctctttcctttcgctttccgatagcttggaggattccttggacat
ctattccttgtaagagactttgaacatctgcttgtggagggtttacacttgttttctgctttgcttgactctcgaatgacggctgtaattgaggagcag
ctcctgagctagatgagaccttcgcgtcctttgattgttctaactcttgcaagcacgtggcatgacaaattcgaccatccttttccacagcgttcata
aatacccacatctccgcttcttcatgccagacactacggaatttttcctggcaaactggacaaggctgagaggcagctatcgggtcaagaggagt
caatacatactgtgaacgtatagactccagttgcttttgtttttgttcttccagttcggcagcagtttctgttactaaatcctccttttcatcattatgaa
tccattcttcctcagtaataaaccagcacctgttgatgccgtgcaagctactttcacgaattcgtttgttaattctaaaatgccaatcaaaatgctgg
tccaattcttttctgctttcaggatcattagcatatctgcgaccgcaattggcacattgattgggatatgaattatataaaagagttgctaattcggg
atgtggagttgataaagaggcttttgacaagtcaatttcggaaataggagatacaaaattggatgaagtactgagggtaggaggaggagaacct
tttggtgatactagtcctgccgcttgtaaattggcaaacaaagagttgatactctcagactcttctctggttaaattattcgcaggattactagcacc
gccgaacatggaagaagccaatggaggaggagaaacggaagaatttgaaacttgaggacgatatgggtatgctggttgaggcggaatgatag
gtgtagaagcataactggcgggcgcgatattatgggatgcatgagagaacatatcgggcgtttgagtgacaggaaaagtagtaggaggctgtg
aatttggaaatgatggagcaaggggttgaggaggaggcttaggagtttccaaacttgtaagttgctttttgataaccaacagttggtcataaggg
agattggaggtagacaaaacctgttgcaattgagaaagtatttcaacccgcttctttgctagaccatcaagagggttccgcatacatctggcctgc
tcagtgataatcatacgagcaacgtcagcaagtagattttcgagagttaagaccttgctaacgggagctgggactcctgaactaggtagatcaag
ggctggagagcctgaagaagagactggatatgaaggtccataagagctccgcatgttagggccagcgtacgcaggattaggtgggacagacaa
aagcggactttggtgacgtaggaaggaagatttatactttaaaagggcattttcaatttttcctgttacagatggagggaatacaggatcgggag
aacttgaattaggggaacgctgtttccaggtagctaataattgatccaacttcagtcttaaagaaggctcaacaacggtgtaggcattcataaacg

tggtaaaaagatgacgaccaaaaaataatgtgtaagggtatcctatgtttttgagatagaatccaaaagatacagggcaggcaatttgcatgac
ggggggcactaaggtcactagttagtattcacaaccagagaacagatatccgcctggaaaagctcactttttgtatatgtttttcgagggcattca
cgacactcaacgcaaaaggttcattttcttgggcaacataggtcaaagtatggattataggcttactattaaatgtcaaatcttctaaagcgctcga
ataatccacttcaatcaaatccatttttccaatcaaaattgtgttctttttatatctggtaataagcaatcactatctaacaagagtgataaattatt
atttagatactttataataaattaaaactctgtttttttctttttatatacaaaaaaaagcaatattgtaagaggaataaactattaaatttaaaaa
acactggctacacgattaagcaaactgcaagacaagcaacaaaagagaactagattacaacgtaaacggacgaaaagtttggtgcactgttta
attacaaagcgccaaatcctttttcaacaacaaaacaaatgaaagaatatgcgaagaataagtagctgttgccatgatcgaagctatatatattc
ctatataaggaggagcgcttgtatttccttaaatatatttgagtagtctaaagtaattacccaaaattatgaacaaaacaaaggtatacaaataag
aagcatggtatttgtgggaataatggaatgctaagaaagaatactccctaatgaagcgatcactagttacatatgaagtggaatttatcatgatta
ctcgaaagaacggttcttccatgctactactatttcgtctatttagtagacatcaaagtcgtactgtattaatcattatcaaaatttccccaaagtttg
ttcagttttaataagccttatgtttctattttgtgtgtgcttgagccaaagttaatattgggaaggtttagcattcttctttcaatgtttttgtttacttac
catataaacaaataggcatcagcaaccataattctcccacatgcttccttggctaagtgttttattgcttgtcatatagaattttaagaaaggaaaa
gagattttgctttcatttgttctataaaggcgaaataaagcatacaatttattactgactagccaagaatgccgaagggaaaggaggataccaaa
gagattgaaggacgtagccatgatctttccagaattcttgaaaaagagaaggatatgaacaaccttacgtcccagatcaagtaccttgaatattt
ggtaaaggaacagcagaaacaaattgcttcttcagctattgaagttcagaaaaagggagatcaagtgcgggatctacgaaagaaactaacgtt
agagaagaatctatttcttcctcgccaagcatttactctcctgaacatcttctgccaaaaagcttgtcagctgaaatcaaaggagcggcaccaaa
aagttgtgagtcgagaggcattgaaccagtagcctcttacgatctggagaaatttcgaaaagacggaaactaccttgattccaaagttcacggca
aacatccaattggtgaattggacgaatcattcgataacactcccaggccaccacttgtcctcaacaaatacaagtcaaatctacgactgaactca
caaaacgtatcgtctgcttgggttcccgaccattcagtcgaccccttcagtcttggtcctcccttttttgcaaccccatacggcttcttcacaagcattt
tatggcagtcagtttttttaagaagggtttggaaaatccagttcgcctatctgataatgcttgcaatcaggaacttccgaacaacggcattcgtaaa
gggacagagatgtcagaaaccagttcccaaaattacaaatggaaggtatgatcttagcataacacaaaaaattctaacaaaacagtctgaaatt
gagcaaattatcttcagaaacgatcagtcggcgtccttgtatctgcagcaaaaattgaagtcaaatgaccaaaatatgaagcatgcgcttattca
gtgcatacttcccctgtcatttacacttatgacaaacaaatttggcaactttttgatacaaaaatgccttgaacagtgtgatgaacgacagctggag
ggatttgctcaacagctaaaagcccatatcaccgaactttcaacgaacgcatttggctctcacgttgttcaaaagtcatttgagatctatccagaa
aattttgcagcttccttaattgatcaattaatggagcagttgccagagactttaatgcagagacactcatgccacgtatggcaaaagtttttggaga
ctagaagaaagtcatgtgcattagggacaatggatacattcaataaaaaactaactggaaattggactcaagttgctacgagcgaaatgggcag
tttagttgtgcaaacaatttttgaaaactgtaaagaacaagaaaaggtatgcggtaatattttacattcagtattaacaattgcagcgcccttgtct
taatgaaatcattcaaaatactcgttccattatcagcggacaatggggaaactgggtgattcaacacatcattgagcatggaagtgaggatgatc
aggagagagtgtttttggatattcttcagaatgcagaattttacagtacaaatcaatatgcatcaaaggtggttgaacgtgcgttacgagtgaaca
ataggacctttttaatgattacattgtcctaatcacacaaccgcaagatggatggggaagaatcttgttgatggatatagcttctagccaatatgg
aaactatattgtccaatatttgttacacgctgctacatcgtcccaaaagcgattactagcagagcttctcaaaaagcatatggtatctcttcgcggg
cataaacatggacaaaagattgcgctcttggtggaaaaaacaaagacataaaaatgactggtttccttaacatccggttactgagaaagaacaa
attcctaattccgatgaggactaatctacctagatttttgcgcttattccgtccattggaatcttcaatttgtttggatattgaaagaatgggaattttt
tatatcttaccctcttgaggagggacataatcttcgtcgtcttcttcattctctaaatcctcgtcttcttggagggctttggaaaaatctatctttttatt
ccgtgttctggaagaagaagtaatgttggtgggatcgatagctgttaaatcttctggttctatagtttcggaattagaaagtaagctcatggactca
ttgcaagacactgtttttgccctaactaaaacttaccatcggcaattccttcttcatcttcttccattgattcttcagaaagttccccttcggatgaact
catttcttcaaaatcaccatcgtcttctacatcttcgatatctggaataaagtcctccgtgactcttcccttccctttactatttttttgttaaaagatta
tcgaagagtttttggcttacatgtcagctttttcattattctctggcatttttgtattctcgtaggtacagcgtttttggtaggaagtaataatactacg
gtaaataaaaggcaagcctgccgaaaagtgaatttttagaaaagatttttttgcttgttcatctttctagtcatgaaaataagtttattattatcctg
gataatattcgtatgtggttttgttgtttattcttcatcaaaattggtaagcaattcctagtctaacttgaatggaagacttgaattaccaagtcaata
taacaaaagtatgggtaagcttcaaaatgaaaatttactgctggctcttttctttcacagtatttgctgtttcctactatttgtgagatgacacaagc
gcgcgacttgaacagaataagcaataacccatattgttatccatgttaaccatatgatagaatgattttccaatctaaacaaagtatgaaacgcat
tatacgcattgcttatgaactgccatctttcctatagtgtgtaaacaaatagctcataaaatgacaacgaaattctaaaggaattgatactgcgttc
aagcgaaacaaattctccagtcgttttcttttaaataatataaattttcagatgaaaataatgaaaaacaagcagagcacaatgcgttcgtttaga
aatactttaaatcatcactgacgtagaaatcgctgtgtcctatatcagatgtactataacatagatcgttattaaataaaacctgtagtcaatagaa
taactcggtcggtattcagtaagtaattatttgcttgtatttagaaactcacttttcctttttgtttgtttatttaccagaacatgtttttctcgtgtgaca
aaagaaagtaatgacaccgtcatataaataataaacagtataagtagaattcaccttcaagaaacatatttcaacataattgcattagaagaatt
aagcactgacaatccctgttacaaatctctcatttttgcgcttaaaacgaagtaagagttcgcatctgctccgtttgtagttcatacgtacattcaga

aaagcaggattgaagaacatgattgaaagcaaaggcaaaagcaaaatcccgtctcatctttctaagggtaaagaggaagacagacagtctgcc
cagttatcagattatgatgtatatccgaaaatacaaaatatgtccttccaacggttagataaagaaaatcctaattatttaaaaaaattccaatgt
aacgtttctcctcgctctacgacaggagagaattcagctgaaacaattaagtcggtacctaacaacttcagccttttggaatggttaaatcaatgt
ttaataccgtaggctgttgccttttgggtcaatatgaagaggaagcatgggatgagttcgtcgacgagtctgttgcctacaaaaagtatgacctaa
cgtcaacaggggaaatcaaacttccttccttccctgtccgcgaagttccctactttcagcacaatgttccatatgatggctatctgagtcctctcagt
taccaaggttggattcgtcatctttctcaaaacgcaggtgtagaagtcagttggaagaaaacgtattgcaccgttatgaatcctttttctaacggaa
tgatctacaccatccctcataacgatggcagaattctactagagtcctgctactgtgagaaaaatttgggcatgtccacttcaccaaggattatgg
agttcatgccatataggccccttgtaaagttcaattgttccaaattattcaattccttcgaaaactattttcgagaacttacggaattcttgctgaacg
gacgcgcacccctcaacgtcttggttcatcacataatgctctatcactattatcccacttctatgaaggatgttttgtggaaagctgtcgaagtatac
ttggatattcactgcaccgaggatcgttattgcagagttcaagtagaggcagctaaacgtagactaggtgacattcgactttatttggtgtaccctc
gtgatatttacaaatttacaaattccaaagattggatcgcagtcgctacacgagggttcatgtcttatattcaaacaaggaaccctttatgtctagg
aagtattccagtcaacaacaagagcatttccgaacttctttcttatacgacaagtacagattgcatggcttggctatccttacttatctgtgcagggt
ctaacgggtctcgtttcccactgcataagtacatcaatacgaagacacgaatcatgaaaagtaaaacctcaataacggacttcttgttttctgatg
aagacctgcttttcttccaagataaagaatccatcaccgaattcaaacctttacaacagcaacttttaagggaactagcagattgtgatgctcata
cacaggcccttcatgattctagtattactcgcgcagttgatgcatccaagcaccaaatgaagggaattaaattttatgagcaagaaaaaacaata
gaagaaacgaaagaaactcacggcgaacttcaaagcaagcgccaacaacttacaaaacaattggaaccccgtcagccacaatctcaaattaat
cgtatctcttcttacagcagcaccattactcaaaagggttatttgacaccgctgcttataacaagggtcttcgttcaataaacaaggcaccggcctt
cacttactcgatatcgtaactatttgtcttttcagtttctttgttgtatagaaagcacaaccttccttcattattcctttacggcccctctgcatccaaat
gaaaattattgaacacatccatccttcgagttaccaagcataaagcttgtatctgttctattaattcctatttttgactctaccattggttttagccaa
aactggttgatatttagatgacattaaaaataaaatttttagaattcgaagattttcttattatttagtacgtacaagatccgtctcacacaatcaaa
attagtcttagttaagattaagctaactacttaaatagcacacaaccattaaaatctcagaatagcatatggcctctaatggtggtgatagaggga
ggccatttcatcaaggtcctacacatccatttacacttctataaagaagatacaaacggagcgagatagagcacctgattagggcgctcaaacct
tagttttagatactcattcatcatttgattttagttgtgtaaagaatcagagagttggaataacgcaaaatataatttttttagcagtcacaacttta
cagacttctagcttctagctctcttgcttaagtaaataaaaattaacctattgaataatatatataatgtttgaaatgtaataattgctcggttgaaa
tatgttacagttactttattattctatgcttgtaaataagaaaaacccagatttccaaaataccattcataccattttaatacttaaattcatcttcca
gagctaaccttggaacgatgttcatacttagaagttcttgaaataacaacttcgctgcataaggaatagtcattttgaccacgtttttagtgctttga
caactgttgcaccatcctctgtaacccaaaagtccacattcaccgcaaacatcaacatcacaagcatcagaggaaagcatcagtcgttccaaaa
gcaattgagaggcaccgtatgcaattaaacaatcacgttccatttcacccaaacgtaaaccaccatcacgagaacgtccttcagttggttgtcgcg
tcagcaccgcccgcgggccacgcgctcgtgcatgcattttatccaaaaccatgtgttttagtttctgatagtaaataggcccaaagaaaatatacg
cttctagaggttctcctgtaattcctgaagttaacatatctttacctgagtagctaaatccgcgttcaactagaacacgacttgcttcttcaacctttg
ttcccccaaaacaggttccatattccaaagtaccacgcattacacctgcctttcctgacaataactcaatcattttaccgacagtcattcgagaagg
aaaaccatgtggattcatgatgatatcaggacagacaccctggtcattaaaaggcatgtcctcctgttggacaataataccacaaacacccttttg
accgtgacgagatgagaatttatcaccaagctctggtcgtcgagtctgtcgcattaaaactttgattaaagtctgatcagagtccgtagtagtaag
cataactttatcgacataggatggttcaggagctttgtaagtcattggggtaggtttgaatccagattctaccgtctgagattgtcctaaagcaata
gaattatccaaagcgttggtgggagtctgtttattaacatacacctgaccattctgaactggagaaccaacagctgcaaggccatcatcttctata
attttatgcttccaaataacctcaccagtttctggatctcttaacggatcaccaatgcgatcgtgtgttccatttggatacttacgaactagaactga
atgcttgtgaaggacttgacagcgaccaaaaccacggtccatagaagctttattcattaccaaagcatcctcaatatcatatcccgaataactcat
aatggcaacggtagcattttggccagcaggtaacttgtcatatccaattaattcaatcgtcttcgtttttaccattggttgctggggataaaccatca
aatagagtagtgtatcaatccgctgtaattggttatatccgatagcaccgatagcttgcttacccattgcgcattggtaagtattacgaggagattg
gttatgatgaggataaggaataagtccagcaacagcaccaagtacagtaaatggttcaatctccagatgagtcgtttctggtgtgacgtcttttc
atatatacagattaagctgtcattttcttcatttacatcaacgtattcaactaggccttctttcaggaagtcttcaaacgaccattttccttccttcaag
aatttgatgtgttccgaggtgactttagggtaaccattttgtacaataatgagcggacgacaaattcgaccaccatcagtagatataaaaacggct
cgttgatgagtattaataaagacaccgacaaaaggcgagattttgccagaacgacgcaagcgtcggaaagacgatacaaacaatgttggatatt
tggagatacctagaatcataccatttaaataaactaggtatgtgccgctactatgtaattcgcgtccagtgacaacatgaatatcttcaacaccaa
atgcataagctaaacgaataatgggttcttcttcctcatctgtagtaatatgagtcataagagccaagtttttaactaaaccacaagcttcaccttc
aggggtatctgaagtgcaaagcataccatactgtgaaggctgtaacgaacgaggccctgaaacctttcttgtttttcaaattgactggttatccgt
gtcatcatacccaaagcactaatgaaactgagacgtgataaaacatgtgtgacaccagcacgttccattttgaaacgtttaagagaccagtttcc
cgtagataaagcacgaaccataccttgcgtaatttgatcgccgtgtacagacaattgattgtaagcatcaaattcttgcgttctgtgaggtttcttta

aaactttgtctatgtttagcttcatatcactattaaatttttgaagagatcttcaaaaagtaatgcaagtagttgaccggccaattctagacgtttat
ttcctacataatcacgatcatcaactaaagtagggtcaaccatagaaatcaaaacacgacgagtcataacagcgatataaacagctttagggtg
aaagtctaagttggaaacattgatatgagcaagaaccacagcagcaaggacctctaaagcttcttcatgagagggtaatcgagctgcaccagca
cgtcggtttactttaacacgagctccaatgtattccaaagcctgctgagtagtgaaaatattaagtttagcacactcttcaacactaggagcaaat
aaatcttggtatgatgcctcattaccggcgaccagctcaaaaatttcctgatctgattgcaagcccattgcttttaacacagcaacaatcggaatat
catcagcaacactgttgtgtttcaaatacagcttcccatttttatgaacaacataagttttagattttcgctcatgtgtagaagaagttactgaggcc
tggaacattccctttttcggttcagcttccacaataatacgattcttggataattgttcttgaaccaaaataaccttttcagtaccttttacgacgaaa
taaccacctggatccaaggggcattcgttcaaagtggccatctccggttctgtttttccgtgtaagatacatttattacttcgcagcatgacaggcat
tcttccaatgggaacatttcttctacggacaacttgtttgcctctagtatattcaacatcaacgtaaatgttcgcaccgtaagttaagtctcgaagac
ggcattcatggggactaatggaagcttgaatcgcatcggcatcagtacgtacaggagcgccgacccgaatatcgaggtattttaaataaaaccat
ggttcgacatctgaagtgactttctggtttgcttggacaattttttttaaatcgacatcgacaaagtaattataagagtctaaatgttgctttacaag
acctttaatcttcaaaaatgcaggtaaaagttgccatttatcttcaattgtaggtacaggatccgtaagctttttttcctttataatcgggctctaaaag
agcagaaaaacttttatcgccttcaatcggtcctgtatttgaattcaaatctttttttccagactcctgcgacgatttcaaccccattgaattgctggt
attagtaatggtggacggcaagattgcttcgtctagcaggaagaatccacaatgactcaaggggtcgagaatcttaaaagaaactaaaatatta
acttagatagtcgcgttttattttttctcggctcattaaaatatatttataggtaataaatgaagaggaatcgtttgtgccacagtgattacaaattcg
caaagcaaaacatggagttcattttaaatcaaaaagggcaaaattgagtcattttatttattatattgtcttgaaaagacaataacaattaaaaaa
gaaatcaaagaacaaaggtctaaaataattgtaatcagaaaaaggaataaaatttaaactgacaagagtaccttggaagcaaatttataaagg
acatttaagctcctcaacaaaaatgggtttgctgatcaaaatcctaatgctttaagtcaattctaaatctatgaaaagaggggaatcgaatatggt
cggtcaagaggtaagtaatttagtccaattggataccagcgtattcgtcgtaaccacatgccaagttgagattttgaagggcttgagtagcggcac
ccttaagcaaattgtcaatggtaaccacgataacagctctcttgcctccagaatgaacaccaaagccaccgacagaaacgtgattcttgaaagag
atatcctttacaaggggggacatcaccctgaacattaatcaatggttcgtcggcatagcgctcttggtagaggttacgcaattcacgagaagtaata
gacttattaaggggaacattgagagtaagagtaattccttggaaccatgaagcaacgtgagggacgaaggccacgggttggttcaaatggtgag
aaatttcgcgttcgtggatgtgatcagtcaaggaataaggaattaagttattagtcaaaacgttaaggtcgttcttgggggaaggcttggtgccag
caccagagtagccagagacaccgaaaatggagggttgtccaccaatgaaagacaagataggagctaaaccaacctgggaaccggtagcatag
catccgggattggaaatgcgtttagagttacgcaaagcaacacgatcgttcaactctggaagaccatagttccagttgttctcaaatcggtaatca
gcgctaagatctacaatcacgctcttaccattagcagaagttaatgcatcaacgtaaggtttgcaaacgccgttgggcaaagccattacccaagc
gtcaacttcgccttcttcttccaatttcttaacatcatcaacggaaagatttacatagtgaatttccttcttactgtaaccaggtaatttggtacccttg
agctcacgagaagaaacgtgagaaagctcaaaatgggggtgagtattgatcaaactaatcaaatttttttccagtatggccacgagcacccaata
gaacaatcttagaaggcttagaagagacagcctttaaaggcttatgaatctgctgagcttctaccgaatagaagcgtttagcaacgttagaaacg
aatgggcgaatcattttattagaattccaacgagaaaacgtagaaaaagaacgcttttgagcggtattagggggattgggaggtgtggcagagt
tggtggttgttttagggttggcctcggcaaggtttgaggataagcaggcagaagcgaagggctgatcagatttgataaaattagaaattgtattca
aatcttttgctccataccaaaaataataaagattattggcaaaaagggtgccttcacttttggcaaagaaccattccaaattcttggtagagggat
gaagacgccagagcaaagatggaaaatctgtacgtaaagtagaaagaagactatcaacaacattgttgagccagttgcggtcataagcaccaa
aagcagctaagactggtaattcaggattggagagatcgacaacagcgatcgattcgttataggaatcgctgtagacgcgaagtttagcattggca
aaagatttcttgaagtcttcgacgctagcataggtagatgggagagcattgtgatgtaaaatcaaatcttccaaagccttactggggagggaatc
caaagaatcagcgtgattgattttgaagccacggcagatcattgtaccagcaccagattcagtgaagagttctttttggaggtccttcgtactgatg
atggctactgaagaagtacgaggaagagtatcaagtaactctttaatttctctgattttgagtttggtaccatacttgacccaaggctctttcatgag
gtcgttatactcgcgatctaaatagatactgctaatcttctttttttgtgacaccgtggattaaaccacccttttcgttcaagtaaacaaccttcaatgg
tttcaagacgcgggctagctcaccagcggtaatgtcagcgtttacattcaatgtttgtccggaagcagtttcggccatagaagtcaagatgggcaa
agttccagctcggatgctgtgttcgatgggagccttattaacgttgacaattttaccaacataattgtacttttccttgtccagatattcggcttgaaa
aacacctccgatgataggacgagctcgtgtacccaaacgctccaaagcttccacaagtttggcgttttcttctaaaaagactttgcgagcgacggc
caatgtctcggcgtcggtgatgcgaataccgtcgctgtattgggcttcaatgccacggctagctagaattttgtttaactgagggcctgcaccgtga
acaacgataggatagagaccgacatggttcaaaaaggcaagactttgggcgagtgtgtccaattcatcggtaagaatggcaccaccgaccttaa
taatggcaaaacgctgggcttcaaacgaggtaaagtatctcaagtactgctcaacctcacggcggctgccaacagaagacaaaatacgaacaa
tggcatcccgatcctgttgaatgtattttggtgtagggacaaacggctgggactgtccaactgccaaattgctttgttgacgacgagaaagcacag
taggacgcaaataagcacttccctttaaaatttggcgcttcatcaagcccttgagagcagatgacttggcagcttgctgcagttgtacaagcattg
ccgcgaatctgtctttggtggtggtggtttggagaattgggtgatggatgccttttatcttgtgtcaaacgtgcatgagatgaatgatgaaaatcgt
ataaaaatttttttacactgataaataaaagtttataagaactagtaagaaaaaaaaaaagaaacattcgttgaacaatgtggaaaataaaaattt

attgaatgagcccttttcgtcagctcttcctgttgcactgtgtagctactatgttagttgtatacataaaaaaataaagtaaaaactgaagaaaaaa
gagtcggaaggagagacgtgtataaaagatgtaacgtaagaagagtaaaaattgcttgctctgttcattttctttattccaatggatgcgaaatga
aacccgttggttcaatggacgcttttgcaagatgattgaatttcaaacaaacgtaataaaagagtttgcttctccaaggtaagcattgattggcatt
ttcggtttgtttactcaatcatggattgtcatccggtgtatcctttgcagacattaaaaacaggaagaaaacgatataaaagaaactagaaaagat
tacaaaactcacaataattaaataaagaaggctacttgtcttattctagactttttatctattcttttatgaattgatctatcaacctacaaaaaccc
aagtgtttgtaaacaagcataaattttcaaaatctcggatttatcaaatcatgcatcttgatctttaaggtagtaatgttagtgggttttatttattca
ttttcgttaaataaaaaagaacaaggaaaaactggaaaacaatttccgaatcttccaaacactgatggtatattagatcgcttggaaggacctga
acaactgcgaaaatttttttgttctttcctgtttacatttttgatttcttctatctttgcatttcccaatatcgattccttaaagtaaaaaaaataaaaggc
gaaaaaaaaaggtatggaataagcgaggacgataaacttgcagtgacttgtttgatattttcacctattatattgagtggaattgtattcttgttga
cgtttttttttcgcattgctagattctgctatttctaacgtttggttcagtagctgtttaatcttttagtttctttttttgttgtttatttcaatcacaaaaca
taatagttttaaattatgtcgtttcaaaaattcccccgtcatttattatcaatccgtgatttgagccgtgaagagatgttaaagctcatcactcgttcg
gcagaaatcaagcatgattacaaaaaaagatttgccaatagggaaaacgttcagctcggtggactggagggtcaaaacgttgccatgatcttca
acaaacgcagtacacgtactcgtgtctctgtcgaaaccgctgtcaacacccttggcgggaattgtatgttcttaggaaaagatgatatccaattgg
gtgtcaacgagtccttctacgatacctccaaggtgatttcaagtatggttgcaggtgtggttgctcgtgtgaataagtattcagatgtcgccaatttc
gcccaacatgcttcttgtcccgtcattaatggtctctgcgacacgttccatcctttacaagctttagcggacttgctcaccatccaagaggcctttgg
cacctttaaaaacttgaaacttgcctggatcggagacgctaacaacgtcttgcatgatctgatgatcgcttgtgcaaaggctggtatcgacacaac
catcgcaaatccaaagaacgtcgagatccgtagtgatattcttaatcttgtccgtgaaagctctaaagaaacgaacgccaaacacattatcagcc
atgatcctgcggctgctgttcgagacgcagacattgtggttacagatacctggatttccatgggccaagaggccgaacgccaagctcgtctcaaa
cagtttgctggcttccaggtcacagagaaaatcatgcaactagcaaaacctcattgtaaattcatgcattgtatgccccgtcaccccgaggaggtc
tcagatggggtgttctacggcccaaatagcttggtgtttcaagaagctgaaaatcgaaaatggaccacagtcgctgctcttgaagctttggtcgtc
aaccgtggtaatatttagacatttcctacatacttcctgtttccagcttccttctccatattttcaaatgaatttgctaatgtttccttttatttctttgga
cgcgtgaatgaaagataaaaactgtatgttctacaaatgtcactgtcatagttcttacgtatataaagaatgaatgcccataaaagaaacggacg
aaaagaagaattcatagtagaatacattcatggtttatactacaagacgaaagagcgtatacccaatagactagaaaatctgtgggatttacga
ctagaatcgaacaataaacaagcttattcaatagcaaagcaattatcatcatcatttcgtaaatctatctgctcgataagcatgggggggtgtagg
tcgcagaagcaaatcaggctggaaacagcgaaagaaaataattttaacaaacggatgctcagacgtcatcataacttatcaagctttacttgatg
cgaataccagtagcctttaaagcttcatgatattctttgaggctttccttggctttatcgacgtattgttgtttcttttgatcagaaagttttctccaagc
ttcactaaccatgcggctgttctcggtcaagtaaggactaagggcagggacacccatttcattacggagtgattcgtttttcataatatccaatagg
aagaggttgaaagcaccagcagggcgcttaggaagatcaggatgacgaagcttttgcaaattgacacccaaaggcttcagctctttcatacggc
gacggttttcctcacggatttgggcaagagagaactggcggagttggtcgtcaaaagccttctttttagcagcgtactctctttcaaattcttgttta
aacttttgaacctcttcaggagccatagtactgtagacatcacgaatttgttcccagcgatcggtgacacctcgttgacccttggttttatgcatcaa
catattccatattgtccttacacgaggaggttgtaaagtgcgtaaaacgtctgtatctgcaaaagaaacagcgcttgttgaaaataaacgataaa
caggcctaaagaacatagttcgctacgtgtttacttgaatgggaaatgacaagtagcacagcaacaaggttgcgtaaataaagtcgaatttaca
agaaagatgcaagcaaaaaagttagtaggagaatgttaagggaaaactattaaatttaaattacaagataatataagggactagtgtatttttgt
ttacttttgtttttgggtcattggaaacatgatttcatcctcttatccttgcgttcggatttctttaaataccgtaataaagcttacatattagaatgtc
cttcgtagaaacaaaatagtgcaatgaagcctctttgaaaggatccgtccgaaaggaggaatcataaccatattgggtcattaccaagtgtgtcc
cttgatggaaagatgaaactaattcactctataaaacagaaagtaaaaaaggaatacaatatgaagaggatcgaaaagttaaatgaaacaaa
ataaatagcttaaggaaaaaagtattgacgacaacggcaggattcgaacctgcgctcccaaagggaatccgctaggctactaaatagcaggcg
aacgccttaaaaccactcggccacgttgtcttttcgttatttagtggaagcaaataagtattatattcctgtaaacttatattgtaatgatgaataaa
actagagagaaccgtggtttcagcctataggatggaatcgaggtactatcatttttaaagaaaaagaaaataaaataatagagtaattgttaatt
gaagctattagttgttccgataacgactttgtacttttgacttgtttccgtaaacatctcccattccctaatttgtcacgttaataattctcaaggggta
tgagaataaatatcgagtaaaagtaataattacaattgtaggaaaaagtacgctaattagactctgttgtactcgctagtaatgtccagggatttg
gttagctgtattgaaagtatacaggcgatcttatttacctacacgtctataatagaaaaaaaaaaaaattgatatcttatcaatctaaatagagcta
acttaaacgaacgctcctttaactttatttctttcaaagttcgttcacgatgcgttcttctccacaaatgaaaaacgtctttgtgtatgacatctcctct
ggtgtttcatcagacgtaagctgatcctgtttcctttttttcgatttccatcgtaatcccgttaaggttccctttttacaagtcaaagacactgctgttg
gtttaaacatacgcgacagaaactttttttcttcttttctgaacttgtctttagccatcgtattcacgttttcgagattacttatttactatttttttaattttg
taattttgtaatttggattatttctgattccaaactacagtatttttgtaaatatgtcgttaaatcctcatatgctagaaaagaaagccattttctttat
catcggtccctctggttcgggaaaaacaacaatggccaagtttttagcagacaaacttagttttgaatacatcgagggtgatgatttgcaccctaa
agcgaacattgagaaaatgggatcaggccaacctttaaatgacgaagatcgttggggttggctttacaattgcggaggtgcatgcgctatgcagt

tggacgatcctaatgtaaaagggattgtgatgacttgttcagctttgaagtgtgcctacagaaatatcctccgtgactctttgaagcatcgtcctgg
cagcgtccattttatctacttgatggcacccaaagaggtgctgattgagagaaccagcgctcgaaagaatcattacatgaaggccgacatggtgg
agagccaattacgtgatatgcaagcccctaaggacgatgaaaaggatgcatataccttgaatgttaataaaccacctgaagagtctaaggatga
ttgctttgctttggccaaggacattttgaagaaggaggagacttacaagaactgaaagtaaaacgcttctgatgaaactatttttttctgtaataca
attcttctgtaattactttctacatctgcaacgttgtctatcctgtattctcacctttcttcatttcatgataaaagaacatattcattagtattagcagg
aaaaaattatcgtgaaaagattgtaatagtactttcatagatgatatgatcacaaggtaagaaaaggggaaagttatttggctattgtcaatgctt
cccctttggttttctttgacaaatttgtgatgtttcgagtgtgcttcctattcagttagagcaccgccaactcaagatgcaaatattcatgcaatgatt
ccttgttcgtctttccgtctccataatgtaatttccgattgtttaaagtaacgccgttggtcttcatcaacaagaatcaagctcaagtagtagcgtacg
ttgaactttttgttgacatcgcgaaacgtaggcgttagcgagtagccattcaaaaacatgcgcaaaggaatcgtttctccacgattcggatttccat
ccatgacctgaaaacgtgtcacggtttcactattactgtactggtttggtggagttccaatggtctctcgtcgtataatattcacttccatgcgctgga
ctttgattcgaacaaggatgaaataaattttccctactatgacatcgcgcaaatgatacttgttcttcgtgtattcaaactcaatatgaagacagtc
atcaataccgacatccatcttgatgaggctattcgtttcgggctcattctcatagcgatacacccataaatctttttctcgaatcacgtccgtcattttt
cgtgtcacggtaactctgcaaaagtaacgtagttttacatttttcccaaagtacgattcatagggcttttccacatgcttgaattcaaagtcaaacat
ttgagcttgtcgcatttctccaggggaagcaagctcttgcacactccttgtaaactcatggatgtttcctttgtcatatgtattttctatacagccaac
aaactcaatcttgattccattgtgatccaactttcttccatctttcaaccgaatcataacctgttcaataataattcattagtaactaaccaaaatga
aaaacagtagtatttccttaaaagcttttctatacatgcagagccctcagatcatgaaacgtaccgcgccttttacagattcatcacttccatagac
tggggctttgtccttccgtccttgttcaaattcgatatctacaaacgagcgttcttcttcattttccaagtggatatcaacatcaatcgggcttctaaa
aaggtattccattgaggctaaatgcgtcaatgtacgaatcttctcgtcgcaaaagttgcagctggaacaacgtactcttcactatttaagataagta
gaacaatcttcttaaagaaaaatgcaagcagaaatcctggaggaatgaaagacacaatctggctgctcttttagtagagtttttctctccattcag
gtttgcgagtaacattaaagttacacaaaataagatacagtataattgtgaattgccgtaagctgtaaacaatccttgcagtagacatggaattag
acacaatgaaatggaatgatataacactcacagctttttatgctaaataccaaaggatgttcttctccctcggtactaattgtgaaaagcttcagtt
ttatagtcaaagcttcaatatctgaatcagcgtcgcgtatttcctctctgcaaaaaaagaactaaaatgacttatataaaccaaattccgataagc
cgaaagtcctcgattccttcatttagccaaaatcaacgatgaatgtttattaacgctacgtaatgaaccttccatcattttacctacaagtaagtacc
aacattcttgttcatcaagcactgtagaaacgttaaagaatgtttgtagaaagatctatgtaagcatatctttatccttgtaactgatatactaatcg
ctctagtttgtatgggttaactgtgtgcacatgtctgctgttcttctttgtataccatttcaacgccaaatatgaacttcaacctattcttactttgggat
tattaaatgcgagtttgggagctatatcagatttgttagcgcaggccctagattcttacaagtcgatggaatttcagaagaaacgtgacattacttt
agaaaaatacggaaactcaattcttttgcctgctagaacagctaaactggactttcacaggacgattcgttatgcggcatatggtaagtaaaattc
aaaaagctgagtgcttgtttcgtttgggagttgagctaatctaacttttgtaggtttatgttttacaccaatccaggtaggttaggtcgaaaaagatt
ttggctttttgcttagaaacattactgacgcgacagtaccattggtttgcaagtttgaaaaacttggtacaaggagagaatgagtttttctgcaacc
ttttccgagtagctattgaccagttgttatttgctcctcttggtcttggatttttcttttttttatggtaagccatgctgtgctattctcggctagaaagc
aaattaaattgataggtttctaatcattcacctatagggcattaccgaatgcaagtctgcagacaaaatcaaagctttcttcaaaaagaattatgtt
cctactcttaaggtttgtccttgtaactgcggttgatgcgcaacaaatctatgcggttcaacagagcttgatgaaaatactaattctactaggcgaa
ctacatcttgtggccagccgcacaattcattaacttcacttttattccgtttgtttttcaagtcttatttgttaatgcaatcgccatgatttggacaagtt
atctaagtttgaaaaacagctcttctaatgtgagcctacatgattaatattattgaacattgtaatgaattatgagaataaagttttcagtgatcaa
cgaacatatttgacgctgaatgtcgtctaaacatatcattctcgtttttcaaatgggacacataaacatatgtgatattctaataccaaaaaaggga
ctgacctatgttttcttccttgttatgcttcgtttgtattttattctgcaaaatacttatatctctcttctcttactttgataatcgttgtgaatatggctta
acagtagaaacgacaacaagccttcacagaagaatcatggcctctcgaaactttcatagtcaagtgtaaaacttacattgttatgttcgcttttgc
aacaaagcttccttctttcatttacacctgcctattacactttcttttctctagtgatttgcacagatacttttcatagtcattaatgtatatcggttttgc
atacccaagaaaaaattgggactatacctttcgttacccttctttagagcttgtagtttacaaattaccgttgtcaatcatcgatttttactatttattt
caaaccattgtttcgtaccgaatcgctccttggcatgttcaaaactagaataacgacaaccttataggtccctattcaggctaactaaacaaaaac
gaacaaactcagagaacgccaataataacattgcagtatgaatgttgagagtaggtcttttggaagctttactatatgcgagaacgaatgctaat
ccgcttttagacgtcgcttcaccttccggttgctctacgacggtcaccgtcttttctctctttggaattatatgtactttattgaaagcgaaatcgtttat
tcggttcctttataacaactactttgctaaaggaacaaaggtattttattggaaagaacgacttattttagttcgtatcactaactgatctcaatagc
ttactgtttacggtgccaaacaaggtgcttgggcaatcgttactggtgctaccgatggcattggaaaagaatatgccacgcaactagctactgccg
gattcaatgttgttttaattagccgtactcaagaaaaattggatgccttggctcacgaactcgaaacggttgctaaaatccaaactcgtaccatcgc
tttggactataccaaagcagataagacttcgtttgaaaccattgctgaacaactgaagaaacttcctatcacggttttgattaacaatgttggccaa
tcccacgttatgccaaccagctttgaagaaactgctgagatggagatggacaatattatgcatattaattgcttcggaacccttcataccacgaaa
gctatcctccctatcatgatggagcaacgccgtaaagacaagaagggcccccgttgtctaattttgaccatgggctcatttgccggtcttgttccct

ccccttacctgagtacatatgctggctctaaggctttcttatctaactggtctgcatcaatggctgaggaagtgaaacctgaaggtattgatgtctg
gtgctacaactcttatcttgttgtttccgccatgtcaaagatccgccgccccaatatgactactcctactcccaaggcatttgtccgtgaagctttaa
gcagcatcaacgttcaacgcggtggaagcgctccttacattagccagccttatcctgctcatgctattatgagctggtccttggaggaattccttgg
aagtgtcaaatactttgtagtaagccaggttgccgcaatgcatcttgacattcgcaagcgtgcattaagaaagcaagctcgaattcaacaatctgt
ctctgctcagactagtgcttaattaatcgcgaggtcttaagatgcttttcaaaacttactagactttcccgattatactaacagtacatgctaaatat
gcaatattagtcatcgaatactctacagaatacaatataatctactaataaaaggtttatagactgaaactaccgagtcgaatttctatatgcaca
aaaaataatagctcatttttaacaaggcgctttgctttgaccaaacaagtcgcttatgtattaagagtctactcaagttcggaaatgttaccacgtt
cagaaacgtagataccgtccaaaaacttacgaatatccttgttgcggacattgcaaatttgcttgatgttggcggcagattgagaaacgttttcca
aagagttgccctcgatgatcaattcatccttgacgttggcagacatggaaacggtaacaccaggcaagcacttgatgacacgagtaatacgttca
cccaagaagttacggatctcaacaacggtaccgttttcggacaaattgatgttgatgggaaagtgtgcgtaaacaagacgcatcttgtaacggaa
accttgagtaacaccaacaatcatgttgttgatgatggaacgaacggtacgaaggcaagcattgtgcaaacgagaaccgtgccagacaatgaa
cttaatagcgttgccttgcttcttcaattccaagtcaacatggcgcaagctcttggaaagagagccacggggacctttgacggtgacattacgggc
cttaatgtcaacagtaacacccttggggattggcaaagtctcgtccttgtaaatatcacgtcccatcctgcaaagtagtgttggtggtgtcgttccct
ttttccagtagataatttccgtttcgagttcctgtgactgtacttcataagtagggtagggttattatttttggatagcttaatgcattgtgtagaaaa
atcctattaatattatggaatcaaataataaagtgatcgttaattaaatataacgaattttaaaaattgcagtaacgtgtatgcaagaaaatatat
gagaggaaagaagggggagatgaggaagcagaagtagaaaacgagggattaaaagcataatacgcggcagaagtaatcagctcataagtat
atgacttagcgggagcctttgtaatactcacgttgaacatcaacaattttaagaccacgttgcatagcaatgtcttctggaagagcttgctcatgta
atgcttcaaaaactgctttaacaacattcatgccatttttgagcccattatttcaccgcttaaatccttgatacctgcacatcgacaaatctcaaac
aggatagcgttgcaacgtaagccaaaacctaaatgacgggttagttattgttcctattgtgaaataaagccactgtctgtgacttccgaggatgaa
ttagacttaccagcaggacgagatcgtaaagcaaggcggacagcgtgaaactttttatgcaaattgccatataccgtcctgtcttcgtatcgtgga
atacacttcatgcttttgacggcacgagcacaagactgtttgtaagataatgaatagtttgcagcttttccttcgccataaccaatgaccccattccg
gttaccaacaacggttagaacgtagatactgggaattttacctttgcgggtttggttaacgacacggcgaatgaccaaaggaacgaagacaaat
gattgtaaataagatttcgggacacctgtcgtctccactaaaaagtcagtcattgtagctccctggtttgttgttccaaaactgaaaggagtataac
taacgggtggtttggcgcggaaaatatccgctggaggtgtatagtttttaaacttttcttcattaaacgcattatattcgtcaaagttttggaagctg
gaaaattcatccaagggacgaacatacttgtcaaatggagaaaagtattgcaaatgtaaattagttttacgtaaacccttggcattttccaatgcc
tgcttggaaagcggatggtttcggatatagtcttcttgcaaagaagcatcctgttgaaactttttaaccatttcttcccaaggactctttgcatccaat
ttatccttagaaacaacttcaggagctactggagaggatttatgctcagttgatttgggatcgaacgcgtatgacggttgggaagcagcattcgaa
tctttaaaatcaccagaagcgtcgattttcgacgattctttggtatcatccttcggttgctctggtggagacgcgtcctttgaagctatatttctaacc
attccatatgtatgaaaggaagagcgtcctaaacgatagctcaaccgcatgtttgccgaaaaaaagtgtagtatatgatttgtcattttgggttgct
atttatccatacaaagtaaaacggaaaccaccgactctaatacaaaaacacgtaaaaaaatatgttctttcaagcaaagcaattcataaaacgg
atgaatcacaaacaagttttcttcgatttgtagactctgcgaccaatcaaaaatttatccttttattcaaaattacaagttcaaaattatttttctgta
aattaactgccaacgtctaaaagaagactctcctttttacttcctctccttgcgcgttgtctctgtataaactcccaaggccatgtattctctacttaag
ttttcgttggagtctgttgttaatttttataacgcagcacattctaaatcggtagtatttatatatattagtgaaccaggcacgcgtaaacgcgtaaa
caaaacaccacttattgtgtgtctcgagtatataaacaggtcctagtgaaaaaaatataccatatagagtgagtgataaatttgacaaataaaag
actcgtgattggccttgtcttttgcttatttgaatatatttccaaagatgttttatgtgagagatttttaagcattttctaagccgaatcagattaagaa
tttgcaaaagtagaaaaaaagcgaattcttatcttataaaacatcaatttgctcatctgaatcttttgccattcaagcattgcttcattgcttactata
ggctatgataagcagtgaaagctgtttcctactgactgcaaaaatcatctgaaattgcaagtactcattaaaatcatattatttttatgaagggtgt
gaacgactggctttgtttattgcccggataacttatatcttgtagtattttatctcttttcaattctgacactgaacaaaacacagatattgccttggta
caaagcaacagcaaaaagctcatcttacatttacttatcaccttagttacgtattttcgttcattcacttggttctttttaccggctttgtccaaagaa
gccaatgttgcaatcatgaggtgctttatttctcaattttctccgtggaggaaactcaactggtttcagagaaatgctaccacgtttacaccgaaaa
acttaaagtctttaactccttatgaagtaatggaacttccacgaacatgtactcccgctgacatcaagcgaaaatatattgaattggttaaggtaca
tcacccggacaagagtaagagcggttccgaagccactgatttaaaagcacacgatgagaaaaatcagcaagaatactttcgtctcctggtggct
gcaaaatcgttgctaacggacaaacaacggcgctatgagtatgatcgttttggcttccattggaatcagtcttctccctctcaaccgtttactaaac
catctgcaaatccaaggtaccctatgtattctcgtgcccgtcgctcttctggtatgggaacatgggaagaatattactacaactcctttgattttgaa
aagaatcataaggaaaagggagattccagaaagattgacgacgagggtatgatggtatttgcaggtattctttcttttcttgtaatcttttcggtat
actcaaattacagaaattcaaagtactaccaaaaagctcgtgttgctgcaattgaacaatcggaaaacgattttgattactacagtactggaatgt
ctgatttaagtaaggatgaccgtatatcacgatttttgattcttcgtgatcaaaatttaaagtctgacccttcatcctcttcttcgacttcaagacttcc
tttacatccttcttcaccggctacttctgtatcctcttcttctgagcggtcattgcctgcacctgcttccccttcctcttaatgctttcttattttatgtttc

gtttacgttcccctttatttaacgtagcctaaatattaccactacgaattttctttgcttctttatttaaaaaatccttcctttatccatcaagtaaacaa
taaactgtacagctactgtttaaacaaatttatttatgttccattatagttcttgacctacccattcaagaaaacaaaaactctattaaaggtatcct
aattataattaactaaaaaaaaaaaaaaaaaaaaaaaacaattatccttttaccaaagcagaagaggggaaaaaattattaaagataaaatat
gcttaaagatgtcgaacactttgagacacagtctcatagacataaaaggtgacagcattcgcagtagctgcacgtatcaaaacaggaatgaaac
ctctgtaaaaacccatcataccggaactagcatagattcctttggcacattgccatgcactcttatagctagcttttgacaagaagccatcagtctg
tattttagacttgacaatgtcaaaaggataggcaaatacccacattgagtatccgcaagctgcgccaaaagcacatagtttccatccagggatttc
agcacgatccttcatttggtacttctccatgctatgctttatcaatgcttcataagtcaaaaaatatacgcccaaaccatgggcttcacgaacggca
gtaggattgaaacccttcataattcctttcattccatattgagaagaaattttcttgatacaatccaatggaccattgtacaacttgttggcgccggt
ttgaatctgaagacgaatacgaatatgttcgacagggccaactaaaaatgagttggccaaaccagatatcgcacctgcaacgtagtattgaggc
ataggcacgggattcccattacgattaaaaaaacgtttggcatactcaaacgtagtaaattggatggagacacaaaatcctattcccaagaggg
gcataacagtacctttgtaaaaagcgccgacaccctcgtttttggatatttttttgacacaatctaatacattatcgtataagggagacccaatgctt
tgagactgaagacgaactttcacacaatcaaaaggttgaccgactaaaacttgtgcgacacctcctgatgcaccagctaagaaatcctttgccta
gattgcgttgttagttttcaatcttccacgatttaatgactataagttaaatactcgaaactatcaaatgaataacaattcgtctgggctctttaaga
ctttgtatattatttaaatgtctttacttactgactgtgaaactgttccttctgccattgcaccaacctgatgtagtttgcactctgaaaaagaattta
ctgttgcacctatatgagatgttaactaatcttgagagcagaaaaaatgaatgaaaaatcaaaaaaaaggtaaatataaataatacttatttcta
catcaagattgtcggaagttaacttccagtcaaaatttctttctttgattgtgcatttcaattcaacaccttttatcaagttctcgtagaggtcagtaa
agcttttatgtttgtgaattttttttctatatctattaattgaagttttgaaaagacgaaagaaatgaattgtctatattgcatttgcttgtttatagaaa
tcgaaatctactatggaagagctaaataagaatagaaagaaaaatcaagtttaatgatagactgtttatatttcatgaatgttaccgcttcttgta
agttattcgaaggtagggaatgtttctcataaaagaaaaatatgttcgattaggaaaaaccatgttggatattgaaatggctatatatattactggt
tacattcatcaggatcagcagaaaatattctgctactatcctttcatacgatggcagagaaagactaaagaattataatgttagtacagccagtgt
cattcagaagctgtcaaaatcccccaatcaatttgttctctttggttcaagttagcaactgtcttgttgcatatataaataaaaagatttttttttctt
ataaaaattgtaaaattgtatgaagtatagcgggtagaccaccttgtacgtcgagtaccgaaaaatgctcctttaaaacagtgaaaggtttcattg
agagcatgattttaacgtttacttgtaatgatctgtgtgtaacgtaacttcgcgaatgattttctgtttgatatagttgcagcttgaaaaacagacttc
aatatgcggctcgcccggtttcacgatggacgaaatgctgaaatatctcaacccaggctattcacttactttacaaaagatgttaacttccgatgcc
taatttatgtaaggcgcacgtataaactcatggcagtaaagtgaatgcagaacgcgcaattgagctttaagctgattatctttgcagaagcccttt
caatgccactaggcgataaagtgaagaaaataataaaagtttatacttcgtaatgaactcgtaaaaccttgacttatcttatatgaatccagctat
ataaaaagaaagcgcattaatcttccgaaatataatctccgtattgttttgcaaataaccctaaagcgtggatagaaaagtaaaatttgtaattcg
ggtaggcgatggccacattcttgttaaagattccttcaattgacttttgtttccaacttccatcggcagcttgtgatttcattatgaactggatccctct
ttcaattggctttctatgcggatacttggcgtgcattagagctagcaaagcccaactggtttgcgttactaggctacgatctgtttccgtgtaattccc
ggtgacacatgcctagaaatgttagaaccgtaactgaaaataaaattactctacttaccatatagctttcactccaacctccatctaacctctgttt
agataaaagaaactcacaagctttcctctgtgtatcacaattttcgaaatatctaccaaccgtggctaaagaatccgttgcaaacattgccgcgta
tgtaaagcaaatagcccaactaccataccagcttccatcaggtcgttgcgagttaactacaaataatagcgcatcatgaattgtttgctcgatctct
tcacgacggtacgagtcatcataacgagtaaattctcgtagtgcaagaaccacagatgttgtacattcagggtaggagtattcaaccataatcttg
ccaaaaacctcagcaggatttagatgttccagccattcattcgctcttgccggttcataggaagcaaacccattatctttattttgcatgagtaataa
aacgtcaactgcctcccttaatcgacaaagacttactggtttctcaaattcagggagagattgcacggctaatacagcccgcaaggcctccgaag
ttgtatccgaaactgtgtacccctgtgtaatattgctaaaggccatgcacctttgctgttgtgcctatacccttttcctgcatactcgtttcgtatcta
aaaagttagaaaaataaaaaatttgaaaaaaaatgcatacctgttgcctatccaaaaaattcaaagcattgattgcatctttttgataaacctctg
attcatacaacccagattctactagtgcactcaaggtaaaggatgcttcccatacttgaaggccattagttccacgcatcaacattccctcaggttg
cacccacatgaactcatgtaagcgacgaatgatttttttgaaacgccttactacttggaccttcctggtaatagacacagagagtattcatcgcagc
gttcacgggacctaaacaagcataatctgtattttcattctcaatttgaatcagttcgtaactctttttttgttcctaactttcttaaccacttggggcga
aaaaatgtgaaatatttatccatgatccagtttaaagagtctaaaatcgtcgtatgtggatgatacaaatcaatttcactgacattgtttcgatgttt
ggaaaaatcaatggacatataatcagccacataaagttcttcacgaagttcacgaattaaggaattgacagggcaagataattgttccccatata
aataacccataggtagatatacttgccggacgtggcaccaccattttcctggatggattggaaaccagtctggcaaaagccatagttctggcgga
attggattcacacctttccaatcgtaacagttaagcgtagctaaccaaaatttcccccagtgaggattagcaatagcaccgccaagcttgtgtaaa
caagttcgagctctaacagcaacagggtgatcccgtttcatgcctagaatacgaagaactgtatagtttaaggaagtaccaaatactgtagatac
accctctgtatggataccccaacctccatcatcgtttgcatgatttaccaagtactgagtaatttcttctggccatccctttggaaaaggagtttgtgt
aatataaaacgcaaagatcacgcctggaagaagaaacataggtccctcataaggactagcccaatgaccatcgtttaattgcagacggcgaaa
gaattcgtatccatgaatggcactttccaaaggagcttttgctagcttaaattttggagcatcatctaagtctaaaccaagaaaatatttttctgcga

agcttaaggggcgtgcttttgcttcctcttcggtttcaaggtattcccagctttcgccagcatccaggccaacatttaaccgccaaagacgcttgtct
aactttggaattgttttcgcccattcaggtcgactgcggcagggttccatgtttctaccaacgaaatgtagggatatttgtctgttataggtttttcac
aggtaagaatttctacgtaaataataaacccaaaaacgaatggaaatgattcaaagtaaaactattcaaagttaaactaaagaaattatttgctt
ttaagaagattaaataagaaacagttacagcatccgctgtcttttatccggaataaaacgatcatccaccaatttttgataaaattactgtacttcc
tttctttgaaagtaaattaaatttttctttccgacgtatactactataattgattgaggttaacagagtatgctattgattagattactgtaagactag
gtaggaacaagaagagttagattggatgttttgatattacaaagaccttaattaaacacaaaacagctttttgtttacttttaatcgcttgcattcct
actcattgacaatattagaatatactatactgaacctttttaaagactctcgaaaatgttctatttaaaataaatcatattgaattaaaaaatcatta
gctaaggagaatcaaacccataaatattctgtggcataatcatcaataacatattgcacaccaaattaaaatattaaattataaaaaacatgcaa
gctcattatgaattaagttccttttcaaataggaaataattcagatcccgtcaattaacagacaaaaatgactttaatcgacttcttcaacttcagg
accttggtcgccgccaggggcagctccaggagcggcgccaggaggagcaccaccgggagcaccaccagcttggtaaaccttggccatgatggg
gttagcaacaccctccaattctttttgcttggactcatattcgtccttggcagcagtggtgttgctgtcaagccattcaatggtctccttgacagcctt
atcaatggcttctttgtcggaggcctcaaccttgtcctttaagttggggtcatccatggagttgcgcaaagagtaagcataggattccaagtggttc
tttgcttgaatacgactagcttcggcttcatcctcggccttgtacttctcggcttcagcaaccatacggtcaatatcctctttagagagacggcccttg
tcgttggtaatggtaatcttttgagtcttaccagtacctttttcgagggctgaaacgttcaagataccgttggcatcaacgtcgaaggtaacttcgat
ttgtggaacaccacgaggcgcaggaggaataccggaaagctcaaatttaccgagcaagttgcagtccttggtacgagcacgctcaccttcgtata
cttgaatcaagacaccgggttggttgtcggagtacgtagagaaaacttcagacttcttggtgggaatggtagtgttacgcttgataaggggagtca
taacaccaccagcggtctcgatacccatagaaagaggggcaacatccaacaacaagagatcttgggtcttttcagaagtatcaccagttaaaat
ggcagcttgtacagcagcaccataggctacagcttcatcagggttgattgacttgcaaggttccttgccattgaaataatccgaaacaagcttttg
aatacggggaatacgagtagaaccaccaaccaaaacaatttcatgaacggaagacttatcgacctttgcatcacggagaaccttctcgacaggc
tccatggtattgcggaacatgtcagaacacaattcttcaaagcgagcacgagtaatagaagtgtaaaagtcaataccctcatacatagagtcaat
ctcgatactagcctgggaagaagaagaaagagtacgcttagcacgctcacaagcagtacggagacgacggacagcacgagcattaccactgat
gtccttcttgttcttacgtttgaattcttggataaagtgattgactaaacgagaatcaaagtcttcaccacccaagtgagtatcaccagcagtagac
ttgacttcaaaaataccttcttcgatggtcaagagggagacatcgaaagtaccaccaccaaggtcaaaaatcaagacatttgtttctctttgggtg
ttacggtctaaaccataagcgatagcggcagcagttggttcgttaataatacgcaaaacgttcataccagcaatggtaccagcgtccttggtggct
tgacgttgagagtcattgaaataggcaggaacagtaaccacggcatctgtaattttactgcccatgaaagcttcagcaacttcacgcatttttagt
aataccatggaggaaatttcttcaggagtaaaagtcttggtttctcccttatactcaacttgaatcaaaggcctgtcatccttattgactactttgaa
gggccaatgcttcatatctgattggacttctgcgtcattgtaacgacgaccgatcaaacgcttagcgtcgaagacggtgttgtgtgggttcattgcg
acctgattcttagcagcatcgccaatcaatcgttcagtatctgtaaaggcaacataagaaggagtagtacggttaccttggtcgttggcgataattt
caacacgattattggaaaagtgtccaacacaggagtaggtagtacccaaatcgattccgatagacttgctcatttttgcaaataatggtcgttaaa
agtaaaatataaattcgtaaaacaaaatcaaaaagacaaattgttcaaatgcaaaggaattcagcagcgtttaaatcaaataaacaatgcggc
aaataaattaaaaaagcaggaatgaatgcaattgtatcgtataaaacagatcttaaagaaattcgtgagtaattaacggacaattccaattctaa
gttataagacactggtggttagtaaatgacagacctctatatataccatattcgtcgcttctcgaaggttcacaacccttgcccttcagagacttcg
agaagagacgagagattacagggatgggggcgaaccaaattgcgtagttccttctacgacgttgagatgtaaacaatcaaattgaatcgaaa
gaattactagaaagtgcgagaaacaagtgaattcaggctaaagaatagcttggctcagttagattcaggaaacaatataaaataaaaaataaa
ataaaataaggaaaaaaaatgtaatcatttcatcgttacaatatcacaagatgttcgtttatcttactttgattaagcatcctcagcaaccagtcta
aatccttgaagcattaccattgatttacaaagacatcgtatattttctcaagtgtttacctttttttttttgaatgttggattgctttacaggtctatgac
gaacctttttcatcatgtggtgctgaaagaagtaagacacagagtttctctccatacactttatttatttttcaaatgttttattatccttattgacac
aaatgtagtaactgaaaaataatatcctctttcctacttcttcttcccgaggcagacgtcattccacttttgcggggacttttagcgaagtgtcattat
gtgcaaagaatgcgacaaaaaagtcctttataaagcttttctcacaaagacaaagatttttaaaatgggagacatgggagacaaataatatttc
gaaacagaatgagatgaaaaaatttttacccatatggatttctgcaatcgaaacggattcagttcaattgagctactcgagaaagaatcgaaaac
tagttctctattcttttcgttcgtttctttgatttcaacgcttgaaagcatactgaaatcttctacctttttatccttcagtacggcgacacctgggaact
attattttgagtagtgttgtaatatttttcaaatatagatatttacgttttaaatgatttaaatgaccagatgagcaaaacagacataaataaatga
aaagacgttgaacactgtttcaatttaatgatcttggtttcctttccaaatgaatgtttatacatatatatttaatcatctgagaaagctcgcattcag
ctggaaggcaaggctttccttcccattagtgttcttttcctttggtatatataatcataattactttcttcgttatttacaagaaatggaaatccgctctt
cccattttgttagtacttttggtaagtatatgatataaatgaaatgctttacttaaccttaatcttttattcttatttttatttttttatttttctaaaattga
aaggtttattttttatggtatttctttcgtaatagtccaacgaaagaagctcccgtaaccaatattcacttagtcaacaaaacattctttgtaacaaa
tttccaggtagatttcatatgataaacttgatttttgaccgatatttataattacagttcgattaaaatccctacttctactttggttgttttcccttagt
caccatcaatcctcttcacttacagtacaaaatccaattttacatggttcctcctacatctggaaaaaagcgattaccatgcaagatacttgttcata

atctccccgaagggcttccggaagatattttcttaaagtccattgcgtcgtttcacccttttataaattggcaccggtatcatcaaggaaaaaagca
aaacccgtaagtgagctctacatattttttcttacttcttatataattaatcattttccttctccttgctagaccccaaccaaattttccttctttcgcata
tttaaaatttcgatcagaagcacacgttcatgactttttcgcttctatcaggggcatgcctttgtcgacaaagatggtaagaaaacaaattgttcct
gttcttcttattaatttaccagatcatgtctaccgtgctttggtactagttgctccttttcaaaaagttccacctattaaactgaagcctgactctttca
acggaaccatcgaagacgaccctgtttttaaaaattttgtagataagtggaatgagagcttaatgcctcctcctcctccttcgccttctcactatgca
tacaatgcttcaactccgttgcttcattacttagctgaaaaacgcggcacaactggtacatcaacgaaaccaaaaaaatcaaaaaaggaaaaaa
agcaaaggtccaagaaagtcaagcaagaagcttcactcccaaagtctgctgtgacctcagaccaatctacgggccatagttcctctcaatctttg
gagccaaaagaacaactgaaacaagaactaattcccccaaaacctaagagcaaaaaaaaggcaaaaccaaaaaaacccatgaaagcttctg
cctcgtctacaaatacagcagattcagtgcctaaacccaatgaaagtactaaagtagtaccgaagaaaatagctccaaagaaaaagaaacctc
aaacaaaaggggaaaaggactaaattcttcgtaataaacagttctttttcctttttacgagcctcatgttcaacctgttttcattgttgttatttatac
tataaatataaaatattatatcttgttaaatagaatttacttgtttcacatatacttcttaaccttttcaaaatttgcataataaatacattaagcaaa
gaacagcatatatcctttgattgtctattataaacttcgtacaagcatctaataattaggttacacataataggaaccagccgttgggaacggctg
gtgaccacttctctttttcttttatctaaggaaaaaaaagaaaaaacaaggttatagtaagaagtaagaagccttgatgtatcacaagggtaaca
aatagaaaaatactttctatctgaaaagctataagcttcgaaaaaaacttttcgaaagattgaaaagagcataaaattagagattcaaacattat
ttagcgaatcatacgactcacaccatcgctccgtcggtttttgcgtccacctcgaacgggaagttcaaagtcagcgttaccggataactgatccaa
agtacttgaaactggtactaaattagcagggtttcgcatttccgctctactcatggggggttgcattgacggaccagagaaagcagagccaagac
tcgtttgggagggattttgatttctctcgagtgggcttctagaaggttttggagtaggtcgtttgctgaatggacgaggaacaccggtaacgttgga
caggtcattagacgaaccgttcaatgatgtaggtggtctgctgcggtgggtagtaggcatcttgagctgagctgatgagttaacgttaaagttatct
ttgggtaaatctttattatgtatgggttcatttcccgaaaaaagaaggttttgaacttcacggctttcaggatgaggaaactgcgtgtcaacaggag
aatcagatacatgagcttgttgcttcatcggaaagctggtatctggttcacgagttaaatgattattggactcagtaggggcaaatggcctagcaa
tctttcgagaagaaggttggtcattagcggaaggagagagtgaatcttgatgagagctttcgaattcagtagtagccatattaggattaacatcaa
agaatgcagaattcgtgtcctcactactggtcgggctaggattagatttggagaatggatccgaattaaacgcaggactgttaccgtttctatttgt
gtttagaggaggaaaaccagggagtctaccagttttgtgaggagaatattccttattgtcttctgcctgaacttggggtcttgcaaagttgttggat
gaaatcaaagaaggggtctttatgtatcggccatcatcataactaggaagctttgagagagtatcacgagtaggtgatttgacagatcgagaggg
ggttggggagacaaagctttgctcatgaaaactaggagacacacgtttagaaaatggagagtcttcctgctcggaaggaggacccaattcagaa
ttggacgttaaagcatcattgaatttcggcagtgtagcgggaacgttattaagcttatcatccatcgtgaggggataatcctccttttctttataaac
agagaaactttcaacggtgtcattggaaggagtggtttcatcatttttcttgtgcatacgaccgatcgagacactacggaagcaaagatgcaagat
tgcaagtagatcgcttccaatagcgccggcaacaacataagattgccaagtattgtgaggcaaaaacagagtaagctcgacgccaaatgcaca
gcaagtcgtcagagtagtaagaaaaataaacagaatgttaaagatatttaatccctgtttcacaacgaagattgaaagaaagactaaacaagc
acttataaaagacataccagcgctgatgatatgggttatcaaaactttggaaagaacattacgtttgtccccccttgtagtagaagtcttggaccaa
agaatcatcgacagtgtagccgaaacttgttttggtgcaatgatcgccattgcccgagcaagtaccgaagatacccaagtaggtgtcgttaacctt
acctagcatcatctttgaggtcgcaggaacagaaacactgccaataatcaataaagcagtagaaattaaaagaaagataatcgtgagaaaagc
aagaagagaacgaatcatattgaggtattgattgtgggtatagattattagagatgtaaaaaaccaaaagcaagcttgaacgaatgtaagtttc
acctttccaacgtaaacctaaaaatcacacaaaaaaaaaatctatcaagaacatgcgttaagcaatcaataaaaatctttccaaatttagcaaagt
acgatgacagagagaatccacactttccgcaaaaaaggagagaaacgaagatataaccgggagaattaggattataaacgagattataaacaaa
ttaaatgtacaataaaattaacgtatcacaagtatacaaaacagattttaccttacactttggcgaatgctgaatatcgatgttctcaaatgaaatg
aagagatcttcaaaataaaaattaaaactagagaaaaagaaggtcgattagaaaagaaggaaatttgagaaaaaaagccgcccaaatggtgtt
ggtagagggaagaatgcggaagtaaataaataacttaattatagtttagggtaggcttgtctgagccactgataaagcaactttgcgaagtacaa
ccattgttgatacatgacgtaaatataaacaaacaaacaaacacaagtgccaaggtgataatggcttgttcccgcaaaaatgaccaaatatgga
tgataaacgagttggggctggcaaatgagatcaaatggatcaagcagatcgtgctacatgtggagacaaccattggcagttttcattgcgaagca
aacaaacgcaaatcaaaggtgtagttgctgaactcgcttccatgtcatgcagtaaacataaacgcacaatgagtctagctagtttggggaagca
ataaagcaggatatcctttgaacgtatggtttcgttggaatcgtttcgtataaaaaaaaaaagacgaaacagcttgtaagcgatatttgttattgga
caccaatattgttttgtttttgctttgttcattctgttcagtttactttatagcttcttgaatgacgatgaaggcaaaaaatgacatggcaccaaacac
gatgaataaacgcttcgctcctttatacagttttgctttagaaaaatctcaatccaaaaaaagcgcgttccttcttttatttttctgtcgattgtttagt
aaacagtatatacgtacgatgttggcatgaactcaaacgacttgatggtaacgtcaccttctttcctgaaaaaaatgatcggactataatttgatg
acttccccaaagcgtttttttttctttttcttattttgtttaattcgacaacagacctaacattcgttgattttgatagttgaaacgtacacaactctttt
tgccggtaaattattcttcctgttgtaaaagcctgtggtactttgagtttttgaagaccatgagtgagtcaaacgggtctgtaactccctcacctgg
aaacgatgttaatgcaattataaatatcgatagtatccatcctactctaagtctgttaaaggcgttgccagaatgcaatttggttataaaaaagga

aaaagacaccgataagaaaactgtcagaactagctttcccttgaaaaatcataaagacttacttctttctggaagagtgtaccatgattatgatttt
aagccccgaaaatcaatcatggatcgtgctactgctccacaagagttcgcaaaaagtgaatttcgtggattttacgttttattttggctttccatgtt
tgtctggattgtacaattatacgcaaaaagttattggttacggaacaccattctcggattgcctattgccaaaattgtctttcgtcagttctttgttcta
tttggctctgattttatcatggttcttttgaccttcttttcctttggcttgcaatggtgtgtcgaaaagcgatacattgtgtgggcaaatactggctatg
ctattcaactaatctggcaaggatgcttcacgtcactagctgtctactgggtaattcatcggaaattcccggtcatccaatgtcttttttttaccctac
attgtgccgtcctggttatgaaacaattctcgtatgcccaacatttgggatacatgagtaaagctcgtgagttacatgaagcttatggtgatgtttta
cagctgattcgaaatatcctgaaagaagattcagacgagggacaaatgtataggtttgaattagaataccctgatcatactgaatcaattgacgc
tttaaaagccgaagaggttgttgctttgaccatgaaataccttagtcgccaattgcggagcgagaagggaaacgtggcttatccaaataacttga
ctcttttaaattacattgactatttacttgtaccctcacttgtctatagtttggaatttccccgcgtcagcaaatttaatttgaagtacttattagtcaa
agtagcttctatatttggtgtcttaattatttgcttggctctcgtggactggtactttttacctaccgctgaaagtgtccaaggactagattttatgaca
aagcttcaccatgctccatttttgatgagcagaattttgttccctgccgttattctctacttgctattgttttaccttacctttgactgtattttaaacgcc
tttgctgaaataaccaaattcgctgaccgggaattctatggtccctggtggaataccgtgacttgggatgaattcgcccggctatggaatatacca
gttcatgttttttgatgcgacatgtatatcactcttctagacagagaatgaacaagacatgggctgttttcttgactttttgttgtctgccttggttca
tgaatttgtaatgcttctagttactggaaagcttcgttgctatattctatttatgcaggtatgtgaattgaattcagtttgtacgtatgtactaacataa
gttagctccttcagttgcccttgtatgatttgtgtcaaatgtctggattaaaaaacagggcattactgggtaacctcttcttttggattggaatttttac
gtatgtttttctcaacgtgttgaattttaactaacttaaaaatttagtggtccttcgtttctttgtatcctatatatcgtattttaagaaacaaaaaaa
ggaggaaggtaaagataaaggctactttagaacgtattagagattcctcgtatgtttactattctgtgtctatgtcttatgatatttatgatgacaaa
tattggtttccttaaggagatatatcagctccttgaatgaaagaaaaggatctgctttctaaatcaaattaaagcttacctatcattatttttattgtt
gttattattactattttatttttcgtatgaaatgcaatagacagtttcgacttcgttttctatccacatgtcacatatacaggtcaaaagacaatgtag
catctccacatcagatttctagtaagcagtgaactgtattcctttttgataaaatgtaaacaaataaaagagcacagttcgagcggcaatcatatt
gaaaatttatcttataatgatatcctataaaagagattagaattgcacaatatccagatgaatcagcatatgcacaaaactctttccatttgcttgt
atacctttccatatacctataaaacaccatagtgtgttgatatttgattgattttattctttttttgagaatcgagagacactttaacttgactctggga
gataaagatattgagtgcctacttgtgttttacaggtttttatatttttgtttaactgagaccatttttgttattgctttccttcaattacttaattttatct
ttttcgttttaaaaaatggatccttctcaagcttcgagtacacagttaagtgcaaagtctttgtttaatcctgatcacttaaagttcataaatcaaca
actttcccaattgagtcctcaagatattcttcgttggagtagatggacaattcctcaactgtttcaaacgtctgcattgggtctttccggattggtgat
tttagacatgctgtcaaaaatggaagtgaaagttccaatgattttcattgataccctgcatcattttcaggaaaccatggatcttttagaaaggata
aaaaagacgtatccagaggttcccgttcatgtatatcattgtgccgaagcatcttccgaagaagaattcgcaaaaaaatttggcgagaaactctg
ggaaacagacgaaaataaatatgatttttttggttaaggttgagcctgctaatagagcttactctgatcttggtgtgcttgctgtttttacaggtcgtc
ggcgcagccaaggaggagaacgtggatctttggatgttgtacaggttgatggacctgttattaagatcaatcctttggccaattggtcctttgagca
agtatactcttatatcaaggaaaataacgttccctacaatgagctattggatagagggtatcggtctgttggtgactatcactctacccaacctgtg
aaagagggtgaagacgagcgtgcaggacgctggcgtggtaaagaaaaaactgaatgtggattgcatacacaaagcaagtttactcaatactta
cagagtttgaaaaaagaatgaacaagacgtttaaaactcaatgaagaaacaacaaagatcatagaacgtactctatatggctggacggacgta
ctttagtgtgaaactgaattaatctgtttctgcttagactgttcttttcttttaagtaattcatattatattataaatttctttgttagataatccttggaa
tcaaccaatgctgctaattctataaaacttatttagaccgtggaaatgaagcaatcataaaataaagaccacgataaaaagctttgctatcttcaa
acgtgtacgatgaatagtgaataaaaaaggttgtaatcgtatttttcatttttttaaatgttgttcaggaagcaattctttagtttcctttcgataggtc
ataatactatctgactcaaagtctggtacgaacagtagacaaaggatcctacagccatgtttcgtaagaaaagaacagcctcaactccgaaagc
ctttaacttaggcaattcattagaagacttggggctccaaataacttcagattatcggctacgtaaacagcgttctccagatgaattgtacgaattt
tctgtatccaaagacaaagcatataacgagcattattatcaagccgttttaggtaattaagattcatgtttaattctattaacactcgtcaaaagag
catatccacgattggacaatagcgacttgtatgcttcaggccattccaatttctgcaaaggaaaacaagacagacgagccttatgcatttgtttac
actacaaacaattggagagaaaatacgaaaggcttggtaataatcgttcagtctttatcgcatcccttaatttggaccaacaggaacgttaagga
gcatgatttgagagatgcaactgttgtaaacatgatctgcgggtgtgtaaacatgggatatgctgttgctattttcacaccttctgctttattgtggg
atacacacaaaaaaaattccaagggtacaagtaaatcggtttcagctttctgctaaccttcgatactagactatttcttctttcatttctctgggagac
cagattacaaagcagaattatattccatgtacgtgtagtgaagcaagagtttatagacattcgtctctgacttttacagccattagatcacccgagg
agtatgtgactaaaggattaaattacattctatcgcaatccaaggccttaaagatatattacattggagccgaatatggttctcaacccttaaacat
gtacttagaccgaaattgggaaacattatcatcacgagctgcatctgtcatcttacttatgaatataacttcaatatatgagctcaacaaccaaaat
tatttaaattacttgtcagaggttcgtagatcttggaatgcccagtttcaagtttgcgaaaagcgctaacaaattgtctagaattgttggaactaca
acccatcgaatagaccaaagcaggaattgctggtggatttgacagcagctacaggattgaagactttctcatgtggtacgcaattcaaactagaa
gtaagcaatggcactaacacatcaggtgaatttattgaatttatttatgatgacattacgaatattttgcttgataagatgaaaaatgaatcatgaa

ttctcattttacaaagagagcgtaggcacgtctgccaagctaatggaatcatatgtagtttaacaaattccttattatacaggtaaataaatcaaat
gttagtatctataacagaagaaaccatttttccaaaccgatttcccaataatgtaatcaaccatatccgagccccagttatttctgaagatatttttc
ccataaagcttgatgaacacctcttaattctataaacagccttcttgtttctctgagttccatctctgtagattctgctttcgcacgccatccagattcc
gtattatgagtctaaaaacaagtgttagaaaaatgttacaaactactatatacataccattaattcagaactaagacacaggatatcaggtgcttg
catcaaagcttctacgccatcatcatgaaccatcatattcacatctgaatataagtaatcgtccgtaatcatgcgttttaaaacaggaaacacgttt
tggacgctggtgaagaaatttattttatcaatcaagcacttcaagaatgttacgggagagggatattggattagacgccttaaaacttgtcgggat
acaatgtcccaaactaaaacattaccgtctttatcgccagtaataattaaagaagcgtcaaatgataaagcaactgtcgtgataggagaggaact
agacactgtatgagcatccttactttcgttggcatccacaactctgcccattccacctaccgcagagggaacagaggaaggaagggtagcaccct
tcgtagtactatagttgttctcgggaagggaagttgaattcgtgtataaggggatccaaataaggcctttttcgttaccgacatatatgatacgttc
ggctggatcaaccgtcatacacgaaggcatgctattaagagcaattgtagtcaaaaggttacctgtgctaacatcccaaacacgaacagtgctgt
cttcactggcggtgtatagacgactagatatagaaggaccggagccattgataacagagacgatagcccgtttatgtccagaaagacttttaaaa
ggttttatcgtagaacctcctgttagcccagccatgtttgaccgatcaaccaaagaagacattaaccatacattgatgtctccatcgttcgaagctg
tataagctaacattccatcgcctgaaaaagaaacgtgagtcaacggctggtaatgagcacgaaacgcatgaattaaagctccggaaagtatcga
ccaaatatataagctgcctttctctgtaccgccaattagccaagagtcacatggactgacagcggaagatataacgacttccggaaggatgatag
tctgatctaagttctcctataaattcctgtattagttttcaaatcattggatacacagcttggccaagatgtcccttatatttacagtaaaaccacgtg
tgctatcaacgccaacaaaatgctcgagaacttacctttccaaatccatgtacatttagttgtggtctgcgcctttgaaaggacaacaaatgcgttt
ttgtagaacaagctgcttgtggaaatggtgtactttgacgaaacgtgcgaatcaatgtcccagtatgtaaattatgagcgattacattgctcggttc
atttccagttgtctcgtagcctgacaataaaagctccatcaaggatagtcaaatgttggtttttctatatgtgaatttctgctattcagcttgattttc
aaataaggaactacagtaaactgcggtaaatagtttggagtgaatagtatagacgaatataaggcagaagaggagggaggtatcatgaagaa
atacaaaagaataaaccataaattaagtaaaaattagtcctaataatacatatcaacgaacatttgagcattttttttgttagcattgaactgcctg
atctagtttcgtcggtgactttataacttgtagcgttgaaaggaacgaaaaaagaaaagaaagcaaataaattactgagcttgtaatagttaactt
gaaagaattcccaaatccaaacgccaaaatcatctagaaggaagttgaggatgtgaaagtgtatgaacatctttagccttattttcgtttgggaac
tacagatatcttttagcctaaacacagaaatgtacttggcaattaataaataaaagtgaatatgaacccgaaattagaaaaatgcagatccctag
tatttgtagccattagaaaacaataaaagccgtaaatgcagagccaaagtagtctttgctctttgtttttaaagcgaaacattgaatgtatgtttca
atatgaaaaattacttacacatcacatcatctcatgaaagcccgtccatccattctcccgtaaacaagtcaagcctttcatgaatatctagttcggtt
tatggacagctcgatgacgagcatgacgctttccatgaacagattctctcctttcttgggaacaagaaaggttctcttcatcctcacaacttttcgat
cgttttcgtgtgggaggaggtgatacatcgcgtcgtctacttaatcgaccgctgggagaaggcgaaacctcgcgtcgccgttgctttcctttttctgc
tcttctggatctctctttagcgtcgtcgcttctattttccacgttcatgttgtgagatcgaacattgcgcatcatattgtttaaaaaatttgtattgggcc
gtagcttttgagcattggaggcttgagagctcgattccccacgttcatttagtttattcttcttttgggcttctaccactaataacttcgcgatatattc
atcaaatgtatagccggtcatatttctctagaaaccccacctcctaaagaaatattttttgctttttaaggataaagaattactaaacatcaagaaa
tcattcacgagatatcccaagtctaattattctaacttactatctgaataagaaaaatggctatgtagttgcgttggtttccggtagcatgaacttgg
ctacactggatcctttttttgatggaatgccttgcacttatcgttctttccaaaacatataaaattagataactaaataaatgcaataaaatacaattg
aaaaaaaaaacagagatgttgccaaaaaaagcaatgctggtttcgtctgttctctacagtaaataaatcgaaaaagtgtaagcaaaaaaaatta
ctagacgaggaatgagaccaaatcactttttgtaagatctgaaatagaaagaatgaatctggtaggtttggaaaaccactgttgcttcttgatata
atttcactgctcacaatgacggacaatgatggacatgttgatttcgacttgaaaacaagactcaattttctcaattcttatgtttccagtaaaaatga
ttgaaaaactattgtcgttcattcacatatttccagattccatatgatgacctcttgtttacgagaaaaacgtttccaagaaccaccatgcaggtttc
tcatataaaacctcaattttatttttttgcttctttatatttattttaattaaaccaaataaaaggaaagaaatctgttcttcccactcacaaacgaca
ctcccatagggaagccatggcaaataacacccccgcatagtatagtatagcgtaccaaaatgggggcgaaagtggatctgtagctcacatagaa
acttagcatctgcctttttatttggcattcctttatttttacgcattatttattaaaggaaatagacaaaattggctaatataacgtttgaaggaggtt
gaattgaccatggggtcgttaaatacgtcgaaaatgccgacggagttgatagacaaccaatgcgtcgatttgttaaatggatcagttttgacaga
agacgactcaagaaaaaaggacgaattacagggatttcctgtcccaagagtgcgaaaggttacgaaaactcggaaaccaagagtaaaatgga
ctgaaaaagaaacaaatgaccttttacgtggttgtcaaattgtacgtatatacaactttgcaattcttttgcatttctatttaataattgcgactttta
ctgactcataaattagtacggcgtgggcaattggaaaaaaatcctgatggatgagaggtttcattttgtgaatcgctctccaaacgacttgaaaga
caggtaagttttgtggcaggcttatttatgcgtgtttctatttctacgcactgtggtacaccaaacagcgtatactaaccaggatggcagatttcgag
ttattctaccagaagattacaaaaagttttaccctaatgcaagaacgcacatggggaaaaaccaaaagatcccgcatacgccaggaatggcaa
aagcgtctcgtaaagagcgaaaacagtttacgcccgaggaagatgaacgattactggagggtttctttttgcacggtccttgttggacgcgaatca
gcaaggacgcaagcttgggattgcaaaatcgccgtagcacagacttacgagatcggtttcgcaacgcttttcccgagcgatatgctgctgccggg
tttaagttgaaaaacaattctggactgcgtgcaaaatatgaccaaagtaattatttaatgaatgatccttcttcgacggaagctgctgctgctgccg

ttgctgcagtcgcagccgtcgctgccgaccgtcaagaagcaccatcccaaaattctagtaaggactctacacaagatccttcagcagtatccgtca
cttctgatgacttgttagagtggtcaaaccaaaatttgaattcttccttttataacgctgatcgtcaacagcctcaatatgcgaatgaatcctttttac
ttagccaagcattacctgaaacatttccgaccaacgctttacattcgtttccaccttatgatgctctatttcccactgggaatccttcttccttgccttc
tgagtcacaatcttctgttcagtcatttccgttttctgtgcaacaacctcctgtacacttggaacccccgctagagtctaatcaaatccactcctcatt
gttttcgacacctaatgttgctgatctgcattctttttcacaattccagcaagctcatcagcacccttctctccctcccggggaattaccgtgggttaa
ccgaggttagttcgttttcccccggaacactaagtttcaactccatactggtaaactaggaaaagttcttggtttttcttttctttgttacgcgtttcat
atctatatatatttatgaaaatgaatggcataaaaatgtcgctgttctctccaaagtggcattctgccaatacaaatttggcaaagaatgtaggaa
cattgagaccattggcattttctcgatttatgagtcaacagcccattagaatgctagatatcccctttatgagtatggataagtacctattctgcttat
ttctaacctgcttttcatttagattgctatgtagagccaaaggatgaatactcggtatttacacacccttatttaaagcttcgtcaatggtggaggtct
tttcgttatagaaatataaatatttacaagagtcggtatttttttacgccgaaattttcccggtcgtgctttcaatatgaattccatagtcggagaggct
gttcgtttacactcgtcagtcaattgtgctttagctaggtaagcacacttgaacacttatgaagtgacataaggataatattatgcggtcggtttggt
tactaatctactttttagccatgatagtcaggttttagaaaataactgcactcttcgtgcttcccaaattttacgaaatcaattaaagcaactaccga
agtgtgaatggaaagttgaaaaacacctaagctctccaaaattgctgaatttaagttgtgctcaagctgatatgcaaggaaacgaatttttttattc
aagctattgttcgactacatactctgcaatcagtaagtatcgatcaagcatctccttcatcgaaacgtttaaaagaagaaatagagaatgttgttat
tcaacaatgttcttggatccctcctgtacgctggcaattttggggtcttgtctcctccacggcagcaaacgcagtaaaaaagaaacttcctgatgga
caaacggcttttgttgaactaaaaataagccatcgtcaatgaaaccaaaatagtagcttattttgagtttcttaattttgtttatcatatacaaaacg
cagcattcaaaagattagtgtaatttatacatttagtagataagaatcataataattaattagatctcaaacgttagtattctcataaggataaatg
tcagccctgaaccatcgtaaatagtcaattaagcaattaatcagtttagtcataagaaaatgttgaacgattgttggcattcagacccaaagcagc
acatgtctcgtaaaataaattaaacttttccatactgtagttgatataatcaaaggaggttgagccgatgggaggaacgttggaagaggcaatctt
cgaagggggagaatccgatttatcttctctttcactgcttacgctaagatcttcccagtcatcgtcggtttcttcgcattgcataacaccccacaagg
cccaaacggcattcaccgccggactaatagctcgaactgcttcctcaagctgctgtactgaagtgtcaaattccaatttaagagactcgggaagg
acgctggttaattctgaagcgtcttttactactagtttttttgaatagaacagcgctttgctcgacataagcacgataaaaccgtttacgagcttcaga
actgggatatcgagaacgatccatcatatgactttgggtagggtgatgataatttgccatccattcggcgaaataattcgcaatatcataggctcgt
atattaggacccgcatactcaaaatcgacaggaacaagtatgtggtgtctattttctggtttaaaagagcgacgtttagttttaatctgtagtatatt
cccgtattgagtatcattatgggagaagacgagctgcaaggagggccaatgtttttttatcacttgcccaattattaaaccattgaaaataatcatg
aaaatctttttcaacggcgtctaaaaagtttgtagttatagataaagaatgcttacggttttgtacatttcttctggccaaaggaagccagctgaag
caaaacttccacgcattaggcaattcgtacagttcagtaggacttaattgtataacataatggagttcggagatacgacgcgccacataaatgga
acatgtaggatctcgaattgtctcgggcgttagtgttgtggattccatatactgttcaaaacggccattaccaaattcaccaagcaagcggggacc
aatgttatgtttagctagacgtttcaagttttccagttcaacgaaacgattaataaacatttctacatttggtccgtaaatacgcaataagagtttga
tggctttacatccttcaggaggagcgatataatagacagcgttcgttagcgcaccagaaattcttctaattatgagagtcggatgagaagctactg
gtactcgtgaccaggtagggatagctaatttgtgaattaccagtaaaaccttatgtcggagaacgtgatcaggatcttcagtgtgtaaaagagatt
tgcatttttagaaacgtatgcattgtcgaaacagaaatcctcttcatctgatagaacaggcattttatagggtattagggtaaaagtttagaaaaa
gggtctaaaggattatggatgaacgtattgaagtaactcagagcttattgtattctcaacaatccaacgggtttctatacaaagtcaattcaaggc
gtcagtaatgctatttggaatccttttatcaaagtaggaattaagtgttcggaaattgttagcgacagcgtaaaaaaccggaaaatgtaagaatc
gagttcgctacgatatatctatactgacacgtagtggtaaatccaacaatacaaaaccaacttgctttgaagagaaaagataagcgaagaaaaa
aaaaaaacctaagaaacaaaaaaagaaagctcgaatctagatttctatatgtctaaaagcatatattacagaaaaataaaccaccgctaatca
caacaaaatttgctaaaaaagagtagaaagccaattaaaatatttctctttaaacaatcaaaaaaggaataaaaaccaagtatgaaataaaat
gttgtgaagcgtgtacaccaaacaacctgatggtgaacggttaagtgcagccaaccctcctgcaaaagatctaaattttgtacgattatcgctacc
gtaaatatcgcaaggaatcttagaatatttactcactgtatataaaaaaaaaaaaagaacaagaacgcagatagcattcattgatacataatgc
gtattgtaaaatatacaaggagctcttacttacaatttagatagaaaagattgagaaatgaatttcatttattaaggatcttgattaaaactaaacc
ttcaaaaaacgggaatagaatagaatatggctactatagctgcgccatatgtttttttatttttttgccagtttaatgcgcaataggcatctgtgcgagt
taccaaaataatgaatatcgacgataggacttactcagaaccgcttatatgagaagcggaagaagccccgtaataataaaggtttccaggtgca
taacaaaattcgggataagggtaatatggataaacagactgcaaaccaacagcggtagggcttacgggagtcatggatggatgaaaaggataa
gtcaaggacgaatttgcaggatttgcagcaggaaacattgctcttggtgaaggaaggaaagaaggagaaaacatgggaacctggtaagaaga
aaccggataagtagaagggggaagaactcgaaatttgacacgtagaggtttcttttggaaaaaaggatggggtttattgttaacggcgttgaaa
gcagcttgctgggaatcaaattgcagaaaagcaaatgcaggtttgttcgactggcgaatgaatttgcaatccaaaactttaccatattcagaaaa
gagttcaatgagagagccacgcgtgcaatttttagaatctaaatggtcgacaaaaattgaaaacggatcgacgaacggcataaccggttggaag
tatgggtgagtcgagggaataaaggaaggatatttaggtggagaaaaggttgacttggaaatactcggagatttgctgctgtactttgattggga

gtaagggctggtgcagggagggctcatggatttgttcggtgtagacatattactacaattatcagatttggtagctgaattaggagtagtcaatga
agaagaagaaaccggggtagggctagccaaagatggcatattggaataataggggaggttagtagatacagtagacgggttcgaagtttcttg
agagggaggatttctgtgagattttgaagagagaccgagttggaagaggaagaactttttagtggacggacgtgattgaaggaggaattagga
tatttttttcggaagatggatttagcagagacattctcggcccaaacgactttatagtgagaattccgcaggctcatgaaggcagagatggcatca
tctcgatagacgaagcgaaccaaaaagcctgagcgatcgtttgtttggacaagctcctcaacgagaccatagggtttgaggagttggtacacatc
agtggtagaaaaactagatggatctgatggataagccagatagatgctacgatttactttggctcgttcgatacgaatgcgacggccatcaagca
aagtattttgagcttctttcagagcagtagaagcgtcctcgacgttggcaaactgcacgaaagagtaggagcgttgaagccagtcttttagaacct
tgacatgaagcaagggaccccatttttgaaaataattctcgacggaaacagtgagctgagattcagtgagggaagaattcaagctaccgacaaa
caagcaggcaatgggttttcctcgtgggtctgggagctttttagaagggttaaggacggtgcattcggtaagctgatctgacgaaacgttagggg
atatagcatgtggggttgtgtttgtagagactgagggtgacgacaaaagagggtagcaagcaaatttgtggcctggcgagccagatttggagat
ggatgatgtcggagagagagagggagaggttgtgacagaagctggagcattggaagaatccatgataccaaaataatattaggaaagagaga
aagacacttttagagcccgcaccaatagagaagagagtttaaattattttcaaacgagcaagtgagaaagaaagaaaatgaaaaggcgaacc
gatggaaaaataaagtgatgatagaaacgagagtagtcgagtgggtggatcactgttgatggtaaggatcactttttttaaattaccaaagagaa
aaaggaaatcaaaggtaacagacatacaggaagaaaaaaggtaaaaaaaccaaacgtcaagtgtcaaacaatttaaagagggaaagaaaa
aagattaaatgcaagcgaaaaaccaaagtaaaaataaaataaaatacaataaaataaaaaacaaaaggtaaacaaaagtgagattctataa
ggcggaatagaagcagaaaaggaatgataaaccaaaaatggaagggggcagagggaactgaaagattccaataaaacgtgtgtgtgtgtgtg
tgtttttttagtctgataaggaaagaaataaggaaggaaagagagagaagaaaaaaaaaaaagagaggtcgaaaggatagatagagagcagg
aatggagggatcagattcaatcagataatctaaatttcgtataccttgtacgtagtaagtagtagcaaggcggaggagtgtattgatttgttgtata
tagggaactaattatatactataaatggttttaattggaaagaaaaaaaaaaggaaaaaacaaataaaggagagttgaaatttgtaattcaatga
gaaaccgacaaaaaaagaaaataacccaaaaaataataataaagtacaacaaataagaatatcgatccaaaatttaaagaacaggagcgta
attaaaaaaaaaaaaaaaaaagaagaaagaaacagtaagtgacagcaacacactaacgatggaagggagcgtgcagcatattcgtacatat
atatatacaaaaaaggaaagggtaggggtatgtatgataagggaaaaattctagcccaaaagcattaaaccaaccaaagattaagaggagag
cgaaaatgatcttgatacaggtagcataagttacagaacctggttcggtgaaatcagatgcgtgtgtgtgtcaatgagagataaattgagaagtg
gtgaaaaagggacaacgtgataaagttgaaggggagggggggatagggggcaaagtgtggaaccaagataaaaagaagagggaaaatccac
caacaataaaaaaaatggagaataattaaaaaaaaaaacccatcatttcaaaggaatcttaaggctagaagagaagtcaggaaaacaaaaatt
gactaaggatatctagagggtaacgatggtcacaaactggtaatgagatcataaacaaatgaaaagtatatgagaaattgtaatttgcggttctt
taaatggaacaagagaaggagaaagagtcaaatatttattataatggaatctatttccactgggttcttaaagaaaaaaaaaatgttaaataaag
aataaagaaggtatcagtgcaatgtttgcggttgaaaaaaaaaaaatatatatatatattttgtttatgtttgttcgtattgttttgtttctctatggca
aatctatctctgacaaaatcaacagaagaccgctcagggaacacacatgtctatgccatgtagtggtgaacccctccaaagtcgaggaaagggg
tgggaaaattgacaggataaaaatggacgaaattggattaggaggagatgtggttggacaatgacatagatagcggagaaaaagaaacaaaa
taaagttcaataaaaatcaaatcacgaattcgtgaacgaaaaaggtgaaatcatttttaatttttacaatttccaaaattcgagattacataatttt
cagcgatgcgtgagatattttctaatgtttttccaagctgagtaagaggtcgtttgaatggaataaggaaggaataaaaaagaaattttttgac
aagttctctttttgtcttgtttatttttctgtggtgtcttggttttctattttagttttcgtttgcttgaacgatgatgataatgagtctatcgaaacgagcc
gattgccaaaaaaaaataaagacaacgaacgaatgttcaacttgaaatctgtcactatctgtctgtcacaataggtgtcttgtttatattatacata
ctacaaactctgtgatataaggaaattgttgaatgttaaaaatcaccataagactaaaggatgatgatatattacacattgtaaacaaacaatta
aaagcgtgatacaaatagagattgaatcatcagtcataggatcactaattaatctgattgaaaaaaaaaaaactaaaactaaaactatatatactc
atcagcctatagttagattcttcagactagaaggttcgaccggtatgactgagaataatactcctatttatatataataatatgatgatataataaa
tcaatagattcaaatctctttttttcttgctgtggactaattctactccccccatctgtaatcctgactagtgtttttctacaccagcaagatctaccaat
aatacatatacgtagtactatgacctctttgtacagtagttattatctatttttctataattttaactggataggaaatgctaaatgcaaacaataaa
aatgaataaaaaacgtgttcttgttttcttattttttcttatctgtaataataataataataaatgaacttcaagtcgagaatacgtgcttgtaaaga
gaggggagaaaacaaaacgttaagagaatccaaattttttatatcataatttaagaagaaggtgaccaaagagattgacaagcctttaattgttc
taaataataagaacagtgatcaaagccatgttgattcatacactcagcaaatgtcttggcttctccattgcaagcattcttggactcttcagcaccg
gcatcggaaaaggcagtcgttgaaccaaatccggtttcggcttgagaaggagccgctgctggcgcagagttgtcttgtgcagcaggagctgaact
tccaccaccaaatgcaccagtaattaaattgcccatcgtatgaccaattgtactaccaatacctacaccagcagctgtactaaccaaattaccgaa
aaagcctgtaccacctgaggaagggatggccgtagggggagcagcaggagcggctgcgggaggttgtgcgtgctgagcaggaggggcagctg
gacgagcaggttgggcagcagaaggagccataccaggagaagcagccatggtagaagcaggtcgtgaagaaccgggcgtgcggcgttggctg
gaagcgggagctgctgcagtacgtctacgaggcatgatgatgatcaaaaataatatgaaccaagctgttggtttccaagaaaaaagagtgaata
aaaaagaactatgatatccttcaacgtttcacaaacaccaaggacgtgatatggttgatggaagaatcaacttttgccatcgacctccattataaa

ttcgttctctttcctaggctttcattggtgttttctcttctttccattgttccaagtgacaacacaatgtaacttgaacttcaacaacacaaccaaaccg
gaccaataccttttcaaacagccttgtacttgataaaacaaggttttactatgaaatttcatacaatgcctaactaaacacatattcatagattccat
catatatcggtacgaatattagggaaagataatttttgtctatttcattcgtgacacagtataactctttcaaggaaatacaatatatccatataaa
agtgaatacatgtttcttaatttatacattttctttttgttttgcctttcacctgagagattcgcatcagtgctgcatagtggtagatatttgagttaaa
gctacgaataccgaaaattgttgcgaaaccattaaactctataaagaaatgcagcgcagcatcattaaatggatacagataatgaggtatatttt
ttctataacaagataacaaatgcaagagaaatgagatgaagagatcagattgaaaatttttcacagattcggtaacctacgtagacaacaaattt
gttttatttcatttcatttccctttgtggtaaaaataagtaagaaatccaacacggtagtagacatcccttactaaattacgaattgaatcccaagag
ccaactatataagagataacagcagaaggttctactgcggaaggaagcgaagctttatgatttgttagcgacaattgccgtctttactatgcaacc
ttcttggataagtattggattttgctggccgaggagcggtagagtgacgtgtgccctttggtccccacaccttaatatatcccggccttaaagacgt
atttcgtttacagaagcatattcgatttgtcctttacataacccattttggtttcgtctattgtttcacaaaggtatgtctagttaataaaatgcaccaa
attatgtaagaaatgaatttcagtgaatacagagaaacggttttcatacgcaacaatgtttgattctttatcatcaactttttctctcacatcttcttc
agcggatttggacttttgaataccacacgcaatctttggctggttttattacgcgtttgagaaaggaatacatttccattattagtggagttgtgctg
gtgaaatgattagaccatctaaacgttatcatagtgatgggaaaagatagtttcaggagtactactcttcacgactttgttctggagttgtaaagtt
tctaaatggttttatccaaaaagaagcttgaatagtcttccacccattggtttttgttttcaatttcattcaacaagacaagacaagaattgtattttc
tttttgtaatttgcttcctgtatttcttagaatttggtgcagcattctcatttcttgcgcgtcctactaacctttactgttattaggatgctcattttaacc
gccccccgatttatgaacaacaccaagctggccagcacccgcagactcgcctcttctctattctcgcaggcctcattacgctccaaacttcctgcta
cagctgctgcctcttactctaccatcaaaggcggttctcttaaagaccgcctcaccgaacttatccccgaaaagcaagaggaaattaaaaagttcc
gttctgaacatggcggtgatgttgtaggtaacgtcactatcaatcaaatgtacggtggtgcccgtggtattcgctccttggtttgggaaagctccgt
cctcgaccccaatgatggtattcgcttccgtggatacaccattcccgaatgccaaaaacttttaccctctgctcccaacggaaagcaaccccttccc
gagtctcttttctggcttctcgtcaccggcgaaatccctacccttacccaggtgcaatctttgtctgctgagtgggctgctcgctctcagctccccaaa
ttcgttgaggaactcattgaccgctgccctccttccctccatcctatggctcaattctcccttgctgtcactgctctcgaacacgactctgcctttgcaa
aggcctacgagcgcggtctcagtaagcatgactactggaaatatacttatgaagattgcatggacctcattgccaagaccgtccctattgctggcc
gtatctaccgcaacatttaccgtgacggtgttgtcgctcctatccaacatgacaaagatcattcttacaactttgccaatgtcttgggttacgccaat
aatagcgaatttgttgagctcatgcgtctttatttatctctccactccgaccatgagggtggtaatgtctctgctcatactggccatctcgttggttctg
ccctctcatcccctttcctctctatggctgcttctttgaacggtcttgctggtccccttcacggtcttgccaaccaagaagttttgaacttcttaaacac
tatgaaggaagagattggtgatgacttgtctgatgagcgtatcaagagctatttgtgggatctcttgaaatccggtcgtgttgttcccggttatgga
catgccgttctccgtaagaccgacccccgttacaccgctcaacgtgaatttgctctcgaatatctccctaaggatcccatgtttcaacttgtcagccg
tctttatgaagtcgttcccaatgtcttgactgagcacggaaagactaagaaccccttccccaacgtcgattctcactcgggtattcttcttcaatact
acggtatgaaagagcaaaacttttacactgtactgtttggtgtttctcgcactttgggtgttgcttctcaattaatttgggatcgcgctcttggattcc
ccattgagcgtcctaagtctttctctaccaaagctttaaaagagcttgttaatgctaagtaatgacgatttgggtaaatacggagtttcttgtatatc
atggattcttgattagaaaacgactagttctatgttataattatctttttctttgttggacatttcagatagagtttgaaatatgagaaaaatatcttc
aaagactttaaattattttataaccatatattaaatatactatatgatgagacactaatctacattgcatagataaagcaataatgaaataaaata
gaaaatgtaaaacacacatagtcagacaaaaaccattctatgtcggttactatcccggcttaattggtgtttcttcttctacatctgtgcagcgtacc
aaaaatcaaagcacaatgctcattttagttacacgtggattcacaaagccaaaatcagtcatttaaaaaatgaccaacttcaaactttcaatcgtg
ttgcatagatcctttagtctaaacaaacacaatcgcaaagctatttatgttatcttatagcatcgacagtcacgcttcgttatatcattttcttttttttt
ttttgtttacaaaatgtgtcgctgtaaagagccatggagaacctaaaaaacttgcaaatttacatgaaatcgctaaaataaagggaacaaggaa
gaacggaagcttatgattttcatgaaaagtgaggaggtttgtggctgcttaagaaaaggagcttcactatataaaagagtgttataaacttatgg
aatgaataaaatacttctggctggttaccaaatcattttggcaagtctaaaaagtaatactctgacgatgcaaccggaacatattggaggtaattt
ctatgttttgatgtacatataattcttctaatatagatcttatgtatacaacgaatagttataaaatctttatctatgaaaattgttcatgcttcctctc
atcaccttgtgcaggtagtccttatgaaaatagcttctttgccacacagagctcataggaacgcatttctgtttagcgactagcagctactaactca
attcttcatcatcctttgaccgagactttgaaagagttttctaccgcaatttccaacctaacttatttactaatgcagaacacaggaatgcataaaa
gcaacggttttattccttctcggtaacaaatatcatattaaagaacttatgctaaacaagtgcttgagtttgcgattgtacatagtaaaatcaatag
attgaaccgtgtcttttatttttaggtaagcatttgaaagaaggaaaaaccaagttgaaatttcattgctagcgaaaagtcgaaattatgtaccga
ggaagagaacgaccgatattctctgaggtttgtgtttcaacggataatcaaacagttgaggaatgtatgttctaattaattaaagagatacaattg
aaatgtttcctgttattgtttcctttaactgattgttcctgctgtaagtaatgtaaacagacgcggcgcgtttgattctcaacatctaccaacatcgac
gcgggaacacgacaaacttagcaaacgcaaaaaccattaaagaaacgctttaattcgtataaattaaattatataaaaaaaaatgattattgaa
gctacacatgtcaatgtaaagcatgagcacgaaggtcatttgagaggcttgttgaggagacgaaattccatgaaacgacctatgattcagcttaa
atgtgaaccaagccgattgaaacaaaagccaacggaagaatttacaattcaaagtgtcaaacagattcgtagcgaattaaacaacatgttaag

aacgatcacggaggcaattactcaccagttaactttgaaccggcacattcacttggatggtgtcaatcagttgcagaaaaccctcctttctgtgtta
aaaccatttgaacaactggatctattgcaattaaatatgatcgcggtaaattattctttaaaagatatattctcttccttcgatgacatttattcttttc
taatgatgtattactctacgattactttgtacgaaagggtggaaatctttctaaagaaaggcctagagttagttaaccaattgtacgatctcctata
ctttcaaacaacgttgcaatttaccatggctcgtgtccctctttcagacactgggcttcataatttaaaccttctggtttctactctcgaaaaacaatc
aactagcctgagcaccacggcaacagcattgattcctttacaagaagaacttgagcaagctaattcttactataagtgctggcaacgagcacata
cgatccttactatctttgcagagccttatcatcttccgaaacacattcgtaatgaaattgcttcttggtttcaaaatatgcccagctgtcatcccaagc
agctcattttactaaggttatatctttaatgctcacaattaatgcaaacgaaatatactcaatacgagcgaacttaataataaggatgcaaatgta
caaatcacgcgaacctaatatacacaatgacatattcagtgtaaacatattatgcggaacaatattaaacattagtacaccctcatctttgtaattt
tgcataacttgaaggtttcaatttttgcttcaatgctcttccgctccaaacaggtaaaagactgataaataatataagagctgatacccagtaaaa
cttgactcccagaaaaaatttcagatctacgtttgttagcgtaatccaatcttagacaagtgctcttaccaaagtaatcagttaagaaaggtatact
cagagtatagatcattaaacttagcatttccgacatgacaattgtttgttccctaaattagttattagcattgcttatttccatgaacagtcttaccat
ctattaatctgaagggctaccataatgagttcgttcaaaattaaacaagaaaaagaaacagctagcatttggccttcgacatcgaacccgacaag
gacgtaagtgatcacttgtattatgacaccttaaaacagtcagtaacaagcatctatatgaataacataccttggtaaatactaattaacacgcaa
gacataaatgacttgagagagaaacatttatcttctctcatctccttataaagctctggaaacagaaaaactagtttttcgctcacatctctatcata
aacaatggaaaaaacgggcatcattgtatacatcgtcgagtatccaaccaacagtaatccttgaaacaaagcaataggctcaaagtttgagatt
atactgtacaccacctgacagatagaaataatcaaaccacgatgtataacgaacattgctaattttgatgtttgtttataacttatacggccatgcc
acaagagtaagcgagcaacgtgactaaattcttttatgctatagtcagcagcaagactggcctgttgaccttcttttccggcaacaccaacaccaa
tattcgcaacttgaatcattccgacatcgttacctccgtcaccgatacagcagactgaagcagatttcttcgtttggattaagcgcgtcatatttgct
ttctgtgttggtgtacacctgcaaatcaccacgcaggataattcagaaactgcttctataaattcgtcctgtaaatacccgatacagaattccatgg
attcaccatctattataagacaacaatctggttttccaattagaatcattaattgattgtgcgcatcatctcgagaattaagtttggaaatggtatga
atgtattgacctcgagaaacgagcctagacgatatagcaatacacctagcagtttccactttgtctcccgtgagcatccaaacatgtataccagca
tttctaagtagttccaaagttactttgacatccttttgaagtttatcttcaacaccggtaagaccaagtaactccatatcattctccaaatatttcgaa
acaacttccgtcattttcttatctctcgaacctgaaaaagacacgctcgcttctgaatacaccgtagcgaaagtattatattgttcattagttaaatc
tttctttgcaattactaaggttcgaagcccttctctagccatatttccacattcttcatcaagccagaagcttggactgataaattgttgcatgaccga
atctgcacctttaaggtaaaaagtgatttgtccttctggcgaacggaccacaatgcccattctctttgtttcagatttaaacggaaaaagttgtaga
atttcgtacgaagacatattaatggttacggctcctcgattacgatgtgtcaaaaccaagcccatcaaactagtccaccgaacaattgcaacttca
tcagggcttgcggcttgaaagctaacagaaccgtcagtagtttcagatggagttacattgtggcataaagaaagggccaaaacagttgttcgaat
caaatttttagtatcatcgctcaacggaatttgggccgtaggatcattaatacaagcttggattacgtccattgattcattggagaatccaatagct
cctaaatgtagtttttttcatttccatttcattctgtgtcaaagtaccagttttgtcagttaacaggtactcaattctaccaagttcttcagggatattact
gcttcgaacaacaacatctggtaaattaggatctgcctcggtatgtttagaatgaacaattttaccaatatctagattgacacgcaagttaatcgg
aataatgctggaaaataaaatcaagtatctgaagatagcaatgtaccagttttctcgtagtccgtggctgaatgtaagtatggtggaaagtgaaa
gaacaatcatgcacagaattttagaataaaagttaatttctttctccaacaagccgacctttgttttagctttcgaactatttaaggactgacgagta
tctttaccagcatagatgacaacaccataaaccgcctcactggccaggacagtatttgcccaaagtgtattgtccacggtaagtggtctcctattgt
cattcaaagtaaaggtaccataaaaatggtggacagacttgataggggaatctgcatgaatgattccatgtgaatattgatttgaacagggtatc
cgtaatttccaatcagtttctccatcgagctgatctgttctaatgaaggcttcattcccaacagatgtatgcataaggatcatgtctgcgggtactctt
tgatcctttgagatgagcacaacatcgcctacttgaatattctgagcagagcagggatggttattaacaatatagatttcattattagcatacgaat
cccttcttcgcctctttaaatcatcaattgcttcttttgtcaaggtaatcatcaagacaaaaattaaaggagcgatataagtagacaggtatccaat
ttttagaggaggaataagctgggataaggacaccaatagaaaatacatgttataaaaatagcgaaactgttcgtacaaacacttgggaagaaa
ggtgaaagcatcatactttgtgtttgttacagcattgttgccatagagaggattaggtcgacctacttcgatcattcgaaaaccggattcaaaatcg
gtattgatatcttcttgagaacccccatgaattgaaattaaagaatccgtttcctccctgtcataagaaagccgcgtttgtcctagtgatgcagaag
atgttcgttttctttcctgtgaattggagagaacactcaaaggtataatggaactcttttcgtctttattctgaagccagcttttcattcttaattgaac
gccatttagtctgggcttcataatggatattgcgatcaataattaatcagacgttccagcactggctcaatattaaaacttttctcctataattttact
tttaattctataaattagaactctcaaagtccacctaaaatgattatttctttctaagattaaaatattcgcattggtatatgttcaggtaaaacaca
gcataattataccacgtcaaaactaaaagtctaagaacagttagtttggtaaacaaatctgattctattattattaaatacaaaaaaaatctatttc
tattactttctcctttgtttttgccagtctatcagcaaaatggtagagtaagagaattagacgtttttctgtactaatttcgcaagttttactctaagta
taaaatctttggttgttttgcttgggaaaacttactttgtcacttttaaaagcaaagcataagatcaggcattttaaaatgaatgaagcaaactata
tttgagagagtgagcagccgtttggcatttccttaggactacaaacaaatttctacatctcacaactttcttccaaggttacacgtttgttttggtaca
gtgaacgaaatataaacaaacaaatcgttatgtgagtgttgtttttttgggtgaaataaacaaaattttaagtatgatacgcttcataccattagac

actgatgttgtctatttaatatcaattatttcgactggttttgtcaaaaaacccccaaccgtttgttgcaaaaatggtgaattcaacgagttctttaaa
tgtaggatttgaggagatccagaaattactttcgctagttgagctgcaaactaaacgggaactaaggcaggtgtaccaaagatgtgaggagcaa
gaggcattacttttagatttggcgaacagcatgcaaacgtatttctccgtagctaatccgcagcaacgaaaactggaagctgtgtacgaaacaca
actcttaattattggcgaaaacagccatgctagtaaaagttatcagcgctataaaagtgatgttcatctatcaaaaaataaaattgaagaggtctt
ggataaattggaggagtttttttctctcgatagaatgattataaataactctgttgatctgtctgaaggaaaatcaccgaatgtatcttttaaagaac
aaagattacgaacgaatgagatggatagttttgaactttgcagacaaccggagaatgaggagtcttctattgaatgcattacccaaaatttgaag
tcgtataaaagaacttcgagagatacttcgcctattcaacgggtgattgcttctcgcaataatgattctctatatcaaaaccgtcaacaaccttttat
atccagtaattcctttaacttaaacgaaacttccaaacaaaagttcaatggaccgagtgaagacttgctttcaagcacggacaataaagatgata
tccgagatttctcgtctttttttgaaatctgacaattatagtcctttgttaagctccactaagaacgtaacacttataaaggatgaaaagcaagcgca
tgaaaatacgcttcaaaatgaagatgatgaattggaaattccttcctctgataacccttctaatgtatccttgagtttggatctgtctcaggaaaga
atgactgaacacagatcaaatacaaatgaaatcatgacaaagcaaaagcagtttcaggaccatcaaaagaaaaatgcagaaataaagaatct
ggtatacgaaaaatcgccaaaacttgaacagaccgttaactatgcacgcttagcaaagtctgtttcatttcttccttttaaagacaaaaagaacag
aatggcaagaaggtcaaggaagaagaaaaccccgccagaaagaagagaaaatggagactcatacagtaatactaggtctaaagatgaaagt
aatcctgctgaaagacacgcgagtcctattagacattgcgaaaggcgcttatcgaccctggaaggtgatcaagaagcaacttcagatgataaag
aaaatcagcatgtccaaaatccgaagctaagaaacattgtgtcagaaaattcaagtagtctttctcatcattgtgaacgcataaagcctcgcttca
ataatgaatattccaatgaatggaatttcgacgataattttaaagaaagtactactattttacgtcgtggccattcaaatgattctgcacagcaaat
atacgctgcagaaaataatcacaaaaatatggttactctagattttaaagattggaatatttaattctatttaacttgcgtttaaagcttaaatctat
cttggcgaatccccttcacttgttgaaagcgcaaattaaaacacatccttttgtttacgcactcgatcctggagttcccttttaaaagatttaatata
aatgttcttatagattatccttactgcctacaactgcggtatggttatctatagtgtacttcttatgcaatgaacttttgaatgaagagtaattaacta
tataactgaaaacgtcttgatttttctagagcatcacatgtaaaattaaaatattcctcttttaaatattttaattatgtttttgtttctttttttgaaag
gtaaaccatgtttatcgtcttacagacctattctgtaaatgatattacagtatagagtacaataattatataagtaccgaaattactatgccaaata
taaatgtatgtaagaaacacaaaatagttgtaatttgccagcaaaaagcagcaataatatttaaactggttatttaatttctctcataaatttcata
aatatttgaaaaatgtatgcaagtctaggacaggcacttcaagaaaactgtggaattgaatatgtgcaaaggacaattacagaggaccggaata
ctttgaatgctcctgatgcggtaagagagttatcgaagttattttcaattcagttggaaaatatcttgtcaaatagactcaattttcataacttacta
acctgatggattgcaggataaacgaacgccccttcattgggcttgttctgtaggaaaagtagatgtcattgaatacttattaaagcaaccagacat
taaaatcgacgaaaaagatgaagctggttggactcctttgatgattgctatttgcaacaaagctattcctgattcattaattgacaagttcctggga
cgtcctgatgtcaatatcgagatgactactcgtggtggacaaacatgtctccattatgcttctggaaaaggtcgacttccaattgttcgcaaaatat
gcgaacgtaaccctggcgcaatgagagtaagagatctccaaggccaacaaccattacatcgagcggctgcagtaggacatctgaacgttgtgaa
gtatcttctttctcaaaaatgtcccttgaatgcttctgattcttttggatttaccgctctccattttgccttagcagaaggccatccggagattgctgtt
gaacttattaaatctgatgcggatactttacgcactgacaaagaaggtcatacagctttagatgtctgtatggaccgaagcattcgacaagagttc
attgagatgtgcaagcaacaaggattggatatataagttttctaattcaatcataatgagcacaccgaaatgtccttctgatttcaagcacaaatc
gtgctttcagaaaatcctttcaataaatttaactttttgagatttatccaagtactcaaacttctatgcttcctatgagattctttaaataataaaaag
cattaaaacaacaaagcgatccttaaatgaaatgtaaattcgatgttgcgaattgtggtaatacaacagtttaaccttgtactataaacgcgaaa
aatatttgagtattcagcatagatacaactcaaagtttatatctattttccttgacatgaaaactcccttcaatttacttcattcctccctcacgcacct
tctcttctagttctggctagtaacgtacttgcttcctacttgaatagttgtggattaagtgattttggaggtgaaaagttacggattattagcaactta
caaatgaactcttctgattgtacatttactgaagaaacgggagatgaaataaccaatttgtcttatgcggaaaaacaaattgaacagcagcgcta
tcaaacatccgtacgtatgaggcttctttttttcgtaaaaatctaacaacgaataggttgatttactgcttcccatacaagaagagaccgatgtcatt
gcaaccaaaattattaattttgtagacaacctttcgtactacgaatttaccggaagccctgatatccttctatttcccgaattgtatagtaatctcttg
cattcaggtataaatttctattataagctacagttcagccttgatgtctaacccatgtaggaaaagcattccttatttctcgggtgccttatggttctg
atgaccaaatttcagttgttaatggtaaggaattaactgaagttgaaagcatcctaatatgagcaattaggaaatctactcgttacgttaagtaaa
gactcatttttgcgaacaggactggatgctcatccaacatcttcaaaggcgaataattctgtatggcttgcttcttatgacttgaaatcgacattttt
agaacctcatcgaaagggctttgaaagactgaaaggtgcactgggcaggttcaataatcagtggacatttttcttgggaattcctccagaacactt
agacaaatcttttatggattcgcttagacaatcgttcccggtaccattggtacagcggcctttacgtgctgtaagaaacgtccgttcatctgttatag
ttcctcctcccataaattctctgaaaaaagatagcaaggatgacttgctagaaaaatctgtcgacactctagaacacttttccttattgcttctgaat
gctgatgttatacaaaaagactattcacctgatccttatatatgctcctgtaaacctaattctaatagtacatctgacatattggctatttcaatagtg
ggtctattcgactcgtctgtttccatgcatgtttacaatttactcaggtaagatataattttgtttctgattaaaggttaacgatgatagggaattgga
ctggagtttttgtgtccatcgtaatgaccatctttcgattcctgattctttagtcgtttccggcaaagccaaccgagctttgctttgcttaagccaaaa
aaatcaaaaattacgctggacttggaaaataatgtgaataactggtgatattgggggatatcgtgtacattattcttaacttgcgaataaaatagc

tacttcgataggcaaatgccatcttatataacttatgaaaaattaaatctccagaaaaaccatatccaaaattcattatactaactagcgctatac
attattccttaggaaggaactgcagtgttcccgtgatgtcaactttaggttcaacatctattgtatgaatttctggttcatcctgtgatgcttttcctgt
tctcaatctctccagcatcaaattggactcaaatggtttgatacaaggtgaagtagtacctaatgttatgtgtagttgaggattgccggtttttccaa
accacatgctctttgaatcaatatctactttaaagcacatcactcttccgtcccatacgaggcttctcagagtaaagctaacatgatcgataacgtt
gcttctcttctttttaagctgattaaggtattgttgccataaatcagccatttcgggacccagcgtgatttgcgacctgtgtatcatcgtcacatgaaa
agaatcttgaagggtataagaagaaaactgatcatcccagcttatacctttttgacctaataacatcctttaatatggcttttactttatctgaagga
ataaaagctccgaaatattgaatttcgttggaatttctttcttgtttcttcaaggagctttcccccataatttttgtataagtaggcacataatcgttga
cagcaaattctagagctcgctgaaattcatcctcagaaggatagttggttatcgagtcaggaatatgttgttgcatgtaatcaactattaagcgtgc
attctccaaagatccaacatgtggatccaattctataacatcatcataaccttcatcatgagaaaataattcgggatcaaacggcttgtattgtttg
taaaacatgtctagaatagaatttaccttttctacaccttccgatactttgatggattggtgacgatcacctcggcttattactcgctcttgcacaaa
agaacgagtactcaagttttgtacaaaaggcaaagctacatagcgaaccccttctaattcagccgaaacatcacttcgaagagcacttctcatatt
cggaatgtgattgtttcggtcagcgaatacaatcggatgaccattttgaaactcgttaacaattgcacgtgcaaatttcttaggtcctcctttaccgg
aagggatattgtcgttctgtacgacaggccaattgaataatttctccaaaatcttggcaaccgttgtttttccacaaccaatggtagcaattggtac
gagtaatgtgtaaggcttctcattgctagactcgaccgaaatcgtcatagcgtcgagtttggagagtagcaaaaactcttctcgcagagatattatt
cctttgtttttaagatacagagcctttaattcaggcttctcttggaactttttctacaaaaatccacatactcgtttgtaatctttttgtacttggcgta
attaagaggcttgtcatggattagcatttttgtaacctctctccattgtcgaaacatgccgtagggctcttcaaacttatacttgaaaaagaaatcat
ttgttgagttgcgttttccagcttgagcagttgaatgacaacgcacaacgaatccctcaacgctgcggttattcaaccttccagtctttgcagtctctt
ccaaaaagtgcttcatgtcttcaaatttgtgaatgtaaaaagaatcagttttcaaaaaaccccaaacctttgcgaaagcggatacgtcttctgatg
acgccgtggtaaattcaggaatattcatatttagaccatgtaaatacaaaccacgacgatcacctcgatatggaagaatgtgttcctcaaaatcgt
catcacataactctgcgacagctgtagcttggcgcttaagaagctcctgggcaagctctttttttgatttaccaaccttcttcagatgatcttcaagc
catcgctctccagctaacgcatgggaaacgctttggccttcaacagcaccaaaggaatgcttactggtgatgatgagctcgccgttggataaagc
actgataaaaataatacaaccgttctccttgactgtcagctcgtaaggcccctttgtgtgttccctcagagagttccaagtagtgagaggaatctca
tcaatattgaaaaacttgtcataaccacggatagcaatcttttgttctttcgtcctgggatccatataggtgaataaaccacgagcgcatgttggta
aacgtcttttcaaacctgtcacaccatcatatgtcttcttgctgtcggacacattgatacgatatgcttgctcccaaactctccaggacgttaattcat
aatcggtacctggaacagcgaaagagacagagcggatggtaggcttcttttcaccaaatattcctacttttaaatcacgcaatctacaaagttctt
ctacaagttcttttggagtagcaaacgtattcgacattgtgcaaatcaaaaattcctgaacaacttacaagagtcaatttcaaccctggaattactt
aaaatcaatatttaaaacataaacaaggtaattagctgcaaaagaaagagcacaaatcacaaatcccttgtcttaagtgaggataagtgtttggt
tctttataaaaacaaacgaacactctgtcattcatttagcactgctctgtattctttcttatccattcactgttctctaacaagagcacgctagtgtttg
ctttacaagttgtttcaacgtgaacgtaattattgtttacagcggtcttagaagtgaccgtaattcctcgtaaaagttcactcctccatccttcacctt
gtcgaggaggagctcgttgacaagaactacagacttgtagcatataaatgagaaatttttagaaaaaaaaaaaaagaatttaacttcaattgaa
gaaaccttcatttgaacagcaggctgaacggacagcatttgcggcgtttgtttacaaactgacagtacttcgtaacgatatgatgaacggcggca
gaagaaaaactgttgatgtatggatcgattgcgaccctggacacgacgatgtcgttgctttgatggttgctgcttgtgcggaccattgtaacattct
aggtttgagcaccgtgcacgggaacacggttgttgaaaatgcaactcagaatgctctttcagtgatggagctgttaaaaaaagatgttgatgttca
ttcaggagcttcgaaacctttgatgagaccgtctaagcttgcaacccatatacacggtgtgaatggactggcaggcatcgctcttttaccagatca
accccggaaacgggcttctcctgatgcagttgtagccatgtatcaagctatttctcagaatcctattccggtaactcttgtcgcgacaggccctctg
acgaacatcgctttaatgttggccacttaccctacagtttccgaaaatattgaacgactcgttttcatgggtgggagtacggggattggaaatatta
cttcacaagctgaattcaacatgtttgctgaccccgaggctgccaaactagtccttgaaactacttcactctccggaaaactttatatggttccatta
gatgtaacgcacagcgttctaatggattccgcaatcatggaaaaattacggcaacatggaagtcccatcagtaataccctagtcgaacttatgac
cgttttccaagccacttatgaaactgtttatggattcaaaaatggtgttcccgtacacgatgcctgtgctatggtttacgcattgtggcccgaaatta
tgtcttcaagaatcatgtatgttaccgtcagcatcgaccagctgactcttggccgtactgtctgtgatgtatggtcgcaacaaactcagatgcttcca
aacgttcatgttgtccttagtgctgatgttgttgctttttggaatttatttatgcaagttgttgaaaagcttaacacattgtgatttggtgaagcgttgt
attttatgatgaaacatgcttccgttacttttagcagaaatgctcttctttcgtaatattacgcacagtgtttttacaggaaacaataataaggatttt
ctgactttgttgtatagagttgctcttcatatttctctatgcaaccttttttccgaagtttttacgacaactttttttgaagattatctttcattcaactgaca
gtcatcatcttttcgtagggattacattgctatcattttaactattatgattattcgtcgtctaaatttaccattgcataacctccttttaatgagaattc
ctatcgttattaaatgcaaataagaaacaaaacctgtagccaactccaatactgagagcaaaattaaatagtatagtcttagcatataaagccta
aaaaagagcaaaaccagacttactttcagattaagtagagaaagaagtaatccaaaacatataaattaccatgaaacgatatccaaattttaaa
acgcttcatagaagtattaaaaatattaagcttcaccagaagaatttgagttttcgctggctgtttttttcagaagtagcatctgcctcagccgcttct
tcctttttaacagcctcaccttcaccttctgattcaactgccgcttcttcttgttcttgttcttgttcttgttcttgctcatgctcatgctcatgctcatgttc

ttcttcttcttctttaactaattcagccgctttttttagattcctttgtctttgattttttcgaagcttgacgagttgtctctgtgttttcaggattctggggg
gagcgacgagtacgagggcgttttggttttcgttcggctttgataggtggagcgtcagggatcttttcaacaatgtcttgtaaaaagtcaaattgtt
caacacttttcaccgcatgtttcagatgcgaaacggttacacgcttggcttgatgcagacgagcttgctggcaagactcatttattatgctttgcata
aacatttctaacgctttggacatgattactggagtaacttgagctaccttgccaacatcctggtcagcttgcataatctttttttatacgagcgacagg
aaatcgacttttccaagaagcagctgcagcggcggctgccgtggaagaagcattcgtaggagattcttcagctacacttgaagaaacggtaggct
ttgtctcttcctcgtgagcaggctcgacttcagtcatgaagaaaaataatttttttttagcaagattacgcgaaaggggttttaaaaaagatagtact
taatggcgaaaggtacccgagagatgtaggaaagttgctactaattgctttgtacctattctggtcgatttttcacaagcagcaaaaatttgttaga
attgaaagaaaaaaattacaagacccaaactttgcttcacaaagttctaaagatatttaaagtttgtaattttaaaaggaaattgtatttttttcggt
gccagtgtggttggtaaagagattacgcacagggaatcttttctgattatttatacgcaaaacagttgaattagaatctttgttatccatgtaaaca
atctatgagaataaaaagtagcaaataagatgtacctggattcaaaggatgtcatatttgcacttattttgaatattcttgtcaaatatagtgccgc
cgttctactaacgtatcagtataaaatgataatattataaactattattaggcactgcaatttctctaaagttgaacgcttgtatttgctctataataa
ggtatcaacaaacttcagattaagtataagggagagagattggtaatgcgtttacgggtttctattgttgttttcctgtttcatgtttcatgtcttaaa
taaaagtatttcatgtgtttctattcagtatggtagtcaatacaaaaacttagaattcgtttcatttggaagcgggaaagttccagtaggatggaca
caaggaattttccaatgcttttattgaagtgttattatagtgaaattctgcttatggagtggacttttacactttttctcccagctaatagtgttgagta
aacaaattcaatttgattactttctttattaccagatcaaatatcttttcttagatctttatagtaattttggcttccttataaatgctatgaatgtccta
ccaagacacctaccaattattgcgagtgctgttgaagctgtactgtactgtccatgcaaataaaggacctttcaaagaaacaatccagtaagtaa
cccttgtcctttcagtagcatccaaacattcatttaataaaattgggcttattcttgctaaaaaaagttttaagcgttgatttcaatcacggttgtatt
gattgaaacgcatggaagcttcctgtccaacttttgacgagtaagtatttctttcgaaaatttgaaatattttcgttttctacttatgaaaaaagatta
cattaaaaagtattttattatgtttgtctcctttctgtatcgttacgaacaacagacgttaacaacgttgcagaacatcttggacgttgaattgcaat
gagttatccaaaatcaaggatgaaacgtatgcttcggcaacaggccgagaggcccattgatgactctgctgtagacctggtaaggaacagaaac
tggaactggaactggaagtgaatgtgaatgtgaatgtgaatgtgattcctttgttcaggctaaaatagaaaaaaaaaaaaaacataacaaaaaga
tttgagtttctaactactgtaaattgttataggtttatcttgattattgtctttttttacaatcactcttgaaagaagccaacattgaagcagaacgaa
ccggggaaaagaaaattcagccgatccatattcaaaatgttaaacgggtatgctgcaaaaagtagaaatcattcataattcttctatcaaaagcg
aattacgttcatatctaaaagaggaaaagtagcgaaactagcaaggaagcgtgtttttgctaacacataatttagaaaattttagcccgctaccg
aggatagcaaacaatcatagcagtcataaggaagaatcaattaagaaccccgatagaaacatccatggtcgctttgaaatgaaggtaaaaaag
gcgagcgccaggtttcgcttttctgcttttcatgtttttatctgaggttttgattcgcttgcgttacatgtcgggtctgctgtttaatacacgagtgatgc
atccaatcccattgacttgaacaatctaatatttgtgtcagttcgtttatatcgaataccgtgaaagagtacatacctttttcatgccagtttagcaaa
acagagggattataggcttgaggatgtgcaagtgcacgataaataaagttcatcatgttatctaaatcttcagtactgcgttgttttaaaaatgcta
taatatcagtggaccggatgtagctcagcaagtcaacaaaggcggataagtattgttgctaggcggaggttaatataattttcaaagaaaagata
aaacaaaaaggatgaagtagtccattttaaagaaacgtttgatcccaccgactatggaaacaaagtcatgacagaaattgctttcttaataacca
ccagttacaaaaactaccacaatccattctcatctcatcatcaaatgactttacttactctagcttcctgaacatcgtctccaaaaggtacataaga
caataaaatccgtaacccttccacagcatttccactgtatcaacttctgttagcaagcaaagaaaggaaaaaaaataagttttattattctattttc
ttgggcttacgcttgaatagtcgatcgagcctgaggaactaactgagcaatagcagcttggacctgttccgaagattcttgacggggaggaaaca
aatcttgctgtgtaagtacaggctctgtaacggaatctacatcttgcattcgaaacgacatgattctagcaacaattacaagtttgaatggatttgt
caaatactgctctttgaagagcagtagagtagaacagacgtagtactaaaacttgcttttagctgtaaatcaatacacgattttggcaaagaaaa
aacaaatacaaaggatgaaatttatactttcttttatttcccacttttcctgtcaattcatttcctatatatagttgaccatgcactcccaacttctcca
gtactccatagaccagtattgtagatggttgtttttttcgtgtttacagacaatcgtctctacctgtggtcttaagaataatttctgaatatcttgtaggt
atatgagcttgctgtccgccctcatagacctatggtgattcagaagatatgttttccataaatatccagctgtatgccggtgaattatgaactgaaa
aagaataatataaagaaaccttttccttgtaactatgaatgtcgatttgtaaatttttgatgcataaaggcaatctttgggaccattctcgaagcaa
attaaaattgtaagtatagccgtagaattcagtgagtcctgggagctattctgaagccgattccaaaaagtagggaaatctaatcttggttatgttg
tacaaacgagttcaaagtaaactaattattggaattcatatgtttactgaatgtagaaaaaatctcatttaacaacactattctccaaaacagtagt
aatgtaatactaaagaggccactagtaatgtaatacctacactcgtttgttaagaacagaaacccatatcatctgtattatgttgattggtttctaa
atggtttcaatatatttcaatttatcctagttattattacttgtttttcagtttaagtcgccgttacatcgtacatacaaatattttccaatatgccggtt
catgggtaagcctttttgcctgcgatttctgcttgggtagtcgcttgaaaccccttttggtgtttgcttctattctcttttctattttttctaatgcattagg
cttgtttattatacgactccttctatggctaccttcttgggacctgcacaagactatcttgtaaaatcatttttagcccagtatatgagtatgctacca
atttcagtaaatatcttttggtatggtaaactaactcgccaatagaaaaatggatcgttcagtataaactctatcggaatgtcctttccccaaagag
tttggaattcttaaagcaaaatatccatccttatatgtatgcgtgtgttgatgaaacgactactattgatgcagaacccgaattagaagatattatc
gtacgtacaaagctgtggacatttcgccaaacgtttaacatagagggagcaatttatgaggttggagatttcactgtagctgttgccaatgttcttc

agaaatcaacatggaaagggatcgtttttcacgttacttacgatggttctgacgatgtcgatattgctcggcccatcctccaagagttctttaatatt
tgtttctttaaaaattcttcgcctcctcctcctatctatgaaaactatttctaccatcacaggcattccattgatcctaaactctttctccaaatttttaa
gcatagagtcgaaatttctaccccgtctagtttacctcgggtgaagtctgaaagtactcctgctggatgaaatttcttcgtatttttttgtaaattagg
tcaaaaacgaatgaatggtacgctgcatgttgttctcccctcctcattttactttttttcccatctgttcctttgctatctttatttttattcacgcttaccc
atagaccttttaattaaatgaaaaattttattaaattacgtgtcccaaacaaaaattattaagtccttttgttcttttaaaacaattaagccgctgatg
taggcagttcatttgacagcttcaataacttagtccaaagaatcggtaacaacacccttagaagcagtgctaacattacgagcgtacttcaaaag
agtaccttgttgatacttcaagggaggagctctccaagcctgacgacggcaagccatttcttgctcgtcaaccatcaaattcaatgtattctcgacg
gcattgatttcaataatatcaccgtctttaacgagagcaatgggaccaccagcttgagcttcaggatcaacgtgaccaatcaaaaatccgtgaga
gccaccgctgaaacgtccatcagtcaaaagagcaacgtccttaccaagaccagcacccatgatagcgctagaaggcttcaacatttcaggcata
cctggaccacccttgggaccttcaaaacgaataacaataacagtcttttcgcccttcttaaattcgccacgttccaaagcactgatgaagtcggctt
cggcatcgaatacacgagctttaccacggaaatgcagtccttccttaccagtaatcttggctacggaaccctcaggtgccagagtaccacgaagt
atacgcaagtggccttcagtcttcataggagcgctcaaaggcttgataatttgctgaccttccttgagaggcttagcctcgcgcaagttttgagcga
gtgtcttaccggtaacagtcatggtggaaccatcaataagaccttcagcatgaaggtacttaagaacagcagggacaccgccaatgttgaacaa
gtcttccatgacatatttaccactaggcttcatgtcagcgatgacaggagtacggttactgatagcctggaagtggtccaaagtcaaggtaatacc
tacagactttgcgatggcaatcaagtgtaaaacaacgttagtagaacctccgagtgccatggtcaatacaatggcattttcaaaagcctttcgggt
cataatgtcactcggcttgatatcttcaaccataatgcgcttgatggcctcgccggcagcttcacattcagcaaacttttcagcagagccagcagg
gaaagaagaagagccgggcaatgtcatacccatgacttcagcacagctggccatggtattagcggtgtacataccaccgcaagctccacctccg
ggacaagcgtggcggataatatcatggcgagttgtttcgttaatcttaccggtaataaattcaccgtaagcctggaaagcagaaactaaatcaat
gggggcattgttctgacaggaagaatgacctgggcgaatggaaccaccatataacataatactaggacgattgatacggcccatggcaattaag
caaccaggcatatttttgtcacagcctggaatggaaatgttggcatcgtaccattgaccttgcatgacggtttcaatggagtcggcgataatttcac
gagactgcagactaaatctcataccactagtacccatagagataccatcgcttacgccaatggtattgaactgtaagccagtaaggccagcctttt
tgacacttgctttgactctgcgaccgagatccaacaagtgcatattgcaagggttaccctcgaaccagcagctggcaataccaacttgaggattgt
gtatgtcggtatcagtcaagccagtggcatataacatggcttgagaggcaccttgagacttgggtccagtgatagtatcactaaacttgttaagctt
cttctcttcgagaactgtagcagaggttttgaaagtacgagtattcaaaacggcacgctttgtagccgcagcagaggcacccgcgcgaacgagtt
tgttcaaaatcatactcagaagctgaattatcaataaatacgaccaaaaattttaacaatgattaattatcccgaggattcagatgagtaagcaac
aaaaaagaacaggaaccaaacaaaggaaatccaaaaacaagcaattattatggaaagagaagcacgttcttccagattctttactatttttcttt
aaaaagtatggtcctggtcaaaagtaaggattacaagatgcaaatttatacagatggccatcgcgatcaaaggtaaaatcgaattctcatgtcag
tgattggctcttaaatcggtaacttttagagttaccttaagcgaaatttgaacaagaatgttttatgctgtaaatgaaaatgtttgttgtagttattct
ctctaacaatcataaaaacttgctattccttgtatatttatgaacagatgaagactgtgttgcatagttgtgttgaatggtccaagtttagcgctttgc
tcttatgcgcttttggaacacttgggaagcgttgacagggaagggtaagcatggctataagcctttctatagcttgcctattgattcattgaatcag
gaataggagtgtacatatgcatagaaggaatcaattgctttgtaaggtgaatctttcattgtaaactgaacagaaaagtgctttgaatccgagga
gccaaaaaaaaaaaaaaaaanaaaaaaaacaaaaaaaatagagagatgtaacttgattcttttataatgattatatttcttgcttattattttca
ctttctccgtcaacgagtatttacaatattgtgaagtgcagtgatggcacaatacgcatttgcgatttatcgttaaatgaaaaccacttgcgagctg
caacaccagattcttgaaaaaattgactgtcttggcagcggttggtttgtttttaggaaagatttatgtttctgctttcattcttataggaaacgttcg
gtcgctaatataatataaggtattgtttaatcttcaaagaaagtttgcctcgatctcattattgttcttgactctcataatttataataattcgttttatt
tatttatttacggctaatttggaagaacgtgaaaagagagtgtcttggtacaatggctccaggaactcgtgtcagtaaatgccaaatatctcgtcc
aattctaatcggaaacgatgcaaagccattaacgccagaggaacgagttcatgctcctccagaccacacgcatacttggaggattttcgtcgaag
gtgttgacggagaagatatctcaaaatggattaaaaaagtagtctttaagcttcatgatacgtacaacaattctacgcgaagtatagagtctctac
catttgaagtaattgaaactggctggggagaatttgatatcatgattcgtatctttttttgtaccagaaactcatgaaaagtcacttaccttttatcatc
gcttaaaactccatccttatggtcctcgcatggaagaagaaaaagccgctggcggcctagtagaatctgtacagtacgaggaaattgttttcaac
gaaccttttgaatacacatataaactcttcttagaaaatcccattggagatgggcatggtttagctgttgagtcggaaccagatcatccattctctc
agcaattggagcaggacgaagctgataagttggacatggcaatggaaaatgtgaaaaagacaattgacacttacaaacagcaattacatgaac
tacaaagccaaaagccgccaatttcttagactttttttttttcagaacaaaaatctcgtccattggacgaacaagttaaagaaagtttcaaggtttt
tattatcagtctgcattaagtttataaaatatatattgtcttttattaatgggggaaatcaatatgaaagtcagaagttgtatcatacattttaggcc
atattagttttgacaaataaaaaattatgtgatttcatcctccttctgccttcataaccataaacatacacctgcaaatgtcaaatatcgtcgagcat
agcaattgtaccagttacgtacacaactgagtttggtttcataaaggtttaggcaactacgtgtgcatcgcaaaaacccaaatttaagaaaatcct
tgataaaagtacaccaagccaaaaagtataaatagacaccctccagaaagcaataccttgtgcatttgaattttagttgcaagatactttccaga
aagtacagccaggcccgtgcaaaaagcatgacctaaattggctcctaaaaatacaccaaacatattatcactagcagccatagcaatggttgcg

atttgcgaacgatcaccccattcacttacaaaggtcagcgcaaatgccttcacaaaaagaggagacaacaagaaactaaaaagattttttaatc
catctagagtcgactttagataattaacgctatcatcttcggtttttcataaataggatctttaggaacctgaccttcttccataagctcttcaactg
gtttagcagtactgatctcatcttccacccgcttgaactcttcatcaatagattcatgagcgttcataacctcaacaccttcacggatcattttgatac
caaatattacaaagagtaatccgcctaaaagatccgtacatcgtttagggaaaagcataggagcagcatggcctaaaagaacgccgagtaaag
tcatgaaaaataaggcactaaaagatcctaggaatacgatgcctcttgaattttcgaaagaaagaagggctgccacaatgaatgttttatcacca
agttcgcaaccaaagatcatggagatggcgaaaagcatacttctcagcatattcttctgagtagacccctctgagggatccacattttcccatgctt
ctctgtcgattgaaagattggaagaggtattctaatacagcaattagataaatgaacttgcaaaagcacataaagactgagcaggcgttcagcat
tcagtgaaagttaaaggaatttatgtggaaaatccaataatgaaaagcaggcatgtactagctcacgatactgcatgtacgagacgaaaccaca
caaaggtgtctgtaatatagaatttctgaaacgcccggaatagtcttttattcataaaaaattttaagaaaataaactacttacctcagccatagcc
acgagactcatagtagccagcaaaagcaaacccaagttccgtttcatcatcaaagtgtattacgccaaatcgagcaaacagtagtaatcaagaa
tactcaaaatatttctagttctatatagttattgaattgtagcttctttataaacaaggatgatggttcgagtgtggcgcttggtaaatgttagtcaag
catataataaaagatgagattagagtgtggtcgaataggacgagaatttattttaatttttctaaagtttacttatatcatatgagctttttatctaag
aaaaaaatattcgaatggttgtgatcatctatgagtactttgtttaacattgtacaatttggcgaggcgtacgattatattggcgaaataaaagttc
actcatttttcttgtttaaggaatttaatgaattgcatagctctgtttggatgtcttagtcgtaagcaatcgaattgattgtctgttcgcccattagata
ttgctgtatttggtagtgccaagcaatacaaacatcatctttatgaaaataatgatattttggccatttccaacaaatcattgattcaaacccttgga
tttgatactgaaaacttgttgtttgcttgatgccagtatgtttcatataggaggactcgggatgactcaaagaatcaataattcattaaataaacatt
gggtttctagcagcttgataattaacgtacatgaaatcggaggtttcaaaggtctttgacattgggcttttgaacaagccatggatatttgagtaca
aagttgtatcccaattttatttttcgtataagacaagctatacattgtttcaaaatacatatttagaaatacctatttgaaagtcttgggaaatttga
cctttaccaagaaccaactttcaatagagaaatatgcttttataggataaagggtagaaaaatggactcaaataaaacggtaaacgtggatgatt
tagtgacaagattcaaaagagaaggacatttcgatcgtttacgaaagcttgtcttggaaacattcaaagaaaaggtatgttaaaaaacgcaacc
atatatatacttctaggaagaccacaggaagaagaagctaaaaaagaatctattttcttccttctcatgggatcgacctcttttaatcatagtcatt
gcgttgcgtctaacaaggtttcttttaggaatcggagggattcgcagagcaacttcgaacaattgctgaattagaactagagaaagatccatcttt
aaaaaccaaagatcatttccgaacagcgcctttaatcgcaggcgccgttgatcggtatgaacatagttggtttcagtcgaacgaatggctaattga
aataggacatcgctttatgaaaataccttggaaaatataaaaaacaatattttgtctcaagataagctcagagtaaatgtccttgagacaataaa
gcaattgtgtgacaaagaaactgaagggcatcggggatcataaaggtaaagctatactacaacaaaatcaaaatcctcttctagtttggttataa
tggttgattcttcattttattactatatatattttggagcatgttttcaagtatttatcgcaaactggggatcgatatgacgttgaatgctggataggat
tcataaggcaaaatatatacgccaaagcaataacaaggaagtttggagaaagaagcgtgttggcgtcctcaaaggattcaccaaataatacga
acgacttggttgaattgtcgccgtcgtttattacgagatcgattatttaacaaatataatctcgacgcacacataaataaaagaacgtgcgtttgttt
gatcagcttaattggtctgctaaacaaacgtgtattaaaaactaggaactccgccaacagtaactgaaagacaagctgattagaagaaataaaa
ataatggacaaaaaggtgaacaaagcgcacaaagattaagccgaaataaaaacaattgcattacagttcatgaatagtttaggtcgtcatcatt
aaggatgtaaaatgagtgcttcaaataagttatgctttggttaacttcaaagtacaagaataaataatcactctttaacagtggtatgtagtagta
ggtcattcaaacattcgtcagcttcctcaactggcgcttccaaatcatggtattttcggcatatgaaacagctaaagtatgggtggctttctaccag
agttgggcgtatacatttataatgataagtgtgagaacaaggagcaaaaaagagtgcttgacagggtaaaacaggcattaaacaaatgcaaca
ctcgggagggccgttatcagttgaaccagaaacggctagttcttgaacacggcgaaattcgttcaaattgtatggattgattcttattttccaagaa
ttattgagttcaacccgcgcacggacacagcgataatgcatttcataaccaccacgataatcggcaccgagctggagaatatcattatttgacatg
gcataaggcttactcgtcttagaaggtggagataaacgaacgtgattaagaaatgttccggaacttgaacccatatcttggatataccaagtatta
ttttcgtgatatatttgagcatgcttacgactcacaactttcgagcgaaaaactatagcagatacgtctcctccattaaacctctcggtgtatcgtcc
aatatagataggtaaagaatcattctttttatcccaacaccgaactagtggatcaaatgaaaacgagtgtgcatttgggcctacaaagttggtaag
acgaatagaatatttacattcatctttttctgaactagattcagcaagaccttcattttctttccgacaaccttccacagcctttgtcatcccggaatc
tttgggttctgggaatgaaagatgcaaaatttacaagaaaccgagatacgacagataaataggaacgccttttaagcttaaaatctagcaaatg
ccaaagttccgtatcacaaagaagagctgagaaatttcaaagcttgcttgtttaaggaaaagctaatagaatgaattgtttagatctaattttcaa
agatttctccagcttttatggcttattttttagcaagatattggttttgtttgtgttgtaatggagatatccaagacctagtccaacaaaaacaatgt
agtgtaaacaaagcagggcttaatcccaatcaacgcaatgctgtttacaatagaaagaattaaaaactctcaattttataaataggttcctttgac
aaataaaaattggcctttcgaaaggcacgccttctttaagctctgtatatctcacagatacaaagcgacggaatgtatgaaacaatgacagaaaa
gaattaattttttaaacagccaactcttaaatagaaacgaaaggagcaagagcttgaaaaggtaatgaaaagatatgaaatggattgggctttta
aatttttgtcactagttatcgttagaggtcgttaattaatttgatacagaggtgtttcttggtatactagtctagacgaagcaagaaatagtaaaaat
gaaaagaaaaatttcagacacactgctgaagcacttactaaaaagtgaataattgggcctacaccttctggaggggaatctatagaattttgcaa
aacgataaaagttcctcttcttcttcaggctagaagtattaatttccgtacaaagaattttagaatgtaaattcataataacaatttagctaaatttc

atttaagataattaatataaagaatgtaaacgatcgctagataacaaacataagagaaacgaaagtattggtttacgaataaaagaagatgca
aaaaaaaattcaaacggtaacaacgaaaacaatagtgacagcaaattaaatcttttacggactctttggcctcgtttgagattcgaggtcgaatt
cgaccatgaatggtacagaatctgaattctgctgatcgctgctttcatcagtggaaacgtattgcttaaattttctccaaggagatgatgatttactt
gaaaagggactaccaaaagctcttctgctagatcgagagactctggcagcaggctttagcgaagatccaaataacaagaaaacgataaagaaa
aataatacaaaaccaagtatagtccatagtaaccaatgtcggagcatataaaatacaaacccagcgtcgttatcatgccaggatgatccttcaaa
aatgttgaagaatgaatatttggaaccggtgtacacatcagaggtcaagacccaaacgtggtgccaggcttcagccatggggagacttcgccac
gctctccaaactatagaaaagaataaagggcccgtcgaatacataactgttagataagggaaatgatagttgaaagcataagatggtagagtac
ggatgacttggagcagaaaaggatgctttttcctgattccaataacattgttgctaataccactggggcttgtttttcgaacaaacgcaggatattt
cagtaaaggctccatagaacgattacaaccaacatccaaatctaaataaataccaccgtaatggtataaaatgaagtagcgaacgacatcagc
gcgttctatgttataagggtacgcatcatactgaggaaggaaccagggatagttttctttcataaaatctcgcattgcctcatccgtccataacttat
attcaaagtcgggatgcaggttgatgcaacttttctgagcatctttccatttatcaggaatatcttcggttttccaagtctgatgaattacgcgcggg
acgttttctgtcgcattattgataagcggataattaaactcgtcgaacaaatcgtttgctttcaaaacgtctgtccggtaatctataaaaaataactt
gatgggtccccagaaaagatggagcgtgaaaacgacaaccagggacaaaacaaggaaaaaaacgagagccggcttattcatagcggtgtatt
ctatgtcggcgtaaagttgaattaggatttagattaaaaagcgacttagaatctcaaacttaaacacaaacgataacgataacaatttaagtccg
aaattaaacttcagggatttctataagggaaaatcggaggggaggcttagtaacgaaggatggattgtgccctgttggtgaaaaatgtaaaagc
tcaacgaaaccgaaattaagcttcacaacaagccaatgggcaattgaacttatcgaattttcagataaaactctcgtaataggtcttgcccagttc
tttgtctctttccagtgactgaatgtgcaaacaatgatgcaattcaagcagtccacgaaaccaaagcccttgttgagaggaaaaggtaagtcaaa
ttttacgttaaatgatcacttataagctggaaagcttcttgcaaactttcgttatcacctctccagtagaaagatgatggagcaacaaaagaaaca
attaaaatattctaaggatgctgtattaaatagaaacaagaagtcagaaaagtaaaaagtaatgataatccttcaaaattgtactataggcttta
tgatcacctcttgtaactggttaaacgctgtcaagggattctaaggaaagtgtaaaggccacagacactttcgaaacgtgaataattctaataaat
atgtaagtattataacagtttttttggattcttcatgagaaactgatattttgttaaaggtaggtgtcgtttgcctctcagtcctgaatctagataacg
caaagtagcatcgttacctttatgtcctttgggtacggctacgttaataaacaaatatgtttttttacaaaatttagttgtttcaaacatgctgtcgtca
attcagtaagattttagagaatcgataaaataatgatgaattaatagcaattcattgatcctatacgttgttgttttctttctaaaaaaaattgaatct
gtcaaagtaaaaggtggataaatcgaaaaatgattgaagcaagtcaatagaatttgagaacttactaaggtatatcgctctactgttttgttttgtt
atttctttttctctttagtttcgaactaaacaaacaaaagacaaatcttccaagtccagctaaaagcttctacaatactccttgtccgcagtagcat
agcactgaattgaactcgtaagggaaatcgtgatcacctttcggactcgtatattggtagaattgctccatgaagcactagtgaatatcatgatgc
ttagcatcttaggagtacggtattgttcttattctgtcatccattttcccgtatattgttccaattgaaaggaatacacgaaaaatttgattctttcttc
ctatacgctttctgctcctaccggaactatacgttaaataaatgcaatgaaaaaacgcccagagatgctaaagaaagtttttttaaaatatttattaa
atattttagacagccatacacttcatttattgtatcattaggcttctaggacaaatttatactttgccttctctatgacttaggaccctcaaattcattc
aaagattaccaattttctgaattgagcaacatattcgattatcggaagcttttttttatattggtataaaaacgctcactagtagcactgaaaagtaa
gtcattgactcatatgctcagttgtttgagtggattccttctattattataattcttatcaagactgacctcaactttaaaggtcatcattccatctacc
ggcttttgtttttatttcttccttttccttttatttattaattcattcatctgtacaaatattacaatggacgatcttacaggggaccctgtagcggcag
aaattattcaagctgtgaacctctcccattctgtggataagcaagtaggagctgaaaaggctatttcctaccttggcgttgaagaagagcgtggtg
tcaatgtcgttgggtccagaatgaagcctcgatatattgtcctttcatatgacgaaaatacccatgttttctatattcatcgcttaaaggcaagtgac
aaaggaggaatgactgtagtgagaacttggcctcttttcgaattacagggaatcactgttattgatgacagagatgtcacgttcacaatcaataaa
ccccatcttctaagaagcaatgatacgctcgcgagaaacaagttcatattttcacttgcaaaacagttcatgcattacatgggtaaacctctgagt
gttctgactcccgatggtcgaaaagtagattgggcttatcgtaattcttttgcggaggacgcagcaaagcctttgagtatccccagaaccagaaat
tctctaccagaaaacgccatgatggctcctagcccctcatcatcttctaaagctggtcacttgcgcaatttttctgatggacccattgttacaaacct
agaaacagatcttccttcttcaaatgataaaggagaaacctcaggctcttttccaaattataaaagttataagaatgatcaaactcctgatggaaa
acttatgtctcattcgaagctgaagcatcaagcaaaaccccttagcagttccatgtctggtaagtctagctcctccgttccggagtctccttttactc
aaaaaaatgaaacttcttctgagaaagattttccaaaaaaacctacgtataaagataattttattcaggaacaggttcaattatttccaccgaagt
ctcctttgcgaaagggtgcccgcagagaaagaaatttcccctctgaaccaaaaaagaatgctgttcaacctgaaaaaaaaactacaccctcgtct
tcatcaaaagatttattgtcgcaagacccaatgtcattgttggacgaaaaatcatatattagtcgttcgaaggagcacaatgttttactatctgatg
agcctaacttatttatgagtgattcgagtggattaaatgaagatgcttttcattcaacactagatccagcttacagttcttcagctcagcaactgcct
cttaaagaaggttcatcacctatccgccctccttctatttcagctaaaagttctttgagaaattctccagtttcgtttcccaagttatctttttcttcatc
ttctgggcttcaaataccggctaaacatccgtctcgccaaagtttatcaactgctgcggatacgaatgttgttaattcgtcggctttaagctctctac
ctttagaatcgcaagaatacactactccagcgcgtcccaagataggtaccatgtctaacaatcaagtctcgagcgaagctttacctacattgaacg
tattgacacccgctttacgaggttccatcattgaagaagcatacaatgtgcactctaaaatcagtataaatagcctaccaaaaaatgtgactcagg

aaatgtctcagttgaactggagtaacaaaatgacttacaaggattattatgattctatggaatctgaatttaacacttcaatgaagaagagtgtcc
aaaatctactagacgaagagggtagaatggaaaagataaggaaaaacattgacaattgtgtcacgggctgtgattctctaagttcaactattaat
ctgttttcactcgaactctcagaggcattaaacgatattatgaacagtaaccagcaaataaagcacaaataatgaatgtttatgaactagaatgat
ggagtattatcaggttaataactattgtgttagtaatgtgagcatagaccttcactaagacgatttacttaaaggaagtaggatatcctcttttgaa
agcaaatccgatatcttggcttcaccgatatagggagtccatcgtaagtaagcgggttttaccctttgaggatgcttttcatcccatttttacacac
atgatataggttcattttgtcaatgtggaggacgtaaattttcttcttcatatcatactttaagacagataaagcttccaaagctgaaataacgtcgt
cgcaaaccatggaagtcgcttgagctatgttttcgatgctgacgggagatctcatactcaacaaagtttcagcaacacgcttcttccagtaactac
ggtacgaaagcagacccaaatcagagagtggcttttcaggagaccccaatttccgttcagccatactcaataagtaactgaaatctattagaaaa
gcaccgtaaccccttcgctgatatatcggtaaagttaaaatgcaagatacattgtaatttgaggctgatctttttttccttactgaaatagcccaaaat
tttacaggaagtcccgtcgtattcagtcattatgtaaaaaagaaaaggttcaacatcgtaacatagtatttttgattgcaaaaacatttttgccaaa
agacataaattttgacagtaaataggttgtcgctgtccgtctacttcgaacattgaaatattttgatccctataaatctcgtcaccaggaggatgcct
ccaagcacatttcattctgtgccgttgcagaacataatcgctgttcatgtatttgagacaagcattgcataagtataggtttcttgagcaactatatt
cttcagggtagggagaagtataccatggtttcactttgtagttaccgaagtaaacattgcgaatcgtcgtaggctgtggaggtggagcatgcagtt
gattggaaagaacaggatcagaaaaagcatgtgtcacttctttagttctcgtaactactttctttgctttatcgaacaattgggtatcatccggtgtt
aaatctgtcaaagatgtatccgcctcttcttcagttaaaaccccaatgaatgttttttcgcattccaagtctagtagaagctttttataattttcgtaat
gtatagatttagtatcaaatttgatgactagcaaactttttgcttcagtaggagacacagctacctttgctggcattgaagcattaaaatggtaagt
gatcagttttatgactttctatttatcaaaaaatttcgtttttaaacggccttaatgactggttaccgtattgaagttgtatttccaggaatgcctgtttt
caaccttcatgcttacaatggtagctaccaacattcgacaaggttactagcttaaggagcctcgatttgattgaactttcttatagcaaaaaaaaa
gaaaaaaaaacaagaaaaatgtttttaaaaaaaaggaggactgaacaatcttatgctgaattaaaaaatttgaaattccgttttcagaaagcat
tttctaaatattcaagaagaaaggttaagtactttttttcgtatagaaagacttcataattcataggtatcataaattttcatcattccgtctaatata
aaaataaggcaacaataacttagctaaataggtctattaactcatggaataatgaagaaaaaagagaaagagagatagagagagagacagg
gagacaaggagagggagagatggacagaaaagcaataatgtaatatctatatcaaccttcacaaaactccatgcgtataacataaacaaaaat
aacaaaaagaaaaagaatcgatcctaagcatctacatctgccatgctagagaatccacggaaacattcctgctgttgtctcgtcaacacagtgtt
caccggtgttagatttggtgcagcactggtaaattcttcatcaaagttattcaaatcatggatatcacttgctactggtttataaacaggatcatact
ttaagtaataaagatcagaccagataatagtggcaaaaaatgggtgattcataacctcaagggcatcagttgggccacttcctaatctcttttcag
gatcgcgagttaaaagctaaaatgttagttaaatatttacaagttatgcgtgcttacccctcgtaatagcgaaacagcatctgccggcatattgat
aggaaacaatggctcatccgttaaaatagcatcaaaaatttcttcttcatcttctcctttaaagggagactgacctaacagcatttgatacataaga
acaccgaacgcccaccaatctacatttttttgtatattgctgttccaagaggatttcaggagccataaactccggagtgccacaaaaggttgaagta
gtgttaccaggagacatgttttctttacatagaccataatcagcaatcttaatatgtccgttggagcatagaaggatattatctagctttaagtcacg
atatgcaataccgttttcgtggaagtatttcaaagctaagcaaacctcagcaccgtagaacctcgcccttttcacactaaattgctgtctctgaacg
tgaaccatcagatcaccaccctgaatgtactccattacgaaataaacacgagtactagtttgaaaactagcaaacatgttcaatagaaaaggatg
cttttctctgtttgcgacgtcaaaaacatgtttttcagtacgtaaactttctagttcttcgtttttcaaaataaactctttttttcaatacttttatggcata
gtgtttcttagttggtttatactcagcaagcataactttaccaaaattacctttaccgagtacagcaataaaagtaaaatcgtctaaagtaatgcgc
tttttcttctctctgcgaggactgggcgaaagttttcctgtactttccgctgtagttgaagtgctagacggaggtgtaggagttagatgacaagggg
agggtgcgctcattcctttgtttctttaaaaggttcatcaggtgtagtaacaggagaggcgttcaatatagtagtaacaggcttggtagctgccat
aaggccctgggatatcgtaggtaaatgttcattagatgaccttgtatcttgcttgctaactccctgtttttccctcgataatttattgttgacatcgagc
cccgccaaaagttggtttttcaaatcattcgacatcccacaaaaatctgggactaaatgggcacattgcccatggcatgttaaattgcattcttcac
acctaaagcaatcttttcgtcgcaaaggtaaaaagaaaccacagtgagcacaccaatgagctcctaacgatgatagtggttcgaacctatgggg
aatttggtatttgagacgaccaaaatcagctttttctgcattggataaagctatgcaacctgcaaggatcttgtgtaagcactttttatggcatacaa
aactacaattctcacattgtagaccaggagaataagagaaaagttctccacaaacagcacaacgcatgatttgataaaattgtttttgaataaac
tggtgccctaaatgacctgcgacaacttcatctttcttttggcgaatagcaccttgacgacccaaaccagtctcaagaaattgccgtttagttaattt
cttcgaaaatgacatagaaacgcaaatttgacctacaggttcaagggaaagccatgtacgagataaaagtgtgtaagatgaaggggcagacgt
cggtgaagaagcaagaggatcatagagagaatttcgaaaagatgccgtagaagcagccgtagttggacgagctgaagtgttattataggcattt
tgatccgccaaacttggcttccaattggcttcagacatttcatgtaagtttctcttccgacgcaattcctcggcgattaaagaagcaggaactaaaa
ggaatccaataggaacgtcagactctttttttgatcataaatagtaagctgtatttctttcgcacgatgaacgtcgaaagtaaacgtctcgtcccat
tggtcgcccggggagcttcgtgaacgagcaacgtgggtatcatcaacatagcatacagcgtaagagaatgtaggagataagttatgctgcaaaa
aagaagtatgttctaaattgcgtacggaatgaatggtaacttggagtctaccagaaactggctttgctaaaccacggaaagcaatttgaaacgga
tgatcatcctgtccagcgataggttgattcaagggaatatgaagttcgttgtagcgcttcaaagatcgcttcaacagattgatttttttgtttaccctct

ctcaacttgacagaaacatcgcgggtatctttcttttctttggaatataaatgtaagatcttttcaattccagaaacacattgttgctctacagataa
ccgcatttgcaagtgctgtagcattgtggaaatcttttgtggagttaaaggcttctccgagctgagtacttcaaaagcgcttctactttcatgtaaaa
gttgtttttgccatcctgctgcttggaagggtacttttgcaaagagtcagtttcttctgtagaggcgtcatctttcccattctgatcgagctttaagga
ttggattctctcttgtaaataagtgatattgttctccgatacagcaatgtttgtttctaaccgtttttttacttccggatttttggttgatgctaccattga
ttttgctcccatgattagcgattcctctcgctcaatcttcttgtatgcatcatgaatagcatttttttagaaacaccatttttcatcgaaaatgttcccttg
gaagggtttttgcctaagtgaatctgggatatgcaatggcgttgatatgtaaacaagtgaagtttcccactactcgattatcaagggcaagatagt
gaggcgcagggcaaagttaggcaatttatatctgtttactcttaagtaaataaaaaaggtataaaattttatttcgataaaaaagcatggtacttg
ggcaaatttgggaattaattaaacattgaatgaaaggtcaataacttgcttaattctcttgtagcagaagctttcttaagctgattttcaaagggta
aattcgctatgtggccatactttacaatagaaattatttttggatacattgaacagctggaaaatcctttccaatgaacctggtaattatcaagatg
aatcttttccaaatatggagaagaaatatgtaccgtagctgagttgtgttgcatgctatacaagatgcaaattctcgtaaacacaaaatagaaaa
caatgatactacagtccttttccacaaggaacttatctaattcacctctatgtttttaacaatctgaacctggtgggtcatttaagttatttatttacat
ttttaccaagaaatgaacaacaataacaaacagagcgaaggacacaatgcttgattgtttgaaaacgatggcttttcagaagaaattcagccag
catattatattttgattccaagctttaagagaagagcagttatttagacaatagcaatgaacatgcttttcaaggaataaacaattcttttctagcct
ggttcaattggctttgtaatttcgtctcaacaaaattaaaaaacaatcccaggtcacaaaaccaccatcaggaacgtttgcgaggtcacgataaa
aagcttaagatgatatagtgataaattcctttctacgaatcgcagaccccaacatgagatcttctaccaacaaccaacattgaagcatatttttagc
cttttgttctagagtgtatcattatgacctatttctacaagccttataacttttatcaaaatccttctcctggttatcaatgtacgtcgagtgcttatgca
ttttgtcaaaggactgtgctaatgaacatgcttattagtacatgaattccgttcttctttcgaaaaagcacgaacttttgatgttgaagatgatttag
aattttgtcctgccttatctgaagaagaagtatgcttatccgggacggtaatgggctaagcagttgcaactggaattgaccagaattcttcactctt
aaggaagtttctgtgagtttgttaattctgcttaattcaccctcttaaaaggatccgttaaacaaggtttatgtattaacatgcttgtatagttgaccg
caatttaccagatgaccgggctttctccgacaaacagttccccatctttgtctccaatgactcccaatatgtatccttctgttatgccgtcgattcaaa
atggaagctacgccgggatgcagtatgcaccctcgtcattccagcacaatccaagcacccgccagaaaaaatcttctagtattccaatcattgac
cctgtgactcgtgctcctgcagttttgacagcacctacagcaaattcccgttctaagccttattggtaatgaggacttggggctactttatattttgca
ttcatgatggttataatctttaaagctctttgtacaattactcattgcgtttacccttgtatttatccatttttcatttatgcagccaatgcgattgtgtgt
atttcaattattgcaccgctcttctttcatgttttgtctttcctatcaccgtcacacgaacacattctacaaaacatcgctttttacgaacattgttaaa
atattttgagaaccttttatcttgtacaatacttgtttctctgttctatccttgtgttgtttatgtcttttgttttgatgaagttattcactatcaacattcat
ttttcttttttttacaaggaggctttatccttcttcggttgtatatctatagttgctaaactttacgaattagtttatcaatattatcagcattgtaatag
ctttaatttgctacacgaaaaatatctaatgccaactgtaatctaaaacccatatgtatacagtaagcatgacccaacttgaaattaattacatgta
tgtatttttcacactaataatggttgatttcatccattctttatttttttgccccagaaacctattcaatgcttgccaaagcagcttgctgttctgcagta
agccttgaagcatattcgttaaagttgttaaggttgattgccatcgcatttttttaaaaagtcaagtagatagaactgtgaagcattaattaggctttg
ttctttcaatgcactcatatcacttacctgcaagtctgtcgtgtcatcctcaccttcagcggatgatttaatggatggaaattcatctttagcaagttc
attgacttctaatttttgttaataaacaataagaaaaaaaaagagttttaccatccgcagataagcccaaatcctcaacgttcgatgggccatcgtc
ccaatcatcagcctcatcattggaaatttcttctacaacagattctctagaaagagacatatattcatcaaccaatagcttaaccaacttttcgcgt
acgctgatggctgtatattgatttggatctaaatataacgttagcagtgcgtttagatatataaagaccatacgtagacgagcctgagaacgggta
acgattctatcggattgtgatattatatcaccttttacttggatcgtatccaaaagacttgtttgtgctttgtaaatgtttatcatggcaatacaactg
agattatcggttagcaacagtagtaaacgatgaaatcatacatgacagacaagttcttaaaattgctgaagtaaacataattatcacaccaaact
tgcattaacgcttctaatgcgaatgtccctctctcatctactgtagagtttgtcaaaaaatccaaaacagcttcagcatttttgttgatcatttttgag
aaaacgtatattatgctctataattgttagaaatattcgttgaaaactgtattacctgaataaaacgaggttgttcagcaacaattgctctttgtaga
caagaaagcaaaacaggttgcaaatctacggcctgatttgcatgttccaagagttctaaaagaatcgttcccacaagaaaacatgaagaatcat
cagatttgctcaataagagcttttgcaatatatacagaatatattgcagtcctgttgctccggatatttctgcttgtaatacctgtgggcagtctttac
gcaagagctctcttatgatttcttgtgagagttgaagcagttccaaatcatcattatgtgtttctgtaatttggtaaataggggggcaaaacgtaattg
ataaaaccagctggaagaggagaaggcccggcacgaattaaagaagaaagaagggtgcatccaacatttaccaaattggggtctggatggctt
aaaacctgtagcaattctggcatagttatttcgcaaacactttcgtaactagctgcctcactagtgaggtcctcaaacgtatcttcgattagtccgg
atatataaggatcagaaggattattagctataaggttgaataataccggaataaatgaactatctggctccgcggctttttgacagtccaatcgag
ccaacgccgtgattgcttcgaccagaagaacgagcgattcgtcgtagctgttttgaacatactgcctaagaacttgcagcacaacaggttgcacc
gaaggtgatggttttatttcactaacagagcataatctctcaattcccttaatggcagcaaattttactaggtctgattgagcagcattcgagatac
aattcaaaaatatttgaaaaaattcaatggctaggggagtactctctgaaaagtagccaacgaaattaaagaaactagccatcaatatctcagat
acatcagaagtttgcattcgaacagatatggctttatatactggctccaaaaagtcgtcataagtatcatctgaagcggcatcgatggagcagcat
gcgtatagcaaagcttcttgatgaatccagtttatgtcattttccaaaatgtaagtgctagccagttcccgaatgtgttgttggagccttaaatttgt

atgctgttcaaaagaagaaagtacgttaacagcggctccacgcactgtatcaacaaatgcgtcataattcgtttcgttgattatatactcgttaaca
tcgttttcgtattgttcagtttgacctttagaaagctgggcatattgaaccaaaacagatatacaaggagggagactagataaagaagaattactg
gctaagaaaagggcttgaacagacttgttctgtaaagctacaccaacgaactgaagtagttccattagataacgaagaggaaaattcatgccaa
aaacgttctcatccgaacaactgtcaaagactacatctttaagataaatgggaagcaacttttgcaggatatcccaaattaaatcaactagtctta
caacataggttgataactttgatggaaacaattcccttaatttcgataaagacaaggaaatttctcccattatacaaaatatacccgtagagtcac
ccaaaagttcattaccctgaaaaagaggagtctgtaatttttgaataaacatgtccatccatggaggtaatagtcggtcaacaaagacgcgaact
ggctcataacgggtttctttatacatttccaataattccaaacatgagcgaaataatcctatcgcacgggcttgcagttttcgtaaagagtcattag
gaggagctagcaaaaccagttgatatagagtctcagaaagacgtggtgctatgataaaaaattgttcctccaccaatgactcatcaatcaactca
cacaagacatcgagtgcggcattgatagaattttcattgtttgactcgagtaactgtaaaacacagggaaaaacctcaggccattcatccggaaa
gtcaacgtttgcaatcaaagatactgcataagcgactgctttattaagctggtagttttccaaggaaaccaattgatgaagaagggcatctctcac
cgtacgcttaatattttcgtcaggtataggaccttcttggaactgttcaaacaagggggaccaatggtgagtaataaaccgctgtaaatagattaa
ggatcccttcatactattagcacctatcactgaatgtactaaaacaacgtacctgtcgcaaagggattgaagctccagaagctgcgatgttcgtta
aatacagagggaactctatttcaaaaatggttaggctttgatctttcgtatactgttgtaatcctttcttctcagctccataccaggcaaattttcaaa
attcttcaattcgagttctgcttgtaaacgaacatttgtttctgggtattgtacaagctcaagagcctttttgatattgctttctgtcatcttgaaggca
ctgatatatatgtaaccaagctatccagatttttgaaccgggagttataacagccacctatttcgtaagtaaaaatatatagattttataaataaat
tacttggatcaatacaagctggaatatagcttgattatttatttggtagaaatatcaacgcgtttcttctgtgtgctgctgagtaacagcttattttta
ggtaacagctgcttttgctaacaatgtcaatgtcacgataggtgttttgttagtattatagatatcactatacactaccataagaacatatttccgaa
tattaacaaacgttataagaataaaggttgagatattacaaatggtaaacaaaacaaagcaaaacaacaacaatttaagatttaagaaacctat
ttcaaaaagggaaaccagtaaacttaagaaaccgttccttacagagagttaaattatgctaacgtttttagcaaagatgtatatactcttgtgtctg
cagtcagaattttgagattggaagattcgactggtgtggctaaataagatcatcgaaacacttccaatttagttaatcagaggacagtacaacag
aatccaattaacgtaatcctacaaatgtaatcattacttccgctcgatatttattctcataacccgtgagaattacttacatgacaagattagtgaa
gaaacctgtttgttttgtataatcttatagatccaattacggtttagaatatgcaagggaaaggatcagttttgtttcaattgcataggcacttaatc
gtattcgtgaatactcatttactgtacaatctacttaacgataaatgtagcactactcttatatgattgtatacaaggacttatatgtttttttgttatca
tacaaaaatttgcatacaggataaaaatggaccattctcattctatttacgcccattataattcaaatgccggtagaatcgctcaatccgaacaag
tatgtctcgattttcttgggtgatgaagtgcattacgtttatttccacctttcgtctgatcatatcctacatgaattgatggatgaatgaaatgccaag
caagcctttaaattaggaactcgatacagactaaaatgaacattacggaaatgcggtgaatggatccccaagccaacaaaacagatttacagtc
ctccatacatactttacaaggcccttctacttgtcaaagttaaatctagcttttaaagaccattctctctgaagaaggaagtctccacgaatgtggg
agcacaaagcatccaagagaaagacttcaaacatctccaaagaccaccacaacatactaaattggttctaaaatcttatcatatatcaatgatgg
ctttcactagtatcatctgttgggtgttttcaccgatcaggagtgccgtgtctgttgataatctttcttatttcatcactatcgccgtcgttagtctactt
gttcttaagttagtgcttcattaaggcggtacactcgaaaggaattgtccgtgacacattgcattacattatgcaattagcacgatatcatattaca
ctaataatttataacgatattttttagcacttttagtaaacgacttaattaggggtatattaaaacagcatgtccatttataattaggtatgcctgtca
aatggacaacagactataaatagcttgacttcctttttgagtgacagatcccaaggaaagtaacaatgtcacttcatgtctgtttatacatcctccgc
atgctgaaacgattgtatatttgaatttgatgcgactatttggattaaatataggaggtttttatgtatttttttaattttttcttgaacgtttttatttatt
aaggatattgttaacttcaaccgctttctttgcctgataggctgaaaatcttgcgcgaccttcctttttccatttgtttattaatgcttaattttatattta
atattctgcgtcttgttgattcgaaatgcttgatttggacgaaataattgagaggctttacaaaaagcaaattattgcagaaccggtgattgcatac
ctctgtagtttggttaaagaattattgattcaagaaagtaacgttgtccgtctttctacgccgattactgtagttggcgacattcatgggtaagttgta
ccgataataaaaatctgtgtaaaagcccaattatgagcatgactgctaacttcgacaaagtcagtttgaagatttgttagagatctttcggattggt
ggtccttgcccattcacgaactatatattcctgggtatgtatctccttagctgagcggcacctatgaaagcttttcagaagaaatctatattcatgttc
tttgctaataattataggtgactatgttgatcgtgggtattatagtatagaaacgatcactttgctattgtgtctaaagctaagatatccaaaaaga
attaccctgcttagaggtaaccatgaatctagaggaattacgcaaacatacggtttctattcagaatgtcttagaaaatatggctctgcaaacgttt
ggaagcaaattacagacatatttgatttcttaacactagcagctacaatcgatgatagtatttttttgcgtccacggtggactttcgcctagtattcaa
catattgatcaaattgtagtacttgatcgattccgagagtttcctcatgaaggagccatggctgatctggtatggtcggatccagatccaagtatcc
aagagttttctttatctcctagaggcgcaggatattcattcggtgaaataatagtttctaaatttcttcacgagaacaatatggatcatattttacga
gctcatcaactttgcagtgagggatatcaaattctgtttgaagaaaagctatcaactgtttggtcagctcctaattattgctatcgttgtgccaactt
agctagtatccttcaaattgatacggataagtcaaagtttttcaatgtttttgacgcagcccctaaccaagagaacccttacgttgaacctgctgcg
agggcagcggaatactttctctaaatacctctttcttaatttttgttttctatacaaaatgctttacttttagcgtattgcgtttgaaatttcccttactat
tgatttgtataataggattgctttcattcattaacatttactacttctttctttgcaatagatgaatctatttctactatatagtaagcactgactgttca
aaagtgggaattcaatgttacttaattaactttgatcatataaaataattaacctctaaaactgttagtataagtaaagagcggaataagatatttt

ttaaagttataaaacagttttactcctgccatttgcatgttcccataatcaaagcattacttgggagtcgatctggtacaggagaaagaagggtcat
tctgtttttacatcgtccacgtcggtgacgaagacatatccaagtatacaagaatctataattgcagtttcattttcatcttcattcacgctcgaaat
tgccaaaattgaattttgtagaattgtcattgcttcagtcgtgatcctttctaaatattgacccttcccactaagagctaaagacatataggaacctt
gtttgtctgcttgaccagtttcttcttcaaagccaattgggagcaccgaagatttcgggccgctttctgatgcttccagcacgcgataaactactagt
tgggtggaatcgacgattatcgaaagtggtgaaagaggcattcgttcatctccatagaaatattgttttaaacatttggattgccattttgcaatcc
attccggttctctgtcaatacatcctccactcgacggaagcttgacgacagaaagccaagaggcatcactataattgcgctttatagtgctgtaca
atcgctcagagcccagtacaaggacaatattgatacggaaatcagtaataatagaatgaagagtaccctggtagcgttcataattctcttgtaaa
gaacaggtgtcaacaaggcaaccagacacagatgcatctgagctttgagtcattcgtgaattaacagcaagtgctaaacgagagcagcaagatt
tatatagtttgggattatcatatggactatccaatccatagttgtataccagaggagacttggcaagaatatgagtagggccggtggacatactag
caccgaaaccagttgcttcttggatgtccaaaatggtggcattatgaaaagcggatatggagcctggcagagcaaccatgggttgactaggatca
aggttaacacaaacgggatgtcgagattgccttaatgcatatgattctaagattttcataacgctcgttttgccagagcttttgggtccaataagac
agaccctaggacctgaaatagagggatcgaccgaagcatcgtcctcagctttttttgcgtatattttccaaagcaaaatgcagatttaaatatgtac
acataggagtttcttcagcgacatactccacagagggttctccttctacttctaaagtacatccctgccaagtaaagattgctcctttgacttgttca
aatttatatggaggacccaaagcgagttctgtaccaaaatactcagcatttccatttgcaagctaatcgacaaaggttagtcatctatggttgacg
aagacatcagaataacaaaaaaaactctcaagaaaagaaaaaggcataaaagaggaagaatacctcttattatttacgaacttttgagaaaga
tcgttgactcaacacatacccgaatttgaattcctggttcctcaacttcaaaacgccattcacaccctttgggaacaactatttctttcatcatttttta
tgtagattcagcagccatagactgctactaaaaagtaaaaaaccaaaccaaaagacaatcctgagcgaaaaacgcagctaggatcctaagaa
agtaaataatgagcccaaaggatgctcctttttccttattgcaaacactagtgaaaccgttatccaagcttataatactttttagtatgcgtaatgaa
aggtaaataaagaagcataagacagcctcgctatactactttcaccatgggtatcaacgtcatataggcgaggaaacgttagaatgttccatcac
gataatttgatttaacccagagcggcgaacgataacaaacgtcacgttagaccacgaaaccatctgctgtgatgtaacagcgttcttgcagctgc
agatcttcgtgcaaatcattatgtgtattttgtacatgtgccaaaggaaagcccctgaacaaacccggttttagcaataaaacggacgaacaagtt
tggttttataagttgaattaagatctaaacaatgtaagaaatatgtctcacaaagcaatgctatgaaaaaagacaacagcggtcaggcgaagga
ttttccgtttacataaatgtagatctatttgttattctttggaaccatagaatccaggaccgccaccccccttatgtatgattcaaacaacaaagaaaa
attatataagaccccttgcctcaatctctttctttaaccaagcaatttttactaagcttaaaaggtaatattcctttggtagatgatacccatgatttg
gagaattactaactattgagtaggattatattataaatttaataatggacaataaaaactgtggatgtggcagtggaaaagcttgcacttgccaaa
accaatgctcttgtgaagcatgcaagggtaactgtggttgcaagagtggtgacagcaaatgccaatgtgaatcttgcaagtgtggaaagtaatga
ttaatcgcggggtttccctttcgtttcatacctgctctgttaccataaacaacctttttcgcatcccttatgtgcaactttttatccaagttggcgcattg
acgaaccagcataccttttcaggaacgacgatggtaatataatgcaatgaatgatatatgtttatttcattttattttattttcatagtttggctgcat
agtatgaaaaacaaacatggaatgagataatcaaagtattgaaatttcgtgagcttcaattaaacaaggtatcgaaagttgctgctcccgtgcttt
ataacgtaaacagacgtgttgccgttgagccgtggagaaatgccgttgagacactttttgattcaataacagcattccgtcggtgcctcctaggctt
gtcgcaaggatcccatttgaccaatataatgatattgagatactgcctagtttgagagactgaatggtataaacttttttttttctctccccggtaga
atagtaaacaagtatacatccctggaacaagctatcattgttacttaaatctaaacttttagttagcgaacatgttgaacgggaagtgggttcatg
aagagcaaaggattggtaagaagagaaattctttctttgcgaaactgatttttattgattttctttcgaaaccatttgtgcattcagaggtaactttg
actgatatattgcccaacaggtctgtaggattatattacaatgaccgaaaacaatggctgcaaccgtcaaagcacattgttaagtaacaacgtcc
agggtaacatgaaggaacattactaggtcttgtattagggataacggtgatttctccagtacgtgtcgcgtttcaacccttctttctttgccttttcgc
ctttaaaagcaaggagacaagctatttttatagctttgttaaaaaaaaaaaagaagagcaaagtaaaaataagaaaggttttcgttgcttttacgtt
tcagcgttttatctctctttttttacggttcattcttttagctctctttcacggtggtcaaattaagaaccaaaggaatttagcaacttatatttccttttt
ctttctttcctttttttgacatatccccttgaagtcactcagaacattctttccctcgtcattctttttgttatctactttttcctttgtttgttcaggtaata
cagtcagtcattctttaattagagactttctcaaatacccttactcaaatatatttcttcccacggattccttttctctaatctttatataatttttcttttt
tatttctgctaaacgtaatatctattcaataaaccctttgttaattgaacacgggcacaatcatcgtcagtctattcatggtgaaggtattatctttttt
tggttgttgtttttaattttcgtattttttttcactaacctggactccagattcgacgttcttggaaaagccatagtcgcatttcgaataaagatccgtttt
ccattgaacccccctagaaaggtcatacgggctttgtatgattacacagcacgaaaagcttctgaaatatcttttacaaaaggagatttttttccatgt
aattggtcgtgaaaatgaccaactctggtatgaggtctgtaatccggccgcaggaacacatggatttgtccctgtttctcacttcgaagaaattgg
gaaaaatgcaagttctatgagaaatagcagcggtagtgaatccactgggaatttcgtggatctaaccaactctacaaattctcctcggatttcttta
aatgatttgcaaacttctactcagcctctttttggtattgtgcagttcgattttgttgcagaaagggcagatgaactagatgctaaagccggtgaaa
caattattattattgctcgctctaatcacgagtggttggtcgcaaagccaatcggtagactaggtggaccaggtcttattcctctcacatttattcaa
ttacgagatttaaagacaggtgccgtgatcaaacatatcagcgaagctattttgcgtacctcttgcattccacgagtagaagattggaaacgtgct
gccgctgattacaaaaaaagcagcatccctttagggacttttcagtctccattggaagactcctcatcttcagttggattcgaatctaaaagtacct

taactgatccagtgaaggagtcatcaccttttttcaacgccttcttcatacgtaagctcttcgaaacttagtccatcaaacaatattagcgatggtca
atacatatcctctgcttcatcaaacactatttctcattttaatgttccttatgtaattgctgcaatggtggaagcgtattttgctcataataatcaatttt
ggtacttggtacgtgcagtaatgagtgatggccgacatcgaaacctttgccgttattacgaagacttttatcatcttcaactgacattacttgatgca
ttccccagcgaagctggtcatggacaagctcgtcgtatcattccctacatgcctggccctgtagatgatgtgaatgaattgatttcatctcaacgag
ctttggatcttgatgtctatgtaaaagaactttgtcgacttccttctcatctgttggagagtgattatataaaaaagttctttttacctatggaaggtg
acgttgatgcttctcatcctaccagttctatgcctcgtgttgtagagcgcccgttcatatttttagaatcacaaaaggcaaaaagccttactgccccc
aatacgcctgtttccgggaacggtcataataatactaccccgatgttccagaaggtaaaaattcgttctggagatgagacatttgccttgcgaatt
gcctcggatatagcttacgaagcatttattgataaactgaaagcaaagttgggtttccaatggtacaaattggtttatagagacgaagccacaaat
gatttcttacctatgtgtaacgcagatgatcttcgcaatgcattctctcagaagtccggtgctttattgcttgcagagagacagaaagtttgacttttc
aattttgaatcattcatactcaactatttgaatgaaacgcagtattttatgatggttttgtgcaacttacattcccttctttttattgcattcatatttact
gatatttcttttgatggcatgttttctataatttggctatacttttgtgttgacattgatcatgttttctagtttttgtactttatttcgggttcctttcgttct
tatttaatatattccattctttcacgaattctcacaaatgcctagtttaacttgtcatcttctttaaggtcttacttagagtatgaaatgtaaaattcaa
aaatagtttatatgcgtacaaagtttcaatttgtatttaataaaaataaaagtttttagttccatgatgagtatacctcactatggtttcttctgcaag
tcgtcagtgtaccactgcaatttcttcaccatcaattcagaaagtgtttggacgcccactagaaaaacatatacgcctatcagctaaaaaataaaa
gtctgaagttactaaaaactttaattatctataccttttttgtactttatgttgtcccttttttgttaagaatcgcaataattttttgttagactatgaacaca
gaagagttacgagagctatacttatcatctattttacctgaaaatatacaggataaagatgtagctactgaaaatgctccttctttcatgtcgcagc
gcagtaaagttatacctgtacggctagaataccaagagagaagactacttcatttattggttgctgcgtacgtatatctgctactttttctattaattc
actaatgaatcagtttggatgtatgcgattacacggatactgtcgataccttacaattttcgtctaaagctaagcggactgctcagcaattaaaag
gcattacctctgtattagcaggaattatggtagcctatgactataaggctgggcaggagttattagaaaacagagagtttgaaaagtatgcgagc
tttttccaagatgtctttgaaataggccgtcggtacaaggtcttgaatcccaatcggttaggtacttcctatggtaaacttatgtactttgttcaggat
tccatgtgtccagaaattcaggaaaatctagggttcaatctatttaagccaattttaactgtttacgaatttttgaaagaacgaaaagctttgaagg
cgcttgaggatccttacattacggttgctactatggaaattgtagcaggcggtcgggcaagatctgctattcaaaaagatattaaagcaaaagag
cgtgcggtagaaactattgcaaaaaggcacgcgagctcaaccataacgaaggatcaaatccgctggtgcttatattccttagcggatagtaatag
ctaccttcgatttaaccgagatccggttgctaaattgatagatatggttaaacaaaacttttccgccgataccgtggatgatgaatttagtttggcta
tcgatgccgccaacggttctcgtttgcaacataatcattacaagcaataccattatgtacttcaaagtcttaacttgtggctattaattatgggtgaa
atcttctccttatgggccttgagtgatttagagttgacaaatccagatgtccggtacgagttggtcaataataatcaaggtgttcacaggatacagg
cgtgtcctaacattcgaattgctatggaacgtattttgagcagagctcagaaacaagccggctcatggatcggcgactcaacaattcatttgggtg
acacggctgtcccaaatgctttgttgtttattgataagtatatgcaagtaccacgtatccttactccacttgttttactatttgaggaacttgactctct
acatgaccaggctttactccgctatataaacactgcttttggtggaaaggaatatttaaaaaaaatggtgttaacagatttcatgcgctttggttttg
acggttctggcgcagacaactggtttgatgtaagtaactttgtttgtgttcggtataataatgttctgaacaatatacactggctgcatttgagttat
gctaaccatatataggctggtagctgtatcgacggtcgccttactagcgcctggaattgggccaacagcatccacacaaaagattattatagagt
actattgatgaccggatttttaagtttcaatggagattaggatgtaaaaattagattcaaaaaaaatcaatgattttatatataatattgaattactt
ttactttatatccagagtcaagttgatagttttattaatccttttagtatcaaacatgctgaactaagtatgtaaactttgacttagcaactgtgaatt
aagacattcttttattatttatgtattattttgaaaatataaaagatatatataatttagaatgcatttctcatcacattttgttgtgctttattgcacta
ctcctagcctacatctcattaatcagtatttccatagctctttccctttcatataggatgtatcttatttatggtgtcaattacagtaaacagacataa
aaattatcttctacggacgtcaaaatcggtcatttcttgcggcataacaacaaatcttgcttgcaattatgtgtatcaaagggcgttgaatgtgttag
gatttcgaggaggaaatgccaggttcataaatgattatcattggtatccgaataatatatcaccttacaaacacttcaggaagaatggagtctcta
atagctcgtttgttaatgtctctgaaaatcaaggtagaagccgtgaaactttggaactatccatatcaaagctatacaatgattgtatcggctattt
aaatccttttttataatatttatgaagctaatgaagctttgtatttgcgcttaattgaattcttttcagaaagccagacagtattcattctcttacaaca
agaattcgaagaagaagcgaaaataatttttgaatattttcaaaaagtagcttgtttagatcctgactattctcgttcatccaaggttacagaaaac
atattgaatgacttatttaagtattgtaaactattgaccgacaaaggtcaagaagacccgaataagtttattcatgcatgcgtcaattgccttctatt
cctatcttctgatcgctccttttctactgtctactggcagcatgggtctcaatatcagcagttcttaaatttattaagaaatgcttcacgttctgttcttc
aaccgaataatgatctttataaaagcattgaaatattttttttactctattcttcaatcttacgcgctacataaaggattcattccttcagccaattctat
tgatcgctgtctacccgaaaacatgcggataaaagaagaagaatgggcaagaatattatcattcaaaagggatccttcccttttttcaaaccctg
agttacctaagaacttctcagattcctctttgctttccattctttatagcgggaattatcaagctcttagtgaatggcttatttcagcttgtaaagagg
gacaagtttctaatttaaggattcttaacctgttattgaaaggggaacctaaaacgataaacctaagagatactgtttccttgtttatgtattcagta
aaaaatatccccaataacagtcggttccttgaggttaaagcttctttattgtcgaaattacttcgagttaaaaaacttcaggaatttcaagccgtctt
atctactctcccaaacatccagtcgatgattgaacaccgaagatatgatgttctgcttccaataatagattatgttacctttacaaaggatatggac

ctatttttggagcttaacacttctcttcttacgtgtacaagggaaataccagaagaattatacgtgaaagttttgaaatgctatggtcaaatgccga
aggatgtaacctattacaaactgtttaaagccttcttggagaaaaaccctattttagatgaaaaacaaaaacgttatgttaatagcatcttattaa
agagctgtcatactcacctttctcatgtactgtatcgggaattcccacacatattgaggccaaaaagcccgattttatttttttgctattgctttagga
aaagggggaacgaatggattctaaaatacttaaacatcgccggacatcctaaagatgatatatggaatgacatgattacgaaccccgatgcctc
taaaaccttactaaaagctttttgttccaaatatgattcaagtcgagcaataaaatttctaaaagcgtgttctgctgaaggaccttacccttcaaatt
tattacgggagattcttcatcatgctcaacttgagaataattgcgagaccataaaatggctgaaatctcaagagacggaagtttctcctcctgtact
tgaaaaacaattataaaaataaccattgtcgagtttactctataaacacttttggattttttttaatcaaatgttaatagcccttgttttcatgcttgct
ttcattaaaaatactaatatcatttcatttttaatctgtattgtgatttgtttgattgaattaagtattaagccatgtcttatgtttgtaactgtttttttag
acatgaaaggattatgcatagtaatttctgttccgaaaaggctagttaaatgtaattgtctatataaatctgaagctttatatattcaattaacccaa
ggctgattttaaacatatattgaagctttcgatatggattaaccaagatccatttccaccatgccaaaatccttgaaacttttaattggcaattaaaa
aggtatgaagagaaaatttccacgaatatcacttctttagcatttattaacaaagtaaagatgaaaagtatttcggcttcagaatacgccagtatc
ttaggcacgcgtattgttcttgtactagaacatcaagaggatagtgatttcaaaattattttagacaagaatgtcgatccaaaaactttcgtaaaaa
aatgctggcgtcacaaattacctatgtatgctttgatattatgaaagtctgtttaaccctgcagtattgtaaaaaatcgaagcctctattgctcaaaa
gaatttccatttgacagaaaaaaatcttttctagatgctttatacgaaaagtatggcaattcttcaccattaatttcgtcgttaaacttgaagaatgg
gaagcaaattgaattttgcggatttcaggacatcaaaagcttgcaagcggactttcagagactaaaaatagtcattttagaaaacttgcagatctc
ctcagttgatactgaagttgatcatggttccattttgtcaggtatacggaaagtgacacttaaactcgactaacccaagtataggagtccaaaaac
ttaacctatcaagtaacttacttaattctatgaatgatgtactattcttatgcagtcagctgccgagtttgaaccaattgattttggagtacgaaact
agcaactattcagtaagctaactactccagtaaaaataaatacctatctttggatgaatttaatcctaagttctctaatcctcaagtatcgagcctat
ttttaagagattgtagcattcgatcagaggaacttggatggatacagaaggcattcccttgtcttaacgaacttttcttggactataatgatgttgaa
ttaaactcaaattgctgtttgatgtcaactgagactcttagtctatcatataatactcatttagtaacagctgatcttacttcttttcctgtgtttgaaa
ggtaatacaattttatgaacggtgcaaaactaacggattacagaacaaaatggctgaatatttcctttaataatatttccgatcttaatatcttacct
ttagaaaacatgcagtacttatctttcctagatatatcaggaaacaacatcaattcctgggatcaaattgctctcttacagacactaaagtctctga
aagagcttcggctttctctttcaaaactccacgataagactgatgagactagaatgcttgttattgctcgcctccctagcctaatgaagcttaatgat
gtttctataagcaagagtgaacgcgaagattctgaaatttattattcctcttgcattcgtaaatttgttttagaaaatgaaatttctaaggctgatac
attggataacatagagcccatttggcgatacatctggaaaaaacacggtttgtcagaaccagaattttatcggattcagaaaatcgataaaaaac
ctggtgtaatcaaagacaatattatacgagatgtatgcgtttcgtgtagtgaaggcaatgaacatgttgttcttcagtttcaccaaaactggacggt
attatcagttaaagcactaattgcatttcggtttaacttacgcgccttcgaaaccatatatcaattcaaaggccaagaagacaaatgctttagaat
aattgactctactcaggacgagaagcagctatatgaattaccattcacaattacagaagttagaataagtcccaaagattgcgtcggcgacatat
cgagaagcgaataaatacaaaaaaaaagcataaatatttttcaaagaatatatagataatataagcgatctattttgatcaactttgtttaagcat
tttcaattaagctgaaggaatgatagaaacaaacagaaatacgcacattttcataagcattaaatatagttaaaaccagccagacctcgatagat
agagaacaaataacaaactgaacaacaaaaataagacaaatcaggatacaaatagacaactcggttaattttcttcgtttccctttcaaagaaa
aattgtggaaccataaacgagaaaactcgaatatgcatcatatacccgatagtaaaacagaaagacgatgttttattctcacacagaatgatgta
catcaagtcaatctataagttaaaacaacagttgcattactaaagcaactttcacaaaaagttacagtagcaaaaatgaacaaatgtattaagta
aaataacccaaaataacccaaaattaagtttaatacccagtagattccaggacttcaaaaagcggagataactcaggaccaagacctaaacca
acagtttcatagtcgttatcaaaataagagccatcagttcgatcatcttctaaagtgctttggaaatctgaatgcaattgtcgatatgtaattttttt
gctttttcttaagcgcgcgacgagctttgcgagaatgctttggttttgcagcaatcgagcttacattgtttagggtagccgagcgttgcttatgtctg
cgcttttcacgagttttgatagccaacgtgcaccattcatcacgaattagttcatctttccgagccatagattttatgtactgattccgtcggaaagta
ccaatgggaattctctcccagcgactcagtagatggtcctcttggttggttacagattcttcaggtttagtatcattattagtcgtggcagcttgtaga
agcgatgtatcaaggatatcatccaaagatggttcttcagcacctccggtgctgtttgacgatcttgcgggggcgagaggctccacatcgccttttt
tggatgcgttaaacttgtcttcataaagagagtgggaatgctttccgtcgataaaaccaaataaatgatttggttcacttttccacgtacctaaaac
agggggtttaggctgagaaaaatccaatttcggatccaattcaaaggaaggaacgtcattcagttcagcagacgtgtcaacaaaactagcaaga
atgggaccgtttatgttatcatggttttcagaataagaagtatctttaaccaatgaagtctcattggagtgaggaagttctaatccagtagctgcag
tgggaatagaagaaacctgagagaagggagagtcagaaaaagcagaagtgagggggtcgtgaatggtggaggaagtattagaaggggcttct
tgcaaagagatagatctagaaagcttgggctcacaaagctgaggtagttcttcgtccgttgtgtcgccgtcgtcttgaggacgacttgtagaaaag
gaatgccgacgctgagtagcaccacgggaatttctagtcaaaggatctaattcgtcagaagagtcatcgaaaaaacattcccatccaattaggtc
cacatcgtcgcgctcatcatcatttaaattttcaacgctcgagtcagaactcaaaagaccaagcaaaaattcatcatccgagcgatcatccatgtt
gacatgatcaggaaaatatgactcgttacgatgactgttgaacgatgccatatcaatatcgaggttatccacccaggatgcactaactaaatcttc
ttcagcaacatcctcgtcgtcatcagagcttccaaagctttcgccagcatccgatacgacagcttctttctcgggatacgtttcggagttcttacgag

cattgtttacgaaagacgcggattttcggcgttcttttaataagtcttctttatgtaaaaattttagatcttctaaactcatagcctcctctttgactgc
cctagatccttcctggtccttgaatccgttggtctttatgggctgaggctcggccgtgcttgactcggatttttcagggaaagattcagttaacccgg
tactattttcaggcgtatgactagttttccaatttatttttccatcgtctaattgcttactatggttctttgcaatcgcatgcaaatcattgtcgccgtcc
aaaaaagtcttttcattcacaatcccggtatccaattccgatccaaacccgagtccatcattgatgccatccaaaactgcgaattgattttcgtcaa
gcgaaatactgtcatttagtccaaatccactatcatattcatgatccttgcccaagaacggtgtcgccaaatttcccaagttcaacgagaattgcaa
ttcttcttgaaattcatcctcttcatcctctccttcacttccatcaaaatcctcgcttatcccaaagccatatatcctactatttgctaattccattttcaa
tcatgcgtgaatacaatttggttctggttgagttgaatgagctcacttttgcacggaaaccctaatgggtcatgcgcatgtgttgtttctcgagttgct
acctcttagaccctaccctgtaaatatttccttagcgatgcttttcgtctttgaaaatagaattttttccccttgttaatttttcttattcttgcttaagc
gaaactactttccacttccttgacatagtcatcgtaatatgtttcttagtagttcatagtacttttgtttaataattcattccttgattttgtgttttcagc
ttgcaaaatcttctcttaatattaacatgttggtgcctgtttctataaatgcaaagttgacattgaggttttctatttttacaacttgatattgggatctc
gcggcgtcttggaaactcatatacaccaatctcgttaatagaagagatgaatatatcttctttgggtttgtttctatcatctagtcattctttcctttca
ttttagggcgatctgaaaagctaggaccttttgatggtttaaattttcggaaactattctttgtatattttccgttgtttttaaagcgaatttaattattt
actgctaaaacggcttttatatgatccccatgttcatattgttttcaaggggttctcttcggagggtcacagggtgaatcagaaacaatagagccttt
tgcttagttcctcgtataactctttgaaccatctgcataagtcaacagatactattgttcctcgaggtgccttgtttgataaagaggagttaaccaag
atcttcatcgttcgttgaaataaacctgttcgaaaccattttttttatattttatcgttactgaatttcgtatgtgcctttgacagtaccaaaaggtgat
tatctcgagatcgtaggaaaaaccgaagctaaatatcctaaaagtcatcgccgtctgttcgatctcttaaattctcttaagtacatattgttagttta
cgattgttttatttatatgatattacaagacttcttccattcttgttttattttctccgccagatttacgctttatggccgcccatctcgacggcggtaaa
tccacctctcataccgaacaaacctatcaagttaagtctattgcatcctatacatgtgttgacactatttgcctaatactacagcactgccgatctta
acagggctaatctagcagcgtacatatccgcgacccgttctgagctctgtcttatatggaatagtagagagtgtgccactgtggatatatggactc
cattgtttgcagtttagacaattggagccgtaatggcgatgtacacaattcggcttttgtaaattctgccatgccgtgcttttatgtgtccagattttct
ccacagagttaggtagtatagaaacagtccagtctccgttttgtgagaactctttagttgaggttacacccaggttaccagcaaatacgttggcaa
cttcaaaatataacccaggatcagaagatcaagctgtgaactatgattatggttgcgtggaaagtaggctaatagaagaataagtggaagagat
agacacccactagccggacaccaatcctggtcccatagacctttaccgaccgcttgtcgctgagtaaaccatgctcctagaggtgcattatgagca
ttccaccgcttaccgaacccagtaagattacttaggaactgtacatctgttactactgtagtgatacctatggcgtaataaacagattcattctaaa
gtgagcctgttgaaagtcctacatgagctagtccaagtgcgtgttcaacatattgacatttgttatgttattgacacaactatgcccactgaccatca
agcccggttgtatattatgcctgacctccatgaccgctacactgttcagcagtactaaaattagcatgatccaacgctttgtactatcgaaattactg
aaaagaggtgcatctacatctgcattagcagtgctgggtcctaaagccaagcatataccagtagtctttgtacagccagtagagaaacggaggtc
atagcaatgtgaaaaaggttgacaggttcataacgggcttgaaggtaggtgttttcggcaactggttgtatgatagattacaaattgtacgcctaa
tgaatttggaattttcgataataaaccggaatattgctaagggtcataacgacgtaaacaaaaaaaaagttaatattatcgttaatatttgcactg
aaaagtgcattatgtaaggtagcagcaaccaagcatagcaggcatagttggattccttctgtatatcaagaaacccttcagtagcgctacggttta
gaaaaagcaatcatcttttgatgtatctattaacaaaagaaatacgctatagtatacgctgtattgaaaaataatacatttatcaaagaatcttata
attgatttcttatatcgtgaatgcaattcttgtactcaacatcatcatcgtgtagacccgtgactatctactttgtcgtcttttctcctctgtatactcat
caacatcatttttaagacacttcggcggtttctctcttcttcaatactaaatcattgcctgtcatacgcttataaatatcacgaacatcgcgagccatc
gtttcaaagtgcgaagtagcatcgacactctttgtgataaagcattgatccttggtaccatcttccataggctcgtacaattcttgcacccgagaga
atgactccacaacaggccccaataatttcaagcctggtgcaatttcagataaaggattggcagtttccacaatggtagaaataatggccaaatag
tagcctttaggacatacattatgtacagacgaaatacatgcaatataaatatcgtgctttctcttgacttgattctgaggaataataatctgcactg
aatccaaattgtctgtattaggaatagggtggtctaaaatacaaatggcacgcaccagtcttccaatgcagcgtaccttttctcggaaatacgaag
gatcaccaacgacttgcttcgccttggcaacttgttctccactgcgaactccaactgcaacaccgtttccatcatactcgatcccatcaaccggttg
accaagcatgtatgttccgccataaatcgcgctcaatcgagcaaaaccctgaggaagctcgccaagaccatacataggataaatataaggggat
ttgccaaatttggcgatcgaagaggcatacaatagaatacggtctcgtgtttcacgtgcaggcttcttgatatatgattcatctaaataaagcgcca
tagcatgtccaataaaatcctgggtacctgactgaaggccaaactttttgtacactgtctccattgtatcacggtccaaattgatatctttatgtgta
ctaggatcttcttcgcgatagttgcttacccactccaaaaactttttggctcggcgcttctcaaataaactcatcagtgggctcttcaacgcttctatc
tcattacctggtacttttgctattcttccatctcggtatacatagctgccagcaatctgcttaaactcaatatagcgagtaacatcggtataaaccaa
gatatttgtcaaatctccgttggccatcagaaatttgggaaccaaatctacgcaccagtctcgatcacggccaaggttttctggtcgttcttcacca
gggcgaaacatagcgtacagttgacttaaattcaagctagcagaatctctgtatatttcctgttagtttctttgttcaccttttttcatactaaggtatg
tatccatgcatttacatacgctccatagtagtcatttctatcaatatgtaggactttcttcccgtcaatactcagtaagccactcaatatacattctgt
aagccctgttcctaatacaattacgtcgtattcctcatccattgtgttttgttgttgacaagcaagcagatgtactgaaatcataagcttgtttatcac
tgttacttgggctaaaaatgttacttgtatgccaaacaaacgttcaaatcctacacccataaaaatgatttttctatgaataaacaaagaacattca

ttgaagaaattaattaaaccgtgtttagtagaccataaagaatgcgagaatattacactagcaaacactaagtaaggtcctctaacaaaagcatt
caaaagttgtgccctcgttgcaaattctctgttcgtttctaggtattcttatgcatgataaatacgttccttttaatttttattttgtatgttccattataa
aattcgtgtcttttttttacgtagctgtttaggattatgaagagtaagctcaattcactcccaactaccatccataatcccaatatcaagtttaaccatg
gataaggtagaatcaacgacgattcccactaatcataaagacttaattaatgatgtttcctatggtaggtgaaagatctctagcatggaattttata
cctaacacaacgtgtagatttctacggtcgaaggatggcttcatgttccgcagaccaacgagtaaaagtctacgattctgatgaagatagcggga
attggaaaatcgcatctgagtggagggctggagatgcaagtcttatgagaatcgaatgggcacatccggaatttggtcaagtcctggctgtctgta
gtttagacagaggtgttagagtttacgaggaacaaaaaggagtatcgaatacgaaaacatgggtcgaggttgctaaactgatggatgctagaag
cgcagttttagatataagcttttgtcctattcagcatggttgtaagctcgcagcgatttcagcagatgctaccttgcggatttatgaagcacttgagc
ctgaaaatctttcttcttggactttgatggatgagattaaattgcttccttctcctccttcccgtaatgaacaaccttccttttgtgtaagctggtgccct
tctcgttggcgtactcaatgcatcgccgttggttgtatggacaaagcttacttgtacacccaaaatgctagcgcaaagtggaagcagattgcttcct
tatctggacatactagcctcgtccgtgacattgcatgggcaccttctatgggacgcctctatcatttactagccacagcctgcaaggacggaaacg
tgcgcatttttaaagtttcgatccctttgcagccaaaccaaacgaattctctcgaatatgagtctccttcagagactactgaagagcccataaaggt
agaattgattggtgaatatgataaccataaatgccaagtttggagatgtcagtttaatgttactggtacaattttaagtacttctggcgatgacgga
tgcattcgactatggaaggcttcatatgctaacctctttaaatgcatttctgtggtatcattagaaaggtaagtgattttttttgtaagatagctagc
aaaatgctaataaagatctaactagaaaaccggaacgttctacttaggggttctttcttcctccaaaaatgcacattggattgagtatatcgtcttt
ccaggtttatgatatttttaaatagactttcatattacaggtttttgctcacccaaaaatgttttgttcttttatgtcttcgattagtaaaaaaaaaggt
ttaatacgcgtctcttgtctttgaaaattcactgaagcttttcttatttaaaggaaaagataaattcatgacccgaaaatgattaaaaatgtttataa
aaagccaacttaaatttctctatctaaccataattgagacggaaaaggtcgttcaaaacaaagtaattaccggcgttgttaacaaggtggaaaac
ttggctgtagcgttgtgcaatttgttcctcatccaactagtatgtaggttagttaaatcttatttttgagtattcaactcatatatcccaaaatgtatat
acaagactcaaagtttctttttcaacttaccaagagttcaccagtaaccataacaacaacagagccgttagcaccagagggttgtgcatccaaag
tagagatacggtgttgaacacgttggaaaggaaggctctgtaaatcaatgttagctttttcttgaacagttcggttatcaattttgcataccacaag
cttttcaacaatattcttagcacctaggagttgagcgccctcaaagctcaacatggactcatcacgctttgtatagatcgttagttactttccttctac
aaataaatccgacttgacttttcttttggtggtacacttacgtataaagttgagagttgagaacgatcggtgtcgaaggtttcataataaaactgag
taaattgagtagccagagtgttaaagtctattctcgttagctattagttatttttcttctgctaataaacttaccagccatactcgtagatattggtaa
aagaaaactgtagtatgggagaagtcatcgtttccccaaaaaatagcaattaagaaattaaaccgaggtacactaacggatattatgaattaaa
ggattcgctaatttattttgcaaagcggatggtatcgttaatatgatatggtttacgaaagcatttgttaagatggaagaactaaggctggaagga
gtctaagcatttctcttctttctttattcttcattcgaaagtatatgtaacttgattctgtattaaatataacaaacttgaaaatgaaggacaattttct
ttaccttttacattacacggattctgttcgcttaaagttatatggttattaatacaggtcgtcgagaagtaaaatgaaacttcgttaacaaatagaac
gtataaaaattaataaagaataaatcaataacaatgaaagaaatgcaataaggagcggtaaaaataaaaactcagaagtaaacaaaaatgca
agattagaggtcatggtacacagtagctattataatgccgagaatactggttttgaaagcttagccctcaaatgtttctccaaagacagaatctgc
taccggatcatctttatcaaaacggtttttttcattagatacggagtcattcacttttggccgatcattttcatggaattccccaatgttgacatcttca
aaacgttcttctaaagatttatcggtactgcaaaaagttagtgaaattcatcatcaactagaaatttcttccgttatgaaataacttacaaagtaaa
tcctttgaatcgatcttgaatagtatttgagaggggtgtagaaccattgactgaatcatgagaactatccaggttcacattccgaacattggtgttc
gtaaattcttgatcaaaattggaaacatccaaatcactctgaacatgaggcttgaacggaggtggtaactttttcttctccagcaaagtccagttaa
tgtcgtcaaaaaaaggatgtgcttttagttcctctgcatctgcagttgctcctatccgatgtctgggattccgattcagtaatccacgcacaaagctg
cgagcttcatgggatagcacgcctttgggaaatcgaacctttccaaaggcaatatttctgtacatttgctgcacatcaggtgcataaaaaggactc
caaccacaacacatttcgaaaactaaaaccccaagagaccaaaaatctacctgcttagtataccccttatcgtccaataaaatctcgggtgctag
gtattctgttgttccacaaaaagtatttgtagtgtcttcggacgaaagatttgctttcgacaatccaaaatcacacaacgcaatatgtccgcttgcgt
ctaatagaatattttcaggcttcaggtctcgataaataatatcatgcttatgaagatgttccaaagccaaaacgagttctgctatataaaacttggc
ccgatcttcgcgaaaccgtccctcatgttgtaaatgccaaaacaattcaccgccgctcatgtagtcagttattaagtacaggtcagaagcagtttg
aaacgaaaactttaatccaacgataaacggcgactcgtgaagcgaagtccgaaccagaatattcctttccccaatagtatgtgtaacttcttttttt
ttgacaattaactttttagaaatctttttcatagcatatattcggttcgtgtcctttttccgaacaagataaacatgaccgaatgttcctaaaaacctg
ttagtacttgaatattcccacaaaaagacaatgtaatttacctctcccgatgagccggagaacactaaaatcctctggtccatatcgtgttattttg
actgttcaaaaatagtttgaacattaatttcaccttcaaaaggctgagataagtcgaagggctccagtttataccagttctcattcaagtactcgttt
aacaaaacaggcacaactttaacacttcctaaaaacatatcatcattcatacggtcgtacacgctgacagaaatggcggattccggattgataac
gtcaaagacagtttcaaaatcccatcgaggggctcgagcttcagcaaacatcaattcagactgcacaagagaaggagatacaggaaaagtttc
gttcacagggcgtgatttagaagggatggaaattcccatcccatttcactgactttctttggagaaggagtaacgacttcggttctgtcataattta
taactgcatatggttcagcatgcatattggaaattaagagattgcgaccttgacgaagacgtacaactaaacgacccagaatttggttggatgtc

attggttcatgttgctcatgaactatgcgattaagttggcaatgtttagatggaggagtttttaatgtgtcgatttgaggctcgaggtgttcagatttg
atatccgcagatgtagacgctgtagcttcggttgtatttttcctagaatggtcaggctgttcctcagaatcttttgtttttcctaaagcaggttagtat
tagtcaataaataattatagtcgaggattatgcgagagcaaacaaaaccaagcagaaacaacagtgacaaaacaaaggtccactccatattttg
aaagccacttttcttatgaatttcgttacagcaatctatcctttctacatactgtttgtttcacttgagtagttatttcttcgtaaacattcttcaaccgg
tctggtaaaagaacggggtcatagcccgctgatacattttgattttcatattcagtcgtcccttttgcttccgctgtctccacagaaacggaaggagt
agaagagagagaaattttaaaagaaggatctgaagatgaggacggatattgattttggctatcagaattggatcctcgatgtatttttttcgaatat
ttcggtcattataagagagcctggagaatctacttgtccacaaaaactccacagcacctatgacgaaaaaattggctcttgttagttgttggatga
aagatgcgtgcagtcggcaaattcactgtcaaccccttcaggcactgtttaccatttagtgagtaagagggagtaaaattgaaatgcttgtttgcc
acgagtaggtgagttggagtggatatcttctttcaaaatgtttgaaagcaagttgttttgaactctactttcgacttcttaatggattacgaggacag
aatccgcactttagagaattatttttcaaccgctgaataacaaagccagaagaaaggaggttttaattggagcatttaaactttgttagaaacgta
gaaatgatatacaatgaatacattttataatgcttcagcgaaagccattaagatatatttgtttttgttacttgtgttacctttgtaaataaatgcgaa
tcctttggttcatcgaatagaaaaggcgggagcatggaaaacttgaagcgtgttgttctgactgtactgaagctttttgtttagaaaagagaagcc
tgtcgtgcagttgttccttctggtgcttttagaaaaagttgtacgcttcctacatcttaccaaggaagggtgtactaaatgacggcttcgctcttgca
aggcgaatcctttcgtgaaaaccaccaggttggtttctcatcgcctgcaattattcgttctgctttccagtcgttgtaacctttctacgcttacaaagg
ataaactcccccccccccccaactggatcagggaagcatcattcactgaagagaagagcaagacagcttgtgcacaagttctatatgggggccc
attattgaagctaacgaagtcgtactgaagaggatgtgacggatttatttccgtacaagaaaagagacaaaaaaataatgaaaacaaacacgc
attcaggaaagaaatcaagaaatgatgaaagcagatagaaaaagtttaaacagtgtcacagttataggttataataattcatgttctttttgaact
gaataattaaacaaaaaaaatttctagatgtttgaggttaactctgagatgtcatttaattacatattcaaaaagacaacggtccttttggaggaa
ttgcagtaaatgaagctcgtattggttaactaaatataaacagtgaagtgatattcactgttcaacatactttcaatgttgagcttgagtatttctgg
gttagatatccattgattcagagtaagaaaagtgtgaatctattttattgatgtacaaatagaagggtaaaggtggcatgaatttataacggagca
ctgagctattttgacaagaaaggaaattaggaatcctctaaagaatgtctcttcagatcaaagcaaagtacgtacgaatgatggacaaaaaatg
gctttgttaatatattgatttcatatctacgacttacgatccagttaaaaactcagtgaatcgacatgaacagttggatagaaaaagtaaataaat
aaacacaaaaacaagagtaaataaagacaaaaaaatttcatcaaatttttccgcgagatgaaatgactcgaaatagaattttattttagtttttat
ctttgaaaaagtaaaagaaaaaaaaaaaaattgttgaacgatctctacaagaaaccctccacttttcgaagcaagcctgtgtgttccttggtggtg
gtacaccttttttcaccaactggaaattaccatcttttcaccaacatttgcaatggctggaattggcaagaaacgtgtatgttttcgggatctttattta
atttttagtagctttgaatattaacattttattttaggtgaagagaaggactcatttaaaggatgatcctgctcgagaggcttcaattcccaaatcgg
taagtatcacccatccggcagcttgagcctgaaaaaaaatgaataaattctaacaaacatgtagatggtcgtccgtgcgggtgcttctgaagttg
gaaaaagtctttcccttcttactcgtgatcttcgacacatgatggaaccgcatacagccgtgcgcttgaaggtgagcattctaacttttcgttgatgg
attttctgactgtttttaggaacgtaaagcaaataaactcaaggattacctcacaatgaccggtcctttgggagtttctcatttgatggttctttctcg
aagtgatgcaaatgcgaatttacgaatcgttcgtgctccaagaggcccagctttacattttcgtatccatgagtatatgctcaacaaagacgttcga
aagcttcaaaagactcccaaatccccgaacgccgagtttttgacgcctcctctactcgtaatgaaccatttcaatacaaagtcctccaaggaaact
cctcacgaagctttgctgacagcaacattccaaaatatgtttcctcccttgtcgctccagtcaaccaacatcaattcagtaaaacgtgtgcttctact
caataaacgtgatgatggctacatagatcttcgtcattacttaattaccaccaaacccgtaggagtctcacggccgattaagcgtcttttgaaggg
ccaaaaacatccttctgaccttcctgatttgagtggagttcgtgatatctcagactttgtcttgcatggagaaggaatgtccggggtacccagtgat
tctgaggctgaggaagatgctacagtcgaagtccttcgacctacaccgacaaagatcgaagagaaacttctcagcgaatctcagctattaaaac
caaaacagcaggcaatcaagttaatcgagattggtcctcgtttgactttggagttgacaaaagttaccgaagaagctatgggaggcaaggtcctt
taccatagtcatgtacaaaagtcggaggatgaagttcgtcagcttgacaagtttcatgaggaaaatcgttatctcaaagaaaaaaggaaaagag
aacaagagcaaaatgttcatcgtaagcaggataagaagaagaaaaaatcaaagaagaatcaaccttcagatgagcattctggatctgaagcta
gtgaaggtggtgaagaagaaactgcagaaggtcatactgcagatgtttaattaaaaataattttggttttggcacacggtaaacattagaaaatg
gaaaaagttttgggttttatatttataggaaaagtcaggacaaataaataagagtttgtttttttagttttgaatatatacagccgtagcagtacatc
ctttattaaagtaaaataacaagaaagagaccctatttctaataaataattatgaaatgctctaaacgtagtgataaattaatctatatatacatgt
catgcaagtcgttatcctcaacaatccaaagatggtctattctggcaagaatatcatttacctggaaaggtaaacttgacagagaattatgatgta
aacgcacagttttaaaaaaggtttctagttctggttccaaaatatctcgtttccttttccatatctgaacgaaaagctctattaaaccgttccacttcg
tatattcctgttcgacaacaggcagtaaatgaagcttggtctttctgactttgtgaacgatgttatcaaaattttcataccaatcagcagtacaatgt
tgctcaatcgcactaaaaagcttagattgtggagaaacttcttgacaaaaaggcggcgggtattcctgaccttgtaatatccaacaaagctccaa
caaaaactcgcggcttatttttcccaaatagttttgaagatccaagacgaggcggttttctgtgtttactaatgtaaacttcgctatagccgaaaaa
agaagatagattttaccaacaataacagggttcgaagaagagtacaatagggcaagattttccaacaccgacatagatgtatagacttcttcgca
aatcttgatttggtgttcttctcttagataaatatattgaaagctgttataaacatgggttaagcaatcaagcagtaaagaagaatagaatgatgct

actaaagatttgtcacttaagtaattcaataaaagttcttttaagcaaaatcttcgcaatgcggagacttcgaaatcccaataatcattaacatctt
gactgtaacttcccatagcaagagttaacagattcgttatatctttctgcttttgctctaataaagcaatatcaaaattcgctttcagaaaaccaaat
aaagcacttcgagaagaatttgatgccaaatttttacgggcataactgcgtaatcgaaatgcagcgtactctaaacgctccggtggttgaggaaa
tcttgcagaatccataaaataacgaagtcctgccaaattcttttcattagcaaagggactgcaatattgaagaatgtcgcccacaacccgctgggc
ggatgaaataaatatggaatgatcatcatttgaataaacaagactttcataactgttttgcatagatgctaataatgaacttaggtattgaagaaa
aagtatttgatagtactgcagacgttccatatggttattcttaacatcgtcgtaaacttttgccatattcccaagaacactggaaatcaagcttaact
gaattgacttaaattgacgcaaaaactcctcttgattgtccagtataattggtaagttgaatagtaaagggttattcttatctttttccaaaagaagt
aaggtaaaggaagcagcatatgtaccatttaaagcacctagtgattcgaaccaaacagtcataactttttctcgttgatcaaccaaaaaagaaac
gtttgtttctattaggatagaataaaattgactacttaatgaccgcttcaaccgtgtattgcgaatccgttcccaggattcggaaccaaaatccaca
taataactccaatcattaatcttagcttcaaccgcaaagctagcgcaaagcgccatgcattcgattaaaggaattaatactggacgaaaaaattt
atattcactttcatcgcttcctactaatgtagatatagtttgatatagataaggagatacatgcttccttaacaatttagccagttcacagatgcgttc
aaatttttcttgctcgatcattaactctgtatcgtcaatgtaatcgttctcaaagtaagcatcttgactatcagtaaagacgacgctagttactgtttt
atcatgaggtatttgacaaatagagtgataggtaagaaacttcttcaaaaaataagtacattcaagtataacccttgagggtaagctcgacgaat
tttgaaatatgaattgactagagtgaacggtcagtagacaatttattcttcgaacgtaggtcgtatgatttgggactaaagcttgaagcgagtaaa
aggccactaataaaaaattctccagttgaccttgggaataagcgataagctcaatctcatcgagactttggtcggaaagtatcaaagataaatat
tcattcagagtgacttccattaatgaatcaagccaatacatacaagcatctaactgaacatccgatccatgtaataagatatacctcatcaaatac
tgccaagctttcaaactgattaagcgtgctttgagatgtgaaccttcaaatatgacattgtcgcgtatcctatttatgcttggcttgcagttttcggga
gcaacccaaaacaaagatattaatagagtatgagtatgctcaagagacatatagtctttaatggaaaaaggaatttctcttttatattgtcgagca
tgaagcggttgaagtctcgctaccattcgacgaatgattggtgtattattagagtcgcatttcagtctttgaattgaaataattactgttttgagaaa
aatgacaaaacaggtatcaagagcacttatgtgcaaatcaggagacctgtccaatgatttaataaaatcaggaaaatctgaagctacatattca
gatgattcacaagaaaggtcggtaaatttacgatgactaaaaaagtcaaaaatagcagttaaaacagatgttacctctgaccaaaaccaatcgg
aaatcaaaaggttaattcgtaataataaaacccgtaaatagcgctctgtgactttaagatcaatgcccttcttctgtctttcaaacgtacttcgaca
agtttttagtactagaggccaataactctggctgattggtcttccagtgataccaaggtcattaaattgaaacactggagatacggtgataatgat
ataccaaatttttttccatttctaaagttgatagatcgggaccatttaagctcaaaaaactatcaacctgctcccataagctttcatattttccagtac
agataatcaaaagtttattcaggacaatccatgtttccaaggtaatcaccgaaaacgggtatcttggctcagtcgaagaccttagacaacggtag
cattctagtaaatcagactgtccgtaactaagcagcttgtttatcagttctcttgccaaccgaatcaaatttctagatgaagaacttataatctcgta
ttgtgcgcagtatataaattgaaacattaaaaaaagaatgttcaacttgaagataaattgattttctctcatagaaatagtcgacatacatcgatc
aaagaacttggtaaacatttccaaaaaagaggaactttggctcgtacagtttttctgatctgataactgcaacctgagcaaataactagagcaga
aagcaaagagtatatacacatcattagcacagttttcattgaattccggaaatagatcgactattaactcaaaagcttcttctaatttatgctttgtc
ttggatatttcgtcggaaatgttgaaatgacgagaaaaagcataaatactggtagtataatttgtttccctatttacaatattttctaaatacccatt
ccgaagaaagcttccttccggtaaattaataccagatttaaagggaaccacattgaaggtaacagaaaacgagccaaccttaaaaaaatgctct
atgttagacagactatcagaatcctttgaagagaaaattaattgaccgtaatcattacgaaggattctgtttgccttttgttttctcggtaatttattg
aaaatggagggcacagttttcaagtcattttggctaggactaagcaagcgattagagtccaaattactgtagtccttaaaagcactatcatcctctt
ctaaggaattttcaggtttatcaacgaaaagcttatccagtgaactttgagagaaagtatacgaatgctgcttcctctgtctctgagtcttaggata
agaaaacaaagatagaaacttttctctttttttgtggattttccctttgctttcttaatgatgaatcgaatttcgaaaacttttcagtcggcaacttaac
attgtcggttttagtgcttaagcttcttgatggatcaatattttgtcgaaaaccttttttgtacatagtttttggcattttcgtaaaagtatccttaacca
tcaaatgttcaccccgatgccatttatccatcacttgttcaatttcattttggtcagacaacgaatcaaataaaaaatacttgctgctatttctttggg
aaagtcttgattctttctttggtatttttgatgctgctcgagaaaatatacgtagaaactttggcgccggtttccccgtcgcgttatgagtatgtgttgc
taataagcccaaatcaacataattacgtttatttttttcttttccttttcctagaaaagtttcgaggaacctgagaagcaaaagtattggacttttcca
acacttccttttgattttgagggaagctagctgaacgaaaggttggaatgctggaagtatttgcaaaccaagaacgatcagtgtcatcttgatattc
cgatttgctttgcttatgaagagagtttgtgaaagcttccttatcaaaatgtgaaacttcgggagacaaagaaggaggactatgttcatagtagtct
tcatgatcattagaatttccaatagagaagcttttttttcctgttctgattcaatttccgtctaacgattcccttttttttttacatcaaccgaatctatattt
cttttatgaacatcattacttctcaagtttgcattttctttatctaacttcctttttccctgtaatgtgagaaaagacggaggcagaacgcctttcaatc
ttttcttgaagacattaagcgatggtgacaatttagtactagaattcatagaagagtaagaggacgatgattctccacctgaatcgtcatcttgatc
agacacatgcgattcaattacatctgaaatatgagatgcttcgctggcgtccacgatactgtcattagactttgaggtctggggacgatggacgttt
gaaggctcatcatcactgtcggacaaaactataccatgccgcttccttaatctgttaggactttttttccgtttttcttgtatcgtttaccggggtttcttt
atccttcatgtttctcctcactatgacattgactggtctgtgggctactgatcgttgttctgttgtatgctcgacgccatgaggaacagaaatcggag
ctaaacccattttcttaaactctaatttatgccttaaaaaatcatacgtgtaaggcattgtttgttcaggacgtcggggacggaattcacgtctcgaa

gtagaagcttttggtgaaccaacttcctcattgctctgtatagcttgtacatcattttgattttcagtagccccaacatttttataagagttatttcgcg
taagactatcatatatgggatttggtaaggaagcaacagaactgttggaagataatgaaaaactgttcagggccttcacgttattttcagaagccc
caaattcatgattgtttttggaaatgctagttttcgtctttacgacagaaaccgaagacgagattgtacttgaccattcacttcctgaaccttgagaa
tctgatacatggtcaagaagtaagaaatcattcatactagtaacccatctggaaaaagtctactcatatggttataatacaaaagggagaataaa
ttgctggaaattatccagagtgggatataggtggaatcttcagtattgctagaaacattgacggtgaaaaatcaaaatagaggtaacaaaacaa
gtaacgcttatggactaaacaattatgagtaaaatgcattcatagactgtattaatgcataataaatcaccttaatctgaaaataaatttcctcaaa
acgaattcctttttgtttagatgatactgtaagtggttacgaatcgagattatgacacagggtcaccaattgtttggtttaagatgagagagcgaag
ccaattcagtaagattacctcagattcctctattggtttcacgagaaaaatgatagatatcatctgtaaaagcgatactcgttgagggttgttcaac
ttgggcaaataggtagcagcattgctaatttttttcaattttgggatcactacaaacgttcgaaaggcccataaacaaagaactttcacattaattt
gttctgatttttttcaaccagtagcttttcattggctcttcaaataactgtgcaatttctattctttcgaaagtgattggtaaaaaggtagcctctatata
cgggagagattccgagtaaaacattttccactcctctatgagttcaataattccgtgatattccttcttcatcccattgatcaaagaaatatccaatg
ttaaacttacacgggctgtgtcgacaaggtcatcaaagtcacgaatagcttcacaagcactcacacaccgtagtaaaagtagatttagctcctca
acaacgattccttttctccagtcattcacgtcatttccgtttgttcgatgttgaggaaacagtggcttcattgcgaggtttaattctttccaggctaaa
agtacagtgggaggatctttttcacttcttttgcatttgatataatgtttatattgttgtatgagagaactggctttacttcctcttggagcaatggagc
tgacagatcccttcgaaaggataaattcattcgaaatggtccttcttctctttgttgtaactttcgacttctctgggttcgaagtgattattttatttcgc
tttcgaagaaatacgtttatggagagaccttctgagatttcaacaacaatgggacctatacgtatacttcgagtctttgccatcttcttcattcttcaa
ttttttatgttaaaattgaggaaatttaacattaacaaacgcaaaaaaaattggcaaaattcaaggattaaaaccagcagtacacatttgtttttgct
gaactgagttgtgttttatgtgttttctacgtaatcccacatccccacaaaataagcttaaactacttcggctagcagtgataattactagaaagctt
attatataataacacctttcgacgaattcagtaaaattaaagacatataattacgattcaggtactaccattgctctgaagcaatatccaattgaca
tgagcaagatcttcatgtaatccgccttcttctcttcaaaatttcctaaatcctattttgcccttcgtttaaatggcaggatatttgtgtgcagcgttaa
cgacggttagaagctctttaaattatatactgagcgtgatcaacttatcaccaaatcagctagatgcaagctttgggtctgagttaatttatttttga
acaaattcatcctatacataagtaaagcctttatagaaaatatgcctgatttaacgctcacatataccaacgaacttgattctcaacactcttgcttt
aaggaagtaccggtagaggaagacaagcaaaacttttatggctccttaaaacaccaagtaagagcattacaagagtactcgaatgtgtatttta
cagagaagatgaagtttgagacgaaaaacgatgttcctgatgagcaacccatcatgctataaacattgggggtgttactactcgttgatagtgatt
gaaagtggactggctaggctgcctgtgtttatttgtgagtttacatgaggaaatctgaaacttcaatgataatgaccaggtggatttttcattaagg
aaaggaaattcatacatacacacttaaataaagttctttagcttctgtaaacgtttaacagcttcttctagtgtttcataagttttgcaaaaagaaa
aacgaagataatcttcggccaaaggggcatcttcatctgtataaaattcggtagggggggatggttgcgacaccaatttctttcaacatccaaaagc
ataaacgaaaatcacgtgggcgagacaagatatgttctgggaaaggataatcttcaggtagttttaccttggaaaagttagccatcacatagtaa
ctaccatcagggatcgtataaggaatgccgagttgatcaaaggcctccgccaaaatggaaaacctctgtttatacgttgatatatattcttcccagt
agcccaccttgtcagcctcctcaaaggcaatggctgtggcctcttgtaatggactgtttacgcaaaaacagatccgtgtatgggcagtagcagaat
acttaatgatgctctcgtctccaatgatccagccaacccgccagccggtgcatccaaacgtttttccgccggagccaaccgtgacgacatgacgaa
acagctctgggcgaagagtagcaagacgaacaaaaggagtgtaagaaagacgatcatagacttcgtcagagactacaagcaaattatgttcta
gtacaatgtcggcaatttcgttcagctcttcttccgaaaagatctttccaaggggattatgaggagtattaataacgatcatttttgtcttgtcagtg
attgcagctcgcagtcgatttatatctagcctccattctttagcagacacagggcgctccgctccttccttgggggggattaaaggcacataaacag
gaacaccgccattcatctggatgttggatacgtattgatcaaagaaaggttccatcaaaatcacttcgtcaccaggattcaaaaatgctgcaaac
acactgtaaaatccttcgttcgcaccagctgtgactacaatttccgtgtccgaattcagcttccgattgaatagaggagagtaagctttcgacagg
gcgttccgaaggcttatacgaccacgcgtgtgggaatattgattgcaagacacctcgtctatacatttcttcgcagcgtttaagacaaatttgggtg
gactatagtcgaagaatccctgacttagcgaaactgggggcactttacattctgctgttgccttgtttaccatagtccaaacatctggacgactccc
agcaacccgaaaggaagggactatggtatttgctgtcattgcaaggattcttgtaggtcgaaacaacttggaataatttttttttttaaaaaaaaag
gagctttttttatatcaatattgctaacaaagctgttaacgtacggtttacggttgaaaaaatcatggcgaaaccaagttttatagaaatttcgtaat
cagataagctaacgaaatattggatgaaaattacgaatgcagccaagctggagataaaacaaaaaatttcatagggaggaaagaaaaaaagg
aaagaattaagaaagtattaacaaaaaagaaaagtaaaccaaaatcataaatatgatttttaaaaatggacgaaagcagagacccaatttctg
cttttcttattcttatcctttttactcctctaaaacgaattctatgactttggttctgtagggaatgaatgatttcatagcaataaaaaatatgtagatt
gtagatgcaaaaatcatgaatgttatcctcattaataaccttataaaagattaggtaaaaacatgtacaagactatcattgggttcacagtttcatt
tttggtaaaatgggtacaacgaatctggtatgatggccctactaccctcttggttgttgatccaacgaatgcactggaacggttggtgcatggatac
tctggatttttttgatggtcttcttgggtgtgcctgaatgatcgctggttaccatcggtgtacgaccacgtcgaggcttgtcggtattagattgcctcttt
aaaagtcgattaatagtttccatttttttcttcctctaacttttttttcgctttgattctttcttcttcttgcattttctactcttcgtaaagccatttcttcctca
gtcagctttttccgacctgatgtttccggcggaagctccaacatgtcatcttcttcgccctcttctaatacgccttggagagctcgttgtctttttgtca

ttctggaagaatccgttgctccagaaggcatgggctgggagtataaatccttcaagtcagtgaagttctcttcctcatcgcctaattgctcgtcttcg
tcaagctcatcctcctcttgttcttcttcatcttgatctgactctatataactaacttttcgtttcgaagcgtttctctctcttctgtctcttttgcttctgaa
attgacgggtggactaacgttgctttctgtgtctttttcttcttcctcttcttccgcttcctcttcttcttcgtcttcatcttcaaagtcgtcatcgcctcctt
cttcctcctcgtcgtttccttctaaagtttcctccagttcctgctcgttcaaaacagagtcaccctcctctgctgcaagagcatcatcacccaattcctc
ttttgtagagacgctcaatgtatctgaatctggatagtttttattaaaaggagtttttctggaaggcttctctcccctagatcttcgacttcttgcaagc
tcccttgacatttttgtttttttttttcgtttcctttttttagaaataaatacgagaataaatattatgctccttcaatttttttaagaggtggcgaaggtcg
aaccatacacttgtagtacagttagaaagttaacaacccgtgcttagttggcttttgattccgcaatttatttacaaactattcagagtgaaagcaa
gaagaaaattcaagcacaacaaaaaaaaaactcaaattcagtatgacaaacgattaggtttggtggttgaaagatgtaagtgcacctaacttagt
ttggtttaaccaacaagaatacttctatctagcttataataataacgaaccgtttctaatgaagttggaaatcgatatacgttgtgatgaaactttct
tgtattccttgagatatctgatttttttctgcgtggttttaaaacatgaaaggggttttccacaatacaaaactttattgtttacacccagtaaaatata
aaataggagaaaagaaatatattctatcctgtagtcgtaatgtacatagttttactctgtgattttgtactttagttttgtttaaagtcgttcataata
acagtctgcttccatctttcgatttgtctgtatatttgtaaagtggcttaatcatgtgtataaacagtaaaattaaaagcagaaggatacgttcacag
aaaccttcatgacaccaaaatgcagcattgagaaacagacagatactgaatcatctcaacatggataaagaagttactcacgaacttttccttgg
ccgatatgcgaaaacatagtactgacgtcttgaaaaaacatagtgaaattacagggtacagatcaaagctttcgccttatagatgacagaatgat
aaacaaaacttgaaacccagcgatgataatttatgatatgatattgagtgtcttttggtctgaattagcggacatgcaacccaacataagttaaaa
gaggggttatttcttgcgatggagaaggtatgaccgggagtcttaagttttcgtttcatcatattgatctgttgaatctgggtactttgaccttcgttct
ttgcgcttcaattctttttcacttggtacacgctttggtgcttttagaatacgacgaatactttcaggagaaacaccatacaagttccccagtacgga
gcatgttaatgaaggatcatcttgcttaagttttcggatctcattcatggattccaatggtaatttttttctttggattccaattgccctgtatttttctttt
caacgctagcttttgctttttccagtcaggaactgattttgtttcataaaacatcctttgaataattccatgacgaaagctggctctattactatggtg
tagaccacgtatgaatcctagaaacctcaactcggatagtaacatcttaagctaccttgacaaaaaaataaacgatagaaatatccaatagtgta
acgtcgaatgtgttaatattacaattcctacgaacaagacaaaatataaatcaacatataaagcaatttgggaagaggtgcaaaatcgctcttca
gttggttgtcatccttggagcactactaactaactactgctacactagatgatctaagcataaccacaaattttatattcatagattgctttacttaat
aattatttagtaaataacaaaagtaagaatttcaatattcaagtaaaaggtaactaaacccataaacccttttagtatacagagctccactactgg
acgcaaaaaaattcatggactataccataaaataaagcataattgaaataattaacaaaaacaggaaagaaagacaaaagacataagagataa
ttaaaatgtgcaaaagaaagcagatagttcagcacacatttaaattttcgttaaagcaactctgcacgcattatgaaaacagaatcgctttttcaa
aacattctggcaagccgaaacatagctgtttctgtaataaggggtgcataaaacaaccttgcctataatgcaaaaataaattaaggtaaaaaaa
aaataaaaatatacacacttttcaataaagaaatagatgcgcttcacatgattatacacgcattcttgtcggaaatcacacgtacggatgtagaa
ggaccagtaaatagatcttcctcgttttcgacccgcttttgtttaatttcgcggggaaaaaacgtcttcataaaatttaataatgcttggtcaatattg
cgggaatcggctttcaatacttcttcagcacgacataaaggacatagattaaccttttgtctctgtaaaataattaagcaatgcaaacaaaaaacg
tgcgagcaaccaaggcgcacaggcttatatgctatgttggaacaaattgcacactcaaagtcctgtagttgcgggattacagagaacaatttgga
attaagattaaagcaaatagcctttgcaacattatcggcaacgagtagagggtcggaatcaatgactttgaagaacaacggctgagcagtaagc
gaagtacgtttatcaaacttctttaatattttcgttactgccagcttgttcatactctggaaaaaagtcattttgagaagattccgattcagttctaca
aactgattgtagaacacccagtcatgatgcttcgtagaagaaggaaggcaatgagccttccgaacttgttggtcaaaccagctcaaatggcgttg
agctaactctggagatctttcacgatttccatcggcttccttactagacatgaagatatctgcatccatatacagtctaaaaatggttctccagcaat
acagtgaattgtaatgctttttagagacaggtgacgcggtcaaggaaatatcatgagtaagattttcaaaagcggaatgcaaaatttcacgttgct
gttgctgtaagtgttcgacattctgcaattctgtagtaagcttttcaaaaaactcatgatcgaaattcaacaagagttccatttgttgaacttcggaa
tcatccgtatatgtattaatttgaccaaccttaatattagagcaatgattatcattaccacctttctcattgatatgaaaagttcgggatgttggagta
tggaaagaattagcgatattagaagaagcagaaagttgtttcaccattgaggctgcaaattcatcatcctttaatttctcaaaattcacattcacaa
caattttgggaaccacaacgtgaccctctttggccaaagaataagtcatggtgatggctcgctgctggttttcatctgatgaggaaatgagacgcc
gaattgtttcagcacttaagcccagagatgaaagctcgtacacaaaacgcttaatgcacttcttaagagctttatactgaattgctgcctcgctcca
accaggaggcatctcgttttccaacgcacgctgaaagttgtgtccaaatttcattgtaaaaggtacaattaataagtttccaaaaaaaaaaggggg
ggatttgatcaaacgtgaccgacgaatgtatatatgcaaagaccaaatagactcacttgagtataatataaacagaagatgaatactgaaaatt
aatagcttctgtaataaattacaaattagagattatttcgtttccaaattgaatcatggaaagaaaaaattatgattagagatgcctttttttcccacc
gaacacaaacaatcaaggttgtactataaagtgagaaaacacaatagcaatctgcaccagagcgcacttattaataaaatctgaaacagcaat
cgatatttgctattatcaagattaaactaaagaaccgattcctcctttaataaacgtcaattagttttaagaagaaagaaagaggaattgtaccaa
tttctcggtataaaagatcaagaagacggtcttctagattacataaataattatagtttattatacaataattccagattcaagatcaatgggtttta
taaaaaaaaaaggagtcctgcataaaagttttggtaaaggacaacttgtgttgtcgttggtatgtcgctaaggaatccagcggacatacagattt
cttctgtaagtggcggttaccgttgtaaagcttacaatccgtaatataaattaccgaatatgcgttgcaaaaaattggtttctaatagtgtaatatgt

ttattatacgcaattattgctcctaagtcacacaccttatatactgttctttaaaacaaattttatcttctgtttatttacactagaaacaattacgttg
actaaacatagatttgtggttcttaacattgaataggatttcttaagaactaaaataaatcattcatagaactggcaatttatccgtagaatgttatc
agtaagctagaaaaacagccaaagtacagaaattccaaatttcattcttgctaatcagattgttcatctggctcaaaacacctgttacattatgtgt
catttacgatgtgtttctaaatgtttgagtcagatgaaggtttatttcttattgaattatactcaatgtccatattgtaaaagcgaatactgctatgta
gcaaaacaaaacaatggataaatcaaaagaacggaacttcaaatgagagaattgatcagggatagttcaaacttgaagaaacatgattgatcc
atgagtgagtttttgttaaacttagaatagccaatttagccagataatatccagacaaagttttcagttaggaagattttgctggttacgcctaata
aattgcgctaaaaacaccttcattcgtaaaactaacagacgctcttataggtatatatctagatatttaggaatcttttcagttgctataaaaataat
ttgctcgataaacgacatggattccaccgtattaccgtgacaataatcatcctcgaagcccttcatactaaagggtatattgttttataatgtcatct
gaaacaagacaaaacaaataaactagatggaaatgttttattaaagataagtctgaaatacaatggtgaataaattgaggattttgtttatttac
atcggctctttcctgacaaatccttcagttaaaaagattttccttgtcgaacaaaacaattgtttcttggtacattattgtcgtcaactaggtatgcca
gagtataatgttgagaaaattcacctcaaatccaatttagcagttttatgctcactttcaaaatatcatcgagtatcattcgcatgacattacgcag
ctatttgataaagcttgaatgtaggaaaagtgcatttagctggtgttgtaactttcaagaaaactcgattgatgtcttcgtaagcaacccatgaata
agtaattaaagataacaaacaaagatgaggtggccacgaatatttgattagttgtttagtcaacaatagacataaaagtacatattacattagaa
ttcttcctctgttaaaagtgactacctgaaaaacactttactacatcgtcgctctctttcgtaataccttcattttcattaccattgagttacaaataac
aaagacagctgagcaaccaatacggctttttcttatcccccacgcattattcatcttgcttcgctacggattctgtaaacatttgtggaacaaggcta
attaatcgacaattatatccataatactaaatcactgaaatagtcaatagtgagaactactaattaaatagaaaagaaacatatatatttcttgaa
gtttaagaaaaatgtcaaaacacatctttgtttttttgtccttcggcgaaatgtcataatatttcattgattactggaaaagaccccattggttttaaa
accttactgctttccttttctttccatttagtaaagtataaattcaagtatattatgatgaattacggattgtttagcacataaacatccaacgatattc
aatacgaaaacatcacagacgtagatgaaatctaaccaagtaagaaaaacaagaattttcaaatagaatatcactattggattcaaatatcaaa
tcaacgtttcgtatcatattcgacaacttcatagctttacctatagttccttaactctatcttatagagaaaaagcttacggtcatttagggctcaaa
agtacctgaaccaatactcacccattgtctatgttttgagtgaattttaattgtttttggttgaaaagggatattttcttttcttttgtctacacgacaa
cgatttcatacaagttaaattgagactttactcattttatctttttgggatacaaggattgaacgaaaagacaagtatcaggagttttaagttttttg
aacatcaatttgatacttttcatgaaattgctttctgttttcttgataattgcttcaacgacaaaacgtaaagcagagtgaaaacgactttagtcgac
ttgcatttggttgttttcctttacgattttcatttccgaaaagatggttaaatcctttgaggatatggattgctatgaaatccttcaggtggatcgcagt
tctactttacaggagattaaagcaagttataggtatgaaagaaagggaataggagcggaaaaatatttcttcaacatactcttattatccaatgtt
cagaattagtactaggaattggttgtggttgagattttggtatatttactgacccctttcgtcttgtatagaaaactagctttacagtatcaccctgat
agacatcctaacaaagaaggatatgatgagatatttgcaggaattagcagtgcctacaacattttgtcaaacgaggataaacgaaaatggcatg
agaaggattatactagggaccaatactcagtgtttactgatgaaattcaagaaatgcaaaccaagttagagaaagtcaactttggagtatgggta
aattttctaaatcgaatacgaatagatgaacaaactgcacgttctacagttgaatggccaagccttggtgatattacctggttatggacacacaca
aagccattttatcaaaaatggctacgattttctacatcaaaatcttttgagtgggaggcaatttacaatgaagaagaagaaacagaccctggtac
gaaacgattaatgaaaaacgaaaaccaaaaatgtattaaatactctattcgacagtatgatgaagcagttcgtgcactgattgcaacaatgtgta
aactagatcctcggcgtcaaaacgtgaagaaaatctcagaccctgaacgttatgaatctttgcaagctgcatctcgcaaacaaagcgagcatgat
cgccgtaattaccaggattctttaaaaaaacaatcccttgcttcatggacagttgtagatcaagaattgaattggagcgaggatgaggatgagga
tgaaagttcgtctactgatatgacggaagcagtagacatcaccaattgttccatatgcaacaagtatttccgctcattacagcagctgaaaaatca
cgaaaacagtaaaaagcacaaacaaaactttcgaaaagcaagaaaaaagcaagagaaggatgccaaaattgcgcccagacctttcgaagctt
caggagaagcttcggattcttctaagaaatctgaacctttatatgaattctcttctaaaagcgttgaagatattgaaagaagttttacatttatcga
gatgtctgacgatgacttctttactgcccatgaatttagcgatgaagaggcattctagaaagtttcttagccgactttattttccagtctttatgttat
gctttggttatttattcgtaaattttatgaccagctcgaaggccctcaatcagcatttgcagttttctctgatcaaaaatctctttctgcctcagtgtat
ccatttcttctccaaattcagtcgtcagttgctccatatactcagctaaagagggcttttctatatgtttttgagtgtctagtttaaatccagaacttgt
atttgattcaaccccgttcactactgtattcgcaagtttgccttcttctgctgacattttaatccctgttttatgccctggaatgtgttacactccttaaa
ctgtcagataataagatgaaaatttcaaatgtgtggtcggtttacctttgaggtgtgtgttggaattttgtcgtttttggtcctcgctaaacccagctc
tacggtttttttgaatttttgggaagctttcgagcctttgtatcaatacctggcagtaattaaaaattaaaaaattatacagacgaaatgtcaggtt
ctgtgaattttcctaatgctgcaaacgcattagctggatctacgactaattctgacaaagagccggttctggagcgaagagaagaagcagagccc
ccaaagatgagcaaatctgcattaaaaaaacttcgtcgtcaacaagaatgggaagcaggacgcgaaaagcgcgccggaatacgccgagagaa
aaagaaacagaaaaaggaagagagaaagcgcaaaattgatgctggtgagaaagttcctagccaaaagaaaaagataagagaaggaaaaat
aacaccttctgaaatgcgtgtcgttctggattgtggatttgacgaattaatgaatgaaaaggaaatcgcttctttgtgtcaacaattcaccagatgt
cactctgcaaatagaactgctttgcatcctgtggaattgtatgctactgactttggtggtcgtttggaagctagacaagaatacgttttacgcggtca
acaaaagaactggaaacgctatcatcctacaactaacagttatttggaagaatttgttaacgagaaggaaaaaatggtgtacctttcagcagact

cggatgagactttgcacgaattgaaggaagacgaaatttacattattggagccctcgttgataaaaatagatacaagaatatctgcgatgaaaa
agctaaaagtcaagggattcgaacggcaaaacttccaattggcgaatatattcgcatgtctgatcgaaaagtcttgactgtaaatcatgtttttga
gattttgtctttatggctagagcatcgcgattgggaaaaggctttcttggaggtcattcctaagcggaaaggtcttgcttctttggacgaagaggaa
agcagagagcaaagcctcgaacaagaaagcgtttccgaaaaggctgaagaagctgaagacgaagcaaaaactgcggatggagttgagatgc
ctgccgagaaaaggataaaaattacggaataatccttcactatgtgtaatttagaaattctgtatttttgttctgttattttggaaagttttagataat
tataggttctaaaggtaatgctaatttgtaataaaagtactgttgataaaaataatttcagtctaagctaattaacgtatttgaaggacccggtaaa
ataaaaattaactgatacatgcattagaactctaaaggatcggcctttcgtttgttatgcgttgtggatttggagagattgttactaaaagatctaa
aatacgctgtaaaaggatcaagatctgtggagatagaggctgtcgatgaaggattagatgtttcaccagcagatggagaagagggagcattctg
taaagagtttgaatgagtaacggattgttgatcaagttgactcaacagagtatggacagtaggaacacaaagaggattaaagctgctcatatctt
gaatatcaatgggaacacaaacgcgtccagttcctggatgaacgcaaaagggggattttagcagatggtttaagtgcttagagacttcaacatct
aatcgtggatatagatatgtcagaacgatatcttgtttcgcgtgagcaataacatttggggagagatcaaagtttccagatgcaattgcagcatca
atgtctgaccatttctttatactagcgcgtccaggatccatctcccatttttccgtagtgcagatgctaatgattcactaggtaatagtttcaaaaga
ttttctgcacctacttcagaaacccatggatcttgatcttccaaaacaactttagcaaaaacagatttgagaatgtcgagcgaacgagacaagtga
ggatgtagagggcgctttaaattgttaaggattcgtacaccgccttgaggatttcctgccacaacttgtagatagccagcaatcatttttcgggaac
gatcgtccattgaacgagccatttcgtcacaaatccaagcgtgaataccacgtcgaccagagtaaacccagagaatatgattgaacccaaaatct
tcacgtaaagcaacatctataatttctacagcaactgtaataaaagaccagcacttttcacaaatgtttgttttggagcaacatgtacgaacatcgt
catagtcggtcatatcgatatcgaaaactagttccttttgtaatgggcgaaacgaagctttccttaaggtttttctatctcttggatttacactgtaaa
ctggtccaatttcaaatctgcttggacacataccgatggcttctttctttaattcttcccaagattgaaatgacaggtatcgcacgtatgcatcattgg
gtaaagtaaatgcaaactctctatccgtaaagtctttttggggaacaggaccatgattcagccattgaaatagtaaccgccagggaaacaaatgg
cgatagtactgaatcatggtctcaggatcaacattttcttgcttcctaaagcatagttggttaacttgttgtaatcgaaacaaatatcaatataaaa
gaagcatgctgattgtatacataaaagctttaggtaaagacgcgtagagcttcttttgttgaccgaacacctttatacttacaccgcatccaaggat
ccattagcaattaattcatccaaatccttgtcttcaagatcctctacagcaaccgtcattctgggtatcttagtcaaagtaaataacccaatatatca
agaagaagaagttgtttttcatgaattgtaagaaaataacgttggttgatagaatgcctaatagtaaataggagctttgggttatatattcctatgt
gaattttaacgaatgtttatgcagtccctcattaattggaattgaaaaacaattcattgtcaacaaaaaggttgaaagaactacgtctgtcttttga
ggagtggaatgaggaatggtagttgaaattaatcagcattattgaaaacaaaaaatattatttactttggatcaatataatctctgatcttttaccg
ggatttcaaagaaaacggttttagacgatagtttactaaaaaaatcgaaaacctaaggaaaaaattatgtctaatgagccatggaatactcagct
ccaaaaactcaaaatccggctgaaaacttgggaacatgagttttcgcataatcagaatcggaagccgacgaaacaagatgtgaaatcaaatcc
ctctatttgtaagaatattaaactacccatttgagggaaagaaacgtacatgttcagctaacaagtagctctactctacaaggaatactataattta
aaacaaaaggttgccggagaacctgacgaaaaatctagtccaaggaagaaaaatgagaacgtggaacagccagaatttcgaacaccaacaa
aagtccgaaaagttgaaaaacgttcgttcgagcaaggaactccgacatcgaaaccattgcaggtgacaccaaagagcgctgacaaaagcttgc
tagggcctacaccgcagctttcgcgaagggtgattaatatctttgaagatatatccccagacccatcaccgaatgcgaaaatttcccagacagca
gatagttctatggtaagcattgctgaaacttccttacagtccatgacacctacgacaccatcaaagcttattcgtccgtctatagatttatcatcacc
ttcttctgccatctttgatacgccttccacatatcggacagaggtcccgtcttctccaaactttttgcgagtatcttcgagatgtcggaaaagtctgag
tgaaatgattaatgaattaaaagaaatcgaagacgattacggaaacgatgaagagagaatcctagcggaggttgaggaaaatgagagttcatc
atcctcagaaccagaagaaaataatgcacagagctcgctgaagccattcaagcgacgaattaagagacaaactcgactagtcaaacgtatgta
tttatggatttctcattttatttccattaacatctttagtgcctccttcagtcagtaccgaaaagagtcatctggatagcatgccagagattgacgag
gaggaagaagccacagtgaatgcaccagtagaagaagaagaagaaatccctacgcagtcggctagaggatctgcaaataaggaaatgaactc
tgacgagtcgaaacggcgtctaaaaaatggacttttgcttggtaagcaagtgagtgaaaacttttcttcatacaaactcaatcgaggaaaaggga
aaggtttccgttctcgtaggtgagagtaaggcacttttgaagggtctatggttatgtgagattcctctcaggcttcgaaacggacgtaatttttaatt
gattgtatactaaggatcataatgcttgaagacgctctttcttagaagctcaggaccaaaataaaattatgcttttacaacattacgatgtttcaaa
tccaaatagatttgttacgaggctaaacatcatgtacattttaagagttataacattgtttaacgacaatacaatacatttggctataccaaactat
cagttagctcttgtagctcagtggtagagcgcttgcctagtaagcaagaggcccagtgttcgagtcactgctggagcatctaatactttttgatttt
tcttccttagtgcaattttcaccaacgtggtaaatatgataaaatcttgtatcagcgatttaatagagcgatggaaagaccccgaatgttataagct
gctgcggttatgccaagaaagacggccaacctggtatcaagtatacgtttttgaatgagaacactatcacagtctctaattcttttcaatgatcaag
ttaataaattacaattcccttaaataaacggaaggctactgatcctagcttaccgcactgcaaagcgacatcgaagaaggatctataaggagaat
agcatagcattggacggcgggttcaagaaacgtctagtagaattgtacctcctaaacataaccaataccatgtctggtatacccaaacctggaag
tggctctccggccgcatctgctggtctatggcagtccgtttcaacatttgtacggagacatagaggtgcagcatatgcgatcggagctgttgcagtt
gttgcaacagccggtggaatttaccatgtgaagcaacagaaggctttaaaaaggggaatgaagcctcgtaaaattgagaaagaaacatctgaa

aagcaaaaagtcgaggaggaacctgaatctggtaaaactgagggtgacgagcaagaagatgaaaaagctggagactctggcaaaggttctga
tgcagctgctggctctgcagccgcttctgctgccgccgctaagaagaagaagaaaaacaagaagaagcttaaaaacaaaaagcccggcgattc
aactgccgagaaaattgctaccgaagaatcggtgaaatctatgtcaaaggaggaacgtgtaaaccttgctactgaactcaagactcttggaaat
aaggcttatggacaaaaagaatatggaaatgctattgacttctacactcaagccatcacttgttcacatgatcccattttcttttccaaccgtgccg
cctgcttcgctgctatcggagattttgttcaagtcgttaaagataccactgaagctcttaaactcgatcccacatatgtcaaggctttgaaccgccgt
tgttctgcttatgagcaactcgagaaacttgacgacgctttgatggacagtacagtctcctgtgtttttgatggcttcgcaaacgaaagcatgactg
ctaccgtcgaacgtttattgaagaaggttgctgagaagaaggccgctgctcttatggctgaacgtcctcctaagcttcctgctgctagcttcattca
aacttatctagactctttccgtcctcaacccaagcccgatctaaacgaagaatatgaaggcgaccgtattcttagctctgcttacgatgctcttgac
aagggtaactatcagcttgcttacgaccaagccaagaaggctgccaccgtttcctgctccagtgcagctatcagcgctcgtgcttataacttaata
ggtactttcaaatttgtttctggtgattctagaggatctatggaagaattcaatgaagctatcagtcttgattcttccttcattcaaccttacgttcgtc
tttcagctgcctatcttgacgaaaacgaccatgacagcatgtggggtatcttgaagaaggctgagtctgttaacgacaaagactcagatctttact
atcatcgtgctcaagtacattttgtttccggcgaattcgctgaagctattgctgattacaacaaatcgatttctctggacgactcgtttatctacagct
acatacagcttggtgttgctcaatacaagatccaagcccttaatcagtcaatgaagacatttgaggaatgcaagaagaaatttcccaactgcagc
gaagtatacaactattttggtgagattcttttggatcaacaaaagttcgccgaagcagttgaaaattttgatcgtgctatctccattgagaagagag
atcgcttgacgattatgagtgctatgcctttaatcaacaaagctctcgctgtgttccagtggaagaaggatatcaagcaagctgaaagtttgtgtcg
tcaagctttgtctgctgatcctgactgtgacattgcaatcgcaagtatggctcaattcttgctgcaacaaggcaaggctcaagaggccctctcttac
tttgaacaatcggctcaacttgctcgtactgaagctgaaatggtgaatgccttttcttatgctgaagcaactcgcacacaaattacattgacagaa
aaatatcctcaattggtaggccgtttaagtcaatctatgtaaattgagaaaaatccagcacctatgtttgattcagatgcgaatgaatgtataaatt
ataagaaaaataatggtacattaattttgcaatccttattacgggtttttctatgttattatgtgacatggcttttgcttgctattacttaaaaaagtat
gaagattatgttttgaatattgagcgactaataaaactccaagtaatgttctttgcgtcaaaatttaaggagttttgtgtgtttgtaatttgtttttcca
gttttgggtgatctttaatgaagccaatgtttgaactgacctgggtttttttaggtagggtagaagatgtaattagaacatgtagcaaagtaagaga
aatataacgttacaacgtaattctaccatccgcaaaaaaatttgattactcccaaataagaatttttccaatgtttttcataaacctagcctctgtat
gaaatgaaaacccagctttgtaagttttatttatttgatcctttcttgcttaaaagcgatactcgtataattattacaaatcgcttcgtctcgttaaag
catcaagggcatcggagtgagagataacgaagcaaaaccatacaacagttagtgaatataagcgtaaaaactcttctatttaccaatttaggaa
tagaaatttgcatttctatttgtagattcgtcagtaatttagtggcttttgactcttctttttttaagcgctcaattgcgagcgagttcgtttgggaggta
attggagaacgaaatagtgaaatatacatagatctttttcgaggatggaactaccctctaacaccgattactctggatctcgcacgattacgacga
acgagttgccttacccagtccctgaggaaggacagtttgaagaacatgtattacgattagaacccgagtctcaaggcagacgtgtaaggtgggc
aaataatactgtcgacaatgagaatttgaataagaaaaaatctaaaggtatgcttaaggatgtgcaggtactgattattgggaacggaatcatca
aggatgatttgaagtctttttctatgcacattagggtatattgtacctagtcccacgtttattaatatatacagtgagtcggctaacctatatagtatg
ttgcatttaccataagactagaaaatttgatgaaagttcatcggaatccgacagcgacacggattcttcaagctgtagccgcaatgcttatgaacg
tgcgtgaggaattaggttgtaaggaatatagagttcagaaaaatcataaattctcttaaaagctaaatatttttcatgttccttattgcaagacagc
tgtaagtgagccatgtccaatattgtttcgcaagcgagaatcacagatttttttttaaagatccctcgtaattggcataactcttttagctgttacct
cgtaccaaatctgtcaacctcaattacttgcccaaatggatgacattctatatgctttcctgaacacttgatttaagtatttttgaatattaaagaaa
gaaatttatcttaatcataaaaataaaatgaaaacgggaataatataaaaaatcatcaagtaaaaatgacacacagattagaaaaattacaca
ctagaatgagaaagggaacataaaccaaagggaacaaggagaatcaatgaatatagacagacaaataagacattaccttaaaaaatataaat
aggatacaggaaaaccaattacacgcaaaaatgaggcttatcactcaagagaaccatacaaagcgtagaatcaatcaaagcttatgagtactt
gcattcaccgacgatttcgttttgtccatgtgccattcaagtcgaaaactcttcgtttgtgaggagcttcatcatcgtctgaatccaaaacgggccga
cctgaacgagctgtgcgcaatgcagagaaattaagatttgaatgactctgaggaacttgctgtacatcgtccatatggatagggtctccattcccg
cctgatgagtgttgaaacgagtctgccacgaactcagggtcataaatcgttagtttttctttgcaatattgaaggacatagtcactagaaacaccgt
tacattcaatcagttctgcgatttctttcaaagcctgtgcataaccagctttttgggaacgaagtacctctgcagtggaagcttggtacaactgtgta
acgcttaatgctgtttgtttgaaatgagataataaattttcttgcccagacattcctattaaaaggtgcgacttccagttcgcataccctgaatatcc
ggtaatttaattgatttgaagaaggcaggttttgtgaaagggttactgattccccacgcaaattgcgaagtagaacgcactgtacaaaaaccgaa
cgttttcaaagtcttgttgcttatattgtaaacaaagcatctataaaaatgaaaatgaagaatactaaacagattatgaatgacattgtcacaaaa
tttaacagacgtcaatgagcacttatagttgtagaaaaaaccacattgggaggaatttctcgatgaacgaacaattagctcctttgctgcaataga
aaacaaatgttttgatgtaccaaattcaactcgtaattggatttttcagtcttttacgaaaccaattgaaggattgatttaaactttggtaaattttta
cttctgatattgtagaagaaacaaaaataaaaacacccttccatatgaatactggatcaccgaaacgaaattagggtaattgtacattattgaga
caatgtgtataacaaggcatttcatcttgttcaactgagttcattcatacatgtccaacttaatatgtattttactgcttgactgttccctttgagatca
tattactggtagagaatttgaagaaaaaccggtgtctacactaggaaatcttatgcagaagcgattctaaagattttaatttaaactttaatagtat

tttttggcctgctttaatccacaaaggattactacaatatgaccgatctcatcaactacagaaaaaaacttgttttcgggtggacacctaatggtaa
agcattatcgccgaacacatacatatagaaattcagcttacgataataacaactacgacttgatatggaaactctattacaagaaaatttggtgta
gcataccagccctaccctaaattccccgaaaacacagtcacatcacatatagaacatttttgtactaaatttacttttccttcaagacatcacgacc
agtcaacatgccagaacaacttgtgctccgtgcaactctcgaaggtcactctggatgggttacttccctctccactgcccctgagaaccctgatatt
cttctttccggttctcgtgacaagtccatcatcttggtaagtattactacggcggctgtgttcatattgtgacggtgaaacagaaaacaaactcata
aatttttcgaacgattagcagtttcgccagctttcttggtaaaacgacctttatactaatattacccgttttagtggaacttggtccgtgatgatatga
actatggtatcgcccagagacgtttgaccggccacagtcacttcgtttccgactgtgctctttccttcgacagtcactatgccttgtctgcctcctggg
ataagaccatccgtttgtgggatcttgaaaagggtgaatgcacccaccaattcgttggtcacaacagtgatgtcttgtccgtttccatctcccctga
caaccgccaggttgtttctggttctcgtgacaagaccatcaagatctggaacatcattggtaactgcaagtacaccatcaccgacggtggtcattct
gactgggtttcttgcgttcgcttctctcctaaccctgataacctcaccttcgtctctgctggttgggacaaggctgttaaggtaagctcactatttttat
cgcatttactattaagagtcttaaaaacgtcttttgggtagatgatgctttagactatatttgctacttcggaggtccttcacgggctcgatgagaca
aaacaatcaccatagctactgatcccaattgacaagttttattgcttcaacggttttgcgattttattaattctttttcattcgcttttctaactcatata
taggtttgggacttggagtctttctctctccgtacttctcactacggccataccggctatgtttctgccgtcactatctcccctgatggctctctttgcgc
ctccggtggtagagacggtactttgatgctctgggatcttaacgagtccactcacctctactctttggaagctaaggctaacatcaatgctcttgtct
tctctcccaaccgctactggctttgcgctgctactggctcttctatccgtatcttcgatttggaaactcaagagaaggttgatgaacttactgtcgact
tcgttggcgtcggtaagaagagctctgagcccgagtgtgtttctcttacctggtcccctgatggccaaactttgttctctggctggaccgacaatgtt
attcgtgtttggcaagttaccaagtaaaaataagatcctaattgttgtcccatttggcgaaaataaataatgttagtggttataggaaggatcattt
taagtatgaatcgatttagattccaccatttatatatttagcggtttgtttttggttttaaaaaagtattgtctgtgtttcatgtaaatttctttggtgctt
gcaagtagatgcggaacaagtatggggaaatataaataatgactgatctcaagattgttcagttaaggacaaggtgtatttcgtatttttgtatttc
gtttgtttttattcaataaaagagttgcttcggccgggaatcgaacccgggtcgcttcgatggcaacgaagcattataccactaaaccaccaaagc
ataattaatttaaataagactctaataagttgcatacgtacgctgccaaagcgaatgaattcttgtgcagtctcaagagttgctagcttttactgaa
aacactcgaacagttactgatcagaaaaaaggagtaacaaaaagaggagcctacaacaccccggattcccatgttgtctctaaccatagtacta
acgaggccctcagacgcttaattgcagtgatcggacggaaactggtgttttcgcctaggtatggccgtagacgttcagcttaaaaagcttttatatt
cttttatatgatatgagtgctatattgttggcttttagtttcatttatttatcaatcaattttattcaagccgcacaaatccacttgcgacgcggtattg
atgatagccctgtatggcaaactatttatgactgcctgtctaggcactttgacggtaaagcaattgtggacaggattttgatcaatcgtgacaaag
agtacaccgtgtgggcggacgttggcaataatgttgaacatacatttgacgaaagtattgcagatgcaataacaaagtgtgtcagtctacaacac
ccaaacattactgtcaacttgaggtcccacaatcagtttgctgtgagaggttcaaggtatactgaacacgtcgtagatgatttctatgatttcttcgg
gcatgggtatcctgcggactacactcctggtaattccagtgatgaggatgatgatgaagtacttcatgatgactatgtcggcttggagagaagagg
gatcttcctgatttaagaggtagacctcgtcagtatcgcaattctctcacagtggcttggtatggtcatggcggggcaaccaatctcatcagcagtt
cagttgaaactaaaaccacattacaatgtgggttatgctatagcatacctggcaagatcgcggacagctttgtattttgaacttaaaaatcatactc
atgttacaattcctcattgacctttagagtcaaaacccaggttcaactcggccctgcttaactttgatgctctttgaattactcacaggaggatcatc
ttttttagtcgataaaaaaaaaaatggaaaagatctttttcaattgatcccgcagcccctacattgaaacaatgcgaaaaggaaccttgttacttg
tcattgttagactagtcttgaccgaaaagttgcgtaagcgatgggaaatcttttagtgaattttggagtcaacattatttattgtcttcatccttgtaa
ttgaaggtttttgactttctctgaaaaattgggatttttctgtgctaatttcctttcctttttttttttttctgcgatttgtgagcatttgtagaatttttct
tcattacggatcaatagctttttcactctgttttgcaaagtcaattatattagaaatggccgaagagaagctgaaattggattcaagcgattttact
gcgcacaatcagtcgaatgtttacaatgttgatttagagaagggtctttgtttggatacacaacaggacatcaacaaatcgtcctattcaacttcct
tgaaatgcaacaatagaaagaaaacgatcatgaatgaatcacaaaacttcatcgccttgataaagaaatatcatgaggaaattttaaaacaact
ttcccttcgctttcctgataatgttgtcattaaagatcgaaactgccatctaacagtaatattattttattcattgatgtgtacattagcaatttcaaac
agtttcgaacttctttcttggctggaatacagttggacccttcttggacctttgcttatgatatgggcaaccttgtatgctctattttattgtaagtgaa
gcatttttgataattagaaaaaaaaaaaagttgcggttcgtttgctaacaaaaagtagtcctttcttcactcgctgtcgtagtagtaagaactgctg
cacaggttataatgtacatacttataatcggaggtgctttcattggtgctctaattataggtgttggtgctttgattggagctgctggagtttttctcat
tggtcttttgtgctggggattttataacattcttgtttttgccttggaatggaatttgagtgctcagctgaaaacactggctcgtctactcttcctaga
tatttagcaaaaggatgatgtactagctcctccgccgacaactacgaacgaagaaatagagctgcaaagtaatcctaccactcaaagttaatgct
aatttgttttctgtttttctcataattctcaactcttctgcttaaaggtagaaatttgctttcagtgctttatttatttatgaaatggtgcttctttcgtata
ggctcaacttagaagcaaaatttttaattaaatcacgaatgtcaagcccgatcgagctaatttatatattcatttcatgtttatgctctctgttcata
tatactcatcagtctatagtcagaatcttcagctagaagattcgactggtctggctaaataatatcgtcacccattaaattaacccaataattccctg
taaatactagtgtctctaccagtagcacaaccgcgtccaattcgcgcataaaacaatgtaactaaagttatttactccgcttgattatacattctttt
acgatgtgaaataactaccgcgacaactacccctgtctatgtaaagcttaccttactttaactttttaataagcgaaagtttctagctgctgccaggt

ccatggaattttactttggtttttcatgttttctttttgtcaatgagatccttatttaatgatgttcgctcaaatctttttttacattcattatgggtgaagc
tgaaatatgtcaatgtacacagcttcctcctcttaatgagttattttatatattcatattcataaaccttattatatgtactgaaaaaaagggagaag
taaaaagtctgcttctcaggtaatgcttatccattttatgcttcagtctattaggaaaaataaaaaaaaaaaaaaaaggcaagcattatagcacat
ctttttttctgttgctgggttcctttagagctttgtaaacagagaacaagtcaatcttgattaggattctctaatagtttaaaataaatcattaaccaa
acaagtcccgtttagctcaatcggcttagagcgtccggcttttaaccggaaggttgcgagttcgagtctcgcagtgggagttcctttttggcttcag
ttttgtcgcttcatgggttcgtattaatatgctgtatgaatttaaattactctagtttttatacaaaagaagatcaaatataaattgtgcagtgtaattt
gtgaacggaaaagggctttctcaaagagccctaaagtacaacaaatgttccgcagaataagtagcgaaacccatttccttgcttgaacaagtata
ttttatgccttttatgtatctcaattctattcaaaaccgagtctatgaagtggaaagccttttacttactgaatgtgattatgtaactgtaagcaagct
aaacctgtgatttgacagcatgatttgcagaagcagtaacaaaagcaatgaagtgttaaactcattaagaggtttacaaaagcttgtttcaatatc
ttgattttaattacttacattggctcttcagcgacatatagcatcgcttgaaaaacagcagccctaataacatatgttgtttatttaacaagagtatt
aaattgctaaactcaattcgaaaacaaactccagaaatttttattttgtttttctacttctatattacaaaaatccattttgtttatttacatgttggcg
cacaagttacctgatgggcgttccatgtgtcgcgaaaaggatgctggttgcattcgtcccttatccattacctctttcttggtgtatccctaaacgga
gagagtggtctactacagaattaaaataagattattaactgttctagtgaatagaaattgccaaatgagctacaacgatatagaaaaattaatct
acttcaacaatcactttctgtctgaattaccagaaccccacgctgtttttaacaattctttatgttttgaaaactttaaaacttcaaacacactaccat
ttgaaaacccatcagactgttactctctgcatactgcttgggataataatgcagaatttcatgctgaggcaggtggtatcgaaaattcgcgtgatgc
cagtggtcctctacaggatgttaaagaatatttgaaagatgatttacatgacggttcggaggcagaaaatggaagaacaagcagcaaactatta
aatcataataccatcgaagagaatcattccgaaacgtccatggacagagctcttcaatcaaacaactccatttataatattctttcaattacgccct
cgaattcaaagatcgccattgaaagtcaagtcggaaatcgtcctcattttagtgatttcccaaccgccaaagaaaatcgatgtctcttactaacag
ctcccagtgaaaaaccagtcagtgatagcaatgaatcatgcccgatcactatgaaacaaagaaatgcctgtacccgtcattatctaccatatacc
ccgattcatccaatgttcccttcattttactctcaattacctaagctttctctgttccccccttcccaaatatcctttggaagaagatgtcaaatacatca
agtcctgtcccatagacgattactatttgcccagatatacccgtggccagggtaagaaaaaagaaggcctttgcccaatttgttgtttccagaaga
aaatcgtttggcttcgcacaaaaactagcgcattctggtatcacatgaactttttacacggtattcatagtaaaggaagaccttatcatccgcccat
tgaatttcgtacaattcctgttcgaaaggtaaagagtatcgtcggactacctgacaggcgtcaaatcgttcaggggaaatgccatcaatgcaaac
gttggattcgttgccagggtacgtctgaaacaaagattgcaatctacaattatttgcacagaatattctttcacattttcttttgtttttttacctttttta
ttttattttgcttatttacatgacaaaggcacattactgacaatagcaggtcgaaaagatgtcaaagtaaaagttcctgagatattttggtggaaac
atgcacacaaatgccattcagtagacccgaataacttaacttaaaattaaaaaattttcatgaaaccaagaaaaagatgactgaaatatatgaa
cagctattgtttagtgaatatttaatttttttatatatactatgaaccttaaacccacgataaaccgaaaattatcatttcgtaatagtacacgaatat
atatgaatttttttttatctcaaaaaatctaactaacgtgctttcttcttccgatttgggcttggtaacctgtcttgatgggctgcacagaacgaacaga
tccacaagattcacaagtcataaagaaaattctgttttccttcgtaagaatagtatctggagacttgcaggtcttgcaagtaacgtattcaacaata
taacgacgaagaacgttttccatctgcttttgttggaaacgacctttaataattaaacgagaagaaccatcaacactaccactagtacccaattct
gcaaacaaaaattgaatcacatggtctaaagaacgatgcatacgcttggcgatatcactgatgtttgcaaaatttgtctttttaccttcacggtgaa
cggaaggaggcacaatagtatatttacgcttttcaccagcaagctcagggttattagtacggaggaggttgaagaatctgttaagcaattcagga
tagtaataatcgcggtcactcttcaaccaagcatgttcgcccatgttttcagagtcaaaggtctgcttgctagcatcggcagcatcgccggtggaa
gaagcttccaactccttatcgaaggcagacaagtcagaggatttcttcttcttttcttcttcttcatagacgcaaagtctaattcaccattatcattg
agaacttcctcgcctgtttcaccttcggcatcaccagaagacttctttgacttctttttctttttcatactggcaaacatgtccttcaactcgtcaacttc
gccctcggcgacaggctcagtcaatcctttggaatctttgccatcatcatcttcctgcttctcatcagaggattcgtcggcaggagaggattcctttt
ggtccggggaattcacttcatcatcaaaagcaaccgatttttttcgtttttttctttttttcaggaagagtgccgttaagaccaacctatcacatttatc
agtaagtatgcagttcaaaggataaattattccaaaaaacaaatctttatttaacaaaatccatttttttagctagtatttgacttacctggtcaatt
acgggttcttgttcagacatggctcttttcagaattcaaaggcaagtttggataatattagccatcctggtcaaaaacaaaaaggctcgaatttgg
aatgaaaaaatacataaggcatattataatattctatttgtgaattatatttatttttttctctgcgtattctgtatcttccacaacaagggttatacttc
gaccatccgttgctttttctttgttcctcggatgcccagatatataaaggacgcgtcgcttcaccaaattgtgatttcaacggaacacgtagaataa
ccagcatggattattggaatcaagacaatattgatttggctcaagtagagttagtatttaaagaattggcgtctcgccttgaacatttaggtattttt
tacagtttctttaaaacatcttttgaaccttcttttataatggtaattgtatagaaattaacattaattactagaaatggactctagcaatattctattt
cggattgctgtaggatctcatcccgacaaatactgggaatccatagcgtttatacgcgaattttacaaaaacttagagaaaaagcataaaactga
accacatgaaatacaaaaccaagtgcgactgttgagtgaaaccagtttaatatcctgccttcgttggattcttcatcgtattccaggaggtgtaatt
acatggcctacttataaacttttccaagaagcagaagcaagggctaattttccgagttctggattcaatattttcatgaaacatgcaaccaagaat
acccaccatattcacgtgctaaaatatttttttaaaactgatgatgagtctctccgcaaagatggctactagcgcacaagtccatgaaggatcatctt
tggaattgatagcccaaatcgctagtatttgggctttcgaatggcctcgcatgtcgttgcaggatacactcgaatactggaataagtaagctttgaa

tatttttttattgatacaaaaagcctcccttacgttttgctaacaacagtagatgctccaatgcttgctttcatcttcttctttgttatatcagaagcat
ggataaacatgatcaaccagggttttcctgcttgccatataccttgcaaactcaagttcagaattgcaaatatcctcctcctgctacaaattatcta
aatgagaaagcttctttagtaagcttctctcttagttcctatttttcaagggatcctttagaagtcatccagctgatttttcaaatgcctgtaccacgt
aagatctcttcgtctttacctcgtcgctttgaagacgtgcagactaattgtttgtccaccttgcagcaagttagcaagcgtacgtcctatcattccgta
ttctccattcaagaatcggcatggtcgaattttatcaccaatggatttgagcatccaattgctccaactaagtcccaagaaacagtctattcgatatt
atctggagaaatttcaccttcaggttttcctcatgtggcaccgttagaccaggtttcctctttagatcagaacatcgtcaaacaatgttcttttgaaa
gtataacacctgtcaaagcttcaaaaaccctttggtggctttgggttgaatctcgttgccccgaaattcctgaatcgaagcgaactgccttcatgaa
ttgcactatgatgctggataaaacggataaactattgattttcgaacagagtcttcctactacaaacttactaactcagcaggatgattatcatgca
aaaagaacaatcttaaacaaaattaggtcctcttttaaacgtgttatgaaaccgaaaaagtctcagaaaaaaacatctatgcttactaactctcca
agaagaagctgccagagcgtgttgtatgaaagtaatcgtgctgttttacttcatttggccgatcaaatgaaccaattatcatcaaatgatgtttaca
agaatgatgttaaaccattaaaagtcatttctgaacatgaggaagattgggaccctgaagcaacgggatacgtgaatactctcttaaattgggca
aaccaacgacagtccatatacgatgaaccgattaaactgttgtacccaaacaatattgattctcgtcaatcaaatgacggaaagtcttcgtcttcg
aattcttctaacaagtatatgtcttcatcattaactgtgactagtgataactcttcgatcactggaggttctaccttttcttcgattgagagtcaaaca
aaaaccacgcctgccggctgtaaagttcagcccactgtcttacacagaaacaacaaacctaaagggttgcctactatgcttgcaaagaaaatgtc
tatcagcaaacctcatagtgtatcagctataaaatctgaaaagactgaaaagaatatgcgaccaccattgaaagattgggattcagctgagacg
ctatcacctatgtttaccaaaaacaatggttcagccagaagcagtcctttttctctcgatcgtacactaagtacacgtgcataacttatgaaacatt
aaactgatgttgtcacaattaaggataccaaaggagtatataatttggttgactattgcaaatcttaatttatgtataccggcgaaatttaaactaa
aaaattttacattataatctaagaaatatatctgttaatcactataaattttaggaaaatagaattaatataataaacaaacttgattgaatggtttt
cttataatcaagtcgaaattatcgaattataaaatgaataaataatttttgaacaataatttgaaagtaattttacaccaacaatataaaaaaacc
aataaatcaagtcactgctgtttggtgttcgaaaccaccgcgctttctggtaggggcatcagtatgatggtacaaaacttctggagaacgttgctct
tcaccaaaacttgcaatagagtggtcagtcatactgtgcctacgagtgcttggatttcgaggaagtgggatctcagtgacgtttccaagggccattt
gatcacttaagattgtttggtttaaactagcattcgtaggaggctgttccccagttgaggttcctgctgttgtcggtcgattcttagaggagccacgc
tttgacatcttagaacgtcgtgaaccgcgtccagcggtaccgacagcactggcaccacccatagtacctaagcaatcatagatagggggaatgcca
agaagaaccagaaagagccaatttccttaggctttcggctttctgtaaatcatgggaaggaagaacgatgccgctaagtatgctattctgttcgtg
aagagcgacgcgaacttgagttggtccagaagctttagagcgttgttgcgctgctgtcaatttagaatatacagtcttaccatatacctttgttctag
aatcctgctcttctttaggtcttcgagattgcttttccaattcgttttgggcttctttgtgaatgtcatagcaatgtctatctagttgctcaacagatcgt
tggatagagacttccatagaccttgctacggttcttcggatataagtcatcattgaaggcttcagaatattcaaaagagctttgtgtcttgactttct
aaccttaagcttaatgttatgaacatccactttgacatggtctactcgaaagaacgaaccaggaaggtcgcgattggtagctttaggtattgctga
caaggcaagatcaacaaccatcccttgcttgtggatagcaagatcaacgatgccttgatcaataaacttaggaaatccttgtttacgcctaatgta
gtaattgacatttctgagatcagtctggatatgggcaaggtgaaggtgaactaagtgatcttgaatagaggagacatttccataaggggaaaatc
tcatgaggttattgttacgaatttccgcaatttttggaaggacgttaaccgtttgaaggttaaggttttctaggacaatgtcaaaagcaggttgctcg
atttcaaggcgaggaataggaaagtactgaattctggcgaggatgacgggtatgcttaattgcaaaatctcatagaccatatcctttttcaaagat
gtttcaccaccctttcgttctaccagctgattatacaatttcttgccatcatttcctaattgtcgataacttttatcagaagctgcgctgtctgcaacgg
atttaccttcagagcagaattcatcccaaattcgcttatagcgtccttgcaggaatccatttccgcgatcataaagctcattatactcgtccgtggca
ccctggctgacggcataacctttttcaaaacaaactttctcagaaaagcataacagtctcggaaccattgcttaaaatcttcgtcttgttgagcac
tatgatggaatttttgaaaatgttaataagaggcgtgagggagtgattaacaatattttcaaggagctctcgaagataccttatggcacgctcgg
tattttcattggtttcaatttgcaaattatcttgcttgtcaccaagctcattaacaaatctttcaaccagaccaaaaaggctctgcaaaggatgttga
taagccctatcctgttgtaattcgacgactaaggacttcagcttattcataatttctttcaagttggacttcgtttcctttttaacttcttctgtttgggg
atcattcaaaatctttctaccctgttttttccttcctctttgacgttagaggacgagggaagttggatgccattgactttcttggtttgcggtaactttg
tgggttctttctggccaattttcccttgctgtggatcccactggttgaacttttgctctgttaacttcgaaccttggcgaccaaacagtgcaagaacat
ctcgcaaaatctcacgcaagtgggtgttgttaaaaaataaaagaaaaaaattgtttaaggtcttcacgattaaagtcttgaccttccatattttgact
catgtcgttccaaacattttgtgcgttggttgatgcactggattgcgtcaactcataaacagcattttgcaaaagatcacctcggttctttctttccaa
catcaaatatgtatcatctaccagctctgcaaactggccaaagaagctccgggcatctttggacatgtttcgttttacgtcctgagcaattgattga
gagccctctgagatagttgaaaataagccacctaattgctggttattcggcattttgcccaccgttagcgcctcccaaagggcaaacaactgtagc
ccttcttgcatatgctcattttgcacgtcttcgtttgtcggataagctactgtcatagtctaatatgttcctaagtaaaatgcctttccttaaattgaag
agttgttccctggatctttgacagccttatttcttcaagtgtttcttttacgattataatatgttccacccaaaaggtttttcttcagtcttaattaacttc
tcttatatctaagcttttcaactttgaatcaattataagaacttaaataaaaaaaatctacctgggcctcctttcctgtttatgtactttgtttttcgtta
cgtttgtaatgactcacagtgaaatgataatgacgttccttcctgtgcaatatgacctatgattctgtcctttgtttccagcattctataaaaaaacat

tatactcgcaatatgatgatttcataacatcggtataacaaaacggcgaagaaacttttattgtcgcatctccaatgttgtaagaaagatgacgtt
gagaatcattgttccttttcccttcgtttattgaagactttgatctgtaattaaacaacctatcggtataatgcattctggggtacttcgcttcctgagg
aacattaaattgcgctaataaacacgattctgtttttgcgcataaaccaagttttctcgtcattctaatggatcttttattttcctttctctcaacttttg
attaaaaaaccgagtgctttggaacgatcttacgagacttgtatcgttacatgctaaatatgaattaataagtcatctcattgctggtttgaacaag
aaataaggaaataaaattaatcaaagtggaatcgtccagcgatataaataatcataatatcatgcatgcataaaataaacacaagaataaagc
atcaactatataaaaaaaaaatttaaaaggtcgcattctatttcaatccatataccgttcctccaagcgacagacaacctaggccttttaaattata
tcttctgttgtcattatttctttctttttttttttaataatgatgcacctgaaacttcaagcgaatatcgacaaaattagtactgttccaaggattccttc
atggtctattggggaatgtttccctgaatactagatgtcgcttcctcttgtttaaccgaatcagaaggaatcatctgcgggtacaatgaagtgctttg
ctctccttcatctttaatcccagtgttctctggtactgtagaagcgctaggtatcctaggcgtctgcagatgcagctgcggagctcttccagaaaacc
tggtttgaattttcggatactcaaacggatattctaaaggaatttttgctaaattattttctccttccacaactccaacacctgattccattccatacgt
cgagttttccatgcctgtacccgcaggcaatgtatccactggcgaactatgttgcataaagaacccattttcagggaattggaaagtatcagagga
attaacctctctcttttcttctctttcgttccttgtttcagcgttcatatatataacaggatttaactgagaaaaacttcctctagaaaatggatgcattt
cagatggtgctgataatggaaatgatacatttgaattggtttcaaaaggcctagaaggctcaatagcgcttcgtacattctcggatggaaacgaa
gaatcattaaaggacatatctttttgtgttctttcggaagtatccgctttcggtgatctctgaaaatgcaattcttgcatttctttttcaagaattacat
ttttcttgttttcctcttcttcgttcaatatcatactatgctcatccttggttattccatgttcctgttcttgatagtttgaggggggatttaagctttttttgc
agtttaatcacagatcccaattctggtgaagcttcccgaatccggtttagcattaaaaatatactgccggcgggcccaattaaagaatgagatagc
acttcagaggcttggtttccttcggtaaaatcagtacagttttcctgtccctctgtagttatcatatgaaaaacaggaggaattgtcaactgaatgtc
aacttgaagccaagattcatttggagcaggtctttctcgagcaggaaccgcaacgttcagctcactcaacaaaggatcatcgacacgggtcacat
gtatagattcaattacaaaaaatgagtactctatacggtattggattccttcatattgtatcaggtatgcaaacagtcgcttgggaggcgagtaaa
aacacatcaaatcttggggagctaaacgcaatcgtttccataaaccaataatcagaagagcgcatgaaaaataataaaattcagaggaattact
attggaaaaagttgactggtggccttgaatggattgcgaaggaaataagcgagtagaagaatatcttcttgtcgtaggcgtaggggataccttgtt
gatcatatttgggttattatactgacttccttgtggaaaaccctggtcgaagataccagtcgcataagccgaaggaaggcttgctgaaactggtga
taaagatgggaaagaagaggaaggatatttcgaaatatttttttttagcggcatcaggaagagatgatggattcttatttggcgggtaggaaattg
aatatctttgctgaaaacgagggtcgttgcttcccgggaatgcatatttgtatggaggatttaccatggtgtgttgcaaggtaggattttctaatgaa
gagggagcaggtttaaatgatgattgaggtgaggaatacgtattccaacatttcctacttatttttgggagcaacgttgtcatgaatggtaaaggt
cgaccgtacgttaaaccggagctggattcgggattgttttcagaactggattgtacttcacgcgcaaaggcctgggaaacttgttgatttaaattat
ctagttccttttgttcacgcaacagcctctgacgttcttcttcttgacgtctggatattaactttgcttttgctcgacggttctgaaaccaaatagtaac
acttcgttcaggaaggttaagttctcgggaaatctcttctcgaatagaaggaggcggatttgcatccttcgcaaactgacgtaaaagatagtttaa
ttgttcagggaccagcctttgtttttttgccttttgagattggagagttaagggacggttcaaaagacaattccttttctggaataattgaactagata
ctggtagccttggttcagcttcttctggtagactcgtatctgcaaacgtctgattaattttagacgattccaagggtttgtcgatatttgaatagactt
cggtatcgagtgtaacagttggagcttcctgttgtggaatttcttttagatctagtccacgtagcattgattcctttggaaatccttgcgcatttggaa
gattggatgattcttgactggagaaggtcggtatagaagagttgcttggctgagttccagcatgattgttgtaagaaggagcaaaaaggaaatct
atatcgtattgcttcatggaagaatctgcttgtttttgaatgggaagaatggaattttcggcgactttggcttccattccttcatccatttgggaacga
ttaggttgctgaaaatattgctgcatgacaaccagcagaagaaaaagaaaaggaactgaagaaacaataattctcaactaaataaatgcttttc
gtcttcttggtatatcgaattattgaatactaatgggtgcgatataagcatacaaatttgattccttcgataagattaaagaaaaataaggcataa
agtcgatgttttatagaacaggcttctttcctatccaaaccactgtaatacgcgattcaaggaatttcttttataactaaaaccgaactcgctaggtt
gaatattacaacctaaaacgcaggcaaatacaaatcacttaaccaaaaagagaaagaattaggaattgatcttttgacaggcctgtggcacctt
ccaaaacagccaattcaacgaattgctatatccaaatctagattaacgttcaaataattctataacgagagtttgtatctaggttgctttgaatattt
cctctgttcttataaatatgcaatgctaataaacgtcgcaacttaaggatttcaaatgaccttaagcaagtcaatgatttcttgggcaaactcttctt
caaagccaagggaatagagaccgttagacgggtttcatgaggcaattgaaatttttaacaatgcatgcgatgggcagatccttttcgtcgaaatg
agaaacctggctttttgtactgaagcatcatcctcacagtaaggatacagatttcataaaatgacatgaatatgacgcccttggagtttgctagact
tccgataactttgtataataatatcactaaaaaacaccgcccaatatattttaacgaggttactgttaaaaggacactgaatactttcatcaagtttt
gcagttcaaattgtatatttccgagagtatctaatgctaatatgtattattacatcgaggcatttatacctactagtaaataccaaaattagacaaa
aaacagctttacgtcgataaatgaagtagttaaaataatcaattgatgcaaaagaaacgtttaggaaaggtagtaataccggatttttgtttatac
gtcaaaaataaaagatttcaatcttttaagtagcggtgtaataagttttcactcattattcattttatgctcttcagccgaagttattcttactagcctt
actttcatatacatatacgaggtttcgttaaaaacttgctttatagagaatagatgatatccacaaccaaagttttattttatatatcaagaaatatt
ttgccaaaaactctatcatctaccaggaaatttttctacagaaaattgtctgctgtatcagtattaacgtttaacatttagtatgagagtttttcatta
gtcaaaaagcatcggtttatttcatatatgtattgatgcctttaaataaagataacaacatcccttcaaatgatcgaaaaagactcacaaacttag

aagcgaaggtcccatccaatttgaggtcccaaagtaggcaattgctcagcaacgctttcaacgtctttatcaccacgaccagaaacacagatcac
gatatccttgtctttaggaagactctttgccaattccataccaccataaatagcatgagaagattccaaggcgggaataataccttcgcaatggca
tagagcacggaaaccttcgaggcattgagcatctgtaactgcgatgaaatcgccacgtttggtttctttcaattcagataactcgggaccaacgcc
aggatagtccagaccagcggagatagaatgggtatcttggatttgtccatcttcgcgctgtaagacataggtacgaacaccatggaatacaccaa
ttttacccatagataatgtagcactatgcttgtctgtttcaacaccagagccaccagcttcacaccccaaaagctgcaccgatttgtcttccttaaaa
ggagaaaacataccaatggagttggatccgcctccaacgcaagccacgacggcgtcaggaagcttgttacgcttttcctttagttgtgctttggttt
ccttgccaattacagtttggaatgtcttaacaatagtagggaaaggatgaggtccgatagcagatccaatcaaataatgagtagtatcaatttgtt
caacccaagctctaagagcttcgtttacagcgtcacgaagagtttgagtaccggaagtgacaggaataacctccgcacccaatatacggatacg
gaaaacatttagagattgacgacggcagtcttcagctcccatataaattgtgcacttcatgccaagcttagcagcagcgatagcagtagcaacac
cgtgttgtccagcacctgtttcagcaataacacggttcttaccaagacgtttggcaatcaaaatttgaccaatagagttgttaatcttgtgagaacc
accatgattcaaatcttcacgcttcaaccaaatgtgagcaccaccgcagtattctgtaagacgcttggcataatcaagagaactgggacgaccca
tatactcatagtaagagcggaactcttcccaaaacttctcttcatttatggctttgtaaaaggcgctttcaagttcaatcaaacattgagtcaaagct
tctggaacgaacatacctccgaaagaaccgaaagtttgaggaaggtacatttcgttagcaggttcaaggattgcggcaggaggagcagtattaa
cggcagcatcagatgcaaagcgtttagcaggaggagcagaggtatcttgttgggtcaaatattgacagtattccttagcatcgttagcagcctgac
cggattcagccttaaaaatcaaatcaataattttactaccgacgacaactccatcagcaacgctacccacatatcggaaatgttcgttggtgtttac
accaaatccaacagccaacggtgtatctccagcatatttgcgaatgcgatcgcacaaatcgggcaaagcactattgatactaccagtagaagaa
gagccagtgctacccatacgggagacgacgtatatgaaagaatctgcaatagaagccaaaaccttcatacgcttatcagtggtagagggagcta
caagggggacaaagctaagtccttctttagcgcactcagctctaaaaccaacagcttcctcaggaggtaagtcaacaataataaagccattagc
gccaacctctttagtcttttgaatggttttagtatcaccataattatagatagggttgtaataacccattaaaatgataggaatggtaacaccagctt
cacgagcacgcttgacggtatcaaagacggtcattaaactaactttatttttaagagccacctcattacccttgcaaatggtgggtccatctgcgac
tgcgtcagaaaaaggaacacccagctcaatgactccagcgcctccagcttgaagacccttcataatatcaattgtttcatcaacacttgggaatcc
acaagtaataaatgttaccaaaacatttctattctcctttctagcttgtaaaaaggtattcctcaattgttctgccattgtgcttgtctatactgtactt
aatggtgttggcaaagagatggttataaacttaggatcgggagatcgaaactgattttttttttgaccaaggtcatatcttttatatatctattaaaa
aaatttaagctttaaagatattttctagcaatagtttaactcttttgggaagattacagaaattgaaaagatcattacttgaatttagaaacacgcc
tctcgtttagagatatatcatgaaatacttgctttcttttaactaaattgccaaaaatattataatttttgtgtcctatttatcaagccataatttccaa
caaaagttgtcgtcaaggagaaaagtttactagctcagagcatagtattcctcaaaaaatgtcaacgaacagctcattttaatctttgtaacctcct
ttctgctaaataatttcaacccaataaacatttaaagaaattgaagttaatatgggggtagcagacaaataggtttcttggcaagatttagaatttt
tcaacatgtgggtgacaaacatttactaaaagcttgaatatagatcttgcatgtacataggatttttcacaaaaactcacaaactcagttcaaaat
gtataattccaatgtcaaatttttagtagaacatagcaaacgtccgcaagttccaagtacacagttaaaagttaacagagtttgactgtagtccaa
atatccaatatctgcataactcttgactgaaggtgaaagccatcaataagtacgggggatcacacaatatggaaatatatatattccttgtcttatt
tactttagaaaagcttaagactgcctgtaacttcattaattacgctcaccggacctggaccaattcctagaacggttgcggacccagaggcaattt
gagtacgtccagcatcatgaacaattctggcagaaaggcctagagacatagcttgagcttgaagaatttgaagctgttcttccgattgtccctgca
atgtaatcttggcttgaccagctacctcccatagttgtaataattcgggatctgtagctgctgcaagcttgtaacacgctaaggctgcatgagcaca
ctgagcagctatcttccccttcgtcatgcccaaatctgtgcggacgacaagcaccattttagtttgtccctcagccaaggccaaagcagcaaggct
gctttttcctttcagaattgcggccaattctgcttcagaatcttcctcctctgtaaaactttcttcgccttcactagtgtcttcctcctttcctgacaaaa
cattggtttttcgctttttgaggtagattcttctgttaaagcttttccagctccaattctaaatttttgataggcatttttccagcctagaaggaaccca
caagaagtgcctacaagcacgcctgttgtgaaatatgaaagggcagaagcagtaaaagaatttttttactgaatttatcaacgacataggtatcct
taagttaaaatactagggcccgatattggcacgaacgcttaaatatgtttatatagaattggtgggaagtaagggtagatgaagatggtaatcttg
agtatagaatattaaaacatgtcccataaagaagaggaccatggacaaaaaacgaaactccttctttaaaaattgaaatagttgattagacact
gtaaaaatagcggtaagcttttgaatttttaaaaagcactatggagggaatttcacgtaattttaactaaattctaaaatagattaaaacgaaggt
atccaaaaacttcattatgggttacagtcattacggatcatgtcaggaatggaaaaataggtaatcgcgcctatacctcatatcgatgtttattcca
gtcgtttcttattttattggtacctcctgcgctttggaaaagaatcacgcctgtcataacccaaacctgctgtacgagaagaacgagatcctgacat
tctcgaagacaacgaggagcccccgggaggaggaccaccgcgaaaaccacgatagccaccacgaaaaccagtatcccctctatatcttgagct
ccgttcacgagggggacgctctttagtataatgtctaccgcctaaaccaccgcctaattttcgaggcaaccaacctttcacagtccgaccacgttca
acatcaacgacaatccttcttccttgtaatattaacccctcagcagcgcgataagcagccttcaggtcacgttctcgttcaaatactacaaaggcat
accccttgacttaccagtggctttgtctctgacgacgcgaattctctcaattggaccataatgtataaattctttttcaatgtctccttccctggtatc
atagtttaatcttcctaaaaacatagttttataaggatcaccaataacatgtcggtcatcatcaggtttccattgtcgtatcatggaccgtaatttctc
acgttgttctaatttcttttcttctttacgacgaactcttctctcttcaagagtttccgtaggctgataattatcattgtatgattttgctaattgtaaata

ttttgctactccagaaaggtgaggagtagaccgtttttctggcgcagcatctgaaggcggtaagtaccttaaaggaggcctaggagcaaacaatg
ccaataccgttgatggtaacttttcggtcatttttccaacattcccttgattttgctgatagcaagcttacagtatgtatcttcttaatacctcggccat
ctactattatattatactatcaaatatttataatataacaaattcaaaatgactagattatttaacaattaattgaataatccaaggaacctgcaat
atatttattctttttgattatacaaatctcgcatagtaatcctctcattctacaaacaacctcttactagataatcactaaatagcctcatcaattata
attaattcatgcaatacattcacaatcaaaatatatacacctctataccacttctgtataactttcaacaggattaagcttaagagcgtaactaaac
ttttctttggttccttactacgctttgtagataagaaaggaagtattcatcggatgacttttcaacagtctgcttgcttttgtcatcaacttctgaagat
ggagcccgcttgaggatctttacagaaccagggaaggtacctggtgtagttggttcttgaattgaatgatcagcttttgctgtaagctgtatgggca
cctcagttgatgtattagccataggctggactgaactacccagtaaaacgttcttcagggcttccgtctttttttccttgtagcatcattatcttcagc
ttcgctcttggcgtccaactgaacacctctactttttggtttcggtgatgcttttgtttctaaaggttgggctccgtttaactcgttattagtaatggctg
atgaagataagttcgaaggtagagagaaggttgatgggttcaacaacgcttcaagcagctgttcagaatgagacgaaggttgagtagagagctt
ggaaacagcaggtttatttatcgtctcagccttttcagggacattgacattattgtcgggcttaggagtgcttagtaaaacgttgaccagcttttgag
aagaaccatctcgagaagttggaaaagaatgagccggtttaggagaagaaatcatatctaaaagtttcctagaggaagcgtcattctgtgatcg
atttaaagaagattcatcgtgaaagcttttcatttttgccgatgttgtatagtaggaaacggatcaatcatttgattcgtatacgcaggaatctgca
tgttcaagcttgggtgagtatcattataaacttcacgcttttgttcaggcttgaaactctgaacagatgtagtagttggcggatggaaaacctcatg
ataaactgttttgggagaaggaagatccataggagaaggcatagttgcttcaggacttgagttttccctgttttgaacataagggctagagtagtc
cggaaatggcggtggagggggaggatggctgaaagaagtcgggaattgtagattggaagcgacattgggcggaggaggaccggtagcagcga
ccctggggaaagattcagaagggttgtgtgggtctaaaccaagatatgcaaatggatcaaatccgtaaagagagggtccgtttggccaagaatg
atctagttgtcgagggggctgttgggggagagatgagactggtaagattggaagggactgtttaggctctaaatttccattgagtaaagtaagtat
tttttgtttaatgtcaactggaggaatttgaggaacagcctgctgttccattggttgcgagataggtaagggtatagtggtaaaagccgtggatgat
ttcaaaagagagaaaagttgggaagaagcgtcagcatcaacgtcaacagaaataccttgttctttggtagtctttgtagccatgttgcgtttcttga
tccacttcttaaggggagtcaaaaagggaatgaccatgtaaaacttgtttttaccgtatcggttttggactttttgtaagtgggcaaatcagccaa
catatgccactcaattttgctaatttctttgcgagttctcgattcaaatactgtatctaaagacatttctggaataatgtacaaacggacattttgtcc
acggattgtcatttcgatgtactcattaggattgatgtggcccattaaatcgaaaccagtttcttcataaacctcacgaatagcacaatctacgtct
gattcatctttgtcgatctttcctttaggaaacccccacccagaggaggctttccaccctttcacgagaagacagcgttgcaatgacttgtcgagca
aaatcgctcctcgaacgggaatacgtgtcttgtatcgtaaaaaatcatcaaaagcttcttcatgaactttactccatttccatagtaagggacaatg
ggcaaataaccttgcactgaatacgcgtagtcctagggatggtaattgatcattctgtgcacgaataaaatcttcataaaaccagtgtgcttgttca
atctgaaagcaaagcctttcgacactgctttgttcttcagccggtaaattcaaaatgaaccgtgcagacagatcatcgagcacttgcgaaaatgta
gcgttcttaaaggacatctcgcatcaaatctaagaaattaaattaaataatacaaagattaacaattaaatcccggttaaagcaccgtctaaactt
taagtatctattctttaaattcgtcgtggttgtgttactattagtcgtacataacacgacatataatatctgagtacacgctttagcgtaggtaaatg
aaggttcatatttatcccattacgtgcaacaacgtttagccttaagataattaaactaacgtcgttaacccggcttattctagaaaaaatagctgta
ttagagcatatgctgtgtgtaaatttgggtagacatagaatggtttttatgtacgttctctctgctatagctgttaccttgtctaagtgttctatatata
ttgactttgaaaatactaggttcctcgaatattctgtgttatattgttcaatagaagatggattcaaaacgaatcctaattatagggaagccaaaat
ctggtaaactaacatttgttaaaggtagctcagcaaaataattttatataaaaggaattcaagcagaatgctaaaacgtagctttgacgggaactt
tacctagaggattgagcttgacatcaagcgaaacatcacatgctggtttgacgcatgaatatacattgagaaataaatactttcaaagagaggtt
ggaatatggattgacgaatacacggacttggaagaaacactggaagcatatgcttcccaggatgctaatgaagttatgaatgccattagtgtcat
cgtctatacattccgcagatttgaaccccaagaatggtctttgtggaataaatttagtgaaaatctggaaaatactattcccatcattggacttcat
atggatagctcatccatgacgaggcctcctgagagcttaaaccctgagtatgtagtattgtcccagacgggaaagaatgagtttggaggtatgtgt
ttgagaagttacaggctctgaaaatgactgcttacagtctaaagaaatgttgggttttgatcgtgttttggatattttcaattgttgcgaatgggacc
tctatcataatgagaataccttagaagacacggaaaaagaagcatccaatacagtgccatatagagattcctcgatgtctctgaatgattcagatt
tgaattctctcgttaatcaagcaaaacagcttcgaggtatgttagtcattaaactacactgaccaatgttagcgttaaatttagatgtgcaagaaa
ggaaaaagcaggcgatgtcaatcatcaattctttagtggatgaagaatttcaagaatcagatctagattcatgttgacaattcggctcattatatct
ccattttcgctccatttacccaaaaatagaaatatatatatatatatatataacgagagtaaacaaataaatattaacataaaaccaatctatcaa
tcgtaacctagaagtataattcccaaattgcatttttttgtgttcaaataacgattacaacccaacgccgaacatttggcttctggtcgtcgacttgttt
tgagcactttcgcgcaagatccacaatagacatggccacatttggtagcccagacgcttgccttttcaacgcttttcgaaattcccaattcttcatga
caacgaggacatgccatggccgtttcagagccaaagcttcgagtatatccctgtgaggcaggctttgggggaacataagtagcctttcgttcttct
atggaaggtgaatgagtttctgtttcagggacataatcagaaaaataatcattaactccagaggcactggtttctcggccaggatgagaactttcg
tacctcattctacccaacagcatttcaaataaagtcggggacattgcgttcgctgatcccaaatcaggaaatgaacgtgtcatgatttcaagtaga
ctgcgagaacctctggaagaccttcgggagacgacttgcggcctaaatacaccatgtcttcaagataagacaatgggttagatgccaggttacg

ccgtctccgaagtcgtcggcgactacgccccctttggggtcgctcttcagcaggagtcaccgtaaatggatcatcgggtatagcatccacatcaat
tactgtaggagcagacgattgcggtggctcgtttataggagaaggggttcttgtcaaatcgatcacatcactcgaatttacatctatggattcattt
aagggcatggatattccaaacacaaattttacccttatttataagctatttggcgcttagcaactttacttttcttgtgaggcaagcttatattatagt
attaacgtacccgaacgtactgtttgcatcgataaaataagtaccattatgctggataaatatcgtgtactatatgaaatagcgcacaactacgaa
aacttttagtaaacaaatgagtgtacggtatttttaagggcaccggcttttgaagatttagtgtagtgttatattaacgaatacaggtaattgaaag
cattgttcatttcaatgtatttttaagcttattatgaggcatcctctgcaatattgaattcatgctaatattctctgaagtattcattaaaaggtatcgt
ataatggatctcgccatataagaagaaatgcagcaattaaagagtacataagtagaaacaaacacttagatagaaattgttggcgatagtgatg
tcctttctgctgtttattccatcggaggaacgattcatagctcataaccccacgtaaatatctaactcgaaaagatagatcccaaaggtaagacttg
cataacgtctaggatgctttaaacaagaaccttaaatatgtatgattcatgatccttatagtaaggatatgcaatccatcccatatttgcttagccc
aaaatcaggttgtaaataataacgagcgtattaatggcaattaatggaactacagaattattagataagagtttccatctgaaaaatgttacatac
aagtcataacagttcgaatagctcgaattagatggagaattctacttttgatggtatcctggggttcgccaaatccataggaagtattctgagacc
atccaactgaataaagtaattagaataagttttattttcttatttttcacttacgtcttcctaaaaaacgatcttcagaaagaatcgcgactgcgttc
aacaaaagcaaaataactaagcaaagttatcttctcattctcttcattcattccttactataaagtatatttccaaatccaaacataagccccattat
taaaaaataagccttttatataggattcaacaaactgtcaaatgcaaagcagtaagtgtgagcttcagaatgcatatgcggaagtttcttatagta
ccttagtaaacaaatctataatcatataagtgataatatgtttaagaatttcctatgtttttttataagttttctagtttttatagttacagaaaccaca
gatacgatgtattaaaagcagacattcattgtttttgttaacaaccggtaaaaaaagccataattcccaaaactagtcatagtttagtaccatctct
ttcatgcgttttcgttccatcaattcttctttttgtcgcgtcttggtagactgcgctagtaccgaatgagacggaacatccaagacctgcgatcgttgc
ttattccctttcttggttaagagagtaaacttcacgttttcttcactttccattgacatactatttagatctgaaaatgacgtatctccagtcgaacctc
tcaatggaagaggaacatcgaaagcaatgcctctctcatgagccctcgaagataaactatcgttcatcatacggtttaactcttcatcgatttcatt
tgtatgaactcgctcttcgtctagttcttcgtcgtcgctattgaaaatctcagaatcatccaaatctatactgttttcctcttcactctctgaatcttctg
atgtatcttcattttcttcgttatcttcccaatcaacagcctctaattcattttcactttcatttgacttttctgactggagattaaagctttcctcgtcat
cttgatcatgatcgtccactccactttgtagtctttcagccaaagcagtccatgcttcatcaaagtttttatacacgttcatactaggacgaacttttg
gaaccacatcctcaaactcaaattgcatatctaaaggtacctctggctgtgctagaaaataacattgataagccaacatatatgtcagcatcgtttt
tctagtctttgagcgtataagtgaagaaccacacgttgagagaaagagtgcaatcatgcgaagtcgaaataactcgttttctttacttattgcagc
agtcggagaaaattgaaggatgtttttaaacttttccgggggaagtaaatagaataagattccgatgattgaccttacatctagtagtctaacaca
ataaagctcagctaaaaactttgcttgtgccaatcgcttttggctatcgccaaaactgctagagtcgatgtacgtcaaaattgaatccaccgtgtca
tcaatcacatagatgcaaaaatcaggatggaacttatataaagaagtcactaaacttgcaaacgaatttaagcactcatatttcatattccacact
tccataaacgtcgagtatagcatatcataatcgttgttccaatcgaattttcgtaacaaggctatagtagcagatagattcgtttcatttaaacgaa
catgtaaaatatatctcagaaactcttcttttaaggaacgcttcctagctcttgcagagctcttctttgggggattcacaaagtacaaagcgttgtct
aatattagctgttcctgcgaaggaagagcggcagctaattttttttttctgtacagcttctagaaatgaggacatttgtaaagaagatttaggaaatc
taaaaagaaatcgaccacagttttctaaaagcaaggacaaaacttccagatcaaaagaagtaaaatctaataaacacattttataacaatcaaa
cacataaggaaaagacatcaaccgaaatttggtaagttctgaaacgtagcgaacaatcaaagttctagtatcatattcatgcttagctttcctatg
catcatgcgacgaaatgcaccacgggcgtgctctacgagagcagaagcaatttcagaagcaacaggtgctacgattctagcaaagcggatgta
ataagggaccaagaaggagcttgttctaggtaaattacaaagagcttttattaatcggtttctcgaagccttagtattcaaataaagaaactctag
agcagcctgatcaacttgagttacagacataaaatttggaagcgtctgcaaaagagtatcaattttgacgctgacagacttggatggttttgtaaa
cttttgagaatcctcgcttgaaccttctgattttgattcctgatccccgttaatctcgaggcttttcttattggcgacatctaaatttggtaattcagga
aacgtttcatagaattgatgctcttcttctgaatcccatagttggacagcacctgtctgaactagagtttccgcatttagtatgttattctgtaaactc
atgacttcttctcgtttttttatagaacgattcttgcttttcctttatgctcaaagggaatggttggtctaatgcttccgatatagaaacaccattcgtaa
tatcgccttcgatagattttaagaaatcctcagccttatttgagatatcctccaatgagctccccaaaaagtcttgctcactaaggcaatccctatat
tcttcatattcatgttggcgatgctttatgtagacaagtaaatcttgataataacggtctagctgggaccgaagttgaagtcgccagcgaggatcac
aaatactatcaatgtattgatttatctgactatctcctggtaatgactcgtgggaagagaatagttcatacctgtatgacctgactaatgaggagac
gattggcaataaagagaaattggaggggggcgcgagcacatcttccagtaacagaataactattggggtttgaagtgctttctcatcaaacattg
ttttacggtatttttccccgaagcgtagacaacaggaaggagatctttgaaatcttctggggtgcggacaagtccatgaagccagaattcaataa
agatcctcaaaagcgcccttgtttttgcaacattaagactagttcctttattagtaactgattctgagatcacaccagtggacgcagactttacaag
gtttcctgagatggagtctgaaggcgaaggatacagcatggaatataaattcgccagtagcggtctgataaatgaaatagaaaatctttggtaaa
ggaaaaacacaatcttcacagcggcctggatatccttcactgacctgcactttgagagcccttcagctatggacagagtgatttcaggcacaaatt
tttctaacgaaagagacttaatttctttaataaataaatcataattatcagaggttaaagaagctctacatcttttcataaaggcagtgtttttttca
aactagagtccaatgattcttggacaggaaaaccgtttgcatctccattctttgcctgtaaagcaagttgtctgtcgttcaaatatacattaagttta

tccaacagttcttcccttgacatagcctaattcaaagttacaagccaatacgactatatttcatagcagaggtttggctgtacagtagggttgctgt
aaaggttttggatatgaaacaaggcgactattacggtttctggttgagactttttttaggatgaaacgagataaaggagtatataaccgacttattt
ttgagatattttggtcgccacgagcaaagattgatcgaagtcaatgtacggtactttctgtccggaaaacaaaaatatcaaatttccctgaaaatg
tgatgaatgtcaaatagctgtttatttattatttaatcaacattcaaaataaaaatagaataaaagaaaaaaatctagtcaaatagattcccttca
acagaaatatcagcaaaaactgtataatattacaatatgctataatactaacaaacataatagaattagtactaccatacgagcaaactctttcct
tatgaatttttgtcaacgtggtttttaaacagtaatggccgggctttctatgctgtataatgcagccattgcaagtacttttatgaagatactgctttt
tccatgctatcgctccactgactttgaggttcacagaaattggttagcaataactcattcattacccatttctcaatggtatacttcagcaatttcaga
gtggacattggattatcctccatttttgcatatttcgaatttgttctttcttggattgcaaaattacttggttttgacacagaaatgctgaacccgtat
aatttgaactatgcgtctccttcagtaattatatttcaaagaagctcagttattatttctgaattggtacttctttgggccttacgagaatatgtctatt
cttaccctgaaaatagtagaggaaacgctgtactcacggcaatagacacgtttttatctcctggatttgttttgatagatcacattcatttccagtat
aatggctttctttttggattattgatctggtctcttatattaaccaagcaaaatcgactacttatgtcagcagcagtatttacaacactcctttgcttca
agcatatatttttatatattgctccagcttattttatataccttttacgagtgtattgttttagcatatccggatttcgcccacaagtactaaacactat
gaagctaggactcactgttagtagtattttccttttagcatttggtccatggatttatatgggacagacaccacaggtcttctctcgactttttccattt
tctagaggtctttgccatgcttattgggcaccaaatttctgggcactttactccttctttgacaggattgcttatgctttcttgcctcgtcttggatattc
ttttcctgaggtaccttttacaaacgctcctacgcgtgggctagtcggtgacacaacttttgcagttttgccaaatatttctccctcttttactttttatt
tatgcctggctctgcaaagttgcgtattactcaagttgtttttccgtcctacgtggcgcgtatttgttggagccactacactgtgtggttggatcagct
ttttgtttggctggcatgttcatgaaaaagcaatcatgattgtcatcattcctttcaggtacgttagctttctttttatttatcgtcttacattaacatga
tgactaacatttgtagtattctgtctttaatagatcgtcgttatttggaggctttccgtccgctggccgttgcgggttatatttctttgcttcctttgatat
ttacccctcaagaagctcccatcaaatatcttttcactggcgcatgggtcgctgttctgttgacttttgataaatgtgctcctgttcctcttagaaggg
tattcttgcttaacagagttaatcttgcttatgtggctgggttcgttcctttgtttatttataacatatttttttcataaccttgtatttggtgggcgatacg
aattcttgccattaatgcttctaagtacttattcagcttggggagtttttttgggctttcatcagcctctcttggctttatttcacggatctaaaatagctc
tttctgaaaaaaaaaaaaagaaagaaagaacaatgatatagaaaaggttcggttattgagtttgatgattcttaatgatatattcttaatgtcgcta
aatggcaaaaaacttgaagagcaatcctattacattctttttttttctcataattggacagcttgagttccattataaacgcttaacatctgtttaca
atttgctaacaaatatttatatactgattacttctttgtctgctcatgagttgccaaaaagacattagatgaatatttgggaaagtaatcaatgtgct
aaaaagtacatctatcggtttcctcgctcgatgtactcactcttctaacaagtttgggcttgaaaagaaaagcaacccaattacttaatttcttaca
ataacatagacagtgaatgaaaagaaacttctatagcacctttccttcatcaatcacccttctttagtacagtgcaaccattgtaaacaaacggtt
acccaagtttccatgttacccaccattcaaaacatccttgaaataatatttaacgaacgttcttttagcattttcctgtatatggaatcatgtcaagta
aaattttaagatctatcggcctgctcgcagtcggtacaatatcgggtgtttctttgacacacttgtatatcaaaactgcgcaagaacctgcgaatgc
tgttgagttttctcgtaggtcaagaacggaccctacggtacgtccagatgatacaaattacgactttgatccagcgaagtttctgcagtatggagtt
cctggtcccgtagcggatcaaagaatcgctcatgggtacatgtctgtgtttgatagaagaacacgcaacccttattatgtaagtttaacaaatcat
ataaaaatggataacccgtatttggaggaatgaatccaggttgtttttggtttggcttgtttcactaacgattgatctttgactgtagaccgccgaaa
caattacagaggaattgttagagcaaagaaaaggaaatcgcaaatactctgagtttcatgttgattccaatattcctgaaatgtttcaagctaagc
tttcagattacaggggtagtggttatgatcgtggccatcaaactcctgcagcagactgtaaattctctcaagaagccatgaatgacacatttttgtt
gtctaatatgtgtcctcaagttggtgacgggttcaacagaaactgtaagtgaatctttacatgccggcatgtctttccgggtagctcaaaatagtta
ttcctactaactgataaattagactgggcatatctcgaagaatggtgcagatcccttaccaaaaagtacgagtccgtcagtatcatgacaggacc
attatacctcccacagaagaatgaaagaggacaatgggaggtccgttatcggatgattggaaatccccccaatgtagccgtccccactcacttttt
caaagtcatcattgctaaagagaaaggtaaagacagcatgaagcctgctgtagctgcctttgtccttcccaataagcctattgctgacaactttcct
ttgaagaacttcttagtccctgtggaagttgtcgaacgctccagtggattggaaattcttcaaaaggtccccaaaagtaatcgacaagagctttgc
aagcagattgaatgcaaactcaacgccaaagcattccttgaagaagttcagcgaaagcaaaacaagtcttgatatgctctactagagaaatgag
gagcagctaatgtttcaagcaatattgtattagatattcctaaccaacccccctctttttagtatacttaagtatatataaagatactcttggtcagtcg
actataacccataaaaaaattgaaagcatttattgactagtaaatatattgagcgtatgtcctatgaagctactgaagatttttttgttcatgttgcat
acgagaaatgtaagttcgagaaacaacgttccaagcatccatgtatcgttccatgcactttgaaacacaagccttgaaaaagttaatataacatc
ctagtacacttcagtgcagaatcagtttcacttaccttttcgttaccactgaaggagcttcccggttgaggaacacacttgtcaaagcagctttcgtt
gattttctagcaaaataagttagaataagtttctttttctacttttcttatccttcagaaatcgaagaagatatctgacatacgctgattaattcaccg
gcttgagcaacagcaagctcttgacggatttgtttcatgaagacatttttcttgtcttctgaagacactgaaggtccactgttaccaccaaaaattcc
cattttgatctgttgaagttgtttaagaagaaaaccttgtagatgttgcctttcgtattccttgcgggtaaaagggcaagttggtatgtactggaattt
gaaagaaggtaccctaccaaggggaaggattccaaaaggacggaaggatagccttgaataggaaatatggtttctttttaacttcttataaagca
agttgtttttttaatttgctcgatttgaaaactgaagcttttgaagaattgcaatcttcgaactcacgattttgttgcaatatattgtaaacgtcccctca

tgagttttttgacgtccttaggacttattataactgctttatgaatagtttctgaggcatgtaatatgaaagcttcactaacggatttctatgagttttc
tgaaaaatgaaggcaagctggcattagaacaaggttgccaaagcgtttaaaagagctaggtactaagttttgtatttcacgagtcgaattgaaaa
gcaccaaaacttacttataactacgggaaaggtgtctactagtcgggaacaagaacggcgatgaatcccaagtacaacggttttatgattttcaa
aatttttattcatgttctcttcattctcatactttcctgttttgaatttgctggataaaaacgcagcctgtctactcagttgaaaaaagagcatcaacgt
tccaagcttacttactcgatttgtatgcgaaatacttttagaattgatgtttttcttcaattgttgtctattggtttagcatgtttatgaatgttgaaaa
agtttttcatgaatcaagaattttaacatctgaatcatcaagtccatggctttttttttttctcacccctgcctgtaactaaatttcttggaatttatctt
gtctgaaataaggcaatcatggaacgcttagcgatatgtagacattcatatctgtgctcttccttacatgcctactactatcatctttttgttagtgga
atactaacaaaaaataccctaatatatagttacatatagtatttgcattaaattgtttgcatttctactactgtttacttttcctggcaaaaaccgata
gatatttggtgtagttttcgttttgccctttgtgtgtgccaggacagcgatcagtgctaatttcttgactgattcaaaaaatttgaagcgattgtagca
tttactttgcattggacactctcaattgttgatagccgttgaaattgccatttttagtttgttcgttatccaagatgtttggagtccaattgtcggaatc
catattgaatttttagttgacttttcttcttttctaggagcactctcattaggatgctgggttgttttgttgattccccaattgctggaaaattacaaga
atcaatcgagtgatagcatttcaccattgttcctcattctatggttagttggtgatgtattcaacattgttggctctatttggggaaatgtttcttcaac
tgttttagtactcgcgatttattacattggtgctgattttgtcttgctcctacaagtgtactactatcgttggaaagcgtttcgcaaatccagaaatga
gcatgagccgttattgcaaagccgttcactagaagaaggtcttcatgagccgttagataaaaatttgatctggtgggatcgtctttcccttcgcggt
caatttggattcatggcttgtattgtactcataacgacttttattggaaatgttttaatcaacaatgcctcttccgataagagtgacgatcaagtaaa
taaatggccttttatagcgggttgtattagctcgatcttatactttgtcgccagattccctcaaattatcaagaattacaaatcgaaatctaccgatg
gtatgtgatgaaacaaagcttgttccagttttgactgcagattttctaactcaaactttaggactctctataatcttctttattctttccatgctaggaa
atacggcttatacgttgagcatcttggtgtatcccgctgcagattatgtgaattactttaagggtaatctcccatggatttttggcgctttcagtacga
ttttcttagacatattcattttctaccaatttttgctttaccgtcatcactcgcacttttaaacaaacgttggaagaaagaagaaacccccaatataa
atatatgttacctatagagtttatccattactgccagtgtggtaatagtacggcagagggaaagatgtctaatggttttattagcatgtactctaga
gataatatgaatatattcctttgcgattgcaactttgaattgccaataacacatacctttcaattgagaaatgggacgatcgaaaacatcggcgcc
gtaggttgcgcgaggaaacgttgtcataaaagctgtaggcaaaggcacttgaagtttctcagaaacttgctgcgcgacatctcgtagggactgct
ctgctggaatgacaaggttgcaaacttggccctggtgacgaatagagaaacggcctgaagacggaggagcacgagaaggggctgcggtatccg
cagcagcagaggtagaagtagaagacggctttttttgtagaagggacggtagtagaagaagcactgttcgccgctgacgaggtagtagcagcac
gtcgctttttatcagcctcaatttcagccaaaattttttttcgctgctcttgatcttcaagcttttgttggcgcattttctgtagctcacgaagacgtgttt
ggtcttgaagctgtcgcatagcctgagccgtttcgtcattggacttttgaaggattctgaaatttttcttcttatcttcttcttcaagagcttcctgttgc
ttcttcttttcttgagccttcttcctcatttcctctatagctgcctttatttcctcaggagatcgttgtcgaacctagagaaaatcacaaagttagctag
accacgaaccatactataagagagccaggattgtccgtttctttccttacagctcagcacgtacctgttcttcagtttcttcgaaattttcatggcca
gacttgacgccatggaattctgccatttcaattgaagaaagcaattttccgcaatcagagcatttcagtgtcattcttatttgctggatatttataatg
gagacaagaaataagacaagatgcaacgatggaacaatgtgggatttgtttgattacagacttgatgacaatttcagcgtacaatcaactgttca
atactgctaagtctaaaaggtttccttctgattaacgtgttttggaagcggttggtctttggttttattatgtttggttctttctagaaacccaaaagtt
gccatggaatgatgtagacagcttagcatgcctctcgtaaatccaattcaagatgaatgccacacgttcattttcaattgaatttctttaaaaaaag
aacttttacagtggatgcagacttttaaaactcgtctatttctctgagaacacaattttgaagcaatccttctttttattttcgcactacttacggtgtg
atttttttcatcttgaccctttatcattcatctttctttaaaaataaattctcctcttgatgtattgttttggttttatttgtttttctctctttattggtttgt
ttatttttctttttttagactttttttgatgacgtaaactttacgatatcggttcgtttcttaatgtaagtttttcattcctccttttacttccgtaaatgatatc
actccctcggattgtttctctacgagtgactctctccctttacgattttcgtcttcactgcagttccagataatttatcacgattttgctgttcttttgaca
gtttcctttaacgaattgcttgtttaagttctattgaaagaaacgtacgatctaaagacgctatagtaatttatcgttgaacgaaattgtagttttgtt
ggacgaaaaaatcactttctttatagtcagtaagtgtttgtgttttgtgccttgttgtgtgagcttcgtgtagactgtgttttggatttttatataaacgt
ttctcctggttgtgatttctcggctccgcatcgtttaatatgtcgattgaatgcagaaactcaattttctttccaagaccacttactcaactggttaaac
gtgattttcaacttgataacccgtcctcggtacctattggcttcaaagtcaagacaacggctcctaaacagtattgtgtgcgtcccaatggtggcag
aattgagcctaattcgtctacgacgattcaaggtaagcctttttttctgttatcttatcctaacaatgttatatatagtaatcttgcaacctttggatcac
gagccagcaccaaataccaagtgccgtgataaatttcttgttcaaagcgcagaacttagacccgaactcgaagggattgatactactgaaattgt
atgcactctcattaaggcaccgccttttctctcgatatgtacttactaacttgcttatcgtttagtggagtcaagtaagaaaagaagacgttcatgag
cgtaagatccgttgtgtttttggagatccttctgaagaagaaacttctcagcacaatcgtcacaaaaattctatatcaagcactacaagcaacgcc
gctttcgcttctcaggctcccaatgctgcttatggcagacaaggccagccttctgcaaatgccgcttcccctaccctcacgcagcctcctcaaatgc
cccggtcctctgttgatgctccagctaccccctagggtagctggaaaggcggctccacctatttctacggatgaccctgttgccacggctcccaacgt
aacgtttcctagcccttctgctcctacatccaatccgattaatcctcggttccaatcggctgcagctacttctcctgcagctacttctcctcctgaacc
aaaatcaactcattttggaacatccaacaccacccctttaccagctacaaaatctacgtccacttttggtcgacctaccagcggtgccaaagttgta

cctcaaattcacaatactgtcactgtacaaactgcggttttattagccgttatttgcttcttaataggccttctattctaatttagatatattgatgcat
gttcaaccatccaataccgctttcgtttcccatgtttataccattcgttgccccataataatgtaagtgattttgtggtgcctgtatttgttgaatagca
acgtttctattttgtaaaattggctcgttttctcgtaattcctttaaatatacaacagcttcttgtaatcggttatgggatttttgattatgttcttcaatc
tcttcgattggcaatagcggcaggatcggccgctcttccaattgtttcaacaccactaaccacgctcctgtaagctcgtaataagcctgacttttgta
aacattatgattggggttatgaaattctgggagctgatgaacaacgttggtagcagcgtccaagaaggcaaaattatgttcaaaacaagtagaat
gagggggtaaataacatgggactagatcatttagagcatgtaaaacagccatatacttttcatgaattcgattaaatgcatactctccttttttatc
accaccatagggaaaagaagagtgcaacgttttggatagtttttgcaaagtgtcaatgcatgtttccaaggaaggtacgggtaatttgtcacgaa
tatcgcgtgccaaatctggatgcttttcaacgcactgaagtagcaaggatagcaactgttgcttttccgtatactgaaacaatcgaggtaaaggca
aatgatttccaacgggcaatggacgagcttttttttagtacaggttcatcagaatcagctttacgctttttcaagtaagaataagaaatcgcttccatt
tttttaaaacaacagcgatgagggaaaagttcaaatgcaaacgagaggaagaaatgaagagatattaaagtttatgcaaaaagaaacagtca
aacaggattatgaagaacaaataaattaagactggataatgaacaatatctgaaaatcaaataagccctcttaattacggatagaccgaaagg
aatttgcggaatatcagatgaatcaaggaaaaaggtaaataacaatgtttcctaaaatacaaagaaaagccgggatattaaatatatactacttt
caaacttaaccaaaagaagtccttattatatcgccaaatacgtgaaaggaagaaaatgtctttcttacgagaaaatgatggatgaaaagagcaa
tcgaacaacctttccttcgatttttatatataacccaagtaatggaaaacggtagatggaaggagtagtttgcgagtggccccaatgtatttaggg
aataaggaactttggcgatgcgatagactaaatttagcggagtacgctaacttcaataaacttttaggttattagagcgtataaataaataaataa
aactagaatgaaaaagtcactgtaaacaaacaagcaaaaatctggaaaacaaatgaaacgaaaatagaagcttgaaaagacgtcaagaactt
ttcatctgaactggatgttttctgcgcagttcactgaaggttactcgataaattgaatatcaaattgattgaatcaattgtaagccgaaaattgtgta
gacaaacaagttttgattaatcaatgtgcatccaaccgacaaaaccttctcttttgaaagaaactggattgtatcctcgaattcaaatagttctctg
ggtaacagaaagttccaaaggacagaacgaacggactcaagaagaaaaaattatataaaaaattttgagcagctttttatagaagtttaatgaa
agttctttcttcgattggacctcacgaaggactggttttctgcctatgttttatttggtgtatattctattaaagaggtttcttcactcaagaaaattga
cctggacgaacaatattcagacaaaaagcaaacaagaataagcttcttgagaatgccaaattctagtcaagacgtgcatggcgtttcaaacgat
tccaaaggaaagaacgaagtaaatgaactcgatggtatgtcgcaagtaaaagtacaaaagttcgctcgctgtccaaaagtttgtactgggggtc
aaactgtactaggggtatcataaaatgttacaaatagaggaaataagactaacaatatgaaccactagacattctagacgagcttgatttacctc
ccaaagtcgacaaggaagaagggaagcaagagcataataacaatgataatgacaaagaacctaggaatcaccaaaaaggaagtgatggaaa
tcttgattctttgattgaggacatgatggataagttccaaggatcgggaaattcggatgcatctgaaaaggaaaaactagcatcgatgggtttaaa
aaacgataatgatctttttcaatctatttttgggaatactcatgaaactgatggagcctccaattcagaaggtataaattacgatgctttagagaat
gctctcaattctctcatgtctcaagtgacgagcaaagatatcctttacgaaccgctcaaagacttagaaggaaaatataaggtgtttttttgaggat
gagaacaatgcaaagaatgaagcgtacggaaaacaattcttaaagatccaacggtgtatcggcatttttgaaaatcctgattaccaagtccaac
gtgattctccacaaattgcaaagctagttgaagaaattcaggaagaaccgcttccaccctcccttatggactcttcgttcgcagcggccggttgtcc
tcttcaatagattcagcctgcgaatatttttgactactgcataaaaactttcgcatatacgcttttcaaaaggttgcaaatgctcacttttgtttacctt
aagagtcctaaactacatttgtacataaaggaaaagttgaaaaaaaaaataatgtccgaatccattcattcatgcaattgtaaataaataataacg
aaaaaaaccatgatatccacctcacctcaaatacggaataaatccaatgtgtttttttatatgacggtgaaaagaccaatgaccagcactttagttc
attttcaccatcaatacgaggataaagtatcaaatagaggctgcatactcaaattcaacaggaacttcctgcaactcctcttcttgttccgcctcta
attcggcaggatccaaaacatattcttctgatgccaactccagttgcttttcataatcctgctgcatgttttgatctataaatcgattcatttctatatt
tccgcctcggtattcattccatttactctcacgtcgcatagtttgtaggtttcgattttgtgtacgcgtcatgtcgcgaaaatgcgcccatgcactcctt
tgagctctcgctttttgcggcgaaatccgtacagggctacttcttttgtttaccgcatgcatatcgatatcagacttatcagataattctttgagctctt
tcatttacattgataccatctactctatgttctgcttcagcaaccgaatatgtatgtgtatagggaaaaaacaatattcagtaccgtaagcatttacc
ctacgaattgtatcgttggaattcactaaacgtattcggcaataattaccttaatgcttcaagagctgatacgtatagctcctgtggccaagctggtt
aaggcctcatgcttatatgatactcctcagaggaggtgtgttacgtggaaatcgcgagttcgatcctcgccgggagcatttttttgtactttaattttt
aatttttttaaatcatcttttattaaattttaaattactgtcagataaaatcctagcctgatcaattcatacttggagtctatcatactaaagggtatatt
gttttacagtgtcacctgaaataggacagacaaataaacgttaattatagatgtgagtctgaaatacaatgtgaatgaattgaggattttgtttatt
tacaacgactttttcctgacaaatccttcagttaaaatgatttttcttgtcgaacaaaacaattgtttactgatatattaaagttcgccaactaaatgt
aatggaatatacagttaataaattcaaaaagacccaattcgaatttggcaatataatcatctatttctatttcttaatctattctcaattctattcata
atttttttttcgtgaaaattccttagtttcttttttttttttcaacaacggcaacttattaacaacattaaaaatgtgcggcattaactaaataacggc
gaaataaacaaaaggaattttcacaatttgtatatctgtatatatcataaaccataacaacaataaaaaccatattaatcatataacgttttcatta
cttcattatatcaaaattaagaagctgcgaattttgatcttgttcttaatttcgtacccatcttgtataaaacaaagggtatgggaatcattatacaa
gatatgacgccaaggattgtacatccccaaccgacgggtgcataagagggtcctgtattattaaacatagcacgagcaaagagaggaaatgca
gcggccatacctgatcgaaataaatcgtttcctgcaaatacggatgcgacgtattttggataacaagaggcgagatattgaaacgtagtttgaa

cattataaaagtacccatggcatagaacacagtactggctatcggcgacatccaatgaacagatgcacgaccggtccaaccgaaccagaaaag
acagataggaaaacagacagtagctgcaaattgaaactttaaaatctcttcaggtggaactttgccattggaagccaaatactttggaattgtaa
ctttatagagcccaatataaaagagaactagcgcaataaaaacgcctaccataattcccatatatgtaaggccgttttctataagcgtgaaatggt
aaaccccttgaaaaacaattggaaacgcttcaaaccatagataaaggatggcatagatcaaaccaatgtaaatgtttaacgataaaacgattgg
ttcagagatactaaggtgaatgggacgccaaacagtttcaagggctaattgcgaaaattttgtttcacgagacgctacttcaccttctgtgacata
actcgggttgccccttgattctcttaagccttttgcacgtttgtatagcaaataggattctgatgtttccggcattgaaaaaagaaggaaaataaag
ctaaaagcacaaagtatcatcaaaaaccaaaattgccatctccaatcttttgccacgaccattgcagcgccgagcaatggacctaaaacagggc
cagctacacagtaagtcagaaaaatgcaccttatttttacttttcatatcgatacttaccaacagcgcctagagaccaaatacatattaaataagg
aagaagcgttggctgccaaatatcagccaaggtagcacctccagtagctaaagcaggtgatccaaagaacccagccaaaaatcgcattggaag
tagcacaccaaggctgcttcccaaagctgtaggaatctgaaggcaaataaaaacaaagagtgttgccaaataaatccttaagcgaccaacctgt
ggtagttctgaaagaggtgataaaattaaaggccccacagcataaccaactacaaacatagtcaatcctaaggttataggaacctcaccaacat
gcatcaccttcatcatctcaatttcaccaggtgtaaatatagcagagccaacgtatactgaaaatgtaagaaggcaaatctgaatggtaatcaaa
ctctttaccagtacaggccaattttgcgggtgctcaggatcattttctccgttccaagtaactatgaattccttttgatttacatcattctctttggattt
ccttgtataacttgatatttgggactcaactcttgttaattcgtccgcatctttatcacgtttccttttagctaaatagcaaatggcttctccaaacagt
gtgtatcgaagaaagttcatttgttggtctaataaatttttaacgattttttgtgtaaacaaaaattccctctaaaatctaaaattaattacagtatt
atttagacgtcaattctagttcttcattacagcgctcctactatataagcgtattcattcgtaaagatgtcatacaatgtttagtaatcgtttcacgaa
atattacaaataaaggaaaattttagtcatactgtgtattcgcttatgttccacttattttagttgaagataatgtcgatgaccttcactcggaagtgt
ctgtctttattaattaaaacagaaaaaaagaaaaacttattgtaagtattaagtaacttatcactttccggcagcaagtctagtcaacgaagctag
tatgaaagagacaatttcatataattacctagaaaactgataattttaagaagagctctacatattcaggttcgattggatttcactttttgatttag
tgccacaaaagcttgcacgaagattaggcttattccttaacgaagatacgaaaattataaagctcctttccatagaaaaatttcgccaatgaattc
gttttttctaaaacactatagaactagggtcgccgaaggttacttttggtttccatttcaaatccatagattgtttacaaacaatttactgtatgtctg
ggtatgactaaatgacacttgtttgtttataatccagcttcaacccaaaagtttggatatttgatcgttggatccttatgggatacttttaaaactttt
gtccattgtttacctttatgcgataagcttaaatgaaagtcaacgaaaccatttgtagcacaagcgaatcgttagcaataacgtccgatgtaacat
gtcagggatagcttgaccaatataatagcacgtattttgatttttaataagggattgtcatgtctatgaattttagaaaatcatttcggagaaatagt
acatcaaactttaagctgatgatggcttgtttattctaccgaaaaaaaaaaacaaaggtcatcagctaattggttcattgcccgccacaaaggcat
gcttcaatgtaaacgatgtcgttttgtttacagacaaagaaatgcgttggcaaaaaaaaagtgggacgcgccaagccaatctatatatttattggct
tttttcaatttgagtttctgtttattgtccccttgaggagaaaatgtggcgaaaatgtggagctaggcgaatcacttgctttacttttcataaacaata
ctgcatcatccatcattagacgaaacttaagcgacaagtaaacaaacaaacaatgggattttatgttcttttcgattacaaaacattgactcaattt
ctgttttgaaaagcataaataccctccttttcttcgttcttttccgcatcttacaagcaattgatcaacttttgtttaccaagccttctttatcgtgcgac
tcactttacttttctcttttatccttgtgttcttacatctttaattctcttacatatatataacacaagaacaacaattatcttttctagcctgttcatcaa
gctttctatccttattatttttattatcttctcaaacgctattttatctgttctttccaacatgcaacaaccttctcagcccatttctgctccacgtaaca
ttcgcattaaccgtcccacggatggccctatcttctctcctatgacttattgcaatccttacatgcctcctgtacctactagcattcctatgggaattat
gcaatcgcatatgaaaccaatgaatgtaagttttgcacaaatgccttatcccataccttttaatgttctctgccaaaacagttctagtgaacaagct
agtattttagccaactttcgcatggatatcactcaacgacctcctcatcctcaacccgctactgatactgatcgaaacaacggaaatgactttacca
actttcaagttcttccttatggttactgggattatcgtgctgtccttattcacaatatgccatctctctacaatgtttccgaatttctaaatcttggacga
tttggaactctcgaacgtgttgtccccatttatgaaagaaaccaactcttccttgcttttctttccgctgctgatgctcttgacttctataacgccgtcc
gttacaccgattttacttttaagaaacagcgactcattgtctcaactatcgaccctatgcctttggaaccatccatcattgaagcctgtcatcgagga
tttgttagcagaaacgtccaaatttccgggtttccttttgattttgacgaagcagctttgattcaacttttatgttctctcggtaaagccgagcttattt
cagtttgcacgaaaaccgactctgtttacatccactacttaagcatcacagatgcgctttattgcatcgattctcttgcttcccacccttattataaaa
atttgaattgctgttgtttttatgaaagatgtgatcgctactacgccactgacttccaagtcgagaaaactgttgctggttctaatttcactaaacctg
aagttgaagaatccattattcataagtcaactcactcttctactcaaacgcatgcaaatactttccctaaaaagtccccagtcaagaaatctattac
caaacttgagcaaaaggaaattccagctaaccctccttgtttgtctgaaaacggtgtcctagataaagaaactactaattcaatccctaaggaaa
aaacagataagaattctgtcgatactggaaaagcaaagtctgttcatatatctgagactgaagtaattcccttagagaaccataccagtcccaaa
aaaccgagcgaagaatcttttcctatttataaaaattctttacaaaagaactctaacactgattttacaaaaaccaacgtctccatttccgttcttga
atgtcattccaagattgctgccttacaaaacgtcaccaatacctcctcctctgtttgccattcatccaagtcaagcttaaatgaaccttctaatgaat
ccactaatactacgatttttcctgatgctgatgtagatgcatcatcttcttcccttcgcgtaccttcagaaagtaattctctacaggacagaaatata
cccaccatttaccctagctcaaatgattcgaatgcaatattttctactattaatgatcaggcatatgacagcctgtcaccgaattctaaaccacatg
ccctcgttgactatgaggctaaagataaagatctacaacaatactctaatgcaacatcaaattccctccaatcttctgaagaaaccacttctggtgt

gaaaaactattctgataatgattctaccccagtgacaaagaaagctttaccttttgtttcctctacttcaattgacacagatacacaaataaagaca
gctcgaaatacggaaatcaccgaacaaaaccttagtagatatgaatccttctccgaggacagttcccttgcttctcttcccatgcaacatgccgat
aaaaaccctgcaaatgacttgagtagtcttccaaagctaacaactgagagttgctcatcgagaagctcctctgccctcacttctcctacccttgttg
gctcgccgcttgaaacttctgcaaaatcttttactgatttctctgaatacaatggaatggatattatatcaaagccttccaacgatattcaacttgtct
ctgatcttgacccttattcgaaaaaccgaactcttttcttaagcaatattcacagagcaaccaagcaattagagattagcaagttgttcgaagggtt
tcctttagaagaaattcgctatctttcaaagaaaaaaatatgttttgtcacttttctggagtcttatgatgccaagattttcttgcacgatcatgaaaa
acagccgccttacctacatggtcgcccaataaaactagagtgggctaaatttaacaatccttttactgatcaactaatatacgcaattgctaatgg
agcatctagaagcatttcctttgaaaaaattcacccgactattactcgcaacgaacttgtctctgttttcagactttatggtcctatagaaagcttcc
atttctcaaaaccaaaaagtgccggatctattacctataacagcatatactctgcaatgaaggcaatggagtactttcaagctcatccaacatttg
aaaaagctaccctttattactcttatgatgatcatcatggtcgaaaaaacgttcttgtcgataaacagccttaccttaaaggaaacaaaggtgatt
gtatctttaaaaaagtttcctttcaagagtctccgaaactttcctctcgctttcacgagtatgattcgtattttccttctcattcaaaaagtaccaaatt
tcgtccaccagcgtgtaccatttctgtcaaagcataattctcctgcattgtttttgaaattcgcctttctttttgtttatcctttcctaatgttattacgtc
aatgtttgttgttactgtttgtttatgttcacaaataaaatccattcaaaaaatcaaaatagttgattgcaagtacaattgtaatcgttaactatatat
gaaaatgataatgtacaataaagacatttataaatctgatcttgacaagaaaattcaccttatcccacaaagggtagtatattcaccaacagaaa
taactattattttcattttcgttaacttaacataaaattttttttatgaagtatatatctctttagtttgctttccttattgatcgcatataattatcataag
actcaattgcagttactcgtctagctatgtacagttaattatgcagtagagggagctttatactcatttgttaaaatttccagacaaaacgagtatct
attatcagtcatcctttagtattctaaaaaaaccttaggtaccaagaacgttggacatataagtccttttgacttcaagctgcaaaacatatgaaaa
gtttccaaacacgatgatacctagattgtatcgatgggtcaaactcaaatagctgtctagagtcggtcagcggttcatatctatactcaacagttta
ttgagtattgatggctatgaagtagttttaaccattttaatcaagctgcttgctaagtctcactttttcaggattgcaagttggactttgtgttcctctc
gcttacggaatagaaaaggcctgaaacggaatattatttattaaaacacaagcttatatattcgttaagactacaatcgtagcaaccattcataaa
caactcgtaacaatcatcatcataaagcatacgaactcactggcacaagcaataagaacaaaaacctgtgtcagatttctcttcaatactataag
cttctttctctgtaaaatgactagcttggaaagcatgctttggtttagcacgaacgctctttaaaagcaattcgaaatcgccgctttgagaatcaaa
aaagcttcgaatcataaatgaaaactgtacgatttgactagtatccaagggaggggctccttcaactggacgacctctaaaggtaggctgaaact
cggaaaatggcatgtaaatactttggtcctctttcgaaaagcctggacaaaagtcatattcatacgataaagtagagcgctcatgctcgtccttatt
ctgatgatcatctttaataataaatgtgtatttgaaattgtcgctctttgcaatattaatctctattccatcaaactctctcaagttccatttacggtta
gggatgtttgtagcttgacttgcgaatccagcaccaccaagagttttcgtgtccaaagtaccccaaaatctagcataagagttgctatcgttatcttt
ctcagcagttaaatggctggttgatgatcctccgcggactcgatcatcaactgcacgaaagtcttgctcgtgccagttttctggttgaccaaacaat
actaacattcttttatccaaaaaatataataattaatcagtccaaagatacaaaagctatcgactgcttgttgtagacaaatttgggagtgtgcaa
gaaaaaacgcctatgtcagacctaagtatggaggatggaagagcaaatacaaaatcgcaagtaaaattaaatacaattttgagacaaaaaga
gatattataataatgatttagaagtaaaatattatataggaggaaaacgaatggatggcaaagcatcaagagtaaaagcattattacgaaagtt
atcaaagcgtcattctattaacagaaaagaaaaaaggtaaagatcgttgcgtagcatgtaaaaaataaaaaaagccataagataccataatat
attcttacgatttgagagcatctagctttccacgaatgtccagtaactgttcgttgtcaatttcctggtattcctgagtaccattgcctccagatacgg
atggcaatgtttgctctaacggacgaacaggctccggttccggttttaatgtttgattaggaacaggtacagatggtttgacagaggacggctgga
attgtgataggatttctttgatttcttcatcttcgtcatcgagctcttcaaggccttcctcttcgacgggaaaaatcatttcgtcacgcatttcttccaa
aataccagctctcgtgagttccatcgataaattgcgcatggtttgggagagctgaggaagacgaactaaacttgaaacactttgcatgatagagg
tgcttgattgcatagctttttcaactttgtacatcgcaatttgatcattgagttgcatactaagacttccaagcatggcttttgactccgctagcttttt
gcgatgtcgattggctcgagcgagttctttggctaagacccgcatatttgagacatcctgctgctttgcaagtgctttcatttgtgcctcagctttcttt
ttaccattttgtaagtacaagacttgccggtcaatgtttcgttgttcctttcgaagagtcgactgccattttcggttctgctcttgcggtgtaggacca
aacaaataggaacgtacagtctgcattttggtaagaaaaagtgtatgttcgactccgttcacgcatgcacggtcaaaaagacacaatctccaaaa
aaagaaagctaactagttttcgggctgatatgttataacgtttgaacctgaaagaagctgttcagagatttgttttcggaagcaatcgtaattgcta
taaaaactccagttacagctcaatttttcttagatccacttcacttcttccaaggcttcgtaagaaaaagaatctatctatatggaatccccaaaca
caaaaatcgtctcgtttctcggtttttttaaacagcgcttattctggatatgatcttgttgcaacaacagccgtttgaaggtactgcaaagttttacag
cgaaaaactgaaaataaaattaaaataaaattaaaataaaaaataagtaatagaacaatgttttacacacaggattccaaatacttttctagtac
aatcgtgaaggtgctgtagttagttttctagaaaatacctgtagtggtgacataacccttactatatataccaagaacaacccttgaacttagcaat
agcaatttactggcatatagaatcaatccggttagaaacatacgatttacattatttcttttctatttaactcgagaaagtatttgatcttataaaaat
atgagcactactcaaattcctgaaggtatgtgtacaaaaccatggcgtagaagagatgaatcttcactgtatgcatccgataccattttaatttga
aatggatgcactagagttgcctaatggttggtttcgaaacttgaaacctgtatgaggaagcgatttggtttactaactgcattagttctctgggctca
aagatctcacaaggagaaccccgacaagaatgttatttatttgactgtagtcattcctgatgctgtggaacctaaaattaatttgacatctgataaa

ttggttatcgacagtaaatccggagctgctgctcattatgctgttcaaattgacttctttaaggaaatagacacagaaaacagcaaatactcactta
ccggtcgtaatattttctttgttctctacaaaaaggaactccaagaagaattttggcctcgtttaactaaggagaagcttcgtcttcactggctccgt
accgatttcgaacgttgggtcgacgaggatgaacaagaagctcagcctgaagccgccgctaatcctttcggcgctggtggcatgcctgacttgag
tgccttaggtggtggcatgggcggcatggaaggcatggactttagccaattgggtaacatgggtggtcaaaatgacgaggaggatagtagcgaa
cccgaactggaagaagccggtgaagaggaaggtgaagagaagaaggagtaattctggaacgtttcccccaaatgggctgcgttgcaagcatta
aaagttaaagtttttgaccttttcagtaattcgttcaggcatattttctttctttctctatcccttctcttctctgcatggttaatgctttatgtatgattta
ttaccatgtgccgtataaacgttacgattttgtgcatagaaagagtgaacaaatgaattctattatgatttgatgaacctatgtcttccttctggtcat
cgtggttttatatacaaactttttaagttatgtttcgtagctacgtataattctcatttcatttttctaaagatagatactttctatttgtagtttcaaag
acagtatcaacggtttcttgcttttaagggaatattgacgtatcataatagaaatttttctgtttgtttcaccctagctaccgaaaggtaccaattccc
aaaagtattcagattcctaggatttcccttgatagaatgcctttttttattcaatagcgcctaaaaaagtcgtagaagtacttgttttatatgctgggtc
tgattttctgctcgcacgtttcattaattcattcagggtgcagcttcatatcaatgtgcgattcttcttccttgtattctattctatgaaaattaactcgt
caattcatttcttttgaaaatatcgacgacaatttgctttgtttacttcgaatctcgtttctttgggttagccaaatacagtcgtctttctagaaggaaa
ttgatgatgtcctttcatttttcctttccgaaaattgaatttcaacgtattcttacatgtctacttcgacaatggcaactacactttctcctgttcctattt
actttcacaaacctttttcttatccgttgtatattccaaccaaagttcctatccaagaaagtcagaagagtcttttcaaaacatagatttatagaaat
ttcccagatctttctttcttgtatcgcttgcgttctttcattgtggatccacccgacgacgccctggtttcctcttcaagtattgcttcatatagagttatt
ttgcttttacgttatttcatcaagggatcatttacaagccccccctgttctgcaatttaagcatactttattgtttcagtctgtttacattttctgttttaaa
cgtcgattatttgtctcgctttgcaagtgaacaactacaattatcaacttttcctcgattactgaatattttcggtaaccatattcatgtttcttatata
gcggttttgtttcttctgaatctcttcccattattcactcctcgagtatggtatccaattcgaccaaatgttcttcctgacatcccttctaccgaacaaa
catgctccttattttcattaatatttacctatggatggcttaataatattatctggaaggcttggaaaagccctgtctcccttgcagatatgcctgcgt
tgcctaatacagatcaaactttaatatggttttcggttttccaaaaatatcggaaaaagggacttttaaccatggtcttgtgctcattgcgatcgaat
attctttgccttgttttgctttctgtaattgtcagcttgactacttttatttctccgtatgctataaaaaatttacttcagtatcttcaaaatcatcaagat
gactcctcaaactatccaattcatttgcttttgatgctattatttgggccgtacgttgcttctgtcattaaagaatacagcgttcatatctcaagacgg
tttatgctgcgtactaaggcaggcatatcccagttaatttatgaaaaggtaatccattccaagatttcccagattaccatggatggttcgcaagcta
atatagatcatatatataatctgcttgcttcggatgtcgacaacattgctaacatgagggaattcattggttttattgtccgaggtcctattgatgtag
gaatagcaatgtttttcttataccagttactcggctggagtgcttacgttggtgtacttttggcaattgttagcgctcttcttcctcttttgatatcctcc
aagttagccgagcttacttctatatctaatcgttccagtgatgatagaattcgatttactacagaagttttgcggagtatcaaagttacaaagttctt
tggttgggaaaagccaatgctccaaaggattcaacaaaaacgcaatagtgagatagcaaatatttggaagttaaatctttttgatattacttttaa
aagctgtatgaaaattgccccttttctaagtatgtttataacatttgcaacgttcacgaaaataatggaaagggaattgtccccttccatagctttta
cttctatttcccttttctctgtacttcggaatcaatttataactctttcaggtatacttcgacaaatgattcagtttcgtgtttctcttaatcgcattagttt
gttcttggcgaagggtatagaaccagagcaaaatccgctcgactttgaagggatttcattcaggcatgcttccctatcttggtgtccatttccttctt
ctgctcaattccagcttcgagatttgaattttacgattccacgtagtcaatttacgttggttgtaggagctacaggttctgggaagtcaacactagct
gctgccatcctcggtgaattgcatctcacgagcggcgaaatgaatgttcctttgagggaggaatgtgttgggtatgtcccccaaacatcctggctta
ggaatgacaccatccgtgctaatattcttttcggtgaaacctttgatgaatatagatattataaagtaattgaggcttgttgtttagaagtggatttc
gaaaggctacaagcgggtgatttaacttttgtccagtcaaatggattcagcctttctggtggacaaaagcaacggatttcgctagcccgtgctttgt
attcgaatgccaatgtttatatctttgatgatatttttttcagctctagatattgcaacttcgaagaaagtttacaaaaattgctttgattctgggctctt
aaaaggaaaaacagtaatcttgtttactcataatatcaatatgtgtctttctatagcagatattgtcatcttgctggagaatgctacagctaaacttt
taccttcgaaagaagtaaaacagcagcttccttctagcctttttttcccaaaataatgacaatagtcattattcttcgcaaaccacctttttgtctgatc
cttacgagaatttccatgaagaaaatctcgtatcttcagaagaggaaatctcaccagaagtttctaatgcgattaagccgagtcttccttccttagc
gtttcagtatatgcgatgctttggatccacatcttttcttaccctttgtgcgttgtttgtagtattaactcaggtgatgatagcatgcatagacttttgg
atggccttatggtccggtcaatctcataaaggactgatgcttccttttccaatgtctttcctgcaggggtatgcatttctcttaatattttacttctttat
agatttaatacgtggaatatttttttcaaccggaggccgtaaagctagcagatctattcatgaacgggtctcagaacatgtttttttgtagtccactac
aatggtttgagaaaaccgcaataggaagagtcataaacagattttcaaaggatatatcttctgtggataactatttatgggtttcgattgaaaata
ttttatgctgtttaatggcattgttagtgggccttggaaatgttacaattgttatgcccgtatttattgctccagccatttttgtttgcatagttgtatat
ggatttgcgtatgtttattcaaaggcgcaaaaacagcttactgcttgttcttcttcccaaatatcgcctgtgtttagcttgttaggtgaaactctttctg
gattaactgttattcgtgcatttcgaaaggaagaagcgtttgaaatagagaacatggctgtccttgacaatatgcttcagtcccagttcgtttcgtat
gcgattaaccgctggctagcgattcgtactgatggcattagcggcttggttggatttcttacagcgtttattgctttatggaagcagaacatacctac
agaacttgttgggttttctctgaacagtgcagttggacttaatctttgcgttttggtttttgttcgagctagtaatgagttgattacacatctgaatagt
tatcgaagacttaatgactatattaatcttcctctggaggaggattcgaccgcatgccactcccattcgcaggatgatgaatggcccagattgggg

tctctaaatgtgcaggaattgacagttcaatatggcatgaattcgcccccggtacttgaaggggtaacttttgaggttgtgccaaaggaaaagctt
gccatcgtgggacgaacaggaagtggtaaaagcaccttgggattgagcttacttcgatttacaaatcaaacaagagggaacatagaaattgacg
gtgtcgatgtaaatacactacaattagagactcttcgccagcgagtaagcctcattccgcaagatcctgtcttgatttcaggaacagttcggagca
atttggatccattcgaatatgtgagtgatgaagaactaaacgctgtactgaaagtaacgtcttgtgagactttggtgacatcggcttcaggaagtg
cgggatcgtcgtttgcgattcacttggacactcctgtagaaacgggtggtaccaatttttcacaaggacaaagacagattttggcgcttgctagag
cgcttgttcgaaagtctcgcatcattattttggacgaaagtacagcttcagttgacgacgctacagaccgccgaattcaagagatgttaagggtcg
ttttccgggacgcaactgtgttgtgcattgcacatagaatcaaaacggtattagattatgacaagatcttggttctagactcaggaaaagtaatag
aatacggatctccgaagtcactgtttttgaagcgtggttctttttggaaaatgtgtcaacaaagtcaaatatcgctataattaatgattgtttgatta
cgagccactcatatgaaataaaaatagaatacttttaaagggaatatctttgaaaggactgctgctgacaaccagaaaggtcattcataaccaat
tttagcatttaagataattagaattagaatatcgttaattaacaaggatcatatatgaaaggacgaagaatgtattgtaaacgctaataacatgaa
tggctacagtgtaaaagaaaaagaattagtttattatccactactaatatccactactaattcatgaggtaaacaagattgcaaaattggataatg
agaaacagggaaaaagatttctacaaaaaataaaaatctattaagagacgttgcaagaaatgaataacgtgttaacgaacgcctaacttgaca
gaagattattattgctttgaaagcattattcatttattaatgtagtataacaaagcctgcgttataataattaacggaaacgtattggaaatgaatg
aattcgtgcttgggttgtttctacgaagaggctgacgaaaggcaaagacataccacatgtgcaaggttatggaaggaggatttcattcgccaatg
tcatacctttaattttcagttctttttctgctttgtattgatcgctcgtctgactttatcggggaaaaaagcttcgcagttttaaaccatcccgtcgcact
ttattgttgctgactaaccgccaataaacaccgagcaaaactggacaaacgttacaaagggattcgatcgtagcaaattaactttcataagagag
aggtctgtttctgttcacttttgcacttaagaaacgtgatcatatcattcgaatccccgattctggtcaaaatcaggcatttctttttatgtgaatttaa
taattaattataatactattaatttcattactttattttaatgttagagtttgcttttctggaatttctttttctcgcagttctttcctcacttcccaacacg
ccagctatcgtagaaaattatgcatcgctttctacaaaccttaagcagtacggacgaggctttcaactgcggaagttcctcgcccgagcacttctct
ttgctcttgatagtcgctacctataaaccttctcctccaccaaaagggttacctccacctttttatccacatatagcttaatatatcgacatgaagtca
ccggtcaaccactaatcccatagggatcatgtatgttttgatgattggctgtagttgctgattatcattggctaatgtattcgtcatattacgtaatgt
aactgtggtgtcaggcttcgccctttgaattatttcatctcagacatctttgtccctcctctgtgtcatggttggtgccactttttctacaattcgtttg
tatgctgcttgacaaaccagccaatcacaaattgtcatagatgatatataagacccatcggcgccgtcgaatatagttactttgcaatcttcgttctt
atttttatttaattttcttgtattaaagagtttcgtttctctacttctagcagcgaattttgggtatccgtattcgttgacttattaatttcttatatcttat
tttgcaattgccttttctgttaagagagaaggataaaagagaacaactttttttgattctcttgagttgcttacgttgcaattgattattcgtacacctt
actatcttttcgtttctgtttcttcgaaaggtttctttgaactagctatatagacatcctttctacatttttcttttattttcttgtttggttttgttgtggaat
acttttaaaccttgatttcttatatttcttttatctatttcgtttttgcactactattatactatcattatgtcttctgttcgtgaagttgcaaacgttcaaa
tttctattcctactggtaagacgtatgaacaacctgttggtttgttcattaacaataaacacgttgatgccgtcaatggaggtcgcgtgaaggttttc
tctccttctaccgaaaagcttgtttgcgaagttgttgacgctgacgaagaggatgtcgatattgccgttaaggccgctcgtgctgcttttcaacctga
tgctccctggcgcaaattctctagtgccgaacgtggtcgttgtttgaccaaattggctgactttattgagcaaaacttggaatatcttgcttccattg
aaactcttgataatggtaagagtattactttggcacgaggtgatgttcaagcagctgctaattgcttccgttactatggtggttgggccgataagga
ttatggtcaaaccattgagaccgatacttctcgctttgcttatactcgtcatgagcctattggtgtctgtggtcaaattatcccttggaacttcccctt c
cttatgtgctcttggaagatcgctcccgctgttgcctgtggtaacacagttatcttgaagactgccgagttgactcccttgtctgcacttgttcttactc
aatttatcgaaccctctggcttccctcctggtgtcattaacgtccttagcggtgatggtcgtcgttgcggtaatgccatggcttgccatatggatattg
acaagattgccttcactggttccaccggtgttggtcgtatggtaatgcgttctgctgctgcctccaacttgaagaaggttactctcgagctcggtggt
aagtctcccaatattatttttaatgacgccgacttggaggaggctgccgtttgggttaactttggtatcttctataactctggtcaagtctgctgtgct
ggtagccgtgtttatgttcaagagggcgtctatgacgagtttgttcgccgcgttgttgctaaggctaaatctttgaaggttggtgatccttttgatga
ggaaaccttccaaggtgctcaagtttctaagcagcaatatgagcgtatcattggctacatcgagagtggtattcaacacggtgccaagcttgaggt
aggtggtaagccccatggccatcttggttactttgttgagcctaccattttatctggtgttactgaggatatggccgttggtaaggaagaaattttcg
gtcccgttttggccatcattaagttcaagactgttgaagaagctgtccgtcgtggtaacaacagtacctttggcttggctgctggtgttcacactaaa
gaccttaaaaatgctcttaaggttagcaacagcttggaagccggtaccgtctgggttaactgttataaccttttgcaccaccaaattccttttggtgg
ttacaaggagagtggtatcggtcgtgagctcggttcatatggtttgaccaattacactcaaactaagtctgtccatatcaaccttggcatgggactt
cccatctaaacttatttagaatgatcaaaaattgatttttgaggacagtatagcatgtattatcatcctcaaaactccccttttgctttttatcacca
aaccttctttcatcttttgtatgtgtgcgaggcatgatcaattaagttttattgacaatgattgcactgcgatgtccctttttttatgatctttattttattc
tagttataatggtttaggaacaaggtaattgaataagaatcctcatttattaacgatatgaatcttatttttttttattattttacgagaaagtaagttg
tttaaaagttgagaaaggaaaagctattattttcaaagaaagcaaacatcaaaaattattgtacagttgctccaattcttctggggacaggcagat
ttccaatagatacgaacccaaagaagcggtttctgacgcatcgtcgccaaataaatggttttccaaatcgccgaatgtacatgaagccaaagcat
aaataaaccgcgaatgtagaccgtttccggcactcatgagcctggaggacaaatcagcaggagcaattagatacaaaagtcgaataatggaca

ctagtatttcagcagtatccgatgtatcgaacatagcagacagaagccgttgatggtaccaacataagccaagagcaaagcaaggcataattgg
aaaacagttccgaaggatttccgtggtgatcgatcggtttttttgcaaaaagtttgtgaatagcgatataataatacgatgaatttccttccacttca
taatggaataaacatcattatcttcccgtgttggttctacgaatatatgagaaatcgtagttatcaatcgcaaaattgaatctcgtccagtttcgtct
gtatttacaaatttgattgatgcacaataagaaactgtcgtggaactcatgagtaacagtaagccttctttgatgagaaatagagagtcatgaagc
agaagacaatggtaaaagacttcgtgttttatttgcccagctacctcctcgaattttgatggagctaagtcggatacgattggcatgtagaatcgta
aaaactggactagtggttgaagtactcctggggaagttgtatggtttttagaagcaatggcctcgatagcattacttatgagagccggaatgtgtg
tatgtaaaagtaaaacgcaactatcaggttcaaaaaaaacaagaccctctaggagttgaacaaggccgcgtaaaggcttgaaaaactcgtgtgt
aacggccattttgaaaagactgacaatagcttgaataacagatttttgatattcatcatactctgatgacaaatcagagttatatatgtagttgcat
aatgattcgtaaagtttaaaacaaggctgaaaagactgaaactcctcaaacaaaggtgaataacaattcagtatgttcgttaaatttcgcgacaa
ctataaatttattagaaaaaaagtactcaagagctcttactaaaaaagcttacaaataaaatttcttctctcttttggaagatgaataactcctgta
tcatactgttcgaatacatcggaaaagacatatcatgttgcatattcgattctttcgacttggaaccctctacatattggggtaatgatatttttcgtt
ttttcttcagcgtcccaggtttcgaggctgattttgccttatgggacggttcagcctgagaagccgaaggaaataaatggtctacacttggaaaccc
atctttgttaggcttttgggaagacgtcgataaaacagaggtctgtttcggatcccgttgtagtttcttcatagcctcgacggcttcttgggcttcacg
ttcatgaaagagagatttcgttttcgcatattcaatctcattttgaagctcgtctagcttttttgatatgattcttgctgttgttgaatagacctgttaa
gtgactctagcgttaagttattttgttcggcctgtttggcaatgttcgagcgtagaattgcattctcaccgcctctgatgagtaattgctgttggagat
cctgagaacccagatttccgtaatcgaatacttgagacagcgggggttgtgagcccaacggatttacaagggtcgtatgaacagaagaaacttg
ggtaggtttctgggaaagcacttgttgttgagcttgctgctctagctcatccaaatttacgccttgaaacagatcatttacgttaatgtctgcaaattc
taagtcatcatctgaagacatgggcaggtcaccgaaaatatgttgaattcacgttcatggacgtgtatccaatgaaacagataggagaaaataa
aaaaattgaaggaaaaccagttcttgtccgttcttctactcgtaagacagttctgaaactgagtttacatcgttagtaaaagatcgctcaaacgcg
gaaaaatagtacctaaaaaaagatcaaagcaaagtatcgaaattcatttattcgaataaaacaggtgtttgaccaggaaatcctccctattttat
atgcaatttaatgtagaggtcgtttgtttacaaaacgcgttgagacgcgtggttaatgaatcttagcgcgctatttagtaaaatttggtttatggttt
agtgctgtgctgcgttcttacactttacaaatatttgcgcgactttgctatttagtaaatatttacgaaagaaagccaataaaaatttaaacgagatt
ggaaatgagcatcatttgtcaatcgtgatggcttcaggttttagttcttttatttaaagcagacgggtaaatgaaattctcccgattttttttttttat
atctacagccagtttttttatgcttccttacatgaagaaaccgatttcaaaagccgtagattggaatgagagaatcatctctattcatacgttatgc
acagtttggaatgacatttatttatcctgagggcaaatgtaaatacttgacacaaacagttgaatacactgcatgtaaacacacttcataagtaac
attgtcctttcgcctcaattatcagatcaacactcaatgagcgtgaaaacgagggaaataacaaatgacaaaaaaatccaaggcattgtccttgc
ttttcagctactttatcattgtctagtaaaataattatctttcatgctgaagaatctgtaggtacttgtccttgtaagttgtgacttgaaggattacaag
aaaattgagcttctcaggctaaacttcttggcaaacataaacattaattcgactgactcgtgagtgaaatcaggtacatattatattgacaaagaa
gatgcaaatagtctgctctatagcatgcgcagcggccataaatgctacttgtctccagaaccgttggtattacctatggtccttggacttcatagaa
gcaactgaagtgtgccagaattcacgtcaaagcatttataccccttgttgtagtcatagagcacgattaatggacgagaaatcagaatttaatgaa
aatatgttatgtttttacagatgcctttttatgagtgaaaagaagcgttcgaattgaccaaatctcatttacagcaagtaaacaatgattaaaata
cacaagtaatgtaaaacacaatattttcagtaattataatgtatcaggatggctttcttgcatccactctttcagagcttccagggactcccaccac
cgttcattttgtcaaaagcaactctcctcaatgtcaacaaaatcctacgattatttaataaaattgcttcttattggagactcaggagtaggaaagt
catgtcttcttctacggttttccgaagattcctttacgccatcttttatcacaacgattggaattgacttcaagattcgcaccatcgaactcgatggta
agcgcattaaactacaaatatgggatacggcaggccaggaacgattccgtacaattacgactgcatactatcggggagcaatgggtattttgtta
ttgtatgatgttacagacaagaagtcgttcgacaacattcgaacgtggttcagcaatgtagaacaacatgccagtgaaaacgtttacaaaatttta
atcggaaacaagtgtgattgcgaagatcaaagacaagtatcgtttgaacaaggacaagctttggctgatgaacttggagtaagattcttggaag
ctagtgcgaagaccaatgtcaatgtggaggaagctttctttactcttgctcgtgaaataaaaaagcagaagattgatgccgagcatgaattcaac
aatcaagggaataacgttgatttaggaaatgaccattctgtaaagcgttgctgttaattccgcttttctgtacatggcactgttcttattatcttgcgt
ttccctttttgatacttcattagcagcatcgtctttttattggcttggcattgtttcttgctcttaatgaattcattatgatactgataggttggctttgttc
tattaatccattaggcttgccagtcggattctttttattattattgtttttcatttttatatggtgtttcagcttgatgctttctgatggatgatagata
gttcgcgtcgtaaattccgtttctttaatgcttttacatacataacataaatctcatgtctaatatcaactttctatttttgtcaaacttctggtggtacc
ttcgtttttgcggtatttaaaaatcctcttttgagttaaactgctcgtaaaatgtctctaatcctctctcccctttatatatataatggagtataaagaa
agatatacaacctgaagagcgcaaaatagatagtgaatagcaatcatttctgctggtgttatactaatactgcaagaaactttgttttgtactttat
ttacgcattactgaaaaagcaagcatatagaatacataatcttctattagcctgaaaacaagcggcttgaatgagaattgtttaaaaagtggttct
tgtaaaaggaagaataatggatctataatttgctttggtacagcaaatagtcaatgtgatcaaatggaactttttttttgtttataacattacggtaa
gcaatcaagtttttcccacttctctaatacgaatcaaaaaataaattttaaaacacttttacacgaaaacaatcttttggaattgtctcggtcatgaa
tttctatttgtagataatcaaattgtgtttacctgatcttattaacgagttataatgattgatcactgatagcacagtgcaacgataaactgaagatg

gtttgcccacaacgttaactttccatgcaaaaaccaaccttccaaaaaccaacaacgtcgagttggaaaatcaaaatgtcaagccaagttactta
tttgtctactcgtggcggatcttcaaacttctcttttgaggatgctgtggtatgtgagaatgggtgttcatatgtggttttgctcagtcgctaatgaatt
gtaaactagttgaagggattggccagtgatggcggtttattcatcccaagcgaaattcctcaatttcccgagaattgggttgaggaatggaagaat
aagagcttttcagaagtcgctttcgaaattatgtccttgtacgttcctcgcgaagagattcccgctgatgctttgaaggatataattaatcgctcata
ttctaccttccgtcatccggagactactccaatcagaaatttgaaaaacggtcttgatgttcttgaacttttccatggtccaacctttgctttcaaaga
tgttgccttacaatttcttggtaacctctttgaattctttctgactcgtaaaaatggtgacaagcctttggaacagcgcgatcaccttactgttgttggt
gctacttctggtgacactggaagcgctgctatctatggtcttcgaggaaagaaggacgtttctgtcttcatcttatttcccaagggacgcgtttctcc
tattcaagaagcccagatgactagtgtgttggataagaacgttcacaacattattgttgacggtgtgtttgatgactgccaagacatcgtcaagca
agttttcggtgacgttgaattcaacaaaaagcatcatattggtgccgtaaactctatcaattgggcccgtatcctttctcagatcacttactatgtct
acgcttaccttttgacagtcaaggctggtaaggctgaccatgttcgcttcgttgttcctactggtaattttggcgatattttagctggttattatgcca
agcgtatgggtgttcctgctgataagcttgtcattgccactaatgaaaatgatatcttaaaccgctttttaagtccggtcgttacgagaaagtcgat
tctagcatcgctcccgctaactcttctgcttctgccaaggaaacttattcacctgctatggatatcttagtttcctcaaactttgaacgttatttgtggt
atttagcattgacttgcgaagctcctaaccatactcatgctgaagcctccgatatcttggccaagtggatgtcagaatttaaacaaaacggctctgt
tgctgttcgtccagaagttttggagactgctcgtcgtgattttatcagcgagcgtgttagcaatgatgaaaccattgcttccataaaaaacatttatg
agactgaacaatatgttgttgaccctcataccgccgttggtattgaaactggtttacgttgtttgaaatcatctcccggtatcacatatatttgccttt
ctactgctcatcctgctaagttcgataatgctgtgaatcttgctcttagctctttctctggatacgacttcaacagccaagtcttacctactgaatttca
cggccttatggatgctgaatgtcgttccattgctttcggcaagccaaacattgaacgtcttgagcaacttattgaaaccgcccttgatgaagaaaa
gaaataaaggaaaaggtcgcttaatacccaagaaccaatatacctttgtagttagaacatccctttttttgtcttttgtatattaattttgttatagttt
tggtacctcaatgagagaataaaagattttcattatatagaattgagcaattaattttctttgtggagtaaatacttgattgaagttaaatagaaag
ttgtataagagacatggtaactatggaagtttgtattatgaatcaatttcataatctggtgctgctacactttctttatggtgaaaaacaagcatgaa
aataagatcagtttttttatgcttttaagggcgaatgaaacacagaagttttcaaactgaacatctttctaattgaaataggcattgcctaaaataaa
atatattttcaaattacaataataccaagcaaaaggaagttacaaagattaatcaggattgaacttatgaatgtttaaaatcaaaatcagttcaac
agaaaagaaagaaaataaataacttagtgaatgaattttctgatcgccgaacctgaaaagacagcattgaatcaatcgtcaaccacctgaaact
tgtattggtctatttgctgaaaatactgtctcaagtcttccatacgcttttgctgttcgtcgtctagtactttttttccctcttggtcttcatcgctagaag
agccatcggttgatctcttattatcatcatactgtgaattgagattttcgttatccaatgtaaacgaagagtccgactgcctacgagaaagcttcatt
cgtttcgactgtttccatagtgtctctgactctcccgactcggattcagatgctgacggtaagggtgatgagttaggaacagtgtgtagaatggaaa
gatccacctcattattcctgcgagcaggacgctgtcgcagtcgctttttgaggttcccatgaggaataggaacaaaacgttccaactgccgagtcg
tcgcttctttcgggaacgaggaccgtgaggatagtttggatgaagaaggtggatgcatcgaagaagttgtaacagaagacattggaataggaga
ggagggggtggagctaaaattgggaggagaagaagcaggtagaggtacttgtttcggtgaagaggcagataaaggtggaggatgtcttgaatg
aaggttgaaggaatgagggtcttctacatttttcagaaattcgttctcgtttacaacgggaggagaggaagatagaattctttcaaaaccgtcttg
atccgttacgccaacgttcactgcttgatttgcactgcgtaaaacagaagagtccgattccaacgtcgaactaagacggaccccttaacagaat
caaagccaaaaggatcatttctgattggaattggctggggaacctgtatgctcagtaattgacgcgcatttagttttgtattcgacgtagacgactg
ctctgtcgaattcaaacgagaagctcctgacgaattacgttccataaaagaaataaatatacaaagtcctttctatgatttgttacaatatcatact
tgaaggtcctttcaggccgacgcgttttggattttcttaccgtagaaaggcgattttgttttcttccaaagatgctacctattcgttacttaatataga
aaaaagtttttttaaagtcattgaaaatgttaggattgtatagtgcagtaattaaagtggtaagttgttttcatttagcatgtcaaaaataaacaatt
cagaaaaagaaacagagaaaattatttcatgtgtattttcatgatggaaacgatatcggagaagtgtagatcacaaactttattattcagttatgt
caatttaaaatacgaaaatcatagaaaacttggctatatataaagcattggttaaaaacaagatgataggaacatataaaaaaataaagacag
aataacgaccaatgtagaaattcattctgactagacaacggcttcttcaggtgagtaaggaaatttcctaaaaaagtatgacataacgaatgaat
gcttacaaacagtactatcattaaatgtctttctttttctccaaagttttggtagcctcagcgtatttatcagacaaagcagagtactctttatcacga
gattcaagtttggtgcgcaatgtatcgagttcttctaaagacttagtgtcagatttgttggatttgagttcggattgaagagcttcaactttttcttca
gaagcaatcagggttttgagagagaggtaataacggttaacgaccaaggaaagaaacaaagcagatccacagagatacaagtttctttgtgca
taaaattgaccagccttaaacccagtacgagcattttcagcggtagtagaaccggctcctgacatatcaaattcggtactaatgcgaaccactcta
cgaacagagtcggaaaacaggaccaaaatacaaatgatcataatctacaaagcaagcccattagtataaggttcaaaaaaagaagcagaggc
cagcccttcttgatgatcaacatctctaaagtatttttttgtagcatagcatggcaagctagcgaacgggcatcattttaagattcaacctgacaaat
aactagtctatccagtttcgagaggaacctatcgcttcagcccaatctgcccaagctacaaagtcactcacttagagatgcgacttcttccattcct
ttgtcttacttacctttaaagtatgctcaattttaccagcaataggagagttactgatggtgttcaagatagcttttctcaccctcataggcaatggta
agctaagtatcacaaatgatactatttctagcatcagcagaataaatacaatcatataatagatcgtcattttggcgatttttcctaatccaaacga
atttggctttggtaaaagttgactgtgaattccttcaagattccctaggtctcttgtagcactaattaatgacgtaagcttaacgagcaatgaacag

gttcataaaatggttccacgaaacaatagaaacaggagattcagaataaaagaagctctcacatttgttttcgtagctttagaaatgtaaagtac
attggagttgatagcagataaaagtgttgtgtggtaaatacaaagacaagtaaagaaataaaagccgtcaccatttctataaagaaaacgttgt
aattgttttggtcttggacgatggtactggtttcaacttgtacctatttatccttttcaaatgttcaattataatttgaatgaaattcctcaaacaaaat
ttccacgtttcaaggacttaatcactctacgaaattacatttacatttggacagactccttctcacaagataacctatgctttattaacgacaatctct
atgcaaataattattaagaaacatagtgaaaacatcaaacaaaataaaaaatcttcaatcttttaatactaaatatacattcctttaccagtcact
gcgaccactggtagattgtttaggtcgagaaggactcaggcgagcacggctacgtctgatgttcatccaagggaaatttttctctctagcggattc
aaaagcatcaatctttgtggcctttttgcaaggttaagccaatgtcgtcaaggccattgatcagacagtgcttacggaaggactcgacttcaaactt
gatctccttgccgtcataacgaatgacttgattaggcaagtctacagtaaattctaactccttctcagcagcagccattaattcattgacttgctcaa
caggtaagggaataggcaacatgccgtttttgaagcagttattaaaaaagatatcagcaaacgaaggagcaattataacccgaatgccaaaatc
attcaaagcccaaggggcatgctcacgtgaactgccgcatccaaaattctcttgagcaaccaggatgctggcattacggtatggttcacggttcaa
aacaaactcgggcagttcattaccttccgcatcaaagcgaatatcataaaatgcaaattttccaagaccagtacgtttgattgtcttcaagaactg
cttaggaatgatcttgtccgtatcaacattcgtaacaggtaaaggagcagctataccagaaacgacagtaaacttgggaataccagcggtagaa
gagttgggtccgtcagcgacacttgtagtcataccagcggagtcagttacagcatcagtagcatcgtctacggcaagggtaacaacgctaccagc
atcatgagaagggtcataattcctgttcgtaatcacagtaggagttccagtgttggtatcaccaaaaaattcacggacattgcagagatatccctt
aacagaggcagcagcagccatagcaggactaacaagatgagtacgacctttggcaccttgacgaccttcgaagttacgattggaagtcgaagc
acaacgctcataaggcttcaattgatcaggattcatacctaaacacatagaacaaccagcttcacgccagtcaaaaccagcttcaatgaagattt
gatccaaaccctcagcttcagccattttcttgaccaaaccagagccaggaacaatcatggcatgcttgatgttgctagcaattttacgacccttgac
gatggaagctgcaagacgtaaatcttcgatacgtgagttggtgcaggaaccaatgaaaattttgtcaatgggataagaagtaataggaacgttg
ggttcaagacccatatattccaaagagcgctggatcgaagtagagcgaacggtgtcattgaccttggaaggatcagggaccaagccgttaacgg
gtatgacatcttggggagaagtaccccaagtgacagtaggtaaaatatcggcggcatttatttcaacttcaatgtcatatttagcaccctcatctga
acccaaagtcttccaataagcgacggcttgttcccagtcctctcccttaggagctaggggacggttcttgacatactcataagtagtctcgtcggga
gcaatcataccagcacgagcaccagcctcgatagacatgttacacatggacatacgagcttccatactaagagcttcaattgcctcaccgcagaa
ctcaataacgcaaccagtaccaccagcagtaccgatgacaccgatgatatggagcattaaatctttagaagcgatgccttcagggagcttgccgc
tcacatggatgcgcatatttttgctctttcgttgcaaaatggtttgggtagccaaaacatgctcaacttcactggtaccaataccgaatgccaatgc
accgaatgcaccatgggtagaagtatgcgagtcaccacaaaccaaggtggtagctggcagggtaaaaccttgctcaggaccgataacgtgaac
gataccttgacgtttatcagtcataccaaaataacttaagccaaattccttaacgttgtattccaaggccaaaacttgagtacgggaatcgggttg
atgaataaaggattcaagatccttcaaatctttacgggaatcggtggggatattgtgatcgacagtggccaaagccagctctggacgacggctt
ttctaccagcagtgcgaaggccctcgaaagcttgaggcgaggtaacctcgtgaatcaagtgacggtcaatgtagagcaaacaggtcccatcttct
tgcctgtcgacaacatggctgtcaaaaaccttgtcgtaaagtgttttagggataatgtacatattccacaaatcttatgaacagatcaaataaagtt
aatgtgctcaagaatagaaacactggctaaaacaaatataaaaaagaatgtaaaaaaggaaaagaagcaaaagtcaactgaattagggaat
gcggtcctataaacaaaacaagacgcaaatcaaacaatctcaaaatccaaatcgaaatgcgtttacagatatcaacaacccctccgtttcaaga
agcaaatctcttctcaaggcactacttatataaatatccaagaagccttggtgtttggttacgacaaacttagatgggcggcgatctcaaaattata
gaaagcagcttaattaaaagacttctgattggcaattgataagatctggaaacgatggacttaccgatgactaatggacgccacctaaaaacata
catcgcagcgcaagaccaaatagagatatcacggtaatttctctcagagcttacataacgaagttggtcgtacgaacgtttgctaacaaaagatg
aacataggaaaaaataagaggaaagcaaggtaagaaagaagagcaagtggctagctgtctgcaaagtaccgaaacaaatttactattactctg
taagattagtttttaaaaaaaaaaaatgatattattcaaaagagaacacatgtctttataaaattgaaagcgaaaaaaagctcgatagaacca
atagcgtgaatggaacgggttctttctttgtttacaagtaacgtcataaaccaaaggaacgttggtgtgctacttgaacagtagagtgttggggag
tcagaggtctagcgaaagggccaatagggatgcggcccacaccaacgattttggagagaactcgtaccaatccccctcagttaacgaaatcca
tacaacataagcgagtatattatttgatgggccaaaaactacgaagggaagaacttatacataatacaaaagagagaacgggagaaaacgtca
gccttcataagggttttctattatagccagtagaagtagttattttcaaaggaaaaacgctcgtcctaacaaccgcagaaggtattaatttgagaa
acgaaaggaatcacattctacgaagtttgatgtactttgagcctaagaggattcagtcttaggagaaaaaaaaaaaaaaaaaagaaaagaaaaga
aatagaataaaatagaattgataaaatatttaagatttattaattcgcgtaataattactcccttaagagacgagactgttaacctcgcagtcagc
aacacttaggaagaaatataggaatttggaaatataatatacgagtaataatggaaaagtcaaaagataaatacctaaagtaataatgggttgg
tttcttaagcttatacagagtaatagtaaacgcaatgcggaaaaagtcattggattgaatggatcgcctatcgcctatcacatgagtggtaagtta
atgtggccaaaggccaccaaccaagggcaaaagtagtagtaaccaccaggaaagccaagttctaatggccgaaacatgcatggatgatggaa
agacggtcatatagagtggaattggccttctaaagtattggttaattatgaattgtacgcagagaagcaatgtgaatgggtttcatcccaatatat
aaatcccatttctctatttcttacaattttcaatcaaaaattacggaaaacattgatttgtgcttgcgacaactgctaccttcagataccattaacata
acaataccttttttttggaaaaataaaaatctcacaaaatacgtaggacatcaagagtctcgtgaagaccaagaaaacaaagtggaaacgattt

catctttattcacaaatatctcataacttcgctttgcattgattcaataactttacaatacttaacttttgttctcgtttatcggctaaaagcctttcttc
aagcttttcttgcgtcccttaatctcgccagcaagtttcgtttgttcacagttttgagtgccgaacatttaccccaccttctcctacttttccatcacttt
tccattgtagataggcattagattcctatttgtaatccacatcaaggttccttcgcttcatttcggaatttggaacctaaagtatcagtctcaaaagg
tcaataatattttaaagctcagattgtagtcttattcaccttttcgtgctttgcatctttttacttgccttgcctttatcgttttattattcctctccgttttt
atttttgaattaaatcaggccaggtcagtcaactctgtgttacttcccccgttcaattcagttacctaaacgtcggttttgtaccagtctagctgagtc
cctgtgcgtgtgaaagtatcttggtcattcggtttacatattaagtaatacatctaaccgatcgttatcctttctggtgtccattcatccccgttctcttc
tgattcctttgtcctaattctacttattagtaattttaaagttctaagctccttctgaccgtattatttattacatcgtctatctttattgatttatttcttag
gctttgtttgccatacctcttagtcttactacggctactttcctcctcttgatacttccttaaatatcgttaaactttgtcaaggacaaagtacatcgaa
tctcaatctggtatctaggtatcgagttattattatttgtgaaccccgaaatactgatcagtgattagctgtaccatagtgacttcaagtgctaccttt
ccaagcaagtctccgtaccgctcaaataacttttccgaataaccgtggcaaacgactacacctattggctgtttaatctatctgtcgtgaaagccac
cgaaactattattggctctcacaaaattcccgacgttttgattgacagcgcataaggcgcacattttgtccattttggtttgtcccctgggctgttgtt
actcacctgcttgttacgtgggtcctactagctcaccttatccacgcaaccggcggattacttaaacgctgcattcccgcctgcgacgacctgtcta
atatcccattacttacatagtacgaataccattgctatttggaggctttaggaggagcctttaaaccaaactcgctggaataagcaattggctttttt
aaaccattttctccttttgtaccttagttctcctattacctttttcccatccaacacacttttgcaacatttggaattcatcttttgttgttgtagttgactg
tgtgtcgttttcctagtggttcatccctttttgtgtttgagtcgtaggattccttattgaccgttttcttctttttttctttttttggttaattcttccttattat
ttgttcattactgcttcaggtttgaattatcggtgtcggcaaattggaattgttcgacttaactatttgttaatttttttggtgttttggtatttcctggatt
ttttgtttttttgtcttattttgttaaacccgacttgtttttccattcttaacagcgctgtgtgttggaatttaaactctaagtcctttttgtgattcgtcttag
tataccctggttttgttctatataaccttgtctacgataccttgtcaattattaggtattgtatctttcttccgtgattccattattttgtctggcgttttgt
cttttctagttcactatgccgtcccttgctttgccaatcaataaaccttcccatcacaatgcccctagctctgggaactcctttgccagtccacaggct
actccttttggttatagcccgcagtctttcggaaacagcttctccgggcatgcctggcttcgagatgcaattccctcactaacatcctatccggaaca
tcatcgtccttccgatgatttctctactatgtatcctaaccgtctcgaagaggcattctgtcgcaacttcaattgctgtggaaagactcttgatgatct
ccatcagcttatccatcattacgaggagcaacatgctgttcttccttctgacgtaaatctctcctcctctcttgacaatagtaactctgctagtgttgc
aaatcacctgaatcgcaacaacgtacaagtccttaaacagcgagagcgtattcgtatgcacgacttagaggaccaaatggactcgtccccccttt
tcgataattcggctgtcatgccctttgcttttccggcaaacgagggtgccaacggcccctatcgtgtttccgtcgttgtcccagcagccgctgctgca
gccgcgactgctgctaacccggatcttgctgaagagacttcgatgcaaaacgagccttcatctactcctactcatctccctgaatctatgattatgg
atgatgctagctcccctcttagtgatatgagcttcaacatggatatcagcaatagtaatcgctacagtatgtttggagtgaatcaaaaagctgacc
cagaaccagcttttctttccccttttcattacggccaagatatcttttctttccgtcctgacactcctaataccgtccattccgaaccttttcccgataat
attatgtcacctactccggaggtagttgcgcctgctgctactagcacagcacctagctctcccttcataaaaaattctagtaaggactttgaatggc
cagcaccgcttaaaaaccagcgctcaatatctccggaggattcttctgaatcccctatgaccatcgattctcccggttctagtctggttgtcgttgac
aaaccgtataaatgccctgttcccaattgtgataaagcttacaaaaatcagaatggtcttaaatatcataaattgcatggtcattgttcccctataa
ccacccctaccccggcgccggttcctcatcaaggatacgttgtagaaaacaaaccttatcgatgcgatacttgcaataagcgctataaaaatttga
atgggttgaagtatcatcgtgcccatagtcatttgcaagtttctatggcccaagcccagcgcgaggtccagatgagcttcatgcgcaacgcgtaag
tcgtcccatgcatacactatttatctttcactatatctacgaggtatttaatattttatttgcatcatatgatttgaacaaatttgctttatataccgaac
attcttttatgacttagatgtggccatatcgcgttggtattcaatttggtaaaatgtaaactgccaatatttctatttaatcattactgttaagtattttt
attttcctatcatttgaggttttataattttttttttgttgcttttgtttgattaataatttaatttagttttcgggtctttttgggtttaaaataatttttagag
cttggttgtgattttaggagtttatgttaatgaatagaaggtttttatttcaaaatttcagttaaatctgttgttgtctaattatgttcaagttatccaaa
actttgaaagtaaaaattacagccaaattttatagaattaccataatgtacatgcaactttttttctttttatgtaagtaaacctctttaaccctccgc
ctcttctacactgttacttttatgtttaggtgtgaagttaaatttggagggtgtaggacccaaaaatctttcactgagtttgacccagtcgttgacatt
cgtgctagcaagcatgccttccatttcatccctggcattgtaaagaggaggccgctgattcagtcttggtctcgcaacttcatataaagctataggg
gtgtaccgatgaaagaaactcataaattccaaaaagaacctcctggtagtgtttataccttgggcatctgagccccaatattgtaatccattattgc
aatattcctgcaaaatttccaatcgttgagttgatgatttatcgatgtgttgacgagcctcaatctcttcaaaaatccaaggctttacgattgcacca
cgggctaccataattgtgtcgactcccgtatccataccctcataccaatcctgccagctgtagcaatcaccattaccaatgaaagcaagtggttga
gtacgaagactaccttcgggtaattcgtcaattccttcattcaatttccgaatattagaagcgcaatctttcacatattcccaatttgccaattttgag
tatctctgctgacgagatcggccatgaagcacagcagcagacgtattcgtctcaagatgaatacgactcaatagtttatgcacgacgggatgatc
ctcacggtttcctgtacgaagcttcactgtagttggaatggtaccagaaatagcatcaataccttgcaaatttctaataagacgactggggttctcc
agcaaggatgatcctgctccttgacgaaagacaagatcgatggggcaaccacagttcaaatccaaaaaatcagcaccatcacaattttcagcaa
taacttgagcagtttttactccttgccaaagttttgagcaagctaattggatacctaaaattccctttcttccgttaggcatcgtttcccgttccgtatt
gataccacgaacaagagcccattctggctggttaccttgcattaagggataacacattgccatttcggtataaaatgtatctgcacccaaagaccc

gcaaagctttcgaaaaggaggattaccaactgtggttagtggagcgagaatttttctacctttccagtcaattctcctttttttcttgaggtcgcaatg
tcggtaaaaggacgtcagaacgagcttgtttatggatgtcatccgtagttactttatttttgtttcctcctcagtttgtttatgtgatgtctcgatggtt
tgtgattctggtttatcatccatgtcgactttttgtcattgttggtatcaaaaggtggttgatcctgctcgcctttacgagaaacttcttcttcagctcg
gggttcttgggtattctgatcatttgactgaggcttttcttccccaagcacgatagagatcattctttcagctttactcaagtccaattgctttgttcga
agcttcttctgaatatttttgtctacagcattcagcatattaagtccgaatttgctatcaggtttttggactagctcgtgttttccatcctcgttaactct
aacatgacctgttaaccatcgacacttatacccggccggacagacgccataagtctcgtaatttatgcatttttcacctaaatcaggttccttttgcg
caaggtaaatctctacgtcgtgaccaaatttacagttctcttgaaacggacattcctttccaacagaaatcgagggacaaagtgcattttcctcacg
tacggagactttactcctctttttatgttgcccacgttctttaggcttgcgctttctctccgcttgctctggtttagcaagaaatctaaaatgaattagc
aattgaatgcaagactcttttcacatactcttcacgaataggagcaacaccggaatgtggatctgacattttccaagcaatttactagacaatgctc
cccctcggaattgactttattgctgaaaacaaaaaattcagcccttcatactagtaacctgggtcaaataacgggctgtgaatgaatacacaaaggt
aatataaaggattgcagtgagagagtgttaaaccgtaaacaaatctttttcaggaactgattgctttgttggaaaagcaatcaaaatgaacattg
aattgagcttgaagctcatcaaaggattagtttattactactttagagaggtgaaaaggacgttgttattttcttgatttcatcaaggttatgaacaa
accttgagtgatagattcaaacactattaagctaaataaaaagaaagacaattcatatgatttaaagaaaaagatcgcacctttttttcaaaacatt
tgaaaggatcagacgtgccctcgattcgttcctcccaagggttcatcatccaccaagccttccctgaataaatctatcattgtttacaaatgacttta
agactaccagggcttgcagatatgcatgttcacctacgcgatggggaaatgatgaagcatgtcgttcctactgtaaatcaaggaggtgtgtctgtg
gcttatgttatggtatgtgaagtacctatgttttgaagcatggggagtggaaatgaatgagtaatggaaagctaatttagagtaatagcctaatctt
gttcctcctattacaacagtagatgcttgtttgaagtataaacaagtaattgaacagttggaccctaaaacgaaatatttaatgtctttatatctgtc
tcctcaaattacacctcaagtcatctacgaagccgctaaacgaggagttcgcggcgttaagtcatatcctaaaggtgctactaccaattccgatgg
cggcgtagaaagctacgaacccttctatgctacgtttgcagccatggaagaaactggcatgatcttgaacatccacggagaagtcccctcttcaa
ccagtaatgatgtctttcatgcagagccaaagtttttgcctaccttattagacctgcacaaacgcttccctgatttgaagatcgttttggagcattgt
agtacgcaagcggctattgaagccgtattaaactgtggtcctagtgttgctggaaccattactgcgcatcacatgtacattacgcataaagattgg
gaaattgatccccattgcttctgcaagccagtagcaaaaaccgaaaaggatcggctagctttaattgaagcagctacaagcggaaatccaaaat
tcttctttggatcagattcggctcctcacccccaatcctcgaaacaaaagtctcctcctgccgctggtgtattcactcaagcctttgaagcatcttatt
tagcagaaatctttgataagaaaggatgtttagacgctttggaggggtttgcttgtaaatttggtagacatttctatgatgttcccattgaagaatca
acccctacgctaattttggaaaagaaacctttcaccattccacaattaataaacgactgtttagttcctttccgacctttacaaatttcgaactggca
atgccgttgggaggaaggcactttataatccacgtagaaatatttgaatggattactttataacaaactttatttgcttccatgtagctgtgtactat
caaaagcattacagagttcattaagttataggaagggcaatctgagtacaagtgcaagtcagccgatatctaaacaattgtataaggtttaatcc
cattatctggatattgagtttttcattcggagaagcaccatggagtcagtcttgtgttatgactacaatattgaactatcgctcaagaaaggctctaa
tgatgagacggaaaactccaaaaacggcctttctataatcaatctttgtatcattattttttaaatcaagagacgttatgaatgtccttgtgatccga
tgtactgttgatagaatttgggtggacaggggtcttatcgttttatagacagtaaatttatcctacactcgctgtacgcattcaatctttcccatacaa
cagtttttttaattcattaagctactgttcatgaaatcatcactaaaagcttcttgttgttgttcctgatacaattttcatgaaagaaaaaaagaatgt
agaaaaatgaataatcattatctaaatattataaaattacaatttttttctatagatttcacttgcttacttaacgcctgctatactactaccagctttg
gtgtagacttgtcagcacggcggttagttgtcctttaccatctcagtacaaactacaccaaagtaaaagatgtctgaaactagattgtttatagggc
gtattcctcctcaaggtaatataatacaaaaggtagacgtgtattaacgaaattattagctactcgtgaggacctggaggactttttcaaaggcta
cggccgtatccttgactgtaaagttatgaatggttttggattcattgagcttgaagatgctcgtgacgctcgggatatagtgaatgatttcccagga
aaggaatttatgggcagtcaaattgttgtagagcctgctcgtggtgaaagaagaagaagagaaaacttccgtgaaactgctgcttctaagtaagt
acatttctttttgtaaaatctatacaaagcgctttgttgtacctctttcgagttttcgttaagcctatgtatttgaggagattgggttgaatcaaagttt
gcccattcggattttatgactttttttttcttttcaatgactttaagctggaataataataactaacatcttttagataccctcgtccacgtcgtactggt
tatcgtctaattgtcgaaaacttatctgaagaagttagctggcaggtaatgctttatacaaaagttaaacgaatgcaatattaattctatatttttag
gatttgaaggatgtaatgagacaagccggtgaaccaactttcactgacgctcaccgccaaaatcctggttttgggtacgctttaatgtcttcaaagt
gaattgattactaacaatgatagcgttgttgaattttctactgaagatgatatgagaagtgctcttacaaatctcaatggtgaagaaattaagggt
gctgcaattgctttgcgtgaggaccctgtatgtcttatcttttttttatttttctagtacactgtactaacatatatgaatagaatgctcccaatgaacc
tcttcccgaaggtccaggtttccgctctaggtctcctcctcctcgtcgtcgttaccctaccgataatcgtagaggatttgcccgtcgtgatacctatcg
ccctgccagagatgatgatcgtcgctatattcctcgacgtggtagccgcgacagtcgcgacagtttccgccgtggaggacgtgatgattacagac
gtggccctcgtcgtgatgattatcgcagacccagagccgatgaatatcgccctggtcgtgatgattcttatcgtcctgaacgtgatgactcttatcg
ccctgaacgtgatgattcctatcgccgtcgtgatgactatcctacccgtgatgaagaatatggtcgtgatgcttatgagcgtagcccttctccccgcc
gtgaacgcagtcgttctcccagatatgcggatgaatataactcttaccctccacctcctgcttcggaaatgcagcaatccaacaaccctccaagct
atcctgaggaatcagctaatccagtctctgctggaaatggtgaaggtcaagttgctgctgaatggtaaatttagacatttggtttcattttaaaatc

atttatgatacatttgttagtattcctacttgttgctctgatatccttattttgcttgaattaatattctcttgaggctggaatattggatgtttcactcaa
aatgatcatggattaatcgtaatatttcattcgtgtagtacctttataccacttcctttgtattggaatcttcaattttggttactcatttatgctgaacg
ttctttacttctttacttcatacatttctcatggtataaaagttttaattgtcaagcgtatgttgcatgtttgtgtttcttttcgttcttttctgtatgttgag
ttgataataaaattttggagagtcttgtgatattcggaaagtgcattcttgtgttatagagcaattcgaattgtcgtagcgttttaacaagttgctttt
actagcgaaatttttacccattgttcatatcaccacctttattgactactatctacataaaatttgtaataaacgttttaatattaatgtagtaagatc
ttatatgccttgaagtcgtacgcctaagacaagtaaatttagaaattcgtgctgagtttttttaaagtgatctgtatcctagaattcgtccccttcggta
tctatcgagactcacaagcgtttcgaatagcgtatactgcaagacagaatacgaaaggagacttaataaacatcattgtaaattaggattgatca
tttttttgtttcggaataaggatagtatatgaaacttgcattcttttacaaaactgtcgctaatgcaaaaaaggaagtacgtgtacgcttgtaaccgt
gtatatatgagtatgcattacatatacattattgaaatgctggaaaaaagaaaaagaaaggaagaaataagtggtcagaaggagaggtttttaa
cgaagcatattataagaattggaaaacacgaaagggaaaaatgcaaaagataaaaagaaaacaaacgagaacatgggagagaaaaaacaa
ccacaatgtaaaaagaactaaaataaataggctcgatgatgcaaagtgggagaaataaggaaacgggaaatgaagcaaatatcaagggcgt
aaggaacaatgatgatgaaacttcaaagcttcaaccattagaaatatactgcagagccattaatgttataagtaataggattataaggtaattgc
gaaaaagacagctaaacaataacgcagcattttgtagagcttttatccatttccaaatcgcgtactgttaaggcagcacgagttgcagcctcaaa
aacatcatctacgccatcacctgtcaaagaggaacattccatgtactttcttgcgccaatcctttgtgcaacaatctcagcttgctgtggtttaacaa
agttttgatttcgccgccgcatttcttcaattgcgcctggatcggttcttaaatccgccttcatgccaacgagaatgaaaggtatattagggcaaagt
gtattaacttcctcaatccactgtatacatgttagaataagtaaaaaaataaaaaaataaataaacggcaataataaaaagcatttaccttcgta
gaaacgttttccaaggaatccggagaatcaatagcaaaacctataagaataatgtgtgctttggcataagacattggacgtaaacgcttaacga
attagcttcttgttacaaaaacccaacaaagatctgcacatacttcgtattcttcttgaccactaatcgtttgtaagtaaagtaaagaaacaataat
tattgacttacgctgtatcccacaatgctagctggacggattttccatccaccttgatgaagagttagtagaatttaaaaagacacttcacgtactc
tacaatccgaaacgtaattctcaaacacggtaggaacctgatttgcgtgttaatgaaggaaccaaaagaacaaccatacatattcctaaagaat
gattattagtaaaatagaaataaaaaaatgcaataaaaccggtaaaagcaaaacataccgtaggaaaataacctaaagtgaatactgacagc
agagacgtttttccacatgcaccatcaccaacaacgactagttttctgacaaccgtcaatatttaacttcctttacgttcacaaaccttcgaataga
ctgagactgtagcatgggaggaactttttttatgtaaaagaacgatgggaaacgaatgtaaatgaaatctagctcacttccacaagtccaacaca
gcacaaacgatcgcggcacaatctattaagtctatgtaacacagcagttctaacaagcagacaagaacgtcaacctatggtactcttcttttccaa
gtacgtgcccaaattaaaacgaacgcaataaggactaattccagggttagacactatttatatgtgctttctcgcaggatttaattctctgtacgta
ccttgcaaagaaactgatatgaaacttgaatatgcttaggattggtttcgtaggtcatcaactctggaggtagggagacagaatgcgaactgtga
cctttagcgctgcacgcgttacgtatgtcgtaaaagagtttgcacactttacgaaactttacaatatgccttacttgttgttaacgcactacgctatat
attccttgggatcattatattcacattatacggaacaaagctatgtcgttatgtttccgtaaaagaaattcaaacactttggcactgaacgatttcct
cacttttcgctttcgctagcattgaacctgaaaggagtcgagtattcctgttttggaagaagataacggtttttttaagtgccgttaaaaagtacgaa
aagctggtattcgagcgggcgattttaaggtagtactgggaagtgttgaaagaaaaccaggaatttactctttaactatcgtctaaatcgacacat
gactgggtcctctttgttataaagatttattttagaaccttttttgaagagcaaaagctgcaagctctttccttttgtgtatcgacgaaagtatactata
gcaccaagtctagttgtttgaggatttctttgaagaaggagaacgaacaagaaatacgtccgtatttgcaccatgggtagtcgagagctctgtatt
gaggcgctgaatgcagggatagagcctccgagctttaaagaccttttacttcctgtatcaaacccggctgcaaatgcaaaaaaacgaaataaga
aaaagaagaggcaaaaagcgaaaaagcaagccgaagtttctgctgccccgtctggaatctctgaggatgagggacttagcaaaatcgagcaa
ctttctatgcatccatccttagacaagctgatggcggaaaaggacaaagaatatatggaatatagaagaaacatggagagtaactttacggcttc
tcccaacggatcttcgcattactatccatcgctggatgacatgaatgcaatttcctcaacggactggcaatttacgttggagtcgaatgcagccgct
gaaccttctgatgaatgtattatctcttcttctcatatcgtcgatgaggcagaatttcctatccattttccaactttggaggaagctgcttcgccttcca
acatctcctcaaattctgtacataataacctatcaccttttccgccgatagaaccaattatgcctcttcctacaacgacatcttcaaaaaagaagaa
aaagaaaaagaagaaatctaagaatgcaaatcccgccatgaatgaacccatcgacccacagacggacgtgcaagaggaatgtgatggtaatg
cagatgagcatgcctttccgtcttttccaaacactaataatgggaaacctaaagacaagatttgggcagcctcaaatactgaagaacgcgaaca
aatccgtgaattttggttatctcttagcgaaagtgaacggagatcacttgtcaaagtagaaaaggaagctgttttacaaaaaatgaaagagcaac
aaaaatattcctgctcttgctctgtatgtggtagaaaacgtcttgccattgaagaagagttagaagttttatacgatgcttactatgaggagttaga
acaatatgcaaatattcagcgtaatcgtgctttgatgcaacagtccaaagcactagatgagatcaatactcccatcgcatctgcgcctaatgttga
aatcactgaatatattgatgaagaggagattgaaaaagatattgattttcaaacagagtcggaaacccctgaggaaagtctaggggaatatgttt
ctcatcaagatgatacctctgttcaagatggtcaagggctctctgttgacaaaaatatttccgcttttactgagcaattggattcagtagatgtcgat
gcaaatcaagtggaaagtcttccttataccccctacggatccttctcgacttcacgaaaaaaatagttatcatgcagatttgtataatttcggtagca
gtcttaccgttaagggaggtattttgactgttgctgatgatttgttgaaaaacgatgggaaaaaattcattgaaatgatggaacaattggctgaac
gtcgcatgcagcgcgaggataataatacttttcatgaaccggaaatgtacgatagcggtatagagtacgatgaaaatgaagatgaagaagatg

aagatatagatgaagaagatgaagatgagcttgacttgatgacggatgaacaaaggatggaagaaggtagaagaatgtttcaaatttttgctgc
taggctatttgaacaaagagtgttacaagcttatcgagaaaaggttgctcaacagcggcaagcaaagcttctcgaagaaattgaagaagaaaat
agacggaaaaaagagcgagagcttcggaagttaaaagaaaaggagaggaagagggataaaaagaaacagcagagacttgctaaagaaga
ggaacggcagaaacgtgaagccgaacgattagcagaagaggctgctcagaaggcagctgaagccaaaaaagctgaagaggctcgtaaacga
aaagaggagcagcgtattaagaaggaagaaaagaaacggcagcaggaactggaacgtcagaaaaaagaggagaagcggagaatggaact
agaagaacggcagagacaaaaagagttagaaaagaaactgaagaaacagcaagaagaggaacgattaaacgttgtacgtgaacagcaatta
cgtttacagaaattagaagaaaagaagctacggcaaaaagaacttgaagaggaagaaaagcggcgaagcgaagaagaacaggaacgttcat
caatagcacaagcgcaggaggcagatgaagcggaacgaaaattacgtgaagctaaaatagccgctttttttgcaccttcaaaagaagacactat
gaacattgctactcattcttcctcacttcctcaggccttaccaaatacttatttacgaacagagccacaattctccccttcccgtgcttcatccgttgc
accaaacttaccttcaatacggccaagagaaattaatactcttcctttcggaacgcctcaacctattccgacatataaaaatccttctcaacttcct
gagagattggcaccttcaccaagttttataacctctccctcttttccttccccgcttaatatacctaatagctctctttgcttagataataacggaaga
tcgaggcaacttagtagcggtaacccaaatttgctgaattctaaccgtaattctaatggtctatattctccggtctctcttttaactaatcacagttct
tttggtttaggctcaagcactcgtaactcgctaggccaaacggcttctcctgtgcattattctcgattttcaaactgtatatctcctttaagtataaaa
ccttctacgactaagacaccaccattgttatcaaggttgaacttgcaatcaaattctcagcaggatccatcggagttgaacgatataattggaaac
tccgtacctaacatgaatatggctacttcgaaagaaacattaatgggttcttttgggacggaagccttgggattgggatctccgacacccatgcgc
agtagacctgcacctattcgacgaccgagtcaaggtagttcgctgttaatgaagaagacgctcaaggaagaaagactgggtagtcgtgctttgct
agatgaaaactcggatacagttttatctcaaaacacgtcgttgggtgcgtcctatgacggattgaacaggcgagcaagtcatggatatgctaagt
ctttgaattattctaattggaattcacctaacaataccgccttctacaatccgctctccacaggcccatggagttcagcgctgggcgcttttactcca
agtactttaacatctgatgcttcacgcagacaacgtacatgggcagaatgaaggatgatattgagtaatcttctctatgattgttatcagcagcatt
gtacataaatgatttatatgtttgttatgtcttttgttgttccttgttttggttgttaaattatattggtttttttttcgctaatgctgggatgcgttcggttc
tagctttattaaaacagagtcatttaataatattaatttttacgttcaacatttgtaagtgttaaatgaataatagattaagcaaacatttattcattt
ttcttatactatgatagaaagcgatcatgtatctttggcacaggggaagtaggtggaaaggacggactgtaatgttttagaactaaataaatctaa
aaataaaggtagattaattatgaaatattcaaaatctcatcgttaaaaaaggttaaaagacattacaacagattacctacgctcaaagcactatg
aatcaaaaagtggaggtaacgaatcagatgtaccaaggtcttctaccgaaggtaaatcattctcacctgaatacgaagcaaaagatttttggctc
caactttcaccttcaacgttaagaggagttgtgccgtactcacgattaaaattggtaatgttgacaccgtgatctctgaaagattgcgattcttcttc
ttcgagcatcccaaaaggattctcaataaagccgccaccatcttcttcgatggaatcattcaagttcattttctgattgttttcgaaccattggaaag
gaccttctaaagacacagattcgggagcaggtacactattagacagaatagaggcttcctgaaggaaagcagatgtggaatctgatatattttga
gaatacaaaggagttgtatgaattgtacttacagcaaaattaccattaatggcttgccgcaattgaaaaaatggttgctttcctgactccatggaa
gtttgaacagagttcaatataacctcttttagccggtcacatagttctgctggctctttcaaaacttcttcagggaaaagaccatggtacttttcata
ctttggagatgaaagtcttattgcaagttgctgcagagaaatctcttgatcttctccttttaacaggtcagctaaaactttttctattttctgaatatcc
ggattcgtttcaaaggtctttctcatacagggatttgatgctatttccgatagcaaaggctcatcgcttgttgctcctttataaaaagaattgagcgtt
tctggagctgtcttctgtttctgttctttcaaaacttcttgtaattgaggttcagcatgttcagacggccgtgctttttcatcctcagctttcagtttcttt
atccatggttcttgttccaaatagtccccttctaaggtgccttctattgaatgctctctttttagtacgtgtttttctggggaatagagtaaataattta
aatgctcttctaattcgtttccttcaggaattgcagaatccagctcaaatgccatcaaactttcagtttctaaaaatatatgaagacgtttcaccaaa
caacttctctactagtactcatctattcatacagataagctcatatattttgaataatacaacctcctgaccttaaaattttcttggttaaatttcgtca
aatgaaacataccaacgtcgaacgcgaattaggtatcgcgccgtactcaccttaaacttatatcttcgttcacacagttccaatttcaatatcttga
atccactctacggctaatatttactcgatctcctttccaatttttcaataattttattaattattcaatatttctttttttatacttcattagttaactattatt
tattattacaagaatcatgaataggaactatacaaagcaacaagaataacaacacttgtagcaaaaagtttgttaagtcaagttgcatagacaa
atggcaataccccacagaagttacttccagctaagaaaccttatgcattgaactcgagcttgaaagcaatagtctcaataccatattgtccttcgc
ccttgacttcttcaatttaaataaatttaaagagaggacccacaaagaaccgtttgccatttaggcagaaatgtgtattgcgtatcattatcatcat
caaaacgcagctaatcgccagatgctgccaaaaatgtttaaaatttaacaatgccatcaataagatgttttctttttatattgcaatgagaagcag
ctttttcctaagaaaatcttatcccttccctgttttacaagggcctgaaaaaacagtgacgaacttatccaccgaaaaaaagaccaagatataatt
cccaactgcaggaagactccagtgacattctcaatagctaatctcccaagcacaactagaatatgggaaagcttattgaaaactaacgatttaca
atcgttatagacagttatttcacttgacctaacgactagcgatatgaaatattcagtctttttttttctgcctgcttaaacccacatataagtttttgatt
accaacaaattcaccatataatttgtttcgttgaatggatttttggcaaacatttttctccaaagaataccacttcccatcacttttagtgtttacgga
attccgagagtacaggataaatagcttcaaaagcctggtaaatttcctctcgtactttggcacccgttaaaacgattttaccagatacaaaaatca
gtaaaaccactttgggcttaaccatacgataaatcaaaccagggaacagttcaggctcatactgtttgcaaccgttagtatcgtttgtttcagcaac
atttactaaaaactacatacggatgagaaagttccgtgagaataagccaagccctccaaacggattgggaatttcacatcacaacttccgacaat

attttgaatcttaaagtcggtaaactttgcgttaaagccaagcttctggatgatacgagcatatttccgagatgccaatttagagtcatcctcggatt
taccacccaaaacaaccattttgcctgaagcaaagattaaagcagtggatttgggttcacgaatacgcataatgacagctgcaaaacgcttggg
attatattctgcattacgtgcatgaagggcaatagttttgagatcaagacgacagctataggaaaagtattagcatttcataattagattgtctatc
aaaaacactcattggaactgctgcaaccatgacccaccgtcgccaacagtcatcgtcacggacagtcttcacaaaggccctactgttttcaataat
cgaatgccttgaaaacataaaggtaccttgaaaaccatatccgggactatgagacatacggcaggcctgttccctatccatgagaggcacgactt
actctaagttcacagtagcaacaatgttctgaagggtcggaacaattccggaaacaccctcagtcttcgcaacttctgtatcaccggaatctgccg
tgtcatcaactgtttcgttattattagttggtaaaacagggaacgctaaagaagaattgttcataaaagcactcgcttgcgaggccgtggtgggca
aagcaaaatccattttctgtgcactaaattgacctaacaatctataatattattcactggcttgcgttatcccaccctactggaaaccctagagctca
cgggaggccgtctcatttatataaaccaggatttacagggttagggtttctcgtgtgtaacaatcagccctcagtggcagatagatatacaatattt
gtcgaataaaattctagcactcgtttttatacattcttttctttaatttcatgtattttttaatgtaaagtttgagcttgtttgataacgtccgttttcagt
gagtttttggaaaaaactacaggagatacgaagtaaattttgattataatatggttttaaaaagttccactaaattcaggacgtccttcctttcaaa
atgcttttttttaggtgttgttggttgctggcggcttgcctgacactgcggccagcaactctcatttgggaggagcagaaggattatgtaatcgttg
cttgaacagtcctaatgcagttgctgtgtttatcgttaaatgctttacaatcctttaacatcatatcggtatcggtaaagtgggggtaaactaagcaa
gacgatggataggatggatgaaaggatagttctatagggcagattcttcgattggatttaccggaaagtaaatgaaaggctcgcttcaatttttta
cgattcgtagtagataaataaataaataaagtttagagaatattaaaaaggaatgatgagactgtacgatactaaatggtttaaatgaggactca
agcgctttatatggggaaaaaagcataaataggtaagaaaactacttccataaacagaagcgtctagataattctctgccatttctataaaagat
aatgatttctgttatgtcggtcgacaaatcccgtttactttttatttgtttatttatttgttttcctatactaaccaagtaacatgctccttagactttata
aaagcatagtctcagtctggtataaatgcttatagtataagagcatataagcaaagtatgcttaatcgttaatgaaagcaaaacctggcttttgttt
gcacacccaactcacgttacaacccagaccaaattaacaaatttccaaaacgtttttacaaaacgtgaaagtgataggcgtcaagttgcttcggg
gtttttcctttcgaaattcaattagaacgcgttgtttcaactcaaagccatccatgtcctctgaaaaatcgcaactgtcaccatggtttgctcctactg
gacataagacggagttaaacgttttcaacaccattacccattccaaggtcccttttaagaccaatggctccatggtcacttggtactgttgtggtcc
aaccgtgtacgatgctagtcacatgggccatgcccgtaattatgtgactacagacatcttgcgaagaattttacagtcttattttggctataatgtta
ctttcgttcaaaacgtaactgatattgatgacaaaattatccttcgtgcccgtcagcaatatctttttgagcaatacaaacagcaaaacgagcata
attatgcttctccaactgtgaaagctaaagttaccgaggcgtggctagcttatgccaagaagaatctccccgaatccccccaatccgtggatgaat
ggtctaattggttggctacccacgatattaccactttggcacttgcagctcctaaggttcctttgtatgccgattctctcactcaaggtgtagaagga
ttaaagatcgctgaggaagccaaatctgaagatagtgagcttttctggtctaaggtccaagaggtgcttattccggtccttgactccgaacttgga
aataccgttacggatcctgctattttccgtaaattgtctgcatactgggaaaatgattataacgaggatatgcgtgctttaaatgtgctaccacctac
cacagttactcgggtttctgagtttgtacctgaaattgtcgaatatgttcaaaaaattattgatcgtggttatgcttatccagttactgatggaagtgt
ttatttcgacactgaagccttttccaaaggtggtcacttttatgcgaaacttgagccttggaataagggaaaccgtgatttgattgctgaaggtgaa
ggttctcttgctgctctaactggtaagaagcgtcccggggattttgctctttggaagtctagtaaaccgggtgagccttcctgggacagcccttggtc
gaacggccgtccaggttggcatatcgagtgttctgtaatggctagtgcagttttaggtcccaatgttgatattcactcaggtggtgtcgatttagcct
ttccgcatcatgataatgaattagcccaaagtgaagcatatcatgactgcccacagtggattaactactttttacacaccggacaccttcacattga
aggccaaaaaatgtctaaatctctcaagaattttattactatcaaagaaattctcaagaaatttacacctagacaacttcgacttgcctttttgttgc
aacagtggaattctcaactcgatttcaaagatgctcttttggctcacgtgttgaatgtagagcaaactttggaaaactttttccaaaatgtcagagc
tttaaacattgaatctcaaggattaagtcaacaaggaggccatgttactgaaaaatatggagaactcgaaaggcaacttttaaacgatttgcaaa
ctgctcagcgcaatgcccattctgctttgtgcgataactttaatactcctttagttatgttccatatcgatagtttagttggaaaatccaacatctaca
ttcgagatgttggacaaaaaccgtgcactcgtttgcttaacgaaattgccatgtggattaccaaaatgctgcaagttttcggtctggatgatgaag
gtcgtcctaacgccatcggttggtcgtccaaatcctcagcttctgtatccaactccaatgatgctattccatatgtacgagcggtttctaccttccgtg
atcgcatccgtgaactatgcattgctaaggctcctatccaagaaattttgcaagcttgtgatgtctttagagactatgacatggctgagcttggtgtt
agcttgaacgatcgccaacaggggccagccttaattaaacttgtagatccacaagtattgatccaagcgcgtgaccagaaaattgcagaagaac
aagaaaaggaagccaaaaagttgaaggcgaaagccgagcaagaaaacaaatatatcgagcgcattatgaaaggtaaagttgctccttcagag
atgtttaaactatttgaggaatttttgtcgtttgatgaaagtggtttccccactaaagtccgtgcagaagatggttctgaggttgaattacctaaaag
ccgtaaaaagaagttacaaaaagaatataccacccaagagaaactgcataaagagtacttggcatacatagagaaaaagaaccaagactaaa
ttttatcattctttaatattaattcagtttaaatttttggtatgctaaatattctggctttgtatatatgtatagattagttacgagagtattcttaattat
atcctgctcaagctagttttatgctatttctaaaagcttcactaacttcttgtaaaatttctatttaaataatgagtaaaatcacattcttcagaagca
aatcttgcataatacaagggaccgacatactatatactattaataatcaattcatcatcggaccagacaactattggtgggttgccgatattcactt
ccactcttctactacttgaaaatttccttatctttgttatactaatactaattaaagaacagagaataccaggttgaactaagagaatgaagaccgc
aatttttgctgcaggttgtttttggggtgttcaagaagtctttcttaggaaatatgtccctacagccgcaattttgaagacttctgtaggctatattgg

aggtattactgataatcctacgtataaacaagtttgtacaaacagcaccggccatgcggaagccctcaaggttgatttcgacgagaatttgacctc
ttatgagaaaattgtcgagtttttcttcgccatgcatgatccaacaaccgtagataaacaaggtaatgacattggaagtcagtatcgctctgtcata
tttactacttcacctgaacaagaaagcattgcaaagaaagttctaaaggaagtacaagaaaagcacttttccaacaagaagattgtaactcaaa
ttgttccttcaggacaatggtgggatgcggaagaatatcatcaacattaccttgacaaaaaccctgaaggatatcgttgcagttctcgtaagtttcc
aaagctcttctgtgaggaagatagtattactaactactttatatagacttccttcgctggaatccattcgcttaagatcaggttcaacctaatgaacg
taaatttcctgttatatctaaagaaattatagtcattgagatgagaatgaatttgttcgagaaatgaattgaaatgaatttaaacggctagaaattt
tggtttaacaaattacattgggtaggtttcaaatagacgagattattcctatccgtttcacgggtcaagtagtaaatttatcacctcccacatgagg
ctcctctttttgataaataaacgataagagagaatccaaattagtgagcacatttttttgaaattcctctttcgtccatattcttcactgtagtttccaa
ctgttgaagaagctgctgagctatatgaacatattcctgtgtttttttcctcatccgcctcgattttctgagacagagtttggttctcaagttccaagtct
tcaatgtcctctttcagttttcttttaaggttctgtttgtgtgtatagagatgagctttaaggatttcttgaggctgcttgctttcgatactatagaacgt
tagaaatgctttactgttgagagttttcttactcaattgggggttctcctgacccttttcggtccttagcttccttgatacattgatccaggaagtcaag
tttttgatgcagaccccgttcctcaatcagcttggcatactcttgctaaatttcagtcatcagctgctgttcttcaaacgttccatacataccttgcag
gtttcctcaaaaaagctgataagttgctgatgcatggctgcaattgcctttcctttttgaatgttggaaaacattgtgcaaacttttctaaaggaat
gtgtgcaactgtttccgataaggtacgtcgtaaagatgcttccaattgctccttcctagtcatatatacccaaaaatcaatagatcactcttaacag
acgactcgactcgcaaactaaacactgtttgcctaaaggttgataaaatatagaatgcctaattattagatcaaatttttactatttttagtaaaat
aaatccttgtttctcaagatttttcacgattgttctcatagaaaagattttcccaaacacatgaaattaaattcgtgctaatcctttttgactattttta
tgaaataaataaggataagaacaataaacttataaagatacgttggttcttttctaagcctttgaaaaggcagtttgtttacagcctttagtaagtt
caattctgctcctgcctataactacatactgaacttgcgtcttggacctcaaaaaaaaaagatcagaaatttgttgaaataagcaaatatatagac
ttcgcattgaaatttattcatggatatccttcaaaaaggttacaatgtgatagtagcgcctccaaaagttcaacaggcagatgaagcaatctcaac
tctttgcgatcgacttgaacatgcaactctctttgaagatagaaaggccgctgctctaggaattaaaagctttgctcgagagtttaaagaagtaag
ccgttgatttatacactagacagtactgacaaacgtttgctatagttagtcgctgctcacgggctcaaagggattattcgatctttacaccgggattc
atcagatcccgagttactgagagtggctttagaaacctgtctgattctgacaaaatcttcggatgaaaaaacttcgtcagacacagctttatgggtt
tctgatcagtttatcttaaatcatgagaacattcaatgtcttctaaatagtgttcaacatgatgatttttacgtccagttatactccatcgaactctttt
catccatactggcctgtagaccagctgaattgaaggattgcattcaaagttttccttctgccatctcaacggtaatgactccattaagagataccag
tgagcctatccggaatgctgcactatatttcctgaaagacctaaccatgaattgtgcaagccttcagaaactagttgtttttgaaaatgcattcgaa
tctattttatctattttagatgatgaaaatggagttgagggtggacttatctctgccgatgcacttgtctttttagacactcttttaaatgacaatatta
ccaatcaacatttttttcgagaatccaatcaaatacctaatattattcgtctattacgtcctgattcgtttatagatgcaaactgggattctcttcgtac
tcgttgtgttaatcttgttctacatgttctacagtctttgacacccatcggtctttcatcagggaaagcaaaccaaaatgcagttatgaaaagcaact
catttgatttcctccttcgattggcaacaaatccagatttattacatttggatgtcccaaaggtcgcttggattacccttgctcatttggtttacgcaa
ataatgcttgtcaggacgctttcgtcaaaagctcttatttcattaaccaaaatacagatgctttatcatgtcttctaaatcagatcttccttccttcaat
ctctccttcacatcgttattctgttgcctttttttaagggctttggttgattctaacaatcatttggctgagaccttcctacaaaggataatagatgctt
accgaaataaagagtcatcaaagctaaatcttttagaccagtacctcgacatatccatacttgctgacaccgaacaatacggacattggttaatta
gcgttattttaagttatttggtggcaggcagtgacgagagaaagttacagttatgcaaaataccactttatcaagaaatcgcagcagatgaagaa
gatggagaagaagttacttttattcaatgtatttccaccaagcttattgcgacattgcggcatgatcatgctcttcataactgtgttgggtatctgac
cttattaatcattttagttgatagcaatgctcactgtgtaaaagactttctatctgaaagtagtatactccaaaccttgttgacgacattaatggacg
aatcttcttcagcaagttccattattcagggtatgatcgccgttttattggctctggtgtaccactattgtccccttgattcttctgtgccaaaatctag
tatttatggagcaatcaattcagcagttactcgtgatgtttttttaaatcgccttcatagactacaccagcttgatcaaattcgcagtcatttaccgtt
aaacgaaattcctgacggtaacttcaatacattactcttcgatacattgtttgttgatttttttaaagacaatttttatcgcttaatacattctattgat
gatgcttctgatgcttacaatgcaagtgatgatgggattaacactctacgtgcctatgaagaagctcagcaaaaactaatcacgctaagcgacga
acttaaggaaactaaaagtctcttgagctccatttcaagcgacaaagaggaagagttgcgaagctgtagaaatgagatggaggaaaaacagaa
actaatcaatgagtttcaagttcttaatgacgattatcgtcaagaattgagttctcttcgacaaagcttatatgatattaaaattgaattggattatt
cgaagtctaatactaatagtatggaggaggaaatgaaggtgcttcgcgaaggtcacgatatggaaatcaaagactttaccgaagaaaacgaga
aacttcgaaagcttttggattctaccaaagatcagtttaaggttattagtatgaaaaataaagatcttcatacgacaatgcagggtttagaaaagg
ccaataacgaattccaaacattgaaatctgagaattcctcattacagcaaaaagttaatgacctcacagagaaaatagaggggctaaacaaga
ctactatttctatgaagagttctttgaacgaatccaaaaacttagaggaatccttgactcagcaaatccaaaagaaagacaatcttttgtccaaat
ataaagacgaagaaaacggatacaagggtcagatcaaagaatttgaatcccaagttagtagtttaaggaacgaattagaagtgttacgtgaaa
gcaagggttccttaatggatgaaataaaaaccacccagaacgtggtgaaacaccaatccaatgaacttaaacttgctaaacaaaagtatacttct
tctcaagaaaagctgaacgcaaactcacagaattcaaaaacaaatgaaaagaaactctcctcattagaaagtgaactcagggaagaaaaaga

cgcagttaaaagattacaggaggaactggataatattaactctgaaaagcaccgcttacaaaaggaagtaaatgaaggagattcgtttcgaacg
gcttcgcaagcagctcaaaatgagcaaagaaaagaattagagaaattaaaatctgatttggatgagcttcaaataacacgtgagacggctggtg
aggagaatgaaaagttgaaagaaatgtgtgaagaattaaagaaaaaggttgccggcctagaaactaaggatgatcaaaatacagcggaattg
caaaaacaaaatgctgaattggaaaaaaggcttaaagaagccgatgaatactggttacttatcatcgaggagttggaaacaaaacgctcgagg
gataaaaaagctttaaaagaattaggacatgaagtttctgaagatgagaatgaaagtgatgaagagtaacggaactgcaatcttttggatataa
taataataataaacattaactattaattaactaatatctaactaaagtcttctcctcgattccacgaattagccaaaacccaaaaatgctcagcatt
acctagttgttcaagagattgccgagcttcattctccgttggacattttcgaaatgtccaatatgagaaatttcgtttgcttaaatctccttcccatac
caaatcacatttgttctcgttatagggtatgctgcttccattcatatcagccagcttatttccttgagctaatatagaatttcttgaggtatctgtccaa
tctatacgatttatcattaatttttaaaatgcttaatatttttcttccgccttcaacaataaccaagttaaatttatcacttaatatacatgctcctgt
aagacccaattgtttagcatttaaatcaatttttaagcgatgtggtctgtgtgcaaggtatttaattctgaaatatgttagaataattcaacttattca
taaaaatcacttacttgaaaacaaggcactgcaagccacttgcagcatcttcttcgagctttttttagagttctatccctcctcttcgggagtaagt
tttctttcttcgttcatttgttcgtgacgaagacgtcgttcttctacttgccttctaacctcggcctccatctttgttggatccttaatagctgcatcgccc
aatacgcgcataagatttgaaagcttcaccttaggaggttctggaggttctaatcctaatagttgacgatcttgtttttctttacgagcttcagctcg
agtttgcctcctgattttcttttgttcctaaatacattagtaccaatatttgtatgttttactattacctttttttgtcaagaaaagagatttagaggatgg
ttgatttttaaataagggggaagaacaggaataggatgttgtatattcgatgttatagcctcgctattttcgaatattttccatgaagattgatcac
cgtaagttgatccatcggcaacatacggcaaatcccaccattcgactgtaggaggctccactcgatgtatggatttactagcgattagaagctcat
cattaagccctgccttttctgaatgcaaggcaacacgtttttttcaacttttctaattgagattgacgtcgaaattgttcagcttcttcgatatatttacc
ggaagcattcagacttagaacccttggagctctagtacgtcttgattcctttgtccttgggtcataataaggaagattttcaactttgggtacttcaa
tatcttctataagtggatttctcttactttcagtgggacgctcctgtgaaagagcatcaggactatcccgtacaggcttattagatgctgaaggttctt
taaaccttccattggatgaatatatatcatcttttaatagagcaggatgaattccaacccttaagcctccttttgcgacatcaccctcagtatgttga
gatggagcagaggatggtgagaaagaattcttaacaaagttagaaaattaatataatatatgcacttacaaatctttttgcggttctttctcttagt
tctgctatacgacgctgagcagcacctggttcagtgggtctattaccatcggaattcttgtctgaactgccaatgttttgagaagatgttctttttttcc
aactctaattttcttcttttcacagcttccatctgtatttacgattaggttgacgttacaagatggaacgataggaaacttactatgcaattatcataa
acagttagcaggacgtgtaaactgcttcaagggcagttgcacctctatatgaatgtataaaaactataataaaagatcctaaggaacttttcttga
aatagcaagttacgaaagcagttggtgttgtgatgagaggtccgaagacggtaatgccattaaagatttattagctattgtacatgagatttttat
aaaaaagatcaagaaaaataccacaattaatccaaaattaagaaaccacatgaaaaacctttgatttgaagattcagaaaaaggtacagtcaa
tcccataatcaaatctccaaacaaggatctcactgttatgagtacaagctataatacgccgatgatcacattgtagtccaaatactcgagatcttc
gatcgttatgtagaacgcagtatagttctccggtttcaaaattccatacccgtatagttccatcgtagcctccagagactactttttcattgtcaaatt
ctaaggttcggatgaggtcttcatggccttgaagtatacgaagtagctttccggagttcgcttcaaaaatgcgaactgtggtatctgaagatcctga
aacgatgtattttccattatattgagtacaagcaatacctctctgatgtgcatgtataatctttaagcactgaccactatggacgtcccacatcctta
gagtacgatctccgctggctgttacgataataccagttgactcattgtattgaacagaattcacagaagcaagatgtcctctcaatacatgtatgc
aggactcttccgcggatgaagcatccaacttccataccctcgctgtgtgatcacgggaacaagaaattatatagtttccagagagaacgacacct
agcacattgtcagtatgtccagtcaaggatttaataggacaccttcgttcccaactccaaattaaaatcgaagtgtcggatgaaccagaaataat
gagatcacgttctcgatcaaattgcaagcataagacactgcccaaatgaccatgaaggaaatacagtaaggcacctgcgtgaatatcccagata
cttattaaacggtctttggatccactgacaacaaaatcatcgtcgtattgtacgcaatatacgctatctgttgaaatgcgagagaacctccctagtc
gagttggggtttgcatttcacataaaacaaatcggccgcgacgccaattcgcttcaagtcgagcgcattctttgtaaatgtgagcccaattgagca
aaggacgattctgcgaatcatacacgtattgtcggtcgtgaaagatagttccatgtggacctaggaaatgttcatcgattcgaatgaaggtctcttc
agacaatgactgatccttgatacgcatccattcttcgaattcttgtaccaccttggggtttagctgccaacctttccgttcaaataacgtcttccatat
gctagggtcttctagcagccttttccaccgcctggacattaatttacatctgcaaagatcattttgccctagatagctgaatattcgcaaaattatttc
ctcgggaaagaactaccaaaccgattagaaacataaaacataaatgtgaaaacacagcgtacatcagtaaagtccttgaataagagagtttga
atataattataaattagaaggattccttcttttggcaactctgagagagtatgttggatgatatctgatcgttaatttttgcttccaagtttatattagg
agggcataccaatttttttcgctttccagcggggtaaaagaggatgatgggtttcctttttcgttatcgaatgaatccttttgttcagatactttaatcc
ctagttcattctgtgattcaattttactgtagttcgttttagggtcatcagacatttgaataaaaaaaaagagagaagaaaagtttttctgtgaatat
caaatataaagacttttgtttacacaatacaaatcctcagctcttgaaaagctcaagtgaaagcatgctacatgaaccagagaatccaaagttgg
gaaagtagcctcgcaaattcaaacactttctggcttgttcaatttaaataattcaacctgtttttaatcgttaatttgcaatgaaatacaacaaaaat
tcaaacaaaagctgctacatctcttgaacaggttgggttaagcaatatcgttctacctcgttggtaaataaataaataaatgaatgaatgaatgaa
tgaatgaattaagataagaaggaaaaagcgacccttctacccttatagtttcaatctttgtatcttattttgttagcttgtcgatgaaggtggaaag
gaaagctttaagatagatttccatattgcctatttaaaagaaagacacgtttttgattcatatatcaaaatagcctaaaagaacctctactaggtac

ctattcgtgtgtttgattattttcaacatgtttcatgacaatattcaactcatattttgttttctatgctcaaacaaagtgtaaacaatcaatctgtcgct
accaagactgactgcttcagcgaggccgggcctaattgtttatttccacgtgaaaaaaaggctaagaaagaaaaagaattatgcacagttaaag
agtatcccttttcaaatataacaaacaatgtatgtttaaacaacaatgtaatttgaaagaaagaataaaaaggtaaggtttataactattttcctat
ataccgagttataagtatgtcgtaaagtatattgttggcagtttctctcttcaatgaattgaaggaaaaacgtcgtatttgattcaattacaataatg
atgcgacttacagactacgcttcgcaatcaaccaaagcgttgtctcgcttctataactaaatcagtatccttaacttatcaccataaacctagaatc
tataccttccatattgagtatcagtcttggaacacgtgaaactttaactttggttcctaatagtaggatcttcaagaacttttatcaacaaaggga
aacggaaaatctgtaacgaaactttcctttatttatattttttttggtttctttttgcatagaaacgtctttctaaaacaaacaaagaatttctccggtc
cttctcttatctctacttgattgattatcgcaaaaagtaccagactggtgtaaacaaacgatgagtaggtaacatacgccaatatcatgtcttgaag
actttaagatgggaaaaaaaaaattttgtaataaaaaaaaataaagaagaaacttcaaggaagattaaaatcctttgtaaaagagatcgctcaatt
tttggagtgacattttcctagaaacacaggaaattatcatatccaggttgtgtttcctgtcatttttttgcagtaattcgtccaatgtatggtacagaac
gatgttcttattttgtctcttggtatgaatgcatgctctagaacgagcctgtcaatttggatttacaatagtaggtaacaatttgtgtggagcctctag
atacttgaaaaggacgtcatacgaacaaacagcgctcgttatccaaaatttataaaacgatatagtcttatcttacactagaaatagcctcctttta
tctgtcaatatatagctcccttttttccacgactgactgcgtttcacccaaaaattgggattacggcctgcttctctgctctttcatttgcaacaaacag
ttgaagttaattacaataggatttcataattactgaaaaatccatttgcttgctgttaggaggtactctactactgccataaaagcgcaatcctacct
ctcgtttttttcagccactcaatccgtttccctctgttttcttcagtcaatcagctcttaattccctttaatctgtctttgcttttgaggtcttgttctcacatt
atttttgtgtttttttcgttcagcagtcctcaaaattcacaggacaatccggtctgattgcatcgcatttttttattttttttaaaaagtctttctgatttgtt
aataattgttttgtttagagaaaatttttaaaacatggatgcttcgcgaccctctcctttcttgcctgggtcaaacagctttaattatattccttatcag
attcgctctataattggagattctttttcctttttttctgaaaggaagtgctcatgattatcatgaggattctctcttgcctcaatcgtcatacaagtacc
tcctttccggaggtcttgtttgtacttatttatacttactttataaatggctctcaaactcttttatagtcaaacgtcccgtgtctgctgagttttcaggg
ctgcggcgtgttcatgggctttgtctttggatgctttcgtttctctttctatgtttaagtacattcatgtatactgaagggcattttccaaagagatttat
tggtcatggtgtcaaccatttattgcttgctttctctatacactcctttcttcttcattctctccgcattcttgttccccgggcgcgtcctaccttgtatcat
tcttcagttcttttaaacctttccatcattccggtgtttttcgatttttatgtttatcccttccttttaatccatcaccctcccttctccatatttccgcccct
aatgtgggcctattttaccattggcgtatccataaacatcattccctggttcacaccacgtgtatggtaccctttatttcctgaagaaaacgatcacc
cgtcggaagagcaaatctgcaattactttagctatatttgtagctataattggttaaacaagcttatcaatgtctccacccgtcgtactgttgaattc
gacgatgctcctcatctaccggattatgacaaaaacaaactgtggtctaatgtttatgagaggcttaagcgcccttctttatttataactctgcacct
tatctttgcatatcaaatgataggtatgggtgttgcaagctttttcattagtgcttcacagtttatttcacccatggccatgaatcggttccttcagtac
ttagaaaatccaaagagtgaatacatatctccctatgtatggattgccttgcttttaattggcccttttctgaattcattggcttttcaatcttatttatt
tttgtctactcgttatgccatccgttcttattcggttcttgtatatcaagtttataaaaaagtgcttgatttccgctttgtacagaataaaaatgagga
atccaaagtcggccgagttaacaatcttatttctaccgatgctgatgatattggcgcgttgcgagaattagtccatgttgcagtaagagcacctttt
gaaatcggcatttctatctatttgttgcagaaacttttgggatggagtgcttatgttggtcttgttcttgccgtcttaactacatcggttcctattctgtt
aggtccctttgtggctaaactaactttactctctaacaaagcaactgattcccgtattgaattaatgtccgaacttcttcaaagtattcgtattacaa
aattctttggttgggaacgtccaatgctcgaacgtgtgcgtcacaaacgccaacacgagcttaatcgaacctggaagttgcttttaatggaaatat
taattcaagttttggttgaatcattaccgttctttagcatgtttgttacttttgtaatttttactacggttatgggacaaaaaattacaccctcaattgc
atttacttcaatatcccttttctctttggtgcgtaatcagtttgcatggattgcttatcttttaaggcaattagtgcagattttcgtttcaattggtaggg
tttcaaatttttttgaacgaccccaatgaagtggatcccacagtttacactgactctgaaagtgaagacattggctttgacaacgccactttcacctg
gccttgtaatccagttgagggagatttccgccttcgtgacttgaacttcaagttcccaaagaaccagctttcaatcgttgttggtcctactggctccg
gaaaatcttcgctaatttcggcattacttggtgaattaagtttgtctagcggctcttactccctacctcgttccaaaggtgtatcatacgtccctcaag
ttgcttggttgcgtaatgccaccattaaagacaatattttattcaatgctcctttcgacgaggcgcgttacagagaggttttgaaatcctgtggtttg
cagagtgatttagaaaatttacctgccggtgatttaactgaaatcggtgaaaaaggtgttaccttatctggaggtcagaaacaacgtattgcgctt
gctcgtgctgtatatgcaccatcctccatcgttttaattgatgatgttatgtctgctttggatattcacacatcaaattggattttccatcattgcttca
aaggtcctttaatgaaaaatcgtactgttatccttgttacgcatagtatacatcttttcttagatgctactaactttgttgttaccgtaaagaatggat
ccgcttttccaattaaggataagtcaacacctattttgcaactggatgaacaggctaacacatccgaatcttctgcaccggaaatgcaagttacgg
agacccctcttgattcctcaatagacgcagagcaagttgctgaatcgggtaaacttgtagaagaagaaaaaagagccaccggagacgtaccttt
gtctgctgttttcgaatatttccgccattttggctcaacattttatgtatgcttcattcttgtattactagttgctacacaaacagtatctatccttattga
tttgtgggttgcattttggaccaacaaatcaatagactctcctgacatgagcaataccaagtttttagtagtatatggatcaatgctcatcttttactg
tttgctagactacttgcgcacgtatacatatgacagaggctccttgattgctggaaaggctattcataattcaatgctagagcacgtttttggtactt
atgcctcatggtttaacaaaacacctactggaagaattgtgaaccgttttgccaaagatattcgatctttggacttgaacctgccaggctggcttttc
tggtctgtagaatgtttcctttcaatccttgccgggttaatttctgtttcttcagctatgcctattttcttggttcctgccttatgtatctgtttaataggat

attatgttggtatgctttatactaaggctcaagttggtatcaagcgattaatttccgtttacaattctcctattttcggccttcttggagagagtatttct
ggacttgctgtaattcgtgcatttaatatgcaaagtacatttaaagatgaattctcgaagaaaattgacaacctactgcgtcttcagtctacatcgt
acaatctgaatagatgggttgcagttagaacagacggcattagtggattggttggtggattagctggatttattgctcttaaacaaagcaacatta
gccctggtcttgttggtttctcacttaaccaatctgtaatctttagcatcacagttctttattttgtccgttcattcaacgccttacaagctgagatgaa
cagctaccaaagagttaaagagtatactgaactagagcaagaacctttaccaaccaaagaaggagaagttcctgtggcatggcccaaagaagg
agacattaagttcgaaaatgtcagcgtaagttacactccaaatggatcgagtgtcattaaggatttaaatcttcacatccatcacggtgaaaaggt
agcgattgttggacgaaccggtagtggtaagagtacactgggacttactctcttacgattcacaaatcgtctaggtggatctttacgtgtcaatgat
ttagagattgaaaatgtcaatttgaattctcttcgtcaacgaatttcattcattcctcaagatccaattttgttttctggaaccgttcgaagcaattta
gatcctttcaatgaacttgatgattctttgttgaacgaggcgcttcagacaagtggagcaaataatatggctattgcctattcggaagaccaacaa
ccaattcatataacgctagatactcaagtagcttcggagggctcaaacttttctcagggccagaagcaagtcttggctctatcaagagccatcgtt
cgacgctctaaaatcattatcctggatgaatgtactgcatccgtagatgatgctacagatcaacaaatccaaaaaacattgcgtagcgcatttgga
gatgcaactatgttgtgcattgctcatcgcttgaaaacgatcgttgactatcataaagttatggttatggacaaaggtgtacttgttgaatatggac
cacctgccgagttatatcgtaagggcggggtttttcgtcaaatgtgtgaaaagagttcaatttctttttggattctacagtaaaatagacttgactc
attagaaaagccatggacttgtttagttttatagatatgtcacaattattataacttggttcatttgttagaaaatgcgtgtgattggactgattatga
atttaatacgggactaatgcaatttaaatttttacatataatccatcttttatacgtgacatactacctcaacatcttgttatacggtttgcagttagt
caattcggtactataggaaacgtagagagctcatagacagcgctgtacaaatattatactacagtattttatacaatacataacaagctggtaagt
caccaacacacatttacaaattcacagaaaaacgggacaatggataagtcaattttcaaggaacttcaaacaaaaattgatcatgagacttcttt
gcgagacgtatgggttatttttcaccggcaattactaaacaaataaccaggaattaagttcgcgtgttgaaactattgatcaggaaacaaaagttt
tacgtctcttgcttggagaagttgagatttgtatggcatcaaatctgctacattttagtcaaaccctgttaattaacttatagcaaataacgaagtat
ccattgatattacaaggagtacagaggttatcaaagtgaaaatccaagaattagctaagttggcatctgaacattcttactacaaatatgttaatg
tctgggacagaactattcaagaagtcgcgtgcttgatcttgttagcaacttggaggggagctcttcgtggacatgaagacaagaaataccaaatc
ttgactttggaagaagtcggtcaaattttgaacggtatgctatagtgtaaatatacgcaaaacttaacgaaatggttctagttcctgtatatcccga
agaagttaagtttcacatttctatcgaggagtatttgcatggtgttttgtccattagttctgaactggtatgtttaactcttgtataccgttttctaaac
attatactataggcaaggttttctgtcaatgctgttatccatggcaataaagaggttcctttaaaatcacttcaaactattgaaaacgtacattcttc
ctttcaattgttatctttaaagaacgactccttgcgtcgtcattttgacagcttaaaataccaactcaaacgagcggaagatggtatgttaatttcttt
atataagcacccttactaattgctctagtggtttacgatatgagaattcacaatttgctagactaataaaaattgttctatgacggaaaatgtaattt
cattagttatttattgacgttttattgtttgtacttcgtctttgcatatgtaatttttttcttccttggtttatttactaaggcactctagcaatggtttccta
aatcctggaattctgttgcaaaaataaaactccaagtacaatttgttcttagagtgttaaatctgtgtttttttatctttttttacaatcgaaagaaagt
ctatcttatttttatgtgtttgtttctatcgtcatatttgaattgaaatgccattaattattttgtctggatatccatcctctggaaaaacgacgcgtgcg
aagcaacttcagaaagctttagaagagcgtatagaaaacaatgttgatggaaccaaggactatcgtgtagtcctcatcaatgatgaatctttgga
cattgaaaaagaagtttacagaggtacacaataatttttttaatacaccgagacaacaatgtctcttcccattatactagggaaggagaatcgtttt
tttgaaagacatactaactggtattaaattttagaatcgaaaacagaaaaggctgctcgcggacttttgtattccgccgttcaacgtgaactctcaa
agagtactattgtaatttgtgatgccttgaattatattaaaggatttcgttatcagcttttctgtgagtcaaaatccatgtacacccctcactgtgtgg
tatggcaatacaaactttttaccatatatactaacaggatttcctaggttcatgtggctgtccccaagcagatggcgaaaaaatttaactccacctc
ggaacatccatacccagaagatgtcttagatggacttatttttcgatatgaagaaccgaatgggatgacccgatgggattcgcctctctttacagtt
atccatgatgatcctactcctcctgttgatactatttggtctgtgctaattcataacaaatccgtgaagccaaacttggctactatgacaaaagcac
ctgctgaagtaaattatttatacgaattagataaagtaacgcaagacgttatcatgctaattctcaatcatcaaagtacagagggatcttcttcgtt
gcccatccctggaagcagtcttcaagtcgaaatgccaagcattactgtatcgcttcctcttttgcagcgttttcgccgtcaatttgttcacatcaatcg
ccaacagtcatataacaccaatcttttaaaagatatgtttgtcgatttttttgaatggacaatttgaaacaatggagtaaaggctgttggcttattgct
gggcttggaaaaaacgaattaccatttttaggagttcttgtatgataaaagtaactgtttagaaaaaaaggatgattgaaatttttttattttgttctc
ttaactcaaataaaaaggggttttctatgatcttattcaaaccttactcagtttctttaaagtgaacaggtagctttttactgtctaagctatctccttt
cttgttttagtgttggattcatttacagactaacaatgcttgagatcttttgcctgtgttatataagtttttgtagaagataaataacccttttctattcc
atatttttctcaaaggctacgctaccttctttaaatgacatttttaaaacagcagcttataattattaggcagtcgtagaatatccaaattactaaaa
aatcaaggacataaattacttcacagaaatgttgatttcttcatatgcttttatgaacatagagaatcagtatgccttaacaatgcataaaaggat
atctttagcatactcaagtatatgcacttacttaaagtatatcactatatctttcattaagaaacaataattgtcatatggcattataaatgctttaaa
ctacaatatcataaactagactgattcctatgtattctgttaatgttattctgcttcgtttttccttcgacacaactgttgcttgtttttttcaccgacttgt
aaacataaaacgacttggagaacataatggattaaatgaccattcgatagaatacgatgaacatatatagaagtcgcatagggtagaatagca
atctttttattgcttcttgtaattccagcaaccaccaaaattaatttaaatttaattaaaatatatcgaaagtttgcgcatctttgggactccttataat

tgataccggattggaacgcaattaggggttcaagcatttccatctccaatatacttctaccatcgtcaataatagtatcgtgtttaagtcatagtaat
tggcttatagaaacctgtaataaaatgcaggatgattctgggaatgtggcagaggtacctgtcgtaccgaatcatgaagagatgtccagtgagaa
caaggtgtatccagctgaacagaaagctttcaaacccgataccgaaatagactcgcataaggaacccgaaaaaggacctcgagtttatccaaat
ctagctttggagcccataaatgccgccgaagatggacgggcaccaggtcagggcgacttgtcttcaccagctacgttaaaccaaattgtgtacga
gcagcaagggcaagaagaaattcctgcaaaagtccctcttgataataaaaacttaatgagccctgtattagaagaaccacgacctgaacccaat
gaaagggtagatcaaaatccaccgactccttcaaattgggaacgatctgcacgtctcgtgcttataaagtgcattaatgattttcaacagattcgt
gcatgcgcgaagtttgatccattacgtaaagtaatggacgacctaaagcccagtcttcaaactgacaacgaatacgacaaaactcttcttatcga
tttttttcaacatgcttttgaaattgctcaagatgacgtcttaaatattgctctaaacaccgtatcaaagttagcatcttttgcttacttcccagctgac
aaaaaggtttcagcttcaaatccttcctccaaatcaatgctacaatgcatcgtggatatggtatgtgattgtatcaatgatgaagttgttgatggaa
cattgcaactcaatgttgtcaaagctctttctgcattcatcctttgccctaacaagaacgatttacttcatggagctattcttctcaactccattcgca
agctttataatattttccttcttggcgatgatgattccatacaatctgtagctcaggcatctctgactcaagctgttgggattgtttttgaacgtttacg
tatgtttcatccttccaaaccatctacaaattctaggtttaacgacatcgccactgcaagcactgactctttacatgaaaatgtctcaaatgcggaa
gaaaaaataactctgcaatcaatggcttccaacggaacgtcgaagttggattatgtgaaaatggatgttgaaaattccaagactactagtctgga
gcatttatctctgcaggatgcatatctagtattccgtaccctatgcaggcttgcagtacgccatagttcttctgaaaaagttgacaacatacgttctc
aggccatgagatcaaagcttataagccttcaccttatttattttatcttagacaagaatggtgacttgttcacggaattgactttccctttttcaaaac
atacctgcattagagggattaacactagtacaagcatcacgtcaatacatatgtttagtattatctcgaaatgctgtaagcccaataccccaagtg
tttgaaatttgctgcgatatttttggcttatcgtatccaccttgcgtatacattttaaacaggaaattgccgttttttttcacgaagtttattttcctata
ttggatttaaaaaatacctcttacaatcaaaagcttcatacacttttgattttccaacgtatttgtcgaaatcccagggcactggttgaattatatatt
aattatgattgcgaccgcaattcgacaactaatgtttttgaacaacttctattttctatatcaaaaaccaattctatcactcctttagatccaaacac
atatcaatatgaagacttgcttccttcaatggaaacttctaacagaagtgctactcctttcttgactactaattctagctctcttaattcagaagtagt
acaattaaccaattttagcgattatcagttaaagctgaaagcattggaatgtgtcatcgatgttttgcattccttgatgagttgggccgaaagcgga
ttttatttagctaggggcaatatatccgcagatgaaaatacgacaggtgctgatgatgattccttagctcgttcagacacccctaatacactccctc
tacacagtggtcgtccatctgttgaaacgaactcatgcagtagcagttcattggcattagctgatccttcagaatttgagttgaataagcagcgaa
aaaatttgttgcgacattgcataaataagtttaactataaaccgttgttaggaattaaacacctctctgagaatggatttgtcaacactgacgatcc
tgcatcattagctacattcttatttaacactgaaggttttgataagactactctcggtgattacttgggagctggagaggagtacaacatatctgtta
tgcacaactttatcgactgttttaactttgccaattaccgctttgttgatgccttacgtcgtttgctgcaggctttcagattgccaggtgaagcccaaa
aaatcgatcgtattatgttgaaattttctgagcgttatatgaaagaaaatccatcagctttcgcaaatgctgatacagcttacatattggcgtactct
attatcatgttaaacaccgacttacattcgcctcaaataaaaaataagatgtcgaaggaagatttcgtaaaaaacaatcgaggaattaacagtgg
cggcgatctggatgaagattaccttggttttgtttatgaggatattttaaataatgaaattgttcttaaagacgaacaacaattggctgccatagca
ccattgatgacttctaacaacaatgtaggccttttcgctgcttcttttactcagacgggtcgtgacttacagaggactgcgtatcttcaagcttcaga
agagatggctaacaagacaaccaccgtccttaagaagctcatgaagcagcagaaacataatccaaaaaaagagaatgtattttacaatgctaa
acactttgagcatattggaccaatgcttgaagcggggtggatgcccattttggcagccatatcaaatcctttacaagaatctgaatacttgtatgaa
ttatcttggtgtttggaagggtttcagtatgtgattcatatagcttgtcttttcggccttgatttagttcgtgatgcttttataaaaacactggtcaattt
cacgaacttgcattccgcctatgacataaaaactaaaaataccatggtcataaaaacactgcttaaaattgcatgtactgaaggcaacgatttaa
ggtcttcttggaaagacattcttaccagtattagtcaattggaacgtgttcaattaattggcgaaggggtcgacgaaacagagattccggatgttat
aaatgctagagtacgaccatcgaactcaatgaaaactcgtcaaatgagtggaactcttcaaaatcaaacgcctagatccacttcgaaacaactat
cctttgatgttatttctgagttgatgtctactgaaactatactctccattgacaaaatttttacgcagacttcttccctatcgggtgttgctataaaatc
tttttttgaagctctatgtgaagttgcatgggatgaaataacttcctctagtgattccgaaaatccccgcctatttagcttgcaaaagcttgttgagat
ttcatattacaatatgggaagaatcagagttgagtggtcttctatttggaatgtgctgggaaaattctttaatttggttgctagccacaggaataaa
cgtgttgcagtgtttgctttagactccttaagacaactatccatgcatttcttacaaattgaagaactttctttgttttctttccaaaaagaatttctca
aaccttatgaatatgtcatgtcatctgatgcttctgttgatgttaaagagctcgtgttgcaatgtattcgccaaatgatacaagctagagtttccagt
ataaaatcaggatggaaaacactgtttggggtttttactttttctgctaaagcccgagatgatactttgcttttattgacttttgagacactcttggac
atttttgaacaccattacaattttatagttcaacaaagttgtctgattgacatggttgtttcttttaccgaattatgcaagaatggtgttaatcaaaaa
atatctctgcaaagtcttgacgttattaagaagatatactccaaactggttttagaagctaaaagcaaaaagaatactaatgaggaagaagtacc
aaaatctgcattccctattttattcgcttattatgatgttgtagtttctgctgaagacttggaagtgcgttctagagctcttcaaaatatgttttatatat
ttcttgaagagtccgatgaatttactgagcagatatgggagatcgtttcgcgaaagttcatttttcctatttttttcaacgtttagtttagattctgatga
cattttgctacgtgacgaggaagttcgtacttggcaatcgacgactctggtagaagctctgaagggtattgttgcgttaatttctcgccggcttgag
aaacttcataccctactcaaaggttattttcggttgtttagcacttgtatatgtcgcgacaacgttagcttgtctcgaattggtactagttgcatgcaa

caggttttacttgaaaatgcacataggtttacaaaaggtgattgggatttagtcgtcgagctctttgttgagctttttcgcgaaactactccgcataa
attgttattggaagaagccttttggttcgatgacgaaaattttatccgttttaattcagctcattctcaagccacaaggactgaggttgacaaaagtt
ctgtaaatcttattgcagaaaagcagaacgaatttcggtccatgattcgaaaatgcatactgcaacttttgctagtcagtattgttgctgaattactg
gattacgaaaatatcttcaagtacattccgcaacatcaagttttaaaactcacacaagcagtttatgattcatggcagtttgccagaaaatttaac
gaaaacaaatccttacgtgtttctttacttaatattggctatatgaagcagttacctaatttattacgccaagaaacagcgagcgcattattgtacat
cactttgctctttagattattgaatagcgaaagcttaccggttgattcaagttcttgtactgaagttaccaacctactttttcctgcctgtgcgggatta
ttagaactttatgcttccatgaccattgagaaacacacacggaatcacgctagctggcagcccgttattgtaaccattcttgataatacattaaagc
ttcctgaaagtttgttcattggctccatgcgacaattatattactcttgttgttccatgatagctaaaggaagtcttgatgatcagcttcgaggattgt
taaaagcttatttcaatcgggtgggtcgtacttttcttgagaaagaaccgaaagaaccgaaagactaactatagctatgcatacacagtgcctcct
atttaatgagtaaatgatgtatttcttcttttatacgcttttgtttatacggttttatggttgtgccaaactatttcttctttctatcgttgacttatgatgt
cttatttttttgaatgtttgttaaattagagtgaataaataacaaccattactcatttttttttttttccgtaacttttagtattcttaccttttttttatattt
attgtacctatattatctaagcagcctgtgcctttttttaaatatttatcacaacatatatccaagtattttgtcttatatagttctaaagactaagaaa
cagaagaggagggctttaactactattaagagttgtaaagccaactaagtgtaaatatagctttgaatttcgataacggaaatggaatgctatgc
tgacgaactccacagaaaattatatttgcgaatttgaacaacttaatgaaaccagaactttcgctaaaccacaacaatgcctttgtaaaacgcta
agacaaaagatacgcttattcagaaatccaaaatgccttccttctaattgacgacaattcacctatttcgaaaacttaaccttgggcaaacgaaaa
acaaacgaagtattcaaaaaaaaaaataagccgataaaatccagcaatccagtcgttatagttatcacctcagaatttttttatttcttttttttatttt
taattttaaaaaaatagtaatcaagcattgggttccgaaagcatcaaattatcctttatgtttccagacaaattattcatctctacggcagaaggag
caatagataaattagatatcgaacttaccaaagatgagttattagagttttgctgggaagcagtagaattaggaagttcaaacagacgttgttctt
ttggcaaaacccaaggctcccaagtttctgaacgacgaatcttagccataataggagattcaaaatgagggttaagtgtaacaacggaggaagc
ttcacaagcacttaaaagaaggtttatatcgctagtcaaacttttaattctattgaaggaagctaaaaaagcgagggaaacgtaaccttcgtcatc
catatgtttacgtaaaaacatgtccttgcataggttttctatggacatatagtaatcgatttgagaagtaaggaaagcctttacatcataaacatat
tgaacggttccagcgggatttgtgacaacaggcacattttgtactttaggatgatgataggagcgattagaacgatggttgggattcttgctgcga
tatcctttacgatagtttctgccacggaatccactattgttctgagaagcctcgttgatgcctctttcttggagggtgtcgtcgattcgacgaggatta
gggcggaagtggttattggcagcactaggctgttcgctagacttcgtggtaccagtagaggcaacaccctcgttaaaagattgagcagtatttcga
ggaacattctcctgacgacggttttgattttgagaataattttgggaactgcctttacggcgtgcactaccagaacgacgatttccatcgtttctgga
acgattgggtttcttagaaggatgaataggaggatgacttatttcaggagtgataactatccacttttctttaccgtttgttttaggggccaaaggct
ttttcacttctgggtcatgctttttttcttcagaagacttttgttcgactaattcaggactaggccagatattgggatcttcaagtcgagcatttttttgat
tgcttttgttcgtcttgagagccactagccttttgtgacagttgttcacgacgaacagcccaaacattgacggacgagtgagtagagctttgatcat
tcgcaaccggcgtggttgaaccagaagctaaggaagaagcggtagctgaagcaatatcacctgcagagtcctttcgattgttagagaaatccgt
atcattcatcttcgaatacacatcactgggctgtttctcagactcgttcacaggagcggcttgaacaaccttttgatctttttcttgtgccaaagtttgt
tcctgaatcatttttttattatctcttgactgttcttgcagacaatgaaaaaagtaattcaacctacaagtttgtgtatcacacacagaaaatcttgcg
aaaaccagaattagattgactcagtcaaactgattctttgtacgttgaaaccaataccaaaaccaagccgattgaactttgtttcagcttgtcaaa
aattcacacgttcgtttctagttttaagaaaaaaagtcacaacccctgtatcgtaatgaacggtactgactacgataaatgataaaaaaaaaaaa
aaattcgctacttcgagttttttccaactcaatcaacgcccccctgtgttgtacttggaaaaagccttttgagttcaatttggcattcgtattactgcgc
ttttttttttccttcttttaacgaggtctacttgaaattagtgaagtcaacaatagcatatacaagaatatgtaccacttcggtaaacgataaacaaaa
cagagaaggcaaagaaagtaagaaaaaacagaaagagtaagagcagtagtaagagaaatagagcgctttcgacgtataaaccatttctttcc
cgccagaaaattattacttttaacaggtgggttgcatataaaagtgttgtgtctctttcatataaaacctatagttatacatagaccagagagaaaa
gagaaaagcataaagcaagaaacgatcattctaagtagaggaaagtatataagccatccatacctatcataattacaattgctttcctaaggca
gccagttggacatagagcgtgaggccattctagctccaaacgaaactgttcttgtgccaaatgggaaaaaagcaattaagtaagcgataaatgtt
agtatatgaattgctccaaagacaatacttagaattgacgattttaagtaaattgtagatacaacagttgccgctaaacttgcaaagtaacctaat
gtaatcggtagtcgttccttcgttattagttgttcaaagtgagctttaaatccaagaagaattgcaaagctaaatttccgttagcgctgaataggaa
aagattaaacaaaacagctctgatttttgacctagcaggtattgtataacaactgctgtactattaaaaaaaatcttagagtatgctgtcgtttttttt
ccttaataagcaagacagacttacccgaagacagctaacagactgcccatagtccacagtaaaacaaatcttcttggcttcagaattagtaccgg
aaacataaaacaagcaattgcataacaagcgaagctgcctaacaagcaaagtccaaacaacatgtaccgctcccaacgagacagactgaagt
cagaactctgaaaatatgagtttccattagcattggttggtacttcagagtaatcattcgtccatggaagtgagcttttgacacgctcgtaccaagt
attcgtcgattctgacgcagcttcaccagttctttctaacacagactgcaaacgcgatcgaaaactctattaatcaatattagtttcataaacactc
attgtatacatttttatgtcaaattcgcttcttacatcttccattttctattctcgtagtgtatcgggtttctcaaaaacgtcagaagcctcaatctacca
agtacttatttataccttagtaccaaaagttattggttttgtagtaagaaatatttttgtaagtgttttgaattgatctaaacgatttaaagattcccgt

tcgctctcactgcatccatgtaacctattataaccatactcttataagtcgttactgtacatgttttacacagactatattataaagaaaaaaattaa
atttattaacttctataacttattaaggtaggtatcaaagaacgagaaggcattagtattattcttatttaaacttcgtagaatgcccaaatttagta
aaacatgatgagctagtatttccatccatccacgattagtttaaggtctcatcaagttttctcggcgaaagaagaggtaatatccaacaacaattc
caagctggcatattaggcaaatccaaaatggcgcactagctaaaatacgtgctccgtccttcggttgccaagacgtaaaatcaagaaatacatct
gcaggtccaattcggcaaggtgaagtagccctgccaatcatgcttgctatggctccaataacctgaccaagattaagaacaaggtttatgtgctct
cgtgttaatgtttggagtaaaataattccaataccgacggcctggacagtatccagcaacccaagccatagccaaagcatgcgtgacatatacgg
cgcagctttcccaaaatacggaaggtataagcctatattcgaggttccccataaagtttgtagccagcgtggagcaccaaagcctagtcctaaaa
ttggcaatatccaagaatgcttctgcgaaagtgatgccaagccaccaatagtacctaaccatattgcaacatagaaaaagagtaccaataaaaa
tgtcacccatctgttggttgagcgtggtgaccaaacaaatgcccatgagcgaccctgtagtgtcgacatccaatagttttgcaaaatagaagcaa
aaaagaaccaaaggatcaaccgtcttcggagaacggatcggtaaaatgcgggaattttaggaggaatttgcctgtaaaaccttggtagtccttta
taaagcaataccgaaaagcacaacatcatcaaagaaatagaccatacaatagcgtatacccaacgaacagaatgtacttgttctgaaactcca
aatttattaggtttggagttcatatatgaaccccaataccataatgcagatgaccatagttgctggcatccttgtatgaaacaagctcgaataaccc
aatacaaaacatcatagtttccttcatcgccaaagtttaatgagaagaataaagcctgacttcctgaagcagctgcgtagatgcaagttgcaatg
cgcgctagccaaattctggtgtcacccaacttttcaaagctaaccagtccgacgaagaaaaatgcaagcccgtaaacaatgaacggaagtgcca
agcagtgtatactaggaactaaacagtgcatgacataccaaaagaaggtagacacaacataaatcccacacacaatgtatagaaaatttaccg
attgttctgagcttcctgataaaagacttagttgaaacgaggttacggccagaatctgccctaaagcaaggattatcgtatatataggccaaccaa
agacttgatactgtaatagcttgcgacaaccatagtattgctcaaactcgtagacatattcagcttcctctttgtcctgtggttgtaatgcttctttact
tatttcattctcgttgagggaattatgaacatcttcacttcgcaccaattcaggcaaactatttatggaactagttaccgttacatcattaaaaaagt
gaagcttttctggttttaaagatccaagctgtaagcgctttacctccgtaaagtatttccgggtacatttcataataaagtgatcaatgcagagctc
agttaaagaactttttggatttaaatattgcaaccgttcagcaaagacgcttaaagctttaccatcctcatcatcaaattttctagggacctttgtca
aggaaaaatcacgcaaattagaccgaacagacgcgatgctaagctgagagctattgtgtggaagaaacatgtccgaagattcaaaagccttgct
attagcctcttcataagattttgatatatcatccagcttcaaatcactttctgagatgatatttgatggattcgcttgatctggaacatcgacaatttc
atcattcttagcattatcacttaaggtatttgaatgatgatagttttcctcagtactctgctctgaaggtggttgcattaagtcaaattctattagttga
tttcgatattcatcacgacgaggcagatcatcaaaacttgaggatgaaaccttttccaaactctctgaagacacgtttaatatattgtcattcttcga
ttcttcccctatactgaatagttcgttttcatgtggtttcatagttcccattaaagcactacttaattcttcattccccatttgatccaattgctgtatttc
aggttccacttcttgcagatctcgttcaaatccttcgccatcaagagaagaaggctggaagttattttgatgttcagaatttccatgtaagatttcag
ggttgtttttttatacagagttttatgcaagtatcgatatgcttatttaaacgaataacccactcggaaatgggaaacctctgaactgcagagacagc
acgtagctttgtacgagtctctctcgtagaggataaagcctgacgacaagcttgctcaaattgtttcaacaaatgtgaggttgtgtttgattccactg
cataccaccagccaggcatttgaccaagaccgcccacattagctcctattccaagagtaccttttctcccgaactcgacagcaacaagaccgaaa
ggttcgtctcttgaaggaattaatgcaaaatcagcaccactaaaaatatactgcggaagttgtgtaaaaacgggccgtgaaaataaccgtcgtgg
atatttctgcatcaaagcttcaaacttttcagctgcaaacttaccatatagatcgataacaggaccgattacaacaatctgtgcattatagttttcaa
ggatcacaggtgctatatcagcaattaaatctattccccttttggatggaccatcgcccaacgaagattaaaagatccgcatccgggtctaattcca
aacctgcccattcttgagtttgtcttttatgctcctccttcgtaagctcaagagcctgcagagactcatcagttacctgaagaatcccctcacctttgt
cagacggatctggatttggaagcttgccaatctttcgtagtccccaaaaaaataggatatctcgcccaagaacgctttccgtacttcttcgatacacc
gacaacaccgtatcctttttgatggattcttatataggacgcaccagcatgcaataaattaaaaacattaccaaactggacatatttagagcacac
atccaggggaatattgaaaacggagcaaacctcttccacctcttccggacttcttagaggccaaagtccttgaaattcagcgttatgcaacgaaa
gagctactgggataactttcggcagcaagtaaactggagccaacgctccatgataatcgtttatgtgatacaaatcaactttcttgttcctcattat
attagctatacattgattccatgcagaataaaaaattgccgaactcaaatcatcgactcgagcaggataaggctcagatgttgtctgtcttctaaa
aacaggagcatccaatagcaagtatgttatatttcgatgtacatatcggtatacgttaattaaataggattgtcctataattaccacagtaattgga
tcgtctcgttccatgtcaggatatatcacatcaccaacacatggaataacccataacagatcttgatgttccaagttcttaaccattaactgtgccat
aacacccaatccaccaatcttaattctgattttcaaagttggaatatcgtattcaagagtagcgagaagtattgtttttctattaccttgcgaaacac
ctttctttttcatgcccatcatttttggaaaatccatcattttaaacatgatattactactcttctctgcctcaggaaaatcaacaaagtatttatcgat
gtgtcctttcccaaagtcattgaactttacgtgatagaacagccttttaaaaagcaacacagaaactaagcccgaaagaataggaacaatgatg
aaagagacgtaaattccgatggaaacatacatagatcctgatggatgggtgtaaagccgacgtgcttgaggatcaaacgtaactttccacgata
agctttttttgggaggtgcagtttgcacataaaaaacattgtcctctaatgcggaaggacttgctcgtgacagaaatgaatcatttttttaaactacc
ataaatgaaagatggatctggcttacctcttttatccaaaccccatacattgaattgaaaagccgaaggataagagtcagatataatatgcccgat
aaaacttccattctcctgcatggccatcctgtttcgctcatccgaatcgtacgcccattgagttatttcactgtccaaatacatctgagggaaccatc
ttttatgaggaaaagatacatcagattcttgcatgtggtttgcagaaccagttaactcactccagtactgtaccataacatgatgacccgtccatttt

tgacgattagggcccgaccagttggtaagagagcatcgagtaaatgtaccattgtattctagccatgacgaccagcttgatccgaaatccagtga
gtatcgaaaaagatcagcaccatcagcagaatgatttaaaagcaaggaatctccctctagctttaacaaatcaaaagagtaattggcagtagga
aaaattaaaggattgttaagtgacccaattcgcagcaaaaaatgatcaactgaatcagtaaatgctgatccatcactggtagtaacgtttgaaat
agttatttggtgtatgccatcctgaagattagatagcacagctgaccacctgaatctgctatcgggctgtcctgtaaagcgtaattgatcctcggaa
attatttcttcgcatgcaatcgaaccgtaatcaaactctgccgggacatgatgtcgactcctagatgtaacgtatatactttcagtaactgactcac
agctcattttctctgaaaattctacaataacattaacccttccatgagtattaacaattctggaatcatgcttggggaaaatttggtaattataggg
cgccgagggatatattcgtcaattgggacaaataacttgaaaccataagcatcaagtgaaattttcggaagacaaccagcaaatgaatcacttg
aaggaccgactgtagtagatgattcttgtagcacatattcgtcatacggaaaaacgagatttttttatcgttgttccagtctcaaatggtgctatattc
gcaagctcagacttacaatccagattcaactcgacggaatggttgtgattagtaaataacatccacacatcagttaccgatattccatgacttgag
gaagaggaatactcggtttttaagtcttgtataggcaacaaggaaccccttacaacactaaagataccaactgtcgtcggattcgctccactgttg
ggcatatattcatcataagtccaatttcctaacttttcagacttccaaccatccactaaagccggatatctccttcttatatcttggaactcaataaa
gacttgcctctcaactttagcaacgtcaaagtgatctagcgtattccacgcatcaaagcaaccgtgtcgggcgggagtaagatcaattgcctgata
agtagaggaacttccagtatagcacccatggatgtaccaagcaactgaagaacacataggctgccgactatataaataattgtcggctcgattgt
caagaacatacaaaccctgttcatctccataatatatcaatggaatgccagggaaaaggaaaaaagatatcattgtaccgagaattaatcttcca
gatccatccgtaaccgaagaccaacggaaaacgtcgtaatttgagacaccgtataaatgccgtgggtcatgcttttcagtcatagcgtttattgaa
tcctcattaattgcaattgtttcccaagcttctgttaagtctgtaggcaaatcgaatgatatttccatcaagccatccattctcaagaatcgaagtaa
aatacgataaaaagagtaatgaaaagcagctgcatccaatgcatttttgctcttttctcgtaaaaagaaagagtcatttgacggtgtaatactaaa
ggcattacgaatatctgaaggtcgctgatcgggttttctacccctcccgtaatatatagaaccgtaacttgaagatcccgtaacttcacctggaatg
aaaaagttgtgcttattatgctttctagcacaatcccgaatactatcagcccaatcagctaaaaagtcaacggtcatttgcgtagctttatcaacac
ggaatccgtccacatcgagcatagaaataactaagcaagaaagcctttttagtttttcggatacagaaggtttccactctctcaatcgatcctgtac
cgaagcaaacttcgacagctgccgttcccagtcagggtggctaccaaaggcttctgtatctccgtattgatcaaaatcagaatcatagcaaccttt
ccattctaaaacaacagggtatccatcttcaccccagaactttggaagggtacactcaaagtcccactcattctgaaacttccaatctcgataacg
gtgctcgcctttccatatggcattatgctccattaaagaaaaaggagttgtagtattaacatagttttgaaatccaacaaggtcggaaagcgttgc
aacagtaaggtctacaataatatacatatctcttttatgcatttcttcaatggtactcctccaatcggcaattgttcccaagtgagggtccaacaaa
gtaaaatccagaggggaatattgatcggcaccccaaggtaaatttacaaatggcgtaccggcaagatagacacctttgacacccatgccttgaa
gataatctaatgatttctttactccaataatatcaccaccatttcgaaattgtgtttcatacatgtcatattcgaatctagtgccattgatgtcgttatt
ggttggatcaccatcaacccatttatccaaagcaattgtgtatataggaaatctccatgattcaggactagggaaaaaatcatgattctcccattct
ccagagtaatcaatgggatcggttgcaacttcattagtatttaaattccatggtactttagacaaatcaaatggtgaagagcttacacgaaggcaa
caaaatataaacaagtagacgaataataaacagtgaaataatgagaaacgtttaggaatcattcttttctcgcttataagactctatggagaaag
aaggaaaacgtttcttacaaagaacagaaaaaaaagaaaaccactcttagcaaagacttgtctaatactctttttgaaaaaaataagagaaag
atttaaataaattccttttaccataagcaggtatattaaatttaataatttcacgatacaaaaggaggtttctcaaacgaaagaaagaaaggaatt
ctctacttatgaaaaagatagatgcttttgtttggttgttgttagaggaaaataacaatttgtagtaggtcccaatgtttgtttacatttgtgcatact
gatacacaaataaaaacaagcagccattgaccatacctagaaccatccagcatagtagcccaataacaaaaatagatttataagaagaatgga
cgtatttataattaaaatgtccatcttttgtgttttcttgttcagtcttcttcaactctcgctagcaggacacagcgttgaagaatggaaacaaagatc
gatataccagattgttaccgatagattctctttaaataaagatacgcatgagcggattccatgtgatccagctcgctttatgtattgtggaggaactt
ggaatggaattcgagatcatcttgactatattcaagacatgggctttgatgcggtaagctcaaaacaaaccaagtataataaggctgacatttag
atatggatatctcctgttttcgaaaatgaagagggaaataagattgatggttggtcctatcatggatactggactaaaaacctgtatgaactgaat
caccattatggaacaaaagaagattttaaaaatctagtagaagagctacaccgaagagatatgtggatactattagatatagccgtaaactctat
ggcactgaatggaccccttgagaatataacgtttgatggagtaatacccttt aacgatcagtcatactttcatccattttgttgggttgattacgaat
tgaacgatattgaaattgtgcagaatgtaagattattttccagtaaagttcgacttgaagatcgaactacatatgtttggtgttgtttgtggtggtaa
catatgaacaccactaacgaaactgtaatgaatacaggctaaccaaatttatcttagtgctggcttggagatgataatctagttttagcggactta
gatacagaaaatccaactgttgtggaaacattggtaaaatggattcacgatgtggttgaggaatatgatattgacggaattcgtttcgatgctaca
aaacatgctcctatcgagttctggtcgaaaatggcagaggctgctggcgtatttacgattggcgaatactttacaggatctccaactgaagcctgc
gattatcagaatgcaggtttagattcctttttaaactttcctctttattggcctatcacttgggcttttaacaatactggtcttcaatgtgaggcacttg
caacggtaattagccaaattcattctcaatgtcgggatgtgaatgccttgggaacgttcattggaaaccacgatttaccccggattagtcacaata
acacagatcaagcccgtgtcatgaatgcaatctcctttgtgatgatgtgggatggaatacctataatttattatgggacagaacaaaactacaata
cctatcatgatcctttcaaccgagaagcactttggttgtcgaagtacgacacagaaaacgtttactataaattaatcgcaagcttgaatcagtttcg
aaagtctgtccaaaagaaagacccagaatacgtaaatacccaatctactatcctgaaagttcaacttcattatatagtgattcagaagcataaag

ttatcagtgttttaggaaattggggtgttcatagtcaagaaggcttgacaatttcatttacccctttgggtgctaacactgaagatgtatatttggat
attattagcgataaggagtacaaagtgaatcagcaaggcaagctaaatgttcagattttccatggatttcctgttgtacgttctcttcaaataaaca
aacattatgtttgcacactaactttcttatttaggtgttgtatccccaaacgcaggatcggattttgcttcccaacttcactcctactctcttaccagag
actacttttattcctagtatcacgattacaacccattatgtggagcccacgtacgatgtcccttttggttatgatatgcacgatcatcccggtggcca
acgattctggaagtccattatttcgcaaaagagctcttcaactgaaaagaatccatctcctgggaaaacacttcccattctcttatcttttttacctt
cttcttgcctatctttgttcttgcatgaaatatcaaatacttacctaaataaatatccttaactaatgactatgttaatctttatataattaccaacaat
atttctaagcttattctaaatgtataaagctttttaattgattctctttctttcttatttattaacgaaattataacgtttcctaattatcgattgcgtagt
ttaacgaactttactaaatactaccatccgagttccttgtttacttggttgcattcctgccaagttgttgaaacctttatcaaaatttttgatgaatctt
tatgaaactctaatttccttcttgcaatccatcagcttagattctcgtaaaaagaatgcagatttaaaaaatgtttgtctccgaatttgaataaagtg
aatgttgttgttctaactttttaggacgctgaatcctcgattaagctcttggaaaagattaaaacaaattcggatcatgaattggcgttaggtatgta
ttacgcgaagaagatttggttgttatatgtatgttaatttctccgcagtattgaagcaagacgccctgtttttcgaccttttctttccacgcttgaaag
gaagctagaccgttacattattcctggcttaaactcggtccagtcactggtaattcatggtgccctatctcctgtacgtcgaatatagttcttgagctt
ccgtatagtgctaacgtacatttagtcaattctgtcagaattattccctgtactagataatatctcttctctaggacaggatgtgcagttgagaatac
ttcagatacttcctatcgtttgtatcaagtatgctgagtatatgcaactctctttattggtttctgtgcttcacacatgcttttctttacataataatagg
aatgtaatcgtttctaatgctgcttctgctacacttcgtcaagttgtgatattggtgtttgaccatttggatcatgaaacctcggcacagagaattaat
aatgtcctattttccgacttgcttactattttctctgatctatgtgctctaatcagttcaggaaactctgaattactaaaggttaatggcatatccgaa
actttcggtttggaattgattgaaagtattcttgttaatcaccatcatttattcgaacgaactgaatttcaagattcagttcgaaaagacttacttcca
attataactgcctccctcgcctctatgtctaatttttccgtagctcttcgtgttgcccgtattttatccattcttttccagcatcatacagcttcattaagc
atagatgttgaagttattttgtcctttataatcagttccttggagaactcggaggctccatggaaaaaagctctatttttggaagttttgcggtgtcta
ttcactaattctgaactcatatttattatgtattccttatttgatggtgtagaaggacggaaacctataatctccaaattaataacttcactcaatcgg
attataaatgaaaatccttctatgataggagtaggatcacgagtacttctacctgattttatagacgattacccgtctggtttttcatctacacttact
tttgaaatggaaaaaataataggtacattcactggttcaaaaccggaacaagtaataggtatatgccgtgctacaattttgaaaactccatgcatt
gagcagtttgacaagcaagacccgcccatcattcccgctgcttacttagtttacctggctatgtgttccttggtatctctttgcaaatctactgctgag
tttgtcatggacttccataaaactacatcaaacactgaattcttgagtttgctacattcactgcaatctaatgaagatataaaattatatccggatac
atatccaaagttaaaatgctactacggcttaatctctcagaattggaccgccttcctagtctcgtattctacttttatgtcgtgtgctttgagcacgga
gttgctaaagtttagtgttaattcttatgtaaaattcatatgtgtctgtaccatttttagattggaaacccctcgtgacgctttgctaacaactcttagc
aataaagcggtaccttcaatcctgttgactggaaacttgcccaataacaattctaattctgtatctaataatggacgtttatccacatccacgatgtt
tagtgttgaaggattaaaggaagcagcaacgaccatagctgggatggcaacttatgatggtcatgacgataaacaaaagactttttcattaaga
gaattaatatttttacgaagtctttcttttgttgctcaagaagttggcgagctattgggtaaggggtggaaaatattccttgaaacggttgacaaag
cagacatcattttaaacagatcgccatcatctaaacatttgtccacaagcagcttaagtcgccttggctcttcgcattctgttgggcatgagggcaa
agccatatctgggttaatggatcaggaaatgatatcgtataaaagtgaactttccgagcttatgttagctacctctaagtatgatgatcaaccttttt
atggaatttcttgaatctttactggcagtactcacgtcctcgtgtatagatcagacgaataatttgactgtaccccaactgcctgaagagagaactt
ctatttcatctcgtggatcttcagcgaggcttgcagcgaataggtcaatttctggaggaattcgtcttcttagaaaaagctcagaggtcttttattca
tttaccattttggaagtgataagttcctgtaatttagatcgcttaagcaacacaaagcatgtaaatgacatatggcctctgatctcttcttccttaag
cgatattttcaaaaattcaacagtttcccaggaacttcgaataagagctgcgaaatgtttggccaatatattggtggaattctgttcatgcatctca
aacgaaagaactgatgagaacaagaagtcccaagaacagtttttacagtcggtcagcttactagagccgtcgacggaaatatctgatactacgt
cgaaaaatgtagagtacgatatttcttccataggtctcgaagctttagcattgattctagaaacggtaggtcataatctgtttcaaggatggcgtta
tgtatttgacatgcttcgattcagttgtgccaagtgctccgatctgtttggtcaggataaaggggccaagatagtaagattagcgttttcctgtttac
agttaatctgtaccgatttttgggttcactggatatggacaactatctagatttgctcgatactttacttgtattttgtcgacaggaaaaagatgcca
atgtatcacttaccgctgtcggactattttggattgtttcggatacattaaaaaatatgtttagtaaatctgactgttcttgtacttatacctccttgga
agaattatatttgtttactactactaaaagaagagaaattttaccggaagtattatggatcatgctgcttgtgcatctagcagatctgtgcgaaaac
tcatgggccagtgtaagagatggggctgctcaaattctgtttcgaattttcaattcccaatgttcgaatctaggaattaatgcatggcatcctgctg
tcaactagttatcttaaaaatattgtcatcgcaaacaaaactcgcgaagttggcgttaaatagcgatgctcatgatgaggatgctgagcaaacta
gcgctttaattatcagcggtatagcgaatgtgttttctgcaaatatggacatgcttttgaaagtggaaggaattttgaatgtcgtcaaggaaacgtt
caattttatgcgaaactttcataatggtaaatatcaacgcatatgtatgagtaattttaaagcaatgagggagctggccttccattcgtctcaatat
aaacagaaatctttgatttttaatgaattggtcgaacttgaatttgaaaattggatatcattttgtgaactttctttagtcaatcctgtcatcgctggct
tgcctcaggaaggtctcaaattacttgttgaatgtttgttatacttattaaaggcatatagatttgaggaagataggctctgtcttgcaataacgcgg
atacgtaaagttatattttatgaagcaagcccatcatatgcgttggacattgactttctatcttcattgcaagaagcgacatatgcgttgttggattg

cttaattgaagattataaacaagaaggtttagtcgtggatatctgggctgacatattattttatgcgtttaatgaggaaaagttatcacgaaattcg
ctaccgacatttatttgcttttcaaaaaaaattttagaacaagtgaacagaattactgaagacaaaattttgccgatgctaaggaacggtgttatgt
cgcactttttttaattcgttattagtacctatgaaattgaaatatcgatgtccaaggccgagccgaatcaatgagaagcaagcacctatctggacaa
ttgcaagctctgtattcgttgacttaactacgaactgctttgttgaactggaaagaaaagatgctcttgaagaaaatggtcaattagaggcattatt
tgactttagcgtgaaagctatgcgatctgttgtgtttccagatataaactatgaatctgtgtggagccttgaaaatacctatgaaaaagaagatgtc
aattgtattaaagtgctgcattctctctggaagccttatctacattcatcttcgttgtgctcacagattcgggaatattttacggttatgtgtaaggga
tccgtccaataccaaatgcggggaacagagcctatggtgatatgttctaatgagatagagattcaaaaggaaacaggatactcttatttcccttca
actgagacacctatagcaaattgccgtccgcaagtcgcagagctttgctattctattctgttagaagagtgtcaatcatcgatagaacaagtaaga
caggaggtattgctcattttgcagagccgtatctgttggtctttgaaaagatacatggcagaccgactagtatcaggcttacttccgttggcagatt
gtcaaaaggaggatatcgataagatcactgaatgtatatcatcaaaacctgttttattttcaagctctgttcgttctctgttacgccgtgcttattata
cgatgaacgctacttctgtatctagtaatatgagcttaaagattggtggattgactttagaaaacgaggattggactggaatctaattgcttgccta
atgaatgagttacttaacgtgatgcgaatttcgtgaactaaaaaaacttatgaattgattctgtacatttaaatcttcgacaagtgaccattgaaag
aatgtagactgtgaatttttttaatttaatttaattttttattttattcctatgcttaatgtttatgtttgtttaaatttctatttacgtacaacaattgtagct
ttctgtggattggcttgaaattgggacgggaatcctcagccattgtcgatgcgatgtcattcccgttgaaaagagatgccacaagaaagtaacca
atgaacacaaaaaaaccataagttaaaatacagatatgaaaggaagccttgaagatcatagttaaagaagaaaatacaacaatccttcactga
acctacttacttgagacttttgaattcattttctttggaacttctgctacgatgaaggtacctttccggtaacaagcggatgtggatgggatgacaca
atatctaacctacgattaactaaccgagtgcggagaaaaatgattcaaagcctaggtcatactgagataaagtcattagaaaatgggaatatat
attagagcgactcgacgaacacaaattcacatcaaatccacgagataacaactttcactaaattctttctttgtttacaatttttttgtaagaaataa
aaatcaaccttgcaacatttattacgttgagagggtaaaatgagtccagctatgaattctgaaatgaatgtgcctcctcctcttgctgaagccaag
cacgttggaaagcgcctcttttccaaccaaagtgacccacgaaatgtgcgtaacctaatgacaggcctagtgtttttttaggagtgttccaagttga
aacactaaccataatattttagacatatccaaaagaggagcttcctggttcctatagagttttgcctcccggaacttttagcacactagactatcgc
gatgacaggtacgattgagaaagacacaacagcaaagacgagtttggttgaagtgtccttatgtttgaaataagaattgagtgactgactaactt
ttttcagagtcaactttcacaccgatgcagacggtactattacctatgtaactcggggttaagaagcataattgtgaggagtagcttacctttgcca
tgtctttctttcatgagagttcaattgttatgagacgcgttatagtattagtaaagaatttataacatttatcttttttttaagaaaaaatagatcagta
aaagagagaagctagtagttgacatagctctgagttctatgtataaaaaggtagaaaaatcatttttattcaaataatggaaggagggacgtttt
gtttgtgcaggtcataaatacaagaaatttgctttctttgtttttttggatcttttcttgtgtacaccatgagcaaatcagggattgttatatgccaaac
caagaacgtaggattctttttcgcacaccaaaatctttttctttgtgtttggtatatataagacttctgaaggagctgaactagtccattcacagacg
aaacgttcttcttctggaaatatggttccggcaactataacagacgatagattagctttacattcaaatgagtttcatgattaaacagtgaaatttc
cccagcgtataaatgcaatcacctcttctattactattgaagcatattttctgctgaaggaggatcttaaattacctacctgcaattgctagtccattt
cgagccaacacagctccggaaaatttctcgtttacactgtaatagtttagttatactgttcattttgtaatacatagaacgaacttccacctaccact
tgttgagttcttgaatcagattcccctccatactacggttgcaaaaaaaatcaaggaaaaaaagaaacgaaatgaaaaaagaataaattcctaa
caatcgacgaatttcatgtaacttaggaaaccgatgaaaactctcttatgctttgtagagatgaactggagttgagacgaggatttcttggaatgc
ggtatatagctttaacgaaacgggccgtgaaacgaattatgaaatattgagttgttatatttgtttctctcgacttactcaatttgtttaccgtataca
atgatgaggaaagcatctctttgtttacggtgaggtacgattttgatgtccaacatggaactattaatagtagagaaataatatataatctaataca
acaaatataacaaggtgtaataaaacaggatctatttgattgtaacttggaaagtccagaaaatcctaagaactgactgactatgctggcgtggg
cgagatgaaatattatggatggaagacctccttttaaagtaaaactcctacgaatagattcaaaagacctagacctagagaaaaagctattgag
atttcatctgtaatcgcataaacccatactctctttctttcgcatcataaataaagttgatttttttgtggggaaataaaacttgtcgattccaactat
aatgtgtccgcttttcctgagacatgtggagaaaaaggtatgacatatagaatcagagcagataatacagcttgcagtctcttgccgagaaactg
taaacattgggagcagaacagaaaatgaaattttccataaaaaagtggctttcaaggtaaaataaaaaagatttataaatataaatcgtaaacg
ccaggtatttacaaccatttcgtcaaaaaagtcagtatcctattgcgcagtaagaagaaatattaatccacattttactttctatttgttccatttata
ttggtaagctaaagccatcctccagaaaaatgtaaaagcggcatagtatggatgttctgatcccaccgttcgtttaatcgatgtcgttatcgttgtct
atagtggcattagcaggtaattttgccataaaacgaactggcaaagtaaatccttcaatagggaagaaagaagccatacgaattaattgactat
gctgtcctgcccaaagttgagcaggacggactccttttctgaaggattgataccaacgccctctagccgtcaacttctgaaacacctcttccgcata
ttgttgagcagacatggtagccttgtccccaaaactcggagcagacaaaagatccgttctgtatggataataaatggagtttactgagatgctctc
ttcgttcaattttgagtttggtttttttaaagacgctcgtcgaaatcaatcccgtcataatgcttgtcacttgcactccaaaaggagccagttctacgcg
tagtgtattggcatatgctagcaaggctgccttggacgcattataagcagcctgaaagaccaatggtacatatgatgctattgaattcgtgtgcac
aatggttcccttggcacgaatcaattgtttttgaaaaaccgtgtttgttcgcataacaccatatagattgacctccattagattatgtactataggaa
tttccagatcaatggctggacttgctaagggctgcccagcattattgatcaggtagtgtaactttcctcccgtttcatttcgaatcactttttctgcttc

agaaactgaagtatcatctgtgacatccatctgtacgatctgaatacccatatcaatcagattgctcatcgactctagtctgcgtgaagtagcaaa
aactttataaccctgtttatggaactctatagccaaagcgtttcctattcctccttcactgcatcctgttatgacgatattttctgtagaattactcatc
ctattgttttcactgttcaaaaattctaacactcaagcgctgatggaatgcatatcaaaatgtacttactctggatttaatgatggaacgtaatactc
gcgtaactacaaggcaaaaaaaaacaaacaaacaaacatatcaatgggtagatccaaatttctaggacttcaagatcaaccaagtaagcgagc
atttaaagtaattaagctcgcattaaatcattagcaagagttcaatggaacagtttttatttatgtaatactacaaagagaacttaatcattctttat
tttttttgtttttgcaaaatactctttacttttcactggcttagttttgtttgttgttcaccattttgattttccgcagcgtgaaaatgacaaactctatcgt
ttctacatctgccaattgtagacaaacaacaaacagaagtcttctttgtttgttttcttctacattgccgattaaatgaagatcagtaacatcgggcc
tttaaaatggacatatgcgatccgtttgtttttgtttacttgtttttctgttacgtataccacaccaaacagactatataaggcagcatctcagctttcc
ttttaatcagagccaacctcctcctttcttttcaactttcattactttctttctgaactcctttcaacaaatcagtctatttcagaacatttcaattcacct
tcacaatcagaaactttggttgtctggtgattgttaaacgaattagacatttgttaaaggaattcgtctatctttttcagccagttctgcccaaactcg
gctgtaacttttgtcatcgtcccatatcatattgattccaatcctccggaaccaatacaaaaagcattcatatatcctttactttcattttaaggaaaa
acttttttttttgattttcgcttaatactttcaacaaactaacacaatgagcgtcttgactcctattgctgatggtgctcccagttctactttccctaag
agagaccatcctctacaaccgaaggccatttccgtatctgatcaaccaatccaaacaaacaaattctatggtaaccttttcttggaagatcaaaag
tttccaagtttcccccaaccatatacgttgacatggcggaacaacgatgatggcttctctggtctaggcatcagtcatacagaagccagccagtat
acatatggaccagatgctagtaaaactccgtgtgagttctatatcaatccagcaggattattctcattgattatttctgcgaatgagttttctaaagg
aaacaaactttccgcgagtaactctcgccatttttccgttcaaactgttttggaaccctccgattcatctgacggaaacattacgattcctattgttgc
tggaagtgcgttcatcagtgccgtttacaagaatttgaccccggtcttcaattcgtcaatcatggtaaagtcgcttgataagagtgacttcagtggt
ggttctaagtacaaggtcacattgaacgataacaaggtttggctcatttatgccttcccttccgactctagctcttttgatctaaaaaaagacagca
acagtaaattaactgcctcctccaaatttaacggacttgtccaaatttgcaaagttcccagttccagcgtcagtgacggcacgggtgacaaaactt
ttgacaagtatgctggtgtttatgcaactggcatcactttaagtgccaatgtttctaacgatactggtgaatactggtttacttttgataaagctggtg
acaaatctaagagtccactggtgtatgctctccctcaccatcaaagtacttttggctctgatacccagggatcaaagactggtatggctttacagag
taccgttatgggtgttatgactgcgtatggagcagataaatggcatttagtggaaaagaatcttcctacggatgttgagtttcttcccattccttgga
acggaggaaactctaacaactctgataacattttgagtactatcaaatctgcttgccaaaatgacataaattttgatgtcgagagcgccacagata
ccaatagtatgtatgcctctggtaaagtcttcgctaaatatgcccaagtttgtttggtggcatccaaagtattgaaggatgacaatttgactaagac
tggtcttgacaagttgaagaaagccctccaaagatatgttgacaacaaggaacagttccctcttaattacgatcaaacctacaaaggtatcatca
gtacggccggtatcaagaccccccttgctgattatggtaatacttactacaatgaccatcatttccattatggatatcatatctatgcaatggcagtt
gctggcttattggattcaggttggctttcggattcccaaaacgaatatgttaatgatttgatacgtgatgcagcaaatcctgtggaggatgataaca
atttcccttttttccgatcctttgactggttcagcggtcattcttggtctaaaggcttgttcgcaagtggtgatggtaaagatgaagagtcaacctctg
aagattacaacttttactacgctatgaaactttggggaattgttaagaaggataataacataatcaaccgtgcaaatttgatgctggcggtaatca
gaacgagcatgaatgattatatttatataactcccgacaacaagattcagccagaaaaaatacgtggcaattactgtgcaggaatcacttttatg
aacaaggttgactataccacctatttcggtactgatgagtacttaaaacaaggaatccacatgatcccaatcactcctatcagtggttacgttcgta
ctccttcttatgtaaagtcaagctgggatgctaaaattgctccaactatcaagaacgttttgaacggttggactggtgtcctttattccaatttggcta
tcactgatcccactgctgcatggaaagagtttagttcttctgactttaaggactcttatattgacaatggtgcttcaaagacatggtatttggcattg
gcagccggtatgggtgcctcaccttaaagctgtgctataaagtatggaaatatgtttggcatatatgaaagaaagtaatgaaagaaaggacagtt
acgaacaggcaggcttagcaatctctttacagagagtgcttttacgacaaacgtttagaagttagtctaaaataaatttacaggctacttgctacg
gcaaatgtagtggttttacgagtgcattgaggaagtaaaattgattacaacttaattgcattcctctacatttttttgtgaaaataaaaaaaatata
atataatattaacgacattattaccagctagtttatagaaagtttagaaggactttgctagttgaagtaggcgacgttaagaaagcattgcactat
acataaacgtaaatcaaaagtggttttaaagtaaaaaaagagtactacaggccaaagtaatttttaaaagagcttgtttaaaagaaaaggaaa
aaagaaatattttacagcttttcaagtatacgaatgagtagaaattctgctaatcttggtaagaaaccatacgtagcgacgatttgccggaagatg
gaggatgctaacagtaagtttagttgaaataccggctttagtcatagatcctggaacatgttggacacgctttgggttcgcgggtgaagaatcacc
agatgttacactcccgtcaaattacggtgttttgcaagacagcaatgaggaaaaaaattatataatagatgaattgcagattcatgcgcccattcc
tggtatggagattgccaacggtacaaatgatgggataattcaagattgggagtctacgttacaaatatggaagaggggactgaaagacaaactg
aaagtcgatccagccgaatatgcaatgatgattacagaaccgagctggaacacacaagctgttcgtcaacagacaatggaagcagtatttgaac
agcttcaggcacccgcattctatttgacgaaacaagctgtttgcgctgcgtttgcaagcaataaagcgacagctttagtagttgacctcggcactg
agaatacaaccgttactcctgttgttgatggcattgctgtgcgaaaaggtataatgaaacaacgtattgctggaaaatttttaatgcaaacgtca
aaagtatgttttctaaaatgaatataaatgtcgtgccacactaccgggtagccaggaaatcagttgttattgagtcttcaggtcccggtaatccag
aagaggtgaggccaacagttttctatgatccaattaatggtttaaaagattcgtatgaagaatttgaaaaacagaggataattgaagaatggaa
ggaaagcgttctagagacattagataccccatttgatgaattaaaagcgtctacaaaaaatcccaagcattttgagttcccagatggttcaaatta

ccagttcggcatcgatcgatttcgtgttggtgaagtactgttcagtcctaccttcgctgctaatgattctcctgaaagcacccttccggaaaaggctg
tcgggcttcacagcttggtgtatcaaagcattgttgcatgcgaaagcgagttaaggtctcagttgtttaataacattgtactaacgggtggcacttc
tctcattcctggtcttgctgagagactacaaaatgagctccaaagattagcaccaggaaatcgaatccatgtacacaccagcggagattctactta
tcgaagtaatgcttcatggctgggtgggtctatcttggcaagtctcgacaactttcagcatttatgggttacaaaacaagagtatgatgaagtggg
agtagacagagctttatttgtggaaaagagatgcaagtaagggtaataaaatgctgcaaaacaaaaaaagactttatctttcattttctataaca
ctgacattctttcgattgttgatttacgatatagatagatcttgaaggtactagtatctttatcctttttgtagtttctcgtttcggtatcttaccgtttaa
gaaaaatactaagcaaagcattggaaataataaaacctacaaagtatgggttataaggtggagtaatacaaggatagaagtgcaacgaacga
aaaggcaaaagattcgttactttttcttgtctgagattccaaaagggcataaattgtcataagagcagtttggttcgattcagaagttcccgtaac
agttagcaaagtcacatttgattgagaattaccttgctcaatatcaatatggacattagttgactgagaaatttcttggagttttgtaggctgataat
aattagcttgcgaaatcaaaagttgacaggtacataccacttgagaaagaacagcttcaagaacgttagatggcacagataacttttgtgttatg
atttgattagaggacgcatttaacggaatagaaggtcccgaatatggatagtattgagtcgctcgcaaaggatctgagttgcccccggctgtagtg
agagcaatcggagtgacaccatacgggttttcaggtgtaaccatagtgctattgggatgtaatataccaaacaaagacggtgacccataaacac
cgtaagcagcatctgggatataaggaacgacatttatacctgaaatgatacgacttcttgacaaaagggcatttaggaaagtttctaatactttgg
acgattcttgtacaggtgtctcaaggattacagagcgttccattgaccgcggcaaaagagaactacttaaaagtaaacgcgaagaagaagatac
ggaatgaagctgaggtattaaatgctggttcatccaagtttttgagccaagtaacacatttgaaaaagcgaaactgaatatcaaaattgaatcatt
ttcatttgaggatctaatagcttgagcgatagccggtaatgcttgcgaaatattcgtcaggttagaatgacaaattaggacctttctagagctattc
gaagaaatcaacgtttccgcacggatctgaccatctcgcaatatatctttttgttcctcttctcccaagttcatccataattcatattgttgagcactt
agaagcaatttcactgttacagagttcgcgtccgttccaaatgcttgctgcaggtttaacacatgaggatgtaaaatatctacaggtactgtagaa
acatattttgacccagattccataacaaatacaccgaaagaatttcacgtttttctttgccaaaagcaaattgtttttcagtaccttcctagtttcaga
aacaaatagcacagcgcgctctattgcgttatgatgatttccaatatggacctatcatctgttcaaaagcgattcagacagtggttatatactcata
tagtttatttaattatccgaatagaatcagacaaagctttttgcaacggatatggatatgttgcatagaaagcagtcggctcttacggtaagtagcc
aatatatgcgtaagatgttctattaatcattaagaatgaatatatttctatgaaaactagagggacatgtgggaattggaaagctggtgctttttaa
aaggaaagcaaaaaggaaagaagtcattctcattacatagaaaggtagaaagatagcgagcacggatgattttcaaggtgaaacgtcaattct
ttgcatcctagcgtagcgtcataaactaaaaataagaaaatagcactgaatataaattcagctgaagtataaacaagctgcaggcatttaaaaa
ctgctatactctttttttcgcaaagaaacctcgacttttatatgtcgacttccatttcgtccagcgtttcgactgtgacgaacttgtgaatgtcttcccg
taaaatggctgtagtaccacgctcgtatttgtagttttgttccttttgagcacgttcgttcggcttagaatagccgcggtagggggtagccttttcatt
atcaatgacaaccaaatcgcaattggaaccagatccaagatcattgaaaataccagcttcaattgcctcacgaacaagctccatagcttcatgtc
gttctaagttaggtttgtatttggtttcaaggactgaaatagcagcaagactaccagatccaagagcaacataaggtaatttgtcactggaaccat
gagctgcgatggtgaacagatgtgggcctttacaatcgtaaccaccgaggacgaggtaagctccaatataaccttggtaacggaaaagatgctg
cttcaacattgtcaaggcagttgccactcttggctttcgattaacatacatggagtgcaactcaacgttagaagaaatcatcgaagtcacgaattc
ggtatcagcagcagtaccagcaccagcgcaccatatattaggagcaattaaatgcaactttctgcctgtaaaagtagttagtaactgaaaagaac
gcaaggtgcagaagtccctttggaaaaaccatcaataaccattataatctatgatacatgtattcgattttaataaaggtcaagtgtaaccactgc
ctagttgcttcatcacgtacagtttttatcagcaataatcggtccagcagttgctctagtgtctgcacccaaaacaatacaatctttagcaatcacac
cgacaatggtggtaccagtagaagttgccttgggagaagggaatccacgctcctgcaaaagtgcatttctatgcctgttagtcttccaaaaatatt
caatactctctgaaacgactcatttgcatttctcaaacacttacctttgacagtaatcaaagtcaaatcctttttctttcgttcaatcccttcatatttgt
agggttattgatattgatctgcaagttgtatttggcaaagggattgtctaacggtgtgtggtttcctccatgaagacagtgctcgctaacgctcgttg
aaatataaataccatggtgcacttcattgcactaaaataaaaataataaaatctgtatacttccgtaaagtgtatttgccaaatactagtgtcatca
ctaaataatataatgaccttgtttgccattgcagagaagcgactagtcttaggcattgggaagcaagtaagaacttactactgggaagtcattaag
gtgaaagttaaaacttcataactttagtcaatcttaaattgctgttataaggttttaaggtgcttgatcctcatttttttgtaaatatatatatacatat
atatatatatctttttacacattgttgaaaggagtaggcatttatttacgagtcttgcaagtagtttaattgcaacaatcaagttctgataaaaggat
cctatttggctctatttatttatatatgaaatgaacactgacaatccatttgctgcgcttactccaagcccagcgccttttgctggtaatacacatcat
gcggaaattgaagaagcagatacatcggtatgtcatgtttccttctatttaggctgttctaactttccctgtagagctttttgttttctgctttaaaaat
tggtactacagtttcacttgctcaaccgacgtcctcagaccctccagctaaatcacgtttgtttgccattcacaatttaactaaagtgtttgtagctgg
tgtccctaatggtcttgttctaggttcgacttccatattgcagaagtgtcttgatgaggcctcgatgaatgcaggccctcaacaacttccagaatcgt
ctgaagtagttcaccttgccattccaaactctaacttttctttcgtcgattttacttctcaaggaacatgtattgtcgcatacgatctttcctctcaaga
aattgtcgtctttaatacaacctcttgtttagagggtaatgtcagtcctcgttggaaaacacaattgccgaatttggatttcgttcttccaaatcctga
aattgacgatcttgttgcgcttcatctaactacacaagaattatgcatcttaagcatttctcaggcgaccatacatcccccaattgctagagaagct
acctgcgttgcttggtcaagacgtggtaagcaatgtgctgtaggatttttaaatggatctatagttcagtacactcccgatggacaagctaaacag

gagattgctgctccttctgacttacaagacttttatgtccaagatattgcgtggatggaaaaccgagagttcattgtttactatagccctataacagc
gttggcaagtagtgagcctgtacacgagtcagtttgttatataatcagtttgggaattgataaatcaattacttatgctgttgcgacagatccaact
cctccgttcggagccgattatcgtcacgatcaccattatttctctcttcttcagtcttggcatccaaatctaagatctttggtgattgtctcagcaactt
caagctcagatattggtctgtttgcccatatggccgactcaaattcatggaagagcttggttattactgaagaaactaaaagagcttctcttccttat
tctcaagcaagagagcaagatacttctcctttgtctctcagtttagatcttacttccacagagagaatcgagaagccactgagcccagtagaagtt
cctgcagattgtccccctcttcctattctttgggtttatgataatgactatcaactttcgggattccgatttatgtatacagatgctattatggaaaatt
cactctatcccgaaatggtcgtagccaaaagcgtatccggtactgagactgttcgaacctctaataatgaaaatgtcagttcttttgcttctttatca
gaacctcccaaattcggttctcagatgtcttttggtgcatttcagactcaaaaacccgcacaagcatcagcattcggtcagagccagggctctcctt
ttacagctccaagtgcaactccaaaatttggtgagtcttcgttttcatttaacttcaatactactcctgcaaataatgcttttaccccaaataattcttt
tgctggttttggaacgaagtccacttcttccgcattttcttcttttgctagtgccttaactcccgctaatttatccaacgttccaaaggataatgaagc
atccaatatttttggaaatgccaaagttcctgagaagcagccatccgtgtttcaatttaatcccgtcgagaaagcagagcctgaaaaagatatgg
aaaatgaaaaagttggtacacttgccaacgctgatgttgagccctctactactgatgataaagctggtaacagtaatattaattggggaagtggct
tatttggtaaagctgctcagccctcattttccttcggtcttagcttaaacgataaaacaccaaatgaaaacttctccgttttcggtgctaagtcgaaa
gatacaactgcaccatctactgatcaagcttctactaaaccattttcttttgctccaccagataaatcggtttttacctcttctataaagccaacaact
ccagcgagcaatgctccagaattgccaagaagtgagtctgtctctatggaaagcttaacagaaaaacctgagcaaaaagataatactgttgaag
tgcttgagagatatactttaaatgtttctctagagaataaggaaacagatgagaaacatgcagaagctacagtagatgaaggtctattagcatct
gacaatgtggaaactgaaaaggcaaatagtatcgacaaaagagatattcagaatgatcgacagtctccttttatagaagaattgaaagatgatg
ataccgatgaaattgctttagaagcacagaaagaatctgttacggatacaatgccgaaggaaagtgttttggagactgaagataagcttctagat
actgaacttagtgaggaggaaaatcttttacgtacggtttcgcatgagacaaatgaaagtgatgattgggaaaaagttgaaggtgaagaagact
ttgatgaagctgcaactacaaagcctgaggattttcatgaggaaacgccggaagtctctgcagctgctgaagaaaagcttacagaaaaagaagc
agataaggagtcatcaattgacaaggatgcgaaaaagcagatctcgaagaacgagccaagtgattcatattccgaagatattaacaaagagga
tgttgctgaaaaggaaccagaaccagaacgtcaaggagttccttctctaaaaggtaaagtgagcgaaggagcctttgacttaccatcagctgaa
gaaagaagtataatagcaagtgacctcaaagaacctgaagaatctgtcgttagtgaagctcccaaacttttagagaagaaacccgttccccttga
aacgctggatttgcttgaagatatgcaagagatagtcagtactgaatccaacccaatggtcaatgcatttgaaaacctgtacttgcattttgaggc
agagatggatctggtaacccgaaatatggaaaagttggtaaattatgtaaatgaccaatctaatactggatattctaatgatatttatccaaacat
gacgtgtcttcctaatgatactcaaacactggaaagtttgctcgatgaaatgaatttgtggacctctggcaaaaccgaatacgacaaaaaactag
aaactttacgtgtgactctcgttcgattaagtgctaagagagttcaaattcaaagacttgtaaatgctcacacagatcctgtgtttatcaatcgtctt
aaattacgacaattaggtcctgaagcacttcgacgacaaaaagaactacgtgtttccatggaaaaaattcaaaaactaatgacctcggtggaaa
atatggcatttgatgtacgcttaagtgatgttcaaaatcgcaagcgtttaagtttggcttcgatagaagttgcttgtagtcgtgttgctactgttttag
ctcagcgggagagagaattgttgaagcttgaagttgaagttaaagggttgtttggctcaaaccccaatggtccttttataatggatgatttgagttc
cagtttaataagttcgcttgaaaaaggatcagattcccttttggattacgagaaggttcaaggtcatcagctaaaacggaagtgcaacgccaag
atgagttttgtgacataatttttaacagccttcttcttttaaggaaaagtatataaaagaccttcatgaaaaattgttttagggtggtgtccaagagg
atattgtcgcactgtatggaataaaatagatttgtatttaagttgcttaatttatcttgtattctcaacagatgtattaaaaaacggaaagtaatata
ttaaaaattgaagtgaatgatatttctcattacctagtttatctccatggattctgctacattgatctgtagacgatctataccaacctttttttctgag
attcatatatattcaattttggtagttagaacttatctaaatataaagacaatctttagtggtaaacgacaagagtacttctgaatattcctgaatct
ttagactctcttttaattgggatactaacttacagtctgaccaagtttcgagaggctttcttacaaaactattggaatatagtactacaaaaatctg
gtgtattatttgcatacaaactattgaaatagatctgtttggtataaatacacaatatgagtggagcttcctttaattgtctaagtgctttgtcgcata
ctcttcaaaaacaaatcacatctaagcgtgtttccgttggcgttattggctctggtaattggggtactgccatcgccaaaatatgtggagaaaatgc
taaagctcatccggacttgtttaatcctcaggttcacatgtggatgtatgaagaaaatattgatcaccatggcgaaacccgcaaacttacggatgt
attcaactcgactcatgaaaatgtgaagtatttgaagggtattcaatgtccctctaatgtatacgctaaccccgatatccgtgatgttggagccaga
agtgatatccttgtatgggttcttccacatcaattcgttgtacgagtctgcgatcaattaaaaggctatttgaaaaaagatgccgttgctatttcttgt
atcaaaggaatatcagtcaacgctgaccatgtttgtcttttctcagacatcattcaagaacgtacaggtatgtactgtggtgttctttccggagcca
atattgcaagcgaggtagcaaaggaaaaattctgtgaaactactattggatatgatcctgacagatctgtcaatcctttgtatactgccgaaacca
tacattctttgttcaaccgaccctatttccgtgttcagattgttaaagacgttccaggtgtagccttaggtggtgcattgaagaatattgttgcagttg
ctgctggtatcattgatggacttgaactgggtgataatacaaaatccgccattatgagaattggtctagcagaaatgcaaaagtttggagccatgt
tctttgattgtgatcgtcttactatgagtgaacaatcttgtggtattgctgacttgatcactacttgtcttggtggtcggaaccgtaaatgtgctgttgc
atttgctaaaactggtaagcctatgcatgtattagaaaaggaacttcttaatggccaaaagttacagggtgctactactgctaaggaagttcacga
atttttggatcatcagaataaggtggatgagtttccccttttaaagccgtgtatgacattgtctatgagggcttacctgccaataaattgctagaag

ctatttaaatctctaagcatcgtttgatgaggtttttttgatattttttagtttcatattacgtcctgctggtgaatttggacttagactcgaaactttaa
tctgtatcaaattgcgaaactaattttgaaaataaaaaaagtcgagttttgagttgattctgttccccttgtgtaagcttactactaatgctggtttta
attcttcgcgtccatcgtaacttttggttattaatatagttaaaatagaagttattgttttaaacagtgttttaagaataccatttacctagtagacca
aaaaaggtagatgatcagaaactgacttgactataatcctgacacttttactattccttacatttccaagaatcgctgggctacaaataaagatctc
gcatagaaaagcagtcttttctttcttaagtcgataaataattcgttaaaaagtatttattcaatgtacatttttgtctaaaagaaaaacttaaaggg
aaagttatctttaggcaataccgtagtgtcaatacctgtgattccgacaacttttcgagtttttttgacatttttatttacttaatatccaaaacaccctt
aaattacttgggtttcgtatacctgccggccgaaggcttatacttccttttcaaatagacaactactgcattctttatattttgcatttcatttatactgt
ctaaaatatattaccattctctataactggaaggtgtcgttcagttttaatctagtttttttttaacgaagaaacatggaatcattcgaaagcgttaat
accaacaatggatcttcagtaaagaagaactggtggtggggcacgaaaggaaaaggatatcaggtgccaggtccttcaagaagcagttcatcg
ggccgacgctcatcggatgaaattattccagatgaattgattgttttggatgactcgtctttatgcaatgttcccttggagcttcctactggagtatcc
agagatacagcttcgaaaagtttttccaaactcactcgagaggaccagatgcttagcttacgactgaacagtgttcaggattctatgtttaaaaaa
gccccgcgtcaatccacagacgctcttcttgatcctggccctgtaagtaaggaaaagcaagctgttctttctgtcggtagaccctcttggcttcctcc
taagtcaaaagcagaagaaaagcagcacctccgagaatttgaacaaattaggaaagctgctctaaactatgacaaacaaagccagaaggaga
aattcaagcaaatggagcggcaacgcaaaaagaatcaatatttagttcaactatgggatcgttacatttttcgatactggcctgatagccttgcat
cctctaaagttactggtgtttggcgagagggtattccttctcgtgttcgcggcaaggcttggcaaaagataattggaaaccatttacagttagatta
tcaatcatttttccttgcatgcgagcgtgctaggaaacttgggtattctaatgaaaatgagcaaacccaaaatgttaatatgttttgccatgataga
agtgcacttgaggtagatcttgaaagtacaattcctcagttacatttatttcaaaaaggagaagcacttcacaatgatctagttcaattggtattag
cttattccgcatatcgttacaatgtccgttatgtgcctggtactagcttcattggagctttgttattactgaatatgaatttaacgtctgccttcaactc
gatggttaaccttttagatcgtcctttcatgcaggccgtatacactcaggataaggaagcattggcttctttcacaagtttgtttttaagttcactgaa
gaaatcacttccagatttggcgaatcattttaaatatactttgcaggctcaaccagatcagtatttgtatccgttgttgcagaaatttttcatccccat
gaattctgtagatatttcttctcgcatcatggattgttatttgttagaagaagacccattcttgatccaagtgcttgtggccttgttttcacagttgaaa
ccgaaactattggatactgattttcgtttaatgcaaaatactttgttttatcatccttggaagcttggtgaagaggatgagttcatgaagaaggtgtt
tgagacatctatctcttaaacaatgaatctgcccgttttaagatttagcattgattaagtctttatttatttggtagggatagcaatccgcgatgata
acaaaatagagcaaaaataaaaacatgtgatatgggttttattctagatatataatagcaggtatataaaaaaaaaggtataagatctatttgaa
aattactttttgcgcaacattaagaaacaacagagataaaggagagtagatctagtaattgaagggcttcatccgctccatcagaaaggcgctta
tcgatttctgataattttatgaaaatcttgtgttttgctggactgctaagtgtttcttcatccaaaataacatcatgcaactgtgatagaataattcca
gttgaatagccttctgcttctacttcgcgcgtcacagcctcgatcttcaagatatctttggtataggctgtatcaagcaaagtacgaatggtgtttga
aggaacggctcctgcaagttcaacaatactggctacagtgaccggcattccctgatgaagagtcgcagcactttgtaaaaatgtgatagcttttct
catatctccgccagaatattttacaagagcatcaacaacaccgggttccatttcgacattttgatctgtagcaataaattctagccttttagaaatat
tttcagcatccaaaggtttaaaacgatatttactacaacggctggctagaggatcgatgatcctagtcatataattgcagattaaacagaatctgg
taatttttgcgtacatttccatagttcgacgaagcgctgcttgagcatcttgggtcattgaatcagcttcatcaagaataataattttgaaaggagg
gcagggatatccatctaccgaatttgaaacggtggtcttcgcaaatgttttcaccttgtctcggataatggagatacctcgctcgtcagaagcattt
aattccaatattcttgatttcattagttgaggcccaaagagttctcgagccaaagccaaaattgtagaagtttttcccgtaccaggagagccatag
aagagcatatgtggtaaattgttggacagcaatgtctttgttaatacctgaatggtagcttcctgagaagaaacttggtctaaggatttgggacga
cttcaagaacgcgtcagttttcagtaccgcatcaaattgtcttttatttacttactataattctacccaaggaaccgatttcttcgattcttgttcagtt
ggcttggaccttggaacaaagaaggacatatttattagaaatatatagaatgaatgagaattcctatatatacagggaaaaacaaaaagagga
gcttttgttggtactagacgcgtttcgggaataaagaggagcaacgagcgcgcaaaatctaaattgtgttgcaggtatataggtatgacaaaata
gtaaatataagggaatttgtgttgattaggaaaagcgtctcagaaggatgagtgtgagtaaatataactaaggatgaagtttgcttttctcaaaaa
tggaaccaacaaatgagcaaataaacataaagcatgaggtactcgacactagggcaggcaatgatgagtgttgtgaagacttatgcaggaatt
gtaggattgaccacaatttcatttttgtttagcgggttcgtttcaagggtagataaatttggtttgttcaacagtttcagaaaataggtaaataagac
gtggaatctgtcaacaaagtggtatcggtgctctaattgcaggtctgaaagttcttttaattgagaaaagagttcgaaaagctttgtgttttcaatc
aactctgcgtttaagcctgaaaagttctctaatttttccaaaatggaatggacatcagtcatttcggattcggtaggataatgattgaaacaaagca
ggattctttgtagtttgtaacaatagtacattaatatcttcgctcgagcataataggcttgagcataaaggtccgttgtactaagaggttgatccag
ctgagatttaaaagaaaatactgggagctttggtggagagagactagcccttgctgcttccttttctgactctttctttgtaagttgctttcctggtga
tgcttgggttgggggaacagtcttgtttttatttcgaatgtgccggtcaccggaatcaggacttgtatatcctatcccttcttttctgttgcttttttttgt
acgttctctctatagtagtgtctttcccttcatcctcagatgaaggaatttgtttcgtcccatcttccttaaattcctcgcctgcttcatcttcctcctctt
cttgttcatcttcttcttctgtttcctcagcatcagaaaacgagcaatcatttttgattgagtcggcaataatctcaatggtctgtcttatataggaact
aaatcggtttgattctaaaatgtatatacattcatacagatctaggcagccgacattgcccatttgtataatcgcatagtctctagacggaaggaat

tgtactgctagtcctttgtattttgctaatcgtttcgatatatcctgaggtatctcttctctacggacaattctagagggccaccatggatagaaactg
tattttactaaaacatatttgccttcttcaaaatagctatctggctttttattaggtttatactttttcaatgaccatcgtcgatcctgcctaacttgcttt
tgaagattttccagcgagccctcgctacttacagtcttttctgccttttttcttctttgatgcttcctttaaatccacttgtttctttgatctttttaaattcg
tatcgcgacttcgatttgtagctcttgttgttaccatgacgaatccctgaaaggccagtaagttgggctctgttaattattaacagctaacagcttga
aacctacgatactcagactttcttaagctcataaatctggcattgaatagatatatctattttcttatttctgatattccttttgagaatgaatgaaaa
taatgcgttgttgtaacattgtaatttacattagtgacttacgttagaattgcttagtaaacatagaaaacgattatggtagctttcaaccagaaac
gacattatgtagaacattgacattcctaattgaatatttcttaaataaaacccaaagtaaaccggtgatactaaataaaagtttgtatatcgataat
tattcaatgtttgtattcaaagactttttatcaattagtttaatgaatttcgctacacctcattaattaactatacacgtcctcttcatggtgagttaact
aaacctttgccaacaatatgtcgagcggtaagttggaagaatactgtataaaagcaggaggaattaacaacatgacagtagaagatgctgagct
ggagaacctacgagctcagagagagcaattgaggaaacaacgggaagaatctaaaaattcagtgcttagtaaaacaccaagtattgcaggtac
ccttgattctcatcgtgtacaatatgatactcatatatataataccagcaatgtaacagatggatacgacagatcaatcgatgtccatgacgatgc
atatgaagagaatgaagaaggcataccagatggaggcgaaactagtagacctttagtgcgtcaatatgaagctccgaaggagcttctgactgaa
tatgccgatgtaaattatgaccctatggccgagatgcaatcgaaaaaacaaattcaagaccgagaatcggactatcaaaagcaacgattccaaa
gaagattagcaccaacaaggaatgacgcttttcgagaagatggtaagatagatggtggaaggtcatatgcagatgcgatgcgtgaggttgagtt
agagaaggaggaaagcagagtgtatcgtgaaattaatcaaaggcgtcgtgagggaaccttagaagaaatagaagaccacaaattacctgaag
agaaaaaacgtacagttgaaacaattccctctgaacccgctccgaagcccaaaaggtcccgatgggatcaaggccctcctaaggtagctgaaca
agccgctgttcctacaaggagatctcgatgggacaaagctccggacgcgtttccaatttcagattacgttgcagaaaacggtgtacagaagttgg
aatcggtttcccaggaaaagcaagttcctattcatcctaggattactgatgaagaattaaattcactacttccttcagaaggatatgtaatcttgga
acctcctgaaggataccttgagtcgattcacccccagttgaatgaaaaggaaaatttagtagatacttatcacattccccaagaacaactattacc
tcaacacaaagaattgcctgccaccttacccacggaaattccgggagttggtgatttggctttctttaaacaagaagatgtgaaatattttggaaa
gttacttcaaacaagagatgaagccgaattgtccattgccgaattgcgagaacgaaaaattttaagacttctgttgaaagtcaagaacggtactc
ctcctatgagaaaaagtgctttacgtcaattaacggatcaggctcgtgattttggtgctgctgctttattcaatcaaattcttcctctattaatggaac
gaactttagaagatcaggaacgtcatctgttggtgaaagtaattgatcggattttgtacaaacttgatgagttggttcgtccttttacacataaaatt
ttggtggttattgagcctcttttaatcgatgaagattactacgctcgtgctgaaggtagagaaattattagcaatttggcgaaagcttctggtctggc
tcacatgattgctactatgcggccagacatagaccatgttgacgaatatgtgcggaacacaactgctagagccttttcagttgttgcttcagcgctt
ggtgtccctgcgcttctcccttttttgaaagcagtatgtcgtagcaaaaaatcttggcaagcgaggcacactggagttcgcatcattcagcaaatag
ctttgctaatgggatgctctgttttaccgcatttaaaaagtttggttgaatgtattggccatggcttggaagatgaacaacagaaagttagaactat
gacagcattggctttatccgcattagctgaagcggctacaccgtatggtattgaagcttttgactctatccttaagcctttatggtcaggcgttcaac
gtcatcgaggtaaaagtcttgctgctttttttgaaagcgaccggatttataatacctttaatggaaccagagtatgctagtcacttcaccaagaggat
tatgaaaattgttttaagagaattcagttcccctgatgaagaaatgaagaaaattgttttaaaagttgtctctcaatgtgcatccacggacggtgtt
aacccggaatatttaaaaacagaagttttgccagaattcttccattgtttctggtctcgaagaatggcttctgatcgtcgtagctataaacaagttgt
cgagacgacggttgttttggctggtcaggttggtgttttgcaaattgtagacagaattgtgaataacttcaaagacgaatctgaaccttatagaaa
gatgactgctgagaccgttgataaggtaattggttctttgggtgtatctgaaatcgacgaacgtatggaagaattgctgctggacggtgttctttttg
catttcaagaacaatctgttgaagaaaaagtcatcttgaactgtttcagtactgtcgtaaactcgttaggagtacgatgcaaaccttaccttcccca
aattgtatctactgttttgtatcgtttgaataataaatctgcaaatgttagagagcaagctgcagatttagtttcaagaattaccgtagtgatgaga
gcttgtggtgaagaggctttgataaggaaactaggtgttgtcttatatgaatacttaggggaagaatatcctgaggttttgggctcaatattagggt
cactaaaggctattgtctctgttgttggcatgtcctcaatgcaacctcctattaaagatttgttaccccgtcttacacctattttgaggaacagacatg
aaaaggttcaagaaaatacaattgatcttgttgggcgaattgcagacagaggttcagaatttgtttctgcccgtgagtggatgcgtatatgttttga
gttaatcgatatgttgaaagcgcataaaaaaagtattcgaagagcagctatcaacaccttcggttatatttcgaaggccattgggccacaagatg
ttttagctactttgctaaacaacttgaaggttcaagagcgtcagaatcgtgtgtgcaccactgtagctattgcaatcgtagctgaaacgtgcatgcc
atttactgtcatacctgcattgatggcagattatcgaacaccagaaatgaatgtccaaaatggtgtcctgaagtctttggctttcatgtttgaatata
ttggagaacaggctcgcgattatgtttatgcgattactcctcttttagcaaacgcgttaatggaccgggatgctgttcatagacaaactgctgcttct
gtggtaaagcatttgtcattaggttgtgttggcttgggggttgaagatgcaatgattcatctactaaatcttctttggccaaacattttagaagaatc
acctcatgtcattaatgctgtacgagaaggaattgatgggattcgaaactgtgttggtgttggtgttgttttggcttatttattgcaaggactttttca
tccaagtcggaaagttcgcaatacttactggacttcatataattctgcatacgtccaaagtgctgatgctatggtcccttactatccacacgttgaa
gatgatcagttcaattcttacgaattaaaaacattgcatatttgcatttaacaaggagtggtgttacgaacggttttaatgttatctaaattggattt
gagaaggtctgtgtatatgtataataagatttgttattctttaaatgctagaaaagaaaaaaaaagctgttagtacaaaagttaacatacataata
gttcgccttaatttagacaatgcttaagaataaatagtgattagctcaataggaaactcccattcctaaccaaataataagaagtaaatttaacgc

agtgagaaataaccatattaaccaagtaagtatagtcatgggccaacctaagcttagatcatggacatttctaattacttgaccagtttcttcatca
accgtctccgagtaagtagacattactttacgagaacacgtaaacattacaagaggaaaagttaagataggcagcaaaatggaaaggcaaact
tgacttgcgttcaagacttggtttagtccgctttgacctacggcagccgcgacaactaagcaagggatgatcgctatagatctggtaagagttcttc
gaatccaaggacgtaaattccaattgatgtaaccctcactaaccatctggccagctatagtacaaacatagccggcactcatgccagaaaacaa
aagcgctacagcaaataatttaccacaagagagagagacatatttggtaagtaagtcatagatagaaaataggtccgaggtatcagcgttgcta
gtgttataaaatacagctcctgcaacaattaggatactagaattagtaaagagggcaaatgtaaagagagaaagagttacttcaacaactgagt
atgtcaaagagtgcctaattgtttggatagaaggcttatagtccgaacagtcatctgtaggaacataatttccagttcgaacatcaaaatcctttaa
acgggtctgaacaagaccagatcctagaaacaaagaatgaggcatgacagtggctccaaggattccaatactactgtataagccttcacgtgaa
aaaacggtggaatttggaagaaagcctttgaagacttgacctgctccaccaatatgaacacggccaagaacgacagcaaacgaaatagctaca
acaagcactagcatagcaacagcagcttcgaatatacggacgccgagcatagatccttcaggacgccaagcaagtaacacaagtaatacgtcc
aagatagtaatgacaacaccagcgacaagcggaattttcattaaaatctttaaagcgactgcggtaccaatgacttctgcaatgtctgtagcaac
aattgcagcttcggccaaaatataaaagactatacagacgtatttatgataatgttctcgacaattccttgccaagtcaaagccagtgacagagcc
caaacggcagcataatgcctgtaagtaaacagcaaagagattcgataggaaaacgatgaaaagaagtttatactggaaacgggcaccagcgt
ccaaatcagtagagtagttacctggatctatataagcgactgcaattaaaaatccgggtcctataaacttgcaatattctcgaatttcatagctaca
tttttccaatacttcgaaagtttggttttttttccgttgagtgctcaaaggatccttcttcatcagaaggcagatatgtggaacgatcattgcttgca
aagttataaggtcttgtaacagttaaagattctaatgacttcatttctatcatgtccttcgagttggcagaatgagaggacataaaactatcagttg
gttaaggaattaaaagacaaaagatataacaaataatcaccaatcaattcttaaggatgtccgttcctccagaattccacaaatgccaagcaacc
tcagtatgaagtatctacttatcaatacaaagtaattgcaaattaaataatagtaaatatatcaaaaaccaatcaactttcagctttccttcttcgtt
ttcacaacaatttcagggaagagtacatatgaccaaaggttgttagcccgcaaaacagtcacttcagttagtctttgaaattgcaagtttaataat
gtaaatttttccttttagtccccttgtgaacggtcaatttcacttcattgacctttcgaagtttgggtgagtaaaggtaaacttcttaagtgtttatgag
cgtaacttgggaataagctgccttcgaaaagtgacgatgttactttgattccttactgatctcaactgaaaaatcaagtaaaaatcaaaagagaa
aggcttaagagtagttttaaagtatgctgagaacagaaaataaaatttaagaataaaaagagaaattaggttaactaaagtacggaaaacgaa
gactttttaaagtatccttttggagcagagaatagaaagatggtttgaacaaagtcatttaagggattcatttataagtataaaaaaaatataaa
agaatgtaaatttgggtttatttcaaaataggtttttataaattaaactcatgaacttgtgcaaaagtccagcgttaaacatcataggtgaaaacca
aagatttaaaaaatcatttacgtgcgtatggaaaagacagaactttcgtttttatattctttaacggaccactccaaaagtttaaggggcccaaat
aagataaacagaggttgcaaggatcagaagtaatgcagtaatgattgcaacgcgcatgtacataacggaattatcaaagctgtaagaaatctca
acaatctgagatggatcaccatcaacgacattggaagcttcaaaagtgtaaacttgtcgaccttttgtgtccagaaatttgtgaacggtgttaaca
ttcgaggagtcagcctctacaggcaattttacagaaatgttggaagcgccttctgggaaaacataagaccaggaagctttttcaaaaaataactc
tccaggaacccaagtgagaggaagttttatgaaaatttgtccatcctcgtgagtagtgaaggtggaataaggcatagaccagcccaagttgtaca
gatagttccaaccaccaaacactgggtaacgaggaagcagctccagcactgtttgttgaggctcagtacgcatatgagaggtggtaatgtttcca
acttcatcagtgtaagaaacatcgcttacgtcgctcggcaaagtaattagaaaacgattgatggcggtggagatgggattgtagaagttcttcatg
taccatctagcacgatcgaaaggagtcttcaatttagcagagcggttttcgagaacaattcgctccgaaacgtccatcttttggcggtactgatga
aactcaacttgcatgtgaaaatcgatagcattgggaagaggtgtggtatgctcatagcgaacttgaatttcttccgagttgtcaccgggcaaaacg
aaggagcgagtttcataaacaattgtatttccgttacgagcaggagattcatcataagtcgtatacgactcaacgttagaggatggaaggataaa
gcgagtacgctgttgttcagtagaatacaaactgtcaaggaaccgagaggttttatacaccaaaaactgagagtcatcttgatcaatagcagagg
gaagaggacgaaatccatgaatcaggctggttcggacggtaatagtacggctttcacctggctggacgggggtttccaagaaaatatcaaagga
aacatcatccacttgggtgagttgagccttctttttagaatgatcctttaaaacagcttcgatttttgcgatttcctcaccacgattaacatgttgatg
atagcgatagacggtttgaggctcatttccttcattgacaatgattaacgaagtcgtttccttgacgaaggtagaccgcaaatcgattgatcgaatt
gcattgctattcttccaaatttgttcagcaaaaacataacctgtgattaatatccataggaaggccttaaagaaccacattttgacgggttattttgt
acagagtcttcgtatttagtgcttgtaacgtttaaaggactctaaaaatagagttagatattcactcttttaaaaaactaatacttcttcaaaatcct
cttacaatcctttcgaatattaacccaagtttctagttttatgtagtatgggacaggtaaatatatgaagctgaagtattactttaccatttgccgaa
ctttgctggaactctgtaaatgaataatgatatttaagaaatccttacatgctgagtgaagaaaagaatagaaaatttataaataaaaataaaaa
taaaaagagaatccaaggaattttaatttacgatagaaaacaaaacagctgaaaattccaaagaatatgtgattttcattttgctgttctaagagt
aagcatttctttgtcagttggtaccatcctcttaagaagaattctgtcgtatgtagacaccatcacgaaaataaaaggaataaatttcaacagctcg
taaatgatttccttttcatttaaaactctgggacgaaattccattgctttttgtatcgtattccagatttgtttcgctaaacaaaaccttatattaagcg
agaaataaacaacgctcttcgagagaaatgagaattttcacctttcctttgtactacagtgcaataacttagtatctatagaatttggggtgaaaa
ggtcggaattggatcattaaaacaaaaaatgagtgttgagagaactatttacagcgttaagattgataaagagcgcgtccctctattcgatgaag
acttttaccaaggttttcgcagtgaaatgacagtccatttcaccatggctgctcttgaccctgcagtaaagtccaatgatcctgttacagtgaaggt

aattactcgcctccagcattctgatgatgaggatgaagaagatgaagagaaggatgatgaggaagagcaatttgtcctttgcaccctcaagaag
ggagcaatttatcagcaaccaatcaatcttaccttttctccaggcgaagaggtgttcttcgaaaaagcaggtgatgctactgtctatttaagcggtt
cttgcacggtcaccaacgtccccgaagactctgaagacagcgacatggatgatgatgaaagtggagatgatgaagacttcatctttaatgacgat
ggcttgagtgaagatgaagactttgactttgctgcttctgagaacgaggatgaagatgagagcgaagatggtggtgctcgtattgaggaattagg
tagtgatgaagagcaacaagaagatgaacaaaagcaagggtctaagttggagtcccctcaaaagaaagagaagcacactcaggaaaaagaa
gaagaagtagaatctaaaaagcgtccttctgaagaaactcccgacacagaagaaaaggagaaaaagcagaagtcagagaagcccgcgaaaa
cgtatgccaagaaaacactggaagggaatgtagtcgttcaagacaaggttattggcaatggacctgcagccaagaaaggaaagcgtgtttccat
gcgctacattggtcgtttgaagaacggcaaggtattcgacaaaaatatcaatggcaagccttttgtctttaacttgggcttagaagaagtcattag
aggttgggacattggcgttgccggtatgcaagttggtggtgaacgcactttgcatattccttcacttatggcttatggagccaaacgtctccccgga
attcctgctaactctgatctgatttttgatgtgaaacttttggctgtcaattaaattcagcttggtcggtaaaatgtaacaaaaaaaaaattaaggta
ttttggtcaattatttctaatatggtttggtttggttttgtcttaggtaaaactttggacgaatatagcgtttttcatagattttattgaatgatgaagta
taaataaatatatataaagatttatacatataaacctgaaaatgtatatagtcaacagggaggagagaaactaagctggaatcaatctctcatca
gcatgcaaaataaaacgtactaacagattcgtgaattcattgatgatcctacaacaaagcgaccgctgcattactagatactctcttacaaagatg
tttgattcttgcaaaccgatttgtttatcaaaccctaccctactctctgaggaaatcccatggttgctgtatcgaatcggtgaacaaggccatcgtta
gctttccccggggcgtatgtaacgaatgctttctttgtttacactctatgaagtaaaaaatttaaacaagcaaagcatttgttgccaagatgtttccc
ttaatatgctggttaaaaattttaaatataaaaataaattaaattaaattaaacaatgaaaatctgtaaagttctttgttgctcacatttgctagtac
aagtcaattatagttacataagttattcatggtttttccctacagtcgcatagttttataatcttcttagggtgaactactgatttactcgtagcaatg
agattacctcttgccgaaagaagcttccttgatatcgctgtcgatgaacccggggtattatcatgccaaaccaggaatcttactatcctgtcggcgg
agatgaacggtgaagtatcctgactatgacaatgatgtctttcctttttccttttctttttctatatatttgctcctgttcctgttgagattctcaagtaa
atgaattgcaagcttactgtcaaatcaaaggtatcgtatgaaatactcgctttgtcttgtgaaggaaatttaattttgcatttgttgattcggtttttt
gcactatcatgatcgactttacttctacttctgtcaaagctaataacaaggtcttggaaactaaatccagtacgaaagactatcaatccgccggcg
atcccaactccaaggttcctcaaggtctgtggtcctttgacgacgccatgaaaatggcacctgaaacagtccgaaacttgcaactcgctcacctga
acaagctgcgtactatccgtggtcatgctcgaaatttcgtcaaggctgaaaattctcgatttatgatgataaaggacagcaacatcttgatttaatc
ggtggtgtcggcgtcgttactgttggaaacaacaatgaatatgtctggaattgcttacaaaagtgctttaacgaaaaactttacatgatgggctct
atttcgtaccgtaatcttgctgctgcttttggtagcaatatggcattgctttcacctggtcaaaagcttactcgtacttggactgctaccggcggtgct
gaagcaaatgagggtgttatcaaattaattcgactggctactcgctataagatcaacaataaacggtttctctctaccctcaactcgttccatggca
aaaccactggtagtgtcttcttgggcggtaaggaaaaatggcagaaataccaaagcccttccctgttcgacgtggattatgttccttatggagattt
tcatgcacttgaagttgctctgatgtctggtatttaccgcgcatttgtcgtagaacccattcaaggcgaaggcggtgtcattgtccctcctcccggct
actttgccaaagcccgtgaactttgtacaaagtatgatacctatcttgttctggatgaaattcaaacaggatgtggtcgtactggcaaattttgggc
atgtgagcatgaaaatatcattcctgactgcatttccttcgccaaaggtttttctggaggcttaattcctttcggcggttacatcgctactgaagagc
tatggaatgctgcttacggtacagttgaaactgtttttttcacaccgctacttaccaggtaaacacacttggtcttgccgccggcgttgctaccattg
atttcatttatcagaacaatcttcttgacagagcgaataagcttggtggtattatgtttgaccgtcttagcaagcttcaaaccagatatcccgatatt
attaaggaaatccgcggccggggtatgatggttggtatcgagttcttccgtctccctgccgttgctcaagaagtattcggtgagttttatgcacttcc
tattgttaacgatctcgccgataatcatcgtgtccaagtttactgctcattaaataacccttctgttttccgttttcttcctcccttaacaattcctgaaa
aagatttagatgaaggtttgaaatctgttgaagatgctgttgcagccttcagtgtaaaaattaataaagatgtcatgggacaaatcttcctaattaa
tactaataatttttttcataactcatttcgctttcattattatattactgcttttgattctataatgctatatctattgtttgtctcttttaaactggttattt
caagaataaagtatgtattacaaaatacatttaaatatatgatttataaaactgagtactctcttgattatttagataaatagttcgtttcatcttaa
actctaaaagaagctttgtacaagcaattttcctaggttcatgattttcattcaatatatccatataatcctataacccactgatattttctagttttga
ctgttctcttttatcctttttttttttcgtggcatttttgtttcttcttatttttatttatttattacttaatttatttgtttttctgctttacacgttcatgtcgc
gaaggtacatttttccattattagaatcggaggagattttggaatgtatagcgggtttaggaattacgtttagtaaaaagaactagttcagccaa
catcacaggcagtcatttccttatgtgaagaatttcttgacatgtttatggggctgactagtcaggatttagaagacaaggcgaattcgctacagg
atacggttgaaaccttcgatatattacgcgaatcgttacggtttatgatttttataaaattgcaaaccaattcatgcaaagcatttgtgtcgatgac
ttttccgttcaagacctattaaagcccacttcaaatcgactaaagcgaattctgtcgggtgtgattaattttgcaaagcttcgagaacagcaattatt
actcttcgaaccggacattcaaaagcgagaaagtttgatcgagaattattccctacttaatacccaaagacaaactcttgaacaagaagttttgct
gagtcaggaaaaacgagaacaaaatcaaattattattaaacaaaatgaagaaagaaatgaccaactttatcagaatctattggaaataaaacg
aatttgttcgcaaaccaaatctgaatacgatcaaataaagcaggaagcactcgagttgaaggtaaagcatcttgaggtagagtctttgatatcaa
atacgttacacgaaacagagcagctacaatcttccattgtgcattctcccgaaaaacttcaaataagaattttggatatgtcagatcgtgttcagtt
agatcgaacgcagcaatttgaattggataagaagtcaaagtaccttcagtctaaacttaatcaactatctctgacggaaacggacctaaatgctt

gtgtaaaggtactagaagaatgtttggtcgaagtggacaagcttgagcacgcttccaacctttt atcatcgaatcaggaactatgtgaacaagttg
aaatcaataaaaggaaacttgagtttcgtaaagaacagcttttaaagcagttgtcaaacgcacaagaaaagttgcagcatgagcaacaatcca
gaaaccaaaagttagaggcggcgaaacagaggatggatcaaattaaggaagaatatcaagagctactccaaatccggtctcaaaaagcccaa
gaggctgatcacaagcaaaccataattgaaatgacggaaaagcaaatggcggatatgaaaagagaactggactcacaaacttccttagtaact
atcgagtttgaaaagctgaaggctcatgttgagctttacatcgctgaactcttgcatagcattcgctgttcgcaataaggccttttacatcgaatcat
aagcatattgtaaattactcatggtatagttctgaattatatttgcatagaatgaaaaattaaattaggtatactagaaatacattccatttttggaa
agatgaaaaactacttctatacgctctcatagtatcttgcaatgcccaaatttaagcaaggaatgaagccatattattttctctactaaactccaatt
cattcaattattaaatatttaggcattctcttgtggaaaatatcataggttttatggatacataagacgaatttttaaactctcacatcattctaatctt
accaccacaagactaaatctacaaatattcgaacaagatgtggaagccattagaaaataacccagaggttttggagccttatttaaggaaatta
ggaattttgaatgcaagtgtgtttgatttgtttacgttggatgaaattccagaatacattcctcgtcctgtggcagcacttttgttggttcttccatctc
ctggaaaatttgataaatcccaaaaagaatctaaacttctctcagctgattccaaagaggtggtctggtttcctcagctggtatccaatgcctgtgg
tactgttgctttggtgcatgctattgctaacggaaatctttccaagcaaaaatacaactctaaatttacaaaagatctcttaaaaaatgctagagaa
cagacagctgatgaaagagcaaagatgttaaaagactctgaagaggtagccaaacttcatgcagaattcgccggtgaacccgatgaagaagaa
gaaggagatgaagaagatgaaacagatcttcattacatttgttttgttaaaggaacacaggaacatgattgccatttttatgaattggacggtcgt
cgcgaaggaccaattcaacactctgaaattgaaaacgacgttttggaccctaaagtgttttctgttattaaagactatgtgcaaaagaccaactct
ccttacttttccttagtagccattacgacaaccgacgacaactgaatatgagttgagtgatgaacacaggggtaatgaagttcgagttatttattttt
cttttatgataaagcttactaccttactcttcttgattcaatatgattgaacctgcccaagcattccatatgcaatttgctaataccatagttcctcatt
catgaaacttataaaatcatttatgaactactactcgacggttgcaagtctgccttactcggatgtcaatgcttttacattcatcatgttatttgtttta
cgttattatatgaaaaaataaaattgaataaactgtgtctttaagataacctataacctgcgtccaaagctatccctcaaatatcaaaaactcttca
tgtcacaaaaagttgctttcaactttcatagaggaccatttagagcctttaagtctgtaattgaaagcaacgtttgtgtaaggcatgtttctttgtttg
ccattattcttttgtcttagcattgtgtcgtacgtatattcatcacccttaccttaagaaacgaatataccctgaatctccatctgtccacctccaccaa
caaccaagcatagacagaaacgatcaaaatggataagcttagagaagtatgatacagaaatattgaaataagtatcaagctgctaacagaaac
agaaaattaacgctgctcgcgccgaaactgatgaagccgttgcacgcgccgaagctgcagaagccaagctgaaagaactagaacatcaaatgt
ctattaaagaacaagaatatgattccctttctagaaagcatgaaacttccgagtctcgcttagaagaaattgaagaggagatgaaagatttacgc
ctaaaagcagacgatgaaactggccaaaagagtgaaatagagcaagttactcggaaggtcgagctattagaagaagaacttgagacaaatga
taagctccttcgcgataccacagagaagatgcgccaaacagacgtcaaggctgaacactttgagagacgcgttcagtcgttggaacatgagcgt
gatgacttagaacaaaagctcgaagatatgactgacaaatacgcaaagattaaggctgaattagacgaggttcaccaagcattagaagatttat
agattccaacgcgtaatgctccgaattacagcctgcttttgtacgtacaaatctgtttccttgttcgatttggttggctgctcattggactaattgtgc
caatgagcttttggatcttacatcggcatgtccgtcgttaatgtatgaactattaccttctttttgatgtacctttcatttaccaataagtttttttaacga
agcttcgcaatttgtacttggtcattctatcttgttttcaacgttacgttttattatctttcgattttgaaatacaatcgactctttaagtctaaattttttt
ttttactttttattatttctaccgttctttatgatactatattgaagagaactgcgattcatgaagtattccaaattctatatccactctctttgcacgttt
actgtgtcaagttccactcttcatgaagtatataatctgcaagttgtgattccaatctttcagcaatggtaaatgaaatgtatatatgcagaatagta
gcgatacacatactagaagtgttagccagtatgacaagagccagatcgattgtttcctaggaaaccatagataacttcagattactctttctttcaa
gcgttatacgtattggtaattagaaacaaaaccatttatccacctagagaactatattacgtaggcttgttaatttcataattacagcaaggtcaac
aatgctctttattgtaatgttgctttgtatgtccatgttacctcgagtatctctcgtttaggtcgttcttggaatcctcacctcaacctacaattctttgt
atattccataaattcagcataagcaacgaaattggctgtaaaacgacgtgtatatatttattaatttcctcattaacgtcgtacaatgtcatatccga
atccgatgaacgattcaccgtacgggtatggcacgaacagtggcatgcagccgaacatgactggtcataccatgccaatgacccaaaataatat
gggatttcagcaaagtagttatccttaccaacagggttatccagctggcggccttcctggaaccaatccaacgatgccctttcagcaaaatgccat
gaacaccggttatggatatccatcgcagcctatgacttatgcaaacgcacctgcatcatctacttttccatctcaaatgactggttataatggaatg
atgggtgcaggaatgccgcctatgggttacgtgcaacctcaaagaaccggtactgcttcagtaccaccaatgggtggtttcatgcaacctcagag
aactggcagtcaacctttttatcaaatgaatagctttattcaacctcagcaaactggaagtgctttgcctcaagcaaccggttttgtgcagcctcaa
agaactggaagtgcccctcaaggtttctttcctcagcaaacaagtgggtttatgcaatatcctggaaatggcggtcagctttataataatccaatg
aatggctatatgcagcctcaaaaaactggtggtttcggctcttttatacaaccacaaaaaactggtacgatgctctcccaacctaccttacaacctc
aacagactggttcttttgtgagccaacccggtttcacacaacctcagactatgggtgtagcacctcaaatgactggacttattcagcctcaaagaa
ccggtactggtttcattcctcaaacaacgggttatcttcaatcacaaccgacaggcccttttgctgcctttgtgcagcctcagcctacttcgatgcca
acatcagccgcacctttgaaacctcaaaaaactggtcaaattcacattagtcatgctatggatactagactttcatttattacagcttcggaccaag
agaaatttgagaaattgttccgctctgccgttggtaacgaagaagctatgtctgctgacatcgctaaagctatcctcgttcgttcaaagctccctac
acccatcctttctaagatttggtctttatccgacactactcgatcgggtcatttaatgtttcctcaattcgttttggctatgtatttgtgtaacctgggtc

ttactggaaaacctattccagataaggtgccagataatattcttggtgaggtcaattcaatggttgatgcaatatctttctctttgcatgatagctct
agtcagtatgctcatccagtttctcagaataatgctcaacaaatggctgctcaaatgttcaatggtgttcagcaagccgctggtattccgtcccaga
ttacggggatgtttcctcagcaaaccggaatgcaacctcaaatgactggcttccaacaaccgatgatgccccaaaggacaggtggcatgccaac
acaaatgaccggaatgcctatgcctcctatgatgcctcaaagaaccggcggtatgccttcacagatgactggtggtatgcccccgatgatgcctca
aagaaccggcggtatgccttcacagatgactggcggtatgccaccgatgatgcctcaaagaactggaggtatgccnnnnnnnnnnnnnnnn
nnnnnnnnnnnnnnnnnnnnnnnnnnnnnnnnnnnnnnnnnnnnnnnnnnnnnnnnnnnnnnnnnnnnnnnnnnnnnnnnnnnnn
nnnnnnggcggtatgccaccgatgatgcctcaaagaactggaggtatgccttcacagatgactggtggtatgccaccgatgatcgccctacgaa
aagaaccggcggtatgccttcacagatgactggcggtatgccaccgatgatgcctcaaagaaccggcggtatgccttcacagatgactggtggta
tgccaccgatgatgcctcaaagaactggaggtatgcaacctatgatgcctcagcagactggaaatcttatggctcaaccaactggagttcctgga
caatggggatttatcaatacaccattaagtaatcttccaggtattgaagcgttgggagaacagatgatgccaaatgcatctgacggtagcttaact
cgcaattttacaggaaaaaaagaaattccttgggctataagcaaagaagagaagcgcatatatgatcaaatctttgatgcttgggataaagaaa
agaaagggacccttggaggaaatgccgtattggaaatctttggtcaaagtaaattatctcgttccgagcttgagcatctttggaatctctgtgatca
tggtgataaaggctgtttagaccgtgacgaattttcagttgctctccaccttatttatagaaaacttaatggcaacgtgatcccatcacaacttcctc
ctgaattaatcccgccttcaaccagaaatattgaagcttctttgaatcaagtgaagaatcttattaaaaatgatacttcatcacgcaaggcatttag
taacgaaaatgtaagcagattgaagaaaaattcattttatgataatgcgagtaccttaagcgaaaaggatgctacgctttatagattcaacgattc
caatgtgccggcttatgtgtctaattccagaaggagagcttattctaaagacaggcctgaggctttaccgcccacagctggttctcttaacgagatc
aacgatgaaactgaacttttgaaaaagaatattcgtgagaaagaaatattatataatgctttggaggataaaaaagcagcccattcagatgtttc
tgacaacgcttctctaattgatcgcattaagtctattcacagtgagattaataaaacgctggccgatcaaagatctccggaagcggatgtttttatt
tcacgccttgacgcattaaatttgaaggttactcaacttgtttctgagatcactactgctgatgacagattagatgaactttcctcaagcattcaaaa
atctagtggaattattgattttgaaaacgtttctgcaaagataagtcagattgagcagcaaagaaatgcgtcaaaggattcattaaagcaactta
aagcatctatcgatgacatcgagttagaagcccgtaagcaattgataccgtcgagcaaaatggatgatgaatggatgaataaaaatatggttga
tgattctgttaagaaggttatgaaggatcttccaccagctgttcaaccgcaagaacctgttgctcaacctaagacgggggctgcgcatcctgaagt
tgctgcttccaccgccattgagtccaaaccagtagctgaaaaagaaagctttactaacgttcccgatgtagaagatagagcagcaaggataaag
tccgaggcccagcgacgtatgaatgagcgtttagctgccttaggtataaaacctcgtcaaccgagaagtgccagtggtaataccacggctcctcc
tctttcagcaagtccttcgatgattactgttacaaacgaggcccctcaacttgaaaaacagttttctaatctttcggtcaacggcaaaaaagaatca
ccagttccttcttttaatactccttcattggaccaagaaaaaccgaaagatataagtggaaatcagactaccgagaatcctgtagaacgtaaaca
ggctcaggaatcagaccccaaagtcgatgaagtaaggaaggattctgtttcttcaatgtcgtcggggtccaatgtttcgtctgtgaaagaggccac
atcagcttcccctcaaagagtaccttcaagagatgctaagcctgaatcatcgcttgctcctagtgcttcttctactcaatctgccacacagtcacctg
cacttcctccaagtccttcggctattgctaataatacattgaatatctcacagccttccgcagcaacaccgcaacctcctcctgcacctccaagtcct
ccacctacgcctgctggtacatcgagtatctcccagccttccgcaacaacgcagcattctccatctcctcctgtaaccaatcctcctcctcctcct
gcgcctgctgttcatgccccatctttgacacctcatattcagaaacaagcaaatagttccgctatggggtctaccttacagccccaagtaacttcta
gagctccttcctcaggtactgaaacggctcttcctccagttcctccccttcagcacctccttcagcacctcctactacgactggatttaatcctttttc
gaaagttccacctgctccacacacgagcggatacgactcggacaattggagccagcatgaagaagaagaggatagtgaggatgatgcgcgatc
tagtaaggatgcagcagctttagctgcgaagttatttggcggtatggctcccgttcgaccactttctgcctctcctggcaaaccgcaatcttccagta
caacaccatcttcagttcctccagcaccacctgtttcggcatctccaatgcctccagctccgcctgtcgctgccccgccagcagccccagcacctcc
aatgcctccgtcaccacctgttactgcaccaccagcagccccagcacctccaatgcctccggcaccacctgtcgctgcaccaccggcagccccag
caccgccggctgctccggcacccatcgtagctaatccagacgatagaggtgctttgttgaatcaaattcatacaggtaaaaagttacggaaagcg
gtaactaatgacaaaagtaagcctcttggaggtcgtgtgttagatggatctagtaatggagataattcagcttggtatggcaatttgtcctaacgcc
tatatcctgttgtttgacgatcatactttgaagccttttttagacgatctttacgaattttgactttgtttacccttttattttatgttttgctttcgtcatca
gttgatttagtaaatcaagcttatttaacatcactatccgaattgtccattgatatagtgaatattcaaaagaattacaacattacaatagatgaca
gactaactttgattatacagcaagaaatgcaatctcatttttaaaactatcactaaataaattcacccaaacttagagcaccaatcgaacaagtaa
atccctaatagttacacgactaggatggtagcaggatcatgaaaacacatcaagaaacgttcgagtaggcaagaaacccatatacaataaaca
agccaaaaagaaggagaagcctaatcttcaatagtttcaatttgttcagaaataacctcttcaatagtttcttgaataacttcttcttcgtcagcag
gaagaacagagtgaagaggttgaccaacagcagcacgctcttcagcgacacgtgcagtaacttcttctctatatctctgccattcactaataaggt
ttcttctacgagcagccaactcacgattggcagaactggtttcggcaatatcctcttcatcaaagagacgattgtaatctttaagttttcttctaatgt
tcttcatgtcttccttagacaagagggtaggaggacgaggtcgccatatgaattgcttgaagtctggaatcatctcctcgcgaagaagggtacctc
tgaagtctgaaattctgtagccgttttcaagcttgtggcgccagacagtactgcttgtagtcacatagcgtccgcttggatcccactcgagttcagtc
ataccaaagtgttcggttgatccagtttgttggacattagccaaagcatcaggttccttcttatcagtatcgaaatccatatcataaaattccaaatc

atactgtgtagcagaaccgagagtggcagctaccaaaaatctaccattaggagcgaaaaagattgagttgagagacttcttgtcaaacgaggtg
atgtgacggaatgtactagtagtgttcttcttatgctcaaatccatagaaagaaagggttgttttaacattggtggaactcaagatttgatcgttag
cagaaataatcgcaaatcgatcactcttgggttcccaagcgaaattgagtacaacatccttaagatcaacgacttccacgggaatgtttttctcgc
ggatgcggaagatctccaagttggagaaagtagactttttagtcttggtatggcggtcgactttcacggaaaggaagtcaccgttggattgccaat
acaacttgcaatcagaaacattaaaaagatttttagtacgaatagtctgcttggatggaacactaatgagtgcaacacgggcaggttgatttaag
acttcgggagtccaataggctaaaagttgttccttgtcgtctccaccgagagcattggaaacgggacaccattcaaagttctgaacgccgtcaatt
ttaagagcctttttatcgaccaaagaaagagaaggagtttcgtaaatagagatcgattggccaacattaatacgtgcaaggaaacgatcatcag
cagaccacttgaaaagaggccagaccaccttcttggcatctttaacgttatcgacctgctgaggaatcttgaaagaacgaaggatgcgacctgag
gcgatgtcccaaacgaaacaatgctttccttcatcttcatcggtaaagggcatagtctcacgagcagggtgcccgataggaggaagacggacag
gttcaaaagaccaagagacgaggtacttttcgttggggctgaagtcaataaatttgacatacgggtgcaaaaagcgagcacacatgttccacga
ctctccaccccaaagttggataccacggaggtgcaaagaaactaaataagtacctaaaggtgaccattggacataggtttcagtccaattttcac
ggttgacagcttgctcaggaacatcgctcttgcgattccagttaacagaaacttggttgccatagtagcttataaactgatcacggccataatagtc
agcaagccagctacgcaagtgttcgcgctctttaaattcacgctcctcataggtgaattcctctggagtgttcaaagccttttcaagctggtttaact
tgcggacaacaaacacatgactttttaaaacttgttttccatcaagttcttgtacacagaggtcggcaaactcggagttttcgaagtctgcaaacac
gataccaatcgacatcttcttgccgcctttgtcttctaaaggcatgtagagaccattctccttgaccttacctattttcgcaaacaatctagtggctaa
gaaacgataaaaatcttgaactttggtttcttctacaacaggaacgccttgaatcacgaccaccgtgtcgtaacccacgggtttcgcgacatttag
ctttgcttccaagtcggagacgttaatttcgtcgtctcgaacgacgatttgactcttaatctcctcaacaaggctttccgacattctggtgttgtgccg
ttgtttctagtcaagtttcttctccccagtcactaggagtacaaagaccgtagatacgctttccataaattctttcatatctcggttaaaacgaaatat
gtttacatgagtagtcatagaaaaaataaatatgtataatgtatgtttaatttggtcaatgagtattttcagagaaagctgcttttaacaactaccgt
ttagagaggaaatccttctcttcaggaaaagcgtttttttacagtgaaattttatcatttattaaattttttttgtctttactaaccgataaaagacctt
tttaaggatctggaagtgcatatacttgaaattgcctaagtttggttaccttgcttggttctcccaaggccctgtgtttacctttttacaagtggaaac
cccaattcacgaatttgatagattcctaggctattttgcacagaagttttctttgtttttgtattttattttcttccattgttcttctgtctttcgtttatacc
cttgtctttcttttactgtctttcccattagtttctcattttcattatcaatatgccaccaaggcctcctggaggcgggcctacacaaagcgcaagctc
aaaaatgcagtcaaggattggaatgaatgcttcgagagttcccaatgccactattcctcgtaaacgtcgtgctgatactcagagtactgttggcta
tacctctgcatcccaaagctcttctgcggaaagtgcgcttcatgatgaaaatgaaacaaacatcaacgtcgccgtccgtgttcgaggccgtagtga
ccgagaagttcgcgataatagtgctctcgccgttagtacgtcaggcgccgttggtgctgaattagcaattcaatctgatccctcttctatgcttgttg
caaagacttatgcctttgataaagtatttggtcccgaggcagatcaactaatgctttttgaacacgctgtgtctcccattctaacccaggtattaaat
ggatataactgcactatctttgcttacggtcaaacaggaacaggaaaaacatacactatgtctggtgatttatctgattcgaacggtattctgtccg
agggcgctggcttaattccccgcaccctttacagcttattttctaccttagataatagcggtcaagagtatgctgtgaaatgctcgtattatgaactt
tacaatgaagaaattagagatcttttggtagctgacgatctgcgcaagcgggctcgtgtttttgaggacacttctcgtcaaggaaatgtagttgtca
cgggaatggaagagttctacataaaaaatgcagcagaaggcctcagattgcttcgtgaaggctcttaccgccgtcatgttgccgctaccaagtgc
aatgatctttcctcacggagtcattccatttttaccgttactattcatacaaaggttccctcttctagcgctcatgaacctggatttaccaatatgtctt
tttctgcaaacaacaattcagatgactatatgcgtgtcagcaaacttcacatggtagacctcgctggaagcgaaaatattggtagatctgggccg
aaaataagcgcgctcgtgaaacaggcatgatcaatcaatccctacttactttaggacgtgtaatcaatgctcttgttgaaaaatctcatcatattcc
ttatcgcgagtcaaagctaaccaggctattgcaggattcacttggtggaaaaactaaaacgtcaatgatcgtaactgtttcctctacaaacaccaa
tttagaagagacactatcaaccttggaatacgcctcacgtgctaaaagcattcgaaacaagccgcaaaataatcaattgatatttcgtaaagtttt
gatcaaggacctagttcttgatatcgaacgtttaaaggcggatttgaatgcaactcgtcaaaaaaatggtgtttatttggcagaaagctcttataa
agaattgattgagggtgcgcaaagtaaaagcactctttgtcaagaacaggctcggaaattagaggtgctagaattaaatataaaaaatgcccgg
gagcaattgcaactggtctcaaagtctaatcaagaacacaaaaaggaaatagataatttccaaactcagctcacatcttcaaaatctgatttaga
ggcggtaaggttggaaaatgaaaaattgcgggaacaattggcttcagaaatggaaaaaagaaaggattatgaaacaagtgagcaaaagatta
cgaccgttgcaactgatttgtctcaatattatcgcgagtcaaaagagtacattttccgcctctttgaaaagctcagtaggacggaaaagataagcg
aagagaatgagaccaatttttggaatcttaagcacgaattgctcacaatgctctctagctttcaaggaaattttacggatgggacaaatggctact
ttaatttattaaacaattttaacacttctatggaggagattttaaataaccacggtactctattgaatacttctataaaagaagtgaatacacatttt
attaatttagaacatctattaaaggaagcacggtcttctacaaatccttcaacaacacttgattctctagtttcggacttgttagagtccaggaaaa
atttactgcttttacttgaaaacagtcttcaagaggttagcatgtcttcccaaaaattgggtaatggaatatcgactgaactcattgaattacagaa
agagatgaaagattcataccgaaaagttgttcaggaccttcgtacgctctttaacgtccaacaaaaccattgcaaatcacagaaagaattaatat
caactgccaaggatgatattactgtcgtactccaagcttgcgcaacgtctataaacgaaaataatagtagtattgaccaatttttagatgaatata
aggttcaggaagaatcgaaacaaaaagaattgatgaatcgaatcaattcagttgttggtcaatttttgcaggagcaaaatcagctgctctattcta

atgtcggagggttccagacgagattaaataagtcaaagtctgagattgtcgattcgaatgaacaattaaaattatattttgataagctgaacaac
gactcagacgtgtttaacaaggcactattagaaaataaaggaaagttacaaagcaccatcacgcagagctctgggctattggatgcaaaagga
aaaagcatccatacaaactctcgctccgtttatgatgaatgcatgacattggctgaagcacagaaggagaatgtttattctgaagttcgaacgctg
gatcgtttgttacaaagtctccaagctcattccgatgatcatcttcaagaaaagcatgaaaagcttctactgcgattgaatgatttgatgaaaaag
acgacgacggttagaaactctgttacagcaccccaggataatctaagtgcgcttactagtcaaatccaagagggaacgacatctttagcacacca
cactaatgatttactcggcaatggaacagaaaccttgacgaacattgaaagtacattagaaaaggtgaatttcggtaaggtagagccaatagca
gatacattcagacaggagtttcctgagacaccttcttggtcacgggaattagcatttgcagacgatctatcaaatctatttatcaataaggcagag
ctccatgaaggagaaaattctcagccagcggtttcaccgataaaagaaatgagctctcaactttctcaacgcttatcgtcgaagccatcgaagttg
gatacttcgaaacgaacaaataatgctgtcaataagcggttttcttcacgtggtaaacggtgaaagaactggtgttttgtgatttaatgattctatg
tactaattttctgtgcttcttctttaatgattctttggttatatagaatatttagttgtaaccttaatattgaaatgtgttatatataacatgaacatcaa
atatagcaggtatacttacaagctgaaagctttttgtactgcgtttacaaatattactaaccttactactccaacaagctagaaattttagctttttgc
tcttcatgaagatgatgtatttttatagctctgaaacaaccaacgtcaagccaaagctaggtttagcgcaatcttctagcttcacgctcagattcaa
gaagaaccaagatgtcatcctcacggacaggtcccttgacgttacggatgatggaacgagaagtgtcatccataaattcaacacggacttgagt
aactccaccactgccagtaagttagtaactcgaagttctaatacttccctatggctttttattatatatgactggatgcttgaaatggtatcgtaaat
aaagcttgaaaaagcttaagatatgcttgcaagcatctttccataggatgggaaaaaacataataaaaaaacttaccgagagccggtgcgacct
aaaactttgatgaccttagccaacttaacaggaacctttgaagaatccattgttctcttaatctgtgtagatttgcgtggtgtttagagaagaattgg
ttttggtaccgttagaaattttggggtcccgtgactgcttttctggggaagggtgctgtgtttaggagaaaaaataaggaaacacgagaagcgtta
aagaagcagtgaaacaccaaataaaaaattcataaaaaacaaatacaatatggatttggtcaaccacttggaggatcgtttgctttttgctgtcc
ctaaaagtaagtgacaataacaattgtaaaagtgaaaattatctaaggctgaatgtacgaaatgaaagtggatgagcgcgaaaacggtagact
ttttaggagcgagcgtggaatttttatagtctatgatatttactttacaagttgttgtctttaatatgttcgaaagtattaatacatatgtctagagggt
cgtttgtatgagtcatgtgtgaatgttttaaagggcgctgatatcaagttccgccgtaactcccgcttagacattgctttggttcaaaacttccctatt
gctttggttttcttgcctgctgccgatatccctcgtttcgttggtaccggtcgtgtccatctcggtatcaccggtcaagatcaaattgctgaggctcgtc
tccgtatcggcgaaaagttgaaggttgaggaattggttgaccttcaatttggtggctgcaatctacaagttcaagttcccgaaaatggtccaatcc
aaaatgctaagcaattggtcggtcaacgtatcgtcacttcctttgaatatttggtgggtgattactttaaaaagttagaggagaatatgaaaaagg
aaggggaaattagcaatgaaactgagactgaaattagctttgtttcaggcagcgttgaagcctcctgtgcgttgggtattgctgaagctgttgttg
acttggttgaatctggtgaaaccatgcgcgcttctggtttgaaggccattgagactattatgagctctagtgctgttttggttcgatctaccaattgtg
ctcctgaactcgagcctcttttgcaaactatcattacacgcattcgtggatatgtaattgctaagcaatacgtactcgtcaactacaatgtttctcgt
gatcacttgactgatgttctcaagattactcctggcaagcgtgctcctaccatcacaaccttggatgaacccaattgggttgctgttagcagcatgg
tgttgaagaagaatgttgccaaggttatggatgagttggcccagaatcatgctactgacattcttgtcctttccatcgacaattctcgtccttagaag
tggtttttttttgttgttttaaagaagtcattatgaaagggaaatattgcagaatttttgttgtgtgcgtgttcgtacttgtttcttatagacagttttaat
tacatcttcaaatagtaattgtacatcgatctttatttgcttttagtttttatggataataattatagttaattatgaacactcttccctttcgtaatgtct
aaattgttgaacttctttttggcttagcatttgtttatggtgtaggtaacaatgtttctttgtcatgaaacacgaacgcgtaggaattatacaagttac
caactgcatactactgaatactagcttttcagcaataattatttatgtgcctaactattgaaatatattgacatatatttactaatttatcttcataata
aacaatatagcatatcgcagcttctacaataaaaaacttacttgatcaaccaactgaatgaattgctgctgttcaacgcagaaatggatcatataa
acgaatttctcaacgcaataataaaaggaaagcagttaacaaaagctttgcttgatagaactcttaacgctgtactttctgtcgatgctagcgaag
tacgcgataagcagactatttcaaatatatcaaatgctttttcagtcattggggaaagagagcaatatacggaacccaaaatcatttcgtgtttga
aagtccttttacatctgtgttttctagggaaacatccagatttaggtgacctcttcatagaaacatttaccgggttaaatccctctctcaaagactct
gttaagaaatctattggctcattagctttgtctttaggttgtcaattcttaaacgaaacagactttctaaatgtgacaactacctttgagttgtatgat
gaatcagaatatcaaatgattgtttttgaacgagttgttcttcaagtaatgtctctcttcattgcaaaatgtgaagacaataaactttacttaataaa
taacagaaaagagctgaatttacaggcgattggcgagcgattaacccaccgtgatttcgcatgtaggtttctgtgcggactatttatatcacggttt
tcggtctctggttcggatgaggaaagtaatagacaaaatgctattcgtttaagtcttttctgcccgagcgattcttcaaaacttactttgaccatgca
aatgctaaaggcggatcaaatctttcaaacctgcgtcgcgaactctaccaaagtttataatggaacattctcggtatgttcagaaacacctgtaca
tgctactatgatcattgaaccaagagatgttatcatttatagaaaggggtttactttgttgcaactatcatggggatctatgaaagacattacgatg
acaagtgatggtccttacagattttgctttactactgatataccacatttaaaagactgtatctttgaatgcctggatgattcttcctttgctgatttaa
gctcaacattgcaatttgtcggagattcgttaacaccaaaattgtttgtttctccaaaagtagccataaatcaatcaagtaacagtgctaaaaattc
aatggaggaagtatctcagcattctgcgcgtgacgcaatggaatttgacagtgcctatgaaaagtttgaaaacgagattcttgacaaaagtttgt
gtgtagaaccaacaaatgaaaccactacagggaacaatgataaatcgaccatcaagatagacgagcctgaaaatgctttgaatcatgaggatg
ctgcaaatgaggaaaccaaagaaatatctgaaactgctgaagttacaacagagggtacaaagcaagtagatccggaaaatgatactctggtta

cccccctaagaaagcgtacacgtgctggcaaagcatctacaaagagtagtcgtccaccaagagtcaagaaaccaaggaaaactgagaaagtta
aagaagcagattctaatttggaagggttgaaagagcaagagactccttgtcccaaaaataacaagcagatcaaagaaccattaaggtcagaag
aagacaactccgaacactctcttttggatgacggggaaaagcaaaattcaaaggatgatgaacaactaagtactggaatgcaggattcggtaaa
accagaaaaaaatactgcagaacctgtgaataatcctaaatcatcaatgcaaacaatgggaaaagttaaacaggagccttcaaatcaaaatat
gaatattttagatcattcacaccaatcagttttgaatgaccataaatcaatatggaaacagatgctgaaggataaatcgtggaagaaagagttgc
ttacaccccctcctcaaaatagaaagcgacaaattgatttagcaacttttacgaaacaaggtcactcaacacttgggaatacaccttccctgctta
atgcttgctcaagtcctcccattgaagcttcgtccttgggaccattcgtgaagcatactgatgatggattaaccagtagtactcttatatcggatacg
aatgattatatgaacgatgtaaacttacaatctgttaaacaggaagaaaagactttaaagcctccgtttgaatttgataaggtgcaatcaaaaag
ctttggccaagttgatttgccaaatgcacatggaattgagcgttgtgtttctttaatcggaaaaaatatttactcaattttaattaaacgtgaagaaa
aattaaaggatcaattaaatgtttttcatttaaactcgactaatttggttgaaggatttcaagagagacagaaggctaggtatacatctatacaga
aaatgcttacttcggtggatacgagtctattgcatcgagtgaaaaagctaatctatgaataagtttgttaaacaaaggtttaggagtattaaatgg
aatgagtctgaaacgattatgccaattttaaaccacaagaacaatgacaattggaacagaaaaatgttgaataaattataaaaaataatattac
agtaaaataaaataaaaatagaaaaaataaaatacgtttgattgtaaagtgataagggtatacaaagcatccattcaattagcaaaatagtacc
aaagcaaaaacaattattcgagaattagagaaatacacgagtatttttatggaaaataaagttgaattatcatataaacgaaaaagtcggtcaa
acagaataacaataaacaaccgacccgcacaacctgttggagattgcacttaaattaaaaacactatctgtacatgtatgcaaataatgagaaa
ccacaggaacgaaaacaaacaaaataccagcgaaatagagtaattaatataaaccaaagcaaagataataaaaatttaaaagttagtagctg
ggcggatgagtccttttaaccaagtgttttccatttccaactcgccaattcgagcttccaaagcagccatcttttcagaaagctcttttgcggtacgtt
ctaattgttgttccttttgctttttcttgatacggaatcttgcacttgcagcagtgtttctgcgtcgtttatcctcttcggcagcattacgaatggctgca
tctgaaaagtcagtcttgggacgcttaatggtaggtgcagaggtagtaacaggagttgaaggaacagaaaccaatgactgttcttcctgtgtatc
actttgtgaaaactcggacagttgagcagaggtagagtctctcttggcagcagtagtagaaggagcagatccttcgtttcctgcagacaaggaag
ataaatccaaagaagaagagtcactagcgcgcttagaggcaagagagggggagaagggatcttttgctgattctgagaggcatttgactgga
aaaaggaaagtacgggttcggtggcattggaaagaggaagcaaagaagcagaatccttacctcggagttgcattacccttgaagcattaagca
agtcaaagttatcagcagccaagtagggattttgattgccgccttgaggaagtttgacagaaatgtcagaatcttccagtggttcttgtttgggaat
agccgagacagggacattagaggaagccgtaaattttggtggagtagccaattcctgttgggacggatatttggaaggaacacttatagagctg
gaattcacttttgaggtagagatccgtaaaaattgttaggaaacaaattgacaggctcaaaatcaaaaaaataaggattgctgaacatgttcaa
ctgctcagccaaatcatcattggaaaactcctcaacgtgagggctaccctccaactccaagttcaaattattaatggattgattcggagtaaagtc
catattcagttaaaacaattacaacaagcttcaaagagacaaagcggaaattcgcttttctgacaaactttggtcaaattttactggaaaaaaaa
ggacacccacctttttattgcaaagaatcaatctatcacacttatttacgtgattctaattgatagaatggcttttccagcaatgttcatctaacaat
gttgtacgttattagacggttatttccgcctgccacgaaagagataagatcggctgtttgtaagagttgtgggagagctttccaaagcgcattccga
ctcgttgcgctatgttaattggcaaagatagcgaagtagtaaagagtagtatcattttaaatttttattataatataatatcatattaattatatcata
taatgagaaaagaaaggcagaaaagccataaaaatcgatttttcaagcgtataatagtgctattaagaaaatcgaatgaaaagggagaaattg
agagttggtaaaggcatggaagccagtccatttgctgccctcatttattatccagccgttggagaaaatatcgtaaaataaccataggtaaaatg
aatgataaaatataggtttttaaagcgaagttacattcagactcaattaggttatttccatgctattcctgatatcgtatttctttgaattgtttacat
ggctcctacaaaacttctttcattaagcggaaatttttggtatcaaggttttcaagaatacggataaaatttttattcattctttcaggttaagaaact
cttcaagtattagttgaagccttattccacttacatgggcttgtaactgagtacgatttacgaagtactcttttatttcattcaaatttctttcagagtt
cgttgccattcaacttgctttttttttatttgaagatttatatcgtgtaagatgaactattcgaaattgcaaacgttgcaacatatttgcaaagtcagt
ggtttatccaggaaaggtcgaaaggcagatatgttgcagcgaattatgtatgctccgagctatcctacggataatgtgttatcaattgatttaggg
attaagaattttgcatactgccttgctggtcgcacggaggatgccaaagttaatatctatgaatgggcccacgaagacttgacatccccaacaaat
ggcttaggtattcagtggactgaaaactatcatcctcaatacttggcacgacttgcgtcgcaaatctataccacaataacttcacgctttcatccca
atgtaatccttttggaacaacagcgctatcgttcaggagtcaaaaccattcctgaatggactttgcgagtcaatacgattgagtccatgctgcatag
cttacattataacagccaagttcaggtaccgccggaatcagagtcctcttcctctatcctactttcccttgcacctaaatctgtgtactcttacgtttct
acgttgcctgagtttgtaaatttgaacaaggctactaagacaaaatctgctcgtgttaaattcctaacctctatgttgaagaatggaaaggtatttg
tttcaaatgatgatgcacggaaaacattcgaaactcccgtgaagtattcaagtaaaaaagatgacatgacggatgctgctatgatggcaattaca
tggattgaatggcaacataaacttagacaatataaacatgaaattcgtagctatgttgagcggacttcacaacagtaaagcattatacgatgata
tagaatattaatggcagattcagatgaggaaaagttattaagtgtgtttttgtatgaataaaaaatgttaacgttttaaaatgaaggctctaagttg
aacgatcgatttttctgaatggactatcattatgcaaataagatgaaacctattctatctgaaaaactcaatttcttgtaacgaatgaacaaagga
attatattctgttttgttcggtagaatcattaaacatgaacgtgcgtcattaccaaaataaataaagctttgctgttgcaaaaatttgcaaatggac
attaaaaaaacacatatgcgtaaatctaagcttgtagttaggcatcaatcataatcagtacataaacaacaaagtttgtccgtcaaggaatgaaa

cagaggtcgttacgatgcagggaggaaatctcgtaaagggacgcaaacagcgaatattcgaggtataagaaactgaaaaagacattattataa
gaatccgtaaatcataatcatatgaaaccatagatagcaaagttggagacaatcacaaaatccagtgatatcgttattgaaagatggaataagg
tagttcctttaatatatcttaatcaataggacgtcgttcgtccatggaaccaaccgatggagcggcagttgaataggatctttcttgttgtgatctgt
cattattgtaggaacgagagtactggtccttcgcatcccgagggtgttcatggctgctgcgacgacgatcattataagggcggtatttattatcgtt
attggatcttctaggacgtgctgatcccggagagcggtccctattattatcatagtttctgtcaaagccagagcgataagagttttgaaatctacga
ttatcgcggttaggacgataggaagaagacatcctattatttccattgtatcgatgaagtccctgtcttccctgattcatctttgtcctcattggtccc
atatactttccaggcgtaggagagcgaggacgtgatcgtttagccttctgtacacttaaaacacgtccaaagaattcctgggaatttagtccttgta
tagcggcatctgcttcttccagtgttgataaagaaacaaatccaaatcctctagattctttggtagcaggatccttcaagatattggctcgaataat
ggtaccgtacttgctgaacatttcatgtagatcctcttcctgaatacgaggagcaatccctgtgacaaacaaattcgttccaggattctcaggttcg
tctgactctctccctacagtctcgtgggtttgtgtttcgggttccctaagatgtgtattccagcggttgtcatcaatggactctgcatgagcaggagat
ttttcacgagagaattcattcctagtgagttcttcgtttcctttctctagacgttgataatcatgggtatggttagcgttcgtgttgaacggtactggat
gtgattcattcaattcagaaagaacaggagcagtctgattgaaattgatttcttccgaagccaggtaaggatccattggtgcgtcaatcatgctga
atttcgagaaagcgagaatccgaaaatgattacaagacagacagaatgttcgatttctgtggataaaggtatgggaatctttacaaaaacgttct
ttcgctaataattataaatcctacgggttctttattcaaaccttcaaaaaagtgtttttctaaagaatttggtatttgtaccatcggtcggtcctccag
ggaatacacacaaagtgttgacaccatgttctcgtttagtgtaaagtaacgaggatacataatctcgtaatcaggtaaataaacaatccaagatt
gaaaaaatacatttcattatcgcataaagttttatatatattttaatttccatggctttatcattactcttttaatatacaaatatagaagaaaaagg
atttggctgttgttttgttaggcaaaagcattctacttaatgtatggatgactggtttcacaggtagtagttgatgaacatgaacgctaattcattttt
ttcaaaccgttgtacttaaaacatgtttacaaaaaaaggctagattcattccgctttgactaatgtatgtttccctttcctttctcataaaggacatgt
ttgcgctacttttagatatagaaaaataatcatattaaggcataatcacatataggcggatgatgttcacattgcacaaaataagtttgttgcttat
aagcgaaattgaattgtttccatcatttcattgaattgtttattccttcgacaaaccctaagaatgttcgtcttcttttctctggagaagcacaacattt
ggtaaaatcgaaactgagaaataattactttatcctcgcttccttttattttcccggtttccttcttctactactttatttctgatttattgttttctttattt
ttgttacccaagtaacatgttcaacgcgtcatcacacattacaagaaaatacgttcccaactttggtcctaccatcattactcaaaggttgtctttta
tgaaacatttttgcctttgaaactaccacaaagattcgccataattcccaagatcttctcttcctctgtatacctcgcttcttaaagaaaagacttctc
aatttttattcattagtcaatagatactcatgttattgctttctttttaaatatattcgagcaattgaaatttttagtatctattatatggtttattttaga
cttgtatttttatttttttgcgtagcttttctttttttctttttttcttttttttcttttctggtgaattcctttcttctcttttcaggctattttctatctaatcatcagt
atttgcatttgttaattatcattttcggggtttctgtcattcttgttaagaatgtcgaccatcgatgaccaagaaaagtactttcgcagtggattgaga
accgaaaatgtctttgataacaccgaaactatgatgaaagagatgaacgaaacttctattgaaagtgacgatgttatgaaaaagcaaaagtcca
agttcaagggaatctactatattaccggtttttacaaatggttcatgaacctctctttcatcactcgttggttggctttatggtttcctttggcatgctta
ttggtcattcccttggccgtaggtgcctcaccaaaataccgctatgcgaagctaggtactgtccgaattttctgggtttttctttggtttgaaattgctt
ggggtggcttttgggtttctcgtttggtcgcaaagttcattccttattccttatacacaattgttggaatttttcctttcacagcttacaaactctctatc
atcatttctgctattgaattgcctttggctttctttttcacctgtataattggcatctgcacattcccacctttaatgataaacaattccgctcctgtgtc
ttatggttcgagaaatacaacggtaacaccttcagcttccggcgccaacaatgtcaaccccaatatagatttggccaattcgttttccattgcctct
aacgttcagccttggattaaagtgtgtacaaaaatcatcggtgcctttgttatccttagtatcgttcttcttttggaaaaaacatgcttacactttatt
ggcttccattaccatgaagtacaatatcagtttcgaattgctgacaatagaaagcatctttccatgctgaccaagctatttttagcatcaatgaaag
ccccatacaaagattctccctttgttttacaacaagatgatatcctcaacttaggtgcctctgattctttaatcaaggaacatacacctcgtcatattc
gacaggtaaagcgcatcacaaaaaatgcgaaaaaagcctttaggaaaacaagggtagtagttggtaccgtctttaataatatcgttggtcactc
gcgtaggccgtttactccagaagaagagtatattatcgaaatgcttcgatcccgaaagagaagtcttgctatcgctcgaaggatttggtatgccat
tgtacctgcaggagaagactcttttaaaaaagaggatcttgcaggtcttatttctgataacgatgacattatttctatatttgaggttttagacagag
attatagtcgtagtatcagcttggacgaattagagcagttctgtagagaaatagctgacgaattcttttctattcgggtttcgttacgagatgtggat
gtagccttatccaagttggatcgcgtgggacttgcagcagttggtattatcggtattctcatttttgtttccttcttggatacatcctttgcaaccattct
ggcggcgtatggaacaactcttctgagtttgtcctttattttcgctaccacggctcaggaattgatgtcttctatcgttttcttattcagtaagcaccca
tttgatatttcagacatagttgtcatcaacgggacaaagtatgaggtggtaacattatcgttactgtacactgtttttcgtaccatgagtggctcgac
agtacaagcccctaattctttattgaacaccttattcattgagaatttgcgacgcagtttgccccaaagcgagtcaataacattgaccgctcctttttg
cgacggactttaagcaattggaacacctgcgtcacctgttgcttgggtttgtaaaggagaatgattgtgattttcgacctcttttggacttgtcagtg
caagatttttcagccttggattcactgaagtttacgataactttttactacaaatcaaattggcagaatactgctttacaagccatacgacgaaata
aattcatgtgtggtcttatcaatgcagttcgagacgtgaatttgccagctgtgggtgatccagttcgtggttctcccgattacccatttgttattgagc
aatttacaaaagagaacctaaagcctctttcggatcatagcatcaggccttcatttactgacgtatcttctacctccagctctcccttattaaaacgt
acgccaagcgcttctttcaccaacaataaaacagagaagcaagaaagcacacccccaggtgacgaaagactaaaaaaagaagcaactgcaa

agtccgctaactctaacactaatgcggctcctgtaggctacattcgtcagacgctagccacttggcaagtccccgaacttatgaattccttgaacgc
tttggataggaatcaggagatttcccaagaaattcgaactacacagggatcccaaacgtttaccgctgatgaccaggctgaaaaccccgaagaa
gtcttggcaaccgatgaacatagcagttcttctgatcagtgattatttattaccatgtcctttaaaagttcttatatttattttccagtgtaacattaat
tgaccgaaaacaaaatagataatctgtcaatgttcaatagtatcttcaaaaatttaataaagtctaatatacgttctacaaggttgactgatctag
aaatcataaaaaacattcacgccatgcctttttccttcttgattcttgacatttagaaagccactactaaatatttgggagccgcagcacattcctgc
gatgatatttatacctcattaaagcacgttactagtatcttttcagttaccgtcctatacggtatattataaaaaaaacagtcacatgtttcatccaa
cacaaacttttcgactgcgatctctaccaccaccaatttaccaacatgggaattaccagagactcacgccacaagcgctctgcttccggtgccaag
cgtgctcaataccgtaagaagagaaaatttgagttgggccgtcaagccgccaacactcgtattggtaccaagcgtattcatgaagttcgtgtccgt
ggtggtaacaagaagtaccgtggtcttcgtttagaatctggtaacttctcctggggctctgagggtgttgccaagaagacccgtgtcattcaagtcg
cctaccacccttctaacaacgagttggtccgtaccaacactttgaccaagtctgccgttgtccaagttgatgctgctcctttccgtgtttggtatgaa
actcactacggcatcttgatgggctctaagggtaagaaggccacccctacccctactcccaagtccaagcacgtccaacgcaagcaaagtgctcg
tattggtagcgctaaggtcgaccctgctatggagtctcaatttgctgctggccgtttgtacgcttgtatttcttctcgtcctggtcaatccggccgctg
cgatggctacattcttgagggtgatgaattgcacttctaccttcgtcgtatggccccctaagaagtaaaaaaaatgctattctagccatcttaatgca
aaacatgtgcccgtaaggaatttggattataaagtgacagtagattgagtagcttaactcatgtaaagtagaaattgtcgtaaagtgttcattattc
attaactcgtcctttctaagtattggttatttgcaaagcatttggaggtatttgagatttcaaatttccatgtcgcttaaattctgaagctggataaatt
tctatcttttatttagtataactcttaaatttataagtagttagttgtattcatatacatttaatgtttcctttaattattgctgatgtatgtgccaagtgc
ttctcaggtatgtttttatctaccctcaaccacacacaatcactttttataatcttttcccatggaagaagacaccagtaatttagtagcaactttttg
ctctataacaaactcaactccagaaaaggctcaggaataccttggagttgctgatggagacttatcgactgcagtgactcttttctttgaaagcgg
aggcgttactggtgattatggtagcgaaagttcaaatgctgttcagcctgaacagactgaagaagtccgtgctcccattgctcctacaagagaggt
attggtggatccaatggcagatactacacctgggctaggatttgtaggtaacgaattcggttttgggtcaggattacctagaatgaaccgcagaca
aagacgaagagttggaatttttgatcaatctccttcagaagttcctgttgcgtcttctgccgatacctcttcagaagaatcagaaagtaatagtcgt
gcttctcgccttgcaaagcttttccgccctccttatgatataatatctcatcttacactggatgaagctaaaatgaaagctacatatgagaaacgat
ggatcctaatcaatttacaaacttcctcttcttttgaatgccaagtactaaatcgtgatttatggaaaagtgaatctgtaaaagaggttatacgcgc
ccattttctatttctacagttgttggatgatgaagaaatgggtatggaattcaagcgtttctaccctgtgttatccactccccacatcgccattctaga
tcctcgtaccggtgagcgtgtcaaacaatggaatggaggaattactcctactgaatttgtaattgaattaaatgactttttggaacgctgcaccctg
gatgaaaccaaagacaggaagaatcccctcggtcccaaatcgcaaaaaccagctgaagcgatgactgaagaagagcaaatgtcaaaagctat
tgctgcttctttaggagttgagcaagaaagaactgagagtactggtgaatcatcccaacctattcaagaggatgaggaagaagaacaggacaat
acggttcataaaattaaatcagaagaagggaaagacgaagagcctagtcctggacccggagtcaccagaattcaaatccgcatgccaactggt
gctcgttttatccgaagattttatgaaagtgatctcgttcgcaaagtatatgcctacgttaagggagttgctgaaggagctgactctcaggcgttttc
tttgacatttcaacgtacaagtttgtggaattctttggataagaccattaaagaagccggtatacagaatacagcgcttcagtttgagtttcaataa
ttcgcacgttaaaacgactaaagcttgtaaatgactgtattatagataggatattattcccttgcctttaaatgtccctcgcgaatgtattcttatgtg
cgagctttatgtttataatgcctaggactaaaagcgtctacggattgttagagtgggacaatgactgtttgatgaatttatgaaatattttttttgacc
gtcatgtaaatagtctaatttaagtattaattcattccatttagtcaattcactccattaatccctgagggtgcaaacgtaaaaatttccagtagaaa
gatatcagacatttttaaaataattcattacgtcctttaaatcatttcctgcttcttaagaggaagcaagctttacttagaaaaaaccaactacctg
tctgcatatcccaaccattcgcttgcctacgataatttcatctactttaatcggaatgaattaaaaaaatcatgcaaaagcaacattattgtgatttg
gagctaataggacaaggtagctttggatctgtcttcaaagctataaatgttgaaacatccaaaccagtagctcttaaagtagtagatttagacgcg
accactgatcaggttgagtctcttactcgagaaatcaattttttgattggactgaactcgccatttattactagatattatgagtcttttctggatgga
acacgattatggataagcatggagtactgcgatggaggaagctgtacagagctattgaagacagtaggaaaatttccggaacaggtaattgcag
agatccttaagcacatattagaggccttggtgtatttgcatggtcaaggtaaaatgcatcgcgatataaaagctgccaacatcctaactacgcagg
acggaggagtgaagttggctgactttggtgtttcaggtcaattacagaatttctacgataagaatgatgattttgtaggcaccccatttggatggc
tccagaagttgtcaaacaaacgggctataatgccaaagcggacatatggtctttgggcatcacggctttcgaattagcgcatggagagcctcctt
actctggaatacatcctatgaaggttttacttttgattccaaaacacgctcccccatcattagatagatcatcctttagtccagcattctgcgattttg
ttgatttgtgccttcgaaaaaacccacaagaacgccctacagcaatgaagcttttggagcataaatttataaagaaatatagctccaaagatact
ctgaaaataattatccaaacctactctaaatggaagtctaccgatcaagcttccgactatgcctctccatacaatggttcagatatatttaaaaatt
ctgctaccgtgaataatgtggtatgggattttggaacagtatccaggtagccatttacatgagttgaaatgaccatgcaataatgattaataacta
ctttagttttctttcttttctagacttgagttaacatttaaacaaattcatttactgatatcaggccctaaagcgctgaccttaagacattacaatata
aagaagctttatgaggattagactcattaacctttaattattgagggacttgttctataaaattatgagttgtgtccgctcagcgaataagccttga
aatagatgctgagacagagaagtattatcgtaaaacaaataaaattcaacgaaaacaaggccattctcttaagtaacttaattatgacttataaa

agaaaggccattagaggagtgcaaaaagggacgggcggttcaaactctgaggatgaattcaaggaaggcatgagagagaagaagggcttga
aatttaagaaataaaaacattatttagagattcaggcaaagtcaagggctttcccgcctactaacaagcccacgatcaagccgaacagacccaa
aacacttccaaaaatttcgacaaccaaaacttttacaaacaaggaagcatcttgtgcatcagctagggcagcactactaccagtaataccaacgc
aaacaccacatattaaattgcagagaccaaccgtgagtcctccccaaaagatagcgaaaccagtgtaataattggatttggtatagagatggga
agcactaacagcgtttagcttgccagagaaaacaatggctataatcagggaatagatggctacaacttcgcagaaaatgatgctgatcaagttct
tagttttaattcggggggctttaacagcgccgcccaaaatagaacttccgcaaataaaaataccccaagctgctccaataataccgaaagcgacg
cagctggcaatgcctagtaagccccaggtataggggctggtctgaagaagaaagcttccaaaatcaaatgcttctccattgccatgaaacaacat
atataggccgactaaggttgaaaatccaattgtcgtccatataaatgactttaatacggacatttttatgatttagctattttgggacttttctcaaa
atggaaggtgagggctttaggaggactaaccagaacactgactgtaaatagtcagaaaaaggctctcttctagtcaattcgtttcaagacttgaa
aagaactgctttattttgtggcttacgctttttgggtagaggaataaccctgcgctttttcctttttgaatagctgtgtcaaaaaccaacccgcttaac
tgatcgtggtttcgttggagaaaatatttaaaagcaacttttcttttgaatttacaaactatttcaagagtcttttcttcagttttatctattactgttta
tcttacctgacattcaattgcatacgggtttccctattattagacgaaactaaagccacgaggattactgaagacgcgtatttggttctatattgcag
attattttaagctcaaattcgtataatcgcgtctgaaggatattattctataggatgaccaactctgctgaccaaaagaatcttgtaaaatcatcca
agggaaagagaaaacaagaacaagatcccatgatccggttttcaaaagcgctttccaaagttcttcgccataccgctcaatccaatgggttacga
cttcgtcatgatggatttgtgcttgtagacgacctgctgagactgccgcaatttaaaagcctgtcgctagagatgctgaaaaagattgtggatgaa
aacgaaaaaaaagatttccttacttgaagcagaaggtaagctattgattcgtgccaaccaagggcatacaattcgtacagtcgaagtacctatg
atacgtattaatgatgcttcttctttgcctaaagttgtacatggtacaagaagagagctgtggacaaagatctccaaagaaggcctttcgcgaatg
aaaagaaaccatattcactgtgctacaggcttattcggtgaagccggtgttatttctgggattcggaaatcatgtactttgtacatttacattgatgt
tttaaaagctatgcaacatggagttgagttttaccaatcagaaaatggagtcattttaacagaaggcataaatggtatcttgtcgcctgtgtttttc
aaaaaggtcgagaccaatcttggggaagttttatttgacgcaaatgcgaatccagagacacccaaaactgaaaattcactacagaaaagcacg
gataccgacagtgttgatgatttagatccgtttagtgaggatatggaagcgctaacaatgcatgacttaaaccttgtgcaggaaaagcatagcaa
ttttggctattcagaaggaatcgttaagggaaaaatcgaagttgcccaaagcgggtttgatgatggatataatcgaggggctctgtatgggtttga
aatgggtaaaaaactaggagaactaaaggcaaaattacttgtggtgaaagacgaaaaggaacaagacactttgcagaaaaaattagaagaac
ttgaacatgaaagtgagttttctaaatttgtggttgcaaacaaggagtctattcgattacaaatgaataaaaagcctacttcgacataatattttga
tgctttctggacattgttcctgtatgatgattcaacttcatgcatactcataccattggataggaacaggcgttcccttcctttaaatttcaaggatga
ttttttatgttcagcttacttatacctaccacctacttatcttttccatcacttacgcctttttcccatcggttttttttctatttcttgttcattatttctcttt
ctaccgcgttaatgtatttagtgatatattatgtgtggttaatttatttaacgatattgtagcatttcgcaattttaacactttcttgcttcctttctctcc
actagaatttgcttcttttaccaaattttccaaaagagatgctggcacctacggcggccgtaaatgacattcctccaaaagaaaatggtatgaaaa
agcgtttgaatggaagaatggagctaacaatgccaataggggacgatgtgatgattgatacatccggcaacatgactgaagttgctgaaacaag
aggcgacagcactatggacattgaaggtgctcccaaattcgctcctattcaaccaaaaatggaaaaaaagcgtggtctgaagcctcaaacaagg
aaggttcctattcctcctcacagaatgactcctcttcgaaatgtgtggcccaagctttatccaccgttggttgagcatttattccttcaagttcgtatg
aacgtcaaagctagagctgtggaactacgtgagagcaaagccaccacggatcctggagctttgcaaaagggtatggactttgtgcaggcgtttg
ccctcggcttcgacgttgacgatgcaattgcgctcttgcgtctagatgatctttatatcgacacgtttgagattaaggatgtgaaaaccttgcagggt
gatcacttgagccgtgctattggtcgtattgctggacaagcaggaaaaaccaagtttgctatcgaaaatgcatcccgtacaagaattgtgctcgca
gatagcaaaatccacattttaggaggcttcagcaatcttcgtattgccaaggatgccatcgtctccttaattcttggatctcctcctggcaaggtcta
tgctaatttgcgcagtgccgctgctcgtgctaaagagcgtttttaaccacatatgaaaacgatatatctgtgtttttcataaaaacatagcagacat
tttacgtggattccctcttttccttcacaaccgtgcattgagttgtaaaatgaatgaactggaaatcactaattacgagccggataaaaagtattca
ttctcatttctttaaaaatgtttttattctattttggacagggttgattttattatttagttttgggtttattaatgttaaaacggaatattccatatagtt
aatggatttttgaataatcaattatcgacaatttcttattgtaagcttaatactgtaaatctctctttctttcttgtagtttttatatggttttaatccttc
attttggtttcttgtttttatttccatcctcctattgacctttctacctagtaaagccactcaagttcaaaaaacaagctggtgctgccgtaagagaag
caagcctgtgtaaacatttcccttctttcgttattttatctacatttattctcttgttattatatgagctttcttctttgaaaagacttagagctactaggt
acagtttgttggaattatcgtgtcgtgtaaagaatgagtgatctgaatcgggacgagcagcttgcatttttagaatgtatgagtaaattgcaaaag
gaattagattcaagttttcagcgttatgaaagattatcgcatacgaccaaagaactccaggtatgtttgtttgtccagcttatcttgggaattctgtg
atttaatcgtcgtctaaccttccctaaggaacaattggatcaagcaagagtaaaatctcagcatccatctcgggtatcttcgactggaacactatat
tcggaagaaaagttgctcccaagtatggagcttatagagctacgagcagaaaacgcatgtctcaagaaagatgcttataattttgagcaaatatc
caataattatgcaaagggaatggaagaaatggtgggctttgtctcttcttatacagtaagtttgatttgatgcgcttgcgttacagaagcgacgctc
taaaacaagcttaaccaaatatgtactaactatggaaggagaaaatgaatacaaagatgaaactagtacatgatgaatacttaaaaatgtacg
atgcgttggctgcagagcatttgtctctatctaagaaaaatcaccaaaataatcaatcattaaataaaataaaaggatggttgcttcaggattag

aagaaaatccttaaatattacagcaaaatttaaagggtctatacgaatgggtgatcaagcatactacattggttccttcacaatacataattaaca
tctcgtttctctagtttttccatgataattcatgctaataaatggatagctttcgaaatgcattcattactattcataaagacaaacgtatactcaaag
cagcacgtgaaaacaaacagagaacatgtcatctgtgagaactagtcatattccatgcaagaaaaatttaaataattaatcgaacatatgcgag
aagtaaagcgcccgatctagaaaatgttaatagctaacgaactgaaatttcaagaaatctccagacatagagtaaagattcgtttcataccctgt
tacaatccccaacaaccattgaagggtttgtgttttaaatgtggctaactgagtccggaaattcgacatttccgtatcgatttcggcagacaactct
cgaagtttaagctcctgtaaagaagcttcatcatagtgatttaatcgctcaatacttaattgtaatctagcttctgccgtcgaattatttagctgatca
cgaaaggatgtttttaagcgttcgatatccgaaaataattgttccgcttgggcatgtaaagctgccatttcttgacgctcgatgctttgtaactcact
acgcaaatgagcaaaatcgaccttttgttgatacattactcggtcttgttgttctttggtcaccaaatatccactcattttcttatgactaaaaatggt
taggatcttgtcttttggtcctcaagttacttacgaattcgcatatacattagaaatggcttccataaatatttctgcatttttcttctcaatgccgacc
tcctgtaaggcacggacaaatcctagtgtattgaatccatgatatgatcttcgtgatataagtgaaggagttttcggctttataaatgaaggtttga
ataagtaaggatgtgaaaaataaggaattggtgagattcttgaacaaaatggattccttgaaaggatgcgcattataagtgaattggcagagtcc
agggaggattggtcacattggaagaaaggaaagaagagcaattcttcatataaaattatcaaaaacacttttataaatgataattgaaacataa
aagcaaaagtgactaagtttcagtagcgatatctcgaaacatcgagacttcaaaggcagcaaccataagctgtaaagcatcagaggtatacttg
gaatcattccctgcattatgaagacaaaaaggtcgtatttccagaagtttcaacatacttcgaagacctacatgaacctctgaaagattcgaattg
tgcttggacgctttgaagaaacaaaatatgttttgggtgtccaacatgatgactttaggagggatatccactcccaaaatttgaaagtatctcaatt
cattagaaattccatgagcaactagaattgaattcggaagctgaagtatttcgtctaattttctctgcagtgcagccttcgttagaaaaacggattg
tccaaagcgaaagttatctacttcatcaggaacatatttaccattgcgcagatgcaaataattagtaatccgatagtgaaaggattgccattgacc
gttcactctagtagtaatgccagcttctgttatgattttatgatctcgttcatatgcttcaaaatccactactatgaaactccactttttatctgcaatt
gcctcaaaagccagctttgcctcccttttaatttgcatatctcgtgcatattttctttctttgacttctaatagtcttcctgctccatgcgttgctttacct
tcttccaacgtcatttgcgtaaaggtggtctaactaaatcttatgaattgaacaatttaattttctttatgaaagcttatgctcaagaagattttacta
tcattgaaggacttaaaaatgaagaaacctacgaaaaggtaacgctgttctttccttgaattttgtgacctaatgacacttgaaagtataaacaaa
taaaaggagcaaggacaaaaggtttatcctatcataagccaaagcaacagtaaagatctcaaacttcagaaataataaaaggagaaaggtttc
gataccgaaatttatcacgattctacactttcacgcggattccgtttatcaggctttatattcctagaattcaagaattcaagaataagagagcaag
ccaacagaaaaaatgaaagaatatactcagagtaaacagcttatgagatggattaagcaatcaaaatttcaattcaaaatcgcgagtccggtct
tgtttactaaggcatcgcaacataaccttgggcaaggataaacaaaagtgtataaaagttgaatacaagagactagttcaagaggatttgaaac
aaatgaatcagattaattgaaaattatggtccgttgtttcgtctcaacgtctagaaagctacttgttcatagtggtacatggatcttatacatcgaat
tttgtacaaggactttgaaaatcatagagaaatagtcaaagaattggctctaaaatgtaaaaacagaccttatatagactattttgactacctgca
gagagaatgctgttcaggtcataaagttgattaaaaataaagtgcttttattgctaaaatattttacattaatagtaaaaatattttaaaataaatc
atgtcaagaaatgctcatatgtattagcaaatatgagagaagattcaaccaattcctcttactcttctttcgccaagaacatccaacattggaaga
atgcagaaattgcaagattccgaagagaaaatggatggaaagcaaccaaagataaaaaataatacaaataaaaatagtccacgtaaaaaatc
gacaaagaaaaatgaagaattggcatcagtggaaaagaagcaaacgtcagaccaacccacgatgggtttaggtgctggtttgatggaaaaggt
taaggagattgttcaaaagtctgatttattgggctctttaaagtcaaatagtaagaaaaatcccgcgcccaaaaagcaattggataagccttccg
gcagaaagcctgattcgaaaaagaaagtatcattatcgcccaagacctatccttccgatgagaaaaaaggacgcgtgcaagaaaaaccgcaag
aaaaactaaagggaaaagaaaacaaaatatcaaggtctaaaaaacaaaaggagaagagtccaaaaccaaaaacaccgtctacaacgatag
aggaaaatgctttcatgaaacctaaaaagtcgaagaaacagaagtcaaagaaaaaaaataatgataaagaaccaattactgagaaaagtgag
ggcaaggagcttcaaggaccaactccaacgaagaaaagcggtaaggccccgaaacgaaaacaagtctacgaaaagtattatacccgtcaaga
agtaaatgaaggcttgaagtcaggaaaattatttaaaggaaccttacgcattcttgacaaccatcgaagcgcattcgtttgtatggaaaatcgac
ccgacttttacattgatagtcctattactcgcaaccgtgctctgcacaacgatatagttgttgttgaaccaattgaagataaagataaaaagaata
gtagcaacgatgatgaacataacggaagctcgaacgatgatcagtctatggcaaacgaagacggcgatctttccgaaactgaacaacaacttg
aagcgttggagttaaatgaagagcctgtaccaaaggttgataatcccaggtgtcgtgccaaagttgtttcaattgaaaaacgaagtccagtaaac
aagatcgtgggtattatccgtcctcctggatggtcgatttcaaatcacgagaatgtttccaccaagagcgattatgccatttttgtacctaaagaca
agcgaatgccatttattactatccacaaaaatgatttgggtgttctaaaagttcacgactggatcgaaagcattttacagcatcactcaaagttatt
tgccgctgagatttcaagatggggtacttattcacggtatccgatgggagtcctgaaagaagagttaggctctgtaacgaatgtagaagactata
caaacgcgttgctcttagaaaatggaatttcaagtgagccttttagccgcgaggtattagaatgtttgccagctgaggattggagaattacagaga
aggaattggcaaagcgacgagatttccgaaatgacatcgtcataaccatagatcctgatacagccaaagatttggatgatgctgtctcttgtaaa
gatttaggtaatgggacttatgaagttggtgtccatattgctgatgtaggtcactttatgaagccagatactcctcttgatacggaggcggcttctcg
tgccacgaccgtctatcttgtgcagaaagccattcccatgttaccctcattactatgtgagcgtttatgttcgttgaatccccaagtggagcgacttg
cgtttagtgttacctggaaaatgaacaaagaaggtaaagaagtcggcaagcgctggtttggtaaaagtgttattaagacgtgtgctcgacttgctt

acggcgaggcacaaaaaatcatagagggaaagtcttgggccgaaggagttggtaaaccaattgcaggaaatcattctccggaagatgtggaga
aaacaatactggtgctttgcgaaatttcaagaaatttacgagcagaaagatttgcaaaaggagctgtagaaattaactctacagaattaaagttt
cagttggatgaatatgggctaccaacaaagtgtgaagtttatgaacaaaaagaagcgaaccacttgattgaagagtttatgttatgtgcgaatcg
atcggtcgctgaaaaaataagcaatacattccctgcaaatgcattattacgtcgacatgcaagtcccaaagaaaagcaaattaatgaattttgca
aatttatggagggattgaattttcattttgatgcttcctcttcctcggccttcaatgactccatgaatcgtttaaggaaattctttaatcctgacttggt
agagcttttcgaaaacatggctgtacgttcccttaatcgtgctgaatacttttgtacgggtgattatcctgagcgcagcgattggcatcattatgcac
tttcttttaatcattatacacacttcacctctcccatccgcagatatccggatgtgattgtacatcgtttgctggaaaactgtttaaaacaaacgtcac
ctaatctgcccaaaaaagtgtgctcaaaacacgcggagcatagcaatgaacgacgtgaacaagcaaacaatgttcaagaagatagccagaatt
tattcttatctgtattcatttcggagtattgtaagaattgtggtgtgtcttccatgcccgttactgcgtttgcaacgagaatacaagatcgttctattga
tgtttacgtctctcaatatggcatctctagtcgagtggatttactaaacgaagacaatataaagagttatacgatattaccggatgactcagctgtg
gttatgaagaagcaggatgactcagagtttaaaattgctttaacagataagttccaggtccacctgtattctgattacagtcgcacctttttttaatgt
tcgctgttcactgtttccgaaaacgtaaagcttgtttgcatacagaagcgtactgagtaaatgcttttcaacaagtaccttaatgatttttggttcatt
caatttttttcagcttaacggatttgtgagttattaaaagtacaatttgacatgttaattttaaatgatgaaagcgacgttttgacgatttccgttgc
atgtgttatgtccagtcatatatacgtttgattcactatacattcctaagtaggatctttttttcctttattatattagttatttatcttaaataggacaaa
agtctcattattaaatatgaatgaataaataaattttgaacgtaagtaacaaatgtaccgttacccatttttagttggttcattttcaaacttggtctt
ttctctgaagtgtaggaacgaggacatggacttttatattcatgctgcaacgattcttgatgatttaagtcaaaaaaaaggttctattaagcagctt
gtttttaattcaaaaaagcacgatcctaaacggacgtatgcgctagtctgcgagactttaaaatgtaaataaaagtaactgaagtttttacatgtct
aacagaaaaataaatagataaacatgttttggacgaggtcattcaatgttcaaagctattggatttagagaagaaggtatgcaaattaatttgtta
ggatgcttacaaggaaaacgaatctagataaaaaagaaccttgcaagagttttagttcatgatttgttaatgtcgaaacgtggaattagtgcatc
gacgggttcaataaaagaagcaattctgcgacataaaactcgcttgaatgctgaatttgtaaagctaaaaattcgcaataaagtaaagtcgcatg
aagaattggcattaaagaacccagttacgcttcctcgttggattcggataaacacaattaaagcgacaaaagaagaagtactcaagcaattggc
actgacccgagtatcttcgctgaaagaagtaggaccaggaacatattatatggatgaaattatagataacttaattgcacttgatggtgaaacttc
cttggtcaatgaaagttgttaccaggagggaaaagttatcattcaagacaaagcttcatgtatccctgcagcgattctcgggaatcagcgacaga
caaaggtaggagacataattgacggatgtgcagcacccggtaataaaaccactcatttggcagctttatttcccaacgcaaaaatattcgccttcg
agagaaatagtcaacgagtgcaaacgcttcagaaaatggtagagatttctggagctcagaacgtcgaaattttacaccaagatttcactcaaagt
gatataatggatcctaagtttgcgaatgtcacccatattctgctagacccgtcatgctccgggtcgggaattgtaagtcgtcaagactttttacttga
agaatctgagcaagatctggtggaagatgttaatcgactcgagagcctgtgtagcttccaatctaccattttgaagcatgcattgcagttccctaa
atgtcaatatgtaacatattcaacatgttctgtacaccgacttgaaaatgagcaagtcgtttgtgatgttttgtctcaagagccccaatggaaatgc
aacactatagaaaacacgttgccccaatgggaggaccgtggtataccagagtattgcacggatcctatgatggctaaagcaatgattcgttgcgt
ccctgggaaatttggaacgattggtttttttgtttgtaatttgtatcattcgggacgggtaggtaatttgcttggtaacggcacctcaaatgcactag
aaagcaacgcaaaacggaaaaggaagaatcagaaaaagaagaactataagaaaatgaaagaacgaaagctgggaacagctatggattcca
ccggtcatatttccgaatcgaaaacataggtcttttctcagtctggtaccttggaaaatgatatatatggggtttgactattagaaactaaaagattt
caagcgcacgaaaaagaaaatgtatgggttagaaaacgtaacatctggataaactagtgttttacttgagttttctaatgttatttttggaagagg
tcgaaaagtttacgattaaagagtttaaaaaacactgtcatgtaaacaaatgaaaccttcctatcctaccagttctgcaaattacattgagtaagc
atcataggaatgtatagtactacgaaataatgttacacactcatcatgggtttgttagatttccatactttctgtggtgtttttactttgtaccaataat
cattcctgtaactacgcatgaatacaattaattctatagatcaccgaaaagcactccaaaaaatagaaaacacaaaaagtggaagcatgtaacg
cccgttcggtcagacttcctcgagaatcccgtgaaggcgaatcccatcgcaatcgcactgcccaggacccgggcgtggtgtgcgttgttgccaacc
tccaccggtacaccgcggtttaatcaccatctccgccgaagattcctctccgggaagttatggtgaatcgagggatcgactgcgagactcgaaccc
atgaccacgagacactcgacctcgaagcactcaaccgttcagacagagccattacagctagaagtagctatttataaaagtatatatagcaaga
aagttaaagttattagtgagaggattcaaaacctgtgacagaacgaaaagttttgtatttttttgagtttccaaggagttactcattaagcacattg
tggatgaactactaaatgagaaaaacaatagagagtagacaggaaagtaaacgctccgaacagaatactactacatataaaatattaaaagtt
ctgattgtaaaaaatggggagatgggctacaaagaacgaaaagtaagcgaaaaattcttttatttatccaaggcataaaattggggtaattaag
gaaacacaaatcaagtcaatcctttgtgtacctgcttacgtacccgcaaacattgatgtagaagcaaacacaacttatagaaaaagtacattagg
gaatataagaatcattgcctttcctatacgaagagaaaacaagcctagacatcaaaaaaaagagattatggcaataggtcataggtcattgtac
aaattaacctacaaaatgtatggtaaagaaaaaaaggggaggaacactagggattttgcaagtgtcgaaagcgaagataaatttcaacttcaa
gacttggcatactttttcattaaatgttgcttgaaaaacaggttctctttaatcaacgagacggctgacaaacatgaataagtaagtgaagaagag
aacggagaaccaggcgagcttaatgaaggaacgtttttgacgatttgacacatccttgaagatggtagtggagtcgtggagatggttatgagtca
aatacaattgaacgttccagaggatcatagggagattgaagaaaaagcaaatccatgctcccgaaagcaataaaagaacagaactaatggcat

gcaataacatttcaggaataacataatgattcaattttcgagacactagaacaaacgccgttagttttctgttgaagggaagcagaaaccgattc
aactaggctagcaggtaaaccagcaactaaccattcactacgtacagtcaataggattgataaagtcatcctttaaatctgagtacatgacagta
aaatacatgtgcaacattatgcttgcacaagtcatcattagcgatgtaaagtagatccatggagaagccattttgaacaatttatataagaaaaa
gaagcaaaaatacctggaagtacaaacagtaaaaagggaaaatcaaagaactttttgtttggagaggaaagagaaagatgatttgtttgtcata
gagtctcatccaccagtggtgctaaagggactgggttcgagttttggtacggataatataaacaaacaatataagagaaatgacaagtattgata
tagaaaatgttaattgattgagaagaatagaaagagattacagaaagcgaaaaaaagagcgacttcaactcttgtacaaccttttctcgctcata
agtcttggaacttgatagcaaggaagcatgatggaaattcaaaaaacaaactgcataaaacaaatctagataccaatttttccttcataaaaaag
aataaagtgaaagatctcttgaatttgcattgaaagtatatgacaaatcttagtagcaaaaaagtacgtttgtttacattctgtattggaacaatga
gatgcattcgtgacagtacagcattcgtcacatagaaagacaaggtgtttccgttcgttttccttgggtttgctaacttttaatgaaggagaaactc
acgcatcaccgcagaggacgaaactagcgatccactcctttcgttcactcccagcataataggcaaagacgaggcgagacggattttcaaatttt
tcacaaggaactccacaaagaaggaaaacacttgagcagtctcctctctagttagaaaaaatgctgctaagataaaatatgtaaaagtaattatt
ttcattaaaagagcaatgacttgttcttttatgaaaatattcttgaataatttatttttttttttttaaaaaaaaaaggaatcaaaagaataaagaaa
aaggacagtccccccttaggaaatactttcctgacgctctcgcttttcctttgcaattataaacgtcagtagtaattattcttctagggagattgcagct
aaaagttgattcaaatagtactgttcttcgtattagggaagtttacattgagaaattgctgaatccttggaactcatttacattctagtatcattcact
tccaactcaaaacagaacgatggctactttaacagttcgcataattcgaaattttgaatatcgtacacttaaaaatgtggtctttcatggtattgatc
tcgctacaactactgttggccagctcaagaagataatccaagatcgaattcaaacagatcctgcgcttaagatttatcggaccaccaagtttgaca
cgttgaaaatttatgtgaaagctcaacagcacaagacgcaaaacttagccattaacttggatcatgatgattgggttctcacagacgatcaccaa
actttggctttttgcggaattgaaaacgaaactgaactttcctactttaatttggatgcctaccgtgcgtaccaagagaaccccgttcaaaaatgga
tgtaaagaaacaagcggccaatggaattctgttgtccaccatgaaaacgaacgtaaatgttcgtatgcttggatccttctgtttctgccatccttaa
ttctaacgaaacgcaaaatgatcctgtaaatctcttattttcggaatatttagagttcacagaatcgctttcctagaaatcaaccttttatcactcag
ttcatttcgtctaccaaccaaaaaaaaataggacaatagtataataaatataaaaggtaatagaccttctcattccgtctatttcatctcatacagtc
attctctttcctctcaatcttactactaattatgtattccacttcactgtcgcccatacaaacagacccttcacttgttcgttctccagaacactgtcga
gcttcacgaaaaaaaaaaaataaaaagaattgcgcggccaggtttcgatcctgggacctacgggttatgagcccgtcggagttccactctcctacc
gcgcacattcgtcagcagcaggatttgaacctgcgagcgcagtgcgcattagatagctcgaatggtgtattcaagtctaactccttaaccactcgg
acatactgactactgttgatatgcttaccataaaaagcattgtatacgagtttttagaaaatttttgtcagccatattgattgttcacaaaaaccaat
ttttcaagaaaacaatcaccaccataataaacaaattgctacaaacattccgtcaattcattctaaaaaataaaagcagttttagttgacgaaag
agaggtcggtagattcatttcttaaaaaggacaaacgtagataaattgaaccaccaaaaaggaaatgctcaactaaggaaattaaagttaattg
attggattttgtgaataaacaaaaataaaataaaaataaaaatcgttaaagatcaggcttaaaatacatatatgcctcaaaaatatgacagaac
gcgtacgaccatgacccgtacttgtttacatcacagaaaatgaagaaaatgataatggtaaataatatcgccttactctgatatgtactgtagtct
gctactttctagtaaatccctatcattcccggttacatacacaagaatccttctcaaccagaacctgactaactaaatcatttaataattcttcagta
cacaaccttacattatacttctcaacatttacctcgcaaagttcaacttttaagcgggaagagccaaattaattcccagctattcaatagtatacta
actatcgtctaaattaatcaaaaccgtaataaaaacaacctaaatgcccgcaaagattattgtatgcatgagtcaatttagagctcatcatcactg
acttcaaagccagccttacgacgagccatacgctccttcttcttcttacggagctcagcaccagtgagcttcttgggcttcttagcctttttcgatcttg
ttaccgaaggcatcaaaggtatcaccctcatccttttcttgaatacgaggaccagaaccttggccttggacccagttgtgaccggaaggtgtcattc
taccgtggttgacggcccagacttcttcagtcaaaccttcagtgaactcacgagagtgagtaaccaagacgacaccaccaccgaaactcttaaga
cccttagaaagggcaccaagagagtcacggtcaagatagttggtgggctcgtcaaggacgataacgtgaggacgcaaccaggtgcaagcagcc
aaaacaagcttgaccttttgaccaccagaaagacccttgatacgggagtgagaaaccaattcaggttcaagacccaaaagaccacagtgttctt
cgatctccttacgaaccaaaggacggaattgaccgctcttcaaagcctcagcacggtcaacttcagcaaccatcttagcgtggctttccataatttc
accacgaggtaaccaagcgttatcagaagacatcatgggaacccagcgttcactcttcataccaatgttttcaccaacagtgaaagagcactcgt
actcgtaagagttcttaagctttctacggctgtggatacccaaaatcttacgaggggtaccctcgatcttgaagattttgttcttcatagcctcctcat
cagcttcattgatgacacgggaagccttgtccatagcttccaaatcttcaccagattggaaacgccattggatgtactcggaaggagacttgtcag
ggtgaagaccaagatgagcgaaagcagcttgagcaacgtaggcaatacggcagttctcgtgttggtaaatgtcaccagtagtaggcaacaattc
accggtaagaaccttaatcaaggtagacttaccagcaccgttaggaccaatgacagcaatacgagatgaaagtgaaacttggaaagaaatgtc
aatcaattgaggtgaagaggtaccggggtattggaaggacatgttgttaaccttgacaatggcacgttgcttggtcttgacaccttctaagtaacc
gggttcagggaatctaaattccatttcagaagcacccaattcatagtaagacttggcggaaggaaccttttgaacgaattcagacatattaccgat
atacttacggagcttgaagcgctcgtagtgaatgatggattgaacgacgtggtccaagaaaccggaatcgtgagagacaatgatggaggaaac
attcttttgaccagtcaagaaattctccaaccaggcgacgttgacaacgtcaagatggttagtgggttcatcaagcaaaagaatgtcagggttctt
gaacatggcacgagtaagagccaatttcatcttccaaccaccagaaagggagccgatgggtttttcaataagttcatcagtgaaagagttttcctt

caaacccttaatgacttcttcacgagtcttgatggagacactagggtccgacataatgaagtcaactgaaggagtgtcggcctcagattcatcaat
gtcgtgttcgacataaacagtgcggaggtgagtagggaaaccctcaacttggccattgacaatagcacgcatgagagtagacttaccggaaccg
ttaggaccgcagagaccataacggcgaccacgcttgagacgaaggcgggtacggttaagaagaatcttagcaccataggcaagggagaactc
gcagttgcaaaggtcttcaccttcctcttcttcttcaaggtgagcaccaccaccgggaatcttggcgatggagcgagcgcggaggtcttcaatgac
gacttcaacgttagcttgctcgggaacgaaggtagcgatatagggagcaacattttgcatccaagattcactgtcactgtccttctcttcaacgagt
tgagcagcaacagcagctgcgtacttggcaatgacttcctcgtgatgaggaaccttcttatcaccgagaacggccttcaaagtctccaagcaaac
ttcagggttggcagcagtggagacttcaggaatcttgccgtcgacaacgttaccgacgcgttctaaagtaccaatggcacgttgaacgacggaac
ggcactcaggatcaccaatggtgtccttgatgtggtacaaaccaggcaagagcttgggcaagaaaggagcaacaacttgagggtcttctacaag
cttggacatgttgtcaataatgacagcagccttacgcttgataggagtggagcgttcgttcaaaccacgagccaacaaggggaccatgatggaca
aagtaggggcttgaacttcggtaacgaaagtagtggcacccaaagagtggacagtctcggggacctcctcaggatgagcgatacagttaatgag
ctcgggaatgaagcggtcaatatctgcgttggcaatcaaggtacagaccttagtcatggtttctctagacttcttcttaatttcagccttagtatccc
acatagattcggaaacaacggggataatggcgggaagagagtaggagagctgagagggggaagtctcaacaaggagatccaagagttgcaa
agagttcatcttttcagtccacttaccagtgctgtgaatagattcgagaacaataggaacgatagcctttacagcataaggagaggcgctacgaa
cgatagcgcgagaagcattaatagcagcatctctaacgctcatttgcttgtcggcaactttttcgacaacagcaggtaaaagttcaaccaagtag
ggttcgacatcggcagcaacattgggaaagttggcgacggcctcgatacccttcaagacgcgctcgacaaccttcttgtccttaagttgcttttcga
tgtgcttaaagaccaaagaaggggcatcttgctcctcaataggtccattgacaaaggatgccaaagccgtagcggcgtcaccctcttgtgcggca
tcgcacacggtaaggttattcaacaactcatcaagagttttgactgactgcttgttctcactctttgcagacatcctgggagttggttgttggtaaga
tggaggttggagatttgtatatgaatcaaaattcaaactcaattttagcggaggggcaaaaaacagtcacaatatagagacaattttaaaaaaat
gtttttatgaattggaattgccgttatgagatgtgactgtctttttgtctcgtcccagttcggtggtggcaagtggtcgcttctacaatgtaaaaaaat
ggtagggaagtgatttaacgatgagtttgtctggttgtcttatagtaaatcaacgtctaaatattcatattcgctattgtagcttggaattcattgtta
ccgttcacaaataaataaataaacgaaagtaagcagaaaatttgaaacgataactatttcgttcgaatcgttacattctacatagagaatagcga
atgatagcagcgtagaggttgttatgacagtaagttttattaggcgcacacaaggtaaaagtcgttgtaaaatgtgattgaaaagaagtcgtaat
cgttcataaaatttagtcttcctcaagcttaaaatgaaaggatgttctattacttttatctttatttgtaaaattcagcggtgaaaggtactagttgttc
gtttcgtcaatgtaaacaaaagaaaatacagtcgattgggaactttagaaaaggaagacaaattataaaaatcattcaaataaataaaaggtaa
tcttaattaagagcaatcatttactccatgctgatttattcaccttggtatttttttagtaaataaaaaaggtattggcagaacagcccttattcctcag
caataataaactttctttattctttgaagtaaaacaagttctcttttataattttgttcgtaattaaaggtcgatccaggatcattgtacagcttgtagt
aaataaagaataacatttaattacgttttctgtatcctttgttttaggtaaaaattatttttttagtaatataaatttattcttttactacagatgctttat
ttcttgtccccaagattcaaatgtccttagtcgcatccaccgttcgtatcatatcgtacacctctggggaaggtaattaacgcagacaacacttattc
aacctcattcaattattgtttatattccatttttttaaccaaaaaagtataaaaagtttatagagataagaaggagtccatgtttatgtgcgagttttg
atatatgaacgatcaacctctctagtgattatggaggaacgttcatcaaactcagaagagactgatactaaattccttcaccttttagcagatgatt
ttgctggactttctttaactgacgaagaagttgctattttacgctcccaccttccctacagaaaggactcacatacctctaatagtgagcacagtca
attggagtcagattataatgcacagaggaaggaggaaaaaggaatagaaaataccacttccgatgagaatgccatacccaaatccacactgtt
agaaaaatcgacacttcctgaacctttcaaccaaggggaagaagatgaatcgagtacacaggaatatcatagagaattcgatcgatttttatcct
ctccttcatcagaaaatgcttcggctactccgagaaaacagcttcctaacaattcacctttaactcctttatccttgatatctagagggatttctttga
atagcgatgatgaaactgatacggacgaacaactttccccagtgcttactgcaaagaaaacttacggggacctgaattatccatcgactttgtacc
actctgatgatggatcttccaatgaatcattgcatcgtgaacagataatcttaagcgatgaattggaagatgcaaatgatgaaaaccccgggtat
cttactcccataaaggaagaaggttcctccgcctattcatcgaaaataccggatactactgtgttgagcagaaagatctctgatatatccatccctg
catctgcaatgaaagaattagaagaaaaaaaaggccttttaagcaaggaaattcgtgatgctccgccaggagcgtctttgacgttaaaagaaca
agccaacgttatagacaatcttcgtaaagaagcatttggactcaagcttaagtgtttttttcttgtatgaccaactaaacaaattccatgactctgag
gtccaagacattatgaagcaaaatatagatttgaaaacgcttacgatgcaactgcaacgtgcagtagccacttatgaaaaaaagatatcctctta
tgaaaagaaaccctccactgctgcttctgatacactgcagtcgaatctgcttgatttacagaaaaaatattcaaaaacttattctgaattagagtct
acgaaaaaggcattgactaaagcagaaaaagagcgacggaagcaaccaaattctgtttatgatccatatcatgggaatgagaaaagcactcttt
cttctttactagatagcgagcgccgtgaaaaagaagcattgttacaagaagtagactctttacgtagtttgcttgcccaaaaaagtcagattaaac
ctcggacacctgatgaaagggttatacagagtttacagcgtactaatgatcttttacggaaggatcttggtgcacagaatgaagcttttctatttca
aatgcaagagaaagacaagcttatgcaggaaatagaagaactaaagggtaatttagatgccgcaaaattgaatgcagtcgctgaagctgaagc
aagtaaaaatgaaatatgggatttaatgatggggtgcaagatgaaaacacaagagcaagccgttgagcttagtcgactttataaacagctcgaa
gaaattgaatatgattgcgaaaataaacttgtcaaaatggaacagcaatggaaagatgatgttgatcaattacagcaatatgtagaggagctta
ctcaggacctacaaactgcaaaaggcgagctaaccagatccgttagtgactctcgtgcatatgaacaagcgctcaatgatttacgaagggaaac

tgaaacagaacttaatcaatttgacaaaacaatacgtgaaaatgaagaaagtattggtctgttcaaagaagagattgaaaaattgactgacgag
ctttctcatctttctgaaagttaccgtgaaaagtccgatgagttgaaggattctgaaattaaaattgctgatttccgacaaaagttagaagactcta
agatgcagtcctcagaagtggatgcactgaacatccaattggagaattccaacaagttatggcaatcagcaactgaagaaagaaattcattaaa
agaaaaattagacatcatgaatgctgaaatcgagggcatgagaagtcataaattagagtcaagtgaaagggaaagagaactgcaaaagcaac
ttgaggaattaattatagaaattgatcagttgaagcactcacacgaagatgaaagtaaagagatttatgttttgtctactcagttggatgaaaaga
aaagtgaattgagtgaagttgcccaagaaaaggcgactattatgaaaaaattggaagcactagagaaccgatatacagttctacaaaacagttt
tacgtcactgcaaactgctatcctggaaacctttgacaagaaggtggaacattgttctgctgagttactagtacgtcaagtgaaaaaattaaagg
atgaaagtaaacgattaaattctgagatagagaagtctactcgacagttcagcaagaatgagcaaattataagagaaagggaaacttctatgga
gactcttcaatcagaaaagaaagagctaaaagccttacttgattcggaacgtcgctcaaagaaagtaatgcaatctgaactcgacgctttgaact
tgcagaacactcgccgtagtctcagtagctcttcaagtccctcggagcgatcgcaatctagagaattcaaattgctacaaacttctgagaaacgtt
tgaaagaacaaattgatgagagaaataaactaataaagactgtcattagccgtttagctcaactatcctcgtctagtgcaataagtggaaataat
aaacctgattctttaactacattttctagtatgaatcaggcaatgcatggccaactgaaggaattagaaaaatctactcaaggatttaagaagaa
gtgtaaagctatggaacgagacttcaagattgagattaacaaacttgatagtaatttagaggcccgaagcaaaaagcttagtcaattggaagaa
cgtttcagacttatgaatgtctcaactttaggatctcgacctgcttctcctatgtcttcaaaaaaattaattgctgcagcagaatcaccagatgacaa
gaaacctccgcctgatagatcagcggtacaacgtggtatcaccgcattgaaacgcgacgctgaagggatgagccatatttggcaattgagattgc
gcgaattggagtttcaactcaaagctgagcaagaaggtcggaaacatgataaactaggagctcgggagcgccttcaagatttagtcaagcaaa
atcgaagcctctcccagcgaatcacgttggataaaaacttgcaaaattctgtttcttcgcatgaagccgatagctaacagtccaagacattcattct
tttattgtttatggtttattttctctcttttcgttctctttcaaatttttgtatatttagattttataatattatggaatgggcttaacgtgcttacttcattc
agttagttaatggacaaaataagcgagtttgcgagtatttttaatatctacgaatatagtttaattaatgcgttcacggagctactttaatgcgtac
tagtccttaatggtcatcttagaacattaatcataagttttggggctgtgcccaataattctgagggttttactcatttttaaaacaagatgtttgttat
gtttaccttaacgtagatgtaatgacatttcataaagtttgtttgtttatattctgatgattgataaatctcaattaactgaagaaataatgcttcagc
ttcattgcaattatgagaaaaggtaaaaaagaagccgataattatgatattgatgactctaaatcaggacatttaaagtttccaatgttgaatagc
tacgacaaagagcttaaagatccatcttctaaccagttgagagaagttaacaaaaataaagtaataaagaagaaaaggagagaatatcaaaat
gagaatcaaggaaatcctttcgtcgtgtcgaatacaggagacgacgatcaagaaaatgctcttcctagtttgtcatctttaacccatgataaggct
aatgtagccgatgaaaggaacaaagcagtttccaaacggacagtacgattaggaactatacgttatcaactgctacgacctagtcaagttgaaa
tattgggtcgaccggaggttgcacgaaaaagagttgcctgccaactctatttccttaactattatatcaacttcatagaatattacaaattacggag
agagcgtttaaatcagtttactgaatgcactcaagatttgaaaacctcgaaacaaaagaaattatggaaagagcaccgcggacgagaaagagc
ctttttaagaaaaagaagagcaaaaatccaaagcaaccatttcgatctactcgtaaaacttggacaaggcggttatggatccgtttggctagcta
agaagagagatacccatgaatttgtagcattgaaaatgatgaggaaagcagttttacaacaaatggatgaagtaaggaattttctaaattcagat
taatttctaacctgaaataggtgcggcacatcttgactgagagggacattttgaccaacacaaattctgaatggctagtcaaactattctacgcatt
ccaagataaaacttctgtttacttagcgatggaatacgtaccaggaggggattttcgaacgtacttggctatgcatgggttacttcgtgaaaagca
aatccgattttatctagcagagatgtttatggcagtcaatgaagtccataaattgggctatacacacagagatttgaagcctgagaactttttgatt
gacaagagaggacatctcaagctaggtgatttcggactcagtacaggacttgtgagtagtgtacaaatccgaaagctcagaaaaaaccttgcta
gcgcagagacaccacaaaatgcttacttaacccagaggcagagaaggaatatatacaaggccctcatagagaggaatgggtgtcgggtatgaa
ataatgctactattatggtctaacaaggttttaggcaaattccatagtcggttctcctgaatatatggctccagaagtaatatatggcaaaggttat
gacaagactgtggattattggtctttggggtgcatttttatgaatgcgctgtaggttatcctccattttccggggcgacaacacaagagacatgga
ctaatctttatttttggaaagatgttttgcaaagaccggaaaagataaggaacggccagtacgacaaaacagcagttcgcatttctgacaatgctt
ggtcgttcattctaaagtgcttaactgagccggaaatgcggtttcagggtatgtcagaagttcaaaagcatccttatttttcaaaaattcactggaa
tcatttgagaactcatggaagaccaccgtttgtgcctcatttgagtaaccagctagacacctcgcactttgatgatttttcgaacgaagctgttatgg
aagcgtataaagatgtttatgataaacaaagaattatggaggaaaaattgcgtaagcgacaggcttgcatcaaccagcgtccctttcaagggttt
acatataagcatcgtacgaatcatctgaagcaacccaagccggaagagggtaacgactggaagaaaaagggcgagaatgtttctagaaaaaa
gaaaacgaaggattgcgtttacaggaatgaattggctcttgcgtccgattcatcagaaaccgttcccttatataaacacttgaaagccgagaaaa
agggaaataatgaaaaaaataaactgcatcctaatgactatttaagaagaaaagaaaaggattactatgaagtcttattttgattagttttcaaat
ccttttctaattatgggcactctttccagagtgacaaaaaaaaacatcttcaaagcttagaaggatatagcttataaagagaaatactatcacact
ctttttctagtcttttccctctattcctgtattcgacaagtggcatgcgtttacagttttcgttgcaatccataccttaccgaagcttaggcattataaa
gttagcgaatttgtaacaaatccccatagttaacaacttaaagtcatggggtcatccagaaaaagagcattatctgtttgttaatttagataaccg
attggcacaatagcaatgatttttatcaggcatatactttgtagaaaagaaaactcgtagagtaacttatttattgaacttgttgatgcaaatcatg
attctctcattcttttccgtatgatttagggaaataatatgagcataggcaattttgtactttttagtttccatcgtcagcaaggttaaaaaggatttta

actttttgataatacaaacaaacattgaatataaaatttgatttaaactttttcaaagtttgatttgttgtgcgattttggttcatacttttgcatagct
gaattgtttctaggcagatttgacatgggtaaaccgaatatccaagatccgaaaataaatccattaggttcccctctgaaattaccgaattgttga
gaatagaaataaaaaacagagtgaagatcaagaagcccatttgttgtcgttgcgaagtttgtacctttcgaagcaaaggcacatgaaattcaag
accatcatgcttgcagaagagcagtacaatactcaaaaggaaaaccaaacaacgtaaaaaaaatcataaaaagacacctttagttttcaaatc
agacataagtatcataataacaaggggttgaatatgatatcaaacgaaggcaggtgtgcttgtagtcaacttggagtatgacacaaaattaaca
agctttttgaaaggaattcattaattcattaattaaagaaaacaatcaaaatcaaaaaatgttttctttgaattattataatagctatgtatctcgtt
cagataacattcagattttatggtataaggaattgttgaatacttgggttttaaaaggaacaaagaacaggtaataaaagtaagaacaaggtttc
tctttgcatgtctcttttgtcttccatttccagcattcaaactgtaagagtcaattcatcttgtcactgttgacaaggatatcgtagaacaacaaataa
tatgatacaatgccctctttcgtctgcatcaacggggtaaaccatgcgaccaagacgtaaaagtaaaaggtcactatgaatttaacaagattatc
gtacaaataaaactgtacgatattactttcacctcctttgtgtaatcatattttctcgttagccaaattttgtaatactgggtcaccttttttatctaata
cgaggtagagggacagaggaatatagtaaggaggagtaaacgtttaagacagataaacaagatacgtaggaaaacttatactggcattggca
atcatcaaagtaagttcaaatggaaattgttgataatgtgggtgagacggatgttgaggagcctagctgattgttaggcgagaggcgaatggaa
gggagacttgcaaacaaagaaaattagcatttctatagttctaaagtggagtggagaagataatgaagaaagagaaaagatatgttttgaaga
gaaacaattgtcctcaatcttgaaagaaaagcagaggttttctactcaaagacgtaaaaagataagaaattgtatgcttattgacaacatagaac
tactagatcgaagcaaagcaaatccgtcgctattggttgtctgcgaggaaagaaatcagaaacaccttatcgtatcacgtttatcacaaaaaccg
ccccatagcaatatcaaaagaatgtatcgagggaatgaaccatgtcattaattgtgcaaaaacaatggccttttggaaaggaaatgtaatggaa
cagaaattgcaatcaatccgtaaatgattagcaatggattatcgcaatataaacatgctcacagctcatcatcatcatcgtcatccttcccatacat
taaactcagcggaaaatgttaggataaacaactctctcttttgggaaagtttggaattttgtagatagccttatccaaaccatagttttataaatat
aaaagaaggaagcgatcatgacatggagactttcagacactctttcagacaacgtctcatcaacaaaacaccaaaagaagaattccatcatga
agtaagaagaaaacgaaacaagaaaaaaaataaattgaaggttcccactagacaatgattcctaagtgtgctatgagcgcttgacgatttccat
tccatatatttaaaaggaaaaccgaaaaattaaaaactagctcatgtctttttctattcttctcttttttacctgttctcattcatcttggttttgtgttcct
cttgcactactttatgctacctttatttacccattcaagtttaccctttcagaaaacgcaatctaatttactttggcactccatcccaaggcagtcgcg
aaaaaaaccatcatcaaacccaattggtctctttctattcatcttccaatctctttgaaagcaataaatcaaatccactagactggactagactaac
taaactatatatatgctactaactgctactatcccaaaggataatttaacgaggcaaagatccgtttcactcaaaagccaaaacctcatttaccac
attacggaaaagtgcgttatactagattttgtcattcttttgaaaagtttgttggcataatatacatccttcgtctcccatttatccatcttgcttggttt
gtttaccttttttcgaatccaatatgcgtcgttctaccattgtaccgaccgtttcgaatactacaggaaacgttccttctgcaaattcacataataat
tttgctccttctcctaatcaagtgaaaaccattcaaatgaatcgtcttgctgaccagctttctaaattgcaggccaatttgtcccatctagaaaatca
tgttcatgtcacggcaattcaagctgaagccatacgacgcatgggtgctctgcaagctgctttgtttatggcttctggccgtgtcatgtctgaggcca
gtatgaattcggttcctccttcttcggcctcttcctctcatccctctccttcgtccttcaaccatcaagatccaatgtgaacggataattagttgccttg
attaatcattgcttatggtactccttctcgctatttgttcaagcttctttcctctagatcccttcttgtactactttttttcgcttcactattcttctttaagct
tcattataccttttaccacatcccgtgttccacctctttaatcattgtttccattgtacgttacgattcaacagcacagagtgagtgggcttgaccttat
cttaacgcagaacttttttttttatctccctttccttaaaacaattgtttgtaaccccccctgcataacgttgcaactatcagttcgatgatgaaagtat
gaatcacgagtggccaataatcattcgcacaaaaacgattaccagcgatcggatcatttcccccagccaatttttttcaatagagattacgcctcgc
ttcggttgctgtgcattaaagcatataacgacatttaactaaactccttcgcttcgttgtcagctttgtgaactatagtccttctaccactgccaaaac
caacaagctcagccttgtcgagagtggagcctcaaattcaagtaatttatattggtttctatccttttcgttaattctggtatcttaattgataattttt
ctccatcttggttcattggatcgagttttgtactcaggcgatttcaaaaagtatcgacgcgttgaagcttgccaccgacccgtgtatgttctctggac
cttttgggaaattaaattaaattaaacttgtttctttttctctttacagtttttctttggaggttgaagaaccgctcaataattagaattgcctttgtcta
ctcaattgttcaaagctgtgtttgttttccttttcttttcgttaatcgctaccacatactatttatcatcttccttctgttttctttttttaattcaattttctct
ggacaactttacggctgttcggtctgtcataatccgtgaatcgactagattacagtttatgaggattcatcctctttgtatctatattctctttttccttc
aattttaatctaataattgacatctcctttccgttttaccattgtctttgatttttaaattttgctgttatcccttttttgagtatactttgaaagccaattct
ctatacggaaccttttttatccttgcttttttttgggctgcttttctctcagatttctttttctgtttccttttgaatcgtttctttgctttgtgtggtaaaatat
ataacctttatacacctcgtggttgaaagctagcatatctaaaccctattaaccgttttgcttccatttgttttctttctttttttattacgttggtttaac
tcaagtttattatataatctctatggatctgaaattcaatgaaatcttcaaagagtctgaccatccagctcttaaggagagactcgactctgctacct
caaaaccaaacactttctcgcaaaaccctaccgatatggcctctgatctctggaaaatgatgtccaaggccaaatcagatatgcaaaatggacgt
cgcgtcgaaaacttatcatggcgtcttatgtctattaatcttcaaaagagctctaatttttccagtagtactttatcccccaacgttcctacatacaat
aagctttcctctagtcccctgactcctcactccaaccctcatagttccgataatgtatccttaacctctggtacccctgaccagaaatccgaaaccaa
ttgctttatgaatacccttccttcctacagtgttccaactgataccacgggcagctccatgatggaacttaattatcttcaaagacgtgagcggaaa
actagtttcaatgaagcctctgcaaaaagcaagaagcgtcccattactagcactcattttcccgctaacgatgtagatttctctatggatcttgactc

ccatcctttcagctcccctaaaattcatgcttttgattcttttaatgccgctaaaaagactgttggtagtgctaatgcttcttcttttccggcttcagtgc
aatccgattattttgacaatcagactccatcctttgcttcgggccttcaatctgtgcccatcccttcggcctctggtatggtaaatccggcaaaccga
catatggattctacctctgctgccgtttctaggaagtctgatttggatgttttaggtttggaattcgacatgactccttcagagccatcctcatcttttc
aagataacagtggattcccatcttttgttgatgctggaacgaacaacgacgacacgttattttcttcctctgcggctacttcttttagttttgaacatg
gaccgtccagtttcccagttcctggcagcatatcaacaagctccttgcgtggtcatgcaccttctgaagagggttatggcaattcttataacagcca
aagtgcttttggtatttcttcccctttaagcggcggcgtgactccgactcaatctttcttccctgaacctgctaataataataatgtttttgagataccc
aaacaagcttctgatatgtcttcccctttgacccccctcttcaggatcttttcctactatgtcctctttgcatatgacgagttcattgcctaattctgcttc
tcatgctggtattcgccgccctcaaatgttccgtaatgcatccactaggtcgattggaaatatcgctcacccagttgacttaaaccaagatcaaaat
gaatctcatggcggatctttccaacgtagtaatgagtggaccggtaatgagtcaaatggtataggctcgaatagttccttgccaggttccgatatgt
tttctccaccatttatgcgcgttggtactgccatgggtgctgctcctgttcgtaacaattcggtttcttctttcccccataattatttacatcagtcaagt
ccgcaatttgctgccgtaccccatcggaaagttaattctcaagagcctagtctaatgggatcaagtcctggtatatacaatcacatgccttatctca
accgctctacgtcttcgacgtctgtcgccggagcgtcagtggagggcatgccaactggtatgaaaaagcgtgctactgctaatgcttctgttcccac
tcttcctctgctaaatcaaaatggcgatcaaatttccagtccaagcataaaggaaaagtcagtagaagataaaaaggaaaataatcaaaaagg
agtaactggtacacctacatgtacgaattgtcaaactcatactactcctttatggcgtcgcagtcctgatggtcagccattatgtaatgcatgtggc
ctgtttatgaaaattaatggtgtagttcgtcctttgagcttgaagactgatgttataaagaagaggaatcgtggagctggaagcaatgctagtgct
cctgcttcgcgatctgcgagtacccgaaaatcggcaccccgaaaaggcagttttaaatcatcaacctctaacaaagttaacccgaaagaagaat
caaagactagagcaactagtattcaaaacttttcgcccagttttacgcaacaacctgaaaatcatgacgatggtttgatggtggacacaaagtcg
tcagtagagaacccagctgtgaaagatccagctttgggaccaccttcaagtatgttatctcctgagtttgtgcaaaattctagtgatttcggactctc
gtttaacgaggatatttcttctacaggtcatatgcgtgaatcctcggtgggaaatatcaagtcggaatcttccaaggctgtaagttctccgttgtttg
atacgttcgatactgatttaggaatgccttcaattagtgagccacaaacgattgaagttgactcttctgacttgaacagggtttccaagtcatcatg
ggattggtatagtgtcatgtaagttgtgcgagaaagtaatttcgccgatattttgtttgacgtatgccttttcttaattttgtattatgtacagtcaaac
gttcttttaatttcttatcggtcttgtctttagatggacttaatttgatttcatcgttcccgatgaatttttattgtacatgtttagcatatttaatcactac
ctttttaattgctgagttgttttgactaagtttaattgctgcgccaaacttatttttgaagcagtccggcgacgacgaaatttgagaaatatgtttgag
taatggagcgtgttaatccatttaacatagaggtccgagatttctgcaagctagaatagacctgttttattttgttttcccttctttgtttaatgctttgt
tttagctgtgaaggctttgtaatttgtgacggactgacaattattttctagttcgaataaaaaaatgaatacatatatcaagtttagtttgcttttttcc
cttataatttgttgttggtagctcaggaagaaagctcgtttttcagtaatctgtgagatatgtaacttttgacgaagtcaaaacaaaccggtcgaat
aaagaaaagtgcatcagttgattatatattaaaattacaaattaactatttataaaggagtattttcaatgagtgtgaattcaattggagagagaa
agagagaggagggaggttaaaaagaacatgccaagttgtaagcctgatacggttattataaagaaactaagtgaaagctgttagcgaattttta
gttaaaatatttagtatctgaagagaaaaggaaattcacgaagtcagaaacatacaaatataagtataagtaaaaataaattcaactaactttta
tatttaattgaaagttgtggtaacgtactaatagtctttgtcttattcagtccccccttttgctacattcacatacgcacctacctactaccaaacactg
aacacttcataacagagtaaataatttggaagttgagcgatttgtcgctccaagtttaattatactgactttcactctcttttaacacattgtatgaa
gtttttgcagtcaagaaggtagattttgggctgtagttgaatgcttaaaatcaacgattgtaaatactagaaacgtcaatccgaacttcaaaaagc
tttttaattggagatttaaaagaaaaagttattctatttatttttacatacattcattctttcgtaagcattttgtgctatccttctcgttcctttgcatca
acactgaaaaaaataatcgcgtataatgtcaatggattcaaaacttcgggttttaagcggtatgtaaaagatatctttgttaataaaaatgacaac
ggaactatggagtcgctttttactatctgaattacaatatactaaccgaatgctgcagttggtagcaacgcaatttctgcttttataggctggcggtt
atcagaaagtaaggcttgtcatacaagtcttgtttggcgtaatcgaagtgaaagcgtcatgtctgagggcatccgtatccgttccagtgtatttggt
tcaactaaatggaagcctgatgttggtacgtctttataagcgcttatctttgtttgatgcgctagtgccgttgttctaattactaatgtgtttttttaag
tcgtgcctacgattgagcagctcgtaacaaataacgaacctttcgattatgtatttgtgtgcctaaaaataattccttctgtttatgaccttggtactg
ctataaaagatgttgttaccccctggtcatacctgtgttgtattaaatactaccggaattgttggtgtcgaaaaagctttacaagatacgtttccgaat
aattctattatctctttcgttattccggaccaatttgctcaaagaggaccaatgcagtttgagcatactgcattcgctgccgatactgctagcagcgt
catgtatttaggattaactgacgaggaagatgaaattccagactcagttcaggacgctatgattgaaacactaacgttaactctggaagcaggcg
gtgtatcctgttcatttttacctaaaatacaacaaaaacagtgggaaacaggaattggccatatggcctttttttcctatttccattctaaatgacgaa
ccaaatttgtctttaatatataaagccagttcttttgtaaaagtcattgatggtattatgaacgaagctttgtccactgccgtgatgcagggttgtgat
tttcctctggaaaaacaagattcattgaaacgatttatagtaaatcgaatgttagcagctcctcgtccctcatatatatatcaggactatctagctca
tcgccctttggaagtagaggtgttgttaggataccccgttcaaattgccaatcaatataaagtatcagtgccatatatggaaaccatttttgcattgt
ttgaagcgaaagctaaaaagaaccttacttccgctccagctgtccctcatccaatcccagctgtcgcctctagtcgaatatcaccagttggtttaac
ttctcgatctccgtctcgctctaccgtcggtacgtcgactagaatgagtagtgtggatgatctcatgaacaagcgacagtttgcctcttcaccagctt
cgcaagcgccctctactcgatcaatgtataagattccaagtgcatccatggtaaatttgtcttcacccgttgtcacatcatcagcagaccttggagg

aagagcaatgccaacgcgatttacgggaagaaatatgcgtggaagcccgtttaccatgaacaaagctggatcggtgtcagatattctaagtatgt
cggatggattggtccctaatgatcctagtgaaactgcgtcagttgcaggaggggcaccttcttttgatatgctgacattgacgcaacgaaggaatc
gtagaaactcacagatgtcctcatcttccatgggtcttccatcgagcgctccgtcttcgggtgatcgccgttatacttggactggccgtccaccacca
cccaacaaaggtaagtgatagaatagaagtctattattcattcatagtcttgtaatgattctaattactaactcgttcgttgtaggctttactgaacc
cgtcattccaattttagaagacccaatgacggctttgtatgatactagtcgttacccaacgagacctccaggtagtagcgcatcatcaataaatgg
caagtctactcctaaagcatcccgaccccttcgattgcatctacgtcgactactcctcgaagatttggttaacacagattgctcttttgttcgcttct
ttcctaacctaacctatcctaacccttcattattcattgtttaattgttatgatacgttcctcttttactactcaattgctgtactattgtctttcattactt
gctaatgcattttgtttacaatttagtcgtacccttctctttgtttatttacgcttcaagtcacttgttcttttaactgtataagaaaaattgctcttaag
acatgtcattcgattttaaataacgaatacgctgctcatcgttatgtctcattcagtatactcatctttcgtcattgccagtttcaagtttcccagcgca
aatgctctcttcctacgaaattataataatatagtaagaatggacgatttaacatcaacattgttgcttcttgaactgcagcacgagtttagtggcct
aaaagtacctggtaagtaggattttcctgcgcttcaagtcatgtctgtcaatttctttttgaacgatttcgaagtgagctttttttctttggaggaattac
ggttgttttagatattaatgaataagaatgaatcattttgcatttattttctggacaaaatggcagtagaaaggaataatcggccattatactcagtt
cggtgggtagcaacaaggaagaaggctaactgagtatctaggtcgactagcgaagtatggtcctcctttcaagcctaatgaagagttttcggatg
cggaagtctctccggtgatgagttttattgtacaaagcttcatatttacacctacactgccaggtgtcaacaaagctagcaaagagttttggagcg
accagcttcaacccttgctggaaaaaattggtaaacataacctttcggattcgtatgaccatggcagggttagcaaacgaaaaatgattgggtttg
ccgtagtcgttgttttgcacgtacttgctaaggggattttgggaaaggcttctgtggatcctgaagagaatgaaaaaaacgagaatgaagaaaac
gagaatgagaatggggttaattcggctccaccaagttcaaattcgtcccctcaggccaaagttttagtgcctactgaaagcgatgctacagagcc
agtacaaaagactcttgcaaacgtaaagttggaagaagattccaaaaggattgaatatccacaaaagcaaaagttaagagaagcgtactccct
tttcgtttatggtgatggcttagacgaaattatccgccgtcttggcgagtctgaagacattagtagctgggacgaaccccttaaatctttggcaaag
aatgttcgctatacgatagcggctcttttgcattttgtttttgttactgtagacagaatcccccaatcacctcatcttgcaaataatgggaaaggacc
agaaggaatcgttgaacgtatatataaccgcgttccatggtttttgacgcgtcaagttcttcgtgtaggaaatgctggccttttgatgtcaggtctta
ctcgattatttttgatgaaacctctctcatggttcggtgaaagtcgaaatttattgcagacaatgctttctggaatttttggtgctgatttggaacaca
ctgataatgttttgcaagaaatagatacacagtttaaagatgatccggagtgggcacaaatgagagaagcacttcaatgctatgcacagctctcg
cgaactcgtcaagatgaaattcgtgaccaaagtatccaacgcggtttgaatataaccgttgctatcctaaaggaatatcagaatgaaaagaggtc
ttcaaacacggaaaatactagttccacagaggaaaaatttgtttcggaagacaatatcgactcttctgaagtttatacggttcctctgtctagtgat
ggcgaaaaagatactaatacatttgatcctttttcagaagttggggtccaaatggcaatgaagtattttgacgctgttttgactcaccgtgatcgtg
aatgccttatttatgacttatgtgaaaatgatcagttaactgatatagtaagagaagttatgaactgtttttatacgattatttatgatgctcatcaa
gctgcagatttttcgcaggctatttatgatttccagtgctttttgtatgatcttatcaacctttcaaaggctactgctcctttatcgagcttcgttgagct
ggtggagaaatatcaaagttgcttccttcgttttgttcatcgtatagtagttaatgctcctgatctatgtgccgaatggtctgtttggtaccgccattg
cttagaccaatgtaacatcaattctcaatcgccgaatgctgtcaaggccagtcaggacgcactgaattctttggacgatgatataaaacagatggt
tttgcaagaaataaagacttacgtaaacgatttagaagaagaaaagaaaatttctattcaaaataatgattgtgctagtgaatggcattttcattt
atccagatttttggatctgcaatcatcaccccaattaaagctaaaggtgctccgcagccacaactacccaagtgcaatttgaatttaacaatgac
aaaagaacatttattaacccctttccgtaagtgtctcatgcaacagttgaatacattatacgaaaggaagaaaactatgcattctacatactaata
gatatgctctttaaaaggctttttattggtttcttttgatttttatccttaacgctgtttgataaaagtaattagcctttttttttaagcacttattcttg
accccatattcataactgcaaataaaatggattaagcatattactaattaaatggaacgtacatatctaagtttgtaagaaggtcttacctttaaga
tttaaaaataatgaaacttttcatttcgtcagcattaccattattttacagcattccaagttgagaagaactttgcttaaggaggccgaaaatcgtta
cgatgcggtcataaaggaagaattcagtttaaaaacaatagaggtcttgcttcggttcgatttcaacttgatgaaaggaagaaaatggtatgtgt
attaatagcgcttttaacgttgatatgtggattgatacttccttcagaaggattaatccgaattgcaaagcgtccagatcatcgcctttacactgctg
aatatacttcatgcatgcaagattccttagtgactttcgacagatttgatgtgagttactactcacgcaacttgtctatcgttttaaatatcgagggtt
attctaacatagatgcaaatgtaacctttgaaataacggttgatgcctatggtagccaacgtgctagattcacgtttgatccatgtgatatgaactt
aggaaatatttgcccattgacaaaaaattcaaggattggagctcatggtgtccttccagttagccttcaggatgtcaaaatggttccaaatctagct
tggacgattcctgattttgaaggatttatcaattttaggatgtatcgttcagaggatttagaaaactcttctataaataaggcgaatcctctttcatgt
gccatggcatccttaaataatggtcacacgtttcaaaacagagtaattttggcactttcaattgtttcttttttttggccattgccggtttcctcggttt
ttatctgatcggcatggagcacagcatttatatgttacggcctgtaattacaaactccatgccatcaatccaatatataacagaccttttacagtgt
atcgtgcttactgcagctgtaggagtcaaacaaccaaaagttttggtatcatgggctagtaattttgcatggattataggactgattccaaacgga
cagctcgacaatagtatagattctcttcgaaagcattctggtggtaaactaaattcacatcaggacggtatacattgtcgcaccactactgcatcg
aacccggaagattcaatcgtcggtacatattccggactggtagggttagcgaatagacttcatacctgttccacgaatttgtttacactttcgctgat
atggtttgcaatttcaattgggattatcgggggattgtgatcggtttcgtttgtatcatcccgctcttacgaaaatctcaagtaatttgtccctcttct

tgcatgtatgagttgaagtattggaaaagactcctttttaatgcacttatgaaatgggtatgttctttatatatgctaaccggcttctaacatgtatca
taggtttttatttgctttccggtcctgattgtgttagctgttttccaatttacattatcagatgcatatggacccataattctcgcggctacgttgcttgc
cttgtttgcaggactatttgcttggtgtttattcgaggtagccaatgcaatccataccaatcggtttctaacaaaaggttcaaagggaacgatcaat
gacccacatgtatttacatggtggggttggatgtatttgaacttgaaggatcgccattggtatttcttcatagtacgccttgtactagtcgccatttca
ggtctcctattagcatttcttcaaaactatggaaaagtccaatccatcattctcttcgcaattgaggtggttgcaattagtttattaattggacttcgc
ccctactttggaacttgcagcatgatgtgcgcgcttttgattaacggaacacgccttatatctatggttttgttaattcccatggcatttgatataaat
atcatccgtaaagtaataattggaattttgattatcatcatccagggactcgtattttctgtgctaacgatttggggatttttgaatttcttgagtatg
attggaacattatggcctgcaatcaaatatcaatcaaaatggcttggtcaaataattcagcaaaatgaacaagatactcaacctgctatgggtga
tcacaaagtatacgagaaagaagatccagcattcagttcgttcatgctggaaaatgtttcttctgaaagagttaaagaagatcctgagctaaaaa
atggaaatgacctttcaaaagcaacagaagaatcctcaatacgcataccaatgcctatgcatccatcaccaacatcctcatattgtttttcggattt
tagcgaaatccagcgaactccaatagacttttacaaaacaccctaacgtccgttcgattaaattaccgttgcatatcaaagcgcccaaattatttt
tagcaccaaaaaggcatccataaacaaaagacggagaaggaaaaaaaatgattgagttagacactattaatccaaataacctgaaggttttag
aggtaattaatcaaaatacgattccagaaggtgttcgctcgttttctcctcagtattataaagaaacagtgcaagttggatccttagcacaatatgc
gtatttcaacaacgtttgcgtaggagctgttcgatgcaagaaggagactcataataaaagcagtaaagtgcaaattctttctttgggcgtgttgcc
agcataccgcaagtatggtgcgggaactaaacttttggagcatgccgttaataccgctaaggatattggatcgaaggagatttacgttcaattacc
tcagaagttgaatgctaaagattggtttttacgccgaggattcacagaattgagtgagtccagcaacgaagacacagccgttcttaaccatcctct
gtactgaagcagtttttctttcccatcaaaggaaagaagaacaaaataaaatataaaaggagaagaaattaactttcttatgtcttttggtttcacc
aactcttgttttctgtccttgtcaagttcgaatgtaataatataccgataaaccaaaaccgagtggtatttcgccggtaatcttcatttcaaaaacag
gaaacaaaaaagaactacaatttctgggatctgagtgatttcattccttcctacctttgtcagcaaggaatagaccatgcaaattttctgcacgaa
agcgaatgctaataaataaaatgtatttgtaacataaaacatggacaattgttatcatcccaactcattataaattcatccggaccgcttcatcttc
aatagaatcaatcgacttaataagtattaattccatcaaaattcctcatcgtcatcgcaatactgagagaccagggatggaggatgcattgcattg
tttgtttttcatacagtttgagttcagcttctgtaatagcaaaagtattcaaccaatccttatcagatagaagttcattgtatgaagctaaaaaagc
agcttgaatcatggttatctgaaaggattgcttaccaatgtcgttatccttatccaatggatcttctatggataacaaagttggtttagcttcatcgta
ccatcctttttgtaactttggcacgatctgtccagtcaatggacgcaaaacgaaaagctcatagcaccattcagcgccccaaaattgaaaaaaag
attgtaatagagatcgtaaaggtcgctctttcgatgatatatcctttttgtggtatcgcatttcaagccacccgattagcatgtaagagagtgcaca
gctgctaattccaccaagatgaacacaatcgagatctctttcataagtccaatgttttaacaacatcagtaatgttccatactcggggtttttcctac
aataggtggactgtaattcagcactaaggcaggcgttttcatttgcaaatgtcaagtctatggcaataccgctcacatcgtgtaagtagacgcgtg
gaactcgtgcactaaaatggaactctgtattaggatccatatatgtacgcaaaaacatcgtaacacgtcgtttgtccatttcgccaacagcgggac
tctttaaagacaagtcgatatcactaatgtttaaagaaagtccagtatataagcttccatattcttgcagttctgggttcgaaagaactctctgcaa
atagtcctgaagcttctgtacgatttgctttcgatactctaactctgtgggtttcggtcgtataaagtccaaaaaaagacagtattcgctcattatcatt
caacccttctttttcccatggaactggttgtaggtttaacaacaaacgacccattcctttaggaatgaatctttttgcttagacgtgatattgcacccg
agttttgttttaggtgacgacagtgtaagagtgagattgaatgtaaacagaggatgtaaaaatacaccagaaagaaaaaaaaaaaacctaagt
attgctttcaagttaaatagtaaagtatgctaccaggtctccgaaggactgtcgtacagctcgactgagagagctcggtcatttaaaagttaatga
agaataatgtcaggcagaacagattttgaaaatgacttatgaaatgaaatcttaagattattgttaagaactagtagctttattcaatagcctcag
tatagatacggagacaagtctatcataattagatgtgttttattcttttcaggtttatcgtcatatttcaataagaatagtgctcattgaacgaaaaa
gagcttacattcagaatcagcaaatcgccattatacatatcgtttgctcatgaaaaccgaagttgtagacgagggcttagggtcataaagctccac
caagttcttgataaaacttactcatttcgttgtctggatcttgatttttgtttcttttcagcatgattttgagcgactgccgattcatcttccctagacttg
cgcttttgactgcgcacagctgcggggagaaactgagtagcttcctttcgaagatcgcgaacaattggctgagcagaatactctgtaaccacatct
tctatttgagcctcagctttatgggaatcttctgcttctgcggtattcgatggttcattaattggggttgccgctgtcgatgggggttcatctcgccgtc
ttccaatgttacgtaccttgttcttatccttcttcttcttttctacaaaagggtattctccttctggcataggaatatcaattacggattcatctgtctctg
agctttcttcttctttctcgcgatatggcattcccggaggaggtacgccatacggattaaacactggatcataataagcacttttctttggatttttgg
gaataaatggacgtctaggcttgaactttcgctcttcttcctttgtttttcagactcgatcctacccaccgaatgaccatgtatatttttctttcgtatg
accgccaaatccttttgtaagttttgtaaaatttgcttatcatgagcattaagaccttgggatccttctaacgcagtcaattccgatatttgacgctcc
agttgtccgacatttctatgtgatagtttttcttcatagcgcctggctcgttcttcacgcagcttgttttgctccctttttcttcttctttttgtttctgcttg
gaccggattatacccgcgtttagacaacatttttctgtttttgaagagttgcctttttccaggtaatggtttatcaccacaccaacgttcacatcatca
gaacttctgttgtcgagtacatgataaaacttgatttttaaagctctttatattgaagtatatattggctacatgatatattttaggaatttacattcct
tgttccctcgatattgttgcacagcttttcaaaatttacataagtgttagacccacatcagaacaatttacactagaagtaaaataattcacagtcttt
acaggatatatagtgttaatccttaagtaaaaggattttggacaatggtacaatacatcttgcatataaatattcaaaaatatcaaacgttcttta

tttaagtttttaccccaattataaacatcagtaaatatacagaggttgtattaatagcaactataaacaaatggatttacatattaatagatgtagc
catattaacgaaccaagttcttcattaaaagtctcaggttggcagatacaatggcggttcctctaccaccaaactaaatgaccaaaattcatagaa
cggccagtaaataaacactagctcacgaactatatcggcctcagagagtggtggagttgctaaagtcaatttcctcacaacagaccgttttgtaat
ttttttaaaaacaagtcacactttaagcgaaccttatcccagttttacgttatcttatcttggctcgaatgcgatcttcagaatctatatgtacatgtta
cgttctctgggttcaatgagaccttgaatttgacgttgctacttgcatagcatgttgtaaaaatcctgaggctccatggttttcgtctattaatttttg
agaacttcagtaggaaattctgtatcgttggatgaagcattttgttttgaagatcgtccaatcaaggcataatttcgagtactgggaattcgtaaag
gttcaatttcgatattccagtcaaaccgttcggaaatttggcggatccattcaatgtacatagcataacggcccccgtgcttcttgtctcgaggtttc
ggaagggaccaagatatctttgcgcctgtcaaatcatgaacgcagcacggtattgacatgaaggaagaatttttatttaaacgggccaatatcgg
aatgtagggtgtcaattcatccgcatgattaccgattataaatgaattcacagggaaccttccatcatggattgtcaaactttcgacgggtataaat
ggaagtggttggtcctcaatttctttaagaaaaaatggtactagtactttttgagacagattttcttgaacgtaatcaggatatgttttccacgatcg
tctttcacgagcgtcaaacccatagccaagatagccttccgaaagaagaatatgcactaaaagcccatttccgcacccaacgtcaacaaaagta
aatgatttctttgatgaatacatctgcttccagagctctatcaaaaaagctgcaatgcccaaatcttcaaaaacgtgcttcccaggatccgttttctc
aacccatccttctatcagtttttttgagtatttcctctttagttctacataagtatcctgaaaattattcctttcaactacagtgtcatgcagcatccgtt
tttcataaccttccacagaacccttgcaatgtttgtgtaaaaccatcaagagcatattagctgttctttgtagtctcgttgaaatttctttttgatgtat
tgcattcaataaataggcaacacgaagtttggtagaggtgtattccagggcaactcctgcgactgctggatgataatagggtaagtgattatacg
gttcctccaaagatagatcaagcttcggtaagtacagtagaatcctcttgtgatcattgcttcccttcaataaaatagtatcttgctccatacaagcg
tctaattgttgatttcgagggataattcgacgaactatactacgatagacttggtaactgtcacttttaataacatccttggattctttgcatagactc
gaatcattctgtgagtctcgtaaaatgtccgtccgcataatcacgctactggtcctttcaggatggtacacgagttccatgaccatcttgtcaaaga
tgctagcttcgtaacaaacatcatgttccaacacatatctccattgataagaatccagagaagggattataggatagtctgtaacaacagtattgt
taagctctgtcggcttccagaacgccggaaccttcatgccaatgctctaaaccaaagacgcgaattgtcttattatattaaaaaaataatatatgg
agatctcaaaaagaaacaaagcatacaagttgtattttatctcaaaaaaaaagaggatctgcaaaaaaaatgagaaatatgaagaatttttca
gtgtttacatgaagtaacattgtaccttgatatttgaactgtaagttgtttgacggcataaacgatacacttagagactttatatataatttttgaca
gcatattcatttgctttttgaagttttgtatccagtgctgaaaatgtgaccaaaaaaggttgttgcagtcaatgttttcttgtgggtgtaagcaaaac
tgtctacctgaagaacagagtagcagagtgcagattcagtgcagatttagaggagcatgtacgatataattataattgacacagttgagggtaaa
agaacagaaagcagagtcataaagtgagggttatcacaacgcagaagctgagcagagcatcttgtggtactttaccgttctttcatgaaatccgc
aagaacacttctttattgtcatcagttctcaataggcagcattcgcccatactttttagcaacaaaaagtaaactcccaaggcgagactcgaac
tcgcaaccttctgataacctgcttaagagtcagacgctctaaaccgattgagctacccgggactttgctagtccgcaggtttcattaaatatgatat
gtgtaatgaattttatatcagctagaaatacagctgtgtaaattcaaaacaaaaagttgggctgttctgggaatcgaacccaggaccaccatcac
cctaagatggtatcataccactagaccaaacggccgaattgttatacagttgaaaaaaaatatcaatatccgtaacattcatcagaaccaaaata
tgcaaacgatgcccagtttacaagtctttcacaaaggtatggagaaatgtcgttgtccgcacttatttaaaggtaagagctgtttcaaatcatgac
agatggtctcaatattccaagaggagttggaatgttgtacgctccagtagattaggatagtttagcttcaacagaactgatttaatatcgacaaga
tattttcaaattatctatttcacagttttatcttttgtctctcggtttcttcaaaaggaattgataggattgagagcgctgttttacctttcacgatggaa
aatgttttaattcacaaacaagagaaatgctattagaataagggattatgaatgaaatcgttgaacaaaaagtaagaaagaccagatttggacc
ataaactcaagaaataattcgtaaaaagagtgccttgggcttaagcgacaaattcaagagttatcttatattccttatgaggcttcttctggctttg
gccggttgagttttagtaattctgcttttatatcatcggaaatagctttggaatgctttaacttcatgccatagctaaacaatgggccttcaggcgtat
gaatggaaaaagagggagctttctcatttgataacttgtggtcaacgttaaacttgatatgctcgttgtggtatttcaagcttgggagatactccttt
acaaattttctagtatctagttagtaaatgacagcaacgtagaagagatggagattccatcgaaacactgtccacagcaagaagcattcggaga
caaattttgaacttatttcaagctcaatgaaatcaaatttccaatctccctcgcttttcaggaaacatacctagcccccatatgaccatttttactatt
tagagaatactggacacgcacctcgacaacatttttttggtaaaggtagagcaagttgtcccgcgctaacgttttccaaaatggccttgatttttgtg
aaggatttaggcattgattactgtaatgctttaataacttctattcaatcgaaaggtgatgcaaattccaactcgtctggattttggtgaattcaagg
aaagacgcgttttgtttgcgttctcgttcaggaaataaattaatacaggtagcattgatactttttaaggaagttttacgcactgcgctctgggtcac
taaatgatttgtttagactgtagtatgcttcgtgtcaagttggccttgaccagacattccaataaaaaatgaatgcagaaacgcatacaacaattc
gaaatataagtaaaaactcagtggattttgttctcacaaacactgatttatcgtaagtaaagaagcgggagggaaagtacgttaacaagtgcca
gaattgcaaattctttgcgacgcgtcgttctcgcagagattcctactgttggtatgagataaaaatagatctagataagaacaatttttaacagaa
aaagcaattgatttggttgaaatcaacgtaaatacgtctgttatgcctgatgaatttttagcacatcgccttggtatgattcctttgaatagtaccaa
catcgatgaacctccaccagtcggattggaatacacaagaaactgtgattgcgatcagtactgtgctaaatgttctgttgaattgaacttgaatgc
caagtgccttggtgaaggtactatggaggtatatgcaagagatctcgttgtgtcttcaaactcaacacttggtcaacctattctcgcagatcctaaa
gctagaggacccttaatttctaagcttcgcaaggagcaagaaatttctcttcgatgcattgcgaaaaagggtattgctaaagaacatgctaagtg

gtcaccaactgccgctgttgcctttgaatatgacccctggaataaattacggcatacagattactggtttgaaaatgacgctgacgctgaatggcc
taagagtaaaaatgcagattgggaagaacctcctcgtgaaggagaagcaatcaattaccaagaagaaccgagacggttttacatggaagttga
gggcgttggctcaatccctcccaacgaaattatggttcaaggtttgcgggttttacaagagaagcttgcggttttggttcgtgatttagatgaagaa
caacctgctcaaatgactgctaatgagatccccatggatgagaatccggaaatgaattggtctccttatcaaaatggaggagatgctgcttggta
gttttacgataagagaaggcacacctcaattatctcatttctacactaacgtttgtagaaatttcagatgcaggacttgtattattatttttgacttttt
ataaaatgtttaataaaaagcttggacgctttaactcatttaatatttccctctttttttagcctttgttactccttagactaacactttttttggtttcctt
ctttggaaatttattagatagcaattcacatggataccctattttcgtcttatctaaattcgaatatatatcggtcttgctgttgttcattcatatacttt
atgggttgagtgggatacataataaacacatggcgcaccatacagcatgctttacattgttcctgctttttacagacggtaacatttctacatattct
ctgtgtttgtacttgaattacatttgaaaatagaatctatgctattgaaatttaaaatgaacttcgaacatgtacttttatcaactctatattattattg
cattgaaacttcctaacactgagctttttttatttttataacgtactcattctttacctcattgctagtatttactcattttgcgacttcgcgtaaagttttt
ctgcttcgtcaattgctgtaacgttagacctgagtttattgatagtctcaatatactcagcttgctcatcagaatcaaacacgatgccgccgccggct
tgcaaatgtaaaactccatccttgtataccatggtacgaatagcaatgcaagtgtccatttgatcctctttgtatccccaatgaccaacggcaccag
catatataccacgcttttccttttctaagccagcaactagttgaatcgcacgcactttgggagatccactaacagttcctgcagggaatatactacg
gaaggcatcaaatctggtttttccttctctgagcacgccagaaacctgagaaaccaagtgctgaacatgagaaaacttctcaattgtcatcaatcg
atcaacatgagtagtatccaaattacatacgcggtttacatcgtttcttgcaagatctacaagcataacatgttctgcacgatctttaacactggaa
agcaactcatttgcaagagcttcatcttgttcttgagtctttcctcgaggaacagtaccggcaataggatggttgataatgcggccatcttccgattt
gaccaagagctcaggagaggcaccaataatatcaaactcatcacaattaagataaaacatataaggagaaggattgacagtacgcaagtgac
gatacaaattgaagggatgtaagtcagtaggccgagaaagacgctgactaggaacggcttggaatatatcaccttgatgaatatgctttttcaaa
gtagaaacaaaattcttgtagccttcttcgccagtgttcgatttggcttcatatccaagatggatgttttttttgtggaggtagaggaatttctgaagc
atcaagtttcttgtgaatcatattgatcttgaacattgcggcttcataagcttcttcgacaggccttccttgatcgatgcagatatgagagatgatttt
gacagtttgataggcatgatcaaaggcaatcaaatcatccgtcatgaagaaaagagcctctggtaaattaagagtgtcttgagcaggtacattgg
tagtaggttcaaagtacttgatacagtcataagaaacataaccaactgcaccaccactaaaagaaggaagacctgggacaggagcaaccttta
cctctttcaactcacgttcgagacgagctaaaggatcttcaaatccattggccatgagcactctgtatggactaccaatgaagctataacgagaca
cattttctccctgagaaacactttccaaaatgtaagaatagcgcttgccctgagaaagctttaaataggcaatggatggagtaagcaaatcagca
ggaataacaccataaatgggaaccttggtagcattgtgttctttaacaagttgctccgcttgctctaaactagggtaaatctaacacgtgaatgtta
gtaagaccattcgcaatgctagattgtttttttttttaagaagacacaaatatcccaagccctttcatgtataaagtcaagtggtgaaatagacag
tggacgaaagataccgatcgaaacaaatagttccgccggacttctatcgctttggctcttggccatgaaaagttgaatagatccttaccttcattgt
ataactaaaagaaattccaaaactgtacgagagaacgaggtttcgtcaaaaataaaccctttaccaaaaagtgcctgaaatttttcctgtaaaga
agaaatgttcagcaagagcgaaaaaaaaataaaaaaagaaataaaagcgagaaaatgccctgtagaagtagcaagtccaattgcttaaaatg
agatttgaagaagacaactgacaaatccaatggtgtattgcagtaaatcactccttagagttctctatccatgagatgcattgttcatgtcttagtat
gatatatgggaagaaatcgcttcagagactgtaaagatcttacaaaaatgcaaaggaagctattcattcaagagctttcaatgaccacggcgttt
tctttggctttctctcggattggaagagtgaatgagaacgcaattacgccttgagtaagatgatactagctttcgtgtgacgggtaagaaaggaaa
cagaagttcagcatatgttttgctttttttattcagtacattccatttactttcagcatctcttgattctagcttactgttcttcaattacttactaccgaa
gcacaaggaagtttccttagtaaacaatggagaatgaaaaacaataagaacgtattggcaaaagcaagtgcaaccggtttatgaagatagaat
aaacgaaggatgtggatggtgatgatgatgagcatgacgatgaggataaggagaacctgtattcagtggatagaagttgaagcccttcgaccca
gttgaaagaaatgcaagtgtggctgccaattggatcctgtgaaatgttctttcttaaaaaatagtgtcatttttagaattgcaaactttagaatcctc
ttctattttgaaacatccaaaagttccaggatgcagactgagttcacagccaagcagatcaacttgtatcggataggaacgacttgagatgtaca
gaggagctaaagattccatcttttcttgttttctttctaaagaagaatttaataacgaaccctctgttcctacctttttaaagcaagagcgaggtgca
caagtcctcttcgtttcactcggaataaaggaagaaaaccaaagaacttaacaatctcgtctcagaatttctgtcttggagaagctttgatctatgt
atgggtatcccggtacggaaatcaacagctcgttcttgaaaacaaaaaaatacgaaaacataaaggatttttttaacgtggaataaaagaaaatca
acaagagttttcgaaaggttctttttttatcctcaacacccttattgtgtaggcttctgccttgaccaacctatcctctgctaactccccccttcaactctt
ctctaagaaaaacaagatggctgaacaagaagaacaagaaactcctcaaagattcaccctttggaactggtaagacttttgttaggctattaaatt
gatttctaacttggtgtttagttggttttgatgcaaggtatatctaatgttagaaaacacttgaagtttggaatggaaatgactaaacaggacgtttg
ttttagattccccaatacaaatcaaacgaagcgtatgtcttgattgatttagaatggttaagaggccggatacgaaacgttgaagatacttccgcc
cttttgcagaatagtagaatactaactaactcttcaaatagactgcttccaaaattacattgattatcatcgttgcattaacgctaagggtgaggac
tttgtcccttgtaagcaattttggcatgctttccaatccttatgtcctatgggtatgtgagaacgacttcattttttaataattcaagctatgtgcggat
aattaacatggatttagactgggttgagcgttgggatgagcagcgtgataacggtatttttcctgtcgatttgtaaatgaaatgacaatgttgttcg
aaagtacaccttggtaatgatgagagaaccgaatttttgataaaaaaaaacgaaaaaaaatttcgagctttttttgtgcactgtataacgaaagcatt

ttctatcaacaatctcttctacatttaccaaggagaagaaccttgctactctctttgtcaagaactcccctttcattagtctatcaattttagcttaatg
aatatcctgaatatgatattcaacacttctttatacaaacttcattgaaaatctgtgtagtgattgattgatacagcttagtcatacgtaggctaaga
ggtttgtaatctacgcaaattgtaatctttgtttgtctaccattattataaaagcttactgatcgacttgtttattgtttctttcatgtatattcgctcgac
tcactagagaccattttatcactcaacaatgcgagttaattttctttacgaaagaatcctctagaaaaaatatccaacatttagtttccagtattgtg
gctattcttacaattggtaagcaaacgcctctatccttccaccatagcttagtcattcaagtgttacattcctttaatatatctcttgtacttttgcttgt
atattgaaggtttgcaaaatttttgaagaatagaattcatccaacagtgtaaatttgtttctatacacttgcttttcaagtatggaattgcgatttac
gtacgcaggaaatgtcacttcactttcttttcatgaagaagatactatttggtcagcaaaagaaaaaattgagccaggaaataggattttcgccaga
tcaaatcaagcttctttataaagggtcattgtcaaacgaatccaagttgcaagatgttgtcaaagacaaggcaaaaattatgaccattgtaaata
aaaatgcaaacttgatcaacgagactatatcgcaactcaattctcctagaaaaaatgttgccagttcttatgctttgaagccaaagaaacctcggt
cgtcccctaaacaagcatccccatacacattcaatcaactccatgtgcttgattatcctttttaaagacagagctttgaaataccttgaacgtctgcgt
gatgatgccggtattcaacgaattatgaattcacatcaatggacagtctctctgctaactgaaatggatcccgcagagcatacccaacatgactcg
aaaacattgggtttgaaccataaccatggtgcccatattgaacttcgattgcggacagatcagtatgatgggtttcgagactacaaaactgtgaa
aagcacacttattcacgagttgtcacacaatgttcatggagaacatgatgcttctttctgggagctcttccgacaacttaccaacgaagcagatgc
cgctgacgttatgtcaaaaccaggtcgttatgtgagtgaacaagcaacttatactcctcaaatcgatgaagctacggacgaagatcagagagac
cacaagagagatttgctattagcagcagcagaacgaagaaaaaatgcaaaaaattaactattttaaagaatccctcccgtgtgttttatgattta
caaaaagaatgacatgtatagataggatcggttactagttgttactacttcaatgaatagatgatatatggacagatgggcttatgtatgttaaac
aaatatataatagatcaaagctttcaattgagaatagagggaatcgtaaatctatcatatcatggaaaataaagctcaagataaagctacgcaa
gcaacgtatctcgaacattcctgagccaccccatcaagaaaaataaaacgcataaagatatgatgctacataagagtcccaacacgaaacgcct
tcaaacaaatgaagaacaacaacaaagttgaaaggactaggatccttatccggcatgaattcatatacaggttgactagtgcaattagaaagga
tgtgtagagcaggaaacggagaaggagaatgtagggtaatagcgtcaatgcaagcggcgaaagcgtaagagatttttttattaagaagaatca
aacaaaggaaaggcttcaaagaggatacaccacgtttatacttgatcgaggtaatcacggatttcagattcagtcaatctgtaaaaatgaccacc
agggacaccctcaataccgctttcagtgggtctgttggcggcgatagctatttcaatgctgtcttcatttaactcgccttcaaacgtttctttcaaggc
aagaatagcagtatggacagcatcatccaattcaatctcattgttgtagcgtttctctaaaaaggtcttagcagctgtagaagacttgccaatggc
cgtagctttccaagcaaagtaagtgccagagggatcaacttggtacaaagcagggccgttttcgtccattccggcaactaaaagactgacacca
aagggacgaacaccaccagattgagttgactcttgcatcacagaagcaatttcttggacaaggattttcgtgggtgggtatgcgttaaagacattt
ttgtaagcagtttgggcgacctttctagatttgtctacaagaacacggaaatcaggacccattccactgtaaacctgttgaacaacatgttagcaa
ctatagatatagaaaaaaaaaaaaaaagtttaaactaattttccatgcaaaatttatgactctttcatacgaaaactcacgcagagctccgtttagc
ttttctgcaaagaaaagaatatccagtcctgtccttaccattccaatatcgggagtgattttacaaactttctctaaagaacttccaatagccaaatc
cgtagtaggtttcttttctgtagctaaaacaactccatttgtagctaaaacaatgcattagcatcctctgtcccatatacacattgacaaagtcaagt
aattctcattataaagctccatagctcttctactaaactgaatatcaaaattatttacctttgataccaactgacgtgacaccagcatttacagcatt
caatgcatattcaatctgaaccagtttaccgttaggggaaaaggtagtcaaggaaaatgcgtatttgtccgacatgtcaaaaaatcgtaaagatt
cccttaaaaagtctaaatgcgtgagtttggtgaaggaagagttttctgatttcttttggggtaagccagaacgtatatcttctccttcacttttctttgt
taaattacatagcgtactttacggagcgtttacgagtcaccttgcccttcactagtaaataaacttgtgtagtggtagttctgggggataaatcaaa
ggagtatatttatgtaagaaaggatgttgtgaatgcaatcttgttcctttggtccctctgctcatagacctttaaaatacttgtcatggctgattgctt
cttctcgaaaagtagaattatcccaaatggagtaacgcatgctttcataaacaaggctttttgtagtaattgtatttttgtttattcattcacgaacag
ctaaaaaagaaaaacgacaccagcaggatttgaacctgcgcgcggagaccgcaatagatttctagtctatccctttaaccactcaggcatagtgt
catatgttgaagttaaaaaaaacaaaccaaatttataaaaaattgtattttgtttacctctttttatgaagttatttgatcttgtttcagaacataaga
aaaaaaaacttaatgtattgaagataaattttctatatatttaaaaataaacaagttacctttggtcaaggaatgcaacgttgagcgatcctcaaa
taaataaacaaatacaactagaaaagcggcctagcgacaaagagctactcgatttgccctaggatacttttggaattacttgtgacgtctcactta
ttgacgaaaacacaatgctggattccctgagaaaaggaccaacctgtttttagaaagaagcgaattgtctttcatacccagatgcagctcttcatc
ttttggttaaatcaattctactcgattcgttattggaacacagacccaaaagcttgttgaaggtcgacagattcaatagcactattgagagaaatga
ttgagtaaggaatgcaagcatcctttgagtctgttctcaatccctcctatctcatggtcgttgctgaacggacgaattcaacacgtacgtaaataaa
aatagcagatacgatacagtctcttgcatgttgcagttgcattttaggatagaaacaaaaatcaaaagcacaataaccttgcaggagaagactat
gaattaaaaagggaattaaaaaaggaaaggaccagaacaaaagaaaagaaaagaaaattagaaaaaaaaaagggaatcagaggaaccgaa
ctcaaaaaaagattggcgaaaacaccaagaacaaacaaaaaaataatcctttttttctagagatgcaaaaggaatatggatttaatgggacatg
aacttgatggccaataagaataacttttgatggaagttcaatagaggaaaacggtaacaaaatgactgggaagcagtttcgtaaagcatttgat
acatccaaggatactcgtttcatctttgctactctaaactgaattgagatcctgtttagcattccttcatatgaagcgaagctcgggagtttagaaag
aatgctgtttggaatgcttctgaaatgcatatgtttgcctaatcgaaaaggaccaaaactctacctcaatttacacctgttcaggatgtatgtttgttt

acacatcaagtaaacaaacatgaaacgagtgtagaagaccatccacaggagaataaaaagattaatctgatatcttttaaatggtaaaaataa
aacaaaaagagaagcctacagcaccccggattcccatgttgtctccaaccatagtactaacgaggccctcagacgcttaactgcagtgatcgga
cgggaactggtgttttcgcctaggtatggccgtagacgtttcagacaaataggaaaaataatatatacgttaggctgtataggtaactttcatcaa
tatacctttcgaaatttataggattccttgtttacagcatcaattgtatttatttgcatagaaactttaaaattaaattgctacttacgttactgagaa
aaacgctgaaaccgctttgcctaacaatgacaatggtttctatgcatccaaactattatcataagaacaaagcgaaaaagagaggggaagtgtt
atgtataccagaaagatagaaaaagataaagaatagatcataacagttgatcatcataccagaggccatgaacgcatggcaaggcaaatttac
caagtagaggaaagactaatgtttataaatacgatttactaaatcctgatattggttttgagctagttcagcgtacctattcacagaggtttcagta
ttttgaagggtttggcgagtttgctgacacagttgctctcggcgttcacgtaacgcagggaagcgggtctcagtaaatactcccaactttcttgaag
tctttggaaaacaatagacaaatgttccagcagtcgccaaagtagtgacaagccagcgagtaggggcaatcctattcctagcaaaaatatttcct
gccataccaccaaccgcgacataggcaattccaggaagcttcctttcatctgaagaaaagatttcttgcttagcgatggagagtgttttacgttctt
tcgacaaaatccatccaaatgcgttctccaatttcaagcctgcaaatcccaatgcacaatagaccttggaacgaccatcatgaatactatccgcca
accaaggaaggtcatttttcaattcctggttattttccacttgttcttcttggtaataatacgacatatttgtaagttggttttgggaattgttggttcct
tgccggagacaggaatagtgtaataagaagacactatgaagctcggtaaattccactatagcgtaaataaatgaaggaagaagaatacagcttt
attcatgaagcatttatgatagcaagaagaaaaagaaagtcaaagaggtaaaaaggtacaaggtcgagttctttttctacttttctcgtaggcaa
aggaagttactcttacagaatatcctcaatggatattcataatttcgtttgatatgatttgctttctacaaagtgcaaagaatttccaaaagttcgat
aaagtactggttttctatatacatatggactttccttctattttaatgtcgagtatttctttctacgtaagaattggaattatccaaagcaatatagca
agacaaaagtgtaatcaaagcagctcttgaatcgatagttctcaataaaaaatgacttgaaatgtctgaaacaattgctacacttcaattctttca
aaagaagtactacgtaaagatttgtcatgtttatttactaacgataaaacaaagaatacgttagtcgatttatacaattgtaacgcattaccctata
cagcttggagcaatttctttagtaagaccaaccatctagcgtatgcatagtaaaaagaaagagccagaaaaagatagccgagaaaagcaaggc
aaaatcgcagttcatattttacacttctaaaaggaacatttccagggaaataagacgcatttctgtatttttttaagagaagagcctagatttttct
caagcgtcacgtgacttgtttcggttgtgctgacaccttggattttgaacctgtccttcgccaaccacaacattattgctgggatcgtaccagaacat
gagcggcgagtcgcacggtaatgatgtcaaatccgaatcgaatgaagaacaattttctatggaaactgaggccgtaaacgatgataatagcggt
tcagaaccgtcgaaattcccagtaaaaacagaagaaagaaatactagttcaaggaaacgagatgtttccgggaaacaggtgaatgaaatcgct
gaaccgccgaaagactccgtggtaaaatcagaagaaccttctgaatcccagttccaaagtctatcgcaatcccaggaaccggttgagaaaaagc
agcgaggggaaggaatgcctcctgctcaacaaaagtattgtgccgcgattgttcgtcagctcaagcgaaccaaagattcagcgccttttcgtgcc
ccagtagatcccgtcaaacaaaatgttcccgattatcccaacatcattaaacatcccatggatttaggaaccattgagaaaaaaataaacaaccg
catatattccttaccgcaagaatttatcgatgacatgaatttgatgttctctaattgctttttatacaacggtacgaattccccagtcggatccatggg
taaaagcttgcagacggtgtttgaacgccaattgaagcaattacccgatcctgatcaagcaccggctccggtgtccaaaaaacccaagcaaaag
gcatcaccggctcctgctgctgttgcagcggccccaacatccgccgcttctacgcgaactcgaaggagctcatctgtctcttccgcgtctcgtccttc
catttcttcggctcctccttctaccattccctcgaatactactccaccgcttattgaaggcaagccgagacgccgaaagaacaattctcaaatgcgg
tactgtacaacaatattaaaggaactttataagcgacagtacgaaccgtttgtcttccctttttatcagccagtagatccagtggttagtgattgtcc
cgattatttcgacgttataaagcatccgatggatttgggtactattcaagcaaaacttagtaaaaacgaatacgaagttcccgatgaatttgagtc
agatgtacgattgatgcttgaaaactgttttacgtataatcctcctggaactcccgttcatcttatgggccggcaacttgagaatctgtttaatgaaa
agtggaatgctcgtcctaagtatgatgatgccactcttgttaagcaacaagctgctgagacagctgccttattatatgagaatggcgatgaagatg
cttttctatcagaagaagaagttaacggtgataaatttgccgccgtggataaacaaatttctatgctccaggacactttagaagctatgaaagcaa
agaagatgaaacgcatgcgtaagcctcggcgccgtgactctaataaagagtacggccctatcacatatgctatgcaaactgagttggctgagag
atgtaattatttaactgcagaacagctgtccgcagttgctgaaattttaagagaagaaatgccttggttacgggataccgatgaaattgaaattga
cgttggaaatatgaagccagaaacttttcatcgcatttatcggtttgtctgtaaacctgaagcaacttcctctgatcctccttcacctacgttgatgcc
atccagacacgagaaaaagaaaggccgagtgctatctgaaactgaacaagcagacaaaattcgacgtttacagcagcaattggatcgttttgct
ggcaaagcgtccgatacacctacaggacattcccaagagtacgatagaacatccgaatctgctgaaagcgatcatgaaagtgagagcagtgag
tcagattaaaaggtcatgttcgttcgtctaagcacaactggatcctgccaaaaaatgcatttgtttgtttactctggggaaagcgaacaaatgtttg
ttgagcgttgattatgttatatataattaatatgtttaataataggaaccgtctttatggtttattatgttcattcagttgaagaacgttataaagccat
gaagtgacccctcgccatctccacattagcaaaattgctaaatacgattttgatgaaacaataccctaaagttgtacaacttcagctgaagctttat
gagtttgaatccatctgctcataaaatggaagctatcgttttacttaggggagagaaatcagttaatttttgtccaattcacaacatttgcctaatat
gttgtactgtttatgactttttttttctttcgtgtataatgtcttttctccttgatgattgaatatccctttcaatctattccctgatggtataccaatcagt
ttagcaagaggctttttcttgtgaaagttttgtacgtttcatgtcgctcttttccgccctttaccgcatacattgtgcttattgtacaatataaatatcaa
ttactctgtcatttttgtttataaattcactgtaaataaatattaatgttttcatttcagagtgtaaactagatacaccatatttttcttgtggaaacaat
gattatcatacgaagtatataaataagaaatattacaacttaatcgttaacactatattctgtcacgggaaaatactccgtatagtaagaagagtg

tgagtggaatacagttgtaattgctttaaatgtcaagccgaatattttaaaataagttactaaatatctggagtgaaatatccttagtaagtccttg
gtgatacaaatgaatatgttatcgaacaagtttattagctctgccagcaaaatcttcctaactgaagattttgtctgagaagaacgggcgttttata
tagtaatctaaaactagggtttaacaaacaataaagcttaattaatccttgataattgtttaatctttcagtctgtttgttcattaacattaatataca
gtaatatactccatagcataattattgccaaatggtttataatatatattagggatttgctctgacagcatcttgtgagcggttcgaatcatggtgaa
atcaaatttcaggggaaaattggtaagtgtttagacacgttggaaaagttgcgcagtgccaacctcagagattcacaaataagcaattatggatg
cttcatctgagcaagtaattcggctagaagaaggacaagatacaaatgcattatttgaaaaatgcttaggatgctctgacgttggaatcgtttctg
gtactgttcgaaacatggatggaaccatggccgcggagcttgtccgaaaattagtgacagaaatttcatcacagatgcgtcctaccatggctattt
ggttgcattggattattgtgactcacggtggatatttggccagtgtccaagatttacaagaaccactgaatgaattgcacgccaagctgatacacg
gcggagatcttcttatgaaaatttcagcgctatcaggaagattaagtatggtacttggacaggctgagatccgagagtctcgccgattagatcaag
aagaaagtagtgaggaggaaagtgaatacgaggatggacaaaattctgactatgatattgaggtagtggacgagaatgaagatgaattggatg
aaaatgaagatgaggaagatgccgcagctgcagcagcagaaattacgaaccagaaccttgatgaagataaggtaaacgcatctctttctcccg
atgcagagttgtcccctagaagaagttctcgacgagaacaaaagaaagatcacttaggtgatgaagaagaagaggaagaagaagatgatgat
gagccagcattgcatgaagaagcaactgatgaatccgagtctgaagcagaagtggaagctgctagacctactccaatacgcaaggatattcgct
ccgaaagtccaagaaaatctatgcgcaaatctaggtaaaagcagatttcccgtgttgttaagacagctagtggaaacagtttttttgaaccagtatt
ttaatttgttgttaaatttttttgaaaagaacgagactgaaacacctagaaacttagcttttggaatgctttcataacaagcggtatagagaacaaa
agcgaaagttttgtaatacgacttttcgtctgtcgacggaaaaaagttaacgatacggtgatttcaaaaaaggatttcaattgtttatggatctaat
ttatttcaggtttagatgtaccataggttttgggtttggctaataatactgtttggttactataaattttattattaagaattgcctatgtaaacgtaaa
gacgcttgttgtttaacctgtactgcacttgaatgcaaacttcagatccaacaaatactttcgcatcatgtattacttttgtcttcattcttttacatat
acgttttacgatttgcaatacgacctataaggcttcgctaggtaagcggtatctatctataacgatgcgaaacgtattgcctgacgaattagcaaa
attaattaaaaaataatttttttattttacgctacaaagctagttacctgtccagctccgaaaattttttttcttgtcaacaaccaactacaaaatgggtt
tcactaatctcacctccgacgctggtcttaagcaactcaacgactacttgactgacaagtctttcattgaagggtaaggaaacttttatggagccat
tttcaagcataggaaaattcaaagaaaactcgacatgtttcgtattgactcggttctcaaagtttgttcggaacgatgaaagtttctaatgaataaa
aatggcgatttcgctatgcagagatagctaacattggatttagtcacgctgccagtcaagctgatgctgtcgtcttcaaggccgttggtgcttctccc
gacgccgagaagtaccccaacgctgctcgttggtacaaacaaattgctacctacgacgtcgccgccctccctggtaccgccaaggagctctctgc
ttatggccctgctgaaggagccgctgctgaggaggatgacattgatcttttcggctctgacgaggaagatcctgaagctgaacgtatcaaggctg
agcgtgtcgctgagtacgagaagaagaagtctcacaagcccaaggctgttcaaaagtctcttgttactttggacgttaagccctgggatgatgag
actcctatggatgagctcgagaaggctgtccgtggcattcaaatggatggtctcttgtggggtgtctctagacttgttcccgtcggtttcggtgttaa
caagttccaaatcaacttggttgttgaagatgacaaggtctctttggaagctcttcaagaagaacttgaaggtttggaggactatgtccaatccac
tgatattgctgctatgtccaagttgtaagctcgttccctactagtagtaatgcataaggacgaagatgaaagattgggtatatgcaggaatggtca
aataacaatcgatttataaaattattaaattgtaagaatgattgtagcgaaaaataaacatgaagaatggtaaacagtaataatgaagtatcaat
tattaggaagcaaggatgttttatactaagtcaggtagaataactctgatagatgaacgagaaaacacaataaaaaaaaaagctaaagaaata
tcacaaacttttcataattgaaaattaatatggacatgattgtgaaatcatcaaaaactgaccagctaggcaatcgccagctaaacaagataaaa
acacttactgaggtcccagttttatcctttattcaagatcttcttaagcgtacagggcttttaggctgtgaagaatcccgactgccagggccgatag
gtgcatcgctgtctaggccaaaaactttcgggttgaaacccggaaggatgcccggttgaacttggctaaaggctcgaataatctgctcttcgtcga
tagggacgtcttccatgataggattaacgagccattgttttagtgtgtcgtctttaataatttcattttgcccgggcagatcttgaatgtatgccttgt
acgatcgacgcattttccgtttatttccatgctcatcaatgcgtccaaccttcttggcaagctccgtaagaccatagagttctgtcaagttctcatgcg
gcgatggctttacaggttgaaactgtgtacttcctacatagtgatacttaggtgattccggcatatgctaatgaattaaagtcttttaacaaaaaaa
tataaaaaattccgacttttgcaaagaaaaaatcttcaatgctgctagatatgaataattcctttgtagaggttgttaatggtacgagaaaatgtag
tagtcatagcataagtgaaattgcattgcttttatgaaatgtgggcgtcggattttaggaaggagataatgaaataagaagaaataatcaaaaga
aattgaaccccagtgaagactggaatcgtcgacacttgtgtatagacgaataagacgagtgtgtgaaagttaagttcaagccaaagaattaatg
gttaggcctgctgaatgggtacaactttgacttgattgtatgtccaacgatctaattcgccttggacttcattctcgcgatataacttttgcaagtcat
cgtatccaccaatgctatgtccaccgaaaaagatattcggaagcgtagaaacatcactgacagaagacaaccacgagcgcaattcatttatgta
ggtgaactctgtgacttcgacgatgacaggagcaggttccgtggtgagtgtttctgtcaacatggacttagctgccgaactgtatgggcaacctgg
acgactaaagaccatgacgggattttcaaggaaaacttgaaaatccttttcttctttcgcatctttgctcggcaatttaattttgaaggaagacgaa
gaaggatggctttgcgcggcaagaggactcgacgagtttttttgttagcggtatactaagaggtgtatgtagaaataattgaagaaacaataaggt
aatgagtatggatacacataggataacctttgggttcagcattgtggatattcaaattgggcaattttcgcagaacgtaactcgtaagatatgtatg
aatgaagagataaccaaatgaacttatacaaaaaattttagatgccgataccgctcgaaagatgaagtcttcttactgtttacggtggatggggt
gtgtagaaaaatgaacgacacatcagaaaatttagatgaagcaagccgttattcaaggaagactgcgaaaggctttcctttcttttctgttgaggg

cttgtctttactttgggggtgttagcacatcagatattactttttacactgccatctctgcaagcatagaaacaatagtatatattatattaacgatgc
gattgtttaattatagaagaatgtaacacttaagtaggaaaggtcatgaaagtactgcagataagtaaaataaaagagaaagcgactaaggaa
atagcgtatgaagttataaaaagtaaaaggtaagagttgacaaataacatgaacaattgagtactgcatcgtaatttaagcaaagtttataaag
ggaagggccaacaagactgaatcgaaggccataccatatcctgcttgataaccagtaccagaggcaccatagtcatgaatcaaagggaatttgg
tccctggaatgaaatccaattctacacgagcacccccgttacgagaagggcggaatccgaccgcttcaaatatgatctttgcagcttcgggacctt
ggttatgaaacagttctggcatgagagtagcagtacgctttaatatatcttgagtatcttcaggatgaatttcacgatcccagttgcccttctgcata
aaacctcctaaaacgacaccaccattgaaaggacgaggaatgatgtaagtatcggaaccaataccattacggattctcgtttctttgatatgagg
agcgtcgacacagacgatttggccgcgagtaggatacacgttttgatcttctactcctccaagtgtacaagcccaaacgcctggacagttaaaga
ctacatttggattaggaacaaaattcaggacgtcgctaatatgtttgatttcttcctttatgatggtgaccccagaatctttgacaatattgtatatga
agtttagatactgaggaacatttataagaaacgtagtgcccgtgtggccataaatgcatttttcaggaagctcttccttcgggatgactctataattt
tttacataagaagtccaagagccaagcttgttatgattaggctcataatcccacagctcgcgaagatcagcaaagacgacacctgagtcctttcg
agtgttcgcaaggtaagcaagacgattgtaggtaatcttgtcccagcgcaaggctttatcatctccacttgagatactgcaaaaatttgctccggc
ccatggtgaagtgtaatcgacagaacgatcttcaggtgtatatttggcgataattttaatacgaggacccaatccgaaatcgcaaaggatccatg
ctgttgtaagtccaatcaccccagctccaacgattgcaatatcctgtacttgtcgtgctgacatagtgtaagagtaaaaatattaactttagctcaa
aaatataatactgaaacgaaaaattaattagttcctacattcttgaaactcgaatcccttgctcactttgtttatttatgtacccagactttactcaaa
gtaacctttatgacttacccagaattcttggaggaaaacaatgtgcgaatatacgctgcttggctgaagtcaataattgctgtattgttgtttttatat
tgaagtaaattgtaaaggttgaatccttatcgacgggtatttctttatatctgcggaacccaatggtataacatccaactgatgacttatgatagcg
ttcaaatcattatcgttgcagtgcaaacagtttggtgcatttggtgaggctttacgaacataactacacaagttgtttttccatactgtgaatatca
agtacttcgaggaagccaatgtaagcttctgcttctcttacacactaacaaatgtgatttaagacatcaagcaaaggtcatcagtttttgatgaag
ggctttctacaattcagtcccaattgctttcataatcattgaacagatgtgactggttactttcacgtactcaggtaccttttactacacacttttataa
aaaatatgatgcttcaatccttatactattaataatcattaaatcattcccacatcctagatccccaaaaattaacagatgttctgttatttttttttt
accacttattggccagcaataagggattagacaaatccgtttccactgatcggtaggtcgcttgcaaataatttggagccagctgtggatctcgaa
tccattgctcaagaatatcatttacaaaagaacgatcaacctctttttaccaggagaatagtacagagattccacttggttcatccattcccgact
ggccaaaatccgttcaccagagggaaatggaagctgaccacctccattgatacagcctcctggacacgccataacctcaacatagtcatactgtc
gattcctctgttgtattttctgagaatcgttcttcatcctccgtttcagtaatacttgtcctcgacgattggagttcttcccattcactttgcgtaccaaa
ttctgaatgtttcgaaacccatagcaggtagacattgtcaacaaagtttcatcgcttcgaggatctcgtaatatgtattcggttagatcaccgttttg
gtcaacctttgtaacgaatgagttagcatcatgaataccaaagagcatttcagctgcataagacataacgtagctcatatatccaccagagctag
agccaccctgttcttcaaacgtcaatcgtggataccaagctggtaacacctcattcgatattttgtagggaatcctgttctttacgatgcttcttggat
ccaaatccaaatacttgaacatctcaacaatctcttttggtgtcaatacagcatccacgtctcgaacaccatcttcggaaaactcatcacgacttgc
ttctagctttttgtcaaaacaaggcataaggctcaaatgccaaacttgcttgcgttccaatgaatattgtttgctcgcccaatctttgagtagacgtc
cacatgcttgttgtggtgaacgtactcgagataagtgcggaatgagtgctgagtgtgttttctcaacgtaacaaatccatcctgggcaagatgatg
acagcattggcaactttgcatgtgcatccggctttgaagaggcgacattatttatggagtctttttgtaggtcggacgaattcaaaccaatagctttc
tcacttccattttcagatgaattttcttgttgtctttgcttccatgactcacaaaactccaatgcgcactgctgtaaaacaatttcccgagctgcattcg
tatccaacacggtacagaatcccagtttcccgacaaagaccatttccaaaacatcttgcatttcttgtaacgacagaccataatatgcagccaagt
ttgcacgtacctggggacttgaagaaacgtaaaataactcttggtcgtctgttctggcatccaattgtttcagcacttcctgatatgactgcaaattt
accaacaccgtttctgccgaagtgatgcatccactgcaagccaagcaatcattcaatgagatgctcgctacctctaattccttcgactctcctgtat
ctttggtaacttcgaaataagagtttccttgaacttgaatttcgttctaataacaatctattagccctttgtttcttgaaatgccttactcgcttccattc
acgtttgctatacttacatcttcatgactttttttccgactttttttacttcagctggcttgatgcaaacagctcctgggttcaaaaagtcatttagatcat
tttctgaaagtttagccatgcttcaaaatgatcttaatgcttggtttttttccaagtttaaatctttcctaaggggcaaggcctccttgatatgagtaaa
cataatttgccacattggatagccgtatctacttttccgcattctatcacgctttaagcataatttgtaatcaatcattagatgtccttatattccttttt
tttctaattcagctggcattctgtagttgcaatgttttgaatttttagtaattgataaagctttatttgaatgcaatgtacttataatacttatattaaat
tttctttgaagtcttgtgatattttcaaatgtggaagattactacttttaatttttagtactttatcaatgacggacgttcttaacggtttttttggtttttgt
tttgtttttatttttttttattactttttttttgggaaattcagaaaatatcatcccgagctttagaacatcatgatttcagtagctggtttcatgagaacta
gcttgacaggaaatggtcacataacagatgtctctgaagatcaagatatatgtaatcaaagtctacaagaggacatgattattttaataaatgga
actttccaattattttcaaccaactataagaaaagcttcctaaaagatctattagttgcttttcatgaagcaactccttttgcaatttttttgaatatatt
ctaatttagtatgtaacagtcgaatgaacaaaaaggacgcataaaaggaagattaaaaaattgaaacaaacaagtaaacagaaaagatggct
ttgttatactgaatagaaactcaatgaaaaaatactggaaatgttgggaagcctcaacatttcagcatgaatgaaatgaatgaaggtaataaata
ctataaacacaaagacaaccatacaaaagaaaaaggaaagtcagtcttggaacaattatcaggagaattgccgataataccagcgacggtttc

ctttataacgtcgctgattatgtcctataaaatggccaacaaatgtgtagcctatcgataaataaacggaaatagataccactgaagggctgtatc
acttccaaaataagtcaatctatgcctttgatacttgactttaatattgtctaaataaaaccgatagaatttttttttttttcgaaaatttcaaatgctt
taaactatccaccagagtaaacttccttgagtatctagggatcttcttcatcatcgaactggagaccattaaagtaagcaaccattcttgtatggtt
caagtcatttgcgctttccgtggaactcgtatccaaggaaaactcgtcccttgatcgggttcctacaggcgtctccatgccagcaactgaagcacg
gcgagatgtatgttcgcttaaataagaattcgacgtgtatctatttttgaaatttgtcatatactcgtgcatatcttcgatggaagcttccgtaggtgt
gttattttgatgctccttcttgagcttctggatggtactccgattggctttgtctaagatttctaaattacgatagctgaaactcccaaactctggtgcc
gaatctacggtattacttctcattcgatttttcctttcactcaatttaagatgctgaggaattgaagaagcaatagtttcagagagaggttctttgtga
cgagtttgctcagaaggctgcgtgtagtaacgactatagtcaagctcatctaatcctctagaatcaaaataaattgtatcatcaggcgcaaaaggt
ttcggaacaaaaggaggttcctcgtttactacattgtcccaattgacacctctaaaaaatggatgcgatttaacctcttctactcctcccgctcccag
tctctcagatggttccatacacagcaagcgattaattaaatccacagcatcgcctaactcatgaaagttagtgttgctaggccatgcaattcttcga
gcgataatgttcttaaaaatttcagttgggctatctgcattaaagggaggatagccaaaaagaaactcaaatagaacacagcctacagaccacc
aatcgcttgctttaatcccaggattacctaaaataacttcaggggcaatgtaatctggagtcccaataaatctcttaaaggaatcatcattttcaag
aaaaggttgaatcttaggtgaatcagtagcttgatttcgattgtcatcattactattaacagcattagacttcctaccagcaaggagtaaatcttcg
gacccgttttcaaggctaaaactttgagaagcaagtctgatacttgttccatctaatgatagtaatctttcattgagtgtaggacgtttaggcgactc
cgaagcacccacagaatgtgtatctaacaaagatgaagcagtacttaatgagaggtccgacaaagaacccgtacattctgctaaattaggtgca
aagctcgactcactatcaatcctgttttgcctattcagataccccactctgcttaatccgaaatccgttagcttgaggtggcctgattgagcaatcaa
caaattttctggtttgatatcacgatgaacaatttctctgttgtgtagagcacccaagcatagaacgatctctgctaagtacttatgaatccaatcta
attctagcacgcccattgttttcaaaagcgatccgcaatcacctccatttaaatattccataacaagataaagataatctctagactgaaatgtata
atacaatttagcaacaaaaggaatttctccttgagccattaaaatggcacgctcagctcgcacattcaccacctgatttttagcaatcatatcagat
ttctttagaattttgacggcgaagtaatcaccggtcgttctttttgggccaaatagacagaaccaaaggcacccttgctaattggtttgagaatttt
gtaatcattaatcgaaggattatgatgagtagctgaaggcagaattgtatgtatgcgaggagatggagagggaagtcgtaaatccttttcagata
ggttattcgaccgtccacgtgaattcagcgatgtacgagcgtttagcgataagctatgacctgaagcatcagaagccaaaaaccgaggactgga
atttggagacaagaacctcgattcttcaaacttgtgatcagagcaacatggctctgacactttgagacgctgaaaaggtgaatgaatgtcaggca
cctcattcaccggagcagaacaacaatcggaatcatgagtgaagcaatacggatggcattgtgtaagtagagaacatgaggaggacctagaaa
gatctgagttcgaatccgagtaaggtgtaaattcagtatcatgtttgaattgtggattaatttctttaactgcatttacatcgactcctggggcagct
atagtaggagcgtctaatgttagaccctccacgtaatttgagtcctgcaacgacgacaattcagaaacaacattatcaatcacttcttgaacatga
tagtctacttccaatcgaatccgctcagagtagtacacagcgttctttagacgggtaattccaataactttcatatcgaatgcatttcttaaatctttc
caaagatttataactgcctgcgagcagttttgaagagagggtgtgaagttttgcaattcgtctgtagaaacttctgacttaggggattgtaaccga
agagattccacatcactgaaatttacaatgactttcacgggaggtatgcttatgtctataaccaagttcagatagtatgttatttgatccaggaagg
cggacaagaaggagacggagaactcatttcttgcatccttaggaatggataaagatgacatgtcataatcaggatataaaatcaacggttcatct
ttataaagtggaattaaagaagggttttcgaataattcattttggatctgtaacaaggtagatcgaaaataaagcagagaatcttgggcagattgt
acaatagattcaaaagtgctggtaattaagcaaagcttagaatgaacctcaaagaaccatgactgaatatttctttcgcaaatttgacaaaactc
gggccgcggaagaggtaaagactgtaaatcaccagttttaaagaacgattcacgcaaggaagataaatactggtcgagaagaccagcgctcag
accaatgacttccaataccttatttgcgtataacttcgagtctatagtgttattggtaacggggtgagccacccacattgtatggacaggcgtatttg
tatgagagttacgaatcaaaatacctactgcatcaaattgctcaacaggaaggtcactgtcatcgtaagtgagaggggaagtatgattaggtgca
tgagggtgctgtgtaagagaaattctcgaaggatgtttcaatcgaacatgatagctatgagaatcatcttctaacagacattttgcagctttagtga
acgcatccaatccggttttacccaaaaaatcttttactgaatggccaacaacttcgtcctgaggaacatttagtaaatcagaagagttctcactaa
cgaattgaaaatttcccgataaatcaagctcgaaagaaacgggatgatgggaactttcgaaggaattgccgttcgttagagaagccacgctaca
ttcttgctgttcatttgaagcttgttgagaatcgctgaggggttcagatgtgccacaagcagtaattggcttcattgacactcaaaaaagccttgaa
ttatgctgtactcttaatcataaccaaatagcaataattatccactatcgacaaaagcctgtgtaatttattaaaagttttttttttattctttcttaat
cctcgtaagcagaataaaagctttgaataataaataatttcctgctattgtgtattctaaagaaggactgagacctacaaaggacttttcagcgga
ccaggagtagttgttggtggtaggtaactcatcactagagaccctaaaatagtaaaagcataaggatacaaaacacgaacgttgtgttgtgatct
aattagtaataaaaaataataatgatgatggttaaaaatttcagatttgacatgttatcttgaaagaatgttcactttattttataagaaaatggg
gcaatttttttgtgtgatttttcttccttgtcttttgcttccaacaattcattcaagaaaattgcttgcttcgtggaagtaatgaggagattcttcctgca
ttcaatttcatttacttgaaaagttcatttccaagaaaaccggcttcgtaagtaaggaaatggtattggagcattgtatcacaaaacgtacctggta
gcagtttccatttatctaattaaataaaagaaaggttatgaaactaaaaactaatctaaagctttttcttataattcatcaggattctgattgtgtca
tactctatgaaaaaggattgaaatcgcaactaatttttcataccagaaaaaactcttcgattcctacattgtgcaagacactttcaaaaaaaaaaa
ataaaaaataaaataaaaaaaaaattactggaggtccctcaatgatttccatcaaggcaaaccatttgcctataaattgggagaacaaacatctc

aaaccatacctgtttgttaaccatatttgacctgtgattagatttcgttagattacataactttgacttacagtcactctgtatctacactaatactatt
attattattactactactattccagcatattgcttctcattcaaccatctagtgtttatttacaaaattacggcgtaaattccatttccttattcttattac
gagccttgttcgaacccacaatcctcgatatttgaagcgaacagagtagtagagggtacaaagtatgatttaatgaaaataacacttacccttaa
cacgtaaagtttactatggaaacatcctcttcagacgaacatgcttctttgcagagacattatttgaagaaggagcttctttctcttttagtcgagga
cgaattgaagtttattggcgatccctcgaatctcgttcacttaggaacaccatttatgaataaggatgaacccgttatcacatttcatgagtctaaa
ataccgattctaagattcattttcaaaaaatttgttttaacttttcctttcctcgacccagcaagccaaacgcaattatggaatgtgagctttcgtaac
gtacgtagaataatttcatacgctcttttaagaaactatggagggggggcacattctgactatgttttctagtttttaggtgcaatgtacgaaaaca
gcgtttctgtactacctgatgacccagatgcgacttatatgcacaaagtgggaatgaaattccaaggcatgcttacgcttctgatgagtcgagcca
tttgctgtattcaaggagaaaaaagtatcggacacgaacagcttgatatgtcggatgtgaaggataattcttctcagaccaatttagaatcccaca
aagaagaattcttgacagccttagttgtcggtgttcgtgaatttgcttatacgacttatttaagaaataagaaaacttatgtaagtgatttttatact
acttggctagctagataccaatgtttaaaaaaaatatctgcccttttttgctttgcgactggcatctagctacagccaaagtagttgtttttttctttcaa
ttcacatgttaacaaaaaaaggaatatatcatcaaaataaaagataaggatgctatcacattcgtggcacgaaggtattctgaattcaccaaatt
acaccatactttagtgaaaaagtttccacaagctaatgttcctcgtctacctgctaaagatatacatgacacccatcttcacgacggttcaaatctc
tcatctttagacactacccaaacgtcctctagtgttgaatctcctaaagaaaaatcctctctgattttgcaccaagaaaggatgagaattggattga
gtcattacgtaaatcatcttcttcaacaagacgaacttcgaaatagccctgaagtacagacctttctttcttctgatccctttacgcctaatgaacaa
gaacaaagagacatacattttcgtcaagagttagacactgttcgtcgcatagaaagacagagatttatagacattgcttctgagcgcgcaaaagc
actcgatatttatatggacgaatttaagcggtcccttacagaatccggtatgcttagctacttttagtatacattttccccgataaccttactaactaa
aatttctaggaggattgacaagtttgtttacagagttaaaagagaaggagagcattagagacttatctccaaccttacaacaaaccatggaatgg
gttcgtgtaaatctagcttctactatttatgatgtatttatcgccaaggaaaagtctagtgtcacgttttctcagattaagcgcatgcatgcaattttt
ccctatagaattatcaaaaatattatgcgtttctcaaatccactttccgtcatgaaacgtatcctagacacacttttggctcaaccttttggaatgaa
gtctctctttcaacgtctgttgagtttgagtcttagcgataatgttcgtgctattcataagttaattgacaaatatgaaagtcaagttgataattcaga
tattttgcaaaaaattcaacaacaagttgagaatccatgcaaagcagctcaagaagttttacagaacgatgaaatgacgcctcaagattatttatt
atatcttcttgtttctgacgaggtacctcctaggctgtctgatgcatcaatcaagcaggtgattatggacggaagctcctggaaagaagctatgga
gtcagaagaatatcccgaagataaaacgattcaagaacgagcaagaagatttggattcacgttgaaattaatgcatctgtatgcgaagaagtat
aataaaaagcgatctatttctttgattagtgaaggggccacgggagaagttatgaaaagcatggttgttatattttacaaacctctaatgaaagta
tataaagcagcaaatgtttatacttcgtatgttataaaggctataaatacaaacactaaccttatacagagtggatgattttgaatcgtttatgggg
gatatgttacggcttgtagaaaatactcaagaaaatcctgaactgaatccgaatacactggtagaacgttatatagatttggtaaagagacacga
agacgcgttttatgactttgtacatcgggtatatatgcatgacagtggactgtttagctctcttatggaatggattgaaagcataatctcatttttaca
ttatggctcagcgtatccaatagacatggacgaaattattagagaactgagtccggacgaaaccaatctattatcagaagaattgacgagactta
ttgaatggaactatacaaagaaaaaaattcttatttcggaagaaaacaacaaaatatacaactggggatgaatccactcttcccgccgtgaatatg
gatgagcttggacttgacgaagagattttggctgagttacgaggtgatggagacgaggaagaagagaatgacagcgatatagagcaggacga
aagcaggataaatgtaagttttgagtttgatcccattatggaagaacggcaacgattaaaacaaaaaggaaaacattcaatcaatggcgtcccg
caaaagccagaatttaaggtcatcccgaaattgcttcctgcatttcgcaatcttatttatccaattctagctcgatttgcaaatgaaagttcagcttg
aagaaaaatttaacctattatattagaattggcttattttatttttattttttgatacgaatgcatagtttcatgagttaggaatgaaaaataatacga
aattagacgaaagctggagatggagtgatgtatatgtaactagagagttcgcataaagctgataatggggattatcaaaacttgtagctacactt
tagccttcttaatccattctttcacatgtgcttccaccagcttctccgtgtactttccgagttgatctacttcaacattgactaggtcaccaacatttttg
ctgggcataacaacacatgactgtgtgtagacgatcatcataatgctaaaggtcgcatcatcgacgtgcgtaactgtaagtgagatgccatccaa
ggcgatgtaacctttgtaaacaatgtacttgagaaccgaagagtcacgaggacggaacgtaaaaacaatggcctcttggtctggtttttttctac
aatttcagctacggtatcaacatgaccttgtacaaagtggcctcccatacgagtggtactcagaacggcacgctccaagttcacagagtctccttc
cttcaaggaacctaggttcgtaaggcggagagtttcgggagcaattccaaccacaaagcttgtattaccgatactagtaaccgtcaaacatgtac
cgttgacggcaatgctgtcgcccacgcgacaatcttggattatttttggctcttcaatatcgactgaaaatccctcaggcgactcaatgacacgacg
aattatgccaacagcttcgacaagacctgtaaacataacgagaatttgggatatgtagtaaataaatgtcaaactgatctgctcctttcagtaaat
tcaccctcggttcaatcacaacgaagacgaaaaaaaaatacctaagctgagctcctgattaaaatgtctggtgcaaagtaacgaatgtagagag
ttcgttgttgtaacttagttgaattttataagtgtactgtactgtagtaggggtcctttaagtcttacagtcggtaaacatgcgttaaatccaaacttc
ttaataatacgattatatcaccgggataccaatttagagtcgtcctcggatctgccatccaaaacaatcgctttttttgcttgaagcaaaaatgaaa
gaagttggtttaatttgggttcacaaaaacgcattatgatagctgcgaatgcttatcaaaagtacagaaaccaattgaaagttttctagatttttttgt
gtgagtgcatgtgttttcttagaatatgaatatttttggaagaccaaccaagcgatttgtgtctcatttcctcgttgaggaatgtcgtttccctttccta
agacaaatttcgtaccaccatcggggtacaatgaaaacgaggaaagtgtatatttatatttactactgctgaagttttcagttagcttctgtagctc

agttggtggtagcgtccctctcatatcatgacaggggaaggtcgtgagttcgagcctcaccagaagcatctataggtcggatagcacagttggtta
gtgcgcatgactgttaatcatgaggtcgagggttcgagtccctctctgaccgtttttactgttttggaaattattactttttcttcatttacgatgatgct
cttgtggtaaactgaatacgatttctattttatcatagactctccgaagaaacactcgaatattttaagagaaagctcgatttatagtcccagacat
ccagatttgttttcgggatttttcttattcattctaacgtttcctttcgggttgacctgaatagcttctttaaagttcattcccattactaaatgatatgct
tttcaaaagagaaataagaatcaaaaaaatgcaaatttttcattatcatccaatgctaaaatactctatgaaaatttttaaactgtttttcatttttc
atcatcactcatgcatctgcagtacctctcatcaagaatgcttcccattatcgaatccatccttgtataaaactcaaggtctaactgattttagagaa
aaaaatgaaaaaaagatttagatattacctttcgtaattcatcattatgttcccaaaagagtacatccagatcatgcttcatcctaacaacaatgg
gccgtctcttaaggcaaccatctgctaggaatatatcgtctaatagaattgcttgtatagttcaataaaggacttgcgctcgaaaaaaaataaaac
tgaaacatacttggtaactgcaaatagctggataagacataacggaatccttctaagacaactctaaattccatccaaaagtcatggtaaaaaat
ccgtgctacccagcacactttttttaggaatggatggaagaccacaggcacatgctgagaaacaaaatgtttcacgctgttcaaactttccgaga
cctggattcgggttgatgttgaccgacatatagttcatggaacttttgaatatagttgatggagcgggaaagccgatcaaacgtgtttactaaagc
aacaatctcagaacggcgtaactgtaaaggagtagatgtggtttcgaattgatcaatttcgttctccacatccggcacttcgaaaagaatttttaa
ggccgtaccaaatccattcgtctgaattttaccccacaatcggcagcgttcacaaccaacacaatccatcaaagccgatactgtacggaaatgct
ctcgaaactcctttttcattgccgacgaaagtacaggttctcctgtaaacaaaacagattcatcaaagtaccgaggatgtctgtcaaccatttctac
caaatcacggattttatgacgaacttcggtatcctgcaatgcactgtcatgacaaaacgtaagtgtatccaggtgtccgttcatcttatccatagcc
cgaagaagcaaagaataagcaaagtataaattttcaagtctttctggatgtgacaaaactttttccataaaacagttcaagttcggtccccaaact
ccggttgactgatttaaataattataacataaatgcgtagaaatactggagtgcattcctgaaagcacacgataaaacacgcgttgctcgatgctc
cacatatccatacgttcgttccatatcttttcttgagaatgtggtgggggagccaaagaaccctgcaaaaagagggaagggaaatctatcgtggg
ttcggattcatctggcaaacactcattgtaaatcatttcccaaattcgtgtagcatgtggagccgcgtatccagtaaatctttctggattgtctagta
aagaaatatacacgcatccactatccaattcgttatcaacgtaacagtattgacggtcctgatcatctaatcgttccaatagacacgattgcgaaa
tcttgtctacattctccatcacagattggtccgattcaacgatctgacgtgagagctctggcatgaacccttcgagttttccaagagatttcgagctc
caaatctctggaacactgctttcctctgtaatcactacattacaggcgttacttccgcagaaaacattatcgtccattttatacttgcactccttatta
aacaaattcaatcggtagaagcgcatgtaatcagatttctccgttaagtctttcagtaatggcgatatgcgctcgttcacatatttaatgtctccaaa
ggttgcttctgatgtatcaggaattacatgtttcgtatcgttttcaaacttccatgggtcatccacctccaataccgtctgtttcttaccaaacgcatct
tgcaaattggcaagcagacacaataaagaaaataatgaatcgcgtcctaatctcatcccgagtatagaataattatgaacgtattattttctccta
aaatatattttctgaagttggctctaagatcgtttgtttacaccgtcttgaagctttcactcatgtcacctcaagatgtcacctattctatctctacttc
cttggtaagcttctcattactaattaaatacgagctgttcaatttcacttttctgtaatacgtgtaagaaataagatggggttttggggaagatgcag
gtttgaaaccaacggaaaggtaagtttaccagcagtacataggagatacagtgctattggcttttgcttcactcataaacaaatgatcaattccttc
atatataacaccatacgcttctcatatatttgtctcagtgatttccttaggtttgtgaaaagccattggatttccttgagctgaaagtattattttaga
agctttctcatatccacatatatttcatttatttactttgattttgttgattaactttactctgctaacccgcgaaagtagcatattctagtaaccggcg
gatcccaaggctttgggcaggctcttgcaaaggaattcgttcttcgtggcggccatgtaaccattgtcgctagaacagagagtaaactgaaggttg
ccgttgaagagctctcaaaattgaaagttcacagtgatcagattgttcaatatagaagcgtagacttgacgaattatgaagatgtcaaaaccatg
attgatcaattggaagtttgcccagatcatttggctcactgtgctggttcttcttatcctggctttttttgtagaccttcatccaaccgtgtttgagaatc
aaatgcgccaaaattacatggcctctgtctacatcactcatgcactaattaagagaatgaaagatatttcggtgccctattcacgaagaataatgt
taacatcttccgttctgagcgcccttccccttgtaggttataacgcatatagcccttcaaaggcggctgttcgagctttagccgacggtttacgacaa
gagtgtattctctatggaatagaagtctccattttcttacctgccaccatcctttctcctggttacgaagaagaaaacagactgaagccttctttggt
attggagatggaaaaatcagataaagcacaaacatgcgaaaccgtagcatattattgcatgaaaggacttgatcatggagattttgttataacca
ataattttgtaggagatatcatgaaaaatcattctcggacttccagtcctcatgataatcctattttggaattcctttattgcatgcttactttctttgtg
tggcctatcgttcgagctcaacttgattacgatgtttacaaatatgctctgaaacatcgtttaagaactgccattcctagtcagtcaaactcattcat
caccgtcttcttgacatctatacaattgtgcatttttttattaccttattccaactctcgtatctaatccttgtgtgactttgttaaaatcttttccactatg
gtttttattgcaaacgatccaaacatatttccaaagacctcgtgtttgtttttcctttactaatgttatccgttctattggcactatgtgtttgggaactt
ttgtcatcgctttcgttctatttgcctttggtgcacccttggttagcaagtttcagttaagctttctttgcgctgctactctttcggtccttacaatatatc
cactgtctttttctttcaatctgactacatgagatggggccaattcctcgctttcaaacaattcaaagccttgggtagtttacaataccgtgcatggg
gtcccataattggtgcttggttaggtgctattcctattcccttggactgggatcgaccctggcaagcatggcctattaccattgtcgttggatcattca
ttggtcatcttgttgctacaataatcggagaattattacagtaacactctcatgtttacgagcatactttattagattaagaaaaaacgaatggaac
tgaaagtaacatgtcaaaatgcaattttatataataaatcatcataaacccaagaaacccggcaaaaaagatataaatatctactgaaccttgaa
atttttcaacagcttttcttcattttcctccaatgccattacagaaggggttaaggttggttccaaaggaattttagctgatttgctaaaaccgatttg
ttcgcatatacaatttaataaaaattcacttttacatcgtatttgcagcagtgggatagcttgccaagtagcacgttcgcctaccaatatccaatatt

gaatatcttgtagagagggcttcgtgaaggcatgctttatatcggtaaatgagaaggtaggagtgagcgttatgtaagcacttggaataaaatcg
tccactaaaaaacgccttaacctcatagggaggaggccaagaatatccatttggcgaaaacgataagccatttccaaaccaaacagacgaatta
tttttaaggtataaccagtatgcgtatgatggtgcagttcatcgtggaatgttggaaaaagcgagggacgagactgagtgattataaaatgattta
cgttgaaaagctcagaaattctagcataagaattgggacgggtagggtgattcatgctcgcatatggccatacgaaatttttggagtacacactt
ccgtagtgccgtcaggtaattttgctaataagctggttgaacggtaaattgcagcccaactgttactggcacaagcagcactccataccaaaacat
ttggagctgtatgataatttaaaacctgggggaaccagatgtaacagagggcggggcgataatgttgatagcacgatttgttttctcaaaagctt
cttggaaagttaattctccaacacatttatgaacaaaagaagcaatcatcgagatgtctagcatatttccatattttatgactttagctagagaata
gtcagaagtatttttacaaatatcccacaattcttcgggaaacgtttggaaaaggtgatctagttcatcatcacgatgtacacctaacaaagcagc
aatcaatgcgccagacgcggaaccagcaaggacccgtggtagtaaggttctttcccacaagacttttgctacgccaaagtgatataacccaaag
gcagtgccaccattgaggtataaacaagtggtgccaaaggatacttttgcagcggaaaaaaagtcttgcttttcctcgagggatagctttctcgta
aggaaaaggaaattcaagcaacgatttacttcacgaacgtattcctctattaggatctttgttcccgaataattttcagtgtacaggcctgaatcac
caatgttgccgaaattgcgcagcaagctagaacgcaagaacatgagcatgggataggattgctttttcgttcgataaagcttcaaggtcgaggta
catcgttcaattagggaaaaatcgtacttgttcgaaacgggatcaaatctccatttccaacgacctgatttcttgtcggtcgctgttgccagctgctt
ccaatcttcccaggtacagcaatcgcgcttcaatactgttttccctttccgataattcaaaccgcgtgcttgcgagatggaagttgctgaaaacaac
cattcaaaaaaagatagaaccagcagccaaactgtgcacgcatttacccttgtggtttgccaagccctgcttaacgagttcatgataattgtccaa
acaaaccttttataatacatgtactttgaaatggaaaaaactgaacaaactcgtctattgacgtaaatcttccaaagctttaccaattctacctatt
ctcagaaaagaatcttcacggaaggtttgttttcttacaaaatagattcaaaaaaaaattctatagtatcaattccattcgcacgaagtcatccatt
tgtacgtattttttgcaagcacctatatcaacaaatacaaggactccatatagcaaactacagcgcgatgcctaaactagcctatagcgtattgca
atgatagagataataaacgaaaatgtcaactatgaattagcgccccatatggatacctactaagaaaaaactcaatgatgactcctattatacag
ttgggcctatttttctgtgttttagcaaagtcaccgagacctttttgactgtctaagcctaatctttaagttcgtgacagtaatcttgttaatactgtta
ataaaaattcatatccggattgttcaagcaaataacaggaatcagttgactttgtctaaggtatcacaacaaataatttagacgcgtcgcgcggtt
ttttataaaacttcattttacgagcaaaaggaaaagagggaaacgaaataaataaaagtatttttttaatttatttcttttatttgtgtataatttat
aggtttctttttttcttttgccaagcccattatgggggataatggttctttgggctattgaatgctcttttggtctattgaagacagaacaaagagaag
gacccgtctaccccatatttgttctgaaaaatgagattcacacctatcatgaacaagaatcctcacagaaggagaaatcaattggaaacgctttta
gtttattaacgagcattcgaaaccagaaagcaaactactttgatgtgtttgtattattgaaaagattcggcacccctgagctccttactgtctatata
acttgtggttttaggataatattattttcttttctatcgttttcttctctacattcctgttctgttctgtaagttgcaatgggcatccaagcgattgcgcgt
caggattatgtgcacagctcagaagcttctcatctgtcattccatacaggagacacgattattgttgtcaacatacttgaaaatggttggtgtgacg
gcatttgctctcagcgtcgaggttggtttcctttgtcttacatcgacattcccggattccaaggtcaagcatccaaaaaatcgagaatttgcagtgat
caagattctttgaatgattcaaggagtagcctagacttcccacctttgccagaggatgactctgccactaaaagtcctcctgctaaggataacaat
cccgttcatcaaactatccttcaacctctcctccgtaatactctcgaatatgtaaccctcatccggaaatccgtttattttggccgtactcaagtgttg
tctgaagcattagatgtaatacgtacaggagcattaactattctcgtgcgtcttggaagtcttttagacgaccattctccgtttacaaaagagcttgt
gtatatattctccatgttagaaacattggtgacgctttccaggaggaggaacctgtacaaaagctcttgtatacaatttacgcagcttttaaaaaca
cttgcatgtttgtttgtggattatataaaaagggaagaaaaggaaccgtcttccgaaacatcgaaagaagccaaagatgtctgctttttaccttcg
agtcagtctttggtgtctccaccttttcatcagcttcaagataagtcgctacctctatgtacaaccacttatgttgctatactttcccttactcatcacg
catatactttttatcgacaagccatttctggcaacaaaataacaccaagcaactcgatttacttttccagcatatatcggaaggcaagcggtccgc
aacagaatacttggatacagtctttgacaatttcaccaatatactccagtccttaaagcgcgtccactcagcttttcagaaagtggattatgcagcg
tttgacgtatcagtgccttcaattacgattcatcagatttttgggtctttagacaattattctactaaaattagtgatctattcatgacagcaatagag
cttgagcaagcatttgatgatgttatctataagaataggagcatacgaacactcactccttacttgctacttattgaaacaaattttcaggatgtaa
aggatattcttcttcatttaacggaaagattccttttcctctctcttgaacatcgctacctaacccaatttattcctgggcttgaaagcgtagagagta
agcaaatagaactcatggatatggatgacattacttggtacgattcaaaggggctcgtccattacttattaaaacaatcgactatagagccaaag
tccattaggaattggttgtttctgtattggtcaaatacaacctcgtacaacgatgatggaaaagtcaagtttgcttctcttacttctattttaaacaat
attgttcgtgtagatattgacaacacgctatttataagaacttttcttagcacacatgctagtatcattagctcaggcgatttgttttctattttatcttc
gcattatttatttcttggtcagtcgcaaagcttttcaggccgagttgatgctactcttataaaacgcggccgtaaaaagattgttgacgttatgctca
cttggttagaatcatactttattgaagaattggaaaatgtacatacgattttgttcttgacgaatgcctacttattttgtgaagaatttatggtgccta
ctttctctccttcacttgagttgctgcaacatatttctaatatgttaaagcatccctccacaaaacttgttaggtcattgacaaatggccttgaaatgc
aagatcctgaggataataccgcaatttcctgtcctttggatagcatgaacgttaaagatgattcccttttcctttttccgcccgatgagatcgctaag
caactatgtcttattgaatttcatactttttcaaacatcactgctgttcactttcttacaaaaatatggtcaaatttgaaaaacatcgctacgaatgaa
tcgagtactgtttgttacatttctaatcagttagtcaactttgttgccgaaactattgtgagagaaagagacttgcgaaaacgtacacatttttaag

ctttttcattcaagtctcacactgtcttttcaaatacaataactttgcgagtttattttctatcatttctgctttaaattcggctccagttcatcgtcttaa
aaggacttggtcgaacttaagtaacaaatattcgatattgtttgactcactgaattctttaacagatacaagcaaaaatttttctcaatatcgagaa
tgcttggagcgttgttcactgccgtgtgttccatttctaggcgtttactttaccgatttgacatttctaaaatcagggaacaaggatacaaacaaaa
acttaattaatttcgataaacgcatgaaagcagcaaaaattttagacgaggttcagcagtttcaagctgtcccttacaaatttgatgctaccaatg
atttgcaagagttgttacatgaggtcgtatctaaagaacgagactctaactttatttacaatgagtctttactctcggaaccacgagaatctgaaga
ccagaccctacggcgactacttgtagattccggtatattttaatggtttatgatttaacgacgactgtatgatgatcatttgcataaaattttggtaat
aaggtgaattgttggataatttaatttaacatagaatacaataattaaaaaaacttctttttttcatgggtgtttgcaatactgaataaattcaaatat
taaatctgatagctcacgacttaaccaaagacatagatatcatggattggaaaaaatcggtccagaaagctacggcattgacgattgaaacaaa
tgactatgtttcatattgcacaatggtcgaaatgctactagaaaaaaaatttgacccggaaagaagaggatcagttagatttattacgttgcttaaa
tgatgagcttcgaaagccggaatatgtatgcttcattactatcaattacgttactaactggatacttttaggaccaactcacgaaagagattgcatg
ggatgtactaggaatgttgctgccatatgtgaatggttgctcttcagaggaagcagtaaaatttatgcgtttttttggcagaaaagggaaatcctaa
agaagtttttttaaagtctaatgaaatccttacgaatatgatgttccagaatctgtctcagttcgaggtgactgtagactcgatgaattcagctatta
acagaattgaaaccaataaaccattggtttttcttgatagttacatagtttctttgtattctgggattactagaatcttacttgtgaaggaagccaac
agatttcgcatatgggatatgtgcttggattcaattggtaaaataacacgacgttttgggaaatattcagagtgtcatttgaatcttatggcagcttt
tacaatctttcttcaactgttcctttcctctgaatgcttgtttttctctttccgtgaattattgaactcgaaacatcctcatgtagaatttcgaaaaaata
gattttcttatgatgttcaacaactacaagaattggactctttttttggtacgattgttagatattttcgatgaattatgctctatgggcgaaaacggct
gggaacagttagtaaaacgattctatgttcagattatgaatcttgatagcgctgaaggaagtatttgtaatcaggaagaggaagaacaagaaga
ggatatgcatcaaaaagtaatgatagacaacaaagggtgtgttgctttgttgacgattcggagcttttatagggaaagaagcttcttacaggattt
gtttttcaatagcaaagaacacattctattggattcgttatttaccttgatgtccatcgaaggaaatcctgaagcttcgggtttcactgatttagctct
ttatcaaactttgttactctccaagtttattattgaatgctcagctgaagatcatgagcggtttttttctctattacaaatttatcagtacttttctgcttt
tagctcagaaccgtggattcgactgatcgcgaatgacattgtgtttcatctgttagattctgtgtcccctgatatccgttttaaatatgttttagacac
cttggaagaatgcccttttgtaaatgttaaagctagcattttgaattactatcaaaaaaatggattactttctaaaccggtcgaaaacaatccttttt
tcctaaaaaagaatctttccacagtccttaaaattctctttgacgatttattcgaccttacggctgaccctctttctttgatttatttgcagcaagcatt
aatctttctgtactatttgaaattccaaaattgtttggactataatgatcaagtccttcaatttttaaaaagaatagaaaattttgttcagcaaaacg
aagagaaagttccaaatcagaatttaacttattatctggagcttttacagccaaaaaaattgattccttgcttcgtatcatgattgcctgagcaccca
aagatgtaccattcgttcactttatattaaaagacatattttaaaaatcgtttctttctttcttttttctctcccagctacaaagtacacgaaaccaaat
atgattttcacacaacaaatgcctaaaaaattgctatattttgaatatagaactgaaacctatacgttatccagcttgataaaataataatttacttt
taacatattacacttactaaatggatgcattcaatagaactttagaatacttcattatgaaatcaagtaacattgctctcctactaagtacaggttac
ctttgagtaattctgatgagagcaaacactaaaaattcattttgggagcaaacttgaaagtgtaagcactgtctttgggataatatagtgtaactta
ttgctgtaagtctctaaaagtcttttgtagcctgattgaatgaaattgctctctgtggaaaagacgctaggaaattgaattggtttaagtgctaaag
aggtcggagcttctaattgaccatcactaagaatatcggaaacaactactatggagcctggattccctcccatgatggaaagaagtccagcctac
agtcatattctgatggctggaggaattggcggtgctacggcagacttattgatgcattctttggatacagtcaaaacgagacaacaaggagctttg
tatacagacaagtatagaggaatggtccgtagttatcttacaattttcagagaagaaggtctttttcatggtttatatagtggagtgtgcccaatgtt
actcggcagtcttccggctacggcattattctttagttcttatgaattctcaaaaaggcatctgatggacacctatcatgttccagaaactctttcatt
tttactcgctggttttgtcggtgatctttttgcatccgttgtttatgtaccatcagaagtaagtctcaattctcattattaaatatcgaaatttggaaaa
gtttttagttttcttctaaccggtctcaataggttttaaaaactcgactacaacttcaaggacgatataacaataaatactttaagtccgattacaat
taccccctctttccgaggcgccattaagcaggtacatttacatcctaataattaaaatgtagttttcatacatttttttactaaccggagatttagattg
cgaaggaagaaggcccaagaacatttttctttggttaccgcgcaaccgttctacgagacataccttttttctggtttacagcttctattttatgaaaag
ctccgtcagggtgtgcaaaagtactctggtaaaaaagatataggtgttagatatgagttgattattggttcattagctggcgcaggagctggatttt
taacaactcctttagaccttgcgaaaacaaggcttcaaaccatggttcgaccgacaggcgtttcggataatataaaaagtggtcgttatttctatg
gtcaaatggaaaactctttgggaagttctgcctcctctactagaaagcaaacaattggaattagtaaagtcttgtctagcctctacagaaaggagg
gtgccatgggattgtttaaaggtgttggccctcgtgtgttctggacttcatctcagagttcattgatgtttgtttttttacgagagcataataagactttt
taaacaaaaggaagctcaatctatgttggattgagttcattatctatatatgagaactattgcactattctttcttttttggcgctcaaaaaagcctct
caaaatccccctcatccaaccgtttttagcttttatcagcaatgatatgttttataaagcctattagactccccttttgttctttttatgttggctatatc
attgttgtttgaaaacacactgctttttgaattcatagaatatttgattcatttttacatttatttttgtcttctgactatctattccctccttaaaaatgtt
gaaaaaaataaaaaaaacaaaaaaaaaatctctccctcaagaattaaggtctgcactatggccttatacctttaatgaaatagaagatttagtc
actgatcccctttttttgttttcttctttagaatacaataataatattatagtctacgataacggcatgacattcgaaatattagcttgatttcttccatat
aaaaatcaggaaagctacagccattattcactgtattaagaaacaaattatttgaatttattaatctaaaactaacaaccttaaattaaaaaacta

tcgtatagcaaaaatattatgctcatgcaaaaagatggatacttttaagtgtcgtaagcagaaacatcattgaaaaatgacaacggtttaacaat
gtaagtactgtacaaaagaccaatagtaacgagggcattaacaatggcgtaaagaaaaaccaaataaaataaaataaaaacacagtcgggttt
ccacaaacctttggcgttacccttaaaccacttatccaaaatttgaaactcgtagattcccatgcatctttcatctgacgattatcattcaattgtaac
gttcgttcaaatgcagatatactcaatcaagttatatctatacgcgaagaacgttaagatgcttcagatgatgaagcgacaagtttcgactttttg
gagaagtgatatgagttctatcctcgccctcatttaccaaatcctcaccaagtatgttatcgtaatcatctagtctccgttttccactctgagtttgac
tttcatttattacatcaagttttacttgctcttcatccagcaacgattcaaacggagtcgtaccagatcctgctatttcaccattattgtttatgtcttcc
aaaatagaagtcagagcgctatcatccaaattttcagcattcgggtcattatcatctacccctgattgctcatttgctgctatatgctcctctgtaga
aacttctatattcttagcatcacttttgttcgaagaggcttccccttcatccaatacatccttagcagcgtctatccgatcatcattactagtgtcagg
aggcatattttcttccgattcttttgaaagatctggagttatcaccgaatcctcattgtcttgttcggctaaatccagttcacttgcactagcactaga
aacatcctgaacaagctcgttgtatctagcaataaatcgaggttgagtcgtaaataagacagaaaatgagttcccatgactacaatgagcagata
agagttctagaatatcgactaagtgaatctgattggcataaacattgtcctcagaaatttttaaattcaagtctgaaaattcgcatactagctctaa
atcagagcttaatgtatccagggattccagacgtgctcgaacaagcccaaataagtcttctaaagtattatcgtttaaatcatcagtagcggaata
aatgaatgaattgccgtcattattgtcaataaaagagcaaacgcttgaatctggaaatagaaccaagcctttagatatccactcaagatctaaaa
tatcattgctaggtgtgacgtttgcctcttcctctttttttatttgctattgctttattttctgttatatccgtgaatccagataaatctttagattcttcctct
tctttattaccatcaatatcaccgttttcaacaaaagagtgtgaatcatcagaatctactacattgtcatcttcttgcacttgcgtttctgttccttgat
cttgaagaaacatagaattggtttcctctttcgacgaatcacccaattgttcatcttgctcaaaggatttctcagctacatttgattcggctatttccg
tttgcccttcactttctcctgcttgagatttctcagctggatccaacgcactagaaggcatattatcattttcaatatctggctgagacatctccgtttc
tttgactgccgtatttggtgaagcttgctcagtattttttattgtctgtttgtgatctgtttttgtatccataaacactagagtttctactggtgcataaa
ccacacttccttcaaaagcaacttcactcataaatatttcgtttaaaacaattcctaaacctgtttttgttagtagttttctgtagttaccgtttcttta
aacaaatgtagttggcaaaaggtaagttgcattcaagaaagcatcgttgcttacgacgctcgctcgcgttgctacgtggccttcctcgttatcctgt
tttacgtgacttcgtaaatggatttcatcttaccatttatgataataatgtttcgtacagcgtatagggataatacatattggtctcacgcacctgaca
gtgaaattcttttattgagctttttttcgtattgagaggtgatctgtttggttattcctttttagtttaaatatctagtacgcttagtcaaagtaacctaa
caagtcccgggtagctcaatcggtttagagcgtctgactcttaagaaggttatcagaaggttgcgagttcgagtctcgccttgggagtttactttttt
gtcataaggtactctaaatggtttatgaaccctgtaagacatatcagacgaattaaaaataattaaaataacttgtaggtgccgttgacaatcctc
tattagctaaatctttagccattcagagtaaggaatggtagaggcctgaaagtgttgcagcaaacgtgtgtacaatcagtccgttcctttgttgaat
taacttagcaactacgtaaacgaaatttcaaggaagctataagaaatgggaagaaataactttaatcgttcgtaccttgtttcttttattgtaattct
tatgtttttgaatcattactaagcgtcagtggccgtttggtctagtggtatgataccatcttagggtgatggtggtcctgggttcgattcccagaaca
gcccatttttttaatttttaaacacttttttgaaaaatggtttgtgttttccaattaaatatttaagccagaaagcgtctttgctttgttgaacagtcttctt
acgagtcggtatggatagctcttagcaaaggtgttttcaacgaaatattccttagggaacgtcgttcttgatgagcttatactttaccggattttgta
ccgataccgtaaccatccttaaaaataatagaatcgcgctgcatatccctatactgaaagctgtacgtctgtcctaaataaatgtttcgctatacca
aaaacaacccaagttaaaaaatgtcaaaactaaggtctacagctgggtcaaaagattctatagttgatcccttagaaacaaatgacaaggagac
aaaactacttgcttacgtgaatgataaccgacacttttctttactaaggtaggattgtatgaactcttcggtactcgaaaggcatatgtcatgagga
atcagttgggtttatttgatagatggctaatgaattgatagagcccttcatcttgctgatctaattactctcttcaatggattttgtggtgttatgagta
ttttttctagtttgcgctactgcttgagtggtcaaactagctccaagtaccttgttcgggctatgtattttatgccgtttgctctgttctttgattttctcg
atggaaaagtggcccgttggcgtggaaagtcttctttgatgggccaagagctggatagtctagctgacttggtaaggaagagaaagcttacgaa
atgaaaaggcgcatatggctaattatttctttcagatatccttcggcgtatgtccagccgtttttgctttttgctgcggctttcaaaccttcattgattt
aatgattcttaatttcttcgtcctttgtggtcttacccgtttagctcgttttaacgtttctgttaattccattcctaaagatgagtctggaaagtctcaat
ttttcgaaggaaccccaattcccactacccttgccttggtttctttacttgctttttgtatggtcaaaggaaagattcacgaggaactccctggtggtc
tttggtttccagacactccctttgcaattcatccgatggtatctctttatgccttgagtgggattgctatgaccagtaagaaactttgtgtaccaaagt
gtatgtgaatgcatcaactcattcttttcattctcgttttctaaccttgctagtttaaaagcctcttcttagatctcttcttgcttaattatgactttccat
aacccatttattcaatctgttcattgtacgtttgctcatccatattgattttttttggttagctagactttcaaaaatagcttcaactctatttatctgttat
catgcgtcgacagtttttttcagtctttattattatttgtttaccttttttttactgctattgctaggcgaatttccttcagttctaccctctcatacatattc
ccattttaaccttcgctttttccttaatcccaattcttttttatgtgggcattacagtctgtgcaattaatagaaaggttattacgtactaaaactatgg
ttacacgcagaaatacgaatgcagacatgggtaccctcaacggtaaacacgtttttattcatctcttcatgattatgattgatgatctctgtttccttc
attaagcagaaaaccgaagaaaaaagaaattcataatataataaataaatacactatcctatacacagcctctgttcaacaatctcttgcggtag
gaacactatttttgaaaaagggcataaagcacttccataataatgtaccagaaaactttttctcgttactctaatccgcgtttgttgtttttcacgac
agcagcaacaggatctttgataccttgattctcagatccaagacccaaacctcggctccaacccatcatttcaagtagttttttacctttacctgctt
caatggtgggagcaaaagaaccaactacttgaccatctaattgacgcttggaagtaagacttgtctctttgctaaccgactttgttttgaactttctg

tgctttttagttaaagcaggtggctcttgaagtttgctctttctctttttagacattttcacctctttttcaagagaaaaatgacggtcgtgttcaacaa
gactcaacatatcggcttcatgatcagggatttgttccatgtaatcattcctagcgctttttttgccaaataaggctttaaacttcacaaaagcatctt
catcacttaaatgccttttccctttctttcttttatcgggaacgactttgtttttttcttggaacaggttttggctgaacttttgcaggttcagtttgcacat
tttcccattgctttgaagagatgggaactccgtcttcaataataactgccatactttaatctaaactgaaagcgaaattctcaaactttcgattaacg
ccacggtgcaaaaatattttgatactttccagttcttaggtagctaaaaaccgtaaactgcaaatagtttatctcaaatattggcattgattatactg
aaatcacataaaactcgattcctcccaacatataagagtttcccaaaaatgggtttttttactctgtcgctcctgtgtaaaaccggtataggtagcttt
tagcaacaatgttacctcaacaaggacataaccttttggtatgcaaataaataaataagcagagataataataattttcggttaaaacgagctttc
taccttcttttaaattagatattcggtagaataatgagaacagtttggaattgagtagacaaattttgaagatccctgcaatggacgaatccttaac
agagtacaacagaaaagaaatttttcttcatttaatttgaaagaaagtttgaacacaaccactgttcatggacgtaaagtgagagatttcattacc
cagtgatgtgctgaaatttaaagggctagtttttgagccgagtgttcgtttgatatttaattggcactatccagttgccatataagattacctgatca
gtcaatatttgtacaaagacgatcacatggaagaccgtagtatataaagctttgtgagtatgttttcatggttcaacaaagcaattgagatatata
cagattgaacttagtaaaaacagcccactttgcaaaataaacaattcaaactgttttcgttttctggaaaattactatttgaagaaagcggagtac
aaacagcttccaacttctcactgtattatctcgtgagatacgacggtgagaaagacagattcagttatcttataacgagaacaacaagtatttttct
ctcatttatcatcaccaacatcaacgcattacctagatttcataacaacaggttactgcaaacgggagcgaatttcgttaccgagatccatcaattc
ttccttggtagtagcataagtgggccagcctacacccaaaccgtattcctcatcgggtttgggaatgatgtgaaagtgaacgtgattcacaaactg
atgagcaatacggccgttgttctgtaaaaagttgtagttttcagccccaatggccttggctactttcttagccagaggaagaatatcaatcaagctt
tcgtcactcaagcctgttaacttctcagagtgttctttaggaatcacaagagcatgacctttggcagtaggggaaatatcaagaaaagaaagaga
ctttgcggtctctgcaagcttcacacatgggatttctccccgaataattttacaaaagatgcagctcatttttagttgttttaggaaaattcaagtaa
ctccggtcacccaatatatatgtcaaccttgttacttatatgagcatcgaaaatattgtcttaaagcaacagcttttgtttatttatttattttttagaa
aaataaaataaatcaacgaaagagcacctcagctacgatcggccattcatagaaaaaaaagaaaaaaaaagccatagacttcgtttaaaaagt
taggtgtttttatgatttggctgtacctttttgggacgcaagactaatcgagggtctccttgcttaaacatatttcgtaaacaaatgttctgtgcaagg
gagtaaatagcagaagttgtccagtataacgataaagctgtcgatgttttagaagccatgaatgacactacaaaggcagataaaataccagctg
tgttgaaaattcgtagcagccgggaatcctgaggcctccgaagaattgacatattggttagataggtgcacgctagaatagctggtagaataccg
tgtgggtcaggcatagtcaaatcgggaaaccaaagcatgccctcagtagacatggtatccggagacacttctacagccttgcgaagttgaaaag
atgcaagagcaaacaagggaatttgcgtcaatggcaatgcgtaaagcataaccggatgacagttaaaccgtttatatattttgcttcgaaatgttc
ggtatgctttgtcgttcgggcatcttttttttgcttcctttaacaaaggttgaacttgagcatacttacgtccagttttaatgctcgaaatagccaacg
gcaatgtaattgtagaacgtagaaatattgccattgcaggaatttcaaaacaccatggcatatacgcatggcagcttagtaacgtttgtaacatttt
ttcacggttcaccaatgaagcagacttcctgtcaattattatttagctaatggtcttttcgaaacttttgttcgcgtttataatgtactaaaagaaggt
attctaatattcaattcagtttttgaacaacgtgggtaatgtttgcacttggctggagacgacggatttcatttttttgaatttaatggaattttagtatt
tgtttttcagtatcaatatttaagtattaataaagcttttatctgtatcaaaatttaatttaaagtatttcatccgtaatgtgttaagaagacgaccttg
aaaacatggtttcatatttccatgaccgtcgacagtatgtttaaacgatgagctgcttctgtttctgttaaagttatacacagtaagctcggctggtc
gaaaaaacaatcagaaaaggatagaatttgtattacattttatcgtcttgcttcaaatcaaagcaagttgtaaataatctaaataaaactttcata
catagttcgtcaaagtggcaccatcaaatacttctgtacctacctgttcaagcatccatcaagtcttcattacaagaaagcaaaagtttaccagttg
tagctctgctttgaaacttatcatgagcttctttggcttcttctaacttccaagtatgatgaatggcgatattaagcttgttctttaatatttcctcccaa
agtttgcttgtataacggtcaaatgtctcttcagagtctagatacccgaaaagggtaggacgaacgactttgatacatttagaggtaatcatactta
aagggatagattctatttttccactagcattaccgtagcttacgaaggtaccgccgttacggattgcgtttatggaaatttccagggtatctttgccg
actgagtcaaaggaagcatgtacacctttaccgtttgttaattgacgagagaattcaggaacctcatcataagaacaagcaaattctgctccattg
tgaagagcaagtcgtcgcttctcaggagttgaagcggttgcaataacatgcacacctctacgattcaaaatttgacacaaaagcaagcctactcc
tccggcggcagcgtgcacaataacgtagtctccactgcggacgggataggcttcttcaactagagttaacacagtcaatccttgaagtaaagaag
cagcagcgagcttgagagaaattccctctggtatcttagtagctaagacaggagaaacaagattatattgagcgtacccgccggacattgtcaaa
tatacaacacgatctccaaccttgaacttgttacattcaggaccaacatcagtgacaataccggatccttctttaccagaaacaaaaggcaaagg
gacaggatacactccacttctgcaataagtgtcgatataattcaagcctgcatatgcatttttcaccaaaagtttgtttccgctaactttagggattt
cgcgtttgacaaattgtaggacagaagagggtccgtatttagagaattcaacaagatggtttgacattatagttataattactatgaaatgcgaaa
aaactaagaatagctcagaaactgtacccttacgaaaataaactaggaggatgtagagttagaagaatactttcgaagaatgatttaaaagcgc
acgtcattacaacaatctattattaaaaaaagagaataaactttttttttaaataaaagtagaatattgaaaaaactgaaattgaagtaagcagc
gaggtaggtgaatcgtatataattcgtatctcattcattacgtacgtacaggattcaagaaattgaatatcaagctaactacgttttaaattgtcta
aacgagccgctagttcttcaaagcttggtggcttatcgggcggatgtaaagcgcttttagcgacaggcggaacgtgagttttgggatcaggttttg
aggttaaaggagaggtagaagtcccgttagaaggaccgttagtagcagatgaggtagattgacggagatcccctgccttcgatacctcgtcttgg

gttgatgaaggtgctgacatatcttgagatgaagacgctaatggcaatagttcctctgcttcttttgaagaaggaatctcttccttctgatactcttc
aggaaaataagttcgtcgtagttcttctatatagccattaaccaactcctcagatggaataggagggtataaaagcttgtaaaactaaaatttatt
aaattgttagtataaggatgaggaaaggaaaatctctacttacctgctcatcatttttggataagtcaggatccgttaattgggtaaattctttgcc
atagagtctcaccaagaaatcatgtacctgttgtttaaaattagtttttagttttcgtatgttaatgacttacagatttaagttctttaatgggtagctg
aggagctgcagcaaggacgtgatacaaagatgatttccaaggagcttcagcattaaatcctcgtttttccaaaatagaagtgcgattggcaagaa
tctctgcatccacttgtaaaagctcaagcagttccatgtatacatcctgcgtaattaagggttctactcgagctcgagctaaagttggtgaatatgtt
tttaagccatatgctacagttctccttgcttgttttgcaagagcctcttctttttcctcaaaatttccattctggcagcagccaatttgaactgaagtt
gaagtctcgacatgatggttgcagttgacttagcaaggcaccgatggccacgttttacttttctttttgactggtaaactctattatgcttgttcgtat
acaattgttagcccgtcggcttttcaagcgagtctccttctcgttgtctatacggtttggtcacgtacatgtgttatatccaacgcttttccttgtttga
aatatgggaaatataagatcgatgttcatctatgaaacttctttaattaaatctttaaattaggataaatggggtgctcttgttcagactatttttaa
gaacaatcaaagattcaaaaatttaacgatggcttatgggttgcaggctgtacatttgtttttaaagcttctcaaacttgtttaaagtagtttaaggt
gaaaatcgagaaatcattaacaggctttgttcacattcctgatgtcgctttagtatcgaatattcacaaaaccattcaacaattaaaccgagcgct
aattcagttaactaactttttaataagaagaaaaaaaaaaatagagacgatattcgttgaatcccttcgtgactctaatgttgacttgaaccatatgt
gaagaacaaaagtaaaatgaaactcaattcatttgaccaataagtaaaaaaccttaagaaacttcttatggttctttgtaggcgctctgtcctgtct
cagattttccaaatttttttgtcgagcataccaacaactgcctttcttaccaaaaacttatttctgggtaaaccagaatgtccgtggaaattgatgga
aacccatcttttgatgataaaacagtaaagaatgaaaagataaaaaagcggaaaagaaaagatgaagagaaaaatacaggctctgatgctgc
cagcgctgcaattgagcgtttaaaaaaatcagctagcctggcccaagcggcgcaacagaaaataaaacaagaaagcaaaaatgaagaagaa
aaagatattgaaatggaagatgttgaacagaaggctgcactacatggcctaacaccaattcctcaacccagtatcgatgagtatttggaagcaa
agtcttctgctgctccctctattgtttcctctcttccaaagtggctttcggagcctattttggttgatcctactacaacaatccaattttcgtcacttaac
attgctagtaagctggtggaaaaacttcaaagtcaaaacattgcccaaggatttgcagtgcagtcgtctgttcttccactgttactacaagaagga
agagacggtcctagctactcttatggaggtgatctttgcgttagtgctgcaaccggtagcggtaagacccttgcatacgttcttcctatcgttcaatg
tctcagtcgccgtatagtccctcgtttacgatgtgtcgttattgttccaactcgggaactaacaattcaggtttctcgtacgtttgagattttcgcttcc
tgcatgggacttggtgtatgtggatggactggacagaagaacttactgcaagaacagtatcagttgcttggaggttcagaggaaaatgagagca
cagtggatattttagtgacaactccaggaaggttggttgatcacatacaaaatgataatatctttagcttacagcatctacgttatctagttatcgat
gaagcggatcgacttttggatcaaagttttcagagttgggctgatgttgtcatggccgaaatagatcacccaaaatttttttcaaaaggataacgag
ttatcaaaacaaaacatagctcctttttttcctctcctcattacctacggctttacctcatcatttggattcccctgtacaaaaactgatcttttctgcaa
ctttgactcgtgatccatcgaaaattgcaacccttcgtcttcgaaatccccgcctgctacttgtgcaaagcgaaggtgtagagttagatgatggaga
agagattgagggagatgccattatcttctctgtccctcctacattacaagaatatcgtgtttctgtcagtgccgagaagcctattcttttgcatcacct
tattcactccaacaatcttaacagtgttctttgtttcgtcaagtcgaatgaaggtgccgctcgtttgcaccggttgcttgagatctttaacggtattga
agggaaaaactacccgtgtggtttggtatcaggcaatatttctcgtgatgaacgaaaaaagatgcttaatggatttgtctctggtgaactgaagct
tttggtatgttcagacttgatggctcgcggtatagatgtcgccaacacagagcatgtaatcaactatgatcctccatcgagtcttcgtcgatacgttc
acagaatcggtcgtactgctcgtgctggtagatacggctatgcttggacacttgttcaagaccatgaaggtcatcacttttccaagttgctcaagca
tttaggaagaacactccctctccagcgcctccgtcttcatttgaaagacttgcctaaagagtatgtggaattgtatgacaatgctttagaaacgctt
cgcacggaggtatatgagtcacggaaaagctcgaaataattcaatttgtttcgagaagaaagagaaaagaaaaaaaaaaattgtgcaagggct
ttcattctatttaggaaacatatttaatggttgatatggtttattttgggaaattagttttatttgctaagttttaaataactaattatattcaattcttg
gacggatctgaagatcatgcctttctcgctaaaacttcttttcaattaaagatgaaaatcaataatgaaagggatcatttggcatcgttctttaaac
taacgtaagacaaaaagcttgtccgctttcttcagtatagtcctggccggccctattatacattaatatagagtattgaaactgactatattctcaca
taataataactactaataataataagaagaaaacagcatgcgtgaacctaaccattcacctacctccctacatttcggagacattacaagaaatg
acatttatcagtggaacagcatgtcacggtagatatctttaaagggacacgagttgacgatggcttattattggaaaagttggaaaagaacaact
caccttttgctatactcaatctgactaggttaaaagaacttgcttgctttactgagtggatctatcaagatggaacagccgacattcacagcacatg
ttttggatacaactagaggtgttcctgcagaaaatatttcggtttcggttttcaaacttagtactagttcgggtactcttacacaagagcaacttgct
actgcaattacaaactccaacggccgtgttggtcaatggaacgtacccttgaaagaagtcgaatctggggtttacaagtttcggtttgatacgggt
ccttactatgacgctatgtcggttgaaaccttttatccgtatgtcgaattaactgttcaaataaagaagggccagcattaccacattcctctccttct
atctcctttcggattcactacctatcgcggctcctaagtgctgaggactccaaacgatatagaattccttgcccgagcaacaacagcattgaagaa
tcagaatgacaaaacataagtatgccaagaattaaaaatagaaataaatgttcagaaaagtttactttgatggtcgtcacaaatctttaaactat
gataattgcaagttgaatgaatcaacaacatggataagcattacccccatttttaataagtctactcttgaatttaaaggcaattacttaaattcac
gaaaagctaaagtgctaccgttgatgaagtatatttttacgatttgaaaaaaactttacgataaagcaaccattcataaaccctttttttcctcgct
tccacactaggcaatcatacattggattggattaggaacgactagtttattgtgtatgatgtaagttgcgaaacgcaagaaaaaaaaaaaaaac

aaaacaaaaagatgatgatgatgggtatgaatatgaatatgataacttacagcgtgtataaactgcaatggatctgtgaatctttgccggtcctg
aatccttcaatgagctagttacgaaaccatccttgcagataaagggttggatttgaaacagacgagtttactgttgcgatgaactctcaattcgag
tccctgcagatgagtcaaccttataatcatgcatatctcaatttctttttcttttctttttttctcctcatacaacaagccgaggaacttttgagaataag
ttagagctgtttcgctcgtttcgataaattttttttttgtcatttattgggtgagaagcaattacagtcactatcaaacatccaactttgcttcatagggg
atgctccatttggtggaattttgtgttcgaagataaaagcaaaaaatttttttaaaaaaaaaagtccagattgggacctcctcaggcaacggggtta
tgataaagaagatgaaccgagatatattgtaaacaattctgtgctatataaacgggcgatgggaaatgctttcttcattgagaactttcttttttttcg
cacaaagttttatagaagaatttacagtctttgtttgtttatatctttgtttgtttatatacaaggaaaagtctgtaagttcctttcatagttccttccct
ctttaaattttcatttttttttttttttttttttagacgatggctttcctcaaatcccttgtctctgctttggctatgacctctgctctcctacctgcttctgca
ggtcttattagtaaacgtgatgtggtcaagcccaaagtcatggttgtatcaatgtttgctcctgaagccaatgcttggttaaacccttggaatgactt
gtatgcaaacaatataaccgttcccggtttgaataccctctttcccgaagtgcactgcaatagtgaagagactgtttgccaggtcactactggtga
aggcaagtcgaatgctgccagtactatgacggctttgactttaagttccaaatttgacctttctgagacttactttttgatttcgggtatcgctggtat
taacccatatgcagcttcccttgcttctgtcaccgttgctaagtatgccgtggatgtcgatttggttcaaacaatcgatctccgtgaacttcccaaac
agtatgaatccgcaagctgggaaattggtactgatccttataatgatggttcttctaataatgaagttgtttaccccaagtcatttccttaccaaacc
aatgtttatcaattgaactccaccttggttgaagctgctcttcaagtcgttaaagatgttgtccttgaggataatgaaaaaactgccgattatcgtaa
gcattaccaacaagctgccgctcaacgggctcctttttattgttcaatgtgatactgctactagtgataattattgggctggtaattatatgggagact
ttgctagcaacatcacaaatgtccttaccaattacacaggtcattattgctcctctcagcaagaggataatgcttctcttactgctattaagcgtgct
gctagtcaaggtttggtaaacttcaaccgcatcattctcatgcgttctggctctgatttcgaccgtggtagcgactcggtttcttctcttgacaacctt
attaaggatgatggtcatgtccttcctcttgctgctgctaacctttaccatgctggcaaacctttggtggatcacattgtgaacaattggtcttactgg
aactaagaatggtcttttgtatatcttcattcactcgttaactcttcacctgaaacccttctcttttcacctttaattattccatagttattgttaataatg
ttttttagacgtatttttttggatttcctaattggatttattttcttatatattatttgtatacacttgtaaaagatgctgttcactacgaagaaatttattg
ttttgaatatatttgagaacttatataaaccatgactttacagtcgaaatcttcaggctggaagcttcgattggtctggttaaataagatcgtttacc
aattaattacatagacctctaaagttgaaaacgtttagaaagtcattaataccattttataaggccactctcgaatgaaaccttcaatataagctcg
aaggtcgtaatcgtgaaaatagtttcatcttttcaaacttttaagtcctcaccatcgtcaaaagtcaacagtaaattcatcgaatagctaccaatgg
aaagcaaaactttaacacctttctgtccttttcaatctgtggttactgttttagttcagtgaacttttcattatcgctgcataagtcggaaaaatcata
gatggaccagcccgttagcactcaaaaaataataacaaaggataaaataaaacattggtatttggagattttgcagtaattaactataagatgca
aaagatactgtgctatggaaaaccttggtccatcttgaagtgtaggtagggaagaaaacaggaaatattttatacgtagtgggtgcaataaagat
ctaaggcttttaatagttgagtgcaaattaatacaaaaataagacctaacatgaacaacattgatgctttttgaaagctaatgcatttgataccaa
aaatttactgctttctgtattcttaatcctttggtttgtttacgcgtcagcttctgctaattatcagttgtagataatcaatcctacgcgtaatcctttcg
caaaattaagtttggggatgcggagagtgaaaagcattcaccaataaaaagtgaggttgtcggggtttctaatatctacgcgaatgtgaacatta
caacaagtcttctttcatgcatcgagcgttcaatattgaatgaattttatattgtgtaaatcaagtatttgaaataagaataatttttttttttctccgag
taatatgaagcatggctctaaaattgaccagtcgcaatccattgatctctacctttgtaaatactgcacgttgcgcttgattgacttgacgaagctg
attctttttgacattttggtttattttacacgtgactattttctctataatggtacaaataaacctcttcaaaatccattaccatttgccagatacggtta
ttaaggattgaattgtcatattttctagcataagtcatatactacaaggtttaaatcgaataaaatgatatatcatcgttgggatatggtactagac
aaaaatatcacgaaatagcccagtagttgctttcattgcatctttttttgtaaaaattgtatccgttggattagagatctaatacggtatttccatactc
ttaacagatttagtgactcgaccaaggaaatattaataggaaagtacgggattttcaggcctatgcacattggaaaaaaatttcgcaaaagcgatt
ccctgaacatttatctttaaaagatctttcctataggagtttcctttcgactttcggaatgatcttcagtaggtatttttttctttaaagatattgaatga
aatctcatatccaattatcgtatatctgtctactataagaagatactctttaaacgacctttgtggataccgtaagaatgatgtaaatttcgtaaatt
aataataacgtagcatagggagaaggatgtggagattttggaattcatatcaacttaacctttcctattactttgtgcgcacatctgataccctttttt
agatatattcagtctttttgacgtagttttcggtttcattatttatttgttatgaaatttggtgaaaggtaggtctatccttgttataaagtttactagga
aataagcattcaatcgtcaaatgttatgaaactagtcaatttacaaagaagaaaaggatctaagaaatcgacatgttgtcgttcatttttctttgtt
ccatagtctttaagctatatggggttaatatcgtgttcattcgatgagacgaaaagctaggctattactttgttgttttagattcataacactaattac
ttcctttttttatccttgttcctcgctaacatgcagtctccaattggccctaatctctgagttcgctgaagaatatgtagattatcaaggaataaagcaa
agaatacaattcgtggcctctcttcccgaaaatgagaaagaagcatatttgcaacgagctccagatggatcttggcctaaggattccccagacga
gatgttagttgcaaatttagaaaaagtagtcaatttcttctctacaaacatatctcagctgaatgcttatataaatcgtctctcgaatgaggatgatc
cggaattagctcgtcagtctggttctattaacgaacttcaagagcgacatgagcaaataaaggatttggatttacgacttcagaagctttcttctttt
gctgagtggaataagctggcattcgaaaaaattggcggctagaatggacaagcatcttggtactgaccgacaaaccacattttatgaacggaaag
tttccaaactcgattttgccaattctgcaaaaatttatgaaacgctcctttcattgaagaagttggatagttcgcaaaaaagaatagacactctaaa
aaagctagagcatatatcttcgaaacatccaataaagaaagatttgcttacttacatacagcaggatgacgttaaagaagtggcagagacttgc

ataaatgctcctgaaactgttttgattgatatccttgctgccactatagtagcagcagctaaagcttgcatgagagaagtcgttggaaggatggga
cttcgatctgaatgcctcgcttctgcattacgtcgtgcgataaataatctagaaggacctgaccatggcgtgcactgctttttagaagttgtaaagg
atatatgctgtgttgtgtatctagaaaaagaccatcttaattctctcttaacaagaatcctcgctgttgagaggaatagcaggggtcaaactgtaat
gcactcagctgctcaaagtggctaccatatgttgtgttatgaattttgtaaaattatttcgtccatcccttcggcggaacctttgaattggatgcgtcc
ttattggtttgatgttatgcaagatacccctttaagtttagctattagaggaaaccatgtaaaagtgttacatgcccttttatcttgtcaagacaggg
gtacgccttcgaccaagcctggccctatacctccgttggtgttggtatgcttggtaggagacaatgctggaatgattaaagaacttttggatgctgg
ttttaacagtaatgagcatgatgcgaagggaagcacttgtttgcacgctgccgttcgaaatcatcgctcaaattgtgtagaaacactgatcaaaca
cggcgtaaatcaaaatatacgtgaggctcctttctcttggacgccgctgatgcttgctagcgctcttggattttcagacattattaagatccttgtgg
aagctggctcaccagttgatgcagtcgatgcttccggttggactgcaatggagcacgctgtcgttcgtggaaatttggaagcacttgatgtattgca
gacgtcctttgctttgagcgatggtcctgtaaatcttgattctttatccataaaccctctggagcagaggtcagaagccgaaaacgcgccatgga
acttcaaccgtcttcgtctatagtgattttgaggttaagtggcttaaccggccgtatacgtgtaacacttgaaggtgaaggtacacagtacctctct
ggtaaaacatcgtcgtctgctgtttcctctaagtcatcaacaacagatttactctcaactagtcttgcgactaatatacaaccatcttcagagtcatc
taattcgtccttgcttcgtgaaaagtctgcgagctccactgtggtcattgatgtgccccgttctcattttgatagtgtcggtgaagtagattttgaaga
tgtggttgaacctgatgaaacaaatgacgagtctatttatatgcattatgatgctgctcaggaagtgagtttaaaatccaccgttgcctctcctcctt
atgaattaatctttataactaaagacgttgacaagacaactgtttgtattgatgccttgggccatcgatccctgaaagtgattggtagagcttatgc
tcaattgagcgactttattccaaacttgggtaagggtatgcaatcattgaagccactaccgtcccttactttgttatccaataaagggcttaaacct
attgctaaaatacatgtggatgcgcttgtttcgactgttccggatgaagatatcttgaagttaggttctgagcgtgttcatggagcgtcttctttaga
agccgtgtcgaattttgatagatcgacattggaggataaggacgaattgtctgcaacatcgtcgaaatctccttccgaatctggaaaagtaagtag
cgtagaggttattggacatcgaggattagggaaaaatcaacctaatagtttgactttgcaattgggtgaaaacacagtacaatcttttatcaaggc
tgctgatttaggtgcttcatacgtcgagttggatgtccaacttacgaaggatatgattcctgtggtttatcatgacttcgctgtaaatgaaacaggaa
ccgatgcgcaagttcattctttaactttggagcagtttttgtcagtctcacatagtccttcggataagccggcggaagacgtccgtgagtatagtctt
aaacgtcgccctcgtgcctatagttcttcagtatcaagatcctcagatactcaactgaatttattgaatttactgaattctcaagaagatcccgtgag
tgcgaagcaaaaagtttataaagggaatgctttgggacatagcatttgtgccccgtttaacactttgcgagatattctagaaaaggttcctcaatc
ggtaggattgaatgtggagtttaagtatccaatgctaagtgaagccgaggaagagaatctgttacctgttgcatatgattacaatgctttcgtgga
cactattctttccgtgatacaaaagtacggaggtcatcgacgatacatttttttctagttttaatccagatatttgtattttactatcacttaagtgctcc
gtaccggttctatttttaaccgaaggtggtactgcttttcgcactgatgccagagcagcttccttaaagaatgctataaaatttgctgcccaatgga
atttgcttggtattgtaagtgcttgtgagcctttaattcaaagtccacgcttgattaaagctgtgaagcaattgaaactggcttgctacacgtacgga
gtcttaaacaacaatactgacaacgtaagacgtcaagttcggttcggcgttgatgctgttattgtggataatgtgttagccgttagaaaagcactc
aatgaaggtgagaggtgatatgtttatgtgatgtgataatgttgttagaaatgagaaaattatgagaaaactagcaatgaatttatttattaaata
gtaaaacttctgaaaagaaagaaaaatcaaataagatgcatattggtatttatggagcatcatctgcactttttctgcctaaaaaagtattgtatat
acgacgggataatgctgttccaagatttctctcgctcacagcagtagcaatcataatattagacaaagattcttcaccatttgcttctaaatattga
accagcttgggaatggatggaaagtgatcgcagatagcattcgaaatattgggggtaactctcagtatttgccttagcataaaattcagtgtattc
tcaactcggtcagctgattttacgtgtcctatttctgctaatgaagttctagcagacaagtgggaggattgtttatatcgtgatagagctatgtcaga
agtaaaagacttaatccagtcggcagtttctttttcatcattggaatagttcaatagaacattatgctgaacgttcatcctgcatacttcattctccag
tacttcttttgtgaccgtctgatattttgaaagggaaccaaaaagcaagggttttgtacctgtgtttacagcgttagcgtactgacgattaatgtcag
acttgacatttttgaaatgactgttaacgccttctaatatgaaaatgagtttgcttttaggaaactgaagcttgcaggatttaacaaaatcatctact
atgttatcaacaattttcgtcacgaaatcggaacaggggatccgataaagtgaaaatggctcattgtgtatgtgttcagtgacgaattcgaaccga
tttgatgaatgatcaaagtaagtgttgacctttcttttccaaatcacggtgcttttaagctgatctttggcagttatgaactcacaagagaagggttt
caaagactcacacagtgaagctccccataaggaaaacaaatcttcatgagcaagaattaaaatcatttcggataaacagtcttctttagtcctctt
taccttgttaattctctgtaactctctattcttctttctttcggcctcatcgatcttttttttggacaagccaacttttcccatagtttttgattttcgtagg
gtattatggagagcctcccggctatcgtgatcgttaacacagctttgaaaactccttgagtaatatttttttcagtctttttagtttttgtagatcgttac
tctgggttgagtctgttgaattatgtcgattatcgggagtagcagaggatgcatcgagagcgcgctttcgagaaattgttgtagatattgactccac
agaaggcaattcaaagagtggttcgtcctgatcatttttgagaggattgggttttgaagaagaggaaggaggtttggtgctttcaacgttagagcc
tccagacgatcccaccaacccattagctacatctgtttgttcggcgaaagatatgctcgaatcattatgagcttgttgaggggagcgtgtaacttgg
tttgtatcagcttctgaatggtgaacggatgcgtttcccgagtcagcaaagcggttagacagggagctgaattgcagtaaattttttttcgtggagaa
gagacctgtcaagggagttggataatatctcaagagaatcgggttgaggaagtgcgttgtgggaagcatccaaaatggtttctttaaaacaggac
gctttgtcacgtggggaatcggcgagcgctacggaatgtacagctttgttatgcaaaggggaagatccaagctcaagagttccattttccttttga
aaattatcgatgacctgtaggttagaaggtggggcagatgcaaataaaggacttggctgagagatggcagacggaggagaagaggatagaac

gatgggatcatgaggttgcgagggagtgttagggattttttgggaggagaagggaatggtagcgtagggggacgagtagaaacggggttagat
tcgcaagtaatgccaagagcatttctggggctaggaagcaagtcatctgagtctgagtaagcagaggtgctgtatgatagggaaattagtttaag
gaaataggtgcagagaattctcttacaaaactgagtgaacaaacgttcctagatgtgcatcttgcctaggcgtttcagatatcggaatcacattcg
ttttagaagcgagaggagtctatacaggtggcaaaagttagaagcaagcaatgaacaaggtgcaaacctttgtatgctgggaagatgcatctag
ttcgtcagtgtctgtctctgaaggaatgaggatgatatcgttttgttttgacattcaatgcattaaaaaagagcgaccttctgggcgacagggaagg
taatgattatgaaataaaataaataaaaaataaataaagaaaacagttgcagaagaacagaaggagaaaaaaggaagaaacagtggaaag
aaaagaacagaacgaagtccaatctaatgtgatacctctttaaaaggatgttgatttcggtaggatgcagaagggtgaaaggataaaattccaa
atagtggaaaaaaaaagtattgcaaataatctacaagtgtattcttttccaagatgttacatgatgtattttagaatgaaaagtggaaaatttaatt
catgagtggatggaatctttctccattcccatttttccctttgcattattgtcattaccacttcctgcagtttctttgctgtgcgcacttattttcgtcttt
ttatgaggaagatttttatatgaatttttatttattttttttttggatgtgtttattgttgtttccaatgactcgaaaatagtatcttagtgatcagttaa
cgaatcagtattcatagtttttgacaaaggaatgcttccagcttagaagtcaaaagagtcgtaaacttatatttatttgcttacttatgactcaattc
gtgttttatattttcagttgtttacttttgttcgaatctattgcataaaattcgcttaggcaagaactttatttatacccaaacttttaatccttttgttac
cgtactgtcttttggaaacaaaaggtatattctcggattcgtcggcaggaacgtgccaaatcaaagttaagggaaggggacttctgagcaggtaa
caatcccaccttttagaaataagttgttttaaacgacaatttgaataacacaaagacgctttaaaagataattcttcccgtttttagtaaccaagta
ctttatatataagttttatgtttctatgaagcaagagactcctacgctaaaataaaacattaagtttttcttcctgctgttcttgattaaagagagaga
gagcggttcaatgaaacctcgtcctattacgttacgctgtgctaccgcacaaggagattataggctcaacttaagcgttatcagaggtggagtcct
taacaaataagcgaaccaatcatattagttacaatagtccagttatcgatcagttctaatctatatatttttttgttggaagcaaaaaaattgaaccag
tatcattggaagaaaaacacgtcatgagcgaagaggtccatccatacaaaagtccactatttcttttatttaagcctctcttttgcaagtcagcaag
aacaaagcttggaaagaaaaaggccgacgttgggatcgagattattatttttttatttaatccaagaaacattgcttgttttttttttcttgttaacta
attccttgccgttgaactctggcattttcaaaaggtctcctttgcaatttcctcattcaacgtccctgcttacgttttctttcacccgcggaacatccca
aggaaaacttcttctcttttccctttttgacttcattacactgctttcctttttccatagtcctttgtatttgctggtagacctgaatttgggttacgtttttg
ttctttcttgttattatatttatactctctgatctttgtctgatttaaaatatcaggtatgtatttatagtaatgcttgcagcatttgcatttgttcattttct
tttcagattttggaaaccatggaatacgatcttcaattttccattgcatctccattctccacgttctattgcttccaatttggctatttagcctctttttct
tttctaggtccagtcactaactcatttctagatatcctacaccttttttttttttgtttgaattacttggcatttctgttcattgaatagttatggctgtcttt
tcttcctcatccaagcctactgaatccatcaatcaaaatgttatttctgtagatgctttatcacaagatgccaacaatatggcgctctctgatgagta
ccctcatgcccctatctatccttatgaagtagaaggaaatgaagcttgggctggctctctcccaaaagcacaggctctttatgaccctacttttgag
aaagactcatgtggtgtaggcttcgtctgccacgtcaaaggccaaggcaatcacaaaattgtcaccgacgctcgtttattattatgcaacatgacc
catcgtggagctaccggcgcggatacccgtgacggtgacggtgctggtgtaatgacttctatgccttataccttcatgcaaaaagaatttaaacaa
attcttggccacgatcttcctcaacctggtcaatatgctaccggtaatgtgttcttttctcctgagcccgaggtatgtcaggaaggtattgaatttttt
accaagatcgccgaagatcttgacatggcaattcttggttggcgtcccgtaccttgcgacaatagtatccttggacctgctgctctttctcgtgaacc
caccatcttgcagcccatgattgtatacagacctgtataccaaggtgatgtcgagttcgacgaagtcctctttgaaaaacaactctatcttcttcgc
aaacgaagtacgcatcagattggaaaagaaaaatggttttacatttgttctcttagtcgcgacactatcgtttacaaaggtcaattggctccagtcc
aagtgtataattactttttagacttaaataatgccgaatatgtctctcactttgctctcgttcactcccgtttctccacaaatactttcccctcatggga
ccgtgctcaacctatgcgtttggccgctcacaacggtgagattaatacactaagaggcaataagaactggatgcgtgctcgtgaaggcttattaa
aatcatccaaattcggcgatcagttatcttctttgctgcctgttatagaacgcggcggttccgattccgctgccttcgataatgtgttagagttacttt
gcgttaatggtacagtttccttgcctgaagctgtcatgttgttgatcccagaagcttggcaaaatgacaacagtctcagtgatgaaaaagcagcttt
ttacgaatgggcggcttgccaaatggagccctgggacggtcccgccttgttcatttttgccgatggacgttattgtggcgccaatcttgaccgtaat
ggcttacgcccttgtcgttattatttgacttctgacgatatgatgatttgtgcctctgaagttggtacagtcggcgttgagcctgaaaaagttattcaa
aagggacgcctttgtccaggccgcatgcttttagtcgataccaaagaaggtcgtatcgtagacgacaaagaacttaagcacaatatggcaaatc
gttacgatttccgtagctggattgatcaagagcttttggacatgaacgacatcgtagacactttagttgaacaaagcaatatcgatctcacttctgg
tgtagacaatgaagctctaggggatgacaagcagctcctagcttttggttataatttagaacaaatcaatatggtcattgggcctatggcgagtga
tggtaaggaaactcttggtagtatgggtaacgatgccgccattgcatgtctttctggtgagccacgtcttctatatgactactttcgtgagttgtttgc
tcaagttaccaaccctcccattgaccctatccgtgaagcaattgttatgagcttgcaatgttatgttggtccaagtggcaatcttcttgaaatagacc
cttctcaatgccgtcgcttacgtcttcctacccccatccttactttggaggagttgaatgctatgaggaatgtcactcacctccatcctgattggaaa
gttgcttccattgatattacctttggtaaaagcgaaggaccaggcggatatgctgctgctcttaatcgcatttgttccgaagttgttaatgctataga
aaatgattacaaagttgtaattctttccgatagaggtgtaaactttgagcgtgttccgttgtcatctgttgctgcttgtggtgctgttcatcacttctta
gtccaacagaagttacgttcacgcattgctcttgtgtgtgaatctggtgatgctcgtgaagttcaccatgcctgtattcttctgagttatggtgccgat
gctctttgcccttatttggctatggaagctattatcaagcttcaaagacaaaatgcgttaaaacctggtgttactgaagaaaaggcccttgaaatt

acaagcacgcgatgaatggtggaatcttgaaggttatgtctaaaatgggtatttccactcttcaatcttacaagggtgcgcaaattttcgaagctct
tggtattgataatgaagtagtcaagagatgctttatgggcactgcatctcgtatccgtggcgtcactttcgaacacattgctttggatgctttggcttt
tcatgaacgcggttatcctagaagtgactctatccttagtttgaatatccctgatatgggtgattataactaccgtgatggtggtgaagaacatgtc
aaccatcccaaagcagttgcttctcttcaagaagccgttcgcaacaagaacgaggcatcttatgctgaatttgctaggactcattatgaacagacc
aggaaatgtactcttcgaggaatgcttgatttcgatgtcgacccatcccaatcgatccctattgagcaagttgaaccgtggacagaaattgttcgc
cgtttctgcactggtgctatgtcttacggttcgataagtatggaatcacactccaccctggctattgcgatgaaccgtattggtggtaaatctaacac
tggtgaaggaggagaagatcctgctagaagccagcacttaagcaacggtgatactatgcgttctgctatcaagcagattgcgtctggtcgtttcgg
tgttacctcctactatctaagcgactctgatgagcttcaaattaagatggctcaaggtgctaaaccgggtgaaggtggtgagcttcctggtaataa
agtcagtgaatctattgctaagactcgtcactctactgctggtgttggtttaatttctcctcctcctcatcatgatatctactccattgaagatctaaa
acagcttatttacgacttgaaatgtgccaaccctcgtgctcgcgtttctgttaaattggtttctgaggttggtgttggtattgtcgcttcaggggttgct
aaagctaaggctgatcatattgttgtatcaggacatgatggcggtacaggtgcctctcgttggactggtattaaatatgctggtttaccttgggaac
ttggagttgccgaaactcatcaaactttggtattaaatgaccttcgtggtcgtgtcgttattcaaactgatggtcagattaagactggtcgtgatgtt
gccattgcttgcttgctcggtgcagaagagtggggttttgctaccactcctctcattgctttgggttgcataatgatgcgtaaatgccacctcaatac
ttgtccagttggtattgctactcaagatcctgagttacgtaagaaatttgagggacaacccgaacatgtcgtcaactttttctattatgttgctgaag
agcttcgtgaaattatggctaagctcggctttcgcaccattaatgaaatggtgggccgttctgacaagctgaaggttgctgatccagttaatgctaa
atccaagttacttgacttgtctcctttattgacgcctgctttcactttacgacctggtgctgccacatataacattcgcaagcaggaccatagacttta
cacgagattggataataaactaattgacgaagctgaacctagtttagaaagcggtatcccatccgtcgttgaatgtgaaattataaacactgatc
gtaccttgggtgctactctttcaaataaaattagcaagcgttatggcgaagaaggtttaccagccgactccatttgtgtaaatgtctttggttctgct
ggtcaatcatttggtgctttttggctcctggtgttaccttacaactagaaggcgattgcaatgattacgttggtaaaggcttgtctggtggtcgtcta
attatttttcccccaaaagcttctccattcaaacctgaggaaaacgtaattgttggaaacgtttgtttatatggtgccacatccggtcaggcttttatt
tcaggtgtcgctgctgaacgtttcgccgttcgtaactctggtgttgtcactgttgttgaaggcgttggcgaccatgggtgtgaatacatgacgggcg
gtcgtgttgtcattcttggttccactggtcgtaactttgctgctggtatgtctggtggtatcgcttatgtctatgacaatcacatggacttttctaaaaa
ggtgaacctagaattggtagaactcaatgccatctctgacgctgctgaaatagcctacttgaagggtctcattcaagaccatcgtcatttgactggt
tcgcaggtggctgatcgcattttgagcgacttccctcgcaacttaagtaggtttgttaaggttttgcccagagagtttaaagctgtcctggaacgtg
aggccgctaagaaagaggaagctaagcgtcttcagtaccctaaggcttttatgcctggtaatcctatccgccaacaactggaggaatcgaatcgt
gatgccgctataacagatgttgaggatactttaggtactcctgtcaaaaagagccctcctcttgacaagctacgtggattcatgaaatatcaacgt
cgaagcgaacactaccgtaacccacacaagcgtaccaatgactggaaggagatttctgtgcgtctcaaggaagacgagcttcgcctccagactg
ctagatgtatggactgtggtactccattttgccaatctgattatggatgtcccgtctccaacaaaattttcacttggaatgacttagttttcaaaaag
caatggaaggaagcgttggtgcaattacttatgaccaataacttccccgagtttactggccgcgtatgccccgctccttgtgagggttcttgtgttct
gggtatcattgaatctcctgtaggtattaagagcgtcgaacgtgcaattattgataaagcatgggaggaaggttggataaaaccccaaccccctg
ctaaccgttctgataagcgtatcgctgttataggttctggtcctgccggtcttgcagccgctgatcagttgaatcatgcgggtcaccatgtcgttata
tttgagcgtgctgatcgtcctggaggtttattacagtacggtattcccaatatgaagttggataagttcgtggtagaccgccgtatcaaattgatgat
tgacgaaggtgtcgagataataactaacgtggagattggtggccccggtgatgtttctcttgaagagttgcaaagtacctacgatgctgtaatatt
agctacgggttccactgtgcctcgtgaccttgccattcccaaccgtgatgccaaaggtattcattttgccatggaatttcttcatcagaacaccaag
agtttgcttgacagcaagcttgaaaatggtgaatacattaatgctaaggacaaggatgttatcgttattggcggtggtgacactggtaatgattgc
ttgggtacaagcgtacgtcatgttgctagaagcgttcgcaacctcgaattgcttcccatccccccctcgcgaacgcgcctttgataatccatggccac
aatatcctcgtgttttccgggttgattatggacacgctgaagttcaagctcattatgggcaagacttccgtgaatactcaattttgacaaagagtttt
gagaaggatgaagatggaaacgttaagggtatccatactgttcgtattgagtggaccaagaacagtaagggccgctggattatgaaggaagtca
gaaactctgaagagtttttcccagccgacttggttattctggccttgggtttcttgggtcccgaggaacaggctacagcccgtatggacattcgacg
agatgcgcgttccaacattagcactcctaataagtcttaccaaaccagtgttcctaatatctacgcctgtggtgactgccgtcgtggccagagtttg
gttgtttggggtatccaagaaggccgtcagtgcgccagagaaattgatttgcagctgtctagcaattcttatctgcctgctgacggtggtgttgttaa
gcgtaatatagccatctaaatgttgtcaaataacttaaattaaaagatgttgcaagtcgctggtcgagtttcgcaaataaatacttatgtttttacat
cagattatacgtgaatgtgttgcaggtttcatttgaagctatggatatatatttaattaaccatcttgaaattcaaggtaattaattattccgcgcgtt
aaatgtagatgttttatcttgatagaatagatcttttcaaaataattatatactttaatttaattactcttataatataacttgttaattaattaagtta
agtcggttataggcagatataataagatgacttgttgaccataaatcgtaacatgggtgaaaagttttagaagcttgattttccccttggttcaaac
ggaaggaatatggttaaaagaccggcgaatataaatagagctccacttacaaatattggagcagaggttttgagattcgcatacatagcaatga
tgggagcgaatacaccaaggacccgatttgcagaggaagctaatcctattgcagtaccgcgaactttggacggaaaaacctccggtgtataagc
gtataatacgccatacataatgtctgaaacaaaagtgaaagcgcagttccacccgagatacgcattacttgtttttgccgttgttgaagcaaataa

gaaaacacccgtaagcataagagaaagagtaagggtacctttacgaccaattcgaagttcaactagtaaacctgctagtaaggaaccggggat
gcccaaggtggaaacaatgagcgaatttcgataagtttcgtaaacacttaaaggcttattagcatttcctcttgattcaagatagtaaggtagaaa
cgcattgtataacggaaatgctagaccgatgagagcccaagaaaatattaccaacgtagaagataggctaagtctttttgaacgaaaacattga
cgaatggtctgaaaattgaatttttttagtacttcttgcagaggagaaatttttgttgcacctcctagtgtgtttctaccttgagattccggatcattcg
agggagaaggttggaccatgcagaagtcttgcacagacaaagaagagattttgttgttatatctcgccatcttatgcactgcttgaacagctttttg
ttctttacccttagctaatagaaaaccaggagactccacaagaggaaataaaaacctgacaaggaacatgaatagggtcaaaccacccatagt
aaacaaaagatatctccatcctttgttattggctttatagcatggttcatccgaattagaacacgaaaaatttgcaatcaagccccatgcaataag
gtttaccaaaacttgtcctaacgcccaccaagcactcatgacagtcaaaagccattggtaagagctcggaattgcctcgaggaaaattgtggaat
cgacaggtaaattacctccgacaccaaaactccaaaacgagttgaatactgcaatagcaacaaaattgggggatgccccggcaacaagggaga
aaactccagtgatgagaaaggtgtaattaaaagcccataaacgaccgaatatatctgaggacaaggaccaaaaggcagcgccagctaggagc
cccaagttctgagccaaggtcaacaaaggagcgtcctttgcgtcaggagggtgaacgccgtctacctcattgagacgagacaaaattaaagaag
tgacaacaggccaaatgttatcagacatccacccaaatccagcgatgaagagtaatgcccattgatatttaccaaacccaatttcgtcaatggctt
gactcaagatgcgagacttggccaagtaaacttgatcaaagtccttcgtatcgaataaatcatcattagaaggggaattcgtagtaaagtagaga
ggaagagtctctgtctcaactgcaatttcaccgtctttgatcgatgccttgtctttggtgaccttgtcaggaggatgatccatttaagatagcgaaag
agaagaaaagaaaaaagagcaatttttcaattggaataggggtaaattcaaaagaaagtatgaaatcaagggaccttgactatgagaaataat
tagttaaagcatcaatcctactttagtaatcaaccagtttctgaaacaaatgggtaaagtaatagcagaatcactatcaatttcatatatctatcaa
gaaatagaaaaatggatgaaaaatatgttgcacgaaattgcgaagactcacttgccttgaacccatgaataacagatgacgatgatgatgatga
taccttcatcacataattaggaaggttcatctccaccgttaaattggaagagcgattcctagcgtagattgttttctacttcagaaagctgatttatg
tactcttagctctcgttggattttgtactttggtcttccatggtccatatctatagaataggtgagatacgaacaccatttttttgaaatcaaatcggt
agaatccacatcaatggaatcacgggttgttcatcagttaccatagaaagatacaggaatgcagagagtctcataatgtacgaaggcttgggaa
gcgagttatgagaacgctttgatggtttgcttacaatataccatcggggatggagttttcttaaaagcagtaaggttttgtattttcataagatggtt
gtggtttgtgtttagcaaactagttcattaatagaaaagcgtacaaagtctttcaaaatattaagcagccatcgccaaaaaaacagtgaagcaag
tttaagcaagaaacacaagtgaaaaaaaagttaaaaggtcacgattgaattgggaaaagggtcttgctcgttgtttgccaacgaatggatttctg
ttgcatagcagtcttgtatgctgaattgttgtctttgattgaagtgatagcattcagttttcgatgttcgtttttactatgcttgtaatggaaagcctat
ggttgtgaaattttaaagatttttgtgttttcctgtcttgaattgacgaggactttactagttgagatatttggttatctgaggaagactatacgcatg
gtttcttttctgtaaaagagtggcctaatggatccctgcttgacttgactctacttcattcacttgtttctgtactattgttcgtactttcgggaaaaatc
atcaataaaaaatgaataaataaataaattaaccacgaaacccactgacctaacgacacctatggcatgaatgtacaaacgagtgaatacacg
gagcaaccattcaactcccgagatatattctacaaagaaagacttttgaaattgtagctgttccctccctcaaaaatttcaagttcgtattgctcgat
tttccaatctttgtaccatcctcgaacggttttcaagccttcgcgatgtgtttttcctgcgcctccaagctttgcacttttcttggaatcccaccagcat
gtcgctaaattttggccatacgtatcgtcaggcacaccataccagtatttcagtaagcctccagaaacgtttgcctccaagtgtgatcccttgtcaa
tttctgctagtttgtggcgcatttcttgggtagtttgtacttcaggatgcaatacagaacgaaacgccacgatatatgcagagaaattaggaactgg
cttaaggcattgatgtgcatattttcgtactaattgtacaatttcctcaatatcaaatgcttcagcgtagggaaggtgagcataatgaatcgaatctt
ttggaacgaaagtttgaagggccagtgataacaaacaatggccaaatgttaaggactttaaattgagccaatgctctttatcaacgtacggggaa
gattggatcaattctgtattagccgttttggagatatctaatgaacttaaaatagggttaaataagccatggtcttcttgtatcctagctgttttatgt
tgagcgatcatccttgtcgagtgaaaccaagatcaattgatgacaattgcaacttgtagtggaaaaaagaatgcgaaagcatatttataggaag
gattttcttgctatcgccaaaacgtttgcgaatcctgctcctatttcacgccagcatgaacgagaatcattggttataatagaaggctttgatgaca
aagcagtgccaaatgctcacataatatactttttgtttacgctgaatgagaaatgtatcgtaattgcgtgcgcattgcaaaaaattgtttatattttc
ctaaagtatgatattcgcaaaaattcacaaaatggtaaaattaaagtagtgaaattgaaggagtatgaagtcatggaaaggggttccttaagga
ggcgaaagaaaccacccccctgagaaagagagtatattatataattcatatcgatcattacgagactacggaaatgaacaaagaagcacatcttt
tttttatttagatatgaatcgattgatactatttattaccctttcgaagcttgaaatatctttaagttagctctcgcctaaaattacgttttcattgtttca
ggtatattttccttatcataggggatgttaaacagagctcacatggtccagtggttaagactcatcgttgtggccgatgcgacccaggttcgattcct
ggtgtgggcactttttatcgcaaataaaatttatacattgcattttagcttctattcctttctttcaaagaaacttccggtttagcttctaaaagagtca
ctagtcatcgtttcttattttcaacgaaaacccataatctgtgtttttattaacatccttagggcatttacagctgaacttctttgaagtgacagtcca
aggagtttgtcaccccacccatttttgtacctcgtaaacccaaagttggtagatgtcctttgccaacgtggaaaacagagaaaaccattagaagg
aattcgtctcatttaatatatttatcgcttcattttgacacaggttcgtttctcctagactttctcttgagaggacatctgagcccacattggaatcgtg
aacagaggacgtttgcgtaagctattctagtttggaacataaaagggaacgattttccaggaatttttaaaatatgtccgttgggaagaaagaga
attctggcaagttgcgtcaacgttttccaagcaaaaaccattctcctgagatttccgccgttgcaaatgaaaaggaagatttgaaaaaacaagat
gacaatacttcttcagtaaagagtgactctaagtattgtaacctttttggatatgtcttggtccctcttttattgacagtaattgctggttttgttcgtac

ttggcacattgccgatagtcccattgtggtttgggatgaagctcattttggaaaatttgcatcctattatctaaagcacgaattttatttcgatgttca
tcctcccctaggaaaaatgctgaatgcagttgctggaaagttggttggttacgacggccatttcggctttgattcaggtgctacttatccggaaaat
atgaattacaagtttatgcgactttggaatgcagtttttggtaccttttgcattcctttggcttattattctgctattaattttggttactctttttggtctg
ctagcttaattggcttaatgattgctttagacaatcatttagcaactatcagccgtttcattcttttggatagtatgctttttgtttttttatccttagtacct
tctttgctctttcccggtttcatgtttatcggaagcagccttttttcgttttactggtttaaatggcttgctttaactggcatttgcgttggctgcgtatgca
gtgtaaaattggtgggtcttttttattactgccgttgttggcatctacacagtagacgagcttttcagacttttaaacaacaaaaaagtcacatggac
agcttacgcaggacattggctcactagaattatctttttaattattcttcccttccttatctatgctctaacgttttggatccagtttttagttttgagca
agtctggacctggagatgcccagatgccctctcttttccaagctcgccttgagggctctccactcacaaagaatcccgtcgacctcatgtatggaag
taaatttaccttgaagagtaggaatccagctggagctttgctacactctcatatccagaccttcccgggtggtagcaaacagcaacaggtcacgg
gttatcatcacaaagatggaaataacgaatgggtgttccttcctgaacatggcgttaattacaactttgaagaaaatgatcctaaaattcctatta
aggaaggtagccttgttcgccttgttcatccttttacgaaccgcaacttgcatactcatcaaatccccgcacccttgaacaaacgcatgtatgaagt
ttctggttatggattaggtgatattggtgatgagaaagactattgggttgtgaagatcgtttcagatgccgctcataaagatgcttcctccgtccgat
ctttgtctaccgtgttccaactatacaaccctataatggagtgctatttgagttcttccacttcttcacttcctgcttggggtttcggtcaaattgaaat
gtattgtgatcctaatacagatgcaaaggatattgacactcattggaacgttgaagaacacataaatcctcgtctccctgaaggaagtttgaagg
actatcctaattcattctggaatgactttttgcaccttaaccgagctatgttacgtgccaataatggtctcgtccctgatgaggataaactagatgct
cttcgcagttcagcctaccaatggccattcttactggctactttacgtatgtgtggttggggagacgaacagattaaatatctgttaattgggaatcc
cattgcgtactggtttgctacggctagcttgtttgggtttgctttatttattgtcgggtctattttgggctggcgtcgtcacgctttacgatggaatgaa
aagactttcgatgcagtccattttgccggattctatccctttttgggttggtttatgaattatctacctttctatattatgggacgagtgctgtatgtgc
atcattatgaaccctcatatgccttttcaacatttaccgctggctttatggtagactggtttgctcagaagcttcctcgtgttgctcgcatcacagttta
tttaatgctttatgtcatcatcactgccgtctttatttatttcaaggatgtaactttcggtatgcatggtccctcctccaatttccaccatttacgctggc
tcaagtcttggaatgtccacgattagttatatgaatgtgaattttcttcgccgaatctgacacctaaggaaattactttggcttttcgtaaaaaggga
cttctttttaatgcgtctggatactcattataatactgaacgaaattcaatctttacttgtcaatagtgaatcttaaaaaaataaaatgattatttatgt
cttttgagaaacacaggtgaaccaaaagttatgaaggacgttcactttccttccattgtactaccattacgcgttaagcaacttcatctcattcaggt
gaatgaggtagcattcgtttttacatttttatagaaacccaaatcttacaataattaacccaggatttgtaagctaaatttacagaaaaatatatca
ttcttcgcatccatttcttctaattaaatgtactttgctaatataccactattcacatccaaatgactacaaatgttaaaaccttctcttgcagtaagct
tcattcaaatggaagccactgccttttaggttttcgtttttcatctactgctgctccgtaagtctaaatgcgtcgaaaaaacatagaggctcaataaa
acagcagcgcaacgtgaagagatttcggcaggtataccttccttatataccaagcatttcttaaacagtcacaatagggtagcttactgcactaca
aatcgttaatacattctaagatattgtgccgcgtttcgtaaaacaatgggtgaaactcaaacatgtattataggaaaagcttgcgtttgccagaag
caaatgccaatttttagaaagaggacgattgataaaaaaaaaagttctaacattttgagatgaagatattaaacgcaagagatgcgtacttgacc
aatgccgaggtttatttgcacctaaaggacatggaagaagagcaaaaccaaagaactcgagaacggggagcagcaggaggattagtaagtga
aaatttgagaactatacaatttgaaatcctgaaatatatgagcacgcaaacgaattgctcccaaatggatgaagaaaagtttcttgcatgtctaa
aggtgttggatgaatatgaattgaccaaagctgaaaaactggtaatcttaaataatcggccgtcaagtattccggagatttatgcttgcattgaag
ggattgaggagcgtcttcaagaagagcaaatggaggctctgatcgagaaaataaattcggtttatccataaattccaaaatgtgttaatgatttct
tgaaaacaaaatgtacatgtaattcttagcggataatattattatgagagagggaaatttttgagatgcgaacatctctattgtaagctgaatcaa
ctggggagaaaggaggtgaaggcttgtcatcagagtccgtaatttatcaaccaattaagcggtgctcttcttttttggtaactttgaagacagtttt
gcattttcaacagtagtattgaaaaaattgctgacaaagagtaatggtaagctaagatcaggagagttagaagaatcttgaaagggtatcccgg
agattcctctagctttgaggcgctcttcaggagattgtcgtgtgagagacgaaaggctttttatggtttcccatgaatttcgatgtagaagacgtaa
aaacaaattgggttttggagtaatgagaagccagtcattcgttggcgttaagcgtaacaattgtttactctcaaaagactttggtgaagctggcaa
tgcttccttgttcatttgtataacaccatccacaggaccttgtcgtcgcaaatatctgtaaagttcgtcgcttgtaacttcattaggaacgtactgtaa
taaaagagatttggggtatgcactcatccgaaattgggtcaagagagctttttggttttcaaattcctgtaactgctggttatctgcaaggtaccaa
gtgcctattccagagaagtcatcagaaacttctaagggagcacgtggagggataactcgtttcacaaaagtcgtcggttcagagctatcatctctc
gcaaagtatgtttggggaagtgtttgagcatcttggttggctgcttcttgagattcgtctgggggaaaggcgggaggagaggtaggaatcggcaa
gcctggttgaatataagcacgaacaaaccattcttttgagcggattttcgcgttgtagtacggaaacgcctgaagtttaaagaagcgtctcggaag
gcttcctgttagcatatcttctgcatagctaactggataataggttttcacgtggaaccaaagaccttttttctggttgctgccccaatgctctagcatt
caagataagccttttataaagcgctggcaattccgaacgataaaatcttgttgttccaacagcgccagaaaacagacctgattgaattttcatgat
gatttgcaaaggtagaagatgaaatcgactatctttaaagttgcttaattcagatatcaacaaagaaacttgcgttcgatgcgtcggcacgcgtca
aaagaagttgcgcgtctcacaacaagtgaaaatgaatgctccaaaataaattcttttttgatgttgaaaaacttaccataaagagacgtaaggca
taaaaagcgactgaaaaatgattctcctgttacattcatctctctttgagtttgtgtacatcaattccactcgacttagggagctatattgtgttgcgt

tatctttattagcgtagtcctaggaacagcatttatttatctctgagggtagggttgaaatgtttagaaagaaatgctgtgactgtttctttgaaagt
gttgaagcaacagaaagttgcaacagcaggaattctccaaccgccacgatgaagctgaacatttcctacccggcgaacggtactcaaaagctaa
ttgagatcgacgatgaccgtcgtctccgtgttttcatggacaagagaatgggccaagaagttcccgctgactctgtaggacccgaatttgctggct
acgtcttcaagatcactggtggtaatgacaagcaaggtttccccatgttccaaggtgtcttgcttcctcaccgtgttcgtcttttgcttcgtgctggtc
acccttgctatcgccctcgtcgtgatggtgagcgcaagcgtaagtccgtccgtggttgcatcgtcaaccaagacttgtctgtccttgctgttgccatt
gttaagcaaggtgagcaagacattcccggcttgaccgacgttactgttcccaagcgtcttggacctaagcgtgcttctaagattcgccgtttcttca
acttgaacaaggaagacgatgttcgccaattcgttattcgtcgtgaggttcaacccaagaaggaaggtaagaagccttacaccaaggctcccaa
gattcaacgtcttgttacccccagaacccttcaacacaagcgtcaccgcttcgctctcaagcgtagacaagctgagaagaaccgtgaagaagcta
ctgagtttgctcaaatgatggccaagcgtgttgctgagcaaaagcaaaagaaggaagttgttaaggcccgtcgtgcttcatccatgaaaaaataa
acaaccctgctttttgtagtatcctttcaacgagttttcccttcctttagatacattttgcttttggtttttcttttcttaaatggctgtagtctgaggtta
ggagactgcttttgcggtaggtagagatcgggagtttgtagctttttttgatgaattccttaaaccaacctttctattggtcttagcaacgggtagcct
atagtggtatgtatcatacaatggtaatggtttacatctaaatagtagtagagaacaatgttgagaaggttcattttcaactcacttcatactgtgta
tggaatgtcaaattttagtaaatgctagtcatctactctacattgattgaatttatacaagataaaacagaatgatccatatgtagtacgccaaact
tgttttgtaccaatttgtttggatccgtacttcatattactaagtgaaaagtctgtagcaaatttctacagtcgtaaacaatcataaatcatagtctta
gtatcgatgcatgtttctttcttattgtcataaatgcaatttgtaacaagcgcaacaaatatcattcagtataatcttacatagtgaattaacactatt
agactgtaaaggagtattccactattccgaatcgatctttatttacttaaatatacaaaagatatcaaacaattttaactcttcttgtaggtgcctgtt
tacaaataatctggatatttaattgctttcaagggtattgttagagcctatttcgttcttgaaggcgaaaattgattggattcgtaaaccactactaa
tcataacaatggcctcccaaaaaaagcaaggagagcgtgttcttcctggtatggcataaagaaagaggttttcgtgcaatgttgctaatcttctag
tgcgatcgactagaaaacgtcgccaattacctgacatgctttattacgatgagaatactgattcttatgttatgcctgagcataagcaagagtctcc
aaattccaagcagaccagtcaggcttcctctctcacttctgagcaagttgcaaacttgcaagtcgagcatgctcgtgagcttagagttcaagagct
aatggaatcaaagcttcatcatattcgaaaacagcaacgtaaacagagatatcagagattccaatggatgcaaggtacttatgttcatttacagc
tttatattttaagtgcgtgaaactaacatggtaaagattatcaacagtgggataaatcagaagaccaagaccagtggaattcagataccgaaact
atcggtcccgccgagcaggtttcagcatttgtagacgctctccgcttttctgagcaatttatgcaaattgaacaaaactaaacgactatgaaacgtc
tcagaaaaaattactgcaatccacattcacgctctatgtataggtcagagagattattttattttatttatgattttcaaaagttcttaggatgattgg
atcaattgttttgacgaatgaaagaaaggaaaagtaccgatttggagttagaagattgaaaacaacaagaataaataattatttgatataagcta
tacttttgatgaagaaattccatctttatgaggaataatgtttgggaagggatgggcttccgtattgaaagtgtattctgttaatgcgagtacattgg
gatcatcttcaaaaagcaagtataggtattttaatgtttcagcaaaccaaaaagattcagttctatctctgtggtttgttatgcctgtagtctcaaca
ttgttcaagcacgtaaatgcatctcttcctggtaatcgagcatgcttcagaaaggcctgaaaaatattccaaccccattctctgtagatttcttttcc
agtaattctatagagtataaaaagactttcaacggtttcgggcctcataagattgtgtctgtcctgtttgaaggtatacatatcacgatcctttgagt
ccgtagcaaaataaacaatctcaggagataagccagtagctgtagagctatacatctcataacaggttctacagagatcttctgacaactttacta
tttgttcctgttgctcattccactgagtaccttctcttgcaacactgagacttgtgccgttagttgcgccccacataagcgtgccggataagaaacat
actagatgatccatttttggatgctgtttcttatttaaacccttcggcaattcagctatgtaagcgtaatgttcatgagctgtataattaactagatga
ttcaaaattccgtccacggctatgttgtaagcctcccgataaattggcacatgatcctttaattggaggctttgcttcaagaggtactcataatacga
gtcaccgtgcgaaccaagtcgaatgttagcgccaatatacttgcccgtttccaagttgaagctgatgggcaccagcccaatatgagaccaactag
gatcatttaataacatagtagtaacttgatcagcggcatcccaataacgatgatcgcctgttaaataagacagatagcgtaattccatctgaacag
taccacattcagaagtgctaaccatgtaacttctagtgcgtttttttggaagagcgagttttaagattcacattcgcacgtgggataccggtagtcgt
attataagccgtaagtaatcggtcacctaaatcaactgcctttttctaaatacaattcatcgtttgaaagatgatgagcagagagtaatccacctag
catgcgtattgtcgtctcaaaaacattcatctcctcgtccccttggtcccacgtaagtgaagaattgatccaatctcgagcatgtttgacctgttcgtc
taaacgcataatcatcatcgtatccaaggcgtcaataatcatccaaccgatgcctttcgaaagataatttcttcccttttttcttgataggataatact
catcaaacccccatccataagtttcataatctgaccagctttttttggaatgtttcacgtattatgttttgcttctctgaagccgcactctgatgtgaagt
ttttttgtagcaaaatagttagcgcataaataaatagaagtaatagacaatagtgttataaatgcccgcttgaaagaaccatatttaagacgacg
cccgatcattattcaggacaaactttagcaactatccaaaaataaaaataaaaacaaagttaaaagagctactagatggggccaagtcaaatct
cgcacctaatgaaaatcgagagatctagagattggatttttagtggcagccaagaaatataattatcgaaataaaacttctctcacgaaaagtctt
ttccaaggtaatatagtatcagccgcaaagcagcctttttggtgagttctttactttggtacgaactgtttctggcaatttggtttagttgtaagtgacc
tttactcgttgtagtgaaaaattgaaacattcttgtttaggtactgtatgaagaaaaggaaattaatacttggatgaaagagtgattgatgaccttt
ttttcggtttataattgtacgatctggtaactatcaaaaatggctaaattctggcatgaacacatggcattgtgaattcttcttcatattgatttattgc
ttcgatttacaagtttcttaaaataaactagaactttattaaggagaggcttcattagatggaaattactttttgaaaaaaagtgatatagaaactt
aaaaagaatgttgacagccttgtgtgttttcgagtcaaaccaacagtgcaattctttagcaggaaaaaaaaacaataaggaagggggattaaaat

ccttgatcagtttgttgattatatcttctcagtacgtaaaaggattcaaatggactatactttcatggtgatattcgaaaggaacatctttacaacaa
ccctgcaggattttcatgcgaggtgagaagcgcatgtgtaagtagaacaccagaaacgagacagagaggccataaaaggctgctaaaaggtac
caaaaagaggacggccactatagttagagaagagatggctgatacagtataccttggttttacctgaatactctcgttaatactgatgtctccaaa
ggaatatgctataaaactaagaaaaacgccaattgtcatgaccaataaggaggaggggtaccaaatgaatatataaatccaggcagcgcaaaa
gaaggctgcataataaaaggcatttttcttaaagttcctttgcatacgagggtgtacggcatccaaccgtggtacagagacctttcccatcctccaa
tactcatcgtgcgaagctgtagagatgactcgtttacttgtagaaatagagtcaggccctaactggtcaaaagctttacgcattctgtaccgaaaa
tccatttctagaatattttgggaaaggcaagtctaggatacttgttcattgtgattcattcttcactttgtttacgtttggtgttttgacgaagtacttac
gcatgtaaacaaataggcaaagtttatagtaaacaaattattattaaacttggactacaactttggagaataaaataaaatgtcatgaagtaaat
tgcactcgtatactttaacctgtttgtaatcattcagggcgtcgttttctttcttagtaaacaatgctcttttttcatttaaattttgcttcatagaaaat
gaatttacaaagaaattacaaacgagaaaaaggaatacgcattgaacagcaaatgtaaaaggtacttgcaacgaagatttgactggaaaatat
tttttagacattcgattgattgtttagttcgcattcctgtttcttttttctttttatttcttaatacttgttctcgcgactacccatagtcttttaaatataa
cctacttaatgttttactcttttcacattgtttgtttactaaaacgctgcaacgagacgtaccgtattcatttcgtttacttaatgattttctctgcttgga
aacctttggtccccttcaaataactggtaaatttaatgatgataaatcgttattttttgcttttcctattcgtgggagtatgtctagcatggagcgcgt
ccgatattgaaatctttcggatcgttgattcattaaaaactatctttaaggaaaaaaattacattttatgaattattaggagtttcatcaaaagcttct
attaaggaaattaatcgcgcatataggaaaaagagtattctttaccatcctgacaaaaatcccagcacaaaggaactttataccctactaggctt
aatcgttaatatcctacgaaattctgatacacgtaaacgatatgaccatttcttgaaatctgggtttccacgttggaagggaactgcttatttgtatt
ctagataccgccctggtctcggaaccgtccttattttacttgctgtatttattacatttgcccatttaatgatgcgtctgttgagtgtcaaacgccaga
aaaaaatcatgcaagatcacatcgacgtggctagacaatacgaacaacattccaacgccgctaaagggaccaagcgtcttgttacagtgcctga
aggaaaaagaatctatacggtggattcgttcacgggacaagtatgcattttagacccttcatcaaatgttgaatatttagtatctccaagcgctgtt
cctgacgtgcgattctccgataccttattgtatcggattcccaaattctttttacaaacatttattttacgttggtttacaaagaatgacgatgtccag
gatgccggtgtgaatttctccaatgaagaagaagaaaaatcagagtccaaagaaagattaccagaacaacaaaccaaacctgccgagggtac
agccaaggctggaaagcgtagggttaaacgtcgttcaaacagggttcctcttcgtaaaaacgatcaaaacacttaattaaaatcaaccatcttat
tacagttacatatcaatcgttggaatattagttgctgaactatatagtctaatgtttatcttatgaatagtttggcatccgtcttgttgggagacaaat
ggcatgttcaactttagggaaatttgtctcgtcattgaatgccaaagcgtacaagcccaacgaagaatgtaatacgtctgggaattcccccgctac
ctttgaaaatccgccgaaattgtgttgcgtttggtttaatagaaaactctttacattgttatgagaaataaattgagatgcccctaacaattgtagcg
aaccttgaacccagtaagaatagcaagtgtcgtgatctttattcgttcttccgttaaatccgccagaaatctgctgtctttttactaaccatgtaata
agtttcttcgtatcgggaatataatcttgaagggcgtagtgttttgggatagagtcgttatccaatgtagctaacttgggctgcttttgaatagatat
gatattaacaagcagtctcaatgacgccaacgcgcaaaaggttgcaccggcatgggcttctgcaaacggtattaaggaaaatcctccttcgtaat
tctgacaatgtaatatgtactgaaaagataattcgatatgaggaaatgagatatcgagtagggtagctattgtagtagctaggtataattgtcgca
tatcttggtcatagcatgttgatgaagctgtgcttttgaagtgaccgcccggcaattgacataaggaaagaaactgttcaacggcgtgtttatcaat
tcgagaaagatcgtcgcccagtaggaggagattactaatagcaaagcttgtagctgctagctgaggctcctgttctactgtaagggaagtaccac
ccgtagcgaatccacgaaatccagcgtatttacagttggagtcttcacaattcattaggatgtaatttctataaatccactcaatccatgattctcga
tcttccttggaaaccttgtctaacgagttaagtaaatccaatccaagtagacaaaaaaatcccaaagccattctttccgaatcatgttcctcataag
gattcggtaggaggatcaaatgtcgctgaaagaatgcaacatgttttgatacggtcaactgcatgtacggttattaataccсttcaaggcctaatt
ctatcggtttttttgaatcaatgttctaatacttgttattccatctacgttaaatcctttttttatataatattcattcatatattcagcaggtaagtccg
cctcggtaattattggtaatattcggtgataagtttaccgatataattggggtattcggtcaactggggattaaatcaccggtgttttttcttagtatc
ttaagaagtagtttagtacaaaactttcaggaatccgagaaaaagccatgtcggaccctttttcacgcgtccggaacatagaaagagaaaagc
gaggaatgcagcaccaaagaaatcggctgcagcaaaaaaaaagccacaaaatgaaccaaaagatgaggatttagcatctatatcttcagagtc
agatgtagaaagggaacaatacgatccagacgaagaaaatgaagatgctcgagaaaatgctgctgagaagcgattgagaattgctaaagact
atttggaccaaataaaaaatgagattggtgaagacgtcggtttcaatgcagaagacgtagatcgcgagttgctctctgagcggttgcaagaagat
gttttggaaaaacaaggaaaaatgtatttagattatgcaactcgaattgagccttccgttcaatacacttgcacacaactgcgtggccgacactcg
aagcctctcgttggtcttgttatgaatgaaaagtatttatacagtgctgataaatcaggcttagttcaaaaatggcaaattgttaatgaagatgccg
aagatagcacgaattcgacgtccgaactgaagctgaggcctgttcgattttctgttccccagggaggcgaccatgaccatgtggcagagattaca
tgtttggcaatcagtaataatggccaatgggtagtgacgggtggccttgaccatcgtattgttgtacgagacgccgaaacattgactgctcggtatt
gctggaaacatcatcgagatgtgatattgtctttggcgtttcgaaaaggatcaaacgaaatgttcagtgcagctgccgatcgttccatcaaagtat
ggtccttggactcaatgagctatgtcgaaaccttgtttggacatcaagatatagtttacgacgttgatgcgttctcccaagagcgctgtatttcagtt
ggtggtcgagaccgtagtagtagattgtggaaaaatagttgaggaatcacaactcgtttttcgaagcggtggtaactcggtcaagatgacaggtgg
atatatggaaggcagtgtggactgtgtcaccatggtagatgaaaacaattttattacaggtagtgataatggagttcttgctctatggtctttacaa

aggaaaaagccagtttatacttaccctttggcacatggattagataccccgttggctcctgaacggcaatctgcggaaaagacaccagcagatag
ctcaactcctccacaaccacgatggattacttcgttgagagctttgccgtatgcaaacttatttgtctcgggttcatgggatggaactatacgattat
ggaagattgccgacggacttcgttcgttcgagcctgttgagttggagaaacctttgacaacaaaaagcattataaactctttgtcgctttctcttcg
aggaagaaagggtcatgaacaattattggttttggctgcctgtggtcgggaacttcgctatggacgctggaagaatattcgaaatgtggccaatc
gtggatatgtcttccaaattcctttgaaacctgctctgcagatgtcgaacggaaccgcatgagcgtaaacaaaaaaataagcgcgtaagctaggt
actggttttgttttgtataaaaaaagagtcttataatctctctacaataatgtatggattggaaaatcggttggattgttaatgagtacaataaaatt
tttgtatgttttggagaaataaaacactgtaaaggtgtctgacgattgtttattggtaaatattggtagatgttatggaaacagcacattcattatcg
aaatgcgtcttgtatttgaaattggtttggttttttttttcgtattctgtcgtaaacgaatggaaggacatttgaacctctcaaagttttctccttgcatt
cttttgtttccatctagcatttccgtataagagagtatacagagtgaaatctctttctgtttcttcttggatgtttcaactttacatttccgtttttcgata
accatagtaatagttacccatgaacatacagatattcgtaaaccccccatttcaatatgtagcgaacaaatgaaatagacttcttactattcttatta
attttttcttatgcgtccggcttttaaaaaataattgaatttggtttatcctttatttttcatttctttttgttcgtttatgtttgtttattaccgaacacgaa
gatctagtattatgtgaataccttaaaagaattagcaacgtatgtgcttccttttcatattaccgggtatgacttataatagctatttgttctttgttac
gactctgagcggcttacgtgatgattacagctgaagggagaattggaactaaccataaaccaacaaccaaagaaacccataattgggtgttcag
agtaaattacaaaggaaacgtccattttgaagtcgggaatcaacgagtaagttaaattgctagaaaaaaatgaaactttctttttttttgggcagca
gcctgtgttttgcaagctgcaaagaaccagctttcttctgccgcaattgtcgatatggatggcagccctggaaaatctgtcgttttgggattgcaac
attcctatgctcaagatgattctacaaattttgaacgagctcgtgtcttgaacaaacgtgaggactcgggttatcctcttctggatttagagtatact
agttcaggaagctattttgctagcatcattatgggtcataaaaagagaaactactcattgacactcgatacaggtagcccttacacctgggttaca
gcgaacaatgtgacagctctggacccttctgagtttcagaacggaatgcctgctggaaagtctacatctgacattcgggagatttgctctaactac
acctgttatgacttctccgacgggagtgcaaaacttaccagtaacagcgtttttggtttcctcacctcatatggtgataatacaactgctattggata
caacatggtagatactgcttactttgctggattatctcttgacaactttacttttggtgttgctactcgggagtacgattctgagcagattagcgttac
ccctggtattataggcttgtccccaggaatgaccattacaggtgtctccaggaatggacgagcggtagcttacacacctccaacgatcgttgaaca
gctgtatacttctggcttaatcaattcctattcctttggtatttacctcaatgaaaatgaaggtgaactgatttttgggggttataatgaagctagaat
tgacggcgacgtccgttggctaaacgttactggcacagatagattttactctgtttatttagaaagtttaagtttccctaactcatccagtacacaag
actcatcgaaacttagctttgagaaacgtgacactggtaatatccaagtcgaccaaaacgctactgtggacacaggaactgttttgttgtacttac
ctgaggatgctgttaattctatagctgatcgttacgacggggttgtaagtaatcagggatacccggtcgtttactgtagttcattctcagattctgatt
atgttcagtttaatttcgagaatgacgttgcattccaaatccctatgaagcaattggtgattttacgtagacctgagcaaggtcaagaggtttgttat
ttggctttcgttccttctgcacccggttcttacattcttggtcaatactttttgcaaaatgtttactctgtatacgactgggacgctcgaagaattggttt
cgctaatcttcgtcagaatgcttctaattctggcgttgaagttatgaacatcgcttccgatttgagtggagtacaaaccagttccaactctgctacta
ctaccttttcagatggttataattacggatcctcgaccattcctttaaccggtactcctggagccaatagctcagccccttcccaggacagtagctct
aatggaagtaatgagtctgttgcctcggcttctagcaaagttggtatatattctttgcttgcgtttatgagttcttcttttcttttcttcgtctttatttag
atactcgtcctttttatacgttgatcctttctcgaagtcgttcgttgtcttcctttgtttctgcttaaaaacatcgaaaaagaaaaaagtataataactt
atctattattacatatatatatatatatatgtatctgtctcatgaatccttgaagctgtcttgattatttcttatattttttcttagttacctatcttgtgtt
acgttattagtttatttttgtcgatattcattctgttatgtctttaacttttcttttgagtattatttgtaagaatagaaatcaatgaatactttttttttctt
ataatagtgtgatgagaatggcttaattaaccctttgtaacatattttctgggtgaaacaaacgctcgccgctgagttattttccttaatagctatgtt
gctaattagtaaatcaaatatttatctacttacaagcgcatttatgctcttaaaagcttgatatgtttgaaaaaattatatttcgcatcgaaacataa
tactgaaaagcaaccaaacttttcaaccaaccaatattcttaaaaatagactcagcaactcatagtgacttgctgcaccaaaataaagaaaaag
ctcatatatttatagatttacaatatattttcatttatatgctgcagagatgcctaaccattctttaaatttacattcctacaatgaagacgagataaa
gcaatttcgaaaacatttaatagatttttatgaggaaacaaagcgcgtattgccatggagaaaacaaggatgcgatcttccacacaaagacgca
cctttaaacgaatgggaaacccctatacaacggctgtatgaagttttggtatcagagattatgttacaacaaacgcgcgtagaaaccgtaaagag
atattataataaatggatagaaactcttcctacgctcaaagcttgtgctgaagccgattacaatacacaggtcatgcctttatggtcaggtatggg
gttctacacccgttgcaaacgactgcatcaagcctgtcagtatttagctggactgaaaccaagcgaaatccctttaagtgctgaaaagtgggcca
aagatatccctggtgttggtccttacacggctggtgctgtattgagtattgcatggaagcaacctactggaattgtggatggaaatgtgatccgtgt
tttgagtcgaacccttgccattcatagtgactgtagcaaaggaaaaggaaatactttaatttggtacgaactttgattgttcacaacattgtttacta
acgcatactagggaattagcgaatgcattggtagatccacagcgtccgggtgatttcaaccaagcattgatggagctaggtgccgtcacctgtact
gctcaaagccctcaatgttcgatttgccctgtttcggatatttgtaaaggctataaagagcaagtcattatttcagataaacagaagctaaaatatg
acatcgaggatggtgagctaaattgttgtttctttcatgtaggctaacaaggattatagggccctgtaacgtttgtgcatcagatcccccgtctgctg
aaaccatgcaaaattgggtggtaagtaggtacccaattcgacctcaaaagacaaagcaaagggaagaaagagccttagtcgtgatatttcaaa
agacggacccagaaacgaaagaaaaattatttctgatacgaaagcgaccaacagtaggtcttttagcgggattatgggattttccaacaatcga

atatggtgaagatgcctggccaaaagacatggaagccgaattccagaggtgtctaaaaatatggttggacgaagaaaacttatgtgtaaaaaa
acatcaatcacgtggaagatatcttcatatttttagtcatataagaaaaacaagccatgtatttttttgctatcgtgaccgctgatactaaaacgaaa
gaagactttttttggatatctcaatcggatttggaccatgtaggtatgtgtgagctcggactaaaaaactaccggggagccttggaattaaaaaag
agaaaacttactactttgtctaatttcaaatcccctacccttatttcggcaaaacagcagaatgtgaaaacagaagcctaagcctttcttttgttcgt
ttccaattcttcctttagttcttccattaatttatttggaaagtattcatgattccactcatcaacctttcctcctaatatctttgtattttcagaagtgag
aagcagagttcccctctttatctgggcattctgaatgatgagctttgtccccaggtctgtgtcgagttggattcccggaatcggcttccattccatac
cataaaatctttgtccgtttgcgtcttctaagaccaatcggcacatacacttgaacccttttccgtcgaatatggcttcttgcgcctcagcaataccg
ctgtcatctccttctttatcttcttgatcatcctcatccacttgccgaataatttgctggccttttagctttttccgttcttccagctggttcaaatactca
agctggcttacgatacttttcccaatttccctaattctgaccacttgtaccaacagcggttttgaaagtgtaatattatgctggtcggatatagcatat
ggtgcagcacctgcactagatgtagaagcacgaatatcggatgctacaaaaaaagggagaacgagttcggcggttatgttttctctaacatattt
ccgttgatggactaggtggttcacgactaataagagccacgagtcctgtagtggaatacctctttgttccaattctctaagcagcaatgtatttgtc
atccttgctttttgtttttctactactttaaagtcaagtataaggtactgcgggagttttctcagagaagtccaaaggaaccattgaattgcaagtag
ctacagcaggcgtggtctctgataaatcctttttttgaacaaaatttactgtacaataactatgtatcttaaatgattgtgctgtccccactaatata
caaataaaaaaacaattcgagatgaaatatataaggcgtctacgacctcaatttcgtaaaaaccaacttttattgaattttctttatgaaaattatg
taaacaaagagtagagaatagtggtgaatatacgccgtagtgcttacgacttcagtaaagtgcgttgttgcaattcgcagtcacaaactatacctt
gtacacagcaacaaaactttcgtagacaacaccaatttggggaaaatggcactcaaggagtatcaagtcgttggccgcaaggtcccaactgagc
aggaacctgtccctaagttattccgcatgcgtttgtttgctcctaacgaatcggtcgccaagtctcgttactggtacttcttgaagatgattaacaag
gtcaagaaggccaccggtgagattgttgccattaaccaaattcacgaacctaagcctcttcaacctaaggtttttggtatctggattcgttatgact
ctcgctctggtactcacaacatgtataaggagttccgcgacaccacccgtgttggcgctgtcgagggtatgtaccaagacatggctgctcgtcacc
gtgctcgtttccgcagcatccgtatcctcaaggtcgtcaccgttgagaacaaggaggatgtccgccgcagctacgttaagcaacttcttgatccta
agttgaagttccctctccctcatcgtcgtactggtactctcggcttggctggcaagaaggtctttgctcctcaccgtcccaccaccttctactaattttt
tgtatggttttcatagacacagcaactgtgtgttgtacaaaatacagtttcttaacatataaatgggataagtcaggatgcatttcgagtcgtaaac
agagtgaaataagaatactgtaactttctgtaagtaaaaacatagactcaagaaaggatattttatagactttactaggtataaggcagcagttgt
taggtcatttatagagggctgaaaccagtccttaagtctcaaaataagaactttacagtaagaagaatatgcaaatacgtgcgcttccctaaattt
gatggtttattatttttatgaacaagcagtgataaactggatcaggggagatagttaacatgacttttcttttgttcaggtttcttgttattgtttttcct
tcttttgtcctcattgtgtatttttgtggattactcacttagatgatattctataaattcatattgtagagctaagaaaatgcagaaggcttacgattca
actgtagtcgctaataatgtagaatgataaaaactagaaaatgattcatgatatatatgggaaagagtagtctccaaattcacaagtgtgtaaat
ccaaccaagcgcactgacagaagacaaacaagcctataccgattttaaggatcgcaaagtaaaaacaaaataaaacataaagaattcgctatc
ttggctatttttgttttgtttttttctttgcgattcttaattttacagaggtctgcctcacccttcctcttgttcgcgatcataagggtaaggaacttggcgt
caccagcccctgcgatttcgaacttaccgtctccctccttctaccacgacgcattgccagccgtggatcaaaaagatggctcaacaattaccaata
cggtttagtgaagttttacaggtatgttttggtaataacggtcttttgataaagaagctaactttggtattttagctggcttccttgggagttcagcctt
cgtttggcttcgccaatgttactcttcagtcagaaaaatatgtttggtatggttttgcttgtgcgctgctaaagagaagctatggcgcagttgaatgt
gagatctttggactacatactaatacttttgttgtagtattcgcgataattccaacggtgccaaccaagtggtgatcgttgaccttgaaagccccga
aaatgtggttcgacgacctatttccgctgattcagttattatgcatcctaagaaaatgattatcgctttaaaggcacaaagacaacttcaagtcttt
gatttagaagccaaggccaaacttaacagctatgtgatgaatcaagacgttgtttattggacttggatcagcgactctaccatcggtattgtcacc
gaaacctccgtataccattggtctctttctggttcggatcctgtcaagatgtttgaccgccacagctctttgaacggtacgcaaatcatttcttacag
aagcaattataatgaagaatggttcacccttgttggtatctcttcacgagataatcgtatcgccggtaacttacaactctactccaagaagcgaaa
agttagtcaacctttagaaagccatgcctctgcttttgcagtcatccaaccagaaggccttaatcatgatgtacaagtacttgcccttgccagccgg
ttaccgactggctcgaaattaagtgttgttgaaatcgatcgtaatcccaataaccctgcttttgctacgaaaactgtcgatctcttcttccctcctgaa
gctattaatgactttcccattgccattcaaattgcacccgcctatggtgtcgcttatgttgccacaaaatttgggtttattcatgtttatgatttggaaa
ctgctaaatgcatctatatgaatcgtgtatctggcgaatctattttcgtaaccactccccatggatccgtgaacggtgtgatggctgttaaccgtaaa
ggccaggtcctcagtgtttccataaacccagacacaatcattccttatgtcctaaacaatttgaacgatgcaggattggctgtccgattagcctctc
gtgccaatttacctggtgctgacaatctgtatgctcaacaattccaacaattaatgatgcaaggcaattatggtgatgctgctaaggtggctgcctc
ttcaccccgcggtatcctacgtacagctcaagttattgaacaatttaagcaattacccgccattcctggccagattgctcctattctgcaatattttg
gcaccttgttagataagggtcccttaaatgaacacgaatcattagagttggctcgtcctgtacttgctcagaaccgcgtgcaacttttagaaaaatg
gttcaatgaggataaacttgcttgtacagaggcactcggtgaccttattaagcctgtaaactctgccttagccttgaaggtgtatgaaacagctaat
attcccaacaaagtagttatgtgtctgtctgagcttggtgacttcaaccgattagtttcttacacaagccaaacaagcttcactcccgattatgtctc
gcttttgcaaaatttggttcgagttaatcctgaccaagcggctgatttcgctactcaaatttacaatagcgatccttcggtcaacttagaaaagatc

gttgatatcttcatgtctcaaaacctcgtacaacaggctactgccttcttgctcgacgctcttaaggatgataatcccgcacactctcatttgcaaac
tcgtttactcgaaatgaaccttattaatgcccctcaggttgcagatgcaatccttggtaatcaaatgttttctcatttcgatcaccaagctattgcttc
gctctgtgagcgtgctggcttagtccaacgtgctttagagctttacgagaaacccgcagatatcaagagagttatcgtgcatactaatttattgaat
cctgaatggttaatgacatattttggcaagttttcacctgatgaagtttatgattacttaagagagatgcttcgctctaatctccgccaaaacttgca
agcagtagtgcaaattgctactcgttattccgacttagttgggtctcaacgacttattgaaatgtttgaaaagtttaggacttttgagggcctttatta
ttacctgggtagcattgtcaacattactcaagatgctgacgttgtctataaatacattcaagccgcttgtcttatgaatcaattcaccgaggttgagc
gtatttgccgtgacaataatgtctacaaccccgaaaaggtcaaaaacttgctcaaggaagctaagctagctgatcaacttccattaattcttgtctg
cgatcgctataactttgtaaatgatttgattttctacctctatcgcaataatcttcatcaattcattcaaatttatgtgcaacgtatcaatcctttgaaa
accccacatgttgttggggctctgttggatatggattgtgaagaagagtttgtgaaaaacctcttgaactctgttattggtcaaattcctattgatga
attggtagaagaagtagagaaacgcaaccgcctcaaattgttactcccatatttggagtcactgctgcaatcaggcaaccaggatcgtgctatct
atgatgctcttgctaaaatttatatcgattccaacaataacccagaggttttcttaaaggaaaacgatatatatgacactctttcgatcggtaaata
ctgtgaaaaacgtgatccctatttggctttcatcgcttatgagaagggtactaatgacacggagctcattactctctgtaacgaaaattcaatgttt
aaacaacttgctcgttatttgcttaagagaagcgagcctgagctatggctagaagtattacaagatcaaatgttccgcagacctttacttgaccaa
gttatcgcgacagccgttcctgaatcttcagacccagaggccgtttctattgtggttcgttctctcatggaagttgaccttccaggtcaattgattga
gcttttagagaaaatcgttctgcaaccttcatcctttagtgaaaatgctaatcttcaaaatttgctgtttctaacggccataaaagctgataagtcac
gtgtcatggaatacatcgatcgactaagcaactacgatgttgaagaaatttctgaaattgcagtagagaatggcttgtttgaggaagcgttcaag
atttacaagattcatcacaagcatgaagaagctatgaaggttctcgttgaggatattgtcagccttgatcgcgccgaagagtatgctgaaattgtc
gaccaacctgaagtttggtctcgtttagccaaggcacaactaggtggaattagaatttctgaagccatcgcttcttacctaaaggccaatgaccctt
ccaattatgaggaggttattgaattatcttctcgagccggtaagtacgaggatttaattaaatacctgtttatggccagaactaagatgcacgagc
ctgacgtcgatagtgccatattggttgcttatgctagaaccaaccaattgacagagatggagacatttttgattggttccaatgtggctgatgtgaa
atcagtcggtgatgaatgctttgacagtggtaactatgaggctgctaagattatgtattctagcatttccaactggtccatgcttgcttcaaccttgg
tttaccttaaggaatatcaaggtgctgttgactgtgctcgtaaagccaactctattaaggtatggaagcaagtaggcactgcttgtattgaacagc
gtgaattccgtttggctcaaatttgtggcttgaatttgattgttcatgctgaagaacttactttccttattcgtttgtatgaagagcgcggctattttga
tgaagtcatttccttgatggaagctggtttgggattggaacgtgctcatatggctttctatactgagctggctattctctattccaagtacaaacccg
agcgcttgatggagcatttgaagcttttctggggtagacttaatatggctaaggttgttaaagcatgcgatcaagcacatttgtggaatgaagctgt
tttcttgtacgtccacgaccaaagttacgataatgctgccgccattatgatggaagaacctgaagcttttgatcatcaatcatttaaggatatcatc
gtacacgtggcgaacttggagttgtactatagagctctacattttatttggagcagcaccctatgctccttactgatttattagctgctttaactcct
cgtattgatcattcaagagttattcgcatctttgaaaagtctgaaaatacaccactcattatgaactttatggttgctattcaacatttgaacattga
agctgttaacaacgcttacaacgacttattgattgaaatggaagattatcaatctttacaagactcgattgagaattatgaccgatttgatgccatt
gccttagctaagcgacttgaaaagcataccttgcttgaattccgtcgcattgcagcttacatttaccgtaaaaatagacgatggattcaatccattg
aactttcaaagcaagatcgattctacaaggatgccattataaccgctcgtgattccaaccaaactgagattgctgaagatttgatgaaatacttcg
tggaaatcggcaactatgaatgctttactgctatcttatatacttgctatcatttgcttcgtaatgacttagtaatggagataagttggcgcaaggga
ttacaggactacgcttatccttacttcattaacttccaagccgaaatgttttccaaaatccttactttagaaaaggatataaaggaaaagggggtca
agtcggatgaggaaccaagcactttaggtactggcatcttcggcaacaccctgatgcttacacaaggacctatggctacaggaagcaacaatac
tggtgcttctggctttggattttaagcaactttttttccattatgcgttcgttattaagaaatttaatgccattctttttgaattactagaacttgaggtt
cgtagcaagaaatcaattctattatgatactatagacaatgttagtgatttatgttttaaggcatctcaatctcaactgcattactcaaatgaataat
aacttatggcctatataacaaggcacgaaatcccataaacgagttctaacgtactacgctatttcagtctcattaacagagaaataggttggatgg
tgccaagcaagttgccttcagcgtcagtctattcattctcttaagtatgtcaaggcccgagcatatcgcacctcctgaaatcgtaagtaaaaaaga
aaaagacgagactgttaacgttgagtttagttttacaacgatgaagaggcaggcaaatataacaccaagtaagtaacttgtactatgaaagctcg
tcttggaagcatgaatgagcaatctacgaaaagattgaatggtaaaagcggcccgtcatgttgatttgtagaaatgatttttttaagggctttca
ttactaacacagaacatagtactcgtattcagtctattcaagctgaaatgacagagcgtgcaattgaattgcttgatcctgaaggcccttccttatt
gctagacattggtagtggatctggaatttcatcaagtatagctgaaagtttcggtcacgttgctgttggtatggacatttctccttcaatgctttccgt
tgccttggagtctcaagacgttgaaggcgatatgttgttggctgacatgggtaatggtgtcccctttcgtgcgggtacctttgatggtgttatatcga
tttctgctattcaatggttattaaatgctgataagacctgtaatgttcctcagagacgtttgaatcggttcttcgagacattatacagcagcatgaaa
cgcggaggtcgagcggttatgcaatattatccggaaagcgatcgctcacagcagatgatcctcgacacagcaaagaagtgcggatttgctggag
gaattgtcgttgatcatcctgaatcgaaaaggcagaaaaagttctacttgattttacaagccggtggaatgcgggcgttgaatacgagtgaaatg
tctgttgaagatgaaaaagttgatgctaagaagcgcaaatcgaaaaggaaacatgaaatgaacactcgtgagtatattgaacataaaaaggac
ttaaaccgtaaacgtggccgactttccgtacccaaagactccaaatactctggacgtcgcagaaaggttgcattttaattagggtattacggaca

tgtataatatagggttaataaagaaaaaaaataaaatatgggttttttgtttttggtggtcaaaacgcttgccttatagaaacgactgtattacatg
caagatactgaaacaacaggtgcaccttttcataacttcaatacatctgcttacaaattacttttacagtcactacaaattacacctacgccaaga
aaactactagcacactgattaccaaatcatttgctaccattcaccattataaaggtaagaatacaaaagagtaaggaactttaaattacttctcc
ataacaacagcatcatacttttgcttgaaccaattgatggtgtccccaggagtaatcttgtgagggaaaccaatacggctacgttgggccttacga
cgagtgacacgcatgccaggacggtccataacgacatagaagtccataccataaataccaatggaaggatcgtacttgatgcccaagtcaatgt
gctcttggataccaaagccgaagttaccagtggcagaaaagttgcgacgcttcaactcatactccttgaccttgagaccacgttccaaaatttcct
cagccttgggaccacggacggtaacatggcaggaaatcttttcattacgacggatgccgaaacgacggatggtgtaacgggccttggagaaaac
aggagtttgaccagaaagttgttccaaaacctttgcggcacgggtgagacgatcaccagattcgcccaaggagatattgagcaccaacttggag
atacgaagctccttcatagggttttgtgccttctctgccatatttgtatgatttttggtggatgccaacttttttgtctaacgcatttctcattctcacaag
aagtttcgttgtgacattgtgactgttgctttgttgccagggtagggttaacctcaccaccatcttgccagtgctcaagttgacagagctcatattcct
gcatcaataagcgattcgctgtagggttttttaatataaatttaattcaaaatggtaagaaatactaggatgatgagaatttttttcaggatacgtt
gctaaacccatcttgtttttagggtacaacaaagatggtttctttgttgaatcattctttaaatgtaactacaaaagatggacgaacttttgtaggt
caattattagcttttgacggccatatgaacttggttctgagtgactgtctggaatatcgtcacatccgtaaacaaaataatccttctaactctgtgta
cgaggaaaaacgcatgctaggtcttgtaattcttcgtggtgagtttattgtctcactctctgttcaaggtccaccacccatggatccatctatgagag
gcggtttgcttagtggtcctggtgttgctcgtcctgctggcagaggaattcctcttggacaagcacctgttggtttagctggccctgttcgcggagtt
ggtttcagcgctcctcctcctccagctggttttggacgaggcgctcctcccccaggtttccgtcctgtataaagatgtcgcaagcatatggtattttttt
tcttttgctagacaacaggtactcgcaaggattatgtccatttatcatcatccttggaactactttacgtgaaacctactcttccggaatggcgagtc
gatcattgtattttaaacggcagaagtcttatatgctcgtcttcctcgatcattgtacgatactttatctgctttttagtccatggataatgatgctttc
aatgatgactcttacattctttagtgcatgatactagcgcaggaaaaagaaaaccgatctttgaatcatttctcttgtactatagattatatttacca
aaaaagaaaaaaaaaaaaagaagaaaatccctacggttatactagcttatgtaaaacatacttataactgttactcacggactgttcatgttaga
gaatttatgttaacgcaaatatcatcaattattattgaatagaaacttctaaaaccaaatttccgctatatacaatgattaatgtagttttatttaatt
taaatttttttaaccccaaacatatttgattcataaattaaatcataaagacaaatgctaacgaacgataaggcgaaaaatagataaaaaaatccc
aaacgtacgaaaaaaagagtgatgcttatatcttgaaaagaaaaaagagcgatatagatacatattaatcaactagattcgcatttgtttgacga
atatagtcagtgaaaattagctcataattttcgatgagcatattaaaaacttcagaaggaatattcaaagttggagaaaaaacgatcccgatgttt
cttgcgttcattttgttatatttttcaaaagcaataattctgcgtaagtgatgtaaaagagagtctaaaagaacaaagttttcaacgggtaattcccc
aagtacccgacaaagttcatccattgagctctgtgtatgggcaattcccggcattaattcaatgattttctgtagtgaattatccagcaaattagttg
gcagctcacgaaggtatagttttaacaaaccagcaacagcatgaatatcaaacatttcatccgaagataataagtcgtaatcaatgcctttgttaa
actgttcctttaggctttttattgcagaagaagatccactcagtcgatatataccttcttctttttctgcgcgcattgcttccaaatactccaagcaccg
atatatcacaatgggaagcccactttcattaaattgcgaggcttgctctactgcatcaaccagcggtaatccaaaaaccgaggcttgctttgttatc
ggatgcgcatttggagctggtaacgacacaggttctgaaccgttcgccttatcaagggtttcaaaagaatactccttcctgttctcttccaaggtatt
ggcatttgaagattgatcgtgcatctgaggtatcttattcccagaaatatttattcgtttgaaatgattgagaaagtcttcttctacagatgcgttctt
atcctttttcggtaactgttctttattgaaactcggtacaactttcgttactctgggatcagtttccaaatagctattcccagaggtacttgcaaagga
tagttcggattcgcgttcatctgttgacagttcagctgtatcactatgtccagcagggagcaaatcaacatttgaatctggtctattattaatgttttc
tttatcccccattacttcgaaagcactgtcatccacataatggctttgtggcagagagtcatcatccaggctccattggctaacaccaggtaagaa
atccacctgacttaacttttcgttttgctttttggtttgattccgacgagatccaggaaaatccgaagcttgaatttggaaatcaacaggggtagacg
acgtagaaactggaggtatttgataaagaatagaacgtaaccaactttgctgatcttccatgtccttcgccaataagacatggatagtatccgttc
catttttttgttcagtttgcttttcctgtataacaaatgcagactggtaattttcgccagttaagaattgatgaggaggatcaataatggtgaaaaca
ggcaaatttgcgctatatcggtggacttgagcctcctgcaactgaattgttcctagaacaggtgcattgcggttttctgcataatacaagacacggt
ctttcaagtaaaaatagcgagaaacaagtccgatattcttaatatatctaagtaaaactccttctttttgagcatgatctggaggaagaaagaaaa
catcattaagatattctcgtaaagtgtctttgcttaagaactgaattaacttaactttctgttctaacgtaagatcagcttggaaaatataacggaaa
tatgtgtcgacagaccgacgaagtagttcattttgtatataagaagaatcgtttgcgaaccagtcaagtgtgggaaacggtggagcacctacatc
caaaataaatgatgaatcaaaagctcgacattggccaatactcctggaatagctccacacttcatccttccattgtgaagcaagaggcggtacag
cggtgacacctaccacaaaatgtagaatgatattatcaaactccgcagatgaagaagacagagttgtcttcatgagatcaaatttcaggtatcgt
accagtgcctcatccaacctgaaactgagtttgctgttgtataaagtttcagaattgtttgaagaagttaactgtggattactggctatggaatcact
ttttgtgcgattttcttgtttcgggcgagcaaatgccgtcaatgaagagaaggaggacagaaaagatttcgtagaagacttcttggattctttcgat
tgggcctctgaactttgttgttgtgattgctctgtaggagagtgaggatcaatgatagtccatgttcgattagagtcagagagacgtcgcgtcaaac
agatatcactatccggaactttcaacattgataaaggttcagatgctattgtttcgggtgagttaacaactgaaaggtttatagctttaggcaaagg
ttccggataagctaaggaaggagtaggagacaaggagtttgaattaaagttcttattaaatggaaacccggattttgaagttgcattatcgttcgt

atcaagatcagtaaccgctgcaggagacgcaattgtgtttttcataggagattcgtccaacgtggaatttatgccgcctttcttcaatgctacagag
tcgttgggagccgaaaagttttcaagaaactgttttgcaaaatcgctattacttttgctatttgatactacactcggatcttgaactgaggaaaactt
aaagttagttgaagtaggagaaaggctttcagtacaatccaaatcctctttcataaaccatcgagtctttgcagcttcattccgcaaagctgagtat
ctttcgtgcgacacaggtgaagaaagcgaagaacgtgggtcatatggaccagaaatcgcgtttacaggaaggcgttttgtgttcaatagaagttg
aggcatatctttgtaaatattttgactccgttcttccaaagatgaacctccgtgcgtaagggaagagaatgacgatcgatcgtctttgctggcactc
cctacgaaacttggggataaaccatctctttgatccttcgaggctaaccttgcaaacaaatttgctttattgggagaagggatttcagcgctagaat
ggtactcagacaacgaggtttctagattttccagtttacttaaacgtgatcgcagaacgccattctcagaaagagcttgctccaaatctctacgtaa
atcatagatagtatttctttggttatttaccaggtcaaaaagtttttcctgttcaacccctggagtcgcattaccatcactgatttgaagttcgtcctg
agagcccatatttctccctaacttgaatgtattttcaaaggaattgcttcctttcattgcttttaaacatttaccctacaattaaccatgcaaattactt
ctttgacagccttcctggaggtcggctgggataatgaaaaggtatggtaatatcgttggtagcgttaacttaccatctccatatacatgtctctcga
gctagctttcggactcaaagcattaaattgcatattctaattttataaacaattttgaaaattttatattcagtgttgtcttttcaccactgtttttaatt
tactgttatgaacggaaatgatagtatgtgtcaattaactcattctctttgctaacttttaatgaattagcctatttattatcccttgagatttccaata
ctaccgatggatctattttgtctttgattcctggaaacaatggaatgctgactcctgtttctaataccaagcgaaggttactagatatgacatatgta
tgtttatagggaataggaagaaagtttagaagaacatactttttttatttgactttgataaaaaagtcagttgctagattatgcttactccaataagct
tcatagtaaaagcaacggaagtattaagtcttctcttcgtttctttttttgctacgtaacataaaggtactgtttaacgctaacataggtaggatact
ctgacgaagcttgtactcttgaagcttgaatgtcaaaccattcccagttttcgactgttctttaccccagaggttgctaatataaagcaagaaaaca
aaaagctctttttactcaattgtgaatcgatcgcgtcgttaaatagtttttttactcgagtgatatcttatgatgaactatgcagccaacagaacgttt
atgaagtgtctacgttagtcgagacaaaaaatgatgactacggattctcctatgattggaagccttctgttcataaaactttagaggatggctgctt
taactattcatgcctagctatttattcgttcgaaaccgaatgtttggtttctacaaacaagcttttctactacataaaatttgactcatcagattttga
agatactcctatgttattaaattcgcctacgaaagtatcgcccaaaactactgaaaactgtttgtctcagagtcctaaaccttcccctgagccagct
atttacagttcgaaaaccgctatatatcctgaaacttatgaagacaatcctttttttcgctctatcttgcatgaattagaacagaagtcatcctccat
gaagttgtattttaaaaaaataatgaaacgaatcatgcaggtgagctctgctttcgaagaatccgagtatgcttttaagcgactgtctgacactctt
tatgaagcatccacagataccacaatcgcttcgcaaccgatgctagatgaatttttgcttcgctcatttgaacttgaaatgattgttttacggcatat
gaaggctgactttatgaatcatatgtatggacctttacaaagaatatatagtacctctataaagcctattgatgataagaaattggagttcgaaga
acaaagtaaaagttattatactttactgtctcgttttatgtcttgtcgtaaagattctaagttatccgaatcggtctttttcgaaaaagaaaagcgctt
tgctttacaacgttatgattattattgctttatgcaagacctttataatggctccaccatgagtgaaataagtgaggttttttttaaaatttgtaaatcg
acgattcgacaaattgtccgactatatggcttccttggaatatttactccctcagctacagcaattgatgtcgaaactagaagcttcgaaatgggat
ctcacttttcaagaaaaatgccgaagaaagcaacgttcggctatgataacgaagaaagatcgaccaaaatccatgtttgctattccatcctcccct
ttatctgatgtatcggaaaggtctgattatacaatgtacagcccaccacattcttcttcatctgaagacttccaaaaagtaagcagtcctacatcaa
acgttcataatcctggtttgatatacaaggaagggcttttgtttgttttcactgcctctgaattaggagccgacttagccgttcgcagtaaagcagcc
tggcataaatactgggttgccatcaaggatggttatatgctggagtatgcaaattggaaagattctgaaaagattagtgtttattcaatttcactgc
gaaatgcttctgtgagaaaattaagaaaacagccaaggaaatactgtttagaactgatttctccaaagacaaagcgcttatatcaaacaacttct
gaaactgaattggatgcttggattagttccattaatgattccataaaatctttaactaagcaatcttctttagaagattcactcacatcttcagatga
acagccagtaatcaaaggggtttcgtcgccgcctgttgagcgcgctcgcttgactcgcaagatcagtggctccggaatcagaagagcattcaccc
ggaaagggtcttggaattttcctcaactttttcgaagtgcaagcacaaacacaaaaactattgaggatttagaaaaatgccatgcaagtgcaagc
attttcatcgaaatgttgagaaggatcgatccagaaaactctctctgtgctgactgtggctctacgaaagacgtgacatggtgctccatcaacattc
cagtaattttgtgtattgaatgcagcggtgttcatcgttcgcttggttgtcatatctcaaaaacacgctcccttttatttgactcttttccccaacagtt
aaagcttttgttgtctcgagtagggaattcagttgtaaaccgtgtttttgaagcgaggataaattcattctctggtaaacctttttcctgacagtgata
cgggtatgaaagctgtgtttgccaaaaagaagtatgctgaacatgcctttatggatctgtcatcagtagacgcataccagtatcttattgaaggtct
aaggatgaacgagacagaaaaagtcttgctagcacttgctgctcaaccaaacttcaaagagcaccgttctgaatttctaaaggaagtaactcaa
gatgcaagccgcttacattatttggagctgatgcttttgaacggattgttattgccctcctctgaagaaatatccaagtatgcttgtaaagaaatgg
aagcattattacaacaaaagcattatgtcaaatatcgcgaggaagaagtatagggaaagaacaaagagcagatgtttccttactataaaaaatc
cattcttctttgataaaacaggttttaatgaaggaagcagagcctccacattcccaaccgtaattcctctcactaatagaagtcgtggactctgaat
tgattcaaaatattttcaagcctttatctcatacccgtctctgaaatactgctctttctttgtgccgtcttctctgttgtatatgcagcattaagacagtt
atttgaattccgttcaacgaaaagtgtaatatgaacgacaatcaaagtgaatcttcatcatcaaggaaagacacgttttcgtgtctgataaaccttt
ggtaaaagttgtcatagtcatttcgtttgttcgaattgaccagttattctacaaaattcttaaaacgcaattaaaagaaaaaaaaaaacgtccatcg
ctcgcttaaaaggttttattttttctcattaactttacattactcgctttattgaattgcgagaactatttattacgcaccattccttcttttcttctgac
aatacacttacttcttggacactcattttggtactaattcactaataatagctaatttgactgtgccctcttttttttttagtcgacttgttatccgtca

agacttaaatccatttcaaacaaaaaagttcataaaattacaaaagaaaaacagactcaatctcgtttatgctaccaaattttactcattcgttact
tctttccacagatattcaccacacattacctttgtaatcttatcaatcatcacgaaggaagcacccacttttatatgtctaaaaacgttagtacttcat
ttattcaaggtattcgaaaattcagtccatttgtattaccaaagttttcctgtagaatgaaccatttcaatgcttctcttgttccaaaggactttcgtcc
ttttcggattggactcgttcagcttgcgtcaactgccaacaaaaacgagaacttgaagaatgcgtgtgctcaaatttcagaggctgtgaacaacgg
ctcacaaatggttgtcttgcctgaatgcttcaattctccttatggtaatcagtatttcaatgactatgctgagacgattgaggagacttctcctacata
ccaagcattgcattgcattgctaaagatttgaaaatttatctttttggaggctcgattcctgaaagaaaagatgataaactttacaacacttccatg
atctttaatccatctggcaacttgattggcattcatcgtaagattcatttatttgatgtggatttccctggcaaaattacgttccgtgagtctgattcac
tgagccctggtaattctatgaccattatttcaactgaatatggcaaaatcggtttgggaatttgctatgatattcgtttccctgaattggccacgattg
gtgctagaaatggttgctgtatgatgatttaccctggtgcttttaatcttactacaggccctttacactgggagcttcttgcgagagctcgtgctattg
acaatcaaatttatgtcgcttcttgcgctccagctcgtgacatgtctgctagttatcatgcctggggacactctacggttgttggaccagacggcatg
gtatcggctaccatggatgaaaagcctggtattgtatatgccgatattgacccggagcatatggcatcaacccgtaaaagtatttccttgtacacc
caacgtcgttttgatgtctactctgagacaaagcctctcaaacttaatgaaaactaggattttgtcgtaatttattattaaaataattaaataaatca
actatatgagtttatatgaatccaggggtaaagtaattctaaaagtccaaaataccctaacgaaaaccgtttgatgtcatttatagtattaagttaa
tgaaagataaaatcatcgatgagcgccctttccattgtagcctgtagctggagagtatccgtagccataagagttgccacgattattcatatgaaa
gttgccatttgaagggcctgattgtccatatccacctcttgcactatacgcattcgcggatcttgggaagttaccaaattgcttccacggcggttgac
tacccgtattcgcaaatccattggtaactccagtatacgtttgctgattgtaactatagttctgtgattgataggacgaacgttgagcaccatagcct
tggaaacctcctcttcgaggtccacctcctcggaaagagttattatttctgcgtccagtattatttcgttctgcacgtacgatgctaacatcttctgga
gtcaatactttgggggcattttaacaccgcgaagaagcattgatttatgaacaaaatttgatttggggacttcatattgtaagctcaaactttgatt
gttctcaatgttgccgtatttttgaagcgcatcagcagtggtaacgtctactgggcattgtactgaaacatttggtaatatagaatcatcattgctta
cctttccaaaaataccatgtcccacttttccggataacttcaaaggtttcccttgtttctttttgtgtacaattgctttacgacttctgggtacatggg
atgatgctcagaaacaaacagcaatgtagatccgttcgtatttcttctagcttcctcttcactcagagaaggatagaccttggaaactgccgataa
caatcggttttcatcaataaaaggaagcaacgcaacaccttgccattcaaactttttaccgtttaaatctataacaaagtcttccggataaaaatc
gatgatctccgagtcattttgagtcatcaaagactgcaatttctccggtaaattgtttttacttgccgcgggaagtacacccaataactgctcatatg
gcctgaacggttgattcaagtcaaatttgacgtcaatagaagagagatttttaaaatcagccgcaaagggggcatagtgatatggataataccaa
gtccatgaaggacaaccttgataatagtacagcaaaacccaacataatccatgaacataatggcggattgcctcccctaactgatccgtatcatc
cacggaaaatccaaatttttgttcataataacgctctcgatatccagcttcatgtaaccgaactgtatcagtagtggcttcgtcgctggttgaattat
cttcagcttttcgtttcatgccagctgaaacgggtgctggttcaataggagttgctaaaccactagggtctgagcttgctatgtcatccgtagcacta
ggggtaggggacgaaaccacgtcctcaatagggacagcatgaagaacttcatttgaaatcatttgagactttaagtttgcagcagcaattttgtta
atagaagaggtagtcgcagcttcattggaaaggccaagacggttggcggcacgattattaatgagatctttgtttgtagctcctaacgatctattg
gaggctctttcagataaatttaccaaatcctcagggggcggaccaccggttttccgtattttattaggtcggccatcgtcttctgtgcttgtctcaaca
gaaggggcaaccgttgcttgtgaagattcaacctttactgagcgttgtataacagcctcatccaaataagatgaatcattttggttggcttctctttg
tgctctacgcttctggttttcagttcgacggtcttcttgttgcttaagacgcttaaaaatatcgtcttcttgatcgccgacagcagtcaaaatcatctc
ggcacgggttaaattaactgatccatctagggttaagtaacctcccatatgtggaagacaggtacgccaaatttcagtcagacgttctactgcacc
atcacgaatatccaagcttggaagatggggaagaaagtcgttaccgacgaagaaaatgaaaaacacccagtcatcaatggctctttcaagatcg
aaagggaaaggcaggttaggaacatacagctcaacttccaggtattctcgaagaatactaacatgtaaccaaatgaatggcttcttcacaggaa
ccttattagtttctgatgtttcatctaaccgcttcaaacctaatcgttcttccttcgtttttcgagtactgccttgttggaaaaatacatcttctctcagt
acgcggaaatgaggttcatgagttgccagaccaagcataattaaatcggcatccaatccatacacaacgtgatgagtatttgggtcatattctggt
tttactcgttgagaacgaataaattccataattttgtgctcaccttcacctggcacagatgcatctgacagaatgaagcgagtgtttctccagcaag
gatctgtgtttagtttatttataatgtagtatcgtagcgatttagctaatgtatccataaacggtgttcctggtgtaatacaattgctatcccaagcctt
tttcttgcttgcctcttcatcgacaggaattccctgtttttttgcttcctcaatgcaagtttcaagctcagcttctttttgagcagcttcacgagaagatc
gaaatcgacgtgatcgctgctgattcattttggcacgaggggctacaccatcgatggcaatgtaaagcaactttctgggacgaaccattgccaaa
atcctatctgtatactcaaaaacggcaatcatcatctcatcctcagtttcaggtgccggtcgatcttcaggatgcgaacaaggatggacaatgcca
ttcatatccaggtataaattatcgcattcttcaccattgggatttggaaggctcagatctggtccaacttctgtcccatcaggaagcgtctgagtcgg
tgcctcaactacaggcgtaataactttggcaaattttctcgacaaaagtcgaaaaagtgctggaacacccatcgtgaccaaaaactctgaaggag
atcaacgctacttccttcttgcttctgaaatcaatccgattctgtaattaaaagaatcaatgatcgaaatcgtttgtgtggtatttgacagacacgaa
taaggcagtataatttatcattctcttaatatttacgaccttaatatatattactctaaaaagtatataattctataatcacagttgttgagatgcgtt
atactattgtataacgaggactagattactttcattgataaacaaataggtagaaagtgaaaattaatatatgattttgagctttgtacacataatt
atgtaagtatgtagtgtaacttttcataatattgattgtacatagtattttcttcaattgcggtattctctttccatggactgaattacaatgtttatttt

gtagttttatttcaaagggaatgaggaaagaataaaggatcagtcagtgatgctaagataatagaggaattcatggaatacggaagaatttaga
gcattcaggtagattgaaatcataaatgtccgtatatttacaactcgttcttttgaaattatgtctgtccaaatcctcttttgtaaagcatgcgtttaa
ctttaaaataattaatttattatttaaatacgaagcaaattatatttttctttctaataataacagcgtttgagccttctcgttttacgagtttttgcttg
ctggtaaaataagttcaacatatcttccaaagttaatttcaaggaaaaacacccttaagtggactttcagctgctttcaaatataaatttcgcatctt
tgtgaagactcaaaacatgattttcaaatatgcgagacttaacaatgagctgaactgttgtgagaagaaaaccattgacgttagtttctaaagaa
aaatatggtgaaaatttattgtttatgtggatagaccaccagagcttaatgattctgtagcatttcatgaatatactcctgatcatgcatccttttctg
gcccataaagatgtagaaacaagattgaaatgtcctaagatatagaataaaatactttctttatatcggacaagctgctcattagtaagaaaaag
atttggcaagaaatgaatgtgaggaatgccaaaaaagactcaaaacttattaagctctttgcatgtgcaatttatagctgtagagaagctcaatat
aagatatagaggaagctattcatttaaacaagcctctgtaaaacattagtcttgaccctatgagtatctaaagcagacgaatgactcacatgggg
gactttttaagatttatttgggattaccttcccaataaaatcttaccttcggggactatacgatcatcttcgatgacgcttacttaaaacacataag
agtacagggataataaaaggcgtcaattacttaattgcaatcctactgttctctaaaacttaagatataacaagagaacggcatgtcctctttcttg
tggtgaagctgggacatacagtataagatacatatctgaatgagtattaatattaaaaacatatgattcaagaaaaagaaaagaaactttccgtg
cataagaaaacattgaggatctgtcaaaagagggttcaactcgtcaaagaaaactgacacgacaataaagagtatgaagcaatttatgagcaa
attattaccgcgaaagccattgtaagagaaagaaaaggaaaaactatgctccaagatcacactgacagataaagcaaacctaactctctaaag
aaaatagcgttcagagcaaacctaggcttcggaaaacattcttttacaactttttgaaccaatttgtagaagatagaaaattactggattgatcga
acgcccattcgccaaatccaaggtatttttcaacggcgagcttcacatcttcgttatggactttctcaacatcaaacctggttcaatgtattagtttcg
gttttgctaataatggagacgaatttataaatgaatagagtgataacttacgagacaccagaagaacgacgaattcctgcgatgcaagtagaaa
ctaatgctaaatcaactccgtaatggaacaaacgagaaagaacaggctataattagcttttgcgaataaaataacataccatttttaaatttatag
ggctgctcgtagttttcctaatgttactgaagagaggaggaggtttggtgtagcttcgttctatgtgctatacatacagtagcacacatggatactat
gagtaaagcaactggatataatagtgaggacttttaaaacgtattaaaaataacagaatataaaaaggtttaaaggaataaaatgtacctgatg
gccaatttatatgtacaactattctcatattgcttatgtctataaatactagacttatggataccaaagaaagagagtaacattaaacgatatccct
aattaacatctgtagtgtcttttaatttaaaataaaacatcattgctcctttttttgacactttctattttttcttccatttggaatatcctattttccaccc
atcccaaacttgccacttgagcattcagcaccttaacgatttcagaaatagaatcatcgggcttcgagaagtcattcatttgatttattacagttcct
aaatcttcgccgagcctatcgaggcgactcaaaatatcatcggcaacgttaaacgcacgctcgcgctctacgttaggtgcagtaggtgtgcgacc
atcaaaggattcaagctgtgcttcataagaatctaacaatttaaagagctcctgttggctagaggaaacatattcaagtccatcatcaactcgattt
gataactgttcagcatcgacagtttctgcatataatttggaaattaacgctccgttatctattaaagccttatcccagtcagcgacctgatcgcaaa
gtttgttaaatactgtggtctgagtagtgaggtccgacgaccatttgttaaggatttcttgtaaggttttatgcttgatagatgagggtggagattcc
accggtacggtagcggtagacttagaagcagtatctttagtagcatcaggtttggttacattagactctgcgggtttggaaccaaatagtgagcca
gtggttgttgttgttgtaggcttagtggtagagtcttgcttaccaaaagagaatccactggaaggctgggcaggggctgtagtacttggtttcgtgg
tatcagtcgatggtttattagcttcggttgaagcaggtgtagtagtggtagtacctaatttatttccacttccagctcctcctccaaaagaaaatagt
ggtttgctttcagcaggcttggcggcaccagcagtactggttccaccacctccgaaagagaatccgcctgtcggagcggggcttggtttgttgtca
gaagaactttctgcagcttttccaaagttgaatcctccagcggcaggtttatcttgggtcggtgctgtagatggagtggctgcagcattagtactgg
ttccaccacctccaaaagaaaatccgcctgtcgttgcgggactaggtttgttttccgaagcacttcctgcagcttttccaaaactgaatcctccagc
ggcaggcttatcttgggccggtgctgtagatggaatggctgcagcattagtagcgggtttctgtccgaataagccaccaccagaaccaaggccac
ctccgaacgtaatcccgccaccggtgctggaagctgaggttgttggagctgctggcttggcaccaaaagaaaagccactggtaccggatggttga
gtagtcggctggttagtattggcacttgcagtaggaccagtcggtgcttctgccgcaggcttattcaagccaccgaataatggagcattccccgat
gggttagactgtccaaaggaaaaactgggcttattgccagaatcagatgcgttggagccaaaaccaaagcctcctcctggttttgcggtattcga
agctgatccaaacaaacctccagtgttgcccgaatttcctccaaacaaatttgtgttggaagaggcaccagctccttggttagatgtattatttggt
gctgaagaaccgaacgaaaaccccgttccttggttgtttcctggattaaaggctattgatatggttaatactaaagctcgctcaatttaccttgttca
tttaaattacttacccattgtatctggggtttggaattcgcggtgcttccttcacgttgcctttgtgatgtctttctctgctctcggtgaacttcggtaat
atttcgggtttttacttaccgggatttatggagtaacaccgagattaccgaaacttgtattctaagagataatcatagaagaatgaaacataccaa
gtgcacctaagatgacaaacatttcgttaacgattaaagctgccaatgatcaaaaatacactgtatctgttgacagtgaagcgagcgttataaatt
tgaagcagactattgcgcctgtagctaatgtggaggttgaacgccaaaggctaatttacgctggacgtgttttgaaagatgaagagcctttgaaa
acttataaaattcaagatggccagagtgtccacttggtccgttctttagggcaaaatcctacggcagctgccagtaatgttcaggatcgtacacaa
caagtacctacaaatattcaggctggacaaggtgcaaatgacccactagccaacctcacaagtgctcgttttgctggatacaatataccccctccct
tctgcctcaatgtttggtcctaatcctgagaatcctgtccctccatctaccgaagatcttgctaacatgttgtcgaatccaatggttcagtcatccatt
aatgaaatgttttctaaccctcaaatgctggacatgattatcaacagcagcccacatcttcgtaatgctcctccctatgttcgtcaaatgatgcagtc
gcccgagttccgacgtgccatgacagatccagatacgatgcgtcagatggctcaaatgcaacaacagcttggtgctgctggtatagacccaaca

agtttgatgggtgattctggtcttggtggtttaggcggtatgaatcctggtcttggtggtttaggtggcatgaatcctggcggcgctggtttgggagc
tgacaatgcatctgttgatccaaactcagctgcaagtacaatccaaggtttgctaaacaacctttctggaaacggtctcggtggtgctgttagccct
gctgctactgctgccccatctcaaagcacccaacctcctgaagagctttatgctcaacaattgagtcaactcaacgaaatgggatttgtggactttg
aaagcaatgttcgcgcccttcgtcgaagcggtggtaatgtccaaggtgcaatcgaatccttattaagtgaattttaatgtattgctccaacaaatgt
tagaaacgttattagaaaaatgatgtgaatgtcgttcatgattcgtttatattactggttttgttttcgtcctacgtacgagtttatgtaaaagtattcg
ttaaatgcacgaattttggttttgttgccgtctgctccgcgtagaatcctacgtatgatgctctattaactaaaacctctatttttgtatgtatatattat
agctcagtatgtgaagtttgaggaactgaccttttttccatgaccatggaatcaaaatttatgaaattttctattatcgaagttgtacagtctgtaga
taaaaaaaaaaaagaatcaattacatgcacatgtaaaagaaagtatgaaaaagtctgacagtaggattccttccctcggaaaggattaagcca
agatattttccatatattaataaatgtggaattattttttaacgtctacactgaataaatccgccctcatatacccttattttatttaaaataacaaaa
aatacgtactcatgtgttgacaatttataaatcgaaagtaatgacaggaagaaagtagtcagacacattccaataacgacgctttgcaccttccc
attcttcatctgtactgcttgttggcaaaacttcgtaagtgcattggaagccgaaggaagcgagatctataggaactaatttttatacacatcgta
gaaggtctggttatcagcacccaaaccgttgaaaaatgaaaatttagagtcagtatcaggatctaaaataccaactgcagagtcaaaaaattttc
gtagatcttcgtagctttcagcaaaagcgtcataatagataccatcgaacacgtacttagaagcaatgtcttcgatagcgctttcccaagtagtctc
ataaatgacaacattttgacgatccatccagccatttttccgcatgaaggccaatacatgtggatgaggctcgatgattacatgcaatgatggttgt
cgttcttgtagaaaagtatcaatgatgcccaatccaaaaccaacgtttaagactcggcaaccaactctgggtgcaataatctctgcagaccgttgc
atgattttcttttcccaagacatcattacagcatttgcatcactgtccaagagactaatactgtcttcagtaggttgggtatatgaaagagtgctctg
taaatacttttcattggtgtccaggcgttcattgactttttctttcctctccagaagagacagaagtagctcacagcggacgcccgcgttaacaatcg
tctcgtagagttcatttaaatttttgcgacgagcaatgcaaccaggagtttcaccagacttgtcaatgccattccaaactccaccgttacctagcat
ccatttagcaagttcaacagccttcttctcgttttcggcatttgcatgtaaggccacgaaatgcaaagcgtttttttccactgtcaacatccaaagcag
cagtgacagcaccttgatcaaccatctcttggacctttgccaagtccaactgcttagcagcttcgattagttgcaaggattcaggagattcttgaaa
agtatcagaatctgtgacgataggtacttccattatgatggatggtgattttgctttgggaaggaaataaaaagacgagaaaaaaaagggtcca
atattaaaacattaaaagcaaattattgagaatgaacgttataagaaaaaatagagaaaatcgtacataacttggtgcaaaaggaaaggtatat
taagcctgcaaacgatatgttgcaaaagttttgatgagagataaagtaagctgaaatttgaaataaatgaaaaagaatgaaaatctttatttaaa
tataagcaaggattgatgagaacaatagttggaaatatgaaacttaaaaagcattatcgttcattaataggaaattattaagaatttgtacgtga
aacaaaaatgacacagaaaatctttaataaacaagaaagatcgaaagtgcttaaaacatacgaagctggataaaaaagtcaaaagtcccaga
aagccaaaagaaaagggctgatttcttgaaaggttacttcaaaacggccattgtcaaagtgaatatattaacgggaagtatagccaccatcaac
aacaatatccgtacccgtacaaaaggaactggcatcagaggccaggtagatgtaagcaccgaccaattcttctggtaagccgatgcgaccctga
ggggtatactctttccactgcttacggagcttttcatctgcatagagagtcaagtcggtgtcaatgtatccaggagagacagagttcactctcgcaa
agggagcccattctacggcaagcgcacgagcaagatgttttacagcacctttcgtagcatgataagaggcccattgttgtggccagttggcaatat
gagcactcatgcttgcggtgaaaattaagctgccgctaccttgcttcttaaaatgatgcccagctgcttgtgcagtgtagtaggcgccattcaggtt
gacacccaccaccttggtccaggtatcttcatctttgtcttctaaagcaagatgcggaatgccgattccagcattggcaaccatgatatccagacg
gcctccgagttcttcaactgcacggttggtagtgtcaatgacagcactgcgcttttcaatagcgcaaggataggcttcggctttgacattgtattttt
cgcgaagctcttgagcatagtcgagagctttttttgttcttaccatacaaaagacccacattggagccagaagcggctaaagccttcgcgatagaa
aacccgataccaccggatccgcccgtaataagagaagtcttaccctttaacgaaaacatggggagcgtcataataaaagttgtatgaaacagga
cgttatggagacagttcgtaaaaatataacagcaatagtgggtttactttttttgagaaagtaagtggtagtaggaagagtttggaaggtaaaaat
tcacagtgaacaaataatactttcaaggttgaacctaagtgaagcgagcttcattcagagacaaaaatgaaagaattgaaactttattgctttatt
gtataaaaaaagcaaaaatcctgattctatctttttcactgctagcagactacgaatcaataattgtttattatacacaatttctgaaatttctcaaa
aggttccaaaaaagtcaatttataatggctttggaagtgaaattttgaaaagacatagaaaacgaacgatgcaataaatagctggcctcgtttgt
tgatagatcttgattgatcaaagcacaagagttatgtaaacaaacaaactgattgtttcggtggtgtttgttttcgaaaacaataaacccaaatgtt
gtagtaacaaaaagctcaaaaacacaaaatacagaaggaaagaagctatgaagagtaaaaggtaatgaaaccaaacattacggaaatgaga
agcgtaatcattcagaggactcagaagaagaaaatgaaagccgtattcgaaaataaaaatctgtttgttttctgattaggtatttcatagaatgta
cggcttttacgggtcgccatttacaagatttcatttacaattgtttactacggagggaatttcagcctacgcaatccgactattaagaagcgagacg
aacgtctggcaaacttgcatgttttcaacttcgattggagaacaataagaccccccttccccttttcacatcagggggtttttgttgtcagatagtaat
ataatatttagtgaatgtatgtcacgtacacattttccaccgtataagaatgtgtttcgagtggtaataaacaatttaaagctgtatcgttttgtacgt
gaattggacttgtttttaccacgcttatgaacactggtcatgtacagggatctgctgaacgaaatgaaatgctgaaagataatcacatttagccag
accagtcaaaccttacagtctgaatattcgtccgtgaactcatagatttttatagttttgtttatgtagtgagcgtagtctgtttctttgtttcacaagc
ttctatttctaataatatcagtcccaatgtcgcatctatttaaggcttatgaatattagtaaaattcactactatgtgttacttactcataattactgag
actgtatttcttgaaattctgcaaactgtacttagagacacaagcagtgaattgttgaagctgaaaatgggtcaaacataagcaggatcatatttg

tatagataaaaaaaaatagtatgaaagcagttggacctgaagtgaaaatgatttgcattacaaatctccaaatttttgtttattatttatcggttg
aaccagttgacataacatgttcctccaaggggtatgcagcaatggtttcccaaacaggaatccaaagtttataaaataagaaaaaaaaataaaa
taaaacaaaacgaaaggaaaggaaagaaaagaaaagaaatagagaaaaacgaactcgccttgaataaagatatttcacagctttcggtaaa
gtagatagcatgggcatgtagacatactgttagtaaataaaggctgatcagtcatttaacttgtattttcaatttcaatggaaaggaaaggacaga
cattcatcaataagaatatatttgtttttgggcttttatagagcttcaaggaattcatgtgctttataacgtaaatggaatttataggtgaagaaat
gatgcgatccggtggattcataagcaatcaagttatgtttcgtttgctttttgttattattaaaatctatgtgaagtagttcaattaaatactgttttca
cccatctcaaatgtcgtcttttgctttgacttaactccaaaatgggtatcctgttacttacttccaagttgctggttgccttgtgcacacaaaacgaaa
tttgggtcctccatccttttcccttacctttacaaactcagacttaaacaagaatgtcaaatccaagcagaacgttaaacataaaaacaaacgtcgt
gaaacgcattcttaaggatgttcacgtatgtagtgacaaatcttttgacaaaaattcgcagcaagtttgctgaccagaaccataggttgctcacgt
cgacattcaggatgctaaggaacgcttacagactcgtattgacaatggagaagacgaacatgaaattgagcaccaggtatgtcatggtctaaatt
taggcagatttcctattcaacgtattcttcgtgatgaattcttctctttttttatcgctttgtcgtgataacaaggaagctaacagttggggtagaaattt
gtgttgaagcagcatcttcgtgctcttccagatgctcttaaccgcttacagcgggctgcagaagaccttcaatccattgttgataatccagtttatgc
ggatttaccggaattggaaactgctaaatcagttcttgcaacggttactgaagtcatggaaaacgagcgatctactgatgctcctaaaaatggac
acacttgaatttccctgtccttttaagaataccattattatgaatccatcctttctttgttcgtttttattcctctgcggtttgctcttcttctcatatattgt
ttagacttccttttcaagttttcctattatctgcaggaggttctgaatttatatgtacgacagctttttcgtagaatgatcccttcactataaaaataca
ttacagttatgtagaagtcaacgaatacgttcaacgcaataggtatctatagtctcacatacaaaaaattccgaccatattggaagacttgcggat
tgcagttaatgttccatttcattctgtatatcagatgctttcgcttttcagtaagatagactctgaacatttgaaccaacaatggcaacgaacaatgc
aaacaagctattttaataaatgaaaccttctaattactatgatttatgttatagaaataacgcacttccaacctttcatattacaaacatatggctac
atcaaaattaaacaacgcaaaggtgactttttctgaaagaagcaatttgaatgaaaagcggcgacatagggaacaaggaaaattcataaccct
cccctggtgttttcattcgataacgttttgtgatcataatgcaaccagtagaaatccaattcatgaaaatagatataaccgaatgttcataaaataa
atataagcaaaaacaaaaccgttaaaagaaagaaccgtcttcaactcctttttttacccattctattgcttattgtatctgtttatttacatcaggact
atgatatgagtttgtttacactattcgtaaatagtgagatattattacaggctgtttctactgattcaccgtacttcaacatattactaaaactctttat
cattgtttccttccatctccgtatttcgtaaacgaacgcgtacttacctacagacagaatcgtcaagatatcttgtctgtattcttatgaatctcgata
cttcgtcttttcgacggtcttcagacgaagaagataatcaagacgccgtaaaagatgaggattctgactttgaatcgtcacccactactatgaatc
ctaaaaatgcagaacctgaggatttgacaggaaatgaatccagtgactcttttcaggagaataaggaaacgaatcctagctactccttggatgga
tctcaaaattttggaatggacgagttcaatacaatggaagaagcaaaccataaggaaagtgttattcaagaaaacgagtcaccgagctcttcat
atgaagcagaacagcatggattggaggaaagtaaagattttgatgattttgatgattttggcgaatttggcgaagtggagatggaggctcctgttg
caacattcgaaggtgataactggatggaaagctttgaagcttctgttcacgctgtctttggtcctccaaaagaacctaaacctcttcccgagtcccc
tcttgatcacgaaaggatcttatcactatgggataaactcattgaaatgcctgttctccaacgccccgactggttacggtctcacattcgacatatat
tcttagtcacaatgggattgccggttgatttggacgagttgttaccgacgcaatccaatagttctgcttcttcctctttcaaaaatatccttaaaattt
cgacaacttctccgcagttggaaaacgagcctccattaaattatgccgttgctcgacgtttatgctctactacacatgaagcacttgcatctcgatct
cctgaagcactaaccgagcatattgaaatactggaacaatatgtcatggaggccacttctctagcacagtattggactgataaacgggattcctcc
ttgaatgacaagctgctttatgaaacagtagttgacgaccttgttcagcatagtaaacgcttgcgcaaatccgctcgataacccggatcgcccgct
ttttctctgacagaatgcgtttctttttttctttttggttcaaatcataatagttatcgactgttcttagctataaataagctttacaaaaacctcgttat
caatctttcacgattaatatgtttcttgttttgggcgggatacccaagcttgcctttcattctttttttcccttgttaatatgagcgtcatggagcccta
ttgattattatcctaattttcgttcgtgttcgtgactctaacactccatccatgtcttatatgtttaatgacttacctaaataatcggttgtatgttttgtt
cattattagtaaaataatgaaactgaatatttctttggcgtttagtgaaattgattctttatttgcataaaaacataagtgtatttttcttaatcttaaa
atcgtgtttacatttatttatttatatcaggtttcttgcgaaaatatataaagtttcgtcactattgctcgctaaaagtataaagctaaaatgcaaga
aaaaaatacaaggacggtattttaccaaatactctttgccttttctttagattctttctgcaaatccttcctcccaactttaagtataatggcttccaa
aataacatctgcaattataaatcccagcttgacgcataaagcaactgtcatctttttttcatggtatgatcctggcagtaatgatagcacagtgattg
ctagcttttcttgtttagcttagtaaatttcagttactaacgctttaggccttggagattctggttacggatggtcatttatggcagagcagttcaaat
cgatgggtcatgtaaaatggatatttcctaatgcgtacgtacgttaagaaggctcgcagacgtattaacacgaaactttagaccgaccattcccgt
cacagcaaacaataacttcaaaatgcctgcttggtaagtctcgatttggatgcaaagcttcttttaagggacgaaatcatatgctaattaatactct
cattaggtttgatatttattctcttgaaaatttgaacaaacaagatgaagaagggattcttcattctgtaagcatacttcaccaattaatagacgctg
aagttgctgcaggaatttccagcgacaaaattttgatcggtgggttctctcaaggtactttttcttttcttttttttaaaaaaaaaatataagagcatt
tctctgaatgcttgcttcaaacaaatccgcaccaagtatacaatactaacgtatcatccaggttgcatgatttccctctttgcaggattaacttatcc
aaaacgcttagctggtatcatgggccattctggctttctcccactcgagcacaagtttccagagtctctttcggatgcagctagaagcattcctattt
tcctgggttatatgagagatgatcccattgtcccttccgctctctcaaatgcttctgcagaacatatgaaacaaaagctccatttgaagtgtatgaat

aatccttacgatggggatatgcattctccgtctccagaatctttcattgacatgtacaaatttgctctttctaccattggatcctcttagccatttgatt
tgatcgtttgatagactcagttgtaatatctatatctctttcgttgacatgttgaatcagtaaaggcaaatcaataaatatttaaaaagatatggagt
atcgttaaattaaatttaaaagagaagcttatatttttaaatttaacttctaaaattttgatttaaatttcagtactaatgctgtacatagaagttttgt
ttacgcgacgcgtctgaaaaatggaaacaaaaatgggcgtatgcgtgtgcatgtagcgcgcatatatagtttttttgaatacataaaaacattattc
tttcataaataaaagagtaatgtgtattcattcagtccttaatttacaaattacaaaattgacttaagttatataaagcgcgtaatgaatttacggtt
ctctcaaaacctgaaacgtgaatatttatcttaaagcatacttcaaacacattcgtagtagaattagtatattactcatttaaaactagttatatcaa
aacaaaatatgagtacgatggcagatgttgttgacacagctggcgaagagcaaatgcccttcagtgatattgaagaattgacctctcatggtacg
cgacaaaaaagaaaagaacaagagacactaacgtttttctatttaggcattggaatgaccgatattgtgcgtcttaaacaggctgggatctgcac
tgtacaaggagtccacatgtctaccaagagatttctattaaaaatcaaaggattcagtgaagctaaagtagacaaactcaaggaagcggcttca
aagatgtgtccggtgaattttgctaccgcaatggaactgagtcaaactcgcaagcgagtatggaacatttctaccggttcggaagcattgaatggt
atcctaggaggagggattcaatcaatgagcattacagaagttttcggtgaatttcgatgcggtaaaacacaaatgtctcatacccttttgtgttacg
gctcagcttcctaaggagatgggcggtgcagaaggaaaagtagcctttattgatacagaaggaacattcaggcctgatcgtattaaagcaattgc
tgatcgttacggcatagatcccgatctaacaatggaaaacattattgtcagtcgtgcctataattctgaacaacaaatggattatatcacaaaatt
gggaaccatttttgccgaagatggtcaatatcgccttcttattgtggattccattatggcgcttttccgagtagattactctggaagaggtgagctgt
cagagcgccaacaaaaattaaacatcatgttagctcgattaaaccacatttcagaggagtttaatatagccgtgtttgtcactaatcaagttcaag
cagaccctggtgctgctctcatgtttgcttccaatgaccgtaaaccggtcggcggacatgtcatggcccatgctagtgctactcgtctgttattgaga
aaaggacgaggagaagagcgtgttgctaaactaaatgattccccagatatgcctgaagcagagtgtagttacgtaattacgcctggaggaattg
ctgatgtatcgtaattctatcaaaatcgactttaaaaaaagaggaaagaggaaaagagagaaaataacattgaacccatacaatgaagcatca
gtctccgaaatatcttagcaatgcaggcattcaatccagaaggataaaagcaaaaattaatggtgcaaaatattatatttcccttgactctcgtgtt
taacacttctttattgtattgcagttattcatcctaaattaccaaatccgagataatgtacggcaccatacaccttttttaagttttgatgaagagatat
tcggttcaattccttctttttatcccattcaagaattacgacaataggttgtcgttgtaactcaatttttttcttttttttttttgtttacatttcctctttcctc
ctatcttcaatcatcacttctgaaagtgtactttgcgtcatgtgggactatggcgcgtgaacaaaagctctcgaatgaagatacaatcagacagctt
attcaagtatgagaagcgtgagtcctagtgttcgataacgttctttattaccgaatgttgtggaacatttgttattatttccgtaggaaaaccctatta
tgcaatacgcagcgttacgaaaatttgctgatgaagcagtcccctattcaacccacacaacactccgaaacctttccagtgccaaccacaactaa
tcacacattttcattagttagtaaagatgtcgactacttctcgtgaagatgctgtttacttagccaagcttgcagagcaagctgagcgctatgaagg
tatgtactaccattttagcacatgacgctcccaatatgtggaagaatcgattctattttgcaccaaatttgaaaacccgaagtgtcttaccatagac
cttggccgcttttcaaaatatgatgaatgaagaagaatgccttaggagggcggttattagtcgcaaatatccagtttcagccataaaagcgagag
atgatttacgaaagcagaatggagcgtcatggttactataaaacaataatcttattttaatcctctatatattgaagctaacaaaagataggcatg
gtcgaaaacatgaaaagcgttgcttccaccgaccaagaattgactgttgaagaacgcaacttactctctgtcgcttacaagaatgttatcggtgct
cgtcgggcgtcatggcgcatcgtttcttctatcgaacaaaaggaagaaagcaagggtaacgctgctcaagttgaattgatcaaggattaccgttg
caaaattgaacaagagcttgacaccatctgtcaagacattttgactgtcctcgagaagcaccttatccctaatgctgcttctgccgagtccaaggtt
ttctactacaaaatgaagggtgactactaccgttaccttgctgaattcgccactagtgacaagcgtaagcaatctgctgatcaatctcttgaaggct
acaaggctgcttctgagtctgctcaagctgagcttgctcctacccaccctatccgtttaggccttgctcttaacttttcagttttctactacgaaatctt
aaactctcctgaccgtgcttgctatttggctaagcaagctttcgatgaagctatttctgagttggatagcctttctgaagaatcttacaaggactcta
ctttgatcatgcaattgttgcgtgataatctcacattatggacttctgatgccgaatacaatgcagcagcagctggtaatgccgaaggtactcaag
agacccctgcttcttccaatgctcctgttgaaggagaatccgaacccaaggctgatgcataaatagctgttacttatgattgatgccatactttcatt
agtatgaactatgctcgaaaaaaggttgacttgatgaagctctttttatttttacattagattttgtgtatcgcccgatttgatagcttttcttatttac
gtacatttttacctgatctttttacttattctttcctctttttttgctggtgtatatcccaatccaattctcacataggtagttactgtctttgttagttggta
caccattgaaacagatgatgctatcgctgatttatatataaaaatgaagtgaaagcaataagctaatgtttatttataaaaatttaaaagttaaaa
cagagtaaaatctgagagtatgaacaaacaaaatatacacgtatatacatgtatatgtattggcaatagtttttttttcaagcctttgtgttaggat
attgagtcgcttattaacagctctcacaagtttcgtattctacatccatctgacaaattttctgcttgttcagaacgtacgagcaaaccatttaaaaa
agcagcgcatctgtgaaaacggcttaaaggaaaagttttaacgaaaaattggccaaatatcgaaagagaataacatgtcagctcgtccttgtctt
cattttacaccgccagagggatttatgaacgatcccaatggtcttgtgcagtccaatggattatggcacttgttttaccagtggaatccaaccggaa
tggaagcaggaaatcaacattggggccacgctattagcagaaatctgtataattggaactatcttcctgtagcacttttacccaatgaggacgga
ggcctcatgttcagcggaagcgctgttgttgataaaagcaatactaccgggtttttcaaagattcacagggaaggattcctacaaacactgatggt
cgtatcgttcttatttactcgactcattacgatgaccgcgagactcaaaatatagcgtatagtcccgacggaggcatcacatttattaagtatgaaa
acaatcctgttattgatctcaatgagtctcagttccgcgaccctaaagtttttttggcatgaagagacaaatcattgggtcatggtcgttgcactcgct
caaaagtttcgcatagctattttccgttcccctaacctgaaaaactgggaaaaggtttccgaatttggtccagccggcttcactggttaccagtatg

aatgtcctgatcttttccgcctccctattgagaatacggaagaccatcgttgggttatggttgtatccattaatcctggatcccctatgagcggagga
tctgtcacccagtactttattggcgactttgatggcattacctttactcctatcgataacatggctcgtatctttgactatggtaatgactgctatgca
ggtcaaacatattccaacgtcctcaacggtaaagtgatttccatgagttgggctagtaactggaattatacgaatgatgctcctgcgaaaaagtat
cgtggaatgttgacgtgccctcgtgaactttctcttcgttctatgcctttgaaccctgaaacacaagagcttgttctagttcagcgtccctgtaacatt
gaaactttgaagcatcctctttccgaacctgctcctttagccttgcaaaccgtttctgaatttcctccgacatttgtctgtggcactagattctatcttt
gcgctaccatgcctgcctctgcatccgaatcagaaaaactggaatttctgcgtctgcgttggtcagcaacttctgatgtccttgatagcaaacaata
catcgagattggttatcgttttagcgatggtgctgtctatattgatcgcggttcttgtgaagcaagctggaagaatccactttatcctgaacgttgca
tttccagcttacctcccattgttcttgaagataaactattggtggatttgggaattcttatggaccattctattgttgaagtttatgggaatcacggag
ggaaggtctttacgaacgcctactgcttttcagaaggatctgaagattctcctcttaagtattatcatttggagcttcctaaagctgccaaattagtc
aaagctaatatgttgcctcttttcacaagagttgcaaagtaaatgggcttaaggcgtgtaaactttttgggtaagtaagctttacattttatttatgg
cttgttataattataaggtattgtcgcaaatggaaattggtataacgatctccttttctttcgtctttctcttataatcataccgtattttatggtttcaa
ggacatgacaatatgagtttaggtgctcaactttttttttttcacttcaatttgtactactatagaacgttctttatttcgtacttggtgatcggttttgg
attacattctacagtagacaaatcgaccaactcttacaaaatagacatctctgaagtttgatagcttcgcttatatttaatattaccatcatataaat
tccacatcattttctgtaaatgttagtgtttctgccgaaacttggacacctatggaaagttaatatacatagaacagcttacactcaatagaaagag
tttttgcgatagtcttattaaaacgaaaaaaatatatttatcaaattgaatttcttgaaagttttctacctttgcttcattatactaaacaacttacatt
gcatttccaacttagcgactaccaattattccaatgatagctgatcctgaccagttaatgcaaaaagtaagtgcaagcaatttcaatatatatattt
ttaacatataggctgctaaaaaggccagcggcactagtggcttcgccattttcggtggtggaaacaaatatgaagaagcatctgagctgtatttgg
atgctgccaacggttatagacttcaaaagcaaggtaaggaataaacttgtattcaaaaatgctgatgcaatatatagggagtgcagctggcttcg
cctttgagaaggctgctgaaatgcagttgagaactgatgaaaaggatgatgctgccaatacatatgttgaggcctacaagtcttaccgccgtgaa
aaggctgcagaagctgcccgtactttacaaattgcggttgacttgttcacgagacgtggtaacttccgtcgtgctgccaactacaaaatggacttg
ggcaccctatttgaacaggatctgcagaatcctaaagctgctcttgcttcctacgaagatgcaggtgaatggtacatgagtgatcaagctgaagc
gtatgtattgtaacaagttgattgataagcgcatgactcaaggcattatacgtcttcttagtttttagtcgatgtcagataataatctgtgctctcttct
ttggtttgcttaaatactaacttttgcagtcttgctaataaggcgtatttgaaggttgcagacctagctggtcaagcaggtgaatattctattgcaat
ccaaaaatttgagcaggttgcacgtgctagcgttcaaaacaatcttctaaaatggagtgttaaagattatttgctcaaagctggcttctgttatatg
gcccaaggtgatgagattgctacacgtggggctctcgaaaatttccttcaaatagatccttcgtttgctactactcgtgagcatcaactactgatgg
atttgcaaactgcaattgagtcttctgatgccgaaatgtttgctagcaaagtctttaattatgaccaattatccaaacttgacgcctggaagactaa
ccttttgttgctaatcaaaaacaatattcaagaggctgaggacgatctgacttgagtgatgcatatatttttttctttatgatgttaagctccttgagg
aagccatttccgttactcaactcccttttcctgaattcacgttttcttactttttgattaccattccatatgtactgacaaagtcaataggtactgtatg
aatagtttattggcttttccgctcaagtctcgtttgtcaggttcgaaggagttcaatattgtagagtcattcttattctttgtttcttttcataaaagcat
tcgagaaactggattatattgtgtatatttaatactgaaatgaaattacttttataagtaagttttagtttatcccttattctgtaaatagatttgttat
attgtatattttagcccacataactgatgaaaaattatccaaaaagtattatacatatatttattttccccgagcaaaccgcgatagtgcatagccc
catttttccttttcttcaggcaatcccttctctcgacgaacgcgctgttcccgcatcttcttttcaacatcaagagatttttcgacattgagacggcgtt
gatccactacgttccgagcgtgcttacgcacataacgacgcaaagccgagtttttaccacgagccttgttcttaggaagccatttttcttccggagg
cttttcttcttcaacttcttgctttcgaatagaaggagcagctctatccacgttaccaataaattcaggacgaagagaaatcatctcaggacgaagc
ttttcaagaagttgacgaacttcagtttcttgtctctgtttcttggaagcgaaaggatcattttcgtaagaatcataattaggttcaccggatccagg
aatgattatactctcaaatcccttgtcatgaccaatgcccaatatatcctcataaggacaatagtgaagatcaacaacagacgaagaaggaatta
aatgattcatgtaaggagaactttgttttgtacgtagagcatctttccaaacagtggcatgaggaccccaaccaacagtgagtaaaccagtatca
ctcaaagtcaggcgttgggccggggtaggagtataatatgaatggacttccttgtagtttcgaagatcccagaccttcattaaactatcagcaccg
gcagttaccatatatttaccttcacgatctacggccaaatcacgcacaggtccacggtgggtaagcatcttagcaagcggtgtagtagacgtggg
agcccaaagtgtaacttgcccgttggcatgaccaacgtgttccacggcattgtaagggttttgatgaagaacatggcaagctcccatacccgtacg
aagctctgcaaccagttgacccgttgaaacatcttgatatttcagatagccagcattaccaatgctagttaaaagaaggtgatagggaagaaagt
ccaaggcatttacatctatatgacgctttaagcaatgaatttctgttcccagattatcgtatacgtatacatatttcttttgagcaacggcaaagtatt
ggtgattatgaaaccattttacatcacgaaccgtttcttgcaaatgtaattctgttaataatttaccattcctccagtcaaaagcagcaacatggccc
ttacgacctcctaaaagcatcatgcggccatctcttgtataatcaaaattgtagctgccgaatttatccaaaccaagagaaaaagacttacatggt
tagcacaattaaatcacaaaaagccttaattttccatacctttgtagcagtttcaagggcaacgtttggagcaacttggttttgtgtgaatttgtatg
ttttttctaatccttcagcttccaaataacctggttcatcgacacgtaataactctgtttttgaaagatctgtctctacattttcaatcctttcctcgattt
tttgtacattgcttcgaagctttttgtctttaattttctatacgaagagttagcgagaattgtcatccattttgctttgcttacctttggattgagctttcg
accacgagagtatttgtttgtcttcgccttaacctcgtccaagttcagttgttgaacagacatgacgcgaaattaataagcagaaataactgttctt

gctctttatttataaaggaaaataacaaaagcgacccaaaacgtcctgcagaaccctttctagtgttcaatggctacaaaaatttaagacacgtcc
aatgacaaaaacctcgtttcgtacttttcacgctgaagcgtatgttctaagatcgatatgggagaataaaatgaatcaattaatggttttacaatct
tgtaaatgtcaatattcaaagagtttttgccctggagtgtatgggtaaaaggaagaagttggctacgagtaaaaaatcatactagcaatagacga
tacgcaataaatatgagtttccaaggctacaagtagattttaaaaggataactaacatatatatatataaataaagtagtttttattaaccttgctc
gaaggatccaaaatgcaaatttacaaaattaaaacccaattaacccaactacaaaaaagaaaaacttgactcaactgaatgaagaaatgaaac
gctaaatttgcattattgctttttattcatagacaaagtctcaattgaattagagacttgatcgacattcgaattggaattgtgggcaattctcagaa
cagactgtgtcgtcgtatagtcatcaagattttgtaaaagcagattctgctggtatttggtgcttttttggaatccttctttgctttagagcgtttttta
attccctgtgaagtagatttcggcttcttttgttggtttgctcgtttttgagccatttggtaaaagttggaatttttggttagcgacgaaaacctatac
gatacaagcaaatacgggccataagagatcataagacctgtcttaagaaaagagagaaaatgaacaaagaatcattcaaaccaattgaagttt
tgcatacactggccacttatcctttttttggagctttgaaagcagtagagacggatttgcgactaggtcagcaatgggacgagctgaaaattcaca
tgcttaaaatgtattcagagaaggaacggccgttgctctctggtatcccgaaaacgggcttgttacagaaaaagttgcaatatgttttcccagtact
tctgcaggaaacaatttccattaagacgctaactgcaatctttgattcaatgaaaaaacaaaatcttctacttgtacaagacgaacggggtctatc
aggaaaagaatcttacatatacttgggtatagtttgttcggattctacagtcgtatattacaaagtaacagatggacttatgaagcctcgccaaaa
cgatgaaaattaatggacgtctatatagtattttttttttttggaacagatacaggataggttggttgcatagttcacttgactggtttgcgtgttcat
ccaagacaaatggagtagaagtcatccttaacaagtcatagtcttctcgagtgagctacacttctatatagcatgcacatcttccagactcaatat
ctgggcaatggtttgcatccgggcgaaaaaggtatccaatagtactcagatggagagttggagaatcgtaaactttcagcactagagaaagtat
atctgattctgctatttcattgttagttttgtaaacatttcaacaaataataataaggaaaggtgagatacatactccatcgatgataataaatgcct
ccttcgttgagcaagtgttgagtcagctgaacaagcgaagaattctctcgaaattttcgtaaagcaattatctcattgtaaggccagtatttacagt
atacatcttcgttaaagtactcccatgcttgacggggtcgatccggtatccattcttgctcgtacttgtccaaagcttgttcaagctcgaggattcctg
tatgtctcgtttcgcacacatggttggtgaatcctaaggttgcattattttgttccatctcatgaaacaaatctctagacaaactgttaaaaacattg
atcgtgcttacgtgcatttttcacatacgaattttcttcacaattaaaacgaactcctggctcaagccgccatacatagtcaaatcgaagctgtgga
tggtataacaaggtatgtaaaatccattcctggtatcgatgaaaagatcccgatctactgtagttgactccttcatccgaaagctttcctgattgctc
caagtagtatatatcatcttttaagcctgtctccaaatattcggtagagtcccattgaactagactttgttaaatttcttcattttcctcgtaatttcctt
accatattcacaatttccggatgctacagagctcgtagattctataaaaaaaggagaaacttccttcgaactaaaaattgtccagggataatgga
atctcttattccatcgatcttccacagttcggatagtttgcaaaacgtctagcaaatctccatctctaaaacataatgataagtatttgtttaatagta
tgacaaacaaccctgaaaggtaaccaatttttacatttgctcggtcttcttactctgcaaacattaaaagagaggcattcagtagtgttttctgtgg
aaaacgcttcactctcaaaacgtttttaaagaaactatgccagattggaaacgctgaatgaatactgggattgcaatactaaggagtaaacaca
aaattaccaatttcatgagcaattgcaaacgaagtattccttttcacacagtttattagattggaaaaaaataactacaaagctgcttcgtagaatt
ttcaacaactgaacctggaaacgagcgctactaaaccttgctagcaatcaagaaagccttgcttttactataaaacgaaaaaggatatcgagctt
aaagtgtttaaatgaatttgcaatctcgttttccatttgttttcttctcaccttcgctctaaatgacttgtttgcataggagcatatgtatgcttctatgc
attaatggtaatattgtttagtgaatacaggcaaattgtcaaggatcgttcattcatttctttttctagattttttcaactatccattcccattactttc
cttttaaagaagatagtaaacaattctctctcatcattattacaacgatttatatgatattatattatttgctccccttattcctctataattcgatactt
ttctatcgtgacctggcagtcacgtacagggttagggttgtattagagaaagcaactataaaaggctgctgttggtcttctcaatttcaccaaacaa
atctgaagttacgctgttcagttttcttcggagaacttgcgtgtaacgcggttggcgtgtagaggttgacaagagtacgtgtagggcgttgattgtc
cctgcaagattcgtcttgcgggttgagagtcaatgtcttactacgaaaattgggtacttgatgctgcgaagcctagccaacagatttgagatcgat
cggagtgatcctgtgcagtgccttcgggtgctgtatggttgatcctctgacaactgtatattcctaaatctttcgctacatggtccctggatccttgca
gttgggtaaaaccatacagttgacatttctaataattgtatatagatgatagatgagatatacttggtgtttgagggtcgagttgttaatgaattctt
ttgatgataaaatgtggtggtcttatgaagatatactgaggaaacttaattaatttgaaaatagtagtagtatattattagtctgctttaacagtaa
gtagaggatgaaaatccataaaaccaagaatgggttgcgtacggggaggttgtgatcgacgacgcttttgtatgagtaaaataatcatgatgcat
gataaaaacaaaattaagcaataaataaatggtttgaagtaaacacttctattatatgccttccctccacaaatagttgctttacccatgttttggt
gagaaattgtatgagcgttgtcaagaatcgctgtcaaagtagaaaacaaagatggctcgtctagttccaagggtctaccgtcctgtccttgggtgt
aaaagacttcacgttcattatctatgataaatacggtgactttgttgatcaagttctctgagtatcctaggtttgtgagtaaccaagtttgccaaaca
gtggagtcggtccatagaaaggatactgactcagtgaagattggatgttctacgacagagtacttgatacgtttgattttctttgaatccttggatct
tttagcctttgttaaggctgtatatttttttcactcgctgatgggagataaaattgcgttcccgcctcttttgaaactggaaccaatgttttccaagtttt
aataaactggctcgatcttccagataagtttcggactctgggttgagcaatgctataacgctcaattgtcgacctagcattttatgacaattgcttgg
tgacaattctgaaaccaatggaatggatgcttgtcgaatccagttacggaccctttcatgggaacgaaattgacgcggtgtattcgcatggtaaag
aaaaggttcaccgtttctcaccgaaacgaaagccgggaaggtatggactccatatctattagccaaggtagtggaatcagactgaaacaaagga
gcattgccagcaagatacagttccatacgcttaatagactgctcatcctccaacgtaagagcgtagtcgtgaaagaagatgaaaaacgttggata

taagccttctacgagttcgatttcttgtggttgaatatgtaacgttttgtaactagccaattggttggcatagtgcaatagctctcgataatccagtgg
accttcgtatttcaaagcatccagctgttggaaagctataaacgtagggaatttattcacttcataataagcacatagttcctcttcagcatcgcag
ttcacgtatcccatagtcaactcttcagcaacattccgggtgatgcttaaccagataggctcccactgaaaacaatccgggcacttgctagttgagt
aaaacataacaaaagccctatcggaaaggatatttgcagtatctgcaaggaaatctttaaaaattaaacttaccggttttctcaaagtgttgactc
tcttccaggattcgtcttcctctagttcaaaaaccgcttcctgagaatctaagtgctcaccttcgtatttctcttccgttagacatttaaagccagactt
gaagaactccatttcacaaggagtcacatgtgcttcgataaactccagcgtcgaacgaactgaatgattactgtcgtccaaaacatttctcatcca
gactccgttacgtaaaacaacaatctgaggatactcaagtacgccttgacttttgcaaaattctatagattgttagtccatttatccttttagcttgtc
cttaccaaattccaaatcgcacagtaccttggcatgatgaagatttggatatgcgctagaggtggcttcccacatatcatcaaactctttaaaagcc
tctccgctttttgtaaaagtcacaaaccaaatgtttcgggtttccagaatggaggatgcattgtgcgtaccaaggtaaatatcctcatttgaaacaa
gcgactcagctaaagcaaaggcaagtacaaggaaaccgaaaaaataagataaactagagaatatcatttttttctttttcagattgctgtgtggga
gtttccaattattctttcttttggaattcccctatatttcaacgcccacaaatgtaaacaaacatatacagttgggtggacaaatgcactttttcctatt
cgatattaaaatatatatatatataacgaaaataaattcgatagaatgataaaaaaatataaaagcgtcctaatgatttgatttttttaacaaatcgg
ccaaagtactttgcatgaatttaggaagactgatccctcctgtcataatggaagaagaagctatttaatccattcatcgagaatgtgggacagctc
tttctgcttctttgaattcaatatatgagagcagtcctgcatctcaaccattgtagactctgagcaatgagtctttaatttagagaaaaacgagtgaa
acgttttcaagctggtgaattcatcattcacaggagcgatatacaggatagaggaattctgaggaaattcttctgcttttacattccagtaaccagt
caaaagcccgctccctagaccaaggtatggagctaagaatatgtagtatatatttatggactcgagatttagtcctttccaaattgaaggatgcat
cgcaattcttcctccataagagtaaccgccgacaagaacgtggctcggtgaatgagcctcaactatatgatgtgtaaatatagaaaataactttgc
gtctcttctaccactaaaccaggttgaaggcagactgccaaaatctaaaatgaaaacactgtaacccttttcttgcagcttttcggccataataacg
atgcatggatcatcaacagatcctcctaaataagcatatggatgtgctaatattgcaatcttagaattgtcactaggatagtgtttatatagaattg
tctcattggcaatagttacactgtccatattggaaattcagtaagcctcaaaagagtcaagcaatactgctaaattaagagaatgagcgaaaatc
aagaatgaagtaggcggtcaaaatgaaatgctttccacgtattacttcaacgtagggagtaatcatggattcacaaaagtgttagctaacactat
gaggataagaatagcatagcagtgtaaaaaattaaaaaaataaaaaaaaggtaaagaaaagaaaagattatttctgagattatgaaaataaa
aaaaaacaccctcttaactggttacctttcatatatcatataaaagtcaattctaccaaagacattaactgaaacagatttattgaaaattaattga
gataattaataattgctttgctaattaaagaataaacgacgttttatggttcctttttacagcaaattatcgcaaacgtttgttgtcgaaaaacgcag
tcacacatttcctcctgagaaactgtataggcaaagagcgagttttaaccaagacgatagacaatggttcgcggacccaaaaagcatttaaagc
gtgtcgccgcccctcaccactggcttttagataagctttctggtacttacgctcccaagccttctgctggtcctcataaggctcgtgaatgccttcctt
tgatcgttttcttgcgcaaccgtctcaagtatgccttgaacggtcgtgaggtcaaggccattctcatgcaacgtttaatcaaggtcgatggaaaggt
ccgtacagataatactttccctactggtttcatggatgtcattagcattgaaaaaactggtgaacacttccgtcttgtctatgatgttaagggacgtt
tcaccgttcaccgtatcactgctgaagaagccaagtacaagctttgcaaggtcaagcgcgtgcaattgggtgccaagggtattcccttcctcgtca
ctcatgacggccgtaccattcgttaccccgatcctttgatcaaggtcaacgacaccatcaagctcaacttggaaaccaacaaaatcgaaggattc
atcaagttcgatacttctgcccaagtcatggtcactggtggccgtaacatgggccgtgttggtaccatcattcaccgtgaacaccaccttggttcctt
tgaaatcatccatgttaaggatgccttggaccgtgagttcgctacccgtctctccaacgttttcattattggtgagaccaacaagagctggatttctt
tgcctaagggcaagggtgtcaagctcagcatcactgaggagcgtgaccgcagacgtgctcttaagggaactgcttaaatatggttaatagtcaat
tcgtcagtacaactatagactcctatgtgtcaaatttcatggttgtattgttgtggatttccatcctcctttttttagttgatctgctattttatgatcttg
tcattctgggaatttagtatatatttgcgttttcccacctttggatgtttttctgatgggtgatcaataaattcaacggttgtttagctggttattctata
aacaaggtgcataaatctcagtaattatgaaaaacacctacctgattccatagaaaatgtactcattcaaacgctcaataaaccgtatggtattca
acattattttgcatctgattagatcttctcttgcatcgttacattacggttggtaactaatttagcgtatcgcaacataccacaaaaagcagtcacgg
tagtttctacaccacaaagagaaattgttcattcatcctgcgcttaaccaggaacagcaatgcctcattcattcggtctcagagctcgtacccgtta
cactttccaacgtggcttcagagagcatggtcaaattcgtttgtccacctacttgaagacctacaaggttggtgatattgtcgacattaaggccaat
ggtgctgtccaaaagggtatgcctcacaagtactaccatggtaagaccggtattgtttacaacgttactcaatcttccgtcggtgtcttgatttacaa
gatcgttggcaaccgttatatggagaagcgtgtcaacgtccgtattgaacacgttaagcactctaagtgccgtcaagatttcttgaaccgcgtcaa
agccaatgaggaaaagcgtaaggaggctaagactcaaggtaagaccgtccaactccgccgtcaacctgctcctcctgccaaggctcacgttgtc
agcattgagaacaacatgcccgtcactcttcatcctgctccttatgatactttcatctagatcaaagattgctgttcctttcagttatattatattatat
ttgatggattgatcagtccatgtgtagtttgaagaaagtaacttgaaagtagagtatatttatgtcatatgattttaagaatgtaactcattagtaat
ggtcgtacagttgaatactgggcataggggttataaaacatctagtcattgctcttttaggttgatagaacaatctattgttgaattatttgggttgg
actcggagccttactcgtttagcatgaaaaactctctgtaatactgttcggtgtaacgacttgcctgattatcttttatctgcttcagaaaccggattt
tcagaagtgctatccgttgttgcatatctctcatatagcagcttttgtaaccttttgtaagttagtaatcaagaaaaaacccgaaaagagtgtttcat
accatcatatgcgttgtctaatagctttgtaataaatatccttgatggcttcaatttgtcataattagatatctgaatggatgtgtatgcatagaagg

ataatgccatcaatgccttattccccgatgcaatgatctcaggaattaaagaatctagaagctcagtagacttgatacaatccaaattgccaatca
aacacaaaagacataaagcttccaataaataaagtcttaaaaatgaagacgaggaaatctcaataatatttaatagttcgaatatggctcggtg
aagtacattcgtgttctaaaacagttagaagtcagtaaacttttattgcacatacgacataaacatcaactttgaggagcatcaactttaacttcca
ttcaactgtgagattcacatcatcttccaagagactttcaattgcatctatagactgttccagttagtagttgactaccacatcatcatacctgactta
aattgcctaagcttatgtcaagtttaagggtgtaataatgggttttgaatagggattccttactatgtcggaatccattcaagctttttaagagaaaa
atagctgttttataattttttctgtaaaagaaacgttagaatctcatggacaatgtgaaaccttactcatttaatgctttgtccaaatacagaaagca
tttttcacgaccataaaagtgaacagattgaaaagaacgttccgattccatttttgccatttgtaaagtcgaagaaacatccggccaattaccattt
tcaaatagtcttctggctttattcatgtagcttttaaccaaatcaataatagaagcttcctgaacaaagctattttgataaagactaatataattaaa
gcataatgagaaacagccgagtcgataccatatttcttgcgtaaggtccatgtatctcgaaaaacagtggaaggattcgctataacattgcaaag
caaacgattttgccaagttggaaaaaacgcctaccaggttattaccattgtccaaataatgctgagcttgcattaaatggactgtggattgtaaaa
caggtatttgagcaccctgactatttatagccaaatgctccaaaatatggttttcctcttcaggatttttgtctttagaagaaggaagctgagatcttt
taaattcatacatccaagccaaagcaaagtttagacaggcaatatcaccgctttctcgtgcagtattaattgtatcttctatagcacgcaaggcttc
tttgtcgcatccaaaatcagcctgaagaatgcttaggtttaacaaagcgtattgataggagactcgttcattagaatgcatgacatggtcaaagta
tctgcaaagattctctatagattgctgataatcacctttaaaccaagcatttagaaacctatttagttagagaaataagcgaaagaaaacaaatac
ctgaagtggtagcttgtattcaagtggtttccttcggcttctaaaatatcatggagttttcgtttcatttcatctggtaaaaagcagccataacctaag
gaaattatagttagaaattttttttaataattggaacacatacgctgcatgtgctggatttgtaattgaatgagcccttcaatatcttgggttgcttga
cagcctgttcaacattagctactgctgtgggtgtaaagagagccaaacctggagtaattccgaatgccaattgaagtaatgtttgattacttgcga
gacccaaaagttcatggcgtacgttaactttacaatactctacaccgtctttgcgaaaattcatggacggagaacggtattcaaggaaagatttga
aaaactcaactccctgttcaaaagatagctgttcatattctaccacacattttcggaggaagatagacaaaatcgaagacggtgagaatagcata
tgagagggaggttcttcatcaggctcatacaagttcgtaatataactccctaaggacataaaaatttcatgaatatcttcataggagtgtaaggac
catagctagagcaatggctttgttagtttcatcacgattcgttatagaagaaatgaaggcatacccgttcgtgtaataaatcgtaaatactgtaacc
atctatagtactcggaagatttgttgttacattatgcaggtcgatcaaactttgaggcagcgaattagaagactaaaagatcaaagtcgttagaag
acaatagctgacagaattgagtcattaaagaacatacatcgaaatactccaagattatatccagaaaggatgtggaataactcggagcagtagc
atgctgagcgtaaaattcaattaataaacataaggcaaatttatgcggcgtaaggaactttgtgtagaaaactgactttttattatgtttgtctaat
ggtattgggaaaactgaataagctgaaacgcccaaagggtcctgcatttccgtgatgtccatagtaatatgtcgagaatggtatcggaacgtgat
tcagtcaataagatcaattttaaaacacaactgatgaggtcacaaagtaaataaaagtatattaatctatttcaaactgactgaagcaatactgtc
agcaattggagtttcccaacaacagtagtgttcaggaaagtagtaaatacttacaataaataagcaagaaaaaactagtatataaaaaattgttt
atttattcaatataactttttcattaatgagttagcaatggtagtttactttcgtagaatccaaatcttacaaataaatagcttttcatttaacaaattc
tttacaggaaattggcttctatgattacctactggcttactcattgatataattattttacgagttgtaaaaatcgacagaaccgtataaggacgag
aatattacattttattgctgaatttctactagcttaaaataaacaagttgcctgttgttgagaaacgcaacgttgagagttcctcaaataaacaaac
agacacaagcagaaaagtggactagcgacaaagaggtactcgatttttcctaggacacttttggaattacttgtgacgtctcctatcctctagtttt
taacttctactcaattcgttattggaacatagacttgaaagcttggctttggaaacaatggggattgagtctgttcttactccctcctatctcagtttc
tttgctagacattgattatttaacatgtaggtgaataataatagcagatgagagagagtcgctctgttgttgcattttggaatttaaacaaaagtca
gaagcaacagccctggaaggagaagactgtattgaggtcttgtttagcaattccttcataagaggcgaagctagggagtttataaagaatgctgt
ttggaatgcttctgaaagcatatatttagtcgaaaaggaccaaaaggaccaaaactctacctcaatttacacctgttcaggatgtatgtttgtttac
acatcaagtaaacaaacatgaaacgagtgtagaagaccatccacaggagaataaaagattnnnnnnnnnnnnnnnnnnnnnnnnn
nnnnnnnnnnnnnnnnnnnnnnnnnnnnnnnnnnnnnnnnnnnnnnnnnnnnnnnnnnnnnnnnnnnnnnnnnnnnnggaatt
acttgtgacgtctcctatcctctagtttttaacttctactcaattcgttattggaacatagacttgaaagcttggctttggaaacaatggggattgagt
ctgttcttactccctcctatctcagtttctttgctagacattgattatttaacatgtaggtgaataataatagcagatgagagagagtcgctctgttgt
tgcattttggaatttaaacaaaagtcagaagcaacagccctggaaggagaagactgtattgaggtcttgtttagcaattccttcataagaggcga
agctagggagtttataaagaatgctgtttggaatgcttctgaaagcatatatttagtcgaaaaggaccaaaaggaccaaaactctacctcaattt
acacctgttcaggatgtatgtttgtttacacatcaagtaaacaaacatgaaacgagtgtagaagaccatccacaggagaataaaaagattaatct
gatatcttttaaatggtaaaaataaaacaaaaagagaggaaaggagcctacagcaccccggattcccatgttgtctccaaccatagtactaacg
aggccctcagacgcttaactgcagtgatcggacgggaactggtgttttcgcctaggtgtggccgtagacgtcgtaaataacacatagagacctat
atgcaagttatgagtgtaatgattgaatcaatgaaatagcattcttataataagcaatagtcattgctaaggaagcaatgtttaaaggttactttttt
cagtcatttccacaataaaaaaaaagcacacagctacttgctttctgtattgaggtttcaagtaaaattaactaattctattggcttttcttgatatat
tatttattttcaccacaagatactggaaatatttgtttttatgatgtaaactaattgagattgtattgtttgtttacttttcgttgtcgtgacgttcgcgt
atagagcaacgaatgtttacgcgtaccgcgtttcaatttaatagcgacccatcataatgaaactcatccactaccgtacacgacctctttcaaaac

aaaaataaaatatcccttttattatatttactttcaagttgtttcattcctttgtttattcttcactaatagagaggtcacatgttctattctgaggcgat
tctctcaaaaaaggtactttgtttacatcctaaaaatggtttcatctttactgaaaactaactatttttcgcgtttagggtcccttggcaaaagtatgg
ttagctgctcactgggaaaaaaagctgtccaaggtccaaaccttacatacgaacatcgaacaaagtgtacatgccattgtcacggaagaaacag
cgccgatggcattgcgtctttccggccagcttatgcttggtgtcgtccgtatttactcaaggaaaactagatacttattggaagactgtaatgaagc
tctcttaaggctcaaaatgacttttcgccctgggcaagctgaacaaattaatgcccctcctgttacctcaataaaaggaaaggatgccatggctca
aagtgcaaatcttactcttccagataccattactgagtttgatcttttggccccagatagctcttttgatatgcaatggtctcaacttattcaaacacc
taaatctcgtcccattgaattacataccctacctacttcatcttctccttctgtcgtttcttcagaacagtcaattgaggctgcgcgaaatgctcctcat
gagagcaactttttctccgatcctcttcgacgtagtagtattgatatgcaatttcaactacctatatttcagtcaggcgctgatacgcctagaagcga
gcaatctattgaagtaggaagagatgcaccccatgatcctactggtgctgctgaccttagtgcacttgttggatcacaagttggaaaatcgcctgc
ttcttccgctagaagttttgggaccccaagtttacttcccgttggaaacaactctttggataatgaattgctagctcctgtcgatgatttacagttaga
tcttggtttggacgatctaatgaatgatactactgctccaccgcaacttccagctgaggaaacatatgctgatgaagtttctcacatcgaaattcctt
ctgataatcttgaccaggtatcatctccacaggaagaagctcctgcagagataaacgaagttgcagagcaacaagtctcaccttctcgtgtccca
cgccgtcaacgtgctgtcattgatccggttacggaattatcttctcgacaaatgaaaaagcaattgtcagatacttcttctatcacagcaccactttg
tttgaacactaaccccgtggtattaaatgctacagttaatttcatgcgcaatggtaacattcaagcttctttgtcttcttccaacttaaatcctcattta
gctgaaattttcaaaatggacttcttaaagaagctccaggaagatgtaaaaaagcgtaaatcgtcccctgcaagtgaagaggaggaaaatgaa
gtaagtaaacatcggaagattgacgaaaatatcgcgcaaggtcaggaattagaagaacaacaggttccagaagagcacgaagagcttcaagc
cgagagaaaccctactgaggcttctgctttagatgcagctaatgtaactaatgatgagcaggaagtgctagcagctgaaagtccctcagttcctcc
agcggatgagagtattgacgatgattctatgatgagagcaccacctgtcatgttggatgataatgaatggaatgctgataatacactaaatgaac
ctccgtcttttgattctatgcctagtacgcaagtggctaaagattttgttcattcaaaatggacccctcgcgtacaaggagaacgggcatcatttaa
agaattgagcggtaacacaaagcgtgacgatgctgtacagctattctttgatgttttggtattagcgacgaaggatgctatatccgtgaatcagga
cgaatctgcaaaaggtgacattatccttactgctaaacaaaaaatgttgttcaattctctttaaagaagtgaactgagatatttttacttgagaag
agtgatggggatattttatatatttaatcgcatatatgtcataattaaaaagtcctctgcttcttgctgttttcagtcatagtcttcctgacattcgctc
atatttttatcttttaaatgcttgatggtatacattgtgtatacatttgtttgcttgtttatatattttcgctaacatgtattttatttttgattaaggggc
taaaatatttttggactaatgaatctttatgctggattaataaattttattttattagtatttttggtggattaagtagatatgtcatccaagtaacca
ttatatttccttttcaaaaaatgcgtgcgaaaatatttctagtttggcttcagaaaggatgatcctgctatgaagaaatatacatgcgagcttatttt
ctaaattcaaattgtagtacgttttcatttcaatactccttttgtttcacccattaaatgtaagtttgaagaaatagctataccactaataatttatca
aattgttatgtgtttcattgaatcttctaataatccgaataaccataggagaaagggaaagttaaatgagccgaatccgttttgtaccgcttaaaaa
cccgttttgctgatttgaaacaacaacctcaatacctttagatcgcgctgcatcaattgcatctttagatacagaatcacaatttataatttcaattct
ctctaatgaagtcagacctctcaaaaggcttatgaagcccgaaccagttatcgaaatgcaattagaaaagttgacaactcttaacttcgggcagc
aagcgacaatatgggaaagatgattattgtttagttcagcattatcagacaggtccaaaatttctaaatgaggaaaggaggtaaatagctccatg
aatctcgatgagattggcaatgaatcgcgtaagatcaatgactttaagtcatggcttgataccaggaaattcgtggtattttcaacctcttgcaatg
ttaatcggataaacagggttagctctatactcttgagcgaagtaggtcgatggggcacaggaaataagtatgctgaagcaattgtcatagaagg
actagaaggaaggatagataaattttccaaagaagagggtaaatatattatatctttcacagtttttttgctttgtatcgtcaataatctgtaaggatt
taagattcggcttagaatctttcaaagtaaccataagatttgattgataagtaaccacgagctgttgcaatttagggctacatgaaatcagactgg
caagaaactcctgctccttttcatggagcggatgcacctggtttcttataaattgcaagtttcttaaatttagaacactatgcttattatcaaattgct
tgtcccagtccatcgtatcgaaaagcaaatcaggaacaacatcggccaattccaaccgctccaacttcttgcaagctgatagaactttagatacg
gtagaaaatgtaatagatgtagcacaagaaaaatattttaattcggtgaatggtgaccatataaggtacattttgggcattaaatgtggagggctt
aaaggtgaaatagttttgtaggtgtgaatagagcctacgcatcgaagcagacccttagtaggcgttaaaatccctaacttttccaaaccgattact
tcttgtatattgctttttgcatatgtagaataacgagccaaagtcatgacatttcgatctctgaatttgctgcttcgtgaagaagcttcagaaaaatc
caagctgtgaaataaagaagattctcttttgattgagttcttccaattctttgaaacttgcatgcaacggactatcgttcgaaattccacatactgaa
aaatatgtaaaagaatctcccgcggtaaaagagtgaaaatgtcttggactggcttagacttttgcattcgttttaacaattgcaagctagcctctttt
aacttctgccgaaatgggtccagtttgtgaacatattttaaaccctgcatgtaaacatgatcagcttttgaatcaagaccatcatattgaagtagct
gagccaagcgtaaatatcctctcgcattccttgagtttaagttaatcattctttgagcatcagcttgggctttttcatataaacccatctctttcagta
caatggccctaatttcaaaaagatctataacgggctttggttcttgttcaatgcatgatgttatataagcaagggcgtcttcatactttctcttcaaa
aggtactgttttgtcttttctcgccaatgttttatttggtagttattcatctacgaagcaaaagagacagaaacctttaccaatgccaaaaaagaag
gttatgtttccctgatccattagttaataacccatttccttttcttttaaaccatacctttgaccacgagtgggtttggtaaagtcagttagctggttaa
agcattctttgtatacaacactgaacgtgaaaattgaaatacaacttgtggttttatctttatttggcttagctttaattactcaatactcaaactgtg
tacaaagtgattattctcgtgttacgcgttattgagcttgttatcatattcgttgtccagcgtagacagcttgtaaaaaaccagaactgacttgaaat

gttcactgatattaggatatgcactcaattgacatccatcaaaacaataatgaaagccttatttagcatagatacaacaagaaagacgaattcag
aagctatacacgcattattacttactagaaagaattgaaaagcaaacaaatcataaagattgattgacataagagaatggctaaaacataacta
agtgtttaaatgaacttatacatataaaaaaggggttcacgagaaagataagaaatttttcacttttgctttttttgaagaagcctttggaatttgga
caagccaaatgccgcaaaagattaaaaatgcacccaaaaccgcaaagcaggagacataataaggataggcatgagggagaaaacggggata
ctcattatggtagaactggcgaatcgtgaaagtggccttttcgtctataggattcagaccgggccgattgtaggcgattttataagtaaacacacc
atagtggtcaggagcagttaaaacagcctcatagtgttgataagtcttactatcagaagccacaggaaccatgtttacgcgataataaggatcga
gcattacaagctccatttgaacatcatcggctacataaggttgaggttgaccatcattcaaaattgcaatatcaatggaaaaaacctaggtttatt
agcaaatcagcaccaatttctttccacttaccatctgatccttaattctgtatgtagaagcgttacgaagctcataaggctcgccaacctttccgtaa
gtcatatcagaagccttcaaaacggcaacacgttggaaaacccagttcgttaaatcacgagcaaatagagcatttgctgcggaataggagggag
aattgggcgaaaggtacgtgttggtaaacatttcgggacttccgctaaaaccaatacgctcgccagcatcgttctgaaagacactgaccaaaaac
aattgagtaccggctgcccaaggatcttcggaaactttaccttcagatttttttgttgtatacatatgtagaggggtgtccacgaagtacaggttgag
cacgatgattagtacccaagtaatggccatttcctttatagactattgaaccagcttgacgggtctcgtctgaaataatataaggattttcgcttaca
gcatccaacaagacggattcgtcgttggagctttcgacagaatgaaaatgatcaacaactgaggaatgacgctcaccaatatatatatctaattg
tcttcccaattcacggattccttcagggagattggagcctgcagcaaacagaacgttaccgccagcttgtgtaaactctaaaatagctttaggaga
gaaagtagctccaaaacctaaaaagtcaatgtcagcctttcattcttagtgaatcatagcttcttagtcgtctaaagacacgtacttttgctttggga
ggagagcaaaataatattgtcatacatgcgctcgccgtattgaaatagttgcgacttttcgtcccttacgttggccgtagtaacatcatatccattct
ctacaatcattatgttagattttgccagccttatagaaactattacctgtcaatgaattcagaaatgtgctatagtcttctaaggaatgatctgctac
agccaaaattgtctgatgcaagccctgggcaaaaagcgaccaggacaaggcaattaaaaggcacaatgcacgtttcatcgttgtgctttaccaa
gtttcaccttttgctaaggtcacgttcgaattctccatgcgcagtagaccttcctgtttagtgagcttgaaggagacttctagcaacagaaagcatc
ggaaagagattgtattggcatccgccttagtacgataaaattgagttgaaacaagacgaaatgcaagagggagggctcaatactcccgaacag
atgttgacatgaatgacaaggacatttggtctttaccagaaaaagaactacaaactgtacaaagtcatggcaaaaaaaggattacaaaaaaag
taacacagccgacttgaggtatctcaaactgatgatgactttgtttattgagtttattcaagtatttcgaaacctataaagcccagaaacttattgat
tatatgtaacagcgatatactaaagagctacctcttttctatctatatatgcagttaaattaaggaagaggatattgctttttgggaaggggaaggg
ttgtaccgcttactttgatacattcttgacccaaccagtggccgagagtaatagcatagtcgataccgtgttcttcagcgagagcagcgatgaagc
caccagcaaacgcatcacctgcaccattggtgtcgacaatttcctcagcagggacgtgattaggcttgtaggtgttcactttaccatcttgaacaac
gatagtggcatcagcaccttgtgtaataacaacagcacggctgcgcttcttgtttactttctcaacagaagccaaagccaaggcaacttcgccaac
atccttagtttcaatgccgtggtgctctccatagctgagtatttcggactcattaccaatgatatagtcacagtaagggacaacagagtccatctgtt
ccttaaagaattgagagaggaaaggagcactgaggttcataatataaggcttgtttttttcaacggcatgcttagccaattccaacatggattccg
gactgacagtcaagtggtatccaccaacgtaaataactctagaaccttcaacaaggctccaaacttcgggagcctttaagttctcaagcttatagt
tattagcagctcccaaatttgtgcacaaagagcgacacttgttgttgttactcaaaacaacggcacaaacaccagtagttgcggaaggatcaaca
gcaaacacagaacgcaaaccagccttctcattggattcacgaagcatgcgagcaaagtcgtcatcaccgacacaaccagcaaaaacagtagag
ttgggaggaaggacgtattgagcagcacggcagctgttttgagcagcaccaccagctccataactgacacagggctccttgtaaatgcccatttg
attctcagatgccaaaacggcatcgttgctcttcagtccatatttttggagtgtagcatcttctcctccaacgtaataatccaatagaggattctcta
atccaaacaagacgtaagaggacatgacgaaaactaaaaaccttttacaaaggatataggaaaaggttaactgcagttccacacgactttctat
gacagaagcttccataactctacttggaatagcgaacgtttgcgcattgctatcgttcatagcacattcgcaccagtaccttaaataataaacaat
gaatctatcaaatgtatgaaagtggataaaaggggctaactatcttttcctcacacttcttctttgtgtggtaagagtttctcaagaaatcatctatc
tagtcttcattacctttatcttaagtaatagttgcgttttctattaacatgggaattactcttcattagtctctaattccttcttcttttcttgaatagctgc
aacaatatccaaagaaagcagccgtagattcaaatcctcagtaacttgagccggagaagcggattcactctcccataccaataataataagagt
tcctgcaatcgttcaaaattatcacgtagttgaacctgctctttagaaagcacatgaatgcattgtgaaagatcgttctcaagtgccatagcaccgt
actcgttccattgaatcttataaagaacgagatgctccaagtaactagcagctttttcgcaagccattctagtcagtataaattggtttttttggagtg
aattcctcaaaagcagttatcctattccaaattactcgaagcctgctcacaagtgaccgctcgcgagcaagttcctgggtatagtcatcataagaa
agcacatattcacagtccataaacacatcgtcaatgctttttttcgatttttggaagcatgaagatagggaaataagtattaaaggcataggaacat
aatttatcaaatatgttgctgtccttccgaaatttatccaacgttgttatcacttgttctttgtcttttgaaaatgggtaaacttcattatctttttttgaa
atctcatctagaactgtgcacaaatactctttgctcaaggaagcactattcgttaggacaacaaaacgctcgtataatgacttttggattttcccag
cgtctttaaatttttgaaactgaccgcggatgattccgcacaaattagtcaagtttgaagaaatatatacataatagatgctttgaaatatttgaca
aagattttcacggataaacttatccaaatatgcagacgaaccagaaaagaaaatacgctgaacaacttgtttggtaacgtacataacatcatcta
caattgagctggtccaaggaacaactcttgtataatgttcctctagctcaaaagctgtttccatactgtgttttagataataagtttctaaatgcagt
agagagggtattagttggttagaaatatcttgttcaactttcgagctcaagaatacgtttgcacactcaaatgaacgttcatcaggggacttaagg

gtagagggatcaggatttagcaggcggtgaataaatctgctgatgaacaccttatacatattccattttgttataatagaggtgatttcactcaaca
agggatgtaagttctgtaaacttactcgctccatttcatgctgatcggaagggagattacgatgcttgattttggcattcaagagcaggaatttata
attctttatagaattcaaaagggaagtgatacgacgaacatcatacatagtgcttatgatgattgaaccttgtctatcacaatcatactgtaagtttt
cgataactgtaattgtattttttgccttataataccgaagaaccaatggagtatgtgctcggactatacttgcaacatgttctaccaaaccagtgaa
ggcttgtgaaaaaaaaaaagaatgagatgagggttcatcgagcgtagctctagcttttgaagcaatgattccaccgaaaaagtgccaatacgac
tctaagccttctttttcttttcctattaaaggaaaaagcttaaaatacctagttatttctttttgatccttgtcacgagcagctttttgaaattctctctg
aaagagtgcatgcagtgattcgataacttcatgcaaagtttcggtaggagcgagtggctgctcactggttggaacaactgctaaagcaaactttc
cttgaataatagaggaagaggtacaattcgctctgtgtacgaaatctgcagccttttcccaatgttggcgttgcatggctcggttcaattctataag
gcattccttaaagtcacgcacttgccgaacatacaaaagacattcttttaagcgattttgctctctatccacaactttgattttgttaatgacctgctg
atccacgattgggatatcacaaaattcgtgctttaattcatcgatctgattgactaacaaaatcaacctttctgcaagggttttaaacgtggatttat
aattttcaatgggttctcgactatcgagaagtgattggagctgatcatccgtttgttttgattcctcctgcaaagagtgaaatgatgcttcaatctga
ttaatactcgtacattcgcatatagatatatttgtcataatttatgaatgaactaaaaaaccccaaatagcatatggttgattagagcagacctatg
tcacgcgtttggtacaagaagatcgcgtaaacaatgaagaaacctagcagtaatccaagatagagtgactgaaaacagaaaattatttttaaat
aaaagtgttagtaacaagattgacccagtaacactgtgttttttcaatcttctttaaaattgtgtcaaagcaattaaatatggtccatgcatggtta
gtctctctagaaagcgacaatgtcatcctttgttcatattcgctaaagcaccctatcttacataacgaatccttgctttcctccttactaaataatcaa
tgattacgttacattatacaaaataaaaacagagttacgaatcttctcacctctaaaattaaattcatcagaatctcattagcaaaagaaggatgt
attgagtatacagccatcctaatgtgtcattatatttcatgagaaagatatcttaaatcacataatactatgactgaagaagtctgaaactcttcta
aaaagaaagagacggttcaagttcttaatccttcttcatggtatgcatttccttatcggcttcataatgttgaacttcaggatgagcatctagagaa
cttacagcattctcaggaagagtaacactaaatcctttaaataccgtatcatagacatgaccaactttagcacccgatgcttccaaatcagctttta
ccctacatcacacacttgttagacctatacacatattcataacttgctacgtacttgttaacatcggtggtttctttcaattgtacctacaatcgttgtg
ttagtcgtacgactctataaaagcctgaaattgctcgcatagccactccgtttcgcttaatctatcgattcatgcatcaattcttccgggaaagtcta
acgccagcaagatgtaaggcaggaacgctataaagaatatccaagaaacttacgatgtaggatttttcactcatattgagatttcgaaataaacg
tataaaactaagaggctttaaaaatcttataactgaattctgttgaaaaaatctactttccagaagaattgtaatcgttaaagacaagttcccgaa
tgtgctgctatatatttataagctttacgtaatggccttacccttcctctttgttcatcggacatccactcttatcatatgaacacataagctattgata
ccaaagcaaagattctttttggtgcgctgtactacgctcgtaaatttatctacagagttcaaacctcttttccttacatgcaatacgcaacttaggcg
cgtacttcattaaagggattagcgaccgagttccaagtatgtaaataaatattgataccaacgggataaaagtagaagaaaggaacatcagaac
acgaaacagatatactcacattgtttcttcttaaaattatacacaagtgctctaaattaaggagaaaggacaaacacttgcaaagctttcgttttct
ctcattccgggtaacaagttacttctataaaaaaacaaaaaattgtgaaaaccaacacgtacatggaaagcgaagagacgaaatagagcatgt
tgtacgcgtaaattattgctttttttgggtctatttttaaaggttggtaggaaatcatcatttggttatatttagtgaaatttttaatttaagcaacaagt
actataagaagtatcgaaacaatcaaaatccaatggacctccctctaattaaagctcatccttcatttcaagaaataaaggatgtgttaaacgga
atcccatctggagaccatgtttgggatttagccggcgacgaggaaaagcctgttttgaattggctggtagggctcgtcgcgacaaatttggaatgg
attgaaaatgtggatgagaaagaagaattactagttattatttgtcaaatgatagctgaacgcagcggtcgtatggctgctcctacaaaagtacg
aactttcgttatgaataatagcaaagaaattcgtattctagaaccaagtctcacttacgatacccttggatttaagacatggggatctgctcctttat
tatctgctcaccttcccgagtgggaaactcctaacccgtcccttcgtgcacttgagctaggatccggtacggggcttttggggattagtgctgcccat
caactaggatggcagatggtttgtactgacttgccggaaattgtcgaaaatatgaggcataatgttaccgaaaatgaagatttgatccatgaaaa
caatggttctatggagtgtcatgtattggactggttgaatcctcctcctgacgatgaccttccaccttggctttcccagccgtttcaacgaattattgc
tagtgattgtgtgtacgaaacccatttcggaaaaattgcaatcgctttatttttgaagtacctagcgaaggacggtattgcaatcacagaatatcct
cttcgtgagacacatttagacgagattgccgaattcgaagaagggatgcaacaggccggcttttcaagggaaactggtgaagaaattgggcaag
aagattttggctcgcgataccccatcacttgccgctggagtcaatggaggtataataggcctcaatgatgtaagtttcgtgtcgtccaccagttattt
cctatcattatcctcaaactatgtctttcattttcataaatccttgtcctatgtgcgtgattcaattagttcggcaatgggtaagcatctaaacatacc
cattcctttcaaatttgcttttactgattacggatgcttttcttcccataaaaaaaaaatatcggtatcttagaaataaataaatgaacaagctagtttt
tttgaaaactggtttctctatatctcttttctagtttttttattctatctgatttactggtgtctttgtttacttagccctaccctaacagagaagatatggg
gaaggttaacacccaatgtccgttcgtacaactgatcctttcccatccacgaccaaccctcctctcattagcaggaacagagttttaataactttca
attctttcaataatgttatccggaaaattgtttatgaagcgacttcccaagcagttacttggtgctcgtaacgcggctaccattggtgccaataccac
tacctctttacaaaaggaaactcccatggccgatgccatgattgctcgtcctcgtctcgtcgatttggaacaacgatgggatctaatgcctcaagag
gaaaaggatagtttaattactgatctgtatgctcgtcaaaagcttccttgggcggacttgtcggttgaagaaaagcgtgctgcttactggattgcttt
cggtgaacatggaccccgtgcttacagccacattaatcaaaagaaagtcttttggggtaccgctgctggcgttgttgctgccgttgccttgtttgctt
tgattcgtactcaagccgctcctgcccctcataccatgactcgtgaatggcaagaaaaatccaacgaatatttgaaggagaacaagattaacccc

atcagtggtgaagctagtgagggctttaccggacgtggtcaaatcagcggtggtattttctctccttctggcaagcaagcttaatttgtctctcactt
ttgattatctctttcttttctgtactctcgtaaccgaagatttcttcttggttttaggtgttgctttgttacactcatgttctttttatttttactcgtaatt
ggagcttagtatcaattttcttttcgattgtttctttacgaatacagaatgaagacaattgtcttggaatacaaccttttctttgttttattttcttcctta
atttcaaaaatctgatccccttacttgacatctaccacatttttcctcattactccatccgatttgtagagccattatgaagtaagtgaataagga
agaacaaattcatgaatttaaatatccaggtccgtgtatggtgaccagccaaatataaatatgctatttgatgcaacgaatagtagtacattgaaa
tagtcatttttacatatcatcattcgttgtcatcatttgtaaaactaatctgctgtaaagatgaatgctggcatttgtaacccttttctaaatacgaat
ttaacacattataagaattccatactcaaacatgattgtgaattcctaaagacatactaggtattcgataaagattaaagagaaaattcattatat
atgacattccataatgatacctattaccatatctataaatattaatctacgaagtcgctttcccaaaatccatccgggatcctttttggaggcaccag
acgtcgatgacggccaactttttgaacaagtgtactgtgtgggcaagtatgaggcaagctatttggttttcgattctcgtcgttccactctagtgcaa
cgccagtatcttttatagggccgtgtaacttgtaaaactaaaaaaaaatttggttagataaatgattttttttacctaaaaaatcttactttttcgcaat
caggacaatcaaacgctggtaatgcctttcgtttctttccacgaacgacatcatcaaactcatcctgcgagttcttctttttgctaacaaattcaggtt
gtttgggaacttgaaattgaggcaggctgttgatgtctaatggagccaaaggctgggtctctcgtaacaaagccatgtcttctggagcacgggctt
gcaaacgggcttcgccgggagaactttgataggaattctcacttgataatatggtatcctcattaggttcatccttaaagattgaaagagatatatt
ttggttctgctcgggcttgacattagactgaaaagggagtttgggagacataggttcctgctttggtacttcgtgacctgcgaagtcttcctcagca
gtctcactccctgggatttcatcttcctcttcagtcgtggaagcttgagaattgggcagaggaggtaatgaaagggcaggtggggcggttgtcttta
tgggagatttggtagcaggaagtgtcttttttttgagaacgctcattactaagctcttgttctaattgagcacgaattctttgctcgtaagtcaactccg
tttttaagcgaatattttcttcctctaaacgttcaatggcgtttcctaattcatggtagaggctgctccagtgtcggtaagttgagcttgtttttttcagt
attcataagtgaggtaggacacgtttaccttgaaggagacggtgatagcaagcaaacaactatgcggaccgtttttgtcgatgcacgactgcttc
gcctaggacgtgattttgatgtgaataatattcttcagtaaatttggattcttcttgctctacacgctgaatccgtttttcacgttccacatgcaaaga
gtataacaaagcattggttgcagtataaggctgaaattcgggttcaaccggctttggcgattccagaggtcttatccttttgaatccacgttcctca
aaaggagagtctgcgtcaccagaactttgtttccgcttaatttttaaatcttcaacaagaatggatcagatagtctccagtcccagttggcgttcaa
tcctcactctctcagctggtgattgggaaaagtcaagtgcttgccaactataccaaatgtaaacaaaaagaccgcgacgcgtcgcgtcacgtctcc
ctccagttcactgcagggtgttaagcgataaagcagtcacaggattaatgctatatatagttgcttcaaaacttacgaaaacactacaatgtcattt
gcatcccacagagtcacgaacttggttttctccataccgagaatagcagctgtcaactgctcgtgctacgggatgcaaagaggaagcgcttgaac
tcttgcgacttgtattatatgtgcaaattacgccttgcaagatgagaggatagcctacatgtttatctcatagtatttgtagtatttagaatagatatt
tcgtgctaataataaggaaaacaatttatcaaaatcagtttatatgagtatccttcgtatggtaatgtagtcttcgaagagaaaagctatgttctga
gtagagcacttgtttcattagtatcgagatatgaatgaagtctatacaagggatgtagttcggatctggaaggcataatgagctatcgacaagaa
ggagggtacgaaagaagaggaatttgttacacatttctcttcagttgtttgcaggaaacactgggattatgcagctaagttgcaacaaaccccttt
gcaagaggaaattttcttttacaaagaaaaaaagaatgcccccactcggaatcgaaccgaggatcttcgcattacaagtgcgatgctgtaccact
aagccataagggctgtatctgattgcatgtaaattaagagatatatgtaaatgaatggaaggtatatgtttggataaagttgcatatggccgtgta
tgaaatgtgggaacaatgtagtttttaaaataaacgaggatgaatcatccttgtacatctgccttttgctgacgatttaatgtgtaatgtgtctacga
gaaacccttgctttttttcagggtagccaacgaatgttggatggcttcctgtttatgggtatggacagtgtattccattgtgaatcgctttcagtttgag
tcagaaaagatactttgtttatggcatggaactattcggatgaagataaatgtacagaatttgaaaccatactcaacgtaatgaacacgaaaaac
aaagttattttgcttgcaaagaaatagtatatcgaacgaaacattggaaacgaaatcataatggaaaaaagtagaaattgaacagctggtttggt
tttctcgttctcaacgagaaacaaccatacaacaagaatatagccaaaattactgtctgtagaaactaagagtaggtcacgtccaacacaatttc
aaatttgaaaacttaccttttgttctcaatccctgaaagcatgtttatttcttatatttcaacgcttgttaaagttgcttcgttttgagtctcgctagcca
ttcgtttcccagatttccaagaggcctttggcaatttcttattgaatatggttttctcttgtttttcagatttcagcaaaagcttcgcacagagaggagt
gatacaatggaaaatgtgcttttcccaatttttacattctgtttctgttgaagaacggaatccttaaaatttttccttttttctttaaatttacttacagca
aatggttgaatctttgggttggaaaactgcatacacgaatggttctggaggaactggttcttagacaccacctctgtcctcaacaagtaccctgga
atggccaaaatacaactgagcccaaaggatattactcaatttcgtactgctttagtaagattatatacaaaagaaaataatataatgaaaattatc
gctaacgttcatagaaatgctatgagactaggcagtacaaaaagggtcttaaggctattgagtctgtaattgagaagcatcctgagcatggcgag
tcaatcgccctcaaaggtattcttcttcattcgctaggacaaattaaagaaggttatgaaaatgttcgactcggtcttcgtcacgatgttgcttccgg
tgtctgttggcatatatatggcctcgtttgcaaagcagataaagattatgtgcaagctgctaagtgctacattaatgctcaaaagttggaaaaagg
aaatactagccttttgcgtgaccttgcccttctgcaaagtcaacttcgacaattcaaacctcttgctgatacaaggaactctcttttgcaatcaaacc
ccggtgtccgcggtaattggagcgcattggctgttgcccaatatcttcgcggcgaatatgctagttcattcaaaattttagatgcttttgagtctact
attagccaaggaattcctgttgattcccaagaggagtctgaatctcaactttttatcaacttggttattttaaagaaagatggagctagtgctgcgt
atgatcacctgctttccgaagaaaagaaaattatagatcgtactacatttttggagatcaaggttgaatacgagttagctttgaaaaaacttgagg
aagcaaaaactactctcattagtttgctggatcgtaattctgataaccatggttatttccataagttgcaacaagcctatggctatgaagatgaaaa

gcacaatgtgctcaatctcgataaatggctctctttgtatgataagcttgctgcgctctaccctagatcggattgtcccaaaagaatgcctttggatt
cgctaactggtgacttgtttgctaagagagcagattcttatttgcgtaccaagcttaaacgaggaattccttcggtgtttgtagacatcaagtctttg
tataaggatgcttctaagcgtgaaaatgttgaaaagctggtttctggctatgcctcttctttgtcggatcatgcaaagttttcgactacagatactga
agtgcaaattcctactaccttgctctggacatattactatttggctcaacactttgattacaaacgagaattaagtcaagctgaacactatatctctt
tagcaattcaacatacacctacccttgttgaactgtacatgacaaaggcaaggatctttaaacaccaaggaaaattacaagaagcaatggaaac
tatggattttgcccgcaaattggatttacaagatcgctttgtaaacaataagtgtgcaaagtacatgttgcgcaatcatgaaaatgaacttgccgc
aacaactgtcggattatttactagaaacgaagctgttggtggttctgtgggtgatcttgcagatatgcaatgtgtttggtatttgttggaagacgcg
aaatcgttttatcgccagaagaaatatgctatggcactaaagcgattcagtaccgttttctcaatctttgaagcatgggctgatgatcaatttgactt
tcatttcttcgctttccgtaagaattcccttcgtacttacctggatgttctttcctgggaagacggggtattcaataatcctgcattccgtgaagcttct
caactcgctattcagatttatttggatctcttcgatcatccttttgctcgttatggtcctaagcctgcagattatgacaagttattgcaaggaaatttg
gacgaggaagagagaaagaaaacaatgaaaaagttgaagaaagacctctccaagcgtcaagagcgtgccgagaaaatcaaagaaacagat
aaggctcgtgcaaagtcagaggatggtacgcctgtgaagtttgacgaagatcctcttggtgaagagttggttgctacgaataccccattgaagga
tgcccaaaagtatatggataaattactgtcaatccccatagaaaattcagaaactttacttttggctgcaaaattatatggtcgccagggtgactta
gaagctgcgaatcgctacttggagcaggcaacccaaattttgggtcaggatcatgtgtctatcatagaagtaagagaatccctgaccgctagagg
agattagaatttaagaaaaataaatgtttttatactataacaacaaacatagcgctatgtattattctatagtaagtattagggaatgagagaaat
gtaatacaggactatatgatacctctttgtttgatctttattgtagatttttagaacgttccatattaatcccgtgtgattattatatatatataccgaa
gttgagttctggaaagcagatggaaacatttagaatatctttactacaaccatacccagcaagcattgagacaaacgtaaaactaactaagatta
gtatcaattactgctaacaagaagaaattttgttgaaatgttgtttgatgctttcacaaaccgcatttttgatgatatctttgaccttcatgaccgttc
gaagcaagcaacgtctgcttggtttagttgttgggggcctgcctttgaattaagggaaacagatagcaccattgaagtcgacgtggaagttcccg
gaatccctaagaataacttgaacatagatttggatggtcgaaaattaactgtttccggagagcgcaaaccagatgaatctcaatctggtcctctca
ttcgttggagtgagagatgctttggcgctttttctcgaaccatagctctgcctcaacatgttgatgatcggatggtacaggcctctttaaaagatggc
atactcagtatcatcatgaacaaaaaagaggtagagtccagtacaagaattattgagattcagtgaggtatgtaaaagtagaaactttttctctt
cctcctcctccttaatgatcattgtccgaaaaaagtaaggatggtataaatctatcacttgtttatcggtatgaatgaaagcttcaaattttccttaat
aatggcttaatgacactcgcttctataaactagctacggagtatttctgttgaagactaagaaagcacattatcactcgaatatttataaatatcat
tagagtgatacacttggatagacagagatgcagttagctgcagatgcgatttttattattttgtgtatttatcttcgtcctatgaatagtttacgaaa
aaaaaaaaaaaaaaaagtacgaactcttactgcaaaagtacacttctcttgttgaaagcacaattcggcgtgtaccgtttgcatacctctttacaa
taaaaaagcctataacatgctttgattcatcaaaaggataaactgtcaatggaagaagcccaaccagaaaaaatagtatcttttcattgctttcaa
tgactagcttccatcgatattctggtgtttcagtatctgttttaataaaaaaatttttatcattataattaattctataaactatgagcttgattttctct
gtattgtgaatttaataagtttctctaataatctagctttttaagacagccatgagtaatgatcaaagtaatcaaaatctcatattgttgtttgtttttt
gtgaaattgagcattctgaagattaatttgaatcccgtaaagttcagatacattgcttcatcttgtcatctaccaaacactgcaactatggctaaag
tgtaagaaacttcgaaaccccaattaaacgctaatatcatgtatagtcctcgtaattttcgacttttagaagaacttgaaaaaggcgaaaaaggtc
ttggagacaattcatgttcttatggattgacaaatgctgatgacattagcttgtccgactggaatgccaccatacttggccctgctcatgtatgtttg
aatgaaacagaaactgtttgcacttattctctttagggaaagttcagtacatatatggtgcatggtttactaacgatgaccttgtttctgtcacttttg
attattgaattatactaacagttcgaaccttagagtgttcatgagaatcgcatttatagcctaaaaatccactgtgatgctaaatatccagatgcac
ctcctcgtgtaactttcgtctctaaaatcaatctaccaggtgttgacggtgaaaccggtgaggtaagtaaagaaaaacactcttcttgtgactattc
tatgcagtgaggaaggaaagattatgcacataaagaaaatcgagagtcttagcgttttacatcgatatttcagttgcacaaagatcattactaact
atttcttagatcaatccaaacaagattggttgccttcgtcattggaaacgagattatacaatggaaacaattctattggagttgaaaaagtaagtg
gatagatggatgtaatgtaatggaatactaacagaaactagagagatggcttcctcatcgaaccgtaaattacctcaacctgctgaaggtagcac
ttatgagtaggtgagaataaaacgatcccgtttcaatgattctggatttcttttaggaaggaaaaattttagattctatttggatgtaggactttcca
taatgcaaagcttccacatattcaattttcagcaacgaccttagttatgtctattaagataccatctcaacatcttcaacaataaatatactttacct
aaagaatattcgcctctttggttttcttaagatttgaatgtgagctctgtgaatgacaaacttgtaaatgataaaataaagatatggatagcaggta
gataaaaatgaatgaggattagcagaacgtgtcgtggtcttggaattttacggtttattgaaatatatttttactatagtccgcttcccaattgccaa
acattcccgggcaagcattcttaagcgtttcccaagtctggccccggataatattttcaacagcttgctcagactgaagcgccattcgaacctcttct
ggtggagaagttcttatttcttgtgttttagcgtagggatctaaccgttcagaaaccaacggttcttcgggagaaaatgttgagctcttcaatgaag
gggtatccttgtgaatttcctcttcagcaattgagatgcattccttcaaaaattggtctctatttagccagctttgaaatagctctcgtttgacacgat
agcatcgctgctcttgagattccctggcttggcctggtgattcccatttagcggaattaagagacagttgaattgtatcgtccatcttccttgtctttct
taaaacatctacataattacacctgttagtgacttctgcagatctgaccttaagtcataattccaaaagtaatattcatactctttaaataccgaata
ttcatgcaaagtcctctcttttcttcactcattattcttggaaaataaaatctcacttaaactctagatgatttgattaaaggaatttgttctgaaaag

ggagccttattccagtggtagaatgtaatgaatcggaagagtactcagtacttgatttgagaaaaataagctgcctgttgttataaaagattcttcc
tacaatctagaagcttacaattgttatttgttctactcttcataactttggcaaagggaaaggcattgttgaatttgtgaaaggcatatggtcttggt
gtaagaaaacgtccaggatatgctttttatacgtttttctataaaactatttaaaatgaacgtcaaaaagtaaaattaaaggatgaacggttga
agctggaactcgaaaagtattcgtcttttgtttgttttccctatatctatggtatatgtaaggagttgtggattatttggccctttttgaattctttagaa
agcgaaattccttttatagaaatgttggaattgcgaaaattggatgcatctaccttgatggcgattggaaccaagcagaaaacttcgcttcgctttt
ctttcctttctttctctattggattatgtggattttaaattacgaggagactcacgttcagtgcttggctcacaaacctccgcatgttgtggtatagtgt
tgttgcagcttaaaaaaacattgattttctgagactttacagaaagcgaattaactttttcattttcagacattgctgtaagagtcctttcttcgcttg
catcgctcggcagagttaatgccactgcagaggaatcctcagagagaaagatcagcgatcgtaaaccaatgtcatcttgaacacaattcgctgct
ggattacttttcttgaaaagccatgcggtttttgtgaataagttctttacgatgcagagaatccatagaaagaattcttttggactcatcgaatcgaa
tgaatacaagggataataaaagacagtatagtagatttattataaaggcttaatgtgtgaaaatcctttcaataagctgactctcttatctctttac
caaatgaagaaattgtgatctgaataccagatattcttaatggcatatatagcctcaatatatatatttttacttaagaaatctaaaattcaatcggt
tgaatcccgtttggtagaagcaacgacctcttccccattgattccaactttgggtaaacaaataaacaaatgatgaggatacacaaataattcttc
tatggagtatcatggcggacgattcagatgatgaacccattaattatcctctgtactttttattcattcttatttttcagtgttgaagattttcataata
gatagtcattgaaataagcatgcaagctccattgtctacttgcagatttttacgaattttgtagcttagcaacaaagcaataaaaaagagaataga
aagattataatatttctattgacttcgtgagtggacaattggtaaaataattaccaaaagtctgccttcagtatatccaaaatattttccctatatcg
attttaagctctttagctcttaagcacgataagtcttgaatttgggaatcacatagaaaaacatattctgaaaggtcaaatcaaatcattattccaa
aaacataaagctatatattatgcacgtacattcaattacactaattgtttttttttaaaaaaattacaacagggttaaccgttcacaacggtaccgc
cattaggatggattgtttgacctgtaatgtaaccaccgtcagagcaagcaaggaaaagataacaagaagcaacttcagaaggctgacccatgcg
acccataggtacttggttggagacttcaatcatctctgaaggaaaagttgagtggacaaggggagtataaatggggccagcagcgacagcgttg
gcacgaatgccacgactggcatactgattactgagagcccttgtaaagctaacgactgcacccttagtggatgtgtaatcagacaagtctggtcgt
ccaacataggcattgatactagcagcattgacaattgcactaccaggcttcatatgtttaagggcagccttggtaatccagaagaaagaataaat
attggttttgaaggtgtgatcccattgttcatcagatatatcttcaattgtctctgcgagtttttggtaggcaacgttatttacgaggacatcgatgcc
tcccatcttttcgactgcaaattgaacaaggtttgtacaggtttcagaataacttaaatcggcaacaaagatatgacactttcgaccttctttttcaa
ttaggtctttagtatgttcggcatcaggaagctcttgatgaaaacaagtgataactacatcactgccttcacgagcaaacataatagcagcggctt
taccaattcctgagtctcctcctgtgataattgagcgcttatttagcagcttaccagtgcccacatgttctctcccatcacggaatttcaataaatca
ggctgaggatccaaatctttgtgttggccgggatattcttgagtgctcttcgtagggacaggctctgtcataatggataagttaaaggaattaaata
agtatccacaaatttcagttgaacactgtgtttcacattgtctgtagttatatatcttagcaaagttctctgatgtttatccacgtaatgagttagtcat
taagtcattgatgaaggaagagtcatcgaaaggcacatgatgaaaagcggcatgttggtttttttttttattattaaaaagtatttgtggcaagcga
aagttttgaatccgaatccgagtttgatttggacttggacttggaattgtactgaataaaactcaaaacattcgaaacgaaaccagatggttttatc
aatttactttctgaattaaaaaaaaataactatcgtagcaaaatgtaaatctcttgtttttttctttctacctttcgatgtcttaagaatcatacttggga
ggattcatgaaattgcgactggaacaaaaaaccaaaaaacaaggaagttgagatggaattgaactagttatatttataatagtaggaatgcttc
aatacgcttgcttaatggattgagatacgtgagcagacaagtcaattcgtttgaccagcaaaatcatccctaaagaaagaacatttttggaatca
gtcttgctattacatcgcttttcgtcctaacaaggaagtgagaatttccaagttgatcaacagtggatttggttttcttttcttttttttttttttttttttct
ttcttttttttattagtactataattagtacttttatttttgtattcccccatttgtttacttgttaatttatctaaaaggtagcgtcgtaaaaacatactta
ttactgttaagagtattattacaaatgactgaatacttctgacatatgtaaacttttattgtttacaggccccaagtgcctttcactcaacaacatgc
caacagcaaacaagaaagaaagaatctaaatagtttatggaaagaatttttcctttttttagagaaaccgatccatccatcttgtaaattctgttgat
tggttttggttttagctgttgttcctacctaccgttaacgtattaacgaggaactgcaaaaatttgaacctctgtataaagctgacttgaaagaaatt
cttgccatttttgacattgccaaaaaataaacaaaccgagttccatgaaaccttcttccatcaaatcctgatacatttctatgatctactctaagcct
atcaaaaaaaaaaaaaaaacaaatatgaatcgtcgatacaatacatgaccctaatactaaacttaagtcagcttgtatttcttcaagatcccttcct
acgatccttctggcttctttcgctttgcttctcgtttctctttccccttaaattcaaacgctactttcaggaagtaaacactatctcctgttacctaaagt
gatttgcacatgttacaatgtgcttccatgccctctacgtcattatgacttgaacgaatcggaaagcaatctactggtcgtcacaaaggctaacccg
accttcctataaactgggtaacgatgtcgtttaaagagttagaagcagtaagaagagattccatcgacagcttccaatagtaggaatggagtttta
taagatttctataaacagcgactttttttttacttccaacgaaataaaatgaaattcattcgacttgacatcctgccttgcttaaaatattcttttcgctt
tcttttacaatgttttccatagcttctatcctatctatatctagttagagtatagagaatgagattagaatgttcttacgtttgtgtgtttattaccatat
catatcacctttccctcaataaacttcctacaagctctttaacaaaccaaatttacaagctcggtataccaacttctccgctttttctgttgtcaggga
aacggggcgtaggttacattaacaaaatgaactattttattcctttttattgaaaaactgtatgttggctatgctctcatctattcttggatattctggt
ttccaccacaaccaaacactacaaaaatctatacaaaggataacggcgatttcaagaggattcaaacgatatttctttgtttttgaaactctgccgt
cctaagaaaattagctttctttctttactttttttcttcagcttagtggtttctttcgtttcctcaagttgagaacctttgctcctgcaggacgaagtattc

ctcctgcacatatctaattttccggttatttctttgcatagatatctttcatctttttggattctttttgttttcatattttttcattggttgttaaggttttt
tcctttttctactttcctttctcttttattggcaattccttgctgatttctcctttgcattcaaaatactaacaggacgctcaagcgtcgattgattgattc
ccttaatatctctcatctctcttttctttttacaaaacgttttctataaagctttggaagtaaaatctattccttcccgttctcctcttcctttttaatag
atttcttttcttcctccccttttgatcgttggtttgttacaaatagcaactagcattgtgttttgcagctcaactttgtataaaagttacggttactatt
cacttctaaacgaagtgctatatctttgtctcatcccttttttcattttcgggagtttgcaactttgtttggttggttgtaaaccgttctgcatctgtcctt
tcactttcttttcattactttccttctttttcttttgcatttctttctttctttctttcctatccatatttctttaaaaatttggggtttctttcgagttggaact
atataccttttgcatctaaaaagtctgttcgtcttaatccgtcaaatcccaatttcctatatttgaatcatatatcattctactctcgtttgctatttttt
taaactatcattccatatttgctcctaatttttgatttactatctccgttctttacgaaattagttcttatgtatagtcttcgcgaactatggatctgtaa
agctatcattgcttttctacttctgcttcttcctcttgcatatgctgcgccatactctaaggaagaagaaccgtggaacttaaatacaaacaaacatg
ccacggatattcttgagtattcaggtgtgtggtccgatcatacctttttccagagccctgagaactggcgtatgaactttattgctattatcctcgatc
gttggtccgatggtgacccaaccaacaatgatattttaggtaccccattcgaaaatgatatttctgaagtttcttttcgtaatggtggtgatatagtt
ggtttggaacgttctttagactatatccaagctcttggtattaaaggtatctatcttgctggtactccctttgaaaatcaaccatggggttatgatcag
ttttctcctcttgactacaccattttggatcatcatacgggaactattgatcaatggcgttctgccatcaacgctattcatagtagaggcatgtatttg
atacttgatcttaccgttgcaacccttgctgatttggttggtatagttggtcatcttaatgataccggcggtgtcccattcaacttgaacgaatataat
gccatgtggaagacccatcaacactacgccgactttaactttaccaatgtctataacacatcatgtgaatatcctaaattctggggtgaagacggt
agtcctgtcgttttgcagtataaaggctgttatgattctttttttgatcaatacggtgatactgaagcttttggtgctcactctgactggcagcgtcag
ctgtccaagtttgcttctgtacaagatcgtcttcgtgactggaatccaaacgttgcggtgcggttacaacacttgtcttgcttggtcatctccatgttg
gacgtggatggtttccgtattgacaaggccactcaaatgaccgttgattttatcgctgattggtccatgcatgtgcgtgaatgtgcaaaaaaatacg
ggaaggagaactttctcatcgttggtgaagttactggttcttccagctatggttccatttactataatcgtggtcgtcagccagaccagcgtcctgcc
aatggtacaatcgcatataaccttactgctaatgataataagtattccttgcgtgacagggatcatgcaggttttgatgcttctgccttccattattcc
gtttatcgcgcacttgttcgtttcctcggtatggactcaaaggtcgaaattgggtatgatgtcccttctaggttcaccgatgcgtgggaacaaattca
ggtaaacgaagatgccgttaatattaatagcggtataattgatgctcgccatatgcttggtataactaacaacgatttgttccgttggggctcatta
ttaaatggtaccgaaaaggccattctgggaaccatgattaccacgctcatgttccctggtattccattgttatactatggtgacgagcaaggcatgt
atgtcttggataatagtgcagataactatctatacggccgtcaagctatgaatagtgccagagcatggtttattcatggttgttattatggtaaggc
ggcttcttatccgagagctgattggactcctgccaataatggatgtaaagatgagtggaactatctcgatcactttgatgtagcttcagctcatcac
aatatttatatgaatatgaattctatccgccgtcattattacgctttgagcgatggttatcgctttgagatgatcgggagttggaccaatgattattat
ttccctgatagccaaccttatgccactaccatgggtttatatagcgtcttgcgtggtccaatggaaggcattcagcatcaattttacgacgatatagt
tgagactgataagaatagaaagactatagtatggagcctgtttgctaacagaaacgacactacgacttggaatttctcatgcaaagacaaggat
gcgattattggtccttggaaggctaatactatagttaagaatcttatttacccttatgataagcacaaacttcaagaatcttggaacgagacatggg
gttgtctacctggcgttacattcgatccttatcagttcaagttgcttgtgcctgaagaagattttgttgaaaatgatcctgtgatcacaagattgaatc
cttcacatgactatagaatggattataatggtaccgaacttcccatcgaaattgagttttcccgccccatggattgtgactcaataacaaattccctt
aagatccaatcgtccactcttcccaagaacaagaccgctcgttatatggatggatctgcttcctgtagaaactactctgattcggagattaacaga
aacttactacatgttcagtcacctggtgtcttcctatggtctgccaaacttactgacatttatgagggaattcatcgtgtttcagtaggaccagtcag
gacttctgataattcttctgggacgttggctcgagataattttctgtttcgagttggcagtagaaataatcctcttgtgttttatgctgcaaattattca
tctaatttgcttacgaagcaaaataacagcctttacgttaatcatactgcatccggagctacaaaattccggtatagtacggactttcaatccagtt
ggagcgattggatggactataacggtggattgacgaaattaaaaccagctaattggactggtaccaaaaaacaagcctgggagggggagcatg
ttcatgttcaatactggagtgacttaaccgcttctgctaattacatgcaacaaggcgattatgatttcaagtatcgcagactattcccccacatgttc
ttggaaggtgattttaaccagtatggttatgattccggtgttcaaaatgagtttaaacaagtcgatgactcattatgggaagtgggttttactagcg
ctaagtatcctgcaactgtccaattcaatgtctggggtatgaatccagacggtaagcctgatagaactcatgtttacggaagtcaaggtaagtcgt
ctgttttgagtcgaagtgatcctgcttccttcatatcaaacaatgttacaattaaggaaggtcctaatggaacacgtctcagttacaaaattaaatt
cagagatgatgcaatgtttttcaactgtatcctgctggcgaatggagcgtatcgattgcaatttatgttttgtgcattgtggtacctccaatttgcgc
tatttttgtttcatggatgttcaaaaattctttttatactgtcaaagttaataagcatggtaacggggactttggaaaattttatcctcttaaaactttg
gtacctttcaagaaaaagaacgattctgaattaactggatcttctgaaactgccggaggtggtttagcccggaaaaagaaatgtgttttgattgcc
actttggaatatgatataactgattggcaaattcgaatcaagattggcggtttgggtgtgatggctcagttaatggctaagaatctcaaacatcaa
gatttagtttgggttgttccatgcgtcggtgatatcgtctacccggaagctgaggaggctgctcctatagaagttaaaatcattgatcagacttaca
ccataaacgtttatcatcattacttggataatatcaaatacgttctactcgatgctccagttttcagacgacagaccactgtggagccatatccagc
acgtatggatgaccttggttccgcgatcttttattctgcttggaaccaatgcattgctgaggttattcgtagaaatcctatcgatatttatcacattaa
cgattatcatggcgcccttgcccccctgttatctcttgcctgatgtgattcccatcgctctttccctgcacaatgctgaatttcaaggtttatggcccctt

cgtacatccgaggagaaggaagaagtatgtgcggtatataacatttcccaaagaatctgctcccgatatgttcaattcggaaacgtttttaacctg
ctgcattctgctgtttcttatattagaattcatcagaagggttttggtgcagtaggtgtctcaaacaaatatggcaagcgttcatgggctcgttatcct
attttctggggactgaagaaaattggtaagcttcccaaccctgatccaactgacactgatgatgtttcggatacacctgaaaaggtagttgatgtc
gatccggatatggagaaaaccaaggttgaacataagagacttgctcaagagtgggccgggttagaagttaatgataaatatgatttactggtgtt
tgttggacgctggtcttctcaaaaaggtgttgacttgattgccgacatagcccctcctttattggaatcgtacaaggttcaattgatttgtgtcggtcc
tgtcattgatctgtatggtaaattcgctgcggaaaagcttgaccttcttatgaggaaatatcctaatcgcgtcttttccagtccaaagtttacccagc
tacctccttacatcttcactggtgctgactttgctttaattcctagtcgtgatgaaccttttggtttggttgctgttgaatttggacgtaaaggtactttg
ggtattggtgcaaaagtcggtggtttgggtcaaatgcccggctggtggtattctgttgaatctagtgcgactccccatttattgaaacagtttgagc
aagcttgtcagcaggccttatcatcatcacagcgtactagagctaggcttcgcgctcgttctgccaagcaacgtttcccagtctctcagtggattgg
aaagttggaagcacttaccgatggctgtataaaggctaatcagaagtttgggaagaactcacgtgctcgttcttctttctattccttaattcacgagt
ctttcagtcgctcttcagatgttttgcctcaggctagtgataacggtttagctgctaagcgagcagaagaagctgaaatgataatgatgagtaatg
aggattctgcccccacaggtggtgctaagattggaagatcgttatctttgggtagtcatcgtggccctggtcatacgggcaatgatgattctagtga
tggattggataccattcaggaagagagcatgaatggtggtgattcccactctggagactctggtaattctcctgctggaattgatgatgagaatct
ccattcgccttctgagtatagttttgattctgctgattacgaatttgatccccagcgctcctactattatgatgatttattcgatgatgaaactacaatt
agaaacacaccctccttccgtccgcaaatgggcaacttcaacccggaacatggagttggtgctactttgagtcaggatgatttgattgatagttctc
accctagagactccgaagcacttagtcctccagtagctccttatgccaacaattcacgaaacaatccatattcttatggcaaccttcagacggattc
cagtttaagtcttgcttctgttatgtctggaaaagaaaagcgggacttctcgttaactaaagttgaagaaattttcacagacgaggatggtaaagc
tcttaaagcattccaagataagttacagaaactcactgctaagaattccaaggatgatctctgtattgagcagttcattatgaaatctgagcggat
gttctatcacgaaaaacgtgcaatcaaacttggtttgcaaaagccaaataagttgcatgtgaatgccgcttcgtctcaatctggcactgatgagga
gtctttaagcaatggtcatgcttcttatgatgatattatcggtttaactgatgaaaccccatcctatactcaactggaagaggagtcagaatacaag
actattcatgggttgcaaaagttcatgttgtttaagatatacgactggcctgtgtattccctttttattgctttaggtcagatccttgcgtcaaccgcat
accaattgacccttttcactggtactagcagtataaaaacataccaattgtatgctatatgtggatttttttattgcagcttctttcttttggtggtatgtt
tattctaaggttccggcttactatgttttgtcctgcccattcttattttacgcccttgcattgtttttggttggtctacctgcttttgaccgcatagcaggt
gggagagtatggattacaaacgtcggtgcctgggtttattcaattggttctgctagtggatcaatcttcttctcccttaacttcggtgaagaaggca
gtgttcaaacacgcgtatgggttttccgtgcttgtttagtgcaaggacttcaacaagtctggtcagcagctttgtggtattggggtgcacatttgaat
aataaagtaaacgaaggtgatgcgaacaattatagggtaagccctgctatccctgcgatcacttggcctttgagtgtggtttgtataataatctccg
ttatcctttataagggattgcctcaatattataggcaattatcaggtactattccggcattttacaagtctttgttaagacggaaacttgttgtttggtt
ctgcattgcggttttccttcaaaacttctggctttccacgttgaatgctcgttcatggtcatacttttgggatcttggtgaaattcctcaatggcaaatg
ttcttgttaatgattgctttctttattgttttatgggcgttgttacttggtgttttagcttggattgcacgtactcactcttggattatatgcgtttttggtg
ttgggcttggtgctcctagatgggttcaaacattctgggctacatcaaatatcggtctgtatttgccgtgggctggtcgtgcgggaccgtatcttggc
cgtactctttggctttggttaggtgttttggattccgttcaaaatgttggtattggtatgattttgctacaaacacttacgcgtcgccatgttgctagca
ctttgatgaccggtcaaattattggtgctgttgctacgatgattggacgtgcaggcgctcctaatcgtcttggtcctgctaatgtctttattgatttcac
tcaatggtctcgaggtgatggttcggcgattcttgcaagcgctcctttctggatttgcataatttgccaacttgcaatttgtgtcggatttttagccttc
ttccgtcgtgaaaaccttaaccgaccttagtcttatcaaatgatgttagcaagagatgatgatgcatgaagacgaaaataattcgcttgtgcctaa
gaagcagtacgctacatgttacattcttatcattgaagtttagtggaaggaatgattgtcttcatgttgagatttcctattaaatactgttaataagg
ctatgaatggtaatgattaactggtaagttaatcaagaatttaaatttcattggggatttgttcatgatacttatgaccttgttcgttgattttgagat
atacttatgatcgttgtcttacaaacaagaagttttctacaaaaccaatgtatgtatgtttgcccaaggattgagcaaacaattaagttactttaaa
acattttcatatttttatatatacacatatattatcataatttgcattagaacccttgttttagtcaataagcattactttgcattggtataatctggtca
tgatagtagcgtatacgaaatgtattaaatagtctttttatttgttatggtaattggtagttaattgtaaaggacatttgcagcaaatgaaaggtggt
gtgtttcaacaatttttacaggaagtgcaacatgaaaatccatacagttcgtgaaaattacagatgatttcatgaatataggacacaagtaaaac
gtacatattgaacccattaagaaaggttgggtttggtttgcaaataaatgcatttatgattgaacgtaaggcatgcgttacaagtaaacatgctat
cttcatttattcataatcctcatcttctttatagttatacacatgtttttctcgagaagccggtttggtggctcctcgaccgttgcccatgaatcccactc
gagcagcagcgtcttcaggactacggaacatactagtctctcctatttttttccgtttaccgacacggcccgtttttacacctcgcgaggcaaattgt
ttccaagagtttttagaggcctccagagcagcattgggtttaagttgcgtctttggcttttgtgcaataggggggaggaggcgtagaaatagcagga
ggttcaggaacagtggtgggaggaggaggtttggaagcggtatggaatgaacctactggtgcaacctttgcggctcctgccgcagtagctggag
aaaacgcacgttccggagaagctatagaaggatgaattctggaatttgccattggcgagatacttcgagaaggagccatcgatactgtaggattc
ggagaaaagccagcacgaggagccttgttcaaagagcttgacatgacaagcatattcctgcgttttgcttccggcattgcctttacgttcttagaa
gacactgtttctacttcagggtaatccaaaaattgaatcgtatactttttgttcgggccgtatcctgatacggctgttatttttccagcgtagaatgca

taatctcctgaagtccaccttgccattaccaagtcaccaggaaaaaattctaaattttgagcgttcgccatagattttgagtcgaccgctgagaatc
cattttcttccagttttgtttgcttttgggtttcttgaccttttcgtttattacttctcctgtcaggtcaactagttcttccaaatcattctttaaagcaag
caattcttgattatctgggtctttttcgagggatatttttactaacgcgagttggctttgatactgtttatattcatcctccatttagaatatgtaccaat
atacttaggaaaagataataaaagaaataaaagtaattattctatgctgaattagtggtgttcagggcagatttgaaaattgtctatgatgagtag
tagtgtctttgagtttttagtccaacaaaaaggtgaaatgtaatgtccattctcgaacagtgatttaaagatattattactaagatttataaatttag
ccttcatacagttttaagaaaagcatttttttgtataccacggcatttatgcaaagtattaggaatataggatcgaacagtattgaaagtcgctgaaa
atgacaaattaccaatgtaagaaaaaggcgaagccaaaagttaatatcagagagaaataaatagcattgaaaaagactataaacaaagcagg
tttcggtccaagcaagaaaacgaaaagacaacctattagaaagaaagggctttgtatagaacaatgtttaaggtaatgaaatttaaccttggac
gcttaagaaggaatttaggaattaaagacaccttccataaagtctgaagagacaacagatcataaacaaactcagttgacatgaataattaaca
aacaagtaagatgttttattttgaaggatgaaacgagcgtccaagaaattgtttgggattttcccaagcaagccaatcactagttgtagtaggtaa
tagacccattttctgtagtttgtaaataccaatagccattagcagacattgtgcgaggatgtaagctatcatcggtggccacatggcattgggatgg
atggtgcgaaaagatgagaaggcatcaccagcagcagagattgctttcagtgggttcaagaacaacatgaaggtagttattatactgaatatctg
caaagagttacctgacatgtaagcgaaaaccaaattcatcgggatctgtttgagtggagaatacgcaagctcccatgctttttcattataagggc
cttttcggcgttttgctttcggacgtcaatggcagaactttcggacgaagtttgatccctattagatttccggagagagttagaattcacttgaaagc
ctcgaggggatggatacttttcagaagtgtccacacatcccttggaagtctttagtatggcagattgaagcactggataccagtctgtttcgtcgtt
gataggcatgctaaaggtgtaaatgaaaataggtatgttatattttctcgttgaaatgcgtttctccagtagagaggattttcaaatccagccgagg
aaagaatgtgttatgaagaaaggttgttttcaaaagaaacgaaattgcgagtctatgagttacgaatgaatccacaatcaacagatagaaaact
cgacgacgaaatttttttctccaaccacctgagtaagaaagacaaagtttcacttctggaacgaactcttactacctttcaagtttgtatctatataa
atttcaaaagccgttcagctctagctgtttgtttctacagatggatttaccttatgaggaaatgtggttgagtctggttgaagtattgcaaagaattc
ttagagtagaaaatgaaacaaatgtatgaaacttttaataaaggataaaacaatagctaagttgtgtttcattttcattattcagttgcatgtccttt
attttcactgttttacatgaagcacaggtttaggaaatttcgcaaaagtgtattttgagaggactgaataaatgaaagaacttaaataatcataac
attggaagtttttgaaagcaattgcttggctttaccttcttatttctgaatggcaagaaaagggaaatcaggccggttcccttgtttccatgcaagcc
gaaaaggatgctcaattaatgcatagtcatattagcgaaaagaaaaaaaaattggagatccatgctccttcactgttcaaggtaatgaagaccaa
caggaaacagaaggagtaagttttgcaaataaagagtgtaaagccatgagatctattttttaatgaggacgcataggttctaaaagttatagaaa
tatttcttgaaagacgtgtcgtgtctcccctcgtttgcgaaatatgtttggtaggaattgacgcagaaaagcaacgctgtcatccaaagcaactgat
tccttcaaattaatttatgaatgattttgttatgcacgcttttgtccgttataactaggaatttggccccttgttcctgtaattcctttcctttttcctttatt
tactaaggcggaactcgtccatctctcgcccaagtacgctattaagggctaggtcatataatttgatgaacgcgttcttcgtccatagcttttgtgac
agaagtttaggagaacttcctcttgacaatcgattgatacgaaaaaattgtagctcaaacctcgaagagttcgtggcctgctctactcttgtttacg
actctccttaagaacagtaagggtaaggcctctacgacctcaccaccgaacgcgttctcaaagtaactgtcttcatcgatttcgctacttgagaact
cttggtttaggaggctcaattattaatgcaatggaatcaccaataggatcagatcagtcaacgccgacgaatcctaccgtcaactcgaaaaaacg
tagaggacgtcctcccaaaggaagttatgcggcgtttggcagtgatgatgaagacgacgatgaatattacggtggtcgttcgcgtaggaggaca
aatcgtgctccaagacccaaggttgcaacgcaatcctctggttctaccgtcgttcccaaagacctgtatacacatcgtgcaactctagaagacgac
gaactcaattttggtgtggttgatccagaaggagaaaaaaaggtgaacgagcttggttatttgaacggtggacgcgagtatcgttgtcgaacatt
cacttgtctgggtagaggaagcaggttgtacatgctttccacagaacccgctcgtgccatgggttatagggactcatatttactgttcttaaaacat
cgctcactgcataaaattattgtggatgatgcagaaaaatgggacttaattgaacgaaatattattcctcactcatacaagggaagagctgtggg
aattgtcgctgcgcgttccatttttcgtgaatttggtgctaaaatcattgtcggtggtcgaagaatagtcgatgattattgggaaggtgaatttcgtg
ctcgcggttttaaagaaggagaactagcagatccagatgacaagttgccccctccgggtatgccatataatcgaaatcaatatgtcgcttggcat
ggtgccagtgctgtatatcattcccaaccctcttttgaagcacaattaccatcggcagtcagaaaacgtaaaaaggaccctcccaaagatgctac
ctggttgtttcagcatgccaaagccacttctgcttacaataatgatataactaaaaatatcgcacgaaaacaagcaaccggttgttttgagccgca
tacaaatttacttcacgtttttcaaaactcgcagccatcgagaaccgagtggactcaagttacttctaaccaagaagggtacattactccaaagat
ggatgtattagtagctctaaatcccgatatagctcctcaagtctcgttaataaatgttccctcctctgtacttgcttcttgtcctcctcatatacaagac
gcgattcggaggcgacaagatcaagaacttttacaagccagattgaacggtgcattgtggtaagttggaagaatttctcttccaatgcatgttaat
tctccttttaaagttcgttttataaagagcctaactgaaaggatatctggtttagacttgatggcatcattacttaagattagcaatgagcatgctttt
ttgttaataattgaacgttgttatgtatggaatttggttagtgtatagttaatgaattctttcagaatttttggttataacatagcagaatgtttcaata
gaaagtgtaaattcaatctgtacgttaagggtttgcatatacctttttttatcgacccgttttcctctacagtactgtcttacaagggaaccaacaca
cgaccatgaatttagcttgtgaattataatttattaaatatgcattatagtattaacgtagcatcacctcccaaggaatttactttcaaattaagaaa
aaagagaaatacaagacatttcaattagcagcagcggcttcaataggaggctgaaggcgagtaggttgatcaccaacgcgaacaatcctttgac
cgaaagtatctttcataggtatcttttcaccggcaccagacttccaaacctcaacttccataccaccacggacaacggcaatacggtaggtggag

agtaaattatgagcaccgttggtgtgttcatatacaccaccattgaaagcagcagtggcatccttaccaaaggcagaacgcaaaagaccttgacc
gccggggtgttgtaagccggtcacgtcgtgaacgacaccattaatgaggataagaggacgagtcttgctttgctccaaaaattcttcaaattccat
gacagggagaacttccaaaggagtaccccagttaaggcgagcacgccagcggtccaagactttttgctcttgttggacacgacccttttcaatctc
attttgagggaaagtgttaagatcataggcgagaccaacaagactgcaaagccagataataatcttggtggggtcgtattcgtaccatctcaaac
cattacggtagtcgttagggaaagcgtggtgataattatggtttccctcaccgagagtaacgagggcagtgatgtagtggttacgggcagagtta
gtatcgtcaaaaggttgagaaccaatcaaatgagcaagggaattcacgcagaaggtagcatgatgaacaaacacgagacggcagacaccagc
gtagaagtaaccacctcggaagtcaccccaaagaaaaccacaaacaagggaaggtacgacaaaggccataaaggtagccaagtaaatgaag
taacggtggttccatacaacaaacttgtcggtgttcaagtcagagatgtcagtgcgaccaatacggcgtgggttttggaggatgatcatccaacct
atatgggcataccagaaacccttttttaacgttataagggtctttgtcggtatcagtataacggtgatgagcacgatggtcacgagcccaccaacgg
atggaaccttcgaatgcagcggcaccgccggcagcaaagaaccatcggaggggttgcttggccttgtaagaacggtgagcccaaagacgatgg
taaccaccagtgataccgagaccagagaagacatagtaaatgaaggaaaagataagagtctttgtgtgcaagggagtagttaatataccataa
ataccgatacaaggaaaactaaacagaagagtcaaatgaagccagttaaagtgacgccaaaagttcttaagagtccatggctgttcctgaatgt
gtttgggagcctttggatcgaaacgacgttcagggacaacgggaaccgtaggaattttacgttggcgaagagtgttttccgcaccagaagtaggg
ttcaaagaggcattggagccttgaggaatcacattcgaggacataattttttttttacggttttaaagagcaaagtggttgtaaacacaaaacttg
aaagagataaaaaaggaatcaacaaaccgtagagagatgaatcgaggaaacaactccagctttttatataatctaaatcaaaagcttggaatc
gctttccaggaatgaaagcgaaaatgaaacaaaaaaaaaactaaagggataaaaggaaacacaagacacaaagggattcaaatctagaaaat
aggaagatagtacaagacgacaacctagtcgcaactacacaatgccaaaagagcaaaatttgatgctatcggcagtagtttctgatatgcgaca
agaaatatacaaggaggttaggcgttttggtgtggtcttcaagttgcggatctcccaatcttatatatccttaccgtgtgaaggtgggtgatgtata
gggtagggtaagggtagggttgggtcgtacccaaacgtagcctgggaggaatgagcaacagacagaaaaggaaagagattgctggttgggta
atctctggggaattttaaccaatcacagggcgtctagattaaacgtcaagaataggaaatgtgaaacgagaaaacaagaaagagggaaccag
aggagacaggtgaaaaaaaaaaaaaagaaagataacttcatagaaaaaaacgaaatttataaggaatctatttcaaaacataataaaaacga
aaaaagcacaaggattcgtctcaaaatcaccataaaccgacaaaacgaaaacactgagagtatcatttaacgggcctccttgtagaatgagcg
atgctaatttaggctcggaacccaatcggaaggcgatgcgttgcaagctacctaagattgccaccgtgattcgtcagtagttaataccaccgtgag
aatagataccggaaaagaacgaatagaaggacgttcctcaaaaaagattgtgctactacaccgaggccaaaagtaccgcagtgcatttttaaa
ggctttggtaaacgaactccgttgcataagaggcttataaggatttgttcaatgaggataaagcaggattggtggattgcgtctgactgcagtaat
caataaacaaagaagaatccaaagtcgagattgggtataaaaaagcggttgtaagatgaatttaaatgcaagtggcttcatgtgattaagtatat
gaatattaaaatgcttcgtttgctatgctatttttgattcagtttatgctgctaatttttgctttgcaaagaggtttattctatgtgaattttacattactg
tcggatctgctgaattctcgaatcatgcaaggcatcgaatccttggttgttgttcctgggaattgtatgattaattagtaagaattgtaacatggtta
tgtaccttttcattagatcactttgagttatgtttgctttctttctaatttctatgaataaacaagctactaaattccttatgtggaaagcttgacttaag
gatcatagattttatttatactggcaaatccagcttcctttctttacaaaggctcaattggaggcaaagggcctaatggagtaatgcggagtgggtt
gatttgagtatggctacgagtataatgccacttgatatggtcaaagtcggtagtctcaaaaaatgctggatgacgccaataaaggttacgcaacc
aagtatggatgtgtggatagtcgtggcgaatggtaccaatattgcacttgaaatgttgtacataaactgggtcaaaacgaattatcgtggtgtaca
aacgcacgtcggtttctgttaagcgatcccccaaaacgtatggtcctcgagtctgcttcagcaagtcctccattttgtccaaggcttcaaaaaccac
ctttacattcctttcatatgcttctgcggtggtagcaaatcctgatttgtacactccgttgtttagtgtgttataaaaatagtcattccatttgtcaattt
cagaacggaggtcttttggatacagatctacctccttctttttctggtcggtaatcaagtcattgaattcgtagtttagaaaccggatgatctctgaa
ctttcattgtttacaattgtatggtgtttgacatcccaaaatacaggcacagtgaaacgtgcattgtaattaggatcagcgcggaaatataagttcc
ttagaaatggacttttataaagtggttctccagtgcttccatggtccgtctcaaaatcccagccattaggtcccatcaaccatcccacaatatgtac
gggcaaagcttcttctagtcctttaagtttgcgaacaattagagtacggtgcgcccaaggacaagccaaactcacgtataaatgataacggccta
tttctggttgaaacacaggatgttcttttgaaacggactcacgaaaggatgaaacctgtcgtcgaaattcaccatcttggccagcccagtttgtaat
tgtacctcctgaagacattttcgcaaaaattgaatgtttttgcctctttaacaagttaccataatttcgaagcattcatgcagctggttttatagtcgtt
ttagtgggaattactaatgaatcagcattgtgtcatgagtaatcaatgttacctctatttttttatggaacaatagaaaatgaatttatttctatactct
atactttatatttgataatactttaaatacagtccagtctataaaattttctcttgggtctttgataatgctagaaaaggtacgtaaatattgaggtcc
gtgtagagaggaagtagtcgataagaataaacgtaaacgtaatataatagtcatccaatctggtttataacgaaggaattcgagggttttcttaa
actcgcttttctgatactttcggtatgcttatttggaaacgatagacaggacgttactttcagtatggtctgtgggtttttttattatcaaacaatggct
catactgtttgaattagaaaagaataccaagaatcttgtgaagccattatacttgaatcaaccaaatggtttgtttttgtttaatttaaataacataa
taataatagaatataaatatataatatatatactgtaaatatgaagaaaaaagcaaagaaagacaaaatgaaaatcaaaggagtagaaattca
agcatctagtttcgtctgcttctttacgatattagcaatgtgtaacacctctcccggtccacatttgtcgccttgtttggtcgtagatctgcattcatga
tgaggccacaccattaagtcaacctcataaccttgataatctctgatctctagagagctcatgattttgccaagtatactgtaacatacaccacact

taagttctattagctttcagtttttactactccctgaccagtactgtaatgtaatagcagcgaaccatgctacattaagggaactcctgtatcgaggt
gtctcgctgcgccagtcaggcttatcaacgactcgtttagacaatactacatcatcttggtcttctgagccatactcctcacttgtccacccgggatc
ataatcctgagggtattctagtccaaagaagtcgtaaaactcttcaacatcagaatgacagttccgaggaccactcactgcataatcattgtgtga
tcggatggtggttgtggtatttgttcttgacctggagacacagtcttttattctgtcggttagagatgtgtttagctcatgactaaagttgttgccagc
gtctagccacacagtgaagtccccgtcagtgcggacagtgttgcgttgtatcacagccttatcgtcagtatttagggtgatacatcgctttatgaca
ctagcagtggtattactgccgatgtcttcgtaccaggaaatgacattatcagcttgagcctgggcaaagccaatacagatagcagatagtattag
aaagaaaaaaaatatgcatctttagttaaaatgatttttttaatatattaatcaaacacgttattaacgagatattttgaggaagaggatgatggt
gtagacagcacatgaaaccgcccttgggaatttagatcaatattcattatgaattcgtaaactcgaaacatcagcaaaccgacagacatagctat
ccttgtgtctgatggcaaaaagcagaatcgaaattaataggaagtaagattgtgatatttattttatttaatatatattttttttcatatacttactac
caagcaatgggtatgtagcgtaatggttaacgcgttggttttcggcaccaaagattgcaggttcgagtcctgtcatactcgtaaagtcacttttttt
aaaaaatttgattttatttttttattttttttatttttaggaattcaattgttaaactagaaaacttctgtatgctaaatgaaaataaatcatggaaata
aataaaatatatatatatttctttttaaacaaaaaatcgaaaaccgaaacggcggaaagctttaaccatttttcgagtcgatgctgatgattgata
atgtttaaccgttaaacattctggaatcgatcaaatttatttttttcataataagtttctaaatgaaagaataaaaatttgagtaatttttagtcattg
aaaaccggttttcaagaatgaggtttttatgagacttttccaggtatttgtttatttacatcttccacatgcactataccaacttccaaaacgaacta
gcagacgttgcaagttcagttacaaatctcgtttccgtttccgttttcattttcatttccatttcatttttactatttatgtacttttactggaatccttttc
tacattcaaacccctaatatacaatggatgatgaattttgttgcttcacatttgcacaaggcatacgattttagaagctgttatatatatacccacaa
aaggaaattatattaaaaaagaaatgctcattaatatacatacataatcatctagagctccaacatgttctatgatttttttacaacacagatacagt
gttttaaaactcttgactggggttagccttgccggattgcttgggcaaaaggtgagcgttgatgttaggaacaacaccaccctgagcaatagtaac
atggcccaaaagcttgttcaattcttcatcattacggatggcaagttgaagatgacggggaataatacgagtcttcttgttgtcacgggcggcgtta
ccagctaattcgagaatttcggcagccaaatattccaaaacggcggccaagtagacaggagcaccagcaccaacacgttgagcatagttaccct
tacgcaacaaacggtgaacacgaccgacagggaaggccaaaccagccttggcagaacgagattgagcagacttggcaacagctgccttacca
ccagattttcctccagacattgtaattgtagaaaagtttatatgaaaagtaaacagaaatgtttttcagagacaaagttagagaaacaaggattct
ttgagctcttattgtaatccaattctttctcaagataaagtgaaaaccttaaagtcttggacgcaatacacagaaaaagctaattacatgggcacg
gatctcctatatatagtaacagtcctcatttagcataccacgccgcgacgcgttaaatgacgctccacgcgacgcgtcgtttacttgagggttaggg
ttcatgatgaggatttagtatacatatatcgcgtcaacagcgaagctttccaagaaccaaccactataatttagagcgctaaacgttcaattgtgg
aatatatctcactttttttaacgcgaacaacaaaacacgaagattaaccatgtctgctgaaaagaagcccgcatccaaagcccccgccggtaagg
ctcccagagacactatgaagtctgccgacaagaagcgtggtaaaaacagaaaggaaacctattcttcttatatttacaaggttttgaagcaagtc
caccccgatactggtatttccaaccaagccatgcgtattttgaactctttcgtcaatgacatctttgagcgcattgccactgaagcctctaagcttgc
cgcttacaacaagaagtctactatctcctctcgtgaaattcaaaccgctgtccgtttgatccttcctggtgaattggccaagcatgctgttactgagg
gtactaagtccgtcaccaagtactcttcttctgctcaataaatgcgttcagtttgaaactagcaaattgaaaaatttgttgggtagataaattcgca
actctgcggtttctaattatgttactacgtgtttacaaaacacgttctttggtttacggtttgccttgaaaagttatcttggttttgaatgggtttatgat
tggataaacatggcaggttcgtgtggaagcccatagaacaaattatattcattcaacgctgcataattacaagacgcttgcaaggattattaattt
ccccgttctgctttttccctttctgcatttcctttttattattttttagctacgttaccactggttggaaagatgagtatttgtttctactgtttgtcatttc
ttgtatataacctaaagcacagatgaactttgcagctaggtttcttattttaattcccttttacttcactagttgatgatcccttcctaccttggtttatg
tttgcatgattttgttgtgctgtgagtcatcaatgggctgggtactacggtatttgcttgcaaatgcttttatatgctgaaagattgtaaagtaataat
atattaatttgtttatgaaaagctcatttccggaaattgaaaattgtatcgtgaaagtagattcgtaagtttagtttccgtagtggttgatgttccgac
aacagagacttttccttgcatttcttgtagcttgctcatactggaagtagacagacatttttgcaatatttgggctaatacaggaaggtcttgaagtg
ctgctgttgtacactgcacaaggtaaagcttggttagacttgatgttacataggacatccatcgatgagcttcctcggcaggcaacgcttctttaac
ttctaaatacacactttccagaagggtgcagagctcaaacgtaaacggatgaattgaattcgagtttgaagaaacatgagtttgctttgaaagcc
attgcgaagacataatctcttgtaaaagcaccagggcatatagttgcatgagtgccccaagggaattatgaaatgtgttgtctagcgaaggagct
gcttgattcaatgggaagctatggataaggtacgtttgcaaagatggtaaacggtctttaatggaaaatatacgactcgccaattcgaaaatgtcc
tctttaaagagggaaaacgtaagattaggtaatttctctcgcgctttttgtgtaagggatgccaattcagccatgttgccttgttgtgtttttttcaaca
cagctgtcaattagagaatccatactagaatcccagtttgcgaagcttgaagtagttgacgtatccaatggtggaatatatgctgctgtatacagc
actttatagaatgcctcatgaatttcttttacaatctcatcgcgttggggagcttgatataagttttccaaacccttcaagatatcgataattagcaat
tcggctttcaaatactggctattaggacccttcaaagtgaataagctagaaagatccgatgactgtgcatgagatgcatgttgtaggatccaagtg
tccacgagagaacgcgtcgccgacatgctaaaaattgagcgagctttcttcggaagtaaatcggcaactcgttgaaagtagtgcaactgaatca
aaacataagccccatccaattctaatcgctctcctcgttctccaacaaaactatatagcaactctataaattgaccattcagcttttctataatgact
cccatttgtttgggatcatgaccataacgtttggcatcttccggataatctttttgtattgtcccagccaaaaactctgtagttaacagttccatttctt

tgatttcttgggtaattgtaccaacgcaaaattccgcccattcaattaattgcatacctgcaaatacctctggtctccccctcaaagcataatccaa
atcaccacctctagacacagctttccaaactagcataggagaagcccgtgcataggcgatctttccagattcctcttgtcgagatttaatgatggat
tcataatatgagaacttctctttcataaatgcgtaaagatatttttgctggctaaccatggcatacaaaacgggatcacgaccgcttatgggattcg
gtttaatctttaataaatccgctaaggattcaataatatatgaaaaatcgccttctgtatgtaagatacgattccatatggaagaagcaagactgtc
aagggacgattgagcaacatgccaagcatgttccattagggtagccaaagttttattgtttcgtaaagctctgtcgtgttctttttcataatgcttttt
ggctttttgtactcctgcaataattttgaatacttttcctgttcataaaactgctgaaacttttctggaagggaataaaaggctttacattgttgtaa
aacaagaagtttcccataatcaacatccaactgcttcatagattcgtcattaagtccgtaaaaatctttgattgcttcattaaggctttcctcacata
tttccattgtatttaattgaccatgttcgccaagtgtagatactatctgtgcataaacattatcaattgtctcctttgttgtgataaatttgttaaagtta
gtctccatcagtcctaaaagcaagcgtgacccattcgatggctttttggactgtcctttgccgttgatatcagttgtcgcagaaggcgatccgcgatt
caaatttgaagatggagagctataattgcttctttccaacattggcatagtcatagatggctgtgcactcgtttgacttcttttcattgctcgatgagt
cataggttttccatgaacactattgggatagtaaggatcagatcctgagctatattttcttaataagtttttctattttctattccttcttacttacccat
tcattcgaagaacatcgtcagaacgattgcgtccatgatgcgctgagtttaaaggactcgacaattgagctgactgacctcgcagttctaaatcgc
ttttctctgtccagtactcttgatgaaagctttttaatccataatgagctaaaatttcttcatcctcgttcattttaccaagagatacctcccctgggta
atttggtagttggtagggatacgaatgatttaatttctgctgcaaataatgagatcgtagcatatccaagtcaattatttactacaatactcaacatt
catttgcatcttttcattctttatgtcatcttacttcgaacatgcagcacttgtagttccactgttcctttcgccaaggttttcagcaaaagccgccttg
gattcatttatttttcgctttcattccattacctaaagataatatcaagggaaaaaagcgtttgatttggaaacataaacataattgacaacactac
cttgaaaccagaacttattcatacaagcaattgaggatggaatggaaacactaagaaagcagaaacaaatatttattctaagattagttttaact
aaaaagatcaacattttctctcgtatgaaaggcgtgtaaacgttcatgtttacacgtatgtaaacaaacaaaggaaaataggaaatcatcaagtt
tagcaaaagttatgcatattgctcaagatttcaaccttttttatagaatgcttctttttcatctatttttaccttaagaaatatcattaaacaacaagg
gttgaacaaaccgaggtttctaccgtttacaagaactaacacatgcctttttactcaattgaatgtacgaacaagcgagagttaaaagcataaac
gtacttccaaactaactactcgcatcatggtcgcgtactagaatttggacccgttcatagtagtctttatttagagtcaaaaacgtcctggatatag
aaacgcaagtaagaacttattgtttcttgcattggtttatgaagattggaagggctgtttaccctttgttttctttctccgtaaatatcataaatatgat
ttccctttgaacgatactacttttgttcacacccaacatgctgggccaactgttgtttttgacagtaccggctttatggacgattatatggatatataa
atattctcaacagcattcgttaacctggatcactgggacttttgtatttctaggtctttacatacctttctagtcgtgctactagttcctcttgatatcg
tatgggtaagtgaatgaattgttaaattggtttagtaaaagaaaaaagaattgtatgacaggatgtcttgtggaagttgggattgacattttgcaa
taatgaaacagtcgggttctcataggatgaagggcaaacaaggaccgtcaacaaatcagcattccttattctcattatattaattcaaaaaattttt
gaaactaacgttcttttgtaggacgtcagcattttcaaatggcgagttttatattggactacattcacagcgacatggttcatacttccatttatcca
agaatatatcgagagtcaattcccaacggttgaaacgagggtaaaagactctctgtacaaaaacttgcgctattatacgcttttgacattcctatc
gttggtggtgatagcttatttgcgctttacccttcgtaccatgtcttttgcaaacttcaaggaattgattatttcccttacttatttttggggcttgttgtt
tgttatctttctcctcggtaatggactcgtttacgttcctcggaacatttggaggaaagcctttcttaatcaacgagctcgtttgttagaaagaaaag
cagtgaacatctattctaggttgcaagaacggatagacgaattttcggcttattcgagctccagcacgctcaacatggatcaatcctacagcctag
gcattcgagcttcttatgccaccgacagtgactttacctcttctgctgtatcacaggccatgtctcacgttttgcctttacaaactacatggacacaa
atggtgaaagaatataacatgataaacacaatacgattcgcaaaagcatcctcctcctatcgactatacttgcctgactcgtatattcaaatgcat
cctgtttttgcttacgcgctttatgtttgggtaattcctgtttttcggatctttttagcttgttttcttgctttattaagctttgttatcgttgtgtctgaact
gtttttacactcaaaacactctcttgtcggcatttttttaaatcgtgttcaagattctccttccctttgtgctttcgtatcatttattgttatcaattatatg
agatattgcacttataaatctttgattaacacccaatttgcatcttaccaccattatgccgtcgttcctttccgtgctacagggccagcgtcattgctc
tgttttgcttcccaactgtgccgactaactttgcctttatgctataattttacgtccttacagtttttcctacgcagtttcacaaattttatggagaaag
tatagatctattacctcttggtaatttaattagcaagcgctacccagtatttattctcatgcctgttatttttcgttgttttctttgaaatattggcttcg
acacattatttattcttatggacttcaaaagtcacctgtgattgattcacttgaaacagaatcctcaaatacggaagataactttctcgatgaaaca
tctccatcgtatttggctgaaggtagagctcttcttttgagtgcaaattatccatctctctaaaaaattttatgttctttcatgttcgggttttaatttgg
tatgattgcatgatataatattttagtactcctcctttataagttactgacgcgccgtaacttttactttatatttaaaacttatgaattcattgatcttt
atgtgaagagtataatgagaagtaagaattgcattcaaaaaaaaagtattcatatattatttattcaatatacaaaaatgaaacataaaagagtg
ctgtatacgaatcaaagagaatgcatagtcgtgagtagcgagtggaaaactggggcatatatggacagggagtttatgagttatgtataagaac
ccttttcatagcataaataaaacatataaagcaatcattctagcaggtactttaccaccagtcagcgatagcttccatagcacgagctacacggac
gaaaccagctagattggcaccgtcgacaagactggggatattattaactccactttcacagtatttggaagcagcatcggttgattgagtaaagat
ggaattcatgataccagccaagcggtcgtcaacttcagtacgagcccaagtactaaattgagcattttgagccatttccaaaccggagacagcaa
caccaccagcattggcagccttaccaggggctaaccaacaaccttcgccgttagcacgagagttctcaaagacctcgacagcttctgcagaaga
acccatgttagagccttcagcgacgtaacgacaaccggctttaatcaaggcttgtgcttcttgggcgctaacttcattctgggtagcgcaaggcaa

agcaatgtcaacttcgcctacatgagtccaagggcgagcaccaacaatgtaactgtacttgctggtaagctcaggacaatcagagatagcgaga
cgcttttccttaagttccataatcttgtaaatgacatcaggagtaaggccctcaggagaagtagcaattaaggagcccttagaatcacttaaagat
ttaacaacagcaccgagctccatgcatttgagagcggcatattgggcaacattgccactaccagagatagcaacccgcttgccagtgatactcct
ggagcccttagtagcgtgttgaatcatgtgacacacatagtagacaacaccataaccagtggcttcgggacggatgttgctgccaccggtaagca
tgtgcttaccggtcaaaacaccgctgtactcgtcacgaagacgcttgtactcgccaaacatagcattgacgacgaaaccagtgacaccaatgtca
ccagcgggcacatcagtttgaggaccgatgaaacggaagagttgtcgcatgaaggcttgggagaaacgacgaatttcagagtccgacttgcctt
tagggtcaaagtccgaaccacccttaccaccacctatgggaagtccagtgagggcatttttgaagatttgttcaaaacccaagaacttgagaata
ctcaaattcactgtaggatggaaacgcagaccgcccttgtaaggacccaatgcagaattgaactggacacggtaaccagtgttgacacgacaat
tgccttggtcatcttcccaagtaacgcggaattgtaaaacacgctcggggacagagatgataggtaagactcgttttagctcaggatggacttcaa
aaagcttggaatcttcaacagagttaataatttccttgtaagcttgttcaaattcgggttcgtaaggagagaccatattgcaaattttatgaattag
gatcgaaaattatataacaaattatagttttaaccagacaggaaaccttttgttttatattctataaaaacaacagacacaccgaaacgtgtaaac
aagctgtttttaagacgtttcaagacgagcaaacgtagcctctatatacggatcattatctaccatccctagacaaaacagtcacaatatccgcttc
tttacacgctgattgaagtagaacatcggacggcaacagtgttttagcgttgatcttcctgattccggcgattgaacttttgctcgtgcgtcatatgt
ggatccgtcgcttctccaaccaaaaatttgcgggactatagataacgaatgtttatctaatcagatagaccaaaaattttcttcctcataccaaccc
catcatagataaaaaaaaaaaaattgcaagaaggtgaatccaaaacatatagcttaatatggacaacattgggatgaagattcaaatcatctgt
agtgaaaaaaattagttttatttttattctgttgctttcactttttatttcaattccatagactgtcagacataggccagaaaattaaattaaacttaa
catccttaacatagaaataaatatagaaacaaaacttcactgtttttcttcctcacttcttaacttgcgcaggaagtagcttttgtttgcaaactattt
ctttacagcattgaaatcaatggtttcttgggcttcttgtctcgaggatcttcagcgaattgaccttgtagtaaatggttaagcgagggtaagtgatc
tttttcagtagtcttttcctcagcttcctcctcccgctggctttcgtctctcgtcattgccgtaggttgcgttcgcaattcttccaaaaaggtcgctggag
atgctttgtcatttggttcaagtgtagctcgctcagatccacgatacataagtggccgtctttttagctcgccttgtctcagttttaaagttcccgcctc
caaaagttcttttcgatgtctgaccacttctcgttcataatacactgcatcgggattttgtcgtgaaaatgcattccacactgattccacaaattcccc
atcatcactataagtttgtggcatcttctctaaacatcgcaagataggcgcgataaggtctagcaagtctaaatataccgacggaccctgccggct
ttccatacatgcaagtcccacacatgctatggatctctgtaaacgatgctctttcgaaaagggaggtgccttctcccataacacattctctctcacg
agcatcctcaatctgggaattaggtctttcctaatgcgcgatcccataaactcgtatgccaatgtgcacatttgcgcaatgcttcctaaagccagtt
ctaccagcactgcctcgcctgtgttaagttgcgttatgagcaatggccagcatgtgtgaattgtggggtaaaaggtattggggtcctcaacaagta
cttgacttgcataccctaaaagccttatcatctgaactcgaatcgttatttgctcatgtgtcaaaaacagttgtgctttttctgctatttgttgcaccat
cttgatcatcccagtactttctttctgttcctcttcatctcccttagatctttccattaattcttcattttcatccccttccttttcttgtttctcattttcttct
tccaaatgctttttgaaaccttcctgtccgctttccgccgcttctcctaattccattccttcttttcttggtggaaaagggtatttttggatttcgttgaca
agcttccaaaaactcttggatatccttggaaatggatccttgagaacccttttcttctttatctgtaatttccttcgacggttgataagtcaattgctgt
ctcttttcacgcgattgtgtactttgttgcttaataatttcatacaggatccctaaaagtccctctgtaagtctggtgtacccatggtaattatccagg
attgtgaagatagactcaataacgtccccaatatactgtacgccgctaccatcgtcatttctaatcacataagccatcaccaaaggaagctgtggt
gtaatgtccaacgtatttaattttaatgcgacagagtttacaacgtagtcaatgttctctcgtagcatttccgcagctgaaccatatttgcaattgta
aactatggattctatgcaagcttcagcaaatgtagagacaaacgaagaagcaaacgccaaatgttctaaaataggataaaggacattcataag
ctttggacggaaattttctccttgttcagcagctacccaactaatacaatccatcgcgatacaagcccgagcacaaagtattccaaactgttttcttt
gtttttcttccttcatatttttcccgtgaaaggtatttaactgagatatttcttgtaaaatttctagagacctttgctcataagactccaggaataatac
cttaacgctcgtagaattagtatgcttcaaaatgtgcaagccagcccaaaatgacgttagggaggaactatttacaagagaagtagatttcaaaa
aaagtgattgcaagatatatttggcttggggcgagtcggcaaattgaagcagaatggacctgacaacctgctccatttcatgatctccaaagaaa
aatccgttgattatatttttttcctaaagaggttgtggtaagaactttttgatggtttgcttctatacctgaagcatttggggacagctgactcagaata
tcttcaaagagaggctcggtcgactgtaagacactgggaacttgtccggcaaatgtcaaaaggcatcgtaattgctttaacttttccaacttaacgt
cctctgaagctaaatttgatataccagaccaggcagatgaccatgattgcaggcaatcggataggacattttccataaccagcgtattgttttgag
aagaaatgaaattcgataataattcgattccattattagcaagttgtggattttctttttttatttaccagcagaagcatagtttccagtaaatggatg
cgacaatcctttagtgactctagacaattagtaaagagtgaaaagcaaaaacggaagagagacgtttgaatgtgcaagttttgggaagagcggt
gatgcaatatcgtttttacagcggtgctaatctgttgacagctagctcgcttccaagccgcagttcttttggtgactcccataaaactagaggaatc
cagagactctttgacttctggtaaatcttttgtgactttatccccgataatcgtgacaagagtataatccaatacttgtaaagcctgggaaacgcac
tgaaaatatcggcatggtcctttaggactcagcccttttgctaatccagaaacaattccaggaagaaaagtagttaatatgtcgagagaaactgg
cagacgtagtaaagcgtccatgcatccaagggttgctttttgtaatcttacagaactattgcttttagaaaacgaatccaaactataggaaaaaca
tttagcaaatagtaaataatgcctttgatcctccattctaggacgaaagtcgactattaaatccgttagtacttccagagcagtttcttgtacataag
gatcttgctgtgcatgttcggacggagacttccagccgtctgcaatgttcataattacaaaatgagttgaagagtaagtgtttcattatgaatatg

tttccacccgtgattgtatatcaatcgaatgcactctaaagcgtattcaagaccagtgctaggaacacgactccaagatttcaaaattggagctat
aggaaaaaataaataatcacataaattagcagataagatttggttagggtcacttaaggcttgtgctaaagcatcttttgcttctccaagggcgtt
ctttaggttttctttttgaattatcgacgcggatgaggataatggaagactatattgacttaatttgcagaaagggccgcgaattctgctaaaaata
gtctgaacgtgagacatgtaaggaatctgggaaaatcgatacaagccgacgacttgaaataacttcagtcttttaaatataccagaacaagatg
attaaaatttctataattcgtttcccttgttaattcctctaaagttgacctaatggtaaattgctgtcagtatgctctcttcaaaggtcatagtttatga
caaaaactaaaactcttttggctatagctttaattcaaacctatattaagaaagaaaaaacattcaaatattttattcattaccaaagttggttaag
ccgcttcctgcatgtcgcatactttctatgaagcatgtcgcatattttcaatgaagcctgtttaacgagttcatcaatagtctcaaacataatttcaat
tgatatctactgctattctttaatgaatttgcatgcaggaactgaaggacagctctcgtttctgccacaattcttcaaagggagtttattttcaatttt
gcgatagcaaggcgaaaaaaaaagaagcgaagaagcaataaagaaaagaacaaactacaaactactagatattcatttgtccgttcaaagcc
ctcatttgtttatttactaacgaacttagactgtatttactattcatatggattccaattcgaaacagaacactgatttcagctatgaaaatacattag
atagaagagctcaatgtcataattcatctttgatgattccctttctcatatctagaaagcaaaggtctgtgtaatgttcagtcatatgcacgttgcaa
tagtgcatttgtgtcacacaattgagctgagaaaaggtaaaataaaagcctgcctttctcatattcaaacccacatgctatttccttttactattaag
gtctttttgctgcaaacgcattttcaatttaaaaccgtctataatatgatattactagaatctaaagtactattatatagtttgtcaatatataatatta
ctgttttgggcccaaaatccagttgtcgtcgtcttctctacacagttttagactaatttacaatttacaatcatgtcagcatccttagaagaaattgcc
cgtcatcctgcaaacaagacatgctttgattgcggttctccaaaccctcaatgggcttcttgcaaccttggtatctttatatgtttggaatgtagtgg
acagcaccgtggtttgggtgtggagaagagttttgtacgcagtgtcacaatggataattggaatgaaagacaagttacaatgatggaaaagggc
ggaaatgatcgtgctcgtgcatttcttatggctgaccctgagtttttttcgaaaactattcgcgataaggttggatgaaatttataggcttaaaaaac
taatttcttttagtataacactgatattgctgaagattatcgagaaaaactacttgcagacgttgaaggccgggaatggataaaacaagagcgac
ctaaaaagcaagaagctgagtcgagcgcagttcctattccaagtaaggaagataatgaatcttatttcaaccgattaggctctatcaatgcccaa
cgttctgctgacttacctccttctcagggaggtcgatacgctggatttggtagtactaactcagtatctcctacgtattctggtcatggagacagtgg
tccttccttcatggaggagctttcaagagatcctctttctgcctttaatcacgggtggagcatgttttcaaaatctgtttcacaacaagtgaaggaca
ttaataaaacatacgtccaatctacctttgcaaaggtaagcgttttcttcgttgatgattattttctctaactttcataaggctcctgaaattcgaaaa
gaatatgttggtttcgctaagtcgttatcttcctccgttcaatcgaccgctcaatcgaccgctcgtcagacctaccaacaggtccgatccatgattga
tgaaaatatacatgcaacagatgcctcttctcaacagcctgcttctgagcatcatacctctatggatgctaccgattctttaatgcataacgatgat
catagcgataatccaccccccttattcttctgtcgtcaaatcagaagaaaccgaacgtaagcgggattaagaagtaatgcaaactcgggatttctct
ttcggctttttttaatggatatgttgatgatagaaaccattaggcaagctattgcccatgagaaatgcaaacataccatacttttttttatcgtacatac
gtagaatcaagactcattgatttttttactctttaattttagaaatagacattactattaaattattcattgtaaacatattccgtttacttttctaactc
ttctttttttcttttttttcttttttttctttcgtataaggactaaatcctacaagaaattgggaaagggtgtaaatattatttgctgagtacgcaaaacga
aattctaagatgcacccattcagtcaaaacgtcatgaaaggttaagcccctttaagcataatggcatagttctccaagtattctgtttatcttttttttt
gatcttacgagggctttgatccgctgccctgaccattgtagtattggttaggatcatagtagtaaacataaccataaggtggggtagaagtagaag
tagatggagagaatggacgaacattatctgtattcatccttgacattaaattcgtatcattacttggtaaaggaggagcaacgttgggaggaagc
atggattgagtagagtgatcgtaaacagcaggataaggccagtaagcaggcatcataggataggccgggagaggagcagggcttgaggaagg
aaggaaagcagaattaggataaactgccccaagaggagcagtagtcggctggtaaaaagcagaagggtcactaccaacaccaagagcagga
ggaggtgccgggggggtttaaaattccattacctgaagaaatctcaggacggaaagttggtacaccaggaatggagggaccaaatgacggagag
cgacttgcagctccgtgagtttcttgggaaggtacctggggaaataaggtcgggtcaaacgggggtggaaagtcatactgaaagaaattcgattg
actaggatttggagggatagcgggtgcaggaaaaaaaattaaaagggggtacagggggggagattcgatgcataggggataggaggggctgcag
ttggaaaagaaggcatggggacaggaaacgtgttcatattaaatttgtttttcgactttgagggaaccgtgatcggagtggcaccaggtaagtta
acccttaggtttttctgatgaagacgggaattatatacaacatgtgtgatacgctttcttgattggagaggaacggctttggaaggagaagatttcc
caaaattcaagcgaatttcaatacttcgctgaccccttttttccatcccaatgttttccatctttgttgcgcgaagggccttgaattgggacgctttcct
cgaaatactgagtcctaggaatctgggggcgacgataaccttgaaaatgcggattcatatctggcttcataggcataggcggagggccaaattgt
gcaccagccatatacggatgcaacatggggtagttgaatgaatgggtaagagggctattcccggtaattggaatcccttctaatcctccgggaac
atttgggaacggactgactgtctgctgatggaaagattggttggatccattgcgaaaaaaataagaatcttcatgctttcccctacgtcttccatct
gagagtggttttggaaatccagtaacgatgtgaaggccttttttgcttcgttgactgtgccgttctcgacggagagtaacaatcactttctgatctgg
atttttcatcctgttcggtagtaactgacgtatcttcaaattcctcctgcacgtccaattctacactttcttgcaattgactattttcttgaaataaatta
gatttcttcttttcgatagagtcagtgtcgtcgttcgtttgggttagagatatgttttcgttcatttttgtctcattctttaacggctcggactccttctttc
gcatattctcacctgaggttatagatacattttcattcatattatttttcaaaaaaaaagaacgtaaaaaccagcaatttctacgcaatgaactatcc
ggtttctgtgttgtaaatgttagaagtattacgtacggtagtgcaaaaattataattccctctttaagcagccttaatgtacttaacgaattgtaatg
cgtcctaaaactgtaaatatagcgatgatgaaaagtttggcaaaagataaaataaacgaaaagttgaagatgaaatgtttttttccaaggatgaa

aggaatttcttttgctatgtcgacttttctattgtgagaacaatttgtaagaaattacaagtcattgcagaatcatcaggtagatgctacatatattct
cctgttctattgataggcaatatcattagaataatggggtaaggcgaattttagaggaagaatgtagaaagttacttcgtatataattctacgtaa
agtagactatggcttcgtatatcttatttcctttgccaaattgaatgaaaagaacgaaaaaaatctacgagtctaagcttgacgataaacaaaaa
aagtcaagaatgaatcctgcaaaatcaaaccggcatttatattggtgaaagaaagaagaaatcactgttcttcagatacttttgtaaactattata
agtgacaccaagaaaacttatggctagatgggtagttgtatttgtaaacgtaggataaagaacgacatactcgaaagctacgtttcctcgcttac
aatggtggaattaagttttattgaaataatacgaatataaacctacctaaaaacatttgtattcaagttttatgtttatagcttgcaatttgtttatgt
cacaaaggcgccatagggacatctgttaccgtatttacaaactgaacgctgttactgttacttaataaaaaaaaccgaaaaacccaaggattata
gatgaagggagtttttatacacatgggggactgttttattttatacaaaagaaacattgtttcgttgacatagtgtctctttgtttagaaaagtgatc
aattactcaaagataatagcagtagtagtaacaatttccaagaaatggaggtgatcagataaaagttaaaacagatctctctctagttaaatgga
aaactttgaaaaaaaaacaaagtgaaagcatataattacaagaattcaaaactatgcctgacaaaaaagggtgaggccacagcacctctccaa
ccatagtacaaaccaggcttgtacaaactgcttcttgagtaacccacccaacagagataacatgctacctggtttgtacgaggtagcagactatc
aatataacgacgattagaaaaaagcggtaagtgatatagcgaaattcatcaaaagagcctgaatggtcgatgacagtttcaccttagcaaacct
aaagtcattgccattgcgttttcaattcgcgtctgttggagcttttatggggtttctattttttcgtggtatgagttgagttagtcactctctttcttaga
aagtcccacgcagttcttgttcgttgctaccaacttggatcattttcgcggttcatttcagactttttagtaatcgttggaacatgtttgttttttttttc
tcttaaaaatgaacgaaaaagagcaagaaaaacacttcgaagctgtcaaaccagccaagtcttcttgctgggagtctgattgcaacgacagtga
tttaccttacagcgatatcgactattcgtctcatctggacaatttggaaaaaggtcgtttatgtttacgtccaattcgcgctctatcgcatgttgaaaa
gtcaagtagcgtccatgaatgcttctgaaaattccatcaagtctgtattatggttgattatctgtccagcaaaggttgctaggattccacaggctact
gtcaaagtggtaaatgaaactgctcgacttgtctagtgctgcatttttgcatttgtgctggattttgtattttcgtactttcttctttttcctttttcaaga
agtcaatggtcgaatttcttacgtcaaatactcagttggttctagcgggtcttcttgccattttggctttctggatttgcttattggttgtttcatgtgag
ttgattcatagatatttgttttgagacttttactaatggatcaattatagacttgttggatcttttcattctgttaccaaatcaacttaatctctggattg
cttcaattttcggtttcgacttgacgttggaaagaaacgacttgacgaatgataatcccgtcccactggatacaatcccaagcgagtttcaaaacc
gatcaaatgaccctaataatcttgcagacgaacaatcatcttctgaatgcgttgaacaatttccgcgaaagacagcagaaaccaagtcaatctag
ggacgttgaaagcactgggaatttggaaagcagctgattccttggtttcgtacttttatttttcaacctcttaattcttctttcgatattttaatcggcg
aggtgagaaattgaaaggtttattctctctttcatattcagtgtttttatgttaaagtttcttcatttcaacaaccttttttttccgttttttttttgcataa
attatttattttgtactttgcattgctgatgtcgttaaatacgtgataatccttgatgctacgatgaaatcgtattgactttgaggcaagtgaacgttt
acataacatggtagcgattagatggtcgttatttcactggtatcctttcgctatgtgatctgtactttagatcgggacgacacctatgagatgactttt
ccatgtcccctaaacgagtatcacttttcttttggataacatcttgcatctgcgtgacacttcattccctctgtaaaaaaaaaaataacattgttaca
cttttgttcttatttgtttgtactggaattttagatatatgcccctttccgttgtattaacatagcattaatgaggtctctgattgatgtcttcagattcct
gctatttatcggtttttctgcgtgcacgttcccttacatttgcagtgtttaatcaatggacgtgtgataaaaagcacagctatgtgaatcttattagag
gtgaagcctatagcactccggattcccatgttgtctcagagcatattacttacaagaccctcagactcttaattgcagtgatcggatgggaactggt
ggtttcgcctaggtatggccgtggacagttccttcagttcaaagaagtaatttataggtttttttgatcctgaagaacccgtgaaagccgctttttgg
ttgtaggctgttattcaattgtctaatgaaaagcagttagctaatgcgtctgcagtttccatgttttgtttgttacttcttctgtgtgaataaattaagt
acaacgagaaattagtagctcactttcaagtaaaaagttgttaacagttcacagaaaagtgagatctgctatgtaaacaaacgcgaaactagcg
aaacttgttatttgttccttgtgactcaaacgtatcttcaacgatttattaacaataatcgctataagagatagcgtaactgaacccatcactatcct
gagcaaaaatgttgcgtaagtgatgcgaaatttcttgctggagccaactttttatttcttcaattcctgccaactgggctgattttgattaaagtctg
agctattttagatgaaagattgaaattgagtctactttgagctaaatccctttcaatttcgcctttcttagcagcttctgaacatttgcgaaatttttac
atcgaatacggatcgatagcttttatttgacgctcttttgctaattattcggtgaaatttctttttatttatttttaggatgtcggaaaaggagctaaaa
ttcgattcaagcgatcctcctgcatacagccatgctgatgtttacaatgttgaccttgagaagggacttcctctgtatacaccaaaagacataaaca
aaacttccaattcaacttctgtcaaagccaaacaaagtaaaaaaacgataatgactgaatttgatagcttcaatgatttctttaccgaatatagga
agaagacagatcattatttccctgaaaatgtagtcgttaaaagtaaatcgatgtttttctgtttgatattggctttatcattcctttgtacattagcaat
ctcctatcgtttcgaactattttcttttctgaaacaagtatatatggaaaatggatacagtttggcccttcttggacctttaggcatttggcttgtgata
tgggcaatattctttgctcttttctatggtaagtggagcattgttgttaatagcaaacgttattagaatgtgtgttactaataaagcctagcacttatt
tggcctttcatattgacaatacggattgccgctaaagttataatctttatcgtggttggtcttggcaacggtcttgcatggttcgttagtgttggcttcg
aaggtctcgcatggttcgttggtactggcttcaaagttcttggtgaaggtctcgcatggttcgttggtgctatcttttggggaatttataacgttcttgt
ttttggctttggaatgggatttgagcgctcagctgaaaacaccggttcgcctacgcttcccagatatcaacagagagatgatgcattagctcctccg
ccgacaaacacgaatgaagaaatcgagcttcagagcaatcctaccactcaaagttaatgcctacttatttgcctttcttttctcacgagtcttaact
cttccgattgaaagtcagtaaatgaaaaagagaacattacaagcggtttattccgatgaaggtttatttctatgcttttgatttgtattaatataaat
ggaaatgacgaagaaaagtggacaatagaaaggcatgcatacaatccgctcattgaaaggaaccgtaatctgagatcattggcatcatactag

gttcttaatgattattattactattattattaattgcaattgaatacgatcgttccatttcttttcattgaacttgtaattagtcttttgtcacttgtaaag
gaacagcttgcaaaaattctatcgagaaaacggttgcaagcaacagtagtagacgtattaaaaaagaaaagcaaaataaaaaaagtaaagag
caacaaaagaaaagcctacagcaccccggattcccatgttgtctccaaccatagtactaacgaggccctcagacgcttaactgcagtgatcggac
gggagctggtgttttcgcctaggtatggccgtagacatagtttatgttcttcaaagtgtatttatcttggtactttcggtagaatcatgaaatgatgct
tgggagccaagagacttctcactgactggaagaacctttcttatgtactatatcttgttgattgggttctatctattactttagcaaattttcacgcatt
gtatgattataatgtgagatttaaacaaaggagttttattcattagattaaatttactataaattaagcaagcagttacaatgttagctaatggaca
agaattgtagcatcatattaagtaaacagacaattggaaaatacaactttgaaatcgagaatgtgaagttaagcaataaattgataagcaatga
tactatcaattactaagaaataaagtatgtttactaggttagcgaaacttttatatactcataggttctcagtcgaattttcagtctaaaaattcgac
tgatctagctcaataagattatctttcagctttcttttcgacaatctgattccttgacaatactagcgtctatatacaagtgcaactaagccaaattca
cgtatacaacggtattagctatagttgtttattccactcgaaattcataattatacagtgtgaaaattctgcatgaaggtacaacttcaagaagtta
aggtttctcttaaatagataacctggagaagagaaatggtatgagacaccaaatgacttacaacagacaaaggtttgactattccatgatgatta
tattcttgttcataagaaaatcagtagagactcgcttattcaacttactgaataaaagttcgctatacctcatgaagaaaaaaaaaagtgactaaa
ttaagattaaattcatatatatatatacaacaaatcatcgtgtataaacatacattaattatacaataataataaaaaatcagtaataaccatacc
aacccattaaactcataaactgaaaatgacaatattgtatgcttctccaatgctgtaagcatgcaacctaattccatattacacacaatcgttcatc
tattaataaaaattgtttcgtgcttcatgtcctgctaaaaaggtactgcatagatcgaacggaaagtccctttacattatcttcatacggattttggg
aggtggttcatccacaaaatcataaatgttatcttgatcttggtcatctaagaattggagattcttgctattccgcttgccaagatgtcttttctttcta
ttctcagattcgattatatgcttgtcatcctttattgatttaacaaccggtttcgttaagtgaaaatgagaatcaattgccgactgaaggttcttagag
tttttcatttgcgaactgcacgtagactttatcctggccgatgttcttgctggacgagttgttgttgttgttttcaattgcttttttcttgattcgtatctt
gttcatttagtttccgttttgaataattctgtttacttgtggaaggaatcgtgtcagctatctgaacataatcttccaggaacgagctatcatcattgc
ttgatggtgatgagaatagcgttgagaaccccgaaaaattatttgtaaatctagtttcgtcgtttttgacttcttcatcaaaagtcatactttcctttg
catcctgaatttgagaacttgaattatgtgcgaatgctttttcttcgagttggtattgcaaaagtcgttcattacggattttattgcaatgaattctgta
aaatcctttcaaagaaaaatacttagtaagccatagcaaattattgaaacgcttaagaaaacagtgagggtgtaatattaggtcacgtccctgcg
acataaaagaatggtcattaaaatcagtggatgccttcaattttagctttgtatttgtattcgaaaagttgctagaattcttaaagaaatcctgaga
aaaaaatattagcggcagcaatagcctttttggaatgaggtttttcagaaaccgaataactggagagtttagggagatcacaaaagagctattct
gaatcataccttgaatgaaattcacaactactgcctttgaaaaggttgaaataaaaggactcgtagcgttcgaaatatttatgttactccttgaata
aaaagaacgatttatgagtttaccataattccccttttgcccttcttcctttttgtcaaatttcctttcccttttcatgcttctttcttcttctgatttgcaa
gcttcaatgaaggacttgcgagtgtgaaccaaaaccgttgaaaataagtgacgcttcttaggccgatgacatgtaataataactttttcaggagtg
tttttcttattattcttcctagaattcgatttttgagcgctatagagaaaaggcatatatatactacgaccatgatggtaggtaaaccaagctccgaa
ttttttaacaaaatcaacgcatatttcttctaataaaggcggaatcatagcccatgcaatttcaaatccattattttcctgaatttgaacattcttttg
gtcaatatctacaatgaagtcgcattctcgaagtatttgagcacgtatatatatatagtttagtaattcttcaaatccatgcaattcttgtgatgtaa
acttttcaacttcaaataaggggcctgtttcatatattgagagcatgtcgtcaataccatctcgtggtccactatcaggaaaagcaaagtagaaat
gaacagcagtataccacataatgctttcaaaacatggaaattgaaactctctttcaatacccagattgacttctaactcattgatataaatttgaga
ttgaatatggagaaaagtgagaaaattaccagaaattgcgatggtatcacatggggtttcaacagcatatatccaaccagacggaattataata
gtttcttttgcgtgaacttccacacgatagcatttatccaccatatctggtaaaaaagaatctcgcttctttccattgggaattaaccaagacacgta
agcatcccagtgtttttggctgggagggatcaggtaaaagactttataaccatccaaaatattataataagcactcgatccgccaaattctatatg
gaaatcaatgtatgagttttcggctgacatttgacaataagtgtcaacacggggaaagtcccctgaagcgaaagcgctgggaggccatacgata
ttaacaagatcaagatcgcgtacaatgtcgggctttcgaacataaagagcgagctttgtagttgatacttcgaagcttaaaacgtcgtatattcta
gatcgttctgaaggcttctgtctcatatactgctgccattgcttcatagtcattttgttggataactgtttttgagtgaagacatcaactatatgaaga
ggacaatcattgccaacgagaatggcaattttactaagagttaattggtcggtattttgagtatcaaccgaaggaaaaataataggatcattgaa
gccggtttgacgaaggtattctaggtttatgtcttgggtagacatgcgaggaaaagcatctttatcaaatgacttaaaaagaagtgcataatgatt
aggatgaagtttgtaatgaaaccgattcttcgtgtgtttattcttcaaatgttgtccttgagaatttgatatttcgggagtagaagcttctgacaacgt
tggattgggacttataggatagtagcactcagaattcctagataaggaagaatattcaaaaggattgatatggcatggattcacacaatccaaat
tttctgtgagaccgatagagtgattctgcatattattaattagtgacgagccattgtcttcaggagatggaggtaatatgtcgaacggttgaaatgt
agtttcaaaagaagctaaatcatcctggacttctgacacgatcgggcaatcagcattttgagtgaattcctgggttagaatatcatcatattgaaa
ccaaggatccattccaaaacaagctgacaaagttagaatgaataacaaaatggatggttcgaagaaaaatctacgaaagaaacaagggagtt
attacttctataaaccaggggttgaggcgtaatacttccttttctaatagtgcaatgctgttataaggaattactagaagaaaccgccgatgtgatt
gtattgtttggcaatctggattcgcagaaataaaaccaaatatatcttaagcaccaagagaaaacatgaaagaagtaaacatcaagtaagcttct
gcaaagtactgccttcaggtttcgagaaacgcttcaatcgatcaagctcggcaaagacgagagacacttagtattcttaacgtaataaaaaaaa

aacagaaaggaaaaaaaagcaactgctcggtttaaatctcaaaatgaaatgcagaaaccttttttcggtttgtatacattattcgtgaaatagag
gtctgttctcgtttctgttactcgttgttacctcttaaatttggcttcagtttctactgttcctagtcaaatgatccaaaattaagaacaatagggcgat
gtcagaagtcaaagaagtttatgaaacaaaaacagtagctgaagtcgaagttaaagaggaaaagcctttcgttgctgaggaaacgactgcacc
agaccaaatgatgttaggaaagtatattggagctcatgtatctgctgctggcggcgtgttcaattcgttaattaatatgaaaaacataaagtatgt
gcagagtaaagttggtttgttggatgttattatgcgttggtacgctaagaatcggtttgtttacacatgtaatgaacgattttatcagtctgcgagga
tggcaattccttttgcatctatacatatataagaaacgatactgattcatgcataatttatccaatccgtagatatacaacaagacaaaatgtttag
ggaattgtttttactaataaagtttagtgctacgagctgcgcattcttcgtcaaatctcagaggaaatggacatctgctgatatgccagaagaagtt
gcggaaaagtttcgagagttggctatggaatataaatttgatgccaaaaaacatgctcttgttcacggtagttaccttattaatatggcaaatgca
gatcaagaaaaacgtgagcaagcatattattgtttcctcgatgatttaaaacgatgtgaacgtttaggtgtcggccattataatttccagtaagtaa
acacagaaaacaagaaaagacggccatgattttcctttgctttaggatttgtttctaacaaaatgatactagtccaggaagtacttcctcttgtaca
aaagaagaaggcatcaataatttagcagaatgtattaatcgtgctcacgaagctacaaaagacgtggtcattgttactgaaaacatggcagggc
aaggaaattgccttggaggaaattttagtgattttgcagcgttgaaagcaaagattaaaaatctggatcgctggaaagtgtgtttggatacttgcc
attccttcgcggctggttatgatttgcgcacaaaagaaagttatgaaaaagtcatagaagagtttgacaaaacggttggtgtgcaatatataggtg
gatggcatttgaatgattcaaaagccccattaaaaagtaatcgtgatttgcatgaaaacattggtttgggatttttgggtttagaaccattccgatt
aattatgaatgattccaggtgggatagaattcccatggtccttgagacacccgccaagacaccagaaacatgggaaaatgagcttctgatccttc
gtacactggtcggaaaatctgctgatgactcttatgttcttggggagagtaagcgactagctgacatgggtgagacatcacggaaagaccagttg
agtaagtttgaaaagaaagaagcaaaacagagtgaaaagaagcgaaaagatgagacgactaccgcccagacaactataaagacagttaaac
gaacacgaaaaacggtacaagcatcagaatcatgatccagtacgaatttagggataccatattagacatgctttgatattgcactctttttgactt
ggacatgtattaatccagccgctctgactatggcttgccaattggctgctattcaactgccattacattatcaaatttccaagcttctttttgttcatta
ggctttatgcatcgaatgtaatgagcgttagtctaaaagtcattattagtcttttataaatacaaaaaaattagttagtaatttttatttttttacacatt
ttcttccattctacacggctctttcgcttttctctcctaaagtatgcctatacgttcgttaaacctcgatcggtttcttaattttcttttacatagccttcgt
tcaactttcttatacttagccgacgatttagggttatttgcacaatagccctaacctacagagtattatgtgactgttcttttggtttcactagatttcc
cattttaccaaggaaaaatctctacaccacccaaaaactcaacataagagagatgtctacagtcaaggtcaacggagccaagaacggcggaga
acgtaaggttttgcctactggtgaagccgccgctaagtactaccctgttttacgcgaaaatgtccctaagaagactcgtaaggctgttcgccctacc
aagctccgtgcttctttacaacccggaactgtttgcataatgttggctggtcgcttccgtggtaagcgtgttgtcgtccttagccaattggaagacac
tcttgttgttactggtccctacaaggtcaacggtgttcccctccgtcgtgtcaaccaccgttatgtgatcgctacttctgccccctaagcttgatgtttct
ggcatccctgtcgagaagttcaacaaggcttacttcgctaagtctaagcgtaacggtcctcttaagaaggatgaagctttcttcgctgaaaatgct
cctaagaacgctctccctgccgaacgtattgctgaccaaaaggctgttgatgccaagcttctccctgccatcaaggctattcctaacatgaaggaa
taccttgctgcttcctttgcattgtctaatggtgaccgccctcacttgatgaagttttaaatccgtaaactttggcaataggtaaatcgatcattgtttt
tggcacttgcaggaccttatttatcattctggttagttctggtgcttttgtagctatgtagccaagtttttatggatggttagaacaaggatggtttcat
actcaaatagttcactaacgtaatttggtttattggattttgtaaacttgaaaccttgcaatacaatgtattctcatgtatcgatattaagtagtgtat
taaaggtggtttgttttatgcatacgaaaaagcagggaaatattaacttgtgtaaggatatagtatgtatgaaactgagtagaagctcaatgctca
tataattcctagggctcttttcatctctgataataccgaggattttcaatttcaaccttcttgaatacttcttagaatttgcgttctgacgactaatata
ctacttctcgattattttcaccaaagacatcggagtagagcttttcatgggtttatattattgaaaatcaaaaactatgtcctcttccgtttcctcatac
agtggtaaagtaaaaaatatagtagcataatattgacattcactaacttgcttttttctaggtttctccccgaaggaagacgataagattcctgtttt
ggataatgtagatcatatttctcccgaagaattttataaagattatgtttcaaagcgagttccggtggttataaaatcttctctcccagaatcagatt
ggaagggctatctctggaaacaagaggattaccttcttgaaaagattggtcaaagcgtatgcaagattgaacctatggatccttctactgggactt
ttggtcaggggatgagtcgtaatgagatgaccgttgcagaatttctacaacgcataaaagcaggagagcgtctgtatttgacaactcaatacgat
gactcagatgatgttctggatggtgatgacgaagtttccttacttgttaagtcactttgcccagcaccaacggacaaattattgtctgatttttctttg
actcctgcgttaatgggaaatcttgttccccagcaatgtaatatctggattggcagaagtgagaacggcacgtcttctggtcttcaccatgattttca
tgacaacatctataccgttgtgaaaggacacaagcgatttgtaattctttcccctgatagtgcgcttcatcttcagttggctggaaaaatatctaag
attcatcaaaatgggcttatttcttatgaaggtgaagatcgtcctcaacgtagtgatggtttaacagaactagatgctgcgatcgctcacactcagt
tcctggaaatgaaaatttctgacttgaaagaactccaagcttctgaagacatcattcaaaggttagaggccgagtatgaagcagaaatggacaa
tgtccttcagatgcagcttggtggagaagcacaggattgggatgaattgcaagaaggtgatgctgatagcttactggataaatcctcgaacgaga
atgatgaaggcttctcatctgcagatggcggagatcaattggatagcgaagaacaagtttctgagtccggaaatgagaagccatcactacctgat
cattttacgaaaattacagttcctggattacacaagtacatgggcttcgacaacgccaaggatattataccttctgaagaagaaagaaccgctcta
tcaaagacacatcccttagtcgtagacttggagcctggagatatgttttatttgcctgcgagttggtttcacgaagttacgtcttcaagcgcagaag
gtgaaagcagtattcacgttgcattcaattactggtttcatcctccagataatctgcacaactttgatcatccatacgaagataggatttatggaa

ggcaaaaaggtctatggtttctaaagctatacagcgtttgtatgctaccagtggtactactgaaaaaagaaattgggaaggagaatcatctacgt
cagaaaagcagacaaaaaaaagggtacagtgattgaacgtatttgactaatataaaatttgttaaggtaaaatattcataaaaatccaataaaa
aaggaattgaaaaagtaaacgaatgaatattgttgaaaattggaggtatttaagttataacaatccataacatgcataagaggggaagaaatat
ccagtatatctaatgtgggaacataatggttgttaatctgctggttcatttcgagattcaggggataaagaaacatctctaattgtataaagaccga
taatcatcagaacggaaaaaattaaagagacttgaaacaggtccttttgagtagaagaatatgctgcattaattaaatttcgtatatgggagcctt
gcgggtaagacagagcagttcgtaaatctcggaagatagattcgatttcaggaacgggtaataatccatctaggcttgttaataacctgtaggga
agcttcttgctccaaacaccacctgctacagcagacccaaaggcaccaccgacagaagaaaaggttagatacagtgttaagttttgacctattttt
gtatgagaagagacggattgaaccgccacctgagcactaagagtaagcaaccctcctccaacaccagcaagaaatagcataaagacgatcatc
tcaacattggtattttcattatcgaatccaaaaagaatatatccaacaagtccaagaaggtacaagggaacagaaaacttcaataatattttaaa
ccttgcaaactttttcattaggaaaccggagaaaatagcagaagtggtcatagaaaaaacaaagacattgacaattaagccactagaatctata
gaaaagtcatgcgttacctgtaaatacgaagtgaggaagtttgaaaaaataaagtaggacataaagtacaggaaaatcaatatgcatgcgcct
aatacagtacggtctttcagggaacgcaacgcaaaaatggggtaatcagcgaacttggtatcataaacacataaagcaataagtaaaaaaata
cccaatcccatgctgaggacggttaatatactcgaatcgggagaaacaaccctgctagaatgtgaaaaaggaaggagaaacagcccaaatccg
gaaataaatagcaatattccgacgccatctagttcatgccaaaagttataaaaattagactggttcatgaattgatggtgttctctgtacaggcctg
ctgcttttgctttttgctgattaaaatatagtgaaagtaaaagaggaagagatactacaggaaggatgatagcccaaatgccgtatccaattctcc
aaccaagggttctctcagggtgaaatgcttgggcaaggacaggacccatccagacagtaagaagatagggctgctcaggaatagcggataaaa
ttcccctgttcaataaagaagatgtatctgcaataataagctgggagagaatcccaatgccagtttgaccacaaatgtagatgatataagcaagg
gtaaacattccaatgtttcgtgagcaggctaagaggatatatcccgaaacagtcatgcatagagctagggttaagctttctgtacggccaaacac
atcacctgctttaaccataggagctcgaacaacggccaagagaatgttgctaacaagattgatcgaagccaaattagaatgtgcagagaagttg
ctggtggcatatctttgaagagaaatagtagtttgttgttctaatgaggaagcaaatgctaataaataaagactgaagcagtgttagcgcgttgtt
gtataatcagataaagtaaaatcctattgcaggaaagcaaacctaccccagatatgcaaagataattgattttcttgaccaagtcgcggcaatgg
cttgtacttttgaaactccttccaaagtgatgtctgagtgttgatcgacttgagtccttggtgaaactgcttcttgtctttcttgattgattaaatgactt
tctaccaaatgattactgtaaaagccatcagcagatggatcatttctctccatacttccataattctgattcattcactagatcggttaaggacagat
ttgggtcggacaggaagttgtgagtcttatcagatcatgccatttttctcggattaccgtttgcctaagcgagtttgggattgtataaacattgcgaa
aataaatgaaaaggtaaataggaaaaagtgagagagaattaatgttattcttttcttaaaattaaggatatgcatttttgtgctacttgctaaattt
atggactatgaacggctgatagcaacatagtgaacagtctttgctcagttcctttggctcatctagcatcatcttgtttttttcattttagaaaaggaa
agtgtaaatacctcgcattgacttgaaaggccagaaacgaagttttctacagacaaagcttcctcattgtgctagaaaaacggtgttagaaaata
caatgtaggtaaatatgtgactaaagtaattttctataaagcaaatatagttcctgtgtaccgaaaaacctctgaagttgttatactacttacagctt
aatttgttccatacgtgcttcgtaccaactacactagaaaggggatatgggaaataggtttgaaagctaaaaatagaaagaatgagtaaataca
gagttgaaattcagcaaatgatgtttattttaggagaggtacaggatcctctcccagagactattcaattagtggaagaattaattcgaggacaag
tcatggaaatgctgattcaagcaaacgaactttctatacgaagaggctcaagatccatcaacgttgaagatctattctttttgattcgacacgatcg
cgcaaaagtcaaccgactcaagacatatttgtcatggaaagaagtccgaaagaaggcaaaagaacaagatgctaacgccgctgatacaaaag
agctttttgaagaagtagaatctaaaacaaaaaccttgactgctcccgtggctatgccatgggaagtgaaaaatatgttttcagagccagtgcca
gaaactgaagagtttgaggacgatgatgacatgatggagatggcatatgcgactagagagcggctgaaatcagcagatgaaagaactaaaaa
gatgacaagagaggaatatgtccactggagtgaatgccgtcaagctagttttacataccgaaaaggaaagcgcttccgcgaatggtgtggtatgt
ctttgttaacagaaacccgtccagataatgatttagtcgatattttggggtttttgacttttgagatcgttgcgaccttaacggaagaggctttggct
gttaaggaaaggatggatcaaatccaaaacaatacaagacatgggagttattctgtccctaatcatccaaaggagcgttatttgtttgatggatta
actgaaggtaggacgcctttgcaaatatttcatgttttggaagcttttcgacgactgcaagttcccaacaaaaaacacactgctatgagaagcttc
aatggaggtctggtcaaatcaagagtctatttaatttaggccttctaaatctgctgatgaaatatacacgaatacgggaatgactgaaaaagctg
attttctaatgtactacttatgctcatgaccctactaatagtggagccattgaagggacggcggttttccttgatgttaatggaccttcctttacactt
aatattattattgttatcgttattattcctaaagtccaccgtagtattctacgggtttttggcgtacactaaaacttcaacctcagacttttaaaacaa
aaacaaaataaaaaaagttcctataatgtttgtataagaagggatccaaccaaactattaacttcaattcttagtctgtattgttcttctattgcag
ggctaatcgcttcactttatgttggcttgttttagactttcctaacaagtaggacaatatgacagacaacctaaggaaggtatcaaaaaaaaagag
tttgaagcaaaccattattcagcattcttaacatgtctttctacagaaatccattttcatcatgaagatgttccacatcatatatacgcaaatgcttcg
gtaaatgtcctacttatacccatcaactgaaccagattgtaccaactctcagcttttatttctttagtaaaacctttggatttgtattttctttttctatt
ttactatattttaaatcagtttgagataaactctcagtaataaatcacgttcgcaatgtcatcacctcaaagaggaaatcttttgactcgtgtcaagc
gtattataccaccattgtctggatcggcgcataagggacaagctggccgagtgggtatttttggtggatgtcagcattataccggagctccttacta
ttcttcgatgagttctatgctctttggtactgatcagagtcatatcttctgtgaaaagaatgccgcaaatgttatcaaaagttattctcctgacctaat

tgtacatcctttcttgcgagaaaaggaaaacaaaaatcctggtgactccattgatggtatcctggaacttgttaaacctatgatggaacggttgca
tgccatcgtaattggtccaggcttgggtcgtgatgaatggatgctagaaaccactgccagagtaatccagtatgctcgagaaaaggaaatgccta
tggtaattgatgcggatggtctctttttgattcaacagcgccctgaagtagtgagcgggtatcataacgtagttttgacgcctaacgtcgtggagttt
aagcgtctttgtgaaaaagtaaatattgaaagcgaaggccctgatgcctgtcaccaattggctaaaaaactgaatcttttgattatccaaaaaggt
aaagaagatctaatttccgacggaggtacgacttacgcttgctctgttcccgggggattaaaacgttgtggtggtcaaggcgatattcttacaggg
attcttgttaccttccttgcttggcgaaatgcctatttgaataagcagtgggatatcgaaggcgacatggatgctaaagaatgcctgtttttggcag
catatggagcctgtgccaccactaggtggtgcagccgtgtatcattcaaagaatgtggaagggcaacacagtcaagtgacttggttcgtcatgttg
gacatgtttacaatgctttaatggacgatgaaattccttcaactgagcagatggtcaaggaatagatttgaaacacattatgagcattgcaacgat
ataaggattattaaattttataatgatagctacgatctttagatgattatgcaagttttctcttgatgttttgatactttggttaaaaattgatgtttttt
aaaatacaaacgtgtggtagtgggatgtattcactatggacccctctttgatgtcttaatgagcatagttaacatttaacccttctgtggactttgca
ttatacatgaaagatagcttgtttttttcgataagagagttacctgccgaatatattcatatattgtaaaagtcacgtatctgtcatgcaataattta
agtctttaagtgttcaagagatgggcctatacgtggatagcagaaaaatacgaataaaaaggacataagaatcgaatagcacattacaaacta
gatatcatgaaagatcggtaaaccacacatcttcaaagtaggaaacacggcagtcttttgcgaaacaaagggctaaataaaatatgataacaa
aatgactgctaaggaatccaattattttgaaaagacagtaataacagcaaatacagcgtaaaatagagcaatgggataggcgacaaggaagcg
catattgttgacttggagaatggcaacaaacatagcactagcagcatatgtgcaccacatagatgctaaagcagcaaaggcatatcccaagatt
ctgctataacagagttagtacgtgactgcatgaaaccattttaaggtgtttcccacatacttaaaagacatcaagtttttgaaaaaggctactacta
ccaaagggagcaaagagtatcccaagacgctgacggtacgggtataaaataaattcttcgcagacattaaacgtaaaacgagatgcaagctaa
gtgaaccaagtagagctattccataaatatatccaaaatgggatcgaccatgtaaggaaaggaaagtactgaataaaagacagaataagacgg
ggcccgccatatctgtatcgtccataatatgaacatcaacatgtttaaagggattcagtacatgcgtggtctagtcaccgagttagcagatattctt
ttaaaagcaaaatgggatagaacatatctctctaatgagagtagacacacaattcacgcagattctatacgctaaactccctttctcaagtttctcg
cattggcaaactgacagtgtacaaagtaaaagcatcgaatcacttaccttttgttttatatgactgaaattaatgtctagttcctcaagtaatgagg
gttctcctgggtatccactcgtcgaaaatgccgctagccagccttgacttagagattcacttaatggttcatcataaaccccgaagaccgtgcctg
tgcagtataagaatttgtttcgggaaaactcccttgataatattgtaaatttgccggattgttgtaataagccatggattttgttttcacagatgcat
gctctgggtacgtagtcaattctctccccatatgagtatacggtaagggttatgcttgtatagaaaacacaaaaaagatggatatgaatagcaaa
ctagacagttgaatgtttttaaacaggttccatagtatttaattcgtctcgaggtatcggtttttctttgtgtgcaattgcggtgaaagttgagcgaaa
cggttggaaacgaagagtagtcgatataaacggccatagaatctctatttccattaagtttgtctgtcaattatgaaagaatcaatggtttcctcag
cgatcgcaaagaataattcctgctaacatgaaaagataaatctctctgccagatttgacaatgggccttttcattttcctcaaagggcttgaaacat
taaagctaccagcctaaattgatgtcttgttcaaaaatatgtatgtagaattgcattttgtacgtggaatttgcaccaaaacaaatatcatattcttt
tcaaaacaatatgatatttgaacgatgtaaagttcatacgctgaagagcttaaactagaaaaaattcatattctctttatgagaaacttttggaga
ctcgttagggatgtaggctcgaaatgtctacgctctatttcatctgctgactttacctatacaggaatatattctcaatgttctcaatttataaaatag
ctattctataagatatgtcaaattacatttatgaatgctatcgtcataaagcttttgagattcaaatcatcgtccatcatatcgaaaccgaacaaag
cggtttctcaattcctatacgaagaattattttctttttttcgtttttaaaagaaactacagagggatctctccaaaagccatagaatcagaaatgta
aataaaagatagtttagcgttgaaaatttcagagcgttagtaaacaaacaatcattcacctttgtcaaaatgcgttgatttagagaaatgaacatt
ctcttctttcccttcatgctctctttttactatttccttcaatatcttctttttatgacttgttctcgtgaaaaacgaataaagtttttttgattaactttttta
tgcgaaaaaatgaaaaacaattgaatcttccacgcgttcataggtgtagttttactgtaatctaataaaacaactaaaaaaattccccattctatgt
ggaactaggaaaaaatacacagaattattcgttctgaagttgggattgtttcactcgctattaataaccaccaacttggcctttacagcaatttcca
tgttgttcatctttagccatctttaatcatagtcaaaacaaatactttctttttccttcccttttgttttattttttttttgttgctccaaagtgttgtgcttttt
ggacaacgtgaaatagattcagtaagaaggaagcgcattctttgccttcgtccagggtttttctctttttttctttttttcattgaatccttctttttttttaa
agaaataaaatcctgatcctcttccccaaaggatggaatatggtctggggtaaactaagtaccgtaggtaaagagacacccgatttgaaaaacc
gaaatggcatgaaggacaagacgtataaacgatgtttttcctgtaatgaatatgtaagaaggtcaaagaatggatccataaaggagtggacttg
tcctctttgtgaagctccaaaccactttgatgaaaatggagaggtcatggattatcgtcctccaacgccgccatcgtatgagacgtctgctcctaat
tttctctccagaaccgtagaaagaaaacagatgagaagccctttttgcgagaactgcttgaggaaccagctgctcgtgaatcgcatgctggcgga
ttttcttcccgagcataaccatcccgattataaggcttatgaagctgcctttcctaagtataaagaatctttggaagaaaagtttccaattgtttgtg
ataaatgttatgacccagtccacaaacaacttgaggcaaacaattacgaagcaaaaaatcatgtgttaggacagtggctcgttcaatccaagga
aaagctttattcgacccccctcttcaagtaaaaattcattttctgtcactatatgggtcctgagaggaatagggtttctcctcttttattcacacgcgat
gggatggcatctttaccattccattatacccactttttttcccattatttgctcaaaaaatactgaaccaactttctaggtttttttttgttgagtttaagtt
tgagtcaaggtgcaatgatcacttggctgggttttttttgttattttttggaatccttactggcttgttcttactcgagattcctctactagtctctatgga
agagagcagtacattcgttctcagctcatttctattctaattcgactaacttctgtctatattctgaatctttacgagtttgctattaaagctgaatatt

tccgagacgctaataacgttcgttccacaatctctcaaatccataccatcttattctttgttactattttctttttaattgttagcttttcttgcctagatt
tccgttctcctcgaagaataaatttaacagccacgacgcatccaataaagtcaaggagaagcaagtctactctaaacacgttgaaaaaaaaatt
acaaggaaagtcattcaaaagaagacaaaaggataatgaaaagcctcctgctttccctcaaacgacacctttgtcttcacattttcgaaaattgtc
agagatgcctaaaatttcaccaatctcaaatctacagaaaaagttggaagctcttccaacctcaagcccatattctgcaaaatcttctcctttttcag
catgcttccccgaatattagaggaaaagattccctgagaatgatgccaacctttgctgattcgaaacccgaaaccaaaaacttggatcaagataa
agagactgtaccgttatcgggtaaacagggagatgatactaatagcttcttcaagccttctggaattgaaaatttattttctaaaacgctaaaccta
accaacgaacatgttgaagttttagaagccaaaactgctatccggagaagaaatgggcttttagccacgattatgagtattttcatcatttcattttt
gttgactatacaacgactctttgaattaaacaggatgaactttgtttgtatgcttcttgcaggactaagctcttgctgtatttatatgtatcgcaatcg
ctatgtttttaaggcctaaggacttccaagcgttgtcgctatatatttgctattaacgcatattaaaacacttatgagaatgaagttttacctccaag
aatttaagagtgatcatgtatttgttaaatttattactcaatttttgtcaaagtcgaactcagaatatctataaaggagtgatttcatagaaaaatta
ctctatccttattaaattctcgttttagttttatttttctatttattaatatattttctataagtcgttttctgttatctttgtaagcgctttttataaccaatt
ctttcgcgaattgtttgttaatttgtttattacaatcaggtaacaacaataaacagaccaagtgacgatgttcgtataccataccaataagatacttc
tgaacttaccatctgccaatctcacggatcacaattgttgcaaagaaaactcttgaattgaggagtaaatagtgtgaattaggtaaatatttacttt
gtattaccatgcagcccatctctttagccaagtacattcaaaaatctcccttttttgtacaagcttttgaagcccgtttcgcaagcctatattaaccttg
ctggctatcgtaaatttggtcttcgttacgatgatttgacattagaagaaaacgaagtcgttcaaaaggctttgaaccgtataccggaacaagaat
cttttgacagagtttatcgtatccgccgcgccatgcagttgaacattaataacaaggttttgcccaaatctgagtggactaagcccgaagacgaca
ctctttatcttcgtcctgttgtccagcgtgtgaccgaggagaagagagagcgtgaggcttttgactctctcattgtcgacagaactcccaagccatc
tgcaacttctcattaattgggatatgtttataagaagctggatctttagttccagtaatgcataaaattgcgaaacgtgagcagtaagatgcagtca
atgacaaaaaccttctttgctcaattcagttgagtgcctgattgatcacgtacaagattttcattggtttttctcagacctaacaatgcaattctggct
gtcccacttcttactgtttatccatggtgatacagtactcaacttcctcccttctctatgtaccagtcttttaacattatcctcttttgtttctatttgcact
taatcctttttattactttacagttttaaaattgaaaagtttgggttatgcaatgaccatcttgaaactgtttttcaagaactttctcatatctcccccttt
ctttgactgtcattcgatgattactcttttctaaataacagcccatttttttcttttgaaatttaagcgtgtcatcccaaagtagctctcttcattccaata
ctacaatttcagttgttttgtagcaggccttaaacttacatgcaaaaaaaaagaaaaaaaaaattcagcattgtatgataagcagaaaactgtca
tgcaaatgtttaaataataaaatctgtttacttataattctcaattgctttatctcgatagattcaaattatagaaatctgttgtcatcgcttcttcgct
caaatctaaaaagaaaagaaaaagtaatagacttatattcaatttaatttggctcgtttgaatcattaagaattactgaacatagcaagtcaagc
atagaacttttctcttatatcctctaagactgctttgagtttatgtaggaaatgtttcagaaacaatcgcattggatattcggtcgcctacaggtttat
gcctttttttgcaataaaaaagagacatgagagaaattatagactataaaaatccaaaatataagcaaaagtacaaagatttgttccatcgattt
gtcaaggaggaaaaacaaatagacgtggagaggtgcagtcatagtagacagcatgacctttggagaagatttgttaaacgatggaatgatgga
aaaatgcgtcaacaatggtattccctaatgcaaacttccgaggccggtcataatgatcaatcaccggtaggaccaagcaaaccaacggttgatg
acttgtacgaccgacaagagctacgcgaacagattcgagaacagcaacagtcttcaaaacgttatgaaaccaaactgcgagtaagacgtgaaa
ccgaactgttggacgaagttttgcctaaagagccagctggtagtaaacaagcgttattgcagaaaaagaaggagaagcaacatactttgaagca
ataccgtgagcggtcggatgaagaaatggaattgaacgagaacgacttgtttggcgaacctgactcttttaccaagcaggttgaaagagagcatt
tacgaaaaaaaaagcgagatgaaggtcgacagatgcgtcattcccaaaaagacgcagaaatccttcaaaagatacaggaacgaaaagaaaa
ggaaaagaaaacagtagagatgttacgagaaatggctcagagccgctttacaaaatcgtcctaatatatacatcttgcttctatattatgactagt
aatagttttgatagcaaacacaattacttttattttaatgaagatatattcatacttactatgctcctaaagaataagcaaaaatataatcaagggg
taaaaatcgcaaatcatgaactttatttgattaagaattaaaaacttcatctccctttacctttaaaatacatgaaaccaaaaaaataaaaaacatt
aaacatatttttcgtttaagccgaaattcgtgacgaaaaatgcacaagtcgatcaaacaaacagttgtacagctttacttattttcataagaaatat
ccggtgtgggtgctatctcaaatttttggatttcaaagcttctatcatgtaagaaggacgtagactttccaaatatacgaggctttccgttattttttt
cacataaaagtaactgatcgaataatgcaatgtttggatcattgcgattgcactgagaacgatcactcacaaattcggaaaatgcttgagtgttac
aaaaactgaggatccaatttccatggaccttggatgaatgtcgtgcaaactttttaaaattgaatgattcaaatcttctttgaccatgttcgtgatttt
cttctttttctgaaaaaattgcctgtaaggaccgagtattttagtgaaaaactttaagaagacagtcgacaccaattgaccattaaaacaaacgg
gagtacaaggcatacatacataccgaataaactcaacgttgatatgtaagccacaattgtcacaccgtagatgggtttgttctaagggagcacca
gaaacagcacagatggtaagagaaccaaccgtatataaatcgatggaaacataccgaagtttgtgcccttccttatagagaagatgtctgcttgt
tttctcggcgacattgtttaagtgaggaatgttcacaaacgatttatttctcttaaagggctcttcatgttgtccatcagaatacgtataagctgctgt
cattgaatttctccgactgctttcagtggcagacgaaaacgacttattagtttcaatcgccataggagaaacagatctagtcaaaggcggggaag
aaaagtgacgagtgtgttcagaggttgagcgtccataagaagaaagtctaggcaaagattttgacacatccagctgtttcggaaaagaaatactt
gaaacgttttcctggaaccttaagaagccatttaaaatcggcggtattacatacgaattcctattaacccagggatgccgagaaaggaagaatgg
aatatggaatctttctcgcaacggaaaactcgttgttgtgctcaaaggcaccgttttattaggatacgcttcatatgcatatcttggaacaccgaaa

ggaatatcatattgagagtgcaagggggcagcagtatttaaattcgcatatagtttatgccgtagagaagcaggtaaagtgaccaactcgccata
tgtcttaacccagttgttatccaagtcgcataccactagaggtacactatccaactgcaaattattcaaagatatagcatcgctcaaaataccgat
aatgtatgagcaaggtgcttcaagacacgatatcaacctacgtggcaaaacaggaataaacagaccttgccaaactagtggataaagtaaatg
aataagagcaaatgacgcataactaagcatgcttatactcttggatataaacagaatttttgactctaggagagctgcttcaaaaagagttaaga
tgttccacgagtctaaagctcgaaaaagaggatacaagtccacatcgcaccatccaggaatttcattcaacgggctacgaaaaacacaaagtcg
gagaggtccaacttcaacgcgagtctgagaaaggcctctaggtggtaatggaatttcatagcatatgttctttatataagcctcaatagacatattt
cgtaagaagtcaatgtcaaagggaggaatgtaatcaagatcatctacaagaagaggcgaacatataattcgaagccaatcgcggcagaaagat
tgcaaataaggacatctggtcagaacgccgtatgatttcggcaaccagatgtgctgaccttcagtgaatacgtcagttaatccaaaaagtatattt
ttcataggacgtaacatctctttgtatagatgtatattttcttcgcaaatagccatttgatctgataaatgcgcacgtgaaaagtgggtttgctcagt
gctttgcgccatctcaagtaactgagaaagacgttttttttctgcatctagcttaaatagcaaagaatcaactacttctagatcctcgggggacgcg
tagtgatttcgccaaacatcacaagccttcagcaactctgattgcatctccaaagacatttttacccaaacaaccatacaaactccataaatccta
gaattatcctcgcttgtcaaagcaaaggagtggtatgttgaggtaggacgcgtgcaggactgcttgatagctatgtcattaggaaacacaaacat
tggcacatagttgggaaattcacaacgagagctttttgcactttctttgtctaaatctttagtggtcggatatctgagcaataaagaaggaggaaa
actttttccaaggggtgtaacgagggagataaaggaacactttccggataggcaacgtaagactgatatacttcagacagtgtataggtagtgc
ctatactgacgcgattttgtagaggttccttatctaaatcagatatatttggagtaggtatattttgtggtcgtttatgagtgatagagctaacactgg
gacgaataaggcttgaattagatgcagaaaacccgtcctttttcttgatgtaggatctggtgcctttggagaggaagcccaacttgatgcagctc
gcttcggcgatccggtaatagacgaagagacgaaaaggcttcctggctggttctgatctttgttaatggacaataattgtggtatacccgatgccc
ctttcgttgaagtgttctttgccaaattcgctgctaaacaattgctatgttcggagacatcatcttcagtttctggcaatgtcactacatcactgaacg
tttttttcgacaaccctacgagaaagaaatgatctgcaatcgtttccattacaaaagcaaattaaatcatgattcccatttaccaaaaaaaatcttc
cagatctagacacgaaggcgcgagtaaaataaaatcatataagtgcagaaaccaacaatcccttcgatatggtttttgactaactagtatttgatc
tcgtaacgaattctcttagctcgtacaattttattctatatcagtcttaaataaaaccctttctcattctacctaccttgtaggaataggttaaaacag
acacagacgcctctgcctcctcgcctacttccagagaaatcactgaattacgaggtagagcgatactggtatacagctttaacggttccttacaaa
ttttgaaaaattgcatttttgcatttcatcttccaaaaacatttaaaaatttcgaagttttcatcctttagagtgtatcctgcaagcttctgcaaacct
cgcagttcacctctttctaacaagtttctatttatgttacagagtaacacgttactttgaaaacgccgtgaaaatttcaaagtcccatttagaaaag
actgtgtatggcaagataaaatacagcttagtatattcataactcgtctatgtctttttgaagccaacatgaagacaatttcgctctttgaccgtcca
ttagaggtaagttactttgtcaatgcttcacattttcacctccataccaagtacgaaagcttgtcatttttcttttatttttcattactttaactgtgattc
tcttaatttgtttgttcgtgaacgatgtataaaaacaatctttcgattacttgtcgcctgaataaaaaataaaaaaacacccatttcctactgaactt
accatttttaacactaacacctgttagatccgggatacagaaacaaaaggtcgtggtgtattcgctttagaacatttgcctgctcatacttgtatcg
agatcagcccagttttactgtttcaaaaagagcaatatgaaaaccatggtcaatacactgttttaaatgagtatacctatgtttggtcagcaggca
ggcaaggattagccctcggtttaggtagcatgtttaatcacgatcggtgtcctaatgtttattggaaaaaggacgaaccgaaccgttgtatatctta
ttttactttgcgacaggtacatcctaatgaagagttatgtatatcatacggtgatcagttatggtttgacgacggctcagcttctagtcataatcact
atgatcaacctgaagaggaaaacagtttccttcaaaacctgcttctttgattaaaaaaaataaaaacccacaaatccctttctatccttttagtattt
gctcttttacttattcgcgttagactatgcggtataatatggttttaacacatgtaaacaacgtttatgacctttgttcatttctctctcttttattttttttt
ttatttgagatagatagctttattttataaaggatatttaaattttaatgtattgtgttaacttgaaagatcaaaagactcaactcatagaaatatatt
taatttctcagtcaaatcataaataaaacgaatggaatgtatcaagcactacgtaacgatataattcttccataatcctttgcaaaggtctatggcg
aacaaatcaataatagtgcctttcaaaacaagcaaattcagacggacgcataatgacattaataaaaacgaatgacaaggtggaactttactca
ccattgtaaagccaaacatataaaaacacaaatacaaaaacacaatatcaaaaaaaggaaaagaaacgagtcaaggataaagtctacaaag
acttagtgactggaagtcatgggatgttcaaagtaatacgaaggagtagcccgtgtttggctcgaaggaagaccattggaagatagaaaacctg
aaattgaatggtactctggatcatctcctggacgggacttgtttaaacgctcatagtcacgccaagcaaccatcggaacctggtccttctttaagtc
aaaatcgacttcttgggaataatccatgggatcaccatttggttccatggaggcttgactatagacatcataatcagctaccctcgaatgcattttt
gacgaatttggactttggtcttcagaagaagattcttcatcataatcatcttcgtcaccaacatcctcgttattggcttcagcagggtctgaaagttc
actagccggggatcggtcccaattgaaatcaattttaggtttttctaaatgctgaacttcctcgacggaacctttgcccccagatttttcttcggattc
gcttgcatctctctgttgcttgtccatagacatgtgattaaatgaatcagacaactgcgttatggataaagcaagggatgaattactggcatcgtttt
ctctgcgaaaagacgaatcagaaggagacgatgagacttcctgttcttgcgttttcggacgacgaaagcgaaagagcttacttagcttcttacgc
aaacgtcccaccaaactgacagcagaagcaacctcatcattggatacttcaacgcgaccgcttgcttcggggtttgtacccttaatccattcttcat
aatctggcatatcacgaattgtccacggatgacgcttcacttcagccaaagttatacgagtacttggatctttgctaagcaatctattaagcaaatc
atggccatcctctcctatctcaggggtagaagggatttgtaagggttcattaacgatcttatcaaacaactcatattccgtagacgcttgaaaagg
gcatcggccaaacaaaagacagaatagagtggcacctaaagcccatacatcaatagcttcagagattttcggcctgggacggtctaaatccgtc

caacacagctcaggagcaaaaaaggccggcgtacctacagtctttacgagttcaacatcattttcctcatttaaaccactacgcgctatatacgaa
acaccaaaatcggatattttaacgcacgtggaagagctcaaaagaaggtttgcaggctttatatctcgatggatgattccttgataatgaaggtat
tcaaggccacagactacatcgcggaaatattggcgagcctgctcgacagacaaaagaggagactggttctctgtccactgaatttcacctccatt
catgtattcgaggaccaagtacactttagtagaatttggatcgtcaataacctcgcgtaagcggacaacgttcggatgaatacatttttttaggata
gcaatttcgcgacgtactttctccttttggctcgaagaattggcacgaccgagtttaggacgacgctccgtcttggggattatttttatggctaataa
cgccttggtattgacttcgcgccccaacttgacttttccatgcatgccgcggccaatttcgctaacaatttcatagtcgttgatgaactttcggccgg
aaaatctatcaaatcgcttcttgattttctttgtttcttttacttcaccccactcctgtaaacgtttccattgacgctgagcttctcggaaattattggag
tataaaccctgattgtgtcggcctggttgaaacgtccaggaatgggtaagagagcttccttcaccaccatttgccccaggtgaaatgacatagtcg
ccagcactgtaattccgctttcgaaaagagtccgatcggttcgtatcggagactgttccgtttaaagttcctggtaggccaggagaccgcctttctc
tttttaactcattagtttccgggtgccatgtatgctttcggagaagattggtgttgttgagtttttgatcgttattcgacgatcccatgaataaatctcg
cttcgatcaaaaagttagattctttcagctcaaaattaaatcatgaactctgtaatttcaagtaaaacgacgcatgcactcgtatgaaacctttaag
tttcgactattcttcgctgttaatgttcggtaaaaagcagcccaactcaagcaaactttataaattcctttttctttttttattaaaaaacacaagtttcg
tgtatccagctaagttcaaaccctcaactttcagttcttttgagaaaatccgtagttggggaaggtcaagtttgtctattatgatcagctgttatgaa
gtagatgacgcgaagattgttaaaaaaggtttagcaaagaataaccaacgtagaagcttacgtacgtacgttaataataataacagataaataa
cgtagtacgtaatttggtaaacaaagcatttcctagttgaattccagtgttaaataccaagagaagaaagcacaagctgtatgaatttagacaag
aaacgaaatgaaacacaaatatcacttttttttttctaatttacgactaatgcaactatatacgtaaattaactctcaatttgtcagcaagttcatgca
agaaataaaaaaaaaaggaattctattaacaccaaaaagaaaagaataaacgtttgtacagaaggaatgaactcactgcgtcaaaaccagaca
aacaaaaaggtatctacgaaaatattaacgagtcaaaggtgccgaaaaattgagcattgaaatcatctttgagcttgttataaaagataaaaga
tagatatggatatcaacggttaaagggagtacacacggaaccaaaatcaggtatctataacttagtaacttttcacttaaagcctctttacaatgc
attcggcaataagctacgggcagcatttcggctttgttgaatcattagagcatccatgacagccaaggcagccattgtttcaacgatgggaatagc
acgaggaacaacacaagggtcatgacgacctttagcggccaaaacaccaccagaaccatcgtagcgagtggaaggctgctccttgccaatcgt
agcgggagacttaaaaccaactgtaaagtaaatattttctccattagtgataccaccttggacaccgccgctgttattcgaggaagtaccgagctt
gccagtaactggattacgaacaaaaggatcgttatgagcggatccaggaaccttacatccctcacggccagagccgatttcaaaagacttcgtgg
caggaatgctcatcattgcatgagccagcttggcttcaatcttgtcaaaacaaggctcaccaagtccactaggaacattacgaatgacacaagtg
acggtaccaccaatagaatcgtgactgtcacgagcacgagcaatacgttccgacattctgttagcagtctcagcgtgaggacaacgtacagttgt
tttatcgacttcctcccgagtaatttcgtttaaaaacttcaagtactcaggagtaataggggagtcatagtcatccgtctcatcatcgattgcagag
gagctagtctttgtttgatgaagaggaatggcgattttaccgacgcttgacacaaaagcaacgaattcgatgccaaaagcttctcgtaaatacttt
tcagctatggcaccgccggcaacacggccaattgtctcccgagcactagaacgaccaccaccagaggaggctttaacgccatacttttccaaata
agtaaaatcagcatgtgaaggacgtggatagttatccatatccgagtaatcatgaggcttctggtcctggtttaataccatcataccgataggact
tcctaatgtaacgccaaactcggttccactttggatatgaacacggtctttctcattacgaggtgtagtcagatttgattgaccaggacggcgacga
gtaagttggacttgtacatcatcttctgcaagattcatgcctggagggcatccttccacaatgcaaccaacacttctacagtgtgattcaccataag
ttgtaactttgaataatgtaccaaaggacgacatcttgagtttttgttattttctacaaataaaatgattcaacgatcagcaattaccgtttcccaatt
ccaagcaaaaattggcaaaagccaagatccttttggctccatcaaccaaacaccctgtttgattagtgtgatctagcattgtacgttatacgaact
cctcgctgcttgctatacactatgaatgctggtttaataagtgaatctcgtacaccaaagctagtaaaagtagaggcatagatatcgatgaattga
actggaaatatctggtaaacagaaaaatgaaataagaaggtaagacgagacttcaacttctatatagtcagacatatataccacttatttaagtt
tttatttttgttaaatatactaaaggagatccactcatagaaattgaaataacttagcattagtataaacagaaagtaacggccaaaaaagattt
aatataaatagtctagtaataagtggacttttgagagaatgaaacataaacttctcaaaaccaacagaaagcatttcaagaagaacgaatatgg
tcctacttaggtataaataatgttaaaacaaaaagaaacaatcaactccattattagcaaataatcaagcaaaaaaatgcgaaagacgcgactt
aaatatgaccaaaatgtatgtcgcctgtacaggaatgcataaacaactttcataaccttaacttatgttcattcaaattagtagtaatattagtcac
aacaaataaaaaactaagatgaatacgacaacccccctatttccacttttatctttgactaatacctaatgcaaagaccatgcaaacccagaatga
aagaaggtatcatgctgaaatagtagaattggataaaataagagttccgattccattaaatgcaaggcaattacgcatgcaaggaaaattaaat
tggaaggaaacgcttgtaaacgtgaacaaaatcgagatgaagaaactatgtcagatatagtacttgggtgcaatgagtcactcactctctcaaa
acttcgcattttctatagccgatccacaattgtacattatctagcaaccctgttccatacacatagagcaaagatcgctcatctgcatacatatattt
gactgatggcatgctaggcactgagtgtatgcgatttttttaataaaggctcgtcatagaaattagtgctataagactgcgtggatattatagacca
cattgccgaccacggtagaacagagtgcgttgcttccgaaggatccaatatatcattacactcgaatttactttccgtatatccaacttctccagca
ttaaactcatcaataaatgaaaatttttgcgttctcggacattgaaatgctaatttagacaaacaattgaaggattcatgtgatttggtacaagaaa
tagaaactttccagttctcccgtttttttcaacactaaaggcggtaactggacaccggtaatctgactgactaagaatacaagagtatatggagctc
aaaacaactcgttcagtattagcagcaacacttccattccctttctttaactcgtcaaaagatagaatataaaagggctcccatgtatcactagtca

gtttgtgtctaccctgcccattctcagccgtatttggagtatgaatgccagggaagaaatcacccgaacttttcatcgcatacttttctcagataggg
cctgacgcattctgttggtactcaaaagtaatgcattattatgctgagcacggtaatggttgaagagactgacacaatcaccatgacatatactca
cgtctgaaactttggcaagagcacgtttttggcatggttttgaaataacgcccaagcgtactttattgattcctagttgcttctttttgctatggatatg
atttgtcatgctcccctgctcctgtataataaatagttctcaatagggtacttgcccaaacaggattgtattttgagcacttctcttttgagctcactac
ctaccttcaacgtcataataaaatattgatttggaaatgttcgtatcctcagcatctttcactacgtttgacgatataatcatttcgtctgatttataat
taaaccaatttaatcctaaaaaatacaacacaacaggaggacataaatatcgtctttcgcttccataagatttttggcacacagacggcatgcag
agattaaaactacagaggttcgttgtgttatagtacataccgcagtctttgttcaatgaaggtatagtaggaatatatgcgaggaggaattgaaga
tgaaatgactctatcggtagtattttgcggtaatgagttgtcagtcatgctttttctcttcttcagtgaaagaggctcattatgctgagaaccgtatat
attagaggccaaaggttttgaagggattggaggatctgtaacgttagcagcaaattggaagtgcttttgactttcactaccgtaagcgtttgagtcc
attaattgaaaactcagacatggatcatcttgatgttgatctgtataaggacgataaaagaaaagaccgcgtccgtaattttctaacttcaaagac
tcttttctgaaattattaggagcagtaacgtgtagatttagggactctgaggatggttgagtctttgccattaagatcgctttaagacccaaacgctg
ctgaaggtgatgctttctggcaccttccgacgcgagattgtgagatggtagttgactaccatcaagtaaaagatgaattcatgtttgtagtagaagt
ggtcgccaaagacatggaaggcgtcttcggtgaagattggttttgttcagtataaacagaggaggagcaagtaaaatttttggggagttgtcattt
caagaaattgcgggtcaaaactggagtagtaagaatatggatgtggactagggatagatgaaactggaggatctagaaatctataggtagacg
tatttggaaagttgtattctgcggaaggatctgagattagggtaagcgatcaatttttgaaggattcgacgtttcttctgagatggtccgttggaca
ggataagtagacaaattagatgaatgataaaagaaggatgatataaaggctggaaagggtgttttatagtggaataattatgagaaggattttc
agaataagtaagcgagtcgcaaactggttgagactgctgattgggatcggcccagaacttctatggagatgcatattggttagaaatatccccaa
agtttatctcgtttcgtgtgttcaccttttttcgtaattttaggatcattctcttgggtaccttaaaagtatcaatggtaataagaggaggaatcagaaa
cattggtttgagaatcaacagaaaatgtttggattgaagtaaaaggactgtttttctctgatgatgagtgtaaagagttgttagaccgagcccaca
agtaagcaagacgttctgaatagattttgtgcatcataagaagaggtatttaaagatggagagggtattttttttgaaagagtgattgttgaaacgc
ctaggtgtcatagattgtaaggacaccaatatgcaaagaagcaccctgtacggtattcttccttacacttttttcttttttggggttatcttttttttttaa
aaaaaaaaaagaaaactatgaaagcaaattctgaaattccctgcattcacaaggctctagttgttcctaactttagatcatttctttccctcgaaac
aaccacttaaaagtcgaagctggttaagcagtgaatggatgcgacatccaatgcccccccccccccaattgaaacacggatttaaattcatgttaa
agcacaaattctattcgtttgagatcataccattatctaacaggcaatttcgtaaacatggtaaaagtagacaaatgaaaaaaaaaagaaaaaag
aaaaaagaaaaagagaaaccagtcgacaaagcgggcatcggagttagcgatttttttttttcacgctccaatttaacaaacagaaagagtaagg
gaaaatagtatgcaaaaaaatttccccttttttcctttactaacatcaattatgtcaacgcgttcaggcgtatcactagtccatgctttacaatcccta
ataatatgatatagtaatcatcatacaagtgaaaaacaaaatttgacgaatcggcttttcgccagctcacaataaatcatttaggctgaagaagct
gaattgccgaaaaccccggtttgttaattttcacctttttttgcttttcaaactgaagcttctttcataggttattttcttgcaacgagtgtgaacagta
accatatactatagactcaagaatagatgtgaaattcgtctttctttttattgcaagaaatggttctaaacacctcgcttaaataagtatgtaaaaa
agaataaataaacaaatacaaatcaagaacaaggaataagaaagagaaagagataaaatgcctattcgttgtttcgcatcgtttcaaaagtcgt
aagtgaaaagggaattgattgaaaggttgcgttcatctaaaaggttcttgctttctaaatattcacaaacaacctccatcagtatagctttccgcct
aggactttttttggttcaattatttttctttaattttagatctcgtctcatatcgtctgctcataagaacgtacaaacataccaagaacgattcgttgta
cctgccaatccacaacaggagttagaatacatgcacgaaccaaaacctacagtgtagcactataggtttgtaagtacgtagtcaacctacgcagt
tagttgtcgctttcaaatgtccgctgtaaacttagatttttttattttctcgttcaactcataattgtcgttactgtagtcaataccgattccgtgaatcct
acaccgcgtagttcagcgagttctttaatttcactttcatgaacatgttacacagactcattaaacgaacgcagaaagtagcctccattcctacaac
gagtgtatataaggtattgtaagacatttcgctattcgaaacctgatgcctaatgcttgttgtaaacataaataaacaaacatttgtgtgtatgacc
gatcggtctttgaagcataggcaggcggcaaagaaaacaaagtcaacaaacatattcaatggaaaagtaggcgtgcatgcataacgcatatag
tccatagaaatacagggaaatgaaaagacaaatccttgttgagcgtttcagtacataaccaagaggctttgaagatttgtttattgtttaccttgtc
gattgcgttctataaaggggcagcatctttatagaaaaacttgcataaaacacatactatttattttgccttgcatttgtattttatcccccaaggtg
ctgaaaaagcgaattctctttgaacataattaacattcttagtatctttagtcgctatcacgcttagatacaactttgcatccttaacttcttgctttgt
atatttgataaagatcgaaaggccgttggattgaaacttctctgtgatttccctactccagttctctttggaatacttcgaaaagcaaactgtttcat
atttcctatcgcaacaataaactcaattgccctctaagctcgaaacaatgaagattacttccctaagttatactctggcattgctggggtcatggat
gctttccactgttttcgcaaaccccacgacatgcggagcaccagaatacattattcaaaaccaaagcgatcttgatgttttaagtaactgtcaagtc
atcaatggttctatcatcatgaattcaagttcagccgtctcgctgtcgttgaatcggatcgaaactgttaccggtgacgttattatccagaataacta
ttatttggcttcggtttctctaaactcattgaaatctgttcacggggaacttcgcctggaaaagctcactagactcggtagtttgtatgctcctgttttg
gaaactgttggctcattgaacttgcgtgttttaccaaatcttcagggaattcgattcgacaagggtgttaagcacgcaacccgtttgcgcattgagg
acacacaattatctactcttgatggtatatctctgaacaagaccgacaccatgattgttgtcaacaacaactatctccgagaggtgaacatgcctt
acttggaagtcgtaaaacataagttttatgtttcctacaatgccagggaaatcgatgttaaattacccatgcttcagcaagtaggagacatgactg

ttcagagagtcgctaacattgatcttcgaaatctaaaaactgtcgaaggattccttggatttttgaacagtactatgaaagaagtctcttgtcccaa
tatgactcgtattggtcaatctttgttctttgttggcaacacgaatttggattcaatcaactttaagcagttggaaacaattggtggtacctttatgat
tttcaataaccaagaactgaaaaaaatcaagggatttgagaagttgcgaacagtgtctggatctattgatttcagcggtgacttggctgaagtcg
atttgcctagtcttagagatgtcaagggaggtttgaatttgcaatcctcctctgatcaattctcttgcccattccgtcaaagcgatggagtcatcaaa
ggcaaatcatttgtctgtcgtggaaacgtagagaatccaacgaatgaggaagctactcttgatgacgatttttggaggacgaaattgaaggaag
ccgcaaggaaccgaaaaataactccaagagctcaaatgactcgaagggaaagtcaggtaaacaagaaagtgctgctaacaagagtaaggttt
cctcctctattatgattgcttctttcttagtctttatggcattttactttgaatatcttctctaaattgcctcagtcgtgaaagaagtatgaaacgaatct
aaaggttgtacaaaatctcaagaaatatgaaccttgcttctatcgtttcttggtgagaatcaacttttatattgaagacaaactgggactaaaataa
gaaaaaaagaataggaagcgtcatccatccggactgcataaagcattatttaatttcctgcatagtatgagtcttatatccaaccaagaatattag
agctcaaacacgaactttacgattatcaattatgaaagcaattattatgagaaaatgaacagtagtaatttaaaataaatcacctgaagtataat
attaatctcttttcctgataacatccatattttagtagactgtaggtatccggagtggttacagccaaagcttggaggagaatgagggtttttttagta
aatgtactcttgctcggttcataaacacatttttagagatgaaatgtaataatgattgcaagaaacaaattcattaatagatgaatatcagagtatt
tcttaaaagttagcaagagaatataggcttttttaacacgctttagttaaccaagtcccgtttagctcaatcggcttagagcgtccggcttttaaccg
gaaggttgcgagttcgagtctcgcagtgggagttcgtttttgcccaagacgagaagttgtatttttttgaaaactagaagttacagatataaaaagt
gctaattttaaatagaaaaagtcttcaaaaagctttctacagatataaccgaagtttgtttgggttttcagattttgataccgtcttctaaatccaaa
ggtatacatgaaagcaaacaaacatctctaatttatgttcttcttcttcctagagaaaaaaaaaaagaaaaagaaattaggctcgaagagttccttc
cttagctatgaagtatttccgagccccatgttcgtgcatacattgccatgcttttcataattgtaaattgaatataataaattgaaaagtgtacaaaa
ataaaacaacaaaaagacaaagtttatccatccaaggcctaatgaattcaaataccatttcggctttatgaaatctattataaataaagatgttaa
atttaacaaacgtaaacaaaatctatacggaagaaatctcagagaaaatgggagtagtttttataataaggactcttcttactacagaagtaaag
gtaccaaaaataataatatacgaaaatggaattcacggatggtttttaaataccttgcttttgctaaatattcaaaaaccgttgccttaatataaac
ctgatgctgatcataatgctttggcattcatagattggaaaacaagataccgatgcaattactcatcttcgacacttcaataggaatcaaatggta
aagctcagtgtagaactaacacaagaattggcgtatgaaagaagcgaataaaggatataaatactccatatgagtattattagcgaggatagta
gaagtacgccaactgaagatcaccaaaggaagagatatataatacaacatcgcataattaaatggaaacattaattatgagtgtattccttggta
aagatccttggctgtaaaataccaaagcgatagaataggaacaacacgcttgtcttctcaccttcttgcttctttttctaaaaaaaaaaaaatttcat
cgaaagccttcaaggaaagaaaacaaatggcatgtcaacagaattacaaagctttagtaaaaccattattaataaagcggcaaaaggaaaaa
gttaagatacaaaaaaagagaaggagagaagagaaatgacaataaattaaaacaaatgacgaggtgagttgggataattaataacgtccacg
tatttataaaagatcaaactgaaaagcatatagaacaacaaaaaaactgaacggtatcaaagtgagattaccctgaagaatgacgaggtgtaa
aattaggtttatattttaatgcaagaaatagcggtcataaatgacagattcatcatcagaatcactatcgtaggtatcaccactaccatattcgtga
cttgcaatgtaatcgtattcaagatgtatatttcgggcatcagctacggtattagggccacgtaattcagcgtatgaagcaggagcacaaggacct
atgccagattcgacacgatggattatccagtcataccattcatccaaagtacgacctcgcaagagagctacaatgcaaatagtcatattgtcgca
gccaattccacaggactcactattgctggcaatacatctatccattaaattttcaccgattgtttccaaagattgtttggcaacaattcctctgcgaa
caaattcaacaacttcctgagaagttttgcagtcccatattccatcacatgctaagaccaaaaattcatcttccgggccgatagtatgaacaacga
cgtcaggaaaggctgtgacgatttgcttttctggaggaaggagactattctttttgtattcgaaatcaccgatagctcgagaaagtgctaaactgcc
attgacccgaccaaaatcaataaatccacctgctgcctttattcgtgccttttcgacatctatatttggtttatggtcaaaagaaagaggttctgaga
ttcctcgccttgagagaacggttcgagaatctcctgcattggcacaatatactacttgatcattgattatcatgactgtcgtggctgtgcagccaga
ggggtcttcttgcatatcgtaatccctcattaatgcctgatcagtttccaaaaatcccgactttaacgcttctccatagtcccctttccagaaggagt
gttgcgattttataatttctggcaagttttgcccgcaatatttcgcaacccgatctcctccatggccatcaaaaacgccaaaaaaactaatctgagg
atttcctggatggtgtaacatatttaaaaccgcactgtgtgcatcctccatcgaaatccgccagccttgcatgtgtgatactccatattgtacaaaat
tatctccgccagaactagaatgtttttctacgactggttcggagagagtttgtcccatttgaaaatatagatgaggattccttagagcgttcaactac
aaatgctaatactttgcttaggcaaagtttttccagtctggattgtatagtttcagtaaaataacgatatatactgtttttttcttataatagctcacgg
aagagcaaagtagctctcgctatctctaagcatttcctatataacgatatcgcgttaaccgtatttttttataactccagtcagagggagtctgtcact
tgtcaccataagcgtcttgttaaaattatatatatattacaaacgcctatcataagagcgtatttctgataagtaacatatgccataagactaaagg
ataatatattacaaatagtaaacaaagcattagaaaatacgatttaaacaaattggacagctttcatagtggaagttctttagacaaagaaactt
attcctacaaaaggatcacattagctgttgtagcgaagatgtataaacctttcagcttacagtcgaaattggcagattggaggatttgactggtttg
gttaaatattcagttggaaatatgctctcaaatgaataaacttgttcacatccttagctggcctatccagaggacattaagtaaatgtatgtaaata
aaaagatctaaaactaaaagtcaaatttgtaccaagtatttactgagctatatcactcaattctgttcatactccacagtattattaccgtgataac
accgaaggtctgagaaaagtagcggttctcaattgtacagtaatagtaagaaaaaatgtattggtttttaagcttaacggcttgaaaggaagtgc
ttttcatcgctgtctttcgagaataaatctttttttgttttactgactgttactgttacctataatagcttgggtctgatggtataaaatgctggggtaaat

tcgcaaagttcatttaactaacaggctaattgaaatgttttaaaataggtctttctggaacacatctcaagtggacggctaactgtctctgcacagc
acacaaacattagtgaatattttcttcattggatttcttctgtgaagttattacactcatcatcgcatatcctgtttttttgttcttcatatagtgaagata
gccagcatgttccagtaatttgtgtcatgcaaacaggaatcaccacatccatatgaaaaatcttttagtttttttacgaaatgaagcaaagcacaa
taacagtatgagcaaactttaaagtatcgaaaaaaatgtagtacagaacaggtcgtactgggagtcgaacccagatctcgagaatcaaaatctc
atgtgctagccgctacaccatacaaccattttactaccacttggttagatgtgtgtttattttgaacttgtaagttactgatagtatcactggttagttt
tgtatatcaacaatttttgtttgtatatgtatagactgaaagtaaagagttgatacttcaaaaacagaagtagttttgttcggcagctaacgctactt
atataaataaggcataaaatacgatcgaccattcaatgagaagtagcaaatgaaacaatacctttcatttaagatcttctatttatttaaaaataa
attgcctttgatcaagaaatacaacgttgagcgatcctcaaataaataaacagatacaaccagaaaagcggcctagcgacaaatagctactcga
tttgtcctaggacacttttggaattacttgtgacgtctcctatcctctagtttttaacttctactcaattcgttattggaacatagacttgaaagcttgg
ctttggaaacaatggggattgagtctgttcttactccctcctatctcagtttctttgctagacattgattatttaacatgtaggtgaataataatagca
gatgagagagagtcgctctgttgttgcattttggaatttaaacaaaagtcagaagcaacagccctggaaggagaagactgtattgaggtcttgttt
agcaattccttcataagaggcgaagctagggagtttataaagaatgctgtttggaatgcttctgaaagcatatatttagtcgaaaaggaccaaaa
ggaccaaaactctacctcaatttacacctgttcaggatgtatgtttgtttacacatcaagtaaacaaacatgaaacgagtgtagaagaccatccac
aggagaataaaagattaatctgatatctttnnnnnnnnnnnnnnnnnnnnnnnnnnnnnnnnnnnnnnnnnnnnnnnnnnnnnnnnnn
nnnnnnnnnnnnnnnnnnnnnnnnnnnnnnnnnnnnnnnnnnnnnnnnnngatttgtcctaggacacttttggaattacttgtgacgtctc
ctatcctctagtttttaacttctactcaattcgttattggaacatagacttgaaagcttggctttggaaacaatggggattgagtctgttcttactccct
cctatctcagtttctttgctagacattgattatttaacatgtaggtgaataataatagcagatgagagagagtcgctctgttgttgcattttggaattt
aaacaaaagtcagaagcaacagccctggaaggagaagactgtattgaggtcttgtttagcaattccttcataagaggcgaagctagggagttta
taaagaatgctgtttggaatgcttctgaaagcatatatttagtcgaaaaggaccaaaaggaccaaaactctacctcaatttacacctgttcaggat
gtatgtttgtttacacatcaagtaaacaaacatgaaacgagtgtagaagaccatccacaggagaataaaaaagattaatctgatatcttttaaat
ggtaaaaataaaacaaaaagaagaggaaagnnnnnnnnnnnnnnnnnnnnnnnnnnnnnnnnnnnnnnnnnnnnnnnnnnnnnn
nnnnnnnnnnnnnnnnnnnnnnnnnnnnnnnnnnnnnnnnnnnnnnnactcgatttttcctaggacacttttggaattacttgtg
acgtctcctatcctctagtttttaacttctactcaattcgttattggaacatagacttgaaagcttggctttggaaacaatggggattgagtctgttctt
actccctcctatctcagtttctttgctagacattgattatttaacatgtaggtgaataataatagcagatgagagagagtcgctctgttgttgcatttt
ggaatttaaacaaaagtcagaagcaacagccctggaaggagaagactgtattgaggtcttgtttagcaattccttcataagaggcgaagctagg
gagtttataaagaatgctgtttggaatgcttctgaaagcatatatttagtcgaaaaggaccaaaaggaccaaaactctacctcaatttacacctgt
tcaggatgtatgtttgtttacacatcaagtaaacaaacatgaaacgagtgtagaagaccatccacaggagaataaaaagattaatctgatatctt
ttaaatggtaaaaataaaacaaaaagagaagcctacagcaccccggattcccatgttgtctccaaccatagtactaacgaggccctcagacgctt
aactgcagtgatcggacgggaactggtgttttcgcctaggtatggccgtagacattcgacattttcaaataaaacattatatagagagtaaatata
tattttgactcttgaaaaatcaacttagttatacaatttttttccacacttcttaaatccttaaaaatacccttttattataattatttttcttcctcgttcg
tttagcgataagcaacaacgtactttaaattaaaccatgcaattcactatatgcgacaggttaagtaaagttgggatgccaactttgactttttcaa
cgcataaacaaaggatgaactctcttcaaaaaggatcttatcctattcttcctgggtctagtttttttaaataattcaaatggattagtttccgttaaat
gtaggtttgcaggtcactagagaaaagacacatatttgttgttgtcgatgaccaggaggttaagttggcgctctatttttattttaaacaagttttcg
atttctaattgtttgttcttagataattttaagccggaaagcgtggatcgtagccgaaaaggcgacatgaaaaaaattcaggatacttatcaactg
aatgtaccatctacccttgtaggttgcaaacccaatacatacatatattgcaaagtgttggagattttctaactgatttttggtttgtaggaggatgg
acgccctcatgtgtttgaaggatcgggtcaacatgcacgaaatgtcgattgcgttttagtatataatcctaaaaacaaggtttgtcggtggtatttc
atttatgttctcactaatcccctcttgtgcgtagacctttacaattgagcagatggatgagattataagagttcatgcccttcgcaatgggggacga
gcggttgcgaattcagcaccccaacagcctccagaacccatgactcaaggaatggcttttccttcagtgtcgtcgtccgttccatcttctataaagg
aaaccccctaataccgctgggttgacgacaaataaaggacctaatcaaagttcacatccccaaacttcgagccgacggtctatacctgcatttgca
ttgtcagcaaaaaatgcagaaactcgccccaactcaacttcaactcgagattttgaaattattgaaatcgagtcaggtgcagaaggcgaagctga
tgatgtgttggagctcgatgactttgcaaaagaacttgagctcggtcttgatcaggagttagggagtaatcctgaggaaaaaaattcatctgcgac
tggaaaaccaatctcattacgcgacttgtcaatgcaagaaggcggacaaccctctgcatcccctcacgacgcattaagtagtgcctcagaagaag
attgagcttgaggatgacatgagcaagggtgatttggaatatcatccgcagcgaaagatggttgtacgaaggattcttttgaaaaaagaaataa
aaaattaacaaaacaaggtttgtttgttttacctttacgaaggagacgcattcctttggtaaaaagtatgtttggaacttgtaagtgaaattcaaat
tccattattggttttatacagtcaaaccttcttttttttgtcatgaatattattttattttaatgctgatgctattcttatggtttctatctagaataataaa
tgaaatgcaagctttatctcgtttcctatgaaaggttagtaaatcagatgaaaatatatatagaaacttctacaatcgttgtcttgctaagctcagta
aaaacaaattgacagcatcctggttgtctagaccatcaggctcattaaatcagataatttggcatcctagcgaagcaagcaaactttttttcctactg
tatattcatacctttctcaagaatagactctaaattcttagcagaaactaccaaagttggttgacagataccactgcagcttgtttgcaaaggacaa

gtttgtcgaacacaagaagcagtcttataagaaataatggtcgtgttacctttgttacctatgacaaggaacaaaaacaatagagataatttttc
aaaataacgaatacaagggaaaagacaagcccaaatgaagtttcctattcaagctaaccacttgaaagcgaggaatgtgttctaatagttgcaa
ttttctatttcctgggtatgtatgtataatgatagtgaattcataaatgctgaaaactttcgcaattccgttgcttgtttttaaagccagattcaccaa
aacgaataagaaaagcaaaacatctttacttacgactcttaggcataagaatctttcgtgcgtctcattacgaacgaccagtatggacagtatctg
tcattaaagtcagaaaatttttttattaatagagtatatgaatttatagtactgtatcgttgatttcataaagtatgggtccaaatcgaaaaaaatct
tgcgaattccaacatcggttgcaacccctgatgaactgaaagattattgaacgttaaatcggttgtaaaaatcttatgagtttttttcccaatcaactt
cgttttttttcactccgatttttttaaccccaaaggagttcggtaatacagttttcggataacttggtaccgccctccttagataattgatatctataaa
gaacgaacggttacgtacgtaattgagtccgaaggtagtgaaagtcggtatgacattgttctaaaaaaacccgtcgggtaactacggtgaaaca
agttgaatgaaagagtttaaacaaaatcttcagcgctttctggaaagaaggatggagcgttcgtggtgtcagggatgttaaatccgactttgaac
agtggacgaaaagaatgctgtaaacaaagttggaaggagaatcaattagcaacaccttaaccagaaacaaggcatctttctggaaaatattttc
aaagcttaatttaaaagttttaaattgctgttccctttcatttctgtctgtttttttctttcctccttcttatgatgctatgccgcctccgatgtatcgcatc
gctgaaacgggcactatctgaaagaaaaggttcctttactaaggaacgagatacgcagattttgaatgcacatttggatagtggtgagaaaagtt
atataaagagaacagacactcaaaaaaattcataaagtgaccacattactacttacacattaaagaagtcttctttttcgaaacgcctttgtaaac
aacattcttttttttttcaacacttaaactatatttttttgtaattatcatgacgataaatacctatgtcaaggaagcctcctatggtaagaccatggtc
agatttatgaagaaagatgtttgtccggaatcaaaagttcataacgtttacgaaatggatgttcagagtatgctgagtggcgagctcgacgagtc
gtacaccaaagccgataattctattgttattcctaccgacactcaaaagaataccatttatgtgtttgcaaaaaacaatgatgttagcgtacttgaa
gtgtttgccgctaagcttgcaaaacactttgttgatcgttacaagcatatccatggagctacagttgatgtcaccatcactccgtggtcccgattgac
cgttcgtggtaaacctcataatcattccttcattcgtaaccctagtgaagcccgcaagacccatgttgctttcactgaaggtaagggattcgatgtt
gtcagctccttgaaggacgtctcagtcctaaagagtactggtagtggcttcaacaagttccacaagtgtgaattcactaccttgccagaagtcgat
gaccgtatttttttctactaacatcgattgtaactactcctttaaccatttcgctactttggacgagcttgcttcttatgattttaatcacgcctacgaaa
cggtgaaagaaatcactcttgacacctttgcaatggatgatagtgcaagcgttcaagctactatgtataagatggctaaccaaattatcaataaac
tccttgttatcgccgaggtgtactatgctcttcccaataagcactactttgaaattgatctctctcctttcgatattgacaaccgaggccctaattgct
ctttgtatcaacctcaagcctatccttctggctatatcacttgcaccgttgctagagattaatgttctttctgcaaagactttcattattatcatcacct
aatttcatgatcccttctagtcaccagctttctattgcatctattcactcctttttgtttactgtaaatggcttacagggtgttggtatcttgttccaattg
atgtaaatagaggtgaatgaacaataactatttcgcaaagaggaacaaacgacctacttgacatcaccttttttcaatttcatgttagcgaaattat
aattcattcgcataatcaagaaattctctctaaagtactgtcttactgatttgaacaattaatgtttcagcaaattttatcacttttaggaaattgatg
cttcctcctagataggacggcttcaagttcatttacaagctggttggaaagcgttcgctataagaggccaaagctaatgaatttcaatatgtcaag
aaagcaaagttcatcaacgatgtaatttggtatataacgacttataagcagatacttttaagacttttaacgcaaatcgaaaacgtataatgaatt
gaagcatgttctttcttcattcacttttattatttttactattcttacttttactatttatttattctctctacttatgagccccaggtagtcatctatcatgt
tcagagaaagatataccatgaaattaatagatgggtttattttggacatataagtatgtgaactcagctccattattgaaaatgaatatctcgttct
gatagtaaaaactcttcaaagtctgttactttaaactcatatcgagaaaatacgtgatgaaatctttaactgtaaataaaccattgacaaaattag
cggcgatattggacatccctaggtataaattccaaaatttcgactatgtcttccaaaagttcttatgtgaacttatcgcatattccaaggctttgaca
ttctcaacaatttacattgacttgctaacaacgaggataggaatagacacgtgaggactttttaaagtccaagcaatagcgagctgagcaggagt
cgcactgcttctctctgcaatcttactaacttggcgaatctgctcgatttgcttcttcccttcaggagattcaagattagcagcatctctgaataagtt
gtgtcaagacgagggtgtttaggaattccattgttgtactttgcagtaaaaataccaaacttcaatagactccaaatagtagcaccatagccatag
atctgttgaagaggaagtagattttctcaaggttatcccgagttacattattgtatgaaggctgatcagcaataggggcaatcaagttgtacttggt
agcaatgtggtgagcatgtttgatttcaaaaaaaataaacccactcagacgtacccgagtagaaagcttaccctcatggctggtttgagtaaata
gttgtccttaacgccaaatttgcaactatgatctatctgatactatttcagcttgattgattatattcctttagggaacaaacagactactttatgaat
tgaaggtaaccgtatgtcttcaaacgttcgaaaccgctttgttgttgtcgtttttgatggatcttattagaagaattttctgaaacagttacctctatta
gtgttagttatttatagtaagtaagcataattaatgtacaatgattccatttatgaatgactttcctacttttagcacttacgtagcgaatcattgata
gacacttctgcatgagaagagctataaacaagtgaaacaaaaaaatctctattctctatcgtctattttttttattctaaacagccaatttggacaac
gcatgagatacatcgtctttaccatacaactcaaactctaattcaacttcctcattcttggatccaacagcctttaagtgtccttcatgcacgggaat
accaacaaacttctcaagttcagcatgcgacttcttctcccatgtgggtgtatccttgggttcaaaagggaagaatgaatcgcattggtattcatgg
taaatgcgagtaaaaattatgttctttgtcaaacgatgataaagagcgctaccgtacaagaaagagcctccgatgacaaagaccttgccaatttg
gatgtcggtgttatcatacttttcagccaaagtatccaaagctgcatcaatggaatcggcagcatacaagtgttcaggaaccttatccttcttagtc
aaaccgaagttgtagttctcagtacgagtaattactacattaatgcgctccttcaagggacggttcttcttaggaatgctatcgtaacatgaacgac
ccattaacacaacgttcatgatcttggattttccttcggcaagctcaatgccattggtggatgtcgtcacatttgcgaaataacccatttcttccttaa
tgtgccaaggcatgttattcttcttgccaattcccattgaaggagaagaaacggccacaattaaagtcagatcacgaggatgcttagtaggctcac

cagaaataggaaccgagtacatgaagtcacggattccagtcttgtaaacagcttgctgaggaacaatgtgcataccaggatgcaacaaaatgtt
aggattctcacaacgctccatcaattgcttggaacgctcaagtggaacaagggtatcggcagtaccactgatgtgtaagttgggaatagtaagttt
aggctcataaaagtgattaaactcgggcttttcggagcggaaacctccaataaatacgacaaactttaagggaggatgttgtacgtaaggattag
gaggttgaccaggctgcaagagatgtgccaacatagcaccaataccagcgccttgagagaaaccaatcaaaccatcaaaaggacccttttcttt
catgtactcgttgataacctgtaaactcttatcccaggaatcataagtatttttgaagttttctacttcaaaccaaccatatttaccaccttggtcagc
acccatggaagcaagacgcttttgttcagcttcgtcattggggtcaacttcttccttgtattctacaggacctgtagggaaatgtaattcagcaaatt
tagataggtatttttgcacagttcccatttttttgagaacgtagggccagattgcacccaacctaatgtaaataattagcatttggtactcattttac
ctatatcgtgcattaaaagatgaaaagaatctttctacaacacaatattcaacccgcaaacataccatgcaaacacaatactttcatggatctggg
catactaattgtatcacaaatcgattaaaaaaaaaaatacacaaaaatcaatcaaagtccaaaaatctaagaaacagaataaataatacaaaa
ccaaatgcaaaatattcaatgaaagaatgaaatcttacaatgcctggtttcagagtatatggttcttggcaagttgaattctcccataagcgaagt
taaaatttctatgagtaacgctcggaatgtaaattttcttgtaccttatggtaaaagcatatatgcttaaaacaactaaaagagtatgatacaagct
tttgtttacacaatatactatttgcaattctttcattttgtgctgcataaactaatattccagaataacagctttatcaaaatgtaattctctcgtaact
tttagaaatttttatttcagcaaaagatttgacaccttaaacgaaaaaggctttgatgagaaagggaaaggattctcaattacagcagaatctcta
ggtcactctagctaaggttttgataagttataaagactttcttcaaacaaaactcgaaccaagacactgagaggtggttcatttctcttggaaaatg
ctactaagtgacgaatcaggacaattatttccttagatgatctatttttgtcaagccaaaagcatgcttgtaataataactaaattaatcgtaaaaa
ctgacaagagattcctaagttcctccacatagtaaaaagtaaatactgacaccataagctctgcaaatcgttgtatttcagggcgttttgaatccct
gtaatcaatatatttatttgattatattcatattatcaaaataatacttgtttctccatggattttacttctactattgtttatggattcattttcaaagtt
tgaaattcatgttttgatgataatgaaatgtaacgatgtatgaatatggcaaagaacgtggagtgttcggtatcggtaagcttgtttaatgatacac
acaatctctgtatctgcaattttgcaccaaacacttgacgaagtgaatagttgggttgactttttccaaacacctaaaaacaaaaaacgagatgga
aaaacaagtacaagaccttacgttagaagaaaaaaagcaagtcgctaaggatgtaatccttgggtaagctggtggcatgaaaacaaagctgag
cctgtgctaatgtattatttcaatagagaagacggccagccattgtccaagaaggctttgaagaagctagagaaggagcgtgaaaaggagcaaa
agagaatagaaagagaagctcgtgaaactgaagaaaaggctaagcgtgaagcttctgaagtcgattattctgaaggacgctatggtgttttacc
attaaaccgttctacctctcgtgaaaaccgtacatacacggatatcaaagatatttctgctgcaaaggatggtcaaaaagtcttggttcgtgctag
agtatacacttctcgtcttcaaggcaacaagatgtgtttcttttctttgcgtcaacagtacaacactattcaagctttggttgtcgtcgataaagaca
ccattagcaaacccatggtcaagtggtgtggcagcatcggattagagtccatcgttatggttgaaggtactgtccaaaagagccccgaagttatc
aagagtgccactgttcaagatgcagaaattcatgtttccaatgtttatgtcgtttctcctgtcaaaaagcaccttcctttccttgtcgaagatgccgat
cgctcagaagaacaaattcgggaatctgaagaaaatgcaaaagaaggtgattccaagttcgttcgagttttgctcgacactcgtctcgataaccg
tgtcttggatcttcgtactcctactaaccaagctatctttgacattcaatccggtatctgcgacgcttttcgtgagcatttattggctaattccttcaat
gaaattcacacacctaaaatgactggtgcctctagtgaaggtggtgcaaatgtgttcaaaatctcttacttcaaaggtgcaggatacctttctcaat
ctcctcaactttacaagcaaatgctcattgccgctgaccgtgagcgtgttttcgaggttggacctgtattccgtgctgaagaatccaacacttaccgt
cacatgactgaatttactggtcttgatcttgagatggctttttatgagcattatcacgaagttgttgaatttttggaaaatcttttcttgcatattttca
agacacttaaagagaagtacagctctcaaattgctcttattcgcaacatgtattcttcagaagactttgtccttcctgttggttccgaacgtgtccgc
tttcatttcaaggaagctgttcaactccttcgtgaagctggctatcgtaagcagctcacccccggccaagagcctcctaaggatgaagaattccgtt
actgtgaggatcctgaatttgaagattttagcacccccgaggaacgtgttctaggagctattgtccgtgaaaagtataacactgatttctatgtcat
tgacaaatacccaagcagtgctcgtcccttttataccatgcctgatcctgaagaccctcgttattctaatagttatgactttttcatgaagggtcaag
aaatcatgtctggtgctcagcgtatccatgatcctgaattgttgactgaaagaatgaaatcgcttggtattgctccggatgctggtcttcaagcttat
attgattctttcgctttgggatgccctcctcatgcaggtggtggtatcggtttggaacgagttttgatgttcttccttaatctccctaacattcgtttggc
aagtgctttccctcgtgatcccaagcgtctccttccttaaaagttcatttcgtttttcattaagttagtatagtcatttgacaaatggaattggattaaa
gctaaatgtacgctccagtaattgcttatgacaatcatatatataatttctagtactaaaactttttttataacttgcaaaaagattgtcataataaag
gcattcaatttgttagatcgtaaagtaaattaacctcataaagaacaagctattaaactaatgctaattcgtccgattgactgcgaagaaggacct
tgataaaattagtctgctctttaattttttttatctcttctgactgtcccaattctgaatcagaagtagcatgattaaataaatggctgaatttttcctct
aaagagcgtatagcatctgtaacgccattaagagtcgacgaaatattagtcatatggaccgcacgattagtttcgttagacctaacagattcgccc
tcgtcatcattgccttccattgattgagaggtttcaacatatttttgataattgctttcgaattcggcaaaacaacgttttactagagcaaatgaatcg
acaaaactacaggtttgtttgaatagctcatgtaattgagaagtatcaccacgtaaaattgcaagtgcctcggggttagcatctttcgattcctcca
atgctgcaatcgtggcttgtagtttcagctttaccacatctagctcagatgctgtattagccaattgcatgcgcaaatttaccagctgcttagcacca
ttgtttctttgttccagagcgttcattagctgaacctgcatattaccattctcctctttcaggcttatagtatggcaatctaatgcattgatttttttcgga
agcttcacgtagttctaatctcaagccatcttcaatatatttactggcattgatcagcttttcccgtttttctagtttagcgttgttgatccttaattgttg
tcgtagaccctccaactgcgagttacggttgtaagaatcattttccaaaagattgtctaaagccgcggtcaaaccgttgtttgtcatagtggaattc

cgctcatacgcagccctatcaacatccaacttagagtatatgttagaattcgaatcaatgatagcttgtttcttggtaagctccgattttagaagtgc
tacttccgcaactaattgagaattctctgtctgcacattgcttaatgactcatgcaaagaaatatttaacatatcggtagcttcaagtctttgtacca
aatctttgacagtgttctcaaacccggtggatcgttctttaaaatattctttttcagatgcaaaattattaacaagcattgtctggtctaccaactgat
cttccagacttctaaccaaagactcacgatcacgataaaccgactgaagttctaacagttgtaaagaagcagcatggtgcttcttttttttcttccga
aatctctttctccaaatcactcattttcttgataagggttttcttagatgaaccaagttcatccatttgctgacttagttctgtagaagtttcccgcatt
acttccagcgtcttatgcaagcttacattctcttgcatcaaatgaacgttactcatactgttcttctcagtgtataaagatttgctagcttcgtcctgct
tgttaacattaggattaactctagacaaagcagcttcaaagagggctgcattcttctcagcatttgaaactttcagtttctcgtttaaggtctcaatc
tttacagtagattcgtttaaaatctcatagagtttctgtttgaaccaagtaacgatttgttcttttgttaaagcagaatattgttccacaagaattcca
agatcgttgttgtcttgcgatcgctttattccttgttcaacactaaaattcgacatcacatcatctgtactcacctcctttggactgattcgttccgata
tctgacggtcggatgtttctgaattcaaggaaattgacctaatcgatttttctggtaaatcgttacccatttcgccataatgactgcctttatgattaa
tagtcgcatctaaggaatgtagagaaggagttattgacgatggtgattctacaggcatctcaccttctaatttcgacttattagttgaatgttgatta
gaaactgacacatcatcttcacttgctttactctcaaaagatttaggatccacagcagacgatgaattcaatttcgccggaccagaaaaagcatct
ggaggactctgatcatttttttaatgaaggcgaacgcctgataggagccttgagattcacagaagtattattattggtagagtggactgcatgtgga
gcagtacgagtaacttttgtatttgttaaagacggtattcgagagggtggactattgttgccagttaatccattaaagaatgatgcatttctctggcg
actcgagttaggaagcagtttatttaaacgaaaccgagtaacgtctagcatatatacgttggaatctgtaatatctttcatttgtttgccgccgatta
gaactaaagttaggccggtggacgttaaggagtgagaggagcgaggagatggtgcaaatggtaaatcgagtaacttataccattgtttagttaa
aattttaaaagcatatagatcattcaaaaaggaaccatcagatgctcttcctccaaatacatacacgacatcatctataattgaagctgcatgtcc
tgctcgcgggggaggagaatgaccgaatgtttctacttttgaccaagcactctgttttggatgaaaacaccaaacatcattgaaacatgtttgacc
atcagtaccaccgaatatgtataacttatcagagtaagttaaagcgacatgaccagcacgagcaggagggggatcattaacaacggaggctagt
tcccatctggattcttgggtatttagtctgtttagatcaaaacaaacgagatcgttaaagtagtaatcgagtaaacggccaccgaaaagccaaac
cttggaccccaaagtgctaagcgtgtgaccatagcggccagatgggcgggctccagacgctgaggctttctgccaaagtaaggacgaggtgttc
aataaatagagggaattgtcttgatggtctatatcctctttattcgtgagaccaccaaatacgataaaagcatttccaacaagaactgaagcatga
ccaagccttgcagacggtacttggccagtggaacgcaagttcgagaactgacttgtagcaaggttcagaacccaaagatcatttttttgcttcaga
gttggaggaaatacctccaaatatatacatctcctgtcctccttcagcaaaaagatgagaagaatgggaataccgaggcaatacgtgtgaagaa
ccgcgtattgtcagttttgaccaaggagataccgtgaaactagagccgttcgaaggaagagctttcttcatttctgaagaagattggctactagttt
ctaaatcagcttcttgagtagaacgaggaggagaatccttcaaggatccagcgctcttataagcagtaggtctattcagcggatttgagccagctc
tatttttcttgaataaaaaagccatatttgttggtaaaattgactcataaagtagttttatttataagagcccgattccgctccgagttgaccccaag
tattgtacagaaaacacaaagcttaagaagcctcgttaaaaagaaggaaaccaaaaaaaaattaaataaacaaaaaaatgtaatatagatatt
aaagcttttgctgaaaagcagacaaaaacccgaaagaaaccagtaatcaaaactccacacgtttgtctttggcttgttgaactggttgtataaga
agtaacaccccaggtgacaaaatgtttacttagaaaaaggtctgtctagaacaagacagctagagtgtttacattgtttgaatatgcataaaagc
gaagtaaaaaaaacaaaaatgacaagacattgggggtttgagtaaaatttagaaaccacagagagattcagttagaaaagacatggtttttccc
tttttttccaaaaattcctttacgcgtattcgtaaaaatcaattcatgtaaacggtttcataaacaagcgaatgtcaataacaaatcttataacagtt
cagtcatccaacttaatcacctagcatgttccgttgatactacaatggaaacaattgaatcagtgggcaacgcgcatgatgaagggtatcccgaa
cgttgcctgagatattagtattcttgcccaatttacagtttaatattgtttcacatattccaaaattaaagagacacaattctattttctcattactttc
atacttcttaaaattcttttaaggctgcatatatgtaggaagtgagtagagtaaacaatagccgaaatcaagaaagctacagatgttactttcgtt
accttacagttaccttgttacaaagtaccataaaaaccataccctaccctgacattcgtgactgttttttttcggtcgaaacatacgcttgagcaaga
acagctccatacctcaacgaaccaccaaaaatgactaagggtacatcttcgatgggtaaaagaggcaataagtctcataccctctgccgtcgttg
cggaaagcgttctttccataagcaaaagagcacttgcgcttcctgtggttatccttccgctaagacccgcagcttcaactggggtgctaaggccaa
gagaagaaagactaccggtactggtcgtatgcaacatatgaagaaggttcaccgtagctttaccaacggtttccgtaagtaacatttgagcgtag
aaattgaatggacaaacatattaactctcaattaggaactggtacccagcgtgctgctgttgtcagttctgcataggtgaatctaataaaaaatac
cttatgtctatgctatgtgttaagtaggaaattggtggtgacaatgtgtgaatactgagaaagatatgtaacaaaagagccagctaaaatgtggg
actcttatgaaagagtttttggctattgggtatttcattgtaagattaatgtttcatgtgtgttcgaaatgtatgtaggtaacgtggtgaaggaattgc
attacaagaaacaccagattttatgaattgtatttccaataaagtccatgagttgcgcagcacaaagtaggatgagaagtgtctgaagaaagtca
aggtaatctaattgtttcttaaaaatctttagaagttgagcttcataataatccataaattcgattcactttaatataatcataaaacccgactgcta
actggtgctggtgcttttacaacgccgtaccaccaaatcacatgtcgtatttgaatgggtaagaagagtaatcccaaggttttattattaacactta
atcagtttagagcaatgtgttgattccttacaaacaagtatagataccgtatgtgatctgcgtagttgctccatgaattcattattaacctgaaccta
gctttcatcaagcatcgtcgccctagactcaggaatccatgattttccaaggatcaaacaagttctaaaagtccaacgacattttcatttgatatct
gagcagcaacttcaagagcgtcgtcaaaaatttgatgaatccgttaagccttacttgttacaggcttttcaaaaattagaagattctataactgcat

tacaacgccaggaagatagtctaagaacaaaatacgagctgcaagaagctcgtcttgatatgctaaaaaaccgacctcccataagtgcgctccc
tttaaattcagacaccactgatcaattaaaaacaacaatagctaaacgccagaaattattatacactttagaaagatacgatttgcaactgcgtca
aaaacgaggatattaagttatcgcctttcatggattggaatttaaatattcaaaaagaagtatcatgggctaaggagcaaggtgctcgcgttcatc
ccctcttaaaattttccgtcatccccaatgccggaagttgcattacaaatgcaagtcggattgaagcaggtgaaactttgctttctttacctaataat
gttttaattaacaaaaccacctgttcttggtgtccatttgcaaaacatctaacttcctttcagcttctatcctggattttatgtaaaggaaagagtact
ggcatggaaaaatggccatattacatcgaagcactaccacaagatttttcatggcatccagtaatgttctctataaatgatcctgtatggtctgatg
tgccgaaagatgtacagaaattggttcttgaaaggaaacaatccttattgcaagattatcagagcatatcaaagtttgaaaagctagactgggca
gtatttcagtgggcttggctttgcgtaaatactagatgtttatattatcaaattgcatcgagttcttctgatgaacaattaactttagcacctgtatttg
aatatttcaatcattcttttgatgcacaaacggacctacagagaagccgtactggtatatctattatagccaagcgtgccatagagaaagatgagc
aactatacttgtgttatggagctcacggaaacgatcagttattttctgagtacggattttgcttgggtgagaatcccctcaatcattttgctctggatg
acgagtttactttcacggaaaagcaggaagagtttcttaagtgtcatgggtattggaaagactatacgttttccatggacggtccctcgtttcgtac
attgattgctttgcgggtcttgttagtatccaagtctactgatttgtttgactctaactatgatgctactagaaagctgttgcaatacatgaatggttt
gtcagacggcagccgagaatcccctatggtccaagcactgtgggatagacatttaacagttctcctgcaaatagttagaaaacaggttgctaaaa
tggacgaacttagaaacaaggattacacgcagaggtgtattctacagctttggaaagatcgtcagatgtgcatagagtatttattaaatcgtccta
tagaatagtttaactcgcatttcattataaaacaatgttaataacgttaattttcaagtaccctttatatttgattgcgaaacacttttaatcaacata
ttgagaaaggatataccggctgtcacgttagtgattctcacgggttatgagaataaataccgagtggaatgaataactacagttgtagaattgtat
acgttaactgtactctgtttaatgtccaggaatctggtcaactaaattatggtgtttcgatgatcatatttagccatatcagtcgaattttctaatttg
atgattctgactgtagacacaagggtatatatagctttgttaatcaaattgacaatagattttatgaatagataatcaactgtatacgtgtctttatt
ctccgaccattcgtatcttgatttctgtatcacgcttttaattgtttgtttacaaaatgtaatataccatcctttagtcttatggtgtttcctaatatttga
taatataaggatactatttcatattgtaatatatatagtattaacaaaatacatattatgacaaccaactgaaaaagaagactttatttttataatg
gacggagaggaggtaaaaaaggttttgcccacaccaggaatcgaacctgggtcgcatcggccacaacgatgagtcttaaccactggaccatgtg
agcttgttgtaaacttctggggtttggttattatagactcaaaaatcagtatataattaattttcattgtttattcgcaggatttaatcttttttttttttt
ttttttttttttttttttttttttttttttttattacatgcaatgcttcgtctttaatattttagtaaatagaaagtattgtgtataggtaatgctggaagaac
tatgtgctaataataaacagatatctccctagcttgatagttaacaaaaagcaaaccaaacccttttttaatggcgaaaaattatctctaaactaaa
tatgtgttagctgaaaactttcgatctactactctgctttacactctcaagatgtctgtttattcataattatttttttatacgcacttaagcttgagaag
attcatgaagccaattcagttttgattcaaattaaaccaacataattgatttctaatttatagttgctgttaattgtccgtttatagcatttatctgcgt
atttgattctataatgccgatgacattgtatgaatgtagcctcaatatattttaaacattgtttatatttgatatatactttttttcacttgcagagattg
catgaactttccgtttgtaacaatttttgttttttcgcaccattaattttgtaaacattgagaaactattaataatatggatgaaagttgcttaaaacg
ggcacgtttgaatttttttcaattatttattcacatatgtatttaaaatcaaataaataagctgcaagctcttcaattttcataatcgaacttgtatgtc
ttttttgaaagagccaaaagcaacaccaaacaggaagaaagaatattttatagatgagataaataaattgtttcataataacaaaaaacattca
cttaaaaaaggcgtataaataaaacaataattatctctagatcaacgaagctcacatggtccagtggttaagactcatcgttgtggccgatgcga
cccaggttcgattcctggtgtgggcaaaacctttttttattttcctctttagcttcagcatttttatgctaaaatcgaatcaaggtacaactcttaatttt
cttataaatttaccgtggtgatttttttattatcctatttagttccgttaaatcatgactattaaaaatatatacacacagggatgataaatgggatag
atataattcattaagaaatactaaacaacctaatgggttcagaattgtagacttacaaattggcaggttcgacgtaccgaagggtatttccactaa
aaactcctccctttaccttgatttcgtcttgaatttcagcaatagcttcttgtttgcccatgggatccttctcatcaggatccaactcccatgccaaata
gctattagtacaatttggttcaaagtcttgggggacgattccaagacgcttcgagtagttggtaactttgttccagtctcttggaacattggaaatgt
cgcgagagaaataggcaaagcaacgctcgaactgctcacggtgcagctcaagacccatatcaggcttgaagtgcgtaaattccttataagcctg
aacagggtcttcgagcatggaaagtgtagcagaacgaatagctcgcaagaaggaaacgattttatccggattctttgcaatgaagtcatcgtggg
caatatagaggatcgagcagaaacaacagcaacccaattcagcgagtcgatcaatgcggagcatttgaacatcagagcgaggacgtccttgag
aaatacaccatctctcgagctgtacttgttgcatacactcaatgccaataccgccatcaacttcaccactgatgatggcaggagcaatgttcatac
cgcaacgaacagctgtgtaatctgtgggagaaagtccaaatttgctacacaagtcgtctaattggatcttcccaaattcaccaacataaccaatac
gcttgcctttaatatccttgaaagcgtttatgccgtttccttttaaggtaattaaaccggtgaagggctcgttaagtaaagagccaaaacttgtcac
aggatatccacgagcttttgcagccagtgtatgaatcatggctttgaggcccatgtccactttaccagagccgattaaagcagtaacgtcggacgg
atcagtgggctctaagatagcaacttcgatgccctcttgtttaaaatagccattactttgggccaaaaaaatgggcaagtggtaaggagtagcttc
ccaattggtgaggaaagtgattttgttatttgacataatgaggaagagtaattaaaaagaagaagtaactttactgtatacgttcgcttggacctt
aattgaaaagcgatgaaacgaacgccttttataattttctagtgccattcgtttgtgtcaatacccgagtctaagtgaattacggcttgccgaagaa
taaaataaaggttatatgaacaaattgctaaatgtatacgtatcccaagacagcagttttcctttcgggaatctgagctaaagcagtatccaactg
aaaggggattgacatcagagtttaataacaatataagactggaatctacggaagataaagtatgacatgatatttcagtggaaatactgaacat

aaaggatgaaggggcaatgtaaacatgctttacttcacatagcaaggaaaaatatccctataaagacgtttggttcatttattcactaagaagttt
aatgaactgaaaggtttacatattttacataccaagcaacagactatgatcccagtggttcagataaaaagttgaatccatccgtcctttgaaaag
cgttacggagactttgcgccgaaacggataatcaaaaactaataatttacattcgcatggttttcccaatggaatggtaaaaaagatgtgtgtttc
cgagcggtaacagaaagccgatggatgacattttatgaaaaatgcgtcagagtcaaaaagtatgatgaattaccgttaagtatccgtctgggaa
catagctggctctgtcaattatccctctaagcgtttcctcaaaaggcaaagcattggctgttaagagcaactctgcataagactcatccggtacatc
aaacgtctcgtcttttacttcaatatctggtatttggttttcagaaatctctacttctagtccatataatccataatctgataatgattggcccacagtg
acatccagatagttggaattcgattagcagctggatggaatcaccgaaagtcgaatacctcttttccttttacagactattcccaaaatctcttctta
tgagagtccttcatctattcctaattcgaagttctatgcaaccatcattcacttcgtcatcttgggaacaagcaagtagcctcatttactgatgcttct
tcgactgaatctttatccattagcagcttttcttcaaaagcacctacaacagcatctagtgatgtccattgttactagcaacagcgtagactcgaat
gtgagttcatctggcaagtggtatttgaccgctttgtcgactttcaacgtacccgatggggtttgcttggtcgaatatcaatggcttttcacttttgat
atcatctggaattgcctataaacaacgcattatggatatgaaagcaacctccaaactaaacatcaagtttgcactaactatttactgagatatatc
attcaattctgttcatactccatagtattcttcccgtgacatttgaaaacaaatgtcttcaaacgaaagatcattccaaagaagtgaaagagagttc
agcttatattagaaataagggaatcggtttctggaaatcttattcaatatttttactatgatatatttggtccaagtctgtgtatcagcagctagagt
atggagatactgttgcacctgaaattcaacctcaagtccggcatgaaagacacagtaaaaataaccaccccgtccgtggtattctctaatgtata
atatgctgactgtttgatatttgattcctttaacttagtctatctttgtatgtgactggaaactgaaaaaaaaaataataataataaatcaatac
aaatagccgacttggaaaagagcaccttcaattcgtaagaattaacgtagatatggagtgtcaagtatcatttcatttctttcagtaccgttgagga
aatagtcgcatgtaaacaaacagtgatgtcatggtatcaaacgcgttgtcatgtttaccatgcacaaattttcgtgtttccagaaggtatgggtaga
gaataatattggatgcaggatgaaaatcgcactttattctattttaaaagcgcagtttacaaagattatttaattcagtatacgaatactcgaatga
atggtgtagcttaatgataataaaccttcacgcgaaagtctaacagaaacacttgaacaatacatttttggaaaccaaccataccagtgaacgta
taccttgcaactgaaagcttcgaatataatcaggtgaatagtagactttattcctggggaaacattcatgactttgacacgacgattgtttatttaca
tttggttgccacagaaaccgacgcgtaaacaaaccaagtcaaaaattatataataagcagtttctctacggtctcaaacttagcgactataaaaa
gactgttattctgtgtgcgtgtgttagaaaataatttaagctttggatccccaattacatttaccacgcgtataggatcttttgtttacttccattccta
aagtttgaatggacaaaggtcattcatagcacagaactttttgtatcttattttatatattattttttggccagttaattttttttttgttcgcgtttattc
aaaccctttttggttgttgcaattataattttctaatgcaatctgtatactctttgctcctactggccattataagccaagtcacttgggcgttgaattt
cagtaaccattctactgtcgttggttactggggctcgaacttgtctggtgaactgggctaccgtgaccaaaagccacttgcagactactgcaagtcc
tcgaactatgatgttgtaattttatcctccgttattgactttagtgttaacggctggcccgtctatgatttttcgaacttatgcagtgacagtgacaaa
tttagaggtactgaactgaaacagtgcccacaaattgaaagggacattcaagtttgccaacaggaaggcgttaaagtgttgctttccatcggcgg
ttataacggagattttactattgacgatgaagatgacggacagaaattcgctttccaaatgtggaatgtctttggcggtggtcaagagttatatcgc
cctttcggtaaagccgttgttgatggttttaatttagaggttaacaagggtaccaacactgcttattctgcgtttgcaaagcggttgctcgagatcta
tacttcggatcccgatcgaaaatactatttatcagcagcccctacttgcatggttcctgacaaaacgctaacgaatgccgttgctcataactcctttg
actttctttctatccacacattcaatgcttctactggtgttggttgtaacggaagccgcaatagtacttttgctgcttgggtcgaatatgctgaggaca
gtgcctatgacactgaaactgcacttctatatggcatcattgcccaaccaaatgctgcggttgatggctacatgaattcttccaacttgatctcgact
ttgcaagcgtacaaaagtaacagctctctttttggtggtgtagccgtctgggatactagcttagctgaaatgtcttacaacacatctacttcttatat
cgatattgtgcgaaatgttctaaagggtggctctgttgcaaattcctcaagattgtcagaattcgcccgcatttctcctgcttcttcctcttcaagctc
tacagccaagccttccacgtcggtttccgttagctatgacaaagtacctgaaatttctacaacaaccgattatgagacgttttatacgaccacaac
gattctttctaccattggtgatagcagctttattacaaacactgccgtatcaacaatgacaaagcccgcttctaccagcaaggataagtcaacgac
catttccagttctaccagtatcccctccagtacttcttcaactagcggtcccttgtcttctaaaagcagccctacatatatgaaccccactggttctgt
ttcctcagataactcaagtacctcggttgcaaaaactagcaacacatcgctctcttcttcagactctatttcaaagagtaaccaatcgtcgacgagc
atagttagtacaagctcaacctcgacatcctcttcaagtattaattcttcgtcaccacccatgcgtaactctacctcccaaggagttacttcagcatc
atatcaaagtgcccctggcagtccactggtcaattctacttctgcaacccagtcgacttcagtttctgctaccaatcactctatcacttctatgatggc
tacaaatagctcagttttggctcctacgacaacatctacgtcttcttcactaaatgcgaccgtgccatctttaagttcgttcgcacattctgaagctc
ataagggcgttgtaacagaatctttcgatgcctataatacctttacaaacaaggtaccttcaccatcatcgtcttcctacacttacttaagcaccttt
gcttcagacagcgatgctgcttttacttctcagtataccatgagtacatacaatactgatccaaaaattccattggatagtcccagctcttcaacatc
tattcccttagaactcgcttcttctgatatgtcttcgagctcaattactcctactagcacaatgccgtcttcaacttggggagctacgcctttatcatcc
aatctcaaccaagttccatccagtgtttttctgaaagtactactactcctattactgatagcacctttacatacggctcatctgatactgcgggcac
cccgccttcgacttcattctctagtacttggtcttcagaaactcccgtttctagttcatacaacccatccactaatagctggcctacagtgacttcttc
ctcgaattcctattttccatcatctagtacttggtcttcagaaactcccgtttctagttcatacaacccatccaccagtagctggcctacagtgacttc
ttcctcgaattcctatattccatcatctagtacttggtcnnnnnnnnnnnnnnnnnnnnnnnnnnnnnnnnnnnnnnnnnnnnnnnnnnnnn

nnnnnnnnnnnnnnnnnnnnnnnnnnnnnnnnnnnnnnnnnnnnnnnnnnnnnnnnnnnnnnnnnnnnnnnnnnnnnnnnnnnnnnn
nnnnnnnnnnnnnnnnnnnnnnnnnnnnnnnnnnnnnnnnnnnnnnnnnnnnnnnnnnnnnnnnnnnnnnnnnnnnnnnnnnnnnnn
nnnnnnnnnnnnnnnnnnnnnnnnnnnnnnnnnnnnnnnnnnnnnnnnnnnnnnnnnnnnnnnnnnnnnnnnnnnnnnnnnnnnnnn
nnnnnnnnnnnnnnnnnnnnnnnnnnnnnnnnnnnnnnnnnnnnnnnnnnnntcgaattcctatgttccatcctctagtact
tggtcttcagaaactcccgtttctagttcatataacccatccaccagtagctggcctacggtgacttcttcttcgaattcctatattccatcctctagta
cttggtcttcagaaactcctgttcctagttcatatactccatcattcacttcgtcatcttgggaacaagcaagtagctcatttactgatgcttcgtcgg
ctgaatctgtatccactagcagcttttcttcagaagcatctacaacagcatctagttcgatgtccattgttactagcaacagcgtagacccgaatgt
gaatacggctgacaagtggtatttgaccgctttatctactttcagcgtacctgatgggtttgcttggtcgaatatcaatggattttcagttttgatgcc
atctggaaatgcctataaaaaacgcaatatggatatcaaagctacttccagtccgctgccgtccgacacatggaacacctttgtcactaaaacatc
cgtcacaatttaattcatatatagaatattttactgttcccgtgcatgtcaatagatccttttacttgtcatttatttcgttagttactttagggatattta
ataatttcaatactataagaactgttttacttaataataggttttctttatttgtttgaaaatttaccctcaaaaaaatacattttattaatcgtcctta
aaaattaaaattaattcgtccaaacgagaagcctgcaataacagcttttcctcatatgagcgtaactttttcaactcgacttgttcgaacttttgcag
gtactcttcttctttccctttaatatcgatacgctcagccttgtctattagttgcgacctttgagcccgttcgtcacgaagtcgatgaaacattcttgca
attacttcatacaagtgatccaaaaagatagaataagcccgttctgaccggtgaaaccagagataaaaagtctttgaaggagcacgatccgagc
tccttgggacttcttgtaattctaatgctcccatttcagccattgattgaaggactgttcgtagatctttttgccgtagtaaggctatatttgctagttg
cttttcctcaatttttcctttatcctttataattcgtaatagtctagttgctctgtcacccagtttttcatctaaaatattttcaaattctacagaacgaag
catatttgttaaataaacaaagtctattttccattcacccatgcccctgttaccgactcttgtaataaatttcagggaactatcagctaataactcca
aatgctgtgccaaaaccttgcttttactattgctttcgtcatcgatattttcttcataatcctcatcttcctcatcttccacttctaactcctcatccagtt
gatcaaatgcagcttttgcagagggcttcctaacgtttgagctttttgcaaaacaagactcaatgtcaatgtctttgggaaagtttcttgtaagttcc
atggtagtgatagtcaaaactatacaacgttagcactcgagatttttatccataaaataaatgaattgaattttgcataccagtagcaggagacgg
agagcctacaaatttagactccagggatgctaatagatgagaatatatgatagcagccgtttttccaatgcgacgttctacccaatggacaagtct
ttggtttcttgctaaaaacgagaatttcgcacgatttacacgtacatgtgcagccggatctggaagcagaatactattctcatcgtccaaaacttca
ttaagatcccccgtcattgaagaagtagtaacgtttcttcgttttcttttcgtcggcctgggaatcgaatctttgggttttcttataagaccttgattttc
cgccatgtcgcttgatcggagctgggccatttccagcgagacagagtcattaacttcacgatttttcttgatttcagaaacattttctgacttacgct
gctcaagatatttaatacggagttgcatttcctggtcaccaaccggaattaaatgtcgaggctgtacaaccacaagatatttaagataaatcaggt
cagtaatattttgttgtagcaacctattctcctcgtctgtatcatctggactctttccaaatgcactgtatacatcttgcacacgcgcacggccctgtg
tactcacatatttagcgatagatgctgattcctgatttaatttttgcgaaactaaataaacgtcctttcctttccttattaaattatacatttcttgccat
tgtgcttcatagtaagtaacttctcgaagctgttcaatggccgtaacaaacaagaccatatggtgctgcattaaacttgcaagagcctgtcggattt
ttgacccaggaagattcgttcgtttcagaatcataggtaaattcattcgtccatgacgaagtaaaatgcttgctatttcctagttacatccatctagt
cagcttctgcacgaacagtcatcaatctttgctttccactctaacataccgagcagcattctccaaaaaactcgtctaccaggatttcgcacagccc
tactgcatactgtgacatccttgcatctactacgactcaaacatgcaaattgcttcagtagtgtttatggtaaaaaggaatttggtaacaaaacctc
gaaaaacaaataacgaagcgatgctaaaatactttaacttggttaacgcagcggtattaaaagaaaatcctgccttagtaaacaaacgcaaaa
gcatcattcaattgtgcctctgactactggttctttataccaattgagctataaaggcctttccctttccttggaatgtctgtagaggtattagggtgtc
taataacatgagccgagtatggattgaatgtcaacataggagcgcttctacattttccgtatagaaagacttcatgaatcgttttcatccattttgcg
aacctcacctttctgtgtttgaatgctatctagctgtctgcaatttcttttaaagcattgtaggatcctttttcagtcgcacaagctacaatccacagc
agagtaataatttaaagctgcagttaataatattagaattcttcgacattatcatgcgttcctgttccctttttttaaggattcgtcttcgttgttgacta
aagagctggaacgatgctctcattaacgattccactaagggttgattttcttcgtctgctacaatggcaggaaatcggctggtgcctcgatatgcgc
atactgacggactaaccaggcttgcgtatacagtagatggtacctttttacttaccgttggatccaatttggtaattcgaaagtttcaggtcggttct
gacgaagaacctgaatccatagacaatcatcaagatcccataactgggattgctgtttctgtatggtttttatttgtttattttactttttggtacatat
tatctaatcatgtgttttcagaaaacccacttttgtacctgttctgaggatgcaactatctgcatatatcccattaataactcttcagaacataccctg
ttagcaaggacaacacttccagtgcgtgacattgcatattctatcgatggagattggattgctattgccagtgagtaagtaatgccttaaatttatc
gacatctttgaatgctaatttggaggaaactaacgatgacagtgaaacagcagtaaagcttgtcaatacgaaagaaccaacgcaaattcattctt
tacgtccttccaaaacttcaaacaaacatgtgtcattcagtccaaatgaaaagtttcttgccatatccagctgcaatggtattctttatttttacgata
atcagtccagaagtcttttaaaatttttaacaaacacaatcttgtccgttgaaggcgattccgacctcactagcaaagcaatttggcacccctctga
tggtacatttgctgtagccaattctgaaaatcaggtagctatattatctactgatgattggcttgctctctacaagctaactccaaaagaaaactact
cttctataacagatatcgcttggtcaagcaatggtcgttttctggctgcttctttccgcgataagggtatactcatatgggatactctatctaaggaa
atagtctctgaacatcattactcgaacgtggttgctttggcttggcagccttttgctaatgtgttgtctttcactacaaatcaaggcgttctttacaatt
gtccggaagtaattcctttttcaggatccattactgaagccgactttcaaaaaagcactcagctcacgaatggtcataagaaacgcgttattttgg

atgaagacgaagaagagttatatagaaaccaaaatactaacgaaaatgctgctgctgctgatatcacaaacggcgaagaagatttcggcg
attttaacgaaaatgctttggatgaccccttggatttggatggtgatagttacatggtaaatgaagatgagttagaagtagctaagaaaaggaag
gcgcgtcccatacatggccatcaacccgtttcctttgaagacggtgcaaggaagcgtcgcctactgcagtttgctgtccataagccagtatatgctg
gaagtacaccgtggcaaggaaacagacgctatttatgtctgaatttggtaggatgtatatggaccgttcaacaggacgacggccataatactatt
accgtcgaatttcatgatgagtcaaatcaccgaaaatatcattttccagatgaccaaaggtttgaactggcttgtttggatgaggaaggagccttg
ttttctgctccagctactgagcaatcaccaggtgttatatactacaaacctcatgtcgattggtctcggcgatctgaatgggcattgatgcttccgat
ggaagatgaaagtcccattagcatagcactaagctctagtgtagttgttgtttgtacaactagcggttatgtgcgtatttattctcgcacgggatttc
ctataagtatttataggagtaagcaactaccctttgttgcttgttctgcttataaagaccttgttctgatagttgcgaacggcggtgccgctgctgat
gggactcctcaattggtctatacaattgaagacataagcaagaattatgtctatcaatccaatgatggattggctctcccacctgcaggcgagcttt
taagcattttcttttcagatgtgggcgatccatacatgtatgattcagatggtgttctcttggccttgcttcattggagaactcctggtcaggctaaat
gggtacctgtgctagacacaaatgaattggaaagacgtaaatcgagacaagagtcctattggcctgtaacagttgcagataatcagttccattgc
attctactaaagggaagtgctcgttatccttacttccctcgtcctatgtttaccgaatttccattccggattccttgcactgtaatgactcctgatgctc
ctacttctgttccttctttagaagaattgcagcttcgaaacgtaattttgttgaccttaaccgaagaagcaattgacgaagggaatgttagtcaaga
acaacagctgcaagtaactaagcttgaagctaatattgacaaagctttattgcaattaattcaaaaggcctgtacggaagaaaagcttgaacgtg
ttctggaattaacaaagtccttaagaagaacacctagcatattggcagctcagaaaattgctttgcatcattcccttacgcaaattgcagaaaaga
tcggtacccttcttcctgctgcttaaaagttctacttttgtacattattacggaaaattactcgctctttttgtattccctgtatagtgctatgtgtaat
ccatattttatttaacgcctagttgtaatattacaatgaaacatttcctttaacatactctatagttttggtctgaacatcgtttccaagttatgcaata
cccaaggctttttttacaaacgttgttccaaccattttcgtttttacttacttctataaattacaaggaatagaaacatttctcataaatttcgttagcc
ctccttataccctttaaaagacaagaaagacagaactgtcgacacagaacaaacatacgtgtgtttaaaatggaacaaaaaaaagatgcatcg
gtgctttccaatcagtttcagtcatcaataggcatttctagaaatataatccgctcctggttaggagaagaagctcattcaatcgataatgaaggtt
ccaatgaagaagtcaaattacaagcaagaccgtcgcgcttaggattgggagccatggccaaggaatcctctgccaattcccaatttgctttcaag
aatgagcgtttgaaaagccttccagctacactgaggaaaaaaattgaacgacaattgcaaaagaaagagtcagtgaaaagcaatgcaggaac
agaacaatcttctcaaaagaggaagagagaaacagaaagagatacattgaaaggtcaagagaatgacgccgttgatgagggcgaagaggaa
gatgaggactccagaattcaggtagcaaataaatccaagcgcaatattaaccaaggatctggattcgacttgtataaaagactcaataaacgga
aataaagatgtgtctcattcgacacattctcaataaggtatttataggggcgacttattatgacatcgggggacaacggttttgtttaatggaaagg
aacaaataaaagatttttgccttaacgagtatgtacagatgaagttactaagaacacgcaaatgtataaactattagagccagtttcaatttaggg
aaatccaaatatataaaaaggtttttatgtgtagggaaatcttatacccaaaagggagggagggttgttattaaataaaaggacaaaaaatttaa
aatgtaattagttttttttttctgattttcacatccaaatagggatttttcaacatgctttttttaagtcacgcaccatctgttacattttgacggtaac
atcgagccatggaacgtgaatcgtacgtataccagaaatccactgtaaccaggaaagtcgaattagcctatagccggcctcactgaattctttgg
gaaaaggctttgggtcaaaaatgtatcgactaaatgtaaaaaaagatgagtcactccacaggcttctcgtattgattaaaatggctaaaataatc
atgcaataaggactttcctcttcgttagcatttataaaacgtgacttcgagtcatagttgtctttgatttcataagttatgcatatacttaccaaggat
attttcgagcaattgtagctagaatgaacactggatagaatatgtaaggtgctgcattagcaactagaagcatgggaataaaatatctcagacat
agcatattcgtatctctttggcggagtgcaagtaaagtagcagcgtatgtacggcctttgaaatttttagctactacttgaggtgacacttgtcgtca
cttaccaaatcatacaaagttgctttctttttattttgctttcatcaaaattttaggagctatattgttttctttttttataggagatgagcaaggatcagt
tttttccttgctgatcgtttcatccaaggatttggttcgggcagtatgcgactgaaaagaaaacgaaaacaatatatgaatttatgtgcgcaatttc
aaagaaagatgttcgctttattaaagagtaagaagtcgcttctatactaatatatattaatgatcgaaggaatgtagagtaaaattccagcagat
aatcttagcacgatattagtaggcgatatcctcgataacacctttaacacgataaagctccattgaagggtttgcgtaagtatgtatgttatactaa
tacttgctactcataacaaatctgagagtaagtcttattttcctttttttttctttggaattgcttccattggaataccagatttattgttcttccattattt
tatgctctttcagcatcctgcaaagctgaaatacaaccacttaaatatactgttttgaggaaaaggattctagcactttaaaataatttattaaattc
atttctctaattctcctgcgacgttcatgaccttcattacgaatgaaatattttgtagcattgcgagattccaaggatgagccgttgatccttatcacg
aattttgaagatgattcttcagtagttttgactgtcatgagcttagacagtaagtttgttttacaaggtatgcaatgctggatttactatgaaagcaa
agtacaggtttaatacaattatggtagacaaaaaacaccagtttcaacaattgcaatccaaccgaactgtcgagtccttaactccagatttaattg
agaaattactttccggagtatcagatcccaaatttaaactgcaacccgtattactagaagatctctgccgagtgagttcctggataatgctgttcag
ctttcaaaatttctttactaacaattgcttagatttacatcctagtaatcgatccatttcctttacggattgcttggtttgaattagccaagaaagcgct
tgatccaaaaatgttattttcctcgttttgggaatgcagtaatgttatccaagtaatagatctggaatcctttagctcttaaaactgacataataattt
ttttaggatgttgcctttgctcgactagctcatcaagaagaagaaatgatacaggtggttcaacatgctcgcgaattagaagaggaatttcgtatg
aaagagcgatttttattgctcaaatttcaagaattgattcggaatgccaaaaagaaggaagctggagagcaaagtgggtcctcgaaagaagag
gaatacgatgaagacttgaatacttcctctataggctctacaccgcttgtgaaacgagaatctccatccatccactctccctcttcttcagagagtc

aacatagccaacaagaagcatctgaggagtttgagacacatgaagcagataaaacatctggagaaagcagtgaaaccgaatcagagtaatat
attttcaaagagtataatctatatactttctcttatgcatttacttgttagcttcaaacgtaatgtttcttctatattttgttggtgtttactaacgaattc
caatgcattgtaccaaagtgtttgcatttcaaagaatccattcgaaaccattgaccctttatgttttaaatttcgtacttctgcattgttctcaacattt
tggacgagaatagccatttcatccgtcagctttcaaacctcaaaagttacttttgggaaacgagcattaaccttctgcgactgtttttctgtaatgtt
gcatctctttccaccgaagttttacaatttactctgaaagctacgtataaacttcccggtgtatgctttttaatttgctgcctcttgttacgaaagattg
gtgctgtcgtaggtccattttctgattctacgagcataaatccgttttcttatttttctttgatattttaactcataagaattgaaatgaattctcatccc
agctcgaatcgaaaatctgtgcaaaggagtacacatcctactcttccagatatcaatgaagcgaacaggaactctgatatagagtttctaactcc
accttcgcaatataaacaaggtcaattttatttaccttctccaattactcccaccctccctgatctacatttagggcgtacgaaagatgagacatac
aatgccttgcccaatcttctaagcccaacacttccttcaccttttgtgtttgacctagattatgaatccaaacagaattcagacgctattgtttctgat
ttcggcagctttacaacgaaaaacattcctacacattcccaggcagaacaaaaattacatgaaaaagggtcttccaaggagtccacatataaaa
atgatacagagctcgaagtaactgttcccgaggaagagacttctcacacatcggaagtaccagctggaaaaactgattatccgattgaaaaaga
aaaactagtaagtcgtggctctactgaatcttctattggtgataatctgaaaaataattctgctacagaccacccggtctcgaaacgaaaagccaa
agtaactttggaggattatagacggatcaaaatacaaaacaaggaaagtggtagcctcggaagttcagcaatttcagctagctctgccagtccct
caatgcctcttgtacaaaatcaaccggatttcgaactcttaaaacaaaattttattagactccttgctgatggtaaatttcagaagaagctggcaga
taaaaaagaaaagtactcatttctcttcgctattgatgctgttttatgttatgtgatcgctttccattttcagaatgtatctaacgttacaaaaaatca
acctgctacaaccagcaattggagaacattacctgcctatattgaattctttctacgttctgaaaatgtgtctctggaacccatttttcaaggtctttt
ccttggttatcttggaattgctttccgagaaatatttaacattgaacttcttcgtatgcaccacttgcaacatttccttatgaaagacattcgacacaa
caattcagaaataatttcgaaaaattctactggaaatgttagaaacctttgcgagaatgctgttcgcttacatgattcctataagcggtatctagct
gcgatgaagcaatgctctcacttgtttagcgacgattcactgaaagggcttcccattacctataaaaaaatttcatgaatctaatctcacaaatctct
ctcctccattttgtgtacagaccagtattgccgattcggttgagtttgggtataatataataacagaatttaaagatcaacatggttttgcagttaag
tcaaaaataaataattcagatatttctcttttacgttctctagaaaaaaacaacccccttgtgttaatattgttaagttatactgataattggttttcgtt
aaggtctcaatctatcgtttttttattagtagattaattcaatgaattgtttttattttattaaaaataattatagaatacatttaatttttagagcaag
agatttgctaggtataaactctgtatgttttggtatcttcggtagaagtgttcaaatgactcaacatagaaaattagaaactaataaacaatcgtga
gatatcaaataagacataaccgtcctttaaactagaaatacaaataagcgacaggtgatgcgcttctatgccaaggtaataaataaagagcgaa
tttcagtacgaaatgccttcttctaattggaatagaaaacaataaaaagaaacaaaccaacatttaagggttgctttgaaactttttagtattatgg
tacaggtaaggttattctttttcctttaaattggggcgctgacgttctaattttgaaatgatacggcgaccacagggtgtgtttttaatgttaggtata
agcggctttatgcgttctgccaactgaatacgttgcttcggttcggcgttatcaatggcagtctggataacataattcgcaaaagaatccttaagca
ttttaactaagaacttttcatccatcaattcagcaatcaacttctcctttaaaggggaaggtgagtaaaatatacactaaaatgccttattagcaaa
aacttcaaagttccaatcgtaccttttctataacattagagctaaatttttgtgtagacaaaacacggactttattaataaagtaattgacaatcgtt
tcagtatgtgaaggattattaagttccaagacgtattgcaacacatagtttccaaaagcatcttggacaagaagcaaggcgtgaggtataatgtg
ttgaacaagttgttcaatttgagttggcgaagcgtggtcaaaacaacgttgtactacacaacaaccgtgtctatgagtcgatacatcgactggatc
attgcaaattgcatcgaaaataaactagtaaattgttaaaaagtgcgatttctacttcagataacttacctgacaatcttcatgagaaaatttattt
aggcatttttgaataacatggtttccattcaaatctttagtaagcattacaacgtttggtctgagtgcatccactatgcatgaaacctgatctggaga
agaaaccaaatcaatgatttttttgaagggcccttgttccgtgcatattgaaagagatggggaccaattttggtgctattatctgaatgaaaacgga
acgttgggcatccgatacatgttcgaacaatttttggcaaagataattgccaaatgcgtcaaccattaaaacaggaagataagcttgtgtttcgtt
aaaaacgacattaacatatacgggatttttcttttctaaaagtcgttgcaagtatcgacaaccatgctgatccttgcaaaatgaaagaatgttatta
gacggaaacgaaaaaggagcaagggtagaagatacagccggttgaacgttcggttcctctggcatgtaagcaggaacacacaatccagaagg
atctaataaagtggatggaattcctgctgccggtacccctaaagaattagggggaagaggaggagcactatcgttacttccagaactggctgtcg
aggtaaaagaagcagttaaggaaggagtaaacgtatcagccaggaacggtgatcctgaagaacgtaatgactggaaagaagatggagtttttg
agggtggaaaagaggtcggatccccctggatagacagatgcagtatttgcggaagttacgggattaaggcgtggactcaaagtaggagtcgaaa
gcctatgggcgtttgcaggggacggagcaccaacggagtttgaggcaaaaggcgtagatatagatttttttatattagagtttgacatggaaaga
cttcttctggatccttggtttgaagaaggagatgatgacggataagacgataaatttgggcgccttaaaagaacaggaatatcatccagtgtagt
gttcatttgtaggatggatgatgacaaatgctttgatccaaatctagaggaagaaggaccagaagaagcgggggcagcaggagctgttaaaca
agtagatcctagtccatttgaaagaacaggtggtgcaatggttccggaagccgacgacattgaggaagtgggagtcgtctgaggaaccaacgag
gatgaagaggaactaagaaattgatagggagcgaacgtaggagttgcgaaagaatagccagcaagattagaattacgcgacaagctgttaga
acgattcccaaaagcaccgtatccagcatagccgtcagaagcagcaatcgaagaaggaaaataaggaatggagctatcctttagtttcgaacca
ttgggagcttttgaagtatcccatgagggagatttcaacgcatgtttgttttcaagttcgggataagcagttgaaagaacgggaagcgagtattca
gtagacataagagacgggtgaggaagagaagactgctcgggcatgctactacgctttaacaatgcgatcgaccaatgtctgtggacatatattta

caaaaccgggaagtgaggaatttatcaaggaattagaaaacagaagaaaccgtttccgaaacaaaagaaaggttgcctcaactatgtaaattc
acaaaaaaccacggtgtcgtccacttaataagaagacgatggtggtggaacgaacgtttagaattcttggaagatttgatgatctctcttcgtaaa
cttagaggtgacagcattaaggtaacaatcgttggaaaatagaagtagaaacaggatagattccttaaaaggaaaaaaggaagctcttttagg
aagaaactgagaaaaatatgaatgaaaattcagtcatacactggttctgctaagaagcatgattgcatgaagcaaacttgaaaaccttcaatgg
aagaagcgcctttgaaaagaatggctacccatactgaagagattcatactattcattacttggctttttttctgttttctttcgttttgtcaggtcctttc
agtcaggaactttttttgctaagaatgctaacccgcctcccatctcaagctttctgtatctacttacgtcctttcctccttagatgaattgtttggaattt
aaaatttggtttttgtgaatgtaattttaagagagtaaatttctcaagctgaaatcatcattcgtagtttccgtgttaaaataagagaacaataaag
ttgaatccgttgatatatgaaatttgaaggtttctcctctttcaagaacgtaaataattgtgtgtcaacataaatatataattttgaattttagatctt
ataaaatcaaatgacgttttcgcttagataaccttaacgtagatcatgcatgcactgtagatctttgttgaataagtaaacaaagctgtacatggta
tttgaataggtaccttttgcaaggcagaattcttctggttatttttaatttactatctttctactattttttgtataaaaacataagacaaacggaaaa
ctgccaaatcgcgtttgttcccattatagtccaacaccatattcctttgttcaatcctttaccgacacgtactttagttcgacgcgtaaatttcatgaa
acaacaagaaaaacaagacattaaacaatagagtctataaaaatcaaacatgtactttttaactacgactttaatcgcgttcgaacaagacggt
gaaaagtacttgacccagttagccatttatactcaaatcccagtttgtactgatccttcttgtaaggacgcacagttcttgaaacgaacacttcttca
aataaccttcagtggtatccagcaactctatactacgtttcctaatgtatggcattatgccttgttaagtaaagggggggaaagaagagtcattaatt
agtgtggaagaagtaccaaactcagagtttcggatacactattgtacgcgaaaacaagcttttatcatgttggaaatggtgtgtttaatacgatgg
aacgcagccattgtcaaagataaaagaaaaaataaaaaagaaattaacttggcatgaattcgaatactaacaatatctatttagatgtgttacca
tggactagacgactgcgaggctatcaaacggtcaccgtccaaaacggagtacgtgtgcctttaatcaaaagatcggaaaaatggagaatactcg
tagaatgttaaaacccagaaaatgcataaatatagcttgaaagcaaaataacattaaaatccttaaccaagattcacactttcgaattcttctgat
ggatctaacttagcttgaggttcaactgcatcttcagatgtcctttctcccgtagacgaatcatgattgtcattaacttggttttcattttcagtttcttt
atttaagtttctgttttcattatccacattcgtatcttcctcctgctttgctttttttcacgctgtccttctcatcagcagtttcttcagcaggctttggatca
tcatcgtccttcaaagaagcactttcctgtatctggttgttaacgtttgcttcaacattattcccttcgtcaacttcgttagaaggacctgctgattctt
cttgtccgaccaaagcctcacgctcaccaacatctgggttcacaatcgtactgtttttcatcgtggaatcaggttcatcgccttcttgaataggttcc
atattgtttcgtgaaacagcaccttgcttggactgctcatattcctttacgttggcatcattcgactcgtctttattgctgatctttccttcctcatttgca
ctattaggaactgtgctttgagacttattgagagtcttattaggtggaacgatatcactggatacatcgacagactgctcagcagaatttcctggat
tcgtagagctagagggttccttagaagaaatgcctgtttcatccttcacctgtgtttctccctgctctttctgttgtttcgttgcatgctcattggccgg
aacttcgggttgcgttttaggaagatcttccttaggttgatccacaggggcttcaggttctgctatatttgcgacgatagaggtaggattgccaggt
ggtttttcaaggttttgagcgaccggggtttgcgtggtaccatcatttacatgactaatatttacagatgaagaacctccagaacggttttccatgta
aaagccactaacattaacaagtcttccttctgactcacatttcaaaagatagtcaacaccaagtacaggaatgttagcggcctttgcgcgttcgta
ctcacgaccaccgacagaagagcaaatgaaatgggttgtatctatttgaacactcgtttgaattggtttggcatgaatcctttcaatgcatttttgc
aaagcttgcatttcctcaggaatcatgttaccaacgcagacttgaataccggtcaaatcaatcattcggagggtcttaactggaatgtgtttgctgc
taaaacgccctgctgtagtgtcgaggacgagcgaaaattcgtattccgtatccaaagataacccactaagttttgcatgttgtgtcgtcaatgaatt
ggtgatgtttagagcacgaacattatttttgtaaagacagagactttttagtctcgaggttcctaactgtagagcatcccactctaaaacgatactc
gtttgggtgacatttttttagcctcaaaacaggtgctgaaggaagattttgaccataggcttcatagatttcattctgaatatcatcaaattccttttca
cgtgcagcttcctcttctttattatggcgcacggacatatcaataatggatcctgcttttacattttccggcagcaaaaccgatgggaactctatgat
gtgaaatgagggtgttagcagaattgccattccagcatctattttggctacagataccataaattgatttgtttccgccatttttttatagatgctcttg
aaagactgagcatgcgcgtttggtaggtgaattctaattatatcaaccaagtaacaatgttactcagttttcgtctatacatataacaaagtagaat
tgcttctcaagttgcatactgaagtttttcgtttgaatatatagtaaataaaccttttaacctctctcaatggtcttttgtcatataagactgtttactct
ttggtaaaactggctactcatgtctccgattgcatatgttgcttcactaacaaagcttacgaatacatttaaaagctgcaaggctcgtttgtaggaa
aagaaaggcagttatcatatacattaaacatcaactatcctcatgccatacgaattgtcttacggaaaagcggcgaacagccacaaaaattgaa
atacacaccgagcaggcttcatttgggtataccgagtcttttcgacgaatgacacgattacactcgtgcactttgcataagtcagcatttgggaatc
ttggtaggaggatatataatctatcaatttcgtactaatgcaatgcctagctgaaactgtaaaacagcttagttaaaagccccaatatgtagaaaa
accaactcagcatgtccgtcaccctcatcaaatgccggggctcatgcgatgagaatgtgaaactggggaaaaagagaagtaattgcttaaaaaa
aaaaagaaaccaatattttcacgttttaaatgcgattctaagcaaatataagtacattcatcatagtatagtatggaatattatcttttatagcata
aaaaagataagactgattaagctcgaaagcgaggaatactgcattccatctattcataggaaatgccctacagcatggcaatttcataacatcca
gagtatgaacccttactttccatgaagcttcgttgcaatgggaacaataataacgactagtaaaataatgataccaatgatgatacataaccgca
ttcgcatatctttccaccacatcttcttgcgcacgcgattggcaccgcgacgaaagccctgagcacttacagccaaattatctgcaatgaacgttaa
aatttcgttggtctgctatcaatatgcgcatacctgttttgtcctgtaaggaatcgaggcgttcaccgcgctcagacaccttggagatattctctctca
taatgcccaccgtatcatcaatttgttgttgaattgcagctgtcttcacattgggagtcgattccgtgttggcattgccagtacggactgaagtcgac

gattccgtcggtatatatgggtcgtagggttcagacattgtttttatgcgctggttggtaaaggaaacaaatactgaagactacgacttttcataaa
ccacacccccaccttttctgggattgaggtactttgatttacaagtatttatagagaaaatagacactttaaatacatgggaatggtaaaaataaa
taatagtatgtttatttaaagtattccttgtatttttattttttgacaatctggtctgaaatttcgactcttcagtgtaaacaaaacctatattcttgcca
aatttagcaggtgccgcctaaaaactctaaattcaccggggtttcttgctgtttggagcttttacaataactagcaattgcaccagtatttagcgctt
actttactttctggatttatgagtgtatactagctttttgctcctacctttgtgttcgtttgcataaatcaccaacgaaatcatagaatagtttggcgag
tattctttactaacttacggtgggtatatttatgaaaaaaaatcctgacgcggtgtcttgatttttctggttctctttttgtttcggttttggtctgtggat
tgcttgcatactctagtcaaaagatttctgtttgcttattgtcttaatttttcctctcttattcgatttttgtttgaaaagttctctgttatccttgctaaa
gaggactcctttcgctttaaaccatggctgaagttgatgatcaaacctgttttgagaagcttgtcctctataaaaatgcacagcattggttttgctct
ggattgcttgaccagtatcttcgcccaatcttcttatggatggcgaagcaaccatcaccatgctttgaatgggtcttaaagtgttataatgagcgctt
gtgctcttgtacatcttgtattcaatcattttatcacttgcgggatgaatctttggcaaaattttcatatcattattctggcgtttctttggcaaacctcg
aaagacaatgggacgagtggatgcttaattgtacactttctaactttaagaaggttgaagagacaatactagatttactgttctaccttctttggtg
tacaatgtcttcttgaatcctcgtcttctgaaagaccacgaggcatacacccaagcgttaagtggatttgttgatctcgtttcggattggtcttctgat
acttttctgccagggtttcttgtcattttttttgaggaatctcattctcaaatccatgaatatattatcgatttcctcaagacaacagaaaactttcacg
tgacacctgaagtttttgacaatatttttctttaattttgtatcagcgtacaaacgaagataaatcttctcttctttcaccatgctctgctgaattttgg
aagcgtgcttctgcactctttcagctactgccactcaactgtgtcacggctttatgtgaatctcatttccgtgactttttatttctcaactcgagcatg
aggctttttcagacgagaaatttgcttgtctaagagtttttccaactctccttccttttggctcgcatctgacatttttgatcagttagagaactattta
aaaaaattgttaaccaatgttacaattgtcgaaaacgttaatgcctcagtgtgtggatgggcttatgtgttggcttcattatttagattttgtttgaac
gattttgtcttggtcttcccttcttggctggaaacctttgttcaagacaaaaaggttgtcgattttatcatctatgcttcattttatgcactctttgatctt
ctttctttagacaatagctcaccgcattctctcaacgaagttttgtcgatgattgacaagaaaaatatcagtttttttagtgaagagtctgattatttg
caaaaactcaaatttcgatctttactttacgatattttattcgttagttgggctcacaaagctctaaccagagatccttcatttatctttgatccaacct
ataaggtctctcctttttggaattcagatgttgtgaaggatgtgaattttacaaatcgatttttttgaaggactgtcaacctgctattttgttcatgacat
tgagctttcagaatcaaccacgcccgaaaagtctgtggttttgttttcagaaatttggcaaactatatcaagccatataagctcattcttacgatcct
tttcgcaaaaggacccttcagaagtagctaattacatgcaaaatgactttatttttgataccctgttaggattcttgctatcaccatctcaacaacttt
atgtcgacactttccgtattttccaagtgtttttggaaacgtaaaaaataagaatgaagtgatgaagaagcttatagaaaatcattttcgtggcat
agttcacggcttggttgatgcaacccttcattggcagtcggccttcaccttttttcctgctcttcgaataatgcgttttgtcagcactgttaacaaatta
ttctcaactgataatgaagtttggagagaaaatgatttatcaagtttaggcgcctattggcaatgtgtttggaatattttagacctcgttcactccaa
tgttgcgaggtggtccctgtgtaattctaacgataccgtaaaagcatcaatgaagcttgccttaaagttaatcacagacctttttcagaatgatgga
cttttcatcaagttccttgccaaattcgatggtttagtgttacttggtgaaacaagtgatgctctattttcgttaactacctggcttaaaattaatgatt
tagaattgagagagcaggttatcggaagtttgtgtatgctttttgtaaagtattcgaactttgactatctttttgaagatcgcactataaacttcttaa
ctgaatttatcattagaaagcagaaagcgcatctttctgctgaccaatgcaagcgccttgcagaggtgatcgctcaagcaagtcctgaggctaaa
aacatcttggatcaaaatcgccttgcagaagcccgtaagagtaagaaacaaacagaattggttagtggtacccaattcaacaagaaaccagaa
ccacaaatgagggaaaagattcaaaatgtttctgttccagctgacaagaaagctcctactcctcgtatgaaaatgttggaccaattacgacaaga
atacattgcacgtcgtgctggacactttgctgcacgagacgctgctgtgtcgtctagaaaggcaacatttgaaaaccaaaagcccccctaccaattt
attgactgaagaagattctgacaacgaggaagttgatgagagcgatagaaaagaaggattgttttcactggctaaggctaacaaagtacctgaa
attcgtcaaacagagcgcagacaaatacaactgttgtctactcccagaagtcagttacatccttctcaagctcgaatgctatccaatcgaagtgcg
gctaatacgaaagctcgtctatttcccaacatgtctgatttttataatgaaattttatcatgggatcccaattacgatgctgcttctccaatactgaaa
tatcataaatctgaaggaaaaattgctgatactttcagaaatattgagcaatatatggatgttttgcagcctatgatttttatggaatgctggagtc
aaattcaagctactaagaagaacctgaaattttcacctgtagagggtattatgattcagagaacggctgtgaataattttgtcgacattggaatttc
agtagcccccaaagatttgtatggctatccacttttcgatactgaaattgtctctctagccttttcaagagaagaagcattttctatgaagggtctgt
gttgttttgctaaaatcgaaaaactcgttcggcaagaaaacggtaccttagtagttgtgagaactcttccaactttggaaatgttaaacaagcttca
aggaaacattcctttatatttcttgaagctaacgaatttgtctacattcactagacagtatgctgcaattcgagggctgccatattaccacctgttag
atgatattgttcaaggaaagccttgttcgcaaccgattaaacattcttctagcgaagttaaaggagccatgagtcgttatcaagtcaatgaaccac
aggcgaaagcaatcttatcttctcttgacaatagtggttttaccttaattcaaggtcctcctggtacaggtaaaacaaagactattataggtatcat
cacggctttattagttgatttaaaccgttatcatatttcacgccccggcggtcaaagtagtactgagcctaagcaacagatgttgttatgtgctccca
gtaacgccgctgtcgatgaagttttacttcggttgaaacgtggttttacccttagcaatggagaacattatgttccaaaagttgttcgtattggtaac
cctgaaacaattaacgtcgctgttcgagacacttcattagaatatcagacagaaaagcaacttcttgaagtcaatcagggagcggtagatctagg
atcccttcaggaacttacacgttggcgcgatgtttttttataactgtatacaaaaaattgaggatcttgaaaagcaaatagatgttgcaagagatat
ggaagaagatacgaaggcgttgggtcgagatttgcagaacagcattaatgaaaagaacttagccgaacagaaaattgaagagtttcaaaagtca

gagctttacgaaaaacaaggaggtcgatcatctacgcaaaaaggctcagaaagctattttgaaacaagcggatgttgtttgtgcaactttatctg
gtagtggtcatgatttggtcgggcacagttttctgaacttttcaactgttattatcgacgaggcagcacaagctgtagaattggatactatcatacc
gttacgttatggtgctaaaaaatgtattcttgttggtgatcctaatcagttaccaccgacggtcctaaatcagaaggctgccagttataattactctc
agtctttgtttgttcgtattcagaaaaattacactgatcagatgtgtttattgagcattcaataccgtatgcatccacatattagttacttccctagtat
gaagttttatgaatctcgtctgaaagacggagagggtatggctgaaaagacaaagcaggtatggcattcaaatagtcagtttagccagtatcggt
tatttgacgttcgtggtcaggaaaggatatccacgacatcctcttcatacaatatggaagaagttggatatgttgtacaaatgtatgaaaaattggt
tggctgcttctcggatgtcaattttacgggtagaatcggcgttattacaccttatcgtcaacaattacatgagttgcgcaaggcttttaaagacaaat
ttggaaaaactatcatgtcctccgttgatattcaaactgtagatggtttccagggccaggaaaaagatatcattatcttttcttgcgtcaaaagttat
tcaaagtattcaattggtttcttaagagattttcgtcgtcttaatgttgccttgactcgtgcaaggagttcactatttattgttggaaatttggaaacat
tgaaaacggataatctatggggaagtttagttgatgatgccttgcaacgtaaactagttgaaagtcctcatctgggtactgatggacaattgatcc
ctgttaaaaatgctagtgagaaacgctcaaaaccaacagattttgaagaatcttcaggcaaaaaacaagctatctctgacggtattttctgaagt
agtacatattactctttaggtgagacatattctctcgctgatagaataagcttacctgacaaaagcttttatttttctaaattaaatgtatatacttgg
aattgtgcaattttgattctagacattgagaatgtgtagagtttatttacctacgacaaatagattcagacaattagaccggttaaggcatttacga
ttactttgtggaattgtgtattgtaatcttcattagcttgaacttactacggatggactcgtatggattctataataaattttataattgcaatggtact
taatcaaatgcaataataatttaattatatataattttgtccataaatactgaacatttcttgtttaaatctctgagaagcttttagggtagggtaagt
gactgtactttaagtgttctgccaagacgtagtgttggtggtagtgaacagttcggtgaactttttcaaagacccttcccaaagcaagccaaaatg
aaggtggaaatctgctcattctctggtgctaaagtatatcctggagctggaagactctttgtccgtggcgataacaaggttttccgcttcgtaaaca
agaagtctgaaagcctcttcttgcaacgcaagaatcctcgtcgcttgtcttggactgtcctctaccgtcgtatgcacaagaagggtctctctgagga
atccgccaagaagcgctctcgccgcactgttaagcatcagcgtggtgttgtcggtgcttctttggatgtcatcaaggagaagcgtaaccaacgccc
tgaagttcgcgctgccgcccgtgcctctgctgtgaagcaagccaaggataagaaggctacttccgaatccgagaagaaggctggcaatgctaag
tcttccgccggcgctgcccgtggtcaagccatcaagaatgctaaggccgccaaagccgctgctcaataaaatgaacgccagcttaatataataag
atgtcattgtgtacacttttagaatgcggactttgccgcaaagacttttattaacttgaaatgcccatagtagaaatatgtctattctgcgtagtttca
caaagattatgtgggtgtttagaattctaatacaactttctattacgaagtatccgtatttttcatgaacttcacaataaacatcaatgattcacctat
cactgaagtatccgttattcattaatcagaaaaaaaaaaatgccataactcataggtaaactagcataaactactctatttttttagcctgataaagc
aaaatataggcactgtctgcttcaccaccaccatctaaagattcaattttctctgcgggtacagtgcttactcgggagtcgttgaaacggaaccatt
cactatgctgatcaggatttcggatccaggcttggtaatgtccagaagaagcacttgcacctgaatgagacagaacaccaattaaatcatacaag
ccggtcagattagcaccgtcatcctcacttaattgtggatctataagcgattgacatgttgcaatttgtgttaatctgcgctccgcctctgattcccgt
tcctcttcagaaggttgaatcttaatacgttttatgtttcgttcttgctcctcgtcacttttctcgatttctcgcagtttatcacgaacaggaattagctt
ttgagagagttcaggggcacaaaattctacagcatctaactcaaaaggaaatttgaccttacgaagaatcttagctttttgaccaagagacgcttt
ccagtaaaagcgaacaaaattgacggttaaatattttggcaagcgagaaatccgtgagactttattgtacgaagcatcacgattaagtaattcac
ttcgcttagtaatttttttgggtcaatccttccaaaatgccttgagtcatatagtttgtatgcatcgatatatgacatggaagactaaggaaagtatctt
gagaaactgtaggttcttcattttcgttttcgtcacatttcattgtagatgtaagttttccagccatatttttctggacccattctgaagataatgaacg
ttgtaaagaagacaataagaatgaccagcactcttcagcatcttgttgggcatatccacctgtttctcgactcatttcagcaaattgaggatattct
gaacgtaaattctagacgtggattagacaaatgaataggaaacaggacaatagacgtttgtacatacctgaagaaaacgagcgggggttatag
aattagaggaatccatagaacgtaacaaactggaaaattcaccaactacaccagaaggaacgctggatttctggactgcttctttaagctcagga
atggcacgcagcaattggattgtggaattcatataacaagtatttcccaaattgacaaggccagatgggtactaaaacaattgttagtgttatccg
ttaaactgtttttggatggaagcaaggaaacccccgttttcttgctttagcaagcatcctaatgacacctgtcatttgtcgtcgttacataccttgtca
tcttcagaatccgaagcctgatctgcagccgctgtgataacaggaattggaatattcgttaccaattcaccaggcgtacccatcatcattactgtac
catttggtttcaagccaacatttttttaaaatttgatcatcttttagttgcccacctttcacaataattttttgtcgttcaggaggcacctgtgtaagagt
agacaatttgctttttaaggaaatgccagtatcttcaggatcatattcaacattgtatgtttttccctgccaacgaattgttacaggaatcataatta
gaaaatgtacgatttagtaaacaaagagagtatcaatcgacgcttttctattcagggagtctaaaaaacaatcgtgtcggttggtattgggagag
gcaacaggttcccaccggtaagccaagaccggtagtaccgcggagatctcagcggtactaccgactgagatgaaagaggacgagtctacgcaa
cacagagccaaacaattgcaacttgaaccatacaaatagaaacgagaagacttacgaaatcatggtaagagagctgatttttcgggtggaaga
atgacggaaactaataaaggttgtctagtctaatacattacgtccctatttgaatgctgttcgttctactttgacagcgtcattggcattggaagagt
tttcgtcggaaattgtggaacgacaaagtcagccagaagtggaagttgggaggtcattagaggtccttttaaaaccactcgttgtttcccggaatg
agcaagaacaatgcttgattgaaagttcagtgaataggtacggtttctacttagacacataaccgagttaattcaatgaatcacttctgattcattt
tcaacgaaaagcgcttttttttggagtgaataattctgacatggcataactaaccaaatgtagcgtacgattcagtatccgtatcaagcaagtgga
tgaaattgagcgaattttggtacacaagtttatgcaatttttgatgggacgtgcagagtctttctttatcttgcgtcgcaagcctgtcccagtatgtta

tttggcaagcgttagcgttctttgctgaagtgctgctatctcgacacatgtagaaataatactaacttgtttttattagggatacgacatttcctttttg
attactaactaccacactgaggaaatgctgaaacataagcttgttgactttattattgagtttatggaagaagtagatgctgaaattagcgaaatg
aagcttttcctgaacggtagagctcgtcttgtcgctgaatcttatttaagctgtttctaagttctttttctcttttatttagaaaaatcatttttgccttat
cttttcttcttgtctcgtttctcccaacctagtcttcttcttctctcttataaactcactcacttatatatatgctacatatatgtatcatgcacacatgac
ggttttctacgttttcaaacgttcgtattgatgaatctatgaacgactgcgtatagtcgtagtcctatttagcaattgctatctcccttttcttttggatg
atacttgtaacttttcagctgttcatatgcgtcccatttcaacgtataaatgtaaatgcacacacctctgaacacctttcctacatgattagattacat
cgtataaggtatttagtaggatagcgtaatgctgcgatgcactaagtctctttgtttgggttggaattttcctcacaaattatggtaaacaaaggaa
acttgtgttttagactatttttttaaccttttttctatttttaacgtagtatatattcattctttacaaaagtacggttttgaattcgtcaaactagcgtttcc
tttggcagaattaatgcatccaaacctttcgtatattgaaacgtgatcccatggtacagtctgctacaccaaaagacgaaacgctggatgaccttc
cacaggggaatttttccttgaattcatttgagaaacctctgaaccttggtaagccttcgacagattcaagagactcatttgcgcgtacacctttgcat
ggctttccaaccaaacaaacaaaaccactggagtttacgcctcttctgaattcagctagacaaagctcttcctatcaaagatcgcattcagatgag
aaaagtccgtatcatcgagacttaacaattctgtctcccgtatcggaaaaagcatcgcaagaaggctttagtattgatcaaaaaagtgatgcttat
gacatttcaaaattagatgagagctcgtttttatcaaatgccgaacgcacaaacgatgagctacccgcaaacgcagcgctcacattacgagagc
aggaaaagattctagagaatgtcggcagagagaattttgggttacgtgttaagattgcttacctcaataagcgtcttgattctatggcaccagaac
agattcgtgacgccgtaaacgacaatgtagatcttcatgtggaaagaacgaaactgcttctacaactaaagaagagtgagcagcttcttacaaa
atcgcactcggaaaacgaggatcttgtcaaaaagattcaatttcttgaaaatgttaacagcgtcgaacaatcccagaatgtaaaggtatttatgg
aaagaatccgatcccttgaagcagagtgctctcatctaaaagcccagcaagcaaagtcttccccaagtccaaatgagaatgaaaatcaaatgaa
catggaatatgacatgagaagacttgaagatcgatatgacgaaatcaatgaagagctcaatgttgctcaggatgtgctaaatgaaaaggatgca
gaaatattttcgctaaaaaagcaactggagaatattccctctgcaaatcattcagaaactaaagaaaatagttttcatatacaattactagatgac
tttgaaactctcgagaagaaatttaacgatctggaagatgaattagcccatgtgaaggatgagcttcagcaaaagaataatgagctagatcgtac
tcaagatatctcagaggctcaaatgctaagagaaaagctacaagaaacggagcttcatatcaaataccttaataatcaaatccatggctttgaag
gcctgaataacgagttagaagagctttctaatcatatagaacacatggagaatgaaaaaaatagtctactaatgaggatagagtcactagaaag
caccaacggtgatttgcgagagatgacagcttcattaggaaaccaggttgagtccttccggagtaaaaacatgtctctggaggaaaaaaataaa
atgcttgagttaatggctcaatctaatgatacagaagaggttttgcaggaactgaatatcagactagaaacgataacgactgaaatggctgattt
acgcactaggtacgaattgaatttagcagatttagagtctctaaaaaaagataagatgtatttgcaatccgagaatgatagacttcgaatggatg
ttgagattaccatcagtcaattgaaccaggatgttgaacacgaaaaggcaaagtcttctcagttgttagagaaattaacgagaactcaaaattctt
tggaagaaagaaatgaatctcttgaaactgctctgcaattaaaggaaaaaaatatctttctatttgaagagcaaatgcgcaacttacgtgcttctg
agcaagaactattgacaaagttacatagtggtcaggaaacaatctctaatttaagaaacagtttgaataagtgcgaacttgaaaaagaggaact
aagccaaaagttacgtgagagaatgagtacatctccttctagaagcagtaatcttgtcgataatttaaatgatgaattgagtgttattcgaagaga
aatatctgatattaaagacgagttagagttgagcgaacaggaccgtgaagaagcgttagctgcccaggcaaagttgtcatcacaagtcgatcaa
ttaagtaatgagaaacagaatatgttgtccgattatacaaagctaaaagatgagcttcttcagacacgagatctactttacaaaaaggaccttga
agtgacagatttaaaccttaagctttcggagaaaagaaatattataacaaataaggaggaatctgaaagacaaaggaaaaaaaatcgaatcatt
tactgaagattatcgacgccttgaagctgataaattaaatctagaaaaagtggtatatcaattaagcacagccatgcaggagtctgaagatgatg
aaaactcattcaaagaaaggctttgtgcttttcttttagaaagaccatctacaacaagtttcagggaagttataaaccaaattgagtacttaataa
ggaaccaaaagcagaaaattcaggagttagagtcggaaatagacaagctaacttatgaaaatttgaacggccaatttaaggaaaattttggaat
atccgaagctggaaagtccgctctttcacctatcgataaacatggagttcaaaaaatgctggaagaatatcgtgagcggcttcataaagaaatag
aagctaagcattctctaagaaaagagttgcagactctaataaaagacagcaaatcatatatcgagaaggcgaaactgcttgaaaaagaaaaga
gctatttagagtcagaattaagaaaagctaggtattctaatggcaactttgacggaacacatgatgaagaaaaaaggctgttataccaaagagt
gaaagatttagaaagggatgaacggattcatgcccaacgctggatggaaaaagaaaacataccatctcctaatgctacaaagaatgaccggca
aacactcgactccctaaaagatgccctttggtcaaagactataaagatggagcaacttaagtcgtcgtatgaaaatctgagagtagagtacaac
attttgcttaaaaataagaaaacagaagatccgaggtacttcaatgagattgataagctttcaatcggatcacagactatacggacaagacatac
ttcagaagtacgagggttgataaaaatgacacaatttttacagtctaaatttcagagagaacatactcttcgacttgatttagctttctccaaaaag
tttattttattgcagctaactggctatgaaacgtgcaacaagctcaatttacgtttgctgcaaaagatcggcatcacgcccgataatgcagtcaga
aagaagaagctgagcttcaagagtgttaccatactgatttgttccatacaacgaatgaaacgaatgagaagcgagtggcagaagcaattggaa
gtaaggcaatatcttcaaaaagcagctgaaaaggcacgtttggaagctaagaaaactaatatctaagttattgtggaatggaatgaatatattat
taatttatgaaagcgtgaggtgaataatggtaaaacaaaaattcattagtctttcgtaaaaagtgaaattaattattagtcaagtaaaggtaatat
cgagaagtaacttaaggtaggcaacgaagtgtgaaatgctaacagattgcaaaaagtgtattagaagtaggtattaaagaatcagggcaaaga
gagcgaaagtacatgttcaaaatgactgccgttataccccaaattcgaggaacatctttgatgttgaattcaacgtattcgagattagtgtttcgaa

atttcgcaatcttctggtaattcggatttaaaaatgaggtcaaaggaatacaatacagtttttccacttcaccaaaaccaaggctagggagattatt
ttggagcgtaaaggcaacataagtatgaattttggtatgcaggtctttcgttaataacggagggattctgtttatataagtaaagaaattcggaag
caagtcgatttcctcataagtctcccgtaaggctgtatagtaatgtgatccgtcagaaggttccacacgaccacctggaaaacacatttgacctgc
atgagatcgtaaatatgttgatctttgtgttagcagtagacagccacgatgctcagaattaattaagggctgtgtcgtaaaaacggtaagcaaact
cagttctctgtaagtttcgaaaatgtgtacttaccattaacacagaagcaaactttgtaggattgtaaggagacgaatattgaagatgagtcggac
gtcgatgaagtagctcagcctgcatttccaatgatttttcatttaaaaacccatgctcgtccacaaccttttcccttgcaagcttttgtagtgctatct
gttcataagattcttctgtatgaatttgaccataagagtcaacctttgctcgtgccacagatgtataggttcgttcagtaccatgcgaattccttcctt
tcgttttatttttcccaaaaaagtaatcccttgctgattccacgaataatttactaaaaagtttggcacggtccgcagccgtagagagaggcgaat
gttctttatagctttgtttgggaatttcataagtaaatttagactttttcaaatcaaattgggaaattccttgtctaacaaatcgttgaaatgtatgact
tctagcaatctgatggttttcattagcaattaataagtcaaataatttactactagtaattaaaatctcttaccttagatgctaagtacgtggtaaga
agttcaattataactttcctaaacataaagaaggcaaaggttattagttacccgaaacattacattgtagcagaagtgttcgcaatatcacctact
gaatgaattccaactgcgaaaaacatcataagaagtgtttgaaatatataattggcttttctaatacattatagattacatccaattaaactccctt
gaatataagaagttgaatgagatagtctagtagatttggctttatttggtttccataaatgaatttgttggcactatctgaatgaaacgtaggagct
agtagatacttgaatgttttcatgcattcataggaacttcataagattcttctaattaacactaatgtacagtgagtagagtacatctacccaaaaa
aaaaggtaggaatatttaaagattgcaatatagccaaaagtataggaggttgatatcgaataaagatttactccattctctcattttctcaaagag
cgatatgtctggccaaagttgacatgcaacaaatttaactgctcgataggataaacaacctcctttttagttcacaaaacccatttattctagatttc
cattttcgtcttatgtttagttggcgatagagttggatagaagtcgtgggattttcgataactgtattcacattccaatacatgtaaatactctttgtat
tttatgctcttttcatatagaaacttcattttcttgttccagttcgatagcgcttttgagtgattctttgatccaggacaataacagctaagcattttgtc
atgcacattttggaattcgagacaggtcttaaggagggtaggaacggtacgacgataattgttgactttttctcttaaaggtctgggaatatctcgt
agaccttgcatatccgtagattcccaattttgtatttctactaaatctccaacgtaggtaactgtaccatcagcggaagtgtaatggggtttttcttca
gctcgtcgtaaaatcgggccccaaccatattcggtatttgtgacattttcaggaagtggatgacaggatgccatcgcagcattaaagttaaacgca
tggatccattcattcaatacgagaatgttgctcacttgaataaggatgctgcattcaccaggaatactaagcgtaaaacaattgtcttttctcgagg
tgcatgatgatggtatattgacacttgcaaaagtattttgtaaatcaagttccttgtaaatagtaccatgtttttcccaaacttcagggttgtaaagg
tctccagaagcaaacatagataacttgtatatatacagctttccctgggcaactttagcatagcgttgacaccaaggaccagccgaccatttgcca
ttcttttcgggaattaatttttgtataataataatccttgacgcaaccaaggtaatttctcgtgggttttctcttctttcatatgaatctcataattgaa
catttgaaaatgtggtcttccgtctatcacgtcaggttcaggaagatctatagttttcgttgcaaaaaagccagcattttcttctgttaatacatagg
atgaattttctttaaacgcgtcattttcgctggcaatattcgggtctgctattttcttctgtggttctgctctgctcaacaattctgacattaaaatcatt
ttatcccgttgtggactaacatggcctctaacttgattacataattgtatataaacagcctttggtacttggaaaggattaacaagaaacaaatcgt
aaaattctctaagcatacactcgagaataaatcctatctcttgaggcatactattccaagaggcacatagaactttagctaaaatcgttttcgattt
cccatacttcctccattttcgtctttgctcaataggaacagcacacacagcttcagggtggattaaaggctgtataacttgaaacgttgatttcaca
aaagtctctgctgatgtgaaattgtacattcgtcgtctagtgtttgtttctttatgtaatcgaaccagggattttaaaagctccagaactatatcttca
gttaaaaacccaaatgttgagttctggcttaaccacttagtagcaaaaatgcgtataagtcgtgagttcaaaaaatcttcagaaccatagtataga
tcgggacaaatattttgaaaggcctcgtaaagactcatatttttagatcgtagtacgaaataaaactcgagaaaacttcagtcttattagagaaa
taatttacgaaccaatcagtgaagttataggatagtaattggttttgttgtagatcccatagacagtgagcaagtttcacaggatcagaaaatctc
gaatcgctgcagacttgtaatagaggaaggtaggatatatctgggatatgcaaaacacggagagcgcagtgagggactaaaaacctttttgttttc
gtaattatctatttcatccgtttctttaacgctggatgtaggggtctcatcgtcctcaaacttcaagtccaaattgtttaatgtttcaatatcatagata
tcgtcaccttcatcggttgattcgtccataaactcttccaaaaacgtttcttgtatatccatggtcttgagatctggattcgcaaataacttatttcca
attttctgtggtttagctcgaacacttcgtaaatttcccatttttttgatttcttcagcgattccgttccagtcttcgttttcgcgacgaatgttattttgt
aattggtataaataacttctttcatcgtaactaaaattctcatgatcttcttttaggaaatttacaaaagacttaagaccttctggggtgtctggcgtt
gaaaagattctatcccgtcgcataagttcctcgcataagtctatttcagacaaagaagcataaaacgttacaagagacgattgaacagtgagtat
tctctccaggggagctttaagataccttgcaaacacaagcgcaaactggtattcagcatgtttcagatcaaaaacacgaagcaaatgcttcgcag
ttctgcaaaaatatagtctatctgaaaggcgttctataagatatgattctggttctgtattttcatggtcagcagtaagtatacactttggctgcaga
gaccctgctctcgtaggctgctgtgaaacctcattctctttgtctaagtcacttttctcggttagaatatcatttggaatattttcctttttgcaattcctt
caaagcatggttttgaattataaccttatcaggggttaccgggggagtcaacttggatattggttcgatttcgttctctttagaagttaaacctttgg
gtttcgcttccttagaaaattgaggggtactagcaaactgttcagctaatgagcgaacatatcccttcggtggtgttgcatcgtctgtatcaatatca
ttcggtatagcatctaaatctcccaaattggtatcttttgtatcatgcttcataaaatttgatgtttttttctctaattgcctgcaaaaatctatgagaag
cagtctgttcttgcaatatgtctgtttcggcaggtttcgcgggttcattgggttgattcctggatgcaaattttgtgaacacatctctaatttgatttac
tttttccgtaacaatgttagaaatttgatcgggtcggttagtctccatttgtaagccattttgattgacctcgggcatcacatctttcctctcttcattgc

gacgccttgcaagttctattctggcacgaacatttgctcctgaaacttgctgttgtatttttcgccgagcagataaactcaaggtaccggatggata
gatttcattcgggaagttggacacaaataaacgaggctccggagtaatgtttcgtttttttaagggttgaagatcttcaatatcatccaattctctttt
catagatctggggctactattggcacttctagactgtttctcttcattggcatcttgtgccatgtgagaagagctgcttacgggagcactagtaactc
ttttagctaacgagtcatgattcggtgatttctttctaaatgttaatgaagctttggggcttctctttccatcatatagagatttgagcttgtttgtggat
ttcttagtgggtgcatcagcgttgagcacttgtgcagttgacatgaaggatgcatgatttgagggttcaaggtgagaagtacggcgaaagttaag
aaattcttgaaaacgctttgatcttggcaagcgttttatgccaaatttgctatgttcacttaattcacgttcggtgatttcaacatcaacttcgtccaa
cttcgacgatagcaagggcttatcttgatggtttttggttaatatgtcttgattttgtttataacgattcaatgcagtcgctgaaagcaatttacttcct
ttgcttggataaggtgaaccatgagacattggatcgaattttacacggccatccctgcaggattcaaacccagccaacttttcagtaaaactgaga
tgagtcggggacgtttcttcttcaaacatggtaatgtctgtgaaaatttccagtgactggagctgacgttgatcttaacttgaaggcttgaatttgct
tggttgagttacacgataagtcgacgacttcagatgccgaaaaacaatgtgaagcagatcttatagaaagaaatattatataaatgattcaatga
tgtgcatacagacggtccggttagtctgaatatagagaatctcctattaatcctccgggtacaacgcaaattcgagtctgcaggaatagtctccttt
tatggaatggggaatggtgaaaactaagatatgttttaaggccaactatatgtatatatcaacaaacaatggctctagcaattttaagtcgacata
aacaaacagctgtcatccaagtgaacgtaaataaacattggctgaaagaagacaaaagcgaaaagacaagtactttgacgaatcattgagtct
atttaggtttttccaagtaagttgatcatgttgattctcaattctcgcagcggactcctacgttcattttcacaacttttataaaacgaaaactttcact
atatttgattatttgtaagtagtaggataaagaaagtatttttaaaggagtaagggtattccgccagaacgaattacaaaataacgttttcaacctc
cattcttttgaagtccatgattagcttgtcgaagcatttataaatctttagattaaattgcattcaatttattgctcaaaagtaaagatgatcccatcc
aaatcacatagttgacgagaagaacttcgtgtctgaatgggaagtatggaggagaagaatccagttgttagtgagcgtacgatattaaggcaaa
gcacagaccaaaaggaaatactctagaatacagtggtgaaatggatacgaattttaaaaagagaataataaaaacattttctaaagatcgaca
gttatcacagaatattttaattgaatgcggtacgaatgagacctctggcgcgttgtagaaggaagctaatatgaagagcaaaatactggtttatgt
tcattgatagatagtatctgcacctattatgaagcaccggcggttaacagcaaaaaatcagaacctaaagaggcaagcacgaaaaggaaaagt
cgactccaaggcgatactggagacttggtttatggcgtctcaggactttcgtttcagtcgcctatgagaaagcgttttgacacatatatatacgagc
gtggtatcagcataactatacccggagaagaaaatttagtggaattatggctaccttggacagatattcatttcgtttgtcacatacctggtcctag
aaaagcaaatgttcagaacaacataatcatatttaggaaaagcaaattcaacaataagtcggttttggagaattcaagctcagaaggaaatgaa
cccatattttttactgcaccacattctttggaaaagctcaacctagtagaaggcaaacgttcgtttactcattgcacaaactcatgggatatctttag
agatttatttgaatatatagggataagcatcgtcgccccatctcaagaggaatttgtatgccctgtagctcaaaccggggacaatggagtctctta
cggggtagaagcaaatcacaaaactaaagatggtatggtttatctgatataatatgaaaaaaaattgctaactggctacttttcttaggctactta
tatttcttaaagaaaggagttctttggggatttcgtaaacccatattgtttatcgatatatcagatattcaaaatttttattacagcagtgttttgcaa
agaaccttttcaattaattttgaagtaagaaacaccatcatatcttttgatatgatcgatcaaagcgtttttcaagctttgaatcaatacgcaacaac
tcacggcttgatggatactagcttagcagaagaaaaagcagcacctcctactaagaatcccacaacctcgtatttaaaagaggctgatgacgac
cacgagaatgggtcggacgataattattctgacacggatggtccgaaagaagaagaacaagaagaatcagaatcagaaaatggcagtgaaac
tttggactcagatgggtaatgcatttggaatgtgttcatcaatatagctccttttcatactttcatttcagaaagtgttttagctatagattaacgaag
tttagtctttcacgtataaagagactttttttcaaggaatttaatcttaaggaataagttggaagtaaaatactatttttttgttagctttctttcctttgc
tactgtataaagttcgtcaattatcagagatcatatttaaagttctttttttagaagccaatacttttttaacacgattactcgaccaattccatttaca
gtgacaccatacaaaatccagtctttcaattcgagaattcctgtcgttttcacgatatactttcatcgtttatttactatcgtatttcaacgtattcgtt
aaaaaaatactggaatgcaacgatagcgtaattcaaatgagacttggaaatttgagtaggaaagagaaccaaaactaaaaccaaaaacagga
tgccgaaaagaaggaaacagcgtggaaaaaagcagtcttcaaaacagaatcctggaacagatgaggtacaggatcaggtagacaatcaaatt
ccttcaaaaacagctgaggaaggattcgaagaaagtggatacaaggtcaatccttacacgaatgagcctgtgcaaccattccttggcgcattgg
atcctgaagaagaaaaatattttcaagaagcagaaaaggcttttgttagtcattttacgcgtgataatgaggagacacggtattttattgatagcg
tttatcgtgaagtaaaaggaaaagagttaatagttttgaataatgtctttggttccaaggtactagagcatatgtttcctttggcaacacctagcca
aattaagtctatcttctcgtctctaaatggtcattatttagagattacacaaacaacgtttggatcttatgcctttgaaaagctcttaagtcacatggc
agagattgttgatctagaatgcagaggagctttggacaatgaagaagagatgttagaaggagaagatcaagtgttcatcacggcagagtcgcta
gtgatgtttatgtgcaacgaaatacgacctgatttctctgtccttatcaatcataaacttgctgtccacgtactgcataagatccttatgttattagat
ggatatcgatttatctacactgaaggtcgacgcgagtccatggtacgtatacgggtccctgaaactttccctcaatttgcacattccatcattgata
gtgcagtggaaaatttgagtgcttcagaactgcgtaactactgtgtggatcgctatgcttctaagatcatgcaagcatttgttcgcattgattttgag
cgtgcacaaagtggaaaaaagaaaaaagctgcaccgctggctgataaattgctactttctcctgataacaatctgaaagaattaccttttcttgat
actctactaaaagacgaagctgcatcccgtattttagagatcattgttgaaaagatgcccagttcccaaattcctaggtttcgggaagcctttgaag
gccgcttttatcggctgtgtgtgcatcccattgctaattttgtcatgcaacaatacattatgcgacttcctttggaagagctcgggtatgtggtacaa
gagttgaagcagggctccgataatgtggtacgtaaatcttccttggcagtcttgaaaactcttctacagagatgcaatacccttcaagcccatcag

catgaattggcctctttaatttttgcttctgccgtggatgaaggaaagaaacaaaacataatacctacccttttgcgctcgaagcacaaacgagat
gtcaacgatgctgataagaagaggaaactggttaataacatgctaggcgcacaattattagaagagtttcttaacggtcctaaggaatatattca
ggtcattcttgataatattttggaactccctcgtgaacagcttctggaatactgtcaagagacggtttcttctcatttgattgaaaaaatattggattg
gccagacctagatttcgtttttcgtaagaagttcctgaatatgttttcgggtgccattgtggaattgtccgtatcggcgccgggttctcacatcgttga
caagctttggaaagcttctcttgggttgcctctttacagaactcgcattgttcaagaacttgcccagggtggtgacgaaatcaagtttgacttttatg
gcaaaaaagtatggtcgaattggaaagtggaactgtttcgccgtgctccagatgaatggtatcgttttatgaaacaggatgaaccagtgaaacga
gttcatgagaaagctcgcagtatgccgagtacgtctgcaaacgctcaaccactccgaaagagaccaaacatggaagaaagtacaagtgagaca
gacaagcgtgtcagggcttagtttgttgcaaactcacgaaaaattacaactttatttatgcttaactcggaactaggtttttttgcttataatttcatt
ggattttggggtgttcataataaatactacgattttgatcatcgaaagtttgtatattcgtatatttctacaattcgtggtagaatgtttaagaatact
gtttttttttctgtagatgtagatgttttatgtaaaaaaatattttttttgcaagctctcaaacttgctacttacagttcgaatctcatctctttcctaga
acctgctagctaactgaaaaccttccaatgaatcgtctttaatgttaccagaggcttgttactttcatgtggcttgctacgattcgttgctttgtgttaa
ccgtttaatgtggctggatgaagtcctgagttattgaaatatatagtgagctgatagctacacctcgcaggatggttttcaaaagcccaccgtgctc
cgtatccggggaagagagtttttttatggctcacttgaacgttttgagttcttcattcatcttgaaccattgcgcggtattcaaggtctgatttcaagaa
aaaagcatggaaattgacgacatttttttctcagaagaaggcgcaacccaaggaacagggtcccgttgagaaagagcagaaccaacaagaacct
aaaaagcctgaaaatgctcgttcttccaaacccaagaaaaatgtcaataaagaagacgatttatttttagaccccaagggggcttcaggaagta
agttctaggcatattgcgcattgtaggaacatgcaacgacaaggaagtttcgtcatgtctatcacgtaaacttctttcagcgagctgtatcttttatc
attttggaaattgaattgagcattcttttacgaaaagaaggaaggatgtcctttgttgacgcttgagtttaaattgttcaacagaattcttgacttgc
tgctgaaatgcgtttaccgatgtactttttcgactcggtatagttctttggtggttgaaggagatacaatatgttactttgcaagcgcattttcctcttg
ttgttaaatggtttgtttactaacaaaaatataggaaaacgaactgaagaaggctttttggtgtacgatgaagatgagctaaaaatgggtaaagg
aggaaccacaccgctttgcccttttgattgtgaatgctgtaaggaaattcttaaggtttcttgtgtccttcattatactaactttctataggttattaaa
actatggaagattgtttgggtcatttttctttaatcaacgctgttgaagggatagaacagtgtccataggatgaaatccttggtagtctaaattttca
ttgttgcgataaaatttgcagacgtaggtttgtttatttacatgatgttatcatccattcaacgtggataccttatgatctcgtaaacagtaaataaa
cagtgacggtttttgtattcctttattgtttatgtttagtatgttgtcctggcaacggaaaacatccaaaaatcatataaacaaatgcctggagcgcc
tttattttcaatacggtaaccagaaaagacattcacaatatcagaaaagagcatttcaaacaaattgaagtttgtttttcttttggtttagatacctg
gcctcagacattccttcctctctccaagagggcattgcctccttaactctccttatattacaatagtagtctgaacgttcgttcgcgctggtaaacaa
caatgtcttacgaaggagctgcatctgtcgcgaagaatcctgcccaattctttgctttggatggtctatctaatttccatgaccataatgaatctcttg
attaccaggagtatcttccagaaatggaagcgacctactcgtgtcctctttcgaccgtaatggagcagcctgagtactctcatcaaaagcaaattc
atcctctagctacttcttccttatctacttcatttcctactagaatcacagatcatatggactattctcctttcttgcatcttccgattgcttcctcacttg
aatcctcttcgtcctatttgaatgttcatacgggtggtatttcagactctcaatcttacagttcttcttatggtccttctctgtctcctccttcttcaccctt
taatacaggtatccaaacctctatggaccccccatcaacgattattgaagaagatgaacttggttccttacacatcagacaacccaatggtttgtc
gtatgaagaaaatttgacatccgcttggaaaaatgacttggcaggtgagagcagtaacccttcttttcctgaagtacctatcaaagaagaaagtg
aacctttagcctattacgataatgaagattactgtccttcaagtcagctgtaccgtgaggaagatctttattcttctactgcgtttcctagctcagatg
ctattgaaccaattcaggaggccatgtcaaatgagtttgtcacattgccttcttctccccctcatcctgaaggaccatatgctcctatttcccctgata
agttgacgttgcaaatcgcaatttgtgagccctctctcacagatatatccacaccagacacacccgctaatgtctcacctgtctctccgtttgccccg
ttgaccaaggtagagccggtctcacctgtgaaaccttcttttgcatttcaaaatttacctaatttaagtataaatacgagatcccttgagcctcaggt
atccctcgccggcgaggtcttgcaacctgaaaatcaaactttgtcatccattagaactctacaactggataatcaacaaaatgattttcccgacatc
ttccaaaagaaagattcttctattcccttttccaaggacaatagccgtaactgttcttctgaaaacaaagagatttcgactatttataatcgtcaag
atgaacagaaccaggagctgaattgttttatctcttctgaaaacaatcacaaagaagctttccatgccgttcaacaacccctcgtgggcgcttctg
aagcattgcttcccgtaaagcaggagcctttggatacccaagatatgcattttgtacgaccattattgacacccaaacctcacatgaaaatcactg
aaaataatggctgcaaaacgtctcgaaagcaatcctcacgaatttgtcgtattcctccggaaactatggccagtctgtatcgtggacctgaagaa
gacggtaaatttatttgtttattcaatggttgctgcaaacgagttcgccgaaagtataatattgagtctcatattcaaacccatctaagtgaccggc
cctatcgttgtgaagtctgcaaagcgggctttgtcagacatcacgatttgaaacgacatctgcgtatccatgagaatggacgtccatatgtctgtga
atgcagcaaaagattcaaccgtctggacgctctgaatcgccataagcaaagaaatatttgtatcggaggcttaaaccggagatgaaacgttagg
cctttgtaatctgtataatgtgtgtgtgtgtgtgttgttttttttttaatttatgatgattaatgttttggaatttggtttttactttttgtaatttaatgatta
tttgctactttgtcttttctttttctacgcttatttatataactattcgtttttgtttttggttttgttgtttgtagatactgagttgggaattctgactttgat
agtaccaattttctagattcttagctacagtcattaaaaagtgtttctgcagcgatttattttgaatttctgtaattttttctatcatatttgagcatctc
acaagggttttgacaaaccaggtttaagtgaatacaaaagtaaaaggacaaaagtttgaaagaacttacacatattaaatttttaaattgcatgc
ataattaattcgtaattgttcaaaacagacatgagcgttccttaagaaaagactgatggtaaacaataaaggctttcattttttgctgctgcgtgagt

gttctgataggaactgtagaaaactgaatctttaatgttattgctatcacactgaacgattaccgagacccgctagatgatgctttttgctgcctctt
ctattcatgctctttcattatttgtttactgttttacgaatgctaacattctcaattagcgcgaaatttgcaagagctagcatcctcggaaattcactta
taatatcgttgaacatcagtaagggaaaataaagcacatatttaacgagttcactacaccctaccctaagtaaagacagtcacacaattatatgt
cgacaaccacaactttctgattctaccatccgcaaggaagaggcaaaactttgctgactatgcagatctttgttaaaaccctcacgggtaagacta
ttacccttgaggtagagtcctccgacagtattgacaatgtcaagggtaaaatccaagacaaggagggcatccccccctgaccaacagcgtttaatt
ttcgctggtaagcagttggaggatggccgtactttgtccgactacaacatccaaaaggaatccacccttcacttggtcctccgtcttcgtggtggtg
gtaagaagcgtaagaagaaaacttacactactcccaagaaaatcaagcacaagcataagaaggttgagcttgctgtccttaagtattacaaggt
cgaagatgatggaagcgttaagcgtctccgtcgtgagtgccctacttgcggtgcctctgtcttcatggccaaccatggagaccgtctctactgcggt
agatgccacttgaccctcaagttggaagcttaagtgttgtaataaaataaggaattctcactataaattgatctcgtattatagttctattataactg
aaacttagtagaaaccgatctgttgattggttcatatattgtaactcggctgttaccggatttgagtaacacagtgtaaggtaacacatacgatgtg
atagatataggggctatttttgctttttcgtctaccatgtagtaaaggcaaaaagtagtgaactgtttcttatcatttttgaataaacccattttgaaa
gctaatttcattgaaaaagaattacttctaaaaggttatcgcagctttgcttcgcatccaactaagttttgtcaggttgggttcagataggtaggcta
atacttgccttatataagtacatatacaaattgttcttttatttagacatccttctcattgacttaatttaagcgttttgctaatgggtcgtagtaatgc
aactcgcggaggcacccgtggtggtcaaggtgaatttcagtgggatcaggtaaaaagcgataaacaaaaaggaaattatttggggcaatcaatc
tatgctgcaagtggtcgatgggcgcaaggacgcgacctagagtggtggtcaaaaggcagaaccactcaagcagagcccaattcgaataacgaa
gaggcttacaaaaaagagattttagaagttaaatataaagagcaagttatgctagctgaaacgctgggccttcctcctccgcctcccctagaagtt
gaaaataactctcaaaataccctgacaacaaaagaaatcaaagagcagcttcctactcctcatcatcgccgaaaatccggtagacgagaagatt
atccgcccaggagacatagatctcaatctccttcccacggtattcgatatcatcggcgagaaagacttaaaggagatgattatcatcacggtcga
catagagatcgtagaccccgacgttattctccaccttccgatcttcctaataaagaagaaatatttgctccatatagacactccccccatcatcgcc
gacatgtagatgatttacctgaacgggattatgaaaaggcttacaatgatcgttcatctccccctcctagacgtaggaaatattaaaatggaagta
tataaatatatatatatacatctctctgagttttggtgaaaaatcttatactagtacttccattcttgtaatttatttattatagacataaacaggcatc
atttatctatttacttaatacttttctgacataatattcacaatcagtgtagtccaaccagtaaaatctttagttcgttggcccacgccggttacagag
tcgtactgctcccagaatatgcctgtacgcttccagttgttgaaaacagtgtttacaacgttgatacgaagttcctcatagatagatttagccaatcc
ctggttaggtccagggacatggatataattttggtacagactgctcaaaatcaagtaattcatattcatccagacagggccacgccagtagttctc
gtccgttccaaaataagggtcattcaaactcaagcttcgaataccataaggagaccacaactcactttcatctctaatcatatgtaacatagttgtt
agcttttcagaatcaggaggcaaaaggcctaacatcaatggtaacaacgaaacatatcccttgtgacaaacatatttaggatcatcgtattcatc
aacgctagcatcacagtaagtctgcatttcctcatcccagtgaagatcatccaagtttcgaagcatagcatattcattgttggttaatctttcggctt
cctcagtttcacctaagaattcagcaacaaaacgtagactctttgtgaaagaagtcatccaactaagcaaatcaacatgcagctcagcaatacta
ggaggttgggctctgggataatcatctaatccactggcgagacaatgtcgatatgtacgaccacgccagcgatagccttccacattagaaaatga
ttcacgatcccatgtgtcaaagtcgccttgctgagtccttctaaaccattcatagtggcgcaacatcaacggaaacagttctcgaagaaattgtata
gacatttcaggatttgaaacagaaacagagcgaaggtattccaagtcttctaaagaataatcggagccaacgtcgtcgaatgatttttataatct
tgttctttaagtctttctatgtaagctttcaaagccaagatgagagtgggggattcgcaatatcgggatattgagtctgaaactcatcaggaacct
tgctgcgagcttcagacccaagtatttgttcacggccaacccaaccatcttcattaacaagagaaaaccaactctttaaaatttccagactgtgttt
attagcttgaattcgtaaaatatgttacatacctaaaatcgttgtcccagaggccaattggcaaaagatggaagccttcatcccaataaaatcctc
gtgggaaatgagatcgactggggatagcagtgagcaaatgacgctccttatcaaagaaagcagaattttcagccaactttccctttgcactttcga
agccttgcataaattcataatcttcatcatcaggttcgataggagttttgcttacgattgagtcaccttcaaaaaatccaacgttaccaaataaatt
ggcaaaagcaaactcagcaaaacgttgatattctggattttcatcataagggtacttgagtggaaatacttcttgaaatctttttgagaaagcttta
tgattttcttcaatcgtcttggtgattgtttcctcagaggccactgcggttttagcggaattgaaaataatgtcgaaaataaactctcccttgaatac
cttttggatgaactggagaccatcaccacctggaacatttggtaaaagcaaggtttcataggcaggaggtaaatcatccaaggttttcatattttcc
ttttcgcttaagacttgcatttttgtgtctagcaaatatagaaggatttcagaagtttgccaaacttcttttgaattgattttaaagccagcaaagtaa
tccttgtccattgtagtactttccaagtcttccactccactagtactaggatgctcgcccatgcgattataagtatgaacacggaatttacctaagtc
aggtgtctgaccttccatgaaaactttcttaggagatgttgatttcaattccaagttgccttctccattcacataggcgtagaaaaatagagctgtgg
tcaggtcctcaggtgctccaggcagaggagttccatgaacacgtaaggcccaatttcggtcaggtaatttaatgaattcggtttccatttttatacc
cataagaagatcttcaatcacttgttgaccacctcggcgaacatcatacgaagtccaaccaaaagcgccaatatcatcgccatgttctgcacggt
gacgaagttctaaagacaactgttaatgttttggaaggataaagttctgtgccaacatactcgaaaatctggcataatcatcgacgtttgcccaca
ttaatccactcattagagaatttgggacctttggacgtatgcctacgtatagatttgggcggtaaggtccccataacaaagagtcattcactacatt
ttcaattgcgtttacgtttgcgtgacaaaggagcgcattccagagtaaaaacacagttcccaataggaaccatgaaattactcgacaggtactact
atataccatcgcgtactctcactagaagtatcgacgatagatttgtgtttggtgtagaatgcttccttgctacacttataatgttattttatttcctgtg

ttacaatacgctagtaaacgaaatatgcgaatgtgaacacgcagcaaagtggaactttttttgttaaaaaagaaaaaacaaataaaataaaagct
tatggacacataacttacatatttctctttaatttacagttcagataaagaggttggcttacatttacgtttaacagtatattcaccttagcaaagtca
aagtccagaatcacaagtttctattactagaaagatttcccgtttaagaactcttagcaagttcagagttaagctcagactttactgcatccaaaaa
ttcaaaagtatcaacccattccttgacatcaggactcaatatgcaaagatcctttgtcataatacccttctcaacgcaattaacacaggcacgctca
agggcattagcaaagttgatcaagggttcattgttatctaagcgaccacgttgagcaagaccacgagtccaggcgaagatggaagcaatactgt
tcgtagaagtcttcttgccttgtaagtgctgcataaagtgacgttgaacagtaccatgagcagcctcactttcaaaagttcggccattgggatggat
caaaacagaggtcataagacccaaagaaccataagcttgagcaacaacgtcggacataacatcaccatcatagttcttgcaagcccaaacaaa
tccaccattagacttgatagcctgagcaaccatatcatcgatcaagcggtgttgataccataaaccaagtttttcaaactcagccttgtagtcagag
tcgtagatttcttggaaagtatccttgaatttaccatcatatttcttcaaaatagtgtttttttgtagaaaggtacaaaggcatcttcttttggagagcc
atttggaaagaactatgagcaaatccgcggatagaatcatcagtgttgtacatagccatggcgacaccaggaccagaaaactcgtaaacattat
gggtctgcttagcagcctcgttgtttttgggggtaaaggacaattccaacaggccgggtccggaggcaatcatgtctgtcgatttgtattgatcacc
aaaggcatgacgaccaatgcagatgggctttgtccatcctgggatgtacttggggatgttgttgataagaataggttcacggaatacggtaccatt
caaaatgtttcgaatagtaccgttaggggacttccacatcttcttcaagttgtattcctgaacacgagcttcatcaggagtaatagtagcgcacttc
acacccacgtcgtgcttgataatagcattggctgcatctacagtcatttggtctcttgttttgtcacgagcctcaataccaaggtcataataatccaa
tttaatgttcatataaggaagaatgagcttctcacgaataatttgccagataacccgagtcatttcgtcgccatccatctctaccattgggtttttca
catgaatcttttgaaatggcttagaagatgagaagcgaatattgcgataaaggctcactgcctttctagcctgtttgaaaggcatatttggacgtat
ggcaagcatcctgtaataaacaaaaaccaactatgtaactaatcaaatacagacaaacaaacttccagttgtacagcgtcttagatagttggtag
tttacaagttgctttgttcactcgttaaacaagtcttcggttaatgggtaagtgtaagattgattttaagataaggagattaatctaatcagatcaag
gttgtaaacaaaagaggtaaatattatagaacatttttaaaggatctatgtatattatatatgagtgaaaggagtattcaccttaatcatgatttca
tgtggattgaatagacatcgtggtactattgtaatcttgcatattctcgaatacttaggttgggtagaactaactgatcaagaatctgtttgatgctc
attataaaaataattgttcaggcctttattgatatttattcctataggcaagcacacgagggctaaaaacccatttttattttagaactaaatcatat
acgctgcttccaaaaaaaaaaaaaaaaatccttaaataaaaaatagattctttcggtacaaaatattgctctttatattcgagaatcagctaattta
gacagtagtcattttcactattatttctatgtctaatcactggtttgaccatatacagactccaaatcattctggattagttgaatccagtgagataac
caaggaatactttcgttttcggatttccgtttgggtaactctgtgaataaaagattcaggactgcttctttttcttctttggattccctgataagttcttt
taagttttcatcatcactgtttacgttttctagccgagcatccatcttcttcaaggacatgtgcattaaggaccatgtccaactcttggcccatattga
tggatccttctgatctagtaaccaccgaaacacagacgtaagactcacaattgaatagttaagcatcttacccaaccatataactgcaatacttgg
ctgtaatctccagcataaaaatagttcatcaattgtttccatctcggttgataatgattttctagaaaaatgctttataacatctaaatgtttttcgaa
agcattcaatgcatgggagagtgatttcgaaccaagactataattacacgccattaagaaccttactccagaagtttcagatgctttattttcctca
acagacgagagtgctgacgaaattacttcaataggtttatgtatccttaaatgatcggttatctctttaaagcttgcgtgcaacggatgggactcgt
tttcatacgggatttcaggcttcggtggttcagcggtcattaattggcgcaagggttccggtagagaatcatggatacgtgaccaatagctcaattc
caactccttggaaattgtttctctcataaacactttttttgggtgaaagtcttctaactccatgttacctatccattcctgccacttccagaaaaagttg
aaattacttaagtggtgagaaaaccagtcaataaatttttcaaaagtttccaatggaagattctcagaaatattaaacacaaaacgaataattct
accaaaagttggtgctaaaattttgggagcaacccgacaacactctgtgagtaacgagtggtaatagataggcttgaaagcgggcttggccgtc
gtcagtagctctgaaaatatagcttctgttactatatcttctaccttccatcgagactcctctggatccagtttgggaagctcaccaaccggagtacc
acgtaaagcaaatgtcttataagtaaagtaaacatcaaggtccgtcaaaatttgagcggcttccatacggttatactcgagatggttgatgatgtc
agcagtaacatcccgaaaaatactggaagcgatgttaagagtttccggagtagtacggatttcagaagtatgaaagagttcgacaaaagggcg
aaaatatggtggcttgagctgttcttctgtaatagaaggattccattcaaaagttttcaaagagacaggtgaaacctgggctaaatctgactcaaa
tgcttcccaagggcgaggtaaataagagaactgaagtccatttttctttttcaagaatggtttgacgaaataaaagatctaattcttcctcaacatac
tcctcagagttagaaaaagggtttttaacttgttgtaataatggttttcttgattcaacgtaggatgcacatcgttgaatccagtctccaactaaggt
gtttctgtcaggatgtttagaagcggtacagtaatagggaatgtttatgagaattattttcagtatttgatctccaaaaatggaaggtgtcgtttgct
cactcagtttgtctaaaaatttggaaaagacgggctctacaccttctgactcgtagatacgactaagacatataagtaaacggaaaacaatctta
gcttggtaataatcgaactgctctaaagaaacagtcagttttgattgtagaatatctacaccacgctcaactaaaagaggtgcttttaatgctgccc
gcatcattagcgttgccaaatgcgctactttgacaggaatagaaagagcactgtatgcatggttagagtaaagcaatttacctcaaaaaaaaaa
ggttaagaaaacaacatacgtttcaagaatgcagttcaaaatggcagacacactctcttcttcatgagactcacggctcacagacatagcaacat
aattaatatcttctgtaacgacagggttaacacgagggtctgctagaaaggtgatatctttttttatgcgaaaaccaagaagttcttgaggatttgc
acgattagggcgaaacccagagtcagattcctctctaccgcgctttcttgatcgtataggtgtgtgaaatgaagacattttgcacgtttcgatcgcg
tttgtgggtttcgtagacaagtaatcaaagatccaagcttaatcgtagcaggtaacaatgaagcaacccaagtatcataaacaaaggctgggatt
gccagaaaaatcagaaggataaaataaagagaaacagaatatttcctgctactcaaatctcgaaacttttaatttttccttctagaatcaattcag

ccatccaaagaccagtagctggtccttgagtaataccccagttcgcatgacctgcagcaatatatattccagagcatgagactgttagaatagga
attcctgttaatctgtttgttgggagaaaacaacttttggaagaaatcaatttggacgtcatctgctctgctagtagcagatctaaggtttcttttagg
tccattaaatgctggggattgactcgaatagcttcattgtctttcggtaatggagtgaatagagtagctccggtcaaatacattttattatctggcag
aaagtgtacttctatgcttctttccgggtattctggattctgtggattcacattgcagatggctacatattcttttgattttgattcagagccttccaaa
tcaaacaaaagagattgagcaaacagacttcctattccagagtaaggaagaatatagccagtccaagcacctgcgctcacacaaactttgtcag
cgtccaatattatggtatcctctttcccttttggcacataatgtacgcaattgtttttttacaagaattgcttttccatacaccagttgtactccacgttc
gtgaattttctcaaaaagcttttgacaaaatttcccggcattgatgaccataaattcatctttttctccaagcttagagcctagttcacaatgttagtt
agagtttttttattgaaaaaactaaaaacagtttacttgcatttttgaaggtgttcatcactaaaataatcgaactcttccggtaattttgcgacaccg
tcattcaactttataaaattcattagccaaggctgtacggaacggaatccccattggtttttttccatcatataaactactcaaccgttggtgtaacttt
acagagattttcgtcaagggttgcactgcttcgttttgccattgagaagtaattaaagcagattgaactgaagaggatccagatgcaatgccactt
gactccaaaattatgatttccacctttccttgtttgtataactcatgttctgtcaagtaaaagcttgtgcaaattccaataatgccacctatagactca
tatggtaaataaagttcatcctatccaataaagaaagcacacatacatccaactataatgcatcttagctttccgttctctcccatatttttcaaagc
tgttctccactttccctggtattgataatcatcaattgtcagaaaataaatatttagtattggtaaactacccaaaaaaattctctccttcttgtcctat
tttaatcattgttcttaccttctcttcatacaataataatgctgagaactattccttgtaaattcattcgatcaagattgtctaaacatgacgacggca
ctcattcactattgcaacaccgtcaaacatacatcaattcactcaaataattcgtttcaattcaatactgtaggatgaccaggaactctccaagcag
gcctttttttttgtgaaaacttttaagttatttagtgaaccaggagagaatccatttcttccatttatttatcagcgaactccaatcccagaaatgttcg
ccaatttaaggatacctaaggaaatacgatatgtttctctaccagtgctgtatttcaaatggtaaggaacaccatttacaaacagtgtttttcgtca
atattatctggcgatgctatggaatttgactttttctcttttgtagctccgtcgagaatacaagcgttagcctgcccatttggcagaattagacgacat
cgctttcattctttccttgaattgttgcgccgcgtcagtcatattcagttaagtgatattcctgtgggccgagcgacgaaaaaatgtatgcgattttga
cttaaaactacttatttctaactttgtagcaagctcttttaatcctttggcgttccccttagggcgcattatttacaacttcaccacaactctggatgat
cagcaatctttattagaagaatatgagtattttcgaagaatattcgtcttcatcggcgtaattgatggttctgagcaacaagactttgcgcaattac
agtcttccttagatgcttggcagcgtcgagttcctcatgcttcagttgcaaaatgtcttgtctttgattgctcttcagaatcggagggaatatatcagg
atcctcgatttgtacttggtccacgaagcaattcatcggtaaactcagttatgaggtcgatactttgtgatattacagctgaattgttagaagaattt
agctctctagaattttctcttcatgctaggtctgtaatcctatctcccatgacagaaatgcctcatttagctcccttacaacgaacaaactctttctcct
caatgcgtagtcctcgttcctcaaccaccactccggtaagcagaacaacctccattccttctgttcgctccgttacagcagttacagatcgctccaa
aagtctttccaaaggtcgtatcgaaaatcaattagggcaactatatctcttggctgggaggcttcccaatgctttaaagcactttgcaaatgccatt
gcaacaacaaaatctacaagtgattatctttggcagggactgtctctcgagctgtttactgtatgtttagtattaatggctcaattacatatcgatgt
tcaaattcctcataacataactgccatgtttccttcctataccgatcgttttaatgaacatggccctttaaagcttgatttttttgttcagtttcatagctg
agactcgaaacgctattgaccaactttatcataaatccactttgcaaccaaatgattcaatacctggtctttgtttttctgaaagcatccttcgatttt
ctcatcttttgactattgtttacgtatgtaatggccttaccgatgtcgctttgaatcatataatctctcagtctcccttgcgctcttcaaagatttctgct
tcttacgtccctagcaaagcaactatttatcaatgggttattcgagcccgtggacagcatttaaattatctttccatccgtgagaattgtcgtatctat
ggtgctatggcaaatatgcttggtacaattggcttccaccgtcaacgatctaaaatgttacgtgatatgttatttaacttaactccatccattgttga
actgagaaaacaaattgcatttaaaattggtatacaccctgaggttgtgaccatgcataccgcttcttctctagataggtccaaaattggcttcaat
atccttcctttgattcttgaagtatgtgaggaatttggtctcttacggctagatggcacagtcctaaatcctgagacaacaaaatggggctggacat
ctcttcaatttgatgttataaatgagcttatttctttttgtgaagcattggcagattaccagacaatactcaaattaatttctttgtttttcacaatttca
ccgtcttattctacttctgctcagcaaaattttctttacaaaattttcagaaaaacttgttcatttgctagcaatctaggaataactatccaaacacct
tattgggatccatttatgattacagatatgcagtataacggaaatcctgattttgcagagccaagacttgttcatgtgaaagcatcaaaagcaaac
tcatctcaaggtagaagccctttcatctataatcctttttttaaaaaaggataccgaccataaaaagagagtatgtgtgaaatccgctaacagttttg
aaactaacttcttttactagaatattttagtagttgatgagcctgttttttttaacgtatacttacgaaatccttttcatttttctgtagacattcaggaa
atttacctcgatacagaaggggttgaagttaaacattcaacatgtatgagtactttacgaccattatctacggaaatggttagtttatcaataattc
ctctggaagcaggcacgctgaacgtacgtggttgtaatgtcaaagtgtttggatgcgaaccttctgtacaatatttttacgataaggaaaaagaaa
aggatgctttgcatatttcactggaaaaaataaagcatgattttgatgaaatgcgtcctcctaatgtcgcaaagcatcaatggagcaatataccca
agctttctattaacttagaatccttaaattttcatatcattgaaaagcagccaacactgatagtttcttctaagaagttttcaataagcaatctcaatt
tcgctgaatatgaaactggcgaactgctttacactattgagaacgtttctaatacaaaggctacacatgttcgtgtatcgtttgatgacaatgcatc
taaagtgtatgagcaacttaaatctgacaaaaatgtcacagcagatttgttatatgagcttcagcatgaagagtacgaactgccaaccttcaatgt
tttaaatgaacaaacctttgctcttcaaccaggtgaaaaaagacaaattcatgtcaatgtctttgcaaaaccgatatctgggatgggtgccattatt
ttcgaaagcagttgccgaaatgacgatgaatctgatttctacgctaggcacttaaagctacctattgctttaaatctgagtaaaagagtgactttaa
gcggttggtctgtgcttcctgatatagaaaaaaatcctaattactgtatgctgttgttgaatttttataaccatcattccgatgctttgagagtttcctt

aaagaaaagggaaaaagacgtaattgaaagccgatttgtgaaacccaaagccgatcacgtcttctttttaagactgaaacgattccgtatgtcag
aggcgcaattaaatagagatattccaaacctttcttttaagcaatttgttctttccagtggaatgaaaagaagtcttgaaaatattacagcactgaa
aagacgattttgggtgaagcagcatctgatggaagaaattcaggctttctgggaaagcgatgaacaaaatcgccatggtcaggtgtatatgaagt
atcattcattaacggacgacatgacccagcagctattgctaccttccattcaaatatcagcgcattctaaacaaaacggagaaacaacttctcgtg
ttttaccgcgtaaaccgtttactttagtattatcgtttgacagctctgaagaaaacttacgctataaattatcatgggtattactgtctcgcgattcac
cgactacagtggatagacatatgggtttaattatggatggacctaatgaagcaaaagtatcagcttcagagaaaagagataattatcttgttattg
aaagaaatttctttgcgcttaccccggtatttatcaaatttatatatcagcgaagccagagagcgatgattccccgttttccaattgcgatgtaagt
gaaccaatcgtgcttcatatcgaaaccagttagttatgcttaatacttctaaaaatttcttatggaccttctgctagactataatgacatttttgttttt
tacgagtaattattttttttttttttatttttccttgttcaccacataaataatgttcattttattaatttatttataagccatttaaaacaagtatatgaa
ataacaatgttcagatataataggtaatagctgtgggtgttttctataagtaacgaaagagcaagtatcatttggagtacgacctaaccagaacc
aatatgtttagaaatcaatatggtaaggaatatggtaaaaaaagtctttaattaactgttttagatggagatgcaactacctggagtcctcaagga
agacttcatcaagtcgagtatgctcttgaagcaataaagcaaggatcagcaactgtaggaattgtttcaagtacacatgctgtcctggttgcttta
aaggtatgtaatgaagtaatattaatatttgctaactctatatagagaaatgctgaagaacttagcagctaccaaaagaagttaattcgcatcgat
gatcatttaggtattgctatcgctggtttagccccagatgcacgtatattaaggtatggtcggcttaaattaaaacggaatcgaataggaacaaaa
gtttgcggtaactaacgatttagcaattacatgaaacaggaagctacgtcctcaaaaacgctcttcagtcgtcctattcccgtccgacgtttattga
atcgtgttgcggaaaaagctcaattaaacactcaagaatatggtaaaaggccttacggtgttggctttttggttataggatatgacgaaactggcc
ctcatttgttggaattccaaccttctggtttggtgttagagtatttgggtaccagcatgggttctcgttcccaatccgctcgtacctatttggagcgca
attttaataccttccctactgcttctagagaggagcttattttatctgcattaagagctcttcgcgatacacttaccaaagaacaagagttgacgga
agacaatgtttccatttccgttgtaggcaaggatgaaaagtatacactttatgaccaagccaacactaaggaatggcttgaaaaattgggtgaaa
gaggaccagcagctgctcgtgcctctagaacaacagccgaggaaccttccgctcctactgcggaagccatccttgactctgctgatgccatggaa
actgagtaaacgacatgattatattgtgtatgagtagagattaagagcagtttgagctatgacaaatatcattcatcaagtcatatagagttttgtt
accattgaagatgatactacttttaattctgagcctaccttttgaagtttgacctaagaaaaatatgtccttgattcactaggtacaggttatactttt
ttaaaagaaactatgaatccattacagtcttttcatttatgaattcagttgaatgtatacaatatttaaaatctaaaaatagaactggtaaatatta
atttccatgtaccaagattctttcctgtttacattttcgagaacttgcttttcatacttctataattaaatgatatagtaatgttgaaaatatataattat
aggaaacatgtagcgtaatatatgatggggaaacactacttccttacttacgtagtatcccttcgccaaattcaataaggaatcattgttcaagaa
agcttcaaatttctgctcagatcaacccattttaagctcaattgctccactatatcaacaaaaaatcaggagattaaggacaacgtctctgtaatac
gagaagtttaaaagtttgtcaatttccataaatttttgttagaggaacgaagattcatttttacttaaagattcactcagaatgtctcaagctaaagt
cgctagtatgagtgctgtatctacttcctctatgtctcagacgcttagaatgtcatggagacagtggcgtacagagagcagctcgacatatgctaa
aagatgtgagatagatgtgttgaattgtgttgattttattcgtgaaaacgaaaaggagccggatagattggttgaaatttgtgatttccctatttatg
agaacgatggcgtaattcacgaattgtgtgttagtgacaaggaaacagggaagcagaataagaagaatgtggtttatttgcatggatatggagc
tggacttggatttttctttcagaacatcgatgaattaacgaaaggaacgacaaaaaggcataacagttactttgtcgattggttgggtatggggaa
ctcctctcgtccgccctttaaagtcaagggaaagacttcagctgaacgagtacacgaagctgaaaatttctttgtagaatcgttggaatgctggcg
tattaaacatagaattgagagcatggttcttgtgggccatagccttggaggatatttgagcgcggtttatgcgatgcgttatcctgaacgtgttgaa
aagttaatgttggtaagtccggttggtgttccagaatgtccgtacgcgacagatgacgacgctgaagaatatccgaatttcgctacttctgcgcttc
atgctatgacggatgataccccctaagacgaacgtagcgaatgaattattacaaacgcaagatgaaatttccggcaaaacctctcctcgttctata
aagccgaagaatccccttccaagatggatcacctttttatgggaacagaacgtgactcctttcgctcttttgagatttagtgggccgcttggaccaa
agctaaccagcttttggacatccagacgcttttcgactttaccagagaatgtctttcaggcgcttcatgcatattgctattccatatttcgtttgaaag
gctctagtgagtatgctcttggaagtctactagcaccaggcgcttttgcaagaaagagcatccataatcgattgcatagaattaaatgccgaacaa
tttttatgtatggtgacaaagactggatggacgacatagctggtttgaaggccgttgaggttttatcacaatttaacgttgaggctgaacatcatta
tatcacaaacgctggtcatcactgctatttagataatccgaaagaatttaacgcgttggttttggaagaaattctttaagaaaactgaagaatcaa
atcagtcggcccggccttgcctcctcctttctggttttgctcattatttattccttatagtttctttgttatttaccttactgttaataatttttgatgatcat
cgtttcccaatcaatctttatgtacgaatttaatttccttgatgcctacaattgctgtgaatatttgaggttaagcttattgtgtgtcagtcactcatcct
tgagattccctgcattgtcgattgttaattatttggttttatgtaactattgctttaatttattgaaatataaaatatataattttagtgttttcgttttgg
taaaacccgaactagaaatcctcaaaaaagttgctgtttttggattaaagcgaaaaaaaaaagatatttataaaaaggcattatgtttatggcga
ataaactaatggtctcatggttgctcgaacgagagaagggttggcagttgaaactgcttccataggtctgccatgaacacgccaatagcaaatga
ttcctgcaatgctatatagtctgtatttgtagagaatttgtagttgttatttatttcttattttttttcttttatatatttttggatatcttcacaagttatata
gagtttagaaaatttggcatcgtttttgactaagtgtttttagaaagatacgcccgatagattttttttttggaatttctgtttttttatttattttataatct
ctcatgagctctgatcctgacttagacgcgaaattacccactccagaatcggcttctccataccatcattcatggagtgttcccaaatttttttgagca

gggagaggaaaatgaagaacgtaacagttatatagatggaaaatgggatttcttttgtgctacagccttaatcaacgagagatatttgtactttca
ctgcagtcataaaagtctgccaacgagaacgtttctcacattgaatcttgaaacttttcaagcaaagtttattgagcttgataaagactttgaacgg
ttcagttacagtcaagcactagttgcgcatgacaataatcttattttatttggcagacaaaaaacagatggtgtagcgaaggatcaacagaatga
ctatttgctttttatttttggtaatagttttgaaagatgcgacattatgaactatgaagactcttcgttttcttcacgatacggttactctttggatactt
tagataccaatattttttatttggtggaattgttaacgaagaatactcaagcgatttacttttcataaatcttcaagggctccctgttgctcctaatg
aatcctccgacgaaaaaaaaaatatagtcaagtggtctcaaatacctcttcaatcagcttcccctcctggccgctgcaatcacgctacaacggtcg
taggatataagctaattattcatggaggaaaaaatcaaacagggcttttaggagatctatggctttttgatttagagactgtgagttggactgaaa
ttcggcctgtaggcaattttccatgcccaagagaaggccataaagtcgtttcttttggaaataaactttacatgtatggtggatttgacgaaagcgg
agccatttgtaaagatttatggtgttttaatttggataaacaatgttggtcgcggacaaagggcccttccattgctgaaaataatccaattttcctca
acatggcttgttataatgaacgccttttcctaatcttctctcatgagacacatagctcacgccgattccatagttatgaatgtgatccagcgagtcttt
cttgggatgacatgggtgcttatggattacctgtctttaatcatgtaagtatgtatccctcaaatgcatctagcaatacaatttcccttggaaattta
aatcgaatcatacaaaaaatcgctcgaacgcatcattcaacttctacgattacttggaaccaactcataaggcggtacagtccacgaggcataga
cactatggaagtttggacgagcagggactgaaatctttgaaagaacttaagaggaattctctacatcaccatcgaaattccgataatctttctcttc
gtgaattcggtcacagtagtcctttcgcatatgacgttgataatacaattacttcacttcccattgcttataacggtgaagaagacaaaaaggata
atcaatcgcttttaagtatacctgatcatgtatcaaatcgttcggcgaatttgcaagcctttcacttcaatagtacagatgatcgaatcgcatggtta
gaagagcagttactttattgtatgactcaaggctacgcgttgaagcctcctggacatttgaaagaaaagtatcctaaagggatactttatcaatct
caacctttaaaaatgcttgagtctttacatgctatggaagaggaattaagcaaaacagaatatgagtttaaacaaaacgcttcccttttaatttctg
aaaatgctcaattgttagcggaaaaggatgctgcgcaaagctctgccaagtttcatatgaaacttcttcaagaaagtaacatagatagtctcactc
agatgttgagtgaacgggtgcatacgctagaagttgatgtacaacattctttagctgaggcgacgtcttactaccaaagatataatgagctaaag
cagaaagttgaggaaatgcaatttaacaatgctatgatgagaaatcatcaagcggagcttacagagaactacgcaaagcttcagtccataattg
aagaggatagtagcaaagctgtatcgctacagacggaaaataagatactcaaactagatgtcagcaggctctctcaagaatcgttggaatttcat
tctaagtgtcaaactttagagtacactaactacgaattggaaacaaaactgattgagttgtgtgatcggatggaaatgcaaacgaacgtaattga
agcatctgccagtgctttagatgtctcaaataccgctatactatcttttgaagattctcttcgtagggaaaaggaagagaacaagataaagcaag
aaaagtacttaagcttgacatatcagagttcccgtctgaacgctgaagtagaacgacttcagattgaaaattcggaactacagaaaacaacaga
ccaagttcgttcagatgcagcatatttgaaatcaattgttcaaaatgggttgtcaaaattactatcctcagaaattgaaacaaaagaaagccacc
aggattccccaagaactaatcaaaaaatcaagtatcttgaatcgcgactagttggtatgcaaaagttgtttgagaaacagcagaacagacttga
gactaccatgctagaaatggtttctgtacaaagaaagtatgccgaaatgaaacatgcttattcagtggcactcatcgaaaaaggtgaattgctca
gtcaaatgagtggctttactgaaaaaagtaaaggcgaacagagcaagctcttcgaaactcaaagagcgttagctgactcaaagctaagtcataa
tgattatgattcaaggtctacgaaaaataattcttatgagcaccttctgcaccaaaatgatttgaattcaaatgaaaagtctcaggagaaggaag
atgcattatcaaaaaatgatgaaataaatgcaaatgatactttgtttaagaattatcagagaagctacaataatttgatatcagcaggacaaata
accatgtcacgccaacaagatacaattcatcttttaaatttaggactagaggaccaaaaaaagcggatcattgataccccgaatgcactttccaa
tgatttcagtcagactgtcaaagttgttgatgacagccaaaatgaaaatgatgagcaactgcgatcggcatctgatatttattatgccaatagg at
taaacaattagaggacgattatcaaaaggcgataacatacgcaaactgctctgacgaatcgttccaacagttgaattataaatttatatagacag
attactttccttgttagtttaaatattatgggtataagggttagttcaattgtagttcatacataagaaaaagaaggtgagtattaaagtattctatg
agaccaaaattaattttcttattactaaatctgattatgaacattctaaggtacaagtactgcataattaaattccaaaacattattaaatcgaaga
atccgtattagagggaggatttgactcatttgaaggatgcaatagctctgtccaacttaaaaaaggtggagcgggccactgtttgtctatatctatg
tttaagcctagatcatcaccagaagaaaaatcattacggaccagcaacgacgatggatcagacaagaaatcagtatttgggacagataatgaa
agatgatctggttgtggcaggtcaacacttttatgatcagctaaagtaccttcatcaactgaaatggcatccgaagccgaagccgtttgctgttgca
aatagtcattttgagacatatcaccaacccaagactgccactttcgaaagctgaattgaaagtcactcgactcaggaacaaaagaaaacgtcttc
ttgtttgttagttgatctgaaagctccctcaagctttgtaattttagacgagttatttcatttctttgagataaaaactccatgactttccatagcttag
atactgcatcgtcaacttctacagaaggttcattgtgagagcacaataacaagataatcccgctacaaatggggagatacgcgtcaaaaaatga
ataactagacaggcgcgtattttgaaacaactggtatgcgatattaatcatacgacaagaactgtcgacgcacatactggcatactttaggttttct
gcacttaaatcttttcctttaagtttatttagcaatacaaatcgggtagcaaggataactgcattgtcataaatcatttgtaattgagcgttcacgcg
aaaataaggattttgtggcgaagactcatcgaatagaagagaatggtgcagattccgtttccaaagttcaagtcgagcacatagcctgcgaacat
gagatagctgcgtattaaggtcggaagaatttttccaatacaggcaatcacatacttcactcgtgatctttgttaactcgataactgcgcgcatgtt
ggttaccatcgaggggataccaggtacttctaattcaaataagacgttcggataaggggtagtcacattttctagagcaatactaaaaggtcgac
ccattgcaatgctaatgaaccggtcgatgcaaaacactgaccaccaaaggcgatttcgaagttctctagagctttgactaagggtcaagtcctca
cagtttttatgtaaacctaaggagatagcggttttaacagccagaccaaagtaagaataactagccttttgatatacggtaggctgaagataaag

tgctattaaaatagcagactgaactgtattggtgttaccctgaagcaaaaccattgggaaaatgctgacagcggcagagaagagacgattccct
attatgcaaacttgaggacttgctgctactggatcgctttgttgttcaatcatatattgattcccaaatgcaagaaccattaataatatgcaaagaa
atccaggatcagtttgatttttcatagataaaaatatttccaaacgtattttaaaaaaggatgcatgatagaaaaataaatttgtttgtatgttcgta
aaaaacgtattcacaagaaactctgttacagtgagagaagggattaggccacgggcatgagagagcaaatactcgtctaattctggattaaaga
aagaagaggtaggttgattttgtagtaaagaattagcggaggaaaagtcgtttaggcgcatatcattctgtatatttttcaagaagaggtctagcg
aggtaggaccaagatacatcgtatcttccattttcccaactagcaatgattcaacaaaagtgatagatggttcattttcgagggattcatgagaat
aattaggatcaatgctattcggctcagaaacgtagttatcatcatggttgctaatatgatttctctttagctcattgtttatactaagaataggatcta
aatcattaaggatagaggagagttgatcgggagttagatgttcatgctcagataatttcttagatactgtaactaaatttttcgtatccaagttaaa
ttcagggcatatcctagaaaaaatgtattcaaagcacaattggcgctcagacaattcgtttaaatattcccgagacggcagcttactttgattttttt
gagtatatatacactccacgccaagtaagcgacagttctcgcaagagtcattaccatcacacttaatctttttctctctgcaatgtttacaagcgcg
aaagactcttcttcgttgttcaggcggtacgcgtcgctttttttttttctcaaatgccttgacatctgcgtataattgagtcttaataaatcccaaattc
tcattcatatcaaatcagtcccgaatccatcgcaatctttacgaatatttaaatcatacaaaccaaactaaacaagccgccttcttttgataattgct
ctttctcagtggtcgtaatgaatcaaattcactggaatgatgctaagttctatcatcagtaatataatacaaaggaggaagcctacttaagcatat
gcgtttataccgtattttgagatatgtttgaaggaagtaggatattttcttaaatatgaatctttatttttttattattaacatatgcttactcatttaatc
tatgctcttatagtacaactatgtatgttagtatgctaaatagcgtagttttctgaattaagataatatgatttatgctctcgtagaatcattgatgtct
cttgtaaattttagtttgctgaagattacaagcgaaactgaaaatagcttagggagagtatgttatgtcggaatagtgtcagttttcttgaaaacaa
aggagattatacatggggctttttattcaagtagaaggaccggtcgaaatttatagagcacctcaaagaattcgtaaaaaataaatactattatg
gaaaaattgtccgcaatggacaaatcgttatacggttacttattaatgattaatactacgtagagaaagacgatatactgtttctcaagacaaaaa
caaaataaaatctatttgttcgactagactcaaattaaaaatacccaatataaacaccaattaacagaatataacatagcagtacccaaaatttt
cttcaaacatccgtaaccataaatgaaagataacaaacacttttcgtctagcgccttcttgttttgcgaacttcatttggatttctccggccggcgcc
tacgctaatacgcaaatgttccttaggggcacccgttaacaaagaagggaggaaatcttgatccatcagatcaacttccttctttcgttgcatataa
cggacagccttttggtattctggctgatcctcacgcaagataggattcttcttgcctttaccacttggtgtaccatgaactggtttcggaatagcgtat
ttcgtacctcgggtcttccactcaaaattcttcttcaagtgtacttggaactcacccttggagttgatgctgcaagtcgtctttatcagcgtatcacct
ccgcaagaggtacaaaactttttcgacatatcattaacaatagcatagcaaccatgacagcgaagaacgaaacgtttaacgttttgaattttcaa
accatcggaagaaaccaaatttaggcctatttgcaacataacattttgcattgcaaaatcagtcgtggcgcatgcgacctttaaatgggagggttg
aacgacactttcaccaacgccatcctgagtttttttcttgcgaatattggaaggtgtaatccagccgtcatcttcctcttctcggtcttcttgctcgtcc
tccgtcaatttaggcttttgattggaataagaagcatccttaatgttttcgctttcatccttcgttttctgttccatcaacggatcttgtttttgcttatcc
ttttgctcgtcttctttattttcctcgggagacgatgaggtttctttcaccgataattcatctttgttaagatgagctggaggcttgccatttattcgtttc
tgaccaggatattgacgaagtctccagtctcccccattgaattcatattcaagctcgtacgtaagggcaagaatgtggatatccgtaatagataaa
gaagcaaagtcaccagtttgctttgcaaaatcagaaactatttcaaaaagttaatacattgtcaatcatgcatgtaaaatgaaagctatgatacgt
cggagaataataaaacaggctataaagccaaagcaagggcaaaaacattactcgatagactttttttgtttcatcttacgcatacatgctgaaatc
cattccattctagcaagtattttcgtttttaaaggaatcattatcagcgaatagagtcttcctcttcactccaatatagcttaatctagaacctatact
tacctttctttatgaactcttgcttaggaacacgctggatgacttgaccgccccaaagctcaaaatttctacgtgaagcttcatctcgaatttcttga
acaactcgaggtgtggtataaaacttctcagcagaattccttaacagagagctcgaacatataatcccaccagtatccaaaacgagatgagtaat
cgctttactcatagtatcatgaaacaaaaccccaatgtggtttaggacaaattcatatacgccctactagaactattcatttcgctctagctcgata
agttttgggtattacggtgtagtccgctactggtttataccgaaatattatgcctcattatttgccaaatgaaaaagtacataaacaagagtctcca
aataccaattcattgccatatgaataagtttgcatgttcctagtatttttttgacgacttttgagtctcctggctaaactagtgtagtagaagacaaga
gtttttttgacagaaacaggtggaagtggattgtttatggcatttgttagttttcgcgtcaagtttttgattagtcaagccaaaggaagagtttatgttg
gttccatgtgaaataatgactaggaaaacattaattgatgaaaatagggaattgattaatgatgaattggttgccttacaatccatttatcctgaac
ttgaattagatggcaacaattgtggtcgcgtttccatccccgtgtcaacagaagacacgttttatttgtcctttccagatctgaaaaggacaaataa
tatagacacgatagcaactaaacatttccctgctatagaactagagtttttttcttcgggaaggatatcctcaaaagtctcctccagtattcttcctaa
agtcatcgtggcttcccatgagacaacggcgtactttaacgtcctctatggtgagattatggaacgaaattcatgactgtgttttatttgatgcaatc
gaacttttgcgctccagtgcatcatctgcgtttcaatctcctaaggagatggttttcccttcggattcagagagtttgaaacaagagttccttacaac
tgataagcatgcaagtttgatggatttcgagaatcgcacattcacgtgcttaatttgctttgacaattttagcggcaaagactgctttcggctagccc
gctgtggtcatgtatgctgtaagcaatgccttaccgattattattccgtctgcattgaagaaggaatgtttactcagttaaagtgtgttgatttagag
tgtgctaaaactgcttctatcttgacactggaggaaatggaaatgatagtcggcccggaattgaggaagagattccaggaattagacgaaaagc
ggaggtatgaaaatgatgcgaacattgtattttgtcctcggaaatcatgtcaaggcccggcaaggcgagatcctggtcagaagcttgcaatttgtc
tcaagtgtgattttgcattttgttcattttgtcaagcatcatggcatggtgacttatctgcttgcaagcttcagggtgattctaaaaaattagtgaaga

tgtatctggactatcaagagtcagacgcggacaaggccgaggaactcgaaaaacgctatggtaaacggattttggacaaactcgttgagcaagt
taaaaacgatgaggaggctgaacgttgggctcttttgaacggtcagcgttgtcctacttgtgaccgggtagttgaaagaatagatggatgctgtca
tatgatttgtctttgtggaactcatttttgttttctttgtggtgcttatctcatggaagccaatccttatatacacttcaatgaccctatgagtacatgtt
atgctatgctgtttgcttctccagaagaaaagcaacgcttttccgaaaattggttatgatacctactgattccattagaagtgctatgggaatttgga
aggaaaaaaaaactgtttttaaaaataaacgcttattaaattatttatttatctattaataatacctttttgatcatctatttatgtcaccttttttggg
cggagttgtggatttcaaggattagagggagtttgagcaggattatggagagattgtagactcatcatgaaggctcgcacaactgaaaagtcttc
agcggaaaaggtataatcaacattgttttctgcattggtgttttcgtttttatttgtttcctgtaagtctttgccttgggaactcggattatcagaaatc
ttctgaggatatgtatatccttcttgaggatgatattgagggatattttcaatctttttaaagctgtttgaagactatgagtcgcattcaactaaaac
aaaaaaaaaattgttagtcgattaaccttaattccgaagtaggcttgacgtaccattctagttgctaacaaagtatatgcattaggagccatgtaa
atattttcattacaaacgaaataagtacttaaaggttttacttcgcagtgttagtgtatattgaagatattggaagaagactgttataacatgcata
ccttcttgctcgttgagtcggtgttgtttttgaataatccataaaaacggaggcctttcatgagtaacgacaaattgtgttccagtcaatcttcttaga
tgattatttaagtcacccaaatcgagagcgttgaattgcgattgcatttttagcatctgattattggatttatgatcgtagaagggagaaaatgaaa
agtaatcaagaactaggactgttaatataagctgaatggaagcatagtataggaacagggtcgggctccactggtcgtgtgggcccagtagcag
aagcatttgcagttaaaacaggagagaaagtagatacagttcagccatacttcttgtaaacataccattttcggtccgcagacctcctacggattg
tacccattcaggcattcgccattgtatattgctgagatcaaaaggagaaggtgtagttgtcataatttttgttgatatagtaaagagtattactacg
aaaagaacgattcaattagaattcctaggaataataaataatgaaagtctttcaacgcagataaattgaggcctgcgaaagagattcgtaactc
gttattttataggtaaatttttgtatttgttctagagaaaaaaaggtagaagatagaaatatatcagttttggctaggaaaaacagtttatgatagc
ggtagcaggtagtacccatagacctgcaactagaaaaagcaatttgaaagtcggtttcgtctgaacgttgtatcatactaatatgaataaatactt
atttatataataacatttcattttgatatctagtttcattcatattaacttgttaataacaaaaaaagcaacgcaaaatcataggtaaaacattatat
agaactaatatacaaattaagcaatagggaagactaagagtcctattaatcgtcttgtcgtccaaaattgttgaaatatacaaatatacaaaaaa
aacgatcggtagggtaatgattcataacagtgtttgtatgaatggaaagccataatggataacacgaaaaaacgaatcagcgaaaggtgcttaa
attcaaattcacgttgggctctttttagtatacaaagtatgcaatcgtctcaaagcatatatagaaattgaggatcatcgtcgttatggggctgctt
caataattcatcaggagtcaactgatccatagaccgcctctttgtagtattcttagctgaataatagaaaatggtcaggatgtgcaaaacggttgc
gatgccaatcgcaactaaagaggtagcatgaccagcgacatacttgggcgggaactgatacacttggccagagagaataccagcagaatttgc
aattgccatttgcatgctaattgctgtcgcacgcttgtaatggggtgcgacattggcactcaaccaggttacgttcaggccagcgccggtgtaaatg
cccaatgaacataaataacaagcaaagtataggacgttgtttttccttgacaagtgagcagcaagatataaccaataatggggaagatgttgct
agtaatcaaaatgagacctcttgatcgaaagcgatccgaaaggaacgaagctataatatagacagcagcacctagtatatagactgggatggtg
agatattgaacagaaagagattggtgacccattcctcgaataatggcaggaagaaaggtactgaagccatataaacaggtatcttgtccaaatt
ctgcaagagaagaaaggtaaattttaaaatcttttaaagcttctttgacgctcttccaatcgaaatggtccgcctccaaatcaaagcgtcgctgcg
cttgacgacgacgaaccatttctcgatagggctcagacaaaaaccaagcctttgctggatcatttggaataacaaagtatccaaggactccagcc
acgaatccaatcataccttcaatgataaacagccaacgccatccagcgtagcctccgacactgtgcatgtgtaaaatggcgtaggcaaagagac
cgctaaaggcaccagatagagctgatgcagcaaacagatatgcaatgcgtggtgccagctcgtctctagaataaatcattgtcaaataaaccgt
gagacaaggaaacagacctgcctcacagacacctagaacaagtcgagtagcaagaaacccggcatagttttgcacaaatccagtgaatatggt
tgtaagagaaaaggttatcacaatgagagttagcatttgctttggttgaaactttttgatcaataaagtcgtgggtatttcaaacaaaataaaagt
aacataaaataaacttgttacaatattgaattggtttcctttaaggtgtatatcttcagtcaagcctgcgatttttgcattgccaatattagtgcgatc
caaaaaggcaattaaatagaatatcataatcgtaggagctaataccaaatctaagcgccttaacaaacgtcttcgttcgcctactgaaagctcctt
ttcggcaaggtcactcgatgatgattttgtagactcgccctctttctcgtctacgataacgtggaatacttttggggcttcttctgactctcccgaagc
cgcgcgcataatgggttaaaagtcgtttctccagaaaacaatgacaaaagtgagtattgactctgaattgtggggttacaaagttcttaaagtaa
acgacaagaaaaagtatagatgacctgtcttttagtaaatacaaaagaagagtttgttccaaaaccagaaatgaagaatgatggcgaaaagtc
ggcaggatcaaaagacaaagaaggggcctttctacaagaaattttgttatactccaccgttgcctttgtaactttttcttataaattgaaaattgta
aatgaaaaaaaggcacgcgtagatgtttataccggatagctggaagaagcagtccatcggaaatttggataggaaaacccctgattggacaaa
aatttaagtgctaaattagcgaaacttatattacaaatgaagtaaccaaaataaggatgaaaattattatttatatagtttgtaataaattatgttc
ttttggatagtaaatttttaaaagatccaaactacaacttgattcttttttttttcatgaatcggttcgaagtgttgaactgttgataattatgaaattt
cgggatcccggttagacatctcaatcggaaattcggcaatacgacatttgtaaataattgccatattaacgaggtgcttaataaaatacttgttggt
ttcaatcgtataattttatgatttgccttgggtatgaagaagataataaagggcttaaagaaattaagtataataagtataataaaactaaagcct
cctaagctaactataacctttataaatccattggaacaaatgcttcaatgaaaaagggactgactgtctttttaattgagagttgggaaagaatat
aaagatcagtaaacaaaattaaggcaaattatatcataacaattgagacacccttttttcaataaaagacagtatcatttcaatgaaatgcatcttg
acttcaaaaacaaacaatatccaattcatgataatttaaatgttcctacggcaataggaatccatacgtaatcgggtacaccaggagaaaaatga

gagccttggggcatggaaaatataagaaatcatcccgtacgaaatttggttttcatcgaatatttctttaactctcgcttgttctatagcttaggcag
ccgcaccatagagtcggtcaccgatgtcgccaataccagggacgaggtaacctttgtcatcaaggctcttgtcgatggcaccaaagacaaattcc
acaccaggagtattggaaaaccggacaaaagcttgcgcgcttgctaaaacgctgacaaaaacaatgcgcttacaaccccactcttggagagtag
taatgacggcttgagcagtaccacctgtggccataacggggtccaagacaatggccgtttccgtagagtttttaggaagcttgttgtaatattcaat
aggttgcaaagtgctttttttcacggaaaattccaatatgataaattggcacatctggcagtacctttgagaaagcgcccatcatagacataccgga
gcgaagaaccgggataaccgagacagaggaattgtccaaggacttggtggactcgtaagccagaaaacgagaaatttcatcaattatgccgcg
aacttgagaaggtttcaaagacagatcacgaaggacagaaattaatttattgacaacagggtgatcagaaatggtaatgtttttcatggtatcta
cagaggatgagagagaaatcgaagtggtggtattcgtcattgtagaagaatcgttcctgcggatataaacaaatatatagtagtgcgtatctatc
agaatggttgggaaacctgttatgagggcagttgtaaacaaagggctggttgcaacgatgagagaagaagcctaaacaaaaatggagagaaa
cggaaacctgtctctaaaggaagaagtatgagtgaatggatgtggtttttttttgactgaaatcctttgaatttatattatttccttctcattgtttta
tggtttcctcggtactcccgcggttcagacaggtctatcagtatcggcttgtagctgatatggacggagagggcttccgtggtattacttactatagc
atatgctttcatttttgaagcatatgcttatactttgggataactatcgccatcagcgattgtccatccatatttttgacgggattccagccgcggtttg
gccgtggtataaataaactccatgaagttgtgacatacggggaaagcgtatcttgaatggaagccattgggtggtttggtatctgtttacatgaaa
attactcacctataaatgaccatcctttttcctttcaggtacccttttagttttgtttttctagtgtaatcatagtactagcaagaaatcttaacatcctt
gtcgatcaggagaacacccaattgcacaaaagaattcaagcatacattctttttcgctttttgtcttgtctctattcctctcaggctaactccttcttt
gcttcttgttatttgtttgtaattgtgaattgattcacggtttcccccaaaatgactacagatacaatcgctgatgttttgtctttacagaatattcgca
tcaaggctcaaagagtttttgccgttgccgaacaaaaccggttgcgcgctttcgagtacgatccttgcaagcttgacaccgtagcagactttgtggt
atcagtgattcaagcagactttaaaggaaaatacgaaacaattcctcctcatgggcgatggcagcactttgaggttggaaacgttgctcgtctcac
ccagctgattagcacctggaaccagcagggggtaacttctgttgaagtctgcaagcgcgtgttggacataacatttcttagtgttttacttgatgcc
ggtgccggggataagtggtcatacttggatggtgttgaacgatatgctcgtagtgaaggtttggcagtcgccagtcttcgttgctttcaaaagggtt
tattctcgagcaatccagagaacccataccaggtggacggtgttgctttgagccacttgaaaacggaagctttggcggatggctttcaagtttcca
aacagaacccaattgctggtatagagggacgagcctccattcttcgtgctttgggaaatcagttaaagagttctcgccccagcttctttgtggacag
cgtctttcctacaggggactttacttctgaaagcctcaatgttgaaaggctatggtcggaattgcaaacggtgttaattcccatttggcccgagcgt
acaaaatatgcgggagaaaacgtcggtgatgcttggttcctacctacgattaattctatacaacctttccataaattaactcaatggctaacctatt
ccctcttgattcctttgaagcgattgctgcacatacaagttgttaacgaaacccttctgaccggactaccggaatatagaaatggtggtctctttgtg
gattttggagtactcaagcttcgacccgaatattcccttactgacgaatacgagccagagagtattgtcattgttgagtggagagccatgaccgtt
gtactacttgacaaacttttaaatatgataaacgaacgcatgaaaaatgaattatcagctccattatcgttggcacaaatgttggaagctggaagc
tggaaatcaggaagaataattgctcgcaagttacgacccgcaaatgcaggcagccctattctgattaaaagtgatggtacattgttttagcttgta
tttttacttgttcgtttgtttttgtaatgaattttctagactatcaagaatggcttattagtattttccatcggttcatatgattagtgatcatccttgtatt
gaaatataaaagtagacacttgtatttattgaatagaaagatattgttaccaaaatctaaaagaaaattttgttagtttctttttgctttttgtttattt
atttcacaattatgtactacgtttcaactacgagtaaccttcgacttttagactatttctcataaaaaggaattcacgttctccttagttattctcttatt
tcagccaaaaacaaagatagtgcaatcaactgtttgtttaccattctcctttttgtttatttacattttccaataaggcgcccttgcctcgtcgcttaa
gcacaattcaaatttagtcttcggtattttcccgcggatgtcccgaatgtataaagcaccttttttatcggtaggcagttataaaactttaagctgca
tcttcaattacgtcttcctttcgacgacggcggcggcggtaccgcgggttcaccgcgtgcactactaaaacaatagcttgataaacaatagataga
gccacaaaaggtataaataaggcaacaacaacccaaaaaaaacaatattgcaacacaattgctttacttattttcatatctaaagaacagtttgt
ttctcttactccgaaattgtcgtcctcaattcgtttgtttacatagaatccatttcttttcttacgtcttcttcctagaaatcacaatgttggttactgaac
aagcaaaacctggtactcatgaacaggaacatcatactttagaagtgaagaagcctctctattcccgtcctgtcgttttgactacttacccaactca
aaacaagtccgacccttttccgatgaattggggagcttcatcaccacaaaagcgtggtcctgtaacagtcgctcgctccattgagacacttcccca
tcgaaacgccattggcgctcatggcggcagctattgtgtttacaactcgctggcagttgccggtggctttttgcctgctgatcaccatccggatttta
cactcacagagcctacctttgaatttcctgaacttgcttcttggcacgataagaataaaattgtcgctatggacccctacggccatgatattcctcg
atgctttaaggagtacttggacaagggatatgatgtccgtccaactattgccattacgcgtgctaatctccgagtaagtgaagtacaacgatccgt
tgataatggatctctagctgttgatggcaaagttgtactgaacaaggagggagatattgccgtcaccaaagttgcggttgaacctgtctggtatctt
cccggagtcgctgagcgctttggcgttgatgaggtgactcttcgccgtgcattgtttgaacaaatgggtggtgcttatcccgaactaataacccgcc
ctgatattaagatcttccttccacccatcggaggtgccacagcttacatctttggtcctcccgaatatctcagcgaccctacgaagaaattggccgt
ccgtgttcatgatgagtgcaacggctccgatgtatttcagagtgatatctgcacctgtaggccttatctggtatttggtattgaggaagcagccaag
gaagcacagaatggtggagctggcttagttgtttactatcgcaaggaaggtcgtgccctgggtgaagttgttaaataccttgtttacaataagcgc
aagcgtacaggagatacggccgataactatttccgccgaacggaagaattagcaggcgttcgtgatatgcgtttccaagctctaatgccggatat
cctccactggtttggaattaccaagattgaccgtatgcttagtatgtccaacatgaagtatgatgcaatagtcaactctggaatccgcatccttgaa

cgtgttactattccagaagatctcattcctgccgactcacaagtggaaattgacgctaaaatacaatccggatacttttctaaccataagaaagta
acggaagaggacttggcctgcgttcacggtcgtacctggtaaacataaggtttattttctgtaacatccatatgtagaacgtcttttgaaattttgtt
tactttctttaattgttaataacttagcatgaatactcgccatttttttagtcaagtccatttcttttacttccatgttaaatgataattatatttttttctatt
cgtctcttattgaaacatgacataggatgtgcttttaaagagatttgaacttaggttgactcaggcgggtttgtctttcgtattcctcctagaatgtaa
agtactcttcttctacttttatattcgttactttctatattaaattatattccctacgtcttgtaaggtaagaagatacgtatttttcgaaggatgatcga
catcgtccaaattttcctttttttgcgacgatatgtgccttatggggaaatcaagcaactctctcgaaacgaatgaaatcaggtttttacagcgcaaa
cttcaagaaaatataaggatgaccttaaattattcatttttttaagtttatttactaaatggaaaccaattgaggtaaaagtagtatggactgtttaa
agaaacgattcataaacctaattttttttgtaatattatcttatcgtatcatgttaagtatattttctacactaaggatttaaaagtacatagaattact
atacactattcgtttgtttgataactatagcaataaaaaaagaaatattactatagttgtctagtacctaaaaagtaaatttctttgcttaaagatctt
cgtttactccaggctcttttctattattttcttcctttaaatagctaagtaagcatctaagatcgctgcattctggatatatactttcatttcccttagcg
aaggctactcctgttttattgtctaatctgttctcatacttgaacaaacccgctttttccaacaaaattttgcttcacaacataaacctgagagtgttc
agttgtattctgttcctagaagtcaaagctctaaacaaattctgcgaatttttttgggtaaacaaccacagtctccgagaaagtattcgactttaacc
tctctgtgttcatactgttaataagagtacttttgttaggtatttggactccctgctaccaatgaccctttcctttctgtatgcatagcttgtctataaaa
catcaattgaggagtccgtaatgaatggaatgaaagcccaacatccatccagcattcttatgcttaaacggaagagagcactagaagcttgcga
ctcttgcagaaaacaaaaaacgcgttgtcttgctggcccaccggatgatgaagatcgcacttgtttgcggtgccggtctctacagctcgaatgcag
cttggccaaatctagccaaactcttcgtaaacctgatggtgatgcgttagaatcttccgcagcggccatcgttaatgtaaatctcgaaaaacggta
aggcctttaccataatatggagcttcttctttacagaaactctatgaagatcgtaatggtcttaatttcttccaattttaacgtgtttttttattaacaaa
attctatagacttaaagttttggaaaaggccgtaagctctctttcaacgtcagcccttcatgcacgaccctcctacccccgggaagacatttttgtta
acggtattctttcccttgaggaaatcgagcttttactcgatatctttattgagcgctacggtaggcgttggctctcagttaactgcggtggggctgaa
tacctcgagtttctttaccacaagtctcatcttttgctcgcaactgcctgcgtaacggctttacgacacaatccttccctcagggctcgtgtgtatcca
caactactcgatatagttgatcgccttgtttccgaagagttactcactgcatcaccctctctccaatttttttgaggctgtctctatcttgtctctttatca
acctcttcgctatgttcaaaaacaagacctttggctcctaagtgggttttccttgcggcatcgcatcctctcgagtactcaaggttggttcaacggttt
tttaggttctcgatctacccttacttacctcgacattgttcctgctcgcacttggaatcatctatgtcacgctcatctagtcatatgcatagggtatcgt
cgacacgcgatgcttgaagagaccactttcgatgaatgtagaaatattttgaccaactgtaaagccagtgaatttgatggaaacatccttggtatg
ctgtccctttactcaatgttatatcgtctacttcgttcgtcatttgacttggaatatgccatctttcagcttgaagaatggcgaaaagaatggtgccat
ttatgggagcaacccgaaccagtatattctcaagttgcttattattacgcttacaacgtagtttatgaagcttgcatccaaatggccaccgatacga
agcattttactaatattgcgaaatatgtcagtatgcttgaagcatatgcgttaaagactgtcaacgccatttttgaactctctgcttacgatatgtctc
gttgttctgaccacgttttatttcatgctgcctttgcttgtaccagcatgctacgactaatctatgctgcgaaaaccaaagatattggttctctagcca
ttcaaacagatcttttgaatgaagttgttacaaagacatggaaatggcttttattgattagtgttgatcagtaccatctggctaccaagtttgctacc
tacctaaaagaataccaggttactgtcaatgaggcaggccctgaagcttttcttcctaccacaaaatcaagctcttctgtcgtccattcaccttctga
tccgatgtcgactgcgtccacttggtataaaggccctcctcgaccggtatctgatgcaaccctacaaggtctaaaaccctataatttaggagtggct
accgtggaacatggctaatccagcttgttaacttttcattaaaatgactaaatttctttgcttataattggtttattttcattgcaaaatgattgtcaaa
aacgcagagaaatatcagcactcgttgctgaagggtaccgcgggaggcccccggaatctgcatggaattgtagagctgaaagtcccggattttgt
tcatatatatatgaaaccaaaagtctctcagaaaatgtcattttctatttaaatttatcgtttttgtatctcattctctcccatcctttttgtgtcatatctt
tgtcaagatatcattgattccttaaaataaatattttcatgcctcattctgctcttgggatggaaaatgtactagttcttcgtcagaccctgtacttaat
ttctttaatgacaataatccgtgataaaaataccgaccattctgcatttgtcagaacatctaatattattatcaccatgcttgttcaagaagcaatga
atcatgtttctatgaagagatgccttattgccagtggttcgggtggtacgtatcttgggctaaagcctgcccaagaaatttgcggtgtaagtattttg
agagctggtgaaagcatggaacatggcttgaattctgcttttcattccgtgcccattggaaaaatgcttgtgcagagaaatgaatctactttcgaat
caaaacttctcttttgcaaattgcccaaaggcattgaaaccttttttggtctttcttatggatcctttattggcaacagggaattcttctattcttgctat
ccaagttctattagaaaaaggtgttcctgaggaaaatatcgtttttctcaatctcatatcttgttctcaaggcgtcgaaaacattcgaaagcaatttc
ctagacttagattgattactgctgctatcgatccagagttgaatgatcaacggtaaatcatatattattttttttgatctatgctaaccctttcagctat
gttgtacctggttgtggagactttggcgaccgttattttggcacctattaaatccgttttaattatacgcaaaagtacacactttctttttgcttgcagt
catcggtgctatgcatttttttaattgtatacataaatatattatttaataaactatgtattgtatcatttatgtaacccaagtggaaatataaattcaa
agaacaagagaattgcaatgattaatgaactaaaaacgcttgtttacttactaaatagaagaatcatgaaagaatatttctcggcaaaatcgtca
gttgggaagatttggctaatcgagctaaagaacttgctattgaatatatcggagttcgtcaaccagacgtattggaacattcctataatgacttatt
gacagaaatttgcaattcaatcgcaactatacaatacagagacagccattccttatttcatgctcaattacattgatatttcttagtattatccttga
cagaaatagacttattatgactaatatctgaagaaaatgaaatgaaataacttaccctgtttacgcaaaaaaaacgcacataataaatagaaaa
tgttattaacatagacataaaatcttgatcatacaaaacatggataataaataactaaaaataaataaaatgaagccttgataccttgcattgatt

caaatgaaagtgctatagctattatttcgtcagtaggtgattttaattaaaatacataataaatcaaggaatcaaggcagaaaataactaatcca
aaaaccaaaaaatcttaacgaaatgactaacgattagagatacataaaagaaaaaaagtaaaacataacttatggcaagcaaccaacttcttt
ggatttaagactgtgaatgtcgcttttatcttcctccttgcttggtaaaccatctatgaagaaatcagaagaaccatcacttgtgtcttcaagactga
tcaaatattcttcagtttcttcaggtgtttcgctcaagaacttttcacctacctcagtaacggggaaaacatagcagaggaaaatatgggtcaagc
cacttactaaaattcccaaaaaataattgagattaaacatagtggtagcggcagctgggactttgttaccaactgaaccagccataccaacaatg
ttaatcagcataccacatatataagatacaaagccacgaatattaaatcccttgtagaagtaatagcagctggtatcatcggccgtgtaaagatg
cgaggttctaatataacctttacggataacaaagtagtcggcaagaaagatgccagcaaaagccgacatgaaaacagaataagaagaaagat
aagtagtaaagttgctgctggagctgagcaaatgccaagggcacatgcacaatgcaatgatagaagcgaggtaagcaccacgtcggacattaa
aaacagcaggaagcaaggcagaaatatcgttaccggcggaaacagagtttgcagctacgttcgtgccaagttgagcaacgcagagaccaaaa
gcgatgaaaaaaacaccagcacgaacaccatgagcgtttgagtgatccaaaaatgctgaaaggagttgagttggatcccaataagcggtgccg
taaatggccttggaagcagaagtgacaaggataccaattaaggaggtaataccgaaggagaggggtacagtaataatctgaggccaaaggttg
gcattgggagtagtagcaaaccgagcaaagtcaggactattaacggctaatgtagcaaagccatctaaaacggttacgatgccatttacaacag
tccagccatgatcccatctattaagtttcgtaggttcgtgaatgacaggaccagcacccttagctttgacaagggcccaaattaaaaaggcgactg
cggcaggaggagcgacgaaagccttgaaggagaaaagatggcgaagttggtgaaccggaaaccagatggagacattgcaaatgatccaaaa
gataaagaagccaacccactgataagtctcggtaccactgttcgccatggtattgtgtatatgagaaaaggagggccagatagatcgtaacatta
atacaacacattcacctccaatccaggtatggacaccgaaccagacacaagccatgacactgcggttcaaaactggccataaagagccccaaat
accaaacgaacaacgggaaattacaggaaaagaaagatgatagacagcaccagcacgaccagttgcagcaatcaaaaaaccacagatcatg
taacctacccagacacaaatccaagcttcccaccagttaaggccagcttcaatagcagtagctgaaatcatccaagtattgatattaacggactc
agcaagccagaaagagacaaagttccaataaccccaaagacgtctgctagttgggacagggtataagtcctcgttggtcaacagccgttggcct
aacgtcaaacccggcttttggtctaacaaaacaatgtctttcagtttttgataaaccttattttccgtaatttttcccaacttgcccatgattataaatt
ataaaaaaaaaagagacggtactttccaaacaatataaaagagaaataataataacaaaaagataaaaaaaaacaataaggagccaacaaa
caaaaattcgtcagcgatgaatgtctatgaaaagatttttttacaaaaaccgtgttcgatgaaaaactttttttctggactctgaatccaaaaataca
agagaaggcctctttagattcggattggcttgaaaggaatgatggaatatggatatttgcccaatattgcagggcgaggtgtttgggaaagacaa
gccatagtgagcaataaaaaaattatatgcagcgccccaagcacacgattttgacgggttcgctaaattaataaactattcataaacgaaggag
agttaccactactccacaaaacgctaaaaaaaatttcgttttccaattgtttgatggaacatagagcgaaatgttttaagaaaagatgagagaaa
gagtagtgaccggtacttgagaattacggtctttagatatagaaaattattatatctagttccgatcgttcttttagaagaatagcgatgttcaggtt
gaaaataagaatgaaatacaatgctcaaacaggtagcaattggaagaagggtccacatgaaagaaaattatcttcacctaggaaaacttagaa
ccacagtttctccaattcaaacacagttgaccgattctgcctcgattaacaaataatgatggtagtgatcattcaatgagcaaatgaatatacaat
ctttgattatcaattttggtgttctttcgttgcaaaatcgctcgctctatatatatatattttcacaggctggctatcctatctaaactaacgtttctattc
ctccaaattgattatcgtagcttgatttgtagtatcgatgcattgaagctatggtgccttgggaacaggattaggaaaagatgcttctttcgtacatt
ctttgtttgtttgttttgttgttatattcattaatatttctatatttacgctatagacgaaatggaatcttatactaccgattagacggttttttttacaattt
cgtagctttcctacccattaaaatggatgcttttcctgcagtggaaaagtctgtttttgaattagaaagcaattgtttctttctgccgttttaaatctgt
ttttgatgtggggttttttttcttttttttcttttctctgttgttcttaagacttcagtcaacgctaaaaactcgctctctacttctagagtcaaacctaagc
taagttaagcttgttttgttccttgtgttcacaacttgagttccaccacaaacattgcagtatggcaaagccttgtaagaaaaaacataccgtagtt
gagattcatttgctaatattatagtcaaaatccagtatcttaagttatacaatgttttatcgtgctttttatggttaagtgtcttgattagagctggttt
gctttggcaactttccggcgaccagcgtgtcgtctttaatgaaaccaatgtcttggttcgctgggttcagacattggctttggcagaaatcttccata
gtatctttggattcgtaaagtcatctcccttcacgactgctatgcaagtaatttctcgtcttatactcgtttggggagtatgctatctttttccgcatatt
atcaacaaatcttccatctatctttttatgattttagcttggtcgattactgaggttgtccgttatgcattttacgcttgtaacttgaccggtgccgtccc
taaattccttatatggcttcgttacaatacctttatcgttctctaccctattggcgctggaagtgagttcttacttgtcctcaaaacttttcgcactgcct
ggaacgccttcttcttaagtaaaattgtgtggaccgctgcaacagtaatctatccattcggtatgtactgttcttctttttcatcttgctctagtcaact
ttacagttgtctttgtttatcttgaccaaacacattgctatatactgacaaactttaggattctatatgttgtacacgcacatgatgtctcaacgtcgt
aaagttcttaaacttacaaagaagggtggtaaggcttaatacgaatgcaatgcttcactacatgattttcaatcaaccaacttgtatcggttgctta
aaagtacctatatcctcactcttttcgtcattgactttgatttttttaagggttatacaatccatgagataccaattctcatgaagcttgtgcaagaaga
cgtttctaaatcaatcgaaggctcaacggccagaaatgcaactgctaatagggatcaatattgcaatgtcttcttaacctttaattaaattaatttat
tcagtaaacaacagaaacctcagttgtatcgtattcaaaaaggcattgtatgaaacttaatcgcacagtatctgttataataaaatagttttatgac
tcatatttttttcaccgtgttaacgtcgtccacgggttggtaatggcgtaccacgagcaggtcctactttaaacataggtgcatttctgtctcagttagt
aaaacatactctggatatataaaaaaagcttactttagcatgtctggaacgatcaaaaatcgaatatgagatccccgaatgtaaacttggtcaag
ttgagaaacacgtccatctctagcggtaacacttatgtctttcatttggcaattcatattatcctccgctatatgttttattagtctaagtttaggaaat

ggttgctttaccttcaattagcttgcctctgtaagtagagccattttctaattccacagtaactatgtgccctagcattcaacgttagttttcctcaatc
tgaaagaaaagcaaaagaaaaaaaaaaataaataccttgggactcatggagcagctttaagcacaaagacatgattccgtttaattgccttgtg
tggtagtatacagtaggtgctatgctatgcctgtaaaaagtttgcccactacaaacgttgtgtaagccagtcaatattgttattataagaattacttg
gttaaattggacttttcattataaaaaaaaagaatataaatctaaagaattggggtcgctattagtatagggaaaatgcagaacatggatggaaa
tgacaacgacaaagtatttctttgtagaaaatgcaagaagcaggcaacggtaatgtctagatccgaatacagttgtgatggatgctttgttcgatc
gacagaaaataagatacgtcgtcaatttgagcttttgcgtccaaatttggaacatcgacgctctcgaaaagctttgctcgctgtttctggaggaatc
tcttcaatggccatgttagaaactgcatattatctttcgcgatacaggaaagataattatcgacccatgttcgatgaactcttagtcgttcatttaca
aagtggatctttacaagacgaagaagggcgcgcaaaaacaaaagttataatacaaaaagccatagagtcttattacacccaatgtgaatttcag
gttatccaagaagatgatttactgaagaatgcatattgcattgatgcaaacggcaaattatctcacgatgattcatctaattgcaatagctttaacc
tggaagctattccatcattggcttccagacaagatttattatatcgtgttcgcgaacaagtccttatctcgattgccgaaaaggaaggctgtgacac
aatcgtttttggtgattgtggtacaacgattgccgctcgagtcttggagcttgtagctgaaggaagaggatttgctattccttggtgcacgtccgttt
attccaagcttcctgattttccagtttccttgttgcgtcctctccgtgaggtattgtcttccgagttaaaatcttatatgaaaatcaaagaacttgatta
cataccaacggtagtggaagaacgccctgctacaatctctggtgtagcagagaattacttcaaaaacttaaacgaccctttccctagtttagtaaa
cactgttgtaagaatgtcttcaaagcttaatgtgccggaagcaaataaaacctgctctctttgtaagttgccgatgcaagaaaacgcaggagcttg
gttacagaagactactgtgcaacatcctagcgaaggacaagaagatgccgttgccaaccctgatatctgttatgcatgttctgtttcccttaaaaa
catgaaacagcctttacggattcctgccgtcaacaaggaagaataaacgaggattgtactgtacattatataatttcgttcggatttcgtaaatttt
gggtgaatacttatttatgggagtgataaggaaagaaaaagagtaaaggattgattaattaataatattaaagtgttttgtttatgctgaactaga
atcagatagctgtccatcaatttttccataaacccggtatgactctgctgtttcagaaaaaggactccaaacaccacgcacagatggattcgtag
attcgacgggttgtttgatgaatcttggttttaaaccatcactatcacgacacaacatgattttatccgtaatctggttggcatccatctttctagtac
aaattgtttttctcgtccagtatctagaaatttagtcagaatgcaattacagttgagatagtaaaagacttgcatgccgcaaaccgttgtacttgtt
gttcagccgaggatacttactgtaaaatgcacgtattaaaggatgttttccttgttgattgtagacatgaaattcgatatcctgagaagttttagcaa
tagcagctaacttttcgataaaaactctttcatcgattttgaacttccgccccaattgcaatatgagagctcgatcctttggcactaattaaatatc
gttagtaacccaacctttctttttctttcaatgctgtatacatacaggacggataaatgatcctagtccatttttgggtatggccgttttctggacgat
ttcaatcatttagacaatggttggatttcctgattcaatggaacgaaaaaagggaaaaacaaaaaaatacaaacgttaagagaacaaaccgga
attcaaaatacagcgaacgcttcggaggacgcctactttacaaaatttgctacgaatgaataaggtgggtgcgtaggaaaggactagtggaaga
agatgcgagagcctaggtataggtatgagcataagttgagtagtgacggacaacgaacaaagtcgtataaaaggatgttcttttaatttctattt
tgtttgtatggatccatttctgaaaccattttccaaaaacgaacgctcgtacctcccgaaagaattggtacattttaggctacatttactttacactat
ggtttttcttttaggtgtttttcctttggaaacactactcctacatatctggtattaatacattctcgttgaatcctatatgaatgacttgccaaagaaa
gaataagcaagaatcttattaaatcagtgtttccaagagcctctatggagagaaaaacctataagaaggaagaataaaagaagcgactccact
atggactacactttcatatgcttccttcgtaagaaagactgttgctcgcttggaaagaaaagaagaaaaatttgcattttctttgataaaatcttctg
ttaatgagacaatacctcctcaacagctctttccaccgtactgattatttatgctttccttgttaaataaaaaagtacttttatttgtttactttgtttga
aaatacggtatgacaacgatttctccatattccacttttcgcatcgcgcaatggtttgttaaaccccgctcttccatgtaacagcgtattgcgtctaa
gcggaccgccacagatcgattccagcctgccatctacaaacatcgaaaggaacaattctgagtgagatcgagaaaaaaacaaaataagaaac
aagaatatatgagaatttgcaacgaataaggcttggagagcattgagttgtccaaagtcttcgtttgacagtgaaacagtgaaaatgtcgtttcga
attagcggttggggtgaacagttttcagatgccttctatgagcgagcgacgacattgctaactgccgcccttaacaagggtgcgaagcactcacta
attgctgatcgcattactgttaaggaattggatatgggcgaacaaccaccggaattggaaatactagaaattggggatctggctcctgatcggttt
cagggtttatttaactttcattattcaggaaatgcatcattggttttacagacaaaaattcgggcgaatcccttgtccgtcgaaaaacgtaaagtac
ccagctttggagctagaaacaccatgcttgcatcttgtgcccctttgacggttcccatgttcttgcgcttatcgaatcttaaactcaatggtattgttg
ttttggtgttttcaaagcaaaagggaattaccatggtgtttcgcaacgaccctttggaatctttgcgggtttcttcttcatttgactcaatacctgcga
ttgctcgttttctgcagagagaaatagaggtgcaacttgcagctttgtttcgagaagaacttccttctatcatttacaaaatgtctcgtttgtggtttg
ccaaagcagatccgtcccttcccttttccgtccctaagcctggccctactgtttttggagcctccagggaagccgtaaaacaatcctccacaaatcc
cgaatttttagacggtcccccagattacatcgaatccactaaggttgatactcagtatccgatgaaaatgggtccgttggacgttcatgctcatcca
aatcttcaatcgttgacgaagttagcattgtcaaggaactccctatatccgaaatcctccaacaacattcccatgtgcatttttaggtccactccgtt
tgataccatccatcggctttctgctcaatctgaggagtcctcttgtactcctctttctacactttccccttctccttcttcagattatttctttacacagca
taatgtctcttctacagggactatatcgacgcggccttctatatttggcagaatgaagaatgctcatcctcatcgtcgtcgtgagcaagtgaagaaa
cgacacgttatacgtttacaatctttaaaaaacacgcaatcctcgccgtcaaatacaattcctgccagtcataattcggacccttttgtctgcgaac
ctattttgttttcgaaaaatgaaaagtcaataatggcagattcttccgaaggaaagccagagaaagtaatcctttacgattccaatgtaaaagatc
gaactgaaaaatctcccctaagcagtcctatccctcattggcgagcagacgaattacaacgaaagctacaaatggaatcaaattctaacaaattt

cccaactcacaatataataccattaagctacttgctctgcagcgtttattgaatgctaatcgctctttttgattctttctttttgtgttatcctttcgcgc
tgtgaaactatcgaccaatgtgtgaaaagagcgatttaaaacttttacgcttgcttgatgcctttttgctagaatcaagctttctcatgtcattgcgta
tattttcttttagccttttgtatattactggtatacgagagaagcttaattttctcttatttataatccgttaatgttttacccttttgttgctaatttcgat
agttgaatcgaataaaagttcttaatttttttcgtacggcttggtatatttttcaaatgttagactagtattttgcagttctttaaatttaaataaaaaaa
ggaatgtaacactgtttagttttctgtaggaccaataataaaacatgagatacagtcctaacaacaggttgaaataacagtaaaaagaatagga
aaagccaacaaaatccaactgacaaaactataaaaaacattgtttaaaatgcgcaatgtaaacgtaaacgggctatgcagtaagagtataagt
catgtacaaaaaataatcaaagaaaccagctaaactaaaaggctcatattgtctatggaaccgaagctggttaataattaaaaatcatcttacag
aaggaagggacgtttcatagttgctctgcgttctcgaagttttgtctgattgtacagaaccatgttcaggaaaacagaaatttgatgggaacaggc
cagtatttcctgttaaatttccatcaccgtccaattgctccccaacccaccatccaacattaactttcttcaggacaagtaaaatgtcgtctttcttca
aagctaactcattttccatctggggctcaaagtcatatttcatccttacagcttctaagcctttatgagcaagtaaaggacttgataaacttttgtgt
ggttgactcacagatatcgtttctttctccagggaatatgttggcataggtattgccgataatgaaggactatggccagattcggtttttgtttcaag
catatgatctttttcattatcatcggacgaatttttagatttttcataagaagtagaatgatttgaagacatgcgtcctatagatcgcggcttcgatga
aataggtatttccggtgacggagactttctaagggatagtgaaggtgaagatgtattatctagctgcaaatctcctagtccttgcaaaaatttagct
ggaagtaaagcttcttccccagcattgataggagttggagtcgtccatgatgttggttctaacctaagagaaggagaagaaggacgagaatcca
ctgcttgattatggttcttacaaatgacagaaggcgaagaagctaatgactttgtagaatacgatcgtcttgaacgattcgtaggttgttgcatcgc
tccaaagcgttgagattccaaaggtcgtagaatattcagacactgttcatggaattgaatatgtttgtgtaataaaagaacaagcttgttgatctg
actggactatatattggaatactgttagcagaagtaaatgaaagtgactactcacctcaaattcccttagctcaatcattgtttcctcaaaatcttct
aaagtattttcgtattttcctctggccatccgcaaatcctcttcaattttagaatcctctttttttagctttctggattttcgttgagagtgcgtcaaatac
caacctcctgttttcaagtttttttctctttagcctccaacactcttctttgtgaaagccatcgctccaattctctcatgtatgattcagtgacgaagcga
acaaagttttcctgtagcgttccaatctgaataagagtctttccatacatattataggtgtcagaatgaacagaataatcagacaaaatgtatccta
gattaaggaaagaatcacctagaccgacaatcggcagcggtttttctttttcttccagagttgacttctttcgttccatggattgtatccattgtctaa
agtcccgatggagcttttcaattccattaaatcgaagttccatttctcgccgtaactcacagaaatcatctgaaagagtcgttttctgtcgatcaaac
ccaaaacgctctccagtccattgagaaaaccttgcaaagtgtttattcatagtagaagttgttcaaattcctccaagaagcttacagtttaaaagtt
tagacttttaatacatttgcgtaattaacggaagagaaaaacattgtttatgaaggaatcgttgctaaaatacgttaaacgagtaagtgtaacggt
attctataaatttgcgcaatattcgtaaaaagaggtattgcttactaaaggcggacttaaatctaccaaacactgccgaacactcataacaataag
gagtatattattttacgagttacttgaatccaaacaaaaccaacaaatgggaatacaaattcaatgatcgcaaacattctttttctttttttattttaa
aaaaaaatcgaaatagtaaaaataagatcgattctaagcggagcttcacttagaaatacttctcgtgatgaagtcagctaacttgttccttaaga
atacttgaactagtcagccagatgtgatggaagtgctgctaaatactgagcacaccaaagtctaaggatatccaaacttagcagtatagttgctc
attacagtgtttattctgcgtctgctaatgatatgttctggcaacatagtgaataaaagataatattattaaagcttaacaacttaaaaagaaaaca
aaaagaaaatataatcatagaaattgggacaatgtttcgtacgtttacgtttataataaattaaagaatgaatgtctcaggctgagttttcagagc
cagaagcataacccaaatcttcttgaacccgtgatccaatctcaatacaggacgatataagatgatgacaaaccttgggatcctctaatcctaatg
aaccaatcccaatgcgaacgtacatggccatagtttccacgacgtcgttttccttctgcgaagtgcatatgggagaaaacgccttcaatgctttcca
accaataacaaaagacaacatactgatatcggaattatcctctggaacgcagatttctctatacatgcgcttggcgacgtcagtataaccaagac
gtaacaatccaagagctaaagaagcgtgtatcgtgaacagtttgctgctgcttaaattcagtacgctagaaaagctgcgcttatcttggctccgaa
gttgcaatgtggtgttatatgcagccaaaacttctgtaagagattctgacgtattgtcctcgaacaaaaccaatttggcgactaaggcatgacgat
acactggagagttcttcggagaaagagaaaggactttatcaatttcagacttttcaatacggtggtctaaactttccattaacaaggcctctagca
aatcaaccgcataccaagaagcagttgtttctaaaggagacatgaccacaacatctgaaagcttgctaattatgctattaggcaactttgcagaa
tcacgagtacatctagccagtgtccacaaacgtttaaccatccaagaagagaaaatatcttctgcatcacatgtctcaattaactttacgacgcatt
caggaagctcttgaagatctgactgcatagatgcactactttgtaggcgtttggcacgattccaaaaaaatgaagcacatttcgatacaaatgca
gatggcataggagagttcgagaatataagttcagagtacattgcctggagagcagacgtatggctggttgtaggcaaaaagaaggatgaagca
aaaaccataaataaacgaaggcggttgaccttttcatctccgcccattaactgtgcatcaagcagtttttctaaatttctgatatctgcaagctgctg
ttcactgtaaacccaagtctcagacgacgcatacaatccactaattcgatgatttatatagttgatagatgatttgacaatgaaaatcaacaaacc
aaagaaaagtgatattgaggaagaagaatgatccaataaacaatatttttcaaacattgttttccggaacaaaatagatgaaggcgatgtagaa
gacatcgctgaaactttgggctttccttttgatttgaaacccttaaaaagccggttatcattcaagatgtaaaacaaccctaacaatacaacaccg
attttcaataaatgccaagcaattgtcgtaaacggaattgaagtgaggaaggaagggagagcgaacaacccgtagatcgattgtgaatcattac
caccacccccgagataattgaagagacccaaaccgaccattccattcaaagcagtgcgacccatagaattaagagttggtgatgtacgtgcatg
ctgtggcgtatggcgtcgggcagaattttgaacagaaaattccgatgaaacgacattgaccggctcggaagctttattgttatcagctgacacggg
agccatggatggatcttcataaaatgctaatcgatcagaaaggcgcttattttctttggtaagctgcttgcttttggattccaaatgtcggatatatt

cagttgcttttgctaaaatagtacctttatttaattttcttgcaggagcaagcccatgtaattcttcttcttctaaagttacaccgcagcgaagagcag
cagcagcgcgtaaagaaggaactgcatcacgaagttcacaaatgcgatcattcagatttgtacgataacgcttttcaatcatgttgtgagatgttt
tctttggtttcccggtggtaggatctatagtttcgggtgatgatgactccttttgaaagttttccattggatcaaggacaggaaaaggtggacttcca
gaagacacagaagagttcacaccagtctcagtagattgaggagaaactttgaccttttcgcgctaggttcttgttttatgtgcgtagtatcggcat
ctaaaaggtcttcacgcttaagatttccaggttggttatcataagacatttgaggaggatgagttcctacagttgacggatcaattgtatcaacggt
atggtggtcaggatccgcagaagactgaaacttaaaatctatatccaaatcattcgcttttgcaaaattgaaattctcattataataggaattggg
cgattcgcagttggagcgtaatggattcgaagagttaccgagtgagttgggagagttcacgccattcgaaggaaactgcgtagagttcgtgctga
aattatcgtcaaaggcaaacgattgaagccatccattcattgacgattccatatcagaaagtgcaaatgagttaggggagaaggaagtattggta
agggtcggaggagaatcttccacaaagtttgcaagatcatacaaaagattattgcagttatctaccaaaggagacgaagagttcgtatcggtgg
gttcagggatcgttgtagtacaattcgagctttccgacatcttcataccatctttcttgttacaagactgggaaggggaagctgaagagaccgaga
cagacggaacagagggtggagcaagcatgttgcaaataaacagaaaaggaacgaaacctcagaggtatactcaaaaaattctaaatagaagg
aaaaaaattacagaaaagaaattgacaatataactgaatacactgccttttctttaagactagtgtttttttataacgtgctggttctttccctataa
acaaataaagcaaaagggtcttttcagcaaccaaagcaacagagcaacgtttctaaagtactcaatagcaaattcaatgagccaggtaattaaa
cacgagttcttcactcgccgagcgaaatggacaactcagcgcaaacttgcaaacagaatcaattcaattccaattgaatttgtttctatttttcctc
gtaattttctgttaagctacgttactattccctttccaaagaagcgatttgcttaaagttgtttgattacgacaaaaggtaaaacgccgcagacaat
gcttttaataaatgtgtgatcaaagtggactgactttaagtgtaacgagaattcagaaatactgacgaaaatggttcgattcattccactaccttaa
gcatattacgataaacgaatcaacaccctatccttctttaaaagaatattcgtactctataataatatcataaacctttcattctttcatgctagaat
gtgtaataatacacatatgaaatgtaacctagtaacccaagaatattgcagaaaaaagtgtataataaaatctgccattatgagaaaaaaaagt
agtaactgattgcttctgtatgataattaaatagcagatgttcatccatttcgttttcttcttttcctaagcgagtaagatgtatcttaggaaggaatc
tttttcgtttcatcattttcagattcactctctgaagatgacggcaagggatcaagctgatcaagctcttcaagttgataccggctcattaatggtggt
agagtgacgtcgcagaccgtctcttcgtttaataaatcatcgataaattcatccatatgtgaaacgtaaaacccattatgatctcttactctcaactt
tcgatagtcatttaaaagcggttccaaagcttgatagacatctggatcaggccatgttaaacgaatgtaaaatgctgctaatgctcgtatatatttg
aattcgggaatggaaaggtattcaagtactatttctttttctggagctacttgcagcagcttgtataacaaacaaataaactcggtaggacgctgat
taccaaattgcccaccaatatactcaagacgcactgcgcgatcgatcaacgaagcagctaaaaaacgcgttagttttgtaaaggcgaaaaaaaa
aaaaaaaaaatacaacaaacgatgaaaagaagggaatgtgaaactacattcatcgcttgtcgagacttcttaccatttaagccaaaacattgtt
ccttccagtaaaaggattccacaattcgttctcgcagaatcttgcccaccaaaaatgttggcagcatctgatgcactacatctccctgcgactggta
gtcccttgcgataaaatccgccatgatgaggcacaatgcttatgagctaaaggtttcttcagtgtttcggtgtaattcctcaaacaatcaattgatg
actaatgctttggaaagaaaagtacagagtataaatgaactacaataacgtccaaatgcgggatttaatttgagtaaatccaatgtaatgggtaa
agacaaaattcaatttactcatattgccctaaaatccggttcttcaccagtgttctttcgccaaattacaagacaaaaacctatagtttgtcaattct
ctctgattaaaaaaactagcactacctattacaaatagtatcggtttaccttttacacatgcatgtcttatagtaaaaaggaaattctacaatcgtc
tacgatcaaggaaaaaacatcatgaaacagcaaatctcattagaaaaaagtaataacgcatacgggtaattttaatgaagcttaccgtctatatc
aatgtatcaccaattcaactgcaggaccttctaatgaccaaaaatttagagggtgttgttggcatagaagtcctcaagggcggtgacaatgcgctt
agtcatggtcttctcaccttcgtggatccagacacggggatcgaacttcttcttgttaggcttatcagggccctcggggttaccaacgggggtcttg
aggtagtcttggtacttcaagatgtagtcacggataccttcgaggtaagcgtattgggtgtcagtgtcaatgttgaccttgacaacaccgctcttga
taccggtacggaattcgttgacggaagaaccggaaccaccatggaagacgaagaaaacgggcttgtcgttggtggtcttaagttgctccttgaca
tacttttggtggtcacccaagatttcagggcgaagcttgacgttaccgggcttgtagacaccgtggacgttaccgaaagcggcagcgatggagaa
gtagggggagacgctggaaagctcacggtagacatcccagatgtcttcgggttgagtgtagagggagttttgttcggcaccggagttgtcgacac
catcttcctcaccaccagtgataccgatttccatttcgatccagatcttcatttggacggcacgttcacagtacttcttgacggaagcaatgttttgtt
ccttgggctcttcagacaagtcaagcatgtgggaagagaaaaggggttcaccgtggagcttgaagtaggcttcgtcagcagcgaacataccttcc
atccaggggagaagcttctcggcgcagtggtcagagtgcaagacaacggggacaccgaagaagggagcgatggaacggatgtagtgagcggc
agcaacagcaccggcaacggaggctttttggccttcgttggaggaacccttaccggcgaagaaaacggaaccaccgttagagcattgcaagatg
atgggggagcgaacttgacgagcagcttcgagggaagcaatgacggtggaagaggaggtaacgttgatggcagggatagcgaatccgtgctc
acgagcgtaagtgaacaacttcaaaacgttgtcaccggtaataacaccagtaggaacaatatcaaggacacccatatttatattttgtttgttaat
cgtgttatgatatgtcctggtgaagggccaagctcttatatattctgcccagctaccgcattcggaaaaactcgtcacctgatgaacgtatcgatgg
tgaccgaaccgttgtagttgcccaatcagcctcatcaacatctttaatccctgacgtcaatggaaaaaaaattgttctcaaagagtaagacttacg
aaaatcgtgtacacaaagctgtcaatgaaatgctgggtattacaacacaatgaattgatcgaaatggttttgaaagacgcgaatttgaagacagt
agaaaagcggagaaaagcgtctgaaatccaacggctacgatttataaacaatgactgttaagccataaacaatgcctaaagtgcgtcgcaaca
gattatgcaacaattgccttcagaaagaatattgtttttgtaaacgcaaggtttgcgtactaccaaagtaatagacttgctttgaactccatcgctg

atgtacaatactgtttttgtaaggatgaaacaagggacagcgaatcattaaactttcattccaaatttttaatggttcctttctctctttaagcttaca
tacgtttgacaattcatgtttcatccttcacatccaacttccttccgcatttttccatactatgtctacgtcctttttcaattcgctactaaaaagtagct
gaattctctattgtttatttactaatgcaccgcaagcagcacgatggaagcatttagagtatgacgaaaatgtccttttttttacgtcggaaaaattcc
cggtcgtctcgtcaccgtatctctcgatcaaccatccctgtttgaattgatccatttgtttatccgacccctccagggataaacaagacaaatgttac
cttccaaatttgtaaactgaaaccttttttggactgcaagtttgtatttgtttaataaaattcacttttaattcattacaggtcttgcttgttacaagtctc
ttgtccttaactcggttttctgaattctaaggaatgtcccattttcattctatgctttactcgcacgttagcacctcttgtttttccttgttctttgtttactt
ttcatctatgtagcgtagacggttactaccgcttctcggtaccagttcaatctttgttgtccgcttccgaaatctcttcgcttcttccagcggacaggc
ggcaaggaaaaatttttccattgctctttacaactcgtccggcattttgtaatggttcttttctttgttctttggttttctaatttgtagcatgtttcatctt
agtacgaaatctcgtcccacattttgtactgccccgccaatacgcacacgtcttctttgttttcctttcggttcgctcttttctagacattcctgtaaaa
aatcggtgactcataaacgagttcttcttatcatcttctgtggatttttttccagccttttccactcagcggaataatatcgtctaaagattgatttactt
tcgcttatgataaccttattagtatgcaaacgattactagagctagttcatcattttttgttttacgacgcaaggttaaaacgtaaacaaagttttctt
gcgtagacgaccctcgcataaaaaaaaaaacaccatgagtccttttcaaaatgtccttacgtaaactttaaaccctggttcctggtttgcctccatgc
atcaccttcaatgtagagatttttgggacttcaattttgactttattcgtggctttgctttgttgatttcatcgttaaccttctaaaatccttcccatgttt
gttttcctatgatataacgtttgtttcgactctgaccgtttgcgtttcatcacggtgagaacatgacttcatactctgtttaataaactctcatccatcg
ctttacgttaacttcgattctcgccaaccattttcaagtggttttatttattgattgattgaatcttaactttcttttacttaaagacacgtttctagcca
cttggaatttttcaatttcttatagaacaccctataaataataaggtcgcatattccttacgttataagaagtttaagagaatgcaattcattagaac
aagtaaatcataaaaaagggaaattaaaagcaaatgtaacacttgcaaaaatatataaataatacaaatacattagcgaataaaagctacttg
gtataagcaagaagcatattaaaatagaggaaactaaacaacgtagagaaaaagtataagggaaccatataaacctatagtgactttcgaggt
gcatctatcatattagggcttcgaccgggtggtgctggacataacacaaatccagaggcatacggagaagcagcagctacgggcgctctctgtgc
agggtatcctgaaacttcagagtcattttgagaggggataggtgtaggaggaaggtaatttacagcttgtgagttatggcaaggccaattcgtgtc
ggttgcaaataatggcgcagtaatcggcacattatactcaggagaggtggcagagtttggcgttaaaaccggaaagggaacaggaagcgtagg
agtataaggggacttccattgatttgggagatgaggagtcggaggccaaggaaggccaatatgtgcattaggtgcatatgccgagttcatataac
catttgcttcttgcagctttcttaattcctgctgctgttgacgttggtaagcaaggtattttgacgagactaaaggagctgggcgcattcttcgcgtaa
acgttttacaattactgacaatggttgagagctccaataatcctattctagttttcggatttcggtcaaatatgcgatttagcaaggagtttaattctc
ggcttatggggagaatggataatagggttgacggattttgtacgtaggaacgataagtgccgtctgtttgcgaacatgcacgcttccatggatttct
ttggcaacacaagttgattaatatgatgcctaacgcccaaacatcgtttgatgctgtagcaaatactgtggaagaattatcggatgattcattcgtc
attggtgtatgaactttataacctgaagacggaagtgtgcttgagttggatgcctcacgctgacattctggagacatgtaaaacaagctgccgcaa
ccaaagtcacttgagtagtcatctgtagttgcaaggccaaaatcagccaagtaaactgtgcgcccctcatcgtctacgagtatattttcgggcttta
aatcacgatggtagatgccaacagagtggcaatgctgaacagcactgataagttgactaaagacgtgttttatcaaggaatcattgcctcggtat
aaattttttcagtaatgcaggtaaataagtcgccgtcagggcagtattgcaaaataacataaatcgtatcttcagtgtccaagacacggtataaa
gacaatatattggggtgagactgcacacgtgcatgcaaataaagttctctggactgtagcagtttttgcttcgcatccagtccgtctttgcacagtg
ccttgactgcatataacgtgccgtcgtacagatcttctgctttataaacaactccatacgcgccaacgccaattacagaaataaaccgcaaggaat
catccaacgtttcacctatacgatcttcaggagctaaacgcatgtttaggcggtaagaaccgggttgaatcggttgttagatagaaaaagaatag
aggttaggaggaatttcgtgaacgaacgtagatgagcacgactgattattgagatttactctaaaatgaaagaattagctagatatcggccgata
ataaaatagagacgggattagattgaattaggatataggatgttcataaagaccagcagaacaaaacaagaatcagtttgaaaaagaaaaag
gaaggaatgagaaggacaataaaggggcgaaatatacgcaatgacagttttaggaagagaggcaaagaattcttgtcagtattcctgccgaat
tacttgtcgataagcgagcgatttcacgaaaaagagaagccttcaatcacaaaagtagactacgaatggataatcaaaaggaaatccttcagta
aaatgaaaaaaaaagtatgacgaagcaaccaagaggaatagaaaactaacgtagagttcaaactcgctgtcaatcaagaatggtttgtagagc
aaaaatgaaaaagaagagtataggatccttttctcaggaagtcaaagtcggtcaaaataataagtagaatgaaacgaacaaaaacacctaaa
gcatggaaaaagggaaaaaccccaaagttgaaaatcaatgaaaagaaaaaaaaaaaaacgaaaataaatcaagagatcaagaaaaagcaa
aatacaaccaacaaggaaaataaaagaatactaaaataaaatagtagaaagaaaagaaaaaaaaaagaaaaaaatttttttgagtacgttgg
gttttatgtaatccgttggatcctagtcaagtctggagccttggaaacgtcaattggagatttgactaaaaaaacgcgtagcaaaaattcgtttgta
agaaacaagaaaatttcttaaaaaatacaatgatgaaaaacaagaaattctataatgaaccacaggaaacacgcgttcctaaaaaaagccaat
gcaataaacgaatgatgggaccaactagtcaagtccttaaggtcagtccagctcaagtacactacacgcgcgtatagaataattatgctttgaat
gaatcgtagttaaaatgatatgtcgaacgaatggaaactgaaaggaaaatccacaatgaatgtcttttcaaacggatgcctttttttttgttctctc
tcactttcaatttcaacaagtgctagagggatgaatggaaggaatgtgtggtgttgcaaattccgctctgagaacaaagaacaccatgaacaaa
gaaattctctaaacaaagaaaacagaaattcgtttcttctctctattctctttttaacgataaccttgcttctctttaaacccactttttgttagtattc
gtagacacaagactcttttcttttctttcctttctttgtttacgtttgtttgttgtcattttcaagctttccacctcaaccgccgattgaatcgaaccgcct

ttttccatatccaccgtaccgtgtcctctctttcaacactctaccatcatctcatccagcattggcttcaacacatctccattatgcatactcttccaac
gactcgatcatccttccttgctctcataaagcaactcccattgcacctcgaaccattgtcccacggacaacagtgcccataaacaagaacccaaa
accaaacaaaggattcgaaacaagacgtaactctgctttcatttcatccatggagtattcctatccatctttgcttgatttgtttactttttcttttgttt
acatgttcttttattgttacccagagaaaactcccccatgccaatgagtatcattctttcaatctcttgttctttttttacaccattcgattcgtctttact
cagaaccctaaaggaacggacttcgacttcctccaatccacttccgtaatattccttaattcgtatcagtgaaacccaaagagaccaaccgaatcc
tttcatggcatccgtcttccacctttaagatgccttcattccgttgcacccccctctttttttacctttttttttcctacgtccttgtactttctcggccattga
aatatgcataaatgtgtttctttcctaaactccctttttctttatcattgcgcaccccattgaactctcgcaattgaattgctcttgagcctttttttggtttt
gtttttttctctgttgtttgtcgtcatcgtcgccattgaaagagattgcacggatatccattcaaaaaattctatcataggcttaatcaatctctcaaa
cagtttgttttgatttggattgcattcttgacgaaatttctttggtttttttccccattgtttcctttgcattcacactccttgtgaagaaattattgtttctt
ggcttctgtaaaaagtctcttcttgtctgttggtaaacaagaattataagcatgggtcaggtttatttatcttaattttaagtttttttgcatttttcctgg
atcacattgagtagtgaaatctttcaatttttaaactttttattttttttgattcttttatttcatattctttgaaaaagtggcatttgtctttttgggatacc
ttttgtttctgttttaatctcggtttcttaccttttcttttcgtttttccctcttatcgcttttcaaatgtccgtcgtaaaccctttatcctctcgtcgatcaaa
agaatacaatcgcatcttcaagggtgctgcggtcttgaccacctttgctgctctatacatcgtcacatcttccaatggttctacaaccgtcaaggca
aagtcatccatcaaaggtctctacaattcctcgggcaacgattgtttttttgaatagtgttcttcaagccctcgccagtcaatcttccttaattcaattt
ctccatcttcgttcttcttcctctaccctgtatgccgcccttcttaactttctcgaaagcctaaattccggttcctccaaacccatctctgctaattctttt
cttaatgttttagaaagaattacaaacaaggtcattttacgctcgttacaacaagatgcccacgaatttttacaatttctattagaaattatcgagtc
tcaatattcttcttttccctctaatccctattcttcttctattcgtattatgggtttccctgcgcagcttccttttatcggaaccattcttcaaaccgtaca
atgctcgtcctgttctgctaaaacttgcacaaactttaaaaataccactttacaattgagtttaccctatgcctatggtgcttcttctatttctttatctc
aacttttcgcttccaatgcatttgaacaaattgatgattataaatgtgatgcatgctatcctaataccaaaaaaactacctcctgttatcgcaccgtc
cagtggaagcatcctcctaccctccttcaaattcagctagagcggtcttgttacactcctctaggtctttcccggaataataccgctgttttatatga
aaagtctttcctactacaaggccggtatcgataccatcttcggtcgctcataacccatcatggcggtgcaaattctggtcattatatttgttaccgga
aaaaagacaatagttggtggagtgccaatgatacactcatttctaaatgccccatcgaaaaggtacttgatcagcaccgccttgtttttatgctgtt
ttatgaaatggaatccccttcagaattggactaactctcttcttaccttacattattcattcaatccatctttccgcaccttttcccagaaattcgttta
aaaaaaaaaaaaaaacatataaagtcggtttggttttttttgtatgaacatatcttaatattgtttcttttgatttcccttctcattcactattgttattcc
ttttttgtatctatagttctttttactacatacttttaatgattatttcttattcttttatttttgttctcttcatgtaaccaccgtatctgtccacaaagcca
cagctttggctttgagattttcaagcggtcctcttttcttttggcttgatagaagtgctgcatggcccaactatccgtgccgtcaaatacttgtttgtag
ttttcataaggtcgcagaagcgactgccactatgttttcttaccagcgtcctctaaactttctattgtattatgtaattacccaaagccagttcgttaa
tttttattctctattctttttgactaatgtcaaggacgactgctaaaatcaacagcaatattatgtttcccttagtgtcctttgcttcctgtagctcaaaa
aagtaatacaagttcgattcttcttcattatttatcccaaagaactgatgtctttccttaacgggcttcaaactgtaccattctcttggttctcattttgt
acatgacacggtgtccgcattcacgacaacgaattacttctttcgcctgaatggtattttttttgcaccgcaatctatatattcattagaaaatcaaaa
agaaaatggattcctttcacgtacctgcgcaaagataaatcattgttactggcctaggtgggttgaaggcggctccgccagcagaagcaccaaaa
gcaccgttcattgtgtgtatgtatgtatgttaaaaaccaaaataaccttccgtgtgtttttttgaaaagaattcccttttcgccaacaaggtgtagtg
gtaaacagtaaaaaattcatcgtctagacagtagaccattatcctctaattaagataatataattaaaagcaggttatattaatcaaggaatattg
agaatcaagagttaggcagttcgtgaaaaggtattaagattaggttaataagtgaacaaagatcagtgaaaataggcataaaaaggtctgatcc
ccaaactcccagttccgtacaaaaaaaggctactgaaaggaaagaaagatatagccaaataaaaattcaatagaaagaaggtttaataattga
ttcctcttcctgtccgccagtactaggatacagcaggtgcgaaagaagccgtgaaacaacattcacattgatgctatttccaagtaaacggtacat
ggtttttttctgaaatctctatttctttccactgaaatgaatcaggaaatcccataatccttgcaatttcccttgcggtaaagtatcgaagtctcaaaga
catcctatctttttcgaattggtctgctatactttcgtgatctgaggtttgtaaaactgatccagacccttgtactaattgtccataacctctagtaaaa
cagcaacagttagtgctcgttggttttacaatatccagttgatgaccataacgaaacatgagtttttcagggagaaagtatgactcaaagctagct
tgcgtttctaagaaactttccaaagtatttattccttcacctttataactgtttttgaatgaagttgctataatttcctcaaaattccaatttactaatga
acctttcctggccagaccataccatcggcaacgagaattgggaatgtttgattgttttggcgttaaattcccctctataaagttgtaattcattccttt
taacacttcacgacatttttgagcagttatactattttcaaaaccttgaacattttcaatcaaaatgtacttcggtggactttccactttcggaagttc
ctctaataaatgaaggaaagcatgtgatcgtggatcttgtacgtcttgctgttttccaatgcgtgtataaggttgacaactgggactcatagtccat
aaatcacaatggtatccatctaattcagatgcattgaccttagtaatgtctatctacaaatattaatatagtttgagtcatttagatcgtacatgttta
gctcggtttcctaaattatgattatacaccgtattcgcaaggggattaatatcaatcgcgcaaactacttcgaatggtatgtttgtagactttaatgc
gtaatgcattccaccaataccagaatacaattccaaaactcgtatattctgtgtccttcttttcatacttgtctttggtagttccttcattattaattggt
agtgtatttctcttcaaatgtcaagaagaaaatttattgggactacgctctctgaatgtattgtaatggtaaaaatatatgaataaccctcatgattt
tattaaatacgaaaaattaaactttcataaaacataccaaaccgtaaaatcccgtttttgaattgcttatgaaatctcatataagaaagcagatct

aattatcttctcttttttgcttttgtacatttcctcaatattctcaggtttgaagtaatgaggacttatttccagtaaccactctggctgaatttgcataac
ttgtcgacaatattctttacttgtcaacacaagttcgtaataaataatgatttttggtttcttctcaaccaaggtagagcttggatgaatataaactgt
ttgattgctcttaacggttctatatgaatctcctgtgcgatccaagcgtgcagcattagagaaatatcctgcagttatcgccttttgaatgggcatca
atgagtctgcagaattagttgtcaattcaatctcaactcgaccacagaggtttgcaagttgatctcggacatcacgagctctacataaacttttgta
ctgtaaaaagttttcccgtgaccactgataggaaaaatctgtatctacccactcattccatatacgaaataatgttaggtgatcacccccaggttgt
acgaagttcatcctggctttgtcagcctcccataattcgatcttttggtcgataaaacagcgaagcagattcacctagcatagctattatagaaaga
acttcttcaacacatccatattttgcggaggctatcaacgatttagaaagcatagggtcagtaggaaattctgccatctgtcttcccaatttcgtaag
ttcacctcgattatttaaagctccaagtgcatataatagctccaatgatcgcatcaaagtttccggaggtggtggatccataaaatcgaaatcaag
taaattattaattcccaatgattttaaaagcaagactatatttgttaaattagttctttgaatctccggcgaggtgaccatatcaagctcattattgta
agtccaacgagtatagagacgaaagcatttaccgggtccaactcgaccagcacgtccagcacgctgatcagcagatgcacgagagcaggggac
ggaaactaagctttccatccccgttctcggattatacatgttttgtttaacaaatccagaatcgattacataatttacaccatcgatagtaatagaa
gtttctgcaatatttgtagccaaaacgaccttccgagcaccaggcggtgtgggttcaaatatctttgcttgcaactccgagggtaagttagcataaa
ttggacaaagaatgatttcaggtattctctttcctaaaatcttacacaactcttgaatattttcagacataacttctatttcatcctgaccagtcaaaa
agactaatatatcaccggcttgttgagttgtgtggatttgtaaaatagtggttatagctgcctgtatataatttgcctctggctgtggagtatagtaa
atatcaacaggataccgccgtcccggaacataaaagactggtgcttcatcaaagtaggaggaaaacttggctgcatcaatagtagcactagaaa
ccaaaactttcaaatcgggtcggaatcgagcaatatccttcacaaggccaaagaggatatcagtatgaagggttcgttcatgtgcttcatctataa
ttatcacagagtaactagcaaggtctggctcagtcaaaaattctcttaaaagcattccatcagttaaatacttaattacggttttctcagaagtgttg
ttttcaaaacgaatggtatatcctacttcttggcccaatcgaacatccatctcattagctacacgtgcagcaacagacatggcagcgacacggcgt
ggctgtgtacagcaaatctttttgttgtttttttgtatatccggcttcatgcagaaactggggtagctgagtggtttttcctgaacctgtctctgctacaa
ccaacaaaacttggtaagcttcaattgccttcaataaatcatccttaaacttataaacgggcaaagattttctggtttcttccatggaaagagccttt
aattcggctgaggaaagtgcaggagtagatggttcagcacctgcgatagatttaccaggtaaattggactctaaaagaaagtcaattttttgtga
atcatcaaaaacataatcaaatctcttttcttggttgggggccttgagattatcttcataaacaagagctttagatatttgttgggattcccactgcg
cttgttcggcagcaccaaacggggcactaggctgacgctcgtcattggcatccgtgtatcgttggtgcaaaacagcttcttttcgtcttctgtctagc
tttccttgttcagtgaagtaatcttcaggcatttgatattctgcagcttgagcatttaaattgatgcgctcttcagcaatccttaatagctccttttttctt
ttccaactcatggatttcgcgttttgttaattgttcgtcagcaaataaaagctcttcatcttgaatttcccttctcaaaagctccaactgttgttgagct
cttggcttcaaataatgctgacgagactttttacgtaattcggcagctagtcgttctcgagaatctgggtcatccgcaagctttcttaaagcaatttc
ttcagaagaaagctttgaagagtaatcttctaccaattctcgagtcgaggtcatcaagtcttttttttcttaaacgctcctcaaactcttctctctctctc
aagtcatgcattcgttcatcttcttcgttaccttttacatttgagtcaaccacagctgtttgttcttccagtggaagtttcgcttcgtgatcagttacagt
attcgagatttcttcttcatattcatcactttgccattcatcatcatcatgcctgcgtcttaaaaaaccctgcttatgttccttacgtctttctttacgttt
ccgtgaatccgactgcggtaaacgggctgaccgctcttcttgttgaggttccaaaagcagttcgtatttcgcattctggttctccatttgcaaaatct
ctctctgtcgctttttttctatagttgtatgcttcatctgatatcctttctttatttttggtatcctagaatatagagaatgagcaaattcacgtactttag
aatcggcttcttccattccaaatcccactaatttttggaacagctcttgtgagctacgcgaattggatgcttcagcgattatatactctacgactgat
ggttctgacatccctagaagcgcaattgcttgatctgaaacatattgttctaaagacatttttcctttattaataccgccgaatacaccttctaacctt
gactttatttattcgattcttaacaaaggaaagattctcaacgattatctatagcatatataactttatgatgtctcaacaattggtaaaatatatttg
gcatttgcatgcgtgtaagtcgctttccaaagactatgcttcttcttgctcattgcttgatcgcattaactggaataacgaaacacatcgtacctctct
atttaattgaaatgactataattacgttttataaattcgaaatatacttgtttaaaaatcaaggattttcaattcatatctactctatataagcagcta
attttctatttactaaaggctttcaccattcgtttgcttagacaagtgaacactcgttgttaaacttccattgtgagtaatagtgattgtttctgtcatt
actatttgaggtacattacgaaaagatttgtcgagccataaggttcttcatcttatataacacctttagttttcactataaagaacatacttgccaaa
attcgttcgatacttctatttgtaaaaaggaataagcaagtaacgtcttacttgcgttctcaacgtcctatttttggttatagtcaccgattttgtacct
ctaaaaaaaatgtcagacgaagtttaccagcaatttttggacgccgctaatgctccagttccgtctcctcctcctaggagcccgagtgaggttccttc
tcgatggatgaaaagcactggtaaccttcctaattcggctccattgaacgccttgtcggaacaggtaaaaggacagtattatatcagcgagtccg
acgaaatcttccatcccatctatttagagaagcttccctctaacttccgaaatattgataaagaagagttcgatcctcataataaatatggcagtat
catgaaggctgtccaaagttgctgcagctcaggctcggtacaaatttttagttacgaagaaacagatgttaaaactatttactatattctaagcca
gctggacaatggcggttggtgcggccttacaacaatggcagtctatacctgatttgggttagactttaatgtatgatgttttacattcctctagagtt
aaactgtagtcatacaacattaaaattcaaaatgttgtatatgataatataactgataatgtctaaatcatgtcttatttctacataacatttagcta
ttcaaagttttcaaaatagcagatccttaagaagccttttcatcagaagtatctgcagttggtgcaggttcaactacttccgaagattccggaattc
gtgaatcggtagtagtaggcacagttgaatcctgattctgaatagtagcattattaccatcattactattattgtggttactgttgtagttttcgttttt
gtgtccattgttttgatgacggttacccctggacgggttgaatgatctttgggaattgcttcgtctagaatctgtatacataggataacgcatgttcat

catcccattgttcatagcatactgtccatacataggaggtacaccattgacaggataagtcatcattggcattcctcccatctgaggcacatagtaa
gtggcaggggccactcctctaggactaccctgtgcttggaacggaggtacaccgtaaggaggcatgactggaggtgccataaacataggcgaag
ctacggcactagaatttcctgaagtaggtgtaccactgggagaagcagtaaccgatgcttgttggcttgcagcacgcatgtacggaggatatccc
ggcattgagtagccaaaaggaatgtagggaacgttaggcgggggatacatcgtcattgctggtgatacaggtgtattaggcaaaactaaaccag
gaattgggcctgatgttgccgaattacgttttccttgacttgggtcagcctcgatttttgattggagacaatatgttgtaggttgttgggaccagaa
gcccaagtaggaggacatgaaaatggtttctccaatgtttctccaatttgttcttgttcagcagcagtcttaataacgttaaagttttccataacaga
tgactctgctggagaacctccatcagggaaaaacgcacgttctttattttcactagaagcttcaatgtttgaagctttcgaaacacttcctgtttctgt
actagaagatttttgagaactcataggttgagaagcaacgggcttcactggaagaacgaatttgcccggcgtaaagacgggtgcaccaacattg
ggcttgaaagacgcagccttggcgttcaacttcaatgcctttgaaggatctttatttgcgggttttacttgctctttaccggactccaaagacgcagc
tggcttaggttcatcttcctttttttcttgagtcttttcctccttattagaaccaactattgggtcaggctctttttctcccttttctaaagatggtccatct
ttgaatggaacttcctccttttgtttattatcatttttgagacatcaacatttggaatctcgtcagctggcttggaagacttttgcttttccttggaagc
atctttgttttccttgacagggtcctctttattttcaaatccacctaacttcaaagactgactgaattgcataaattccttaatctgaccttccttttctc
ctttggataatgtacccatcttacggcgggcattgaagaatttttcagcagcacgagagtcaccgccacgggaaaatgccatgtgtcgttgagata
ctatggcaggatcattaggtagataaacgttgcgtggaccagcgtttcgatagcgatgatcccgagattttccttgggaatagttgtctctcgatga
tgaaccccggttgttgttctttccgggaagagatgaacgctgaacacctgagtaaaggtcttcttcgtcaagtccactatcatcaaccttgcggcct
cgttcttcagcaatgtgaatgttatcggtagcagaagtttcaatttctttggcaatacgatcggcttcttgggcacgttccttatacttggggtgactt
cgattgatggtagaagtgtactgttcctcatcaaaatgtgattgaacaccaaaaagcttttcattggcagcgaattgatcccattctccaccgcctg
tttgattctcagactcttccaaactcatcaaagaggcgctcgcatcctgcgggtcgggcctccaaggcaataattcacgctcacgatgttcagcgg
agcccctgtgcgaagaaatcgctgtatcggttttccatgatttgctattagacgaagaagtcctatatgcattattcgcacgccctgtgggtgcattt
gtggatcgtttgttcgttgaagacatcgaatttacgttactgttgtctttagcattatccttaacactcgcgtctggcacagtccttttcgtttcagtaa
agatacgttcactactgtttgtcgactgggatgctttgcttcctttagcagcaaccgcagaccatttttttgttgtttcagtcttcgtaggaccggcgtt
tcgttgtggggtctgttccattctgacagaatcggttgccataaaaattacttgagtctcctcagatacccttgcaatttcttaatttgcaacctccca
gtacccgactgagatattagtgaattccgtggaggttggaagttgaaggttggatcaattacgatagaacttttggggtttaacgtgtggcgccca
ccgcaaattttcgtaaatcctttaggcgaattaatagcaaattttataaaatactctaattaatttattttctttattaaattcttcaaaatggattttt
gtatttatctatccttctgctttccctggggttgactcaccaccataggagaacaacattatgacagcatgtaattgtgtaaacatgaaaaaaaata
gattcattttagttaacattcatttaaacgagaatatttttttgttagaacgattgtagaatacatgaaacgcttttctttttttgccagggtcaagagaa
tatacgggaaagttgtaatcataaagtaatccattttcttgtgctggtaaattaattaattactttattggtttgttaaatcattcctagtgaaatgcc
atttgaaagcaatataaaatcgtcttcatggaataccttaagaggaacgtacttggctgagttaataaaactacttatcgtctctgtcatatcaacg
aaaagaatgttttgttacaaagttttgcttgcatgaagagatggatcctttcgaaccaataagttacctaggagactcccgcccaaagaaacaact
agtccttacggcactttgacactgtctttagaaatattcttaaatcttcggtttcctcctgttttcatttatctcaaaatggctatgacctttaaacaac
agatgttatctatttttggaattttcatatttggtaagacaaatgaatggcgtctcactggtttttttatgaaacaactaccatttgaaatgcaattccc
atttcataggttatagatgagattgcatagggaggatctactgaattggttgttaactcagtaatttaggtggtcctgtttcgttcatcttactgaaga
accatctatatgaaaagcgaagggaatactttaaactgcagaagcagagagatgctcaaaacacaaaagacaccaattaaatgattaggagtc
ttatgattcagcgttcgtcgactttatattctcaagcgtttcttcctcaaagaggagtttacaaagtgtagatttcccactccccttctgaatccatg
catcatctgcgcttaacaatgtgaaagattccttccgctattacaatataaagaaataaagaaaaagaaaaagcatactcaggacaaatccctg
gtacttctcattttgtctccttggctacaagtataaatgagttgtcatttgaacaaaataggcacactaataaatatttttttgtttaaaataataacaa
gttgcaaacaaaagaaaaatttacaacaaaatggaatacgaaagaacgaattactcatgggaacattcattgcaaaacaaacacagttatctat
atagattactgaaatatcctcgtaattgtatggaaaacaagatgaaggaaaaggaaattaggaagaataaacagaaataattcattgaacattg
ggcacatccgttcggttatagattaaagatacgttcatacacaaaattaaagttgcgacacgggtgaggcatcaattgaaacaaaatttgcgctc
atcttcttgatttccttttcaaaaaaaggttcttaaaacaaaatttgtcaaaatcgccgctaataatacaaaacacaagattgtaggtcctaaacga
tcatcataaggaccacttcctcttcttcgaattgcagatgctctccaatgataaatagctaaagcataacccatcgttcctactgccacaatggtaa
aaagtccggcactgattcttccaattctgtcaccaaagtttagcaaacctacagataaaccacctaaaacaacggaaaagctaagccaagaaag
aaatgtccgttcattagcaaaaaaaaccttaggctctacccgaactgggagcgcaattctctttcctggagtagtttgcaaaagtggctgagtcga
catttataatcactgtaaagcttggaaaaataaaaaaactttcctttctgtaaatgaatgtaaccgactattaaatttaccaggaatccgaatgaat
actcgatttttaaaaacgttttgacaaaggtaacgtacacagatacctcagttaggggaagactttttgtatttagtgatgtgcactgcaacttctta
tcgtatcataaacgaaagctggttcgaaagaaaatataaaatagaaaaaaaatatgcttttgtagaaggaagaaggaagaaggaagaaggaa
gaaggaatcagaaatgaaagactgttatttataagtttttagaatcttgccgatcttgtagaatttttccgtaaaacatgagttgtttcacaatttca
ggatttggtatctcttgttgttcttcgaactgctggaggatgttgatatactgcatagcctctttaattgtaatggatacaacatactcttcctcttcttc

ttcgctctcaaaatcccggtcttttaagaattcggctgcgatttggtcagtaacatcgacatcttcgtccacgacactttcttcaacaggatctataa
accgatcgaggaggtgattttctccacttgtaaactgagaaaataaagactttaactgttctgaagcttgggttgcagaagaagggggttttacgt
cagaagaattttcttttatgcttaatatgccagaacgctgaaaactaaatgcaatcatggaactggaaacatcaaaattccatgcggcaaccatcc
aacgaatagcttttagtatgttgacagctttgagaggatcatgaccatggtttatttggtccgccatgtactggagccaataatttctatagcattcc
ttaaaattacgaaatatacccatagaatacgggttttgtgtcggattggtacttttttggaagccacaagatagctatattttggaaactgcgagtac
gacgaatgttttctacagcgcattcatgagcgctcaatcggtccaacagaagtaaaacctttctgccggacatcctttgatcaaaccaaataagcc
attcctgcattataagattattaatttgggcagtgccatttgatcgccattcaacgtcgagagcatgaatattgactcgcgcagaacggaaagccc
gaggctctttcgaataaccaatgatccacagaggttgtttttcggtaccaagagcattgcaacacattgctattgagacacgagccttttcgcgac
ggataccgtttgtaaactcgacagaaggagggttgtttggaacgagtttccatagaagcccgacctcgtcaaaagaaaatacgtcttctaaggcg
taatgctttacgaattcataaattagatctgtgtcagctagcttcatattgtcttgaggatttaaataatggttttggaatttttccagccaattgctgc
taaattctggagtaggtaaattagaatatgcgggaatctttttccaaaattcacatgctgtttttcgaataacttcctcattcacttcaatatctgattt
ttgtgttctctgaatccattccactagagcactctccaggacaggatatttcgcaggtagggtattcgtttttttcggcggcttcggaggtcgcttcatc
aaggaaatcatattttgaagacaatatctgagaaaccgacggttgcgacaattctttgtcaaaatgttgtctgaaccaagccatcaacttttgttgc
gacggtttttcgacacacccaaagtagtaattacgtagtgctttcttttctattttcgtaatgacttgacgccgtactggaggcattttagatccctag
tattcagcattaagcattttggttttgtgagtactctacgatctcaatttttccttattaccatactgcatgtgggactaggggagggccgtttagccg
aacgaataataacctcgaaataatgaatttagtatggaggttatgcacacaaccatcaatttcgtatttatcagaaataaactcgaccaggtcga
cagacctctttatgaaattttgcatcattacccatacttttattatcttttcaagacatccttcagtattgtctttgatacacgaagttttcatgaaaaa
tatattttagcatttttggattattattttcttcactgtagtttttctcttaagaaagattgttgggaatggatttgatgataaatatgacggtacggtcg
taacgctactgatgtatcatatatcaacaaaaacgactgtagcaaagagttatatgtttttttcgataaaaggtcctcttcggcggcgatgatttatg
tccttttgtaccgcttatcattcattcacgaatttgatctctatattcctttctaaacatgaccattgctgtcctatacatgaaacatttattgtttattct
ttaggaaatactagctgacgttgaaaattcttgaaccttattttccaggggactacttacttttgacgttattacttctggaaaaagttgacttgtttt
agaccctgatgtttatttacaaaacaccatacagaagccgtccaaagaaatacgctagcgtttttagcatcgcctacgaatgatcagatacaaaa
actcattgggaaaaaaggggtgacttacgtaatgctttttcgacacattcttctttttcttttgctcccttaaaaagttatccaagcggtagcaagtc
gatagcttactattacgtatagagatgcgataatttaaaggttgaatcagccaaagatctattgatttaggtgtatcaatattaaaatactcgatac
agcatcatacccttctataaatattgggaactgaatacttttcttcaaatattattgacgttctttattactaagctaactgttttgtaattcgtatcaa
ccagcattttgttctcagattaagcaatttaacaaaccaaattccaaaaaattccatacgacagactgtaaataataaataaataatcaacacaa
agaagtgaacgtacaatcctttggtatttgtcttttatttggtgggttgtttaggaatttattgtttatttattttaccaactatgaccagaatgtatggt
atacggaaaacctaaaccgaaaattaaataaagaagaaatataatattacttttacccctttgtcaagaaatgtggaaccgatacttgccttttttc
cttcaataaaaacatacttgtttgaacatatgcttaagaatccacttcaagcaagccttttaccatcaatcataatactatttacgttattataagca
tgtatcataatgcaacaggatatattttgataaatttcataaaatcataaatgacggtaatttaggagcatatttcaatggaggtacaaaatgatg
gtattcgtgtacaaagtaaagttattggagaatgtaaagtctaaacaagccagctccgtgaggtcaaactattgaagcaatgcaaaatggcgtat
gcacaagtatccaaagataaacagaacttttcctgctcagccagtgctctgcgctttacgtctaacaattctaagggaagcaactgtgtacaaaat
cttagtgaaagatgcatcaaactttttttaaactctaataaatcttcatcgtatgtgtcacctacattttcatagtctcaaactactagttattctttat
gatggtcatcttcataacattaaacattgacaaaaatgattcacagtgaatccttattcttggatagaaacccccgatcgtcctgtttaaggaagtt
caatataatacattaagaaaacgaggtagcgaatcctagatataatttaaatatacatgtttacaaatcatatttgataatcattcaaagcaacgc
atacagctgctttgtgatggcccgagtattggcgaattgtttccccacttgaaagttcccatagacgagaaacatgatcagatgaagcagtaacta
ggtacgttgaatcggctgagaacgcacaatcccaaacccatctttgatgcccttgtaggcgcctttccaacatgaaagacatatcgtcagtgctcc
aaatattaacagtggaatcagcagaacaggtagccaagtgtttaacatcgggagaaagcacacaccttgtgatatatcgctgatgagcttggaa
tttcacaactggctggagcaaggaagctccttgatgattgagcatcctccaaacatagcaattacctttgttatttcctgcaatgagcatacttccgt
cggaggcggctgtaacactactcatcggcacatcttcctcaggaatcaattcgtgtgtacatgaatcttcacccaaatcccatgcacttacccttcc
attttgatcgcaacatattagttctccttggtttggatggatcaaaagatcatttacgggtgatttatgatcgtagtttctttgtacagatggtgcacg
catgtcccacaccttcacggtaccatcttcgcttgacgttgccagccatttcccatcgcaatgaaaagctacagcagttacattattcgtatgtcctt
caaaagtcattaggggcaattgactattagaattaatgtcataaaggcgtacatgcgggtttccagcagcagctagaaaacgcttgtttggcgat
atacaaagtcgattcacttgtgtgtcggcatgctggattgttcttgaacaaataccgcttagtgcttcccagaatcgaattgtatgatcatatccact
acttactagcagaactgaatgctgtggtggatactggacagacatgcggtgagtgggagaggacacattgcaagtttatctcggtaaactttaac
aattatttttaaacattggttgaccgttctaatagagttcttgattcttcttttaaaaaagaaaaaaaaataagaaaaataagggttgcgagttttc
aatatagcaactccttactgcgagcgtttttattgaaaggaagcttagttaacaacaaaagaacatcagtaatataactaataatatatatatata
tataatgatctataaaacgtgcatgattaattaagttacaagaaaaagagaccgcttctatatcaaagcaggatagctaagtagatttgttgactg

ttccacgctaaacttagcagtacttaaatttggaatataaattgaaaataaattaaattaaattatgaagcaaccttcttcgaaatttgatataacc
atgtaaatccaaaataaaaagtacagaaaattggctccattaggacgagtagttgccataaagatttgaggaaccttctatcagaattttcgtgct
gaaaattaatgttcataataagacttcaatgcttactgaaagcattgaaattattcttttttctcggagaataattcaacatatcacgataaaattc
ccactcagaaagtaatccttttagctgaaaaccaaagtgatcgtactcttttaacgctacttcatgggaatcagtattagcattttcttgtaagtata
cactgatgttgccatgtgtttcaagcatggcaaaatgtaaattctgtaaatgctttttaagattattcacatctaaaagatggttatgaggagaatg
attaataagctcgttttctgaagttattgcaccagtttctgatatagagggagagaatgaagatagttgttgggtattttcaaaaatcacagaagta
aaactgtcaatattacgacacagtgttggccaggaagtgcattcttgagggaacgtatgtacagaaaacgacagtatacaacaaagaacttgttt
agatataatcgttaaatcagagtagcttttcaccaaaaaaagcaatgacccattagaatgagagttgtattcaattgcctctttccggagagaata
cagtgaatttcgctggaggttgggattcgaattgtgcttcggtaaaaatggggtagaagtattagtgaaacaattcggcagcttgtagagaagta
gaagatcccttaaaaaaaatggagctgtaaatggatatagccttgacaatagtgcttggtatgacggtaacagctttccaaatagatcggatactt
ttcgggataacaattcttggtttcgattgatttgacaaacttgggtatgatgatataagccaccagaggaacttaacaagtaatccggatggccta
gctcgcagatctgagtgtatacccgattggaaacatcttcgaaagcttgcaaagcatgagttaaacgatatatgctgtccaaactggctatgcgag
tgtaatatacccaaaaatggcaaaatattatagcaattgagaaaagaagataactgaaaaagaccactttgaggggcttaagatatatgaattt
gcaaagtaatgaaaccacgcagtagaagcctaagcaataactaatttgcaagacagattgataagtaaaaatataccataactttttgaagag
aaagatggaggagttgggatcggaatagaggatccaaagttcagatttccttgacaatgcaacttatgatccccgacaagcaaatctgaagtat
agattatatactgaatattatcataaagaagtgttctttctttttcgcatatattaggttggaaagattcctaataagacgaaaacattagcaaacg
ggtaacgacgatgtaaatatggggatgaacgtaccaaaacataaggccaagcataatttactaccagactaaacgaaacacatccaaagtaat
gtaacttagaaagcgcttttgaccagtagcgccaaaaagcagttacacacgtgcctatcgcagacgatagttagtcaataaataatttactcacta
tgagtagtagaacggggacaacaataccatctgaagtagtttctctaatgaaattatttttaatgaatgatagctctctttcatgctagcggcaag
tataaccggatgctctcaaaggattctattgtagcaaatactagtctttggagattgctgatttaagggtatgtcgaaaaagaaatcataaataaa
atgaaagaaattataacctcaattgatttccatttaaatatttctatattagcgagtttcgttcatacaatgagaaactcgcgcgcgtgatgctttctt
accattcataatgtaaagggaccttcaaacctctacacggttatagttatcttacaaacgcgactatcgtatcactgctataatacaacataagag
gtagaaattacaatcttaaatccatccataactaaatggaaaaggcttctgctgaatgctcgctttaatagtctttccggctttgtttttatactggtg
aacctaaacgactattggatttctcgctgtcctatctcttatagtgtggttttgctttcgttttaccatcagttttaagatagaaagcgacccgcgtttc
catggtaaatattgcaaggtataaacagtgatttcaaacccgcatgtttttcgatacacatacgtaagtgtgtttattaaatcacttcatttttagctt
gcttatttatttattgtttattttattcaggggcgtaaatgaacgttgacgacatcttgaaattgacctatcggaattgcccgcaaagccatatagtgt
gtcttcggttgctagcttttacttactttgagtttcaaactccaaaagtaacaatgtgacctcgtctagaatcgtacacagcctcaccttacctcaatg
tttatataacttaccgataccgtacttgctgaactacaaccaacttcttttgccaatagacaggcgagagaatagcaacgctaataggtcttcttat
aggtttatgattaattaaaatttcgagaaaagaataaaggatatcctagcatcgattataaacgtctatatatggataaagcatttttgcattgaat
aactacttgcgttgagggtcagcataggacactgtctaacctttgtctttcatttaccatttgttcattcttttaagttcactcgtaatatttactggtta
ttaactgctttacttttccaatacccagactacttgttgacatactttttaactattcgaagtaatcacttctcatattctggtatttaaggggcatttca
tctgtgcatttttttaaatttcttaatcgcttttttcacttttacgaaggcgctttcatttttcaattctcactcttttgctatacgcttggtttttatcaattta
tatttgaaacctcataattatattttccttcctttttgatctttagcaacgagcatagttttctttttctgcttcgcttgctgtttgtttttataatccattat
actttacacgcgacatcttgaagtctcctttatatctctcttgttatatttcgatttactctatttaaagcctattaactgatttctatcatgcttcccgct
acacattattctcagcaaagtctttcgactgatgacctttccagtaacttggagaaaggtggaacttatcctcaggaatatgagccacaaactcgt
aagttaaatactgatgatgaaaatcatgacggcataaaaaagccctggtatcagaaacatgagagaaagattctggttggtatcattctttttatt
tgtattcttctgatagccgttttgggtggtgtccttggccatcgtacccatgaacgacaccgtcatccctcgtacaaagaaaagaactactctttgta
taagacatacaagggtgagtcatttttttgatggcttcgatttttttaacgaaacggatcccacacacggtttcgttcaatacaagaatcgcgatgct
agtgaacaaatgggtttaattcacgccaacagcagcaatgtgattatggctgctgatagcaagtccaactacacgaatggccgtccatctgttcg
aatctcaactaaggaatattttaagaatgttctgattatcgttgatgttttgcacgcgccaactggatgtggtacgtggcctgcattttggacaactg
gtgataactggcccaaagacggtgagatagatattatggaaaatgtcaatacggctcaaagtaaccaagttacattacataccgacaaaggttg
cgatatgacaggtgttaagcaggtgatgactggaaaagtcttgcaaagtaactgttgggttaatgcaccaggtgcaaataacgctggttgtggtg
taggggatactgcggaaggttcgtttggaagttcgttaaataaaaaagggggtgccatttacgctgttgactggcgaagtgagggtattcgcgctt
gggtttttaatcgtacacatattccttccgacatcacttcaaacaatcctcagcctcataattggccaacgcctctagctgactttcccaacacaaaa
tgtaatatcgataaattattttctcaacagaaaatcatctttgatcttacattctgcggtgattgggccggatctaactcttataatgccgctggatgt
ccttctacttgtgaggattttgttgcgaatcacccaggaaattttacagaagcatactgggatattcgcagcttgaacgtttatgaagctaagtaatt
tgttttatgaagggcgaagcacagcaaaatctttaaagattaagttttcttatgatagcaataataaagttattcatcaatggtgtttacacttgcat
aaggttggtttaattagcgtattgatttatgggtcatttttttactgattaaaaattcacttatagtgaatttgacataataagactatcatttcatactt

catgggcattatgatagcgatcatcgtttctatcaagtagtaacaatatcctaaatgtagacaaatcatttagctgcgtgctaaaaatcctacgttg
gaataaatgacacgctggctagtagcagtagcacctggaatttgggcgggttgaggttgcttactgcctttggcatatctagtgactgtagcaaag
accaaagaatcatccaaagagtgaaggataagactcgtatcagcttgctgatccactgtaagctggttctttaactcatcaggattaccagaagc
cttgtattcagtgtaaacaacgtaatcgtcaggagagtcaccagcgccatttttgttcaaaaagccatcaacaatttcaaagtttaatagttgcaga
ggctcggtcatgggggcctgttcagatgtgagataaccggcaaacttcaggaaaggggaagaggttttgtgagcagtttctaagacgtccttgttg
tcataaatttcaatgccccaaatgctatctggattcgcagcatcgcgagcaaagtagtagcatgaggtaaaaggttcattcttaaggacatgtgta
ctcacatcactcaataaattaaggacctacagggtaagtaaaacttgaactcaattgatgaacctaaggtaaaaatatgggatattaacgccagc
tagaagggataaatccataagaaatggaaaaaaagcttagcttcgttgaatttcgatttgtttcatttgtggccgaactaacgagtaatttacttac
cttgtctccttcgccagctttgaggacgactttaggaatataaacgtgggcatttgcaggaaaactcatattttctagtttatacgatcgcagttggt
ataatagttaaaagtcagagtggacttgatcaggtccgcttatataatatttaccaaagtttgcagtttcgggtaattacgtgatacaaactaaaa
gaaaacttatatgttccatgtgttagcgccaagaataaaccttttaattttatagaatttgaagtcggccgtctgtaaaaaaatgtaaaagtacag
atagtcctatgtctgagactttgtaagctgaatacagtaacgaaagacagctaatcaaacttgtttattaaaaaaactactttttaatgctgctata
acaaaaatatgttcttataaacatatttgttaaatatatatatgaaaaaccatccgtcaagtcatgaagtaggtatattatattatatgttgttaaaa
tgtaaacgaggtaaacaaaaagtatctttctaacataagtaaaaaatataagtgctcaatggctaatctgttaaggatggaaagtaaaaaataa
atacaaactaaaaagattctactctactactttactcctaaataaaatggtttcatatagtggtgtgcacaaaagtgtactgcatttcgcagtttga
atgttacattgactaacgtaataatagcaagtgcatggatgcagttggaaccattctgtgctatagcattgaggtattgtggaatggaattataggt
ttcttttcggttataagattacctttctttttccttaataatcaccttttctaggtgacgtaccctgcttcattatcatttcattaatatattttatgctctc
gaaaccgcatcaaagggctcatggccgtagtgccattgaattaaaagatttagaaaatggtgctgtatcaaacacatttcagatgttgacagacg
atgtccctgatggtagggattcgccccatgatgcatatacaacactatcctttcagcagaaaaagaatatcgtttttttgatattattatgtatgtact
tgttattacgaattgatttaaaggacccaatttccaaatgtgctaaccaaaacgtcagatctaatccaaggtgtgcctatgggattggttaagggta
gtattccttacttcttgaagccacattgtacttattccgatattgcgatttactcgttggcggcttatccatatagtttaaaggtgctttggagtcccat
agtggacacatactactttcccaattttggccggcgcaagacatgggtagcaccttgcacgttctttctagcttgcacattattattcttttcttataa
catagatgtatggattgaaagaggatcttcttatatcaaatccttcacggtttggagtttcttccttgttttcatttgtgcgactcaagatattgcagtg
gatggttggtcattaaatatgcttgacgctcatcaattgaattatgcttcaactgctcaaacggttggcctgaatacaggatttttttctttcatttacc
gttttgttagttttgacttcccctgagtttgcaaattctcttttacgtctaagccctttagatgaaggactcattactttgagtggctatatacgtttctg
ggctttaataaccctaatttcagttattttcgtattgttttgggaagaacagaaacctgaccgtgtagcgagcatgagggatacttttcgttcaattc
gagacatcctttcgttgaagagtaagtttaaaattagtaaagcaactcagttaacatttcttagatatgaaacagcttatggtggttcatctattcg
gtaaagttggatttgtcgctaatgaaactttgacccttttaaaagcaacagagtttggtttaagcaaagcaatgctttccttaattattttaatcaatt
ttccgttgggtttgatattgggcgtatacattggacatattagtaacaatcggcctctctatgtatggctatggggttattggggccgaacatcttcta
tattatttaacattgcactcgtgtatgcagtcgctcattttctgaaaagttttcgatgtttctttccattctcgtatgctacactctcaacgcttccttta
tgacagtccaatttgtttcaattggagtgttccatagtcaaatctctgaccctgccatcgggggtacatatatgacggtatgaaaagctagtgcgat
agcgaaagctaatttcggttatagattctaaacacgttaagcaacttaggcggtacatggcctcaatatattatgctaaagctaacagactgggta
acgttttcttattgttccacagatgatagcctgcaatgcattacggatgacttgaaaaatcagtgtatggattcaggcggtcactgtatatataaaa
gagatggctactattttacatctattgtcggtatattcctttctatctttctttgtattattgcaatattcccaatcgtacgtcagcttaataaagtctcg
gcctcatcttggcatattcacggtaaatattcctcaatttaatagggattctacgtctttcttttaactaaatatatttattaattttgtttcgaattcaa
aatagacaattgatgtttgctgatcataaagtaatttattagacgtaaccaattctctgttaaagggggtattgtaatagaatttaaaagacagaa
gcaccacccgattcaataatattcatttctaggtcgctaactggcatacgatgaaaacgcggtgagagttcatttctatcgcttgcaatgaaattgc
tttccgaggaaccattagaagatgttaccccttagaaaagaagctagaaggtaaagcaaacggaacagcaggatggggatgtcgttcagcag
aagactctgctctgttagtaaaccttctcgttgctacaacgtactggttaaatgtttgacaaattgaataagcgactttctagccattgtgttttgcca
atttcttttaaagataattgccaattctcaagtaagaattattcgctacttccgttaattatggttttagttccgtcgtggcatatcaccccaattgtaa
agtcataatacttcattagtgcacactctgtttcactattccgtatacagtcagtcgtattatgatactttaaacatataacgtgtctctgattttatac
attaactgaactttcaattatacgaatccttgcataacgatactgtttttgaaactgtcgcaagtaggtaggataaagtgctgctagctatatttaaa
cttaaaaaataaactaaagcttgggattattttgtaggtgtttgtaagaagtaaaagatgatggttaggatctaatctcagaaagaggataccaa
aaccgaatcgttttgtattcgtgtttgcaggagaatcaatagtacactttaatatttgttaagagtgaatacataagtagattttcttattaatcaaa
ctacaattaagtatatgaaatcattttattctatcacagtgcgccgggtatcatattgtcacattagtaattctcacgggttatgagaataaatatca
agtggaagtaataactacaattacagggctgtatacgttaatttttactttggtgtactacgtatatatatgctagtaatgtccagggatctggttag
ctaaattgaaagtgtctcgatgatcttatttagccacacaccagtcgaatcttccaatctcaagattttgactgtagacacaagggtatatatagctt
tgttaatcaaattgacaatagattttatgaatagataatcaactgtatacgtgtctttattctccgaccatttgtatcttgatttctgtatttcgttttaa

ttttttgtttaccatttgtaatatattattatttaatctatctctgaaaatctgccggcgcatttgttaatccaaacggcattattgtaattcgtatgttcc
ggtgagagttttaaaggcagttttatgttcatcacctttctaaactctaagcaagtgatatgcttccttcaaaccgatgtactaaaagcttttgctac
gcttagccaatttataattcctataaaatttgataattatccatctttgtaatcgcgtttaaagtttataaccatgcgatatttatctgttctatttttgg
aactactaatggtgcgcacataaagtttgagtaactttcgcggattcggctttgacttggtaattttagcaccttttctttcatagttgataattcttttt
ggttttaaaatctcctcttaatattgacacgttggcaccagtatttataaatgcctttactttgagattgatattttgacctcttgatattgagaaggtt
ttcccttgtgggaagcaaactgttcgttcacatcaactcgatttatagacagtttgatttcttttgtgactgaaaccatcctctagtcattcattaaatt
taggtttcatcattgttttggtgtcatcgaaatttttgattccttcgttcttgacatgtttccaggtattttgctttggcggtacgtattgtcgacgattta
attttcctggtcttgtaggtgagttataaaaacgcttattttgtttccttcatggttttaaaaggatctcccttttcaatattattttattataaactgtttt
cgattatgcattttctgacacaaaaacgcactaatctttatgtttgtttcagcgtctgaacttaaaaaccatctctgtattcttgaaaaacgaaatca
gaattatttgctttatctatatgttcatgtagagtttttaaccaagagtcatcttgaccgctgagaaaaactttttcaaatacattatcaagagttttct
gggctcccggtttcttaatgatctctttaatatgttcccgatggtgaacttgcaaattgatttttttcacgatcagtcgggaataccaaaggcaccgttt
cgtaaacggtgagcaaatcttacaaaaaatcgttcagtgcatgcttatccggcttatagatcgctagttctttcatatttattcgatataaatatagt
ttaactctttcgccatatatactttctaccatttcgataaggtcaattggtttctcagagataagtcttcatcaatttccattcagctcatttttttcttcca
ttctttcttgagttattaaggtgatctgctcggttaaagcatttatttacttttgcatatggcgcatttgtttttccatttgctgagcgatgtatcgctccc
agcgttttcggaatccatatacatgtcttttacttatcttactcaagttaatattaagctatttccacccttcagattggaagacaatataagcattca
ttattaaaaaattgttacaaactgcttggaaacactagaacggcaactcgttgtattcttaatatagcgtttttttcacccaattccttcttctatcgtct
cgtgaattcctataaccttgatctcactagcattttgagaactatgaataaatagttttcagattcgaatggttgattaatacgaatatataattttct
taaacgaaggcggttcttctttattgttattattatatgtcatgtatttatataagcatttagggtagggttcttttgttgtgagcagtcactgtttggcg
aatattcacaagagactaggtgatgccctatttgaatcttccttgaaccaactgaaacaaaaatggtaagaggccccaaaaagcacctgaagcg
tgtggccgcacctcaccactggcttttggacaagttatccggtacttatgctcctaagccttctgctggtcctcataaggcacgtgagtgccttccctt
gattgttttcttgcgcaaccgtctcaagtatgctttgaacggtcgtgaggtcaaagccattctcatgcaacgtttaatcaaggtcgacggaaaggtc
cgtactgataacactttccctactggtttcatggatgtcattagcattgaaaagactggtgaacacttccgtcttgtctatgatgttaagggacgtttc
accgttcaccgtatcactgctgaagaagccaagtacaagctttgcaaggtcaagcgtgtgcaattgggtgccaagggtattcccttcctcgtcact
catgacggccgcactattcgttatcctgatcctttgatcaaggtcaacgacaccatcaagctcaacttggaaactaacaagatcgaaggatttatc
aagttcgatacttctgcccaagtcatggtcactggtggccgtaacatgggtcgtgttggtaccattattcaccgtgaacaccaccttggttcttttga
aatcattcacgtcaaggatgccttggaccgtgagtttgctacccgtctttccaacgtcttcgtcattggtgagaccaacaagagctggatctctttgc
ccaagggcaagggtgtcaagcttagcatcactgaagagcgagaccgcagacgtgctcttaggggaattgcctaaaatttttcaatgtagtatcgg
taataaaattaactttacataaaacagttttattgatcatttgtacaagaaataattttctttctattttctgtagtgattgacagtttggaatcatcta
tctagtttatacctacaacttgtaaatcaggatgtttatttatcatagtaaatcgtactgcggcatgaaactctgctttaaccaacctcaactgcttgc
tgatattaagcttatcttaattgaacattactacgttgtttgagcaaaataatattttattgaatagagcgtgacggaaatcgtttgattcataaacg
tccgaaaggctgaaatatacattaagagatgttaatctatctgtttacggctgcctttatttattatactatatggattctaataatggtaattgtgttt
tttgtttctatttagtaattattgtaaactagccctttgttgacgggatgaacccaacccagaagttcttcttagatcgtgaatgggctatcgttgtac
cagtatcattaatgctgtttggaatttgccttgttgggacattcatttcattagtgatgatcaagtcgcaaagaaagactcacaagacgtaatgcta
cgcgtcgcgtctttcctttgtcttacccattagtctgaaagtcatatctgtacattacacttctgtttgctaaatttattgttctatgcatagaatatata
gccccatcaaagacttcctgttggagtatgaatttcaagaaatttggaactctttggaggaagatattgaaagcattatttcctctccttttgaagta
tattttctttattaatgtactttagttaacaggttatttgaagtttatagttaaagttcgccttaatggaaagcccgtcgattctttaacgtgctattctt
tgggaaatgagatcgctagttttcctgtacgttctatctgtctgaatgacttacttatagtaggtttggaagaagctggaattctccgtactgctgaa
ttctgaggtgaacggacatatggtgagatgtatgtataagcttatactcatgatacttactggttagtcactattagtcctccaaaattctctgttaa
gccagaatccgttcgggagttacttatactaataatgagttgcgactttcgacaaaatgatgtctctgagtttgattgcagttccaagattgtagccc
atttattatatgaggctttgctaaggaacattgtgaatgtccttagccgtaaatgtcctttatcatttcagccgaatctttataaggtataacgcgctc
atattgtttaagatattagctaatgtggaaacatggtttagaaattttcgaacacgatgttcttcgctcataagtactctaattcaataaacagaatc
cttaatagagctggatcgaatggcaactcatctgaaaaaatgttaaacgacctgatttgtcaaaagaccaggaattgcttacgaagaaatttaag
gaaaaatgaactctatagtggactttatgaatacgaaacaatggttaatgaagttactaatcgaactgaaaatgatgagatcgaagaacataaa
ataaatcaaagctcaacgttaaactcgtcacctccatatgctatttcatatccctctttgccgacggaagcctttacaagttatgagtcgagcacca
cgctacaggaaactgtatcttcagatacaattttatcgtcggataattttctgaatgaagtactatcatctcattccaagtgatatttagaccttgag
ctctcatcttcccttaatatgaattccgcaaacgaggaatatatgtcattacttagtctattgtaaattctaatgaaaatgttgtgagttatgcattaa
cgttaagattagagcttcattgaagctgagcgtgattatttctttgttgtaattcaattagacttttggcgcattcttccaattctttatggacgaata
attatcttcgctcggcgaaggaggctgatcagctttggtagagagtaacgttttaatattttcagaactctcggtttttctagtttgtctggccgtttta

ctttccaaattagtgctcttatcgactgtcttcttcaaaaaaaaatagttcggcttctccattctggctttcacaaagagtaagcatcgattttcttttgg
gagctataccttctatttcaaaatccgaacgaccgcggagtaagctgttgtttctacgcagtgattgacgtttattctgaaaccaaatttgcaattct
cgcggagacatactagaatttagtttttttactaagttcttgcctttccaaaagatttggtttaggtgtttttaaaaagtattgctctaaaatagatgct
tcagcgtctgtggtacgccttcttcttcttttggcccgtaagtcttcatcttcctgatccttagcaaaatgattatttagtacagaaggttcaattacttt
tttaggagttggaattgtattctgagacattcctttttttttagatatccattggtaattatatggttaaggataagcttgtatgcttttaggaacagta
gttgacaccgcgacgcgacgcgtctcagttttaatctaagcaaaaataaatctttctgaaagttagaatttattaagcttctaagtttttgaaaata
acaagtaatctctaagtcattatttaaccaatctatcttaaccattgctctaatatggctaatagcgcgaataaatcattattcgcttcaatttcaag
attttcagtaattcggagaacttgaatgtatgattttagtagggttaatgtaagtaactttgtttgataagtgtcataatatatgactatattcactta
ttcattatcagtactacatttttgtacaatactgactataaacgatgagctaaagtcgcaagaatatttgaattgaacttggataagttcgtaaagtt
taagcaaagaagtatacaataaacccttctgttctttatgattcaatcatgcagtaggcacacatttatctgttaatattggtgtatcactaaacttg
gttttctatccggaattgtttattaaactagacctgcaactaggaaactagctataaaaatttgaccccgccaggagtcgaacctggaatctcctga
tccgtagtcaggcgccttaaccattgggccacgaggcctcttgttggtagatgcctttctctatgcattttcatcactagtacgtagcaattaggcga
aaaaaaatcgcgcttctgtttttttgggtttctttttcttgtttttttctttttttttaattagcctaatcagtcattttgcacttgctattatttgattcattcg
ttcgctaattgtataacatttgatttttcagcttttaaagtacaatttatcatgtttttacggagagaagggatggttgccttcttctttctcttggatta
ttcgcttgctatccaacataactcaaacagcaattgtattaacagtgaagaaaaagaaacagaaaacaggtatgattaaagttaatagtagacg
atagcnnnnnnnnnnnnnnnnnnnnnnnnnnnnnnnnnnnnnnnnnnnnnnnnnnnnnnnnnnnnnnnnnnnnnnnn
nnnnnnnnnnnnnnnnnnnnnnnnnntataattttaaggctttgagaagctataaactatagttgtttttttatgaactttaaagataatgaa
ggcagaaaaatagcgattgaaaagaaataataaaccaaataatggaaaatagaagcaaaaaggaaataagaaataggaagaaaacaagag
tttttgaccccgccaggagtcgaacctggaatctcctgatccgtagtcaggcgccttaaccattgggccacgaggcctcttgttggtagatgcctttc
tctatgcattttcatcactagtacgtagcaattaggcgaaaaaaaatcgcgcttctgtttttttgggtttctttttcttgtttttttctttttttttaattagc
ctaatcagtcattttgcacttgctattatttgattcattcgttcgctaattgtataacatttgatttttcagcttttaaagtacaatttatcatgtttttac
ggagagaagggatggttgccttcttctttctcttggattattcgcttgctatccaacataactcaaacagcaattgtattaacagtgaagaaaaaga
aaacagaaaacaggaatgattaaacttaatagttgatgatagcaacagatggtcgcatgatgtagttggttatcatgtcgatctaataagtcgaa
tgtcgccagttcgagcctggctgtgaccatgatttttttttcactttgttttctttttttccctcatcaaaaatttgacgttttctttacgtttactaatagg
tttattttggtttttttcttcctttctttaattatattttatttttattcattggggatctcatccatactgcaacattacaaagaatcctatttcctgttata
atggtggtttttttttttatactttctaatttatgctattctaatctattctattctttttcnnnnnnnnnnnnnnnnnnnnnnnnnnnnnnnnn
nnnnnnnnnnnnnnnnnnnnnnnnnnnnnnnnnnnnnnnnnnnnnnnnnnnnnnnnnnnnnnnnnnnnnnnnnnnnnn
nnnnnnnnnnnnnnnnnnnnnnnnnnnnnnnnnnnnnnnnnnnnnnnnnnnnnnnnnnnnnnnnnnnnnnnnnnnnn
nnnnnnnnnnnnnnnnnnnnnnnnnnnnnnnnnnnnnnnnnnnnnnnnnnnnnnnnnnnnnnnnnnnnnnnnnnnnn
nnnnnnnnnnnnnnnnnnnnnnnnnnnnnnnnnnnnnnnnnnnnnnnnnnnnnnnnnnnnnnnnnnnnnnnnnnnnnn
nnnnnnnnnnnnnnnnnnnnnnnnnnnnnnnnnnnnnnnnnnnnnnnnnnnnnnnnnnnnnnnnnnnnnnnnnnnnnn
nnnnnnnnnnnnnnnnnnnnnnnnnnnnnnnnnnnnnnnnnnnnnnnnnnnnnnnnnnnnnnnnnnnnnnnnnnnnnn
nnnnnnnnnnnnnnntaataaaaaaaaaaaaataaccaataaaatacaaaaaaccaaaggaaagcatatataaacgatggtggtactag
tacattcattgtccattcagtagcgatgagaaccttcaagacatgaaattatgagcatcatatacgaaggaaactatggaatagcatcttattaga
ctagcacattagattaatatcaaatagagaagcagtagtaacaaaggccgtgtggtttagatggttataatttctgcttaacatttgatgttcgcag
acggtcccaagttcgagtcttggcacggtcatccataccttttttttcattttcaatttgttttactaggatgctgcctttctctctttgctttccttttcttt
tcgagcttgcctaatattgctttctttcgtttgtatcacccaccacctactttcgtacacaggtcatacaaccatatgagataaaaatcaagatcttc
aaccagctcgaacaaaagtaggaaagcaggaagaacagaacatacgatattgaaacaaaaaataaataaaaacaatacaacgtaaatcata
aaacccaagatgggaaaaaaataacaaagcaaaaaacaaaatcctctccctccgggattcgaacccgggctgccagcgtgacaggctggtgta
ctacccctatactaaggaagatttgcttgtgttgtttacagttatttttttttatatactattcttatatactattttaatgttaatgttattttgaacacca
tacagtgtagcaagcattgtatctctcattagcttgtcttgaattcttattttacaaagaaagtactattttacaaaaaagtaagtcgaataatttctc
aacggtcttgcatgcagcatttctatggtagtgtatctcttgtcaattcgttacataaatacgtactagacagcatattgcatactgaaacgaaagg
tcttaaccttctaagacaactttgcttatttttgaagccagtattatattttacaagcttaaaggacaagaagaatggattgctttcacacaacacag
ttttgtttcatttcagtatcatctcttatggtggtatatcaaatcttttgctcatctttaagaaacgaatagtaagtataatataaattgagtataacgc
aactttgacgtagtattgtgtactactacgcagtacagtacatatattactttatacaataaattcaatagaataaactatgctttattttgatactca
acctttacaagacgatttattattatacacaatataataaatgagagttcgacgtactattagagcacgctcgctgtatgttttgactaaaaattga
gaagtgagatctacaaaacttttgttgattcttctaatcggattatctgttgaatgttcttttagtaaacgagatttatagaacacagtttctttttttgtt
tttttttccacagtttgagatttttagatgagtagacttataaaggtttttttatttgcatttctagagtagtagtaatatttctttgagtcctcaaaagc

tgagcacttcggctagctttttactcgtatacgacgatggaaagaatatcaaataagtgccagctcctataaaaacttaaatcttgcataaacaa
catacatacatagattgtaggttcctcgtcttcgattttcaatgttttctcgttagtacattcctttacatttttcttgccaataatgatgtagaataaca
tgtattcaagcgaattagattgcttcttttattccgcttctttggctaatttaatgcagaaatggataccttagctaaccatagttgtaaagaagaca
ccaatacatttatgggatgaatgtttgttgaaggctgatgactttttttaagctagctttctaacatctcgaaacaaatactctcttcgtataatgga
acatcttgcgtccttatgacaattaacttattcccttgtttaaaaggatactagcaattaaatgcagaagttaagctatttttagctatggtgaaaaa
gtatctattcgaggtattttcttctgcttctctagtgagtgaccccgttgttcaaaaataatgagtaattttatttttacatttacgtaaagaattaaaa
aaatagctttgaatctaaaaagtatgtactaatcgtccacaccttttaagattacattgaaacttccttaaacttctgtacttatcctatgaaaaacc
acctgtatctacttgcataatacctactgagcatagtaaggtattagtagagagttcgattaagtcgatgatcaattgatgttgttcaaggtaacta
aaaaatgtaacgataaaaaataacaatcttatgttatatcgagggtgctgagagagtcgcacgattagtgacatctgctatgtacataacgtgag
acattcgtttatgcaaaaaatatccacttaaaaaatagtagtaaaaaagtggacaagccagaactcgaatctgggacctcttccatgctaaggaa
gcgtgataaccatctacaccacatgcccgcttatgttaatacagcatttaaacttatatttttggctatatcgtttagatattcagagaaccgtttatt
ttctaaaagtttacaactctgttacatcgagaatacgccgagagtctctagcgcattataaaccctttctgccacatggactgaataaaaggaagt
aaaaaatatataagtaaatgtacaacagccctatttaaactagcatcaagagcgagaatatatttcccttcagatacctatttataaaggtataat
gttgactgagctcaaagacgaagtaaccttttagaaaatttttattgctatcattcatcgaagtaatgtttgatgagtattaatattgttgagtgtgg
atttcgtaattggtaattgtatcttttaagttgaaactgaattagaatatatggttattgctaatcttaatatagaaaaaaattgattggctagttaac
gcaaatatacggacttactcggaaaaatcaccctcgttgatgattttgtttggaaacattctcggtaatttttcctcctctgttcgaatacaattacta
attaatcatgtgaatgtaattggtgtttgaatttgtgtttacacttctcgtttcttttgtctccaattcatgtaatacctaataataattcagcgctacga
atttcggttaatacttaggaatattaggattttatcaataagtacattaaacattctttcacaacttgtagattactttgttttagcacatccttgctttt
taaaacgaacggcggatgcaaaggtgttggaacaattagtgtctatggtaggaaagcgaaactacaaatcttaaaggttaaatacagaataatg
tatacgaaactagtagcatttatagcctttataaagtaagttattagattagaaggtcaggatcctcttcgatcgttttagcattaacacagcttatt
gaagaaactttttttctcataggtataaatcattgttctgtagttattgggatatttggtcataaatgaagcttgtaaggaccttgtaaaagttgcat
tcacagtttataatgctttcgctttaaacaagtcgtttactactaagcttcttttcataggtgcattacgaaagttatactacgaggaactctgtaggt
tcatttaattcattagttctttgcttattggaagctattataggctattagaagctattagaggttctattataacgctttaataatttcccattacaaa
atgtttcgtcataaattcgtacgcatcataaatttccttcctagattcagttgatggtaatttgcttcttcgaacgaaattgttccatcttgggcaagat
gatgcttaaannnnnnnnnnnnnnnnnnnnnnnnnnnnnnnnnnnnnnnnnnnnnnnnnnnnnnnnnnnnnnnnnnnnn
nnnnnnnnnnnnnnnnnnnnnnnnnnnnnnnnnnnnnnnnnnnnnnnnnnnnnnnnnnnnnnnnnnnnnnnnnnnnnnnn
nnnnnnnnnnnnnnnnnnnnnnnnnnnnnnnnnnnnnnnnnnnnnnnnnnnnnnnnnnnnnnnnnnnnnnnnnnnnnnnn
nnnnnnnnnnnnnnnnnnnnnnnnnnnnnnnnnnnnnnnnnnnnnnnnnnnnnnnnnnnnnnnnnnnnnnnnnnnnnnnn
nnnnnnnnnnnnnnnnnnnnnnnnnnnnnnnnnnnnnnnnnnnnnnnnnnnnnnnnnnnnnnnnnnnnnnnnnnnnnnnn
nnnnnnnnnnnnnnnnnnnnnnnnnnnnnnnnnnnnnnnnnnnnnnnnnnnnnnnnnnnnnnnnnnnnnnnnnnnnnnnn
nnnnnnnnnnnnnnnnnnnnnnnnnnnnnnnnnnnnnnnnnnnnnnnnnnnnnnnnnnnnnnnnnnnnnnnnnnnnnnnn
nnnnnnnnnnnnnnnnnnnnnnnnnnnnnnnnnnnnnnnnnnnnnnnnnnnnnnnnnnnnnnnnnnnnnnnnnnnnnnnn
nnnnnnnnnnnnnnnnnnnnnnnnnnnnnnnnnnnnnnnnnnnnnnnnnnnnnnnnnnnnnnnnnnnnnnnnnnnnnnnn
nnnnnnnnnnnnnnnnnnnnnnnnnnnnnnnnnnnnnnnnnnnnnnnnnnnnnnnnnnnnnnnnnnnnnnnnnnnnnnnn
nnnnnnnnnnnnnnnnnnnnnnnnnnnnnnnnnnnnnnnnnnnnnnnnnnnnnnnnnnnnnnnnnnnnnnnnnnnnnnnn
nnnnnnnnnnnnnnnnnnnnnnnnnnnnnnnnnnnnnnnnnnnnnnnnnnnnnnnnnnnnnnnnnnnnnnnnnnnnnnnn
nnnnnnnnnnnnnnnnnnnnnnnnnnnnnnnnnnnnnnnnnnnnnnnnnnnnnnnnnnnnnnnnnnnnnnnnnnnnnnnn
nnnnnnnnnnnnnnnnnnnnnnnnnnnnnnnnnnnnnnnnnnnnnnnnnnnnnnnnnnnnnnnnnnnnnnnnnnnnnnnn
nnnnnnnnnnnnnnnnnnnnnnnnnnnnnnnnnnnnnnnnnnnnnnnnnnnnnnnnnnnnnnnnnnnnnnnnnnnnnnnn
nnnnnnnnnnnnnnnnnnnnnnnnnnnnnnnnnnnnnnnnnnnnnnnnnnnnnnnnnnnnnnnnnnnnnnnnnnnnnnnn
nnnnnnnnnnnnnnnnnnnnnnnnnnnnnnnnnnnnnnnnnnnnnnnnnnnnnnnnnnnnnnnnnnnnnnnnnnnnnnnn
nnnnnnnnnnnnnnnnnnnnnnnnnnnnnnnnnnnnnnnnnnnnnnnnnnnnnnnnnnnnnnnnnnnnnnnnnnnnnnnn
nnnnnnnnnnnnnnnnnnnnnnnnnnnnnnnnnnnnnnnnnnnnnnnnnnnnnnnnnnnnnnnnnnnnnnnnnnnnnnnn
nnnnnnnnnnnnnnnnnnnnnnnnnnnnnnnnnnnnnnnnnnnnnnnnnnnnnnnnnnnnnnnnnnnnnnnnnnnnnnnn
nnnnnnnnnnnnnnnnnnnnnnnnnnnnnnnnnnnnnnnnnnnnnnnnnnnnnnnnnnnnnnnnnnnnnnnnnnnnnnnn
nnnnnnnnnnnnnnnnnnnnnnnnnnnnnnnnnnnnnnnnnnnnnnnnnnnnnnnnnnnnnnnnnnnnnnnnnnnnnnnn
nnnnnnnnnnnnnnnnnnnnnnnnnnnnnnnnnnnnnnnnnnnnnnnnnnnnnnnnnnnnnnnnnnnnnnnnnnnnnnnn
nnnnnnnnnnnnnnnnnnnnnnnnnnnnnnnnnnnnnnnnnnnnnnnnnnnnnnnnnnnnnnnnnnnnnnnnnnnnnnnn

nnnnnnnnnnnnnnnnnnnnnnnnnnnnnnnnnnnnnnnnnnnnnnnnnnnnnnnnnnnnnnnnnnnnnnnnnnnnnnnn
nnnnnnnnnnnnnnnnnnnnnnnnnnnnnnnnnnnnnnnnnnnnnnnnnnnnnnnnnnnnnnnnnnnnnnnnnnnnnnnn
nnnnnnnnnnnnnnnnnnnnnnnnnnnnnnnnnnnnnnnnnnnnnnnnnnacagtatataaaagaataataactgtaaacaacac
aagcaattcttccttagtataggggtagtacaccagcctgtcacgctggcagcccgggttcgaatcccggagggaggtattttgtttttttaattt
ttgtattattattgtatttttttcctatcttgggttttaggttttagatttcatgttttacgttgtcgaagtggttgaagatcttgattttaaactcatatgg
ttgtatgacctttgtacgcaagtaggtggtgggtgatacaaacgaaagaaagcattgttaagcaagctcgaaaaggaaaaagagaaaagaaaa
gaaaagagagaaaggcagcatcctggtaaaacgaactgaaaatgaaaaaaaggtatggatgaccgtgccaagactcgaacttgggaccgtct
gcgaacatcaaatgttaagcagaaattataaccatctaaaccacacggcctttgttgttagagtgagagctacttaattatataatagcaacctat
agatgcggaattgtttctgcacatctggattgaatactttttgtcaatgtcaaagcaatatgcaggtgagagcagaacgtaacagaatgtacaata
acgaattttaagagaaaaataacagatcctagattctagacaagcaaaaaagatctaccttcattacaaaagtgaactttgctttataataagaa
tttgaaacatcagtactctacgttaacgactaatacaggctacataatggcctcaaacacacccatatagtagtatcctcagggaatctgagtaca
gcatcataataattatctctatataaattatgggaaagaagacatcagaaagggcatgtggtgtagatggttatcacgcttccttagcatggaaga
ggtcccagattcgagttctggcttgtccatttttttcagtttcatttcaaaaccttttgtcaatagataactctcaaattcaaatgtatatatatatata
gatctgaaagctatttaggaaaacaatgatgtgtttatagagtcgttgattaggattgcaaaccttaatttagtaccgcaacaatgatttacttcgc
atgttaacttttcaatattttcgaaggataactacgtagctttttagtaagatggcaccacccaaagttgattttacgtcttactattaaacgatttaa
gtgctgtagttagtttggaaataaaaaatgtcgaatgatgaaattcaaattattcaagtatctgatacctagagacaatgaagacttactaaaggt
ataagcatagattgataaaaatgaacaaccgaataagaatctcgactatcgatgcacaacaaaaacagaaaggtaaaaaactacaaggcatg
agcaaagttgaagaaagcagcataattgagtaacctaatcttttctcctactcactagttctttaagctcaatgattagtctaaatcgctcaaatgt
atacactattttggttgagtttactaatgaagaactaacagataagacttttacaactactctcaatgacttacgagtaatatatcaatagttaattg
gagggagccgtcatctcactttcttttcttttgtaaaaaagaatgaaaatcagtctcctgagccagattcgaactagcgaccagtattttgaatatt
aaaaggtttttcgttagatatctcaagaaagctatcagtacttccaatcgttctctaagtttgcttcaattttagattataaaaattatagttagact
gtaagtttaaaagcgttggcagtccatctcatgtcggaaatattgcatagtatagtatgttcgtcttgttaagccgtaatgaatcaaatatatatttg
tggtcaaaagacaacgctgcctgtgttagcgtgcaaaattttcagatttagcattgaaattcattttcatttttccttctgaaattgtaatcctagaaa
caagtgtataaaacgcatgtatttttagaattaagcgtcatcaaaaatcaaagaacaacgagaaaaaaaaagtacgtgaatgccctgcaatct
cttcagaaaattctcaaacgatttaagcagtgattttaggaaagaagtaattgattaccttgcataaccatttacattagcactttgactttcaatga
ctatatgaacagcgtcaacctgaaaagtatatatatgcgaaaataaccatcgtatcctcagattttcccatttataaatttcagtaaagatattgat
gaggtaaaccacaagcgtcttaacattacttcattagacaatcttaggtttagtattctagcaatgccaaatcctaatagtggtaactgaatgagta
aatttggcagattaataataagacatagttaagtacatccgataatggacgaattgccttaacagcaagtgcttaataaaagctatttaattaact
gctagcgccaattaccagcaatcagtaaatttaaatgacggtaagtaatgggtattcaatcaagattgtacaggttcagcagctttaacctactct
ggacttataaggaattttaaacggatagattcttcagatttctactgttactgtaatattctaaacagaacagcgactttcaaaatttgcacagtga
aagcgactcagataaataataagctgaaagaaacaattttcctagtcaaccaatttaacatttcaatgtttagcacgagattattgtttccaaaaa
gatcagaatacttgaattttctatgaaataaaataaaaaataagtttgattataaaacaagcataaatttacttatataatttactgttacttgttaa
tgataaggacctttctcattgcagtagtaattcgtaaattctcttctcttctcttttctttctttttattttatttttttttagttaattgagactttcaga
atcaatttactcaacttcagtttactatactaacaatttacatttttgtcaaccacaagtcacggtagcaagtggaagctgatttactaatgtccaga
tggagagtaagaaggacttgtaggcgaataggaaggactagtaggcgaataggaaggactagtaggcgaatatgatggactagtaggcgaat
atgatggactggtaggtgaataggaaggactagtaggagagtaagaaggactggtaggggaataggaaggactagtgggagagtaagaagg
actagtaggagaatatgatggactagtaggagagtaagaaggactggtaggtgaataggaaggactagtaggagagtaagaaggactggtag
gggaataggaaggactagtaggagagtaagaaggactggtaggggaataggannnnnnnnnnnnnnnnnnnnnnnnnnnnnnnnnnn
nnnnnnnnnnnnnnnnnnnnnnnnnnnnnnnnnnnnnnnnnnnnnnnnnnnnnnnnnnnnnnnnnnnnnnnnnnnnnnnn
nnnnnnnnnnnnnnnnnnnnnnnnnnnnnnnnnnnnnnnnnnnnnnnnnnnnnnnnnnnnnnnnnnnnnaaggtactagta
ggagaatagtgatggactagtaggagagtaagaaggactggtaggtgaataggaaggactagtaggagagtaagaaggactggtaggggaat
aggaaggactagtaggagagtaagaaggactggtaggggaataggaaggactagtaggagagtaagagggactggtgggtgaataggaagg
actggtaggagagtaagaaggactagtaggcgagtaggaaggactggtgggtgaataagaaggactagtaggagaatatgatggactagtag
gagagtaagaaggactggtaggagagtaagagggactagtaggcgaatatgacgggctagaaggcatgtaagtaggagatgtagaataccct
ggggacgatggactgtaactgggagacgttaagccgtatccaggactcatagcacttgaaaatggactggtatagccaggagacttaacagaat
ggaaaggactagcaccaagcatatttccatattcacctaagccatctcgaccttcgggtccgctttgaattaatggtgagaacgcagctgcatcag
aggagctaagataaccagaatcaaccatcggtgaacgctcataaggcgtagcagcaccctcaggaatttgtgattttccgtattcacctacggct
atcgaagcggcgggagcattcagactgtaattcatcaacataccctgatcgagataaatatcaaatgcaccagtacccatcggtgccatctggcc

gagcataatattctctgatattcctttacagtcatccttttctccgcttgcggctgcatccattagaatttcaacagtttcctcgaaagagcatctcatt
aaagcacccgtttcagcacgattaataccgtgacgtgtaattgccattagatgtccgcgagaagtcatcacatcacaaagaagagctaaatgacg
ataatttacataagatccatcaaattcaataacgtttctgagttccttaagcaaggcagacctagtagcctcaattccaagaatttgcaaaatttca
acaaatgaatttgaatatattctagaggaatcaactccttcaacagccattgcttcagtcaaattgattccatcagtttccaaaacccattcatcac
ctctttcaaatataccagtatccttctggcgaataactttgtgctccatcatgtagacacgtgtaatgttaggaacacctctcaaactaatgctttcta
acatgtgtccttcaattgtcttcaaaaagacgtcctcttcaataagattgtcatcctcctcagtttttctatcgtcatcacgaataatacgacagcga
attaccaacttgtcggcattgtcttcagaccatatcgtgaaaagatcacgttcaaaactttctgcgatctttcctgccacgtcactcatacttaacttt
ttgtcaagcatcttagcacgatcaagctccaatctcaataaccatggggactgtttccataaattttcttcaacttcttcatcagggatagcaaaaa
aggcttcaacaaaatctttatcctcttcaattacagtattttgaggatcaggatcataatgaatttcagtagctgacgtaactgtactcagagtggtg
tgttcgatctgagtctgaatgttcttagcaaaatccatgttagctgcgacgtccggtaataaatagatagtcaaagacggtgttttaatattcttcgc
aacattaagaatctctttgagacgaggaacacccaacgtgacattcttcgatgaaacaccagcgtagtggaaagtgttcagggtcatttgtgttgc
aggctcaccaatagattgtgcagctaaagtacccaccatttctcccggacttactactgcttgctggaatcggctttcaacctcacccattacccatt
caaacgcaactttattcaaatgatattcagtgataacacgcttaacagcaaacttcgagcgcaagtgaatttggaaaagcagtgtggcgttattct
gcacatcttgggtgatccggtcatcaccacgaaatatttttagtttattaataagctcatttaggccttgaattatgtctccaggcaaaagatcagac
ggtttgcgagattctaaatgaaaaatttgacaagcattttgaatgattcgttgcacattgaccggcatcggccatcttgcatcacctttagggaaaa
taaagttacatagaagatctctatccttcgagagttgtgtaaactctgcatcaagcaagtcttgcacaactggatcattatcaatagagttttccat
aaactgagatatgctacgatcttccatcaagtcaatcttatatttcctttcaaattggtcgtttgataatcgcaaagaatcgaaaacttggtattcaa
ccagcgtcgcatcgagaccatcttcaccgtaggcaaactgaatgatgtctcccattgcattacgtacggtaccatcataccgaaccatcacatcct
ccattgcttttacaagacgacgttgaatgtagccagtttccgcggtcttcacggcggtatcaatgagaccttctcttccggccattgcatgaaagaa
aaattcctgtggagttaagccacgtaaatatgaattttcaatgaaaccacgagattcaggtgaatcatcatctttaggaaagtgtggaagggtcct
atacttgaagccaaaaggaattcgttttccttcaacaatctgctgtcctacacaagctgacatttgtgaaatattaataaaagagccctttgaacct
gccgccaccatttgcttgacattattagcatttttaaactgtgctctgcggatctaccagcgttatctctggcttggttaaggattcgtgacacttga
gcttcaaaagactcacgtaatgtcataccaggttcaggtttaagtctattgtgttgggcatcctgaattgattctgcaacttgcgaacgagcttcttt
gacggtcctagtcacctcacgcatagtgtcggcatctgctatcgtatcacctataccgatactaaatccgttgtgtaataaccagtagtttacaactt
gttgaataccattaaagaaccctttgcatgtttcaggtcccttctctttccaaattgtgtgtaccaaaccaccttgagaagctcccacagttttcttat
caacaacaccatagataatttctccgttttcaattagcatacccaaatccgatgggtttaacaagccttgtttgtcatcttccctcattaaattgatac
cttttgggattaccaaactcaaaatttgtttgccggtccacagaggtctaggttttataatcaccggtggtggcacgactccgtcccatcctggtacc
caaagcataatattcataacagcctcacgagtcaaaaaattgtcacgtaatgaaaactttcggacaccggctaaggtatcctgtacgatacccat
taccggtttatttgactgaggtgaaacaatttgtttgggaaccatagctatctcctgaatctcagctcgtgtttcttcggattggggaacatgcatatt
catctcatcaccatcaaaatcggcattatatggtgatgtaacagacaagtttagtctaaacgttgaatagggcataactcgtatcctatgtcccatc
atactcattttatgtagtgatggttgtctgttaaatataacgatgtctccatctcgaatatgtctctcaacacgccagccatatcttaaaggtatatcg
ccagctctcttgtgatatcttaaatcaattcgttcacccgtatcgcggataatatatttggctccagggtgttcatctggaccgtttctgacaagttctt
gaaggtaataaatattataaggagtaaccgtttcaggataagttaatgtctttgcaatactgcgcggaacacctaattcatctaatgataaatttg
gatcaccagtaatgacagtacgggctgaaaaatcaaccctttttacccatcaaattaccacgcaaacgtccttctttaccctttagtctagcgcgaat
actttttaaaggtctacccgatttttgcagagcttgcggttggcctgcaatctcattatccatatacgtggcaacgtggaattgcaataactgttcgt
attctgaaacaatatgtgctggtgctccttcttgctcacatcgccgaacattggcattagcttttataatatcggatagtttatgcgttaaatcgtctt
caccacgactagttccatccactgaaatactcgggcgaacactgggcggggggactggtaaaacagtaataatcatccaatctggccgtgcata
ctgttcattaagccctaatcgtgctaagtcatctgaagatatatgcgtaaatatggtatgtacttcgaggggagtaagtaaacgtttttcaggaagg
tcggattcgtcttttcctttttccatgaaccccacaactttaatccatctttgcgaattgtaggttgggcgcttccacagccgccatgacccaaatttg
ctgtaggattactaggatcgaaattatccgagccaccggacaagccagtgtcacaaaccatttttgttttacagacattccataccgcattaagtcg
atttttaggatcacggtatcgttgtgtatcttgatatttcggatttgactgaatttatgttaataattatgtcaaataaaggtgtctttatgtacttaca
gggtcaattttcagctttccgcaattccaacaaacacactctaaaatctttttgattttgctcaaaaatccgatatggaatacaggtttagcaagttc
aatatgacctaaacaaagttagcattcaatcaatatacagctaccattaggaatcttaccaaagtgtccgggacattcagccatcaattcgccgca
tgtttgacatttatattgacgatcttttccatgttaacatatattcgcaaaagaaattaggatctcccatacctatggtgcccaaacgaggatccaaa
agtccaccaactcgaggacgttgaccagtttcgtccatggtttcgggaaactaaaataattaatatgaaagctcgaaataattaaacttacttcaa
tcttcgcgacactcatggaccgaatctcctccggagacaagattccaaattggacctcttcgacccggctataaatgttaatttatcaccctaaagc
tttcaaattaatctcataccgaagggggacagatgaaggtgaaaactgcatgttaataaacaataaaaaatgctaccttacctgaatcccgctca
ttctacctgagttgcaaagattaggtggttgtacgtcttcagaaaaatttaatattgataatagaaacccttacaaaccccgaataacgaactccgt

aaatctcggtaatgtagttatcgccacctattgcatttctgaaacttcttctaccaaaaacaaaacttacaatgatattactttaacgtatggattat
aaatacggagatgagcgcaaccagatccttcataaaaacacaagaatatcactaatcaataacaaaccataaagcatacgtactttacagaaat
taaaagctaaagtaagtcttagtaattaagggccgcaaagttggtttgtggacaattgatatatacaaaatgataaaaacctatgaaaagatttc
ctgtacgctaaaaacgtaacattagcaaataaagttctagttgtaagaaattaaaatagccgagtgttatacaaaaattccccatcgaggaacttt
taaaaccgattaaatagaagctgtagcggcagttggagcactgttaggatttacagagcttttacggcgtttcttcttatctcgcttcccagcagaa
ggagaaggattaggttcagccttctcagcacttgtagatgcgtcatctggttgggtttgagattcggcagcagcgttgttgcttgaacgcttcttctt
attttgttttctagaagcagtgattggagatgacattgatgcctgtgctaaaggtctggtttcagaaaattcaatataattgttattagcaggagtgg
taggcatggagacagcaattgagtttgcttcttgtgattcaccatcggcagctgctgcaggagcgggagaagaaacagattctctagaagcttgg
aaagcagacatttcagcttcataagtttcacgaagttttgaggcttcttcttcaaaaggcttgcgatcagtttcgcttaaagacgtccacttttcgtg
cattgctttgttaacttccttcacatcatttcctttttcacccaaagaatctttaatttcagaacgctggctcttttgaaacaagttataagctgtaggg
ggacgctttggttgagctgggtctctggttttacgcttgttgggagtaggggaaacactagccacaggttcgtttacagaagaaccagcttgcaca
gcggaaatagaagggactggtgcttctacagtagtagtatctttggcagctgctgtggatgattctttgttaacattttgcttggagttttcgttcttg
ctagcctttccttggggtttactaacatccccttttcagcaatgagaactgggaggtattccttgatatcttggcatgctcccaaagccagtttaaa
agcatcctcaagtttagaaatcgagcttgagagctgataggtttgttaacatcgtaatttttgaataaagaaattatttttttgcacaagttgctact
tgactcatctatctttacttatacttatgaaaacatacctttgaagatttccgtgttcttctgccattttcaggttttctatgaaaaaaggtataagaa
tgaaaaaattactattttcttagcaattctttagcgaccaaaagtccgtaaagcaaggaaaggggtgtacgaatttttggtggtggtaaccggttg
tggctgtattcctttattataaggtgtgtaaaaaagcaagttggcatagtaatgttagggacgcgtggcagaccaactaatggtgaagcctataac
tcgttgatataataagtaatgtgaaaattcgttacaaggtttcagtacaatataatcgaatttcactaatagaacaaatgtataaacaggctgaaa
tggtgtaataaagtatttctccagatatctttaacctaattacctatagtttttaaaggaatagtaatccaatctgaactttagttctgattattaaat
tataatcagagaaccatttagtttttattggtttttccagtgctatcaaccaaagaatcctattagacgtatatcgcctcgatgagtttgcacctgaat
tttaaaagtatataataactacaaaactgacaaactctttatgaagataatcagaaatgaaatttccgacagcgaacagtacttcactaatagca
gcttatagcgtagtgcaactttaaaatttttaaaattgtattacgaaatcaaccacgaactgaaatttgttccacagacgccgtctgtaaagaggg
agcagtcgatgtcttaaatttgttgaataagaaaactgttaattgttatgtcatttaaccaaaaaacagttcctgataatgattctgctgtaagtcct
ccagataataatatctcaatcgaatcggaaaatttttgagtgcccttaatgagatttccaattctcgatctgttcccgatgtttctatgctatggaag
ctattatttgagcttcaaaagatatcaaagtttaatgataaatctgaacaatttttttctacatttgaggagcaattgcaaagttctaagtcaccaca
acagccatctactgcttccccaagttcagttcatcgatcgtccgtttcttctgaaatagatcaagagaaagagaaagaaaaagaggaaaaacga
acgttctcctcggcgtttaatactcaggatagtccatcaatattgaaaaagcgtaagggattacccacagacgataaagttgataaacctgtaatt
gaggagaaacccgatgaacagcgattagaaatctcccaagaaaatatagacctgcctgttaaaagcaaagatgatcaagtcggagcttgttata
aatctgcaaatgaacttgccactggttctcttatatctttcatcgtcgacgatcatatgacttatgaagaaaagaaggaactgttatgtatttcttcat
tcccaaaaaaagatgtacgatgcttagttgccggtaccccctgttacagaagacttttccaatagtaaaccgaacaaccaaatctcaatatctactt
tctactctttttagatccttattttcgaagttttgatgatgaggacgtgaatttttacgagaaacttcggatgtttcaaaaagttatcagaacccgc
ctttgggagaacgatatcctgaagttccccaagaagatgatattggtaaactttgcgctgcttcaatttaccagaatctcaaaacggtacctcatgg
ctctttagagttgcttcaggaagttgatacagtaggcgaggatattagctgcgggcctttaacagaaaggctacttgcatcgttgcttcctgatgat
gatgtatctagtaaaaataaagtcaactcaatcgtaaatggaactgataatgctaaggattcccacgtgaaaaaggagagtctagaggagactg
ataatgagaaagaggagcaacatggcatgaatgatgaattatcaattctatgcgaatctgacttttctttttatgaagaaaggcttcgaaatcgtc
ttcggtctttgggtgttatatatgatgatgatattgactggacaaaaaggcaagatgaccaaatatctgaaactttaagatcactacaagatcgcc
tgaaaaaaactgaagatgcaaatcagcaacggaaacaaaagattttagaacttttgcctgaggaaatggcgtttcaagaattttcaagtgtatta
gacgacctcgataagcagattgaaaacgcatatatgaaacgaaacagaactctgaaagtaaagaagaaaaggatcgttaatgaaaaaataaa
tgcatctgcaacaagttcatatgctactataaaatcactaatggataaaagatcactatggttagagaaattgcagcccttattttataggcgacta
caaagtgattgtcttcgaccgccggattctgttttcataagtcaagatgaaaaaataatgaatacaagtccgttatacggcacgaattcattagag
aatgtagactacaacgccaccccctggtgtttgaatgaataatataatgtttacgagtatcaacactgcctagttctcaatttgcaaagataatattt
aacctatataaacttctaattgacaaaaaactcatctattataaaccctttaaaatgcaatatgaggcaattcatagacacaaatttcatatacagt
acgctagacaacttcaaaaatcaaacaagcatcacgaacaagaaatatatcatagtttaaggactaacgaaaaatgcttaaatccactcaaact
ctccattagctttaaaaaataagtagtcttttccattcacgccgacaataccgtcgcgaaaaacgcatttccatttgtttttgtgatgattcaccttat
catataaacaaagtactattgcttgccctatgtcagatccttcttctgcttctggagcttcctctgaatcaggatcatcaaggtcagaattaatggctt
catcggaaggagtgccagggcttggtttttatcatcttcattatcatcaaaagtaccatctatctgtaatacccgattatttgcaagcatctcccta
atcctttcatctgcgaaatgagccaacccatacttgtcctttttggatactgttgactccacatcgtcctctaatatggtaccgtccgactgtggtata
tctttattttcacggctttctgaagcttgctgcaactgtgacgcctcagtcggttcgtttttaccgcgttttggttaggcaataaaagcccgccaaag

gcgttattattagatgaatttgaatctaaaggaatccctctatcagaaggaggcgaaacaacactcgcggacaaattaggagagctttcgggatt
tggattggatggtaattgtaaagaggtacttttgttaaaagaggtaaatggagcactcatattattcggaagatcagaaagcgatgacccttcagc
ggaagtatctggttgatttgtttgatttagacttgacgaagtaggagaagggtaaaaaccagaaccaccgtgttgctgagctaaagaatccatttg
ctgaaccgcccgtatagctgctatattatttaaaataggtgagtttgttgcgccggcagcagcagcagcaacgtccaaagaatcagtccgtaatcc
gctgacagggtcaaatagttgaccaataggaaatgctccaacgggggcttgagcccatggaaaagtagcgacattcgtggctactagttttcctt
gccataactaacaagttaaaatcaataattttctcaaatatttcttacattttgcaattcctgaagcgtggcttcatcgacaccattttcctcaaaatc
tgttcttgaatttgcaattacatcaagaatgacatgctgataaacttcaccctaaaattttgtcaataaactagtcagagttattcatattagcttac
cactatagaattagacatggtggtgaagtcggataccttatttgtaaaaggatacttcgtagatgacggtagtactgccatcgaaaataatatata
ttaccgaaacgaaacgatgcttcgttattccaattttcttgaaatagaagtgaaagcttgactgggattgtatgaatgagcgttaatttactgtaac
attgaattttttagatgtaaaatgtattcatttgtaaatggcaaagattcctttcaattcctcgtgtaaacataatatctttccatctacggattctct
actctatcaatatacgtagtgaatgaaagtccaaataaaagttggcttgcatagtaaaggagtttcactgacgaattcaaacttccatgttgacat
ttaatgctgagtttggagagtgagttggatttttactttgttttgtttacaaagtaacatcgtcactcgcctacatatattcacatcttaatgatgaac
attttaccgcagcagcttacattagcttagaaatctagaaatagcctatgctattattcatattaatattttgtttgtcgtattctttccaaaaatgtgc
gcttggtgaaaggttaacgttctcttacgatcccgtcgataagaaagctacatcagcaaattcaacagaagctgatttatttggagttgcagatga
tattcaaccattgttagatctggttacaaatgctacaacgacatttgttaacaatccaaaaggacttctgtacgatgctaaagcaggtgacccaaa
gtcccaatttttagtgggaatgctgtatgccatgggtcctgatgatcgacttatcattcagttgccccatgatgagtcgttatcaacattatacttgga
gttttcggcaaaacaaaactatacatatgcattacttgctctcggatatcgatattctgcaggaattggcactcccaaacaactggataaaggaat
tagctactaccgtcaagtttctgagcaagtaacctctttagtccgtcctctaacacatttcgctcgagatatggaaaaagattaccctgttgatgttt
atgacctttatggacgatattcttcggactcgagcattttacatataagagacgacatgcttgaatatttaaaagactatgcagtgaacggaaata
atataacggcacattttcatttggccgctgtatatcaccgtggtcatttaggcaaaccaaagaatcccgctttggcagtgaagcactatctatctgc
tcttcggctcgcttatccaggtatttctgaagattataaaaaagcgatagatcagatgtacgacctgaataagaatggtctacccaaaaaggcaat
gaaggatttggacagcatcgccaatactgcttttaatctcggatgtatggaattacatggcgattttggaaaacgaaatctaaccgctgctttcaac
tggtttaattttggaagatctttgaaccattcttcttctctatcagcccttgcatatttgtatgttatgaactatcaagttcctttaaacgaatcagaag
caaagcagcttttgcataacgctttttctcaaatggatcccattgcatatacaattttaggaaaacttgcgttctctaaaaacaacattgaggaagc
gctaatgtatttcaaaaaggcattcgaaggatctcacttagaagcagctttatatcttgcttacatctactcgaatatcgatcaggaaaaagcaatc
ttattttatgaaaactttatcagtagaactttgcagatgtactttgattttagggctttgtctatggatacgaaagtgaagtatttttttactcgactttc
tgctgagttagggaatcccatgtcgcaagttttggcaggtaaaaataccgacccttccaagtcctacttgaagagcgctatttttccgttcaccaat
accacttatcgtgaggcacaaatagctctcacttattatactcgagccgccgtaaggcatcatgtcgattcgatgataaaggttgctgactttaatc
aatttggactaggtacacccagagattcttgtttggctttctctttatatactcaagctacaatggatcgttcttcgttagcttactggcgtctaggtg
agatgcatgagtatggacgttgtgttccagtagattttgaaatggcaaaaaaaaaattacgacacttctttagaattgagtagtagaggtctacttg
ctattctcttggctaaactacgaatgaagttaaccaatcctgatactaagctttccgtagtatatcacgctatgggtttggttattatgggtgttttaa
agagcgttcaaaaatttattttgtttctcaattcaattttctcacgggatcacccaaaaaagaatattgatttgctcgataactcctttcttcaaagtt
tttcccaaaattcagacggattaaagtctatgactccagaaccatccacaaaatcttcggatacgttaacttcgacgttcgatatgaattcgttgga
taggagatttattgagactttgtttgtaactttgtttgtaattatcgtcggttatatcttgatgaagcgtcatcaacaattgagaagtcatcaagtgca
ggtggataaccaaacgcaaaatgagaatatcgataggcttccattcgatggttaaacttatgtaactattctatatatgttttctttttccgttcgttc
ttattctttccttttcatacgtgcacgatgactctgctttacatgattttagttttagaagaaatcctataatattattttggatatgaaccagttttgtt
atacagactattatttcaagattatttattagtatttaaaataaataatatttttagaagaaaacgaaacgtaagtgccttaaattaattttaataac
tgcataggacattagttgggtatttgaagtttataaaataaggctaatagcttttcgtaataaacagacaatcatcagtatcataaacaatatatat
atacagaagtactttttacacatcaatttaacccttccaatcttcaaactcttcatcgtaagcgattaagtcaaaaggtacgccaaattgttgtaaa
aacatacccattgaaaactcagatcgggtttcttctatatttcctttattttcaccttcttgaatcgtatatgtttgagtaactccaccagctaaacgg
atacgctccgccataggtcgatacagaacttgtgctgctctttgggctactcctctccatttaaataccaaaccctccaaatctttatcttcattttta
attcaacttttactgcggcttcagcagttgtcaactgctcttgaaggttgcgtacgtcctttttcgagttttagacgctccgagagaagacgagcaact
tctggatcgttttgtactttattcgttgcagatattggtgaccgaaataccttctttgctcgcttaattactcttggggaaggacgagaaggagatga
aatgggatcagataaaacatcatcagaagatttcaattcgtcagatgcataattagcaactttacaggttccaggggttgttttcggtgtagcagg
gtacttaagagggcttttaaaaggctttaataaaacggattttgcttccctagcacgctttcttgggttgggtgggtttaattgggtatttgtagtatg
tttttctatgtttgataacgatggtaagtcagaactgtcctgtgaagcatttggagtagttggcttaagttcagaattatctcgtgaaggtgtaggag
gaagttcagttttagaaatctgggcttcttctgagtgtatactgtttatgtcgtcaattgctggacgtgacaagtaagagtcagcctcactctctagc
tctaggttcttttcactgttttccattttgatatgtatgaattatgagctgtggttccttatgtatggctctgaatatgtcggtgacgcgacgtgccacg

aaaactcttgctagcaatatagctgcaactacgcggaaagcaatttaagttttaaaaaatattaaaactaacgcacttcctacgggtaaggttatt
attaaagtaactatgatttcaagatatgttgggagagggatactttatatgacctcgaaacccaaacaaatgctacacgctacgatcagaattcca
ggataattccatacaaaagaagcttacaaaaagaattaagagtttttaaataaatcaattgactattgattgagaccttcttctgaatcacatgta
caacaatgccctactgtaataatcttataaaaagccttgaacatttttctcggttcataatattataggaatcataaacataaggtggatgcagag
aaagaaagttcgtataagctaaaatccatatacacgaatgtgttctgtctttgattatacgaaccatctaccttgaaacctcatttattgatttcgttc
tctttccgtataaatcggtagaggcttgtttatagatcctagcttaaagattactgggaaatttcaaatgaagctaaggcttgattgacagtttataa
tttaactcatcaaagtccaaagcccaatgtctattgaatttgaatggtccaagcttgattcagagttagaagaaaagctctttcatatattagaaga
tcgagtttcaaagctagacctgccaagctatattaaaaacctcaaggttactgggtttcatttcggtgacataccacctcaaataaatattcaggat
ataggagacccagaccctcagttttacgaagatgaagttttggaccaggtgaatgatgaactagaaggtaatacttctttcagaacgaatttctca
acgtcagtttctgatttacctccttatggagctgagtataggtttccgaggcttgcttattttcatccagcgattagtacacctgggattatttcagcc
agtggactcacaagtcctcttagaggagaacgtcctatattagcttcggaaagccgtgaggaacgaaaccactcagatgactttcaagtgaccat
ccgtgtacagtatgatgggaatgctagtttgagtttggaagcaagtatatctattaattatcctaattccgagtttgtcagtcttccttttcaactagt
atttaaggaaatatccattgacgcgaccgctgtgttggcgaaaatgggttctcgtacccatctttgcttcgtcgacactttggctcatggttcagaag
aaaatgcttctagcgtaataaagaatctcaaaattgaaagcgttattggagaatcgaatcgacagctcttaaaaaatgtcgccaaagtggagcgt
tatgtttcacaaaaagttaaaagaattattgaagatgagttagtctggcctagttatcttacgtttgaatagaaagtcagtttaatttttaatgaatt
gtatacttttctttttctctttattgtcttagtcaacgctcgtcaatcactcttctacgtttcttatactaaagttcgcgagatccagcgctcgctatgaa
cttgagcaaagatacattatactgccgtagccagcttggtttcggagtttaccaactaaaagagtgttatgaacaagtatatacagcattgctatct
ggtatacgattaattgacagtgcaataagttatcgcaacgaacgagaatgtgaacgtgcaattcaagacttttgtacgaaccacccgtatgtgaa
acgagatgaaattactttagttacaaaaataccagattccttgcaaggatttgacagaacttggaaggcggtttttcagtctttgcgacgtgtggac
cggccttctttggatatggtattaattcattcgcccaaatggcctgagcggcgaacagaatcgtggcgtgcactcgttaaattaaaggaggaagga
ttcattcaagaaataggtgtctccaactataacattcatcatttagaagagattgctgcttctaaacttccactgccagcggttaatcaaatcgaatt
atccacttttaatagcaggcaagaaatacaaaaatattgttcggctcatggaatcgctctcatggcattttctcctcttactacaggacaacgcctt
gacgataatcgtctcttagacctagcagccaaatataagaggacaccatcacagatcctcctgcgctactgcttggaacgaaatatatccccaat
ctttaaaagtgagaagctggatcatattgttgaaaacgctaattgtaaagacttccaaatgaactctaaagacttagcatggatggctacctggga
tgagggattcataagtaagccctcgtggaatcctattattcttccatgatttaataaaaattttattcaagtatttaatatatagagaaaagactttt
ctctctttttttagctgttggctgggacctttattcaagctatccctaaacaccttgtaaagctattaaattttccataccagaagaaagcaatttttct
tgatagaatggagctgcatccatttttagatgcacttccttgtatgtgaaagctattaattgattgtttttctaatattcggtttagattatgaaaaatg
gtaccaagagcaagatcgtggagaagcaatgcagcttgtagaagccgaggtagtaaagtctggtgggataagacttccaaaaccctctcctgat
gattttactacgccattcaaaatttctgagcgaatgcaaaatgctgttcaagcagccggccgcggagagaaattacagtctattgatataaaccgt
tacatcaatttacaagctccagggaatcctggaagcctaattcagtcagcagtagctttggaatacctaagatcacgggaagataatctggagtt
gctgagaaagcatggtgaaagtgcttggatgaatcatattgggtcactggaagagactttgcaaaacatagaacgtgagatccaaactactcga
agggagactcaagcatgcaatcgaaagcgaaagcattatcaaatggaaattggagaaagacttgcggatcatgaaacccagtttagcaacttgt
tactaaactcgatccaatgccgcgtcgctattgctttggcaaatcattagtattttattttcctttaaaaagaaacgactttttaattctaactttacaa
acctaaaggcatgatacgctgacactacgtaaagaaccatttatatgatatcggatttttggtttccttcccttactttactttactttaccctaccat
aaacacaggcattaataagttttaagaataataaatctcggtttcgtctggaaaccgttaggagtttactgtacaatttatcaaggttctctataag
actacgaatgcctatatgtacaacgaatttttagcgactgttcagcaaaatcatatattattcacttttcgttcataatcatcatacaaacagaaag
aaaattagacagtaccgcacttgtcgatggcaatacgagcacgagtagcaccagagttggaacctaagctttcaaccttcttaatgacatccatg
ccactggtaacttcaccgaaaacgacgtgtctaccgtccaaccaaggagtgacaacagtagtgatgaagaattgggatccattggtgttgggacc
agcattagccatggacaacaaaccgggcttgttatgcttcaaagtaaagttctcatcagggaacttctcaccatagatggacttaccaccggtacc
gttgccgcgagtgaagtcaccaccttggagcatgaattgagggataacacggtggaaagtggatccagcgtagccaaaacccttttcgccagta
cagagagcacggaagttggcagcggttttagggacaacatcgtcaaataacttgaagacaacacgtcccaagggttggccgttggcaataatgt
caaagaaaacgttagacatcttaattttcagtatatggttaaaggcaagtcaacttagtactgaaatttataggtaacaccaatatcgtaactagc
aaaaataatacatctaatttttttatacataaatctcttaatatctttatgtttttatatatcgcatgacttttctgtagctgttattctcatagctaacat
gccaaattccatagaaatctttatgaatcaacagatgccaagccaaacactacatggtctctttcatcactcggtttaacgatatgttacgcaactc
gcaatacgaaagtggttaacaaacgttgttaagggtgaaagttttatctggtaaactactgttttagggacgaaaggcaaatactatgcagaata
atttttcattagagaataaacctgtttaaaccttttttacgtctgcatattggaagtgcattccttggttccttgtgggcaagaaaataggaagtatgc
aaaattggaaaattgggaaatttttaccaacatgtgtacggtcaatgtcaatgcaaatagttaagggtcaggaacttcaaaaaaaaaaaccaaaa
atttaatcacaacttgaataaaaatttattttctaatacattcattataacgccgaagcataaacaaacaagagtcgaatttgtaatactttttaag

acacaacaaaatcgctttctttcggctttttaacgctatccgactttccaacactttaattttatgtgcgaaaaaatgttgtcctccatctgtacataa
tgaaaactcagtaggatacccatataaaaagccaatacaaacaacatagacaaccaattaacatcaccactccttatagaaccatagaaatatt
aacaataaaacaaaaaacccatcaaacgaatgaacctattctttttccttcttttaaaaaaaaagagataaataaaagttccagtaaatagggtc
attccaaaacactgcaaccatcaagatttttttaaatttcagataaggaatatcaaaacttgaaaaaaaaaaaaaactgaagtttacaaaaccgtt
tttttaagcggcagacgtagcagtgtcattttcttgagtgtctcgcaaagcctctgtttcacgacgaagagcagaaaagtcccagttctcttcgcctt
caatatcaaggttctctttaaccttgcccttattacgttgaaagaattcacgaccagtcatgttgcctctaccagtgggacggtttttacggcgctctt
cagcttccttagccaaacggtctttgcgcttatcacaccattgtttaaagttctcttcggtaacgcgagtgagattggtacctaagcgataacgttca
atttcaatgaatgcttccaaactaatctcttccttttcattgtcacccttcttcttatcacgggccaaaacgtatccacgaggcaaagaatgcttgtag
atacaagccatgttgccgttagggcactgccaaagccaaccatatttgccttgttcgcaagcttccaagaaaaacttgcaaacaatgtcagtattt
acccaagggcgtttttcaggaggcaaatccgtttgtatgtcacgaacatctgagtaaaggtcgatttttttgagactctttgacttcttctttgcgatta
acatattgttcgccgttattttttcttgggtatagcattaaataaggcagaaacttcagcctttgcctgttcagccgctttcttctcctcttcacgacgct
tacgcatgacttcctggcgtttggcctccttgctgttggtactgtttgcattttgttctatttgcttgattttcgtctgtaccttggtagacctattcttgtt
tttgagaccaaaggtggcgtctgcaactttttttcgaagggtctttttttgggtttgggctctggggcctttccttttccttgtgctgctgctttttttaggtg
gcatcttggatagctcgaattatctttttaaatactttaaagaatatatataaagtactgaatgttttgaaaaagggatgcgaaaagagggtaaaa
tggaagaaatggggaaagtgggtgaagacagaaaaaaaaaagttgcaggtttgcgtatgtcaatactgacagaatatgacgcgctcgatcgaat
tcgaaacctggttctcgctgttaatggcttctaaaagtctcgtttgttaatttaatgagatagaatttcgtatcgttaatcaagttgcttataccctaa
agacaggtaaaacagtgtaagcgtcgtaataggaaggatgatggagaggtgtcagttatgtttttttattatttatgaatttggtgaaaaactccttt
aaatatattttagttattctaacaactttttttcttataacagtgcatatttattctaggcatatgatgcatattaaatattacattaaagcatatattca
accatgaaaagatatgcataatctattgtagcatatgcttattcgcgtttaaaagcatatggagaatgattaaggaaatagctatattgggatgttt
gttgggtaggaaagaaagaaaaatgtacaaggtgagaaaattgttcttataccgaatatcgctcgcacgacaaaagaggaagacgttcattaa
aagacaattacagaaaatgcaaaagtggcttttttttttcttttctttcttttctttcttttctttctttctttcaagtagcaaattctctaaataagtaact
caacggaacataaacggtcgcttcaaggttaataaattgaaataaaagtccgttaaacgaatacaagaataatcacgacattgtgactattcaat
tgcaaaatcacctaattaaattatgacagaatttaatggattgtaagagtatttaattttgggtgttagctctataagaatcgtcttcctttaactccc
tcctttttttataagaatatttttcctttcatcggagcttagctagcagattacgaattgcattctattattaaccctttttaaaaagaatagaaacgaaa
acacatcatgagtcaaactgtcttcaaagatatttttgctagagacgctccttttcgtgagcaactcaacaaggaagcagcatctttgcaaaagat
tattttaacggaacgccaattttgcgatcttgagcttattttaaatggtggtttcagtcctttggaaggatttatgactgaagaggactatctaagtgt
cgtaaataacttgcgcttgaagtccggcgcagtattccctattcccgttaccctcgatgttagtgagagccaagccggtgccattaatgcttcagag
cgtattgctcttttggatcctagagatgaacagacgattcttgctatcctcaccgttgaagacaagtatgttcccgataaggttgtcgaggctgaga
aggtttttggcgctaatgaccgcgcgcatcctgcagtagaatacttgtttgggactgctggtaatgtttatcttggtggtaagcttcaagccgttaac
cctatacgccattttgatttcgttgcctaccgctataccccctgctgagctccgttctgaatttgagcgtaataattggaaccgtgttgttgctttccaa
actcgtaatcctatgcaccgtgctcaccgtgagcttactgtacgtgccgctaagcaacactctgctcgtgtgttgatccatccagttgttggtatgac
caagccaggtgatattgaccacttcacccgtgttcgtgtttacgaagctattcttcaacgctaccccaagggttccgctaactttctcttcttccatt
agctatgcgtatggctggtcctcgggaggccttatggcatgccatcattcgcaagaactatggcgctagccatttcattattggacgtgaccatgct
ggtcctggaaagaacagtcaaggcaaagacttttatggcccttatgacgcgcaatacttggttgagcaatactctcacgaaattggaatcagcatt
gttcctttccaaatgatgacttatttgcctgatgaggacatctacaagcccgtcgatcaagttgaacccggtactcgcactctaaacattagtggta
ctgaacttcgtcgtcgccttctcgtaggtgctcatattcctgagtggttttcttaccctcaagttgtatccatcttgcgtcaatcttatcctcctaaatat
gctcagggttttgttttggccgttcctgctactgcagataagctcgtccctagtgccttgatttctgctctcaatgaagatggtcgtcgtcacgtaact
tctgtctcccgtttggacactacttccttaacttacgctcaggaacttcaacgtgctggtgctgccgtcgttgtttctttggctgatgctgataagtcaa
ttcaaatcccagctaattgggttcaagtgaacattgatccccatgaaaccgtatccgaagtcacttttactgttctttctcaattgtcagatgagggc
tatttgtaaaaacaactgacctagttgaaggttataatctttcttcttcctcaagtgggcaatgtaaatttccccttcatgagatacataagaagcca
tgttaatacctttctttctttacactcagtttctgattcttgtttttcttgtttatcttgtttattcatgttcagttttggcactacaaaatcaatccccttctt
tcataggttgttaagatgtaactgctcaaaatatgattaaaaattatgatatccaatagttttagtgctggcaacttatatgaaacagaaaaaaatt
gtaaacaaaagaagccttccctgaaaattcaataaactgtaaataagtacaaaaggacccaagttttattttgtattattcaaatcaaatttatttg
atttcattaacaatcctctttctgtttgtaatacgtctcgatttctttattggtctttcgctctttcttgaagctaccctttacctgaaacaaggaaactg
ccaactcttaaacaaaagtaccacccgatactatataacaagaatgcaagctgttcagccttctacaagcgataagtgtaagtcagaaataatgt
aagttataattctaacggatttatgtagtaaagatgggagctattatggggtcggctgctgggcttggtattggtttccttttcggtggcatggccgt
gctaagatatggtcctggtccacgcggtttttttaagaactttgggtcagtatatgcttacttctgccgctacattcggtttcttcatgtccattggttcg
gtcattcgtaatgaggatatccctcaattggaatacaaaggcgttgagtggacgccaaggttaatgcaacaacgttcaatctatgatcgaatctta

gcagaaagctcccgtcgtggggagactacatccaacacctcgaattctaagtagttcaatactcgtattgtctaatttttcttagagcagatgaacg
acttgcaagtattagctgacagttttggtttcaaaaaaattagaaaattttgatccgttaattttggattgattctaatatcaggctttgttgaaaaat
aggcactagcactagtatacatcgtgaaagtcgtaatgaattttctaataatgtctacaaacttatatcaccaatgtagcaacctttaatgaatagt
aagtagatctttatgtctggtgtactaagttttgaattattgtatactgctatataaacaaagcgatatatacaagaaatatatatatataagtaaa
aggaaaatcgaaatcaaaatcaaaatggatttcataatagatagagtttaatgctatagaaattgaatgcaagcaaaaatcaaataaaagagc
gtggaagtataataaatgcttcgtgaaaataagttcatcaataattgccaaaaataaaagaaaatcgtcatcaaaacttatcaaatgagtattca
cgcacaagttttatggtcgatgtacataaggtaaaataaaatatttttgccatagctggctcttttatggttttcccccaaaaaaaagaaacaaga
tatgaaaaaaggtttatggagatccaaaatcgtgtaatagaatgaaggagcttgaggaataatgctttactagaaaaatcactggacgtctttgg
caacttccggttctatgcgcttgtctgaagcagaacggatattttcttcttcttcagaggcatccttactgctcaacgaatgttgactcatgatttcttg
acttttcatttcgacttctaaagtttcttcatttttgtgttcattattctcttcgtcaattggtttttctgacgccgaaattaaatcaacggcggcttcttc
atgtatcaactgactgtcatcaacccgtttcccttcttcaactttgctctctggtgaaccaaaatcaataagtccaaagtccatcttcttatgggaatt
taggttagcaaacaattcttcaacttgaagctgtttgagagtaagattgtttacagcatccctgtccttttcctggtgttctttctccagttccaaagt
atttacacgagaatgcaataattcattctcttttgttagaacgtccatgtgtttttggatggcttcctgcttttctccgtccaaagcttcgctcgtgact
cctgattgagtgttcatcgctttcacgacactgagttctgcttgcatggcagttagttcatcacgtaagttgtttatcaacttctttgagaattcattcg
actctctttgctgttcatattttaactttagttgtgaaatttgagcgagaagatctttctcaataagtttgttcgctttcttttcgttttctagttcattgat
ttttgattcaagcagcaacgaggcttgctgcatttcatacttctcaccataaagcaaagaacactgagaatggagatctcttatttcaaaggacgg
catctgcttgttatttttcgtcattacatcgcttggatcattggattcgggaacagtattctcctttatcgactcattggaattatgttcagcttttccta
gctctccctggagagatttaacctgttcactttgagcttccacgacttttctaagttcatcgatcttcttgacattttcccctttagtaaattccaataat
tcttggttagtagagtaaagttgtctaactctttggtattttgttaacaaatgattgagcaaaaaagaaaaagactcaatatcaaaattgggtttca
ggtgcatttgaacctgttctagttcgtgagaggaatctacttgtaagtctttgtgttcattttcctgttgaaataaacgatttaatgctttccaaaaatt
cagtatagaacgaggtgcaatgtctttcgcatctgcttcctcttcgagcttcctttcatgcattccttttagtaaaatctcaagatgtctaacttgctct
aataatctctctccagctgtcatatcaacaacattaaccacagaaggttcatggacagatgaacccatggaatgcactgtttcgagcatccatccc
atacgctcaacaagttgttgtatttcttcatgattcatcacttcttcctcctcgatttcttttttcgtctcatcatcctcgttttcttgagatactgcattcg
aatcactttgttgttcaccgtttcttggctgtttcttgatttctcgaatgcaaacagcaaccatatgcttaagcaaaaagaaataaagctctgatagt
tttcgttcaataacccatatttttgaagagagctctcgtacttttctagagcattgctcataatgctcaatggtgctttgtttatgtttaccttttttttcc
ttggtttgcgcaagcatcagatttttggcagcctcgcggattttagcttccacagcaagtttctttacggacacatccaaatttccctgaaagattttt
atcttccttttaatttcctcaacctctttaccatttaagcattcgaaatcgcttgcatcttttacagctgacgagacaagtaaatcttgcatatcattag
gcaatggttgactcccagaatgttgtttttctctcctattatacttaaaatttgggttgaagggattaaacacctgtgtagttaagagaactcctgaa
gacggcggctcagggtttccgtatttttcctttagctctggttcatatttttctgtagtaatacatgttcctccactcatttccatacttcaatcttggttc
ttagatatatttgtttgtttacgtctagcctaatatataaacagtttcttgtttccatgacaaccaaggcaggtaaataaacaaaatctgttttaaaa
gaaactttgtctatgattgtttcatgaagtcaacgagcgtatttaatttttcaacgggctttaattttctgaatttttttcaacttttcatgaaaaaagat
atcattattcctaaaaacctaacataagctattatttatataggttaaacaattgtaaattgtaaacaatttagcattagcatagttcatttttctgta
atttaaagccatgaatccaggttgaagtcatggataaatagagaagtttcttaaacttgctgcatcatcacttcttgacagttggaagtacttttaa
ctggttgctgcatattggtatccgtataatttctgaaatactccatttgatagtcaaggaaaccatagtgcttgtcaagatgaaccagatgagggaa
tgacctgccaagaaattctaaatgctgcataacaaggagccaattctgagcagatcgggatggtgcggtacttaacaacctcatagtctttgcatc
gtaagttgtaaaatctctatcgacgaggaaaaaagaggttgcactagctgttttaaccccaattaaacgtggctgattttcaacgcagaaaaaag
acgaaatgggcacagtatgaagcttgggatgtccaattcgacaaaggattacttgtcgcttccaaagtgacaataggggatcggtttcttgttttg
aggcaaagtcagcaaggtgtacccaaagagcacgctgttgattcaaaggtagactttccataatatccaattcaaaaagaaatttgacggattt
ttttccagctccattatataagcaatttcatcttccttagacctagacgattgaaaatctctggcccttgctagagttcgatgaacctgatagttatac
ttttgtaaattagttggcaagattaaagcgatgaagcgcgtctccatataagcaagttctcgtacagagaaaggcatctccgatttatcttgctgat
ttgagttatctttaactacaaaaggaatatggttaagactttcgaccttaaaaggaagctgttcccaatgaattaatcgttcttcctgagtttttcga
aacatccagcgggaagccttttgctgaatattaaacaaagaaagctctttagcagtgtattcgtatttttctaaaggaagaagactatcctctttaa
atacaaaaaaataagtatcttttacagtatgtccaataactagatcattgttcttcgccagttccacatacaaatctagaatctctttacgcttcggt
tcatttcccaaatgataatcgggaatttctgaatctatgacgaataggccaaagtcattagcttgctctgaagaagctttgctttcgttgcgtatggt
ttcaagctgagatacggctcgcaaacgatcgtttgtttcttgaacctggacagagtgatttatagacttaacgttctgctcattcattgctgattgaa
cgccttcttcgtttgccgcgttgaaagaacctttgtcaacatctatttctgagcttcctggatttgtcgcaaaatatgctagcttttctgctagagcag
aatgagcctttgcagctgcgactcttggtccatcgggcatgtatttttcatgctgaacaacaaaaccagtgtctggtccatccaaaagatacttcct
aagaaggtacataaactttacatcataatctttaacatcaaaattggagtctgtcaaaacaaggttatccctaatttcgtcaaaccccccaatctgc

ttaagaacaggaatgtatgctgggtcgcttactagctcgatataaggatcagttgagttaccttcagcagctccatttgaggagctttcttttcctac
ttgaaggttttcagaaagtttacgaacccacttgaaaaagcgcagctgatggctgctcttggttgtctgactcagccttgataagattgaggaaga
attgggatctgaaagttgtgaatcggtatcggagaaggatgatcctacatcttgtgttctagcttcaccgttaacagcagtattaacgctgcctgag
gatacggagttttgcaagccagagttctgatcactcaaatgaaattcaccattgttgaaggaattgtcaatccccgatccattcttcatcttttcaat
ccctttggtactggcagaagacaggtataatggttgtttacatctttcttaaatagtgcaacaagcgatattccgtaaacaaaataaacattgtca
aagcaaaaatcaataaaacatttactgatattcaagatgaacagacgagaagaaggcacctaaatattaaatgatccatgtatagattttgtgtt
gcagaaaaaatagagatatatatttatttaaattcttaaagaatccctgaagaatttgtaaagtatcattgtttttcaatctgtactataattactca
cagctgtgaggttgaagagaagtctatttcgtgaatgcttcctcaatgagtcaactggaaaggtattgaagacaaagaggaaactatgcactaac
atttgaatttttttagattagaaagattggaacagtcaattacattagcattgtacgaaattgatgcgaatttcgcgaaatgtaacagaacagtgtc
gactcgaattcttccaattatagagaaatatactaagaactgtaataatatatgggagtcatcaaaggtacgggttgaggagaggaagaaagtc
ttttccacttacgtgcattttactaatgcaaccagttttggaagcaattcttcgaaacaagcgctagtgtctcgctctctggagtcgaagaaccagtc
caagggtctgaaaaaccgaaagaaggttttgtatcctcagaatcgaaatatacagacagcggtgatccaaaaatcgaggaagatgaggaggat
actgaaacgttttatcaaaacattccttcgtctttcgggcacgaagcttttgagcctcaagaagtaaaggatgaacaagttactcagtctactccca
agaaagacacatcctttgaaaatctttctttagaaaatgcgtctttaacaccgatacccgcgcgtttgcaaaccccagctcgaaagatcgacgaa
gctaatctgcaacatactggacgtagtgctttgttgcacagagtactggacacgaactggcacgtccaggtaactcctcacgagaagaagaagta
tgaagacatggacattgattcttctcctctcatgtctccttctccaattgccatgaagatggagaccatctcagcacccggagatagaacatcaaa
atccgctttgtctatgttttcggaattcgagcacgaaagcaacgatagccttatgccaaatggaatgtcgcccccaaaactattcagttcagtcct
cattctttaggcgactccttcaatcgtactgatggtgagcgaagtctttctttgcaaaggaaattcgatgctttaaatgattccaatgaaaatatgat
aaaagaggagagttgggaactttagaaaatttcccgtttcctttgtaccttttttattttcttgttcactactctgtactctatttgatgaatattctgatt
attcttattttttatcgactttttgtttgtttttcctctatttgtttacttgtttgtttataacttagcatatcgcaaagacgcttcattatataagattgtgg
ttcctgctaccaagtccaaagaagtcagtagaagtacagaaagaaggatctgggctttggaaaaaaccaaccatccaaagagtctaaatcaaat
ttttatacttttacgctacgaaatgaatattgactttactggttatttacaatcctacactataaaggattggatacgtatctgcatctacatttgtgg
atatctcttactaaggccctacttgatgaaaattggagagaagcttcaagagaaggatcataagcgatcagaagaacaaggatatgtcgaccct
caaatgactcatggaagaacagagaagtttcatggtgaatttgacacagatgatgaggatgaaaaggaaaatcccgatgctgagttccgctggg
gctattctgctcgtcgacgtatccgcaagcaaagagaagaatatttcaaaaatcaaaacaaatcgccattggatgcatacacggaagatgacaa
tgatgcagacattcaagaacatctccaagactaagtgttcattttcgagtgaaaggagtttctggttcttctaaattggctcgaagaaaagcataat
ggctgtttattaattcttcattttagtttttctcccttgagtgttcaatgtatttttggcgttcgttcgtttctcgttttcttccacctgttttcttatataatt
actctaaaatcgttgaactctcttttgttaattcattatatgctttctttcattatttaataaaagatacttataagacaaggtaacgtttcatcaaatg
aacgactagacctcatctttcgtaaattccactctattcatgtaatgtgtaagaattcaattcatttgagagctaattgatttccttgaggcttacgaa
aacaaaaagacaaaagcgaactttcattgatcctccaaatatccaagccggctttgtttataatttatcgaatttttacgcacactcattatttggg
attgtgcattcgagtaattcaacgaactctacgtcttgtaggaactaagataagttactttactcatgattcgctcttttaaacgattctttttaaattc
agaagagcttcctcgtgaggcctcgtcctcccttttgataactctttttcctcaatgcttcatcagctcttccggtaatggatttcagcgagaacctac
tcaagtagcagttcatgttcaaggatgggttcgagaggatcctaatggaaccccatttggtcgtaaagaccgagttatgttaagtcttcttcgtcgt
tatgttggtcttcctcctcaggaaactgttgattcgaatttaagtcttccagaaggaatcgcatccatgaatgcaacgccttcagtggaacatataa
aaaagtccagggaaagtaaaggacattcgcagagtgaacccaatttaagatactgtgaacaagaaacggaaaatgtctccacgggtcaatcca
atacctctgctcgatcttattatagtctgtccagctttgttcaaaagttcatgatcccttcaaaggagagcagttatatttcgtctataggattaaagc
agtgtgagcataatttcatgacacgctcaacgtatttgttgagccgtccggttaaaaacaaaaccgttttggttcggatttattctacagatgacaa
ctttcaggaacattgcgtagctgaattcaaagcgataactgacccaaatggatactttatattaaaagaggcaattccgtactttaaaacgagaa
acaataaattttcagtggaagccattgccattcgttcttttgcaacgtcggaacaagctcgcgctgtattgaatgaggttcccattctgagtcgtaca
ggtgtttcagtcatttctgatatagatgacacagtaaaaaatacgagggtagttgagggaccaaagaaagttgcagaaaatacgctcttggcacc
tttgacaaatcaatacatagaaggcgtttccgactggctccgtattgtgacaaatttaggcgctactttacactttgtaagcaattcccccctggcag
ctttggcctatgatctctgaatttttttgataccgataatataccattcgtctcttcgttgcagttaaggcattttagcagtgttatacaaaacctagttg
agcctgctgctcaacgcaagcgtgcaggtcttatacatacaattcgaaaccttggcgataggaaggtacttttgattggtgacaacggagaacaa
gatttagaaatctatgctgaaattgcgacgtgtttccccgatcgggtgctaggtatcttcatccgcgacgtgaaatccgactttcatgctaatgtgca
accaaaaggtaccaacgacacatctcatctgaatactattgcaactactgcaccaactggaaaatcaggcgagcatactgctaatgagtcacta
aatacaaagccgaaagacgtggataatgaacaggcttcagagtactatcaactagaacaggagcaaggaaccgagccttcaacatttcaattg
catcgctattatacgtaccgtatcttcgttgaggataaaaaagcaggatttcgaaaacccaaaacacaagtagagcacgcagtgacaaggttgc
caaatgttcattaccttacaccgtattccagtatcaagtactcaaacaatgacattcaggactggtatattcagttggcacgtgctcgagggcaggt

cgcacaaaatatacctattgttatgtggagaaagggcgaggagtgcatttcattcacaaaaatgctaattgatgagcatttataagctaagaattt
agctgtcctgttttggcaaagacgattgcaatgttttgttagaatatggaagggaaatttaaaaaaagaagggaaagtaatatgtttatatttgat
gaccatctcgtaaacgaaacggactttattgcaagaaataatgattgatgtttgaaacgaattattatatcatgtaattttatagtgtgattttattt
atagataaataaattatctacttgaaagctaccaagggaaaatataaaactaaaagggattttagttcagcatgtaaacattcagattttcttagg
gtgtagtattgctgttgcgttaatgctgtataaggatgttgttttacgaagccaaacgaactaaaagggaaacagccctaccctaaccaagaaga
atgtgactgtttcttgacacgacacaaaccttgcatgaaggagaacatgcttttcaccagacccacgacacaaaatgaagttctccaggaatgtct
ccgactctcgccgcaagcagagaaaggctcacttcgccgctccttcctctgttcgccgtgtgttgatgtctgctcctctttccaaggaactcagaga
acaatacaacgtccgttcccttcctgttcgtcgtgatgaccaagttactgttgtccgtggatccaacaaaggtcgtgagggcaagattaattctgtct
accgtaagaagttccttttgctcatcgagcgtgtcactcgtgagaaggctaacggtgcctctgcccctgttggtattgatgcctctaaggttgtcatc
aacaagcttcaccttgataaggaccgcaagaacttgattcttcgcaagggtggtaaggtcgagtaaattttataaaatggatttattttgcactgg
accgtacaatcgaaagttgtgaaacaatggatggtagcgtaaggagtaatagtatgggaatatgcgaggtatgaaaagcaagaaagtagaaa
atagagaagtagatggatagaaagaggtgaaaattcaaaggactgcaggtttatcttggcattagaagcaaactactttttttgtcataaacgtc
ctgcggagaacaatacctttctgcagaccgtgctagatattaacaaatggctagtatggtttgaagaaggggtacaaatgttataaagcaattgt
aatgattatagaaggagatttagaatgatagataggttttattcaataaacgttttaaaagtgattaatcataaagacaggaaggtttacgcagc
agtatgtcacggtgtataagaaaagaacatagatataagttttcacatagcaggttagaagaggataaagactgtaagccgtagaataaataaa
tcacaataaattgctaaaaaacaagtaaaaaagggattatggtattttaagggaaacataaaaaaaagtttcaagaggaaggatcttttttttt
ttagttttgagaaaaggtttgaaagcctactggtaatcgttgtaggggttatttcaatataagtttgattttcttgttttctgtttctgttagaagctgc
acgataagtcgctgtccgacgattctgcagcgtttactgtttgactagtgcacgtcttattggcaaatcgctaaaatcctcgtcgtcaatgatcgaa
gatggtttttctttcgatacgtcccttttccgttggtaatattgacaaaacgaacccttcaaccctgttgacgacttgctagatatcatttcggagtat
gagtcctcgtcattgaaagaaatgaaacgctttcgcttgacttgcatttggacggccacagatgctgttctcattgtagcttgaggattctcatcacc
acttggcgaatcaaaagatgattgtttccttcaattctctgtattatcattatgctattgaggaagctggttttctacctgttcctttagctatacaaaa
gggcatatttaatccctctccttcctgtaatatttccttcaggatgtactattctccttggcaaaacatttttctagtgactttttaaaaagttattata
acaagttcatctgtttctttgccttttatccttagattttgtggtatacgatttcgaacgctgcctaacagcttaagatccagtttcattgtcaatggaa
gctttgtctttttgtcttgcatgtgcatctcgtttacaagtagcgcaactgtattgttcatccaacatactttgtataatttgatccatctcatcccaagt
agattcgaattcttccagttctttcatatataactctctaatatgtaattcacttaaagtgtcaatcttcgtcataacatcatcccttctcttcaaaagc
ttcaaatatttctcgtatgttaaagaccaaattggcatagacaagaggtaatcgaagtcactggaaaaatcatcaactgcttcagctggatcttcg
ccactttctcgagcccgctttgctactatattggcctctcgacccttcttaagctttggaatagactgaaaacctttttcgcgaagttcaagtataagt
atgcctcttttctttcccgagaaaataagctcatttttgataatcatatgaacgaatctcgctttattgaaaaatatgttatagcgacgttccaattca
gccacttgatggtttttcgtctttgatatgttagtaaacggatttcaaagaagtctcttaaaatatcttcaacagtatcgtatttccttattcttcccg
atggatcaaatgccaccatattactggtagcctgggtccggactaatttgaatttagattctaaggattcggccaaagcttctctcatgccttttct
gtgagttacttggaaatgtacatgatcaaaaccaccatagtctttgtaatcttttatgaacggttggactttatcacttcctgtcatacccgcttctag
gaattccttcatatcttgagtccaaaattgagcaggtaattctgttatttccacttctaagtcactttttgattaattaaaccattaattttgtaactgt
ccggagacactttctctatgctaccacggaaaccgcgataccaaggaaatatgtcaagcaaaggttcatttataagaagtcgttttaagttagca
acaatatctttaggggttatattttaatatgttggttgaccaatccgttccaatctccctctgcaccatttatagaaccattggcaaaattgggacgta
atagtcagggttgatctgctgcccttcctcgttctgataattgagtataggatcatcttctgctttgaataactttctggctaaaggagaaagaaaag
ccgaaatgcatctaattgcttctggatgatccagtgcgtccttgccgcctttgaaacgagtacccatgtaaccagtaggcactagaaaattgagat
tattacttccaacaaaattttgagccaaatttatagctgtctgcatcaaggaatcatgttgatgataacctataccattctcatgcaaaacataagg
tgtaatctctatcacccttttgaacgagtaatattttttttttaaacagtaatgaataatctttcactcatctatgttcaaaccgtctacaacagagg
gaatagaatggatattatctgttgccatactcagtagagtgagtttctaaaaacattgattattcgaggaactgtgtaatccatactgatatataca
tatttcttcactgaagtagttgtctgattatgatagtggggtagagcattttatcgtttctaccatctttaatagatttttcgcttagggcgacgtattc
gaaacataatgaaacacaatgctttattttgtaaataaatggaaatattattcaactactctaattttgtgttatgattctgtttaagtgaaacaaat
aaccataaactatttacaatctgaatcatatactgtgcgatactgtgcagtatgattcaatcgaatggaatatcaagtaaagttaccaagtgcgaa
attgaagttagtttcgactattatttctcaattggtactagactagtaatgtcctgtgaataaattaggtaaacatgtacaaacaagtttgattcgtc
ccatcagccaaatcggtttaactgaagatttagctagaaacagttaggctatattaaactattctattttcaatctgtcactttccaagaaacagct
aattgaatttcctggtcgcattgaatatttttttcttttaattgttgacaactttttttgttttgtttacttacaggtaacttacagcgatacgcgttctttag
aaatttatcaaccgagtagtatattattcgataatataataagatcccctgaacaatatacactattatataaaaggatctatctaataaaactaac
agtaatagaaagttggtgcttttcgttcctaaaaatggatgtttactcacatatcctcttcattctcttcgactatgaatgagtcagtcgtctgacaac
tacattcatcctaaaagaactgtataaatcggttgaagatacaaagttgaacgaagcacaacgtaagacgttggaaaacaaatcaaagcttaac

aaactaaatcgataacgtagtagagataaagttgaaatcagcgatatccaaaaccagcgacataatttggctatggaagataggaataaagtat
ctagaaagattaagggattcagagtgaaactctaaacaaagaaacctaatttgctcatgagtaagcgataaaacaaaacataactccagacag
atagcgaaggttcatattcctcatcgccatcgtgatttgcatgagtggaaagaacgataggaagaaatgcatcacgtggataaggaagcgttata
aagtgattatcgaggtaacattgatacacatgcattccacacgtgattataaacgacaaggatggacgtttttataaaaactaaccgaattgctat
ctctgttatgtctctcatctttgtctaagaatagggacaagaaaatacatcatactactcatctatgaagcatattactcaattcttgtgtttaataaa
catgaaatggaaactccaccccttttccctgttctattaaggtaacctaaccttcaaggaaatacaaatcacttcacgtttctctttgtccttcatttt
gtttatcttctatctcttatcgtattccctttggtcaatctttttctcctcctatctcctgtggggtaaacaacaacgtgcctgatagactaaatccaaa
atcccaaggaaaaggcataaaaggcatgggtttggctgtttcctacatcgataaattcaagctttctctttagcgggacatttaatcctttcaattca
tcttcttttttttttttttctttttttcactcctttcatttccttctttgtcatctctttttttatctttttttaattttatttttttcctttttcctttttcttttctacgaaa
ctttgatctctagtccatttcgctttcttttggtttttatacattgacagctgtttcgtttacaaggtctttccgtgtcttccttgtttttgttttctttcttttt
attttcgtgaattctgtgaaagcaaagctaaatcatatgctgattccagtttctttcttgcctttttttctagatatacaatctctttttgtggaagaaac
ttattcttgataccttctgtctggatcgtcggttacgcacttcttttattccttgcttcatggattacaacgctgctgtctccaatggctcgtccataaag
atggcctcctccgggaggttggacgaggaaattcgtgcctcaacttcatccaattcttcaaaatctaccaagcaagccaccaaggaagctgatgt
cgatacttaccccgtaaaagacggtaaaggtcataactcagctcgttcttctgccgacatcgaaaaaggcgacatcgtcgaaagtgagtttgtag
atgtcgaagacgactctccatatcccgaagttcgtacttccgtctctccaaccgatgacccttctatgccttgtaataccatccgtatgtggaccatt
ggttttatcttttctaccgttggtgctgccgttaatatgtttttctctcttcgaaatcctcccgtcacaatcaccgtcttgatcgctcaacttatcgcata
tccttgcctcaagggttgggattatatcttccccgaccgtgtactccgcattggtcgtcttcgcttcaatctcaaaccaggcccttttaacgtgaaag
aacataccttgtcgtcatcatgtcttccgtctcctttggaagtgcttattcgacagacatcatcctttctcagcgcgctttttacaaacaacgctttg
gtttcggttacgaaatctgccttactctcgctacccagcttattggttatggtcttgctggggtttcccgcaaacttttggttcgtcctgccagtatgat
ttggccctccaaccttgttaattgtacccttatgaaaactttgcacgtgtctagcaaaaaaaaataatccgaaaagtgcgaacggctggtccatttcc
acttttcgtttctttctcattgctttcgtcggttgctttatttggacctggttcccaaattatatctttacggctctttctctctttgcttgggtcacttggat
caaacccaaaagtcccattgttaatcagcttttcggcgagtctaccggcatctccctaattccgattacctttgactggaactcaatcagtggttatt
tgtcgtctcctctaatgtttccttctgatgccatcttcaatacattacttagtaccatcattttcttctggatcgtcactagtgctatacattatagcaac
atttggtatggaaaatatgttcccatctgcagtaacacaattttggatcattttggaaatacctataatgtcagtcgcattctcactccagatttcag
attggaccttaaggcttttcaaaattattctcctctttttatgcctactgcatcttccgtatcatttggtctttcctttgcttccatgactgccgttctggtc
catgtcattctctattatggcaaggacatctgggcccgcattcgcaatcctccaaaacccgatgttcatgagcgccttatgaaacaatacaaagaa
gtccctaattggtggtatcttaccatctttattgtattctttggtatgatgatgggcgtttgttatggatggcccaccgaaactcctgcttgggtcgtca
ttgtgggattgattttgccattgtatggttcgtacctatcggtgttatttatgcgattaccaatgtcgagcttggtatgaatgtttttaccgagtttatc
gttggttacatgtatcccggacatcctttggctatgatgatattcaagacggttggatatatcactcaagcacaggcgctgacttttgtcagtgatct
gaagctgggtcactatatgaagatccctccaaggatgatgttttgggctcaaaccctctcaaccatctggtcttgctttgttcaaattggtgttttag
attgggctctcggcaatattaaaggcgtatgtaatcctgatcaacctgatggatacagctgccctggcgctaaaacctttttaattcctccgtaatc
tggggtgttattggtcccaatcgcatgttctcaggcaaggctccttacaccggattgcaatacttttggctcgctggcgcacttgcaactgttttgttt
tggattttatggaaaaaatggccccaaaagtggtggggtaaactcaatgctcctattatttttggtggtacgggtatgttgcctcctgcgacccta
ccaattacctagtatggtgtggaatcggcatttttttaactactttatcaagaaaagattctattattggtgggcaaagtacaacttcaacttgtcc
gctgcccttgatacgggtatggaactggccctcatcgtcatctttttctgcctacaacttcctaatgtaagctttccaaattggtggggcaatgttggt
gcctttaatacaatggatgcttctggcactgctgtattaaagacggtcaacgaaactgctggtgaattctttggacctacaagttggtgataattga
tatcattttgatttctcttctgtttgtaaatggtcacttgtctattgaaaattgctaaatcttactcctaatttgtctgtggccatttttcactcttaatctc
tgatcatagttttgtacatctatttgctttgtgtttagatatatatatatatgtttgtatatgattgattctaacgttttatttataacatgtaacaaattg
aataataataatttttaatagaaatacgtatactgaaagtcgtctcagcaagcatatattattattgttatttcaatttcgcgttactgactcaaaca
tcatatgaacatattggagatatcccataactactttcattgattcattcggtatatttcttctaagttggatgtttacacctagaaataagagaaac
acgttttcagtagcagagcttattctgacagactaccaacaaagtgaaacataatagaacatcgaagtgtgtaccgttgctaaatgaaatattcta
atactactgtatgcaccagataatattttgctcccaaaacaaaatattttaaagcagagggttcaagcatgtaggtctaaaacttgatttttataac
aggaagttactgagtggtcataagctaagttgaaaagattgagctgaaaacagacgggcaggctatatgtacatacttcatccaaaaacaaagc
aataatctttgacccgatttccctttcaaatatactcgttgtatgtagaatttaagactcagtgtcaccatgacactatttttgacgaatagtccgcca
gaaaatgattttaatatttttattcatatactcttccaaaatctatcttctttcgataaatccaagacatcacagtaaaacatcctctattttttttttct
ttaataagacagtgaaacattaaaaattaacaaaaacaatgaatccatcaacttgatagttcaattattaccatacataaagaagtcgaccattc
gaaaacattcataaaacatagtaggcactgttgcgtgcgtcaagttaaaattatcgataaggtctaagaacgagtacatacagcgatacttcagc
aaagattaggatgaactgaaaaacaacgagcttttattttcgagaaccgttccattcaaacattaataattaaaacaaaccatgactaaaaaagt

catttcaactagggaatgccctttctacggcattgctttccttttttttgaaacccacatctaccaactaaggtatagatttctcatcaaagataaaca
gtatgaattaatagcctaaattaattcgttgagacatatcaccatttccgatgattgtggtagcgactgctgcagagttaccttttgcaacaggaca
atctttatgagcaatcaaaagtgttttcaaactgacaatctcttctctgagagcactaacctgtgcactcaagatttcattttcattgccataaaattc
aaccttcgcttgaagattggatagccattgtttttttgcgttgacggcatttaagcgcagcctgacgattgcgttccaaaaagcttttccgcttttcatc
atcagtttcatttttagagtttcggcgaggtgtcgaaccactcttactattgttactttcaccgttaaaatcagtactgtcaaggtctttcatgtcagct
tcgctgggagatgctgaaggagacacattagagtgaccggacatagcacgtggttgagaatcttgacgttcattaacttcggacttaacttgaac
cggcataccgttgctgggcatctctctgttaccaccctgtccaatgtgagacatgttattcatgttcataccatagttgttggatggacgtaagttta
gagaagggtcagcagggttaaccgtctcaccgtacacagaaggctgcatgtcaccacgcaactggttggcattcatataataaggtgattggccc
atattttgggcttccaaagaattattggcattgtgtgtaaaagaaggacggatagaatcaggatattgctgagattgagactgtttcagcaaattc
gcaacagcagctgatgtcgaagggtccgtttcatttggaacggatggctgcatttttttgctgttcaggcacttgagaaagtagacaaagaccatga
acggcagcattggctccaaaataatcagattgttctccattagtaccggcagagactggaggcttgccatctcttggagtgaaacgatactggtca
ggatactgcgaatcaaagggttgaaaccctaaaagattagcggttccaggagaaggagcaggaaaactagcacctgttggggtcaaaagaga
gcgaaatgaatcgttgcctccaggggtaaacccagtgccaaaggaaaaccttgaccccatagccattgaggggtcggaaggagtgttgagataa
tcagggcgaagagtagcatcatagatagcaggagaaagagggttttgcataccaccgcgagtaaacccagaccatgcagggtaggcaagtggt
gaagcaacaccaggagcacccaagatatctggagaagcgatggaagagacgggagggagtagggttcgggagaaagaaggagggatggcg
gagggagcgggacgactggtagagggcccaggctgttgttggtttacagtagcagtggatccaaaagaatgttcaaaaggattaggctcgaaat
ccagcttcaaagaaccaacaaaagaaggagtcactcctgctggctgttcaaggggatcagcaccaccgttaacgttattgggtggtggttgctga
gattgttgcgtggacggaacatttatgttattatcaacagaagggttattattcacggcggaagtagaattagggtttggtggggcttcggagggg
ttataattgttagacgcgttattaggagaaggagacataataaagataaatgagtaaacttttaaacagaaaaatttagatgaaaagcaacaaa
aataaatagatgagtaatattacagattgctcaaaaccaatatgagacgatagatgtcagtagaagaagaccgaaaattccaattcaataactg
aattccaaaatttgtaaagtaaataagaaacgaaccggtaaggatgaagaaaaaacgaattaaacaaggaagcgaaagaaaattaggccaa
ctggataggctaaaagttaaagtactagaaattccaatgaaaacctaaacaaaagttgttagtaatctagacggtaaaagtcatagtaaatttgg
ctcctccgctcataaaagaaaaaagaaaaaaaaaaaaggttatttttcctactaagatgcaaaccggataccgagtcctcgcatcaaccttgcaaa
gaaaaaaacattatagcgaatcagcaaagccaaaagagaccttgtttttttgtttttgcaaggaagaacaggacaaagcaacgtaaagtcggga
aaaataaaacgaaaaaatttttttttactctagaaaataaaaagattgagttttgacaatactcgattgttttcagaaacgttgttcaaattgaaa
cgtttcactttttcccctcttttttttctttttttacaatcatgtaagagggcacacgttagttcaaccttctttgtctcccttaatgacgaaaacttgaac
caacagtattccttacctcaacttaagtaggattactttattatcaagtcaaattatacttagtttatgtccggggtggagtgccaaggtcttcgtcat
gaactgatgaattcgtaattcagatgtacttaagcgacgggcactatataaaaatgatgaaaaaactcggtcaacgctcaaaaactaaaagact
caaagactcgtcaattttgttcagacaaatcgttcataaatagtttcaatgggccttcctttactttttcttctcggatcctttaatgctcaagtgtata
gtagatggcaagtttcaaattcagcgatacaggaattcagactcagtaaattctatatacgtcatgcgtcacttccatatgatgcatgaaccaatg
aaatgtgagtttttgctttaggtaccgattcgattaaggtctcgaaggtagcatagcaaacgttcaatagtgttcgtttggcaaacgttaaagaagg
aaaacgagctcttgcacgttacagtgacgaaagagaggacgtaagcaggtaaggtaaggtaagaaaaatgaagacggtatggtactgtaaat
gtaaacaatgaacgaacacgaaggaactttgaaaacaatgaaccaattaataaaaaaaaattcttagatatgtaagtaaaaaagcattgggatt
ataatgatttactcgtcttcaattgaattctgtattgcttgctacttcataatctggaaatagtacaataaatgtttttacttacatcaatgaatgactt
gagcaaacgcgtttttttaaccaggtttctttttgtccggtttattcttttattaattgatttatttttcttttatatatatggaagtcttttctacttcattttt
ttattttgtcggtttttttatacttctcagttgttttatttacaaattactgcaacgcgctctactactttgattctaccagccatttttttctcctagttagac
aaaggctccttgcagtagtcgctaaaagaggttcatttttctttctttttttactttactgaagaagcatcatcacaggtctcttacatacagattcggcg
ttgaacacgatgtccaaaaaaaaacgaactggggaccctgattgtacgggttgcaaacgcggtaagtattatgttgtattggtttgctcagctgtgtt
ttactttactttactttactttgctttgttttggatgatttgatttttttcttgttctcttactgaccaactcaatctagaaaaatttgcccaacaaagccct
cgtcggtaaacaaagcccttactgtagctgccgtgtcggagcaatcgtaaaacgtactcacacagataaacggagtggacaagctccttcttgga
atgctgtcttggaatttcctataccaagcgaaagttacaatgtcatgaaagtgtatgtgtaccatgaaggattccgaaagcactctcatttaattgg
ggataccgtactttcgtttcaaaaagctctccaggaaggtgaccaaagccagtggtacgagttaaaaaacgactttcagtttgctggcgaaatctc
tgttcaattcaagtttcttccttccgaccccgattactttaaatttgtcaatccttcgtctacttctcttcctttcccccaattcagtgtagccaccttggc
ttcgttgcctgcttcttcctccagaccttccagctcaactgaaaacactcaaaggaagggcaaagcacaccaccctcttcccactcccccggtcatt
atttccaaggactatagcacattcccagaagatcgatcgtccctttcacaccaagcaaatgatttaccttccttcccatctccctccatggttgacga
atactattactaccacgatgacattccggaatccattgacgagttcccaaatgccgaatcctcgatatatcctcattccaatcttccacccatcccca
tcgaccaagaggattttgattattaccaccctcttcctcctgttccccctccgcatgcatcttaccaggactcgtttccctcctcgtcttacaaccacta
aaagcctattttcttacctttactgtaactactatttatcaattcttgcatttcattagctcttcggatttttcgtttggctttttggtttttttgggttcttat

tcttttctctttcattttctcaccccccacgatacaaaccacattagatttctctttcttttttttatacatcgtttttttgattacattccttttgttcaata
actcaccactagcgttccttttctttcttttcattatcctatgtctctgaaacttacaaagcatagaaaattgctacaaaccaatcctttgttgttctttc
ttactcatgcatctacactcgcattagtgaaaatatcacgcgtcatctcaaaaccaaaacattcattactttacgtttactctcaaaaacaaattcc
aaaaagggacataaatatttaaaatcattaaacaaaggaaaaaaaaatacataagcttctttaactcttttcttttttttagttagacagggcagt
attcatctttttcaatggctacgccactccaacaaaaaggggttttctctattttacttctttttgggccacccaaaggagcaccgatattttggcc
acgcaatttgatgcccaaagctcgctccatcttggttaaaacttgttggttaggaatagcacgtccaccttcgtagtcgttgacgacttgaggctttt
cgttgattctttgagccaaatccttttgagcccaacccttcgtttgacgtccctgttgaagagcttgagcgacagaacggttggtagtagatggagg
cttgacctcgttttcacgatctatcttcgtcaagtgttgtccagcaggatcttgggcacgattgccggtggcatatttcttttcagtgccaacgacggc
tccggcacgacgagcactattgatttgagattgtgtcttggcaacgtgggtgcgagcaccaggtccggcgcgactaccaatctttgtaacgttatcc
caggattcagacataatttaaaagaccttttctaagatgagttggtaaacggaagttctgaatttgcgttcgcccatatatatattgacactacaa
cgtaagaatgtgctagaagctttagataaggaactttgcaatcaaccgtaaaagtgttttagcgagtaatttggctttcactgcggtgtgtaatata
catataacgattataacacaacgttataatagaatggttaggaaactttgttaagcttcactatactttttcctgcttctatgcaatgtcttctatgat
agtatattgacgtcaagcaattttatttcctcacatacaacacacgctggcttgagaagggtcccttggaacgtttcaaaaacaacgatctacacc
aattttttgaacttacatttgctagattttaagtgcttttgattgcttccgaattttccggaaaacgctacgaaacgtctgattatcacgtgtttttacct
acgtttgtggattcttcattttcttgttacagaattcttcttcgtggaaaagcctttttgtgccgcagtctgctgaggtttcgtcaaattcattttctggg
agtttcgaatcaatgctggcgaggttttcatctatttaattgaaaatactttctttgctctctagtcttttcttaatttgttgtatctgtctgcttttattttt
atttttattttttttggataaaacgttggttgaagaaatccttttccccttctcctcctcactccgtttacatagcattcaatccttcctcaatcagcaaa
gctgagctgtaaatacctgcccttgctcgtgtagctgaatctaaaattcagcttttggttttcgttcctgacagaacagcccaagtaaacttcaagg
aattgttattttttttacgtattcatagctgcttccgaagtttctgcctggtcccattgatctgctagtttttttgtttcgtattttgattgaaaaaacatac
ttcatttttcatttcttctctacacccattcttattcccgtttcacatacacaatagctttgttgttctttccgtactaaaagctcaaaatatgtacgattc
tcaccaagaagagaagttacaggcacttctagccgtcatccgtggcttaaactatattttggacgataaaatggacgaagctatcaaaatcttca
attctggtaatacctccttccatctgagcggtcaagctgttgtctcttttcttcaagctgtattgacgtttgagccttctcggtttggagagtctcaaaa
caaaattgatatagcaattaaagcactcacagaagacaaggattatgcatcgaaaaatgatctttacctttgtacttttgatcccggaattgagta
tcgtgtatccatcggccttatgctgttgttgtccgctctcattggtttttgctcagagtcgattgtaacaagtgtaaaatcagtatacaagcttcgcaa
agcccacaacatcttcaacaaaataaacaagcgacactttgaagccttttctacgtcttttcaaactacgaataataaagatatcgatacggtgaa
tgagtacgtacagacgggaactctcttgtgtacgggtttatttacactcttaatatctttacttcctccaaaaatgattactattctgagcttactagg
ttacaagggagacagagatatggctctctcatatatgtggatgtctgctctccagcgaccttccagctttttttgctgcagtagcatttgcagcactta
ttcaatattatagtggagctgtgcagttgtgcagcatttataaagtcactccaaccgagcctgatggatggcctgttcaacgctgctttgatattcttt
ccaaaatcagagtaactcatcctgaaggcccgatgtggcctttgcacgaagcaaaactgctttccatggtaaaaaaacagtcggaagcgattgat
gttttgaatgatcttatgtcaaagcctccccctaaacttatgcaattggaagtcttgattgtatttgaacatgctcttgattgcgccttttctcatcgtt
atgttgatagtgcccatagtttttttgaagttgtctagcctgaatgattctagtacggggttatactcttatttcgctgctgcttgctttttgcaagatgtc
caggcgaatgctaatgttgaggctttagagaacgcatctaagctacttgaaccattgggcaatcttttagtagataagactgctcctttggatatac
atatccgaagaaaagtgaaaaagctagccgaccgaagagctagtgttggcaatcaaggcggattggccgaatatgtgggattttctcctttgtac
cagttggtctatgtttggaacggttttcgaaggatgaatgcggaggaactatctcgattcgatgtagaaagagtacagccttggcaggatcaaga
cgatgatatttgtcaagctttagtaaaagctactttatggaggaatacagggaaacttgatgaaagtataaaagttcttcaaaaaatttgtcaggt
tacaaaaactactgaatgctgggctgtcgcttttgcccactatgaaatggcggttgcccttttcgaaactgatagtacatctaaaaactccttgaaa
gaatgtgatagttacttaaaggctgcccgtgattttggaggggataatgaatttgaaagtcgattaatcattcgcgtccaattagcaagacatgtc
gtaagacaatgccttcaagaaggcggctaatgagttcgattatttccatgagtttaaattattctacataaatattaaagataaaaaggcactagt
tttttttgttgtatagacgtttagttaataacaattcacgaattaaagcgaatgaaggtagtaaagtttaagtgttatacatacatacatactaatac
gcatatatagagataaatgtcccaaaaccattatattttattaaatccgtttccttccaaaaacccaagatcatgatctcttttgtttttttagtctttct
ttcatccttcttttcttccttgtcgtttaactgctccatattgcgccaaatttcttcttcctcttctttcgcaacgcgactttcttcttcttctttgatgacttc
ttcatcacggccatcgagaagcaaaatgctagtaggcattacttttacgtatgcattagccacaggctttcgatgaacatcgtaaattcgaagatg
tctgtcgagaccaacaatagcaatcaggtctccaagattatggagacaagaagctgagccttttataccttgaaaacggcccacaaccttcttctt
tataggatcgaatacatagacttgagcgcgtttatctgcaaaatacagcatgtttatggaaggtaaaagcccaatacgagtgagttcagaggtgg
agatgtcagtactaaagattggtcgacgagcctgtttcgtataataaaacctcaacttgaaaagaattaataattaagattcaaacagaattaagt
aaaaacatacctgcccataatgagtgatagtagcaaaatgaaaagctggaggagaatcgtcttcatctttcccgtcgatgacattaaggacttctt
ccgtaaaaacaatgcctgtgacccacacacggactcgcaaatctaaggcatcattctttacatttttcgcttgaaaaacacggacaacctggtctt
gattccatagtgtcgacaaatcaatggacgaggagcaactagcattgcaggaatatatagaaacatcgttttcttctcctccaatggcaacgaca

ccagcaattcgaggatgcaaggtagcacaagacaacggggaagcgacttggatctttttttacttgacctaactctggatttcggaacacgattgtt
ccattactgaaagcaatcataagccatccatgagcgtattgaaatgaaataatagacgcattatctaaaagtttttcatccaattgccatagactg
tctaaagcaggaggctccttctttatgtcccagcattcaatagctgcacttttacgaagtacatagacaaactaataaggttagtatagcgctaaa
aagaatgcaaaattgtaagatttctaaaggaaaactgtacatgctatgctacacatagcacaaatttgcttaatcccagaaaaattaccgaaaca
aaaggtttgcaatacattttcgttctggcagaagaataaaattcccttctgtttatctaatttcccaaagcgttgaatagtaggagcttcagaacttg
gattagaagcatcaattcccctttttatttcaattactagaaaccacaactgtcagcttgatatccataaaaaacgtttcatacttacattttaattga
cctaattcatctccacaaagcaatttcatggtgatttcccctagtaaaatcaatttctcgaaagaagaagatgtgtgctgtgggattgcagaacaa
caaacatgttttgcatagatcactatgtcaagacgtccaaaagtaaaggaaaaagagtttagtaacacggagtatagaaataaaattgttgtgtt
ttggaatcagctttcaaatagattttgttgaaaaaaatcaaatcgaacaaaacttagctttttggctcaataccatcgtttatacagggcacccttttt
tttccaaaaggagagaagaaaatatgaagcgtttatgaactcaccgtacatacttacgaaattggtctctctggcataggacttgaatagaatca
gtgctttgattcatgtcgtggaaggaaaccccagatcatgtacttgtctctcatacaatttcgattaccaaggtaaaaggagttcgcgagccttaca
tttgcagagaaaaggtgttttcaagcctgttcattgaaatcaatatataggtccaaaacgaccaattcctagtgcacctttcttttgctttgaataga
aaaaagttctccttccaaaaccttttttaagcctttcttcaagagcagagtgaattaaatcaatgggggtttgtatacaatcgttgcccaagcttgcg
aaaatttaagtcaaaagtatcgaaaaataaaaagatgaattaaaagatattgaaaccttacaataagcactacaatttctccagtttatgctagg
attcagtcctgttcgaaaggcttgcccttcacacgcaacttgaaaacaacaagtgttctcgcttttcgtgatcctaaaaatgctttggctagaccaat
atcgccctaaaacactttcatctttagactatcatcatcagttatccgagcgactaatttctctggcagctacaaatgaattcccacatttattaattt
acgggccaagtggagcaggaaagaagacgagaacagttgctattttacgagagctgtacggccctggcgctgaaaaagtaagttcattcaggct
tgttgcaatacctaatattgtgctaacacttggatctagttaaaaatcgatcaacgaacgtttctcaccccaagtaaccgtaaaatccaaatcaat
atagtttcaagtttgcaccacatagagattacgcctagtgatgttggaaattatgatcgagtggttatgcaggagttgttaaaggacattgcccagt
ctgcacaggttgatttgcaagctagaaagaagtttaaaggtaaacgaatcattacagagtcttataatggtaagctaacataagacagtggtggt
tatcaacgtcgccgatgaattaacaagagatgctcaagctgctcttcgtcggactatggagaaatattcaaacaatattaggctaatacttattgct
aactcaacatctaaaatcattgaacccattcgctctcgtacattaatggtccgtatcgcagcgccttctgagtcggacattatgaaagtcatgtccc
aaatcctagccgttcaagaattagaagcaccgacttctcttctcgaaaagattgctaataactgcagtcgtaatctcagaaaagctattttattatt
agaaactgttcatgcaaagtcacctcataatcgacagttgacagataatgggtctcagctaccgttaccggattggcaaacttttatcattcaagt
gggaacttctatgcttcaggaacaatcgcctgctcgaattttggtagttcgttctatgctatacgatcttctctctcattgtattccaccaaccatcatt
ttcaaggagctccttggctttcttttactaaaggtggatgctaaattgcatccccttcttattcagtctgttgcaaattacgaacaccgagctcaaatg
ggaaataagtcgatctttcacttggaggcatttgttgcttactttatgaaaatttataccatgtaagtgtttgttggttcaaagctgattaaaggctgg
taaaagaaagatgagatgaatactaacatagaacaggtcgcttctgggaatggagcttccttatccataaaggggttccttttttgctttgtatatat
ctattatagttactataaaataattaggaacaaaatgtcaacgccagtcattgttttgaacttgaagccctggtgatttttttctattttacattttcaat
ttcgaaaagtatacttaacatgtgttcatgatagcacttttctttttgaatgattttacctaaaaaggcaccatctatttatatcactattgtcctgtca
atactatctgaatgtaatacagctagcgtttgaataaaacacgtaatgaatgcggataacaacatgctcaaatgaaatgaatcaaatcaaacag
aaaagtgaatattagattcaaccaactttcagtaagaaaccgactcctttgaaaggagggaaatggaattaatttggtatgaatgcggcaatatg
cccaagaaaaggaatgaaaaggccacggaaaaaacacaaactgtttaaaaagcttactcttgattgaaaaaagagcggatactgtcagctaaa
aagaccataacgacagtttggtaagcaccaaggcccattctaggaataacacccctatacaagcccttaattccgttgtttttgtaaatggagctg
acagtagagatgatacctcgagaggtattttgaggaccattgcttaagctctgcatttctacacgagccacttcgatgggctggttccaacaactta
gcgctccgcctgcaatgctagcaaataatcgctgtcccgaggttaaaggatcagaatctctgcttcctgacaaataacggataggtcgttcgaaa
aagcgagaaagagcaaagcgacttccccagttcgtcatttgacgaagggcaacggcattcactcctcgattaacaccacggatgcccttttccttg
tacaagtcggtaaagacgcggaatgtactagggacatttactccgctggcagcttgctttgcacgagtcacctcagcggttttcatgcaagtacaa
agacccattgtagcataagcttgggcaataccaccacccatgcctgctaaggcaccggctacagttgacgagaacccagcacaacgtccccaat
actccaactcagcggaggtgaaaagaagcacggctcccttagtagaagcctcgatccaagcccaaggtattaaaccctgataaaatccgaagat
accaccgcgagaaagaatggatttcaggacttgctgcatgctttgggtacggttggctgccatttggacctgctttgttaaccaatgaaacttgaac
agcaagagaaaccacgaaaagattagaagaaaaaaaaaagaaagaaaatagctgatcacttcaacaaccttgaatatctctttcgcaagtcat
tgaaagccacttggagggaactcgggaagaaagcaaatcgacaaaacgtgatctggttcggtatagtccagtatgttccgataatgtttggtttct
tttcctttctttttccaagtcttgtttgcgaacactctaaatccaacacttaccttggtaacttccaacggatgacccaaagaagaggctctaaaaac
ttgtttagcaaaaagttccaattcaaaaatacaccagggaatccttccaaaacctaacgaaaaaaccaaagaaaaccaaaacaataagaatgt
atccttcacataataccatccttcactaaacaaacgatattcaaaacgatatttaaaacatactcaaacaagtttaggcctgcgcctaagagcaa
attcgcagcagaagtttgtttctttttttatttcactcatatttgcataatgagcagaaacacagctcaattcaattgacgatttgtataatcaactaaa
cgcgtctacttctacttagactaagaaaacgtctattaccaacgtaaatgacccttaattgtcgcttttcatagaaaacctctatcctagaggaata

gttgagttgggttgtttctaggttcttacaaagccatatatatatatataaatatgtgtgtgtaaatatatataaggcgaacgagaaataaagttca
tacgataacggtctgataataaccttcttgtaaccaataacgttattgtagctaatataaagccaagagattgtaagagcaattacaactgtattcc
acttacactcttattactatacggagtatttcccgtgacagatataaatacttatgtaaacaaagaatagtatcaatagagacaatcaaatagtgt
aaacgagactctggaaggctctttttcaaagcgattggcaaaagaaatattaagggtaaacggagctaatgtagatcggatcaaacgggttcttt
tgattgttcattgtttttatctttaaatgaaaaaagaaaaaaaggaaaaagaggaacattcatctcaaatgtggttaaattattattggtaaagcta
ttgctactaggtggctacgctgttatttacatacacttaaaacgaaagtcatggatgaatatgagttggatgatgaaggttgcaaattaataaattg
ggatttctcttttctataatgctaaaatttgacataaaatcttcgtcgatacctagagcttcttccatcgattcactgaaggtgatttcattaaaagtt
ggaaagcgacggacttgctttcgaggaaatcgaatcgagacacaatgttatagacgaagaaagaaaagagagtcatcttaaagaaagacgca
gcaaagaaaacacaactaacaaagatggaaacgggaacatgaaacaaataaataaaaaaccaaagcagttctgaagagaagcaaaagtaa
atgaattaatagaaaaaaaaaaaaagatcatttctgctcctttctctcttcttttttttaataattttttcttttttcttttttttattttcctttctttctacacgc
tctttcgatgtatgatcattgaacagaccttcttctccatagcgttactgggtttgaacaaagacccagaaagaaaaataaaacacattcagtaca
aagacagttcaaatcgtatcactttttctttttggctccacggagctagttattcatgaataactttttttgttttgttatacacagatacagtgtacatc
tattggtgaaatggtcaaagtagatggggttcacacttcttatccattctaatgcatgcaaatgttaaattttcagagcaggtaatgactctgtggtc
caaggacgctccggacgaggagctttaaatacacctgattgaggtgaatagctaggtaacttggtaatatgcgagtcgtcttgatgagaggaatt
ttcacatcgactaagctccatagggaaattacgggaggagaattggatgggtgcggaattaggttccttggagaatacagaggtaggagtcaaa
ggagaattggaaatcagagggacgacactgtcgaggcgagggaacctgtgtgacaaattcttttccgacaaaggagagcttgtaccactgaag
acgtcagtagaaactctgctagagggttgagactccaagtcaagtgcctttttcttcgaacacttttgctttttttagtatcttgaggcgatacctttttg
cttcgatcatacaaatacagaccgaccgctgtcaaagcgatacccatggcttgagtaaagctcgtgggctgccgaaaccacaaaatagaaacga
caattacaaaaatacgcttaataagactggcgatggagtaggcaaccggtgaaatgatacttaaaagcgtaaaggcaagaatgttttggaaaa
aatgggacaatccgttatgaatgatattgaagaagataggcgatccaacaactggaaggtactcgaatccttcttgatgaagccaaaccggaat
catcacgataaatgccataccggatgagtagagcaaaagattcagtttgttgtagtggctctttctagaaagattgccatgctttttttgcttccgtga
agattttcttaccaaaaatattttgagaaacaaaaataaaagtagaaatcagagcgtaaaacaatccgcacaagtctacagaaagctcaaagg
aacaggccagagttacgcctagagtaagcggaacaagagaaaaataggccgttaaggaatatgcatgctgaaagagaaatcgatatgccaaa
actgtgaaaagaggagaaagagccttgacagtatgcaccgtagaaactggaatctttgttgttgctaaactgccaaagacgtgtcctccaatctg
aaaaacgctcaatggtagcgttgtcgagagtactaattttgaaggcctttgaattccattgccattgccaaaaaaattgccgaatgaggagacaaa
ctgcgctaaaaaaagcaacgaaaccgaattgcagaaaagttaatgtgacaggacatgaaacattgttgaaaatgctttttgaacttgtatttcta
gcccacgttagtaacaactctagtcaaacttcaatctcccgtcttcaaccagctgtcgctccaaaacatacgttaccgcactacacagataccata
gcagacagaggaatacgaatccaatcttttcatggaataaaacctgttcgactttctcctttaaagacatagatccatacacttcataaatggtatg
acggattcaggagtttttagaagcaaaagagcttatttaacaatatattaatggaaagaaaaaaactcttctgcaaaaatttcacttgctcttgctgc
aatgaacaatcttggcttttggatagttgcgagtggctgatgtcaacaaatagtgagacacgcagtgatgctcaataaatccagtcaaaaattgta
gaaaaataaaaataaaaataaatcgtcgttggaattcaattcaggttttaaatggaagcataaagtctgttttggtagtttttctccatgctggattt
tcaactattgtaaacgttacttaagattatcaactggttgcttagttgcatatgtttattggtcattgtgattttataaccatgtcatgaaagagtcatc
aaatattcattaacagttccattcgtttgtgaatatttgatatcacgcatgttttcatctatagactccagttcagttcccatgctcattgcagaaaat
caaaaaaaaaataaatgcgacagcatgcatcgtctaaccattgtaaacatcctgagttgtagccctagaacagtttatgcgaggcgtgtttgtttg
tttttttagacaactttttcatttttctttcaagaatacagtaaataaaagctgataaaggaaatccaataatttaaaaatggtaaaacgaagcgat
taaatcaacaagaaaagtcacattcataaaagaaagcaaaaaccgaaaaagaagctcgtcaatgatgatccttgtaaacaatgaaacgcttag
taatttcattagagtagcagagcagtactaacttttgaagccacaaaaggaaaaccactttttttgaaggttattccaaatgaacgagaaacttttc
tctacatctacaattacgttgtcttcttggtcacttctgattcctgctgctgctgagtaacctccatggatcgagcatgctcgctcaatttgggaagga
caaaaaccataacgacggatgctaatgacaacaacatcattgggcttttcagtagacgaatcaaagagaattttctgggttcctgtaagtattcat
gcttctgcaccgcgcggacagtgatggggtgcgatatacttttcatggcggacgtagagggtctctcttctccagtcgtcgtgtaataaggataaa
cccgagacccgtttacgatgatatgaaattgagcaaagtcatattcaatggtttcaaagcgcaagagatatgttccattctccacgttcgtaaagc
tgaattgtccattccgttgaactgggtaatctctgataagctccttagaaattagttttgcatgcgtatcatacggcagatcattcaagcttctcata
accagattaggaacaatttttccctgcacttctgcttgcatcgacaattgaataaagcaaacgaagcataacgccaatgcaagaaaaagcctcat
cttttaaatttaagtaattatttatgttatgacaatccagaaagtcgatgtaccgtcaagattctatcgcgttttgaacctaagtaaaatacaactta
aaccaagcaatctattgattatttaattccatacaatgcacgtaaattttcaaaataaaaacctacttctagtttaaagaggatgcaaacagtaaa
tctcaatgtcttcttccataggactacttacacctgcaatgcagccaattctaattgcaacttgcaacgtaattcggtatttcccacagaatgctaag
acgcgagaaatggctctgtgtttggtgattcattctgaataggcattttagggcttttaaatttcatccttctcgcagttctaaggtttttttgatgagtc
gtacgtacgaaaggctaaacgcccctagcgaaaatgaagacgatgtatatctggagaacgatgtcagcaacattggaagaaaacgatataaaa

caaaagaagaaggtacgtttatgaatctggttctataagtcattgaacaatcattttaacggtaatcaaaaggatactatgattcggactcagaag
aagaggtagcgatgaagaagacaaaaaacgatgatgatgcctctgacatgttctcagagtcatctggtgatgaagaaaagggaaatgctggta
aaagaactgaggaggaggacccttcggaacgacttgctcaaaagggtttgtcttctcgcaatgaactagacgacgagtttgaagaggacgacgc
agaagccgaagagaagttaagttatgaggatatcgaaggtcaggaagaagcgccagatgcacaagattctggctcttcagaagaagaaatgac
ttcgggcaacccaaaggtcataccgtttaatttaaaagaggatatggaagaaggtgattttgatgaaaatgggaattttgtacggaaatcaaatg
accctagcgatcagtatgatgcttggctaaacggcgatgtgtgttccaaaagaagcattgccgctgcaaagagagcggaacttacacgaaaaca
acaagctcatcaaaagatgctttctgacatagaggaatcgaaacggcttccttttcaaactgttccagaggcgctatcatttcttattatacgaatg
gaaccagacgaaaatgtcttgagttttctacagagacaaagcgtaagtaattctgcagcatctcgttcaaaaaaacataaaaaaccttcggaac
atttttcaccagaacgtcggtctgccgatgaatatcgcaaaagactgattgaaataataacgggttgtatcacgttcctagaagatagaacaaaa
tttgaaagcttgtattcagaaacaaaagaggccttgcaaaggctttttcagcgaatcacccatgaagcttggaggccaccggctgttgatatgga
aaaacctttgttttactttaagtgggaaggcgatgaaaaggtatatggaccttattctggctctcaaatgcaggcttgggatcatctaggatacttc
acaaatgctaaacaagcagctttcgtttccacgtccgcagacggcaactggatgtttccgcaaaacgtttcctacttgagtgaaaactcaacctga
aatttttatttttatttttgtttcatgactagtcttctataacacttcagctatgtccctacaaaacgcattttgttttcatgaattcattttgcaacaatg
agttgatacagattccctattctattaaatacatttttcatcattctccaatcttctaagtataaagtatatgaaagaaaattcaaaaaaaaagaaa
cattaacggatttagtcttcttgcatggccgcgacatcttcagaagcaggggcaggccattcattttcagcaacataattttttcatactcttccaa
atacttaacgcttcgtttttgatctaagaaaagttcgtaaacacgcttaatatctaactcctcgatggtagcacttttgcgcttgagagcaacttgat
tagatgaggagatgagaagtaatgcatatcgtagactagcttcacgtccaatagatgctaaataatccaaagcttcaggtgccatgtcaacgtctt
cctcttggcaacgaatccgcaaaatttcctttacttcctcatgagagtaaggttgggtcgaaataattagcattctgtctaggtaatcgacaggaat
accatgaggagaacggtagttagtgccacggatccgggtaattcctcgatttgaggccatgataacaataggagcaagttcatcttccaaagcac
ggttaaaaaatgagaagcattcaatatcaagcatatgaacttcatctacaaacaaaacgccaggagaaatttcggcttttccttcttctctccattc
gctaactttggtgttaatttgttcacggacctctggtttaatctcaccggtatcaccagcgaacaaagccaaaaatccttgagttcgagagttaatg
acatcaatatcatgcagggaaacagtatgaactatttcctttctcttttggatttcgccttgagggcattgaacaaaacgagtgtccgctcccatag
cgtcgtagtcgcgagcccttgtgaatgaacgacctaatttagtgactttacccatagacttgtcaatcgttacaacatcaccagccaaaaccttgtc
ttttgtcaaagcatcaatcatttttgtacccaaatcatataccgtttccatatccgtagtacggattgtaagtttaccttgcttatttccaccagttatc
gagcgatcaatttgaatctctacgacttcaccttcaattatctccgtttcttctttgatgcgaacgccaatacttttccgtagtgcttgcagtaaagctt
cagtctttgacatttccaaggagtaaacttcactggcagaaagagagacgaacggagtgtctgaaccaagagtctgagacatagccattgcgat
agctgtcttaccggttgatggtggtccagccattagtatagagcgacctgcaattttaccttcttgaatcattcgaagaataacacccgcagcccga
cgagccttcacctgacccaccattccatcactcgattctttgggctccagattatcatttaggccaattcctttaatatgagaatgagctcctactac
aaacgaaaggacgttagcattcaacctctttacttgtgcgtttactggttcaaattacaaccatattcatgttcatctttataattgacatgtcactta
catctctccaatttagaagcttcattttgaggactaacagaaatcgacattgctactgttgtttttggtttttttgcgaactgcctgcgttcaactaaat
taataaaaactatataccgaggttcaacgtggccctggacattatttacgcgtacgctgtaatctttcatttaattaaaagcattttatttttataaat
aagtcagctggattacttggataaataataattttggaaaatataggaaatgatataattgaaaaaataggcagactactttttttattttcaacaa
aaaacaaaaattctcgaagaaaaccaagagaaaaagatgaaattcaaacgagcgacttaatttttaatatacagtataaagtacaaaagatct
ataatagatgtcagcaccttaaattggattattccaatgaagggaaaagagaaaagtcattgacagcctccaattcttcgttcacacataattcat
acgtttcaagggaaacagaaaagtcttctggtgctcttcttgttaaattggaaacagcgtcttgcgtatcctgttctaatccagactgcgaatgaga
ccttcgaaggtcttcgagcctatctaatcgatcttccaaatcatcagtgacctccagaagttctgagcgattttctaatgacacatcttcagtgttttg
ctctatctcgtcagaaacaggagtaccttcatgcattgctggttcagacgtaggaacatttccgtctacaatgccatctggtttggaaggtgaacgt
gtcgccatgtcggaaggaacactctcgttggaaagtttcgtaccgttcacctttgatgactctgaatttgttgattggtctagggcaggtttcgttga
aggaatgaagcgtattggatttccagggagtagctcgggtaggacaaaacggtctaaagaagggagttcatcaaaatcattccaattcactgga
aaaccatcatcataaataatattgggaatttttaaaggtggattatagtaagacggactccgccggccagcggcagaggatgaagttaaaatac
attcataaggaacctctatcgtgtcagagctaccacactcgagtaaaagcatgcgctcacttgcacggtcttcaagaatacgaccccaaaaaaca
tcacctcgagcaaattgagcaggtactggaagccctaggatcgggggtgggaataacggatgattagaagcatgaaggtacactgactcaaatt
gggaaaactcaacattagaattggtcatctcctgagatttttaataaactcggaacggaacttagaattccataaggtaaataaaggcaaggat
gtagaattggtgtcattgtcagtaacgggggacggggacgaagacgaggacgagatggaattaattgctgtggaatcaagttttctatcgttatg
aatagtttcacgcactccattccattttggggttttattagactgtaaatcatcttgtttaaaagggttatataagggtaacgagcctttttcagtcgtt
tcttgagggtcagcgtgagaaacgtcttgacaagacgctttcttgggtttatgtacgtgtggccagttacttttagaagaaaaagccgactccaca
attccaagtgaccatatatcttcataagaagtaagagaatcatgagcatagaacacaaatcgccgtaaagcagtatcaccaagacagaaagca
ctatggtaagagccaggataagtaattagcatttcatttggacgaagcacaattgaagagatattaatcccgctttgggcaagggtagagggagg

aaaaagaatgttttgatgcaataaaaaatccgcacatcgccaatgctcatcaggtgcaagtttttctgcaagtttctgaaacgctgcggcttgaga
agatggaatcaaataccactgaagaggcgcaccacctaaatgaacatgaacggcatataattttttgattcaatccgccaaggtaacgttgtttt
ccatttcccagcaaaagttctaccagaggtcggccatgtattttcaggaacactcgaccgtaaatgaactctactttctaaagaacggcgagaag
gaatgccaaaaatcatagggtcattatgatgagaataaccgtaccatagattatcgtgtgttaaagatcgccaataataagcaatagccttagat
atgttgttttcaatctttggtggctctaaaatagtagacggagatttggaattagactgtgtgcttgtatctgatacaagttcaatttcaaagtactct
ccttttcgacgaacgcttctttcaacccaaacgtctattttttttatctgtagaaatcccatcggaagattcttcccaaggttttgaaacggattctggtt
ttaacactttaatcgctccataacgctctcctagatcgcgaacctcttgaaaaaatgcccccatatccgaaaattcttccgcagtctatgatttttct
cgttagcaaattaaacgatgccttctttataaaaagttttcatatacataccggcttaacgattgggattccatcagcgccctcgacaacatattcg
aatttgcacgaaacgcgttctccagcttgatcacttgtctcgatcggtttgcttttatcattctcttgcataacaatccgaaattaaccgactgatcgc
aagttctattttcctcttacatccactaaatggtggcttttgttgaaatttcctttgcttactaccaactatgtcatggaacacagtaaaccacgcaat
cgacttttctgaatcataccaatgtgttaatgctattaacgagcctcgcaaaagcgcataagtcaacaaaaggtatgcaaaaaagagaagaaag
ggaaacggtttgttccgaagttatgaagagttgagctcttatttgatgaaagcgtctttgctgtggtctatgacaagaacttgtttgttatcgaactg
catcgctcctggtgaacaagcttcagtcttggcataacattcattgtacattctctttcattttataagttattagtaaagaaatgatttctttaaataa
tgataaacttcaatttctgcgttgatggaaggactttgtcttgcctgggatttctgctcaacatctgcaatcaactaccatccttggcagaactttgta
aaacactcgcttccttaattttggcgacgaatctcaataacttgagctgttttttttttgctaatttcacctaattaaaaactcttgttttaccagagg
acctttttctttttcctcaataagtcatcccaaatttcatgttgtaattttgcgtaaagctgctacctacaacactgattcgggtccttggtgaataat
ttctcagttcatttgtgttcaaaaaggtttttcgaatatttctaatcacctttctttaagatgtccttcctttccaggtttgcttggcgcgcttggaagcc
acaattcattcctatgcttggagctatgagattccattggaatcgtaatctaccaatgaaccacgtccatatgctatcttcttccccttttgcatctctt
aattctcgacttcctctcattccttcccaaggcttcaaaacaaaggcttcagttaaaaaacgttgttccagctgttatttagtgcgcagaaaaggcc
gcttatatatcctttgcaaaaagcatcccagacataaaactagacaaggataaacttattgggaatcatttacgtttgggttaccctccgtttccta
cgaaacatattcgcaaagctaagccttaaatcgataatttctttaactttcttattttttttaggatttatattacttctgcataccctccatgttcttc
agatagtgatagttattaactatattcctcattatcatttttcgtatcaactacaactataaataatgattctattctttaatttgcatataattttctcg
ttataatttactcattagaattgagctcagccgtaaatctttcgccgagtttttcattctcctctatcacaccctgattcttcttaatccgagctaaaag
gtaacgagctctttctttcatgccagattcgaagttaccaaggcccttcaatagctgcaattcttcttcatcagtccgaatagctgagttgcaaaggc
gaataatggcattgcatctagcttgactcatagctgctcgtaagatagagtcatttactagctgttgaaacgccgtaacatacacgggcttaccgg
caaataccttgtcaggtctggcattcacacttgtgatcgtaattaattcatcttcttccaaagcacgaactgcatcatcatgacccttcaataaagg
atcaagcatcaatttatgataaggaatgtgttcgtgctgcgacaataaagttattaaaatccacgcttgttccgaagtatatagcggtttcttatcgt
caccgaaagataatgaattagatcttaagaaactctgaagaatgtctgaagtgttttgactaatgattccaccaatcgcatcagacacgcttgaat
tgttcaccctcaagcgtctagcaacataatctaggtcacgaagtcgaccaccaattttaggtagttgtttcttaagctctttctcaagcctttctgttc
gtatttctttcggtattgaatccaaaacataacgttgagcaagtaagggatcagcatcgcctaacagtaaagtacggttttcccacccgccaacat
actgctctaaagttttagtacctcctacatctggtgtcaacattagaacatgcacaactccctctttgactaaatttccagcccattcagcaaccatg
tgttgcatcgggttgtttaactttcgtagttgaaagttatcaaggacaactaccggcaatttatctgcatgaacctctaagtattctgattcatcaag
aacattactatgttcggaatctttatgttcgtctaaagccaacttacgaagcacggtttgcgtagtgctgaggatctgtcgaacctgaccttctgctg
acgaaacaattcctgtttttggccgataagaccctgagctgccatatcaatcatagaagataaattattcaagaaactaaataacggaaaatat
cctgtctgacttgccaaggaactcacaaacattccctcattcgcaccggaaaataatctatcgcaatcaaatactaaaacatgttttcgatttccaa
ccgctcgatctacgaggtcacgcttaccacttcctcgaggaccttggaccacgatgaacgaatgcagtgcctcgtctagccactcttctagcttctg
acaatccttttctcttgtagtccatataggagttatttctaaatcggaatcttggaagggtttaaaaacgatgtcacgggttttttttatccaactgaa
aagttttatgttacgaagtgtttgtccatgtacaatatttgtctcaacaaaaaatctccgcattgggtcgaataaggaagcagtaatgactgttatc
aaagcagcaattaatggaattgcaaatcgcgggtgagaaaccaaccagtcttttatggataaaaatcgattggtgtgtcggtatcgcatatgaaa
cttcgtatcgttcattcggaacccatgcatacaatttaaagctgaagtagcggctctcaacgaggtaaacttcaggatagcaccctctggagatac
aacttccacagaacgcaattttccgtattttcggaaaatactgtaaagcttttcctgtgataagctgggaccctcaaagttcagttcgattgttcgag
caggaaaggcgtacaaatcctgtaaccaaggtcgtccataaacaatatgtggagtaggtctagagaaaggatgtaagagcgatgtgacagggc
gttctttaaatgcatcacgaattatagcatacatttttttcggtatcctcatcagtctctgcataacgaacctttaagaaagcacctccatcttgccag
cgaaaaataacgtcttctagatgaaaagagggcgaagacggaaatgcacgttgaatttggttacgtagattcttctggaataaaaatgcagcat
atcgttgaatattaagaaacgtgggagtgtttactaagttatccaaaaagatgaggctttcgtttttacctaaagggatatggccaatgtcatgggc
aatggaagagtgtgcggctttacgaaaaagaaaaggtgatacaactgtccgaaagcgattcccatttttttaagaaggaagagaagagcaatctc
atacttgaatctccaaatgcgtactaaacatgccagtgtataaactctctaaaaataagtaaagaaattaaataaataatttcaaaattaaatatc
tctgaagaaaacccgtcaacttggaattttacaagatgcaaagcaaaatcgagatgatgtatgcccaaaacatcgtgcctttaagaatggagttt

cgcggtagattgcgctgcatttcggtaaatccaaaccattcaagatttgcaaataaaaggataaaaaaagaaaaagaaaaaaggaaatgaagc
ttaaatataggacctttgtctccgcagttgcaaccttgaagcgtacgaaagggctcatttgtcgaagcataccaaaccattgtcaagcatatttgtt
cgtagccgtatcgttcatagacattgttcttcatgaacattttaggaagcatcatatagacaccaagaaaggattctgagtgattctatccttggaa
atcaaattcactcatatcttcgtaaaattcagaggcatccccatttgtgatgtctgcatctaaatccacatcttgcgtttcttgctcatttggctgagg
aaagtcatcgctcctcagctcttcctcgtcaatctctgtgtccatgttgcctgtttcagcctctcgctctccgagttctgcttgttcctgcattgttttatt
aatccctataggccgtatccattgaaagccatgttgcttaattgaaagttccttttatatctagtcagcgccaagcgtcataaagaatcgtaacatg
taaacaaacctacaaacagacatacctggttttcaagctccaattcatctttttctagttctttcatggacatttcaaaaggactataccacaattca
tgagcagtagaagccattaaagacataaacgacattgtttactttgctggctgcagttgtatttgtgttctgagtataccatggcaaggacatatgt
ttgttgctcgggcaacgccatcaaaggagacatggcatgtaaatagcgaaatgtaaacaagaaatgtaaacagaagcgacttttccaacgagtt
atatgactgatctcaaggaaaacgtacacataattgaatccaattctacatgatcctgttttcgttgaacaccgttgaacgaaaagctacgctatat
cacttggtcagcaaggcttttactagaacaaatgaataaaaacgttactaagctttcttcatcaatataacacttagcgcaaaacattattatttct
aattaaaaaataccaaagtgtttagccgagttttacttctgcagcggtttttcttttatacactaattgtatttgtttacttctatatcgaatttgtata
acgatgtttccttgtgagttcttattattgtgctgttcatgtttaccgaattttcatttctcttgagtatgcttctttacgaagtgtactgcgcaacccaa
agcttaacctaacctaatactcggaagcagtcacactggaatgtcaaacaagtgaaacatacatggcccaggatagaatctgtcaacagcacaa
tgaccaagcgtactaaaaaggtcggtgtcactggcaagtacggtgttcgctacggtgcatctttgcgtcgtgatgtccgtaagatcgaagttcaac
aacatgctcgctatcaatgccctttctgcggacgcaacactgtaagacgcagtgctgctggtatgtaaaactggtgatggctgtagaatttggattt
cttttgttcagattttctgccatggcttagatatctattcaacatttaaggtagtgaagactaacgcaaattttaggcatctggaactgcaacgccaa
gggctgtggtaaggctatggccggtggtgcttggactgttactactcctgctgctactagcgcccgctctaccattcgcagattgcgtgaaatggtt
gaagtttaaatgccaaaagtgttttattgtttttatctattgtgagctaagtaaaatatttatcgcctagaagatggttagtaggtgaaatgaaacat
cgtaacgtatatacttaagctttagtagatacaaaagtagctcaagcttacaaacatacctcatttgcatcgtaattaacagcggtcaaagcaata
aggcttttatctttgggaaagcccatcgatagtagctcttgtagtatttcagactttctctcgtcgtgaacttcatcgctagctgtttgttgagtccgtc
cactgtggctttcaacaacttgagaaggcgtatgactcacagacgacctcgaggaacttttaggaaggggatgagaagtaaaggatgattgacc
tctttcggtacgaacctcttcaggacgtaaaactggagaattaatcccgcttgtagcattcaagcttgctgaatctgcaagaacgtcgtctattcttc
ttcttagatgttgaagttccttgataacctgtctcttatccatagtaagttctccaacttgagcactaagctgttgattcgccattaagtttcgagaaa
ttgattgttgggtggcttctttcagttgagatatatcattcaattcattacgaagacgctcaacatgactttttgctgttgaacactagattcaattct
ttcatagctttgctgaactgtttccaaagtgaacttggaagaactgatttgctgctgcaaattttcaatttctgcagggtcaactgtagcttcatcag
gtttctcttttacagttggttcttccggcttagccataggcatggatggtaaattcatagactcttcactttcttggtgcggcatagacggagaagag
ggttgattttcacgttgaagttcttgtacccaatttgcatggggtggttgaaagtttcctttttcagagtcgttatccaatacttctttcgtaggctcatt
tatcaagctcttatctccaccgggaactaagttttcaattgtttgctcaagagtcggttgaggagccatgttttccaactcatttatttgattattattt
gagcgaaaagactcggtaggttcacgagtgatgttggaaaagtgttcttcttcatctgtaggaacgttagtattttcaattggctcagaagaaattg
ggggataatcgggaagttgactagagtcagaagctttctctttgcttgaaagtgcactctgttccatagaacttgatgagattatgctgtccagagg
ctgacccgaaagtcttagttttatgatttgcatagcgacaacaaattcatttttcataataaatcctttatcgtctttgtcagcaagttcccagattct
ggctagttcatcgggatcaagtcgagatgccataaaaaaaggcacagcctcttctccagtaataaatcctttattttctgaatcaattttcttaaaa
agtacgcaaaaattttcacgatcttctgggtgtatagatttactaacaaaatctaatgaaagagatttagcttctgcgctggaagaaatagaagga
atacttgaagaatttctaccgtgctttgtttcctgattcgatgagaagacagcattactttgattggaaggttccagtttagtggaggaattggcatc
gctcttcgccgagtcaattacataagaaggaagcgaccgaggtaaatgaacaaggttatactttttgcaaagcataatcagatgcttagcaataa
taaattgctcgactctaagataaccaacgtgcccggaatctgctagattccaaatctgagccaacacactagtagaaagtccagattcaagaaaa
agcggtttagcaatagaactaggcataagggttgggtcttctaaatcttcaatcttatccatttgattatagaactcttttcaaattcggatcgtag
atgcgggtccacggtttggcggaatgggctaaggtccgttgtgtctgaagtttgaaggtttgatacctcagtcccatcagcaaatgaaggaggagt
tccagctttttcgaaggttagcaattcattttaaaatagcttcttacctttttgatacgtccaattcctgcaaaggtacacccttttgagcctgcgctac
gtagcgaaggcaagcgtaagcttgggttcgagtgaagtagcctttatcttcttcatcgcaaaagtcccagatctagaaaagtatgttagtttaggg
catcttattcttggtcatcattcatctgtacatactttggctaatgtttttgtggaaggccaaattttaagaggaaagggctgacaattgatcctgga
aagcaattggggtcctctactggtaaagtctgaagcatatcatcaaatgcttcttgctctgtgagcgtcagctgtataggaggtaccatgatttaag
tacacaatacgggaaaagaaaaaaaagaaagattttttaaagaagaattcgtaataagttgcgaaaaagtgtatgatatttaacacgaggga
aacgttggacaactttggtttgtttactaagatattccttcaaaaggctgaaatatagatcagctaaatcgtaattgtaagaagcaattcttgaata
atagaaaccgggctacaaaagacgtaaattgaaatcaactataaggaatttctttgattgaagagtttcggcgccaagtcaaaacacaatgtaa
aatctttcgtgtaataagcaagaaaaaagaataaaatcccaaaaccttacactttttttcaatgatttaaacaacttcaactgcgtagtggttaga
acctatgcatacgtcaagaatacaagactccgtcaagtcgtaaggtatgagaagcgaagatgagggatcctaaaagaatttataaataaaagta

aagaggtaataaattaatacaatgcaagtgttctaaatagtaaaataaagtcgttcaagaaggactgtaattaagacttcatgagaaacactgat
acgaagaaaccaggactcttgtcgttggtatgaacaggtagcagagtcctttttgtcttcgttcatcttgacttatttctttttgtggaatctttgaggt
ctttacattgctttcaaagctggaatgcgaaacttgtaggaacagaagctattccatgatattcatcacttactaaagatataattatttttagtata
atgatttatcttgtatcacctttccaattgagaaacgctattcgaaggaaaagaagtagaaatgtgctgctttccaaaagtcatgtcctttcaagctt
ttaaataaaatgaaggatacaattcctcttacaataggtttaggaaagatatgttgctgtcttaatggactttttataaattcgttggaattcccaac
tggcactcattttcactaccgcgtctctctcttttatagaccgagtcttcaaagtattgattttatagctcaacaataatgcgtcgtagtggaaataaa
gagtctactgttgtatgtagacttcttgtagctaaggaagaacttgtgcattaactatttgactaggtttactctgctctttctttggcacaatcagga
cgtggttctgaagctattgctctgttagaaccattaaagacgactgttattagtagcctgaatttgattgatattatacaggctgtgtatgaggatca
aaaacgaggtttgttgaatattcgaaaagaggtacttcttggtatcaatatgctatgatcttttgcaacgaagacaattctgattcttaagatatttt
ttcagagatcatgctaaactagcgaaaagaaaggacttgccttgttgttttaattgaaagcagaaaactcgagaaatacctctagaaggaaatac
ctgaaagttattcttgctaacaaaaagcattacaggcgaggaatcatttcaattttgggaaaaatttttgcaagctcacggtaaaaatgaaaagc
acctatcagcttattttaaagccgcagtgagaacgaagagtcttattcatcagagaaaggtagggtctcatttatttacttgctacaagacactaat
aaattattataaggccgctgtggaattacagaaactctttccttcgcgtaagcacactctatgggtcctttcatctctgtatttgttatgcaaacgctc
agaaaatgagatggagcagcgtttgttaaaagcacttgccgaaaaaacagcaagcattactttctccgcaccttccggacacattgattcttgtga
agaatgtcatctttatttggacatactttcattggtcgggaaaaaaagcgatgctgttgaactactcttgaacgaagaaacccaaaaatttgtaga
agctgatgccgacttgcttcttcgaaagctccagcttcttgccgactgcgaaagctgggacaccttattctccgttgcttccacatatttnnnnnnn
nnnnnnnnnnnnnnnnnnnnnnnnnnnnnnnnnnnnnnnnnnnnnnnnnnnnnnnnnnnnnnnnnnnnnnnnnnnnnnnnnnnnnnnnnnnnn
nnnnnnnnnnnnnnnccgcagtgagaacgaagagtcttattcatcagagaaaggtagggtctcatttatttacttgctacaagacactaata
aattattataaggccgctgtggaattacagaaactctttccttcgcgtaagcacactctatgggtcctttcatctctgtatttgttatgcaaacgctca
gaaaatgagatggagcagcgtttgttaaaagcacttgccgaaaaaacagcaagcattactttctccgcaccttccggacacattgattcttgtgaa
gaatgtcatctttatttggacatactttcattggtcgggaaaaaaagcgatgctgttgaactactcttgaacgaagaaacccaaaaatttgtagaa
gctgatgccgacttgcttcttcgaaagctccagcttcttgccgactgcgaaagctgggacaccttattctccgttgcttccacatatttccaaaatgg
aaatattgactggaagatttgtaaggctcttctagaatgctcattacatgacagccaaaagtaagtttagctatgtttctaatacaaaactaactac
gattacagactcaaacctgtccaggagctttctcacaatgctttgtctacttttcactctcaaagaaacctccatcttctttggatccattttacatctc
aatttttaaaaaatgaacatgaaaatgctattttgaactatgtgctgaaaatgtgcggtaagcctatagtttttgaagacatagtacccttccttca
aaaattaagcaatgaatccaaagcacaattgctagagaactttaccattgaaagctgtcccgaaactaataaggtaagtaactaattttgtatat
gcaggttagctctaactaattcttagtctcaaaaaatcgacaagctagttgctaaggttctgcgtcttaaaattcagtttttctgctttgagtcttttac
tcaagaaaacataaccgactttttgcaaaattgcttcagtgtttaccaagaaggcttgtctttgagtgataatatattgcctaccgattttactcacg
gatatgaggctttactattggctgttcatgcgctcatgtatttcaaggagtatacatctttgtcaagtgaagtaagtcaagggcttatttttgatgcta
tctgtttgttagagaaaggtctcactagcaaccttcataacttcaatttgaagcttcctttgattcgtttgtatttgctcctggacggtggatttcctgc
tgcttgcaagatttatgacactatgtctattaaacaagtccaaaatgatactatggatcactatctcttgacgagagcaaccacacacttcccttcg
accttggctgcccaatatttaaatgcaagtgtacggatttatgcttcaaatgaatatgaaactcctgaaatgatttgcaatgcatacgaagaggaa
gcttattcgcaaattgagaatatgcgtgaattccgttcacgtttggaccatagtatgtggaagagtgtcgtgttgatagagcgagctagatgtcatt
acttaaataattacaaggcccctgcccaatacctaccaaaatgctcaaatcccaaagataatcgagatttagacgtcttcgtcaactatgcatctc
ctaaattccccactgctgaacatatactgagaaatgccccacatccatccaccgcttgggtgcacttatttgttctgggacacatgcttgttcaaga
ggacattgtgaatggaagatgggaagttgcaagatctcatgttcaggaaatggagtctatttgcgatcgcaataatttgcaagaacaattaacctt
agaagaagtcaaacatgttaatatcttgatcctacttggtaatttgggcttgaccattacttccgataactatgataaactgaaattggaggaaatt
tccgcgttagtccaatctttagaatacgaaaacagcacttctcttagtgtaattacgaaacagacggaagtatgtaaatttattgatagtaaggaa
aactaaccgccgatagattttgaatgatttaataatctgcctcaacagcttcgtttaccatgtttcttcttccaagaaaaaggaatttttgagagaat
atcaagctttaaaaaatctgattacttcgaaactcggtagtatttctgttgtgtcaaagtataaaaagaaaaacgctcgaaaaattgtttctgaatt
gttcagtcattcctggcttaataagtcctctttacaacaggtttgttttattttttctaaattataagtaactaatctttttctattttaggtacctttttga
ccagaaatttgccaagcaagttatggagagtatgatagattcatttacccagacaggagatgctgtcgggaaactttcgagatttgtaaaattta
aattttccataataaataaaaaattgggtttcacctaaacacgaactttactcctttcataagcagatgttaaagagacagtgccagatcctcttcg
gaaatagatgtaaagcatgatggcagacaacaaactcaaagaatacctggattttgttagctgaagtataaaacaaagtaaatgaataaattac
caagtaataatgtattccgtgtgtcggttaaagatttcgacatgatggggatggcccaaagggatgccctttcggactagatcagcttcttcaaaa
ggagggagttcggatcctaactgtgtaatcaaaataggaagggtattcttaagattagcaaattcgtgaacatccaaaaagaaaaaggaacctg
ttgatgggtcattgttcaccatgagctttggtttttcagtatgttttctcaacagtccctcaatttgtactgtgttggtaggtatggcagcgctgtctcg
aagagaaggatcacctaaagatttggatatccaaccccggttaaccaaaacacgacgaccatcctccagaacaaatggggtcactacgtgatat

ccgggacttccatccttcgtccttgggccaacaagcatttcctggtcattacagaatgtacctttcagagcaattttcgtccattctttcgcttggattt
ctttttcacttaaaagtttttgggttagtgataatcaccggttgaatatatagaaataaaaagacatactctaaattagcgggaagtggtatagcat
cttgttttaatttgctagttagagtatcaataatgcccactttccattcacggcgttttacctgccaagtgcctaacccaaaacaaataactgggact
gcagtcaaaagtccaacgataaatttgtttgtcttaggagcaacagaggtagcctcaatcgttccgatagatcgatatattggtgattgaaacctt
gacttcaaagaaggaaatgttctcggtaatcttttccaaagcattctaagtatgtattagataagatcaaagacgctataaaagtagctttttgaa
gatatagatcgtcgtttgccagtgatatttacctgagaatgaatttaccctatactgtcgtaaatagcgaatatgatatatttactgaataataaaat
caatactttcagctaagtaaatatgaattgaggattgttgaagagtatggagtttctgaatagcgatgaagagttacgattgtttcgttcgtagcttt
tatggtggaagacctgtgacataaagtgaattttgaacgaaaggaaggtttgatccgcattgaatgccaattttcctgcgcaatttttgtattagt
agaagaaacaaaaacaatatctcttttacgaccctcacttagttataaataaaacagccagtccagttttttataaagcaatcgcttaatttaagt
ctatgcgtaaccgagaattctccaataagtacaaaaagaaaaagtaaatggatcctgtcctttcttatatatgcatttgtttgaatgaaagaaattg
aggagatctttctactctttgggatcgtttttcgataattcgatccactctgacgattgtatttcagaagcccttgggtcactccaggcaacttcataa
atttcagcaaagaaactattctaggatcccccaacgaattctacgatgcatgcacatggaattacagtcataaaacgcatacaacaaatagaca
aataaaagaaactgtctaaatatactgtgtgctttatttatgtttataatcttatcctttcagatccagcccttatggcttagtggtacagcatcgcac
ttgtaatgcgaagatcctcggttcgattccgagtgggggcatttaattcttttcaaatttttttattttagtgaacttgattgattcaagactaactgttt
aatgttcaagaaataataaaaaaaaaaatcacaggtacgaagcatatctttgatcttaaaaaaaggaattaatatttgttgttcgcatattctgttct
gcctaacgtgcgctttgttttcattgtttatgaacgtagcaacccttactcatgtgtatgcttttagcttcaatttgatcgcttgctgctatctatagttt
gtttgtttgaatgtttattttagaatgagaaccattcgaatttacttaaaaaaagaagtaaacagaaaacacactgcaagaaacacagaccgtta
gtttgtttattgtaaatagacttcctccaccaaaaccacatattaatagtatacatgaaagaaataaaaattattttaataaaattttatcgtttaat
ctgatgtatttatatattccaaaggttacgaaatggagatttcttttctaggaacgcgcctccatatgaggagagagacgcgtcaactagagttgcc
ctttaccaaacacgctatatcagaaccccaaaatcgcaattgctcccaatttttaaaatgctgtctggagggggaaaaggaaaacatgttggagg
aaagggaggatctaaagttggcgaacgcgggcaaatgtcccattcggcgcgtgctggactccagtttccagttggacgtgtaagaagatttttaa
aagcgaaaacccaaaataatatgcgagtcggtgcaaaaagcgctgtatatagtgctgctgttttagaatacttgactgctgaagttttggaattag
ctggcaacgctgccaaggatttgaaagtcaagcgtattactcccagacacttgcaattagccattcgtggtgatgaagaattggatactttaattc
gtgctactattgctggtggtggtgttttgcctcatatcaataaacagctgctgattcgtacaaaggaaaagtaccccgaggaagaagaaattattt
aataattctcatcataatgaaaattgcttggtcgatttgttcctttgatttagatgagaacttattgtctcaatgtaattattgaattatcaacgtcctt
atacgatgcacgcgttttctcacgttatatatgtatatatatatggactcctttatttatagaatcttgcaaaaaaaaacttccttgaatcaattttgg
tattctaaacaaaaacaactgggacaattgtctttggaagccccctgctcctctgtttcgtttctatctgcacgttttacttatccttctgtctttaatat
tactactattatttttatgcctaactgattataatatacttcatgtacgattcatgagtgaactatataagaactgattatcggctgctcgatttcggt
aggaggtgcaatttttcgtcggcctacgtaatgccattgtacaatagatttagttagcggtgaaaagaatagatttattgctaatcaatataaaag
gaaggacagacgtatattgaatttagacatccctgaatattaagtatagactcgtataactaaagataaaagatggtatataaacgatagtaaga
aaaacgaggaaaatgataaagacacaaagattgaggtacaaggaaaaagccatacgatttatcccacttaataaatgagtatgacctagtttat
ccaagtacgagacatattacaaggtctgaaaatgcaataaatcctttaacacatcctgtgttagtaacaggactgtgtggtcagccaaacataag
atgactccaactgcatgataacaaacataagccagaaaagaaatgagggttaaatatgagttaccatacataataaaccttaaatccgcatacg
cataataatagcgggtaataatgataaatatatataaagaatgcttaagaaaatggtagtacagcagacacttcttacacacaagtttctccatat
tctcgtaaatatggctgttgtagagctcgcttggcactgattcgatgagcaggatcgtaaacaagcattgcctggataagatattagtaaagcagt
caaagtggataaaaataagcaacttaaaaatacttaccgaaagtaactcaatcgctgcttcttcaccgttgggtataactctgtgaagatccacgc
gcttccaacgaggaaaggtagacttgtaatcctgtaaaagtgtaacaccaggccatacttcttcgtttggagttcccaacacctgaaagattttga
aaatttcatcaatttcagagtcgccaggaaacagtggagtacggcggatcatttcagcaaatatgcatccaacactccaaatgtcaacaccagta
gagtaatgtcgagatcctagaaggacttctggtgctctgtaccacaacgtaacaatctggttctgttagtcattgctgaacaggacttggtcaaaa
catacctcgtgtgtatagttgcgtaaaggtacgccaaaagaccttgcaagaccaaaatctgctaatttgagatttccttctttgtcgattagcagatt
ctgaggtttcagatctcgatgaatgattctccgggaatgacaaaagttgacgccgttcactaattggtatgcaaatttttgaaccagtcttggatct
aattcagatgctccggtttctgatatcctgtccatatactttttcaaatccatatctaaaaactcaaataccaagtaaagtttggattcagcatgcaa
gatgtccaaaagactatataccgttagtaataaggcaagagaaagagcgtgataggaaggcaaagcaacatctccatgaatcctcgcctaggtt
tttttttttgtttttttttcataccgaacacaattcgagcgattactttcatcgttcacctcttttagtaacgaaatttcccgaatagctgtactaggaa
ctccttcagactcatcttccagacgtatttttttcatagcaacgatgcgccctgaagacttatgttttgctttgtagacaacaccataggtccctacca
aaatgattagtaagaagagtccatcaaagatctgtaaaagacatgtcttcattttctataaaaccgactttaccttcaccaatcttttctacttttac
atagttatccatcttgggaaagacgaagaaggaggtaaaacaaactttcaagtcaaatagctattattgaaaatttagtatcacgttttcttttttt
tggcagtatccaaagcgtatttggtatgtgttgtggagttgctaaactactataataacatactcagaacgaagaatagaaggaacaaagaagc

aacagtgaggaatgtattgaaataatactaacaaccaacagttaattatcaaatattaaatacataatactagcgtaaacgacaaggaataatg
tcatcaagaatagagaagattttgcatggaactaagcataaataaataaaggctaaagagacaacaaatagtatggtatcatagtttgaattcta
cttccatgttgaagatggtaattaagcgtttgagaaaagcatgaaaacaaatgcatcgaaagactggcgatacattgaacaagctaatcgtaaa
taaagagcttcataatttctattgatagaaactgtagaacaagataatcgaaaggaatgaaatgatgtcttgatgcttctctgtatggaaggagtt
ggaaaaatcttacatagaatgctgacgaaaagttgaagctacttgtctgtaacaattaaaattctccatctttatctcattataaaataaacgaact
aggaggctacattgacaaaaattggaaaagcaaaaaatccaagcttattcacatcaacgacagttaatcaagaacatttcgatttctttgcttcac
ttcacatagactatgaaagaaaagagaattcattattcattgggacagcaaacatcagacatggtgagaaagatcacacgatctccagatgtttc
gcttcataatcacatcaaaaagtatcatggcaaattggagaaggagcagaactttgttgcataatagtcgattaaaagaaacctccatcaaagtc
attattgtcataatcaccacctccaaaatcaccaccaccaccatcatatccatcatcaccatagtagttgttttctaccacggtatcattatgatcaa
aggcatcatcaaaagcatgcattgctaaaccaccagctgccagaccaccgagaccagtcaccatgggattggataaaggccaccgccgcctcc
tccaaatctaccggaacggccaccgtatccagcagcaggagcatagccataacccggaccgggaggaccagcagcataaccagggcctgggc
cagcgccataaggaggatagcccccatatccaggttggccaccgtagtaaggttgttggttgttctggtgtccatgatgaagacggctaaaaattc
ctctgtctccagaaccagatgcagcaggagatggagaagggcctccggtagtaggatgagcataaggtggagggggttccgttggaggggcag
agtgttccttactcgggggaggaggtatatttaagttgggtacgggacattcccattgaggttgaggcttggaagctttttcatcaacgtaaaagta
acgctcatagcgaggatcccattgtgcgacccaaccgtcaggcaatccttgacgagtttgataagccattttgtatctgtaagtatgtggcgaaat
ggaagactagaaataaaaggtgtttttttacttcaaaaggagaagaaaaagacaagaaatgcttcgaaagacaaacaaatgaggaaagattt
ccaatcgtatcagtcaaagacaaatcaaaacacacgtaaacacgtttccaagttaaaacgcttgaaaaatccaattgctgattgtttttagtattg
cagacttgttattgtgaaattggaagtctgagaaacgctcggcgaatgtctacctcgttatcatgactactattatataattgtattgctcaaaaatt
actcactttcagcataagaaacttttctttttgatttgctttctcttagtaaattttattttgactctttctttatctttttttccgtttatatcttgtttcttt
gatggagaaaccaaactacttttcgtgaactgaaactctcttcctgcagaggccaagccaagctagtaataaggctgtagaatatggaatgaga
acaatgcaagtattgaaacaattactcagaaggaggtccttctgtcttcgccctagaagccatatgcttaagcaagcatatatcctccccaaattg
tcaacgtcgcctgctttcatcaatgaaacgtactaccaagaaaataatgcgatccttttgctgcttcttttgttctttttcctttgtttactaccttttgt
ttacacatgattttgtcatcgatgttacttgcggaaagcaacaactaaagtagcattgcaatgaggatatgaggttacttactactaccatacttaa
gaaacggaaggctcggttcttgaaaagatcttaattatagttgtcttggaattataatgagtagtagttcaaaaatgaccaaaaaaaaagggcatg
aaattaaagcgcccgtacgtggattgacgaattggaagaatgagaagctaatcaatttcaattagtgataaatcgcgtttcgtacagcaaagtctt
cctgcctggcagttcatttttacaccgtggtctgtgcttcctttactgtttgttttgggcgtcatttttgctcctttgggtggagcaatgtatgtggctag
tcaacgggtatgttttgtgtcgaaatattccatgcttgaaaataattcctatgttcgaagtagagtaaaatatgtcatggggattcttaagtttatga
tgaacgatgttcaccgtttctaacactatcgccaggtaaaagaagtaagactcgattatacgcattgtctggataccgataatactttcacagaca
tgccggcaacgaatattgtttatcaaatgagccaaaataataacgtatctgctaagtggaggtcagaaaatgatcattgttatttacgatttcaaat
cccagaaaagctgggtgctcctttattctcattctaccgcttaacaaatttctatcaaaatcatcgtcgatataccgcgagcgttgacgtttttcagct
ccgtggtgaaccaaggtccccggaagaaattaaagtcggtgaatgtagtccattagacgtaaatgagcataacaaaccatattatccttgcgggt
taatagccaattcgttatttaacgacactttttcttcacttgttcgctatgattcatgggatagcacggaatcattggatgaataccaaatgacttcg
aacggaacggcttggccagaggataaagaacgttatattaagaccagctacaatgcatccgacgttgttccgcctccaaattggtccaagatgta
tccaaacggatacactgatgataatttacctgatatttccaactgggacgaattgcaagtatggatgagaatagcagctcttccaacgtttagtaa
gcttgcaaagaaaaatgctacggctgaattgaaccctggtatttatgaagttgatatagcttataacttccctgttatcgagtataatggcaccaag
acttttatgctttctacaactaccgctataggtggtaagaactactttttagggatcatgtatatggtgattggtggtataaacatcgctagtggtatt
atcctcagtgttgcatgcatgattaaacgacgacgcgtgggagatccgcgatatttatcttggaacaaggagaagactatatgatgattctaaag
agtttctatatatatatatatatataacctttattattcatcatgcattttagtgattccttcgggattatgttatgttcaggtaatgtcgtcttattgaa
attggtttgcatcttcattttctttctttctttgtaaaatagagttttcctatttgtctttcttggtcttaaggcctttgtaaaactatacaatatacataa
atatacaatcatatatgtataaaaattaatgaaattttgttatgaatatacgttcgtgcaagaaagattattatactagaaaatagaatacttgtga
tgtttcaaaaaggaacgctacgacaagccgcataaaggctcgccgctagaagatgtgcacaatacggtggatgtggtgatacacatccacctcc
ccagcgatcccactttttaggatacttttcctgggaaaatgtcgaaggatttttgaaagcattgtatgtaaactgcggacaattacaatgccagacg
tgcaagttcaaatgaatgactttaccggtttcaagggtgcattcccaaagatggtatgacggattcatcggatataaaacatttgaatccatcaga
gccatagccgagtgccaggtattatgaaataggaaactaaggccaagccaaatgtcacctatggaggaatcagaaagcaaaagctcatttaag
tgcaactctcgtgactccctcgcagcatctagctttgcaatgttgatgttttcttctagagttgagaagagatcactcagaatgggtcgaatttttgta
aaaggctttcttactgccgtgtcactgttgagttcactgaagtcaccctccgtactttcttgaaaagacaacgatggaggtgtggtttctttattaga
aaggtcattctgatccttgtctttagtatactgattttccgtttcgttttccatttagacagagtaataatcctgttcaacaaggcgaatccttacgctt
ttaaataaattatcattcaataacgtatttctttcagtttcaatttcacaattcttttgaagcaaaaaacatatagcccaaatcatcactcttcttaga

aactgcttttcaatagagtcatgcagttaaataccatattcatttattgtccagaattcaaattggaatcttttagtaacctcttaagaaacgtaaag
aattccaccgtttatacctttagacctcagatattcactagggtgttgtcacgagttcttccaaagtcactgactatacttaacgagagtggacttag
aggactggatatctaaacatctttcatgattttataataaattatcatttgctaagtttaacaaccttaccaaatctgattttatttgcaggctctgaa
gagactacctttgtttatacacgtccttgtgtttgtcgtgatccttttagaataaagttatacaaacggccgctggaagctttaataaaaacttgtctt
tttaacacttctaactaaacgggacatgacgaggcctgctttctcagaagaattcgtcgattattacgatgtgctagggattgaaagtaacagcga
ttatgtacaaattaggcaacaatatttaaaactcgttttacgttttcatccagacagaaatccaggacgggagcaggatgtgattcctcatttccag
ttgattcaaagggcgcatgaagttctacgagatgaaaagttacgggaattatatgaccaaaaacgactattagaattgggaagattggatggatt
atcgagatttcgccctaaaaggacaagtaataattccccaaactggagaactcctaatgtaacttcaaaagtgagcgcaaaagtctcacaatact
tttctacaagtaaaaataagggcggattaggaaatgaacacaaacatgaatctccttaccgtcctttgtcgtcctctgaacggcagagacattcat
ccaccttcataaagaagagccccaactctagcccagtacctgcaagacatcacgaagatcagaaacagctatattcctcccctaataatacttctc
atgaacctggttcacccaacaatacaaatgcttatagtcgatttcagccactctccaagtttgaagcaaaaatatacttagagtctttacgcgaaa
agcgtcgttctttcagttcaactcccaaatcggacaatacctcttacacccgttctcctactcctacctccagttctcatttagtacattcattttctag
ccatggcaggtcgtcatcgtgttcagatcctccccaaagtccagagtctttttcctttccactcaactcactttccccttcacctcgtcaacctcagctt
tataaaaaacatgatctccgtcaacatcgtccgtcctcaatatcaacaaataacgattttcgctcaaactcaagctattatcctaatccagcaactt
ttgaggagatagaagaaaaccaagatgattctttacggcctcccaactccgctaattctgtttcctcatcagccgaatatcataatgaactacaaa
aagttttacgttctctcgaaagagaagaagaatctgaagatgaaatagctcagttgcttccaaaaccacctaccttccctcaaatcaaagctccaa
ttcctcctcagttaccaacaaacatttcaaatgaaaccatcgataagtactttaatgactttgaagtatttcaaaggaaatggtcttcttattctttga
tatacacccagtttctatttcactggcagatatttaaaaacaaatgctttcagttggatctcatgaatactccaggccacagcagactattgcaaaa
ctggaaagagggaactcgaaacgccagagaatttctagcctatgaagaaatgcattacaaagccatttcggatttacagtccttcaaagaatctt
tattctcgtccctcaacctttagtctcaagcaagttttagcataatatttgtttatttattttattttttttttcaacacaaagtaagttttgtaacttaatt
atttcgttcattaggacttaattacgatctctatttttactctccattcgcatcctgcccgtatacccacctccgcacaccaaccgaacattggaagg
aaggcatgaattcttagtttcttcttcttataccctataatttatattactaagaagtacaatacataaaatgaggttatcaattcgacatatttaccg
acctggcttcttatgcggtcaaagtcaagggctttttggatcttccttcctaaccaatttccatactagttctgtaactaatggactaaaaaaacaaa
aggctagtcgtcgaaggaagctgaacatagagcgtcaagacagacttcagaaagaacgccaaatgaacttgggagattttgtaatgggtaatcc
gaccgaatggactaatcaattgattcgctcaaagcaatttttttcaaaatcaactacaaaagaaaactacacctctcgttccccttaaatttcctattt
ccatgactgaagtcgacaacattttgcaaaaatttgatgagcttaacgtgtctacgttaaatgaagcacgtgaatttctgaaggtggatgattttgc
ggctccaggatctactgacaacgtatttgagtctctacgagacttggatgaagcaccgggtgctgctgaatattttcaacgtttagcacaagaactt
agtaatatgtctcagatcaatgattctagaaaagagagcattcgaagaatctttagcctttcaaactccaatgctaaattagctgaagcaaataat
aagcgcactgccattgaatccttcgcaagaacagataatgacacaggaagtcctgaagttcaagcagcagcctttacaactaaaattttagctct
aaaagaccactccagtcgaaaccaaaaggaccagacggctaagcggaaacttcgattctatgcccatcaaagacaaagaatacttaagtacct
acgattgaaaaactttgaacgttacctgacatgcattgacaaccttggtcttacggatgatgctgttttaagggaaattgttatgtaattaacataa
agcttattttctcataatatcatcttccatcctgctatctatccctctctctttctttgtaatttacccaatatctttttgaaaaggagttccaattatcttt
gtctaaaaatatctagatttattaacaatgcataaataatcatgactagtctataacaagagaaggtttgataaaaagtagcggaattaaaaagg
tttctcttcagcttgtgatatcagcatatcaagccattcctttccgagaattgtataaagatgaatggacatatcagttcctacttttgctaaactttg
gttacaataatacaattgtaaaagtaaagaagaacggaccgtcgtttcatgaactctaggtagaaaagtctccaaaacataacatatctctttca
ctaattttttttacgaaagaagatatgatattttggctaaagctttgcagactttcatgatccagctggtgcagagtagatgcctcgttgaaagaaag
gttggcattgtcaactgtagaatttgctggatcagcatacagagtggttgactcataaaatacactctggtattggaatagtaaagagaaagcac
gatcacgaatcacttgcaaatatctctttaaatagagctctggattattgtaatcaagtaaaggaatcaagctcttcaaatttgactgcagctgctg
ccaagcatagaaaaggaatatgtatttaatttccgcctcagaaagcttgctagtgacacgaagataagttactatcttaattagagtatattgtttt
aaaggtttttgaagctgcagtaagaattttgcggatagcttttcagataatgcctctatttgatataccaaatcctgtatgactgcaattgaagcaa
accgatctcgtaagcgatgtgtaagaccttgaaactccaaagcttctgaaaaatactctttctgaatgcaagaatccattaactcgggaattttca
aaatactagagacttgaggctgcattcgatgcataagcaaagcttgctggtgatgaattgaaatcgacgataaaaattgttgactttgagaggtg
atgttatatagcgcatcctttttaccgaatatattctgggaaagttcctttgtattgcccaagaattctttggtagtagcttggcaatcagctaattca
accagagttttatagtttgactgcaaataagaaagcttctcttgttctagactctcagactttcttttcaatcgttctttttcagcttgaatgtctcgaa
acggacgaccaatgagttcttgcaaatacgattccatatgagaaggatggtaattcacagcttgagccattctcttctgcttgaagcaaagtaata
acgaaaaattcttgtataaccaaacaatccgaaagacagttcacaaattttttgaggaaggaggacaagtgtttgtaagaaggtcctaattttga
aaattctctgatttgcaaagaagtgcctaagcacgtgagtgaagaaaaaaatattataatccaaaatactaagaaaaaaacagttaaatttcag
ctacaggaaaagagtaattgcaagttactagctgccaagtatacctagatgcttggtataatgttgggttttaggattttaaagcatataataagta

tattctaaatatgatagcatagaattactaaagtaatggtttttataaagtaatatttatgaggtgttaatctataacacatgctaaaaataattag
aaagatatgattaaacagtattgctaacaacaaataataaccattaaaagcatatacataacaataagcatgcgtatgcttatctgtgtttttgtta
agggttttaagatcctctggttgacttcactatctcatctggatgtagaaaaaggccatatctatggtctcattttctctttaccaacgattctcaggt
cgcagtcatgtttgatttaaatacttgcattcgtaaaaacattttggatctacagccctatcgctgtgctagagagtatgtattgttgtagacgaggt
ctggacgcgattgactaacccgtatagtgacttttcagagtaagtgatgaacaggttttcatgttttagactttgatttacgtgtgaagcactgtaac
taggtcttcatttgttgctgaatcttaagctagctataagctggttttcgttatacaaggatcgtactaacccaaacactctagaggtattcttttaga
tgctaacgaatgctcttttggatcagttatcaatgattgtggcattgaatataaccgttaccccgaccctagacagaatgatttaaaacggagattt
tgcgatttaaggaataaggaacaccctttggccaaacccttgaagcctgaaaacatggtcgtcggtgttggtagtgatgaaattattgattccttg
atccgtatttcttgtgtccccggcaaagacaaaattttagcttgccctccatcatatggcatgtattctgtttctgctaagatcaatgacgttcaggtc
gttaatgttttgcttaaacccaattttgatctagacatcgatgcggtctacgatactctttctaaagacaattccatcaagctcgtttttacttgttctc
ccggtaatccgacagctaaagctctgaagctcgatgacgtaaaaaagttactggctcatcctacttggaatggtatcgttgttgttgacgaggcct
atattgattttgcttctccagaaaagaccgctatttcgcttgtgaatgattaccctaatttggcagtttgccaaaccctttcaaaatcttttggtctcgc
tggtattcggtacgttgtaattctaattacctttactaacacctgctagtattggattttgcttgaccaatcctgatattgcggcaatcatgaactcatt
aaaagccccatataacatcagtcagcccacttctagtttagctattaaggctttgtctcccgagtccgtttctaagatgtatgagcaccgtgataga
gctcttactcaacgcgaccgactctgtaaagaaattactgctattcccggtgtaggcaaaataataggtggccttgatgcaaacttcattctcgttc
aagttttgtcaaaacccgtgaatggtgtacctagtaatgatgttgctcgttttgtttatttgaaaatggctactgatcacaaggtcgttgtccgtttcc
gtggcactgagcccatgtgtgaaggttcattgcgtataaccgtcgggactgagagtgaagttacagctgtactcgatactttccgacgtactttgga
ggaatattactcaaaaaattaaacccatttttcaatatttctggacttcttcctgtagttagttggctgtgatttttcaaaatacgtacattgcactgct
cttttaatgctatatttaacaattggattttggattttagattaagatagaaaacagaaagagttaatttcatttttgtgttttatttccatttttttgg
gaaaagaatgtatatgtcaatcgaaaaggaacgaatgctaactacaaattaaagaattaaaagattaaatggttctggcatacatagcaactgt
aatgatagaaagaatagtcgcaatgccgccataccaaaggcttaattgagaaactgagtcatcggtggtggtagcaacgccgtgaaaaaagctc
aaagaatagaagaaaagtatcgtaaacatgaaccatacgagtgaagctaatgtcaaaagagcgactgttccatgaaagcatcgcattatgtaa
cgtttcggcgtagaagaacaaatcccagacattatttctgattcaaccttggaagccatagcgtcagcagaaaactcattaattacccattttcttc
cttcttcacccattgctttgatgtcgtatgccgaattagtagcagcttcgtgaattacgtttccccaaattttaggatcgcggggacggagccaaccc
gttttcccatcagatacagtctctaaaggtccaccatttgactgagcaagaacaggcacttctcggagcattgcctctagaggtacaattccaaag
tgttcattttcgggtgtatataggagaatcttagaagtagaaataagcgcatctctgatatcagaaggtacagatagcaaaaacaaaacatttac
atgagactcaacggataagctatcccaatcgtctttcattgtacgataagtgaaacccttctgcttgcataaattttgaagtagctccagatatttac
gattctcagtaacacgtgtgtcatatcctcccgcaacaaccagaaggtagtcagaaaagtgatccgaggataaatcacgaagagcagcgaacg
attcaatggccagagcaatatcctttttcagttcatatctattgacagacataagtactttgcgagatctaaatttatgaatggattggaggatttttt
aatataagttcaaggacaagacaaactaaagtcatccatgaaatccccaaatcacatccaagaaaactcgttagtaacttgaaaaaaaaaaa
aaaacatcaatacgtacgaaattgactgtgaaattttatattgcgaagaatggtcagcagccttgacatctacacagggatgaataattcgaaga
ggctttcgaattttgggaaaagctttcttgaacacggatgctgtgaaattgctattaacaacaatacgatcggctaaacgaacagaccacgcctca
aatgagtcaaacggaacgcgataaagccttttaagtatcccacctcctggctttaccaagtacttatcaggaaaatggcaatagaacaataacgt
acgacaggcgagtaacagcattggaatgcaattagacaactgatctacaaatatagcatcatagtaactgaacttgaaaagaaggactaaggtt
aagtatatttgtcgcacggtactgcaaagaatcgacagcttgccaaaaatggaagaaggtaaccagtctccatatatttctacttttagagtacct
aaaataaaaaaataattagcatcgatgccttccaagtatgtaacatcattcttcgtaccatcccggatttcttcaaaacaatgctgtttgtcacaat
gagaagtataaatgataacctctttcccaagggactgtagtcctacggcagcatctactactaatcgttctgctcctcctaaataaccaggattaat
aattcaatcttttgatctttgttataattaccaattcctaaatcaggatgaacaaaggcaattcgcaccggagcttttccagaaatattattattttcc
tcttcaatcatattttttaatgcttaggccttaattcttataataatttatcaaggtcttttctgctcctggtgttggtggttcaggctgtaacatccaac
ctcaaggtactttactcatgtcgctacaaaactttgattgactgtatatttaggtgaattacgagagttgtagtatgtcaaacgcttccaatgagtgt
ttctagttttataaattggtatttatctgtgtatatcgattttgctaagaaaggatactacttcgtagaattggtaatttcttatgtgagatatactcaa
agcaaggcaggatcctaattgaaacagtgtctaacttccaaagttgattatagaaggtattggaaagcgtaagtatgcgctctcatatcaccagg
acgtttcgaataacaactagtatatttcgaaacgtattttaaatactgactatgaataagtaaactaaaaagaaggaaaaccatgagtttatttct
actaaatttcttcctctatgttgtttgtttatgcctgcctctattaccatacatataaacaattccccctggcgctattatgacttattgagtccaattac
tacctttatgttttctattggtgttcagttatgcaattgaatttgaacaggggttatttattacttttgcggtctagtctaagcttacactttgatgacac
gttttagcagtgatttgacttcaagtttgctgaagacttcatttgaggactgctatacagcaaatgtgttcctcattcaagtagaagccccgcaacg
aaagctgacatcaaaagagtatcattgatgatgactcaattcttacttttcttcatactaggaacagtctcatgagctgttcctgattcctttatctgg
ctgaatccagcaatcctttccaaaaccattcatataactcaagcatagttttggtcgtctttcttttaagtataagggtttcgaatatagcttatacat

cgctccacactctttcctttttctttcttttcgtccgttttgatagtttacttcaaacgaagtttctttcttttgtatcatgctttttaaaggcactacaaa
ttccatttatgttatccttttccttatttcaagattagtttatgctgcagtcttaacggatgagaatgaaaaatggaacttaaatcgaaaccgttcagc
aacaactatcctggatttctatgctgaatggaccgatcatgaatatttcccttctccttcaaactggcgagctcttcctttttacactgtaatcttaga
caagtggacgaatgggttgcctgaaaatgatgttattgagggaacggtattcgagcatgatccctatgaagtgacttttcgtgctggtggtgacat
tgcagggcttaccacacctcgttctttagattatttggaaggaatgggtatcaaagctatttatatcgcaggtacaccgttcgaaaacttaccgtgg
actcccgatggctactctccgcttgatttcactcttttagacaaacatacagggactcttcaagaatggcatgattcaatcatgaaacttcacgaac
gaggattttacgttattttagatttaaccgtttccactttgtcagaactgagtttttttagcaatgctaccgattcttttgctaatgcttccgcccctttta
gtacgcatggatataccatgaagtataagaatactcaccacttttactcagatttccagttatcaaatggaactgagtacccttgttcggggcctac
gttttgggacataactggtctacctatcaatgacactgcagatacggattccattgaccagcttacttgtattcaaggtgattttgatcattatggtg
atgttgaagcttttggtaatcatcctccatggtggcgtcaattgtcaaacttcgcttcagtgcaagatcgtctgagagactgggatcctatcgtctct
aaaaagctgaaacacctaggatgtcttgcggttaaaatgcttgatattgatggaattcgtattgataaagcaacgcagattactgccgatttttttgg
gtagttggagttcttctattcgcgcatgtgctcgtgcagtaggaaaagacaacttcttcattcctggtgaagttacgagtggcgcagactttggttct
atttacataggtcgtggtcgacaagctgatcagcgtcctaaggatgatgtgcaggctctaaagattggctacaatgagacagaatttttcttaaga
aaagagggtgatgcagccttagattccgtctcttttcattattcgatttatcgtgctataactttactacttggtcttcaaggtaagctattcgcaacc
tatgatcttaaccgccatgattttgcatatatgtggaaacaaatgcttattcaagacgatctaattaatgccaataccggaaagcttgatccaaggc
atctctacggtgttaccaatcaagatattttcagatggccttccacttcagatggtcaatataaacaattgttgggatttttttataactcatttattaat
gcctggaattccattagtttattatggtgaagagcaaaatttaaagttactcgacaaccaggctgctaattatgtttttggaagacaacctataaca
tcttcacttggttggcaaaagcatggttgttaccagattggcaatacgcaatatatggaattagatttttcaccggctagcaaagcttgtcatgatg
actggaatagtttcgatcatttggatccaagctcccataccagaaattatattagaaggatgaacgaaattaggagtcattatcctcaagttcgtg
acggttgggatctcgtagcattaggaagatggacagaggatggtgtatttcctggtaatgagatgtttgccgaatccactccaactatgtggggttt
gtggtctttctcacgtggaccattacttccttatcaggattttggaaaacaaaatgactatatgtggcttctcttttcaaatcgaaacacctctaaga
cttttgaattcaattgctcacaaaaggcggagtataagtttcctccttattatgccccattaggaccgtttaagaatggtaccaccatcaaaaatct
gctgtatccatatgaggaaataactctttcagcttctacaatagattcacccgagtttggaaacattggctgcattcctcgcctcgaaatagatgcc
tacggatcaaaaatcttcgttaaaaaggaagattggattaaaccatccttgtacttgaccgatttcttgcctgggcatgaccaccgtatatattcttc
gtctgaattgacgtcggtaaacatcagctttggtttttcagaggaaattgattgtcaatctttagccaagaaaattactattaatactcatacactcg
gaaattactcgcaaccgaaaatattagagtcgagtatctcctgcgatcttcttgagaaacctgccactcaaatttttaccaacgcacctgtgacac
gatggtattttaatgcaactctggaatcagtcccccatggaattcatgaactagtactaaaagatgttaagagtgtttctaatctgtctatgccatct
gaacacacccgacttttactgcgggtcggaaatgaagaaaatccaatggtttacgcaagcaatgctacattcagcacaaaattgttacatgtggc
tgacaacggagatttgtatatcaatcacactggtattggagctgataaatatcgttactcgctgaactttggaagcacttactctccttggaactca
attaagtcagcttccgaaaagcttagtaaacaacattggaatggaagtcatgttcaaggatgggatggggagcatttgattgttcagtattggtct
agcttagctatgtctacggcgcatattcagcacagtgattctttaggtcttcctaggcaatttcctcatctatttgtacagggtgcttttaatagattcg
gttatgatggatcgattcctagtaaaatgcggtataatcctgataatagaacttggacttatgagttaatttccacatggccggcacagattgtact
taacgtttggggaatgaatccagacaacaacgcagatgaaggatggatttatggtgacatggataatgatacaattattgatagagttcctccag
gtactagcagaatttcgaattttattcgattccttgaacctcctccaaagccatttctatcatacaaaatgtatcttaatgattttactcgacaattac
attatgtccctaaaggctcatggtcggtacaaattgtttgtgtggtattgttaatatgtcttcctcctgcggcggcttctttttctgtacttctgtatagt
ggagctttttcacgtgttactatcttcaacggaagtcaaagtaaaggttggaaaggctttaaacaaagatgtaaaaacgcgtgctctaaattgttt
ccttttggtattactttacctattgaaaacaattcagaactaatgaagcaactgccatcatcttcgaagaaacgtaatgtattggtagcaacactgg
aatacgacatccctgaattgagtcttcgtgtcaaaataggtggcttaggcgttatggcccaattgatgtctcaacatttagagatccaagacatggt
atgggtaattccaataatcagtgggcttgagtatccgtttgataaattaagtactgagcctaaaattaaagttaagattcatgatgtcgatttcattg
ttaattgctattcttataagatgaagaacatcacctatatatttgtgcaatctgaagtttttttacatgcagtcaacgaaggagccttatcctttgaag
atggatgatttaaattcggcaatgttttattctacttggaatcaatgcatagccgaagtatggcgtcgttatcctttagacatttatcatgtaaacga
ctatcatggtgctttagctccccttttatttattgccggatgttattccagtagcagtttctctccataatgctgaattccaaggtctatggccacttcga
acttcagaagaaatagactgcgtttgctcaatttttaatgtttctagagagatctgcgctgactatgttcagtttggacatgtttttaatttgctgcatt
ccataatctcttacgttagaaaacatcaaggcggttacggcgtagtagctgtatctaataaatatagcaagcaatcattgaaaagatatcccattt
tttggagtttggttcacataagtggattacctaaccctgatccaagtgacttgaaaatgctcaataatttttctgatgaacacagtgatgtcgacttc
gttttggaggccaaacgaaaactgttcaaaaaacaaacacaagaatgggccggtttggacgtggatccgtctgctcagttgttggtctttgtagga
cgttggtcgcaacagaaaggcattgatttaattgctgacttggctccaaaacttctagccgaatacaatgttcaaattattacgattggtcctgtga
tagatttgcatggtcagtttgctgctgaaaaacttcaatatgttgcaagttgctatccgaacagagttttctgcagaccagttttcactgctgtccccc

catttctttttgctggtgctgattttgctcttattccttctagagatgaaccttttggtttagtagcagtcgaatttggaagaaagggagttctttgcatt
ggatcacgaacaggaggcttaggttatatgccaggttggtggttccaaatggcttctccaaatacaggacatttgcttgttcaatttgaaaatgcta
tatcaaaagctttccaaagcccaccggaactgagagccagacttagagttgaggctttacgtcaacgctttccagtttgtgtttggaaagagaaga
ccgatgatttattaaaagcctgtatatacgtgcatgaggtcgaagaaatcaggaaggccagttttgtttacagaacatatcaatacctgaaatctg
ctggtaatagtattatgtccaaggtgtctcgtcctgcttttctccgtcatagaggatctgctgagcctgtgtttagcgaagacccccaatgcatgat
ggagcaaatgatgtgtataatgattcgtacctgtcagaatcctcgccaaaggtaaaagatgaaactgaagaggtggaactctactctattgataa
catgcttaatagctctcaatcacttagcccttattcttcagtggtgtcggtcaatgagcagaataatagaagctatgacaatttcgctcgtgatttac
aaactcacaattatgctaatgagcgggaagattttgatattcaagaaacatctgctaccgcagaagaggacactataagagaccatgttcttcca
atcggtgattcatccgtagattttcaaaacttgaatgccgataaatataacgctattgtggaatcaagacccgttgtttttaatggatccgttacgtt
taccgatgaagatggcgctacccgaagactgtttttatccaaacttgataatttaaccagtaacaatactatgaagtctttgtcaattgatcatttca
ttcgaaagcatgaacggaagtacttttctggacttaggaaacaagagattgatgtgaaattaaacttctttactgaaaaggataaagacaaagca
tgtacaattacggaaactgtcaaaccattagaaatggagacggttgaaaataccgccatacaaaagataatgcttcataatatcggtagctggcc
tttatacactattgtgcttgctattggacaagttcttggtgcatcatcttatcaattgactttgttgtctggagagtctgcacagtcgacaaactctatg
tacattttgctttctatttttttcagccttctctttgttctggtggtttttatctagattcgtacaaggaagatatatacttagtttacccttttttttctttgg
tctctcctttttcttggtatcgataacgcacttctttcagaaaactaatgcttgttccataattcagcatattgctgcttacatatacgcaatttcgagc
tcaaccggttctttgtatttcgcttggaatttcggagccgaaggtggaattgctacgcatcactgggtcctcagagcttgtttggtacacggcgtaca
gcaaatttggtcagctattctatgggcatggggggacttaatttcaaagaaagatactggccaaaaggtaggaccgggaatatttgcaggtggat
tggtcgcatcgttggtctgtttcggtttagcatacgtaacgtttgttggccttcctacgtattataggcagagtccttctatcattcccgcattttatcgc
tctcttacgaaacgttctattgtactttggtttttttattgctcagatcttgagcaactattggttagcagtgccttatggacaggcttggcggtttttctg
gaacacgagccatacacccttttggagcattgttattttgttattaatattttttattgttatttgggtactggtccttctcgggataaaatttttgtcgtt
gaataatgtctggtttcctgtaatttttggattaggtttattgtgcccaagatggtgcttggagttttggtcttcttccggactgggtttgaacttacca
tgggccggttcaattgctccgattctcactaaattaatttggctatatctagcactttgggacggcattcaaggaattggtatcggtgtgatgttgtta
cagacattagcacgtgatcacgtagcattcactttaatgcttgctcaggtaattagttgtttaactatcatggtagcaaagcctaccatacctgtga
gtgataaagtatttcctaatatgggatcttggaatccgtccgaaggtcctggcccttgttcttccccatgcttttatttggcattgctctgtcagttaat
cgcgattggtggtctttttttactactaccggaagagtcagcttgccctctaatctcacctgttcattgatacgagtgtttgggtataattgtttttttttt
gatgatgaacgtaagctcttttttttgtttcattgatctttatgatagatcaaaaaatagtaaaaaaaaaataaaaattagagaagaaactttacata
tatatttaatgagtttcttgatgatatttaatgatattttaacttagttgaactgtgggaacagagcccttttgtatcgtgtagaaataagtttcctagt
aactggaatattttctttttcaaaacttgagtaaattgaaacatgaaattggcaaaaagtcaatgttaaaaatgtttctcagttaaaatcgcaacaa
tctagaagcatgattattatttaccaatgcacgcgaacgaaacgtaaaaatttgattaagtgtgggaatatggttcgctatctgcgatacggagtt
gtaaagcaattacataatggaattgtcaacggtcgaaggatgatgccagagaaaggacggaacaaatgctttcatggctatcaatcgaaaaac
aaaggactataaagaactcatacatacttatacagtaggtatatatatttgtttttaaaaaacatatatctatttttcgagtatataatttttcgct
atctgtatgtaatccggattcatacaggtcaccactgccttcgtcttaacttaccgtttaccaactacttttgtaagcaatacaaaaaactcaaaaa
ccaaaaccatggaagaagaaatcgcagcattagttattgataacggctctggtatgtgcaaagctggtttcgccggtgatgatgcccccagagct
gtcttcccctctatcgtcggtagacctcgtcaccacggtatcatggtgggtatgggtcaaaaggactcctatgttggcgatgaagctcaaagcaag
cgtggtattttgactttgaagtaccctattgagcacggtatcgtcaacaactgggatgatatggaaaaaatctggcaccacactttttacaacgaat
tgcgtgttgctcccgaagagcacccctgcttgttgactgaagctcccttgaaccccaagtctaatcgtgaaaagatgactcaaatcattttcgaaac
tttcaatgctcccgccttttacgtttctattcaagccgttttgtccttgtatgcttctggtcgtaccaccggtatcgttttggactccggtgatggtgtca
ctcacaccgttcctatttatgaaggttacgctcttcctcatgccattatgcgtcttgacctcgctggtcgtgacttaactgattatcttatgaagatcct
tatggagcgtggttatactttctctaccaccgctgaacgtgaaattgttcgtgacatcaaggagaagctctgctacgttgccttggacttcgaacaa
gaacttcaaactgctgctcaatcttcttctctcgagaagtcttatgaactccctgatggacaagttatcactattggtaacgagcgtttccgtgcccc
tgaagctatgttccagccatctgttttaggtcttgaaaacgcaggtgttcatgaggctacttacaattctatcatgaagtgtgatgtcgatattcgta
aagatttgtatggtaacgttgttatgtctggtggtactactatgtatcctggtatcgccgatcgtatgcagaaggagattcaagcccttgctcctagc
tccatgaaggttaagattgttgctcctcctgagcgtaagtactctgtctggattggtggttccattcttgcctctctttctaccttccaacaaatgtgga
tctccaagcaagaatacgatgagagcggtcctggcatcgtttaccgcaagtgcttctaagttctttcatttttgtaacgattacgtacttttgaataa
catcgattctttctgatatatatatatttgtctaactactcaatcttttcatatcgtgttccttatttcaaatgaccttaaaatgatcgatgattatcttta
actgatcaggagtttctaatcatggaaatgtgcttttctctctgctccttgctttcgtttaactggtactctgctttttttccctttttttgtttgattgtata
gacaaaaaccggagattctacattttttgttcgtgaaacagcagatgaaatgtcttgtccgtgatttgattccttgttgcttccgtccatagaagttaa
aagttcttcaccttggtttttccatcaagatcgatttcgatgtctatccctattgatggcttgattgctacaattcttatttagaatattgtaaatgaat

gatataaacagttttattttttttatattgtgaatattgtgagtatgttaaaatggaatacaaggaagactactctggttgtttctgcagctaagagg
aagtctatcaagtaattacagtaccaaggtatgccaaaaagaatgaatgaaagaatgaataaataaataaatatattagtattcctgagaacat
atgacgatgtaggtagtgccgtgacggataattacggcacgtgcgctggtcaaaactatatgggtagagaagtcagaaaatcagatgataggttt
tccctaagaccatataaacttctatagacgttagagaattaagatcaccagcatcgacatatccttctcaccaaagagttcattaaactataagta
tacttctaggggaggcgttccatgtcgcctaaaatgcataaggatataaatagggaggagcatcaaagtcatgagattattaataaaaggattat
gttggcgttctatcattaaagtgggcagctttcatgataaatcataaacggtctcggcatcaatcaactttgggtcaacattagatatgtacaaggc
ccacaacttacagctttcgaaaaagtataatcatcaaatattaaagaaagtaattcatcataagtcattaatttccgagcccagacgtagcagctg
tttatctgcttcatctaattgttcaagattgctaccagagcattcttcaaccttttttcgcagtttgatttgtctcagaaaaacttgcaaaatcaccatta
aagtaagaggcagtctcccttctttcgctctcatgggctaattgttcagtagccgagtcttctgatgtttggtcaaatgactcatttccttgtttcagta
tttgagaggaatatagagaagtgggagcaactgagccagagtcattggcattactagattcaataactgtctctggactattttgaaagcaagct
gctgataccaaatcgtcaagtgaagactgaaaagaatgatggtcgaacaaggattctgaaaacagctggctggaagtgttccatggatcagaag
catcaagagacggcgccaaatccatcggacaaagtaacgatttaacgtgctctcgatgcttctttaatgaagtcgctgggctattcggagctatttt
ggtacaggggctttcagctgcattatttgaattgtaactggatgaatgaacagcatcaggggcagaataggacgtatcagttcgatggaaagaag
gacatccagggtagtcgtaaaaccgagaaggacgggttagggtggctattctttttgacaaatattgcggggaatgagaattcggagaagcaac
gtatgaagatactaagttatgttgctgggaaacaggaaaatcagcttgtaaaacatcttcggtatttgtggaataggaaatgtcgtttgaggaaa
acgcatggcggaaatagggttgttcgtcatcagccaagctactaagggaataatcctggtaggaggaaagttgggaaaatggagggtcattattt
tcataaaatggttgatcagatgaattggactgctcttgctgttttaaatcagtgaagggggggttcagaagttttgatagtgtttgaagcagatgcta
aggaaagcggtgatttgtttgtgggttttaaactattgtcatagtttaagggtaatgttggctccgtattacttttaacaagtggaggtttagaagct
gcaattgaggaattacctgatttgaaagcattgatccgagttcttttacgtggaatgggagtgtcccgttttaaagatggtttaaaatcattgctcct
cgaggaggtccgctttttagagcttgaatctgaagagcttccgcggtttaatttaatattgacaaacccttgaacgtaatcaggatcaattgtccaa
taatgaccttttgatacctttcctttgcttttcccaactttcacaaaggctttgtttagtgataaattatgccggatgctattttgccagcccccgtcgtg
gtttaagtaatagcgaaaggtatttctaatccaggtgtaaataccattcaaagtcaattgtctctctggggcttgaagaatagctaaaccgattaat
gtagcataagagcatggaggtttctctccagtgtcaacaaaccctgcgatttcgtctccgaaaagcaattgacgaatgctttcgctgttcagatttt
ccttgttttcttctacttcagattcatatttctcacaaaactcgtgctctagcaaagggatagaagtttcagttttgtcaaaatttgacgttgcttctag
caatgggtttaccgtatcattattagcatcatgaatacctaacggcttccgtttgtcattcattagtatgattttttttaattatttatttatttataagag
atcgtttcacctgttcttctatgggttaatagactacccgatgttggtaaaggatagtatatgcacaaggctggtttacccttgaatataaacaatca
atttacgatataacaatatcgccaatgcacgaattacagaggggttcataagaaaataaacaataagaactgaaattgagtgaaaattaaaaa
aacaaaacaatcggtattcttaaattcgcataaggaagggaccagggtattatggaatcaaaaatgaaagttgcttaaagacatagtcaagata
aattaaacaattaaatagaataaaagatgtgatctacttttaataacagagtagcgatatgtatcaaatgcaagctctgaactctttagcacgcaa
ataaggtaaaccaaaaagaatggccttatagaaatatatgcaaccattctatggtaatcattaaaagtaagagaaagatatcaattctttggtat
atacattgacttactttaattcggtcactcaggcattctaagtacttagccaggggaagggggagccggatctcttttcgtattctcagcagaacca
gatgtgttatgcaagttcgaagaagatctgtctgcgccatcgcggttttcgacagagggttgggagtcattgggtttagaactacttcgctgacgtt
gccagactggctgctggtttatgttagaccttttcgtaggaggctctacagaagttcgagaattaatatagccggatcgttgtggtcgatttcttggt
tcgggacgataatgcctgtcatgttcagagccagaaggaggaagataatcacgaggacgttgatacgattctgacctattagacaaattttggttt
tggtttcttcgtgaatactgatgatattgttgcccacctgcttcattttcacgacggtttccaccgcggtaatgcggattcggtctgcgcattccgtac
caaggatcactcgaacctcgagtaacaaaacggaaggaacgctttttgttgtaaggattatcattctggttagcaatttgaacatccctttgttttct
tttgtcatattcgtgtgacgaagtatagtgttgcaactgtgagggatttgcaggcaagggctcagtagtgaaatattcatggtccagtgccatggca
gcagataaccgttcctccggatttaatcgtaagatagagccgcataaagaagtcatcgccttcccgtagctaaaaaaggcagtttccaaagttcta
ggatgtgctggaaatgaccggacgccttcacatcctggtaattttttcccaatttggcatagaggcttgagtgggggatccacaaagtcgaaaaatt
ttatctaattgatccaaatcactggatccttgcataattggtttccctttgtacatttcagccataatgcaaccaacactccacatatcaattgctgtc
gtataacgacgttcattcaataatagttcaggagaccgataccatcgtgttacaacgcaacctgtatactctcgacgattggggaaaggaatccct
gaatacttattggcatacgattcttcgtctataacacgagccagaccgaaatctgctatttttaagaatccgtgattatcaataagcaaattggctg
ctttcagatcacggtgaagaattaagctgtcatgaagatatttcgtaccagcaagcaattgcttcatgtaacacttgatttgagcttctgtaaattta
acgctaggattcttcagcaaacctgacaagtcatgatccatgtaaggtgtaaccatgtaaatagaacctcgttttcgatgctttttatctgctcgtac
gactgtcatatccgttaaggcaatcacattctcatgagaaagtgattttaagatcctgatttctctaattgcagttattggaaactacatactcagtt
agtatttcccggtagaccacacaaatatgctgtggaaaacaattgactatgaaagtcttcatgtagccattgtatcttacccccttctcgttccgtatg
caataatattttttttcaaagcgtatagttttccagtagccctgaatttagatttataaacctcactaggtagtcgttaataaagctgcaaacacatat
cagaatataatagtaagcatacccaaaggttccttcacctaatttttccataagttcatatgaagaaagatgactacagccttgaaatcgcttttga

aagtctgaattcacctttctacgcgaaactttgtcattttccgttgaaacactaccgctgcgtttcatcatatgtaccctattccagcccaaaattgtc
tatttcagcaaaaagtttactattacatacacgttcctgtagaagaaaattatgaagaatgctgaatggatataatatagtcgaacgtctaagtct
ggagaatacagtagtgcgctaggttacactaatgagggagtattagtttacaaaatcattatctaggaagaaacggtttttaaaaggtttcgtttg
gttctggaagtaagctttcaaggtggtgatcataagtagaacatcaaaggtccgaagagtacacatccgggaaaagatatcgcggtattggaac
acaagggaagtaagtatatgaagttgaaaataaataaatcaaataatagttttggggataacgggagacttaaagaaatgtagaagactatgc
agtagtatgtcaagatcatataaactagcaattcgcttctcaaaaagaatgataagattcaattggcaggtatgtgaattgtttgcgttgtttctagt
tcctcattaagagatccttgagagagcataaattgagaagacatatcctcttcgctttccaataaatatgcaattatataagcatcttctccagata
ttaacaccgtggacgagtgaagatgatgagcagcctgaagccactgagctctgcacactctgaagtctgatttagggattgaaattactcgaggt
tcattccactcgtaagaccaaaaataaattgaagagttgagaatggactgattttcctggccggttttaggttttgcagatttcgtctgaatcattag
acaatgtggataggtaggatgccaagagagaagctgaatgggtcgctgctgtacgagtatggtatccaagcattgagaccctagattccaaatc
cacacaagattcttttgttggagtgtgtaagaggctgcaaaggaattatcattattgaaaaaaacttcacaacgttcatgcctatttgaaggtttca
ttgcagtcggccatggagcgcgctggtaagtcagcttgtaatctgaagaaaagacttcttgccagcaattcgtcgcaaagggctgctcaaggttat
gctgtaattgaaaagtaaggttaaatgtttgggtatccaacacatgaatggtagagttttgtaagctacataaagctagcagagaagaatcggga
ctccattgaatttttgagaaacccaattgcaaatcttcttccatcttgtgactatataggaaaattccagggtatgaaaatatatgaataacgggat
ttaaagtaacatcttgaacgacaagccattgaccgtttggtgaccatgagacacaagtggcatctattgttggtagtttgtaatttcgctctaagttc
cattttccatctggcgacgtttgataaaagttgaggtagtcagatccaaactttcgaataagcattgaacaaatgttggaatgtgggtgaaaagca
acagctagaagaaaaggttagaaggatgaattatatagatatctacatataagtcatataccttggtcaatgtcagcctttggatgagaaagtac
aaatcctttttttaattccaaagtccaaatcgtaactttattgtgaagttcagagaaaaacaatagttgacccaaaggactgcattcgatgtttttaa
cggaatcttggttacagtcgatcgtagcataaaaagactcttggtgaaaatctaaaataaatgctttttccaaagaagccacgcatatttgtgtcg
atgtaggagtatctggcttggtcttccaaagcaaatatacggctttctgcacaaactctggatttaaaagaaagacatgctgcaaatcgagggttt
ctgtgcttcgtatcagaacatggccagatcgagaaagggacgcaatccaacaattacaaggtgaaactacagcatatgtaggactggaacttgc
atacaaagcagttaaatccataaaatctccatttatttaccaaagagtttggactaggttctttatttacatatgttgctatagcaacgctggtcaaa
acttctcggtatataatgagaaacagcgcctttcgctttatcaaccaccatgctcactttcttatgaatagatgcaaaaatcgagtgctgctttagta
aaatgcgtctatttgtaggaaccttgttgcaaaaaacggcgaatgtcggtctgtacttttgttggttgggcgtggcagaaaggcctttaggcagca
gctcgaagcagctggttttgtcggagaacgacaagatcatcgatagagctgtacatttttttataaaatattattttaaagcattataggtctttatag
aaagaatatataaaatttaacgaaatatgtatgaagtggataaaagtcaacataaagcgaatacctatttttttatatggttgtctccagtattggtt
gtttcctatgtatttcctctgtaccttatagaacagtggtaaaccaagaactaccagttaatttagaatccttcttactgtgaatttaggtctttgaaa
gagggaagtaaatgtttaatagccaagacaaaagcttcagtggtgaaagtgctgaacaaaagacaacttatataaatgtatttcattccagatta
ccaagaatacatgcattgtaaagtactcgagtatatcctgtgcaataccatatatcaagtaatttatacatttaagcatatgctaacatttattttac
aataaatatattttattttcttctagattgaacaaggccccttaactcagttggtagagtgtgagattccaaatcttgaagtcaagtgttcaagtcac
ttaggggtcatggttttttttggtcattgacttttcaatggtgctgaatagaatggttttgttatggtttcagtgcaattttctatttttctttttttcttttttc
ttttgcaaattgattaaggaccggtgagtagagattcggcgcttcttgccaacttttgccagtatcaaaatgtaagggtagttccttctgctacacga
acattcaaatacaattatgaaattagattctaagtatactagtaagtttgatattgatcccatttgctcattcactgttcaatcctacaagcacgcttc
tccatctcaggacatgtttatttacatgtaagtaaacatgggataaaataaaattatttgacgttcaaatgaatcaattcgtgtgatatgtgaataa
aggaaagaaaaaaaatccttttccatcatcgtattcattggtctttgttagtttcttgattgattttgtaatgactctaaaaaaaaagtttgtttaggat
accgaatgggcttgtactcagtttcttttctttttttcatccaagcgttttccacgatcataaaaatgctttctttttctttgacacagaatcctttgaaga
accaattgcgattcctgtttttccttggaatgaacattttaaaattagcccttcactaaaaccacctcgacaaacaagcgacaagcttgctttcaag
tgagtttaaaaaaagttttacaataatggcattcaagaaaaattattttttattctttgtttttgtgtttatatgtgtcactctatgcttccacaactggc
accgatcgtattcgctgtttctaaaaaaactgagaccattaaatgaaacatatgatgctccttttgctgtgggatgtaaaaatgttgcctatgaagc
aaatacctacccaagaatgaatgctacgttcatggtgcttacgcgcaattctgatcttgatggaatcttaagtggaatgaagtcagtcgaaaaacg
gttcaataagcactttaattacccttatgtcttcctcaatgatgagccgttctcagaggagtttaaggaagctgtccaaaaggttacacaatcgaaa
gttcagtttggtgtgctaggccaagagctatgggactttccttcagacactgatcaaagtctcatcgatgaagctatcgcccaacaagttggtatcg
tttatgccaacatgccgagctaccataaaatgtgccgcttcttttctaaacaattttataagcatcctttaatgcaacaatacgagtggtattggcga
ttggatccagacgtttctttctcttgtgacattagctatgatcctttttattacatgcaaaaacataacaaagtatatggctatgtaattgctatcaaa
gaattaccaaaaactgtgccaaatctatttcgttacacagtcgctcataaaaaagctgctaatttagagacaactgatttatggaagttcttcattg
atgatgattatgacaaacttgttcaacaattgaaagaaaagcaaaagttcgaccatatctatgttctaccggagccgccattggaaaaaatcgat
ggtgaaatctataatttatgccacttttggagcaattttgaaatagcacggctggatttttatcaaagcaaagaatataatgattacatggaggcat
tggaggaagccggcggattctgggctgaaagggtatgttattttatcttggacaactttacttttaattcttcttattttattaaacaattgctaatctt

ttcctttagtggggagacgcacccgtacattcgttggctgtaggcctattattaaaccgaacccaagtacattactttcgtgatctgggataccagc
attcaaccattcagcactgtgctcaagaccttggttgcaattgcgactgtccatttactatgcaagagattgaagctaggcctggatcttgtctcaat
caatgggctgaagtgatgggtggatttctggatgattaaatttatatagcacacacttcaaacttgatacgagctttaccttcatgatatgattacta
tatttagtcatggagttttaatttctttcgggcgcgatgaaaaaaccataaataaacaatcaagatagtattttacaaattagtatatccttaattat
taaacaattatatattgcatagcagacattaggaactttgcgcagacattctaagtcattatgcaaacatgtgattcaagccagttccgtgaaatc
gttatgcttacaaaaatattccataaaagcagattaagtaaaccagagtcggattgttgagtcccaagaaccagttgctaaagctgtgccgccag
gactgagggtaatactgcttacgcgattttcatgacctgagagagaagcaactgatgtagcagcagcagaatcccaaacctcacatacattaccg
gtagccgcaaacaaaaatttaccagaattagaaaagcgaacggaagttacaggatttgatagttgcgaggggtattcaaagatggctgcctttg
cacgaatatcaaagcacttggtactcccatcctcaacaccagtagcgaacgcagttttagatgggaaaaaagaaatagagttaatatcagacttg
ttatccgaaatagtcaaacaacaggagctcgttcgtaggtcccatactttggaggttttatcgcaaccacccgaaataaataaatttggattttcag
gcaaaaagtccaatgccatgacatcgcctgaatgtccttcaaatttcgttataggggtctgtcttcgtatatcccaagctatgcaggttttatcacca
gaagaagttaatagacggttttcgtcaatatatttgcatgaagagataaagccagtgtgtcccataagttcaacaggctcagcatccggtctttct
gtggagattactttacagttattgtttaagcctccagctgctatggtttctgcggaaggtgaaaacgcacatgtcataatccatagagaaggcactt
ggaataaggctagtttattatacgtgtgtgcatcccaaacaagcaaagcatcatcctctgaagcagacaatatatattttccatcagatgaccaat
ctacctttggtattttgcctaactgagtctttaagacacgcggatgctgtaatccaagtgaaacgtggctactcatgttaatttattataatctcgttt
gcaaatgccgtcttcccaacaaactgtcaatgtcaaataacgaaagagaggcttatttccagtttgtttattctgtgggttctatgtcccttgaaaag
cgtaatccttgaaaagctgccaaatctcaagaattcaactaacttccttttccaagctctttctatcgtttgcaaagccgtcaaagctttctaaaata
tttttctataaaaaatcttggaataatacaaacgttagctttgctttcatgaaaagtgatagcaagatgaacgtcagattgttctctcttggttagc
caactgtgacctgaaataaacattgaaaaatatggtggtcgtatcctctaacacttataaatttgcgaatattgaattactctaatttatatttatta
aagaaatcatattaatatacataacattctcagaagatgttaaaataacgttttcatggaagactgaaggcagcctatcttaaagacacttgaaat
gtgcgataatgtatgcttcatcatagtactacttcccgttttattaattgaggtaaaatattctttcataggagatcggagttctcttatagacgtcca
aatttgttgaacattcatagcaagtaaaagagcgtggaagaatgatttatagtaaacaaagtcaagctggtaaagtaatagacaagtctgctag
gcaatcaaagaaagtcatgtctgagtttggtccttgttccaaactcccagaagaatcagcagagaccccccacacctgcagctcctcttctttaac
gtctggaaggtgtaagaatatctgtaagattgtggtgctcatggcatggttgatgaggaacgaaaaccaaggtaaggtactgagcgtttgcataa
ggcgtctggcctggatgcaccatttggagcaaaacctggtgagaagcccggtatagtgaatagattttaaaaagagattcaaatattctagcgaa
agtccgtaggtaccaaagattcgaacgactctggtcggaggcatggaatagatttcaacgtatatttgaggagtcgacattactttaagcatatac
aagaattgatggatgttttgaggacatggatatttggaacgatagagttcctcaggaatcttttccatcacgtattctgactttctacgatctctttcc
ataaactcttgttcaatatcgctaaacggttccctcttttgcaatgcctcttcggagactctttgattcgttcggtagagggactctctacaaaaagtg
agatcttccccttggcgttcacatcgaaatcggcagagacagcaggccgtatgtgctcgcttccaaagaaaaaagccacgaataaagtctggat
acatctttcgtaataaagcaggtaattgttctctagcggctacccgtggatgccgtaagtcccatgtagtatagcttcgatcacatgttgtgtagga
cgaccgcagctttgtaagagccttttgtatggctatattttctgaatccgcatccaaagcatgttcccagtctctattagctaattctggatgaccgat
ccgaaggtatgccagccctcgtctccaagcagctttattgtcatggggattaattatcaaagcaaaagtgcaatctttaattacttctgaatatctat
tatatgttaactcttcatgcatctgagtgacgaactcaattgttatgacaggttattggaaacaagaagtattaccttttctttttcagatttatcaac
gatctctgcaagtacttttcattgttgtacgattcgtctcttagaccaagacgaaattcgtcaaatgcttctgattgatatgactatgcgtcaattagt
atgtattcgtgaaacatgtttgtgaagagcataaaaaaaaaaaaaaagagcttacaccatcttccagatcagagatgccttgaaacatttcaatta
accttctttggaaaaaaagtttgaaaagaaattgacatcgtcttaaaaagaaaggcgggttgtttatccaaatcttagcgttgataaacaaacac
aaagacaaccagatataaacaaaaaagggttcttttgttttcattgacacaaattcaaatgaaatccaaacgcatgttcagcaaatttagcaact
attaaattggcattggttgtatggtacaagtaatgcttcttcgtctatgtatgtagcatggacattcaatttgcaaacgcacaaaaccatcattcatt
attaccacctaaataaacccagtgtatacaaactaaatagaaacccagacaaaaccgagacaagaatatgagcagcaacaaaaataccgatt
atatgacaaaaaacgacatatattgtctgaatgctcacatggataggaatgatatagaaaaaattagaaataagtaatatttatatagcatgata
gccattattttttcttcatgaactttgcgaatgaattttgcttttgacgctttcctttcttcaaatgcttctgggcccaacgatttctgttgttgtcacgag
gaccagaggtggacactttaggctgatcgacatccatagagccacctgcaggagcctcagaagcaatttcttctacagccttgtccgcaatctttt
catcgcgattggcaggagacttagtcaagtcattcacttgatcttgaaggtgggcagaaagtctctttgttctttcatcaactacaggttgaaatac
atgttcacgaagctattacagttagtattcttgaacgactcgtcaaagttgagattcctccatgcttaaaaaaaaaaccagggtttttcgacttttgg
cggtgacagatgatccaaagcaaatagaatagattcacatggaagaataaaatttgtttttacttacagcttttctgtttctgcgaacggtcttgga
acgagcagacttggccatgatggggaattggcaaaagttcgattccctgaaaatcttgaattgacaagagtgtaacgatggttaccaaaaccgct
aacttttattgtgaaatggtacgagctcactaataaacaaacgaacgccgtttgacaagaatcagaaaagaaccgaatgaagaaaagaaaccg
atccaggtacagaatttcatttctctttttaatttcttattaagtctttttttatgtgacaaatatcaaaaaacgacatactgtaccgtaatgaaaata

aagtcaattatttctaatgtatcatgtatcggatatatttcgaataaaaacgtagtaaggtcgtaaagataaatggaaaataacgctgaacccata
tacgtcctcttgtttatccatccatatatgcaatcactttatttctggatcacttgtcaaaatagaggatcttttggactacactggtgaaaaaatgaa
aatccattccaagaacggacttttcgattcgttaattttctcgtatacacacagaataatgtcttgccacaaaactgtttctttaccttgttccttactt
tctttcttttgtttacgaaatagcgaactacgcaggacaccttaaaaagagtttatcattacaacgcgtaaaacaggtatggccatacaaacacca
aagacatagaaacaaatctaccaaaagccattcttcgaggaaagagtgaattgttttcaagtaaaaaccagtcttttatttgtaaacaaaaagttt
gaaaatgggaaaggcaaaggcgacgagaaaatttgctcaggtaaaacgagttttgaatcctaaagatcaacgtgtacaaaagaataatggcaa
gaaggacaaaccagccactacgaaaaatggtgaattagttagagaagtgtatgtacaattgaaaagtcgaacacgtagaaagtagactatttta
atttcgctaaagagtctaagaaagagactaacatagttgttatagacctcaagttgcctcgaacctctttttcaatacaacgaaagtcttggtcca
ccttatcatgttctcattgataccaattttatcaatttttgtcttcaacaaaaaattgaggtgtttgatggtttgatgtcgtgcttatatgcaaaaacca
tcccttgcattaccgactgtgttatggcagagttggaaaaactgggaattcgctacagaattgcgctccggatagccaaagatgaacgtatggag
cgtttgccctgtacgcacaagggtgtctatgcagatgattgtatcgtaaaccgagtgatgcagcacaaatgctatctagtagctaccaacgataaa
aatttaaagcaaagaattcgaaagatacctggtgtaccgattttgtcagtggctaaccacaaaattaaggtggaacgtttggtggacgtggtgga
ttaaaagacgttcaatctgatggtctttggccgttatttttctttagacagataagttgtggattggggatggatttctacaacttgtacacgtttaca
gaacattttgggttattggaatgagcataatttataggactagttaattaaacatgactttttgtatttcttacaatcttccatagattgttgccgtaa
gattaactggtgaaaggacaaaaaagtaaaaaaggaacagtgaacacgtaccaaaattacgacaaatgggtaggaaagagcaaagaggaa
aagaagaggaaaacgagtagttgtaaataaaacatcatctcgaagaaggattaaacctattatcgtaggatgttaattaggggaagtccagacg
ttattagaaagcaaaaattcggcaatgatgtggaagtaaccctcgcctaaacaagtgtcgttatgctttgctttgggaaaaacgcgaatacgcttt
tgtttactaggacagatggagtaaaggacatgcatttgagaaggaggaacgatttcatcatcagcgccagaaaggaaaagaacaggcaagtcg
ttgattttgcgaatggagcgtacgttttcccatttttctgtacaaaaatgagataggagatttccaatgtaagggaagacagtagggatcatgtctt
ttatagaagtaaaggtattttccaatacaagagcagagatgacagattcattctttgaggcaagagacaaggcaacggcccctccaacagactg
accataaacgactatcttagtggaagagcagacaggatgctctcgcagaaagtccaaggcagtctgagcgtcgatgttcatgccagattcactag
aagaaccagtactcctaccgtaaccgcggtaagagataataaagacgttcatgccaagtgcagaataaaagactcgagcaataggaagacgat
ggcccatattaccagcattggcatggaagtagagcagagtaggacgtgactcaggggctttcggctgaagcattaagtaagagtcaaggattatt
ttgtcgcgagttcgcaacgaaatctgttcatagtctatgccgaaatcttttggtgtagggacagtagagcgagagccttcaggaaatgaactagga
taaatcagagttgtttgataacggtacaaaaaggcaagagcaataagagtaatggaaagagagatggcaccaccatattttaaggtttgaaag
atgctttctgcaaaggtcgatgtcatcgcgagttttcgtagaaatcctcaaatatttgaaggatggagaacaatttaagcaaaaaaaagaagaat
gaaacaagaaattaaagatatgaaacttttagggaagaacagacaaccgaaagaagggtttgaaaaggaaaaactttcaaaccttgattcaaa
taaagatgaacctaaaataaaggaaagctctcgaggatgcaatgcgatggaggtttcttcgccgatcgtacaaaaccaaggatgcctaagtgtg
ataacgaacaggcgacgactcctcgactctaaatagggaagcctaagactggccagccctttcttttcttcgcgttagaaaacgttgtgtttatcgt
tagctaaaataacgataataaagaaaatgcattatgtaagaataaggatagcgtggaaaagaaactcatgaaaacttctagatggagcaatag
gtctagggaagaggggggagttatgagcgagtaggggattaatagaaattgtaggaaaaacaaaacaagaaaaaactaacgaagaaagaat
aaaagttttattttacacaaggttagagtgaaagcactagaattcgtcaaatactggtaaaaaaaaaaaaaaaatttttttgcctttctttccttgc
attcattctcgttccggcgctttattggaaggaagtattatctgtgtgtatttcgtgcgtaaattttatctacaatcaaacgctcatggtacgaaggat
taacagaaagtactacacaaagaaaaaaaagaaggaatacaataacaaactataacaagattcaaattactttttttatcgctactaaaattcgc
tccaacaatatagcattctccgcgctctcccatcgcttcttcaatgtcacctatgatttcataaaaaactaccccgcggatatattatccaatcaatg
gattatatgatgatttacccacggtacttagcttttccgcatccaacgaaactccgctcaatcaaagtttctccgcagccccgatgttttaattcaca
tacgcaaaaccgaacaaatttaccggttttcataaatacaagccagaagcgtatgctattctttgggttgtattgtttacactctaattcttagattct
ttcagagtcctttgagttattccaaaagttcaaaaggttgcattttattaaaagggaaaaggatttggcgtttcttgcctctattatattctccatcct
aggcatctttcgtatactgctacgtgtattttctttccgaacatccgtctgttcgttgtcgttctcctttcttctccatacttactctcctctctttgcttgg
gaacaagggttttagtgaacttgactgcagctcattaacttttgtgaaaacaacgtcaattttattgcatttcgtcactagaagaaacacgttctcta
cgtgcgactcgttactgtttgactaccggttccccgcttctttccccctccataaaacactaagcggaagctcctttctaccccaaacctccacaaac
cacgagttcgaaggctaccaacaaattttggatatatcttgttgatagcattcgaatactgtttttttacttttccaacgttggcttttatgccattttac
cctctttttggattcagcgcttgtgtctggtgaaattttcttttcttccattcattagtatcgttctgctcttatctttctatcttctaaaagaaattagaa
agcaaagtaaagcagaaagaaattcaatttcagacgttgtataaaaagctatatctgtaaatatctaacgcgttgaaaacacagaaaaaatacg
acagaatttcggaaacgtgttctttcgttggatactcatatttttcccttcccttagttcgttcagtatttgtttttagtgtgtgctttctcgttgcatgca
acttctgattagaacttacactattggttctatcgctttttttcgagactccagttttatcgatttgttctcatttaggttcccttttttgctcttctaatttt
atcttgcatatttcatcccgtacgtttttcatctcctttttcatttttttctcgttcttttttttttgttttctagtattgttgctatacaataagctttgtcttc
gttatgcgatcaaattctatttttttacaaggagattgacagtccagtggtaaagaaaactggagtcgaacgtccaccatccaatggatcgtcatca

tcgggttctgacaattcacacaaaaaaaagaagaaaggatttctccgctctttgttttcaagtagctccagtaatgacggaaaaaataattgttcg
tctcttcatcgtatttggattgaatactacgaagtctgtcttcgtaaagatgatgtcgatcactttaatcccggatactggatcctagacactaatatc
gatttttattacgagatccttgtccgccaggtactgcaaaaacaacctcctgagaatcagggtcaaatctacttacttcgtcctgctatggtatttttc
ctcgcacaagctccagaccccaccgacattgtatcagcccttccacctgccatgtttaatgcttccttcatttttttacccatcaacgacacgaatga
atgtggcatagaaagtggttcccactggagtttgttggtcgtttccgtcgagaagggtttaggttggtattacgattccatgtccaatggcaacacc
aatgattgtaatcttgccattaaaaatcttagcatcctgtttaaaaaagactttcgcattcgtcacatgaaaacaccacagcaaattaacgattgtg
attgcggcttacatgtttgtgaaaacacccgcattctcatgtatcggcttttgcaaaagccttacattccacgtgtcgacatgagcattgaaaatac
aaaggtagatagccatcgtatccggaaaaacatgatgcagattatcaccggcctcattacagagtatggttctaaagttccaaattcaccagactt
tgtccctgatttggaaaaggatagcagaatttttgctcgaatttttgatactgtactgtatgaagataatttctcagaagaccacaaaaaaccaac
gctgtcttcgtcgacggtgtcgatgtcgaagaagcctaagagtactacctcagtcccagtgaatagacaccttcttgaaacgtcttctccgatgatc
gggagaacagaaagtctgccgcatgccaatcaatccatagaaatccattaatccattgccttgatttcttcgttttctccttccttgcttgcttctgaa
aagagtgtcttgatatcgttacaagacttgtgaactaaaaaaataatttcgtgccgtctttgtgttcttataattactatgctgagcattcatttcctg
ctcgtgattgacgtgttgtttaaacaagactttttcctgtgcaaataatttgctggttatttttcttaaagtaaatacagtgcttatgcgcatcagatct
ttttttaggctatagcttgctcgaaaaaagccgctattaaaagtagagcttgagcagtactttatgagtttatgtcgtgactttttttatcctcatgtttc
tatttttatcgtttagtgatttgttaaattacgctggtcaacccagtcgctcgtttgaatgttccgccattttgccattatttatgaaggggaaatgata
tgattatctctgaatcctgtctccttcctatgttcatgaaaacaacgttcctttaatttcaattatatttatttatagtggactatatctcttgcaatcag
gataaattcaatagcaaacccgttgcatgtttttgaataattaataaagatatgttgcatatatgttttacttagagcttgtattacaataattgtgtt
ctagtgctagtctaaatattcattgtgaatttccactaattcgtagcaggaaaatatttttgatagtaaactaccttctttcgatacggaatgtaggg
tatatagaggaaacgttataagattcctacaaacacaaaaagtgaattcgctgctcttttacctttcctagatatcgtaagcgaaataatgctgcct
tcgtcaacataacagaactgaaagaaaccccttaggaccacaatgaacatagaaaacaattacattttgcgacaagtttcagagtttgatctaaa
agcttgttttattgtaagacaaaagatgtgaattttgagaaaagcctactgaaaacatggcaaactagctgctaacgtgaagaactagtgctaca
aattgactaaatgggtactgattacaaaatcacaatccgatttgtaagtttattactatgaagccaatacagaaagaacagaacagttggtatctg
ttctagcattcaggattagcaaacccttgaagatgcaaatgcttaccaaggaatcagtttttacacttgctttaatcacttagaggatcgaggattt
gcaactttgacttatgatcctcatagtaaaccattatcaatgcctaaagtcgatgaaacttccacgtctaccgaaaagaaccatcattttgaagaa
cagacaaacggtagtaatccaaggaaggatcgggttgagaagtcagaaggagatcatgaatcaccgggacacattttcgactccaatggttcag
cgaaagtatgcgatgtgaatccaattgaaacaaggtctccctcatctaaatcctctccttcaatatcatcgggcgcaaaaatgtctggcaggcaat
atgtacttcatcgagatatttttcaaatgagacttcagtttcatgctaatttggctgcccagaggaaaaccagacagctattgaattctgggaaagg
actgccttcaccaccttccaaacctcttccttaaattaatttccaaacagagatcattttctcctaaacggaaagctccgactaatttcttttattctct
tttctttgtatttcattttacatgtgtcgttacgctattttcgtcgagtaaactgaaacctgtttacattttcttcttttcagctacagtcaaatttattaa
aagttttatatgtccctttgagtcctcaattctcttgttttgaagaatttgtctttcacaatatgagttcaactactgagttgtcaaacaaaactttact
gaggaatgggacaaggatacaaacactatttaatagacttccaaaggactttctaattaaaaactgtttacattggattcaatcacagacatatcc
accaaatgcagcagttaacaactttgatgatggatttcctatggaagactggaatcctctggagttttatctccatcctccgaaatcaatgcttaaa
cgtgccatcgcgtatagaatgattaatgtggattggccaaacggtttttctttaggtcaagttgctcatctagagctttcagctattgctgaaggcat
acttagtttccgctggactgcatccaaagttttccatcataaagggcgttctttgttttcaaatccagcagttattcttcaaatgcttaaattggaatt
acaatctctattcatttaccatacatacatgctgcaacacccaacactccctatatggttaatacgtatccagctacacgagtcgaataaaccctcg
tctctccatctacttccttcctctcgacgaattatatatctgggaattataaaagattccgatactatattgcataatctctttctgaagaacgatgtct
gtcataggttattcttgcagtgtttaacaaggctcgtagatcgtcttcaaccagccagctttttacagccattagaacttacagcaaaaagcttgga
gagtttgtatcaaatgttgggctctcaccgaaaagctaatgctttgggagcttggcaaatttatggaaataacgaagtagatccgtcaccattgga
cttgcgtcctacagatttaaataataagtctcttcatacaaggcaacttgcatcaaatacgctaagtgggtctgacaggaaaatagcaactcaaa
atcgattcggtgacatatccaaccaagcctttgatcgtgtgattattacgctcaaccatgactatttgtatgataaggaagaagaattaagagatg
caagtattaggcgcaaatcgctttctagcatgaatgtccatagacccattttgacattacagtttcgaggtcagcatgtttttcaagggataaaaga
gctttgtgaagaaggcgcaatagatccgattacgattccttcctatttaactggtgaaacaggcaaatctatgctttatgttaacgatggattagct
caagataaaccaactttacaaggctttcaggaaacattgtaaataaaacattttaatgatattacgtgtcattgagaataatcgtttgattaaaga
aaatatttaccgaattatttatacatcgatttcacttacattttacagattactcgatccggacatacctactgtgtgttatgagatatacagtcacat
gcgtaagcttaacgcaagatacaaatcccaattgtattgcattttcagtaaaatatattaaactttttctgcttaggcggcaacgggacggccaaat
ccaccacggaaggaaggagtgaatccaccaggggcagcaccatcatccttcttctcagcacgacggtaaccagcatcaccagcagcgcgctcac
ggggttcagggcgagtagcacgaggagcggcagggcgaacttgacgcttgtgggtagcgggcacgacctcagcaggaagatgcaagtactcac
gcaagtattcaacaccctcgttgttcaaagtgtagtagaagtaaccccagttgtaacgggtcttgaggtaaccacgagagtccaaggattggcaa

gccttgatgacctgcaagttggggacaccaacttcagggtgcttgggaaggttgaaatctgctttagttagtcgccgttccagtacaatcgttggca
tcaatcttacccttcttagcaacgaggacaccttgttggaacaaagtttggtgaatggccttgcggttctccttgggaataagcattttggtgagga
atttgcacggagtagaatgtactttctgtgttgtggacaaccagtgactgctctctatgaataaatggttagggtagggtaggaattatagttcact
tttcttgggccttcgtaattcataaataaggagaagcaagaaagtttaagcaaaagcagtattccaaagttttggtacatttctttggaaatttaat
ataaagaatattatattactatattagtagaatagttgcttgcaataacgaaagcatgtagattaaaatcaaatacatctgaaaggtgtacggata
tatttgatatttggcatgagtgttttgtacatattagattaaatgtttgaacggaccataccaatatgatcatagaacaaaatatgaacaccaaacg
aaatacaacttggaacatcatgcaagctctataaaaagcatctagttttatttttaccaacaaaccattaaaatactcataattcaatttcaaacac
aaaccaaaagaccacagcacacaaaacaagcccgtaagactttatatgctgggattaataacgccgccgcaaaatagaaggtgtccatggtag
attaggatgagtttcgggggtagcattttcatcttgtttatagatttttatactcttatcagcttcacaagtaatcaaacgaagacctgttttgtcgaa
ggtactagcaaaaattccagcttcactatccaaagaaccaggttgaacggtagcttgtaactcttgatatttatgaccagatttccaatcccaaaa
acacatgcttccgttgtcagcaccagaaaacatagtattatccggattaattgacaagctgttgacaatcgcattatgcccttcaaaatttcccatg
aaggccccttcaggaaatttccaatgcttaatattatcagaagagccactggcaaatgtaaattcttgaggatgtagagcaagggctcgaacagt
ttttttatgatgcgttagagtagtcaatgttttaccagctgccaggtcccacaagcgaatagttgaatccattgatccagtagccacttgaggctcgt
attcctgtgtagccaaggatgcgattgtggatttatgaccagcgagtacatgaacattctggcgcgttcgcatatcccagacccgagcaactgcat
cacgaccagcggttactagcacatcgagagtaggatgaagacttaaagcataaactcctgataaatgtccatgataatggcggattactttgttc
gtttcaagatcccaacattttaccattttatcttcaccgcaggaaaacaaataaggatgcctgggtgatacagcaagaccacgaacacctgcaat
gtgaccggttaaagtgagttttaatgttcctgaagcaagatcccaaattttgattgttcggtctccggcgccagtacaaaaccactcattacctggtt
caacatcaacacaacgaacccatcctaaatgaccgcttacgacacgcataagcttccacggaggatgccactctggacggatttgctctgctagt
gttcgctttaccaacgatgcttgcgactcctgcgcaagtctatgagcagcattgggaacattattaatcttctctaatgactgcataggaacatgac
cagcgattggtttgtttccgtgaagactaccttgctgacgaactagctcttgaccagatgggtcggtatggtttgccgttatagtgagagcactgct
aggtgcattttcaagaatttttccttgacagcagcatttttcttctatgattgacatcttctagtatatttccatattcaaatgattccttaaagctcc
ttttggtactttgcccaagggttttgtcaaaggtaggtgcagacgcgaattcagggccaaataaagcctttgttgtccgcaaactatttgtaactaa
agacaatacgtcttgactgtccaattgatcttcgttctctgccataagtataggattcagcggtttgtgccttttccctctgtatggcagtcgctatga
gtgtggtaggtaaggttgtacaagaattcacttacagtaaaaacttaaacataattgtattcataatccattttgtagtagtaagtatgcacaatttt
attaaagatgggtacaaagtgaatttgaatctaaagcaagagatgatgtttagcttccaaatgggatacaatgatgaagaggaagtgaaaagtg
taaatttgaaggtaatataatagacgtaacgatcaaaaactaacaaaaaaggatgaaattaatttaagaaatgttatgataaagccttctgtata
gtagcgacagcccattccacattttgcttaacactcaatttgctggaagggctgagttgtatgcgagtatctaccaatttcccatgaacgtctttgttt
tgaaaaagatgagcatgaccgtttacataacctggccagacgtaattctcaaagtagttaggtggatcctgccaaaaccctgaatcaattaaaat
taatatttgaacataaagtcatttttacgaggaagacaatcatgatataacataccttcttgggtcacataacctttacgagattctctacgagctct
taatgtatcaaagtcgcaaaccagcataagacagagatcaaacgatcggataagctcttcgtttacatagagcataaacccatccacaaatata
attttgtaatctgaagaagaaatagggctttctgggtattcttcaaggattgaagtaaactgtgcgagagcttcttttgccacgtttttattgtctcga
ttgtgaagatgagtaggcaaatcaccatgttcacggatatattttagtgtttccaagaataagtccaagttcaaggactctttgcaatcccagtcgg
caatcccatttttacaggaatatctgcatcggtctacaggaacagaagagcattagcacagatcaagatatacaaattctagcaagaaacgaac
cttgtaaaagtcatcttcgtggagaatggaagatcctttaaagatagcatgaagaaactgagtcaatgtctatatccaatcgtggagaggttaatg
ttctgaaaaaaaaaaaataaaataaaataaataaagaaagagaagaaaggaaactagattaagaaataagaagaaaccccaagacagcaa
gcgtcaaggaagcaaagatcaatatcataccgactttccactgcaagaagcaccactacaaagaaattaatatcaatatcaatacgcgtaagga
agacagtgatattcgtacctaacacccacaatcacagttttaggaggcattttcgtaaaaaaaaagagaaaaaaataaatgtacaagtagctat
ggatttacagaacgttcaaaaaaacaaagtagaaacaatacccaaacactagaaagagagttgaaaataggaagggaaaaaaaaaggtatc
aatattcctgaaatcccaaatgcaccgaaacctttcgcatttattcccagtctaaaagtataagggatatgatataaacttgaagaatcgggctc
tgtggttttccaagacgcaaatagtttcagagaagccaaatatttctctgttcaagatgaatgacaaaggaaagccttcaactgtagcaaatgctg
acacaacaccttcgcagtagcgtagcgactttatgaagcaatttgcaaattccaggcaaatctgagattttcttaagaaaaagaaaataaatgta
agctgttaataaagaaaatcaaacaaaacggttgaaaataagcaattggtggtctgcactatgggttggtatttggtgatggccgtcatgaatatc
attagatcgagttgagttgtacagagagggatgagtgagatatccgaggaaaagtatatatcaatgaagtgtttccataggaagagaaaacatg
tatgaactgaaaagcgtttttccatctgtccccctctctatctctctctctcttcacttaaacattcttctttgaaacatttgttgatatatgcttttctt
gttcattaacaaacatgctggatcaggtcaggcccaggctcaagaaaaacatttctttctcatgagatttctgtgtgattctcataaacattgccag
acgttctagagtaacatttccctcgagatgtgtaaacttttgttttgaggattatagatgcagatagctgtcttcatttttataaacagttgtcctaca
accatagctaagcttaacttcactttggtcctgtacctttatatatagcaatggacaatggaaaacgtaggcaaggggtaaagtggaacctggaa
atttcggtttcctcttactgatagagcacatttacagaaaaaaaaatatatgtgttcatctatttctgctcctactatgctgtttgcttttctttttggc

acacgattctactttcaaaccgtacaaaaacctctttataacgataactatataaagaattgaaagtcttcggggattatataactctacagtatat
agtctttcttatcgaggtccttgaaacaataggaattccctacaattgttactctattgttttcaaaatactaaaatagtcagctggaagcacatggc
tgtagtaagaaattccaaccatccacggctttaacaggtaaaacacggctgctgggtctatcgttccttttaatcttgctccttggaatgaaattctg
cttcaacagagtttaacaaattcattgaatcgtttctcactgatcttaatttaatcctttctttaatttttaatattttttctttccttttaatagaactcgt
attcattaggaacttgtttacgttcctctttctcgtttgtacttgtttattgcaacctttggatcgttgcttaaaagtacaggtggttatattcattaaag
gttccattatcacaacgacaccaattagtatttagctcatccgtgatctataccgacattgtggccttcgttctccttgtatgatttatgctttagcttc
ctttctagctgcttaattctgtattgttcatacacttaactagcatactttcccttctccctcttttcaccttaatttttttggtactcacactgactttctta
gtccattcgtccatctcgacaatatgcgtagaaatattcgtgcgatacacgctggttctgatgatgatgaagatgacgaagacgatgattactact
ccaaaactcgtcaatcccatacaaatccacatgctttttatactcattctgaaatgcctgaacaattcaattcttctgaacaagatgaatcctctgac
tctggctttccatcgttaagaaacttatctcaagtcggtcagaggtctccatttcaaacaaagcaaagtcatccaaagcctactttctccgtcgctat
tcatccctcctcgcaagcagctaaatttcataagcacaaacaacatcctaaaccccttaagactcctttgaaatcagtttccattgccgctttacaa
gctcatcccgaaacgaaacctatttctcccccatatgaggatgatttactagatgatcacttaccgtttaaacgagttattgaagaggatgaccaa
gatgagctcgatatcatctccccttccaagttagataaagcttctcatactctttctccgcctaaatctactaatgatgaagatgatattagcttacat
gatgccattagcagttcgcattccgaagacgaacaggacgagctaattaacgaagggcaatctgagttcttaaaatataattctcctaacgatttt
ttagaatcttcatctcctgtgaaacgaggtaggggaagaccacccaagaaaagacgaaccactcagcctttacgtagaagcgctcgcgttcaaa
ctcgcagcagcgttggacctttggacaagtcatctcataatgttcgtaacagacttcgaaatcaaactgctcgtcctagttatgacttggtagattat
tctgaagaaacgttattcactcctccacctaccaaagcatatccccttcacatttagcttctcttaatgcttttgctagtcttcctttgggtattgata
atgatcaaaaaggtaatgatctcacattttcagactcggaattgtctttagatctcattgctacaccaaagcaaaaagatagtccaatgactccttc
acataacgatgagaatgcagcttcttctatatctcagtcttttggccaactcgatcgtaggtctatcaagaaagatgacccttttgcaacagaaga
agctttaagctttgataatattggtggtcttgaggatgtaatccttcaacttaaggaaatggttttacttccactcttgtatccagaagttttttttacac
tttcatataactccaccaagaggtgttttatttcatggtcctcctggaactggaaaaactttaatggctcgagcgttagcagcaaattgctctattgg
caaccaaaaggttagtttttttccttagaaagggctctgattgtttgagcaaatgggttggtgaggctgaacgtcagttgcgcttactatttgaagaa
gccaagaaagcgcagcctagtatcattttttttgatgagatcgatggcctagctccaattcgtactactcgccaagatcaaacccacagctctatcg
tatccactttgcttgctttaatggatggtttggatagtcgcggccaagttgtagttattggtgcaactaacagacctaacgatattgattcagcttta
agaagaccgggaaggtttgatcgagaattttatttttctttgccaagccaagaagcgcgtatgaaaatactcaaagttcatacaaagcattgttct
ccttctttatccgatatatatttgtcagacctagcagcagctacctccggttacggtggtgccgacttaaaagctttatgcacagaggccgcgttgc
aagccgtccgcaggtgttatcctcagatatatcactcttccgaaaagtacttaatctcaccagataaaattcaggttgatgctcttgattttgaacgt
gctttggaaaaggttaatgtttctacacgaaggggcagcgcgattccaaaatttgcgatttctgattcacacaaactgctttttaaagaaacgcttg
atctactttcgatgaaaatatcgcatttactcaggttagatactcttccttcaaagtcatcgtttttttcaaaatatatcgttgtcgcaaatcaaaaaac
aaaaagaaatttattctttaaagaaaactatggtttaccgccctcgtttaataatcaccggtcatcaagatcacggtcagtcgtatttgagcaccga
cttatttcgttctttggatggtgtatacgtgcaatgtcttgatatttcgcagctgctagtggattcagaggtttctcctaattcttctctaataaatatat
ttgcctctgcaaggcatcaatctcccagcattatctttatcaataatattgaacaatggcctgttctcttcacgcaaaactttttggatgttttcacttt
gttattaaactctttaattcccatggagcccgtactattggtaggcgtagccaataccacctacaacaatttacctgaagtcgttcgttcttggtttcc
tcatcatttatccgagcattttgaactttcattgccatcatacgcttcccgaaaatcgtttttttatggacatcttgaagaaaattctggtactgccattg
acgcatacagataatgatgacccctctcaatgggaacagttaccaaaagcttccacttctcataatatcttttacaatactaaggatcgtgaaaga
caggaaagggtggataatagaagaattaagaataaactgaaaatcaagttattatccatcatggatctgcttcgctctcgttacaagaaattcaa
gaagccaattgtggtatgttatcagtttttattctcaactaacctcgtaggacttggccgaaatttatccaggagataatgagacagagccatcgtt
tgaagttgcacctgaagattttccttttttttataaaaggcaaccgtgtgattcgacgtgaagatggtgccacctttaacatgatgaatttagaagag
gtggatcgtcgtatatggtcaggtttttattgcacgcctcatcgtttccttcgtgatttgaaattaatattggaagatgtaagccattatggtgatcta
acattgaagcagaaggcaaaggagttgtatttaagcgcggaacttaatttggaagagatgattgatcaagttttcatgtatgaatgccaagagat
ggaagaacgagaggccaggcggagaaaggaaaaatctcttaaagaaggcaatgatgaaaacaaagatatgaatcgaagattaagtaatgac
aatgttaatgcgaatgacatgacttccgaaaacaacatacattattctagggaactagaaatcccaagctcttctcccgaagatgaggacggaag
tgaagagaaagaatatatgaagctacagtctgagaaggaaaacattcccgtagcacaaaatttctctgaacgaagctttataaataataagagc
ccatcaaatcttccgggtaaactaaatgaatcttctagtcacagaaaagaatttgaattaatgatgcgattgacagaagatattgcggagcatact
tccaatctaaagatggaatggttggacttaatttattctagattgtcgaatgtaatttgggacaatcatgaagaaatgaaccgaatacgtgttttat
cgttagtaaagcagacgttttcctgtatattaaaagagattcgttcgtcatattaataaattttgaatattttttttgagttaaatgcacaaaacgggt
attggttgggactttctgtgatatgtgaaaggtagaagtttcaaatgctattttttagagcttccaaaatgttgtgttggaattacacaaggtaagat
atttttagatccaccagtaaaggtgccgttagcgtctaaaagataaaagaagggtgtacctttcaccttcaaaaccatgggatttttgtcaatggat

tgtgagagaagggaaacctgattccgaggtaggacaatagacttttcaaaataaagcttgttgttgggccccggaagacccgtcttcaattcaac
ttgacgggctggtaaagtagacagagaatgaatggttcttcgaggcaagaatatgagaaaagcaactcccaaataaagtaagggaatgacaaa
gcaagagcctattcgagatatcttttcaaaataactgcaagtgttccaattctgaagattgatttcattagaacgatacatatccaatcctgtgagt
gtagcaaggaatgcagcatttatagtagtccctagaaaaagcagatataaaaaacgaaactttttgtcatttgccgcctcataaacaaggaaacg
cttttgagtcgtcatcgcctgccatgcgagatccgaatgaagagtatttattgtggctgaagttgctttatttgaagctttttttgcttttcctcaaggta
gagaaggtatggaatgaaccgctaattttgggaaataatagtgatgaaaggacacgaggaacaacaacggagttaaaagcagcatttggaag
cttggggcggattccaacatttcggtacatacatgctgcaagaacggagatatttcgagagccagtgaacggtttttgacgaaatcgttgaagatt
gaccgctagtctgacaggcagcattggaataagagtatgaattgttttcatgaagtgcaaattattttgtttggatttctttccaaaaatgaattgaa
atgctgctttttttcgattttaaatgacaactcagagttgaaagaataacgaagtggtgcctttgtttacgtgtttgccagtaaaagaatctgttcgca
gacaagccgaagtgatgattcagtatgatttgcacatatctctaagagaagaaaagcatttctatataaaattatgagagaaataaaagaatctt
cttctgagaaggtacgtagcagtaaaagcttctttgcttgaaggtaacctctatatcaccagtactccaagtaatttcttttaactaaaaaaaagaa
aaattacagctttaaaaataaaaacttagaactctttcctcttccgttttgcttcgtagaaggcacttgtaaagggtaggttgaaatagtcggaaga
gtcaaggaaagggctggggggagtccaaaatacagtttaatattacaaagagcacttgataatcattgataagcttcaaaatgccaaaggtaaa
gcagtccccatagaaataccaaaagggctttctatgcattcattaaataaaaataagctcaaaataaagttagcaaagcgaaaggtgctgattg
aaagattatgtagttgacgataaatctagaaatgcttcaaaattacagacatatagaataaacaaataaagaatttaagagatgaaagctcaat
tttgttttatcttttccttttacctttttttttttttttttgacatacaacttcagtcgtagaaagaagatttactcaagtctgacgaccagtaagcatttt
atatactacataaaaagcggccagagcaaactagcttactttttaggatacaacatatgcaaacaaacattaaattagtacaatagggtctaaag
gacaccgtaaatacatcgagagcatttctcatgcaaaaaaaaaatcgtaaagagcctgtttccttttccaagaatcggttcttgaaacaattgtta
tacatatatatgaatataaaagaaaaagatgtgtacagggatgaattaatagcctatgcaaagatcgagacatatataaatgtgtattaaagagt
ttgtgtgcggctaaatgggaaatgattgaataaaaaagaaagcgtgcacttcggaaaagaaaccgtcttatcttgtctaaaatgactgctcagag
aatatgcatcatcattagaagaaaaaaccaaaggaacgacaggaacaaggcttatgaatacaaaccaaggaaagaaggaaatagataaaag
atggctgtaatgcaatgaataatgtccaaagaatagggatgattaatcaagcaagcaagagagaagaaaggagaaaacttaagcctcttctttc
cttctttgcttctcaacctctaccgcagacaaaactggatagccgataaaaagcatcaaaagtccacagcttagcaaaattagagttaaaatatta
acaaagccacgaagcgtgaaaatattgaaatctcgtccatcatgaaaatcctcattatgtaaaatatcatccacctccttcacactggggtcccaa
agaagcgtagatgattgcaatggcataggagattccatagaattgagtgacgaagcactgccaaacggagattccgaaggataagagcgttga
gccatagacacttgctcatcacggtaaggagagaactttttctcggacatttccttagtatgatactctgacagcgaatcttccgaatgaccttcttg
cattaccggctgcaaatcttcagactcccaaatgtcatccgcatacgaatcttcatggtcgtctcttgaaacttcgtttgcaagttcatagttaagtt
gttgatcaagcacttcatcagtttctagcccatcatgacttcccaaaggaatccttgacattatgctaaaatttcaatccactccttcgtgttaggcta
ttcacaagggagcaacgccgcgtcctagtagatgctagcctgcattttcgttcgagtgcgttcgatatgttaacgttacgcaattttacaaagtatc
gatatagcactttgtcttacgttattaaccattgctagcctttctttttcgttctcattcctgcccttagtccactactagttataggcattggcaatctg
gaagcgtgtcatgtcgcgaattattatcaaaaaccttccaagatactatgaaaaagaacaattatcaaaatatttaaagtcccagtccaacctgg
acgttgaaatcactgatatcagcgttgcgaagacgaaagacggtgtaagtcgaagatttgcatttattggattcaaaaatgaacaggatgccgag
aaggcagttcgctatttggatcgatcttacatagaaagttcgcgcatagaagctcgtcttgcactggactatcaaactgcaaatgaaaaattacgg
ccatggagcaaatattcttccaagacaaaagaattaaaggctatagaacgggaaaaagaagcacagaagcaaaaacttttagaggaaaaaga
aaaggaacgacgtgaaaagaatttaaaaagaaagttcatagacaccctccaagatgataaggcccgtactttcttgcaattgtccacagccacg
agtaagtctcgtacttgggacaatgaagattacacattgaatcaggagccttctgttgctgaggcaaacgagaaggacgaagaatatcaggacc
ttccttccgctaaacgacagcagacagatgaacagctcgaactccaaaagagtggtgcccccacagtgattgatggacgtcctcaagaaaatct
agagtcagttgaaggcggtgatgaaccacaagctagacattctgattctcaagataatggagatacttctactactgtggagcaaaagcctatgt
ccgatgaagaatggcttcgtcttcacagaactagaattcgtgaaaatactgaaggaatgccaactactgaagatcaaactgtctcagaatccacc
actctccccgaatcaacggtttccgaagtagataactcttttgaaaagagccatccttcatttgaggaagaggctcaacctggtgtttctgactatg
atcaagccatcgaacaaattcaagaaaccaagcggttgtttttgagaaatctcacatacagttgtaccgaagaggacataagctctcatttccaa
ccatttggtgcactggaacaagtccatatgcctatagataagcgcacaaatgttcccaaagggtttgcatacgtagagttccgcgaagcggaaga
cgccgttcaagccttccaagaattagatggaagaccttttcaaggccgcttgctacacattcttcctgctaaagctcgccacaatatccttcaagat
gaatttgctttaagtaagctacctttaaaaaaacagaaggaattgaaaagaaagcatactgctgcaacaagtactttcagctggaacacattgta
catgaacaatgatgccgtggtaacatcattggcttctaggttgggtgttaagaaaaccgatattttggatcctacctcatctgactcggcagtccgt
caagccttgtctgaaactcacgttgttcaggagacaaagcgatttttttgaagaacatggcgtggatttagatgctttcaaaaacagtgctcgttctg
ataatgtgattttagcaaaaaattttccttacggtactactgccgaagagctgacctcgctctttggccaatatggagagttgggaagggtcttgat
tccaccagctggaactattgccattatcgaatttgttaatactccggatgctcgacaagcattttctaagctggcttataataggatcaaaagttca

attctatatttggagaaggcacctaaagacgtgtttcagacggccttcagaaagactcatgaaccggaagttgccgttaaagtggatgcgatcac
tgccgaatctgcgaacacggccaaggaattggagactgaagatgttgattctctcgatactgccacactctacgttaaaaacttgaatttttcaac
caaacaatcggagtttcaaagtgtttttaaacctctggaaggttatctgtctgctgtcatccgtgcaaagcctgacccgaaacgccctggccagttc
cttagcatgggttttgggttcgttgaatttaaaaataaacaatgcgctgtcgcagctatgcaagccatgaacggcttcgttttggctggacacaaac
ttgatatcaagttatctcatcaaggagctgatgctgcagcagaaacgaggaagcaggactcttcaaaaccgaaaggtacaaaaattcttattaaa
aacttgcctttcgaagcttctaggaaagatatccagtctttatttggtgcttatggccagctgcgttctgttcgtgtaccacggaagtttgatcgaagt
gcacgaggctttgcttttgctgaatttgtcactgctcgtgaagcggaaaatgctatgaacgctttgaagcatacacacttacttggtcgtcatcttgt
gttgcaatacgcttctactagcggcatggatgatttgcagcatgcgatggataaagctgccaaggaagctcgtgctgaagctggttctacttctttt
acaggcaaacgtcttatcgagactggcgaaaacgagtgatttttgaatttcagaaaagacattaaaaatttgggataccgggtataatgcgcata
taatttctttttggatttcgattattgggcgtcaaggcgcatgattattgggtatatgtttggtataatagtgtttacgaaatggatttattatttttctt
tttttgaaattgaatagtcataggaccagtttgaatcattgattcctacgttggtgcatccataatgtattaataacaaaaaaataaaatgttaagt
caaaagtagataatattagaatgctacattaaaatagtacaaaggaaaaccggttgaaagttattagtcagattttacttcaggatatgtatctac
atcgtcataaccattcacgaacagacctaagctaccggtgaatatggaaaagcctgatgaaaaaacagagaaaggaagctccacggaaatcgt
aaattctcctgatttcgttgtagaaatcaatgtccaaagtcaggagactgagaagacagttcgtgcagtactgcatgaaatgctacaagctgggtt
tgaagctgccgtccgacctggcacagaatccagcctatttatattgattaaggctccttcggaattagtccttcaattagtcaacaaagatcgttctt
gttcgttcctctacggatcattgaatgatttggatcatgtgcaaattaacgacattacacaggtggaaacaagcgaccgtattcgtgttctttatga
atacattactggtctcaaaaaagagaattgtcttggaattgttcccagtgaaaacccgtacaaagacataatagatatttttccacttcacaatgtt
agtatggacgataaatggatatcttcgtggaagtttaagtcatccttgaaagttcaagatttggataagatcaaatctgagtatggttctcaggtaa
gttgttatgattgacgcgattctttttcattggcgagtaaaaggttaatcatcgttagattgctcttcattttgcatttcaaaattttttaagaatatg
cttgctgtattatccgtttggggagctttgggtcactattcgtttcagcaatactccgttatctactccgttggaatgtctgtatggggtatatgtttcat
ccagttatggaaatataaagagaatcagctgtccaagcgttggtctacgagatatagtcagtttcacgaaaaaagtagagaagagttccatcctt
catcggtacaagtcaacaatatgtccggcaccgtacaatcttactatcctcattggaaagtggttcttcgaagtttgattacaactgctcctcttttc
tagtttcaggcgttattttatttattctaatatcaattgcttttgctttcgacgtgacgctaactgaggtttattctggtccgtttaagccagtggtcagt
cttcttccagctctaatcttccaagttttcactcttccttttaatattatttacacattggttgctcaaaagcttacagcctgggaaaatcaaaggacg
aaaaacaagttccaatcttcgctggcctccaagattttttgcaacattttatgctttcttatacagcgcttattttaatctcatacgtttatggtccttt
tggcgaacacctagtttcgaatgtcttgcaaaataaagctgcgaacctgacaatgaagctttcttttataacagattctaagtttcaattaaaccctt
taagacttcgcaatcagtatttctacttcttaacgaatgctcaggttataaattatttcacaaacctcgcgcttcctcaaattattacttatcttaaat
actacgtgaaatctaagattggcatgaagcgcttgtctattcacgatgatgctgctgaggccgagtccctaaattttattcggaagcaatctgagct
ttctacatatgacgggtacaatgactacaaggatttggtattgatttttggttatcttgttatgttttctcctatttatcctttagctcctttatttatgta
cgcaaatagctatttgatgattcgctcttcagtatacaaactttgtaagttgtcaaaaagacccgttgctgtacgtatcgaatccattaaagattgg
aatcagcgtcttactttcataagttggttgggcagtattacgatgccttcaatcagctttctatattcccctatgaagacaatgacaaaacaaaaac
tttactattctttgttagttggtttcttctctgaacatgtatggtttttacttcgccatacaataacatcgcttctcccaattgaagaaaccccacgattg
cccttccaagaacgtgaacagatactacatgataaatttgaggacagtgtatatagcactggacatctacaaagaacaaatccttttgacgagag
ccttgaagtattagcattctatcactcggcagaatcaaagaaaatcaattaggattttattttcctggtatcatttgctatagtaattacggatatatt
atatctgattagaccaaaataggttatgcaaggcattccatttaaacaggttatcctggataaaattaatgtagatggagatgtaagttataatga
ttggaagagtacttcaataatgcaaaccattgtgcatatattaagagaacgatcaaaaaagaaacagattagatccaaaaaatcaaagaaccg
ataaccaccgtttcccatgtagtcatagctttcagcatatattaaaaatcaaattggcgcttatatggtttatttcctcggaaagactgcttaaaaga
agtctgacggtaaccacgattcttacggtcgggcatacgagaagatttgtttatgatgttcctgacatacgtacttgacatcaaatcttgttttgcag
ctggttcgtcttcaagtccaaacaatttcaaaactaactcattcgaattcttcgtttgtgaacgatcctcgaatatttgggatttcgcggcgtataaa
gtcttcagtgtttgaatacgcatatcactagaaatatcttgcatattcttgttatacttgattatttcttcatctgatagtccatccactgagtagtcttg
aataggcaactcagaaaggcttcgtaagaagtattgctcttgtttaggaaccacaaggtaagactcaccactctttccggctcttcctgtacgacct
atgcgatgaatgtagtcatcaacagctgaaggactaaagatttgcaccacctgagaaatattggggaaatccattccacgggcaactacatcggt
agcaaataaaattcctttgggagctctacgaaacttatcagtaatacggcgtcgttcgcctgtggcaagtcgggagtgtagggcataacaaggga
tatctaattgactctggaaaagatcagcaagaaaatcggccattccgatcgttggtagaaacacaatgattcgaggagatggctgttgagagagt
tcttgtaataaagcaagagagtcatgaaacaaagaatcaagttctgtcttaaccacaaattgaggaacacgttcgtgtgtgggaggctcattagg
atccaagcagttgacaatcttaattttatttccaagaatagcttcaaacacgtctgatactttgggcggcattgtagcactaaagcaaagtcttctg
gtaaaaggacgtgcaacatcgcgatggatatctttaatagtatctgcaaatcccatatccattagtcggtcagcttcatctaaaacaaaagagttg
acagataacgcatttctcacttcagggtttgttaggtgatcatttatacgaccaggagttgcaatcaaaatattagcaaaacctttgcggattgagt

tcgattctgacatttttgagttaccaccaatggtaaaggtagttttaataccggtacctggcgtcagcttcttagcctcctcaaaaatttgtaaggct
aattcacgcgtcggactaagaataactgagtgaattttttcgaatgtaacgaaggctttccctttagcatgtcctttattgcgaccaataagaatg
ctaatgttttaccagttcctgttttagcctggacaaccgcgttttcgttatccggaagcacttcttgaataacgcgtccttgaacgggagttgtcctgg
aaaacccacgatccgtcaggttcctttttaaagaatcaggaattcctctaatttcagaaaacaatacttcagtgggtgtctcagtaggtgtctcagt
agatgtctcagtgggtgcctctttgctagttgaaaccgtccggtggaaagactgaaaccgtaacgaaggacatctttttaaagcgctcacatacct
gagcgatattttcatcattttgatgccaaatattgtccaattctaagaacaaatggatcagaccgaaaatttttcttttggcaagcaaacggcaacc
tggggttttcggttaataagtactaggaccagcttagcgtaattagttagcattcgttggtaaacaaaactctgggatattctttatttcttttatttct
tttaattttttatataaaagaaaaaaaaataagtaaagaaacactagaaatactctgaaatgctacggctgagattttaagtcgcgtttttctatct
cagagcggttgtgtcctagctacagcaacaattggctttcttcgtccatatccgactttcttattgtttgcgatcaaatacgagaattttgaaaaccc
cgtagctcagtaagcctaagtaaactcaattggcaggttgaaagactacgttgaaagaaagggtgtgaatcaaactttgaagcgtgaaggaaag
aactatggaataattgggagcacaaacattcatgtttagaggttttcatttattgtgaattggtaattgtaatagaaacggtttggaaaaagacaa
aattagcaactttcaaagatggttgtcaataaccaaatgaaagggctgaaaacctttatttcagacttaaggaatctaaaggagaaagatgagg
aaaaacgcgtaaatatagaattggctaagatccgagctaaatttcaaagttcgacgctttctgcttacgatcgaaaaaagtatgttagtaaactct
tgtatatatacatgttgggataccctatatcttttggtcacttagaggctgtcaagcttcttaccgctgcaaagtatagcgaaaagcaaattggatat
ctggcggttgctctgctgctgaacgaaaaccatgatttaattagattggtcattaatagcatcaagaaggatttactgagtttggactcatacaaaa
attctatagcattacatgctgtcgctaattttggcggtcgtgaaatgtgtgagacagtatatccagaagttcagcagctgcttatgtctgccgcaact
gagaatattgttcgtcagaaggcggctttgtctctgcttcatatatatagaaagtttcctgatataatggatccagacttgcgagagcccataattat
gctccttgacgacgatgacttgaatgtatcccttgcagtatcaacatttgtgctcttgattgtctgcagagaaccatccctacacaaccttgtttgta
aaaaggcaattcataaactaaaattgattgtttccggaaggggatactcatcggactatgtttattattctgttccatgtccctggttacaagttaat
ctttgccgcatattgctggcttgccataaagaggtgattgatgatagtacaaagcgtactctcattcatgttttagaccgaatgttgagcatctttga
cgatattaaagagactcagcatctcaatgcgatcaatgctattctttttgaagcgataaaacttgcattcgctattgatgattctggttctctttatca
aaaatgcatgaattgccttggtaagatgattgccgacaaagaagcaaatgtacgatatctagcgtttgatactacagcttacttaatttcatgcgg
gcatcatataccttctcttaaaacacacaaagaacttatactatcttcactacgttataaagacgtctctcttcgtaagaaaagtttagaacttttgt
atatgatgtgcgacgctgaaaattctaaatctgtagtggatgatttactgatgtacctaccctatcttgattctgtttctcaggaggatctaatttcta
aagtcgcctctcttactgagatctatgctgacgattataagtggtatgtggatgtcaatttacaactgcttcgtgttgctggaagatctgtgaacgat
gatgtttggcatcagtttgttcgtgttatcgtgaacaatgaaagtactcagcaatattccgtccgtagacttttcaaattactacaatcagattcaac
tcatgagtgtctagtaagaagtggttgctatctgttgggggaatatgggtatttaattgcggatgagccagaaagcggacctctgcagcaatttct
aaccgtctataagaaattgcatgcgtcttctacaagaacgaaagttcttttactgacagtattactaaaattgtcgaatttacagcctgatttgcag
aaacgcgttgcatcagtttttaagcaatattctgttagcttgaatactgagatacagcagcgagcttgtgaatatttacagcttttaagtctgccaag
tgactttttatctattgtttgtgaaaaagttcctgttttcacgatcccagcgaaaagccaaccgtcctcttttattagcaagtcggatgtttcactaact
gattctctaaactcagttaaggaatttgatatttcttctagcctttctggattttaccgtctatgctggagagacaaaggtatattgtatagggactct
caagttcaagtgggtgccgtcatggaatttcatgattcacaagggtatctttcattgtattttgagaacaggcaaaaagaaacgtttagctcgtttt
cagctactttacttaggacatttcccaattttcaggtggatgcttctttcactgattccattttctctccagataaacaattgcagcagaggttttcgat
tgatcgacttgatggattatttgatccccctattttgcgcatatcatttttggcaggtgtaatgagatcagtcaatctattactgcctgtcgttttgagc
aaatttatgctaccgacggtctttgatggctctaccttcttccaaagatggggaaaaatgggtttggaacgagaagctcaattgacctatggcttaa
atggtgtgcacagtaaacttgatgaaagacgattagtgaagattattcttggacttaattggggattatgtccaggaattgatatgaatcctactaa
cattgttggtgccggcattctccaagtgaatgctcagaatattggatgtttacttcgactcgaaccgaattatcagaaaaatatgattcgtctatcta
ttcgttcgacgaatacgactgttgcgaacgtcctggccacaaatatgcaagatattttaaaaaattcatttcattcagaagcatgaaaaccgatctt
ttacgcttttatatttatttaattccaaccgccgagatcatgctgaaaccgttaatgcattgaaataatattctacaatcagtttctcgaaaaaaaaa
aaaaaaaaaagaaaaaaagcagcacatcctctctgcaagcctttaactttatgaatgaatcttccattgtatatcaaaagcgccaatagtatattc
tatatgttcaatgggcaaatcgacctcatagagtttatttatatttattatactacatttttttactgttgaataatagcaacgtttgtggtaaaaattg
aaatatacctatattactgaccttcatttaaatgtaaataagattataccttgtaatattatagttatatctatgtgctaataatactaatgaaatatg
cgtaaaaacaacgtctattaattataaggtgaaaaagaagggatttgagttaggcattagctgttattggcatgaatttaacttttagtgactttatt
gatttacgaattcgacagccttacaaaagagatgaattgacaaagtttgttcaagacattgagcaactgcagctcccagattttgactcattgcct
gaaattacagaagaggaaaaacaaattatacgggaagatttcgagtacaaaaaaagcaagaattggattagaagatgcttggctagaagcgtca
gaggctgaagagccagtgccaattctcagagatacggtagttattttgatggaagttaaggatttcattaagaaagttaaagatgatacttcggat
gataacatatttgctttggaaaaagattggcatcgattgtcaactaaagaagaccaggctggaatcttgccaaactgtatcctcagtatgacaga
aatactttttacagaaaaaaaggagagtgctcagcaaaagctaaaagaaatcattcgtgaaagactgaaacttgatttcgaatggccttctctag

acggtaacaagtatgcagtatcggtatacaaaagcttcgtcgactatcttcgatccgtcaatgctttttcaaatggaaattctggacatcctttattc
ttggaagtttttgttgaaccctatgcaaatcagtttcgctatcatttcatgtccaaaagacaaacaaatttaatctcaaagccagagtggttttatga
atatctcttaaaaacttttcgttctttgagagctttttcttaaatctcagaaaatgtgaagctgactcttttttttttctattctctgactttatccttatg
cttaatgaccttgtcaaagagaagctttctcacattgcaaaatatgatgattatttagttcacctaactcacgaaacccttcattatacagtacactt
aaaacaatattttggttcagatcatgattacttgattgattttctgttttgtcagccttctatttatgataagtggctcaaccttgaagctcagcaaat
gaatgcaaaattaaatgatataaaatcgtccaaagatgcttgggatatggcggttgatcaggctaatgactataactttacggttccaacaaaga
ctactattcgttttcgagacatgatggaatcaacgtatgctacactccggctgttaccttcttttaagtataaaagggatttttttgaaaaggttcaat
tggaaccgttgcaacagtacgttcagtggcttcagtctttctatgaggcacatgagagctcccaaagtgaagcattacctggatcattacaactag
gccgtcaaaatgaaactactgaagtagaacgtatgtgcaaattgtatagcaattttcatctcttaaaaaattggatcgatgattatgaagatgaag
aaatatatattgagattgggagtcattatggtcataaagaacattctgccttttttcgtcatgaagaatagaatcgatgttttgaagaaaggatcatt
ccgactcattttacgtgcagcaattcagatgctgcagcctttggttaatgactacgctgacatcgatacgtgggtaataaaggaacaaccattaaa
tgttgatattgctacatctcccgtatcctcagagatactgaaaattcaaagttctttaaggaaccttataagaacgttagaaagtcttctttccggtg
cctcgttgcgtgaggttatatatcaattaggaataattgtcggaaactggtttgttaaaatcataatgtcccatcaatttagtctccgcggtggtata
cagtacacaagagatataatgcagattctattggaattttcatactatccattactaacttttaagaaattgacatcatctattgaactgctgtcatt
ggatctgggcaaagaaatgaccattagaagttttataaaagcgattaaggatgataatcatcaagttgttgaccaagtattgaaagaaaaaggg
attgagcttcaatattcagaagtgcttgatgttttatatcgacgagtagaggcatggaaggattagagtaatcctaaattattataaattacttatta
cctctagggagtagaagtggcctttaagcgcattttgtgcagaaaagaaggattgtactcatagtgtataccattaccaggtgaacgacgaaatg
gtaaaaaccagtcataccaatgatcgcccattacagatctccagttttttgaataccccaaatcccaaggaaggtcgcccggatcgctttgtacat
gaacacgactggtcatttgctctggaaaaaacacactaaagtgataaaccttcgacttccactttcttccttcatggctattcatgtttaatagtaga
aaaacagtctgtcgaagtaataaagcacccatgaaaagaacgccaaagccagaactaattatgttaaatacgaaggtacaaagtttgtgaaaa
catacgatattaaaacaaagatccaagttccaggaaggcttcgatattctcctttacgagcgtacattattgcagtgctaatcaagacaaaaaga
gatgcaatggttccataaaatagaaattgatagaagaatttgtgattgctaaaacaaatatctttaccaacgctattaccattattagctagaagg
tataatcaaacaaacaacgtacaacgcacagtaatgatcaaatttttgatgcatttcatactgactctgctgtgatgagatctatctggtagccaa
catttacatgttccacatatacgaggggcgccgtttgggccacaaacaaagattgtagttgtatcatcgtagtaatccaaagcttttaagcgttagt
aaatctgctaaaattattttgtgataaagcacttactaatatcaccacaataggacggatttgagaacagaactcgaaaataggataaatagctt
aatcccacgcttataaaaaacaagattacgaatgtaactttctggttaataatactgctatgaaggaaatgaccgatcattgaacttcataaaaca
tacttcctgctcccatagccctgtatccattttttacttttatattggaattaactaaatagataattagtttgaagaaaaattacagtacgttcattta
cataccacacactaaagcgacatagacccatactatataaccggtcaccagtaaaatcgcagttgggtacacaacgcccagaaacttttggattt
ttgaaaatttcaactgtttgtcaaaatctgtcggcggcatacaagacctaatgtcaaatgcagccgtttataaatttgagggctgttgttgtctaaat
caaaaggaatgaactcagtatacaaaatgtagttatttttttataaacctaaaatagctcgaaagtttctctaatagaaagaataactatgattcac
tttaacattaaatgtagtaaagataattgacctaatatactattacgatctctttaatgagatccaagcagaagtcaaggatgatgcactcaaaaa
ttgattaatgaatcatattgtgctgtatatcaacctattgtactatttagagaatggaaataatattagcagcctgtaatctagctgcccatcacatt
ggagtcatacacatgagtaggctggattgtataacaaatgtcaggatttaaaatcttttactccgcttaatgttctttgctatcgatttgacgacaaa
tgcatatcagatctacttgtaccgtcaacaaatccaggtaagaggatatgtctatcgaagtaaaatgagcttagaaggagaggaaaaagatctg
cataagggacattctggcttagttttcgtccagttaaatatacaatgccaacagtatatatgcccgcattcagtcactgtaggtctatgtatccattc
catacatagagcgcattttctagagggagcaggtataaaaggaaatgctttaggatcctctagtgttggaggttctaatgaccaagagtcttgatc
tgtctctggattaagcaaatgcttgcctcgttttgtattcctcaagaaggatatcgcaagttttatgaaatgataaattataagcagcaagaattcct
tacttggaagatttctccttgaggtatctaatgaaatataatgaaagccgagtaagagttgagaaaatgatcgacaatgatgcttgttaattaaga
ataaaaggcggatgatggaaaaaatacgtgatagagctggcatccatttttttgagaaaaaccagtaaccttgtaacaagttcatatttcgtatgc
gagctgtctgaatgatcatgatgtgagccatgtgtggagctcatacgtctgacactatgctgaccccatttttttaatagcaggagtattatttcgga
tcctaaattgaataggatttcataagtttgttgctttggcgataccttttccaaggcagacctgccattagatttcaccaacagtattctagatacttc
attacccagggattcacgtcctaaacttgacaattggtaaagcaaattcgctaaagtactgaaacactcttgtgggacgctggaaacccaaagag
gtcttggagaagtcagtgttttcaacgctgtttcaatctgacttcttaagaattgtgtacataaaccatcaatttcatttgaaaggactgtatttgtta
gttttgggttcaaagtataagacaagacgaaattggaatttaccgatttgaagaggactagcagaactgaacaactccatgcgtacgaaatggta
atttaacaggtttaatgaatgacttggctaataaagaagtatttatcaaactatccgcagctaaatttaaatgaaaatttttgaattcttgagaaat
agaaaaaaaggcgcaaaaattactgaagacaaaacagctctgaggagttaaggaatattcatctccggaaatccagataaccaaaggcaaaa
cgctgacagatatattcaatgaatttaaggacacagttattatctgaaacctcgaaacaatggtttaggctattacatgctaaagttatcgtaagta
agtcagttgacccatactttaacttggctcttgaagactttctgtactccaacagtaaatctcctcggtctttaattatttataccaatacccctagtgt

gattattggaagaaatcaaaacccttgggtagaatccaatgtgagaaaatgcaggagcaatttaatcaacattattcgtcggaaatcaggaggc
ggtactgtattccatgatttgggaaatgtgaactatagtcttctcacagaccgaaacgagttttcacataccaagggtgctgaactgataattaag
gctcttcaagaattaaacgtaggagcagagcttaaccaacgtcatgatattgttttaaaggaaacgaagcaaaaaataagtggctccgcttataa
aatcagcaaagggaaagcttaccatcatgggaccatgcttctgaaatcggatttgggggagattagtaagtatttgaaaagttcaacgaatggca
ttatctcaaaaggtgtttcgagtacccgatctcaggttggaaacgttgaacttgctcgaatagattttatgatggaagcaattttaagctttttacgc
tacgactttgctcggcattcacctaaaaataactctagtatttctcgctttaatgatcttaaaagcatagactctattccggaagacagcattatgttt
gtgaattcagatgatcttactggtgtgccgcatatattgagaactatgcaggagctcaaatcgtgggattggacttttggacaaaccccattctttc
agcaatttttaactacaaaggatttacctattggtgaccacatggttgcacttaatatatctgtacgacatggaataattgaaaacttcagtttttca
agttcaaatgatcaccttcaacaagaaattatgaattatccttggaaaggaatggcatatgaattgggatttgtcaattcttttactataaataaca
ctccaagctcagaagcacttgttgttctcgattgggtgtccaaaaatatctagcccaacaaggaaatactattttcccaaattttatcagctaattct
tcgagatctattccttttatcttggctacagcttctgcaacgaccacggtgttacatggttcgttccttcctttaaccatacatccttctttaaatctctc
ttttttgcatgtatcaaaaacgtttttggaatccaagaaagcatggccagcatgcgatggacgtatctcacaccaaggggcatctgtagaatccag
ttagaattttttttttcaaaattatcaataattgatatagcatagaaaacgtacccgtctctaataacattttgtccaaaggaattgcttttacaactg
caacattttcaggcgtttttaatgagcaaccgttgactccaacatataggtcatgatcgacacattgctgcatctcctcgatcgttccggtgaaaga
atgtacaacacctttttttaggtagatttggtaaatatttttcgaggatttcaaaaaagtctttggctgcattccttgaatgtaaaaataaaggcaatt
gcattttgacggcaagctttaattgttcttcaaagtatcttttaatgttaaaaaaggaacgctaaattcacttaatagtaggttactcacgttttctgt
acttctgctggagcataatgaagtctgtcataatctaaaccaaattcaccaaacgcagccactctccctgttttctttccttcaagtaccagggcttc
taattgatggaggtagtcttcaggtccctgaggattcttattaaagcactcggcctgacaaggatgcactccaattgtacatgtaaaagaatctgc
aagttagcttcatactaccattgaaaaaaacgtcaattttgtttatgtatgtaaccgttgatcgcttaggtttttttcataccatgttggtttgcaagct
ttaaagcttctcttgaatttacgacattgtcaccagtaatcatcatttttttggacacctttcgacaacgcgcgagaaaggatagcgtcaaaatcatc
tacatggcgttgtttctcatgataaaagcctcgaaaaacaggatctgttgcgttgaatccaatatcgtaaaaccgaagactaacaaataaatcagt
attagacacgatcaaattgctttactttttgtatcgtattacctcttttgaattgcctgttttacctgagccatacttgaacaggaaattaaggatgatt
tttgattttttttattccaatgaagtgaagctgaaagaagaactgggcactgacaactacttctattttaaaactagttcatttctcgtataaaaagta
tttactttcttaaacgatatattatttagtaaataaatttctctcaatattaaatttttacaaaatcttattctatttgtgtctcaggtatataagattga
aaaaggaacggacaaatggattaaaatagaaaatgtgaatatgtttaaactaaagttaccaattcctctgcagaagtgggatgaattgcgacgc
agttatcaaagtcggatttggtagcacccactatagtatgttagcaacaaacttgacataaagaaaaacaaaaaataaaatcacttactcttaat
agctacaccaaaaccttgaagaatttcagcagaaaagtcaccaaccaagtgcaagccaacgaccttttcaagaggaccagcacatactaattta
taagtagtaggaaccttatcctcttcttcaaccaaagcataattgagacctctaaaagtggatttgtagaccttaatctggttttcgccatatttatc
aatagcttcttgttcagttaaaccgatggtaccagcttcgggatgagtaaaaacaacagagggaacattaacataatccaaatgagcatccttaa
ctcctccaaagagtcgatcagacaagcgacgaccagcagcaatggctacaggggtgagctcaagcttgccaacaacatcacccaaagaaagg
atagaaggaacatttgtgcgctggtattcatcggcaattacataaccactaggaagggtcttaacgccggcctgatcaagacgtaaaccctcagt
cttgggggtacgaccgacggcccaaaggaggctgtcgacatctaaagtaatttgattgttatgaagacgcaattgagaagaaggaagcttttcaa
ccttactaaactcgtgataattactatggatttggataccaatgtgtttaaagtacttcataatttcatcgctgatgactggatcaaaacggcgcaa
aaacttatcttggcgaataaacaggtgggtttctgtaccaagagcagcaaaaacaccagccaactctatagcaatgtatccagcaccgacaaca
gcaaccttcttgggttgttcttccaattcgaagaatccatcactgtcaataccgtattcggcaccgggaatatgagatggccagataggtctaccac
caacagcaatgagaatgtgttttgcagagaatgtttgaacgccagagccgtccaacatatctacggcaacttcagtgggtgaaacaaagctagc
atgaccagaaatataggaaacaccatccttgtcgacattacgctcataaattccatttagtcttttaatgtaagcatctctcttctttttttataagacc
ccagtcaaagcggccaacgttgctctctgggaaaccttgttggggcgccgttttcatcttgccaactaaatcggctatattccacataattttttttgg
gaacacaaccatagttcacgcaagtaccacccaaacggccagaagcttcaaccaaagctaccttggcaccatatttggctgcacgtcgagcaga
ggcaagaccacctacaagagcatacgtattaataactcctatcaaaattattcaattcaataacacataccgcttccaccgccaataactaaata
atcaaacgcctttgtagtaggaggcatatttcttcgcttaatgtgtaagggaaaggtactttctacttcgcatttacatcgctctgcatgtagcgttgc
aatacaagggtagggttatgatatgttaaaatcgtttcgctaatcttttgctactacggcaaattattagtaaggatttgagagatgttaatgaagt
aaatagatttgtaaaattagatgttgatttcttttctaagcttgttattatacataggagtaaaagtacgagtaagagatttaggtaaagtaacgaa
tgaatgaaaggtttgtaagcgattcctccatctattcacatcttttaattttggagtgtaagaaggacaattatgaatacaatgaggaaatctcaaa
ataaaaaactgttgaaaagagagacgacataaacaagtaagaatataacggattgtatccgttatatgacttttcgaacagcatccacacgaga
ttgcataacgctcagagcaggacatacccactcaaggcaactgcattgcattccttgccatcggtaggaaccaatctttgaattgcacttgggaca
atcgaaacgaccctctaaatttccttgttctaattcaggctgcatccaacgaataggctcaagaaagtaatgagtacagcggacattaagcggttt
attctttggttcatgaggaataatatagtcagaggatgcaagtataaatctgcactttttacaacgaacttcggtttgtcctgcgcgagctcgaacg

gactcagtgtaggttacatcagatggcactctgatgtttaggacagcaaaatcgccgtacttatggaagagccatagtctatacggacgttgttgtt
gagaaagctcaaaatgacattcatagaacacgtgcaattgccttaaaaagctcggattcggagaaatatacaagcgtttggaacaaatataact
taaggctttctcgggattccattcatttttcttcattaaataagctgctactagagtgacggacctagatacgccagcgaaacaatgtacaagaac
accagacttcgaagcaacggcttgatcgataaatttaattgattgcgaaatatgttgcagaatgttttgcgagctcccatcctcgacaggaatcca
cagatggtgactgcttggtaagtttagttttgggtctaaactcatagcactcagcacgtattgtatatcgttcttttccagtaaacttttctgtgaagc
agccttccaactgcttatgtagatatattcatcaattttggatatttcgtctaaagaatctgcgtagccgacctgcttgatagctttttccatggatcc
atcatgttggagggcagcttctgcctcttggacggaagaatgaatctcactgaatacaggctgccatgtcggttcatcttgatgcagttccattttgc
agtacaagtctatttctcttattttaaaaaaatgatgaattacaaagaaaacgcgaacaaaaaccaaacaattttaataatacaatgctccaaaa
atgcccaaatatcccaaaaaagctgtcttttcctctttgcgagttcgttctcttaaggattacctgttacttggtagggagggtgctaaagttgtggct
gttacctggtggctttcgaaaatcaatcgatgcatgcttctatcctctgcatgcaagcataaagggaagtggcattgtacttaaaatacgaaaata
aactcttgttttattaagattcttctgtaaagttttattgcaatggaagcaagcagagaggaagcgcttcggtaagattattttcgcgaaatattgtt
tttggaagctaattgttgtcattgtagctgtgtaaatctggctaaaaagtacttaagtgcaggtgattatcaaaaagcgtccaagcttgctgataaa
tcaacacgaatccttcctacatcagaagcagaagacttcaaaaggtatatggcaaaagagaggacttctggtaaaaaggaagcttctgccgcat
caactggatctaataatgctacatcaacaatgcgagagagaacccctaatgcatcgaaaagcaatgattcttctcagacatcgtatacgcgtgaa
caggtagaaattgttcagaaaatcacgaaattaaaagcgcatgaatattacgagattttaaacttgaaaaaggattgcaatgacatagagatca
agaaatcataccggaaacttgcgctgcaattacacccagataaaaaccacgcacctaatgctgacgaagcttttaaaagtaagtaatggtcgatt
actttgcactttttccatctctcctatcttaattcgataatgtgctaacacttgttttagaggtttccaaggcgtttcaggtgctttcagacccaaaca
tgaaggcacactacgatcgtactgggcttgatccagaatcacgagccagcgcttctagctcttcgttttctgagcgagctggtccaggctttcaggg
cttttctgcctatccacaagcgaatatgtcacccgaagatctgttcaattcgttctttggtgatcaattctttgctggtaagttacatgaataatagta
tttcaattcgaaactgacatatatctttaggagggccaaatacattcttctttggcggacctggagtccgtgttcatcaattcggaggtcgtcctcgg
aggttccgtaggcagcgagccgaagatactagcaacaagagtgtttttttatcaaattctccccatcatcatcatcgttattttttgcatttttgtctaat
tttagttctttggacggtaatacatccgtccgtgctggatactcgtttgtacctacagaaaagtattctgctcaatatgtttctcccaactacaaggtc
ccgttttatgtcaaccctaaacagtacaactctttgtcttctcgagattcttatagacttgctaataagattgagcaacagtatgcggaaaaagtgc
acgctgagtgtattcaagaaagagagtacaaggagaatcaaattcgaaaatcgtatggctggttctttcccgatgcagagaagttaagcagggc
gaaagaaattcctttgcctcgttgcgatgaattttctaggctcagttatcataaaccttttctaattattactactactaagtctaatactttaattca
acactttgattcattggctttttcattcgttatcgttttccctttcgatgatttacttttacctaagtacataatgattgcgaatttgtatagaatagaa
ggacggatagaaacttgagatttccttttggcaccctttcacttcgttttcaagcttaactcttcaagtcgttactgcgactagttcgttctcttttgat
gatttttcccttgttccattaatgactttgggattattaactaatttggaatattgcgttttgcaaccttacattgctattcggttggtcacttattttctt
cggtttaataattccactctataaaaatcattatcgactttattcctatcggttttggttttggtttcacaatgtaccagcattttgtcgaatttgtttaa
tagtttactactttaaagaatctaaatgccatgctacatagatgtgaaactcgtctatctgtattcgaatatttgaatattttcatttctttcttctcgct
acatgagactgttattacaaaacaaacaatgtatgaacctatttttaaacatttcatgaataaaccacatttatatctttacgatatcttagtctatg
aatctagtgctcactccacctccttatatgcttgatctgctaatagattaactgaactcttcataaactttttaccggaaaagaattgcctaaaaatg
gcaactattaaattgcagtttcctgcatccttaaagacttcttatagatttgcaaactctttaaaagttgctatttctgtccatgagtatggtgtaaaa
gctcctatagaagcaggtaagatttagaatacgaaaaggttggtcaataattgacacgaaaatagctgcaaatagctcttccgttcgattggtag
atgccaaagatttagacaggtttatttctgatgccaacgcgatcataaggtaaggattcacttaacccattcaaaatatgtagggatgcttacagtt
acatagttatttatattggaagcaaaaggacgtgtcttttgaagaatttctaaactctaaaatgtctattttggattgggaagctttgacatttggtc
ctttggcgaaagaagctgctgagaagaaaaatttcactcaactactaagccttttacaggacaaaatcaagcaggactctgtttctgaaaaatttg
gtcctgccgaaattgctcttgccgctgacattcatttctgcattgccaatggtgctggcttggaagagtacccagaattgagcaaatggtacttgaa
ggtcgaaaagaatccttcttttgtaaatgctttaagagtttgtcttgaaaaatcattaggtcagcctgctgagatttccgctaaggttcctatttctac
agaaacccgaaacattcaaactagcaatttgatgcgtgaacggaaacaatcggaaaagcttctccccaaggcaaatgaacccaacatcttgatt
acctctgctttgccttatgttaataatgtccctcacttgggtaatattgtcggaagtaccctcagtgctgatgtcttcgctcgctaccatcgtgcccgc
aatcataacactctctatatctgcggtaccgatgaatatggtaccgctaccgaaaccaaagctttagaagaaggtgtatccccctaaaaaactttgc
gacaagtacaatgctttacataagcaggtctacgattggtttgaaattgagtttgatgaatttggtcgtacaacaactcctaagcagactgaaatt
gcccaacacatctttaaaaaattgaatgaaaatggttttatgtctgcggattctatgactcagttgtactgtgaagttcacaacggatatctcgctg
atcgctatgttgaaggaatttgtcccaaatgccaatatgatgatgctcgaggtgatcaatgtgataaatgtggctgtcttttgaatgcatttgagctt
atcgagcctaagtgtaagttagacggctcgaaacccgtcaaacgtgaaacgagccacgttttcttatctttagataaactgcaaccccttgttgaa
acttgggctactgaagcggctgttgagggtaaatggactgttaatggacgagccattactgaatcatggttgaaggaaggtcttcgtcctcgttgc
attactcgtgacctgaaatggggcaccccagttcctttggaagaattcaaggacaaagtcctttacgtatggtttgatgctcctatcggttacatca

gcatcaccgctaagtacactgatgaatgggagaaatggtggagaaaccccgagcaagtcaaattgtatcaattcatgggcaaggataacgttcc
tttccataccgtcattttccctgcctctcttttaggtacgaaggaaaagtggacgatgctgcaccatatcaacactacggattatttgaattatgaga
ctggtaaattttccaagtctcgtggcgttggcgtgtttggtaatacagctcaagatatcggtttatccccctccgtttggcgctattaccttttatctac
tcgtcctgaaacttccgataccatgttcacttggaaggatttcattactagacacaattctgaattgcttgctaacgttggtaactttgtcaaccgtac
cctaaagtttacaactgccaagtacaatggcgttgttccacactatttagaaagtgcatctgttggagctggtaagctgaaaacggactttgttcag
gatatcaacaagttgttgaaaaagtacaatgctgcacttgagcaatcgaagttgcgtgaaggacttcgacttgcgatggagatttctgctcgtggt
aatcaatatttacaagacaatcgtgttgataataagtgtttcttgtacgaaagacagaagtgtgctgacgcaattggctactccctaaacttgattt
atttgcttgcttctattatgtatccgtatatgcctggtgttggggccagtattaataagcaattaaatgttcctgctatggctatcactgatgttttctct
cttcctctgcttcctggtcaccgaattggagagcctgaatatttgtttactcgtattgatgaagccatggaagaaaaatggcgtatcaagtacggag
gtactaatatccaaaatgaagctgcttaaaagattttgtgtgctgctttcattgtcatgataaaatataaatatatcgaagatatacaagtttaggg
aaacccattcataagtttggaagaaattttgtcgtttttggattgcacaggaaacgcggttttatagagcagcaccgagtcttcaactttagacttat
tgatgaattgagccgagtgtacaaatattcgaacaaacttaaacaaaagaagaataataatttgacattgtatacgaatagcatacccaactatt
gaaaaaagatgctactcttaataccaactccattaacaatcattggaatatcttttatatgcatagtattgaaaaaaataaatattgtaactgtattt
tttgttttgttttgggatctcagtaatgaatggtatccctattttttgctgcggcccttgctcttaacttacttaggtaattctgtgaaggagtctgtaga
acagttctggtatgtgttctagttttaaacactcaattaactcactcaagtaaaatttgggaacaactcattatatttgcttgcaatttgttaagaatc
aagcaaatctgagaattcccatagagtactgttttgggtggccattgaagtcttaaaatcgcttattcatacaatttttaggcagttaacagtctatt
tttgaaagaattggtttgattgatggatattgcatgcgatttaaatgtcaaaggttattttttttacggataagctgagaacggaaactcccatgtct
atatcaggtcctttgaataatgaagccctttcagaagagaattttgattgtttacgaagcaaagaggagaaaccacttcagctaaccggtttggat
gaattacaagtgaagcagtttcttaaagaaacaaagatttcatcttcggtcccaatgtcgatccctttaattgtacctgagaatttcggaatagttt
atccaaacataatgtatcgctcatcatgtcccaatctgatgaattttccttttcttgaatctctgtctattcgtaccatcgtctcattgcgtcaagatga
atatactcaagaagaattggaatacatgaaaaagagaaatatagcttattatcatgtcggaatgccaggaagcaagtatcggaaacaagaattt
aaaacagacggagaacagagtgattatatgggtaagtgataatggtgaaagactcccaatttctagtttaattttgcttttaaatatatatgtaact
aatggatccagatattgattctttagtcaggaaatcccttcgcgtaatattaaagaaagaatcttggccggtgctaattcattgcagcggaggaaa
ggtattttactaatatctaaatatgatcttaaaatattaactttaatggatagcatcgcaccggaattgtaataggctctcttcgtgcacttatggatt
ggcctgtgcaggagaggattgaggaatatgtgtacgtttcttttatttttttcaggaggaattatattaacatatagctgtcattctcatccgaaagaa
cgtgaagtagataaagagtacattcgaaactttgcggccgattctacccttgtaacactagctaatgatttgaaaaagtatactcaaactgtcaat
gaggactgaggacttataataattcactaatgtctttgtttaaaatcttattgacgtctctttattttaaccaactcttaactaacgttaatgttgagtg
tacaacgctgtctctaaaaagcatattactaagttttggaagttgagggaattaaaatttgctttatgtaaataatgtgaattgatttttttttcgctat
ctgttcgttcaaacaaaaagtctttcattagctgtacgtataggtcctatgtattagcaggtcatttcgttcttttcttttctattttcttttgaaagattt
agatgtgtatttcaaacccttctttctttcctaccaatggatgcgtcgaggtttgttaatattcctggatcattgtacgcccataaccgatcgtatttat
ccctgcaaatgtacataaagctagagggaatagacgcaatgacttgatagccttctttccatttatcaataaataagccaaagaccatgctgtag
atattcctgagccgttcgctaaatcttaagacacgcacaggttagcgacgagtaaaaatattaagattcacaagcaaatatcatttagtgttatgc
ttttatagtatttgaagacttctcttccgactttctttcacttacctccactggcgatcgcaagtctttatgaataaattagttagggacagtttcaatg
attgagacataatgtctggcaatatcgttttacagactgtacttaccctccaactgctccgaagcctccacatagtaaacaacctggtgcaaaagg
gcctcctactgggcgtttgagagctctaggtacggatgccaaaactggcacattagcttgggaatcgaaaatgtacaccattcaacatacgagca
ccactaaaagctatattggttagttagcaacaatgaaagatcctaccctaccgaacgttgggtaataagttttatagtagcttttgaaggcatcgct
aaagtaagtcttttgttccatctttcgtggaattataagtcaaaatacaaagatattgctttcagtctgaattactcgattggtatatgcgttcattaa
gacgatctttatagtatataacgatattctctttgaattcagagaagcaatgttacgtaatattatatcgactcacctaaaaaatataatcctaaaa
aatatgcaacactatcagatcaactggtaaataaacaattaaattaggataaacaaaaatatgctgaaattatacatgaaacagcttctaatacc
atttgagaaatggctatgaaaaaaccgattttgagggaattatcacagcaaaatttatgggaatatttggccatttttttttttatatatataaaca
gccatgtaactagcacgttgatggctattaatttatcgtaaatgtctacataaaagtgaatggaccattcatgttctcgttattctataatcgtttggt
gcgtcagagtggattctagtgcttcttcttttaaaagattaattcatctaatgatatttattctttttttgtatatgctctttgttcgttcacggtgacaat
gtatcataattagtaatgcacgaaaacaagaagaaaaaggatgggtaagaagaaggaggaaaaactaattcaaaccgttaaatattttttttaa
acactctacgatgatgcagaaaaaccgattttatggattgaagtcaagtatgaccacaacgtaagctaaacaattatcgtcagataattattgtaa
aagcctgatagaactgtctacggccacacctaggcgaaaacaccagttcccgtccgatcactgcagttaagcgtctgagggcctcgttagtactat
ggttggagacaacatgggaatccggggtgctgtaggcttttctcttgttgctctttacttttcttattttgtttttttctttttttatacgtctactactgttgc
ttgcaatcgttttctcgatagaatttttgcaagctgttcctttacaagtgacaaaagactaattacaagttcaatgaaaagaaatgaaacgaacgt
attcaattgcaaataagagtaaggataatagtaataatcatcattaagaacgtaggatgatgccaatgatctcagattacggttcctttcaatgcg

cggattgtatgcatgcctttctattgtccaattttcttcgtcatttccaattcatgttggtacaaggatgagttcgtagtcaaaatcaaacgcaagaa
cgcttaacaaatatatataaatattcaccggaacaaattgcttgtaatgttctcttttccacttattgacttttgatcggaagagttaaggctcatga
gaaaagaaacgcaaataagtaggcattaactttgagtggtaggattgctctgaagctcgatttcttcattcgtgtttgtcggcggaggagctaatg
catcatctctctgttgatatctgggaagtgtaggcgaaccggtgttttcagctgagcgctcaaatcccattccaaggccaaaaacagtaatgttata
aattccccaaaagatagcaccaacgaaccatgcgagaccttcaccaagacctttgaagccagtaccaacgaaccatgcgagaccttcgaagcc
aacactaacgaaccatgcaagaccgttgccaagaccaaccacgataaagattataactttagcggcaatccgtattgtcaatatgaaaggccaa
ataagtgctaggctttattagtaacacacattctaataacgtttgcgattaacagtaatgctccacttaccacagaaaagagcaaagaatattgcc
catatcacaagcaaaaggcctgaaggtccaagaagggccaaactgtatccattttccatatatacttgtttcggaaaagaaaatagttcgaaacg
ataggagattgctaatgtacaaaggaatgataaagccaatatcaaacagaaaaacatcgatttacttttaacgactacattttcagggaaataat
gatctgtcttcttcctaaagtcggtaaagaaacgactgaagtcgtcggactcaactattatcctttttttctatgtttggttttgaaagaagttgaatt
ggaagggttctttatgtcttttggcatatacaaaggaagtcccttctcaaggtcaacattgtaaacattcgactgactgtacgcaggagggtcgctt
gaatcgaattttagctccttttccgacatcctaaaaataaataaagagaaatttcaatgaataattagcaaaagagcgtcaaataaaagctgtcg
atccgtattcgatataaaaatttcgcaaacgcaaaactcatcagaagtagcagccaaaactaaagaagctctacctggtcattccaaaagctcaa
aatggaagtcaaaatcagcccagttggcaggaattgaagaaataaacagttggctccagcaagaaatttcgcatctcttacgcaacatttttgct
caggatagtgatgggttctgttgcgctatctcccataatgacatatagcataaatcgttgattatacgtttgagtcacagtgagcaagtaacaaatt
tcgctaatttcgcgtttgtttacacctctgtaaacaaagcagatcccatttctctatgaactgttgaacaactttttacttgaaagtgagttactaattt
ttcgttgcactaaagttattcatacagaagaagtaacaaacaaaacacggaaactgcagacgcattagctaactgcttttcattagacaattgag
taacagactacaaccaaaaagcagctttcacgggctcttcaggatcaaaaaaaacctataaattacttctttgaactgaaggaactgtctacggc
catacataggcgaaaaacatcagttcccgtccgatcactgcaattaagagtctgagggcttaatctcgtttgtgctatggttagagaggtgctgta
ggctacttctttttcataatttatccatttttcttttaatttatacactttctcgtttaatatggaaaaaacgtacaaactggtttatacttgctgcagg
atgttaccctaaaggtaacgagttatcagagatctcaataaatcaaatggaattacaagttcaagaaaaaaggaaatcaacgatagtattcaatt
tgaaaataaggatattaattgatggatgaaatgaccaacgacccaaaaacgttaggctccagtaatgttctagcaagtttaccaactccatgcct
ctaaaggcgacgaaaaaagcttcaattattcatattaaataactggtttttcattgcaagactttactatcatattaatacaggggcaatttgaaaa
ctaaaataaacttggaaattcgatgtaaactcgcaaagtaaggtagtactgagtaataacaaaaccagcagtttcaagtctccctcctcactcatc
tcataataagatagattgaaacaaatgcaattcttcattaccttaaagtaaaggttcagaaggagacaataacaaacgaatggaactaacgtca
atcgcaggattggtttgcagctctattgctttatccattgtcgttagcagaggaggaggtgtagcatccctttgttattatgtaggaagtgtaggcga
agcggcgttttcagttgagtgatcaacttccaatccaaaacaacagacaaaagcgttatataatgcccaacatatagcacttgtagttaacttaat
tttaacgcttcctgacaacagctatgtaaactatagcttgggcgaagattcctgttacttagacaacggcagatattactacttgttagcaagcaat
tataaacatgctccacttacgatagaaaatagaagaaaataaggtctatatcgcaagtaaaattcctaagtaccataaaaattccaattgtcctc
accttgtcataaattattgataggaggtacatgtttgaaaatggtaatatatcgctagtgtactcagcaatgaaaaaaaattgcgtagatgattgc
acaataactgatcagtaacacaagttttcagtcaaataattcatataaactattctgactatgagacaacatgcgaatactgggtactttttctattt
ttggtttctcctgtaaattgtctccctaactcatttcaggacagtttggctttgtttatttgcaaataagtcaaaaagagctaaaagattcaaataaat
aaataacatagtttgcaatgtttttttttgtttttttttgaaacaaaccaatcgtgatgcttcgtctgtttgttgttctatttttgtttacattgccaaat
gtcaacaaacagacaccaaaaaagatataaataagggtataatattctcctttttaccaaaccaaaattaaatccaaaaaaaagcttcatcgttt
tcaatcttcatgtttgggtgattcagtaaaggtggtcgtaaccccttgcttgcttttcgttccatgtataaaacaaaaacaagcgattcttttcggaa
gaataaaaaagtacatatccttgtatattgtaaattacagttcatctaaagcataccaacaaacatttctgcatgattgacacgacaattgtgatct
gggtatctcttgtgtgatgacagtatactttattttcaatttttatcatgcgaaaatttcgacgtttttgtcttctcagtttcaatttattatcgaattta
aggacgaattcacagttgatttcccttttttttttttttttttttttttggactttttgttgaactggcccaatttgccttttcctcgatggcttcgctca
ctatctttcaatgtttgttttatatcgaaatgaacctctcgagacttcaaaaatcatacagtttgttgcaattcttcatcaaagattgaatgcaagag
gtaaacgaatatttgaaagcgaactcgaatgaaatttaaaccaaaacataaaacatgaaacctctctcaaaacgtcgattcattgaaacaataa
acgaaaacaaggtaaaacataaaacaaacagttttcgtttattatggatttcgttgttaaagagaaatgaaaccattttgctagtgagacctctga
acactagacattcatcttaccgagatttttattttattttatttttctttttctttttctttttctttttctttttctcctgcgtaaacagaaattcatgcgaac
tatttgcgcaccctactttatccccctctttctcgtgtgacggaattctatgctccaatgtttaagtggatgtagagatttcagcaaaagctctaataa
aaattcattatttcttaataattatcgattgtttacgttattatatacgaaagcatacaaattctccgtaaactagtgtttcttttgtaatactatatatt
acagcacataacctatatataatcaaagatgctcgttcataacaacaagcggcgatgcccgagtggtctaagggggcagatttaagtggcttcaa
agccgtgaaccctgctgttgtaaaacgcgtgagttcgaacctcactcgccgcaatactttttttaatccatctgaaacggttcagactgtatcctaa
gattggattagacaactttggattcttacaaactttcattcgttccactgtttttgcaagtgatagcatagcatttcttttcgatttcggtgccttcaat
gccaatgtgcctactaccattttactgtttcttatagtttctattatcggctttcgtttcgttagaacgttaacgaggtcgaatgtattgtttttaaccta

aaacattgtcagacattcacctctaacaccaataaaaatcttccaaaatgctgaaaaggtccttaatatcgaaaagtttacggcttgcaaatccca
ggacttttgctccattcgtaaacagacgatttgcgtctgagcctgctttctccccagagaagcaatcaacgccaaagccggaagaaaacaaagga
aaagaaggaaaaagttcattagcgcaggaattacttgagttgaatgggtcgagtgaggatccctacagcggcggtagcgataaaggtcctgaat
atgtaagttcgacaatgctgcgccgtgaaaaacaggctcgctatgcgttctggggcttcatgggtgcgctcggcggtggtatactttattatggtcg
caaatatacggatgatgaaaaggatttagaaaagaaatttccagctgctggctactcacccatggattggtggactcgtgtgaaagcacgcacca
agcacttctttgcatactatcaagaacctgcatttgagaaacttcttcctgacccgcttcccgaaccttataaccgcccctatacacttgtgttggac
ttggacgatttgttaattcacgctggttggactcgtgaacatggttggcgtaccgctaagcgtcctggccttgattactttttaggctatttgtccatgt
attatgagattgtcatcttcaccaatcaatatttagcaacagcaaaacccattgtcgacaaattggatccttatcatgtcagtatcagtgctgtcttg
actcgcgaacacagtaagtatgaaaacggaaaactcattaaggacatttcatacttgaatcgacccctttcgaaggttatcatggttgacacaaa
ccctgatgcttggtcgaagcaacctgataacgccattgccatggttccttggaaaggtaatcccaaagatcgagagttagttggcttgattcccta
cttgaatttatcgcaattatggatattcaagatgtccgtcctgtcttgaagagctaccatggtaaaaacatccctctcgaatatgctcgtcgcgagg
aaaaacttcgaagtcaactcattgccgattggcttgaaaagaaaaaatatagtaaagcttccttgtttggaggtcgtccagtttctgatcagccac
ccaaacttcttattgacatccaacgtgaacgccagaaagccgcctatgccgaatttaagaaatatatcgatgaacatggaccgaagatgctcgag
gaagaaagagctagagaagctgaacagaagtacagcatgtttgacatgatcttcaatggggataaaattcagcaacaacaattagagcaactt
caacaagcgcaagctgcctctgcttctggatcagccaactgaagtatccgatgcttctaaagtcttctgcttgttttatggatcttgattctatattat
tcttaccttttaggagtctttctgtttaattcttccttttaatgcttttctgaatgaaaactttcataaaataattttggattatggtgaaaaaggactag
cccaattatccattttaaccgttcgctactttacgtttcgagctgcccaaacgctttgataatgaaaaagcttcacaaaatgcctcttatctttcttct
gtctttttgtgcaaggcattttattttgtcctttattttcttggttaattttagtactatttcttttgtataacttcttgcaccttctattcttcgtattcctct
acttatctatgtacaaaaattcaacagaagaacaaataaattttagtttgaggtttatttcttaaacacaaatactcattattccaattttattatgat
ctatctgctttgtgattaatgtttaaaactaattatgtactgttacctattatgttatggcttcttctagaggtaacgtctagtaaattgttaccttattt
gttggacgtttcagatgctacgcatggaaagttacaagccgaacatactcagaaccaaccaccttgaaagatatcataagaatacgaaaaataa
agacgggtatttgcctgcagcttataatataaatcttttaaaacggtcacaaacgaaagcgctttcagaagttggagtgagtaatgaggttaaaa
gatatcaataagagcgcaacattagcgtggtccccacgaggagtaaatgataatcagcctttacttgccataggtggttatataggtaccgaaaa
tgccagagaaagtgacaatcttttggaaatttgggatacttcatcagaatccaataagccaattggttctacaaacgttaataccaagttttatgat
ctcgactgggaaaaatcgttagataaacaatacggtgtcattgccggaactttagaggaaggcggtattggtttttacgacgcctctgccattttgg
attctaatgagaacgctgcttctatagcaacatataaatctgagaacaattctatcctgggtcctttagattttaatccagtacaaaccaatcttta
gcatctggtgataataatggtgacgtttgggtttgggacattaaaaatccttcgaagcctttttccttgcctaaacaaaaccgttcttccgaaatcca
tgtggttgcctggaatagcaaggtatctcatattcttgcttcgagtaattcttccgagtataccactgtgtgggatcttaaagccaagcgacaaatc
cttaatctttcttatatgggtgctactggtacaagtgctacagggggagtcaatagtattgcctggcaccctgaaaatgccactcgtcttgtaaccg
ctattgatgataacagaaaccctatcatcttgacttgggatttacgctatcctacaacacctcagaatgttctcacaggtcatcaaaaagctgccct
ttcactatcttggtgtcctcaagatcccagatttttgctcagtagtggtaaagatggccgtgctattgtttgggatgtagatagcggcgaatctttag
gaaacttccctcgttctggaaactggtatactaaaggttcttggtgtccttcgaattccaatcgcgttgcgacggcttctttagacggcaaagtctct
atcttctctatccagagcaccaatactgataaatcccatgaagcttctttgcaaggcgttacttctatagatgacaacgaattcttcaacaatcttcc
ctcaatagctggttctcaggaacctgccttctctttacctgttgctcccaagtggtataaagttccttgtggcgttcgctttggatttcctaataaactc
attcatttttctcctgactctaaaaacgtcgttatagagcctatagaaattcaaaaggatgaagagcctatagataatttttcttctactactaagttt
cagacggagaatgacatcgtcgctttctgtgattctggcgccaaatctgcgaaaaccgaagaagaagcaaataactggaaacttcttaaagcag
tcagtcagcgtgtatctaggagtgaatttgttaaacttttaggtcatggaaatataaaggagatgcataaggaagacagcaccacagaattcgat
gagaatataaaagcatctagcaacgctaatgacacatcgaagaatcttaataatgagagttatgatgatgatacctctttttatggtaaactttctg
acagcttgcagaatactacagtttctgataagaataaagtagaagtctcaaatgacccctttaagatttttaactctgaagattcagagctggaaa
ctaatattacaaaaatgctacttacaggtgatgtattatccgctatcaaaacctgtataaatgataagaaaatttctgaagctctcttcctagctact
ttcggcgatgaagaatgccgcaagtgtgttaggacagcattttacgaaatgcaagagcataagccatcttatatgcgtctttctgcctgcattgctg
ataatgacttagaaaatgcggtgcaaaatgcagatcttagtcaatggaaagatatttttgtttttatctgcacttatgccactaatgaagcattctct
cctctatgtggtgtccttggaaaacggttagaaaatctcgacgactttagctctaatcgtgcagcagaattttgctacattgccagtaaatctttaca
aagttatgcgaatctttggcttaaagaacttgctaattgtggtactgatgatgattcgaagtcttcttatgtcggatatgttggtcaactcaccaaac
ttatggataaagttaatgcgttccgttcaattgtcgcatatgaagacgatgaattgtctgcaactaaagactggaaattaggtggcctttacgaagt
ttatcttgaatattcaaggattttgctttcggctggtcactatgagcaagctagaagttatttgaacctcgttcctggtgcgttccccggggccaaac
aagaaatcgaaagattatcaattcttcttacgcctgccccaattcctgaagttaaaaggtccacttatggtcctgctggaagcggccaacaccctа
tttcctcgtttgttcctcaaccaattcagtcggttactaatcttagtaatccttatacttctgcatactcgacgaacgtttcacacgttgctgctccacc

ttccgtgtcctctcctattccaccatctgtagtatctaataatgctgcagctggctggaatgatcctccaattgttaatcaaccacctttgagacgtgc
agctccctcattgcctgctgtaagatcaccgttccctaatgctggagcaactttgcctcctaatatgtcacgaactagtagtaccagtacactacca
ccaccaccttctgtaggttcgactgcagccgctcctcctccaccaaaagttggtgaagtacatcagccacaagtacaaaccgcaagtcagcctttg
gtgaatcaaagccaaagaccagctaatccttacacgccaattggggcccagtcgcccgcaaggacattctctggtctttcttctcctgcagataca
caaagtagcaacgcttatgtgcctttaggtggtcctactcgaacatccgctatttctcctcctattcaaccgacggttccattcagtactgctgtacc
acctccaaaagcttcggctaacagagtagttccggttcccctactcctagccaacgtatttcttcatatgaacctgttaatgttggttttacccaatc
accacctacgacaatgcctcccttaagtgctcaggcacctcctagttctatgaatagcacttttccattacccctatggcgtcaggctcgattgtct
ctcctccaccacaaaacctttcaacaaagagacagtcagttgctggaccctcgggtggcgctgctggaatgactacatatcctcaaggtgatcgc
actcatattccacctaatttgagaccaatatatgaaatgcttaacgctgaattcttgcgcgtttcacagagtgttcctccacaaatgtcacgcgtatt
atatgatatggagaagcgacttaacgtgctgtatgaccgactcaacgcaaatgccatttcaaaacctttgattgatgaactttatgcgctggctgtt
gcattgacgaatcgtgattatcaaacagcgtctaacattcaggttactatggttactacccttcaagaccaatgtgagctctggatcgttggaatta
ccagacttattacgataagtaaatctacagcttaacagtgatactacattcttgaaattcatatattgaatatttttattattaaatgatgtaagacc
gtttttgtgagattttatagtgatgatactacttgtgattattgagcactgtttacaggttaatattagaatgaaaagcttcattatttaagtaagcaa
ctgatgctagtatcatgtagaatgtaatgaaactatttaattgcgatgaaatagttaaacctctgttaacaacaacaactgagaatgaatgagata
aactgtggaaaccactgtgaccactattgaatgtgaaataatatcatatatcatcaaatgctgctgtttattcttagttttgtcattaatgatttgcaa
atttgctcattcttgaacagtatttgtttagcctttagaaaatccttcagaagtagagcgattaatcttaaccggtgatgaagtatcgtataccatca
actttgagtaagcttgacaactgattgatctcgaagttcggaagccttgagcaccataattttgacgaacgggtctaggaattaaactatgattaat
acctatgattttgaggctcggtgtatcaacatcttttgagccatcaataacgttgttgaagtcagaagaaagcttgaagttgctgctagatgttaaa
ttcaatgtgagtggagccttattcagctgattgtcataggaaagagcggctttaggcgaactcaactcttcaccagctcggttttcaattcttttagg
tataggattaataattttctgagacttagcaggaaggctatcattcaaaactccaggtacaacttgcaaattagagtgagttgctttttgaggcatc
aaatgagaagaagactgatgttcataagacggcaataagcagttttcaacaagctcataagacaacgcttcaggatgttttacttcggaaggact
ctcatttgagttcatttttcactcagtaaaggatgagacggatgatctactttctcatcactatatagctttattggttttcgaggaagatgagacgg
tggagcatcaggtaatgcatttttcactgcatttgtaaagctagataagttctttttgaaccagctgacccttttttcttgctttttgtctaggataggtt
ttacagatgcagcattcttcaagcttacaggtttggtcagtttttctgattttagctttacttcgtgttgactgcttggcagttcaggaactttgggaaa
gtatctctctttgcataatattgcagtggggcgaacaaacggatcccatgctaagagtagcgcgatcatttcagcatacttgtcttcacagcttact
ggaagtaatttccttattggatatggcttcgtcacagaaaaggagaatccaagcttagcagctaaatctctagcagagggccagtagtgcagtttg
tctttgtcatccaagagagggtttccaagaatttcacaaatcctgtcgagctggtctgtctcatttcttccagggaatagaggcttcaaggtaaaaa
cttcaaaactgatgcaaccgaaagcatacatatcaaccgcctttccataatcttttgctctgagtaaaagctctggtgcacgataccaacgggtag
atacatactcagtcaatggatcactcgaagtagaagatcgggctaatccgaagtctgcaattttgacactataccttgaaaacactggtcgatctg
taagtttcttaaccaaaatattctcaggctttacgtctctatgcataaatccatgcgtgtgtatatgctcaattcctcgaataatctgatttagaatat
gacacgccgtgtactttgtgaatggttttccttgtcttcgcttcagaatttgatatagatttgcttccatacactccattactaaatagatgtttctgtta
ggatccataaatgtctccaataagtcgattaggcctggatgttttgatatttgtcgaagtacctatactgttagcaatatgtttaactcatcatttcct
aaagataataaagtcaaggaaaggctacaataatcttaccaacgactctcgaagtttcagtaactcatgtttcggctttggtttattgctggtcttc
agcttcttgacggcaacctaaatgttgttagaaatatcaattggtttttctcatactatttgcccgtgaccctgctttcctttgctttttgccagataca
cagtgccaaagctaccatctcctagcttctgcttcacataatatcggtcttcgaaacgaaattccttcaaatctaacagcgaataaggaatttcttt
gctcatatcctattatgaattgaaaagaaagctggtttgctttggtgaagagtcttttcttgtttgtttattttgtttgtttacattttcgcaacttcatag
taacgaattcaacaattttattctttattttacacagaaatccatgaaactaccttaaaaacgttttcttttcttctaatacatgtaaattacaagaaa
ctccataaatgtttgattttcatatttaacggcatttccgtaattttattaataccataaggaattttagaattatcttctaaataaataaactttttta
tgtatcctccttctgaaaggaagaatcccatacaattgtcagttcctaattttaactcttcctttaccacgactcaagcaaggtaaattgggataag
acggtagcttttattaataaaaacaaaatgagtgttcagaattataaggtcgctgatatttcccttgctgcctttggccgcaaggaacttgaaattg
ctgaaaatgaaatgcctggtttgatggctattcgcgagaagcatgcccagtctcaacctttgaagggagctagaattgctggttgtcttcacatga
ctattcaaaccgcagttctcattgagactttggttgctttaggtgctgaagtaacctggtcttcttgtaacatttactctactcaagatcatgccgctg
ctgccattgccgctactggtgttcccgtctttgcttggaagggtgaaactgaggaggaatacatttggtgtatcgaacaacaattaaagtctttccc
tagcggccaacctttgaacatgattttggacgatggtggtgacttgactgctcttgttcatgagcgtcatcctgaacttcttgttgatatccgcggtgt
ttctgaggaaaccactactggtgtccacaatttgtataagatgttccatgagaacaaacttaaggttcctgccattaatgtcaacgactctgttacc
aagtccaagtttgataatctctttggctgcaaagaatctttggtcgatggtatcaaacgtgctaccgacgttatgattgctggtaaggttggtgttgt
cgctggatttggtgatgttggtaagggttgtgccacttctcttcgcaatcaaggtgctcgcgttatcgttactgaagttgatcctatcaacgctctcca
agctgccatggatggctttgaagttactactatggaagaggctgttactgaaggtcaaatattcgttactaccactggttgtcgtgatattatccgc

ggtgaacacttcatggctatgcgtgaagactctatcgtatgcaacatcggtcattttgatgttgaaattgatgttgcctggttgaagcagaatgcta
aggattgcgtcaacattaagcctcaagttgaccgttatgaattgagcaacggacgccatattatccttttggccgatggtcgtcttgtcaaccttgg
ctgtgctaccggacacccctcttttgtcatgtcttgctctttcgctaaccaagtccttgctcaaattgctttgtggactgacaaccaaagctatcctgtt
ggtgtccatatgcttcctaagaagttggatgaagaagtcgctcgtttgcacttgggtaagcttaatgttaagcttaccgaacttactcctgttcaatc
taaatatttgggtattccatctgagggtccttacaaggctgaccactaccgttattaatttctctaattaattttgcattggctttttgcacactttcatt
tataaaatttcaacatattccgttgtttgatgttttcaacagcatcatgccacaacgataccatgaaatataataatgatattcaattgggtatacgt
ggatttaggtatattattaatgaaacggtttagactgagtaccgaaagagcccagattaagtgttgtgtaggtgaaaatgatgcatgtatgttcga
agtatagtaaaagtaggcatttcatatgaggttttaagtgagttagctgtattataacgtataatgagattgaataaaccgaaagtatctttattta
aaagtacaataattctattggtttcacatattccataacgaacttagctagagggtttcttttttctgtgattataaggtcttcgctgcttaacgtaag
tactaaaaagagttagtcaatatttgaaataaagtagaaaaaaataaaaagcgacttactcaaaaaagcgctcgcaagatgctgcgttctcgca
attgctaagcaccaagaagtcaccttcctgatagttcaaatcaacggcagagtcagtataagaatttaatactttaggagacacaagagcgacat
gttccaggatagtaggatggatttctagcaagtgggataaagcagatctttcgcctaaatctttcttataaggtttgaacaagggctcattccaagc
atcaagtaaataaggagcaaagtcatcatttctgagaaggaaagaacgaatgctaattccgttatagtcatttgttgtaataagtttgacatcttc
gagattcattttgctgtttgtacgaatatgaggttttaatggatcaaccgggtaattacgcaataagttgttactgagttgctcattcataatagcctc
tttaagtgactcgcctgtattcatgattacggcccttggatctaaaacccaaacccaagaagcttcaggatgttcgaataacgtttcccgaattgca
gtcacaaggctccaagtgttgtcgtttccatatttgcgttcaatgtcacgagcttctttgtggacgagctgatagccgtgacgctgagcatactcatt
tctgtctgaaaggagcttgtcgaggaattttggatcatcagagatctgctcatagcttaaaacaagcattacaattgaattaggatcctgtcggac
aggcttatggggttttataggctgatgagggccatatcgtttagaaggagcttctgtcagtgatttgaaggaatataaacaaaagaggagcaaaa
ccacgacgaaaatgctcaaatatttagctcggcgtgaaagcaagcgacacaagttcatttggaggatggaaagaaaaggatgaatcgtattta
aaccacttggacttaaaaaatgctagccagtaaaagaaaagaagtatgaaacccaaacgagatgagataagaagattatccttgaataaaaga
gcgtatagaattacaatcttccgtatgcagcttatttcaacaagaagcagaaaaggaagaatgggcgaaaaggttgataaatattttgaaagac
cgaaaatacaaatgcgttaacagtaaaaacaaagagtataaatgaatgcaaggtaaatatagcagagttaaaaacgtagattgctccaatggc
aagctgaactaattttgaagtagaatgacctcagatctttggatttttcccgggaaaaaggcaagtatacctttcctgacaatccaaaaatgatga
aacaacttttcaaaggaaaagatgcttcgttttggtatttttgctttctggagaaaacaaagctggttggaattcaagtacgcgagttgcaaatca
ttctctcccctctatcccttctttgttgttaaaattaacgatagtacaaagctatattataaagtatgtaggagcgcgttaagctatttaaggacactt
tctgataaaaacctgtatgtagtaaaaccatagaaaaaggggatttatatttagagatttagtaaaattaagaagatttaatctttaatcttcacc
tcttttcccttgtacttagccgtcttcactctctcaatcgaactgcttgtttttctggtgtcatacatcactatagtacgttagtaaacaaaccaagta
gaaacgttttccttggtaatttgacgagaaacctgtgttgaatcaataaagagaaaaaaaaaggaaaaccgcttagttataataaagacgaatc
gtgtctttggtgcacttacgacttgtgcttttctgccttttcagttctgcaaacagatggaataagtaagaagccataatgctaatcaataggctgta
ggaaactaagctatgcttcagtcatttacaaaactggaacttccagtcgcaaacatccaattttcttgagccgtgattgttgatgaatttcctgttta
ctcttgtgtttgatctaagcattagctcttacacagtttcatttaaaaatgctttgtaagcgttttacttgaatggcttgaaagggtattggacttggg
ctttatgccctgattccctatagtcaactgctaaatttttcatatactcattcaccggtcatttgcgctcgagtagggattctttcttttcatcaatttac
aaaaacttagtgatccttgtggtttaccgttggaattctttcaagggctgcttctattcaatcatcttttgctatttcctgttgtgtttttttatattttgt
ttttcccttcttgcatggaataaattcctaaaactacctaattctcatcaccagaagcttttagttttgttatttgtacagtatggaatccactagacg
atacactgtttctatagccattcctgtgtcttgcttgaatgaggctcacaatctacagctcaaaagcgcccttgtctaccaggtaaatgcgaaaaaa
acgaattcaaaagagcgtttagcttacaaattttctagattgcacgactggctattgtctacgaaattgacgagattatcttagtcgctgatccggc
gaatgtaaaacaaacgcaaaatgccagtgcttcctctgattgctatatacgggacccattgaagttcttttctgacatccttcgatatatggaaact
ccagcctttatgcgcaaggttctattccctttacttccccatttaaagtatatggggttgttccctttaattcctctacgcaacgattctagtcaagttc
ctgataaaaatttcccttacaaagaaggctatgttcttaatcaattgcctcgctccaaaaacaaatacctagttcacgttggaattgagcatcctat
tcaagttacttctccacaatcactttcagccaaggatagagttaccgttcggatgaagccacaagctccaaatgctgaagggcatttgcaaggag
acatagtatcttatacagctcctcgcgaaaaaggaggaaggtattgggggtttcaaattcggccttccagttcgctaagtgatcttattaaaggac
cttataaaggtggatatgatttaaagatacaaataaataattccgcccatgcttctcctttggagttatcgaacaagctccaatcctccttccgtcac
gtattattgctttttggaaattcttttgaaaatcaaccaatagatgatgtgcacaccgtcaatccatttcctttcatgctacctattcaaccacgacta
gaggaaaatcttttagctgctatggctccgctctccagcattctaaacagtcatggacgacactaaaatcttcgttgataattctttgctactgaaa
atttatgtagaatttaattagggttagtatggaattagaaaaaaaaatgattattaaaaatattaggttcttcgaatagtgcagaatgccaaaatg
atggatttatgaaaactcatgacaaaggaacgttaaggaccagctaaagttgaatttcctgatctcttctcaatcaagtttttaatgaaaaattcac
cagccttatagctactacgtacaagagggccactggcaacatacaagaaaccgagcttttcagcaactccttgccaatactcaaacttcttagggt
ggatatattcttgaaccttagcatgacgcttcgtcggtctcatatattgaccaaaagttacaacatcaacaccatgagctctcaaatcctttaatgct

tgcaagacttcctcatctctttcaccaagtcccagcatgatacttgttttcgtaatcaaatgaggaaccgttttcttggcctgctccaaaacattcaa
actttgacgataagtggctctacggtcacgaacataaggggtcaagtcttccacagtctctacattatgtgcaaaaacatcgagaccagacttggc
aaccatttctacgagatccaaccttccaccaaaatctggtgtaagtgcctccacaaggatatggggagccttttgcttgatgctatcaatggttttg
gcaatgtggttggcgcccaaatcagccagatcatctcgatcgacactggtgagcacaacataacctagattccaacgggtgatggcgtccgctgt
tttttcaggctcattgggatctaaaggaggagggcggcgactagttttgacggaacaaaacctgcaaccacgagtgcaagtatcacccatcagca
tgatcgttgcagtagcttgactcttgtcctttcctccccaacattctccaatatttggacattttgcctcttcacaaacagtatgcagatttaacccgc
gtaagtcatttttttattttggtgaaattggacccaagcggcaccttcgttttcaaccaagaaggaagacgcttatgaactgctccattaggaagctc
tacctttctatctaactcaaaggcttcctccccattaagaggcttttccagatttaagaagtctgaaaaagtcggtcctttcgctaaacgctctgaga
agttgtctgagtaaaagcgaatcgacgcactcttctatcttgttaattgtttatttttattgggttttacttactcgaaagagtactctttccgactttag
caaatttccagatactctcaacataatggtagagagttgtttttgacaaattaagtggcactaaattgaagcaacccacatcgggaaaatcataa
caacatgacttggaaatgaattttattagagagaaagaaaaactaacaataaattcaaaacacaacttatgaatggtgctactgaccttgtctgc
aacatcaatagcaggcgagaatgttattatgattttggctgacaaaaatcaatcgtgatagaaaatagaggaccgtgaacctgatgaaattcatc
tagtcttgtttctcagcccttgttgtgaatacaatttcaacacgacacttgaggtctgtttaaaacgaacaattgcgaacgtggatacaaaagtgaa
tcagtattaccctttttcctataaatattttcatcaatagttagggatcaagggcttcatggaggactttggctttgctagtttgacagcactataggt
ttacgtgtggttttagcaggaagaaacgaagcttatagtcgcttttcaggtttattagctcctgaggatatggaaaattgccagaaagtacaaatg
cgcatgaacttacctgctacaggttgaacaatgcgtttctagtttgtcttaatatttgtttctggcttcgtttcatctttatgaatggcccgctaacaca
ttctttgtacttaaaatctttatcgccgcataaacacagaacgatcagctccagtttcatcctacggtgtgttccatatgccaggtgaaaagttcaaa
tcatctacttacctgatacactaaggacggagtatcatcatcaatcaacagtgtaacttgaattctttatataataaaactaaacttgcaagatgtc
gctacaatcaaaggattctcaggcttgattcttacatgtaattactcaactggcagcaagcaattgaaagaaagaaattcatcattatatagttatt
attccgtgcatctatttatgacattcagagaatcatgtaaacaaactgatgcatcctagagtgtgtttactttcactcaaagcatgtccagccaaac
ggcgaatttctttttcttcgtgccaatatactatattttactcttttatttctgctttttcctttaaataagaaattgtaattaccaaatttgtcttgttttat
caaacaacccaagaagacagccaccaccagctttccacacatcacaaggttttctagacgacattgagtaaagcacacactattggaaattattc
atgtcatttcaggatagaacggcagaatttcaagcatgcgttgcaaataccagagctcgaatgagaatttcttcatcaaatgcagcaatgagtaa
tgcattgcaagcaaagcatcagaagagcgagtttactacagtcgcaaaaaagattgctttcaaaatcagtgagacaagtaaacatttacaacgtt
tatcccaacgtatgcccattttagaattcctgttggcagaagaacactaactgttgatttagtcgccaaacggaaaacgctgtttgatgatcggcca
gtggaaattcaggaattgacatatcaaatcaaacaaagcctctcttctttgaattcagatatcgcttctctacagcagattgttcgtcaaagctcta
agggtactcgaaacaaacctgctcaaatcaatcagcacaatgagaatattgtcgtttctctccagaattcacttgctgatactagcatgaactttaa
agatattttagaagttcgcacgcaaaacatgaaagcctcccaaaaccgaacagaaaaatttgttgcttctacttctctaaatactaatcctcttgct
aataataattctgctacttcgccttttaattacaacgaatccaagtatgagccaaatgacgattaccttgctttaaatctaggagaaggcgcgaata
cccgatacgagcaaatggcattactggaaaatcaaactgacgtgtactcccagcaaagaatgtcttctattgaaagcattgagagtacaatttcc
gagctgggtggaattttctctcaattagcacaaatggtgtctgaacaacgtgaaactgttcaaaggattgatctttacacagacgacatagcatca
aatattaacagtgctcaaagagaaatcctgaaatttttcgatagagtatcttcgaaccgtgcgttgttatttaaaatatttggtatacttattgcctttt
tttcttttatgggtccttgtaacttaatttatgtcttacagttggctactcttctaattacgcattcgcttcctatttcctatgcggaatgacggtttgata
ctttgtcatcgctctttttttccaacttttagttattggttatgaactgctggttccaactgcttttccctagaatacctggaataccagtctataccttttc
tcctaatgcttcaatgtttaacattccgtttacttttctgatttttatatgaacctaatcctctccatcattatcctcttccatttgttgaatactttccgct
tccaactcctctagttgccagccaacgtcagactttttttggcatatattccataacctgataagtcagggcttctagcacgttgtcactaaaacccgt
tagtatacaattaagcttttagattttcattcatcttactttgctgtttgaatccaataagcataatcattaataatcatggtattgtaaatgtaatata
catcatgatgttcaaataaagatttcatgcggagtaaaacacgaagaatccatcgctttcccaggctgagaatggcatacgtgtctttccaacatg
tttctgcaagattccaatttcgatacaagggatatgccaaggatcggcggaagcatgcacaagctacttcttgaattgaagaaaaatttgtgtcaa
ggcatgcgatattaggagccatgattccaatgttccagacagattcactgctagggtctcctaatgttgtccggtgatcataagcatatgcgaaaa
ttagtgccaccaggttaaggtaaatcctctttggattttcaataatatatgatttttttcggaagggataataaaccttgctgttcattttctgtgaattc
aaaaggtattggttctccggatttagcgcgtttggacaattcaacataatcaggagtgtagtgaatcaactcatcaatcatctcatgatcgtaaaa
atcagctaaataatactctggatcgaacttctcatcttctgtttgctttcgtatttggctcctttgtaccgggtccgtaagttcaggctctggcaacac
gtttatttcattcccctccaagtatttaagtaatccagtatgttgatcattaaatccgtaatttgatgtagatgtatatttcatctattggcatattagct
tacaccgctggtaactttgagtagcaaattgtttcaccatacctttttatcttccatcctttgaagcaaaccccagtcgaagttgggatcatcaaaac
ggatatttctccaatcgatttctggatcaatgtcgtctgcttgtacctctccttctgtacttgctgcagccgggacatcacccacttcttcaatcaatgc
tttcttaggtgtctttgatttttctttattcacaagtaaagtcgttaataagtctaaattaggaaactcttccccaggagtttccttggcgaatttaata
tgaatcattcctttcccaatgtcataagaagcatgagatcgttcatcatctaatacatttcccggaagctctagtcttaatccctgttaatcaaaattt

atcaaaaaagacttagacttacttcaagaagtacggggcgatacaaaaatggatctctgatcccatagcttcaatttcggcattttgagcctatac
gtgtgagtcgtgatcctttcacaaaaaaaaactttacctttatatgaggtgcatgaatgcttaaataaagaaaatccgcatcctgctgcaaatcaa
attttggagtaatcattgcttaggataggtgtatgcacaccggaaagcaacattcatttcacaaatgtttatgatgttaatttaaagtctttatgcca
aatagagtatgtcaatagaacaagaaaacgctttcttcttctaaagacactatactagttacaagctacaaggtaaggtttagtagggcattcctt
tgaggaaggtcatgaataacttttatatatatgtttctcttaaattcagtagaagtgaaggattgaaccgaaattgttgccaacttgatctttagtta
ctagaaggatttgtcacagacagtaaatcaaatttgcccgttgtgaaggactatcgtgtgaactttaatatcctgtaaaacttgtagatgaatgaa
aaaagcttcagttttctttgtaataactatgttatctcgagtctttcctcatacaagcaaaccattgggaacctttcaaaggcttgagtccagttcaa
ataatttcaatttaatcattattttttttcattgaattaatccacgtgaattcttttcaactgactaaaatctttaaagaaaaatcaatactgctgggtt
tagaaagcctttcgtttcatgtagcaacattgaagaagaaaataagcacctatttttttatacaaaacaatatatatatagaaacttcgtcaccggt
gatttttggaccatgaagtctctcttgtagcaagatgaatgaaactacatactctctacaagaacagttttcctgcgttctccaagcaattgagcgg
ctataaaatagaaaaatatataaataaggaaatatgaatatctcaagaaaccttttttttgctaccgcgattaagcactttactatgcagcaagacg
ttttcctcttcagcctgggcatggtcaggtcataatagtaagtaagaaacaatctcttgtttcaaaaatataccccttgtgtttctactgtagcgtaat
attgagtgctattaagacctggtatgtttgattctgttggtcgacttgtctcattctgcaatggaagcaaacaacttgagatatcaagtccttatttag
agattaaattaggtgctttcttttgtctattattccttggaggctaatccttagttgttaattacattgcactgtttttacatgtgaaacacaagagggt
tcattactgaaaaacccatcacggttctactttatctttctatagtctaactaactttacagaatggtccaaaattaaacacaagaaatctgcaaat
gaccaagcgcgtagtaaacaagtcggaaagtaagtcttaaagctaatggaaagtacaggatgcttacaatggtgataggatttcacagggtatt
acgatgactgtaagaaccgaaggttccaatcccgatatgaatattcggcttgcaacacttttagagcaagcacgcaaaaattccattccaaaaag
tgttattgaaacagcaatgaaaagaggatcgggaggaggaggagcggatggtaacctaatgcaagctcttgattacgaggcaatgcatccatcg
ggcgttggattcatcgttgaaacaattaccgataataggactcgagcggcagccaacattaagcacgttcttaagaagtatggtgcatcattgtcc
aacgccagatttttgtttacaaagaagggaaaaattgaggtaaatcagccggtcgaccgcccttccatggagcttgggaacgtattggacgatgc
aatcgaggctggagctgaggacatagtccagagaccaaaagaattaacagatgaagaagatgagggccaatatgtaatactcacggagccca
cttcgttgaatgaagttgctcagtatttcaggtcgcagaaatacgaaattaaagactctcgaatgatctatgtgccggttcctgacacagcaatcg
acttgcagacaaatgaacaagcgaaagatcaaatgcaaaatctgattgacgagttatacgacattgacgatgtcatgtatattcatacaagtgcc
gccaactctcattatttacccttaaacaactaacattggtttgaaatatctaatcttgtcttttacaacccctctcaaacaaaaaagaatacctttcat
ttgcataactgatactagctagcatgtctgtcaatcgcaaacacaattatccttgccttaatgctataataaatgaatcatacactttttttataaaaa
aacaaatttattaataatattatatctaaaatacaacagtgaacatgaaatacgtttaaaataaggcttaactctgataccatcgacaaaaagta
aaacctctccactttacaaaacatatcaacgtggaatgaaacacatctgaggattgagagcacattcaaggttatcagtttaggtaaagtgaata
ctctcagtttcatcaattaaagggtcattaggatcacgatagtgaatcgttgataagctaggagacaatcgtctttgtaccacttgagctgtagtgg
caggtaaactaagctgtgttcgttttcgttgtcgtaccgcccccgcaacttcacgaactaaaacatcgaaaacacgattcacgacggccgactcag
tatcaagaacatccgcaaaactatcaaatgtgaatttttcttggtgttctggcaaacgatgattgtacacctctagcgcgtgatctaccagcttcttc
gttttctgatgaaaatccgcaacgcagtcttcttgagagtcatactctacaaatgaccgacccatggcttcttagcgttattggcgaaagttttgatc
acgaaatctcgcttgttttcaaacaagctagcaagctgaaagttggaaactttagacttttcttttctaataaaccagtatcaaaagtaaccttaat
ggtaaagagataattatcaaatagatcctaaagttttactaaagtaatggcaattccaataacgactcctagggatcgcggtgaaacaaaaata
acgaattccaaagaatgtatgataaatgaaataagccatttgaggaaatcatagcgagccaaaaatccataaacaaaagctgtgaataggaga
ggggaacaaggtattcagaaaagcaaagtactatccataagaatttatttattctttcatgtgaatggagaaatgactagatacgtccaaaaattt
ataaaaatcataatgactgaaatgaaatttcgggaaaagcaaaatagccgtttctcttgtcttaaagattaaatgcgcaacaatttcaccaaaag
tttggcaatagcttccttaaagcctttctctgtgatttctttggctcgtgcatttggcgctgtctagctcgggtactactggaagtaccgcgaactaaa
tgttgtcctccagcgtgatgacggagtaaaatcttctctttgccaaaagggacccaacgctttttagcacctgcgtgagtcttaactcctcgaacga
tgtcaattcgtataggatttggcatagcagagcgaaacaaaccagatgttagaaaaaacatttttttaaaaacgatcgttatatccttaagatccaa
aagaacaatattactacgaactcccaaaaaccctcaaattgtataaactgttgtagctacacataggttgacagctactgcttgtacaaagccttg
ttacttacccttgctacctccatcgaggtaacgcgtaggacagtgtaaaccaagggttctttctttgccctctgtaaacatcgagaaaaatggataa
tcgcagtgattctgaggttgattgggaaaatgaaggtacggaatttgaactcttatagaaagcagccttgcagggcgtttgttagccttgtacaact
tgtcttgctagaagttgccaatgtggccgtgtttcagcaaagtgttgaggaaatttggagattaggattaaacatgcaattgaattacataatgag
gaatgcttggctgtttatcttggactttgacatttgactctggttgatctttgtacatactaatgtcctttactagtagacgaagtctgggaagaacaa
ctggaattcaattgtttgttctgtaaaaatgtgttaatgtccttaaatgacttatggaaccattgtaaagaaggtaagtagaccatcggcccgaaac
ttatttgcttaccgcctttcagcacacaatttcgattttcaaaaagtaaagaatgatcatcacttggagttctacgactgtattaaacttattaattac
atccgatcccaagtagcaaaagagaagcctattgattttaacaatttatctactgtacttgcttccgatgaatacatgatttccgtattccctgacga
tgctgttttgttttctttgggagatactttggattctgactttgaagaagacgctctcgacgttgctgtagatcataaacacactgattctaataaaga

cgagctcaagtcgttaagattacaaacgaaaatgctgtcaacccagttggaagaaatcagaaaggaaaaaatggaagaacttacttcccaaac
tgcaagctcatctttgcaagaacctggaaaattgatagacaatgattcttattactttgagtcgtatgctgggaatgatatccactttgtcatgctga
gtgacagtgtacggacagaaggatacagagacttcatctacgagaataaaaaccttttttcatggaaaaacagtcttagatgttgggtgcggaac
aggcattctgtccatgttttgtgctaaagctggcgccaagcaggtttacgccgtcgataactctgatattatacaaatggcgaggacaaatgcata
tgaaaataatttggctgataagattacgttcattcgaggaaagattgaggaaatacaagtccctgttcccaaggttgacattatcgtgtccgaatg
gatgggttacgccttaacatttgaaagcatgattgattcagttctcattgctcgtgatcgatttttggctgcggacggtcttatggctccttcagaaac
gcggttggttttgagtgcaaccaccaatagtgagattttggaggaacctctcgatttctggagtgatgtttatggatttaagatgaattgtatgaaa
gatgcttcttataacagagtcaatgtggatgtgattcctgcttcctatgtgaatgcggaacccgttgaatttgctaccatcaacatgcacacatgtca
agtacaagatgttagctttaaacatcctttctctcttactatgaagtcttccggtcctttctgtagctttctggtttggtttgatacttattttaccacctc
aaagcatcaacccgttccttcgtccgtagatcagtcttttggtttcacaacaggaccccacggtacgcctactcattggaaacaatgtacgttaatt
ttacgtgaacgtcctcatgtcgaagctggaagcgtgattcatggatccattgcctttattaaaaactctgtcaataatcgtgatttggatattgaggt
ttcttatcatgttaatggacaagactacacacagtcctttattttgaattaaacaacaatgggggtaatattttttggaccgattctatatattcgtct
ttcagttaatatattttcatcagggtttgactctgttcagccattatttacgggcacaaagttgggaagtggttaatctaagtatggatggattcttac
atagagataaaattttttttttggtattgacacttaaataattttcttttttgacactaaatttaagtaagaataggattctgtaattgctctttcattca
ctctttattatatttacaattctcaattatcgcactccagcgcactggtttcatgtgcaacgtttctggcatttacatttaccagtattcttcaaaatca
caaaacaaaagaagaatgaatcgcaatacactctacgtaaataatttgaatgataaaataaacaaaaatgacctaaaaatatctctctacctctt
attttcgacgtatgggactgttgtggacgtaattactttaaagacacccaaaatgagaggtcaagcacatattgtcttttatgatgcttcagctgcg
gcgattgctatgaaagctttaaaaaatactcagtttttttggaaaggaaatggtaagcaatttgagcaaactttcggtatacgagactaattcttga
catagcggattgactatgcgaaaacacgtagtaaaatgattgaacggatttacggttcagaggtaaggggtcctctgaagcgttcaagggaaga
cgcggacgttgagaatgaagatgctggaatgctgaaaagggcgtcttgatcttgtttccttagctttggaatactttcatcgcatgaattcaaatgg
ttgagagcaatggtcccaatggattatatgtttgattctgttcactttgaaagttagtaagagcaattcacaaacgcgttaaaaatcgtttgctttat
ctgctttcttcatgtttttgttcctttcttgaaaccgatgtttgaaaccgtctttcttaatatgcagtacatgaatttatttggctggggagtatttttttatt
tttgatcttttttttttggtgaatgcctcgtttgtttattagtgagctttagcacgatacacttttctgacacactttcctattacgaccttttttggcaaatc
gaagcgcatgcaataatgccaaaagatgtgtctttggatttttttaccatcaagtttcccgagttgtgaatggtcgcctgacggaaagttagccttct
gtaatggcggacgcgtgcagattctggtaagcaatattcgccgtcatttttttcattattaaccgtaaatagactccttctcatgaaattaataccccc
agttacatcaagtcactggccattttggagaattacgatgagaatgaacgaatgtattcgcgtaggtgtatacaattagatctaggattgtaagaa
aaatccaatcaattatgctaattatgaaaagcaacggatttgcgcataggtgatttgatttcgaaccatactgctcgaaaggttgcttggtctccta
ctggattatctgaaaattatgggtatgtatattaaccacttaaatcatggtgaactatcattgtcttcttttgtactgttaaatccctcaataaacgaa
actaacgcagtcaagatgcttgctgacggttttaacaaatcgatatcaggtttatctatattcatccaatagtaaagttctgcatatgaattggaag
tgtgtatgttgcttaaacaattatttatcaagagaatcccttgcttctttgcgcatccatacctttgcttggtcgcctctcctttctctcccaaagctttc
tccctggggtatttgtttactaggtttaggggcagaagatggaaatttacatgtgatttctttatctaggaactcggatatgcttactaagtccatca
agcttgaatgtggatggttggtacggctttccttttcgtcatggacaatttataatcacgcagcagtctgcaatcttgtttgtatatctcagaatggcg
aaatttatgttgttcgtgtttctgtaaatctcttaactggaagtttcgaaatgcgtcatcaacgttttccatttcaagtcggtcaccggccgatagcac
cagctatagcctggtctcctgtgcttaacgacgaagaattccttgcacttgcttatccaagaagtctttacatttcttgttatagcaggtcaaatgag
aagtttctttcaatcacatcacacgatctgacttcacttgcttcaccggtaggtgttttctttgctagcaacgacataagccaattttcagtgtatattt
tgactcccttagccgaactacaaattgtacctgttaaaatggatccaagcaatactttcgaacttcctgaaaaacaacttctcgacagatttttaac
gcatcgtttacaaaattacggttccacttcggagccatcaaaaagcttacgtatacatgctttttgtccttcgcctttttcttccacagctatcatacat
ttcagcgtttcgtatcctcaaagttttatttatactgcctcggcaatggaacggagcttttgtgctacttttccaagcattgccttaaaagctacgtttt
ctcgtttaatcaattcttttcttactaactgctgttccatacctgcagaaggctgcttctgtgaatttgctttattagaaaacagcagtaaagcagatt
ttgacgtaaataacatgctttcggaaagcttatcccaacttttaatttcagactttgatttttgcttgagattaaaagacacggcacctttttctacttt
aaccagcgtattctttgatgcgtctttaaatgctttaaggcttctctatggatggagtgtctatagtcagaaaagtctgaatctctctattttttcttctt
tgcgtaacagactagtcctttctcttatggttttcgctttaaacaaagtatcctcgaacaataattatctgactgctacttgtaaagcaattttaaaga
attgtgtttctttcatttacaaagaattatctgaagtcccagccgtgctcgaaattgctgatcaaatcgccaagtatattgatgttccttccgcattcc
aagaactatgtcctgcttgcaaagatcatatagagtttacgaatgaagcaatagcaacttgtagtaaaggtcatgtatggcaacgttgttcaataa
ctatgcttttactatatcaacgaactgccagatactgtggaatttgtaaatctgttactgttgattgggcgtcgttgaacgtcgaaagcacatgtttt
acgaaagaaattcaggaagagctgagtatttgttatttttgcggtggtcattacctatcatgattacttagatgtacagaattttaactgaatgaag
ttaaaaagctcagaatgcttccacatgtccttacactctttttaccattttaaaaatacatagatcacccatagatataaaaaatttatattaccttct
gttatccgaaatgaaataggaaaattttcagtaatctaaagcttcagaagactcaaaataaaggtagcaccttgaacatcttgttcttccgaggca

atcaaacctgattcctcatttccatcactttttttcaattccccggcgcaagatcttagtgaacaatgggtcatttctctcaatagcttgctctaatcca
atagtatcattttgaaagcataagtcatgcgaacgtcgtccgttacctgtaattggctctttttacgaagacgctgaacacgattgataacctcgc
gtgccaaaccttcagattgcaactgaggatcattctcagtgtcaagcaaaactatacaggtaccgtcggtattggatttcagatattcatttttgttt
tgaacagaccgaatgatttgcaaatcaccttcaaccaacttgatgccatcaagaagcatctcaccattcttttggaattgtttaacgtcttcagtggt
aacgttaggaagagcctttttaacccgagccatatctttacgaagcttcttacccaaaacaggccaatcagcttgagcactgtaaacaacgccata
tttttcttcatcagcggtgaagatgacttcacggacgttaagctcctctaaaatatagccttctaatgacttagcatcttcaaggtattcaggatcag
taacgataaccacaaggcttctcaaaggtgtcttcaaagagatattgttttgttcacgcacataacgggcaagttcaatgatagtttgcatgcgctt
gacacgacgcagaacagtcgcatcagaaagttcttccttgtaagaagggaaaggaaggaagtgaacggatcgtaaagacatatcactatgatc
aatctccatgtaatttctgagttgttggtagatgttctcagtaatataaggagtgaaaggagccataatgcgagcaagggtaaacaaaacagtga
aaagaacgttcaaggccacaacagtttcttgttcaccgttttcacccttcagtctacgtctgttaaaacgaatgtaccagttagtcatttcttcaata
agaccgagcaattgaggaacgactgtatacaaacgatattgttgcatttcttgatcaacgaattgaataagatattgacagcgagcaagaatcca
gcgatccatgacattcttactaagctcaacatcttccttataaacaaaatctttaccattaaccttcttataaagagcggcttgagcatcaaagaac
ttataactgttccaccaaggaatcataactcttgtaacaacttctctgacaccgtcttccttaaatttcaagacttcagcacgtacgactggtgaatt
gatgaggaatagacgaagtgcatcagaaccatatttttggatgataaaattaggctcaggataattctttagacgcttagacatttttttaccgtct
tcagccatgaccaaaccgctgacaataacgttcttgtaaggagcattgccaaacaataaagtacctaatacggtgagagtatagaaccaaccac
gagtctgatcaacaccttcagagatgaaatcggcagggaaacccttttcaaagttctcaacattttcaaaaggataatgacgagaagaataagg
catactaccagattcaaaccaacaatcgaaaacttcagaaatgcggtgaagggtacccttacccgtctttgaaggaatagtgatgtgatcaatac
tatctctatgaagatcattaattcctgaaacaccagaaagttcttcaagttccttaactgatccaatgcatacaatttcttcgtaatcatcgctagcc
cacaaaggaataggagtaccccagtatctgttacgtgaaatattccagtcacgagcattactaagccagttggcaaaacgcttatcacgaatgttt
tgagggacccaatgtgttttcatcacattgtcaaccatttgatcagtaatacccttgaccttaacaaaccaactgggtacagcacgataaagaaga
ggagtgtcagaacgccaacagaaggggtaactgtgaaagatttgactgtgacggacgagacagcctctgttctttaaagtacggataatttcttt
atcagcatctttgacatattgaccaacaaagtcagtgatttcttcagtgtagcgacccttctcatctatagggcaaggagcaggatgatcagaatc
aataataccgtttgcccaagcagcgtcataatctgcttcaccaaaggcaggagcttggtgaactataccagtaccagaaccttcttcgacataatc
agcgttgtaaattacgaaagcatgatctttgaaagttgaagcaaaataggggaaaagaggcttatacttcattccgttcagtttagaaccgctga
agcgctccaaaacttcgaagctagccttcttggggtttttgtagagaattccaaggcaagcttcctgtaaaatgtatttttttgttagattctttatcca
aaatctttaggtattcgagtttgggattggcagccaaagccaagttggaaggtaatgtccaaggggtagtggtccaagcaagaacaccgacttca
ggatcatcgacaagttcaaaagcaacaacaacggcaggatcaggaacttccttgtaattctgttgagcttcgaagttacttaaaggagtggtaca
agcagtagaataaggcataacgcggtatccgcggtaaactttgtccttttcataaagcaccttgaagacccaccaaatactttccatgaaagaag
ggtacaaggtcttatagtcattgtcaaagtcaatccaacggcccaaacgctcaacagttgcacgccattcagaggcataagtcataacaatgtta
cgacattcagcattatacttgtcgatgccgattttcatgacatcctggcttccagtaatacctagcttcttatcgatttcatgttcaacgggtaaacca
tgagtatcccaaccaaaacgacgttcgacgtggtagcccttcaaacaagcgtaacgagtaacgctatccttgattgtagaagcaagcaaatgtcc
atgatgtggaagaccagtcgcgaaaggaggaccgtcaaagaaagtgaattcaggacgtccctcagagagttttagttgtgtgcgaaaggcatca
atttcgcgccaaaacgccaagaccttttcttcttctttaggaacattaaatgacatgctgaatcgtctttgtaacaggcttttaagccgcttttacact
cttcaacaatcagcagttggtagggttttgtagatgagaacttgtgattataagatggactgcaaattttagtgatctatagtgtacatttgtaaga
ggtttaacgtagttgaagcatgctataaagatgtttagcgtattatagaaagctcatcaattgtaatagcgatgaataaaacgatcgttattctttct
gtcagcgagacgacttctgtaataaacaaaaacaccgttttcggtttgatttcggattttacaggtggaatggttcaaataagtagttgtaaataac
atgagcaaaaatgatgctcaaaatagagacatagatcatggtcgacgaatcatgtctttggagttacgtcgtggtatgaagtgtaaacaatgtct
aatacacgaaactaactgtaaaaatgttaagatttggaaattttacgaagaatattgttacgacttagtgcttcaaaggaagactcttctccattat
cagtatttaaggcatatgaaaatgtattacgcgaaaatgggcttagtccttctgaaaatcgttactatattcgtgttttattccgtttgatttctacta
aacgatactcttgggaacaggagtggaagaatctaatgtcaagaatggggttttctcacctatcatcgagtttgcaaactcctttactttctccttct
aatcatatttctgacgacccatttattgtcagggattcagataaaaaaagaattactcttcaggagaaagagaatgtcaaggacggtaataatttt
gccagcaatgctgcgaaaacaatcaagtcgctttcagcgatgtctcgtttggcagatcagtttcgttctattcgtgagcatcagcttctagcaacaa
cattttattatctcctcgataagcatatgtatcagcaaagaattgctacaatttacgagcgaaaagctactgctgtacatcgtacttttctgctgctc
aaatatttccccgtatgggtgcaaaagatgcgccaggtacaggaggttatgtctacagctgagactgcttataatttagttgtgcaggagaacttc
atgtacgcatggcgtaagaagacgcgagaggtatatgcacgatacattgaagaacaaaaaaaggtatttttgcataaagctattctccaatgga
ataaactagcaaatgaatggaaagaaaagcatgaacgtatactcctgttgcgatttttgactcagtggaaacttcggaagaaacaacagagcaa
cagacgagttgaagcagtgggtttttacaatcatacgctttgtaaaaattgttttcgagcttggagactaaaatccactgttctttctataaaaaaa
gccaaacgaaaaagtgtgctagcaagttcatatttgaaatggcgagatgtttcagcaaagaagcaagagctacgaaatggagcagattttaagt

atatcaggaatgtcggaaaagctgtaatatcaaaatggtattccaaaacctccggttattatgcgcaatgtgaaaaggctgatgaatttcgcgac
agaacattgctcgaaaacatgctttctaattggaaatcgaagacaactacttgtagaaaacttgatgattggatggatgaacacgatgtcgaaat
actaaaatctgctcttgaacgctggagacatcggtattctcgaattgaagatggtttacagcttgcaaattatattttacaagatacgtttttgacaa
gatggcgtttagctttaaaaacaaatatatttcgagaaaacaaagagtatgcgctccttgttcaattttataagttttggcggattagagaaaaag
aacgtgcttttgaatataattataaccagaggctactagtaaagacatgggatacctggatcgagcggacacaaggacttttagaagtaaagga
cgttgcagaagatcttcatcaggagcgccaaataaaggcagccaaaaactgtattttaaaatggcgcatgcaattattggaaaaggaagagctg
cttatccatgctgatggcctttataagaaaaacgtaaaattaaaggtttggagcagatggaaatacatcatgcaaagaaaggctagattacgttg
gcgagcggatttgatgtacgacaagaagcttttaaaattatcaatgcaattgtggagaggaatttgtgaggaggaaattaccataaagttggaaa
caaaggtagttgattatcaatcatcaatagatggaaagctagtggaacgttattggaatttctggatagaaaaggttatgaagctttctgcactttc
gatggtggctgatcaggcacggcagcgtgtactaatcagggcttatgaggcatggtcataccaacatagtgagctaaaagggaaattagagcga
gccgaacgattttacaatgaacggctgttacgttcggctttaatattttggcggtaccaactgcgggcagtacagttcctacaaaatattagtgact
ggaagcgacgggaatttgatcgaggaatcatgcgccgggcgtttgattcttggaatttgttgacgttccgtgtcacgagccatacaatgcaagctg
aacgattttaccagcaaaaacaaagtaaacttttgggaaaaaatgtttcggcgatggatacaacgatatcgaattaacaaagaagactcctcgat
attggagaacactacgggtatggatgaatcttccagtttcatgcatgcgtttgatacaactatgccattgcagtatgatagtgttttagggactccat
caaactttcaacggcaatggaagcaagagctgaaaactcccatttctcgattttccaagtatctgcgtccgattcctccaaagactcagtaagtaa
ggattctatgtatgctggactatttgaacggcgaaagttaaatgaatttgtacgtttataaattggttcataaagcatgtgtaaaaacacactgacc
gtgtcgtgtcgttaatcctaaatacaaagtcttcaattaaactagtgtcagtacaaaagacctatctttacgttacaaattctacacctacaatgcta
aacgaagattcataagaagcatgcataaattaaaccatcaaaccaagaaatgtacattaataaagctttgacgaaatcttctcaaacataaggg
gacgcgaaaagaaagtaagatgcaatcaaagggtataaatagaaagtcgtttcaaactacaagtcttctactttaagcacgttcaccgcgaaga
cgacgggccaattgcatatccttgggctgaatggtaacacgcttaccgtggatagcgcacaaattggtatcttcgaagagagagacgaggtaag
cttcgacggcttcttgaagggcaccaacagcagaggattggaaacgcaagtcggtcttgaagtcttgagcaatttcacgaaccaaacgttggaa
aggaagcttacggatcaacaattcagtggacttttggtaacgacgaatttcacgaagagcgacggtaccagggcgataacgatgaggcttcttg
acaccaccagtagcgggagcagccttacgggcggccttggaagcaagttgcttacggggagccttaccaccagtggatttacgtgcagtttgctt
agtacgagccatttcagtaaaggaattaaggaattaaaaggaaatgctttaaatataacacagagaattaacgatttcttctagcaaaaccgac
gagaatggcaatcttacttatatactagcatatgatcaacaaccctaaccctggcaagaatcagatattggcggtacgcgacgcgttatgaatgct
ataaatacagtaaatttcacctcaccataacaaaacacttcctcttggaattgatttggatattaaacaagaactactaaacaatgtctggtcgcg
gtaaaggaggaaaaggattaggaaagggcggtgctaagcgccatcgcaagattctgcgtgataacattcaaggtatcaccaagcctgccatccg
tcgtctcgcccgtcgtggtggtgtcaagcgtatttctgctttggtctatgaagaaacgcgtgccgtcttgaagctgttcttggaaagcgtcattcgtg
atgccgtcacctataccgaacacgctaagcgtaagactgtcacttccttggacgttgtctactctttgaagcgccaaggccgtaccatttacggttt
cggtggctaaacaatgtgtgctatggcctttttctttacatataggacttttggcaatttggaagcctatgtttcgtctgttttcaaataagttatagat
ggttgcagccacatgaaagtgatgcgtatgcggtgcgctgctaaaattgggcagactgtatgtgcgagacatgcatcaatgcggaattactcttcc
tgacaacaatcttaaagatgctgagcatctagcttatgtcagttggctccggtagcactgattcgaaagttgtttattttacatatttaatgatattat
ggattatgattctattcaatgtgcaacgtatatattaagtaagaagaaattgtaggtgaagaatctagtagatgatttcgatacttcgtatatatga
agcaaccgagtatacaaactgttattaccgtaactagagttgatagtcatgagatattaaaaacatataaaaaactgcgatatgaaatataaccg
tattaattcattttcaataacgaaatgggcatgtggtgtagatggttatcacgcttccttagcatggaagaggtcccagattcgagttctggcttgtc
caattttttnnnnnnnnnnnnnnnnnnnnnnnnnnnnnnnnnnnnnnnnnnnnnnnnnnnnnnnnnnnnnnnnnnnnnnn
nnnnnnnnnnnnnnnnnnnnnnnnnnnnnctgtatgtgcgagacatgcatcaatgcggaattactcttcctgacaacaatcttaaagatg
ctgagcatctagcttatgtcagttggctccggtagcactgattcgaaagttgtttattttacatatttaatgatattatggattatgattctattcaatg
tgcaacgtatatattaagtaagaagaaattgtaggtgaagaatctagtagatgatttcgatacttcgtatatatgaagcaaccgagtatacaaact
gttattaccgtaactagagttgatagtcatgagatattaaaaacatataaaaaactgcgatatgaaatataaccgtattaattcattttcaataacg
aaatgggcatgtggtgtagatggttatcacgcttccttagcatggaagaggtcccagattcgagttctggcttgtccaatttttcccattccatttct
ttgatggggttggttgaaggttacaaagtacgaataaagcacatctgtcaaagcggtttttgtctgacagaagtgttggcaaacgaccaatgtcgt
tagttaatccctgcctttttccttaaaattggttccatgcatacgcttttgctatcgtatattttcaaacatccagcttgttgaaatctcactggataat
atactaacgagaaaagtacgttttcgttacttaaatgtaattttgttcaaagagaaaacgcatttgaaagctcgtttcattagaacacgacataac
cagaatgaaaaattgccagtccttttcatgaaagagcccctttcaatgaacggcacgaaaacccctccgtttttgtgcaccaccacatcaacgcat
tcgtcaagcagtattcccaaaaattgaattcataaagaacattttcacataagaaaaatacaaatagacaatgatcaatttgctttcttctcctcgt
tccctctcgcttcttttaagataaacaaataccttgtttctttaaaaggaacatgttacctaaattgacattatagatgcaatgtctaatatttatagc
taaacgtgtcattaacaaaccacgacggcactttataaaataatcaatatttttaattagcggaagctttctttcacatataggctgttattttgtatt

cttgataagcagtttgctcagaattggccttgttgaattagctttttaaagtgaaaccttcgtttaatatttcatataatctaaagtaccttgaacctg
aatattgaaaatgccgttgtttgatacatctttatttcgttttcgattgtcatccctccatctttcttcaagaaccttaggggtttactttgctggtgtca
tggtaagttgcaataacaatttatttgttcgatttcctaagtggtaagacactaaccaagttcagtttgcgtctgctgtttgggtttttgttgatacag
cactttatagcgctttcaattatggcagcacgttacatattacctttatcgactggatcccgtttttatgctctgtccttggcatgattatgtatgtcctt
ccgcatttcgcctttctcttagagttcaatattttattaactgataaattttagtgtgaattcaattgacaagtctcgccttagtggtgattcctttgcat
ataccgacgaaagcctagctagaaaagctcggttcattctctttattgggtttgctttactcgcaggtggattgggtggaagctttacggtattcatt
ctcaagtatgtcgttcctggttattctcttacgatgggtattgccaacgttatctgcaatgttctctttatgattagtgcaactactctttggattactgg
caatatgaatgatgactatcattacaatttacagctttaataattctgtttctgtttactttcataatctccttgctgtttaattaggatgacacataatt
tcaatgatctctttccttaaattctcaatgcttagacactgttggatttcaagtactcgttggaatctttcatgtcgctttggcaatgtatatatattatt
tgtgaatgaatcaaaatttgattgtttcctcattatccttttctattccttgaactttgaggtattgcatgtacaaccctttttgaaatctgaaattggat
gaaagaattcctatttatggatcagcatacactttgagactttataatcagatatttttttcataactccactcctgatagtaatgtttatatttttttcatt
ttgttttctaaagaaaatgccttcgtgcatcaaccccttaaatattatgctgtgtttagagataaaaaaaccggcacacatgttaagcgtgaacag
gtaaaatataaaacgttgagctaaaacgcagaaagatactttcaaagcatgagaaaaaaatgaatgtatgacacgcaggaaaatgagaaaaaa
ggagtgaggggagaaaagccaaagaaataaaacaccaatatatcaagccttatgaaaatacgatttcgaagctgtgtaaatgaaaggacaaa
atttagaacattaaaaaatcgtattaaaggcagccttaaaaagagagaaaagatgccgcattgaaggaaagtgatcatgcacataagctgctcg
atttatacgtcacggaaaaaaaaagggggaaaatatttcagacatcgatatttgcctgtttgactaaaacaggctcaaaattatttatatccacg
tcgagattttcgtatgtgtggggcggtaatatgctcttatttaagttaggacgaatagaaagctcttccaaagagaaattaaagagcggaggaag
cggcaaatcatgaatatcagaatctcgttcaacactatatctcacattcgggtcgcgcaattcatcaaaaaatggatgagtaagaacctcaattgc
ggaaatacgtttggagggtacgtaaactagaattttctttaacagatccaaagcatttttggtacatcttttggaagaattgattccaggaaatgt
ggacggacatttggtaatgtatggtgaacgtaattaggattcatagaagtgatttcttgttgagaaggtgtaccaaggacctttatgatttcaacca
attgctcaacactactatctccaggaaacaatggacgtcctaaaatcaattccgctatgacacatccggtcgaccagacatcaatcttagtggtgt
aatgatttgctccaaacaccaattctggtgcccgataataacgtgagcaaatataggaaacgttgggctctgatggaactagaatttttgcgcttc
caaagtcacatagtttcaaaacacccgttcggtaatcgactaaaaggttctggggtttaatatcgcgatggcagattccagtgccatgcaaataag
aaagagcacggaaaatttgaaatgcgtatattttgatggagagcatggacagagatttgtgacgccgggtgtaccacctcatatcctcaaacact
gtttctggtgagtattcaagcaataagcttaaatgtgtttcgtcatttgttggattatgggtatgataaaacgcaattaagttaacgatattttggtgt
gatatggcacgcataatttggagctctcgattctatttcaaaagcggttagcataaatatcatcaagaaacaaagaaagtatagaaactaaaaac
tctattggtcaatgcttcattctaggcttgttgtcaaaagaaactgcctgtgaaatcgaattcctaccttgtatcgcttgtcctggaatacccgcttaa
cagcaatgaattgtgagctacctataatctttgctttcatgacaacgccaaaactccctgagcctacgacttggaatagctcataggaaagtgtcc
gttcttccccagtttttgcatccaagactacttttgtttctacgtgcgagtcatctaaagtctgttaacaatccatttcaccaattccttgtcttaccact
catttcttaatgttgtttgtgtttggttcactgcacctgagtttaaaaacatactgttgactatacgtcacctagacttagtttgttgattgctcaagta
aataaaatagagatgcgcatcgtaaagcggtagataacatagtgagatggaaattaactaagaaagcgtgttgaacacaattgtgtaaacaga
aggaattccttcatgaagaatggacaaagaactcttcattaaatttcatcctttgagaataggtttcttagcgaatgggtttgtttcccaaaatcgta
ttttgttgctggtgtgaatgaattcattttgtgctttttgtttgaactgtaatgtttctccatttacaaatcctgacgggtaaatttttgtttataaatcag
tttttatttttatttttatttttatttcctttccatttccatttccatttgtcttaatctgattctatctaatatatttgtttcgttttttttcatttgtttacgtgt
tttgataactttcaagaaacggcgtaaacaaacattacctcgaaaaagataaaatgtctgcttctttctttcaaaaaaaaaaaaaaagaaaaag
gaacaaatttgttacaaacaagaatctttcgacttaataaatacagcccaagtccatccatcatactgcgaaaaagcagacttcataaaaggcac
actcatgtgggggcaactcaagaagagaatagctgtatgcagcaaacaatgagaaaagagactgcaaaataagaattaaatagaagatttata
agtttttaaacagtggtattgacagtggtattgttaaaatgttcttttgcttcaataaaaatttattgcgggcgaatgtcgtaaacaacaatgaccaa
tatgtctaagagaaaaagccatgttttcatactctgaggctttatagtctacaatttgctactcaatacatgctttgcttaaacacttgtcattcatga
tggaagctcagttcaatgaaacgttgcttctccaaaaagaaaactctgctgttaaacaaaggaattcagcctacgaatttcttcctattgagaaag
taaagaaatgaatagtagaattttatagattataaatcctgtttcatatgctaattcattaagaaacagatttatccaagaattttaagcagcgggg
gcagcaggggcagcagcgggggcttgaccagaaagcaactgttgttgttgttgaatcatctcatcacttccgggcaactccatgctcaagccaag
accaaccttggcgtttctattgggatgatcaatttcgctgctaaaagcaagagtgatagcgggagccaagcgacgttccaaataaacaccaacct
tacccttgctgtcaacttgactgcgatagatgctttgagcaaattcatacttgagaccaaccgatgtaagtccttcaggcttagggccagtcatcaa
ggcagcagaacgattcaagccgacaggggataattgacattcaacaccagcttcaacctttttcgctcaacttgcgccagaaagtggcagtaacat
caccttgagtaccattcaagtgagcagtagcgatccagtcatcagcattgtaacgagccatatagctcaaggcggcttcctcagggccgatagag
gtagaaggcttttgccagagagcctctacacccaaggataatttaggagtaagggactggagcatggaaagaatgtaaataccagtcagcttatt
ttcataccaagggttcattgccttaacgctgaaagaaaagtcctttcctctgtggtcatgctcaatttggcacatgctggggatagcagcatcggca

atttgcatttgaaccttagaaatgatattagggctccaggaacagttcaacatggcttggacagcaccgtcattatcaacgcttccgcggaggaac
aatggctcaccaccaaacatggtgctgaaagagtaagggggtaacacttggctgccaagagcaaatgcgtgagaaacagtaaaccaagggttg
gtagagaatccctttgttacatcagctcgaattccggtgaatgcatagttagaaagcaagagatcttttgaaacttcctttgacaaattttcagtagt
acccgggttggtaaggttcaaagaggacttgtagctatcgatcttgtcgccaagacgtccaactgaagaaacagctgtttgaatatagtccattat
gattagctcagatgtgctttttgtgggaactagtttttaagatcttctgtgagattattaagtacacaaaccgtaaagcctatgtatgtttcctttatt
agtcaacttggtggtttcgcttaggatgaactcctttttattttactctgattaaccaccttcacgtacaacgcggttatgataatggtggagagaag
taggtagtttaccggtatttcctaccaccagctgcttgtggtagggaccaatataatactaataaaagcattagaatattacaacgaaatattattt
tataacaaaatcatctttctgagagtcatccataccggtatggcaaataacacattcgtttttttattagttttaaaacaaaaatacattttctacctt
attcataaatacttcatcttcactcacaaacacttactgctagccaactttctcgtataagcagtaacgaacgaacggcttctatgtaactatttaca
tatcgaatacccgattacaatattatacaaacaggctatataatactatacataacgcatcatgttgcattatgctaagtgtttttttaggaaatgtat
tattggtccctaaaagtagatctgagcatcgggtctttgtgtctttccagcaacacatttttttaaaatttaaggaatctggttttcttttccctaaaat
cgtgtctataagtagaagaagtaaactctttcttcttcgaaacaacgattgtcagttaaggtccacctgtttgatatttaaagcccataaagttggc
gctcgtgcatcaaagttcgcaggtctttttcgtgcttctgggaagcgtgaagaagccaccttaatccaggggttcaaggccatatttactccatata
gtcaaacttggcttgaagactttggatctattcctgttttcacatcctagtttcacctgtgattcaagggattaagcatatttagctttttattcctttga
ttcaatccaaaatcagtcccagctgattttattccttttgttttatttaattatttaacatttctatttacctttaatttttatatcctaatgatgggttcat
ctaccgaaagacatgacgagaccagggaagtagccggtccttcccaagcaacaattgactatagtgttaattcttctgggagtgaccgtactccg
cattcttcgctttccacagaggaaactactcttgttcctgccaatgaagcatcaatgtctcctctaaatcatggtgtcaccgaatccgagccgcttga
tttaccatcgatagacactgaatcaaataatgcggcttctagtacgcccgtagcaccaccttcgtttcctacggctgtagcacccattgaaaattca
gagtatttcacgcaaggtcatcatgctgcttcttctaggcgtcatcgtcatggaaagaaaaaaagaaagcattggcgcaaacaggctaaacttcc
ccgtccgcttcctgatagcgaaccgtgtgcgcattgtagtcatgcaaagaagcgatcgtctggcaaacgacaccgcaagcatcaaggacggaat
cttgcttctcgtcattcctctgggttgatacagttttctgaaagtgcagctgatgcgtatcattcttcctactcccagcaccacgggtataatttatctg
gtcaacctactatgaaagcctcatcagagcacctggggcagttttccagtagtgataaaagctcttctgagtctttgtattcaatttcttctatgagt
ataaagaacgactcgaattcagattcattgtcttcgtcgtcctcctctactgatttatcatcgaatcaccgcccgttacttcccgatgaaaactttctt
ttaaggcctaatgctgatgccgatggatcttcattagaaccacctagaagatccagtgctaccaaatcttgctcagctgccatgaattctcctcaga
atttgccaactgagatgaatacgcagtctgatacaaacttagcagaacaaccttcaaggccttctgttaactttgcgccagatacatatatgacaa
aggaagacggatctgcttcagttaaatctgttgcttctcaaaatcgagatgcgacttctcatgtagattcagagttaccgagagatgtcgaagaag
atgtctgcttccccatgcaagaggaatcgcttgcttataatggcattgactttgatgagcttgataattttgcatctgaggaattacagaagagaat
ggcagatatggtaaactgtcgatcgaggcagtacagtgtcagcaaaccttttgaaccgcattggaaagatttgagtcctcaaagagattctactg
ggacctcttcagaagcagtgaatgacgaaaaacaggtaaataaaccattgaagagttatatttctcccaatagagaatctataggatcaaaagc
tcaaagccctcataggtttagcctgttctcttcaggtgaaaatgaaacaattcatgctgctgcgatatccgatttgctagaagacgaggtacatagt
ttccgttctcttctttgcgccgagaaaggtgtctggtggttagattgcttaaaccccactgatagtgaaatgcgaatgctttcaaaagcgttttcgatt
cacccgttaacaactgaggatatccgtgttcaagaaactagagaaaaggttgaattgtttagatcttattactttgtatgtttccggtcattcgaaca
agatccggaaggagaagattatttggaaccattaaatatgtatattatagtatttcgcgaaggcctcttgactttccatttcagctctactacgcatc
ctgcttctgtgaggcgtcgtgctcgtcaactgagagattatgtcgacgtgagttcagattggctttgctatgcgttgattgatgacattacggatgca
tttgtccctttaattcgtgctattgagacagaaacggaagctattgaagactccgtattggtgggtcgactggatgaaagctgtgatatgcttcgaa
gaattggtgaatgccgaaagaaaacaatgggtatgttccgtttgctgtatggcaaagcagatgttatcaagatgcttgcgaaacgatgcaatgaa
aaatggaatatggcgccaacgggtgaaattgggttatacttgggtgatatccaagatcacttaattactatgacatcgaatctttctcaatttgaaa
agattctttcacgaacgcatagtaactacttagctcagttgacctcaaattccattgaagagaactcaagaatgaacagtgctttaggaaaaatta
cattaattggtacactgctagttcccatgaacttgattacgggattgtttggaatgaacgttcctattcctggtcaatcaagtgacacaaacacttcg
ttggcttggttctttggaattattggtgttttagttgccttggtagtgattggatggtttgtggctctccgatataatgctttttaatttaaaacttcaaa
agttgtttaatacttatcatgattgaagaagtgagttttctttttacgatgagatgtaaaaatggttatatttaacaataaaaaaatgaaacaaaaa
agcatcggcttggtataaggtttagaccggtttgtttttttgtttgagttgattagtcttttctaatgttacttgtaaagaaaaagtgaacaggagaag
ctacgtccttaatatcattaattggtttaggtattagacgttttcgttttaaaagttgtattaataaaaactaatcgcatgttacaaatacatagatttt
tcagaatacgacttgttctttggagcctttagttgaagtagaccgattatgaatttcatagatgacttatgagggaagcactccgactaatgagatg
agatataaggcgaactgatcaaaaatcttatgaactatttggaaatatcttttgggttgcgtcgggcttcctcaatcatatgaagcacaccttcggc
cggctaatacactgttaggaatctgcagatgaagaatggagtggatgaaggaagagttccgaaaatcttttcctgttcaccattcatgcaatacat
acccttgagtctttttttcttttctcatagtcagctcgaagctctggcgacaaagaatcgatcacatgctgaggagaaggagtagtgattttcattgt
agcataaccaaaaccaacgattcctattgcaaagaaagagtaagttaacgtctaatgaaagtctatacttgacaaccatgatcgattgttctttct

tctcaacataccaacgccatacaaaagacttttgacgattctgttcattttcggtagcagcttaaaaatgtgtttgtttgcttcgtttacaactgttcc
aatccttaatgttaattcggtcaatgaatcttttccaatatatacgcgttgatgtagctacagtaagatggatggcatctttacgtttcactatttgag
tgtttcgtaaggtggattttattttcacgaacggtttttatgcttgttgaaccagaaaaagactaaaataagcaataagactgatagaactcgtcgt
acatttcacaagattgaccgacgatgctatccattcatatataattttatgtgatttcgccagcgaggcctttctttcacgtttaagtccactgttgat
gaatcaaaagaaaaaaaatcccaaaaataaaaaaagtaacaattaggatatcaaaaaatggggtttacctcgaatactgcctatgcctgtccat
ttttattgggaaagtataatatcctctataatatgatatatttggtttgatcaaccagttgtgaagagtattgcaagaggacgtttgtttttctttttttct
atccatttctctttcttttatacttatgttatcgcattagtgtgcattttccttggcaactcttattatttcaatcaaatcaacgcaaggcgtcatattgtg
gagtaccccacaatgatcagcgcttaaggacaaataacattccaacgttatcatcgtagctttgttttaacgcattacgatcgatatgcttatgcca
attgtgcttgccacgacaatatcagataaacttttcaccaccaaccctccaaaattttcgagcgtaaactgcgtatttttttgaaaagaaaaaaaaa
aagactttctttatttcgaaaagaatcggtggatttaaatcgaagccactagttttgaaacccctatttcttctggctagtcccatcggtcaaagtttc
aatagtcttacataaagaaaagtaaaaaatcgaagtattactaataagaagatattgaataaatcgttttctatcaaggttctggtgagtttttaat
ctttctctttatttttatcatcagtcgagtgtggtcgtgctttatgctcaataaagggatttgaaaagaaacgaggtattgattacggaagttggcttt
ttgaatcaaacttgctggggtttttttgacatagccaatcttttctagatacttgtctgcagattcgatttcgtcgttacgcaaatttttcctaagttttc
attcttccttcattttgtatggaaaacaacttcaatgctaatgaattccggtttctggaatctcagaattccttagagaaatcccatccacctcacaat
tccttatgtacgaatgacttcgacgagcaattctattgggtcaaccagcatatgaggcatctttggactcattctaacactcccatttctgcctctgct
actacctcagcaacagacacaccttatggtacttttcaacattctttccatccattgaacggtaccattagcgataattctattactaagaatcccgt
atctccacatttcaatcctttgcctcctggcactggcatccttgaaaaccttccttcatctgagcctcgacctttgcctagtatagcttctgcacatcat
gcaaacggccatcccttgtctacatctataagtgccgttgattcattaacttattcggatgacccatcatttgctcccaaaaacctacctgtccctac
ggaacattccaatcaccattcaatgttctcatgtcctccaccggcaaacaatataataggcgaacgattagaaagcggcaatccagttccgcaat
atgatcagcacttagatcaatctttattgcaagattcagctccattataccgtgtttgctcagcgcctgatccacctactgtttcgagaccatcagta
cgatgtttaagcatcgagaaccagtcttcttcaactgccgatacatctcctgatttttcaagcttaaaagcgccttcagctatttcaggtttgggagc
ttttgaaaaagttaaagaaaagctgggtgaagtaaagaaaaagcgtcagcaaacaaaacatgcttgtgccaaatgtcagcgtgataacaaaaa
atgtgatgagtcccgtccttgcgtgcgctgtcgaaaaacacaatgtgaagatcagtgtatagacattcctcgcaaaaaacgtcccttaggaatga
aacgaggtccttataaaaaaggaggaagttcaatggcctctacacgacggtctgaccaagaaaattattctttagcccagaagaaaaagactac
tagagtggatgatacgcttgagtcgttaaccatcaaatcttcccagacaaacccaagcaacaacgaatcatctcagacaaatgttttacaagacg
ttcctttacttgatcctgccgaagaaactgcctggcgaaaatcgagagtacgtagctatacaaatccaatcggtactgctgaaaatttgcattttca
gttgcctgaaaatcttccactttacccgaaaaattctccgtccgtcgctgaaagcgattcatccaaaatccatcctgtgcaccctaaacttccaatg
ccgacagatttctcggcctattccatgcatagtttccctgatcagtctgtggattcgacaattggtagaaccgatgagtatgtgactcatccatggtc
agtacatgacccatccgtagttccacctcatatagcacctcatataggatatgaacatcccccggaagtaaaaatggaagacgggagtgcctcgg
taacggatggtccacaaaccggtaatgatccattgcatccttcctaccacatgggctatgaaataagacatattcccgaggccgaacagtatccc
agtacccaccctatgtttgatggaatgagccatttggaacatactgtgtcgaacgactcgagctattcacctcatgatacgaattttgcttctccatt
aaaaatggaaggacagaacgcattacccgaaactcattatccctttcttcgtggaagaatacactcattttcgatagcggctgattcacctcatca
tttggtagagcgtccttgtcttcattgcctcgctgtggccgataaccctaacgagcatgcaaacttggcggatcttcgtgaaagacagcagcgcga
gttggagtttacctctatgttattgttccatgatcacccttcctaccacgctaggcccgatctgaattttcacgaagtcctgcatcctgaacattttgc
atacccgaatatccatcacgacaatagcgacaactatgactatttacacccacactaatacttttataattctggatgctaaatgctctttagtttttt
gtacctttgcttcttaaaaatttttttgtttgcttgcaaaactgtgctgattatacgagctatcattgatgttgcattcgatcattttggctttctatatcc
gctgcttttgctacggataggctgtaaatttgtataagtcatatggtgcattcatgggtcatcgaatgtatttgcatatgcttagaaaagtgtatggtt
taaaagcatatttattcactcttttaattgaaacggaaactgttaatttattttattatgttctttttttcctgaatacttatatctcgttatttacattttta
taattgcattgcatgacttgactcccttgggttattaaatttaaggtttttttaaaatgtgtctttcgtttggagtttttgtgttctattttatttaatgaatt
tctttcttaatatataccttggcattgcgatgaaagcaatttcattttccaatacttaaaacctttccttttgttaatagattttggtaaaccctttctttg
acgtggaaatgatatttttgtttctttggctctgaaaaactcgtcgtactatcgtaacattatctatttatctttttctaattttctttccaaatgtttaca
gcctttttttcacctcatacttaacaccttctgatgctaatttggtgttttcgtttagccaacgtgcaacgtgtttcattaaagataataaggagttgca
acgatagctaatacacaaatctcaacatctaaggagcttccaccatacggaaacctgtctttcacgaaataccaaaatgcaacttacagaagaat
caaaggtatgctgctctggatttttaacgtagacataaggaaaggaagtatttaacaaagtaggagcgtctcatcaaggttttcaacgtcggcaa
aactgtcacccattacggttggattcctttcatcctctggttaggatacacccaaagctctcccaagcctcaattgatgcgtgttattaatcctttggc
ttaaagctggaaaaggcatttgaagattttcaatgttttacctactgaggtacaaaaattcagtcatcatttgaaagtttatgtaaagccattttggt
ttcaaatgaaatgtttatcctatttctcattctttgattgaacgcgacgcctcttgttgcgccgaaacgaacaatatgcttattccaaataaaacgaa
tccaatctgcttacaaaagcctaagttaaacaaatagcatgaatattcatgcaaatgcactttatgtgtttgcagcccttgtgaaatatctaccgct

acttacattaagcgaagtagcttttaatacttattgaaacgaactggggatgaaagctaacataacagacccttagcatcgtgatcgtaatcgtaa
gcgttaaggctattagtagttccttgagctgttgaacttattttcaccattaaacgaaaacttgcttcacctctactatgactgtactactacaaaag
cttcgtaatcaaactgaaatgggtgtacaaaggaacaagccagttgacagaaatagtacgatataaatgacgagtatctaaacaactttagcttt
tcttctatgctcgttttgatcctttacaaaatcaatccttctagtcatcttcttctattccctacccccacatagatttcgtctacttgctttggagatgca
tccgatgttactctgagtgtaacatttccatacccaagacatttcagatactttgtatcgtaaacgctgatttcctattactagagtacatattagga
aaataagtgtttgatatctcagaaataccacgattttcagtttgctttcgggcggtttctaattcgggcaattgaatgaagcgctgaaagaatttaat
agaacgttagtggattaccaaaacgtcacattcaaatatattgttcaatcaaatacaaaagagaagcaagaaagttacaaagcaattcggcgac
gcctatctttttcggtttagtttttggtgttctttttgttttcaagggcttcccatacttgaaaagtatatgtatactacattttctacataatttatactt
aaacacagcgtttggttatggaccaggcgattgccggcctttcagctggcatttgttcaacgctagtcatgcatcctttagacttggcgaaggttca
gatgcaggcttcttcgaatcgaaaaggtatttctttgattcatgtattacaaaataatataaagtacacaggatcaatacgatctctttatcatgga
ctaggtgttaacatgctcggttcagctgtcagttggggtacctatttttatgtctatggtttcacgaaaagtttattcttgggaacaacatcttcggaa
cctgtcccaatcaccgctatgcagactttgataagttcagcgttcgcgggttgtatcgtggctgctttcacgaacccaatgtgggtagtaaagtcac
ggattttatcaaggaccgtgaattacagcaatcctttttatggtgtctatcatcttctgaaaaatgagggagtgagaggctgttatgctggttttaca
ccttctcttttaggtgtttcacaaggtgccttgcagtttatggcttacgagaaactcaaaatatggcataaaggacaaccatcgaatttagattattt
atttatgtctgcggcctccaaagtatttgctgctatgaatatgtatccactccttgtagttcgcactcgtttgcaagtgtttcaaccaggaatatcaat
gcttgaaatgtttaaaaaaatatggagagtagaaggccccttagctttttataaaggatttcaacctcatttattgagagttattccgcaaacttgt
attactttcttagtttatgagcaagtaggtctacatttcaaatctcgcgcttccttataagtcagaagagcttataacgaaaaaaaaaagtataattt
tgataattattgtataaataaacttcctcttatctcacaaacacaagcgtttcatccatgtgtattgtttgtttcctttaaattcataacaaaaaccct
attctaattgagtttgcataacaaaatcttcgatgcatttcctttgcgcgttggttacttgttgtagaatatctttcgtcgtttctggctctttggacatt
aagttaagcaacaaatttccaactttttttacggacttcaggtttctcaatcaaaacaccaaaatgtccgattattgggagagccaagttaatatag
gagggttctatttgttagcaaaccttgaaaattcattcaagtaaacagcatacttttggtctcatcaatgtattgatacatgctcagtaaggacggc
agaaagctttcaagataccggccgctttgtaaaatttcatttttctgaagcattgcaacagaatcaacaataattttccgacacaatgcattcattg
tgttatgtttgctgttcgccaacaataacgaagaagtgcattttgttgctgtttgaaaatcctgtaacaatgttaacgatgatatttaagttagatga
attacctctgttgtttttaaaagatcgaaaagggacgttacaaagttttggattataagctgctctccatctaagagaggagctgctatcgtgagga
cgagcaccatgctcagcaggatgatctcctttttatctgtgctttccgattgactattaaatggttagaaagcgccattttccatttgaacgtactact
tgttttctaacatagaataaagatgctcaaacaaagtgtagcataaggttgaacaaatagagttatcttgcgtaaccattgtactcagtagttttcg
taggacacccagaagctcatgcttggagtatttaaggagggacggatcatcaagtataactttaaatagttagaaaaaaaaaataataaaaaaa
gaaaaaaaaaatttcaagtatagttaccttcgtaaacgtgtaaagcagtagcaaatagatcaactcttacaatggtggggaaaatattagcaagt
tccaaaaaggagtcaaagcatagtataatgaaatggctagatttttcatttccagcagaatatacggagtttgatcgaagtcctggcaacctgac
ggttaaagctaaaatcaatatcttaactaagtcaaacaattgctcaactccttccgaaacagaaggttctaaagaagtaacagaatcactttcgc
acacttttaacttagaatgtccagggttcaccaacgctaatctcttgacaaaaagaacgatactggtttttgtttcccaatcctccgtaagaataag
tcttatgagaagatcaataacctctgggaaaacgttagtgtcatacagtacatttgtacataaatcctcagtcaaaatagaagtcatgattttcatt
attcgtaaaaccggtttctcaaatttttgtaaggtgcttgggataaggagtgactgaaagcagacactgaaaataaagaaattgaacgaatgctt
gtcacttatgtagtttatattatcatggaaaaatatatcttctcctgataacgttagttcctctccatttaaaataccatagacaagatcgggattttc
gtccagaagaagagtcaacgcttcagcgatattcagccatgtagcttcgtatatctagatagttagttgggaaccgaaaacatacttacatctaaa
aggattttaggatttatattttgggagtgggaaaaagaagtatccgcaacaaagtcttttagtaagctaggtccaaaccgaagacgtgaataacc
tctcaaggttaccacccaatgcgcagcaaaacgtctcaaataaggagaaactagttctttcagatattcgattttatgggagtaaccatacaagat
tgcccaggctgataacgtggttactcgaaggacgtagattgaatcgttctgaaattgtttattattttcattgccattttcaaaaaagcttgtatcaa
gtgcatatttcaacatatttactacgcgattcgcttggctagctgtcggaaggagacctgttccaatgaaattagcacctatgtttaatgctagaga
aacaatttctggtgcaaactcctcagttattgcagaagtgaaagcagagctgatttgcgcttgaaattgatctaacaaaggaacatcctcaaaatc
agggtcctttactgtttgaaaattctattgtcagtcagtatatgcggtaatcaataacttaccataattaaagtatccaaaagtttgaggccactttc
ttttaaaaaaaaaattatcagatgtagatagaatgaaagctacacgtaccaaatctgaaattctggaaatcaaataatgtgaagcttccgctttgct
ggacattatatcaaatatacatgtatttaaaatcgaggatgcaaaaagttgagtttgccagcgataattcttattgccagctgtagaaacaaggga
atgggcaagcgacacagtgtcctcatccaagttatgatttgtttcatccccaaaagacctttcgaagttaaaatttcctttacaccatttaaatata
agagaatattaacccaccaggaaatatcgctttccttgaaatctttaaaccacatatgtactatatttctcaaaaatttgttttcgggtgtagcatca
attgattgccagatgagcttgtccagatgccttttgtagcaagactcggaaaacgattgattttgaagtaaaatttgatacaaaacctcaatggctg
aatctcgaatcaatggatgtttaacatcaacaatatttctaaatattacatcgatgaaaacaccagagtttaaatcaagtggtgtgaacagataca
aatgttgataacatttatatactcgagtgatgttgttactatcctctccagcaaaacgcagtagctctctcgaaatgttgtaagcaagataagagtc

ataagagttactccttaaagtagggcccagctcattaattaagcaatcaagatattcaataaataagtgggtgggatagaccaagtcggtttttct
tttcagaagtaacatatctatgtcgacatcagctgaaatcatgtaattaagtagtattcgcatttggccagttaaaacacttggtatagttttcgcta
ttgttcccaatgacacgaaagacgcttctatggaacagtttcttacagtgtcattggagtcagcattaaacactttaagtagctgatacgctcttact
aataatgcttgggaaacaccagaaccaagcttggctaacgtatgcccgacacccattattgatgttgctctacaatttggatttccggattccgcag
cccgctgtatgagaacgtcaagaacaaatgtcggtttattatgataaagcaaagccaaaaatgagtaagattctgaaaacagacggattacggt
catatcgtcagtaccagaatatatatccaataattcaataacaagtgaaacaaccttacttgataaagaaagcttcttcacacaaaagacttttag
agctccgtgaattgaaaatagaacatttgtagaaatcgctttctccctggaaatatccctcgtattacttgggttacagactgaggcaattaagatt
tctaatgtactttccataaccttcggcggttgatagcaaaatattaaagaaaataatatgactgcactatcgactacggcagtggccaaaggagg
aacaaatgggaaagaaaaattgtctgattcagaaaacagaaaaagcatgtcagctgtcgggtctcctattaagtaaggcattggtcctacagtg
atatcattaccatcatcgcttttcttggggtagtatgaagaaaataaacctggtattacctttaaagaatagttgcgttgttgggaatcatcatctag
atattggaggtatgaaagctctgctattattgaaacagttgttgataatagctcttgatcgtctaatgtaacgccatttatgttcgtaagttccaaga
aacattgaaacactttcgataaaagcaacgttcgcaactcgctgtgagaatagtcagaataatgaatagctgtttgaagttctagcttcttggtaa
ggacaaaagatttataaaatttcaagcaattcgtaaggaataaaattatacgtttacgaacatcaggagttagaagaacccggttatacttcaaa
aatgctagaaggcactgaagagcctcatagcgagtaaataagtcagcattagattccataaagttttgagaatgaatattagatgatatacgagg
aagcgcattcttccacaaaatgaacaattgcgaaagttgcattctagtaaaagaaggaccgaatgaaacatacgttgtcatcaaacgccatccta
ctttcaactgagcctgagctgtgaataaatctaaactggtagaagaccgtataagatcgagggaaaaattgaataatttagaaaatataaaaga
tgaaacatacaacggattcttttcagctgcgacatagcatgatactaaagccagagttaaattattcaaaattccgttgtttggtttgatctttgatt
ccttcgttttcgaacgcattagcttaagttcattctctagctttcccaaaagtgatagtaacgttggcattatgggacctggcatcactataagcaaa
catcgcaaacaccatgccgcactattcttcagactgtcattacccaagctaatactatcaaatagtaatctgaggaattgagtatgatgaggagta
atagaaccctttaattttgccagaatatcggcaatggaagcaaatgcgacctgaactaaggtgattgatacttttgtttggtcagctacgaacggtt
ccaatactttagataaaagcaattcaaaagcctgaacctcagaaccttgatccaagaaacgcgaattgattacatcatgcaaaagaaaggtaaa
atgttgttgctctaaagcagataaccattcgcttaagggggggatttacagtagaatcaagcaaaacacttaagatttcagaataatatgttcgaat
ttttggaaagtcttgtaaaagaaaaaaatgcttgattgcaagagttattacacctctaggtgtctcggattttgcgtatttatgattgtagagtccaa
ggagcatctgcaaaactttactgaattcatcagattcatcaactctttgtatttttttgagcattctcagactcttcgttattttcatcatcagctgcaga
agaaccagacgcagcctttaaagcttcaatctgacggtgcgcttgtaacatggtgctagcagtaaagcatttggctaacatgctttgcaaatccga
gttttcaatggagcatttttttaataaataagattttaaccattccaaatccgaatttcgcaaagaagagtttaaaagtaagatgtaagagcaattt
aaagcagaaagaacgacgttcaacgaagaacttcccaaattagatttgatagatttccaaatctccttcaataattcttcgctcaaatggcttcca
gcgtgtcgaatgttttcagaaagaccttgaagagcacatacccgaaggccatgaggataattattcttggagtatttgagaaaaacctttacgaa
cgtctcttgctggtttatctaattatattgtattagaacagcatataaagacatacacataccgtttgaccatacgcttgtaagcaattaccaagaa
ccgataagttatttctggtaggttagcaatcaagtgaagaaaatgctattttcatacagaatttcaacattatctttttttatcaattatattgtcaacg
aatccgaccaatacacggtttatctggtatccttcaactggcagagttagtacagcaaactcgtaatgataaaaagtcggtcatacatccttttaac
agtgcaattacacagtttgataataaatatcgtgtaggatagcctatataacttccatactgcttgattaaagcatgtattgggtctatgaagctttg
aagagagctctctcgaaatgttttgctcaatcgttggagttcctgttcataatcgagtaattttatataaattacttccctttatttaagcctgcaact
ttttcaagattttccaacgacaatggtgccgtcgacatgtggtttgattagcacaaagtattggaattggtaaatgagaaagtgctacctacggcat
ctgaatgcagagataaaaacattccatagaaacaaatattatatattcaatgtctaaaagtaattaacttaaaacatataaatgaaatttctttag
tagtaagcttatatctctgggacagacagtatacttaagtacgtatatcttggcagtaaaacatcaagaatgggaaaacaaaaattaaaaaacc
aacttaagataatggcagggtagcttttactgagtatacaactatagtaattagtcaatacttcctaatcgttagtaaagcaacaaattcattatta
ataattaaaataaactcttatgccaatgtttgagctcttccaaagagtataagcagggggattatgtttggttcatataatacagggtaaactaac
gatccatttcggtatgtatgctagagatgagaaatagtgctgtctggataatcatctctcttcctatcttgtccataatcatttaattcttcaacattat
acgcctgttctttctttttgctgcggaatcgaaggcctagtggaatgactaataccaagaagccagtaaacgacattacaacgactggaatataa
aagttaacaaatttcttatcttaaagatagacacataccagcagctttccgaatctgctcccatttttgcttttcattttcggctcgaagctttcccaa
agaaacagataacagattatgtagttccacaatctttgcactaagctgttgaacatcattccgagtgtcatttttcctattttcaaattccattttactt
tcagtgcttagtttgttaagatgctcatgaacaagttgttctaaaccttcgacctctcgttggttcgaaaataggccagcacgaagtgtgctaatttg
atctttcctcatattgattttctctgaacgaagctctgacaaggcagttcgaaataaataggaacactaaatcaaagaatgttagttgaacaagct
actatggcatatttaaatttaattcaacgtcttgaactattccatttcccttttactcacattctcaaagtcggcttctgataaataggtcatatgactg
tgtcgtaaaagttcagttataaacatgttagtgacatccaagaaggcctctgcttctttttcactatatcctccttcaactaatgtcttaaaaaatttg
taagtattgaaatggtaaccttcaaggtctcgaaatcgttggtcgatatcattcgacctcaccagggaaaggtcagttttggatgtgttatcattttc
cttattagtagaataaaatcgactacaatcgtgcctaaacacagtccttgctctaccttttctagggtgtatactcgatagtagcgtaattatgggta

gtcgattaggttttattagagagcggtttattagaggaaaagacagcatttaacgtaataagaactttccttttactttaatttgttgcataaaataa
cttcacaaacagcatttggcaatctgcgtaattggggttgtaacttggatactcatctgcagtcatttgaagacagtatggtaaaggacgcttatgt
gaaatcaggatcatgtatactatcctttttattttcaaaattagtttaatacagtcatataaatctttattctaattgtaaatcgatttttcgcgatattg
aatatatcattaacagagagcctagtagggtgtaatattattcaggaaaaggaaaacagggagttccaaaaacacaaaattaagataattaatt
gttgagtaatcaaaaggataccgttcaacatgaatctaggagcaaagatcttccataccatgagatgtctccgaaagaaaccagcaaagaacac
cgtactagctgcgattgataaataataaagtagaaaagaagaacaaagtaaaagtaagtttttaaaaaacgtttgttttgtggtataaggctttgc
tgcaaataaaggaagggaaaaacaagtaagaataggacctcccaatgtatgcaaaaacataagtacaccattgataacttgattttctgcagaa
ctggaatgaataaaggcaaagttccaatcaagagaagaaataaccgcctgattcccagttgtaaagaaatgtgacaatcccagaatacttaata
gtacagacgtaaacgttactcctggtgatgactgagagttttctaatcgcaacaaaataaatatctgcaacaggcaagaaactaaggatagctta
ccaagtgggcgttgcagaaacaataataaggacgaaaataatgcgaaaataaagtaatattcctccacggcaatcaatgcttctctgtttttgac
ttattttctcgtttaatatacacagagaacataaaaacttgccaagacaagcaggtaattatgccaattaagaatatatttgcaatatagacaagt
atgctaccatacttgctttcaagagaggaattattagccaaaatatgctgaacaacccaaaaaaaagaagaaaggaattcaaaaaaatgtaga
attgcttgcttccttttcgaaaacgaagtcttcaagactgatactacatgattctttaatacataaggaaggaaaaaagaagttagagttgaaatt
gtcaacgtaattaaacttcgaaagttattttccggtgtagaaatgtaagtgctagtgcagaaaaattcttgctcctcgcggcagttcgttatgtaag
acgttagaagttgcaggataataaaaaagatagaccaaacggatgaactaagggccaatgtagcatcggtatgcgtaaagcttttacaaagcat
taaaccacccaaagttacacaaaaatagtgagtcaatcgatcttcccatatagtgaaagaattagagccaaaactgcaaacatggagtacagct
aatcctaacgctataacgctgtagatgctagggatagtaaacataaattcaaaagatacttttctgcataactcgataataataattatgttcgga
attagcgtgattaatgtcatcggaaggtaagtgagtattgacatcttgagcacgacgaacagtaaagaaaaaagaacgatttctttaaagtaat
aattaacacattcgaggccttctgagtagcatttttatcgttcattagtaaagcaagagataaaccgcctataaccaatgatacgaacccaataac
cataggcgctaggctgaattctgcccaaatgcttttgaaataatcaaaaatacgctgagaatctgtaaagctatttaagaaaagttccaaatagtt
gtcatcggtctgcacagtagagttaacaaaagcatcatatgatgtttcttgtttatcctcataagcctgaagaaatctattaatctgagccatattta
tttccatactatcgtgcaaatatttagctccgtgaaaataaaatgcttccggtattatagttcctaaactgccaaatggaattgcatttccaagcatt
aagcttaaagtcggaaccagatcaacttgatttacggaacgtctttctccgaattgcttgaacggcttgaaagctggcctcttgctatacatccata
gtacggaattgatttcatcaaaagagtcaccgccatgattacccttaccgtccataccatgatcacccataactagtagaagagtatcatcgtcaa
ttaaatccatcgtcttcttgatacattcattcatctcttttaacttttctgtcatggtaggatggtttggccctaaccgatgtcccacatggtcaacgcc
taaataatgggcgataagtatatcccacggtgccttttcgatatagtcaaatatatatcggttcacttttcgatccacgccatgaaggtccggaaca
ttaaaagaaaaagcaggcctggaatatgtatgatttaagtaatcatggaataaagcgttccaagtatcatctcctaataaaacaatttctttaccc
agagatttccattgatataaaagattgtcttcatcaacgttcgtacctgcaaagtttgaaccgatgtcaataaatgttggaagtgagccagtagtta
agcctttaaggcgctgagaagtagtcgtgggtgcgtcagctaggaactgaagtaaaacggactgctcaggatgagaaattgatgtttcatatgga
gtattcaaagcgttaagataataagcagaatcatttgagggtattaaaaaatcatatcttagagcgtctataataattataacagctttcgaaaac
aactttggagcccagcattcagaagtttctttccattgagatatattaagtggtggattagaacaagaggatgttgagtttaaaacttctcttcttaa
tagaaatccatcagtgaaagacttcagtccatataaatttgaaaacagaattagtgcgaaaaaaacacttgctttcaaagcatcccatgcattcgt
caaactttttttttagttaacctaaccttcccttcttcttgcataaacaaatgcgtacgattaattctatgatttatcaatgctcacaaaccgttgtgttt
tatgagaggtggtgaatggtacagagatatacattgacttacattttcatcgacaatattgccgaagaaataaggcagattattgtatgctgtaat
gaatgctaatgttactatttctaattagtggaaaataaagacaaaataaatcccagcagtaagaaaaaaatttaagatcaatagctttcttttctatt
ctagctattaagttttgattaaattagatcatcaatttaaaataaataaaggacattacaaaaagtatcagaagtgttgttaggcttatcaaattaa
gatgtttcccacatgactagtagcatataaatcctcagataactgtatctcgacagttaaaacttttccattaaccaaaactagctgttaaatgtta
gtaaatcctcgtcgtataatcttcttaccaaaattttgaatcgctggaattcatcttcattacctggatacaaaggtgcgacactcatacccaagact
aaagtatcaacgtatccgtttaaagcaatgatttgtggaataaaggaataagcatatatagcttgtccgttgattgtttttctgtcatgatcaacct
taatattgacaattgaccgctttgagtagcgcaaacaagactctgcagttggggcacgtattccatcatatttattctgtgaagtccttcaaagcgt
actgtggatggatcttcgaagcaattcttgcagctaacaaaaggatagatttgagacttacgaaatccacacaagacaacagaatacttagtcgc
gaaaacaaaaagttcctctggaggttctcgaacaaatgtacgaaagcaggattttagattgtcctacatttattcatgttaatggggaatttttcgc
aatgagttaatcacttaccggagattcatcatgcatatgcacataatactccccttcaaacactgcatcatcatcatcaaaatcatcttcgtcagaa
ataaataattctaaatttagggaaatggatggattccaaggagtttgtccaaatgtccttgcaggtatcaacgtttctattttatccttcacagttggt
acaaaggtgaaggctgatttatcaacgaatttaacagaccatatcctgatagttattaataagatagatattaacatttgcataccaaactttagt
atggaatttggacaaacaagatagctgattaacatcccataactgtaatgaccgatcgattgatccacttaataaaaaattcccctttgaattgaa
tgatacacaagggactaatactatgttaaccaacagcttggcgagcacgacttactattgtgttcatgtcctttcagttctagggattcctgtcgaa
accaaggagaacgatagtctcgtttgcatttttttgtaatttgtagataaattgaagctgcataatttgttagcattgtgggaaggttttgttttcgctc

ttacatattgacaacatgtgcatttgaggagatggccaaatacccttgcgatgaagcggctattccccaggcagaagcacttacagcaaaagtca
tgggacttctatggaggttttgaacatcgtacattgcaacttttccacagtccgtcgcagtgattaaatattctaaatcaccaaaaagctcacatct
aacatgattgattccattaggcattcctggagagagatcgccatgttgtaatggcgaaggctcatgagcacaagaaaggaccattagaggttctg
gctcacttgtatctcgagaaaaaggtaaaagattatagacaaaaacatcctctatagtagtagagtagttaataatttttcttaactaaaaatttcc
tttgttattgtacctccgacggctacaaacatatagtcttttaacttgctacatgcaattaaggaatgtctgaaaagctgaaatggtttgtcttgcag
cttaaataatctatagattgagaactggaggcgaacgatattagcaagctttcaacaatactttgcaagtttacatacatctaggcggtttagaaa
ctcactgactaacatttctttctccattattaattggaaacctttcttttaatatctcttcaattcataaaagatacacgtttaaccaaatgaaaaagt
agaggtggtttatgtactagtggtcacttacacacacattactagtatcccagaagaatcacataaaggtgtttcttccatatttggaaacaaatat
cttcttcagataaatgaaatggcagcgactgcgacacattgcttatccttcgcccaattccagaggctgagatactttatggatacgtcgactcagg
aatcaggtcgtattgagtttgaagggacttttaaagatgattgggaagatgatttagactgctattttgattcagaagaagaactatttataacagc
gggaaggatattctactcaaaacttagagtgtttcagaccgtacgaaatctgagtagcaagcttgaatggccagacttacccttagtttctaagaa
agaatttatttctcagtgccgtgaagaacttgaatctgggaaaacacttcgtaatggggctttttataccatcttgtatttatgtgcaggagtgtatg
attgtgtcaaagatccaaaggctcattttcaaagcataagagaaaatgtcgaattactcagaagatttcgaattttagaagtcatatattccatctt
tacaaattggaacgaaaaccgtaaacctggctatatgtcggacttgaaagaaaaggaattgcttgctcttttcttgtctctgatatttttctttttaac
tgttccctcagaaaattacgttgaatggactgaatctgctaggtctttacaaccatcattaatgtcttccttagtacaattatttaatgatacggcga
atgaatatgaaatggaccttgataatgtcagtctttttcccttaagacaaattacgttcctaatttataagacttgtatccgtttatggggacagaa
acggaattccaggaaaagaaaaagtttattgcgagcatgagtaacgcaacttcatctatggataaaccaaaaactacagtacttgattatgaagt
ctttcgtcatgaaattgctacgaagtattcctcttttgcccatcctttttattcactgccattggatcgagaacagacacatattcttcctacactaaa
cacagattcatttcataagtcatattttatatgcaataatcgtttctgttctccggagaatccttccctaccgataccaaactcgaagtccaaagtaa
gtactcggcgcgagcaatttcaaacgaatcaaaacttgccttttgggtttaccccttctataaaagattcttcatctcctaaaagtataatagaggc
ggcggaattgctgcaccgtcaaaccaaacataatgtcgttgtagagcagctacttttcgaacgagaatacttacggcattgcacacttcaatatct
tcttggcactgattttaatgatgcagttagctcattggatttttcacatgaaaagccaccagttaacgatccgactttgaattttgtttcaaagtcata
tgatgacctattccttcatttggatattttcgtgcaattatctatgcatttattttattatacttctaagaaagtaaacaagctatatattgaagcatttt
cttcatcagaggcagcaatgcaaatgcaaagtattaatgatatggcggttgctgactcccctagtgaggaaatcagtgaaccagtggtcaagcca
cttgaaaaggattcgatggatgatttgagcgaattggcttctggatttttttgtttcaggtactatttcgtactttgttgatacgtgtgaacttatcctgt
attctctgtcgggccttttcttaatgatgctcaaatggtttcgtttatctcatgtgttacgatcagagagatttgcgaaattactgtacgataaccactt
tttagaaattctcagtcgctatttatcagataaagagtcttccaatcacgccgaacgtgatatgaaatgccttcgaggtggctttttttcttttacggc
gaaatcttataagcaatttccgaatagtccttatatttactccaagggtccctcttcgagaaatacgttaattactttaaattgccttcgtgtgatggg
aaaggtttgtaaatatcatattaacagaaaagagttgcttgtgaagattgatatgcagaagcatttaaaaaaactgctagaaatccctcatgaga
tacttaaggtgtatacgctgaaggttttaaaattacatataccgtatctaggtataaaatggaaacaggcgaacatgaatattatcacagatatat
acttacattgtcctcttaatctaagagatagttggatctttcaagaaaacaatttggaaacgcaacgctcatcagatcttcaagacaccttttgtgc
attttgattcgattttatcataagagattatatggacagaattgcaaaaagctgtatgacctaaatttgcttgaggaaatccgtttgaagcaagcca
tagaagaattagttgaatctcatatgatggattatctgccagatgctatgtggacctatacaagtagccatgagtctcccgatttttcgataatga
actttcatctgttcttgtacaaaatgtgtcttcgtttttctaatctttactttaccagttcatttattattatctttttactatgaataataatcctacgag
gaaatgaatttatgtcagtttgtatatatttaatcactctctttgctttcacagttcaaagacttctttcacagtccaatattccaagatgtcttgtatg
aacctttttgaacggaaatctggatgcctatttctattgtaaacttagctaattgtttacaaatggggaatagtgacatgtaacgagaaccggtata
atctaaaattatcagatcattttgcttctgttaataatttatatactttcatgtatctgagctcaaccaacactaaactgtacacacgaaggtttata
taaattaggtagaaacgctggattataatcgagaataacaatttttcgattcttttatctaataattgctcttgcaaataaatataagtatttgctgg
gtattaaaataacatcccaaagagttatttgcccctaagcgtaatttgtgtgagaaacaagactatctgacgttacgttcttttataaactaggcaa
atggatgttgttttagaattggcagacaactatgtctttgactcattgtacggcaaagttgtcgatgcatttcacgtgtcccccatgatgggagcttc
gagtgtgaacacgactgcccctatatttactacttctgttagtgcctcctcttcaatcgcgtctgctttactcgatcgcaataacttgtttcgacaagg
ccttagtttgtttttcatcacatggatcatgggaactttaacgtattttgtctctgcatctatagcttattttttttattttgatcgcgaagaagcccgtc
agcatcccaagtttttaaagaaccaagagtttttggagttgaaggttgctttgatgaatcttcctggtatggccttattaacgtttccgtggtttttgg
ctgaactccgtgggtattcttatgcttacgataatccatctgagtatggatacttttatttgggcttttctgttgtcctatttcttctcttttctgatttcttt
atttactggattcatcgtggtcttcatcaccgttggttttatgctcctttacataaattacaccacaagtggattatccctactccatattcttctcatgc
ctttaactttcttgatggttatagtcaatctttaccttaccatttgtttcccttctttttccccttaaacaaatacctttacttacttttatttggatctgtca
attactggactgtccttatccatgatggaaaatatttttcaaataatgccgtaattaacggtgctgcccatcatgcttctcatcacatgtatttcaatt
acaattatggtcaattcttcaccttgttcgatcgtctctgcagctcttaccgtcaccccgatcaagaattatttgatgccgaactcaagagggatcag

aagctgcaaaaagaacgagttggtgaaatggagtttatgttgaaatccgttgaaggggaagacgatcgcacgtattctggcaaaaaaaatgttt
gaaagttgtgtttttagttttgtcagattcttttggtttatgttaatgaagtttttttcccttgcccctatctcttctctctctcttctctctcttctctctctcc
ctctctccctctcttcctcttaaagcaggctaacaagtttattaataagtattgactagataaaaatctagtcaaagttgctagacaagaacttccta
attaaaatttcttgatcaattagtaactttttttatggtaggtaagatagtttgagaaagtatcaataataatttacaaattgttctgtacacgtctcaa
gtttctcgtcaaaaatacctgtccctgaagtgtacgagtattatgttttatttgtgtccaactgttagctctcaacctgtcgaggtttagtgaatgaga
atggcaatcacgaatgccaaaaggacgatttcgaacaactaatactttggtttcagaaacatctaggtcgggaaggtttacagattccagtggttc
tttgaaaaagctatccaaagtggaattgaataagtccaaattagaaaataaaggcgtttttttgaaaggtagtatcaaatgcaaattcgatggcctt
agcttgatcaactggtattaaattaaacatagaacagtacaaaataaaccttttttctgtttatttaaaatttaaccaaatattatagcacgagcact
ataaaaaacaataaagggatagtaaaaggtaataaagagaagcttcagtaagaaagctgtatttttcaatcttaacaaatgatgaagaaaaac
gctgtattacacaaaataaattaaaagctttcgtaaggtgaaaacgaatgtttcgtctttagaaaatatatttttttttcagtactcaaaattaataaa
gtctgtagaattaaatataatgttcttgttcatttgtgcggtactactaagtatttcaataggattgaatacgtgaagcatgcgttgaaccaccaaca
atctgtcactgattcagatatctcgtatttcttgaataaaatcttcctttatgaataaataatgggacttttgatacaatggtcctttacgcaaggatt
catattagggcaactgttaacggtttcagttatttacgtgtttcttaggttttttttgttttgtaatccaccaccacaagacctagccaaggatgaaga
gaagattgaagcaattaaatctcaacaaactatttcttctcctagttcttctgctggcactgccaatgctagtgataacgctagacattattttcaag
atgcccattcaactttgaaatcggagtttttagattctctttataatgtcaacgaacacgaacctgaatcattagactggttcaatgtcttaattgccc
aagctttaactcaatttcggcatgatgcaagctcgaacaatgtggccttaagaaaattagaagcagttttgaataaaggtacccaagacagaaca
atggttgatcacatctatgtgaaaaacctttcactgggtagaggattccctgtcttcagtcattgtagggttcttcgtcgagagcagggctcttctca
gctacgagcggaaatgctagtttctctgaaggataatctcacatgcacagttgaaacgaagctgctactaaattttccaaaaccttcatttgcgac
acttcctctgtcgataactatcagtatgcgaagatttgtcgggaaggtatgtcctactttttgtacaatatttcaaaaattaaccaatcgatttcattt
atcttgtatttatggtctgtttatggtttgctaatacaccatattttagatgatgattcatttttctccaagcaacggagcagatcaaccagcttacttt
aacttctcgtttgaccctgattttgtaatctctttagaagtcaattcccttataggagcacgatcaaaactacaggatatcccaaaaattactcaatt
aattgactctcgtgtccgtcagtggtttattgctcgttgtgtttcccctcaatttcaacagtaagtatattatttatttatgtactttattaaccaaaaaa
ttacagaatcgctatacccaacctttggcccacttctgcaaaagaagggcatgcacgctcacaaacgacgaattcagatgtacactgcaatatgt
aaagaatgcaaatccaacaaaatccttaagtgtttcattctatttctgtcgtcttgacagaaaaggtggatatttgcgaccgggtatccatccatta
ttttcgtatttgagccaccaccgattgcactcaaaaattactccttccatattcattctctttatgatccttgcaacatttctcgaatcgtctatgccac
aatgttggtttccttcaaatcgaagattccatcgttgcagcattctttcaatatttcttcttggaacacggtatacatctgaaaaataggctcggacg
tcgacgaaatgacctttcatccagtctgggatgtgtgtatcactatatttgaactgtaatgttagctgggataacttttcaaagagaaggaaattttt
ataaatcatagtcatgttaattaaacaaatgaagatgaatacctgctttgcaaggaatgatgccatatcccatgggccatcacatgcccaagccc
aattttttggctgactcctaggaattgctcgtgagggtttgaggatttttatttcatcaattggtggttgaagaatagctctatgttgccttaaaaaat
ctgttaaattttcgagtacttgtaggaatgacggtgctttgtcaataactgactgttgtatcccagttaatgacttgcaatagtcagataatgtagga
ttcgaatttggttgaacataagaatgaaattcgtcgataatctcatttgtctccaaatcgtaaagtagacagggaaattctattatttcattatcgaa
agtaaatccacatccttcctcacatgttgcttctacatcgactattaataaatatcgactacaatggaaattatcgtgttagttaattgtaaatttctg
tgaaacacaacaagatgaacttatacatacagaggagttctgtctttgttgtctaaagaataaagattccattgctcttgctttttcttttcttgtaaa
cgctgttcccgaaacttatatcttcgacgcaacttgtccctatttttgtaagtgactatgtctcaaagaaatactagtacagaacaaaatttattttttt
ctattacatacttgcttcctgtagaattcagattaagctcttccagtgctaatctaagctcttccactgttgatggtgactttttactcatggtatggtc
tagttgacattctgataatagctcttcacattcgtttgaacgtattgcgaaattacctatagaacttataatttggattttctcataaaatataagaat
taaaggggttttaagataatttattaaagcttttttgtctataaagtcgttgtatgagaagaagtattggcgaaagaatatcgggtactgggttaaa
tggaagatttttctacattaaagcaacctacaggaacttgggaagcttccgaagaagcgactgatactccaagtttacttgtagtaatattggatg
caaatccagcttcttggtactctctttctaagcgtataccggttgcaaaagtgttggcagatataaatgtctttttaaacgcacatttagcttttcatc
atgataatcgagttgctgttttagcctctcaatataacaaggttgagtatctttacccaaaaccagagactgaagagactgtgcctttgctagatcc
tacaaaagaagcaaatacttacagaaagtttcgagaggttgatgattttgttcttggcggcatgaggaggttgatgaattccacgtcaagcatttc
caaaaaaacattgatgtctggtgcgctgtcccgtgccctggcctatataaaccaagcccaaagtcaaaaatatatccgatctaggatagttatctt
ttcattgactggtgatgtggcacttcaatacattccgacgatgaactgtattttctgtgcccaaaagaagaatattccaattagcatatgtaacata
gcaggaggaacgctcttccttgagcaagctgctgatgctacaggaggtatttatttgaaagtcgataaccccaaaggcttactgcagcatctaatg
atgaccttatttccggatcaaaatcttcgaaaacatcttaatacgccaaatcaagcgaacgttgattttcgagctacttgcttttgtcataaaaatgt
tttggatattggctttgtctgctctgtatgcttgtctagtaagtacaagtgttctgtagcatcaaaaagctaacaattatctagtctattgcgaaccaa
gagatttctgctcgacctgtcaaacaaagttcacctctagcacgatatctaaacggaaattttcgtagtttactttcatcaacatattcatgacgggt
taatgaaccaatttttttgagaattaacgaggcatcaataaaacttccacgacctttggtaagctaatttaatattaatgtataaacagtctgcatgt

taactcataggaagaagggcggctctgacattccttgatgagattaaacattgaaggtaaaatgctgtacaaatctaagaacttcaaattacaaa
taagatgaaaatcgtttagtgaaaacaatacaatagctttgcaggacattgcactctcatgaatgctatataagtttttttaaatattttgaattta
gcttacgactccatctttaagaatgcatattgttcattcaagctttttattttcctgccaaacttcagctatttctacatattgatcgcaaatagcttttg
gttagtacagttaccattataaggataaccacttttatttctaccaccaaattacagagaatttaattgatactttagttattgttcaattttgggttag
cttttggaaaaatatggatatctgatgaaaattgcataatgtttctgaaaactttcatacatatatcaaggagaaggtttagtacaaattttgtgac
aaaccaaggacatgtcgtggtaattggaggaggccacgcaggagtggaagctgctgctgcctctgcacggcttggagcgcctacagttcttttga
caaggtcatacaatgatattgggcaaatgagttgtaatccagctttcgggggaattggaaagggcacgttgatgcgtgaaattgatgctcttgga
ggtgtcgttagctctgtttgtgatgattctgctattcaattccatgtgctaaatcgatcaaagggtccagcggtctggagtccgagggcacaaatgg
atcgttcaacttttaaagcaaatatgcagaagaggatgaataattatcggaataatctacaaatcgtagaaggtagtgctgttgatatcaataca
aaggcagacgcttctggtaaattatcctgttcttctgtcaaacttgctgatggtactgaaatcatagcgaacagcattgttataacaactggtacat
ttttaggtgccaatatcaatgttggaagtaagcaaactccggcaggaaggcttggagaatcaccttcctttcctcttagtgattgtctacgacgtct
aggatttcagatgggacgcttgaaaacaggaacacctccacgtattcatccgaaaacagtcgacgttacaaatctcacagagcaactgggtgat
gaagtacctgaagcattcagttttcgtaacctgaatgatgactttgtcccaaaattaccgcagcgctcatgttataaaacgtatacaacggaacaa
acacacgcgttaatcagtgacaatattttattagctcctcatatgcttgcacatgacattcattcccctcgttattgtccttctttagaagctaaggta
attcgcttccccgaaaaacaaaggcatatagtatggttagaaccagaaggcttaagccctgactctttttggtatccaaacgggttaagtaattca
atgcctgaagatgtgcaaaaaataataattagaacagtaccaggacttgaaaacgctgaactagttcgcccggcctatggcgtaatgtatgattt
tattgatcctcgacaactctatcctacgttggaaacacaaaaaatccgtaagctttttacagcaggccaaattaatggcactactgggtatgaaga
ggctgccgcccaaggtatcgtagctggcataaatgctggtttggctgctctgaataaagaccctatcacaatacctagaaatgctgcattactagg
agtaatgattgatgacttagtaaccaaaggtgtaaaagagccctatcgcgtttttacctctagaagtgaatatcgacttacaacaagagctgataa
tgccgatttaaggctgatgcccctgacaaaatctcttgatttagttgatgatagtagacagtgggataactatttgactattaagagcttgatggat
agagtaatcaatgatttgaagtcctatagtttaagctctcaacagtgggccaaactgggagtttcagtgccccatgatggaaagtatcgaagcgc
atggcaattactaacatttaccaatttaaatccaatggatattgtgcttaagcttccttcaactaaagaaatccctcgaaggatactcgaaagagt
aattgttgaaggaaaatatgcattttatattggtcgacaggcatcacaaaacaagttgctttactatcgagatgaatcaacagttattccttcagat
tttgactttgaaaagtaagttttttttgttattcattatactaacaagtaaactagacttcaatctatatctgcagaagaactaacgttactaaaaac
aattcgtcctgttacaattggacaactaaaacgtattcaaggtattaaaccaagtactgttatttatatgtttcgacacgccagtcatattcctaaga
aagaaacatggatgcaagaagtttttaagcctatgtatgattttcattcttaaattctcatttactttggttaatacatctacattctactcatcagttg
catttctatatgcacacttgattaaatcgagatggataaataaaaaataaagcacaaaattttattaccttaaatacttttccttgaacaaataccc
atcaaagattttctatttcatgtaaagagttttaaataacatagccatacgactgtagaacgctttgtccatcaccaggcaaatgcatgcttatgatt
tgggacattcgatttgcgtaacgtttgttagcgccattcatctctgtagcagcaaactgcttaagatcagtgatttgagggaatgggtccaggcgtt
taccaccactggtagccaatcttgaaaatgaagcctggaacattacagaatcagcgtcaatttcgtctaccataagatcttcgtcagcctcggtttg
agtttgttcaattggaagctcgaacaacttcaataacgctgataaaataggaatgaataagcgttcgtacggagagttatcggacaacaagagat
cactagtgagcaagcgcaccataccaattgcagtgatcttcctatccaaaggaagagatagcttttgggtctgaggtaaaataatcgaagacata
agttgtccaaacactccggtttgcacagaatctacgccgtgaatcaaattgtctggtccgcaggaacctgtagtagaagcggccaagaaaaaga
agaacacagtgcaacgcaatgaaaagcgttcggtacgtgagtttttaagacgactcagcaacaagaaaaagatatgatttatatagggagcaa
ggatattagcatcgaaaacagaaaacacacgatctaataagtcgaaaccaaaatggtcatttattttagaagaaattagcttcctatatattccca
aaaccggttccacaaagttatttgagataaaaatttgggggccacgagtgatcattgcttgcagaagtctcaccaaagcaggaatgtttcctttag
aatcccagagagctggagataaacaaggttgaatcatgtttaccacaaagtcaggcaatggttcatttccacttgcttcgatcagttgagacaag
agttgtaaaacgtaaggaataaattcagtgacatccataattaagacactctgaaaagcaggaagcaaagcagcttctaactgagcaacggttt
gtggtcctgactttgataaacttcgaataaacgcaccaattgactcgaacaagtaatgattaaattttggattagaagggtttttgcatacttcattt
gtaaccttagttagatgagttagagcaagctcggcaaatggtacaatgcgctcttgagacatgataataattcgcataatagctttcatcaaatag
tcattttcagaaagcttttgtggagtagtaccactttcgataatgaaaaataactgcgtcaataccggttggataatgggttctataagaagactag
taaaaatgtgtacgtggttatggcggaccgtaagcaataaatcgatggcaatcgaagcataggtgtaaacaacaaaatttggtagctttaagaa
atcggaaataataggtaagatgtcgataagttgctgagattgtaattggtttctaaaagtaaatatgaacttgatatcttcagccagcaccatagg
atgaacgctaccaataggttgaagtaaatcaggtttgatattgctttcaaaaaagcttacaacgtcaaccatgagatttgttgaagtaactcccaa
tctggaggattgacctttaattgcgatggccgcaaatagctgtaaggcagcatactttttgttccattctaaggctggatttaaagaggattgctga
agactagcatttatatgagtagtaataactgtggtaatcaactgatcgaagtgatctaataatcctcgaaccagcacaattgcagagcgagaccg
agaatcactatttgacccttccaaatcacgacggacatactcaagaggatcatcctcaaagagctcttcatcagattcacggaggcaaatatttgg
aagaatgacgagttcgatgaattgttggagaacaggttcttgtcggaagaattctgcatgtctacgaattcggataactgaagttaagaacgcca

tggcttttcctaccaaaacgtcgtatttctcctcttgggttaagttcgtgagaagcgtccaagtcatatttacaaaatcggttagattggtaaacact
tcttcgtatcgcagggtataaagctcagcaatctcgcaaatgctggcctttactttaattaaagtattgttatcctcatcctccaaagcaggattaga
ataggtaaagtaattaagaaatgttgtcatgaattcggtaatgtgatcttcaaaaaactcaggaatatcctggcaattcaaatcataaaagagctt
acattcaagcaaaataacctgaaataaaatgttaagagaagcgacatcttggggtccatttgtcaagagagcatttgtttgcttgaaaagagcaa
ggaaaggttcacaaaaacgatccaacacatatttaatttctaaaaacaaagcatcgcttcgaaatagagggcgccaacgcttaaatatcgcatg
agcagtagagagaaccgaaatatttacactcatgtctactggggaaagtcgagaaatcaaatcaggcaaaagagagtcccaacgatcaggaa
agtcgaaattggcaatgtagccgacaacttctcctaattgtacttgaatattcggaggactcttgagcataaggttgatgatttcgcgcttgacgag
ttcggctactccgtcgttaatacgaacagtagcatcctcttcagcatcccagtatcttttaatatagtttttaaaatataacgaagcagcaagcttta
tgcttggatcggctgtttcatcggcaacaatgcttaatacctgaagagcaaagtttccatcttgaacttcccatgttttcaaagcttcctcagctgaa
atcaacgtcagtaaatttgcaatatactttttttacttaccagtcctagatgtagcaggatttaaagtacctgcaagaagagctggaatcccgtccat
gatatacgtgaaagggttgttgtggttgaaggtaggatgaagagcatggaagttttataggggatgtcaatagtaaatcagttttgatgggatatt
aagctgttttctttttttcattggcattactttctttgaatatgatcgggatgaaagaaatccaggtgtcaatgtactaaggaaaaaaaaaaaattg
gtttcttatgccacaggctacttttacctcgatggattcagctcgtatatgggcttatattttattacgataactattaaaccgtacataaaaggatgg
gaaagcgatgcaatggatgaacatcacaaaacagaaagagcatggtatccttttaaatcttttgaaatccgactttacattagtgaataccaaaa
agaatacgcataatatgaacttttattttccctagccatgaacgttcctgtatgaactaagaaatgaaaaaaaaaaaaaaaaaaaaaaaaaa
acctatttgtttacgccagcaatagaccgtaatagacagattctttatgcctacgtcaatgaaataaatataaatataacatttttgaaatatttagc
attgctcaaagttctattccttttacctggcaattattttgtaaaagctcttgttctcttttgtttaccaaaaaaaatctctggtattatcgacagtactc
tagtttcaagcgtttcaatcaatttttttgattatttatattgttagacttattatgtagtttttatctctgagcatatataattttttttttttttgcttag
ccgtagcattttacattataacatgatgaagtatgtggttttaagtcctagaaaaaagctgaataaaaaaaaaataatagatatggcatgaaatg
tgaatcattgaacttttacttgaaacaatacgctagtgcaatttatttatttatttagtaaacaacaaaaaagaactgtttactctcttattttacaaa
gtgcgttaatagcgataaaccattgtgaacgtttacgcaaagtagattagtgggaaatagtgtcgccctttatttatttacaacccagtatccaaca
gaaaaccacataaatttaagtggtggatttattacgaaaaagacgtcttgttttaacattataagaggacgttcaagtatgaagtagaatgtttga
cagagagtatgctcaaaaaatgaactttggtggtcctgactcgaaaacaactgaagcatcagacctgagtggacaaggcaacagtggccaattt
cctccctattcctctaatggttacccatactcctcgtcgttgaatacatctatggaagaaaatctaggatatcctgagatgatgtcccaatcgcgtcc
ttcagcgacaattcaaggatttccatatggcccttcaggtacatatgaaggagtatcttcgacgccctcagtttacatggaagaatccaacaagct
tactcctgcttttatggcttctgctagtatgcagcatgcgtcgccgccaatgccacctgctgctgcttcatctattcagcagcagcagcagcagcaac
agcagcaacagcaacagcaacaatttctacagaggcaactttatctgaaaaaccaattggaaacagcgaataatttatttgagccttcttccaga
aaccagccagaattctcgccaaacacacttcctaaccaaaacatggctacctcatcttctatttctcctccgatggcgcctggtatgtctgctaatcc
aaattcttcaataaaagataaaaacacgaattttacagacattgcagataattacaacaaatttatggttagtttgttaagttttatggcgaagcgt
gggactccaatgacgagttatccgacaattgatggcaaccccattaatctcatgatgctttatgccctggttatgcgcgctggaggctcaaggcga
gtcaccaatttgaaattatggccaaaggttgcggcttctcttgggttcacttatccgaaagcagttcccgttttgtttcaaaattacaacaattatctt
tttccgtatgaagaagcgtggactcgagctcatcagcaaaagcttcggctagaaaatcgaattccagccgagaaccaaactccttattcggaaga
cccaaggatgcctacttcaaggcaatccttacccaatacggcctcccacgcacaaataccaagttctaaaaatgaaacgacgtctctgaatatac
ctcctcacgtagttgctaacaacaaacagccaacccctcctaccgcagtatctcaaaagagcgactctatcccacctatccctcctattcatgaaa
aaaacaaaccttcctttgcttccacagttccctcatccttcccatccgcctctaaccaacctcctcctgccgaagctagcgttgaaacccgtgtttac
aatcccataaaaaaaggcctggtaggatcaaccgctgggttccccttgcatctttctactactcatcgtatcgatgagtctttaatgcgcttgaaaa
tacctagtatctcagacctaggctgcatcaacatccatgctctctgtatgagtctgttatctacgcttcctagtgaaattacttacgccctcaatgtttt
gcttcttttggctaatgacaaacaagaagtggtgcatttaggtaaatgcgaggaactgattgatgcattagtagacgttggatggagctgcctaga
tgtattaattgaaagacttgcatccgaatcgaaccaaagcaattacaagcagagaccgtcatatcgatgtctactgctggaatgtatacaggaga
ataaccttgaaaaaattcggcaagattgcaaatctgaagcttatctctctaaggaaggaaaaagctttaatttagcggagcaacatttgcttgctg
tatgtataatatttcgtaacatatctcttgctgacgaaaacttaatgatcgcctctcaaggatctgatttttttgaattcttaatgcgattaattcggtt
actgaatcgcggcgagccttatttacttgaatctaagcgtgccatgctggatctgcataaagatgttttactacttctttcgcaggtatcgcataaca
tcgtattaccaaacatggaatctgcattgcaattattatattttattttggtgttttctcctataacgaagggaaataagaagcgaggggtttttgaa
atgaaagatccacaaaatgtcctccctattgatgttcctaattataccccttcacacacccatatgctgggccctctataactgtatatgctaaatt
attagcccgggaagccgttaacaagatatacttccagacggtctttgaccaaaatcctgcccttctaaattcgacatttctcttccttgcctccgttat
tccaaaattcaatagacagtgccttaaactttgcgaaaagcgcttacctttattgcaccatgcatttttgtgcttagaaaatactgtatcatttatac
gagaaccgtctcaggctatattgtggtgcaacattggtgatggtttttttgcttctctcattcgtttgttaattctactatccggtcatccatctcttaat
cccgtttttcctcccgatgttcctacccgaaccaatgcgaatcccttccgttatatcattcagagtgggctaatctgtgcaaagaagctctttagttta

gcacgcttgatagatttccctattacgtcttttccaaagaacgaaacaattctagcgatccttcttgctcctacaaccgaccgaacgtttttgaatga
tctatcaactatattggacaaagaccttgaggaacaactatcatgtaataataccaactttgaataattatttctttactattcatatggttctatttt
cgtcaattatattattcacctcttagcttgaacaaaagcatcttgttatctaatcgtagtaaatttcatctcctgaattaaaaaatccaaagccaaat
aaacacaaattttaacataaccaaaatcatacttccaaaaccttaaattacattgaagattcaccacaatcttacaaatcaaattcctacagattt
ccgaaatatgaaaataaagacaaaaaaaaaataatattgaagaatcgacaaaatccaacatcagacatccaaaggaaggactatggccgtca
tttgcgactagaatgggttcgtatgtcgctccgtgaaaactgcgaatgtttattggaaatttcatcacttgttgtccttgacgattcttctttccacatc
taactaatcggttctataagcggaactatgactaactgacaaaactttcattgtcaaaacctgaaaaaccaaaactcgctcgtataggagcagct
accacgattctttcgaccatacacgcttatatatcaatacgaaataccgatacatcaaacactttacgagtttgaacattttaaactttcatgatccc
atcgcaaaagcacacctagttcagttagtaacctgttttgtcgtaataacaatatgaggaacgagaaattcagacacctaaaatactggcaagta
ttacctctttcgggaaaaaaaaattgaaagaggctagttgtatcgtcatagtcgataagacgggtaaagaagacgcatatataatatcatcaggta
ttctcgtcctcatataaagatcgaacttacctctacaaacaagttcaccgatcgatcatccaaaaatttagttatccaaaaacaccaaaatttaaa
gtttcaagttcatgaacgaaggaagatacagcttgatccaacacagctggaattgtttcatcagctcccagaatacgcgaagcatcactgtagtcc
ctgacactgccgtctacgaattaccgttccgatacacgattacgcgtttccacgattccagccagacacgtccacttacttcagctacggtgtaaaa
attacaaccagcagctacaaacacgtttcagcgctttaaagtcctcactgataacatcgccgccatcgcaaatggggtatccttcaaacagcgatc
atcactgtcgttgtgactgagaaaacaaactgaagcaaaattcagttactgagaataactccttctgaaaatggcagttctatcaccagtgagga
cttgacaagtaacctgcagataaagtggtttttggcggcccttatatagacgaagaaaccctaaccctgaacgttctggaagaaaatgctgtggt
gaaaaaaagtcctttctagcatcaattacgtcactcatatttctccaacgctgaaaataaagcactacacaatagtatgcgtaaaattaattagca
cactgccgaaagacaaatcggtaattcgagaaagcagctaggatcgtcatcagtacatgtagatgagggaaatagtaatgatttttcttaataat
aataatgctacctgtaacaagtatctcaatagactgactagctttcccgttttcgcttatggaaatgtgagtaagccgatgaaagcagctgagtgtt
aaattaaattttattaactccaactaaaatttagtacgtgatgacctaactgaatcaacactttcctgtaccgcataaatctgttgggttttctttatg
tttacaaagtaagaaaaatagtgaaacataacgccgaatggtattcctgataaacaactactctatgctgcgttattttggtgacagcaaatctgt
tcgtatgcaaggcttcaattttacgctttgcacagagcgattttaataactgctttgagcaaacagatatccaatctgtttcacgaagagacatggt
taccaactatgcatcgatgatgtacaatagcggttccaatatccttgggtacggagttttgagactgttacagtacaatgaacaattgtcctgcggt
tgggaaaacaccatgaaggaagacattgggtactggagaagattcgtgcataatttctttactgagaagggaaccttccggtttaattttgagac
aaatcaacaaaggactcgtgagccaaaacagtttgagttgtcttatgcagctttgcccagatatttttatttaaactatatggggaatatgagacg
aatgtcaattgttttaggtgatacaaaagaatttgcaattcctaataatggatattttgttgaatcctcacatgcaagccttgtttatcagtttgcaag
cggtattcaagtggtcctgtatggttgtttgcgagctcacttctatcgtgcacctcttttgaagctagactcacttgaattttccgcaaccaattattct
gagtatcttcttcgtgaactaataaacagtgcttctcttgctcagcaatatcaacgcgagcatcctccgaatcgggaaaacacatcgtctttcccttc
tcaaggtaacgaaccagcctcgttttatcctccatataacttcgctgccaattcacagctcccctccacgccagttaatgagtatgggattgaacct
cggataatgcgctttttggagatttctgaaactatttctgggatgcgagatttaatagcatttactctggctcaacgttcaggtccggcaagtgctct
tcaaaaattttctacagctttacagcaacagcagcaaatgcagaaatcaacggccgcgaaggtttcctttataaataatccctcttcaaatcatca
atactctaatgtcactgaaaattctcttcccattcctccttctgcagcaacaacaacagcagcagcaacaggacctcccgagtaccgcgttcctaat
tacccagctcattataattatcatcaatatcaacgtcctatgagtacagaccttgatccgtctcaattcactaaccattttccgcatcctaactctcaa
tctcctgctccgttgatgaatcctacctattcagtacctcctcaaaaaagatccgctacactatctccgcttggggaagccggaaagcaggatgtta
atcaatctttttaccctaacgagtctttggggcctcaaaaacgagccaaagtttgattgttacttgtaaaaacaacttgatcctaaaacaatcgttgt
ctttattactcattttgctataattagctatgtttccttattcattctattaatttattctcgcatatttgcttaaaggttttacttaatttcatgttaagtt
attatggtctccgttttgacgcttgatactgtctccttgtcccttcgtttactttatttgtttctatatactcgtccagatgtctatttatggagttattttat
tacaaaagaatataaataattagagattgaaagaaatttgaattgtcttcattcttcttcgaactttcgttcagtgtaattggctctacagtactttct
accagtttagttcaacttgtagctaacttatcgacttgtacaaatttgttacaggtttatcgaaaaatgttttccgttttatttccttatcgtggcaaat
gcacctttttgccctctcctgtgtaaatgtgtaagcgatgatcttaatcttcggtagacgtagaaacctacccttcgccaaacactgtcgcgcgcggt
ataggctattgcatataattttacttggtacttctttttttatattttattctttaaaaacaaattcctttaattcaatgccatcttacggcgacttggaag
tattggatcaacttgatgatgctgacttccttaactcaagtacccggattcgtcgctttaaagggtctcgaaaaaatgggcataactttactcatagt
aagggtactcggatttcacaatggcctcgcatgaaatttgttcgtgccgtggatatatttaatcctactgaggaagtaaatagaatgattgaagaa
gaaaaaagaaaacgagatactacttctgactttgctggaagcgaacgtttaaattcagctcatcaaaaggaacaacctcgttttaaacaatcttct
gagattcatgaagacattgttttatttaagccgattcgcaataacaatatttcaggtagagaagacaacatacgtgagaaaagggaatctgtaca
aagtcaaaaaactctccatgaaaaagatgccatacctgagaatagggaacctgtacatatgaaaaaaaaatatctatgaaaacattaatattaat
gagcccgaaattgtatccgacaagaatgaaaatgagatgttgtcaaagaaatcagaagaacctcctacttctactactgcttcggactctcgtcg
accctccaaacccaaaagattacagaaagagaacatgaatgaagcggacactcaaacattaaaatcatcaaatagaccatattggcgagaaa

aaccgtacattgtctcagattcttacgatgacttacctgatgacgaaagcactgattcagatattaaattcaacattaataagtttgctaacttgtct
atgctatctgataatgatgaaaatgaatccatggaagaagaagaggttgagagtataaaagcagaatctacagaagatgatttattttatagag
aggattcggatctgcttgacaaccagcgtcagcacgattatgaatacaatgatgaagaactagcaaatatcttggcagaaacagaaaacttgga
agatgaagaagtaaatgctttgctcgcaagcgtttttgatgccagtgatgattcagaaatcgaagcctatcttagtgttaaagacacaggggcaa
atgattttacaatatatgactatgatagtgatgatgaaccgtaccagaaagcagcttccgaaacgttatcaaagtctttccaaaaagaccgcaag
aaaaatcagaaacccaaaaaaatgtctaaggaagaaagaaaggcccagaggaagcttgaaagacaagctggaaagtcaataacaaatcgac
ttgctagttcttttgatgttgatgaggatatagctcaagaattagaaatgtactccgatcttcaagaacaatggatgaaagatcgcaaaaagaag
gcaaaaaagcgtaacgagcgtgaagaaaaacgtgcacagggcctcttgggtaaaaaatctagtaaaaaactggctaaaaaagcggcttcaga
aaaaaattcggatcccgatagtcctacattatccaaggtggatcacgattttattatgtacgcatacaagcgcatgcaagaattatctctttcagga
gtggatgagatatctcttcctgcatgtcgtaagtatgtacgccgtttaattcatgaattagcgaatcgtcttaaccttcgttcacaaagctatggttcg
ggcaaaaaacgttacacggttctttcaaaaacacctcgattcaattcttcaggattgaatagcgcgtccttaacaaggcttctgcatcgtgtacagt
tatcggtcggaaaacgatcagaaatatctggcaagccaggtaaaaagcctgggttaatagttgcttcgaagcgtacagttactagtgctacgaaa
gtgttcgaaggtcaggtggttggtggagatgctcctgaaatcactcgtgctaatccgggtcgtcgtatgttggaacgattaggttggttcgccggta
aagggttaggacatccagaaaatgaagggtctcgtgaaagcttgcgtgctatcgtaaaaactagccgcagtggtttaggctaaaagccattctac
ataattcaatgatggcaccaaactattgatgactaacatttttggacacgatttatttattaatttttttttttttaattaactgttatatgaaaagctat
cctttttttctatttgttccgagcctttttataaataatctactgttgtatatgttatagtaagtagtttgatttgaaacttcttcttatcttccttttcgtt
tctatcctttgaagttattaatcattctttttccaatccatgaatccaattctatcaccttctatcctttatttacactactagtaagaatacaatcgtt
cgttgcgttacgctaactcaaaagtacgcgttcctgtacgcatcataaagtttatttaccgaggatcaaaatgtgaggtccgttcttttcgcgatgta
cgagtagttacccttcacacagcaccagaggaagaaggcttttgtcctaacctgttgtttctactcgaaacgcgtacgtttctttatttactttgaaa
aattcatttgattgagcaatatatttgtgtaatttctttttattcttttgagattggtgatttactcatttgtcagtcagttaaggtttgactgcatattttt
cccaacatttcattcttttcgtattgtcgttcgtttttctatgctgttagatactatagaatttatcttcatctagttctcatttacaagtcaacgtttagt
tttttatttggatatccgttcatgcttggataataacaagcttctattcgtcttcgttcttttctaaatagtctctcttttattttccttctttttataactt
ttcactacggttctagcgttttactggggagttatttttccgaggataagtcgattgtctaataatcttattgtttggctgcgataccatgaaattcttc
tattacccgtccttctctgtgtggtttctccaaattcttatattctttcatctgggaagtcaccatgcatatgccaatctaaattcccccctttgggtgtta
ccaggtagatgaaagtttaagtgaaaaaggaactcataaatggcttactcctcagtattgctacgataatatatgtaaggacgatcctgatatttc
ttttgtcgctgttcgcggagtcagttgctattgcggcaatacacttacagccaaaaaggtagattccagtaattgcaacactgtatgtcctggatgg
gatgagtttaaatgtggcggtgatctgtactggtcggtttatttgaccggtaatggtattttgcatacttcatcttctactacctcttctcaaacatctt
cttcaaaggcttcctcgtcgtcgtcttttaccacggcgagctcatcaagttctaatcccacatcaagttctaaacctacatcctcctctcaacacttga
catcttctagccattctgcgtctaaaatttcttcgtccagctctaattctcctacgccaactactttttcttcttccttggtgaagacagtaactcctagt
gcgtcgagctcatcttcatctccatctccatctccatcttcgactgctccaccgaagaactctcaagcctcgaaatcgagtaattcattaaatgctgg
tgcaattgttggtattgtaatcggttgtgttgcttttgcggttgtcattgctttatgtgtctgcttattcttctggtatagaaagtttaaagcaaagatga
atgctaaagatgatattacttttccttcctataaaagtgagctggactcgcgtcttgatcctggtatgctgaataatcgcaattcgagtgaatcgttg
gccgatagtcaagactactcgagaagaattttaagggttatgaattagaagaagaaaatattaatgtatgcatcttttgcttcaacgcacttggag
acagctttatatgtttacccaaacgtcttcatgtgcatttatttcccgcatataaagataaaaatgtatgttttatttctcgctccaatatttatttattc
atttacttcgttgtttcatttctttctttttttatgggatttgtttttcggcttctaatctcatactttttcttcttttatacctaattacaaaatttttattattt
ctattcgacgttatttttacattttcttctttaagttcggtgtctaccatcaggaacatgtgcaaaagatccaaggattacttatcagacttttgcagtt
tcaaacttttattgagaaataagcgaatgcggtaatgacagcaaaacattattgatgtttttgtattttacttatatcctatcctttaaaacgatgttt
tctttcataccggttgaataaagaaatacttaattaaatttccgttagatgtctttacctctttattattagatatatgtttttataattcattgaaggat
aattaacctttggaaaggtatggtttcgtgaatatgtgttttcatataacgctaaagaaagttgccttctatggaaagattttcgcaacaatacaca
tggttggatcttaccaaaagcttcagttggtgtcgcgaccgacctattgcaatgaacttaaccactttatttttttttcttttctcaggttatatcctata
actcagtcactctttcaattgctttaaatgatttttttcgcttgctttcttggtttttaaaggattgattaattacaattcagtaatacaaagtagcaatc
agggcacacatgttgttttgacccttttttactttcatttttattttttttttttgatagggcaaaaaattaaaaaaaagtgtgttatgttaacccttcctc
cctcttgtctcatacaaggctttgtgcaatctgggaaatacgactgcgcacaactaaaaaccaattgaaacattttgtatttcttcaaacccgataa
gagaagaaagcttgctttttatgtccatcatgaatatattgacctaggccctttattttcattcctttcaaacttcctatgctatatactatatcacac
tctccctaccctcttgagctttttacataatactctaaaagcatatgcttatttctttatcgtcttatttcgctagaatgattaacgtcactcggattcta
gaaggtgacgtcatagcccataagagttataaattcaagaatcctctgctacagagttgctgcttgttatttacgaaccaaattagatcattatcag
gtcgtcgcgatattactttaccagaaatactgataaatttctgttcgtcgtcgtgagattctaacaagtgaattttcgaagacagcatgtaagtgttg
agacaagaaaactgtgccacctgaaagctttgctcatactaactttctttacaggggtagaacatttcaagaaaaacagcaaagccgcagagtg

aaaatgagcactggcaacatttttcgagagtctggaactcagtggtctttggtttcggcgctgctttgggtgccactgctgctagtgccatgattgg
tgcctgctgtggttaaaatggatgctatggaaccataatagacaaagaataatgggtcgaggtgctgctttcatttttttacgctggaatgcagttt
cacttcgaagatgtctcttaggatgacacgtaaaaaagtttataatacggtgatgctttattttatctcttgctatttgaaatccctttaagttttatga
tatttttcattcatgttttctttataatttttctctagttcagtcctttaatatcaatacaaatgagaaattcctatggaagtttgaaatcgaatggttat
ggaaacaaaagtaatagggtatattagagatattgtacctgtcgatagtagtattcattcttgcagtcaaaggttaaacctacagaatcggatga
acgtagtaattaataacagtgaacgtgaagatgccttcaatcaaccctgtaggacattcattttcataagatttttatagtcgtttacttgcacaacc
atcacattctatttcccagtttagagttttgtaatataagtattacctggtttagtgtaattcatattttcaactatggtcatcgcctcatcacaaaggc
caacttaaccagaaccaccattcagatagaaacaaaccaaaagaaactctgtaaactaggacaaactggatttgtttgttaaaaaaaaaaataag
attcgtcaaaaatgcaggtgttcgcctctttaggattaaaaacctggatccagtgggtagcccttgtggctatcctttttcaggttgctaccgcttcc
gtacctctgacggatgaaaattaccaaactgaagttaaaaatgggcaatggtttatcaagttctattcgcctacttgtcctgcttgcaaacgcatcg
ctccaatgtgggacggtatggcaaaaaagattgaagagaaggctaatcaggttaatattcacatcggcgaagtgaattgcaagaaatacttaaa
tctttgcaaggatgtaaaagattttcccacgcttcgattttatgttaatggtgagaataaagacgaagtaaattacagcggcaccaccaccgaggc
ttctcttttagacttcgttgatgctcgtatcgctgacgctgaaaaggaagttgacgctgaaaatgcagaggcacagaagaaagtttctgaagacgc
taccacttctgatccttcttttacaacttcgacgagctctttatcacttggctttgtttccggcacctcaacgaatcctcatactgatttcacggacctg
aaaatggtacaagtttctcctaaaacttcttccattagcccaatttcttcctctttgccctcatcttcattcacggtagcacaaagtactggcattccc
aaagagaatcactcaaaggcttctagtacgtctcccattagtactgtttccgcccccatgagtgctgaagaaaagtttacagctacttcagtgtccg
cgtcttctacaacatctcaagctacctatgccatccaaactgctccaaatgttcctgatgaggggggtgacctttcggagcccgtcaatgaagattc
tgaaaaagcagcccagcctaatccaactggccattccaagcctttagtcacccaggaagacattgacaatgccttgtcaagtgaaactggctggt
tcgttcatttctattcttcggaatgcacagattccgcagttactacagctactacagcttggaatgcgatggctggaagaatgaagaataagctca
atgttggtcatctcgattgtagcaagcacaaacctagctgcaacaagtataacattcagcattatccttcttccattttctttaaggagaatgaatat
gtggaatataatggtctctttaaagtcggtgatcttactagtttcgccgaggaagccgccaacttcaagatccgcgaggtggaatttttttgatacta
cggagtctgagaaacatggagacgtttttttcttgtatttctatgacgataagagcgctgatactcttagtgcagttcgtaaaactgctattcaactc
ctcggccacgcccgtctttaccttacaaagtctcagcaacttgtgaagaagtacaatgtcaatagcttaccaaagttaatagtcgtgaaagattcc
acacctagctattatcccaccagtgtagcatctgatatcaccgattacagaagaattttgaattggatgaagaacaactggcttccagttcttcctg
agctccaaacatttaattctgatgaaattgccaatactgatactgttgttttgtatttcctggatcctgattcatctaatttccaggaaaccaaaagc
actgctcgaaagattgcctccgattggttaaatgaggaatctcgtaattatcaaactgcttggaaggaagaaaccgataaaaagaatgctcatct
gaacaaagcagaggaaaagaatgatgccgaagctctagaagctgctcgaaacatgaatgttaagcacaagcctgcccctacacgatttgcctg
ggtaaacggtaattattgggctaaatggtccaataagttcaatcttgatatcaacaatacaggtcccagagtgattctatataacccttctaaaggt
atctactgggatacaaatgctaagggtgaggctattacagttgatgaggacgtagtgttcgaggttctcaaggatgtggaagctaatcctaatgct
ttaaaacctaaaaagcttaaagagaatgctggtttggagtatttggcatcttacggattggatacacgtgctttatacttgatttgcggtgtcgtttg
cgtgggcattattgtttctttatttggtgcaagaaggataagaagtcttgagcgccgacaccgatcttcacctattctgcctatcgcaacaaagaat
acgggtaatactggaaagtttgattgattcattattcatgacgggcttgttattgaaaattggcgattttacgaatttctatctattgttgcattatgat
tattaatctatcatttttatgggttattcgctttagattaagcatttttaccatatataatactatactcttgtttcttaggtatattaacaacccagttat
aaaaagttgaaatcaaaaaccacctatctcatacaaaacgtataacctaaaaaaatctttatttaagtaccaaaaattcccatttataaacagtat
tcaaaattaagcatcatttcaacctttaaacccttttttaagtcttttagaccctaaacaacattagaatatgcacgtttctttccatcgattccgagc
attgaattatcaatatcctcaagagatgttttatgatttagatcccactcatgttttaaatatgtcaaaccagcaaggttgtatcccacaagcgatcg
atcttccactaacgaacctcttagactattgcgaatattattcagcaaaggtcgcattttgatcgtagccgttagttgacgatgatatgtcttcaata
agaagaaaatgattcgagatatcaatgggatagaccactttcggctagcccaaatatcaacaaagcgaaaaagggttaaaacttggtcaaagg
gaagcactaacagagcatcatcaagatgacttgtgcgaactttcttgaaagtatttaagacgtattcttccgccgaaacatttaagtgagctaaaa
gcgggttacgaggggttctagatttgttcgaattgttgagtttctcaagtcgatattggatttcgtcgtctaaatccttagctcctataactaaagctt
ctaaaatttttcaccgtcttttaagctttcaacggtttgcttactcactgtggcaacgttattctctgctgcctcttcgtttgcctcttcagcactataa
ctggaaaccaaagttgactcgtattgttcttcaagttcacgttctcgttcttcttctaagaatactagatcatctgattgagcccatacacggatgga
gtgatcatgtgagcccgtaaaaactgaagttggagccaccgctaacgaccaaacttcagagtggtgaccgcgtaatttcagaatcaaatcaaaa
gtgttaccatcccaatatctgacttccctgtctttagagcatgtaaaaaagtttcgagaaccaggttggaacgctaccttcataacagaatcctggt
gcgcaaagattgatttatgacagtcaccgaagtctaaaccccataatttgacgttcttgtcggcactacaagtagctaataattttgagtcgtgcga
tatgtccattgaaagaacaggtaacttgtgtccgtataaattcaagaaaaacttcaaagtatccaaataatataccttcactgtattgtcaagtaa
ggatgcagtgataaggcgtccatcgggagatattttcgttgcaagaacgtcgtcagcaaaatcaatttgacgagtttgctcgatcctcaaagcga
aaactttgacggaagtaccgggagcataatcgtaagaagattttaaagaccataatttcaaagctttatccgcagaagccgtggcaaaatagga

tccattatgcccaatggctaagtcccaaataggtccattatgcgcctgaatacgttccaataaagaagaactagcaacgtcaaacaattcaatttc
tccggctttattgacggaaaggatttgcttatcagcattcacgaatgcagcagctaaaacgtaaccacattcgaaagtacggatacaagagcctg
ttttatgattccagatttttaatgtaccattcgcaccacttaagaccatttcatagtttgaggaaagagccaaagttcggacatcagcacgatgtcc
cggtaattcgatggagttggtgcgcatagctcgctccgtaagaggaatagtggcatctgcctttgcatccacattatagacttcaatggaattatttt
gaagaacagcgacaaacaaaggactacgtcgatgaggaatccaagcgaaagatgaaacacgtgaaggagcacgaataaccgcaaaaggctc
gaattcatctttcaaagtcaattctacttcctcagtctttctgcgtctacgacgacggttcattattttttccatttcagacgcagttcttatgcgaaaa
atctcaatcaaacgatcatgggcctggaagacgacaaatcgaccggaatcatagaagtctaaggaaactgggcgatcacggctttggcgagaa
acaccgcctaaaagttttaaaactttggtagcaggtgtaatgtttccatcaccaggaaacgagatttcccaaactttaacatctgcatcactgctag
cagttaaacagcgttttctatcaggactaacagacatcgcccaaatttcaccttgatgattgacatgggtctcaacacagtgttgaattgaaagat
cccacagctttaaaaatgagtctttaccagacgataatagatattggtcaatgtttgcggcatcaatctccatgttagacgaggcatcctcttcgat
actgtccgtattcgcctcgccatcagcggccctaggagttaggaagatgagtttcgttacttgatccttatgtccacgaagtctgtataatccagttt
cgttcacgacatcccacagaataatgtcggtgtctttactaccgctagctaaccgagtgcctgtttggtcaaaagtcatagtagaaacagcactttt
atgaccattcaaagtcaagagcaattccccctgtttccatagacgaatagatccatcctcgtaaccagcggcatacacgtcgccatggttagtaag
acaggagaccttgaaggagcaatccgcgtccttccacttggataacaattgaccggtcttcagatcccattctaaaatgctttccagacctccaac
aatcgcattccctacggatttcgccgattcagatggttgagctacgacattacacccggatgaagcgacgaccccaaaaaatccggagggctcgt
aacgcgtgtaacttttcaccatggcgttttcctcgtcctatggcgttccagagttgttggtagaagcaaagttaaaggctgtctcttctttctttttgac
atataacgagcaactttactatacgttcgaatggataacatgctactttttcttctttgttaatatagcgaactttttaattcgctaaatgtaattaa
ggtatagtaacttcctctgttttattgacttttaagacattttgatgcacctcgctttcccgtactctttgcctatacttagtaaaagcaagtgtttaca
gagaaaaaaaaaaaactttgttttggttttctctctcgtttaattccatgtaagagaataattaagggcctttaacatctccttgtttctggattttatc
ctcggttagcgtgaaagacgaagttctccttggttgaattcaaaaagatttgtgggcgctcttgaaaagttggcatttttataattttatttaggaca
ggggctcccaaaaaaggtgtgttctcttattctaaaataccagcgcggaaaagactgaaaatgcctcctacaccacgatctggaagtcaaagata
cgttccttacgatgtaagaatacctcggtcgcctagggaggcagctgcagttgctgcagctcgtcgtcaaaaccgacgatccagcgaggtctcgta
ttctactgaagaagcacctcacgaaagccctagtgagcatggagatatcagaggagtcgcagacagagatgtcgatgaagtatttaataatcac
ggattacagcagaacacatatccaggtttgtcagaagcctcgtcagttcctcgttcagtccgaagcttggcgcataatgagagtttaggatcgctc
gaaagaagtagaaatgagtggtcagcagaattggataacttggatgatcttgatgttcttgaggatatttctcctctgcaagaggtatctcaatgc
cgtgatccctctggtaattccatgtcagcctatgacgatgttattgatctaacagaagccgaacaaaagctgagtcaaccaaaacaaagaaatag
aaaacaaaaaccgtcatcagaagctgcacctggcatagcttccgtaaataactctcaaaggttagcggattacaaatgtgtgatttgccttgata
atcctgaaaacttgtctgctacaccgtgtggtcatatattctgcaatttttgtatcctaagtgcattaggaaattcttctgcgacgcaaaaatgtcctg
tctgtcgaaggaaagtgcatcctaagaacgtgatttgtttagaaatgatgctgggcagcgaacaaaacaacaaggattaagtaatgttatgaac
ataaaataaagcaataatattattattattagttatgtctattcttcgtaaaatcaattattagcctttccagtcagagtcccaaagaaaatatctac
attgaaatggctgtttatcatcatcagaaaccgcattggaattttcaacgacctctcctatcggtcgcgcacacatccaaaattttcgtccataattt
atgcctggctttttacagtcagcaatttacagggctctttatgtccatcacataatggaggcaatttttctgaaacatgcttttccatgcagttaca
ctttccacattagaggaattagatactgttactttcgactcgatatcatcaacttcgatcattggaggtgcaaccttctctccctcatttttatccgtcg
actgaggctttttcatgaaaaaagttgatagctttggctgctgtgttcgtcgacgcttctcaggcggaccattttcaggtacagacgatggcgtacc
gctgattgcagatggtgagtgagacggtgttttagatgaatcttccaaatcaatgtctgattctgatttggattttttttagcttttcaaatttttgaaac
atggagtgtatgtttttttgacggtcgatatgctgaatgatgacatgccgatagagaagggggatcttttgcatgacttagtaattcatacaacggca
cattgtcgagagaatctcgaaagtctatataaatagggcaatgatcagatcccatgacttgtgccatgatgtccgcatcttgaacccacggtaaaa
gatctggagttgccagtgtatagtcaattcgtgtacctaaaaaagttagatactcattattaatatatagcattaccataatttgtaggtctcatgtt
aagtctagtgttccagcaagtgaacatgccctttctagtaggatgccgaatccgcccaatatcgagcaaaagcccagacctgtgtggttttaataa
ctctctaagccatgttctcgattcaaatacacttggaaccaatgactcttttattatatctttttggtctgcagtgtcgatagagtcacaaaggatgtt
tacatcgccaactaagacgaccttccgttttccttcttttataaaagcagaaattctatcccttaagcatttatagaatgctcttcgataatccatcct
atggtccccagaatttacgggacaatagacaccaattagaatgaacatacccaagtcaagtagaatacatcttccttcatcatcaacccattttgc
agctttcctatctatgtctttgggatagtatccaatctgttcatgcggaggagcttcaatgtaggagtatcgttgattgcgaactggtaaaatcccag
tgatcccttcttctgcttttatgggtgtgcacaaactctttttacataaataccgactccactgtaaccttttcttgtagtggggaatgtgaaataacc
ttcatatccttcgattagagcaaattcttggggaaagctatccttttgcatcttcaattcttggaagcatacgatatctgcttttagctcttcaaaaac
ttctttgtaagactttttattgttccatggatagtaattaaatggattttgtataccatttacgttccaactgacgattcgcatactttctgtcgcaatgg
tgcattattggaaaggagtttgttcaattgagcgtcaagaacgtgccttcgtaggagtgtttgaaaaaggaaacctctttagaacttcaaaattaat
agatctatgatatgttggtataaaaaaaatttttgcttcctcttgaaaatgttcaagactatacataggaggcgaaaataaataatgaaaagtcgcg

attataacagtctatctaacttttattaatacaattaaaaaaacaatgaataagttaatgaaatgactgcgaaaagaatggaattccgagaattca
acaaaggcatggttaatttttaaaacttgtgaataaatataaagtaggagacatgaatagatacatagatgaacaagcccaatcataaaatga
atacatgctgaaggctgttttgaaatgcaactgttgacgagcgacattaaagtctttgaaggaaagaaagaatgattagaacaacgaaatgggg
tataccaagaagccaaaaaataaaaataaatccccttttaagattaattgttaggagaaccatggagttcagattctgactcatttacgtcggaa
tctgaactaccttcatcaatgggatttcctgttgcatcagttaagtggatggtatctatataattaggttcagctaaatcctccacaggattttctgct
gtctccatatccatgtcctctctgtcagagagaacatcttcctgagccaattcctcagcaacgtcttcttgttcgagttcatctcgatcatattcttctg
tttcattgtctttattaagtacagctttataccgtggatccatcttgcttttcttcgtagatttatcctcgtctacttggggaagtttcagtttcttttcctt
caaggaagcttcatatgctaattttgtcagactgtatattcgtaaagagataagagatgggtaaatcgaatcataccttgcaaatgagcatccaat
cgattcgcgaattcaggatattctatttcttctagagcacccaaaacatcctgaggcataacaatttttcgattgttcgtagaagagatttctccagc
gctaaacgattgcatgggtcagtaaacatatcattcaaggatgaactttgaatatctaggttatccagtcacacttgtaaaacgatcaaaattaca
tacgcagacgttaaataactaataaaaagagttgctgaattcgtaatggccttgacggcttccttttgtgctgtactcttctggggtaaaacttgttt
aatcaaacgcataacaatggaacgaggaagagctaaatcatctagctcagtatgttctccagtatttgttgaatccattttgttactgttatactcgt
ttcagactgatggtaaaagaacgtgaaagacaaatttctagggtgtgtcagtattgtttatagtaaaaaacaagtttggcgaaatgtgatataag
aattctcctattgagcttttttttcttgtcttggcttcttataattgaattagatattggaaaaaatagaaaaaaaaaaaaaaaaaaaaaaagttta
ctatcctggaaagctcgaatcgtggaaacaaaactactcgaaggtcatacagtcgaatttaaatggagtagaaaaggctttagaaccggaatctt
caagtaagcaaaacactctttgcatgctgttactggtatttcttcccaagagctcaaggtcaatggttcatataaagtgtatgcatataccctttcaa
aaggcaactcaatggatttgatgcacgtgtaacctttctaccaagagctttctgtacactgacagtccttgaaactcctgagaagtagacaattttc
atacgcattgaggtatcttaggcgtttgcttcaacaacaacctatcagactaatgaaaactcagccgactgttatgaattgaatgcgattgaagaa
agcataaaagatcttcttctgttagtcgatgactgttaaaccgagtttgcttctttaaacaggaaagcagtcaatgaattcatcaatgtttttataat
ttacaagtttatgagaagtttttgttactagatttgaatacagaacctgtttctgcgttctgagttgtttcccagaaacttctacttcactttaccaccc
tcgatagtcttttactaaaacaaaaagtaaccaaagtgtagcgaattgatcacctttactcttcaagtaaaagaaaggaatataaactaagaattt
tttgttaatttcggccttcacgcgtacctacagtttgaatgaatcaagcaggtcaaattattccctttacaacatctggctccctggtggattatgttg
accgtatgttattttcattccttttcatattctaatcattcatttgttcaggtaaagtaatagtggttttaagagatggtaaaaagctgattggcattct
ccggtcctttgaccaatttggtatgattccgctttgtgttccttgcttagataatagtgatgttcgagttgctaacgtttatagccaatctgatgctcca
gtacacgatcgaaagaatttatgtggatgatatgtatggtgacatcgatcgaggtgtttacattattcgtggtgaaaatgttgtcttattaggagaa
ttggtaagaaattagaggagttgaaaagtctttactagggcttgaagttccgaattctattcactgtactaaccaacttaggacttggataaggaat
atgaaatggaaaggggattgcgccgtgttccggcagaacatctatatccattgtcaaagcaacgtcttgatgacaaacgaaaatccttacgggaa
aaaaccaagaaactgaacactgtgggtttcagcgtcgattttggtcatgatgatttgtactaaaaattacagaagtgaaccctatttttatttagct
gtctataatataacatttctttgatcgtttcaatctatcgtcttcatcgatatttgttacttcttttaaagaatggaaagagtaagtttacgaatgatat
accgatcgaacttaacaagtgattattcggagcctgttttccgtttcaattattatctccaaagaagtgtaaattattagagaaggaattagtaaaa
tttttctgatgttgaatcattactagacgtcttgaatatatgaatatcatcttgtgtagcagggaaaacaggtggacggtccactgcgaggtcaattt
ctgaagccggccaaatccagtgtaagagcatcataaccaaataatctaacgttattaatatacaaaatagtttaccagcccattgaccacatccat
agtaaagttttaaacgattaatgaggcgcaaatttggctctgcgcatatactatcataaactttttcagttcgttcggcgacgtcaatccatgaata
catctcttttacctcttcatggaatttcaccgttttgattttattattgagataatcagatatagcacttgacaaggttcgtactaaatcgtcttcttcag
gtctagcaaatcgagtcatatgagaaggcaatacctcaggaacacctccaaccttggtagatataacgtacaacccgcaacttgctgcttccacc
aagacggttccaaacgcttctgtcaagcttgggtgtaaataaatatgaccacgtaccatgacatcccgaacttggtcatgtcgtactgaacctaac
atttccactctatcttgtagcatatacttttcgcgcatttgttccaggtcaattgcttttggtccatctcctgcaattacaaatcgaactttgcgaaatt
ctgcacaaattttaggaatcacagcaattaagagatcaattcctttattatagtataatcttgaaattacaacgatggtcactgcgagcataaggtt
aggcataaaggaacacttgggttttgacataaagaagacaaacacatacggtaatcattagaagcttttgaagggtctggtctgaagttttcggg
gactaacgcatttggaataaccgaaaccctttttgggattaaggacagcacgaagaacggtgttttctcgactagaaaacacagttagtaaagagt
tggaattgttaaagcataccatgtatgggaaacacaaatgacatgatcaatatcactcatggtgaacttgagaagcttattggtgataatacttcc
cgcatcagcaaacccaaatagggaatggtcagtaaaacaagtctttaggcccatggtacgggcatgcaaaatggcatcgtggcataaaaatga
aagacttccgtgtccatgcacaatttcaatgcgttctcggataataatatttcgaaagatagggaaaaagtaaaaaaacgaaggaaaagtcgtct
ctcggtataccgtctattagagtaagcgtcagcgaaacatgattttcttgtgaaacgtttatccaaggagaaaaacatacatgaaggggaacata
ataagccgttaaaccattggtaaggtatcggacgccgacgcggtctttgtacgcatgtgtaatgactggtggaataatgtgtcaataaatgacga
attgaaaaatccattgagatgtttgtatacctataactttatggccaagatcaatgagtcgttgagacaattgaaaaatatgagactcaattccac
ctggttggggaaagaaaaaatcagaaaccatactaaatgtaggttagaatagaacagacgcaaagctcaacgattccattcaggaaggagaa
gcaagcattcgaagtaagagcaatcaatccaaaacatacgctattcggtaacccattgtgagaaagaaatcactttactgtatagaagaaaact

aagataaccagaagagaatgcacatttgcatttcgtttttctaatggtgaaaacgatgtaaataccctttcatacacaaggtacgttggttggtata
gtaatatatcaaaataatattattatattaatgtgttcttaatacatcgaaaatcaaaataaggaaaaagggccctaatctatttcactagaaaat
aaaagaaaatcacttgactttgcgaaatttctgttctttacagtgacaattcattcagttaactatttccaagaatctatcatgttttgttttgttttgct
tttacttttgacatatctttcattttgtatccattccagtataaacaattacattcctattgctgtttgcaggcaaaacctaaaaaaataaagcactta
ttgtaatattaattataagaagaaatagttaactcatacgaatggaaacaatgaaaatttaccaaataaaagccgaaaaataaacaaaagatat
cacttttcctcagtaacagccttacatataatctttactttagaagactataacaactggtcgcatgatgtagttggttatcatgtcgatctaataagt
cgaatgtcgccagttcgagcctggctgtgaccatagatttttctatttactggaagtatccatacttcgaatattttgttgacttactttatgtagttt
aatatttttttttatctgtccctatccgtttcgttgctttcctaatctacttttatttccctatcgtttaacttgttcgccaaaataggacaaaactagaa
acaatggcatgtgcaagtattgcccttcggttaaccacactttacatattaaccgatttttttttttcgatttctagtgccagccaaggttacacctag
gtacaacatttttaaaagagcgcacttcaagtacgaaagacatagaatgaaaaatttagtggtttttggaactcagtcacatccaaaactaacag
aagcaatatgtgagcatctttgcttggatgtaggtcgagccgaacttggaaaattcaggtatgtttttgcacaggtgataaaaagcagaaaaaat
ggatttttgctaagcagatttcaataaattgcaaccgttgctcttgagaattctgaaagcttttatttggaaaaaaaaaaaattcccgaacggtttag
attgtcgacaaaagaagtgtctacaattgatttttaaatgacgcagataagatcacaaaggagccatgtgtaatactattaagaatttcttccgtt
ttattgtctactttttctttttctcgctttacctgagacattttcatgagtgataaactaatttttttcttacagtaatggagaaacctctgtaaaagttaa
acaatctgttcgtggttgtgatgtttatattattagcccggcttctgggcgagtcaatgaccatttcatggaacttttggtcattatttctgcatgtaaa
actgcttccgccaaaaaggtgaccgctgtcttgcccgttttcccttatagccgtcaacctgaccaaatttttttcttctcgaggcgcacctcttctagga
tcttcttctgatggcgttttagaaccaaaacgcacttcgtcgtattcgtcatggattgctcaatccggaactttggttgctgatttattgatgtgtgctg
gagctgatcatattatcactatggatctccacgaccctcaatttcaaggtttcttcgacattcccgtcgacaacttgtttggtcgtcctttactgaagg
attatatagccttgaatattcctgactatcataatgcagtctgtgtatcaccagatgctggtggtgcaaaacgagctactgctatagccgattattta
ggattggattttgctttgatccacaaagtacgtttttttttttttactgatgactagaatactaatattttgtagaatcgtcgtcacgaaagtggagtct
cattaatgcttgttggtgatgtaaaagacaaaatagctattctaattgatgatttaattgacactgcttcaactctggtgagtgccgccgaatttgtt
aaaaagcacggagctagccagatttacgctcttgttacacactgtgttctgtctggtgatgctattgaacgtgtaaagcaatcttgcattgacaaac
tcattgtaaccaacacagctcctcaaacaattgaacccagtgactgcttcgatattattgatgttgctcctacttttgctgaggtaagcactgcgatt
tattttgaagaggttaattttaactaactttaataggctatccgtcgcatacacaacggagaaagtatttccattttgtatgaccaaaaccacgcttg
gatataaaatttttggtgttgatttgtacttatttctagtcaaatgtttttttgccgatgccatctattttgctgacatgataaaagcctttgacgtgctt
tacatatgattgtcagattgttttgggatacttttgatttgcttttgttcattatttcttttttttttagggtgagaaaatagatattctacaaacggactt
ttaataataaatatacttttttttttcagattaattaattgcgtcgatttacatatctatactatagtaggaaagattcaaacgaaagttaaataacat
gaggaataaagcggatattatgtagaaaacgtaaggggatatctgaaaaaaaaaaaaggacaagaaagtaaataataaatgcgttaacagtc
aatgtttgctttggccgttttataggcatacaaggatcagtattctaaagaaaaacgttgatacgacaaaaggactcacattattatatataacaa
cagaccggatacaacaggaattaccacattatcatctaagccccaaacgtccatggcttctgcaacagcaccgacaaatccgtttaccacacata
gtactgggaaggacatccaagactgagctgccatactatcggggcccgtccggatgataccccagtataaataagacgccagaatcccacaacc
aaatgcgccaaggctgccggctaagcttttatttttagcgatcttgggagtatactttccatatctacgaccaaaactagaagctgctgtatcgcac
caagacaaaagtaaaatactcatcactgcaatatcctaaattgcgaaaatgttagaacatccggtttcaaaatcgttatattgagtaacgaatac
ggttaaaatattggagagctgagaattcaccaaataccttatggacctgactccctcatagagtttcctccctgataaagccaagtggaaaacaa
aaggatttttttcatgtggttcaccttctgcagcatgcatccctgaatttcttttacatacctctggaaagaagcttagaactatccatgttcctagca
aataaaatataacgccattccaactgtttttttcagattcacgcattaaaggtcccatcactcgattataaaggtggctaaaagccggccattgaa
atcgtataacatctcctgtaaaggctggaagaaaaccccatgccaaatatggagtaatctgctcagcatgaaatccataccgttgaagtcctaac
gtgatgaaacctatcgatacaggttaataatgtacgcctcaatagtctgtcacttaccaatcgatgtatgcagtagtttccgaggaatctcattcttt
ttagaccattgcgaccaagtcaattttgtcgacattggatcgttcttgctctgttcattccactgtagatattcaggttttctttagaagattcgaataa
cgatagtgagaaaggtagaaggagaaaatttcaatacactaataattatagcatacttgtacgagagctcattatctattttcacgtgtacacgttt
aaattattccccttgttcacgtaaataaacaatagacagaatgttttcccaatactatcttaggtatatttgcttattgtgttaaaacgaagttttgcg
catttttttgaacttaattctgttccatgttcactgaaaacgaagtttttatttaagagaaggaatggactttctctgtatccattttaaatgaagaatc
aatcagagaaacttgattcctaaagatcaagaaaacgatacgaatgaaaagaatatgatatagaagaaacatcaaataacagaaaatgaaac
catcttgattacaaaacatcacgcatcacgatccttcatcattgcattttaagcaggtcgaaaatgtaataacgagcaaatacatactttgaacag
gctcaaaactcttttcaatttttttttttctggtaattgtctttgatatgtttatactattatcccattgggacagcgaatttcttctattgtgtattctcgat
tattgaatgaatacacagataatacacatcctttcataaaagattatagcaagagaaagttcaatctatattcagtttagttattttttctttagtaat
gagagtgcgaaaccctgaattttcgtttcatttctttctaaaattaatatttaccaacgacacgttacacgccctcagtattcccacttgctatgattt
ccaaaaacactgagaaaacaagctcgcaacctccatggcggattcacctcgaaatccgtttcattctagatcacaattacctagattttagcaac

tgctccgaaaaagccttcatcaattcctgcaccgaaaaagcctggttccggtacgcgttctctcgcatattcttcacaagcattattactaatataac
agaaaaggatagttctcgggtgcaatcacttgaatttgctttagaaaatgcaaaaaacgatttgaaacaaaaagagatcgaaagtgaccgaga
acgcaatgacttgctcgccaaactaacagaggaacgcgaacaatcgaaggcttttcaaattcgcgtctctttgcttgaaaagcaagaacatgaac
gagaagcaacgaatcatgaaactattgaaagactaagatctgataatgaatcgctcgtcttacaactttcagaattgaagtcgaacttaacccaa
gaaactgctgcccgcgcgactgaattagaaaaaactcagcaagagctaagggaaaaaatatacaaggtggaagatttccaacagagaaacaa
gcttcttgaagagaatgcatctcagatggaaattcgtaattcctctctacaagaacaactaaactcaaagcttaaagagtcactagataaagagc
aacaagtacagactctcatatttgaaatagaaaagctaaaagaatcagtgaatcaagcctctgattctttggagttgaaagatgccaaccaaaa
gctcttagaacgaatcgccgagttggaaaaacttcaggaaaagcatctgtatatgattgaacgatacaatacaaactctagaaatattgaaattg
taaatgaagaaaaagctgatcttgaggctaagctataccaatatgaagactacaaagagaaggtcgctgttttgggtgttgataatgagaaactc
caagcagaactgaatagttggaaaaagacattcatggatatgaacgttcccgagcttgctgcagaaaagtttaagactctccagtcagaaaata
agactctcagcgagcgtgtctcagatctcacacaacaactactccaggaaaggattcaagggcctccaatggtcatcgaattaaaaaacaaggt
aactgacctcaagaacgaaaataaagaaacgagtgagcagattcgacgactccaacgccaggtattcttagccaaccaagaaatcaatcttttg
cgcgcaaacttgaaaagctatgacgatgaggaagccataatgaataatgaaaagtctggccagaaaaagctagaacggatcgaatcactcacc
caattgattgatgaatataaagtcttgtttgaaaattcaaagatctataaccagggagcttccgcagatgaacagtccctaactttaaagcgtcctc
ggaacgaagaccaagaggatgttggctacgtgacagaattgtatcgagaaaatcaacatatacagttacaattacaagagaagctaaatactg
aaggattcctaagagatcaaatcaatggacttgaagtcattattgctgaattaaggcaagaaatcattcaaatgtcagaactaaaatcctcaaag
gtccttcaacatagatcgaaccctacgctcaagcatgaaagggtaaaagcagaacagcttcaccttcttgaaaaagaaaatgggggatacaag
gccatcttagaagaaaaagatattcaaacagtacctatggaatcactggaattagctaatcggagaactttggagctaaagaaagaactattag
atcgagataaaagaatacagagactaaaagagatcttttctgtgaagtctttggaatttagagaggcaatttattctctctttggctataaaataga
gtttctgcccaacggaagcgtccgcgttacttctatgtactcacatgaaaacaatactgcttttgtttttgatggtgaaactagtacaatgaaattag
tgggaaaaccttcaagtccagatttcgagcgattagttaggttttggtgtgatgagcgaaagactatacctggaatgttggctgctttgacattgga
attgattgacaaaaatgaccagtctaccttgaatgagcaatagcgtaatgacaatttcaatgacagaatcaatttatgcagaaatcctttaagtat
ttataggatctatacaaactactttgcattatacatacttcatacgcatcatttatacttaattccctctattttattcaacaaattttgacatcttatg
cagtagctggaatacgtgctattgattggttagtggtcttaaacgttgataccaaaaacgaacttactgtaaatgtattgcttagaggaatggtcca
ccttattaataacaccacgctcggccaagtccttaatggcaatacgagcaagagcaccgttaatcttcatacggtcgacaaggacagagacaga
aacaaacttgaaggcgggaacctccttgttaatacggtcaatcaaggtcttgtcaaagacggtggcatgttgagccttgtctttcacttttcctttag
accatttctttttaggagcctataagttattagtctggctgtcaaaagacatggtaaacctttccagttaccactaaaactcaaacttcataccatttt
tcacagtgtttacctgggattctaagttatgcatggaaacgttttgaccaaaacttgtgtgactgttttccaagtaacaactcactaaactgaacaa
gtaacaagtctatgtgagcaaaggaatctgcaaaaccgatctataggttcttatttttggtagcagatttcttaggtatggtatgaaaaatttaattt
actttaagttattcccattaaggggaaaaaatccaattaccaaattgggtcatcctttgctcaataaattaggaacaactgtgcaattgcggcgtgc
cggccaacagatggtgcgaagaaaagaaaagagaaatacaaatcgtatttctttgaacgttataagttctgtagcggctgacaacatgattgctc
ctttgttatgtgtcacccttgatgatagaaaatacttaattggaaatatgggggagttgatgcaattgaagtttcgaaatcatccacttaattatgga
gggaaattattgcgatcatttatgatgccaggaagtggtcgaacatacaatccatggagtgctaccgctggcctactcggttatgtgcaatcaagc
gaaatcgacactgttttagatttgcatgctccttctagaatagcatcgcttatagcaaattcccgaaatttgacttcaggaagcagtttaaaattaa
atgttataccttttcaaaacaagctctcggaggatcatgaaagctttgagtttcgagaaaaggatttttgtacaatacaaggagaaatgtcaccttc
atggtcatatctgtctttcatgaccgtaccaatgcatggtacttttaattccgagaaagcgaaactcctaggtgttccttttggaaaagcaaatggtt
tactatgtcaaggaaaatcagttctagctagcgacaacaaaacatgggtgcgcccagaagaagtactgggtcctacaaggccggctcaaaagtt
tgttattatcggttgtagcagcccttcggctctagaaaatctcataaattatgcgaatacatgggagcacgagttaccatcttgcattattcatgtcc
ttgacaaaggggtttgggggaatgagtatgcgaagtttatgaagcatccgaagctacgaaattcgacgcatttgatttcatgcattgagcttgcaa
aagattctctcatattcagaagaaatagatctgctaatgtgcttcctagttgccgtggatttttgtcaacacatctctcctctgttcacagtctttctga
gaatgtttttgagttagaagagggcttatctgtagaaatatctgaccaaaaatgcttcgtcaaaaacgaaaacacggaaaatgttggtgtaaaaa
ctcactctttcaatgaattaccgtctccttctgtcggttatgtcgtggatattttgggaacgagtgcaacatcaccgacattgtatcgaagcctcagtt
gctacctggtaaatgtggataattgttttattctactagactgtggcgagggctcttattctcagcttatacggcaatatggaacgaatcttgattctg
tattgaagcaactccgtttaatatttatttcacatatgcatgctgaccattggttagggcttgtgaatattcttttagcttggaatgaaagtactcgtg
gagcagacaccacacttaccgtcgtttgtcccaaatcactacggttttgggtgtcaaggatttgtgattctaccaagctttcacatattgttgagcgt
attcggtttgtgaatgcttctactgtacatgcggggaatgagtactcattgtcgcaaagtttatctttatataccgttccatctattcatatttatgatt
cgcactcagttattttatcccacaaaagtaatggaaacctggtctatagttccgatacccgcccaaacatgaggctagcgaaagcaggaaaaaa
cgccgccgttttattgcatgaagctacctttgaagatgatttacatgaagaagctgtaaatagattccattcgaccgtctcggaggctttgatggttg

caaagagaatgcaagcacaaaaattgattttaacacatttcagtactcgttctattgatacttctttcactgctcccaattggtctatttatccaaaa
cacaaaactatttatgcccgagacggaatgcgttggacgcaatctatatgtcagcctaataaaaacgctcataaatcataatggtaagaagtatg
aataatttctttttaattcgttaaagtggagccgtttcactatcacgtttttgttgcgcttaatatatgacacattaatgatgccaaacttaataatatc
tttactatctgtattaatacttcaccaaatgtatagtagtaaatttactggatactcataagacttaattattacgctcattccatatcaccaacaatc
atagaagcattaagacatgttttccattgtttaatttgcatagctaaacattagtaagtcagtcacgcaactaccataattcaacgaaacttcgcta
ttaattgatgctactatcaaactaaacctccatctgtatgagtaagaaataattattaaattaaacaaggaatactaacatctaatatttagataca
aaacatagaatcaacctgattttcataagtgtttgtagtaattggaagcttctataccttgtatttaattatagttcagatgtatgttgatagaattga
ataaaaaaatcgatgaaccctcgacaaaacacgattttaggattcaagaaaaacaaacaataccaattgatcgcttcttggcagacatcaagata
cctgctaaacttgattgcccaaagatacatgtaaaataaaaaacttttattcttatatattagttcatgttgcgaatcagcaatacgctagatgaca
cagcattaagtgaacccgacttcaaaactaaaaacgactagttcccaacacaaccaattcaggaaccaggaatagtagcgtaaaagattaatat
agacaacagggataaaaggaacaatactagataagcataataacacaaaactgtatcatgcaacattcttatatatgcgtatttacatataaac
atctgcaaaaaaagcagcttaatcatagttggcattggagtaaacaaacattgtcataccgagacgagggctgatcgtgtgaacccaggacatg
gaagtgtcagggttagcagcagcaatttcgtaggtgtgagaatgacctgcgcgagggcaagcaatgttggtagagtattcacagccatagttgga
gcattgaaagcgagcaacttcagaaagacgtgtgggtcccttgttagaggtggtttgcttcttggggttaccagacatgttataaacaacaaactc
agcaggaccgttagaagaagaaacgttcaaggggaagaactcgttagtcaagcgaatgtccaaagcgcatttagtatagtagtacaaggaacg
gccataaggcacgtcaaaggtttgcaagacggattggttttggccattagcactagaaacttgaggaatagtgaaacgctgcatgcgcttgtcag
gagctttgtagttgtagttgtagataccatggggctgaagacgctcagacttaggaggaatgtcagcagaacggttagaatagtattgttcgctgg
aaatggatgtattagaaagaccagtgtattgactacggtagtcagagaggttgaaagcgacagagtcgatatcttcacagtcaatgctacgtccg
gcaccagtgaagacttgagtaccacagtgcataaaacccttgccggaacgcttcaagacataaccgaagggatccaacttaccacattgctggg
tggcagcaaagttgttagcgggcttgcgttgagtcaagtcaacacggtgactaccatcatcatgttgcttcttcttatagtcggtgtggttggtgaat
tggagatcacggttggcaccaatgtaagcgtatttgttgctgtcagaagcaagacgtaagaaagactcctcgtcaatcataaagacggcgggatc
gacggcatcgtcccaagggcgaaggtaagtcttaccagaggtaggggaaacgtgccaaacggtgtagtcgtaaccactcttttcacggaatcca
aggacaccgaaagtgtggttaacaccaacaccattgttaacatcattggaatgagaggccttggaggacttgcggtaaagagtattagaaggag
cagcagagccactagaaaccaaaagggtggcagaagcaaccaaaccaaagaaagagttgagaagttgcatgtttaaaagaataacaagtaat
ccgatatagataactatagatgaaaatatctaaatagataacagagggaaaacaaaaaaccaagttcaaacagaatagaaccgaaaattatat
gttttgaactttttatttatttcggcgattagaatacgaaagaataccgatatcaacgatgagaaaaaatttcctaacaaagttgatgaaaaaca
acgaaagaaaaaggtaaaggaagggagagaaacgagaaaaaaggatttgacttttcttctgtttcttttttttttttaagggagagaaggagtta
ctttactttcgacttgaaaagacaagacagaagagttaaagttaaaacaagagcaaggtaaaaaccaaaacgaaaagactatacaacgagat
agagaaggaagcgtttccaaaaattagaaaagctaccgtagtactgatcctttccttatgaaaaaaagtcaacctcgctatcttggttttcaaaaa
aagctgaagaaggtgggagggagcctaaatatatacccgcttcgtcacttccgtgagcggaggtatcgggatgagaaaataataatgacaacg
gccgatcgcgtgattgacgtaaccttgaatctcggcactcgttggtcgggatagaacgagttggaacgctaactttaccaaagtggacaacatca
ggatagtgaaagtagtctgttcgttttctgaatataacgatagtttatttcataatgcaataaatataggatatggtaagaggggaatagtgcgatc
caggagaaggaaggaaagaatagaaaaaaaataaggacaaacaaaatagtacaggagaaatatagatggatgaagattatgttaattcattt
tatcatctaaattattaaaggaaagtttttcatcatcaaaagataaataaatgaaacttaggttattctttattttggtggtctcttccctctacggat
tattctgtttcgcttccttcttagagagggcttgactttttcgattttcacccctcattctcctcaaccctcccctcgccatcttccactctcatccttaat
ctgtctcattaaagtttggcttaagacatggtactagaactttgctgttcgttataacaaataaggacgaggtggaagtatctcgcaaatatgattg
gcgaatatagagagtaccgaccgatagcagaaatcagttgagggtgtcaaagaccgaacgagaccctaagaaacccgagactttacgggtgac
agattggagagagagagagacagggagagagagagatagatagttaaagatactatgattttcgagactgatacttaaaaggaaggatgagatgt
agagggctggtagaagaagttgttcgcagcaaaaacaaaccaaagataattaaaaaaattgaaaaagaaaaaaataataataataaagtatt
tattgaatagataaataaataaacaaataagacgataacgcgttctcgtttggatgattgtttttcattggaagaaaatttatcttaccagttacag
ccaagtagtccacgtatttaccggtttcggtcatgactggcttataggttaggaggtcatcgctaaaggaattttcatgttgagactttgaccgaact
ttccaatgagatatgataagcaagaaaacctgaccgagtggaaaccagcacataaaagtgaaaaccaacagaggaataaacaaaaaatagat
ataggtgtagataattttcttgaaccatctcttacatcccataagtcctttacgcccttttttctcattttattctcatttaatttttattctagagtgccaaa
tacaaatcgtttatacttccaatatatacaatacaatacaatacaatacaacagtaattcctcgtgaaccattgaaacaagattgtagaaagatac
tatatcttttctacgaattcatctgtttatcattcagtttcttcttcatttaaatcagaaaaattctgctctccatggttctctctttgttttcactccatca
ttgttgctatccataaaattatcttctctaatcaccattctattaaaaatcacagttaagataatattctacattgattttattagcgaggtttctttgg
ccttctctaacgtcatccttgaaccatccttctttcatcagcaacggccatttatcttctccccaaaacctacagcatcgtctgcagattctgattttgc
atttttcgtgttttctttgccatttctggtttagttggttttgcttctcttaacctcttgttttagtttctattgatctcatttttgctattatttcttcatatctt

ctccgcaacttttttttgttgaacgtttgaaccatgtctgaacaacattctctgaacccttttgaacataccggtgctgctgaaaatgcagacactac
ccaaaacgctgctgagcaactcatccagtctctgaacgctgatcagctcgctacttcctctaagcacgacactactgctaccaagaatgaatctgg
tgctggtggcgctgcttctaatccccatttctctgaacacgagactcctgttcccaaagaggaggaaaaagccaccgcttgcccattctctggagcc
aaggccccctctgctgatggtcatcattgccctgccatggcttgtggtggtcctttcacgagcgagtctgagcaaagcgtcgatcctaaagttgctt
cttccttatggcgtgttctcacttggaagaatacttcttgcagcttgtcctctttgttgagcattctcgcactcttctttgtcccctcctggattaacattc
cccgcttgttcttccgcactattcgctacgtcttgttgatcacgtccatcattgaatttggcggtcaatttgcttctagcggtcgtcgtggtgttttatcc
cctttccgcaccaaatatgtcacttgcaacaccaaagctatcgactgcttcgttaattgtactgttgatatcgctaatgtccttctcatccaattccaa
cgtgtcttgtttgccgaatgtcctttgctaacttttgcagttgccattggctctttcattgaatacttcctttctggtttcctctcctacagaggtctcttt
atttggaatatcattttcgctttcactcttcctaaattgtatgagatgaatgaagcctccatcaagcgcatgatcagctctcttgagcaacaacaga
acaagcttaagcatgacgctgcttctgccacctcctctcaaaagtaaaccaactgtgactgtggctttggcttcggctttttttgaaggagaaccttttt
gtaaaatgttctttttcctttttgttgtataaaaagacatttggttcctatgtttgtttttgtaaaagacggtttgctttgtaacgagagtttcctgtttat
ttcatttatttttcgtcattctatctatgaaacgaacgaatgatgctgacgcttctctgatcttgttgtctatcagacaatgatgccaatttattcatgg
tttttactttacatatctacctgattatatgataaatatacttttgttagtaatcaactaacttaatagtgggaaaagcttcttcgtgaagtcatttcag
agttcctgctttttgtccaacaagtttgatccaccttccaccaccaggtcgtagactttgcattcatcaatattaatgctcagcatacaactttataaa
agcttttgaacattatgtagagtagtataaaccaagtaggccgtggaaaatattgaaaggttttctgcgtgtaaacatcaatcatacggttttcacc
atcgaccatgagtgcaaagaaaaagtgggttatatcatcgcaaaaacattactcgccatccgaagacaaaagttcttttacaaaaaacgcatttg
gatttcactcgaaaaagtttcaaaagtcccctcttagggaaaatattgaagatattgaagaccaagacgaaattctacttacctccatacgaggat
cctcgacatctaaaaagcaaaaaagggagcagtttcacgatgatattcaagaattttcgtctgaagaagagccgacttccgttcctttaggtagcc
atgatttccgatattcatcaacaattgcaaacccaagtctccattctccctcctctgctctcacttccaaagccttcgcgttccaatcccccacatctg
tgaaacgagaccaggctctcgaaaagtctcctgtatctgtgcatctagcaaagttttctgcagcagctaccggaacgcctcctcccaaaccagatt
ttacaaaccgctcacctttcttagataaaaagaagaaagagttttccttatcggaatgcacaaaggcactagaaagccgctatgtggcacgcata
cagcatcgcgattttcaacagctaaactttgcctctcaaaatgttagggatcagtataccaacctgttgttggtagaggagaatccagcatccgatt
cacgaaaaaactatctactatgcaagaagtcaaccgtcgaactggatcgattacattcgttttccttgcctcctcccgtgtatagataccagaatga
ttgtattgatgtatcgataattccttgttaaagccaatatctgtaccatagatagttttgggttggctctcttcaatcatttcttagcttcctgctacccc
agagatctccttctatgccagtcaagtaattttatgtggattttatccgaatgctctaatgatgtttgttccctttgtcctgcctgtctaaatcgcgaca
atagcgttacatatgctacataattcttctaacagtttcgtctacatactaaatacttccatgaattcttattatcttcttcctatttatttacagttctta
taaaataaaatgaaataaacactataccgtttccatctctcaagacgcagatagaactgacaacttcaatctacaccataacctacggtactttttt
gagctccctgttttgttcaaatctcactttaatcgtatggaatgagtactttggaagccatctatttaattgatatctatgggtagtaacagttttctat
tttttgcaaaatctaacaaaaatttgacagatcggtcctgcttcaaattgaatccagaggtagaatatcgtcagtgacttcagagtttataaagagt
gaaatatcaaaaagcaagttaagaaatgaagaacctccttttatcatctatcacatgaattacctcgtatttcaacaattgcaggatgatgttcga
atatgtataccagtaacctgcgatacagaacctctttacatacatgatgtgatgagacggattatagaagtagtaaagacttattttggaagcttcc
agtcatccataattgaaaaacacacctctttaattgcacagcttctgatagagatgattgattatgggtatgcaatgaatatggagcctaatgcttt
gcaagacgttattcccactccaagtttaatgaaaaaatttatggaagttactggtcttcaacctaatacaccttctgttcacaaaaatacagtgccc
tggcgaacctccaaagcccgttatactaacaatgagtttttttatccgtattatagagcgtgtttttgctgtatatgaaggtaataatcaacttgcattt
ggttttatgaaagttgacgttgaatgtaaaagtcagttgtctggaattcctgagcttttactatatttgacgcctggatcaactatggaaagtgcaa
agtttcataactgtattaatttaaaacgatggaaagaccatcctctccaattgcagttcataccgccagaaggaaagttcatccttgcatcctttca
cgcagatttatttaaacaaaaatctttgcctcttagtgtagaagcgaggaatatgaatgatggaaattttgaaattcgcatacggaacacaggga
aaaaacccattgaaaatctggaaataagagttcctttgcctgtgtctttaagcagcgtttccgccacccatggtgactatttatttcgtccatcaaaa
ggtttaaacgatgaaggtactgtactagaatggactataaaaaaaaattgaatggacttccccagcctatactttgaagggtgccctaaacaaaaa
aaagggactatctatcagtgatactagttctgaaaaggaagtgtacccaaaacttgagcatctgactcttcattacaaatatccactctccatcctt
tctcattttaaggttgaatctctcaaagttgtaaatcgtccggatatgaagccttttaaaggcgtaaagcatagtgtaatagcccaagatgtttctgt
tcgctttaaataacctacttagaaatgttttcatgatcgttcttagatgctgattactaatgtacactctttatcttcataatatatactgagtttaact
agtataaatataacttttttttattatcaaaaaggccttgtcatccttgaattgctccactgtctgagtgctctaatatataaattaaagtagccaaag
gcttcaggaaactaatgactttttccgtattaagttgctcataagccaaataaagctgtctcattttatctagcaacaataggcgtttgtttttctgta
aactagtagagtctgcagatagcaaagaggaaaaaaaaaaacgtccagcttattaaatgaaagatctgacgatttttctttttcgggataaaacct
cgtgaaagtgaatcaagatgctgagtcgaactatccaatacgtgacgaatatttacagatatgcctgccccttttcattatacaatactgactcaaa
caatatcgaagagaagagttaccaagtgcctgaaaagctcgctgttgtttttgtggaggcaaaaactccaaatgaaagtcgatttggttcaaaaa
atgataggattttgttagttgtacaacgactgtaaggcctttgggaagtgaaagccaaaccaattgaagcaggtgggccgcatacttttctagtttt

tgagtagtctcattcgacaaatatgcctttcccgcggtctttctatgatctttctgatcatcttccgaaaaaggagaatcatatggtcgttgctttggc
agaaaaccagttaagataatctctactgcatcatctaattcacaatattcttcacccaagtattctttcattaataaagaattatttaacttcgcatc
ggaatctaaggccagtatccaaatttctagcaagagaagccatcgattcatatcttcgggaattgcaaacgtgtatgctaccaaatcccaaaaat
caacgtctttcgtaaacagattatgcatagaaaaaactaacaagctttcatcacgaggactatcaaagtcggaaagttccgtctcaaatgaccag
gctactagattgtctttgcatcctttctctaaaatttgtagaagcacccagcgactagtttcttgaagattcacttgtgagtattcaggattaacaag
aagagttaagcataactgaatatagcaagctgggactcttgcagaagacacctgaattatatactgtaaaacccctagagcttttaataagagat
cttcctctatacaaagctccaatattgctttcaaagcataattcatttcgttatgctatcaatactttacagtacagtaccgctttggcccaggtgaat
gaattaagtgttatttttccaatcccactaacagtagcagcaaaacagtctttataaagtattaacaataattaaaaattaataaaataatattta
tgagattgaacttcgatcgatatacttcctaggtcacagtcaaatctctaccgaaacatttacttttacaaaattatgcatatctttttaccatgaatc
caatcacttcttcacctctgtaacatgttcttgtaaggtgtacgcaagttcacctaaacagtagttgctatgttgtagtaaactagtacgggacgtga
acctatccttcaccaacatggctgactttcaagatgaattgttagcgttagctggtgtagatgatgcgggtttgggaaatgatcgtaaaagattgca
tgatgaactgtatgctgcttttttttgtcaagaaatcctgaattgacacggtttttagggacgatgatttgagtagtagttcagacgaagaaaaagg
cgatgaaaatgatttcaattccaaaaattccgaagtaaatgataaagaaagtgaatctgaagaagatttcgaggaaaaataccaaaatccatat
ctgctagaaggcaaatttaaagatgaagcagatcgagcaaggtaaactctttggcaaattgaattcggatagctgactggatttagaattatggc
catgtccgaaattgaaagagaaagtattctctttgaacgtgaagaagaaatatccaaattaatggaaagacgagagctggccattcgtcttcgtc
aacagaataatcaatatgctgcacagacaacaagaagtacgtacatactatttgaagtagagttctttttttatactaacaaattcttcagagtctac
tcgtgacaaaccattgacctcgactgcagccggtaaaagagataaattgacagaactaaagaaaaggcgccaagaacgaacatttaagcccga
cgaaccaacatttaagtcaaagaaagaaggtcttacgtcagattatgaagaggaatccgaaaaaagtgaagcagaagcttctccttattctccta
cagacctggaaggaaaggatgaagtgaaggtcgggttgtcggacctgaatagtattcgcatgggtcgtaagcatgttgctgaatatatgtatcac
cctacattcgaaaccacaattactggttgctttatacgagtaaaaatcggggaaagagatggtaaaggcgtatacagattatgtcaagttaaagg
aatctctgaagggcgtaagccttaccgtgttgaaggtgttctcacaaaaacgtatctagaatgctatcatggtaaatcaaagagagtttttgaggc
taatgttttatcaaacgaaccttttaccgagtcggattttcaacgttggtgtcatcaaatggcagaggacaagctcagtatgccatcgaaatcattt
attgagagaaaactagatgatctcaagcagatgacgcagtatgtattgaacgaaacagaggtatcagacattattcgtagaaaaaaggagcttt
caagggttccttccaatctcgctgcagaaaaaacacgccttcgtcaacatcgtcaagccgctattgctgccaacaacacagagctcgcaaaagaa
ctagaagagcgactaaatacacttcaggaactttccgtgggaaatgcgcctcaaacaaacttttcggcaatggatcagcttgcaaaggttaatga
gcgtaatcgccgccgtaaccaagctgaggttcgtttagctgagaagcgtatgaatgaagaacgacgacgattagctacatctgtgtcaacttcag
ctacaagtctgccatcgttagctattgctgatagtaattctaaaacctcgacgccctccgtgtctgcgtccggtgtttcaacgcctcaaaccgatcaa
aatctctcatctaatactccttcagcgtctcaatctcaaactgctgtctctttcaacactacacctaaattatctccttcggattctcatatatcgatca
acgaacctgttccaccgcccgctgatcgtgagcccgccaccttagctgagaaggaagctcggcttcgtgagaaggctgttcacggtattgatgatt
tgattgctactgttgatttcggtattgatattaacatttgagcaatttcagttaagattgtatacttccactgtttattgcctgtttttatgatgaaccca
ccgtttcttgtacaatccgcatgttgaactccaaaaagctacactaacttatttctatatcgtaatgtaattataattatagtttgcagtgggctttctt
gaaagaaaaaactaaaaatacttgaactactggatcaggaaggttcgtagctggtgtatgcactcaacaattttgctgctttagataaatgtttaa
ctgtaatttttacgactcaaaatatactttttggaacatttatttttaattctgattgcccacaatgattataatgaatacttatattattgtaaactc
cgttataattatgcgattttataataaccggccttaaaatctgttttctaccaacaacgtctggtgttgcaaaagccatattttggcttgcatcttgtc
aatttgattgtaataaataatttatgagttatgaaggatcctattgataatcaggtaaggagttcgaagccattgctcaaataacagataactaac
cctaaaataggtgcccaaaaagatcaagcatttgcagtttggcatcagcgggccaaaggaattcgtcaaagatgccacagtcgaggtttttagcc
gtgatttatatacaatgaccgataggtcaccagccgaacatggtgccctggacttgcacatgggtactagcaataagcaaattacttgtaccacat
gtggtgagtcaatggccgattgtatgggccattttggttatgtgaaacttgctttacctgtttttcatatcggctactttaaagctacaatgaccatttt
acaaaatatatgcaaggattgctcaagtgttttacttacagatcctgagaagaggcaatttcttaaggagcttagacgccctgggattgataattt
aaggcgatctcaagtttgtaagcgcattaatgatcagtgtaaaaagacccgtcagtgtcctgtatgcgattctatgcaggggggttgttaaaaaggc
tggtcctttgaaaattattcatgaacgttttcgttatgctagaaaatctcaagatgatgagggcgagtttcgccataccttcgatgaggctatcaag
actatacccgaattgaaaatgcatcttaccaaagctcacgatgatcttaatcccctgaaagttttgaatttatttaagcaaatcaatccagttgatt
gtgagcttttaggaatggatccagaattcggacgtcctgaaaacttattatggcaatatgtccctgcacctcctgtctgtattcgcccttcagtagct
caggaaggtgcaactactgaggatgatttgactgtaaaaattactgaaatagtttggactagttctcttattcgcgctgctttgagtaagggcactc
ctgtcagcaatttaatggagcaatgggaatttatgcaactatcaattgcaatgtatatcaatagtgaaatgcctggtctacgcccttctgatatgcc
atcgaagccaacacgtggcttatgccaacgtttaaaagggaaatctggtcgatttcgtggtaatttgtcgggtaagcgtgtcgatttttctggtcga
actgttattagtcccgatcctaatttacgaattgatcaagtcgctgtcccttaccgtatcgcaaagattcttacattccctgaaagagttactactca
aaacaaactatttttacaacaatgtataaaaaatgggccggatgttcatcctggtgctaattacattattgatcgtgaaagtgatttgaagagattt

ttacggtttggcaatagaaaccgtatcgctgatgatttaaaaattggtgacatcgtcgaacgacatttaaatgataacgatgttattttatttaatcg
tcagccatccttacacaagctctctattatggctcaccttgtaaaagtgcgtccatggagaaccctcagatttaatgagtgtgtctgtggcccgtata
acgccgactttgatggtgatgaaatgaatcttcatgtaccacaaactcaggaagcaaaaactgaggctttggagttgatgggcgtcaaaaacaa
cctagtaagtcctcgtaatggtgaacctattattgcagctactcaagattttattactgcttcttatttgttatcgttaaaagatacctttttagataga
aaaacatttgctaatatgtgctgttacatggttgacgcttctgtacatatcgatatgccacctccggctattttgaaacctcgctgtttatggacagg
caaacaattattctctgttttgatgaagccaagccgtttcagtcctgtccaagttaatctggatgcgaaaactcgatcatttagcagaattaaatcc
aatacccctgaaatgtgtccaaaagatggctatttaatgattagaaacagtgaaattattgctggcgttgtggataaatccgtagtcggtgatgga
aagaaaaattctttattttacgttatccttcgagattatggctcgatggcagctgctgaagctatgactcgattatcaaaaatgtgtgctagatttat
gggtaatcgaggtttctccattggtattagtgatgtgcagcctggacaaagtctttcgcttcagaaaacggacttggttaacaaatcctatgccacg
tccgacgattttattatgcaatatgcgaaaggtactttggaaactcaacccggtttagaccaagaagcaactttagaagcaaagatttcttctactc
tttcaaaagttcgtgatgatgttggtgaaatatgtatggatgaactgggaccaatgaactcacctcttattatggccactgccggctcgaagggttc
aaaaatcaatgtttctcaaatggttgcctgtgttggacagcaaattatcagtggtaagcgtgttccagatggttttcaagatagatcacttcctcatt
tccacaaaaattcaaagcatcctttggctaaagggtttgttagcaatagcttttatagtggtttaactccgaccgaatttttgttccacgcaatttctg
gtagagaaggtctggttgatacagccgtcaagactgctgaaactggctatatgtctcgacgcttgatgaaatcgctcgaagacctctcttccgcgt
atgatggtacggttcgaagctccaacagtgacattgttcaattcttatacggtgacgatggtttggatcctacatatatggagggtgatggacaag
cagtcgaattttctcgtagctggactcattctgttagtatttctgatgataagcatgaccagcccttatttccataccaaattattaatattacaaagt
ctgctctttcacatccccggtttattcaaaattgcaatggagattttattgaaacaattcggaaattcgtgaccgagctaatcgctaaccaaatggc
caaagttcgagaaaatcgcggtttatctcctatgcttgaggaaccggatttagacgatatggatgattttgagagcgatgatttcgcaccagttgc
ggctagaaagtcagtggaaaatattatacgagtcactgagaaacaactaaatacattcttggactattgctgggagaagtacatgcgtgctaaag
ttgaacctggcacagctgtcggagctgtcggagcccagtctattggtgaacctggtacacaaatgacgctaaaaacgttccatttcgctggtgttg
ctgcccaaactacacttggtgtacctcgtatcaaagaaattattaatgcggcaaaaactatcagtactcctattatcagtggccagttggttaacaa
tacagatgaacgctcagctcgcgttgttaagggaagaattgagaaaacctatttgaaagacgttactagttacattgaagaggtctacggcccta
tgtctgcgtatatcgccgtccaagtaaattatactactatatctaagctccaattggatatcaccttagctgacatagctgctgctatatggagtgat
cgaaagttgaagattccttctcagcaggttaccgtgaataatactcttcagcagatccatgttcatacctcctctgagggtaagagcggcgagagt
gaattatattatcggttgcaaaattacaaacgcgctcttcctgatgtgattgtggctggcgttcctgaaataaatcgttccgtgattaatcaagaag
ctggaaagattgaactatttatggaaggttatggtttgcgtgaagttatgaacattgaaggtatagtgggcacaacaacaacaacaaatcatgtt
atggaaatgaaagcagttttggggattgaggctgctcgtacctccattatctcagaaatcggctatacgatggctaagcatggtcttactgtggatc
cgcgtcatattatgcttctgggtgatgttatgacttgtaaaggcgaagttttggggattacaagatttggtgtatcgaaaatgaaagatagtgtact
ggccttagcttcatttgaaaagacgacagatcatttatttgatgcagctgctcgatttgctaaagattcaattgaaggaattagtgagtgtgttgtac
ttggaaaattggcgccgattggtacaaatgttttccaacttatcagaaggactgaagaagatgaagagcagaagccaaaacgcctcttatttgat
acgccttccttacctcaactaaaactgactgcttaattccttacccttgcattgtataatacgaaggcttttataaatattggttttcaaggttatgttt
tggtttttttgatttaataatgatacatagtattttagacttgttaacatggaatttagttgcaattataaaataaaaggatttccggtattaatttat
acatttaataatatggactaaaataaattaaattccaaaaccgtatcatgagggtcagacgaataaacgaactcagagcattgcatgttgaagtg
ggttaaaaacgtagaattgtgaattcaataataacttaaaaacattgcctacctatgagcggtcaaaattaagcagcgacgtagttcgagtatttt
tcaattaaattggtgcaatgcaaaagcttttgtaggttgcatatcatattaacctgtaatttttcattaagatatgtgtcactagggacatccatgga
atctaaaagccgacaagcatgggaaattgacgacaaatctgtcagggagaactgtgagagatcttcgagtttgtttctcaaatgagaagcacac
gattgaagttttttaacagtaaatctttgagaatttgcatttatagaatcttcattttccgttgagtcaagttgagatagagtttgcaaggcagagga
ataaccagatggatattcagtgatttcaaaaggaatgctagcacactttccactgcgaatattctggatgtcgttgggaatctcatagatcttcattt
cattcaagttgaagttaaacggaaaaagaagtaaaaatttcagctttaaaacctttgagctctattgcacgacgttagcatggcccaatcgttagt
ttagaattaggctggggaccttgctctgcatgcttggggaaaaagcacaggcccattgtttaactcacctgctctaagaatgataagacactctca
ttaatagcatctgcaaacaaagcatatccaataggtgatgatcccaaaagtttgcctgcctgttaacaaacaccaaatgaaaattataaagtaaa
aatcttacaatgttctatcttctccgtaagaaaagatgcatccagatttccatttctacacactaactccgcagactcttgaatggctatagcgaaat
caaaattatgaggtgaagctaagccataattgaagctttttccgacaagtagtaaagctacgtcgggtataggcgcattgttggcttcatacctcg
aatgtatagcggccacttcatataagaccgtcgattctctagttagacattagactttgatcaccgagacagtgcattcatacacataaatcataat
aaacaagaaaacaagagaataaaggaaattcttgctacaacaaacgatgaattttaaaggtagttggaagaaatcacacgtccaacagttcaa
caaaagtaacaaagtaacaggcataaatgtaggaaacattataaacaagtgaagtataacagaagcaggaggatgtaataaataaaaaatga
acaaaatctagaatgaaaagaaaatacaaaccctttcaaaaacagatctgaaagcctttgtagaatacccttgtgcatatatacagaaaaaagat
atcagccagctaacagatacttaagagtatttgacaactcatccgacagaattatccgactacatttcataatgcaggactgttaggataggttct

caaaagcttatgcatgttggcttgtgataacttgatttgttcctttagctgataagctttaccttgcaataaggtaacctgagattccaaagtttggtt
tggcgaatttggaagctgttgctcttctgctgcagaagaagcattcgaagccggggaagaagcagcggaatatgcactatacgggatagcagag
aaatttaagaccagatctcgagaagtagaactgcgaacgtcaggactaggagcaaaaaatccagaggaagctgaagacgaagtcgaagatga
agatttcggtttttttcgaaaaaatcctttcttctcatgtgtgcttcctggagcattcaatggggatgatctctgaagtagtaagtcctttttttcagct
actaacctttgtaacttcaaaatctggcactggagactttccaattttgcgtatttctgttgcatatctgtataggcgttgacaaaagcctcatgaaa
catttttgcctgcttcttgttgaacctagctgtccatcttccaagggcttctgtagttaaaggctcatcggcttgtccaagagattcatctccatgtgcg
ctactttcgtttcccgacgatattgtcgaaaaagaaacgaacgaattccctcgaaacaggaccgttcgatgaggcattacaaaattgcactccaa
agtgcaaggattctcctttggattctattctgaagccaacaagcatccaacgaatgttgtatgctccgcgcaagttcgatttcgtttactcttgcatg
aatcaccgattagcgcattgcaaacagtcgcggtatatggtgtttgtgtggtatagcgtggtcgttaaaggtttctgaaaacgcgttttgtttacgtg
tgcccttgaacatgtcgaaagcaaaacaagcgcttgaatgaagattgagtagttttatattcaatttatcgattcttggagacccgtgaggttttag
tttattcatgcacaaaagagttggcattggcgcgttaaacaaggatccattcaaacaatatgaacaagtaggcaatgaattgatagaacaacaa
gctgaagagattgcttctcaactttccacttttcaagaggctttgaaaaattttgctaaagaacatgcagctgaaattcgcaacaatgctgaatttc
gtagaacatttacaaaactgtctttgaaagtcggcttagatccgctggtaagtggatcgggtgagtctgcatggtcggctgttggtatgaatgagtt
ttacttacaagtcgctgttcgagtggttgaagtttgtcatgcaacacaactggaaaatggaggactcgtctctggcagccgagtttgccaatttttg
aacgaagaaaacgaagcttctgggtatgatttgttaacagaagatgacattattcgtgctgtggaagctttaaaccccttggtccaggctgtgat
attgaagaaattgccggcaagcgttatattaggtcattaccattagaacttagcaaagatcagaactttgtgttagaagctgttgaggtacttggtt
atgtcactgtttccatactgaaagacaattatggttgggatcatgggcgatgcatccatgtgttggtatgttcaatagtcttacactcgatagctttt
actaatttgacgcgcagaatgacttggtatcaaagtcacttttatggatagacaatcaatctgtggaaactacctactggggagccagtacgttaa
ttgacgaaaaaccatttcagatagtagaatattagattataaactctataaattctcttgttttgatgcaaccccattttaaaacttatatggaagcc
ggaacataaggaaaattaacaaaagatgtgaaaaatcataataaaacgaaaaaaaaaaaagaaaagaggtcatgctatgcatacttaaaact
tatgaaaaattagcctctatagatgataaaagtcatgctttgaaggtctttggaacatcaaaagaagatggagagaatggtgtagtcatgaaaac
aaaatgaactggacgatatccttgatcctgcaattcattttcttcactaaattgaagaagatcaacttgcttctgtggcatcggaagcgtcttcagg
ctagttgcagaatacgctctaggaggatgaacgccgaagttatgccggtttgagtatcgttggaaagaagaaggaactcgtttttttctttccctct
ttcgcttatttttatataaactaattttacacggacataagcttctgtttgctctggatgcaattttagaggtaagatcgtttcttgaagtccttcagtc
aaagcttcatccagagaaatctcagcttcacccactacatgaggaatcgttttactacgaaattgaagactaatgttcaaaaaggaagatgaggt
aagagtagtaatggcgaaggaagccttgttgctcagtctatgttcattgactcccatatgcgaaagaggaacacatttcattaccccagataactt
taacaggcactgaaatggctttacttccattgaatgcttgaaaatcgattcatactggttacgatagatcaagcgtttccattgaggactccattctt
cccattgttcaggtgtcacatagatatgcaacctcccgagttctacagaactcatcggtgatatcaatttgtgtgctttttaccctccaaaagataaa
ttggctttatgacaaagggaacaaaaaaatctgcgacatgactctattagacttaccagccatctttattatttttggtattagcaacggaaagtat
cgttgttaccatgaaacaagtaacattttgtttacaatcgctttttttataaaacaatcaaagttatcagattttatttctttaaatctttataaagaat
ttttctttttttataaagatattaagcaatcttggttatgcagatttgcgtatgccaatcctgtcgatgttgagtctctttcgtataaaatgatttacata
gcaattggtcccaattcaagtcatttggtgacatatgcaaagcttgaaagggattttaaccttccgtatctagtggatccgtgtgcttttctttggtat
tcataatgtatcaacaaaactctggtgatggtgtgttaaaatctcaccaaagcctaacaaatggatacctctactggatctataaaagcgggtcta
aaagctcatacagacataggggaacttaattatgattttatgacttcaatgacacttggttcttcctgagtttctctcgctgttgattcctttcggcca
tctatttctctgttcattatttcttataatatttattatttatttgttcttaattttacactgtttgttcaaggtacaaaagacatgccgatgattctttgct
tttatattcattgtcgtttattaatttttgtatttataattatgaatttaacattcgtttttccatttaattagcaataaatcgtagtgatgaatttttcgtt
tgccataatcttcacctcatacctaatagacaacaattagtcagtcagtccacaaatagttgatcagtccacaaacttcacctttgcaacagcttat
tgagtgtttctttttttatttaaagaaattaaatacagatttagattttccagtgttgctcaaatcgattctgtagttgtacaggttttacatcgatttga
acataccttataacaaagctgacgatagtattcctgagaattttgcacttttggggaggtagcggtggacgcataaatgatatgaagaagaaaga
gctgaagaaacggaaaattgacgtgaaagtcgctttgataaattctgacttggatactctatcccaaattggtagaacaggacatggatttcttat
gaaaagctttcgaaagcctgcttgtatgttgttgttagaaaatatttcgacttttgactaattttgatcttactaacgcgtctgatttgcagggactct
ccttagtgggctctcttcacaagatcgaatggaatgttttgctaaaacatccgtccaatcttcctatgcagaccaagttcaagtccatttggattcgg
agagatcgtttttccagtataagctgaatccaatactgcttcgaaaacatcgttcacaattgatcaaattactaagtacaatctttaagcgataccc
agagttgtgctactatcaaggccttcatgacattgcacagattgtgttgcttacacttccttactcccatgctcttcctttaatggagcatctagttttt
catcgcttacgaggtacgtcatgagtgttcccttgcatttttttacatttccaaagttattaatcagcttgttgtttagactttatgctcccaacgttgga
tgccacgatcaaacaactacagctcgttttagagattattcggtatcgtgatttgattctctacgaatatttaaaaaaggccgacgtacagtgctac
tttgcgctgtcttggttaatcacatggtttgcgcatgatatctccgatgtttctgctgtgggcagattgtttgatttttttttatcctcccatccatctgcg
gtcgtgtatacctgtgctcaaatagtcatggatgaccgaaagaaaatcatcgagcttttattggataataatggagcagatattctccacacctatt

tatgtaaactgccatcgaaactcgatgtcgaacaactcattcacaatacttgcgcaacaatggcaagcatcaattttccgtctttgccactggaaa
agcttaggatatccccttttagttcccttcgaaatcagggtcaaccttgggaatattcgagcgacaaaaacggcatggtcgtgtttcgcttactacg
tgccgatcatcatgacagtggcacttccaaacaggactggaaattaccgctgtttttcacgagaatattttcagtggatgcaatatgataacagc
aataacagttattggtgttggtatcgtggcttctcaattaatgcctaaatcctcttccaactaataatcgaggcctctgaccaatcccagtatactaa
catttgactttttgacatccattttctttttttcctaatttatactctactttttttaatttggttcatcctaatcctccatttgatcgatcattgaatgc
gaccaaataacgttggtatttacttttgtttctttgcaaatcctctttttctggaagtgttttatcaaggcttctcttttcgatttttcatcattgttattag
ttttaattcccgtattctattcttcgttctgggaagttataatgaatagtgtccttgatatcctcttataattttgcatttaggatttcattaattatataa
atatatttgttcgattactgtatgacaaatgagaatggggttttgtttttggattggtatactgtgtgctcggatcagtgttcacatatgttcaccactc
agagcaagggttcaatatatatttataatatattatttacatagcaggatatagatcattccttcgaaataagggaatgaattttcgaacatgttca
acaaggtttaaatcgacgttggcgaaaatgagaccattaggagatgcaagatcactgtattgagcaacgaccgttcctacataggttagtaaaa
agaaaataatgttcaaaagcgcaaagcttgtcgacttaccccaaggatcaacaatctataagcaaacgagtgcgttagcaaaccgtaataaata
catatacacttaccatagcgtgaccataactagctcgtatttcattatggctcccccttgagcagaagcaaaaatataagattgagcatcaagcg
cacgggccctaagcaaaacttcccaatgagcagcgcctgtcttttgcgtaaaagcacttgggtaagttataatctgtgctcccatgttacgaagttt
taaggcatgttctggaaatcgtaaatcaaagcatatagcggacccgacttttcctaatggagtctcgaacgggggaagaattgcattacctgctac
agtagtatttgattccttcaaaactggaccatttgcaatgtcgacgtcgaataaatgagtcttgttgtatcgctgaacaattcccttatcgggttctat
aagaagagaactgtttagtagcttgttttcgatagcagaaggctcatgaaccccaatctaaagtacaatacgtaagcaaaaattgaaattgagg
agcaaaaagaacagcttcatacttgtacataaattgaattctgcttagcttcagcgcatatttggtttacaaaggctgaacagtcagggtgacgac
ttaattgtaaagcttcagagctctagttatcatttaggtcagctgcaaagatgattatggggaaaggagtgtagtctgaaaattgaagcgtacatt
gtgagcaataaagtcagaagcttcaggaaagaaaacgcatttcgctcccttggatgcagcctctccaattaggtctttacatattttcgcattttta
atatattcgaagtagaagtaatttgagctactgctgccagcgacatgaacgaaaagaaatatatgacgagtagtaagaggtgaagactttgtttt
ctttcaatctggaaaaggaccaaaagaaaaataggtggttaaagtaaagttgaattcatcatcgtatagaggaacagcacgacaattaaatata
acgacatctgagattgatatgatgcttcaatcactggttctatatctcggtatacgcaaaaaataaatgaaccacagagaatacggagagtcaga
agactcactactcaaagtttgtattcatgggaagatatgaaatgaaatgaattcctagttactatgcgtaaattcacaagttatgctccatcaaggc
gtgaactggaagctacgcataactttcgttctcctttccacccttttacctgactttttctgtgtctttttatccatgaaagtgtttttacttctgttgcct
ttgtacttttcttaattttgtttaccagaagcaaacacctgcctgaaattatgaatatagaaaaggaatcatattaatataaaatgataaaaaggtc
catcaagaatttcaaaggcccctaatacggtaaaagcatacttggcctttttttgacatttccaatcctacgctttttgtttactacagataatttgc
gcagtgcgttatatttattaacgctctttccttctttcgttattaaaataggatgtgtcctggattcgaacttgtgtacactggcaaattttcagaacca
tttcttaagtaccgccgaaaaatcctgccctcagcaaatttgccatctaccaacttctaccccaatggccacggcagttttcaaggtaagaagaaa
caggctctctttttctaaatatcactaacatgagtttgtttagaacatctctccaattccggacgtgaacacgttcttggacgtcgttctttctcgtact
caaagaaagacgcctacggtcattcgatctggctttaaggtatgtggaaggacttctactttgacataagaactcttatgtgttcagtagaaagaa
taattggttgttcttgactctaacgatttgacagatttctcgtattcggggtttctacggcagaaaagtaaaatttactcaagatacttgtactgaaa
agctggatgctatccttcaagagttccctagactcgacgacatccatccttttcacgcagatttactgaatatcctctatgacagagatcatttgaaa
attgctttatcgcagctttctaccgcaaagcacttgattgaaaatgtggctcgtgattatattcgccttttaaagtacggtgacagtctctatcgctgt
aaacaactcaaacgtgctgctttgggtcgtatggccacaattatcaagcgtcaaaagtcttcactggggtttttggagcaagtgcgtcaacatatg
tctcgtttgccttcgattgatcctaatactcgtaccttgttggtatgcggataccctaatgttggtaaatcatcctttatgaacaaggtcactcgcgca
gaggtcgatgtgcagccttatgctttcactactaaatctcttttcgttggtcattttgattacaaataccttcgctggcaagtcattgatacaccaggt
attcttgatcatcctttggaacaaatgaataccattgaaatgcaatccatcactgcgatggctcatcttcgttctgctgttttgtactttatggatcttt
cagaaatgtgcggttacagcattgctgctcaagttaaattatatcacagcattaagcctctgtttgccaacaaacttactcttttggtggtcaacaa
ggtcgatgttatgagacccgaagatttggaccctgaaagccaagagttgattcaatccattttggcagatggaaatgtgcaaatggttcaaacca
gttgtgttagtgatttgggtgtaatggacgtccgcaacaatgcatgtgaagcccttttggctgcacgcgttgagcagaaactgaagggcacacgtg
tgaacaatgtcttgaaccgtattcacttggcagaacctgctgcccgtgataatgccagtcgtccagcaatcattcccgatgctgttaagaaccgtcg
tgcttatgatcctaaagatcccacccgccgtattcttgctcgggatattgaagctgctaatggtggtgctggtgtatttaatgttaatttgcgtgataa
gtacatgctcgctaaccctgaatggaagtacgatcgtgttcctgagttgcttgatggcaaaaacgtggccgattatgtcgaccctgatattgaagc
caagcttgttgctatggacgaagaggaagagcgcatggaaaatgaaggttactataactctgatgaagaagttcctgaggaagaagaggagcg
ccttcttactgaggctgagaaaatccgtgaaaagaataaacttatcagaatggcttctcgtcacaagaagatcaaaaatcgtcccatgttgcctcg
tactgccggtgtccgtactctggatcagttggagtcttctttggagagcgctggtcttccttcccactctattggcgagcgtgctcgctcacgcgccc
gctctggagctgccgaagaatctgctcgtctgtctggtgcagacgtagttatgcaggaaggatctgaagtgcgctccaaatctgtcgctccccgct
cacagagcaacagacgtgatgcaggtttgcattctgaaggaggtcgctcttatgctgaacgcttgaatcgtgtcaagcaggtacaccgtaaccgt

atggctagagcctccgaaagtgaccgtcatgttttggcttcaaaacccaagcacttattcagtggtaaacgtggactgggtaaaactcaaagaag
gtaaagttatggtttggtatggatttttttatttttattttaatatagttttggtgcacttttcattctcttcatagtttggataattcggttggataaaaa
taaacaggtatactatttgtttcatctatcaatgtcttcatttcattatttaatgaattctcaaatacaataattttaaacgatgtaggagtgtagcac
taagtaagttcatggtttctgtttcccttatcgactttgttcagctttcagtatttatttctcaatcaccttagcattcttctaatacatatttcaccttact
tgaggacactgttgaggcgtcgaggttttaacatgaattttggctgattgacctttgtacaaacattagacagacttcagaatttttttaaaaggga
atagaatatgtttaatccctgtacagtacgaaacaaacgttcgactttacgagatattgccccgaaggagagcaatacgtctgtgcctaaaatacc
tttaccggcttcaagattcaaaacgaaacatatagatgctctttgcacattgatgcatctttgtttattacgtgaagattatcaccgagcgtttcacg
ccttctctcttttgttgcggtcaaaaggagtagatatctgtaaattgtggaacatcggtttagaaatattgaatcaaatcaaacctgatgcttcgact
gaatatttggagcgtctcatcgctcgttttcctgcaaacccaaataaaaaccgtacacagcgttccttaacggtaagtgtttatttatatatgattga
ttgattaagtatttctttttcttttaattaacataagctaatgagatctttttagtctgagcaatttttccctgcatacatattactgctaattcagagac
aggaataccaaagagcgatgaatgcgttgaatgaatatatgctttctcctccgtttaaccaaaataaagctttgtatgaatattctggaatgctgtg
ttttgaactcgccaaaaatgaaacttctgatgatgaacgagaccaatggcttcagaaggcaaagcagaatcttcatgatgcgggcatcaatttgg
agttataaataattattcttacaacatacttgtaaattggttggtactgctggttaatacgaaacttgaaatcagtataatttgtcaatttttgttata
cgtcgagatttacgttcgcctattaaggaacaagacaaaagaacaagaaaaataaaataaaacaaaaaataataatatataaacctatacaaa
aacctaataaaaaatgatgaatattagtacaatacacgctctgtatcaagagagattcgtgaatccatcttaaataatttataaagcattttaaga
aaagattgaagtttataacctgaaattttccaaaacgtaaaaatcgtcgcaagttatttagctctagaagaactcattgtatccaacgatatcatat
ccaaaatgtctttaatcttttcttttcttctttttttgtaaaatagcaagttgttttccaagttccatcctttgcctttcgtgtccagaggggtctttttcaa
gctccatacgagagcctgaggaaactttggaaattttatggcttccaactaagtccagttcatcattctctccccaaatctgagaaaaataggaga
cgtacttgcttaattgaagtgcttgaaacgtttctatttgataagttgcagctctcatcaattcaagcaaatgcaataacgtactttttaactcatttc
tttgatttgagaggaagtttggctcattatgggtagcttcattccacaatctttggcttgtttcactttcgcgaactgtttgctctaatacctccgttgcc
aaactgaacgtggtctgcttatccgagagtgagctttcatgatatttcgtcaactctttaattcttgataaaacattcggtaaggcatttttcatcaat
gaatttttcaaaaaagaaggatgcttatttgtcgaagaccgtaaggcaccttctgccgtaatatcgtcgaagaatcccttcgtagcttcttgctcca
aaggaaactccatctcttttgaagttaagaagtcagaaggatattgaccttgattgtttggaatcgttgcgcaagcgtaaaaagccatgagcagc
cttgcgcatttttttagcaccgtttctagcacaaatgagtagagctgtgtctccacgatcatcctgcacgtttagtatttgcgttgcaacttcaacagat
tgtgttgaagttaaattctgaagcaaaatatccatgtaatagcgagcagcttccatcttggattttgaagacgccaaaagtgctatatggtggaaa
acggtctggccaaagctatctgtcatacaaagagcagactgtagaagctctgatacaagctcaaacgtttgaagatcataattcatcgtaaacat
aacacatcgcattaaggaggtttgttgtaagtaattaacagatacgacgttagcttctgcttgtaagagcgcatgcatcatctccagatttcccata
gcagctgcccagtgtaacgcagtatggccatcttcatcaatactagcattgacatcccaatcgggcggtcgttcatagacgaaatctggaggttgg
gtattatttgaaagtaaaaaatagtctaataaggcttgggaataatgctttgagttgggaacaacttccttgaggccgagagcatcattaaaatcc
acagaggagaggatagaactttgagaaacatttggttgaaggcttaaagaatttgacaactccggcattctaggagcagaaggcaattcttgcg
aagaatatgattcggatgaaggctgtgacacgcagaattcattattggaggggtaggaggataccgatttattagatggtttctgcatgctttgag
gtgtatataaatgggcagaattcatatgcgactcgctgtattccagcagcggacttataatggaatatacatcgaactcctgggctagctcaaggg
ctctttccaaaggaacccacgtacctaatattattagctaaaagaaaaataaattgggaaataaactcataccttgatattttccacaaccgcctt
gtactttttcgtgtagccctttttgggcgaacttttccagaattcttgtccgcctaggcttgtctaactcagcaattttgagaatttgagtagcattcat
ccattggtcatgacatcgtttcattaaaaaattgccattgatattgcattcgtaaacgtttacacccgagtatattgttttcttaacatgactaacaaa
catagcgacctttaagccttctaaagctttataaatgccttaaagaaaagggggtacttgaagcaaaactatagcccaaaaaaaaaaagcaaaca
gaaaaaaaaaaaacaaacgaaggaagaaaaaaaagaaagagaaaacaagaatatacttggcggtaacaaactaaaagagagacgtgtgtt
ccaaaagataggtaataaaatgctcctggtaattcgtaaggggagagtattgttgcaatggttattcagagttaattagaggtgtaaaaaccaga
cttcctttctatcccaaagaggtcaataaaagaacaacgatccatatagatataatctgttgaagattatattaaatacgaaaactcaatattttttt
tgtgcgttttcagttctttcagtatgcaaaggcaagtctttctaaacaagtacttcaagttggaaacccttaaatggaaagggtaaagatatgagta
tgaagaaaaggctttccaattccaagttttattatgtagcttcttttactcctcttcccactttttttttttattttttctgtttttgtaagacttttcagactt
cactccaaaaatgattactccttttcagaggtcaagagtttgacaaatcaagaaatttcttctttccacaaaagaaaatcactctaaacaaagact
tcttcgaattttctcaaaaaaacactttctgtttacacctgcgatggaaacttcaacagagggtacaagccttaaaagctgtttaacagggactttttt
acgttttctataaataaaacgataaagggtcaccttcaaaaattattctaagctttttcagcccctttctttttgaagtaaatttgtggtgttgggggt
ctggcagaaaatagatttgtaatgggccaaggaaaaaatggttttgagcagaattaccacaagacacattgtaacctgtcttcagtaaggaagtg
tatatgaaacttgtatatgcaaaatttttacaaaggaaaggttggaaagaatggcttgtttcaaggattcgttatccaactttggtgtacgtttgatt
ttctctatcctttgtagttattcacgagtttgtagcttgtcaaaatgttgagacaatctgtgatatactttcactttgtttttttaacatggagcaaaca
gtcttagtactacaagatttatttaaaggctatgtttaccatgggtcagtgttgcatttgtcgcttttcgtaatctttagcatagaaatgttagaaatg

aaacacataacgagagaacattatgtgaaaatcaatatttgtagaacccggatacattactgtaaagtagtttagtcaaatatcaatcgtctgtag
aacttttgctgtgaaatttcaaaagataattgattaaaataaacaacattctctctcggaaaaaagccatagcacaattatagtcattgattttt
attaagttgccattttacagacgataaccctgcattctggctaaacatttcaagctcgcagaaaaataaagaaaccccccaaaaaaaaaaaag
atacacatacatcttcattcagcctttgttcttgtttgcctgtatacaattgctaacagaaaagaaacacagcgttttgcttttgttcgcagaatctctt
acttggcctggtattgtgtacggtaagcatccattgcatcagattcggtggtcgataattcagaccttgtgaattcgacaatgtcgtccaaagtcaa
aatggatgaaataggaacactgtattctttcttcaattttccaatcgtactttcatttacggctgcctctacccgttcctgtctatccaacagaaggac
aatacccgccaattgaacgtgtttaggttctaaaaacgagatggcttctcgaatagcagttccagctgtaatcacatcatccaagagtaaaacac
gctttccttccatttctgcaccaacaaggtttcctccttcaccatggcttttagcctcctttctgttatatgcaaagccgcaacttttgcctgtcttttcgt
acaatttggcggcagttactgcggccaaagagattcctttgtaggcgggaccaaataaaacatcaaaatcgacattcatagatattattgtttctg
cgtaggagtttcccagtgcactcagatcagccccatgagtaaaatttccagaattgaaaaaataaggagattgtcgtccagatttcagtgtaaaa
gtaccaaattttagcactttattttcaatagcacgatgtaaaagatcagatttgtacgacatcgtcctttttttgcttaattttctaggaacagtacgt
gtgcactggtaaggtgtgccaacgccagtgttccaacaccatcggaggggaggtcaaaacctaagatctagaaacaataccatagaatgcattc
aaacaacgtagcctttatataaatattcttgtatagttatcaaaggattttcaattccaatctttgccaaaaagtacgtccttaagatttcgaccttca
attgactgaaccttcataagcttaatcgtttaaccaagtgggctggatccgatctataacattgagatagtgttcaatatggtcaaggtttttaactt
gaatatccccagtccagaataatctccaaggtgtttttggtgatttcaaagagccaacacttctttcaaagtaccatttgttaacgggattgtccaat
ctagacctccaaatcaattctttaggaaataactctatcattgcattgaaaagcacatcggatattgcagcagagccttgcgcatttttttgacacag
ccaacttatcaaggtaaggcacttttgcttttccatcattaactttctcgtatgtaacaatggccgtaccggcgtaatctccagcaacaataactgca
gcaagagaatttttaaacgcttcaagtaagcttccatatccaatgtgcgattaaaagagtcgttgattagcatccacaatctttcccaagaaaca
gatccatcagtaatacattcagttcccatacatatatatacaggaacacccgctcgaagtaaagtagtcttcgtaataggcgatcgatcacgaggt
agagagcaggaaaaaattgatctttctgttagaatattatggattaacgggttcctgtccatcgaagaatttgtcagcatcgcttcatttggagtag
tgacaatagcagaggcctctttggataacatctttaggcaccgctgagccaaaaatagatttttatgtgatgtttcggcaatgtttttctcaattctt
cgtattcttgagctaagttaatcaacacatggctcgatccttgacgccgtttagcacacggcataccgccaactttatcaagaataattaatctatc
aacattgtaatcatcctctggcttatgcaaacttttcgttaagccgtaaataacttcgtctccatctatactaatctctttcaaatttggcataaacgc
caaacttggaataattggtgtataattagacgctaacgtgcgaaagacggaattggtgtcttctaggtaggcaccattgggacccaattggcaaa
tagaagaataccatggctttgttaatactccagccttggtaaaaatatcagagacacgcataattcggttctgttgagaataaagggagctgagc
atggtccacctcgactgcttttctgctggcattttgaacttcgctgttattcctaaatcagtcccagtaggaacaactaaagtatttattccaaggcg
gatgagtccgtaaatacttctaccaatgccatacagagtctcatcgtcgagcgagtctacatcagcaatctttgcaattgctatacgtttttgaagc
ccatatgataacttgttttctattagattggaagaggcattgttcaacccaagaggagcgtatttttgcaaaaaccctttcgtcgaccttttattagg
gactgatttgagaatccaaattaagtcctgcaggaaagaagggttttgcattcgcgcttcttaataagtcataaaaaataaaactaaaatttaaat
tagagatacttgatcgaatggagagatttgcaaacgtgtttgttcttggcacatcagccataacaaacgtctactacaccaaaacaatcaaaaaa
attagagggttcaaaaatcaaacgaaaattataacaataaaagcataaataatccattttatttaaaaaagataaaatatatattggatatacac
aaaagtatttattggagagtaaaaaaaccgatactatggctttacccagagaattagaggcaacattttcaccagaagagatagagtttttagca
ggaaatgaatacattgatattgttcctttagaaactatggatcaactgcctttagtaagcgcaactatacccattatgaagcctccaaaaaagtgc
agagtccctctatggctggctttggaattaaaaaggcaacatcgagccagaattgcgcctccggaatggttagacctttcgaatttagaaagcctg
cgagaacaagaaatggaaatggatatgttttccaaattgccgtttcgatggttggaaactgcacatttgctgctgaatgcttgccccgatgacattg
aggatgttgaggaagttcgaagaatacttttgactatcagggaagcgagacaaagcaaggcaagagcaggcttggaagcggtaaacgaagttc
agcttgctttggataacttgggagccatggaaattaatgagatccggcccttgttccgagaaacaatgaaccgtatgcgaaaaattgttcaggttt
cagaggaagaataagtgcagatatagtttgccagtcaaaagcaacttccaggaaagtggccacagaaatgtgtattttaatcggcatttagcaat
gacaaatatcatgaaagaaggaatattttatgaaatgttaaaggtggaaaagcaaggacaactagctatggtagtaagtaatcaagacccatg
agtagaagccttttgtttgataactcgagtaggtgtaggagccctggagggacttgatttagcggaatttggtttccgcctagacagcgacaattct
tcaagtttatagaattcctgaatacgactaaaacttgggtcagtaaaaagagatacaagaaggcatgttggaacgtacgttcgccacggagtaa
caggtgtatttgacgacaagaaattctggaaatcgcgaagcttcatgtttctctgagcacgtgggatagaagctacacgcatttctcccctcatgcg
tatggtggatgccagcaaacctgcattggatcgtaatcgccgaattttatcagagactatgtagagattcgttagtaaaaagcttgtgtctagaaa
aaaaaaaggaaaaaaggaaaacaaagacaaaaatccaataaggcctccctaaaaatagaaacgagaaaagtaaaggggattgtttctttggc
tttgggaggcattagaaaagaaaagagcattgtaaagaaataagtaaatttcatacgttcaaaagcaagattttcaagaatgtctgttttgcgttc
ttgcagaatacgctgtcgaacctgacggtgcttggattctccaaactcttcgactctctttcgtttattcatgaatctaagtagaaggaaataatcaa
ccaaggaaaatccaggtagtttccttaaaattcaactaatattagtaacgcgtagtggttgtgtttgagaagcaacataaacaattcagtttagca
agtagcatgttaccgagtaatgtaaacaaaagagcgtgtaattggtttctaccatctagtgaaggaataaatgctatgagagatgaaaagaaga

gttttttatcatgtgattttgttgctcaatggtgtctcaaacaacttgatggtttcaatattgaaaagaaagttacaagtttgtacagaatcaagtttt
cgttgtgtttctctagatttttaccattcaagaaggaaagtataataaaagtgaagatgaagaaaatattaaatgtcgacaacccataaaattgtt
cttgatttttctcaaacgtttatttcctagtgtgacatttaagcttttagcgagcttttagaaccttccaaaatttgtaccatttcttcaaaaaggaaat
tcaaatatgcatttctgagtccgtttccctttgactcaaaaattcctccaattttatttccctctaaaatccttactgtaaataaacaatacaaaggaa
aacatatctcctttcgctttcttgtagttcgttatccgattttcgataccgaaataaccccacaaaccgaactaaagccgcacaaggatgtaactcg
ttattttaactgtccattggcaggcagtacggctacacaaccaaccaactgtacacttgtgagcttgtgcttaattaaaggaccaaccaaaacagt
agacgaagaagttactataacgttcgaaacgattgccatccattgcaaacactcgttctgttaacccaatgaatccatacgaacccgtagaggga
ctgtatgtgaatgctaaacaatatcatcgcattctcaaacggcgagaagctcgtgctcgactggaggagcgtctgcgtggaatgcaaacgacgaa
aaaaccctatctgcacgagtcaagacataaacacgcaatgcgtcgcccccgtggccctggtggacgatttttaacagcagaaaaagtttcacaa
ctgaaagcccaagaagcattagaagagggcaagtcggaaagcccaaacggatcagagactcgtggtttgatggatacactcgatacagcccct
acgacgaccaccactaccaacgaatttttggacactaatgataccgcagatgatagtccgtcttctaccctttcttcccatgttcctaataatgccgc
tggcggcccggcagggttggatttaactggttccagggaagagactcaacataatccttcgatttttggtactactacaaattctaattttaatacc
gtttctgctaatccgaaccctaatgctcttacccatcctggtggcgtagatggaaacagaagtagtactccttctgcagctgttccctcccatacatc
tacagacactacgaatatcggtccttctaatagctcgattaatgctcagatccataattctactgcagctaccaacaatcagcatcatcgacattct
aatgccccctctaatgctcctaccttgactagcaacgaaaacatacaacttcggaatgctagttcctccgctgtttcgaatcctctttcagtaaatga
tccaggagtagatcttaacttgtcgaatccaatagtcatgcaactggatccttcaaatcttctgcattcaccttcttctaatcaaaatactccccgta
cgtcctttttccctccctcaactactactgaagacaggccttcgccaccagtgtctaaccgtcaccattctactgccaataataccgattacggaaat
ctacctactacgacgcatccgaacagtgtctttcataaaaatgctacacatacttatcacactactccgggttccgaattaacgggaccaaatggg
gattcatttgcagatttagacgtataccaaacggacgatccggtaacgacgagtcttatacattctcaacatcccataggatcaattggtgatttaa
gcgatccaagtactgctcatggctctgggatcccaaacttgaccagccatcacatgggtcatcttaatttaacaggcaataaccaagattctataa
ttattgatcagcaaccatatacttcacatgattccaccagtgctcatccatgaatgcttgacagtcagtgattttttttggcatttttttttcttgttttga
tctcccattcatatgctgtcgaaaattcccccacttatttcttagatcttagcaaacacgtactgaaaaccggttgtctgtgccgaatgacagaaa
aaagagcgagaaaataaaaagttacttgatatcattttcatttttatttttaaaataaaaaaatgaaaggtctgtttactagcaaagctaataga
atatgagcaaataaaccagacaaatcaactcgttgcattcatcacgaatgagcacggtccactattttctaatgtttcagaattagacttaagcac
ttaccaaataaccagcatatgccagccaggctagatatggaacaagtatctttgaggccgtaggagcgatcggccaccatgtcttggccaaccaa
cctacgacacctgtcaaagcaccaatattgaggagggcaagttttggcttttgacaacagaaaagtggcatccaagaatagtttagggccaattg
agttagatacaaagccgcacctttgcgaccagcattggtaatcaacaggggatctgcttggtaagccaaatgcgaagcataacccatggcaaag
tacaaaatcgtccaagcgggcccaaagacgtaatcaggtgcttgaaattttggctgcttgcgtgtcttatatctgtccattttgtttttattaagacca
gcagttatccatccgaatgttgtagggacaagaccggcgatccaccaggtttgtgaaatgctagtaaaaactctgtagttcaaatccatagctgtg
tgctttctttaactaatcctttggttaataaacaactaatgatagaggcacctatgcacaaaagcaattggaacccatctactatataccaataact
tcgttaatattgtcgtaaaaaaactaattgcttgatatttggaaaaggtatcttttcctgtacaggaaggaaagaatataaatattctaaaactaag
cttcaacattgcgtcataaatccataatgatttcaatcgaacattaggattctaaataagggatttctaacaggtccagtatattctacgtacacag
ttacacacaattgatgattttaacagttaaaatgaaatgaaatcttaagccctgccttatgtatggcaacctatgtgatagtcaaaacaattcaaca
tgatccaccttatttgatcaagcatatgtatgctattagcattcaattgtgttccaactactctggtacaacgataagagaaggatggttttgaaga
gagcgagcgaatatacgaaaaaagtgtctgcattaattgacaggaaatctagataggaaaacaacgtgtggtagcggacgaacaaaaagcttt
taattgaaatacttctgcttcgatatcgcaaaggaaacatccttttcctttcaattttgcggcgttggattgtttactagcgaagtagaaaattacttc
gttaaaatagatatcacagcggaggcgcacataaaatcagacgaggtgacaactgaagtagcaaggtgattatcaaatggaaccagaggtttc
aatgcatccacttccacattacaggatgagttggcatggattcaaaaaagccgttaaccgggctggtacctcggtaggtaactgctgaaataaga
gtttctaacgattttacttaggtgatgatgaaaactggtcatgttgaacgtaccgtagaccgtgagtttgaaacggaagagcgacgttaccgaact
atggaaactgcggctaagaagctacagaaagaagccaagggttatctagatgcacttcgtgcgatgacggcttctcaaacgagaattgctaata
cgattgacgctttctatggagatgccggttccaaggacggtgtaagtgcctactatcgacaagtagttgaagatttggatgcagataccgtgaaag
agcttgatggaccttttcgcaccaccgtcttggaacccatttctcgattctgcagctacttccctgatatcaatgctgccatcactaaacgaaaccat
aaattactggatcatgatgccatgcgcgccaaagtacagaaattggttgataagccgtctagtgattcctcaaaactccctcgaacggaaaagga
cgctgctacggccaaggacatttacgaaaccctcaataatcaactcatcactgaactgccacagctgattagtcttcgtgttccctatcttgaccct
agtttcgaggctttggtcaagatccagctgcgcttttgccgtgaaggatacgaaaaaatggcccaggttcagcagtactttgatagctcagtccgc
gaagactactctaatggaatgcttgatgacaaagtggaacaggtcctgcaaagcatgcgtgatctttctatcagcggactaagcaataattaatg
tttctctaaccttgtttcccataaccctttgtcctttttggtctatatccttttttcgtcttcgcaagtgcccttagtcctttaaccacttgtatatgatttg
aaatctgtcttgatttcccaaacttgttttcgattatttttatgagcaggataatttcccatcattatagaatgcactcaaatctttagtcttttacgac

gctcctttttggtgataaaaagttggttcagattttttatttatttatcacataaatcttttctagagtgcattcttatgacctatatttccttcttttctt
attcctaagttgtatagagacattgcttggattctttccaacgcacgattgagtctatgatttggtattttacgttttaacgtttaaggctttgtataat
caatcattactgtattaatagttcaattttttttaactcgaaaaacctagtatatgtagcttttttttttatttttatgttgatctttacataagctcaga
aaggctcatgcacaaaaaattgcattgttgctgcttttattttacccttcgtatttacagaaatttgatcctgagataattggggtgtcgaggtttagg
cctatacccaattcactcagtaatccaattcaggaatttcttgcgttggagcatccaaatcccggtaatctttgtattgatcacgacgctctgtatat
gaatcttgtcgatgatacggcttttgatatgaggtatgatgtgaatcctcgaattcaagaccatttcttcttcgatcatacccagagtttccaaagta
agagacaggataacttttgtttgcaagatgctgagcagcataagatggactgactaccttgggatccataacccttcctgggtccaagacgtaatt
acgcaaacaaatagctaccttctttacatgatctagccattctgaatgctttttgctaatatgtttttttaacaaattcaggccccaaaaacaacttag
tacaagctcctacgtggcaacggtacttttggtcgtcctctttgagaattaccgattcaatttcggaatttacaaaggtatcgattggtaacgcacct
ttcgattttaggtcgacagattctggatcaagcaaacatgtgtaatcactctccaaatttgaaaaccagctttcttcctgagaggcttcgttagtgtc
atcatacgatttccttgaatatccagcaggattatcttgcaacaaagcaattagacttgaataactcctgcaagtccaaaaatcgaatgtagccac
ttgacgaagatattccacaagtaggtcgcacaactgaacgttatgcatgcgttctttttctatttcatcctccacgtcaatcggaacggaatagactt
tgtcttgtagcttttcagaaatatcctgtggtaaattgtacatgacaacaaatcgattcattactttaaacagaatgctcaaaatttttttacatatat
cgttggaagaaaaattagaaggaagaggagttgggacatggttccttggaaacttggcgctttcagtttcaatagcgtaatttgaagctaattga
acaccatcaaggatttcctttgctccttctattgcaacgtccggcttcaaatggacccaaacagatcgctcaaaattctttgtaaacttcatgcgtga
aactgagaaatactcaataatgtctatattgtcaaacacctcatgcatgttagacattgagatgtctggcaacacgtgtcgaatcagcaaggtctt
atccttggctaactccctagagtccattaaatatttaaagtatggaacttcttttataaacagttcgggattagtttcattattcatgtttttttacctcc
gtaggtatatcttgttcatttggtagtttaaaagcatcatagtatccgttttcgagtctctccaaaaatatgttatgcatagaaagtcggtatgatttt
tcttttcctgaaatttcacgcaatcgaccctgagattcgtcaaaccaataagcgtcttcgaaccatgcttcatcacaatgatcctgtacaaacttttt
agcggttctcgcatacaaatcctgtttataattattataagttccatgtaacgacttgtacaaatcctctgtcgtagctgaaatcgagttctcttgact
ataccagcgaatgaaacgctcataggaaatgacactttcttgactgtaaggatcattgatactattaatccgatgttagttttcggtttcgaagaaa
acaacaatcatcacgtaccttcttgaatatgtatattgagggccaataactgcatcatcatttttgattagtttgctagtccaaagataatatattac
ggattcatacctcgaggtctttttttcataatgttcgtccctataggtatctctgctgcttacgggtgacctctctctatgggatccagcaccattgttat
acgagggactctgagatcttcttcgaattttgtctaacatatgagtttattagtgtcataaaattaagtctttaacaaatgacctacctcttctccatc
tagtagtctcgcgattatctgtttgttcgatatcgctcatccttgagtttttcgttcaatgaaagttttagaataaaaatcctctctaatttattcaaaa
aaatacaaaaattacagtccttaatgttggcaagtgccaagtaaaagtgcacgcactgtcaaatattttgttcagtgaaagaagagggtcgatat
taatatacttgtttactgtaatttaagagtgcattcatggattgttaagatgttagaataaaaaagaagaaaaaaaataactattattgaaatggtt
aaataaaataaaaaccaataaatattgtctacccctcaacaagaattttgctattgaaaacaaaaacgtaaagattatcattcgtaatattcctcc
tctcctgcataagaatctaatttactccttaattcgtttgtattgaatcccggttttgctaatgaagccaaactcatgctttggtatcccacgagaagt
accttccgtagcatatcccagagttcaaggttagattcacagcgagtaaacaccccatgcttgtcctgaataatgtcgactgcatgtagaagatga
tctgctgcttcctcatagcaactcatatttatgcatgctatggaaaagttggagcgagtacggacatattgaggctgcagctccaatgagcgcttat
aagcttgtattgcctttccttcttgttttgaatttgttaaagaagctcctaatttattccagagaacgcagttttcaggatttctttggacagcctgtct
aaaacaatcagcagcttttgaaaagtcctctaaaagatacatggtaattcccaacccagtctgagctttgtcatagaacttcttggatgacgatga
taaacatatcaagtctaaaaagcaagcttgaatagcttttgtattgtcaagaacatttagactttcaaacttgcgctctatgctggagaaccggtta
gagtattctggatgagatgcataaagccagtcgcgcaaacatgctaaagcattttcacgtgaacctttattcacatatgctactgccaaatccatta
taatattgatttgattagaatccaacatttttatatttaataaagactcaattactcggctttcattgcctagcaaggtatgaacgtaggctagggat
tgccaactttcgaggtgagaaggattttgcaaaactgctttttcaagcaatactgccgcttcgcaaattggacccttgttctttaataacgtttgagc
cttctcataaagatcctctataaagggaagtgcttccagctttttataatcgattgagtcaatttgttcttcactgaatatctccttcttctgctcgttta
tcttgtttaagttttcatactcagtacctgacgtttccattgcttgattagcttcagtatgtgagaaagcatgctgctttgacgcacttttctcttctgtg
gaattattcaattgctctttgttctcaaaaaaatacggcagctttatctttgcttcttcaagaaattcttcccaccttatttgatattcctcaagtgactt
cggatcaaggccgtttgtacgtaaatcagccaacctggcagtccacaactgctccaaggttaatggtgcttgagatgtttgttctaaatttcctttat
ctaaactctttgcttctagggaagcatgattaaacttttccataagctcgtcttcattgacatgagcgacttctaaaggagaatgggctgcagctgc
agacataggtagttgaggtgacgacaatgggactggaaccgttttctctttctgtttccttgattttatggtatcattactgattatagaaggtggctg
ctgattaaattcggatatccattttgtctctgagttctgattttgttgatttgtataggctgaaaactcttgagaccaagctctgtttggaactgaaga
agaatttttcgtggtaaggctgaatagtggaaaattctgatgttgactaggtgattgactgaggtctttccaatgttgaaatgtggaggactcggaa
taattcaagttcggtgccgcaagggtagtggaatgaagcggattttgaaagactttcggatggtaagcctttgatttcaaagccgtatcatgcaca
gatgagttcagttttcgaacaggatttgacgtggcggaacatccaggttgagaggaaaacgacatactcgtgtttaaaaatatagtgttccaaaa
ttaaatctaaaaaaagaaaaatgtaacaaggtgtttagacaagaagacaaagaaagagcataggttgacagccaaaaatgtttataaaaggtt

tgtttcccaaacgttttggatacagatgaaacaatgcaagtatatagaagcaagtctaaagagaaaacaatctgaagtcaggaattaataaatta
tttggtagatcgcacagaaagcagaatccttcaatatatttgttcttttcaagtttatatttaaataaatgtaataaagttactaaaaaggatgccca
aaaactaataatttcccatttttcttgccgggctcttttcgtttgcctgcagtattctattagtaagtttcaagaggtaagggagttaattttattttaa
tctacctagacatgaagatcaagaaaaccaggaaaaaaaagataaaagaataaagaaacgtaatacttaatactcttgttgaaagaaaagaa
gaaactagtggatgcgagaagaacttgcaattagtgtttatttacatttcagacattctttaatgtttgaggaagatagtcaatcgagctcttccatt
aaacacagaatttgaaaaaattggtatattttcgtcaataatcaagaatgttttgagttcaacgatgaaaacacacttaggattgattattcataac
atataccatcatttaccagtaatgttattagtcgaactaccgtagatatgaaagcaacatttgctcaatgttccttaaaggatcctcaaccttcatga
aatcactctgacagttcatgacaatgattctgaagttatgtgatttggttgagtcgtctcctttagaaatgaggaaaccttgacagcattatagaca
ttttaatgaaggaaaatataaataaccgaaagagaatgtcagagtctcatttgttttatggcggcaattgaattcaaagttaatttcagaaagaac
tttctgaacaatgattctgatttattttttttaacacccatccattctagtttaaactatactcgaaatcactaaaacgcacaataggactaaaaactt
agtcggatcaagaggatgtttaacttttcaattaattgtataagcaagggtttcgccgttgatttaatttaagtaaataaagtatatttttacttctcg
agtaatagccatgactgtgccagaggaattaagaaaaatacttcttcacgagctattacttaacatcgaaattaactcgaaaggtgatataagat
atgccaattctgctttctacagatttataggaaaaaaaagtagtgatcctttgctacagacatcttgcaacttttatgactgtattacggaaacgag
cattcctagagtccaacgagcgattcatatgctttttaataaatcttttaaagtcacgcatttccaaacttcgctcttgtgcagcactaacatggtccc
tgatatgtttttaaatagtaaagattatgagacatcaccggaccaagggaaaatcggaatgaagctttattttcgaggaatcttggtggattcttta
gatggtaaagaaactagaatcctatggtctgccaaatgtttatatccagaacgttactatataagccagatggactttgtattattcaacactctgg
gcatcggctctttcgtcttacaaaatcatatagaaagagcccataaagttaaatatgtacacgctcctcctcctgtccctcgcttgtgttatctttgcg
aaaaaaaaatcccagagtggtatttcgaagtacatactgactcttgtttagtatggagcgaactcgtccaagttatacttgatatacagaaacaaa
ttcatataaaaaaagccgaactgctcaagttcagtcaatctatccctcacactactgtttcagtttccgacgaaacatatttaacattgccaataatt
tcagttctgaagaatgggtctactaaatatagacgatttcgtttaaaatcttggcattcttgcgttacgtttttgtatcggcaactggatgcggcaga
gcaaacgtttgaagaactggggaaaagcacatacctgacagtctctcaagaattggctactgatatgaaatataacattttgaagaaaagcatgt
tactctgggaatatatctttttatctccttgtaaaatccaaagcttatttcacgaagtgcattctttgcttaccctgagcctaaagtataacatcaagc
tttgcaatcataacttgatgtacggggtaacctttcaggaggtaaaacatttcttactgagatatatgcggaatttttttgtgttcaaacctgaacagc
attgattatgaactttttttccggacatgcacagctcattagcatgatcagcgatcgtacacacagtaaagaactggactctactaaagtagatttga
ttttaccaaaaatacggatagtgcctaatcgactccctcatttcaaatcggataaaagcctttctattcctctcaaatatacctccagggaaaatgc
agcaagttgcagctccttcagcgaggacgacaacttgaaggagtatatgctgagaaatcttaataaaaagctagcaagcaaaccaagaaaaaa
ttcctttgacgacgaacgggcgcactcgcttcgcatttatcaggagctcgttgaaaaactttattctcctcttatagataacactacagcggctttac
accttcctaaatacaaggaacgaaaatttggagacaagacgccgagatcactaaaagaattcacttttgtcaaagaaataaacagaggtgcttc
tagtagggtttatttggtaaaaaaaaactctaccgggaattactatgcaatgaaagccattccgaaatcaggtctgaatgatcttgaaaagttgag
agggattatgtacgaaaaagccaacatggaaatccaaaagtttggatcaaatattgtcaaggtatattatgcgtttgaatcggaaggattcttttg
tttagtaatggatttcttcaacggtggtgattgccaaactttggttgagaaatttggaccgctgccggaacaatggattcaaaaatatatagctgaa
ttattagaagcggtcgagattcttcataaactcggtattttccatcgtgatgttaaaccggcgaatatgcttatagatcagaacgggcatatccgttt
agcagatcttggattaagtgataataaagaaggtagcagcgagggtgaaatatcttacgaagaacagatcaataaactaaaagagtcaagtca
tgaaggtgcgagaaaacaactagttgaaggccttaaggatcaagctaaaaaagaaataactttaacaagcagggagacgaataaagaaactt
ttgatccctttgagcggctgatagatgttgacacaagtaatttaaatagagccatgtcaaaagtatttagagcgcgcctgaaagttagcttgccag
gaaaactgaacgatgaaaaaaggattactggtactcccaattatatggcaccagaggttctttcagaaaaggaaaatccaatgagcgatatatg
ggcgatagggtgtgtcttatttgaaatgctgacagggattattccatttcaatcagataccatccaaggagtatgggatcgtattcaacgaaatga
cattggatggacgaactgtagccatgagttgcattcaccagaagctatagatcttgtaaataagctgtcggagccgaacccagaaaagagacta
ggtaaaggaggattttggaaataaagtgccatccatttttcagagacatagactgggatcacttgtatactcaaaagggaccatttgttccaaga
acaaaaaatattgaagatctaatttatttcgaacgaagtaagtctacatcaacgtatatcaaggatcgaatgactggcatacataactctcaaact
tcacataatattgatgacatgctcggtaatcatattgatcaggaccatttagttaatgatacctgtataggtttggaaaacagcgaccgaaaacca
catgacgatttagttgagttggctattgaatccttttcaggtatgaatttaaaagccctcaacttttccaacaaaaatgtgctatgggaaatctctga
ccagctagaattcccgggcgcttcatccttttcccatcccccgattcctaaaaaaggccattctatgcgttatcggtttttttgcccagaaaaaaaat
aatcgcaacacgcatgaaagcccctgtaaaggaaattccttcgtccgtacagttcgacagtagaaattcttaatatcaaatgaatgacttttcaat
atccttacgaatccttattcatatagtgaattcttttctgaaagcttgatatcttgaaactaagagtaatacatctacaataccattcttgacttgaaa
caggttgttgaaaagcgcggtacattgtaaggtaaatattaaagctttaagaataaatagccgaaacttattaaaaataagctcaacgtgacgag
cctcaagattgacgaaatacgcaaagatgcgctaaagtaaacaacaataaacaatcaccaaaagatttgtctactaagcgccaaacatctgga
gaagtcgatttcgaaaatgcctggcggcttgttcaatttacgaaaacgaaatgaagtaaacaacgtagaatatcttaaagaaccatcatctcggt

cattgattctgaagcgctatatgctagctgttgcagtgttcttcattgttgcaattggtgtttatagagcggccgtttcttctaaatcagataatctttt
gtatccaaataaaggaattcgcctcagcaatggaacaaacacattccttcctactgtaatcatgatttctttggatggttttcgagcagactatatgt
atcgaggccacacacccaatttgttgcagttggctgaaaagaatgctcatgcaccattcatggttccagtttttcctagtataacgttccctaatcac
tactctcttgcgactggagtttacccggaaaatcatggaatggtgagtaacaacttttacgaccctattagtggaaaatattttgtaaatactcgtc
cagaatgcaatcaagatccatcttggtggacgcaggcagaaccaatttggatcaatgctgaacgtcattcaattcgttcagctatccatatgtggc
cggggaatgaagttgctcatgatggaatacggccctcgtattacacatccttcaatgctagtgtaacccttaaagaaaaaaaggatgaactactt
cattggcttgatcatgcagacgatactcgacctcaattacttatgtgttatgtgcctcacgttgattcggttggccatacctttggtcctgattcatca
caacttgatttgatgctgagagaagttgaccagatgattggtaggttgcttgaaggtatcaggcagagaaacttgcaagacattgtaaacgttata
tttgtaagcgatcacggtatggctcctacgtcaaacaatcgtttgctttggttggatgatctactgaatgtatcgaaaattgaattccgagacggttg
gcccttggctggtttccgaggtgaaactggatcggacgagacgtatattgctgatagtctgaggaatgcaacctcagaatcacttccggggacgg
agtattggcgatctttaaccaaagaagaatttcctgaaaaattgcattattcaaaaaatgatagaatagctcctgtttggatgataccaaatccag
gttggtcaatcgtctcttcgttagaccattctccagatgttgaatatgaaccatatggtgttcatggttacgataatacttctcctctgatgagagcta
tatttacagcattcggtcctgcctttgaaccctttaagtcgaagcaaataatccccttccgtaatattgaagtttacgatataatttcttatatactaa
atatgccggcaaatcccactaatagttcatttcatggtgccatacccaccaaaagaacctcaaattctacaaaggaatggttacttcatgacatta
aacaggcgtataatgagcttgaggctcgctaagtgcccaaacgaatcataatctctctattattattttactttttcaccttctttaacgattcagaat
agtatcctaatcagaaatagcattctataatttcgcatttagcctttcatcattcatccatgcaaaactctacagagaaaagttagtaagccatcaa
gagaaacactcaattaatgtcaaccataccaatattttgtccatctcaggatctaccataacattaattaagcatgtagttccttgtctccaaaaat
cttggatagcatttttcaagtcattgacattcgtagcaaagtagcctttcccaccacacgcttcacatattaggtcatatcgaactgcaggagacag
agccgttgtgggaagttgattgtgagttttaaatttctcataagtcgaatcgtccaaaccgtgatatacaccgttgttgttaattacaataatcaaaa
gatctaatctgcttcgaatggcagtctctaattccattgcactaaaaccaaaagcactatcgccttctaccgcgacaattttatcccaaggattcgct
actgacgctgcaatggaataaccaagaccaacgcccatggtagccattgttccactatctagtctaccacgaggatgagttacttccaggactttt
cgacctttgtccaaagtgtttgctccttcagaaacccaggttacgtttctctgtgtttcgtttaccaaagattgaatagagttattgatgacagttaaa
gcatcttccatatgcaaaggaccatctttttcgatgaacacaactttttagactcatttcgtttgcgatgtttttgaagcatagcaagatacaatgat
tgttcagagcagtagttccaggcttgtaaagagcttattagttgatctaaagctaagttaatatctgaccaaatcccaagagacacatcaccagcg
ttatttcccaattcctcagcagtagaatcgatttggataaatttagcatttttgaccaccggggagagaagccaaagtgtaataaccaatttaaac
gagcgccgaccaataagacaagatctgcatttttaaagctgttgaccttgcagacgaaaagtttagccgatgattttccggcaataatcctttgcc
cattggagtcggtaagaaaggtacatttgtttgctctattaaggccaagagcttttcttcggcatgtgcataagaagccccttttccaatgacaatta
aaggagactttgagcttttcaacagtagcacggctttttggacctggtttggatcaggaccacactccaaggcacgaggaatgcaaggaagagct
tcccaggaagattcttgaatagaggattgaataaagtcagctggaagatcaacatagcaaggacctggggttccgtttatggaaacacagtatgc
ccgatgaatcgatgctgtaatcatcgtcggagcgggaggtcgcaaagctaactttgtgcttgcacttagataggcaacttggtctaactcttggaa
ggctccccgatatcgttgagaagtaccactagaaccagccatgagaagtaaaggccaacgatttgctgaagcattaaaaactcccgccatggca
tgcacgacaccaggaccgccaacaactatacaaactcctggcgttctcgttaaatatccgtaggctgaagcagcataagcagcactctgttcgtta
cgaaaaccaataaactggattcccgctttttggatagcctcacagacctcaattacgggaattccaacaatgccgaaaacgactttgacttttaaa
tcaagcaaagttcgagccacaacgtctggaaaaggaacactcattctaaataatagcttgcatagaaaaggacgaatagatatggtaggcttca
aattatctgatgtttcaagcttctacttttgaggcagcaagctcattcaatggtattattatgccgaaataaggttgcttgaagacgaaaagatatt
gacggtgtttttgtaaaacttaagggtctaggtatatccttgatagctatgcaaatcggaacaaacaaattagaaaagatccaaattaaaatgatt
acgcgaatttcttcggaagagaagagattaaaccagaaatggttcaattgcgttgctctgaacatcaacaatacgatattacacacttactacgat
ggtaaatatgggaagttggaatctggaaagaaagaatacgaaaattacagaatatagccatgaatgcgagaaaaaatagaataaaaacattt
aaatcgtatatgtcaaacttaaccgatatatgaaatttcataaactgggaaaagtatatagtacatacacgtcagattatagagagatcattaaat
aaatgaattagaattggaagcttcgttcatgtattctttgcaagatagcatgaaagtatctctctaacaggatactaaaaagggaacattgtttgc
aggttcatatgtacagtttcagaaggatggcagcacgacaggatggttttaaacgctaactcgttcgccagaccattgtcctgattccaggtgtttc
tcataaatcacacgctttccgggaggaggatttaagtccagaaattcgacaccgtgaacatcgcagatgaccagtgcaaaccgattccaggcag
tttcccagcgattaacaagtttagcatcttctctaccagtaagcgttgcaggaagcgggatgagaggaacttccccttcatactcgctatatgtcac
ggggttagcaaaagaagtacgcatgttaggactgtggttttcccaaatgcgtcgacgttcttcctcccaagaccattcttgaggaatacgtttgttg
ctgcgagctaaatgtgtgtgtaataggtcttcctttggaaactcattcttctctgcgagatcaggagtgtagagccatacttggccccagagacgaa
cttgctgcatggttttaggaagccaactacaaagctcaaatgtattatgcatgggaccagtcggcgttgactttgcttgacgaagggaaatgtccg
ttgctttctgtgatttcaagtccgtagaaaacacaagcaaatctgtgtctaaaggatcatccttactaggctgccagcctgaacccacgaatccac
gaatcgctacagtacgattgcgaggatatttgtccccttcagatggtaatgttgctagttgcatgtattcaggatgaacaagtcccgaagagtcag

cattctctttaaataggttaaccaacttcgttttccagtcgttttccatttctaatgattttcgttcctgtaaactattaaattggtcgctttttttataaac
cctacaagtctgtcatattgatgtcagatgctgcacatgccactggtatgagaaatgtttcgattacgaaagtgtacaagtgtgaaataaaggaaa
ctctcagttaaaattgtagatgaaactctttataagccatatagaccaagtatctaaaaataacattttataaacatattaggtcttcatttgccagc
atctttcttgttaactagtgttggcactcaaggcagctgcttataataaataccaatgagtcagaactttaccgccgtcttttgaagcacagttttcgt
ctgaatatacactgttaaaattttgtttagttacaattttatatgaaaatatatattatcagcaaaacctccacaattaaataaaaggttaacactat
cctatccttgttaataaataaatacgtaaaaaaaaaacatttcaatacttaaagaatggtgaaatcatcaaagtaaaagtaatatacattaatctt
tatgttctcttcaatcttttaaaataatacaatggtaaaagcatttcaaaaggagagtttgagaacccatgttcccaaaaacagcacttgaagagc
tggtcctcagtaagtataatcttactagcataccacatcgtatagcattatcatgttttgcttacgggtaagaccaatctgctaaaccatattctgatt
cgagttcatagcaaattaggtcgtttttcaaccccgtttgataactgttccacttcatagattataaattttgcttgttaattccacaaaataaccaat
aatgaacggaatcaacattccccatgtatcagcatgcttaaaatagtagtagatgacatatctatttaagcccgttacgaaattgaaacgaaactt
cacagcagactttcttttattatctgacataataaaaggttgagaaaggaacctcaaagccccatagtattgtagaatataaaaatttccaagatg
aaccagtgtaacacaaatgttaaaatacagataaatatgtatcgaaccaaatgcgaaatgattcagaattaaattcgaaattagtataattggcg
tatagtttctgaaattccaaaaaacaaaagatgaatattaggcacccattgttgattcaagaacaacggtaataaacagacgaaaagggtaaat
gtactgatggtctgaaaaatcccaatgaaccataaacttatgcaaataataaaaaaaaacttaaacgaacccccttttaatggataatatcctg
gtaaaatttgagcaagtaagaatggacatctttgtaaaaatcctcaacggcatttaaagttttggcaataaaatctagtgctatctcttcagtcggc
agagtaagtgcaccgtctctactccgaagggcaccataattttgaatgcgctcgcaagcctttttcatctctgtcagacccaatgctgcagaggag
ccttttaaaaagtgacccaaagaagacaatttttcagatctttctgctttagtgcctcttgtaattctttaatggtcgtctcagcttgttcaaagtaat
tgaacacgatacttttgctaaactcgtgttgatcatcatcatccatttctaatagctgatcaaaagttgtgtgatcgacgagttgtgggtgtcgaaac
atgtcgtcttcaaaagacatgtcataagcgacaacagaaagctcgtcagaaaggacaccttcgttcaccaaagcttcgagagaagcgatcggca
agtttttcccgcttacattttgaccgttgagataattggatatatcaatgggttcgtttctgtttgtgtttttcctttcattctcttcttctgaattttgtgtc
aactctgaaggaggggcaggagagcttttcgaagaagacatccgagaagtcatagccacgtaggctggatttgaggttactggaattgaaggat
cattaatgtagctgttactgttacatcgtcgatatacatggggtacaggcacacctgcaggagagcacgctctttcagcaatacctgagttctgtaa
ggtcctagctaggctagccgtccaaaattgctcatttcttttggaagccctgttttctgtattgcgatcataattcacgtttgtattcatcattcatcaa
cttttaggataaaaaaaaaccacacacacacactctctaataaaggattatacactctataaaacaacaaagtaagtcaagcttcgaaccagca
aagttgatcttccttcaagagagagagagagagagagagagagagagagagagtagtgtcgttagttttatttaattaattcgtgtattagttggtctt
attgtcttatttcgttacctaaatacgtaaattatttgcacaatgtctcgaccaannnnnnnnnnnnnnnnnnnnnnnnnnnnnnnnnn
nnnnnnnnnnnnnnnnnnnnnnnnnnnnnnnnnnnnnnnnnnnnnnnnnnnnnnnnnnnnnnnnaagagagagagaga
gagagagagagagagagagagttgcgagtttttttatttataaattcgtgtatatttggctattgctatttcgttccaaatcgaaatattttgcacaat
gtctgccacaaaaattatagtgaccttttattatagaaaaaaaagacactagaatagacacagacaggagaaataaaatttacaatgagcctca
aaacactacttatggcaacaaatgttaattcctcttatgtaatcctaggaatgtaaaatttgcccaataatttttgcctgtaactgatgaacaaaaa
ttaggtgttaagaccaataagtgatgatactgccctactaccttgagaaaacctataatgatcttcttgtttgaataatttgaaacagaaaaatcct
atatggcaccctcaaacgaaagcaagcgtatgtttcccttttcttgtttaaatgatttcaaaaagaaataaatggggcacgaagagaattttacaa
gtctcttctaaatctccttttactttttttaatccttttaaattgtaactggtgaatgcgaaagttgagttttcacatttctagaaagtattgctttttcta
atgacgtatgccacaggaaatctaattgatggttgtaaaattgaacgaatctgacaaataacgattatcaggatacatcgctttttttttcgaaacgt
tagatgtaaatgggaacccgattaaacactgttcagtagagatgtaaattaaaaagtccgtaaaaaatgaaaagagacgaaaacaaaaaaaa
aaaggacgaaaaggaaaggaaaggaaagaaaaaaaaaagaaaagatacagttgtttatgttaggtttcattaccattgagacatagagaata
gaggtttatcatggaatgtaagtatgaaaatttttgatgagtaggatcagacttggaaaatgaagatgcgtttgaaggtaatgaaaagattgaca
tagaatgtttgattttattcatgtttgtttgggtaagcacaaaaaaaccgaggagtggaacatataggtctctatatgtgcgattgaccataaacct
tgtaagcaggttcagttagtaggaagaacaggaaaaaagtagcaagcagcttcgttcaaaaaagctgtaaaaaaaagaaaccagaaaaaaa
aaaaaaggacaccgacttagaagaccaaatacaaaaaggtcgacgtttagaggaacactttaaaaatcacaagttatatacaaaataaatagc
atggttgacgatgagaaaggcaacaaaattctatagtcattggattgaaaataggaggatttcttccttaatgagaggtaacggaatgaatagcc
tcggcaaggtagcgaacgttggaagagttgataccagcaatgctaattcggccattcttggtcaaatagatatgccagtccttgctcaaggtggcg
acttgctcgggattcaaaccagtgtaacagaacataccgatttggtcagtaatatgcttccagctgtgtttgctgttaagatcattttccaaaatggt
acgtaaggcttgacgcatggaaataatgcggccagccatgcctttaacctcgttagcccattgtgaacgaaggttcttattcttcaaaatgaaattg
gcaattcgggctccatggactggagggttggagtaggaggcacggaccaagattttaagttgactctcgaatctggcggcttcctccggagaggc
agtcaaggcactaaaacatcctacgcgttcaccataaagacccatgtttttggcaaaagactgacacaaaaacatcgggatattatttgaagcaa
acaattgggtagcataagtgtcacgtgcaaagtcaccgctagcaaagccttggtaggccatgtccagtaatacaaaatgtcctttctcgcgaacg
acacggaggatatcgttccactgagcatgcgtgggatccacaccagtgggattatgagcacaagcatgcaaaaggacaatggaatcgtttgggg

cttgtgataagtcagtaagcataccttcaatatcaaggccttttgttactgggttgtaatagcgatattccttgatatttaagccagcattgctaaaa
acatttttgtgatttccccaagtagggttggatacataaatgtctttagaaggataatatttcgacaaaaatcgagaagaaagtaataatgcgcct
gttccgctaacagattgtgctgaaaccaaccgatcacgaatgctatcgtaaacgtcgccgtaagccatgcgtgtagcttccgtacgaaagcctgc
atctcctgtcataggcaagtattctttatcaaggtcttgtttcaataattcaacttccgcagtctggacgctgggcaagatgtatggctttcccgtgtc
atctctataggcaccgacgcccaagttgattttctttggaaaatcgtccttttttgaaagcttctgaaataccaaaaattggatcaggaggacccatg
ggcacatttccccaaccatgaaatccacgttgcaagttgcatccgacgaatttccctccattcaaagaagaatactgaaaaacagccttactagtg
actctacttgcagtagtatgtgctcgcaaaaccccagaaaacatatttagatacttgatgggtatcgtaagtctgcttagacgacgttactgtggat
tgcttttttttaaataattggactgttcgatgatcacatcacgtctttcgtttataacgaagcttatcgcacgctgacgaaattgaacgaagcacaaa
cccctatcatgaagcgatcaaataagattcgtacgatgatagcacaaatagcgataccaatgatatttgaaaaaaaaaaaaaacaaaaatttct
ttaaacaaaaataaacacaataaacaagaacattaaagcggttttaaactaaaatttttttctttcttttatatttaaatttatttaagagattaactc
aagtctttataaggtcaaacacagtaaggatgatatttgtattttgtcttgtccaaatcttcctaaaatgcgccaaagtcattcgaagcgcattgttt
tcataaaaactaagcaaacaaactaaagaaagagcttttggaacaaaagggaacaaatcaatgaaagaataaattaaatttatatctctataa
aaaactaaccctaaaaaatcgacatatagagtaagtctcaattgattgaaaaaaagtaaacaacgtgattcatcgtaaagtgtcaaaagagtaa
agtaactatcctctgtaattgcttttaaactttttgtttctttccatttgctttcatttgttattgaaacatcttagaagtagcgtctgattcagcgaatgt
gtcacgtcgtgtaattcttctatccaattgcaagctttactgacttcctcttgaggaactgttgtctgtgatgaatacaaacatttcaaaagcatctca
ttacttgcatattgtgtaacttataatgtaaaccaatactctacgactttttttaatctatttatttctcttgcatttaaattacttgtccgtagaaaaac
cgactacgaatacttgaaaccattttcatcctcactcatacatcctactctactccataggaaaacctcttttctatacaacgttcctgaacgtgttcg
ttattttatcccatgaaccataaaagaaacgtttgaaaaatatttatttaataatattgaaattgattaaagcttttttggggggtttcttttttatttct
actgtttttgattcctttttggctttctcgtttttgtactaccatgtccaaatctgatgcagtttctagattgctagcagagatttctgctcaacagaacc
aaaaccaagtggttccagagcttccttatcctacttccagtggcatcatcaacttaaacgaccctaatccatcattgggaactttcgttcgtctgtat
gaccctgcttactcgtcccagccataccaagattccttaataggcactactcctttagccccatctgcatatcatccaccaagttcttctagctcatc
aaaaacttcgtattcatttcccgtcaaaagctgctatgtaggcatgctcattggaaaacgtggcgaaaaactccaaaaaatggaatcagccacaa
agacaagagttcagtttatgcctgaaactccttcaaaaccaggtgagcgaatggccatcattactggccaaaccgaaaccattgatatgtgccgt
agggaaatatctcaaaaaatcaaggattatcaagatgtacttacggccacagcgttcccggctatagcacatggcgttggcggaaatcaccatcc
cacgtatgagaccccctgatccatttgttatacctgagcaccacatcggacttgttattggcaaaggtggtgaaaagattcgtgacctgcaagaggc
aaccgacacaaagattatcattaatacagatactgacttaatgaatcctctgcgacccgtgtccttccaagggtcaccagatggagtttccagagc
aaagtctataattcaagacatcattgtacaggaacaactgaaaataaactctctgtcgacaacctccaacccaaacaaagtacaaactgatgtta
tggctgttccaagagaatccgtgggaatgattattgggagaggcggtgagacagttcgtgaaatctatgcgaaaacaatgtgccaattaaaaat
atctcctgacggtggtccttctgtccctgaacgtgaaatcacattcatcggaactccgtcaagtgtggagttggctaagtgggatgttttagaaaga
ctagagcgagctggtgtgcctcttcgtcgtctcgctcatccgcccattcctcctgctgcttctatacctccaacgaacgttcctggaacggctatgcc
aatgccaatgccagaaccatatcctccttatggagattatgaaaattacatgaaagcgtggcagcaatatcctcctaatcctatgtatcccaagta
atgacgagaactttctaaactaaatgaagaaaaaagctactttttttcttcgcctaaaaatagaactacagattaaattatgcgatgttaatgattg
gcatatgtttataaaaaaggattcttcagatgatgctcatatcgtacatcttatttaaagtagtcagactatagaaatcgctacctttgctagcccta
cttttcgtgatttaagttttgatgaacatatgaagtcaaaagggtaaccgattcgataaaataatattctcctacctattgacttgcctcctatctttt
actaattacatgcaagaatcgttctaatcttccttaatcagtgctaaaaaatgcaaacatttttacttatttattgaattccatagaaccatttacgta
agggaacgaaacccagtgaagtctaaggaatcgcactatactgaaacaactacatgttactacgcagctttcgccttttatgtaagctgaatacta
ccattaccagaccttctttcaaacaaccaaatttttgaaacaaatggtaaggaaactgttatggatctggtttgaatagtaagcataagtaattttg
tagttttgaagtgcttttggcttactttaaccagatagaataaagagtgttaatggaaccacagtattaacatatatgtagggtcaagcacaatctg
gtggattctccaattttggagacggagcaaatgacgacaagaaaaaagaccaaaagaaggataagccaaaatatgagcctccagttcctactc
gtattggccgccgtaaaaagaaagctcatagtggaccagaatcagcaacaaagcttccggctgttttacccacaagtcgctgccgtttaagatta
ctcaaaatgcaacgcatacacgaccatttactaatggaagaagagtatgtacaaaaccaagaacgccttaagccccaagatgagagaacacaa
gaagaacgtaatagagttgacgagattcgtggtaccccaatgtccgtaggtactctggaagaaattattgatgatgatcatgctattgtttctacgg
ccggtcctgaatactatgtctcaattatgtccttcgttgacaaagacatgctggagcccggttgtagtgtattgcttcaccacaaggcaatgagtatt
gtcggtattttgcttgatgatacagatcctatgattaacgttatgaagcttgataaagcccctaccgaaagttatgccgacattggtggtctcgaatc
tcaaatccaagaaatcaaagaagcagtcgagttacctcttacccatcccgagttatatgaagaaatgggtattaaacctcctaaaggtgttattct
atatggtgcgcctggtactggtaaaacactgttagcgaaggcggttgctaaccaaacgtctgcaaccttttttgcgtgttgtgggttctgaattgattc
aaaaatatctgggtgatggtccacgtcttgttcgacaattatttaatgccgctgaagagcattcaccttcaatcgtatttatagatgaaattgatgct
attggagctaagcgttatgaagctcaatcaggtgccgaaagagaaatccaacgcacaatgttggagctattgaaccagttagatggtttcgacac

cagcaatcgtgatatcaaggttattatggccaccaaccgtattagtgatctcgatcctgctcttattcgacctggccgtattgatagaaaaattcttt
ttgaaaaccctgatgaagcaactaaaagaaagatttttaccattcatacttctaagatgaatctaggcgaagacgtcaatcttgaagaattaattc
aatccaaagatgaattatctggtgctgaaatcaaagctattgtttctgaggctggtcttctagcacttcgtgaacgaagaatgcgtgttgttatgga
ggatttccgacaagcccgtgaaaaagttttgaaaaccaaagatgaaggcggtcctgctggcggtctgtatatctaagaaatattccataatgatt
acaaattagttagaaagagatatatatcttgtttcaatgcatgttcttttacgctgtgataatggaagtatttgtgagtcaaaaatgtaaaggaaaa
cttttgttatattgtaaagactacaagcgttgtaaaccaaaaaaataaccttaatagtattggtaaattggtatcgtaaccgcggaaatttttattat
aaataagaattattcagatcaggttccagagaatgtatggaaaacgagggttccttattttacgttttagtatgaaagagagcatgagaaacagtt
ttcacataattttactttttcacgggcttacacaaagaatggtaatcttcgggcctcaaggcacggacagcatccttgaaaattgatagaggacaa
atggtctcatttcccttataaccttgtccacgacaggctcccataacgacatggttggagttgtaagtaagtcgaacataccaatcagatgaatcat
ctgaaggaagagtcgaagcagcgttttcttttgaaagaacgacttgcttactagagtcagcaggactttcggggtttccagcaatggcttttgggg
aagattctttagatgtatctttaaatagctctaattcaagatgagaagtaaaaggaggccattggtaatcaaaagcatcaagtgaagcaaggatt
gcagctaaggttacatcatgagctccatacagagccaaacggtccgaaggagcaccacgtttatcagccttgacttgagattccattcgcatgtat
aggtcatttaaaagacttcctgcgccaagttgacgcattaactttgacactttatatcctccaaaccattcttcagtaactgcgttcccagcatcacg
caaccactgttcatcaactaaattaggatgaagatcaagtcgattaccaatagcagcattaattgtgtccaaaagaccactaagacgaggatgac
tggagacaacacgaacaggttgaccgttcatgaactggctcatcatttccgatgctcttcccgcaaggagaggatcgtatattttggccgctctttc
agcaaattcttcatacaattgaacaaggcgggggcaattattttcatttggaagtaagttttcgtgcgaccaattacgggtgtaaatggtaggcac
gacacccgggcgtttttcatcaggataaagtccagcgaaaacgtgttgcaaagattcaagagcacgagccataggtgtagagcgcatataaaac
ccatcggcagtctctatcttgtctggtaaaaatccgagtttgttgatataccgctctcgaagatattcaccaaggttacgagtggtaatccttccaat
gtctgttaattcaccatgaatacaaacgccgcttttagcctcctcttttgaagaagctgctaaagctgaatccaccggagtctctgtttttcgttcata
tccgagtaccgaccattctttggctcctttgatttgagcaaaaaatcttctagcattgttacaaagcttccaatccttaggaatacctgctgatccta
acctttcttttacgggcgcacgctcaccatgtcggaacacaacctgcaaatatttgagctcaagctgcttaggaaaatccaaaccagcgactggat
ctggataatgaacagaagtagaagacatttgcgacatggtgttgaattatttcttagggatttttgttagtgaaaggtggttttggcattcacatgtc
ctagctaggtgggggcctgtactagctactaaggatgatgaatatggaaaaataaaggagcgactgaagcgtgaaaagaattgaatttcaatgt
tgaaacaaatagaaacctacttcttgttgagtatgctaaagtttgaacccgacagaattcaatgtcaggtgtgattgatagcttggttggtgaaca
agcttccacataacataaatttgtgattgctatgggcaacgaaagctaacggttaagttaaggaatatattgataaaaaattcatctttataaaaa
agaactggctcgcatgccaacaaacaagctacgtttccttgtatcattcaatctgttgctacttatatcgtataatggagtatttcgacaattgttact
tataaagctttctctagtagtaatagtctttcgttgcaattggtgtagattgtatactttcatttaaaaaatcaatcaagaaagcatcaagtttttga
gcatcaaaagacgtaccagagcatcgtttcgtcatatatcacttactcaaaatgagcctttgtttatatactaaaaaatgaaaacaaagacatga
atagtgaatattttcagtctttttttctttttttagaataaaagacatacggaatgaatttaaagaacaacggtccaaatcaatggtcattcttttttc
acgattcattaattgtcctgtttcgtatattcatagactctctgcaaagacctttactatcccatacagaaacatttccattattatcagtttaagaatt
tttcacgtactcgtttctgctatttttcaaccaacattctttccagaatttaacctttcgaagaagaagtttgagatatcttctttagtgacaactttgtt
ttctcttgcaaaaggaagcaaaaattccgtgagttcattctttaatcgattgtaggcatgaactccttcgattgcttgaacacggggaagccgatct
aaaacatgaagtatatcttgcatggtgctagacacaacttcatcaagtgaattcatgtcttccgcttcatcaccgctttcattttccatgtcagaatc
ctcaggttgctcataaatcggttcatcctctattagaataggcatttgacttttgcgaagctcaatacaagaaccacaagggcaaagttgctctcga
aagtgttcgtccagaaaccgaggaactaattgttcttctttctggaaaggcaaaacgtgctgacgattcaaaagacagttcctatacttaaatacg
cagtctttacagattacccattcaaatgactccgaatcaggcaacgacttgccttgattggaaacttccaagcatcgatcatgaaaccaatcttcac
acagtatacattgaaacataactccgctttcttgttcagggtcgtagaaggtatcacaggtacaaaactggccttgaaagttatggttatattgatt
ttccggagcacactcgtttgttgtctttcttatcttacagggctcttcgccggttcttacggttccacaatcacattggaaatgtcgtttatgaaataat
tccaccaattcatgatctgcgtgacagctgatactacaaccatagcaaaccccgttcaaacactctgcttctctttggcaggttaaacaagcatata
atgtctgctttaagtaacctttggaatatgtacattcatcaaatttataagggtatgcctcacgagcttcaagctctaattctctctgtctttcgacat
attgttccgctgttaattcctctaatgttgaatccatttagaaaacttcttatataaccgacgccaatgtgtaaacaaaaggcaaattttgtatgtaa
tttttcatttacagataattagtggagtctgttaatacttgtttaagtaaagaaagaagatttttgaatacggtttagtggtatatcgcaatagtgcgt
taagctacagtcctaggcgaaatgtttgcccaaaaagaaaagccaacaacatttctttcggacagcatctcataatcgatggattctttccggaaa
cgcggccgtagagactcggaaagtcttcctttagggtcggataattcttcaaccgggaacacacctcagtcgcttcctccctcctctcctcctccgg
aattttcagatgaagcagcagaagcgcttgttgaggatgaaattgaagatgttgacaatgagaatgtggatgtagacgatgaagatggtgaaga
tttgtttggtgaagatatggaaaggtaagaaaactatgcagatgattcactggttgaggattaaggaagaaagatcattgagtttttcctcatagt
acagaagaaaaactctttttttttttatttgcttgtccagaaagtgtgcatgaatccaagacctcaataagtgagatatggcatattaacacatata
cagagattatcaacagaatttggagcttgatcgatacgatttagacgagttggatgatgataacgaattagaagagcttgaccttggtgatcgca

gaattatagatgaaaaaatacagcgccgcgatatggaattagaggctgccgctggtcgaacaaagcctgcagcatttcttcaggacgaagatga
tgatttggactccaatatagggcttggaattccaagacatcgtcatcgtgtgtatgatgagtttgcacctaatgcgggagcattagatgaagctgg
cgaacttccactagaatccattgcagatgtaaaagctgatagtatcgccgaatgggtaaccctggaccctgtacgtagaactattgcccgtgaatt
caaaaattttctcttggaatatactgacgaaaaaggcacatccgtttatggtaacagaattcgaacattgggtgaagtaaatgcagagtctttgat
ggttaattatgctcatttgggagaatccaaacccatccttgcctattttctagctaatgcacctgctcctgttttgcggatattcgatcgagttgcatt
ggaagctaccctcctgcattatcctgattatgaacgaattcattcagacattcatgttcgaattactagccttccgacatcttttacgctgagagagc
tccgtcaatctcatttaaactgtttagtacgagtttctggtgtagtaactcgcaggacaggtctttttcctcagttaaagcacattcggtttacctgca
caaagtgtggtgctactttgggacctttctttcaagactcaagtgttgaagtgaaaatttctttctgtcacaattgttctagtcgtggtccttttgtcat
caattccgaaagaacggtttataataattatcaaagactcactctccaagaatcccctggaacagttccatcgggccgtttgcctcgccatcgcga
agtagttctcctggctgatttggtggacagtgccaaaccgggtgaagagattgaagtaactggtatttatcgcaataattttgatgctagtcttaat
acgaaaaatgggttcccagtttttgctacaattattgaggctaatcacatttctcatgttgataacaacggcagtgccgatgacgatttttcccttaa
ccgcctaaccgatgatgaagaacgtgaaatacgcgccctagctaggagtcccgatattcacgaaagaatcatcgcctccgtctcaccttccattta
tggacatcggtctattaaagttgccattgctgcagcgttatttggtggtgtcccaaaaaaatattagtggaaagcacaaaatccgtggtgatattaat
gttctacttttgggagatcctggtaccgccaaatcacaatttcttaagtatgtcgaaaagactgcccatcgtgctgtctttgctaccggtcagggtgc
cagtgccgttggtttaacggcatctgtccgaaaggatcctattacgaatgaatggactttggaaggtggtgcattggttttagctgataaaggcgtt
tgcttgattgacgaatttgataaaatgaatgatcaggatcgtacctctattcacgaggcgatggaacaacagagtatttcaatctccaaagcaggc
attgtgacaaccttgcaggcaaggtgtacgattattgcagccgctaatccaattggaggacggtataatacaacaataccgtttagccagaatgt
agaattaacagagccgattttgtctcgttttgatatattgcaggtcgtgaaagataccgtaaatcctgagatagatgaacaacttgccagctttgtg
gtttcaagtcatatccgatctcatccatcctttgatcctgaccttgatgttttgacaaaagcacctattgatacaggtatcggagccaaaccagttcc
tcaagatttgttacggaaatatattcattatgcaagagagcgtgtgacacctcgtctactacaaatggatgaggagaagatttctaggctatacag
tgatatgagacgtcaatctttggttacaggaagctatcccatcaccgttcgtcaccttgagtctgctattcgtttaagtgaagctttcgccaagatgg
agcttagtgagttcgttcgacctgtacacatagacaaggccattcatgtaactgttgattctttcgttaacgcccaaaaaatgagtataaaacgca
gtctttcacgcacttttgcaaaatatttactgtgagttatatattatttactcggattgtttatctataccttcgttcaagttgatattcagctttcgttta
cccttttcttctttctttatattgtcatttaattccaatcttttgtgtttttattagaattcccctgtctttctctctggtataatagtattgcattcatcaaa
aatatggcatgcatttacctattgtgcgtatatattctattatgaccttttaatttattgatgcaaggtcaatatttaattattcttgcctaaaataaga
ttagatttcgtatatcaaacaataacaatatgtttctgctgtgccatatgaaaatcaaaagtactatttagtctatttagctttttttattttgtaaaagc
aaattcaatccagctatattttggtactctactgacttatgttccgtacccttactgctttcctattctaagtcttcaataacaagaacttaacgaaaa
caccatggcctacagcgggcagtatggacaatatggttatcctatgaatgtagaaagaaatgttgaacaacaaaataatgtagtttatactggcg
aaaccccagatgcagaattggtagcacagatgaatgcgcaaatgaatcttgggcctcaaataccagtttatcctcatgaaaaaagaaggacacg
cagacacagagatgcgcatgcctatgatatgtctttaatggagccagaagctaacgttttaccgccgatgacaccttccactcctttaaccccggct
acacaaccgcttgatacgactggcttaagttcagaagctctacatgaagatacaaatcttctagatccaaagtcattaccttctgttccagatgctc
gctttcatgatcagcaaacatacaaaaactacactttcaactcaatggacaagactaatccacctttgtcaacaaccgactttgttgggtatgaac
agaataactcgtcgtctaaatttgttcaaatgaccacttatgctattccttcttcgaatgatgttcttaacagttcaggtgtaccattgggtatgatag
tccaaccgtttgctgaacttcgtcctgatgaggcccctgttcccgtggtggattatactacatcgagtcctcctcgctgtaaaaaatgcagaggctat
atcaatgcatttgtccaatttactttgtcacagagtggttggacctgtaatctttgcggtaccgaaaatagtctcagcaatgaagtttacaaccctat
gctttttacaaaccaacaaaatgctcttaattcacaacctgaatttaacgtgggtactgttgatttcgaggtcggaaaggattactgggcttccgaa
gaaacaccacaacctgctcaatatgtctttgtaatagacgtctcatatgaagctatctcaaagggtatccccaaaatagctgctgaggccatccga
aatatactatatggtccaaccccattacatccgtctattagagttaccattgttgcttttgatcgaaatgtacactttttttatctaactcctaacttgg
aacagcctcgtatgatttctgttgctgacttggagcatccttttgtcccttttaaaaatggtttatcagttgatccaatggaatcaagaactgtaattg
aacgtcttttggatacccttcctataatagtagaagacataaaagtgccagaacctgccgttggtaatgcaatggaatgtatcaagcaccttctta
aggaaactggtggtaaagcatgcttgtttgttagtgcacttcctacaatgggacttggacgactcagatatcgcgaagatcagagactttacggaa
gccctgaagagaaaaacctctggaatactcaggagaagtggtactctctcttagcggacgagttagtaacctcaggtatctctattgatatattctt
cactcctgctgcttacgttgatatcgctactgttggtagtgtcgcaacattaactggcggacaagtatggtactatggaaattttgtaccagaaagg
gacggaccgcgtcttaaacaagacttatatcgaagtgttactcgtgagcaaggttttcgcgtaatgcttaagacccgttgctcaaacggtctacga
gtcgctagatatcttggtaactttttacaaagaactccaaaagatattgaatttggtggattggatgctgacaaaagtattggaatgctatttaactt
ggaaggaaaattgaataatgcgttggacgctcacttccaaacggcaattctctatacaacaccaatgggatctcgcagggtccgtgttataaact
actgctgtgctgttacttcacgcaactacgacactatatccctcgcttctgttgaaccattggtgggtatattctcaaagattgcttccatgaacatg
gcagtggagtccttaaagacaatcgcgactaaacttgtcgagggtattgtcaagctcttatcttgctatagaaaggttgcatctagtcgtttaacac

caggacagttagtcctccccaagcatctaattctcttgccaatatatatgttgtctgtgttaaagtcttctgcttttcgaagtggtagtattcacagcg
acatacgagtatcacaatttagatttttcgtgcatctcctttatctcaactaatgcttaacttatatcctcacatttatgctttgcattccttacagccg
aatgagtgcgtacctaacaccgataatccaggatgcacacctattgaatacccattgggtgtcagggcatctagaaaatttattgaggatggggg
cgcttttctgattgttactggtcagaaagtttacttgtggttgcaccaacgaacgtctcctttattgttacaggatctatttggtgttaatcagttagaa
gaagtcgacgcaatgaaagcttgtgaattacctgaaatggataacttactttctatccaggttcgaaacttactttcagctttgaagctgcagtatc
ctcaaatgtcaatgcagccacaaattgtccgccagggtatggatggaagtgaaggtgaaatcttttctattttagttgaagatccttcaagagaag
gagttggatatattgagtttttaactatgatccatgaaaccttgcatagccaaggttataagtaggtcttctacgggatttgctttgaattgataact
ctcaaaaggaaggaaatatgtcaaggtttgctatgaaggaattttaagttttattaaaccatatcaatgaatttatagatgtctagatactgcaag
atatcactgtaaagttgttgtttttatgaatgtgtgacattgtatataagttggctacgaaaatcaacctattgaacattgactttactttttgataat
ttatgagccaaatatacggctgaaacgcaatgtttactaattgaagtcagatgctacaaattcaattatagccaaattcaagttttctatttagggc
taagagagtaacgagcttgttttcaataaataatgtctatgttataaactgaattcagcgaattgtacttaggaaattgaaataataagaaaaaag
caatctttaagtttgcagtagattttcagattgattgatctgtcttgctttgacgaatactttaaataaaagaaaatgaaacaagaaaagaaaacg
tttggacgaaatctttacaaacaaggcctatcatgtatactaacgtaattattgtataatattttactaaagagggtatccatacataatagtcacg
aatacatatctcttggttatacaaccaactttccaccttttatgactctaatgaaatatatatatacatatatatattataaataataaagtatagtta
ttctcaattcgtggtaaaacaaaggaaatttatttattaaggcacagattcattgtgcaaaattttacaacattaactgcattccatgggatcaattt
cgattaagggccttgacggttttgattttagaccgtggcgtaaacaaaatcgaggagattacaaggagttacatatttatgattttgataatgtatg
aagacatggagttgatcaagtataggaagctaatttacgtttcagacattatttcaaactcctttacccaatgcgaacatctggagtaatcaagcg
attcgtgttttaatgggatttaatagtatgtatttatgtgagcttgcaaaatggttgaatagacagcggtggtaagattaagctttcgtaaccctagg
aaaaggattgcgtccttaagagtttatagaggaggagtctcatggctttttttgttccttgtcaattagtactcttctacattccttgaaagcttattac
cactgctttcccaagatttattttactcgggtttaaaagtaccattgaatgttctaaactaacagatatatagttctagcaaacggaggatggttctt
tgatcctcgggtgttggcgtctacgggggaaggcgttgaaatagaagagaagcgtgcttgggccggatggtggaatgaaagaatgtaaagtcaa
cgcttctatttctttagattgtttactaaatgtttagtgttgcccaagctagggaatccattcgtcggcaggatgttttggcggtcttactaactggtcg
taatgaacggttcaggaagattaccgaaaagatagttaaaagcaaaggtttgcactttgatggagttgtatttaagcttcaatgtgcgaccggttc
ttggccacagacgttgactttcaaactttggtttttgaacaagtttttctgcagtttcccaatgttcagtctctacgagtttatgaagatcgcccttct
catatagaatcattttctgcttggctagctaaacgagcagatgaagttgaaaactttaattttgagattattgcagttgccgaacctcctaagtactt
aaagtataccacggagataaaattggtacaaaacatgatagcttctcacaatgtgaaggctccttctcttagactgccaatgtacaagtttgtaaa
aaatgttacaagctccgttgttaccgtttctacaactgagctgcatcatcttcgaaatgcgtttttcaatgttcccattacagacattatggaaagtgt
cagcgttaactcttccagggtaaactctcccgataatgagcctcaaattcctgaacccgatttgcctccagataccccgtatagctcttttcctggag
aaaattggattgtaacggcattgggccgctatcgtgatgaatattgggcagttcgtgcagaagcatgggatcatcgctcacaaggaagtatccgt
gatccaaatactgcaaatattccctcactttatggaacgatcctgtacatttccatgtcaacaggaatagtgacatccgagatttctgatattggtg
ctcaacaatggaaggttcttgaccccaacgaacgatggattatgaagaccaatattagacgagaatgtacacatgatatccgttacaagcgtccg
tataaacctgctcgtaaatatacggatatgatggatttggaaaaccagcgtgcttctccgtttactggttctatgcatcgaccaatctagtttggcag
cattcaatgattgtatttatttcctttgataatgactttttggttctcaattcagcccatcctttaagtttggccaggcggtttttatttttttttttttaa
aaaaaaaaacaaaaaagagaagtgtttcggatttttatgaagttttggttcaaccctgtctatgtctactccttactagtaattaattgttctccatt
tttcaataaggatgaaattggaaatcctaagtatagaatgaataccggtctattatttcattaagcttagtttgttagcaactttaaaaacacccaa
ttgtaaaaaaataaaaaataaaaaataaaaaagaacaagtcataaaagatttgaatcatatccaaaagtataatcgtcagtcaacagaagaaa
agataacatgagaaatcatgaaaagtctataagaataagttcagaagtttcaggatttaagtatacaaagtcgtttaaatgttaaaggtttgcgc
ggtttcctccagtaattttatcttgtttctttcgattgtccacaaaccaagatgcaaaacagagaataaaaaaaagaaaccgctagatatccaacc
aaatacagcactaggtacgccgactttggatactgcttggtgtgttaagttcaacccaacgctgtaggagtttgcagatatatttgctaaacccatt
tgaagagcaagcatgaaacaagcagctgcgctggtaaaaaggcagctaatggaccattgtcggatattggatggaaagacgtacttgaaaata
gtaagcacgatagaaatggtccctataaaaacaacaatgcctaacagcactggatgtacttgaagccatgtaaagcgtgccgcatggtaggcca
taccgcttgaagcgtcccaatgacggagctcatgacaaatgttttcggtggcattggaggctttactacaaaccccaaaaaatccaacatctactg
tggacttgggagatttaatttgtaagatcttagagaaagtagatggtttagaaaggcagccacaacagaggaaaatgagaaaaagccaggcga
tccctaaaattatgttcgacgttaaattaggattcggaatataattatagattctagctataaatccaatccaagtgaatttcggtcttattctttgat
cctgtttcatttgtttcaaattctatagaagatttcaagacacagaagaaagtactgatatattccttgagacaacaggtttgatttttctcgtttcg
tttaatgaaaaagaaaggaatgatgaaaggagccattaatgcggatctctcatttatattatacccaagaacaaagaaaaggggcaggattccg
tcatctaacattttctttgttcgaaagtcttttggagtttcatagatatagtagtaatctgactgtaaaaaaatacaatgcttattaatggttgattttt
atacgtatgctcttaagtaaacagaatctcatattcttgttaatgtagtgcaataacccattatagcatgattatgcttacatttagcatatattatgc

ctatatgcttattagttttaacattcgaccatatcatgtatctggttggaaaaaaaagtacttttgcatagtgattatttatattcggaataatgtgag
cagtaaagtagaataaatatggttgtatttcatttttattgaaaatcaatttgaggttcctataatttaagtgaattagccaacgcttgacacatgca
ataggtctcctaagtttctcaaagtaatttgtttattttgaaaacctcgtacccaactccaaagacaaccaaattggagccttgtgaaaacatgcct
cccacatcagcaacgaatgtattattctccgattcgactttccataatccctcccgttttgcccaggtcaatattctttgatcaacggatgcagataa
aaataagttttcttcgagttgaagtattcctgttacactagaagcatgagcctctggaagggagaacttctcaagttcttcaacataaacggttttc
atctctgtatcaatatttagtttcagtgccatgtgacaaacggcaccatcgtcacctcccgtgaaaagagatacttttctgttttgattgttaaaacg
aacatctaaactttttatacaggattggtgtaccctgctagaccagacacactgaggggttttctcaacatcttgtgaatgcaaatcccataacgtg
atgaaaccatccgtagcagttataacagagtatacacgatttttgaaaacaaatagctcaacggaaacaagacaacagcttttgtagtcccaacc
tttcagaaattggaagtcacgctttccctcatcgtataaatatattcttgaagacgaatccgaaagaatagccgtgattaaccaaatatcctttccc
caggatgttgcctttatggtagaaacatcagtgatccgcaaatccccatccattttgctctttgtaagcgttttcgcacaggtagcttcatgtaagat
acgagctgcagatgatttttacctggtccttgtactatccttagcgcatttaattcttccaaccctccacttgaaaacaaaagctcatcctttgccca
gcaaagtgcttgaatcccggaattgtgcactttcacgctattcagtgtatttatttgaccttccttcgtaaaaagggataatattatatttgtatcctct
gcaccagatacaataagagtaccctccgggttaaaactcatcgccctaatttctcgaccatgactcccctttctcaaaaccttgttacactctagta
aatttcgataggaatcgtttacaaaaatttgattggatttatagaatgcaaaagcctgcctcgtttggttcatagaaaacgaccatggcctatgag
aaccaccacattctaaagtgtacacatcggttcccgtagtttcattccttaagaagaaattcgatgcatggaaaccccaaacccacaaatttttatt
gacttccggatatccaaattgaattccctattttgttagtaaaagtttatgataaatgtaaaacataccccacttaggattccttcaattgccttctct
gaagcaatctttctaagctcaaccggattcaatttcaaaaggaacatattcacaagtccatccctgccaacagttttcaaattcataatgttctctat
atcaatttcaattatgatggagtatatggtatcaaactcgtgcaatcttctgaagcaatacaaacaagttggctgcccaattttataaatagccaaa
gaaccacttcgagatccaataaaagctaaatcattgctaattgtgagacaagttggatcaaaggtagcaggaaggtttaacatcaatgacatatt
cattataatacttccgttttctatagacaactgatgtaagtagacctcgtaggcattccttccgataaaggatttagaaacaaggtaatagaaacct
ttatgataagctgtatataaatgctgtattttagaggtatgaacctttttagcaaagcaagcggaaggattatcgtaatcaaatattactagggatc
cagttttggttcctaaagctgcaagcttcatgttttcccaagaacataacgcacttgcttcagaatccaaagaaaaagcaggcattggtttccaaat
cttgttatcgagaagactgatttctctaaagacaaagttagtctataactaaagtcgcatacaacttacccgtttttggcgagacatagcaatttatt
gacacttaagaaacagaaggaagacagagaaacatcaccggtgaaaatagatgaaatataaaaggaataagtgtcggtgtcattgttaacttg
ccattggcgaatagcgccatcgtttcctccagtgaaaagtaaatcatgtttttggatgtagctatagaccatacgtgttttccacgatgacctgtaa
atgtttctgtattatccaacttcacaccgtcgtaaacccattttcgtaaagtgaggtcctcagatatggtaactaaggaagagttagacaaaaagc
gacatttccatacacgcgctgtatgaccaaatccagtagcaagaagacgattttgtttctttaattcccataatctcacggttctatcttcggaaaca
gtgctcagatattgtccgtcatcagagaatcgtatatcgaaacacgcaccctcatgtccacttaaagaatcaaatgtaaaaacaacgtttggagg
attattgatagagaaagaccacaaattaatttcaccaaaagctgatgcagaagcaacagtaatgcaatcgtctgccaagtgtagggcagcaaaa
aatagaagaggactatttcgacaagaaatagtacaagaaagcttccaattcttatttaataaatacgaatatattagcacttcatttttggatgtca
caaaggcaaaatcacgttcggtatttaattcaactgcgttaaaaatccagtcggtagtacgtataaaagaaatctcccatgttttccagtctactag
ccctatattgtaacctccccagatgagcaacccatctcgacatgccagcatgccatgaattcgatttcgaaaaggtaatttcagtactttccagcaa
atgttgacatcgttatgggaatctaagttgtatattcgtaaaaatgatccttgagcagcacagagatacttgtcatggtcaaacagaagtaagctt
gttactggtcctaagaagttaactgaattcaaacaaacttgcgtcgcggtttctgcgctaggcattgtaaaaacaaaataataatttaaatatagc
aatacgacagataattctagaaaagagcgtatttaaagccaaataaaaccgtttacctaattttcccagtataatttctttaataatatcaacgaat
tacaaaatagtttaccaaaacatatcaaaaatggtatctataattaaataagtcaaatgagtaaactttgagttttcttgtagtctgcacctggaag
attcgtttacggtacccaactgcagtttaatggagcaagccaagatttgcggttctgtttagtttaaagcaagtgagcttttgcaaattcgtcaaaa
caacattggaaaaagaatatgactaaacaatttttttatcttgtttcctttcaatgtttacaatttttcgcagaataacaagcaatagaaacaacta
ggaacttgcacatcatttgaagcaccactttcacccaacccttcaacaggttttttggtacactggaaaatcagaatggaagcccgtattacttttg
aattatctgtattctcagatttagttatctgaaatatagctggtttgttatattttggaaattctatattcggcaaatattgacattattataaagattt
aacattaagcgaacgttgtggggacagggtaagaaattaacagaaagacgaaactaagaaaattctaaattagcagtagcggcagcggtaaat
attgaagctaaataaacagagaaacacctctgttggttaacacaaataggctgaatagtacttccttttcaacgctacttctgtcactaaaatgaa
taaaaacaaggtaacttattattattgttattattattattaattataataattattatatttataaagttttttttaccgattggatttgaaaaccaatg
ctcccaaagtatttcatccgcgtcgcgaaattcagctacaccaatcatccttccccaaaactccccctcttttttcagtcaagcaggataaaaagaa
attactctttaacaccattcttgcatttatgaattgaataaaaatgttgaagcttccgattaggcagctagcagggcgtttcccgcaaattcgtaga
agcattttgggatgtcgatttgctaccaccgatagctcaaagccagcagaatcagaagcatctttacaaagtcaacttagtgaaaatgatatgaa
aagattgaaccagattcgcaacattggtatttctgctcatattgactcaggaaagacgacgtttactgagcgcgtcctgtattatactgggcgaatt
aaagacatacacgaagttcgcggcaaagataatgtcggtgctaaaatggattcgatggagttggaacgtgaaaagggtattactattcaatccg

ccgctactcactgtacttggagtcggaccctcgaccaaatacagagtgagaaacaagaaaatagtgttcccaaagagtttaacgttaatatcatt
gatacccccggccatattgattttaccattgaagtggaacgtgcattgcgtgttttggacggtgctgtacttgttctctgtgctgtttccggtgttcaat
cccaaacaattactgtggatcgacaaatgagaagatataatgtgcctcgtgtttcattcatcaacaagatggaccgaatgggtgcagatccttgg
aaggtaattcaacaaatcaatacaaagttgaaaattcctgctgctgcagtacaaatccccatcggtagtgaaagtgaaatgaaaggtgttgtgga
tcttattcacatgcgcgctatttacaatcatggtgaaaaaggtgaaaaggttgaatttactgatactatacccgaagatttgcaagggcttgctcaa
gaaaaacggtcttctttaattgaaatgttggctgatttggatgaacaggtcgcagacatgtatgtaatggaagaaacaccatcttcggagcaattg
atgcaggccattagaagagtaacccttgctcgcaagtttactcctgttttaatgggatcggctttgtctaacattggcgtccagccagtgttggatgc
tgtttgtgactacttacctgctccctatgaagtagaaaatgttgcattaaatgcagctgaatcggaaaaacctgttaccctggttccaaataacaca
aagccactcgtcgccctggcatttaaacttgaagagggacgctttggtcaattgacctatttacgtgtttatcaaggttccttaaaacgtggtggtta
tgtgtacaacgtaaactcaaccaaaagaatcaaagtctctcgtttagttcgcatgcattcaaacgatatggaagaagttgaagaaactggtgctg
gtggaatctgcgccatgttcggcattgattgtgcttctggagatacattcactgatggttccgttcattataccatgtcttccatgttcgttcctgaacc
ggttatttcgttgtctttaaaacccaagagtaaagatacctccaacttttcaaaagctctgaatcgtttccaacgtgaggatccaacttttagagtta
aacttgatcaagagtcgaaagaaacaatcattagcggaatgggtgaactacatctagaagtatacgtagagagaatgcgtcgcgaatacaagg
tagaatgcgatacaggaaagccacgagtcgctttccgtgaaagcttaaccaaaaaggtcccgttcagttatctgcacaaaaagcaatctggtggt
gcaggtcagtatgccaaagtggagggatatattgaatatatggatgctaaggaagatgagggtggtaatgtggtcgactatgaattcattaataa
agttaccggtggtgcggtcccctctcaatatattccagcttgtgaaaaggcatttcgggaaagtcttgaaaagggtttttttgattggacatcccata
aaaaattgtcgatttgttttagaagatggtgcataccatccggtcgattcttctgagcttgctttccgtatggcaacaataacggcattccgtggtgc
attcttgcaagcagatccaatcattttagagccgataatgaacgttagcattactgctcctgttgaacatcaaggtgctgttattggcaacgtcgat
aaaaggaaaggaaccattgttgacagtgatattggagaagaggaatttagtttgttggctgaggttccattaaacaacatgttttcttattcctctg
atattcgtgcattgaccaaaggaaaaggtgagtttactatggaatttttgaaatacttgcctgcaccaagatatgttcaaaaggatttaatctctga
atacaataaacagcagcaaaagtgaatttaagtgtttgttgttgagctttctttttaatataaaattacatcaaataggttttgatcatcgcatacca
aaattatataatttcattcttcatttgataattgatcttctctagtgtgaataatataaattttgcaattttgactggaataaaaaattaactttcatta
aggattttgaggaaacaatcgtctatatagttttcttttattagaccatagtgtggaatgcaaaggatgaacaaggctgataaaaaggttgaaag
gctaaaagggaaggacataaaaagtaggttgagtcggtggtatatgccatgatatatacgttcatagcgatgaacaagttcgctagacaaaattt
tgacaaatacgagtcaaacgaaattaaaagtaaatcacgaaagaaagaaagaaagaaagaaagaaaggaggaggatggaacagaaaagg
gaaagccataatacatacggactttgtctaacaaaaagagtagacggaattcagactatttgtatgacagaacactcaaaggacgcccaaatct
ttttgggcttccaaacggcgttcattttcagcatcttcataaagttgcttctcgctggaccaacgattggagatttttttccaagtccttagggatttcgt
cgacttgctgaagaatttttttcgttttcgatgatttcactgcaactttggaaaatgaattgattccaaccaggttgtaaagaatttgcagtccttgca
atatccatcaactccaactccgtttctttttctaaacggctgaatttgcggaccagatcatcgccaacaccacctcgattcagtaacacgttgagag
ccattctagaggattttagcctcattagctgacgcagagcctccgtcgcacgcaaaaccagtccagcttttatgatggtatcagagaccggaggat
tttgctgcaagagctgaaaaaagtagcttctcgcgtagtttttaccaaaccattcttcagaaacaaattcatttgggatgcttcttgactttcgaaca
aagacagaaacgccgtacatggcgccaattagcaccgcaacatatatcaatggagcataaatagacaccattttccagtaatatatttcctttttt
tccaataaaaatcacccaaagatctcctagttttccatataaaacttacctttgttagcaatggatcttgcaaaggaatgggttccttgtagaaaag
gatagatgatagctcgttaaaatgcggtaaagtgcataacgccctagaatgagtacttgtcaatgcgcattaataaacatagaatcgatgatcgt
aagcgaagcaaaagaaaagaagaaaacccttgatgcattctcgggtatcgtatgaaatagaaataggcacttctacagcgatcgtgattgatta
gagaaaagaatgcttctataatgcgacaccattattttcgtttgtttttgtttgcgtttcaagagcctccacccttaccaaagattgataaaaaacta
aaccaataaagttgaacaataaatatgaataaaaaagaaaaacaaaggattgttctgttcaataaaatttaacagaagtcagtagaaaccccat
cctcacgaaatatgtcattaatgtatttactttgtatgatgttacgagatgtaaatatatgtatgatcaagaatacgcagtgaagtacaaggagaa
caagagtatagagcagcccttgagttgagttgacgtgacctgaaccacaaagttaaaagaaaaacaaaaattaggaaaattcgtcccctatgaa
aaagcctgcttttcttcggtttgttagcgttgaagggttgaaaaacctttctcggcttggtattttaatgactacatcattttgcttggccttattgtttg
tcagtgtaagtttagttggttctttttttttcttgggagacacaaaaacgttggtagcatggattggaaaccatgaaaacttttattgaaaaaaggcg
actggttgtgcttttcatttcaaacatcaattgtagttaccttcggttccaagaaatggtgcttgatgactgtaacatagactataagaagtgaccaa
ctctcgtaatctcataagaaagggcagagcagggatcgcaaagtgttcataaatcaaacccatgtcctagcccattctatacgtcctttcaatacc
caacgatttctccttcccttccttccttccttccttccttccatgaaaaaaagaattgtagaaactgagctaactggtatagagtatcgtggattaca
acacactatggaaaccaggaccagaatccgcgtttactgtggctgaaacatactacacaatgctagccaatgcaagtgcttatcataaatattttt
tctacttcgttgtcggtttaggcatggttttccacctcattcaggccaacagggtaacgggtgatgaccgactgtattattattccagcactcttttgt
acattgcggcgtctgtgatatttatcgtgaatgtttcccccgctatcatcactgcaaaattacgtcgatatgttcaattcgacagaaacatgcatttt
atggttctcggtgcctcccaagttttgattgcctttctcttgaccggcgtccttattctccagcttggtcatctatttgctcttcacgtaaacaatatagt

tcaacttgagattaccgaagaacaggctaatgaggctgccgaagctgaaatttcttctaccgacaaagaatacgtctctaaaagaaacgcaagc
acctctaaatattccaatcaagtaagccacaaaaaggataattagccacccatataaaaaaagttttgaactacttgtattgattattatttttctcc
ttttttggcttctatttacttttctgtcttaggtttttatgtatgaaagaatgaactcatcacattacattcctttttttcgtccttcatctcatcacctgtcc
taaaattccatttttgttacaatctctcctataaaatcactgtattccattatactatttcattttctttttactcatcaactgtgctttctcattatatcta
attctttatagcattccgggtaacagagcattgttactcataccaaaggacaaggtaagtttcttgcaagatcgtgtttcttcctgaattggtcttggt
gtagaaagaatcgaaaaattcccattcagcattctatttcttctactaatattcagattcataacatctgaattttcggagcggttctgaaagtaga
aaccttcaagtcgctaacaatatgctgcagttattcgtttatggtccgggacttggcttaccaagtttagatccaagttgcttagcagctatcttgta
ctgttcacttgctgttcctaaagatgagtttgtaatttatcaaaccgcaaatgccagtatgagtccgacgcaaaagctccccgccttgtgggacgga
catgcgtggataggatcgttaaaaaacgttttattttatttgaagcagaaaggatacgatctggacaacgacttagacaaagaggccatcagtaa
aattaccgcgttttcttgtcttttggagagaaatgcttatgatttatggcttttcaaagcctatgtgtgcgaagacaattatgttcgtgtcacgcgtcc
cgagtggtccaaggtgcttggatttccttataactatttgactccgaatgcatttcaaaaacacgcaaaagagcgcctgaccttgacactaggaat
tagtgatgataaaacctcttacgaagcttcaagaatgccaatttcccatagatggacaaatgcctctcgccagaaacaagcattgttacaagccc
aggcttttcggattagaatgtcttctcttgctcgacagctgtacggctcattggaacttttgattggcgattctgaatttttatttggtgaaaatcctac
ttctcttgattgtctcctctatgccttcctttctttccacacgcttaccgaaaactttgaccaattaaccttacgaagtacaatgcaatcgtctgccccg
aagttgtttcatttgctcgaatccttaaaagcaagatggttcccatcttctccttcaaatggcttactctctaaagtttccatcgaaaagcaacctca
aaaccttctagctctagcgcgacttgcatggaacaacgtgtctacaaaagttgaagaagccaaggcttctttccgtaacgtttctatttctcctcaa
aagcgggtatcgttggctagaaatggtttttttcattctcgcttccacttttggctttgtttggttcgttttagcaaacggcatagtagtcattgaaagc
gaagaagatgcggatttggatgacttgagagaggaaccattccctacgccaaagactgaatttaggtccgaaaaaacagatcaagctctagatg
acgaagttagaggcgaagcttcttcacagccagaagaaaaagagaatgatggcttgcaaacccccttctatgtcagcagaagatcttttattcggt
tttgaaggtgaagacgaagacggttatgacgattgggaagaagaggaaatggatgatgaagaggagcaggattttgatgaagctgatgctgaa
atagcttaattgaaaacttacggaatggaaaaagatctcatttttgggtaatgacacagatttccaggttacttctattttattttccataagagtag
ctatggaattaataacgcttgttaagagtcaaatcaaatataaataaatatatatataaaatataatcgttaataatacaaggtaaaaaagagtg
tacttgactataagagaaagaagaaccgctttgttgtaaagtatgaaagtatcattctaactcacaccatttcttctaccactcttctgctgtccgag
ttaccctcatctatttcaatttgagcgtacggaatgtcagcggaagcgtcatttaacggagggaatatttgccgataacaaatatatgcaatggtc
attcccagtaaaccaccaaccaaaatatcttcttggtgatgacggtagtcttcattacggctgatacctatccaacatgctaaagtcaatggaaga
aaaaacataacgatcgcccaggaagaagtctttttgcgaaacattcttaattgagaagcaatacataaggaaagataaccaagtcctgagaaa
gaaaaagacgtatgtccggaaggaaaagatcgtaatccgtcttgtaatgtactactcttccaaggtgtagtacaaacttgaaggtctacccaccc
cgtcttgggagatgaactgagaggattgcagcgagacaaaaagtctggacgaggtcttcccaccgtatttttgatgatggttacgatgagaccac
aaaccatggtggagtaaagtaaacccgtcaagcttttccagaacaaaagagggcttttcctgtatctaccaattccataaagcgtaaagccagga
aggagaagggaaatcagctaagaagcaatatcagttagtaaggaataaattaaggaaggaatagaaaaatgaactgtatatggctctatgaaa
tgcaactaggataaacataccccctaggtactttgtaggtactcgttcatgctcggcatgaggatgagaaattgttaaatcctctaaagaaaattgt
cgctggaacggaagcattgtaatgtctaaaatgaagtaactcaacgaaacaagaagcaatactgcataatctccgtaaaggatccataattcttt
tattctttcataattcactctattttcagcagacatcttggcaatgcagtactaattgccaagtgtgtaagtggataggaagtatgtcgagtatgaag
taaagtctaaaatgtataaataacaagacaatcaaaactccttttgtaaagatattaagagcaattgaaaaattaaaacttggaaagatccttttt
aaacgttaaagattttgagttgggccagtataggtatgtctatgatttacaagttttccagtatcaacttaacatcatttccatcagcatgttttcctg
taaatttcaatttacatttatattattcttacttgaagctatttcttagagttcgttttcctctaacaaactgattcgataaatatttacactctcaagtt
gcttcctcacttggaatacgtgtagaatgaatgcacgattgaaagagctttgcttcgctaacgaactctatccaaaagaaaagataaaaaatatt
gaatatcatgaagttttgaaacacttgccaaggtttattatcttgaccatctcatcaagaattccccaaatacttcgaatagatattctttcacaaaa
accacaaattgaaatgagagtctttattcgtattcctgttttcttctttcgttggaaaacatattcaatgtctttctgccaacttgtaaacatgtagcta
tttaagcttattgtaagcgagagaaagcttaaaggcaaatttcgtctgaatgtttccatagtgactgatgatccagagaatgtaaacaagctcatc
gtatatacttgcaaaaatgacaacaaataatgtagcaagtccgaagacctctaacgcttttgtatatacgttaaagatttttcaaagagtcgctta
gctctatagctatttctttttgcaagtatacatacgtatgtcgatgttgtcatacaatctcatgtcgcacttcttcttgctcaacgaaacatccccatcc
ctatagaaatttttcaaattgaacaagctcttccaatcactttttcggcctctgcgtagtaacatgaaatgcttcttggttgttggctcacgtaaaga
ggccattgcgattaatggcccccaaacgttgtaaagtcaaaacagtttgagggacagattaaagtgcgacttcgcgattatgaagttcaagatc
atgagtatttttcaaagtccaatgacacatgctctatcatgatagctggacactttgttcctacagaaagtgtaatgactgccgacgatattttgttt
ggtaaccaatttgataaacccttacgagatgttcttcccgctggatcctcttacttaatgaaatgtcttaagctggtggacccagcgcttgagtatga
tttgtacagtgaccaaccatgggctttttctccattttattcgaccatgaccaagatgaaaacggctaacactcagctccctctagagtatttcgatg
aaggttcatccaatagaaagaaagaaatgctagatcctgaaaaccgacaaaaattaccaatcaatccaagtcgatacttcgtcgctgacttttgt

aatcctttttttgaccctagttccatgagcatctccattccatataccaaattgaagttttccatccaaagctattattctgggcaacctttacgatac
gtctgcaaaactaggacagatgaaccaatctttgctatagagtttgatttgtaaatcaaaacaaagtcaataaaaagttaatgtcaccatctagtg
aactgttatccggtcatcagttatgtttataagcactttctgattaattaattattatgtttactttgctccaatagcctcagattgtaagtatactgtgt
acagtagaaaaaccaagatttagattctgtcacttcaaatgctgaatgactgtaaaaatactaccatagtcaaagcaaaaataaactctagttat
tcataactgtaaaagcttaaattattcaaggtcataagtggccttagttcatcttgttggagaagttgactttcatcaaagtcggtttttagtgtcag
atgtagctcttcttttttttcattcaatatcagtctattaaagttttgacctactgatacaagaatgaattaagtaggtgtcaaatttaccgggcacg
cgattcatcaatccattttccatgaataccacagacaaaattgttaggctgcgacacaatttgaaatgtcacgttcgaattcattttaacaagaaat
atcctgtaaatattaatgatttgttattctccgttcagcgggacttgcacgcggttgctttttaaaccaaggaaatatccgtaccatctatggtaaa
gaaatgaacgcattttgtgttggaatgttgactaatgagacagatggaaaagtaatagatgtgtattctgaagccgtacaaggacatgcattggt
agaaataggccgaaaatttgttcagccagaacaagaaggtgaagaagataaagacctaaagccaaagctggagcttcagtatcaagggtcgat
gtcaaataaaatcgttcacgttatgataaagggttctttctcttaatgcgaactaacatatacgtgggtactttttaaggaaataggtaaacatagc
tatgaaagaaaaagaaagaagtaaaaatacatgcagtgcagttgctttacttttcaatttaacataacctacttaataagaattaaccaacattta
gaatttgacagaattagcattttatttcattccacccctataccattactccgtaaatcttttattttatagacaagaagacaccaaaacatagcaaa
aaattttctaacattgtatccgcccctaacccataaatatcctgtagtaagatatgataccttaaaagcatcgtttcaagcacaagcaagtcttttct
ttaacgacgcttacgttgaacaacagtgaagccatcgtcgtcgacaacaggagcctttgattcttcttgtgcgggttcagtagcatcttcatgcatg
gattgttctccttcgacaagttcaggaacagaagatgctgaaggcttcgcagctctattttcttctctctcttgtttctcaatcatgggttttcctaagc
gttcatggatgtcacgaatagggtcaaaattgtcttccaaggccgcttgccatacacgatgtagatcttgagccaactaaattattagtgttagtgt
acaaattaggaacaatcgtgagaaaatggaagaaaaggtcgtcatccattcatatcgttcataaatatcaatgatcgtttgtcattcgcaccgatt
atttaataatcacctaggacttctacaaacgatgtttaaatcccattatcatgtccaatttctgctagctagttaactagctacttacaatgtaaggt
gagtcatcttctacagaacccacattaaattcatcttggagaatctgcaaaatgagctcttcgatatcccatgcatcaacgtcttttgctgaacaga
tgtagtctactagcgcacctgccatccagtctcgtttgtcagctgtatcgacatctgcccattcttcagcgacagcttgcctcatgatgggccaacag
cagactaaaacaccagcagcatattcaaaataggttagcttcttggaaggatcgtcaaacatggtgaaggatgcactgaaatttcgattttggtc
actttctattttcagagaatacgctaaactcgttaatgtcgtttaaataattataatgatatgtttgacattcttatcgtataattcgatagcaaaacg
aggcattataccaagcgtagtgcagtacgcttggttacgctaacctacaatggacttagttgagaactctggaacagagttggcattaattgtttct
tcctgaatctcagaaattgcaatctttcttggtagaagaatgaagttgaattactttggaatcgtcgtctcaaattttatgccaaaaaccgccaagg
tgcgcgttgcaaaggaaaagtttcacccggttatcaagaaatatgtaacaaattaccaaaattacatggtacaggatgatcatggatgtggagtt
ggtgatgctgtagttattcagccatgcagaccacgttctgcaacaaagagattcgaaattataaagatcctacaagaagctaaccgcgtttcttca
aatgccctctcaagctcagaagggaccgcagccgactcaaaatcataataaagcggagttaaatggcttctaaaagatctgcatcaccaaaact
tacatgtcaattgttatgcctttcaaaaattatgggaaaaggggttttgtagcattgagcaagaaaatgtggcatgtgagtcatttatcttataggt
gaatcattaaattatagatggcggaaagcattcttgccgtcttttgcaccttgctcttgactctgaaaactaatcattactccactccacgattacag
tatattgtaattcttctactcttgcatattcaggataattacgcaacctttcgtactttatacctattctctgtgatatagatttgatttctaatgaagt
atgttaatcaactcgagtgtttcttatggacataagttcaaacaaccaacaacaacaatgaagctgaatgaatatgaatttggaaggttaaactta
aaagatgaaagaaaagtgaaatgctatccaaggaagctactttgtatgtataattaaaacatcaacgtttcattggatctagatagaaggcttat
aattaactgaaagaaatacaagaatctgttgctcttaacttaattattgagacttttaagacccatttcaacggctgcggaaccccagtcatcacca
tggttgtggccgccgttaagtccggcacgatgcaaggcttgttcttcatttaaaaccgtcaaaagtcccaaaatcacgggaacgccagtgtcaag
gcctattcgcatgagaccgtgcacaacagcttcagatatgtattcaaaatgcatcgtggaacccttgattaagacaccgatgccaattacggcatc
ataacggttgcttgccatgaaagaacgaataccgacgggcaattcccagctcccgggaacgctcctgatgtcaatattctctggtttcactgcatg
atgattgatcattttgtcaacggcaccctttacgagaggctcgatagcttgcaagttccagcgagcatggacaacgagaactcgaagatcgggac
cctaagtatgttagtacttgtttttcttgctatggattttagaacagcgttcttttgaccgatttcaagatttttttctcggcgctcattttctaaggcaca
gctctctttcatataaagataaagatgttttatattcaagaagaattgacttggtttttccgttcaagtgaacccacagaagatgccttcaaatgtac
ctataacttacttgaatatcagatggattcggtcctttaattccttcaaacatttttaaatacaacaaaaaaggtggttaaagctgcgtgaatatggt
aaaattggaaacactttgtctttaaaattgtgctgaaagtgaagttgatctgttaataaggacaaagcggtctccaaaccaggatgatttaatatg
tgttaaaaatggattggttcataaaaccgaaaaaaaagaaaaaagaaaaaaaaaagatggttttgattataaagactgaatttacaaaagggat
tagtcatcattgtgaatttacaggattaggatgcctttgtacatgtgtataggagaatacaagaagagtttttttcgatgaaaaaaaaaaagcaa
ataatagaaacagtaagaaaaaaagatccatattaagcataaaacaatcagtaagtgaattaccaagagttcgaccctatgcaagaataacag
tagcaagccttaaaacatactcgaaatgtatctgtagacttgagtgtattgggttcgttaactaataaaaggaaaaaaaaagttataaataattg
aagattaaagaaccaatatcaacacttttggttccctgtaagaaattccataaagcaagtgattaggcgaaagaaagtcattcttaagtctgtggt
gaaaatatcaatcagttctgatcggtaagacattcataaacatactttgacataaagtgatttcagattaatgcaggtgtatgttgtcaaatcgcac

cctgtaggcacgaaaataagtctttgccatgaaagacaagtgaaacaacctcctatcctttgattccctaaaccgtccagaatacaaaggaaata
attaaacaattcgtcgtcgatcaagccaccgggcccaaactatgaaagcatgttagtatattgaacttgtttatgggagtcgaaatagagacaatc
tctcgagatacgtcaaagtattgaccaaagatttttctctatttacgcacaaatagaaagtgatttttacgtaccgtgaaaagcgaattttcttcct
agaaacttgccagcaatgggaaatatgactaaacacaataattgttccaattgctctatagattctttcaaatcatcatctagctcgctagaatagt
catcatcctcaacatctgatgctgcaggagagttcaaatgaagcaaatcggccattctaagaaaatcaacaacaaaagttggttttcaagatcaa
tcaagcataaaaacaaaaaaacccttagaaaatatatatcttcgttaatagaaacgatgatgaatgaattactgacgatagaggaaaatgtctt
caacaaaagaggtgagttgttttgaaagcaggatcaggttaagagaggaatgcttcttctactggacgcataaaaccatctggctttaatgaaat
gaaaagaaagataggatttatagatttttacagaagctctgacacgcttaaccatgcgaaaaatattaatgcaagatgcatcatgtatgagatg
gaatgcttcaagattgtaatgtctatgaaagaaaaatagcaaaatttttaagctacatccttgcaaaaacacgttccagaaaatcttttcttttgttc
gaaagtaactacaaaacctttgtttcttaaagctcataattttccatggtaaaaaaaaaatatatataaatatataaatacatgcttgtaaagcag
ttgagagaatgaacttactttgcatctctttctctgtctctttactttttttcctttgtttcaagtgtaaacacactctctttgtttacatgaccctggaaac
atcattcccttacagctagttaatacatttagaacgcgacacgaaacctattatttctttatttccgttatcacatccatagacatcttcttaaatctat
ttcttttaaagaagattattcttattcttaattttatcgtttctattgaagctttgtctgacctttgctgctttttacgacgcgaacgtggttgacgtaca
aatttttatttttcttccaagaggattcttttctttatttggatatttggaaaagaaaaagaaacagacataacttacagtatatgtttcggaatgctg
gatctaaatgccattagaagatgaagacataaacaagcaatgcactactttgacaagatggaatcctatgtttattaacgatgtgtactctatcgt
cttttctgattctatatctagtacgtctacaaccacatctacaggtgttaggattggtgacatgttttattaacatttgctagaagagataggtttctg
gtttggacatcccatttgctggatacaggttgtaggatacgtggtttcactggatttctacgaagataaatacgtttgtacaggtaggtatacaggg
tataggaaggtagacagaggttttgctgcccaacagggactcccatgttgagcgtttcgtaataagcataaagtatttttcaggatcttgaaaaac
agagtactttatttactaacttttctttttagtggatgattgcactagtcagaatatacgcacagtcttttcactgaaggagagacgttccttggcgca
aaaagcaaaaagattaaatccagggtcaattgttcgcgtcggtggaaaaatccaacgagttcattcgtttcaccttctagcatatacgttagatct
acttgaggatccgaatgctgaatggaaggagtggcttaaaaggattcattatagacaaaaactacagagaaaacaatcgatcctaaaagaaaa
gcccgtaaatgaaaaattaggttttccttgtaaatcgtcccattccagtaatcacgccaaggtcctccttcatcacttgcgacgtttatgcaaggcg
aatccagaggcaggcttcacaattgaagaagtgattgggtttttaaaatcaacaacctaagtcttccttccgtctcagttataaacgtggagagtc
aaaggatagatacgttaccaggaattaatgatgaagctatacgcctttcgcttttgtcccttctccgacatggacgagttatttacaaaaagccag
acagtgtatatcgcctgttcttcggcaaagatgttatcaaatatgtgatgcccatgatgtccagtggacgtgttgaggcccatcgtgttttagatgtg
cttcgtcaagcagatgtcagtttccaaacggtccccatctacgccatagccaaacatattcgaaaatttcttcgatctgctagaagtagctgggac
gagacagaaaaatatgtttgggaactaaacaaccgtaaacatggtttttaccaatatagtgtaattaatataacttagtttccgtaagataaata
aaaaataaaaactcaatgggaaaactttcttagtaacattatagacaagcaacttccttaacacctttgtaagaaaattctcctaatcaatcataa
aatcattggttttgcagtgttaatgaaaagaaaagaaaacaataaaatgaaacatataagacacctattcctctgcacctatcttcatcttcacct
aatccttcctgatatttactatagattccatttatttctttcacagtcaagcaagttacgcgtggcgcaatggtagcgcacctcattcctaatcggtat
agaagctgaccaggaatcttgaatcatgaggaggctgcgggttcgagtcccgtcgtgtaagttcatttttgctctttttataaacatttacatgaat
gatttgactaaaaaactaaatatttttcattgtaaagaagtttttataattatacggtccccatggttgattaattcaaatttaatttcattagtaaa
ctatagccaaaaacactatctcatacaaactaaagtctcacagcaaaattatgtttaaagtttcttcctctattagttattctttcaattaattgcatt
gggattcgtttcgtaataaatcgtgatcccctgagtgaaaaattaacattttaatccatcttacttgagttttattgcctgcattctgcttctttgtcatc
ataaggaatatctgtttcggtttgatagggttgaagaaatcgttcagttaaaaaactcatgcacaactgcattttcaatgttttatttttctcttcatc
cataaactgaagattgtctatctccattcctttccttaatggcttttctgtttcattatatgattccctttcgccagaataatgctcggtttgctcgagat
agaatccattatcatcatcaaagggctcataaagtagcttttcgaatttcataactccgttatcatcatcatcatcttcttcttcttctgtactgtc
ccaattaggatttagaggtcgaatattctcgctgtcactgaaatcagcaatcacttctttggcggtatttactccgttcgaacctggttgttgtacatt
gtcatttctcttttctagtcttatttcgtttaaactgttatcttcctccttgtgttccttatcatcaagtccccagttcactttcatatagtctaggcattca
agaatcaacttgttcgtggaaggaaacatgtctttctcgtcgctttttttcttcctcaatggtggcaccttgtgtagactctgacgttgaaatgtcaatt
tggttatcgctttcatttgtcttgccttgtgtttctccgaagatcgaaattattggcagcatacatggatcgttctcttcttcacagcttaattcataatt
ttcataaaagtctctctgattgtaattagcttcctttttttctgccttgctttctgctgctccatagatcggtattattggaagtaaacatgaatcttcct
cttcttcacactgtgttgcatgattttgaaaatcgtgtgtctgtttatagtcagtttccctctctaatgttttgttttgtgcttcttcatagattggtaatat
aggcaacaaatttggatcgtagtctttttcacattgtgtttcatatttaccataatcttcttcaagttcatatccaaggttaaaggtgcagtcttgcgt
atccgattgttttacaaagcttgtcgcttcaattactaagtccctttcttttgttgagtcttctgaaaaaagtagagctggcaaaagatttctattcaa
cccttcgtcttcggaagatatctttgcaatcgtgtcttgactttgatctttataattttcctcttcatttttctcactattgtttagttctgcatcattctcc
gtttcatcatcccattcaaataatatccttgattcttcttcttcaaaaattgatttccaataatctgtatctgcataagtatcgtttatttcgctttcactt
atcacttgatcactagatcgttctatagtttctttctgagcaatgttaagtttgcctgttgatgtttcgtactcgagaagtcctggaaaaaaatctgttt

tgatgccactcagacagtacagcttaaaaatttgattacagcgaaataaatgagtggttgagctatattcattatgaaaatactcacgtacaatct
gttgcattttcgcattctttccattcacattaaaagatattttttcaccatctgcctcaacgtatgggccatcaatgatcggggttaagtgctcgagtt
gtccataacgccaaattaattgcacttttccatctaggcaatagatataggcggtaactgaggggaattttctaaaacagctttcaagtttataaaa
ctttgaatcgtacagtgagctttctttaaattgagaccaaacttgaagcgtttcttggtagcctaagatccaggatgcatatataataaacttctgaa
cagcaacaattgatgcgttcactgataagcttccaattttatagagtagcttgaacggaaaaagaacagtgattataattgtttctaaaaaagtag
ccacggtcaagtatgtaaagatagttatagtagagcaagcataagcgatccaaagaagaacatcttgagagaaataaaaaatgctttcaagatt
ttggaagaagcaaagaacggcaaagatacacactgaactagctagtggtttcagaaagatcttgcctatatccaaataagagtaaaccagtgga
aagttaatgtaaatcatttttatctcttaatgatattaagaaagagaaataatatgaatgagctatatatatatatactacattattgaatgtaaa
caaatagtgaaataagcccaatcaaaacaatgaccatataaacaatggtatatttgcatatgagtagcaagcaacaaacaaacaaacgataga
gatctcaatagagaatgtagaggtcacattatacaaaaaccgatgaaagatcaatagttgaaagtcatccattaagatacgtacattaaaacttt
aagcgatgaaggtcggttcatgtcttttcagcgacatttttttgagagcagctaagggtataagattaattaatgcatagtccaaatcttgttaatag
gaaaagctatgggaataaaatgttataagctaataggaccacttctttccagtaatcatttcataggcatccatatatttttttaccagtagcatgaa
cgatattttcaggaagagagaccccgggcttgccatttagtttgttagaggtaagccaattgcgcaaaaactgcttatcgaagctgtctggcgatg
ttccaactttgtagtcagcagcaagccaaaatcgagaagaatctggagtcaacacttcatccacaagaacaactttgtttgtagtttcatcgacac
caaattcaaatttagtatctgcgataatgattcccttttgcaatgcagcttctctggcaattttgtaaagttcaacagaagcttgagaaacttgttta
gccaagtcttcgccaacgatgctagtcacagtatcgggatggatattctcatcgtgtccctcagcagcttttgtactcggggtgaaaagaggttcag
gaaaagcctctccttcttgcatattagcgggaacttgaattccatgaacggttccatgagcctggtattccttccacgcacttccagttaggtagcca
cgaacaatggcttcaataggaaggattttgaatttttttaacaagcatgcatcgatccttcaactctggatgctgttggatgatccaaggcatttcgtt
ccatttagaggtaattaagtgggtttgaattttacctttaaagagcgaaaaccaaaactctgagatttttgtaaggatcttacccttttcaggtattc
cgttattcataatcacatcgtatgcgctgatacggtctgttgcaacaaacagcagggaagaaggaaattcttcacaatcataaagatctctcactt
ttccagacgcaactttttttgaatggcataggcaaagacgtctctaaaacggtcatggttgtaattattcaaagattatcttgttctctccttaaaccc
gcacaggtgaagtagaagtctatgtacttttaaatttcaagcaggaatgactttatttcaaaaaaaaaaggaaaaaaaagatgctcaattcaata
aatgcagtttcgctgtctgcaaaccaaaattagagggttgtgtgtaaaagattaaagattgatgaacaagatataagatgtagaaaagcgaagg
actttaaataccgtacaaggaaagcacttctagtttgtttttaaaaataacattaaatatagcttgtttttcaaactaaggaatttcattgcaaagat
gaagaagaaaaagcttatgataaatcattgtaaggatccaaaattttgcgaatggatatcaagacaaacagataagtatgattgaattcttccat
ctttattacttaataattcctttagcacttgccgtgctattacaaggaagaattggtgatagaagtcgagttctacgaaacgaatgtttttaaaccgc
tttttctgaaaactcatttctctagttatattcaatttaggatcaacagtaaggaacctctaaaagtaaaaatgaacctattttcgatgaattctagt
catactttgtaattaaagaattcgcatttccggtaacaatatcgattcaaatatcatattatgagtggtaacgtgggtagcaactgtcaacaaaagt
ataacaaaacggtttatggttgattatatctccgggaaagcataatcctaaagagtatagtacagtgagttactacctatcgggaaaaaaaaaaga
aagtttaattaacgaatgtataaacatttaaattagaagttgtaatgctttatattaaaagattgttcgacaagagggtcatattccataaagatat
atttgtatagtcgagcttactcgtgtactaaggtatggatttaatattctccttcgatggttttcgctcatgggatccattttcatttactgaaatttaac
tttcatgtataaagaaaaggttttatatttgtacgaaaattacttctttcagcttttctttgattgtacctgttgctacttcctataaattgaaacactac
gaacgaagatgatgtcctatgtttgttccttcttatagttttaaatacgtcttactacaattaagaatatatttaatcatcccttatgattgctattctg
ataaaaatgtcgcttgtttgtttaccaaggaagtatcttgatattcggtatttgagttgtaaataacagacatcgcaaaacgcgcactggtagaaa
cagtgtgcaatgaaacgaaagcaagaagtgcaggaagggtttacttttgttaggaaaggaaaagaaacgacatcggtgaaacatggcaaatcg
gatacttctcagaatgaagaatcacctaaatccgaaaaaatgattgcactgcctgtgtcagataccccaataattaagaaaaaccagaaactga
gaaaatccaagggaaggagaagtagccttgaccaacgaggaaagcgtgccagtagcattggaacggggtttgaagctcttcctcatgcggacg
ttcctactgacgaatattatcgacacatttcaaaagatttatctgaacctcttcgaatgaaacagctgcttctgtgggcttcctataagcgattggat
gagcaacgggaaaaatacaaggaaacaaaagaatccagcgaagctgcaatcgctaggtctatcattcaagaagttcttaatgagttattgtcca
accaattttctattagttggtaccaaagatccccgaatgagattattccaaataagcctcaccctcagaatttaaaaaatatacagcttttggacga
gttaacggctaagttggctcagctacaagatgaagaagctgcttggagggcggttgctgcaggatcagtagaaaatgaaaagacacttcacgca
tgtcgagatttatccaaccggctcagctcttcaaccgactcaaccagtaacgatcaggaaacatccaaagaaatgaacaactataatcaaagtta
tgttaaagatttaaagatgagtctcaaccctttgcttgactctttatctcagcatatacatgctctccattctttaactcgcattagcccaagtgttcgc
tcagcattcggcgaaagggcagctcataattatcttcttcacaaacaaaaattctccaagacagcaaatcattctgatacaatgaatctcctgcga
attttatccaagagtgtataccagtctcaacgaaataacgaaccatagttatgtctttcgacccatccctatagaaaaagtcctctcatttttattcc
atagcctattttccgcgttctacgattccttgttatagagtacacaaacatgatttttgctgatttctttacgtcgtatacttgtatattaatgtttaattt
atgtataaatgagataacgtcgactattactcccgatatatatatacaattttaaaaaataaatgctcaccctgcacaatgcaacttttgtttaccg
agatgtagaaaaagaaaaacagttaatttttttcattaacttccagtatgaaagttctaattatagaagtaaagaaggcgaaatgactcttcatatc

caagatgctcaatgattttcttttctttttcttttctaaacccaaaatattcataacaaaatcagcttaataacgataaagtttgtgtttttattatgt
gaattaaaatagtggtaaaacacaaagatagcaaagaagcaaattaacatttttggttaataaacaccggcaaatatataaacccaataagttt
tatataacaagccaaggaaggatatttcatgattttaagaaaaaaaggcccagaacgtataccttaaacctttgatcatgtaaaagcaaaattga
taacttctatgatggcgcgtgtaatcgcaattcgtgataaaagatcgtcgaatgaagtacaagaataatattatacgatggctgcctttgattttgc
acgaatctttgcaccataaatgtaaaagatgaagggtatggggattgcagcaacagctatcatacctaaaaggaagaaagcccattgtcgggtc
ataccatccatcatatagtcaataaataaagggaaggcagcaccacctcctgaacgtaccattgtcttagcagctagagcagaggcacaataatt
ttgatagacatcgattaggtaattatttgcactgaaataaaacagcaccattccgtaaccaaacggaactccactgaaagcaggaccgatccaa
attaatctgggaaaacttgtccatccgaaaattaatagaccagcagggatgaaaggagctgcaatcatcataccgatcaaacgccattcaggaa
ttcctttaccaccgttagcatctatgcgattcatatattgtctttccaaaaagggagtagtcgctaaagcaaagacgacacctactaaaacgggaa
taaacatgagaccagtaataccttcgttatagccctttcctttgacgaaaacgttgggatacgcaaagaaatagccataaagcaaagcataaata
agagcaatatagcaggaaatactcatcaaaataggttccgtcatcaacaaacttatgggacgaagcaaattgacttggacaatttccataaagg
ataacatctgcttttcttgtgctgtgtaaacctttcgaccagtctgctttcggatttttgggcgcgccaacgcaaaaggacaggggcataagtttcg
ggaatgggaagcatagtgagatacataacaaacatgtaaatcatattgacccaaaatatccaacgaaagtcaccagtaccaacattgatccagc
cgccgaccaatggaccaatagtgggaccagcataaggaccagcagcgaagaaggcaatggcaagaccacgttcgcgggtttcccaaacatcg
gataaaacaccaccggcaagagtaagcggcgagcaaccaaaacagccagcgaagaaacgacaaattagaagacatgcgatattaggagcca
aagcacagggaatttgaaggataatatagatagctaaagtgaccaagtaaacgatacgacgaccaaacatttcactcaagggactgatgacta
aaggaccgacaccgaatccaactaccataagagaacaagacaaagcagcgacttcaggaccaacctttaagtcgcggctcacatgttccaaat
caccagcaatgacggatgaaccaaacgcactacaaattacgagcaaggaattaatagccgtaatgtaccatttccaaagtttggaccaattttta
gggtttgaaggatcgttcatttcccaggttacaagctcttcgccctctttattaatggtaggatcacggtagagaccccccttcagtctccaaattgta
ctctgctacatcagggaggccgcgagtggtggtaaagggataattaagatcgacaccattaggaaaaagagtctcagattggcgggaggtagta
cgatttaaagggtttccggacatatccaacatacgcgattgcgttaactggacaggattttcagcggcttctagcgcgtctaaatctatcgtatttgc
atcatgggcagcaggattttctctagacattttcaggacggaagcgacaggcaaaatcaggcccaaggaaaagaattcaaggaacttttgtgaa
aagaaaataaagacgatttaacaacaactagataagaccaatgatgtaaggggtagaatgtatcaattggaattaaaatgcaagaaaaaaga
cggaaggagtaattgcaaccaaatagtatagctatagtaatcgacaagcctttttttatgagaagataaattaaataaagttgaaccaccttaaa
ggcattcaaaaaagtacacactggaaaggggataagctgtgtaggatctaattaattctcgtaattaaacaaagttccaacaaaacagcttcca
aatttactagaaagtgaagaactaaagaaccagatatttccctaaaagtatatgctcaatacaacgacaaaacaaagttcatgcgcacatatat
atatgtacgcatttatatcatccttgttcaattcacaacaaccgaagcgaaattaaatttgccgtaaatcatctcattggcttatattcggcattgag
tatggagttattttagggagaaacagcgtgttgggaaacgttaccggaagtatggtaactaataagcacaaggaaaacgaacatggtagtttcat
ctttagatggagattctacactcccggagaagcaaaaccgcttcattcaagcgaaagagaaacttcgtcatatcgaacaacagctacctaagtac
ctatctcatgccactttaaaaaacttttgaaaggacagttgtaagctttttctgttatcccctttgtaggatgagttcaaaataaaagcctactgcaa
aaaaagggggggattatcattgtgtgcattcaaaatgatctcaagtgatggaaacaatcatcattttcatacacaaatgcacccaacgatcggcaa
tcgaaatgacaacctcttctttttgatgcttccttgcgaaggacgtaacataaatgatttgcttactttttttataagccctttccgtagagattggtcg
gcgtcccggcaatctaaaaaatcggcaaatttgccgattttcggcaatgaatatgaatttgtacgatagcactcagtgctcataaaaatcaaacg
aggtaaagctgccgatagaacgaataactttccttaagcgactaagggattgaaataaaaagactatgaaagccaaagtaaataatagaggaa
caacggttgattaaaagattttaatacgatttcaatcctcataaaggataacaaatatgttcaagatgaagttcgcacatttcagtcggttttactgt
catttgtaaactctgtcagaatagcaatcttcatctctgagagtcttttagctttcgtatctttttcgcaattgctctctttttttcaacataccagctcga
gttttgaattatgattattttcaactactgataaaatctgctaaaaggatctaaatgactcgacatcacggcttccagtcagctaaaaaaagaatct
tctatacgtttgtatgtggaagaaaagaaattttctattttcctttttttttttaggcattccctatacttgccgttgcatctattgtcctgcctgtctgttta
ttttattttatcattattttttttttacattcatttcttctctagaaaaagccatcgttgctaaccttgccttgccggtatgctgtttcttttctcttggaatg
tgcaaacttgcagctagggagtagtacattacactttgttaaggaatatggaagaagtttcaagtaaactcgatatccccttttatcgagtttatttt
ttgccctgcagtgcagtatttcaaataaacgaatttttgtctccttagcgttccttaaagttccactacgctccagctatcacccagtggggttttcct
ccacaaacctcaacaaacttcttcgaaaattcctacgttccatttgagatagcttggcatttgctaagcttgtttcaaattcatatattaattccctgg
atcattcaccactcttcctgagttctttatccgatatcccttgaccttgaaaggcaaacaaactttatttgttttaccaatccctgggttattatctctct
aaaaaattcaacgcgaatttttttgttgtatctttagtcataaaatcttctaatcactgatttttttttttttttcactgactgactgattacttcgtttttac
attttgctttttgtttttgtgttcctgttcaacaaccttttgaaaaaaaggtatattagcaatcagaaaaccgttataaaaacgttaaaagtgagaaa
ttgcttttttttagcgtctaattaccgctcacgttttttttggtcatctcccagtccattccattcttttttttatttcttttctggtgtgcatgtaaaatagca
ctcctatttaacggtaatatctattgtagacctttgccaacattggtctatttgaatttgaacaggattaggttcccgttgatcaaaacctattatttca
atcagaaacttgcccttttcttttacagtcccttcgttacctttttttcttgagcttgtcacggcatacgcaagtgatcctttgatattcccctcttctctc

aattctaattctagttttgtttatacccttattctttgatcgatcgtttactatatataaccttttattttaagcttcgttcttccgcttaatttattttctta
aacttttggtcaatgtcgtcgtctgatagaatttctttaccgaatgcagtagaacagctatccttatcatcggatactgctgcgtcttcgcatcatcaa
tcgtctcttaatgcgaataactccgaacacaacttagatcctcaatcgtctttaatctcttctttttcttcttcatcctcgtcctctaattcagattttaa
cgatactaattctttatcgacgtcctccgactcttccgatgataataacaacactgtaaacggcgataatgatacaatttctcgatcttcctctacta
ggtcgtctgatcaattaacatctcgacctgttccgccagtaagattaggacgtcgttcttccagtaagaatcgggcatctcttgcgataaactgcaa
tcaaccagctgaaaaagctggcagtgccctttcttcacccacatccgtcggttctccccctcttcttctatgcaatatcctggtggcttatcctctga
tatccaagagaaagtgaaagctttccatgccgctcgttctaggtcttaccctgatggtggtaatagcacaacatctagtcctgcctcacctatggtc
ggtaatcgatcaagtatttccatccccatcgctcctattcccccaaaaactcctcctatttctggacaaaaaggtaccaatggtactgactcgacttt
agcccgctcgcttgctgccgctagaattccagctcagcgttctgcttcaatgcccaagcaaaataaaatgcgacgtgctcctcccggaaaaattga
cctatccaataacagtaccactccatcaagctccaatgcggattcaaaaggcagtatggcttcacgacgcggtttaaaaattcctcctagtctccg
acagtttgcttctgaaacgccgttttccacgttttctgatattttggatgccaaatctgggagcttgaattttaaaaataaagccattctcaatgctga
tggtgtcaatttttcttctggctcttcatttagaattaatatgtctgaaattataaaacttgctgagttaggtaagggcaattatggagttgtatataa
agcgttacaccgaccgagtggtgttacaatggcagtgaaagaaattcgtctttctttagaggaagccacatttaaccaaattctaatggaacttga
tattttacataaagctacaagcccttatattgtagacttttatggcgcgtttttttgttgagggttctgttttcatttgcatggagtacatggacgttgga
agcatggataaattatatataggtgggattactgatgagggtgtcttggctcgtattgcttacgctgttttacaaggcctaaaaacacttaaagaag
aacacaacattattcatcgtgatgttaagccaacgaatgtactcgttaactcctctggacaaatcaagttgtgtgattttggagttagtggaaattt
ggtagcttcaatctcgaagactaatatcggctgtcaatcttacatggcacccgaaaggattcgtgtaggtggcggtggtaacggtgtattaactta
cactgtgcaagctgatatttggtctttgggtttaaccatactagaaatggcaattggttgttacccatatcctcctgattcttatacatccatttttgct
cagctttctgcaatttgtgatggggaacctccaaaattgccaaattcattttctccggaagctcatgattttgtaaatagatgtcttcggaaaaatcc
ttctttgcgtcccggctatcaagagttgagcaatcatccttggattctcaagtatcaaaatatggaagtggatatgagtgcctgggctaaaactaat
ttagaacatcaagttgcaggagaggccgagaggtaatctagtatgttaagtatagtgttttaacgaaacgatgttttggaaatccatacaaactag
aaagaacgagcttcctcattatttgtttaatacactatactagacatactgattaataaggtcatttaaattagtcactgaggaaagccatcgaaa
gatgacttcgaatgggtaatatatcgcgattttctttgcgaagttctgttgcaaaattgcttttctaatataccatgaaaacatagagcaatgtaatt
tgcataggcggtgcaaatgtgaagtcaagaactcgagagtgctatgaatttatttatattgcgacgaattatctttgtcagtaattaattgtaataa
taagtatttataagcttttcagttataaggagaaatgagtagtagcttattctatatgtaaacaaaaagttgaagaaatcatcttgttttgagttagt
atactggcaatcatacaacgtagtacgttatgtcatttctagttgggtttcgagatatatgcgatttaacataaatgtatgttatcctatcggggacg
aaaaggtagtcaatatgacactattttccgacagagtacttattcttagcttcttctaaggtggtgtcccatgtatttctggtaatccgatccttagcc
aacaacttagataattcaacgattagtcccaaaaagttatgtcgacgaagttgattttcacgtttccatcgtgcacgtttttcatcttcaacagcaat
agaggccgccttttcttcatccgttaaatcaggattgtttaaaatagtgagacgcttatccttggagatgaccatcagattgaaccggatttcactg
gcgtcgtacttgccaattcttgattgtatagtagaaacagcacgtctagggaactcttcctttgtacacgtgccgtgattaatgggtgcaggttgtaa
tccatctagttcgtaaaagatatcgttaatgttcgtatatgcaataaagtggtagacatcatcatcttctgtcgcaaccttagtttcatcactcacaa
aagggtccgatcgagcgaaagaattatgggcacagcgaatatcctcagaattgcccagtgcttctcctttgagatcgggaggtaagagttttgtg
aattctcgaaattctgttaacttggatcccaagtcaatttcctcattgtgattcagcaaaacggataaaagagcttgagtagcacatgcattatttat
gacttgttgaccaaagaaaatacgatctattgattcatagtcgagttcaccatcaggttttccatcaggcttcttccatttgaacaagaaaataatc
ccatagatatctctgacgtaaaggaagttagtaaatattcacctcagaaagtcaactgtcatgtattgtacgtacgaaaagttgcgcaaagtctct
tcatccaacgtgtatagttcttcaacttcaacacctgagactccaagattctcaatcaaatcagtaaacactccagcatctgattctaaggtggtcc
aactcatttttaaggtttaaaaagaaaagaaaagggcaagaacaggtttcaacctgtggaaattctgctaggtttcacgtcaattaacacataat
tacacttcgtaaattttgcataatgatacaaggttcaagctccttattgtgttttatatgttcggatctctcgttaataaacaatccctcatagatgga
aagatccaaagaataaatattgtagatagaatgctatgtaacaatgaatatcatcatgaatgattcaattcagttgaattgagttgatagagttctt
cttaacactcttctctcattgtaggacttgagctttttcgttctcaaactgcttttgtgacatgggtgcttctcatgagaacatcagaaggatatttac
agatttacggtatatttatatatcatattttctcaacgccaagctaatacagtacttggatcatgtattaagctatataagctatcgatttggacagct
aaggaaaatatagagatatgatcaatatattgtaaagaagagcctattcgccttttgctcgattcagataaaacgaatcctacagtaaagacatt
aaataaaatatatgaaacttaaacggatgcatttatagcaattactcggttgcaaaaggactatgaaggtcataaatccttaaagaaaactatcg
cttatgaaacctgccagagtagcgtttgcaaatggagaatgcatatttagagaaaaaaatcatacacataaaaacaatgaaaaaataaacgcc
cgttacctatcctcggcccattcgttttctttacggatttgctcctcttcttcaggtgtaaagtcattggggatattaaaggtcttacgaatgtcttcag
ggctttttccacggatcatatttgctactgttttacagccagtgtcgagtagcggtttgatatcaagataattgctagccaaaataatttcaaaaagc
atttcctggtcaaccgacataaacttcttgtcccaatcatcgatatcggttgatttttttaagccgaatatcgctttcttcctcagtattggagtacaag
tcattcttatggtgttcacaccattctagcacttttcttaaaacattcgaggaaacgtttggcaagggaattggaacattcagttcaccaacatctaa

aagaaacggtcaacgaaagcaattccttttacaattcacataccttccagcatattgttgatcaacatgctttttcagcaacaggtcgatctaaac
ctcacattagtaaagatgatttttcttggtttccaattaaaatgaacctaccaacagtaaactcttcattatcagatgaaataagtttgatttttgac
attctttcttcttgggagtaactagaagaaattctggctccacttggtcttcttctgtcaaaaatcaaatacagtagtgcaccttatagtacagtaaa
cgatttcggtactgttccgagataccataaatgaaattgtgatgctgtacgcgcaaagtaaatcaaaagcaaaacaaacaaaataaaagagaa
gaaattttctttgtgtttcttatagagaaattaacggtttattcttatttttgttttactttctaataactttgattttcttttattggaactcttacttgatc
ttgccttgtaacaagatagtacctagtaccaacctggcatatttactcgacttgaataacttcgataggcattcatctttatagaaaggaaaaaaat
acaaaaaaacaataaaaggactgcccattgtcatgggccaacgattaaatcatcaaggaatctttgtaacatacgctttgaaatccagcttttcat
acaaaattttggtttcctgaaatcattcagaagctcacggttgtaaagattcattgtcaaaaaggactactggaattgtttgaagatgttggttcag
ctgtttgaattcacgcctctttctttcatagacgatgtaattaacattaccaatcagctgttgtacaagactgtcaatggtgttgatcgagcgttttcc
tcgctctcgagcacaagaaagtccccgaaagaaattgaagaaggattacacaagtttgaaatcttatttgagagtatcgttgatcgatattacgat
aggtttgaaatatatgttttaagaaacattttccattatcccccagaactgaagggttacctccgcctagaagggcaaacagcggactacacagtg
acagcggaaaaggatgctaccattgaccaagaaatagaagaggctgctcaagtactcacagagaaactcaaagctcgtcactccctaagaatg
cggctacgaacaagtgaggaacggcggcaacaattggaacgacagttagaaacactttcttttctttccaacatcccaaaagagcaacgaatta
ctttaccagaaacaaccgactttttgttggaccaattggccggactacaaacttcccttcattccgtcacgcaggcatcacctattctaccttcacgc
gaagtagatgaacgcgttatgtatctcgaaaaacgatacgaaaaactagctggcccgatcgagcagcatcgagatttttggtcgcatcaactgat
caagttagaatctatcgcgaacacagaggacgtttcgcacatgagtaaacttttatcttcttctgatgactccagtagcggttagattatttgtgtaa
tgtataatatataagatatgacaagactcgaagatcattccatgaaagtgaaaagagagaaaaagaaaggaaacaaatagcttccattacgaa
aagagagagaatgtgtagagagaaaatgaacgaattgaagagtttgcatatgaaaaacaaaagaagaaatcgagcagtcgctcatagtcatt
gatttgtcttcatctcaaaagccgcaaaccctctactcggccccgtccacgccaaatctctcaaacaactcaatacttgtacatttttcttgttcggc
aaccttgccaatcatttccagagctacatctcggatttcattgtaagtgtgcaataaatctatatgcctttgaactgtgtacttggcgtcaggatttct
gatggcgttagtatcaattcagaaacaaaaaacaatccaatattcgaaaaagtttcagtctatcgtttcacatgattcaaaatagacgcgttggaa
atccatccaacatgacaggcttacatacttgagttttgacaccgcctcttgcagattcttttccaattgctgcttttgctgctccaacaactggacgcg
agcttccaattgaggctaacatggttaattcatatacgacatggacgctacttaaaaataacacgacgatattaccttttccatccgatttaagcaa
agttttcgaagagaaatgatgactagattattaaaaataaaagaatatccgcgatttctatgagaatgatgctttttgctttcaccagtgttatgcg
cgcgcgtcaaaacaggaatttagcgacttcaacctttacacatttccatttttgcgctctctactgtgctactctttattaccttaacaaagtctgcaa
ttatttgcaaactctgttagtgaatacattacgaatcgctattatgaatgctctaaagcaagagaaggttatttgccaatagccatcttcaaatata
aacggattgcttgtttaataattggcgaaaacaaaccatcattataacataggaacgcttggattttattgaaaggtctcacgcattacaatttcaa
ttagaagccactttgtttttttttaggggaattaacagcgaggtttttaggaaaaggctgctgaattaagaagcaattaaggaattgattatgtttg
ctttaaaaagtttacgtttttcactcccaagaagctccttacaaacaggtaaatttggtccgagacttttgcgacgcaatgcgtttcctctagcctttc
aaatgccctcatcgcggagtatatggataagatcagaagaaacgccgaatgagaatgccatcaagtttttacctggactcgatatacttcctccca
ctattggcactttagaatatataagaagccaagaaatcaataactcccctcttgcggaaaagctattcgagatacatggagtgaaaagcatatttt
ttggaaaagattttattacagtttccaaagatgaatctcaatcttggtcacaattaaagccagatgtttttagcaccatcatggagtacctgagcaa
tggaaaccctgtattgtcgggtgaaccattgaatggtcctaccgacacgcaaattttggaatctgattcacaaaccgttgctatgattaaagagctt
cttgaaacaagtattcgaccatcgattcaggaagatggtggcgatgtggaatttcgaggatttgatgaaaagacgggcacagtctctttaaaact
acgcggtgcttgtcgctcatgttcttcgtccgcggtcactctaaaaagtggcattcagcaaatgctgaatcactatatcccagaagtacaagaagtt
aatcaggtcttggaccctgaagaagaaattgcgatatctgaatttgaaaaattcgagagccggattcatcaaaatcagcaaaaagaagaaaatt
aaaggaatcatttgctaaggcatgcatttgacaatattgaatatgatttacaaagcaaaccaaaaagaattaaatagattatgattaatgaatta
atttttacattttccttgtaattcattttaagacaaaaatgattgataaacacatactctagacagaaaaggaagaaataataacacacccataca
attcgattcgtaaaatacaaaaatacatttttcaagaaagaaacaaacatcactatccacgtcatttacaaactcatacgggtttttgcgcgcata
ctgcccgcttttgaagagtcttcatgagcactatattctatagtttctttctcagagatgcccaaaatctctttgaaacgaagactagatacattagt
aatgcgtcgtaaacaaaattcaatctagacggttcatcgtacccaattctttccaaaacttctctgtcattacaattacgtgtccacacagcaatcct
gtaaaaacctcttctcatgttacaaacaacaccggtgacttccttcccggtgggatccaacgtttcacctatcgctgcaagaacctatgcggccttg
ttagtcaatcgatcacagggaaaaccccattccatagttaaggtggtccacttaccatatatagccatagctcgtccaagtttgatcctttgtgcttg
ctttgataagcccattttcctccattcatattgtgaggatcttcccattctgggcgtatacctttaagaaagaatgaatagtctgatttcgcaggaag
catagatgcttttgcaatcgttttgaaaataccccaaaattcttccacatccttaaatgaaataatttcctttaacaaatcgccccattccaaaccct
gcgtaggaggcttcaggaaccataatgtccattcgtgttgaagagaatgcgtttgtataaaagctgtttcagcatttacggattgtaatccttctagt
aagcgagcaggtctcccttctctttcagttctcacttttggagaatcaaataaatccaatgaagcaaacttttggttcatcgctgaaatatcattatg
tccttctccagaacctgtcatatcctttgaagaaagttgaataaactgatataaatgtgaattcttattaaaaaatttgtacaatttcacagaaaa

ggaaggtgtaacttaatgttgtggtgatgcgagtcagagcgatagcttttacaccaaaagactaatatagcgaagcttttaaaaatgcaatattac
attagtaaacaagttatcctgcttataattttactagaatgtagattaaaacattgtttttgatggttttatattttctctgcgattcaccaataatcca
aatgtcaaaaaagaaatgaacaatttcctagaagagagatatgcaaattggtattagcccttatttagtggataagtaatctatcgaaacattaa
acggtatctataacattgaataagctgttcaaatcataaaaaaatttcaacaagatgggatatccggcatttgatagcccttgttctcatatagatat
aattactcagacaaagtaaagtccaaagccgtttttgtaaactagcaaacactataaaattttattcaaaggcagccttatccaaaacgacaaag
acaccgcctgtgagatggagacttccggtcacacaaacaaacgtgctttgcgattcttctttaatattgtgcaccatttcaatggcttcttcgacagt
aggagctacctttgtagtatcgtttaaactggaacgattcttgttccaccaagttgccaaggcttcttgcacgtgcaacgctgggcgattatccgtg
acagtattaacgctcagcaattcgggattgtaagttgcgttcttccaagtaatatttgtcgaaaaaatagcatgcgtaaatggtatatctttccctga
agattcatagccctttaggaaagcttgaatcaaagcgattgggtcatctctactctgctggttgaataccaacactcttgctttggcgtcctgaaaat
gttgtctcctggaagctaaccaagcacccgttgcttccaaactctcctgtgtgtgtgcaccatcgaagcaccactgtattccgtgtatagtctctagc
tggcatctacctggccattttacatcccttaagccattaaggacgcaaggatctttaagaaggttggcgggaccggaatgtccagttttgtgaatgt
actcacctaccattgctattgcgagtgcggcattgctcaactgatgcgctccagaaagaccaatcatttcttccttcaactctaaaggtgctttaatg
acccttaagctagtatgtcgttcgttggcacgctgctctaacacgggaagagcttcaggaacttggttacaggttaaagcagttgctgaactcttgt
agattcctgccttttgccaagcgatctctgcaatcgagcttcctaataaagcagtatgatctaatcctaagctagtaacagctgtggcaatgggttg
ctcaatcaagttggtagaatcatattcacctccaataccgacttcaataatagcagcatcgacaccttctgataggaaaatgtgccaagctagtag
tgtcaaaaaacgaaagtacattggtttttctggattacttgattcagaagcagtagcttctaggcgatcccacatttgaaagaaatatttagtaaat
aattctttagaaacaggtttcccattaagttgaatacgctcacacactgaccgcagatgaggggacgtatacattccaatacatttgggagcaata
tcaggatacttcttttgaatttctaaaagaatagaggatgtaaacgcacaagtagagccctttcctttcgttccggcaacgtgaataacattcaagc
gattcaagtctgatgtctaataataatcgtgtcagaaaatcgttttctaaaatgtataataaatgcaaaactcaactatgccaagttctcagaaaat
aagtttcatgtgattcaaagaaacccaagaaacccttcggctatatgaaggcaactttacaacgtcaggatgcttttgtatcatagattccgtttca
ggatgtgagaagcattttataaattctagtcctccatctcgttgcttactcagaggtttcacaatttaaaccttggtgatatatccatttgcatttgctt
acctggtacccgatcctattgagatattctttcatttcaatcatcgactgatcgttaggaacttttcctctgttgcgaagcatctccaacacttttgcat
ttgactgtaaagaattgagacgttcaatagcatcctgacataagttagcaattacaactgttttggatgatttttttagacttacatcaaaggttcgct
ggtttgtactcatattggactgccctagcctaaaattcaggtattttctagcggttcttgtggtgagcttagccataacctagaggagcaaaggtat
gatagaaattcttgcagattacttgaaacgtatcaaattcagttactttttttagtcgtcaggtttttcaaaaatgagtgatcctgcattcgataaatg
gtatgcatgactacagtcaattctcgatgaagaacatacaccttctggttttcgatcatagttgtaagcaaagacttaaaaaaagagtttcatttga
tactaggattttctcttatttccgttcacatgttagtgctatatgagattcatccattgttattcaaaagtgcaaagatcgtcaagaatgccttttcaat
gtttcttaaatatatttattctccttggggaattccttctgagtatgactcctacatttattctgtcttaaggctcgaaggataagcttttagggaacta
agctaaatgaaatgcccttccttgtcaagtttaagagctctgagctcgttggagattcattattcctaaaagcttcttctaaacctttctatactttat
gaaataaacaaggtacaaatactcatgcgacaatggaaaccagctattgtatgattaccatggtttccctaatgaattttatcaatttgagtttcat
ttgaatggatctaaaacagtggaaaaaaactgttagagaaggagatttgataaagtctaaaaccttctcggagaatgtgaattatttcaaacaag
ggtaaacaggattaagacaagaacagccacaggagatagagctttaaaatggagttttattccttgtcgcagtatgtttctaaagatgttggaag
gttccaatcatcgagttaaggaaccatccctccgtaacaaaaaacattcacctggggaacctattgtaaaggctttgagtggaatacttaatctta
tgcgatgtcttacaatatacactttcgacgatttgtatgccttcaatgaaagcttgttgctcaggatagaaagatatccagcagccaattttgcagc
catagaagagcaagatttgtgctttattttatttaacggttaacgcacaaatcgaagattactctgaaaaaaaaaaaaaattaatgttttccccaact
ctaaccttacttaagcacatttacgagagagcatctgtgcctatattatagcagctggattatgattgctagatctttgaaagttgcttgcctacggc
cttttactcgcaagcgattacacactgtcgattctttgttgtttaagaaagaacatctaaagaagtcttgatttattagttgaataaatataaaataa
gaaaaagaaacttcataagagcacctcacctcaagttctaagaaatcacaaacccttagcgctaccaccaaggttttccaagacagatcaagtct
ggcgtagatttgagcaaaacatgctcaactaccaatgtttagaaacaaaatctctttgtggaacatctcatattgaattggatgggattcacattta
tattgacccgggaagtgatgatagtttggagcacccacctgtatctgaacaaccggatttaattttattgtctcattcagatttatctcatttaggag
gactagtatatgcatatcataaatatgactggaaaaatgctcatatgtatgcaaccttgcctgttattaacatgggtcgtatgaccatgttggatgc
tattaagagtaactttataaacgatataacaaaagaacaagtcaatcaagtttttgatagtattattgctctgcgttattctcagccaacttctttgtt
aggtaagtctgccttcaaaataccagtatgtctcagtcagtaataatactaacgatgtaactttaggaaaatgcaatggcctaacgatcactgctt
ataacgcaggccattctttgggtggtaccttatggagcctcataaaagagtcagagagtattctctatgctgttgattggaaccactcaaaggaca
agcacctgaacggagctgctttgtacacaaatggtcatatcttagaagctttaaatcgccccaacacgctgatcacagatgcaaataatgctttag
tttccatcccttcacgaaaaaagcgagatgaagcattaattgagtctataatgtcttccctttataaaggcggtacagttctacttcctgtagatgct
gcttctcgagttttggaattaggttgctttttagatagtcactggtcttcaacacaacctccgttaccttttcccatcttctttttgtcgcctacttctact
aaaactatcgattacgcaaggagtatgattgagtggatgggagataacatcgttcgagactttggtatcgatgagagcctcttggaatttcgaaa

catacatacaattactgattattctcagctttccagcattggacctggacctaaagtgatccttgctacttcctcaacattggaatgtggcttttctca
gcaaatattgcttaattttatgtctcaaaacgcaaatgatttaatattatttacacaaaaatccaggtgtcagccttctttagccaaacaatttatac
gatattgggaacaggcatgcaagaaatcaatggaaattccttccccagtcggtttatatgccgaacataatattaaagttaaacgtacagagcctt
taatgggtgaggaattgcaagaatatcaagaaaaacagcttcaaaaaattagtcggaccgctgaagatagcgctttggaactccgcaataaaac
aattttagatgaagatttgtcgtcctcttctgatgaagaagacgatgaaatgggattggctcctgaagttccgggagctgcccttggatcagcttta
tttttatctgggaagtcttttgatttaaaccttcgtgatccgtctgtacagagtttgcagatcaagtataaaatgtttccttatgttgaaaaaagaaga
agaatagatgaatttggtgaagtggttaagccagaagattttgcgaccataaatgaaccaatgaataatttcgaagtagaagcctcaccttctga
tagtactactttggatcctacaggaaaaagaaaatggtcagaaattaacgatggaatacataaccaaaatgaggatgaacaacaagaagatttt
ccctcgaagattgtgacagaagaattaatgataaggatatcatgccaagtacagtttattgatattgagggattgcacgacggtcgttcattaaaa
actattataccccaagtgaatcctcgaaaattagtccttatccatgctacggacgaagaaagagaggacatgaaacgtacatgtagtgctcttag
tgcatttactaaggacgtgtacctcccgaagtacggcgaggacattaatgttagtatagatgttaatgcctttagtctcaagctttcagatgatttga
taaggaatttaatctggacaaaagtaggaaactgtgaggtttcccacatgctggcaaaaattgaaaaccctgatgaatcagaaactcaagattta
gaacaaaatgaagaggatgtgaaacagcagaatacggaaaaagaagtagatcatcttccggttcttaatcctgttacgctgcggtcggatttggc
tagggcaccgcgtgccgctcctcttttggtgggaaataccaggttggctgaacttcggaaagcccttatagatcaaggagtatctgcagaactaaa
aggtgaaggtgttttactatgtggtggagttgttgctgtacgaaaacttagcggaggtaaaatatccatcgaaggaggtttgagcaatcaatttttt
gaagttcgaaagatgatatacaatatgctggctatcgtttaattctaaacagtgcattatataaaagcattactagataatcatttttctataggatt
ttccacattttctatgatttttttatgtgattttattaagatattactacaataaatactaaataccaaggattaattaccggcttctcctctttcatgttt
ccaaccctcgctaccagtttggcgagcaggtaaagcagaagaaggaacatctcctttcgttctgtaactcaataattcttcctcttctctcttgtcttc
gtctaaaagtttggatcgatccttttcacttaaagaagaacgaaattcctcgtttaataattttatagcatgcccaattacagattctggacaacggt
ttctagggttgccattctctggattgcgaatatatctattagagacatcacgaacactatcaactaaaaattggtaagcatactttcacattagcgg
tgtgtttggcttacttgaaaacccaatgcaatatgacatttttttcccccatcctagattttaagttagcggtaataagaaaaaagaaaaaagaaa
aaaaaataaaatagattgttaccctgctcataaatcaatggctgatcaaacgattcttcacaagaatccacgtgcacccatctgttttgcttataac
tataaatttcagtccaaacatggtcttctgcattccaaacccagcgagttctggtaccaagtgcacgacataaaaaggtaaagcactataaagtta
gacaacattctctcatgtcagtagatacatacacaagcccattctccacatctccccttccttgaaaaaagaagaggacgaaccttattataccga
ggaaacctctgttgatgagaacaagaattgcattgatatatttcaacgttattacagccttggaagctctcctctaaatttggtccgcaagcaccta
ccaaatttgtatctgactataacaatgttagtaattaattgctttttttactctaatcagatatgaacattacctggcatctttcacatttgggagagttt
acccatgaaaaaaaattcccgtttaaaccaggtaagaagagattgaattaaataatcttgataattccatgatccatcaccttccttctctcttttcgt
agcttcttcaaatagcctttctaatgggaggatggacatggccaaatcttgcaattccgaatcttcataagtccatggctcttgggacaattgtctg
atctgatgatagaatgcaaagctttctggaggtcgggatgcatctgatggctgtgcagccttttggcgcataatattcacaaatcgctcagtgatcg
cttgaaaattcattgtgcaagttttacgtccaactgttcagtttttttttggaaaaagaatttaactgtatatttaaaagaaaaggatagaaaatgag
cggatttgcaaagcaaagtctggaataaagaagtacgactgacaagttttaacgaccaatttttttttttacggaggacacatatattaagttagtct
ttatggtattcaacaatctaatttcaaattgttgttaaaactttgatgttggttaaattggcggtgtttagctcaaatacagcttgtactccacatact
acgaggagtgcaagtggtttgaatcagctacaacatttcaatcactttttttttggaatttaagtacttgcacgaaatttttggcttgcagcaggctt
ccttattataattattttttttggttagtagtccaactttgaagtcatcctaaaattaacgtaggagagcatatggataaactattagcggaagccgca
aaaaacgggtgcaagctacatagtcaagtagagtttgtttctgccgacgatgagctttctacttacggtgaatatgtagctaccgcaaaggctgat
attgaaccaaatgaagtccttatttcctgcccatttgaatatgccatcacttatgaaatagctttaacttctctacaatccataaatcccaattttattt
ccttgtctcgagaagtagttctatataccttttttggctttggaaagcttgaaagcatcggaaagcaaatgggctgcatatattgagtatcttcccaa
aagatttaataccccccatatatttcgacgatgcggataagcaatacctaaaagctacgaatatcgagtttgctatgcaagatcgattgcacaattg
gcgacatgcttacgaagacgttttgaaattgggatcattctctacaaatctattcacatttgatcttttcatttgggctgcaactgtgtttacatccag
atgtttttcttctggcttaattaattcgaaaatcagcaaggaagatgcaagtcctattcttcttcctctgattgatagcttaaatcataaacctagaca
gccaatcctttggaaaacagatgttcgagatactatgtcaatccagttggtatctcaaaacgctacttctaaaggtcagcagattttcaataattat
ggtcctaaggggaatgaagaattacttttggggtatggattttgcatagaggataatgcgtttgataatgttgctcttagggtatctgttcatcctga
tttaccgtatagaaagcaaaaaaacatctgtcttgcaacaggacagcacttttaacttgaataatctgatatttttcttccctaaaaatgcggattttt
cggcgtttgaaaaaagtcttgcaatgccttactgttgttagtgcgtcgtctttggaattaaagcgtattctggcccatcaagctatgtttgggttatctt
cctataactcgtctctgcgcggtaaaatcaagtcatttgagattttactcatgtacataaattcgcgcctgcagctccttttgaacaataacccgaag
cagagtcctacaaacatacgccaacaatgggctaacatatatagaaaaagtcaaataagtatcttggaacgatctttagaaaaagtgaaagatt
tcatggaaagtgaactgaaaaaggtataccttccgctccctaaccttttgcaataccttgtcctgaactccatttctgtttttctacttcagcatccca
acttttctcctctttcaaaagtaattacagaactctacggaactacagatgccgagagcattgctgctagtgaagaacaagataacgttgtactttt

aatgtgtttatattgcttatctgctcaagaaacacttcccttttcaataaagatgcttcaagaaggatatcctgcctcaagcacggaagagggaga
cgaggtctttgaaatttttgatgaaatggtttttcaacattatccagatgttttcggcaataaaaaatttttaacaaggaaaatatgtcatgggcat
tacaattgataaatgaagaatctatagatttttctggatttacatttgtcattgcacatacttgagactttcagtaaaattaaagtaatgtcgcttggc
ttaaccgggctttaaaagaggaaggatttttttaggagaaaacaaagaaaaaagaaaatgtacagaatgcattaatgtgcaaccttcaaatata
tatgcatagaatcattacagcttaataaataaaaaaattagactgtcttaggtagaacccatttggacgaatttaaacggccatttatcagcccgg
gccgctttaacccactcacgtaaagaatcatgaagtttttatgaaatttttgacttaaaagtaagttagcaatttcgaaagggtcaagtttctcga
aacatacctgctttttcggaatctcaatgccataattctctctaatccactgaattgtaattcaagtcgcttacgcattcgatcccttttgttcaatgaa
gctaataattcattagctagttgtatatgggaggcaacataactagttgcttccgtttcaatgccttcaataaatcctagcggaggatcataaagtt
cgtctagattaggttccaattcaacgatattcttatcatgattccaaacccaaaatacgttctttgcaacgtattctagttcgattatccatcttcgat
atggtcgttttactcgagcctccttcataaaatcaacttcctcaagttcttcttcagaatcctcaagaaatgaaaataccttttttagcgccaacctctt
tattggaatgagctttgaggtataggataattatttggatgataacttctcgagtagcaaagtcagagggctgctttttttgagaacaatagttgcac
gagttcttgaacgcggtaggaagatcgtaacaagcactctaaccccaactaagacaaattgttagtatacgaaacatccttagttctcaataaat
aattaaatatgtttgcccttcacaaagtgtagaactcttacctgaactgtacacatggccttaagacagagaagaaattgatccaagagagcatc
atgtaagctttctctccattcaattcttgaaatttttttcaacggtacaaaaaattacaccgaatcccccatcgtggatgaaacatgcgacccacctat
ataagtcagcaataacgaatttacagagaaagaataattgaatgacagtatgagaaaagagaagcaatgaactttaatctgctaaactgcttctt
gtgtttgaaatgattcaccacattaaacttacagaatctgctctttacgaagtaaaagggccagtttgtgtattatagtctcatccatagattcaatt
ctggtattcacgatataggaaataaagtcaattggttcgctgtaggacggttttttctttttccatgacaatatccggcttcgtttttttttgtttgattg
attcaaactattcagaatttctagttttaagttatcgttcaatgatagcaggcttctggatgcttcagtagaaagtacttgggctttaattagatcttc
aaagcattgagggcttgattcttcagaggtcgttaaagacatttgtgataacgactctgattctcgctcaagcgttaatgtgctatttgaatttctat
cagagccttcagaactagagcctagaaatcttcgaaatgagcgtagggtattcattgtctaactgctctatcaatctaactatagagctttgttcca
aattcacaaaacaaaaagagcgatcctataactcaacaacaatgtgttcattcacctaaacattaaaagtgcagacaaaggaataagtaaaaa
tcaacgttgatcaacacatttacatctgtcacatttgtaagagtgggctggtataggaactatgcacagattcaccaattctagttcgctaaagttg
aagctgaagaaaacaataccagtcttaagaattatatagaaacatattaattgtttatttagaaaaaatcgtttcatcggagcatcttgaaaatag
tagcatatcctagttcccatgcttcacatgtttgaagcaatactaaaaaggaaatctgaaagacagtagctaactgactcaatctttaaatgaatc
aagttgtacttatggctccaagaaagataaaggatcgaacatgatctcttttcatgcaatctctatgtcgaaccaaggaaacaggcgaaagaagg
ctaaaagcatcaaatgagtgctatctgaaacttcgaaaaagagaaggcttcttagaaacctatggatagtcgttcacaggcactagtcgagcag
gctcttagcgatcgacatgaccaatttgaagagacaatatcttgagcaggatgccatatttgagctctgttcgtttcaattcctgtgagctaggaac
gagtgtaacgaattcattattcatagttcatagaaaagagaatcgattcctcaaacatgaatcccatcaacatgcgaattcgaagcgatgtcaga
aaatctaagggatcattccaatggagtaaaacgataattacggaaaagctatataatttagcctatctttagaaattatggaaaggtttgtacgag
aaaagtctgatacaagcttcatccttatataaaccaaaataataggaaataaagaatttatattattttagaaacttcattagtctattccaacaat
ttttttaatagaattgaacaatcgaggatagccttctatcattatatgtgcgtgttttcgtctgcgtgaacttgcatactgtctacaacatggggacg
actggcattgaggtttttgccgctctaaatgattttacagatggtaagaaataaatattggaactattggattactaattttagccattgtttccattc
cagagtcagtatgcagtaactttacgtatttaaaagaagttgatgcccagtctcgaggtacgtgtctgttatggaaaaagattataatgaactaac
cgaaaacaagaattaaaatcaaaagtaacagatgaaattgaatcaattttgaggaacgaaagaacaggaagtcttccattaagtgatcgctgtt
tacagctagaaaaccatatcaaagaaatgctccccttttcagattccaagatttgtttggcaacagaagcgatgggaaatataaaccagtgcatt
gatcgactggatgccgattttgaatacgtggagctggagattccacaacaattacgtttaggatatcccgaccggcgtgctttattcaaccatccta
attccggtaatcaaaggatagaaggaaagagagaaactcgaagaaatcaacctcaacagcagcacactgataatcaacagcagcatcagcat
acttcttacccaggacaccccaatgaagagcgatcctctgtactccatggagacgaaaacgtggacgttccccatactacttatagcactcctagc
aagcggcgaaaagcaactgcacaccagaaattatcttctccctctcatagaacaaaacccaatgtgatgaatgttacagaacgaaggccgggac
gacgtggaactaggtatgtatttattaatggtgtagacagccagtaaaattcatgcaaaccagctttactaacgaaaatttagacaagcaacaga
cgatctttcggcaaaaccagacatagtggagccggaggaattgaacgaaggcgagcaactgtattgttactgtcagcaggtttcctacggccaga
tgattggttgtgataacgaaaactgtaagcgcgaatggttccatttaccttgcgttggtctggtagagccgccaaaaggcatttggtattgtagaga
gtgtcgagaaagcatgaagcatgaagcttgatgaagtttggaggaatatttgatttatttattaattgctgtctgttgtttctattttattgtattttgtt
ctttttgtttttgtgtagaaagttttcctacgtctacaagactccatgatataggattaatggaaaagattccctgatggactattacactttgcgattt
tcgattggtattatgtttcttctttttcataacgattagactgatcggtgaatgttgcagacctcaaaactaaggtcataatatgatagtctctcgtttg
aaagccaatacacgaattaatgagatagttggcaatagtaacttgcaagtgcttgagatttaatattatacaatgaacatgagtttgttgatgtaa
accaaagccttttcaagtcttgataaaattgcatcacgttgaaaggacacatacagagggaagaaaaaatatgaattatatacaagcgaaaaag
tcataaaagaaaacaatactacattttaaataaactgtgccatgctgcatggttgcccgttttaatatgagttaaaattgcctttttcactgttccaa

aacattaggtatcttgagaatttcagtacctcgtttatattgagctacaaacaaaacctgttctgcgcctaactttgtaatcttcttcatcgttcgtatt
ctaaaaacaaacatattcgtaagtttgaatgcaacttggagagactaagcatttagaaaaatatgaagtactcattcaatgttgtgatgacttgtg
atggatgcaaaaatgccatcgatcgagttctaaaccgtttaggtaagtgaattatctggagtatatcaaaggaatgactatcatgcagcaatatat
tgaagtcctaagtgctggcatgaaaagagtttcttgtactaaatcagattcgtacggtttcactaatgaacaaacataggagttgaggaaaagac
tatttctttagaatcccaggaagtttttgttattactgacaaaccgtacgatgcagtattggaaacaatcaagaaaacaggaaaagaagttcgttc
tggaaaaactatcgagtaaaatatactggtgtaatggcttttattttcaattgtcttgcctttatattttaaatcaataactttaaaccaactaggaat
gaattagaatttggttacggtactactcttccaatcttctttttttctcttcggtcttttcttagccgtgcttttataattaattcatacaactgactatcttt
gctgctcaaatgaaagaacccccctttctcaatttttttctcctctaagctttaattcctgcctgaatgtgttttaacttttgcttgcaaatgtcactaaag
agctgcaaccttcaaacaagggctcgcgaaaataccatattgattgcacagcacaaaacttttgataaaggcaataagaatccatccgtatgca
aatggtgttctctaccgtattggtcaatgccatcttttttttaagcattggtagagcgtgtagattctaaatgagagctacttctgtaaaacattatacg
tacacgagtagaaaaggaagacatagaatggagtgagtagaatctgaaaaacctagtcaagttatggattcaagactttcttttgtacgttgcta
agaacggtatctaatgctgacacagacttcttgtaaagtacttgcatatggttttcagcgtcatagcaatcatctgttgtccatacccgatccttctta
tatttagcaatcgcctttttttacatcatttctgatagaagacagcgcatctttgcttttctttgcatggttttcaagttctttagctaaccgctgtctgctt
tcttgtgttgtcttttgaggaacaaccatgatagttgatgaatctgttttagaagccttaatcggaaatgtagattcgtccaatgcttttaatacatgc
ttcatgtaagattcctcgtatggatgaatcatgagctgctgggcactttttttagtaagggaagcaacgtcgcggagcaattgacttccacttgagc
ttttcttagaggtagatgatccaaaatgaacagaaagcttttctaattgattgaaaactccttgattcggagataaagccatttccttaaatcccgc
ctctaatttcttgtgaatctgagacatatcattttccatcttctttaagacatcgctgtacgcttctgaatctttaaccttctcagaagataccttttttac
tttttttttccttccattgcgcgctacttatatgaaaattccgcacgtaatttggacgaatccaaaaaattgctagagaacttatgccattgagaagcc
cgtgaaatcatatttaaatttttcattttcttaacttataattaaatttacctttcaaagttttcgcaagtttgtgatagtcgtggcctatctagtaagg
accaaaattgtcgacattcaaagttgtagttaggtaaacattcatatcgaggttactcgcttcattttccttaacacttgccttattacttttaatcttt
tttcttttgagttaataatttttattaccatagttaatagtaaatcttgcttttcgatacgagaaagtttcttaccaatgtgaaagtatttactagaata
cagtctaatatatgggcaaacgcaaagacatataagtagaaataactctcagcattcgcctttgagccttttatattttaaagcgtcgctatctata
caatacaatcacgtaaaaacaaaaaattacaggtagtttagtgattcatccagctctaccaaatcattcataattgtagagatcggtactccacat
ttgtgagccatttcatgtctatgagcgacagacatgtatgtaaaccgctcaattaacgtaccatcaagtatccgatgagcagcattgctcaaattcg
aagctgtggaaattaatcgatgcgaccggggattcaatccaccgactgaatccactctattagctaagtaattttggataatattgagacgacgat
acactctttcagatacaggacttatcatctgtaaacctccatcagcgtttaccatgacgcaagagaaatcatttcctgtatcgtactcatagtcatct
tgtcttgacttcttctcctttggtacaattttcatagctgtaattatatgaccaatatgaaagtcacctctggtaaccaatcgttcacctgaatgagat
tctggattctcaggatcgtaggctaataatctcaaattgcccttagtatcagcaactacaaaataaaggttttctccttggactaaaaagtccgctg
ctgtaactgagaatgcatcttgacccttagcaaaaagagtcattcgataaggctcttccgaaaagccaacgaacgaaacactttgtcgaatatct
ccaaaaagaagtaaatttctcagacattttgcagtaacggtatagcttcccaaatctataaaagctacgccaaccagatggtcttcatcctccaac
gcccgaataattatttttttgaccctgcccactcaataaatagccgtccacttcgcagacaacagctacggtacctttaatctcttctcttgtaaccaa
cttcagtttatgtcgagtttcaggttggcctggttgagggacaacgtcaataacttcaaaaagataagtagcaccgcgaacagcaacatcttcacc
ttttgtaatagaagttccaacagcgatatatggttttctaagttttgtcgtctctgagacttccaaatttacaacagacacagacaaagggatctcat
atggcaggaatccatagctatcaataacggtccaggttaacggtgaaattaaatccaaagagccggtattagcataaggtaggtaatccttatca
tcgtttaacagatataattcattcccatcctcatcagtgacagtaaattcagacggtacacagcttcccacagcatatatcattttagtgggatgat
aagcaataccattaatttgcttatctagttgaatctttttatatgcccaacgagaatcataattgaaatcatcctgaaatttacagatgcgaataaa
agaattttcatccacatatatgtacccatgtggagtatgctcagaattaaaaggcgcaattgaaagaatagaactttcggaagtaacaggataaa
atttagcatttgaatgcttagtactgagtatataaaagggcttactaccagtaacaaacacagctgagcgacttcccaccctctcaatggccacca
tttttgaatgatctattgaactagttgacttttcatacgaagtagacccagcatcacgaggtagtgcccctcccacttggtattctcgagtcattcctt
cttggggcacttttgcaaaagaaagtagatttctctgtagggtctcgtcataaaaaacgaatgctttatataccgttatctcattcattctcgaacgc
aggaataaatggggttccttaaaatcatcgcccaaatcagctaccaataattcaactaattcttgactagattcggaattgaagtaagtagtttctg
actttagcccattgcataaagttgggggagatcaaagacatcacactccatcaaaatagagaaatcgataaggttaaacacttttagtacacct
tctgcggtcaaagcaaataaaattggatgcttttctagtaattgctgatcaagattactgacattcttttcagtgactacgttggcatctgcttcaga
aacgctcttggtttgggtattttcattttgaccatcatcattcaaagcaccatatattcctgaggtttcgttaaaacccgcttgatttcgctcagaatc
attcaaagtccgaaaaagtctagaaaatatcgttgaatttccaggcgcaatcaacgaagcggttttaacgctcgaaagttggctatgtaaatcgg
tcctcgtaagcaactgtgatctcaaatccatccgatacaaggagatatcaccacctaaaaatacaataactatgcaaggatcacaaatactggtt
gacaccacaacctggtttttccaaaattgaaaagttgggtccttttcaaggtagagtcataaacgtgtatgcttgtgggacatatctgtaccattct
cattccagacattagtgagcctacattgatcgttttcgaatgtttgttgaaatccgaattctctacttcgtcaaaagtttcacctgcagaaaacataa

aagattcatcgtttacagataagactaaataggcgtctggttccatattcatgtaagacgtttgtactcttcgacgcgaacgcctttgtctccctgta
aggctcacagaccataaagcttcacagccttggaaaacaaactcaccagcgacaagaggaaaaatacttctacgaaaaacgacaagcccgcc
agctccaccagctccactagttccaaccagctcaagaggtccatggttatcttgtggatagtacgctaaagagccagctttaccaatagcaaagtc
tgttataggtccaatattgagcaagacgtcaaaaatactaattcgaagaggtccgttttcaaaaagcactttaccttcagtatcgttacgatcgttg
gtatcatatatgtcgagcatttcttccatttcagcatcattagtagagtaaaagtcctcattttcattatcttctgtgctttttgataagtttgagtgacg
attccaatgaactaaatagctatccgtcacctggcttcctaaaaatatcatgttttctccaattggtaaagctgtagtcacaccagctctcaagaag
tcatcgtttaaatccaagtttaggttttgtagagagagaccttttactgtctttccatccaaaataaaatcaacgtaaaaaaattgaccggaagtaa
ggattaatagtacaaacggattttccacttttgaattaactagaggtaccgccatagttccctctagttccatattaaagttcgattgatcttgaaga
ggaaagttcgtacacttaggataaaacgtattaacgcctacaccaacagtacgtcctgctgagtccacatatataagttcattaccaccaatcaat
aaagaaccacctagaggcattggtattgggactaactggaaaatatcatacggaaggttttgtattgtggtgatcacggcactagcaccctgatc
caaatctaaagtaacgagagagaaacaaacattgtctttcacaatcggaagggtaactgtggttgtttgctgagggctgtataagatagccagag
ttggttctcggtacccatgtagaaatttaacatctagtattcgtgcaattgtggactctaactgagaggaatctaaaacaaaagaaggtttataag
gagttatagagttcaaaatgcttttaggaccgtctatggttgcttcctccatttcaaggtcgtcaagtttggggaaaggaataatcgccatcatgtct
gtgtggaacctcagcaaacaacaattagaatctggatcaacaatcaattgtgttgggctatgatttgtgcatggcattgttggtttaactacctcgt
aataatgaagactagtagtaaccaggctttgagttttcatgtcccactccaaaatagatattttggcgtaatctgtcaacataactaacagatcgca
tcccttatttccttttccctctagaatggaaagagcggtaactgttccaaaaacttgaaattgacttacgagacgtaaaacgtaactagtatgttcat
gagtacgaatcaaatgcatattcgtctccataaacgcttgactatcgtcaattaagttatttgtatagtcattttgtgtagaagcttcagattcatcct
tttgaactttctcaatctcaaaaacataaagggagtcgactttaccaacaatcaaattattggaagaaggtgaagataattttccttgaacagcat
ttttaacggctgtagaatccgtcaaactttgaaatatagttgacataaagccgaaaacatatatgttgtgttctaaccacacgaagaggttgagaa
ataatggtagtggatgtacgcccgtgtttgctatttaaggtaaattaatttagtagcttgctcaaaaagaaaaatgaaatgactttaaagtaataat
ttagtaaaatgaaatcggtttgggtccaattttaaacaaaaaatttgatgcatgtaatacttgaaaacggacttgctctggctctcgaatataaga
accaagggctaataaaagtttctcagagtcactagaaatgttagcctgagatatgattcatcaaaacgcgttcaatgtataacgtacttgatgtga
cacctgagatacaaaagcgacgcgtccctgtgaattatatccacggaaatagctgatccaaagatcccgaaagattgagaaagagctaaagta
atgaaatgccttaaggttagttcttctagttcaatctccatcgaagaattttggagtctgcaatggttagcaaggagtcttggcactgaaggaaacc
ttacattttcactagtatataataattttcactttttatacgttgatgcaaaaccatgatctgttcaaaaaaaccaagaatggatcaagtaagttcta
ataattggtagtgagaagtttcaagctgtcatttttactgtgcgaaaagaatgttttcaatccttataagctctttttattgtttttttacaaaatcatt
aaaaaaagaaataaattggcctatgaatgaatttttgatatgccgttctctgtctatagaggatcctcaagtttatataacattattaaagcggaag
tcaatcttgaatttatgtggcttaaaagttatgagtaataataatgagaggaaagctttcagtgatattccaaatatatctgttcataaagtgtatct
agcatatgagatgaagaaaatcaccgcacattgagtttatgcttccattctcggtataagttttgcatataagtcatgcatttttctttatcggaagg
aagtactaattcaacggacgtaggaagaatcttttccttcccaaacatagtagtggcacttccccattttatattttcgtcttctttgtgagaatgaat
agctatccataggtaagaaaaagctgctatgtaaggagaaacggtatgccacggactacccaacaaacgacagaaccagaaagcgagcttgg
caaagaccacaataaaaccaagaaaagtttcatacatattagttgtgcacaatcctgggtgaacgacatattgctggtacggtagaccactttca
atttcagcaaaatgcaaaatgtctaataaacgcttcgacgactgatagggtctttcgccgtgaaggcattgtatatcgtcaaagttaagagattctt
ttttagcaacaagacttccagttaaaactattttggtagaagatcccagaacacgaagttcattaagacgtgttttctaactcattagcattcaaca
ctaaacttgatctgacaaacaaataataatttcttacaagataataatggccaaatacattactttgaaaaatatagcccatggaatccttgcttgt
tcgtcccatagcttccttgtagtaagttggataagtaagtgccttcacaggattcattagtgtagcgaatatagcttgtatccaatgaataccttgta
aatcccatgttcctgcattaagataaacgtagtccaatttagggtaccgacttttgatttctaaggcagccgcttcaaccgattccatatctgacaa
gtccaaaagaacgtattccagcctaattttcctttccggaaataactcctttaatcgtttacaggcatcttcagcttttgcacgactcctgcacgttaa
aacgatggtaaacgtttctggttcatcttctattctaggtttatagaattctaataaacgagcggcgataccaaatcctaaaccgctaaaaaaaaa
cattagttaatgatgtattttattctcaatttacctgttagaaccagtaattaacgcatattttcgaaagctcatgcttaggttggttaaagaaaggc
ggatgaaatcaaacgacttttatgtgtgtgccctattgtttgaaaaatatcaggacgagtcaacttacattgattttgactattactgtatagtatcg
cccaggtatattatgaaatgatcaagattttgtttagacaaacatgctacgacatacaagtattaacgcttccattaaaaacgaaatatatatatat
atatcatacttaaaaaagaatgtacctgaaacaaaatattacaaaaagataaagagatactcagatttccattgaagtaagttaacctgcttcca
ggaaatcttctaaaaatttagggtgaggatatcgtaaaataaatagcaagtctgcgattaaaagaaaaaggagtgatgtttaggtagcgacttct
ttattagtgttagagctttgtatttttctgaaaaggatatcgtagatagaatgggactggacgtttggagactcgtcgtctttgctagattcctgttgt
ggaatacataaagactcgatttcagctgcagaaaattgtagaatctgcttatgggcatttaaggcctcgaaagatatagactgtttttctttagaac
agacgatcaaatcagactgactaattttggaggctaaatcatcagtggagactatgaccaaattaattccatgactcctatatttctcatcataacc
ttgatattcttcagatctttcattgtctgatttgtaaacaatcactttggtgaatgaagcagcagctaactgctttatggcattttccgtcgaaaatgc

tttcagaagcttagaaccgttttcaaaagcgaacagaattgacttttcatgcttagaaacagaaacagttccagaagcatcagagtgcttttgctct
aatgcgaaaatttttttaaatcgtcatagctctttgcacattgggcagcttgtctaacgtcttttctgaatgctccttgcatgatttgggttagacaat
acttcgtgttcccaaagttgttttcaacctccaatgcctatatttgttaggaataaaatataaaacaagtaaaaaatctaaaatttttagaaacaca
ccattttaaggtagtccaaagccaaatcatgattagacaaaggaccttctgaacgtaaacaagaaacatttttttctgctgctcgagcaatcaata
caccatccagattgtacttttctataagctttttaccgtcttcatgaccagacacgtctccattagcaagaatggaaacaccatatttctacagacg
ttgacaatatcatgaagataatctcgctttgctggttcggtattccgcattggcgctgtacggcaatgaacagtaattgcacgaatcccagtagaac
aaagacgctctattaatgcaagtgttttctcttttgtttctaaaagacggattttgcaactaatcgaaatgttgtacgggactcctacctcctttacca
aagcctgcaaaattgatataaggcgatctggttcttttaaaagacccgcacccataccagcatgaacagagaagtgcttaggacatccacagttc
aaatcaataccagcaacatcgttagcaacaagctttgctgattccacagctagatctggagaagcagaacctagttggaaaaccaatcgattag
cctctaaaggatgcacacgaaagagaactttgtttgtaggaggcttaacaaaatcgatacaattcactcgatctaaagcataattagtttcttttca
ctagaaaaataaattttaaagagagaagatgatgtaaacattgctagttgaaaaaaggcgcactactttgcgtacacaattggatcaatcgcaat
aggagggacctcaaatataacaatgcattctcaaaagacttaccatttactactctctggacaggagtcccgccaaccaaagctttgtctacaatt
tccggcccccaaaccaagtttgctccataacgaagagctaataatctcgttggaagctctccaattcttaccatgggagctaagcaaatcttattac
tgtaattcagtagtcccattttgtagcatacagcgtcggaatgaaaacgagtgcaacccgtatgtactaaaggcaattcctcgaactgtcagtagt
ataaattttcagtagtggccaattatagtggtcactaccattcttatatatttatgttagcaagcaacttttattcgtatttaaaataattttctttcga
atttttttttatctttacttaacacagaactgtaattgtcttaatcgttttgtaaaaatagtcgaactctcattttattcttgcgatttattaatcaaata
gggttcgatacttgcttctgcaaacgaaatactaacgaaagtcgcggtggtgtagttgggagcatgacagactgaagatctgttggtcaccggttc
gatcccggttcgcgacactttttttgtttcttgtgaattgagaaaagttgtcaaagagaatataggtgtcaaccgtctcttctaatgcacagtacacat
aaagaaaataaaatttaataataataccacatacatgtatagtttataaaccaataaatcctctataattacttttaataatattcatgcatccttac
cgtacaaggtcatcataaatgcttaatgaagtacattgctttctttattgaaactcctaattacagctgctacaaagccgataaataaatcccaaca
ataacatagatcctagtaattattatattttgtttaaccctagacaatatttagtggtatcaaaatatgtcttctaaatcaattcctccaaaactatga
tatatacgttgtatatcctttttaacggagagaggcgaaggccttttgaacggtacgcttaagacgaagctcagacttacggagagcatcagtgtt
agagtcttcaagctccaattgagacatacattcagccaattgttgttcaatcttagacttgtcaccacgcttcaacttcatcataacattaggctcgg
aaatggtggtttcaatgttagaaatgtaggcctcaagttggctcttagcttccaacttcttggcgaagtccttgtcttgttgcttgaacttgtcggcat
tctcaatcatttcttgaatcttagcagaggagaggtgaccaacggagttggtgatctcaatgtgagcagtgcgaccagtagtcttctcaagggcag
taaccttcaagataccgttaacatccaattcaaaggtagcttccaattcagcttggccacgaggcatagggggggatgccagtaagttggaattca
ccaaggggctcgttctcagagcagttgacacgctcaccttggtaaacagggaaagtgacagtggtttggttgtcagcgacggtagtgaaagtacg
cttcttgatggtggggatagcagtgttacgagggcagacaacaccgaaaacgttaccttccatggcaacacccaaagaaagaggaacaacatc
caacaaaagcaagtcagcagtcttgtcagagtcagccttgttggtaaggacggcggcttggacggcggcaccataagcaacggcttcgtcaggg
ttgatggacttgttaagagggcgaccttggaagaagtcagaaacgagtctttggaccttggggatacgggtagaaccaccaacaaggacaatat
cgtgaacatcacccttggcgaccttggagtccttcaaaaccttgtcaacggggtcgatggtcttcttgaaggtagtggcgttaatgtcctcgaaacg
agcacgagtgatgctagaagagaagtcaacaccgttagaaagagagtcaacttcgagggtggtttgagtgacagaggacaaggcacgcttagc
acgttcgcaagcggagcgaagacgacggagagcacgaggatcttcggagatgtcaatcttttgcttacgcttgaattcttggatgaagtgttcaac
caaagcagcgtcaaagtcttcaccacccaagtgagtatcaccagcagtagccaaaacttcgaaaacaccaccttgaatcttcaagagggaaaca
tcgaaggtaccaccaccaaggtcgaaaatgaggacgttcttggcagcgtcggtcttggagtcgaggccataagcaatggcagcggcggtaggct
cgttaatgatacggaggacattgagaccagcgatggcaccagcgtccttagtagcagcacgttgagagtcagagaagtaagcgggaacagtga
caacagccttttcgacacgcttgttcaacttagcctcggagatttccttcatctttgtcaaaaccatggcggagacttcttgagcagtgaattgcttct
tttcacccaagtagtcgacttcaatagtggggataccattgttatcaataaccttgaagggccagtgcttaatgtccttttgggtctcaggatcttcg
taacgacgaccgataagacgcttggcgtcgaaaacggtgttacgagggttcatggcagcttggttcttggcggcatcaccgaccaagcgttcagt
gtcggtgaaagcaacaaaagaaggggtggtacgagcaccttggtcgttagggataatttcaacattggcggtttcccaaacagcaacgcaagag
taggtagtacccaagtcaataccaatagcaccttcgtaaacttcactcattttctggcaacaaaagttaaaactgaccctcgatttttttgagagaag
taggttattgtaaagttgcacttgcggtgaacttacaatatatgagaactcgcagtcacacaaattttatacaataaaattttatgagcaagtagtc
actaatgtacttcattgtatttcgttattatttgcccatctcatgtttattttttttgctttttcttttaccggtaaaattttttttattgtgccactaaaggcc
ctaaattttttcgactttaatttgcttagtggtacaaataataatagtttataaatgccaccgcagtaatctttttttaacatattttacgcaaccggcat
aaaaatttcaatgcatttaaccgtaacatccttaaagccatacaacacaatagaatatgcttaaatgttaaaatactttaacgcattaactgaatg
aaatcgatatttccaccagtttctggagagaatgaatgacactcttggtaagtgggtcaatcatgcataggaaaacgaaatacccatcaagtcat
ggatatacattcctgaaccataaagtaataatataatactaaagcgaaaattacactcttgaagagtgtacgctatagttcgcttttgaagatata
ggcatgatttgtttacaagaaaatgtaaataaatatattacatggataatattcactcatcgacttgaaatgaaaccaataactcttttgcatggat

gatgaagacattaaaaaaatacagataaaataaagcttcaattaactaactgtgtgtaacgtagctgataattgagactatagatacattagagc
gagtttctaatttggcacatatttatataaaacgatatcttaattaaaaggtatatttattaaaaggccgtaaagatcctgaatatcttgacttaagt
aaacgagttgaaagaagtaaatacgatgcccttcctgtttgttttaccagcattcatcacaaattatttcaagtgcaaccgtttgttagaaactcaa
ctggagctgctattctgtctataagaatttcttcttcatagatctgggtatttggattttgaaatccgtctattttgaaatttgaaatttattttttcgtc
cttaatgaattatattttatttatttgtcttggagtatttactttctctcaatgttcgccttcttccacctttatttttataggtttcgtacacttagaaac
gcaaatcaaattacctttcatactacaaaccaaaaagctacacttctaccatttacgaattttgatatactgcattgtattttctctttttaaattgaa
cttctatttagttgtagttagactgaaagttttccatttgtcgagttacatattatgagcgctgaacctgtggtttttttgatgtatgtttaaatgtaac
tgttgttgatctaatttatttttctagattgcgattggcggtaattatgttggaagaatcaaaattagattgttttcaaataccgtacccaagtaagat
catacatgtttttgtatctctttactttaactaattatttgcaaaaaggaccgcggagaatttcaggtaggatctcgttaacctacatatgatgaacg
tgaactaaaacttacatgatagacaattttgtactggagaagcaaaaggaccaaatcgaaatccaatcggtatgacaattattttttcgtctcag
tcccttgatactatatactaatcataggttacaaaaatgcaagttttcatagaattatcccggatttcatgatccaaggaggtgactttgttagtgta
tgattcttcattcaggttttccatatcctttgtacaagggatttagttccatttactacttctctcgatttattatatacgcctcactaatacttgtttttta
gggtgatggtaccggcagcactagcatttatggtactcggactttccctgatgaaaacttcctttaaagcacgaaaagcctggtctactaagtatg
gctaatgctggaaaagactcaaatgggtgtcagtttttttattaccactgtcccatgtgactttttggacggaaagcacgttgtttttggtgaagtgat
cgaaggatttgacgttgtaaagcaaatcgaatcaacaccggttggaactagctcccgcccaaagctgaatgtggttattatggagtgtggtgaaa
tgtaatttctattgcaaatatgcaaaattaaaaaaaatcatttcaaagcttctatggattcatttatgcatagatttttgttttcgaaaattaattaga
cattcttcttcgttataggcgtcgtagatgtaagaacatttgcgagttcctcttgtccaacactaaagagcaacttaatgcagaatatgagtaagcc
aaagccaacaatagcaaaccaaaagaaaaattgaaattctattgatatgtcgtgctttgcacagctttctccagcccattggatcgttctgtttcca
ccattaccagcaggaacgatcgtcgaggcacactgacaaacaaaacaagactctttattcgcatatttcacgcattcacctctaccagagcaaga
gttcgttgcctttctgcaggccgcttctgtgatgaaacagcgtccatcggcatttcggtcattgctggccactacttggtcgacctcttcaccaaatgc
ttccaaatcaagttgacgcttttgatatctagtatttcggtccttaaactcagaaggggggagaaaaacataactaaaagatgagagctgtttcag
ttgcaataaaagcgaaattatattttcttgtgcaatcctgtacttttcagaagttcctccatcagcatgatatattatctgtgatgttagtcttaattaa
taacctctcaaaataacttacctctaaagaaacaatttgaccgagacctatagagggatctgcaacgtttccactcactgatttcatctttagatac
tcgatgtactcatgcagagcagcaatttccgacaagaagattcggtcctgcttttcgctaatatcgaagaatgaggcggaaacttttccaaagagt
ttttcataaagacgtagataacccaaatcatcctccttggtagcatgcagttgaaaaagggcatgaacggatagggaaccgcctaaatcgttcat
gtagaatgttctatcttccttcccatctgcggcactcaattgtctttgaaatcttcgagttaaagctccaaaagagttagtggacggagtgtcttttat
aataaatgttgggtgcatctcctttacaggttcgtccaagtacaagccggaaactgtaagaaacgttttgaatttgaagtcaaaaaaagaagaaa
gagagttttcttgttgtaatagttcctgaagaagctgatcagccttaggatttccctttagattatgaaactgagaaatgcccaacatgtgagcaaa
gatgagtcgcgcttcagaaggattcagtgaaggaacatgtgtaacgtctttttttgtttgatgaagacatgtaaactactgcgttcgaatcccaagta
cctaggtcaactgttaatataccaattcctgcttcgtagctcttttacaaacctgaaacgcatgctacaattattccgagcagcaatgaaaacaaa
aacttagaaaacctcatctttatttgtgtagaaacgaataaagacgacgactttctactagaggtgttggctttatgcggtgatgtgttactgtatca
gtaggtctgagcaatgcctagtagtagcgcaagctgtttgtcaagttgtagtgagatgaaggataactttttaaaatttcttaatattataacgaaa
aaataatatgtcacgtgcatcttattgatttctttttgagtttttttcttgctaaacttatgaaacagaaggcgtactttttttaatgggcaaatgcttcta
gtactgaatttaactgttctttataatttatatctaccgacatataacgttgtttaactgaaatgggaagctgaagatgaatcgagtcaaattctattt
ttgactttgctacatgaaccatagaaaaataacatcgcagttttgttaaaagctaaagatattaccagaagaattaaatgaatatgttttacaatta
tgttcatcttttacactcaagtccatctgattacacagacagaattactccaaaaaaaaaaagtgtaaataataacctgaaaacaacagagaaagc
caaattatatataggagaattttattcattttttagaaaatacaatcagctagttaaggatctataaaaaaactagtaaatagatttttatgggcacattt
gttttgtttgtttacatcctgttacctaattcgctgatacctgatgtgttttagtgctgttcgttcatcatcctaaaaggttttaagagattgcgcagtgg
aacgtatgtcaccggatcaaagttttgcaacgaaaatttacgattttttattaacccacactgtgtctgttgttactacaacacaattcctcaatggc
agacaaaagtactgaagctgctcagtccaaggaggttcaggtacgggctactttgctgttatgttctccttcactaacaatagcttagaattcacca
aattctcaggagcctccaagacctaaaactgagaaagaacgtacgtgacgagagaggttaacggactgaatggatgctgactattgttgaattt
agtggaacgtgaacgtcaaaaagctgctaagcttgagaagtatcaggccaaattagctgcaaaaaaggctaaggaggagtctagaaagccaa
agattgagaagaagtccaagattgctgaacctgtagctgaatatgttgaagaaacaaaacccggtgaaaagaaggtcttacaagacttggaatc
tccctctttgaagtcatataatcccaaggctgttgaatctgcttggtatgactggtgggttaagtctggatttttttgaacctgaattcggccccgacg
gtaaagtgaaagaggagggtaagtttgttatcacctctcctcctcctaatgtcactggtgctctccacattggtcatgccctcactattgcaattcag
gattctttggcccgttggaaccgtatgaagggtaagactgtacttttcttgggaggttttgatcatgcaggtcttagtacccagtctgtcgttgagaa
gaagctttgggctgaaaaaaaagcaaactcgccacgactatgccagaaatgattttgtcgacattgtctggaagtggaaagaggaatatcacgag
cgaattaagaaacaaatgtcccgtcttggtggctcttttgactggactcgtgaagcttttactatggatgagaaccttactcgtgctgtcgtcgaga

cctttgtccgcttgcatgaggataagattatctaccgtgccaaccgccttgtaaactggtgtactgctttacagactactttgtctaacttggaagttg
agaatattgaagtgaccggacgtactgaaatcaaggttcctggttatgatcaccttgtggaagtcggtgtcttgacctcaattgcttatgaagtgga
aggctctgatgatagaataatcattgcaactactcgtcctgagactttgcttggtgatactgccattgcagttcatcctcaagaccctcgttacaagc
atcttcacggaaaatctgtcaagcacccccttctgcaaccgaagccttcccatcgtttgcgacgatatcgctgttgatatggaatttggtactggtgc
agttaaaattactcctgctcatgatcttaacgattatgaggttggtaaacgtcacaatcttgctttcatcaacattctgaacgacgatggtaccatga
acgaaaattgcggtgaattttccggaatgaagcgtttcgatgtccgtataaagattgttgagcgccttaatgaacttggcttgtttgtgggtaccca
agataacccaatggttattcctgtttgcggcaaaacttccgatattatcgaacccatcatgaaacctcaatggtgggttaaccaaaaggaaatggc
ttctgctgccgctgaagctgtacgttctggcgaaattgatatcgcccccgatgtctctcgtaaagaatttattcgctggatggaaaacattcaggatt
ggtgtatctctcgtcaattgtggtggggccaccgtattcctgcctacttcgttaagctcagcaatgaaaagaaccaagacaggagcgatgatcgtt
tctgggttagtggtaggtccatcgaagaagtcgaagaaaaagccaaagctgcttttcccggtaaatcctttaccttagaacaagacgaagatgta
ctcgatacttggttctcttcaggcttgtggcctttctctaccttgggctggcccaacgagactgttgacttaaaaacattttacccaaccgttttgatg
gaaaccggatgggatatttgtttttctggatcgctcgtatggttatgttgggtatcaaactcacaggtcaggttccctttaagcgcgtcttttgccat
gctcttgttcgtgatgctcaaggacgtaagatgtccaagtctttgggtaatgtcgttgaccccattgacgtgattgagggcattactcttcaagcttt
gcatgataaactttcggtcggtaatttggacagccgtgaagttgagaaggccaagaagggacaaaagatgagttatcccaagggcattcctcaa
tgcggtaccgatgctttacgtttcaccctctgttcgttaactagtggtggacgtgatcttaacctggatattctccgtgttgaaggttatcgtaaatttt
gcaataaactttacaatgccacaaagtttgctttgggacgtcttggaccttctttcatacctaacgcgaccgataagcccactggcaaagagtcac
ttgtcgaaaagtggattttccatcgtttgaatgttgctgtggatgctatgaacaagtacatggaagagatgaactttttgcaagcaacttctgccgtc
taccagttctggctttatgaactgtgtgacgtttacatcgaaacctctaagtacttgattagtgatggaacccccgtccagcaagaatctgccaagc
aaactctatacactgttctagacacttctttaagattaatgcatccttttatgccttatgttactgaagaaatgtggcaacgtcttccccgtcgtccta
gcgatactactcccagtattgtgaaggctaagttccctataaccagagaagactatgcctatgaacaagccgctaaagattatgagtctatcatca
cagttgttcactccagccgctccatgatggctgataacggtgttaaatctgatgctgtcttatacattcatcctgggcagaaacagcaagagttaat
taatgctgaaattgccagtattcaatctttgatcaagaaatgcaagtcgcttaaaatcgttgatactgatttcgatggtgagaagtgtgcaaaatcc
gaaattctagagggttgtaccgtctttttggaaaagagggagtaaattatcataaatgaactaagtagaatagaaggtatctataaataaaaata
attggcatttcattacatattaacaataaatatttgagtatacacagttccccaattcatcaatcatcagtcaaattttcgttgaagatatgcaccaa
acgaagcaaggttaacaattgccgaaaagcaagtagaaccgactgttttgagacaaataaaagccgtaaaagtccaaaatacaaattttaagt
agccattattcacggaggcttcacctttaaagacttgaagcaagataattgcaaggatgagattactcaaagcccaaacgagaacatatcttgtt
cgcatatcctgatagtagtcttgactggtacgtgaaacagaattagcgttaatcctgtttaaagacgaaccactctcacaatcgctgtcttcactaa
agtttcttaaggttgaattgtagattgagttcaacatatcagcgtttggtatggctatcatagcgaacttctttttcttttcatttcgataagtatcagt
tagatcattgcttgtagcaacatctaaatcattaagtgtcgaagcatcatgaagatggcataacgcatagacttgctccatataaatgcgagccgg
caatatcaaaacatattgccataaacatgtaagaagaaataatggatccaatgaaattattgaaacaaaaatgtaacacgcaagaactattaag
catggcaacatcacatttgtaaaataattatcgccaaaaatatcatatccatgagatgaatgtatgaagattggaactagagaaacgtaaaatat
gcaaaataagtaataaatcatcactgaagtgaatataagcagactaattaaagaaagcgttttagagctagaagattgttagaaaattgcttgat
agataaaattacttacccttttgaacggtttcccattgccaaaacaaattgtgagaatatagcacacagaaatatatattcaaaaatcaggaaca
agacctgaccccatgacccatagggattaaaatatttatatgaagtactattgcatataaaatggaaagacaaaaagaagtttgccaagctaaa
aaatccaaacaatctctctaatccaaatatgaaaacctgcatagctatcataaggaacctaaaaaagctatgtcttgttttccacagagagaaaa
agtccgaaagaatataaatagcaaggctaaaacggctattaatatcacctcttctgccttctaagatttttgccatagattcaggagccttcacttc
gacttgagcttgaggaacataattcaggtaccatcttgcttcctttttagtaacaaccttccaaaataacaattgttcttgggccacataagcatttg
cttttaatatcccctttctttgtccttctgtttcctttcgggcaaagtattgagttaatggaccaggataaggaaattcatctaataaagcaacatatc
gaaaaccgtagagagcattagacgtattagaaatgtaacccagacagctatcatagggaagcctaaggttattataaatcatttgatcacaatgc
tgagatgctattaagggagaaagaagcttctttccccattttccagttttaatgaccgttttgccgcatattccgccaagtttggaattaacatcaaa
gcttttccaagcatgatatacggatgttgtttgtaatttcgatccaacttttacgaacaggcaaatatttggtcgaacgagagaagaaatacctttt
aaaaaccacctttgggaatcatgcgtcccactctccatttcttttaagcaaacgagtaattgcatgggtacatttccatcatttttttgtagtaatattc
agtttttcgtctaccgaaattgtactggtaaattcatacatatgagctaaaacccttttgccgttaactctacctttggaaatacttggctggtatgct
cctatagaggacagcaaatccaaaatgtttctatggacagaattccggccatcaagtactatacaggttaagactttttttccagctttcatcacccc
aaactctcgatttttttacgcatcgtaagatgcttcacgttttccataacactagatagtgttaatgcgaaagaagcaaggtcttcctgccctacagtc
agacaaatcgcagcttggatgtctcgatcaaatagattcgttctcacttttagctcatttgccatcatatcctcaggattacaggtcaccgcagtgta
tcgtaaagaattcgattcttcagagcttgtgtgaactatggaaccaagcatatttttaagatcaagaggacaaggacattcgacgactaagctatc
gtgcattataggaacttcttgcatttcgtgataagctgagctgttattaaaagaagctgtttcaagtccactgaagccatcttccgacatagcatatt

catcttcatcaatcaattcttccttcttatctgcatatgatccaccgctcaaggtttctgagtcactgcgttctatagatgtttcattatgagggacgta
caaattagacgaattactatgatcaatcccaatccgatgtactgggggaataggaggagctggtgaagcatttaaaacactagagtccacatcgt
atacggaattcgtaaaggaaaatgtatcctcttcaggaacctcaagctgttcagatgaggtaacttcgttgtcccactcataatctgatatatgtaa
attatcttcactttgaaattgatgtgtgttcagttgttcatgtccagaatccaccaaaggtgacagttttccataaggccgcgaatacacgatgctag
gatcattatagtcaggtaagggcttcctcatagcttcaagtctgctaaaaaaaagtagcaagcactaatttgatcggtggatccttgctccctttttg
gaaaaacacaataaaaattggttgaaccaatctcctattaacgtgactttggatgtacgctccattatatgcatataaatccgatatgtattgttgt
aactgacaacgatgatgactgtttacactccctttttgtttatttatttttggttaccccagcaaccgtgcttatttcttaaatggagtctaaaaccctat
gtcatacctttttaagcgaataacgaaccgttcaggatattaagcagtcgactaagaacaattacaggaaacagtaagagccttccatttttattgt
ttaaaaaagatattcctttcttttcatgcaatttttttgtttatctgtataatccgctcacgacctcagtatagataattagcgagtacatccttagtaa
ccggtgtattatatttacatataacctataacccgtggtatatcgcgtaaaaccagaataacagatttcaaaggagtaatagattccagaagcagt
agaagaaaaattgagtatgaggagtattaaaggctaaatactctttattttatttttatttttcaataatcaattcattattaccgttcttttttttaccg
gctaatttctgagttcatggtaaagtacaaagtcgtttaggcagggattggggtgatgacatctactacagcaatcaacaacaaatataactgca
gtctcccaatacgcgtcaagttataaacaagttgccagacaagcagttagtaaattgtttgtgtttgcatttatgaatttctagtaatgaacacgttg
aaagagtctactgacacggaacgcaatcatattggtaagtttactatgaaacttgaaagtttttcgttttggagttatcttttattttttatttttttttttt
tgctagactttccaccagctttatgacttttatttcctgatattaacataaaaaagaaacagagaatgaaccacgaaggaaaaatgtgaatccatc
gaataccccgatgggcaaagtacatctgcaggaactggtcattcattagaccctgctcaaagcaatcaaacttacatgcagcaaggaacttttg
atgtttcttctaataataaaaaaaggcagtttgatgattatcaaaaagagcaagcaattgcttttactaaaccctcttcaattcctacactatctgcc
aacatggagacagacgaaggccttgccgcaatgacttcctcaaatagtcgacctttagacgttaatgatgctctttcgtatctcgaaatggtaagg
tattacttttcagaaaaacgagaggtatacaatcaatttttggaaataatgcgcgattttaagtcccaaaggtaagctataattgacaaccgtcgt
ccatgaataactcctttcatttcccttttttcctctttggggatttctactttatttacctgctagatctgtcactaacgttagtctagtctggatactttag
gggtaatcaatcgggtcgcaagtctctttaatggatatccgcaacttattgaaggatttaacacttttcttccctcaggctataaaatcgaagtccat
acggattcttctaacactagctttgtacggatcggcactcccatgcacccattgcacccaggcacaaatacttcctccacctttacaccttcgaccc
attttgtagaaatacaacagccagccatccctgataaccaatacgaaaaacagtctccatcttctagcgaaaatgtatccgctctacccccaaagc
ctcaagtagattttaactatgctatcgcttacatgaataaagttaaggcaaggtatcctcctaattctgagacatacatggaatttttaggtgtttta
cggacatatcaaaaagctcaaaaatccattcatgaagtgcgtgctcgtgtttccgaaatcttctcggattccccggatctattggatgaattcaagc
ttttcttaccagacacttctgaagctactgaagctcaacaaaatgatgctgctacgactactaacggattacctcctgttggaaacttcaccttacct
tcttccgggcagtcccgtgaaagaaggttacgcccagcacattcagcacagataggacgatccatctctaaaacttctaaaaaccttcgtgaaca
tgccgatgagctaccgagtacttatgcaatccctaattatcaacaaaaaacgagccctgtttctcattatgctgctactcaagaggaattattggct
ttcggctctattagacagcaccttcctgataatatttcttttcataactttttggaattactacatctttatagagaaaagttaatggacaaaacggat
ttactgaattcattctcaaggctcgtcaaaaatgataacttaacattatggtttgcaaacttcattcgatggaatgatagtcctatattggtgaaaaa
tattcctatggatgactcccgttatatctctgaaacttatgaatctgctggttatttataccgtaaaatacctgcagaaaaacaaaaaggaacttgc
tctggacgtgatgccctagcaaattcggttcttaatgacgagtacattttagttgctcccaaattgacaggtataaaaaatgtcaagcattctgaaa
atcaatacttgcaagctctacaattagtcgaagatgaacgttatgaatatgaccgaatcctttcagtgaatcaaagggccataaaattattggcag
agttatgtgaacctgttaacgacgaagagcacattaggtctttaaacaaaacattagaccgtaaagccctaatacgatccgcttttcacactgttt
atggaaaagaacactcgacaacagcttttgacgctctagtaaaaaattccagaagggcagctcctgttttattgaagcgtttaaagttgaaggat
caagaatggcgccgctgcaaaagagaatggaataagatttggcgtcaaattgaacttaaaaattcgtcacttgcattggatgaacgttgttgcaa
aattgaaggtaaagatcgaagagcactttcctataatcgaattttaaatgaaatcgatgatatatatcaacatcaaaaacatggtattggccacgt
taaacaagggttccaatttagccaagtactaaatgatcggcaaatctttttgaatattttaaggttaagtgatgctcaactttctaactcatcctttta
ttcttacgcagacaaaggaagaattagtgctgttcttaaagctttactatcacaatttttcggaattccacttcctagagaaactttggaaagcgat
ctttcctctgaaaacatagaatcagttcaaaagcatcgtgatggattgagcaaaatttttgtacatccagagagtgctgataacagcaacacaact
catatgagcattcagacggatgacacccagacagaggacgaaactatgagcgatagtaatcctattgatcctgatactcaaacaaaaatgaaac
tgcaaggagaagaatcaaaaaatataatcggctataactttttgggaatgcaaccatgtatgtgctatttagactaatttgcgtttcttattctcga
cttgagcaaataaaaatgtttattgagacttctagttcagttgagcccataacttacgatactatactggaaacgtgtgaaaaatatttaaagggc
attctatctagaagtgagtttcataaatatctccaaaaactaaacaatgatgcttgttatatgattagttctatagaacggttactaaaagtgatatt
ctaccgagtgcatgaaattttgttggatcctaaacttggtcagcttttacttttattcgaatctgacggagcacattcagcgacaactgctcgagaac
agatgatttatcgaaatcatgtggaaactattttggcgcctgataccagaatatttaatatgagatggtatcccttggaaaaaaagctgtgcattca
acaattgctctcagccgaccttacaattcataaatttgaggatccggctagagcatttatgtattatgtcgatagctatgccatttcccatattacag
aaggtgttgacttgctgcaggtaaaaatgcctttcttgaaaagatcattgcagttgatctcacagaagggctatctcgctggccgagagtctacta

gtttgcattctctttttaatgagcgattttgcaagtcaaatctacagttgttcttctcaacagatacttatatgatattctttgagtccaatacagaaaa
cgtttatatcaattcgtataatttgtgggttgggcaaagctcgaaacgtcgcaatcaaagaagaatggcacgttggcaaaaatggcttgaacaac
aactcgctaaaaacacaactcaagaaataccatctgctaacaaccaatctgctactttggatcaggttctcctttctctatcatcataatgtataat
aaaatacttttatctcaaaatggaacaaaggaaatgtatatattagtttttatagataagacttctaaaatgagatgactatttagcatgcttaaca
gcgcaagtacagcaaaagtacaaaacgttctttctcaattactgtgaaaccaggttgtgttatatcttcagaggtaggtgggacattggtaggttc
actggttgtcagtatgaactccaagctgttcataccaggtcctaatctgatatcgaaagcagaatttatagaatcaatgttgtaataagttggtccg
gctaactgtaacaactgaggagaagcggctaccggtggtccaatcagttttccattatgtagtacggatatattgtaatttctgatcgacaatatgg
gattaatatttgtaattacatttagaatggcatgttgctgtgccaaattaattacagaaatttctgatgcattagaggacggacaaggaacatgcat
gcagagtggattaggtaacgatgcatgagaaggagaaaatagaaaaaaagaataaaataaacgttctgtatcgcctgtattttcaatcactttg
tttgaggaaaattaggaaacacgaagtcagttctacccaataccgacttatgagtagaatttaagagatctggtactggctttgtagggaaagatt
tcatgagggcaaaggcactatagttattttgacaaatctggtcaataggactaggctttggaggttgctgttcaacaacatctgacatttgaggtgt
aaaatcctcagtaagtttctctttttcttccgcttctgcaggtaatttttcatgaagtaagggatcacttacaacagatggttctcgagtctgtggttc
gaaagaaggagcagcggaaggcattctttgggttggagattcatctttgatattctcgacatctttaggggattccagagaagttttgtacgtggat
gtcaatatgggtgcagatattttcgaaagagcaaccgaatcctcataaacgaaactaccagcttcgttgtatgtttgtgcatttgcatacatttgctc
tagatcgctttcaaagtcctttactgagttgtattcgttctttttcaggcgtttctccatcatttttatcgtcattggcgtcttgataatttcgtaataatc
tggataaagacgtttgctcggtaaatcaataaacatatcagctaaatatctaccggtagagtcttttgcatttttcaaagcatcaaggacattagta
atgacagagtgaacttcatagtccttgccagaagtctgttcttcggtagaaatgggtacttttgtgtctgtggaagcaccgggctgagcaaagtgtt
tagcctctaaatcattgacggctttttcgaccagcaaggcacactcatagacaaagcttcctggtagattgtacgttttagcattgtttaccatgagt
cgtaaatcatcatagaattcacccatggaggaatactgattatgacgggctttgtttcgcattgttttataagcaataggatcagtaataacctgat
agtaatccggaaaatatttcttagaaggtaattcatcaaaaacatcatcgaaagggttccctctatttttagtttgtcagttaatcaatcactactca
tcgtaatcacctgaaaggaaatacttactcctgatctttaaggtcaactaggaattgtaaagcttcattaaactttgtttgggaaaactcaacctga
tgaccgtttatcgaggactgaatattccattagcaatgctgtttgtaaccacttaaaaaatttcacaaaacctaccatgttgtaatccacttctcaa
tatttatgttccaacgtttacagatatattcaaataaaaaaagcacaaatgcattcagtgaagatactaccaaaattttaaaccagagtattcatc
aaaacaacagtataatgtctttatgtatatacaaagatgttttattatatattagtaggccatagaattagttattaactcttctctcttgagatttcc
acaacctggctggtggttagtggtgtacagcttaccagggaaatgttacttgacacaaatgtcacttaaccctactgatggtatcgtcaaagttca
agagtattcccagcctttaccaatatcaataaagattgctgttggattacaaaaggacaaaaaatgacgaagactcgttttcctcgtttgctgaac
gcttctgaacgtgaacgattagagccttttattgatcaaatccactattctccaaggtatgttgattttatgtatacccttttaagaattgattcttgtca
tggtattgatatatgattttctttctgcatcatctattaaattattgaaatgcgaaatatctttatctttgccaaattttttacttatttatttttcaagga
gaaaggttactaacacccttttggtagatatgctgatgatgagaatgaatatcgacacgttatgttgcccaaggctatgttgaaggccattccagca
gactattttaatcctgaaactggtactttacgtattcttcatgaagaggaatggagaggtttgggtataacccagagtcttggatgggaaatgtatg
aagtccatgttccggagcctcatatcccttttattcaagcgcgaaaaagattttcagttgaaatatagccaacaacgatgaatgtctcgtttttatgtc
cttcccttcttatcacgtgtatcttacgaggctttgaaagctatttcgcttctgcctaaaacgggaacgtataccgtagcgtttctacttgctttcttat
gagatgatgattccttagtggcattttacctgctattgcttggtatcttcgctgttcttattctttcatttattcctttttttctcctgttctctagctatcaa
acgatttgggttttgtattctaagttctttcttttctttctaccatttctattaaaaaagccatatctaggtttttatcatctcttcataataacacttcaa
ctctagactttccttacacttcctgcgtttgttcttttttccctatttcataggttttaccgtagaaactaatagactccctcatataataatggatttga
aaaaaccctttctggtattcagcgttaaatataacatgtcggtttcactctgaagtttatcatgtgcgattcagttgttataaaacatgatttttcttttttt
ctttttaagtttcattggttgtatgtatattttctaatatttgtagttttacaaagtgcccatactttttcaataactaccatccatatatgtccttacaag
cacaagcacataagctttcttttcttttttttttttttttttttttttttttttttttttttttcttacgacgacatcaaaattatataaagcatattttgtgatt
aagcgtaggctaaagatggaaaagaaagaagcacatcagaactttacatagtcttgcaaagtaaaaattgcagatttgtgccagcaagagtaa
aacccgaatattgtatccaacgtacgtgtcagcgatgcaaaagcacaagagccttgtttcatttaacccaaagcattagcaccagtaacaatatc
gataagttcgttagtgatagaggcttgacgttgacggttatagatggtggaaaacttgttgatcatttcaccagcactcttgctagcattttccatag
cattacgacgagaagacatttcactgcagtgggcttcagccatagcagcaaatatagcattcgtaaaagcaaactccattaaaggttggtgcacc
tcgtccttagtctcataagcaaacaagttgggagattcggcaatagattgacgagtatagagattcttcatcactgtttcaaatgatacggcagag
gcgaatctgttgtaaacaataacaattctgtcaaactcatttgcattctccaaaaatattgctggaaatttgcaaggcctcttcataggaaggactgg
taccaccaatgtgagcaaatgttaaatagagagaaccagggtaataacgatgaatttgggaacggaccttttctcccaaaacgcagatgctagt
gttttgctcaacgttaggatcaatcaattccttacggacaagacgagagatagaagagtggataccaccgcaaaggcctttgtcacttgaacaag
caatcaaaagagttttcccttctttgggagcttgagttccagcttccttaaaaaaattcattggaaactgattggtatttcttagcgaggtccatagct
ctttgagctctgttcagctttgtctgagcaaccgtcttaatggttttcgtgatcttctcaatgttacggatggactttaatctctgttcaatttcctttaa

agtagcctcacgaggagcactaatcgttgaatcgttagcatcgaagttctttcagcaatcctttcacttcacatacctagcatgaaagcctcgaaat
cctaccaaatttacgataggacgaagcgcatttgactgtttaaccgttaaacgtaacattctgacaaataatgccaaaagttagcaaatttcctgtt
tcgtgtgaaatatgggggagggtaagtaaactaaagtcgagaaacctgatcctataagcggaatgcaaacccgtcaaaccaacacaaactagc
ggtagaacacttagagttttatttttaatttttaaataaaaaattaaaaataattaattagaaggctccttttgagcaggaagcatgcctattttcgt
ctgtcatttagggaatttgagcgaaatggctgaatgcctttaatgtactagaacgctaggtttaaacagaataaggcagtacgcagatagaaaga
attaccagtacgttcctttttcctttttgtttacacagtggtttattctaaaatttcgtataattcatgagatgtatgtaacttaaaatgggattttatagg
gaacgtattctttggtatcttgccaatgggctactgctactgcttgtgggcttttatgtttatatgactccttatacgaaagtggaagagtctttctcaa
tgcaggctattcatgatatccagacgtacaaattggatctttcaaaggtattgtttttttaatcacactctgcttagaagatgatgcttcacgcagaga
aacacataaatgacaaaactaacaagagtcacagtacgatcatcaaaagtttcctggatccgttaagaggagctttatccctagtcttattatagc
tctcgtttcctacttgcctgctaaacacttttcttcccttttggctgtacgatggaccattggcttccttacgtgtgaaagttttgtggtcataggaaaa
gcattaaggaaacgttttgggttctgtgccggtattgcattcctttttatagtaggcgctcaatttcaccttgtgtattatatgtcaagaccgttaccc
aatgtatatgggcttattttcacaaactctgctctggctttcctattgcaagggcattactatccggtcatctctgtcttagtatttgcttctatcattgt
ccgctcagagatatttctattattaagtgtaatttccctgagtttccttgcccaacgtagagtcaaattatttcgacttttcatcattggatccttgtctt
ccataactgcgcttgctctaacagttttcgtggattcatggttttggggaacatggtgctggcccgaatttcaagcttttacttttaatgttatcgaag
gaaagtcttcagaatggggaacaagcccattttattactatttcttaagaattccctggattatgctgaatcctaccttgtttcttttcggctcgtat
ctttctttactgtccgttcgtcaagaatcttactttatgttcctttttgctttcgtatttattttttcatttttgaagcacaaagaatggagattcgtcagtt
acatccttccatggatcgattcatcctgcgcaattggtgtggcatgtctcatacggcaaaggaatcatcaaaggatgaagataagcattaagaag
ctggctatatttatatttttttctggaatttctttcggacttttcgcaagtgctctattactaaggatttttacatttgcttatccaggaggcgtggcact
ggaaaatctacattcagtgaacaaaaactcattcggtatgtatttaattttattgttatttactaatattattttttctattagagaccgttcacttaga
cacctatccttgtatgaccggtattacacgtttcttgcaaaaaccaaactggtattatgataaagaggaagattcttcacgtttaaatgacgatctg
tttcaaacccaatttcattatatcattaccgatactccaagactttacgaaagaaattttgaagttttgcaaactatcggggaaaatcccaaaattc
ctttgatacctgattttatgagagagtgggttacctttgatttgagcgttacaaattctgcctttcccgtgtacgttttagttcagccaccgactatggc
ggatgaagtatcaataaacgattataataaatatattggtcaatctgtgagcaacatacaaaaatggccaaaggtgtttcgaattgttaaaccag
gaacccttctgactcgagattatcgtgaagagcgattgaactttttttactacagaagatggaattctggatcatattacccaaggttaaataaatat
ctgtaaacctctacaacctaacagagttcaattttaattatcgggaacataatatttaagacgaatgtataacgatgtaaatatatttatacataac
agtgaattattgctatatgtggtcattactaagtacaaaacatgaatatatatacgaaataagtatattgtgacgtatattaatgtatagtactaaa
acccaactagacatataccgggcacaacgagcctaacacaagccaataagctagatatccaacaggagatgccaaataacctttataaaaagg
gggaaaggaaaggtcaaatcaaacgatgaaacgtgtttacaaaacacataatggatccagttaacaaaataagctaatgagatgcccagaga
gattaaaagaatgttgtgaagaataccacatgaagtggaaccacttttggccttttttttatttgatcacaaaaagcggaaaccagttgatagtaa
ggaatctattataaatatcagaataagaaagaaggaatgagctcaacgtgttgcgaacattagtttactgttgagcgcattgtacaccagggtga
ccgccgtcatcttcttcttcctcttcgtccatagcgctagcagtagtaggattaacgttgttatcaatgcgaacggcttcagtaggatcaacgttacc
aagaacacactcctcagtaacagcgttcttaggggcctttttcaacacgacgaggaggtaagacctgttcgagtaaagcaagcttttcggcaggag
caaaattgggttctgggaaatcaacagtgaaacgaacgtaaaggttaccagcttggtggtggcgttgagagagcataccttggccgggaataac
cttgagttcattagggcgaatgcattcaccagggatgatggggatggtcaaataacgctcgtccaagtgttcgataacgatttgaccaccggcca
aagcagaaagcaagtcaacgtgggcttcataaaagaggtgatcaccgctgcgcttgaatctaggatgttccttaacatcgacaacaaatataac
gtcaccaggaatgataccaggagcttgttcaccttcttctttgaaaagaattttctggccattatgcataccctttatcaatgtgaacagtgagtatct
tacgttgagaaacaaccttggcaccattgcactccttgcaacggtcttcgttgcggatggtttctccttcaccgttacagtcaggacaggtcatttgc
atacgctggatcataggacccatagcacgggtgatgaacttgacaccagaaccgctacaagaggaacaagaacggacagcaccttccttgcca
ccacggccactgcatttgccgcagataaccttcttttgtaaagcaagcttcgaagtcttaccacggtagagatcttcaagggtgaccttgatagtgt
gaaccaagtctttacccttgcgaggaccacggggcatgccaccgccgaacataccgcccatgcctcccatgccgccgccgaagaaggaagcaa
acaagtcatcagcagacatgccttctccagcaccaccgccttgtaagccttcttcaccgaagcgatcgtaggtagagcgtttgtcctcatcagaga
gaatttcataagcacggctaatttccttgaatttatcaccggcattagggttcttatcagggtgatactttaaggccaatttacgataggctttcttca
aatcgctaggagaagcagaaggatcaacgcccaaggtttcgtaaagttttgtttctttagccatgatgcaatattaatcgataaaacgaaataga
actgaaagaaaattaaaaagacaaaagaaaaatatcttggaaactttaaaaagatgcttgtaaagggaagatacaagcaacaaaaagtgaga
ttattaaaaataaaccgtataaactttttaaatgtacagtacagaaacagatgaagaaaaagagaaagcaatcgagtttctcaatgaatcaaac
accacgaattggaaaagcaaacagaaaataagaaaaaatagaattttcttttcgtttagcttttttttaagaaaaaaataatcctctaccgtcaa
acagactaaaaagctgaagaaaagctctaagttttgcttttttggtgtgtgttgagaggaagtttagttattagcaggtcaaaggccaatgtaa
tgagcgaaatgtctcgtttatgtttgttgtagtcaatggccaaagaacatgggagaaagaaattttctgtcgatcatacagcgctcagggtacaat

gaagcaataccgccgctaagttacccaatccgacacagaagaacaacaaagaacaatttaaaaaagaaaatgcgttggaatacggtaaacag
taagcagtaaacaaacctatgttgaggatatgtaaacaaagggagggaggaaatcaaaaatgctttccggaggaacggaaattttgacgacaa
atgttggatggaatgggtttggaaaccatgaaacagtttccgaaaagagattgagtgggcatcggtgaaataaacaaatacagaaaaaaagaa
aacatgattccttccaatggcaaaagctgaaaagggttaattttattagcgagaatttttttttattctataagaactttagaattcggaaggcaatt
gtacaggaatggtttaatatgtggcacatatcaaagatttgcctcttctctttatttatgagttttgacaacggttcaagtcgattggaaacctcaag
actcgcatcaggttatgtagagaagaatatttaacggcaattacttttccatccacttcatcctttgtttttttttttttttttttttttttttctttttttt
atgctactcaatcctactcttcctcataaatagcactcctacagaacccactaaatcccacatcactattatgtgggggaatagaagcatcgtcctt
agcggaaggtaggcttgaagatagatgtaaagtgcggttgatgtgaggacataggaaaaggtgatttattaatttccacaaaaagggagatttct
atttattgattttcctatcttttttttttttaataaaaaacacagcactataaatggtataggtaatcgatacctccaccttttactccttcgaacaaaa
tcggcgtttttagacttggccggcgtagtcaactactttgatgaaataataaaatgaagaattctaaatgatagcgatacaattccgaaaaaaaa
aaaaggcatcaaattgtctatacttccattcttcattttcctaggtgacatgttccttaaacaaaaatcttgctgcctttttttactgtccagctacaag
caaggcgatgtgatgctgtcagaatatttgacacatgtaggtcgagttaacgtttatttggatccaaacactacgccgcatccaaaacgggttggt
gatgttccgtgttcgctacctctgtccctgagaaaggacgagcctgtggttgccatcaaacgtgctgataccctacaagtagtaattgcttgctctcc
tcgtattcaaaattttgatatcgtcgttccctggaaagccttggatttcgattcaaaacgcgaacatgtcgtccagtgtagctactgcaccgctcagc
tcgcaaccttttcttcctgtcgagacttaccctctgccaattggcaagaactgatggattgctgggcctgtcatgttgatttccctgctgttttggcca
agaacggtggcatgcctgccttttctcctactgagcacatgtcctattctggcacttcttttcttcttttccatccaaattcattggtaagttcaattctc
gttttattgttttattagctttatttcttcttcaatggtattacggacctaaaagaaacccttgatttgcttttctaaatattcacaaatcccaaggaga
tacataggtcctataataaacaaaagaaagttgggattattatttcgtacccctgttagagggttgctatgataatcccttcttacgctccttcttcac
aggtcgcacctatttaagctctgttaggtcgcggtaactgaaagagtacattccttaatcatttagaattcttctctctttttttttttttctttttttatgcg
ttactaaccatctcctcttcttaggtaaattcccagtcttatcaaagtgattcgcctttgtataaatgttcatcctgccaaacagcccttggaatccaa
gatgaacatgatgtttctgaaagtgtccgtctggataagtcttgtattttaatcaatggcagccgcattcatccttcttatgtcgtcacttctgaattat
tgacactccgcgaattaaacgcaatccataaattctgtctcaccgatcaaagtaatactctttatgtttggtgcttcactccatatcttcctgtcactt
tgtttaacagcccaaaattaactgcttcttttcaacaatcgcctatcattacgatcaaaacattctatgcctatgatgcccctggggactttgatgctc
aagattggttagacataaaactacttccatctgtttttaaggagattaaagatttattagagcttacaactcaatgttttccttcttctgctcaaaaat
tcggtttttggtatgttggtttgcttccacggttatgatcttgaataaaataggcaatgttttattaatggtttgtttttgcacaatgaagaaatgactt
tcgaacttacagaaatgtaattcattaagcatctcgtgatatcatcaataaaaaaatgatattttccttaccggttgaatcaaatatcctttttttcat
ctttttgatcatctcttgatttaacagcaggccggctatagacatatgtcgcactagagccaaatactaaacaagcaccaagcacaacagcaccg
cttaatcgattaccgaatagcagtatgcttaaacatagacttacaaactttcgaacgtttagaatgatcgtgacggttaatgcagagctttgggaa
ccaagagaatttacgcctcgtacgcacacgtactgtgccaatgtattaaggcaaagataccaaaacgctggaggcataccaaaaagttcaagat
tagaattttcggaatctgtaaataaaagcatccactgttttcgaattgaactcaataacggtagaaaaaaaggaatggaaaaagcgtgagtgta
gaataaactttctctccagctagtcttgtatttttttgtatgtatcttctagaagtagccccataaatgctccaagaagctgagtgaccattaatatag
aaaagccaattgcaaaattagttgttgaactcatatgcacatgaatatcctgtgcattcccaagaactgcgattacgactccgagggttaaaataa
caacgcttatcatttgggcaattgtatagcttttatttgccaacatccagcctaccgccatggtggtgaggggcccactgcttcgaagaattatatgt
acggggacagaaacgtcaaatcccaaggcaatattgtttaaaacattgatactaaagaataataaaacgatggacaaccaccgttttctaggta
cattaggatttttcaatacagaagggttatgtattaaaaagtatagcagaccctctattgttatgaacacaaactgagaaaatgtaatcagtaatc
cacttttaggatattctctcacaatcgcttctaaggtgtaagctgtaaaacaacaccctccaaaaataaaagaaagagcccccaaaatcattatgc
gattgctgagttctgtcttgcttctgtgttcttttattaaataaaaaaaaagaatgaacccctttagatggacacttgaatatttttacaagttttcag
gtagcaaaagtccaagtgcaaaatggctgcttgcaagttgggattaaaccctttttcgtaaaaattgatttttttaaatactcaagacttaaatcaaa
atactttgaatcgttagttagttgcttgatggaattccaagacaagctaatcctgtttccaattaatttgaagtggatctcaatcctacgccaaacag
gtgatgtacaattatttggtaaaatagactactcaaatagttttaattttaactaattaattcaagagaatgttaatctaagttcatcaaaatataaa
gaaaaattaataaatgaaacatggtaatgcaaacgaaaaattcgtgctgatctgcaaaagccgtacaaaggagttgacattatacgtatattaat
accttctcatttctgacattgattggtcatataaagcttcataatctttccgggcacctagcaatcgctcattaaggtccatcaattcttctttttgaga
ggcatacttttctattaatcggatgagtttgggtcgtagaatgatagcttcatggtatttcgactgcaattcatcatcatcagccgcttcgggtggtg
cagacgataatatgtttaacaaatcatcaatcttgggaagagcgtcaaaagtatactgttcggttttgagcatctgctctttttgctcagcagtcggt
tcaacaattctctcaacataattgacagggaaaattcctacattgttcttacaggaacccttccaccaatctttaaacacgctttcgagaactaata
taacatccccccttttgaaaagacaactcaccgggctcagaagcagcaaagtcgtacagggcacgtacacgagaaattgaggacgctgaagtcgt
agttaaggaagtatcatgaggttggtttggttgttctgaacgctttttcttttcttcagcttgctttttgctttcagcttctttttctgtggcttctgataac
gataaggctaatgcaaattgtaattcgtcttcttctttttttcaattctgagttgctcatttttttgcttgcccggtttctttggggctcgtaaatcaacaat

gtcttgtaacgttagtaagtaaagaattctgcaaaagtaaaccgtacctagttttcgaatttcattgcatgcttgttgcatcagatccatactcggat
catgttgcagctccatcgcccattcattaaccaaaacggcagtcctacttcggacttgattgtgagtgcttgggttctctgctatacgcaagagact
atcggtgaacaaacgggaggcgatttcatgttgtaattttgaaccacagtttttactatagcatctgtcaactatcatggttttgttagtaaatgca
aagaatccaaagaaataaatatttatgtttattttcagtaacagtattatttataatatggcatccaaaaatacttcccggaaatttaaaatgccga
cttacagttaatgtcaacaattggacattggcattgatagaatttaatcgtttattaaggaattgaacagcatttcgacagctattggaatttcaagt
taataagttgaaccgcaattgtcacatacgattcttctgagctacttgacagctgatcgcatacatccataatcaagtcccacttatctttagtgttct
tctcgtcagtagctcgaagaattgccgtttgaatagctgacggtttttctcgaaacatgttggcaaagagtgcagtctctaaatcctgctgtttataa
acttgctatgccgatactcaaatacaattgcttttaattcttttatgaaataaaagaataaagaaattcccaattctagtgtaattggtaaatggca
agtatcaacgaaagcgtttgcgttgtgcgtttctcaggccataagaatgtttttttatccgagtcataaatcataaaatgttgtacagaaaagatgta
tcctagcatattataaacaatcaaatgaatacaatagtattctctccaaaaatatatgtacaatgtacgtttaggaaacgtcgtctacagtactagt
acatgacagatcgctttatgtttggatttaaaaactatttataaatacagaatgccctgcagatttagctagcagtctaccctacgtttcaagcata
gaaaaagttcgtgaaacctcggtttttcttctaaatctgcatccttcctcctaattcttatcataacatttttttattcttttcttttacttagttcttattac
tacattcctatttatttttacggtgcttgtattgttccctataacatatcacctagtagaactcaccaacaagtttctaaaactagaatcgtagtctgc
ctggtagatgcaatttatatccgctaaaaggtattgaatgatacaatattggctagtacatgagctgcttttgaagtgaactgtatgagatactgcc
tgtgaaattctattcaggaaactataatattcagacaagtttgtttcctctttttgctgatctatatgtagtagctcttaatttatttgaaaaaaaaaa
attgattttctttttggtctttgtttagtctgaagttatcgtaaaagggtcttgatagaatggacaagcgatcagaaacgacgacgaacatatcatt
tgatgaaaacatttccagtagctctttacacactggtgagaaggaatctcaacgcacttcgactcatggattgtatacagcggaagaattgagtac
aataattagggaagcatatgttcctagaatatctgtaatatgttctcgtgatgcggatgaatttgctataaataaaggatttccacaggggttttgg
gaattgctttatccttttggtgaccgtgtacgtggaaaagtgcgacgtcgtgatatgcaagatgctatgcttactctggacaaaaccagtttacagt
tcgttccagtagactctttagagtcgcaccttagtgatgttcctgtagtatcaatacgtgatatattgactgcccaaaatccaaaactcgtgcttccc
gaggcgccttcgaattatacattaggtgtccattacaaaaatgtggaacgatgggcttggactttagcgcacgaggaaaatcgtgagatgtcaat
tggtatgtatattcgacaacttttgacgggtatgccggtatcatcttttgaaacattctcgcatcccgtcgcgcatctcgtcgttgttacatcgcataa
cccaactccttttgaatctcttcgagagataattaactcccttcctcaaatatctttgcctgcttttgtttcgcatgatattatttatttttttgtttatgtt
cacgatgaagaccaacatgacattgaattatcaatggctatttttgatacaatgctacagacatttggggaaagaggctattttttgcgactacatt
cacagaaagctacgttagactatgaacatacaattccttttccttgctcttcttggttatctgctgaagagcgactgcaaatgttaaatggcttagaa
aaagcacaacaattactttttcaatcagatgcagaatctttaaaaagactagtaagtcacatagcatttaatggtgtcatccctcatttagacaggt
gcataagaatatgggatgaccagtatgcatctccaagacgtggttttacaggaaggttgtttttttgcttcaaaaaaactgatatcagcacagaata
actcccataattcaaatttttttgcttcttctaatgtctatcggccagactctatagaagcctaccttcgaaaattagcagattattcctttatgctccg
tgattatgtgcacgccaaccaaatctatgaaattgcatcaaagcaatatgctaatgatgacgcttacctatattctgccgctgcttcagaaatgatt
gttataacagaacacatattgcaccttgatcgcccttacatgagcctaacgaacaaattgcgccttaacgaatgcatgcaattagccatggcaaa
ctatttaaacaaaccttttaattcatactatcacgctgctcgctgctttcttttgttaggtcaatacctaagcagcttccctaatcccagagtagacga
tgctgctaattggaacactggcttattgttctcgaagcgattgggtccggtaggtagagctattgtatttcagcagacttatgagctttatagacaaa
ctgggcggataaattcggattcgctggattcaagagaaatgaagcgagaccgtaaaacagcattttggtgtgtaatggcagcagatgcttggtta
cggtgtcatatttccttccgtgctagaccttttatagatcaagcaaaggacttctatactcatgtagcttggcaggatttaaacgaatgcgtagcca
agctgggtgaagtagatatgactcctaaatctcaagacaaatagttttacattcacttacagggtggtaatcaaaaatataataaaatgtatatta
ttatgatttcatattctttttaataaatatatatatatatatttgtatatggaaacaacgacatttaccatggaaaatgtgactagaaactcaacttat
gcatatgctaagaaaagaatgttcataactcaaacatgagttgaattgagagactttagttattagaattatcaatagttgtagaagtagattgttc
attcggtgaagaaggaacagatttttttcacagtatttttggctttttttacaaggtcagcaaaatcatctgcataggtagatccccagtcagtacctc
caagcttttcttttgaaacaggattggtattactttgagtcttctcccatttgcgaagctgggctagaagtgccttctttgaaattggatatttttgatc
caaattagaattatacatagtgacccattgagcatgtcttctttgcaaaacttgtttattaccatcagtagggaggcccatctcggccaatttggaa
cgtattttagattcagagagtaaagcataagtaagttttggaattcgaacacgttctgattcgggtaattctttattagtctccacaaagttattaat
aggattctgatcaagatgcttttcaatattctctggtggaaaaggagcatcttctgttgaagattgatcgggattagctagacatgtatctaaatgct
gatttatttcggactgggatacctccctcgagcaaacaggacaaacgacgggattttgaatttttcttttctttctatttgtagagcttgaggaatgg
ggagtttcactctcgtcccattcttcggtgcttgactgggaatttgggactgaaagtgcaggcgcatcatcaccgtttgaaatgtcttcaggaggat
catccttctttaaaatttgaattaattccatacggatagaaaagaatgaatccaaaatatcttccataaccttattttttcgtaaacgactttcctgct
caggagcacgacaagctggacatattggatgctcccttaaataatcccgaatacagtaagaacagaaggtatgagagcaagatgttattactgg
tcctttgaagaaatcgtgacagataagacatctcatggaggaatccagtccctttagacgtggcagttttgtataattccagtcggaaggatcaga
cggattatggttctgcatagctttatgaatagcttcagagaaaaaaaaaaatttttattaaataaataacatgtgtataacaaacgacttgaaaaaa

aaaagtggccgccgcaaaaagtatttgttaattgaaactttcacttacgcgtttcgagaatgtctaaatgcagatttatagtaatgaataagtttta
attttatgacttcctcatgcccatgaacttcccaaagagaaagaaccaacgaagctggtaattaaaagatgtgtacacacggtcgattcacttgga
ctaaacactgcacataatagtttcggcattgaaggtcacgatttcatttcttaggagaaatccttaggtaatttaaacataaaaaaagtaaatatgt
cggatgtcgataagctcttgaatcgcgttgaaacaacccttaataaggtaagtaatttgtcacagtactatactcaacagttctttgtgaagaaacc
tagatctctgttcgaaaataaaggaatttctcatgatgatggaagatcatttgatgctttattttctggtttcttcaggatcatatcttagcaaaatgg
gtgctaacgggtaacagggacgcgaaatagaaagaataattgcttcttttaaactgaatgcttatgatgttcttggaattttacctggaatgagtgt
tgacgatatcaacaacttatatcgaaaggaatcacttatgattcatccagacaaaaatagggacaacccgaaagctgcagacgctttcaatatat
taaaaaaggctcagtctgatttacttaatgaaaatatacgagattctttggattctgcatatacgacagcaagaaaccaattgattgctgaaaaaa
agttgaaatgggactctgaggaggtgaaatctgaagagtttttatttgacctaagaattcgctggcgtgatatcttaattgacgatgaaattgcaa
ggcggcgagcaagacaattggagttgtcttaccaacaaagggaacagtcaaaaaaagacgaggaaaccaaaaatcgcaaacgaagaatgga
atcggataaggtttgggaagaaactagagatgaccgtgtaaacaattggcaagattatctgcataaaacgaagaaaagcactataaagaagaa
aaataagaaaccaaaggttttgggataaacgacgtacttatgaatgaactaagataatatgtgattaatctttgctttttgtgtcttatatgaactat
ctataaggtactaatgaacatgtttgagctgaagatgaaaagaattaaatatacttacacaggtttcttcaactgtaattgaaaaatagaaaacta
ctccaataatgtagaagccaaaaaagaaaatggaataaaaagcattcatattttcaacaccattctctgagaaaaaaataaaacaataagaaa
aggtagattaactgcaattcagtcggaacataaagataggcaccagcatgagctttttttgttctatacactataaagcgtttccaaaaacctatatt
tttataactttgactttaaagcttgccgtacaatacccaaagtaatagaatatcagatacacatggatacaagcaattaaataaaggcaattatta
tgatgacaagtaaaagaaagactttagtaaataaatgaatccgcttattaacataatatgctggtgaaataattagagaagtaagcttttgggctt
ctatcgcctttagagaaactagttaaagaacacgattgaaggcatttttttatcgtatttaaacgtgttcaggaactgtggtcattaccatgagctaa
aggaccttcttcagctccatcgtccttataccgctgagtcgcactttgagcacgatgagcttgagcttcagcttccctttcagctgcttctctttcttttt
gttccctttccatttcagccataataactttatcttcatcacttggttcgtaaatatgccagataacatagtgaggaagaccaacaacggaaaagcc
catgcgcttagccattttaccaaagccctcagtttcagcatgacgttcgaatgagaaagcaggaaaatgggcaccctcaaaataaacctttgcctt
agacataattgaaacaccgccgataccgtctaaattcatttcagcattaggatcaccattaggatcacgaaggtatgccaaatgaggtcgccatg
tggcatattcggcatacccttcaacaataacagcatcttcatcaagggtttcggcaagctgaagagcagtttcagattcagaccatgaattcaaat
cataggcttgttcagtggtaccaaaccactcaggaagagggcgatatacgttgggtacaataacatctttatcatgacgcatcagatcttccaaaa
tagtatttggggcaattttaacatctacatcacgccaataaacccatgagtgatatggcttgagagcagcggacagcaaccagttacgcgcgcgt
gccataagtttgcgtcttggaccctgagcagcaaatccatgtcgatcgctaaattgctgaccaactagcgcaccaaaatcttttaaataaatgaca
gcactgttgaaacgcatgttagtatcctcatctttttgaagaatttctaacttggattttaaagaatcgagggtgttatcatctgtatcagaaattaa
gaaggccaaatcaatcaagtgatgaggataagttaagttacgtaaatgttcgaaaaacatagtcaaatggtcccgagcgtttctaagaggagca
caaataaggatgtgtccgcttttagaatagtctccggtaaatttaacattcattaagtcacgtaagtcatactccctgtattgataagaaggatcat
cattcatcgtatacaaggaagtgggtatttgttcaacagctgaaaagaaagagccatggaaaaacatgaaaacaataaatattaagccaatcac
accggcaattctagcacccttctgtcgaaacatggaacttccgtaacttttggggtgaaagcgattcggtaagttattgaacggtccaccatccctt
ccgaaaagcttttcaggtttcatcttggaagagattatctgaacggttctcaaatagtttttttacggctaatctgccagaaactttgagaagtaagg
tttcggtgtggttggtagtgagaagacgatgctagccaccaccactttcaacactaggtaggattcgtaaattcaagaaggctaataaaagagca
atatctttttttcgtcgcgaataaaaacaaaaagtagtttatattacacgatttgatgtttcgttgagtagccaattatccacaaaatatacagcaaa
acgaaaaaggatagaaggataataagggggaaagagggacaagccaaaaattctaaaagagtaaaaagtaaaaaattatactatatagatg
gagttaagagttgatttagttaggaatgtaaaataaataaaatatattaagagaatttggtagagcagtatgatacgaagattacgttactctcat
gctagcagtacatccaatgccattctatttttattcgtgtaataagagtgtcatatatgaatctagctaacttaatttattaaagctcggcccgacaa
cgtttcagattcacacatcttgtaaacgatatagaagcaatgaacaaaattcctagattagatgtgaatattgtaacccagagacaagaaatatt
aaaagtaataataataattaatctagaagaacaataaattactcaagcacccagattttcatcctttctctctaagtagacattttaactgcattag
ccatggtaataactttagacatttcttcgacgtcgtgtactcgaactatatttgcacccttcaaaacactagctgttactgccgcagcagtaccccag
attctttttacaggttctacttcattggtgtaacggccagtaaaacccttgcggcttggtccagaaagccattgtagatccataaattcgggtttttta
acgagttcatcaaatctatttaataaagcaacactatgttcaggtttttttagcaaacccaaatccaggatcaagaatgatgttataacgggggatc
ccagctttgatagcagcctgcactctttcattcaattcttcaaacacttcttgaactatatctttttcataattagaaagagagttcatggtctgagga
gtacctctcatatgcattaagcaaattggaatacccgcttttgcagcaaaagaaagcatctctggacatttcgttccagaagagacatcattgata
atattggcgcctgcaaaaaattgcttccctcgcaacttctacttcaaaggtatcaacgctgatcggaacttggatgtcagcattacggatagcacgta
ttactgggactactcgatctatctcttcttggacggtaaccgtcctcgcatgcggacgtgtcgattgacctcctatatctagaatagaagcgccgttt
tcaaccatcctcttcgcgatttcaacaacgttggtttttgtaacaatagatccgtcggaaaatgaatctggtgttatattaatgactcccatggtaac
tgcaccttccttaacagtgtcacttaaatatgtggcgtaaactttaactccctgagctggtaactctttaagttttgtaagtagtagttgttgagtagc

aggatgaacgagagtacaatctaaggctactaatggtctcaaaacaaattcccgttccatcataccagcgtgaggtattgtaagctctttagactc
atagatacagtcttcatacatcacgatatcaagatctatacaccttggtcctttatcaatcatcttaacacggcctaattgttgttcaatgaattgac
atgctcgaagaagactcataggtgacattttcgtctcaattttacaaacaccattcatgaatagcgactgatccttgtaatacataggccttgtttca
tacaacggagagacatctaaaaggttaataccctcaatgttagagagagattttagggcagtagctatatttttcatgcgatcgcccatattagat
ccaaaagatatatatgctatctttggtacgctggactcgtacttgtgaatggaatcgagcaagaaagtttttctggaacgacatatttgaacagca
ggagcatcggcaaatgtgatagcactaggtttttctgctttgatgtgcacacagtcaacagataatccaaagcacagaaatttagaaagtttatgt
accaaagcctcgatagtgaggaaactagaagtttcaacgaatttcgcaacttcatgacaaatatcggagattcccagagagtcgaattctttgtc
gccgtctacagagaaagcaacgtctaaacctaacctttgtttctctttgcgctcaaaaggatgaatacctatgatagttgcaagttctaaatcaaa
gattttgacactgtctcgatggtactgactatgtctttctgcatcatataccaatccacttctagaacgcagaatcttcttgggtaatcggatattcac
agaaaaatcacgaaatttaaactcattacgacaagtagcagcaatcgtatcagccatgtttcgtaagccgctgaattcattgttttcgatggtttcc
aagacaattttggaagctattccataatgaatagaattcttcaagtcgtgattgtcttcgccatttaaacgagtacctagaaccatacgcagattaa
cttccacgggttggagagctgaacgtctccattgatctgtgccaacttttgcattcgttcgcaggttctgtacaagtatgctatcgaaattataatcg
atctgctcttcatcctctagggacatactggttaagtttgaagaaagcattttactcgagaaatgcttttgattacaatgtttgaaacaggtgaatgg
cctgagcaaccttggcgattgaagagtagaacttagtacgtgtccccgacacctaggtaaatttgctttccggttctgtaacaaatgcttagggact
tttttcaaatatctaaaaagattaaagctaccactcatgtgacagcaagtagcttcaagtgagcttctccgtctttacctgtgctgtgtgtaaatgca
atgcttgttactttgaatgttactctttcctgaatgtttacaactcgtattcagcatttgagcatgtacttacagtatcaaataacacaatgtttactaa
aaaaactggtcatgatcaagaaattagtggtagaaggctgcatataatatgcgcatacaaaatagcctgctgttagtggattgttatggaatcatc
atagaaatcgtgccaaaggctcttttattttaatgaattaaaaaaatctactcttagtaagacacagccgctctaatccaaaagttcgtacttgcat
atttcaagtcacaaaaggtcgcaaattcgtattagtaatcataataatacgatgaaaagaattacattacaattgtgaagacttttgctgaggtttc
gttgttgtttgtcgatgtattatgaaccttacagctcatttttttgttttcaatcttctttccgactttttttgatcattttggtgcttaaagttgaatagtta
ggatgcattcggtcaagttgttcatgaaaattcagtttagcttagacatgaaaccgtagataagaaaattaatcataaaaatgaaaactctatctt
ttaaaatccaactgttcgtacgatttctgcttgcttgagcagtcagtatattcaagtcaaggcccagagatttcttctttccttgttggtttatgtaaga
tgcaaagcagtatagtaaacttttagaaacagcaattatagtccataaaaattgactcctcagcactatgaacagacatcgtgcggtgttggtgtg
atactttctagaaagtttatatagagttacataaaactaagtatcaaaatgttcatatttctttcattgacgggttcaaatggtatccaaagaagcg
gtgtttatagctgactttacactacactccctactattaagactccatatatgttaaaaaatggaggaatggagaaaatatttggaagtaaatata
ataaatgattcatcagcggtaaagattccatttcgttaaaggataatggcaattatctaacggtgataggtgacggagacttcatttagtattgatt
caaaactatctattcaggatgcacaaaggtaagatttgttctatgtcttttgttctagtctaatagttaagttatctgaacgcattctatgaagaaaa
caatcaactttatccggtgtatattatccatggtgtaccaaaggaccttagttccctagtagatttggacagcgagtcagacagtttgctgccaggc
tctctttcccagtatgctcttgcaacgaaaaattctttgccggatatttgcaagcaattcgaagaggggtataagacgcaaatctatgcactatcgg
caaatccaatagtcgtaagtctcaaaggataatttgtttttttcttgctaatactgagaaggattttgatgaattattgcccatcatatataaactacg
aggtaaagatgtcatataccagaaagaggattgtgagaaatatggatttatacataatgagaactcgacagtatgttttaaagtactttacgcca
atatgaactaactgtttattttatagcctcgacaattgaaaaccaaaggggcaccatctaccacgaaagctcctccaagtataaaaaaagaaccg
gctggagtgaatcctcaaaaatcaaggacaaatacttctaatctattcagaaatgtaaaacctccgagctcccgaccggcttctacagccaacaa
aacgatggaggcgcctgagactaaaaacataccggagtccgtttctactggaaagccaaaattacagacgcatcaacttgaaaaggagaatga
tgatttgaaaaatataatggaaatggatgatgatcatgatacaatcaacacaccaaaggaatcggcgaattctgttgccgcaagtcccgaaggt
gcaacaagctttgacacagaatctaataaaccttcaactgaaagcgttaccccagaaccacaggaaaatattacatcaatttctaacgggcgaa
aacgtggtaaacgtaaagttactaggaaggtcaccacccacgacgacgaaggatttttgattacaaaagaagaacaggcttgggaatccttctct
gaggacgaaaacgagaagcccaaaaaggttgctccaccaaggcctaaagcatcaaatcctccttcccgtaaaaaaagcggtcctcaacaggct
gcaaatcgtcagcagaaatctatcatgtcctttttttggaaagaagtaatatcatccttgcaaaaatctcatagttaaataaatttacttagaatcaa
tcgagttataaaataataaatgaaatatattttagtcgctgctcttttacattttaaatcattattattggcatcgtctaagctttgaatgtgactaca
gtcatcagacttctttatggatcgcaattgtctcattatatgatgaccagtttaacaaccgctgtagagaattacattttctagtatatacattaatca
catggagtatagatatttactaatttttgttataggttgggtgtaaaagacaacaccaacttacgttctgtttttataaattgttatataaacaaatta
ataaatattttctttcttttacaaaaagaataaatcaaaaaggttactttacttttacgctaatctttaccaaaacctcgtcttttgctgttccaagtat
ttgtactttcacctatacccatacaacactttgaaaaacccacactctcgttataatctttaataatgggggatcaaaagaaaccaattaacaaaa
aacatagtaatggaaagcaaattgttaatgatcctcgttttcaatctgttcattcagatcccagattttcgaaagttcgccgtggcaatttgaaagtt
aaagttgatgaacgtttcaaatcattgaaggaagacaaagattttaaaactacagctagtgtagataaatacggaagaaagttagaccaagaca
aatcggcgaaggaaattgaaaaactgtatgatgttgaggataaggaatcctcttctgaagaatctgaacccgaaaatgaaaacgaaaagaaag
tcagtgaagaagacttaaaagccgaagaagaagaagaggagtcagaagaagaagaaagttcagaagaagaagacgaagacgaagagcctg

tgtttgatccagctcgcggtgaaggtctcgtaagcacttctgagagtgatgagagtgatgaagaaagtgaggctgaagctcagccagaagttact
gagcttgctgggctagaaactgaagaccgtattcctcgcggtaccgagactagcagactggccgtcgtaaatatggattgggataacattcaagc
agttgatctgttcgcagcgctctcttctttttgtcctccttctggaaagctgaaaaaggtctctatttatcccagtgaatttggtaaacaacgacttag
tgttgagcacgttcaaggccctcctcgtgaaatttttaccccaaaaagcgggaactcttcctcgaatgatcttcaagaagctcaagaatatgggttc
gacgaaaaagaagaatctgaatctgacgacgatcaagcagctttggtcgaagaggatttgggtgctgaattcgacatggttaaactacgtcaat
atcaattagaaagacttcgctatttttacgcagtcgttgaatgtgacaaagttcgtaccgcgaaagctatatacgatgcttgtgatggtgctgagtt
tgaaactacagcaaacatatatgatttgcgtttcattcctgatgacgttagctttgaagaagatgagcccagagaagtttgtacaaagattcctga
gaagtatgaacctcgtgaatttgtgactgatgctctacagcattctaaagtaaaactttcctgggatgcagatgacccaaaccggaaaaatctcat
taaaaaagcatttacatctcaagaccttgaagatttggattttagcgcctatgttgcatctagcgaatcggaggatgaagatgtcgatgagttgcg
cacgcgttatcaaaaattgctaaatggagatggaggtgatgaagctgaacgtaatccgtttgaagagccaggcaagccagaaggcctaaatggt
gatatggaggtgacatttacctctgggttagatgaaaaagaagataaagggggtgaagaagatgaaaccaccatagaaaaatacaaacgcaa
agctgctgaaaggaaacaacgaaggaaagaattaagaaaagtcaggcaagaggaagtgaatggagaaaaggaagaagatttaggttttgat
gatcctttcttccaaggtaaaggttctgtaaagaatggtaagaagaaatccgcaaaaccaaccgaagttgaggaagatcccagttctgcaacaa
aggaggaacttgaagctattgttaaagaagatgcgaatgactctgagcacctagatcactttgacatgaagagtatcttgaaagccgagaaaag
caaaaagggacgtaagcagaagaagaaggctgctgaactggaaggcttgcaagaaggctttgaggttaatgtctcagatcctcgatttgaagcg
ttatattccaatcacaactttgctatggatcctacaaatcctcacttcaagaagacaaagacaacagaaaagatccttggtgaaggattaaaacg
tcgtggacatcttttggaacaagcagaggaaggaaaagttgaaaagaatgttaagaagcgtggttccgtgaagcaagataaaggcgaggatct
taacagaatagtccaatccattaaaagagcaaaaaagtaaggaataagggataattagttttttttgatagtttattttttttttggaaaaaggttt
aggagtctgaagatgaagattttgggttttcaagtgaatagacagttataataatacaggtatatggtttaaaaggaataaaaaggtagggtaac
ttttcatttctttgggtgggtatcttcagagaaaatcaagttaacgctcaagaaaattgaaatctattctgtttttggaagataaacataattttatcc
gaattgtaactttgtatagtaagcaaagtaaacttgcttgttgttatcattgagaaagaattagttaagtggatttttttaccttgagtttgatcacga
gcggcgtaaatctttgcggtaaaaggtctataaggttagtggtactgttaaaaatgataaaaaataggacaacttactcttcgaacattagagcta
aacgataaacaagaaagacttctaaagttaagagaagacccgcgagcttaccgtagcgaagagcggtagaaagaaagcaaagtgcagagcc
attgtacattggattttcagttacgctgaatggaaatccagtaacgcgtgttggaagaagaaatccaaagtaatcaccgagataggttccaacaa
tacctaatttgtacatactcgacaaaacaaaaacacttccgacgacaaacgaagctttggaaaagagctgaagaaaagggtttaaaagcaagtt
aaaggtaggctgttgttttaaagcattttggtaaatcaaatcacgtacaattccaccaacgaaaatacaagcagccaacgcgtagcaggccttaa
gagagtttccatttgcaagctttgtcaatgttttcttgttgtattcggagcgcgcagcaatgttccaaagcaaaggattcaagccgatggaaatagc
agcaataagcaaagacttgttgaattcaatgaaagccatattgaaaaggagcaaatgaaaattgaaggacgatcaagagatgttggatgtagg
aaggaactgtgaggcaatggatagtagaaaagctgattatcgccgttgtatttcttgatatcatcctatctgatgtaccttcgaagtaacaagagc
cagtgtaaacaaaggaagaagaaggaagataaaggaagctctattagaaaaggatgaaatggtgtagaaatttgtaagccattctttgttaaca
agccaacttgttcatcgaatttgcttgcgtggttttcgactttatatacacaaacctgtctgactgactatcatcgtctgcttacctctatccaaaata
ctcgtgttgtatttttttcttttactgtacggaatagaaatgaacgaacaacatcgcaagtatctttacaaacaagacaacagcactatagaaaaca
aattccttgtcaacgtacacagctagaaaactgtccactgaaagcctgcttcgtcaattgagtctttgatggttattttgaagttgaagtattataat
tactgtcttcgtcttcgtcattgttgtttttgtttttccttgtttactgtttattctttaagacgatattataaagctgcaaacgaaagccattatcatgct
tcttcaattctttacgcattgcaaggaaggctatgctattttgttactacaagatgtaactcgtaagatggattgattaatgcttttattaccctcattg
ttcattcagaaagtgacgtaggaatcgctcaagcttcttctatctttatcaaaacagttacatttgaagtaaggactaccaacattgttggaaataa
gaccacgtccttcattgctgttgaaagctcgttaaagttcgcttttgggaaaaggattaaagtaaagcataatcaggtgtgtcttctttttgacatgt
tcgtctgtaaacgcctagcttcgtctccttggaaacgagctgtttctacgaatatcggtagatctctatctactgccccgaaggaattgagccgaga
ggactcgaaaactttggaccaggtcattcgagttgaccaagctggagaactcggtgcaaatcagatttatagaggacaaaattttgttctccagtt
tacggatcccaaagttgcacccaccatccaacatatgtgggaacaagaaaagcatcatttgagaacttttgacaactatatcctcgaaaaccgtg
tacgaccaactctgcttcggcctttttgggatgtagctgggtttgccttgggggccggcacggctcttttaggtacaaaagccgcaatggcatgcac
agaggcggtcgaaacagtcatcggtggccattataatgaccagcttcgtgatacagttcatttgaatgatgcagcaccagaactgcaaaagctcc
gcgatgacttggcagaattccgagatgatgagctagaacacctcaatactgctgtagatgattggaatgcaaaggaagctcctgctcatggccta
ttgagtaatgctatacaggtcggctgcaatacagccatttggctatgcaaacgtttttaactcctatgagatcagtggattttagtttatttactataa
cgaaagaatagtaatcaagttttattgttgtcggttacaaatcctttcttcaagataagagtatttccaaatacatacataaaatgtcctctagcaat
tcagcctttacaccaattatctattcttcattaaacaggatgtaaataaattaaaagcaagtcatgtctagatcgaggcaagaaatggattaagta
aaacgaaccggtttttcattgcttccaaagaagacaaaaccgattacagcatatagaaacaaacggattaggggccagtactttttttccttcgag
atatcttggaaacgatgtccttcttttttttttttttttattcttggaacttgcgcaacgtagagagctcatccaatataccctgtaaagtttcatcgtcga

ctttatttgctagactggggatcaaagtccgtgcttcttctgcatcttcacaacacagcgttcctaattgagcgcgctcaaatttgtgaaacctgtttc
caagaattctaaaaaatagagattaacaacaaggccccgaaacagtcgaagattcttcatacctttcgcaagcgtaagtagactctgcagtttta
aatcttccaaacacattaaaatatgcaagcgtctttttcatgacactttttaatgttagtaagattcatgtattctataaatagtgtatttgagcatac
tcagtcatagggatttccccattcgcttctctagttctttgcgccatgacggtatcaattaaaattttcgcctcagaaactgtgagcatatcttcatttt
caaactcttatcacgttagactactgttcatttgttccacttgacaatagcaacacacctggtcctagttttaattgagttgcatcttcttcaaaaatt
gctctaggcatcctgtttcttgttctttcctattcttccctccgaaacgtaacgaaactaaataaaatatacaaaatgcttccttaaagaaaaagtttt
aagtcccctttcacctttctgtttttcttgggttgtgtttgcatgcacgaaaggaagatttgcagacttaacgacctactgtcaccttccgtaattaaa
ttaccaaatgcgttcggccacagtaataaacagaataatggcagttttctatgaaggtctattcaatttaaacagataaataaaaagaaaaagtt
actattcaaggtttctttttaaagcgtttctttccgttgtcgtcagggcatggaaggaatcggattgctttttctttgagcgctcgttagatgacagat
cttacttatatcgcaaaaagcatagtcaaaagtacatgagcaataccagaataacaaaatgtagtatcaaaagattttagttctcaataatgagt
ctgtgatatcccttatattaactcaggtgtttatggaaacatcattcaaggaatttgttttcagagcagcctcctcctaagaatctcatgtttttatag
ataaacatgcgcaaataaatgtgtttttaactttttttctgtctttcttgatacttgcatgaaaaattactagtatttgcaatataattatttctctcgat
agagttataattatgtaggtgtcacgggagaaatctcacacgttatgagaatgtaaatcgagtggaagtaataagtacaattacaaaattgtctat
tggaattggacgtgttagtactacgtagagtaatagtaatatgtccagggatagtgtaacacataccacactttaagagttttgaccttattgttatc
actcctcccatagtggttattaattatcgaattaatataccaagccagtactagcaaattgagtggtcttctaggttggctcctattattaggcttatc
tacaggtatgggcatgcctctcctgtagtaatggtaatcactaacattactggcattactagcagtgtcactgattgagtctgggttatagtcaagg
gggtagccttttgcaaaatagcgataaaagttctctagatcatctggatgttgtctagagccgctaacagcataatcattatgtgagcggatttcaa
cataagcagtaacgttactactgttcaggcactgttgtaccgcattcacatatcgagcttcaatgatatggctataatcgttcccggcatctagcca
agcggtgaaaccatcgtcagtataagtataaatcctttgcaaaactgctttaccatcgaagttctcttgaatacacctgctaatttcactggtacaa
ttgctgcctggatggtgccacaataagacttcagaatgatgtgcatatatttttaaaatagtggaaagcaaaataattcccgttaaaagcattacg
gctacagataaaattgtttctgtatatttgccatcgtgactggcttctctaattaatagcaataatcacgaattgaaaattaaatcggagtttctgca
ttcaaagagaaatttgcattttcaaatttttctttacttattatcaagcacaattctttaagcttatgaatattaactgatattagctaatattccattct
tataacggattgttatattatgaatgctaacgagggacccaattatgacagtaggtatagtaatcgttggtattggtacgctgtttcgtcaaaattat
ctaacaaaggtagaaagaaagtctttgcgtttgtaaaagaaattgaacaatttctaaaggttgtggattgcttatttcgaagaattgatccttcgtt
tagttataaacgtacttgtttcgtctgtcgttgaatagatgcaaggaaagttccaaagtcaccaattggattgaaagagaaacaacagcaacttcc
atctttatttgaatataagttcaatatgaggaaaaaagttcttttgatgggccgtagtggaagcgggaaaagttccatgcgaagtattgtctttagt
aactatgttgccaaaaatactcgaagactaggtgctaccatcgatattgagcattcacatgtacgatttcttggaaatttagtcttaaatttatggg
attgtggtggtcaagaagcgtttatggaaaattatctatcggctcaaagagatcatatcttccgtaatgttcaagtccttatttatgtctttgacgtcg
aatctagagaatttgagagagacttggtcacttttcggaattgcttagaagctactgtagccaatagcccccaagcacgggttttctgtctgattca
caaaatggacttggttcaggaagaccttagagaattggtatttgaagaaagaaaagcaattctactagaagcaagcaaagaactcaatacgact
tgtctagcgacttctatctgggatgaaactttgtttaaagcatggtcagcaattgtgtatactttgatccccaatactcccacattggaatctcatctt
cgagaattctgtaaagccgctgaagctgctgaggtcgttttgtttgaaagaaccacatttttggtcatttcttcttattcttctgacgaaaacatcgct
gtcgatacacatagatttgaaaaaattagcaatattgtgaaacaatttaagctaagctgcagtaaaatgcaagcacaattcactacttttgaactt
cgtggtggaaatttctctgcatttattgtaccctatactgaagatacatacatattagtagtcatagcagaccctgaaatagagtctgcggcaactc
taatgaacatacaaagtgctcgccgctatattgaaacgtccaaatcgaacagtgctcaaagttagtcctgctattttcaattcttgtcatggattctt
ttcgtgttcagttattttgcttcattcattgacttcataagctttagaaatacaacccttctgctaccctagctttttcttttcttttttttatatataaaat
ttttttgatgatgtttctttttaacggctgtttattcataggtatttcaataaagttactgtaatacatagcatcaccccttattaaattcaactcaggtt
ttcttttgttatattccgcattattattcatctcattctatgattaatctatattaaactaagcctaaagcgatataaagtcgatattatcatctactga
ctgattcatatctgccggtttatggtctacaccagaattattatttgctatatttgttgcatccttttgcttgtcagctgtttttcgatcaccactcttctc
aagggctccatgatatagacggcattggggattagcacaggaaccattgatggcgaattcaccgcactgtaatatgtgtttctctttgcacgaagt
gccgcgttcgcaataattatacttggcgaattcaaaacaaataggtgcagtctcagaatagtgtacatgaacataacgacaattcacattattgca
tttaccctgtaaaaagtaatagcaagcgggtattcgcttcgtgtccaaatcatgtgaaaggctacagttatcaccttttgtacatttctcgttcaaaa
actttggacaaatcattcttctggtaggatcatgtacaaacatacatgaagatccgtttgcacagtaaccactggaattataatatcggcaataga
cactagacggagaattgcctacctccttataaatattcttctttgtttgtatgtaagctttgttattccaataaaggtgtttcggagggcttgggtcttc
gtcaacgtattctaaaacacttgtactggaaacgtcagccaaataagtacggttgtctattgataaaagctgtttctttccactctgcagagtacttt
ggatgtagttttggatacgtgatcgtcgttcattttttgatttttttcgcggtaggactccaagctagcttgcctagcctgcagatcaagctcaaaaata
tttttcgaattagctgcatatgccgatctttttttggatacatattgggaagtatcggtaatggaattttcgtcattctttatgttatgatgcgttgaag
gaattgagtagggtgccgatgcaccagatggccgagggacctttgtagaggggttcactacgagcgactgatttcgagatgcagctcgcggaaa

ctttttagctttccatttcgtattttggttttctgggggatttgtaactcctgtaaattaattagcaggttacactcgttaaatgaaagccactatatac
cttgttgattcttgtaagtgttgattgcgcctaataagttagtaaaggttttcctccataagccgtgaagaagcctgtgctgaaagggaattgtttac
actaaagttcaagataaaaccattgaaatactcttaatgagagacgaacacaagccaaaatgaaaaaaaaaaaagacttaccggcaagagag
gctatttgctgcaataattgctgttcgtccatcttttaaaaatttaatgtgagaaatttgaacagcacataaatatagtttaactttgagaaacaact
aaaagatgtaaaacaaaaaaatctaaggtagaaaaaaaataaggccagaaataagtccttgtagtccaaaaaaataaacaacttcttttttat
aatggcttttctaatcaatattcagccaactttcaagttttctattttgttaaaaaaagagaaaatttgttacaataaaatatgtcaaaactacactc
tggattctaccaagaaaaattgggtggtggttaatggcaagtaattcgcagtattctataacccttcatagttacatgtgagtggatggtaaacaa
aagtaaggatccaagtaaatggttttattaggaaagaaaatagataatagaagtaaaaaataaaacaaaaggaaagataatttattcaattcgt
cggtaaattgttgataaaattatggaaggcaagttaactttttatggaaggcgatcccaattgttgagagtttcggtttcttataaaagaaaagaa
acaaaagaaaaaaaaatcatttgtaattgctcttttttccctcaacataaaagcaatgctctgtaagaggattaaaaacttagaactactctgag
tacaaatcttagagtcgcgacagacctgtgtttctgacctacccttttgtttcttcatcgaaatgattgcttttgaatctatttctccttcttaaactgc
acttatctcgttgtttttacaaggtttcatcaacgtttcgggttctttttcgacctggaaaggaaatgtttgttttcataatttccttttctgagactttta
tttttttatctttttaattttcattcgtgaatcattgatattagatgttttcttggtattgtcgcatttaaaaccaaaaaaaataaacaaataaaacaaa
taaacgaacgaaatagtgagtcaaatataaagtactttatactacgttattactctatatatgtcatacggtatggcaatgcaattttattaatttat
tggctgttgaatgccggtaaatctaagaggaggaagaaatttttactttacctaatcagttcaacgcctaacgaatatatacaaaacgggtcagg
gacattctcagttgaccttttccaacttttataagacaagacgagcatcccatttaggaattctttgaattgtcgccaagtctataggaagttttcgc
catatttgctagaaaaagcttggatcgaagatttgatgcaaattataagcatattacgattatttattgataattgaattgtctattttttctgattgtt
ttttcccttttgagtcattactactaatttttccccttttcttttcttttcgttttagcttttgtgctttctattcaaattctctcttaatatgcagtcgtcaac
tttggttttgacttctcctagttttgcctcttccgaacaggaattgaagcatgtctttagccttatcgatgccagtagtgaacgtgaattgcaaatgat
tgaccgtgttcaaacaaatttggtgactttacctgtgaacaaatacgtgtcggtaatagttgccttagacaacgccaactggtcatcagtcttgact
cccatatttccccaggtgtttgcttgcattcaacctggtggtagtttacgtgtctattcttctgtcggtggcaacgacgaatctttcgaaatgactgctt
taatggccggttggattattgagtccaagaatccatggattttatctcgccccgcacaagtcgaggctgtgcccattaaactgaaccgctccaaag
atggagtctcgaatgatatcaaaaagcctttagactctttgaaatctaagaaacaggtcgctctttcctcgattcaagatggaggagaagacgat
gatctggacgttattgacgaagatgagctcattgatggtgcagatcgaactgatgagaacgtccttcgaccacccgagtgtgagcctgctcccggt
aagaagaaacgtgcctgcaaaaactgtacttgcggcatgcgtgaaatggaacaaaaggaatcttcaaaggcatctgctcaactggaacctgtca
aattaagcgataccggtgaaattgactttacagaaaagttaaagagcaagaatgctgtttccagctgtggaaattgctatttaggagatgcctttc
gttgcagcggttgtccatacattggtcttccggctttcaatcccggtgatccaattgtcttggctgaaaaccgtgataagatgtcctggatggcaga
cgacctttaaaacctcaataccgaatagcttttgaaacttgttttatttagatgttcgtttgtaaatttagttaactatattcctttcccctgtctttgttt
acctgttaatttatgattcagtttcattctttttgattaaaaagctgttgcttttcaatttcttgagctgctttaccttgtaaggattaaaagatatcgaa
taataaattcattaatttgtagtacgaactaatagattgacatatcttgaggggcatgtatatatataatcatataagtcacatactctaccaaagg
aagcgtattccttaatatggatatatacaactccataagtcttctcctatgaagtcgaataaatcctggtgtcacgctattagcaaattccctgcctt
aggctgttaggccaaggtatgtttgcttctcctttgccatccaagttatacttatggtggagacaatgatgggactgagcaagccaacacccatgtt
tgcactacgaaaaagaagccattcactcaaaaagtggaagctggccaagtagtaagtgcactgacagataaagtaaatactaaaagaatagtt
ggttagaatagagctcaaaccatattagacttactctggactagacatatgataggcacagtaaatacgaataagcgcacttaaaaaagtccat
agaccaaacgtgcggccctgtagtccattcactataattgaacgttaataaccgctacactctaggatgtaaacacgtataaatggaacagcctg
ctcagtaaaagttgcaatgagataccttcattagaattactatatacgcgttttgtcagtttgggagtaatataggattgaatagtattaaagatgg
cagtgaaaccaaccaaaaggttccatttggctaatgtgccttccggaagattgcttaaaacatcatagacaagggtagaggaaaccatgttgatt
gaatacagtttggtagacttaaaggaatggtgaatgaatcctactgtttcggtgaaaagaagtcgctaaagatccgagtgattagaggaagtgca
tttttcagacttttttgtttttatttatttatttctttttctgttgctttttcttttgttatatgtaaccgcgatttcgttgcaccttagccaacaaatgtctat
ataccatcttataaatttgtaaagatagacatataaataaagatattatggctatgaactttagtaattaaaaggaacaataaccagccgtaaccc
ttgtctactatttactgaaactttttcttaacattcatattacgatgctaatagtcaactatggaattgatagtcgttacatttgcttcataaatctata
atattaaaactgtttgtttcacatttaaatttcctttaataatttacgtattattaagatccttagcttttgctacatcgtttattttgacggtgcactagt
aaacaaataacccagtcacaaaaatttggtccacgaacaacgcgctgaaaaactccatttctctgtctatacaaagcttgttttaatttgctttatg
cttccgttgattctaaatatgtaatatgtaaatgaatgatgggcggtcagtcatttttttgcgagcattcattataagaatattgtcggggttccctgct
ttttacacaggcggagtactgtctaaaagaatattgcttcttcgacgaacacaatttattactaaattcgggaaaaaattgtcattaaatgtcgcgg
atgcgttgtttagacagtcaaactgttagaacagggtaccaagaaaatcgcatcttccgatttctttccgacccttgaagattttgatttatatttatc
aagaaaagcatacctttgaactcgagaatgcctaactctttcgaactttatcattctcgctgctacaatttggtcaaatagcgaaagctcttcctgct
gcgtttattactttaacggtgtttattcgttacgttaagcttaaaaaaaagtgtgctgggatacaatggcgacctatgacgtcactttcaattcgcttc

ggaagagtgccacaattcgaggttttactagaagctgagaacaaaggaagctgacaagcactcacagaagaaagctctttgtaatcaagtctga
aagcatacatcgatatttttatacatgtttatttccaggaaaatgtaaaaatgcgaaatggtgtaaatttttgatcatttagtttacagaatacattt
gacggagaagtggcgtcactatatatataggtattgatcccctcaattgtgaaacgaaccacatccttttaccataatgcagattttcgtcaagact
ttgaccggtaagaccattaccttagaagttgaatcttctgatacaattgacaatgtcaaaagcaagatccaagacaaggaaggaattcctcctga
tcaacaacgtttgatttttgctggtaagcaattggaggatggccgcactctttctgattacaacattcaaaaggagtctacacttcacttggtcctac
gtctccgtggtggtatgcagatttttgtaaagaccttgaccggcaaaaccattactttagaagttgaatcttccgatacaattgacaatgtcaagag
aagatccaagacaaggaaggaattcctcctgatcaacaacgtttgatttttgctggtaagcaattggaagatggccgcactctttctgattacaac
attcaaaaggagtctacacttcacttggtcctgcgtctccgtggtggttgtctataaactgattatggttttcttacgcttacttttcgatttattttttg
acgaggtagttcattgaaatgtatcttttacataattattacaggtgctatatgcaatgtcaatctttaatgattaatcgcagtcaagtaaggaaata
aacatattgagtaaacatgggatactttatttgtatactggagagtagtacaagttcattatatcgtccagatggtagaaagataatgtaatagca
taaaaaatatgtgcgttagcatacgtaagataatcatgaacgatgcataaccgtttttagggatttgaattaatacgtttacaaaagtacctgggt
aatttatcatttcctaaagattttgtgttctgtatgcttgtttgaattaagaaatctgaatcatttattcggccataagaatgagcaagacgctgaag
ctttacgcatatacgtgcgcacctttaatcttcttgttcactttttttatatatttacttgaattttggtaataaccaaagattaataagttctttatggga
gcttaaaggttcaaattggaagatctccaatgaaagctcgtcaggaacattggcaaacttgattcctaaatacgaaaatgaacaggtaattcgttt
ataccatgacggcttagatgaaatgaaagaggttcaatcgtacgccgaaagcagcaaaattgacatctgggagacaaatccccgttatacagat
gttcgactccatcgagaagtcgtgaaggaaataattttgagagttcctaattatagagttttgatatcaaatgtatctatggctcttactaatagtct
cccaaaggaacaaggaattaagaacctgcctcctttagatttcaatactctctcagctatgagccaagaatcaatatcacaattatacctacaata
cacagaggaattttatagaaattaccaaaatattgaagctatcaattcctaccttcgtctactagcgtccatgtatagtgacttatgtgaactgactt
cattagggactaccgctgaagggcgatctatacttggtttaagaattcatggtcgaaagtcagatgggaatgaatttttttaatgaaaataggaacg
aggttattattctgcagggtaatgctcatgcccgtgaatggatatctattccaacattatgttactctgcgtggaaattgttagcccagtacgattca
gataatcgtgtgagaaaattacttgataagtttgaatggatcatcatacccgtgctgaatgttgatggttacgcttatacctggacagaagatcgtt
tttggatgaaaaacagacaaaaaataagcaattctaactgcaaaggaattaacatagattatgattggggatttggtttcagcggagaagaaaa
tccttgcagtgagttgtttggcggttctgcaccttttgaagctaatgaaacattggccatgtttaacttaatatcaagcacattaccaaaggaaaat
aaaaccacctttgggtttttagatgtgcattcttattctcaatcgattttatggccttatgcatatagctgtgatttactccctgcagatgatgaaaact
ttcaagagctggcaatgggtttagcgaaagaacttcatcgggtaaacggtagattctattctcatcagcaagcctgtgtaccttttgatggtattca
taagcattatcatcctggtgctgcaattgattttgcttattttgttgctgaagttcactggccatttacgatacgccttcgcgatatgggtgattatgta
agaaaaaaaaatactaattatttgatttatgcatattaaccaatttatagggatacttgctacctgcccatgagattgtacctacgtctcgagaattt
tatgccatgcttctttattatggtcagttcattgcagaagctgcatactgagaagacgtgatttgacgctatcaattttggtttttataatattgagga
ataatggaattttcaaatgtgttcatctaatttcaaaggttatttattttgtgcgcttcctatcataaacttggtatccaaagcaggtattggtgtgtat
ttgatgctattctcagcaagatttttttgacaatatgaaaaattatattttagtgaaaagttcttaaagagttactttatccatgagaaatctcagcgt
cctatttgttccttaaaaaggtaactacatatatagagatattggaatgaagttgactagagccaatttttaaaacagtcaaccaacagttttggta
aagctactcgcattatttctgaattaccaatcatagggagcaacccccatcattcctacttgccctctctcttcatagtccttggacatttatacgaat
ttttcactataacttacttaatatctattaattaatacagaaaaaaaaaaatcataactacacgagtaactatctcaatatttagaataacaagcagt
gtttcatactattatataatactatatgtttataacaatctatctacaatccacaggtaaccactgcaaaccatatagattcctaataaaaatgacta
cggctcatgtatgattatttattttttttttatacattacttgactactaacttttaatagagacctcaatttgaccctgctcggggttcctcagagatg
gcgcccacgaggattaccgctagtcgtgcattaccagcccatctgaagctcaaatacagaaaatccaatcaaggcacacaagaagagctatcca
ggaaggattttcgagagcaattgctacgctccgaagccgcccattttgcgaatcgtgagcacggggctgctgtcgacaaacatgccgaggcacct
gctgcgattgaggcgaatccggagacaaaaccaaacgaatctcaagaaaaagtggattatgaagagctattgaggaaaacctttgaggaagac
gctgattctagtgaaagtgaaagtgattccgaggaggaagaagttacagaaggagaaacaaacttgcctgctgaaaaacgattaaaaaaatcc
gaagaagagcggtctgattcagattctaactctagttctgaggacgatgatgatgaggaagatgagacagcgcaacttctccgagaactcgaaa
acatcaaacaagagagacaaaaagaaaaagaaaaacaagaattagaagcacaaagagaggaagaagagaaacgtgaaaaggaaatcgct
tatggtaatattctattaaaccagtcatctacgggttcttttcaagttaaacgccgttgggatgaagatgttgtgtttaaaaatacacataaaggca
ttgacgatcaacctaagcaaggctttattaatgatatgttgcgaagtgattttcataaaaagttccttgctagatttgttcagtgaatccttttctgtc
acgatattatccggctttttatgatttatatttcataccaactgtacaatagaaataaaggttatggttttaattaagcaaaataaacatatacgat
ggtcgatgccctctatatgtactttgtaatttattgagcgtaaataacccagtagttaagcatcacaaattgaagtctacctttgtaaataagaattt
aaaaaacctgttcattcgccttgagatgttcttcatatgttgtttaaaatgatcgctgaacagaattcacatgacaaaatatctttactaatcattca
tcaaaatttcggttgaaattgttcagcaaaaggatatgcatttcacaagaggtttgtttacaaagtagggatagctacttacctgtgcgctaaatat
tttatcatccttttgttgttgttcataaacattacttaccgatgtgcaacgtagatgaacgcgaccttacttgaatgaaattccatcggtggtagaga

acctttcccttaccacgaccgcaacctttacgaaaagaaaaagcaatggctttgcgagttcctaaggcctctgggcctcaattatttcgcgaaggc
tatagaatcatgcaaggtgtcgaagacgccgtgattcgcaactgtcatgccattcgtgaattgtctgaaattgtatgttggatgtcttgaaaatgac
cttttattcaatatgaaagcttcttggactaacttaaacagacaagaacatctttgggaccaaatgggaagaataaaattggtatgattacgtcga
agttttgtttgttgggctttgttgagctaacctgatgatagtggtaaaccatcttcaacaaacattcctcacaaatgatgccgcaaccattatcaggg
agctagaagtaatccatcctgcagcaaaacttgtggtcgatgctacaaagcagcaagaaaatgaggtaggtagaaagtcgttggcatctctgca
aaaatgcagcaaaaaacatctttattcctcttactaacatcaatagctcggtgatgccgcgaattttgtgattgtattctgtggtgaattattggcta
agactgaaaatatgattcgtatgggtttgactccttcagaaattgcaaaaggctttgaaatggcatatggtcacgttgcaaaagcattagaagaa
attgttgctgacaaaatcgaaacggtggaaaatgaaaaagatttgatcaaggctatacgcacttgtatctcttccaaacaatatggcaacgaaga
ctttctttcagacttagttgctaaggctatcctcactgttcttcctcaagatccttcaaaattcaatgttgataacattcgtgttgtgaagattatgggt
ggaagtctttacaactcccaagttgtaaagggtatggttttaccaagagagccagagggaactattcaacgagtcaagaaggccaaggttgctgt
tttctcttgtcctttagatatctctcaaactgagactaagggtactgttctattgcacaatgctcaagaaatgctcgacttctccaaaggagaagaa
gagtcgattgaaacccatattaaggagatccacgatgctggcgttcgtgtcgtcgttactggaggaaatgtcaatgacttagttttgcattatttaa
atcgttttgagattgcagtcgtgcgtattcccagtaaattcgatcttcgtcgcttatgccgtgttgtcggtgctactcctttgtcccgtgtcggcgttcc
catgcctgaagaaatgggctccgttgacatagtcgaaaccatagaaattggaggtgatcgcgtcactgtctttcgccaagttgaagatattacaa
gaactgctacaattgtcttacgtggagccactaagacttacttagatgatattgaacgtgctatagatgacggtgttaacattgtcaaagctcttgt
gaaggataaccgcttactttacggtgcaggagcaagcgacatgcaactttgtactaggcttatttccactggtgaaaagacaccaggaatttacc
aacatgctatcaagcagtacggtgaagcctttgaagttatacctagaactatttccgaaaacgctggtttggattctacagatattctcagcaaatt
gtatgctgctcatcagaaagaaggcggcgaatctattggagttgatgttgaggtaagttaaatccctgcttattgttttgttaacctttttttcttttta
gtgtgaggatgatggtgttcttgatgctaaggaagctcaattatttgatgtgttggctgccaaacactctgccattcgacttgcaacagatgccgtg
ttaacagtcttaaacgtcgatcaggtcgtaatgtcaaaacctgccggtggtcctaagcctcctggacctaatccaaattgggatgatgattaatagt
tttgtctgaaatcaaaattacgaaaatttttctaggtagaatgaaagaagtggtttatttttgcatggcaataactttagactgtaatcgtaaagttt
gataagcaatttatttttgaatatccagcaaatatccagtgttcaatgaaatattattctgtgcttagactaagaattgcaactaaagctgttccgta
tccatgaatttttaatatttcttgagaatatgggcgatgatccctatgcacaagcaaagattgaactagatcgcgtgcttcaatccatagatccaa
agagtaggaggctgaattatcgctgagttcatgttcctgctttttggtggtttacatcgaatccaattataagttaaacgaactaacctttggacat
atcttttgatacgtatgtcaggtggatcttcaaaaggtggtatagcatcaaaaggcaaacggccttctatcattgaaaataagagaatacccaac
gaccagcaatcagctttaaatccatcgtgagccagacccatgtagatttctggtgctgagtattcttgactgccacactcagactgtactatatgac
tttccttatcgacgaatttagagaattcaaaatcagacactttaattaaaggaagttggtaaaattgtaaacctttaacttctctgagatcctcatat
ttcttagtcaataaaatattttccagctttaggtcacgatgtacaagcagttgatcgtgtaaataagcgacggcatggactatttcagcaaagattc
tacctacaaactctggagaaaaatcggtataaaagtatttggtacattcgtagaggtcgccccccttcatagaatgattccgccagaaaaccattta
aaggagaaaggtaataattgatgagatgaacaattccacgatgtcgtaaagaacacagtactcggatttcctgttgaaatcctttgaacgcataa
gaagccaaacgttgtgacttagaaacacatgaaagtttctttattactaagtgccctatggaggaggtttttgaattggtaaccaagaagacctcc
ctgttaagtccagacgataattgctttgtaagtatccaatttagctcgctttgtagatcaattttgtaaacaaatccagtttcagatccaacaggtag
agagtaaaacgtcttttgattgcttggatgcaatacggaagccacttcccaaaactgtttaggttcaaaaacatgtgaacagcttgatgcagattt
atctccttgtacttttgactctccaatcgaagtctccttgctgagattcctcgtactagtatttaaagaattgatggaccgcaaaagctttggtttttca
ccaagcctttgttgattttggtcaattttacccaaaaagtttttattctgattcattaaattacattcttttgatatttttatgacattcggacaattgga
gtaagaatacaatctagctaaaataaggatagaatgtctgaagtgtgtcaatgagtaaattaggaaaagataggcaggttcaattccactcaaa
aaaatttcaatttttagtaaattagaaataaaaaatataaatactaattgtcaggataaagtcccgccgctatagagtcttcagaagaagctgacc
cttctttttgaaaagtttgaaaatggtctgaagatacagccttttcctcgtgatcttgctcttcgttcggtatgttatcgtaggagttgtcttgctctctg
tgtgttgaagcaactgagggaggtagaggaggaaagtttggttcgacggaattgagatcagcagaataaggagcatgatctatgggaggggaa
taagagtctacctctggagaatatgcatcatcactgttatagttaggatcttctgaggtattagcatccactgggctttcattgacgggaggatttct
tccttcaacgaccgcgagtatatttgtcaactctgaaacgtttgcttcttcttcttctactaactgtctttgcttgttcaacaatgtctctaaagatgat
attaaagccaggcgggattcaagacaaagacggtgtgtttttttcgcaggaagataccgcgtttataagctgagctttaaacttatcatttagctgtg
gatcatctgatggcttttcatgaaacgatttgtataagacatcaacagcacttttcgttgtagctgaagattttgcatggattgatgtttccacaaaa
agtttagaaagagacatctaggagcgaaaaagtcagaggttatccaaatagtgaaaaaagcattgaaaattcttacccattcgggttgatcaac
agctgaaggttgaccaagacgattataatccgcttccataaatttattctgaagatccgttaaaattttttcgagaatatagcacgctggcaccaa
acgtcgtatacgtatttgatcttagtttttatggtagaaggacaacttgcatatgcttgtggaacagcttctacgacaaactaaatcgaaaatatca
gttagctgaatgaaactttgtcttaatcaatatatggctttcctttgtcatggatacttacaggtgcaacagcgtcaatcaaatcagtgatctttttta
cacgagactgctggacaacctcattaattaaatatagtaggagcagctttttgttgggcggagctgaataggttaaataaatgaatttgaccgtgt

tgttatcatatcaacaaaccttcgcgtagcgattctaaccacagttgaataatttcattcgcatatcgtttgtgataaataacccaatgtgcgattcc
tgttatactctcttgggtctcatttaaggacactaatttcgcagaaacgatttctggagtaagcgccatcctagcttgtggtggtaaaatcgtagcaa
atgtcaaaatatgatacagtcataatggaaagggaaagagctagattaaacaaaaaacatgaagagtcattatatataattttataattttacttc
atcgtttggaccccggaagaatatgtgatactgaatcgcagaaagatatttgtctattgttaaagtatgcacagactatactaccttgtcaattgac
ctttgctttaagaacatttttagaaattcctctttagaaatgaataaagcatcgtttgctagcagaattcatcgcatatgttttgtttactaatgaagc
ccaaggaggctttcagcaggctaatttgtattttacaaatatatcgatttgtagttttgtaaaattaggtatacatttacgattatatacagtaaaag
aacactattcatggaaagcctttatggtactgctttctcattacgcgcaaatggtttgtacggagtcaactatttactctaagaatgcctgatcaaca
aagcctttaaattcagtgattttgtttctccttctttatttataacatttaaacttgctctattaaattactaaagaaatcttagctctctagtacagacc
tttcccaagcattaattgtagtaacgagttataacgaagaaactcataaaggaaatgtttaccaaaagaaaaaaaatacttgtaataaaaaaac
atgtattctttttcatacatatttattgattcattgatgtattcaactcttggtaaatcggtaatacagcactgcgcaacagttccattccccaacccc
agaccatatctgtaccaccgtgccctcagtactcttaccaagtgtgcttgctttcaagttcatagaaataatcaaaaatgtcttgttggacgcttgtt
cagcaagattctttcttagagactccatctgctgatcagttaaagacagcgttggagtccaaaaatgactatgttaaaatcagcgccatgaaaac
aattctacggattatcatcaatggggaccctttatcttctatcctaatgcatgttattcgttttgtgatgccatcaaggaataaagaacttaaaaagc
ttctatattactattgggaaatctgtcctaaatacaataatgatggtacgatgaaacaggagatgattttggcttgcaactcgttccgcaacgacct
tcaacacccaaatgaatatattcgtggagctaccttgcggtttctttgcaagttgaaagaaccagaacttttggaacctcttattcctacggttcgtc
aatgtttggaacatcgtcatgcctatgttcgtaaaaccgcaatcatgagtgtgtttagtatctatcaggtttcttctcacctaatcccggatgctgcct
ctttaatcgaggacgtgctagtggtggaaactgaaggaacttgcaagcgtaacgctttaattgtgctgtgtacgattgatcccaaacttgctattag
ctggttaacgaataatttcgatcacattatttctctcaatgcctcctctccttattgtcattgaattcattcgaaagatcgtcattgtaaactctcct
gacaaagctaaatatttctcgcttctattagaacttttgcatgtaccttccagtagtgttgtttttgaagcttctacttccttaatgaacatgacgaca
aacattcaaatcatcaaaaccgctgcttctcgacttttggaccttgctacccgtgaagccgataacaacgccaaactcatcatgctggataggatt
gctgacctcgttcaccatgaaaagtccggactagatgatattgttaccgacgttcttccgttcctggctaatactgactttgatgtatgcgaaaaag
ccatttccatcattttaagtttggtcaccacgagaaacgttgacgatatcttggagcaacttcaaaaggagctctccaagtctaatggtgaatccga
aaatgatgatgctcgtcgtcgcgctcttacagaagctatccatcactgtgcaattactttccctcaaactgctgctactgccattcaatacctcttaa
gccacatttctgattttcaaagtaaatctgcttcaagtgttctgtcttttgttaaagaagttatggaaaaattcccagaactccgttctgcaaacctta
ctaagcttttactttctttgaaggaatttcgcgccggaaagattttccgcggcgtaatttggattgccggtgagtattgtcttactgaagcggatatc
aggactgcttggaagagcattcgagcaagtattggtgaaattcctattctagcttctgaggagcaacttttgaatgatgtttcagattcccaagaac
aagacttgcttgttgatgtccaaacagcaccctcttcaagccgtaaaattcttgcagatggaacttatgctaccgaaagtgcagtaacgagcgagt
cacttaatgctgccagactggaagccgtgagagcatctaaaaagcctcctctaaggactcaaatattgggtggtgactactatttggcaacagcg
ttagcttccgctcttactaaacttgttatgcgttatagccaaatttcgaatgatcatgagaggctgaacgctcttcgtgctgaagcaaccttgattat
gacatccattatccgagttggacagtcgaagtttgttaaatatacgatcgacgatgattctattgagcgtattctaagctgcattaaggctctttatt
cctttgaaggttttgacccactacaaaaggtcttccttgaagacacaaaaaatgcattttctcacattgtcttagagaatgaaaaacgtcaaaagg
ctgaagcgtctcttgccaatgaacgtaacgccatgcaacctgatgatactctcaacattcgacaacttagcagagcaattgaggaaaaagctgaa
acatcttttgaatctgatgtgattcaggctgcaagtgatggtattgttatggaagatttgtcatctaaattggatcatgttgtatccttgactgggtat
actgatccggtatacgttgaggcatatgtgaagattcaacaatttgacattatcttggatattcttatagttaatcgtaccgacagcactttacagaa
cctttgcctagaactagccacgctcggtgatcttaaaattgtagaacggcctgctccccttaatttggctcccagagcatttaattccgttcaagcaa
ctgtgaaggtttcctctactgattccggtgtaatatttggtgcaatttcttttggtggtaaagcttccgaagaagatcgagtagttaatttgagtagc
atatctctaaacttgatggattatatcaagcccgccagttgtcctgaatccaaattccgcagcatgtggactgaatttgaatgggagaacaaagtt
gatatctcatccaatgaaaatattaacttaatggaatttttgcataaactaatgaagaagacgaatatgaattgccttactcctgatgctagcttgc
ggggagactgtggtttttgagcgctaatttgtacgcccgcagcatttttggtgaggatgcgctgatgagtttgagtgttgaaaagtctacggatgg
gcctatatctggtcatgttcgaattcgctctaagacccaaggaattgctctcactttgggctctttagttgctgcttaaaagatttccgattgctatta
agatcattcacactttacgagaagctttcaactctcacactgacacattgatagataaatagcgtttttcgatattctacactcatcttttgtttttatg
atttggaacactgctagcgcaaatgacgtttagaagaatatattaactatcgttttccaagccttttccttttctcttattatcgatttcgaataaagtt
ttgccaatagaataattactatttgcacagatggtttcaatgaaatatcttctattaattttagtatggtatgtatagatgaaatcacaaagatctat
gcttctaataaggattagcataagctttctatgtactaatttgtagtacttaaacaaaattaacaattctacatattctatagtttgattacttttaaa
aactgcatagggaaccattattgtttgtctaatttataatataatatatatatatacatatattgttaacgccatgtaatgctttacgttaacggaact
gacttgttaccgcgatcgctacttgatttggtaaacgtaaggctggaacttgtcgttaacctatctttttgttaggcctgaagttgtagtttatatggt
atggtgggtaaaatcataattctttagctaatttatttaggccattataaagagagtaaaccggacgaaaattgcggatgccaaagccccaagca
atgctgggcaacccgctggcgttcgaaaagccgtatttcaaggcagccgcaagaagacagtgggcgtggatgatttgacattactctccaagatt

actgatgatgaaataaataaaaacttagagctacgtttccgtaatggtgagatttatacatacatcgggcatgtattgatttcagtaaaccctttcc
gtgatttgggtatatatactatggatgttctcaagtcatatcaaggaaaaaacaggcttgaaacatctcctcatgtgtttgcaattgctgaaaatgc
ctactatcaaatgaagtcgtatcgcgaaaatcaatgtattattatttctggcgaatctggtgctggcaagactgaagcagctaaacagattatgca
atatataactcatgtctcgaaatccgttggtactgaaattgagagggtttcccaaattattttggcgacaaaccccctacttgagagctttggctgtg
ctaaaactcttcgcaataacaattcctcccgccatggaaagtacttagagatgatttttaatcctggcggtgctccaattggtgcaaagattacgaa
ctaccttttggaaaaaaatcgtatagttcatcaagttcgaaatgagagaaattttcatatcttttaccaatttacgaagagtgctcctcaaaaatat
cgtgatgcatatggaattcaggggcccgaaagctatctttatacttcggctagtcaatgtttctcagttgatagtatatctgatgaaaaagattatc
agggtactatcaaagctatgcaagtaattggtattactgaagcagaacaagacgaaatttttaagatgatttcaattatcttatggcttggtaatat
tcagtttcaagaaggacctgacggcggcagtgtaattaccgacagaagtatttcagaatttttaggatatttaataggagtttcttcctctttaattg
aaaaggccttaactattcgaattatgcaaactcaacatggttctagaagaggctccgtttatgaggtccctctcaatccaacccaagcgttagctgt
tcgagatgctttggctatggccatatataactgtcttttcgattggattgttgagcgagttaataaagcactggttacttcagatagatccgcaaaca
cttcaattggtattctggatatttatggttttgagatttttgaacacaactcctttgaacaactttgcattaattatgtcaatgaaaagttgcagcaaa
ttttcattgagcttacgctgaaaaccgaacaagaagagtacgttcgtgaacaaattgaatggactcctattaaatatttcaataataaagtggtttg
tgacttaattgagtcaaagagacctcccggtcttttcgctgccatgaacgactctattgctactgcacatgctgattctgctgccgccgataatgctt
ttgctcaacgcttaaacttcctttcgagtaatcctcattttgaaccgagacaaacccaatttattgtcaagcattatgctggcgatgttacttatgata
taatgggcatgaccgacaagaacaaagatcaattggctaacgatattttaaatgttgttcagtcatcgagtaacgactttttgaagtctattttccct
gttgctcaagaaagtagtgccagacgtcgacctcctacggctggtgatcgcattaaagcttctgcgaatgacttggtcgacactttgatgaaatgc
caaccttcttatattcgtacgatcaaaccaaatcaaacgaaatcacctcaagaatatgatactcgcatggttttacatcaaatcaaatatcttggtc
ttcaggaaaacattcgtattcgtcgcgctggtttcgcatatcgccaggcatttgatacatttgctcagcgtttttccgttttaagtggtaaaactagtt
atgctggcgagtatacttggcaaagtgatgacaaatctgcttgtgagcaaattttgaaagatactaatattcccccctactgaatatcagatgggta
ctagtaaagtgtttatcaaaaatcctgaaacccttttcacactggaggatatgcgcgataaattttgggatagtatggctgtaagaattcaacgag
cctggcgtgcttacgttagaagaaggtcagaagctgcttcttctatacaaaagctgtggaatcgtaataaggtcaatgttgagcttcaacgtgtcc
gtgatgaaggctcaaagcttttacaaggcaagaaggagcgaagaagatacagtatgttaggctcaagaaagttttatggcgattacttatcggct
tccaaaactaatggcacattatggaatacttgtggcttaaatcctaatgataacgttattttctctatgcgttgcgaagtacttgttcataaactggg
ccgatcctctaaaccttctcctcgccagcttgttttgacaaaaaagaacctatatcttgttatcactaaaattgttgatcgtcaacttactcagcagg
tcgaaaagaaatttcctgttactgctattgaatcagttaatctaacaaacttacaagatgactgggttgccgtgcggaacaaatctagcccagatg
gtgatatgtttttgcgttgtttcttcaaaactgagttaataactactttgcgaaacatcaatcgtcaacttaaagttttcgtgggaccaaccattgatt
attgtcgtaagcaaggaaaagttataactgttaagacacaaaaagaagaaacaacaaacgattacgattattataagagtagcaccatccgtgt
tgggactggattacctccaaatagtgttagtaaaccattccctcgtcttgctactacttctcacgctgttaaacagcctccagctcaggcagctaca
aaaccagcacccgttaaacgggcagctcaaccaaaaccgcagcctgttaatcgaaacaaaccgcctacgccaaaacctcaagctatgactactc
aaccatctcctttcccggcagcgaatacatatgatattaaccaaaccacacaaatattgcctatgcaagaatcatcctcacctgcacctgccgctg
ctaaatctcctgctgctactaacccaactgtttttcaacacctagtcaagctcctcctgctcctccaccaccagctcctcctgccgctccccaaaag
cccttatacattgctttgtatgattttgctggccgcagtgccaatgagatgtcaataaagaaagatgaggtattagaaattgttcaaaaagagccg
agtggttggtggcttgctttcaaaaacggattagagggatgggtacccgcaacatatgtaactgaaagcaaggacactactttaactgccgaaaa
ccaagtaaacaatggtgcttcgagccctggtgttaacaatctagctggttcactggccgatgcacttcgtatgcgagcatctgctgtccgcggtagt
gacgatgaagaggactggtaagtagaattgcctatgtacatataatgacttctaaattttgcaattgaaaactaagtactgcttctttctatcatttt
tatgttttagatattacaaagtacagccatgtcaatttatgaaataaatcgttttattttatcaactgaagtatcaataattagcattcagataaagtt
agcaactgaaaaaaaatctcggcgtatcttgcacattcataatgatctgagacaataaaagcatgcaatcattactagttttataaatataatca
ggtgtaaatcgcttggcagaaaaacctttatattcctttggcgaccattgaaagaatagcctgttttacctttgaactacgatcaaagagagacat
gccaagagagcgtacggaactgacaacagaattatccaacgcgtataacttatgaaatttatcagctacgccgaggtaaacatgatttttaaagt
accggcttctatagtaaggctgaagagagaatatactaccggggtcttgaccatgctggatagcggaagaaagagcgtttgtcaaagcttcagc
atcctgtattccagtgttcattccttgtccagcaagaggatgggtattgtgagcagcatctccacaaagggcaacactttccttcacgtactcgtca
acgtgtgccatacgaagggggaatgatgcgcgtgagttaggaacgatttctgaaatttgaggtggcgtgtcaaaagacaaagatcgtctcttagt
ttcaattctccaatccagttgcttatctaggttatttggctcaacgatgtccattttaaagagataatccaagtcaacctgatctaagagaaacgca
gcattcaaaaacttaacaaatgtaggatctggcaaagcgagtaatttctttgctagttctggataaattgaccaaacaaaagtagcattgtttcct
ggaagagggaggtatgctaaaggaccggtaggcagaaagcgctgaaaagcaactgaaggtaaattattttgagcgactcgaagagttccaacc
acaccatgagcaggatatgcccatccaggcattgctattttggcaaattgacgtactatagagtttctgccgtcggcaccaaccaacaatcgcgtg
cgtagtactccatgagttgatgtgtgtatagaaggagcattcgtattttcatctcttgttatcttttctatgttacaaggaactaagaaatcaaaattg

tcttgtcgtatatcctttctctttaaaatggaccgtagtaaaccatactgtaaattcacattttccgccataaaagccatgggatcagtattctttggt
tgatcaaagtgaactgttgaattagttatcccatcataagcgtaaatatgttgaaagggttgtaatctctcttgacgcatatgttcccaagctccaat
tctttcaaaaaagcttcgactacgagccattacagagacacagcgattcgagtatgaagtaggattccaatcctgaatcatcaaagtgtttttgtta
tctaaaatagcaaccttcagcgaccgtgtataaggattcgtttgtaatcccgcagctaaggccaatccaacaggacccgttccgacaatagtaat
atcataatcactttttggtatgctcgtctgagtagcaaataggcgggttaacttgggataaattcgtaaagcagaattagacattcgcatcatgaca
acaagatacagtattctatagacgaagaaaacctttaattaatgatttattttaatattctttataggttattttctccaacttaacacatatcgaggt
tgtaagtttagttctttccaatcgtaagttcgtagtgttcagttcgcctaaacaagtagttaaaaaagaaaacgatatcagcatgggctaccaagtc
acttctagtatgtacataatatatttttagctactattattatccataagatttcgactcttctctgtattttatgttctttacacgacgagtgttacgttt
tttaatcaacctttaatattcaccgtctaattcagagaagcagtaattgttcatttaaaaaaatgtcatagttatctacttgctcttataatcatacctt
aaaactgataccaccctgcttgtacaactttacatttgccttttactactgtacgacttaacattagaggataaaaaatgatcattgtccaaatcttg
gacgactaatacatataggattccgtcgcgtaatttgaaaacatgcgtgtcagaatagaggcttgagagttcttaacgacctaggagggaagag
aaacatagaaataataaatactagctaacataccaatgaagattaaacatcattcataagaatcataagagtcataaaacaattagaaaagaa
cacataaacaaccataaacaagaacgcaaataaggtatctgaaattaaactccacatccataaacaaagattatttgtttacactttttgttttgtt
tatatactgtttaccatagaattcaatcggccaaatcccaacctcatttcaagaaaaaggcaggaacaacctttcttttttcttttttcttttttggata
attttttggtacgtgggctgcaagttcctaaggtatttgggatgtcgaatttggttgttagtaataccgacatgaatcaaaaaactcatgacgaact
acaagatcagaatgaaagtggcttcaatttgcagaccaacgaaggtgatctacctggcttaactgaaaaacagtcggaaaagccattgatgaaa
aattcgtcaatcaaggaagattcccgcgtatctgagaaaccaagtgttagtgaatctgatgaagtgaaacaaccagaagacaacgctcccgatt
ccaacaatcagctcgctcataatttacatggcgaagcttttcaaagacccgatggtgtaaagggacagcatttgcaggtttatactcaagaatttc
atgttcagactcctgtaacaccacaacccatgacaggcaggactgttgcatcaccaaacgatgatatatcccctactgaatcagtagcttcgaca
aagccagattcagttttatcagatagacaagggggattcgcacctgcgtcaagaagtactgtgcgtatctgtctgtatcacaaactttacaacaaa
tggtatcttaacatgaatgaagatgatgataatcagacgattgcagcatacgctcagcattttagcagcgacaaccgtccatctatcttttatttgct
tcaaggtatttcctttggtccagagtatgactatgtgttacgtcgtaaacattggtggaatcctttgagcgaaaaaagtcatgcgaaaagaggtagt
acttccaaaaatcgacactcatccaggatgccgatctgtatatgagcgttatcagaaaagtaaaatttatggtgatgaagatgtattgattgtacc
agtatacggtctaaatatgtgggttgtctccttgattgttgcaagtgctgcaggatcagtgctaattggtttatggatgcggctttcagatcccttttt
gcacacggtatgcttctcaaccttggactaggaagcattggtagcttcttatacttgctttcgaacacttggtgcagataaagtattgttcaaaatcc
acatttctccattgcttgctttttaatagaaagcacttggacagaatcatttaggtaaagaatatctttttgtttaataacatttatggttgatccact
taaggacttatattctcggttatcaagcgaaggatttactgagataagatgggatttcataaagtgatgtaaatttccttgagcttcatcttcagtaa
tgtgaacaggttcagtaggcatctcaaacttggcttccactgaatccaacatccatggcttcactttcatattttgaaacgctagatcggagggtac
caaaatcgtagtatagttatttgtatcctttagcctttctacaagctcaggaaattgcataagaatatctgaaaaaatattgcaatcccttgagcca
gctaatagctcgaataaacgaagctggtgagtagtttgcatttggaaaggtttctgtcggaaggagaatgcgtttgtaatatataaaaacgtaga
gaataagaaaaagtttacgataaacggtactttcatgctaataccttgacgttctatagcgaaaaatttaagagcatataccactgctatagcttg
taaacggagagaattgataattcagctgaaaagaaaacgtctcaaaaactggttttgctaaaaaaaaaaacgtccctattgagcagtaataata
gcttcctagttcagaaaagttagagaaaacaatactaactaaagaatagcaataataactacgcttacttacgatattttaatcaaagtatagtaa
aagtacagatttgttgaggtaagagggaaattgttgatcatttaatagttaactgaaatataatatgatttacagcataatatatcaaatggattcc
cagagaacatttttttagaaattagtatgattatttctttattccatgtatttttaaaattgaatgaaaacgcgtaacatcttaattgaaactcacca
cgaacatctggtatttcaacagaataaaccaaagactaagaggaatcttaaaatatttattgaattctttactgattaagagcttttctttccagag
aagtacattaaagcatcagtgatcgtagttagggcatcgtagcctaaaccatataataagctagtactcgggtttcgcgttttacggcatcttttgt
aagaaattccatttacagaagagttcatttacggtaaaagaggacttacaaatctcaaaagtttaactattgaggaaccacttttgtttgattgaa
gtgaaaactaattgaataattagtaagtgaaattcctggaacaaaagttaacgaagaaacgactaaacaatgtttgaatgatggagctatctcca
aaggatgatatttctgaagtattgaataatggagcatcgcaatctacggatccttctttatgggctatattcggacaacactcaatggaagattcaa
gcgaaaggaggaatgttggtgctgaagtagataatggtatttttgaccttagtcagttaaacgagcatcaattgacctcgtccatgcccaacgcca
tgggtttgtcgggtcagaatggactaatagccaaaagcttcgatggagatgataaccctggcaaggacgatgcatctctgctaagccaacatatg
ctgagcaccgaaatggaacacaaaattatggaggcgaagggtcgacaagcagtgggtggcaatcgtaaaggtaaacgcgtttctacaactgca
aattcacttgagcgtaatggaacacagtatgtctcaaatgaggctccaatcgccgtgaaatccagcatccccgcgcttccgcgtactacctatgaa
agactctgctacgagtctgcaattgccagtggattatctcctgattctctttcaagtgatgaggtaggtttgctatctgaaatcctggataatccttct
tggttgtctctttatttaaaaatccgtaacggcatatgctatctatggcatagaaacccgacactgtttgtatcctttaatgaagccttgggtatcgtt
cgagaaaaaaatggcatttcctttggcgtcattagcttttgaatttttaacgagaagcggctacattaactatggatcactcaatatttctcttagtctg
caaccaggagaaaatcttcctcagaaaactgtagctatcgttggtgctggcatggctggcataagttgcgcgcgtcaattagcaaatttatttgcg

caatatgagcaggactttataaatcgcggtgaaaaatctcctcagttaatcctttacgaggcatcaagtcgattaggaggccacgtttttttctcatg
gagtacctatttatgattctttaagtgaagaagaacaaactggacgtcagtcagatgcacttcaaacctgcgtggttaacttatcatctgactttct
atatggctgtccctttttagaaaatgatccgttacacatcatagtctctcagcaactttcattagacgaagtaatacgccagactcagaacgttgct
ctatatgattctggagatggtcgaatagaccaaaggcatatagacagagtcttaatgctatttagatctttacttgattattttttctcaatgaaaaa
tgatcaacttctgaatatagtattttcccccaaacaaaagtttattcttactttgcagaatttggactggcgtcttgttactgatgaatttcccttgtct
aattacgactcacttaaagacttcttagaggattccttcaaaaatttgaatcaattactacacttttccattgcggaaacccgggtgtttgaatggtg
cgttgagtacttaaaacaaatgtcttgtgtgccgttagaattaataaaagcgtcatctaaatgttttattaacttcccttttggagaagctgttccttc
gtatgctattaaaaacggcatgtcaacgctaattgaagcactagctacgactcctagtcctttaccggttttatttgatcataccgtccagtcagtca
atttagaaggccaaaaagctcacctttcatttacgaatgacacaaatataactgttgataaagtggtaatgtgtttacctattaataagtatcgaga
gtctaacgtttcatttggaccgccaatatctgagcagaaactagatgctttgaataagtgctacttattgaatcgcaaaaaagttatctttctgttta
aaagccagttttgggaacttcacaaaagcgtctttgggtctttgacctctagtaagaaaaactacatatataacgattgttcagacatatatggatt
cccatcattaagcgtgtacgtggatattcctgcgcatcaacgagttgttgacagagatattgttcataatatcctttagaactcagtaacattttta
aaatcgagaatctgcaaccgttacgcactgttattacgaactgggaaaacaatgcatattcaaatcactactcatatgctatttcacctgattcatc
cttagaagattattctgttttaagagagagcatggacgatactatatattttgccagtgaagcgttatcaaaagagtattccggaactttacgcggt
gctttccaatctggtttattggctgcgaaagatgttcttagttctttaattggggatgttgccgttcctaatactttaatagtagaagaaaaacttgaa
ggatctgaatccaaccgtaaaagatcagcaggacaagtgtatggcaatggaaggaaaggaaatgatgacaataaagccctcaattactatcac
gattatcttagattaaggaagcagagacttgaaagagaagagcaagaatgcgaacttttaatcgccgaacttcttggcgctcggcccgttgctcct
acaagaccaagcgcaaacccatacttaatatatcaaaaaactcaatggcatgtgtgtagagcacttgcagaccaagaaaaacaaagaagtact
ggtgatcctgaggcacgtgccacaaaaaatgaaatcagggccaaacttggaaaaacatggagagctctcgatgatctaggaaaacaaccctgg
gtcgatgaggtctctgctaggcgggcgaattattccagtcgtttagaagagtatcaaagacaaataaatgcctataatgttcgcgccgcacaaatt
cgcgatgagcatcgtcgcaagtgtgaatctcagcctcttcccgaagaagaagcaaggttgaagatcctttcgcaggaggaagacgagcgtgctc
aaaagcgtcgtcttctagaaaatacaaccttgaacgacagtgacgaggaatatcgagaagatatggattatgaagaacaaattgatgataatttt
tccgatacgttcacttagattataataggcaatctaaaaatatacgcttgggtttctggtgcacttcttttttttttttttttaaaaaattttaaaaaaa
agaagaaaaaagtaatacaaaatttattcacgtaaaaactagtttatcatttattcccttcctattatagaaaaaatacatactcatattcctgttct
ttagcctttgtcagatataattaggtaaatacagtatttattgtcaaggtcactacacagtactgggtacattctaaaatcaactaaataaggtcac
tctaatagaagtataatacgaaaataaaaaatgtcaaagaaagctttaagtcatctgtcgtttgatcctcctgaatccttagacaaggatcaagtt
gttgctaaagtattgcaactcagaggttcagcattatttacggtttgtgaagccaatggacgagaactactcgttgagatgcctgcaaaatatcga
aacaaagcatggattaaacggggtggatatgttgtaatagataagtctgaatttacagaccaaaacaacaaaatcgacggttctattgcatacgt
ggttcaaaacccattgaaaatttgggagaagcaaccttattggtaagtttttttcataatctatatgaatagctaacaattgttaggcctaaagagtt
taccaacaataccccctgcagatatagaagaaagtagcagtagtgaaagcgccgaggagagtgattgatacgtttattttttggacaaaaacatgt
tttattaaccttaccatatagctattttagttgcttaatagaactgaaatgtagcttccagaatgctaaaatcataacttatagtactaccaatagca
tttgactcataaatgattccatcagtaattttttgggtcataaaggaataatagattactaaattatcaccaagcatttgctttataacaatcctaaa
catgttagtaatttgcttgatcagtgattgtgcaaacttacctcaccataaaaagcaaggacaatagttctatcatcaaatttacagcctcgcatct
cttgtatggctgcttcagccgatttgatatcagcaaatctcacgtatacttttccaatgccaatggaaggagtgtcagataaaccttttcgttgagga
atctttatgtctagaatttttccaaatctagaaaattgagtagaaacggcttcttgaatttctataaaagtgttagaaaaaaaatttaaaagagacgc
acttaccttcacaatctcgaggatcagcagcttcgttttctgttacaacattatgaatttgtaacacacgtgtagcctgaactttgggatccactttgg
ctaattctgtcaaagcgaccattcccacattgctttcgggtatttttttgattcaaacctacacaggaaaaaccaacctttatttttttgtcgttaaattc
cattccgtctaaattttgaatcgccttccctgcgtcctcagagttacggtattcacaaaatccaaaacccttagaagattcgtcagcaacatttttta
ttaattgaaagctcaataactctccgaacggtttgaataactcaattacgttctgttcactcaaattagaagggaagttggtgaaatagattttatct
tttgagtccaatacttcattttgcgtaaaatccaaacttctttggtttgcagtctcaggcataatttgtggaacaatgtagtttttaattctgttaaaac
ttatcagtatatcaggtgcatattgagcggtatgtaaaccccataaaaatgtggcatcttccggcttcgataattctaaaattgcagaattatcttct
ttggaaaaaaaaacattcgaaaaatgactagtttctggtttatggtacgttgtggatataaacaaatcttcgataaatggcttgaatgtatttatgt
caaagtctgacggaagattggaaacgactaatcttcgagcttgacgactagcaccaggttgcaaagggggaggtggcgccatgatacttcctgct
gccgatcgagcaaactcaagaagcttttcaggatcagtagtagcagtacgaggtgcgccaggaagaggaaataaaccagacattttagcttggt
cagcggttactaactcataaccaggaggtttgatatcccattgagagttttgacgaacccattcattgattggcttcacatccctaagttgctctaat
tcgcgttctatagattgcagagtacgagacctacttgaagatggactcacgcttctacgtctagaagaataccttgaagaatgatactcatggtca
tcataacgtctcctctttgatctttcatcatcacggggacttctctctctatactctctgtttcgtcggtaatcatgatcttctcggtgtcgacgtcgatg
atcgtctcgataatagcttcgctctatgtcacgcgaggagtatctgtccatacgtccaccattttcatctcgagaatctcgacctcttctcgaagagc

gagattctgaaattctggacgacaaatccattttagcaatccaaggtctaaatcaatagtatgtaagactagattaaggtttctgagggtataaaa
taagcaatggaagagtaaaaattatagtaagcaataattatacgttgtttgtgcagtcgtttatataacacgacttgaacagcttgcaaagtcaca
atataattcagaaatataatatttgtttttctttcttatttaatagcaagctaatcccaaaacctttctcctgaatgaatttttagagtaggtagagg
tgaccttgagaaccgtatgtctaccggttactacacaagaacaaaactaccaattttgggtttattttaatgcaattgagttagttcaaaaactaga
aagaaaagaaagtagaaagtactatactttcacttcgaaatggtggaagtgtcggtcaagtaccagcattagtctataaaaaaaattttagccaa
cttttcgacttcaaacgcgtctaaaagacatttatacaattgtttttgttatattgagaggatttctataatatccgtgctcatatgaggatgcatcctt
atttacagtctattcgaatcatatgtagaattggcgttaggaatttctgatattcttattaaattaaagattgtcgttttcttattgtttcgcgttttatg
caatcttaaataacgcttttattattacctactctctttaaattttggaagcattcccttaaatagacgaaaaggatgaagcgctatcttcaggaggc
atttggaaatgaacttccaagtgatgaatatccgactaatgaaaatcggtatcctcctaaaaatgtcattaatttgtcaagtgatcatccgacaaa
gcaattcaaagtcaattcaaccgaaagctcaatgtacttgttgggttttaaacatatactttctacagttcttagtcacgagtctcacctcttaacgc
aagaagagttagtcgttttggataaattcaaaatcctagcttgcgacgaacaataccttttttgttcgtcttttcatgagaaagcgcgacaagtggtt
tcgagtcacacagctaaattatcaaaattgccaaagcataacagaagctagtgcaaaccttacgaaactagggtttttttgttgatgaaaattcaat
aagcattgaggaattattagatactatgactttagaagagctgcgtacggttgctcgtttcagtaaaataaacgggaaatcgaggaaagagataa
ttcactcagttatctgtttatcaagaaatcagtccgtccttcataccgatggtcagttacttctttcatttaatggaataggaaacttgaataatcaga
caaagcaacttaaacagcagcttttatatcacagcggatcatgtgtaaaaccagaaagaagtgtcgttgagctttttcatcgaatccatgctgtgt
actttcgttctacaatttatgatgagcattctctaacttctttgatcctagttcgcataaagaaaacagtatatccaggttatcaaatatctcgaacct
caaacgtattcgaatctaggacaatattaatagaatacgttgatgcgttagagctttgtaagaaacttttgccattatttgaaggacctagtttgtc
aaacaaggaagctcttggaactgcccttgatcttttttttaatatctatccaaaatggttgtcatacatccatcaggatatgctaaagtttgcgactg
attttgaggaagataagcagcgtttgtacagttttatattttcttatcgcccaggggccgtttatacttatttgattcataaaggtacgaatgttttgg
gaaagctacgactagttgaacttgaaatcgaagttcttgatactctacttgctcaaaatttatacttgattggtaaacggggattttggtataatag
aaaagcgcttttggaagggaactacaaaactacggacgttagtgtgttacaaaattggcaacgtcgaggactatgtacatgtacgcacggtatcg
aggacaaaaatacacatatgaaataccaatttgtccttcaaagaagactttcgcgactcgaaaaggtcttattgatacctgagaaggacaggcgt
gatttttcatataactttcaaaataaaccctcaagtattacaatctttggaaaaagagtggatcctaaggataaaagtcaaccaagaacactttgg
cttacttcgaaaggtgatgtattaacagttgaaaagtttgctgttgagtactatgcatcttttggttggtctgctattcattctgagggtggaattttct
taactttatttgctttggtttttttgggatattttgtttatggacatcccaggtgttttccaatccgcgtttcagtcagcacctttagatttatcaacggatt
tatttttttcggcacgagaatacgcaattcgtggaagacttgcagaagtcgaaaacggctgcacggaaaagtatgtcaaaatggtttatacccgtg
aatcaaaaagtcagccagtatgcatcgggcttcaatggtcttattcactcgagacgctgctctcagtaatttattgtattcatggtaaagctttagct
tctatatttcatgccatggcccaagattataagaatgcttctagtggtatgccggatttatgcctttggaatactagtacaaagaattttctactttcg
gaagtgaagagtgataatgacaaactgtccgaaccccaaaaattctggatagatttattgctaagtgcaaacttatctgttgaagtatgccatgtg
ctcatggatgctgataaagcttctttataatcatagtcatgttaaggttttggtcaaagggattagacaggactagatggaatattattcttatactt
cgtaatgatagacctgacctttatttcgctcgaaattcgtatttaaaaaggaagaaattatcaggaagtgtgccaatgtttggtatttatttatttac
ctcctaaggtttttatttcagtatgctaggtagtggatcctttcatgttatgtttcctacataactctcagcaacattgacgattttatgatgagacgct
ttatttcctctcttgcttttcaactgatttttataagttcaaaaatcagtacgtagaatactgcatcttttcaaaccgttcctagactttgataaagtgt
cttgcccattaaggaaaattctctgatcacatacatcagatgtctattcttcaacttcagtcccctttcactgtgcgatatcaaatattagcatcttgg
cttgttcaatgaaatcagcctttttcatcttcataactttgatttcattgattttcaatccttatactttaaacctcctcagcgcaagatgctagttcaa
atcatgtacagcctaatgaaacaaacttgaaatttacgttgtcaaagatcttattgttagaaagaattacttccctatttataatattctatactttttt
ctgtgcacgcgaaaatatttccaatatgtattgctgctctactgaagttattatgccttcgtaatacagacttgcatgttcttgatagagcagtacag
aactgttgcatctaatttctccccaagaggcctatgtgggggtgatgaagcgattgatagatgccctctagaattgggatgctgaatattttgttcg
gtatttcactttaggtatttaaacgagaaaatatgtatgaatatatcgaatgtgatttatgattattaacttaaccaaacacatttcctactgttaac
aatttgtacttttaaacgtttttttactttttttaatcgcattttacttagagggaaagcaagctattagaacaattgcaaaatgggaggcggtgatttg
aatatgaaaaagagttggtacgtgtgaccataaatgttgtcgagaatgaataattactaactgttttaggcaccctcttttaatgagaaatcaaga
gagggtgtggaaagaagaacagactcacaaggaagaattaaaaagggtcgagcaattacgaagagaaattgaagaggaacgacagcttttg
gaacttcagaggttacaagaagcagctggaggaaaaaagaggaaagaccatgtggaatggatgtatgcagttcctacagccgaaggacctaa
acgagactcctcggaaatggaagaatttttgcttggtaaacggcggttagacgaccttttaagtgacaaagtggaggatcaaaagaattccctta
ataagaccgaatttatttctttgcaaaatgccaattctgttcaagatacccaagccaaagtccggttggacccccttttggctataaagcagcagg
agcaaaagcaaatgcaactactagcagaaaagaggaagaggaagctagaagaagaagaaagaaggaaaaaaagagtcggagcgtcgtagtc
ggcatttaaactcacaagaaagaagttatgatagacatggccgccatagtgaatctcgaagacgcagctatgctagagatgttacgaggagaga
cgaatattcgccaacagcatatggagaccgacattatcgcgataaaaggtatgtcgacgatcgagattatgaaaaaagacggttttaccaagac

aaacggaaatacgataatgaaagatacagccgaagacaattttcttcaaggtcaccttcccctcaacgacggagttttcatagaccacgtgctttt
gttgatggatcctcaaaagaagatgcccgtgagcgtcgattaaaagaaatgcaagacaatgcgcaaaaattagaaaagaatagggtcgaaaa
acttcataaattagaacaagaggagttagttgaatctgccaaaaatgaagaggaacgatctaagactgctagaaaatggaataaccaaggaga
atttcttcgagaaatgaagagagaggtatattctggtgatgcggtaaacttggctgaccgtgtaagcagtatgcggcatactgctcttaaacaata
aagcttctttaatggatgttatattagatgaatgttttattagaaatatgtgaattaagaagaaagttatgggaagatgataagttgctgcataact
ggcaacctagtcattctcattgatagaagtaaaaagacgtaacatatttgtaccaaaaaaatgaatcgaaattatccggacctgtctggttcttgtt
cctgtatgctatacgctattgatcgttcatcatcgttctgattttcggcttgcttgatttcctgggatcctacgacgtatgtagttgcaatatcatcaca
agttggcaccgtcaggttaaactcttcgttgaattttctataaaacaatcagtaacgtgtccaaaagttaattgcaataggggaatgcatacccgt
gcagtttacatccccaagctgttgctgctttagaagaaccagtttgaggagggtactttcggattaatttttgcgagacaactgagcattcaccgca
agggaacaaggaagcagatgaaacaatataatgaaacaatatatttctttcatatagtgtaggcaagcttggataattggctaaagtagtatgga
aagactaaacaaataattagcatgcgagacatcaagactcatttgggcttacagtccaataatttttcatcatttcgtttctatttagattttataatt
agtaaagtaatgacagcaagaattgtcgtaatgaaaccattgggtatcacgacatagtttttgatcatttcattccttaccttgtctcttccttgtggt
ttaaaggagtgataacaacaacctcttcacttttctctttattttcgccaagttgcctgagttcaggcatgtataggcgccattctttgtcaatcatta
cgctttgaaatacccatattacaaaacccacaattagaactgttgataaaagtatctgtatcctcttgttaaacaacatctttataaaaattgatac
atcttcagcgtttaataaatggaagttgcggttgaagtttaagtcagagggtactgatatttagtttgtttactttaccatcaatactgaagtttgccc
tacagttcattatatgtggcttcgtcaggcctctcattccacaaaggcaattagatgtggatttatgtgaagtcacaatattaccaagtgatttccaa
ggtatattactataaagcgtagatagtttatggccgccacagttgtgatgtcataattgggtccctcgttagcattcataatataacaattcgttata
agaatggaatattagctaatatcagttaatattcataagcttaaagaattgtgcttgataataagtaaacaaaaatttgaaaatgcaaatttctctt
tgattgcagaaactccgattaaaatttacaattcgtgattattgctattaattagagaagccagtcacgatggcaaatatacagaataatatatat
atgtgtgtcgacagtcgaaatctttagatcggaagatttgactgatcaggctaaataagattgacacttagtcgttttcatttagttaatcagatccc
tggacatattactattactctacgtagtactaacacgtccaattccaatagacaattttgtaattgtacttattacttccactcgatttacattctcata
acgtgtgagatttctcccgtgacatgtgaattgtcagagcaaccactaatattattacaaattattttgtaataattatcctatggagtatattactgt
ataccaatgtttataaacaaacaaactgagaaagcagacggaaagtttaaccaatcatcaagaattagctcaaccttattgtttgtttacttacta
gttttagattactatataaacgccgttctcctcagccaaaatcttcagttaggaagattttgctggcagagctaataaacttgttcgataacatattc
attagtatcaccagagacgttactaaagatatatcactccagatatttagtaacttattttagagtatccagcttgacaattcaagcaattataact
gtattccactcacacacttattactatacggagtattttcccgtgacatgaatgtcgtaggactgtataattattttatccatttgtcacgggagaaa
tctcatacgttatgagaatgtaaatcgagtggaagtaataagtacaattacaaagttgtctattggaattggacgtgttagtactacgtagaagaa
tagtaatatgtccagggatctggataactaaatgaaacgactaagagtcaatcttatttatcctggtcagtcaagtcttctgatctgaagatttcga
ctgtcaacacacatatatatattgttctgtatatttgccatcgtgactggcttctctaattaatagcaatgatcacgaattgtaaattttaatcggagt
ttctgcattcaaagagaaatttgcattttcaattttttgtttacttattatcaagcacaattctttaagcttatgaatattaacttatattagctaatatt
ccattcttataacggattgttatattatgaatgctaacgagggatccaattatgacacctccctgcctaagagtctttcacttctttgaattggaaga
ctcttattttcattgcggttcttctgttggagaagcgcgacgattaaattcaatgagctttgagtatgcctttttttcgaacgatgtctctaggaaccca
tgtgactgagcgatagtcataatctgagagttgcgatctatataaacaattgtagtgagaaggcttttctaggttatcagcttctaaaatttcgtcaa
taacgatgttttgcataaaattgtttgagcttaagtccgcgttgtaaggttcgagttcggatacatgaaaactagcaggatagcgtcttcgtatcgtt
tcaggaaaatttaactcgtagttatgttctcctatttttctttttactgaaaatggaccaacaaatgttggttcaagtttatttgattgatggaaaaat
cccactttgccatttttgactaatacctgatcgttaggttggaaaatcggtggtggaattgttttttggttgtaatactttgcgtatttatcatttgctttt
ctgatacgttcacgtatttcatcgatggattctgttctgtccggtaaataaggaatatctagttcatccaggatttgcgtatcccaagcggtaactat
agcttgaataccaaaagcagcattaattggagctatttttgtagcagaatgctgtgaagagttataacataattgagctttgagaagataattgac
ccaatttgttaaatgtttgttcacgtagcaacgaagatatgactccaagatttgattgacgcgctctgtttgtccgtttgtttgtggatgatatcctga
agataacataagatgtattttatggtctttggcatattgtcgccacttttctgaaataaaaacaggtccccgatctgaaacaatatcaattggatat
ccaaaactggatatgattttgtcctcgaatatttcaattaactgatccgtggtcgttgttgtgttacaagcgataaaccaagccattttggtaaaacg
atctacaatggttaaaattgcatctttaccttgagaaggtggtaatcctgttataaaatccatagaataatgtctgaatggcttttctgcaatgggta
acggttgtaattgaccatatcgttgatgatttcgatttttatgtcgttgacatatgtcacaattttttacatattttggatgtctgcggtcataccttc
cagtnnnnnnnnnnnnnnnnnnnnnnnnnnnnnnnnnnnnnnnnnnnnnnnnnnnnnnnnnnnnnnnnnnnnnnnnnnnnnnnnnnnnnnnnnnn
nnnnnnnnnnnnnnnnnnnnnnnnncaagatttgattgacgcgctctgtttgtccgtttgtttgtggatgatatcctgaagataacataaga
tgtattttatggtctttggcatattgtcgccacttttctgaaataaaaacaggtccccgatctgaaacaatatcaattggatatccaaaactggatat
gattttgtcctcgaatatttcaattaactgatccgtggtcgttgttgtgttacaagcgataaaccaagccattttggtaaaacgatctacaatggtta
aaattgcatctttaccttgagaaggtggtaatcctgttataaaatccatagaataatgtctgaatggcttttctgcaatgggtaacggttgtaattga

ccatatcgttgatgatttcgatttttatgtcgttgacatatgtcacaatttttacatatttttggatgtctgcggtcatacctttccagtaaaacttttcg
cggatgcgatttgttgtggacatttttccacgatgtcctcctgagacgggttgatcatgaaactgattgcatatgagttgtacaagttgtggtagttt
agggatgacaatttgtccttgatcattgacgatgagaccatttttcacagtgtattgtgggttacggtttattccattaagaatattctttaaattcttg
tgatggaaatactcctgtctcacgtagtcgaatatgtttccacgactcgattaactatttcagttttgtgatctggacgacgagaaagagcgtctgc
aggttcatttttcgaccaggacggtattggatttcaaaattgtataaatctagttcagttgcccatctcatgatttttgagcgagtatctgtgtgaac
cttagtgaacattgatgttaaattcttatgatcagtaagaatttgaaatggtttgtcagtacctgccaaaaaatgataccatttgcgtaaagctgct
attattgcatacgcttctttttcccatgttggccaaattcgttgccgatctgtaaatgtctctgaatgaaacgcaatgggatgataaattccgtcgtct
tgtttttgggagagtatggctcctatagcaaaatcagaagcatccgtttctaatctagtttctcgatgtgactcataatgtctgagcatagaaacatt
ttcgagttctttcttgacttgtaagaatgcttgttgttgacgatctgtccatttaaatttgttgtttttcttggttaaatcatataaaggtctcgctaattt
ggataaattcggcataaatcgtcggctgtaatttacgaatcctaaaaatgattgtaatcttctaagagtattgggtattggccaatttgcgactgctt
gaatcctctctgggtcaatttcaatacctttatcggagaatatgtggcctagaaactttactttgtttctgaaaaaatgacatttttctcggtttgcgt
ataggttagctttctgtaatctttcgaaaacctgttgcaaatgtttttcatggtccttttctgatgatgaaaatacgagaatatcgtctaaataaacg
ataacaaatatgccaataaaaggaagtaacactttgttaatatatctctgaaaatttgctggcgcatttgttaatccaaatggcatgactgtatatt
cgtatgctccaaaatgtgttcggaatgcagtcttgtgttcgtcaccttccttgattctaagtagatgataggcctcttttaaatcgattgtactaaaga
ttttggctccgcctagccagtttataatttcttcgaaactcggtaatggataattatcctttcttgtgatcgcgttcagcggcttatagtttataaccat
gcgatacttatctgttcccttctttggaactattaatggtgcacacataaaatttgaagaactttcgcggattcgcccttgagctaataattcttgcac
cttttcgtttattaccgatttttcacgttgtgtgtattgtctcaactttggggtataatctggtaattcttgtgttaattcaatttttaaatcactattcga
gtgttggggcaacggtattttagtgttttccgtgaacactttagcaaacggctttaatatttcttgaactttcgcatcgtttttgttatatgtctggtctt
tttccagattcgtttcgccatataaaaagtctaaatttcccaatttttaagaagggtccatccaatgatggcttcattttcggaagttggcgtaattat
gaatgtctcataaaatttccgattatttatttctactggcaattttaaatttcctttaacttccagcttgtcatttgttatccccataaatgttcggttttc
tttgtttgtatgcctactcttcatcttcaacttttgtccagtctgttttctggtaccatctggtgacctggatctacactcatggtgaggccaccacatc
aagtctacttcgtagccagcatcgttataatattcaaagctctttggaattggtccttcaatgctgtagcatgtgccacattcataccccctgtctacct
aatattctatttactgcatccctaacacgataccagatcagttctatcaatggtctgtagtacataggatcgcctaagttatcaggtaccttcccccc
tctaacatggagttcaggttctagtgatggtacgtcggacccttctgtatcggattcaggatgaaatgggtcatactcttctgggtacccacgacta
aaccagtcatagtgttccttaatctcatggtaatcatcataggaagacactatagcataagaattatgcgaaaacacatagactgaaacattttga
tactgctgctgaatgcattcagaaatctgctcactaattgcttcatcaacatcattttctacattgtttccactgtctagccatatagcatatgatccat
tgatattatggctcaatctatcaatagcagccttgccattgaaatacttactgacgcagtcatctatggcagaccacacatgatgtccatcatatcg
gtgacgattccacacttcagccgcttttacaatgctgatagttgatagcaataagcataatgttataagttgaaatttaatcatcttaaagctgacg
gttcgcttttgtcaaacaaactaatcaaactaaactaatgtaatgtgataaaaccaactgaaaatcaaattgtattttatcgtcgcgcttctccaac
agaagaaccgcaatgaaaataagagtcttccaattcaaagaagtgaaagactcttaggcagggaggtgtcataattggatccctcgttagcattc
ataatataacaattcgttataagaatggaatattagctaatatcagttaatattcataagcttaaagaattgtgcttgataataagtaaacaaaaa
attgaaattgcaaatttctctttgaatgcagaaactccgattaaaatttacaattcgtgatcattgctattaattagagaagccagtcacgatggca
aatatacagaacaatatatatatgtgtgttgacagtcgaaatctttagatcggaagataatttgtcacgggagaaatcgagtggaagtaataagt
acaattacaaagttgtctatttgaattggacgtgttagtactacgtagaagaatagtaatatgttcagggatctgattaactaaatgaaacgacta
agagtcaatcttatttagcctggtcagtcaagttttctgatctgaagatttcgactgtagacacagatatatatattattctgaaaattttccattgtg
gtttgcttctttaattcataagagtaattaaattttaaccggagtctttgtattagaaccagaattcgtagtttcaattttctgtttacttattataaaac
acatttctttaagcttatgaatattaactgatattagctaatattccattcttataacgaattgttatattatgaatgctaacgagggatccaattatg
acagaatcaagacaaaaacaaagatatgcaaataaaaataaaaatatcaagaataaaacaaaacggtaatatacagtaagctaatcagttca
atattctttttatttttagatggaaataatatacttaaaagcgattgtttctacataaaatcttcagttgaaaactttttactggtcgagctaaacaac
ttgtttcctgatacatttcagttcgtcaacaggcttatttggaactcctagtaatcttataaacacaaatagtaggtggacgagacttcagcttggca
gtacaattgcctattccgtatccagttaccaattatctatttactcgaataacaattcaaagtacaggaaggatggtttagtaggaaataggtaaat
tatcataaaatggatatactgattgaaaatccttgagaattcctatcaataactgttatccatggcaaacgtgcaagaaaattatattgtcaagca
attcgtatatgggcagttaacctcattcaacagctctctatatttttttagtgtgaacaatgaatcttttttttttttttttttacttagctttatgtttgttt
ttttacagaaaattcgactctgatttttttttggaaattaaccgaaaacttaagctttatttaagcgaaaaaacaatatagcgctttgtcacgcagca
gagtctacaacaataaactacaccaagaaacaaccaaaataatttgttcatatactccaactacataggattcccttttatgtctgataaaagacc
ggctcgtacatcttcggttccttccattgtagatgctacacctgatcgtgctgaaagacctcggaaacaagttcgtcaagtcgtaactactccaagt
cgatcagaggatccttctcgcatagtaaaactagagcaaactccttctcaagggttccatagtgagtttttaaaaaaacgcatcacagattcctta
cgcgagcgtcctaatcttttgaacaagttcatctctgctaatcaagaaaccccttcaaaagcttctgttcctaatccctttaattccagtaacgctga

agactcttctcttatagaggatgcaataactggacaagataatcttgatgtacttgctccaaggcttattgtataccaattgacgctaaaaaatttc
aaatcatatgccggaacacaaataattggcccatttcatccctccttctcttctatcgtcggcccaaatggatctggtaaatcaaatgtcattgacg
ctttgctttttgtattcggatttcgtgctagtaaactgcgtcaaaccaaagcttcagctttaattcacaagtctgctgcccatccttctcttgatagctg
tgatgtggaaattgtatttaaagaagttgatccggattctaattacatagatggctcagaactgtctattcgaaggtccgcttataagaacaatgta
agcaaatactttctaaataatgtcgaaagttctttttctactgtctcaaatgtactgaaagaacgaggaatcgacttgaatcataagcgctttttgat
tttacaaggtgaagttgaatctatcgcacaaatgaaaccaaaagcgatgacagaaaatgatgacggtttgcttgaatatcttgaagatataatcg
gtacttcaaaatataaacctatcattgaagaaaaaatcaatgaactttctactttgaatgatgtttgtaacgaaaaggagaaccgctttaatttagt
cgttgccgagagaaacaaattggaaagctcaaaaaatgaggtcctcgatatcttaagggatgaaaataatttatatagaaagctgcataccctct
accagttgaacctgttcgaattaaattcaaaaaaggttctaataacaaacatgttacagaaattggaagattcttacaagtcaaaaaatgcaaac
tttgaagaaaatgaaaaagtcgtttttgaaatgactgaagaattaactcagcttaaatcaaaagttcaggatttgaaaaattccaatcgggccga
aaagagggaacgtcaacgttatgaacaacaagctgttaagattgaagagaagttgaagcatttatcttcaaaacaaaagaaaactcaaaagca
gcttgaaactttgtcagcaaataaatcagagcttcaatactctctagacacgcatgatgatgttcatcagcgtttaacccaagaaatagaagaaat
atcctcaaagctgtcttctgaagaagaccaactgaacaaaattcgtgagtctcttcgtggaaagaccgaaggtatctctaatgctatcgaggaaa
aacagaaattgatggccccttctgaaaaaaaagtgaatgagctgaattctgtgaaacaggtcacaaaggcagaactagatatgctgtttgagca
agaaaaaagcttaaatgaagaacagcaaaatgcaaagagttcccttgagaatctgatagcagaacagaaggagaaaacactacttcttaattc
aaagaaaaatcaccacaataccattaagaaggattctcaaagtttggagagaaacatatccgatttaagacgaaaaaagagcgaattacatcg
tgtcatcgctcaaagccgagtaaaactagaagaaatgaaagctactcttagctcttcacgctcaagaggaaacgtcctgggaggattacaaacct
tacatgattccggtcaactagaagggtttcacggacgactgggagatctaggtacaattgatagtaagtacgatgttgcaatctctacagcttgcc
ctgcattgaatcatatagtggtcgatagtttagaggttggccaaaagtgcatagccttccttaggtccaataatttaggaagatctagtttcatagtt
ttacgcgctttacaggaaaggaacttgaagcctatacaaactcctgagaatgttccgaggttatttgatttgcttcattttaaaaatgaaatcttcgc
accagcgtttttcagtgttttacagaacactttggttgcagataatttggaacaggctaacagaatcgcttttggaaagacacgttggagggttgtc
acccttagcggacaactaattgataagtctggtaccatgactggtggtggaacacgcgtaaaacagggcggcatggcttctcaattaccttcaga
catatctccagtttcccttagcaatgctgaggatcagactacagacgctgaaaatcgacatcgacaagtatcagaagaattggtcgctcttttaga
aaagagcgacgaactgaagaacgaagccccatgtttggaattggaaatatccaagcttcatttggatttatcagcattcaataatttggttgaagg
tgccaaaaatcaagtttctaatagccgtaaagcgttaaaggaagaacgaagtttacaggaaagaaaacagaagcttactggggacttgacgaa
acttgatcaacagatagataacataaaagcctcaaatggtgggcttgttgctgaagtcagagaacttcaggataaaattatgcagattggtggta
tcaaatatcgtatacaaaagtctaaagttgatgatttgcacgaacagcataagtatatcaaagaaaaacttacaaatgtcagttttgagaagaga
aaaaacgagcaacgtgttcattcgattataggagaactagagtcattacaatctgatttttctacgactcaaaaggaattggaaacgaatgagac
tgacttcaacttacttcgaggaaaaattaaggattatacgactaaaattgatgagttaagtaattcgatccatgacattaatgattcaatcaatga
acttaacaatcgtattgactttgaatctaaagagataaattccatgaaagcagagagactagatctcgaaaacaaattaacggaacaaaaaact
gcccattcggatgttttgcacaatgaaaaaaagttacagaagctgttgtccgaattaatattacatgacttgagagaatatgatcaatccgaggca
cgggctcctgagtttcaggaattttccgaagatgaacttgcctcgatgaacaaacaatcattatatgaaggcatttctgagtttaagaaaaagaca
gaagaaaaagaagtagacgttggtgttttacaatcttatttgagatgcacgaaggaagttgagaaaagagcatcagatttcgaagctgacacgc
gaaaaaggaacaatgtcaagcaaactgttactgatttgcaaactcaaagattagatgaatttatggaagggtttggtaatatttctcagaaattaa
aagctatgtatcaaattatcactatgggtggtaatgctgaacttgagcttgttgacagtttagatccattctctgaaggcgtcttattcagtattatgc
cacctaaaaagtcttggaaaaatatctcgaatttatctggtggtgaaaagacattaagctcattagctttagtatttgctttacatagctacaaacc
taccccctctatatgttatggacgaaattgatgctgcactggattttaaaaatgtatcaattgtcgccaattatataaaggaaaggacaaaaaatgc
ccaatttatcgtcatttcgttaagaagcaacatgtttgaattatcttcacgcttggttggtatttacaaaacggcaaacatgacgaaaagtgtaaca
ataaacaatcgggatctacaagtgaacgcagaaaaagactcagaccattcgccgtcacaagtcaattagaagttaaagatgcgagcttgcatta
gactttaagatatgtactgttatctaagaactgtatttgtataaataatttcaaattaattcaggttttctcatataactatatctctaatcactttcaa
aaatagtaagtgtttagtaaattattatgatctaagaaacccttatatagattatgcaaattaaacgacccttcaaataacataacatgaaagggt
gagtacaacattaaagactcagaagaaagttaattttaaagcaattaacatgcaattcatggactgaatcatccatgctttaaataagtggtgtta
gcattggtcacacaattcgtaacttaatgtacagagtatgcagaaaataggtaaaacaatcaaaattatgcaatttgtaaagaataacttaaagc
caatcgcaaggatggtgttcccatccatggtactcagttagacccaagtgttgaatgttcctttttatcttcgatcgcttgtgtcccattgatccatta
gttcggtcctttacagcggatttaaaacggctataagcatttgacatacgggtaccaacattctcatgttcttttgtcggaaatttagaagcagaat
gagatgacaggtcttctatttcaaggaattccaaatcacggagagatttttaaaggcagcactttgtcgtgtagatcttataccagcagcagagc
tggttaaataatcgccggattttctaaagtcagaagcattttcctcgtttgcaagcgaaaatctctgtttaaccacctttggatgcttatggaggatt
ccagcttctttggaatataattgtgatgtaggaggctgttttcttattttagataatttccttgacatcattgactggcttctggttttgggattcaagg

gtgaaactaaaggaagtaaaggactctcttcatcgtgagatatcacaccatttgcaagcaaccatggggattgctgagaaaaattcttgtcccac
ggcgagctaggagatttcagttcttcgtatatagcgctgatcagcttgtctgaaacatgccaaggagaagcgggagaagatataggactttttgc
atatgtcgaatctttttcagtatctacaaacgttatatttcttggaccttgaagtttcaatggaggtggtttcctcttcgacacatccataattcaattc
gattttctaaagctgctatccagttcaatcaagactaggaattcggaacaattagtagggaataattgtcttatgtgaagcaagtgatttcttttatt
atcgtaatcaattgaacgattcaatttcaatctgcatatagtttgtctataccgaatgctatgaatgtgcttcaatccttttagaaaagaaagtactt
gcacgtcaattaccatatatatatatatatctgtatttgtaacttactttgcctacttaagtttctttgttcaccatttcgtatacttactcgaacaaag
aaattaaactttctttgttatactagcagcaaacaaatgtcttacaaatttagattaactaaacgagaataaacgtatcatgatcttttgttcttttaa
actatgttcaagtcgtaacgagattattgcgaaattacttcatttactcacacctgacaagatgaagataattatttgataccaaaaaaattcaaca
ataaaataaattaacattagtaaattatctttacatacataactattcagtttaacttagaacttaagaaaacacttttataacctctttgcttattta
gcaaatttttctttatcatatatatatattcctttgcataatcctcaatagatacgttaatattctcgatgaagtgtcattatttccttgttgagaccaat
gatgcccacgatgcaaacttatttaaattacaacttgccagatctttgagggattcatagaagaaaactcattaacttcttctcctgtctccttctgct
tagcagttaattgcttttcttgaataaaatcattccaagggtcattttcatcatccattaacttaacgaaagtatcttcttcttctgggactttgtaagt
tcgatcggtattcattgactcatctccaacctttgtttctattccgggttcagccgttttcgtctccccattaacagatataccctcttgaacaccatca
gcccataattcagcatcgtcatcatatgtgaatttgccttccaaggtcaagatatttgctggactcaaagaacttttacctgcattataatcaggagt
tgaagaaggaatgaaaaacgactgaggtgtttttattaccgaagtcggtgtcaaaccgcttttaacggctgaaaactcatcctcgtgatcaagcgt
tacgaatgcgtcaatagcatactttagtgggtttgcagtagcctcttctctggctttatacgactcagcttctttcgctgaggtattatttagcccatc
attcaccaatgaaccagaaacaggagtctcttcagcattcatggcatgtccttctataggtgtctctaatgaaggtttttgtattaaggggcttatgg
ctggtggtgaaacattgtcaagcttcttcctcttgctcattggtaactttaggtcctctggccttagggtgtgcttcattacttcagatattgctttttct
ttcaattgtgcttcttgttgagctttacttatcgcttcataactgagctgttgttcatccaagccagcttcattagtaggcttgggtatatttcgcacttg
aggtggtaaattaggttgaggtttgttcacgccagcgatctgagtctgcatggcagcagcaggctgatattgtaaaggactttgctgaggttgttgt
tgtttctgattcctttgttgaccctgctgtagagactgttgttgttgttgctgctgctgctgctgctgttgttgctgctgttgctgctgttgagcagaaga
gcttctcatggacgttgaaatttgagctaaagctgcaggtagactttgaccgttcacgttaacttcatgatgcaaccgaattaggtgagttctcacg
tatgcaaagtaacgactcgtcttttcctttatctttgccatcatttgaggtgtacaggtaaaaacaccctgctgaagaccttctagctgtaacttaaa
aatatggcgcatttgtattaattgaatagtcgcttcttcattagctgtaagacaataaaataagtatatcaattgattagttctactaaacattggat
atgcagatttcacgagttcagcgacaatcactttttggtcatgggggagatccagcggtataggtcttatcattgaatgcatcactctctcggttaat
tcacgaatccgaacagtcatttttttgctgccccatagttcctccagattctaaaagctgctgcactataaaggcgcttacatttccaggagcggagg
catttgcaaagggcttctctgtaggttgattacctgcttgaggagcacccgtggcatcagtaggttttaaaggataagaaggaggactgaccgaa
gcattaaacgctgggttcacctcagcacttgacgtaccaacattaggaacatcaacaggagataaattcgactttaatgcctggctattcgcacgt
gccttagttggtacataattttgataattggcttttgctggattaggagcagtttcggcatttatcggtacattagtgctttgaggctttatcggttggt
ttgtaacattagcattcctcatctgttgagcataagcctgttgagaagcagaaggtgctacattcggatcaacaggaacatttccaaatttattagg
agcttgaggtgtctgagtgggttgggcatttacacgtgcagattgcatttttaattgtcgatgttgcaacagtacttgcatatgcttttgataaagaa
ttccaatcgcttttgcttgttcaggtgtcaaacgctgattcttcccaagttcagttacatctctccataattttatatttgaaggtaatcctggaatttgt
gcaaatagttgcttaggaacaggaatgttcagcatttccaaattttgccttatattagcgttacttggaatgtttgcaggacgaggctgctgtggaa
cctgaggttgtgtttgttgttgattggccatattaggatacgtttgtccagcgttattagcattgaaaggcacatttccctgttgttgactctgttgctg
aatctgtttttgttgttgctgctgtaattgttgaagcgatcggaatttttgaacttgattttggtaaagtgaaataagtgtcgcaggcggcatactctg
cagcttttgcaaagctataggaggagttccggtacgagctaacaatgttgaaatttgtgcacaaagattgttcaactgttgttgcgaaagtgccgg
tggaactcgaggttgagcagcacgaccttgattcgtatcggttgccggctgaggaggttgctgttgctgctgtatctggctctgctgataaagaagt
tgtctttgctgaggtgtaagtttgacatttccagcaaattgttgttgaacccctcctggttgcattggcatagtctggttttgcatacgcggcaaaacc
cttgaattggctgccattcttccagcagtaacaggaggtactgaagggacagtattcatattaccattgttgctataattctgtgcttgcttcaattg
agctgcctgtaaatgctcacgtatttggtctcttagttgagcggtctttttactacaagtcgtcaagtattcgttctaacgtttatgttaacaacttaaa
acataaagcatcaaccaaacataccttactattggccccatcgaatgcttgtctttcaaacgaaagtgccatattcatcaactgaacttctgaaata
gatggagaaattatccgcagcgtttgggctctaaaaaattattagcttaaatagaaattacttatgctctctcttacatttgtaaagcaatactttgt
cgttcattaggtcgaatttgagtttgccatcctttttcaacatttctgttcattacttgctatatttagttttgttgtctgtatatatttccaaaacttttca
attagctcttaaccgtccttgcttaatagttttccactaaacttgtaaaggctccctgtacggtattggacaaatacgtcctataggggtacacttag
ggaataggagagattcttaggacgaaaatatggtcatgtctcgcaaataacagctgaaaaacaatagaagatattgaaaaaagacactgttgat
tacaaaggattacaaattcatttttctacgattttaagagtgcgcctttaattcatgcagtagaccacaaaaatatctacagctttcaaacctcaat
taatcttattgaatggtttgtacgaaacagaaacatgttattattttaaggcataacattttaattcataactaatagataaggggaaaactaacag
aaagagaaagtggtaacaaaaacagacagctttttgacgaagattcctttttcttcgttaagttataagtaactctaaatcattagatgcaattctt

ttgcatttttccttaaaaatgaaaatccaatactccaaaaagtaccttttggaagttcctaactacaaagttccgaaagcttccttaattcagagg
atccccggtcttctatcctctctaatgcgtagataacagcctgctgcagagcgtgtcgatatgtcggtgaaatttttcaattgtcattagcttacaaa
ggaaagagggcccgtaattttgatccattattatttttccaaagagcttgtattcaagtcattggatttacccagaatagcttcgacaaccatatca
gcacaaatagtttcttcttgaggcatcgaaagaatcttcagcaaagagttataggccagtcgatgagtacaaactcgcacaatatctttcgtgaat
ctctgcgttaaaagcacagctatattaggataattcatgttttcaatcagataattcaacaaaagtgtaccgttaccattcatggccaaatgtactg
aatacacagttattgctgcggcaaccaaaactcgctgctcatcaacaacatcaggactttctaaacaagtgcgtatagcacgggaaccatagcga
ctttgagcaattacccagaattgattcattatagcttcaaaaacgaaattattcattggcgaaccaaatcggagacatgtttgtaccacataattgc
caaattggtctgcaaaaaggagaggtatatatggctgaagatgctttgcgataagaagcatttgagattcagtagaggctacatcaacaattttct
gagctgcccaggtaccatttttatgaattccaatctgcgcaagatgaggagcaatcctttctaacatcatttctttaacgggatcagagcagtaatc
aaatagcttctgtactactgtattacccaggtggtcactagcaagatctgatacttcatctaaccagttaacggcaatttcctcgacttcctcttgag
tacaaagaccattgtcaatccgcttacggacttgccgaagtttaggagcatttaatcggcggctactgaatgctttagagatggatggtatttgggc
tgtaaattccgttaaatcacaggcagtatctaaaataggacgtatattagagagatcatcatacccaaaggatttaataacaccaattatatcatc
ataaacttctcgtaaagatgtaacgaatgacacgttggaaaaagccttgttcaaaccatcgacagctgtttgagcgttttcagaacttgaagatgc
aaccttggcaaatccgatgcagatagggttagaatccaaaaaaatacattgtccattaagctcttccaaaatatactttgctgtattgacactatcg
aaattaataaaagcggaatgcttatgagaaagcattcttatattagtaatggtgccatgagaagcaaaagtagcagcaacggtatctgaagaga
tgccaacaggaagattgccgatccataaagcatgtgtcggctccgattgagtaagatttccaatagtggcatccccaataccactccaacctgtag
tactattggcgtcaaggacactttttgtgctaccaacattattgacactaacgctgcttgtatcaggagtagaggttctgatactgctgttacttgtgc
tgagaggggaaggagcatttgttaaggaagaaggattatacgaaggtgagcctttggcttttggtgacccccgatccgcagggtcaaccattcct
cccactaatgatgaaactggcgtcggagggtgagtaagagtagaggataaagactgtgatcgacgacgagagtttaacattgtctgagatctgg
ggttgagtgaaccgagaggtggaagctctggaacaacgtccatcccacccgggcgttcaggggcctcaaaagccgatcccttaaatgctaaggg
gttgttgggattaagtggattattgttgagaccgtacgcctggggtctttgataagcaaaaccactttgcagatttttagcatgaaaattatgctgcg
aatctaccaggccaaagggagcgttcagagaggagtccaaacgttcagagttgtgagcaacatgttttgaactattaccggaatcggatggatc
agtactcgatttgccagaaggaacaaagccagaacggcccgtacgcaaagtgtgcgataagtgggacaaatcggatgtccataatggggaagg
attcaactggtccaaacttccatgatttaagtatggtacggcagatgaattcaagctggaatctgcggaaagttttgtatcagcaggaaccgatgg
aagctgctgattgtatggtatgtaagaaatgtcagattgattcgcattagaggaagtagaagaaagagtattggggtgagcgagcgaagaagac
ataaagccaccagatgaactaaaaactcggggattccaaggtagcgtgtaagagcgtaatctattagaaccctttgaacgtttattgtatgcagc
agcatttgaattgaccgaagaattcatagaaactactgggtccttgttggaaatgatttcggaagcagcagaagtcgggatagacgttgcactgc
tagaagaagattttcgctgaccagactcggacgcaggagatgtgtccgtagatggacgaagagacatagacgagacgggttgcttcaaagagg
aagcagtagaagaagaaggagcagctgaaaacttagaagtccatggaaaagagggatcgtatttagtcgaagaagaaagggaagatgtacg
agccctgtttgtaggaggagagacagccaaagaagcagagggaggaatggacaatttgggaacattagctgtagaagtagtataattcgtagct
gtcattccactcaattttgtagattcctcggacgctgacaaagacgaacttctgtgtcgttcactagcagaaggttcgtatgtagcaccagaaccta
gacttggccaaatttcgtttgataaatctttgaattgggatgttgttcccgatagtaaagtattattttttagtctataaggatccttattgttactacc
tgggtaaactttctcggaattagaagctccgagaaacggagatgtggaggaagaagtagctccaattccactactctctaacgaagagtcaagc
aaattatccaactcgtttttttaactctggcatgtcgagggacgcaggatccatatttgaacggctcatcgatgaaggcttgacgccatcagtagctg
atttatgcatttgcacctttattttcttggaataacaatttacaatgcctgccaaaggccaatgatgaaaaatgaagaaaggacaagcaatctctgt
attatatcaattgtttccctttaaatttcaattcctaccaaaaaattccaagacgttttctttttttttattttctcttttcttctttgagtagtggtgttgtg
gttaggttcagacggcgcggaacgaacgagcggatcatgatgctcccaaatgaacgtcagatcagatcctacggtgcgatgccttccacaacaa
atccgaccgctaagccaaaatcggtaattctttttttcgccctccttcaaatttacttcttttataagtcttgcccttgaggtattgagaatggtagatat
tccaaatataatccttcctttttttctgttggaagaagctgtagtgtacgaacggtaaaattgatgatctttttatttattctttttgtttttgtttttgcttt
atgaaatatgtagtgagttgaatatctggttatgaatagagtagacagtaaaaagaaaaaaagaacaaacgaaggagcacattttcccttctttc
ctaaatcatttttataaccaagaattcatttcctttctaatcctcaactatttgccagatgttgccttgaattcccctacctaggtgtcgtccatatgaa
ccatcctactgcccctattcctttcgcaaaatttgttaaggcattcgtaaatctttttgaattttgttgaataatattaaagtttttttttatttttaattta
tttatgtttggtttaactgtgtgccattgtttgttcaccactactgccacggacgagatttttgagcatacctaagaacaacaacgttaaaggtcgca
atttttacctggaaagcgccgtacgagttcaaacatggtaaattgatggaatccttgtaaacaaaagagaattttcaacgctgctttttgggatttt
cattattctattgtaacttgaagaattcactcaaagatttgccatgtcagaacacgagtccttgattgctggtcgctcgagacgagcaaatgctggc
aataaactacgggaattattagaaaaagaacatataagggcttctacggaagaaggcgaacctgaaaaagaagatgaagagtacagtctcga
aaccgaagtggacgcagaacgcgatattgacatttcgagtgaaagtagtgaatccgaagaaggtgaagaagagcagcaagtggaattggctg
aaaaagaagagagattgcttcgtcaagaggagaaatcggagaaacgtcgtttacagaaacatcgaattaccaatttgcaaaaatcccttggaac

acccaaaacaaagttgccagaaaaaagcgaacctggactcctaaaaaaaacatataagaaacaaaaagccgacccttcttctcggaggtcga
gctccagaatgcatacaattcgcatgacacaatcgacggagcatcgattgcaggaagccaaaccaagaagaaagtatacagttcatgccggta
cggatcgaacgaaatttggtttgacacaacagcagcgattagaagaggctgctaaaacggaggctttcaacattggctctttgcgaagctacgtc
aattatgaagaacaacgaaagcttcgactgcgaagaagtgctgctaaacatcggcgcttgcaaggtccaataattaagtttatcaccaaaacag
agacgttggatgaccaaaaacaattccgaaattattatgtcgctcctcttgaacatcctctcagccaaggtcctatggaagtacccagcttgccag
aaaatgttattgaggaatgtccaattacgggacatagggcagtctatatggatcccttgacaagattgccttttgcaaatgcggctgcctttcaaga
gctgcgagatgtctatcaccaaaagtatgcatggagtgcgttgctgaatatctttcattagattttttttgttaatcttgtttattatttttaaactaca
agatctgtattatatctttactttggtcatcattattaggaacatgtttatagaatagagttatgaatgaagagaatttaattactgatgatacgaaa
aatagagaacaaaaaagcataacttaagagtcttcataaattgattgagtgtaaagtatgttgacgaatgaaaaggacgggttatttcaccaagt
atttcaaagcagcctgcatacctaaggcaaatgtcgaaagacgaacgtccacggcaaatccttggtgctctagagaaaataattcatcttgaaga
gaagacaacttgatgggaaaattggtaatatactctccttggtccaagcgttgttggggattttggttttcaggcttggacatatcgatgtcgacga
gtataatcttcaggttggcatttgttagacccggatcgttatacataaccgtagtggcttcctttacggatcctacgaaaccagtttcctcgcgaagc
tcacgtatagcactatcttcgggcgattcattcgagtctacgagtcctgcaggaatctcgatacagatcttgccaagtggtggacgatattgcttttg
acacaaaacgtgaggctcaccgtcaattggaataatacctaaaatggcgacagcgtcaataccgcttttagatcgagtagtacgttcagccatttc
ccaagcccttgttttaccgctctgatcttgccaggtaatctttttcaaactagtccattttgcgtctttggtttcgagatcactaacttctaagaccttg
ggttcttgttcttgactcatggtggtgtaatgactataggttgaaggaatagaatcacacgtagagagatatgtagggagaaggtacgtttttcgtt
tttcaaaaagtcgattgcagattcttctagtagacgaaaattgagctttttgcttctgaatacaattcacgaaaatacaacctcgcttgaatggaaa
gctggtagtagtagcaatgagaaacgaagagtgacaaacgtttggtaaagagcttgaagcgtgtttgaaatgaaaccacgacattggggggtca
caatttcttttcaatcttcacgtcccatgttcaaatacttattgacgttacatgacatcagggctttaaactatgaatcgacataattcttccttacaa
actaaactgccttttcgagttcattctttcgttcttttcatatccgtttcagttttattttcgtagtaaggtaaagatgacatccgcgcctatcatgaaa
ctaacagggcttttctcggtgctcgcgtgtgccgcatatatagcgacttcaagattaacatcggcaccagaaatttcagctactgcttcaataatgg
ttatggctttcctctctattgttgttttacctcgcaatcaaggagccatagtacctattatggctttcggccttatactaggcatttctctaatagtgatt
accatcttcagtgaccggcttagaattacctattctgaattccgctaactatgattattttagtgattttattttggttagaatatttgaagtacttttta
ttcttttctaagcatctttgatagacaacttttgattacaattacaataagatgatttatatttcctaaagtctttctttctttccttcctgcttaggtctg
caaagtaaaccattccttaatgtagtataactaactgtcttcccaacactttcgaaagtgatacttgtatgtgtcatatgcacatatgcccttattgt
atacagtgctgaataatatgcttacatataactaatattactatacttagcaactaatgtcattatgcttttattctgtgctttgtttgataagcattat
ccataaagatagaaactatgaaaagataacagacacgttcatgatcacagtgaacccgcctatttttatggcttcatttcaccaaacccttttactc
tcttcttgctttgttttgtaacataacaaacatatttcccttctttataagtaagttattcctttagaaaaatactagtagaagaaacgtgaaggattt
atcttataaacttttctttcatttacattcctttttttgtcttttgttttgttttggagcaggaaatacttgtttgtttcaatacgagatccttcataaacag
aaatcatcagttccgtatttaagagttttcaatcctcatagttttttttttttttattttttttcaaaggatagtacatagaaaacaaatcaacattctag
gactgtttgattgtcttctcaattcactccctcgcatttctattcaactctctttcttcaacaactttcttcaatctcaaatcttacgctatttctctttcaa
gtttttagggttcctttaattttatttgttcagaacctgctttagacgtttcttaattattacttttgcaattctaaagtttccatctccttatgtgcaaaa
caacaactcactcgtccctttcaaaggccaaaaaccctgaattgcatcgtcaggttgcttcttctgtcgagcatcttaataacaagcctgtttttacg
gagcaaccatcttccgttgcttgtaactcatgcttaaagctgattcaagaccaagtttattttcactgtaaggattgtctggattttgatatttgtcga
tcttgttacaataaccagactttttctcattcatgcctaaaagcatctttcgagagggttatacaaactccaaaacttcattccttgtctacgtctcctt
ccgttttaaagtcttcggatttcatgtactctatatgcgatgcttgtgaacagcccatccggaatttgcgcttgaaatgcggcacttgcgatgattat
gatctttgcgattcctgtttccgttctaatgatcattcaaaaattcacaattttgtccgaattacgaaagcctatccttacggaatctctcctttccagc
ttcctccttcgttctcttcagacactgatttcgctcgtacatctctcgttcatcgctcctcgcagtgtgattcttgccaagcccatcccatcgtgggcaa
tcgttacaagtgtttggtgtgtaaggatttcgatctttgctccgaatgtatcagtcatacatttcattttcatcatgaaatgctttgtttgtctcgtttttc
tacttcttttccccgtccgattctaaacctaaagagcttcagtacgattttcagctcgtacgagactaccttcttcctgtaaatcctgggccgaatgt
atcctgtatgaaaatttggcagttgaaaaatatttcattgaaaacatggcctgctcctctttatttgagatttaacggtggtgatagactaacacgc
gatgaacccctttccattttgcctattgcgcatcaagtccaacctggagaaagtgccctcattgctctgtctctcagaattccccctgtggaaacaac
gaagaaaacattcttttcctttttccaacttattactgattccggtgatgttttccacaaaaacctttgcttttttttatacgactccaagaactgatgaa
tagaaatctcacattttgttcctgttttatgttctgaattacttatatacgcaaaaaaagtcgttaatgaccccatgaatagcccaaattaagttttttt
gattacagttatcataatgatagttaatgttccatatggctaatacgtactgcaaagctcaagcttgacaaatgaatcttaaggctaataaactatc
ttcagtattaaattttagaatttgttaaacttaatttattcgttaccgctatcatttaccactagtgtaaagcttagctgtttccaaagactacttgtttg
tttcatatagatagttgttcagccttttaagagccagtatccttgttattataggctctcgagagtacgctagcagaaatcttaaaacattgacagct
ctcttgtctttaagtaagggacaagcttccttgttgctaaagtgaggagtgccgcaaaggaagcaaatctctaggggtactctatttaaggacac

cggcctggaacatgttttatcccacagcatttgcaaataccattattatcgtcgagagaaacagctaaatatctgtcaaatacagctttcgggatgc
tattagcaattagcaagcttttattgctggcggaatcgtcagcattttcagcacttcttataggtagaggcttccaagtaccgcctatattatggttat
tagcagccgcaaaactagaagcagtattcagaaatgtgtatgatttagggtcagacttatcgtaaaaaggatatgtggctattaaagctctcatcc
tgttccattcatcaaagaagttttcaggagttaaatctggctgatcaaatttgataatagatgaagatgggttatcatgattgttaatcatctgttctt
cagcttgtactggattagtcaccagtatatcagatgaagtagattcacgtgatgaggcgtaacttgtattatattcttcatccgaactattcataaaa
ccaccttcatcatacatgactgtattacgtcgtctaagtttccgaccgtcggtatcagaaagtgaactttcctgagtgatccgttcccgttgtttgag
aatatcaagccaaatatcatcatcaggtttcttatcatcacctgtttccatagtatccacttccggaaccgtggtgttttccctattgtttacccaaac
acgagcaaaagcgaaagcattcttagtacttcctggttctttattagtagaagaaacgtccttctgttccgcgtcagtgatcaaattttccacagtat
tttcatcgtaacgtatagtatcaatgtcatcattctcttcaaaaagtgctcgcacgccgtgttgtagaatttcgtgaaggtcaatgactggttcattg
gtctctcccattgattgcacgataatttgatccagtagcatctttttctgtgctgtttgtaagatcttttcttcagctgagtttctaataactaaactaaa
gacaaaaactttcttggtttgaccatagcggtgagctcttgctatagcctgcatatcttgatgaggattaaagtcagggtccaagattataactgtg
tccgccgtagcaagattgattccgacaccaccagctctggtggataacagaaaacagcttttccccgaagttggagcattaaatgaatcaatctct
acctgtctttcatgttctgttgtcgtaccatcaaatctagaatacgctatacctttataatccatccaatcttcaagtatatctaactcctgaataaatt
gtgaaaaaataaggattctgcgaccacgcatgataagttttggaagtaataaccgaagcagaagcattttagcactagcttcttcaagagaacgc
atgcttagttcttctggaacatcttttccttcaacgtcaggtgaataaatgtatggatgagctaaggtttttcgtagttgcattaaaatattacttaaa
gaagcacgtgagttgttgctgttcgattttgtgttgctcctagaaacgatgtttcgaattagattaacgtttttcgagacgattaacttataaagtctt
ctctgaagaggagtcattgtaagaggtataatcgcttccaccttcggaggaagtttgtctaaaacttcttctttaacccttcgaagaaagaaaggtt
tcaacatttggtgaagttctttaacctttttcttcgttaatatcttgatattgtttttctaactcagaagcatcaattttcttcggattcaggaattgtaac
agattgaatagctccctgatattattttgcaaaggtgtaccagtaagtaaaactttaagactagcatctattgaaataagttcattaaacaaacaa
gattgatcgctctttaatctttgcccttcatcaataatcatacattgccaatgaaacttcctcaatagaccaatttcgctatcaatagaactggtgctt
attataagaatatgtgttcctatacctttttgttttatcatttctgtaaatcctgttgttacgaacgatttctctatttccttcagatccaaccaatgctgc
aaccaacaaagatgggtcccatttacgacattcacgttcccagttggcaacagttgcatgggggacgacaataaggaagggaaagcacttatgt
ctgtgatacaaaacactgacaaaggatataacctgtatggttttacctaaacccatttcgtcagccaaaacgcagtgctgtcgctgaaaccattga
tagtacaaccaatttacaccttgtaattggtatgacataagctttcccccagtgatataggacggttgctcacttaactcaagctgattaaaattatg
attgccaatcaaaccggattttgtagacttctctatccaacaaattctaacatattcgagatagccatctttccaagccttccattgctcaggagtat
ttatatcagaaacagattcccatgtagacattgagtaaggcaatgcacgccatgagacataagctttttccacttttgcaaatgcctggatttgcttt
tctttggatttaaaagttcgaggacttttgtattgcacatcccaaaccttgtcaatcattgtataagaaggaggtataagatcagtagatccgttata
aggatctgtttcccgagaatcaaagtttcgctttttttgcatttgagacgcctgctaaccaagaggcagagcaccaaaaacaatgaagatatgagt
ggttcttgggtcgaacataatattccctggaccaaattcccgggcgatctagggataaaggatttttttacgggtttattgtttaataaagaaatggg
taataggagacgccatgcaagcacctcatcaacatcttcagggtaagagaggcattcatcacataaccgtcgttctaataaactttgaattgatga
tgcaaatgtattggggttctcatcatagtagaaaaagtataacactcttctgcaacgccaacaacgaaatctaacatttttggtgtaggatattaa
gcgaaaattatgatctgcagaacaaaaagtggatggaatatccaaagtgtcatccaagccatagcccgacaaaatgcaattatttaaaagtaaa
tgagaagagggctttgaacaagaggagcaagaataaccctcgacagcaatttcagcttgatgaattttattcaaggcagacattcctgaacattt
caaatgaaaggtattcgtacatgttttacaataaataaactttcgatcagcgtttttaccctcatgctcatggcatttgacacacattgaagtatgcg
tgaacttagcaagggcagtcaaaatcaaaggttttggctttaccgtggtctgcttcagagctggtacgccttcgtctgaagaattgaacaattgca
aagaaaggtcagctccttcatctgaagaggattcctgaaataaatctaatttcgatctgttagtggagcgaggtgaagattggcgagaactccca
cggctactggagctcttttccgttttgggaggcgaggatgcataaaggtcggtatcctgactactcaatcgttggagggaaaaacctcgtacattg
cttgatgaatttcgaagagacttggtagattgatgtttagcataagaaccacctagctcactcgcatcacgatcagcgtcataatcactatcatcgt
ctatgactcgccgctttctctttaggtatttttggtagcaaatttcaccattcttgtataaataaatatctttttttgcgaagagacgcctctaagcctgc
aagaaacgttacatataaaagattctcatcttctttcaggactttctctacagtggtttcgtctgaatcataaaatggaagtaaaacatctagaggt
ttctttggtattacaaccttcttgacaaaaccaacagctttgggcatgctttgcttaatgttcacatccgccaatgtcaagttaaagcaagcaatcta
taagccgcaatgaagtacgctagtatgcgttaataaacaaggacaagttgtttaaagaactttttttcaagttctcgatgagcgtgcataggattgc
tttttataatgatagggatgttgttttccagtcatttggggattgttcagtgtccgaggacgtggaccgttatgcgtaaagggctgagcgacattgac
gtcgggtgatgcatttgcaacaggcattgtcgctgtagtagtcgaagcattaggatatggtgtgttgaaggtagttgcagcaggaatatacggcat
aggcgcagtagttgggactgaacttcttggtgccatataaacgtttgctggcacaatattaggcggcggcgggtaagtaaactgaaattgataga
gtttagctatatcagtatgttagttttggtacataatcacatggggacaaatttgcagcgcatgaacgtacgaggaaaagcatatgctaagggca
gctgcaaagagcttgactcctttaaaccgccccgtgatgaatcttcttcaagtcttttctgggcctcttcttgagcaactctcgtataataatcctgga
cgttttgtaaatgagcgcggcctgcattatgagcttttctaacagattgactgtcgtgtgtcaaccagacctgacaatagtcgcataaatctatactt

gttagattttattctacccatttggcgatcttacattttggcatagtttctatctagttggtcgttgttgagtagctttcgcgtacttgggtggtatagca
aacagaaacttggcggtactggtacctgttgaaacgaagtgaacagtaaaaaaacagtgtaaaacaatcacgttcggtttatatagcctcacaa
agtttaatagactctgaattgctttatcatgattctcaaatctaaaaatggataactttgaaaatactctgatatttgtatggttcaaaatcatagac
aaccaaaagattgaagtctctaaagatacgttttaatcttatttaattaattatttaaactctgccataatagaatgaattatctaaaacctggatga
aaattttacaagtcgagaggccgatgcatacagtagtgatagctcgaatttatgaatttgttagaagaagaaaaaaatctaaatggtaaagttag
acttacttagacatactgtttcacataactctgaattaaagtcacattctttgacacaccccaaactgcctttttatgaactgaggtttcaccctgca
aaaatccagttttgtcaccaatgacggtcataaacgaaaatccagtaccggatgatatttcagatggagaagaaacatcgtttatgcagaaaatc
tttgcattaccagtagacccaaaaattcaagaggcaattccagcagaaaaaccagaaggcggcgaaaatgaggaagtatacgagaatgattca
tccactgaattatctgagagcaccttggatgaagtagaaggcggtgaatgggctgatgtacgtagtaattatgcttcttcatagcttaacttgacta
acaactgattaggttagcaggggtcgttattttggagcagatccctcagaaactattctctgtcataattgtaaaggttttggacacattgcgaaag
aatgtccccatacattggtgcgtttcatgaaaaagacatcatattgctaactcttgaaagtgtactacatgcggtgcaatcgatgaccatatatctc
cccgttgcccgagaacaaaacgatgtattaattgtggcttattaggccacattgctgtgcgctgtcctgaatcgcgtagaaaaggaccaagaaga
tgtctaacgtgcaatactgagtcccatacgagctggacgtgccctttgatttggagatactatgtcgaaacagataccggtgtccgagtgaatcct
gatgaagtaaaaaagtattgctacaactgtgcgtctggggaacattttggtgatgattgccatcttccgtctcgctctaattatccagaaagtactg
ccttttgcgaggcaaattgtcccacagggaatgatgtttctaattcagtctttaacgagaaccgaaaaacggaatttggatcgagaaatcgtgatt
ctggtaaatccaaaaagaatcagaaatacccaaaacagcgccctacaagtgaccttggctcgaggattgatctaagtgacagaccgaagcgga
aatattttcctgctgatgacaaaaacgaattttcatttcgtaaccatggtgggaagcctccgcaaaagaaacataaaaaatttaagaaaaataaa
tggtaaataaaaaacataaagaaaatttttgggacattcgaaaatggagatggcagacggaaatgggctttaatattagactgaatgaattact
agataattatgtatatacattgcattgatacgcttaaaatataatgcgtttaccattactttttttctgtttgctaaccttttaacaatgtctttattgg
gtaaaaaaagcagactaaagtaaataaccatacgacacaaaaggaaagcccaaaactattatccatgatgatacaaacccaatatcacccgtt
ttaaaataaagcaagaaataataaaaattaataagaaataacaataggttttagatttccacgaatataatgcaactgttttataaaattttggttt
ctttcctcctcgtgcttaagaaaggctcttgtgaacaatgaactggtgaagagccttaccaccaggaacaaaaggatacacatgctccttcttagc
aaccaacacttccataagaacaggtcccttggtttccaaaaattctttcatatttgaggcaaggtcctccttcttttcgacgcgcaaggccttaagtc
ccatggattcagccaatttggtaaaattgggattcttctgatgggtatgagaataacgtttctcatagaaaaggttttgccattgggtgaccatacc
ttgttcttcattattgaggagtaaaaccttaacagggatgtcatgctgacggacagtagcaagttccataccggtcatagagaaagaagcgtcac
catcaatatcgatgacaatttcatcaggtttagcaacacttgctccgatagccgcaggcaaaccgtaacccatagtaccgagaccaccagaagta
actaaagcacgaggcttcgtccaacgataaaaggtagcagcccacatttgatgtgcaccaacaccagtagtaatggtaaccttgtccttaatatta
acggtttgcttatcaagttcttggataacttcttgaggcttagccaactcattaggagcactaggggtataagcaaatgggaaacgtttttttccatg
cttggatctgagacaaccattcaacacgagagtcaagatcgacctttggtacagctgcattaagcaacttaagagattcatagaggtcaccctca
atggcttcagtaggttgaacaacctttccgatgttcttgggagaaatatcaaagtgaataatacctccacgttcttcggcaccagcgcgtttagctt
cgggagcaaagagagcaatattaccagttacacggtcatcaaaacgggcaccgagagcaattatcatgtccgcttcttgcatagccatattagca
tagccacttccgtgcataccgagcatgtgaagactaaggtcgctacgttcgtcgtaagcacccaaaccaagcaaagaagtagtgactgggatttg
aagacgctcactgaattcacgaagtaacttgggggattcggagttgtaaagaacaccttgtccactgaaaatcacgggcttcttagctcgctgca
ataaagtggagacgcgttgaatcttagcttcgacatttttgtttccaaattccaaaacttctctcattctacggttcaaagagggaacggaagatgt
aacggggatgggatccctcaaaacaccagcagtaacatcctttggcaaatcaaccaaaacaggaccaggacgaccagaagtagcaatttcgaa
agcttcatcgatacgacgaggaagatcagcgatatccttcaccattacattccactttgtacaagaacgagaaataccaatcatatcagcttcttg
gaaagcatcgctgccaatagaagaagtagcaacttgaccgctgaaaacgaccaatggagtaccatccatcaaagcatctgccataggtgtaact
acgtttgtggctccaggtcctgaagttaccaaaacgacaccaggcaacctcgttacacgtgaatacgcctgagcagcgtgaccagcaccttgttc
atgtcgtggtagaataaattcgaaatgaggagaacggtagatagcgtcaaacacaggtaagatagcacctcctggatatccaaaaacatgcttt
acattgttctttaacatcatatcatgaaatatttcaccaccagtctttccaacataagaagtatcgtattttattccattggcttgtgaagcagtggtg
gcagtagcatttgccgtggcagtagccgtggagtttgtgcgggcattttcgcgaaagtttgcatgcttggaattagaaggaacagttttgatactcc
tcctcagcaacttagtattatataaccctttacaaaatccattagaccaaagtatttcgcgaccacaggacgaaaatgccgcctttgctctcaaatt
tttcaagggagctaaagcagtcatttcaaacactctacaaccccgtccttgtttttctattgtaaaaccttttttttttatttgcaaaaagaaacaaa
taatgacgctttttttggtgaaagcgagaggatgtgctcaggttaagttggaagaacagcgactttcaccaccaccatcgattataatatcgtagtg
tcaagattatggagttgatatgatgaactaccaattatagcttatattgaagcccttaagcattgtttttttgagtttcagagtattgaagaaattaca
ctcctaaactagctacaaataattgtgtacatgaaaagaattcaaaataacccttataggaaattggccggagcaaaaaaaccaatattgcaatt
gagcgtatatcctttattctttattccttctgttatgccaaattataatacacgatatttttataaattactttttgaaaaggattgtactggtttgatac
atatcaaataatgtttagcattggcaataatgcaagcttcaatggcagaaagcctcttttttttgcatcaacacgataggcaattgaagaatggcgt

gaaaaacattacttaccctctctttttaacttcaactgacctataccgtgtatttcaactgtgtccaccaaaagagcacgacgaaaaagtcaaagt
agattattgtggaagctgtatcgacccatattttcagcgatttattgtgattgatataaagaactggactctatgcatttcgaatgatgactactgta
caaaaaatatgatttgttatgatatcaaaagacatttgagtaaattcatcaattttgaaagtggatttgcacctttaagtgtgtttgtcagattcgtg
aaaacgagcttcacgtgtgtgaatcctggatgtatcctcctttttcctacaacgggtgatacattctactgggaggatataaattgtcttcgtttattt
catgaaatgaataattgtaaaggctttttacacgttaagcttcccctttcttctgtggaaaaatgtgtcaatctgaccatgatagagcctgctggatt
tatggtcattaccacatttggtcgactttttcatttgtatccaagagatgacatgcgagagctaaagttagtttatcaaacgcttccattgggaggat
ctatccttcattttcaatcaattatttcgaaaatgatttgttatccatatcacatcgtcgccgtacgggctggttctattaaaggcgcctacgaaaggc
ttgtctacggtttattctcaaatgccgatatatacatttgggaaatattccgaaagggaaaataccaattgctccagaaaaaaaatattcgagatc
tcatacttaaaagctcccgtcttgatggttccactgtaaatcgttgtgagttccttgacttttctatctgtaattgggatccttattcgttggtctgtctt
attacatggcatgacaatgcttccagatggaattatgcagtcgcgtctgtttctttcagtaatgagatgataccagagatttcacattttcataagat
ccgttcttatccttccttagctgcctccgtctccgcccgtattttacatccttaccctggtacagtcatttactgtgtttttgatttatcattagttattctt
cacaaagttagggaaaaaaatacctcttattttgtagaagaaacattatttaccaacagttcgtgtaagaaatttcaaatcataaatgttgcctcc
gtggatgctgttttcgagcatggaaacaatcgggtcttgaaattcccggcccttcatctaatgactaagggtaacggtattatatcaatcgaatcta
ccaattgtttaagcgatgagtttaatggtcttaattttctacgaagccgtctttcacaatatgcaatgcagaaccctttatttaaggtaaaacactttc
aatttatgttattaacagatttctagaaccaattttctctatggcccgatttttcatctgctttaccggaaagcaagttgttcccaacactacttagctt
atgtgatgaagtttcaatctgtaaggctttttattctacccgttatccttattaacttttataagcatatcctgattctcatacttcagtgagtgaagttt
tgaattaccagtttcaaaggcttatggaaatgattttagtttctatgaaatatctaaaactgacagccactgaacaactcaaactctggcttaaact
tgcttatgttaactcgctcgtcgacatttatggtcagttgacatgtgaagatccaaagaacaacattcttcactccatccttcaagctatgacagcct
ctgggaatatagaggaccttttttcttaaaaaatccttgaatttaaatcttttgctgcatttattgcatacgttttgcaaacgctatgctaatacgaaag
aaaccttcaatctgaagctagtgcaaataatgataacggtgaataacgtttttggtgtaagtacatacactttatattgatgtctacatatacatat
atatatatatatatatactaacttggaatagatggtatttggaaatgaattggcttaccgccaagaaaaagttataaaatatgtggatcctacaac
cctttttctatcagagctatgcaaccttgacattgaaaatcttacactgctttcaactcaaattatggagtcaattgctttggatcagaaatatattct
acttgaatgtaaggatcctcgtgataacacactgagattaaaactgcaaaagcaaatccttcacttaatagagcatatttgtttgcttgtacatgtc
ttggcgaatgcaattcagaattcgtcgacttcagaatcagctgcagaagtgcaacactttaacatattcgaagtgcaatttcgaaacacaaggaa
aacatggttaacttacctaggtaaggttttatatttttcaaacgaattcacttacaactaaaatttagctcacattggatacttggaacgtgcaatca
gtatatctgagcatgtgcaagattttcattcattggttgctttactgaactttgaaagcatagagaagcagcaagacaaaattaaaaaacaaaaat
tgtacattgaaaagtacaaggataaatttgctactgttcttttcactcatatgattgaaacgggtcaaatacacagtctgctgcatgacttcaggtgt
tatcaaacttatttacgaaactttttgaaactaataaattacataatttcttatgggtatatgaagctgaattggggaatttcgcacaggcagctga
catcctattacgtgatgattcgataaaaaagtcaaaagtaagccaggaaactacacgcttggctttgtcaaaattgttcacattaacgaacaaaa
gcaactgtgatgataattatatgataaacaagaatgaagaaggctaactcggatcacaagattgcttccattcaaaatgagtatgcaaagttact
tggatctacgacagatatggcagaaaatgaagaggaagctgtaatattactgcttgaggtgaactgctctgaattaaacgaaacatcgatacggt
atagggtaacctccaacgttctcactcgtttgctactggaagaaccctcttcgtttgtggagatgattgattttttacttcattctctatttatggaaa
caaatatctggaatttgagttggcatgtgagatattagagaactctgctttttctactgatcaattcgtcttcttgtacctcttgataacgcaaagatt
tttattaaaaatgaattggaatgaaacgctgagtgctttagcagagaaaaatttggattatttactccccctcaaaaaagtctttcaaaagttaga
atcaaaagaatttattcccgacttttatcacccaactcctctcttcccaaagatcctatctttagacttacttaagtccttgtaccctacgttgaacga
atcccagctaaaaggctacaactatgagcttctggcagaacatcgcagatttcaaaggctttatgaagaccggaacctatctctagttcttcctca
attgtcgaaattttataatgaacaacagaacacttaatcaaggaaatgaattgaatgcttgtaaccccgtatatatatttttttattcaattgacgac
tgttttagcgatgatttgagttttaattaacaatgagaactttcaacgacttaatctttctatatgcattattgatatatggataattatgaatgcaaa
tatatgtttaagcaaatatggaaaatctgtaacttttcatatgaatggttataaaatattcagtcactgaatgaacttagaaactctagtaataaag
agaacaattttcaaaaaataagaatagtaaaaaagcaaaattatacaaataaaacaggaattcacgtttgagagccccacccttacgttggtag
tgacccgaaactcgatgagcctctgcaatttcaataaccaccaaacacatcaaagaagccaaaaaaaaaagcgaaaactagaagtctttttgttt
aaaaagacaaattttgaatttcgaatagacaggtacaaaccataaaggcttaatccaagtatttcttttgttagtagcatttgcctacgattggggt
agtctgcgcggtgaatgccgtagacaagattaaaaattctgcattgaagaggtgagcctctggcttaatctaggattaagtaacagacagagtgg
acctcgaagcgttaattatttgaaatataagtcttccggtatcaagacaagtggacatcaaaatgaatacgcaaacaagtgtggttggcgttcatt
accgagtgggacgtaaaattggtgaaggatcttttggggtgatttttgacgggatgaatttgcttaacaatcagcttgttgctattaaattcgagcc
aaaaaagtcggaagctcctcaacttcgagatgaatataggacctataaattgctagttggaagtggtaagtatattcgctaaaagtatcaaaaag
atatttttttcaatttcctattctacagagggactaactgttaaaaccgcagttgggattccaagcgtctattactttggccaagaaggattgcataa
tattttagtaattgatcttttggggcctagtttagaggatttattcgaatggtgtggttgtaaatttagctttaagactgtcgcgatggttgctaaaca

aatgttggctcgcgtgcaaacaattcacaaaaagaacctggtatatagagatattaagccagataactttctcattggtcgtccttgcactcgaaa
tacaaacctggtgtatgttgtcgatttcggaatggcaaagtactatcgtgatcccaaaacgaaacaacacatcccttacagtgaaagaaaaagtc
tttctggcacagctcgttacatgagcattaacacacatcttggtcgtgagcaatcacggagagatgatttggaatctctaggacacgtatttatgta
tttccttcgtggaagccttccatggcaaggactaaaggctgctaacaacaagcataaatacgaaaaaatcagtgaaaagaagcaagccacttca
ataaatgagctgtgcaccggctttcccaatgagtttaataaatatatgacttatgttcgctctttgcaatttgatgaagacccggattatgaatactt
acaggaattatttgacgatgctattcgcaataatggagaaacgaatgatggagtttatgattggatgttactaaatgatggcaaaggttgggagtc
tgcttcatctcatttttccgtcttagcaatgaaacgaagaaaaaattatttaggattaaatgttgtacaaaatgatgacgccaaagctaagcaagg
taatattcagtcccaaaacacgcgactgaattccgcgtataagactggaaccccgaaaaattataactcatttgaatcgtattcaaggaatcaac
aaataacgtgtaccagacgggagcaatctgcctctaaaaataccatttatccgcaatcgtccgcccgagggtacggtcgggtccaaccaatgtgt
gcctctcgtttggggggtcgtcaagaagatggcaattacgctggtatcaatggttttcaggaacaaagacaaacgagtttttgggatatttttgtt
gtcattgcttttgacaatcacaaccacatcattcgtcattaaaacttttcgattttctacatttcctgcttgtatcatcccggtacaaatactcttactct
tacttttgtcatatgcattcgagtaacattaggattgcagggttctgaacaagggtttataatttatcaagaacgcaaatgttagactggtcttttat
aaggaattagatgaaattccattactcacaaacgtgaagctaagctaggtgtacttcatatttcgatggattggcttatgataaggacaaacattc
attaatgggatgaaacttatgtgttgactaagaataaattctattgtatatatataacatgcttcataagggtatttcttttattgtagacattacgat
ggtaaaagaaaagatttcatttgaactacataggaaaaacattcactgtagaatcgtatttccctgctggggtgaatgcataaaatcactaatac
gaccacaatacattcgttttctacaaaatatatatatagttgataactgcaacaaataaagtgaattgtctgctttcaagttaggaaataggttagc
gtagtatataggcaacaccatcaatttaaggaatttgcgtttaacaaccaaacaggtttggaaaaactcactaatagaatgaagaaatgttatcg
atgatagcacttttgcagcaaataaaagaagatgaagcacaattattagacctttatcgagtgtatgttatttcgcctagaagttgacgcttctgta
aacagaatgtctattagtttttcgctgtgttgaactaacgtcgcatgccagatttttcaatggaagggaaataaaatggcaagagttaaaaattga
taaaaatgatcttgattcttacatgatgaatcttgggtataagaagaggtacgtctacacagtctattttgtatgaagtaaaactctttgaacaact
gaattgcaataataattggctaactgcgtgtctagaaatttgatcttaatttcttatgcgcaccatcttctagaactgcttcgtatgccaaagttagat
gcccatcaacttgttaagtgtattcgcatagtgacagcagattgcaaaaagtcttatattattgaccgacaagaagtatctcttcgttataagaatg
tatcttccttgaaacttatatttagcaacatgcgaaaaccattccttaatgaatcaacaaacaaagatttatctttgctccctattgtaagtatacaa
acgacagtgtcaggataatgaaaaatgacacacaggtttgatatacctacgatattcttcttgtgctgaatgatgaactctttgctgtctgttcttct
gttttgtttagttttgttgatatctgatttctaacctacggtcttagtctttcgagcttagtttttccttggtatttggagcatttcttttcagcctgaaaga
agtatattcgttgttctatcggatgtttgaaaaaatgaaggagacgaaaaagtctccttctgccatgtccttgtttggtttcttttcaaaagatcctga
attcagcactcttcgaaacgaatacgaaagatttaattcaaaaatatgtaaaatacttcatatgatactcgaagaagtttcaatttgtctgcaatta
ggtagataatagaggaagctatggaatcaatagaaaggttatgaatttttttctataaagaatttttttcgattagttagtaaactaattagcttttc
tgtttctataaattatgtcttcatagaggaaggaatcttcttgcgtaattgtatgtttgtttacatatcaaagacttcaaatttctcctttccataccac
aacaaacaaacatgaataaatctactcatcctcaatgcacaaaaaatgaggatattttattcatagattcattaaaaaaacggtttctcaagcaa
aatcgtgaaattattaggtaagggtgtaaaaatagaaaacagcacttcgcaccaggttcttgagtctaatgatcagagtaaatacacagttgtcg
gctaaacttagtcaatctgagatagaaatggaaaaacttttacaagacaactgccaattgaaaaacaatctcatcaaactggaatcgagggttc
aaacgtattctcagtcagaaagcttttatcgtcaatattttgaagattttcaaataattcgtaataagctttcagaaattcatcaccttatgattgaa
atggatgaaaaattacttccaacaaacactgctttaaagtcgtccacgagttcctcatccaccctaaacgactctgttgacgaaaaaagagatatt
acctttcgaaagactcgatatcctttattaaaagacttttctgtccctgatctagagaatcctcctggaatgcacttgccaggacgcaaaagcaaat
ttccacccttacctaaattaccaagtaaatgcatccttgctgataaaaccaacaattgcgtatcaaactcaagaactatgctaaagaaaatgaaa
ccaccgactcacaatatcgaaaacattgaaaccactatagagagtgagaaggacttctcagaaaatgaaaataatgttccttcgggaaaagag
gtaatgaaagaacaggttaattcaaaaccttcaaatgttcgtgtttgtattccttttaaagcttttgatagtgtcgctgactctgaaggtaaaaatca
aaacgaagcagacaaccgttacaggggggaaagagtctactcattcagtgacgactgcaagagctggacgagaaaaacgtgcagtaaagtctgt
taattatgctataccggatttacgaatgaagttgagaagaagctttgaattaccaaaggataaaaaacgaaggcggcattgtcgattgcatacaa
acaaacaaaataaaaatcaaatgaagaatccagaaaacgatgatacagattgaggagcttagaacatgatgtatccaggagagagctatttaa
gaatagaaaggaaaccatatctaaattttagctatttaaaggattatgcaatttatcaactcatatgtaaataaataattgttttattttcttcttttg
aacgatctgaaaagccaaatatccagtgatgaaaagacaagctaagaataaaaggtacgaaacacacttttgcaatcgtagaggattctgaga
atttaagccagttacaaaatacaactaagaaatagagcggtatacttcttcttgattaatttgaggatgatctgcagggttaggagaagcctatag
aaaagttagtaaatagttgatagtaaaaaagagaaaaaaccatacttcatagatagtgattacaggatttcctttgaaagggacaaacgatcca
cgaagacctatgtaggctatctgaatgggatcatcggtaccatcgtttcttgagaaaaataaattcaaattccgaagattttggtagaggcgtgttt
tgacggggaattcaaaaatgtggttaccagaaaaagatagaggaaactcaaaagactctgttggttgagcgtcttggatagcaccgaaatctag
gtcagatcgatttgggtacaaggagacagaatgtggagccgaggtgtcagggaatatacggacaagcaaagatttgagggttgcagaaccagt

aaaaggaacgtgaaacaagagctgatcgtcgacgtcgctttcaactacgacggagtcatcgaaccgctggtcccagggtttgaagacttgcttac
ccgtatcagattcagcctcgttcaaagtgacgatggcatctttatgtatgcaggcataaagagtatcgttagggccagattcaaaaggatgatcat
cacattctgcagagcagtgatggcggccagacatagcgatcgcaggaagaaggatatactaataaataaataaataaaataaaaataaaaaa
gatagataagtcaagagcaagaaaacaaaacaataaaaaacaagagcagaatttacagagggtaaacgatgaagaaagtgagaaatttaaa
aagtaaccttgaaacaggttgttacgtgtggtggaaaagcattggatatgcttatatcagaagcgaaatatgcttgtacaaaaaatgatatgctta
taataaggaaaggaaggtgtaagggttattccagagaaaacatgaatgaatccatgaatgatatataaaaaaaaaggttaaaagaataaaatc
ataattgaatgaatagactcaaaagcgacattagatagatgtggatgaattcatggaaagctgggctttcgttctatgaatactcaacaacgaaa
agagggattttctgaaaaaagatctgcaattgtccagtcgatcttaagatactgaagttttattctatgagtaattgatgatgtgctacaggaatca
ttcgtaatgcacaaaaaaaaaaaaaaaaaaaaaaaaaaaaaaaaaaggaaattcaagaggaggataaaaatacgccttaatagttctttgttgct
ttatacaaagtacctgctttcatgtcaccatgtttgggttaataatgaagacaaaacggtcgggtcgatgaaagcataaacaatggagaagagga
gaaaggaggagtagtaggagaggggaagaagaaaaaccggacatggaaacgaagttgtgtggagaaaggatgcaaaagcggaacggtttta
acgaacaaaaacagtagtcataggagaggtaaaaaatgaaaaggatgaaagtaatgaaagaaacggtacgtaaagtcgtcataaacaaaaa
aaacaatctataatggcatacacatcatgaagggctcataaaggcttctattctcttttgctcagaatggaaatgaaacaatggttcccatgttgtt
taacaaaataaacccccccccccccatcgtttgcatcttccagagtccgaaagaacaatcgcattgtttactttagcaataaggtttagtcacgtac
acaagaaggtgattggtaatgcacaaaacaagcatgtggagaaacgccatcttgcatccaatctttgaaagattggggctatcttgtatcatcga
tgagcaattttgatggccaatgatctgatcgaaggaagtaacgaaagggcgttatcgatatctcgtttgttgcaaaaagttacttatttgttcttttt
gagccctcatttcaagtaggaatcactctctaatggctagagaagtttatccaagctataagctgacgtcatacataagtatgttactttggctgcg
agattaatgtccgctttagcattgactgaatgcgcaatttaggaagggtgaggcgtgctgatgattcgagaatgtcagatcattagagatcatcgg
ggttcacgcagtttataatgaccccacttctccaatatgctgatttcttctatgtaccatttttatttaaacaaaggaatcgtcgctttttaggattgga
attcctcaaaaacgacttcgccctttattgtttgtttggttacgcttttctgtaacggtttcctttgttgtgccttttgttttcttggattgcttgtttttgag
ctcctttttattattccctttttgaaataatcctttttgaggtttcttctcgttccagtctcacttttgatttcttcacattctgtttacatttacttactatgg
atttggattactgcctaatttgcgggaaacctaccactggcaacctttactgctctagagaatgccatttacaggactgcccaggttgcgactcgga
atctgaacattgcgcttattctcatgcagctagccttcacatgctctcatcacaataccttgatcacttccgacgacgctcttctatgccatcaccatc
tacctcgtcactgcttttgaatggttacgcttcctctcggctagctgttttataaaatcatttcgccaacaggcaaagctgcaaagcaaaatctttag
attctctttagcgtttacatgaattgagaagactaaagcatttacatttgatttcaacgatgatatacggttatgcaagttcatgatataaaaaaaa
attgatgatttgcaatgaataattatgtaatgaacaaaaagcacttattggttttatttcttttcttacgggttttatgtctttttctctttggtattcctt
ttttcttgaatattttcgtacagaacatccattcttgttttgttatttttttttatttttttatttcctttagttctttgatgtggttttcttcgcttatattactt
cctgggcgtatgtcactgtttacgtttgcattttgttaattctttaattgaaagcttagcttgcttgcattttggctacaatcggttgtcgctctttcttgt
taagagcattcataatcatgtatttttccagcatattgaatttcttctctctttcttttctgttctttctggttctttggatactcataggatcattaaatc
aataaagcaacacgggtttccctgtttctttcgttgcattatttctgtgggaaactatcctctacattttaacaatgttaaaaacaaaaaaaaattct
caaatttgctgtatcctgttgattttcatcatggtaaaagcaaaagtaaattagcggtctacaagatcggtttatagtgtatttttttgtttatttattta
taaattcaaaaaataaatgaatttctattattgtatttttataatacaggtgatgaaccagagtagtgtgtatttttcatgtattccatgaataatga
atcattgatttttttatgtcaagtgtctaaaaagataaaaaggaacctacttatagctaactggattccaattcaatagcagaaagtaaagaaaaa
aaagaaattttcatgcccacaccaggaatcgaacctgggtcgcatcggccacaacgatgagtcttaaccactggaccatgtgagcttgtatgtac
atgataaagaaaattaaaaactaaattaattcactaacacaccttttatttcatgtctgacttagtctataagcaaaaagaaaaatagataaacta
ataaaaagcgatcgatctcaaaagttcaaaaagtgatgatacatttttgagttttaataacccttgagatccatccttatgcccattgtgaggaacg
gaaacatagtctcactgaagagcaagtgaaaaaggattttattctttctttttctttagtatgttagtgtcataaacaccttacgcttaaataggta
atttcctttgcggagccattggatacatacatatatatatatatgtatatggtcttgacttccttccccttcacgcgttcatttggcctacacattatttt
acgtgagtatttttctttaaagactaaaattttgaaagcctataatttaagtatatattataagttattgtatctttaaagtgacatagaattgaatag
ctcgcgcgaatgaattgatattgaattgctttttacgttcaaatcatggatgtccataaaaaagaaatagaagagaaacgagaaaaactactgaa
gcttcgtcaagaaaagcaaagcctgaagctaaaccagccatccagttcgatcaacatcaaggaagaactttcgcattttctgacttccataataa
aacacaaagaacttcatcataatccggttttgaccgattcttcaaggagctcctcaaaaccgatactgaagtttactccaatttgtgaaatcttaaa
tgaacaagcaagcaaccagaatttaccttccaaagaacatcctcagctgactttgtcatccctctgtacgggacttgaggtatcaaacaaatatcc
tagcgttcataaaaatggggatttcccagatgcaacagtgcagtttacaaatgttgaagggagttctaaatgcgacgcgaaagtgagcgatatga
ctacttcggatgagtctgagttaggaagaactggaagatttttgcagagctcttcgaacgtactagaaagacctttatgtggtgatactaaaaaat
taattcgagattacaaagaaaatcgttttcagtctgtagaaagcttgccatcaacagataaaaataagctaattttgcgctcgacattacatgata
aaacaataacatccgataaatctattaatagtctcgcctattctactttctatgatgagctccttttagcttcatactccaacctggttggcggccaa
cgttcagatggtctattgcttgtttggaatcaacgctggaaaaactatccagagttaatattaagagcacatgtacgttgtttatttacaatcatcta

ttaacctgcttagagtgaagttaccgtctgtaaattctccccttctaatcctaatatcgttgccggtggtggttataatggccagctgttcttatggga
tatgagacaaggtacataccctgcgctctcaacaaacataataacaggaggccatatcgaacccatcagtgacctaaattttctagacagtttttc
taccgataatatagtaacttgtagtaccgatggctacatgcatttatgggacagtcacacgcttgctaagcctacagtgacagttccgttgatggcg
gatgcttgcaagcctatgcaagcgttcgctcctacttgcttaagttatacttcttcagatttaaacgcttttatgattggagcggaagatggacgcct
ccggattggtaaacaacaagacctatttgagcctcaaacgaccctgaactcttttaaaagcttagaaggacactcagcttatgttacaggattaga
cgtgatgcgttcgaatattggtgttgaaccactaaaaaaatcggaaaactaccttcttagtagttccttagattggacagtgaaattgtggtcatct
cagaaatacaataatgtgaaacagttagcacttgatcacgaggactaccagaaccccatcacttgtgtacgtgaatttgaccaccaagacatggt
gtttgatgtgaaatggaatcctgttaggtcaggatgctttgctagtgttgatgctgtaggttctctttcaatttgggatttaaaacaaaacctgcaaa
ccccgatagtcagtgacacaccttcaaaacgtaaaacattaaaccgagtagtttggcaacctaaggatgatcggcatctagcgtgtggtggactt
ggtggcagtgtgtatatttatgaatcaaacctttaaacgatgactttagattgaaaagctaacatttgctttgcttgatcacttgcaagaaaattgc
atgaatactatacataactgtagttttttaaaatataaaaaaaggtttattggtctaaaatgattctttgttgcacgacaactttcatcacagtaat
atcatgaaggcatggaaataaacacgtacgtagcagagtagcagcgtattggtataaaaaaaaacacaaggcaaaatcgcttaagaaaagaa
tgtaaatattatctaagattttccatacgaagctcatattaatccttttcagcaatactttgcaaatgagcatcgatttcatcaacagataagagcct
aaatggcttgttctcctctactacaccgatttgcaattcagttgccttgaaatctgtacttaagctgctcatcaaagattgaataccagtttcgacag
tttcatccaaatttggtggaacaccattcttcttgtatcgtttttctaaccaattcatggtttcaatttgcttgggaccagcgcttgtagctttataacc
aatataaaagccggcaggatctagcttgtaaagggaaggtccaaactcatcgtcaacagaaacaagggtcatagcaacaccaaggggtctcat
ggatgcacgttgagtagatacttgagcaatattggccatacgtttagctaaaacgtcacaaggcataggatagccgtttttgtactcaaactcagc
agcttcgctacgagctctagaaacttgagctcgggcatcagcaatagaaccagtcatcacacagccaattccttttgcaatagggaagatataatt
aatggttgaaggatcaattagcttgtctggaattttttttgagaaattacacaaccacaattttgtcctgttactccaacggaagtgattcctgcga
gattgaaagctttaaaagcatattcaacttgaaatagtcgtccttcaggagaaaaaaccgtaatcgtcctgtcgaatccacgagcttgagacattt
tgtagttggttgatggtacctaagagcactcactgacaagaacacagtattctttcaaagtaataccgaagtagaaggagatgtaaatcaaatta
[truncated: 728,069 more chars]
