# Supplementary material for: Assembly of Schizosaccharomyces cryophilus chromosomes and their comparative genomic analyses revealed principles of genome evolution of the haploid fission yeasts
Source: Sci Rep. 2018 Oct 2;8:14629. doi: 10.1038/s41598-018-32525-9 (PMC6168568; doi:10.1038/s41598-018-32525-9)
Supplement: Supplementary file 2 — Supplementary file 3 [file 41598_2018_32525_MOESM2_ESM.pdf]

Supplementary information

Assembly of *Schizosaccharomyces cryophilus* chromosomes and their comparative genomic analyses revealed principles of genome evolution of the haploid fission yeasts

Lajos Ács-Szabó, László Attila Papp, Zsuzsa Antunovics, Matthias Sipiczki and Ida Miklós
Department of Genetics and Applied Microbiology, Faculty of Science and Technology, University of Debrecen, Hungary

Correspondence: Ida Miklós, Department of Genetics and Applied Microbiology, Faculty of Science and Technology, University of Debrecen, 4032 Debrecen, Egyetem tér 1., Hungary
E-mail: miklos.ida@science.unideb.hu
Tel: +36-52-518-600/62056

>Chromosome II of *Schizosaccharomyces cryophilus*

cttcctccatcaccaaccaaccatatcattatcatccttcctctatctctctacctccctcttccaaagtagagtcttaaaggaaaaacgatgatgat
cttgatatgatgatagtgaaaaaaggaagcccaactgtttgagattcaattcttattttaaatcgaaatctccaacaccactttgaattagcaagtc
taatgcctaccaataaaaagtggtataagagattgataataataatgattataataaataattaagagatgatgtgttcctttttttttaaattggaa
caaatcttgggaacaaaggcttaatctcagcagatcgtaacaacaaggctactctactgcttacaataccccgttccacattaaagtcgtatgcaa
tggattcgactccgcacaacttttcaatggaatttcatgggtaagtcaacaagacaacttccgtctcatcaaccgtcacccatcttctacgatgcag
gagtataggccgaaacctatctctatttgtatacacatcaattatgcggaccaacaaatcgtcgctttctggcatggattcgggcttagaggcgttc
agcctttatccagcagatgatagctccgcggcattgccttgtcaggcagccgcaaataccaatgatctgaatcaacggttcctctcgtactaagttg
aattaccattgcgacgacacttcatcagtagggtaaaactaacctgtctcacgacggtctaaacccagctcacgttccctattagtgggtgaacaa
tccaacgcttaccgaattctgcttcggtatgataggaagagccgacatcgaagaatcaaaaagcaacgtcgctatgaacgcttggctgccacaa
gccagttatccctgtggtaacttttctggcacctctgtcctcaaatttcgaggcaacaaaggatcgataggccacactttcatggtttgtattcacac
tgaaaatcaaaatcaagggggcttttacccttttattctactcgagatttctgttctcgatgagcccccttaggacacctgcgttatcttttaacaga
tgtgccgccccagccaaactccccacctgacaatgtcatcaacgcggatcagttcgcaatggaaccttaaggctagaatgtgggaactaaatccc
aattccgcttcattgattaagtaaagaaacgataaaggtagtggtatttcaccggcgtgcagagaggtccctacactcccacttatcctacaccct
ctatgtctcttcacaatgtcaaactagagtcaagctcaacagggtcttctttccccgctgatttttccaggcccgttcccctggctgtggtttcgctag
atagtagatagggacagtgggaatctcgttaatccattcatgcgcgtcactagttagatgacnnnnnnnnnnnnnnnnnnnnnnnnnnnn
nnnnnnnnnnnnnnnnnnnnnnnnnnnnnnnnnnnnnnnnnnnnnnnnnnnnnnnnnnnnnnnnnnnnnnnnnnnnn
nnnnnnnnnnnnnnnnnnnnnnnnnnnnnnnnnnnnnnnnnnnnnnnnnnnnnnnnnnnnnnnnnnnnnnnnnnnn
nnnnnnnnnnnnnnnnnnnnnnnnnnnnnnnnnnnnnnnnnnnnnnnnnnnnnnnnnnnnnnnnnnnnnnnnnnnnn
nnnnnnnnnnnnnnnnnnnnnnnnnnnnnnnnnnnnnnnnnnnnnnnnnnnnnnnnnnnnnnnnnnnnnnnnnnnn
nnnnnnnnnnnnnnnnnnnnnnnnnnnnnnnnnnnnnnnnnnnnnnnnnnnnnnnnnnnnnnnnnnnnnnnnnnnnn
nnnnnnnnnnnnnnnnnnnnnnnnnnnnnnnnnnnnnnnnnnnnnnnnnnnnnnnnnnnnnnnnnnnnnnnnnnnnn
nnnnnnnnnnnnnnnnnnnnnnnnnnnnnnnnnnnnnnnnnnnnnnnnnnnnnnnnnnnnnnnnnnnnnnnnnnnnn
nnnnnnnnnnnnnnnnnnnnnnnnnnnnnnnnnnnnnnnnnnnnnnnnnnnnnnnnnnnnnnnnnnnnnnnnnnnnn
nnnnnnnnnnnnnnnnnnnnnnnnnnnnnnnnnnnnnnnnnnnnnnnnnnnnnnnnnnnnnnnnnnnnnnnnnnnnn
nnnnnnnnnnnnnnnnnnnnnnnnnnnnnnnnnnnnnnnnnnnnnnnnnnnnnnnnnnnnnnnnnnnnnnnnnnnnn
nnnnnnnnnnnnnnnnnnnnnnnnnnnnnnnnnnnnnnnnnnnnnnnnnnnnnnnnnnnnnnnnnnnnnnnnnnnnn
nnnnnnnnnnnnnnnnnnnnnnnnnnnnnnnnnnnnnnnnnnnnnnnnnnnnnnnnnnnnnnnnnnnnnnnnnnnnn
nnnnnnnnnnnnnnnnnnnnnnnnnnnnnnnnnnnnnnnnnnnnnnnnnnnnnnnnnnnnnnnnnnnnnnnnnnnnn
nnnnnnnnnnnnnnnnnnnnnnnnnnnnnnnnnnnnnnnnnnnnnnnnnnnnnnnnnnnnnnnnnnnnnnnnnnnnn
nnnnnnnnnnnnnnnnnnnnnnnnnnnnnnnnnnnnnnnnnnnnnnnnnnnnnnnnnnnnnnnnnnnnnnnnnnnnn
nnnnnnnnnnnnnnnnnnnnnnnnnnnnnnnnnnnnnnnnnnnnnnnnnnnnnnnnnnnnnnnnnnnnnnnnnnnnn
nnnnnnnnnnnnnnnnnngaggtccctacactcccacttatcctacaccctctatgtctcttcacaatgtcaaactagagtcaagctca
acagggtcttctttccccgctgatttttccaggcccgttcccctggctgtggtttcgctagatagtagatagggacagtgggaatctcgttaatcca
ttcatgcgcgtcactagttagatgacgaggcatttggctaccttaagagagtcatagttactcccgccgtttacccgcgcttggttgaatttcttcact
ttgacattcagagcactgggcagaaatcacattgcgtcaacaccactttctggccatcgcaatgctatgttttaattagacagtcagattccccttgt
ccgtaccagttctaagttggttgttaattgtacgccagtaagggcataccccgtggaagagagcagccttaccaaggaaaatcccagccgccaa
ggtccagccagcagtccagacgtctctaccccgaaaggcaaaaacatccagtccgccgaatccggaccagcaactgttcagtttccaaggcccg
acgtacccaacccttagagccaatccttatcccgaagttacggatccattttgccgacttcccttatctacattgttctatcaactagaggctgttca
ccnnnnnnnnnnnnnnnnnnnnnnnnnnnnnnnnnnnnnnnnnnnnnnnnnnnnnnnnnnnnnnnnnnnnnnnnnnnnn
nnnnnnnnnnnnnnnnnnnnnnnnnnnnnnnnnnnnnnnnnnnnnnnnnnnnnnnnnnnnnnnnnnnnnnnnnnnnn
nnnnnnnnnnnnnnnnnnnnnnnnnnnnnnnnnnnnnnnnnnnnnnnnnnnnnnnnnnnnnntccgccgaatccggac
cagcaactgttcagtttccaaggcccgacgtacccaacccttagagccaatccttatcccgaagttacggatccattttgccgacttcccttatctac
attgttctatcaactagaggctgttcaccttggagacctgctgcggttatgagtacgaccagatgtgaaaacaaggaaggcacaagtaccttccat
tcttccgttggattttcaagggtcgtcaagagcgcaccggatccagcatgaggcgctgaactcttccaaacaccataccctagctccggataaacc
gatttcagggtaagttcgtggtttgttaaaaagaaaagagaactcttcccagggctcctgccgacgtctccaacttcacttacgttgccgtgttgatt
ccaccttctggttccggaatattaaccggattccctttcgataggaggcacgaaaaatcgtgcaactttcatacggagcttccctatctcttaggatc
gactaacccatggccaactgctgttcccatggaacctttccccacttcagtcttcaaagttctcatttgaatatttgctacttccaccaagatctgcac

tagaggctgttcgacccaggctcacgcccaaggcttcgtcaccaacctccacgcctgcctactcgtctgggcttctcaattattacataacccagac
ggtgaggtatgggtagtacgcttaagcgccatccattttcagggctagttcattcggcaggtgagttgttacacactccttagcggattccgacttc
catggccaccgtcctgctgtctagatgaactaacaccttttgctggtgtctgatgagcgtacattccggcaccttaacctcacgttcggttcatcccg
catcgccagttctgcttaccaaaaatggcccactagaaactctcattcgatggcccacgtccaattaagcgacaagggcgtcttacatatttaaag
tttgagaataggttgaggaaattccttccccaagacctctaatcattcgctttacctcataaaactgatctgagtttctgctatcctgagggaaactt
cggcaggaaccagctactagatggttcgattagtctttcgcccctatacccaaatttgaagatcgatttgcacgtcagaatctctacgagcctccac
cagagtttcctctggcttcaccctattcaggcatagttcaccatctttcgggtcccaacagctatgctcttactcaaacctttccagtaaaaaccttcc
aggtcggtcggtggtgcgcgccacaaaaaggacggtcccacctgcattcactttcatttcgcggatgggttttcatcacccaaacactcgcataga
tgctagactccttggtccgtgtttcaagacgggtcattgaaaaccattatgtcagcatccttgactttaaaaaagccgcagtcctcagtccccagtg
gatgtattacaatctaggctataaaccctcttcgagcagaacccgaaaaggtaccttcctaaattcttttccacccccggaaactgatgctgaccta
ccacaggttcagagtgcattgnnnnnnnnnnnnnnnnnnnnnnnnnnnnnnnnnnnnnnnnnnnnnnnnnnnnnnnnnnnnnnnn
nnnnnnnnnnnnnnnnnnnnnnnnnnnnnnnnnnnnnnnnnnnnnnnnnnnnnnnnnnnnnnnnnnnnnnnnnnnnnn
nnnnnnnnnnnnnnnnnnnnnnnnnnnnnnnnnnnnnnnnnnnnnnnnnnnnnnnnnnnnnnnnnnnnnnnnnnnnnn
nnnnnnnnnnnnnnnnnnnnnnnnnnnnnnnnnnnnnnnnnnnnnnnnnnnnnnnnnnnnnnnnnnnnnnnnnnnnnn
nnnnnnnnnnnnnnnnnnnnnnnnnnnnnnnnnnnnnnnnnnnnnnnnnnnnnnnnnnnnnnnnnnnnnnnnnnnnnn
nnnnnnnnnnnnnnnnnnnnnnnnnnnnnnnnnnnnnnnnnnnnnnnnnnnnnnnnnnnnnnnnnnnnnnnnnnnnnn
nnnnnnnnnnnnnnnnnnnnnnnnnnnnnnnnnnnnnnnnnnnnnnnnnnnnnnnnnnnnnnnnnnnnnnnnnnnnnn
nnnnnnnnnnnnnnnnnnnnnnnnnnnnnnnnnnnnnnnnnnnnnnnnnnnnnnnnnnnnnnnnnnnnnnnnnnnnnn
nnnnnnnnnnnnnnnnnnnnnnnnnnnnnnnnnnnnnnnnnnnnnnnnnnnnnnnnnnnnnnnnnnnnnnnnnnnnnn
nnnnnnnnnnnnnnnnnnnnnnnnnnnnnnnnnnnnnnnnnnnnnnnnnnnnnnnnnnnnnnnnnnnnnnnnnnnnnn
nnnnnnnnnnnnnnnnnnnnnnnnnnnnnnnnnnnnnnnnnnnnnnnnnnnnnnnnnnnnnnnnnnnnnnnnnnnnnn
nnnnnnnnnnnnnnnnnnnnnnnnnnnnnnnnnnnnnnnnnnnnnnnnnnnnnnnnnnnnnnnnnnnnnnnnnnnnnn
nnnnnnnnnnnnnnnnnnnnnnnnnnnnnnnnnnnnnnnnnnnnnnnnnnnnnnnnnnnnnnnnnnnnnnnnnnnnnn
nnnnnnnnnnnnnnnnnnnnnnnnnnnnnnnnnnnnnnnnnnnnnnnnnnnnnnnnnnnnnnnnnnnnnnnnnnnnnn
nnnnnnnnnnnnnnnnnnnnnnnnnnnnnnnnnnnnnnnnnnnnnnnnnnnnnnnnnnnnnnccagaaaagacaaaatttc
aaacaaattcacagaaaggtaatagagaaatttttgtgtgcttcctcctctccccaagtaggagagaaaaaagaaagacacaagaaatcttttct
aatgatccttccgcaggttcacctacggaaaccttgttacgacttttacttcctctaaatgaccaagtttgtccaacttcttggcatagaatccgccg
ttgccagcagaaacacgccaatccaaagggctcactaagccattcaatcggtagtagcgacgggcggtgtgtacaaagggcagggacgtattca
acgcaagctgatgacttgcgcttactaggaattcctcgttgaagagcaataattacaatgctctatccccagcacgacggagtttaacaagattac
ccagaccttccggccaaggataaaaaatccaaaggatttttttcaactcgttggctccgtcagtgtagcgcgcgtgcggcccagaacatctaagg
gcatcacagacctgttattgcctcaaacttccattggctttatgccaatagtccctctaagaagctaatgatcagtcaaaatgactgatccagctag
ttagcaggttaaggtctcgttcgttatcgcaattaagcagacaaatcactccaccaactaagaacggccatgcaccaccacccatagaatcaaga
aagagctctcaatctgtcaatccttactatgtctggacctggtgagtttccccgtgttgagtcaaattaagccgcaggctccactccattgtggtgcc
cttccgtcaattcctttaagtttcagccttgcgaccatactcccccggaacccaaagactttgatttctcgtaaggtgccgagcaagtcataaaaa
ataacattgcccgatccctagtcggcatagtttatggttaagactacgacggtatctgatcgtcttcgatcccctaactttcgttcttgattaatgaaa
acatccttggcaaatgctttcgcagtagttcgtcttcaataaatccaagaatttcacctctgacaattgaatacgaatgcccccgactatccctatta
atcattacggcggtcctagaaaccaacaaaatagaaccacacgtcctattctattattccatgctaatgtattcgagcaaaacttgcctgctttgaa
cactctaatttttttcaaagtaaaagtcctggttccctaccacgctcaatgaagaacatggcagttctccaggaggttaatgatcccggccacacca
gtaaacacgccttgcggcggaccagtcggccaggctcaaagttcaactacgagctttttaactgcaacaactttaatatacgctattggagctgga
attaccgcggctgctggcaccagacttgccctccaattgttcctcgttaaggtatttaaattgtactcattccaattacaagacccgaaagggccct
gcattgttatttattgtcactacctccccgtgtcgggattgggtaatttgcgcgcctgctgccttccttggatgtggtagccgtttctcaggctcccnn
nnnnnnnnnnnnnnnnnnnnnnnnnnnnnnnnnnnnnnnnnnnnnnnnnnnnnnnnnnnnnnnnnnnnnnnnnnnnnn
nnnnnnnnnnnnnnnnnnnncgcagtagttcgtcttcaataaatccaagaatttcacctctgacaattgaatacgaatgcccccgactatcc
ctattaatcattacggcggtcctagaaaccaacaaaatagaaccacacgtcctattctattattccatgctaatgtattcgagcaaaacttgcctgc
tttgaacactctaatttttttcaaagtaaaagtcctggttccctaccacgctcaatgaagaacatggcagttctccaggaggttaatgatcccggcca
caccagtaaacacgccttgcggcggaccagtcggccaggctcaaagttcaactacgagctttttaactgcaacaactttaatatacgctattggag
ctggaattaccgcggctgctggcaccagacttgccctccaattgttcctcgttaaggtatttaaattgtactcattccaattacaagacccgaaagg
gccctgcattgttatttattgtcactacctccccgtgtcgggattgggtaatttgcgcgcctgctgccttccttggatgtggtagccgtttctcaggctc

cctctccggaatcgaaccctaattccccgttacccgttaaaaccatggtaggcctctatcctaccatcgaaagttgatagggcagaaatttgaatg
aaccatcgccggcacaaggccatgcgattcgaaaagttattatgactcaccaataagagcccgaaggcattggtttttatctaataaatacatcc
cttccaaaaaagtcgggatttttagcatgtattagctctagaattaccacggttatccaagtagttgaggtactatcaaataaacgataactgattt
aatgagccattcgcagtttcacagtacaaattgcttatacttagacatgcatggcttaatctttgagacaagcatatgactactggcaggatcaac
caggtaactatcttgaggtctaaagtgaaagagaaaaggaataaaaaaaatagaaaaagtttttttcccttttttttacactcttttcccccacgac
catctttttttctctaatctctttccgaagaaaaaagaaaaagagaaaaaatcaagttactagttacatcaggattgaaactgaaaagctgaaaaa
ttcatgaaaaaatttnnnnnnnnnnnnnnnnnnnnnnnnnnnnnnnnnnnnnnnnnnnnnnnnnnnnnnnnnnnnnnnnn
nnnnnnnnnnnnnnnnnnnnnnnnnnnnnnnnnnnnnnnnnnnnnnnnnnnnnnnnnnnnnnnnnnnnnnnnnnnnn
nnnnnnnnnnnnnnnnnnnnnnnnnnnnnnnnnnnnnnnnnnnnnnnnnnnnnnnnnaaaataagaaaagtttttttccttt
ttttttacactcttttcccccaccgaccatctttttttctctaatctctttccgaagaaaaaagaaaaagagaaaaaatcaagttactagttacatcag
gattgaaactgaaaagctgaaaaattcatgaaaaaattttccaacttttcaaatttccctgttgtatccctttttcctaaataaaaattaggaaaaa
ggttgtctggaaaactagcactcttcctagttgtcatttccaaaaactgaaaacacaacaccggaaaaatgattttccaacgacaaggaaattgc
cagcttggttcataatttcctcagacttgttcatgaaaacaaatgattatgattcaaaatcattcaaaaatgatttttactacataccaaagttttca
atccaagtcttcagaagcggttcaaacgaaatcaaaccacgtatacatatatatacatacatatacggctgttgatctgtcggtaccgctaaatga
ctttttcagttaaatgttagtcaaacgaccattcatccacagaaaaactcatcgagtctccaccaaacttcctctttccatcttgacgatggatttgt
gaagggcggattctccagcagggaaaaagaaaaactgaaaattaaaaaataaaatcattcagtcaaactctttttcaagcctgctaaaggatag
caggatgcggagtcaagcaccgtccgtagacaactgcaactcgcttcccaacttatccttaaggtatcaaggtaccctttgtgaaggaaaaaaaa
agagacttctttaaaaagttaaaaaggaaaagtgagaaaaaaaaaaagttgaagaaaagaaataaataaaattccaattctcttctttcttttcct
tcttcctcttcccaacctttcttttccttgccaagtcaccgcgtttccatacgatgaggccattaatgacaagaaaaaaaaaaagtacagaaattaga
aaaagaaaaagaaatcaaatttttcctttatttcttcctctcttaactttttctttcnnnnnnnnnnnnnnnnnnnnnnnnnnnnnnnnnnn
nnnnnnnnnnnnnnnnnnnnnnnnnnnnnnnnnnnnnnnnnnnnnnnnnnnnnnnnnnnnnnnnnnnnnnnnnnnnn
nnnnnnnnnnnnnnnnnnnnnnnnnnnnnnnnnnnnnnnnnnnnncacctcaccaccaacaaccaccaaccactactacccact
actaccactactaccactaccaccaactgtgggactttgtttttttcacgacgagtttcaaccaaatggaaaaatggggtttttttatgtttttcccca
tgttgaaatttttttcctttttcatgtttttttctttttttaaaaatattttttccttttttcttttttttaaatcaaaaaattaccctcttgggtatcgaaactttttt
ataataaatttttgaattcttttcttttgtctctcataaataaatacatcttggtttttcctcccctttttcttggtcttcttggtctttttgggtcgtttttatt
tttcctatttttttttttatttttttttctatttttttcctcttttttttatattcctttttcctttttcctttcccttcatttcccatgttttcccttcctccatctccaaatt
cttccctccaacaccaaaaaaaaaaccctttttctctaccttttcctcccttctttttttatcattctttttctttttatcttttcttttttctatttccctcttttttc
tcctcctcctctcttttctccctatttccttttgttttcttcctgtgttttgcttcgtcagttctcactacctcaccatttccaaaaaatcacccccttccattt
caccgtttttggtttaaatcaacacacataacaactcttttcttttaattatttacatatctatttttatttctgcttcaatccttcaatttttcccctttttttt
ctatactacttctcaaaacctgcattacccttcccctttatcgacacatggtttcatgatttcttgcttttctctttcgattacacgtacacattcaaaga
cgtagaaacgtaacaaaagtttgnnnnnnnnnnnnnnnnnnnnnnnnnnnnnnnnnnnnnnnnnnnnnnnnnnnnnnnnnnn
nnnnnnnnnnnnnnnnnnnnnnnnnnnnnnnnnnnnnnnnnnccatgttgaaatttttttccttttcacgtttttttctttttaaaaatat
tttttcctttttcttttttttaaatcaaaaaattaccctcttgggtatcgaaactttttttataataaatttttgaattcttttcttttgtctctcataaataaat
acatcttggtttttcctcccctttttcttggtcttcttggtctttttgggtcgtttttatttttcctatttttttttttatttttttttctatttttttcctctttttttttata
ttcctttttcctttttcctttcccttcatttcccatgttttcccttcctccatctccaaattcttccctccaacaccaaaaaaaaaaccctttttctctacctttttc
ctcccttctttttttatcattctttttctttttatcttttcttttttctatttccctcttttttctcctcctcctctcttttctccctatttccttttgttccctttttttca
gagtttttcgctttttaaactttttcagtgagttacagatttttttaatattcgtaaggtttgttatttataatcctaaagtagccgtgtaagtttcacag
aatgcaggaatcagtctatttgtgtttaaaaaaagataaatttggtattgttatctatggaagaagtctagtaatttgtttaattagaactgtgtata
tactctggtgtctacagtcagcagcttaaaacattcgactggcaaggcttgaataaatttgtcttcacgctttcaatttagttagcagtactgtcgtct
gcatacatagtacaacagagcttaactaaagtacaaaaatacattgtaattgttacttccactcaatatttattttcataacccgtgaggattatta
acgtgacaatttataatatatcatcctttagccttaggagacttgttactatataaatataatcttataatggtgtattgcaagtatactatcaacaa
aacagctatcgtgacagacagtaagagcactctgtatctgaggacacaatgtttttgttgcattcaaaaagaagtgaaagcaaatgtcttttatat
aactcctttctagcaattcttcattgaatcctttacttctcttaacatcttccttttttgttatttcatactttttcacagtctcaattcagcggtaacaaa
ttgtttgtaagaagtctcaacattcacagtaaaaagtctctaaccatgaaccacgcaatatgataaacgtatagaagagtgctttccattgtatac
aatagcttactacatctcgataaaagcccctgtcttccgttgatttttttcatttgaattcttccacgaactctcttgactgttaacctcttcaacatattt
tctctgttgaattcaactctcatccacagtctcttttcaactttttaacaattctgctgctcatcaacaccaacatttgccttcctcaggagaaagggg
agaagaggaaatatttccaccttgttgccccatccgcattggtgagcagtgccggtatcagcagaagcttctaattttcctttgggaatatgacaga
gatttctatctagaaataaggtttaactagcgctatttttctaatgctattgcatttcatttccgttccttgactactttaaatcgttttcacgttactttg

tagcgtgagtttattgagttgcttttttgtgggcttctgcgcatttctggttcttttactacctacttgggtaggttttgcaattcgttttgaccgtttca
atttgttatcataaaaaagaatgaaaaagagaaagaagctggtgagccggccgaatcttctttctatgtttctcctgccagttcagatagctgtttg
ccgcgatatagcgaattttcttagtcgcatctggacaacttggaaaaaggttgctaaaatttacgtccaatttctgctccgccatattctgaaaaga
agaatcaagaagcgtccatgaatgcttctgaattctatatgtcagtaatctgattggtaatctgtccaacacagattgctaggattccgcaagctac
tgtcaaagttataaatgaaactgctcgacttgtccggtgccacgtttttgcacttgtgctggatttttattttagtgctctttactagaactttagattt
attgcccttttcattaacacagctttaacaatctctaattgatgtcttcagaattcctgctacttgtcggtttttttggcgtacacgcttccttccatttgc
agtgtttagtcaatggacgtggtgataaaaagcacagccatgtgtattaaaagttaacaaaaggtgaaaaagtaggaggtggagcctacagca
ccccggattcccatgttgtctccaaccatagtactaacgaggccctcagacgcttaactgcagtgatcggacgggaactggtgttttcgcctaggta
tggccgtagacatttatatttgagacttgtttagtagctagctgtttgaacattcaggtttccaccttactgtttttgagttaccttacaattggatagc
atgctaccctagcaaaagaactaacaatgaattgaacctggtatagcgagtggttttgaaacgacggtaaatgaatagaaaaaggcaccaaag
gaaagcctatttggtcacctcaaacaaaactctagccattgccattgcggttgtatttttcgtgttttagagctgtgatgaaaggattttatttattcc
tataggggggatcaaaagaaggtgcttttactttggatttttcttgcgctttttggtcttttcctatcaatattcgatagttttcacggctgatctctgatt
tttgtagaaagcaattgatggtttgttttgttctttttctttaaggatgaatgaaaagctgctaggacttgccaaagcagccgaatcttcctgtggg
gtttctcccgcgagttctgacagcgatttactgtcttacagtgaagtccagtattcccattaggatgacttggagaagggttgtttatgtttacatcca
agcctcggtccaccatgttctgaaaaccagaaagacgagttctcaaaggatgtttctacaacaattttgggttgctttggtgcttctgcaatggagc
tgactaagttcttgcttattttagttcagcggcattcgcgaggattccacccactgcgaaaaaaaannnnnnnnnnnnnnnnnnnnnnnnn
nnnnnnnnnnnnnnnnnnnnnnnnnnnnnnnnnnnnnnnnnnnnnnnnnnnnnnnnnnnnnnnnnnnnnnnnnnnnnnnca
cctcaaacaaaactctagccattgccattgcggttgtatttttcgtgttttagagctgtgatgaaaggattttatttattcctataggggggatcaaaa
gaaggtgcttttactttggatttttcttgcgctttttggtcttttcctatcaatattcgatagttttcacggctgatctctgatttttgtagaaagcaattg
atggtttgttttgttctttttctttaaggatgaatgaaaagctgctaggacttgccaaagcagccgaatcttcctgtggggtttctcccgcgagttct
gacagcgatttactgtcttacagtgaagtccagtattcccattaggatgacttggagaagggttgtttatgtttacatccaagcctcggtccaccat
gttctgaaaaccagaaagacgagttctcaaaggatgtttctacaacaattttgggttgctttggtgctttctgcaatggagctaagactaagttctt
gcttattttagttcagcggcattcgcgaggattccacccactgcgaaaaaaaaagtagaatgaaggactttgtcattaatacgtgattttgcatttt
catattacgttctctttcctttttgagggataatatgtttaaggtgacttcaaaaaatgttcaaaacagcgtagcagctattcttttcgcattggcgtt
ttaaattctttttaaactcgttttcgggtacgtcatgaaaaataatattttctgaaagctatgctaatgaaaaatgaatagttttattggatctgttga
ttttctcgttcgattcacctcccttgttattttctgcaggctccatagggaggggtttctgatgtttctaaaacgctttttgcgtggtttctgaagggttt
gattggatttcgaaagctattgcctatgtttaatatttgaaggagccgtgtcggcgagcgaaaatcaactttcaattcatgcaataccttttatttctt
tgagtgagtcaaataattttagtagtcttagaaaagagccatgtactgaatgttttgaacggtatctaagacgaacaagaagatcaacctaagaa
tactgaaagcgctaaagtttgaatgacagctgatcacttctaccttcctctttttgattaactttcttttatttttttacttttattttgtttagatttttata
gacaacgtatcaattgtttggcacttaaaatatcattatgcgtttatgattcattaatgtatattcatcatatcaaatcctctatataagttgaacgta
ataatatttcatgcgctgtcttgcccatagtcttcaccagctaacactactttgacttgctcaccatttagaaatctcctttatacaaataaactcaat
acattgacagtccgccagaagctgaaatgacctgtccagtaatccatcggctgttatcatcgacaaggaaaccaacaacttctgctatttcttgtg
gttttgcatagcggttttcataaggcgtaagtttagcgagaaattgagtggtactctcttcaaagtcatttagcatatcggtttcaactattccgggg
ttaatttgattaacggtatggctttgagggcctaattcggcagcccaacatctcctaaagccttcgatagtagatttggttgcagaatagatgggat
actgagggacacccatcctttcagccacgcttccgacatttataattctgcctccggtgccagcattttcttctttctttggaagatgaggaaatact
gcttctgtaaggaaaaatagaccacgaacgttcagatcaaaagaataattaaagtcttcgatattgttttgtcccaagggttttggcttagttacac
gcccagcattgttgataaggatatgaatcgtaggtccgaatgcctttatagtggtgtcaacaataaatttaggactgccaacatggcttaaatctg
cttgaatcttaattgcatctgccgaagttccaaagcctttaattttcttaaccaattgttctgtcgcttcaacactactatctgaagtaaacgtaaggg
ctactttggcgcctcgaagagctagcgcttcagctatagctgctccaattcctcttgaagctccggtgacgattgctactttgttttgaaggggcaaa
ttggtttctgtcatgttcacaagttcgtaatatacactaaaaggagtatttgctcaagtatttatatggatgtcattccaagttggctttgtcatcata
aatttcaagttactaatcaattccttacttgcataagtttttcctttttttttctttggttgaaagtaacaaggtaacgaatgactnnnnnnnnnnn
nnnnnnnnnnnnnnnnnnnnnnnnnnnnnnnnnnnnnnnnnnnnnnnnnnnnnnnnnnnnnnnnnnnnnnnnnnnnnnn
nnnnnnnnnnnaaaaatagaccacgaacgttcagatcaaaagataattaaagtcttcgattattgttttgtcccaagggttttgcttagttacac
gcccagcattgttgataagaatatgaatcgtaggtccgaatgccttttatagtggtgtcaacaataaatttaggactgccaacattgcttaaatctg
cttgaatcttaattgcatctgccgaagttctaaagcctttaatcttcttaaccaactgctctgtcgcttcaacactactatctgaagtaaacgtaagg
gctacttggcgcctcgaagagctagcgcttcagctatagctgctccaattcctcttgaagctccggtgacgattgctactttgttttgaaggggcaa
attggtttctgtcatgttcacaagttcgtaatttacactaaaaggagtgtttgctcaagtatttatatggatgtcattccaagttggcttcgtcatcata
aatttcaagttactaatcaattccttacttgcataggtttttcctgtttttttttggtgaaagtaacaaggtaacgaatgactaaaaataaatattta

cttccttagcgacgtaattttaagttatgtggatgactaatattaaataaaattagccgatttggcactgtatgaatactctgatcagaatatgcttc
catatcttagtcggtctttgcaaagggtaaatgtaattctacaaggaattgcgaagattgtagactgctgaatcagaagacagttggttgagctttg
attacaaagtttacacagtcaacaatcagaacgtattcatcatgactaaaatttgtaataaagaaaaaaaatgttctcaaaacaatattcatacat
gcgtggaagcagactgttttgtcgcaatgattacaaaccctgcaaacctgttcacacagatccaatgattggggtgaatgctttttatttaactcttt
attactctcaaaagaaaaaaaaagatgcactctaaaaaatagcttgaacggtggccatgacgtttccttcgtacatatcttgtcatcattaaggat
tcattgtaaacatttaatttcacaaacatcgtatgttttaatcttcttcattcctttctttgcaaataaatgcctcgattatgaatattgagttgagaag
aaaccaaccataaattacagttaccaagtttcgtaatctttattagatccttcaaataataaaaacacgtacaggataattaatgagaggaattcc
gatagtaaatcttcgtaaaatgactttatggtcggtcacgaactccatggagatatacattagaacttagagctataacttggtgaataaaattag
tcgaccacgtaaatctaaaaaaaatatgctttttgattcagggaccatacgtacattcattactccttgccataacagatttttttcaatacccctttg
acaactaagaaatcctaaaatacgaaaggaaatcgaagactttgattatttcttcaagtattttattatgacaaaaagagcctttggcttccttaat
gtaactcagaatcgtacttgtatacaatctcgcctcgtacaaagagaaactttcatctatcataaggatttgagtcgaatgcttttactaatttctttt
tagtaaaattaaatgtcataaatcatataaaaaaataatcataatttattctagaacgcgtaattaatatgagaacgataattgctgcgcaagaa
attcttatgattatttcctcattaaatttccccaatcaattaatacatagccaatccaccagaagccgaaatcacctgtccagtgatccaccgacttc
tatcttcagcaagaaaagcgacgatttcagcaatgtcagaaggtaatccaatgcgattttcaaatggagtcgttttttttgcaaactgaattatttc
atccgaaagtacccccttaacatatcggtatcaacagctcctggattgatttgatttacggtatggctatgagggcctaattcggctgcccaacat
ctagtaaaaccttcgattgctgctttgcttgcacaataaacaggttgctgaagaaatcccaaccttccagcaacacttccaaaatttatgatccttc
ctccattttctttttctttttgggaaggtgaggcactatcgcttctgtcataaaaaagacaccacgaacatttacatcaaacatactgttataatctt
ccatagttattgttcctataggttttaatggacctgcgtaccctgcattgttaacaaggatatggatcgtggaaccaaaagcttgaactgtggcatc
aacaatatgtttagcacttgaaacatcacgcaaatctgcctgaattttgatcgcatctgctgaggtgccaaaacccttttatttttttcaccaactgttc
agtcgcatcaatgctactgttggaggtaaaggtgatggctactttagcaccgcggcgtgccaaattttcagctattgctgctccaattcctcgtgaa
gctcctgtaacgattgctactttgttttggaggggcaagctattttctgacatcttaaatgggaaaatggtttaagctctgaaaggcttgccattctgt
cttatatagacaaagatttgtaatatcatcgtattagtcatcataattttttcgttaataatcattccatgggttagtcctatctgtattttctctagtaa
caagtaaatttatgtcacttatgtttgcgcggtgttaagtcaatgtgtcacgttaataattctcacggcttatgaaaaaatcaagcggaattaataa
ctacaattgtagatttctatacgttaattggacttagttatactgcgtatctagatggtagtaatgtccagggatctggttaactaaattgaaagtag
gaagacgatcttatttagccagaccagtcgaatcttccaatctgaagaattcgactgtggattcatagtttatatatcttcgctgaaacaactaacg
taatctatctctttgtataccaactttctttgtctataagacttccgttccgaaaggctttccgatttgttgaaatcatattttctttgttttgtttaccatt
tgtaatgtttcatcctttagtcttattgcatttgctaatatttagcaatatgctcttatgatagtgtattgtaatatatataatataaacaagacaccta
tcgtgacagacagacatgacaggcacttacgaaagaatattaccaatcatcattactaatcaaggcaaactgataacaattgtatgtttggtatat
aaagggacgaatccttctagtgttgagagtactactaccctctagaaaatgtcagaatacaatctttctcttcaaaacaaagtagcaatcgttaca
ggagcttcacgaggaattggagcagcaatagctgaaaatttggcacgccgcggtgctaaagtagccatcacctttacctccaacagtagcattga
tgcgactgaacagttggtgaaaaaaataaagggttttggcacctcagcagatgcgatcaaaattcaggcggacttgcgtgatgtttcaagtgtta
aacacgttgttgatgccacacgtaaagcctttggttccacgatccatatacttgtcaacaatgcaggatacttagacgaaccaaaacctttaggaa
cgacaacaatggaacaatataacaatatctttgatttgaatgtccgtgcagtctttgtcatgacagaagccgtcgcgccttatcttcccaaaagtgg
gggtagaatcattaatattggaagtattgctggaagaaaagcatttcctcagtttcccctatattgtggaagcaaggctgctgttgaaggcttcaca
agatgttgggctgctgagttaggccctcaaggccatacggtaaatcaagtcaatccaggacctgttgagacggatatgataagggcaatgcctac
cgaaattacgcagcttcaaacaaaaagcactccttttgaaaatcgctttggacagcctcaagaaatagcagcgatagttgggttccttgctgagg
atagtggtcggtggattactgggcaagtgatttcagcttctggtggaatggaaatgtattaaatttagatataagcaaagaaaggaaaaatataa
aatcataacaaacaattcagtttgttcctttttatatttaatattactatcattcccatgatcatttgcagctggaaacgacttatcttataactgcttt
ccttatatttttaggtaaaaacccatttaatggaagtcatctctggaagcttatgctacaatggggcacaatggctgttaatcaatacctaaaaac
aggaagctttgccgcgaatttagtgatatttctgcaagtggtaattttctttataaagttaagactcgtttgtttataaatagagaacaaaatacgta
agctcttacaagttaatggttttaagagaataaaagctcttgtaaggtgatcgtgactagtacggcaaaaactttgggttgggaagatttcgttttc
aaagtgtacaatgtacaaacttgtaagcttggagaatataagatcaaaaaaataaataaatgaaaaggttagtaagcgattgtagactaattatt
acattaaatgaaatctcatgtaatagaaaatttaaaacaaaaaatgcttactgtatttactctaattagtcaagtaaactgtacatgtatttgaaga
ttcctgcatagtataatgtttcattttaaagtaggacttcaaaattgaattccaaccgaagtgctccaaatagtattacatattatttaatgttcatag
tatcaaggagtatgtatatcacgatttgatattaattgaactaagagtaaacaaaacaattggatatagggagtcaaagactgataagatgcaat
tagttgaatgaaagaatttcagtaaaggacttttaaataactattctataatagaatatatattgagatgctttacggggagaaatgtctcgttcaa
gtgaaaaaacttctcctaacaatcctctaatcaatttaatagctaaaatattttaaaagtagttactaaattctgtttcacattaatagaaagttccg
ttatcgttcctgtgacagtattattttcacgagcagattccttattatgctataaaaaatatagaactacattatttttccacatagaatttgaaaaat

ttagactttctttacctgttgtacatataagctcaagatccatcatatacttgatcccttttttacacatcagttaataaccctattaaattaaaacatt
aagcttcaatttttaaagctacattatttctatttctaaacatgataaactttatctttcttaaatcgattttcctttacattatgctattattcatttgct
ttacctttaaagagccaaaatgtgtaagaagaaatagccaagcgactttttaatttgattatcttgaagttaagtaaattaaaaggtgtctaaaatt
tacagtatagaaatactacgtatttaaaagtagtgaaaaaggttggatgactattaatttgtttattgagggttaaacaaacgttagataatgaaa
ctagttgcataccttctctgactccaaatctgaccattcttaaaggaaccgaatttccagctgaaaccagattctttcaattttacttattacgattgg
aattttggatgtgtttaacaagtaaattgctttcgactttacctttagttctcttgaacttcgcatttagtggaactgagaatatttacattcaaannn
nnnnnnnnnnnnnnnnnnnnnnnnnnnnnnnnnnnnnnnnnnnnnnnnnnnnnnnnnnnnnnnnnnnnnnnnnnnnnnnn
nnnnnnnnnnnnnnnnnncaatttttaaagctacattatttctatttctaaacatgataaactttatctttcttaaatcgattttcctttacatt
atgctattattcatttgctttacctttaaagagccaaaatgtgtaagaagaaatagccaagcgactttttaatttgattatcttgaagttaagtaaat
taaaaggtgtctaaaatttacagtatagaaatactacgtatttaaaagtagtgaaaaaggttggatgactattaatttgtttattgagggttaaaca
aacgttagataatgaaactagttgcataccttctctgactccaaatctgaccattcttaaaggaaccgaatttccagctgaaaccagattctttcaa
ttttacttattacgattggaattttggatgtgtttaacaagtaaattgctttcgactttacctttagttctcttgaacttcgcatttagtggaactgaga
atatttacattcaaaaaggaaacaaaaagcttccgtcgaataaactcataggagaaaaaatccgataaaagctgaatccgatgtcttttatactc
cctgcatcaatatatggaagctaacagattttcatttccggaaaattcaattggttcaagtacaatacgttgttaatcagcagctttagctttcgtat
gtgttgtgtaacattgtaccatttctataatttatattctatcagttagcaaatgggcgaacagtgttctttaatttagctaacactattgattggaatc
ttcttatgtaagtagattaaaaatatttatcagaaaatgttattgatagtgcatttctagtaagaacttgcatttatgtggttttcacttggatagaat
gagttgtcgcgannnnnnnnnnnnnnnnnnnnnnnnnnnnnnnnnnnnnnnnnnnnnnnnnnnnnnnnnnnnnnnnnnnnn
nnnnnnnnnnnnnnnnnnnnnngaatttccagctgaaaccagattctttcaattttacttattacgattggaattttggat
gtgtttaacaagtaaattgctttcgactttacctttagttctcttgaacttcgcatttagtggaactgagaatatttacattcaaaaaggaaacaaaa
agcttccgtcgaataaactcataggagaaaaaatccgataaaagctgaatccgatgtcttttatactccctgcatcaatatatggaagctaacag
attttcatttccggaaaattcaattggttcaagtacaatacgttgttaatcagcagctttagctttcgtatgtgttgtgtaacattgtaccatttctata
atttatattctatcagttagcaaatgggcgaacagtgtttctttaatttagctaacactattgattggaatcttcttatgtaagtagattaaaaatatt
tatcagaaaatgttattgatagtgcatttctagtaagaacttgcatttatgtggttttcacttggatagaatgagttgtcgcgagagtattattctcat
taagcttgtcgcgagattactagtggtatggttttgcttctttgtaaactattattagagtattttccttggattaaaatttaaatgctcaactgaaac
acaacatcgcccatgcttttgagaaatcaacaaaaggattatgtaccattaccactgcgaacgactacgagtaaataattggggcttcagagcaa
tcctactagctcaacttcgtgaagtgcagaatataccgatatcattggctcacgacaaatgcaaatatttggcattcaaaagcagtatgctcaaaa
caacaatacttctacggatgtaattcttcaatactcataatcatagatcctaagggaatccagaaaactgataagctaaatcataagcactcatta
aaccgacaacatcgcggagcctttctttcaatttaatagtttcattagggttaaatcttattccatacccgtttaaataagaagtgcactgatcttttg
ttaacagatcgatatcttcaactgatctaatttgaggtaatttttcagtaggttcctctccatttaaaaatggtacgacgtcgataggaaaccctaag
gatcttcgttgaacgtttgctattctgtcaattcttgcttccatccgatccattttcgtctccattcgatccattctggtttccattcgacctattctgtta
aatagtcctgaaaatccattttcatcattcacccacatctgaaaccatcttggaggtgtatcctcaagagtacgctctctttgtctgaattgggaagc
tacattcagaatctgttgcttttggagacgaacattaacctcattttcaatatttcttccctcattctcaatattccttcccttggcagcaatttcaaag
tcttttaaaaacctcataacatcctcagatgtttcagtcgtgggcggaggaatattgaattcatttacaaaaactcttgcatccatcttaaattagta
tccacttatttgtcactatgctagaaaaaggttggtaaaacttgtaagcttaggataaacagaattttgtagtcggatgaaaataaatagtagatg
tctgcgcttctatctgtcagagcaatccttaataaaattacaaaccatttggtaatgattatactatggagtatatgacttacttgtcacgggagaa
atctcacacgttatgagaatgtaaatcgagtggaagtaataagtacaattacaaaattgtctattggaattggacgtgttagtactacgtagagta
atagtaatatgtccagggatctgattaactaaatgaaaacgactgtgtcaatcttatttagcctgatcagtcaaatcttccgatctaaagatttcga
ctgtcgacacacatatatatattattctgtatatttgccattgtgactggcttctctaattaatagcaataatcacgaattgtaaattttaatcggagt
ttctgcattcaaagagaaatttgcattttcaaatttttgtttacttattatcaagcacaattctttaagcttatgaatattaactgatattagctaatatt
ccattcttataacggattgttatattatgaatgctaacgagggacccaattatgacattagtatcaatgttaataaacaaacaaactgagaaaaca
gacggaaagtgtaaccaatcatcaaagattagttaaaccttattgtttgtttacttactagttttagattactatataaacgccgttctcctcagcca
aaatcttcagttaggaggatttttgctggcagagctaataaacttgttcgataacatattcattagtatcaccagagacgttactaaagatatatca
ctccagatatttagtaacttattttagagtaaatcaacttgacatttaaagcaattacaattgtattccactcacattcttataactatacggagtatt
ttcccgtgacagccgagtattaacaaatgccataagactaaaaggatgcgaaaatacaaatagtaaacaaaagaaaaaggtacgttttagggg
ttaaggcacctaattaataaagtgaagctattgaataaagatctctttggctacaaaaaggggtaataagtaattgcaaattaaatcttacaataa
gtatgatatgaggcgaagtatacaaaattgcaatatttactctatatatagtcaagtacactatacacgttttgagaattcctgcagcgtataatttt
taggtttgaattctaaaaatgtgctaaaaagcattccttatgtatgcttgatttaaaatcctttagtagctttctgttttagaattattgcaccattcca
taacttaatcagcttcatttgtttagcattactgatcaagcctgaatttacagaaaaaaaagttcaatgtaatggatttatcattatcacctatttctt

attaaaataaaaccattagatgagaatattctggtatatagtaaaagcttttagggtaaagcggcattcagaagattgaaagagtcagttggttgt
aatcgagtaattcttctgaatttttattttatggtaattatcacaactctggctaaggtattctattttctaatgatgagtacgcgaggtaaataataa
gtaagtacagaatgattttgataccactcagctcattctcttaattaagaatgttactatttcgtcggaatatatgaaaggagctaaaacgggactt
gtctcggaagagttagcgaggattaaaccttcaagccctcaaataggcgaactgaaaaaatacgaagtggcaagtagtcacgaatgaactgact
ttattataatttagcttactaaaataggatcataatttatctcctcaggcaattatcgccactttggtgaaagtcttaaccatttataaaggacttagg
tagagtactttttaggactaataaagcgcaatgtcggctgttcagattggtcgggacggtattagtattactgcgacaagcgacaccaggcttcca
gtgaatgaatatcatttgaaagaaatgaatgattcgcaaaagcagtatattcgttcttctatccctattcttgaatcatctggggtaaatataaccc
aggctttttatcaaagaatgttggagaattatcccgaggtgcgtccatactttaataaagctcatcagatatctttgtctcaaccacgaattttagct
ttcgccctgttaaattatgctcgaaatattgatgatcttactccactttcagagttcattgatcagatcattgtcaagcacattggtttacagatcaaa
gcagagcattatcccattgttggacaatgtttactagctacaatgcgggaaatgcttggagaggatgtcgctactcctgaatttttagaagcttgga
ctatagcatatggaaacttagcaaaaattctgattgacgctgaaaagaacatttaccagagccaaccatggaatggcttcgctgagtttgaagtc
gtagatcttattgacgaatctttcgatgtaaaatctgtttatatggttccaagagataaaacgttcaaaattgcttctgcttacccaggacagtatat
tagtatctcatggaagataccagggttgccgcatgaaacaattagagaatattctttatcaaaataccctgataccaaaaaaaatcagtaccgga
tatctgtacgtcgcgtccatgagggaattgtctctaattatgttcatgagcatttgaaagtaggtgatatcgttggagttagtcctccagccggtaag
ttcacatacaaaacggttgaaggaaagagaaagcctccagtgctctgttttgctggtggcatcggaataacacctattgttcctatcgttgaaaat
gctttgttggatggaagaacagttgtgttttgctattctaataggaattattcctctcgcccttttaaaaattggctcttacaactcaaggatcaatat
aaagaaagattcaagttgaaagaatttttttcaaaagaagcttatctccgcaaagaggagattatagacgagttaatgacacgtgttttacttcgt
gaagacattatgaagctgaattctgctgaatgcgacatatatatgcttggaccgaatagttacatgcgatttgttaaggaagaactagtaaagctg
ggtgtagaaccgaaaagaattgcaactgagtttttgggccatactctccttgattggctgacatacgcgtccttttggcaacctaagtctaattcc
catggcagatatgacctcaaccgagagatttagaaaattgatgaactcctgttatagttcttaacgaatttagtaatttacaagaaacaaaaagc
aggaacatctagctaatttgatttatagaaagaaaaattatcagaaatttaaaaatgtttgaggacctttatgctgacatcccaattatatgcatag
gacaacatgtgattgcttaatgaacttttacaggataaaaaaccttcttgaacatatgaaagaaatttatctctgaaaaggctatcatctcaacatc
aattatgttgtgggttacttttctgtcgggaatattcaatgcaccaactctaattcggaaggaagaaaaatcaatagtgaaatgatttgtaatttag
ctttgtccttggtctccactgtttcttattattttagcatgtatctaacaacctaatggtcatttgcatttatattactttcacatctgtgtacagactaa
atagctttggcatttgggtaatataactgaaatggtatgactaattaaggatcattatacgccaagggaatcaggaagataattattgttggatttt
tatcacatgaaagctataattaggtgtattctgttgctacttcaatccatttactattcgataagtcaaaaaggacaggatttgattatcgcttaaat
atttaaatacttatagagaagaaagccgaaatctgatggattgggagaatgacaccttggtactaacaatttttttaaaatacttcttggacctcgat
acgctttgaatgttctgcttaaacagatcatttacgcatatttattaaatgacgtatttttagatttaatccagtattcaaattcgctcgcttgctaacc
ttttgcaatcattgtcctaaaattttggtatttaaagtatccctaaacaaaaatgacgtgccaaattccgtttcattgctgtacttaccttagacttgg
tatataccatttaagggagaggtctagcttattattttccgttacttcccatcccattgcaccatacaatttaaattcaaccttacaataattcacttg
actatcttttggaagaagatttatgttggactcgttagatttgagagatgttataatgaatacgatgacagtaatatagtgttggggaggcttgcat
atgttagcatactcacacttattaatgactttattgagatatgccacgtctggatttaaacgtaacctaatttatcaagcattcaaaagagaggctg
attattcagtttcctgtttcctcaagattaacctcaagattaaacaaaaacttgcaacttcatagttcaatatccagggatatttaaactttaatttg
atatatttatttatgttaaaaaaaaaagcgagtgcgactgagcaaatatatatagcattgtgtgggcgcttcgtacttttataaagttcacagatct
caggtatattggttacacttgaggcctttaataaacatatagcagtttattaacattaaaaaaataagaaaatacattctcttcctgaaatcttcac
attttttcctcaatgtgccgtcatgatggttgtgagtagttgctgatatttttttacaaaatggtacttcaaatgcgctgaaacaattaaaccggggct
actcaaattattgagttaaaccatgtcattcttgtgttactgaaagaacagaaaacaccattaactcatcattattatttgagagaaaaccaatatt
atgctacaataataaatagtccgtaatatttaagcggtttgaatgttattgcttcccatggtttatttcatggtcacttacgaaagctccttcttgttca
gaaaaattccagttagagtattttcatagcaatttggatgctttgttttcattaacttttatgtttccctaacttcagataattgatatgatagatgata
acagcctagtgtataaaacatcatgttaattgaacgaaaatagttcagaatggtttatactgtgcacattcttcatattctgagagcatgataaact
cattcattgaaaagcaactatcagcgcattcgtttaaagtataatcgtaaaagtaagacatagttttatagataatttccgaaaatttctttttcaga
aattattctaacttgcctcttttataatagaaagcttttgtgattccttttaactttgataacagtgtcagtaaacagtttcctttcacactggtgcatat
gccagctcaaaaaaaccaagcattcgttaattgtaaaaataaaaataaataaatacgataacgcagttttgagtagaaggtaatgttctcgctat
tgtaaataacaaggtgcaccgtaactacagattacctgaaaccttttgattaccaccaaactgtcacaagcaaacagtttattctcttctatttcaa
ttagctcgctcgccatacatcctaatctgtgagtggtatttcaattatatataaaatagacgtaaactgtacgagtactgaattttgaagtttggctg
agctgtcaaaaatatctgttttgggagttatataaaaggattcggaactaatcattttgggttattcacttctttcaactatttatgcactttttgcttg
ttttcgatgaatatcaaagtttagtttttttccgatcctacaaagcagatatcccgcagaatctgaagttcttttattgtttttcagtgggaacttcctcg
tttacttcgtaacattctccttcctcaaatacagtttcattattttcggttttggttttctaaaatctttgttatttgttgtttcgtataggtttctctttttttg

ttttctcaaatttctggtgctctgtttttgttatagttaaaaagttactcagtttcaacgtttttctagtctttttttttgttgttttttttttttccaaagca
tgacagagtatatggatactgagtcctcgtcgacttccacctcgaaaaatcaaaattccgcagctgcctctacagattcccttcatcccagagaaa
ctttggattattatatttatgattatttcgtaaagcacaagtttcaagagacggctaaagcctttttacaggaatcagagatacaacttccctctaat
cgtgaccaaaatacaatggaagatttcgtccaaaacaatggatataacaatcaacagaaagagtcttttcattacgaccaaaatcttttcgatgct
gcaaacggcaagtctgaacatcttgattcattcaatatgaatattccatccaatgcaaacccaacttctgaatccaagaaagatacaactgctga
agccaacgatttttctatgcctttgcccgccccgtctgttgctattgatattcctgaaggatttttcgtcgaatggtttaatattttctgggacgttttca
gtgctcgtgttagtcgagttaatagctcttcccacttgtatgatcctcattttaacaggtcggcatacagaggtcaaaatcactcttcttctacaccca
ttccctcaaatgtacctaatgtgcaatcatctagtgttaacccattgaattacgctgctactgaattgaaatcttctgaaaacaagccgtacgcacc
ttttacttcagcgtttccgaaatcagatacaatgaccacaggaaataacccgaaatcgtacgctctcccttcccgagctgtccctttttctggtcccc
cttcgaacataagccttgctgatagatcacagaaatacggatctgcgctacctgctggtaatatacctccctctgttatgccttactcgttaggtgta
cctccttcctctgttcctcccaattatccaaatacatccatgctatcagctagtccacctcgatctttggtcaatgatcctaataatcatacttatgctg
ttggcccaggtatgaacccaagctcctccagaaacggattttatcctcctacacccgctcaaatccatcagcttaagtctcagcaagaataccttc
atcgtcagagcaaacaaatgtcagagccagctgtagattttaatccggctacttccaaggattctcaggtacagtctaatgagcaacctgtcgtga
accctgcatctagtacggttcaaaaaaaaatacccgatcctaaagctttatatagtactaacaaaccaaactctaccacaagaagctcagcgtct
cccataattactcaagcatcttctggaatgggtggtaattcgtctttttacccatatcataataatatgagagaggccgcttcaatgcctacatttaa
accttccccgaatatgatgatacaacctcgttatcctgcaaattctgaatttggggtcaatgatatgcagaaatatcgtccccgaaacagtgttgat
ggtatgcctccctatttgccacaaaatccagttgatttgaatggtaaatatggaatgaatccaaatatggcgaaccgacttccttcacagcaggct
catatgcccaactattataggggacaactaccgatgtcaagtcatcatcctcaaatgtacgaatcgcctactgctaatcagggttcggtagagttt
gctaaaacaagagaacttgcaatgagaagagggcaaacaccgaacatgtctgttctttctaatgacattacaatgtctggagaagctagcaatgt
gaacctcggaaatcctttgacagattatcatatgcagctaatgttattggaagagcaaaacaaaaagagattaatgatggcccgaaaagaaggt
gaaagagattctctttcaccggaaacatacgagcgctataaccgtgagctaaaaaatagtcaagtaaagtcaacaactgatggagcatctcatct
tcatcccaaaccaagtggaggaatgagttctcaagttaaaggaactgatgtgcctacgacgggatatattcaaccgcaccaaaaacaaccgagc
cttccggtccaacattcgtttatgaacgttccacaaaatgaacattgtaagtatttgaactattatgattaaaatctaacatttgtttagcgaatatc
atgatgtctcaatatctgaatcgtagtaagcagcatggaacagcggtaaatgctcgagattctagagatccaaatattgcaaaaaagttcccca
agctaatacagtatcgatgagtgaaaaagatagaatggctttagaagtttccgaaaattcaaattcctggcttacgcaaaatcgtgaaaataact
catcagatacaaaagaaatgtcgaaatatcaatcatcatttactcagccttcccagccaaaattgccttctagtacttcagcatctggtcatccaaa
ctctgactcgcaacactttcttgaaccggtgaaagataaacaaggtatgcaaaaagatacggcacgacctcctgatatcgcatcaaatggatatc
cttcaatgaatgatgctgttcaacaaatttcgatgccttcagctggaactgaagaaaatgtagcagtttcttcagaggaaggaatcaatgcgaac
aaagaccttcaggacaacggacgtatagaagagagtgcctctggcaagactaatgcaggaaaagatgctggagacgttactaaggatggggtt
tttaatgaatctacggataatacgtttaattatgcttcaaaaaatgattccaatgcggaattgttaaatgattttgactttgaaagtttttttgaatgac
gctggtgatgattctacgcaggtttactactgaaacccatagaagagttttagtcctagtgaaagctgcaggatttatgttactttaggggttaaag
gtcattgcttttgttgcgaatggctattgaagttcttttatgaatttgtatagtgtatattatttaatagcaaatgtctatctttcaagagactcttcgtt
ttgaaagttttacagaatatgctttcgcagggattatagcttttatacagtaagtggatttggtcagttttctactattttatttaaatccaggcgtttc
agttgttcgtatagtccttttaaaggacatgtgtctggattatacgtcagttgaggtttttgggaaaaatgagaatttaataattttttagttagctatg
aaagtaaaccttatttaaagtaaggatctgcttaatataaggggaggtgatgatcatgagtatatcctagtttatttagccaaatctcatagtaact
tctgtgatattagggctaaaacgctgtattcaagagaagggtttgaatcaatagccatgaaaacacagcaaataagggcaatgagagataaata
cgttatcctctatttatcatccttgggtagtttggagtctacttgggactccaatttatcaaggtcttcgcggtgggatttcaattgctcttttaattttct
gagttgttcaacttcaatttcatgaataaattggttctccttagcctttcctcgtctagcaatagatccttccctagcagaactagcgttgcttaaagt
gggatctgtggagtcgccgtcactattgtcgctggccatggatcttactcctagaattttcctctgagagagatgaggaacaaatgaacgacgaag
aacaagtctgtacatattacaatttgaattagttggctataggaaataagtgagatgggggaaagttgagttgccttaggagcgggttgctatgta
aacgatgtaatacgtaagcaaatcctactttactatgatattctcctttaatatcatatgtttttcgcagagtaaaataggaaacgacaaagatgca
aggaatttcttttatatttcattgaatgtgcatttaagaaagcatactgagttacgaccctttcgaagtttttatataaaaacttcgacttccacttctg
atgtaagtctcatgcataaaagctttgaagtaaaactgacaataaacatagttctttcgttgataagaacttgatgaaactttttcattttacaggg
caggatggctttcataatctcttagtgggaatccattgtgtacacaacgaaggatatatgcaacatatagtgaatttataggttaatggagaactc
gatagttttcatcatagatggaaatgtcttccatagcatcgccgttgatccttgaaatactttctggtttttcaaattccaatacagtaatgttttgatc
ttatgtgcttacaataaatgtcaattcacttgaaagtaacaattcaattagagttaaaaattacgaaaacttacatgtttctgttcattatttatgca
aaactagcaccatatgaaaaagagtaaaaagctgttaaggggtccttgaaagagaaggagaagaacgatgctttcgtttcagctttgtacttatt
tttcttttccggatccttccatgttatgttattatacaactttcggagtttttctaatttttaatttaccaaccgacaacctataaattatttttatacgaa

gctcagcattcttcggtgtcacgattgtaatttttacgggttatgagaataaatgtcgagtggaataatatctacgattgtaggaatgtatacttcaa
ttggactctggttgatactgcgtatatagacgctaataatgtccagggatctggttgactaaactgaaagtgtttcgataatcttatttagacaaac
cagtcgaaccttccaatctggagatgctggctgtagactgatgagtatatgtagattttctggtataatcagcatagtgctcgttagtgtacctcatt
ttaacttcaacacaatataaacctgctggacaatgtcgtaccaatagactaatctgtacaaggagttttaacccttcagaaatcgttaattactatc
ggtttgtctgactatacatgatcaatcggtattgaccatactctggtagtttatgaaatttttctggtcttgtgcacaatgccaaagagggagagat
acctgtagaggatgtgaacgtttacttgggagaataccatggcagccataccgacggttatgatacctagctaccaactaaattttcttttgcacat
cgcttacccctgatccatttgcctgctgcgttcgagtttgacccggaggagtcaccttcatcctaatagttttctctgtatgaacaggacaggcagta
ttaaagacaactcaattctggcgctaacaaggcaaagggcaacccatattattagtaacataactgttctaataggaatagtaagggttatgagt
gttcattgccgcaagggcctagagacgaggactatggcacagcgtgtcatcccatcagtaatggtaattattgtggatattgaaggcggagtgca
cctcacaaccccggactctcaaatgtaagcggacgtgacgatttatacttccgaataaagttgatgtaataaaaaataagtttatttaaatatcat
cgttaaagggtagttaataaagtacatcaattcggaaagaggcgttaaatcgccttaagtgtacattgggaaagtgcagtggttgaatcataact
acaaaagtgctcaaaagacttaggacataaatttgcgaaaaagcaaagtacatcatcgagttttgaaatctttataacttttcttacattataacc
aggttattgcgcttaggaagaaaactaagcattaaattttaacaacttcgttagagaaaaaaaaggaccttcgattgaatcacgttcaaaaaatg
tctgtacaaaaggtagaagtaaaagattataagaaggttgctgctgttttagccgaagcattttataacgatccagttcatttgtactttgctggac
ctccaaatggggaacagtacgagaggctgctacaagattgctttgagtacattgcttatgcctgtttaatgcgcggtcatgtcttccaggttgatga
cttttcaggcgtcgctttatgggctggtccaggtgaaagaattgatgattggtataccgttttgagaagtggtttatggagattattttacaaactgg
gtgccgaaaatcgccatcgtttcttcaatgaattgattccgttacacaacagagtaatgccagaagccatgaaagatcgaatcgataatttttggt
accttatgtttgttggtgtaaaagcaaacagccgaggaaagggataccttcgaaaactccttaaaccaatattagacattgccgatcaaagaaat
cttccttgttatcttgaatctactcatcctaataatcgccccaggtatgagcattttggctttcgcgtcgtgcaaactgttcaattaaaacaagactct
gatatcattccgatcgacgttatggttcgagagccatagctctttggattcagaacaggcgagtagtaaattgaaaaatcccaatcttttgctactg
caaactgagctctgttttctgtcattatctcttaaattatatgaaaaaatactttactttactttattaagggtttctttttggtaaatccacaatttatt
gtctttcctctgtctaagcaattggacaatgagtatgattgttaacatatttatccatgatccggatgtactatattctgttcactttatcgtaacgaat
tttctatttcattattggtgaatttgctaaaccatgtaataatgcgttttaggtaaaatatcttcacagtaccattcatgtcagaacaatatgtatctc
aaatcatctaataaacaaagggaaatattcattttaaggaaataactaagtgtagtaaaaagaagggagatatgtaaaatttagatatacttcgt
tacatgtttcttatgtcgaacagaagatacatacctctgtcagtttcaagaaatcaaactgccatatttttaagaaaaaacttatagggatctcttg
gaaaatagaatggtaataaggggtagttatcatagaagcaattaaatatgtaagcgtcttctcaactcttgacggtaataagctctttcttctgtcg
aacgccctagggcatcaatgttttcacgggcacgtcgaaccaaataaggaagaacatgataaattggtccccaagagacatacttggctatgtg
aaattcagggacttcacttcttggataagctgcaagtgcataagtaatatcatccgacatacccaacaactgagccaaatacaaagatgttttgct
tgtatcaattttctttctttctaaaaggtccaacactttgcaaatagaagatttgttgtgggtagcaaccataataccccatttacctttgcgggattt
agaagggagctgagaagccacgctagttccaggagctgctctggaagcggctaagaacaaagaatccattgcaccattaaaatctcgatctgta
tcctcttttgtgtcatgaatcaaatgacggggctctgagtttatataggcgcctcggacaatttttgcacccatcagccaatcttcgagagcacaat
gtctcatatgctcattcaagattcctctggtctttttcaaataaagttgataagtgttgtgtacgatagcttcctgcttattgtattttctcatcaaatct
aaagaaattgaatggatggtatcttgaaagtaagtttgttcggcatccatgaataaaaggacacctttttgttgggcaaattcgcacactttctcag
aaaaggaccagaagcgctgaagctcttctttttcatatggagatagcaaagggctgccattcaaaatttgggaaaaactagctgcaggttggttg
gatgtattatttagaatagcgttgaaccggaataaaactaaggggttaatgaatggcgttaacttgatcgcacaagaagagttttcaaccttaga
agccaagtcgatacttaacaaataagagtcgaaagcttcttgagcaatatcatgcatcttttgttgaaccgttacattaggggccttgatctcttca
ggcacagaattggccgtggaaacaaattgacgaggatcaatgtcgcttgggagatcaatctctcgagaagaattgagacaaggagatatccca
gaagcttgaagagattgcatgctttggatgagtgcagtcggagtttcacctccacagaaatgacgatagaaagtacgacggcaaaaaggggcat
agaggaaagaaagatggagagtatcacacaaagacattgcagctatggaggtctttaccagccaagctcgagagcaaatctcataaataaaaa
atccgcgagcaacatcgccgaacgaacggtgactcaattccttggaaaagcctttctgaaagaatgaagcttctgattcgttcgtcaaacccatgc
tattcaaagagacggctgcaccagcgccgatacccaaaaggagcttgccattgcgaagaagtcctgaacgcaaacgaaagaaatgcatttcctc
tgtttaaaaaaggaaatataaaataggaataaaaacaaagagaagccaaaagactggagaaaacagataatctcttgttgtttagcagagatt
aacaaaatacaagagtaagaggcaagatctctttttttgttgtagaattatttctttttctctatcttcccgttctgtattttcaaatgtctttgttgaaga
caacaaaggagaataggaatcactattacaaagaaaagtgttatattatttttaagaggtattattcttaaaaagcaatggttcgtttttttatgcca
aatagagcaagacgtatagtaatggtagcagtcgatgcaaggaaaattcgtacgcaattgccttgaaactctgtataccgcacaagaactgact
ctaaatatgtctagttctagttcctatgatcaaaaaagagcaatgattaaaacaaaatcaaaaagaacataaaaaaatataaacaataacaaa
atgaagctcaaccgtggactcttgtacattatttataccttttttctttctttacgggaatattgatcccctatagattgatagatactatcagtgaagg
atggtcattatcagttaataatgatttgtagaatcctctttcttaaaataccgttatagatcgaacaggacgttaaaattagagttcagtagacgag

agtactattttccatattatattgttgtaaggaaagtttttaagcaacgcttctgttttggggtatggattattgatggaatgaagagattcagctttg
gtttgtcatttcatctttgtgttgctgttgttgttttgttctgtgggttaacgtagtgaaaagatccaaatagcgattatctggacgcaagataagccg
ctacgtaaaacatgatcaagatagagctgactctaaaagaaggtgtaaattgttggtaatagtcgacatgatgttttgaaaggttcgccgtgaaat
ggaaatgaataaaaatataaaaaaaatgtaaaaaaaggaagatcttcttctttagcaacaaaactagaaatcgttataataatacgcgttctttc
atccttggaaccatacatacagtagattgcactagcagagcatgattaaagcagttactagttcagtaattgtttagtcgttccgctatcaacgact
ggaagggtgtccgagtggttatggagctagtttaaggcactagtgcgaaagctcgtgggttcgagtcccacccctttcattacctttttacctttag
gtttttggaacctaaagatcaagcagatctgagaaaggtcctcaatctgcctagtgggaaaccttgaaaaattcaagtctgtttacgtttcactttt
ggttactctcagaatcataattatttgggttacttgggttactaaaaggatccttgaaagtctaaaaacaaaaaaaataaaataaaaataaaata
aagaaatgaagactagtagcaaggttatcaagtgggccaatccaaaaaacagcatggatgagtgattgtagaatgagtagaacattgccacaa
aacttggaatacatcgtagtatctagatgctccttgtccattctcctagtcttgacaaagtttcggagggtcttttcgtctgagtctgaatctgaatct
gaatttattaagtagctagtttctattaggattatagcgcaagacacgaagatacggttgtctttgctatatcctttcaaacaaacccgtgtacccat
ggcatgtccatgatgcttgccaattgtatacaaacggcaagggtgataaagaaaggtactttggtaagttttggcttgtctcatcgatgcgcccaa
gtccataaaaaagcttcaacaaccaaatgatccagagtcctgtgctaagaaagagataacccatgtgtattcagtattatgaaaattagcataat
gtattggttattgagcaacttgcatactaaacagaccatttccaagtcctcgcaaaaatcaagacttatgctttaataaaataatttatatactgtca
tcgaagtatttagcatgcatgttctgctctgtaacgtatttatgaacgttcatagaacaagtagctttcaaacgaatagcgttcagccaaaaataat
tattctttaaaacgtattgaaagaaacaatctgcactgattttgttgaggatacttattaccaatgacgcttgaaactgcaaacaaccattttacta
attgtttctattcttcttatattcagcgcttatttaaatcgtcaacataaatccctgatcggatttcttaagtatactgttttcatccttggtgtttgcctt
ggctagcacacgcagatgtttctttgctgtactctttctgcgatgatattacatacaacttcaatttcctgtaataatgaaagaattgagtgaaagtc
agctaaatcaccttagagattacttggatgaacagttactaaatgtttctcaaaactttcaaaaaaagtaggatcttgtaaacgcaagaaaggctc
ttttgagcaaacgttcgattgaatcgtaactaatcaaattcgcagatttcatcctgggggctttcaatcacttgatgaattaattcgtgatctcgagc
catactttcaagtgctggatgctatgcctatgcaaagagactcattcttggctgtcaacgctattctacgagggttggactttttattgatgcgattg
ttgcttttccaccggcacctgaaccaatgtttcgagcattgtcggttttagatggcttgagtctcaaactcgttcagcatggaaaactctccacaaca
gatgcaatccggttaaaaagccttttggaaaattgtcgtatggtggtaatttccaaacttagtgaggtccaaggctatgaacttgaatgctcttctg
ttttcgaaagaacactaaatgatattgacttttaagggtcatgaatgtatgacaatatgtctcgtggacttgtcttctaacgaaaggaaccaaacta
ccgatatttctccaaataaattctattttaattaccttcttttaattcatgcacatattgccaatggaatctttatgtacatggtgcatatttcaccaga
gaacaagccgacattttcttcatttttttcccgcaaaacgcttcaagaatttaaattaggctcttgagccctttttctccgaaatttaccatttaaatct
gtatgagaccctttatttttattctatttttgcaattcatgcaatttttttgtttacataccatgttacctaacgcttattgttacttagggcagtaattgct
tatcgtcatcatagtttagtttctgttcctaaacagaccgccaaagtcaagtccaattgcagatttattcattttctatattttattgaaaagttcatac
tttttgccatttttttttttaaaaaaaacagtttatttgaagataacttaacctgtttgtttacattagagggaatacacctaaaataattttggttatat
ctataatcaagtaagcgttctgcttgcgaaagctctatgaacatattcattccaaaggcacggcgaccaaccttcgaacttcatttgaaaatccaa
gatttggcaaatattcctttggtcactgggctagtctttataaaatggaatgtagatggcattcatgctcgtgattgcagcggacaaactaatggag
aacctgttttggaacataaagtggtttgggattacgacgcaacgacaatagtccgatttgtcgtagacaatactaatacccttcgagaacgattttt
agattttcatattctcagtgaatcttacagcaattctggagcaacgaggctaggtgttttgaaaataaatttagcggaatatgtcggggtcgggtta
gattctcgaaaatatttacttgctgacagtaaaataaatgctactcttcgtattggcattggattgaaacaactatcaggcactgaaaactttcatg
ttgctaaaaagcttggaaaacctcaagtgtttagcggtttgactggtttattgacagaaggaaaagaaataaaacgcagggatgatgaagcagc
tattgctcctggtcttgcctcaaactgggctgagaaaatgcttacttctattaaggattttaatcaaaagtctagtatgtttcatatgcatacgagaa
ataacaaatacgacaccaaacaaatcgttgatgacatttttttttggtggatctggctgggccgaaaggcctgtattatcggaaattccggatgat
acaactgatcctaatttgattgctttggaaattcgtcaaaaaagctgggtattaccaagtgaaaaagaaaaaggtaaaccttttcactagactaa
atattaagataagtgtaatctattccttatttattatttattattaggcacaaaatctatttattattttattaaccccaaaatccgttcttgaagagta
taagatgcatgtatctaaagcaatcaagtaattttctatcttgtaacctatttttcatgaccattgttaaatcattaacaaaaatctggatgagcgtct
atctttctcatcagttcatgcacgccgtctaaagatacatgtacatgcttcgctacctgaatgattctaaaaatcacataccatacattagggtctga
agtacaacccagtgcaccatcaacctgcacgttggaaaagcagtatttgatagcattaatcccatcttcaacccattctttaagttcttcgcttgatg
acgaagcaaaatacaagagatcttgtaacttttgatagtctgaatagtataaattctgacgttcagtttcaaagagtgccgaatcacgttttaaaat
gctttccagttgttcagaagccttttctgagccgttatggatcataagtcgcttgtttacacaagcttggaattctgctaaacccccgaaaacttcttt
ggttgattgtttccacatactccaaacttcgtttatgcagagatccgatacttgtaaaacttcacaagcgtatgctgcagcgtcttgtctaatttcatc
atcatcatcattcaaaagatctcccagtattaaaggtaatttacaaattccttcgaatttaatttcattcaggctgctgggaaaactaatcccaaaa
tgaaccaacgaacttaaagctgcctttctagtagagaatgccatgtattcatttgtaaagttgagaagaatatcaatccaactttcatagtaactca
taaactcattactgccctccaagaacaactgtttaaggtatgaacccaagaatatcaaaagactctcacgtactggcaagctttcgtttgactgca

cgactgcgatcatattggtacaatccaacatcatagatttataatttctcgcctctaaaatatctgaaagtaaagcaagggcgggtgcacgaagct
gactccactgttgttctatagataagtccaccaaagctcgcattatttcgggattattgacaataaccttttcctcggatgatccaatatgtgctcta
atgtagcgatttgtacttcgtaaaactcgtatctgagcaattttgagacgagaaatccacttgagtgggtatcgctgtgtacggaaaattctttcaa
attttgtaagaaaatttccgcagccgtttgatgatgaataggcaatccaataaaattatacccatgaacaggccatgatttcttttcaaaaaggat
acgcttgcaataatccattgtcatccttcgcaattgctgtaatcttttagcgttcttatcagtatgtagcataaagtaattttgaaggatttccaaaaa
aagctttttatatagaagcttcgcttattggaagtaatttcggagaaacagctcagcaaagttaaggataccttgtcataaaaaaggcttttcgaa
tctcctacgaggtttaaaaaaagaccagctaacatcgctcggacagtcaatagacgaccatgtagctcgttttgtgttaaaacctcaacgtttttta
attgattaacaacaaactccgtcaagcgtgagctatcaatcatgcaagttaatgataatgccgcaatctctcggaccttacagatcttgctagccgt
acactggagtagcagaggttcaaactcagctactccttcccaatcctcctcgtttggataagtgtaagcaaactgtaagcgagaaaacaagttta
agattggatatagaccggtgctcgcttgattgctagagtgtaaagtattgacactattttgtagctcgtttagcaaatatggccttaaggaaggaaa
cttgctaaaaaaagttttagtcgaaagccctttggtatttccaagattaacagctcccttaggacgtttattgccaaatgctctattcattaatgccgt
aaagagcattacactacaattcctaatgggccataattcatgagagaacccttcaatagctaatgcaatagctggttccaaatattccaatgacac
agatgaaagcctatgttccgtaaaaattgttttaagggtgttcatcgcatgaacttgtggaagttcaaactttccagcatttgagtcagctatatca
attttttctttagccaaactaatcaatgtcaaaatagtctcagataacaaagctttgtttctatctttaccagctactaaaatgcctgtaatagaaaa
gggaataccgccacttctgcgggtaataaatgacgacttttcttgaacgacgtctatgtttttctgcaaccaaggaatcggcatttgacataactttt
catctttttagcaaagaggaatgaacaaaagctgacgaaggaaggatagacactagtgaaggctcctcgatgtctaatctcccatagccaactc
atcatcaagaacccataatgatccaaattagacaaattgaatacgtctgggtaaaaagaactgcattttataagtatcgtagataatagaaatga
tatttccttaatacttctccaggaataacttagtatcaattgagccggcgtatcctctaaattttcggttacaccactatcatcttgaccttttggtaaa
ttcccttcaggggaatcatcacaaagaatatctttaacaagtaaccatactgattctgacaacaacaatacctcaatgattaaattcttccattcag
tatcatctgtctttaagaaatttgaaattgaagaagttgaaaatgtataggacaactgaatcaagtatccttgaatcggataactaattgccgcttg
aagtaatgactttctagcagctttaacatttctctgtaaaatatctaaaattcttcttaagcttttgtaggtacttccaggctgcaatttttccatgaaa
tgattgcaaagacatattgtctttgcaccaccttcacactcatgaaccctgaccgagttgagcaagtaaagaccgtgattaaacaagaagcttaa
ctcttctaaagtgttataaccaggcaaacccttataggatagtaaaatattcaaactaagatcacggatagtatcatagggatcgattaatcgatc
aatcagaattcgggttaattccttgtttaaaatattaatttgaaatggaaaagtatgctgcctttctttcaacattgattcctctaccaactcgtctaa
tcccaaagtcaccaatgtttcaagaaaaccaagagaggaaagtacttgctgataattactagtgggatgtaaatatccaatacagatggaaata
aagtactccaaaaatttctgagacctttcaataatcttagcatcatcaggtatggaagaactgcgcttgtatctaatgttcctttggagagcatgac
aagatgctcggatccttacgaaaaagtcatgcattgattttaacagaatttgcctggtatcggaatctgctattccaaacgcgaattggatccttga
ttctatgcaatcaaattcttcagcggtcaaaggcatggatatattaggacaaagagcaattaaattgaaacaagcaagctgtattctaacatcttt
atgcaatatgaggttacggaggaaagaatgcttattgtttagatcgagatgctgaataagctttccatcctttgctatcttcaaagcgaacaaaca
ggcagcatctttggcgtcctttgaaacgtcggggtagttctgtaaagaagatagaaatttcggagtaatattaggagaaacacttaataatgatgg
gattaaaaaggaagacattccactttgagtctggcgattgtctccataaaactcagcaagaatttcaggacaccacaaattcatccattcattctct
gcaacttctttgttttcgtatttttcacataaagtttggtaatacaagtttaatacagtaaataccgtagtagatatagcctgcgcataactgtaatcc
tttaaatatttaaatgaacgtgacaagaagtcaggttgaaaggaaaaaacagcttccgctcccgtatatttgacaaggatttgaatagcagtaca
cacaacttttgcatgtctgtcccaaattagagattgctgtaaaagttggagtaaagcgtttttctttagctcttcgtttggacaagcaagcaaataaa
gttgcaatgttttagagaataagtcttttacagctaattgaacgcctgtgactccaggaatagatgtccgtgttgatggcaagttaagaagggttga
ccaaaatgagggttctatcgactcccaaacagtttttttaaataaatcgtcttctttgcaggcgttaaaaattctccgcagccaaacacttgtagcat
caaaacatgcaagttgagtaggaccatcgagcgtaggatgcagtaaggagtgttttaaaaattgataagaccgaaggaaaaggccatcttgctc
tgtccaggagttcgactgagttttaatgaaaaactccctacactcacgaaatactgaggattcgaactgcaaatgcgagtaatcgccttcaaaaat
agataaaacactttttttcttccatagaatgaatcttatattttaagtaaagttggcgtcctattcaagatgaacttcaaacgtgaagtctcatcaaaa
ttttgctttcttgtatgaaaagcaagaagagagcttatgactagcaacactgcttaaggatttttttttaataaacaagaaaaaaaaaactagtatg
attttattttattagacccaaaagcaatcaattggacgaccccagcagtggtggatgcggactgaagttgagaatggcagtccatgaagcgcgcg
ccaagaaccatcccaagcacgattagaaaaataaccatttggttaccaatctttaactgaatctcattttcatccagttctaagaagattatgctaa
aataaaaagtactttaaatttcagaaaagctagtattcggtaaaaatagagggtacagatttttctcttttttttttttgagacttatagcagaagg
aagccgtttaattatattaaaatattaagtttttatcgaaatattcggaaaaaactatacaaatgtcgcaagatttttcacagtttaatttggatcct
agaatccaacgtgcgattcaaaaatgtggtttcaaggaacccacgcaagtacaaaagaagggaattccattggccttggatggaaaagatttgc
ttgttcaagctaagactggttctggtaagactgctgcatatttaattccaatactagaacttttgttgaagcagaagctgttggatgagaaagatcg
tgggatttttacgttaattcttgttcctacgagagaactcgctcaacaggtttttaaagttttggagaagctgactctttattgtggtaaacacataaa
atttgtgaatattgctacaaatgctgctgactcggtacaacgtcctctactccttgattttcctgatattgtaatctccacgccaagccgttctgttgtt

catttttcctcaggagctctttcgctcgacaagataaagtttcttgttatcgatgaggcagatttgattctctcttttggatatcaggatgatatgtcca
ctttaagcaaagcacttccacgcggggtacagacactattaatgtctgccactctttctgaaagcgttagttcgttacggcagttagtttgtcgtgat
ccagttgttttaagacttgaagataaagaagcagggggacagctaacacaatatgctgtaaaaacatccgaacaagacaaatttttgcttgcata
tattttgatgaaattaagacttatcaaaggaaaaatcttggttttcgtaaacgaaattaatcgttgttatcgtcttaagctttaccttgagcagttcgg
tatgaaaagtttggttttgaactctgaattgccaataaattcaagaatgcatatcgttgatcaatacaataagggtctataccaaatcattatcgca
accgacgaaagcgataaagtagctgaaatcgacgaagaaaaagaagaacagcaggaaacaactgacggagctaatgagaaagaggactca
aaaatggaggaagactcacagaataatcaagattctggacaagacgccaaaaaaaccaaaacaaagaaaaagaaagctgtaaaaaaggatc
gtgagtatggtgttgttcgtggtctggactttgaaaatgtctcttgtgttctcaattttgatatgcccacaagcaccaaagcttacattcatcgtgtag
gaagaactgcaagagcaggaaaaccaggaacggctatgtctttctttgttcctaagtcagaggttggaaagcataagcctacaactctggttact
tgcaagaaagacgaatccgtattgaggcgattgaacaaaaaaggcattgagctcaagccttatacttttgataaaaaacaaactgatgcatttcg
ataccgtatggaagatgctcttcgatcagtgacttccgtagctgtgcaaacagcccgtactgctgaattgcgtcaggaaatgctagcttctgaaaa
attgaaatcatacttcgcggaaaatcctgatgaattattgaccttgactcacgatacggcagcaagcgttcgtttagcccgcactcaaaggcattt
acgacacgttccagaatatcttttgccaaaggggcttcaagctgttaacaaagacgttgggttcgtcccatataaaaagcaaaataaccgtcggg
ctgctaagaaaaagaagaacgcaaaacatcgtcatgatcctttgcgctcgatgagaagggttaaataaaatatttatgtaaaggacgaaaggct
cgtcatttttttgttatttcctttcttcattttattttgatcaattaatagggtcatgcggatatgggtcttaggaattgatcctttaggattgtttaatatt
taaaattgttttggaagcatttataatgtaatgaaaaccacttgtaacagtaagaatgatcatactattattgaggtcaaagataagtagtaacaa
acaatgattgttgatacaacacatattttgataatgattaattaaaagggtagtcttcagttcatatactaacctggtttaatgtagagttttcaagt
accttcaatttacccaactttataaaccaatatttgaagaaaagacttaattattttcacatttctatactttattcagttttggtattttagattacata
ttatgtaaaaagcgttagccctcacgacaaccaaaaagctgctccaaaccacaaccaagtaagttattctattgttctagatatcaagtcaaagtt
cttgatggattctctatcagaaagctcgacttcttcgtatcatggtaaaagaccaagatctttaagtgatgattcccaaaattcctctaacatggatg
atggttttcaaaaatctttaacacctgagaagtcaggagaaaatgctcaaaatccccctagtatttctcaacaaaaagaaataattgcagattttg
taaataatcaaccagatttagaggaaggccgtacagatttctatatattatcttacagttggtatgagaagctatgctcatacttgtctgaagaggg
gccatttcctggagagttggaccaagaagatattatgaatttagatgatggttccttgaaaagcaacttgcaagaagaaattgacttcaccattat
ttcaaaggaactatgggatttgctcactgaatggtacggtttaaaaggaaacgaattccctagggaaactgttaatcttggctctgaagattatcct
catttggtagttgaagtttaccctcctaatttttcgttgactttgttgagcttacaaagcgatccttatgaaacccataaaccgttaaagattacactt
tcttcgaaaagtacactgaatgacttatttgaaggagtaaagtacactttgtctatttctggtgataacttccgactttggagagttgacacagaac
taccacttcaacgaactattgatccatcttcttttataaagattagctcgaaagagattgtagaatccttgggaaaggaaaaaacattagtcgagt
ccgggatggatagtagttgtgcattagtcgttgaatgtatgtacaatgaaacatggcctgtcgaccgtgccttaagacttcaatttttgattcgtcag
agagaccaaaaatcaagtgtgtccttggaaaaccgggaaagtaaggtacttggaacgtgcggtttaaataacctaggaaatacttgctatatga
attctgcgttgcaatgtctcactcatactcgtgagcttcgtgactttttcctttcggatgagtggaaggatcaagtcaatgaatcaaatcctttgggta
tgagtggccaaattgctgctgcgtttgcatccttagttaagtctctgtataatcctgatcatacttcttttgctccccgacaattcaaagccataattg
gtcgctttaatcactcgttcttagggtatggccaacaggattcccaagaattcttagcttttctgttggatggtttacatgaagatttgaatcgtattt
atcaaaagccatatacctccaagcctgatttatatgagctggatgaagaaaagattcgaagtactgcagatgaatgttggagattgcataaactt
aggaatgattctattattgttgacctgtttcaagggatgtacagaagcaccctggtttgtcctgaatgcaagactgtttccattacttttgacccattc
atggatttgactcttcccttacccttacaacagttttggtctcatcctgttgtttttattccctctgatgttactcgccgtccgattactgttgacgttatt
ctcgaaaacaaatctgctacaatcggagatctcgtgcagtttgtcgcttccaaagttggttgtgataattataagagaatacttgtaaccgaaactt
atagaggaaggttttatcgattccttacctctctcactaaatctcttttaatggaaatttcagaagaggacgaaatttatctctacgagttggagaa
gcctttggatgaagcatcagatgaaataatagtgccggtttatcacacgataaatccttcgcctaaatcatcaaacagctatctcgataatcgtga
atttggttatccattcatcctccaattgaaagatgcagagattaataacgcgtctgagattgagaaaaaacttaaaacaaaatatagtcaatttac
gactcttgattccttgaaggatgccgaatgtttagagtctctgaattccaatttgaatgaaggtaaggaccttaagtctaatgaatcctccaaggat
aacgaaagaaagacacctttgtttaatactaaggtattccatgatcgttatgaacggataccaaccggttggaatatgcatcctacaaatcttcct
ctgcttacagaccgagatgtggaatctttacaagcgactgtagagccatcgattaatattgatccattcgatggtgatgaaggtgaaaaccaagc
caacgaggccgctcctggttcgtatcctgaccctccaaagattgagaaaactactcagaagaataccaatcttcttgtccaaggagaccttttggt
ttgtgagtggccggaagcttctcagcaccatgtgtttaacgagtcacctagttctcctgatatgggtcgttcattatggttagacatggaaagaatt
atgtctgagacaaaggaagaggacaatcgttcgcatacgataacccttgacgattgtctaaatgaatttgagaaaactgaacagctaggcgaag
aggatccttggtattgtcccaagtgtaaagatttccgtagagcatccaaagaaatgagaatatggaaatctcctgaaatcctaatctttcacttaa
agcgctttagtagtgagagacgattccgtgataagattgatgaattgattgaatttcctattgacaaccttgacttgtctaatcatacggaatcaca
aaaattcgtagatgcagatgaaaatggaaagtgtgtttatgaactatgcgctgttgataatcattatggtggcctaggcggtgggcattacacatc

tttcgctcgtaacccagatgacaatcagttttactgttatgatgactcgagagtttcacctgtgaagcctgaggaaactataacgtctgctgcctatt
tattattttatcgaagaaaatcgacataacttataattcaaaaatacttataccgttgattcaaacgttctttttctttatattatttctacaggctaaa
aagtgtttacttgtttgggtggatttatcaacgtcggttttctaatcttgtttaaaaaggtaataaaaaagtaaaattacatttattatagctcttttgt
tgtttatatatttcaattatacagcaatccagtacacagaatgtagcaaagtctctggatattattgatgtattgaaaggtttgaatcaatttccaga
tcaagtttatatagatgaattgagacaatttataggcattattgcaagtttcaagaacactaaatacgtgataattatcacatactttcaataagctt
gatcaatgccacatttgctaacgaacaaaagtaggaaaatagcaatcaagttcttttattttgtttataactttattccttcttttcttatagttttgta
tgacattagcttaaattagtaacaattattatagcttgcgtttcattgagttggcattagaaacatcaacacaacatattttccagcttagggtgaag
gcccaaagttgacacgaatttagagaaatttgagaatcgtttataaaaggattaataattaatttaaatattggcacaatggatatttgctttgacg
aaataaaaaaggaattccagattggaattataggatttggggatatgggtcgcctttacgcagaacgattcagtcaagctggatggatgtaagta
aaaaaaaaagaaacgttatgttaaaaattgattctacgtaatactcatgtttcatagtgttaatgtgtgtgatcggaaggaaaatttcgaagcagt
aaaggagaaatgcgatggaaccacaataaatgccctgcaagatgggtttcaggtttcaaggaagagtgattatattctatatagcgtcgagtctg
agtatcttgatcgcatcgttggtatgtacggaccagcaacgaaggtgggtgccattgtcggaggacaatcgtcctgcaaatctcctgaaattcgag
cttttgaaaagtaccttccgaaagatgtcgaaattatttcctgtcattcaatgcacggcccaagggtaaatcctcaatctcagccattagtgattatt
cgccatcgtgcaaaggataaaagcttcatgatcgtaagagaaattctttcttgtcttcaatcttcagttgtttttttatcagccgaagagcacgatcg
gatcactgcagatactcaggctgtaactcatgctgcatttctcgcaatgggtatggcatggcgtgcgaacaatcaatatccctgggaaataaatcg
ctggtgcggaggtattgaaaatataaaaatgaacttatctatgcgaatttattctagtaaatggcatgtttatgcgggtcttgctatttttaaccctg
atgctaggaaacaaattaagcaatacgcgacttcagtcactgatcttttcaagcttgctataagtggaaaggcacaagaatttgaaagtagagta
cgtacagctggaaaatttgtatttggcgaacaatttactgcctcctcgagtggattattgttgagtgatgagcttctggatcaatacagtatatctaa
tgttcctaatgacaagtccgtcggtaatagtcacctaagcattttagcaattgttgattcttggtggaaattaggaattcaccctcaaaagcatatg
atatgctcaactccattgtttcgactttgggttggcgtatcagaatatgttttctgcaatcccgaccttttgaccagctgcatctacacagctactaag
cacaatgaattctgtcctgatgatttggagtttgtaatttccgcaagatcatggtctgaacatgttgagcaaggtgactttgaaaattacaaaaaac
aatttataaaaattcaagattatttccgacctagatttgaggaggcaacaaaagtgggcaacgccatgataaaaaagcttcttgaaaacctcaaa
aaaacggcataagctgatttacataatattataagtcttcaatgttggttgttttataggtataaggaaaaaaaaaaaaaaaaaaaaaaaaaa
aaaaaaagaatgctcttaaactagctctgaaaaggttggacagtgaaacacatacaaatcgacagactttattcattccgctatggaaacattaa
gtattaggaagtaaacgatgatgaatataatttgtaattatcaaaaaaataaatataataaaggaaaagcgatgatgaacaatcacttcaacttt
ttataaaccaaaaatgggaaaggatttttgacataataagtaaataatgcagaagaaaacaatggaaatccaaagaggcaaataaaaccttttt
ttatataagacttagttttagtttaaataaaaaaacggagacagtatagaaaattgtaaatttactcggtctttttggagtctttatccatcaggacga
gcaaagccaaaacattctgcttcaactcatgggcatattttggatccttagaatatgattgtaatatcccataagccgaagacaaacgaattgtttc
acacaaaccggcaaacccgtgtttaatcattaaaatcgcaacaaatacagcttctccagtaactatgggataattgttaaccatagtgatggtagt
cagaacgctgtcttggaaaacagagatattatcgtgcaaaaatttggaaatttttgggcatgaattcggcaatattaaagcgtgtttaataaaagc
caacatagccctgttgctttcaagggcaagatttgtatcagggcttttcgtgcattcgcgtaatttttcaaagcgctcaggctcacttactaatttaca
aagaatgtaaggcatagaaagacgaatcatagaaacagaaagataatgcaattgggaatagctgctacgcaacatcttatcttgaagaagcaa
agaagtagcagcaaaatgggcccgggaggatagaggaatcataaaatgtcgtaaagcggcggaacaagcataaattttaccaacattatcatc
aatctttttctctttgagaatgcaagcaacaagaatttcagaaaaattctcgcgaaataaggcctttgagatatgatcgtttctggcaagatttcca
acgaaaagtgcggccatcgttatgcgaggggaatcattagagagtaaataggttttaagaagttctagaaaagaggcgtttttatgaagatcagt
agaaaagacaccatcgtgttcagcgatacggggaaacaaatttaaaataggaatttttgaggaaaatcacgttgcattaaaactaactccattg
agtcaattagaatggcaggattctttgcaaacgacacacaaatgcttggattgtcaaacgctttcgtaaagatagagtatgaatcgatttcttctcg
gatcatagaagggagaacctgtaacccggtgaaaaccaaagcagcatctttggaaaaaggatataacaactcacagatgacagaaagcatgtt
aatgaaaggagtacgtagcttaggctgccaatcaatccagtatgtaatcgcaagatgcagaatttcggtttcagcaacccttgctctatattctttt
gaatctaagatacagttatgaattacagcaaaagcaagagcagaattttcatctgcttttgcacaatacgtcttcaacacgtcaagacccccatca
ctaaaaaatgtagcacgattgtcatcttcctcgctacaagagtttgccaaaaatctcagacattgcttgtaaataccttcatgtagcgaaatgtctc
ttaacttattcaatgcctttggaatatactttcgaaattgagggcgttcattgggattctttgattgatctgctaacgtttgaaagaactgtgcatcat
caccagtcgtcattcttttagtaaggtagttctcaaactctaattactctaatttcaattttctgactggactttccaaaacagctaagcgattcctttc
tgttctttattgttaaaataaataaaaaaattcccttcaaatatcctttctggtcaagccagaccctcacgtctacaccttttatgttggttgatgatgg
aaacgaatcgacaactgatggaagggttaagcattccctccttctggcgcattaaattgctttacataatttagtgtcgctttgaaatttatttacta
caaattatacttatattttataatagaggtgaaaattatgagagtttagacatatataatatatatatacagtacagaatatccacaaatgcaagtt
cgtctgaatcctaattgttcactctaagtaggacgaaaaaaaaagacctgaatagaactctcaattatatatttaaaaatgagtatggaatctcgt
cgtggacgccctcctactcggagacagcatctattcacaaaagactgtaagtaaaaattgttcatgtatctatctacatgcaaagtgtatgcttaa

agccgcattggttatcctcacagctcacggctttctggtgcgatattatgtactctaggtgacaaaaacttctaactcataagcagtaaaatctctta
tgtatgcttttggagatgatataaatcctgctgccgacagtgtaaatgtcttggaagaaattgtcgttgattatataaatgaaatggtaagttactc
aacaatggcgtctgtattctattcaaagtgctaagatatgtacagtgtcttgaagcggctcgcatagctggcaatcgcaacaaagtaaaagtgga
tgactttaaatttgctcttcgaaacgatcctaaaaagcttggccgtgttgaagaattactggttcttcagaagatgattgcagatacgagaaatgtt
atgaagtacaacaaagaccatttttaaatctcaacatttttacctgatactacgcagagttggcatgtcatactttttctagatacttccttgtctga
ttttccttttgtttcgcttaagttttctgtataatatgttcaactcttacaaaacgtttcaattcagccaagtgctccgaatatgttcctctttttgtatat
atagtttaaattaatatttatggactaaatgaatacttttatattagaatataaagaagaaatatagttggtgatgagaaatttataaaatcatata
taatgtaacaagtcaacctggtttttgtaaaaaggaaatcaataattcatttcgaaccccatgatcacatatacaatataatcgtaacattaattta
aacagtcaagaaacgttttcattattttattgtagctagttatggacgagctgctattcttcctgatagggataagaaggcagactgttattactagg
ctccaattgagttcgtggaaccagctatactaaaagcctctcatttgcaaacgatattttcaataaattgctcagtatgtttcctcaatcaatgaaaa
tattgaccaaagtctacctataaaggtaaacagtattctcttgtgcaaaatcacaatggcttcaaaaaacgattagaaacaaatatagacttctt
gatccatgaaaggcccgcaagtgattttttccttcttttatagaatacgtgctaattcaacaagaaaaagcatgtcaaatttttcaagacaaaaaa
agtatgagcatgaagatctcataaactctaatatttacagttttatgtataacggtatttaatagtgattatgacaggaagaggtttggcacatcgg
aggatcgttccattttaaacattgttcaggctcatgcgtggttacctcgtgttcatcacaacagcagcattcaatttttttgtacgtagtccatacttag
agcctccaaagccaacattctttgttcaggaacaggattagctaaacaaaagaaaagacgctgcatagggtccggcaaatcgcaaacagatttt
aagtatggtaaagtcttggaaaccctaatattttgaacatcacatataagattatactcgcgccaagcctttagatacttattgtaaagctgatcgg
tcgtaacggcttttcggaaaggatatgtgcggaagtatatgttatgtaatataatggcgcacgaccacatatctgcatagaatgcattgtaagatc
caggagttaaaatttcaggtgccatgaacggatcacttccaaatacgccgttactttgggacaatgcagaaggaagcttttctgtgttgccaggat
cacataacacatcacaagctccaaaatcagtaattcgaagggcacctttacttgtaattaaaatattttcaggctttaaatctcgatgcgctattcct
tgagaatggatgtaagcgactcctcgtaaaatttgcttaatcatacattcaaaatcttttcggttcagacggtcatgacgatcgctaattaagctta
gcaaatcacctctgggacaaaaatccatcaaaatacaccatgtgtcactataaagaggaaatatatgagaatctaggcaaagatcataggcttt
cacaacattaggatgttccattctacattgcacaagccattcagaagttaattttgcaatataagagctttccgattcatcatcagcctttcgacgat
atgctttaattgcaaattttaatttcttttcattatctttcgggtttttcaaggttacgcaacggataactgcagtagcaccacttccgatatttttgttt
tcatcaagtaaaacatactttgagcttagacgaggaacgacgcgcttggcataaggtggcacatagtttgatcgtaacttcctaggtacgtgttca
atgctttcaggttttacgagcttataatttgacccctgacttgtttgtcccatgccagtaccttgattatcagaatctgcaggagcagaccctgacttg
taaccgggactaaagggattggcaggattaatacattcaaaacctgtcccttgtggtgtcatgttaaggctaccgctaacagcataagtaaaaca
gtcgaactcgggagaatgaggccaagattcataagaagtattatcgtaagggtttctctgtcggggcatgagtgatttcccaggacctgggcgat
gaccgttggaagtaacggaggattgagaggggctcttagaattagaacgaggagtgccgacaggggaggcattggaggtaggggaagcatgc
agaggagtgtcaaaaaaaccaccagcacgagaggtagatggcctggttctcccatttccacggatatctacaggtttcgaaggtgtgggaaactc
ggaggaatgagaaatcctgtttgtatcttctgcggagccaagtggagatctagacggtttcgtgtgaatggccgagctggcacgtttgggtacctc
ttgaccagtcgaaggagtgttgtttctgtttttttgtgtttgtttgcacataatgatgagagctacttttctgaataccttgggaaggttggggcgtcttc
tgatcaggagaagaaggatgagaggaagggtgtttatgaagaatatgctggttaatgaaggagccaatacctttcgaagggctttgcgatgattt
tctcctagaaggatgaatagccttttcaaataaagacttcattccagtttggtgttgcgagcttccagaagggttagcatgcacagggaccggtcc
gacaggattctgtgtttgctgatgtgtatctaagggttggttgggaggagattcacgaggagtttgtggaacatgagccgtagagtcggtcgtatg
gttgtagcgagtttcttgaggagtttgcttatcaaccgagcttgatcccatttgaccagagtttttccgacttaaaatgtttttttttatttttgagaatc
cttttcgcaaaggcgtaggtacaggggtgctagattgatttcgactgttcaaaatggattcagcatcggtaggttcaggaacgcttccctggctacc
caagccaccagacaaaggtttatctccagacattgataactaataatagaaataaaaaaagacagcaagtattaagcaagaatatccaaagga
aaagacttgatagaaagtagattaacgcacgggttgagcaggaagagctagaaaaaaggatgcaatctgggaaatgctataaccgttctattat
gaaatgggggttaaaatttctaaatgaaaaaaaaaaacaaaaggacaagctaaaatcaacaagaggctgccgctcaaaaagaaatagaaaa
cagagatgctttggactgcgggaagagtaagtaattaacaggaaaaaaataaaacaaacaaattgaaaacacaaaacagacaaggacaaga
gacgtttgtagagatttattcaagagaagacaacaaaaataaggagaaaagaacaagaggaaaatagaaaccgaaatctgactttggaaaaa
agtagactggaagatagagtaaataagaacttttggaggtttcgctgctagataagcaacatccaatgtactaattcctgaaagcttgagtaaag
tttccaaataaaaaacagatgattgtgttattcatgcaccaaagatgaataaaaagatgccgcaaatatacaatatatgtatatgcgtgttgatat
atatatatacttttacaatagattgcttaagtccttgaaaaacaagcgacagaaaagcaacacgaaaaagcacgaggaaagcctaatcagcaa
aaaggtagaagcgatgaagaaacgggagtgaaaaagaaaatcgtaaaagaagagcaaattcagcgtttcaatttgccagaagtccaacaaat
acggcaaaccttcaaagatctcagcaatttatggctcaataatcaacaataaggaaggaaaacactgacgacaaatgtgttggtagttttaaagt
gcattcttatgcaaacaccagataattagggttgacgtcttcttggtggaagcaaccaactaggaaagagtagcgttaagtaaggtacggtaagg
aaaaggaaggtagaaaacaggcaataaagaggaaatgaaggacagacagaaagagaatacagaaataaagttgaaaaacggaagttaaa

aaacagaaaatcgaaccgcgaaataaatatgcgaacgaaacaatgacgttatgatataaacaaaagcaaaaaggtcattgctggaggtaaatt
cattaagcttgccaattgaatagtgaaaaataaatttgaaagagatccacgaagtatataggaaaagcggtttgtaacaaggggtggctgaaaa
agtaagatgtgcaaattgacgtagagtgatgagagtgagatttctgggaaaaacgtaagaaaagcgagtggagagcaacaagagaggaaaa
ggaaacaaatagagaaggaaaggaggtgaaagcgaggaagcagactattgcactgtaaaaaattcgcaggaagtaagggttatagagtgatt
atttttggaggagaaagagaaaagaaggagaacgaaaaagaacggtagttggtggattgagctctttttttgttcctggagagaaaacgactag
gagttaactaagagataatctgatagcgatgagcaatccccaaaacaaacacccacaatctgatgataggtaaattcaggatgaaagagtaga
caaaaccaatgattcttttgttagttgattcatgactggaatcgcttctcgtttgtttcaatgaattgtttgtttactgggtagaaaccatcctgttctg
ctacaagagtatgtgacagagatggcatcgtattactccagtattgtaggtaacctctttctaaggaaaaatccatttggcttatatatttgtttgctt
gttcatcgggtagtacagttcaatgaaaaagggaaagaaaaaaaagagacagagcgaactacacacaagcgaaaaggaatcgacgaattctt
aactaaaacaaaaattatggtaatttcttgagattgggattggaagtgctggttcgtattggttgcatgctttttcgcatacctgaaccgttttagtct
gaagaaacatttggaagcaccttttttatttccattttacaattggtaattggtgacaaatgttataactatccttatcgttgtcatatccacgttttcta
ccaaatcgagggtttgtttacgatcccgaaagtatgtatctttcgactcgaatttctgttttcaaacattacttgtagtaatttgaaagaaaaactaa
ggagaaagagaaaaaaagcgatccaagaacgagagccgcatgttgattatttagacaaaaactttgatggcatcagctaaacactcaggcaaa
cacgtcttactcatttggtctttttcgtattagatgcgattcatccttcagtttttattttctgaaggctttttgttcatcgttcggcgaatccttgctgaa
gaattgccgtgcatacgtataaacaatgaaaatttgctttctagcccgggtttttcatgctttaagttctttctattaagtttatcttcttagccctttcg
aatgatcttacaattcattctgcctaatgataatacgacaccaaggaacctaccaccgagttctcccatactttttcaaacatttttcttttcttttcc
attttcatttatttttatttttatttccttctatagaccgtatgagcagtaggaaaaaaaaaagccacctacatcagctgaatccataccacctgttcc
caagctctattctctatagaacttgaaaccatctcaattgtttcgtatttttcatcggtaattatttataatctcattttaacttttctttttggatttactc
gctttcctagacttcttctattatttgacgcagttcatacttggctgtctcatacaataagtggtgtcttctcatctatcataattgtatgttatctagaa
aattttttttagaaataattaggtacgtttttgaattttgatggcctgaaaacaagcgatagtaaaagagcatgttcattggatttcttgcgagacg
caaaagtcttctaaacatataagaagcttttcatttatttccttctctttttcgcgtcatctgtccataggtccgtttcctgccggttcagtattcaaatt
ttggttttccaaggggggatactgtgtgctctgttgcgaaatctctttcaagatttatctctaacatcatttaatcctaggaactgtatatgatttctacg
aaaaaagcaaaatcgcgacctaaaccacccatggaccgatcggattctgttaaacgtcgggctgcaagatcagctcgtttttcttcggggactga
tttgaaattccaagctttgcaaaaaaaaaaggattaaagaacaagaagtatttgaagctcgacaatccaagcattggaaagaaagcactattttg
gtcggcacttgtactcaaatgtgtccagaatttgaagttgccgaaagaacgattcaaaagtccatccatccatatgaaagacatccagaaacaca
acagcctaatcccaattttactgtcaaagcataccatcgacctgctgctggaaaaggtcccattttaccttccgacgttcgacctccagccattttga
aaaaaacaactgaatacctttttcgagaaatattgggaaaacatcatctccctgaggctcacgcgtttgtgcgtgatcggactcgcgctgtacgtc
aggatttttctgttcaatcttcctttaccgacgaatcaatacattgtcatgaacttattgctcgatttcacatcgtttcgttgcatgaactgaaggatc
aaccggcatttagttgtcaacaagaaattgagcaattatcaaaaggtatgaaactataccctttttctttacttaactaaacaattcactagttctcta
tacccttgggcagttgtatgattacaattggttaaaaaaccgagttagtctgcatgaacccgagtttcgcgcgtatatggttttgctaagcttaggtg
atccagcaatatctatggagacgttaacgtggtctgcagagattttagaaaagccaattgtccgaacggcgttgacccttcattcttatgcacaaa
gaaataatcatactgagcaaaaatcaaaaaatattgatttgtctttgatatcttctattaacactgaaggtgctccgaatttctatacaaggtttttc
aaagttactaaaagttttaaaactacctttttgatggcttgcataatagatttgtttcttccttctatacgtaccggtgcgttgaaggcaatgaaaaa
gtcatatctttcagctcatgctaatattccttttactgacctctcgcgaattctttctgttccttatgatgaggatctcataaaaatttgtaagagtcat
ggtcttcaaattgcgtttaagggcgagcaaccggtgtcggtggctttgaataaaagaatattaatacatggtaagttgtgtcctattatgctttggtt
aacccgctgaatagatcctttaccaatggacaatcaattctcatatatcgctaataccaaacaccctcgcataccaatttcaaatatcatatgcgtt
gcaactgataatgtaaagctacagaggcgtcattcaaagaaatacaaaagaagtgctcttttaaacacgggtccaagttaggaagttttcatatt
caggtctccttgtttgttgtttacggttttaatctgggatatttaaattattattgaatttaaaccacgctttaagaacacatcaatttaatgggcattg
taaggcctagcaaaccaataaaaagtcctttctatgaagatgaagatgaaaatccaagtaataatcaaaggtaagacaacattattaaagagtg
tctaatgcttttgacgtatctattttatctactaaattcataatttgagaatgaactgcctccacgctagtagaagcatccaaggtgataaaagccat
gccttcttccatatattcgcgttcaattcgttcaaagtttttctcaacctttttctgtatttctactttttcgtaacgctcctctccatactgcccgcgtttt
gcagcaatctcaggacgaacatgcaaaaaaataactagatctggacgtggtaaaccacgatcagggctcttgcaccattcccaattgagtcccta
agagagttagtacaatgttttccatagggcgtacaattttggtagggcaacatactttggctgcagagaaagcaattccagaaaaggcataccga
tctaaaatgcacgttgttcctgattgaatttttgattggatatcttcaataacttcccatctgtttgcggagaaaagtaagtgaatgacttgatcgcta
agctgactagtttctttcaaatatgagtcaattttcttgccaatagcagtagttcgatctaaatattagcaaaaacttccatgtaaaattcaacttta
gagaaatatacctggaaatttatagagctggacccttttgccttttgcttgcaaatcttccagtaataattggcactgagtagattttcctgagcgat
ctaacccttcaataacaattaaagagcctctattttgaatactcatcaccaaaagtttaagtttgtaatgttagttattttgagacagtcagacccgt
aaggactatataataaaaaaaataaaaagaacttgaaaaagaacgtataggaataataatacaaagggtcgtagtaacctagattttagatga

gtttaaatagtagatcgtaaaaggtcacatggtaataaataaatcagaggatctgggatttgacacttaaaggtacgggtgcttatagaatacca
aaagttaaatataacgatttgattatatcatggatatgaagcatatactttgcagaagaggtttattcatgataaatcgttctaggatactgatcttt
atgacacccaaatctccacataattgggattatcttctatctttaaaagtcatatattccataaaaaaaacgtactactagtttgttacgacaattgt
caattcagatgctcaaacattcgatttcacaggtaggtcaaaatgaagcaagactataattcaattaattcttcactgccacgaagcgtaaacgac
gatatagtaagtaccaatctccagtttctgtgcaattcgcccatcgcaaggcttgataaacgcgctctcgaagagtttcctttaagtcaggaaatgc
atcgtaaaaggtatgcgcaaatgtgtccagccattggcgaacgtcgcgatctaaataagttggacgactgacgttttccatgaaggtgatttcaaa
cccagcttctttcagcattttcgtatatgctatttcatagttagtattgaaagacccagaaattaatatgaacgaaaagtcatagaagagtaaaagt
tatatcattaccttgctcagtgccaaaataccaagggtttgcttcatcgatctgttctttagggatgccgtggtccaatagtacagtatagatactag
taattacttctgaaacatttccaaatactaaaaacactctaattagtatgtggagtcgtgatcgaaaatcactcacctccgcattctgcaacaaagc
ggccacctggacgaagaacacgagctattccctgaaacacagtacctctttggttgggattccgcaaaatccaatgtagagcggcgttactaaag
acggcgtcaaacccttccgatggaaattcaattgcgcctgcaagattttctccaggaatcacatatgcttctaatcctttttcctttgcggattgaatc
attgatgaactggaatcaatcccaatgatgtgtttacactgtgatgccaaatctcgagttaagactccatcaccgcatcccaagtcaaggacaaca
tccgatgcagttaaagctagcttttggacgatctccttagtgagtttaggaacaaaagcagcgttttttggtacgcttcagctgacctaaaaacca
ttgttagaaactcttgcgccatttaattgctctgtttttaccaaaagtctcttcccattgtaggcctatacttgaggtgaatgtaagttacgttgagaa
tatgtgcgtaatgttttattcgagttcctttatggttatcaaaaccgagtataagcatttaagatcatgaacgataaagaagtgcaataagaatcct
taagaaaggcaagtcattagctgttagtatttgaaaagcaaaagtaaaaaatggaagagttgaaaagagagaaacatgctaataaataaaac
aaacttaaaactttataaatattggtattctctaagatggagacctggacttgtgcttgcaattattgaaccgggcaaattgttgattggtgacccat
ttctcagtaaatactgcttttcaatgctaaggcaccaaaaaaaatatccttttatattcacatagtgtataaccatctatagttcaccaatggcgctca
cgcttctgcaaaagaggaataaacaaaccgaacaactaagttatgtttacatatctgttactcaaaaagtaacggtaaataaactcatctggtac
tagcataagtcggttttaactgcagtgctttttgaattatgaaattactttctttttaagtcaattctcttatggagctatttgttatcgaacttctgctct
ataacccaaaatattacttgtattggcgagttttaaagatatagcattgctatcgacaagtaagcaaagcactattccaaattaaatgcattctgta
gtaaactttgagatattaaagtactaaaacatttttagtttttaccattcgattgccatgtccacaatcattgagaggataacgttgtatatattaa
aacaaagtttcaccagcgagatgttgataacgagagatgcattcctttcttccttatttcagtgagctatcacagagttatgatccaatttaaacaa
aaaaatccactagccaaacattctatggattcaagcatatctatgctggagtgtagtgcaagccccaacaaaaacaagtcattaaagaaagaat
aaatagatatgtgatcgttagaatccaagaaatcttgttattatgagctagtaaacaatagaacaattaatggtttccaaacgactttgtcctgtga
atgtatttcagcatcctactaacaattgaagacatttctcgaaatatattattgatcgtaatttcattacaatacaagcaacattcattgaactgctt
gcttctattcattatcacagtgcgttgtaaccttaagcgtatcttgacatatcataacaacattcatatcgtctagttcatcgtttaatggaaagacca
tttgaaaacaaacttgcatatgtgcagcgcagcggtttatcaaaattccgtttaaaaataatcccagtttcataaatatctcgtttctacagcaaaa
attcctagatacttaatttaaaacattttgaacaaattgtttcagcttatgttttaactcatgcttatgtggaggcggtgcgaccgagtttaaaggca
ccaaggattgatttaaaacagccggtttgcggaagtaaccattgcttgctgctgcacatagcatgtcagaagaaatagagcttgaagcaggcgca
tgctttccctcttgtttcttttccacctgctccggtgtcttctgacaacagtcatgattgtcaatagacgcaacagagagtatcgggctcaatggcctc
gaaatgcttttaacagagcaatgctgttttgcagaagtcaaagggtcttgttgcttggttatagctggataacgatcttttaaagaaggactcggtt
tcaaatttgacattcgtgctgcatgcatattttggtcaggctttttttccatcgataacattattccatgcacgtatagtttctaaaggcctttcatatttc
caacgcgtcggattattggagtccggttctaatcaccatttcacggtcagtacacaactactcctatgctttcttcttcaaagaaaaggtaagtgtct
tacggtaaatacttctccccgagtagtctcgcttcaaatattgtttacggattttcgtagattcacgatttacacgagcatacggctctaattctacta
atggaagggacggattatcgaattcacaagattcaggtcctgtcactctcatcattttcttctaaacaactctcttcaactttttttctaaaaattctac
aatgcaaagatgttggtagttggcaagttgaccttcgcgcgcgattcctttgtatacaagattcatgcgtcagccaatttttaaaacaaagaagca
gaattttgcaacttgatcagaaccaaagacatataaagtaacatttttatatatcaagtgaatggaataggatcctataagtttcctttttttttgac
gcgatttaaacttgagatttcatagtgaaacgaattgacaaggctttcaaaggacctgaaagaagccaaaaatctctccaaagaatcgttgtcttt
tttattaagaggaaggaaaaaatgatcagtggactcgcaagacatgtgcttcacgaatgcttatggacggttgacaccaacggacaaagacagg
gaagggaagcatttttcttctgactctttcttcagagaatttgggccacaaagattgaaatctaaatggaagattatatgcttgtatgaattacctgc
tcgtttacgtcaaagaacatctatgtgcgtcctccgtaagcgttcaattccacaagcgttcgattgttttacttactatggctcttctctactttgttca
gtttggcttctatgttttcatactttgttgataagttcgtcgctatgttattactaagtctgtcgtttacccttaacgcctctcacttcatcataacaaaa
aagcgagcaaaacagcataagcgctcatttgtatacatatacacttcaatttataatgtttttctttgtttttgctggatttgctcttgtagcgctcgta
gtaactattgttacgatgattcatagctatttcctaaaacgtcgacaaaaagctgctgggcaagccaaacccactccacgtggtagagccaccga
aactggcaatatgccgaatcgttcgccgggttctccattacagacagatactgtccctgggtttcctgctccgccttctgtacattattatgccacat
accgccatccacagaccaggacaacgacaaccgttactgaagaccaacctgatgaagttcctcctccatacacagcggctatccgagagaatgt
ttcggagacgtcggatcatacgcagagtccaagaggacaagaagaaaccactagcagaaataggcacgaaagacctcggtctcctcctccagt

ttatttgcccccagaggaaatggtttaaaccgcttcaacgatgaattttcctgaatctttacctttattcgattactggtacgggtattatatgtacga
agtctttacgttgtttctatatgaactgttaatttatttaattattttacaccagcaactctagctctacaatcttcgattaatagtatgaactcgagct
agagttgtttgttcctttttatgacagagttacagacgattcttaatgatatgaatattttgcgggtttttggtttttaatgaatttattaatgtatttatt
ttttccgcttttgatcaaaagacgtttgatttatttttttattttattctatttcatttatgcccgtctaatttagtacgactattatattatttaaatctattg
atcataatttaatttgaatcggcatcgagtagctacaagtaaaagtaataaaaggtttatcttcatttctattgtttttgcgcattcacttacggttatt
cttgaatagtcaacatttcacatatatttctaaactatagctctgtttttttctgaattataactgtaaactcgcgaaaaaacgtaatctcgatgatgc
gttaaagggaaatcacacgtctcaaaagtttcattgtcaacggagtaaaaggagacccatttcaaacggcttcctttgacttgtaagttttgcgcgt
atgtataggctcgaccaacgggatgtgaaagcaaaatttggccagctggagacattgaccatgtagccatgtaagcgacttcaccattactaaaa
gtaggattcgaagaagagggtgtaaaaaaagcagacgtgtgtcggccaaagctcctaacgatgtatcttggttccgtattggttcgaaaaaccat
gcaagtgccatctggatagaatcgaaggtaacgataataagagattaaatggacaggttgattccaactggtatcactaacgcctggtcgaaaa
tatctgcataccgaaatataacagccatcaaaccgaatccgtggtttttttaaatacatctccttccagcttcggtgatatacagacgaaaattcttc
ttctatgtcttcctttgtttgctgccactcggattgttggtaactgaaataacaaaattggcgataaagtgaatcgactcggcacgccttggcaaac
agtttacagctttgagctataagaggaagatacgacaagtcatttaggttttcaaagcattgttctactataagaagcaaaacctcgtctggaagt
ttgggtatgagtgatggtgtcacggagggttgagttaaatctttagagagttgatctgcttgactgctcttaatcgattcttcttcggaagctcttttct
gttccccgtcgctctgctctttttgttttggaacgccaaacgctctaatcgacgataaattacctccacatcctcgtgaactttatgagccaaacgat
acctgtttaaagaggtaccaatatcaccttcctttttcagattcaacagcttctttataatgacttaaagctttcttgagaatgccactgtcagggcaa
acttcaaggtcgttcaatagacgcttttcgtccatttaatcaaaaataaagccctaaaatatcaacaaaaaatcaagttacttgttcctcaactatg
tacaatcgtatattatacaactctctaaatacttaagcaatgctagaggattccatcccaaaacagttatttagtgattttcttatgtaaggttagttc
atgttagaaaaattgcttgcaatttctcttctaatgcaaaaaatcagatactggggtataatacaatgggagggaatgcaagtggcgctcaagaa
tgtgaatgtcaaagccataagctaacgaaatatatacctaatgtattttattaataatgttttactattttaaaaataaaaggcttcgacctctgtaa
gtgtcaaaggataaaattgacttctttcaaaaattgcttccaggaatgaaaccaagtacggtcactgtaactcattaaatagaagctgaatcactt
gggttacttgaaaaaatctagagaatattacggaccatgactgaaggctcgacacattttcagttttcgtctcttcgagttaagccgtaaagggcct
tgcaatttgattaaaacttatatacaatcaaaagaaacatgtttcattgtgtgtaatcttattattattgaacttgtatgagcatagatgaagcacta
ttcttcatgaaaaccttgttagctatatatattcgttacatacacatgttaaggttttctgctgtatgtataaacctttgtagctcatagttgacatcttt
aacagatctatataggagtatatgctataaacttctaaaaaatacgaacaccacttattaattgatattgctagaaccattcctttacctaggtaat
atacaatctatataatttatagaaactcgacgaatcaagccggttcgctaacatttgaaaattgtctttatactagaccaaacatatcttctgggct
ggcgaatgtgacatttcaattcagatccagcagtctaagctttagatcttttctttagcatgctttttcccttcgatcgttaaacaaactcgaaaactt
catgtcttccatgacacatgttaggaacccgttggaaaccaacaattccttcaaggctaatggaatcatattttgaaaaaagtatcctacattttgc
agcatcctgatctaactttccttcggtaattgaacgcattggcattctagctaactccacataatactgaggatttgtaggaggagcatacataaca
acagcctttactccacggatatcaaaacgacggaaatgatgagcacgctcagtgtataacatgatcttactctttgcctgataaaataaagtacga
gctctagtaagatcggatgtactagagtattcagaaatggcactgtaacttaccgcttcctcatccatatgatttcgtattctcacaaaatcaaagt
aggaaggaacatatactaaaatgccaccctggggacttttgacgagctgaggcaagactgagttgacaaagtaggagaaacgagcatcaggca
gctttataatagagtcactagatacacgaacataggtctgcttaattttgtaacctaattgattcaaaactccacggtgtaaagtacgaaacttgac
ttttcctgcaacgttcaacatgtaatgagaaaaaaaacgagttgatatccaaagcattgtattgagaaaacaaaagagtttgtcgcatatatttggc
ctgttgatccagataccatggtcgtacacggctgaaatcacaatcatgagcttctctgggcaagttattgatttcatcaaaaatacattctatatgct
cccaattttgcatgtctaaagaatcggcttgatcaataatcgaaacctcaatagagctcaaaaaatccaaatctcgtttcttatcacctttatttcca
attgccaatcgtaaacctaatggactagcaacaataatatcagagttgtaaaattgagaaaacagtttgatagttttccttgtaaacttgacgcca
atacgaaacatatcgtcagtattacctgcgaacaaataacggaaatcttcgggtttcttttcagagataacttccttctcaattgtgaaatcatcctc
aaagcgcttacgattagccacttgatcagctcctgagtaagatataagagtatttataaactctaaacagctatttcttgtaggtaaaagaaccaa
tacctttggtcgagtgtatccttgatctcgaaactctaactcagggttttgagatagtctggcactgttctttaagatgcggtctcttgtcttgaaaac
atgattgagagcatgcaaagcatacaaagaaacaataagtttatgactcttaatgttcatgttggtgaaaaacacatcttggtaattaaacatgtg
tttggcaatttccgtttgcatagtagttaaatgtccggatggtttatgcgacttataagcttccaagattcgctgttttagacccaaatcagaaacgtt
atcgctcttgggtatgcaataatttgaaactttctcttctatatctggtgtaaaaatttgagaaatcccgagttttgtttcctcagacgactgagacaa
cttcatggcctttatgcaagaaattgagtcattaatgactttcatatcttgagaatcaaaatgacgataaaaagggtcttgagatgattcattttcct
tttggtcatcaagtaaagagttcggattatccacaccctcacgaacaagagcatcaattatttccttttcttcattggtttcattttcttcttcgtttgtg
tctggctcatccaacttttcaatgactttgttctttttttctttatgctttggcaagtcgtcgccttcagaaaccttctcgggttttcttttctttttactatt
catcaagtataataaagcttcatatgattttccagccaattcatcttttgacatctcatcaatgttgccaaaacctgccattgtgatgatttatgaaa
agtaaaattcccttcaattttttttatctttttgaccacggttccaataaatgcaacgtttgagtaattttataaagtatttatttcatagagtccctgaa

atttaccttttgaaaggttagttcatctataggttttggttgaaattgagaaaggtagacgaaaagatcgtaaattaagaggtctaatttattacgt
ttctttttatatttttgagtaaaaaattagagagaaattggtaaaagactataattattaaaattaaatgtaactaaatgttacaagagactaaagt
cacccccaaggatactacttaagagacactctcctgacagaccctacaattagaaggagagaaaatgaacttttaacatcaaagctgtaaattca
aaggcggaaaaaaccctccaaaactacacaaaaaaacaacaaccgctggtcaactgagctgctgtattaaacaaaacgtgaatgagaggtaa
agccaatagtaaggtattaagtgagacttaaggtcactagaatgcataaggtttaatatccggcggccatataaaacgtaagcagattatggaat
gtctcataaatgatagtattagcatgaattcaaacaatcgttatacagttcagacaactgggaatagtaggacaggatttcatgaacagtaaagtt
gcatgattacaggtcaaacccatactgccgccacattccatattaacatatagtaatagtaatgaaagataaaggtaaaggtaagtcttaatcgt
ctaatagatttttaaatgtattaaggaatggtcgagccatcaatcactagagaaggccaaagctttactgattgtgtttaatagttacagtatcat
cattttgactagatggattagacaaatcggcttccaaacgcaaatcatattggctacttgagcgacgttgcatccagcgatttaggccgcccaaaa
agctattttggtgatttccagggccagggctaggcggtgcgcttgagccatgcaaaggggtatattgcaattggtaatcagtagtagccattgaaa
ggaaagattttatgtctgcctttaaatcacgactagtagatacccagtggttacgaattgtgtcacgtagcatcttgatatgatggcgaaagtccag
ctcattctttttgaacagagacacattcaaaacctttctatcaactcctcttgctaggttacgcataacatattgatcgtaatcacgaataattcgtgt
aattaaatcagaggtggaaacaccctccgtacgtttagtaggtatgaacttgcctactttttttaacgggaaggtagatatcaccactatcatcagat
gcgtaaggaatatcatcatgagcgacaaagtcaatttcatgttcttccaaaaattctggtgtaatcacccaaggagcgctttctacgacctcatcta
cccatttgcaatggcgaagtgcttcggctcgttctttgtcattcataacggttaaaccctttagtcgatgagttagcatatcgttgggaagtccaaca
atcaggtgaacgttgggaaaaactttttttgcttgctccaattgtctcatatggccaatgtgaaataagtcaaatacaccatctgcataaacacgca
cgggtctatcattcggcggttcattgatgggataattaaattgggaatattgttgttggaatttcacttcagtaggggtttcatcttcatcggtatcag
aaactggagaagaacgaggtgtttcctcttcattggactcttccttcaaattttcgcattcatgattactttctgacttttcttctggttccacaggatc
tattatggcctttttagcatgttgcttcatcgactcttcatcttctgaaaaatttacattctcaataggtcgctttgcattctcgtcctttttttgtttctatt
tctcccataataataataataatagtaaaacacgaatagtaaaattttactgaaggcgacctatatttgtttcaacaccttttcagaggtgattgtg
gtgaggaagttctcctcagtgatgaaaacgacgaaaaagggtctctacatatataaaaaaaaaaaaattgtggattgaacactttaatactatttc
aattaatatagttagagaagaagtatcataaaactgaagtgagacggtaaaaaaaaacaaaatttgatcattcttatatacacgaaaatcacac
ttattaaagtaaaagcattacatcttggccttggggtgaaagaaaaaggcatcagcaacaaggcgtctttgcacttttcctgtggcagtcttgggg
atctgatctgtaaagtaaaactttttgggaaccttaaattcagcgatcttttgaaccaaaaagttttgtagttccttgacggtaaccgacttaccaga
tgccgtatggatggcgctttgaatatcttgtccgtatttttcatcagggacagcaaagcaaacggcttcagcaacactaggatgctgcatcaagac
agcatcaatctcagctggagaaatcttttcacctccacgattgacaagttccttaatacgacctgtaatgaaaacgaagccttcctcatcaacttta
ccttcatcaccagtacggaagaagttttcttcggtaaaagaagacttgtttgcttctgggttgttaaggtatcccttagtcacatttgatccacggac
acaaatctctccctcagatccctgaggaacttccttacctttaggatcgagaatctttagttcaaccccaaatggcacaccaacggacttgggtttg
tggttaaggggaggaagtggattcgtggtcatttgatgggaagcttccgtcatggcgtacgcctcaagaacgggcgctttgaaggcattctcaagt
tttcgcaaaacaggagggggccaaaggagaagagcatgaacgaataaagcgaatatgaggaagtgggttgggaagaggagtgcgcaacaaaa
tttggtgaattgttggtacggcagtgtaccatgttgccccatattgaccaaactctttccagaatgtttgagcagaaaagcgaggagggacgacgg
cagagccaccagatgcaagtgtagaaagaagtccacacaggagaccatggacatggaagagaggcattaccacgtatgaagtatcacgtgaa
gaaaggcggtaagaagcactgatgttttgaatgcttcggcagaggtttcgatgggttaaaggaactactttgggacgtcctgtagtaccactagtg
tgcaaaacaagtgtgatatcgtcaggatgaggaagagcaaggggctgaggtgaagaagacttagacccttcaacacgaacaatcttcactaga
cgggattcgggacaccaagcaaattctgcgactgtaaccgaaagctttttggctgcccgcacagcaggagtattggcttcgatagagcccttgttg
accaaaacaagcttgctctttaggtcgtcaatataaaactcaaattccgattgcttgtaatttgagttaagcggtccacagacagctctttgccagg
aaacagcatagaagctgacgacaaattccaaaccattaggaactgcaatgtttacaggatcatttgccttaatacctaaactggctatttgtctttg
aagatccataatggcgactcggagctcactgtaagacaattctgccgaaaccgaaggagcgaccaaagccctggcgtgagcatctccacgcaa
agccgtgtagagtgtagcggtagacatgacagaaacaatctaaatttttatataataaacagaaacaagtcagttcagcggcaaccaacgaaag
agagttgaataaaaaagtaggttataaagtaagaaggaaactgaaatgatactgtatgttgaaagaggaaaaaccaagagagtatcgtttgac
tatgaatccaaatctcgcgcaaaataaagataaattttctcaatatactgtaaaagtattatagatgctttcttttgtccaaaaaaaaaccaacaacc
acaaaaaaatcgggttgttcttgtttagatgcttcaagtccgcagagatgaggtagttagtggttagaaaatgaatctctacgaataggctaaattg
ttacactcggtacggcacacctttgattcaccgtaaattgtgtactctaatgaattcctgcttcttatcttagctaaggtttctccataataagagaag
atagtcaagaaaaccagcaagcgagagcgtaaccaaattattttatgaaattagacgtttaataaaaaagacctttactaatctttcttgctttgt
cctttataaccttttattgagagaaaactggctttgcacaaaatttattgacaggacagtttcattgcttatattgtggtagagaaatcaaattacag
aaaggaattgtttcattcaacaaggagtagtatattcatatatgaaggtaaaaaagattcatttggaatttacaagtgcaacgattcaacttccttt
tactgaaaaaattcatgaaggttggtttgatttgtttataaaatataataaaagaaccgaataattcatacttgatttgtctgcaagggtgaaaag
acattcgttcctaaggctttatgaagaaaacttgaacgttttcatattatcccatcatccgtctctatacaaaacagagaagagtacgaatattcgg

attttccatgtttttctttttaaaaatgaatgtaagcaaaccatcgaatcctttccttggcagcattagagatcgatgactattgcagatcaaaaca
aaatctacaaaacacgaaaatcgaaaagaaatcagtagtaacatacaaaaccaatttctataagttacatataggttcagaatgagagaccaa
aagaaaaggaaagataaaacgtatcacaaatatatataaatatatatatatatatttatactcccaaaccatgattgatacagtaaacatttttat
ttcatcaaaaattggaaaaaggcgatcaacttttcatagccctcatcataacgctttttattgttttcgttagaaaggtcagcacgaccggacataa
agccatgatgcatatcatgataagtttggaaagaagattgtttataggcaggattgttcttgaattcttcttcgaaaccatcaatgatgttggcatcc
tcatctttagaagataagaaaaaaactggtacgtgaacattcctggcatcagcagggtcaatgaatgctgcgtgacaacaagcaatattaacaa
attcagaagtgtgaggataggtaactaaaagcttagcaccccagcagtagccaagtgctccaatttggacatcgttaccatgggtgctcttgatag
catccaaaaccttttctagattgggcaaatgatccgcaggagagacacgagttgaaaaaaactcgctacaaactttttttgctcggaagtaatag
gagggtaagattctacaggcaaaggagaacctttgaaaaagtcaggcaaataaacagtgaagccagcctgggctagcttgtcagcgccaatttt
gatttgctcagaaagtccaaaaacgtccaaaacggaaagaatgaccttttttgttggaaatgctgccaacggcataggtgtttaaacctccaaaat
ttatttctttaccttgaaaagtgtactttccgctaggaggaacagcaccaatgttagtagggcagcaggaagccattgtacaatagtaaaacgcga
tgattcaggaaatgtgctaaagacaatttacgcttgacctaaattcacatttatattgattagtcataattagtaattattacaatactgattagtca
taattgaaaagatcttgaaaaaggaacactttaaacgaagaaacccatacaattgatttttttatattatagtatattcaatcgtcttgtcttcataaa
gatgcttggtttcttttattttttttcaaagcctttacggtaaaataacgtatagcattgataaacaaacacggctgactacgttgtagctaagaataa
tcaattgaaacaagacaatcgaaacagtttatgaatctaatttttttcgtttttgcaatcggtcgattaggaagaatacatttcatttcctaaagtgtt
catatccatatactcactctgtctttctctgactctacttccgtatgcttgttcctatatcgtaaggagggatttcactggtaagaagtatctttacagg
ttctcatggaaagtatacttaagatatagccaggaaaaaactgacagtgaaatagaaaatggtcagcactcatagggcgattctgatgctcctgc
aaagataaataggaagagaaattggaaaaaccaggttggcaaaattagaaataatgatgagaaatgtttcgtttttatttatttattttttttttttg
gtcacgcagtaagaagttaaaaagagtttagattcctattgcgtttttttttattactattgctttgaccgacattctactggcgaagttacagtaaac
aaaaccaagagaagaatcagaaaaagcaaaagttgcaattctacatctctaaactcaacatatggtttttaaacataatgagtacagaccataa
tactaaacacttttaaaaatcaaatcaatcctcgaaattaaaaggcgtttacacaaactcgatttcaaaaacccttttatatttaaaaatcctttttt
tttcaaactccatgatgagctccatgatggaacaaaaagaactcccaaggcgagactcgaactcgcaaccttctgataaccttcttaagagtcag
acgctctaaaccgattgagctacccgggacttgcttataattaacactaagtgttagatatttcttcttgtaggttatttattttgaatagacactaga
gcgagaaaatgtatgtaaagacaagatgtgaacttttaattgtcagcattcaaatgatagtcaatgtaacaaaagaaaggcttgtcaactgtaaa
agcttttgtagtccatagatttgcttttgaaaggccatagtgaagtctcgacaaacgatgcttgcttcacttgttcatgctgtcaagatccatgggttt
ttcccaaggaggaaatccaacaggacaccgagcttcataaatgcgaataattgtatgtctttacaaaatcagtaagaataaacagatgaagaag
taataggtcgatgatttgtacttgaggaacataacttgtaagggtactatctaaaaagacagaaattagtcagttcgtttcattgtagcaaaactgt
tgcaagaataactcctcaaagatacatgaattcccctgtttcatagcaaaaggaaacgaaatgagagtcctaaaaccttaaagcaaagatgtttt
cctgatttcggaacccttattaattgtgggtccttgggttcgtaaagccaagagagggttccttggataagactatcaaattggtaggtagagcaa
gaaatactgtttcgttatgttctgtcaaacgaagggctcttctccctttgcatggcaactaaagccatgggtgttctatagtaacgaatttatgggat
aggaagatcataagagtaattgaaagacagtttttttttttttgatccaaccctcaagtcagaggaatttaaattttacaaaagcaccaagttgta
ctcgtcaatgacttgctactgattgactcaatcaccttcagtaaaaaaaaactcaatctcactgttagagacgtaattcaatttgttgtttcacgtcga
ttatcattctcttttttgcccaagtattcttccaatgtgcttctctatttccaatgcaatcaatgtactttcatttttatttttatttttttatcataaaaaga
aaatgagaagtggccatgcacttttgtttaaggctatttataaccaaatgtatacgtttcgaactgttacgtctttccaaaagcaaacactccacta
gttgtgttgacggcctagctactaaattcgataaagtacgtacatactataccttattatatatataaaaaaaaagtagcttatttaaactttataatg
agtctaatgacggagaaggttgtgttattgactggtgcttccaaaggtattgggctcgcaactgctcaggctcttcgaaacaaatccaaagtagtt
gctgttagtcgaagtttgacaaaagaattggaagctttgcttctacaacatccagattccttcgttcatgtgcagggtgatgttacaaagacagctc
agtctagtgtagaaacggctatttcaaagttcggccgtcttgatgccattgttttaaacgccggtcttgttaatcccatttcgaaaattgaagatgca
gatgtaaatcaatggcgcactttatttgaggtcaatttctttagcgtagtcgagacggtcaagcaagcgatccctcatctacgcaaaacaaaaggt
tctattgtcattgtatcttctggggccgctgttcgaccctatccagcttggacggcctactgcacctcaaaagctgctatcaacatgcttgttacgagt
cttggaagtgaggagccagatataatgagcgttgctgtacgccccggtgttgttgacactcccatgcaagaaatcattcgtgacgagcaaaacaa
atcagccatgggagatatgcatgattattttaaagaactcaagtcttccggcaagctggtgtcccctcatgacgttgcgaaagctcttacttatttg
gctatgaacccaagtgataagattactggtcaatttgttgagtggaagtcctttgttgaatgaatcgttccaacacgttcctattgacgattaagcta
gattatgattcttttatatctttctcaggtaatttgtgaatgaacactgttctacttctgttctttctggcttatcaaacaacactgatcccttaccaacc
ttaaaccaaaaaagcaaaacattacaagtctttacatggttcaaagctactcattgtaaaagtgttcaaactttacattcaacttctcgattccaaa
aaatttcaagcaaaggtttaattcgctttcactattaaattacgttttctttattcttcaaaatttttctgttaacatataatgacctgggaaaatctaa
cttgatataacgcttctttatagcaaacagtcaattttattcaaactccacgaaactagcaagcattgatatttattggaatttatcaagtatttctct
tttatttttattctttttactctaaaaaaccgatatttatacgattcaatacttaatcagtgaagaaatgcaagacatccaaaagtctagtattttcac

ccttccagacgatttactttcgtctatcgatctcaaaccagatctttcatctaatattgtgacacctgatgcaaatgaagaagcagaaaatgagaa
agaatctcggattactgattctacccaaaaaaatgttttgtcatgtaccatatgtggaatcaatgatctaagtaatttagataatcgaaagcaacat
gtgagatcagattggcaccgctttaatgtgaaacgtacaggggccggtctttcatctgtctctcaagatgaatttgagcaatttttagacaatctaa
ctgaatccatatctgggtcagaaagtgatgatagctcagacgaggaagaagtcaataaagcccagtgggaaaaggcatttcagaattctcttcg
aatcaaagagaaccctcagcaagaaaacgaagaattaacaggtcacaaaaaatcaccaattgtttggtttgaaataaaaaatcaagacccaac
aaacccgctgttccttggtatttaccgacacttatttccattagattccgccattacaaaagatcaacttttagcagtccaaaccgagaatcaaaag
aaaccatttcaagtagccatgttcatgcttggtggtggtcatttcgcagcaatgatagctaccaacgagccttcagcgaaaactcctcagtcttcta
ttattcccaaagtcttggcccaaaagactatccatcgatatacgacaagaaggaaacaaggtggatcccaaggtgccgcagataattcgaaagg
tgggatacattcagctggtagtagccttcgtcgatataacgaattagcattgattcgggatatccaacaactatttaaagagtggaaatctctactt
gacaagtgtgatttgatatttatccgagcaattggtgcatcaaatcgctccatattcttttctcatacagatcctttagttgatcctaaagacaatag
attaaggacatttccttttactaccaagcgtgctacacattctgaattgcttcgatgttataaagagttaattactcccaaaatcagtaatatcgaca
ctcaagcactggaaactcaccaacaggaacttaaaaaagagaatgaagcaaaaaaaattgcagagcaagaacttcgagaaaagcaggaaaa
cgagaaaaggatattggcaaagcatactgatgcattgcttactagtttgaaagcagataatctagaagaagttttaaagtatcttcgtgcaaatg
gtctatctatcaatttcagatttcaccctttgaatgttcatatccatacttcttcccctttacattatgctgttgctcaaggaaacacaaagctagtagc
gaaccttcttcgtgagggggcagaccctactatacaaaatggaaacggtaaaactccatatgaaattagctcgagcaatcgagaagtgcgagat
gaattttcaatagctcgacatgagcttggcgaaaatgtgtacgactgggagatggccaaagttggtacagcaaagtcacgagaccaggtagaaa
aactgagacaaaaagcgaaagctcgtttagaaagccaaaaagaagagcgtgaacaacaagaggcacaacgacgtcaggaagccataaaaa
aaattcaagaacaagagaagaaaggttacgattcccaatttggtaaaggacattccttgggaattaatcaattacgcaaagaagatgatctaaa
gtctctaaatccagaaatgcggcgaagaatcgagcgtgaaaaacgagcagcggcagctatgaagcggttgcaaaaacaataaagcaatggaa
agatagattttacaatgaatgatgattttatgaaaaagatactgttataattataaatgtatagaaagcaaagataagagagaataaaagttcac
atttctattatagggagggagaaagcacaaaaaattataaacctcaaaaaaaacccgattacattaatttacctaaacctgtttgttttgtttttct
tcttattagacttcttagaaggagcagcctttttgacagcgtcagctttagcctgtgttacatctgaatcgagagtaataccaagttgttcgagctttc
gtttctttaactgtaactttcggttatgacgagagaccagcttctttaccttttcaccggttaaaggtttctgatgctctaaacgtgcaatggtagcac
ggggaatacgtttgaaaggttgggaagcacctttgaacatttcttcgtgaatttgatcagccggaatcactttgcattgcaataatcgtccatataa
gaggtagttatgcatagtctcggcaacaacgttggccacatcaagagactcaaactcaatgaaagcatagtgtttgctattgccagtctattatgg
ttagcaagtgcccaactttgttatcatataacgtaaacaacgtaaaccataccttttactacgagataaacggagacgtagaacggtaccaaatt
gagagaaataacctttcagctgcttttcgtggaatccatggggaagacgaccaacgtataaaacacctggctgtttgttcaaacccttcttctctga
aaccttttttttatttttttctccttttcgtctcctaatgccataattggctttgaagcgccattcgaagtatctatttccacatcgctttcctcatcctcgt
ccccagaagattcaaatcccttcaaggtttctacgttctcattttctccatctacagattcctgctcaacacttccgtgagaaggagtttgctctgcct
tctcagcattatccaccttcttgtcgtgaagagttgtttttaactccttctgttgctctattcatcaattgcgttaatatcgttaatcgtgcaaaacaac
ctttttgcttactcttcgtagcccttgtaggctgctttgttttcgacatttcttcttttaatggtgttggttctaaactgaattttttttcagtaaggacatac
cctccttcaaataacggacttctttatcatacgccaaattacgaagagtctcattaaagttaactatgatacactgggatacgctatatcacatcctt
actcttgagaagcatacctaaataaaattgaaatcggtaggtttgccttttcaatttactcagtaatcgactgcttctctatttaattattttaagactt
ctatttgaagttatttatcattgctaatcgatatctacataattcattcaacgctattttttttataaaaacctgaaaaacaagacgggaatcaaaa
gaactaaagcagtgaaaacactcacaccttttctaataaaaaaaggtaccaaaaagacaaagaataattaaacgaaagaatgaatgcatctaa
gaacaaaagggatgtaaatcattttacaaaagacccttcttgacagctacttcggcattcaaaataccagcaccggcagcacctaatacagtgtt
gtgaacaagagagacaaacttgacgtcaaaaatagaatcagaacggatgcgaccaacggagacagcgtatccttgctggttttcacgatctaaa
cggggttgaggacgatcaggacaagagtcatcaaagacaacaatagcttgcttaggagcgctatggcagtcaagcttttgagcttcagacacat
actcattaagagccttatggacatcagaaacagaaggagtggcgttagcaaatttgacagagatgcacatcaagtgaccatctataacaggcac
acggttacactgagcagagacaacagtgtccgttaattcaaaagcagagacgctttcattaacaccacctaaaattttacgggtttcccattcaat
cttgtcttcctcaccactgatatagggaacaacattatccaacatatcgaggctggaaacaccggggtagccagcaccagaaatggcttgcatac
ttactaccgataccttatcaatggggccgaaagcatcttgaagagccttaagaggcacgacgactgctgcagtggaacaattcgagttggtaatg
atgcaaccatggtctaagttgttttccctgcgctgagatttaagaacatccaaatgttcagtgttgacagtgggaacgactaaaggaacatttgga
gttctacgatagttcttggcattggaaacgacagccagatttgcatcacggaaattcttttcaatttcaccagcaaaatcagagtccaaaccactg
aaaacaatgtcacattcagtgaattctttaacttcgcagggtttgacagtcatgtcagcaaccatcttgggcataggcgaggtttgcttccatttggt
tgcctctttgtacaatttgccagcggagcgagcagacgcaccaaggactgcaatcttgaaattaggatgatcagcaaggagggttaaaaatcttt
gaccaacagtacctataaacaattagcaattgaatctttctgacagttggtaaatcttttagcgaattctccttctcaaaatacaggttcagacaga
aagcaagtcataaatttcctttcatcttgtacaattatctttttttgcctctataggcgtgctttcttgctgtaacaagcaattttatgcaatttatatagc

gagaatgagaatcgccttggatcaaacagcagccaaccctatcaatcataagcaattgaacttaccagtagcaccgagaactcctactttcttaa
caaccatttttctgtctgtgaatatttgatgagtatatgttctgagcaggtttcacaagagctgagaaattatttcaatatcgagatggaatcaaag
atcaattacaaatcgttacatcgcggtaagatattttagcgaatgcttaaacgaacgttagtaaacaagacaatgtttattaggatgaaaaatata
aataaacaactaaacgaaatgaaatgaaatgaaactcgaaaagctttctatttcaacgaggaaaaattgtagctgttatagcgcacagtagaaa
agaccatttctgactggcaattaccttttcgatcgtcggcatagtaggtgccatcgtaggcaaattttctgggtatgtttttttagagaagatgcagg
taggactcttttgtatcatccttttaatataaacttttataagtaagatttcgaaccaagaatgatatctgttagaaacagttagcatggatgattac
ttggaatcttcataagcgtgattgggtattccgttaaataaaacaaagactaaagtacaatatcaaaggaagagaagatgtagtatttaatgttca
agtctcatttccgctcgtggatcgtaagactcgttcatttcggtgtgatctaccgattgctcatccatggaatcgtcatcgtcaccgtcactagtctga
gtttcccttcttctgattcctggctgcgggaatctatccgcaggcgatttaatgaaatccaactttaactctcgagcagcacgcaggttttcctataa
aagaaagttagtagttgcataacgaagctattgacagtaacatacacgattataaatttcttcgttctcaacacgcaaaacattccacatgcatcg
tcgaaaaacttcggcaagcgtgaagaaaaaaactgagacctgaagaatgctgaatatgattaggaaagatagagtagaagatgaaatagtttcg
aatcacgaagttgattatcatcgctaaaacataaggccatatatttttaataaacgatgcttacgaaattcccagcgatgatttttgcggataaaa
agagaccaatccatggttatatcccaagtgtatgaaaagagagaattaattgcggcaactactgtataacaaatttgatactttaaaccagcattt
cgtctccacaagctcaataacatttgtgtacaagctgtaaacatatacttgagagcgttgaccaaatgaggaaacatctgcaaggagtcggcata
tcttcgaaagcattgaaaggtacgtaaaattgccggaaatgccgtaaaaaacccaagaagaggggaatgtgatgaattgcaaagataaggttc
attccagtgccttttatataaacagaaaaaaagagaaacgcatccatttgtataaatcagacttaccatttggtcagcgaaaaaaaaaatcctgg
aagtggacactagaaagcccagataacaagacccgaataaactgagtaaagaaatattttcttgtccgccaatacggaacaaaccgaggaaaa
atgcaaatcacgcctgccacccctaaataaagagccggaaaataattcgtaaatcctggaaaaaaaatctcgcatgcaaagccaaaagaaaag
tgcataaataaaaaacaggagcgagtaaatctccatatgctgcctccaatttaaagtttctctctgattaaattcgaaaattaggatatagttaact
ctggttttctcccaataataacaatctaatgaaaacaaaacaaaacttaagataagtaataaaaatccaccccataattgaagaagatactgatg
ctgttcatcacctttgtaataacaaactgcttctatagctaaaggaattcctgctccataaaggattccgaatcgcatcgtattaaatgagtaatcct
gaaaaagtttagtatacatgctgaatttatattagatttccacgcctatgtcgtacctgtttttgacgtaaagatcttagttgttcaacagcaatcttt
ctgtctccctttgagaaaagattggcatatgtgtcttcaatcttactttgccattgctcggttacagaggtatttgaaaaagaaaaattggaaaggt
atttcatccatcttgtttgtaaatctgtatgaaaaatcttatcatactttttacaatctttcgaaaagcttgttcattcaagctttggtactgtataatc
agttttaaataatcgtaaaaatccagaagtacctacagtgttagaaaattctgctttttattataaaaaagcttaccatttttagtttagccttcaat
ttacgatagctcacaccaggagtatatgcatctgtcggtactccctttcgagcaggaatatgaaagactggctgaaatctagagaatctccgaag
aggttccaaatactgccgaagacttgactgatatttctgtactattacatttgtttcgttctcattctcgttttcgttctctttcttgctgtttttagctttt
ggtaaatatggatttgatcgtaaatcaggtctaagcgttctcgagcctcatttgtcaaggattcgtagaaactttgaattttcgctaactgttgatca
agaaattcttgaaaaatgggatgaaacttaaagtcagccgctaagcatgattagaatggactctggtataagcaaaatatagtgttctcaccaag
cctgcaaatcccattagcaaacaagaaagtataaaaatctataaaacactacctttggattgtcttttcataaaaatcggtgctatctgcagatag
accactcggctttgcaggcaatccttcaccggggtttttcagaatcgacccgattatctttttccttttttataatcaaaataagtagatgcccattct
tagtttgttagtttcgaattgaaccatcattgccaataagcactttaccttctactaattcgttttcaataaccctgttgttgttaatagctgtttcatat
gaattcagtttatttactttccaaatttcattctactaagatttcaacgacattggacatatgagtaaaggaggaaagagacctgtcctttcttcagc
caaaattaacttcttaacggtgaatgatagctttttacgcttggataaacgtttaaaaggagaaaaaatgaagcttaaaacctgaattaaagaat
aataacttttctatagagtcttgcagtagtgaagccaaaggtcataagagggtaaactctgaaaaacaccatattcaaagttatataagctatata
ataatattgaagtaaatgaaaatggaaagagattcttgtgctcaatattgtctaaaatcaccatttatgtgtattctcaagggaattcgtcaactga
atcgattactgtgaaatttgaaaagacctaaggaaagaagaagaaaagaattggtttggaaaagatatctcctgtcccactatgaaaaatatga
aactctgcagaatgtaaacaatctatgggaggcctttcctatcaaaagaattgggtcattgaaaatagatggagcacaagttaaagcaaagcat
accatggtgagtagaagcgcatctaggttttatgaataattgttactggagcttcatatgtcggattcatgatatgtaagctatggcttcaatgtcac
caaagtaggacctccatgggtcctacaaagaagtctatgctctgacttgacgaaggagtgcattcttcccgacttctttttacatttatcaaacgtg
aatgagccatcgttgactgtaatgaaaatatttccaggggcattcaattgatgcaaagggaagtcaaacctagcaaagactcaaggaagttacc
agtaagacctttcttttcaagcagaataaagatcattcagtatctccacaagaaaagcctctctagtaatcattttgtagtcgacatttgcttatcat
gggactcacgaaaacatcctttttttaataaaaataattttgaaaatagtttattttgtagaagagcaaggcttgtggcttcctccctaatgcttgct
gcttaagcaatgaatttctccttccacgaaccacgaactggctggtagaaaagaaaagcccgttgttgtcaggttttacgatgaatccatcccggg
atccttctttactccctaagacgcctcttccagcaggcttattacctcccggctataagtttcgagtggaaaagttcgatgttactatagtacgatac
atcgctgaaggtaattgcttcattcaatacgggatttttgcttctaaccatcatgattaggtggcttctctcatgtttatctcgtaaaaatgtatgcgc
ctggtaaggctactttggaagctgtgctaaaacgcatatttgctacggatgctcttgcttttaaggctgtttatgaagaagtcaatacaatggtaat
atcattataagcagtaaatgaaaactaatccctccctgactagaaactggtttcgaatcataaacgatgcgtttcttactacgggtctgaatttttct

ccgtcggaaagagccagtatgagtctcttgtattattagagtactgctctggaggaggcttaattgacttcatgaatagtaggctgcaaattcgatt
gactgagtcagaaatattgaaaattgcttctcatgtcaccgaagctgtggcttcaatgcattatcttcgtcctccattaatacacagagatctaaaa
atcgaaaatgttttgctagcagctccaaactcctataaactttgcgattttggtagtgcttgtcatccgatttcgagccctaaaacagctactgaaat
tcggcaagtcgagtataatgttgagaaatatacaacctggcaatatcgatgtcctgaaatgcttgatttgcgtcgaggatttggaattgatgaaaa
aagtggtaagtacaaactttgaaattatattgcaagattatctaatgctttttatagatatatgggctttaggtattttattttacaaattgtgctatta
tacgactcctttcgaacagcaaggtcctcttgctattttaaacgtgaactacatgttccctcctttgccttcctattctactcgcttaaaacgtttaata
tctgctttactccaacaaaatccatggcagcggccaaacatatatcaaacattggctgagatttgtcatatgcaaaatatgcccattccaataagg
gatatttatagtggtaaaaattcgtcaagttgtaatccttctggttctgaatatctgcaaatgacttcaaatctggaaaatggtggactgcaccta
gcgagtcgagtggttcaatacacacaagattaccgcctatggcccctcacggccttcccaatgctgcacctgtcccaataccttctaactcagaac
taaactcaggaaagccacccttctcatttgcaaaaccaatacaggtaggggcatcactcccaaatgttccacctaaaccatttcaacccgctcttg
gtaaaaacgtttcatctatttatcctgacagaaattttcacttatcacctacaaacgcgcaggaacttacggatgttaaagctgccaatcctcctaa
agcgactttggatatagcagataatgtgttttcttccacgtcccgtaagcctacggaaaagaaagaaaaagaaaaggatataggtgaaacaatg
ttgaaaaatccaattgatacccagaagtcttctgggttggtatcttctttaccggtttcaaatatagctagatctgagtctccaccaaaggtaaactc
acttatcagcaaatggaacaatgtttcacgtccacagtcattaccaaacttacaaggtcggcgatccgtcccacaagttcctcgtaaacctggtttt
ttatgcggaagtagtcggtcctcaatgagtacaccgactcctcaatcttctgtgccagtaaacctacttattgagcctaaaggacataaaaaaata
tctttcgaacctaaaacgcaattgcaaaaaggaaatattagtgataagggtttagaaaagtccgacgaaaagtttgcgtctaccagaaacgaag
aggagactgatcctccattcgtaagtatcacagaaagaatgaatcaattattagcaggtaaagagtatcaaaaggcatctgatgacggatatag
aaaatacacggatcctaataaaactactaaataatgattttgtcacgatttgtttactgaaaaggataacaaaaagtaaaaatgttatctttatttt
atgaataaaattcacgttctttattttaatttacacacatcatctattagcatatagattgttattgtttccatcaatgtttatttattttggttatatagt
taaaaatctgcaaacaggcaaataggacttataattagcctaaaaccacgtaaatataatgaagattctaaagaaattgcctctatgctttacaa
ataaaagcgagtcagtgatatctgagcaggaaagcaacagtgaaacttgttcattgggaagttcaaaatctctctggggactatctgaaaccga
aacagttacagacgggttttataatggtgaaaaggatacaggcttcggaaaaacaaagcgtttttcttcaacaatagaatggagagatccgtttt
atactggccaaagagaaaaaacactattcccaataactaagagtccaattaggaagtcaaaacctcaacttttagacttatcgagaaatccattt
gtagacacggaaaatttaaaactcgcaattaccttacctgaaggacctttgcacgccggcacgtttattaaaggacacgtttgggtatcttatgaa
ccgcttcgcaatcaagatgattctatctgcctaacacaactctatgttgatttatttggtacattaaaacttaaatctgcaatagagcccatcttttct
gtgtccgaaaagtactctcttcagcatgcattacctgcaactccaacaaacctaggtgcattttcctcaaatgaattcggattcttgctgaaggagc
cttgtaaattcagctttccttttgcgtttgttgttcctttagatttgggacctggatcgttcagaacgcaaaaattacaattttcttattccttttctgcgt
aagcaaagcttatatcaaaatttgatactaacaggagcagaactttatttttctcttctctttcaggcgaagctcaatttactagaacatccattgaa
aaaacaatactcccatccatgaacgaaaatgttgcttcaatcaataataatatcgtttgtgaaaacacagtttattcaaaaaaactcgacacccct
aaaatttcactcagaatatcaatatccagatcattgtttttttagtgggcaagacgttgggctaaatatacagtataatagcaaatcgcaatccatcg
tatgctattctgtctttttattccctttatactaaccatccaggtccggtatattaacgtttgtttgctagagtctattcaagtgttgaaaaccgactca
agccaataccacgcttcacaagcagcaccaaaaaagaagacaggaaaggttgtttcaaagtggaaagtaaatcctcaagatactggatatcat
atttaccctcaattgggaaaaatatacattttcctaaatttaccagggtcctgtagaaccatagaaacaaacgcccaggtccggatatcttacaca
ataaaggtttctttaaaaacggttttacattcaaagtaagttcaaaactagtccttgatattgcgaagctaacaaacgttaagattgacagaggctt
atcttccaataactatattacatgcgcataaatgatatgaatatgaaagcaaatttcataataaaatttacttaatggaatcgtttaatgcaatcatt
gaacctacttggcttgattaggctgcaaatcaaaaggaatcaaatcaggaagaagagttagagaagactttactgaagccattccctacaggca
tattgtacttcaagtttaaaaaaccaaaatgaaagaagtccatatgcaaagcataaatataatagaaaataaacccaacgtgcagagattatag
aatgaaataaaataaagatacttgtaatagaagataaaagtaaaaatgtaaccacaaaaaaaaaaggaattaacgaaaagagcacacatatta
aaaataagataagtcaaagataataaaatagataataacttaataaaagccctgagttgccagatatgaatacatctttaagactttcgtctgct
aaaaagtctacgaaacaagttcatttttttgggttttgattgttctttggccgtcaagggaagagatgtttgtgactgggtatgtgctggataagggc
gggtattcaaaccaccattccgtctctttggtggaggaggcgccttgtttgatgagggttttgtttcatgacttcgtgattgggaaatactatcatca
ctgtcatactcgtttgcattggaaatagggttcagagctcggggagatgttataggagatttcctaacttccagagcagaccttgaacggtaattac
tgggcaaggtagaggaacgagaaaggttgcctgaacggcctaagttaagttttcgcattaaagagttctttctgcgaagacctgccctttccatgg
cctgtatctcttcattgcgagctatagcacttttagaagcaagggcgccttcaatacgggtcgaatctgttaagccaggggcctcagaggtaccag
cgggaatcggatcatcttcgtcataagacatagtgctgaaaggagactgaaggatttttttcgtacggagggtatcatggatggcctttctactaa
aatcctttggagcagaaggatcattaaaattagaacccactttgggaatggaattggcgacgctatctttcggaaaagcagccactggagcttttt
tcacattacgattgcggtgaggtcggcaagcatcaaagggaccgtcatgatggaaacttcctgagccatatattcccgttacatctaaacgatcaa
tctggtctaacggctccttcgtaaagtttttgtttcgttggccttcacgaaccgttttatgtcgacgaattttacccttggcgcgggaattaccagattg

aatggatggagaatactcattggaaggagaccggtagtggccactggaggatcctacatgagtgctacgagcacgcgacaatcggtacgaagg
aggtcggtcagcactcatggaagagggaggtcgagtagtgctcatagatgaatgaggacggaaatctaatagatcatggcttgtctttacggggc
tagaagatgttgtagaaattggtgaacgcacgggtttcttttgcaaactactgaactgcaacaagtagttagtatacatcatgtaaacacaagatg
agagaagcttgagttttgtatatataaactgatgcctactggtcatatcgagtttataatattcctgtttgtttcatttcaacagttttaccatatcatg
ttctacccacctcaattcctcttctattcaaagacttaccaaatcatctgccaaagactccatataaggatcgttcgtgcgaaccaaatttggagac
aaaaacggattcttgctgttataggtctccatgtttgatggatcaacaggcgactcttgtgtggacgattgacccaaatttgcattcctgagaaatg
gattctcagtcgccatcatcccgtgctccttccagaaaaataaattacgagttaaaagattgccaacttatgaaatgagcgtttaacttgtgctatt
attgtaatttcccctgattctaggatatatgttcttgtgaactaacatatgtgggaggtgagactccgtttttacatttttcgttatttgatgttaagtg
gaataacgagtttcaaagatatcgctaaatagttcattttataatataggctggttgggagtccagagcaattggtagagtacgtttggtatcgtca
ttaacaaaaagaggttgatacagtctatgttagtaattccaatagccttcaggtttattcatgaatgagaaaagaagctaaggattcttaatctata
ttttccggctagatgtgctaacaacttatgaatacagaagtcttttataaacatgccatgttatttagcatcggagaacagttgaagtaatgcaaag
gaacattgacataatatcagtacaagtcgtctgttgaaatataatcattattcatcgtaaattttagtttttcatttatagaatgacaaacaactctta
cttaagagcatcgtattccaagtgaagaaggtacaagaaaatgtgatgcacgctcatcgtgtttgcaatcctgtgaaatccagcattcaaagttgc
gtatctttagtataaaatgtagagaacaccaatcagcgaaagcccaattattatagaaacccaaaatgaaacgtttccgagcaatcgatgcttgc
gaatatacttctgtcgttggatcaacatgtaaggaatttgagtcatttgaaaaaataagccataacctcgaattttctttgtgatgcatcccattact
agctcatgcaaaatagaggagaccaaaaaagttagcgtagttgacgtaaatttggaaaatttattattaaaaggacagtaaacgtgtcgaagca
aaaaatagtggactggcttattccatgttctagcaaattgatcccaagtccaacagttccaccaatcgtcgtaaaaattcctgtcagcgaaacgag
tgatttcagcactgaagttacagatgccttcaaaaataacccaaaatactataaggaagctcagcatgaaaggaaacattagataggtgacagt
cttgcctaattgaagcacaaagtagtaggtagatgcgtcttctggagcttctaaaatccattttatagcctgaagtaagactggaatcatgtagttt
tccgatatgactactaataagaaaatgcaaccgaaagtcccgagtgcacattcaagtaaatatgtaactcgaatatggggcgttctaggataata
gagctgataacataagataggcataaataaaaacgttagtgcattgctaaatgtcaagttattcggatagcgttttccatgctcaactatcgactct
tcacacagcttcaagttttgcttttctggttccgaaagagaatctgacttagctcgaagtctgcgggcttgataatagcaatacgaaaaccaaccgt
ttaccgtgttgtatgaatgaattttcattaaaatgtccatagaatgtaggataaagacagcacgatgagtccagggccagtcacttaagcagcatc
gcaatacactatgcatgagaaatgccaaaaccaaagcactgtaaagaaaaatacccgaaccataccagcgtagagtacctgaagaaaacagtt
tctggaatggaaatgaaacaaaaaacaaagaagacataaataagtctgcctttgccagatccaataaattcatcttaaagaattggatgatattg
tttccaaagagcttacctgtttttccatattgtccaaagaatgtattaaaatcattgtgccaacagatacccagaaaagaacaaagaatcctgta
aagtctacattactacgctgatgcgtgcgggcaaaaatggaaggagtaggactgtagtctaaagggcggtaagctaccttgcagtgtcgatggtc
tatatttttagtctcagatatcccaaagtccttatctaagtgattcgaggaagactcgcttcctgctggagttgaagcaatcatgatgaagaatcag
cgaaatagtgaaaagtataagttgaaagcagataaaaccaaaaaaaaatcaaataattctaaagttagaaatggatattgatgcaaatataat
acgtgagcccaatattaatatactaatgctgacgttatttgtagaacactttgacttccgaaaatttttccgtccaaagcgacacctagacagtcga
agatacagttgctttcactagcttctttagaataagcgaattttctagagaaaaactgagtaatgaaaacacggttagacaagtcaatttggatat
ctcgaaagagtaatgtagtctatcgcgaagaaaaaactattttagagacccttttagcataaaaagaaaaatcgaaataagcaaaaaattatta
ggaggttgttattgagagatgtgttgtcgacaagtgtcttcgtaataattataacatcgctatgacattttcaacgaagccctacaaaaaggcaga
aatgtctttaacacagcgctcgtaaagaaagaaaaatcgaatacttacaattgtcaaatacttttctcctaaactgacgaaaatactttgttcacct
aatagttactttacatcgttgcgaatgaaatatgcagaaaacttttaaaacattaaaagcaggaaatttggtaggagagtaatacacaatgattt
aaaatggtaagtattctcgtcaaggttgaatcttaggaaacaactgcagaaactcggctactaaccatagtagcattgtaagatgtcaggattcta
gctataattttacgttacattaattgatgctataatacgggcatgtggtgtagatggttatcacgcttccttagcatggaagaggtcccagattcgag
ttctggcttgtccaatttttttctaaaaaatacaaatttatgtaagttataattactcttttttttgttgataccaacaaaataagtaaaggaattaata
aaaaatactttaagtagctacaattcactatacatgataaacaatcgactttagaatttccttcatacaaaatagcaagtgcaatcaatacaggag
gatagagacaaaaatagatattgcttgttcttgagggatccaccttgctaactcaaaagtagatgtttgttaaacgggccctttgtttatggtcgctt
ttcattttaggattcttaaaaccttacaacaattgatgaaaagaaacaagtataagaaattttagtaatatacatacgaaatgaatagtttctataa
atatgattaacgttttatctgaaaacactaacaaataagttataaattctattgacttgttaaagatgaatttaacatatataaagtaagcgaaaat
aaataactgtattgaccaaataatttgttccaaacaatgatataatgtttgaaaagtcgttaaactccacgaattcccatgatatcttcttctgaata
aactcgataatgtctgaatttgatggtttcagctccactgaacaattacctatataagactagtccttttcgtttgttttgtatccttataaaaatttac
atcattctgagatttatgtaaacaaccttaaatagaatattagccgtaactggaaaagtatataaaaaaaaaaagaaataaataaaagattaaa
aaataaaagagcaacaaaaaggaaagcctacagcaccccggattcccatgttgtctccaaccatagtactaacgaggccctcagacgcttaact
gcagtgatcggacgggaactggtgttttcgcctaggtatggccgtagacaattgtaatgatcgatagtcatatacatagcagtttttttatgggtac
tttgttaatgtttccaacccttagtaaatgtgtttttcggatttattaaacaatgcaatttgataaataaataagaccaaaaaactatgatagtgtttg

aacacactaggatttgaggatattaaataaacaatatctatttaattaactgaaacgatttaatatagctgctgcgaacggtcatggacataagca
taactatcaacgcttgagattttctgtaccctccaaaaagattaccaaaaaaaaagcaaaagaacgtgaaacatggactaacacatgattttatt
taattatcgggatcattagtcttagtaagactaaaagctctctctcgatagaaagacctgttcaaatttgattcctcattgccccatatcatcattatc
caattttcaaaggttcaaagtggtttcatttgttgttaggtaagacatcatgtttgttcaaaacaaggaagaaaagaatttggaggacaacaaggg
ccaggttaagtattgatcttgatttttatttcttccctttctgtgtttacgggggacaaattctcacattagtcgaagaaattggtaagaaacactggt
ggttagtgggacaccattgatgtcaaagagtttacgatttcgcattttgctttctgttttcggacttgatattgcctaatgaaatcgtaatgcgcttac
taataaaatgcctgccctgctatcttcttagtcatgtattaactctttattgataacccttcatttcttaggaattatatataaagagccattaccaga
acggtctcatcactaacaaaaaaaaaacattatgcgtgtatttgttactggagcaacagggtttattggctctgaaattgtgagacagcttatcgc
agctggacatgaagtggtcggtttagtgcgttctgaagaaagcgctgcaaaactcaaaactgctggaggcatacctcatcttggtacacttgagg
atctggatagtctgaagagtggagcagcacattctgacggcgttatccatacagcttttgttcatgactttaacaagtatcaagaggtctgcgatat
tgatgttcgtgcgattgaaacgattggtgaagccctcaaaggatctgatcgtcctttcgttaccacttctggaactgcggtggttccccttactggta
cggtaggcacagaggtaacccaaattagtcaatcgccaattccacgttactttggggagatagcaactttgaaatttgcttctcaaggagtgcgtt
cctcagttgtccgccttccaccaagtgttcatgggaccggagattatcagttcgtgccaaagcttattgatacagctaaaagtaaaggtgtctctgc
ttatattggcgaaggtgtgaatcgatggcctgccgttcacagaaaagatgccgctaatctattcatattggcacttgaacaaggaactcctggaga
catcttccatggtgttgccgatgaaggtattccgaccaaagatattgcaaccgtgattggcaaacgactcaatttacctattgtctccaaaccctct
gaagaagcaaccgaccattttggattccttggcacattcttttctttggataatccaacttcaagtaccttgacacagcaacgtttaggctggaagc
cttcacatcccacacttttggctgatctggacagtgaggcgtacttttaagaactagaacagaatcgcctgcctagtaaataatgatattggcatca
actcaggatcgacagtcaaagatatttggttagaagcagcacataattccaaagccgtcgtcatactgctaatgagaccatcctaagcaagttttg
ccttgattattgattcgtgattccgaaaggtaaaaattgtttccaaggtatctaatcacgaacggaataggatcaattagtgtatatatgaatattct
gatgtcgatatatatcccaatttatgagtgttgttttcgtatgtagaaacaaaaaaaaaccaacaccgatagtaacgtacattctcaaaatcacac
agctgatctattttaatcaaacttttacgaaggtaatgttgctggcgatttgtttactaacgaactgttacggatcattagtaaacattcttttgaaaa
cgacaaatcgaagcagtggtgctgtacgtataccgcagttttctttaagcaaaaatattcctcaaatctattgctgtaataaaaaggattgcttcgg
ccgggaatcgaacccgggtcgcttcgatggcaacgaagcattataccactaaaccaccaaagcagcatgacataaacagggtctatgacattaa
taaaagtactttggactaaaatgaataagtaatgtagaaagatattttacttcaatagacagacgctgtaaaggtatgtaacgtaggcaaatgga
ccgatatcttttaccgaggatcgcttagatggaaaacaacaaaatgaacataaaatatttatatcgggaataatctggtcaaataagataccggt
taccgtttcagagcattgactagaataaaggcatagaatcggattggttttgatgaatcaataatcgctgaaggctggacttgggttctcaaaacg
tcaagtacatgtggagaaagggctatctaagttcggtaaaagcaagcttaaataggcgctataaaaaacaatggaataaagtataaagtacagt
aaatgtagttgtaaatgagtagaagtcatattttgatgatcatcgtcgaccaaaagatggaattcataaaattccaaggaagggaaaaatgaatt
tcaaattgccatagactagaatagcaccagaaagagttgtataaggcatagcttgccaggtcagccaaaagtttcacaataaaattatgatgaat
ttactcgcattagtgtcaatgctgaaacacaatgtctaagacaccatgacagtacaatattttatatacgatatatcatataaaaacatatcatact
atataaatagcacacttcgttaattcattaattacacattgttcaatacggcaggatacgccactatacttcgttaaagtccgtgcattgctactaca
ttctggtcctgtaaactccaattttctcaaagcaaaacacatctgcaaaaagaagggtaaagaggagtaagggggctgttttctttataaatttac
caagttattataaaggaaaagaaaaaatgtctcttaaagtgcaaaacccgaacaatgtacgcgtctacacagtatcaggcgaaggcataacac
aaaaactgcctgagtggatcagcaagaaaaaattgaaaaaggactatgctttgtcacatcgaattgagcttcttcaagagtttgattatccagaa
gctagcaaccgcatcaaagttacacgagatggtaaatatgccatgtccacaggcgtttacaagcctcatatcaaagttttcgattttgctgaaatg
tctttaaaatttgaacgtcacacggatacagaaaatgttcaatttgaaattttatcagacgactggacgaagagtgtccatttacaagcagaccgt
acggtggatttccatagtcaaggtggtatacattattcaacaaggattcccaaatatggaagagatttgaagtatcattttcctagctgtgatcttta
tttggctgctgctggagatgaagtttttcgtttgaatttggaacaaggccgattcttgaacccattcaagattgaaagtgctactagcgaagaccct
acttctggtgtgaatgtgttggatattaatcccatgcatcaattattggcatttggtaccgatgcgggtactgtcgaattttgggaccctcgcgatcg
ttcgcgtgtcggcattttggagatgccctctaatgttccttcttccccatacacaagtgccaaccgtgcagtcactgctttaagttatcgtaatgatgg
actcaatcttgccgcaggtttgtctgatggtattactctgttgtatgatttgcgttcaccaaaaccgtatatgactaaagatcaaggttacagcttgc
ctgtcaagaaccttagttggctggacagtgccctggatggtagcgcccgtgtcctttctgcagattctaaaatcatcaagatctgggagaaagata
cgggaaaaccgtttacagctattgaacctactgtggatttaaatgatgtatgtccagttcctaactctggcttaatttttaccgcaaatgaaggatct
cctatgcatgctttttatgttccttcgcttaatcccgctcctcgctggtgttccttccttgataacattacagaagaaatggaagaaaaccgtactccc
acaatctatgacaactacaaatttgttaccaaaaaggaacttctcagtcttggtctggatcatcttgtcggaacgggtgttatccgtgcttatatgca
tggatttttcattgactcacgcttgtatgaaaaggctcgtttgattgcaaatcccttctcttacgaagagcatagaaagaaagccattaaagaacgt
cttgagaagaaacgtagcagtaagattcgtacccagaatcggcccaaggttaatgctaccttggcaactcgtcttgctcaccaagaagccaagct
acgcaagaagactggagacgaaaatgcttccagtattctcgaagacgaccgtttcaagaatgctttcactgataaggactttgaagttaatgagg

atacaatagaatttaaacaacttaatccaaccaaatcaactaaacctcaactcactgctgctgaagaatcggatgaagaacgcgagcacacaag
aggtgtgcaattgagttctgatgaagaagtgtcgtctgatgaagaagtggaagagcatgaaacttttgataatctattgaataggagacaaaccg
agcataatgataagaaacaaaagggaactgaacaaactattcgcagtacgccttccggaatggaaatgacatttcaagtcgaaaagaaaaag
aagaacaagccaagctctcaagatgaagaaacgtccagcgggaaaaagaagcaaactactgaaggtcgtcgttctgctagcaaaaatgttttc
cgaaaattgtaaacaaaaattgggttttatgttgttaatactaagcttcttacaggctttttttggttcaataattaaaatggttatttgggataatcg
gtgtttcatatgcagttatcaagtgtatgaaacaaatttaatagatttcattagagaataatatttattcatgctttcaaaagttggtctgaacgcag
attatattttattatttaataactaacagtcgcttaatcaagttttaaagaaagcgtcgagtgctctatgagaaagtacgtttgatgagtatgttgat
aatactgattacattttattgtttttaacttaaatgcgtgagtctactttgaaatcatagtagcttgattaataagtacttgtgccatttcaccttgaat
cgcatactcttagaaataaccaaccaacgactcaatttttgatactacgcactattatccttaaaaaaaacgaaaaaaggcgatttagagttgtgt
tttatatagatcatttaaaaatcaaatgcatattctatagagagatatattcaaaggtatagtagcgtttcttatggaaagagatctaacgtataag
tcaagggttgaatagctcataaacatatcttattattgaaaatgaatgaaggaaattcattaagaaatgaaaatattaacagagaaaggaaaag
caacgaagaacaggagatacccagccgaaaacaggcacaacctagcattcgaggagagaggagatcaggccaatacagaagaagggactac
tatacaaataacgaagaagaaacttattcaagggcagcagacgatcatcctcgtaaagatgtaagagatgttgttcgaacacattataatgctcg
acccgatatgggtactagaaaacgacagttttcacctattatccagttgaaacgttttaacaattggattaaatctgtacttattcagaaattcacc
cctagggttgatgataggcccctacttgtgctcgatttaggctgtgggaaaggcggtgatttaattaaatgggataaagctggcatcgatggttac
ataggtgttgatatcgccgagaactctgtagaccaagcaaaaagacgatatcgtgaacttcaaccatgctttgatgctctattttatgcaggagat
tgtttttcccgctccgttagcgagttccttcctccagatcagcggcaatttgatatagtatcattacagttttgcttgcattatgcatttgaaagtgaat
tgaaggctagaagacttttggaaaatgtttcaaaatgtttgcctcgtggcggcattatgcttggcacaatcccaaactcagatataatttctcagcg
cctaaaagagctaccaccttctgaggtttcttggggtaatgacatttacagcgtacgatttacacagcgaccgtctcacaccttccgtcctcctttg
gcattcaatacttcttttacttggaagatgccgtaactgatgttcctgagtatgttgttccatttgaggcatttcgtgcaatagcggagacgtatgatt
tggaattgttatggcaaaaaccctttgtggacatcttcaatgaggaaaaaaactctgaagcttttggtaatttgatggagcgcatgaaagtagtag
acagtaacggaaagagaggaattgatccgcaagagttagaagcagccagcttttacttggcgtttgcttttgaaaaaagggggatctgaataaa
cccaaactttactttcacacaaaaaatcaagcctatactctccttttctcttcaagcgattcagtacctcctaattagttttcctttataattatgattt
aaaaattattgctttttaattcaactataagcagtgtttatgttcatttttttattatttccccataattaagcgacagcttattctgagcttgtcgcga
actcgactgaaaatgccttacttcaggatacacagacacagcatccttttgccatatcgtttacggacagatcagttagaaaaacactggtggcaa
gttgaaattgggcattactattttgtaatttgtgttataaatgcatactgaattgcttccgtgtaattatagtcgaattttgttaattcaatgagtcattt
gctttttgcctactacgtttacaacgacatacgctcttttcagacgttattgaaacaagatgatataaaattacgagaggcaagaaagggtgcatc
cgagaaaagttcgtccaagttgaatgtaaatacaaaagatcgttatggacgcaccgttcttcatatagctgcattcgacaacaaaaacagctttgt
tagagccttgttgcgacataaaaatatagatgtgcttatccaagatgaagaatctggttataccgctttgcatcgtgctatttacgtaggaaatctt
gaagctgcctctttgttactctcgaaagaatcttctaatcttttgaaaattaaggatcatgaagggttgtctcctttttcaattactgtatagaatttac
cctggggctcatccacaaattaattatccaatcttaggaaatgagctttacagtcttgggaataatgaaaatagtattcttgggtttacggtttcgg
gaagtcaggaaatggctaaaagagtatttctttatcgtaagcaaacttccgattcattatctggtcaactttttgccccagataaaatcattgacatt
cagtcctcaaaatttcacagtctggttatcacggatgagccgagcccaaatgtatattcatgtggtattggagctggtggtagacttggtttcagaa
aagacgcccaatatacttttactcctgtgtcagacttaccttataaagtggtacaaatatccgtaagtcaaaatcactctcttgctttaacagatatt
ggaaatgtgtacgcatggggcttaaattcctattctgccctaggattcaacgttgatggttcaaaaaaagaggattctgttcaagtatcccctaaac
gaatttccgcattaaaagacttgtatatcatcgggattgccgcaggcgacacatatagcgctgcatggacgaattctgatttgtacacttggggcc
aaaatgaggggcagcttggtcatccagatgattccttcataatatctgctccacgtcgagtggccggtttaacatcctctgtggtgaaggctacttg
tacttcacatagcattatattattattggagaataacagtatactaatcctatcgaactataagcaactgaaggttcccgtggctgttgatttagaa
agtccattaacttttactaaacatgctttatcactatcacggtttaatgtaagaaaaatagtggcttgtgaggataatctggcgatacttactgagc
aaggagaggtttttgttattgacttaatacccttgcggtcaggaaaagagcaaaagttggcttctttgtttatgaagagtaatagcaaaaattcact
caagattacttcgttttggactgtattgtctccagaaagtgcagcgaatgatgttgcttgggctgacaatagtagtcttttgttatgcctgaaaaatg
gcacgtgttggatacgtcaaattcgttctaagaaacgtgagaaagcctccttaaagcaaagcactaaaggaacttataagtactcttgtattgaa
aatatacaaatgataacgaatgttcgaactaatgcttctggtggaatattcacaatcagaaatgattatcgtcctcccccctattcattttacggttcc
agaattgaacgatgtattcagttctcttcttccatataagaatattttgcataagagaaaccctacatttaaaccagcagaagacgaagatagccc
accttattttgacgatgaccgtgatcccacggaagatgagattcaaacattatttttttaatccgggaataatagtaaatagctataggacctttaaa
gcttcatcagctgatgtacgtcttatatgcgttaatgagactttttggagccataaatttcttttgtcagctcgttcatccatacttcgtcggatgtttc
gtacgaataaggatgtaaaaaagacaactttggattttcgggttgataaagaaggggaatctactttaattttcaatgatgtatctgctacaagcg
ttgcggtacttttgcactatctttactcagactctcttcttcaaccctggcaatgggattcaaagtttttgaatgttaaagaagaattactaaaactca

gcaggtctttagacttgccccacctacaagaggttttaccattcacagtccctcgccagccttcaatgtctttagccagagacataaatgctctcttt
ttaagcaagagcatctttgatgattcatctgatacggttattaaactggaggatggtgaattgaaagcgaacagtttattactgaggattcatagtg
attacttcgattcgttttatacattcctagaagagcaagcacaacataaagcagaaaaatatgtagttaatttaccagataagaatactttccattt
tggactcatccttcgacatatatatggaaatggaagtttagaaatttttaatgatcttaaggatcatgatttttacagctggttggagacgatgacc
gttatgttgagtatatctgatgaacttttattttatcgactaaaggaaatttgtgagcaatctttgcttcaatttctaaatctgaaaacactgaatgat
atgctagaattatcttcttgctatcacgcagaatcattatatagtcgatgtgttgattatgcctgtcacaacataggctattttttggaaaaaaatcg
attaagtgaatgggagaacaaacatcttgccaatgtatcaaagcgtttacattcaagtttgcaagaacaaagattccacacacaacctagtaagg
tgcttaataagctgcttttcagaaattccaaatatttagaggaaaaagcatatgaattaaaggttttgcgggagtttctcttttcaggtgaatcaac
agaattttgggataagtctccttataggacaacgaaagaaagtaatgcttctcttattcaaacgcagccgccaatcgctaaggacttatttgaaca
aaaggttgaaccagtcagagctgttgaacttgagtcaagacaggagaagaatcttaatcctctgctggacaagggaagcccggaaacattgga
acctccatcaagcctgttgccttcatacagtggaaaaatcacatcgccatggacacccgttgccggaaacattaatagatcacctgtaatgaatga
tgatcatcaaataagtccttcgaataatgagagaaaagggccttggaaaactccaaatttagttgacaatgcaaagaagcctccgaatcgagcc
aacctgagggagttgcttgaagaagctaataacgtgagcccgtctacaactaataatgagtttcgtggtatgaaaggtttaatgaagaggtcgca
gaaagagaagaaaaaagagttatctaagcagcaacagccaattcgcaagggaacagcgcacgatttgagtgtggaacctgttgaaacatcaa
caaacgcttggtcaaccaaaaagcctaccgtttcttcggctactaaaaaaccgtttaatgggattcttcgagaagtagctagtgatgaaacaccta
gccaggtattttataaggaaccaaagaggcgtatatccagcggttctccgtctagttggagtttattaggaaaaccgcccattcaacaaccttcat
cattgcctaagagtggagcccagccaaattccttgatggcgattatgtatgagcagcaagaagaaattgaggagcgtaaaagacgactggcaa
gcagaaaaacgatagaggaaatccaacaggaggaagaatttcaaaagtggtgggaagaggaaagtcttagggttcaaaaggggctcggaatg
ataaaatcaaaggaatcgaatgaaaacccttctcaaagaagaaagccaaagaaaagcgacagaaagaatggcgcttccaccggtaaatcaag
ctctcagcctattgatattcctctctcaagttttaagaagggtaaaagccgaatatatcggtaaataagtctgtaatttagaatcagaaggtattat
ccttataaaaaaatgtgcatgatgagaatattcataaaatacttatttatttcgtgctaggttgtctggcaattacttaatggatatggaacgaaatt
tatttaactatcgggaaaggttgtaataattcaaagcattatttttacaccatagatgagtagtttcgtgaaattacatactctcaaagaatcagac
atagtattcctaaaaaaaaaaaagagaaaaaagggggtggagattccttaccacaagactgatataaaacagagtaaaaaattgtaaaatatga
aatggggaaggaaaaatatagcaattaaacatttaacgtgaaaaaagcacttactagcttcataaaacaattaggaaacgcaagtacgaaaga
gatgtttacctctcaacaagcatgtatgaatttacacaaaagaaagatagccagaagtcaaaacggaaaggataagaacagcatagccagtac
gatagacacccttaagagttaattctttcgtggtgtcccaaaccaagtgacgaagtccattgtaaatgtggaaggtaaaaggataggagacagc
aaatttggcaggaacctttatccaagtaggaacttgttgaagaagacccgaaatggtttcggtatcaagagaatagccagccaaaggagcaacc
aagtaaccagtcgcaaagaggtaaagagcaccagccaaaacaacaccagtaacacggtgtatactggacaggtaccaagtcaattgaggttca
tagatggacaaatgaggagagttgggacgatgaagacgctgattagcaagacgggacccgctttctatttccgtcatgtgttcagtagcgacgga
tcgtttccatacatttctcaacaatgaagaggaagaagagatgctggaaggacgcaacaattgagaagcagggcggaagaagcccggagaaa
gagaatggaacatacgggtagcgagcatcattcaaattttgtcgatgaaaaaaggtagatatggtaaccaaacttcaatgtttctttttggcgttt
taattagcaaaaatagagcctttatctgcgaatttgaccctgttttatcctcaaattcacaaaaacaaaataaactagtggtttgtagcgaatgga
attccaaacccagctattggaggatataacgacagcttcggaacttgccaatcaagcgagatggagcgtactacaacgagaaaataatgtcgtc
aagggttcgaatctcaattaggacatgttttatgaatttaaccattcactttttctgaaagtgttttgcaaagtgggcatttcgtgaagaaacagtta
atgaaacttgaatgatagagaagaaatatatactttgagttgttttggggtcttttgtggaaaaaaaaaactgtttgaacgacagaatcctggtaca
tctgtggggacagggtatatacatttgcatagggtaattatttgattgaaagaaaaactattaaagggactattactattactataaaaaaggatg
tctgagcaagatttacacatgaatgaaagaagcgattctggagaatcacagtatgatcctgtcgtgcggactctacctgtatattattcaccggca
cttcgaaaccatttactgttgaatcaatatcctttaagaccaaagaatcgtgaatacagcagccggaccggagaaacaccgatgaatgcacgcat
caagcctaaaactgggtggatggagatggaggtgccaattcctaccgcaaagtattttgacgaagataaagcaaaaaaatacagtgccgaaga
ccagccattgaggacacaagtgcttgctggtcgtcttcagcagccgcaaacaaacctgatggtaggcttaattcgaaatggacagcttcatatcgt
tcctttgcgtggattaacccaattgcggccttctatgcggcatgtagatgaacatatgcaaagggtaaaaacctccagtacatcttcgacaacatc
acaatcgaacgcttccgcttcgcgtggacccgttcgtgccatccaggtgacagctaagcaaaatacggaggctcccaaattgtcaacgacacata
ttattagggcaacggaagaggaagagtggcaatccattgacatccgggagaatgaagaggccaacaccgttgcaaaacagttggagtgcccct
tggaacatcagcctaatgagtgcacaccggcggacgtggagtactcgtttctttaggattaaaagatataaaaaaatggttttactttcatgtttct
aatggaagtaacataaatcaattttagaatacctataatttgctatccactttccatgtaaagttgtcgtacaccttggattgcggtcgtccacaaat
ccctccgtaacggataacaaggctcgacgtttagtatttctactactttattggagataatggaatgatctttctacgttgtgtacacctacaagtat
aaactcaaataaactccataacccaagaaatcatgcttattgatttttaaacaatctactgacgagcagcagccttggcgttcttgatggcttgacc
acgggcagcgccggcggaagacttagcattgccagccttcttctcggattcggaagtagccttcttatccttggcttgcttcaaagtagaggcacg

ggcggcggcacgaacttcagggcgttggttacgcttctctttgatgacatccaaggaagcaccgacgacaccacgttggtgcttgacagtacggc
gagagcgcttcttggcggattcttcagagagacccttcttgtgcatacgacggtagaggacagtccatgacaaacggcgagggttcttgcgttgga
ggaagaggctctcagacttcttgtttacaaagcggaaaactttgttgtctccacgaacaaagagtcttccagctcctgggtaaactttagcaccgg
aaaatgagcagatttccaccttcattatgccttgttgttcgtggtcgttatagtcaaaaatatttccctaccgggtttgtgtttcgaatatagcagtca
ctaaccctaacctgtcctataacttaggagtcgattttcttttcgcaaatcagaataacgaagtgcgtaatgctttttccataaataaataaataaat
aaaaaagcatttgtaccttatgtcagatgcgttactggaataaaatcgactctataatcaaattcatgctaaaatttatttatactaaatatacgtg
cccctgtgttgctatatagcaatcacgtgaagattattgaagtgaaaacttctctttcattgatataaacaccttttgtttttaagcttgaacacataa
aaagaattgtcataagaagatatgaagaaagtggtagatagagcgcaaagtctttgtattgttttgtttacatggaagaactttgaatttcaatga
atgagaactaagcaacatgtcaccgcagaatgtaaacaacaacaagtaaggaagaaagcagttgaccaaaacccatcatcaaggtggaagaa
caggtttcggaagtatgagcgattcgaaacttgcggggtctgtaaatcagctttcaatttcttcagggtcgaccttgccaagcagtgcaaatcccg
gacgtcagcagctgttaaaactggcagtttccaatcaacgccaggtaaactatcctgtgacaaagaaagacagtagagtattagaaggaaaac
ggtactcttcttctaagcaaaaagatgagccaattgcaggtgttccttcgccgatgggaccacaatggcgggactttcatattggcatgtttgaaat
aggaaagccacttggaaagggaaagtttgggagagtctatttagcaaaggaaaagaaaactgggtttgtagttgcattaaaaacgctgcacaa
atctgaactcattcattcgagaatcgaaaagcaagtgagaagagaaattgaaatccaatcgaatttgagacacaaaaacatccttcgtttatttg
gtcattttcatgacgaaaaaagaatatttttaatcctcgaattcgctgggagaggagaattataccagcatcttcgaagagcatctcgatttcccga
aagcacggctgccaaatatatctatcaaatggcaaacgcattagcctatttacataaaaaacatgtgattcatagagatataaagcctgaaaata
ttttgctcggaatcgatggtgaaatcaaactatctgactttggatggagtgtacatgcaccttctaatcgtcggacaacgctatgtggtactctagat
taccttcctcctgaaatggtagaaggtaaagaacacacggaaaaggttgatttatggtcattaggtgtgcttacttatgaatttatcgtgggcgcgc
caccctttgaggacgtttctggtcactcggctacatacaaacgtattgcaaaagtggatttgaagattcctagctttgtctcgccagaagcaaagg
atttgattactcgcttgttgcgacacagccctgaacaacggatttcgttagaacaggtcgtcaagcacccgtggatgctaaaacataaagcttctt
ggagctctgatcaataagatggaaagaaatgggctttttaggttgtttaaattattttgtgtgatgttttatcataactttccttgatcacattaatat
tcgaatttttaataatgatttttgatactttttcaatgagagcttgtatttcggtgtttatatagacgtatatctatggtggagatgagcattatatttca
aggtttgtaataaaaggcgaagggaggaaaggtggaaatgaaagtgaaggggacttgatggtataaagagagagagagagagagagaggaaa
gaaggatgtaagaaaaaactctttttttttgaagtgatggagagaaatagagggatactttttaatttgaggcagtcaaaagactactaactcca
aaaagcaaaatttgcaaagttgaaaaaatggttttgaggattaataaactagataggttaatggaaaatagaaaaaaaaaataacaacatacttt
tttcaaaaggtctggtatcacgaaaacatgaagcaaatacttcctgaacaatcatttctgcttgatgcttgctttggcagttaggattaccttggac
agaaagagctgctctccgttttacgcaatgttgatgatgctttcgaaatgtatcaatgttgccataaaacaattcttttgtccaccggcattcaccgc
tgaggctactagcacgaatttcagagcatgcatgatggcgcaaatcgttccaattgacgtgaaaacgaccgtcatcaaacatatgaatcatttcat
gagctattgtgttttcagccatttttcggctgtaaagtcgattctcgcatagtacaattccttttcctggtacgtacccgcctgagctcgtttcatcaca
gggttcacaccgaatatcggatgcatcaagaccggaatttagatattttaaagactttttcaaaaagacgagaattggtgagtctgacaagagag
ctttcttgatacgttcacagttttgtttttctctactcggttcgatctttgtcgcttctgacatgctgcgctgcgctttaatgatatattctaggatccagt
cttgaaagcgactgcagaaatgagtatgtaatctatgaatgtagacgttagttgaatttgtcgtttggaaaagggaaataaaaaagaatggcttt
cagacacgtacgtctcagttcaaaagaaaaaataattcaaaagtacaaaaggaaaatttggatacctcgttgaattctttttcagtcaattctaac
tctttaatttggaaccgacctgcttggataggatctcaaccagtttgccatttcgagaaattactacgatatcatattactcccggttcaaaaaagg
actaaagacagtagttggcagcaaggaaaaacaagccgataaagaaagaactagattttaaagatgcttgaaaaagtttgttaggtgaaactc
gagcccttgctggtgctttcgatattgtttacactagataactggtatgaggaacaagattgaagcaagaatcggtccatcatgcactaaaggtat
gaattcacaagagattcatttcattgagaattgaccttaatgggaaaattacgaagtagcaaggagaattacctagcaaacaaaacaacaagga
acgtgaaatccattccatgtttgacatgcttcgtctctagatgacggaacgcaaagacaggcttgtattacaattgaatcgttggatataagtcata
attcgcaaaacgaatctgaaaattgaaggaaccgttctacatactttgtaatctcgctagtactttaagagcatattatcatattagcacatatata
tagtcatagtagcatataagggtattgatggtataagctttttttattttcatatgcttcctcaatggtatggtaaatggttatttaagaattagtgtgg
ctggcctcaaggaaggactgttctacagtatgatagaaaaatcaaactagtaacccaagtatttttattgaatgaacgagttgttgtatttgtagcc
agtttatgaactggatggagtcgaaactggcttgtacagcaagttgtttttctcatcgggaaatgtcgatcgtcaatacctgcaccggggacgtaa
acaaacaaaacgtaactactgactacaggattgaaggaggctacaatccattcttgaaaaccaaagtagcaattttaccagtggataaagatac
gaattatggtaaagtaaaatattgaaaataacaaaacaaagataggataggataggatatttgaatatagaatgaatttcattgtggcatttttgc
acttaataattttagagaaaagaaataaattggttaatccatatagatataataacatctattaagaaaatatagaatctatttgaccttgaagaa
agttccacttttaggatattgtcatgtcatgaaaggtgttttgtttgtattatatattatagtatgtataaatattagtatatcgcgagataaaaacca
aaaggccaaaggatgattgataccttacaaaaagtaaacaaaacaaagaaaatacgatttcaacaaatcgggcagcctttcggaacgaaagtc
ttatatacaaagaaagtttgttatacaaagagatagattacgttaattgttttagcgaagatatataaagtatgaatccacagtcgaattcttcaga

ttggaagattcgactggtctggctaaataagatcgtcttcctactttcaatttggtttaaccagatccctggacattactaccatttatatacgcagta
taaccaagtccaattaacatatactatcgattagatatttattatcataaagagtgatgatttttaacgtggcatcttccaatttcaagtgaaaattg
gtttaacttgagcaacataagtaaaaacatagatatggattctgaaaacgcagggagtggatatctcgctcaccaaaaggagatgcaaccggaa
ttaggaaaacaattaaaggctttatccgatcaagtcacccaaatgcatcaggaattaggtaaacaattaaatgctttaactgaccagatcacctta
aagaaccaagacagaatggaagaaaaaatgagccgtatgaaagttgatgaagactttattcccaagcaacctatggacattatagcattggtat
atgaaaaatatgggccaatagcagactcctatctaaaaatgattcatccgcttaatcctggaattggaatattcgaaccatctgaacaatctgtag
cagtttttggaaaaaatctgcaaagcatttttggttacctacctaaagtatttccatctgatcgcgagaaaattgcatttttatattccaaatcccca
gaaaattaccaaagacagatacgaaaattggtgaagtttccaggacctcagaggaccttcaagacggtttttgaacagacatttctaaatgaga
gcgagaaatattggtttcatcccattcctaaatatttggcaaaaggagctcgtggtgagttagatgttgatgaaattcacaaatggctcgacggat
tggacatgaacacaatcaagaaagttgttgtattcgcatttcattcaaattattttagaaacgaactcctcaaacataattacgagcatggcaacc
acgctgcagctgtaaaaaccgtaaatgaccaggttcgatatacaaggtccaaaaatcagagaaaagttaccgtccaaagttccgacctacaaaa
ctttattcccaaggaagaaggagacaaccatgctgataaaccgaaaccaaaagcacaagtttaatactagacaaaagggataagtctaagaga
gatgaagtcgtctctataaacaataggtgtggaaaatttttcaaaccaccaggagaaacaaccttcccaacatcaagatgttaaacttgcaagct
caaggtcaatgcatttatagatactggcgccaatgtatccatcttgagagaagatatggcaaacaaattacgcgtaaattttataaaagaaaacc
gaacgttcctaggaataacgcatgacaaacttgaagtcaaaggaaatttaagtctaccggtagaaagaaataatcgaagattttacgaaacgtt
cataattactccaacttccgacaatgaagttctcatcggatggagcctttttaaaaaattgggaaatttggattttcttatatggcgaaacaaatct
ggagaaagatcagacatatagcaaaaacgatgcgaaagttcaagaaatattaaagccattcaataaggtatttacagacaataccaatatacc
gttgccccaacacttcaatagcgatttagagattaaactgacaagagagctaccagattatgtaccaaaattaagactttataaccaaaagaaaa
atcgataatggcagaaaaggtgcaatattattaagtcagaatcgaatccgcgaaagttcatcaacatttatttgcgcaccattaatagttccaaag
aagggaacagataaatatcgcaagtcataaactataagccattaaacgcgattacgaagagggataattatccattaccaaatttcgaagaaat
tatcaattggctaagcggagccaaagtgtttagtacaatcgatttgaacgaagcatatcatctgctaagaattaaagaaggcgatgaacataaa
actgcctttaaaactccctttggagcatatgaatacactgtaatgccgtttggattaacaaacgcgccagcaaattttcagagatatatcagtaag
gtatttctacctctgctcggcatttatgttatcgtttatttagacgatattctcgtataatcctcatcggaagaggaacacaaaatacatttgcaaga
agtatttcacagattagaggaagcaaatttgtatgcaaatcgagagaaatgtcaatttctcaaagacaaagttaagttcttaggtcacatattttct
gaaaaaggaattgaagtcgatcctgaaagaataaaagcaatcacaaattggcctgtaccaaacactttaagaaggttacagtcatttttagaatt
tgtgaattactcgagaagatttatgccaatttattcaaattggcaagaccattatacgatctaacaaaaaagaacgtaaaatataaatggaccga
tcgtcagcagcaggctttcatacaagttgagaaagaacttaaaattttctatgttgaggcattatgaccccaaaagagaaactagattggaaact
gatgcttctgactttgctatcggatactctcacaaaggcaagacgatgacctttatcatccaattgcttttcactcagagacatttacagatcgaca
acgaatttggccaacatgggagaaagaagcgtacgcaattatagcagctttacgcaaatggtatcaatttctatcagatactgaaaagccattta
caattttaacggattataagaatctaacaaccatgtttaccaacatcaaagcagatatccgtgcaaagataaatagatgggcaactgaaatgga
actatataattttgaaattatcgaccaggtcgaaagaatgaagctgcagacggcatatcacgacgtcctgattataagacgcaaacgattaatcg
cataatagcaatcgattatctttccaacattttcgatcatgtgagacgagaatactctcattataaaaactttcagaatatcctcaatggcacagat
cataactctcgttatattgtaaaaaacggtcttattctctacaaacgaggacaaattgtcataccaaagataccacaaatggtacctctacgaagt
aacgaacttccaaactagctcaatctaaatagaggtttataaattcggactcggaatcaccactctcgctgtcttcctcgaaatcgtagacataga
acctaggcttatgtggcgcgaaagataatgtaactggtcctggtagtctaatcgagaaatagctaggattataccaagacctggaccgcatagcc
tcgttgacctcgtcgatatgtatgttggttctaccgtcagcactctcaatacctgcaaatgtatcattgacaatgacagtcaatgagtgaggtagctc
atgaacattcctgtccgtctgcatatattgtagtagcgcacgccctgtaattttctcttgtataggtggtcggtccctacaacatttgctacattatgtt
ccctgagaggtacggtaacgttaattgccatagttcaaccaagatagcttaagagcggtaaacacagtattgctttctgtcatctcacgatatgtgt
cttgtttatattatatatattcaatacactatcataagagcatttgctaaatattagcaaatgaaataagactaaaggatgagatattacaaatggt
aaacaaaacaaagaaaatacgattttaacaatatcggacagcctctcggaacggaagccttatagacatagaaagtttgttttacagagagata
gattacgttagttgtctcagcgaagatatataaactatgaatccacagtcgaattcttcagattggaagattcgactggtctggctaagcaagattg
tctttcaatcatttcatttagtttaccagatataaccctagatattactagcggctatatacgcagtataactaagtccaattaacgtgcacaaacct
acagttgtagttattacttcctctcgataaatgttcttataaaccgtgagatttatcaacatgacttgtttattgaaggagacgtatttagtcaaccag
gtataggacattgctgagtaaatagtctcatttggtacttttatttcaactcaaaaacgctgacgctttaggcagtcaacagttgtattccactctca
tcaacataatagtaaagtaggataacatcaccgtgacaattagctttttttgagtgatcacataaggatacggataaaagaaagaggatttatgg
aaaaggaattcgtactttttgattaactctcaaaattcatggggtatgaataaggagtatggatccatttcataaaggaatagattcattgaagtct
ttaataaaaattcgtattgcgaaattgattcccatttcatttaatcataattaccggtagcaacattagaatataaatcacaatagtgtttttaaaga
tttcagttgagatgtatcttaccttgtaatttatgaataggagagtcttttaggggagttgaagccaaaaaaataattagtaaaataaaaacaaac

acgcactcgtctttagcttgttcaagagttcctggttgatcttaaaaatcgcttgttccatcttgtgtttttgaattagagcttgtattctattagctgct
agaccgatgtggatggaaaccattcatcagtttggaattgtcttgatactggatctgctgcaagcaaataaagaactgttctacaatgacaaacca
gtcctcgtatatgaaaaccctagaaaattcatttgagcagcttccacacgatagaaagaatgcaaatgaattgaatttttaaaaaaaaatagcgt
taattttctgattgcatttgattttatttacttctaatttattttttacatttcttttctgtatttgtttatgattgcttgtttcctttgcatttcatcactcgtt
ccattacggtcatatattagataacaacgcactaccgaaatccagcgaagtgtggacttgagaattagagagaaacttcaccttggaatttacca
aacatacggcttaggaaatcttgggaattgggagcattttctgttatttataggcaaaaatgagacctttaacgcacgaggaaacaaaaaccttct
ttgagaagcttgctcaatagtaagtacatggaacagaaaggagaaggatcacggtgcaattaaatttttttttttttgccaattccgacatgaaac
ccaatccttttacctttagaacatgaaaaagaatctccaaacggaatattaaccaatgattgtgtctagtatcggtaaaaatatcactcatttaatt
gatcgcccagacgatccccattgctttcgtttacaaaaagaccgtgtttactatgtcaggtaagtagtttttcctctgttctcaaagttttacgccttg
cgttgcttcattttggcgtctattttttttaagtctattcactaactctcgggatagcgaaaatgctatgaagttggctacttctgttgctcgtacaaa
tctcatgtccttgggtatttgctttggaaaattcacaaaaacaaacaaatttcgcttacacatcacctccttggattatattgctcagtacgctagat
acaaaatttgggtcaaggccaacggtgaaatgcctttcttgtatggcaaccatgtcctgaaggctcatgtgggtcgtattaccgacgacacacctc
aacaccaaggtgtcatcatttactccatgaacgatactcctttgggctttggtgtgaccgctcgctctactgttgaattgtcccgtttagatcccaccg
gcattgttgccttccaccaagctgatgttggtgaatatttgcgcgatgaagatactcttttctaattaggaactgtatatgtatatccctttcaaacga
tctctcccttccttttctttcaactagtccactttactagatgttcttcctcgaaacatctctcttccaaaacaaagacgtttcctcttgttatcagctag
aagagtatagttttgctccaatctttttattctgttttgagttttttttttggtataacttcaaggtgttggatttttttgttacgttctcttacgcttaaaa
aatatcttcaaacaagaacggttttgggttacacggttttataattgtagaaattttaatttaagtgaaaaaatttaatattctaaaaaacgtttacc
tagtaaattcttattttcgtagaaacctttttatatggatagaaccattttgcccatcaacagtgaaactgagaaagaaaaactacatatattcatga
cataacctggttcttaagtagcaattccttgcatgtttctgcctttgtttttttatctttacatttgtccgatctcgtttgggaagccagcgttgaggatct
gccgctgtgtttggattgaaattctttggcgtaggctttctgcgctttctcgttttcttcgaagcgtcattatttgcttcacgcttcaatgatccaatagt
agtagatactggaactcctttggactcaacagcatcaatatcaacgcttcctatcaaatctgacacagaaattaaatggctttcaagtgatttacta
atggattcatcaatatagcaaagggcagccacttcgctggaaaccaactcactaacaggacccctccaagcaattaatttctcaatagtcttcaa
accgctttgcacgacatccacacttgccttctcatcaatcgaagctaataatgaacgtgtgcataatagaagtttggcgtggcaatttggtttaccc
gtccagtatctagcagcagcatgtaaattttcggtacttggagtttgagaatgttgaaccttagaaaggagtgcatcgcctaaaccggccatacca
ggagaataccgcaatgcaaaattttcagaaccttctaatgctccacacattttcttgtacacagctaatgcgccgcgataattctcacatgaaattt
tgaattgaattagcaaaagggcgctacaagcatcagcaggatttttttctgaacttattaattaaaaatccaggaagcttcttggaagatgaaagtg
aacctttctcgtgttctgacatgagaacaccataaaatagtttatcctcctgggcacgcctttccttctccttcttgtgaacagcccgcaccttaccaa
cggccatgtccagcagggccagatttcgcacaagttgtaagctttgagaagctaacagctttgtaaaggccttctcaacaggagtctcatgaatat
tacgaaatgcaagatagggattttcattgtttaggcataagacattattaacaaacagaacgcgtgaagtttcatcgagactaatttccattgtca
gtaaatgttataaaggatttatgatgaaacataccttgctgaagctattttcttggattcttcttcagctttttgcacctcaccagccattaaatgtgc
ataaaccaactgcagacgacacaagtttacatgagattcttcttctgaagacagggaatcttttgcttctaaatttccggttaaagaagacagcaa
atcaatggccttgctccattgttgaagagtcaagtaacgagtagctaagttaaactgagaatcttgatcttccaaagaaggaatgctttcaatttgc
catccgggaagctgagaagccacagcaagcacattcacttgaatatcagattgttgttctggctaataagagttagtttagctcgcttttctataga
aggaaaagaaatattgcattgtcaattcaatatattgaaaaaaagcggttttaaaacctgatttcgagaaaagaggttcgtaacggctgggttga
tgaaattatacttaccagttccctttgaagcttttcgtaaatttcaatagcttttgagaaatctgatttacggtaagcctggtcatgttaatataatta
aaatataaaatccatcagcacaagaaacaagcgtagatcgtgcgtgtattgtgaaatttgtgtttacctcttgagcttccaagtgttggataaaat
gggaatcatttattgcaggataatcttgttttaaatggaaagcacaatagactctttcatacatagcatcctgcccgcctaaatacttttggataac
atccaaggcttctacgtagcattccacatcaatcaagttcaatacttttcgatatatatcaattttggaatcgcctacttctattttaccaacacgatc
ggcaagagtagtaatttgttcttcagtatccgccatgttggtggtgtcttgctcttggtaggtctttactaccaattttgacattcagtatcgacacca
aaattcacttgtacagtaaatgaatagtcataaaagatttaataaaatatctacacgcgtaatgtaagcttaaagtgcgtagaaaccatgtttttttc
tttcagagatgatcaacgatatttggctacacaaatgggaagaagaattgcaatgaagtcgttctatatgtttatttggttctagtattcttcaaact
tttcgtttctgttcttctttgtcgatcgtaagtcgcctgaagtgagaggtaaaaacataaagaatcacaaaaggcatctactatcctgtaagtaatg
agtagcaaccagcagacttaatgtaccatggtaaatactatttctagcatgaataagaaagcttcaattaaagggaaactaaaacgaaggccat
agtctagtaaaaaggtttaaaaaagatccacagcagaggaaaacactacataaaaaataaataataaaccaaaaattagtcaagggaaagat
taaaaaaagctttaaaacctctggaagaattcaattctcttttggcaagactcattcgtgcaaccaggacatctctgggtaaattgacgggaatga
gcggtgttcgtttctaaatgaagcttcactaaatcggccattcctctaatagcgatgggattatcgttaatactgggtgcgcgcttaactcccatgat
gcctttgctcttggcatcctcaatgtaatcttcgagttcctttagggtttcaatatggtctgaggtaaatgcgacaggaactaaaatcatgttcttgtg
gccttgttttccaagttgttgaatcacgtcttccgtggaaggagacatccaaggaataggaccaactttggactgccaggagtttataaatctgttc

ttgaatcctagtttttgcatcacgccgtaagtagtagcggcgatctcattcacgtatggatctcctttagagacttgagacatgggtaatgaatgag
cgctgaatataatgacagcgttatcccgttcttccttaggataggtctctaaagttttttgaatattctccgcaaaagcgtctaataaaccttcctgta
aggaccaacgatccaaaacagaccactcaacatcttgttccatattcatttcaagaagctgttttcgaagttcattcagagaagcaccactcgtcg
aacaagaccattgaggatactgactaaaggcaaccgcacgcttgattttggactctttcagttcttttagcatttcttctgtcaaaggaggggcata
acgaaaagccacgaatggaacatgaggagccgattgaggagaggtttcatctaggattttacaaatttcttttccttgatattcagtccaatgcag
gattggacttccacctccaatttctttgtaatgctctcgaacagcgggagtccttctcttagcaatgaattttccaagtgagttttgaaagtagccta
gtggaataatatcaccatcagcaaagagtctttgcaagaaaggtccaacctatgtttattaatattgacgtttcatcgaccgtgaaaaataaagtg
ctgaagaatcgtacctcatctaaattcgatggtccacccatatttagcatgacaatagcctgttggaatgttagtaaccaacgaaggaccttaaca
gactgtacttacggttggcccttttttggaagcaccgtcctgagacccgataggttctgctgttttcgttgccaacgaagaagccatccttgcaagc
gacgatggattctggaagccagagtggaaacttagtggaaaaaatcttggccttataacgggacatcgtgcaaacggaaatcgaatcattttctg
aatcgtctggtgaaaagcggtcaggttgttttactttctatgagtttacctttgagaactttctcagttgaaaaatttctcaaattggatgaggagaa
taagaaggcaaaattttgtataatcggtgctgttatttttagctcttctgacaaataattatttgtaagaggaaaaccaacgactcttctggggcggt
aactttttcgctccattaccaagcgattgcaatccaacttattttatgtagaatattaattgaatgaaacaaaatcttcttcattgtgaaaggtcatta
tgctagaacatgtattatatgtgtttgccaattcactatatctttgaacactttcgttagtacgattaacctaacatattaaaatcaataatgcgattt
tatcaagaaagcctgaattaaaggaaatcgcttaaatgacttcagagcatttattttattttacaaactaggaccttgtattcttcatgttcttgtaa
ggttgttgagcgcgacggttttagtattatataaaatcgtctttgaacgaagtgctacgttcgtttttctggtaaaagattctgtctacgccttttcaa
ggccaatatgtataaaacaactcatatctgagtgcttttcttttcaagtttttcaactacccacaaagtcacagttgtcaacaacaatgacatggac
cacgaaactacaagaaagaatgatcctttttttgtataccctctctctttagtaagtgagcctatattataccacttatgtgatatcttgtacccttata
ctttaactcaattgaataaaattaaattagactatccagcaccaaaagaacaccattttgaactaaccgataaacgtactttttatcttttgtctttt
attttttaacaaataaatgcttcatccaactgaaggaatcacgaatacgcttgaaaaactcgtcatctttatgcgcagcgacaattacctcgctctt
gcccatatcgtttttcatggaaaacagagaagaagatggtcgacgaaccataccaacaccatccacgccttccccttgcctcagccaaggttttga
tgccttatcagccgtatcaatgaaaatatggtatacgaaaagacctataataatcataacgaaagcgataggataaagccaatagacgtgatttc
cgaaaacatggataccaataatcaaactccaaaagtctgaagttaacactgaaatgttataaaaagttgctgaagacatacggaaacatatagg
agcaagcgaatagagaataagcataacaataatgaaaccagctagatatcctcccatagcacgagtccaatgcaaatgcaaaaagtctttgcg
atcgaaaatgaaattctgcacaatactgatgatggaggcataaaggctcatttgaccgacgactacatataaaggcagtttagaagcaaagtatt
cttcaatcgtgtttgaaagaccgtaacaggttgcgccaagaatcatgtaaccgtcacctaaaccagggttcttcgcactttttttcattgctgccatag
tttttcaagtcactgacaacaagtaaggcaaggccaccgatacagaccacaataccgagtaactgagaccagtggtatcgtacttttaagaaga
aaaacgaaagaatgacaactgcaaccgtagcccaagaatctaaaagacttgcactgatcacactggtatattggtaagcgagaacaacaaaat
aattcccctctacatcaaagaaagcaaacaaaatgtacttccaaccatgacgaagtaccatttcaaagtaacttttaaaccccattcgatagatgg
tataaggcgtaaacatgagagtaagcgcggcataaaataagaagatttgaaaagctggaactttccccatctctgacaaaaagccattaaaagt
attactagcagttaagcataatgaaagcacttgacccaaaataaaaacaatccaaaaacgtttgtttgttacaaacgaaaaagccctggtttttat
tcgctccaaacgacggggattttgttctattgaacgttcttgtgaaggtggtctgaattgtacgtcgttcacactagaagcatcatctaagggtccat
ccaaaggtttcgtagagacttgctcttcgtctttatccattgatgttttgtgaagaccctcattcgacttgttgctgactggttgagttgccacaaattc
agcttctggtaacttgttatgattcttcacagtacttgcaatgctttcattatcaaagttggattcctcgacccggggagaaatgttcaattggtttga
attggaactctgaagcgaacctgtttgcgccaacacatattcatcagagaaatctgtgctagaaaaggaggattgtatccctcggtgatccctaat
atggatattttcgctcatttgcaataatatatgcaaaattatgcccgtctccggctaatgtaaacaactgcggctgtttgagaaggtcatgtaaaatt
cgaccacgattttctcttccctcgttattacagtaaccgttgatataaaggggcaatttcattgaccaatagcggaccacaaaaacaagagaaga
gaaaaccgaattccttcttctctttagcgctttatttttttacaaaatctaactttgtcagtttttttgaccgagtttttttacctctattgtttatttaataat
tcctgtggaatgctgtatctatttctatcaacagataatgaagtatactgtatgtcattattgaatatacggcggagccataacgagcttaaagtct
aaagcaaataagccataaagcagcctcggcaaataaagtttatgacgctataaaaaagaaccaatcaatgacagcatgcttcgtgatgtcacaa
aatatgatgtcaagcatttccaataaagcttagactaatttagagagaaagggtcaggcatgccaatctggggatttagagaaggaggaaacgc
tgagttaaggtacgaagggataaaaatgcacttcgtgcgtcactgtataaaggctgtttgtttacgtcgcgaaggatgagaaaggagaaatacc
aaaatcaaatgagaacgtgtcagaaaagattcttccagcctcaaaaactggttccaaaacgttttatccagataacccttaccctttctcagttttc
aaaggagaatctgagtgagctgtcttggagtgatgcttacaacacaattactcaaccggtagaaccaatctagctcttttgggctttaatttggctt
gagattttgtaatgctttcgtttcgcagtcttttaggaattgataagcggggttgtaggaggaaaagtatataaagcctacctacctctacctctagc
aaggagaagtctggtattcaaatctatatctcgacatagatccaccaattttttttactagcctttgcttagtgattactcatttatcgaaacaatcaa
cccccccttttttgcattaacctgttctttgcggactagtgtacatcgctcgcatcgtccttgttcgtttatatattcctggtaatcaatccttcctttcctt
tttgtatcagtccccattaccattcacataacatttgccttgactctcctttgcctgaaaatttatataatctatcacattttgtgtttcgttttggttaga

agtttccccttttttgtttccccataaacaaacactctacttatacagtgtttgttattgaattggactttttgcgtgagcatatacttcagtcagatac
cgaagttccactcattcatttaataattaattttctatattcgtggaatccccatattcttttcgttttcccaaactcttctttctatccccattattctctt
tctgcaattcctttagttccagaaaaagtatcgatttcggattttagtttgttgtttctcccattgttgcagcttctttttggttctcttctttcctttcgat
agcctttcttttttgattgcaatttcagttaaatatttttcctaattatttacctcttctctttattgtttacctcaatcctttagagtttgtgtacaaataa
acaaacaaacaagcagaaggtaaatcggatcaagccattctttttaagacattgaaaacctcatattctgaaaagtctttacgaactcgcttttac
ccatacatatttgtttcctacttgtcatcgggtatattttcaagttaacagttttcaccagcaatcattcatccagctcttatccattttcattctacaga
ggggttctagccttcgtttcctattaataaacttttatccaatccgtaggttttttttatttgagtcaacaaggcgatctcatctttcacaatgagtcaac
tgtctacactcaacaatcatcagaaacctgctaaacctgaagatattagcggacgaatgtctgtctctagtctttcttctaatccttggaacaacgg
gtctgaacaaccgcagacaagtcatacgcttgatggaagttctggtagcttgctcaatcccttgttatcaggaatgtcaaatggtgtatcccatccg
ttgaattatctctatgctccttacgcccaacctgcaaaatctgcggctcctccttatctgtcaaacatgaatcctataggaacgagccccacgaaat
tgccttccttgtctgccacaaatgcttccagtgcagcttcaggagtggtggatgcttcaagcttcccagccaatcgtccagtgcaatctcgtccttct
ctctggaatccgacttatgattcttcccctagcttagaaggaataggtaattcccctccaaagttccgtggcaatagttgcagtgtttcggatggtcg
ttcatctgtgaatactctaaactcgcttacctcctttgacctacaaaatggaggattaaacctttctcttcggtcaagaaccccaagtttggttcctga
acatatatttgatgatacagacagatctttacaatcgaagagcagcagtggttcaaattcattcactgactttactcctgaaagctttaactcttgca
cgctccatgtcctcgatggtcttcctgatgatattgacaaccgagagttcagtgggatctttacgttttgtgaaggttatgttcaatcaaaggttgta
atagattctggtcgtcgtaaaggaatagttaccttcaataacccgagtgcagctaccaaagctaagcaaatgctaatgaactcgccaaccaacac
atttacaatcgttcagggaaacctcccaatcactgataatttgcaacaacgagcaactccttcgttcaatccaggttcaatgccaatcaacaagaa
gctcgaccctttattaggaaatatacgtccttctccaaatatggaaaactcccagtaccttgattcaatttccgaaagcaagatgtcctcttctttcc
caaatcttgatggcttacagtcttgcgagcctttgaaaggttcaaaaaccgattatatgagccaaccaagaacatctattaggagtccttctccatt
aacttggaaaaaccagcaaccttttcagaatggagggataggagataagacttggtatgggcctcaaactgctaagaatgggtataataatacc
acggccaaccctctactttcaaccactgcttccaagaaaattacaaagctaccgacaacactatcagatattagcgaagctaatcccgcgtatgat
atctttggcagtcttgcacctacgattgaggatgaacgtttacctctaccgcaatctatctctccgaaagccagataccatgccactgaaaaacctc
catatcaaaaagagaattctttttcatcttttcaagggcttgatcgctcaaacccgtttgaagaggccttaaggatggaacgattgagtcctgtcaa
agagctgccagcaattcggcctcgagccataccccttaacggcgtagcaccctacgaacatgaatctcatcgtcctccaaaatggaaacctttgc
cagatttaaacgttgttaagtcaagaccgtcccttagaaagcggccaattccaaatcctcaaattactgaaagcaacttgctatcgtgctttcaggc
tgatacacctaaagtagaatttgactccgatgccttagaagcaaatgctcacacggatcataacactccctgtaataccatctatgtcggaaatat
ttctgcttatgaccaggaaaaggagttgcggaagcttttctcaacacagccagggtatcgcagactttctttcaaagtaaagggtaataacccaat
gtgcttcgtagagtttgaagagatctcttatgctacaaaagcattaaacaaacttcaaggagctatcctaaaaaatagccctaaaggtggaattc
gcctaagttactcaaaaaatccgcttggagttcgatcatcagaaagcttgcccattgttacggcggcctcacaaagaacctcccctgttagaaacg
gaagtgcttattctgcaaaagcgcaagcatctttggatagaaattctcctttcgcatcaagtgtttcaagacaaacaagctcaacgagtcttccga
gtaaacatgagagtatgagcgatttgtttgacatctttgaaataaacaccaaataaacctcaaaacccaacatttcatactgatccttcattctctc
cagtcagaaaaaaatattttccattggggattttcagaataaattttaagaatagtaattctcataaaaaattgtttaatgaaatgtcataattcttt
atcttttatttcgtaagcagccatcagtacttcaattcaacatttggataaatcaggaatttataagatttatttggtatagaagataaataacgga
aacagtttcaccattcacaaatatgtaattatttacaatagtaacagacgagtcaattgctccggcgggtttttgttcacttttggtacttgcattagg
ataaaccttcccgtcaggcttgacatagaagtagaattgatattgtaataagcacaaattaaatgaatccaatacctcgcaaaagtagaagcatt
gatattctttggacattcaacatccaaacggacgtagtggtgtcgacgttgacactgaatattttcatcaatagacctagagtcaagggaggcagt
aacgaaaaaactgtctgaaacgtattggtatatttcgtctggctgttgactccacgaggctaacttttcatgaacgatattataaagataactatcg
agcaatataaccaactgcgaggcattgctacgtttcacttcatggcggaccttactagcaatcgtataataatgaaaatcttgctcattatgcttca
tttgtgaagagatgtctagtaatggatcctcgatataattatcataaaatgcttgatgtacgtttgaaaacccaaatactggtctagatgagcagta
ttcctggtatatactttcttgttgttcttcaggaatttgactaaaaagatgaaagtctactgcagcagttagctgagatcaaatctagagcaaacgc
gtaccattcaaataacgattgcgttttcgagaccctacttgccgtgaagaatgagttggtcgccgtctatgctggttttgatgttctaacgattttgtg
gaatatgaaggacgcgattcctgggtggaatgcctaaatttcgcttgctgaaacgataacaggtttatcctacggacaccattgttaactgtattta
ctgattcatgtaaattgtgctgtccatagacggcattaggaactaaaaccatggttggttatcgttgaacttcagtctcactcttggatgcaataagt
tgttacttacttcctgtcacctgtcttccaaaattttataatcgttaatcctagaatgtaaacaaaaaaaaagaattgtgtgagttataccaatagttt
aactagtgctttgcattacttaattttttttttcaagggaatatactgaattcagcgacttcataaattgatctttattagacgtataaaaatcattaa
gtattacaagaaaacgttagatgcttttcaataaataaaacaagccaaaaatattaatcaggaaaataggaataataagagttggggcggaaa
cctttaacgaaacgtttgtaaggctcgagcacgatgcaagcctactgaagtggaaaaggaaagagttatgtaaggcttaaaacgaatcaaggac
aactagaaggtataatgacaaagagtaataaataaaagagaattttttttaagaaaatcccgtctctaaatcaggtgcagcatccaatttcttcatg

ctttcaaagacatgattgaatccgccgatgatatcaataagaccgtgcttttcaaaaatccgcagaattgaaggatcaacagaaacaaggtacac
cttggtactataatttttgtatctaatcaagattcgacgaaaggtaaataaagcactcgagtccatagaagaaacgccaaccatatcgaaaacaa
tgacctcgaggtcttcaatacagtgataaggagtttcaccaggatgcatgcgtggatgtccatatttttccaatcgactcaatcggtcttctaattga
ctaacattagcaaaaaaaaggggttcagggattctgaccacaagaacacgaaggcgatcatcggcatcaacagcgaatgtattaattctttgag
aacgacaagaggcatgatcttctaacaaaatggcatcttcatcttcatcaactgtagtccgaaacatgatgcgagaacgtgccgcatgccgtatta
tttgcaaaacggtaagaccaagtccaaagataattccagtttccaagccaagcattaatgttatggtagctattgaaagtataatgaggatttcat
aaaatgcatgtagctttaccaacgatgcaatttcataaatcacgtctgttaatagagaaagagctaaagaaaccaccattgccgctaacaaaca
acttggtacatgggaaaaaaccggcaaaatatatagcgttgctaaaagtacaattataccgcatgctatagcagaaacctgagtacgagcaccg
gaaagaatgttgcacttcgaacgcaaataaccaccgcatataggtaaacctgaaaacaggcttcctccaatgtttgctatacctaatgacaataa
ttcgcgatttgacctaattatctggcttctttgagttgaaagagccttgatagcgataactgtttcaataaatgacaggaatgcacatgtcaaacca
gtttgtaaagattcgctaataaacttcagcctgtgtacttcaggaaaaggaattttaggatagataacctttgtttcaactttacctagaattggtac
tccatgaacggaatgccaatgaaactgtttactaagcaatgctgctcccagcaatattaaaaccgtatccggaatggagacgactctgggaaagc
gcgtagaaagttttgtctttcctagacgaaaagcagtaaataaagtaatcccaataattgaaatcacacaggtaaatctgtgagattctgagatgt
tgcgcaatatgaatattactttttcaataggaagatctgacttgacaggttgaatgtgagttaatccaagaatgacactggcttggttgatcattat
aataagagaaatagataatatgcacccacgaagcaagacaggagacacaagggcgtcaagaaaaccaagccgaaaagttcctgctgctaaa
gtaaaaagccctgttaaaaaggtgatcattccggtaatgagaatagccatttttttcaggactttcgctttcttgaactatacgttgatggatattacc
cgcgattagcaaacatatgcccgcttctggacccacactaagcaagggactagccgtaaataagctataaagtataggacttatggaagcaccc
atcaacgagtaaactgggggaatccctaagaatgtttgagagtatgtgagagcaatgggaatggaaagacatgcagtagaacatccggccaaa
atatcccaaataaaattcgtaaatgcgtaatttggaagccaatgcaaaacgggaacatagtactgaagccttcggaaggcagaaggcttttgtgt
aaggacattggctcgcttccaagttgaagatcttctagccaaagattgatatgaaggattcgttctcagaagttcattttgttctaacaaaggtgat
cgtaaactgcttggtcgctgaccgtttgccatagcggagcactgccaagatccgcagggcaacttgaatattaaatctgttagatgtcaatgaaag
aaaataacagttggcataagctgctgaaatagcttagataggactagagcagcaagacagtttgaagcaatatgaggttcaagatataggatga
gcaacttttctttagaaaccaatctacttagataaattgaaagcaatgcaaatactaatatttgtgcttcacaacgtgatgggctctagtgaggata
tactcgtttcttaacaattttggttgatcagatgtccaataccatatcataaagtaaagcgagagttaatcatcgctcaccttaaggtgatattctta
actgtaatatgatatcttgaaaagaaacaaaaagaagacatagtgattctccagtcaaggattctttgagcataatctccaatgtacatgttttgct
tgaatcaaggctagtggatctcttctgtttctgaggattgagtatctcaggcgtcctgtacaactctttatcaacaaaggatccagatcaagtgaaa
gcaacgaagtttcagtgattttcatctatccgtattggtcatctagtcttcttgtttgaaaaaaaatgcgtataagaaacgtgaagcattcttggctc
aatcactctactagtagctaacgaaaggactgcagtgcgaaccaaacgtttgacgataatagatcctgattggtttttgatctaaaccgaatttga
catcgatatgtaagagtacttatcgtgtgtagttcctctttaactgacgaaaagcgacgtgaggatgcggcttcaagtagtaggctcaatgaaaca
catatcccgacaaagaatatcattctaatagaagctaatagcagctgaatagtagatataagcgaccgaatccatttatccctcagcaaaattca
caaacagaccagtaaattttgatcccagttcgacaagtttgggacttgaccatgattgttagaattcagcagttacaaaaaacgactaaaatattt
gttatatttgtctatttactccattggaatattcagcaaatttatctgtaagcatcatttgtctactcaaagacagtctcataatccttaatccattaaa
atatatgtcacaagaaataccttagagaaggaaatgtaaaagattggtggttaggggacctgcttttaatactgactctcatgtcgagctggttac
taccaggcattcttagatgcagaatgtaaagaagaatttcaggaacttagaatgaaagaagtcagagtagtcatttgtggtgaccaaggggtcg
gaaaatcctctttaatttcagcgttaatacaagaagataatgttacagctatcccaaaggtaggcatgatttgatgactttccttaacgtcctaacc
caatcgcgtaggtatttccaatcatatcgattccgcctgatccagaattaaacgacaatgtttctttggtactagtagatacacagtgtaagggcaa
aatgcgatttgcacttaagcaggttggctaaccgttttaatgaattttagccggcagtaacgaaagagaatatttggcttcacaattaagaaaagc
taatgtcatatgtctggtatattcagacaattactcttatgaacgtgttagcatcttttggcttccgtactttcgatccctgggaatcaatgtatgcaa
gttctcagattattactttttactaatgaatatcaggtccctattgtcttatgtgcaaacaaatcagaggaccttgacaattaccaagggcttcattct
atcgagcatgaaatggtacccttaataaacgagttcaaagagatagagtcctgcattctttgcagtgcgcttgaaaaaataaatgtgaatgaggt
actatattgttctctgtatttccggtttctttagcagtctaaccagaagtagctattttacatatgccgttcttgtgttatttatcccatcattcctcttg
ggatacgaaagaaaaggtactcgattaccatgagaggtttttttaaataaatattataaacattctctaacggccttattagaaactaaagagcgt
ggcggttgatgccttaagccgaatattctgtaagtttctctaatcatttttatttttttgatggaacgatttcttaatgctaactaatctagtcttaagc
gataagaataatgatgaccttctatcgacaacagagctcgcacatttgtctgagaagtgttttgaaaagcagctcagcgaagcagaagcaaagg
atataatttatacagttcaagaagtcttcccaaaggggatagttgacggccagctcaaccttgctgggtttttagcatataatcaaaaccaaatcg
agaacggaaaacaggaaagtacatggggaattttgagagcgtttcattatacagatagtttatgccttgatgattcttacctaaatcccaaattag
aagtcgctcgaggtcagagtgttgaacttagtccaaagggtaatctttatattgatatgacatttgctaatcataattacaggatataggtttctagt
tgacttgttttatcagtttgatcgagataatgacggagcgttaaacgatgatgaactctccgacctctttcggtatacaccaggacttcctgaaaca

tggattcattctcagtttccaaattcgaccgtacttaatgaacacgggtttgtcagttataatggttggttggctcagtggagtatgattaccctattt
gattacaaaacaacattggcgtacttggcttatctgggatttgataccgaaggtcgtggtcatacaactgacgcactcaaagtcacgaagaagag
atccattcaacatcgtcgagtttccaagtatgatcgaaacgtgtttttgtgttttgtggtcggatctactggttgtggaaaagtatgaattaaccttga
atttttatattcagctaacagaaagacttcattattatctagtttcattaacaaaattccgcatcggttgcccgatgatttaattcccaacacagttgt
caacagtgttgaatttcaaagcaagcaaagatacttggtggtatgaaagtgttagttattgtgaaaccctgctagctaaccaactagctctctgaa
ataggcgagcaagaccttgacatacttgccgaaccaaaatcattagatgcctgtgatgtgttgtgcttgctgtacgattcctctaacccgaatagtt
tttcttttattgccaatcttttggtaaggataaaaaaacaggagtaatatatattaacttcgtgtttttttagagtcgttatcctgcgcttcaaaaaatc
ccttgcgtatttgccgcaacgaaagccgatttagatcgtcaacaacaagttagttaaattttcaaattatttgttttattgttgctaatcttgaaatta
gagatatccggttcagccagacgaatttacgacacagctaggccttccatctccaactcatattagtacttctgctatctggaatacttctaaagatt
tcttcattcaggtaggtactttgaaaagttttgtgacatgctaacataattatagattgccgaatcagctcagttccctgcttcttcagtgattcaggt
ccccgatgaaaactccaataaaattaattaccaactggtggttgctttaacagcctttggtgccttattactttcggtaggaggcagccttgcttgga
agattgtgaaacatcgcaacatgttcaaacgatagttgtattaagaaggactgctgataggcctgagacttctcaatataataaatatgccgcttt
gaataaacacctttctcccagttaggaatgcgtgcatacgactaatcatgtgctcaacaaagaaaagaaaaatgaaggtagataaaaacagac
gtaaaaatgcaaatttatctatattagatttcccaacttacagactcaaaatacatgagttatagaaaagttttacagactattaatagacccaata
tcaatcgtttatgaattcctgacttttaagtttatgttagataccgtttgctgaaaagcggcattcatcaaggcttatattaaaaatcctaagtaacc
caaattgcataatcttgtgtacaaatatgcatctttaaatgttacagaatcaacctcttgactattgtatgaaggaattgaagatgaaggaacgtg
agggggagcaggaggatccgaagcaatatgcataccttgcattttagcatttatcaattttaagacgacgtaatcccaatcttcatgatcctttcta
ctgacaatgtcccgccgcttttctatacattctcgaatgtattccagtccctgtctagcttcgtcttgtagctctgggatatcatagacaccaattaaa
acacaggcagccaaactgaaaaagtgatgattaaggggtgtcaatggagtggaagaagacgaaagaatgttagccattttcatagcgggagtt
aaaacagcggaaggtcgagcatttgtagataattccaaaagcaccttaacatgtgtgacagccaaggtcagtaaattcattcgaccccaaacaa
catcaacgctttcacggaatgcgtcaatttctgaccttaactgttgagtcacgattggattttcccgatcactaggatcaggagattgaaagacatc
cgaaatgtgaccaacgattaatgacaaacgaactaactggcttggaaaacttccaaggaccttttgatcttggatcgaaagctttatgcaactgtc
aggaatgaacaaaggagaacatgtggacacagagtgccatctgtctaggatgaccagaacccaccaggccctacgacataatgcttcattgcca
tccatatcttgatttgttgactgtaaggatatatgggattgtatatgcaaacgcatattacaagccaaaccaacggcagcgccatgccacatactct
gaggtagcccatttcgtccagtaattgtcgtaggtcctcgttgatcactttcgaccgccaataaaatcatggtctgcaaatgtaagattctattaga
gatatcctgcacatttgtacatatcaagttaagtaaaccaattgccttcataggatttttatgaaagatacttccgagagatgaaaacggcgggcg
atttactaaggaataaatcgcatgtaaacaacatgatcgtaaaaactttggagctttttctagttctaattttaaacgagtttttgagtgatgcaaga
ttggcaaggtagaatgtataagtagataatatctgtcaacagcttcgtcatcccattctatgtcttcctgaaagactctcgatatttcatgtatgtcat
cgtactgtgcgttttcggcagttctcgactgctgtacattgggaccagcatttaatgtagaagatcgggcttcttgccgaattgatgagggtgcgctt
tctggtaattggttttttgaaaagcacgcccaaaatttggtaaatttccaggagaattaacaggaacaggttgcatgggaatgggaggttggtag
gacgagtttggtaatccaccgtattgagaaaccgtattaagacgagaattatcaaatgaagcaatagaaggcaacggcagagatctaggatctg
atgacagaccatttgtgagtgcaggaggatatgctataggcggaggctgcatcggcttataaggcgattcagcaagaccaggatggggggtatg
agtagggttagcacgtaaattggaatctggcaaataagccgtgttcatgtttgactgaaaaggtaggctgctaggtacgaacggttgtacacttgg
agcagtataaatggagggctgcgtaccagacgtggaagaaaaagatttaggatggtggaagccggcaacggattggctcgagggagcggata
ggtaaggatacgattgttgaaaaggaggaatcgttgaaggaactggctgagactgttgagaatgaaagggcgatggaggaggatggcttgatt
gttgaggatgccatgtagcagtaggctttgggtgctttgacagaataggttctttcgcgacaggcgatacagcaggtattgcagtatccataggaa
gaactttactaggattggaatcatcaggggaggaagaatttgatttaacatcgaaaggtaaggtatcagggagagtagaagaaggagaagcag
cggcatctgtatcaagcggagaagcagccgccgtggtgctattggaacgtagacgtttggttttatcttcagggttgcgtattagacctttgaccgg
gccacgctttaattgtgttcttgtatagatgcattgtaaattgtgctttgagcaattgatgcaagcacctgtggatccttccaccatgtcacagcgaa
tctaaattagttagagaaagaataaataagaaaaaatcatacctttttttttgcgacagaggtcacaagcacgagaaaccttttttctcctcttctcg
tccgaagccgaagaagaaacgcggacagaactaggacgtacagaaacctggaaatcttgagggttactgttggatacagggacgttcaccggg
cgtgggacggatggagaagaagaggactgggaagcgaaagggtcgaagagatatggataagggtaatcattaggacctaaggaagggaccc
caggcgcatgccgaaagaaggaaggatgggatggagcagaaaaggaaggagcatgttggggaggaataggggaagtcgatgagttggaagg
aaatttagcaggaggaacaggaagataagaaatggtattaagatccatgaattagtcaaatcattaaatccaagaaaaagcaaaaagtgaaa
aggaagcggaataaaaacaacaatcaatatctattaacggcgaatgaatggagaaccagatataaaattcgacacagaggaaggaaagcaat
gcaatgaacggaatgaaaaagggaataataacaataaataaattagaagaaatgataaaacaaggagaaaggatgaaaacgaatagtctct
ataagaaattagaccaaaccaaaggaagagtcaggaaattgtggaaatgaaaaataaaaactatttatacagaacgaaagcaaaacgaaaa
ataaaagtaataaacgaagtaagtaggcgaaagaggaaaatcgatgtgatgatggcaacttataaataacgaaaaaaggcaactgagacgaa

aaaacaaaggatgcaacaagattttgagtaaaacaaaatgatgaaccaaaataaatgcacaaactaacgagaaacaaacctaaaaaacaca
ggaaaagaggagctgggagaaaagggaagatgagaaaagaaggctagaaaaaaaaatctagatgatgaaagtatatgcggcgattcacgtg
ttcgttcttcagactagaaggatcgaattaaaaagaaagaaattgaacaatctaaggttttgttgtggtacaaattgaatccacattttctaagga
gccgaaaaataaagtgaaaagcactaatcgcttgaatatcaattctcgtaaaattgcgaaaaaataaataacgcattcgttgtcagaggaagca
atggaacactaaataatttcctcggtgaatacaaatatcacagctgataaaaatgcactaaactgcgatggtcaagcaccttaaaggaatacag
caaactagcgaaattagaagggttgtttccgtattctaaaaaatacgaattgaatcaattcgctataaaaagaattcggcgcatcaagataaaaa
tcaaaggaaaagaaggacagggttgtaagataaacgaatttaaaggagtaaaggtccatttgaaaaaaacagtgaaccgttaagttaacatcg
tatacgggaacatagaaaccaatgtaaggtgcaattagaaagtaagaaatcagggaacgtcgtagaagaaaatgaatgacaaggaagtaaag
aataaacgaatcgaaaaagaaagcagaaatcgatgaaaaaaaaaaggcccaaaactgtggagacgccaaaacaaacggaagaacgggtat
gggagaaaacaaaagtaaacaaaggatgaagaagaaccggaagacaaacgtcagaggaagagaaaggcaacactatactaaacctcggta
cccaacaagcgtgcgtttgtatgaagggaagtacataattggaggccacgtaaaagaatttaagtttacgcgccaatcgcttagttgcaaaccga
gtccgtcgattacaattggaaagctggtctgacgtaaaaaccttaagaatctgatgacgtaaaaaagggcttagttcatatattctgatccaaact
tgggtacactattatgagtaaaaacgaagtaccttatcattgatgaacgaccgtcagaggaatcaatatagaatagctatatccaagtataaaag
tgaaaactgagttgtaatttgagttgtaattgtgaattccagtcaagaattcaaaatataataatgacttacttaccaaactgcgttcgaaagtggt
atccagtatgtaatcagatcttagtaatgactagaagttcccttcttttcttctctggagaaggaaaaattaggtgagtggggaaaagatattttca
ggagagtgtacgaaagtgattatcaaaaaaaaaaaaaaaaaaaataatgtattcagattgaataaatcttgtatattgcgaatgtttgattacgca
ttctattatgttctatgttattcttggtagtggaatcatctcgctgttgcaattttacgattcttccatttcgtaaagtgtagtacgatgctgtaagcaa
agcctcccccgatggtagtgatgtaagaaaatcaagcaatagtgggaagagctacacgagataggatttgttatcgaacgttctagcctctcgaa
gttcctcaagattatcgctaactgaatagtaaagattattatataagtgggacgatcctgctgttatttcgactaaattaatagatcgatatcctcgt
aattttttgcattctctttctttttttttttttatctttagttctttttgacttacatatcccacaatgaacatggacaacttgaagaatttagccggacagg
cttatggtcaataccaaaagtacaataaaaatagtagtcaaggcaatggcaattctggcaacgactcttacggaaatgataattcttatgacagc
agcaacaacaattcctatggtggtaatgacaattcttatggaaatgacaactcctatggtggtgattcctacggtagtggaagcaataacaagtct
tcctacggcggtaacgacgactcctatggaagcaataacaagtcttcctacggtggtaatgacgactcttatggaagtaataacaagtcttcctac
ggcagtaatgacaattcttatggaaatgacaactcttttggaggcaacagcaattcctcatatggaggttcttatggtaactcagacaacatgtatt
aattgagtggttattaattcccttaggtaataataatatgcttaacaactctaagagccaaaagtcttttgatcaaacttcaaactcatctaaaatg
aattcggataaccactcggcacccttggatactagctcctttatcaatgccttttccaaactaggtcttggtaaagataacgatcaaaagtcttctg
ggcaaggctcggatactataagttatgcagctgttttcagcgccgttcaaaagtacttttctaagaatggcgatgcgttagcaactggccaagttc
attctgagcagcaccaacaagatttcttatccatggtggagcaagaggctcaagggttgataatgaaaggtaactcttcttcaggacaaggccag
cgaggtgcaggcggatttgatggggatgccaagtctgctgtacaattggcgaaaacgctatttcaaaaccgtaatatgcttatgaagttggtgga
gtctggaggctcgtctcaagctagcggtaacagtgctgtattggcaagcgttgttggctctttcctcggaggttcgacttccactaactctaattcgg
gcaacaacaatgctcctcaagctaatgcacatggattgcaggacatggcacagagctttctgggtccaggaaaccacggatccagtcaaaatgt
cggtaattcgcaagcaagctctggtttgggtgggttgcaaaatctagccggtagtttgcttggttctggtaaaaattccactagtgattctggtaatc
aacagcaaagtcacggcaattctcagtctggattagctagtaacttattaaatgcggtgactggagagaatggtcacaatggaagacctcagga
gcaatccaagcactcggaagatggaggtggtttgatgggaaaggctattaacatgtttttgcagtaaatgcggcattttagtctttatttagtcatg
gttcttggactaaagtaactttctgttatgtacatattaggggtattaagttttgcatgtagtcttttaaatgaactgactgacaatatattgaaatag
aaatgtaaaaaacaaaacaattgtaaatcaagaaaatcattagaagaggtttcatgcataattctagtcgtaaatgataggaaccgtctatttac
ttgttgagcatatttcctttattacagatgattgaagtacagagttcattagcattataagtataccgagtacagctactgatgagtaaaatattaag
gcgtaaaaatgcttggcgttttacttgtatgattaagtgatatgataaaataggacaggtgatgaacaatggaatgactagaaatgacaattaga
aaagttacaaattaagtaaataaatacaaatctttaaatgccgattgtttgatatccttccctttttggcaaatggcatagaataccaagtgctacg
cgaatacacgtaaagtagaatatcgaaagttagcgacgcagacagttagcttactagtctgcttccaccaataaaatatgtaagagattgattga
taaacaccttccccgccaagtctatgtgtagggggatcgaaagctggcttttactataccgtgaggacgcgtaaaaggacgaaatcaaagaattc
aagccttgctttgagagtttcattgataattacgtgcaaatttacttagtgaagatttccattgaagtttgctcaataatcgggtgaaaagttgttga
aattgaacatctttagtaatttgtagtactaaaaccagcaatgtttggagccataagacatcaaattcgtaaatcttcgacttccacagtggtacga
aatgactggacgcgtgaacaaattagtgaaatttatcatttgccacttatagagcttgtatttagagcggtatgaaagatttgtctgaattgagaaa
accaattgaatctaatgaactggttttatttaggctactatacatcgacaattccatgatcctcgtaaaatacagcaatgcacacttctttccattaa
aacaggtatatagaactgtacatgcatgctgaaaatactaatgctcttctctaggtggctgcactgaggactgtaagtattgctctcagtcttcacg
ttacaacaccggtgttaaggctacgaggatgatgaaaatagacgatattatggaaaaagctcatatcgcaaaagccaaaggcagcacgcgtttc
tgcatgggaagtgcttggcgtgatttgaatggtcgaaaccgtacattcaagaatatcttggaaattattacacaagttcgtgctttgaacatggaa

gtttgtgtaactctaggcatgttggatgagcaacaagccaaagatcttaaagaggctggcttaaccgcctacaatcataatctggatacttctccc
gagtactaccctaagattatatctactaggtcgtatgacgaacgcctgaatacgatagataacatccgtaaggccggaatcaaggtttgcagcgg
tggtatcttgggccttggagaaaaagaacaagatcgtattggccttattcattctttggccaatatgccatctcatcccgagtccgttcctattaatg
ctctcgtccctatccccggaactcccgttggtgacatggtcaaaaaacctgttggctttcactccttccttcgtgcgattgctactgctcgtatttgcat
gcccaagaccatcatccggttttctgctggccgaactcaattctctgaaacagagcaagtgatggctttcatggctggtgccaattccgtatttact
ggcgaaaaaatgcttacaactccatccgttacctgggaccaagacaatcaattgtttcacaaatggggtttccataatttggaaagtttcgagtat
gaagaaggggctgaggagccaaactttactcttcccccaaagaaccgttttcgtcctgatcatgctgaagttcttaaggctgcgagacccgaatta
gaggctcaaggattggctgtttaaacttcctagttttctcattctttggttttttttttttcttttatctttacctgaagcaaccgtcttgtcttttgagtat
cttcatattctacaaagttttggacaagataaatgctagattaatgaattaattattcgttcttttgaatcatcccttcatacgtttcgttgttcacgga
acaaacttgactttctttaagcgtgtatcggctctaatcaatctcttttttttagctaattacagttaaatatattttcggcattgcttattctaataaaa
cattcacttgcgtttctcaatccttttgtttatcatttaccgtaacaacaatttctatcgttatattatagtaaaaattgccttgcactttgttaaatattt
tggcattacatgtattgaaaaaacgtagcaaacgcgacttcccatgaacaatatatttttccagacgcgaacgcaatccaatgccagattggcgtt
cgcaatttgaggtaacgtaaaaaagaggtaaaatttgctaaatcattaggaacattactcccagtatttggattcagcacttgtcttggccattgtc
aacgactgcgaaaacaccaatgatgctcgagaagtattggaaacgctgaaaaatgacggccaatgtttgtctcatgaagatggatggaatcaat
cccaggcaaaggaggaagaaagtgcgattttgcaaacattagacgatgagttaaatgcagagcacgaggttgactattcggaagaattgatag
attgtccttatgatttggatggaacagtatacaactttttgcggtccatgtttcatggtattgctccacaacgagttcaatacgtcctttccaaatgtg
gaaatgatcttacgcgcgccagtgatgaattgttaaatcaggaagtactcgaaaaagatgaaggacttggaaatgctactgatgctctttttgcgg
ctccaaatgcctctcatcgttcacgacaacgtaaacgaaaaaccaaaaaacctaatgcttcgcgaagagccctctcgatgcgcgatctgaattca
gaagaaaatcactcagaggttgaagacgaactagtacagttcacagttcaaaagctttctttctgggaaagaaaccaacagctttttgaaaagat
atcttcaattttggatatcgcgaactctcgagtaagccatgaattttataaaaactctggatctttatactttacggtaaacgctttgctacaaagtc
atcctctgaagacgcaaatctttgagaaatcttgtgaagataatgcatctgaactgtcaaggaacactggtctttcgcttcaattctgttgtgagctt
ttaacctgttgcaaagacttgcaaggtgcaagatggattgcttatgctgtgaaacaatctcacccaaaagatacttccaaactagagctcggtcta
cgtgctggcaacgaaaaactcaacataagcactccttttgctttaaagcataatttgcctgaaagagacaatgagacggaagcatactcgacag
aagactgtaaccgtctggcggatgaatatttagagtgtcgaaacgctgaatatgctgcttcggccagggattaccggcgaagcaagagtgaccg
attgcttggcggatcagcaacataccatgctcaagttggaagagagtatcatgaaaaagctatgaaatggcgaggattagccatgagatctttgg
ttcagactggaacaccatattcattagatttgcatggcgcaactgttcgtgaggctaaggttatcgtggaagaaaggatttcaagttggtgggcaa
aagaagcagattcttctccaaattgcatacatccttttagaattattactggaattggtaatcatagcgtaggcatggaggcgagacttttaccctc
catcgtacgatggctacagcagaatggttggaggtttgaagtaaatcatggtcaagtagaggtttatggtgctattcgcaaaaaagtaaaataag
agcatacatcccagtgggattttggaaatttggtctttttgttgcatgcatgcatgcataataagattgttttggtttatagtaacgttaaaattcgat
taaaatttattttgagcctatttaatttgattgaaactcatcatattagaaaaattcttcatgtactataaaaattttacaagctatactagtgtcgtt
aatgaatcattaaagcaaggaaacaaaaaaggtatctaaaatcagtagtttaccggtagagaaaagttaagtatggaatgaagttaatgatctt
cgggttggtggatgctgacaatttagttctttaaattccttgccgaagaagttaagagacttcagaaggcgcatacgacgagggaccactttcata
cagataacgagaattggttcgttcaaaagggacggtctgattacgtccaaaccgttccaatgaagcatccgggagtaaataagcaggagaatag
gatccatctttattcttaagagcaggattaggagatgtatcggaaatgccacccagggaagtataacccttatcatatagtatacagtatttcgtttt
cgtttccaacaagagctaaacaaccaatttgttagtaaaatgaaagaccacagacataaataaaaacacgtacgtcccaaacctcagagtaagt
ccaatctaaaattggctgaactcgcataaatttcggccatcctttgtcggtcatttcaaaagcaacacgatgcaagccatggggatccaaccggcg
aatgccaataagtattgcttggatatttgggttgtccgctaggaatagcgtaaatgcctctttcataggaagggaaatgctgataatgttaagacc
gtaaaggttcttgcaatcttgaacgaattcatccatttcataaaattcatctttcggtcgtacgaacacaaaagggatctctgctaatttttgtttggc
catcgaaccaccgtatttttcccttaagcagtatatacaaagcaacaataaaaccaggcaatcctttcctccattaaatgacatagcaattcgtga
ggccctgtacaatatcgttagaaaaaagtaaaaaaaagaaatcaattccgtcaaacatatatccttcatacccataagattcgaatgcataatca
agaaatttcagcgatgttataagcctttgctttaaaactctgtttttctcggaagaaggatatgaagtataaatacttttaattttattatatacgtctt
ctaatgcctccatgaagcctttatttcctgtaaagctgcagcaacaatgtaccagtttccgatgcacctgtgttgggttcaaaagtttacgaaacaa
aagaaacacagtattttcttctttaattctagcttctctttaaatacacgcacaatattaattattgatattttctataagtgtaattcgagaaggatg
gaggtggtggaattattattcatgagatgcaatgaggtagttagttgtgttcttcgagataaaacttttctatttctttttttattcaatgattttttttt
gagtagattgtaattctgtttttcatatggacttatgcgtcgacttctttttagagaaacatctttgaataatgtggataatgagattcactaagagg
caaagagcaaccattagtttactttacaattattttagtcaaacaaaataatccaacattatttccctcattttcagaaagttatcagtgcatccgaa
aaagacaagtccattttcattatttctatatacatatacacttctaaattaatatacaggaaaaagctccaaataactaaccagtatcacaaaaaa
ccctttaagtttgtaagttttatatccactgtaacttgaccttaatcaagaaggaacgcaaaacaaaaaggcagagtccgattctttcgcaagact

gactgaaaacaagactttcttattaaaacaatcgaataccttcagtcttctccagtttacgaaaggtattcccataaggccttataaatccaatcaa
tataaatgattataacatgactcgcctattttatttttctatccatcctttgaggagtactgaaagttgcacaacagcaatgatgacccgcataataa
ccaaaaggggattcaagataaaactcttttattcaaatcccaaataaagcatacaatgtgtgttgaagataaatcgtatccccaataggcttagat
aacgacacaaaccaattttttttgcaatgttctaacagatgctgctcaaactgtcttcgattgttcatcttcaggtgtctgaaaaggcacatcagctgc
cgatggtttctcctctgaagttgctaaagatgcagtatcatcactttctttgtcttcttctttcacattgagggactgtgctgcctcactgtcattcgac
ggatcggttgaagtagcatcgccctcggtttttttacatcagctgtagcttcttcttgtttcgcgatttctgattcagccttgtctggagtcgtttctgg
tttaaggctttctgatccttccttaggctcttccttaggtagatttgttttagtattatcagttttcttgtctccgtcatctggattttcttcgtcgagcata
tcttgaaccagacgctctccgagttcttctcgatctttaccatcggagctaagactcatatctaaggctaacttggtgtttgcaagtctttgaatatat
tcttcaacagtttttttcgtaatcaaccttataactctcacgtcgcgcatctggcccacacggtgagcccggtcttcagcttgcaggtcatcaaaagg
attataagaacaatcatatagaatcaccacgttcgcgcaagctaggttaataccgaatccaccagctcttgtagataacaaaaataccgtcacat
caggttctttatggaactgatcaataatgtcttgacgagtttctacttgagtagatccatccaaacgaacataagaaatttttaaagaatccaaaac
ttgctctagaatatcgagcatttgagtaaactggctaaagagaagtatacgatcacctttctgtttcatttcaggaatcatttttttcagctttaggat
tttacctgaatccatccaaggctcctctttcagcatatacgattgcaatgtggggaaattgcggcacagacgatgaagttcaaagtcagacataat
ttccatatcttcaaagatgtattgctcattagcatttttgtactgttcttccttcatgatatctttagccatcttacgcaaagtgttcgtatcgtaatgttc
tcggaagagtaaagcatgattggcggcctttcgcaattgcataagcgtgtgtccaaccgatgctggcttgccattttcctcgtttccttcgtctttctt
tactctcttaggtcgtttatcctcacgctttagttgttgagatttttgcacagctgcataccgattatagatggtttcttgttcggcggacatatcgcaa
tattcaatgtgatgactcttctgaggtaagtcacctaaaacttgagctttacgtcgacgcagtacgaaaggcgtcatcattgttttggctctactgat
tctctgtttcgataaaagagcttgctcgatgtcagcgtcggcagtaggctttgctttaaaaatgacatccaagtcctccatgtcgctatcaaacacg
ttcggaaggatgaaagctagcaacgatactaattctttaagattattttgcagaggagtaccggttaatagtaatcgaaagttagcagtcagattc
atcaaatttttatatcgctctgacattcgattcttcaaatagtggccttcgtcataaacgcaaacatcaaagttttgatgcttaaggaatgagcgatc
ctctttactgctggtggctagttggtatgtagtaaccaaaatgtcatatgctacattattttcttctatagcttctcgaatatcagcgcgttcctgttgg
cttccatagtatggttctacacggagacggggacagaagcgagcaagctcacgtaaccaattttcgagagtagaagaaggaactacaacaaga
tgaggacctgtacggccttgttcaagaagcagagcaaaaaaggcaacaacctgacaggtcttgcctaaacccatttcatcggctaaaataccag
aaagacccaatctatacagtaagttcatccaattaacaccaatcatttggtagctctttagcttaacatcaggagcaaggctagcaggttgttcgg
tgataaagccaggatagttcgaggcattaaaagaggcattatgtagtgaatccatatcaacgatgttcaattcaccataagcttctttcaattttat
accccaggaagccataccatctgatatacgcttacctaaatcttcacaacgggcgataaggttgtcaatagcatagtaaccttccatggtttccat
gcagacattgacaatttttttgacccatagggatttttggtcttcttcggcctctcctaccaggtttactaggaacatcatcaggatgtttaatggcac
aagcttcggcaagattattgaagggacgatgttccagaagcaacttagagtgagcaagtgggcagctggcagtatccgagatttccttagcaga
actagcattgatgaaattgagaacagttcgctccaaagctgaagtgtctctttggacaactacatctgattcttcgccggaatcataaaattcatcc
tcttcaacgggtttctttgcaggttttcgggagtacgagggaattggtgtggtttcttcggcaggcgccatgggatatcctgttgaaggttgggtata
cttatcacgtatagaacgtttttgggaatgcacagtacgatttgaagtctcaaaatttggaataaaactggaaagatcagggaggccatgagtga
aagcctgatgctgttgtaactgagctgatacatcataattcaagtattgacgggacaatacagacaaagcggctatttcttcccaattattttgtgc
aagagcattgatcgtagttcctatgtcggcctgaggaatgaaagaacggatccattgaacacagagcatagcatgattgggatttttttcttggatc
ctttatagaagcgtcggccgtcatgggagatgaaagggaaggtatattctcaggaggggtagcagaagtatgttgaacggatgatgatctagga
tgactaacggaccgagactttgagcgggtatagctaggaagcatgtttgctgcgggagtagccgaggcagaagaattgtaaagggtagagttat
cgacggccggcgcaactggattcgatgaattcaacaatcctccagaactgacattagacaaagcgggaacatttcccatgaaattgttattgcca
tgatactgtgaattgtaattgggatatgcaggttgctgctgcaaagactggatactggggaatcgggaaggagcattggtcattccaggagaaat
tccagtaagcggagaagagtttgatcctagattgtaagaagagttggttgggtagggtgaaacaggttgtggaggaataccacccatgtaagca
gcagttgaaggtgcagacggcgcaaacccataaggataattctgagtgtttggcatcatgtaagagctatttaagctcggggtcggatcactaag
caacgatggattggaagatgcatacgaataatattggtgaccaaagttaggattctggaactgactatttgcagcagtaggtacataaggagact
ggggaacagtcgaaggattcactccagaaacataaggatactgagaagagcttaccggtgaagatgacgatgtattagcataaggagtattga
atcctccaccggatgatgcaacttcaccagaaggagtctgattctcctcgaattttcgttttctgttgtccgataaataatctggaagcatctattcta
tttagttacatgaagtcttttgtaacagacaaaatacaggaaatctacggttggtttttttgtttgagatttttggtggctgtttgcagaagtacgcaa
aaccaaataaagcagtttagtccaattatcgtaagatacacaacgactaatagtgagatatattttttctcgttattattttgtaaacaaacaaaag
caaagtaaatatagctcaattcaagcagaagaaaattggaaacccccaaattggagtttggaagatccatacgaaatatgagtttatttatgtta
ccaaaaaaaatgttttctgcgagacgaatgaaccctaggaaagttacttccaacatcatatccgacctgaaataaacgagaagtacaggacctctt
tttcgtctttcttagaattgttgagagttattcgcgtcaagaagaaacttaattctttcccctttacgaaacgcttccactagtaacatcaatgattttc
ttatttcctaaatgaaattgtgagctgcttttcacgaaaacaaatcaaaagccttgtcctgtttttaacaaacatagtatatttggccacgacagatt

gcagcgtcagtatatgtttagtctatcgagtaggagtaggaacaggaaagtaaataatagagacttagagcctgttatgcttaacagctatagct
ggatcttcaattagcacgagtttgctagctgaaccaagaaactttttgctcgtacaattttatcagtccgctaggcttgaacataccgcttataactt
cataaatagaaataatcacaaatgaatttccgactttctgccgtgtttacaattgagtatttcataccaatgatacttataacccgagatcggatttt
cgtgacgggcaccttttcagaatagattcgcataatttttattcctcagcagccaaaatgaatgaatgggctcattaaacaaagagtctccaaact
tggtggttcgaaacttttattttcatagacaaatttcgcaatatatccacgccctccaacgaaacaaaagttcaatgaacttccaagattatttagg
taggagataaataataagaatcttccataaaattgaaagtgatactacaaggtatcgttttgctaaaaaagcctatcccaagaatatcatcatatt
aatccataaacttttcaacgaatagataataataatgattcaattggcgtaacattacctagagatattttcaaggtgttcaaactttagaagcttc
caattccatcctcgaaaggtagcaacgacatgggtctatgattgatttttttctatatggatgtcacagaatatcaacaaaacaaaacaaaaaaat
cttttcgaactttacttcgtacctacctggcttctgaacgcctttagtgtccatgattcactaacagcaatgtggttctcatgaacgcaacatgtcagg
aaaaacagaagaagagaattccatccaaacgtaagtgataatatcctttagaatcatattcgatggttgccatactggaattcttgatttaataga
ttcctattacattggtcaggcatactttttgaacatttcttgtaggaagaattatcaaagggcttcctattttggaaacaagaaacactaacttccttc
tcccgtaaagatttgctactaacctcagatcagtcgtattttggtaaaattcattggatttagtattgccttattcgcattacctttactgacttattatt
ggactctaaagcactggttttctggtatgaactttacgatatttatatttttggttaaattaggaaccaaagctcgaaaaatctgagaagtgtacta
acgattcattcttgtaggctacaatactctgtatgcaggaatagcagctgttgtcgttgtgaatgttgttttagctttctatattgctgttgcatttttgg
aaaaggatgatacttctccaaaacctgcgaaaaaacagcaataattgttaatgccaacttgagctgctgctagcatcagtcagttcaactttattt
aaaaggatcgtaggaatataattcacattcaaagaggaagaaaagaaacttacacaagctcctctactgtttctgtttccatgaccatagaagga
ataaacttttgagagaaggaaaactcaaggaatagttggatggtgcgtaatgtattttgtaaggactagattcatctacgctttttcgtattaatga
tttctgccagaaaaagcactagtcatttcagcagttcgtcttacaataaatccaagaatttcacttttgcatgttagacgatctctataaattttacg
acggtattataaaaatagagatttcaaaagactggtacagtttaaatgtttatgaaactacccactctgcttgtatgtaagtttctaccagatgata
aaattgagccttttcttcataaacagcgtggtcgaggaaatcatttattataattttatttattcttgaaataattaatcagggcataggccataaaa
caaaaaacggtttgaggtgcattcctcttgaatgaacttcctttgcaaacactctgtttggaaataatgagtgacctttttctttacaatatatgtgta
tataaataatgttttgtttattacatgaataatctgaaagttgcttcaagcttcgatttcaaccttaactcatacttatcctttccttcatcacatcgaa
attagcttgtaccgaattttggaacataggtaaattagaatggtttcgtagtttattaactaattttcttttcaggttttccaatatttaagattaaact
atatccttttggtaacattagctaaaaatgtcatctgcagctgaacaggcaagattacgcagagagcgtcgcatggagaagatccgccaaggag
gaaattctagaattcacagaattttgggtcaggaacctaaagaagatgagcaaatgccttctcatgtctctactcctgaaactgttgcttctccgag
cattcagtcaacttctaaagccgagtcagttatgccttcggacactcagatttccgaggaagataaaggtgatcctagagatttacataatctcttg
aattcacccttcaatttacagagcgacactccatctaatgttccccctgagaatatgttcgctgaaaaccctgattttgccaaatttttccaagccctt
atgcaatcttccaaggaagataacaatggaccgtttgaaaggcccgatgctactagcaaccccaatacttatgtggaaaatactcaaaaccaaa
cattgcgaaagtatgtccacttacttttcgttgcagctatagtttgtttctgctactttcgtcgattggtatgtatcaaatatttgggtttatttgtttcgt
atgtaagctaactgcaaaaagcctttacttccttggatgtttactgttgaagcatgcttactttccatccggttgtttctggacagaaagaatccacc
atcatcttctttattaatgacgctggcttctcaattgccggctccttatgcgtctatgattagacacactacaaactatgcttctttagtatcccagttg
attactgatgctagtgtagcgattttcgtgattggaatatgttgctatataagcccttctttactttaatgatgatgaatataatagtacaatgatcgg
agtcgcagggaaaaaaaagcatacggtaattttgagagtcgctacaaagtttttacaggaaaaggaattgaactgaatcttctaataaatatata
tgtttatactttttcttgttttccttatgagttgaatcatcgcaagttacgtttgatgctgaattcaaattagctagctagttgtctattttaacgagttta
gaaccatggcactttataaactatttcattgcttgattgctctcgcattgaaaaatatactgccctaagaaaatatgagtttcgtaaatttgtttagta
aatagtgatcaacgctctgaaatacagaaatgtttttttcataaactaaatctaaagcatatttcaactattgcctttcaaatacttgaaagaatctg
aactttatataaatccaccttgcgactcttatgaatttacactcttgaaaatacaaaccaaaccatacgaagtaattcgcatgatactgggaatgat
aatttagcataaatatttttttcaaggactgtagaaaagaaaaagagaaaaacaaattgaaagaagaatagcttggattatctgattcaatttcag
gattcgagttgaaataacatgaaaagaagcgtacgagcaattcataaggaagagatccgtaagaagtggcgtcctttatcagaaaaacaacat
gaagagattatgattgttcttcgtacttgcgctaggtaagaaactctatagagaaatgccggtttttgaagcttttggatggatacgtataactacct
ggaaaacctctatgtctttttttctatctttcttggaaaagtttataaaagaaaaattggttaattcctaagccctatgtgaatatgcgaactaacttg
ttaattctagattagtattaaacagcatcaaatccgaacatagaaagactgcagcagaaggatggatgctacaatgtttaacagggatgaatca
gtcactcaaagcattgcccgttcctcctagtaaatttggcaacggtgccttttctcaagtgctaactgcgaacgtatgctgtttagctcattgcaacc
agaaaggaaatattttatttactaattaaaaaattatagaatcaactagagcgacaattatatggtgacttggatcatattcatttacttcaaaaag
aattacacttggaaagcatgagattagagcaagaacaaaaagcttacgagcaaatgaagcataatctttcttcaaaccaagcaagatcacaaa
atatgcggaccaaggtacgtggaccctgtctactatattcatatagctgaactgctaatatacttacctaactagttacatcctctattgaaatcttc
ttttacggagcgttcgctgaacaatgatacaattatggacaacaaagatgcatcgggtttgctatcctccaagacaacagctttggaaaccaaag
ctaaaacaaaatacaacaaaaagctacaaccattttcaaggacattacatcgacatacaggtcaaacagttcaattaagtcaaaaggtacaga

aagctattctactacttcaaaggcttcaacagcaaatgcctgtaggccaaccgcaaccagaacgtccatcataatggccttctactttcacctcttt
tctgaaaaccaaaaaaaagatggaccggaaagacacttttgttgcagtcagttttattttatggccctggactaatgcaaacccaaaaactggatt
tacagtccatctactctcagcactcagactaatcacctcgtcattggtaattctcacattaaagtgagacgacaagctaacttaatggagtgcttgg
ttaaggaagaaaagttttcataaatttgctacctccattgtcctctacccaattagggtagggtaggataaataaaccaggatcgaaaggaaatc
aattgaaatgaacaggttgagagaaataatgttttcgggaatcaacacttactttataattcggtaaagcctctccaaattggttttacgagaccg
agaccaaatgctctttctttagacaagcaaggttcttttgggatagaagctttccaacactatttttaagtgccgaaaaccgccatttttctgctata
atgtggctttctaaaacgaggcaactttccacaatgaaagccttatgctaaatttcaaggcctacaactttggaaatgcccataacctatttggcac
ttagaattataaaggagataagagtttgattcaacgtcaaaaacagaccggttgatatgagctctcacacaatcgatacaaaatctcatacagat
aatgctaagaacctctgtagagttggacatgagtgtttttccagtattccagtattacagagatccatgtcagaaaaacatacattatgtgataaa
gaagctttttttcttaagcgttttgatggcacatattgaagtcttgatatgattacccttggcaagagtctgagacaattcatgaatgctctctagga
acctttctcaactatgagaaataaattaattaatttatagttatacctgcaaatttcgttcttgaatgatgatgatgtttatacacatgctttttaaacc
atgcgtgtcgttaacaggcagctttctctctattaactttacccattttcctaatgatttctgaattggattaaatgatactctattggattctcttagtt
tgctttcaatctacgtggaaaatttcttgataccactttagtcataatgttttcataaaagacgaataatgagatttctttttttatatatataggcaa
gtcttcttcatatatgggaccgtcttgtttctgtttcatagtctctacaattctagttaaactgaaaattacaaatcatattcaaatagtaaaagaagc
cattacttagttaacaagtaatgttaatgaaatcttttcttcaaattactacaaaggattaccgttgtaggaaaagttcgccgtcgtcatttatacga
gcttcttgtgaccatagtggtttacctttgaaaaaaaaaccaattcaatgaatctttactgaaaactaactaaaggataactttcattttatttgtta
ctaaatatcgttcgggatataatctgattgctacaattagactagtagtggtagaaaccattaaaaacagtctgcatacaattggtggttcttaata
cttgaatttaacttcgttatggatacgaatcaaattaattatataatttggagatatctgcaggaatcaggtaaaaatcaggattcgtttgtaaaatt
cgaccatatttttgaccaacacaatcatcactaacaatccaggttattcacatacacagttcgcttttgaacatgaaacagatgttcagaaactgg
acaaggcatggggcctgacctgtcctgtcggaaggcttgtccaaattttacaaaaaggtttacagtacatggaaatagagcagcattggatagag
aatcctcccccaagtataaaagatggaatattaaatcaagaagacatgcaaaacatcaaacatccttgcaagaatgattttctcttaaccgttcct
catttctgttacgattccaatgttgatagtgttcaaattgctaacgcaaacgaaaatgaaaatgacgggcgtaaggattcgggtttattagatactt
cccatgaccaggtaattgctttggatactccggatattcacatggaagatcagtcagctgaaactccttctaaagaacctttactatttacagaaaa
agaggcgaccgcaaaaactggtattatagacgaagaaaatactcaaccgggagtatcttctttgggtggatcattgtcaatttcggatatcatgtc
tatgaggcatgctgtaacttgcgcagattggcagccgaccaattctgaaagattcgttgtgggaatgatgaacgctgacttagttatttgcaatctt
ggtgtcgttcctagaggcttttacggctcaaactctgaaaaacaagacgttactaatattgcgtggaatgtaagaaagtgttatacagtattgaca
ttaagcggtagctaacaagacagcattcgggatcctattttgcatgctcgtttttttaatggagctatcgaattgtttaatgattatggctcccaacaa
accataattcaagaaaccaacagccctgtactaacgctaaagtggagtgaaaccgatacatacatagcggctggatccgctgatggtcgtgttgt
attgtatgattctttagaacgttcacttgtatacttagtcgagactttttctgcgattttggaaattgaatggatttctgtagatacttttgtcactgctg
atgcagatgggaatatgaaaatgtttactgttcggagcatgaatccgatttttaatgttttaaaggcacatgaagcttccattttgtctttgcattatc
atccacacttcttgttattaatatcttcttcaagcgatgctaccgtaaaggtacgtgtatattataaaaaaaaaaattgctaatttttagttatggtcac
agtcgaactctgaatcattgcagtgcatgcatactttccctttcaattctcctattgaatgtattagttggaacgtaggaagtgggtctcctatttttg
gaattgcttctaatagcattgtcagtttatataacgccatttcgttgcaacaactctccgtgtgtatgaggcatgcgtctcctattacttcgatttcttt
tagcaataatggacgattgttggcaacgggtgactcaatcggaagtctttgtatatgggactgtaaattaagtaagctagtacaagaactggttcc
caaagattcctcattagctactcttacagatagtcaatccgaggaacagatcaattttatgaagtggagtcctgatgatacgcatttaattattgga
aaacaaaaacaggagcttaatatctgtgcacttgttgacaagggatttgagaaataattggattaatgaattttcatcgttatgtctttagaaacat
taaagtttttatctcgtaaaatatgtatcatattgaatccttcattattaatttccataaattactaataaacatatgcgcctttcgcaaaatcattat
aaatatcataaacaagtagaagtagtaatttaaagaggtgttgcattaactggcgtaatttgagagaaaattgttggcttgttcgaagaataatgt
tgaaccttagcattttcctgtctaacaatctcaaccatttttcgaatggttgtccggtcaagatcagtcctgtgtagaagaatcatttcaactaacga
taatggagcatcccaataagattctttcagaagatgaaaataatctgcaacacttcccaattccgcatctagcatgccgaggagtaacttttctatg
ggttcaaaggtggcttttaggtgctcagcagccatatattcaccaaatgcacgaatagcaagagcgacatcagatcgtatttgttgaatagcactt
ggaccttcgaatgtaactcctttgttataaattccacgaagataattgatcgtgagtgcgttggaagattcaagcaagaaaacatcaaataggcc
aggtaccatatgttctatgcaatccacaatgtagtctctaaatgtatcgacaatcagcttcatattgctggattgataccatccagggtggaataaa
gtagcaagagcaggtttcacatcgttgctaattatttcaacgatttcattcacaccaatatcgctaagtaaaatgaaaccatctactgttttaccga
aagactcacgaaggtcttcttcgcgactatccgtcgccaactcaaatgtacgtagaagcgtattgtccatgaaacctgcacatttaaggttgtcatt
agctaaagctaagaggtattccattagtcccggaggagcacttgaagcatccgacagatgtttctgaagttctgttttcatcacagttttccaatgc
tcttgcatcgcctgcattacatacataatggaccgtaaaacagtactaacaacctctgggttattcgtttgtgagattatggtgacttgttgagtaat
catttgaaagaagataatagttccctgaataccgtaattaccgtcactgtcaagctcgggttcactctcacgcttaacgaaagcctcgacgctactt

tggaataacttatctgaccattcttggattttctgagtaaagagccgggtatattctttcaccaatataccattacttccatcttcaagaggaggttg
aagctcatctatggaaaatggagcatgttcttgataaaatgtacggtactctttgtgaaaattgattagattcaaaaaggtagcagcaggggtatc
agaagagacagcatcttttacaaggtttacgagacattgctgatagataatagcaaacactttggcaatatcgaaggatggagaagcaagctct
ggaagaactttgaatgcatattgcaaatcgtctaatacccaatccaattcaaggtcggcttgctgagttaagaaatttgaccaagcttgatgaaac
cgttctgcaattgatacttggaaaatttctttggcttctgaccaaaagttccgaagctgacgcgagctgccttgaagattcacgaatggagcttcat
tagcatcggtaagattagattgagcatttctaatgctctttagcatttcgtctgaagcttcttcggcttcgatgattttaaacactcggacaattgtctt
cgtattcccaatccttaaaagttcaagtaaattcctgcaatagttaaatatcaatcgatcaaacttctcagaaagagcattcaaatttgaaaaaga
cgattgcagggtggtagacacgtcgcttctactttctaatgaagcttggtgcatggcttcatctcgaaaagtacgaagcttagagagtttaaagtgc
gctttgagcaaattcggcatagtgggatcctgctccaggtcctctcgaatcatatcttcaatgatgtcggtttcctgcatcagattccgaagctgtat
aatcatttggcgagtagcataacaatttttgtaaatacgagaaacttcactaatgagaggaaattgctctaccatctcatgagctgtcatgcataa
ggatcgcatctaagaaaaccctagtcagtaaacccgttatatacgaatgaacaaggaacaaacaatcgatctgttcttctcaagcaattttattat
caaaattgcccatttagcaagacagactatttagtgtaccgtacatgatttagttcgcgaagtaacttttggcgctttagatgagaatcttgaagag
aaagccttcctttcttgaaacaaattagacataaagttctgtaataataagcagactggatacctgcaagctctctgcatgcttttcaagctccgttt
taacgtgcaagtcaaacgtatctttttctcgttcaaacttctcgacatgggacgaaagcttataaaagtcctaaacagtctgttagtcgattttgctg
actttcttttccaaattcgagtatttacctcgctctgtcgcaggatatcggctactcttaaaaagttttgttaatacgaatgaatcaccagtacagaa
agagtatctgtttcaatagaaaacatacttattataaacaagttcatcagaagaagccaaagacatttcgattataaaacaaagaagtcgctttgt
ttatgttgttgactctggagtgtgtgcgcatttaacaaactactgccaaaccggtaattggatatacggacttggaacgtatcgcagaaaggtcaa
caaaacgaggaatggaaactaaacaaggagcttgtgtcaaattgttttaaggtttacaagattattatatccttgcttatgtttcaaacaaagtccc
tataggttcgaaggttggctgtttcctcgagtgattcgattcagctgaaaaaagctgaacgagtcagtctacaaaggttctaatgaagcaatcaat
ggttaaaagagacgcaaaaaaattaggcgaccaaatttttcactatctcatagttactgatgagctgaaacaggttggtaaagacataaagtgct
aatcgtttacttattcctacggtttgtttactacggtatgcattatattctctattaagtattccgttccaatcctctatttgggaaaaattgaaaatgtt
ttttttttgatattatggatgataaaagagaattgatattaaattttgaattctttttaaataatgactgttcagttcctttgcttttattcgagaatttct
ggttagcaccgcgcggatttatcttctcgtccatcacactctccgaagtagtactaatagtccgaatcctaaggaaactaccaactacgagtatcc
ctaaaatgtatgaaaagttgtgagctattggaagctaatgacatcgaattgtaggaatgcacccgaaagattgagtcaaaaagcctcacaaagc
gtgatgaagcggtttggaacatgggtacgaagcccagagtttcgtaaatatttgatgagtactcagtacgtttgaaattcgtctactttgactagat
gctcaaaagactctactgtcttgtgttcttttgcagttgtctgtggttgatatgaatactggtttcattttgctaacttagatcctaatagcttctgggg
ccctttgtcaaattttggtattcctattgctgctatcctggacttgaagaaggaccctaaactgatttctgggcaaatgacttctggtaggtggctata
agcgcccgaacgagtattggacgggcgaattcgatgaaatcgttctgggttttgttcccataggaaaaatttgtttatgcttcttgaggaatttctta
ctgacttgcttttagctcttcttgtttattctgccgtctttatgcgttatgcctgggtaaggaaatctgatttaatctgacatttttttcaatatggcaaga
tttagctaattagtttctatcattagatggtaagccctagaaattatcttttgatgggctgtcacgctttcaattgcaccgtacaaagtgtccaaggg
gtccggttcacaaatttttggtagtatgtatattgttgagatctttgcatgaaatttgcatgaaattaaaaaagaaaacatactaacgttataattta
gtggaaacggtgaccaaaagcctgttgtccaaaatgttctgaaggctgctaagaaggaaaatactcctaaggattaaatttaaaattttttttatat
ttcatgctcccttctctaatcgtataaatttttaaggataagtaagaatttgtatgttaggattcatatgatctggttctacgatttcgctttacatttgc
agagatgagtcgaagttactatattctatgattttagaatagtttcgggatatctttttttcattcttttctttgtcgtgttttcgatcatagatttaggct
ataactgcatcgtttttaatataagaaaatcagatatttttcgctcaagagtatgagtgtgcgtgtgcgtactgtttttttttttttttccttctagtaag
tagtacattttcggataaaaccccatccaacttttgtttctgtaaacaaaatccagtacactacgacagaaatgatccatctactcacaagttaaag
tcaaaaaaagatacaaaaatcgaaatcgaatttgaatttgagaatttagattacttgttattaagtcattattttgaaattaaaaacagattcaatg
taagatgagagaaattagtccatgataggagagagctaggctctagtagcaaaaaagacagcaaaaaaagaaagaagtcaaacagatagat
acagaattagtagttttaaattatcataagaaaaaaggtaatattatacgcaaataccaaaaccttgaagttcgactttcactttgtcaaagaagg
catcattttggtattttccacaggtcatctctggatatatacgaatggcagtttgcaggtctgctagtaaaaattctaagcggagaagcaaaataga
aggatcatcgaaagagccactatttagtgatttccacatgttctccaaatgctcaatgtcttctccttgcacgacctgagcattggatctttgttctgg
gtttttcaaaagctctttcagaatggaaaagttcttttcaatatcagaacttgagtagctgagtaaatcttcatcctcactatccatggaaaaccaat
caatattgatattatcttcatgcttatccttcatagtgtcttgtttgtacgagccagcatctttatgagccgtacatgatattgaagaagcagtatcag
gaaagctgtatttggtgcaagtggaagattgcgaatatgacgatttgttgtcgtcgttgattactgtcatggatacaggagattgcatagcgtcagt
gtcaaacatgtcaagcgaactttgtctttgtggattttcatctttgtgtactatgtcaataaccccagtggggatatgcaacaaattctttgtagtaat
tgctcgatgggtatgctgttgttcgcgacgagcgatataattgtcaggacaatataatagtcgaaacaacttatgcaaaatagtatctcgtttgcga
agattagaaaagggggcttttgttaataggttatacagctgtacgcgaaggccttcgagctttgaatacttatccatttcgaaagattcaagcttcg
tatgttcagttgaaacagaaaagcttgtcgtttgagcatgctcacgcttggattgtatggtaggacgattataagagactttggagatcggagtcat

gttgtgaattatttaatttgagtaaggatagaagacgatagtaaacaccaaaaagtagggcgagattaggcaaaaaaatatattagagcagggc
caagaaatattgatggaaaaactagagaaaactcccaaaatatatagcagcgcatgaatgatcaagtcatttcatggtcatgcttctgaaatcaa
atggacaaggtgttttcgagaagttgtctaattaaagctggacatgtaatgaaaccatattcttcccctctttattttgatccagccacctgactatt
agcacttttgccaatgcaactgctaaatgatgcgtattcaccccttttggctaaactgaataagctttcccccaatatgtccctcaaccttagcatttt
caaattacagagcaatgaggtgtctagtgttgaatggtagaaacattaagatgatgaacgcggaaattttttttggaattgatcgtcttttgtaat
gagcatgttgataagaattaactctcataaattcaaatgaatcatatcacgcctttgtacacaaacgatgagatctccttttgtttgtttgtttattga
cattagcgactcatgcccaaatcctaaactaaacttaatttagggatccatatgattggataatgacgtacacacataattaaggtcatgtaatcct
tgctcgaccaaaagttaattgaataaaagtgagtaaatgcgacaagcagtagctcacggatatgtcacaccaaactcgtttacggcaggcgtatt
accgatctcattattgcagtattgtttgtgatgcttcgatcataacttgaaatggagatgaagaaaggcagtgaggtaattagtagatacaagcaa
tatatagttaccatagataagtagaattctttgagaattctaaactagttttgcgcttcgacagtttaaatcaaccccacttagtagacacgaaaac
atcgttcttctctcaaacctttcaagctttgtctatcccatattaatttcttcttttttacgcttcagcaaaaccattgaccagttatccaacttttgaaaa
actgaatacataagggatttatcgaaatagcgctctcattaattgtttatttcggtcgtttgttaaaaaaaaaagaattccttccctccacgataaac
ttcggtaaacatcaatatacatgtaattggttgcgcttttgtttgtttacattgaaggtttgtgtatataagcaatcgttatctaccaattggcatctaa
ggtatattatccaaagcagcattaataaatccgtcgacttcttctattccttaagtcacaatattatatcttataaaaaatcagtggtttattttccta
gtgggacttttactcaatatctctcaagatgggaaaaacaatcacaactttgatgttggtcttcgtgtccatggccggttggatgttcggtgctgaca
caggttccatcggcggtattacaaatatgagagatttccagtcacgttacgcagatcggtatgatccggttgccaatacatattcatattcttcagc
ccgtcaaggtcttttggttggtatggtaaacgtcggtacaacaatcggttgtttactttcttctcctttggctgatcgttttggtaaaaaaaaatgcat
catgggttgggctctggtttacattaccggtatcattgttcaactcaccacggtcccttcttgggtccaattcatggtcgcaaaaatttggactggtct
tggtattggtgctttgtctgtcattgcccctggataccagtctgagtctgctcctccacaaatacgtggttctattgtaaccacttaccaacttttcatt
actttaggtattttcattgctgcttgtatcaacattggcacacaaaagcgtcatgcaactgctcaatggcgcatcccaatcggaattaatttgttgtg
gggtatattgatgtttttcggtatgattttcttaaaagaatctccacgcttcttggcaactagagggcgcaacgaagaatgtatgcagatcttatgtc
gtaactctggacttactcctgaccatcctttaatgcagaaagagtataatgctatccaagctgatgtggaagctgaaacagcaggtggtccctgta
gatggatagaaatttttcaaaatccagtccgctaccgtacatttctgggcatggcagttatggctttccaacaacttactggtaacaactacttcttc
tactatggtactcaagtgttccgtggcaccggtttaaactctcctttcttggctgctttgattcttgacgccgtcaattttggctgcaccttcggtgcta
ttttcgttcttgagtactttggacgtcgtatgcctctgattattggtggtgtttggcagtccatttgctttttcatctacgcctccgttggtgatcgtgcct
tgtaccgccctaacgggacctctaatcatcgatgtggagccgttatgattgtcttttcttgtctcttcattttctcttttgctcaaacttgggcccctgct
gcttacgttattgttggtgagtcgtatcccattcgttaccgttccaaatgcgctggcgtagctacggcaagcaattggtttttttaactttttgatttcct
tttttacaccattcattagcaactctattggcttcaaatacggttatgtttttgcgtcttgtaacttggcggctgcgatcgttattttcctctttgccaag
gaaactaagggtcttacgttggaagaaatcaaccagttatacatgtcttccattaagccttggcaaagtggttcttataagcgtaatcgtgaagag
gttgataaaagtgaaatggaaaaacctcgtggaccaagagctcaatttaatgaatacgtccagcatggacccaacatttattctggtgagaattc
agtgacaagccggcagaaccaccctgagcaggttacgaatcctgttggtctttgagactaattttgtatgaggcaagacctctattactttcttttcg
ttctttgggttacttggttaaagctattcagattcggactagggtaaagatgtaaaaaccaaggtgtgcatgaaaaccttaaatagatgcatatcct
ctttttttgttcagtatttaaaccctaactaagctcctgagcaaaaatagtcctttgtttatagtataattggattatttctcataagaaagtaaagtgt
ccgatagtaacgcacaaagtatatttctaaaagaaacttctcagtaaattaaaatagctagacgtggaagggccatatttacatggaggtgttaa
aatatgaaccctgccatgatatgtgaaattgtcaaaagcaaccctaatattatacgaaatcataatactaaagagagtattgtcgtaaggcgcta
catgggatgaagacaaaataaataaacatttggaaatacaaaagagcgatcatatacaattgctaatgcattggaaatatcgccttatctatatg
attaggctagattttgagcaactaaaagtagtcaaactactcccagacagaatctttggttgataaaatgttatatcagggaagtattaagaaaac
aaaaaagaagttgcaagggataattcctattaatttggtaaagtaacagtgctataattgagtaatattgattacaaaaatagtatatgaataaa
gggcttatagctgtatctggttatttactgactgtaaagctcctttggaggaaagtgtcattgttgtcatttattctttcagtggttaagaaaatgttta
tgccatgattaaataaccaacaaatcacactccaattctattgtcaaaaagaaaaataaatagaaaatacccatctatattttcgtttaatatatta
ttgtaccctaatactcttccatgcaaactttcgctaatccttatatacgaatagtctgcatatttttcgtactagaaccacttcattaaaatacaaaga
tgaaatataaaaacgaaaacaatagcttttcaattttcctgcaaccaaggaattattgaattgctagaaaagtcttccgatgatttttttatttactttt
tcttacaacagtagaaccgaagtttaccccatttttttcccctttatttctcgtgaaaccaagcctcaaccccctcacatgggagattgtcaaccaatc
ataaatcctgcgaaaacttcccacaaatcttatttttcccaggttgtcttgttttctctcgatttaccccaacattatctaagagttgattggctaatgt
gagaaatttctggtagagtgcgtggcgaaagcttaactttttttgaaaacacgtggaatagggtcgtcatcaacatgggagttttaggctgggtgcg
aacgagcctttttaattatttgctttccaatttttaaattaacgcgtttattagcttcttgaaaagtaaattaataaaatattgagtaagtagcacaac
ctttctccattcaatataaacacctagtaactcctcttgtttgcgggattacggctttgcattcgtactgtccataatgtatccctgttttactcgcactt
taattagattacagtttaaacgtacctggcagagttttgttatttggccaattgtaagtttgcagctcgtcatggtggtatttgattggaagcaatccc

ttttctccctacgtcgtcaagcgtctcgtttgccaaatccaaaaagggccatattggacttttttggactatataaacagtagtaattcgtccgtttc
agcatcatttaatactaacaacaacatccctaagcattttctaccctataattttacgaggatctgcgctttctagggatccggttctttcaattgcga
tatatacttatttgaatactatcaatcagttctttggttcctagtctattctttcttttctaaactgccaattcacttttattctttcttaatccttctttttc
ctttttctttgtcttcaaagctttttgtgtaaacccatttttgaacagtagttttcacaatggcaaggatactaacaattgttatgttggtcttcgtgt
ccatggccggatggatgttcggtgccgacacaggttccatcggtggtatcacaaatatgcgcgacttccaatcacgatatgccgacaaatatgac
aaggatacacatacttactcctatagttctgcacgtcaaggtctcttgaccggtatggtcaacattggatccatgaccggctgcattctctcttcccc
atttgccgatcgtctcggtaaacgtaactctattctctttttcacctgtgtctacctcataggtatcattgttcaactcaccacggtcccttcttgggtcc
aattcatggtcgcaaaaatgtggactggtcttggtattggtgctttgtcagtactagctcctggttaccaatctgaggttgcacctgctgcgatgcgt
ggtgccattgtcactacttaccaacttttcatcaccgccggtattttcattgctgcttgcatcaacatgggcactcacagactcgcgaagagcgcttc
ttggcgtgttcccatcggtattaacctactttggggtcttattactttagttggcattcttttcctacccgaatcccctcgttacttgatttctgtcggca
gagatgaagaagctttgagtattatgtgcaaaaataacgaccttcctcttgaacacgaagttattcaaacagagtaccatatgattaagtcggact
gcgacgctgaattggctggcggccctgctaagtggccagagattttcaacaaaaatattcgctaccgtactttccttggtatcggtgtcatgtctctt
cagcaacttactggtaacaactattacttctactatggtactcaagtgtttcgcggaactggtatgacttctccttacctgtcagcgttgattcttgac
gctgtcaacttttgctgcacctttggagctctttatgttatggaacactttggtcgccgtatgcctttaataattggtgcactctggcaatcactttgct
tcttcatctatgccgctgttggagatcgttgcttgtatcattctgatgggtcttctaaccaccgggctggtgctgttatgattgtcttttcttgcttcttta
tttttgcgttctctcaaacctgggcccccggtgcatatgttatcgttggtgagtcttaccctattcgttatcgttccaagtgtgctgctgtagcaactac
tggtaattggttttggggattcttgatttctttcttcactcccttcatcactaatactattggttttaagtatggctacgtctttgctgcttgtaacttgtg
tggtgctattgtcatctttgcctttgctcacgaaaccaaaggtcttaccctcgaagagatcaacctcctttatatttctggtcgtaagccctggatgcc
ccgtcccaagagtcttgacaattactctggtccccacaaggatgctttggagagaggcaagcatgatgaaatcgaccacattgagaactttacttc
tgacagtatggaaaatacttcatcttctcattcgaaacccaaggcgaagtttttttgaccatattcaggcagtttaaagtagcaaaacattccaaga
gtcttcatggcactcatgtttggtgcttattcaattctctttagacatgcatgccgagtttttttattcaagccaccttatttgttctttcacccttaaagc
ccacagtcattctttattgaatggtttcaaattttgtgtttctctacaattttatgagttatgacattctaataatcgtatggatggatctttgtttcattc
aaaacctacctaaatatgcatcgacgttggcattacctttcattatgaaaaatagactatgactcaaaaggaatcgatggaaaaacccttgatatt
accaaacatatcttgcaagaattatagaatgaacttaactaattagaagttgatgaacgaagtcaattaatgctcgtttataaacaattcccataa
tggacaggaaagaacctcatgcttaattcttcttacctctttaagcaccttatgtttatttattaacggacctcttttgcatcatgatttgttgattgag
tttcaacaaaacaatagcaaattgagtcaatgacatttccaacaacgaatattattaatgagtcatgcgaattgtatctagtagactgtagtgaca
aagatagcaaactgaagccatagatttaaaatatctaaaaatgcaaaaatgctccagcagtgactcgaacactgggcctcttgcttactaggcaa
gcgctctaccactgagctacaagagctaactgaaatctttagaacttaaaaaaaacaagtagaggttgttggatactttttgttttgatatacccat
atcactgatgatttatttacgaaaatatttaggaaagcaaaataaaagtaaaattggtatcgtatgccaatgaaaggcaaaacaagggtcagtat
tgttgcaatttcaaaaagtataatactaacgaggtattatgatgacaagagtaaatactgaattctattacatcaaaagtatccttccactaaccac
attgtttaccgtccgttggattcaattgaaggtcaacattgtaaggacctttgaatgtgcatatcatgcaaaaagaaactaaacccgaagactcta
tacccggaattttagagtccacaccagtcattccgaaacacaagaaatcgcatgtgtctcgtttgcttgagactttagccgttttccttcatagtgtc
agcttgacgcttactgcatcttggtttacaatattatggatgtttttaccgatatggcctgtaagtaaataaaaaacagtgattcatggcatagtgga
catttaagttttggagtatggggattggaaattgcatttccatgttagctgccatacttgtttttttcactggatttatgtaacgcttgagcacaagaaa
gtgtaccaagcatgtttcgttaattgttatttggttacgctgatattaacatctacagattcttttggtatatataggatggctagtatacgacgacgc
tttcacgacatctaaggatcgtcaaagttcaaggttaagaggagctgctccttatcactggttttgccattatttccctattcgtcttcacaaaacag
cgaatcttgacccaaagaaaaattatattttcggctaccatcctcacgggattctttccctaggagccttcggcggcttcgcaacacagggtatgtt
ataacatggaatctgtattcttgctggtctctaatctaacactttatcactcaggtgctggattcagcaagctatttcccggcataaacacttcagta
ttgacgctgaattctaatttttatgtacctgtatatcgtgactatttactcgctttgaacgttaagtctgtttcaaagaagtcttgtgtaaatattttgac
cagaaatcctggcgacagcgttgttattgtaattgggggagcacaggaatcgctgttatcccgccctggccaaaataatttggtattgaagaaga
gattgggatttgtcaaattggcctttttgacagggtcgagtttggttccttgctttgcttttggtgaatctggtatgttttattttttttagtttcaacaat
tgaaatactgactggtttagacattttcgagcaagtggacaacaatcctcaaactcgcatctatagatttcaagaaattgtaaaaaaagtagccg
gttttactatccccttttttctatggtcgaggactattgaacaagacatttggccttatgccttggagaaagcctatcaatattgtagtcggagaaccc
atcgacttgcccaagaagtcggaccccacaagtcaagagctctatgaagctcaagaggagtatattcgccgtcttgagggaatttggaacaatta
caaagatgtatttgttccagaaagagttaaagagttacatttggctgcttaaatgtcatcatttcaggttttctcataattctttttttcagcctttcagg
tcctttctaactttctacatatttatattcttcccctcttttaaattttgttactcttgtttaattggccttttcttcatttattccataaagtttgtcactata
cttactgtttcttctttatatataaatacttctcttacctaacttcacctatgcttacaatcttctctcctttctcgaatacataagaattcaaacatgct
ctcgaatttttctcttcccgttgatgtatgaaacagaactgctattttgataaaagatgttcaagacgagcctttaggcggccaagtaggctttctta

cgatgccaaatagactcgcttgtagaataaccaatcttctacaaacttaattaccagaaacagccttattcatgttgctttaactacaagttgtgcct
gtaattaggataggatggctgtaaagtagaaaacactacggcgatactggtcaacaatagtttataaagaagaaggtaaatcctactgaattatt
gttgttattcattgcttttctaataaaccccttataattttgctttgtagtttatatatatatgtcattgttgattaatgcaagttttgtttacttcttaaaa
attctttcaacaaaccgtggtattatgcactaatgttatttacgaaattgagtgaagttgaaatgtcaaaaaagaagttgcagcagaagaaaaga
tcaaacttgaaaaatggctattaaagttgaattactgtatgttaaatgctgatttcattcttttattgacgggggaattgcagtggaggattagacat
gctctttaggaagcaaaaggagctctctttggagcttgcagaactaggagaagtcaagacattaagatccttgatccattttatggctgaaaaaat
tgaaagcccgactcaaaaggaactttttttacctggatggcacggtgtatgttcgatttatggtacctttatgtccttgttgatgctaacgaaatgact
agtcgtcctggtattatcgtcctggtgaatgatcaggactgggagcttcttgacaaagaggattataatctcgaagacaaggatcaagttgttttttg
tgtctactttacatggtatgcttggtgtttactttcgctaaaacgaatgctaacggtacattaggaggttaactcaatgaaaaactaaatctagggt
agctgacttatattacaaaaacaaaaataccaagggagattctatacagcagaataggctgtaacgtgttctacgtagtgatgttttgagataag
cgattgtagcgaacccaacgacactgtaacttaagtagtaaatttatttactgaaacaaaaaataatcataataatacagtatacataaagcaga
caaggatgaacagagccattaagggaaacggtatctagtagtttttacatatgacattactcttcttcatcttcagaatcaacgtggatcgtccgcg
tccgacgtgacggttttcgggatggagcagctgaagcagcataagaagaaaaggaatcggtgacatcttcgtcaacatcaaatctattttcatttc
tttccaaggcagcattcaaatgtggcccaaattgaggcatttcttcgtcatcaacgtcttcttcatcatcgtcaattaacattgatgtcgatctaggtc
gagaatgtcgacttctttttaaggggcgatccccttctgttgagtatccatgtctttggtaatcgctgctttcatcatacaaatcatacagctccctag
ctaactcttcatttttctattttcaagctcttctgtgtcgatgtcttgatttgaagcctcaccatcacttccagacctttcgacctgagatgccaagttg
ggttcatctacttcttcgtctggaacaataaaatcatcctcattgttctcaataggggacaactggctcgcattgcgattatcaatgaattcatcatc
caagtcataatcacccgcaccagaaaaatcagattctgcttcttctccaaatcctatccagtcttctggaacagctgtaatcggcggtctccgcgaa
agatttgcccgtcttcgggtctcttcttcactgcctccactttcaagatcttcattatcccagaaccattcccggtcggagtcggcctcatccgaaatc
tgaaacccacaatggtcacaatggtaaggattttccaattcccagtggcagcgtgcacaccttaagactccatcttctgcatcctggagaatgccc
gtagggtacagaggctcggtaccttggaatattccatcaaacaaaacctcttcctgccgttttgatggatcgtccctgatctttagcaatgataagg
tcggatctacactagcaatcacgcccatcaagtcatagacaagatacgctggagatggttgggtatgaatcttttgacgacaagtgggacatgat
ttcgattcctttaaccagctttttagacactacataatcccattagccactgttttatttatagaagcagaattgatacggctgtttcgtaaaacagca
atccaaacacttcctctctccaatccatgacttacctcgtaacaataagtatgtccgcacgacgtagtaaaaggtctttgaagtgtttctgtgcatat
aggacaatctattgtttttttttagttttgccaaaaatgagggtaattccgcgtttgacggcatctgaattgtttcttttatgtcatttttttgaattaaaat
ccatgtatccgttttcattcccttcttcatctgctttttcgtttgtgagcaatgttgaatcgtttactgcttcgttcatgacttccgagtccttttccacatc
ttttccatgaaacaacaattcagaatcgctctcctttattaccatcacctttataaaagacagttctccactcctactttgttgtttacaaacttgacc
aatcctattcaagtaaataaaccccttttctcagcgtcgatggaagccggtaaaagtactcaatgtcttcttatacctactcaaaatgcttcgtcctt
tcctaccacgtttctttccaaatagcgatctgttgacgttgaacctaaaatcgacaattcttgctcatgataagtttttatgtgccaccgaaaaatcct
ttaacgttacgcgtttggacgttatataaaagattatcgatatttagcgacctctttataagtctccaacaattcaaaggattatatcggtaagtatt
tattactataacaaactcggctcgataaaacttaattataactttaaggaattactataaatttcctttctggtttacggctgatgattacttattcaa
aatacgtatttttacgatcgacaaaggttctcacattaaatgcacttttattttcgttcgattcaatccattaaccattccaaaaagaatgcaaaaaa
atctatgaatcgattttccggtaagttcattcgaacgagtatcaattaaacaaggaaacataaaatatcgcaaatagtatatctgagcgttttgtac
aataataagttttgagttacatgcaaggtttgatgagcaagaattaagggcatgatgaaggagaaatgagaacagcaaagaaacataatgaac
atttttcagaaaagggtgcatgcattatgtaatgaattaagcataacgatccggttgatcaaggtgagctgcttcatgcttgtgatggagagtagg
gctgtttcctgtagagctttcgacgctctcattagaagtttgggcatgttcaacgaattgtgagctttctccttgaacaccacgagacttctccaaag
cctctttttggtgtgcggagtactgaccaatgttcttgggacggggcatccaaggtttggaaccggaagtgtaaacgagattgatttcttccaaggt
aagacccttggtctcgtgagcaaacaagaaaatgataatggcaccacataagttgcaagaagcgaagatatagccgtatttgaagccaattgag
ttagtgatgaatggagtaaagaaggaaatcaaaaagttccaaatccagttaccagaggtggctaaggcagcgcatttggaacgataacgaatg
ggatacgactcaccgacaataacgtatgcagcaggggcccaagtttgagcaaaagagaaaatgaagagacaagaaaagacaatcataacgg
ctccacatcgatgatcagaggttccatcagaacggtacaaggcacgatcaccaacggcagcatagatgaaaaagcaaattgactgccaaacac
cacctataatcagagggttacgacgaccaaagtactcaagaacgaaaatagcaccaaaggtgcaaccaaaattgacggcgtcaagaattaaa
gcagccaagaaaggagagttcataccagtaccgcggaacacttgagtaccatagtagaagtagtagttgttaccagtaagttgttgaatagaca
taacggcaataccaaggaaagtgcggtgacggatttgcttatcaaaaacttggggccaagtgcaagggccgccagccaactccgcttcgcagtc
cgacttaatcatatggtattcagcttgaataatttcatgttcaagaggaaggtcattatttttgcacataacactcaaagcttcttcatctctgccgac
agaaatcaaataacgaggggattcgggtaggaaaagaatgccaatcaaagtaataagaccccacagtatgttaatacccataggaatgcgcca
ttgagctgtagtatgggttttatacttgtgagtgcccatgttgatgcaagcagcgatgaaaataccggcggtgatgaaaagttggtaagtagtgac
aatggcaccacgcatcgcagcaggtgcaacttcagattggtaaccaggtgcaatgacagacaaagcaccaataccaagaccagtccagatcttt

gcgaccataatttggacccaagaaggaacggcagtcacttgaataatgacaccaattatgtaaattatagaccatacgataattgaggtacgttt
accgatacgatcagccaaaggagaagaaagaatacatccagtaaaggaaccgacgtttaccataccagttaacaaaccttgacgagccgaag
agtacgaatatgtatctgtatttttgtcatatttgtcggcgaaacgtttttggaagtcgcgcatatttgtgataccaccgatggaacctgtgtcggca
ccgaacatccatccggccatggacacgaagaccaacataacaattgtcaggatctttgccatcttgtaaataacttaatatgatatacaaaagga
taaggagagtaagaaatgggataaaaattaaaagtgcgaatcgaccaaagaaagatattaagagaaattaaggagaaactgaaaagggattt
aggagcacaatcgttctactgacgaattaaagcaaaaatattatgaatctgaagaagaacttaaaaaattaagttaaaaatttcgggcaaagaa
agctcaaattaagagttacagagaaaggaaataggaaagagaatgacaagagctgtatccaattgttgttctccaaaaaagtttcgagttcaac
aaagttagatatattgaatgtttaattgctgaagaatccataaccaagaaccttcatatatatagtaaaaaaaataaaggaaaacttctacgggg
cggagtgcggggaaaatgcggagattatctggagactatgcaatgcatattttccgaacgtgggggacaaaaacaaatactttgacaaatatg
attggtcgaaagagaaacgttttcagtacattgttactataaacgaacgtcgcctgtgtcaatgtgggtctagactatgcgaaggaaaaaataag
caaataaacatcgaatcttctgccgggaaccctatagacgttccgcaggaatatctggaaaaaatagaaccattaagagatttttagagaaaag
aaaaaacaaagactttttgatggattattactttttccttaatgatctccttttactgggatatgggatcaccagatagtttgtttactaaataaaata
aggttgtactcgtcctttttccccaacttatgggatgatacgacgccacatgcaacggtataagggttgggattaggttgacgaacttaagcttacc
caatcacagtttaagcggctgggttctccgcatgctgggagaaattaaagacgtaaagtgatttaataatgccttgcgaattgggtgggagaaag
gtatcaaagtagcaagcgaaatttcgttctttgtacattaaggtttcttataacttggtaattgatgtctacgatagctaatcttatgtaactggcaa
ataaattatattctttatgataacgcggtaagctgattatcatacgcaagaaaatgagggttagcaatcgcataaaccagacaggggcctccttac
atcctaattcttataaagtggtatctgttcacgattatagaatatattgttgtatccctgtacgatgtaaataaacaaaccgaggtcatgataatttt
gaaagcgacgtttttgttagggaaatgaaattttgaaaacatacatggtttttcttgttgttacattcgccacctcttctctattgaatttcgttgtcg
gctattcatcatgtcctataccgttaaaaagattgattggttttaaattgagaaccactggattcttccagttgataaaaaaaaaaaaaaaaaaga
tgaatcgaaaagaaagatacttataccagaaacaagtaagaacatacattttattttgcaaaaattattcgctttagagataaatcccattaggtg
tttcaatctttattgagtctcactaaaatcatgtacgaattcgccgcttatacttaaaaatatcctaccaaaatacatttagattagctctcattatttg
gtatgtcttctacggccatacctaggcaaaaacgccagttcccgtccgatcactgccgttaagtgaaaatgtctgagggtctcgttagtactatggt
tggagacaacatgggaatccggggtgctgtaggttaatattgctacttaatttttgagacaattttctccaaaggtataagttttctactacgtttttc
ttttcaacttgctgtttcgtatattaaaatgatacttaaatactgtttttcagcgatacttttcttatacttgcttgtcagctcttagtccttcttggacaa
ccttgaagatccttgttatgaagatttttctacccttacggtttcgctctaatcattgtatttcggttaaaaattaaaacatcaaaactaaaactataa
gaagagaaaagaaattattggactctctttttaggggactatcatttttttgcctttacattaccggttttagtatcacgcatatacatattttttttcttt
ttttttattaaaaaaaaaaaaaaattatcatacgttcaagcatggatttcaagaagtccctgtccaagcaagaagaaactgaagaaattaaaccca
tcgatgatcccttcttaatcacatgggattccccaactgatcccaaaaacccaaaaaactggccctactctagaagatggattcagctagtcatcg
tttcggtgtttgcactgttaggccccatggcatcttccatggtagctccctgtcttgaccaaattgcagaacggtttcatattgaaaactctactgag
aaagcccttatcctcagtatatatttgctggtttttgcaatttcacccatgatcagtgccccactgtcggaaatattcggtcgtcgaatcctgctacaa
acaggaaatgtcattttcattggtaagtttccttataattttccatctttttctaactattagtttttaacatggcttgtggcctcgcaaaaaccaaagc
tcaaatgtacatttttcggtttttggccggctttggaagtgcaacaccaatgggtctcgggagcggaacgtatgtcagtttttactcgatctcccgttt
ctaacactttgtaggattagcgatttattcactccagaagaaagaggaagagctgtcgcagtcatgtccctggctcctttgttgggtcccactatag
gaccagtaattagtggatttattgctgactatacaacttacaagtggatcttttggtctactaccatttttagcggttttgtatttgctctttcgatacc
ctttcttgcggaaacgtatcccaagaccctcctgggccgcaaagcaagaaagcttaataaaaaggaaaatactttaaaatttcatacggaatggc
gtttagaacacattcctcaacataagcttgtcacccctgccttgatgcgacctctgaaaatgttgacaactcaacccgtggtaattttgtgcgcagg
ttacatggctattcagtatggtaagtgagttatttagtgaacttgctgggatgtttttctaaccgttaggtattctttatcttgtattaacaacttaccct
gttttgtggacacatgagtatcatcaacgtccttccatcgctggtttacattacattgctcctggacttggtttgatctcgggtagccaggcatcagg
cgtttttatcgacaagacctttcgtttcctaaagaatagaaatgacggaaagatggcaccagagtttcgtgttccggtaattttattaggtacaata
ttttttccagcgggcctattcgtgtatgggtggaccgcaaagtaccatactcattggattggccctgatatcggtgccgcaatgtttaatgtcggcct
catgttgggttggcgtggtattcaaacttatttaatagataccttcataatatatgctgcatcatcaactgcagttgcttgttgcgtccgttcccttgca
gcatttggttttccattgtttggacaaactctttacgatcatttaggttatggatggggaaattccctcttgggttttatgtacgttgtatcactttttttt
tttaaattgatactaatcgattttagcgttctggggtcttctattattacctgttccgtcctttggtttggaggaaagaagttgcgagctatgtctaata
tggttgtgttcaaggatgaataatttgcacctatatttcgaagtttttttgttgtttatatattcactgtctggaaattcttcagtggactttttaacgct
ggtatacagttagaatattccaggtagattttggattataatattttttgtaactatttagaggttgacttactttctctaattaatttgtcataatgca
gtgacttaatcgggaataacatgtgactaatactttacgaagaaattgagaagcaagttatatatctaaaaaactcatagagcaccttaagaaat
ttatacaaactactgacaagatgatacccatgcatttcatcaaaggacaatgatcttttggggatttgggaattgtctttagaaacgtgagttacaa
atgaatcataattattgggacaaaaataaaagaaagaaaggtctacgtcgtcttcacttttactctaaatgagtcttctctgaatacatattaacga

ggtcgattttataaaataccatattaaagctatatgaagggggttttacttggaaaagcaatagcagaaggttgtcaatcagtttcatatcattaag
acaattagactggctccgatatttccccatggatatcaattttcggatgcaggggcccaaagaacatgatatacatagaaagaatcggaagtgaa
aacattcgttgcaacgcggtatacggtttgtttacattcataattatgagtgaaaatacggaagaaaagagcaatggcttgacagaaatagctta
caatttagagaaagacagcgtagttcgagtgctaaatgccgacggccaaggcttagatgaagatgagaaattgctcaaaacattgggctaccaa
ccaaagctacatcgtacgtacgagtttttttgaaaactttgcaagctccttttcatgctgcgactttatggcaaatattcggggctcattttatgtagga
ttgttaacgggtggcccttctgcatactggattacttattgcattgcgattcccctgagtctagtatctgcgtcatccattgccgaagtttgttctgcat
tgcctactgctggatcagtgtacttttgggcttctgctgctggtggtcgaaagtatggtcggcttattggcttcttggtcgcatggtgggcggtcgtag
catggacctcctttgtcgctgttagttgtcattctacatcaaaatttatttttggtgagctccctgtgtttaaatccaagttttcagtcagctctaccgat
gttaaatttcgagctgtgcaatgggctgtgggcgagggcttgcttcttatttccgtgctcatcaacagtgtaccaccaaaatggtttcgctatgtattt
cgtgcatctgtatttgtcatcctgttggactttctgctcaatttaatttggcttcccatcgctacttcaaagaagtacggatttcgagaccaagaattc
atgatgtcgacgaattacgggattgggaaggtaaatcatggatggtcttggtgtctgactttcttctgtacagctcgtattcttgtcggttacgacgc
tgctggacatgtagccgaagaaacaaagaatgctagccaaactgcagctcgaggtatgttttactcggctttatcaaatagtatcttaagttgctg
tataatgatcgtttttctatattgcctcccgccaagccaaatcttgtatccactaatgaaaacaaacgagcagcaaccattcgtaagcttttatgcct
tagtattaggaaaaagagcacatgtttttatgaatgttgttggaataattggaactattttggatacaaccttatccattgtagcttcgagtagactg
gtatttgctgttgctcgtgatggtgtcttgccattttctcaatggcttagcaaagtggacggatacggccagccaaaaaatgctatctatttcatata
ttttgtagctgctgctctcttgtgctcaaacttacctagttctgttgccttcacgtctttaatgtcggctgccgctgtacccacgattctatcatatgcta
ctatagctttcgggagattgtttctttccaaagataaatttcccacatccaaatggtcccttggatttctatccaaacctttcctcgtaatcacctttct
gtggaatttatttaccgcagtcatcctgttttcacccaaggcctatccggtaactgccaaaaacttcaattatgcaccagttatctttggtgcaatta
caatatttggcttgattagctggatgttaataccagcggtgaaatggaatacttttcatgaaccaaaagatgatgacgatcgttcatcccttcaaaa
caaaatttaaaaatgcatataaaaccaactaaagatgaaggaaaaatttaatttattcagtgttgagttgctttattctcgtcgccttaacctaaac
ctttaccaatgcttttcttttccagtagcgggcattcttgtacaataaaatcttctttttcgcaagccaaagaaacatagaatcgcttgtagggagaa
aaggatagttgtagccttctttttactgcttcgaacctgcctttgggtttctaccaaaccaagtgattcactctcaaaatcagatcacttgcttatacc
atcaacagctaacagtaatttaaatgaaaaccctcctctctgccataagcccattcattcaaactgtctgtttacattctgttacttggtgtaggtag
gttgctactcgtgtcatattacgaattatagtaattagctgcctcaacgtatagacgttgaactacaaattcaaaggatcagagaaccttacgtttc
tttaaaattatgaaaaagtcaactgatgttcctcctccggcctattctgctactgaaaagcctttcaagccctacagagacaaatcagaggatgac
gaaaatgactatacaccttatgagccgccatctcctccttcatttgtaggcaaacatactacagaggttgcgagtatttcacttcatgaacatgata
aacttcgatttacgaattttccgtctccgcttttggatggagctgtgattctaattcaacaatattggccccggggagttcggagccagcggaagtat
ggcccttcacatgaaatcagaatgtctggcgctccttggcttgcaagcggatttggtacaattcatgtccaacgtatagtgaaagaactcattgctt
acatgatgagtcatgactggaaactttacatattatgtaatgtttctgagaaaatgggctccttggacagttggttcttcttacacatccctggttta
gctgctgctacgataactcctcaaaaggagtggtcggtcattacattcgaacattccgatcgcattcgtatcattgatggttcccaacgcttaatttc
cattgttcacagtgcttgcctgcaacattggtatcgtggtttgcaaacattccgcctttatcgaggtacagtgcctgaatgtaaacttcgtggatatc
catgggctgcaaatgggcccgagaccatgtacgttcgtctcttcatagcgacgcttattgaacttttagaaactgcgggttactctattgtcggcac
gctcaatatctttcgaacagaagattactcttttcctgattcttgggtccttttacatccttagtatgttcatgccattctttccaaattccattcaattc
gtaacttggatccctttttcttcccatgtccttaataggttatgcaactacttattccttcctttgaatagtaccaccctaaatagaccccttttttcaata
tcaaagatttatttatctatcaatcctctgaattttgatttagaatccgtccgttgaaatattctcgaacttgttcgtttttgcttctcgttcgctataca
aactatgtatatatgtttacttttgttttgggttctcttgatcacaatataatcagtaagtatacgatttgacttgtcttattttggaggccatatattaa
tgtatcttatgttttgatttgtttaaagactaatttacttttcgcgattcgattcatgcgtcctttgtcggtttaaaaacactacttactacttaatattga
atttctcaagcaatctaaaaaactaatataaaactcttaacaagtctagaataaaatgtttttttacttcagacattacaaagaaaatatttttatttt
tataaattcatgtttagctcttttgcatttcgtagagcccaacatgcttattctttttttggtaactatataacaacttgcaaggttcataaacatacgt
tcaactttaaaaaagacagtagaaatcattaatcgtagtccaaagcaaatttgatgagaggaccacctaacatacggacctccgttatataccttt
tcaaagtatattaaaagagattataaaacaataggatgatgatcccgtctcatcctcaatttataaacacacagcccaattttatgggtataaaac
tcaaatttgttctctgagaaactcaacaatgatacttcgacctcttcaaaccagtcgaaaactccgaagaccttgctaacttttgacagagaaaac
ataaaaaacaacctttacatggaatgcaggaactataatacacttagaacacccagtgttctttttagtttaaccacccttgtaagcatgagcactt
ctttgacgaattcttttaccaaagaaatagaagaggaaaggaatgggaataagaccacaaccaataaatgctagaagtgatgtagcccagtgat
agtttaaattgttgtacataggacgagcaacaacagtcataccaccaccagcagcgtaacgcgtagtggtaccaactgccaatgcactagccgcc
agctcatggtaacaatcgacaagataagaataacaagcttgcaagacatacaaaaatccccaacccaaaaagacaccacttactaagggggct
gcccaatgaaccgatgggtaacaagtccacgctaaccaaaacaaagcaattggaatgaagaaagaaccgaaaaatagaggccatagacggtc
ttcaggacgaacattaccgttgttcatatgaaagcgtttttttgtaatgccaatgaattaagggagtaaagcaaaagccaacaaaaaccccaacat

cagcaccaagcagggcaagaccctcttcaccggaattgaaattgtattgtgcaaatatcataggataaccgacattgaccatatagagtaggac
gttaatgaatacaagatacaaacagatggcttgaacaattggttcagtacttaaaagcttcacaccacgcgtcaaagaagtacggatggcattgg
taacactacgttgacgttcatgttcgttataccacaaactacagcttgtttgcttgcgaaggtacctagccttaaagtcgagaattgtagcaccatg
actttcgggcatcacaaagataacgataacccaagtaagggcagcccaaatcatattgatccaaaagtcccatctccagcctaacttgctacctg
tgataaaaccaccaagggtaggtccagtaagaggaccaacgaaggcacaagcacaaaagaagagcaacgctgaagtacgctcaacgggcga
aaagagatcgctaacggtaccaccacatgcagacataggcgtacaagcaaagaagccatggataaagcggaaaatgaccaaactccaaatat
ttttggaaaggccacctccaatctgaaagagggtaaagatgatgagcgtacaagaataaataggaagtcgtccataaacctcagaaagaggag
caaaaatgacagaaccacaagcgaaacccaataaaaatacagctgaacacaaatcaccgactgcagcacctgtgtgaaactgttttgcggcga
caccaccaccagcagagaaaatactcgaattagcagtaattacaatggcaatgaaggaagcgatacttattgtaagaatttttttccacatgggc
caattttttgggttcaacggatcgtctacgccggcccaagcaacttgatactctgtagaatctgattcctcttggtaagatttactgtgagaggaggt
aaaaccatcatcttcatcactggagtgggcctgaggctcaactttttcatgctgctgatgagtaggttcttggtcttgcggagaagatgctttcctgg
attgaaccttgcttatgtctgcattaatgtcaaggtgacggaacttcctgcccgtccaaaggaaaatgtagtccattgtttttttttataactcaagaa
aacagtgttaatgttacaagaatatatagcaaaacaatctttcaatataaacaggcggtttaggctgaatatataaaggaataaaatagagtag
aaaattaaatgcagagaaaaaaaaaaccaaacgttaaacagaaagaatggactgaacacaagacgatatggtaaaaccaagcaacttcgtcac
tggaaaagaaatgcaaagaatagaagaaaacgagatagaaaagagtgaaaacaaacacgtaaatgaaaaaacgaatgtcgggtgaaatta
acaagacttgaaaggataaactaacttttgataagcgatttcagtacggctttttttataaattgtctcaaatgaactgagataaatacctagataa
aagtattttaaaaacgaggtgatcaaaatcaagattcgtcgcttccgtcgaccttcgaaagaatgttagatcagttatagagaaacaaggtgcga
gggacaaaaaatactattacaaagaaagctgagtcaattcaaaaagaactaatcgacatatatttccaaatatgtagcgtttaaataaggggaa
tcacgtaaaataaagggaaacaacgcttcaaaaaggaagtaaggggaaaacggaagaaaaaatagcgcgaatgcagatttaaatctacaag
ctcgcaaaatacctaagtagtcaatgatacagtcggtcagacgagatagaggtcaatgcagaaccaactaaggaagaagtagcgaaggaaac
tattgtccaaacaaagcaaaaaaaaaaaaacaaacaaattcaagaaataaaaaccgaatcgatgtgaatgtcaccgactatagggaaagaaaa
aagaaaaatatctcgaggaagcgaaactcgagtaaaacaacaccgaaggagggatacggttgacgtgggggaacgaggcctttataaatgca
agaaattacatgcccgaatgtacaaagttctttgcgactcaaataacatatacaaaaaaatgacgcaaaggatggagtgagagcgagatagta
agaagaagaataagttcggctacgtatcaatgaggagaaaggtaacatcaatgaacattataagactcgaggcattgtgaattggcaaaataa
agtgcaaccacacacgagtcacgtttcttgtttattctgattggtttaatgatagggctcataaccgttctagaccttttgctacctttgcaatgccga
cgtaagtgtggagagccggtatatgagatcacgagatactttttcattgccggtatttgacgtaagtctcgaaccttgtaggagtgtttcgtgagga
tgatggaaagtgtaagaggagcttgaacgaatgagaaaacagacgaaaaggaggcgccaaaaaagcaacaaggcggccaaaggaaggtta
caggagctagtgaaaatgcataacgggaacatgaagtgcgtaaacttaattatttctaagtgcgcacatgtctgaatggaatagacggggtaaa
tcaaagtttaataaattaggggaagaaataaaggttaaatgcttcggatgcagtaattcccgaaacggcatttgaggagtcccagcaagtctttt
aaagatgtacttggctgaccgaccactcggcggcagttgaagagggaaccagagggtattcgtaaaaaagataaaaaaagggtaagcgacga
atgattgggactcagtatcggcaattgcgacggctggcatcggaagcaaagagcaccccagctcaggataatgaaaatttaccatcacagaagt
tcaacctctttccgtaggagcatgtgattatcatcagcatcttctcaggtgaagcctagtcatctctttgaaaagtttccctcatgtgttacttttcatt
gttgtatgatgtcgctgtatatctccccttagcgtctccgcatctttttcgttttaaatgggtttctttttcgtttaactgaaacttctttccttcttgttta
taccactctatccggcatacccgagttgccattcaaaactttcatacagctcacttacaaatatttagtttcaaaatataaaagcttttgctgtatgt
gatacagacgcgctctccatcccttcctttcactcagcaaaaaatcccttgggaagttcgtttcactcagtgtctgccttcccttttatataaagagg
gatttgttctgtctcttcctatgcatacatcgaaatctctttccgttccgtcctacctccgtaatagatgcatcgcgacccttgcgttccttttgaatag
ggatcggtcccttagcgtcgttaaagaatcaaaggtaaggtacatagaaccctcggtattacttttccctttaataagcgattggaaattgcttcttt
cagagaaattatttcactacagatagttaaacatctacaaatctttaattctgatgactggtatcctctgcttcttcccttacactacgcagaaatat
accggagttaagagtctcatttcggtactattggatgctattgccgggtacacaatatttgccattgaaggaggtgtaacgtgaacaaaactaaaa
agcattttctcgcttttaatgaaggcagaaataaaaaagcaatacagaattgcataaaagggcatcgttagagagcagccttttacataacctta
gttttggtttttcgaggattttttttcgttaaaagttttctaagtcctgttttgtgttgattgattgtttattcttttttttttctttatcgctattttttttcttt
tcatttatttattcactaattcgttctgcatcttttggttacattagattcattctcttctttctaattgttctttgcactatattgaatcatcccgcttgttc
aatccgctttccaagtgctgctgctttttttatttagttaatgggtctcttgaagcaacgttatcatgaacttttggacttgcgcgtacgtggtgaacgc
cttcctattgctaggcgccttcaacactttacgtgggcgtggtttgcttctactatgggtactggtggcattggcatgatcacatctttgtattattttc
gttttcatggtctcaatacccttgggaaaattatctttgtctttcaactttgcatttttttctctttttacagtatgcattctctttcgtttccttcgctaccg
gggtacgttcaggaaggcttggaataaccctggcgaattagtctttatgcctacctttctcctctccattgccactataattagtaacttttacccata
tgcctacccttacaccggcgaatggatggtttggacgattcgcatcacctactggatttatgtcgcttgcgcttgcatttttttgtgtcagctccttttat
acccttttttcgcaatcatccatttcgtcttaataccattattcccgctctcattcttccaatttttccctgtatgatttgtggtgttgctgcgagcgccatt

gttgagtctcagcctccagaacaggcaaaaaatatgattgtcgctggtatcgccttccaaggcttgggtttttggatctatcttcttgtttatgggaa
taacatacttcgtcttttcacagccggtcttcaggccccggctgatcgtcctggcatgttcatctttgttagccctccttcatacacgggtctaactctt
ttggatcttgcttttggtgctgtacacaagcatcaccctatttttgtcacaaaagactcacccgaatggctacttttcatctgcagtttctttgctttgtt
catgatcggtttgggcatttttaactttgtacttgctttggtttctgtcatcgccggcttttgctctcctggtaaactgaaatttaaggtttcctggttcg
caatgatctttgcgaatggtggtcttgttatggtcatccaagagcttggcaaagccatcaattctaaagcccttgatgttattggtcaagtttgcggt
gtcatcataaccattgtttggatttgcttaatcattcttgccgttcgtgccgtatatgttcaagaactcctctatcctggaaaggatgaagatgtcggc
attcttcctcctgatgttcttgaatactatgaaaaattagaatcctttgagaaggacggcacctccaaggatgctcaagtgactctgaaagaaatc
aatcataatgacaaaaattcctaatactggttctgttaagctttttctttggctctaatttcgttgaaaaagcatggtaactcttggtttttttcttttg
tattcttgctttcttcttttttttttttttttttttggttcctttccataaatgacctgtacagctgatgaaaaaagttagactgtttgattgtttcatgttc
ggttatctatttatttttagacgtttcgttttcatttgccatgattcgtgataaaaatggagatttttcaagcctggtacctaattattttatatttttctc
tttttaaatgtaacgagctttcaacatgaatactgtacctcctttctcacctgtttcctattatcatttttatttatcaattaagttctaaacatacttctt
ttttggtagagttgaatggaaagctaatgctgtgtctaatggaacccttatgtcgctctttttaagttttacattggcaagccgattttgaatatatct
aattataactacttgatttaatcgctaaggacttaaataatgcatgcgctttttttaaacgatcattctttagtgttttggttcaattgatttaaattcat
attaatctgtagaggtttgggtaaatcgtacagtatgaacttcatcattaagttctattaaataatacataacgtaaagttactaagccttttttgaat
atgtgtacaatatgattgcaggcctattgatgagcagttagaattatacaagaatatcaacctgtaatgataatgtgaacttttctttttaaacgca
gtctttttttgctatttctcatagtgtttgctaccatattgaagtgcacctcataataccggagggggacttctcacccttagggaacctcagctttccc
tctaccaacaccataattctttgagtttttggcaacgatcagcgttgggaacggactgaaaattttaatttgacatctttgacgatttgctaaatgtt
gtctggaagcctcgttcgtaatgtgcgccatggtattgctgtaacgaaatcgttgcaagtatctggtgggtaagtaaaaagaaatcccctttctatt
tctgttcattgtatttgatagtaatgactaacctattagaacaattacagccaagcgcgccaatctttacccattgattagtagaatggctcggtttt
atgccaccaaaaaagaatatccccctcacacaattttaaatatgccagccttgtctcctacaatgactgctggtaatattggagaattccagaaaa
gcattggagataaaattgagcctggtgatgttttgtgtgaaatcgaaactgataaggctcaaatggactttgaacaacaagatgagggttacttg
gctaagattttaattgagagtggtgctaaggatgttgctgtcggtaagcccgttgctgtcactgtagagaatgccgaagacgtggaagcttttaag
gatttcagtattgaagattctgctcctgaaactactgagactcctaaagaacaacccaaggaagcgaaggaagaaaaggctgatgttactgctg
aagctgagactgccaagaccgagactaaggaagccactgccgtccatgatgatcgtatctttgcatctcctatagccaaaaaaatggccgaaga
gagaggtgttaacttatctcaaattcgcggatctggtcccaatggccgtattatcaaggttgatgttgaaaacttcaagcctgaacgtcctgccgct
ccctcaaataaagctgctgctgctgcgactagtcctgccgcttctgctaaggatgctgctgctgctggcacctatgaagatttacctttgagcaatat
gcgcaagattattgcttctcgtcttactgaaagtaagcaaattaacccccactattatgttagcgttgccattgatgtagaaaagattgttcgtctcc
gcacagcccttaactctattgctgatggccgctacaaactttccgttaatgatctcgtaattaaagctgttactgccgctttgagagaagtacctga
agtcaattctgcttggatgggtgactttattcgccaacacaagaatgttgacatttctatggccgttgctactccttctggattgattactcccgtcat
ccgtaatgctcatagtcttggccttcgtgaaatttccaatatggcaaaggattacggccatcgtgcccgtgccaacaaattgaagcctgaagagta
ccaaggtggtactttcactatttccaatttgggtatgtaccccgttgatcaattcactgctatcatcaaccctcctcaggcttgtatcttggcagtggg
tactgttagcgaaaatgttatccctgataagacctctgagaagggcttcaaggtcgctcctatcatgaaatgcactttgagcgctgatcaccgtgtt
gttgatggtgccatggctgcccgtttcaccaccgctcttaagaagatcatggaaaatcctttggagcttttgctttaaacggtctttcctgttcaaagt
tcaagagagtggattcagcgtccttccttcgaacgtagctggtgactgtgtaatatttgctgtgtttttaaatcttcatatataaatgtttcaagtcga
ttgtggaacattatcttcatgaggttctatgcatttatttctttcgtttaatctaagtatatgtagtgttttaaaactatatatttaccataatgcaaggg
ttagtacaatgatttgggtgaataagtaagaatagtaaatgtatatatatatatatatatatcacccttactaaactttaataagtgaaataact
ggttcgcttagataacttaattacgtttttatcacatatttgcctgcttaagagattttagtacaccaccaccttacttcatccttttaccaactcgaac
gccatttcatgactgacctttcaattggcttctatgtggcttttatttcgctctaagcacaaaaaattggttctcaaatgctttccttcaagaaaatta
ggaggatcagaacctaacggctcacctttagcatacttgagctactatgccgcatcaaattcagggaaattgaagaaggtcattcggtatttaagt
cgtcttactcaacacatcgtatataggagaagggacaagtatgggagcttattttgaacgagctatttactaacaggactagtgaaacggtcatc
gctttgaagatttgtcgaactttgattcaaaagtgcccggagaatgtcatgataatggcttcagagattgttaaaatattgttggcttctagcaatct
caaaagtttggacgttcagaacatgtcggtggattgctttgctgccttttgcgaacgtagcgctaaaacaaattctacagcttttggaaatgaatttt
ataaatctttcgatgaattggtttctttttattttgacctggccagaactgacagtcccccagatcctcagcaatcaagaatgttaggcttgaaggca
tttcacgcattgacatcttgcaagctttctgatgttgaaggcatttcacgtaaccaacctcattttgttattcatgctttcctaagtaacgcttggagc
aagaatgaaatgactcaacagagttttattgacatggtgcgctcaacagcgcaaacattcaggccgttacccccctagtactactggtatatcttta
ccggagcatgatgaagttggcgacactgtagagatcgcgaataaaaaactctcgctgagggttttatataatatatatactcagacgaatcctgtt
ttcatgatagaaagcacgaagtctctaattcactttttcgctagtaatcctaatactcggattaatcaaaactacatctctgttgtgctcaaccaaat
agttgagtggacttcagttgaattacgccattccatcttttttgctgtttacgctgtctccaaagcagccgcatccaattcgcaaataccaatgtttt

ggttccctatatgattcattctatccttaactcgcaaaattccattccaagtctttctgtgattgacgtcctcagagagctagagtttcatctactagtt
gcaattgagaatacacaagctagtgattttgaatggtacacgttatgtgaatttccggctgagcgattgcctcaacgtttatatttacttctgttatgt
atcacttccttaatcaaacatcaatattacaacgaagagtttgctgatgtttggagagagatggattcacttttaagcgaaaaagctacaactgca
actcagctggttgtgcttacggctttgaatacgatgcaaatggaacaaatgagaaaggaaagaccatcttcagttggtacactttcgtttattgctg
acttcttgtaccgggcttggccagtatcttttcagcgagtatatatttcggattctccatttgttcgcttgaaaacagtgaatgtttttttgagtttttg
aatattctacctacttccattttgactttcattcagtttttggaaaaagtacagtatcttatttttctgctttatggagtttcatctacgaaataagtgg
acaagtggagaaatggcttggaaaattctcctccttaggagagtttgaggctgtacgtaaattatcacgaaaattttatgctctttatggacctatt
actgtcgctgcttttctccctttactttatcgcgctttaaatgcgacaaaaggaaaatatttggttacccatcaagctttaattaccgattatcttctgt
acgtttctgattgtctgagcaacgggactttaaaaatgatgagcttgaggtggaaagagtctcttccctctgattctctttcaattttgaattacgat
ggcacggtgaccagaagtttatgggagccactaccctcagctgctactagcagcgtttttctttcacttgaagcggtcgtttcagaattattacaag
aaaacccttcagtctatcctgaggaatcaaaagttttatttttggaaaaatcttggcatccgaaatattcgagtttttatgaaaaactacagaaacc
tacccaaacagttactttgtcttcttcatccgaaaattcattttctaatggatcgccgatgtcagacttcaatccgaacccgattatgcatcacaaaa
accaatcagctgcaaaagtcgacaatcagaatcctagaaacgagaaaattttgacgttaaagcaagctttggaatgtccaccaagtgcccgaac
atcatcgggtttgcgtacgccgtctgaaattacactatcattgcggtctgctagccctgttccgctgttggatatctttaatataaataattctctctca
gctgcgactatccaacatcctccttatgttaacatatagactttgttttacatgttttgtatatgtgaaattatcataaaattattgctttctttttgatgt
cgttctaagtgatcgatagaagttatcagttatccggttaatatgtatgtacctataattatcacttcaaatacatttgtcatattagaattatggtttt
cgcttatctagagatgaacagaccatctggtcgtgtgcatagatatacacaaagccttctttgtaagtgatgttatgtttagcgggtttgtaatttgc
tttacaatatttttgaaaattcttcctctccataaagcaccactttttagccttttcagaaccttccgcatacaacaagctctttatagattatttgggt
gtccatacacttacatcaactataccataaatttttttttaaaatatcaattactaaattaattcaatcctaaacaaccaactgataggagtgactag
taaagacctgatgatggtaatcatcaagtactttacttcttaccaccagccttttgggcagccttggtaaccttagcttgaccaggggcagtcttctc
gacggccttgatgacaccgacagcgacggtttgacgcatgtcacggacggcgaaacggccaagaggagcgtagtcagtgaaagcttcaacgca
cataggcttagtaggaaccatttgagcgatgcaagcatcaccggacttgacgaacttgggagattcttcaagcttcttaccggtacgacggtcaat
cttctcaatgagctcattgaagcggcaagcaatgtgagaagtgtggcagtccaaaacaggagagtaaccagcagagatttgaccggggtggtta
agaatgatgacttgagcggtgaagttagcacagcccatagggggggtcattcttagagtcaccagcaacgttaccacgacggatgtccttgacgga
aacgttcttgacgttgaaaccaacgttgtcaccagggagaccagcttcaagagattcgtggtgcatttcgacggacttaacctcagtggtgacacc
agaaggagcgaaggtgacaatcataccgggcttgatagtaccggtttcgacacgaccgacggggactgtgccaataccaccgatcttgtagaca
tcttgaagaggaagacggaggggcttctcnnnnnnnnnnnnnnnnnnnnnnnnnnnnnnnnnnnnnnnnnnnnnnnnnnnnnn
nnnnnnnnnnnnnnnnnnnnnnnnnnnnnnnnnnnnnnnnnnnnnnatttgaccggggtggttaagaatgatgacttgagcggtg
aagttagcacagcccatagggggggtcattcttagagtcaccagcaacgttaccacgacggatgtccttgacggaaacgttcttgacgttgaaacc
aacgttgtcaccagggagaccagcttcaagagattcgtggtgcatttcgacggacttaacctcagtggtgacaccagaaggagcgaaggtgaca
atcataccgggcttgatagtaccggtttcgacacgaccgacggggactgtgccaataccaccgatcttgtagacatcttgaagaggaagacgga
ggggcttctcgacgggacgtgcagggggctcaatggcgtcaatggcttccaaaagagtcttacccttgacaacaccagccttggtctccttttgcc
atccttggtaccaggtcatgttggtggtaggctcaatcatgttatcaccttggaaaccagagacggggacgaaaggaacggtcttggggttgaaa
ccgaccttcttgacgaagttggaagtctccttgataatttcctcaaaacgagcttgagagtagttaacggagtccatcttgttaacggcaacaatga
gttgcttgacacccaaagtgtaggcaagcaaagcgtgctcacgagtttgaccgtccttggagataccagcttcgaattcaccagtaccaccacca
ataataagaatggcacagtcagcttgagaagtaccagtaatcatgttcttgatgaagtcacggtgaccgggagcatcaataacagtaacgtagt
acttgggagtttcgaacttccagagggcaatgtcgatggtgataccacgctcacgttcagccttgagcttgtccaaaacccaggcgtacttgaagg
aacccttacccaattcggtggcttccttctcgaacttttcaatggtacgcttgtcgataccaccacacttgtaaatcaagtgaccggtagtggtagac
ttaccagaatcgacgtgaccgataacgacaatgttaatgtgtcccttttccttgcccattttgttatttaataaaagtggttggtggatggaagcttg
tatggtcccatgttctacttagtaggtcgggtgtgactgtttttttcttgatggaaatttttgtgacttgtttttgtaacgttacatttcgtaatgttaactt
tccgcgaatgtttgaaaatttgctaataaataatttggcccaggataaataaatctagagtaaacagatgcatgaataaaccaaaatgaacctgt
ttatgactgtgaaagtaattgaattatgaaattaaattttgtaagagggtaaaccacgtttatggaagagcaatgttgaggtgctgtacaagatgt
ttttgcaagctttttgctcatagaatgataacaaattaccaaaaaaaaaaaaaaaaaaaaaaaaaggtaatgaatcattgaatgaaattctaaga
ctaaacttctctcacgaataagaagaaagaagttttgaggcaaaaagtcgtttactaaaagattgcccattaaaaaaaataaaagtaagaataa
aagaaatcagcgacccataaaattcaataataaccatacaataaaacaactacttagctagtcttgatataaaataggagtccttctgttttgaga
atagtgtttttatcaaaattccttacataaaaagtgccatagtcttacatattctagctaaacaatgataaaaaaagggttttctattatgcagttcc
ttttatttaaatcccaatttccttaagtattagtattacgctagttgtctttcttaggtagaaggagtcaattctagcgagttgaaatcgctaccttttttt
cgtatatcgaacaaatacctttattggaaatttcattgtctgtggatgattattgaagacgattttccatagttatactcagcttaatttttgttgttac

cactggcatcagcatacgtatttcaagtatataacactattacttcataaggaaccctttattctgttattattcctataagaaaatttgtaaacaaa
tctagtctttcagccttcacaacgaggatctgtctgttactttctaatactacaagttttttttgttctttgacaggttcaaggatgtcaagtaatatact
agacgaaataaaagcaagcacaaaggagctagtagataagtgcgattggatatccatctcacaatcatctttgccttccttgattgatgcactac
agaataccttacaggatccccgtccatcaaagtttcccttaaatttcaaaagtatagaggatgaggttaactttgttgctctctatggtctcgtaagt
tttggatctggctttcgaaaccagcttcacgaagcgtaagtctattccttaggtcaatccaccaaaaactattatatcctctaaagttcatgtctattt
ctaacttttaatttttacagaattggacgtggtgcccaggaaacttcgctgtttttaatgctttccatatatatctctaacgaaaccggaataccaga
tgccaattacttaaccaatataaaagagcacgatgttcagtcttggatgaatgttcctgctattgaagaaaaaccagtacaatcacttccaggtgt
ctttgcagtgtcttctagccacttgatggaattagtgaaaaaaatgacattcgtctttaactctaccggtgaaatactgaaaaaggagaatttctctt
ctatgggagaattctgccttcgatatggcaatacaaaggatccctctatacttgtcaatgctctggttagcacatttccagcatttcatgatgtcgaa
caagtttttggcgtagaagtatatttactaaagaaggctcaattaaccgcctaccagctttctcttttgtttccagataggtttggctccggagggtt
gtgcattttcgctgacaatgttataccgaccattttatgtctctatggggtcatagaaatctctaaagaattaagaaaaaaaattagagaataaagtt
cttctagaaaaggaagaaatcacggttcttcgagcgtcttctgtcgttgtctgcgagttaatttccgaagcaacaaaaattccatcacctcagttag
accaagctctctggaagctaggcaaatccactccagagctacgaaattctccacgtttcttgtaccaagacactgttatgttttaaaagtgaaatgt
tctttcctcattacgatacattagtatcaaaccttccaagttttaaagctttgtataaataggaattcatttctatagcctttggttttcttaacagtctt
ttgttgatttttgtatttttctttaagatcagataaaaaaatcattcaagtgggcttattgcaatacgtagttctgcaaataagcttcggtaagcaacat
tatacgctataaataaattcaaagccaccggaactacgtttgtttataccgaatattactgttgttttcttctgcccttaaccagcatcatttaaaag
agaataatgctacatcggcgagttcattttaagtctcagtccactttggactttgatagtattgctgttagtatttcagctagcacaatgaaaaatga
gctcgataagctgctacagaactcaaaggttactgatcgacgaatttttaaccttcagatggaaaactttttttgctctgtatcgcagatatttattac
acacagtaaaaggatatgagtgtgattgggattcgattcggcctttaggcccgaaggatgtcacggattacatggatttacctccttgtgagcaag
gatcacagtaccttgatcggatagcagttctaaaattgaatggtggaatggggaataatatgggtatcaataagccgaaagctttaatcgagctt
catgacaatcaaacgtttcttgacttaagtatccagcagattgagtaccttaataaacgatatgatgtagcggttcctttttttgttaatgaactcttgt
gttacgaatgacgaaacatgtaaagttcttcgaaaatatactggctacaatcttgatatatctaccttcgagcaatctcgtttccctcgtgtcctcgt
agactctcagcttcctattcccaaaactgctccttctcccattgaggaatggtatccaccggggcatggggatatttttgaagcactcgttcactccg
gaatcatagaccgtttacttgcccaaggaaaagactacttattcgtgagcaacattgacaatttaggtgcctccgttgatcccaaaattctttgtca
tgttatagaaaaccaaatagagtatacgatggaggtcactgataaaacgaaagctgatgtaaaggttggtatcctcgttaatcaggatggcatttt
acgattattagaacggaatcaggttccagaaaaacaccaggaagaatttacctcagatcgaatttttaaaaatatcaacacgaataatgtctggc
tttatctgccagctgtcaaacgagtagtcgaaaatcgggaactgaacttggatgttatgcccaacattgaaactatcaaccacaacgatgagaca
gctagggttattgagttcacaacttctattggttctgccatcagtcaattcaaaaaaaccgaaggaattaaagtgccacgtcaaagatttatttctg
taaaaacatctgcagatctgctcctcgtacgtggtgatttatattccatagaccatggatctttattagttgaggattcaaaggttggatttcctcctc
ctgttgtcaacttaaccaatgaatttgttgacgtagcacaattatttcgtcggataccgcacatgccgagcattaaagacttaacttcattatcgata
tctggtaatgtttattttgggaaaaatgtagttttgaaaggcaatgttgttattgtagcatcaccaaatgcgacgctgaatatcccacctaacgctgt
tttagaaaattgtgtagtatctgggaactgtaaaataatggaccactaatgttcttttgctaattgcaaaaactttgatgtatggatttttatgattag
atatggaatactttaaatggcttattaatacaatttcattatttcactataccgttcagtaagcattatttattaaattatatggattttaatatctaga
tatccaggtagatcctgcaaaatatatataagttgctttacgtacaacgcttccttacgattggcgcgttttcacccaacaaacgaaagctttctttt
tatgagtggaagactaaaatacatagttccagcataaaggactgactatccacgtcttctccaatattgtgcataaataggatacaaggcagcttg
tagcaatcataaacaggaatgtgaacagttagtttagatcaaattataaaaaacaaaaacgacttgataaaggagggagagaaaagtatagca
agcttcgcaatattatcaaagaaagaaatgaaataattgacataagcaaagacaaagtaaaatcagcgggttagttttcatgagcttttagtgaa
cgcggcaaagtcatttttactcaatcatgaagcccaattcagaatgaacagaagaaacaaatttcaagctaatgtgcaaacttcataattatcaat
aattagttggggactcacagaagaaggttattgtcgtcactggagagaagattggaagtagccggaatagacgatgtagagacggcaggagcc
attgaggattgtggtggattagtacccagtttggaatggtcatcatgctttactttggttacggcagaactccaaaggctcccaaaagcatctccac
cagagctgccagctttagtaaccttagctgtttgggtaacttgattggaggagggaaggttttgggaggagagaccgacgaaagtgggagtacc
aacgttcgaagaagcaccgtttccaaacgagggaccagcaacagaagaagaaagagaaggaagggaattgaacgaagccatatttcccattc
ctacagatgcagaaggaactgagggtgcactctgaaaatcgtcaaaagcatcatcgtcatcgtttccaggaactgtagcggcagttggaagagc
ggaagacgtttgaaactcaccaaatccatcatcatcatcctcaacagcagcagtagtagaagaagtaggcatggtagacgaagggggcacagt
agcggcgggctcatcgagtccaagaagatcagctacggcagactgttgctcttgcttaggtggggaagagactgaagtagcattcagctcagcat
tcctcgtagcacgtggagttgccctaggtgcgagtgcatgagggccggcactatcgtgattttcattatactcgtcgtattcggaatgatcatgtgta
tcgctatacgaagacgagtcacggaaaccaccgccactactaaagtctgtgaagccaccgccatccccatagacgcggtttctataacttgcggt
acgaggagagcctccaaagcctgagaatctcgggcgggatccagaagacactcttgcatcaccgtcgctacctacaccgaaatatttaccgcgc

gtctgtcgagcctttcttctttcttgacgaatgcgatcattgtcgttgagtaattctaccaactctttcgctctcgttctcacgtttaatccttggtctctt
tgataacggtcaatataatgaaagttgcgcaacatctttattgttgcctgatgtgctctagcatcatcaatgactctctcggagccattctttactaa
aaattccagcagttgaagcgctttgtatatttgtcgccattcttcagctgtttttttcagtgaatctgcggtaaaccatcggcataatctcatttaactg
agaatagttatgcgttccttgtgcaatttccatcatcagagacgtacttgcaccccagggttcgttgttggtggcttcacgcactttggcttccgtcga
agtataattcatcaccacattttgcgctttgcgaaccgctgctttaacgtcatacagattaatgtttttttatacttgattgaagtccgtccatctgaat
gctcgttttctccttcaatgaattcacaaattgcccagtgaaagcctcgtccaaattttcacaactctctatttcttttcttttcaatttctagaagcttc
ttttgtcgtttttggctgcaacgactaaatcttccttttgaaatcttttccttgtgaattgtactctgcaagtgcttgcgagttcaattcaatgaaaccgt
agtggttgcctgctgtaagttgaattacgtcgtgtagtgatgctcgttatataattagtaagccttttccttctacgttaataaacaatactctagaga
tctagcgatattatgaataatcgagtaaaagaaaagaattccagtgaatcttgtaagttttaacactttttttatgaagaaaacgaatttgtacttctt
tcattttaggttcccaatgtactcttgtgtctgacaactgtgtactgtcatcctggatcgtttctggttaatctttttggactaatctacattcaaatttg
cttaggtgtagacttttggtggtaatttctttggttcttttcgctgaataatcaatacataaaaaataattttccatacaagttaacaatgtttcggat
gctgtaccgacaagttatacaatatagacagctatttgtttgagtcatgtatgaagaacatgaagaccagttttaaataaaaaaaagcaaaattgct
ttacaaaaaaaattgatatatgaattccaaaaaaagtgagctttaaaccatgaatgatgatgatttacattttcttttttccaaacttgttcgtcagcaa
gcagtgggaatcgcatgtaaaaatttgcatagtacctttgggctccttgaaaattaaacaaactttttgtaacaaggatcccattcttggtttcgtat
ccattcgctcaatgccttgtcttccgttgggaactcagtggtattcatgttttcttttctggcttgcttcatgacagctagagtaatgtgacgggaaatt
tcacgagcattcttcagatcaggcaaaagagcctcttccgacttgaataaagtcttgggaacagtggctaaggcgtcagaagctgctttcatcata
ccattggtcaaaaacttgcagccggagagcacacagcccacgcccaatgctggataaagaagagcattattacattgagaaataatgtaatcctt
accatcacgcttgataggaggcataggtgatcctgtagcaagaagggcttttccatttgtccattcattgatttgatccggtcttgcttccatcaactt
cgtgggattggagatggggaaaataatgggctgttcaacatgttttgccatttcgcgcatagcgttttcggtgaacttaccactttgaccagaacag
cccaataagacggtaggtttgacacgagagatagcagtttccaaatcaacagaatcttcctccttaatgtcccgaaaatcattgcttttctttaaga
aaggtttttggccttctgtagcaacagtggcgtgcttttccagtaacaagccatagcgatcaaccaagtagatatggtcaatagcttcttgtttgcta
agaccatccccaaccaaggcactaacgatttgattggcaatacccataccagccgtaccggcgccaaaaatcattatgcgttgttcggttaaagg
tgattttgtaacatgaagagcagccacgatggcagctaaagcgacggcaccagttccctggatgtcatcgttaaagcaagcaaattctttgctgta
tttgtccaaaattcgtttggcgtttttcaagccaaaatcttcaaaatgaataaaggcatggggaaacacctcttttatgcaggttaagacagtatcc
aaatattggtcatattcttcaccacgcacacggtttcttttaagacccatgtattgaggattggaacgatgtttttgattgtcggtaccgacgtccaa
cacaatgggcaaaaagctattggggtcaagtccagcacacaaagtcattaaatggcccttggcaacagaaatcaaaacaccaccgacaccttg
atcgccgatacccaaaattccttcagagtcagtcagaatgatatatttgacggaatcttttgttccaaatgcgccaagctgatttttgatatactgtg
aatccttatgatcgatgtccaaaaagcatccttccgggtaacgatatacgtctgagaattgcataatagcatccccttcggttggtgtatagatgat
ggggatcatttctaccaaatgctggctaatcaaagcataaaacaaagtctgatttcggatacttaattgggacaaataaaagtttttttccaaaac
tctgtcgccaaatgacttgtattgctcgtacgctctgttaacctgttgctgcaaggtttccacaaccggaggcaagcgagatgtaagctcatacttg
atacgctcttcttcggtaaaagcagtatccttgttcaaacgaggattcgtcaacaatgcttcacccttcaaagggcacttgatttcgtccttcgccat
agagttcatagtctgtttgcttattcgtttgtttttattttcttaaaaaaaaaaaacagtaaataagaaaagtaaacaaaaacgtctattgtttgtggg
ttgttttgtttgtttgttttcggaacaagactttttttacttgtttctccaatcaaaatatagatattgctatctttcaacctatacttatattatcttatatc
agtattatataggtatacccagaaatagaatcgaagtctacttgtgtgtgtatgtatatacgtgtacgtgtatgcttgaaggtacgtttgtgctaggt
tgtgctatcgccgctgtttgtgtaaccgtaggtgtagctgtgtgtatcagtagcctagtacaagggtccatgcactgtttatataacacgcgtatgta
tggattgttcttatgtagagataagaatcgatgtttctatagacccagccagacctttgtgtttaagatttggataacgtagttcaatataatcgtcg
ttgaaaccatccaaagcttatcgataatgatgacttcaattaacaagctgaaacgtatcaaaacttacagaaactgtagcgcttcaaccctctcac
ccacctttctagtcaccgtccgtcatactctttcttccctttgttcttcttcttcgactttggattgactactggatcagatcggatcatccaaccaaga
gaaaaaattttcttgtgcgtgtttttttcaaacttgagggtaaaatcgtccaaggtcagccaaccaatcatgaatccgtttcttgtatatttcctaatca
actaggttggttggttcaacggttatcatgaaatcttttgtttttatcaacacacataaacatataaacaaacatataatattggaaatgctaaaat
aatggctaaataataatttaatgaatgatggaaacattcctttttcttttttaatccttgattctactatttgttgattcactcgtcatcctttttccctta
tcacaacactttacatttcgttaacaagaattatagctactctataaaatactttatattaagctaccaactgtggactttttcgttaaagtactttttg
aacccaacacaagggcccgccttatagaggctgttgaaaaagggccaccaccaccccagggaaagtcagacaggcacacataccatgtcaac
acatcctttctccctactaaagaacaaccaaacaaacaaacaaccatcctcaactcaaccggtcaacttggtaagaatgcagtagaaaaaggat
gttccctttgtttaaattttttttggttcttttttttttttttatactaacaacgatttaggatttcaaaactccagttcaaatccattggcaaacaccac
agcaaacaattgctggtcaatctttttgtcaacatcggccaacacggacgatttgcatctcttttgaggattctttgtttcagttcacccaaaaaaaa
ccttctttgcaaaatcccaaattgcgcaaattggaatcttgactttcgcttgatcgcctttttttttgtcgttgttgtcgttgttcttccttcatcaaagga
aaggcccagtttggtttggtttctttagaataaaaaccaatttttgaattccattgatttcactttcaatggtcataacctaaaatatttcaatttattc

atttttattcttcgttttgcattccttacttctttttcctcttttattatcctcttctccttgcttcctttttttttttgacgttcgtatctacttttagatacattt
ctatgtcaacatcaaaatgtccgacaacttggaagcttcatcccacttgtttgctcgctcttccgttaatgatacttcttcgcaattgtctcgtttgga
agaggtagagcttcttgagggtgcccttcattctccttatagagaagagtttctaaacgaggaggcattgtatttgcctccaaagcctcagtatggc
aatacctatttggctgctggcccttattacgccaacaaaatgacccctggatggcctctcaattatttcattggggtggaaagatctcggttcgaaa
aaagactttccaacttttggctccgaaatttcttgctgcaggttttcgctccttgtattgtccttatttggtgtgcaatacctatgccattgtaccagga
taatcaaggcattgttcgtataaaattttggtttttctttttttttattacggtatctataatgccgtcggtttattgtggattacaaaactttttcatattt
attctgtcaattggtgttcaagtaaactcggtggcacgatgagctatttattgttttgggcttttagtatcctcatcggcagcttggccgtttattttac
tgattggcgccagattacattcacttggatcaccttaatgttcatttccatgatcatccccatcggagtctcttttttcaaagttgtggaaaaaagcattc
acgccgaactactcaatttttgtcccagttgtctttgcttgaacccagcgttcgaagcaacgctgtcttggaaaccatcggctggagagatgcctac
gctcattattcttggtttatcattgtcctttgtgttaccctcatcgtttatcttgccggtgaatatcttaccaatatttacatgtctacacttccccacagc
tccagtgtcgctattgcatatgtgtattcttggatgtgtacggttagtttgtgcaatctgatttcatcttggatattaaacactaagacccactcttatg
cgttagtatctgtgtttaaactttattttgagcttactcttcaggtttacctgcgtaatctgtatgccagactcgaatcgcctcaacagtttgttcttgta
caaattgcctcttctcttaccatgatgaccatcttccctttaattttgatgtcgcgtacagcatatcactttaataagtctctcacaaaaagcctagat
ccctacgcagtgtatcgaaaaaacatgggcagaaatttctatgtgaagtgcgttgcttccaaccttagtatgctttcattcttaggctggtctcttat
cctacattttggacctaacgcggcgatttatccatattttacgttcactaaggaaagtccatacagtttcaagcttacattctatgcttcttctgccgt
gtgggcaagcgagcttatcgcggcctattttactcggctgattatgagtaaatattatgatttcgaagtctctttggaagctatccgtgattttgttga
ataccctgacatgataccagctttcatcgcagtcggcattcatgttttgcaaaacgttttatttagcattattcagttacgattttaagaccttttttaat
ctctcgttccttctttgttcattttcttcgttttagattgattgtcgaaccataggtatgtatagtagcatatgggtcgtatatcttatttcgttaaacgat
tttcatctttattcctcttctctcttttcctgtgatatgatcgctattcgcaatatcttgtgttgtttacggttcttaattttatttcttttgtatcaaactaat
gacttcaaatgcaagcatgcacttaccatactgtaaagtcataaatagactcttacgagtttcatatgtatattttccaatgaacaagaaaacgga
atctttaataataattatattagttttccattcgctctaatagctataaaacataattggtgaagtcaaaatctatatatcctttcttctaaacggctat
gagtgttatttacggaatatatcaataatttacattatctccaaaagttttagtgtatgaagaaagctctttatcaaatgacaagagtggcgaaatc
attgctaacacactacatgaatataaagttatcagttaatttgtttttttgttttccaacgatcctgttttggtttttgaggtttgtttattaatcgttact
tattgagtagcaaagtaaagaaacggtgaatttatgaccaaattgtttgagcaattttacaaaggatttatcaagctatattctcaattaaataaat
gagcaaaataattaaggctccaccagctctcaaattgcttgctttccaaatgcattaattgtttacttgctttactatgcattttcagccattacttctc
aaacaaaaatgtaataatatttgaccttgttaaaagactttcttcgaacaatatacaaaagatgtgaaaataacttactaaaagcatgtaaaacc
taaactctaagcggtaattaaatagtgtgtttgctggactgggcgacttgttaacgaataactgagattggtgtttatgaacacaagaaaagtgaa
acaaaatttgttgtttgttttaaaagaatatatatttacgaaggcatcagttgacttgttcacgatctatcgtttatttgtgctatagttattctgaatc
acccataaaaatttctaggtttgtttactaacataacgcaaaaggaacccaaatgttatcttagtataacaatgcactaacgtcaacgcagcatttt
gtggcgccctaaagacttcaccagcgggggcgaaccgcaatattccaacttgcccttagctaaactctgcgttttctttttatcacgaaaaataag
aaaatgtcgcaatattacgaactctacagacgtagcaggtaattttttcctttttaatttctcttttgttttgctaactttcatagcatagggtatggttt
ttcgttggactgctgctggtaatcatagatcaaacgctaattgaaaatatgttagaatttcattaacagatgctttggatgatttgatttcccaaggg
aaaatatccccacaactggctatgaaagtcctcttcaacgtaagtttaatgtgcctagacaagacccagtcttgatttgacattgttagctaacaag
gtcattattgcagtttgataaaagcatgacagaagctttagctgaaaaagtacgggctcgtttgacattcaagggacatctcgatacataccggtt
ttgtgacgaagtttggacatttataattaaaaatccaaatttccgtttcgataacgaaactgtgactagcaataagattcggatcgttgcctgtgca
acacgtgattctgcaggaaatcgttaataagttaagaatataaaaatggacagtttcacgattttactttgtcatgaactgtcttgcgcttgttatga
tttttcggtattggaaacgaaattattgatattgatagaataacgatttacaagtttttcttgtcctttcgttcaatcgtgtctttctagctgcactgttt
tgaacctttgatgccttttggataactcattttgagatttttcttttcgtttttttttctttcatgcttacatgatgaccaatggcttgttataaccttagaa
ttccctttcttgacattgtaatcttttaatttctacatttcctctgcactgccttacattattttctttaatattttcgatgcacctgtggatatattgtcgct
gaagttcataatggtttatccttcctattatgtaagttaggggcacttaatttagatacgatctttctcttcgcgtaagttttcctcaggacttttggtat
cttatcccgtactaagcttttcatatgatttgtattataattgattgaatatatgaaaatgttgttcgaacgttcaattgtccgtgcttcattcattttta
atgtatatgctactttttctttccggtagttcattattatgcgtttagttcgttataatagttattagagtataagcacataagatattaaagtgtaaac
gaacaggaacaataaataagaatatcagcacgcaggatgaattagagaccttcattgaacgttgtcaaatgacatatttttagtatatcatgaaa
aaagttgctcctccgactaaagcaattaagcttgtaccaatcaactcgtgcattgaggaagcaccggattcaagtgaagtggaaccagcagaact
gatactactagagttgctttggtggttagtggaattgcttgtatttccaaacgggctcgaaatggcgttagcactagcaatgggctttgaagacaca
taagcagcgctgttcaaagaagatgaagaactaaagtaagaagaagttgtggcagtaaagtctgaagctttagttttaggagtgctgttttgaga
agagaaagttgtagtgctagtcttataggaactactaggtgctgagctggttgaagacgatgtggaggagctgctggttttggagctagttttaac
ctgtttaatagaggatgaactactgcttgaagcgcttggtggtaagagagtgaattcctccgattgagcataaatttcttcaggagattttggatttc

ccgcattgatgctataacctccaccagttggccaatttgtagtatcagttgtatagctgccgtccgatacagaaaccgtcgcaaggagctttgtaaa
gggaggatacctaaaatagttgcttaaatagattccaacaacattcttgtctgtattgacataattccaggttatcgtgtgatcctcaccagtttccc
atttttcaaatttcgaaggacccgtgatttgtatagccatagagtgggcgataaaagcgaatactgcaagttttgatagcatcatcttaaatattta
aattgggacgaacgagtttttggtaatgtagaaaatacttttgtgatttcaaggcaaagaaaagaatcaacaatattataaaaaagactggtcgc
ttgaagctagcgaataagaaatgcaagtaaaagttcaacaaatagatgaattaaggaatatagcgcaaggcttttatagtaaacttctattatttc
tttagtgatacgaatgggaaagaaaaaaaaaaggaagtaagactatatataagaaaaaaaaatgtttatgtttgtagattcacgaactttatca
gcctaattataaacaaatattagcaccccgcccaagttataatataccccgcaaagtttgctatcataaaatgaaataaaaaaacacaaaacac
accctttaatcaattcctttttaatcaatgtatttcttaatgtatcattattttcaatgctttaaaaacatacccctctttaaattgctcggctttattt
atgcgaaagcacgctgttcgtttgtgagaataggaggtaagttaaagttaatgcttattaatggctcgctttttttgaagcgaaagagacgaggct
ttgagttttaacttaggtaacattatttatgggaatgtttattatatgctttccgtttgttcgctatcatatgaaaattatttttatgtttattattatcaa
ctgttgcttataagcagtaataattagcgaatcgtccaatgacgttacctgcatcagggcagaataaaccgacattgtgaatttataagccgataa
cagacgattggcttttagctagagataaaaatagagcatttttaacaataacagctctttccaaacaaaacacctgataagaagattctactgttt
gaggtaaacaactgctttatctaagaacacttcaatttgtcaatcagacgtatagctgtaggatttctgaagtatatataatgaatagagtctcaat
tcaaatccaatcaaacataaaaatacccataccttagtctattctcacttctaatgatacccttagtattatttcagtaacaatatgacgctgtaa
attaatagaaaccatctgtaggataatagtataggtaacgatagtgagtgacgctttttgtggcctcgggaaatattatagttaataatgccttaat
ttgcctttctttagtcggttagtgaagtttgctgcctaatttcttacattaagattatagttataatcgtatggcgtcaactccttataactgagcatga
gcatacaagagaaacgcgtaactttttatttaaatttttttttttttttttttttttttttttccttcaattagaatgccttaaaacatcgtatattaata
aaatagatattgattgaaatgttcttcaattacgttgctcgagcatatagcaacatggaacgtgccctgactgcactacctatttctcctctacacca
taggtttgtttaagacgcgtaaacaaacttttatttcttgaaaaagggacttttagtaatatatagagggattttaatttttctttctttaatttttatat
acaatattatttcttttgcttgactagaggttcaacaggcttgacggtaatttaaagaatttttttggttcatgatggaatttcctccagatcaagcac
ttctcgaagagaagaatactctacttcaaagttgcggattagatgaacaaatgatgttctataaatgggagtcctggtgtctccagcgggataata
agcctgtcctaaacttagacaactttaaagcatttgccgcagacatgagaactcaaatggagcgccaggttagagctactttacgagacaagcca
gagaagaaaaccacaaaaacaaaacttggaatgaactttgaaaatatgtaaggcgtttggttactttcgatgatatctaactttttttaggcttgg
cttacctgctaaatcccatgactcgaaaaatagcaactcaaatcatcccgtgcctcccacttcagatgcaccaagtctcactgatgagtccggccg
tgttcttgaagctctcaacgcacatataccctcggcatctactaacgctaatggtaaaaaaccagcatcaactgaaataacatcaagtattgatcc
gagagcataccattataggatcatgtttcaaaaactccaagattcgtctgaagttcttgacgaccgaatcgatcttttcagcgctatagtttgcgat
cactataagattgctgatgaggatcttgcaaatccaagtgaacttacacaggaaatggttaccgtggtcggtagagtcgttgttgatagcaacact
gttggaggacgccttaacactaatagcattttgatagaaacttcaagaagactaggcgctggcgccagagtttcccttaagttagatgaagtttct
agttactccatctttcctggacaaatacttgctcttaaaggatccaatcctagtggtaaaacttttgttgctaaagaagttttacctctacctcctcttc
cattctctgtttcatctaaagaagaaattgagaattttaacaacaaagcaaacaaccaacccatttctatatatgtcgcttctggtccttggtctctt
cgagatgatctttcgtttacccctttgaaagccttagtttcatatttgctagaaaatcctattgatttggttattttaacgggcccgtttatagatatca
accaccctatcctttccaccggaaatatcccagacacacaagttacttctcttgaagatttttttaaagaacgtgtttctccgatcctctctaaaattt
cttgcaagtgcattctcctgcctcacatcaatgatgccatttctaatcaccctgcttggcctcaagacacattcgatcgccatacactaggcctccca
tctgtacgtccaagtttgattcttttagttagtattaacattatagaactttatatgtttcccaaacccttgtatcttttcagtgaatgaagttgtattcg
gcatatctactaacgacattctgttacatacttccaaggaagagcttttccgaacaccaacccatgggaaccttttctcccgattagtagctcatgt
aatccagcaaaggcatttttaccccttatttccaggtggttctttggacagaggtaatccttcgaatatcgatatttcacatcttaaacttggagagc
tatttaaaaccgttcctgatgtcctcattttacctagcgatatgaggtattttgcaaaggtaagcttgttcatatagcttgtcaattttaacgtgtttat
tttagactgtcgaaaacgttatatctattaatccaggaaaagcgacgaaaggagtcggtttgggatcatttgcaaagctaactattcttccttatcc
ccttgattcttcaactccttctagtgaccttctacacaatgctcagtctcgtactaaagttgagatactaaagttataaacccattacttccgttgttct
tcatatcacgatttgattttatagctacagtttacaattataatacatggatatgcaactatgattcatttaattcattagttacaatcatcgttctttt
aatttatctgtaagtataataattattaattataatattgtcccgcgagaacctcttttatattatattttagtattcgcatcattcctatcacttcttgc
aacctcatccataaattagtttcaggatgcctgtgattcctcctgaacaattagcacctcttcaaagtaaccaaaacaatataagaaactttacttt
gttggctcatgtggatcatggaaaggtattgaacgattttgctacatttttcttacatatactaaccaagcatcaattagacaacgttagctgatagt
cttttggctacaaacggaatcatttctagtaaactagcaggaacggttcgatatctcgactttcgtgaggatgaaattactagaggaattacgatg
aaatcaagtgcgatttcattgttctttaaaattattagtgaacacagagaaagggctgttgaaaaagattacttaattaatcttattgattctcctgg
tcacgtggacttcagttcagaagtttcatctgcttctcgtttatgcgatggagccttcattcttattgatgccgttgaaggagtttgcagtcaaacaat
caccgtccttcgtcaagcttgggttgatagaattaaaatgatattggtcatcaacaagatggatagacttgtgaccgaactcaaattatcacccatt
gaagctcattatcatttacttcgccttattgagcaggtcaatgctgtaataggtaccttttatagaggggagttaatgaatttagatgaaacggagg

aagaaactagtgacgagggcatttatttctctcctgaacaaggcaatgttatttttgcaagcgcctacgatggttgggctttctttctagaccagttt
tcagatttttatgagaaaaagctcggattaaagtctagtgttcttacaaaatgcttatggggcgactattatttggatccaaaaactaagcgtatcc
ttcaagcaaaacatcttcaaggtcgacgtttgaaacctttgtttgttcaatttgttcttgataatatttgggctgtgtacaattcagccgttgtagacc
gtgacttggataaaattgaaaaaatcgtgaaaacgctaaacataaaagttcttcctcgagacatgaaatctaaagatcctagaaatttgctgcta
gcagtttttcaacaatggctacctctgtcaactgcaattctactaacagctattcgaaagattccttgtcctatagacgcgcagaaagatcgcgcat
tgaaagtgttgaagaccactccacactttgaaaatgttgatcaaaacatagcgacggctatgagtaactgtaacgctaacaaagaagaaccaat
gctagcctacataagtaaaatggttgctttctctgaaagagatcttccaaaccagaaacgtcgacagcttactgccgagcaaatgcgtgaactga
gacaggctaaattggctggttctcttgaagactcggtgggtgtgttgtcaattaacgaaaatgaagaatcaaatgaagcagaagagaattcgga
aaatagatatgttaacgaggaagaagaaaaagatgtttttgttggttttgctcgtatctacagtggaagtatttctgtaggtcaacaaatttacgttt
acggtccaaagtacgatcctgcaaaacccgatgagcatattaccaaagtcacgattgaatccctatacttaatgatgggtcaagagctttttgcgc
tagataccgtcccagctggtaatgtatttgccattggcggtttggctggaactgtattaagaactgccactctatgctctgtaccgggaggaccaaa
tttagttggagtaactcagcaagcggatccaatcgttcgtgttgctgtagaaccaatgcgtccttacgaaatgagtaaattggtagctggattaga
aatgttaaatcaggcagatccatgtgttcagattgcggcggaagaaaatggtgagcatgtaattatgtgtgctggtgaaattcatttagaacgttg
tctcaaagacttgcgtgaaagatttgctaaaatagaaattcaatcttcgcaacctattgttcctttccgtgagacgacagttgcggatcctgatatg
actgctaaggacaaggatacaccaattgggtttgtaagtacgtccctcgcgtctggcggagtaaagatcggaatttctgttacgccattatctagc
gatttagtgaatttcttattgaatcatgaacacacaattgagttatttatgcatcacattacaaagaaaactagaaatggcgatattagttttgctg
atctacagaaacagaaagaagaaattcttagttctgaagactttataacaagtattcatgacttattagctaaggatgcatcagataaaagcaac
cttaacgaataccttgactcaattatgtcttttggtccaagacgcgtcggtcccaacatcctaatcgataaaactggcttaatgaaaaaggcacgtc
atgaaagtgatggatcaagacttattgcctttgatctcgctgagtatatcattactgcttttcaaatttctactcaacaaggacccttatgtgccgaa
ccggtgcaaggcatctgtgtttctgttgactcaattgaaataggagataacggagaagagaatgaatcactcacagtcaataatccgcagatccc
tggtcaggttatttctactgtacgtgaggcagttaaacaaggattccttgcctggtcaccaaggttgttacttgctatgtattcatgcgatatccaag
ctactgctgaagttttgggtagagtgtatggtgtagttgccaagagacgaggtcgcattgttgacgaggaaatgaaagaaggaactccatttttta
atgtaaaagctgtgattcctgttgttgaatcgtttggtttcgcagtcgaaatcttaaagcgtacatctggtgcagcatatccccagctgattttccatg
ggtttgaaatgctagacgagaatccattctgggttccgactacagaggaagaactagaagacttaggtgaacttgctgaccgtgaaaatatagcc
aaacgctatatgttagatgtacgaagaaggaaaggtttactagttgaacaaaaggttattgaaaaggctgaaaaacaaagaactcttaaacact
aaaaataacagtgatttttttgaaattacaagtatgatttggaaatttatataagcagttactatttcgtgatttttttgatgtgaaccaaatcaattta
tttatgttaagaaataaaatatttttaataatatttgttgacttatgctcaattgtattccaaacagaaaatacgttcatgcttctaaaacacatgaa
gcatattaaatggaagttcataacttaaaaagcctccatagtattataaattataaacgttattaatttatcacaaatttaattaaagatgcgtcgc
gcttaatgccgtaaacaaaaaccactactacgctgttagaaatttgcttttaccagcggtttatcaatgaagcaatggaaaagccaaaatgaaga
tattgagaaggaaaagaacatctttgatatacataatgacgaggatgaattggattatgaagctacattaaaacggcgcttggctggaccttcag
ttctaaagtctggtcaagagagtgtggatcaagaaaggattaatgagattatttatgaggcatccaaagggagtaaatattttgaggcagaacag
aaacgagatcttgaacttcgaatgagaattgagcgtgtacgacaggaggtaaaagaatattaccgtaaacttgaacacaataatgcttttcaaa
aagaatgggctgaacggactgatcagattgacacctatatgcaagagctccaatcttgtcgtgacttaagtcaagttattgtacatgtggactgtg
acgcattttacgcgtctgttgaagaactgagaaatgaaaaactaaagaacatcccaatggccgttggtaaaggtgttttatgtaaggcttatttta
ggaaagcgaattaaagcaacaagctaattacataggtactgcgaactacatggctagaaagtttggcgttcgatcagcaatgccagagtttattg
caaagaagctatgtccagagctagtcgttcttcctttagacattccagagtatgcagttaaatcaaaggaaatacagaatttacttctggaatatg
accagaatctttgtccagctagtattgatgaagtgtatatgaatttgactccgtatatgaatcgaaataaacttaataaagatgaggataccatgc
agcaagtcattatggaaataagaaataaggtcctgaaaattactggtgtcagcatcagctgtggaatcgctgctaataagttattggcaaaaatt
gctagcaacatgaagaagccgaatgatcaatatctggttccaattgaagcagaagtgattgaaaaatttatgtcaaaattgccaacaaggagag
taaggaaataaattaagacctttctataactaattactaacctaacaagatcaatggaataggcagagtattggaacaggagcttctcgggctag
atatcgaaacctgtggagacatattaaataatagaactcttttgtcatacatctttagagaaaaaacctttcagaatttaatacaatgcagcttagg
gattgggtccactattgtccaggattttggtgacagctatagaaaaagcattggttgtgaaataacattttcgaacgctattgcttctggaggtctcc
tagaaagtaagcttcaagccctatgtcaaagtttagcagagagtttatcaaaacgagatttgattgcgaattctatatcagtaaaaatcaaaacc
gatacctttcaagttcatactagacagaagaacttacgagaatacatattcaacgatacagatcttttctgcgaagctctgagattattgaggatg
gaatatccactacgaatacggctattgggtgttcgagctagtaagttaactaataaggcaagctacatagcttctcgcattgtatgtaaaacgtgc
taatcttataaaacattttctaacgttctagaaattttcgaaacaaaacagtgtttcgtgtcctgtttgtcgtatagacttaaaagatgacttggctgt
tgtgaaccagcacattgatctttgcctaaatacggaaactgtgaagaaggtgatcgacaatgaaaagaaagaaatccctctaaaaagaaagcg
gaacactatagatgcctttttagacattaatttcacatcaaaagacatccatatagaatggagttaatttacgttttggatacaaaaaggacagc

aagtgtacaatggaatgaaaacaggtcaaaatttatttacgttcaaaattaaatagaattgcttatccattggatttcaaacagactgataaccag
attcgtaataaactcatgaaaaacaaagaaatctaaaggtcattcttttcaagcatcattattacaagagctttgcgctgataacaaaggcaatga
gtataaaaacactgtaccttataaattggaaaatggttaatgatgtccagtaaaagaacaaaagaccttcagcggtagcgagcgagataaaga
aaagaaagtggcctaatgaagaagaagagacagaatatacagatgtcttactagagaactgcatccaaacgccgataaggacaacaaaaaca
gcagcaacagcgagcgagaacaaaaggctcaagaatttattagcggcttttggtggagcagcaaatgtatgaagtatggtaggttggggagac
gttactgcttcggtcgatgttttagcattaaaagcaggaagggaacctgagtcaaagacgagagatcctaaggaaattgaggaaggagatgcat
caccgaaagaaccgatcattaaagtgctcagaagtccgtcctcactttggactatctcagaaggaagctcagacaaaggctaaaagtttgttaga
agattcatatattaagaaacctgtttcacattaattcaagcagattcgttcatcgtaaatctgtgaatgaggctttttctaaatacgtaccaatttta
agactgcatctccatttcccttcactttcacggggtaaacgatcgaaatactaggtttctttttgtcactgaatactatatgggcttggtgagggaca
gcagcttcatcatcaatggcacaggtgaattgtactaccaatgtatcctccagagaagagtctatctcagtaaaagtgtttaattcatcaaatctaa
caaaaaacaaaattagaactcgtgtctacaagcagtttctcaataatttaccttttctcggatcctgtctttaaggatttggtagggctgagaagctt
gagaacaccatccttagtattccatgagctgcaaaaaacacccataaaaaggcataacaaagaaacgaatgaaattgccctcatttttgtgtatc
cttgggatttgtagtgggtaaaatgaaaccgaatccacatactgcttcgagtataaatagcgagtcctaatgtatcggtaaagaggatatgatact
ccaccataaaagaacgaaaatacctacttccttccactgctaaactattcgacacgcgaaaacccgactttttacctcagttttgttggattcgtcc
caaggacccatttttttgtttttttgtacgtttcttgaacttgttagtttgttgtctgtgaatttgtggccttctcaatgcaattgcactttgtattttcaca
tttgcaaaactcattccgtgctggaaaggcaatgacaagcgttcccaattgcagaagcgttttagaattgagtgctgctttatatcatcaaggattc
attagcgccattcaacgtggtcatcttcagggacctgacttggtgcctacaccaacaacacgaggcaacgttgctgaaaggaggctatggttgac
tttgaagtattttgaagggaagcctgttatgcaatatattcgaggagtatccaagccgagtcgtaaagtatacatgacccccaacgaactaatga
actttgcaagaggaagaacagttagctttgttcacggactagagcctgccgagatggctattgtagaaacaaaaaaaggaatcatgggagtcga
ggacgctattcgtgaacagctgggcggacgagtattatgccgggtaaagtaaaatcaatcatggaataaagaaaaaaagcaactgtgttgccct
ttcttactttctttttttccaagtggtctcatatcatgtttatttctttatgagttcttatgtgaattcttctttgatatctctgatattatggaattgatccg
ttaaacttttctatatatttttctttgtacacatccccttttacaatactctcgaccaaacataagacactgctttgcttttatattatacttccttgtatc
tacctaattataatggaagagcaattttgcagtccacttcactacgttctttttctctacatgtcaggtaacttgtataacagttaatttgaatattata
taaacgttcgattaaacagccttccccttcatggtgctacagcaattcaataaaactacttcaacactcattcggaagctctgctaatccaccaaaa
tgcatttgctgccttactttatataaaccttacacatactatcttaactgttattgtatatatatatattattgttcctaacttacaacaagtcgcggtg
gtgtagttgggagcatgacagactgaagatctgttggtcaccggttcgatcccggttcgcgacactttttatcttagatatctttttcttggactttat
cccattttcgtcgtgtttcagaaaatgaacttaattgttttcatcgcatttgcttactgtagaacaagtgtccatcccatcgtatacctgaagatagcc
tagtctaatttgaggttgacattcgtatttcacttaaatttgaatcaagctaaatttattatcaatagtacttataatgtaatttgttcgctttgatcttc
ctggccttttttattgtgatactcaacatcaccgtttgagtcacctttgaatggcacgaacccatcgtacgcaaactctggatactaatttattatttgc
tttactttttggaacttaaacaaaatttgtaagctgtttttttctctattttttaacagttcctctacgtgaatttgcatagtttacagcaaacatggaag
ccgaatttcaaaaatttcagcgtgtacttcaggagcaacaggacaaacgagaaaggattatccgaatttctcgggagatcacaatccaaagtaa
acgaatgatttttctgcttcatcaagcatcatcgagtgagtttcccctatcattggattttgacaaagcaaagcaacataaacaaaatattcaaaa
acaattggaactgttaaagcaagaactagccgtcccggatgctgaacgatatgctccttcttgctctcctggaattcaagagtttgtcgaagcttat
gcatttgaacattggctgcaacatcagactcttatacacccaaaagatagtaaattccagatagatattggacttcttgattatgtcttaggagtttg
cgatatgactggtgagatcatgcgttttcttataactcacgggtcaaagttcgctgttgaaaatatcctttcacatgttcagtttcttcgtgatttaaa
tttagaatgcacaaaattaaacaacttgaatcgtcgttcacaaagtgaactaaagcaaaaacttggtgtaatggacaacagcatttcaaaagttg
aaagcatatgttacagcaaaattctcagaatgtctgaccaaacatccatgaagagggacgaagaaattatagcttctaaacagaaaaagcttcg
tattcattagatccacgatgactatcattgctttgcatgctgagagtatattcaatggatgagataaataaaaaaaagactcatattttcttcaaaa
actatgaatcacctaactgaaaatctacaatattcaaagaacgagaataaatattaaaacataaaagacggaaaagaatgtcggaaggaggtt
cgagtggttaactataaacttcacttcccaagggaaagccatggagacaggcttatgtcgcatcgtcccgatacaatggtgccgtctggaaaaac
aaataacaggggaagtgtttcgcaggtgatagcttccgtagaaagctggatcaaactaaccagaattgtgacactcctagataagctttttttctgc
tttctctattttcgtgtaattcaagggccctatattgcctagccatatctgtatgtccggagaaaacccttctatacttaactctaatgactgattgctt
ggcaaataaataggcggggcttccacgcatggtaaatctaaatgtcgaagagcaaactgactttgtactggtcttagaattgtacaatcacattg
ggaaattccaacgatagtccaacaaacagaatcacggatttttaatacttcatcattataagtctctattggttcccattcaactggtacaactgctt
cattaggtaagttcttacattgagcggaagttgcacccaaaaaccatcgaggttgtgtgcgatcagccggcataaacccacttctggcgttttcat
atccaagtgctaaatttgttgaaaatgaagctgagctttctaaaactatgcggtgcatttgattcacgtatccatcatcttgagcaatcccgtcttttt
ctactcggcggataattaaagggtcactcataatttctgaaaattcatcttgtagttggataacagaaccatacctaattggaaccgcttctacaga
attattcaagatcccttctgtggtgctctcgggtgatgaagaaattagatgaatacgtaagggtgcccagccatcactccttaggcaaaattgccg

attttctaatgttgtatattttgtgcgaaccgtttgcgaatttattcggttgtaaagacttatgacggacccggaacgcagtgttatatgactcacctt
agattttgtgcctttttggccggctttgatacaacgtcaataggggaagtcaccaaatgcgatactctttggtccgtaactgtcgaaacaaaatcg
atggaaagccttaagattttcgactttgcagctgctagactagatacatgcaaagatcgaaaaaccatacactgttgattattgtatatttcactga
ttgcctgtgaatactggccttcttcattcataatagagatctcaactcgaaaggataagccaagcaaagattgaaatggaccatctatcatgagga
cgggaggagggcaaagaaatctcttttcagaaccatacgatttttggatgacggaggcatgtctacatgagatttttatcatttggcttcttccatct
ttcattcccttgctagcatcattcaaccaaaggtcagctccttgatttaggttgttttggcagagcaactcgatatttttgctaatgaggtcgtttata
gagttttctgaaacattttcatcttccatatttttttcgttttctgctttgctcattcatctgaatatgtgaaaggctgttaaaaaagtgaccctttgggtc
atcttcgagttttgcttgtacatgacgagaacctgtttctgtcttttccacattagtaagcgctgaattaacagcgctaaataaagaaaggacttga
attggcatcgcctcagtatttacgttttggtcattattttgatccaaattcaaatctccgcgaaggattagttcattttttaagttttcaactaataaag
caagttagaatcgttgcttttgaatatacaattgaaaccataatatcgactctggtcttttgatgaataaccagtctatgtgtacatactttttagatt
atccaatgtgagagagtcattttcattggattcaggtatattccagctgccaaaacctgccattatatagtctattttggcagtttaacccgagtcga
ttattctttagtttagaaccctcaataaagcatgtgcactttcataaacttcaattgtctataaattgtctataaatcattaatatttctatctgaaacg
agagaatggttgcttttgcaaaagcgaacgaaggtagtagttcgtaggcaatttacaaattacccaagtcaactttttctttcaccaactactcgac
agacaagaagccattggaagcggcgagaaagttactttttaatacagttttcgtttaaaaaataaggaaaagatttggttgtactttttttggtctta
attctcagtgacttttggattacgcagtttaagtgattagagggccattgaaatttgcggacgatggatagtgaagaaatactctctatacaatata
atcaagatgtatataacaaaatagaagacatatctagacacatttctcaaacaattgagttttgtaaacaaaaagctggcgaactcaagtctcag
gacgattcttacactcatgttaagtgtacgtattttatttatttataatcatttctttatctaacttttggatctagtttttacttacttaaatggattact
aaaacaacactcggatgatcttatttctgcagtagaaaagaaaaaatcataccttttggtgagaaatcaggaaggactgcctattgtggacattc
acgaggaagttgatgaagatggcaatgtaataaaatcgaatgtaaagcctcaaaggatttcttccgttacagattatgctgatgtgatacgaagtt
tgccaggatttcaaaatcctaaagaaaaagctacaacgccttcgaagccttctgcaactgaactaccggaaacaaggattgaggaattgccagc
agaagcggctgcagctactggatcgctggattctgatgaacaaattaccaaaaagaacaatacagaattccaagatagagaaaatgcatccca
atatatacagcaaactcagtcggattatgtcaaagagaaagcatctaccatagaaaacaaaaccgaccttacatcgaaaaagatagaagaaaa
ggaaccagaaaaaaagctagatgaaaagaaaaataaaaagtctgtcttcaaaaaggggtttctctctaaccctaaaaacggatctcaaaatcg
ttctacaaaggctaaacacgaagaagaatccaaaaagcaaagtcaaagagaagaaaagaagcgtatagatgtcaccgataataacgctgtaa
aggaagtggttatggaaaaatctcctcaagaatcgaatagtcaaaaacagttaaaatcagagtcacccattaaatccaaacccccctaaaaaact
gtccaagtttaaagaagcgaagctccagagaagttcaccaggaagatatacacattcggaggttgctgaaaacgaatccacaaggtcagaaac
agatggtacaaatggtgctgagcctactaaaaaagttgatagtttacatgaagaagataaatataaggaagtagagggtgctttaaagcaagtt
ccaacatctactgaagatagcattgccgcggaacctaatgacgaagatccggttttattgcccgtaccaacggatgccaatggagaaagtataat
gcatgccgttcaatatgacggattaaattctttagacgacatggagcagctccttcaagatatggaagaagccggtgaattgcagtctgacgcag
agtcaggttcagatgaagacgagcatggtatgagtttaaatttttctcgaaatttaatacctcctccaaatccaaattattttggtgaaaatgaatc
attagctccaaaagttgcttctggatcaaacgagcataaagagaatgctttatccacaaaagaaacgcaaccttctgaaaagcatgtgcatttttc
tgacacgttagaaattaaacatgttagcagaaatggaaaagcacgcatcgagaatgtacctactcctacagaggaatatgacaacatgtttgag
cctcatgaatattgcagccgcatatcagcgtttcgcaaggcgcgaagcaaaataaaatctgaacacaaggataaagatttcgagtctcctataga
aacacacaagggagacaaaagactactcgatcatgacactattgtggagcgctcttctgataatccaccaacaaaatccgatcctgaagaaaaa
ctttccgatgagcaaaacgaagaaaatttagtggagaagtacctgaacgaagtccctacggtgaaatctaccgttgttgaaaggcaacctgcaa
gaagtgtagagcaagcgaaagaggattttgaggatcatcaggatttcgtaaaggataaaagagaaatctctcaacgatattacgaactccggcg
aaagctagttaacccagaaactgaagacactgaagaggaaggtgttgtaccggtggacgaaaatgggaacgagcaaccaaaattaagtaaat
ttatggccgctcgactgggtggtaaattatgaggaaattaataatgaaagcatttattacggacttttggattatagatttagattttgttagttatct
caatataatactcttgtaactgattttgcgttactgtagaagggcttcctattaataggtcggtgcctgaatatgtttttggaaatgcgataacctctc
gtataccattggatttacaaggagagcaataagtcgatcaaaccctagagctattccgccatgcggtggacatccacttcgcaaaacgtcaatta
gatgattgaatgtgctcagttggtattgtttgagttttaaaacatgttctaggataaaagattgaagttcaggattgtgtatgcgaatacttccgcct
cccaactctaccccatttacaaccaaatcataatggagagctcgaacattcaagggattggaggagagattatgtaaatctttaggatgaggagc
agtgaagggatggtgagtagacgccagggactccgagtttgagtcggcaggcgagaaaagaggaaagtcaacaatccatgtaaattctaaagc
atcatcatctaaacgtggacgctgacattgacttaccaaaaggtcatgtaagagtggtctaagcctacccaaaggggtcgtacctgaggacatgt
gaacagggcgattcgctatgaaaagtgtcaactcgggacacagaccaagttcttcatttattttttttatgtcgagatgcaggtgttttagttgtgga
accttttgagtccattcggataattgagaattagaccgaattgtcacgaaggaaacgccttgccctacttgttcgcacaaagagcgtgcttccgcat
tgcttagtaactttttttacatttcttaagaccaagacttcagtgcaagcgtcagatttaggaagaaaagacgtcaaattttgcaaagttaaatcgta
tcgtacgtcaggcttgtcagaaccataaagttctatagcgtcagcataagacatacggggaaaaggagactttaaggatttttccccagatctct

tagaagccgggagaataatctttcaattacgaccatgacctgttctggagaatcaacaaagctcatttccaaatcaatttgggtaaactctggctg
cctatcgaaacgtaaatcttcatcacgaaaacaacgagccaattgataataatttccaattccgctagccattaatatttgtttgtattgctgaggg
ctttggggtaacgcatagaatttaccaggtccttgtcgagaagggactaaaaactcacgagcgccttctggtgtagatttgaaaagcaatggcgtt
tctacttccacaaagtcattcatatgaaagaaatcacggacaatatgtgctatattagatcttacccggagataatggttatattttgggtttcgaa
gttggatatgacgatgagttagttgaacaggtagactagtattctcatcattcgggacaaaaggaagtgaatgagccatatttagcacttccaagt
tctcgactttcaactcaaactcatttcctggtctgcgatctcgttcaggtcgatactgcagtttgccggtgatcttcacaacactctctggagaaacg
gtttggtgagtagtttcattcaaagcggaagaaacaaatttcgagcgatcgacggaatcccataagctttctgctagctgtttcttgggaacttgca
agggctcttgggaaaatagctgaagggatttgccttgcacatcacgaagctgattaaacttgatggattttgagatatttcgattcgtgtttacaag
ccaaccggaaatgctgatcacctttcccactaacggtgctaatacatttgggattttatgaaactgcattcgtagaaaaaaaggtattattaaaaa
taaaaagacggtagggaattctttccaaaaaatgagttctaaaagaaagccttgaaacctcaattgaaaatcacgacaaaagtacactttttat
aaatagtttacgatagcattcacgaccaaaagctttttaggaagctttcaatttctttttaaagattaacaaatctacttattcacgaaaagcctata
tagctaggctcattcctgtaaaatcaatgagctttggttagtatggacttgaaggatgaatccaatattcatagtgaggcctttattacataacatat
gtctttcaaaaaaactaagtataatgatattttgattggttttttcttatggaagacgagtgattcttttacattcatgtttaaattgatgaattgtca
tttatgccgagatgctgttacaaggataaatgcacgaacatgatggttaattttaagaatagaggaaggaaaatggttacggattatgaagctct
caagctaatagctgaagaaagctaaactaagagttgttgaaccataacataaattcatattttaaaggcgaacaacgacatgtagtatgaatag
aatatgttacggaaagaaagatgataaatgaaatgttatatatccttaaaataatgatcacatttttttgcgctttatgatacacagtagttgatata
tccataatgcttatatctacttgatatgctttatcgcatattgcattactaagtttgcataaaaacatatgctatacaaatttaaacaatatcgttacg
tgtttagaatatttaacataagcatattcgtattttagcgcacgtctgaatcaagctaagccaatcacaaatagcatgtaaggaccagctacgtatt
gtggggaaattcccatttcgaacttttgtttactttccattgggttaattatcaatggagtttctattttttttcttgccttataatattttatctttttgata
aagctaccatataataacgaaaactagtttcatataaatcaagctgccgtccgtcgatgctttgtcctaggcataggtaacattcgataacgtttag
gtagcactgaaaacaaagcaaccgaccttaaaagaactgcaaacttgtttccctccaccaccaaacccatcagttttgaaaggatattcctttttttt
ctttcttttccgttgaaaacgtcgaatgcgtttacgaccttcaaccgtaacttaatttctttcgtttttttttgtcccattggttagttttgaatattttcgta
ttttgtccagtacaagaattttgcatttaccaccttgctgaagtgtccattgattttgatcattccaaagctgatagctttttttttgcattacttagattt
ctttcgtttctggctcctatcgggacgctttgttgccattctttaaagaccaccgaatatttgcattattttctaatcatcgcaaagtagtgtactatta
ttctttattttaaagcatttagacattatttaagctatcttttaggttttccttgtcggttcttttttttcaacctttttttatttggtatatttgcttctttttttgtt
atcatgcgcgccctgccatatatcgctgctgccaccgtgatcgtatttatgattatcggaggtgcttggtggaacacgagtctggatactcgcctgc
agtcaaaagtagagaagatactcgacaagcatagttcgatttttgtgacacccactgccacagtcacgaaccttgagactgatacgattcgtactt
caatccatacaaccattagtcagactgcgactaccacccaaacaatgacttctactcctacgacgacttctgttattcatcgattggaacaaacag
atcctcatcccgacaactctaaaattgtcatccttatgggtagcaatttccacaaagaagatggccctcttcatccatatgctaaatcaatcataaa
aaaccgccgcgaatatgccgagcgtcatggctacatttttgaattcttagatgcaagcgattatgcttccaaagttgccggtcacttgttcccatgg
gttaaggttcccatgcttaaggatactatggacaaatatcctaatgctgaatggatttggtggttggatcatgatgctcttattatgaacaaggacg
caaacctcgttgattacctccttgaccacaataagcttaatagtcttttaacccgtggggctgaatataagagtggtgctggagtagactctgatgg
ttttcgtgcgcctgaacaacaagaggctgaggatgttcacttcattatgtcgcaagactttaatggaattaacgcaggaagcctttttcgtccgtaac
agcgaaatgggtcgctggatcgttgacatgtggtttgagcctttgtacttggatcacattcaagggtatgctgaacagcaagcgatatcacatatg
atttattatcatcccagcgtccatcaacatgttggtcttgttccactccgcacgatcaatgcgtatgactttgcggatccctggggttggcaagacgg
tgatatttgcatccattttgctggttgtaactttttccacaattgccctgaaaaatttgagaaatttgccaagatactttccagtaagcaaggtgatg
attggttagatcctgaagaaaaggagttcatgaagcagaagatgtctgctcatgattaatccccattaacattgagagtttggattaaattgggtt
gtgataatcgttcatatttaatcttgtcttctataatctacaaccttcctatttaatgcatgtgtcattcgaaatcctaaagttttcctttgaaatattgg
gtttctttcacttcgactttatttatataaccgtccttatccttagaccatctcttgatttgaatgcttcttgttcaatactctaataatatgctattagttt
ttagtagtgtacaggcgtcttcgatatgtactgacttcaaggtgaattgcaacgagcgtctaagttgtctttcgtttctccagaatgattaatttgaa
atacgtcaagctttttgtaaccactaaagcaaaattggtttataaagcgatgagaccgaaatacttgatctaatatgactgaagaaagaaagttg
caactgggagcgttcaagatgctcgtaaaactttaaaacagtttgaaaattagtatggatctcaatgtatgcaatacgatttgagaaaagccaaa
taattgtatcatgactttcagaaggaatacttgagttttcagcttatgaacttagcattgtaactatagtcagaaagtcaatccttaaagtcaaatat
tgaatctattagattcatgtcttttaaattaggaattcaatcaaattccaaaacggtaatatacaatacccccctcccaagtactacagaaggaaatt
aacataatttacttgtattattataatcacaagggatttaagatgaaatttggcgttttctacatagtaactgctcagacttgttccccagagtctaa
gctctaatgttagagtgaataatgcttattttatacaattccctttatgttgtcttttgagtcgggtctttataaatctcaattcagctacaaaatagaa
caagattcaacactgaaggtttacaatatttccttgaataaaagataaattgatattcaacatcttgaatgcatcaattcaaacaagagacgctttc
tcttaagtattttggtttctcattaattcgttacgaacttttcaatgaaccaacaagcaacaccaccaaaaaaaaactttctcgcttattatattattc

ccaagctttgaatctattaataaaatcattttgataactccttctttttatttgtagcgacattttgcccgactactttggatggactattaatactttgc
taccatccatacttccagaattgcaaaatcggcatcgcaacttaattcgaaaaacctttccatttacttttgaacatctgaaagccttactaaaaca
tttttgctctgtctgtcttttcaaatatcttcgtcttcatgggatccatactctctttcaattgtaaaaggtattcgctcctaaagtcaccaaaaataatc
tttaaaaatacctataaaactttagattcctggtctaatattcctgaacacataaaagccaaacagggccggaaattgtttctgcagaatggacat
cctctatgcagcctccgggaattattacaacgtcagttcagagataaatcaataaattgcgttgcaaaagagtcacctgtcgtttcaactatcaca
aacttcgactccttgggaattccaaaaacgcatgttagtaggagtaaatctgatacctactatgtggacaaagacacatgcttgcgcacgcatac
atcggcacatcaattggaagaattcaaaaatttggcggaaagtaagatgtcgaaaaaaggcttcctaattaccgcagatgtttatcgacgagac
gaggtagacgctagtcactatcctatttttcaccaaatggaaggcgcttatttatgggataggaacgataaagcaggcatgttaagagatctaga
gcaagtaaagcttgatagcgatttgcaaaagcttgttattgacgaagcaactttgaaagatcataatgacttacaagatatatactctttacaaga
aacccagattgcttctatacatttaaaaaattccttagttctcgccattcacaatttagtgttaatgaccccagacaatggacttgataaaagtcaa
gttcgctaccgctggacgtatgattccttccctttcacgcaaccatcttttcagttagaaatttttttggaaaggagcatggctagaaatcctgggttc
cggtgttgttcgtgaaaacattctaacccaagccggaatccaaaatcatgttggctgggcttttggtataggcttagaacgtttggcaatgatactt
tacagtatacctgacattcgtttattttggacaactgatgaccgattccataaacaatttactccaaacgaaatcactaccttccagccttacagta
aatatcctttatgctataaagatatatctttttggattgatgagtcttttgcagctaatgacttttatgaactcgttcgagacgtctgcaaggacttgg
tggaatctgttgacttgatcgatcagtacacaacgaaagcaggaagaacaagtctctgctatagaattaattatagaagtatggaaaaatccctt
aaaaacgaggatgttgacttactccaagttaacttacgaaaagcacttgcctcttctctaaacatccaacttcgctgaaagcgagacaaagaaga
tcatcattactcatattaatctttgtaataatatatatttattaattttttttatacattaaccccaaaacctctaaaaacgaaagaatgcgtatgca
gtaacgagcgatcattaacacaaaaataaaccgaagtttttaacaaaaacgaatgtaatcattataccaaccacatggtattagaaatgaatag
aaagtaaaagttcgcaattggctattttactgccagagttcactactagaatttaagtagagtagccaactgaggaatagcaccttttcctattaga
taatgaacagaattagttgcttttctagaaacagcatactccaaagactgtgccaaaaagccatcaatgcgaaactttgaccaacgcatctgagc
aagattaaaccagaagctgtagacagtatctagaagctccacagagtcctttattatacattgttcaaatgcgggctgaaaaactgtttcaacatc
gttttcttctttactcacaacttcttgataaacaagatgaccgatttgcatgagccgattcgaatctttcatctttatactaattagaaacccattcgtc
aacgtagaagttttcaaagaaaatcttcgggtttttagatcttcgataaaccgccgagcattaggttgaaacaaaatacgccgtggaatatgtcct
ttatgtcctggatggattacctttccgtttatgatctcttctttttgactctttttgacatagccagctgctacaagttcatctattcggttcaatacttgc
aatgtaaaataccggcgaggtagagagcgttgaattgcaaggatcacgcttttgggattcatgcatagagtaaagtcaatgtacaaatatgaag
gtattttctccttgattccaagtcggttccacagaacaggagacacagcagctaaactatcatttttggatagcagaaaaattctcgacagcaattc
gtagggtaaacgctcgagcaacgtcaattttctgtcagtttttcttccaacagtagttagcccttgttttcgtattttctttttggactcatcatgtttat
taatgtgtttacgctttttctcaattggcgacattgtggacgttcacagaggagtgatcaaagtagaagaatgcattcaagtactaaaaagaatta
tatatacttggttttatacactttaaaaaagaataaaatttgcaaaatccaatttcatcccactaatttaaatatatcagtatgctactttacatcctt
caaactgtctattatcctttatcaatgtccattggaataacaataaaacacatttgagttaaatttaattattaatcaattcaaagaaaatcatataa
atatcgaacataaaatgaaaaacaaaaatcttgtaaaacatacacatgcataaatagtaagaaagaataaatgtacattgtttggcgtcttcctg
acccatgaacgaaacgaatgagcaagatgttacgttttccccttttccttccttccttaaataaaatcattttcttcttcctcgatactcgttgtcgtag
gttttgtgttacggcttttaaatcgaagcatgctcaactttacccttttaattttacttgtggtagaggagatagcaatggaattgacaaagggtaag
tctttgcattccgaagaaaaccgctgtatgggagaagaggtatattgtttacgaagggaacctgcaggaacttttcttcgtggagaagaagaagg
agatgaggttgtaacgttgtttccttttttaatgagcattgctgtcgagtccacctcttctgaccgttctgtatttagcgataaagtattatgaaaggt
ggttaaaggattcaaagaatttgcagagatagaattattcaccgtttcagtttgtaaaggagaaacaaagtttcccgacgcacgagataagggtg
ccgtgaagggtttagaagttgtttttttgactagttggtaatgctgatgaaacaaagcctttttgcgaggtctctacctttcccttgtcttttgcattatg
aaactgtttcgagtattttccgtaaacgaaaagtccggttcgaagagaaggtcccgaaggtttagtcaaggcggaatgtaaatgagagactggtt
tcatcgtggaagagtcaggctttggtacagaacagggggaactaaactgattcctcgtattagaatctgtaacagcattcgggaggcttggctttg
aaacaaaactaggtggtcggaaagttgctggcttgaatattttattatttggcaaagtaataggagcagctgtttcgggatttttttgagtttgtcctt
tccgtatgtgagtcttcttcgttatcactttccaaaaactctggtgagttaggtatttcaggatccgtactttcaggtaaatttggttgagcagctaac
atcgatgtactacgcatagctcgttcataaagatttttggaaggatccgacaccgtaacgttactaaaattgtttcgctttccgactggttttcgtaa
gcgcggcataggcaaccgaggtacaggattcctctttaccgatgcggcatgagtttcatttctatttatgaaggaattttccagagaaggaaagga
aagagacttttcaaaggacggagatgtatcagtcttcccatcacacggaggtagcgttggtcccgaggctgatcctgaactggtcagcattggttc
cgacaaagatgaagaaaaaagaggattggaatttcccaacgggtggccagcatccattggttcattaagaaacgggggagtagactcgtaatct
ggggaaggagaagaaaaaaaatcagagtcaatattatcttcgtcaaaattttctaaggggtcatcttgaagaacattggagggagaccttggtct
tcgaagaacgggaatacacgcaatcctagttggtttcgaaacttgatgtttcaaagaatcaaggggaaccgtagtagttgtcaaattatttttgaa
tggcggtttgaatgatgtagagttatgagatggtttcggcagtgaagagtaagttttctcagaagaaataggtgcagaagtagaagacattggag

gcacgaagttttttttgcggccgtcgacacaccattttccgcacttttattaccatgcaaaaaaggtttcgaggatgaaaatgaacgatggggag
aattatgttcttgaaaagatggaaattcttcttgaagcaacgaagattgccctgcttcattcgtggacttgacaacggaaggctgttggtaaggag
gattgaaggaagaagatcgggaaggaccatgaaatggctttgtcaaatacaaaggacgagcggaaatatctttgtaagcggtggctgatttgga
tggaagcggaggaatttccttcgatgtcgttgcttgcaagtcctcgacataaattagtgtacgattcaattctagatgagcatcgatttgtatttttg
gttgaatataaaagactcgtctatgacacggttttgagaatcgtataaaatcccatgattgttgaatgtatgaaaccgcatagttccatctttccat
actttggatttctttaccttgtcggatgtccataatacttggtaatgcaaaacatttgcaacagtggccatagaaccatgaatctttttcttcgcccg
ccacgtaacaagaaaatcacttacttttaaattcgttcatgaactcggaaaaaagtcttagtgacaagcgcaaaatacccttttagaatactgag
atatagaaaaagctagtacttaacaaaagtaattatttagaggaatttcacagatttgctgaactggtagtagtgaagactaaaatggctggtat
gtgtctaaagctaaatctcttgaaatccattatttaagagttctatacaaataattttaatgatttcctctatttacgtttcaatttaaatttcaatacg
tgtttgttttcattttcgcgattttctcgttttgtttacatgaggtgtgtaatatttgatttggtttgtttcgtctcataacaactttcatagtcaaatgcag
atagagtatgcttagagcgtgtacggtctcatatagttaatgaaaaggctgcaaaaccagaaaatgagacagtagctttttcaaccttcatagatc
acaaaacctttgaatacatattcttcttgatttatgtacttcttttagatatttctacagaacaatatgcaaacatcagtgaatgtgttcgtcttttcct
gagtgttttattttcctctcaaagcaaaatttgggagcggttttacttcaagaatcgttgtcatttcgtaaatatgcaatcaattataaagctatgac
agtcagtctctccaagttttgcctcttctactgttatttcattcatacctcttacatccatcatctacctcataacgccaactactgtgactacgtgaga
cgaatcatcgttggaaacggaaattttacaacccagttagttataattttggaacgttttatataaacatcttctcttcttacaagaagaaatttggt
aaacagtaaagatggagcattacgttaccattgcatcctggtatgcagaagagcttggagaagaagcataggagcttctttactcatttttaaag
gattagaattctcaccattcattagtcaactgaatcaatgggcccttgattttgaagggaaccgtgacagaatcattgaatctatcaaacaagcaa
aggaacaaaaggctcgtcttcgtcttggtcccgaattggaaatcacgtatgtcttatgggtcaaatgatgacttggtattaaaatgcaacaatgttc
ttgtgaactaacatgctgcagtggttatggctgtgaagatcattttcttgaatccgatacttatacccattcatgggaaatgctctgctggattataa
aacatcctgattgtcaaaatatcttattagatattggaatgcctgtcatgcacaagtcagtacgccataattgtcgtatcctagctttggatgggaa
aattatattaattcggcctaaattatggctttgcgatgacggaaactttcgtgaatctcgctggtttactccatggttacgtcctcgtgttgtagaaac
atactatttacccaccttcgttggcgattcactcagtcaaacccaagttcctatcggtgacgctatccttcaatgtaaagataccgttgtaggtattg
agacttgcgaggaactctttactcccagtagtcctcatgtggatatggctttggacggtgtggaaatatttttgaacgcttctggatcccatcatgag
cttcgaaagctatctacacgttatggacttattcaaaattctacacaaaagtgcggtggtatttatttgtatagcaaccaaagaggttgtgatggtg
gtcgtttatactacgacggttgcgctatgatcctttcaaacggagcactactagctcaaggtagtcagttttgtttgagagatgtagaagttatctca
gctactgttgacgttgatacggttcgttcaagcagatttcaaccctcacatggattacaaggtgttactcgtccttcctatgagcgcatctatcttgat
tttagtcttagctcattcaaacagaactttgatatgcataacaagcctactgatggacaacctattcgcttagtgtcaccccaggaagagattgcat
atggcccagcatgttggctttgggattatttgagaagatctcgctcaggtggattctttttgcccttaagtggtggtttagattcagcttctagtgctg
tgattgtccatagcatgtgcagaatcgtaactagtgctatcaaagataacggtaagctttggaatgcatcacaattggttctaacaattttcagatc
aaacagttttagccgatgttcgtcgtatagttggtgatcctaattattcctcaacttgccccaaggagcttgctagtcatatattttacacaagcttca
tgggttctgaaaattcctctaaagaaacaagaggtcgtgctaaggaacttgcttctcgtatagggtcttatcatgtggatatgagtattgatacaat
gactcgtgcgattgtaagcttattcgccatggtaacaggaaaaactcctcaattccgttcccggggtggctctcaggctgagaatttggctttacaa
aatattcaagcaagatcaaggatgcttttagcctatttgtttgcccagttactaccttgggttcgtggttattcaggatctttgcttgtgttaggtagtt
ctaacgttgacgagaggtatgaaaaatttttaatgataatttatttttactaacattttagtttgcgcggatatatgaccaagtacgattgttcaagt
gctgatgtcaaccccatcggcggagtatcaaaaaccgatttgaaatcattcttggaatatacaaaggatgcattcgatatgccaatcctccaagat
ttcttggatgctactccaactgctgaattagagcccactactgaaaattatgttcaaagtgatgaagttgatatgggaatgacttacccagaactta
gcatgtttggaagactcagaaagattgcaaagtgtggtcccttcagcatgtttaacgaattgattcataaatggggtgatcacctttctcccactga
ggtttgtaactttgtatgtatgttttcttactaatggctttttagattgcttctaaagtgaaacgcttcttttattactatggaattaaccgtcataaagt
gacaaccctcacgcctagctaccatgctgaaacttacggggttgatgataatcgttatgatctaagacagttcttatatccttcttggacatggcaa
aacaaaaggattgatgaagttgctgctaaatatgaagaacatgagagaaggaataaagatcagtaaatgaatcaatctatgtcgtgattaaatt
ttgactttattataaataaatccattaccttttttattttctattaagaatgttaatatctagcggcatttttgaacttgcagctcagtaaatctacattt
tttgaagtagccttttgcaccagcaactactaagtgtgttcagttgacgaacctttcgttgatgaaaacttgaaatctgatattcctttcatgaattgt
ttgatttcttttaaaaatacaaggtcatgtttcgttaatataccagattccttgctattacaattggatgggcctatacaagagtatgcagcttgcttg
cagatttcaacgattgaaaaagaaagaaatccgataagttatttttgggtatctctggtttgccttcttccttttcaacacatgacactattgaaatt
gatcctatatatgctcatactctaggattatctgagtctgaaagggtatacactaaagattaagcattgattatctaacaatagtaaaggtttattta
tacttgattaaggttgaaagtataattgaaagactcgaaataagacccgtttctcctgacgattgggaaatcgcagaacaaaatgcgaattggtt
agaaacaaatttctttaatagctaccgcattataagtagagaaaatttccccatttttctaccttcagggactgcaattacattccaaatggtgaat
gctgaacccaactttcatgactgtggacgtttgaatccctcatccgaagttctggtatctccaatacttagagagaaagtaagtgagagcaattcg

ggagttactttacggtgtgtaattgaaaacgatcttttaccgagggatgctatcatcaatcatcctgaactgtgtgtatacattcctcaagtgtttgg
aagtttagtagattctgtctttatagagccactagaagcttctttttcttctcctttagtggtccgtgctatccaactcccttttcaatggttgggacaat
ttttcgtgtcgggaagtttagcttcgtcttatagttttgtcaccggcgatttttttaaaatctggcctgctgatgttgggacgatgtcatcaaaatttat
tttacgcccattgaatcaagagagtatggaggaatttgttactaattcagttgtttcgaggatcaaattttcgtatttaatcaacggaatggaacta
caaattcctgatttaaatgctgtgtactcggtggaaggccgcacaggttacgtagaaaatgaggaaagtattaccgtggactccagtcttttgtcct
ttaatgataaaaaacgaattgtccataataaaacaagaataacaccaaaccctttccaaggattgatggatagcattctaacttcggcgaattttg
gatataatgttttggtgaatggtattaaggggtcaggtaaaactactttactgaagtctttacttcaaagttgctgcttttctacggtgtttcacactg
aatttgtttcttgtacggacttaaacttgtcttcgtttagcaagtttgaagggttttgggatcaactgatacttagatctttgaaatatagtccgacca
tcctgtttttggatgattttgatagtcttttggtttccgaaaatgaaagtgatgaattgacttttgtttcagaaaggcaagctcaatacttagttgaaa
ggttatcatatctaaagtctatagaaaacatttcaattgttgcgactgttaaggatcttactaaatatcctactgatttgaaatcacgcttttttttca
acaacgtatctcaatcccagcgatgacatataatggtcgtgaagagatgctgaacttctttttcaaagaactgaatcagattcttccaaaggagtg
tttgcaaatcttgtcaaaaaagactgatggttttacactgttggatttgtcgttagtaacgaagcgtgtatatagtgaattaactttgctagattcact
agagaattttggtcgagttaatgaatctataaatactatcctaaagggctatgtccctctaaactcaagaaaaacaaagtttgtcaagtacgacat
tgattggaatagtatcacgggaatgcgggatgctaaatcggtcttgcatgacattatagaatccccagtcaaatatcatgctatctataagcaatg
ctctctacggcttcctactggagtattattgtatgggtactcaggctgtggtaaaacacatttggcgtcttctatttcaaatgcttttccagtgcagtta
atcagcatcaaaggaccggaaatattggacaagtatattggaagttccgaacaagcaattagaaatttgtttgaaagggcgcaattggccaaac
cttgcgtattgtttttgacgaatttgattcaattgctccacgaagaggccaagatactacaggggttacggatcgagtggtaaatcaacttcttact
caattagatggtgctgaacaatatgagggtgtttacgttgttgcagcaacaactcggcctgacatgatcgatcctgcattgttacgtccgggccggt
tggataagttggtattatgcgatcttccaagcaaaaaggaaagaaaagatttttttcttaaaatcttggacaaatatcaggttgctgataagtcggt
cgtgggtgacctagtacccaaaacagagggttattcttatgcagatctatcatctatattcacggaggctcatctgattgctgtacaccaacattta
gaaaaagaaaattcatttccccgaaatgagaaattaaggtctttgacaaatttgtcaacagaggtcacggcagacaagacgaccttagcctcga
gggacacagagcttacttacggcttaaggaagctcgagattagtaaagaaaatattttaaatgcgttacggaaaaccaactcgagtgtttctggg
gaggagtacagcatgttgtcatcaatttacaaacattacgcacactcaagtttaacaaatcaaaaagaagaaattaaaggaaaatctggatata
aaacacggcaagcataatatcttagctttctcactgatggattcttctcaaaaaaatgggcctcacccttcctttcgctgattcacttgacatacctaa
agtgcaacgtttggtggaatcgcttgaatccataaaatgaaagatgatcgagtatcagagaatgcaattcaaagggatgctcctccattaaagaa
aataaaaagtaagttttttttcctatgcctgggaaattgtctaactgtctaaattagaacaaaatcagttttcatcattattggaaaatattcctaacg
ctgcaagatatcgtcgttcctttatgcacaaagacacgttacattcatgttattctacgaaaagcaacctcattttaacgattagttatgatggacat
gtcaagttttggcataagacaacggatgggatagaatatattaaagattttcccacacacaatgggaaaatcatctctacttatttaagtaaggat
gaaagaatccttgctacttgtgctgatgataatactgtaaaggtatttgacattgacagcattgatatggtaaacattattgatttagattttgtacc
aaatgatttggttgcgtttagtacaccgtctatgaaggaagagatgctggctgtttctgacgccaatacaccgttagtgtatttctttgatttagaag
gggacggtaacgttctgtatagtgtaaaaaaacattcgttcccggtacatgtattggaatacataaacagcttaaactgtatgttgtctgttgataa
aggtggcatggtggaatactggtgtccagaggagccattccaaaaaccgaatggacagggaatatttgagctaaagagtcaaacggatttgtat
atttttaagaaaacaaaaagcattccatcatctatccagatcagtccttcggaaaagatttttttcttcaatttcgtatccggattggaaggtccgagt
attcgatgtaaagagcgggcgaatcttgctagaatttgatgagcatcctttaaatgcggcgaaggaaatggagcaggcgttagaaaaggaagat
gtgaactctttgtattttatgaaccaggtggagtttggaagaagattggctatagaaagagacatagagcgatctgggtggatggctgataccatt
gctatatttgatgaaagtgaaaattatattatttatggaagtatccttggagtgaagattcagtcgatttcggatggatcgctggtacgagtacttg
gtcgagacgaaagcatccgttttgtgaaagtgtcgctatatcagggtgcgcccaagaaaggaaaaatggcgtctttggaaattatggccagcaat
aatcccttggttgaagaaagctttcgcaaggatccaactttattctgtactgcatggaaaaagcagcgcttttatttatttggatctgctgatgagaa
tattactttgactgatcgagatacactaaatgagcatacagttggactcacgaaggaatcaaacaaacctgaagaaaggactgctttgttaggga
aagctgctgtactgcacactacccaaggagacatcagcatcaagcttttacctgaagaagcacctaaggctgttcaaaactttaccactcatgca
gtcaatggatattatgataacacaatctttcatcgtattataaaaaattttatgattcaaggaggggatccattgggtgatggcactggaggagaa
tctatctggggcaaggattttgaggacgagttttccccaaacttaaagcatgatcgtccttttaccgtctcaatggcgaattcagggcccaatacaa
atggctctcaattcttcattacgactgacttgactccctggttggatggaaagcatacgatttttggtcgtgcttatgctggattagatgttgttcatc
gtattgagcaaagtgaaacggacaaatatgatcgtcctcttgagtccacaaaaataattaacatttccatcttgtataattaatatgaataatcaa
ctagaaaatttggggaatatcacgacaaaagtgttttatttgagaccagttttgctgtccgtatgattcattattcataaagtaatattatttttggat
tttttaataatataaatacactttttcaatataagaattccactccttttatgcaaacttaggttgtatagtgtttccgtaaaaccttagctaccgaac
aacgctgtactttcagggaaggcaactctctacactcacccttccatagaaaagttttagttttatccattcttgagtcttattcttctgatgcataat
gctacattgtaatattgtaacattgtatataaaggctcagtgcataacgtttatttacattgttacctcctgtgttatttatgtatcgcaaaaatattgc

tatactaaagttgcacgggatgttatcccgttttactattatgttgatgaaagtggaatacaactgttcattgcccaaacgttggcagtttgagttgg
gctcaaaagtactgaatgcaactatttacttggtaatatcctatacctggttgactaagtacatcgtcttcagtaaaaaagtcaattagttcgagcg
gtcgaatcttccaatccgaagatcctgactgtagactgatgagtatatgtagctttgctaatcaaatcaatcagtaaaccttgtcaattggtaacgc
atcgcatacgtgcttccgttttaattgtttgtttacttcgtattatgcgacgactccttgagcttatgaatattaactaatattccatttttacaatttatt
gttatataatgaatgctaacgaggtatccaattatgacaattgtgacagatactgacattttgacatcaccctgcttccttttcgaatttcaagcaaa
tgaattaactgaactgcaattggcatacaaaacttctaccaacacactcttttttatgaacatcttttaagaggcattgttagttttctgtgcatgcc
cttttttcgttgtttttatttttacaagtttacaaaagtcgttgattttgttacaattgtggtaatttgtgcatagctaatcttcattttcaaagattaa
atcatgaagaataattacgctcctattactgaaaaagatgaagcacataattctatgattgacgatgtgtcgagcagacgtctttttgtgagaac
agcatatcgccgcagttgccgcctccatattcaagtaagtgcttattgtaatagctgcgataaagtttgtttactaagctaatggatgattgttgaca
aactaattcttcctgtctgtcaaatgtaaacaatcaaatgggcacatatggatccctctctggaccatacattgatgtctatttaaagaacattttct
aattaatatgtagattcgacgaaatctattgatttggaaatggcagaacctgcagttgaaaacctttctccatcgcattcaccgttaccatcatcga
ttgaaaaaaaaaactttttattatctttttatagccatccatgtttatattatttgtttattctatcaaggattaatgaagaatcggatagttttctgga
ctgtaattaatttttgggttatacttttcttaggagtactatacagtaaggattgatattgtttacaaattgactgacaaatgttttagcgacatacga
atcttggacggaaggtgcttgtgcgactgctcatggaatcaagattggttcttgtgcaatgcaaaatttcttcaaaaaatggtttcaagccatgctt
ctggcctttgccaaaatagccattctcgatatttttattttaagtgcagctttttccgtggtaaagacgacagaaatatttgagggttgtgatccaaa
gtttttttggatactcttcggcctacacaactcgagtatcttcttaattgaaacaagtaaggtgattactaaaatacgattgtactaacttttagttta
tccaaattacttcagaattctaacgcttaacgtaataaaatcactgattggtacgtgaactattgatgaattatgtttatgaaaccgttcttgtttatt
aactgagccagaactacacgaaggagagaattattggcgtccaagaagagaggcttatgaaatgaatgagcaaccctgatgaagctttaccact
ttctctcgtttatagatgaataattctaatgttaataatttttttaggttttgagacggttctgtgcagccgaacctttctcgaaaagagaccaagcatt
atagtaaaagttaatataggatgcaatctacattgctttgtttaacgtttatgaaattaggtgctgctaatgaattcttgctttactttttttggattat
gaaactcgtacattgttgcctgtcatcaatattacgtactgcttgatggaaaagttatcttatctgatcccgatcactgcttttttttgaccgtttctact
ttatgctaattaaagtgatgaaatagcagaattccacatgaagacaagtacgcaattaactaattccccttttttaatagaaagatcggatagttc
ggtcccttgataattagagtaaggattgatcagacaagtgaagtgagaaactttttcccctaaataagtcttctcttcaatggtctttcacaagtac
aacacagtccatttaacgtacacaaccctacaattgtaattattacttccactcaataaatattcttataaaccgtgagacttattaacgtaaaaat
atgctcttatgaaagtgtatggtaatatttattacaccagcaagacacctatcgtgacatgacatatgtattatgtttaattccaccctgtgtggcca
tgtaactactggtacgaagttggagcgacacttggatatgaggtaacaccctgttgaaagcaaagtttactttacaatggatatctaatattattta
aaaaggaataaatgactggattataatataagcctcgattgcttatctattgcgttgtttttaaaggatcaaagggcagttctaacttcatcagaac
aaatcacaacaaaactaacactttctcggatcaacgacatgtcgtagttatggatgttacgccttataaacttcctacagattccgcagtccgtcct
ggttacggcaaactaggcaggccagtaaatctgaaagcaaacttttacaagatcactgtgcttccgagacacacaattaaccaatatcatgtaag
aaatagagtctaaaacgtttatgttggaaggaaaggttggtatacataacaaaagtggatgcaattctgatcccgtcaacattggcactaattgat
ttttttcttttcatcggttatgtgaacaggtgatgcgatttctttctctatagtaggaaacgagctataggaatgtacccacaccttccctctttcggcc
tctgggaatttgtggaatctttcccatgcttccttatcacttgtttagttcttctttgatgattcaacctactccttcctatattatttctttttttttgtttt
tttaacaaagtttaggttatcatcggtgatgggtctcgtctgcctcgtaaattagtagaaaagatttggaattcaaaggaactaaaggataagctt
ggctcttcatggcgttcctgtgtgtatgacggaaaaagtttactctggacgaaggataatatccctgatttcttcatcaaagtgaacatcgggaatc
tgaaacgtgaacagttcactgaatttggcattcaaaagacgacaacgattaacttgcagactctgacggaatttgtcaatagcaaatatgctttgg
atcctcatatactaaacggaattatgtttcttgatttgctgttgaaaaagcaaccctcagagaccttatacggatttatgcggtcctttttttactggtg
agaatactttttttattaggaaacggagttgaagcctggaaagggttttaccaaaccattcggcctactcaaggctgtatgagtgtcaacgttgaca
tatcttctagtgcattttggagagatggttcttttctttccttgttattagaatacacaaattctaaagatgttcgaaatttgactcgcgtcgatttcaa
cactttatcacgaaaatttcgtttgctgaaagttacctgtaagcatcgaaataatcaggaaaggcaattgtccaaaaaagtatattctatccaagg
attttcttctaagagtgcttctgcttcaacttttgtccgtaaaaaggatgacaaatcagaaaccatttctgtttacaggtacttccatgagcatcaca
atgttcggttacaatatcctgacctgccttgtgttgtcgtgaagaatggtgcaatgctgccaatcgaattatgctatgtagaaaaaggtcaacggta
tactgccaaattaaatgcagaccaaactgcaaaaatgatacgcttcgctgcacaacgtccactcgaacgcgtacagcagactgagggattcgttc
gtcaatcggcatgggataaggatccttatttggcagagtatggcatgaaaattgcttcttccatgatggacgtttcaggtcgtatcttgaacacccc
ttccatctgttatgggggtaattcaattgaacgacctacaggtggtagctggaatttacgtggtaaacgtttctttcagcctgctcgtacacccgtta
agtcttgggcagtcatgtgttttacctcgactcgaaaattacctactgtagtaattgaaaattttttaaatacttatgtgcaaaccttgactgggctg
ggtattcagttctctcaaaagaagccaccgattatgtatgccgacgtacaggcaaacattgaagactccttgaaaaataattatcgaaaggctga
gcaagtcagcaagtgccctcctgattatttatttttatcgttgataaaaacgctccggaaccatatgggtctataacgcgcatctgtaacactatgt
tgggtataccatctcagtgcgctttgagtcagcacatccagcaggcaaagccgcaatattgtgctaatcttggaatgaaagttaatgttaaacttg

gaggagtgaacacgattctacaggcaaaatcgaatccgcttgggaatattccaaccctgatcatcggtgcggatgtctatcatcctggcgttggat
catcaggagtatctatcgcttctttagttggatctctaaaccttgaagggagtaagtatacagcaatttcacgttctattcctcgacatcaagaagtc
attgatgatatgaaagatgttgtagtccatctcttgcaagggtttaaagctacgacaggtaaaataccgcaacggatcatttattttcgtgatggta
cctccgagggccagtttaagtttgttgttgagcaagagcttgctgaacttaaagcagcttgtcaaaagctttcgccgaagtacgatccaaaggtta
ctgtttgcactactcaaaagagacaccatgctcgatttttttataaaaaataagcaggatggtgatataaatggaaatcctcttccggggacaatta
ttgagaagaatgttacgcatcctttcgaatatgatttttatttaatttcacatccaacgcttcagggcgtttctgcccccgttcattacacggttctcca
tgacgaaatcaacatgccacctgatcaattccaagcgctgtgttataatttgtgttatgtgtatgctaggtccacaacttctgtgtccttggttcctcc
ggtgtactatgcccatctggtaagtaatttggctaagtaccaagattcgacgtatgatgatacggctagtgttgtttcagaagaaattgctggtgaa
gctgtaaacccattacttgaggtttctggcaagctgaaaacgaagatgtggtttatgtaatttctatataatttctggtctctcacaactattataca
atttattgaacaactagtaaagaataggtggtattggaaaccctttgttttgacctagcatagtgatcaccaatttaatacttattcaaatcaaaga
tgagctaccggtacctctcttgagcagaatctttatacggttgggttgattcaggtacctttactgaatacttacattacagttcgaatctgaataat
cgcagacatatgattgatgatcatttggcaaagtattacagaatcaagggacagccaatgttgaaaagcaatcgcatacttgatattgattacatt
catagcacaacattaatatatatataaaaaaaaagaaaaaagaaagaggtaaaaagaaaatgaaaaagaaaaaaaaataatgttgcaattttc
atcatattctctatttttttaaccaagaatttaaaacttcgaaaatgctgagaaatcagctttgagaatgtttactgattcagtaaccaaaacttttc
attaagtacgaagttaaactaggcattattaaatttgactgattcaatacctaaaagcgaagtaaatccgatttatgaatttgtaagatataaattt
ccctcgagtgcaaattcatatttcacgaataagtatctaatcaatcaacgtttttgaagcgagtttcatttttttttacttgcactaaaatttttatataa
aagttgaaactttgacgcgtaaaattgcatcagcagtttaacagatattttttatgtttacaaacccattaccgatcacgtccaattcgcttcatgat
ggccgagttaaccaaaatacaaaggcggcatccctcgtcgtacatcatttccattccatctttagcagtcattccgtgattcattcgtccaacgatat
cagagatttcagcagagaaaagattgcaactagtacgttagtaaatgtaaaaaaccgaaatacagtcgctttattcaaacatacgttttaatccat
ttcacacgaacgcctccggcgtatgtagcattatctgcccaaaaagggagttctgcactgttcaaatcactcacgacttgagcataaccaagagc
attatttgtcggctttgcaataaaaataaaatagatactagccttctgttataatgttagtaaaataagcagcgtcaaataatatatgtcagtatta
ccttatatgcatcagatacacgagaagctgcttcgtgctttacagcccaaatagaggttttctttgcatgggcaatcgctgtttcgttgtcacaaagc
ataataaaataacgagagcctaaatggttaataaatatgatcgaaccaatcaacttttacatacgatgtgaaattccactttcgtcaagaatcaca
cgagggttaatcatagatatccgttcgcctttgtcatttctatattcacttgctctcgcaaaatttagcggagggggagtgggagcacgcggcttag
gtttttcctttctgggaggaggtgctgaataaggctcagcgttcactgagtaatgagggtgatccgcataaggaacatctgcaacagaacgatgg
gcataagggtcaataaggctaggagaatgagaagcacgaacatcactagccatcaagggatatctactagtggtaccacgcaaatcatcagca
ccaagctgagaggaagctttcgcaccatacggaggatagccaacaggatacatgggagctgataatggaggacgatcgccagccattgcatat
ggtgattttgagtagggatcatcctcagtaaaacgaggaccatatgggtcaacgattctccttggtggaggactcaatgttcttcgctttgaagga
ggttgcaattcaaaacgagcgcgatcataatacgcggcaggtgcatcagattgggctggatagtatttggattgcagcgtagctgactcgggagg
atatggtgggaaaacaggagactcaggaggaacagcagaaggcggtccatacattaacgattcacgacgaacagagcgaaagtaaggccact
cataagaatggccataatcagtacaatggcgagtaaaatcatatttcactggcttcgctaagcgtggatcatcaatcataattcttcttccaatggg
ggcctcacgagaatcgggagccggtctttttaaaggagaagtaaaactattcgcagtaggatttgaaagtacatggacgggttccgctggtctgc
ttgaccagacacttctagagtcattagattcgtttgacggttgagaagctaaacaaaaattagtccatgtgttcaagcaagcaaaacgcatatttc
aatcacattgctttgactattcaacaagcggaagttcttccactttatgtaattaataacttacgccataagcttcgcaaaacctccaagtttggga
gttcaggtaattgtttagaagactgagaattaaagtttgtgtttgacattctccaagaattcgtacaatgtaattttcaagctttcaaattaaatgga
agttttgattaattattccaatatcttcgcaatctcttgaattagaaaacacagttcaaaattgaatttcgtcgacggttaatcttgcaagcgaatac
caaaaataaaactggacacaagttttaaatgatcaaaaagtacctcaatttagttttcctttttattacaagaatcctgtaacttttttttttacgcca
aaaccaaattatgaaacaaaaacgtctccaaaaaataatatatatataaacaagaggaagcagccacttctagccaagttgccaaatggaaaa
ttaaagttgaatagctgattaattgtttaattagttattagtaagaaagaagcacttgcacttccacgactttgctgcggtaggaagagagtaggtt
ggaaaggggagaatgggacatatcaatcaaaaacatgtgtatgcatctatatcgctaacggagccaaatatatacatatatctctaaagaaaaa
attatcagcacatgagatctatttttttattggagttttgtgtaaaattacttataaacctatatgaagccaaatgcttcgtctattaaatgatcaaaat
gttactcaaagatgtgtttactgttactcttgagaaggattttcatcttcaaggattacttgatagccaaataaatggtagaatctactattttcaaa
cttgaatgctctttgaaagaccggttacgttttcagtaaagcagcgcttcagtttcagatttactatttggaagtcattcggcataaacaaagtttgt
acataaatactataaacaggaaaagcaaaagctatttaaatagcttttttttaattacttatatttgacaaataatacacgtattgagagttgtaag
ataaacaaaaaggattctcacttcaaatcgtcatctgtaggtaagtactgtagcttttcaaatatttggtgagtgaactcccatagcttattcacaat
gaaagggtcatgatttcggtaacaggttgacctctgggcgacagctcggaagaatgctccattcagtcgctcttcagagatggtaggtgacgtgg
cggcgtacaatgttgtgtaagctccccaaacgggatcaagaagtagacgatttaaaggagattgtatcattatcttccgaatatattcttgggaat
attgcaagagattcgtcttcactacacctggatgtaagctgacggcgtagatgccatatttctcgagacgatgggcaagcgcttgtgcatggagga

tattggcatatttcgattgtccataccgtgtttgttgtcctcccaaaacataaggaaggttaatgtctggaaagtagattccactgtatggagccgta
aaataggacaaactagagacattgacaatgcgtacatcaccaggtcttgaattctctgcggcacggcgtaatgcaggaataaggcggtacgtaa
acaagtaatgggatagataatttgtctgaatttgagcttcatagccattctttgacagctgatatttgccaaacatgacaccagcattattgattaa
aaggtccaacttcgtttcctttgccaggaatacttctgtagcttgatagacactgtcgaaatcgagtagatccatatgtaagaacacaacgttggcg
tttttgcattcttcgcgtatattcttgatcgctgtaatgtatttttcttcattgcgaccggctaagtacaccttcgcacctttaattgccaattgcaaagc
agtgatatatcctataccaccagaagatcctgttacgatcgctactttgccgtgtaaatcaggaatgtcttgaaacgaccaattcggggtatttaca
ccgaaaaactttgctgaacgatcaattaaattatgaaacatacttcttgaggtcagtattgtcaaaaaggtatatatgaaagacgactttgtagtt
gacgcagcatataatagtgatgtcgttgaaggtactcaaatacatattaaagaggtaatataataaaacaaaagtgtagaccagtaggaaaatg
ctgctttttaatgaggttttgttttacactatgaatcgccttctcaaaatctgttattgacttttaaattcttactcgtcatgatgattctcataaatgc
acctacagagagaagattttcccttcaaattgctttcaatagcatactgagtttatatctccctacaaatcgtgtctggtaggagctatttggtgttat
gcttggctttactttcgttgttctttcatctacgaatagctttttcgttagcgataaaagcatctagagcaaatcatttcaaatagaatttcttctttcct
tttattttcagacttgtagacaatgtcctgtttgtttacatataaattctgccttccttgctggagaacaatgttacctgctttttttcaactttcaacac
aaactgagacctatagaacccaacttgtttggtctgtcctcgttgaaacagaagatttcatacatgtctttctttttataacactctttatgacagggt
tccttttttttactagcgaatgttgagtaaatcgaacaaatggatatagacgagtttaacgaacggatactgaatcaaatttcagaaaaggtatttt
ataaaagtatatgaaaagaaggaaaacaaagactagctggatcaaacatagaactggttgcttatttcctttgtacgcaagcaacgtagcacca
gttataaaagacataaataataataatttgtttttaaatgcgaatttacttttcttttctgagaatctattttctttctatatattctaacaacatc
taggcatggaaaaccagactcacagcatatgaagaactattggctgcgtttactagggcttctgatgataacgatttatgttttcagccttggattc
aagatcctagtttatggaagcgtgggctctgcgagagtaatgttcctactcaagaatgcgctgtaaaagttctttatgctttttagataaatcgcgg
aggaagggtgtctccagttctaaaaatattgttatcccttcattacttgaaaaatgtcttccttcagcaagaccttctattagagaccttactcatca
ggcacttctacttttggctagcgctggcgctatagataccgttgttgatggccttctgagtagttgtcaagtgaaacacccaaaacaagccatggcc
tctattaaagaactatgtctatttattgagaaatacggtatacctaccattcctctttctcctttatacaagcttcttcctggtttattcgctcaatcaga
taaaaatgtgcgtcaagaagcttccaagcttgccgttcaggtttaccgctgggtcggcgattcacttaaaaccaacctgtttccccagctaaaacc
aattcaaatatctgaccttgagtctctctttcgttcagcacctcctttagttacgaaaagctcggtatcggctatcaattgttcacccgtttcccccgtt
gcaaaatctcctgtttcaattgaaaatcgctcgctggaacagaaaccatcttcacgcaatgttagccaaaaacagaccaacacctccggcaattc
ccgtccaatgaaatcatccttgagcattcctcatccatctcaatcttctccttctcctataaagtcatcaacttttgacgttccgattgacgttctttcta
aattaactccagaactctataatgccttgtcttcgcccaagtggaaggaccgcaaagaagctcttgatagcattcacgaagtatgtaaatacccct
gctatgaagaaggagattacgatgaactattccgtgtaattgctaaatcattgaaagacgccaacgtggttgtcgttggtacctctgcgcagttgtt
gatagctgtctcaaaggcgctgaagaaaaatgaagttccttatgtacctattgtatttataccattattcgaaaggtttaaagagaggaaaccttcc
ttggtaaatgttctttttgacgcagcaaatgctatgtttgaagcttgtggtctaaatgagcttgttgatcaagccttagaatttttaggacacaaaaa
ccctcaagttaagacagaaacactgcgttggctaaaccattgtcttcaacatacagagtcatgccctccgagggtttctctggaagcattatgtaat
cgttgtttagttctagttaatgatacgtttgaacctgttcgaacggcaacaacggaggtgttagctactctcatgcaaatgtttggtcaaacaatctt
atccaagtatattgtcggacttgatccaaagaggttgcagaaggtaaatgagctttcagaaaacattcaggtacaagcacatccaaatcaacccc
ctagaccaaaacttcccagagtagcttctcctttgaagccatctcctgtcaaaccaattttatctcctagctttacgtcttcacctttggcttctgtaca
gacaagtgtacctgaagaacctccttctcttaagtcctcccctgtcaaatcactatcttccagtctccgctcgcaagctatgaatcatagaatgacc
aactcggctttgaaaccagcccctaagtcggcgtctattggtgttaccaagcagacaaacgaagttttgaccccgacattgactaaaaaggtaga
aacgaaccgcttgtccacgaagactcacccaagtctacttatgaagtccacattaaaagagcatgtctctccagcaagtaaacaagccggtgtaa
gtacctctaatttggtgaccattacgttgtccgaaaagattgagttggatcttttaagagaagagaaagctatccgacaagtacagcagacagag
gatgctttggaaagagaacggttattccgggaaattaatgatcttcaaattataaacgcagaattacgagaacagcttgataactgccaagggat
gatcagtcagaaagagtcgaaacttttctctttgcaagaagaggtgaaccgactaagtaatcgattacaggaggtcttgttggagttagagcagc
gacaagacaatgtggatgaaaattccatggacattgattccaaggaaatggaaagtacagtaaaaaaagaagacgggacggattcttcatata
ctatgaaaagtccaattagaaacccagaagaacctttaagaaatattcggcgatcaacactgcttccagcacgacggtctcttgcaggatcaatg
ctacaaaaaccggcagctttttcacgcccatcttttttttccaatagcaatcaatcaaatggcttctcttcacattccccctcgaacagtttgtctcc
gtggtcagaggggacagatatctccacgcatttgttggaacaaattcaaaggatgaagcgaagctaaatcaaaaaaatttttaaaaagattcaa
aatgctgatttttggaaccttcttgttcgattgtttgctgtatataatactcttcatgaagaattcaatgcactgtccggagaaggactatggatatcc
ctgccgagtttgctaacgaacctgcagacaagaaaatgctgaatggtaatagggttttcttcatgcatggttaaataccaaattttctttacaggag
gattcgcaggaaaaatgccattcctttattgattctatggggttttgtttacaactgtaaacgaaggtagatggaagggtagtgcactatttactttt
ttttaacaaaatgtattgatatatgggttgcatacagatataagaacatgattaaagcttagattggatttgatttttgttatttaatctttcttattgc
gggcagtagcgaaagatatgattcgtttccctataaaacccctcccccgggggaaattgcaaagtctagcactaacgagcttctttttagtctc

ctaatatggtaggagagtaatttgggttttaaatgaatatcaccttagaacgaacactttgtcgaactatgatatcaactttatggaaagcgaagg
ggttcatgcaacacaataagtggccaaaacaataaacaatcacctaaaggagcagtaaaaatcatttgatttgaaacatttcgatgactggttaa
agagaacaatagatgcataaggaatggacaaacgaatttaaccagtcaaatcaactatagtgagaaattcggtttcactttcgcttattttccaaa
atcttttaaactcttatattcttcgattaaacttgtttctttaaatccccttacactactaccctgttaactacgatcatattattccttactactgtcttg
ctgcatgcatatggagaagattctcagtattagcatatccctagtatatagctatacttataattatatctcagaaatccaaaattgtgggaaagca
gctcggagtttatatttgctacatacgacatgacgttatattgacctcatgcaaatgatagaatggacgaacgagttgtaagtttgttgcactttaa
agcaccatcgatgatatcattttcccatcaacgaaaattatataaacgcacgacctctttatcgtctcggactttacaacaacatttgtgaacatca
aaacactatcttttacggatcgtttctagaaacttatcaatcatttgaatttccgaatacgtttacatcgatcttttgttattgttgaaactacaacgta
ttgataccctttctttttctgtataccagttataccagctaattatttatctgatatttatttgggattgtaaagatgcaccgttcttactctttaagaaa
tagtcgtgcccctacggcttcccagttgatgaatcctcctccacctgtctctagtgcccgtaatagtcgatcctttactccattcagacattccatgcg
aattcaccaagctgttaacttctcccctgatttggctaagcgtttagccgttctggtcaaaatggagaagaacgtcatggctagcatggtccaagtc
actcgtggtcgtcgtgacgctgctcgccagttgtcttactggggtgaagattgtgacgatgacattagtgatgttaccgataagttgggagtgttgtt
ttatgaagttgccgagttggaaaactatttgattgatcgtcacgaccaatatcgtgttaccctaaagtccattcgtaacatcgaggcctctgtccaa
ccttctcgtgaaaagaagcagaagcttttggatcacatttacatgcttaagcataaggatcctgaatcacctaaattggttacaatggagcaagag
cttgttcgtgaggaagccgcttgtttggtagctgaagctcaactttccaacaccacccgtgaaaagttcaagcaagccatgacctacaatttggat
gctctccatgagcatgccgaaaagcttggcttgattactgcctatggacgtcatttgttgaacttgatcgatgacgctcccgttactccaggtgaag
ctcgtcctgcttatgatggctatgagacttctcgccaaattatcatggatgctgagcatgctcttagcacctggatcccggctcatccttcagtaaac
tttgccaagcctcaagaagaagatgttcagagtgacgctcgttcttggaatgactatgaggcccaaggtgagcctgttcctgttcaacaaatgact
caaatgaatgattctcaatccgataccagcgaaatggctgagaatgaccctaatatgcatcctcacgttcgtgaagaacgcattgctcgtatggat
ggtgttgaaaacaacgctgtcaatgataccacaagcttacaacccaaagagcaacaacctgctgtacaagttgcttaaacaacaaatggaaga
aaagtttcgaacaaagaagccaccatggtaatttttgaaacacttacgcgttgggttcatttgttgtttttttttttttttgattccaatgtaaactgct
taaaataatttttccgtaatggcagggaaaagaaattcctaataagggattgctaattattaatcaagcatacaaacgatacaattatttttaccg
atggaataatctaagaagttggattgagcaaagcaagttaatacttaatgaactagtgtgcctattattttttttttaagagcgttttgtgcgtttcaa
ggaaagttagtcgctagatggaatatttagttagtttgctcaatccatgaatgtttttgacaatgtggagtactagctgattttgaaaatttaatttac
cctttgaagtatttacaatgagaagtagaatgatagtggtttatttatgttttgggataaagatagtgaaatagtgcctgtaccgtctattgatgaat
aagtgcgtgataagtagagaagaggtatgtaggaattacaagactgcgtacatgcacgactaatacaatttctatggacaaagtagagtgcata
aaagagaaaagtgagtgatgtgaatggaagtgaactccgaattatggttgaagtatgttcatccgtaaagaaatgaataaaaatgaaaacaga
gttgagcagaggtacgcaagaaaattttaaaaagaacaagttcatggtcgtacgaacttatggcaaattaggaagaacctaaggatcaggaga
tgattacggctagcaacaaaattcttacaaaaaatatttttggattttacatatatttgctacaaccattcctgcggcatttgttgtttttttacagtaac
tttttacatacaccctatgtaaactatgacggagagcttttatgagcgaatgaatacgagaactattaagaaaattgtaggataacgctggccatt
ccaaaagtggtttaggctcgaatcaggccatgcgtaccatcgcgttacatataataaggagctcgatgattgtagacagtatacggaagaaccct
tacgtcgctgagagaagaaggtggaacttaccttcagccaacatcctcgagtccctttcaaaaaacaacggaaattggaaaggacaagggcgg
aataacccgtaataatgaaattaaaggtcaatatcggctcagaccctgggcacacgcaaattgcttgggtgaatcatgaaggacgacctacaga
agtcgatgggccgttttgggttggtagaattctagtacgagtgcgtgactttgatggaatcacacccgatggtagccctcctaagtcggattctatc
tattttcgtggtcgttcaagaaagtttcagatccaagctgaaggtcgtttcaagaaagaatacaacggcaatcaagtcatatttggcacacaattt
gatcatatgatttctacatttccggaaagtatgttcagtaattgattttatatataatggtggccatttagcttgctattgacttgtggaatgagaaga
ccagtagtcagttagatggtacacccattgttttagctttttttagataacggaacgcttactaaccactttggtataggcgctttccgtgccggcatg
cgtatagccaagtacattgacccggctgtttattatgacaagtttgcaaaatcaccctatattatgtctccgtatgtattaatctattaacaggaaag
ttggaagaacaaaaacaaaaaaatttaaagaataaggaagcgttctattgcaaatatgcgtgacctgtcttttcctcttttcctgtttcattttttttt
ttgaatcacatggctgaattttgataactaacattttagattcgttgcatgcgttaacacgctatctgcttggcccgcgcccagccgtttggatgacg
cggtcatatccttagtggaagctgaccctcaggaatcagatacagactctttaccagatatgagtgagtctctggatgatgatctccagcctagca
tatctacgcccaacaaatctccggtcggtaaatcaagttccacgagaacccctcctcccccataaagatcaccgctacgcaatcaccctctcctc
ataccaacgttccttctgggacagatcacgataatttaagtatagtatcctctgattctggaaatactacggctgaccctaatagaaaccgccatcg
ctattggagctttgcgggatttaacgataataccaggttttcacatctcaattcggcttctcacacaccggaaaccagtacatcacgaaataaaca
gcgagttgaaactgatccatctctggtggattccattggttcatcacttattcgtgtatctggtgaagatgcagataacgatgatgatgaacttattc
tgcatcggccgatcaccaatcgccacaaagattttagccgtcatgtccagcagttgggtctgttgcataaaccgagtttagatggggatggttatgt
taatgagttctatgataacaattcttttgacgacgaaaacgaagaggacttacagcgctatttcacagctctcgaaatgcagtccgataaaaaac
actatagaaaatcaaaaaacaaggatccctttcatcggattacgcatccaaatttacatatcggaaagatttctacgccaaaaattattaaacgg

atgtctctccgttcgaaaaaaggacttaaaagtcttaatcccgaatcttcttcccaaacagaagcacaacgtacttctacgtctagtatgtctcccc
cgcctgtttctaaaaatgaaaagaagatgtctgcacctcgtcgttcattggataaattaattcgtataggctcgttacatcgacatcaccatcatca
ccaccacaatcagggtcctggatccaaaccggatcaagttgaatccgactctggctttgatttatataagagtgaaagcaagcatcaccctgaatt
attattgcaaagaaattctatgtctaccgatcgcccttcttcttcccagtctactttgacgtccccggatgactccctatctaaccccaaagtaagtg
gtgagaccggttctttaccccctttacgcgaggatacttcgccaggtgaaagcaacggtataacttccaatccggatcataaggaagagcctgat
aagcccgggaaaaatatgggaaccccatctcttgatatttcatctcataacccgtcaaatgatgatagtaacaaaaaacccggtgaagttgacaa
ccacttaacagtaccaacaaaattggcaaagcccagtccttctcgaacgcagtctagtcaatcaaagatcccaaaaccttgtgtacacattccttc
tgagtcaaactctcttgatcctcagcttggtccttttagatttgccaatccgaaagtcgatccaattgaagataattctttcatttttggtgagcacaa
atctgttaaagatagaaggaagtatttttcaagctctacggatactagagctgatttctattacgataaggatgttgtatattgcatgagtttcttctc
accgcatatggatttcaacagattcaatttgaatattggaccaattaagctaaatgtttatagacatctgaattcggatggccatcaacctattcga
tacatgatgcgcgaaacggaggacgaacatgcaattatgttcgttgtggaatttgatttggttgaggaagacgatgaggctgttttattggaacaa
gctaatgaacgtgaacggagaaaacattagcaaagatggtcaagtatttaatggctaaaacttatggatgttatttattattaatgatttttctttct
ataactaatttatgtggacaaacgcagtcatttccttcgtcacaacaataatagcctttcgttttcatatgaatgtcgtcgttctctcttcctctaataa
gctagtgtttttttggactcatgcatagtgcgataaatcaatcaattaaggttttgcccattctatctgtcatggaatatatattcgacagtggtaggt
tcccttatatatttcaccttactgaagcttttaagtttatattctcagtttcgtcagcttataatctataacagctacgttttcggtcagacgactgtaat
agctcgcattttctattcagtatacattccataaattcaataatttggacgcagacgcgtttataattttatgtttttgcttcgtactcaatgtcaagat
cggtttattccacgtaagatgaagcaattatatatttggaacactgaaaatttatactgactagaattgttaagttccaatctgacaactggcgattt
atataaaaacttaatgattaagaaaattttgtttcttttatttccaatatgatcctaatttaattaaatttttttacacatcatacaagctgaatatcag
gtcataaaaaaatgccaatttttaactgacttgaatagttaataaattgattctaaacttctagaacaaaaacaattaaaaccgtcagtctataaaa
taataataccgttatcccacattaattttgtaagccctaagcttaagatgttgagtagccccagaaaagcaaagtattaaactaagtaaatatccc
ttatattttccctgtttcacaaaaaataattttagaaaagtcaaaaatgagcatcaaatgtattaacaagcattagtaaacattaagctttgattac
ccattagtttggctttttggtagtgacacttgcattggaagaagcagaatcatcaatctcctcaggggcatcaccatttacactgggtgctgctgag
tttgcaacagaagaagaggtaggagcaatactcggggctgttactagtgagaagccgtcattatcagagctatcgaaagcactattatgggaat
atgagttgatcgaactatttaaacttccattgacaacagtggtatgatgtaccatctctggcgaagcgacaggcgacccgatttcggtaggatgat
caacagcactgtagagtctcctgttatctgagtggacagcattgtccgatggaacggcagctgaagattcatacacactagtagtgtgagtgttta
aaactggatggggatgaaaatccgtaagaggttgttcggcaggctcaagcacttcagccgtcggcgattgtttcgtggagggcatgttttcgttttg
aggagctctaacagtttcagataatccctcaaaagagctgatcggagcatccacatttgcttcagacctggaacttggagtagggaagtgattga
cagaagatgtaggaggagcgggcgtcgccgatgcacgttctgaattagacatatctctggtagtagtcggaaaggcaccaccagcaacatttctt
tgtgattccaaatctttatcacttgcaatttgctgttgatttttttccatatactgataatgaccatacatgtacatgtgcaaaaacgcaatccatggc
gtaataacaaaacccaagacaccgccaaaaacaagcttaaccacttggggagcaggatagttattataatagtagtcattgccaaagtggtttcc
tggagcacatagaataccgatggaagcaggccaaagtaaaaaccacagagggatagaccagaacaaaccacgtaaggcggtaaaaactaac
cattccatccagcggaagacaataaaattaaagtgataatttggttttgctcgtaggatttctcgcataaaagtgtttttcggccaccaaccaaaat
aaacaggccgtacaagacctgagcgcaaatcagcctgcaccaatgcagattcaatccagtaagtgacgatggcttgggcaaagttggtgactgc
taaatcaccggaaagtgtgtcaggaagacgccaaaggcggactctgtcctccgtatttttatacataccataagcaatagcgaattctagacctcc
agatatgatgaaggaaccaacagaacagagcataagataaagtatgcgtggcatatgaatgtttttgcggttaaaaaaagtattcggatcagaa
cttagatcctcgtcatacgcatttttttccaaagcgtgccgttttaaatcaaaatcatcagcgtttttttacacttgatgtttcttgatctttattagaaa
aaatcctcttccaaaagctaacaaaagaagaaggagccatttagcaaataaatcacgattggtttagatttaatatcctgaacactttgaagaaa
aataaagggaagagtcgataaatacgaaaatcgtttttacaaaagcaaagacaatagactaggattaagtgaaacagctgaagtctgctaaat
atcgcaaagacgtcctctgtacaaaatcaatgagaacccacttaaaataggtacaaaaaattagaccaataattagaaaataaattaggaatat
ccaacgagagtttaaaaacaaaaaaaacgattgtaactcgtcgtaacgaagcctgaatgaatgacaatcagatggtaacgagggaaataaagc
gcaggaaacgctaaaaaaaaataatatggtatagcaatcaaaaaaagagtatagaaaaaaaaatcacaacggatgaagataaaaaaggaa
aagcaacacgtatgaaaaaattagggcgggtttcgctttcgtttcagtgattgaaactttgaacgtaaagaaaccgaagtggttagaaatagctt
aaattgcagacttgtttgaaagcagtctacaatgggaataaatgaaagcttccgcaaacagcttgtattcagtaaacaacctgtgcttgatacaca
agtaaaacacaagaaggtaaaagtagaataaaacagcaaaactatcagattttgcagctttattttacgatataggttccgttttctgagtagatc
ttacacccgaaaaaaaaattccttgggggaactgacctcgccgaaagcgataagaatagtcacaaatccgtaaaacggtatttttagtcaaaaa
tcaggaaaatcaactatttcctgtgaattttttttgtggatgtatggagacggaagaaacgtatttacgataggtttgtatgtttaccttcgatgtaag
caaccgtccatgacctatcggtgaggtcacttgcatactacaacgtatgctcgtaagtccatatatcgtgctttactgtagtttttctacctttataat
gtgtgcatatggctattaaaacttgtaatttctatgaaagtacacaatgtagaaacaaaatatcatgaaaaccatctttagatcttcgaccaatatg

agccttcaaaggttttaggatacagaaagtatttgtaaataaacaatgtatgtttactactgcaaatatgacatgttgttcgctatgttattttaaaa
taatttcgctatacaattgtgtactgtgtcatagctcgtaatgattatgtgtaatatgatgactacaaataaatgtaaggggaaaacatccttcattc
acgctcgaatacagtaaacaacaaggaagaatatttctctttcatgaagtagtagtagaaagtagcaccaaagaaagtatttttgatggttccaa
ttcgaatcagttgcatatgattcaaactacggtctaatgcttgagagaagttccgactatgacttacaatagttacatattatacggatacaaagta
tttttttttctctatgctttttccgagtcatgctaataaaaacatttccaaaattcaattcatactattacatacatttacatataaataaataggcaac
acagaagcttaatcaaaacttcattacgtgaatctttatatcttcaggaaaacataaaggttctttcaggaccgtttcaagtactttaaacaccaag
agatgcttcattcactgcttattctgttttcatagaagaatcttcatcgataagctcccagtgagagccatcaagccttggaaaaaatctaccaacc
tttcgactgtacaaagagtaaagcggatacttcttggaagagatagtttccgttagccttgtcgagccttgaaacacggcgataagacccaaggg
gcccagaattgtccagttaatccaagtctttgaggcaatagccccaaacagatagaaaacgacccaaataatttgttcagctaaaaaattagggt
gtctcgaccatttgaaaagaccacttgtattaaacccttttgcgagtgtcattaaatcaaaattcaggtgtgggggaagctccttattctttctgtat
agatctcgtgcttggtagaaatcccactgttgctgatcagcaaggatttcaactgcaaacgtaaacataaacaattctaaagccatgcaatctaat
aaaccaaagggtcgttcgttagctgccagcattgcaatatatgtagggcaagcaagccaatataggtgaagaacctgaaatatgtgaatgtaaa
agaaatgtacaataggatacaggtaagaaggtatattgttacgaaccataagccaccgatagtcttctgctcctcgtttgtaacctcctttacgata
gtaattatatgttaatcgaatactccacagcaattgcaaaaacaccataatcatcagccgacgagaagcaatgttaaataaaatcccataaagc
agaaaatgaactgaaaatagagtcggtaaaattggccatgcgcgatccacttgggacgtgtttcctgtttggacagataccgcccacaggaaaa
ccccaaccatcaaagcaaggattaatccagtgacaaagggatttgtttcaacataaaactcacgaaatcccatattccctgacagaagcaacttc
acattgctcgcttgctccaaaaagggagaaacggccatcgtccaactggcggaattagaagccgagtaaattgtcggtaaactagatatcaatgt
catatcttcaaattggcataaagtaaatccgttttcagacgttttggaaaagactcttctttaactgacttgctgcctcaacgtgacgttactggaca
cgagaatagtgagacataatgagaaagacatttcgtattcacagattcaaaaattattctcaaaaggatgcctgtttaatttggttttctctttggat
ataaacaaggtttctcaattgatgttcgtggatagaaacttgcaatttaatttgtttttaccagtcgtcaacgaggatagcttcttgagatttacacg
acatatacttcactctttatcgtaaaagttccatcattttgtctgttgataactgagaacggtggaattctgcgtgaccttcatgctcaacatccaata
ttgctctttccctttgacgatcaactagacgttgctttgctgtctccatctgattgattttatgcagtaagtttttttcctcctggttaagatcctctgtaa
atatgatttgccaatttccatgttttatgcacctgcgcttctcctgaaagcagacgctatcagaggtagaatgtatttgtttaacttctataataggaa
cttcaggggatatggaatgccatacatctcttaagtccctatcatttaataagagccgattgtcaaatccacaaacgtcctttttttatcccttcccgtt
ctttgaattcttggacgagaacccgtgaagcatcctttataagctgaaggaagtgcttcttttcttgagtaaaattaaggttctctttcagcaaaatt
agattacggtctaaaacttccaaatcttctcgttcaaatgcgtacacgttctctgggataattgtgtctggtaaagaaggcgcctggttaccaagat
tgcgaaattcttgaaagttatcggcaaccatggtcaatgtctttaacgaagaaagatcaattgtagtgttcttaagcttagatcgcatgaatagga
caccgtgttcatgacaacaatacttcgatgagatttcagcgggctttgagcattccctcagtctacactttcttttccatgcagtcaaaccacgtcca
ctagctgtgcaacttggacaaaagtactgagctgttaattcatcaaatgactctggaatgttgacacattctccatgaaaccagtcttcacaaccat
cacatcccaacatccatctaccatcatcgggacctctacacagacaataaacagggccttcaaaatctgcatttacagaagatcctattcctggttt
tgttcttcgaggtcctttacgggttacggttcctgttggcttctttggaattgtcgtacctagtcggtgtaattctgtatcagtttctgtacttgatgatt
caggagatttggattcttcttgctttacttttgatggttctggtattgaaccatccttaatatcatatttgccaacttcctgaaaattctggttagatgct
attggtaaaattgaatcctctattttaacacgttcggattcatcgacattcattgaagtttgattaccatcgctggaaaacaccatgcctttgaagttt
ttatcagctttcaggtgttttgtttcataccacgtgtttaaggcattcaaacgctttctccttatatagatgtatgaaaaaaagcataaaaaacaaaa
ggaaacgtatttcaatctctttaaaatctagaagacaataaaaaaatatataacaatcattgttcaaagagaggacttatgagttttagcaggatt
tgatcgagtatgattattaagaaattgaaatttgaaaacgaaaatcaaatgaattcataaacgtgattatcctcattagaccgtaaaggtgattaa
tgatcaaaaataaaggtgaatttcaacatgaaatagcctgtgaaatataataagagattgaaagaataaataaataaatccatcttttgcttttct
taacctagtcgacgaagccgatctttagtcctacgcattttcttgcggttttgatcggtatcatgtgacaaatcgtccaacatggcattctgctcaat
gagttcattgttaatagtcatagaaagctctttttgtcgctttattatcggtagtaacgtttctgcttgagagtcttgatttgttaaagcttggttttgaa
gttggtaaagaccaacattgtcaagctttttagtgactggcgtctctagagagcggctttttcctaaaactctattggaagagggagtagaacgaa
gagaaggctgctgatcagaggaagaggatattggattgaacggtgagctaggactggagaaagcagtgacctcagcagaggatttaggagag
ctcgtagggtgagtgatacttttcattatttttgataagaaaaaagctctgaagatagttgatctaacatatcacgtcttcggaacacctcgccag
aacctagggcattcatttgctctagccgattgagtgaagtagctagaagatctatagaccttttagtagtatacacactttttgtctgtccagaaata
gaactagtttttcccgaaagtaaatcaactctcgaagcatgcaattctcgcttgcattcatgaaacaattgaatccattccacaggaccaaggctg
ctttctttcgtaatgtccttaacattatcctctatgtccaaaaactttttttacttggtccatttctatccacgggcttttactcaattcttttacatactcct
gcaaagctactcggcgagattcacgaaatttctcgtttaccacggttgattgaaaccaagactttgaaggaagattcggtaacgtgtctttaccatc
aaaaggtctaatcttttcatccaaactgacaaaatcgctgaatcgtcgaaagacaattctacgtaagctattgggaaaggatacttcaatgtgata
tacggtccaccgcgaattttcgtcttgtttctactatatgttaactattttcttctttttttcctttacctgtgatgtttttggtattttcaattgaattggcgc

catactggcaaaagtatacatttatttacgtgtacttactccctatatgactgattttaacgaaatcaaccgtatctggatattgagaacaacattct
acgctaaatcccattggtaaacaaataagtaaataaaaagaactcctgaaaataaactgcaattcaaataacaaagagtagtgcagatgttata
tggagtttgtttaagaaagtacattctacttgcggactggttgaattgactttttgttcatgttagaatgctaaaagtttacaccatatatacaccag
aaagaaaagaattgtactttgcactcttacatgatgtataagcatgatgacgatacttataacttatttttggatattcagtgagttttgaatgtcgc
agatggaggctttggagcaatgaaggtttccatgtttatatttgactttctagtaatatgatttgcatttctgttatttacttcattaaaggtttcattat
attgagtttgtttgtttttttctacacgttggactatgaaggagttattgagtttctatgaaaaaaaaaaaaaaaaaaaaaaaaattgttatacagac
cagcttggaacgtacatgcataatagctgcaaactcggtattacaacattataaacgaagggaaaatcttgattatgtttcgtcaaatcaggtacc
aaacataaaatgaattgatatatttcattgagacctttccaaagatgtgttgcgggttcttcctctgaaagttatagaatgcaatccgatcgatgat
agttcaaaagttccgcaaacgtttcgtagtagaaaatcaagtcatctctaaaaaaagaaatttagaaaaaatagaggaaattaaatactaaaaa
ggcttaaactaacctactttgagtttgaaataaaaatatatatataaaattcacaatgaatattaatatggaatcattaaagaataatacacctgt
acacatttagaaaaaccaagaaacataaatgcttcaataacagcattttaaacaattcatagacagcaaacacaccatgaaaagttttcaaata
accgtaaaagggggataagaatgactttgatatgaaacagaaaatcttaccagggaagaaaggtgaagacaaaagataacatcgacttcta
aatcccttgccgctcatagagaaatagcccagcagacaagaatcacaatgacggcttgggtaatataagtaaacaatacctgacacattgggat
ccaatggggacaaaacaaaatggtaaaggggacggcgcaattccagatggaaaagaataccattagcataagagattgaagcatgtttcgctt
ctcaaagactagcttttgtaaaacatagccaataggaacatgtgcaatgtactgtaaaggaagaggaggagcaattaagctagtaacgacgtca
cgaacattaccaaggacaatcgagttaaggtacagcttttgggtagaataattaaacacactaatcaagacactggccataaatgtaaacaagg
tggtaatgctggcacgcgtcatgcgttcacgagagagtcgaggatgcaaagcattggtgcctgcgcgagcagcgagcaaagacagggcatctat
agcaaaagaccaagcgatttgccgaagagacacgagatagtatgtggacaataaaatatacaagggatactttgcaactaaataccagctgat
accaaggttgaagtaatacgtcagaaacgactgaatcgcatgcatcaaaatatttacatagcactaaattttccaacggttaggaaagataatat
caacaagctgcatcctctatgaaacctaccatccaatcaggaatatttttgtttacaacaccggtcgatgccagcaaacgaggatttaagtgcata
tttaacgctccatacgtgattgtatgcacagaaacataagcgatggctcgaagagttcgagaaagacgcgaataactttttctatcatctcgata
atattggtagtttgcaagtgcatcatggtgtctattggaaatagtgaacctgaacgaactaaatttcgatagctgtcgcaaggattcttcaaaaaat
gtcctgaaaaaatgaatggaacttgcttaataaaataattgaaagaagtaaattttattcaactttaagcctcaggaattgaattctttacaattga
acctatcgtatcattcatctttgtcccttgttgataacatatagaagcctattcttcaacaaacaatacgatacaatacatcttttaagtatcctcata
gatgtaacatatttatttataccccttgctatgttataaatggaatattacaaaatttaccaccctacgcgttttatttatttaccatttaacgaatttgg
ctaatctcataatttggtattagaatctctagcaaaattgttaaaattattttcgttcggaacgttcctacacgctatcccgtaacctttaccttgttta
ttgctgggatataccaactctcaatcaaccaatactccccctctcctcgatggtaagtttataacaaaacaattcatctgttttcctgtgttaacaaa
tgatttaaggctagttctacgatctataatattttttcagaaaaaactcttccttctacgctactattttcgtgtcagctttttcgctaaagtgtatgt
gttcttggaagaaaacgcgggatttttggtagagcttgtttactaacagtatagtggctttgatatttttactgacaaggtttgggagaatgcaaat
gctggtgtaagttgcctttttcatctttgtctcccttcagtcttctcttttctaacttcattattatagatgcaatggaaggatgtcaagcctcgcttctta
aacaacgatgaagaagaggaataaatactgttcggggatttctttcttttgtgtttctgtacagtccctcccgactgtcgtaaacaaattcaatactt
tttgaagctgcttcagtgatataaccctcatttccctcctttatctttttttactttcagaacttgcaatgccttttagtttcaattcgttctcttttaacatc
tttcaacgaataaatttaatttcgccaagtcatccttcttcatgcgactgtatctgtcttttgtcttgaatcaaattgaaatttgtagctcaaagtccat
tcttactaaatatgtacactaaaatacatgcaaaagctgctatactgtccatattgaaggcttcagcgattcgaaaaactaatgaaaagaataaa
agaaggtttaattgccgacaatcttaaataaaataaaaaaagtagtaagaaaaaaaatatgtatacaaagacttcacctgtgtcttcccgtccttt
attttgattctataaattgatcaaaagaatgaatagtagatgacaaaacatatactttaaatacggtagatcatattttcggaaaacatgattttgt
tttgggcgtccatctctttcaaggcagagattgcctcttctcttgtaaaggaagcgtgatcttcaactcctgaatttatcgcgcttagaacattctcca
gagacgcacattcttccgaaaacacatctgtcttggtcaactccgacaagcgagacataaaatttgtcatcttttccctcgataaagcaacaccct
gagaagaggaagtattactctctgaagccgcacttgtatctgcattgccactacgtgtacttgctgccattgtctctctagcagttgtcggaaaggt
cgaatggctagaaggccatgataactgagtattggatgcatttcctatatgtaattgactgccatcactggttgccgtggctgctatagattgatttg
ttgtggaagcagcatcatcaatatccatttggctttcttgactttgttcgttttgtacttgagaacgcgtctgtcggtttgaaccagacgcattcggttt
ggtagcttggaatgtgggatcatcgttcaagtcatcatcagcccctatttcctctgagtccatgtcttcctcttcggaactttcctctgcttcaccttct
ccttctttgcgttgctttttacggcgatttgattttggcttaacaacttctctgaacaaagcataacgaaggatcttttcagcagccttggcatctcgt
acttcaactgcattgcttaatcgtgctttcgcatgcgcagtactcaaacgaatcaacgtttccaacgtacgagccgtaagaggactagtacggcgt
tgattaccttgcaagtcatcgtttcgtaggccacaatatatattagtgatgtactctgcagtagcttggttaagcactgggtttattcgagatttggcg
tattggatatatttacgaacaaaatttatatttaataactcctttcgctttgttcgagcgttagcatgtagcaaacttgaaaatgtttcccaaaccggt
gtttccgattcttgctctccatttccggcagaaacccctgcagcatttgtcgcaccaacatttaagactgagtttaatgagtctctaacaggagtgcc
aggttccaaacccggcggtaaatagcgatgcattcttaaaacatgttcacttaaagcacggtccttcttgtcatcaatatcatctgtaacaataaat

aataagtcgaaacgagaaagcatggaatccggaagagctatattcttggtaggatcttttcgaatatcatattggccataaattggattcgctgct
gccacaacactgcatcttgcgttcaaagaggtatggataccggctttagcaatggtaactgtctgttgctccattacttcatgaatagcgacacgat
caatgtcactcatcttatcgaattcatcaatacaaacaactcctcgatcagctaaaaccattgcaccggcttctaaacggcgttcaccggtttcctt
gtcagaagttacagcagctgtaagaccaacacctgatgaaccacgtccggtggttgcgatagctaaaggagctgtatttaatacaaagcgaagt
aattgagatttggccgttgagggatcacccaccattaaaatgttgatatcaccacgaatatgtgtaccattggaaagatttttctctgtaccaccca
aaagtaaaagcaaaattgcctgtttaatgtgctcaaatccgtaaatgctaggagccaaactggtggaaagaagctcaaaagcattttttttcctag
ctaacttattgatattacgaatatccgcgtcggtaattgtcaaagcccctccaccaacgttcccactacctggcttattcgtcaggagtacaacgtta
tttgcgagcaaaacggtacgaaacgtggcacttgtacttccagagtttttggatcccatagaacggtattgcccaacaatatgtactcgatcacct
ggctttactgtatctaccaaatcatcatcgagtaaaatatcaatggatcgaggtaattgacccggaggtgcgcgttccggcatttcttgaagggaa
attgtttgatgatctcgaaaagtgctatagccgaattcaatagaaagaggattaccattttcatcctgcgttggataaacagtagactgaaacgaa
agaccaccgttcattgtagaatcagcatactgtttgaaatgatgacgcttagtagcttcacagtaatgtatagacttaataactttgggacgaaca
aacgaacatcttgtcacaattccttcgagagaaatcattttgttcaaatgcaaagcccttaaagtacgaggtgttacatgtaaatcaccaaaagag
ccacgaaatccaatataaaagggtttatcctttaaatccttatggacgataggatcgatcagtgtactaatcactccctgaagagcttcattaaag
ggttcggcaaattccatgggctggtagagaattccgtctgctaactccttattataatcgcgaagttcatcgatgttaacaatcaagcgtctttgccc
catattaagcattctaagaatcgcttcatgatacaatgttgcattcgcatcgtcggaatcgtgctcaagatattcttgaaaaatccttacacggtcct
taaaaacctcatccgccaacaattccgacatggtgtgttcttaagcagcaaaccagctaacgtttagcgacgcgtctctttaaatttttatcttccc
ttaaaacttcagcctaaagaaacctctttaaatcatatactggtacaatagtaattagatgatgagaaaatattaatttttgggtttcttgttcaagc
aggttgtttacagtaattgtttagcttggagtgtgcatcttctgaatcttccctaaagtatagtaacatgttacccataaaacatttgtatgtatttatt
tacaaacagaaacagtagactaaatcgaatagtcagcttattatgatgaatttactttgataaaattaatcttacacttgaggctttcaaattttgtg
ggtttttgaatcttcaaaaaacagatgcaagaacttaagtgctaattgtcaacaacgtccgtctgaagtaaaccaaatctatctatgtaaattaaa
agtaagactgcaaattcagtatatgattgtaacatgcacatcccgggagatattaaagatattaatcatttgagccttgttcgtaattgttcttcgcg
ttaatctaagatatagggaattatttttcattcatgactttaccagcttatgaatatctgacataagatcccacccataagttgcgataccctctac
aagctgctcatccggtatggtcccaaaacgacctcgtataatttcaccatctgacccatatttgattttgtaacgatgctttgtgtctaattgaacttt
cacttctttatcattcgaggtaatgactcgtcctgatttttcagagatttccggtgtgtatgtattggcattcaagtccatgatggtaaatcgcagtag
actgccaggaggaaactcaacatctaaatttgtacttttggatataggaaccgcattgtaggaatgatcaactttgattgtatctgaatcgaaagt
agaagtttgtccctcactcgaaactttgctctcatccgttatatacgatctatccgctaaacccaaatcgcctggcatagactctatatcttgaccat
atttttcagtaatctggatttttacgacggagatttctagatttcgtagcagagcttccagaaccaggaggctgaactgaagttgaactattcag
gtcttcattgtcgagttcatgaaaagaacgatgattggaaacgtcatttttatcttcagcagaccgaaaatcagaagtactgaattcaactgaatc
ggttccgagatcaccgaaacgcttatatacaatatcacttgcgtgctccaaaccaggttcagctttatcgttaggggaggacttcaggaaatttgc
agagtcaaaaagtgtcgtcctttcaccatccattgtgctcccagaacaaagggtctcctggttggttgtatgcaaagcagaagaatcacgaaaat
aagacatttcgtttgctgtgtttagagaatgagaaaatccagatgtattgctctttgtcacgtcgtttgattgtcctatatagtgatccaacttttcag
aattggaagagtcactgtcgctggatgaagagtccgagtttgaagtgctttcgtcagaagaagaactgctctctgagctttccgtctctgaagaag
tggcagctgtagatacagaagtagcatcagaagaaacagacacagaaacggcagatgaggattcctcggaagaagcatcggattctgaagatt
cgctcggagaagatttttttggtgattccttgtttttaatgggtttgtgttgattctgaagggctagtggacgaaggttgactttttctttcattgggttg
aaagtaatttcacttcctgtagaatgattcaacttgcttttctttgatgttgacgtgtcattccatgccgatggggcagctgcatcacgctttttcgtgg
aaaagtcactattatttggagttgcgtagggtaaagaggaactaactaacatgttgactggtttcggagcttgagtgttttgaaggtgagctttact
tcccctaccctgaatgttgttgctccattcttgaagagctgtgtccagagaagtggatgagctcaaccgtaaagccatgacttcgcagcctcgcagt
aacgcttcctccctaggaagggcacgcaattctaatacttctgaatcgctcaaaaataaatgatatgccatagatatcggtaaagcaaagtcatct
tttaccagttgtaaaaaaagttcatttcctaatgcatatttggcttgattccctggccctacaaggatagatcgtccatccttacaatctccaaaatt
aaataacagtatactatacaacaaatcagcaatagagctagttttatcagaaagattcaattcagcagtgtggatccactgctgcatagggatca
aaggctcacagcttcgaattcttagaagcatcgtcaattcttcaaattcttgattttatgcgtctcgtactataccataaacgttataaaactcataa
attataatcctattagaacgatttataaaaacctaagcttttatttttttccctgagtatttcgctacttttatcatatagagggagaaagtatggtttgt
ttactgttggactatttacttccaaacatacgtggatagcgcattacggttgaatatggtatcatctttattgaactcgaaagttgtcggtattttgta
aatagttgtgaacagacagacaacaaacctgcctttttctaatttcaatgtttgcaaacgcgcgagctaaaagacgtcccagagctacttctccta
cgcctgcgagattaaccagttatgctccagctcgtgtagaaatcacagaagaacaacgacaagatatccatgaggctttcaagttgtttgactcg
gacaaagacaatgcaattgattaccatgaattgcgggctgctatgcgtgcactaggatttaatgcagaaaaaccagaggtacttaaaattctacg
tgactttgataaaactgggaagggataccttcaaatggaagattttgttcgcgtcagtaagtttcctctatagcatcttgaagtatactatctcatga
atattggctaatataatacagtgactgaaaaagttgttgaacgggatcctttagaagagattaaacgtgcttttgagctgtttgacgatgatgaaa

ctggtaaaattagtatacggaatcttcgtcgcgttgctaaagagttgaatgaaaacattgatgatcaagaactagaagcgatgattgaagagttt
gatcttgatcaagacggtgaaattaatgaacaggaatttataagtataatgatggatgaagcctaaaatatacactagcaatcgaattactggttt
cattcctctcggattccctatacaacatggtccaagcgaatatttttgtctttctaccttctctcgttttccaactctttgtgcgtggtatataatctttat
ttatataaaacattaatagtaaatggtttattattttcttccaagtagacttcgaaagaaaccctttgtcttgctttcggatggaagctcctctgtttcc
tctttctgtggtttggcttttccaaattctgtttgctgacccaggtatgtaatagctaaatcaaagaaaagtggtttggcaggaatagggcgcaattt
cagctgagtcaaattcaagggtttctccgattccgtagttacatactgatccatagactcgactagcgataaatccttaggtttttggttaatcatttc
cagagaagccaaactacgcaaaagcaaaattcgtttcttcaaatcattaggccctgtttgtaattccatgacaatatcgaaagctatttccagagg
attttcctcaaaattatgaaggtaatcatttgcttgctcataatattggcctgcacgaatacacatagcaagtcctttggctttgtcttgcgattgata
ggcatcggcaatgacttggcagcgccttgctttaacaatagcaatttgagcttccagctgtaccatcatgccattatcttttgcgatacctggtaagc
ttttggcagcttcaatgttctatattatagttagacagtttgtaatacataaaaaaacagcatacctttataattgtatcatacaagctttgttgagct
ctaaggtttgctaaagaattcttgacggctggcagttcctgagtatgaatgtcccgttgaattcggagcacgataaggttgtaagcaagataacta
tagcagattgagagttcttgatactcattgctttcgggagaaatactatctaaaacgctttttgtggttttctcggcattagcccaagcaccaagcaa
gcgatcatcaactgtagtagaaacatccatacctgcttggacgtcatggataccatataacgccaacacaatttctgggtgtgtaatctggatggtt
tgatcgcgccaagtgattcgagtgatggacgaggtaccagcttgatcttctgcattcgattctaatacttgagggtttagaacttgaatatgttgaa
caattaagtcttcttttgaaacatttgcaagcataaggatgtctaatgattgcgtttggttttctaaaccgctgcgctgggcgacatagcgaattgta
gagtcgatttgtgctagtagctcattgaaaactccccgttggtgttctgataatgcttcaagagatcgctgtaattgttggaatgctaaacgagagg
aactgaatgaacgcaaagagtcctcccatgatcgcttttcatataaaaatgtaccttcaatgtatttcataaacgcagcagcctctaaaataaaaa
tatgattattatgatcctttgactcacaaaaagaaaccaattcctttgaggtttgcagagcccgttttaagcgtcgaagagcatgttgacgttgaga
agaccgtaagtatgtatattgctggaatgcgcgatcagcatcaaacagttggatttccgcaaaccttcctaagattagctgcttcattcaaacttat
ttcctttctctagtttacgtacctcttatcagccgacgtctttggctttccacctttctgagtaatgtttaaagcttttcttaaggaatgaatgcgctgtg
ataagtacttcatatactcatttccttcataaaaatgatcagagcgagcctctaaaagcaacggtaacagataaaaacctgctttttccatgttttg
aagaatttggctctgcacacgcgattagttcaacctaagttctgcttcctgcacacaatacatcactaagcttcgaaaagtcgtttaaccacaccgc
tgcaatatcttaccttaattatggtaagtctatctttttgtcttactgatattttgtggcctttgttgctattttctatcttaatttacgaactgaaatgcct
acgttagtaaacaaccagcccggatttatggaatagtattctagcattgttattaattttaaataatttagacaagttattcagctagtatgtgttttc
taagaggaagtatgaaggattatcaggaggaagggtaagcatctttcgagttgtattgtttggtgcttgtacatgcatgccttccattttgtataagt
ttttgcttcttggcttattcttctagtttcaaggagactgtttattcttactagtcctctacgaatctttataagacgaaaacattgttgggtagtaggtt
ctgaaagcttcgactttatcgctttcatgaaagaaagcatttcagaacatgttaagtaacttgcccataattttatacttaatttaactaatgtaaca
gaaataaaaagttccaatctcagaagcccacaaaatatccaactattcaatttttctattcattcgctatattgcgttagtaaacaaaaaaaagc
acaggttctttctataatcgatatacaaatgctagaatacaaattccttaacaatattgttgctttggacgactataggccaaagagcccacaaac
gatactatgtgcattttgtttgtcactgaccttgcaagtcaccacttcatcaaccaacaagaaaagccaacaagtccagtgttttccaaaacacag
ataagtggttgtttgaatggaagaagcaatatggaatgaatgttggcaggaaaagtaatttgtttcctttcgaaacttatacaaaattgcaactcc
ctctgttagagccgactctcttgcatgaactagggattttcaatgaaaacgaaagcatcaaacgaaagtatttggatttatcagagggcatagac
gaggtttctttagcgaaggacaatacactgctgggaaaagatgtcttcttaggtgtttcaaactcgtctggtttgatcaactacaaggctgaaggg
aaggatgaacatagggatgaaagttatttcttcgaatcttctacagacaggaagcggataaaatcacaaagtccggatttagaatcttcgtcctat
gaccaaagcgctttcagtgattcgagcgttttaggttattattcgaattgggaaggtacaaattttgtcgcgaaagaagcaaagacaccattcttta
cagaatgtcctccttttatctatgacgagtttttacatgaagatggctctcccgatgttccatctgttcaacggatttcatctcaacaggtttatttatc
attatgtgctttgattcaaggattcgaaacttctttattcctttggaatgatgaaaatctaacctttcttgtgcccaactgtgtttctatgtcaggaaca
tctcagctttcctttacgagtttcgctgcaaagtttgtttctattggcaatttaatacgcaaatctttaaagaaaattaatacgccagtgtcaattgct
caattttacttgtttaatttctgttccaacggtattactcagtatcgtaataagttacaagcttggttatttgaaaatcactcaccaatgtgtggaattt
tatcgcttatgagttttatccaaaattttcatcctttgttccatatacttgaatatgttttgcatattccgccttctcgacaagatgctgtatcgataatg
aatgctctttatgattatacaaatatgtgccagaataccaaagattatccggtagctttgcagtgtcttgtttattgttctgacccctttttctaaaat
taaatacgacattggactttactgtttcaaataagcacatacaaaactatatcaagtcatttccaaactttttcccttctgatctcgctttgcttctttc
tgaatttttaaagtctttcactatgattcaagaatatcctgcattctatgaagcattacggccaaagttagctactcctttaaaagctggtttctccttt
ggtgaaaactttctacatctccaaccaaaggcatcactaaatttatcagtaaacgagttttcaactgagaaattatacctaggtgaagaagatgat
tttgaagtcatgctacaaaagatgaatatgacaccaaatgtcgatgatgaacttcctgcattgcattttcgagaaggtacagatagaacgttacca
ttgaatcttataatgcagttatgtgtctataatccgattcaggactacattttagtacttatgcgaaccgtgtatcattccttattcacgacgtgttttg
ctgctcaggtattttcattactacaaaacgtatgctgttttcagagctcatcttttgttgacgaattaatgtcctatatacgggacgatttcctcagatt
agatgattcaagctcatttgaacatgcattgttgtcgtttgcgtcttcttctaaccagtttacaattcacgaaatcgaacgtcttgggttagatgtatt

tgcgagctctcatattcaagtctttttacactacaacaaaggagaaacccgacctaacacgttcggcgctttaagtctaaattgccggatttcagga
ccgttacaaatgttattcccagaaaaagtattgtatctctattctgaaatattttccagactatcctatttctttgaacaaaaagcgtccatcgaatat
gaatttcaacagagtcagcagctttgggacaagaaagccagaattcaaaaatggtttttttaccagaatattaaaaatgtcgcttatgatctagta
catgttattattccagaagccatgtcttcctttaccaagtatttcctatccctttccaacttttcgtcagacacaccgatcagctattttccttctgagat
aaatgagaaacatacaaaaatcctgactaatataatgaaagacatagaagccaagatagattccttcgacaatgcaaaggaaacgagtgttaa
ataagaattttctaatgctacacttagacaatagactttcgtagaaatatgatttgaataattattaaataaacaatcagtaactataaaaatattg
tagatgcataaatttaaaggaaatggaaataagtggctccaagaacttgaatcactattgagagattaatataaataaaacaagtttttctggaa
attgagataatttggtttttttctggatataaaaaaaaaaaaaaacttatatcacaaaatgaattaagtatgcccaaaaaagaaaagaagaatg
acaaaagagaagaacaaaacctactaagaagaaatgaaacggccactgatcataataacgtaaaaaggccaacaaacccaataaagatact
gcgggaaggtaaagtatatttttgcagcgattggaaaggttaaaaaggcaagggtatcgattaccacagaattagatgagtttaccaaacgaaa
gcgtcattgcggtaacggtaaatagcggttttgtttaggttaacagcagtgtcgttactgaggtttgttttggaaagagcagcacggctgtcattgg
tatacacacaaataccttggttctggtagccaacatttcggcaagtagcaccgccaaaaggagtgcgtaaagcagaattgcagttgactggatat
tgagtaccattgaagcaagtattaaccaagtagggatactcctcgttgcagtaagtcttgttgcaagcccactcgtcaagggaattgcgagcaaa
gagaagaggtttttcaacaacgttagagtagcttaaagagagtttagggaaggcactcttcttggtgtttttgttggaggatgattcagtatcgaaa
ttggaagaattggtctttccggaataatttgaggcaccgatttggcagagaccacgggtaagaccgtaggaaggttctgtggcaatgcaggaggc
accttgaccagtagggtaaccaagaacagtgggacaatttaaaaggacagtactatggttggcacagatagcaatgacttgaggagatacgca
aagattggatccagaaacatggcagaatgttccttcagatatgttatagtaagaagtactggcgattttgttgctcatgtcatataacgcagagcg
tttttcgagaccatcgggtttgaaagcagcacatgcttgaaacaaaacaaggactagaaagaccgcagaaagaggactagtgatagagaatga
tgccattgtagaggagaaaagaagagataattttctgaaatttggcaatgataggtgaaggcggcaaaaactttttttacgtaaaaacgaacaaa
tggttataacaagaaaaatgggtaacaaataacaactcagaagtcttttctgaacaacaattaccttttaatggcttttaggaagattgtgtatagt
tggaacgtagctgcaaggttgcggaggcgtgtatatataataatagataagtaattttgtttacatccatcgtaagaaggatctacgtggcgacca
agtttacgagccaatcaggtcccgtaacgggcgttatcgttaacctagcaactactcttacgtcagttgtaaataaataaacaatagtcatgcgtg
catgtaaacaagccacccggtacatgaaccctgatgaatcaagcagcaaaatgtgacaattttcatggtaccatgaaaaatccatttctctatttt
aatttctagaaagtttaaaactcccttactcgattcctaggcttgctggaataacattattatatcatcacttggagacttattagcatgaacaaatt
tgagaatctcttgctgattagacatagagtagtaacctcactttgtttgatactgtttgcaatgacctttgacaacaattggatcatccatggagatt
cgtctcgatatggaaactgaaaattaaagtatgaggcgggatcgtacataaatgaagagcgatcgaagtctatgcagggtttgcgtaatgtgtgg
gtgatagacccggtgatgtgtacagtagagtggagtaggatggaaacgaagtcgcaaattgaaataacaaatgcgcgctttttcttgcaactttc
cagtctttaaatacctcagttgaaatcgccttttcaatcgttgtattgaaatttgtaacttttctgtaagtgattctttcgaacatacatttgctgtaca
atcgagttcctcatacagtgcgcttttgtttatttagttataattcgttgaaaagacacacacacacacatgtacatacaaaaaaatggatactg
tagttttccctatatgaaaaaaattatttgtttttatccatttaattatttatttgccattttgtaagtttttttgatttgtatagccataaacaacaatatt
tgcgtggatggaatcaattgtgaaattccttccttcctctttttacctttgtcatcgatttttttgtagtcaacttgagcatattatttttcttaagaaggt
taattttcgtatagtcattttcatctatggctagtacaattcttatataattgcgattcatcccgttagagttagaaaagcgctccatcctacataaat
atgcgttcacagcacaaccctttcaaaatccccaacatgcagcttttattgttcctagatcagaaactaatgtagaatcaccttccgtagtatcatg
atattcccatttccattaaaaatgaaccctttgtttacatccagtagcagggtacaatatttccttgttacttggaagcacctacatgacgctgctcta
gtgaaaggacacgcaccttctgccaacgcttcttttctttaaagatgtctgacagcatcaattttcgtgacgctggatataattttactacgattctta
aacggtaagttcgtagcgacagcgcatctttttctggttgtcattatcggctgttctccgaacccttttgactaatcattgctatatgtagattggaag
cagcaacctctcgtttagaggatctagtggaaagtggccatgaacctcttccaaacttgcatcggccttcaagagatcacaacgctcagactcata
acatttcgtttggctcctatgcctcccagaatggtggtaccagtagccctgccatcgctgctgttaaggaaaatgcttctcctagcgtaacacctcgt
cagggtggtagcagctcaagtactgattctgatttaaattctccctccatcgctgcatatgatgagttttgttccaaacacctgaatgagtatattca
attaagcaagaagctcggtggacttgttgaacaacaaagtaagcatgttgaaaaagcgttcaagctgttacggggtatcttggtagtggccatga
aagcaaagaaaccggactatgagtctcctgaatttttcgagtttttgaaacctattcaatccgaactccttagtactacgaatattcgtgatgaaaa
ccgcactgctcctgagtttaatcagctctctaccgttatgagcggtatcagcgtcttgggttgggtcaccattgaacctgcacctgtttctttcatgtc
agaaatgaaggattcctcccaattttatgcaaaccgcgttttaaaggatttcaaggggaaagacgagcttcaagtttcttgggtccgttcttacctt
actttgttgggtgagcttattaattatgtcaagtcttattacaaaaccggtttgggttggtcgaacaagccagatgcttctcctttgaaggagattat
gaaagcgactactagcaagcaacaagatgaatctccaagcaattcaacttccggagcaagtggcttgcctcctcctccccctcctcctccaccat
ccaatgagttttggaaagacaaggaagagccaaaggaagaaggaaagactgacatgggtgccgtctttgctgaaattaataaaggagagaac
atcacctctggtcttcgaaaagttgataagagtgaaatgacacataagaaccctgacttacgaaagaccggtcctactcctggtcctaagcccaa
gatgccatcttcggatgccaagcaagaagcagctactccagctaactcaacaaaagcaccacgtattgagttggagaataccaaatggttcgtc

gaaaatcaagtcaacaaccatagtattgtattggaaggcgttgaattgaagcacactgttcaaatccttgactgttccaactgcactttaatcatca
agggaaagctcaacgctgtatccttgtcaaactgcaagcgtacaagtgttgtcgtcgacactttggttgccgcgtttgaagttaccaaatgtagta
acttcggctgtcaagtcatggattacgttcctatgattgttgtcgatcagtgtgatggaggagccgtttatttgagcaaaaattctttaagtaccgag
attactactagcaaatcatccggtttgaacgtgaatgttcctgttgacgatggcgactatgccgaacgtgctatccctgaacaagttaagcacagg
attaacgaaaagggagagcttgtttccgaaatcgttcgtcatgaataaatgtgggatgctatgagaagacgaaatgaggatatggcatgaggtc
gattgactaaacctttcatcactttttcttgagaagttacgacatgactatgaatattgtttggtttttcaaagtctgcctcattaaggtagcatctca
aacttccaagatgatttctaactggtattgctgtcatcgatttaatgatgtcttgtattttatctttttcgtgtgaacagaagaacgtatacaacacctt
ttgaaggaaaatacatgcctctacctatgctgatgttttacgatgaataatgtgaatgatgtgaatgatgctgatataccttgagctatacgtatttg
atataccacttaagaggaagaaatttggttgtttgtttctatttcttcgttttgatatttgtttctattagaatctgaaagacatccatccatccgttcc
atcgttaagcatattagcttttgagaagctttagatactgcttaatcattcctctttattatagaatattacaattttgtttgttaattttagatatcttg
attctttacttaattcctaaaactgtaaatggtgacttttctctcaaaattcgttcctttgaacaaaaaaagatggaaccgaaaagaaagtaataat
caagaaacagaaggtcgtcaaaacaagagacttgtctatcaggttcggcaataactactacaatttcattaggcaaaacaagaaattcgtaatt
gtataaaatgagtattggggaacgaagccttcggctattgaaacaaactcttgttggtcagtttcttttgttcgggtattgatcgtatgggggaatac
gtaaaacgtcagtccctcagttcctggcggatgtttccgtgagtatatatacgtgatggaagggaaccataaacttctcctttaccaacccccttc
aaagtaataaacgcaggcagaaagaaaactaaagttttcaaattcattcaaattaactacgaaacacatattcgaagtaagttgctattgaattg
ctgatgaaaaatcattcacgacgggctttgttgtaatctacgcgaacttgaggttggaaaagagcatactttaaaaaaagagagaattatctgt
gggaataaagcaatttcatagcaagataaattgagaagatgaatatagagtgagataataacagcacgagaaatttctgagcaatggagttca
atacgccatattatttcttcactattggtcctcacctctttttatgcccatttgatgtatctatattccatcaaaggtagatgtcagcatgcctagtgat
atcattctatttagaaagtcaagacaaagaagtttaactaacacccaaaatagaaaagctttacgagtccttggaaaaatgatgcataaaacagt
tttacaaagattgggcatgaagatctgtatgcctcgcaccttttcaactagctccagattaaattacaaggtagctgttttgggtgctggcggaggt
attggccagcccttgtctatgcttttaaagacaaacacgaaggtttctgaattagctctttttgatattaagggtggtcctggtgttgcagctgatgta
ggccacatcaatacctcttccaacgtcgtcggctattctcccgatgacaagggtctcgaaaaggctttgaagggcgctgatgtggttgtcatccccg
ctggtgttcctcgtaaacctggtatgactcgtgatgatttgttcaagaccaatgcttctatcgttcgggatttggccttcgcctctggtgaagctgcac
ctaatgccaagtaccttgttgtcaccaatcccgtcaactctactgttcctatctttaagaaggccttggctcgtgtgggtgttaatcagcctaagcac
ttgtttggtgtcactactttggattctgttcgtgctagccgtttcgcttctcaagcaacaaatggccttgctgaattactcagtattcccgttgtcggtg
gtcattcaggtgctactattgtgccccttttgtctcaaggaaaggttaatttgaccggtgctgaacgtgataacctcgtccaccgtattcaatttggt
ggtgacgaagtggtcaaggccaaggctggcgccggaagcgctactttgtctatggcttattccggagcccgtatggcctcctctgtcatccgtgctt
tggatggtgaatccggcgttgaagaatgtacctttgtggagtctcctttgtataaggatcaaggtattgactacttctcttctcgcgttactttgggca
aagatggtgttgagactattcaccccgtgggccccattaatgaatatgaggaaggtcttctcaaggtcgctcttgctgaattgaagaagagcattg
aaaagggtgaacatttcgttgcataaactatttctgcgaaaatttccgattgcttttccatcattcttttaaccttcaacgcaattcgtttatgatatct
atgcctagattaaatccctctttttcttttaatacaacatttagtttttattctatacttactgtaagggcttttcgcttgcttgcaatttctactgaact
gtaaaaacgaattgacatcgaagtacgttgtaaatgatgagaatttttgcaatagagtagcagatttttatttgaagaaaatttatgatttcgtattt
gacaaacaaaacatttatttttagaaccatcctttttgcctttacttactcctaggatatacgacgcctctagggcgaaacttacgaaattctcttac
caaaccggttggaacaccaaagttctgtagaaagtaaaatatacgatatagaatgccattgaaaagaaaaacagaatataaatcaatagataa
gcttgatcagaaattggtatttgcatttcttagtgaaaaagagggatgaactaacaaaatctcgcagattcaaaaggcttccgcagctttattaca
atacgtgagacaaaaaggttacaatcctgagaaagaaaggatatatttgcaaataaatacatttcatccgcctgagaacgaatcgttaaggagg
cccgacaaaatgtaagtaatttatttcaagagttgacagatggatgactttcttgccaattgaaagttgaatttttcaagccgttttcttcgtagaaa
tggcagtattgttttccagttccctaggtattggaaagtttcataaggatttgtttgactcttcgggtattaggccaaaaagaactgtattcactaac
attatattagttttcttcctaatcgggttttacatgcagaagatgcctgcttatttgtgaaaggctctcagcaacaattgcaagacgaggtacaaaa
gcaagggttggacgagatcattacaaaagttgttagcattgcaaggttaaaattaaaatataaaacagtcaaagaaaagtgtcagttacgtgatt
ctcatagattttttttaatggataaacgagctgcaaaagacgtgacggcattgatgggtacggttcttgcccaaaagaagataaaaacatttccaa
tttccgtcgatcaaaagaaagacacaattcgccatcaagtcgctcgttgcttacattctacttactttcagttatccgccggtacaagtcattccatt
ctatgcggattggcttctcagagtagtgagcaattgcttcaaaatatcgaagccatacttcgtgctctgctaggaaaatacattcctagaggatgg
aatgcgatcgataatatcgccataaaaacagccgacagtgtctctcttcctatttggaagtcggaacaagcggctgctgagtataaaagaagtgt
tgttcacatccaagatagccgtcctccaaccaaaaaggagttacgagcaatgcaacggggtactcaagttcctaacaaacctctctaaagctgcc
ttactatgctatcgtttggttcattgacgtctaaggagtataatgtctgttcatctctgtaattattgactgtgttgaacgactatattatattatattttt
attttattttatttttggataaataaaaggtcattggtttggtacactacaacaatttcaagagtttccatttttcaggcaagtatatggtaaggagtt
cagttttgcattttcgcaagcagaattcccaatcagtattccttatacatattcaaaatataatacctaataatatcctaatccatttgatactaattt

gttttgctcatccaagctgttttattatttcctcctttatagtaatattaaaataagggagctttgatttcatatattgtacctcccatctcaagcaagc
taggaattcttaaggaaagattcgattttcctaaaaatcgagacgaagtcaatccattatgaatatcctgaatctttctctgtttttctacatttcggc
tgcagtcgctttccaggatacttttccattattcgcattttcgtcatcttcgtaagtccaaaatgtagcaatgtcaaggctcccaaggctaacttttatt
taaagatctgtaattgatgctaaagaaggtcctgtcttaacctcctatctcaaattcaacaataaggcagaacagctcctgtcaaattgcgatgct
actacggttttcatttaccaacctggtcttcaggcagaagactttcattcttctgctttatctcatatgaagcgtatttctttggacgctgtctctcaat
atatcttaccttattccaaggagcgtttgcaacctgatcgacttattcacgatgcgaaaagctcttgctcttcgaaattagtaaacattgaccctact
catgagtcatttcccactttacaaagaaaccaaatcagtgtggtctccatgaaggatttgtctttagatgctaaatcacgcgaacaagagttatttg
aattggatcaagttatttactcgcttcactctactatatctgatgatactgacttcaactttttcctcacttcttctccaaagcttgcaaacatgcagtt
gttggaagatgacaaaactaaacaaaaggaagaccaagaagaaggagaagaaggaccttcgactatagactcaaactatttttaagtatgaa
tcgccttcgccatgcacaaccaatctcccttcccgtggaaagcatgaatgttcgcggcaattctctagttcgtaaccaacaagaacagaatgtaac
tagtactgaaggcagtttatttgatcgctatcaatttttaccccaggaatttttatgggatatctagctctagcagttttggtgcctactctgatcatt
tcttgcaagttgctctcatccattcagatcagctaccatgcgttcagcagccctcgtaaaaagaatttatctgaaaagtaataacttgttttgcttaa
ttttgcaaagtgcaacggatactgtgttggtttatgtaaagcaacaaaaagtgccgcttaaaagataatgtagaaagaatgggtccttgggtccta
aactaattcttgtgagatgatttttctgttaataggataaagtgggttcaaactgaacagtcgagaaatatcatttggcagctttgcttctataaaaa
gaaaaccccaatatgaaatcgggcaaggaaatgactgactgttttgttccttctaattcttcgatgaaacagtatgttgattgaattgtgaatcact
ctgactattgttaatttagaaagacgcgaaccattacaattgaaaagccccctatgcgattacaatttggttccttgatttttttcactagagattata
ttttatgaatagtttttgacaacgttatttgaatcaagtacatgttgaatgagttagaaagagaatcagatgttggtttttttacagctaatttgtttca
tattaattaaataaataatatcaactttataatgcttcatttcacctttgaaacaataattcaagtatcagcatcctgtcacccctattattgctaaca
gctggaatacccttcgttaaccaaatttgagcttgattgtgtatattcctataacatccgaataaattgttaattaattaatttaagaggtagaagaa
cacgagaggatcaatatggcacctgataatatatatcgacctcatcctcgatatgctgaagggttccctaatcaaggaaacttgaaacgctccatt
tcggtgttatcctttacttcgttggacaatatgcgatcaacgttgtccgggttattcactcctttgaagctagaagaagaagaagaagaggatgatg
atgaagaaccttccattcatgattatgaagactacgcaagtaagcagaaggacgacgatctcaaaaaagaaaaaggaagaaaacgggttatca
gagtacccaatttatccataaagttactgcttttcaagtttttggcaatcttcgggttaggagcaatatttacttacatggctgaagggattgtacaa
gaagccaaattatcacttttgactgtacgatacgggaactgggtgtttaaaccaaattggtcagttacttttggacttgtagcaatagggcttgcag
tctcatatcgtttaatggatgtacgatatccatctggttcttctggaacaaaaacatcaaattcttctcaatttcaagtttttttcaagatatcttgctgc
ttttgctactctacttctcactatgaaggtaagttgaatccgcgtttccgttctgaattcgtgaagccttacaataaaaagaatttttccgctaacaac
ttgttcttagagattattgagcactcagagcacacattcttttgctgctttaattgctagcgctattaccatctggtatgtgtttgaccgttcaaaaaat
ggatttactgtatcttgtgtaatgagtattgttggcagtataacgtattatgtttttgttgatagtgctcccttcaggtaagataaaataatggttgatt
ctgtttgcttacgatttagtctgttcgaaactaaccctccggaataccaatttcgttactggatccccatgattcttttctctgcctcaactattgtagg
aaacgctggtcgtcttctcttttaagtttttatgatgatttataaaccttggattctttaatgtattattctttctttttttatgtttgtaattgttatggcatt
ctagtatatgatttatatggcaaatgcttaaaacactataggatttctctttcagtctactttattaaatttttcattctgtctatggatacgttggtcaa
aagacgatgattccacatgcatactcggaaaattgcctaatttaacaaacagaaatggaaagtataactttcccgtgcctctttcgtccagttcatt
tttttgattgtcgattcgacgaatgcttgaaaattttggtttatcattttgaagacgtattattcatcctaaaaagcacggaaaggtaaacgaatact
cgtgtatgaattctgttagtctgttttcttcttctaaaatgtattctcgtacagatttcatgctaaacgtttcattgtctgtagtacttgaaatgtaagtg
cattgattgtataaatacaacgagcaaggtggtgtatccagaaaagtcttgatgaatccctctgaagatggatttcatttatttatctatgcgtaatt
gcattaaggtaaaaacaaacatagaaacgtatctattcccaaaagttggcgcgtgacatactccttaatttcttttaaaagataaagataattattt
attttgtaaaagtgttacattcactccatttgaaactacaatgtttaaattcatttcttcctttgtttattatttaacgttctctctacgcttcactattgtc
tcttggattgggaaacgcggtgcactactccaccacgattttacctttctcctatatcttacaagggaaggccaggcaaaaatttatatgtgattgtt
tcatccttttttaataaaaaaaaattattgctttgtaagttttgacttgacaggttcaatttcggttgtttttttgactttgtttacgtgtcgctacgaatca
aacgctactcatgagcatctttaattattaacaccattgactttaatacctgaatgtatcctatctgaggtctgcaaatatatattacacatgtctac
atttcgcaaagtatcttgctttaatccatcacactgatattgactggcctttttttaatcatttgatttttctacatttctgatcaagtgtgttacgaatttt
gtgtataatttaatttaaacatcatggacagttttccttcaaaaccgccttctcagaagcctaatcctccgcttcagagaaataattacaaactaat
atacgaccccgagctaggctttaaagaatcattaggaaaaaaaccagtataccggttcaatggcgttgcaaaacctccattggtagttcgagacc
cgagacacaaaaatcccctctattcgcgggggataccaaaaagtgggcgaccatttttaaaatcattgcaacaagtcagattcgaatataacga
agattctttagggcctgaaccccctactcaaatattcgtctataatctctctcctcttgttacagcagaccagcttcgatatcacttcaaatctttcgg
ggacatatcccaattggatttgcaattaaatccgtatactggtacttcattaggcttgtgccggatttctttttcaaaacatgagtcagtatcgaagg
ctcatttggctgctaagcttgctgtccagcaagctagtggtctcaaactttccggaaagcaacttatggtcgttatagatgatgatgggtcgaagtg
tgaagctgctcgcgacaaagcgctgcaagtgattgaagccgaatttgctgaagaattatacaagcgagagcaacaaatgaaaaaagaagaaa

aagaaagagaagaagaaagagagaaagaaagggaaagagaaagagaaagagaaagagaagaggaaatgaaggctattcatgaaacttca
aatgtgcctccgtggagacgatcacaaaagaaccaaagttataacaaacagcagaatggaaactttgatttttcaaataataacttaaagacaa
acgatacttctgttccaaaagatatcgaaaatcttatatatattttcatcgacgatagatttgttcctcccgatcgcgtctactattccgatataaaac
accattttcgaaaatttcggtatgaaggtatatatatgaatagagatgggttttatattactttcaatagctaccgcgaagctagcagttgtttttgg
actctcgacaaaactttcgttcaaaattgtcgcataaaattgaaacttcacgatgctcctcaatcgaagaagacggatctggcgcctactaataag
aaaaaagagttaccgaaagaggatgcttgtacgacggcgatttctgtaatcctaaaagacttggaagaagtattagtacgagacattaaaaaca
agattgcaggaccatgtatttttcagattttggaatcatttccaaaacccgcacctataaaagaagaagaaaaaggaccaaaaatttcgactctt
gaggttcctgaatcactgaaaacggaagcaatatcaacgttgccccatttacccagatttaaaaagcgagcagatgcccacaaaataaaactgc
caactgagagcaaaattaaatcaaaattgcaaaggagacgaaggaaaaagattgaggccagacctttgcattatcaactaaatcaacaaactt
acgattcttctgattccgaatttgagcaagaagaggcactagatgaagaaaaggtttttgacggtaaatctgatggcttcaagcctagtaaggttg
aggaatcaactcccttcctctcagaaacatctgaggatgaagaatatttggaaaaatttcaacggacgcgccccaagtctcgtcttcctagacgg
aaatctgacaagcttgatatagactatacctcttctagcgaaactgaatcggatgaatttgtttcccctgctgttatctcaaaaccaccttttgaaag
atacaaaaagcatgctttagtgaaggaacagcaggatatttccctgctaacgactaagcataaggagaatgagtttagtgaagatggcggtctgt
tttatggtacgctaccctataattatcctgaggatgatgttttggttgatctggacggagttcaatatttgttaaaagacgatcaagatcttcaatatt
tacgtgaagcactcgcttcagaggatgccaatgacattggggatcccaattattgggcatttagacgaaaaatctgtaaattcaggaatggcgac
gtttctgctgaaggttctcctgtactaccagcttctgttggttatgaacgaatcaattcaactggatctgcaagaagtgagggctactatattatacc
cactgctgaaaaatcccaatacttacctctccgtaatcgctcaacagtggaaggtgccaaccaatcgacaagtagagttacctccagaatgcatc
gtgttaataaccgtagacttgcagccggggttgaaaaatcattacttccagctgaagctgatttacttcgttttaacgcattgaaagctcgtaagaa
acaactcaactttggtcctagtcgcattcatactctcggtctctttgcgatggaaaatattgacaagaatgacatggttattgaatatgtgggcgaa
gtgattcgccagcgtattgctgacaatcgtgaaaagaattacgttcgggaaggtattggtgacagttatcttttccgtattgacgaagatattatcg
tcgatgctactaaaaagggaaatattgccaggtttattaaccattcttgtgctcctaattgtattgctcgaattattcgtgtagaaggaaagaaaaa
aattgtcatttatgctgatagggatatcatgcaaggagaggagttaacttatgactataaattccctgaggaggctgataaaatcccgtgtttgtgt
ggtgcgcctacctgtcgtggttatttgaattaatagaccagctatactatagaagataatttgcttacgttctttttctgctaacgacaacggaacttt
tgatcgtgctccctcagagatctaattttaattgttgataatttcactttagaagataatggaatttttttggtttaaatttagcatttacaattagtttc
atatagcttttatatatttgtgcaacttttacttatccatttggcacaatttagaactacataaatacaaaagataaaataaaaaatgaaatcacag
ttacactttatgtctcaggttttacattagctagaatgaagcactgttgttcaatgtgcctgtcaccgcccttccaccagaaagctgtaaacatcaaa
taataaatgaagcgcgccagtttagggggccatacgcctgtaaacttaaattcattgaatgatgatgcattggagaaaaagaaggctaaagaaa
aaagcaggaaggaacgtgaattcagaagaagctcagcgcttcattctattactcccagaagagaatctttaggaggaccaggctatggatcacc
ctttaatagctcgtatcaagttcctgttttgtctaactttgaagaatggatcaaactagctaccgataataagatcaattcaacaaacacatggaac
tttgcactcatcgattatttccatgatatgtctttgcttcgagatggagaagctatcaactttcaaaaagccagttgcactctagatggatgtgtaaa
aatttatacatctcgaattgattcagttgctacagaaacaggaaagctacttagtggactcgccaatgattccaaaacccttggtgaaattgaaga
atctagcgagaacgtagaagaggaagaaggaaggaagaaacgtgaacgaaaacgcgcgaatcgatctgtcaatacgcttgctaaagatttcg
aaagcttacgggcaaaacggttcgagttagaaggctcttttgatccattatttaaaaaaatgtgtgcagattttgatgaggacggtgcaaaaggg
cttttaatgaaccatttgtccgtcgatcaacatggtaaaatagtttttgactccagtgacgctattattaaggatttagaaggaaatgatgatccga
aggagaatgttttggaaaataatgcttctgaagatccagaaactggaaatgagaataagagtacttatgccgatggtatttccttggaggccctg
ggaagtattcaaaaacgttatttcattgatattgatcaacttacgatttgtccttccttgcaagggtttgaatttgataataaaggtaacctggatgtt
tcattgctaaagtctttaagtgatgaggtgaatatgattgcgccgaattcattgttagaagaacaagcagctatgaatggagatgtggaaattggc
tcattaccgacggattctgaggaggatgataatgataatgagcaaggacacattgttcatgcacttgaagacatggcttacggtgatgaaaacat
agacttatctgcgaaaccattagatgagtttgaggaaccaaacaaaagtatacacgatgatacggaagcaggtgaaactatcaatttagcgaat
aattcagttgacaacttatcatccatcaatggaatttacgagtattttgacaagtcgataaagaagaactgggccggtcccgagcattggcgtatt
caagctctacggaaaaggagtgtttctcaggcccctggaatggattcttccaatgtttctgagggcgttgacaatactcgggtatcaaattctttag
aacgtagacggcgtcgtgaagcagataacactatcgacttttcgaaggaggttgatctcgattctttgtttactcctgcttccggtaatttgaagctt
ccgaaatcgcattggaaaaaacgtaatcgatgcttactaccagatgactatcagtatgattccagacggcttttacagctctttcttaaaccaaaa
gtatcccttttaccgtttacatcatttaatggagaaaccggtgttaaacaggtgtttgaaggacaggatgaagcagatactgctcccatataccctа
cgggatttgacgatgacagcgatggcggagatatggatggccaagctttgcatgaatttgatggaggatctatgcaagaatttcgggaaaacac
aacatcacctacaccaccctcctctgctgggtttggagaaaatttattactgaatgcacgacttgccaaaccggatagtattaattacgctaaacg
agccaaaagagtagatgttaaggtattgaaagaaaacatctggaaatgcttagaccttgaagaaaataattcgcgtgaaacgtcgaaatccaac
gtcgaatctaatccagaaggcgatgatgagctgaaagaagaagaaaaagaagaaccagtgcgtaccttttcctcaactgttgctcaactagaga

atatgtatggtcaaaaggacctgaaggatatatctacaagctttgcatttatttgtttattgcaccttgctaatgaaaatgctcttaatctgaccagt
aacgaagacttttcagatgtattcattcaacatgaccctaatttgactagcttggaagaaatggaaaagaatatgtaacatcgctcattttatgatg
gcattcatgctggtcattagtgtcactttcttttttttttacggaattttaggttgtatttaatgaaagaagaggtttaatggattacatctccttattgt
ttttatcctttgtatttcttttgacttgggtaatgattcatcaatgtaagtaatgtccttaaagtggaaggaccctcattagtccctttctgtcttcgcgg
cgtaatatcatgaaatatttatattaataaaggaaatcaatttatgaaattgccgtcaactacgcgatcatgtgaatatatttcatacgttcttcttg
ctagtaattgcggtccttcccggtacgctcattcctttccttgtgtaataagcaggggattcagaagctctactagttcatgaaccgttatagttcttg
ttaaaggtgcctaacagaaaacatttctttccccaatagcaactcgaagcatcattttataactcttaaggctatatgaccggacaaggctgttttc
gctttgaggatcataagattctcattccatatatcaaagccatatctcattcagctcaatcttcactttctttatttgtttactatttagcgcacccgat
ggataacttaaaggatccatagttactcagccttagtcgtccttaagccaacgttgttacttgccgtatcccaagcagtctgaataaacaagcccc
atttcttgtttgtttttagaacttacctcaaaccaagaaccgacaagatgtcaaactatgcctatactaaaattagcgaaaacgttcaagaattaat
gtcttctagggtaagattgaggagtgtagaacaaaacatcactaacgaagcaacagtcgcagtacctgagtggattcaaatctttaggcgattttt
tggattttcgacgcgtttcaagacccgaaaactttggagaagcgcagtcaagggtccgttttaatcttagacgattttcttcaaattatttagcgata
attgctcttctcgctgtttattgtctcctttcaaacccttggctttcttttgttattgtagtgggcggtgtaggagcgtatggaattagcaaacgcccaa
ctaacgaggtgcaaattggatccagggtttatagcaagtcaagcctgtacatcactttggcttgtattttatttccttttggactttttgcttctcctatt
caaaccatcttttggttagttggagcatctgctgtatgcgttcttggccatggtgctctattggaacccccctgttgaatctgcttttgaggctgttgag
caacaagtataaaaaccaagaatgcttattttcttatcttttattcaagaagcttatttttcgtcttattaggtacctcttttggtccgcttttctattac
ataggctgacttgctttttgctctccaaattaacgattagcggtggtatgcttgcttattaccttatgacgggtttgtcgctctcctgaacatcaatgat
ttttggggttgatagcttcatccaacctccctttcttctttacaagcactaatgcgagtgaaaggattggtaacaagcgctcaatttataggctatat
aggaatgattgtgtgaagttgattgcagtttcgtcttatatttttcatcttctcgtctatacttctttctctccaacgagacatgaattgagcgtttcatc
ttcctgtttttagtttcctacatttactttgttgctccgatgcgtgtttgcagtgtcatgtatattcgtttgtaatagttagtttagagttttgtcgagttttt
atgacttactaaacaataagaaatccacaattttgtttatgtaaattatacataggttagatttgatattactagtattttttttattgtatgtttacata
catctcagcaatttcaaggttatgcatgcttattctatcactcagaatcatctatatcgctataagagaattaaactatcataaagcaagtcgaaac
ttcattgacctggctaatcatatcatagaacttttttttaagtaaaaaaacattaaagtttttgataatcaacggtaccttcgagcttcttaagatcag
cgactttacgggctgcagacattaaatcggattgtacaatgtaatcacggtcttccttaatggctaaaaatccggcttcagtcacaacgtttcgcaa
atcagcaccattggtaccatcggtaagcttaacaatagcttcgtagtcaagttcacctcgctttgacacgttttcaagatgaattttcaaaatttcca
tacgaccaacttcattgggaagaggaatttcaattttacgatcaagacgaccaggacgcaataaagcagggtcaagggtatcaggacggttggt
agccataataattttagtttgtcctaagtaatcgaaaccgtccatttgattgagtaattccatcaacgtacgctgaatctcacgatcagcagaagta
ccttctgaaaatctccgtccaccaatagcatcaatttcatccatgaaaatgacacatggctcatgctctttagcataaccaaacatttcacgaataa
tacgagcagattcaccgatatacttatctacaataccactggatacaaccttcaagaagttaactcccaaagaagctgcaacagcacgagctaac
aaggtttttcctgtaccaggaggtccataaagcaaaacacccttaggaggtttaataccaacacgtaaaaagagttcagggttctttaggggaag
ctcaatgacttcccttaactcacgaatttgttcatttaaaccaccgacgcctgcaaaagacacatcgccaggatcttcaacggacatattataaac
caatgggtctacttcccgaggaaggatgcgcataatcgttaatgtagtcatgtccaaagaaacacgaacaccctgaattaacttagattggtcaa
cattattcctacatccaacaacatagcgaggaccagaactaaactgttagtagcttgtttcaattatatgagcaaaatactattatccagctggac
gttgttttattctttttcttttttttttttacgaaaccacctcgtacacgccatgaccgtccatgtggtaacctacaaataccccgaagaacagcaacc
agtacgagctttgaccatcataacatgtgtgtggattctccagaactgaacctagagatctgcagattcacttacctagcctttacaataaagcgct
ccggatcaagttgttttaagacctcgccgataatttggcctacggactgcaagctcttgatatcatcttctgtcttgtcgtatttcttcaccaaatcgc
gaattccaaaacgcaggtcttttaattttgcatcaaactctcgatgctgcagcaatgctgccttatatttctctaaagcaacttctctttccgacattc
gggtttggtaaaggaattgttggtttcttcttaaggactcttctgtagcctttctgcacgctgcagtgcgttgatgagtttgtttatcaacgctgtaaa
gtgtgataaatcgtataccgacgtataacccaaaagctctattttaataacgaccttgtaatacttcgctaaaccacaaatacttcctgtttgcata
agaattgaaaagagatggcaggaaggtaggccttcaattattgctagttacataataatttgtacagaaaagtcataaatatccttacttatggta
gaaaagctttctgtaagagaaatgtaaaaaccagaattttcaaaaaagaaagcgactatggtattaactaactgaaaaaattaaggtccaaact
aaatggaagaacaaaaatatcttagatctctaataattaaacaaaaaaaatagaaagacataaaacctttaattattaaatcaagtcaatattg
gagcagacaagacttatattataatagtcagaatataatgcacttgaatcagaaatcctgtctgcaataggacagataataaattgtatgtaaaa
tagtggatagcaaacattaaatcagtcactaaataatgctgtgaagcgcattactgagtacgtgtgccaaaaatccctcaactaagtcggattctt
gaagcaaatcaaacaaaagtcgcaagctgtcctccgatgtgaaaggtcctaaaagtgaggcactatcagaatattcgttgttaagaaacagctc
ttgagcttgctctcgtatctcgtcatcactctcttcacttactgcaaattccgaatggctaaaaaacaaactgttcttcaaagcttcctccaaataca
gttcatcattatctttgctcatggatgaaggattccagtctgcacccctctcattatcacccgcacagtctttatttaaattcttcaaatgttcttgtact
ttttctttttcttcgggccagatactgagaattaacgaggtaaacaggtcaccaaccagttgcgtatttgacagctttgcaaacacatggtctttcag

catcttctccactaatccagagaagtctaaaggtccatgcataagtaacgattttagtacagataaaactgtccttcgaaaaaaccacgtttttgg
gttcaagcatgatatctctccaaaaagagcaaaaccactatttatgatttccttcaaagtttcctcttggatattaaatgcagagttctcatccacat
acagattgctctcagaaaacatagaaccatttttctcttcgtccttgttttcgaaagactgattatcatcttccaaagatgatacatcatttgagggt
aatttggataactcggtatcctccaacatacttttctcaaatttggttttccaagatacttccacatttgacgaaccttctgactcttttgagtgggag
gattttccactcttattactcacaatctttgtaacccctaaaagcttgtgtgaaacagcagacaaagaagagttcttttgcttttccatatcatcgact
acatcatcggataacggttcaaaagacatggacttacagaagaactttcggactgcaacattattcgataactgagcattttgtactattaaattta
aaaatgcagatagttccctttgtaaatggccatatgtctgagctctccattcaggaatttttaggggggtcccaagtgcatgcaaatattcttttctt
atatccttatataaggacttcagactggtataaggacgatagacaatccaagggggatatctaaatataaaggatgcaccgtaataacaaactt
cagattttttttatcctttattggggatgaaaggtctttgggctcaagcgaacgatattgtatatttatctctgcttcaaaaagagcattattatatga
agaagaggcttgggcaccacctgtgggcaaagtcgccgatgaggatgtgtcttttagggtttggtttaaaagacatcgatttatatatgttttcttgt
ctaaacattcgtccttctcttttgaaggagaaaggaaaagaatgatacaacgattgacaaaccttggcttagcacaatgcgttgtaatttttttaaa
aacttgcatagcaagaacttccctcaataaaacatgtagaggaagacaagcggcatcttctggtttgcaaaattttgttaccatcgcagaagcttg
cgcgcgtaaggcctgttcgttcgcagccttatccagcatctttgcaacagccgaattgggataacggacagcaaattccgtgatgggcattttttg
ctcaaaaacatattcaagtcagaaagcaaaaacgtaatcgtatttagaacatttttaccatctcaaaaataaggtaggaagagtcttgggtactc
aaataagaatagaaattcctgaatatataccctatcgtactttccacctctgtaacaaaggaattatcataactcacttgtttataccaaccatatat
gtaatgatgtgtaagtttcgtgatgagaagaagaagtggagcttccaattcttctgggagagcgtgttgagggtatctctgagggacatactgaga
cggatcctctatattcggatgtttaatatttaggataatattgccgtttttttgaaccaaaccctgaaaagaaacgccaacaaaaattcctatggca
aagtaaacaaaccgtccaaagaaaacatgaagaacataaaacgatatcaagcacaaactaaagataaaaacatagcttgatttattttctaag
cttaaaaacgcttttccagctttcgataaattccattgcataaacatgtgtaacagccaaataacaaattcgactcgataatttcaaatcctattcc
aacgcttatcttcctctaccaacacagcgattgtcagaaaagttataaaggtataagggaaaccagcaaattttttttgaacgttatatgaagcaa
ttgagcataaatatcgggtataagaaaatataattaattgataaatattaaacggttgatcagcaataaaaataccccatatacagcaactgaat
ataaaacatggtgtttgcagagaacatccattgtctcagcctcacagcaaaggctttagctgacataataatgcacctactctccattttcctaacg
agtttacggtattacaacacatgatcttaataaacataagatttctttaaatggtaaaacccagtgtttttttataaagcatagaatatagcaaata
tattgaaagatgaatgcatcatgaagctaccgtaaacttagactaatatgatcatttgttgaggtcttgccttagaatggtaaatcatcaactaaca
cctaataccgtcgttacgtttctgatgactactatctttgtgtaataaccacatttcataccttcatctaatatgtaaatacaaaccgcgaattattaa
aatttacaatagctactcacatactcctcaaccatagaaaattaattactgccttaaaaaaccaaaagtagtatttgtaggtatagaacagaagc
gaaatcaaataaaggcatctactcctcgatcttttcagcagcggtgtaagcttgctcacgcttctcgccgacaaccttagctctacggagacgggc
agccttggaacgggcacggcgagcatcttgttgctcttggagtaacttagtacggttggcttcagccttagcacgttggatgtgctcaatcaaagc
acgcttgtgcttgaaggtgttacccttggcagccaagtaaaggctgtggtaaaggtgcttatcgatcttgccagtttcacggtacttacggagaag
gcggcgcaagacacgttgacggcgcatccagacgacagcagagggcatacgagcctcagcagtacccttacgcttaccataaccagtgtgacg
accaagacgcttggcagcatgagtcttgcgaatacggaagcgagagtgcatcaagttaggcttgcggatgagcaaaccatccttaaccaactta
cggatattttgacggctgttagcattgctaatttcgctaagctcgttggggtccatccaaactttacgcttgccgcattctaaaacggacgcagcga
gacgcttctgcgtactgaggtttgccattgtgttggtcggttggaagtggagtaactctacaagaattctcatactcaaacttgccttcatatcagca
gtcacattgtagacctttaccctaacctggaagaactcgtttaggtgacacgcaaattcaagtaacaatgttaaacaaagcaagctggctcttgct
gtgtatgaatgaagcaagtcatttgtcttgcttgtgcataattctatcgtatataaatgaaaagcaaaaaataatttagggatatattggctgttta
aaaaaaaattggtggcaatggaatttcaatgaactatctgcttcaaaaactttggaccataaacaaaagttttgtgaattacgtagaagatgttat
catatctaacattttgcaatagaacatataataaagggagggaaagcattaaggaagaatggaatgtctacagagtgtaaacgttatccagttca
ccatattaagaatactgaactttttcaaggtatcatgcgtaattttattttttatgaaacaccttctcaaacaaaaaaagatcgaattatgaaagat
gcaggcgtgggttttatcgacatcccaataagcaatgaccctagtagtaaaaggcatgggaataataaataccaaacgaacgtcgaaattgtac
tttttatatatataatcaaagtataaaagaccaaaatggattcaaaatgtttatccaacaaccaaaccaattgtaggtgggtgagtatgaaaggatg
gaagagtatacacacacagacaaaagcgtcattataatcccaaaaaaagttctacaacataaccaacaaccaaaatccaaaaaggaacgaat
aggaatagagcatgcaattctctcatcctaaatgagcgagggagaggaggatgactgacaaaaaagcttttcggtaataaataagtagaggtac
aaaaaaaaggaaagtatacggatagccatcggaacatgaatgatagaatacgaacttttagtcatcaaaaaaaccttcttcatgaaaagctttc
cacaatcgactagccttgttgacatccacacgactagcacgctgtgcatctgttttccgaaaacccaatccctgttgctttcgataatgccattcagc
aaagatacgtcgtttattgtctagataaaccatacaaggcaagcgaagggtagatgccaaatggacttcggcagggtgcaaaagatggtaatga
gggtcgtttgaaaggtctagaggaggacccttccattcagcacgcaaaggacgagggtgagaccgtttatcgagcacagacatgtcgggacaat
aatcaggcagggcttcgaaaggaatgtttgcaacgtccataccattgtattgacctgctccacgtgttgatttccttccccttgtttcaacaggaga
agctgaagattccggggttggcaatttcgaattgcgagtgcctccgttacttgatggtgaaggttcccgagatgcattcttagacgaagtctccgtg

tctataacagaagagacaatgcgattaccacgacgcccaacgaaaccttgacgacgcgcttttgtaattggttttacgtgttctttgcgaagccaa
atcctttcttgctgaagccaaagctttggttgcgttttgtatacctcatatacaattcccttcatacaagcgcatgaattttgcttccttaacgaagaa
acatcccgaactccctttgaatgaaaactcaatggtttcgtcttaggaggtgaaagaggaggtgttataagaatacaatcatttccatttaaattcg
aattatcctgttcttttaagatatgcattatatacttttttttgttttagcactcaatccaatggccctatactataccctcaatgatgaggttttggtat
cttgctaaagacacctttctgtgggaaaccctcttttgtaagccctaatatggtaccctaaaagttcaccaccatttcagctagtcaattgcgagctt
actgtgcgaagttgttcgcttgtttttatttttaagtgaaacatttttatactattcattgcttgaaaggttggacgatggttccctaaaatgatggtct
gtagaaacactcactgtagcaggaacctgtcactttcggttttattttctttttttatgaaagaaaaacaaaaaaattctacatatatatatttttttcca
taaatccaaagaagaccagaatcttttgccgaagggctttttctatgaagagcgcaattgtttatgtaaaaagctgaatgctttcggtttaagaac
aaccgcaaaggaagactatattttgaagagtacacataggaagaaggtttttgaaagcaaagacatacttccttatgagttttctacgaattgata
cttaactacttgttggtcagtaaaaaccatcaaggccccgtaaatttttgagtcaaatcaattgttttgttctccaaaactcgtcaaaatacacaaat
aaggaaaaaaatttgattttgatcatatggaatcctcttccaagtagcaaacaccagcaaagctaacaagcactgtacaattgagaattcaaaa
agcaaactatcgatcctctattgaattttggactcttttcttcatatggacaaaatgtttgctgggattattttcctacacatctattgaaaaccattg
attcaatttcagtatacacatcattcataatattaactttaattacttttctacttttttttaattctcggacttcttttctactttttttcccttgcatagatt
catatacttatttttctcttttcgatcctccatttgtttgtttacaaatggacattgctacctctgtgaccggtaacgtatttaccacgttgctttatgctc
cgagcgtatggaccaatgaaaaggctcgttattctcattcttctcctttttttccctttttttctatggttccattcactaatttttcctttttactttacgctt
tttctccgtaatcgctttagcgtacacattccaagacgatatttaatttacagaaatggaacatccaacgcaaatgtctggaaaaatttcgcatgttt
tctttttttctcttcagcaagcatcaagacaaaggaaaaccacgaattttggggttcttttctggggtgaacgggctcttgttgcattttttttggtagct
tctttcatttgcggggttttggttgttttgacaagaaaactcttttttctgtgcctttataacaatttctatatggtagaaatttcttgtatttgaattgga
aacggtttgcatgtttccctgcctatttcttgttacattcgacaaaacttccaattgatttatctaattccctctatttgttagggtctacagcaataatt
ggttgctttttgcacaacttcatctgtattatttaaaatcaatttcattctacttattacggattttggatcagtgtttttttaggcttttgttcataagagt
ttgcatcggttgttcttttgttttataaaatatttggcattttttttttgcatcctttctattttgtttagctttatagtatgtcgttagcaatgttgaatggt
tcaatgaattttgctgtaaagcattcgcttgacgatctcttgggcaaccctgttcgctttcgagctgcttctgtcagtactggctctacacctgaagga
ccatatgttcctttgaatgcacccaaggacaaaaaggtgaatgatttacaaaattctaaggattctgaaaagagtgcatcgaacaaggccacttc
agtcagtgttccttccgctgatcgtaaaaaaccttctggtacccgaggcgaaggtgcggatgactttgtcgacgaagatccatctttcattccccct
gcccgtattctatttccagaagaaaaaaatttctttggattggcaatcggttctccaaaatgctcctggtcttgtgaaccttggcaacacttgttttatg
aactctgtcttacaattaatgacccatacacctcctttggtacattatttactttctgcccaacattccatgtcctgccggttaaatgcatgtgtcttgt
gtaaaatggagcagcatgttgctcgcgcctttcccaacaagggcgcaaagcgtgcttctgcatttaagccatcaggcattcagggtatgctaaaa
gtgattgcaagtcatttccgtccttaccgccaagaagacgcccacgaatttttgcgctatttagtagatgcttggcaaaaatcttgcctacaaaattt
caaaaatctcgatcaccctagccgagaaacttccgtggtccatcgcatctttggtggttaccttcgccagcaaattctttgttctgtttgcaaaaagc
cttccaatacttaccaagcattgctagatttgtctgtggaagcaaaaggtgcttcggtgttggattgcttaaaacactttgtccaagctgaaaaatt
aatcaagcaaaatcgctatcgttgtagccactgcaagaagttagtagacgcaactaaaaccatgactgtttatcgcgctcctaacattcttaccat
tcacttcaagcgttttaccttcaacggatacaattctaccaaaattggtaagcacatttcctatcctgagcgctttgatcttggtcccttcatgtcgga
ctcttcacatccttgtcgatatgagctgattggggtcctcgtccatgcaggtggaagtactcgaagtggtcattactactctttctgcaagagtagta
acggtatgtggcttaagtttgatgatgaatttgtttcaaactctagcatcgatcgcgtcctgaatcagcaagcctatatcttgcaatacagacgcatt
cctggatcttcttcttccagtaaaagaaaagctagcgatacgagtaccactgcaccgtccaacgggcagaaaaagaaaaagaaaaagaatgta
tctttttaaattaacaacaaagtcatttaattcgattccgttctttttttcattcgttaattaatatttaatcccttttttttctaatatgtatgtcgttttgttt
gtcttcaacatcaatctctttcaatgtattctttccttcatatagcatttagcatcatgagcaagtctttcagtttcaatcgtttagcatcatgatgtgc
gtataaaaatttaggtactagtaacctcgcttacaattaatatattcattcatcattttgtaagcttttgcgtccaatattcgggtttgcgaacccatt
ccaatgctcgaataatcacaaaaaaaaagactaagaaaaacaagaattactaattctttttttttaacttctctactgtaaactcctgctctaatacta
tccattttgtcagttacttcggtgaatactacgtagagcatctgcgaaccagcgacataaccagttgctcaacatcgtttttaaagttgtttcagtac
gtgaggcaaatatatgtttagtgattgtaagttaatttttagaaatataagactaatcaacagatgcttcgcgttttcttgcacaatccagattttctt
cagccggtcaacaacattccggttttcgatcatcgaattcacagctagatataaccaagccttcactaccaaggtcaattgagaatgagcaggatt
ttctaaggacaaatgaagcatcttctgctggaacgagtggattgcattctctcttgaactcggactctataaactggcaattgagagaagaagagc
agtctgatggctctgagatcctttcaaatgatgattctagagatcagagattcatgcatatgtcgaatggcatcgaggaatcatctatacaacccg
aaagcgagtccaatttgtcccctagtggtaacgacgaatttatggaattgcctaatgagcgatatctggcaactgagagaaaaataccaaataaa
aaatggggcatcttattcggttgtctctgccttgtgatgttcacctacagtatacttactatatggcgaacgaatcctgagtctcctccgcagacatc
accatatgccactattcaaagagcctttccgttgtttcaaaaggatgccgtcgtttgcatgtttttggctgtcgcatggctcttttgcctagtcgcaat
cccccagttccttgttttccttttagccgctgcacctatttccatgtttgctttttctatttatttatttaacgcttcgagaattcaattagaaaactccat

tcaatccagtgttatgcgaatagtcggtttgattcttcttttattcactatgatttgttcttcctatatttggcggcattggagccgatttgaaacctctt
ttaatattgttaggcttgcatgccgtgtaatttctgacattccccaaatagttttaatattcataagtttcttagtttccttttacatattgatttttatat
gggtccgtctgtttgctcggctgttcttgcgtggttcttcatttgtaggttcagtctgggttttaccaaaaagttcatggatattagcttcgttctatgct
ttccattttctttggctctgtgtgtttctaaactcgctacaatggtaagcataaaagatttcaaccagaaaaagctaacatatatatgaagttctattt
tatcgtcaatcgtctcacaatggtttttttatcgaaatacaagaatgaccgcaaccaaaaccaatttggtttctcatttttttctacgacgtcctctttta
tcaatacgggttgtgctcggtttcttccttttttaaccatctcaactaaaatccctcttcatatctttccaacatggatgaggcatacctctcacctggtt
tattatatgtttacttccatttcggtttcctatgtggcttctcctcttacgcttgcttacgcatcaatatattcagttccatacatgaatgcctcgaaaac
gctaagtcaaatagaacaattgaaccgagtaaatttacgtcgacgttgctattacatttctaaatacaccctacttcttgctcgtactatactcgcca
ttggggttggcataacagcgtggaatttctcggtccactctaatggaatttttttacggatatttggtcggaattcttggtggattcttggcgtggttaa
ttataggagtcattgaaggaggtctgagtatgctgtatgtattatgaatttccaatgttttgcttactgactattgattagggttgatgctttgttaata
tgtactataattgatatttccagttgtcaaggcgatcccaatggttctcactgttatgaagcttggcaacttttcgaatccaacggattttaatacga
cagcatttttattcttttttttgaaaatatttttacccagtttcatcacggtgcttttacttatgtttcatatcggcaaagtttagtttcgtttcttttggtcc
gctgaagaaatactttaatcgcctattttatagcttacgttggagagtatttctttaattcttcttttgaagtaatgtattattgtaatgatcatctatat
agttcattttctatccatgcctttatttttcgatgaattctgtaaggttataagccgtttaaagaccgaaaacattcagaacctgcacatctctgtaaa
atataatttggtaaaactattctaaagtcattaataagtatcagcagacagtcaaactacgtttcaggaatcataaaattttagcaccaaattttta
agatgaaacaacgtccaacaaagaaattatgacttctgctcagcattcgcagctgcactttcttcttgttgcaaaaggggtttgatttcttgttgttc
gacggttttgcttgcagcaagagaatccaaaagatcatgtaaagcaatgacagcacgaacgatgctagaaatataaatactcattaactggtcat
tggagttgatattgaaagcacgctgggcttctgattgcaaaacttgttccgaagatacaggtccggagaaaacgttaggtaaaagattaaatacg
ctttgaagctcagctaaaattgcgtggttgattggaagctgaccgttcacaactttcctgaggtagtcagcaatttctgtcaagcgctgaccaagac
cttgcagggattgagcttgctgagtgacacgagtggcgagagtaccaacagagacatctcttgtgtcacgtagtaaatgttcaacaccaatttcct
ctgcctcttcggcttcaatgcttgaaggaaggtgtacaaacgttctgctggattttgagccatcctccttgatttcatcgatagcaaaataggcattc
gttggcaatccaacagatttaggcatgacatcaataattacaagtacaggattgggaatgtactttttgagtaaattattaatctccaagtcagatg
gacgtaattgaggacccgtgtggtaccaaccgatcagtttttcgctggcgttgattttttttaaacatttcattcatcgactccataaaattgtgatcca
aaaaccatactgacgagttcttttcatcttcttcgaatgggatcgcgtaactattggcaacgttgacaactgtgccattattttgtcctaaaagaatg
ccaacaactctgcgtttcgatccttgtgcagaacgaatgtaagaatctactgcagaaagcaagaccaatgggtgaacaatctaatttccatgttac
tacaacatcttcatttccttctacttacaacatgctgaggaacaaccgtagttccttcggaaatcacagaaggcgaaggtggcattgtcctttacgt
tattataaattcgcagtttatactttttcaaaggtgttggtaaatgaagagtttttttgtaaactggtgcttctttacataaagaacagtgccattttac
gtatgcgaacgtatcggtaattattacactgtagtctgtaaaactggagtgcaacatagcgagtaggaaattgatatcttgaagggtatacacaa
acgaaaagtagtttcttttccttaccaaagcctttccttaatcatgacgaacatggccatttctcatccaaagtagctcaaattgaaaaaattatata
gaattcagctatagattctcttaaattcgcgatcaattacgtggaattttgactgaatggatggttgacgcgtggacgtgacgaaaagactcgattc
gcgctcatattcctttttatttcattttttttaaattattagatttcattcattgcttgaatgcttatgaattctttctaaattgcacgaacacgaaacgc
ataatgttaccttgatgaattgcacaaggtaacagcagtaaagaaataaataaaacaagaaagacgtgaatgggtactgaaaaacaaatttca
aggtggttttgaaatatcaaatctttttcttcttgttttatttattattttataataaaaagaggatttgaattcattctgatttgaaaggacggaaaat
agaaacttactgtgcgtgacaaacttttcggttaaatttttgatttttgatttttttctattttaatactgttttggcaatagtggaggtgaaatccggct
attttgattatctttggttcaaatcaaaaaaaaaaaatttcttaagcgatcaccgttatctcttttatttccaacgatcttttaacatatacatacaggt
attacttgaacttatcttttccccaaactgcatcagcacaattgctgcatctattaatacatattttactcaaaaatggcacccgttcaaggaaaaa
atacagaatctcaaattgctcgtgtagttggctctgcttcagctggtattctcgagctctctcttttccatcccgtggacacaatctctaagcgtttga
tgtctaatcatggacgcattacttctatgaatcagctgaatactgttcttttcaaggatgctgctaatgcccctttgttgaagaagcttggatctctttt
ccctggtttgggctatgccgcctgttacaaagtcgtccaacgtatctacaagtacagtggtcaacctattgtaagtttttttttctcgtcaaattcgt
ataatttttgtttcgcgctcagcaatgtttgctaacaatttctttgtaggttaaagacttcttggctactaactatggcaacaaatttgatcaaactttc
ggaaaaggctccggaaaagccatcatgcacgcaaccgctggtagtattgtaggtattggtgaaattttcctccttcctttggacgttttgaaaatca
agcgtcaaaccaatcctgaagctttcaaaggacgtggagttttccgcattttagctgatgaaaagttcgctttataccgtggatggggctggacag
ctgctcgtaacgctcctggttctttcgctttatttggaggtaatgcttttgctaaagagtatatatttaagcttaaggactattctcatgctactttcgtt
caaaacttcttcacttcaattgctggtgcttctgccagtttgattgtttccgctcccttggacgtcatcaagacccgtatccaaaacaagagcttcga
caatcctgcttctggctttacaatcttaaagaacatgttgaagtacgaagggcccacttctttcttcaagggtcttactcccaaacttttgaccactg
gccctaaattggtcttttcattcaccatggcgcaaactttgattcctttctttgacaaagcttttaagtaatccgaaaaatctcatcagcaccaaaaa
aaaaatcctctttctttatcttccctacccttcacttccccttcaaactctccccttttttgtttcctttgtgacaaatacaattcttttttttgtcaaagaat
aatcgagaaatccatttttttttctgaaaaaaaaattcgttgataatattttttactactctaatagatggaacttacattttttctggcaatctataattt

actttattttgttttgctactttgtttgggtagatttatgtttgtttgttattgtttactctcgtcatcgctggtaaacactatttgtttacttttcccagcca
tttgctttctgtaaaaagcacaacatatttcctttctgtttcctttcccgatttagcatataccttgaaatctttgtttcttttgtagattgataaaaacc
atgcaaaccattgaacttggaatgccttcaacttctaacgcatcttctgtttccagcagagtaaatccatcaaaggtaactgcaaactttgatgaca
gtgcaattgaagaacaagatccttactttacggtgactagaaaacttcatgcactggccgatgttgttgacgctcagcaaagggagattgaaaac
caaaagaaggacatccattcgaagacgaaatgtattgaagaactaaagcttcttttagggaatacgaaaactcctaccgaaatagaacttgtatt
aaacaactatgcttccgtaagcccttctttgccgacgccatctgtaccttccgcttctgtttcagataatggtctacaggctccaaagttaatgcatc
ctaactccgtaggctctggtggtatgggtagtctctattcatttcccaacggggcttcgtcggctttgagtgatgtttctgcgtccctcaattttcctcg
aaaacctgaattgccaagcaggaagtcaacttcctctgcttcattttcacttcctgtgaaaggattaagtaatgagaacttttcgtctgcttcatcga
catctgatacccttgagtcatctaaagctatagacaggaaagctaaattaacatctaccccttgggatttatgtaataacaatcattctgatctttct
ttggccaatatacaactagatggatctaaatcaaccattgctaacaactctttatcaaactcatccaagcacggtatgcaaaagtctgtattttccg
caggcacggatgtatccggtgctgatcaaactgcgtatatgcgtgatttatggcctaatgaaacaaatgcaagtatggcctctgcaaccttaggat
caaaccttgtatctgccttaaaagctccttcattaagctcctctagaagctcttcttacaccaaagggccatctagctcgatctgggacccatctagc
aatgtatctctgtcaaatttaaccgattgtccaaatccccgcgaaaatgccatccgttatagaaagctctccgaaaggaacgctgtttacgattgg
aatataattgttaacaagattatattaagcaatgatcagcaatcctctatttttcttcaacaaaaactgaaggtggcttctcctgagattaagcaat
caataataaactgcatattaattcaggcttttccattgatgctgaatcgttttggtaatttcttaatccagcgttgcttcgaacatggaactcctcatc
aaatccgaattattggatctgctattctggggaatatgtacaaattagctaccgatcctttttggatgtcatgtcgttcaaaaagcatttgacaatatt
gacgaagatattaaatattccatgatggaagaactgtttgtaaccattgatgagacgataatgcatcattatgcttgtcatgtttggcaaaagctct
ttgaaacacgttggtttaattatcctgtgaatgttatggaccgtgtgaatatagctttgaaaggaaaatggcatgaagtagcattaggagaaaatg
gaagcttggtcgttcaaaacatgtttgaaaattgtgttgaaaaggataagcacgattgcgttgaagaagttatccttcatctggacttgattgtacg
gggacaatggggtaattgggtaattcaacacatggttgaaaattcccaaggcgatgttttacgacgcgtagtcgctgctttgcttctacgagctac
ggagtatagtgtagatcaatatgcttccaaagtaatggaaaaggcaattaaatttggtccaagggattttatttctctgtatctaaaacaaatcac
gaatgctagaatggaccgtacaaggcaaccattaattgatattgcatcagaccaatatggcaattatttaatccaacaaataattcagcttgctca
gcccagtgatcgctcgactgtggttacccatataaaaaagcatatggtatctcttcgcggaagcaagtacggtcaaaaggttgcctttctagctga
gaagtggaaaacccagaatccgcagtttgtttcgaacgttgactttgattgagctcctaatctctgaaatataaatatcgagatataatgtttctaa
attctttttacattctaccatataagtcgattagattatggcttttctattgtagtataaattttatcgttacaggattactattataaagcatagaaag
agaaaatagaacaaaccatacccgtattatgcgtaggcaggataaaacagcagagaaagaaaacgcgcgtgttcatgaattcattatttagttt
cccgagcattggcagccaaaacattcatgtaattgttcatgagcaaaaatatcacaatcaatgcaaaaatgctgcttacaggaaggacactcaa
atcgcattgaagatgccatttgttcctgattagacatttctgaagctttaggaaatggaacctggcaagcaaaacaatgtgatgaagtaggttttg
cagaccaaggtaagtctatccaatttttagaggaaataaatgatgataggatctagcaagatgagtggataaaatcaaaactaatttacaactg
ggacattctatgggtaatgtgcaaacttttgctttgcaacgaggacaatgaaagccgccgcgggagggtaatgagtgacaggtacataaacttgg
ggattgctcaactacctttgaaggaaaccccatcattactaatgaggagtcagcggtctttgaagaatccgttgcagggggggattgtgctctccaa
gagtaattcatgaaaatgttgctcacttaaaacaacaccatacgcattcgtagaagcactattagttcgatcgcaaatttcacggcaaatagcaac
ctcagcagcaagaccaacaatccgaacacggatttgatcagcaacaagggcgtcaattgttcgaaaaatatcaccaggatcggaagaaaagat
cgaaccaaaaataattaacacctctctcgtcccatgagaagcaatgtgagacaaggaagctctggccatctccaaagcattttgaagggaaaag
tttccattacaatcgcgaagtgactttatttttgaatgtgagattgaggattgccatgcaaatcagatatacgttgagcaataccgtctttcacacc
aattattgacagctgggaaataggattctgctcaaaatactccatgacaaactcaactgcatatttaatttgtagttcaaaccgtttatgatgaaaa
tctctttcttccattgcggagcttaagtctaaaaccaaaaccatgtgacgaatgattccccttcgtaaaggtgttgtgtcacgaagtaatctaaacg
agaggaggttaatatgaaattcctttctctgaacttaccgttttctttttcctgcattaattaatcctgcaatgacaccaattaaacttccttctgcatc
ctcctgaacaatatcccatgagcgctgatactctccttcccaagtgtatccttcattgtcatcatgtttgcgacctttattgctcacccttccattttgct
gttcatcctcgctttcagacttgtcagaatcataattctttttcactttactcatcgagaaaaccctgatataagcatcaacttttagcaaaaagatc
aataacgtttccaaacgaacaaagattatgaaatttaaagctctagtaattcagtttctatgaatattctaactttcttatttgaagtctatttcaatc
aggggaaaaaaaggactggagcacagactcagttcctttatgtttctacaataaactttttcaaataatcaattttttttgttttctggtcccgccaga
gggtttgaacaaacagcctgcattttaccttgcatttgagacataggacgaagatcgtttaacacatcgtaccaatgcactgtaatcttacgtagcg
aaacaaattagttctccttatattaaatggagaagcccatggttgattcagtgattattcttaataaacaaaattagtctgaggtgatgaaaattat
caaagcaattacttaacaaagccttctattttacttcgttcatagagttcagttagtttaacaaaaatcccattgaatattatcccaaaacatatgta
atttttcccgtatagatatccttctacactttaatcactgtctgaatccaaattgatgcttggcaaagattcattgtcttcatcagcaaggccattgtc
attagaagcattatcctcagaagtgattgttacttcttcatgctgctgagcatcctgtactccggtactttttggtcaacagtgttggaacgatgagt
agacggtgaatcatctttacttacaacatccatcatctcatcagaaaggtcttgtttctgagtggaatgcttcgtcgtggttattatcacaggtttttc

tccaacagtgttagcttgcgcgttgactgatgaatctacttctttattatcagtaatcgcatttatagtttgtttagcttctgcattttgatcagatgag
acagtctcctccatggtttgattcgattcatagacattctgtttgttttcgtttccttttttgctcattattcttcaaaacggccgactcaatatcaattga
ttctttataagtctcattaacggtcctttcagaattcagatgtgccgagtacccttgataagaagactgctctttgtgctctaaaagatcttgggtccc
taaatcaccttttctgccttcatcacttccaccaacaatgttggaagcgaagctggtagaacccagtggctgtaaacctggagaagttcgagaaat
agcagatataggctcgaatcgggactcaaaagcgctttctctttcatcgtatactggaagccgtttctgaagaggtggaaatctggggtgcaataa
cacatcaaaagtctttatgtactgtcctgcatgagaagaaacaattggcgatgcatgcattaatcctaagggacctgtaagaacttgtaaagcatg
tggtaaaattattgcttggcaatcaccaggtgctagaatggacgcaattaacaaatcgtgtaaatgagtaaggacatccggtgttgcttccgtgag
ggccaactggatgacgatacgatcaattttagacctaagcattctaggtaggctgatggtattttttaaaacagaactcaaaaagaaaaagcaat
ggcttagacatttatcattcatagaaagaccagaagagactgacgagatcaaagaatcacctgtttggctgcccgctaatgttttgcgtttcttaga
agcgcgcatttggtcagcagtaatctcaacagcttcattggaagcacgattctgggactcgaaaatccgttctaagaccaaagttacaacattttc
gtactttggaagaggaatatttgcatgcatcttctccaaaattccagaaagaagttgaagagacgtgcaaaaaacgccatcatgcgtcgcttccg
aaagcagacaatcatttatagaatctaaaatgggtgataagaccagcataactgagctagggcaaacagcaagcaattttcgggtcaactcatt
cgcaagcgtatgcaagtgtggtaaaaccatgaataacaatgcttggacggaactttcaattgtagatttcactgaggaagatagctggacactgt
atatacgtcgaataaggtctaataagtgacgaattggtatagaaaccagcttttccgttttcgtagataaaaaggacatgagtatcaaatgcaaat
ttctacatctttgcaaagccatctgataagattcctcataatggcctttaatctttggaaaacttaattcctcatctcttgcagcatccgtatacgtttg
ttcctctataaccaaatggaaaaggaaattcaatgcgacgtggtactcctgtattgtacttatacattccttttccaaccgtccgaaagatttccttt
tcccagacatcggtacaaagaggcatacaaaacagcagccttctttttgaggggtgaatcgacgatttcgtgctcttctaatacttgaacacaaag
attctttacagaggcgtaaaaaggacggaatgtaggggcgtgatgaagtaaaatttgatagaaacatgcaaaaacagtttccaagcatttacca
gattcgcttactcgaagacaagcggttataaatgaagatagattaggagttacaagctcccgagtgattcctggcttacccgccaaagaagtgaa
gatcctagagacagtactgattgcactttccaaagttttagggagttcaggtcgatttaacaactgaatcaacaacttgacccacgtagcaccatg
ttcaatgagacaatcccaactttgagacaccgtgcaatctatcaaaataatggcagaccagcgaactcgaaagtctttgttatgcaacatacgag
taagactagtgcaccaatttttacgaaccataagagcattttcagttgaaccaagctttttaatacatttttgcttcaaaaggatatcggcaatgag
gggtatctgtaatggtaaagtttttttcgtttgggaaactggttgacaaccaagattttaccaaagaaagcgatcctccctccaaaagatccgtcatt
gagttttgcatagagtcctgttaatgataagtgtatacactgctttctcctcttttcttatgcccttttcagagttcgaaaagttaggaggtggtaagt
gtagaggtgtacataaataagagtgaagcgttgaattatgaacgtatagaaagagagaaaatgtgagattactgattaaatggaggataatata
tgaatgatagtgtaatttttatacaaatactaacgtgtaaactaaggaaaacaggtttgtacgcatactttcaaatacattattatcgaattaaatc
cgaaattcttactctttttttgctaatactcttctaaaagcgaaaagcgattgaaaagttttaaatgaaggactctaaacaagaaaagagtaggtat
tttgagggtaaataatacatgaaacacgatggcagatgccagcatattttcttctagatacgaacgatggttcttttttttatagtatgtataaagac
agagggggtaatagtttgttcataacgggtaaaaggtgaacatcgacaagtcctttctccgtgatcattttccatgataggcaaaaaagggacttt
cttataatacctgaaaggtgcatctatttttctaacttgccttgtattgttttccagaaagcttcacgttcagtcatgagatcttgcaaccaacctttca
cctgagaatgcttaatcaactgacttgcccaagaaaattgactaaggtcctttgttccctgatatttcttttcatactcaaacatctgagcaagcatt
tcaaacttgtcgatatccttaacaaactttgcttcagaagtcgaggcatgctcatactccaaaaatagttctcgaatttcttgggcattcatcgccaa
gtccaatggcaacatttcctgggtaattttattcatggcttccatttctagacgatgcttctcttccttagacacattttcatggggtgtaatatcaccg
acaatagactcagctaaatcatggacgatggcaatcttaacacaacgatctttattgattcctggatcattgcaaagtagtgttaggacgcccata
cggtacatgtggtcagcaatggattcaggagcctcaataccatggtatagccaaccggttcttggagtagtttttaatttagaaattgtttctaaga
aaggaacgattgattgagaaacactcatgttcatatattgagaaatgggcaacgaaaaaaatttcaaataagaatcaagcaagtgaataagcag
ccaaaagacttcgtctttaaagaaaaaaacaaaaccaaaaaactaataagcaaaggattaaatgaattcaaagcccctttccgaaaaattgaa
accccaacgagtatatcgtagaaacggtgttggttgctcttggcaaaatgatatttatcaatcttgtgttcatgctgatacattttctgaatgaatgt
caaatcagttttatcaatcttttgactgaaaaagaaaacttcaatgaacaaccgtttaatagctagaggccaaattgtagtcattcagatttatcgg
ttcgtttatccattcaatcagccgagtgaaggagtttaatgcggaaatagtctggcatttaattgacaaaaggagtacgaaaatttagtaattcag
cagcgtatacagctcaattactatgatgacctcgaacggcttccttgaggcattcgtcttggaaggaaagctcctctataacatttacaccagaatt
ctttaacagatctactccttcacacttcacgaaaatatctggttctctagagccaagcacaactgttgatacattttgactgataatttgatcggtac
aggatttttttccctgaaagacgcttactacagggttccatagtactataaatcactgttcctttcaaagaagatccgcgaggttgttgcgacaagta
tttcatgatggcgcattcttccgcatgggtattgccaggcaactcacgtgaatatccggttgagactatttcaccgtgttgaacaaggactgatcct
acgcaaaaagccgaatcggtaggctcgcatttctttgcttcttctaaagcggcatacatcaaggctttatgattatagggtttcaacaagaaaaga
gccatattctctggaaatccctcctgttcgtagttaaaaagaaaaacctggtaatagggttgaatttttagtaaaaaatcggtgagtgtttcagggt
cccttaattcttgacctacgagacaagtgaggtcaggaaagaagcgcgcaaatgaggccatagaagatacaaggtgagtattcaaatttgggtt
gtatacgcagtcaaacaagagcaaaatgtctaaagaggcatctttccactcttttttcagaagggggggtttgtccagtccagttcggatacctctac

aacattagctccattctggtctagattttctcgaagcccttttaagtaatctttctgatccgtcgcaatatacttgccaacccgtggacctagaagag
ttcctgctatcccacttataccggatccaagctctaagacacaaacgtccgatgaaagtgtgttagcaaaccaaggttgttttatcaaccagggaa
caacgtttatagatgttttccagactacagaaccagtagaaccagggagttggtgcaacgattgacggttttgtttaatttgaagcttgtactcatc
aagttctatattcactctgtccattttagagtcaaacattcctaaatctgcatctttcttcttatggacagcgaataaaacttgcacatcttcttcaact
tcttcaattggggcatacaagtttaaaggaacctccatcctttctttttgcggggatacgaacttacagaagtagttaattttaataaagatttagca
aaataagatgtttttgtttaacggaaccgatcttagtagacttgcaaaatgcgtctgactcgtttccaaattcttaaacgaaattcttcctaagctaa
aattatcgaatcacaaaatgataaaagaaataaatggcgcttttaataatgctttgtatggattagagggtggaagtagtcgaacttgtcaaatat
gtctaatactatatcatcttgtctggtcgagaacgcacccactacacaaacctcggggtctctacataaaatcggatttttctaaaaaatgagcctt
tccagtagactttcgagtcttcaattgaatggaaatgatgatttgaagccggcgtatggttcgatgaatgcaagtccaattgaaaaaccaagtatg
gatttctataaatatgattaggtaaaatggggggctaatcattgtgcaatgtagattcttttggaaatttaggtccatctagttcgcaatcgtccagt
acaattgctgactatgctgcaaaggggttgctttcttcctctactcacaatgctaatgattcccatactgtacaccgaaataatggtattgtagatgt
aaacagtcataaaaacaagcttttaggggtcattactgggaagtcgggcccaaactcaccttcttttactgataccacggaagcaaaaggtgcaa
aaatatccgagaatctggattccaccgctcccgcagatgagtctgcaaattcatattcccttgaatctcttctgcccattattcgtatggaagatacc
gatttgtgcatgttacaactgggttgcgatttatctgccttaggctttgatctggcacctgtagagtaagtcctttagtaatggttttagctttcaagtg
ttctgactctttttctttagagaggatcggttaatttctactaacatgtcttctccttgggctgagcttaacaccaaaaaaccagtttccgaacctcc
attcaagcttccagaatgctacaaaagcgttaatcctcccctcagctctctaaaattattcaatttgccgatgaaactttgttttacatcttttacac
aaaacctcaagacatattgcaagaggccgctgctcaagaactgtatgtacaattgttacaacccattgtctaactctctagtacaaatcgcaattg
gcgctttcataaggaacttagggtttggttaacaccagtccctggtacgaaaccacttcaacgtacgccccaattcgaacgtggttattatatgatt
tttgatcctgttcattggaagagaattaggaaagactttttgcttatgtatgctgctcttgaagatcgttctcaaaatgctattccaacatgaagaga
aaagtgtaaggttcgtttgctttcgagcagcttcaatgtcttgtacaaaaggatttatttgggtttggtaatcattatgatggatgattagttttaata
tcgcatccgaagtattcatgaataaaaaattttgatgttttatacagagtatttagaatttaatagcctcaacgatacatcaacaatatgattatgtt
attctgtatctttaattcttcttagcagaaacaatgaatcgttatttatctatctaatttttttgtaaaaaaaaattaccaccatgcacatattttataac
caaaacaaattcatataaccttcatcgacattaaacatccacagaaaagccatcacgactttccagctattaactcaaagtgtagattttgtgtttc
tgactggttactttgagtcttttgagggactgtcgctaccatcttcatggtgcaaacgacggaacgcccgttttaggaagcctcctactgagccgtc
cgatttagaatcaccagatgaagtgtcggaagatttatctttagttggtgaatcagaagatgcagaggattgatcctcggttgaagaggggggag
tagttctacgtgctgtagtatcattaagagaatcagctagctgttccaacaagcttcgttcgtgaggtgaaagtgcctttggaacgctgacctcaaa
atcgacgtaaaagtttccataacgagcagtagccgaaacgttgcgaatacctttaccagtcatggtaatgcgatcaccggaaactgtaccaggag
gtacacgaaggtccacttcaccagccaaagtaggaatgcgaattgtgcctcccaaagcagcggtagtcatggggaccttggctgtatataagac
attgtttccttggcgcttgaaaaattgatgcttgcgtacacgaatagtggcatagagatctccaggacgggatttagcatctggggcagccttggc
ggtagcagcatcgtttccagcacctacaactcgtaatacagtactgtcatcaatgccagcaggaatatccaatgaaatagtcttacgttcacggac
agcgccagagccgctgcaagaacgacaagcagatgaagaagggataatggtaccacttccaccacaaacatcacaagtggattgcatgtgcat
ggagggtgggatgaaatgaagacggtgaccgcttcccttacaggcaaagcaggtactgcgatgggcgccagtttgaagaccggaaccgtgaca
actagaacaggttgaagagactaagtagatgacatccttcttgacaccatttacagcttccatgaagtcgatgttaagacttacttctgcatcttca
ccaacgtatacctcaaaagggctggcacgacggcctcgtcttgctcctccagtaaaagacgaaaataagtcttcgaaattaaaaccagggaagc
cagacccgaaagctgattcaaacccattttgaaatcctccaaaatcattacctgtaaattcgccatttttgaatgcaccagagccatacgtatcaa
aagcctttttcttttctggatcttgtaaaagctcataagcttgcttaatatctacaaacttttcctgggcgcttttatctgggtttgcatccggatgatat
ttttttgctaacttgtagtaggcagacttaatctctcgcggagaagcagtcttgggaacgcctagtgtcttgtaaggatccgtcatttcacgagcgac
attggaagcatgaaagaaacgctgaaaacgctggggtcttggtgcattctcattctgtaaaagatggctagccttggaattgagagcatgagaaa
caaaagcacgtcgaattggttgataagagatatttttgcatgctttcgatatgcgatggcaggaatgtcgggaagtaagagaatgcaaagcaga
aagatttgaccgtacactaaaagggatcattctgaagaaagaaaataacgttcgcagtctttttttagatcgtgatgcacgaagttgttatttgatt
aaatcttcgaatcaagcaatttgagacggacttttaagggcaaatcgagattataaattaatacaaaaagccgaaagaatccaacaatctgactt
tctcctaaaactcttgatataggtgaataaagccaaggggggtatgagaaatcaccaaattatcaaaaaaatacttcaatgacaatttaaaactaa
tattagttcaaactcttgacaggaaaaattcaaaatactactttttgaagaagaagagaggatggttaaggaaaacggattgtcatggaaatat
gcagctaagaaagcggtgtagaagctaacagaacttcaccagagcccaaccccaagaagaagcaaaaagccacaaaacgaaaaataaatat
agtttataaggataattaataatttcttcttactcttctaaaaattaacattagatttactgaattaacgcgtaattggactagaccggttctttcaat
tgattgacatttcaagcccaaaatgcaaactgagcaacttgcaggatgtgcagtgctaataactgttgccgtttttacgattcgatggatttttctg
cagacaaatcttctcacacaaatgttcgtaaccttggtgcatcagaatccgcccatccagtaaacagtgaacaagtgaatatgattaaaacagttt
ttcctaatattgagtctgttgccatcgcttacgatttgcagaagactggcagtgccgatgctaccatcgaaaatgcgatctcttcgcaaaatttacct

ctacctccgcgaggatccgctttgtacactcgtttccctctgtcacctggatcagatttgagtactcatacctcagccgctgacagtgcaaatcgtaa
ccaagttgcccagactgaggaaggtcgaaatgttcaagggaatgcatcaacatcaggtgcctctaaaagccttattgataagtatggtctttcttc
tcggctaaatgactcagatatgtccacttcgtcttcgtcatccaataatgtagctgtttctgttcaccgtcaaagatttcccaattcaaagaaagaaa
gggaagagcttttccgtcggcgcaaagaagaaatgataatagctgctcgtaaacgaatggaagaaaaaatgatggtagataatgattcttaaat
gattgtttaaagaagaaaactattagttgtgtttcattatttgaaaacggctgatgaacacggtcatttccttttccatctgatattgaggagcttcta
aacgttaccgaagggaagcctttcctcttttcccttttttttgttggcgcatgtcttcgtttaattttcttgtatttaatactagctgacaaaataagcat
tctgatcaatttcaatgatcttgttggagcgtacgcggttttatgaaagttttcctcaaaaatatgcactataatttttattatttgcttaaagttatca
tgtctatatatatactatttttttttttttattgtacgataattatttactaacgcacggttttaccgccatacttctcaccagtgggtttggactaggaat
gacgaaaatgactaaatttactatacaattcgtttgttcttgaaaagctatctttactcaagatgtctgaaaaaagcacaatagtgggttcggaaa
atgatactcttgtgggtaagtaagctgttctggaaattttttggagcgcaatctgctgaatttaaattatgctttgcagagaaagcttggtgattat
gattaaggcctttttactggtttcatatattcttttgcaacaatattgttaaaagagaaagtctttgaaggttaaattccagcaatcagacgaacgtg
cattgttgtataacatcaaaggaaacaaactcaacgatgtcttttcattatgaaaagtttattttaaactgtattggaacagaaatagttttctatag
aaattggtatattagaccgtcgttttccaatacttttcctatatttgtgtcctagcgtaatataacaatagcgggttacatataataattcttacataa
ccgtatataccccaggtttcatgatttttttatcgcttccaattcctatactccaaatttgatgactgagaaataagtaaatactttgttatttcgtgatt
ttagatttcgttatttaagtgaaaatcattatctaactgagaagggtttgtggtattttgaaatccgcaagtttcgttgacctaaaaagaaaagcca
ccgttttagcattgttgaccatttttctttttatctcgatttgaacgttaaacgttcgttatagggtagcttgaaagcttctgactatttgtgtgctttgct
tgaattgaccgttattgtttgtttacgttcaggggaagtttcgattagcttatcgctgtattggcaaacaatcagtttttttcttttagattcaagttata
acatagtctctcctcctttgtaggctctagaaagaagattttgagccttcttggatttgtagaaacaaaaattggaaattcatgggacaagcaaatt
ctcgtccgcaactgccagagaatcatgaccattactcaaccagaaattacaatttcttgcatagattcaaatcattttggaaacggtacgtttcacg
agatgacacctcttcgttaagcaaccaaacccggtctactgggaaccaaggctctccacaattacccagtctccatcgtacttcaagtttatcaatt
ttagaacaaagatcacttcatacagatggctcgtctgttcctgacgagcctgtcaaatcctcctctcagcagataccgagaaacagtattcctata
gggaattcatctttaactccgcaaaatacagaatcaagtatggctgaccttttacgcattatgccttcaaggccgcctgacaattctataaaccag
aggttggctacaggcagtgatccccaattacattgctccagcccttcaataagaaatgatcataatcacgttataacttccccttttcatgaattca
atggatccaattcatccatatttgacactgctaatgaaaccgcgagtgatgaagcgactttccctacatccgatacctcgattcctgtacatgatca
ttcacaagcttctgctcaaaatttctttctaacaataatacacacaatcaacgttccacaagtcgatcgccacttattgatcatttacgacaatattt
gacacaggatatcaatcaacaccaaccgcaccgccattccgtttcctttcgtgacggagtctcatcaaatgttggtgatcgctctcattcctctgttt
cttcacgttccgaacctgaaatgaattccaacgtgaacatggataaccgtgcaaattcgggtttgcctcctgaaactggttcggcatttcaaggatc
caatagcgagaatgctaccagtatgcttagtagattactgtcggcagccgccattgaaactgcagcctccattatgaacaacgaagctcatggac
ctgatacaaacgctcattctggtgggagacagtccacagttcgatctgtcgatggatcatttgaacactttctcgatgatcttcgtaatggtaatata
accaatgtcttgcaaaattctaccaattctcggagagatgcagcgacaaacaggaatcctgaaggagacaatccagaggtgcagtatttgagaa
tgtttcggtttccatcttctcgtcagtcaagcggtccgaacaatgaccaaaatgtgcaaggtaatgagcaacccggactttcccttgtacctgtgct
gattgtttgtatgcgtaatatcactgacagttctgaggatcctacgcaaaacatgtctcaagataatcaaagttatcatgaggaatttcgagataat
atttccattaacgatattactgaacgaatggcccaatccactaactcttctaacgaagctatagcctcaagaacgttacctgtggatcgattctcga
atatatccaacacattcgatcattatagctcagcgtcagcgagaagtgaggcttctttacttggatcacctcgccattctaatgtatggccttcttta
catcagcaagttcttcccttccttctgagtttaggggtcacaatgtgctcagaagagcaaacgagcgactcagttcagattttgacacagtacaga
ctcgggaccaatctgatcatcatcaatccacttcagtagcggctgcggacgagactccatcattacatattcggcgaagtcatattggttctggtga
tgatgccgagaatagtgacattgataggttagaggaaaaccagggatggttaagccataacagtggagaagcgttgggttttggaagtgtcgaa
acgtcgacgaatcagctttatcaagattccatttctttggataataaccgacataacacgtctagagggtttggttcagcggatgaaggaaataat
atgggtgcagaacgtgctggggatgaagaaacgtccaatgctaattcaaacggcgcccatggcgactggcttatttacgtttttggaggattgttt
ccagagtaccatccgatattaactacagtaagcttgttttctgacaatcctacatatgaggatttgttggcattgacaagttatctgggtcctgcaaa
gaagcctgttgcaaccgcggaggatttaaatcgtgcaggtgggctatttctgtatctggaaaatgacaatattgtctctaataatacgtgtctgattt
gcctggaaccatttgaacggaatgtattatgcagacgactgaaaggttgcaaacatttttatcataaagattgcatagaccagtggcttactacag
gcaacaattcgtgtcccttatgtcgttctcgaggtgtaaaaaccagcaatgagggagcaaatcaaaatataccggacgtttcttttatttaactatt
gcttagaatgtttaattttcattgattatcatgttgatagttctattacacttattatacttaatgtttttttcttttttttttgttttgtttttatattatactgg
cgtttgtttctattactctaagggcgctcgcattcgtgatagattattcaggtcatcgattataagcgaatgttaatgatttggctatctgaacataga
catagttttgttgtaacacacatcttttatgtactgtgtgtcagtataaggtgcttaaaagagcttaggaagcatttttctggacataaagttgttag
aagtgattgtgctcataccatatccatatgtttggtgctccctgactttattcgtaaaagatgcttcgatcactatttacattgctatttttttttagcta
tagaccggcaattatgaaacttatacaatttctgcaagttacttctgagacatgagatggtcgactttctggatatcatgaattctatactctttttc

aattttccttgtcttccatttggcttgtaacacatgtgggctttatcattccactcaaaacatattgtatatatctgtactcatatccatcgtaatgtca
gtctgttgatgtacagaattagagaacataaaggcatttcgtaccatcctcttgtttacttttttttcataatctacttcttgttaaatatttttaatgc
ggataatcgttttcacctatagttactcctatgatgcaagcggcacaaaaggtttgatttaaatggtatggtgttataatgtgtgaatttagaaatg
aatcaaccgggaaaaaaaagtaatgcatatatgtttggggcactgtacccactataattatagaaactcttgttcacagaaacatcaagataaaa
ataaagacaaactggatgatgtttttgatcgaaaaagttgatagatgaataaagctattacttatgacgatcatagggtacaaacatatgtagac
attgtaatttcgatttcaatcattaggctcttgttttcgagaacggtcattagccctcaaagtgtcgccgctcaggtgtaaaacgggaaaatcgtttt
aacggggagttgctttctctaggaaccgacgcggcaagagaatgacggtttcctctccggtatgtgaaaagaggactaccaggtgttttagggct
gcgaacagagggtgaggttttgtgttccaaaaacgcatcgattggtcgttttggagaataatactcgacagcagcactcactggaccataactag
tgtttgtagcaacgtctcttttcacgacggatagtcgaggctcaacaaactttttgtcatattcatagaaaacttgctgatgaagacaatccctgtcc
ttcatcatatctttgaaccttctagcaatgaaaaaaagttgaagagaaaatataacgcttaaaagaataatcttaggagatagaatgtaaatcca
tagaatcaatagatggataggagaaaatagacaggaaaagtaaagagagaatacgttaggagaccaaacgactaattcaaaacttctttttca
ttttcatttggttctgaatgagcaggccgcaaccctggtgttgtttgaacattcttatcagattgagggagtgttctatatgtcttttttggaaagag
aagcagctgaatatattgatgatactgaaggccacaagacataataaaaagaatgaaagaaatcctctaaaccaagaactcgttgctttggctg
atctggtacctataaacagttgactacgatttaatcgcgtcttggtgttttccaggtatgcacaaataaaaatgaatagtatattagacaccagact
taaaggaatggaccacctctccgataccttatcccattccacactatcaaagtcttcagagatggaaattaaaaagtcaatagggaaattcagga
ttctagattttagagacgagcgacgaattaactttggcattttctcagatattgtgaaagaaagacagttgaatttgtcctcaactttctctttcggtt
aacacaaataaataaattttaaggaaatatttgatggatgattttaggatcgacacaaacttcttttctaaaacagtttacaatcaataaaagatg
gcctatttccactttcaccttaacaagccacaggatttaaagggtaagaataagagctcatttactgttatgtttctactgaattccttcgtttgcttc
aactttgtcaagtccaaaaggtaaattcatgcaaagttgtctaactgtaaaacttggtgtattgtgtgttccttactacaacacaaacacaccagta
cttcacatgaagccttttatttcctctttagtagtctttaaaaattaaaaagaattgtaccaaatactgtttcggttagaaattgactatctcaagcttt
cttaggatatcgcgacttgcttgacattgaattctctctaagtgttttttgtcgaacgcttttagtgatatttttttggattctttttaagtatagcaaat
aaagttaactctttatttattgcatataaaaaaaatcgacagactgaaacgtctttttggttctgtgcttttcacgtttttaagttgcttacgattttaa
cgatttgtctgttcctgtattttgcgtttcatttattctatttcatggtttttggatacaaaactatctgttttgtttatgaggttacttggtatactggaa
accagggttaagataatcaaatgcgtttttagtgattatgcagaacgaagtcaatttcgatagataaataaatataggtccattatgtccggtaca
agaagtactcgaattaggaaaacttcactaaaaagagaattggaggaatcacagaatggcaatagtccaaaggctcctagaaatgaaattgga
tctcggcaaactagagcctcttccagaagacagaatcaagcgcatatgaaggctgatcaatctacctctgatgaagatgaagaaaaaaagaagt
taacaaaacttgaggaagaggaagcaaaggaaaacgagaatgcttttagtttcttatcaacgaatgattcttcttcatcaggtgaagaaagcaat
aaaaactcaccgaaaatttcagaccaagaagatgaatcggatgaggactctgatatatgggagcaagtcgatctcaagccgaaccgtgatgaa
aaaaaggatgaagagaaggatgtccacattagtgcttcaagtgaaactccgtctattaaaacgaacaagaagagatcatctggagcagttgaa
aaggcgatccgtttttctattcatgtcatgcacacatgttgcttattttaccatggatgtcgtcgcaaccagtggagttgccgacgagagcttttgga
atgtttacttgataaaatctcagaggaacgtccttcagtagtagaagattggattaaaagtgaacgaacacatgatgatctgttgagactaataa
acggactgcggtcttggtggcagaagaagtttagcataaccaagcatgggctaagaaagctgggttaccgtgatttcaagcacgggtttgaactt
ggtttcgaacctgagattatcgaaaatgaaaaggtatttattgaaaaaatcctttcattcgaaggaagccgagatttatctgctcaaggatttactg
ctttatgtcgatccttgcagcttaacgtccgtttaatattttcccttcagcctttaacttactccacggctgcttatgataactggtctcatcatgttctc
cctgatgaggctacaagctctattgatggagaccttcgttatcctatattttggacagaagtgtatgacaatgatcaaaagcgttggataagcgtg
gatgccgttgttttaaacggagtgtatacaaatgacatgtcttggtttgagcccaaaggagcttacgctgagtctaagcacctgcgtatgggaatt
gtaattgcgtatgaaaatgatttttatgctaaggatgtgactttacgctatacggatgtgtcatctaatcgttgcaaaaaaattagacaatcttattc
accggagaaaaaggacgatacatacgtgagaatttttttctcagtttacaaaaaagaaaaaaaatgaacaagatttgattgaagatcgtgaactc
gagcaaaaagtgccagtgcgcgagctaaagagtatcgctgattataaaaatcatccggactttatattgaagcggcatcttaaacgtgaagaag
ctcttctggatgatgcaaagcctgttgacagggttgtctttggttcgaagaagaatcctattcaagaagaggtttaccgccgaaaggatgttttgac
atgcaagacacctgaaaattttcataaggaaggccgtgtaataaggcctggagaacagcctcgaaagatggtaaaagcaagagcagtaactat
cacaagaaagcgagagcatgagtctaaagtcatagagtccaaggagccagtcatgcaaggattgtattcttcggatcagactgaattatacattc
cacctccaataagagatggaaagattccgaagaatggttatggaaacatcgactgttttgttaaaagcatgatccctcaaggagccgtacatctc
ccgttccgcggaattgcaaagattgcaaagaagctgaatgttgattacgctgaagcggtaactggttttgagttccgaaagcatcgagccattcct
attacaacaggtattgttgttccagaggaatttgctccgcaagtactggacgaatttcgcgtgtaccagagggaactggtggaaaagcagcttgtg
aaagaacgaaaaactgctattggattgtggaagcgtatgatcaatggactacgcattcgcaaacgaattactgaagattatggctagatcctata
acaaaaattcctacgacgtgatataagttacttaattcttgttcaactagtataattattaaaactattgaattgcactggattcccttcaaccatga
ctaaaataattaaccgctctctatgcaaagttcacaagtggcgttgaatataacactttttttacaaaagttaagtgtaaaggggaaatgagtcatt

gctaaatccggaattatttgaaactatttagtcaataaagtcgaattagaaatgtaattacgagtgtaaatttttgtaagggtagggcaggtttggc
tggggtcgtcaagtttaatatgatacaagctgtcgtttaccagttacactaattaccaaacacaataacgtttagcagacagagaccttttttctaa
tcaataatctagaaatttaccttagttaagtcaaggttatcgaatgcgcatgcaattgatcaatagtcttttatgaggacgagaggaaaaactgaa
agaaatcaggtagacgtaataacgaatagtattttgaacagcttcgcatgtcaaattactcatagtacggaaatctactagcaatagtatcaaga
atgagcgatactgctgaactcgcggggatttccagaattgggaacacggaggatcaagaagctatattatcagatgagtcttcagcatttcagag
ctatgatagttcaaaaatctcatcggagtatgaattagatgatgttttagacttgtataacgataccaccgacgatgctgaagatacagaggacac
aggcccgtatatgctttcgccatcttcaagcatttccagcgggagtagcaaggacttgaattcgtttatatacccaatttaccgacggcaacgtacc
ttggtggaggggaaagaagcagaaagtggaggagaaagcgattttgacacaattgtaccgaacaatcataaagtttactctacttgtgcctacat
caaagacgatgcttctaaaccaactcttccaaagcataatctgcaggataatttgacgctttcatgggaactttcggagaacaacgactcactggc
aaaaaatgaatttgcagctaccaaccatgaaaatgtctcttccaaagcttccaccaagactaattttacgattaaatataatgacgataaaaaag
attcttcctccaagatttatagtgacaaccttccgtttaaaatcaactctattaattgcgatatcaaaagtgatgacggaccactttctcaaacattt
cgctcctttaataaatttaaacaatgtcattcagaatgggatatgtatgggtttaaaaaggatagtcagtttacgtctattgatcagtatgatacttg
gttcgaaggatatagtcaatatttggaccgtcgtgaacagaaatggaaactgttgctttctgaaaatggaattgattactctgcgggcactcctgat
atctttcctagtcgtagtgccaaagtgcagaggtttatacggaaaggtattccttatgatcttcgtggaaatgcttggttttattacagcggtgggta
tgagctttgtcaaagaaatcccaaactatatgaaacattatggagatgttcttgtattcgcaagccttctgattctgacttgattgaacgtgatttat
accgaacatttcctgataacatatattttcgtggtaaaacgaatcagcctcaaagattcaccggggtgtctgaaaaggatcctgacgtcgctatga
ttgctaaacttcgccgtgttctcatgtcgttcgctacctatcttccagagaatggctattgccaaagccttaacttccttgctggtttttttctgttattt
atgagtgaagagaaggcattctggatgcttgttattacttgtcgaaaatatttgccgagaatgcatgatgccaacctcgagggcgcgaatattgat
caaagtgttttaatggcgtccgtacgggaaagtctcccagctgtttggagccgtattaatttaaacttcgatggtatgcctgttaatgatattgttgc
aaagcttccccctatcacccttgttacggctgcttggtttatgagtgcttttgtaggcatattaccgactgaaacagctcttcgcgtatgggattgttt
tttttacgagggaagcaaagtattatttatgaccgctttatgcattttgcgtttaggggaggacgaaataaagtcgaaaaatgaacaaactgatgt
ttttcaagtgattcaagatcttcctaaatctttactggatgccaatgcttttgtatcgttactttttcgaaggaacttccgaaggagtccgtctcagaa
ggacattgaccgcaggcgccagaaaattgcaaagaagcgcaagaaaacggtaactaaggtagaaggtcaggaagctgaaacgtcaaaacca
tctttggctagatcatcttcgcgctttaaaggttcccatttaatttctcaattacagaatcatctgaaaaaatcatcgtaatttgtactttatgttgtga
tgggaatgcttaatatgtcattcgttaaattccctattttcgtccttttctcaatgctctcttttgtttttggttttgctctttaaactcgttctgttgcttat
ttacagattggcacattgcatctatgatatttaacttgtatttatctataatgattctacggacttgctatattttctattcattcgtttattaccaggga
attcaatctggttgtttttatcttcaaattcatcatcttgcaagtttaatatattattacaaattactgaactaaagtttatcgttcccttgacattagtt
ttgattttcactttcacctcaggacaattttattttctatagtattaacagtatatatatatgtattagaaagtaattagaaaaataaaggaaaagta
atgctgtctttacgatatagagaatgattagatgtgggtaaaagaatgtgaataaggcaaacactttttttaaagggaagagctaaagaaaacac
caagaaaacagtaccagattccaattcaacttaataataaatatagattccctaagcccataacttcaggtacaaaagttgaatacttgctaccgt
agtattattcggactcaaataacaaacgagcattaaacaaagaaaagtctgaataacaacgcaagcaacacagacacaatgaagaatattagc
taaaattaatgaaatttggagaagcaacgtaagtgaaacctcgaaaagccgaggaatgactaccatctggaagattgtcagaaaaaataggag
tgactgagataggcatttttgtgaattcatcagaaaaattctcagcagctgctggatctgttatgcaaggaacaatcgggggaggtaactcacgat
tttctaatttcgcccagttgatttttctgtacattcgatgctttttgatagcttcatagtcagcaacggaattattagcaccaagacgttgcttggggtt
tttctttaaatatttgttgatcaaatcacgtgcgtcaggggagacgtagaagggtatatttgcctttgtacgaactattttctccattatttttttatggt
tatttgcagtaaagggtggggagccaacaagcaagtcataaattaaaatacccattgaccaccaatcaacaccatagtcgtaaggttgttcaagc
aaaatttcaggagcacagtattcctccgtcccaacaaaagaatggcaattcgcacctggttctgcaactttggaaagaccaaagtcagttaacat
gacatggccttccgcatctaaaaggcaattttcgggtttcaggtctcggtatactataccaagcttatgcaggtggatcagagccgaggtgagttct
gcagtataataagcaacggcatcctcaggcaacattctccgctcagccaaatgagcaaatagctctcctcctggtgcgtattggagaatcaagta
gagacgatccaaatcttggaaggcataatacaatcggcaaataaatggatgccgtacttcctctaaaatttggcgttcatttttttgtttgctgaagc
ccttttgctcgaagccttatactggctttcttcaactgcttctgggcaaacaagcgacccgtttgttcttgcttcactaaaagaactttaccgtaggat
cctctaccaaggacagcgatgggatggaaatcattggggtgcatttttttgattcggcacataattgttgttacgatctgaaataaaatctccatcttc
gcccaatttatactcgactgtaccttccttttttttcacgtcttttgcagaatcttcctcggaaatactgagctttctaaagctcaatgggtcgatatgg
ccgtccatcccttcaacgacttcatcgtcgctcgacaacccagagtgtgcatcctcgttcaagtagtcttgctcgtcgaataaaaaaacaggcattc
gggataaaaaggttcaaaggcactgtaagggcaaactccctttcaaatagtccttctttgttttttttgtgggtttgttcgaagcagtaaatttttagct
gccaacagatctctagttgtttgtattcattgtgattaactttaagctttcgctaatttacaaaataaagttatctttgttttctttgattgagctgctaa
gtaacaaaaacaaaaacaacccataagtaacctcaacacgtacaaatagaaaaactttttgagtgttgtgttttaccagtctttttacatacagaa
agcaatggctttaacaaagtgtcaaatctcgtatcgtatgtacgtgactttagttagggagcgcgatgttgattgcttcgaatgactcttcgtagaa

atctatgcatctgaagaaatttaaaaatcaaagctttcaacttgtacgtttgagaaacatttcattgccttcaaagatatattgaaggcgcgcagct
ttacgcaaagtgtattacaaaactttcttgtcacttccctcagaaaagtatggtcaattcctgttataattttgtagcaaatggacaatgaaacgtg
aaaaaccgaagcacatattcaaacttcagcattcacacgatttcatcttaaacggatgttccgcggtagatggacggtctaaccgagggtaaaga
cggccacaattcatataccttttgaatgaacacagtagtagttactaccatattaagcttaaaaagaatccaattgaaatagattaccgtttacta
actttttagtgatatcacttcttttcttgaaattcatagaaccttggtacggttttcaaattgccgccaaattctcttgttcgcgtttgacgtgtctatga
agagcaaaacgacgggttcatagcatatgaattctttttttaattaagaaaatgacagaaaaatattttttccaaacgaaaaggaatatgatgtg
aatgactatgcaaactttcattttgggactgaaaacgacaaccatgcttccatgtctcagaaagaaaatcctcaaaacttggtttctgatagaggc
tcaaatatacatccttcttctgtcgcctctacttccttaattcccgacgttccttcattgagtccgaactttcacaatcttcagcaacatgtctctactcc
ttatggtgaaatggccatgccggttccctcttctgctggtatggctgtagtagccccagaaggacccgaaagcaatgtgaatgaagtccctatgac
ttttaatagaactatcactcagccgaatacatctaggttacgtgacacgtcttctcgttaccgagcatccaatagttctcgaaaatcaatgtacaat
gaagatgagttagatgacgaatctgacttgtcggcttcgatgatgcaagatgattttcaagtagaaggcatgaaaaccaagtccggtcgtaagat
tcaacggcccgttacgtataatccaagtgcagcatctctaaaaaaaaggacaagaaaggcagatttggtttcgctgtgcatagtctgccacaggg
gtcattctccagcttctaaccagctggttttctgtgacggttgtaatagtccttatcaccagctttgtcatcaacctcctattgatgatttggcagttca
gattgaagattctgaatggtactgcatgaactgccagtatagtcgggcaaaacagtttccattggaaactggtgctacttcacaagtattaggctt
ggatgcacagcaattgcgtgcatactttttgagcctaccagttccacatttagtagatctaatattgctttatgagaaatctttccctgatctacgcat
ctatagtcctcaaacacgcgaaaacttgggtgaaatccgtcgacaagtgctcatcagtgccgaacggaatcaaagcactttacaagaaaagaaa
aacgccaagcaagatgaagtagctttggatcttcccccctcatgtaccatacactactgagtatgtatcccgttccggcattctttatgattatccgac
tattttacgacttgcaatacgaaacgtggatgttccttcaaaggaaaacattttcgcctggatgtctgatcatttgcctttaacaatttcgttccgtga
aagtgcctctgatgctctacgatggatggtaacccatggacagctggttcgcacaggatatctctatcaaattactagtgtagatgattttttttcttt
acgcccttcgttgttacccacatttcaaaaaagaaggaaagtaccaaaagttatgccgatgtcttatccaacggatgaatcccaaagcctttctttc
actgtattgtaatttaatggaatattaatgtatattaaactgttttgtctttgttgattgagttctattatcaaaatattattgttgataatttatcgccta
tggcactatatggctaccgcgtccttacaaagaagccttaataagtttcgtaagttttagctttcaaatcttctttcaattgcttttttttctcacttgtcc
aaatgaatagtggggcatgttaaaaggacacacaccgtcgaaaagggcttaaaagtaaagtttatgctgggttgagtactagttagattccaca
ggttctcggtatcgcaccttctaaactataatctgacttgttctcattgtaattagttacaagtgagtaagtattggtccgcaccaaagtaatataag
gaaagagaaaatccattcatcttttattaccaagtataaaaagatttaaaatacctacatattacgaatcgtaaattttggtagtgttcatcaactt
caaacacatgcctcacttaccaactaccaaaatgaggcccgccctttcactcttaaagcaagcctctttccgttttccaaagggatttagtcttcca
attccgaaagaaaagaaagtacctcaggtttctgatacccatccattatggcaattctttcgaaataaaaatgctctttctccccccgatgaagagt
ctaaatatggccgctcttggacggctgaagagcttcgatggaaatctttcgacgatcttcacggtctttggtataattgccttcgtgaaaggaacct
tttgctgacacaggcaacggagatgaaacgtctcttaataaatgtccccgattattccaaggatcgtgtgaagacggtaaataaaacaatggcca
ctattaaatttgtattgtgggagcgcgaacgtgcttatcaagctgcaaaagacttagaaaaaaaagagtctgaaaaagcgaaaaatcccaatca
agccgtataggttcaatattctttcatctccaacaatccctgtatgagaatcttttttcttaatgttacaaatccctttttttttaagcaggtactttttg
gatttgtttactttttttttgtttacatttcaccatgcgtttggttaccatgacgaccagcgttaaagagaagctgacgacgcgtcaagcttctttttaat
atttacatgctatttacaaaattaaaagacgcgtaattttgaatgaagagctgctaatgcttcgcttatgagttgtgggaatcctttgtatttgcaat
ggattcaagaatggatggaagagtctactcgacggtatccaaagtcttaccagacatggagaaaggtaggatagaaaaacgcaattcattgtct
cgtttctttatgaatagcttcttaggtcgcttaataaataaaagctagatgtgaaccatagatcgttttatgaattccaaactatgacaatgaaaag
tggtaaatcccatattaacgttcttaggcatttgattccatgaaaaactgcccagttactttgcatcatccatcacaagcatctgcgctaaaaggaa
ttgggcctacaatttgcgccaagcttgaaaagcgatggttaacatactgtcgagaaaacaatataccaatagctcctccacctcagcaaggaaat
gtgaatgatacagagaacacaagtgtcaatcatgcaacatcttcctccaatgccgtaaaaaagccagctgctcgaaagaagcgtccatatgtac
cgggttataggtcaggcgcatatgccatattatgttcattatattctcttccaactcatgaattcgctacgaagcctcaaataattacttctgcgcag
ggtttttgcgattcgtcctttggaccaggtgccgatgggaaccaacgttatacagcttggagtgctatgaagacactcatcatgaaaggacttgtct
accaaactggccatccagccaaatattgtcttaccgatgatggcgaagaagtatgtgatcgtttagcaagggttgatgacgctttccggaaacag
ggtagccctgtaagattgaaaaaaagcaactccactccggctagctttcctagttcaagtacgcaggggaaagctatgcagaacgaaaacatgt
cacagcagcgtttaggaaagaacatgacgttgaataccatctcgcatcacacaagtgttgtggaaaagcaaccggaacttgtttccttaagtgaa
gaagaaatggaagagatggaagaggatgagaaaacagatgtggatgagcttcaggaagaacctattgttgagacaaattcaagtattccaaac
atcgatttgactgatggtagtgcaacgattttggataaagacagagagaagaatgttgacatccctttggaaactattccattccatcgatgcactt
tggttctattaattgatacccgagaaattcgaatccgtggtgatcgaaacaccgttgctgataagttaaacaacgactataacgttctgtgcgaag
tacgagctttggaactcggtgatgcgctatggattgccagagatagagagacgaatcaagaaattgttctcgattttgtcgtggaaagaaagcga
tatgatgacttggtagcctcgattaaagatggacgatttcacgaacagaagcgccgtctcacgaaaagtggaattagcaccgtcttttatatctta

gaagaatccaactttgatgaaagcttttctgaatccattcacacagtcgtctcaaacatacaagtcgatcatttatttcacgttcgttgtaccaagtc
tttagatcatactaccgctactttagcgaacatgacaaaacatatccataatctatatgaagctcgaaaaaccttacaattaattccagatttatcg
gtggaggcaaagacatttgaatccatgagaaagtatctagagagactagacccgtcagttacctatcacattacttataatgctttctcttctgtctt
gtcaaagtcttctactctaactcttggtgacatattcattcgaatgctgatgacaatccgtggtgtcagtgcacagaaagcaatcgagatccagaa
gcgattccctacattcattgatttatttgaggcgtatgaacgttgttcttcaaatcaggatcgagaaatgcttctcgcaagatcatttcaaggctttg
gatcacaagccattggtccagctctgtcagcaaaaatagcctatgtttttcatccaaatcctacataatagattgttggcatgatctcccaaggcat
tttaatacctcttattctgaattatcatgaaacgaaattggtttagcgaagtacagtacgttatccttgtacaagcgactaactaactgacatttaaa
tcttccatagacagatacctcgaaaaaccctttctttgaacattaactatctcattattaatatttatgtctgtgcgtcgttcatttaattgcggcaggc
tcgctaggcagcttggattgcttcgccccggattctgtggaaatacagtctctttagccagatacaatagctctgactcaaaaaaagcagacgaaa
gctatacacattttggttttaaacatgtaccagaagacgagaaagaatacatggttaaaaatgttttctcttcagttgctaaaaagtatgatcaaat
gaatgatgcaatgtctttgggaattcacagactatggaaaaaccaatttgtatctgcgttaaatccagggaacagtactactcctatgaagatctt
ggatgtagctggcggtactggtgacatagcattccgcttattggatcatgcaactcaaaggaacggtgattatagtagcaaagtgattgtcgcgg
acatcaaccctgatatgcttgctgttggtaagaggagagctaagaaaacgccttatcttgagtcgggacgtgtcgatttcattgagcagaatgctg
aggttttggacaaaatccctgaccatagcattgatatattcactattgcttttggaatccggaactgcacgcatattgacaaggtattggagcaggc
ttatcgagttttgaaacctggtggtgtttttagttgtttagagtttagcaaagtttatcctcctttgcttgcagaggtctatcgtcaatacagcttcaag
tttcttcccttgcttggtacggttatagctggtgatagtgaaagttatcaatatttggtggagagtatagagcgttttcctgatgctgatacttttgca
agtatgattgaacgagctggtttttactttagctggtgatcgaggatatgaatgtcttacctttggagttgctgccatccatactggtgtgaaacttta
agtaatgtccacagaaatttgagtttacgatgcttgaagtttgactttttctgccattcttttcttcatctttaggaagcggtcagtagtttattctctta
tgtttgcattaaccaatttcctatttaacggttgtaattatttttactaatcatactattatttttgtgagaaaacaatcaatattaattcactattatca
ttcattattgaccattattatatgcgtaaccaaaattttaaatatcattcgcatatcgtcacatctcattcaaaaggcattgtaaacataatcaacaa
gaaaagaaattttagcattaaaacataactttaaaggtcatcatcgatatatcaaaatgacaacggaaattttgcaagatatattatcatggtgaa
tattgcataaacattaacgatagttcttgaaacattatgatcagcctttccttgtctttggagaaacggagtttagttacatttttttatcttcttcag
gcaaaggcgtaggaaggaaacgatttccgactttttttgtcaatattccattcatataagtgagttgcagaagcaacatataccgatgaaccatcg
tccgcaaagcatgagccacagattccttgagaaactgaataaggactatcgtcttcgtttggaagaactaattcaacatctctattggtgcgcaaa
tccaacaaatgggataattttgtgccttcagtatataacagtaggtccaatggaccagcgttgggaggagtaaagtggcagctccgaacgcttcc
atcaggctgacttcgagatgtagttttagttaacatagggaatttcatatatcgaacgtcgaagacgctgaggcttgagtcttgactagcacaagc
aaacatctcattgttctgcgaaatgcttgtgcaaatagaagagtcctttgtatttgcttgcgttgtgtgtcgaagaatataatctccggatcgggtaa
tttcgtaaaaaaagaggtctggtgaatcgccgacacaaaccatgtatcggccatcattgcttacagaagcatgattcatatttacagggagacga
agattccttgttagtattcctgaggcgaggtcgacgactttgacggagcgatcatttgtggaaatcaatgcctcaggttgatgatcggaatttcgttt
atgcacctcgataccattgttatagtatggtcccagttccaaatgcttatgttttaaagtgggagaccagtaatcaaactggccggccaatccgcct
gacgcgatatagccatactttccaagcagacaaacaggctgatatgtcagcatgtgtccaacttgaaaaactttttccgagttggggtaaatcttc
caaacgaagaaatcattagtgtaataaataggattgaattcttccccagtatattgggtagagattaagttttttaattgtggatgttcagaacaca
ctttagcccgatgctgcttcgggatagaatctcgaaggcataagctagactgatttgaagaactgacgaccgtcataatatgcgaaaagcaaact
tatgagtcgataacttgtgaaaacaaaaaaaaataaaataaagaaaagggaaaattagagaaagcaggataagatggatcaataaattatta
aataatagacagcgatagaactttctttttttgccaagaaagttgaataaatgacataaaagctcagagaatttgaataaacagtgaacaaaag
acgctactagcgaacaatatgcttcagagaataccaattactcaaataaattcgaataacaagttggaatagtccaaatatttaaatattaaaaa
tggaggatgtcaatggctggcaaaaagcttttcacaaagaatgcacagtttgaatccaaacaaataattgaacccaaaagcgaatatgggaaa
agagaaaaatccaatcagctaacgttctgaattatcaatgtcagggaaatcttggaattgttttacagactacttcgttcacgcttaaactgaaatg
cctgtctgtatgacgaaaactagtatgaaatctgatattgcaatgaaagttgttatatcacagaaaatatgacaccgactgatgaaaatcaaatcc
ttcttcctgttctgatcaagaacctgttaaagacaagttgaaagatgagaaatcaagcatgaaatccgcaggtgcaatggttgcgtaaacagatt
cgtgagcgtatcatattgggtttggtgatggctgaaaatatcgaataaaagggcatccatggtgtcgtttaatcaacacttataagaggagtgtcc
aatgtgctttgaatgtggggaggcgctaaattgagccattgtaagcgcaaaataagaggcaatttcaaggggagaaatgacggatgtatgacaa
gagaaataaaaatacaacacagttgattaagatgaaccaaagtaaatttcagaaaagagcatttgtgagactgcagcaaaatacggtccacctt
gattaagaaacgttaaaggggtcttcgttagtaaacattgtcatagagctaagaacaaacaagtttgaagcatattgagcaaaagaatgaaaag
gatttcatgaaaattcttttaaagattgataaagtatcatggcacttgctgaatatatggtgtctatgcgttgagttgagtcgacttgagttgacttg
gcaaggagagttagtggggaatgagaaagcaggttttaagggaaacaaaatatcatttgaaccgagcatatgtatcgtagaaattcttgttgtta
gggtaggattcaacaaatttcgacacatgagaaattcatataaaggattgtcgaaaaaacagaatgcaatggaaggatttaccgtgtgttgtagt
ataatatacttttaagacataattgactcttcaatcaatcaatcctgaaagaaagaacgtaaccaggttgcttcttcttgcataacttccggtgtac

ggggatgacggtcttcatccttgggaacgctcttcttcatagatgtaggaggactttgatcgccaatgatggtttcttttcctgtggcttgtaaagat
gcaaggttggcctctgccttctccaaatgttgatatacctcgtcattctcgttaaagtcgagtttcttacgagggatgaggtgcacgtgaaggtgag
ggacagtctgaccagcgtctcgtccgtcctagcaatattagcgattagaacgcttgtatttacctctgatgaattacctgaataccaatgttggaag
cacttgctgtgtaaactttttcgagcacagtttcaaccttagcgacactctcaaaaagatcactaatctataataaaggttaaaatgactgccttag
ccttttggacttgcctcttccgaagaaaggtcgcgaagacgaggtacaaccctgcgagggattactaaaacgtggccaggcaaaatgggttttag
attaacaatcgcaaaagaatgctaaataagtacaagtcagcaaatacctctaaacagatggaatcgcgtcgtgttcattggtcaccaaaaatctt
tatgaacaacaacgctgtttcctttcacttttgctcttatttacttacttttgcttgataaaacacctggttggtaacgttaaaagcagaaaacataaa
gggtttcgacattttgaagtaaatgaaaagatttcagtagttggaaaaggggaaaaatactacaaattgaaggtaaaattaatggctcttcatttg
cttgtacgcagcaaaccaacattcatctacaaaaagagtcgaattctaccactaaacttacttgttttacaaagccttacagagaactttattacgg
tttttcgttgtcaaactttgaaggagaatctcgtatgctgtgttaatatcgattaattcatgaaattaagtttttttaaataaaagtaacacaactttgc
tcataatattctgactgtcgttgaagaacataaaccaggataaactgttttcataaaaaattaaccatatcaggaaggtattaaagattcgtacaaa
gcagcaccatttaatttttcctatttgaaaacaagaatggaccagttgaaggatccggtttcttaatacaaggagaattcatttttggattttgtgtg
taccatggaatgttgaatgaaagaacttattcagttcagagcaggtaagggcgaactcggatttttaaagaaaaagataatggcgaaagaaaat
ttttgggttgtagctggacgatgattcccattttaatacaaaaggcgagtattaatcttccctggcaaagactctaacttagtatgcaaagtttccaa
ggaactaggggaacaatcgctgatatcggcaaccaagtaagcaatctcaccacgagagtccgagaattgggctttgatgttgtgattgacaagg
atttcattgacatgtcgaaggacaccaggcacatttctatgagcgaacaagacacggacgacattggtatcagcttcggtaagactgcgcaaag
aaacttcggggaagttgacagcactgacactgttaccctcattgatgtagcgaatcagtgcctcactcacctcaagaccaatgttgtattgagcctc
ttcagtgctaccgccgatgtgaggagtcatgataatatttctgcaagagcataactcgctggtccaagcgttcaaactgttggcgaacttacccttа
ccattaccagcaggttcagaggggtagacgtcaatagcagcaccggcaagcttgccgctcttgctagcagcaaccatggcagggatgtcaacaa
cagtaccacgagaagcgttaatgagatagctaccgttcttcatggcagcaaattcagcactagaaaccatgtttttcgtttcgggggaagcagga
acgtgaaacgagacaaaatctgcgtgatggagaagctcatgaagggagctcaattgcttggcagaacccaaaggcatgatagggagaacatca
tagtaaatgacgtgaaggcccatggcttcggcaagaacagaaagttgggaaccaatgtgaccgtaaccgacaatacccaaggtcttgccacgg
atttcccaacagccattagaaactttattccattcaccattgtgcaattccatagagcgatcgcctacttgacgagccaagcagatgatttcgccaa
tgacgagttcggccaccgaacgagagttggcataaggcgagttgaagacggcgataccacgctcggcagcatattccaaatccacttggttagt
accgatacagaagcaaccgatgacgatgagattatcggcggcctccaagacacgtttagtcaagcgggtcttggaacggataccgatggcgtga
acacccttgatttttcaacaagctcatcttcgctaagggaagactttaagaattctacttgatagcctcgttgtttcaacttgcctaaggcagattg
gttgacgttttccaagagcaaaatcttgatgtcctctgaagcaaatggcttcaaggcacgttttgcgggttggcttaagctacgaggagcttggaat
tggctcgaaagaaaagagggcttgtctggaggagacaaggaaagttgagtcatgctttcatcgacagtttggtgagatttcgttatatccatagtg
aagtgatgcaaaattgggtatcgtacagtatgaaaaggaaagcaagaagtccgttgttaatgatctctcaataacaacaaattcagaattcgttt
ctaaatcacaaataaaacctgtatacttaaacgaatgaactggtaatttacctacagtcttttctaacaggtataggtgaaagtgttgttggttttg
gtaaggaaagattcaccctaagagaaaagatccttcgctcaatgtatcgcacgcaactttggaataccgagctcgtccgtatataaagtgaaaaa
tccaacggttgatagaagagattaaaaaggtgattacgaatttgatatcaaatcagataccgaagatgttaacaagagtcattttgtgaaaatttt
cccggttgcgaaatcgttctatgttgacttgtgatttgtaaaaccaaacgtatcaacgaaggaaatctataagaaaaaaaaaaaaaaaaaaaaa
aagaaaaacgtgagatttcttaagagatccttcaacgagatttctcaaaagatggacattgtgaatcccattgttggctatggcaaaaacaagaa
aagtcaagcactgtgcattgcagcatccttgtaaatataatgcggcgaggagagagagagagagatagagagaagaggaaagttttgttttgcg
actgtgtgtttatttacgaccacagtacagaaaaaataatagagaattcgaaaatttgtaggaaatgaacacagaaatacagaaaaagtaaata
aataaatcggatgaattcattcgctatgaatgcatttttttgaaccagcatcgtagagaaaggaaattgtcacatcatcgcatcacatagaaataa
atgtttaaatatcctctgtatccttgttttctctttcttctttcttcaaatggacacattaaattaattgaatagaagagtatcgcagttctttttgttca
ttacttttattttttcctacccaaacatgtaaacaaaccaaagaatgacaattaggtaatgcttgtacgatttgcgtaccgggtcctgaactattgt
aagcagaaattactgaataaaaattagaagcctcctgttgtattcaaagcaactcctcggttgagaattgtccttaacaatggcacaaaagtgttt
gtttgtatttcttaatttgttgctcaagatacattgaataaaagagtggtggacgaaaagagatggatttcctctgtgactttcaaaacaattgtatt
agtaaacttcttttgtttgagattttgcattttggagaacgtgtcggatcctctgcattgaataccacctattcttcaaagtttgatgctccaagtaga
tttgagagctgtatatcgtcttgtttcccttttgcgatggcttctaggtccacacgtcttgataatatacttcaaagggtgtcataaacaggtgaattt
ttactgttcttgtataataatacgtagtaaacactttaataaagaattcaattggtacttgagattatccctcaataaattgaatgtgtttctatctaa
atctagtctttgtattttattagaaaagagaaccaaaagccattttatatcaaatactaaaacaaaagaaatgcagttttatttgttagtccgtcaa
cattagacactatgcctgagtggttaaagggatagactagaaatctattgcggtctccgcgcgcaggttcaaatcctgctggtgtcgtttttttggaa
ttatttttagttcaatacttgaaatattaccattctaaccaatacctttttattttttatttatttttctttctttcttttatcaactattaaacgtatagaaa
cgagatacattgctgacggtttcaaatcatgaagaattcctcagtacgctaagctaaggacgttcaagtacattttcactgaatgcctgtagaacc

accatatacaagatttatcaactcgttcatacctttatttacatacaacaaatcagtaaaaaaagtcattgagtcccgttcgtaacttactcaaaaa
atactcagcatatcttgagattacacttgcagtaccagccaacatttggtttttttgttaatacatccagcgatacaaacagaatcttgattgacag
atgagtaatatatttcccttaagcctaacaaatacgttcgccacactgcaacattcacaacaaactgtatctaattcagtaacgtaaatatcattca
aacatttatagcagctccatataagaagatgttcttcaacactgcttgcaaatggctgctgactttgccccagaaaggcgtttgcgctgaccaaaa
tttaaattctgttaacatataattctgtaaaatcttacagacaaaatatttagctttccataatgttcacatggcacatttattataaaacaaaagat
tagacaagttggatgattcgcatgcaaagctttttactgctgacgacactcagcagggttctgcttagagtgggcagttcgatcttcatcctcgttct
cggaactatcatcatctgaatcggaaaagttttcattggaagaagactcggcatcttcatcgtcatcttcaacatcaaggtcaccgaactcatcat
aatcctccaaggcttcacctgtaaaccaatcgaccgctcgaggaatgagcttttccttaaaatcttcaccaatttggtaatcaagttccaagagttc
atccaactctggattttcttcatcatcatcctccatctgaggagggttaaagaagttaaagaaactatcccgaggttcggaaactttgacagtacg
agtttgcttggtgttcttattacgttgtttctttactacagttcggacggttaagtctgcattgggatgccattgaatttcgtctcccttggcgctgccgt
acataaacacattggaagcaccagtctcttccaagtaataataactcttcgtcaataccttatttgtaaaaaacggattttcctcaaattcgaactg
tacttggaaaccaggcttttccatgtaaacaagacgaacatcaatgagatgtcttaatgccttctcgtcttcaggcgttatcatctcagagagagat
aacacatttttcattgcagtcaaccaaaattcgggtatacccttcattcgttgatcattttgaccctttttcttctaggaccttagaaatgtcttcacctt
cagggactcccttctttatttcttcctcggatggctccttttcaccgtgaacaagctcacgtcgtctttcaaaaagagggccatattttttagcaaatg
tcttttctaattcaaacaactctcgttgaaattggagctccaacgaggagtattccttttgtaaaccttttaaacttgaaattctttttgaacgggtgt
gggcaattcagagactcgttgcgcagtacgatggcgtaaagaatccaacctaccgtcaatagctcctaagtcttcatcgccctcgtcaatagtttta
aattgaggttcctttaccccgaatgtagtattgtttgcgcttgaaggtgtattctggggagtcggtgctgctgacatctttccagaccgatttttatat
ccactttttccaccattttttagcttattttatgagtacaaaaactactcactctacagcttcaactagaagggcgttgttggctaatggaaagaatc
catgctgcctttccaggaacttaacgcagctacggagtactttgttattctggcccattccttaatatatatgaaattgcaatgatttatgaatcatta
ctttactaaaagatacatttaagttctataagaattcatctttatttcttttttttacttattattagcttattgacattactctttgagagtgtttctctc
accgacatttagttacaaaagcgttattattttacatgtatacatttttctatcctcaatgtatccttacattccttgtaattgtttttttagagaagttc
caagttccatgtaaaactcaattatatattcttgcacctttatttatctgttcataatcatcgatatattagctaattatctttgcacacattgatatgtt
tcgttagacattgctgtgatttggtggtttcttcaatgtaacatttcttcgattgactgccaaaaacaaagattctgcttctcgtgatatactttttgcc
aaagcagtctttacgtgtgattcatgtaagcgctgtgcattctttctttgaaggacacataaataattagcaacacaacttaagttccaatcatcgg
gaattgtgctgatcacaaacgaaagtgaatagtatgacgccctgcggtataacaaatctttaacatagacaatgcatgaaggtccggtggaaat
gctttttccaagagagtgttccagcaactagagtatacgctttcaatctgctgacagtaaagctcagcacccttataatcatacacgcattctatc
aaaacagccaatgccttctcgtaatcaccctttttatggaaaaacctatgctgcagttgtggaaatagaccgagttcttttgaagaaccttgttgta
aaacaacttcaaactttgatattgcttcctgatccatgttttctattaagctgatagaatataaataataaaatgagagacgactgtcacaatcatt
catataaccaaactgggtatccaaatactgtgcaaaaatgggctttggtaaacagggaagttttcgataattttcaattagatcttccaagcgagc
ccggaattcttcatgctccacaaagccatctagcagtttattcacgcaaagttccaataacaatcctcgatgtgatattgtgtttgacttgactaagt
attcaattaaaacactatcattcgcgggatctaaattatttaagacatcatctcccgatacagaaccagaaagcgttttgtctaacaaaagcttggt
tccttcatcagcgttttcagcacatagccaattagtatacttccaaaaaagagaaagatcgatgtctttcattaaacaatttcgtaatttgttatttc
atttcgagatcgtttgtcttgcatttccccctaaaatcaacttttttccacaattctaacgtttcagagtctttatgcaaattcatcagcaatataaccag
caagtaagtttcttgttctccagaaagtaaacgttcaatgtcatcagggttagccaaaccagactcaatgagttcttcagcatttttagttccttctg
cgatgtcttccatgctcaagtataatttcaataatacatgatccacaacgacagcgatatcacttttcaaagaaacaaagtactcaaataaattaa
catttcgcttataatttttcaagtaccgcattagcatgatgtatgcgttttctttcgtaatctcaatcatttgcttctgagtatactcatctgtctctcga
aacaaatcattcaaggatagcatagaaacaatcgcttcatctactggagaaaaagttacaagaaactcttgtataccttgaaaggtaacgtagg
cgatgcggtcatcaaatacatcaggatataaagagattattactcttggatcaatttttgaagtttcgagtaaagacaaagaattttcatattgtcc
ttgcttccaatattgcagagctgccatttgctccagatatatgtactgcaatgcggccgcgtcatggtccacatgatacttttcaggatacttcaaac
gcttacttaaccgacgaagtggagattcaatttcgcctgaagttatagtcatttccaatctttcaaagaaggatgattcagtaccaaatccccatgc
attcttcgatgcaaatagaataattggcttcaataaacaacacttcttggtagcttcagaaggaacttcctcggagagactctcgtatttatgatta
agggagaattttttcaaaattttctcatcacaaaagtaagtatttgatgcaggggagataaatgaaaaaatttggttttctccatcaaaagcaaga
tttttcactaaacgtaatgtctgtatatcaataatttgtaacatattaggggctagagcaaagacataatgagaggtagcaacgacatctttagga
tagaaagaaaaagtgagtgtacttttagtaacgttgccattcatatccacgaaaagtccgatagcatctttgggacttcctgtgatcagtagaaac
tcgttattttgactaacaatcatcggtttgtatgagagctcgtgactttcagaatcattttttattataggaaacaaaggaatgttttcacctgtaagg
atgtccacaagctcaaatgactgatccgtcaccaagcaaactgtctttccaaggagcgttgccgtttgtacattaggatacttcaattcctgaactg
gttaataccgcaagagacagcaaaaatgaatacatacgttgcgaaggaaaaatgtaccatctaattgtaatgacacaagttgaatatgagagct
cgtgacaacaagataggtggatttctcctcaatgaaatgtccctcatgactaatttcaaccaggtggtcgagcgaagaggacgggtaaacgaaat

cgaggtttgtagcatgataaaaaaccgctcgatcatctaataatagtatgtcaataatcttgtatacttcactgtgaattcttaccttgatgaacaa
ggatagcttcaagaaagggaacacaaaatattctcttgaccggtgattttgacggtaaagctgcagacttcagaaacttgcaagggttcttaatct
atcagctcgtggattagcataaaaccttctacaacgtctactaaaactaaccctcagttcagaatattcgtaaaaaaaaattcctccttgcttcgta
ccgaaataagcatgaccatctaaagaaagttagtctgaggaaaatatacatcccagaaccataccatgtaaagctattgtagatatttgatctttc
aattcactatcaagctcataactgctgagatatatatccgacattagtctttagccacatgaatcaaataaaataattataatccataaactaaag
aagaattgctgtttttttatagagaactgtaaacttcaacgacgcagcatattccaacaagttacatcgacgtttcaacaaagcgtaccgaaatgc
tgtagtaaacaaacgctgtcttttgaagtttgcttatgaattgatgatgaattttgagctttgttcgatgatttattctaatatgagtgagcattcaca
cgaaataaaaagatcaagtccaagtaagtttctgttcataatgaatcaacttgaactttgaagcaaaccatccgagaataccttcatcatgcacgt
tccgattccgtttctaagacttttctgaatgaaaaaatagatacttaaaaaaaacacaaaagacaacgtatgaaccttcatacatcttcaagttac
caaagtaacttcacatttagagagctaaagcatacctcactatttccaaaataggtagtacgtggtgtgcaaacaattactactagtccatatttat
cttgagaatagtaaagacggccaaaatggctgttgtgtcagacgtagtacttcaaaaattaattgattcctggaaaagcatgtgatatctaattctt
ttgtacgaaagactaacgacaacaccagattcccggttcgaggaactacagaaagaaacagatgtagcagccgaagaagtggaaaagacccg
aaaggcttctttggaagaaagaaaagatctttctttaaaaactaaaggtataaacgaacgtggcagtttcatggtttaattgtatgcttacacgca
ttgctattcaacagaattcaaaaaacaaacagatgaagtgaaattggtagagatgaaaacccttctgaaactatatcaaggaggaatagattcc
ctcacaaagcgtgcaaagtatgcagaatccatttttttccatctgtacgaaaccttaggagagtttccagatccatatcctttattagagcaggcag
ctgagattgatcaaatcaagcaagaactagaatccaaggagaaagaagaaaaggctctcagagaagaattgaaacaaaaggagtccttatca
accgaggttcgaagtttgcagcaagaattgcagaaaacaaagaattcggttggagcagaaatacagaaaagggtatcgaaatccgttgaggaa
tgtgaaagaaagttctcagaaaaagaaaggttgtttaaggattctgagcaagaacatgctgagacgattaaccaacttaacaaaaaagttcgcg
aattacaagctacacaaattgatcatactctgcagtttggacaattagaagccccaactgaagaagctatcaccaaaagctcggaatatcaaagt
ttactaaacgaacttgatgaagcaaataggacggttggctctttagtgagcgaaaatgaaaggttgaaacaaagctctgatgagtcaaagcttg
aaaacgacaactccgacaataatttgaaagaacatcttgatttcatggaattgagaaatcgcagtcttactgctgagctagagcaacttcatcaat
cctcccaaaaagagctagaaactttccaaaatacccagctagatcttgagaagcaacttgaacagaaacagaaaactattcataatcagcttga
gaccctgaaaagctattcggactacgaagaaatgaaaagagaactatccgtattaaaacaaattgaattgagcgctgactcacagtcgaatgag
tcaacttccctggaagcccagatcctgaaacgcgaaaaaaagctatctaatgagctttcgaagcttagaacccaagaagcacacttacaacaga
agcttgtcgatgaaaaaacgcgtagtcaaaatttggtaaaagacaatcgtgaacagaaagaggctttacagaaactggagcgtgaattggcttc
cttaagtgccgaaggaagcatgtcctatgtgaataatggttatcgtgaatcaagcggtcgcctttccccaacttcttccattattggaggtagtccta
gtgtgtttggtgggagcaccatgtcaaaagtcactggtacagaaagtagttctacgattatggatattgttaagcagcaaagagatcggtttcgac
gtgccaatgtcgacttagtaaatcaggtcagttcgagtaacgaaaagattgctactttagagagtaaacttagagaaatggagaaaagcaatgc
actgttatatgaacaaatgcgcttccgtgaacagtatcaaaaccatttgcagccgagcccttctcaaattgaagcagctactgcatacgaaaata
atatttctccgttcgcgtctttcagaaaaaaggaagccgaacgagctttttcaagaatgggctcatttgagcgtgtgttatatgtgcttttacgtaca
attttattgaataatggcaccagaactatatttttgacctatttgataattttgcacctgtttatattggtcgttcttttcaaattaggcgtgtctgtcaa
cagactatatgccttaccagaaccgacactaaaacccgaatgatgatttcttaacgtcttaaatttacagcaacttttatggcttatacttaatctga
atttccagtgtacactaatgaatcatttgaaaatagcgctaaataagccaagtaagacttcattgagtcaactcgtgatgtccaaactcgaatcag
tccttgtaaatcaagatcagacaaattatacgcatgccatcaactacaaatcagttacattacacctttacatatacaaacgactgaagaatacaa
aaaactcaaaaccatgaaaataatttgaactctacagaactaatgaaactacaataaccgaatccattcgttaatcaatagctccgttccttttac
taaatctaatttaggcagaggtgatggccttgcgagcagccaagttacgagccttagcaccttgtttcaaagaaacacgggcaaccttttcgttag
ccttaggaacagcgtcttcttcttcagcaacaataatttcgatatggcaaggatgagacaagtaagcagtgacacgaccgtaagcacggtaggta
cggcggtgttgcttaggagcaccattgacttgaatgtgcttgatggccaacttgtccatatccaaacccttagcctcagcgttagcttcggcattctt
caaaaggtcaaggataaccttgacggacttaacgggccaacgagcttgggtgacaccgaattccttgccttgagcagtacgaccaacgccgcca
ttgaagcgacggaaaggaacagcttgcttatgttcagtgacattttccaagtaagcaaaggccttcttcaagttcatgccattgatggtgaaggca
acttcacgggagttcttaaagtgagttctcaagtaagaaccacgagccttggcgcacttggtctcagatgcaggagctgcagagtagcgtgccatt
tttgtttttggattattttttaaaacttgcgttgaggaaagcttgaaattctgaccagcaccaaattttcgacttgtgactgtttttctttcagggtag
tggtgcaaaatacggtttgttattattgcaaatacgtgttagcaacactactttaatacatcataaattacatgtcctgtttttataaatatgctatg
cataatgaaaagagaagtttttgtgaatttgttaaacttgatcgattctgagctattgacattcgttgagttaaagggtagtactgtataaaaatga
agagtaaacagaagaaatgctttcatcgtatctgtttacaatgaacgcaaaagccggttgaactaaaaacttgaatttgaatgtaagtacctaaa
gatgttctttcctcgagatcatgctcatgtaaattaattgaagacgcttaaatgaatgaaagatgaaaagactttctagtgatattagctgaaactt
tataatgacaacaactttcctacagcatcacctagtaatttttaggttgcttggagacatttttttgagtctctttccccgatcggttcagtgtactcag
gttaagagtagctttcttgagagtgagggttcgagggaacatttgtttctgcttttggtaagtgttgcttcctatcacgcctttccttttccattcttggt

aatcgtgaagaagcgtccgtggtgttcatatgagtataagacatgaaccatataatgtaggaaacaaattggaaccatgccgccaggtctaattt
tttatggtattttaaggccattcacggcactaatgcattaacaaagtttatgaagaaaagaaagcaaatatgcagaaacgtttaaaatttatagcg
tactatttcttggttgacatcctcgtatgaatcatgttcaggtcgacgataatcgtacacaacctgcggttgaaacatgttgaatccgctgctttaaa
attggggatgattctttgatgagtatttaatttggcaatgtctacgaaagagaaaagaagaactgaaatttctattactttgtttccataagttctatt
ctcagtaccttttattatatatttcggttacttgggttatttaaaatctgtttcaaatgattctggttatgaaacagaatttagagaacgtattgcttat
catttaccggttgtggttgtttaatatttttctgctacttttgacgcgtcgttgttaaggttaaagagattcagttcctatggaagatttgtcaaggaaa
ggcgattaaaaggtaaagtagctttaccaacgtcttaactatagtcccagtcaaagaacagtttcttccagagaatcataaagattttagaggatg
caccctcgggtgtccttgaaagaatcgacaacattgctgtaatgattggaatgtctacaagaaaacttacagaaaatgtgtgaaatatatctgac
aagcgagcgacatccttgttgaacacgaataatcatgtaaatacatagagcttcttatgcagcgtaataaagcagaaccatcactactaccctaa
aatgattgattagtttacgggtatagcaaagttgcagtaaccagtatatctctaattagctctaacgaagaatgttcctcttcaacgaacgctctca
tgaaagctacagcatcaccatatatgagtgactgtacagcgtctgtgaacctgacatgaaatttcaaccttgctaacaatttgcattgtcgataatt
tagcccgttaagctcattttattgtactgtattcttacggcgacaaagtttattacatttcgcataattcacatcctttttctttcctttttttcctttct
acacaatttaaaatccaaaaaaaggtaaaacataactttaggtatttgtcttgtccgtcattttgcttgctctactctcagacacttttacactgtgtt
aactacagctaattcatgtccttgacaaaatcgaacaatggagacaaacaactaattcaaaaagaagataaatacactttaggagaaattaaac
ccattgctattgaggaagatgattatattgaaggcttaaactcaataattcaaaaaacctattatccagacctttctcgtttgaaagcccattcaga
aggatttacatatgaatttgaagatgatattatccaagagaaaaacgacgcagttcacgaacagctggcattactcccgaaaaatgatcatcttg
tgcagaagctaaagcgtagtactcagggagtaacgcttgctgattatcagaagaagttcacttctgaagacaatgcctccttttctgaactgtttca
taatgaaaagagtgtcaggaaagtcttactgcaaaggcgctatggtatgctacatgatgaaaaagctgtcgacgcagattcctcctctgcaacaa
acattcaaccaagcaaggcaattgcatggaaggagaggcccaatactattcatacttggaatttccaaccgagaaattcgttaatgttttatcctc
aaacctcagaaccgtctgctttaaccagagcaagaggtcagcatcaaaaaatcgatagctcatcaacccggcttgatgacgagtttataaagaa
atcgaatcagcccagccaggatcctttaccggaacctgcaattaacaggcatgaagcctcaattaacggatatcccttggttggttcaaattatgg
ggtgacctctcgaaaatcatcgacaaatttctatattcctgagacgtctcgaagagaaaaactgcatgatctccgtttacatagcttaaagtctcag
aataattcgccttctactcctggttactccgttgtctcttctacggctgaacaagcatcggcaacctcagggaagcttccttctagactttctactttg
actccagcagctaaacgtcttgtagcgcagtcatatttgacggcttctactagggactccccaatgcgtcgtcgcttgacggctacttcagtaccaa
agttttcttggacacctacacctcgtactaaaaaccctgccactcctaaacgagtctaaaaataacttataatgagcaagttatgaatgaatgaat
aaaatcgataatgcttactttgtggaatgtagaaaaaaaggaacgtatatcgaatggcatgcaaatctataatgttaatgaagtgtgaaatgatg
ttattttaaaaataataaaaattgaatacataaacaattatttttaaccaaaacccttgttattcctttttacaaatatttaatctagataacactca
ctcttctttttgaatttcaatagaatggttttgttctgattcccaaagagaagaaagcgcaaaaagcctcatctcataaatgactcctggcttattga
aagaataccattgatgtaaaagcgaagatcgaaggaccaaaatcattccatcatgcagacgcacagaatgctggatggcaggaagtttaagttc
ggcctcttgtacatcaccaacaacaataattactgtaaaaccatactttatagcagggtcagaattaagaaaagagactttgtcttcagtttcgatt
agccgaacagaaaaattaggaaaagcaccaaatcgagcctgctgaggagccacctctaaatttaatagagattcgtgaaatttaggaacatga
gcactcagaattccacttgcaaatcgaaacaaaggggagcacttttgaaggaaatcactatatgcaagtgaacgaatgctatcagccgttttaac
caacgatcctccacgcttgtcgtaacttgttccaaaaatgtaggaatattgtttagattcctgattctgagcttgaaaaaactgttcaatggttccaa
acaaatccttagtaaatgatgtaccagctgtttcgcctaatgcatataacaacaaattgccacgtttgtcaccaatttggtagccgagcttctcttgg
tcttttgtttggcttgaaaattccttcttctgttcttctgagatttctggatcatttttcattaacttcgacagagatttcagggacaccttttctagattg
attctcttatttctttgttcttcaacaatttgtttagtcttttgtaacttttgggcctttagtttctctgctcgagcttcttttcttttctttttgggaggctcg
ttttccgcatgttcgttgttaacaggctccttctcaatctttctatcctgtccgttttcttcagccttgacaaccgttaaatttaatgtccacaagcatca
ctcatactttacttaccattttgtggggtttattgaaatattggtatgggggacaacagctcaaactggaaatgtcgagttctcagctaaacaaaat
tttctggccttgtagtataagtaactactaacaagtgaaagagcgaacgtttaaatcttcaagcgtgtttataaattttctctcatctttccttctgcat
atcattcctctcagatccttcaccttgtctcgctagaaactcagaaacgaaagaaaatgtctatttctattctacaagaacatttcaagcatctatat
caaattaataaattctttttttatgtaaaaaactttttttggaaacatcatactcatttactcgatagtatgattttagaataatgatatgtaatggaac
ctgaaaaatatacgctaaatcattaacgaagccaagtaccatattctaaacgttcgctaaatgcttacagacacaaggtaaacgttacgcgacct
ctgccaatttgattgaaacagttctacgtgaattgttcgtgttttgttggatttgaatattggatttataattattttataacaagggtatctaggatat
atttttgaacggtggtcccttcgaattctgttaatattgaaagcatagaagttattatctttcatccttattcgttttgttataaggctcataaagtcttg
gatatttcttatttttttgtgcatattttacttgtatattactgatgcttgtgctagttgacaatgagtgaaaatgagtctggtacagggcaaacgcttca
tcaaaagcgtaggttttctgaacaagacgaaggtttctatgaggtaaataatgtttcaaaaaagcaacttttagaagaattgcatagtagcaggc
gacgctatgaagaagcatccagaagattggataaacagaagactgtaattaatgatagattacggcacatcgctttttttcctcagttggtggccgc
tggtagctcagcaattttattgcatcttccagaatcgcgagcctgctgattttgggaccgaacagtccattgcgcttgtggagcagattcctgatttg

gaacacacaagaaagtcgtttcaaaatgaaagttcgggaattcgtacaatgttgaacgaatcattttctccagaaataatgaaaggactaatgtc
aacgttagaatctcaatccgccttcacttttcctttaaatgacgattcgcaattaagtcaaagcaagcaggctctcgattccgaaatagaaaaagt
aacgaacgagcttcagtccgcaaaagatcatctgcattgtattttaaagcttcctgatcgatctagtagttatacattacgatgtatcaatgaatct
gtaagtttgcatccccctacaaagacagaaagttcaataaataaaagggagcaagatgcttctgtgggagaagctttgaaacagcagtctcaatt
gcaagagctttccagattacaagaagaccaacaagccctaatgaatctatactctcaacaattatcctctgtcgatttcaagttgactgaagctga
aaatatcgttattcaaagaaaaaatgagcttgggagcgttgactgggaacatcttcagaaaagcccttccttattgaacttaaaacacgttttgtt
aatgacgtctgctcaatatggattcctcgaacaactggttactgaatcttgtgaggagaaggaatttttgctacaacagaagaggaactttgacga
ttattctaggtcatattatcaatctattttatcggattttgaaactcagattgtctcattacaaaaagatgaacatagaattgcccaggctaaaacag
atctgttgaaagcaagcgaaaagagacaagcaggtttagatgaaaaggcgactacattaaaagaaaagaaagagaaactcaaggaacttgat
actgaagtggaggaaaccaagaataaaatagagtcaataaatcattccgttggtcctaagttgcaagaaggaatgaaatctgtactctttcaaa
aagagttatccaaatttttgacccaagaattagctcatacggagcgggctttccgtttagctaatcaacaagtattaaaaaaaaatgatatgcgga
tggaggaattaactaataagtacaaactcgaaaaagataaagcagaacagaagtactttgtcacgatgaaagctggagataatttacatgtcga
agttagagcacttcgtgaaaaatacaagaaagcggatgaaattacctcaaagatgctacatgctcagcatctaactattttagatatcattaagtt
agaagaaagcatctctgtactatctgaggttcgaaatacatcttcaagatattccctcagttttcaagttaaacaagcaaagcaaaatttttcgata
gctagtttggagaaaaaagtgaataccctggatgggataattggaaaagcaaagtctgaactatccgacttgaatgaacaactttctttactatcg
tcaaatattgtttccttggaaaaacgaaatgaacatcacgaatggcaaaaacgaaagttgagggacaatgcagtatcatcatctgaagaggaat
tgaagatgtacagagctatgtgtaagtgttctgtttgcaattttgaacgctggaaaaactctgcaatatctctttgtgggcatgctttctgttatcaat
gcatacaaaaaagaatagaaacacgacaaagacgttgtcctatatgtggtcgaggatttggagcttctgatgttgttcctatgcaattataacttg
ctttccttgctaatgtaatgtaaaatactaaaatgcgatggtcgctaatttactgcttgtggataattaaaatcgggttatttaagaggatattatctt
cattctatttttcaattacatctctgcacgtagttttgggattgtaataataattaatactgcgttatgctaagcatgcataataatcgtaattcattaa
acatcttccgtagtctatatgataaaacccaatggcttatttttggtcagcgataacggcattctccagttgagtcattatcaagcccactttttaga
atactgacgctgaaggtcttctcttccacttttattggcatccttcattaatcgctgataataaatacgaaccatttgcttcacatctttaattaattca
ctggaaggagtagccataaagtgttcaataattttttggcaccattttgatatattccgaaaactttcgaagcctttgggaatgaaacatcatgca
cgtttggctgcagagattttagcacctggattattgttgtcatgtttttaagcatctgtaaacaagccccacgattcatggattcaagcttgtagaatt
tcgcatatagcagggaatccaaaaatccatcaataccatggattacaaaactagatatttgttagcacttgagattattatcactttattacttactt
ttgttcccatgcaggtagacagttctttagcttcaaaacagtggacacgatggtagcgttcaaatccacaatagaaacatcaggtcgacctctgta
ttcaactacataatcaggaagagtcactgtttcctgaaaagcattcatatatcgtagccgtatttccattcttataagaagtagagcatcaaactta
gtttgttgaagcttttcgaataactctccgtaaacactgaaaagttagcatgaattttggtttcgatttacaaacctttttgagtgaattggcttctgta
cgaccaaagtccatttcattaaaggaaataatgtttccatttaaattagcttgtgatcgtcgtaaaacattcgaaaaatcatcctgttcgagataaa
acgagaaacacctttgaagaatccactccatggaattgtagaaataagagagtagctcaggtaaatccgaatctttacttaacacagatacagg
caatttcttgtctgcgattagttggccttcgtattgtaatagtcgtttattttcagaaatgcatatttcatcgagatcggctgaatttcttttggtctcttt
cataagacgaattaattggggataaattaaatcatcatgtagtactcgaaaacgaacagtgtcatctacatcctcattttcctcgtcaaactttcta
tattcttttaataaagctgaccgacataaatctgaaaaggttaatatcgatttatctgtacattcaaacatacctgatatatattcagcacaccgaa
gctgaaagcgacttaataaacctttcaataaatcataaaattcatccaaatttaaagatatagttggaagataatcctgcaaagcttgtatatattc
aacaacacttatatggcactgctgaattagcaatcaatgatcatttaagtattcaacgtactttaaatatgggaaacttcgaatattctcgccaatt
cggatatactcgaaaagaattagcgtctcctaaaatgttttcgagatggctagacataaaattgaacagctggggcacgtaatgtccttgtaaaa
attctttaagaaaattaacagcaaattttcgagacagatgagggctactacagttagttgctaggaatgctaaaaatttgaacatactcctcaagg
taaatgatggcatcttctaaaaacctcactgtagaagaaagcacagaagaagcatgaaatatcgatggacctaccaaagcagggtgacctgcta
agcgagaaatatcttttgaaccagaaaagagttcaattgtcgagacagaagcattggagcgcttggctaagaagccaggagcaatcgaggtca
ccaaattgctgacatctttatgaaaactgcttccattttcaacagctacttcacttgtaacgtcaaagagcttatcagtctggttctgtcccggaagg
ttgtaaatggacagttcattgctttgctcgatacttccaattttctcagtaaattcatcctttgataaaaggtaatcacgaagtagcaacctaagttc
gccctctatggcttgcactaaaggttcgtaagtaaaagcctgaaaatccgccgcttcaactttagggctttttggtattgaaggagttttagaaggt
gtaatattagactttaaagattttgaagcatctatagcacttagcgtctctggtttcatcaagtattcataaattccacgatgatgcataacaatgca
tctcaatcttaaaaataattcttcaaaaaagtcagataaaagcttttgctccgtgtaagattcggcatttaatccaagttcgaataaagaatcagg
aaagttaccggcacctgcgtctttggtatttaaagcatattttgtcataaactcacgagatacggtatccacggtggaaaatatttcagctgatgta
cgagatttcaaagcagatattgcatttggtaattttccgagtaagttgagagactccaaaataatacgcagatctcgaaagctatcctggccgaat
ttcatttcgatacattgtgaaagctaaaaattagcctggttcatgtaaaagaagaaatcttacctgtaacgataaagattttttaaataatttagct
gttcgtattgctcacgcaccagaacgagttccgatttggttagaaaattacgtaaaaaaagagggtcagaaactaaatgcggttagtcaatgagc

tgtttcataaaaatttagcatacttcgtagggaactagagcaacaagcagacatgatgggataggctaaaggtctcttcagaaaaacaatggaa
ataaggtcatctgacaagacatcttctaaatgagataccaatccatttaaacggcttttaaattgctccacaaccaaaatcccatcgaaagcagg
gtgcattaagcgctcgcgaacacggtgaaccaaatcacagagattatagtagtctttagcttgtaacagctttggcgccgactcaaaaaccctgtt
tgcctcgttgatctctttgaggatttcgatgatttttcgatgctgtgcgcttcttgtcattacttgctgtagatctttgctgttggtaacgcctatgaattc
tcgagagttttcaagcgaacccttcaaagtgtttatgcggacaaggcatctttgtattccatcaatgatttctccatatgcagacacacttcgagca
aaatcatccttgtgctggtcggcaatggcttgcaaagaagaactcactttatcgtatacacgtacaaaagatttgtaatctctaccaaaagagtctt
catctagcaattcgagggccgtagatattggacgatactattactaaattagctattgtttacagcaacagtgatacttacgtcctcgcggcacaa
atctttccattcatcctcgatttcttgaaccaaaactgatacttgttaaagccctattctttcaaatagttcacgtacattttaaatcatgcatttgcat
tccacgcattgcactttcactgcgcatttcgcgctctggtggtgaattcgagttttcatgcttttcaggcgatattaatcttgaatggctacttgtagg
atgccctttctttttggagtttgagtaaaatttggcatccatcaaatccaacacttttgtataaaaacgaggttttatttactcttccacagatggcgt
atgtcaatggattgccgaactgtaacaagtctcctaatcgatttagcaagttttagcattaattcaaaaacgaatttagcaaagtacggtagtctac
gcaatttaatcgtttttgaaataataattgaaaacaaatacagaaagtcttgcaggtgttcaatgtatagttacgagacaatagtattatatcaatt
tgctagcataacccgtataccttaattgggtatgaagacagcaagttagtaataataatgaatacatgcaaggcagaaacgtagggtaatagaa
ttagaaacagaaggaagacggactataaagaaatcaaagatttatatcacgggaaacatcgatcaatataaatgtccaaagtaaaaaaggtaa
taaaaaagaaagacgaatctgaactagtttatctatgtaagttcctatttccaaaagcatgaaaaattattgtaaaccaaaacaaccatgcagaa
tgtgcaaatcatcaaacaatatggaaggaatcaaaatgtcataaactaatttaaactcatctttacaacgcttaattctgcctaaagcagctgctct
ggattatacacagtactttgatagaaaggggaaagggacatagaataaacggtgaatataaagatattataagctttcgcattaaaagttattag
agatgatattttagctttaacgaagaaaatcaacgacattctgttaatgctttctcaaaatcatgccaatacaaaattgaggcccaaaaaacgtac
gaaaataatttttctaaacttcttgccagcagtctaatcataatcaagtagaagcatctccgcaagcatcagcgtcttcatcaggaaagtcaagct
cttcacctagctcatcgcaagcatcggtgagattatcttcataaccacgttcagcaacgcgcattccttttgcctctcggtatattttagaaaaataa
aggcgtaaagtacgcaaacccctaatatatcctttgtcatacgaatcggggtgttgaggaggacaagttgcttccgagagcatgtttttgtcttctg
caaaaacgcaatttgcaaaatcgacaattctaatgtcaatctctcttgagtttgctccttcgtgatctaaagaactacttgatccgttgttcggtgaa
ggttcaccatcatatataaataaaagagagctcgcatacaatctagaacctcttaacaaacgaacaatctgttccagatgtcttaattttcgtaaa
atagcagggatatgatgtattagtaaatgttcattatcctcatcatcctccgtccatcccaaaaaccgcattaatgcaagctgaaactcctcgcctg
cttttatatctctaccaacatacttgtcctcaaacgtgtatgattgaaaccatgaatgccacacttgcattccacatattctgacacctaaaacacga
gacgtagtcattgcacactttttttgtttgagaagctttcttcttttcagtggccattataccatattgcctagttcccattttaacatccagaacgcaag
gacgattcattccactcgttaaatcttcaatgacaatatagcgttctatttgagtcgctctatgcaaggagctgtcttcttccttggaatgctttggta
catcttgttcttcatgtgatgcctttggcaatatcgggaatgaagtggttctatgcggttggtcatctgatatgttattgtcaacagcttcaagcggag
gaggaccttcatataagtcttcgttgtccatggcgaacataggcacttctgatgcatttttttgagcttgcaatttcttggaattttcaatatacgtag
atgatttcggcaaacccggtttgcattcgtaatcgcccaacatctcagataaacgggggaattcgctggattctggctcctcccatacagcatcag
aagagtatcgtctttggtttcgagaacctttgttctgcttacttaaaggatcatagtccatttcttcattttcctcgtcaacagccgtgcacatatcaca
agacttatcttcaacactacacgacaagctcttattaggatcagcctcagaatttcgtctggagcgagagttatgatcaaaatggtaaatattaac
agtgttatctctaaaaacagagggtctgctcgatcggtgcgacttccttaacttgtagcgagatcctagtgattgtgattgacgagcatgtttaggg
gcaaaaacttcttttaatacttgttcacgtagctttctattgatcaatgtagtaccccaaggcgtttttcgaggttgtgaagactctccatcattagaa
gatggccgatcatcgatagagctagatttgttgtgatcgctttcagtatcatgtcgaatctggccgggtagcatccattcaggaaaaatgtgccgat
tttgttccacactgacttccgggatatatccacatggttgtcgttgagacgattgtccatacacgtttttttggcagctttagaggaagactgggta
acgtcacgatcattttcagttccttctgcttcgtctttcttctgatctgtttcggtatgagtgacattcaaaacaccgatgtatttaggaataaatggta
acaattcaggatggcaggtctcaatggtttcataaaaagtattttcgttgcgagtcaacggcttacaaacagctcttctagaaaaccggaaaaaa
gccgtatggccaccaacttgatgcttgaaaggttctaactggacagtaattggataatcaacattccaattaaaaaagggattgtcatccggagg
agaagacgattttgaggaaaatgaagaacggggagtagacaaggggctagtacgagggcgagatagagagcgatcaaattcaaacgatgcat
caggtaagagaggtaaatgttgagatgacctagtagaagggtggtgaggaatgtatgtagcggtggtcttggaacttgtggatccttctttacgac
ctcgagaagcttttggagttgtcgttaaaattccttgagggtgtgcactgcccacattaggttcaagaagggcgggggtagagtttgaatgataat
tttgatgagagtatttcgtgatttgatcagggactgcaggggaatttcgtcctagagaatcattctcaggaactggatgcaaggtgttattgttggc
atcattagggtttaaagttgaggactcaaacacacgcagtgatttactggctttccttgcagggtgaacgtcttgggatgcttgagtttcatttgcat
ccggttccattgaggatggttcctcaacatggattgaaggttctgtggctgagaagggagattgacaatgcatatcggcaaaattagtgtcctgaa
gaaggtcttgtaaagaatcatgaaaagcagtctgttgattgttaggagtaagttgctgatagtgatcggaagaacgtcggctgagcggagcggta
tgcgattgtcgttcattaacttgcggatgagacaaatttgatacaggggcatgataaatgggggctaaaggttgatgtacagtattttcaaattca
gaatgtggaaactggtcactttgtcttctacctggagacaggacctctaaaggacgtgtaggtttttgtagcgaaaaatctgaatttctttcacgatt

tttcatttctttcgcaaacgaaccgttttctgggcattttaacttttccaattttctctctggatacttggcgtttctgccactatacgaactaggcaggc
aacactttgatgtgttctgcataaattcataagttgagatttttcgcaaagcaatttctgctataatatcccggcttgtaaccccgaattcgtcaaact
cttttgtttacttcttcatacagaggcgtttggttgttggtaagttctttggatcatgcttcgctacgtctgagtaacatcttccttaagtggggggtaa
aaaaagcagcaacgcttcacatccgtattattgaattacaattgcgttacgtaaacgttataaactttaccgtactctttgtatttcgctaaattgag
attcaagaaaaatgaactctgctaaaagttctaaagtatatcgtgaacagtgtatataaaaacagtaagtttaatttacaaagcattacataatcg
attgcaaataaacttgcagaaaaattaaaaaacgacaccagcaggatttgaacctgcgcgcggagaccgcaatagatttctagtctatcccttta
accactcaggcatagtgtcgattgttgtcagcaattagagtattccatattagtaagttcaaaagttgtatgagttttaatgcatctatattcaaaag
aaaaaattacactgatcaggagtatgtgaaagcatggtcatttacttgaacatcctctttaacagtaatgcaaggttcaattggcaaaacacttttt
actgaaatattatattacacaagagcccgtaagtctcctatctatacgagagacaacctaaccagttcgaataggatgaactgttcaatggatgg
aattctcctcgcttaagttcgttaagtacttacagtagtctaccacaattcgtacagttccattaaattcatgttcatttgtttacaacaacattgtag
gagggcgacttagcagggacaaatatgcaggctatgtaaagggaatttttgtgcggtctatcaagggattgttgttttgataagaaaaatgcctgc
tacatcgacgagtagaaagtcggttgacaaacgcgagacacacatgttacaacatgttactataacaaatgagtacggggagctgtccgacctt
gaagacgtaatggaacacgggccctttacactaacggggctattggtaatgagtaaaagggagcatggtgggtcaaacgaggatagtacggtc
cttggccattccattaaaatctctcctatatggacgttcagcttgagagatgagttcaaagaggaaacgacaaagctatggattatcacgtcaaat
cgtcgctatattatcttgtcttcttctgaagagtaccaaccatattatgaacaaattctggagaaaaatcgattatttttgtcttaaagaataaattc
aagcaggacatggtacggggacacttgcaagactacgatagctacatttctactgtgagcgaaaggatgaaccttgcgtccgagtatcatgccat
tttgcttattcaaaaacaccttcgatttttgctgctacaaatgaccagtgcgacttctcttcatatatgggcggaatcaccttttttacaacgaattcg
ttcttcatacgagcatgtaatatttgagatcaatgcaaatattcaaaaagtccgttcccggaggaaaaatactaagcgtccgtcagctacaaaaat
agagaatcaagatacttatcagctaaaagaccaaaggccatccctagcttttcaaccagttcagcctttccgtgtgtctcccttgaagatacagca
gcaacattattgtcttacacaaacgtttccaatacatataccccataatgattcctggcttccaggtcttcggatagtcgatcccaatttcgatgctat
tacactgtggaaacggatacaagcatcgcaagaaaactcaaactttcattccatttctctggaggaagcatcccgcgtggttgctgaagaatctca
aagtacataccttgaagcaaaaaccaaaatcactgggcatggagaagcattattacagattatgtattttacccccctcctggcgaggatcagcttt
gtttaatgatttaaagcatgctgttggctatcagactactatataccaagcgagagttcagttccggacccgttgccaacagctgcttcaacgactt
gattcccctttcgtcaaacccgaaaaagagaataccgatattgacctattctccagtaaattggagcctagcaaagtggctttacctttatattttac
tcttcttaaacaaataagttgtagtttgccaggtgctgattatacttacgctcagtacgtgcatcatctaagtactcaagctgcagtgcaacaagaa
aaagttggagatttattaattagtatccttcctgaatttttgcaactttttttctcgagaaattggttactggccagtgttctctatctatagttacttata
tgaatgtttgaaaaaatcatatcccaaggttcgttctaatattcctaatttttggaatgaaatatggctcaacaaaaaaaaatacgtttgcgcatcta
gttaagggctccccaacaaccacttcagtccctgttccttctacttctattccctttaatcctcgagagactacccctaaatcaaccaaactgcgcgt
acttcagtattccgacgttgaagctgagaaacaaaggcgatatgtgctttataaagtgcaaaaagtttatccagttcagttagaatccgatataca
gtcaggtatttggacttgccctgtgaaacattgtttgtatttctcaattaacgataatcccttaaagcccaaccctaatatttatgaacatattatcgg
tcatatgaaatctacgcccatgttagaaggtttacccgcaaaaaatagtgctttgattgaagatatcgaaatggtaagtcatgaaatgctgtgagc
tcaaaagtacgcagacgcatgatttacatattgtttatcaatgcagtgttacatgactatagacaatattggttagaatgatattatgaatctataa
taatcttaagggaagtcatcttatgtgaaaagaagaggaatatagaatataaaatgaatattaaatgtatgtcagataagagacattataaatca
tgagcaaaactagatcaagtcatactagatggatgcttaaagaatcataaaagcatgtagcaacctacatgaaatctcaatgctgtagataatca
tacgtatacgtctagatggcgttacaaataaatcaaaaagtaagtaccaacaaaataggtactgatagcggcgtatggtcacgcgatatcgtctt
ctttttttcttttttttttctttttttacggttgacaagcatgctaaagcggtacgagaccaccaagtcctatggcaagaattatgcagagggaaagtcg
aaaccatataatagttttcctgggtaatataaaaaaaagttgtgcacataggacagaagaaactcaatctaatatatctttttcttttcatcaacaga
aaatttaccaggaccagtattaacgagatataacaggccaccaatgatagaaagcgtctggaagaagtcatagcggtaaaaatcacgatgggg
actatttctagcaatggaccagaaactattgataataaagttggagactgaaaggattaaaaccagtatagcggcgaaatacttagctttgaagc
cgatgacaaccattgtacaagcgattccggacagaagatgaacaaagatacgagtgtatgtgatgccgcttccttccttggctaaaaggccaata
aacatgaaaataagcaagacacgaccggcaagctggaaataagtacgtttattgtgctcagaaacggcgggtaagccagcaaatcggttgaga
cgacgatgaataaaagtatcgctagccacaaggcaaagtccaccaatgacactaacgtttcggaagaacatctcgccgctggtaagtagtccat
aggcaaaagcttgtagaacagttacaaacaaaagagaaccaatggcataggcttggcgctttctaaacaccaccattgttgatccaatgaacat
gaggaccacacatacaaaaagcagcaagggagcagtaccgaatcgaaagcgacgataatcccgcatataagaaacctgttcaggccattgtgt
tacgattcggattgcgtcttcaaaatacgtagcaacaataaggaaccgacccaaaatgggcatatagggacctaaaggagacaaaaaaatctc
cgcctgttccacaatctgagaagcacgctccgaaagtgttctttgtcgaagtccatagctggaacgtgttgcataagaatcttgattgaccgacag
aggaattgtcgaatatggagcacgtgaggtcattttcttgttttgtatgtcgccgatgataagtaaaatccttttgtgtatctatccctctaattttta
gtcaaattcccttcttcagtgcccgttttgaccttgatttttgtattatgaatatagggaaagcgtaggttacaatagtctccatatccttagctcgtta

ttcccatcgtcatataaggaattcttacccaaattttagcgaaactccctaaacgaatcggtttgtagaaaggaaggattcaagtcctgaagaaag
aaaaaacaataaagataattcgctactttatagccattttcaaggatgaaaatgcgcatcccttaatgcagtttcgtacctattttagccaatagct
tgaattcaatgtaagcgataccataggtagtaaatgctttgatagaaataagaaataagagaagaagaatgcaaattgttgattgtattcttaac
aaacattagccgaccctgaattgaactggagtcgtaaaacaaatttgttcatattaatatttgaccgttgtatgatattaaattagactcgtgatat
gaattaatgatatgttaatagttcaaatagctgctaatatagtcgaaaagtattcctcaaaaacttcatttggagatagaacgatgatactattga
aagaattaccaaaaagaaaggggtggactgtgttcccatagttgaagactgttttttttattttctaatgctttgtatcgtagaatcctgttataaac
aggaagaccaaaaaaatatctcctgagccagatttgaactagcgaccaaccggtatcctttaaattacagccgggtgtgataaccaactacacc
atcaggagttatgttgattgcatatgtcacctattattatttatgtttattttaaggcattagaagtaacacaaaaaatgcataaacgaaaaggaaa
agagaagaaaactatggctgatagatctgacttcgattatcctttgaatttttttttctttttttttatgaacttgtatcttttcccgcgttggtcaaatctt
gatacctctgaaaagaagaattgaggttgtttaactagaattgaaaaaaaaaaaaaaaacaaagcctgggaaattttgaaacgaattatatattc
caatttttttttctaatttctacttcataatgaaacgcactacaacaaaaaacatccattcgtacgtttagcaattgttctcatatagggatcaattga
tgaacaaacaaacaagatgcggtaaacaagtatcatgagatttaatcaaatattttatttatcgattcatttttctagttttcaagattgttttgtacc
tttttatttattattattattattattaaatttttcctttttataagaacagcaaatggacagtcgcatacaaatatgcaactttgtatataaaaaaag
tttgaagaaatgagttaacaactttacgcatcacatacttctttcatgattcctttactctcctcagtagtattctcatcattatattgaatttttattttt
tgctgcactacaaattattatctttaaattatctaatgcgacttagcataataagcaactctcgatggtgtagttggttatcacacccggctgtaatg
tctaagataccggttggtcgctagttcgaatctggctcgagagactttttgatattttttttagtcaaaagccagaggaaaccttgcgatagagcggg
tatttttcaaaagagaaaatagcaactcaaaatttactcatacacaattcccgctggtttaatcctcggttgaatccccacttaaagtaacgacctt
tcctactacgattcaccaaaatccaggaatacaaaactacaaaataaagttctttacaatatacaagtctacatccacattgcacagttttcaaca
gaagtaaattgtaatgccttttgattattacaacacataacatttcttttaatgagccaaacgagagctttgaatgacaccggtaagctcttcaatct
tgcccttagcacgcttgagggtaccaagctaaagacggttagtaagaagaatagagaaaatttcaagcaacatactctcttcttggcgagcttac
gagcacgcttgtcttgagagttacggatcaattccataacacgacgttcatagggagcgaaaccagcaacctcacggacgacagagcgaacaa
aagaagtacgcttcgattgatgtcctttacgacgagaaggacgctcaggcaattgacgcttggtcaaagcctttcccttgtttaatccaacgacaa
gtcctggtgccatttcgttttggttgaagagcaaaatctttggcgttcattttccctacagttgtgcgtccgaaaatgcagtcacatgtactcgaacc
ctaccctaaggcaaaactggttgcagttcggtatttgcatgaacgaaacccaaagcatgttcagtaaaaaacactcaagtcggaattaggtaga
acagaaagaacagaaccaaggacatactgattttcttatttcttttttttgtcttcttaaaagggtaatggaaatgagaatctcttctcctagtctac
gatccttaactctccttgctttatcatgtgtatgtttacccaatacaacgtagcgcgttcttgtaccttgataaacaattcatctaagaatcaaataga
ctcagtcagtgccagagagacttgtatactaaaaagcgtaataaagaaataacccaagtacttcctaaaaaagaaccaggagctacaaaggcc
agcgacccagtagaatgaattttgaagtaacacatcgtgcaaatgaaccctgtgatttacacgcgttccagcataaacgattgagcattccgttga
aaaaaaaaaagaagaggaacaaaagaaataaacatgtcgaaattatcaaattctcgtagtgcgtaggacattcgttatgtggtttatcacgtctg
ttaattctctttgcgtcgcactattttcatgctatttacattctgaggttgcttgattcaacttgactttccccatgtttttgttgaattctttaagaggaa
ttactcgcggcaaccggtcgaaaaaacatcagaataattccaattcgggaaattcaacatcggggtcaccatttaaaaagagctctacggttcaa
ttgtcaagaacaaattctcctagctcgaatcaacccccctctttacttgctgcaaccatttgttcgaaatcatctagtgaaaggaaacttcctcacaat
tgtatctttgccgaaatatgtggatttggatgaatggcttgcattgaatggtaggtactacttaaaagacacgtagattattgaatgagaatggcat
agaataatctagcttctttgttgctttcagtgaaggactaggactttatgttagacctctattattgaatccatatttcgctgttttcgcgatgcctgct
ttcctaaatgttgagtcacttctcctgctttcaattttttttttcgtgtcttcttgttatgaaatttaaatactaacacatttatcttagtttatgagcttttt
acttatttgaaccacttttatgatgtctttacttcgttctgtagagtagacacatgtcccattatgctggcaggatccaagtatgtgccttcttcatgca
atttataattatttttgacactaacttttccttgtttagttttgattacaaatggttagataataaccgtaagcctctctctttaccagcacctcagtata
tagaatatgttttggcctggattgaaaatcgactgcaagatcaaaacgttttcccaacaaaagcaggcttaccctttcctacgaattttcatgcaat
tgtacgatctatttataaacaaatgtttcgcgtatttgctcatatatattatgctcattatggagagatccttcatttatctttagaagcacattggaa
ctctttttttgctcatttcattgcatttgcaaaagaatttcatcttttagatccccgtgacacgttccctttaaaagacctaatttcggttttagaagag
caaggaaatatttaataccgtattattcctctatcattccggttattatgattttatagtttcttcgtcttcactttttttttcctttcaattaagtttcctac
ggaaacgatccgattttgttttgcatcatgtttcactcctaattacctacttcattaccattaaaaaaaaatgtcttccttatttgttttgatgcccatt
catgagcgcctttttttgttttctttttttttttttttggatgctccctcttcatcgattgtttgtctttccgcaatgcagtcttctttgttttactaaaccgtcgg
tttagacaggtactttctatatccttggctaaaaatgtgttggcctcagtgtattttgacgcttattatcatgatttcattggttcgcattttgagctatt
tttgtttccaatacctatgaattgtttggatttttcattgttataaacgctgttttttgctttatttggtttactctactaagacagttcagtaaatctttgt
ttcttacaagtggtgggattccaaaagttatgatctttaatttataacatgatttagagcagattaactaatcttcattttaagaataaaattcttgat
ttattgcggtaagtattatctacttgagatcataaaggttggtagcaatggcacataaacgatagtatttctatagacaagtaaagaacaaacaa
atttaaaaaacatacacctgatcttcaattttggaaattgcacgagccttttcgtatcccaatgcaactgtagctttacgaatagcattgaccatgg

gtgtaggtccgcaaatgagaactttggagtcaggagaaggagcagggaagtggtgcttaattagctcttcggtaacataaccaacggcgccggt
ccaattttctggtggattattaagaacatggtgtaccgtgaatctcggatccttttcagctaaggcttcaatttcctcgcgtagaataatatccttctc
agagacatttgcatacaaaagagtaatttgagtaggatcttcagggttggaaagaacagcacggataatttgaagcataggagttataccagta
cccccagcaatcataccaaagtgacgagcaagaccaacctgatgtttccaattgcccttggggccacggatgccaattctatcacctattttaagt
tcggaaatcttcttactgacaacgccattgggatatgccttgacaagcaaatcaaagtagcccttatcaacatctgaggacaaaggtgtataaga
tcgggaatgttcttttccatccacctcaacaactaccgttaaatgttgtccaactggcaagccaaggacatcgttagagcgaggaagtcggaagc
gataaatggcggtgttatggtttaagacagctttgtcgctcaattcaaagtattgaattttactgttcaaaactcttcgaacacgtccacgccaaaa
cttatgaaaccctagggccaaggcaagtaagatcgagtatcccatccactcgcgtttcactacaaaagttccgaacacaattaaaaatgcagga
atatatacaccatgtaaaggagtactcaatagtgtttccttcgacattttagctttttcggatttaacaactctatttcatgttagcttatgatctggtt
aaatactcatatcagatgtcattcaatcagctaaaaagccattccatattaagaaaatttcttctattaaggaataactaaagattgatgaccgttc
aaccatatctacatgtttacttaccttatgaacaattggtactgagtaagttcatatttttaggattgccttaagtagaagtgcagtctcatgatccta
cttaaaaaaaaaacactcactgcttggaaatatttactaatttgctaactttatatacaatataaatttccatttgtaaattcattctcttataacctg
aaatgaaagcatgctgtaaaattctatataaacaaaaaacgaaggaaagtagcataataagtatatccaaatgaaaggaatatatgcctttgaa
agcaaatagagtctctactcaaagttgtctgcaattttcctaggcaccgaaaagaatcgtttaccgtaactaaataactttaaaacactactgatgt
tggaaacacgatagtgaaagtactaaagaaagccttcagagctccaaagaactacctcagtaactcaaggtgtatagacaatcgacgtctacct
tgaaagaaacattttaaaatgctacaagtaacaaaagaacaaggctaataataagaagaatccaatgctgcttttcaatcgtgaacttcgtcata
gcaaacgatactgtcggcgtgtacaagccagccataggatccccagtagaagacccaccatcggtgctattataaatatgaatgtttttaagtaa
caaaggcgcctgtattgcctctaatgcagataattgttctcctaaaccataaagccctgaaaaaagttaatacatagcgtaaagggaagatttca
cttaccatcccatgtatcattaaaccaccaccactgaaagccacaagtaactccatcatgacctccgcagcaagcttttgctgcagcaatggctgt
cttgtacaaaagaggctcaatcttaggaagcatataactggccatttgcgcagtgtacaccataaatcgagccaaaaatcctttaaaggatgtttg
gtcatagttacaggagccattaagctcacaaactggttcgtataaaacatcgttgtgaaagaaggaatcaatcgccttatttacaaaaccttctgt
ccttgttctccagattgacgatcctgtctattccacgttagaagaaagaaataaaaaaaacgtacatgattatacagaaacgctgcacccgccat
gtacaagccgctgttgtaggtccactcggtagtttcaatggaagagcagttgtcttttgttgatgtcccgtcaaaaacggtataatcctcgtgaaca
aaacctactttgatgctccaattgtacactttttcggcccattgcgtataagtgtcgtttccagtgaagcgcgccaatctcgcagcaagctgaaaaa
aggcaccgttcgttacggaatttttatagtcgaaaccagaattaaattctgttatttgccatcttattcctccatcacatttactaccgtcccagcgat
taacctgttggttaaaaacagattgagccagttctagccattgaggctcatcagcgttcggattagaaaaattagcttctgcagctgaaattgcag
cgagaccccaaaaagcctgatcgtcgttaccttctgaggttgaaaaattagaaggcatataatcagaattttcacccacttgatataacatgctcg
tctttacgagatcattataggttgaatttccagtagataaatatctatttaaaagaccctgaatagttagatcggtttcgatttgtaaatttaattccg
tacattccaagcagcacctgcttcccaccaatacgcaggcggtaaaaacattccaatggtccctcctttttgtatccagcataataatttagcaac
ccatcagcaactacacctgcagcttcatcaacggaagaggtatcatttgggtctaacatgaatcccttgatacagagaaacgaaggaaaagaga
gtaaaaagattaatagactatacatgtttttatctcaaaaagatgttgtaaagactcaaaccatgaatctaactttgtccgatatgatacaaggat
gactcaaccagatgtcacatttgaaggaaccttaattctgaccattactttcttgatgtcctcaattacagaaagaactatttgctgattaaaccga
aacgatattcagcacttatataccaaacaagcatatccctactacacttgtagactcggactcgaagattctttgagtaaaacttgtgtaatcgtta
cagatgctactctgtaatgatacaagaagcacattagcgtgaataagtcttgctcaatgatatttcgatgttggtgaacatctattttcgagtccgct
taattcctctttttgcttgttttggtgaaagatcacggtccgccttcgtttctaatggcagttgctttataatatcccagatagattcaatagactctga
atgtgattgacatttctcaaacaaagatagacagaaggatttcagctaaaaggtacatcaaaattagttcaataaaatagtgtaacaatcgtacc
ttcaatatatcttctttaatctctaggtctacctggtaccgtgttaagccttccggattcacagttttagcacctatttcatagctttttacgattaacg
aatgcattttaagaagtatacatacgccgatattttatccattgtgagagtgtacatatctctgtctatgattaatacttgatcaaacagaaaatgg
gccattataccttatatcgtccaagtacctaggagtcgaacgaaatatacaacctatagctgtattgcactttgtacacttaattccggaatatacg
ctgaattagtgttagtaattgctagtaagcatttttggacctaccaaaggccatcgtctgaagtccttaaaagctcttcaatatcgaaagtattttct
gctaagtctaaaactatgttaaaaccagttttctctaaaaccttaccactcaatgtaaagctattcatttcacgatgcgatatcacccatgcattgga
atcgcctacaatttgaagacagtttttacattgaaacaccgttggaggggattcaaaatcgttatcaacactttctacagcatttgcttccgcttctg
acattgtctatattgggcatatatcaagacagttcttggtgttcgtcttagtctactaagagaatgaatactgactaagatagaaacgaggtttaaa
cgtttattatatttccttcattaaataatttttattcataatcctttaatttctttcggaaaaaagtatacagtgaacgtcctgcttcaggtcctataagt
tcagaccattttcgttcatctacttcgcaagcgtcctgtatatttatcacgtcactataaataacattttgtaatttttcatagatatatagggaagtc
ccattagtaagtcaactggagcctgattgtaagtgtttgtggaatcttgaccagcttccaaaccaatggaagctgcaacagcgggatctggctcttt
ttccaaggattttaaatcttggaacatcagagacgtagcataagcgctcgatgaccaaaggattctcagcttagggaacgcaatagttaaaagca
ccaacttagactgtacatctgatttaccaatctcagtagaaagatccgaaaacggctattatattagcaaagtaaaatttaactagatttaaattac

ttacgggagaagtgaaagtttgattttgctcgaattcgattagcagcacgggtacctcataatgctccagcatagcttcgcactgagaatagagtc
tcccgctacttaacgactgtattaagtcacgtatagacttcctttcaacacaaatatttggggaaagcacataatcgccaaccagtagttgacagg
gaataacagagaagttgtttccatgcagtatggacggcaaagagcttcgaaattctcgaagatccaccactatctaaacgcgttagtatataaat
gaacagcgctttcttatacccttggtttttcatttgtcactgaaacatgacctccaccagcaatgcgtgtattcacatttcttaaaaactttgtttcctg
gccttcgaatcgttcattttctcctgtaagaacaacagccatattcttaaaaaagattagcgtaaccatttaaatttcttcaacttactgatcgttcac
gaattaacttggaaaaggagtctttttctcttctaacggtataaaggtacttttgttcttcaacggaccctccataatatataaagtatactcgtgttg
ctctttgtggataggtagctttatacacttcaatacgccggataaagtcaggatcggaatcgaacatgataataaatcgtgggcgcagactattta
aaagaagggtatccatttcataattgtatgtataaatataaatggaattagtgtcactcagaacctcgaaagattcattttcttcctgtgtataggtt
gacttcgatatttgagagagcaaactatatgttaataacgttaaacgacgagagagatacatacagtttttccaagcgtatatctttggagaaatt
atcttgagccaaattgctttctgttgtagaccgagacgcagctatgcctcctcctcttactcttctccgtttagatgcaggagcaccttttctgctagtt
tcaggggtgttttgcttctctttagaatttctttcaatcttgctaatcgaattactcatatttctatactgttcacgccagtggaagtagtccatcagtc
gactgttcatcattttaaaggactctttagtgtcgtaatgtatagtacttaagtaatctcgtaattgtaagcaggtccgttcttctgaacacattataa
gaattgagttcgtggaaaattctaaaagttagcagtaggcttttcatgaaaaacataccagggtcattgctttcatggagtatattttcatggctaa
tttcttctaaaacatcgcgcaaaacagaccacttcggttgttcctctaagacaggataatattcatccatggatccagttggagaacggtaaacac
gatccctagctattcgaatcattgtatttgcggaatctaacatgagccatggtgagggttgagcattactggaaaatcctccggtgcgaactgaaa
gaatgatagtgtccaaaatcttcaggaagctgacgcagtcgtaagaaattagtgcattcaagaggaactttaaggtggacagatcgcctactaat
tgcttgctcttgggactcacgcggtgccaaaccggatccagttgacgacgaatagtcacatcaaagctcctgtgtaaggcagaatcgatgttccat
tcttccatgtctaggtaagctgagttgtatctgcgaagttctcgaacggtagcttcaatacaagaaagtagacaagattgaattgacttctgagaat
ctgtaaagtcaacatttaactcaataacatccgctggtgtcttttctaaagattcagttacatcaacatgaaaccgaggataaataaagacatggc
gaagaaacatgcatctcaaagcattgctgagagcattgattcccattaaaaatcgttctgggtcatcgctaaaggccttaatgaatccagttttgtt
tttctctctatataaccgtataatgaaagcctctgttcccgttgaaacaattctgaaaaaggtcagtttagtgtaacagaggaaaaacgaaaatac
ctgtctgcatgcagtaatacgagcccagtaatgttttctgtgggaattatatgagtcaataaatccattaccaaaatcctgctggttacagaaaaa
atgcctccgtcttgatatgctttttctctataatacaagagtcagcttattaattgaattaatctcacatgaatgatattttgcttaccgtttgtctacg
gacatagtttctgtgtttacaatttgtagagtttttcccaattcctcttccaactctcgctgcattagttcattatcattagaattggctccgaccagca
agactaacgaccttggaacggcaaagtaagctagaacatttgctgcaatttgaagtaaagaaaggcctggtgccataacacagagtccgtcctc
attgataacttcattaaaaatctttttttggtaggagagtgttaaatggataggtatgtccatgtttttgaatgcgagaccttgaaaacacaatcagt
tgcaagcttttaaatacggatcttaagtcccttcaaaattgtgctttgagcaatgtaactgttcatgtattcttatagaagctgtttaatttataattta
cgaaggatacttgtgaaacttgggttttaaagagaaatcttaagtttttaccgtgtatatggtaattagcctgaattgttcacgtatgataaaatact
taaaacccggttgcggataaatactttttaatttcttttaattaaattagtaaaaaggctagttttgtacatttagatgccaccatagccttctcatct
atgagattcctgtagcaccgaactaacagagaaccactacatatacatgtcaagagatactcgatcaaggtcgccgccaagacgaccatctcat
agagatcggtacaattatagaagctcaaaattttacgatcgctatgacgatcgtcgaagttccaattacggtcaccaaagtagatcggattatcat
agatcccattctatacgaaggaataatagagaggatagctggaagcatgatcggtacaattacgatgataagaatgaaagaagaagcgagact
acccttcctagcaggagctctcatccttcacggtataatgctacctcgactttagaagattcctctcgcaggccaaactatgtaactgcttctgaaa
aaaattctgaagagagaaaagaacaggagcgtcttccaaggagatctcggtttgaccagcctgccaaaacagaacgcccaccgcctcctccttc
agcagaaacaaatgactcgtctgaatttatacctaccattaataagccccgtgtttctgatgaagacaaaaaacccgaagataaggtatcagctt
ctccgacaaatccgcctcttgatcccaaaatccaaacacgattggaaaaagtacgtgcttggaaggaatcaaaagctgcacagtctgttaataag
aaagactcaaccgctgcaattaagcctcctcataatcttttgcaaaccgacaaaaacgctatgccttctaaggggatctcggggtttgaactagga
cgcttgaacaacaacaataaagagcccaaaagaataaatcgcgtgcatatggatgatgacgatgcacccagacgactgaatttgcaagattac
gaagacttatgggataaagaagacagggaaggcatacaagctagctcaaacccaactgccatggacgaagatgaggttgaccctttagatgctt
atatggaatcgcttgttggctctactgatacagttcgaccaggtttattgaacactgaggtcattgactctcatgcaaacgaagatgaacgtacaat
taaatcagaaactttggaagaagaggagaacttgctcgctcttgccgctaagcgatcaaagaaaaaagatgtaataagtgtagatcattcgaaa
atgaattatgaagattttcgaaaagatttctatgttgagcctgaagaactcaaacatcttactcctagagaagtcgatgaattgcgtgcttccttag
aaggcatcaaagttcgtggtatagattgccctaaacctgtcaccagctggagccaatgtggattatccgcccaaacgatggatgtaatacgaggc
cttggttacggaaacccaacatccattcagtctcaagcaattccggctatttcatccggaagagatgttataggcgtggctaagacgggtagtggt
aaaacaattgctttccttcttccaatgttccgacatattcgagatcagcggccgttgaggaatggtgagggccctataggtattatcatgaccccga
cgcgtgaattagctgttcagattcatagagaatgcaaacctttgcaaaagctttgaatttaagggctgtctgtgcatatggaggtgcgcctattaa
agatcaaattgcagagttaaagcgtggagcagagattgttgtgtgcacacctggtaggatgattgatgttttaagtgccaatcaaggacgggtaa
ctaacttacatcgctgtacttatttggtattggatgaagctgaccgtatgtttgatttgggatttgaaccacaagtaatgcggataataaataacgtt

cgccctaaccggcagacagttctgttttctgctacatttccaagggcaatggaagctttagctaggaaggtgttaaagaagccaatagaaatcac
ggtcggaggaagatccgtcgtagcctctgaggttgaacagattgttgaggttcgctctgaagaatcaaagtttatgcgccttttggaacttttgggt
gaactatataatacaaaggcagatgtccgaactttagtgtttgtcgatagacaagaagctgctgatgctttgttcgctgatttaatgaaacgtgggt
ataccagcaattcaattcatggaggaaaggaacaaaacgatcgcgattctacgattagtgattttaaggcaggtgtttttgacgttttaatagcta
ccagcgtagctgcccgtggtctagatgtgaaatcattacaacttgttgtgaactatgattgtcctaatcacatggaggattatgtgcatcgagttgg
taggacgggtcgtgctggtcataccggcgttgctgttacattcattactccagaccaagaaaaatatgctgttgatattgcaaaagctctgcaatta
agtaaacagcatgttcctgatgagttgcgaaaactagcacagcaattccttgaaaaagtcaaagctggtaaagaaaaggctgctggtggtggtt
atggaggtaaaggtctgtctcgcttagatgaaacaagaaatgcagagcgtaaaatgcaaagaaaggcctacggcgaagatgttgaagaagaa
caagtcgaagaggagcacgctcctttggagaaactgcctgctgaaaagtctactggtgattttacacttgatagagttcgagcagcaatcggtggt
attactgctcgagcattaagtggtcaaaatggacaagcaaataagttgacacaacccatctccatcatcaaaagtgatacggatgagtttaaagc
gaagatggaaattaatgattatcctcagcaagcaagatgggctgttacgaacaatgcaaatattatgcatgtaacagaattgactggaacgagt
attacgacgaggggtaacttttatcttccagggaaaaatccagaagccggcgaggagaaattgtacctgtggatcgaagggccatctgaattggt
tgttagtcaagctgtcactgagctcaaacgcttgcttttagagggtgttgaacatagcctcgaagggtccagcaaggctgctcctaccgggcgttat
acagttgtttaatttacctttatttaattttgtatagtaatagtcttgtttcagataaataaaaagggaaggttcttgagtacaaagaaataccgaat
ccgataaagatggtttcattatattacttttgatagaatagagcaattgataaccatccgatacctcttttgtagtcactgtaccagtttttattgcac
aaaactatatattaacgatagaattaccctagtgaaatttcagatttgacaaaaccaaataagttattagacatccggtgatagttcagaaaagct
ttcgcataatatttttctatttgagccaatatttttcacctctaaaaggcaacccaactcgtcaatttggggtgttaaatataccattgaatcataccc
tactcctatgcaatactgacaacccaaacttctgtataacatattctttttctaatacgtgtcagatatctaacggaatatctaggatgactaaccaa
actgctcttgtcaataaatgagtcgactagctcagatccagagaaatcttgtctattcaagatcttacctggaaacagctgttgttgcctacaattgt
tctaatatagtaaattaaaaaaaccaactccccaaccaattgcattactttttttttttttactgtatttccagtttgtttatatttactatatgggtgtt
accccaggtaacgaataataatgtaaatatataatcgtattctgaaggcatacaggctgcaaaataagattagtgtaccttaccgatcacgatgt
actttatttgcgtactgtaccacttcgttatactacacacataaacattcctacgagtaaaagcttgacggtttgacatttgcctttccagggcgaaa
atataaaatttaggtaaaaatgggattacaagaagaaacaattgattattatgagcttcttggactatcagaagatgcacaagatgcagacattc
atcgtgcatggaggaaaacatctctaaagtaccatccagacaaaaatccagatgatccgcatgctgctgacaagtttcatttgctacaagtagcct
ataatgctttaacagatacaacaatgcgtcaggcatatgatgctgagcgattagccaagcttgctcgtcaaagacgcgaagaagcatttgatctg
aataggaaaaccatggctgagaaattacgggagagagaagatggatttcatttttttgaaaaacaaaaagatcaagagaacaatcgtttacgg
gaaaagttacgttctttacaagagcaaagcgctaggttacgacgagaccgtgaagagagactgtttcaagacaaggattcaaacaagagaaaa
agagaaacaagcccggtaaaaactcggtccatttctgaccttgatcgatctgtaaaggttcgttggaagaaaaagtatgtcgaccaagtggatga
atccttcctacgttctttttactcaaaatttggacaaattaacgatgttataattcaaaaaactgttaacgatgataaaaaatatgtttacgcaattc
ttgtttttgatactctggatgctgcttactctgctgtaacagtagaatctccttctgtcattaaggatacccaatggctaaaacctttatcggaaaggg
ctgagtttgaagaaaagaaacctaaaagtactatggattacgaagaactaacgatgttgcgaatgaaacagaaacaacgcgaaagggaacaa
gaaagaaaagccacaatggctgagacttaaattttaagcaattacaatatggctaaatagtatagaattctcttattgttgctaggttttgagaca
gaattatggatgtacttagattgcaagactaggatgtggtaccttccacctcactgaaggtaaatctgatggaagtttcctgaattgagtcagactt
ttgccaatgaaggttaatgctcataatttattgtttaattcgtcaaaatggataggaaaggggactctcaattgctcttcaaggcatattcatgaggt
gccattgataaagccgagccttaagttttataaacgtaagaaagcaaaagagaacaaataaaaatttttgctaacggtcaaagagctacttaaa
caacctcaaccaagtttaaatgtggataccgatgcaaatgtttcatttttacctgctgaagaaggccctgcttcgttgcgtacatattcaattggaa
acgttagccacgtgtataacggcatccgagttcctcccaaaccagaggaaccattaaattgctgtcagagtggctgttccatttgcgtctgggatgt
gtttgctgatgacttggaggaatacaaccaagctcggcggaaggcgaaacgaagtttttttagaaaaacatttgactctccccaaggaactggaa
agtgtctcattagaggaaaaaaagctcgctacaagaattacctcctcagttacaggcatttgtacttttagaaaaacgtctgcaacaacagggcaa
taattaaacgtttaatttatgaaagctatggcggttatgataaataaatcattttatggaaagaaattaatctatctgtttttataattgccgctttgc
tactggcaaatgaaaagagaatgctttaaacaactcgaacaatcatttctattcgtacgtaagcaaacaagaattgcgtttagtaaatggtttgtt
ctctttatgattaaaacttaaatatgaaacagaaaggaactgaagaaaagctcaatacattcatcaaggtctttcaaggatcttctactttcgtacc
agttgccgttaatgaatctgggttgggattgggaatttgataaccaccttttgccagccattcataatacatttcgtcgttcacctgcttagacaagt
ctgctcgagatatatcgatgcgaaattgtgggtttttttaagagagtcaacatatgcaagcaggtgggataaatgataaactttacatagtcaggtt
ctctccaatattccaagtattctaaatactgcaaaaatgtttcgtcctcgaagtatttatgctgagcaaggaaattcaaataccaaggattcgacag
catttggacaaattccaactcaatttcaaaccgactcttttcatccggtactcgagaaagaagccattttggttccattgaaaatactgtatcttaac
ctctgtactttttctgctgcagaaattcaactctccagatggaacagaccaatatgccatctttgaaggtgtaggtatcctcataacactgtgttaag
ttttttgtgcggttaacagttattcggtatttggtaaacaaacaaaaaggagtctacaaaaagtgtgcattgaaaaggttctatggaggaaagag

gaaaaaggaaaaaaaccgagaaatggattactattttcacgcttcaaatccatgtgcctcctttcttttgtattaactgttctgaacaaacttattga
ttgaaaaatatctcgcggatacatgcaagtacttgtttctcttgtctttatttactaatgttttcatttcagtaatcgtaaagtactgaatgcctcgagt
tttgtgctatagaatgttcctaacaaaattttgtaaacaaagatagctgttgcaaaacgagaatgtttaattaaaagattgtataagtataaagaa
taaattatggacatatttatttacctgaaacagaaaaggtcgctgttgttacatatgtagctgattttacttcctactcggaacttaccactgagcag
aaaacagaacgatttgaaaacatatacttttttttttctgcaaaccatacttctttccttataattattaacttaactattttgttttcattgcaggtttct
taattgaaagccagttgtttaggagttttgttgaatcctcaaccaaaacatcgcaattttatgcgtgaaggaatctgggttaattataaaatattttc
gtaatgaagaatctgatttccatttgttctattttggcttactttgttcattactgctatgcggctaccggtatgtcttgctgattatgagtctcaattgt
ctgtcgattttaggaaaaattatggcttttctgaactgaatactaatacgattagctacaagtaagtctgaagcacttcctcctggagcgactgcca
caaaggtatacacgatcactagtggtacacatgttgtaggaacagaaacaactatgtatcagccttacaggacttataaggggctacctacagac
gcctattacaattgccatcctcaaaaatattttcaaagtaatatgcctatttgctttcctactcctgactctcgttgggttaaaggaaagccctatag
ggtgagttgggatccttattattttggggtggatgaattgcggcttgttttgtcgtatcagaatcattctggcttaattgctattgacaagaaaataa
aaaataaagatggtatgactgttgaacatcaattattataacgatcggcgaatcactaacacaattgtttaggagagattagtataaaagcaagc
gataaatggctgcatgatgactttgttcagaacatgactgttaaccttataacaaacaacgagggaaatgctacattcgtgtcaggtcctaatatc
actctggtaaaatccttagaccctcatgagctgtacatggaagaccaagaattttatgcacctaagcgtaatttgaaggcagctatagctgttcct
agtatcttcttgggcatcatactcatgttactttgtatcacacatatcgaagggatacttggagaatctttgtcgctaaaatgcgtatacgaagctc
aaacgctggttatggaattcggagaagcaaacgacagcgtatgcgtggacgaccaactgtcggccttgcttctagaagtgaacttgtatacgacg
attccgaagacgaagagtattttaacccccaacttaaaaaaatgtattaaattggaaaaaaagtgtgcttcatcataaaatcggatcaattcgtct
tcatcttttcaacatatgacatacgaggatcatagcatgccatgccatatgttattttctcgggtttcttttctaattctgttgtatacgaatcttagtat
tgagttatatactctttagactttcattaattttttttaccagtgtctgtcgcaaaaggaaagcgtcagaggcaaactccgcacggccgccgttgact
tttctgaattgcattttgtattattgtcacaaaaaaaaaggaaaagcatagagcagtaaaccttcatataaatgaaaaacacacctactcccaatt
ctcaaaaataacacataacggaacaaatttaactaacttttaatttcctggtgtaagcaataaaattattattcaaatattgaacatcaaaatgag
aagtcaggacaaaatattccttccaaaactgctccatgccataactgaatactgttcctttttttggctttagcaatacatatcctaaacatatccaa
aaaaaagttgctaaagaattttgatttttttcatgatagtttcatttttgctcgtaaaccgtgttttgaggacgacttgttaataacatattctttttctac
ccaacattcataccattatcaacgacttaagaagaagaaaaccaacaacccctttatacaaacggtgaaacaatgtcaaggttgggttctgaca
aaagaaaagggctaccagaagaaagtatccaaactatcgactctccaatctatttgaaccaaatatcgtctttaccaaaagaagataatgtacat
tgtttatccttaaagcaattgattgggagcccccaattgaagcagacatggcaattcaacttttgtgtagacttaaatttcatccttgaaaatatcca
tcccaaggtttttcggacggttgatatacgaatcactcatggatatgacagaaagtcagatagcttggcgagactgactgctcaaaaggagcatt
gtcctcttcaagtccacttatacagcgtgtatgtacccatgtggggaactcatcattccaaaattatggtgaattttttgaggatgaatcatgtcaa
attgtgattcatacagctaacatggttgagcccgactggatcggaatgtcccaggctatttacaagacaccgctgttgtttcccaagttaggagga
atacatctagagcatggaactactaatggcgaagaaggatcattaagaaaaaaggtgaagcaggaagaacaaaaacaagtagaagttgtgga
cgtcgatgccgaaaccgaagacgaaagcccactagaaagcgaaatgactcaagtagggcagcagtttcaaaaagattttctggattatctacgg
aattataatcatacaaaggaacttattaataaactagaaaaatatgatttttcaaatgtccgcgctgtttttctcgggtctgtacccggaaagtttat
gggtgacaaggaatcgcaatggggacttggaagactcaagcatgtacttggagaggttgaaaaaaaggaaaaagaaaaaggtgaagaatttt
ttgacacggatgtctgtgtttcacaatgcagctccatgggatcgtttggctccaagcaggaatacttagaggaattaatggaagctttttgctgcaa
gaggaaacaatggaagtttatttttccaacggtgaatcaaattcaacagtcgatgttgggatggcagtctggatcatccattcactttaacatatta
ggaaagacaagtatggagcaagtcgaggatctgcgacaaaaacataatttgcacaagtggggatcaatgaaagctggaagagaacgaattgc
accgcacattaaaacgtaccttcgtttaagtgaatcgggcgacaggattcgatgggcactagtgacaagtgccaatctgagcaaaccggcatgg
gggacgatggaaggaaagggcgtaaaatccaagacagctcgtggtctaaggattaaatcctatgaagcaggcgtactattatttccgcaactatt
cgagagtgccgggaccatggattcgtctttttgcagtatggtaccgacttacaaaaccaattcaccgacgtcggttggtattacccaacgtgaaag
gcataatggctcgaaatctcagctactagttggatttcggatgagctgggactttccacccaaagagtatgtgcccaaggacgaaatttggtctcc
tgtagttccacgaaaggagaaggattggctaggatatacatggcctcctaattggtaataagcaagtatgccctagagaagctgttttagtgagg
agaatttgttggcaaacgatgccaattgaagacgagagtaattcattactaatgaatactttttctttagttaaaacaagctaccttatctggtcaat
tgaaaattattacaactactttttttatttttttatttttttttttcgttagctttggttttgtcatgtctatcagcgttcttcataggagctacaccatcc
ctatattcttgagctaatacctttcctatcagaaggacgaacctttgcttgcctctagtatacatcttgcctttctttgatcatctcgacttttcattcttt
atccctctaaaaattcatttatttacttgaaggtggaattctcatagtcgaatttagactactattaagcgactcggctcaacaaacttgaatgcca
aaacaggtttacgtaaaaatgaccgcaacatttaatcaaaccgtacgtaaatatgaaggaactttacttgacgcaatttagtgcagcatcaattc
gatgattccaatttcatcgattgcttaacttcagtgtatacgttggctgagcataccaagtaaatagaatattaactaactgttattcatctattcacc
tgttaccaaacgcttagcactgccgtttacaagtaaccttcagacccttttcgtctcactagatatatgcaaaagatttcgaaaaactaataaagcg

aaatttctgttttaatacctgctttttactgttcgtattgcttgagatcagtttgtattatttcattgtgaatctatatttacttctatatccgaatatttat
tgccgattttttggaagactgacaagtgcatttgattcattgaagaagatttgaggacatttatcatggccacccacgaagaagaacatgagcgt
atcccagcaggtgatccctactcttctcgtcgtcctatccctactatcccaaggttctttcgtgaaagaaaagaacgtgcggacgtacgagaaaaa
caaaaggctgaaaagcatcgaaaggaatccacacaagaagaactgtttgatcctatcactcagcggaacgttgagatctctgatattaaatttgg
tagccaaaaagcttatgaaaatcccagctacactgtacccaaccaaaacattcaatctgggttatccgccagccaagagccttatttgagcccaa
atcagtctcaatctacctacaaacaacaccaagacgaccttgcaccaccagaggcacatgatgatattactcgagatgtccccattagcgatgag
aaaaccaatattcttttctttcctacgccggccgtcgatctttcgtacgtttcaaaagagattaagcaaaaaacaaatcactacaccctgttttctttt
ctttttaccctaattatcacttggattatttcccactccattattctttccaccatctttcccatcaccgccgcgtcttgcatttttttatggatggaacac
gttcgcactgttgctcaggccgccgaatggggcgctgaacagaaacgcggtgaatatgcccgtctcaatctcatacctgaaagtgccgaatggat
gaatgatttttaggaaaaatctggggtctcattaacccagaaattttctcctccatcatcgaccaaatcgaagatgttatggaagcttccatccctt
cttttattgaaaacgtccgaatcgcatcttttagccagggttctcatccagtccgtgttatctctattcgttcgttacccgccgacgaaacccagcac
acttttaaagatcatcaacaacccaaagatgaacacaaagatgaaccagagcagaaagcaaaaagatattacaatctcgaagtttgtgtcgctt
accatgcgatgcctatcgaaagtacttctacaacagctcgtgcttccaatctacatatgcgcattgtttttttatcctggtataaaaggcaccgttggt
atccctctccccatctgggttgaaattaaaggtttcgtagccagaattcgtcttcgttgtgagctcatgcctgagattcctttcttgaagaatgttacc
ttttccctgatgggccttccacaaattcatgttgctgcagttcccgttgccgaaaacggtcttaacgttctcggacttcctttgatttccaaattcgtta
atgactccatagcagctgcggccaatgagtatgtcagtccaaagaatatgaccatagatacgtcaaaaatactcctaggtgacggcataaaaaa
ggaaaccagtgccattggggtcattcacattcatattcagaaggcggagggtttaagcaaacaggatgtcaatggactttccgatgcctatatcac
catcagttattttaagtttggcaaaccattattttgtactcgagtcgtaaaacaagatttgaatcctgtttggaatgagcatgctataattgccgtgtt
cccggaacatattaaagcgggtgaaaaagtctccgttgaactttgggacagtgatcgctttagtcctgatgatgtggtaggacgtaccaaaattga
gttgcatactttgctgcagaatccggaaaaaactattgatcgctgcgatgaacttactggcattagtgaggagacgtctcttcctggacatatatat
tattcaattgcttactttccgaaagcagattttagagaagagttgagaactggtggacatgatgtttccattccccgctttctccgagaagatcctac
tttccagaatcctcaagggactctgagtacaaaagaagaggacgccgttgttcacacagctccagattcagagtacccaagcggcatattgtcat
tcatcattcatcaggcggttaacctagaaatcactcatcctaaaggtacatacggaaaggtcaaaggagcttacaacaatactacacctcagcag
gtaggagatacgaagtcagaagaaggtacagacttaccttcttcatatgtttgtgttgatataaatgatagtctggtttataaaacaagaacaaag
gtttattcctcaaatcccatctataatgctggaagtgaaaaaatttattagggactggagaagcacgactatttcttttactgttcgaaattttcgactt
cgtgaacatgattctattctgggtactgtaaacatgccaattgctaaaacgttgacgagcttttcccaactcaccaaatggtatccgattgaaggag
gaattggattcggtagcattcgtttatccattttattccgaagtgtaaaacttcaaatccctcgaaatttattgggatgggatattggtacccttgtat
ttattgataggcgcttgattgctgaaggtatccacagtgtctcaaaggttaacttctcttatgtaagaattcatgttggcgtgctaaaaggtaccgca
aaatataccaactcggattctccggaaaaagttgcttgggatgttcgtagcgataatttgagtatgcctctcaaaaaccgttatagcacgccgtta
gtttttgaatttcgaaatcatcttcgcaggaaaagcaatgtttttgcaatcctatggcttgtggatcttgtggacaacgaagaaacgactgtgcgta
ttcccatctttttttagtgccaaacctgcgcacgtcttgcagaacatgattaacattaacgatccagacaagtattcaaagctcgaaattatcggttat
ttgacgactactctgagctttcaacgtggtttaggtgttgctcacgaaagattaattagtagtgatgatgatagcgccacttttgagacgtatatga
gcttgcgttctcaaggagcgcgaaaaggatacgtgaaggacatgagtaatcctttatatgacaatttaccttgtcatgaggatgagcatcctgcat
caagagttagtactatggaaacagcggatgaaaataccgaaaatgaaagtgaggaaaacccaaacctgttagcaccgtcgaactcaaatcaag
cgaacggagtagaacagaccgttacgcttgagaacaatgaatttaattcgcaaactacagatggccctactcgtgatttaaaccctgacgaaaa
gatttttgaacgagaattcatctcacttggtcaagcagcaagccaaaataataatcatactcaatctttaaaccagtcaaatgttgctagacaaag
caagaaagaaaacaagagagggtctcaggtcattgatgaccaagatgctgaaacattgagcagtgaaatctccggtgatgagcaagaacgca
agttacttcatagggacgacaatgagctggaacgtcgtatgcatcgcggaatctacaactacaaagctgtacgtacgggcaactgggtaaaaga
cggagcgaaaatgagatggagaagtctgaaaaagcgcttcagcttaaatggccgtgagccagatgtggaaacagaaatccctaaataaattca
aaaacaaaacctacaacatttcacggtttatgaattcttctcatgatgttatgactgcaaaatctagaaagcatttacgattacaaatgaaatgga
caactataagtttttaatctcattaatgtaggtaccttatttactcttccttactatttaacctcttcagcaagtatattgggtacactgtaatttgactt
acacattatttattttgtgatatttcgatgatagtgttatttcaaatatcatattgaaacatttcgatgatttcccgaactgatgaaaaaaccagtccc
agtaagtcttagggtagggtttatatgatacagaacagacacgaaatttttgggttgcgaaacacaaccagaaccaaccacgccctcccaaatca
gttgcaagtatggtcgcgttcacacctgaggaggtccgcaatttgatggaaaagccatcgaacgtccgtaacatgtcagtgattgctcacgtcgat
cacggcaagtctacgttgactgattctttggttcaaaaagccggtattatttctgctgctaaggccggtgatgctcgtttcatggatacccgtgctga
tgagcaagagcgtggtgtcaccatcaagtcaactgccatctctttgtttgctgagatgtctgaagacaacatgaaggatgtcaaggaacccacta
ctcaccccgactttttagttaacttgattgactctcctggtcacgttgacttttcttccgaagttactgctgctcttcgtgtcactgatggtgctttggtt
gttgtcgacaccatcgaaggtgtttgtgttcaaactgaaaccgtccttcgccaagctttgggtgaacgtatcaagcctgttgtcgttgttaacaaggt

tgaccgtgctttgcttgaacttcaaatttccaaggaagaactctaccaaaactttgctcgtgtcgttgaatccgtcaacgtcgttatctctacctatta
cgacaaggtcctcggtgactgccaagttttccctgatcaaggtactgtcgcttttggttccggtctccacggttggggcttcactattcgtcaatttgc
caaccgttatgccaagaaattcggtatcgaccgtaacaagatgatgactcgtctttggggtgaaagctacttcaaccctaagaccaagaagtggt
ccaagtctgctgttgatgctgatggcaacgaaaaccaacgtgctttcaacatgttcattttggaccctatctaccgtatcttcgatgccgttatgaac
ggccgcaaggacgaagtttttgctctcttgaccaagcttgaggtcaacctcaagcccgatgagaaggaactcgacggcaaggctcttttgaaggt
tgtcatgcgtaagttcttgcctgccgctgacgcccttttggaaatgattgtccttcaccttccctctcccaaggctgctcaacaataccgtgctgaaa
ccttgtatgaaggtcccatggatgatgagtgtgctgttggtattcgcaactgcgatcccaaggctcctcttatggtctacgtttctaagatggttccc
acctccgaacgtggtcgtttctatgccttcggtcgtgtcttctccggtactgtccgttccggtatgaaggtccgtattcaaggtcctaactacactccc
ggtagaaaggaagaccttttcatcaaggccatccaacgtaccgttcttatgatgggttctaagattgaccccattgaagactgccctgctggtaac
gtcattggtttggtcggtattgaccaattcttggttaagtctggtactttgaccacctctgaggttgctcacaacatgaaggtcatgaagttctctgtc
tctcccgtcgtccaagtcgccgtcgaagtcaagaacggtaatgacttgcctaagcttgttgaaggtcttaagcgtctttccaagtctgacccttgtgt
catgtgcaagacctccgaatctggtgaacacattgtcgctggtgccggtgaattgcacttggaaatttgcttgaatgacttgcaaaacgaccatgc
tggtatccctctcaagatctctcctcccgttgtctcctatcgtgagtctgttagcgaaccttccagcatgactgccttgtccaagtctcctaacaagca
caaccgtctttttatgaccgctgaacctatgagtgaagaactctctatggccattgagactggtaagattaaccctcgtgacgatttcaaggctcgt
gctcgtgtcatggccgatgagtacggttgggatgttaccgatgcccgtaagatctggtgcttcggtcctgaaaccactggtgctaaccttgttgttg
accaatccaaggctgttgcttacttgagcgaaatcaaggactctgttgttgctgccttccaatgggctactaaggaaggtcctatctttgaagagaa
tttgcgttcttgccgctacaacattcttgatgttgttttgcatgctgatgctattcaccgtggtggtggtcaaatcattcctactgctcgtcgtgttgtct
acgcttctacccttttggctcaacctattattcaagagcccgttttcttggtcgaaatccaagtttctgaaagcgctatgggtggtatctactctgttct
taacagaaagcgtggtcaagttttcgctgaagagcaacgtattggtactcccttgtacaacatcaaggcctaccttcctgtcaacgagtccttcggt
ttcactggtcaacttcgtcaagctactggtggtcaagctttccctcaattggtgttcgaccactggagtcctatgagcggtgatcctactgatcctac
ttctaagcccggtgctattgttgctgaagctcgtaagcgtaagggtcttaaggataatgttcctgattacactgagtactacgaccgtttgtaagag
gtatccattgaacagactaggtttgggcttgcggggaaaaatcttataattatttacgaagatagagattacgagaaagtaagtttttcagcgaaa
taatagtgtcgatatcggatattgaaataataccggtgattgtaagcagtaataatcgacagtaataatttagtatgaggattaagacatggggct
aaggttgctggttgtagcagtttgcagtgaaaaagcatagctacatttgagcttggaaacgccagccaaagtttcaaggactcgggtactgcgag
cgtctagtttattgaaatcagagcttttacacaaaatggaaagtatacagagcactactaattacaccttacataaaaaaatttatagacgaataa
aatgcctcagaaaggactcccaaattttatttaaatgttagaagggtaaagcatgactctaagggaagagccaaaggtcttggcaggcaagttat
gagagaaacgagcacgaacacagccagagttaccatgagcgcgagcaatagtactaaatgtttgttagttatatgaaggaaggataaggacta
gagaaggaagactactgaatacggttccagagctttgaaaaaaaagccatatgaactacagtatcgatgacagtcgattacttacccccagatg
acacgaatcttagagccgcggataggcttggcacacttgtagacgtagcaaacacgcttgcccaagtaaaattgagcattttccttagtgtcacat
ccttcgatcttgacaatggaagttccagggtggatgtgatgcttggaacgttggtgggaaaggtgcttagccttaacgtacagtctatgtccttgag
caggcattttgatggtgatacgtcggtgctgaatgaatttgagtagggacgttagtttctgtatcgaaactttttatggcagtcacaaaaaggcaaa
ccctaccctatacgaaggcgtgtggaactccaggttaaaaggaaatagcgaggtaaaaggtaacagcaataggttggttgatgatgggaaagg
agatttgtagtctgcgtatgtctacgaatcctgtctttttgtgcaggatgaatcaaggttttgtaatatatagttatgtatccgatgaaaagtaaaat
gcaatcattgtttataatatccgtacggattgtgtcgtttatttgaagaagaatcggcttcctaattgcaacggctccgtatagaatctgtaaaatag
cgacttcggtggcagggcgttacattcgtagaaagataatccctaaataaaaaaaagaagaatcactgatttgcatgggaaattattcttttatct
ctttcgtattttagtgtaatatttcctcggctaggagcagtgacttctaagagacaaaaggcatcattctgccacgaaataattatatggaatatac
atatgatcaagtggaatatcttattaacatgtctagtgcgaagttgactttcgtctggacttttatttacttattaattactagtaatgtcctttggata
aactaaactaaaggtgtacaaacaagttacttcgttcagccaccaaaatttttctgactgtaaataatgctggaagcacttaggctagaaacagtc
ctccgctccaatcctccatatataaccttttcattgaattgtaatggcatgggtttttacatagaatatcagtgttcttctgtttgctaattctggtttg
gtttcttcaagtagtactgtcataatagctggtttgttgaaattatacgtattacagtaacgggtaatatgaggatattgctgaatattagaatacac
tataagactaaaggataatatgcaaattaaacaaattggacaggtttctgagcggaagttctttacaaaaagaaaccgttccttacagagagtta
aattatgctaacgtttttagtaaagatgtatatactcttgtgtctgcagtcagaattttgagattggaagattcgactggtgtggctaaataagatca
tcgaaacacttccaatttagttaatcagaggacattattagcgtctatatatgtagtacaacagaatccaattaacgtaatcctacaaatgtaatta
ttacttccgctcgatatttattctcataacccgtaagaattactaacttgacaagcatcacatatatattctatagaattattcatattagaatatcga
agtatttcttaatgttcggactttccctccatggcttcgccaatatgatctaatataccagtgatgtttatggctttctatgtaacttgtaccaactaca
aggatcgagttgaaaccaggaaatcacggttgtacagtggtttttgaataattgacggaactttataagaaagttcattttaatttatcgtttcttag
tgaagcgtacgtgtaataggttaaaccatttacctcggtgcaatttatctgccccgttttatagtgtttaccttcgtagaaagatatcattctttatttc
tatattttaattcttttgactctatttctgccagcctttttgtgtgtgtgtgtgtgtgctttctttttcttgaaaagcagtaaagatggaagggaacca

accttcagctgcggagcctgtcatatcccaatctacgaaagttgtttcggaatctccttccttgaaccatcaggataaagtggtttctacggctaaa
gagacgagaccagttccgcaagttcctggtaaagagccgggagaggtaagtattgatttttagcgctgtcgatgatctttttttgttgtcgaaacgg
ctttatttgtcctccaagggagtttgacgaacaagcttttattcattataacgacgtctgacagggattcttccgaatattgtattaacaatggttata
ggttacatcaaccgttagagatgctgctcctaccaactctagtcagtcttcaagtcctagctctccttccaatgtgatgaagggagtaccagcggaa
tctagagctccgcaagcactcccacatacaagttcatcagctaccactgaccgcaaagagcctgcagctcaaaatcctgtgagtacttctccaaa
ggttaagaagccggatgagcctaccggacaatctgctcaaatgcccgctagtcaccctttagaggctacgaatccccaaaaggaagaaccggat
gctcttattggttggagatctttgcataagacaagcgtagaacaaatgcgtacatttgttcaggatgctttgtctgtgtttgaatgggatagcggtcg
tgtagagcgctccgttttggacatgtatttacctggtaaattaaatggtcaatggtggcaaagctcactgattttggcttctatttccgttctaagttg
gttggcttccaaattttcttctgtcgctggcaaattcttatcatcatcattttcctcagcacctttgtttatggctctaatgctatagcccttcgtcgtac
ggttcgtgatgaagtcatccgtgagatgatgaagaatcccactaatgttgattacgaaactatggattggtttaacgttattttccagcgtctttgga
tgttaaatgaaccaatgatttcccgtatgatagtgactaatgttgagcaatccgtcgctgaatatcttccatccttcgtcccagaagttcgcttttctt
ctttcacactaggttcaaaggctcctcgtattgatggaattcgaacccaccttgccactgagccgaatgtggttttaatggacgttgattttagctat
actcctaacgatactttggatatttctggtgcttcttcttgccgtgtaaattcagccgtttctcttcgtgtgaaggttggtaaaggaaagtttatgttta
ctcttcctgtttctaccaaagatattttcgtgtcaggacaaattcgtcttcgctgcaagttgtctgaggagttcccatttataaagacggcttcggtct
gtcttttaagcactccaaaactttccgctactgttcgtccatttgactttagcttattcgatgctgatgttttctacatacctggatttaatcaatttttg
catgagcaaatgacgacgatcttgtctcctctagctatgtggccaaatatgcttgatttggacctttcttctcttatggctggtattgctccttttgctc
ctgcaggtgtaatagctatcaaggtctactctgctcgtcgaaacgaatatcttaagtctaagactaccaaaccttcttcttatgttgcagctttaact
aatggctatgaacatggacgttctcctgttcgggctaacacttacacacctatatttgaacatacgatatatgctgttgtttcctcatttaatgatcca
cttgagttgtctttgtttgatcctgaaagtgctagtttacctttcggtactacttacattaatactcgctctcttgttgagaagggttttattagtgatat
acagccgttattgtttaatgctgtaaatcaaggatctgttgcttatgatgctaccttttttcccttctcttttcccaaaaaagacgttggatggtaattta
attgaaccacctgctacgcctaaaggtattttgcaattgtcacttggttgcattaacaatattgtcgagctttccggtcttgaaaagaaaacacaatt
gatatatagcgtttctgttgattctaaagaaatcggatctaaaacgtcgaaatttgttgaccgtgcccctatcaatttgtctgcgacgacttttattca
aaatcgtgaaactcaaaaaatacagatcactgtttctaagaaaataaaaaaggagcctaaccagcttataggaagtatggtgattccgcttaata
aactacttgaggctaatcaatcaacttatccattcaaagaaaagccttcctctagtatggatatttcatcttcttggattcccgtaaatgttacggaa
gaaaagaacgctgaatcataccttaaggatctcattggtgttatgcgtattagtaccataaaggcgaacaactgcgtcagtagcgattttgctttg
aagaaaaatgatccctatgttagagtacttgttaacggttcagttgttggaagaaccgttcatgctttgaataatacaaaccctatttggaatgaag
ttttgtacgcacctgttttggcagataccgatgtaattgaacttgaagttatggatcatgaggattctggacacgataagtccttagggtttgttgaa
attccagttaaaagcttcaaagacgcttccagaaagacagagaaaactgcattggctaaccaaatctttggcactgatgacattgagacactgaa
gttgatttcccgtaagggaggctcaacgcgtataactctgacagtcaactgtgatttccgtccttgtctaaggattgcttcggatgatgaaccgact
agtaagggagaagggaacaaggctgctgtcaccgaatctgttaatgataaagccgagacgtctagtataacctcgttaatcagtgttagtgaagc
ctgcaagtatccctctggtctttctatgatgtcggttaggtcggctgaacttcaagcagagggcgttgaattaagaattttcactgataactgcgcct
tcccattcttgacaactggaatagccaggagcaaaacacctcgctggtctgcttttggattatctatgattagagagcttgaatattctgaaacaac
atttcagttgactgatggttcgtccaaagatccacacattattgctgaacataccgagaaaacgtctaaacttattagcgaatctttgggtcatgta
tatgcagtcgatattcaagggccgaatggtaaaataaacagagttcgtatctcgatgaattatcttccagtacctatgctattgaacccagttgaa
agctttatcaacacaggatcattgcattttaacttgtataacggaaaggatcttcccgtaggtgacattcgatcctcggatccttacgtggtattgaa
gatgaacaatgaggaagtttacaagagtaaggtcattaagaaaaatttgaatcctgtttgggatgaagaaaccgatgtaataattcaaaaccgc
ttgttggatacgtttgatttaatttgctatgactgggatctgggtgaggccgctgatgttttaggcagtcatcatatagacctgaattcattggagcc
gaatgttcaatctcaaagtgaattaaaccttgaaggtaaacgaggaaaaattaatgttggtttaagatttgaacctggttggtatcgaagatctcc
tgaattgcatggttctcttcccgataatttcttttccgttgctaataaaggtgccaaattcttaataggaggtattggagctgctggtggttttgccat
aaatggtgttggttctgctggtgatcttgccttaaacggtgttggttctgctggtggtcttgccttaaacggtgttggtgctgctggtgatttgggtggt
aaagctgtatctggatttaccgatacaggaaagaatttatttaagggtgttactggaaaaggccctaaatctgctcgaaacaatgagcaaattga
tgctatgagcacccaaactgcttcatccgttgcgccaagatcatcatcagctgctactatacctactacagctggcaagcaagagctctcaaaatt
cagactctttgttacaaatggtagtgattgcccccataagcttgtgaaggttgtaatcaccgataaacaaggtcgtacattcaagacaaggtcaca
caaaggtccttctcctaagtggaatgaacaaataccgattggatggaatcttggtgatgtacttcatgtttctttaatatcctcgaacttaattggac
acagtaaatttggtgaagctacttttagtgaaactgctgtaggtactttcaaagttccgtttgaaggttctactacttctgtggagatgagagtgga
agctgtatgatttataattaacatctgaattttattaaacaactttttgtagcatcaacgatttgctatgttattattgcttacacagcatattgattat
ctaattgctaacatagaataaaatgcaacttcttaaagtagaaaaaaaaaataactctatttgtaaaattaaaaatcgtaaaatacccagaataa
ccaaactgtaagacatgaataaaaaaaatgcaaagtacaatacatttaagcaataacttaattataaattttgaatataatcttccgccctcttcc

cttctttaagaagccattcaatctggcgttcacattcctcttcgaaagctttagcacatctagcatactgatatttaatccaattctctactgtcatatt
taattcttcttccgtcagatcctccgattcacgacgtttgtttgcaggggtttccaaaaggctagaaaaatcaactggttcccatggcggaagcgaa
ggctttatagacggtggagcaggtttggtcctcatcggtgtacgaggagccagaaaagaagattgcgaaagaggatcgaacgggctttttgaaa
tatcatcagggaagtgctttgatggagaagcagaaaattcgggaagttcgtctaaatggtgatgaccttttggagtgctatgcaaagaatgttgg
acttcatatatatctgattccccgggggaattttgtaatggagtcttagaattccctaaagaacctactgcttccttcgaacctacactctcgttttctt
ccgttttgaaaagattgtttgatagttcgctatttgcattagtgggtttgtgttcttcgcttctaacctcagaactcgatccttccaattccttctcagag
ggtcccaagcttttggaaaaggcattttctgaatcatggaatatctcctcattggtaggtgagttcgtgtttacgtccattcgagtatccttccttagt
gtctgttcttcatcttcttttgaacttgataaagtctcggtttgcgtatccaataaacctctgctttcactcagtaatggatcctctgatatagtagaag
gccgttttaaagcggttctctctatatttgtcagatttcgaagcggtttggaatcctgatagtctcctttgatctctgaatgtttagtttcacttgaaca
tcgttgacgcgaatcctgtttaggagttgaatttatcaatggatcggaattcattgttttattgttattgtttttcaaaatttcatctttggttggagtga
tcgaagacgcggttaaggtatctgattcaagtgcagaatctctatcaaaatctgaagttggagtatgtacagcctcaagactagaggacgaattg
tccttcaaagacgaaacggaatgcttttcacttctatttttcgaaatctctggacttacatcttttccctctgtcaataccttgtgcttaggagattgaa
gcaatttggctaaaggttcatcgtcactagttataaaggaagaggcgtgtttattgatcgaaggattttgaggaaaatcttcgaaaatacttgtttc
cgcagaaaaggcttctacttctgattcactagaggctagctggtttttcgtatcattagctgttgtgctttcagtagacagctgtcgttttacggttgg
aagagtaagttcggggacagtatccatctgctgctgatgagaaacatcttcaatttcagatgatttcctttttttagatgtctgaaagctttcttctgg
gtatttggaattaacgatcgtttcagcgggaaaagtgatgtttgataagctcatagtaggggtattcgtaggttcagcataattggaataatttaca
gagttttctttgctatttgattcaccggcatcttcttggatagggggtagagcggtagccacaggagaatgaattgaatggtaactttcatcagttct
attaggtgaaggaggagttgaaaggggtaaagtagagttaaagggaaaattggagccgttgggaaggatgtgtgatgaatcttcttgttgttggt
tatgtttttttaggagtgttcgtagaaaatttgggagggcttgatctgaaatcaggctgttcgtcttcgtcttcatcagagaatataggcctagctggtt
tgaacactgcatcttttgactttctcctcaactgcttccctcttcgtctaggagaagtaactacagcaggctttgatggggatgagccatgattttca
gactgtacgagagaagagtcgtgattagaattggagtataaagaaaagggatgagattggcttagagaggagattctccttcggggagaagttt
ttgattcgtttgccattttggaaggatttttttctgacgaattagaaacaagctcagatacggaaacatcttccagatcaacatttgatgaagaaaa
caaggaaagcttggttggagagagcgccgtcgggtctgtccacgtaaaaaacaaacaatcaggacgtcgacgttgatgctctttaaaaggatca
tcatcgggctcccagtcatgaaggtcaatattacagtacaagcaatgcgctgcatccttggagtctgtactaggagtaaaaacgaagccagctgc
agccatcgtattcggctggcaatggaaatctgaacgattgatatatggccatactttatcaacaaacgtcagttctctacacttcgttaaagaagg
agcccttggatcctgatgaggattatttcgagccgacaaaacgtaagcccatgggcaactcggactatgtgttatgtgctcttttaaaggatcatca
ttttcttcccagtcataaaaagactttgaacacatgaagcatgtcacattgtcgagcctttgttggggcctttgaattggattgtagtagaatccaac
ggcagacagctaattatagctatgttagtatacgaaagaccatggatgctatcatgtggaagcattttctcacatactttttctggattcggcgaac
taagtgaccatgagattttcttgaatgtttcgagtcttttggaaacattacacatttccttacgatgcctactccatcttcgtttagatgatggttctac
aggcttcatcacggacaaataatgcaatgaatgaataaaaacaaatgatccttaaccttttgtcgatcctgctaaaaacaaaacccagtaaaacc
gtagaagcaacgcctcaataattaattctaaattatacaaagcttctcataaagcgaaaatttatcccaagaaatttagttcgatcttgtattgttta
cgtccaactgagtaaggaaagtaatgcaaatacggtattggcttatgtatcgttattgagacaacaccgatgctcttgaatctgtattagttttcga
cgtttaggtacttcaggtcgttatacgttaataaacatatgcttgctgtaaccgtgcgatctcatggtagaaaatttggaatgacaaggattcatgtt
gcatttctattgagctttaccttcctcttccgttgattacaattagttcttgtaaaaagattttgctacaccttcaaaaattggttggcaaatttgcatct
aatttataaacaatcattatcgtcattcatggtattttacttttcttgaaagtaatacgaaaattaagtatctttcgcgcaaaaagtcaaaaggaata
aataccctttttgctttatcgtcgaaacaggcaaattaggctgctaaaagatgatagcacgacgatccattacttcagttatacaaacaagaaatag
aatacgcacaaaacaaacatggaattagtaaatgaggaaaaagaaagaaaacaaaacaaacttaagaaaaaaaatacacaattcctcaaca
ccgttcagaatgccaagtcattacatgggcatatcttcgcctttattctcaaagttgaagttgctagtcggcaactcactagatccaaaagtgaatg
tgtcccctgcaacatcaggttctagatcctcaacaacatcttcttctccaaaatacctttcaatgatgttatatgccttctgatatatttccgcatttgc
agaattttgcaattcgtagataaggtccataccgccagcatcctcaatataaacagcgtataagttaatcttgtcgggattctcggctcgatcaagc
tcgccaacgcgaagtatgttttcaattccgtctaaagcaacttgaataatcttattatcctgacaagcaagtagattacacaagggcttgatagca
ccttgagaaacgatatatcgaatttgacttggttgatttataccaccagaggtagcgttggagacagcccagcaggcttctttctgaatcttgaaat
cagctgttgcgagaaggttgatcaagggaggaataatgttggcttcaatgacatactgaatttgtgaggcattgcctgctgtaatgttacttattgt
ccagcaggcctccttacggataccatcacgaggatggctgagcaatgacaatagagcacttagagcaccacaattaatgatgacttgtgtttgaa
gatcatcaccagtgacgatgttaccgacagagcgaagagcgggagtctggatttgagggttaggatgcattaaaagctcaactaaccttctagg
gattcctgcatctataattgcttgaatttttttcattgggcccgtccgataaataagaaatagcccaaagggcatctactataacatcatcatcaagt
gaatatattaatttcgacaaaactgggattacgcaagagattacattccagtctggttgagggtttttaccacggcacatattcgataaggtccaa
gtacagttgcgaagcatactaagacgcttacaagatccaatgacggctagcaaaggttccaaaactccgcactgaaggacgtgatcacggcaca

tgggtgaatcaccagcaatgttaccaagagcccaaacagcttgctcgcgaacatccttttcaggagatgaaagtaaactgacaaaaatgggaac
ggcaccagaatttacaacggtatgagtctgatctgaagacccgcttgcaatgttggttaacgcccaggaagcctcaaacttgagtaaattattttc
gtgtcccaagaattctacaaaacggggcacaacaccagtgttgatgaccttttgaataggtgggtcatgctctttcgataaagctttacgaaactt
ggtagtagcctggatttgtatttcaatatcatcggtatacaaagctcttaccatgttaggcaactgtaaatccaactcggagtcattttccttttcatt
tactatgggcgtaacttcttcagtaggctcttcaactacatctgctaaattacgacgcttgttcagattctcttcgcgcttttgtttacgaatttcaattt
gctgcgtttcacgtcggcgacgcaactcatcagcttgaaaagctccttttcctttgtaattttgtctcctatgttcaggaataaaacgagaaggcgta
ctcatgttcaattccagatttccctatattctttctggatggaagtggtaaaaggctgggccagctaaaggtacgaaatgtgaagcgttctttcgaa
aagggaaaaatgatagtcgtaaataaaatatttaagtttaaatcagaaaaatgtacaaaaatgctttctattactatattttcgtgtttttaaaata
gtctgattttgtctgtttttggaaacatgaaaatataaaatgcaaatattgatgaatgagatttatatttcttattaaaattatatagtaaattaattc
atttgggactcaaaccacttaaaggagaattccaagattaacataagacaagcttcttataagacaccttcagcaaggttgatgtgctagcttcgt
gacaaatatagtgtttttgtaagaggaaatgaaatgtatataggttattgttgaagaaattgagatatttatccatccctttatactttatactttgt
aactttagtctgcttttgaactagtagcacatagtgtatacaaacaagagcttagttgaggtaaatgtactatagtattaactatcgattgagattct
atcttttcttgtttcaacgttaatctttaaaagtgagaaatttagaatctgccatgtctgacaaaactgatctcaaaactcaagattgcaaaagaaa
aagaatttcggctgtagcaattagtaagataatattaaatcttgcaacctctactttttacctctcacactttgatatgcgagaaggaatctttcaac
atacatataaaataaatagaaagaacaaaggtattcttctggacagccagataaatattttagcaatcaaagccaacgaaagtagttaacaaga
gaaagaaaactgccatcctgatatctaatctcattatacactcatgttgcgttatgaggaatgaaatcgaaagtgagtcctatgtcgactgttgcat
tccttgcttattcctaaatatatgactcatatttattgggatactgctttttaaagcactaatttcatgtcgtaatgtggaatatcgtcctcaagatagc
tatcactttgaatcataaatccaaaagattggtaaaacgctttcaaataagcttgggcctgaatatttacgacttttgcttctgggtatttacctctac
cggcagctaaagcttcttcacaaatcctgcgtccatagccatatccccgcgcgtcaggtgaaacgacaagccgtccaattcgaaacccattgggtt
cagatattactctagcataagcaagaattttatcctcttcatttttaagcatcaagtggtcagatatcaagtccacttcatccacatcttggtaagga
caatgttgttccacaacaaacactgccgtccttaaatagtatatagagaacaactctttaggagttagttgatcaaatggtttcaggaccagcagc
attgtgtgataacccaaaatatggatttccggcttcctttttcaatcaatcagtttaatactactgtccccaattccttttgtattgccaaatacggtgc
tttgtgctccaaggtcatatactaccttacgggattctaagcttgtttcttaatacgaatagagcacgcaaaatagctgtgtatcgctacgttaaagt
ttatagaaaagtttccttgttcatgtgactgttttctcgagagaaaccattttgatgaagggtcaagttcaggaacttgatataaaccagttacatac
cacaatgtcggaacacggagaacaagttgaagctgagcaagttgacgttgttgaacaagttgaagaacaaactgctgctcccgaagtttctgttg
aagatgctttgaaggaagttttgaagcgtgcccttgttcacgacggtcttgcccgtggtatccgtgaggcctctaaggctcttgaccgtcgtcaagc
tcacctttgcgtcctttgcgagtcttgcgaccaagaggcttacgtcaagcttgttgaggccctttgtgctgaatctgaaactcctttgatcaaggtcg
ctgatcccaaggttttgggtgaatgggctggtctctgtgtccttgaccgtgatggcaatgcccgtaaggtcgtcggctgctcttgtattgccgttact
gtaagtttattgatagaagaagcagtgccaagtggtgttgctctttaaacttgtgtttttacgttttctaaagactaacgaaatttaggactatggtg
aggactccgctgctcttcaaaagcttttgggtagcttctctgcttaaaaactgtttcataataaatatgaaaaggcaatatagcgctcgttcattctc
tattattgtagattatctttgtttcgtaagcacgaatacggttatggatgaaggccgccagtcataggttcagaaaactgatattttctgttttgaaat
ccaatgaagcgaaccgaggtagtgtgttcaagtagaagtcgtatggactagctcaaaaagaaatgatttgtcacaggtaatcctctagaaatgcc
atctcatccgtataaataagtctacaatggtgtaattgctgtatgagtatatatgtcataagaatccgatctcaagattttttttataattcatgcatt
cagtgatgtgaatatatgcaatattgaagagggctttgcaactaatttcaatgatccattggatatttttggtcaggtgcacttgccctttttcatatat
tgagaaatcccaatggccttttatatttagcattgacaagtatctttcttcccaatagttatttctgcttttctctgtagctaatagtcttaataatcctt
tagaaataatcatttacgggttcattccccattcatcctttcattttttttttggaatttctttgctgattgaattcagttcttaatgggcgttccccgttt
aggtcgatttctggagccgtttgggaaacttattcattatcaagtccatcctcctgtctctcctggcaatatagttatagacggacctgcatttgctta
ctggctttggtttgctgtctctatttcaccttcaaattatcgctcgtttcaagatgctatcctcagtttccaccaatttctgttaagcttgggatttcttga
tatgtatgtcaccaaattctttgagtcttctcaattccttttttgattgtgatcacaaaattggttgctaaccataatagagaatatgtatttgatggtgg
actcccttattctaaacatcagactcgaaaagcaagatatcaacaaaatatctcagatcccatggatgcttatgttcacctttgtgttccgattgca
catcatgtccttaaaggaattgcgggcgcaaacgttgtcgtttgtaatcaagaagctgataagtattgtgcaattcaagccagacaattagatggt
attgtactcagccaggattcggatttgcttctctataatctagaagcccctcatactggttatgtgcctttacattcaatttcaattacttctgatggct
tacatgggaagctgtataactttcaggaaataaaaaggcatttttcatttgatatccatttactagcggcttacctgggagtcgaaggccatccgg
aagaccaagtcatcgattacaacagcagttttcaacagatttgtaaagtacttgaagaaaatgtgaaaagtgggactctggaaaagctcgtttca
gcacaggaattacgccgtgttcacaaattctattcattagatgatgttagtttatcttcttctcagttaaatgattttatgtggggaagagtgcaaga
acttttacattctacgttagagcctccggagatgtggcttcctcagttgcctgaattaccagctcgtccttgtagttggactgagaacgcacctctca
ggctgcaggcgtatgcaaacttcttttccaaagaaaaatcaaaagcatatgtgctagagtactttcggaatcacggacagcttagcaaaaaactc
gtatctattaattttgactatgcttcggtcatagatacaatgcagaacaagtttgctagttggcctcttcagcatcgatttgtcttgtggactctaagg

tgccttaaaaatcttacaaatgttactgctaccagctttctattgatgcacgctagtctctacctaaatacacctttacgtttgcagtttgttacacca
actcaagaaggcatcgcactagtttcacgttatattgctacgagctactcattgtgtatgttaatatttagtgaatttgataaagaccgatccatcga
ccttcgaagtctctcgtccttcagtcctctttcgagcactgttaactttcgtttattccacgaagcaatgagcaaactgaaaacaggaaagagtcca
ttaagtattgtttcagacaaagccactgctgaactgacttacagattgtataaagaccttctcgcagactataaggatatggaaatagttatcaat
atttggaacacagagatcattaaaaagaaacgtaaaaaataaaataaatatattgattatcacaaacaacaccattaatcacctttcttttcttta
aaaagactagttatacacgtcaatgccaaacaaaaaccattatcttttcgaaattctgtaaaactgtatgttggtattaattcctttaaggattagg
aggagatgtgcctggaggcatgggcatcccaggaggtgcgccaaagggaatagactggggcatcccaggagcatttgctactcctggtgcacct
ggtaacggcatgcctggaggagccgcataagcagaaaatgcagaaggcataccttgcggaggagccattcctggtggaagcataggaggctgt
gggagattcatcataggagtagccaacatatccggtgctgtactggctgttggtagtccagggggagcagtcgcaggaacaggtgcgggaggaa
gaccaggaggcatgccgggaatgggtacacccggaagatttgaatcggatacaactggttgggtattctgttgccaagggggcaacccaccagt
tgatgtggaagaggcaggctgagcaggtaccgcttgttgccaaggagcaggagcaggagtagaagcaacgctcggggtagctttcgcccaagg
agcaggactagaagattctgatgatggccctgtgttggcagcccatgggggtggtgcagctgagtttgtggtggaagtttcagtatattccaaagc
gtgttggccagcagaggaagtaatagagccaccacccaattcttgcatgaggctttgatactcacggtcagccgcggcatcacctacgggaggct
ggttctttacaggacaatcccgtgcaatatggcctgcgtttccacagtgtctgcaaacaatatttagggtatgattaattcgttcgggacagtcata
acgacgatgaccaacattaccacagttttgacaaacctgattctcgtcatctcggagagtaccattgagagtggccagttgacggagttgatttct
ctttagatcattctgaccttcaggagtggaagcagcagtttgaatcacaatatcgattagtcgaatggcaagactaattttatcttctgaatcggcg
ctcacaagacaatgaaggtcttcttccatattgccgcgtacagatggatcgcttttaccttttccttctttgacacttcctttaccacgaattgcaattt
tggcacctgatttcgactccatgtctttcaatgtatgaccacgaggaccgattaaaagaccaatgaagttgatttcagggtaatcttgcacaggaa
cgtaaaccttttcttgtgtttttgctgggcggcgataatcgctaggagccttaaaattgggaacagtcttcatagccttttcgataatatgatgacgc
tcgtcctctagctttttcttgtagcgaatttctcttgtattcaatcgacgaccatggttgtcgtattgaggaggaggagaaggagatcgttctctatca
cgaggaacaacgtttcctgtacgaagtttttgggtgatttcttcaagtcgtacattcatagcaaacatttccacttgttcattcgtcatacaagattg
aatacttagttctaattccatttgagaaggcaacatttcatcaataggagtcggtttgccccaataatgtcttctgtaagaatggcgtccacgatctt
caggatatcgattttcatttcgacgataggaagattctgattgatcagcttgatcaaatctgcttgtagagcttacagaatcaggcaatggcggca
aggagtcgggtccttgatcaaaccgcctccgaccaagaggaacattgttagatcctgtatatgttagcaatgtaagctgtttcattctttttcttctta
cctgtagaccccatagatctcgctttcagcattttgaaggggttctgttttgtgaacacgagagtggtagaggaagaaattgggtgtcggaggagg
caacatgaaaggacaccaaccttcatacatggaagcaaccattttctatttggaatgaattcattcgtaaaatagtatctatataactctatctcaa
cttatatatcatgaatagtttgcttttgggccatgaaaccaagaaattttcattgtaaggaggccatacaaaaagatgccatgaccatcgagatgg
ttatcttccatataaaagaatcgctagaaacaaaaatgacattagaaaacaacatcattaattcatacgagagccatttatgatcctcttttaaga
tcttagaaatgcatatttgcttactactgaagcctttcgtagtacttaatcctatttcgtgggatttgttaataaatatttacaaggcaggcacacata
tggaaaaacagaatcatctaagaaactcacaaagattcgtgcaaacgatctcagccgtaagattcatttcgtttttatatgcatacataggttcca
aaggtcatcatcctatggtcggatcctttatggtacattactgcaaagatggaagagagcactgctcgcttgtattcaccgaattcttttcctaaaca
attccttctttatcatttggtattttttagtaataaatgtggtatagacaaaaaaaaggtacttacagtagatcctcgttagcacgattttctttggctg
atgcactagatctctggaaaaaagtctatctgaagtgttaattaggatatagaaaacgaaatctaaaaaaaataaatgttcgaaaagacccttg
atcgaaaacatgaactctacagtgttcttgtagctaggaacttgccagtcaatttcgcaaaagttttccagcagctagctgtctagttggttctgag
aagccttatttcttcttcaaagggataatcgtaaaagtagcttcgtttcttctttctataagatccaattctgtttatttactattataccgcactttagc
ataattgaaataaaaaatcgttactagtagtataggagcagaaaaaacagaattcctcgaaaccgaacgatagagagaagaaaagaaaaaga
aaaaacttaaagagaattcaatttgccaactaccatcctcccaattgcatttccccattgtcgacccattctctcaacaggaagcccttttacctgttt
tgatcaactggtgtgaaaaacaattctagctaaatttgaaactttttgaacatcctagaaaaacgaatagctcttgacgctctgtacaatagactc
caggtacttacgaacatctgtatatatagaaacttggacatttgagaactgtttttgttttttttagacgcttttctgtttgattccctttttgtcaaatc
ttaaaaatcacgaaacatgcccatttctagtcattctcacagtggacagtaagggccaccataatgcgttgtcaaggtattaacggatccagattt
tgtttacacgctcaaggaaccctagaacaggttatccaggaagctatacgacagggattccacacgttttcattcactgagcattgtcctcgaaag
tatgctgaggatttatatccagaagaagtgagtattccattcttttaagcttcatacctgaattctcttttctttcccgcctcctttctttcttctagata
aaaaagagccagaaacgaaacggattcatttgacatatctcagaaaaaagaaacggaactaaccaatcgaaatgtagtctcatttaactcctca
agatttgtttaacacatttgatgactatgtccgtactgctaaaagcctacgagaaaaatacgcagacaagattcagattttaattggatccgagac
agaatacattcgtccagactctatcgatcttttacgggatcttgttgtcaagtataatctggattatctagtaggcagcgtgcaccatgtaaatacg
attcctattgatttctcatcagaactttggcacaaagctctggagcatgttgatggcgattacgagaagctgtttttagactatttcaatcatcagttt
accccttttgacagccttacagcccctagtcgtcgctcattttgacttgatttgcttgtatgcgaacgaagaagccatgcaagtattcatgagttctac
caaggtttgggaagccattgaacgaaacatacagtatgtagttaactatggcggtgtttttgaaatcaacacgtctgctttccgaaaaggatggtc

agtcgcttaccccaaacctcaactggttaaagtaagttttctatatttcactgtgttaacctgttggcagttgatgcaaaaacataatggtcgcttta
ccttgtctgatgatagccatggtcctcaccaggtaggtttaaattatcatcgaggcaaagcgtatctagaagatgaaggcattcatacgcttgcgg
ttctttcacgtagctctgagggaattcgtactactttccaaatgatccctgtttcccaaatatggtctacctttgtagggaagaatggcgtcgctctat
aaataggtcttcatttttacacgcaaacactttctttttactgtctcaaaaaaaaacccagtttttagaagagaatcctctttctttgtcgttgcaac
acatgtttcacttcctacagataagatggcttacgctctctacattcttccaacttgaatttttatttcattacgccgaatattgatttcaatacacaaa
tcatcgataagaagcaaaaccaaagaatctaattatatcgacattacaacagacttaaaagatatctattgtaacaaaggaaaaacaggcccaa
aacaggttgcaaacagctatgttctttctctagttatggaacttatattctcatatttgtgaatcccaaagacttgaacagatctaggaaaaggaag
tcttttttagaggactgtaaacgtgaccacaaacgaacttttattagcatattcactgcgtcatggccctatgttcggaaaggaaactacaataca
tcatgaacggctcgtaacggatgaaaggggaggacaaggaattaaggagctgttggaaaggaaataaaacaaaacgtgaaagaggaaccga
aatatgcagcaaaatctcgttacaagctaaattaggtttgcctattaagagaacaaaaataagaatgccacaaaccactgaaatccgcaaggaa
ccggacaagattaacaagaaggaataagagagaaacaaaaaatataacacagcaaagccatacatccggacaaagaacgagggtaacaag
aaatcactaggtaaaagaatcaggccattcaattaatgatcaagattaggggcaatgctagagtgaagaccgctgcgaatagcattcatattag
gactcatatttgtacccatgtttgtacccatgttcgcacccatatttgtacccatattaggggtcatgtttggggtcatatttggattcatattgacgct
gtgaggtgggccataaggggagtgagtttccatttttgccaagatgtcattgtgatcgagaagctgatgaatacgcttgttatcggtgtacagttct
tcaacacgagcgctgagttgcatgatttttcttcattgtcatggagatgtttcgacaaaaagttaaccttagccactaacatgtcaacattttgttg
atcgtcagtgagtttttaaggcgttcgttgatctcatcattacaatcacattttcgcttgccgaccaaaaacttttcacgatactgttgtaattcactt
aatcgggccttccactcgtcacacagttggtcaagttcatcttcttcaggagtagttttcatgccgggttgacgatgggcaagacgttgttcgtaaa
cacgcataattgagatgatttgattacggcaagcagattcagtatggatagcaagtccttcgttctcataaccgatgcgggcaatcttagcataga
attcgctagtcttgcgatccaccaattcatgcttatgttgaacaaccaagcgcatcaatttaatcttttcttccaaagacaaatgagtacgctgagc
ctttgcgggagaggaactaggagagagagaagcatttttcgtttataactcttcaactccattttaggacctgcttccatacctgcaaataatgag
ggatccgtcttgggcaactggggtgaaatttggtgcttgggtagcaagcgccaaacttcgagaaatgggggaaaccgaaaaaaaaaagcgggc
gtagagacgcgcgcgtaaatgctataaatgcgaaagggaaaaagcgacttgctggaaagatggactacggctacaagaaacggcgagtccaa
caggaaaaccaatggaaaatatttcctaccaacaagaatgatgctactgaaccgtaaagggagacaaagcaaaggttcacctcaagaacgaa
gattgatgaacttttaagcaatcaattatatataaaagtccaacagctaatcaaacaaggactgttgaacgattaaccaaaacaaatcaacaag
cacggtaatgcaatgtaatccacagattcaagaagggaacgttgtaagaatacaacgacaagcaacgaaagctttatattgaaaaaccaaagg
ttttatagcgttcgcttcgtagagaaaaaaatagtgagacgaagaatctgaagagcgtaaaaatggaatttcgagtacaagtgaaaccactaaa
agaaacacttttcagaaaacaaaaaagatgctttatagcgtaagcttaagagtcaaaagcaaacctaacgaaatagaagaaagatgctttagg
aagaagaaaaaagcaaagcaaatggagggtgatgcgtggttgaggacgagttcgttgggatacgcagcactcttaataagatagagtgccacc
aaaatgcagccaggttcagtaatgatgacatacggcggggaaaagaagtggtcacaagggaataaacagtggccaatcagaaaaggcaaaa
agaagtgagagaattctcgaacgtatgataacggactgttgcgaacgctaaccaacgagtgacgttatatagaaggattgtgattggagaatgtt
agaaagtgcattaggtggatacggaaggacaaaatttgcatttaaggagacgtaagaggaaggagattgaaggaaacccttgataagccttca
ttacacaatcgaacgttactcgttgaggaaatagtggccaaaatgtagggtaggggctagttccccaacaaaagaaattagcgcagttcagaaa
gaggcgcaaaacaaaaaattcgacctgcaacgagggtgagagagagaaataaatcgcaaattcttaaacacgagttaaatctcctatggtgtc
atggtaaggtggtttagctctggaatgaatcatgaagatgatgtgatataaataaacaaataaacaaacaaataaaaaggaataagagtgtagt
ttcgatgtaatacagtatgataatcgttagcaaccgatatttacaaaggctttgttataaagaatataagaatcggtaataaactcgactttagcac
catgagagaagtgtttttatttacgaattctgactagcaagagacaaagaacggaaacagaaaaataaatatgtcactaacttttggttttcgtag
gtcattccattaatatgcaggtattttcctttttcactcattttatagaagtatcgatatagtcgatagaaacgccctccattttagcaactcaatcttt
tcacggaggcactggctttacaaactttttttatttatttttgtatttcgttaaaaaaacgtgtcgtacagatctttgtacatcatgttaatagaaaagc
ccaatagcaatgtatttaaacgatagataaaaagaaactaccttctagattgaattgaaagttactcgttctaattcacctttacatgttgcattac
attaaccaagcagttattttgctattgctgcttgtatccaaaaaaatacctgagtaaaagtaaacaaatactaccagagtagagtgagagtaaatt
ccttcattaaatattcgtagagcttcaacgtttttaaatttgaaagattaatgtaggcatatgttttttttttgtttttaattccaaactatcaaattttg
actatagggtatgtgaaaagcaggctagcagagctttaacgtttgagaaattgactagaagttaccatgtgtttatatagtattaataagtaataa
ttaatcatcaattagtatagtctcactctattgaagtatgcctaccaggaaggacgcatatttttttgttatattagaaaagattatcgtacttggaaa
tgggaggatccgacttaatagaactgtaatctaatcagcgtcctagtgaatttacaaaaaatttgggttgtacaattgtggattttgtgcaacgcta
ttgatgaatttttatttctatttattgttgaaaaatgatatatcaaataagatgattcataacgcaaggtttttggtaccgattgtttaaaatcatggtt
tcaacgcatattaaccaatctttatttaatagcacagaaaagagattttattttgaccttaagtattttgatttgaatttatacaagcaggattacttg
ggaatgacaatctgccttctatgccttttatgttcttgctaacttgaaaatatggacgttgtgtaagaagagtaagattcgaaaaattgtttatagga
gtgtctgctttttcaggtaagtactatattgaataaggaaaacaatgggatattccttttccttcatctacgaatccttcattcatttcaagttgttacc

tacaaagtaacgaccaacaatcaaaaggtgaagtatttcaaaaacatgagattgagaatagtttcttggcgatatatcgaattcaaattgtaattt
tttgtttatagttggaatcttgtaagttcatactgttcaagaagagaagagagaaattaagaaccggagtcaaacgagcagtctagctaaaaata
acttcaaagaaactactgggtgtaatgtagttgaagaataagaacaaaactgcatcttacataatgagattttaatggaattagcttgtctgtcttt
tgtaaagacaacctaactattcaggtaaaccattgttcaacgttatataactttctttgcagttcatttattactcatcatataaattgcctttctaatt
ccggtcaaattgaagatttatgaataatataaaaaaaggcgttactcatcttgttcttttatgaatgaggacattctaatctcctctacgataacgtc
tgagaggtacataactgttctaatatacttaccagatactttctatcatcataagggttcttttttatcaattcaataatgatcaaccttttttaagc
agcacttgtttgtgaaaaaacgtatataactaatatagtataaaaaactgtttgaaaaagagagcataactagaatgaccgtttattaatcttttat
ttacagctatatgagaattcatcaagcttcctcttgtattttgagttttccctccttaggcaaaatgatcaacaccaagctccaatcttaacgctcata
aatgaagaaccatttgtgccacactgcatagatttacgcattaaagaacaagtccgttaggcatgattagaaagattcgaaattcgctggtcaca
aacaaatcctgggtcaagggtactaggactgagggttgacaagttatttttgttttatagattgcttatgcgggtcagtacctcttgattatcttttaa
ccattcgtaaaaattcattgcaattaactgaaaatatagctgtataaataaatcattattcatatggttaggagcctctagttcctagtgaacgaaa
gtgcatgctaatcataacaaagacgtagtctgacaataacaaaaaacaagaactaaatgagagtattaccgaaagagaaagtgcatacttaat
gtttaggggacaagaaagcaattggaatttcagatcaagtacttcttaaaaactgcttcctttcaaaggacttaggttctatagtatcttcgcgttca
accgagtgcttccatggttccgcatgcactttaccatcacttacacattgtgtaaccggtaaagttattgtctttgtgaaagattccagtatttcctga
agacgaagggatttttctcgggtgaggtgagcgtattcatgtagatagaagcaaggttcaaattcagaagactagtgtcttcaattttacattcag
tgcctgcaataagttttccattcacccaaacctgggcaacattgcgatcatcaccattaaacacccatttactcaagatgttttcccaagtatcacgt
tcgtaaatatcaacgggagtatttgtttcagaggaaacatctacgacaagagcatcccaggcctttccttcttcgaagctaccgacctcatgccca
cggcccaagacttcggctccaccttgtgtagcaaggtacaaaagttcgggaagctccaaagcaattcttgagtcccttaaaactgaagcaagag
agcgtgatgttagagctgcttgacgaattgcaacaagcatgtttggggtgtaacccccagagacatctgttcccaaagaaactttgatcccggcat
ctaaaagtttgcgaatatttgccataccactggccaaaacggcgttactagttggacaatgagaaataccgctttgtcttttataaatagtttcaat
ctcttcgtcttctaggtagacagcatgtgccaacacagttttcggtgtcaataagctgtgatgatcatagacgtcggtataagatttccgatcaggg
aacaactcacgaacgagagcaatttctcctaaattttcagatatatgagtttggataggaagatcatgattagcagcaagttctccacaggctttc
agcaaatctttggtacaagaaggagcaaaccttggggtaatgatgggagaaacaaactttcgattgggatcaagttgtgacataaacgaaacaa
gtacgtttgtagcctctacagaggacttacaagacttttcgcaataatgagccggtgaaagggtgttcatattgcatttaccgatatacgcccgttg
accaagactaaaacagagttctgcaagtaaagccgaggcaggagtatgaagagacgaaaagtagctagcaaaagtagtaccataagaaagtg
tgcgggtgacaacacggtaatagatttccaaagccctatccagattgcggaaagaagactctattggaaaagtatatttttcaagccatttcagca
agggcagatcaatgccgaaaccagcatttgggtattgcggggcatggatgtgcgtgtcaataaatcctggaaagaaaaactgtaagggctttaa
tacgtgaacttcggcgttcttcaacatgtgatgggattttgcaaaacgaatggtttcatctgttacggattttgcaagaaatacaatcgtaccttgat
gcacgcctactgtcgcgtcttcaatctccagtgtgcctagtgaaggggtatgaatgagttttcctacaaagatgatatcaatgacttgaaccatttt
gtgagaatttcgaaccaaagacaagcctcaaaatatagtttttcaagagaggtttcgattcagcttcaatatatattgatgtaattaaagcgaagc
cgacaactgtagacagcactagttgtaacctgtagatctattgaaatacaaattgcaatgcttttggaaatagtaacaagaagtcgagcttgtaa
agatcgtcaaaatagtaccttttgtatgtataatgccttggtaccttagaatttgatgaattttctcaccaaaacaaaaaagactagtgttagtagtt
tgtttgtcgatattcttgcaaattcgatataagtagcttttctaaagtctatatatgaataaaaacacctaaatctcgccttgcagcaatctacactc
acagaccagatacgactatgataaaacaatcctatccttcgggtgtgatataacgaatttggatgcccttaaaggtagttcaaggagattcctacg
aagataatataaccggcggtggtagaaaacgaaaatttagcttcaacttcgatccggtgtttttttgaggtcagcgaacgtcctatacgaaaatta
taggacgaccacctttaggaaatccttaaattttagttcaaaagcaacccgaaaaatcgttatgtaccaaatctgtattgacgcctttcttgctttat
ttttcacttctttgaattgtatctctcaagagaacgcactttctgcatttaaagtaacagttaaatttgtcacttgatttagaaaaaaaaaaatttgcga
agggaatgagatgtagtagaaatcattgctgctttcacagaaattaaatgttttatagctctttaaattttaaatatgagaagccgcggctgttata
gagtaggaatgattgcgatgaggaccatatgttcatgtatgccataggtaaaagagaaaaagataaaaaagactagttttctaatcactgtttac
ttggatctgatctttctgcttcctcatcggtaaaccccaaattgcgcatgtcatcttgtccgaaaagacctgtgtccatgcgacattggagatactttt
tggcaaggggtcgacattcctgttgttgactacgattcgttttgatgcattccaaatactctctcatgacatacgtgcattcacctatcgttaaaaaa
gattagctcattgcccataaaatcctttcaatgcataccaaaatgatccaaaggaaaactgcctcgctcgggtggttccttacttgccataggaata
gagccaccagctaaagttcatagttagtcaatcattctttcattaaaaataccgaaatccgagaatgatgaaagaaccgcaagctcatctctttttc
gaagccattgctgattagatgttccagcacagcataagagaaggtaagttaagaaaattacttattgagtattgtttcccatcgtatcggaaatgg
aaaatttagccttcaatgttactaaatctcatatgatccaaaaacgattgaaaacagtatacaagatcttcacaattcaatcatttccaaaaaaaa
gtctagaatgccttcgtggaatttgttcctctcatcttgaacagcttcgtccttctctttctttttacttacctgctccgaaagacatcttgtaatgaatt
cctttatttttttaaagaataacgtatataaaccaaaaacaaagtagaagattttatagaaatatggcttcttggttatgtacaaggatttttttcgt
agttgtgttctgttctttaaatttacgactcctcaaatgccactatttcaactctgatttccttctcgatccatgcattaaactctcattcagaaaagta

tcaccaatatgaaatatctcttgttttgtgaccttacttattctaatttttctgattacttatacagaatagtgcagttactttgttctactgaaatacca
accgtattagctcgtctagacttcaggtttgaattgctttaaatataaaattattctacaaaaagaaagctttcggctagatatccatttctaatgtc
caagtattatcagaaacaaagcatcttttcttttaccattaccgcgtttcaaactctaaactaaaactacacagtgcaacctagatccttaagaaaa
actgttctgatactacaggcttatgctttttgctctgcgttactaacagatttctcttgcgaggattcagtagcagcttcagtggaagattgtgtttttt
tcagtctcaggctgcttgtttgcagcatccttcttagcatttttcttcttgtctttcttagcctttttctcgtcctttgggtcaggataagccaacttaatg
actccttccatatcaggatgagacttcaattcagcttgaacagtggcaagtaaagcattgatggatgattgtaatccatctttcaaatcttgaggag
ctagagtctgctctacgtaggctttctccacttcctcatatgtgttaaaaacaatcttaccaccatacttttcaggacggtcaatgacaaattcctttt
caccacgaaacttcattgcaggaaaagaaacatgcttcaaaaatgcaaggcatccattgtcttcaacaacaccaggctcacaaaaagcactacg
aacctttttctttacgacattgggttcgtcgaacaaatcaattttagcattagcacctgcggaagcagacattttaccgcctgctaagctgggtacc
atagggctcatgagatgtgagcgcttcttgtaacccatcataggaagaagcttctcagccaagataaaaattttacgttgatcaacaccgccaaat
tgaacgtcaacattcaaatattgctcatcaagagcttgcataccggggtacatcaagctagaaattaaaggactggcaacctgtttgacgacttca
gcaccagctcgcatagcatcgtgctctgtagtgactgcacaagcacggtagttatccaaagcatattcttctttcaattgataactagatccaacaa
taaagtgcaacttctcaatggggacattcaatgacttcaaaattgcctttatgactagttcataatagcgaacacgatgttgaactagttccattgg
tgctttcatattatctaagaaagcatgaacatcagccaaaagcactttcacgtggacaccggcttgcaaaaagtccgctagtttcatcaaaggaa
caaagtaaccacagtgaggacgaccagtgggagcagtaccaaaatatagttcgagatcacgttcctttaaaatttcacggatagcatctccacca
agaacctcatgtaaatttcgagtaatcaactcgtacttttcatcaacggaaagagccattttctgactgaatcccctattctacgaaaaaaacaca
gacacaggatgttggttaaggccgtccgatcgctgccctctttttctccccggtcttcttttaccgtgagatagccaaagatggattctcaacggtat
ctcgattaacgaaagctaacgaagttcattaagcataaatcatatcaaaacaaattgaaggacccagaaattaaggatttattaacgaagagcc
caaacctcatgctcgtttaatttttacggcagtgtttttaggcttaatttatgtttacgggaatgaaacgtttgggtagatgcacagagttacaacga
tcttcaatgcttttcacgaaataatgaagatccaagtgcctcgtcaaggatcgccttggggcgacgctaatgcaatgtttggcgtccaatttcatat
ccaaaggacattgagcataacatcctggagaaagcgtaattcttactgaaaactctttgtttccggagggattggtacgctcaaacagttcaaagc
agatttgtgtaagatagtccaattctggaatctggtttcggggcatcttcataggtaacccagactcaaggatacaatttaataatgtatatatatg
cgattctttggtaaagtacatccttgtggatgcgtggtctgagtctctcgctaatttgatgtcatcgaggacttgtcgtaaaggggtacacttgtca
gtaatccaatttcaagcttttcttcctgttctataccatactcctgaggagaaacgaagtcaaacaaaaccttagcaagatcatataattcatatag
cttctctaagggtctttccgtctttcttgcagatttttttcatatttgaaatctcattgttgttggagtcatttgaagaactagaagaatctctgggaatt
aaactaggatcttgaggtagtcttaaatattgatacggagtaaatattctttccaaaaattggcgattgtgtaaggcgtcatatttaagggtatcat
agagttcggacaccttagatgggtccgctttctcagaatcgcaaaattcattaaaaagcttttcccatcgctccctaaacaaggcagggttttcact
gcagcaccagcgagactgtacttgctcaaccccaggtcccaatatgataaaattctcacgcaaaatggctcggtgatacttcattaactgaactac
ctgttgcataacttcacaaggtttaggcatgttttctggccatgtaaactctttccttgccgtatcactcacccttaataaagacttcaaatgtttcttg
accttatccattgtatctttagcagcatttgagtcgtccaaaagatcttttctgattttaagcaagtcatttgctatggtttcttgctctaaaaaggcca
ttgcaaaaatttcagcagaagttgaaacacgtcgttcggatgaagtgaaaatttcaacatcttttaaacattcacggttcataatatacaaatcttt
acgaaattgttcaccaagatccttagattggtatcgtgcagaatgggtaaattcaccaccccatttaatgataagctgtagttttgtgagctttcctt
ctgaagagtatgctggtttgagctgaacttttgtgccaggtaaattttttttggtttctaaagcaagtctaagctgcttcagtttatttaaatcttcaca
tttcagctctgatgctatattcgtagcggccagaacgctatttaactgttcgtggcgcaatataacttcttccttgtgaccttgaagcagcttagcaa
aaggctcagacgtgaatgaaaatttaaatttttgcttcggagtgcggtcggcatgacgaagtactacaaccagactcttaagtctccaagcttcac
tttccctgggagggggattaagtacttcttgaactgagggaacacggtttcttctatggcgttcagcaacatgaaacatctgttttagaatacgggc
ggcgttatcataataatcattattatcttttacaaatgaccagccatttacatcaatcacataggattttccggatactcttagcaagtcgaaaccac
aaactggctgttcgaaagcaatagatatctttgaagccataactttttcctcatcggataaattggtaatgaaccgtatttcttttccatggggattg
cgtctcacaatgccatctacaacgggtgacttccttgtttcagcatgactataatgagagccaacagtgtatattttaacatcctctgcgttatcgac
attcataaattgttcataaatatatgatccgtctgtacgagggtagcaaagttcaggatcgtaatcagaggacttgtttgcaattttacgaaataat
ttccggccaccaccacctgcagatttgggaaagtagatataaatattatgatcttcaccatatgtaggcttctccacaaatggctttcttagtatttg
accgtccacacagagtgtatcgtcatccagcatttcaacctttggtaagggttcctttctcaaatggaaaccaaactttcgtttcaatttttccgcca
atggcttattcaaatttggaccgccatctcgattacaaatcaatcttcgaggagttgaaacgctgattgagtctaaaattttcaaaactagacgcct
atcccatagcaattcttgaaatacaacatcgttcacacaaaacggttttctcaaatcaacgtatcgttctgctttttcaagaggaaatccgtttgaat
agaaacaaattaaatagtcacaagcaggccaattctctacttcttcgtccaaaatcatgttgtctccgaaaacaatagcctcaaactcgccttctg
cgataattcgattcaaaatattacggcatggcttcgaccgcgccttagcatccatagcacatacacctaccacattatgctttataggaggctgag
aagttcctgaagatacaggcgatgttgttctggtgaatggttctgtctcagaagcagcgtgagatgctcctttttgagacattcttttggaaagttaa
taaatttttttttattacaaagtagaaaagcttttaagtaaacaagcaccgagtttttgagtattcttaaagtattcgttcttgttttgtactagttgcat

ggttcggctaaatcgttcatgttaccttgtgctatagacaacttcattcggacgttattttttctttaaagaattacattctgtaaataaacattggcct
ttcctttactttttttgtattccttaagagcctgaatcttttctttgacaaaattttcctttttcccctatgcttgtacttgcgaataacgttcaatttttctc
tttacaaagaagctttaagataaaaaacataccgattgaacgtagaaaactaatttgatggaagattttctgttgatattgttgtaaacaaaagga
cctttcaactttaacgcgtctttcggtatgtcttgtaaaagttatacgatattacggcgaatctcccaaatatgattaacgttctactatttttcgcgca
aaaggtagaaaatggaggttttcattctttctgcctttgacaaatccattgatttgcacttgaccgtaaaagcacctaattgacggcttttggtgcaa
aaaaaaagttcaaataaatataaaatgccgtttgctgaaaagaaaagaagaactcaagatgttaatgtgaaaccgttgagaaaatcaaggattt
ttgcgcgtatgtacttttgtgtagcaaaatgcgagacttgttgctaacaaatattcagcttttcgatccattggttatgtttcgaaccaaataccttttt
gatatagaagttcgtggaactcactttcttgtgacaacatgtgttggaaattcctttcagacatacgattgtgaacgactaaatctgttgtttgtcgg
taaacaactggagcgagaaatctcttgcttgaaatcgtataaagattttatgctagcaggatctggctctaaaatttttgcctacaagcgaggtaa
gattgtttgggaaatagagctggaatcgtctgctgatgagattttgcatttggatgcttttggtgaatggattattgcaataagcagtactcgccatg
ttcatatttggaagcatgtttccaagtatgaagtaccagaattgcatacgatttttcaacctactgccaatgcagacataacggctttgatccaccct
agcacttatttgaacaaagttgttttgggtttctctgacggttctttgcagttatggaatatgaggacggcaaagtgcattcacaaattcgatgggtt
gagaggcgacgcgatcacctctctttctcaagctcctgtacttgatgtcctcgctattggtgctgcaaacggtcgaattttgctatataatttaagaa
aagctgtcactttaatggagtttcgccaaagcggccgcgtcctttcctcttccttccggacagatggtgtacctattctagcgacatcgaatcatcaa
ggagacatctctttttatgatttgtctaaacgtcgtgtccaaaatgttgtttatggagctcatcatggccccattcccaagcttcagttcttaaacggt
cagccattattggttactaccggccctgacaactctcttaaagaatggatctttgattctatggatggtgttcccagagtcttgcgttttcgcaatggc
cattacgaaccaccttcttatattcagttttatggtcaaaatgtacattttctgctttctgctgccactgataatacactacgtgctatcagccttttcc
aagataaccaaactgttgagctttcccagggctctgtaacttctaaagcaaaaaagctaaacgcccgtccagatgaactaaagcttcctgaaata
gtatccatgtcttcatctaatacccgtgaaaaatactgggataacattcttactgctcataaaaatgataatgctgctcgcacatggagctggaaa
aacaagactttgggtcagcacgttctaccgacgacagatggtacccctgttcgcgctgtttgcgttagttgttgtggtaacttcggtatggtcggtag
ctccaagggcttagttaatgtttataacatgcaaagtggtattttaaggaaatcctttggtcgtgaacagcagagtactaagcctattacgtctatt
atgcttgataacgttaatcgagtcttagttacatcttctttggatggtaatttacgtttctgggatttcaataaaggatctttaattgataccgttgaca
tgggtagctctatcacgaatgctatttaccatcatacatcagatcttatagctatcatttgtgatgactttggcgttcgcatcgttgatgttcaaacaa
ggaaaattgttcgtgaattatggggtcactcaaatcgattaactgaccttgatttttccaatgatggacgctggttagtcacatcatccttagatgga
accatccgcacttgggatttaccaacaggtcatttgattgatgccattaccactggtagcgtttgtactggtttggcctttggacctaccggtgatta
cttggcaacctctcacgttgatcaaattggaatcagcttatggaccaatctttcaatgtttaagcatgtatctacgaaatcgctacgattagatgatg
taaccgaggtttctcttccctcaatttctggcgaacggggcacttcggtagtacaggctgcattgaacgttgaagaagaggaggtagaagaagat
gatatcttacacactaccgctgatcaacttgacggtcaattgcaaaccctaagtaaattgcctcgaacacaatggcagactcttgtaaacatggaa
ttgattaaagctcgtaatgctcccaaagaggctcctaaggcacccgaaaaggcacctttctttttgccatctcttaaagagcaagctgaggggctt
gtttcgaaggctaatgaaacaaataatgatgaccaacaaagaccaaaattggcttcctcagttcaaactgttacaaccagattttctgaacttttg
catggtggagatggtaagtttattaaaaactgtgtgtgaacgtttgataagatttactaataaatttgtaggcgatgcgtttttttgaatatttgaaga
ctctgccaccagctaagactgacttagaaattcgttctcttgaagcgtacccaccatacgaagaattcattttatttatcaaaagtatgacatcccg
gttagcgagccgtcgggactttgagctggtacaggcttatatggcagttttcatcaaagcccatgaagatgttttgttgtcttcagattctccagaag
aaaaggaaaatgtcctgaacgcattgaaagcttgggatgatgtgcagaaagaagaaaacaggcgtctgctggacttggtacattattgctctggt
gttttgagtttcatgcgaacttaaaatgtttttgcgttgatacaaggggaaaaaacgggttaaaaaaagtgtagtgtgtatgcactaattgttttat
aaacttataatttttgggtcaaatagaatgcaggtttattagttattatcgacatgaggtttcttgaaaaactttaattgctaatttggcagatatttg
taaaattaataaactttaaataaaacagaagtatgctagaaaaagacacagcatgtatacaaaataaatcgaaacagctcacagcagatatat
ataaatgaaaactaggataaaatagtctcatttgtttacaatagcaacagcagctcccgtggcctaatggctagggcatttgacttctaatcaagg
gactgcgggttcgagtcccgccgggagctttttttatgaacattcattctctctcagagggtgtgaaataatgtaattaatttttttacacttaattgaa
aacagttttattataatagttgaggcttccttaaatgatcactaagttctattcttcctcgcttattagtgtaaacaatgaaagcgacatttttcacga
aatagtgaattcaccaaaacaaatgaaagtctatacattccctaattctcgtaaattgttgcaactaaactgttgtaaaactgacaatactgtaca
atatttaaaatttttttattaaaaaacaacatgggtaatccccaaaaaaagacatcgaacttcccaccatgcagagaagattgagccaacgcaatg
atcgatgcaaaatcattgatccagaaaaggtgaaagcaaaccatgaagacacaataaagtatcgcttaagcaacatctaaggaatgcaaagtt
gtagacattattattgtttcatagagctgtaatccctaaggatattaaggtgagtatttgtaatttttccacgtctagtttttggtttcttctgtttctgg
gcagtaggcgcagtcggtctcttgatggttgaaggatctacgcttgtcggcttcaagccatatttcccaagttcggacgccaaatccaaggtagga
ggaatgggaatttcatgccagacagtttggaattctttgtcgataaagcaattacagcttttgtcatttctccaaaccatcctaggaattccgtccttt
cgtgttcttaatagcaaaacctcccctagtttttcaagctcttctagttcagctactacattcggccatccgtctctaagctctttaatactcattcctg
tatgtgtcttctgggaatctaagtaagccaagagaccagcggcagagcgaatgttatgtaaaggtttaaatgcgaaggtttctgttcgttcatcata

ataaatacggtcatttttcttcaagagttttaatagcataggcgtcagcggcgtcgagaggtacgaagcaatctcttctgcggttttaggttcatttc
tctctttcaaatactcgacggcataatgaagctgagataaataatgggttccgaccccagaatctgctggttgagagtacactgaatgacctagtt
agtaagaaggacccaaataaaatgcgtatgttcgtcttcagactttacatacccaaattcgccttagagcgacgtttcttcaacgtcaatccaggg
cttgaaactgccgaagcaggatcatttggagtatttgagcggacactggctgtcggtgatgctgatgtatggacttgtggttttgcatagacaggtt
gattggcgactttttcttaaaagaagataattgttcacttaaagaactcataacagcctagtaagaaacagaaaggtatattgatcaaaacttct
cgaatccaccaattttgtcaatatcacgaccaaaagaatagtctactaagaatcctcttctcagctattatttcaaaggaaatgttcctcaaacctg
ataaaactaatttttagtcccaagatttggtgtgaatccagggatatggctaatggcaagtttcatttaccattccaccattaaaatgcaaaatgct
tcgtttaccttttataaggcgcttaaaatggtacgcatgcttaaggaaacttactctaatgttttaggaacgaatatgtctccctctttttaggaattc
tattcgttacgtttgagaaattactctcgggtgttgtgtttgcaattcacattatacttaggcccatatggacaaattatataaaaagaatgacaaa
aagatatccagaaaagcatcaaagtcccttcgtcgaacgagctcgggctattcacgatgccgacgatattcgtcagatgtgcaacatctctggat
acaatgttgaggaacaccttgtaaggactcaggatgattacattctttgtattcatagaatttcaaaggataaaccaggaaaaattggcatgcca
attcctaaaaagttacctgttgtatactgtcatcatggactattaatgaattctgaggtgtgggtttgtaacattgatcagaggaattgcttagtata
tcaacttgttgaccaaggatatgatgtttgggtaagtatttttctttcgcatctggcagttgggcttgataagttcaaatcatcttttctagcatcgac
atcactctaacaatatttctgtagcttggaaacaacagaggaaataagtacagtcgccaacatcttcggttcgattctacagacgtggacttttgg
aactttagcattgatgatttcgctcattacgatattcccgatactattgattatatattgaaaacttctgaacaaaaacaactaacatatattggatt
ttctcaaggaactgcacaagcatttgccgcacttagtgttcagcctcttctaaatgaaaaagtaaattcttttatagcattggccccagccatgagt
ccgaaagggttgcacaatcggattgtagatgccttcgtaaaggcacgtccctctattttatttttcctctttggtcgaaagtcgatactgccgtctgct
ggcttttggcaaagtatattacgtgagtgagaaaaattgcaattttaagagaaaaacttagttctaacctttcattcagcgaatgtgctttttaatcg
tatcttgtctttctgtcttgcacaattgtttaactggagctgtgaatccatgtccccgttgcaacgacatgcatcatttgcgcatttgtattcgtataca
agcgtaaaatgtcttgttcattggttccaaattatgcgcagtgcagaatttcgaatgtacgacaatgatcaaattgtccatgattacttttccaaaca
ttatagagctgcacgatttccgaccaagaatatccgtactccaattctgttgttttggggcgattcggattctttggtagatattcaacccatgttgaa
ttctctacctcctatggctgagcaaattagggtaagtttactttctttctatactgctgacattctcaatagattcctggatatgagcatttggacatg
atatgggcagatacgactcataaatatgtgattccttcagttctggagcgtcttagtatcatctacgctggaacacagtctaaagaaaatgctgat
gaaaaggctgttaatcttcataaaccaagaaaacaaaaagcacacgaagaagacctagattttcaagaagaggaagctgaagaagaaagaa
aggtggaaaggctctcaatacgtaataagaaaagcaaaagaaaatggggatcgaatttggatgacgaggatacgaagaaactttatcaatcta
acgtgaattccaatatatgaaagatgtatcagaaatatagttaccctgtgttattgctcgtccacctatatctaatgttcttgctggtttatttgtatat
aatattttaatgctaatgaataaatacatgtctttatttcgatcatgacaatgtttcttcactttatataaatgtgagttttccatataaacaatccata
ccacttcctagatggaacgcagaatccttgaaacgtcctttgggtttcataatcaagacattgatataattagcgctaaatactccaacgcttctct
cgtaccaaatgtgtattgcgtctcatacttacatgtttatcatttgtataatgtccatagttgaaattcatgaaaacaaatgcgcataattaaagaag
gcaaaggttcagtcggcagaaaataacaccaaacttgtattcctaaattaaaaaattaaatttttaaacattcttttctttcctcattcaaagagc
aagcagcaaaaatagacaaaggatttgaattattatttatttgtttatttttacttctttcttcgtttattcccactttattaattcactaagatcaaccg
aaatgttcataataagcgtctttgtttacattgatttgctaacgaaaattttctttagcggtcacaatttagctttgaacgtgggtttctctaacactcg
ttgaacaaaccctaccctcagtagaaaaaacagtcacttgtttttttctatataagggaagatgaaactctctaattcaccatcatcagtaggattt
aacttactttgttatataagtaaagtaaattcaaacctgaagtcaagtatgatggttggagtcttaggacgaagaaaatgcatccgttccgtcttca
gtgatgaattgagtagccatcccggtcgttgattgtttcccatccttcaagggtggggtggtttctcgtacatttttgttttaatttcgtttgttattagt
cgttgaatgtttgttatttagaagaaataagagtgaagattgtgaatgaaagttgtattgtatgtagactattgtattggaacggaagttgatctgg
gaaacgaagtttaacttttaatgatttgttggtgtatggagatatgtgttgtattatgcttggcaaagaaatttagttctttctgggtggtgagattat
gagggctttgtttgtcgattgtgtagtgatttgagtgcacaagcttgatcaagcgtcgtgaaagcaccattaatcgtgtttgtattgagacatttcaa
tttaccaaaaaccatgtcccaagaagaatctgttgcaacagacatgcaggtaggcgatgagcaacagcaagttgaaaaagaagttgagttaga
aaaagtaattcaggaaggtatgcttcacttacattagcaatttcctttggtatttgtttgcattttatgataaagcaacgttagctaatgcctcctttttc
accatagaatataaactttggaaacaaaacgtaccctttctctacgatttagttattactcatgccctggagtggccctctttgacggttcagtggtt
accagataaacagacgtatgtagaaaacccatcttaatggtgtaagcaagttgctttttgtatgggcatggaacgtgtaggttgggttggtgttta
actctaatccaacacgttggcttccctccatttttttcttatgcttgagaccacttcgctcatcttttccgtatttttggcttgtacattgtcgacctgttt
ccatctttattaagcaagcatcttattaaccatgtgactatagggtatcaggcaaagattattctgttcaacggcttctcctaggaacccatacttca
ggccatgatcaaaattatctgcaagtggcttctgtccaattgcccaattttgacgaggatactatcgacattacgccagctaatgttcgtagagccc
aacgtgaaggctcttacaacatcgaaattactcaaaagatttgtcacgatggcgaagtgaatcgggctcgctatatgcctcaaaatcctgacattg
ttgctactatgggcgttgctggaaattcttatatttttaacttgaatgatcacggcgctttaccaactaacaattctttaccacaagctatccttcacg
gtcatacttccgaaggatatggtctttgctggaaccctaaccaaactggccgtctagcaactgccgctgaagatcagttcatttgtttatgggacat

caacacatcctccttttcagctacccagtcagtggatttatctcctaccgcaaaatttcatcgacattctgatattgttaatgatgtccaatatcatcc
tcaacatgaagcccttttagtttcagtgtccgatgactgtactttacagatacatgatactaggttaaaccctaacgatcgcgctcctaaggttattc
acgctcactcaaaagccattaatgctgtagctattaacccttttaatgactacttgttagctactgcaagtgcagataaaacagttgctctttggga
ccttcgaaatcctcatcagcgccttcatactttagaaggacatgaggacgatgtctatggcctagagtggtctcctcatgacgaacctattttggctt
ctagttctaccgatcgccgtgtttgcatatgggatcttgccaaaattggagaagaacagtctgcagaggatgctgaggacggtgtccctgagttgt
tatttatgcacggcgggcatacaaacagaatttcagaatttagctggtgtccaagtgagcgctgggtccttgctagtttagctgacgataacatcct
tcaggtatggagtccttccagagttctttggggtcgtgacgatgtacaaatatcatctcgagatttagagtaatacaaaaaaagaaaacatgactt
attataaaaagttgtatacattttagtttcaccatcttttcaaaatagcctacgaactaaccaatacatgattcctcctttattgactatttataccgct
cagtagtactttttagaacttattaaaagagtgttaactatcatcactaacaaaacgtgtaagaaatagaatccaaaaaataaacaaccaaaaa
tataaaactttaaaggaattaacgatgtttcttataccagttcgttttgcagatttgtataaagacgtttgtaaagttttttcgaggatttccttttgttt
tgcaaccgttgataaacccttgaaatagtattagtcaaaccagcaaaaacatattgctgtccactagcagcacaaacataataaattatggcaga
cagtattataagattctgaaagctgtttgacgtaggcgtaagttgaaacattctcataaaataattgttttgatcctgtagcttgataagagaaact
gtaattcccaacaaggcgaaaattgttacagtaaaagacacatttctccacattctctgtctatagggaggcccaacagtcaatacaagccctatc
aatatatactggaagcaggaaaataaaaacaagcttgttacatcgctgttggcaatgcttgatttttcggatccagctgcagggggggatatgtac
caaggctgctggcggacgagtctaagaatcatatactgaaatgcgcagatgaagaagatctgtccaattaaaggtgatagaatccgttttgaaa
ccaaatttgctgtaggacgtttccgagctagcctgtgataaggcttggatcgtcccataaatacggcaatcggtaaaataattgccaggtcaataa
ataagaattgaaagtcgcctaaattagaatttgttgtatacaacatactgacggtaacaaattggatagcagaatataaagccatgtattgaaaa
caaccaaaacttgttaccaatgctgcacgaccgtctttaatgacatctagtacgcaagaaatctcaaaccaccgactggtgaaaggagcagcca
cggaggcttcagcttcagacagcgaaatgccaacgtcagcagcttttaatgcaccgcagtcatttgctccatcaccacaaaaaccaacgcagtaa
tctagcttttggaagcatgagaccaactcgttcttttcggaaggagacattcttgcgaaaatttgtgattttagcaatatgacataaaaatgctcag
cgggagcataatcaactaaccaccggaagacatcacctgttatagccatgtggtaatttttttaatcgtttgaactcagttgataataattcacgatt
tgcggaattcaccacaggatgaagggttagaggatccaagtaaacttcgttatcttcaattgactgccatataagttttcgagatgctatatcccca
atggactcttcttcctcaaaccgtggtacaaagaccagaccatcttctggaagcataccgcaccgtttacccacacatatagaggtcaatacgttg
tcacctgtacacatcacagtccgaatgtttgcatcttctaattctctaataactggaacagtagtagactttaatttgttttcaaaaacaataaaccc
acaaaagtctaaatcagattcaacttgtgtccgtttcatcctttgtgctttcgcccatgtacagttctccaaatgtttggatgcacaagcaataactc
gaaaaccattatgggtatagtaatctaatacctcttcgtaattttcaggaaacgactcaggcttgcagatcgaagacataacttcaggtgcaccttt
tacataaatatccatttcttacattgcccatgtttcactattacagccatgcggcgaagttgagatacaaactcgaaagttcggattatgccaagtt
ccaaatcgctttcggaataattgattgatggatcgttccatggaggtgatacagtaggtggtattaattgctgggttcgcatgtaagcggcatcctc
agattgctcagaagaagtcatcttgttcgtaaaattctcttccgaatatgtccaatccgtaaactcaaacattttcaaatctaacggatctcctagca
gttcaccgtccacgcgacgaagcatatgacaagtagccattgtaaacagtaaattagaagggctatcatcagaaagcaagtcttttgaacataat
ttactaggattagttcgtagatcacttaattttgaatcttcaataacagaaacacccataatatcaagtccatcttccgtaagtgttccagttttatca
aaacttatgagatcaagctttccacttacattcacacgttgaggactgatgcaaaaaatcccttgccttcttaatcttgaaattgcaaaagtagtac
cgattgtaagagttgccggtaaagcaggaggaacgacaattgtgattaagtctaaagcacgaatgatgatagttttaacaggtacacctatagtc
aataaattaatagaactaaaaacaaacccgacaagagcgataataaacatagcggtgataaatcggaaagaatcacgataaaagctaaagtt
tgtcggctttggaaaaatcatggatcgaactaaggcgcctttggtggtattgaaaccagtgcgaacgaccatggcgagagaagcaccttcttcttt
ctcattaacaaagggcttacgcacttgaataatttttgttcctccgaaaaggaaatgtttagagagagaaggaggtataattctagaaaatgcaa
aaagatctttcattccttgatctgtagctggaatcttcgaaacgggaatggattcaccagttaacatactttcatttacaatgcagtcacctgaaag
caaaagagcatcggctgggaaaaccgttagttctggatccgagatttcaaaaacatcaccaattactaagtcagtagatggaatgctagcccaa
aagccatcacgataaacacgaacgggacaggtaaatcgggacatttgacgcatacgtttcaaagtcgtttgagtttcaattaagcttccaatgat
agataccacagaaatagtgaaaatgcaaatagcatagtaataataagcatctaaagaccaaaggataattgagaaaacttgaaaaatataaa
aaggatgcaaaacttcatccaacaaaagctgaataacacctttgaattccaaatcaatcaaattttcgccgaaaatttttagacgatcattgatag
catcacgttccaagccttctttggcaacgttggtatcacgaatccagcgcggatccttccaatcttgttgaataagaaaccgttccaagatgggatg
gaaaattaacttcatgtagcgatagtttacatagcgaaagttttcaacgaaaggatcattatcagtgtcttcaggtgtagccacacgaacagagg
caccgaaaatagatgaaagagaattctcataaggttgaacctgaatgtcatacctcgagatctcaccccattgagtttcaatagttaaccattccg
cagtagcgagcggttcacgtttaccaatgaatcgaatgtaatatctagggagccatctcattataagataaacaagaccaaaagtaaggatacat
aacatgttgtatattctagcatggagtttgttagtcttgtatccagcaattccaacctcgagatcttcttctaccaaatacataatttggcagcatcca
tcatcacttaagccagcagcttctccgggagattcacgtcgcaaaatagggtgaaagttaagttcctctagcatcatctctgtaatttcttcttcatc
actgtaaactgcagaaccattttcacggaaaaaatttgggtagtctacgctgctagctgcggcgatagagtccgtgtcattatatcgactcaagtt

gtactcagattgcaaatagtccatgctgaattgactaatactagcatcagaagaacctcgttgagttttgtgatggaaactagaaatactagatgg
aacgcttgttcctaagccaggaccatcaaaaatactgtcatgactcattaaagaactcgccaaataagtatttagtgatttctttgaacccagcga
ggtcactgagttctttccccgaattgatctaggagaagtcatttttattttccgttacgatctctctgaaccccctattacaatcaattctttcgatcttt
agagattcgtaggatgctgaatgtgaggaatattgaaccagtttacattgagaaacatcgttagcaaaaaaatacaattgtaatatacgcgattg
cataatgtatatgattataaagaaactttattaagaaaggaaaccatagaataattaagatttataagtacttctcgctttttcgtttttgagacttt
ataaatgaataaaacgcctttaaatgttatttgaatgtaacagcttatcgatttgaaatattcgcattatataattaaaaccattagtcaaatgttac
ttcgagatctcaaagaagacatgttccccaatagtcacttatatcgtttccatttggtatcttaaaaaggtacataattctttaacaataaggtttctt
aaagtttcggctgtaaataaaacatcctacaggattcatttcaatggcatttttgtctgttgtataatttttcaagtaatgtataaaatgaagcaac
ataatatggcaattatattattgttgggaataaggaaggcaacatatccttgacagtattatatttggccatttgaaacaagaatgcgtcgactaat
ttgagagagaatatttatgactaggaaatgctatactagcaggtaggataaaaactttttattccaataccgaaaccattgagttctcttcaacgta
aaaaagtgcaataaaagcattcataaacaaacgacatttcaagaaaagcatgaatataagaatccatttcttaactaatgcagctatataagta
gaagagcatataaaaacatttccatttttggcacgtaatgaagctttcctaaacagctataaacattggtaacggagaaaaattcctatatatttg
tttttactccaagtcaatagcatttcttcctcctgaaggattggtaaacagggaactcatgagattagccaagatattgggttgtgtattgacagga
cgaatgccaaaataaacatcttcaagttttgcaagagcctaacgagttgttagagatttttgagacttaagtttctaaaaaaggacataccgact
cccattctggcttctttgggtatttttgcgaaagcgaaagataagtttccttatcctttcgataagcagcaagaacgagtaagtgtaagtagttgag
aaaaggataagaattgaaagttggaacccgaattccgtcaatggagaaagtctcttctggtgaatgtccggagcgttcaaacaactgtacaaact
tggttaaagattgaagggcatcagctggacttccgactaaaagataattgagaacggcacgtccaataaacaaggcaggatcttcatcttttgaa
gcttgggcccaattatacagcatttcagcataaagagcaggacatttttcatttcctaatacaaggtgtcgttctgcggcaactggatctccccctat
ctccatgtcagtattgaaaccattaagagaaataaagaaaaataaaactcacatttcataaacataataccaactgatgcatgaacatcacgatc
cccctgaggtcctgtagagtttgtccacctgaattcttgttagctaccgatatatcgtttgagctttcttgaacttaggaaaattcaagggcccttcta
ccgttactgtcaacatactcaagaagcttcttcacgtattgctttttacttccctcctcggctggaaagcatccaagaagagctaacgcttttgctag
tgaacttggttagatcctattttcgtagagtaattctgcagaaattcaaacctttgttatccatcgacacagagctcgacgcctttataaaaacatcc
accaaataaattgataaatctccagcgcttccagtttgttcgagttcgaaaagagtctttgcgccggagtatagtaaagcaattgagtcatcatact
tcttagccataacttgtctgatcatcggttatttacagccttaaagaaacaacgaacctattaactaaagtacgaatcatctgatgaccttcataag
ggtcttctcgtaatctatatcgcgttaactttcatgttcctggttttataatgaaaatataaaccttgcttctgctctttttatttttgcttccatgacgct
catatttaattaaataagaaaaatatcctaggtaactagcacagcgacaaatttcctgagaattcgtttgttgtagaaggtctatcctcaacgactt
ccatattatttgaagtcatttcggcataaatcgcaggaattctgaaagccttaattatatatataataccaatgtattagaaatggactttgactgtttg
gaatttgttaataaggtatttaaatccagtgttgtttttgctaatatatgtagattgctactattgaatcgaatggagatactttagaccatttacctc
gtcttataacgttagcttgtcagaaaaatcaagagcagcaagtgtaccaaagactcaatgaactagccaacaagaatgcaattcgcgttgaaca
gctatgcgccgaaaataatcaagtatatcaatcgtttttcaagtacttttctaacaatattaataggactttagtaaatctgttcatcaactttccgtc
gtgagaaatgagcttcatagtctgcgttctcatattgcagacttgaatactgatattcaagcgagcggtaaggatttgaaaatcatggtaagtaaa
gaaattgaattagaaaacaagtattatggtcaattcactgagtgcagtacttactaatctacatcagaaacaacaactgaattcattgagggactc
tgaacggaagctatatttatatcaagacttgactcagagctgtcttcagatattttacaagctgaaggatgctcaagaacttttatcaaaaaagca
atacttaccatctttgaggatatactctgaagttcaaaagattaacttaaaacggattgaaggacttacgttacactccaccgttcagcaaatgatc
tctcgaatgcaaaattctgtatgcagttcggcactagaggaccttcatgaatggttgtttgctttaaggaaaaagctaccacttgtaggggaaattt
gctttaaagaaatcgacgaagctcgaagaagatgggcaaagaattaccgagaggacttgaccaaccttactacgaaaacacctttttccttggat
ttagcaattatggaatatgtagattgtaagtaccatttgcctaagctcttgtctaacataattagttgatcctcttaacaatgatttggttcaggttga
ctttattcctttgtatgtttgtgtacaggttcactcgtatttgggaatcttaagttcctttcaggcgtcatttgagaaggaccgcaaacgacagcaag
aatttttagctccaaaaagtctagagtccatcgatttggacgttattagtgaatggttgaaatcagttgctggatacatgattgtcgaatattatatc
ctgcaagagattcccaatttccgatcttacgaagaagtccaaaatatatgggccattatttgtgataaattggtcgaaagtatcttacgagtggcat
tttcagagcaaagtacaacggcgattataaagatgaaaaataatattgttttactaatgcacacaatggaacgttttggtttcccaacgcataggt
tacatagtctttctgtggagttgatagacgcttttggaggagcactgatattaaagcactccgtgtctttcgaagaagcctttgacacagatttatac
gctccaatggtgatcaataacgaaaaggagtatgatgaattgatagctccttactggactttaccaccggccccttttcctcgaatggtggaatttt
ctaaaatgtgctctttgtgttgtattacactttcaaaattcgctcggcactttttatgttctctaatgactctatcacgttggcgttggacgttcagga
aaagatcccaagggtaagaagaatgccaagattaagtgctaacaataacaaacaagttttatagaagatttattttgcacagcttgcttgataga
ctcaaacatctttatccaaaactaaatttgtcacagatggctcagttagtaaaaaatttttatgcttttgaggagcccttgttacaaatggagaaga
cgttggtgataagcaaatctacttctcatgctggaaatgaaattactaatgtgaatatcaaatcttccgaactattggaaggtttagcaaatgcacg
caagtctgccttacatgaaatttttgttaaaattaaccacaaaattgatgatttcattggtttggctgaatacgattggtatgttgatatccttgaaca

tttttatgaaattctaataagttcattaggtcgactacagtgcctcgcactgctccaagcggatttttgcaagagatggttccgtacctacaaaccat
ttacctcgattccctagcaggtcttcctaaccatgacaagtcttatgtgtacctggaaactctagaccatttatgcactgccatgattgacttactttc
aaatcctgctattcggaaaatatctactgcagcagcggagggatttaaagtcgatgttgaatatttggaaagctttgcttcccaggtaccggacca
gagcattgtgaacgcagatagctttatagagctaagacaatgtgccaatttactgttaggagataacatagaagattatttggacaccgacaaat
tcactcgtgactttaatcgtttgcatccatcgacttccgtgaagctattagagaggtatttgaatttttgttgtgagagaaatactaattaaccaattt
ttaggctcattagcagttattctgccaacccgttaagtaatgatcgaccgaagcgtagagctatagaaaacctacttgctgtatttcgacgaaaaa
caaattgaatgcagggaaagggattttcgagttttattcgtttcatgaatggatacgatttacgtctaagattattcctaggatatttatgagcttat
gataccataaaaaatacaatgaatttacttgcacagttttctcgaacaagttgtaatagaatttcattgaagtcttatttgaaagatgaaaaagaa
gaaaaaaaaaaggtaattttcaatgaatagatttttatttattactgaacacaaagtactttgtaagactctagtcgcatacctgtcaaattatata
tattagttttctaacttcagggtctccgaccatccactacattcatagtagagtttcggtttatataaacgagaatgtctttaggaactttatactcttt
taaggacaatactagaactgtttgtttgcttgaactagctagacgtttgaatttacaagttgaccttgttgaaacttatcctcacaagttccctgccg
agttggctgccaaattccctcttcaaaaggtaagaaatgacgaggaataaaaaataaaataaaagatgaaaaacttgtgattggtaattttatat
gaacaacaaattcttataatcagtgctccattttcctttctcctttctccttgaaaagtttactttagcgctttatttgacattactgtatcttaagtgtg
tgctttttcactgatctacgtggaaaaatggatgcattgagaatgttcttgtcgtgtgttataatattgcaggaaaagcttactcaatcgacctccc
ctacatatttttgttttgagatatgctaacaatcgcagctccctgtattcattggttccaatgggctcgaattgagcgaactcgtagccattcttaagt
acttctatgaagtgggcaagcagaatgataccgagctacttggtgcaatcaatgcaaaggaagaagccgaaatttggcaatggattggatatgt
gaattctgatatcgtaactccttctgtcattcgtccctggttgggcatgtgccgaggtgcgactccttacgaagaaaagcccttcaaggagtctagc
ggaaaggctttgggtgttttgaagaaatttaatgatatgttaattgaccgcaccttttggttggtaaccgcttcacaattgccgacttagttgttggc
agtatgttacgccttttctatcgcttcatcgttgaccctgagcaacgttctaagctccctcatttgaccagatattatgttaccatgttccatcaaggt
aacttatctgaaattattcccttggaattgattccttctgtagttattgcaaagaactaggcactatttttacaaaagcagtgaatttaactgcgtat
attgaatattgaatataaatataaaattaaattgattttgttaataactatgaaatttgtcagtaaaaaggacgcgtatcatttgtttttagtctaag
agacttgtgagatcgtgaatcgtagggaatgtattttggggaaacaaaacacaggcatgttcattttagtaaccatcaccgtccttaaactcaatc
tttttagaaaacgcatctcctatttcttttgctaagatatcaagcatggtaccttcatcccttgtggacttccaagtttttacgtttgaggcccattcaa
agtgtttaggaccactagacacgttagcaaaagaaaattggcaaccataaacatcttgattgaagactagagctcatctggtctttcgaaagaat
tactaaaaagactctgttggacttgtagttgaaataaaacaaccaagacatatactaactctgcagaaaccttcaaagcccctccaaaccttttttt
gtttcatctctttgatacccatttgttggctattcaaaatgtagtttgacttacctaactggggatgagagccagacctgatgtgctggtggttgttta
ttaataacataagtaccatgatctccaagattcaaagttattacgccgctctatgcggcttcagttagtattatgcttcctttccaaaaaaacaacac
ggacttaccgaatattcaatgtcataatctgttcttccagattgttctagtagatcttcaaaggtattgtttaaaacttcgagcgtatcgtcagcacgt
cggtggtattccaagtcagacaaaggtccattttgatttacagttgacaagaaccttagacgaaagttccagccagcaagtgagcgacgaagag
gtcgcaaagaatttaaagaagcttttccaaacaaatttcctcgtaaagattgcatagaaacaaaaaagtcgagtaaaaaagaaatcacagaat
atctgacctaaaaagtgatattacaaaagatagctctgggtaattcaatttttaaaaaaacaagaaagaaaattgtaataggagtcgcggtatt
tgggaattgaaacaattggtaaaactatacgcttatgtagctgagtttagtactcagtctcttataaaaaaagcgtgattacttcaaagaatgagc
aaccctttagtagaaaggtttgaatgaaattgttacaaatgaagaattggataaattgagatagaaaaaaagagttttatttgcagctgttctgta
attcaaaccaagaaaaatgagcgctacttctattccaagataaataaagaaagagaacaaaagttcagattattaaattgctttctctttttctgta
cgtgttcgaaataatgctttgatgtattgagctatgcatccagtctatagagttttagccaagaaatctatataaaaaaaaaaaaaacaaatatca
atatcaatatcttatttgatgcaacttggaccaaccgatcaaaacttcgaatcaggtatataccaaggggtttgaaagcattaaaaccatgaattt
ggagggggaataatatgaataaaagatgaacgatcagaaaaatagctgactcacaattttcagctcggagtctctgagtttgtctgcctgggt
tttggctttctataataccaattaagcagccgatagggaacaactctaggctttatttctcccttcatcatattgtaccttaataataagtattctttaa
acatggctctttgattgaaaaccggatatattttatatcgaattgtccatttatcgtctttacggagcttttgaatgaaaaacctcctctttccattgac
ataattggtaactcgaacttggaaagcgtaaaaacgattataattatttacaggctcagataaattcacttcttcaagattggcaggattgaattta
tgattctcattgtcgggaactgcttctacaaagacttgcaaagctggattctttgtattttcttgcgtagagaacattagtcgtatagactgcttggga
aatttagaagaggcatattgtagtatagattcaaggagctgaatactaagcttaaattcggcgtcacctatatttcttccccaatggattgctttgtc
catctttttcagactaacaaattgaaatccaaaaatttgacacgtgttatgatacgcgacaaagacgccttccatgcgacctaatcgagcctgtaa
actgtatttcaaaaaagcagaccgaatcatatcataatattctcgttcataagatacaaaatttccgtaatatttgtcaagagtataagaatcatag
acctcaggttgagtttgattaagacgtactccaagtacagctcttgttttcaggtcaaagaccccagaatttggcaaagccttgtcaaagcaatcta
actgagatcgaataaggaggctccgacatttgctatattgaaatacatctatttgaggttttggtttctctggtaaattttcatttttcagataacgac
cataatcttctttttctagagtgaaaaaagcctctaaagacttccctatactcataagaattgtattctcgtttgtttttgatgataagctgtgatctct
gtcgatacaatacaagccatttggtaggataaatgaacggagctaggtgagcgacataaacgagtgtaatgctgttcgtttttttgctacacgaaa

cttttttgataagcctaaaaagctcggtggtttccacttgctaaggaaaaaatgaatttgtgagagtaaacctgtcaaagaactcgttgatccagc
gaactttgaattagtctcatggccagctttcttcaatgctgtatctttagaggggggataaaacttgcttaagaggccgtagttaaagtcgtcaact
ggtgtcaccttcgataaatactcgtcccagttaaattcttgagtagtagggtccctcaaaaatttaaaccgattgttattcaaaactgtattcaattg
atgctgtaattgatttacttcttcctgcttgacgttgtttacttctacatatgttagcaaatcatcaaattgaatctaataatcttacttggaaaaatct
cggcgcgagaatcaattacgccggatttgaagctttttgaataaacatttctttttagtatgtatgaacctgaaacctttgcgctccacaatgataat
agccgcaagtttgtttgctcaattaacagcaacattccatcagtttcgttttttttgatacctgatttccttttcataaaaagaaaaattaagttttttt
tgcttcaaggactcttactttgcgtccataccttttattcaacaatggtctattgcttggtatatgttaaaatgtaggctatatggcaaaagtgcagg
taaatgtgagtcgagtcttaaatatcattaaaacgttcagagttataaaaaataaataagtttataaataaaaaaataaatgaatattaatcattg
actttccaaatcattgctgacttttacttccttccatagtcgtagaagccattgataattcacaatttcaggattgatttgatgactaaaggttagaat
agtttgatagattgaattgcaataccacatacaattcacatgctcgtcgtaagtatgcgacgcgatcctgatcgctgctaaacataattatatgagt
tagttgtttggagaaaaaagtattttgcacagaagcgccgttccacgtcagaatagtttccgccagtcgatactgtgatcttttaagactgctgtctt
ttgaaatttcaaacccactgtcgtctaagtaagcagaaatatgagacagagggaatgctccaggaaacgatgtctcccttgctaaaaggtgcttc
gaactaaatagatttttctagtattctctgggatataaatatttaaatctaatcgtcccagtttagtcagtaattcagaaagactagaattggttaa
gcttcctaaatctttctcccaagaatactttttctctttttgaaatagcaggcttctatgttagattcagtcattgggtttctttctacgtactatggtcgt
tcagcaaccttctttcctttaattatgctgtccataatccattcaggccgaaatatggagcacattcctttttctcttccacaagtactcttgttgattc
tagcgcatgtgaataactaaaacgtgacgaatcacttactcttatccgctactaataccattttcccttccggaacggaagtaactgcttgatggat
tgtgccaccattggaagcaattatgctttccaattcctgctttgatttctcaagctttgacggaggaaggacgactgcttgttaatgaacgagcagt
atacaaacaacttaccaaaatgtagcccctgaaatatgctagacaatggagtggactgctttagcggactattaatttgatgtagaaactttttttc
cggctgtcttttccgactgacttttcttttttttcaacatgactaaagcagttagttattcatatcacaaaacgctggtaattttcatactagacatctt
tttcttgattttcagcgtaacttttcagttcaaaaaactcatttatagacagagcatctttccagcttcgatccaaccgaattttctccagtcggggaa
atcgcaacgtatacgtagtcttaaatcgatcttaataaaattagaagtaagaatttagaagcaagtagattataacttactagaaacgacaacttc
ggcagctttaatttgaagtacaagtgaatctctaggatgaatccaaacatcgggtttctcaaactcttgtctgctaccagctaattcaaaatactct
aatgcttctgaagaccatagctgccattttccttcagtctcctttctaatataatttagttagaatttgaaaaagtatttgagatccttaccggataat
atcacgatcgaagtaagtaaacccacctccaacgcgaacgaatgattgaaaccttggttattagaagttagtccttaaaaaaagcttacctttgat
atggctcttctcctggagcatgatcaactcgaagaccacacaaaaaagaggaagcctgtttggattgcttaccacgtccaaaataaccacctgat
attaattaataaattttttgtgccttgatattacctagaataagacaatccagatcctattatctccttcgttaattatatacttcggaaaagagtaa
actcacctctccaaatccttccaaatagtatggttttactttgatccaatcatccattcgttctcccagtcgataagttccagaaggtttctataatgt
aaatatattctgcaaaccaaaagatcttacctttattaccagaccctctgatccctcttgaattgtttgtcgtagttctcgatctatatcactaacagt
ttgtccagttttatgagaaagtatacacattcttcgagattcacgaacgatcactttcttcagaatcttatgtcttgcttcaagggagtgtctgattaa
cgactttccattcaggtatagtatatcaaacacgatgtctataataagaacttaagttagctatactatttcaaagcttccagttttccttacaataa
gggtaatatgtaggatgagttgatgaagaatcaaatgtgctccttaagctaccataaggaatgatagtttctaatattggatcccaggtaaccatc
tcgccatccaaaacaacttgcgagacccgatcatcaaaagctccaatgatatattgagtaagctttgagttttcatcatggtatgaggagccatag
acatccgtatatgtccttgcatttcgactgtagaattgaaactttcccatgctcatatgtagctgtatgcgctccccatccatcttttcttcaatccaaa
aatgttcactgtgcatggcctctagcacaaaatcaagggtcgatttcttaaaattggccaattgtggctgaaaacagctaaaaacctcaacatcgg
tatccaattcactaagacttctactaggatcatacagctcccaagcaatacgctttagagaactgcagagacgatacaatcgagctgcatctgggt
gaaaaacgcttaatataaacttttccgaggttccgtattttctgactaaaaaaaaaaaaaanaaaaaaaaccattagcaaccaggtttacaaag
aattacatacccttaagaagtataacaattaaccatcgcatttcaagcgggcttaaggaattatagaatttttctagaacagatcccctttcttctct
taaattttgttaaacactatcttatgcaacggtcgtactctgaagtagcttctgctaagtggtctaataaattattgatatcgccaatagtgaaggag
ctgggaaaggtccgataagcccgtctctctaaaacatctagttaagtagttaatgtagaggttcctataaaaattgagcaaaccttgaagcatcgc
agtaaaattcccaacgtgtcgggaatcagaacttcgccagtttctaagtgctttggcatcttcggagtttgcgctcaaatgcatagcctgttaagta
attcattagagtacatattatggaatcccttacgaacccttatgaaaagttttcccaaaccaaactctttgaatccaaatgaacctctttctctgtcc
aactgcagacatgttagatgcatattctttgaagttttagaacttgtcagcttgtagaaaagacagctgatgtgcacgtacatcaggcagcactaa
aaaaatattagttgacaaccaactttttcagtcttacttaagcgtagtaaaggatatagatcttgtcctacttgttgtctccatttggtgaaataacta
gaaatgatttttcgtttttgttcgtaagggtcgattcctgagccttttgactttctagtttttcctatcttttgcaaaggctctaacaaatttactacgaa
gtcataaaacctcaatggctcagaatgattgctaggtttaactagccctgtagctgattcaaacgactccatactcttctgacataaatacttgcga
atgtaaaaatataatattcatagaagcagaaagcattcaaaatggatttatttactcgcagatgtccaataaatattggctaccttgatatgagat
gtagcagggttggcggttccatatttcaaatccttacttggcgacactcttttcctgtgaaagtgaaaccagttttttcacttaactattttaaaatga
gcgtactggaaaatcctgaaaaacgaaaagttgaggagcctattgcaaatgctccaaaaaaaaacgaaacagcaatgtttagataaattcttcac

tgcgaaacctaaaatacaaccgaaagctcctgtttctaacgccaatatcatttctgatggaaataaaataaaatttgataaaaagaaatgggctg
aagaactgacagacgaccagaggaagctgctacagtatgtaaatactacattatcagtaatgaaaggcgatgacaagcaagtataatgatgct
aacttctcatttttatagactagaaattgataccatggaagattcttggtttgaagctctaaaggatgaatttttacagccatactttttaaacctga
agcagtttcttcaaagtgaatggcagggagagaggattttccccctaaagaagatatatattcttggtcgcatcatactcctctgtccaaagttcg
cgtcattatattgggacaagatccctatcataatgttgggcaagctcatggactttgcttttccgtccgaccagggattccctgtccacctagtttaa
tgaacatatataaagctataaaagttgactacgaagatttcgtcataccgaaatcggggtatgtatatctcgttttagtttcagctggtttttgttta
ctgaagcaatgttattaactaattgacattagatatttaataccatgggctgatcagggagttttaatgctaaatgctagcttgactgttcgtgctca
tcaagctgcatcccattcaggtaagggatgggagcgatttacatctgcggctcttcaaattgcattaaataagcacaaacgtggaattgtagtgct
tgcttggggaacgcctgctaataaacgtcttcaagggcttcctttacagtcgcattgtgtactgaagagtgttcatccgagccctttgtccgctcatc
gcgggtttttgaatgtcatcattttaagaaaacaaatgagtggttagctgaacgttatggagctgaaaattctattaactggcatgctgtttctctt
cctacgccttcaaaatctgcaagtatgaacaagcctttaggtaacacggttgaaggttcttcactcatggcagaaaaaataaagcaggaggaac
aaaaggcatgaaaaattcgagcatggatttctgttattatatattactaaacgtagatttcgttcattttgattcattatctttatcttttccaagtgttt
cactcttttatttcgatttgaagaatatctatggttttgatttttgatatcccttattggtatggcgcttcataggaataagaaaaaaatttcgattttc
cattgaagttcgaatagcggattgaatatttttagcttttgcatgcgtctttcttgcatcttctcatgatagtccagttcacgccttaatacttcagttt
cttgtcttgctttggcgaccatttcatttgcttcagtaaacaaggaaactgaaagctcttccatttctttttggcttttatccaactgactttcaagctcc
tctcgcctttgaacttcatgggataattgttccttcatctgactcagtgccatggaatttgctttatttgtcggatttttttgtttttctggtatcagttttt
cgttaacattctctttattagacattgaggtaaacagccttgttatttctgtctcgttagtccatctgtcattttatgctttcttcgcagtctttcattcttt
tttttctaacttacgagatccactcatcataatttgctatttggttcattacaaataacttatgttgctttcatctcagcaatattgttgtttggtttgatt
gtaaacataaacaacgaacccttgttagtacacatactaccacatttctctcttttcaatatgcttccattcattggaatatataaagatcatttata
gttaagaaaaagaaaaagaaaaaagaagaattattaattgaataccaacctagccaatttccggcctgttagtaaacaatttaagtaaaattat
aaacattttgataaaacctttctgagaatcattgcttctgtttaataatctcccttttttccattttcagctatttacactaaataattgcgtttaatcttg
gagaacaatctaataattagtacattcttcttattcataaatcccaacttatttactacctaacgaaagaaagaattcaaaaagccatttgaacctt
cagattgcattcaatacaatgcattttcgtttcatacctgtctctacttcttatttactactgtagagaaacgttttcgttatggttatttaacgcgggtc
aacattactccgtcttgatgcccgacccttcctgaatatttttcgccttgtagctacaagagactaaaaaattaccaacaaccttttttgttgagttctt
tgttccagtcgaatttatttgctaatatctcctcaaatttataagagataatggctggaaataagagaaggagggagaccgtttcgaatggcaccg
ataagccaaagaacgataactcttctcaacaaaatggaactacatcgtctctttctgctatggaatcctctggagatttccctcgtggaggaggctc
cacgttgacacctcttgaatataaagaggctgtaacagaagccaaacgagaggcccttatgggtactgatggagctgaagttaccaagacttca
accaaaccaaccaaaaagaaaaagaaggtggctgctaaggccactccttctaacgaagaagacttgtcttcagtgcctgtcgaaagcttgaagt
ataaaaacattgtaccaggaacattgcttttgggacagatctctcaaattaataccttagatatagctgtatccttacctaataaccttaccggata
tgtacctattaccaacatctctgataagctttctcaccgtcttgaaaatttggatgatgctgcagctggtaccgagaacgaagtcgttgatcagatc
gacgaatttaataactttcccgatttaattgatctttatcatgttggacaatgggttcgtgcatgcgtaacgaaagttggtatggaagagtcaaaaa
acaataaaaagcatattgagttgagtatgcgccctcaagatgcaaatggttccacagaagatgctaacaactttacgcgtgggtccgtgattcaa
gctgcagttagcagcgtggaagaccacggtattgttttcgatgtgggactgaagaactttaccggctttttgtcaaaaaataaggtaggagaattt
acgtttgcagaaggtcaaagcttgatttgcaccgttctatcaaaagatcaacgtatttttcacctatccctttccacgccagcttcggctcctctaga
tgttttgccttctgttcaagctattcttcccggtaactatattaatgtgctaattactgatctcaaagataatggtgtcgtcggaaagtatatgggtgt
cgttgatgtaaccagtgacttgtaccattgttctgctaatcagacaaaagatttggaaaaacaattcaaacttgccaaatcttcgccggctcgcgta
cttttcgtcatccctggtgatcctccaaagattgcaatttctttcttacctcatgttctgaactttaattttgattcagcaaaaccccaaaagcttgacc
agcttgatgttggatttatcattgacagtgctaaagtaacatacgttagtccttctcttggcgtttttttgtgatgtaggcgttcctggtatctacggtttt
acccatatctctcgtttgtcggataaaaaggtagcaaacgttaactcaaataatggtccgtataagcttaatagtgtacaccgtgcccgtgtaattg
gctatagctacgttgacaacttgttcatcttgtccttccagaattccgttttagaacaacccttttttacgtattgaagatatacaagttggtcaacatg
tggagggtactgtgtcaaagctaattccacaaggtatcgtcgtctccgttgcagatggaatcaatgcattggtgccatcatctcatatgtcagacat
tgctttgcagtttcccgagcgacgttttaaggttggagctactgtcaagggtcgcgtgttgacgacgaatgttctgagaagaagagttatcgtcaca
ttaaagaagtcccttttaaacagtgaattgcctccaatttgcacttatgaagaagcaacaccaggtactcgtgctataggtgttcttgcccgtgtctt
agaagatggtgcggttgtagagttttacaacagcgttcgaggtttcttgcctgtgtctgagatgagtgaagcttatatacgggatgcgagggaaca
ttttaagattggtcaaactgtttctgttactattcttagctgtgaaccatctgaacgtaaaatgagagtcagctgtcgtgagcaaaattgggacgaa
aaaagacttgaacaatttaataatattcctttgggctctgttgtttccggttcggttttacagaaaactgaaaattctgttattgtcgatttgggtaac
aaagtctcaggcgtcatcgctttaggtcatttgtcagatggcgatcttaccaagtgtactaaggttttgaataaacttcgtgtttctactaaactaac
agatgtgttggttcttcgaaagggccaagacaaaaaaaaatataagcctgtcattaaagaggagtcttatagaagctgctaaggaagaacgcttc

cccaaggctatagaggaaataaaagagggtatcaaatatgctggtttcgtccgcaatacaaccgatttcggtgttttcgtcgagttttgtgatggct
tagtagcgttggtacccaaagcttatttgtcagaagactatgtacctgtaccaggagcattatttaaatctctgcagtctgttgaatgtgtttgtctct
ctgtgaatcttcagcagaagaaaattttcatgtctttcaagcctttatcttctaataaacaaagggtaactgaagttttggatagcaaatatgacatt
gaaaaacctgtagatgagtctattaagaagacttataattatacgaccggaaagatcacatggggtattataacttcagtcaaagcttctcagctt
aacgttgaattggccgctaatttacaaggtcgtgttgaagtttctgaggtatttgaccgacttgaggatattgagaatctcaataagcctcttaaaa
acttcaagaaaggtgataaaatccgtgtccgtgttttaggtatccacgattcaaaacatcataagtttttacctatctcacatcgtgttgctcccaaa
caatttttggagttgagtatgcgtccttctgtcatgagtatggagcccttcaaattagaaactccctcgttcaagaaaggagattctgttgttgcttat
gttaacaatacgtctcaggagtgtgtgtgggtttctattacgacgacaattaacggaaggatcatggctatggacttggataatgatgcggaagtt
atcaatgctatacctaaatactttttggggggtaaagctttgaagtgtacagtaattgatacctcaaaaattctcagtctttccctcataaaacctgtt
gacggttatgaatccgtcaatgtaggggataaattcgtgggaagagtgacaaatgtaaatgacagcggagcgattgttcaccttcctggtcattta
tctggaagagtttctcgtttagacatgtcagatgattacgatattgctccgagtgaaaaatttgttcgtaataaatacatttcggtatatgttttggac
gttgatgttccaaataagaaaattgccctttccgctcgtgaatctagagtaaagccacaaacagctgaggtaaaagatcgagaagtggaatccat
tgaagatctgaagcctggtgatatctgtcgtggatttgtttccaacgttgcagatcaaggtcttttcgtttcattaagtcgcaatttggttgccagagt
aaagattgcagaactctttgatactttcattaaagaatggaagacacaattccaagttaacaagctcgtgagaggaaaaatactcagtgttgatt
acgataccaagcgtatcgaaatgacacttaaacccagcaaaatagatgaatcttcttcgattacgactgcgttttctgatatacaggttggaagta
acgttgatggtactgttgctaaagttgaagattatggtgttcttattcaaatcgatagcactgatggcattttaggtttgtgtcataaatctgaaatcg
cagacgctgttgtcaatgatataagcagattatacagcgtaggtgataaagttcgcgcccacgttttggatgttaatcctgagaaacgacgtatct
cgttaggtttaaaatcgtcatactttgatgcagattcagatgttgaattatcagatgaaatcgaaatgcagtccgccgatgaaagctctgaagacg
aaatggaagaagatcagcagtccgaggcagaatccgacaaaagttcatcgtctgaagatgaaaatgataatgacgaaactgctgcattgaacg
ttaatggttttgattgggacgataactctaccgtcttcgataaacctgcccatgaaatatctgaaagcgaaagtgaagatgatgaaactcctaaac
gtaagaagtcaaaatctgaccatttcaatgatgaggagcgcaacttagatgaagctccacgcttacccgtggactttgagcgtcaattgctatcat
cacccaactcatcctttttatggatcagttatatggcttaccaattgaaccttaatgagattgagaaagctcgtgagattggccaacgggctttgac
aacaattaattacagggaagaagaggaaaagttgaacgtttggatggcattgcttaatttggaaattgcctacggaacgacagattctcttcaaa
agactttcaaggatgcttgcgcacactgtgaacctctggttatatatgaaaagttgtgtggtgtcttgatcaagagtaacaagatggatctggctaa
tgaatatatggaaggtatgctgaagaactttaagcaagtcccaggcgtttggattcagtatgcttctttcctttttgacaaacggtaacgaagataa
ggcaagaggcttgctgcagcgtagcttgcagagcttacctaaatctgaacatgttaaaataattgagaagtttgctatccttgaatttaaacaagg
tgaccctgaacgtggtcgcaccgtgtttgaaggattgttgagcaattatccgaaacgtctcgatttatggaatgtcttaattgatatggaaatcaag
caaggcgatactgctatcgttcgtcgtctctttgagcgtgtgttagcatccaagctttctacaaagaaagctaagtttatttttaagaaatggttgac
ttatgaaaaggaaattggcgacaacaagggtgttgaagcagttaagcaaaaggcattggaatatgtgtctggaaatcgggaggattaatctctc
aatccaaatatatattcttttgggatatcaacgttagcatgatgattgcgcctatctttgacagtttcaaaagaaattaatatgtatcaggacagtg
atacagtcaatggctttttatatggtttggtttttggatttaatttcctctaaaatggatgataagattgggaagacttatggttagaagttatatctt
atagtatttcattgaaaaaaaggttatttatcggatttaataaaagtgttttgaataaaaagttatagtagttgcacagtaaaaagtgaatatgaa
gggggaaatttggaagtttgctacaaatgtactgttctattgatagctaaacgcagacggatacttatagaaacatttgattttgaatcaagtgaa
attgattgcatgtaagaaaattatactagaaagtgaaacaccaaccccacatgcctttttttagtagagtctgaaaggcttacccatggtagactt
gacgaccaaggagccaatgttttgccagcccttcttaagcaaagagaccaagaagttgacggcaagcatgatgttggatatcaattggtcttgag
acatctcaacgtgaccaacggcaacaccaaggcaaaggaccttctttaattggaacttgatggtagacttaacttcaacaatcttaccgtaaaga
tcatcgttgtgagagacaggggtggggaacttaccagccttagaaagaccgggacctaacaaacgaggaatttgcttgataaggacttcagaag
caacgaaggcatcgtacttcttagccaacttcttaacaagcttcttgtttttgttaagcttcttcaaatcgtcgaccgacatagcatcaacaccgcca
tgcttagcacggtccaaatcgtgggcatcaccaagaatgcagagagccatgttagggcgagggacattgggcaacttgatggtaccagagaaac
gcttatcacgttgagggtcgtagttcttcaaaccgatttgaagctcgacggtctcagtgaaatggcgtctcttttcctgagatccctttaggatttgct
cgacattagagcgaaccgtggcaacgctgacctttgacatggttcaatgctggtgtagtgcgggtacgtgaataaacgaattgaaatttcaccgtt
tctttgtgattccaaaaaacagtcacatgtttagtggtaccaccagtaggttagggtgaatgggaaaagagaagcaacgtatgaaatgcaaaac
cattttactgtgctatgcatacttgcggtatataagaataaagacgttgaacgatgcacggcaaccaagggaaagaaaatggtagcagaagcag
atgaaatgagaaaaggaagtaaaagtaatatttaataatattgtaaggaatagttatcaattggggagtctcgaaatcgaaatgaacgagaag
gtccgcagcgaacagacgatgttaagttttccatgaaaagtaactatagcaatttggagacggtcagtagcttagcaagaataagatcgcatag
aaagatgataggggcctgtaaagatgagttccactgacgatatacatccttaggattggtgttgatatttgttatctacaatgaagaagttaacag
aaatactagccaaggcggcaactaaggcgtaccattaggaggaaaagtagagtaagcaggaggatgggtagcggagttagggccgaagtttt
gatgagatgtgggtgaaggaggaggaatggaggcgtattgaccgcgttcagtgtcataaacaggataggagggatacttcaagacaatgtaga

tagcgtggattatgccgggtaatcctaaggcacaaagacaaatgttaatgagaaaactagaaagcgttgttagtacagtacggcaatatgaaca
cagggaatggcataacagacaactgccaaagatgggataaatccaagacatgaaggacatactctgcactacaaaatcccctacgaataccga
cgacaataaagggaacaaaaatagaaagaacaattaacaaaaaactagtcaaggtgtgaggcgacatgatggatccttataagagtgatata
aaacaatcttaaggaatacagggaacgattattttagtcttttccagaataaaattgcaattggatcaaacaagcgttctcttagggaatggggtt
aggaaagtgagacttctcagaaggacaggcttaagtgacactgagaaaggcatgacgatcgggtaacagtaaacaatgagcaattagtacaa
gagggtcatggaaatctttcctggatctagctattactaaagaagaagtctttttgtattcgtttacgttggggtgcttaaggtccaagttgtcgacc
ccagctaccgttgttcacactgtggctattcgactataaaaggtaattgaaatggcctttaatggttcagacaaatcagctccaagtcaatatttctt
tatttagaacactaataatgttaatcatgaaacagtgattgtgaagccacgccgagaaggacatcctaattagcaaaccaaaaagcaacgaacc
ggaccttgttttctttttgggaaccagagtgaaaacaatatagctgtaagctcatgatccgtaagaaagagacaaaaatgatttattcaatcacta
ttctatacgagttacaggggtttgaacaaccacagttatcgacaatggaacatcaaaatttcggtggatctccaatacagataacatacattttaa
accgctttagtatttatgtttattgaattcagcgtcaaattccatcatacaaacaagctggtctgtttacgtttttattcaccccgctaaacaattcccc
tgaatcggaaattatctattgaaacgaggtcgcctaaataaggatatgacatcattaatattcttaagtaaagttaaatttaccacacctttgccac
ttagctaaaaccagttagtttagagaaaagaggtatagtagactttggaaggcagtaacacgcgtttcaaatccataaattcttttgtttatttgtta
gagacttatacttctttcgttttgtatattctctgatactggaatttgggacgtaagttaattgttcgggtctttaccacttttatggtttttttttagag
aatctatatccatacttttagcatatatagagcaggatcctttttccccttttgcgttaggttgttgcgttccacttcactcctttttcgaacactataaa
atctccttaaataaagtaagggtggaatcattatctttgtttcattgtttcttcatccaactgctttccgtctgctttgtgcttggttttaaaacttttgtt
ttcttcctgttgttttaggtggttttcctttggttttaatttcctttaatatattgtgtctctaatagactctcatatcttgatatcccttctcttatagacgt
tactcagctttctggtttaatttttctttacttttactttttttcgtgaagtataaccaattaaccttatttatacgaaaactccctttattcttggttgttt
attcttcctgcaattcgttcccttggtttactatagaaaatgagtactccttcgaataatcaaggggctgcgaaaaatggtcatgagccgcatcact
ctgatgaactctcacttcctgaatataccaatcaggatcccaatccccaatatttgccggagcagcaaactattgaagaagacgttcctcctactc
agcatgagctagaaagtgagctcgagagactgcaccccgatgaaagtccaagctcgactcctgctcgcaatagtcgccccgcatctacagcgta
ttcgaccgcttctacggcctctcacatgagtattgaagacgaaaatcaaattttgaacgcagagatgtctgttcgctctcaatctcttcgtcgtcgta
gcacaggtagaagcattcgtagttctacccgtcgttctaaaagaagtgttagtcgaaaatcaagtaaatcgaagaagtcaactgacaatgaaca
ggaaaagtctcctgttgacgacgaacatcgtcagttggataactttggtcttgtcgagttcagcacagaggctcccattagtgctcctgaacatcct
attactattttcggccggatgttcaaggttgttcaaaaccaaagcttctttattcgttccatgatttatatacttcctatcggagtcttattgctgattcc
catctttgttggtcggttctatcaccatgatacaggcgaagagtacggcaatcagactcgttggcttcatattggtggtgttcagctgatgtggatgg
gcatttggtgggaaatcatatggcttactttgtgggcaggtcgctttgctgcaaagttacttcctttcatttttgcatttttcgtgtcttttgttagtaac
aacgttaccaagtggaaatgtatggcagtagctttggagtttcctttgacgctgttcctttggatgatggcttgttatgtaagttttctcccaatcatg
actcgacaccatgtcggagacggcggtgtgttggatgcgcaaaagacatctaaaggaaagcgaactcagttagattgggaaaagagtgcgaat
aatgtacttatttctctttttatttgctccattatgaatctgatagaaaaggtcttgatgcaactgattgcaatgtctttacacagacgtcagtacgaa
tctagaattctgtttaacaaattcgctataaatgagttggctcggctttatgaatatgctagaactcggtcctttgatttcaatgatgccattcatcgt
gctaagaagactggtgtcttcaactttgcagaaaaggatcgcggtgggaagacggtgaatactgccgctcgagttgcacaaaatgctttgaatca
gacaacctatagggcaatgggtgctttcaattttgctcacgatatgataaataaggttgctggtgaaattgctaatcgtgaaattcaaggttctact
tcacccagaaatgttgttcagcatttgctgagaaccactcggggttgccaatcgctagctcgttgtttgttccaagctctggtgaaaactggaaacg
ttgatattgtatttgatgatttcgttcccgtttatacaaatgaaatcggtgaagtagaagaagaaaaattaaatgcatgttacaacatctttgatcgt
gatttgaatggtgacattacctgcgaggagattgaactagcaattgtggagattggaaaagaacgtaagatcatttcagcttctcttcgtgatcta
aatgtatccattggcaaattggacgcaatttgtatgttctttgtggctttgattactttgtttattttcctatacttgattgctcgcaactttagcggtgt
cttgacttctgctggtacctggttattgggtctttcttggctttttccagttctgcggttgaattgcttaattctattatttttgttttcgttaagcatcctt
acgatgtcggtgaccgcatcagtgtcttgattaatggtgtggttacgcctgctatagtgaaggaggtcgccattatgtcttcagaatttcgtttgcta
acaggtgaaatcattcaagcaccaaacaacttattaaacactttatggatcacgaacatgcgccgtagtgatggaatttccgatccaattactgtc
agtttgaaattcggtactactttgcaacagattgaggcgcttcgtgtcaaattaattgacttcttgaaagaggaaaagcgagactataagccggat
ttgcttactgaggtaactgatttccccaatttatattctatgtcattgcttgttgttttcttccacaaatataactttcaagatgaaattctgagaatga
gacgccgtaacatgttcatgtgtgctttgatgacttaccttcaagagttggatattgtgtctcctattaacaactctcctggtaagacaaaggaatca
cctatgttcatcaacgttggtggtggtaacgttggcggtagtaccaacccattcggatcgaatccattcgataaaaagtttgacaaaaggccaagt
gatggcccacaagggattttgagaaatccctcaataggccaaccttttcaagaaggatcatggagctccactaacgtagctgatgtggaaagac
aaccaagtaagaagcgcgttgatttctcattaggaactggtcacatagtgagtgctatggatgaggttgctgatattggaaggacagataataat
cgtgaacgtctacccgatgttgtcattgaaagtgccggtactgaagctatgcgccgtgaagctgaattacgtagggaacaagaagatgaagagg
aagaagaggaagaggaagagaatgatgaatctgaaaaatcttcccgtagatctggtcgcttgagtctatcgtctcatggaaaccgaaactctgg

ccaggctagtgcacatacttcacctgctctacgcaacattgctgttgatgatatgaggacttctctgatagatcgaagcacgactagatccgctcat
atttaaagattgtatgttgatctgtctgactgagcctttttattattattttttctaatttgcttgttgagaaatttgagcttttatttattttatttctaat
atttaggtatacattttttatgaatttcgtatttacttctaatcatatttaacggaagaatgttttataataaaattgaaagccattctactattctgta
aatgaatttcgttggctcttaaccagaagtgaacgtgaacgttggaatgtctgctgaaattgaaaacataacgaaactaagttattgtagaaatg
aatcggtagtccaaattgatggttgttaatttgcaattaagaatataaaagatggatttggattgtgtataaagatgatagccttgtatgatgagct
taaagtattatgaatgagtacatgtcactcttccgtatttcataatgaaccattccggttttgcttttaatgaaagtagctgtacaattgaatatgca
aagagttataatcgacatacaccaacaacttgttcgtcccaaaattactgataactgacgttatcatacaacatactctttattgttgtattgaatgt
gtagattagatagtgttaggacttacttagtcagcggacatgcgatttatcatggtggaaaagaaggcaactcactataaaatgcgtctcgtttcc
acaaactaatacagtacgattgaaatttcataaagtagagcagccgtaaataaattgcaaaaaggtaacaatgggtgaaaaaatgaagttata
cgggtttgaagtttctggacatgttcatagagtccgacttttgctctcgtttttgaagattccatttgaatggatcattacacaatatgcagacatga
attcagaggaaataaagcaactaaatccatggaaacaggttcctattctcaaggatggagatatcgttatctcggatagcaatgccattatggtgt
atctagtcaaaaagtatggaaacgaagacttttggttgcttaaagatccattggcatcttcttgggtccaaagatggttttcctattctgcaggaga
gctacgttatggaccatcgactgctcgaaggattcagctttataaaacgaacgaagattccgaggctcctaaacagattgcagccaaattatttaa
atttatgaatgattatttagaaggaaaggaatacttagttaatgatcacattacgcttgcagacttggccatgtattcctacacatcgcacgctcca
gaaggccacatttcgttgcagccatatcccaatattgtgtcctggttgaaacgcattgagagtctccctgagtatgttcctctcaaatggtcctaccc
atctatgaaagactattaattacgatgactacgttattgccatgaatgaaaaagtacagatatgttcgttaattatgggaacgaaaagcttccata
tcatagggagttacgtatatcaaaggttattacacacgttttccatcaaaagattatttagcaagagcattatatcgtaaatcaattatccaaatct
caatcaactttatgcctttataatcaatgtcgaatttcagcgagacacacaaccggcccagcccatagtccaatgagatttgctcagcgtgcaggg
gtctcgtttattgatagattactattgttgttcgtgtaagaagtttcttctttctcctttggagactctgcctcggtaatgacattcaactttactgcttct
tggttgtcggtctggaaagaacgtagatatgtctttgagaacctgtgaataccgaccaaatcaatcatccaagaaaatatagtgccaaatactgg
aaattctaaaaagtctaagacactgtagaagacagcttctccaattggtaaagggtagccaatgccttcacagaaaccccagctgacaaaatac
aaagcccaaataacaaaaaagtaaatgatggagcataaaaatccaacctttgctttgcgttttagaggttgaatatcatgtttataggtcaaataa
gtatgcaacatggcacaggcataacctaccaacgaaaaagcgtagaatccccatcggtgaatagagacagtcatagctgcagctaacaagcat
gcaaagcatagccaaatgtgaaacacagttacaacagttcgcataaacgaaacgccagccaagtaaaagatgctagaaatcaccaaagggaa
attaatcatccactgtatgtatcggacaaagtacactactcgaactccacgaaaccaagttgaagtagttaaattgaccaacgtgtaacccagttc
accagccatgctaaaataaacgatcgcagaaaccatgcatgttatagagtagacgtagtaaaagaatctttttggcggactggaaaagatggat
atgataggatagacgatagcacaaaacaagaatatggaaaaggcagcccagtaatagtctgttgcggattggatcaaggttagatcaaaatact
ttttgctccacacatccgaaggtatttttccaacattgctattcatttttcttcgtgcacttccgttgcctagcttgaaaattccaaacctcaattattga
gcaccgaaaacagttcaaaaacaatatgttaaattaatacagaacaaaagatacgtatacaccgaaaaaataaagtatgctgttaaaaagaaa
gattcatgatggagtaaacatgcatctccttataatataaacaaacaaacaatattgacgcaaacagtttttaatcggtattcgtcagcaaactct
aaaagagaacctgtatttgtacatgaattgaacaagcttccatctgcggtacctagtaaacatgaatgtttaaaagactttttgctgaagcacaca
taaacgttcaaattttgagttaggccttcagataagaaaatatctgtaatagctgttaaccatccaatgacggaactcgagaattgtgcatacgat
tatagctttaagaactatatcattaaagaaagatcgctaatacaattaggacattagcaaaatacaaagattaaaagaaagtgaaataacgtag
attgttcagcaagtcatactttaagatatgctgtaacatcaaaaagatccaacgaacatatattaagatatttacatatttatctgaaaggcattca
agttgatgaatagaatcgaatcatgaaggtactttcataaggatcctaagtaaacatgaggggtatctatgcctttattaaataaagggcaatgtg
gtaaagtgactagctgataatgaagtgaaatcagataaccaatccaacaaaagttgaaaacataaaccaataaataattaatttttagcaggtg
tgctgaaacgttcagcacgtttcttggctttttcggcttcaacaggatcttccaaaatcgagcctttgcgtttttgtccggcctgctgatttttggtgctg
ctggcgaaacttttgctttccccgcttatctaaacgtcctttatgagaacgatcctgttcctcattcttttgttcagcgagaggagcggtttgtacacc
aaaccgattagctcgagcagcttttttttattttatcatcatcaattggtaaaccaaaacgtttagcgcgttctacgagcttagcttgctcagctaata
atatttccgaatcttgtgaatcttgtgaatctttcgttgttgattcctcagtggtattttctggcttttcactcggttcagaagttgctggttctaccttttt
gttgttcctcattcgattccaccttcgcaggctctccttcggtcttgggagcctcctctgatggcttggaagactcttctacggcagcgtcattgtccat
atctccccagtcaatgtcatcttcgggaggagctaaatcacctaaatctgtggcagcgtttttgttagaaccttcatttgtggcttcaaattttccctc
ttccgagctagtcaatctagcaaccaactcattcttgtttccattagtggaaaggcctttctcggataacttttcgcgtaaatcagctacctttaagtt
tttgaggtcagacatatttctatgttcgtgtttatcgacgatttttttgtttatttacaaatagcgtcgaagttgtaggtggaacgtagatgagcctgga
atgtggtcagcgtttggatatagcgagagtttacgaagctccttaaatatataagtgtgtatatcagagcatactaaagtggcattaaataaacat
aatcactaataaaaaataaaatcagtcagtgtaggaggaaagttgatctcaccgtgacatacacatggacaacgaaggttaagcttacacacaa
aaaaaattgtttacgatctttaatttaatgccttgtaaaacgtctcattgtagatgaaaccatatacactatcaacatttggagtatgatccacgcat
agtcacttgtttattactcaaataccatctacctatctcatctgtgctatgcgataatctttattaaaccattgtgtgttgcaaccaaaacaaggcatc

caattcaaggaaaaaactcctactttttataaataccctggttctattaaaaacaaaaaccatatctttttagtccctaacaaagaacatttttttact
cttgagggatctcaatgtcaccactgtcgatctccttttgaacatcgtgagggttcttaccttcaacggtgcaaccaacggagaaagcagtaccaa
ggatctccttaacagtaccagaaaggtccttagcgagggacttgaagcgcatggtacgagcgacttcaatgatctcgtcaatagaaacgttacca
gagtggacaacacccttgtccttctttctgtcacgagcaggttccttcaaagccttgataaccaaagcagaagcggaaggaacgacggagacgg
cggcttgacggttttggatggtcaatttgacggtaacacgaagacccttccagtccttagtagccttggcgatatcttcaccaaccttcttaggaga
caaacccaagggaccaatcttaggagcaagagtagaaccaccggcaacttcaccaccgacggctctcatgataacggtcttcacttcattggggt
cgaactaagcaacatgttaaaaacttgattctactattgcaaatttcataacaattcgtgataaaaacttactttaggaggcatcttgctcttagtta
aggagtatggtagaggtgtgaatttcgtgttcgtgttatgtgactgttagtatccctcgtgactattctctggcgtgttacctggtcgatactaatcca
agtaacagtttatttaaagtaaacaaacaagaggaaaagcatgaaatgaaaataaaatgaaaaattgtaataaactgagataaaataaaggg
agtaagtgatttttttgaacaatttacaagactatttaatttgtaaaaaaagggaactggattaagaattgatatctgtgtagatggtagaatcctt
cagttatattaattccttgtcgatttttagtctacatctattttatcaagcaattgtagtttgcatgatatatcattcatgtacttttgttgctttcttaac
agctttaaaactatatgaacgtacgacctgattattcataccttatttcctaagaacttctattgttagtaaatgcttttgtacccaaacaagttctca
agattatcaaaattgatggaatttgattaatcatatatgtaatttttgtttgtgagcctaataaatgaaaccaatataattatatgtaatcctgaaag
ttgaaggaaatgtccataaaacagcgtacttttacaatttatcagttatatgtgaaactggtagcatactatgatatgtctttctattgtaatatcta
aatgactgagtcaattcttctaacagtccttttaatagattaactcaacctgcttcagaaatataaaatttgacgggctataagttgtaaacatcag
acaggcgaatttgaattcgataagctttgaagacctagctatcgagtttcaaatagcctttgtgtcataacggttagtgaatcatccatgtagatat
ataactgcatgcgatggacaaggatagatcagtgaatttcacccattctttgcttaccagttcccactatgaatattgaagattataatatcctttcg
aattacaagagggattatgaccaatttatcaggccaaatgagaatttacggaatgcagttgatgctcagatagatacaccagccgttacttgggc
tattattagtatggtgattgacatgttaggtgaccaggcaaactgttggaattatcaaattcaaggggaaaacaaccatgttagcgtttggcctact
gaagcaagcattaacaaaggtatcatatccgaattggaggatgctgctaaacgtaatcttggaatacatgggtactctatattagagctatataat
atagaaaatggccctgatgaagaaacaagtcaaaacgggcggccccttaccgctatttctgatgatgtttctctaatctgcaactgctggctggat
tataccacaaagttggggttaataagaagtggaaactttatcaagaatgaagcatataaagacagtgcccgtgacttagaattgtggcgtttcgg
taatgattttgacagaactcaattgaaagatggagagatgttaatccacatgctaaaaatgtaagtgatcactagtcatcttcatcgctcaatata
gcatctcaatcttttcacagcgtccatttattgcgggaggtgtcatatccaggatcctgtttttataattttcgctataatcacggtaataactataaa
gaattactatctattctatatgtaaaaacaaggcggaatcagcgattactcaactcaaatagaaacttatgagacaaagaaataactatgacaa
agaatagatcaagtttactacattgaaataaatatatgtagcattagtctagtcgaaatcttgagattgaaagagtcaattggctggctgaataag
attgtttttccatttatcgatttgttttaccaaattcttagacggatttagtatcctttcaggtaatatcacatcgttcatttcaatgtacaatcctacaa
gtgtgttgtaattaccacatcgtgtgaggtttctgtcgtgacaaaaaattagtgccaataaagatacagtaacttaacataactttctatactctatt
tagcacaacctaaagctaaaatctttggttcatatccatggacgatgattaagatagccattttctaatacgagctctagttgctctaaccaaaatg
cagtaatattaagagatatcaattttgattttcatgcaattgatttccaaaccttttttatgagaaatgattcttcagtcttggacaggtaagatacat
tctagtcaattctgcaacgtcttttcatgcatgaccaagataaaaaacgtaaccaagaaaaaagttactcagataatcttttatttccttattctaca
aatcctatttccagaccgtaaagacatcatataggatcattgatatattagttcctaaaagctttaacaattatagaccccttattcacaaagtaaa
aattcaaacccagtactagtatacaaccaaaggaaatactttttttataattaccacaaagtcaaattgccatttccctaaatacgtataaaaaaa
aaaatcctcacgttgttgtagacatctttataaaggatcaactcctgtatagtttcttagttatttttcgatttttttaaagccactcatgacagatttcg
gttctttgccggataagggatggttgcgagcacgcttttcagctggttttaagatctaaacgtgtttagtcagcatagttccttaataaactttcatca
tgtaatacctgaaaactgcaaagtaaatcttcattgacactcatcatagcgccaacgttgtcgaactctccacaatagttgggagcgctgaaaatt
gtcacaagtaaccgtttagcaaaaaaattcataaccatcttcaactacttgatgcgctctacaaattaggtccatgtcgtgtttttgtaaaaaccgatt
tacgacatcagcaccaaatgtatatgaaactcctctgtcattatctccccaacctgtgacatctttttcaggatccgaccataatagatcgcacaat
aagcctgtatcgggaatatcggtaggacgaataattcgttgaatttgctccatagcatttaaatctggggaaaggccaccatgcatacaaaagat
tttttcatcaacaattgcagcaacgggcatgcagttgaagcaatcggtaaaaattttccatattttaatgttgtagcgacgcttgcattcgtcataga
agccataaatccgattgatactagcacattcatggttgccacggagaaggaagaagttttcgggatacttaatcttgaaagcaaataaaaggca
aataacttcgagactttgctttcctcgatcaacgtagtcacctaagaacaaataatttgtatcgggaggaaatccaccgtactcaaatagtcggag
caagtctgaatattgaccatggatatcaccacaaacctataaaaattgttagaaagcttagtccatctttctttcatccctagaggctctcagataa
gaatgaatgggccatgagtaaggattgagggttatcatgcatagagaccaagataagtttgaccaaatttcaagtcggcgaccaaggtcaaatc
aaatcgtaatttccttcaacattacgatatcgaatgtgaacaacacacagcagttctttagtattatctttaataagaaacccattccattcttaaaa
aatggctatcgaataacagtccaaacccacccgaagagagtccaaggaaaaaagatgtatcgaatattatacctttaaaggagcctccaattcc
aacagcattggctgattaagaaataaagagcgggaagtcgtacataaataacgaatttctgcttccgtcaattgagtttggcggcctggtttcaca
tgctggacttagtttttttttcgttagacaacaaaccatcgagattgtaaaagtccgtagctacatacctttataagcttttctataatagtatcaat

atcataatccatattttgttctaaattcaaccgcaattccccaagaataaaaaatccacccaaatgcggtgtttggcttgtgttgagtatatcccaat
attaaagaagccaagaagtcataggtcgaattttccctcttttctcttttaagcgttaaattccattgaaggaaaaagaatctttcagtcgtcttaat
tccccgaaaaggtgcgatttcatttttttacagattcacgttgaactactcgaaaaatcaattttcttcatcggcagacaacctaaagaaatgcga
agtagctttgtttagttttatgggtttaggatttgaaattgtacgtttagaaactcagagaataacttttctgaatttccaaaagcatatatacgtgat
caggaatatagaagtaaatttctaataattttaatttaagactcggaatattcttacgtttgcatcagatgatagaaacaatggattaaaatacag
acaccctttcgttttcctctttccaactatgcttgttacaccgtatgcttactaagcatttgcacacgaaaataagtcttttcaaatgaaaaagatga
actctctaaccataattgattttttttgaagatgggtggaactcgtcaagattaattgattctgatatgcaaaggttatcccaatcttatatttgatatt
ttaagcaagaactattgaaaatgatcttttgtttcagttctaggtaaattctttcggtactaagctttctacagctgtcgttttcgtgcccttttaccca
acccgtacgtaccatgcacatctgatcgaaaggaagctttgcttacaagcaagggaatttttgttaacgaataagaaacctaaatatcccaatttc
tatactctttaattcgcaggactaaaactcaattcttctttcaccactcgtcagcaagaaagaacttatttcctacatttacaactccattgagaccg
gtttgggaattttagtagcttggtaccttcatttagattgtgaaagcaaacaaatcgataaacatgcttcatacgaaacctgcttccaaagccgtgt
acatatggtatatccttgatgtgactagtctatctatggtaaatgtagcttgtttgcaactcatacaaatgctactagcatctcattatcatgtgccta
tggaagaggttcgagaaaagctcgtagtagtgaccacctttgccagcatccttcaatatatggcagctactctcgttgtccccatttattcgcttgtt
attaagcgattttcgccatgggttatcattttctttacttggcttggagaacagtatatgcttgttttgtcaattcttgctttttttgtaccccgcaaatgc
ttctcaaatcgtccatttcatcgtttactcaatttccttactacagggattatcaggagctggtccttccattcatgccggttggaaagctatagccca
tgcctcgtatatcgatgaaccatttgtccttttttattacttctattccgatattttcttttttttttaggtcctttttttcgcaagttatttgctctttcacatatc
tctctttcagctatacctagtggcctggctgttgcattttgtcagcggggttgccttttcattgattttctatcattctcctacttattctgcttcctcttta
gaaactcaacctctctttgcttcttttgaggttgccgaagaagtaaactgtcccgggtcatttccctccgaagaccaagttcacaggcaacacctca
gtcttcacctaatttccagtttatacaaaaagtatattaagaatattaatgatgtactatttaactcctactttttccaattgacccctacctgtcgctg
gttgttaatgtccttcgtttttttacatgccttcttcggtttttttcaactactacgttcaatatttgcttaacgttaccaaatttcccttggaaaaagtcac
atttattagttcattgcaaacatttggctctttggtgacttgctctatcttgacatatttgtctgcttttgatcatttgacaatccaaacatttcactatat
cttaattggaggcacacttaccctctcaatactaactatggcatattacacggctttaaattcttttattcctttcctagcattcttccatggaatgtct
cgttccatggagccaatttttcattccctgctgaccattcacgctcctttgagttctttacaggcttattggagaatatctgccatctttgaagcttttg
ctacttgtttctcctacatatgtttagcttacttttatctacaaggcacagaaaggagacatttactgtttttcctcggtcctgctcttctcactcttcttt
cttgttttagttctcttttatttcatagatattcatcttaatcatgcactctgagcagatgaactgctgaagagtaagactttctcaggttgatgatatt
tgtatttatttattttactagttgtttgaggacactatgaaggattctagatacgcaatatttattattgtttactaaatattctctattcttttagaaag
aaaaaaagatatttgcttgtagaaagagtcgtgaaactaacgttttagcagtcagcaagacggtcattttcagtcagtcaattttcttttccttcttt
cggtatttcatacatgttctccaatgatattttattctagtaacaatattcaaaacaagctactcgatcctttttttgtttagtgtgtgattttgccgtgtt
accatcaaaaaccttcccactatttgattattaaatccaacacaatcgaataatttaatttattaaatattgatactaagtattcttcattgattattc
aataccgtctttacccaacactggaaattgttatccatagcctaaacacttacccactgcccacctccaaaacggttgtggttcttaaaaacaatga
attttgaggaaattgaagaacgagatggttcgtattattaaagaaacactttaaatgattattaacatgcttccttagggatccgttttacctggaa
cgcctttccatccactagaatagaaagctctaggacaatcgttccaatttctgctctctacaagcctttgaaccctagacctgatctacctccggtcc
tttatgaacccgttacttgtaaagctccatgcaaagcaattttgaatcctttttggtaagagtgctcatatatggctcaaaagaattagaaacagca
ggctaacaaaatcatagccaccttgacactcgtgctaagttttggatatgtaagttcctaattatatcaatatggttgtcaaggcgactccataaac
agaaaacgccaagagaaagaagagagatctaactagtattttaggtccgttttgcttgcaaagaaacatgctacctccgcaatacaaggacgta
tcaagcaccagtcttcctctggagttactccctgaattctctacgattgagtatactctacccagacccccgcaacttcctcccgtattcctttttgttg
tggatttatgtcaggatcaggaaaatttgcaatctttaaaggattcactcatcattagtttgagcctgcttcctcctgagtgtttggttggcttgatta
cgtttggtactatggttgatgtttatgagctcggatacactgaatgttccaaatcctatgtattccgtggctcaaaggactatagttccaagcaggtt
caagaaatgttgggattgcctgtcagttctgctgctgctgctgcacctattgctttacaacaagctcgtgctgtttctggaggtatgactgcttctcgc
tttctcttaccaattcagcaatgtgaatttcagttgaccagcatcctggaacagcttcaacctgattcgtggcctgttgctaatgataagcgccctca
gcgctgcaccggaactgctttaaacatcgctgttagcatgatggaaagtagctgtaataacagtggtggtcgtattatggtttttgccggtggtccc
agtacggttggacctggcatggtcgtttccactgaactccgtgaacctatccgttctcaccatgatgttgaacgtgatcaagctaagcatatgaaga
aggctttacgtttttatgaaaaccttagtaagcgagtcagtgctagtggtcatgccgtcgatattttagctggttgcttggaccaaattggtatgatg
gagatgagaagcttgtcggctagtaccggaggttatttggttctttctgattcctttacaacatccatctttaaacaaagtttccaacgtatcttttcc
cgtgatgcgtttggacacttagcattaggttttaatgccagcatggaggttgtcacaagcaaggaattaaagatatccggcttaattggtaatgctg
tctcattgaacaagaaagctcaaaatgtgggtgataccgaaattggaattggtaatacaaatagttggaagatgtgcggtctctctcccaagtcta
cttatgctctttattttgaagttgctacacaaaccgctgccgctcctcaaagtaatgataaaggcttaatccaatttctcaccttataccaacattcct
ctaacactttccgcttgcgtgttactacttcagctcgtgctttcgctgatggtggaagccctttaatcattaattcctttgatcaagaaactgctgctgt

tgccatggcacgaattgctgccttcaaggccgaggttgatgatggaccggatgttcttcgctggactgatcgcatgttaattaaactttgccaaaag
tttgccgagtacagaaaagacgacccggctagttttcgtctctcctctcaatttactctttatcctcaatttatgtattatcttcgacgtagccctttcct
tcaaattttcaacaactcaccggatgaaactgccttctaccgtcatatgctgaatattgaagatgtaaacaatgctttgattatgatccaacctactt
tactttcgttttcttttgagcatcctgatggtaagcctgtgctattagatgcagtttctataaagtctgatgcaattcttttacttgataccttcttccat
attcttattttccacggtgatacaattgctcagtggagaaatgccggttatcatgagaagccggaatatgcgaatttaaaagaattactgcaagct
cctagagttgaggctgccgaattgcttgtagacaggttcccaattcccagatttattgtttgtgaccaaggtggtagtcaaggacgtttcttgctttc
gcgcattaacccatcggaaacccacaactctgctactatttatggtgctccttctgctcatgctgttttaaccgatgatgtatcattacaagtatttat
gagtcatcttaaaaagcttgctgttgctgtttcttgaacgtaatgttatgctggtaaatagctttacctctaatgatctcggctgtttatatgcatttac
ttagggtttttctatctaagtaaacgttattaagatgtttactatgatttttttatagccttttttttatgtcttagaaaaatcgactatctttttacctaat
agtgcgttcgtttcaatggctttaggtaaaactcaatattttaggagctacgatactttgcctgttctaataaaaagtagtatttaaaggaaaaattc
tttactgaattcattatattcgagtaagagatacgaagagtaaaaggactttgtttagattgtttatactcactttccctgcttaacgtttgttttttg
gctggaacttaaactgtttaaattgctaatgtaaacaagcgaagacattctttggcaaaagccttctatccttttattatccaacatgactgtagtcg
aagaatattctgaaattaaacaattaaggaaaccacccaaagcgtatactggcttacagaaacttaattacttaccggtgccgaaacctaagac
attccagatatacaatacgcttctaaattctataagatgtccatgtggagtaagtacaatacaagtatgtgtttactatagacttcatgggaaactt
gttcctgcatttactatagaaattgatccttcatttcaaacacgccaaataggagcgttggttaataatactattcaagattattcgaataacctact
accaaaagaattgaaagagaagcagattgtagtctctccattttatcgagaaagctttgaacttcgcccgaaggaacagcgggtaatcgaaaac
cttgatggacctaactacattcttgttgaagtctcaaaggctaatggggattgattcaatgtacttcttatcttgataaatcgttaaattgtctgtata
aacgtttgatataagcacgaactagctacttagtggaacacacaacttataatttcctacagacaattaaatattcttactcgtaaaagatagtcta
tttaatgtcttacatatcaacccaaaaagagctcatttacaaacaatcactttctgtcattgctaaatttaggatttgttagaggtgccttctcctgct
tcactttaaactcggccagcaagcaaatgtgatcactagggaagtgtgcattcggaaacccgactatgcttgaaaggtaattacgatctacccctt
tcaacaaaccagttgcctcaaggttagcattggtataccatatgtaatcgatcgtgccactgaaaccaggcgtaaagttcgtaaacggaagttctt
gagtttcgctatatacggatttcaaattgaaagggtgagatctaccgttgaaagtataatcaccatagtcattattcataaaatcctcatgatgcgg
ctcaaccttgcctgatgatagaaaatctaggacgccacttccaggactagaattaaaatcaccgcaaattaataccggaatctttgtataatctga
ataagttggtcttgagtcttttaaataatctggaggaagtttcgagggcatcgaacagaagcgagtagcaacctgggcgagttcatccattaacat
ggctacttgcataagcttaacgtctctgaattgaggatcccagtgaatatgacagttagcaacgatcaatcgatgtccagtttccttagattctaac
agagttaagacgctaacgttgtctttggtcataacgcgattatacatgttcgaggtaagtttaatattttgtcttcgaagcgaaggcgcttgattgaa
ctcaataatggacttttcatgcatataaaactttgaggttttaaagaaggtagcacaaccatcaacaactcggcgctcaacctcgttcatagtacgt
acacgactttttgggaaatgaactcctttgtatcctttaatactcatctcgggagcaaagaaggtatcaaagttttcaacatcaacttcttgaagac
agataatatcagctttatagccagcaagttcttgcataatcaaatcctttcgataagaccaggcaagagcccaagaaggagtatatccatagagc
gtggacgtagcataacgttcacaaagtatattatagctcatggccgtaaacttaaaattagtatttgatccatttaattttggaacgctagacccct
cggcgtcagaaacaaccattaaagacttttcattagcatcatcattatcagaagtaagcttttcccattcacgctcaggaggaggaggaacaaca
ggacaaccatcacgaagagctgtaattaaaccagtcgttcctgtttccataagctgagatttatacacatcttgtaaaggatttccttcaacaccta
agattttaagctgatacagtgtacctaattcaggaggaataacggtaatcatgttatcaaataataacacctctcggagttcaacgagtaatccga
gttcaggtgggattgctttgatgttgtttccagacgcatctaagattaccaagtttcgtaactttccaatttcagatggaagccgtgtcagcttgttat
gattaatgtacagttctgtcaaaaaggaaaacttgaataaatcagtgctgacgttgcgcaatccaataccaccgagatccaagcaagtccaatct
tgtcgcgcttggcgatgagtggaaccgtttttctcttcagtcttcgttgtagtagtcgtgaccatcatactcgatgtagacagattgtttccaacgttg
ttactagtagtagttgatactgtggtagagttcattcttccagacaaggcatttgaggcattagcagcagctcctccgtaagcagcaccattgtttct
ggcattgtaattcatcgtacccatgccacttgttcgagctaaagcggcggcattatgagcacgttgatgaggagagctgaaagctcttgactgagt
tgcgagtgctatttgctgtttccaatgttcagaaatttgtccattttctaaagccggcgaattatgtactgacaaaatggctgcatgttgttgatcagg
cgtaagcagccctggatgcgtccctgaatagattgttccttgcgtatacctaggattgaacatagaaggtatctcgcaaatttactgtgtcgagatg
tttaatttttttacaacattcactaattaggcaaacgaagtgaggttgttggttgatggcaagagtttcacccggtagcaagtctgatagcgataacc
aaacctcaccaccataaacaagcataaaaagaagaaaatttagaatgagatcttttatcactatttttatataaaaaatggtttgtttaagtattct
acagatatatatggacaaggtgcatcggtatgaatggatttaagtgaaggtttaaccgctattcttatgtttgaaataagcaaacaacaattgtat
acaatagaagattcttataatgataaaggtaaaatatttcatattcataatacaaaacttgatgatattagtacatacacgaagaagtaattaaaa
aaggtctgttacgaaaatcttctctagctaaccaatgtgcaaccaccgaactgacaaacccataagttgagggttgacgttgagaaaaagaaaa
atgtgcgctgtatttcttctgacaatggcaaagaaacttaattaactgacgctaatcgacatgcacaaatttcttctagtaaaagaaacatggtag
ataggcgtttgctttgtattcaaacggaccatttgatcaggactaaaaattttgttaatttatttaaatatgaacaacgcttaccttgttgcccaagc
ggcagtcgactccaaggcatcagactcggtgttggtataaggggtcactacactaccggccagcgagctagataccgaatcagaataattggttt

ttaaaacatcccaggctcgcacggtgtaatcagctcctccgctaacaagtactgtggactcttgagaaaaggaaagtgaataaatgcctccttgat
ggcctttcattgtctttatccttcgaccagatccaatatcccaaagatgtataagtccatcgtaatctaagaaccatgttagtaagcgtctttccaac
tatttctttttcaaaaataaaataaataacataccagcactagcgacagtatgaccatctggagcaatggctacagcagttaccggctttgtatgg
ccattaaaaatacgaaccgaatgccctctatgaacatcccataacctgcaagttttgtcattagagccagttaaaacataagccgagtttggatga
aaagtaacgcactacatccaatcagttagtatcattaatctacaaagctgtaacctacatcaacatcacttaagtgtccagcaaaaactcgcaaa
ggataaatatggtcacagctccacaataacgctgtttgatcatgcgaagcggtagcaaaataatgtccaaacggaccaaatgctacatcccatat
aggaccactatgtcctttataagcaaccagtgcagttttcgtgtccacggaccaaagacgagcactccgatcttcggagcaagacaaaagatatt
tattgtccggagagaattttgtaccataaataggtcctgaatggcttaaaagtcgagtagagtcactcgctggctcatttgccgtccgggaagcag
attttttatcaggcttgagtgaccagagccgaatataactttcttggaaaccggaagcaatcatagttgtgtctggtgagaattctgcacaattcat
agcaccatccgtatcatggaaggtgtacatacaaacactgggaagtgatgctcggggtcccaaatgcaatcttttactccaatctttcactgcttca
acttcggaagttatatcggcacctttatgaggaggcaaaggtatatagtcacgagaaggagcatcttcttgattaggaggatgatttttttcgaaac
tcacctagtagattagatgtactttgcttggtttcgggatccatatgcatcatatcttcttcttccagatccatttccacaatttttttccatttccttatcc
atgggcatatgacttaaacggaccggctgcaactgtccttctccgcgatccaaaagttgaccggtgattccctcttgttcattgaggataccataac
ttgcagacaaactaggccttcccgggacaatgtggatgtcgacgtgtctattaattagttggataataaccgtgcctccgttagagatgttttcgaa
aagaaagtgaagtagcaagtcaaaggtaattcgagaaaaattcaattgatatttgtttttttcggtattgttgagcaacaaggtcttcatctacatgg
ctaggaagactcagtgattttaagcacttgacatcccaactatgcaaatcttcatggtctttcataaacatatcataaaactgtttggctaattttag
ttagcaacatacgcaaagaccgaacgattgagttgtattctccctaaatttctttcaggcatctcgatctctacacgtagaaacctatgtataaata
cgcagagaacgaattgttcagcagtacttacctgtatcataacgatctaaggacaatagatccaaaaaggagtgaacaaaaattggaaacaaa
atccttcttagctctgatttatacaattccagtgaactatccacccaatctcgtaacagtgtgtacgtatgagcatacgcgtctggagtctcccttata
tttttcagtttttcttccacagatataccagaatcagaagcctctaaacgaagcatggcttccgttctggcatagcctttttttggccaaatagtccag
cacaatgcgatttagatcctgtgcttgagttccattgccggccgacattttttttttataaatcagcactctcaaacttgaccttaattccaaattgaag
cgcttttccttcaaaaaacgcgtaaattcatatgaaagtgctacaatggcttccagcaagtagtttttacagggactttgggggcatggtttagcga
aaagcaacaaagtgcggtatccaaaatcttttgtttacatgattacatgaatgaaaaacatttaattcaagcgtttttgtgtgtagttttggatatca
ttcccgtttatggctgtttaaatgtgaataaaaaaagcagcctaaatctttaatgaaagtcgcttgctttttatggtgaacgaatctacgcttacagaa
atattagtcatcattcattcatttgattttttttgtagtttcctgtctactgtatgtgtttttcttattaaaagaaactgtccagtttaattgtatatatata
gttttttttagcatgccatccctcagatactgctatgccttttgtttacatgcctcggaggacttcttcagtctatcctaaaccagcactttctttcctta
cattcttattctcatttggttaattcaaatctacaacaaaaattttaatggattaatttttagttaatatatagatttggcatgctgtcttgctttcgttt
acgaaattacccaggaacgttctaaggtcatcaagatcattcacactcgttgattcgatcactgttttatgatcgcattgttaaagtcttttgacttca
ttcgtatcactagctttcgtttatctaattcaggtatttacaacctacttgtccctgtccttgcaaatgtccacgaaggcttctaatgcttcgaaaacc
atttggacaacggacaaaattataaaatctcttacatatcctgagcattgttctgtatcactcgcggaacgtcttcgctctaacttaaaaacgaaca
atgtaaaacaaacatcttcttctcaggcgtggacgtttgatttgcttcttaccgctttaaaatgtgctcttgaaaatatatcaaaacgatggacggat
ttgcctagcgaaaacagatcgcaagtacgccaggcattttcttctcttcatcgctctgcctctatgagaagtcaactggaaatcaatttactttatga
ttgttgctgctatataatggataagctgacctctttttgtgaatcagatacacagatttatagttgcgtacgcgccagattagctattattaatagact
tttggacatggaagttcctgaacctgccatatttcatcttggaagggtccactatagcttgcgttatttgtttcaattaacttcagctagtttagtgcat
acttctgcctattccccttcaagcacttctataacgtcagccgcagaactgcaaaattgcctattgatccccccttccttttcactcactcgcccatggt
gcttgttctctcatacttacctttcaacttaatgttttaagatgtcttaatttgattccaaatttctttctttctctgcctataattcaaaatttgttaaag
gactctggcccttatgcttcgtgctctcggttatgctcctttaatgaagagttggcactatccaggatcgaaattttggcaagattgctcgctagattt
gctcttactgagcagaatgcattgcattccttatacttatatgctggctctcttcaatcttggctgttcatagggaagtgtcacctgttaaaaaggtca
gttggcttgccagagccaatagaattacgaattgtcaatttgtttcacaaaatgctaaacctttgtgatccttcgacggtagatcttactgatttaaa
agaaatatttgaaagtgttttatatggtcttaatttttttaagcataagcaacagaaacttactgaatataagaatttctgacacatattacaaattca
atgacgttgcatcttgttttctgcggctaatggatggtgttgattcttcccttggtgacagaattgttaaactaaagtacttactcctcatatttcgcct
ttctctgaataaagaatgtcccaaagtgtcaattgatgctttctcagaaatcaacacttccatcgagcttttaagtccagaatctatctatccggatg
atgttatctatctgcttgaagtatttgaaagtatactctcaggtccttcacacttttcccttgatgatgataaactaagaacctttttgctgatggcag
ctttttcaaaagccattttactcgttaatcaaagctcagtttcagattctgatgcaataaggttatcaaagagggtggaacggttcctttcgctttcat
tttatctgctacgctatttgaaagagactgtaattcctttggatacccttcgatgcttcgtacaagtcgcaattaaattccaagaaaaagagttttcc
caaaaactttcaaatctttgttataacactagtgtaaaatctgtacatacggaactgtcttttcagtatattgacttaagtgtttcgatcacactgtgg
cacaatcttttggatgatgaagctttcgtcttaaaagttgtgaaatggcttcaattattggacgaaaagtctatagaaaatgttgattcattcaaaa
aactttcaaatatgctagagtttcattttgctcaaccctcatttaaggttactacttctcttagcaaggtgttgaaatggattcttaaaaatttatgca

catttgatgaaagccatttgggagcttacatttcatgcttcagcagtaacattctcaaaaccttaagcaacgagatatccaattctaaagaacatg
gttcgagtgcaatttctatacggattcttgaagaaatattggatcgagagactgatttattagaaaaagcaaatgttctctgtttaatatcttgtaca
atgttcaataatgtgaatcttacttcatattacttggcatctgtaaaaaacaagtccgataaatttaaaagttactcttcaaaagatccaaaaataa
atctttacaagataattctcgatttatgggatttttttactaaagtatcaagccgaccagtgttacagtgtttctttaaactctctaattttacggctgtc
taacttcgtgcttacttctcggttacccgcatctccaggaggattaaatgatagagacatatatgctttttttaactgcttctttttgtatattacatgta
actgaagtcttagacgactactgttctgagctcttattgttggagactgtatatgaacttttgaagaactacttacttaatcctgctacgagattgatt
gttgtaattgtcactctgcttgcaaacaaatacgcatttctgggattttcgggtaaagcacatctttgcttttctagggcttacaattattttaaaaaa
tataatagtacggatgaatgttttgaaaatttttggagggtttctttcgctaaatatttaatcattactggcaacactgaaaaagggtaagtcataat
agatagtatttacgttctaacttaacagagtctcgcaactcaagaactataatgctacaaggaaaagactgtttctgggacaacagagctttatta
tatctgattatttaatgatgtatgaaaggttccaattatctgaagcagtgtatctattaggatacactcctcaggcactgcacttaataacacagaa
cctacgggttgtaaaatcgatttttttctacttcgacgagaaaaagtgatcttaaagattacaaagcagatttaccatggcaattggcccggattgct
gtaatgtcttatgatctgactgctcgaatctatgaacatatgggacagttgagggaagctatgtattttaacaagcaagcatgctgcctagcaaag
agtatttttcctcctttcagtgctagctgttcagaattatcgttatgttctttactcgctaaagcagggcaaatttttgaaagtgaggttattattcagg
aaacagaggatgtgttacaagattctaccttctatcacaaatttctttggaatctggcgagtgccgagttatattacttaaaacgggaatatactgg
ttctgtcagacattattctcagtgccttgacttattagaaatgttaaagcaagaagttttatcgtttattcaatcctgcaaaaatagtcgttctattac
gaaaggaatgaaaaggttaagtttatctaaccctgagaatccagctgcgaaagaaaaggcttttgacttggattgttctgttttaaatgataaagc
tgctaaagtactgcaaaagattgccttgatagaagtaacacgtggaaatataaatgaagcgaagtcattgatgaagacatcatcaggaaaattt
attggagacttttctaacgttgtttctctacatatcgcaaaggcaagaatagctttaagcgaggcaggtttaacgttgcaaaatgatccagttttga
gtacccttcaagattctgttatatctttaccaggaataacccaaaaggaaacagccgatgttattacaaaattatcttctgcagcatcagaaaatc
agtcgttaaaaaaacagaaaggtcatcttaattacttaagagagaagttcaaaacaatcttgttgaatatccgtttaaactgtgaggttatcttctt
taatagttttgaacgaagttcagtccagacatgcagagtcgttaacagtttgatgactttttcatcaatcatgcaaagtgctttaacaaggttagag
gagaagaatacaattagttctattgctgcatccttctttatggaaattccccgggctttaggcttccaaagaaaactattagcttcaaaatcaagttt
tcaattacagcctaacggtgagaatgaaattgctaatatactttccgaaaacaagtcaaggactccgcatcttgaggaatttaaaggttttataaa
caagttaccttcaacttggaacataatctcaattagtatcagtgagtcgaaggaagaactatttataacaaaatttgcacacaaaagttcccttt
aatctttcgattaccttttaagcgacataacgcccgagacgcagatgaggaaattttgagttttgaaaaagcacagtcagcatttgaaaaaataat
aggaaaaagtaatgagacggcgcagaacgggaaagattatacaacgaagcaagaaaaagagtcatggtggaaacaaagaagagaactaga
caatagtttagccaagctcctgagatacgtagagctttcttggcttggcggatttaaaggtatattctcaaatcatatactagatgctagcctcttct
taagatttgttcatcaattttatgacattatgagaaagaatttgaattttacgggtctggagaattccattgaaatgtcacacggtattctggaactt
ttcatcactcttggaaaacctgataatgaggatgttgaaaaactgcttgaagatcttttatatttcgttctcgacatctatcaatttcatggagtaca
atttgcttacgatgaggttgatatagatcagattgccgttgaaataaaggacgcattaagaatgtatcacaatagttataaatttggaaatactact
agtcataccgtccttttgctggacagaatggttcataaatttccatgggaatctttgccttgcttgcaacaacagtcagtatcaagattgccttccatt
tctattttaaaaagcttgctaaatgaaaattttcatcaagaaccgtttgtgaaggttcatattaattcaggctcttatatacttaaccccagcttaga
cctgaagcatacacaagatatgtttcaaggtcagttatctacagctggttggaaaggagttatcgggcgaagtccctctaatgaagagtttaaatc
catgttaacagaacatgactttttcctttactttggacacggcggtggtgaacagtatacaacctccagagatttggcggcacttgacaaatgtgca
gtaagtatattgatgggttgtagctcaggagcattacatgagtgtggtatttatgagccttgggggacgcctttgaattatctcagtgcaggctgcc
caactttggtagctaacctatgggatgtaacagacaaagacattgatcgatttagtgatgccatgctttcttactggggcgtgttttctagtagaga
aaatacagaaaggctcaatatatgtgaagcagttagcaaaagccgtaaacattgtcaccttcagtttctaaacggcgctgcccctgtcgtgtacg
gaataccagcgtatgtcactctgaatattaatgatgtggacgatgtaagtgaatgaatctttgggatggggtttttttttcttcttatttatttatacaa
atttaatgtgttgaagttctagaagtcataatataaactagggacattcatacattagtactaaaaagcaaagtcagttcagtgtgcggaatcaac
cgttgtaaacgatttatgcggtagatttcacatcagaagcagtagtggcattaggagcggacgtagtagctgtaggaacaggattttcagcggttg
cacgtttatgaatccaatcacagacttgatttagatagtctgtcctgtcaggctcttgctcaaggtgaactaaagaacaattcgttagttaaggtta
gagaaaacttcgagaaacttacgtgcatgctggcaatcaggaagagacaggaaagttttgtctgttgtaccaacatgctcataaaattccttgga
cgcatcataaaagttgatattatcgtcagtgccatgagcgattagaagaggcaaatggaattgattagcttcatcaagtgtttttgagcactaaat
gaagtgttaatgtaatgattcagccaaaagaaaatcatgtccatacccattgataaggtcgcgcaacgtttctagagagccgacagaggacgta
agaggatcctggaaaagtcggtccatcatttctttgcttctggtgcccttctttgtctccataatcttagtattaaactgaaaatttggcatcactttgc
taactgcagaaagactgaacaaaaagttagtattttactcttcgcaagaaaactccaaacttaagcatcattgcttacgctttatatacgaaataa
ttaggctttgtatcaggatggctttgcaacataggggctgcagcaacgacacccgtcaatttattgcgatgagctcctgaaataccgtattgaagt
gacaaaccaccgcccattgaatgacccattaaaaacaaaggaagattcgtgtcgcttgcacgctgtacatggtagtctatgtcagggagttgttg

cttccaaccaccgctacaaccttgctgtttcggggactgtttggaatagccaaatcctctctgatcaaaagagaatacctcaatatttcgctcattta
ggaattcaaaaaattcgggataaagatctacatgttcaccaaaaccatgaacgaacaaaagtcttgctacagcagctcccttagtgttcttccaat
cctttacgtagagatctgccataataaactacctaacgactactcttctttacttcaatctaccggtgatatttggaaaatggcaaggaatacgcgt
tgagaaaagtcattaatcatagtgggtgagtctcgttggaatatgctattttggtgtaaacaaacacagaatacatcagagtataagggagaaga
gaaaaaaagcaatcttgatttttaattagtaaagaaactcaccacagatagaattttcaagatacctttaaggctatttattcatgtcttccgctatg
atatatacatacatctccagagtgtgacaaatcaacaagattcgctcgcaaacgtagttcaattgagttttggaaaaggaatttaaaaaataaatt
tctttcatttgtgcaatgctcccgtgaatagctggatgtcttacttggcaaatatttgtaaagcggcagatatactgacataaaaaaatgggattaa
gcaacgagaaatctcaacatttcagcgcatgagttgccaaaaatttagaaagagctataccagacgacatcgttaattacaagtgtttagagttc
agacttgatccttcgctttctttgaaagcaacaacgactgattagaaatatataataggctctgcaaaatatttccgtatgccactcaaagaaaaa
aacgagaaacaaagtgaatagctagcttccattcaatacttacagtagaaaactgattacatagcatatcataaattgaatcccacgtcaaaact
atgcattatttggctaaatggaaaaagcaaaagagaaggtttcatcctagtttcgtaaattccgacgatgttccttgcgataaatctatgaagtatg
ctctcgttgacgtcgttttttttgaaattccatgcttgtgagaaaaagatccccttttcttttggagaaacctgttttgcttcacgcctttcctttttctttcc
tgaccttctttttttctcgttttccctctaaatcacttttccccgaaagaagtgactccacaagcgacataggatcatcacgggctgatctctttatggg
tgaagtctgattttcccttgtctctaaattttgtttcatataccaaggaagaggtccttatgaattaattaacaaatattaaaagagaactcatacct
agtccatgcttttctttcataatttccagctcgtgcccgtgctttttgtaaagttcctgacgatcatgttcctaagtatacactgttagacgataatact
ccaaggaagtaaacaaccagtcgtaaataagtacccgtttctcttcttgctcccagaagtttgcaaacttgccctttggctgcggctcggaatccgg
caattccgtcaagcctctcagtctccgaagtttgttctctgattcctaaaaaattggattaggtatatgttacaggattacaagctctttaccctttta
gcatgttgttccccagagattcgagccagttcttcgtcacgtcggacgcgctctacatttttttcgctataaactgattacaagttaacgcaattcctt
ttgaaattgatattcatgacaccgtaccattccagcttttgtgttttagcaaattcaatggcattgtagttttcaaggtagttgccaaaataacttgta
ttccaattagaaaaaaaggttccaatagatctttagtcgatggaagtctggataaaattcaataaatgtacataattcctgaaactcggttcctgg
aaagaagatttctcgaaagagtaaggtttgtaatatacgatcgtgttagtccttcctttccaatttctaaattaaagaagcttgtatattttcaaaat
aagaactttcgaataaaagagtatttcgtctgttagtcggttgagtcttataagttaggtagctgtgggagagctctaggaaaggtcaattgaaat
gtaccatatatacttctgaaagacaatcatcattagatatgatttcttcctataaaatcatttaaatagccatactggattcgtttccctagacgtttt
cgtttcgtcagccctttatgaacaaacttagtcaaaacatgtcaatgtaaatttagttaaaaataataaacaaattgtgggaaatttttgccataga
tactcttcttaaaaccatatatctcttcgtggttgaattggataaacacaaggattttattcacgaacgcgtcgtaataaaacctattccttcccaaa
caccattatttttcctacactgctgccgcgaagggagtaagctgattttacgatttttttgatattttcgtacttttcttttctttactatcgatttgtttc
tctcaaaataggagggaccttttcggtatcttcaaacacgactactgagtcgtccttttgggacaacctttcatgagttcagagcaaataggtgatt
ctctatatttggggaatgaaacagcgaataattttgaagaaaacactcaaggaacgactcgttcctcaccttcttctcctgaacctcctaacgtagt
atgttttgttttctctgttaaaatcaaaatttcacttattaactagaaatcattagaaaaatactaaaagaagacgtgtcgaacatgaacagaattc
ttcttcaaaaaatacaacccttcaaagatctgcttctacaacattatctagaaatgagactatagattcaattggagaggatacttttcaaggaagt
aaccagccgaatccccagtttgaaaatcgcgtcggtgttattgaaagcatccaccttgtaaactttatgtgccatgactcattgcgtataagctttg
gctctaggattaattttgttatcggtcacaatggaagtggaaagagtgccatcctaactggacttactgtgtgtctgggggcaaaggcctccagca
caaatcgtgctccgaatatgaaaagtctaatcaagcaaggcaaaagctttgcaaggatatctgtaactattagcaatagaggatttgaatcttatc
aaagagatgtttatggtgattcgattaccattgaacgcaccattcgtcgggaaggctccagtgaatataagcttcgggctcatgatggaactgtta
tttctacaaagcgggaagagttagacagtatttgtgatcacatgggacttcaaatagataatcctatgaacatattaacacaagatacggcccgtc
aatttctgggaaactcaagtccaaaagaaaagtaccagctttttatgaaagggattcaattaaagcagttagaggataattattctttaattgaac
aaagcttatatcatacgaaaaatgctttggaaggaaaaaaatcaggtctccttcatttagccaaaaaagaagaagggtgtaaagctaaatggga
tcagtcccgtgaaacagaaaatcttcataacattttggaaaaaaagaaagaggagatggcctgggctttagtcgttgaagttgaaaaggaacta
ttagctacggaaatggagctgcaggcagctgaagctcgcgttaatcaatgcaacgaagaaagaaatttgtatataaaaaagtttggtgagttcg
atgaaagaatatcacttaaaaatcaagctatggaaagtctcaaatcctctattacagaagctaaagcaagatttgaaggcgtaattaaaaccttc
gaagcacatcggttggaaatgagcgatgtagacttacaaaaaagagatattcaaaatagtattaatgctgcgaaaaactgccttcaaatttataa
ggaccaactcgcttctgaacgcgaacgccaaaacaataatgatgtctccgatcaccaaaaaagacttgatgatatttctgctttacaaaggcaga
ttgccaacttgagtgaacaagcagtggaattagaatccaaaagaagcgagcttcatacgacttcccttgaaaaaggaggtaacctttcttctcttc
ttgacaaaaaggacgctatagctaagcagttatccgatcaagccgaaaagattcggattatggaggaagttcagagagacaagctgacagcttt
tggtaaaaacataccgtatctgctacgattaatcactcgagaaactcggtttcagcatcctccaaagggaccaatgggtcgatttatgacggttaa
agaagaaaaatggcatctcacgattgaaaggatattaggaaacgtaataaatggatttattgtacggagtcatcacgatcagcttatacttaaag
aattgatgcgtcgtgccaactgtaacgcttccattgtcgtaggtcgttatgatccgtttgattattcttctggagagcctatcacaccgtattccactg
ttcttcaagcaattcattttgaagacgatgaggtactgcatacattaattaatcacttgggaatcgagaaaatgctcttaattgaggacaggaaag

aagccgaaacatacatgaaacgcggtgtagtaaatgtgacccaatgctatgcattggaccctagaagtaaaggatttggatttcgtatagtaagt
aaccaacgatcctcaggtatatccaaagttacaccgtggaatcgacctccaagaatcggtacttccaattcaatggacgctaaacgagaacaag
aacagctagaggatttaaagaagctttatgaaaatgcaaataatgatcttcttgccgcgaaacaagaacaaggaaagtacagaagagaagaac
aaattgttattgagaaagttgaggcgattaaaagagaaattttggataaacggagacaggttaattctttagagtcagtagaaacatcggtgctg
gacactgagaaaattcaaagtcttgaaagaaagatttcagaaaccgagaaagagttagaaagttataccggtcaacttcaagatgctaaaaat
gaagagcgaaggatagtggaggagcaaaaccccgttatagaagagattaatatgttcaaggaaaaattacaaggtgaaaatcaaaaattggc
aactcagcaagtcgaaatcaatcaaatcagagatgaaagaacgagattggaaatggcgcaagaggagtacacgaataagtcggatcatacctt
gaatgagatgcgagaaaaagaggctaaacgaattcggtgttcccaagtagtggcagaatacagtgccaaagcggctactcgttgcgagcgagt
tcccgtggaacatacaccacaggagttggacaatgaaattgaacgactgcaacttcaaattaccgaatggcgaaatcggactggcatctctgtag
agcaagcagctgaagactatttaaaggcgaaagaagaacacgatcgatccaaactactggtatcgaaactacaactgcttttggagacattgga
agaaaccttgaggaagcggaatgaaatgtgggctaaatttagaaagctaattacactgcgtactaaggaattatttgagctttatttgagtcagcg
gaattttacaggcaaattagtaattcgacatcaagatgaatttttagagcccagggtgtatccagccaacaggaatctctcgatatcgcgcgaccg
tcaaacgctgtctcaagttagtgtgcaaggtttatcaggtggtgaaaaatcatttgctacaatttgcatgctactatctatatgggaagctatgagc
tgtccccttcgatgtttggatgagtttgatgtttttatggacgctgtcaatcgactggtcagcatccgaatgatggttgactcagcgaaagactcttc
cgaaaaacagttcatattcattactccacaagacatgggccaaatcgcatttgacgatgatgttgttgtttttcgtttaagtgatcctgttaaatcct
caagtgctttaccttcctctacagctccttaatttatatgttttcgatctacgacttaactttatttttctaatcattttcatcttttgcttctcatactgag
ttcgttcacatgattgatttttctattttgcctaaaaatttgaatgaaaaggcacgtttatgttacttaatagtgttccttgttactttcaaaagtaccct
aggttagggtatgtgactggttctgtcttataccaacataaccgaaaatatgaattttaccaagtcaactcaccgcaggacataaaaatgccaact
cacgcttataagaatcgtaagcttcgtggtcacgtttccgccggtcacggtcgtatcggcaagcacaggaagcatcctggtggtcgtggtcttgctg
gtggtcaacaccatctccgtactcacttggacaagtaccaccctggttactttggtaaggttggtatgcgccattatcacctccttaagaaccccttg
tggcgtcctactgtcaacttggaccgtctctggactttggtccctaacgagacccgtgaaaagtacttgggtaagaacactgaggttgcccctgtg
attaatgtcctccaatctggctatgccaaggttttgggtaagggtcgtcttcctgagtctcctgtcgttgtccaagctcgttatgtttctcgcagagct
gaggacaagattaagcaagctggcggtgttgttgaacttgttgcctaaacaaaaagtaatatataaaaacacctgagtcgctaatatttggtgtgt
gtcgccgattgtagaggtatatatttgatgcatctgtaattgtttgtagtgtattgtagcttgagagcatattttagtatcgaaattgaatcaattctg
ggaacgctgatggcaatttcatttaagtagctgttgattcggttatgcttttaatgaagaaggtgatgctctaatctagaaaggagttaaattatgg
taaaattatccaagtgtttgtagatcaggagtacgagtttgtagacctggcaagaaccatacccatgaatcgatcgtaaaggaggaaaattacaa
actacagaatattttctttatgtacgagtttgtatgggaaggaaacattagttaagttttcagcttttagcagttggttgcgtttgaaaagtgtgtgca
aatactttcatgtacttaagtgaagagtggatttgataatggactcaaagcgccagcatctacagtacttgttaaatctatcagtctctacgtatcc
aattagtccgtctttgtcaagccattatttgcgaaaattatatgagttgatcgaagaatgggatttgccaatggaaccaaatacgctgcttaacgga
tgtaaaaaatgcggaagtatttttatatttggaaaaaatgcctctgtgcgtacacgttcgcaacatcgaaaagaaagatcagacctaccaaaagg
acaaattgcaaggatggaaacaggctggttcatcacgtgtaatagttgtaccagtacatttgagttttctgttggtgccccagcaaggcttcataaa
agtagagccgccttgcatgccagtgaagctgctgatgctagaaggacagaggaagccagtactcaacaggcaagtttcgatagtgccaagacat
cgaaaaatatgaaaggtagacaacgacagaagtttcgatcttcaggattaaatggggtcttggctaagaaaaaggagcgcgaggcacaaaata
agtcttcttcctcattgagcctctcagacttcatgtctactatttaaccattttatcttctcctgtacctttcccttagctaatctccagtaacgatgtat
aatttggttccttttctgtgtgaaacacctttactcagatacgtgtgggagaatataactatagatgacttaatatatgtaatcaaattgtacaatta
aacaatggttttctttatataaaggacagaaaataaactgattcaacccctaactttggaagtaccagtatgtttgagtagtgcaagtatcagactt
tttcaatcgatgctagacaaaattgacctatgtgaaatttggatatttcttcaatgtttctatcattactaaatttatttccgcttcaacggaagcacc
ctacccttacacattcactcattcactcgtgcaagtaacccagcacccattccctaccacggtcatgagtgcaagaattgggcctcaaaggtctac
aaagacggctgcaaagcttagattattaccatctacagaggaattcgctgattttagacggcaagacaccggtcgtgaagtatactcacaacttc
ctcatattgaaggatccacagctagaaaagatgcagaatacctaggaaaacgacatcgtgagcacttacctcgtgttactgcctattgcacgtgt
gatacttttcgggtagatttgctctttaagtttttccaatctcgtcgaagcagtcatagaacaaggcctaaacaattcgacgaatggtaagtgaatg
atactaaaacacatttaatttcctttctttaacaccatattctgtagtatttatagcccatacagctacggtagtgaagaaaccgtagatctgcttgg
agacgaaattgagccagatcgaagatcatcaattgctggatccaatagttcccatgataccattcacaatgacatatttgagacagggttagaca
gaaaccagcctttgtttcgagaagtattttgctttagctacggagtagttgttttatggggatacactattgaagaagaacacagatttttgcgaga
actaggtcggtttgagattgagaaattaagtatgtgaattaacaattgatcttttacctgtcattcatttggctttcattcaagcgcggggaacgctc
tagctaacttgttctagaaatggatgatatggaagttgaagaatttaattattatgttacgaatctttatcaaccgagagtaagctttttgttttatttt
tgagacgatactaatttctcgtgggatagatttttaatgactttattgctttgagagatgcctctaactatatgattcggttgtctatatctcatgcaat
tgcacaatctgtcaaaatttctctttttgaagagctcgtcaacgaaaccatcgatgctaccaaagatacaccacagatgattgccgagtctggtag

aatcaaattaaaacgagaggagattatgatggctgtcggtcaattatttattctacgaattaatataaatctacagggaagtgttttggatagtccc
gagctgatgtggacagaaccacagcttgagcccatttacacagcagcaagatcgtatttggaaatcaatcagcgtgttgccttgctgaatcagcg
tgtcgaggtaatcggtgatctgctgtctatgctgaaagagcaaatcacacatactcatgatgaatcgttagaatggatagttgtgattcttatggct
ttgcttgttctcattgccatctttagcattttggtaagttcgagttttcttctttctaacaatataattccttccgtttctgtgctaacattctatgatccag
gttgacttgaggtttttcaagtagttgggtacaggttgttttatcagtaatttatttaatttcgatttcgatttcgattcaaaaacaagcttctttaaga
atattagctttataaaaccgtcctcgaaacctaggtcatgtacgcggtaaggaatcgtaaatccttgaaaattcttctttcatataaacaaatggaa
tcgtagatatgatcgaaattgcatgtgctatggttggaagaaaaagctcttcgagacctgattagaaacgcatcatgaaatcatataaaattacat
aaaaagagagtttcacgtccctcttccttcatcttgcattctctgttctttttttgttcttgttctcatgaatatattcgtttggcatctggttctttctaatc
ttgcataggaggtggattggagtcctaagtagttagttgcttattcatgcagcaacttttgtagctaataatcttaccgaaacttccaaagatgaag
gaacagcagcattggacatttctggaaattcgtgatccctctgttccttcttatattctgtatattcgttgaatgtatctttgagcatctgcgtttcatat
cgatatctctggatatctttctctacgtggtaaactctaatggcatattgtacacattctttctcaacctctgctactttcttccggatccttgcagtctt
ttgttggtaaacattttctggttgtatgtgttagatatgcgaaaaaagcctcaacttctgcttgttcacaaacctgaattgtcattcggatcctgaaac
aaatcaatagttagctcataaccaaaaaattcatcattttctcgtacctgagaatccatttttgtatacacgcgtcgtttgtactaagaaaggatttat
cgccgtaaacgacgctaaaagaaataaagataccgcagtccgtactaccattgttattttcccccttttttttttacgacttctgttactctgcgtaa
aacagtaaagaactttgactctgtaaacaaaaattcgttgcagtttattcaaaaaaaatcaacatgaatatttcggtcatggtaatggaaaggtat
taaatgttgattaaaactaggtagacatatttactgattataagacttaaatgatattttgttctagttgttcatcaaagacgaattcgtctttgatga
acgtggaggtttttcttttttttttgtctttctttctttctttctttcatgtatggttgagttactgggcttactcatattcaactaactggtatccattcttg
aatgtttgaaaaatatggtgcgtgcatacattttataagtttgggacactcaattctcgaaagttgacaacatatctgcttcgttatttcacattgctg
taacgattgaaattccctaatcctcaaatgaacatgtatgtccataaaattttagtatacgattccaaacaaatacgttacagtggtatataaaca
aaaaagacatagtattaacagtaatcacaaacactaatgatatcaaagactaaaagcattatgttatacgcttaacaaatgagaagagacataa
taactgtagtgcaacaaaaaaatcaaaaaaatcaagaacgttggaaatgtaaaagagatcttttctctgacattgaagaatgttttaaaacatcc
catcccattttaccacaccaacaaactggagaggaatcatccaaaaataaaaaaaattgataattctaatatataaatagaacaggctcttccag
caaggaacctataaatattaactgtcatttcccattataataaaagcgttctcatacgacaatgtaatttgtaattagaaaagcattaaatagtcca
ttaagatctgatcgaccagaatcattgatacattactataggggtaagacaaagaaagaataattccaaaggtataacttcataatcgtaataaa
gaaagtaactttaacaaaatcacattttatgaatcatgaagattcatccaacatatccttccacaaaggatgtctaaacaattcatgtttccagata
gaagaagggataattgacttcacttctagcaatatataaaagtcttggaacgcttgagttgtcctctcatcttcagccatctcttcaaataaagcttg
ctttgaattcattgcttctaactctcgattacatcgcttgaaatcttcccatacaatttcccaacccttcacattttaaatgctttaaagcaaatttcct
cagaaagcatgttgggaagagccagtccaatccacttggcatattcaactccatttttccagccatacttccaaagtgccatgaatacatccatta
aaccagaagcctgcttatattgactatcatcttctgtaagccaaagacaaagagtagcaatagcgctaacaacagtgacgtgaacagcaaacct
ctcgtgatgcctcatattatcccaagcatcacgaataaattccattgtatctccaaaaagctctgaaagagtgctttgaagttgaaaaaactttgtg
ggctcaatagtttgagaaagcttatcacagttttcacagatatagtgaatcaaaattccaagtacttcgtagcatgaacatacactatatgagagg
ctctcgtgcttttgtttttcatcaagagtagacaccaaaaggtccaagttagcacggatttctgcagcacagcggccaatcaccaaaagcaaaga
ctctttattgtcttccattacttcatcggtaccaacagctttcaaaagggagaaaagcataacgcaggcatttgcaatagattcttctgtaatgaatt
cttccaaaacaaccttggtgaatggttcaatacagctggagaggtcatttgaataccacagatggacgggaaaacgtgcgaaaagatttcgaaa
aaacagagaagtatcaggagacttccatccgtttctcttatcgattccttcaaaaattggtttcatatatagctggatcttctctagtgcatacaaac
catacgacagaatctcaagagcctgaggaaggggaaactcattgataataaaggatgtgtaatataaaagagacttcgctgaacgtggattaga
cgatagagcacatataatgggaaggcaaagatcgtaatgtcgaatggcacaggtcaacaaagctggtatgcgtttttttcattaaagagctacga
aggatcttctcatcactgcaaaaggcaaataggacagaaacacccaaatctgcatattcgtaatttttcgaccccagcaaccgatcaaggaattt
catatccatgttttcccagaaagcaaaaaggaagtcgggattgtcatttagcaatcttgcaagcattgttaacgaaacaaatttgcttgtgtcatct
ttggcatgtaacagttcaaaacaattttctaaaagtcccgacgcatgatccttgccttgcttgttcatgtgatctttttcttcttcctccagttgatcttg
taatatatattctttttctatactattcagctcgcgcaccatatcttcgcttattttttctataggaaatgagtcgtctgaatctgaaaacatcgattta
gcggaatcgcgcgtctttctttcgttttcctgcattttattccagcttcaaaacaactctaactcatttaatcgactcaatcaagatgtatagtttttct
cggtcctcaacgccttcttcaattgcagtgtattattaactttttcttgctacaaagtgaacccaacagagtcaatcgttttctaaacagataaataa
acgaaaatcgattaattattgctgtttcaaattggtttttctggaaataagctattggttaaacgtagagtccatgaagccaccttttctgctcgaa
caggttcctacgtgcattacccttccatctatgaaagacatatgtaacacacgaatatgacgttggcatagcaactgatttatttatttacatttcttt
aatagaacgtcttcaaaataccaaccacgaaaccataaaaacgattcctttttgcctccaatatcaaactttttttttctgtagcattaaataaatta
ttcttatctcttcatactcaatatgtcaaataccggattgcctgaaccttggattttgaaaatttcgagatcaagaaaccgtccttactttttaaccc
acagactcatgagtctttatgggaggctcctgcctctaccaatatggaggctctgcatcagtatatgattaatgaattctctgtcccaaactcgaca

ggtttggaaacaccctcttctccaaagatccgtgttagtcatttattagtaaagcacaaggaaagtcgtcgacctagctcttggagagaggagcac
attacccgaaccaaggaagaagcctgggacagaatccaccatttcgagaacttgctcaaagagggtgtcgtcaatattaatgatcttgccatgca
agaatcagattgcagctctgctcgtcggggaggtgatttgtacgtagctcgatgaacggaaggtgtttcctagcttattcggcgaattgggaatgg
acctcacgttcaagaattatagatagaaacagacactaactatatatttaggggatttttggaagaaacgaaatgcaaaagccatttgaggacg
ccgcattcgccttacagcctggagaattgagtcatgttgtcgaaacaaacagcggttatcatctgattcaacgtaccgcttaagtttgcgttaccctt
tctgtttatattgttctctagtgtacggagacatgtgatttcaaaggtatatgactatggtttaatgaataaagctttttttctctcagccagctcttg
agcttgaaactttttaaacagatagaacttgcttcgtcctttttaaaaaaagaggctataacaaatgtaagctatgaaagcaatcgtcgcaatga
aacctagaatggcctttaacctttcggtagctactcacacttcaattaaacaataattttttatttcgtttccgactgatttgaaacctcattcattta
aactttgtatacagaactaacagttgtttccttcttgacctaattgatctctttctgaccttctcttgtttacacctttgggaacctcacttatagaataa
aaaggtatttggaacacctgtgtttatttcaacaccgaaaaaaaataatatttagaattggaatggaatattttccttgctcattctaatccgcgtat
cttttagcatgaaatgagtttaatcgcttcccccattatgtatcattcccctgtaggatatgcctataaactttaccttgaaggaaaccttgcttcctct
ttacatgttttagaaaactatttacaagaagacttttcaaaagaagggaatatcaattcagcatgcaacttgtatttgcgggtctctagtgagcttg
gacattcatggcagaaaacacagcaacggttgaaacttgtttacgaaggtgcagttcctaggtcactattaaacgaattgtacgtttcctatcaaa
aggccttttgattgaccaattgttctagggtaattacctattgttttaacaaaacgtgggagtcgagtgaacaggtagctgaatttctttcactggcc
gagcaatcaaagtctgagcttttctcagatgatcgaagatatccaatagagaagcagcttgtggtattagcctgtgaatggaagttatttcctcttg
ctaaatcgcttctcacagtcccataccagaagaaagagcaagtaagatctgtgctttatgagagaaagagaagtgttactaacattacaggatct
tttcaactacatttctgaacaagagttacaacacgaagttaacagaaatctattagcagaaacacaacctaacattgacgctactaaaaagagta
attttctgataaaattcataaaaaacctaacaaggatatactcacaaataatgcaaaaagttggtttcttcaagaaagattctacctggttttcagg
aaactcaaaatttgtgttcgtcgctttcacttttattgtactgcttttcaagcttaaagctatcattcgaagaattcgtcgttctcatatttcttgggaa
ggattccaaagcttccttgaaaagttcgcattcttcatctcgtgatcctaactcttgggtttctttcaaaccaaggagttttcttaacacggctggaat
taatcgtttaagaaaacaatatattgcaccttacataattgtcttttattctaattcatttcttaaacttctacatacctagatttgtttacggtttagtg
cgttttcttacatatcactaaagaaggtaccaaggcatgctgaactgcttgaaaaacaatagacaagaaaacaagagttttctgacagacttcta
tgctaagagattatgaagaagtagaatgaaaaaacagcaaaataaagtaaagcttattttggagaatccgttaaatgtgccatggtaagaaaga
atgtccttggggtgacgttgctaatgctctcaaagggtaaatatagatgtctcaatacaagaaaaattaacccaggcgttaatccaatctctgcct
acggctaaggaagattacagaaaacatggtttgacgattggtttgaacgaggtcaatatgctattagaaagatgctgtcaaaatcctgatcaaga
ttcattacctcgagtagtttttgtgctacaaagccctgaatccatacttgttgcacattacccacagttaatcgctaatgcaaatttctattcgaaaa
acgaagggaaatgtttattggtatgcctgggtgctgaagcacaggttggaatttcgaaaaagcttggacttagccgtgctagtgccattgcaataa
gtgtatgtattcttctactccatctattcccatatagaagctatactaacaaaatataaagaatgactctttcatactgtcacaggtgaatcctttatt
aaacgggatatcagctccttcggctagttggctggcccaagcgtctagttacgagcccaccaaggttttacgggtgactacaacgcaaggtacaa
aggataaaaaaggatcaaaagaaggaaaaaattgattgtgctatcatgacggtattctagacaggttagctatgcagtgagtgaaagtaaaca
aaacctaaaacaatttttttttatgtagaatcaaaaaatgattactggtagctttcatttgatggcgtctgcatggccgaagtaaattcatgaagta
agcgatcttgcttttgcaacatgaaaaattttgtatatctagtggtgttaataagataaggtaataagattttacttacgcatctgtaaacacaaagt
tctctagcattggtgttcgaagataatgattcgcaaagtgagccatattatcctcaaaagaagaccattctaaaggttcgtagtcaaatttttcgca
gcgattctgaaaagaacgaaaaatgggctctgctagaaggagagtctcaggggggctttgggaatatgaagacgaatatgattttggaatgtt
gctgggatgacatgagaggcatttgaaaacggataagagcgtaccaaataaataagaacagaaatcatctttcgaatttgatgtttcataaaag
attgaccgtgaaacgtaagcttcaaccattgacgattatacttatcaaaatggatagcatcaataccgacatgaagcatataacgccgtgcacttg
gatctccaaactttttttctgtagtataattatggaaagaatgcgttccttgaaataaagaacatgcctctttcaaacattccagtttcgaggaaga
aatccggaaaggagatgatccagagttttcttcagagtgaatttttttgtatcgtttccaagatgcttctgtgtgaagtatgaggtagttgaaaatgct
ttgtaaacaggagtgaatccagagaagaaggagatggagtcgcgagagcgaaagcaggtacccaatactcataaagacgactatgacacgcc
ttatgaggagaaaatgagtttaatgttctttctattttccatacacggatagactctggcagttgctcgttgacaagcgagacccaaggctggaaa
gagccagcatgatccaaaagctttaggcccaaaacgtttacaattgcatgcacacccttgtctgttcgtgcggcggatcgaacacggacctttctg
ggattatctgcattgaaaggggaaatagcaccaacgttacagaacgcacgaaagatctccccttgtattgttttcgctgcatcgttatattgaagtc
ccgcatacccagtaccccgatagctgacaatacagtagaccattcgcttctgttgtctgggtgaaccatcggacagaggcagtttcgtcgtttctgt
cgaaggatatgccacaaaatcactacctaaagaagaagaccatcgcttccatataccagcataacttcctttcacgttcaagcttctaaggaaaa
agtacatccggttgattcaacagctgccaaacagccagatgactgatttcaagagcaatatcaaaccttggcggcaaacaacgttttcttctttca
atatgttagtccaatgtataagagaagctactagcgtttctctgcaagttcgcggatacagacgaagcttttactgaaagagaaacccttctaatg
agaaaataggaaaatggaaaaatgattacgaaataacgaaattaaattgaaagtacaaaaaaagaatcaatcaaaaaaaagaaggtaaaaa
aactattattgcttgattgacatggaaaagaaagtgaagaacgcatgttcctatgaaaattgacgcctacaacttagtcattcggcaaatcttattt

taaggatgagatgagggtgtgtttctctttaatgttgtaattaaatgctatttgctaatggttatcaggtcgagattcattttgcgagactttcatgct
cctatcctgccatctcttgaaattacttaccaaccacctccatccatcaccagtgaaaaagttaagacttttgcaaaaaaaggctttcaacagttgg
aaaaataattccaatagcatttttgtgcttgtaaagcaataatatcaagcagtgaggctttcttttacacttgcttacggtttcctagaatcttgaaa
cggttcttctcgattcgcgatagcgtgtttacgttcatatcgtaaagaacgcgtctttggctttgaatctatcaaattcatttcctttctagaccctctc
atttttttaggttgtccgttttgttcctttccgggacttaccagcctctctctcgtttttcactcttggagcactatttgatagtggacagtcttgtgtatt
aatcaatcttttttttttactcttttcgctccttgccaaagcaacatttcctccttgccttgtttttcaagctttttttcgttttgattttccttctttctttttc
gatatttttatgctttttcgtgtttttcttctatagccctttacctttttttttgctttttttcattcatttgcattttatttccaataattctaaattttgtttgctt
tatcttacttcgtttcttttgattttatttttttgttgcttttggattttgaatcttttggacttgttgatttctataatcaaagttgtcttctgtctctactttt
tattgcacaagtattctgtttgtgttgagctatttcgattaaatcgaagctttgattgaaaaaaaaaaactttttcatactcatttttcccttcataaagt
gtcgctcttttgatatttcgcaaccctgattcatacattttcttcccctcgaatcatacaattagtgttactaatttgaataggcattttacaaaatcaa
ttttctttgtaaatttcgggcatctttgcattgataatactatactttattccggtgaattctcattttcttacctactatcctttgacagaacttgagtca
tcttttggcatctctcatttttggctttgtgttttatttaagtttctcccgtccctcttttatattgtgttgcatcttactttccaattttatctaatattttgtg
attattgggttcgtcagattttgttttcaatttttgtctattctaaaattgctttggatacattttccatacccgcgttataagattttcgaaaggcatta
gctaattgtctattaacaaagcggttgctttcatcaagattcgttggttgttgacaattctatttctttggaacaatttacttcctgcttttcagttctct
gcaactttctttatattatattaagcgaatatttgacacaacatgttcgttggttcacccagtattagcaagcaacctcctttgctatctaaaaaaag
gcttcctgagtcgttgaacattatggacgacactattcgtggttcttctttggttcctgctgggattctaaatggcgaagacgagggtcaagcgttgc
atatgtctccccaaaatccagaaccccagttaggaaaagctgatcctgattatttgccgtcttatgcttctttgaatccattgggggagaaatccac
cactcccactggaaggctgcttggtggtttgaacgggggtttggcgtctgcacctcctaaaatgagcattaactatccacgattctttgctataaaa
attgaagatctacctcgaaatttaactcctcgagaattttcttgtactttcttgtttgctaacaacgttatatctatggaaattgtccctcctacatctg
ataatgatactgcttatggattaactgtgttttcttccagagacgcagctctcgcggctcgtgatgttttactttcgagtgatctctatggtctttgcaa
tgtgaccgtcattgataattatcgcaatctcagccaaacaaatgtaagcgacgggacagaaggctcagaaagtagtgctcgcttttctagaaacc
attctcctattcgtcaactatttggtaatcgtgatattttacgccgtccaagcaatcatatttcgaattctgctgtcggccataatgatgttgcttacga
tcactccaaaactcctttgaatgaatcatttgcaagcgctcctagtagcactttgcaatctgatcggctatggtcgtcgttccctgtgtcatatccatt
atcgttagccaatgctcttacgaaggatgatgtctcttctcccacatggtcccctaccgccaataaaagctctattttacgacaagataccgtccctc
ccgttatgagatttaactcactttcgataaacacaaacgtttctcggccctgtctcccttcagataagggaggttatgcacattcactgagtgccca
atctcctcatcccagagttctttcagccaatggtgcctttggtgttaacagtcctcctttaacaccctctaataccagagactatcctctgtctgcttca
actgttccaccttccactccgttttcaccttacgcatcgcatcatggtattcatcaaagaattcccgctacaacgcctaccaacattaatcctgccga
tcagaaccctccttgtaatactatttacgttgggaatcttccgccctctacatctgaagatgaattaaaagcacttttctcaacgcaacctggctaca
agcgtctttgtttccgtaccaaaggaaatggtccaatgtgttttgttgaatttgagaatatcccttgtgctatggaagctttaaaggcacttcaaggt
gtttgtttaagcagtagcataaagggtggtattcgccttagcttttcaaagaatccgttgggtgtacgttcgtccagctcttctcataaccacggtaa
tgttcgtaaccttcattctggatcgatgaacaattatggtgctggatttttccttaatgatacaggtcagcatgacactcacaatagccccagttggg
ccaataatttaatgtatggcaaatagattctctcagcatttccagattcgcaccgttgcctataactgccaattagaatgccttgtacttcatgtttca
tatgtatccttggttttttgatattcttgcatgttgatttttgttattggttcattagattccttctaattcgtttttttaattgccagaactataaaagc
cttcctctatactaagacttaatgaatttaaatggcgaaaaataccgttgagagattaggagaatttttcaatatttgtagtaattggaaggctttgt
ttttgaggtctcatagtgggttttatgtaaccatacactgctgctctgaaatattcaaaattttgctatctttttaagttttagttgtttgcttttcaattg
tttaatgaataaatatgtagagttccataatatgtgaaatataaaaaatatgtgtagaactaaatttaaagtattggttacgtttcatagaacgtttt
gtatgaggtaaaaaagaagaatttttttgctttcatattatagtacttaagacctatcgcaaacccaaaccaaaaagaagatgtctaataattcga
caagttcttttatttttccttcagtatcacatctactcgactcgcgcacgttttgtacgatcagaagtggttgccttttctgtcgactcatctgaagact
cactgctatccgaatcagactcactttcattggattcttcagtaaatttggcagtatccttaacagatgcatttaccgcagattccattgcaaccatg
caagtgattctgttctcttctggagcttgtaattccattactttattaaaatctttgtcccaaatatgtacagagccatcagagctggcagaaatgag
ataatcagatacagcataaacagcttttactctatttacaatgttagtattgagaaaacgataagaagaaaaaattaagtgtacctttttttcatgag
caacaaattcctttactacatccccggagctcacatcaaaaacctttatggaaccgttgtcgcaaccaacaattaaatttgaatggaataaacaaa
ggcaattaagctgtgtggtagatttatgactgaaaaaggagcttagatcctaaaagaaagaaaaagattagtaaagaatgcaaaattgctttgtt
taaaatgtacatacaagcttaaaaccctctaaaccacgtcgagacatgacaacaaaagaggaatcattgagaaatagaatcccttcagggata
aatgacaaaggcaacacctttccacctttaccacgaataagatcccaaagtcgaagtttgctgtctcctccaacgctgaaagcaagcttttcagaa
ggatgaactgcaattccactaatgcctttttgggaggaagacttcaaagtatgaacaagtaaccaagaatctctagaccacatagtgatttgacc
atgatcgtggcaagctaacaagtgtttctttgtaaagcacaggctacgagaaatagcatttggcaaatctgtagggatggctaaatcagcaatttg
gatattgcgactatgatcgtaaattctagatgtgttaatggggaaattctatgaaagcaaagcaaagcaaactagacgatcaaatggcttacttg

atagtttcatctgagctagtggaagctaattggagtccattcgtagctaaagcggtgagagcgctttcatgagcctcaaacaaccataaaggcttc
gattctttcttctctaggtcaacatctacaccatacaggaggtgagaataggttccaatgacgattctaagcattctaacgaaaaaggatttcaaat
ctagtacacagtatttgaactgaatgaacagaaaatttcgtttactattttctaaaattcttgcaaccgagcaaaatatgaggttttactttttataa
ggtgtatggtggaaataaataggacctttcctaaggggcctgtaactagttagcgaaatcgacttttcgacaacactaatacatgtggtttattgtc
attttcttcctcgtaaagaaagaaacatctcgttttaaacgctcaatttgaacattagtacggtcaggaagaaaatgaaaatttttcaagttgtgat
ggaaattctgtcaaagcctctacttctcttctatgaaaagtcaacgtaatttgtttccattcatagaagatcctcctttttttttttcgtaaagttttgg
catttttcctattagttttcttacagtatctatttcttcactactatttactagtaaaatgtttattagtgaagtgtattgtaatacgtaaacgaaatgta
tctagttgagtaataaatcaatggatggaagtgtcactggaaagatccaacaaagtacttttcatttaattctttttacatttctatttatttactaat
agttattagtattgtcctccgagtagactagaaaagatgcacaaacacgtttactcgttcggccactgaagattttactgggaacagttaggttaat
atgtaaacggtttaaattgccaactgtttttattcatacgagacgttgcatttttactttatctcatctctttggtttgttcatcttcgtcgtataacaat
atactctgtagcattatgagggttgagttgtcacggcaggaccttcacgccgtatataattatataaatcgagtggaatagacaaactaactctag
ctgtatcgttgtatacgtgaattggattgctgttatgctacgtgtatagacgctagtaatgtacagggatctctccagtgaactagatagaatgcaa
gagaaacgatctcatttagccagaccagtcgaaagattttgaatatagactcgtttttcttgtctgataatcttgtgctttccatcccaattgtacgtt
tctgtatcactttttttattgttttgcttacttgatataacgcgatgattccattaacgttctattatactaatcatgttaacgtggtatctgacttcgacg
ttggtagaatagaggaataaacagaagactctcatggccttttgaagaattaaatgtcttgaggaatctttttctgtttttgatttcttaaaaatcgc
tagttttagaatctatattattcgggcaaagttgttcttgtggaattagtgtgattgctgtatcatcttacataaagcctgtgtacgatgcatagattc
accacttgctgttggacttgaggacggttacgaggagaagagactcttgaaagtaaaaaaaaagaaagaaaaaaaaaaaagtaaaagtaaata
attggagatcaattctcactttctgtgttttctttttcttttcttttttttatcttctcttttgacttcttttgtccctctatggtttctatttacttgttaaggg
tttgctaccgagttttaaaggctttacaattgagcttataatgtttaggcttatgttaatgtttgtgttgatttcaaacagacttctgtaaagcagata
aaaaccttccttgcgattccataaattgtgtgtaccaaccactagcgatactctattcaaggaattcccttttgtctacgtttacgtaagtactgaag
ttgtcatctgcacacaccacgcaaattaagacgcgaatggttttcgctataacactgctgtttagcttttgtaagatttttgtatattgtattgaataa
aagattcagtatcactgtattccttactctattcgggacgtttcttgagattctctgtttgttccccttcacattctttttctctctcgtttcatacccatca
ccccaatttattttcttttcattctttgtttgaaaaatccttctcctctgcagttttacgatcgccatattcctttcttcattcccttcttaccttttatctgct
ttcacgacctcataagtctagtttggtcttggtccggtccttccacttccttatcgaactttcggtagcggaataacgtatattttatacatcactaaa
cactgataaagagtcgtaacaacttcttaccaaaactttgttaaacagaagaaagcaatcttgtgaattacgtcgttcggtttctctcgggtgcagc
gaaaaaaaaaaaaatcctttagcgaaaccacctgcaactaactaatcttcttcttcccattcacgcctactgtgttttctttatttttgggaaattttt
gactcttcccagtcttatatacctttttttggtgggaagaacatcacgttttaactttgtcttgtattttcgtttttctacgtctcttgtttcgttttggtac
ctttttgttggccctattattcatctcattctcttacatctttccttcgttttcttgttaatcctttattaccaaagtttttacccactaacctactaccgtct
gtaaaaatccgttatcaactggacaaagttcgtctttcttcgttttcacctttgtttaatttattgttcgtttgatagaaaaattttcgttctttgcttttct
tttctctctgtttttccatcgtttttctcctttttctttttttgtttttttctttctccctagttcacttctgtcatgggtgaagattttcaaaatgacctttccta
cagggtaaatcctttacttcaggattcgaccaatatacaaaccagcccgactttaatttccaatgctagttctgctaatgtaaattctcggttgggcc
ccgttcaaaccgctatcccttctactgcttcttccatgaatcgcccaacgtggctccccttgcagcaacatattcatcatttacgtcatacaagccttt
taccggctgtagaatcttcatttgttcatggtcatggccacggtcatcgtcgaagtgccagtgctggcataaatgtgaacacttcaacgtccaattct
ccttccttttcccatccttcctccgcaaattttcaccctcaggtgactccaacttcctctttttatccttccagcgtcttttctacgcctccatcttctgctg
attcttttacagccagccctacggtgtcctccaagtatagcttggcttcgtcgaatgtttctcctatatcaccaaatgttagtaatagtcctcgtcgac
atgctaaaagtcacagtgtagctaccgtctcttctcctgccatgcctcccttggctcctttatccgcacatgctttgcctcctcctccctccgtaaaatc
ctctccgctaccgactttgaattccgtcccccctttgttgcgccctcaacctcggattttggatggtcgatggcgcccttccacatctcataataacag
tcctacgcactcagccaacccttcgttttctggaaacattgttaatcaaccttcctcttctcttcgtcctgacggaggtggtcatcgacatagacgttc
tactggaagtttgagccttaattcctcaatagcttctacaaataattacccagctggtggatctggcaatacccgtagaaatttattttctccttatct
acctcaatcatcgatcccatcgttactggctgaacatcgcttagtaacaggtgtacttgttgttagcaagaaaaatagatctgatgcttttgtcaatg
tcaatggcttagatgccgaagttttcatctgtggctccaaggatagaaatcgtgctcttgaaggtgatgttgttgccgtagaactcctggacgttga
agaagtgtggactggtaaacttgagaaagaagaaaatcgtcgtcgtaaggacccaatttccaccagaggttcttttgacaaccttgcaaatgatt
ctcttttgttcgaggttcctcagcgatcagcgataaaggctcgtgatgacgaacaagtagaaggccaaacactgtttttgcttgaccaaaagcagc
taagttctgaagaaaagccaaagtatgccggccatgtcgttgcagtcttacagcgggctcctggtcaggttttttctggatctttgggtattttacga
cccagcagcgctgcgaataaagaaagacaaggaactactaatggaggacaaggtcctacaatgatctccggagagaaacctaagattgtctgg
ttcaagccttcggataaacgcgtcccattaattgccattccgactgaacaggcaccctcagactttttaaacaacgaccaaaagtattcccatagt
ctctttttggcctcaattaaacgttggccagtgacatctctacaccctttttggaatgctcgtaggtgagcttggtcctatgaattatatcgaatctcaa
gtggctgcgctccttcatgatacaggtgcacatactgagccttgggagggttctgctgctgcttcagcatcctcttctttagatgcattatcaaatga

ctttttaaataatgcgaaccgaagtgattatcgttcggaagatgtctttatgttcactggccataaacgcaataaccagcctgataattctgataac
aaattagatgatttttcaagtccctttgtttcttctgcttttcatattcggccaactggcaatggctatcatgtcggcattcatattacagatgtttcgc
gtattatcgagccgggctcaccgcttgaccgtgaattgcaacgtcgtggtaatacagtaagtttatgtcaacgaacggtttccttatttccaccggc
cttgggagacgcccttgctttgaaagagggtaaagattgttacacaatatcattattgctagacgtgtcttcttctggaaagattaggggcacttgg
gtaggttgggcagttatccatcctcgcaaggcttatcctattgagaaggcgaacgaactcttggaatctgatcctcgtttgaaactgtttagtaccg
ttggctctcgtatacgagttcatcatctcggtactgatattcctttgaatcgatattgcaatttagtccgacgatgggatgaagaaaactccagtttt
aatcccaaccaaacgaatctttttgcgtcatcaccggtggaagttttacgtgaaacctatcttgataccgccaacagagctgtagccagccattta
agaagagaatttcgtgatagtgcatttttgagagtacaaaaaaatgccaagccgtgaaaattgtcgaatgttacagagtatggctgttcagatggg
ttgcgtgttagatctttcaagtccaaaatcattgcttcggtcgttatgcttgattgaagatgagactattcgtaatattcttcaactttactattacaag
attactcctcgggcagtgtatgaaatgcccaagtaccgccccgatatagctatgaacatgctaaacttgacattagatgatgagacagacgatct
gactcattttacggaaccacttgatagatatgccgatttagttgtacattatcaactgcagttacttttgcaaggagaaactgcatcagaaaaacgc
ctacgtgtttggtctcaagcagcaaatgacattagtcgtcgactagtgataagtaaatttgctcaagaaactagtgtacatatcaaaatatttagtg
aatgggtagagaagcatcaaccttggcaagatggtgttgtctgcttcattgctccttcatatttcgatgtatttttccctggtttggggatggaaaag
cgtgttcatcttgatctattaaatttaatacacgttcgttatgaagaggatcagggaatattatcattgtatggtgatgatggagcggtgagaattgt
gaaattgctttctcccgttcgagtgaagttgtttgctcaactttctactcctcctttgataaatgtctccaaagcggaattagtatgatgtaaaaaggt
attatttcttttaagtctgaaaagctacatcacagttttaattccgaagtttgttggttctgaattttaccatctaaagatgcagatgggtactcatttt
gtttatacacctattgacattagccaacacaacttgcgtgtgtctaataaattataccgcattaatttttcttgtatgttgattataatgataatgaat
actgaagaacaaaatatatatatacatatacatatatatatcaacttggatgaagaaatcggtcgcgtttcatctttatctgttatcctgcattcttg
aattgtctgtcatcctacgtaaagtgagccttttcatcaaaaaaatgagtttgggatcatttgaaaagagcaaaagggcagcaattgtgctattaa
gaaaaatttaagaatatttatgttgattccagttgtatttatgtgtaaagcataagcttttcaagtagcaggaggaaactcatattatatttatatac
accagcatggtattaaaaacttgcagaatttggcaacagagtgggttgctcattgaagtttgaagcataatatatataaaataaaagctttttttt
tatgaatatgaataataattataattcaaacatatacttaccagttctttgcccaaaccaactagttttagtaaaatagtaggtatgttactcgaggt
tacgaaatgaaaatacgcgtagcgcgtctcataaatatttttcaacaaacccactaacaaatacaacaaagccctttttttagaaacagtagataa
tggtttctgagatttagattgacttttaaagcattaaatttgatcgtcgactatttttgtggttcctttatccaacaactcactgttcaagttacctcgtt
tgtcaattgtataatatggaaaggcctcttgggaaaaccgtgttgccattaagcagggtaaaaaagattataaaagaggacgaagatattcattc
ctgttccaacgcttctgctttgttagtgtctgtcgctactgtatgtactgttcagtggaagatgtgataatgctgactgaattatctgtaggaaatgttt
attgagaagctggcagaccagtctttccaattggcgaaattacaaaaaagaaaaggtattcaatatcgcgacgttgccgatgttgttcgcaaaga
tgatcagtttgagtttttgtcagatttattttctatttaactagttggtgaattcatgctatgttgtgtttggaataaaagaagaaaaataatgtttcat
tgtcgataacaagtcttcataaaacatgtttaccctgaaaaggagctgaaagattcaaaggcacgatgtgtgggaaagataaaaccatgataat
gttcaattttcaatttgttttgtttaaaattttattaaaattataaactgaaccttccataccaacattatactccttgttcttgatcccttacatatgta
attaaggaaggaaggatactgatcttaatgcttctcagacaaaatctagcttgagaactgaataaacatcggtcgattcttgttttattttcacata
gtgacatggtattacctattcttcaaacgaatactgttccctattgcaagtggtaaacatccatcctgggttttttatgaaatttagacagaggttgt
actgcaaaatataaaagtcttgaacacaggagcctcatgaagcttctttatcctttgtaccctcgttgtttttatctcattatttattttatttttttcaa
ctatcaaactctcaaatgcaacaaagttttcctacagctttccttattttttcaactcttttctttatatttccctacagatacttttaacagtatctcttc
aacagtattcttagcggactggctctgaagaccaaagtggctcttcctaactgagaatcctgtttccctgacaggatcgtgttttgcaaccaccagc
aaacaagcttactatagaaaagaaattacaaactttgatctgaaaactatcatcatctttaatttcctcggtatatcctgtttacagcgacaaatta
catcataccttactgagtgacccaagttataaaatggacaggagccgaattctatacatagcaaacttactaagcagtcattatgtcggtttactc
gttttctgaaggtagttccgttgccaaagctcttggacaatatgtaaaaaataagtacgatgctgctatggcaaagcatggaaagtttactttggct
ttgagcggtggtagcttacccaaagtccttgctgaaggattatcccaacagaaaaacaatattgacttctcaaaatggtaagtataaattaaaatc
tcttttccttcttccctcttaaccgaacggaggagaatatggaaaagagcaaagtattagaagtagagtgctatatatcacagtacggtgtatcat
ggaaaagccgctttagatcttttcatatagaaagatcacatgaaagctcaagcgaatattttcattaattcgcgtacgatgctcaaaattctgctag
ttttgcctatggtttactaacaaacaaatagggaggttttctttgctgatgagcgtattgtcccgttggatgatgaaaattccaactatgctctttgca
aacaagttttgtttgatgccctacctggattcgtacctgaacaggttcataccatcgatactgagctcttgaagcaagaacccttggatgcccaggc
ctttgctgatgaatacgaaaagcaattggtgcatgtctttgctaatgcctccactgttaaagtacccgtatttgatttgatgttgttaggttgtggccc
cgatggccatacttgctctttatttcctgaccatgaacttttggaagaagatgttgcctgggttgctccagttactgattctcccaaacccccccaagg
aacgtattactttgactcttcccgtcgtcacccatgccttgtctgtcgcctttgtaaccaccggttctagcaagcaagacattttaccttccgtcattg
aagatagtacgtccaagttgccctctgccttgatagctcgtgctcatcctgaccgagttagctggtttttggatgacgcagcttcggctaaacttgac
aaatccgttttacgtgttttccctttttcaataaacgcttacggataatttgatcaagatgtaacggtattttctcctcaatggatttctttagttgccttt

gttatttttacgttttatttttgttagaaaaccaagtttttccataaatgggtataaatattaaattttaacgcttaagaattgaataaaggttttgggc
tgttcctgtttgaatggttttccattcagtttttttttatttgtaggacttaccaaatctttgttagaaccgtgttgttcctagatattagaagttgtaaaa
aacaaacaaatatgcaccattctttgttgttttctctcctttatatatactctatgtagcattgtcgcttatttactgtgattcatcttattttatatgaac
cttgtcttgcaaacattcaaaaatgagatttccatgttccttaaaaaagtgtcctaaaaaaggagccaaaaatttcataggatgcaataagacaa
aaatatttatcacctgtagctttgtttagacttcgctgtttgtttagtatatcgtgtttcctctcttctttgtctttcactctaagtgctttcttacaccttg
actaaaccaaagcaaaaatttttgctatgtcctccgccatgaactatcaaagattagaaaagttaggtaagaatgaaagaagcttcctttattttg
attcagaaaaaggctgaccggacaggcgagggaacttatgcgaacgtttatcgagcgcaaaaccgcacaacgggcgaagttgttgctcttaaag
taatccgaattgacccggaggaaggaacccctagcacggctattcgagagattagtcttatgaaggagcttcgacacccaaacatcatgtcgctg
attgatgttttacatacggacaataagctgatgctcgtttttgaatatatggaaaaagacctgaaaaagtatatggatgcatacggaaaccaagg
agctttaagtccttccgaggtgaagaatttcacaagacaattgctaagtgggatttcgttctgtcatgaaaaccgtgtattacaccgagacttgaa
gccgcaaaaccttctcatcaataatagaggtgagttgaaactggctgattttggactcgcgagatctattggtattcctgtaaataccttctcaaat
gaagtcgttaccctttggtatcgtgctccggatgtcttgttgggttcaagggcttacaacacttccatcgacatttggtccgttggatgcatcatggct
gaaatggcaacagggcgtcctttgtttgctggttctaataatgaagatcagctacttaaaatctttcgtctcctaggaacacctactgaacaaacat
ggcctggcatatctcttcttcaagaatataagactacctatcctctgtataaaagccaagacttaaatcatttaatgccccgttttgaccctcttggc
ctggatctactgcgaagaatgcttcgtcttcagccagagctccgtattactggccaagatgccttacagcatgcctggttcttaactccttaaaaga
tgtcttccttctcattggagagtgtccgttcttcttggtttttatttccttttcatgatttcctcaatggtagatttgcgaggtacaacttactcattcaag
tgtacaatttggcttatctatcgcgagcatacgactttttttattccattttgtgttttgtaaatggattttatttatacatacatacatatatatatattac
gccaaggctttatttcatccgtgtttttaataagtacatccatcgtgtggtcacttaatgtctttcaaaagaatagattacaagtttttcatttctgttt
gatatcaacgttaccaaaaaaattccacgaaatcttcaattacgttagaaacataatgctttcgactcatggtactcaaattacgagttggttcgtaa
ccagactcattttaaggtctttactatttactgtttgttgattttggcatctttttcttttccggaaactttatgtggtaatttgagaataagaaagcatg
aaacccataattgatggtgtttgaggaaaaaccagatatttatgttatacctttagctttttttgttaaggaggagaggtttatagttaatatacatag
ctgggattcactaaattgattctattcacccgacgacgagtagcttttggcttgattgggatccccagcagatgctttcttttcttctactgcaatttat
tattcttttcgttgttcctaaacttttgataatgaattttggatactagttctttattaacaaacatgtacctataaatgtatttcacagtacgcattcac
attcatcttctacatctcagactgaaaaaagccttaaaactttttatgttcgtcaaagacaaaagatggtttctactgatcatgaaaccgaagcttg
tgtttttcctgttataactccatctcactttcacccaaaaactactcacattatagaattagcaaagtagtaggcatgtaacttctaataatctaaac
acttgttcctattcctgtcacaaaaagttcgtattgtgtaggtacattgaatctcagctttcataattgtcttctattaactccaacttgcagatttaac
tgctatatctcgaaaatggcggatactcgcaaattcaaggctacagatttatttaaatttaataacattaatttagatcctctaacagaaactgtat
gtggataatggaagtgaaattttcgaaataattgctgaccgtgccgtagttcaatatctcgttttacttgtcatatttgaaccgatggccaagtctat
gcgtcgcgcaagaatcaattgcctcagaaagtagctctttaatgggctatattattgggaaatcagaaggaaagggaacagattggcatacccat
gttacagctatcacagttgcacccagttctcgaagactaggtttggcaaagactatgatggattacctagaaactgttggagattctgaaaaagcc
tactttgttgacctgttcgttcgaaccagcaatgctttggcaatcaacttttataaaggattgggttattccgtatatcgtcgggtaattggatattat
agcaatccttatggaaaagatgaagattcatttgacatgcgcaagcctcttgcgcgagatacgaaacgtcaaagtgttcgtgaaaatggagagt
actgtaattgcagtcctaacgacgttgtttttaaatgaaacaggtatgatggaagtatagcaaaataccattgtcctttagaaattttgaaacaaa
ttgaacttttccctaagtatttgatcgtacacatacttcatcctttcgctcttttatggttgaacgttactttcataaattcacctaactaaaattccgct
atccgctttcatatcattgtatattttgaacaaaattaatttacttctaaattatctgttgctgctcttggtagaccttataaagcaatcagaagtcagt
taattgacctcttcagcttcctaatgttgtaaccttctaataacatcaataaaaaatttacgtttcctttctcaagatatgtctgaagcatggaaacc
gacagactttacgataaaaaaagcacgtcaaattccactccttcttaatctaggttggtataaaattgaccagagatattatgaaaccatctgatc
agagtgttccaaccatatttaaactttttaaaatttcattaaattaaatcatccaaaagccttggttttatgtaagttaaggtcgccctttaattata
aataaaagtttcactacaaacacttaaaacttacgacaccgaaagatggaacatttgcaagaatgatattttgtaccaaagtgcctgctttactct
tgagggatctcaatgtcaccactgtcgatctccttttgaacatcgtgagggttcttaccttcaacggtgcaaccaacggagaaagcagtaccaagg
atctccttaacagtaccagaaaggtccttagcgagggacttgaagcgcatggtacgagcgacttcaatgatctcgtcaatagaaacgttaccaga
gtggacaacacccttgtccttctttctgtcacgagcaggttccttcaaagccttgatgaccaaagcagaagcggaaggaacgacggagacggcg
gcttgacggttttggatggtcaacttgacggtaacacgaagacccttccagtccttagtagccttggcgatatcttcaccaaccttcttaggagaca
aacccaagggaccaatcttaggagcaagagtagaaccaccggcaacttcaccaccgacggctctcatgataacggtcttcacttcattgggatcg
aacttgggaggcatcttgggttgtggctatgggatcaaattacccttgtgtgaactttgccaaaggtaaagcgaaacaacagtcactaaccctacc
ccaaaattcagcacatataaagaaataaaacaatattaggcaaacgaggaagttataattgaaataaagtaaacattcctgtcaatttccatcat
agtaaatgatgatgaaaaaataaactattgcaaatgattgtttctttaaattatcttggaattaattgagagaaataatgcaataataaaaccaaa
atagatatgattaaagtataatactaagaacaacctcaattgtttatgatctcttaaacctggaatattagtaggctaaataccgtttagttacttca

aaggccagattcaagtttccacttgatgtatatataagtcgaaggttgtaagcagcttcataggagaaatcgtaagtagagttaatttgctttccct
ctttctgctgtgcttgaaagttgtttagggctcccataggcgataactctaaaaccttttcataatactgtacagcatggtgttccaatcccaaaaaa
tgataagcttttgctaagttatagagggcttcctgcttctctcctacgcctgctttggatcgtaagtcataatagtggtacaaaaatgtgaatccttgc
aggatttggtagtgtcgattgtcagaaagcctctgcatagcacgatgaatataagccaagccaatagagaggtttgtcattggacaatctggattc
atcgcatatgctcgactataataattgattgctggaatccaactcctactacgggccatgatatgtccatatagtaggaataaaatagggtcgtag
gatgtaggaacagtcgccaatccgtcttcatttttggataaggtagcagcaccaggaacagaagacttgttaattagttgatccattagttttacca
gacgaagcaaaaacttttgattagcgctgtccacgaacgttcgagagcaatcataacctgaactcaatatacctgaaaaaagtctgtaagtgtct
tgacggaattgaaaagtcgtgaaagtccatcggagaggaccaagtgctgtttgagaatctcccgcatgaatggcgcatgccagcatgcaccaac
gcaaattctggcgttttattggatcctgttcaaacacaggagtcgccatacctgcttttaatacctcataagcttgtttcaccgttcctaatttggcttc
ctggagtgcgtaaagtgcaaaaaggtgaaaccaagagtcgatagaaacattacgaaacatctttgtatgtaaaaccttctccaaatcgacgtca
ccccattttgaatgggtaattgagtcattaagcctatttatcatgttcataagctgatcctttagcgaatgaattcgtacgtgccttgtcaacaagct
agttttcgatctagctttccgttcattaggatatagcgcttttgtatttacaaattcatcaatcagctcggatgcgatgttcagccaagaattaagcgt
ctccgaattcgtcggatcgttttgcatattttcgaacaattcactaagtttcccaaaccttctttgagtatgttctgttttccaaatcttgtactggcgg
gcttcttctttagaccggctaatctgagcccgcttttcccatccgtcttgtggtaatctctgattatttattagtaatcgagaaccaagatcactctcgc
gaccttctacctccatgtccccttctcattttcagacgtccctttctgaagttctattatactagtaacaattgccaacgctgcatcgcgattatcctg
taactcatttatctcggctaactttacaagcgcgttgatattgttattgtctataagtagaatagcttccatacattgttgtgcatgttcgaattctttc
aattcgagatagcaaagacccatatcaaaccaaaggtggatgttttgagcaggaccatagctgcttaaaagtatatgatattctaacgccaaatc
gaggcgttcaatttccatataggcctttgcaatgttatttaatactcctgagatgtacttaagaggaagggttttaattatagaaaagtggtgggca
gcttcttcaagttcaccaattttcatccttgcaattcccaatttagctcgtagtagataaggtaaatgataggtttcttctgaactttcttgtacggaa
gtagcatcaacctttctccgtttttcatctacgtcccactcgcgatcatcatcgtagtcgtcccagtatgtttctgacttccgtccgcgaaaccagcgt
gcacctcgattgataaggtggatagcactagaccactgatggtctaaaagtaaaagttcagcataaagattcagagcgggtaaatcaaaaacac
tcttgccaataggtggcactgggtactggtaataatgtgtccaagcagtttcaaaagcattaagagcttcaccgggagcacgaattctaatataga
cctcagccaaatgctttagaagtgatacgtcaaaaggagaagaaggaagcagttgtcgaaacccctcagctgctttcttcaatgatccatgatcct
tatttaaaatagatcgattaaaaatatactcgtttagtaaattttctttgttcggtttcgctaagattgcacgagaataacaatagtctgcttgatccc
aaaattctagactttcattgagatgtgcacaagtagcccacatttcataatcttttggtttaagatgagctgcagccatccaagcaatcaagcactt
ttcaatattgaccctctcgttgcccctttgacgatggcattcacccaacattttccaagcggcaatcacattattatcgatgcgaacaatttcttcggc
aatttttgagcctcagcaaagtttccttcctgagcgaataatcgatttgcaatactcagcaattgttgaacttcaacagaaggaaccatgtctgca
cgactcacacggccacgaccccgtgagctctttttggcgcgtctaaacccaacagctgctttcaaatcttcctcccattcattgtcgtcaacctcaga
gcctttttgtgtagatgttttgtcaaacgtggatgacgcttcttcttcgctctcagcatattctgactcttcgtcagaccacagaccagcggtatttag
agcagcagtatcccctaaaaatgattcatgattatagttaaaccgtccagactcatcaacgaagtcattaatatatccattaaaatcatgcacctct
tcatccatcgcttctacggttgaactgttcacgccaccaaccgtagactctaaaagggtattactttcattttcgttcatgatttgcagaattatgtat
gctgatacatacttttgcaatgttggttgttggcaaaccagagcagacactacatggtcttatagcatatacatttgatgccttattgaacaaaaaa
agatgatttttcatcatttaaagaacttaaaattaattctctatagagaaacgccaagactctttttttctttagttcaaacaaaccagaccgtgctt
gaatcgttttcagatgtaagtggtagaatcatgattaaatcaaacttttcagcaaatccaataaagaaaacatgcaaaaaacccattgatttttag
caattcttaacttcgaagacactgtttattagtgtgcgttaatgatattcgtatttttgatagcatagagtcttatggtatgttaaatagctacaccca
aggaacttggtttcagaggttgctttctgccttcataattaaaacgacatttccatatgttgagagtgtcccgattttggggaactataactccgcttc
ggagctatccaaggatttttcgatatgcctctacagcccccaacggtacagaatcctttgctgacaatgagcggccaatcgacacgagtttattgtt
tcgagaaaaaacaaaatttcaggtatagattatctcttcatcaaccctaacgagtgatgctcataatgcgctttgtgatttgattgactaaccatta
ctttttatagctaaaaaagtataaaccgatttcccctggtttgcgacacttgcaacgtcccattactgagaatttatggaaaaaaggaccttatcgt
ccattgacagtggccaagcgcaaaacaggaggtagaaataataccggtcgaattactgtgcgacatattggcggtggacacaaacaaagaatc
cgacttgtcgacttcaaacggacttctccaggaccttgcaaggtgttacggattgaatatgaccctggaaggagtggacatattgccttaattgaa
agcctggacgaagaaaagcggaaaagttatattctagcttgtgatggccttcaagctggtgacactgtcgaaagtttccgtaatctcatggcgttg
gataagaatggaaagcttcctgatttgaatccagttttaattgctgaattagaggacgggacgtttactgcccgtaacctgaagccgggaaactgt
ttccccttgcgtttgatcccagtgggtacattaattcatgctattggtcttcaaccaggccaaaaagcaaagctgtgcagatctgctggctcaagtg
ctcgactcattgcgtttgatgacaaaaactatgcaatcgtgagattacaaagtggtgaagaacggaagattttagcctcttcctttgctacaattgg
aactgtcagtaatatatattggcaacaccgaaaattaggaaaggcaggacgttctcgctggcttggcatccgtcctacggttcgtggtacagccat
gaacgcttgcgatcaccctcacggaggtggtggtggtaaaagtattggtaacaaaccatcacaatcgcctacgggtgttttagctaaaggaggct
acaagactcgtaaaggcaagaatatgaataagcttcttgttcgtggtcgtcctcgtggaaaagataagcgctagacaatgacatatatacgtcctt

gtataaattaaaaactgcttgtttgatttttgtatgtctgactttccttttaggccgggtgttttacatgatgtttgagctttggcagttgaaggatcag
ctttcatttttattttgtgatgcattctctccgaaatttttaatcacttttatactctttttaataaattatattatatattccctttgcattgaaatgagt
aaaccttgtatcaatcaaccacgctagtgagtagcaattctatatagacaacagtgcaaattgataccattagaccgtaatttttgacgctaagaa
tttgttttctttttgatcttctatttcgtctatgttttcgtcctgctgctgggctagcggaaattcgttctaagagagacagctgagaaactttgggccc
aattcgtttcctcaaaggattcgatgtttcttccatatcttctttttcgggcgatttccttatgattagttagtaacaatatacatattcaaaagagtag
agaaacaaggtacgatacctttgagaactagaggattcggccttctcgtgagggtccccatggaacaggtagtaacgggaatatagatgagcac
tttttactttcttatccgagtatcttgcataacggatttgatgtgaagaggagagaaagcttggacaaggcttcatacggtattcagcattgtcatca
aaatcagaaaatggctcagatacgagtaggaaaagagctcttcgagcgacatcttcatctggatataccaagttacctgtgaaggggaaattag
tctcttcctgcatagacagcgacttttaggtttttattaattgttccttacattcgttgtcattaatccattcaatcacgcattcatgctcgggagcata
agtggaaacaaactctttaatttgcgcttctgacatattatccaccccagaaacatggagtgctgtttttcgcaatgtcaaatcatccggttgctgag
ctggattctcgttcaggccaacagggctctctgtaagaatccgttcgttcattacggacgcattttgttcctccatatccatgaatgaatcgggaaac
gtttcgcgttttgcgaatcgtttgtttttgtaattttacgaaaccaggtcaaatagtgaaggatggtccgtattcttaaatgggtttgtttacaataaa
agtagaaaataggaaataaaaaactgctatcataatacaatacaccctttggacttgaaaagtacgtttcttcgtttgtttgtctctttatttctgatt
tttcgttggaaagaaaatcagccatgttcaacagtagaagctggctgcctggttcaagagcttggttttctcttgtgatacgtttgttatgtcaggtt
ggttgaagcgtcgattcgacggttcattggaaatgaagtcccatccaatttgagcaacacttacatgggtaggacccttaattggtgaggttttttt
gctctttttccacagaacagtagtatacaaaagtgtaaacaaacaatgggctaaacccttctatttttgtttaccttttaacaaaccccaacaaaac
ccccttatttgtctttacgaaagttgacaaagccaagcaagaaaagcccatgtaaacattgcaagaacaagtccattaaaaacctcgattttttta
ctttggtgtttgtactgaattatacgtgaaatactctgctctatttgatcctctttctgtgtaaaattaacatacgcagcgaaaggagtgaacaagat
tccaaaccattggacgcattttatatactaaacaacacatgaatttgtaaggattccattgaaattgaacaaaataatagaaaaagtcggcttttttt
aatttattgagatggaatcgattattggtagacatagtcgagatgaatccttctcgcgttcatgcatcgctactccgaatgtcctcgacgaccaaga
ggaaaatagtgaacgtccgttttcatatgtacaatcattgagaagagcgagggctaccgtctggagtgatcagggtcgtgcaaagcccttgcagt
ctactccttcctggaaaagttcctctcagaacagcaaaaaggattctagcaaaggattgggaggcatgcgttcaagagtcttttcaagtcaacatc
acggtgtatatcatactcgaccggcttcgttgaaatctcgaacaatggcccctcaacataccatcctaacacctcgactctctgcaactgaaggca
aagacgacgatgaagacgatgtagacatttccacttccaatacagcccctacttatatgtctatgatcaattcctctcgtgctagctccacgcgcag
tggtactgccccatctgcaccttctatcatgggcatgtctattcattctcaagctgatactactcactcagaaggatacgaatccagaagcggctcc
ccaactcacgacattcaatcctaccttgtcaatcacagtagtgattcagaagattctgactgggaaagtggtgaagaaggcgtaaagcgtctagtt
attaccaatatagctaatgatgatgaactggatagtgactagtcacattgaatgaatgtgctccatttttttatacccatgctcttgatatatatgacc
ttgcatcttgtttcttttgatgtcccttatgcttcttcgactttgcacttgtgttcatcttcgtttctttttttcattttctattcttttaagttatgtttcgaacg
gtttcatcgttccttttctcatggttaggtagatgactttccgctttttttcttctcccctatttattatttgaatctatagttgataattattcggttttcatt
tgtcatcttaattcttctaccgtaggaaagaaaatacggaccattcatttattattcataatacagtttattatatgtatgtatatatttacagaaaag
acacaaagttccaaaataaagtagcgattatgccttctgagtcggattgttttcatccactaaagctaaaatgagatccttatctcctttttccatta
ttttttcagtcattagattaccaacaatttttccaaaagaccccgagcattagggggggacagttttatccgtagagagttgatcatctctcaaaagc
aataatgcatcatacaaagcatccatgcagcgatttgcttcatacttttcatcttcgttgcctttttcatgagtctttctaaccaaaatttcatagacct
tgatattaggaactaaaagcacccgaatagcttcttttcccattgttttgagacctattatagctccatagtgagttgtataaggttttgtattgtcca
agaaagcctttaacactgttcttgttactcttggcttcaatgtgtagtatacattgccgaatcgattacaaacaatccctaaaagataggctgctaa
atcccttaaagcataatgatcatggttatgtggatcattaccaaggcgtttggcaaccaggcaagttaaaatagagggcattaactgttgaacata
tggctcaacaaacaaattgggattatcaaggagagcccaggccatttgcataagggtagtaagaataactagattacttaggttatgagagatg
gaatcagaaagaaacataataaaatagggcaagagttgatgcaaaccgggatcgtctcgaaggcttgataacgcggcatttcgaagttctgagt
ttgacacatcgagcaaagcagcagtaatacgctcaaaatataattgaagctctttcgaaagcacgtgacgaactaatggtttcacttcaacattct
ccatagatgaaataccatttgacgcctctttcgtagcggtagcagctcctggagtaatggaagctggacccttaggagcccactctccagttgtgt
ggtcagagggggttgggttttgtggaatagcaggttgaacaccctcaattgctaaccaatgtgcagtataagaaatatttctgggtactttaggta
gaggaaaattaataatttttcaaaatcaatttcttcatcatcgagataatagagagagttttgcccagcacctacggcagcttcatgaaattcaac
aggccgagatgtattgaagccgtaaagtggctcaacatttaaagttcttaaagcggatgaaatgtcagcatttgagagtaccgttcgtttcgaatg
gaccataaacttcattgcttcctggatttgacgttaacatattgtactctgacgaaataataagtgcaataaagagcaaataatcttttattaaata
aaatagaagataaattgaaggatacaacagcaatgtctgcaaacaacctagactttcttaacatacctgtatgacttggtggatgcgatattcaa
gatccattgcgattgcagatgctgcttcatcacctaaattgctaataccaagcatttcagcgacatcttttatggattctacgttccatactgtcaaa
gacatgttggtatatggagtctgtagagtttcatcgtcgcgtttaggaatggaaatagagagcttgagtgtgttgcttgttgtatgattacgctacgt
tgtggttctttaaaatacacacttttggattataaacaaaacattgaaagaccagttgtaaaacaaaaataatgaataaataaaataacttttcca

atttttggcaaaactattggaaaactttttgtaatgaaaaactagaaagtaacgttttgaagctataggtgtaaaatgaaggaaaccaatggga
tcttaaatggaattcagtaggatgcaaatgggactccgttgaaaaagttaggagtataactgtcttattcatcctcttctggttactgtttatgaaat
ataggaagaatcttaactctagaaacaaagcttcgtaaaatgatttgtcaagatattaatataaaaggagaaactttatggaatggtgctcttctt
cacctatatcagcagttaggatcggaagctttttaccactgaaagtctaaataccctaaaatagaggaacgtgcacccttatgagtctacattcg
gaaatgagagacgagaaaaatcaactggtctgacttgatatgtttacttttcgaaatcccatttagttcacgagatccttggacatgacgacttaat
ccaattgaagcataaaacgatataactaaagttgtttacttcgctcgaaatacaatattgtacggtgtagcatttctaccatgacacttaactgtcc
taatgattgtcaattgtcactctactggtaacgataaataaaaacaagacatatatatagagcatagagtggtatagagaaatactgacaatgta
atataaaaaatcaagtaaagaaaagaattttacactaataatagtctataaaagttcgttgctatgaaacgactagggcttgaaatggattaatg
cagacgaataactccatatttggatcacggtaaggactatgaaagatcaaatgttggattcttaaaaaaggtgtacataagatgcaattttatgta
aatatgtttgttctctataccatatcttataacttagtgaatttgttaattttccaattgcgattccagaaacaacttgttaatagattcaagctcatat
atctatagtactattcctatacttcactcagaagaaaagtctttaagggaacgttcttttccttatgtcgtctgaaagatagcatatgtctttcaatag
aacagaggaagcgcatgaaagtagcaaaaaaactctgcaaggcaattcagtatggttttcattgagagtatccttaaaagtgacgaaatccata
gaaacattaattgaagcatgttctactgtgaaagtttggatcatgttcattgactctggatggaaagccaaacagatccctataaataattactttc
taaagccttaatttacttcaattttaaacgcaaagacaagggccctatgatttgctcattctttcctatatttgaggaatgaaatgcagaatggtgtt
atcgccattaaaagaaaggaaaaagattgcaatggaatgcgtccttaccgtatgtacagtcctcattactagatttttcagcttctgttttaccatta
tagcacacaccttttatgataagattttgtaatgatgctctcataaaatgtgatagcaaggaaaggagcaaattgcaaaatctacagtgagtttaa
gtttattagaggatactctgaaagtagcatgaatacaaaaaatttgaaattacttttgttggtacacgcacagtaaacaaagcgaaatttcgtatt
cggaagtttcgtagatcaaattttctcaacactattccaagttgagcactattaaaactcgttcagctccgtctcttagagattaaaatctccttttta
agtaagtttatttttgaaaaaagtaaaaagtttgctccagcagtgactcgaacactgggcctcttgcttactaggcaagcgctctaccactgagct
acaagagcctgattagttggacataatgtaggtatataatcactgggtaaaggaaaatatatcgtcgcaaccaagttttggttggaacgggatgtt
aggtttctctaatttctacttggtttgattgtttataaatgcaggttttcaccctcattcataggggcgttgaatttaccacatttcgttatgatctgtta
tctttcttaaagagttcgtacaaccagatagtatcgtataccaagcaaacaacatccaccccaaaaccatcatgcttgctgattccttatatgaagc
agacgatgaattcaatccttttgcagatgctgtttccccttaaatccacagtctgccaatgtacagacgtctgctgaacccgcacgtgaaatttca
tcaaacgcgccggatgcaaattcaaatatccgcgaagtttcttctccaggaaagtcagtaaatgtgaaatctcttcctttggaaacactcacacta
gattcagctccgttagggccattagaccattccacgccttctactgtaaattcagaaaataatccttcaggatcgagtgttcatgaatcctttgcaat
tcctagtaaaccttctcttttaccaaaaaactcttttggttatagtggtgtacatcctcacggcttgcatgtatctgaagtcaatccgtactcttcaga
gccttctagtattacccaagaagaattacgacacaaagaagcatccgatgcttctccaaagactatgcttgctgaggctggtccgcatccttctgc
acccaccatcgcgccatccatgactcaaaaacacgaagacagccaagtggtggcggagctagaggtgccttctgttccacctctaaatattcaag
tacatgatcctcatacagttagtgagcttgctaagtctcatacggtttacttagtcagctcgcaactggaaagtgattctgtccaatccactgaagtt
actgttcaaagacggtacagggattttgactgtctctaccaacttttgtccaacaattatcctggctgcataattcctcctcctcctgaaaagcaggt
ggttggccggttagacgacgaagtagttgagcatcgccgagctgctttagaggtcatgttgcgcaaaatttcctctcaccctcttttaaggaatgat
tcattctttgaaaagtttttgcgggtggataatttcgacactaagttgacaaattacgttactcctattgatgctgcttcttcttccactgcttctggctc
gtctggcttacttgactctttcacgtctgcttttcacacttcttcatcatctaaatacattgagcaagatcccttgcttgccgaaacaaaattatcgcta
gatgctcttgaaacccagctaagatccatgtatcatgcccttttgctttgtatagaccaacgcctgcaacttttatcttcgatacacgactttggcga
atctttagcaaatttgtccttggttgatcttgatccttctctttcctcccaacttgacaatctatctcagatacaaatagagcttcagttttctcaagaa
agaaaggttgctcaggataatctaaccctgggtgtaactatcgaagaatacatccgtaatatagaaagtgctaaaaatgcttactctactcgaca
aaaattatggcaggtttggcaatcttctttgcagtcactttctcgtctcgatgcccagttggataaatgtcgcaagcaatcaaaatttcagcaaaag
tctcttccttacctagagagtcaagtggaaaagtatcgagttaaatcttctcagctagcgcagcagtttgagaactcaactgctttgttaaaacatg
atttgaaacaattttctaacataagggtcgacgatttgaaagctagtgtcgaaacttggctggaatcagcaattgaatcgcaaaaagagttgattg
atagatgggaatcgtttctagaataatttgctgcatatagtttgtatttctttcttcggcttatttatttcctaatgtgatataaaagttattttctcccct
cttttcaacttactttttgctaccgatgttgtttcctgcaccttcattgatgtcgtcggactttgtttaatgattttgatgctaattacatattttatgaga
cgttaatgaaagtattttatgtctttaaatgctacgcttatcaattcaatttgtatatcatgcggtaaacgctaataaaataaaataattcttatattc
ttcagcacgtcctaaaaaagcatatacaacagatcaatacaatggtaatactacctacttgaccgaatccgagactaattcatccatgttttggct
acgatttcatttatcatcctgttccgactgatgaacagcaatctcctgtgcttctgctagatagggcaataatcgtccataaatatcataaattgattt
ttcaggcatgcttctaattaattcgatctttttgttttcatcccaggattgctgaacgaattcatcaaaactaaggttttgctgattcattgcataagcg
gccttgacggctccctaaaatataagtgtcagtaactttccaattgcaaaggatattcaaaacataccccaatagcgcaagcattgcttgctccttg
cttgagtcgataaacatcacaacccaaaatttcactgatcatttgcacgatggcttcatttcgactagcaccacctacaacatatactctttgaggg
cgagatacatcggttagtaaaggggtaattctattgcggatatctaaaaattggctctcgactatagcacgagcatcatcatcagggcttgtccat

gattctgatgattctgtcactggttccagtttatcatttcggatgttgaagcgccatgcaccgggtccaacagcggggataatttcccgtaaaggat
agtacagcccgatttttgatctggaaaagaacgtgaatccattcgttctttaagggtgaattcattgaacaattcccaagagcttttgtcttgcact
tggtatttttcattgatactatcgcgaacagtttctctagccaaactgccgttcttatagcaaagcatgaccataaaatgctaaacactggttagtaa
aatgtagcccaataagagtaaaagtattttaggatcatgtccaagacttaataaaagcaaacatacatttgtgacgataggatgaccaaacatat
gatattcaggagaacaaacatagttggaggtagccatgagagcggtggtacttgtgcctagcgacaaaagaacatcttttccgggtcgcaacgg
caaggaaagtagggtggcaggattgtcgccggtgattggaatgatttggcaatgggatgaaaagccgtacttttcactaaagtagcttccaatac
gaccgagctgttttgcaccgttcatttcaacggatcccagcttgattgctaaatctgagccttgaaaaggaccagcaacgtcatccaaaagacga
gagtcaaattgctcattttgtatatcccataaattcataccacaaacatcgctaatttccaaaggagcttcgcgttgcaaaaacacagacatgagg
aagcttgaagcgagcccaattcgctcggttttctcatagatatctggatgtaatcgacgaaaccgcttgatttgaggacccgtgaatctaagatgc
gctttggagcccgtcagatcagcaagtttgtcaggcccacctacgacagcttccatttctcgacattcacgagaagtagtagcatcttgccagtttg
gtgagcttgaataaagatacttttcgagttgacttttcaatgtagagtttgtttccaatgaaccaagagctcgttcggctccctctaaccaaaaaac
accagcatgctgttgacccgctccacagattccctgaatctcgcttacatccattatctttgcaagtctagcacacagtaaatctatggcatccaac
cacattgccactggtgctaccacctcatgaccatgtttgtagacaccctttgtagtgttataagagggaaagtctttgtcaaaatccacaaccacct
cttggacgatgtccagcttttcgttaatagtaacgccttttaattgttgagtggaaagatctaatcctaaaaacattttgtaaaccctacgttgtcgt
tagattccagaaaaggatcttggtacaagaattctctaggaaagtccgcttaaatatatgaaaaaaatcacctcttgttgtcgttctttttaaccgt
aaaattcgagattcgtacaactggtcgttgtaagagcttacatcaatcgtttctgttgtcacaaattttctattaaataatctttcatcacatcataac
ataacagaaacgagttgaacaggtacgcatttgtcacgttaataatactcacgggttatgagaataaatatcgagtagaagtaataattacaatt
gtaggattgtatacattaattggactctgttgtactcgctagtaatgtccaggatatgattaactaaactaaaagtaggtagacgatcttatttagc
cagatcagtcgaatcttccaatctggagattctgactgtagattcatagtttgtataccttcgctgagactgctaacgtaatctatctctttgtatctc
aaaggtttctttgtttagtggtttccctttgtgaaataggtttctttattcttaaattattgttgttttgctttgttttgtttaccaattgtaatatctcaacc
tttattcttatgacgtttgttaatattcgaaaatattttcttacgatggcgtattgtaatacttattatataagcaagacacctatcgtgacaaacacg
tacgaagtagtaacttttcttaaagtgtcataattgggtccctcgttagcattcataatataacaatccgttataagaatggaatagtagctaatatc
agttaatattcataagcttaaagaattgtgcttgataataagtaaacaaaaatttgaaaatgcaaatttctctttgattgcagaaactccgattaaa
atttacaattcttgattattgctattaattagagaagccagtcactatggcaaatatacagaataatatatatatgtgtgtcgacagtcgaaatcttt
agatcggaagatttgactgatcaggctaaataagattgacacttagtcgttttcatttagttaatcagatccctggacatattactattactctacgt
agtactaacacgtccaattccaataaacaattttgtaattgtacttattacttccactcgatttacattctcataacgtgtgagatttctcccgtgaca
taaagataaaaaaaggaataacaagaaataataaaatattataaaacagaaaaaatagaaagaatgggttggcaattggaagggccttaaaa
ttgactgcttattatcctatgcaaagaagaatcttactttttattccatttatagattcagtaaatttatatgtctatgtatcctactttcgaactgcata
cacatgtgtttgactctggtagacttcttgtagatgatgaaataatcttatagaccctcaaagctagtcaacaaacgaattggtaagaaaaatgta
cagaaataagaaacgtagccttctattatacgaacactctctccactctttcctaagtaagaaggatttctattttggttactcttactgaaacctct
atcatcaaaaatattagcaaaaaatataattatagggcataaattaaaattattataaggagcttgccctgcatactcaaaaattaaataaatac
cttaaatacctatatacacctagagatctttcgtttcttcttttaggcgcttcttgtaatctttctgtaaatccaggtactcttgatacgcataacccgg
aaaatgtagaataataataacacagcaaagacattaaaaaagacataaccaataaatataccaaaattacgccagcgatggtcaaacttcata
ctgaattgctccaaaaattggtcacctgtacggtacctacaatactgacaattgctcgtatcattagggttcaataaattaccggcattagcacttt
gcatgaaactgttcaggtaggattggcaagtctgccccgatggaggattcaaaatattcagttcttgaggtttgcattccactggaacattgtgcag
caaatcactcaataaaccttcaataatgtaggtaaagggtgtcaaagcgtgcatccaatgccaaaatttcatcaaattagaaagtggttgcatga
caccattaaacgtaataataaaagtgaacgccaaactgttcacgacagatgcagtttgagcattcggacaggcactagcaacagcttgaccgaa
cgtggaatagtacatttgaaatatcatgtacattaaccaagcataacctgttctgtcactcggatgttccaaatgtttataaaactttacaggataa
aaccaacataagaaaaaaatcgtaccgaatacaaaattgaatggaatttcaacgagaatcgcggaaataacaaaagagatccaactgtaaat
attggatggtttttctctcacttcaaacacatttcgaaggtcgatgaatcttggttgtaagccattaataagaggaactgcaagcacggttgccatg
aaaactgcaaaaagcttgttttgcatgttttgataacccaaaccttgattatagaaagtgaaaccaatgaacaaccctgcgaaaatattcaagag
catcttagacattaatagcgaaggttctctccaataagaatgaaagttcctagtcaacacaaatttaatttggaaccataaaggcatagcatatgt
atgagtatcttctttttttagcctccctcttcgaattcgctttagaactttcaatcctatccaattcttcagttgtatgtttcctttcttccgagttattccaa
acttcatgccaatcacggtcagtcttcgcagtagcaccggcgccaatcacatccagaatgtactcagctggattggcatccggagggcaaggagt
ggctccatgattagcaaaataatccaacaaagtcttggaatgatcaccgatatcaccaaagtacaccgtctcaccacctttctgaagaagcaata
acctgtcaaattgatcaaataacacagcactaggctgatgaatcgtgcaaagaattgcttgaccagcatcagccagcttacgcagaaactgcac
aaccgaccatgccgattgactatccagaccagacgtgggttcatccaaaaacaaaagaagagctggcttggctgccaactcaacgccaatagta
gcacgcttacgttgctcaacattcaatccagaaccaggggacccgatgatagcttcggcataactttccatttccaataatttaagaacactctcaa

cgtattcatacttttcagaaagaggaacagaggctggctggcgaagtgcagcactgaattgaagtgcttctcgaacagtactttcagaaacgtga
acatcttgttgctggacgtatccggtacgacgctggaatgtagagtcaagaggattaccgttaacaagcatattaccagtgacaacaccagtatc
aacacgctgagcaagcacgttcaaaagggtagtcttaccagcacccgactcacccattaaggcagtaagttttccaggctttacaaaaccattaa
ctccgctcaacaatcgtcgcttttcgcctttaacagtaatatcataactaagatttctccagctgaaaatgtcacgtccactttcaatactaccatatt
ccttttccacttcttcaggatttcgagcttctttaacaacaccaccgccttgagtatcttggcctgtttctaaatccaaaggttttccgccttcttcaac
agcctctttcacagtatcaggtgcataaccccnnnnnnnnnnnnnnnnnnnnnnnnnnnnnnnnnnnnnnnnnnn
nnnnnnnnnnnnnnnnnnnnnnnnnnnnnnnnnnncgaagtgcagcactgaattgaagtgcttctcgaaca
gtacttcagaaacgtgaacatcttgttgctggacgtatccggtacgacgctggaatgtagagtcaagaggattaccgttaacaagcatattacca
gtgacaacaccagtatcaacacgctgagcaagcacgttcaaaagggtagtcttaccagcacccgactcacccattaaggcagtaagttttccagg
ctttacaaaaccattaactccgctcaacaatcgtcgcttttcgcctttaacagtaatatcataactaagatttctccagctgaaaatgtcacgtccac
tttcaatactaccatattccttttccacttcttcaggatttcgagcttctttaacaacaccaccgccttgagtatcttggcctgtttctaaatccaaagg
ttttccgccttcttcaacagcctctttcacagtatcaggtgcataaccccgacggaaaataaggaattcacctttaacatcactaaaattcaaagtc
tcgctggcaacaacatttacaaaaatcaagaaagcgtaaaagccaatgataattgcaaggtttctccatatttgacgtgtcttgtaattaaagttg
acaagaaggtagtttgaaccgtcaacagaagcttgaccaggctgagcactggtgacaggacagactttatattgagtagaaacattatcgtagtc
acttccggaaggaatcatgtcaacacattgaaaagtacgagccttgaactcgttaatcattagagactcaaaaccaaactgtacaggatcaacat
atgaaatccaacggaaccaccaccctacattaatattggggatggcataacccgtataaatggagatagccaagacacccataccacccaaagc
agaagcggaagctatattaggcatgatcgcagccaacgctctgaagaaagtagtcattgtaagggcagaacaaaacaaaaatacgtaaaatgt
ccaaaaaccgtcggcagtacgacgtaagtcagttagaaaataaagaataatagagaaaacagtaacattgataaaacgaaaaggaatgtcaa
cgataaggctactaatgacatcggccgctggatggtaaagagcggaagaacggtgcttagcaataataggacgttgagcaaacatactggcaa
tttcggaaagactttgaagagccgtaaataatacagcaaagaaaagtacaccaccgcgggtgaacatatcatttgtattatccttgagattgtaa
aagatagaacctatgattagtgactgaaacgtaaaagcaaaagccatggctccaatataagcaggatcattaatgaatcgttgccacgaacgaa
ttaaacagtagcgtagttgcatccaaaaagtgatagtgtaaggagacttcttggataaatgttttgatttttcagaacgagcactttcacgatacaa
ttcatgtttcgtggcagcggacatatcgtcattaacttcccttgaggagcgagtagtagacgaaggagagctttcatcccattttctgtcatattcat
ccatttcacgaagaagctgggaatacaagggatgctcatgccatatacgttcaaagtcttcagcagtacgtggaacacggtcttcccaaccctctt
taggtgtacgagcattgggatcggtgatcgcggtcaaaaattcaggagtggtttcacggggatggcaaatgaagcccatatcaagaaagtagtc
cttagccttggaggcagggccatagtaaatttgacgaccagaataaaggacacaaactcgatcaaaaagctcataaatttttttgactagcttgat
aggcggtaacaaaggaagtcacctgaagttcctcagcacaaatacgaagagtattggtgaattcataagcggtactagaatccaaaccacgggt
actattatcccagcaggcgatggttggtcgaagagcaaaggcctcaccgatggtaacacgtttgcgttcacctccggagacaccacgaacgaatt
cgttaccgacctttgtcttgtaagtgtgtgtaagacccaaagttgtagcaacgagatcgcggactcgggccatgtattcgctgcgtgagaggttgc
aaggacgattacggggagcacgcacacgggcagcaaagttaatggtttggctagtagtaagagagggaaagtggacatcgttttcaccggagt
aaagaatatcacctgggtaatattttttcatttcgacggcatcaataccatcgaaatgaagagagccttctacaccattatagtgttctgtttctcca
gttattgatctcaagtaagtagaacaaccggctccgggttgacccaaaaccataacaagctcaccagcattggcgagggcatggctatgagaaa
ggatggcctttttgacgacctgtcgctctttgaagtggcggtaaggaataagaaagatgtcaatgaaggtagtgagatactcataaccagagccc
aaaccatagatgctggtatcacgcatacagacaccagttgtacgggaatgaatgccatctcgggcgagtgcatctttatagccacgaaggtagcg
tcttagatcaaaatcctcacctaaagcaaaaggatcgccggtaacattattatcggaggagacagaagaggaagtagtaggagcatgagcgga
ggaagacatgatgtcaatgaaataataagtaaaaaaatcaatagtccatacaaatgtggtagaaatgaataggcaatcaaaagaataagcaat
cagcagttaaaagaaaaaattagcgaaacccgtaaagatgtctctttgttaactagtttaaataatccaattcaacaagaacagatccaagatat
aaataccaatgatcgtaaaacaaactaatataataacgactaaaaaaaaggaaagaagaattaactgttaactttgtcaattgtcgcttttctca
acaattctgcctgaaaaaggcaattgtttgggaaagtaagcaagacaaagaaaagaaacttattaggaacttcttagcatagaacagaattcaa
taaactaaaaacgatcgcaaaaaaccaaaattacttaatggattacctaatgggtcttgttggagtgaccctaaccaaaattggaggaagagaa
ctctcgaatagtgaattacagaacagcaaggaacagcgtcagagcgagactgaacccgttaaaattcaattacaaaacatgcaaataaaaacc
gcaatcataaaaaaaaacaaaagaatatatatattaggcataaactaagatcattattagtaccttaccctgaattactaagcattaaacgtatat
atataccttcctatacatatttatgcatactatttttctctttttaggcactttttgtaatctttctgtaaatccaggtactcttgatacgcataacccgg
aaaatgtagaataataacaacacagcaaagacattaaaaaagacataaccgatgacgataccaaagtcacgccagcgatgattaaacttcata
ctgaattgtgccaaaaattggtcagctgttttgtacttacaatactgacaattgctcgtatcattagggttcaataaattaccggcactcggactctt
caaaaaatcgttcaagtaggattggcaagtctgccccgacgggggattcaaaatactcagttcattaggcttgcattccaccggaagtttatgcag
caaatcactcaataaaccttcaataatgtaggtaaagggtgtcaaagagtgcatccaatgccaaaatttcatcaaattagcaagtggttgcatga
caccattaaacgtaatgatgaaagtgaacgccaaactgttcacgacggatgcagtttgagcattcggacaggcactagcaacagcttgaccgaa

cgtggaatagtacatttgaaatatcatgtacattaaccaagcataacctgttctgtcactcggatgttccaaatgtttataaaactttacaggataa
aaccaacataagaaaaaaatcgtaccgaatacaaaattgaatggaatttcaacaagaatcgcggaaataacaaaagagatccaactgtaaat
attggatggtttttctctcacttcaaacacatttcgaaggtcgatgaattttggctgtaaaccattaataagaggaactgcaagcacggttgccatg
aaaactgcaaaaagcttgttttgcatgttttgagaacctaaaccttgattatagaaagtgaaaccaataaataatcctgcgaaaatattcaagaa
cattttagacattaacagcgaaggttctctccaataagaatgaaagttcctagtcaacacaaatttaatttggaaccataaaggcatagcatatgt
atgagtatcttctttttaacctccctcttcgaattcgctttagaactttcaatcctatccaattcttcagttgtatgtttcctttcttccgagttattccaa
acttcatgccaatcacggtcagtcttcgcagtagcaccggcgccaatcacatccagaatgtattcagctggattggcatccggagggcaaggagt
ggctccatgattagcaaaatattccaacaaagtcttggaatgatcaccgatatcaccaaagtacaccgtcttaccacctttctgaagaagcaaca
acctgtcaaattggtcaaataagacagaactaggttgatgaatcgtgcaaaggattgcttgaccagcgtcagtcagcttacgcagaaactgaacg
atcgaccatgccgattgactatccagaccagacgtgggttcatccaaaaacaaaagaagagctggcttggccgccaactcaacgccaatagtag
cacgcttacgttgctcaacattcaatccagaaccaggggacccgatgatagcttcggcataactttccatctccaataacttaataacactctcaac
gtattcatacttttcagaaagaggaacagaggctggctggcgaagtgcagcactgaattgaagtgcttctcgaacagtgctttcagaaacgtgaa
catcttgttgctggacgtatccggtacgacgctggaatgtagagtcaagaggattaccgttaacaagcatattaccagtgacaacaccagtatca
acacgctgagcaagcacgttcaaaagggtagtcttaccagcacccgactcacccattaaggcagtaagttttccaggctttacaaaaccattaac
tccgctcaacaatcgtcgcttttcgcctttaacagtaatatcataactaagatttctccagctgaaaatgtcacgtccactttcaatactaccaaatt
ccttttccacttcttcaggattttgagcttctttaacaacaccaccgccttgagtatcttggcctgtttctaaatccaaaggttttccgccttcctcaac
annnnnnnnnnnnnnnnnnnnnnnnnnnnnnnnnnnnnnnnnnnnnnnnnnnnnnnnnnnnnnnnnnnnnnnnnnnnnnnnnnnnn
nnnnnnnnnnnnnnnnnnnnnnncactctcaacgtattcatacttttcagaaagaggaacagaggctggctggcgaagtgcagcactgaa
ttgaagtgcttctcgaacagtgctttcagaaacgtgaacatcttgttgctggacgtatccggtacgacgctggaatgtagagtcaagaggattacc
gttaacaagcatattaccagtgacaacaccagtatcaacacgctgagcaagcacgttcaaaagggtagtcttaccagcacccgactcacccatta
aggcagtaagttttccaggctttacaaaaccattaactccgctcaacaatcgtcgcttttcgcctttaacagtaatatcataactaagatttctccag
ctgaaaatgtcacgtccactttcaatactaccaaattccttttccacttcttcaggattttgagcttctttaacaacaccaccgccttgagtatcttgg
cctgtttctaaatccaaaggttttccgccttcctcaacagcctctttcacagtatcaggtgcataaccccgacnnnnnnnnnnnnnnnnnnnnnnn
nnnnnnnnnnnnnnnnnnnnnnnnnnnnnnnnnnnnnnnnnnnnnnnnnnnnnnnnnnnnnnnnnnnnnnnnnnnnnnnnnnnnn
nnaccagtatcaacacgctgagcaagcacgttcaaaagggtagtcttaccagcacccgactcacccattaaggcagtaagttttccaggctttac
aaaaccattaactccgctcaacaatcgtcgcttttcgcctttaacagtaatatcataactaagatttctccagctgaaaatgtcacgtccactttcaa
tactaccaaattccttttccacttcttcaggattttgagcttctttaacaacaccaccgccttgagtatcttggcctgtttctaaatccaaaggttttcc
gccttcctcaacagcctctttcacagtatcaggtgcataaccccgacggaaaatgaggaattcacctttagcatcattaaaattcaaagtctcgctg
gcaacaacatttacaaaaatcaagaaagcgtaaaagccaatgataattgcaaagtttctccatatttgacgtgtcttgtaattaaagctggcaaa
cagataacttgaaccgtcaacagaggtttgaccaggttgggcaccgacaacagggcagactttatactgagaggaaacgttatcatagttacctc
ccgaaggaatcatttgagcgcattcaaaagcttgagccttgaactcgttaatcattaaagactcaaaaccaaactgtacaggatctaaatatgaa
atccaacggaaccaccaaccaacatcaatattgggaatagcataaccagtatagatggcaatagccaagacaccaataccacccaaagcaga
agcggcggatacgttaggcatgattgcagccaacgctctgaaaaaagtagtcattgtaagggcgcccataagcaaaaagacgtaaaatgtcca
aaaaccaccggcagtacgacgcaagtcagttagaaaataaagaataatggagaaaacagtaacattgataacacgaaaaggaatatcaacga
taaggctactaatgacatcggccgctggatggtaaagagcggaagaacggtgcttagcaataataggacgttgagcaaacatattggcaatttc
ggaaagactttgaagggccgtaaataatatagcaaagaaaagtacaccacctcgggaaaacatatcacttgtattcttcttcatattgtaaaaga
tagaacctatgataaatgactgaaacataaaagcaaaagccatggctccaatataagcaggatcattaatgaatcgttgccacgaacgaattaa
acagtagcgtagttgcatccaaaaagtgatagtgtaaggagacttcttggataaactctttgatttttcagaacgagcactttcacgatacaattca
tgtttcgtggcagcggacatactgtcatcgaattcctttgcagagtgagtagcggacgaaggagagctttcatcccattttctgtcatattcatccat
ttcacgaagaagctgggaatacaagggatgctcatgccatacacgttcaaagtcttcagcagtacgtggaacacggtcttcccaaccctcctttgg
tttacgagctgtgggatcggagatagcggtcaaaaaatcgggagtggtttcacggggatggcaagtgaagcccatatcaagaaagtagtcctta
gccttggaggcagggccatagtaaatttgacgaccagaataaaggacacaaactcgatcaaaaagctcataaatttttgactagcttgataggc
ggtaacaaaggaagtcacctgaagttcctcagcacaaatacgaaggttatttacgaattcgtaagcggtacttgaatccaaaccacgggtactat
tatcccagcaggcgatggttggtcgaagagcaaaggcctcactgatggttacacgtttgcgttcacctccggatacaccacgaacgaaatcgtta
ccaaccttcgatttgtaagtatgtgtaagaccaaaacttgtagcaatgagatcgcggactcgggccatgtattcgctgcgtgagaggttgcaagga
cgattacggggagcacgcacacgggcagcaaagttaatggtttcacgggtagtgagtgtgggaaagtggacatcgttttcaccggaataaagaa
catcacctgggtaatgtttttcatttcgacggcatcaatgccgtcataatgaagagaaccttctacaccattatagtgttctgtctctccagctactg
atctcaagtaagtagaacaaccggctccgggttgacccaaaaccatgacaagctcaccagcattggcgagggcatggctatgagaaaggatgg

cctttttgacgacttgtcgctctttgaagtgacggtaaggagcaagaaagatgtcaatgaaggtagtgagaaactcgtaaccagagcccaaacca
tagatactggtatcacgcatacaaacaccagttgtacgagatcgaatgccatcgcgatcgagggtatctttatagccacgaaggtagcgtcttag
atcaaaatcctcacctaaagcgaaaggatcaccggtaaggtgatccacggacgaaacagaagaggaagaagtaggagcatcagcatgggga
atataagaaccggactcatggttttgactagcgcgatctctggctgcaacggtagcagtggaggagatagaagagacgtcctcatttttatcttgc
gcagtatcgtacgaatcaggatgaaaacgattggaattgttatccaccgaagcagcagatgcgtaagaggattcagagggagctggggtatgat
gagcggcggaagacatgatgacaatgaaaaagaaagtaaaaggaattcagtactccttacaaatttagtagaaatgaataggctatcaaaaga
aataagcaatcagcagttaaagaaaaaaaaaaaaactgcgaaaccctgtaaagatatcccttttttttactagtttaaaatagtccaatccaaaa
gaaaagattcaaaatataaatactaatgaccgtaaaacaaactaatataataacgactaaaaaaaggaaagatgaattaactgttaactttgcc
aattgtcgctattctcaacaatgctgcctgaaaagagcaattgtttccgaaagtaagcaagacaaagaaaaaagaatttataaactaaaacggt
aaaggataagaaccgttcagcgaccctcactgaaaaggactcctgcaggccttataagtaaataaatttgtcggtgtttttaacatccgcaatgat
atcaccgagtgaagaagcacatccccaaacaccgagatggaaattttcagtagcgaggtgaaatactttgataggcccgaacaagcatgattcg
gagactagaagaattgcgcgactttgttgcgttgagcgaaaaataaaaccctttgaaacttctgttttgccacttcttctttgtctttttaatctataa
aaattgtcttgtaaacacatggtggtcttttttttttttattcattttgtcgcttgtgtttctgtctctctcctactgagtttaatcagaggctaaagcttc
tttcgaatactgcatttcaccgcgtcgctaaatgacggagataacaacgaggagaatggaagagaaaaaagtaggcagggaaaaaaaataaa
cgatcaagctgtcgacaaaggaataaaaaggagaatgaaaagctataaataagaatttcttgaaaggaatttgaataggatcatttgagtttcc
aaattgctcttttttttgtttactttcctctctcgcttcttgacacgtcgcatccgaaacacctttgctaaacacctcggctcggggttatcgacaagaa
atgaaaaccactgaaagtaccagaactatctccattagtcatccttgagaaaagagatatgtgcttttaattgatttgtttacaaggaaagtttaaa
acccatttcatggtatattaccatacggattgttacaaatatgaattccgaagaatccaaaccccataactgacgaatgttcaatctttgtttctgtt
gtttcttctcggaaaaatatctctattttctctctctctctctctctctaccttattccttatgacttgcttctttcgtctctgttaactttcggttgcgaa
agactgtattctaatcctaaagaaccttttaaaatgaacgaaaaattgcgctattacatgattaaataaaccaacatgggaagtaaataatgaa
gactaaaaaaaaaaaaaaatagagatatataatgcaaattaaaaacaattagttgatcattatgtatacgtatatttcttcaataaattacattgc
gtctctctatcgtgcttaacatactggcttcgttctctttaaccttttttatgaatctctctccgaattcttattcacttcgtcaacgttcgctccttcccg
tcaacgcccattttgcctcagtgcaccttttttttgtgtttcactcgttttttttattgttttgccatttttcggctgtttatacaatttccttcaaggaaa
actttctgtttatttctatctctctatttttttgtttgtttctgctcccgtgtttcttctttgcgcgtcttcgtttctttgattcacccgacgttgcatcatcctttt
tattcatacacaattgctttctttctttgccatctttctctttttttttcgtttttatgcttctcttcctgtagatctttttgtttgtgtgtacacgtttggttc
cttcctactctttttcgtttgtcgtggtttattctttctttttggtgttccttttcttctcttttagtctctttttttttcgcttttggctggtatcctagttgcatt
gattccctttctccgctatgtcctcctcctcctctaaccggaggccttatgcccttcggcgatctcaacgttctatgaatcttaatcaggaaaccttttt
ccctccttcttcaacttccactaatgcttctccattttactctcctcaatctccatcggctgcctcttcttcttccaccgctagaccatctctccactcttc
tccgttttctcatccttcccaaaacacctctaccatgaaacgttcctacctacctccctctacagtttctcctttttgcccaaactgctgttccttcagttt
ccgactcttctactacccttgcttctacctctacttcctctcctttttcgctctcttctccctacaagttttccggaagtgctcccaaacgcacctactttt
cctcctccctcgcaaaaggtgcctcctctcctcctcctccctctctcctttcctctcctgctaatctttctccttcctctcctcctttttttatcaccctatcg
cactccctacactcctgcacaaaaacttcggaaaaaggatcctttttcatcaatcaagcatccttccaatatgtccgcttcctctcggccttcttcctt
ttcccctttcgctacttcttcccctcagttcgtttctacttccacacccggtgctactgctgccgctcctcctcctccccgctttccatctcactcccagc
accttccctcatcttcttcctcctcctcctttttttttcccttcttcacatgcttttacttctcctcaaaagtctcctcaccgtttctcctttcgcccagcttcg
tcttcagcaacacattctcctcctgtctccctgccgactcccaaacgtccaacccctcgttcttcttcttttctgtttggtaattcgaatcctgcgcctac
tccctttttcttccttaaaacataatgattctcctaattttcacaatcaatctcgtgattcatcctcacatactgattctaattcatcttttaattccacttc
gaccctttttcccaatgcgactctcccaatcccaaatcctagtcataatctttttactaccccgtttcaacaagtcaaaccttcctctcaggcttttctg
agtacgggtttgctttccaaacaaagtaggcctcggaaaacctttaatctctctactcctttaccccaagcacaccttcaaaaccttcttctattttc
cgccctaacattaattcagccgactctcctccatctccttctacaccttctaacagcaactctgcgtctttttttcttacaaggacaaagcacacctac
gcatcaaagctctttctcttccgcaaaactgggtaaatcatctatggatttttttgagatttgaacctccctcgtctactgtaaaacaaccgcctgattc
ccaattaattgatccttctactcctactcgtaatccgtttgattttgagaactcttcaatgtgtttggacgacattcattcatctgctcaacaccgcaat
aactgtgaaatattagccgaaatggaagaggaatctgcaattttgggaaacccatccattgcaaactcaaatttatcctttccttctgctcttacctc
aaagtcccctgagtcgcctgcggatgacttacataaccggtttcgaaacattactttgcttgggagaggtgagtttagcgaggtcttccaagttgaa
gatcctttggagcgatctttaaagtatgcggtgaagaagttaaaggtgaagtatagtggacccaaagagaggaatcgtttgttgcacgaggtttct
gttcaaagagcgcttaaaggacacgatcatatcgttgagttaatagactcttgggaatttaatgggtatctttttatgcaagtcgagttatgtgaga
atggtagtcttgatagatttttggaagagcaaggtcaactttctcgattagatgaatttcgagtttggaagattcttgtagaattggctttaggactg
cagtatatccatcataaaaaatttttgttcacttggatttgaaacctgctaatgtcatgataacgtttgaagggacattaaaaattggtgactttggaat
ggcaagcgtatggccagttccaagaggtatggaacgagagggagactgtgaatacattgctcctgaaattttgtccaaccatatgtacgacaagc

ctgcagatatttttagccttgggataactgtattcgaagcagcagcgaatattgtgctcccggacaatggtcaatcctggcaaaagctccgttctgg
agatctttcagatgcgccgcggctttcttccaccgaaggctctagtaatacttcatcctctcgggatgtaccgttaaacagtataattggacaagga
ggactcgaccgtgttgttcaatggatgttggcaccagaaccacgcaatcgacctacagtagatcaaatattagctactccagaggttagttggata
gagcttcgaagaaaagccggagccatcatttatgaaggacttcatggatcgtcatctgctcctcaaagcgatcaaatgatggaagactggcaagt
caatgtataattgcttgatgggattacaggatcgtttatggttatttattcttttcaataatttttattacttattcggtgaagtttagggtcagagcat
ccctaacttaacctagaagatatacacttgttttatattcatgctttttatataacatgtatactatgaactaatataagggaacaacgatgattttt
ggcctttttttctaccaaacagattatttctgaatgtatttttgggatgtttttattagttttattatgaaggaaacgaaaggttgagctaatttttttt
ggatatggataagatgatcggtgattctggtttagaattgtttgtaaatgttacaacggcttttaaaaaaaacccctccaaatggtttagagtatac
agttaatttggatttttcttctcttgctgcgttcccctttttggaaaggtttatatatatatctacatttttctttcttttttttttttttttttttttttttt
ttttttttttttgttttgnnnnnnnnnnnnnnnnnnnnnnnnnnnnnnnnnnnnnnnnnnnnnnnnnnnnnnnnnnnnnn
nnnnnnnnnnnnnnnnnnnnnnnnnnnnnnnnnnnagtagtatactagttaatttaggttattttcttctcttgctgcgtttccgtttaggt
taagttatatatatatctacttattttcttttcttttcttttttttttttttttttttttttttttttgttgcatggttgattgacctgcatacaatttattattacc
acactcgtttcaatttgctcaatacatatttttgctatgattcctttttgattttacaaattcccttttattttattttatttctttttaacttttggacatct
taaaaatgttctacccaggtaatttgataatgcttattttcttcgtcaatgtttgttccgcctccacctgtgaggtttccttttctattttaatgttttaat
tgcttttgatgatatttgattgtcttattgggatgacttaccgtttaacccgtctttgacgtttagtgttgctttgtaattacgagaaatcaaccatcaa
aggtgggatgaactaaatcaaagctatatataataagatcctactgcaaagatgtgttttaatatagacttatctgatttacgaatataagtaaac
atatgcaaagattcctttcctcaaatcagcttactttttaggaaatagtgaaggtttattatgatacgcaatagtgaattttcagaaatggagggag
cgtccagtaaccggatttaacagtgtacagtaatgaaatggatctcttgaaagagctccaggtaaattacatttgatcaaaaccgatcagaaaaa
ggaggcatgtttacacggaaactcttcagttcacttgagaataattggagatatgtatctcaaagtgttaacttccaaaggaatcgtccgttctcta
ctggcttgtccgttaaagtaccaaacacaccgaattttaatgtgaagaaaaactctggtttccaaaggcggagtgattcgtttattccaccttttcta
gttccaaacgtttccgagggtcaagtgtactctccaagcgatttgaaatttgaaacggtggaagcacgaaatcaagccaatttcttcaatgccaaa
aaaaacgactgctttaaggctgctaataagagtccaatagaattttggaagaaccctgttgtattgtccagttttattactgacctgggtcgtatta
agccaagagcggatacgggtttgagtgcaaagaaccaaaggcttgtttctcgtgccattcgtcgagccagggcagtgggagttttacctaccaaa
cataagtctgtatatgcagaacgagaaaagtaataaagatacatacttcccagtgtatatgctgggaacctattgcaatggcccaagaataaaa
ggatggatcagaaaaaattacgagacgaaaagagctagtaagatttccaaagttggaaataatggatttggttgggtttccaaaacatatgcgt
gtgttaaacgtgaataatcttttgtaaagcacccggaatggtcgttttagctttcgtgaaatcttttgaattttgtaattgatccctatggggtctaaa
gagtattacaattgactacttttcactaataggtttgaccttcgtaccatgagtttcatagatttcgaaagaaatgtcaatgagtttaaagatcaaat
aaaaaatttaggaaaaataacaatgataattaggttaatctaaagcaaaaaattagtaacagcagtttgttcgtcaaacataaagggtatccctg
tttagagcaggacgtaagaggcaataataggcatttatcaaaaagcactttattttcataattttgtttttgaggatgagatagatgaatgcaattc
ggtggtagcagtggaagatcttaaatgaaaggttacgattgggttggaaggggaacaagaaggctttattttagagaataattgagcagatcgg
ttaatggatgaggtatcttcttgtgtattgagcaatgcacactcaatggattgcattgcttcttgaatagcttctataggaggttttcccaatgctaaa
tttgaggcaatggctgcggttattgaacaactagttccatgaacggctttggaatgaagtaaatcatatggtttttgaaacacataaaaagtctttc
catcaaatatcaagtttatagtaaaagagttggctgttggcatgctggtaatctcaaagttctcattatatgcaacatgataaccatttagcacgat
aaatttaggccctgctttatggattattgtaacaagtttctgaatgtcgtcaaaggaatgaagggcgaagggcacgtgtccaagagcccgctgaa
ccaaaacataggcttccataatattagacgaatagacgagtacatgagggaaaagatgctttatagtggctgctattgcagttcgttcacacata
atgtgcccagcgctgctaatgatcactgaatccatgactatatttttttacttgatgccgttggagagtctctgcaatacggagtacgcttttcgaatc
tggtaacattccaatctttaccgtatggcattcaatatcgctgagacaagcatccaattgcctctggatgagtagagggtgaacggaatatatgcc
atcgatcccttttgagttttgtgaggttagcgccgtcaagaccgacatcccatacactccatatgcttgcataactttcagatcagcttgaatcccag
cacttccactacaatcggatccagcaatagacagacaagcaggagacttaagaaacttcatgaggaaagaaaaacaaatgcaggaatgggtg
agtcggattcaaatcaataaacagtgaattccaaggaaggatgagcaacgccaaattatatcaaaaactgtcgaagaatattgaaactttgaaa
tatggaaagactctttttatgaacaaactctttttttaaaaaattgtttgcttttgatacaatcccagatctgcttcctgcaaattaccattcatccatcc
gctgcttgtgcatctgggtgcaagtatgcgagcgcgattcaatgatcattttcgtaccttaatgtatttttattgaaaaagaaactagaagcttaatg
ttattgtattggtaacagaaggaaagagaaaaaaaaaaaagagtaaagaaacaaagctacatccggccaaggcgaattacggactggtaagga
tcaaatgcgcactgaacttaacgttttcgtcttctgagggccttttgttgttgttgtttttgtgtcctcttattattttcgctgttatgttgtctcgaattgc
atgtggggtagattaacttttatccccacagtgtaaacaaaagaaagaaatgaacaatcagaaagaggatgctgaaactaaaaaaagaaaaa
aagaaaaagagaaagagaaagagaaaaatccatttgtttgagtgatgtctcagtgatattgcgatgtctatcaattctagagatttacttgaaaa
tacgtaactcgatacctatcaaacagcgtttgaacaggcttcctcgcccgtttatgactaataagaagcagaaattctcttctactagacggagca
catgtattacaatgttatgtttgacttgatttcatttttttatagaggaactcatttcaacaaatgaatcgtcgtgggattttttctaaaaaggatctgtt

catcttcattctttcgtttactagttttatttttatttttatttttaaaagaaaattccatgatataaaggccttcgtccccttgccattttattcttactct
gctatgatgtatttcgctccattccttgtttttttccttacacttttaaaatgaagctgattcacaaaaatgtagaaaaaacagggtctggagaaatt
acaatgtatccagaagagcctgaagacatgtggtatgcttttaaactgttcttactttgctagattttgcatagtaacattttaggcacctttacaat
atactccaaattggcgaccagctgaaggcttccacgattcggtaagcatgttttgaaagttcataaaaattggctaataattccgttttagtcgtgtt
gtcaaggttggagccacgggtaccacttctaattcaagagaaaaaatgaccttgaaagttttggtagaagctacagactttgatacaaaagccgc
tgaattacatatcaaagggaaaacaaccgaataccacccactcgtaaggatgggttcatatcacacactggatttggagttacatcgaaattta
cactttacaaaaatgaatgggactcgttcgctttagatcgagtggatgctgcctgtaatccatcaaggaatgcagaaattggggcagtggttctcg
aggaaggtatattctctcctgttcacggatttattatcttgctttgctacagaaatactaacaccaagttataggtctcgcaaatatttgtttaataac
tgattatatgactattatgcgccaacgaattaatcaaactattccccgaaaacgcagaggggatagcagcgcttatcagaaggtatgcatatgaa
gttgaagaatgctgtactctcatactaaactcttcagggtttaaacaaattttatgatactgtttttcaagcaatttctacagagcttgattttgacaa
acttaaagtcgttatcctagcctctcctggttttgtcgcaaaaagtttattcgattatattatggaaatggcagtaaaattggacctaaaacacattt
tcaaggcgaaacaaaagtttttgattcttcattccagtactggccatatccattctcttaacgaagtacttaaaaattcaattgttgagtctaagctg
gcagatacaaaatacattcaggaaatccgcatgctaaacaaattttaccaagttatgaatgatgatgacagacgagcttggtacggcccaaaac
atgtcgaaaaggcctttgaatttggtgctattggtgaacttttgattagcgattctttattccgaagctctgatattgctactagaaagaaatgggtt
ggcctcgtagatgcgttaagagaaaccagtagtaccatatacatttttagcagtcttcatgaatccgggaaacaacttgatttactctccggaata
gctgctatcttaacatatcctgtggaagaagaagatatagaggaagaagaagaagaagcagaagaataaagctaacgaaaaaacaaccaca
aaaaactcattcttcacttttatgattttcaatgtaaatgagggttgagtcgaggattttagagtaattctgagctggttcagtgtaatcaaaatata
ttcttttcgtatctaattaattcatttttacaaaaccacttaaagcatgcccatccagtatttaagtgaatcaatacactcttaaaatacaagccaaa
ctcatagcaaagtctatgacgaagtacacggccatcttccagtgtatactacttataaaagacccttcttttcgttcaaattttttactagttgctcag
ctgtattttttttctttaacgcgaaccaaataggttaccaaagcttttccatcttcagaagctgctggaacagtcaaagctttctctccagccatcttat
attcaaagtctttacataaacgaacgtttaaaagaagcttaccgttaccatcaacgcgtgcaagaagtctggcggcattagtagttctatcaacat
ttaattttaaggggccgagaccaatgtctgtataagactttgtagtgctattaaagagataaagtttggccctcgtttcgaaaatactgtcttcattc
tcttcaccttgacctttactaatcgctaaagaactgtcctgcctttcttcaatttttttatcctcttctttgtcatcctcaacggtctcagattgctgttctt
cctcattctttttatcggaacctgcttcgttatttacactagcgggagatttagctgcaaaagagaatggattaggattagtaccgaaagagaagc
caatcgatgcagcgctaggaggattcacttcaggagttggagttggctttttagctgataaagggtttgtaaaagtaaactttttctcaggagcttc
aaatttaataggcttgctgggatcccatgaaaaattgaatcccttcttctcagcatcctcctcctctttgttttcggtgcttaaattagtgcttggcttc
ttttcttgttgctcttgtgaagtcttttggaaggaaaatgtgggagtgaaagaaggtttttgaccaaaagaaaaagggttcttggtctgagcttctg
atccaaaaggtaatttttttctcttcatccggtttactgatttccttattgaaggcagctaaaggacctgtaggagtaagtgaagaagggttgaactg
aaaagatttgctaagatcaggagcggagaatccaaaacgtggagcacctgggacaggtaaagtagattcggatggtttagtagaagtagtttca
gagggcttcacttcattatccttaggactcttgctagaatccatttgagaagattttacagattccgcctctgaagatggctttacagcttcattttgt
agtttatctttgtctgcagaagatactgagctctcaatggaaaaaagatgctttttgtattcttccattaaaacagaaagatttccgaatggatcatt
ttctaaactttttttgtacgctacccaaaaatgctttgttcaatcctcgtttctttaataatgtgtcatatttgtctgtcgaggaagcaacatttgtcggtt
ttccaaatgtaaagggagaggcaacagatttagcagcctctggttttgccaatcctgaaaaaagcccagcctgctgagacattccagcaggtcgt
ttccttgttttaggtttagcaattctaaactgttagcttcgacagcacttcatcatcattaattttcatactttctagaggcaatgacatcggcagatg
cttcggtcgctagttcaacagaaccactgcgatcatcatcagattcatcctgatcctttgtaatttgatgatcagcaccgcgtttcgacatttttacaa
tttggcaaaggtcaaactcccgtttggtgaacacactcatataacggataacacgaagtctcactattttggaaggaggattattagaaaataaa
gaaaggatattatttaggtatcaatttaatagaaaatgaaatagtctaagttacaaaattaaattttactcttcgtagacaaaacgatgtatttatg
gggacatgacgctgttgcaacgttggcatacttctccttctgaaactgtatgcatccaacatctcaagcactttttcaactgcgcaggttgcatccta
atttgtaactttccgagattagatgccgcagtattctgcagagtccacaaggaggaatttttttgagtcactagtaatgtccagtgtatgcaaacgga
cgctagaagcacccaaaatcaacgatagatcattcccatatttcttcagaacattctgcaatccttgattatcaaccccaattatggtctctgtttcc
aatggacttcgaataccatgctgattccttgcttcttgtagcaacaaattaaacgaatactttatctttagcaataattgatgctcctccaataaaga
ttctttatcttgcttgacgaatttctgtaaatcttgaagcaaaaagtctgtaccagcatgaaaaggcgtagaatacgaaggctgtaaagccttggg
aaagctcctccatacctcttgagacaacagtggtacgataggggacattgaccacaaaagggttagcagaattttatatagtgtagtttgagcact
tcgcctagaaagactgtttggggcatctgagtacaaccgatctttgattgaatcaaaataggttgaagaaagaaaactgttcatatattgaaaaat
actttgaacaaccttattgaaagcgagggagttgtaattctctttgacttcattctgaaattcacttgttttcgcaacagctaattgatcaacacgat
gaagtcgattataaacaacttcgtctgccaaagaaaaatcgcccagattccccaagcagaatcgacatgtaagtctccatttttttagcgattccc
caacttggtttagtaagttgggtccgagatttatgtcgttagtactatcacaactgccaacccatagtctcaacaaatctatgccgtacaactgcttt
tttgaggtaatatttttttccatccaaaatatcttctgggtccgtaacatttcctaaagacttggattgtttcattcctttttcatcaaagacaaaaccat

gagtaaataaagactgaaaaggtgattcttttatgccagtaactgcaagataaatcagaataagagactgaaaccaacctctatgttgatcactt
ccctcaaggcataaatctataagaggtttatctttacgaaatggtactgtagtaaaaccactcccggaatcaaaccaaacatcaagagtttctttc
ccacgaacataatctttatttctgaattccggaggaacccaatcaatattttcatcttcgttgaaccaggcatcaactccttttactttcatcttgtcta
tgatataagaaatacttcttgaagtaagtaatgattttccggaagctttctcataaagtacaggaatcggaagaccccaagaacgttgtcttgaaa
tacaccattcaggacgactgttcagaaacccggaaagccgtgactttccagttaggggaatcattttaaccgattgaagagcttcaatcgctttat
ctttaacagcttccaaagaaataaaccattgagcagttgctctcaaaattaaaggcttccgatttcgccagtcatatggataacggtgtttataagg
agctagctgaactaccatgccattggctttcataagctcaagaacctgttttcctccttcatttaacacatttaaaccaaataaggatccacccaga
gcatcttttgtgtatacgccattatcgtcgacgggagaaaaagggggaagagaatgttccaaacctagtagataatcatccattccgtgtcccgga
gcaacatgtacaatgccagtaccagtggtatcagatacatattcggcaggtagaaatggaaacactttcccgggaatcaaaggattttgatatgt
gagtgatcgaatagtatcataggtacatgtttcaatttcttcaaaactttctagaataggcaatgaatttaccaagctctttgctacaatatacttct
gttcaccattagaaaagattccatactccactcctggatgaaaggcaagtgccaaattggaaggtacagtccacggtgtagtagtccaaattaaa
gcctttacattttcatatggttttccttccaccgtaaaattattcactggaaatgagaagtaaagcgaaaaactaacatgattatcattatattcaat
ttcagactcagcaagggcagaacgactggatggactccagtatacaggctttagctgtcgaaaaaccatttctttctctaccaatgtttggaaaac
ttgtaattctgaaatttgaaactcatggctcatagtcgtatatctggaaaaccagtctcccataactgccaaacgacgaaagaggttcatctgacc
attcatggcattttctgcaaactctttggccgtttttcgtatctgacatggggatccttctttatcattttgttgtaccgctttcaattcaataggaagcc
catgacaatcccatccgggaacatacctgatttgatagccatttagcagtttccagcgattcacgatatcttttaaaattttattcaatgcatgaccg
atatgtaactttccattcgcgtaaggaggtccgtctaaaagaacgaaagattttgaagaggggaggttcttcttctgccatgagtataaatcatca
gttatttctttcatatacttcttctcattttcacgttcatcacttctaatagggaaatttgtttttggtaaacaaagcgaattggagtatttcttagcatt
cgcttgttgactaagaaaacggagtccaattttcggtgttactagccgattttctatataggccttcataaattatatttattcaaattagcatgtgtg
tgcgctacttgttatagaacaataggatccatccaaatattgtggtagaagagatacaagcttaagacttgaaaaccttgttgctattatccttctt
accacatcaagataagaaactattcattcagttattttagtttttgtttctttgatacccttaactacaaacaaagtagaagtattcctattgctgaat
tattacaaagatagaaactagcatgaagcctttcaagtctaaattccaaaaactacaagtatagtccttaaaagagattgctatcagtgtattatt
agcaggttctttcaaaggtgtcgtatatcctttgatcgtgaaaactagtaccacggcttcaaaaaaaaaaaaaaaaaaagaacccttatcctata
ggctaagaaataaatagataaattactcaaaaactttttattagaagaaaaaacctttaacaactcatgactctgttcgccgttggatcagcctct
cggcagcgtccatgtcaacatgaattcttttctttttacaaagccgaagaatttcattatgagaaacgtaaattggctttacgaccgcgttgaattg
actgccaagacgcaaatattccaagtaggctgattgagcttgttcagaagaatcaaaagttaaatacactctatgatcatctttccatgtgattatc
atggattctacaggaaaatatttgcgtaaaagagattccgtcagataataagcatgtacttgcaaaatgtacgctgtagatgtcccacacgacga
tagtgagggttttaatacttcgacctcatctttaccaggcattgatgacatgaaaactctgtctatgattcaaggtttagaaattcagcgatttttct
gtgtcagaatgcagtgtcggtaaagggtgagtgcatgagtgtaaatagcagcatcaagataacttaaattatggcataaacgaaaatatatcca
agatgttataattttatataccaaaaatctttaagccactgctggtaggctcagtaaaagcatatgctagaatatgttttgtaggattccaagcaag
cttgttcaaaggaccgttcgtcggttgcttccaaatttgttcaccagtaagagtatccgcctaatcattagcaaaaaataaataattttgtccttaca
atatcgatataacgatcttcagatccacttgccaaaaagcggctatcaaaactgaaacccaaagaacggataggatatctaatagaatttagtaa
atactgttgcttttaaaatccttacgttgatcttgtaatggaactttggcatatccattcatcggtatcccataatgtaacgatcgcatcggctccgcc
aacagctatgcgctgattgtcaggactaatgtctatacaataacaatttgacgtatgagctttgatttcatatactcgttggagagatggccattca
atgacttggacagtgcctaaaccggtagtcatgaaaaacaagtcttcactaaaggaccagcagcattcatttgtctttgataattagcttagtaaa
aagatagaatatgaaatacctcacaaggttgctggaatgtctgcataatttgccattccctagcatcaatgaaggaaagcatatcatcctatgtttc
ttagttagccaaagacaaaaagaagcattatttttacccgacttccaacgcagcaatatcgcccggaaggactccaactaccgtagatgttgttgt
aatcactctgaatttccgcaatgggtttagcacctagtcaaacgttagcaaagaaccaatatatcaagaggatacatacttcgataatcccaaaa
gcgaatcgtttttccagagtacacggtcattaatcgatcagaatgagttggatcccatactacttgttctactaatccactgctacgtgatcccaatt
ccgttgaaaacttaaagtctaaacgatccaggttccaaatacgaaccgtgctatcattggaagaggttgcgagacggtgacctgaaacattccat
cctaacgaccgaactgaaccttggtgaccatgtaggtcgcgagttcttagttctgaaaagtttggctaaatcaaattacatgtcagctaaatgaaa
aaagagaaagtttcttacaactggctctataattgaggagctcatagaagacaagctatgaaccgctggttcgtgacaagcgtagtataggacac
tctctaaagtaaattttggaacttacatacgacttagaaacccagcagcatacaactgttcttcaatgtgaattaagaaatctaattgaaaatata
attacaaaagataaagatatatgcatctatgaaggtgattgaatttgttcagtagactcatgaccacattctgaaagcgatggagaatccaaagg
atttatagataattctttttaaaccatggatggtctaatgcttccttttcagaaatccgcttctcggggtctagttgaaacatttgcgtgatcaggtc
gattccttctggtgatatttcattttccagtagtggagacatagggaaatctgccttattaataagtttgatggattcttcttgatttggtgctgcaaa
aggtatagttgcggttaatctagtaaggtattagcatctgccggaagcaaggatatcacatagatagatcctagaaaagaaagatttatatacat
acgtaacatataaaacacatccaagactccaaatgtctactttgttgtcatacccaccctcaggaggagcagtcttagattgcaatatttccgggg

ccaagtaacccatagttccgcaaaacgtttcaaagaatgtgcctctgccatgaataactttagctaaaccaaaatcagaaagtttcaaatgaaag
tctttggtaatcagaatattctaacatgcgcattagctctatactttgaaggcatatgaaactgttaaatatacctctggtttgatatctctgtgcgca
ataccacattcatgtaaataaataagcgtttctaagatttctttcaataaaggtttacagtcttgttcatctattgaaccgtttgcaattagaaagtcc
ataagatcccctccttccacatattccatgacaataaatagctcttcgtccgtttcaaaaacttcccgacattgcacaacaccaggctagaaactat
tagcattataaaaatactttgaagcaaacgtcgaatacttacatggtgtaatgttttcaaaatttcaatttctcgttgaaacatttcggtagcacgtt
tttcggaactggtgagtaggattttttcttgttgataactttgatcgcatagcattcgccgctctctaaatcaactgccagcttaaccacagcaaag
gtgcctgtgcccagggtacgaagaacttcgtatctatttacgttcgaagtaaccagttctttcctcatactctctgaacggttgtaaggaatttgaca
taaaaatcctgcaagtgtcagatgccatataactggtttccccatggtcaaagcataatctatctcatacaccataatatattctttttcacttacga
atttcatcttttggtactcctaatccaacccgaatctcatccccatgcgacaaaatactgcgaccatttttcggtaacctttcctggtatgatgtcagt
aaaaagcttaattataatacgattcgcttaccaaattcaaaaatgtgccgttagaagaatgatctttatagactgttagaacataaaagagcaaa
tccttgttccattcaagtccttaccgtgtagaaatataacgttatttcggttttcagtttcactagcatgaccctaccttgtttagcattgaacgcgaa
acacatacgaactgacgccaatatcctacctcgtaaatttcaaagtgaaaattgctaatcctaggacctcctataacaacttcacaggaacggtgt
cgaccaaatctccagaagccattgtgaatatctcgcgtaagaggtataacttgtgttttcccgtcgcccattcgagtcattaccaactttaaataga
cattttcagtttgtgttgccaattcagagggtttgtcggactcattcacgggagtcacttgtgtagcctgcgtagcatcatctacatcttccattgtgt
acagaaggaaacttttactaactcaaaaatcctcactttaaattgcaaaaaaaaacaaaaatttacaatgcgagaatgttcagaatctcctgatg
atgatatgatttttcaacgtggatgtcaaagatgaaccttggtctgaactgaccacatattgagatttactatatagagaactcctgttgcaaagc
tttatatacgataaggcatcctttaaaagtgtctaatatcacttaacgtgcttgtccacaaaccagcgatgttattaagtatcaagaaaagtagatt
tttcctatattcaagtatcctttttttcttcattgttatatattagtttctttttccaaacgaacttgttgttcgttagttgatggtttataattcttgcgaa
ctgcaaaccctttaaagagtagaattcagattgtaacactcattgctttttcatagtaaaaatcgcttgcttttatgttaattataggattgacagga
tccattgctactggtaatgataaaattgacttcaaaaaattattaatatggagtgtgtttagggaagacaacggtttccaagcagtttcatgagaa
ataccatgttccggtaattgacgctgatattcttgcaagaaaagctgttgagccgggtacgaaatgtctacggcaaattcgaaaagaatttggcca
acaggttattctccctgacggtacactgaatcgacctgaactaggcaagattatttttgagactccttcaaagcgtaaacttttgaattcgattgttc
atcctgccgttaggaaggccatgctgaaagacatactttattattacataactggccatggcatggtcatactcgatattccactattgtttgaagcc
aaaatgcacactatatgttggaaaaccatttgcgttgcttctacgacagaaatacaaaaacagcgattgttgaagcgaaatcctgaatattcttct
gaagacgccgaccggcgcattcaatctcaaacacccattcaagacaaggagacccttgctgactttgtcattgataataactcagatttcaactct
ctttctcaaaatattcatgcaacctttgttaaaatgcaaccttcctccatgtggaccctaagctgccttataatacctgccttgcaacttcttatgcaa
ttctattattattatcgccaaatgcaaaaggcaacgaaccagaagaaaatccatttaacccaataaacatagaccaaaccaaactcgatcacta
agaattcaactcccttaaacctgggttgtttctagtgccgagcgagcgtgacttgataggacattaggcgtaaataaaatatgtatgcagcttccat
cttgtatggagaacttgtagacagtaaagaatgatacagctaaaaaaatgattacctagatttctaaatctttttaagctcattttgatatttcttttt
ttttattcattagtcttcgttttatatttaaaaggcataaacgatagtttatattctgtctctaaaaggcatacatctctccctacccttttccaacacc
aattagtagacggtcttatccagccaaattttggcaaaatggatgcttctacgattgatttagatgctggttcgtgtctcattttataattgtatacta
atttcttcagaaatccggaatttggggaaaattctcattgagcatcctgctcatgtggaaaaagttcttcacactttagagttggataattcggctct
acggtacattgatgcgattctaaacagccatataaagcaactgtatcgtaatttatcctctaattctcctctacatactctctcaaccttgagagcaa
tggcctcatggaacaatggggttgcttgtagtcgagttttccaagcctttgactggagtagtaaagtgtttacgaagatgttgcaactttctaatcaa
atgaactcttcaaaacgaaacgtcaaacgtatgcgttttgcgtcttccaagaaacgttcctctttcatcttcctgttaattacattgattcgatacggt
actcaagaggtgcgcacggagctcatcaacacaaaaaatataactactcccttatttaaaggaatctgtaacgatgaaccgattgttttgaaaga
tcttttacagacttggcttacatacataatcttagatcgtaatgtgaatagagctacaaaaattttctattttaatgagtggtctttgaactgcattct
aagtctatattcgcgagaagaagaaatcaatggggttcctttgtacgagcttgttcacaaatttttttcttcgggcttgcactgttcctggagaaggtc
tttgtttttttctcaatgggatggcacgctcctctgaaatttgaagctaataagagcgtttataaccgcgttctccagtcccttctgccttcactacatat
atacaccgatcctttgcagcgggagctttgtttaaaaatattgagtgcatgtcctgatatatctgcctccttttttggaaagaaatagcttgaatactg
aggcgacactaagctctcaatggatttgcttcacctccttccttcaaacttttctaaacatggacttgcctgaaccctttttttcatcctgaaaccaagt
ccactccttctattcatagtattgttgagaatgttttaccggcatcgttttcaaagcctttaatgttgaaagctttgaatagttcagataaacttgtctg
ctacctaggcttgaatactttattatcggccttcaagagattaaaagcagttttggcgactattgacaaatctgaattaagttctgaacgaaaaatc
tcgttaactgcattaattactgatgttatctccaatcgccttcctgatgcgcaattattcattcagctttacacgacaagtaagcaagaactgttgac
agatgctgtttcgaaagttattctatatttttatcttttttcccagaaactgtcttgaaactcaaaatcagcagcaatgttttcttgggcattgacttct
catcattttctatcgatcgtcctttctcccgttctcacttacagagcattgttcaaatcctacagtttctacctgatattaagtggtttgagctgtccaat
tccccgtttattttcctgacttctctttacctaaggtctgagtactctcagttgaaggatgctgtcgttaaagttctggaagatgctttagccgaaact
gatattgtctcgccatgtcttttagtatctcccattatacctatgttacaagctttggaagctatacctggtgaacatgttgactctgtcctccagtacg

ccaatgcatgcataaaccgttgtacatctcgtgtttatgcatatttagatgcatccataagctattgcaaggaatcactgttagattatcaagatgg
aatggtcccgccgttttctccgatttcgatagtgcttgttgagcaggtaccatttctcctgaaagacacctcggttagtcaagatgctaagttatgta
ttttaagttggattttcaaatccttaattttttcggctgctgtggtgagtctttcgaatgcctctcgaaattatttaaaggattaattagtgttatggct
gactatgactttgctgcaagccataccaatgcttttagccaaaccatcgagaagttggcagggaataaggaattggaagagtcattaccaaattt
agacttgctatcatcgcttttgttgactaacgaggattcattttggaaaatcgtttcgactcagttttctattcagccaaggtttgctcttctttattttt
ctattctgctaaagaaacttgctattttgttgatcaacggtaaaccagacatgaagcctcttctcggattactttcagaaaatgtacaattatttttgg
atttagtggatgatcatttatacttaaatctgaaaaatattttttctagtcctgtaaacttacaaagttttgtcatgcatcctgattacactgaatttaa
tttgcagtatgccgatcttctttatgtaaccgatgcgtttaatactgaaggctacattagcaaattctcctttcaagaaacgaaagatctattcaatc
atccacgagaagacgatctatggaaaatgcctctccttgctaagtatatggagctgtggtctcacgatcaaattggcagtgtctgccaaactatcg
acttgttcatccaagagcacactctgttcgtcaatcaacactgctgcagcatttcaccagtgctgtcaaagtatattagttatgtatcgacatgtaaa
caagaattgtctgagcaaacttttgaggtgctgctcaaaaattctttgacgcaattcacggaattatcgatatctttaagtgaacatatttgccagtt
gaaggaacgttttagtgaagcttgcatgataaaggcttcagagacattagaatctgtattacttgagggttgtgacgatataagagtaaatattat
atgctctcttataaacacgtcatctgaaattgcgtctaggttatggaagcttttcctttctaacagagcgctcggtagtcaactctgtaaagaatata
tcgggttatatagcgttttggctcagaaagttgataccaatcatgttcctttgaacgaagttgaagaatggtgtacgtttatcctcgaaatgaatag
agaatacattctaaataatttatcgatccttgctattgcgttccctatggtttcgttgggcagggtgaaaccctccttgattgacgaaactatgtcta
agctatcggaggattacctaccgtatgaaatttttgaactcttgagaattattacttcaaagaacgaagtctcttcatcaaaagtcgccaacgtatg
ctctgatattttattgttgttgactcgtgtcttttcaagtaatgatggattggacaagaatgtgtcttcatttgttgattactttggtgtttatttggttga
caattcagaaagtgtgaaaaacgtaattagcaaaacagttatagacaactttttggaatcaactctaagacatcttcagtgtaagtcaatattgga
attcaatcttaaagtcttgttttctgtacaaacaggacaatgcaattcctcaaagattttgcaaatgatattggcacatgagggcaatcctttgttgt
tggaggattcttctaatgacaataagtcacttattgctgctaccatttgtaagcttacgataatgggcaataactactcattatcgattatggatcag
attattcgcttattcaaaggtcgatacacactccatgactcaatcctctgcgagcttctgcgatatatggaagaaagacttaacgttagcatcgcca
gtcgttacaaaggttgggttatcacgaatgatgaggacgtaataacaatgtctataaaggatagatcttcggttttagagccacgagtggctttgg
aaagtattcatcattatccttcagattatattccgattcaactggaatcgttacaaaatttggaaatatgcgaaaaacttttgaaggcatcagaattt
gaatggaatgaaaaaatttatcatccactttttgcattgggtgttgttggagcattatatcatacaaatccacaagtctctaatatgcaagttatatt
gcagcaacactttttggattaatattaatgggtctttcactgcataatgaacagttacgagctgctagttttcatttcattcagcatttccttttggga
ttagaagatcaaaagcttcgtgaaaggcacctcgttaagctagtactctcaagtgtgattgttaatgtttcttctccaagtcagagattaagcacat
taatggcgaatttttatgctcagagtattagtataattatgcggccatcccatcacatgtttttaggtatcaatcggtatttttgcaaagacaacac
attgatactgcggatatccctatgttttatgaattattataccctcttccgattacacaaagtgtctttcttggttattgtcattgttactggcaggctt
acagaaaggtgaagatctaaagctataccttcgtcgtcatgtattcgaaacaatatctacattgtatgaatcttgtgtggtgaataaagagatacg
agaagccatttcgaaaatccttttaagggtagaggcattaggtgctggtggtatcttaaaaaagcataatgctgtgtactccttactgcagttactt
ccgcagaatgaaaatacgctacaatcgaaatcactaaacatagcagaagcctaaaaaggaaaggaaaaatctaagtcgttttgttatgggaat
ccggttacgtacaaataatagaacttgaaaaaaaattttgcttactcaataaaagaataaaaggatatggaaggatttattatagaactacaaac
gtggataaaattcatgaaaccatagagaaatcgatttggaagaaactagtgtatgcgcttaattctatcattataaaagtaacaaataaggccaa
aaggaagacagggaagtttgagagaaggaaaagaaggaaggatcatagaagtgtagaaagctttttaaagaccgtaacgagcagccatttcg
ttttgaaaagtcatttccattggggcttggctagtgtatatatcagtttcgtctaagcaagcttcacggttggcaaggatatctgcatgaaagttact
aggaccacccaaagccataggtcggacgctctaaataaacaagttagcaaaacaagttttgttcctagaagcaaacaacgattccataaattct
gcaaaaaaatttccaagtttattaaagttaaacgtaagttttaagaacattcttcttagaaattctggattcaatcactaggattcaatcactatttt
atttacttacctgagtcgacaagcgcaattccatttcacgacgaacgggctcgtgcaaaccatagatacgacgcatactatccaatcgaacttgtc
tttgttgagcttcccagttggcaaggcgattttccagaggatgtttattttctgattcgttaactgtagggccgtgagggctcaaaacagacagtttg
taatctccagaaacattggagggcacatttgattcaggaacaatctgcatggttgaattcttaagttacagaatattctttgaacagactcgatggt
ggtggttattggagaaattcacagtacgcttcgctttcgagtcttcagaagggcttgtcacttgttgatgatgtctgttacttggttcgttgagcatta
cccttcctgtacaagaacgcgaaaaggtaccttgacacaaacagataaggaggaacttggccacaaccaaaatagagacgaattttaaagtag
agtatttggaaaagaaaggggaaaggagacatcataatgtctcaagtgattcagtattctgaagcggatttgctgtatctgtctaaatcgccgctt
ataaaaaaaccagaaaatttaccagaatggatactgtatgtagttttgtgtaatatttttgagaatcaatatgctataaatataaaatgcatcttttc
cgggctttattaacacatgtttagtccagaggctatgaaagcggatcgtgagcgacatattcgtcaggagaaggaaatgaaaaggtatgaaggg
attttagggtcgtgcaaatattgaattgggatttgctttccgtaagattgaatgcagtaacaactaacttaagttttcgcagtaaacatgatgatga
gggaagacaaatgcaagcagaacgaaaatttgccaaatcaagtaagttttatctttttttaggatggtgtggtgaagagggatgatgatcttttcca
cggtgtctgaatgaaccatcctttttctttttcataaaggtgggacaagttaaactaacccgtagaacacgaagatatcatccttgggcctccaaaa

ctcagttttgcaagcagcaatgaaaatggccgctctatcggtcgtcatgatgatttattatctttaggaaacgtttatagtggatctacatcagtgcg
ccatcgaaatggaatgggagggcctgcatcaaaaaatggtgctttaaacaatatcgggtcgacgtcatcggcttggacaagtctaaacaagcat
ggtcgtaaaaaggattttgatcgtcgagacgataatgaaggtttagagcggttaacgaaacaaagagccagtgctagtgctgcaagtcctttgat
ccccaattccactgaagaatttgaagcatggaagcgtatgatgaaagcttcaacgaattcagttgagtctccaatcacttcgggaatgccagctgt
tgcccacgtggcatcacaaaagcctgttccttccctttcgagggctgcttctggttcaggatcttcgctttccgctcgtggaaatgttagcgttgactc
tctttttggaaagcgctcggctgccccgtctgttgctaccgtcgcacctcccgttggaactcctggtggtacgcccccgcggtttgcctctcctgctct
tggattggaaacagcagctcatgttgctcctcctactacttcatcaagattcagcaaattcttcaatggactgagtggtccggaagtatcaaaaca
accgacgccatcagcatcccacacggcttctccgaaggttaataatgctcctgctgttccttctgctgcccttgataaagatgcagaggggttccaa
cgtgtccttgcaatgctaggacgtcaaacacctagtgcatctgcctctcctagagttcccgtgactcagccatctgctactcctattcctcctggtgct
aataatgctaatccagttgccatgtctacaatgtcttctcttgcttctcctactgcttccgttgctcctcctccgggattgggcggtcatgctctttctaa
tgaagatgcttcgtttttccgttctcttattcattctgataccaggcccattcctccaccaccgggattctccacaaatgctatgagttctatgaattcc
cctgacgaatttgtctccaatcctcctggtttttacaatcactatccagtgggacccgcagcacctataaactctgtaccagcccccgctcctcctcc
tggttttgggtttcaacagcctccacttccatattcaccaggattttctcctaacatgtatggtcaatcacgtatgtcaggccatcctctccctgacctt
tcaagaacttctcactgaacctttgctttttaagacaaagaaagtttgtaagagttagatagtaatgaagtcgcaaagaaaggttttatatggatat
tatgaaggtattttagggaattattaaagcaatgaaatctgcacatgtcttgaagtatgataatatctagtagagaactactggtttccttaataag
tggttggtatatctgttttcaccaaaggattacttatttgcagagataaacaaataaatgtagaataatttaatgttattgaagcgtacttgagctaa
gcatgtgggaaagtaatctccatataaacgtgcacaaggaagttgtgacattgtaacatatcgaaggtttctttacatttcttacgttgttttcgttta
taattgtaagaagaagtcaaggaaggaatatgactatggatagttgtcggcgaccacatatttctatttcttatcattatcgtactaagcagtctttt
tctgttgaaaatcttagaaataagctacagattgaaacgtcgtgataatattatatttctacatggtatatttagctacttactagaatttggagaat
tatatatagttcttagatcccggatcttgtacgtaatcaagccttgatatttctttctatttggaagtgtacattgatttaccacgtagatttcaactga
agtttaacccttcgtttcgatgaaaactgtatggaaagctactgattccacactttgtttactagtgtactccaaggcaaaatgcgactaacgttgta
gtaatctggtaacgaaacgtaagcgattcggctactttgtgtatgtttacttgagttccatcaagagacgctacctacacttagattagatgcaaaa
tccacctcaaacggaaccaatccatcatgttcagtggttctatttatccaattttgacctctacgatgaaacatcatgcatggaggcgcctgagccg
cagcgctggcgtagatttttgaatcatgacaatcgcgcgttggaagcaaagcaccaggaaatcaacgatgcgaatgtttcgcaagaacctaatc
aaaaaaatgtaagaaaaggtagatgcaacgtttgctgactattaggtaattgtgggagtatcaggtctacatgttgtgaatttaccaacactaac
attgaaacccttgtattggcctagtagccgtagaaacgacactcttcgtgttgtccgcgggttttggatgtatgaggatactggattgccagtgatc
gatgagttggccgaaattttggaagaaggatatcagcggattaagccatataacataaattgggacaagttagaaaatgatatagtagatgaaa
accaagagagatcgagtatttttttcttctggaagacgaaagtcttctaaggacacgataagccatttgcaatgggcattgaagcctcattcttctgg
aaagtatgtcttatatgaagatgggtatcgagcactcttatgccagggaggattgctttcttatttctcgaaaggaacgcagtctcagcgcactggt
ctgaaaggaattccggtaattcgagacttccccgtgaaagaagacgatggcttccgcggtttaaagcaggtaactgatctctttttggttgtccatg
gaattggtcagaagagatctgaaactggtaagacaatgcttgtttacaatgcgtactgtttactaatgatttattttcgcaaaagaggaacgctatc
aatttactaaaacgtgtaatgtgtttcgatcgttaattcaaaagcaaaaactaattatgaaacaaaatcctttgttacgagatgattatgaacctca
attgctgcccatctgctggaggaacaagattaattttgactcgttcttccaggttactgaggacgacgaagaaagcgacgatgttgatggtaaccg
attccacattgaagatattgaaattgatagcattccaactgttcgtcgactttttggggatgtgatgagtgatatcccttactatatgtctcaccataa
agattccatcattcgctctgtcattcgagaagcgaatcgagtctacgatttatggatagattgtaatccttattttaaagaaaataaaggaaggatc
tttattattgggcattctttaggtgctaccgttgtttttgatattttatcacttcaacctacgttcgtaaaggagcttggccccgaaatagaagacgat
aaatattttaagtttgacacaaatggatttttctgttttggtgggcctataggatttttccttttactcaatcaaaaaagtatgattcccaggagagga
cgcgggtctagccattttgctgataatgcaaactatattaacaaaaatgtttcggattcttacgcatattccggaaacgaccgctacgggtgtttgg
cagtggattccttttacaacatttacaactttctcgatcgtatgtgtcttcattttaccattactaaccttgaaacagctatcgccatgcgtttaaatcc
tacagtcgacgtgaattattcgaaaggtatccatccctctaaactcgtttttcttcgaaaaccttccagtatcttaagagtcgtttctcggccaaatg
atgcacctgttcgactagttagttatcaacaggcactcaatcagtcaaactctgaagataatgaaagctcttctgtattgatggattcagaacggg
atatagaactcgaaacgcgaaatttcaagcaagaaaaacgagcaaaatggagaatgcaagagttaaatgagaatgcccaaattgactacgttt
ttacaagtcagaaaggtatcatttcgaacaaatatttaacgatgttatccgcgcataccagttactggaataatgaagacttggtctgtttcttagtt
gtcgaaacagccaggaaatttggaatcgctaattccattgaacaatttcgaggaaagcgtgtatctcacgataatgcttctacattgtaacaccga
tgacacttataatggctgatgatgttttcaaatttacatcttgcttttcttctagcgatgggagatctaagaatgaggcattacttactattgaattgtt
taacgagttataagttttcaatcactcaggatatatttttgatgatgtttttttcaagctacatttatccctatgtcaccctcaaatcaaggatgagact
tttctaaatttgaaaaatgtttgctaaagtgcacagatatttattacctaaattgtatacgactagatttaatttatgagataattaatttgacaatca
accatatgaaaattggactgtgctttgaaatgttgattcaaaattcagagaaacaaaaaaattttaagtaaaattataataaaggtagctttgatgc

agtgtttgaaacgaaaggaagtcttcgtcaatttaggatacgcttatttagttgtgatataccaaagtttacaacactgttgctttttacaaaattca
aggttatgagcggttatgagaatgcagatgagttacttgatcttttaagaaagcaaactactggcttagttcatttggaagattatcgacgggtaa
aacaaggaattgctgaaaaaaaagaaaaggatgcactttcacagacaaatcaaaagatgtatgagcaaaagcccaaataatattatttggaaa
aggttaaaatagtcctaacatagtctagcaaaaagaaaaaaggtgttttgagtaaaaatgtgaagaagcagttgcaactaaataagggcaaatt
gtcatttggacaggaagaaactgaagaagaaacagacgatagaattcaagaaataactttaaaaaagaaacatattggaaaggatcctactgc
taatacctcgtttttaccagacgctgaaagggaagctttagaggaagcaaggaaagaagaataccggaaacaatggttagaagaacaggaga
gaatcaaggaggaagaaattttgattccttttctatactatgatggtaccactactcgatatagtgttaggattcgcctgaaagattcggtaggtca
catactgtctaatatgaaagatcaaatcccaggtctaaaacgcgtcttggatatggataaattccttttagttcaatctgatttgattatccctcatgt
acgtcaatttgactagaggacgacttgttaacttttacatttagcatcacgagttgtattacttttatctgaacaaagtccagggcccaaacggagc
tctgtttgactttgacgctttgcgatgccaaaattcagaggtggcagcaactctgcagttgccagaagtacgtgtatatgagttttgaacgtgatca
ttgaattgttttggctaattttttttttacagaggaagatccctcatttagttcttaagtcgttttatatggaatatagacatgtctttccgtgcaattgtt
gggagatgtttgactctaggaaaaacgtaagatgaatgacttctttttggacgatttaactaatacatttagtattctcagcaaagaaatgaagtc
aacccaaatgctgcattattctacagaacaggttgacaaagttgacgaaaaaactagaagatgatgtccgttctaattatataagtaattatttatt
gctattcctttatgctttttggatgaaacttgaattgttgaaggataggtgaaatcaaaaatatctgaagttaattatacaaccactgcagcaaata
aagatatgaaatgaatgacaccaaagtatttgaatagagaatttttaaactttggcaagttcttttcactgctttgtggattctcatatggataagca
tgtatatatatccatgtgattgaaggacctcgaggtgaatgaatcttcagtttgtatcatgtctactctttgaggaaagcattttacttgttcttaattg
gagctctctccttgatttttcagaagaaaaaaaaaaaagttaaagccaacaaaagtcagaaagcgtccattagctttcatgataacttaatacaaa
acctttgctacgttagaagcaaaaatatatgcaaacattgatcaattagagattagacatcaagaatgtacttacttttagaacaattgcaataag
agcatgcttcatcgtttgcaatctttgaatggaatctttacgaatctcttcaattttaaaacttagatgcttgttaaaccaaccttatgctgtttttgac
ccttagcaacaaatctaaacaaatatcatattgtctgaaatgctataccgatatttttaagacaatcgagtacaaatcaagctacttactatagaa
cgataccagtttggccaaagtaacctattccattccagctggttgagatgcagaaaaacatccatccgctcgtcgctgaaaatgcggctcataatc
aaagggtatgttggctatataatttcttctatgctaattctcatttcatgcaggtggtatcattcattcgaaatatgacattaagcgtttttggctgttc
tgcgggcattctaggcttaacttcttattctggcgttctgttttacctcttcagctatctattcgtctcgtttctaatatatgtgattaagatgggtagta
aaattggtgagtattaccaaccagcttccaagttttggtatgcaaaggtattcgagggtattccttcatttgtcctgttttggactcttttctacagcct
cgtttacggtatgtttttcggctgatgcgaatgaatggttacaaattccatgtcctaaacatggatatcctattgattctctatcactcaattgattga
gttaatcttcatgctaaccatgaattagtttatgaatagagttattctgaagaaattaatacgcaaattctttatgatgggacccttgaatactttcta
tcttcactctttatcggacgtctgatatagtttgtacggtcaatccattcattgaaacgacatttctgcattttgaaaggtacttctgttctttatttaca
tatcttcacctttcttttcctaagaaactcaatccctttctaaaaaactatgatgaatatgaaatactttgtcgaaattcataaatccatctactacgt
tttgcccacagatgaagtaaatgaagcgcaaatcataaataatattcaaagaacatgaaaatgtatctgatttttcttaaccagatagatttataa
ataacattatcttaaagtattaacccacacaagacttcttttacgtttttataataaaaaaattgaaaagacaataaagtggatcgataaaaatcc
attaacttaaatagcataatgattattaataatgtcccatccgaaatagatggtcataaacagaagaaaaaaaaaagcaacagaaaaaaaaag
ggacttaacagaacgcggtaaattacatataataaatacctcttaagcaaccattcaacccgtttttcagttatgtgagtgcttaccgtttgacatg
aggatatggagagccttcgggagacacaaggttatgaagatgggtaggagaacgaatcactttgttattttccatatagcacatggaaatgcttg
caacccactcatcagcgaacacttcatggacatcatatcttttaatcggatccaactctaacatatgcaacacgactgcacgagtatcgcggggca
ataaacgcagcaacctccatggtccgtacacttcttctttgccgctgataaccgcatggtgttcagatgcgcgttgctgtacatctggcgttttatag
gtcggaggacccacgtcgactaatccactctctttaatttctttcaaagcttcatcctcagaaaaatttccttgtgtaggaggtcgcatgcaaaata
aacggaaggaattatcggttagttttgggtatttccacgggaatctacgaagcgccatacagcaaaagataatcgcacaagaccatatatcaac
agaacgaggatcgtacaatttttttggttaatgtttctggagcgagatacggatcagaaccaaccacaccagtcgcctccacaatatccgcttcgaa
tgggtatttaaataccacagcacttccaaaatcaatgattttcacgttacaattttcatcaacaacgagattgtcaagctttaaatcacgatgggca
agtcccatggaatgcaaataagcgacaccagaaagaagttgcttaaaacagcaaaagatctcaggcattgtcatttgtccactcatgacaatcg
aaaacatatcgtacggtgcatattccataacctcataaaatttcttattttcttctataatatccagagtttcaatgatatttgtatggtgaagagcac
tgccaacacaaaattcagcagtaacctttctggcatactcacgctccgattcggatggtctgcgagaacgaaattccttgacggcaaatatctttcc
atcagaagaacgtttcataatgcgaaccgaaccaccagcacctgagcctaagatgcggccaaatttgccatacttcttaaacaaaccgaaatgat
cgtcttcgatattactgcttgatttcgacttggaaacgcttttcttatcagacttttggtgagatttgaaaaaacgacgtaaatctaatgaactgtggt
gtttttcatgcttgtcatgtttatcgtgtttgtcatgtttatcgtgcttgtcatgcgaatgcagtaacgatttcagagacagcgagctcgagctgttgtt
gtggtgattatggtggtgatgatgatgatgcttgtggtctggtttttttgaaaataaactgggacggaatgtcgcgaggatcacttttggtcggggct
ctacgggttcgacgataaggatcattgggatcaccaattaaatcctctgaagatggaaattcatgagcaactttgagataactgggagaagaag
gtttgtactcatgatgattttcatcggaatcatcttcattactcggtgataacaaaccggacctagaaagagaaggaaggaagagggggctatttc

cagggcgaaagacagctgaggactgagaggacgtatggtcgttctcatccgcctcatcgtctgaaggaatttgcgagttgggaattgaaatcggt
tcaaagtttttgcggaggaggaccggtaacggtcttcggcaagtttaggatcaatgtggtcttttacttgtataggagactgaacacggggcaaa
ggatgattaggctcatgagagttttcaacagagttttcaacatcttcgaggccaaatggtgcagtctcgctttgaattgaagactttgggatgtcctt
tacttgaaccttatcttggaacgatttttctaaaggggaagattcgtctaagaagggttcgttagtataaccagaattaaatatacaaggaaaaaa
acgtaccggaatgttcatcctgaagagagaatttcggagcagaccgtatatcagacatattgtagacagcgatcatatatgagtggcagaaaat
aattactgcaaggaatcaattgcaacaaattataagtcgcgattcagagaaggggaaaaagtaatctaaggagagagggaaaatcaaaaaaa
aagtcaaaatagaaaaaatgaaataaataaacaaaaagtgtttttacggtcaagaacttcaaacacaaggtatcaacaaatgaaacaaggaa
aaacaaaaagaaacagaaccaggtgtgcaaagataccgaatctaaatagtgcacataaacgtaaccgtttttacaagtcctgatttgttttgaa
tgaaatcgccaaaacgtaaacaaaggtataagcaattgaagacgtcaaaggttcccctgaaagggagggattattaaagtaataaacgccgttc
aattcaatctattgtctaacgatcctgagtttttatgaggatacagggatttaccctatttctactacgagaggaagagtttgggaaaaggtaaatt
cagttaagtagcaatcttaataaatatatgatccggtgcggtagctattgagtttgatgaatgacgatcaacataacatatgcggccattgaacga
atgatgttgctcgtttaatgagtgctaccgaagtccggaattgaattcagccatcagccggcaattgagaatacaaatcggcaaatgaaaggtaa
caaaaccaatcaatcccacatcatacaaatcttttcattaagtaaacagttggtgtgtttacatgaaaaaagaaatgaatgaataatacaaggaa
ctttcatcaaaagggaatttgggcgggaaaaaatatataatgaattgatgtttgctttttaaatatttctcagcgagtgctgctgcattaacgtcaat
ggaagggaaacaaaaaaaataatgcgcttcattaaagttcgaaaagggttcagaaaaaggtagaaacctttttgagatgatttgtttatatttttt
tgcaacattcgccagcgagctttatcttttaagttcgctcatcctttttgtggagttcggtaaccgggtgcaacttcggcaattgacggtacattgatg
gtaaataataccgagacaattgggtttgccgtttgaagggatagaaaacatgggggagggggcggggaaaatgatggaaagcaattataattc
attgtttatttacaaaagtaggaaaaaagaaaatttcaatcaaaccgttttcatcaagatccattcatagataattgaataaatataaggagaaat
gaaagtatttattaaaacaattaaccattctttaacaatttgtaaaaagccattctttttcgtttacctattatacccatatagatacattcgagtcat
tccttattctaaaattcccaaaaccttaaaacatcctatgctatctaagaatttctcataattcaagcagcaatctctgaaacaggtgcaggcgccg
gggtcgttcctggctgacctttatttttctgtttccggacaaatcgaacatcttcgggactaaacccaccattaccataataggcaaagtctggggt
aggagctacgtaatctaaaggatcaaaatctttttttcgaacgccataactcaaaaaacttgggatagtcaatgtgcgtgtaccatacattgtccttt
ttaaatttagtttgcgcaaccaataaactgcagctgtgagcatgctcagcggcaatttcatacttgacacctaaatctcccaagtacttatctatgtt
ttcaattaacttctttgtgaagtcaataacttcttcgtagtatggtacattcttcatcgttaacggcgactggtccgaatttccactgtaagtcactcct
ttaatctcaataaaacaaggaatgcccaaacgaataagttcagtatattctttaatttgctccatgttaaatcctttcactaaagtcattcgatatac
cgtcctttgcttcttttcacgcaaaatctccaaacacgtcagcaaccgctcccaaaagtctttaaacaagggtcgatcgactgacttaagactttgtt
ttgtgctggcgtcaattgaaacatataattgagtcaccattcccatcttctggaatgcttctgggtgctgagcattcgtaacaaggaatgaagagat
ctctcgttcgtgtagcattcccacaaattcattgatgtatgggtaaaagataggttcaccgaccaatgacaatgcacaatgtctcacacgagaagc
ttccgcaaaacgatcctggcggactccaggtacacctcgcattaatttcaattttgagtaatgtgactctaggattcccttcaaaattaactctgga
ggatctactttccagcgccactccgtaccaactggattcgtgccgtggcgccaacaaaaagtacacttgttggcacaagctaaactaggcgtagct
tccatacacaaatgactacggatgccatagaagctgtatttatagcaaaaacctcgtccacgaatagcagatttcgtccagcgacaaattttaac
accactatgagagccaacaacactgtaaccctgctttgtcagtgctttgtaagttggagatacggtagaaaccatttccttggtgccaatgggcaa
tgaagcttctgtcttggctttggccatcatcggacccatatcttcaacatccattacgtctgaattaccttctccttcatccatagggcgcaacaatgt
tccttttctcatatgttcaatgacagaatttgtccattgagttaagacgtcaatttgagttgcagaagctaattgtgtattcataatacccataggag
caattcgttgggcaccaagcttgcttaaccatttatcggcttcaatggcttgatagcaaaacatatcaccaggataattgtccatgtcaccgagtcc
aaacacggcatacccagacaattttgtaatggatttttctgaacacggaaatcataaacagtctcacgtaaagtagacaagtagaaatctaaac
tactttcaatggcataagagggcaaaacaagaagatagacggcattctctgggcaataaaggaaatattcttccaaatcgatatagtccaaactc
aacaattgcaaatcatctctttgaagattcgtcgataacaacttgtgtacttcattagcatatctctgagctgttcctcctaaagtggcatagaagat
gcaaattggcgaattttgcaaatcaatctcgttctgagacttcgaagttgactgaacaggtctagtcttcatattctttctagtaaacctctttgtcgt
tttcttttgctttccattctctttttcatttaacacaattccttcagctggaacggccaattgcctcttatttcgcgatataattagatgagctgcaacgg
gcaaaaggaggactaaataaaaaactaaacctctgtaagtcccccatttttcttccaaaaacgcaagatcaaaacacatgttggcgtttctttcta
acgtctctggtagttggaagaaggaaaagttcaataagcctgtaagagggtaccgaccaaagcaaacttcttgccaatcagaggcaagaaaaa
tcggatcttctaaatgaaaaattgagttttagagctattgttagtagttatcaagtagttgttttctgtgcttttgttatggataaaattttacttttctg
gatttatgcattttcctttagtacaatatgtaattatcagattgccatgaagtattgttgattttagctggctttattcctgttcaaggatgacacatga
ttttatagaaaaacagaattgagaaaaaaaaaataaaaagaaaatcatttcattggaactttgcatcattatccaaaagtggtcttcacagacgta
aatgtacagttttagagttgagaaaacaagtctaactatttttcaggacttacaataatgaaagaaagaaagccatataaaaagatagatttaca
atttgcgcaagtctatttccctgagggaagaacacttgatgactcccttgtcggcagcttgctcaggagacaaagagaaaaaaggaagaataga
aggatcaggaacccaaattactctcatatcagcagctcttcctgattcaaggcctagaatggagtcttcaaaaacaagacaatccttagcctcaat

ctctggttgtccctttactctgcgcttttcattaatgattttcaaagcgataaaccagatatcgggattcggctttccgcggccaggaagaagacgat
cgtcatctcccgttacaatgttattttcaaatagttgaaacatttctcctagatgcgccgttttctttacaaagttatgagtatcactagaggtcgcga
gcgctatagggatgtcaagcaacttgcattggtgtaaaagctccaatgctccaggcaaaggccgagtttgtttccaaagctcagattgcaattcac
gctgcatctggatgtactgatcgctagatatgtctaactgggaccatttcaaaaaaatttgacaagcttctcgagaaggacggcctttttccatac
gattgttagctgtgtcaaatatacaacgtacacatacccatcatttgtgctttgatttccataggaaaagaacctttttggtatcgttccaatatgatg
tcttgtcgtaaagattagtttcagcaagaaaggagtggaaacgagaaaggcatacttgtgctttttgtataaatggtttccgtgtctaataaaaga
ccctagtccatgttaatatctcttgctcaatcaaccaatttgtaggccagtgtttgaataaggacgtgagtttaattaactttaaaaaaaaaagtatt
ctaggtaataaaaaaaaaaagaaaaggaaaaaaaggcacgatatatcacgtttctagcacaggaagaaaacttacatccatatcgaaaaggc
aagctttaggaggcatcttgatgtttgaaaagattatagattgaattcagtgggacaaaagacagagaaacaaatgaaaaagttgtctattttgg
atattttcaattttataagatttgcgtttgtgggttggtcaagatacaaaactttggatagctcaggtccttaaggcaacaaaaccaagatttgagt
atcattcaaggcttctttatgaaacattagaggataaaaagaactatggcaggattcaactttggtttgctagaagggactttttctgtgaattgaa
tcctttgaatttccaaaaaaaaaaaaagaaaagagtaaacaaaaaatatatatacaacaataataataataacaattcttttcctttataga
aaaatcgaaagaaacaatgtgcgttgttaataaatttatagatttttggtttctaggtcgttgtaaacaatcacttttgtttacatgtgatccatcct
aagaatatccttatttctgcgtccaaaggattcatatagacaataatttgttaaaattagagtgaatttctactgagaaatccttttatccttcctttc
cactatttatgtcagcttgaacatataaacatcaaaccgccttgatcctagtaatgaacaacacgatgtagcaaagaattctaatacattttcctgt
taagtgtagagaattcaaaccccataaggttcctttagacaacttttaccctaaaggcaagtatttctcgaactttgtaatctgattaactgataaa
actgatcagacaaacgtgtgtatgtcacaggaagaattctgtggagtatgaagcgaaatttataagtaaagtatttaatgcaaagttgatgttaat
cagatcatcattcgtttacatgaaacgaacaaagcaccgatgcctgaaaagcaatattttctttcgtattcaactggcctaatgttacatataaaaa
aaataaagaattacttatagaaacgaaaatctttacccttagtctctatgctagctagcggttattaaaactattccaacatatctttgggattgtta
tcctactaaaaaaagtatcgcacaattttcgctctatgactttttgttctttaaatttgtttttttttttttacaatcttctcatcttttgctttccatgaaa
gcagatccaaacgatgctttactaaccttctatacccaactagcaaatacataaggaaggacaagggggatatgcatgttctggaacaaataaa
tggttggtaatcacgtttcctttctttttttttttttcttatcttctttccttctcgcgcgaacgggtttggagtaaatttaacataagctccttcaccctc
cttgaaatccataagctatatattttccagacgcgtacgcatccaacgtttaccacacacgtatcactatacccgaaggtagaattatctgtaagta
actgctggttttatcatatttttcttttgtttttttgttgttttaatttcacagcaaatgaagatgatgttaattgtgccttcagtaaactaaccaagact
atgtagtttatattagtcagcaagcatgtcttcttctttagaacaactcaagagtaccggaactgttgtcgtttccgacactggtgattttgaatcgat
tgccaaatacaagcctcaagatgctactaccaatccctctttgatcttggccgcctccaagaagccccaatatgctgcccttgtcgatgccgccgtc
gaatacgccaagaagcaaggcggctctctcgaagaccaagtcgagaatgcttttgatcgccttctcattgagtttggtactaaaatccttgctatc
gttcctggccgtgtttccaccgaagttgatgctcgcttatctttcgatacccaaactaccattcaaaaggctcgtcaattgattaagctctacgaaga
agaaggtatcagccgtgagcgtattttgatcaaggttgcttccacttatgaaggtatccaagctgccaagcaattggagcaagaaggcatccact
gtaacttgacccttcttttctctttcgttcaagctgtcgcttgtgccgaggctaatgtcactctcatttccccttttgttggccgtattctcgactttttca
aggccaagtacaaccgtgaatacgcttctcacgaagaccctggtgtccttagcgttagtcagatctacaactactacaagaagtacaactacaag
accattgtcatgggtgcttccttccgcaacatgggtgagattaaggagcttgctggtgtcgacttcttaaccatttctcctgctctcctcgagcagctc
aacaacagccaagagcctatccccaagaagcttgatgcctctcaagcccatggtcttgacatggaaaagatttcttatcttgatgacgagtccaa
gttccgcttcaacttcaacaatgatgaaatggccgtcgtcaaattggctactggtatcactgccttcgccaaggatgccgataccttcgtggtcttt
tgaaggaaaagcttcaagcttaagctgcttccttgttatgtctatttctttttcagaaattttatgtttccttagatttttcttcgatatagaaaataata
aactcagtatacctcaatttgattattcggttaataccggtctaaaaaatgattgtttacagtacaaatgaacacatgactttcctctgtttttaacct
atagctgatagtttatataacgttactctatcactgtcaaaaagcaaattatcgctgagtgaatctaataaataaaaaaccgaatgaaaatgggt
aaaaagaaagaagtgccacaatattcactacacatcaaaggtctgttcatcaaatatctttgacctatcttctggaggccattccaaacgcttcca
ggtgcgaattgtccgtttcgaagggtttaattcccatagtctggcacgtcgaatatatccgccatgtccaccgtaaccgccaaacccagtgcttcct
gccatacagaatcttaccttgtaagtcgtatgaattccacagaagtcatttacatgatcatgaccggctacagccacaggaatttgcaattcgctta
acgtccttgctgtccctggatcacaataggatgaggaacatttctctcgatatgatccgaccatgtcctccacttcgcaaaattcttttaatggtata
tgaaaaaataccatttgaataggatcgttttgaaattctgtctttttttgatcgcaaccaagcaagttggtctttcttgatagcatcatacacgggaca
agcccgaccatttgtagttcctgcatgtgagtctagtacgtatatagaaaatctttttgggcttcgcaaaacgaagttcccaaccccagaaatattt
cccattagtccgatatttccagggacctggtgaacaatttgagccatctcatcattagccaaatctcctaaactgtcatgatttccataagtaacag
cgaacggtatatcgtaatctactacggaagccaatgctttcataagagacgttttggaatcgtccaccgcgtcaccagtaatcaagtcccctgtaa
agaggacaagatctggagattctcgttttaaaatatccgtaacaaaagcatgagtcgttgcgtctgccatacagtctgtgggacgttctggaggat
acgaatctcgacattttgtacggtcagtcgcataatgcaagtctgataactgcaagatcttcaacgtttccctcctatcgtcaaagggcacatctac
aaatgcgggttcgttcttcttaagatcttcaatagctggaacaaggggatacgagcaaaatcgttgggtcagcaaaattggacgacctaggtaaa

gaaactgtgattgcactggactccatcctcttcgtacttcgctggcatcagtaccgaataaaccttccacatcacatacgtccccatttggaccgtat
tgaatatgaaggttatgtttctttcctttcatcaattgatggtcgatcattaaattttcgacggttggtgtcacaccagcaatatacctgctaggccttt
ttacgaacacgtacgcccgtttccaaggaagccatgatcgatacaaattatggctagagcgatcccatcgccaaaaggaaagtacaggtctgca
aaagggagagaaaagcccgcattcgacgatgcgaacatcctcaattggtgtttgaaggtaagctaactttgaattggaagatgtagggaattgc
gtcttaacaagccagtaatcaaatataagaaggcaaaagtaaagaagcacagcataaaccaagttcttgcgaatcaattgaaccgttcttgtta
aaatcctcatttttactaacgaggagattataacaaatatacctttaagaaagactaaaaatcgccctctctgcaacctctattgactgatctctgt
cccagctttcagttccttgcaaaatagccagtccgctgaatttctggtgtaagatagagatagaactatgaataaaaaccagtcgtagaaaacttc
ctttttcttggaatttgcatgaatacagtgaaaccactacatgtttgtttacgttcagtatttaaaaaaaaagaatagcgaactttcaagatgattaa
aaagttgttgtgtacaacgtttaatggtattgtgcagtacagcgctgtgccttattatggtaaaaatacaatgttacctgcaaataaacctgagaat
ttcaagaaacctttgcttctgcccattttcctttgattccgtaaatctgtatatttaaacaagtcaatatgtctgattacgaagaagatgaaggttttg
gtatggacggtgccgttatggaagaagaagtggatgaatttcaagtggcagacgaaaatggcgaacaacaaaatggcggtttaggaggagaat
ccactgcaatattgtcagaagacgttgccagtagccgtgttggtcctccttccaaagccgttgaaaaagaaaaacgtatgacaacgccatacatg
actaaatacgagcgtgctcgtatcttgggtacccgtgccttacagatcaggtaagtatagcaatcaaggcgaaagaataaaaatggaattatga
atggttggaactgagaaaatgcatcagtgaagctgctggcattctactacctttgacctgtgtatagaaacatgttctttcaaatggaaacaatcct
tgtgatttttcatatagttattctcccagaaaactaaccagaatgtatagtatgaatgctcctgttttagtggatttggaaggcgaaacagatccatt
gcagattgccatgaaagaacttgcacaaaagaagattccccttttggtccgtcgctatttacctgatggaagttatgaggattggagcgtttctgaa
ctcatctaatggaatgtgtggcatattaataaagttatttaagaaaaaataagaacaataatattggtggtttttggatttctcctttattcaagaaa
gaagtttctttcctttgtatagttttgaaactatatacctggagttgggttattgatgatccaaacgtcaagaacatacgaaaaaacttcactcttcc
ctttgttaggatagaaaagttttcatagaaagctctttaaattatgagcatgtcgttctttttctataattatacaacctgctgcggttgacattgtatt
ctgaatagcaaacatatagaatttctgtgtatgattgtatcacggtgagttgcatgttttaagtttctttaaacaatcaactttgttttctacaaagta
gtatttcttcagcaaaaaataaatgtgcctagctctatgtttttagtgagtgtgttcatgatgctctggtacaggccccaatttcttccaatcttggatt
tgatctgatgtggaagagagaggtgtaacgatacagcttgaaagagcgggcatggcatctagtataatttgttcagccgatttcgccaactttgat
gactcttgaattgttaaatctggtgccattgacaaaatgacatgaatcgcatgagtatttcccaaagtcatagcaactttatgaggaaaatttgctt
ttttagatagaacatcaacaacagttgtttgcaactttgtggatggagctttgtcaagtatctgatggaaagctttacttgaagatttaaaaccagc
tcgaaagacgactatactgactaagcacccaaaaagaggatctaaccatggcgctttcaaaaaatatgctccggaaagtgcaatgagagatact
aggccagtcaatgcatccgcacggtgatgccatgcattggcgatcaataaatttgagcccgttagttttgcaacttcttttgctataacaatcacgtt
aataactttaatagttacaaggcttggagtctatacacttacttctttgaaacagccactccttcagaacaacgcttccaagagctattccaattgct
atgaaaggatgctgtaaaattaactcggaataatcgtgcgaatgtcctatatgagtactcgtagctgctccatccacggcgttgtgcgaaaccgaa
tcttgaaaaaacatggtatacaagcgactcacggaagagtttgcaattcctactgatacagcaaggagtaaaccagaaacactaaaagtcccta
aagtttcccattttccaaatcctgttggatagtctcgagttggtttcttattacaaattttcaaagtaaacaaagtcaccaaatctgaaatcatgtctc
ctagttgatgggccgcgtcagcaagcaatatatttgattgtagagccaatccgccggttcctttagcagccattaatgctatgttactatatctgaat
tatggttagaaatgccatcgatacaatctagctcaatttctgttttgctaacagcatagaaaggagacttacaaaccccaccaggtcaatttcactt
cgggtgtttgctttttttgtttcaatgctttgacaagctcgaagtagtttttctcatcttcgtgattatgctcatgagtatgaggataaagtccatgttg
acggcaaaagaaccaaggtcggtatatagaagaagagtattttgtgaaagattctttcgttaaagcgccagaacaacctctcaaagcaaacgttt
tggtagtacgtggaaacatctttggaagctcaattgctggaattcacagtatgaacaatcctttatttgaggcgtttaacactagaaaggtatcata
aataagcacttcgtattcttcctgagtgtgataattttcgtcttcctttcgtttctataataaatatgtcttgttacacaggcaatcgttataatttcttct
caaataaatatttagaaaaaaatcaattaaacaatataaatgtgaactgaatgacttttcatgaagagttctacgccaaacaaagggacgtcagt
gtcttattacagaccttgaaagcgaaggtatgtattttaagggatcagctttaacaagtaatagatgccactaaaaattccaattaaatcctgcttc
tgacagttcgtaggagtcgttttctttcaaaagcagttgcagatcaatttataacttaatcttatttccggatctgcatgatctactcgaatgacttta
tcagatcaaaaaaaggattacgattttgtatttatgtaatatttttgtggacttcttttctagctttcaaattctatcaactttattatttatcagagtta
cggttcaataaatatatactttaacagaacaaatacgcgcgttcaaatgctagcacaaccattgttagatgtataattacttattacagatttttagt
acagagtttaggacaagcatagaataactaaaacaaacacgtgacccattattattaaatataaatttagtatacacgaaagttttgagcatttcc
actaggcattaaggggcagtaaatgtgaaatcataaaccgacacccaatatacaaaaaagcggattatgtagaatatagttttaatttttatcatt
ccactagtggaaaatgctcaaaacctcaaaacatgcaaatgaaattgcattcatagatactgaatgtagcttttccaaaagaacgctcaaaattt
ccgattcttgacttagaccattatcataaattgaatgaacccttacggaagttgcttgataaacataactcgctccattttgcttcattaaagccgac
tcgggaatgctaaactctattaaaaacttcgttgccctagagtgaagatacatctccagtcgaattacgacacactggttttcttgataaaatgaca
cataaggccaatataatttaagcctttcaaattccagcaaaagttcttgtaattgactccaataaacagctatttcgtccaattccggtttcagtaat
ttgatgtttttcttaataattttgcttttgttgttgtgaaacatctcagtaataataccaaacacatcagttactgtccatgatcgctttgctatttcagc

tcttacattaagatccagtcgttgttggctcacttgaagcaaaacgtaaacatgaaagggttgtaacgaatgattccaaattagtttcatttcatta
ggtcggatagaaactatggtccagccaagttccctttgtaattctgacattcgttgctgatatcctgtaatttcatcatactcaaaatcctgattcgca
ttataaaattccctttcttcccgacactgcagttctagaagtttgtatcgattacttaggtcatccatctctttccgcttatgctgctccttaatttgttgt
tcaagtagtgctttctttttcaagcttagttccctttgcaagtcttcaaatttattttgagcttggagaactaatttttgattatattgtccaaacatttct
tttctctttctcaatgttccaacttttgatgacaccgaagacaaattctcacgtaatccttcgaaaaagggaagaagcttggatctggtttccgaaa
cctgtatcaaattttttgaagtttcatgagattctcattaagttcctgcttaattcctagcaatagggcctcccgccattcataccaatatccctgcgc
ttgtaaacgcgaaaaggttttaattatccgaaactgtgaattcattaatgctttcatctcacttgacgcttttctatactcataaaacagcaacggat
tattctgataagcatcttcagccatttttgttacgaaatcctttccctcaataatatattcttccaatttttgacagctaaatttgtacaattcgagtatt
gggaattgcaaatagtaggactccaaaagttgagcagtcgttaattgaggactttcagctgaaccagatactattgtttcacgccttttttgaactcg
ttatattgtccagaaactcaatttctgtcatattcaaaaattcctccaatgtcaaagccggcaaaacaggatcaaatgcggatgaagcaacctcct
gttctgcttcctcattttgtttttgttcatctgtaggtacaatttcattaacggtctctggcatatcagcggcatgtaagacacttccttccttaatatcg
tttacccccattgaagaactcaaatcctgcgtttcttctaaggtaaatgggcgtaatttgactgctattggcgatatgaaggaatccttttccatttct
tgaaatctgctatttgcaaaatcatcagaatatgaagattccggcgctaaagatgcagctgcagggccaggatgcacttcatgccttaaaggactt
gcagccacagatttctcaattttattcgggctatcatttttgtcatttttttgggatgaaggacttgtaatagattcctgctctaaagtagaggaaaat
gaaacaaattccaaatcattcaaagtagatatagactttctggcatttcggattgttgacagtcgttgttgccgtttagcttccatatccacggttga
tgtactgtacctttgtttacgacgaaaactttttgttgagctcctattcaaattacttttaggtgaaggagaaaccgaagtagctttcttttctaaaaa
gggactttcgggtttactgggtgtcttagcatgaaggttgtaacttaaccgttttgcttttttatgaggaggagtttcttcatatgcaacagctctattt
tctggagaaggaagcttctctattggtaacgtgagatccatccccacttcctcttgctgttccggtacattctgattttcatttacaagagaagatgg
aagattgacgacacctgttacatccatcggttcttcattatcatttatctgtatctttgaaggagaaaattcatcaaatgcttttgtcacattcatatc
atcttcaccagcaccaagagtttcgttgttttccgacccttggaaatcagaattataatcgacttgattgatgggattgacagataaaggaagagg
atgaacagtctgagtgatgtccatagcttcctccccagaggaagattgaagcgaaagctggttcgcaggaagcgacgtcgttaaatccatatcag
taggattttctgaaggggaagcaggaatattaaaggcggtaggagggtctatagatgacttaagatccttactaggtgtaccaatggtatcatcta
tagatagttcatgagtgggacgaattggtgatatatgactggaagtaagctccatgcttatgtccatctggttcggaaaatcttcaggaacagtag
gtggtacatcaattggccgagtgagcatcatgtcttgctcttcttctatgcctcgagccagcgagggaattgacttaggagcagacgaatcagcag
cattataaagcggaaaatgattagagatattcttgacttgtcgagaaggagtttgctctgttgagctggttcccttaacagactcaatgccagaaa
ggtaagagttatcatcatgaaaccgcatagtgacatccatgctcatttcagtttcctcttcttgattgctatttgagttactttgttttattggaggtctt
tctgagactccataagaagctatttgagaggaatatgggtgatcggtagctgtctgagaaggattcggtaaaatggacacagagttggataaag
gaggtgaattcgtaacatccatgtcactatttgtctcattctcttctagctgggaaagggaggaatgattgggagcattggacgaaagggattctct
tgatccctgactacttctcgattcattttcattatcagacaattcattaggagatacattcccttcttcttcggctgaaacagctggcgttatggcagc
agatggaggagaagaaacaggaggagagtctgacgaaaaacgcgatcgatccgactcattttgataccacctcacacgagcgtggcttgcaaa
agatacacgacttcttcggccctttgaatgagttgttggttcattctcttgcggagtggtcatttgtttggatgcaaacattatgctattaggaatacc
aatcacggtattattcagtgtgtcaaaatcctcgttgtaagcacgttcatttttgctagcaggatcaagtccaaaggaagaatatcgtttaagaatt
cctttccctgggttagcgcgttccttcaattcttgcacagcatgggaccctcccaatgaatatgaacgtttcctagtctttttcttttcatacaaatcat
cagtagccgcaatactattacggcgaggcgatgttgacatcgtcaatgaaaacaatacgtttccaagtataatttggttgtgtttaggtttcaagtt
tccaattaccaaactcgaatgaagtgtgcaatcatgcaacacacgaaagaagaacttaattacgcactccaacaaagctacgaattcctaatctt
taagcttcatttagttaaggtagtgaaataagattagcagaattgaggattcaactgattcaataaactaacgatatacagcgttcaaacaaacg
gtgttcacaattgcttaaacaaaattgaatatcgtaaagatctgtcgttttagtaaacaattgctcttctgagttcacaaacatgcttgggatattat
cagattaggaatgtaaacaattgatgaaatgatgggcaaaagtaacttaaggatatatactcaacgtcttaattaattaattattaaagaaaaaa
aaataaaatgctttaacttgataaaagcaacgggcaagtaatggacttttcgaaacgaagcccgttccatcatttacaaataagcagcatgaaa
gatgaaaacgtacttataactaaactgaggtgatttttttcttattttacctcatatttttccttttccttgtactattggaactacattagtgtgtataac
ttttagagttcacacatttgtcttgctaacacaaaaaagggtttacctgtaacaacagtttttttattttttttttaaaaaaaaaataaagagttgtagc
agccacataactatagcattcgtattgcactgtttacgtttgctctccattttgtaaacaaaagacacctttcctcagcaagatacaattgaatgcgg
atggcgcataaattgacttcccctcaactaaaactaaaacgaaatcctcagccatataaaccttttttttctctacaaaagacactcatatgccaat
aaatgctgtacacgaaatgtgccagtcatgacttatgaggaaagtgttacgttgacgaagagcagatccccaaaacatagtgtcaacgcctagtc
attattgaaaatgaaccaatttaacagtcttgaagagaaatttccacgtaatgcgacaaaaatagcaagacgttatcgtcgtaatcctgtaaaac
agtcaaatactaagaacctggaacgttctttccttcttgcacaattaaactataaaagaagcacctcctgctctcttttgctaagcgccatcttgccc
ttctttcttttgttgaacatagttgcatcgaaagacacactcttttgtaacacctaatcattcatctaacaaagtctttcctttcaaccgtcttttgaca
acatgcaaaatcgagacaacgatcctgatccaagtgggtcatatgttagtcgtcaatacgagcatcctcaaacatctcgacagggggacaatac

atcttctcgtgactggaataccagaacatatgtatatgagggcaacgatcccaatgcttctcagcaagaaactgataggaccagcgttgcttcttc
cggcaatgaaagatacactgatagcgatcaaggaaaccatgactccacagctgtagactatgacaatgcatccggcgccgtcgctccaaatgca
cagaatcaccctcctcatccttctgatgaaaatgctgctgaacaacgacctgctgatacacaacctacacagcgacagcagcaacctactcaaca
agaacatcaacccccctcttctcctccagcttctgctcataatgaaaaggctgctgaagcagctaatcaacagcaagaacaacaacaagaaggt
aatgaggacgaagacgaagacaacgaggacattgatgcattaatagaagatttatactcccaggataatcaagaagaacaagaagaagaaga
agatcatcacgaacccggtggtgtcaaaaccgttccagaagaacttctccaaactgatcccaagcagggtctttccacctctgaagttgaacaga
gacaaaggaaatatggtctaaaccaaatgaaggaggaaaaacaaaataatttcaagaagttcctttctttctttataggtcctatccaattcgtca
tggaagctgcagccgctcttgctgctggtcttcgtgactgggtagactttggtgtcatttgtgctcttctaatgctaaatgcaaccgttggtttcgttc
aagaatatcaagccggttcgattgtggacgagttgaagaaaaccatggctttaaaatccaccgttgtgcgtgatggacataccgttgaattggaa
gcccaagatattgttccaggtgatattctacatcttgatgaaggtaccatctgtccagcagacggtcgtttaattactgaaaattgctaccttcaagt
agatcaatctgcaattactggtgagtcccttgctgtcgataagcatcacaatgataccatgtactcttcatcttcagtaaaaagaggtgaggcattc
atggttgttacagctactgctgattcgactttcgtcggcagagctgcatctttagtcggcgcagctggtcaagctcagggtcattttacggaagttct
taacggaattggtactgttcttcttgttctagtcatcatgaccctcctctgtatttatactgcgtcattttatcgtactgttcgcctcgcaagactcttag
aatatactttggcaattactatcattggtgtaccagttggtcttcccgcagtcgttacgacaacaatggctgtcggtgctgcctatcttgccaagaaa
agagctatcgttcaaaaactttcggccattgaatctcttgcaggcgtggaaatcctatgttctgataaaactggtactttaactaaaaacagacttt
ccttgggtgagccttactgtgttgaaggtgtcaacgccgatgatcttatgattacggcttgtcttgcatcttctcgaaagaaaaaaggtctggatgc
catcgacaaagcattcttgaaagctctaaaaaactaccctcaagccaaacaaagattatctaaatacaaaattttacaatttcatccttttgatcct
gtttccaaaaaagttacggctatcgttgaaactccagaaggtgagcgagtgacatgtgtgaaaggtgctccattatgggtatttaagactgttcaa
gatgatcatgaagtttctcaagaagtcagtgatgcctacagagaacaggtcaacgatatggcatctagaggtttccgttcactcggtgttgctcgt
aaagtagaaggaaaacaatgggatattctcggtattatgccttgctcggatccacctcgtgatgacactggtcgtacaattcacgaagctatccgt
ctgggtttgagaattaagatgcttacaggtgatgccgttggtattgctaaagaaacagctcgccaactaggcatgggtactaatgtttataatgcc
gaacgccttggtttaggtggcggtggtgatatgccaggttcagaagtttacgactttgttgaagctgccgacggtttcgctgaagtcttccctcaac
ataaatattcagtcgtcgaaattctccaacaaagaggttatttagtggccatgactggtgatggtgtcaacgatgctccttctttgaaaaaggctga
cactggtattgctgtcgaaggtgcttctgacgctgctcgttcagctgctgatattgttttcttggcccctggtctttcagctattattgatgctttaaag
acatctcgtcaaattttccatcgtatgtatgcatatatcatttaccgtattgcattgtccctccatttagaaatcttttacggattatggattatcatctt
aaaccgtttaatgaatctagagctaattgtcttcattgcaatcttcgccgacgttgcaacgctagcaattgcctacgacaatgctccgtttgcaccg
aaaccagttaaatggaatcttcctcgtctgtggggcctctctacggtagttggtgtccttttagctattggtacttggattgtatataccacgatggtt
gctgcaggacaaaaccggggtattgtacaaaactttggtgtcactgatgaggtactcttccttcaaatttcacttactgaaaactggctaatttttat
cactcgttgtaatggacctttctggtcttctatcccttcttggcaattgactggagctgtattagtcgttgatattttagctaccatcttttgtcttttcgg
atggttcaaaggtggccatcaaacgtctattgtagccgtcctccgtgtatggatgtattccttcggtatattctgttttatcgcaggagtttacttgat
cttgtctgaatcgagcaaatttgatcgctttatgcacggtaaggctaagcaaggctctactcgtacacttgaggatttcgtcctacggttgcaacgt
acttcaactcatcacgaacaagaaggaaagaatggttaattgccagctcctttatttttggtttctgtttccctcaatcctttttgatttatatatatag
cttgtagatagcatcacgatatatgagtataatgttctaaataataagattctattcaaattttcgcaaaagaaaaagaaaataaataaaaaaat
aaagaagaaaatcacttcaaatgtaagctagaaaataccttttttcttcgagcaataaaagataacagcgtctataattccaagtaaataataccc
aatcataacatcctaaaaagcaactcgtcaaagaaagtaacaataagcaaatttaaatagcaaaggtttttactgataggaaatacttactttaa
ggcagaggaagtactattaaagtaatcgattgatccggagaggaattacaaacagtttcataaactggtaacgaagagtttattttagcacgtat
agcgaataaaaaagctgcattcggtagatatatatcagtaacaagagtaaggattggagacatcgataacaataatttataacaaaggaagga
cgttttaacaaataataagagagccgaacgcctaataaagtaagactccttaagctatacagctaaccttttgaaagcctttttctccgtttagcca
tttccagctctctttctctttgctcttctaattcatcctcatggcgagcagcccgagcagcagcttgttcttcgcgccatacatcttgtccggtagcctc
catatcgtcatcatcgctatacaaagcatcacgggagacataatcttgtttccgttttccaaaaattttccaaatctcagaagacacgtctgataca
ccatctccttcatcatcagatacaacaaagtcatctctaggacgatttttcgattttaactttgaggcacgagatgaagatgaagcaacagaagga
ggaaccttcgcgggcctatcggcagcatttgcaatgttttgtttacttacacctgccgaagacctggattttggtagggagcgcgatacaccactag
cggttttggacttcagcgcgagttttgtgtcatttggcatacccgttcgttttcgcataatttcatcttgtacttctgcaacagaccgcttgtcacgtttg
gggccagcctgcaattggaccagttcaccaggacgagagcctactttatttcttattccgctgaaaggcgcatttgcagacgaagaacgagctaa
attagaggccggcccagtaggtctaggagagtctgcttttaaccatgctaaggacgcaggagttgatggattagacgatgtagcgccaatacgat
tactcacaccaaaaggatcctttgtgggggatttttcaggagttttagaagaccgcttaagagcagaagaggtattcaccttagtattatcttttgt
gacggaagatagtttttgagagtcagacgcctgacgaagtaattcattgtaagaaaccggtttcttcttctctaatcgctccttgtctttttgctctcg
caacactcgagcctcgtctgctgatataggaggacgttcagatcttttagcattttcggaggctcgcttctgttctttcgtttgaagtcgtttctgttct

tcttcgtattttatacgcaactcgcgttctctttgagctcgtcgtgcccggtctcgctcttcctttaattcaattgccttcgcacgaacctgcgccttttt
caactgctccagttgcgtaacagtttgagcagattgactatctgcgagcgccataagcttttggaaagatggtgttccggccattactaaattccttt
cttgcagtcgattttttgcaaaaatccaatatttccttaaattagaccatgccacttagttcttctcctttaaatttcctataaatagaggtagtcgac
aagtttatattcttggtcagtataacaacgaagatcatctctaggcttcactaaatgaaattgactagggtagggtaacgtgactgtttatacgcga
tcatcctacagacacgaaagtcggttttggcggagttttgaccatcaccaactactgttttgcaaaatggccgaagaacacgatgaagccctccgc
gctgctgaattaagaaagagaagaactttccgtacctttgcttaccgcggtgttgagcttgaaaagctcttggatctttctgctgaggaattgattg
aattgttccctgctcgtcttcgtcgccgcatgctccgtggtcttggagccggtgcttctcgcttcatcaagaagcttcgtaaggctaaggctgaggct
cctgtcaacgagaagcctgccgttgtcaagacccacttgcgtagcatgatcatccttcctgagatggtgggctctcttgttggtatctacaacggta
agcttttcaaccaagttgaaattcgccccgaaatgattggtcattacttgggtgaattctctatctcctataagcccaccagacacggccgtcccgg
tattggtgctactcacagctctcgtttcattcctctcaagtaaaaaaatggaaaagagggtgtgcgaaaacatggaaaaagctggatatatggttg
ggtatgtccgtgcctttgcttcctatacgcctttccatgtattgtatattcgcataaaaatttttcccgttttctatgaaatggtaaagttggctcaagt
ttgatgtatttgtgaaagtggtatatgaatgtaaaagttggagggtagtagtattgcaaaagtaaacaactagctggtaacgttttgtatcacaaa
tatgtaagcccgtagggattgtaaggctaagcatgaagggatgtttgataaactataagttcgtgccgttgcggttctgcatcaccagtataactat
agaaaacattcatagaatgctttaattagcatcatttaaattcgggtcgacaatattttgtcctggaaatatagtatttacgatcttctcacacagac
tgccgttattttcaaatgaatgatttttgacttaaaaagttcagaaaatatgacttgagtagaaacatttaagaatatggaaatttatgataaaaag
aagtcatttttgacacgccagatccagcttttgtcgcaccctttacaaattcccaaaaagttgcatacacagtcagaatcaaatgatttaaacgagt
cggttgtagaacgggttttccaaagtcagtagataactggtacgcctagactatggagacttattaactcaggtaataggattacatttgagagtg
aaaagacacatacgattcaactactcacagcaagcaactcaacatgttgcagctcaaataaggaagctttacgaacaaagtactcaacttgaag
acttatcctcacttcctcactatttgtatggcgagtttgatttgcgtgacattgtaagtttgcaagatttacctcacccttggccatttcaaatagagc
aaaatgagcaagaaaagcaaagtcagcaggatcgatacgacatgcaaattggagaactaatttctttattgtctgctctagaccaaataaaaca
agaacgaaaagtcttagaggactaccgaatgtctctagagccgtttctagatacttctcaatcgcctgaaagacaacaatggaaaaagaaccca
gaattgtcggcccttctttcctcagtcaacgtcgcaattgctaaaaccagtgctcgtctccgggatgtcccttttatgccttcttcaaattcagcaact
agagatcagatttttccccttgaaaagaaactaaaggaccagcattttcgcaatccttgattccgtgagcacaggatacgctgaaaagctcgttga
tgaatgacattctgattataatcattgatctttaggtttttaattttgagtatcctatgaaaggaaaacctggtcccttaatctaaaattacctattcat
gattcaaggattttgcgaaatctagctgtcaattttaattcgcacctgaaacctaatctcatattttttaaacaacttgtacattaagttttgtatattt
gcgattaacggattccgactgttaaagcaaagcgaatccacagaatgatggtcgactacaaattacaaaattatgtaaaaaaaaaaaaaaaaa
aaaaaaaaaaaaaataagaatagaataagaaagaacnnnnnnnnnnnnnnnnnnnnnnnnnnnnnnnnnnnnnnnnnnnnnnn
nnnnnnnnnnnnnnnnnnnnnnnnnnnnnnnnnnnnnnnnnnnnnnnnnnnnttagttagttagttacgttacgtacgtacgt
aacgtacgtaacgtacgtacgtaaacgtaaacgtaaacgaacgaaacgtaaacgaacgtaacgtagtaggtacgtacgtaacgtaactaacta
aataataaataaaaataaaaaaaaaaaaaaaaaaaaaaaaaaaaaaaaaaaaaaaatagaaatgaaatagaagaacaggttacatgattagt
ataaaaacaaacaaatagtgtgcttgataaactaacctgcaatggatgtactgtacttatcttgactaaaaccatcataccaatattgcattcgtg
aggaataatggttgaacagaaaacgaatcaagaaaatggtactaatttttttttaattggtttcttacttaggtttaacgacattttatttacaatcg
acatatgttctacatgatgaattcgtgaacaaattcataaattgcattaagcaagttgcttcattgactatctctggctaacaagagaagtacttctt
taaacggaccgttaattatagcttatgatgcttcttcccaagtatgttattctgttgtttatcccaaataaaacaaaagttgagttgacaagtcaaga
tttttgaaattcgcattgcagcagaaggccgtaaacaggcaattgagcagtaagacgtgtttgtacttatttggctattaaccgtatacgatactac
agctatcaagaggttgtcgagagatttcgtcaggaattgaataatgaatcattagtagacacacatccatttggttgtatgtactatacatcgaatg
aaattcaaagttttgaagagttttctttacgtctctatggtttccaaacttcgaatgacattaaggacccgagcggagtagtacttaatagctccact
caaggagttttgaactcggaagaagcctctcctaccttcaaatcaaaagaaccaattatcaaagataattccagctctgatatacattcatttcca
aatggtctccaacaaagtttcaggaaagtttaaaacgcatactaatccttccgaaccatataaacttattgaaagaatttggtaaagcttcaaatg
tgaaatacagaatctgtgacgaggaaaatgtacgctgtttgaaagtgaacattcatccaactaaccgcgaatagtatgttacattgcgtgcatcct
caagaaaggacttaaacaaaatgttagatttacttaataggctggattctttacttgctcaagaacagtttgaccatcgcgaaagaaatctcttttg
tgttggacagtttcccaactgtgaattaaaatttttttgctaaaagccgatcatcccttatttaaaattgctttgcgaacccctcaggttcgtaaaac
cacgatgaattttaaagagtcatgtttcttcattttactttcataccatgataatataaccaatgcttataaggtctttccctggttgtataatacttca
agaaaagaaaattcttccaatataaaatttccactttttgactctcacgatgaaagtgttgtaagtacttcttcatcggtgcctcctgataaacaag
atgaaatttttagtgaaaagagaggattagacacattcgcagctcggtcagcatatcctttgtcttcagaatggaataaaaaatcagctgaagga
aatcaaattgtttcacaagtggagagtaccttggaaaaggttcagaagctaaaaaataccagtaccattactcattctgatgtgaatgatactaa
agagaaggtgaataaaaacgatcaaccatatagaatgaaaaaagttaagagcaggcctccgaaatacaatttcaagcttcccccgcgccctact
gattcttcaatagtcagtcagtcagattcagatgagcttgttgttcgtcgtgcagtcaatgctggaaacgacggcaatgaaataatgtctcaacca

aaaaaccgcaagcaacaatcaagtacttttgtctgttcttcccttccggcaactttggataagctctccttgaatgatcctccccctcaaaaacag
tggaagggttttctttggtctctactaatccggtttctagttgtggtattcgcaatcttcgtctttgcttaaacaaggataattgttctaaacagttaga
aaatcatgaagaattcgacaaacaaaatcaaaagttattatacatgaccctctcgaaaaggtaatcagttcctttttttttaactaaaaaatattaa
cttgatttagtttgcatgttctgcagtcattttcaggagaaatacacatgtctttaaagtatagaatatgatagtgttttattttactaatgagtttgca
gaattggaaaaatactttatccaaatttgaaaccagagatatatcaattttctcaaagtctgtctcgcttttataaaaaagatgatttacctaaatc
attatttgcgaactggtaatttataattataggaaaaattgctaatcgcatttagcattacaaattcagttgaagaaatcaatcacttcctgaaaag
gcctgtaattattcgatatagtcattctgaaatttcctatgatcttgcgaaacctgaggctgtggaaacaaatgattactttttattccatggaactgt
taggaatgaaaatagaaagaaaggagaaatttttttaagtccttctttatgagatgtacctctagtctacaggaatgttgtttcttcgaagctcgtg
atgcaatcaccgtttgtaatatcaattttcctgctatgtaagttttgctattttttaaccaggctaatgtcacttttaggccttgggatgctcgcttaga
gcttcatgcatggaccgtgttagataatccggtattggaaatgttttataagtccttatgtttccggtacgtcagtttgaaaacaaagatgtatacaa
tgttggctaattgtaaaaggaatgaggaaaatagcactgttttaagctttaacaatgtggataataaagttactatactgtcggttcagcgaattca
ggaaaattcagtactgtttaacaaggacgtacttccgcagtctactgatttttttgttgaagtactctaagattaaggattttgagatctattcttctat
aaaagatccttttttcacagtacgtgtgtgtgaaccaaactgagaacgcagatttttctacaaattatacggttaaaatagagagtccgttcatgca
ctctcaatttgaacataacaaatttatcaggccaaccgaaacggcaaagtgggacttgagtaatcctcaatttcttggaggtgcaatgatagaga
actcccaggattggtacactgcagcaaagattcttgtaaaacaattagacagcattaacacaatatgttaactcaatcagcagtccctttaagtct
acattacttattccgataacccaaatcttattaaaacagtgttaataaaatattgacgaatgaacttcactcattcttcttccagatcttcaatcttct
ttttaagacgctgaaactgattaataatgcttttccattcttgttctgccccaaaattaacctgcaataaccccaattcacgatcaactttttcgaaa
accttcgtatatttttttcttcaacttatcttcttcgactgaagacgtgaatatataagtcattagtatatctaagatagaaataaccgacaggcaaat
cttagaagaaccggaactcgtccgagagtctttaagtataggacaagcatcagaccaagcatccctgacaaaatctaagagtcctggaataaac
ggaaccatattttgaaatcgaaattgaggattatcaactaggtcatgtttgctggcactaaagagtgaaagatacgaaaacgaactcggcgtcct
cagaagatcaaattgtgggtgtaccactatgttgtgttccatataaggataatctaaatgaatagcggatcgagcagtctttgactgacggtttgg
atcaagccctaaatgtaaaaattcactgcagacgcttatatgacgaattaaattagaggtggattttttagaaaaaacggacgttcttttaactgat
gctgtatcagaactccaaccaagtaaactagagaagcattccaaagtctttttccagtgcagagaaatcacagaaggattcagattatttcggca
agcttttagtatgagtgaaagataccgagttgagtcatttctgatagaaggagtgatatgcgtcattcctgaatgggtatacagaaacataatacc
aacattgggctctaaaagattggggtctatggtgaacatgacttttacgaaaaattggtatagactttctcgcacttgagctgattcatctagaatc
agaggtgccgtaacctttgctaacaacgacgttgacattgtcaacaatccaggatgagacaaaacataatgcgatagtttgtccagcgattctagt
aaaccgttagtaaaactccacaatactagacaaaccttgtctttgagaagcattatggtgcttaagcatcccaattagatgtgaaaattgcgcacg
gtctttcgcatcatgtgtaaactcttctttttcaattgtagcttgcgtaggtaaaacaagagctaaagatgtattagtattcaattattagatatttttg
cttagcttactttttgatcgaaaactcgtgtctgtaaaattgtttggcaattgtttcgatttgccaactttgagtttttgtttctgggataggttaataat
acttgttcctaaaggttttcatacattaaaatcggctcttttagcctttgctttcttggattttcccattataaaatttatgcttcttatgcgagtcgctat
ggtagtaagctaactacacaccacagagactacggtatgttcttgtaacaagtcaagtggattaaaagcatagacgactattgcaatttaatatta
tccagcatattattgctattatacagtcaaaataaatatgagaaaatcaatccactaatatattcagtaggaaatttttcaattagttgcatcgttttt
cttgttgaaacgcaaaaatccaaacgcattttgaaattgctgcttttcaagctccaaactagacagcctcgtgggtgtgctaatatattcgatatttg
tacagctagtaatagagacagttttgtatatttcttttgttgttgtttgatgttgttcttgcagtttctgattaatcgcttgaacttgtaagagtttaactt
gtaagcttttcaaatcttcatgtttttcaaaattctttggtaaaggtgtagctaaaaacgaggcttgatcagttactgcttcaagtggtaaagttagtt
gatatactagaagtaatgaaattccgagacaacttaaaaataaaagcaaagattgtaaacgagaacggaagatttgccaagaatcaggagca
ggatgtacaactttctcataatcatttatcaacacactagtttcatcaaagttttgttcttcaaattcaaaacagaggattaagtccgacactatttg
actattccatgccagttgaatgattgaaacgggttgcttaagcattgttcgtttgctattaggattaatgtacccagcaaggaaatctaatttagtta
gcaaaagcgcgctttaagaaacatacaagatgttgactgaatacaaaccgtagttgggacaagataggacacatttggggtgggaattggaagt
ttaagagatgtaaagtttgagcatttttcatacacagatttaatgctccagcaaatgtcaacggaagaagttggataatgaccttgaaataataca
acctcaccactctcaaaaagaaccaaacgatgttcagttttcggatgaccattaaccatgccccaacttaatgaagagtatttaggctggccaaca
cggatgagttccatccctggtttcaaggaagaaacaaggtttagggacaataggacgtccataccaccatctgagggctggattgaaagagtgg
aagaactgatccaagtatactcctttggccaaggaaattcgggactagagaaggaaggcagtttgcttacataaaattggaagggtcgtataca
aggattagaacttttccgatgataagaaaggacattcaattgtttcaaattgctgggaatccaggagtctgtaaccgcagcatgtgggacgatga
cttcacaaggaaaaggaggtcgctcaatctttagactacacagagatggcaaaggagacaaaatcattggcacggaagcttttgcgccagattc
aatgacttgctgaaacataaaagaataggagcagctagaaaatgggaagccagcttcgaattcacaaatacgaagacctggcaaactaacatt
tgctgaatctagagaagttagaaaacaaaaaaacttattaagcgtcacttactaggtggatctgtgccaataagccgaaatggggctaactttga
taagtcttgattaataaaagattcagagacaagcataaaaaatcgtggttttaagttggaaacattggatgatgatatttgagaagaagggagaa

gtttaaaatatttaaaagtttcacaaataaaagtaagtttgcaggaaatttcaaaggaggaggcttctggatttaaaggaatagtttgaatttcgg
cagtaagagtacaaaaggagctttcgggacgccaaactgaaaggggcttttggtacaagcgtaattgagaaccaaatttaaaagtaaaatctga
gactgaaaatggcggccaacaaaaagtttgctgagcatctgcagattgtatttcacgttttcctttgttgaacgcctttaaacgtgtctgtgtagatt
taggaatggatgtattgacttggtccagggacagttgcagagtgcattgaggagaagaaacgggggattggaaggaaagggtgatatcaaagt
tgtgaggaaggaacgaaagagggacatatttttctagggaaaggaaatcacgaaagatgggaacttgcaaatccacaagaagtacagttttga
ttacctgatttgtatccgagacagttaaggagaaagagtcgtccacaaggtcgaggcagacaagagggtcctgagtatttgtaagagtagtataa
tcaaaggacgcatcagacgactcaaacgaggcatcggagcttgaagaggcaggagacgaagaagaagaattatcggaaagagagataatgg
acggatgttcttggaaatggggagaagtgctggaagagctgtccaaggaagctgataattgaaggagttcatcaatgcaagaaaagcgaaggc
gcggaggggcagaacaatcgtcttcgcggtttagataagcaaggatagatgatgaagcggtatcacgcacaaattcaagggagcatacgccat
ctggagaatacagacgaaagcaaagacgagggatagcaaaagggcaaaggaagagatggagcaagggaagaggaagtggagtcttgttag
caagagggatgatctatgaagaattagtcgcagaaaagaattgaaagataggaaaagaaaagaggacgtactgtatattcgagctgtaaatgg
gaggtaaacgtaggaagccaagtaccacgagtttcaagttgtaaagaatgaaaatccgggtgtcgtgaagaagcagaggaacatgaagaaaa
aacccgaggaatggcaaatatgcccattgtgcctaaaaaacaaagaataagaaacccaatctaagtagaaaagaaaatagacacagtaaatg
tatgggagattgggaaaaggaaaaagaatcgatagtcagaaggatagagtaaacaaagaaggaagacaaaaataaacaatgaaaaacagc
caacaaaaagaaatgaatgaagaaaacttatttgtgaataaaaacctgtgtatatgcaggaaaatccagtgcttatacgtaaaaggataaacac
gatgcaggatgaaaccacacaactgtactcgataaagggagagtaatgtttctactcagtttgctgtttgctaaacaaagacaaaaacatctctc
gagccagattcgaactagcgaccaaccggtatctttgaaattacagccgggtgtgataaccaactacaccatcgagagaattgttgatagctaac
tatgctttatacaatattagaatataagccaactttagatatagatattggtttatattgagtgcaaggggataaattgccgataatcggacaaacg
acttttgaaattctatttctttctgatgtttattttcctgtcttagacgtttccttattctttcccagcctttttcctttttcggttactccttatctttcatatt
tccgtgactgttttgaactgcgatccctatcacaaaactctatgtagcacctaaccgaaaggcggtactactatccatccatgctaatggggaagt
gcatcatcgcgtcacagtttaataatccataaaaatatctatgaaattttaaaaaaaactatttatttcaacggtaccttatttacttcctttccttttt
tttggaatcgcaggttttcgttggtctttttatataagtcgtaccgtaacatttaatcgtgtctatgaacaacctttatccatgttttcatataccctatc
agtccaaaattgcgtccgtcgataacgatcggaaatgacgtcagtttcccattgatatcgaattgaacccctcaagctagagataagctttttggt
gcgtacaagtctagacgcttcgatcctacctggttgtcgccagaagggttcaccttatccaatgaatcatcccaggtttgtctagctttgcaatcctc
ggacgcgtctttttaggtcctgtggcccgatttctcctatttaagagtccttcccccacaagcatacctcaaacaccacacaatttttacaaatcca
tttcaaattagaagacaagatactatctattctgtataaagattcattcattgattctttctgtatatttcttgtaagctttataagaaaacgacgact
gcaatttcgtgtgttttctcctcttgtattattttgttttgtttattgaatttttgctgtgcgttgcccaaaatgtgttactaaatttgtaatacgtgaatc
gtattgttaactcctgttggatttaattgatcgtcgtttttttgtgtgtgttccctatgttttgttgagaaaattatcgccttattggttttggtgtcttttttt
tataaaaaaaaagagaatttctgtttgtttgttcaatatgttctcttaaggtttttcgagtaaggttattttcctgtccatttggtccatccctttataat
agttctctataagaatcggtcttctcccgttttctttttgccttttataagtttattttctatttctatttccagatatcctgtggcttaattccatcttttca
agatgtctcggtgtttttctatatattttgattaaatccgtgctttctgtttttatatatataaaaaaacaaaaagtgtctcgttggtttatttcctattct
aactgatacccgtcgaaaatcgttcagtacccccttatcgtatacttatacactattgtgttttgtttcgttgtgtcaagtttttgttataatcgtttattt
tttttgagtttaatcatgaattactcttctcagcgatctcagttcccctttggtcaatctcttccttcatcattggccgaagggatttttcagggttcaat
gccccctgtcgactattcttcgttaattacctcctctcatctttctgatgatgcctttttttcctttcctccgcagaatcattcaccttcttcagaaaatga
cgagtccaacgaccttcattttgatcctgagctaatgctcaaccttttatcggatccttcatcgtcccttaatacatcaaaaaaatatatacctcaaa
agtctgcttcggcaaacgttcctcaacctaaaccagcttctcttcctgcttggagttcttatcccagtccaccagtcaactctaatcagtctacctcta
ctgctaagcctcgtaaaaatacttctttctataattctccattgtggagtggaaacttagctgctggttcccatatcgataacgatgtcttttccgttc
aaaacagtttattatccgagcccacacccccattcacctttgctcctacgccgagctctgctttttttagaaacaccattacatggatcctcggacctt
gattttagtttgggtgataaccagatgcctcttttccctgcctccgatggtgactgtcagcgcgcttttagttctatatcttacctcaacagcaatgatt
ataagctggcaaatgtaggttttcaatcttcgcagaataaaagacctgcatatgattctccttgtgatatgcctgactcaagtttgcgaacagaag
aaagcaatagtgtttctgattattcagcggcggtgtcttccatggagcccttcttttcttatgaagcattcggcgaagcttccaaaaataacttgtcg
gcgacacctaattcttttacccacgacagttgtggtgaaagtccgtccttgacttctccaaggaagtctgctttgaccactggtcttgtgtgctcggc
tccgtctaggacacctgtaccagctactaagcttgctcgagtggtaaagcctaaaagagctcgcgaacatatatgcttcaattgtggtgctacaca
aacacctttgtggcgcagaaccgccgataagcaccattatttatgtaatgcatgtggtctgtataccaagcaatatggagtgatgagaccattggc
tccgaaaaacaaaagcaatcaaaaaggtcctgagaatcttacgtgcgctaattgcagtgctacaaagacttccttgtggcgtaaagaccgcgtg
ggacagactgtttgtaatgcatgcggattgtatgctcgtctccatggccacaatcgacccttgggcttgaaaaagaacaagctaatacgtcgacgt
cgtggaagattatgtgatgaaagtcccacttcggattctgtgaagtcggatgaatctcctgagctttctcaatctatctcatcctccgaattacatga
ctccaagttcttgaacgagcctatgtcattgttcccagaaaatatgatggaaagcatgttgctgcctgtaaaagcagaaccagaagcatcgttttct

gcttccttataatcaccttttttgtataggaactatcagtgattggtcgtatgggtcgggtctgtttgctgcatttttttgggatatatcataataaggat
tcggaaaaaaaatttgtacgcatgagaggatcagctaaattttggtagctgtgtgaaatatcaggcaatcctcttgatttgcccttgttcaacctaa
gggtttttggttatatatatttatattgttatttgtttaattagtatatctaattcgtgttagattattatgcaaaaaaactgaaaggaaaagtttacgt
tttcggtttttggtatgcactcgaaagctcaaaagaaacccttttgggttactattggtttactatctttttgtctcatttaaaagtcctttttttctttgtt
tttttttagttaattgattttttccgaatcttaaaactcggatttttaaaagacatagtagtagtgatcgtatatgaagataaaagtaaatggttta
atctgacagcgattgagttcaatcaaattatccttgaaattatctgccttgtaaattttggttacagatgaaggttccctcgaatgtgtagatgattc
aaagtttattagttttgtttcatagttgtttggctatgtgggcaaacatttacgttgcaaaagtaaccaacgatgtggtgtatttatggaggtatttac
tgggaagattcatttgagcttatattaattggtagatgtatcgttggttattctatatcatataaacatccagctggttctgttgtagcatagagaatt
ttgtttttgctaattgatttttccctttgtatgtcaaagagaagagtcagaaggtttgtagttttactgctaattagtttcttgatattgttgaaaaaa
aaatgataggaaggatccttgtataaaaactaccgtcaaaacggatagtaacatagatttattaacgaacaagtgctaattgtttagcacgctcc
cagagctttaaggcagtgtcaatataggtttgcttttcgctgtaatactgcaaaccaagggatatggaataagagttgtccgatttatagtgcttgt
cgttgaaaggaacatcagtgggttcagaaatgacatccttcatttcagggaattcttcacgaagaattttacaaacgtcgttagtgataaagggac
ctttggatacaagcatacgaccattactcatttcgggcttttcaattgcaacgacatgagcattggcaatgtcacgaacatcaacttgtacgtgttg
gaaagaaggctgcgccttggaaccattgatgaggctccatagaagacgattagaagcattcaaggattccatagtcttcataggatggattgggg
gtccaatcaccaaagatgggttcacggtgcacatatcataatgaggctgcctctccttaacaaagttacgagcagctatttcagcaaactttttag
aagcacaataagcagcgaacccatttttagtctctaaagcctcctcatagcttatgggattccagtctttttcagtataagtagagccagagtatgg
gtctttcataaagtcgccgatagcaacaaaggaagaagtaataaccacacgttttacctttggttcgttcaaggaagcctccaaaatattgattgt
acccttatgagcaggatccaaaagttgggtcttattgtcggttacattttcataaaagaaaggagaagcaacatggcaaatataatcgcatccttg
aacggcatccttgaacgcgttaggcgcaacaatgtctttaacaaatacaaactcaactttgtctttaagctcaggattcaggccgacaagttcttca
gctttttccatggaacgaacggcaccccgtacacgataaccattgttaaccaatgtaacagcaacatgagaaccgatgaacccagtaacgccag
ttactagaacaagactttggttcggcatgattcttatatttggtaaagaaacaaataaaagtacacacactcaataaacaaaaaaaaaaaacaa
aagggggaaaaggaaaaaaaaaaaaaaattaaaactttcgatagtttccttctttgtataaacagatttagtttagttccaccaagtcagaacaga
atgtaatgtaaaaaataaaagaatatcaaaaaagaaacctcccagcagccaatctctaattatatagtcatttactttctgttgttactaatcatat
actagccttccttgacgcaacccacttgactgttcggtataaactattccgttattctaagaggtactctttataaaagttttctctatttctcttggcc
tcgttgtttcgttcttcgtttgcgagtcaatatttccgacttgattcgtaaggtaaaaaattactaagagcattttataaggaaagataaagcataca
tacataaatagaactgtttgtttcccttagtagtcttctcttctcttcttttctcttggtcttgttgtttccagtagtatcacattaatatcgtattaacga
gttggctgtgctgtcttaatggcagcatttgtttgctttgtttaggaagaataaacagagaaatagaagtaaacagtatcgaaatcaactttgtacg
taagattttaggtaactatttgaattgggcaaaaaaaataaaaaaacaaaaagtccagtgctattttttacttgtaggtatcgtttcggtaatgtatg
gtttgtatataacatttttttggtaaataggtttatgaatttttcatagaaactcgagaacaatcagaaattctgctttttacgggcgctttgaggaaa
actgtagaacgaagcaatgaagtatacgctgcaccttgggcatttttaaatcttttgtgtttgctattttgaatcaagaaaaaaataaacacaaagg
gaaatataaaacaaacgaacgatagaaaaacagtggtttaataagttagaagcgcttgttaatttgctagaacataaaagacaaacaaaaaga
atatttccaaagagggaattaataatggtgacttcgatgaccttttgttgacatagtcaacctgcgtaaacaagtcgacgaatttggacggtaaag
ttatttttttcatttacaatcaatactttttaggttttaacatttttacttacatttgaactcgtaatatcaatcaaaacaaataagtgaggctttcct
aattgctcttcatgctcttcatgctatattgtgtccccaaatgacatactcctgatccttaaaaccttttttttaaagcagtaaaatcaataaggaata
aaaatatgatttagcattgaatcttttgaattttcattcttaagtgaatgaatataatgaatatttggatcggcctattagatctatgtcgaagagaa
aaaaaggctaacattggaatactttttgttcgttcaatacgggctttctaccgtttcaacagaatagaggcacatggttctgaacgcttaagatatg
tgtctagttgtacaagaatttaaaccagatgttctattcctgtaaaataaccaaataaaggatattatggaatcctcgagtttggaatgatgaaata
ataaaaaaaagggaaaattgaagaaaaagtgttcaatagagcaagactcaaaactggttgacaattcaaaaacttagaaacaagaaaagaat
aaatatatatctctaaaagccttatagatgatcgtaatcttcggaatcgtcaaccttgaccatgttcctttcaaaaagttgacgaccggtcagtttct
tttcaagaattgccttccgtgcattagtgttagaagtagcattcaatgcttcgcgaagcttttcttcattctctttacgaatttcttcctctctccagata
tcaaattccttcttccatttcatgaacgattcaactgtcacgggggtaccctggaatttttcgttctccagttcaatttcacgattttgaatatcttcaa
tttctttttcttccttttcacactgttcagaaagaacgatatttacctcttcttttagtatggagcaaagagaaaagatcatggccatgcccaagcatt
cttgcgcctacaggttgataggttagtttaacgaaaaacaaaggaaggaaggaatcatttaattaattcagaaccccacgctacttacattttgat
tgacgacctcttggagacgcttcaattgctcttctccaagccaacgatgaggcggactaaagcggatttttgtgtcagggatttcattaggatattc
atcgcttaaggttgcgataaaaaatgacggaggctacattgtgggttagcaaggaaggtataccaacctccattcagaaaaggaaatccatacga
gggtctgagcatttagattcttccgggtctattgtttgagtgatctgaaacgttctttcatccaaacctgtttcgttagcttcttcacgttcatacgaaa
cccataaattttgcaatttccggtttttcatataaaaacgatcccaccggagccaatagaatgatatttctttttttctacttacactggaattcatctg
gataaatggactctaaaatttccttttcctcttcaatagctgaactcattgttctgatgggaaatttttgttccccgtcaaggtatattcataaaggttt

acgacgtacttcacttttcgcaacatgggagatagaaataactagaataataaagagtaaaacgacaaaaaatgattaaataaataaataaaa
atagaaatctactaccttcgaaatcaagcgatgtgaatttgtatccatttcatcttctgcaaacgtagctcaaaacgatattataggatagttgttta
caacgaaaatggtttacagataaaatacgtatatgccttcactttaatcttcttccttgctcgttcttggctctataacatggcactttctcttttgtttt
attcaatttcatgtaacaccatgtgtaagataaggcttattaagcataattcttgcatgaaattttctacggccacacctaggcgaaaacaccagtt
cccgtccgaacactgcagttaagtgaaaatgtctgagggtctccgtctcgttcatactatggttggagacaacatgggaattcggggtgttgctgg
cttcctttttctctttttctcttttatacgtcgactatttcccgtaatcgttttctcgaaagaatttttgcaagctgttcctttacaagtgacaaaagac
taattacaagttcaatgaaaagaaatgaaacgatcgtattcaattgcaattaagaataagaataataggaataataataattaagaacctagtat
gatgccaatgatctcagattacggttcctttcaatgcgcggattgtatgcatgcctttctattgtccacttttcttcgccatttccaattcatgttggtac
aaggatgagtccgtagtcaaaattaaacacaagaacgcttagcaaagggatataaataaatacttatccgaacaaattgcttgtaatgttatcttt
ttccacttagtgactttcgatcggaagagttaagactcatgagaaaagaaaggtaaacaaattagcattaactttgagttgtaggattgctttgca
gctctatttcttcgttcgtagttgnnnnnnnnnnnnnnnnnnnnnnnnnnnnnnnnnnnnnnnnnnnnnnnnnnnnnnnnnnnnnnnnnnnn
nnnnnnnnnnnnnnnnnnnnnnnnnnnnnnnnnnnnnnnnnnnnaatcgttttctcgaaagaatttttgcaagctgttcctttacaagtgac
aaaagactaattacaagttcaatgaaaagaaatgaaacgatcgtattcaattgcaattaagaataagaataataggaataataataattaagaa
cctagtatgatgccaatgatctcagattacggttcctttcaatgcgcggattgtatgcatgcctttctattgtccacttttcttcgccatttccaattcat
gttggtacaaggatgagtccgtagtcaaaattaaacacaagaacgcttagcaaagggatataaataaatacttatccgaacaaattgcttgtaat
gttatctttttccacttagtgactttcgatcggaagagttaagactcatgagaaaagaaaggtaaacaaattagcattaactttgagttgtaggatt
gctttgcagctctatttcttcgttcgtagttgtcggcggaggagctagtgcatcatccctttgttgatatctgggaagcgtaggcgaaccggtgttttc
agctgagctctcaaatttcattccaaggccaaaaacagtaatgttataaattccccaaaagatagcaccaacgaaccatccaagcacttcaccaa
gaccaaccacgataaagattataactttagcggcaatccgtattgtcaatatgaaaggccaaataagtgctaggctttattagtaacacacacac
attctaatagcgtttgctattaacaacaatgctccacttaccatagaaaagagcaaagaatattgcccatatcacaagccaaatgcctaaaggtc
caagaagggccaaactgtatccattttccatatatacttgtttcagaaaagaaaatagttcgaaacgataggagattgctaatgtacaaaggaat
gataaacccaatatcaaaaagaaaaacatcgacttacttttaacgactacattttcagggaaataatgatctgtcttcttcctaaagtcggtaaag
aaatcattgaagctatcaaattcagtaattatcgttttttgctttgtttggctttgacagaagttgaattggaagttttgtttatgtcttttggtgtata
caaaggaagtcccttctcaaggtcaacattgtaaacattagcatggttgtaagcaggagggtcgcttgaatcgaatttcagctccttttccgacatc
taaaacaaataaagagaaatttcaacgaataattagcaaaagagagtcaaataaaagctatcgatccgtattcgatataaaaatttcgcaaatg
ttcagaagccactaagaaaggcgaaattgaaaggggtttagctcaaagtagactcaatttcaatatttcatctaaaatagctcagactttaatcaa
aatcagcccagttggcaggaattgaagaaataaacagttggctccagcaggaaatctcgcatctcttacgcaacattttagctcaggatagtgat
atgttcagttgcgctatctcctataatgacatatagcataaatcgttgaatatacgtttaagtcacaatgaacaagtaacaagtttcgctaatctcg
cgtttgtttacacctctgtaaacaaagcagatcccatttctctatgaacttatgaacaatttttaccttgaaagtgagtaacagcctacaaccaaa
aagcagccctcatgggctcttcaggatcaannnnnnnnnnnnnnnnnnnnnnnnnnnnnnnnnnnnnnnnnnnnnnnnnnnnnnnnnnn
nnnnnnnnnnnnnnnnnnnnnnnnnnnnnnnnnnnnnnnnnnnnnnnnncagaagccactaagaaaggcgaaattgaaaggggttt
agctcaaagtagactcaatttcaatatttcatctaaaatagctcagactttaatcaaaatcagcccagttggcaggaattgaagaaataaacagtt
ggctccagcaggaaatctcgcatctcttacgcaacattttagctcaggatagtgatatgttcagttgcgctatctcctataatgacatatagcataa
atcgttgaatatacgtttaagtcacaatgaacaagtaacaagtttcgctaatctcgcgtttgtttacacctctgtaaacaaagcagatcccatttctc
tatgaacttatgaacaatttttaccttgaaagtgagtaacagcctacaaccaaaaagcagccctcatgggctcttcaggatcaaaaaaaacctata
aattacttctttgagctgaaggaactgtctacggccatacctaggtgaaaacaccagttcccgtccgatcactgcagttaagtgaaaacgtctgag
ggtctcgttcatactatggttggagacaacatgggaattcggggtgttgtaggctcctctactttttttgctgctcttttttctgatcactcactgttcg
agtgcgttcataaaagctagtaactctgagactccacaaggattcatttgcttttgcagagcatacatttacatcagcgtatcattatttacatatct
tgcttagtatcaacgactgctttggtggtttagtggtataatgcttcgttgccatcgaagcgacccgggttcgattcccggccgaagcaattcttttc
attcaactgaatttgactaaaaaaacccacattaattcttttcgatttattttttttgtttttagtaaccaaactgctaccagttcagtacctaaaaaa
ttatgtaaacaaagcagaacttatggtattaatggttgttcctcaaagaaaacagtctaatcctgaagcagaataagtagggtgaatcgcttggtc
tctatcgtcttgtaggtttctagtagtcctatatttaaatttttcttgttttgcatccttggtttcaaaactgctgcagcgtctatttcatctctggtaatta
caaaccaacaatccttgtgaagagtctttatagaaaaataatggatggcacgagattggtatttataaatcaattacaagactgtccagctggga
aaaaagttcgctttctgggatacgttacggcttatgatgatggaattttatgtcttcaagatggattgagtatggttgaatgcgatctgaccgccgta
cttgcaaatgttaagattcaaccacgagaatggttggatattgtaggtcgtaaatgctcagatggaaagatcgatgtactgttagtgcgttcagta
gtaggaattgatctcccaaagtatagaaaaacacttcaatggcgacaggaatttgcatctgaatgggattccctttaaacgctccccatcttcaatt
ttgtaatctgttccgagctgggttcaggtcaaagtacagaaatgaacactataagcgttgcattgttgattatcttacaatataatactttgctcatg
aagggaggaacctattaaaaaataaaaaaaaataaaaaaaaattggatgactcaatgaatacctaaattgattctaagtatatatcataaactaa

taaacaactaaattttaaagaacgaacaatgcaaggaattatcaataatttttggaaagcccggttaggcatcagaagggtggctatccgtttttaagcccagatcctttttcatcggcgacatggaccactttgttaatttgagaccttgagactgggtatttggtactgacagtgtccgcggcaaacagaaaataccccaaaaaggtaccgcaaaaaagaaacaatataacatagccattgtagtacatagccaaaagcatggcaatataagtaactatgaattggacaatgtagaagcaggagcgtataaagtgaagacatacacgggtgaaccagctaaagccgctaggagaagaagcgacgaaggagctttcaggagaatgagggcatgaagagttaagctgatctcgagaatgtcgaatacaccaacggtcgaattcacggtgtaagcgacgaatgagttccagagccatcatcataaaaatgataccaaaaatacttccaacaaacatactcttgctacgtatatgccagtgtttgctgataaagcaagcatcaattgtgtaccagttccaatacatggaggttttacaggaagcaccggtggaagaagcagtagaagagctagaagtagtggagttcgtcatattcgacattccacccatagtcgtgctactcattttggaggctcctctttggaaaagctcaacagcagaagcatcctttttcaggatccaacaacgattttgaattttgtttcaagcttccgcctaacctaattaaggaattacccaggttttctttggcctgctgaatcaacgacatatcaattaacaatgaagcaacaaaaagcaaagggaaattggaattaaataaagaggatgaaaagcactgaatgtgatatgaagaaaggaaattaatataaagggaggaaaaataaacaagttaatttttatctctttgaaaaggatttttttttaaaaaaaaagagtagagaaaaaaaaaacgaatcaaaaaccgaaaaaagtctgagaatcttcaattctactaaaaggaattttgacgacttttaagcctaaggcagctttacttttggtctttcttttcgtattcgtaaacaaagtcgctccaaatcccttt
gttgaaatcaaatggtgttaccgatggtaaacagagaaatgaaggaagaagaccgtataaataccaaaatccacatttgaggtatttttgatgtttccactaaaaaatgtctcaaggaagcacaattgtctcgtattgttacatcgataaccgtgcgcgctgctactcgtaaacttagcgagtcgcttatactaactgtaaatgaaacgggctaaatgatgtgtaacaaatgcgatgagtgagtatgtatagacacaaaaaggataaataaaagaacaacgatgagaaagaatatattaaataaacctgatcaaacccatcatgtccttctttacacgatacataggattatgagactccttggatgaccagaggtgcattctagctcgtctcattcacaatttttattataacagttactctcatacaattttatacaagacataaaggaatacaatccatgtaagctccaaaaatggaaaaatttaaaaataatggtcacagccaggctcgaactggcgacattcgacttattagatcgacatgataaccaactacatcatgcgaccattaattgtaaaaggagaaatgataactaatatgttccaaagtatgaagcttgaatgaaattactgagctggtaggcttctgcaatatgagagttttccactagatacacaaagaaaaattacaaatcctaataaaggccaacttgatgaatcaagtgagaatgtcaaaaaaattgggctgttctgggaatcgaacccaggaccaccatcaccctaagatggtatcataccactagaccaaacggccaattctagtaggatatagagttatacttattaagatagttggacatacgaaactttctcctcgacgagctccttagttcacttttctcctgtaagtatatttttatttatttactttttttttttcgtcttaaccattggcttttttaaataccggtagcgtggatgaattgctttaaaacaatgataatctatgcgtttatgcgcttacaagaatgtttctcaggctgttcccccttttctaaaccgtaaacaaagtgaatggaagcaattaactaagttcttcggctggatgtatggttttctgtttttctttttacaatacgttttctcttttagtgacagttcaaccagtaggactttagactaacgaaagtttgatttctttagtacaggtttcattctttgtgtttgcaattctcctaaaggacttcatgattagattatatttttgctgaaagtaattaatataaaaaaaaaagaaaaaaacttgaagataggaaaacgtctataaaagtataattaaaaactgtgaaaaggtgaaaaaaaaaatttgcttcggccgggaatcgaacccgggtcgcttcgatggcaacgaagcattataccactaaaccaccaaagcagtcgttgtatttctcgtacagttaagacataattaaactgtaatatcttttgccttaaagagtcactagagagaaagttcctactacttttctttcttctacagtctttggtaacgttgacaaaacgcgtaaatccaaggtaagagatttcctgaagaatttctaaagagtcataggtgtctttgcatctataataaaaatttatcaacctagaattggctggaagaggcaaaaattacatgctcgtgaaaccgatttttgaattttgaagcagtcatctcaaactggttattttgaattgcattaattatttttaatatgtttcattttataaagcagagctggatgcggcaagggatccttatgcagatccgaaacataaatcgcttatatccaaagatgcggatccctaaacgaccagaggttccattaggaacaaaaatcgctgaaatggatgcttctgcagatgtctactataatccaccagcatcttcgccggacacccgaatcacaccgctgttatttcgatatgatgctccatcaacagaagaacgggagacattagcagagaaaatcaaagattcactttctccagttgtttccagaaaagccaaatcaaagggaaaccttcgtttttcggttaatcacttgtcagatgaagaaaagaatgcaattcacgacctgcggttgtcggatcccaacgagtggacgaccggagctctgtcaaaaaagtttcgagccactcgtcttttaatttctaggatttgtgaagctcccaaagaacgcctggcagcggtggaagaggaactggaccagcaaaaacttcattggggaaaggcgaaacgccaagttcggagagaaaagctttctcgaaataactttcgtgtttcagagcgtgtttccaaaatttaatactttgtttaggttcataatggactgattgatctatcaagatgattttgtatacctctcatgattgctttttgtttatcaacaagaattcatctatatatcagtaaagtatgcttctaatagatttcttttagcgaattctcagagtcacaaaccaaaaattactcaagcttgacttttcttttctattaaaaaccccccaataatctactgaagtgcgatgaataaaactaaactttaacttcacaaagacattttaaaacggaaaaattaaaaacttaatccaaggatttttttatcttaatagattttgctttttatatttactatttttgaccaaaaaaactctaacactctcttacaatgttttaaatgattctacatcacatggtatattgacatctcaaactgtcttgttttcgagttatccgcaccgtcctgaattgtataaaatatgatgatctacaacaaactgtagagtctaaaacaaagttatgccacacgatggcaagtttttgggatcaacgaaattaatcattctgaggaatattcaaaagaagcaaagagaataaggatctctttctttccgcttcctccgtatcatcgttaattttgttctcccttgttcaataccattcagcgggatctggcaaagtgacatcagcacgatgcaagctgtagcaggtggtctaaagaatgatttagtacgtacgagaatgatctcatgaattcaataagttacgtttttccttggcgtaactctatttattcattttgtatgaaataaacgtatagcaatggtgcgtactatataaggaagcggtcgtcgagaatgttggtacatatattccttataataaaaccttgagttgtgagtcaattgtaatttatcgaattgactaggttgctattgaatactactattgagttactggtaccttttcataagtaaaagcaattatcagacaaagaatttcacgaatctacataatataccaatcgaaacctttccttactgaaagataaatcagttcaatttcctttttttttcatgatggacagacgtgtaaagattggcggtatagtggctgctggtgcagctgttgcttacatgtttatgaatcgcaataaagaaccta

aaaacaaattggggaagacaaatgcagactttatgaataccattgaacaagaaggccgccgctttgatcgtgctgtaggcgacactgcagataa
ggccaaaacatttgtacaggatagtcgtgacaaggtcgcaaacgaactagaaaagcctcgtgaacaaccgcaggatttacagagagatgcaaa
gacaaaaacagaagaccttttgcattctcccaagaaaaaagatgaatccaaatggttttaagtcgaaactgcgcatctcgaaagggaccgactg
atgttgatatgacatcttcttcaatccgttagcactgcgactctaagattttcgtattgatacttctgttatgctttatggtttatgtcattatgtttaaat
tacggctacttttagatcaacatattgagtagtggttccaagttatgccaacaatgttaatttttagtctacctattcaaaacaaaacaacaattgt
gtattgtgccattgtagtttagtaatctgtactattattcggttataaaatgtacgaaaggtatcctttgagtataatatccgagtaatcttgctttca
aaaaggaattgttacattagagtactttaatgcaagatatgaaatttcagggaacgtatttctcgattcttcagtctgtccttatacttcgtactgga
ccgatttccctttcttaatgctaaatgaattctattcaactgtacagtaatacgttagcataggaatcgtactgaaagtatgaatacgttcagatga
aataagcaaataagtgcttgtatttgtgacattagagcttatttgacttgtaattgctaacagttactattatcatccactgaatcgttgaattaagtt
gcataaacaaaagttgtttcgttcgaccagtaaataatgactgcaaaacagaagacaatatttactattctaattgttccaaaatcactcattcatg
aagcgccatttaatgatcaattctcaagcattctcacaaattgcgcttcatgactttatttcttgtcttgatttaagtaacaacgtaaatctatatact
ctgtagtattatggatgttaaagaaagtacaataatattataaattcctttgacttatgaattgtcgcggtgtcgaggcagatatcttacatcgtata
atatgtaaatcgagtggaatgaacaactctagctgcatcgtaatatgcgtattgaacgcttgtactacgtttatagtagctactgttgtaaaggaat
ctgattagattaactaaatgagacgcttgaaagacagtctagtctcatttagtcagaccgatcaaatcttccaatctggctgtaaattgatgcgtat
atagctcttttgaaatagcgaacgtaatatgcttttttttttaatagtttcccaatgtgctcaggttccttatctcataaaccatactttccatagcgat
atcttaaaatcactgtgtacagtaatacttttaacgttaacaaaacatctatcatggctgccatagctaggtttttacgctgtataattataagactg
agtgtatgaatttgagggttgtaaaaagttctctttgtatatattgggctctacatagtatgtactataagtattttcagagtccactgactagacta
gctaaataaacgacaactagatttattaggccagatccgtcaaatcttccaacctgacgattcgtacttccaacgtttggtaaatatatcttctaaa
acaaataacgttgttccttttgataagataataagacctctattttattataataatacaataattcggccttggcagtggcagagacgagacgcta
tcgtattagtgtatctttaaatatcatcttagctttcatacttaatacgttacttcgaaaataaggcacccttgtgcaatccttgacgtttcaagtactg
atggctttgcacgttctttcgttaaccaaattcaacttagtaaaacacgtccgtcacagtctattcgacgcttgatttacttactgatttgtttgcatct
aaaataaaaaagatgaccatgaaattctcgcatccagcgaaacgtatgttgaaagaatcaatgtaaacaaagcactgctttgagccaacaaga
gctgtgcttctagatttcgtttgatattcaggatgttcttttctaatctttcactttttagcctattacatgacaagcgatccagaatcgttcgcttacga
atgcgttgttcggagatggccagccttgctgtccaacgtcgttgacgacatttcccgccatgtttctaaggctctggaagctggcatgtcagaaaat
tcttcctatgttacccaaggaaaatccatcatcagtcagctgagtcatctcaagtatgacattcagcataaccgtcccttattacctttggagggag
aaggatccgacttggatgagtataatgaagaattgagtcaaatcggccccttgacttggggggatgctccttggttgtatgctgaatgttactttta
tcggatcatgaacctcttcttacaagctcactctgagtggaagcgccatgatcccttttttgaccaaaaagattctaccctgaagtctagtcatgcg
gccattgaagacttagctgctcgctatgtccaccttgcccgtgagcttgaatctgtccaccaaaaacacgaacccgaggtggcctacatgattttcg
ttgaaatggcgcaggttgctttatggggaaatgcaaccgaccttagcttactaacaaatttaacctatgaagaattgcagaaccttcagggccga
aaggctgtagaagaatctcaaaaaaacattttggtgaatgactttccagcggtctggcaaaagcttcaaagtcatcggggtggtcgtatcgatttt
gtcttggataatgctggatttgaactctatgttgatcttgtgttcgcagcttacctcctaaaatcaggcattgctggcacaattattctacatcccaag
gatattccttggtttgtttctgacgctttagtgaatgatattccgcatatatttacttctttgacatcttatttttctggtgataacattcaccaactcgct
tcagatctccaagagtttcatgctgaaggaaaaatcctaatccgccctaatcctttttggacaaccgctcattattttggtcgtatgcctgactttgct
ccggctttagttgatgatttggctcagtcggatatggtcatcttcaaaggtacatttgtctcattactttacttccctactaacgattttaggtgatttg
aattatcgcaaattgactggcgactgtgaatggcccaagacaactcccttcgaagttgctcttggaccccttgctggaaaatttaatatcctagctc
ttcgtacatgcaaagcgtaagtaaattttcgaaaagagctactgcttactaatgtttagtgatgttgtcgtcggtcttgaagaaggcttatttgaaa
agattgctgaacaactccctcattgggaacgaacgggtaaatatgccgttgctcaatactgcccaagaaattgagtttacatcttttgaatttacgt
aatcttttgactatttatacagttcgatagcatagaaacaagatgcaatgatgaaatgctattctcaatgaaagtaaatctatgcaccaaactattt
tccgtttcctaatctttcaatcctttaatactcgtttaaaaccaagggttatgaagctttatttttgctctgcttttattaaacaatgcatgttagacacc
cactcccctgtcgcaaatatgagtttcttttgttgtttcattaacgcccacgacgtaaattttcctacttcattgcttatcgtcgataattgtaccagttt
tttgtttactactgcagtgcgcgatttaaatttgaatcggagatttactcttgaatcatctctgatactgataactttaccgcactgcaaagcaatcca
gtaaaaatttttagcgttatcaaacctcggcttaatgtcggtgaggttgcgccgtagcatataaatcacgtaaaagacggaatctgtcctttgcact
taccaccactttctggtacgccattacaactcaatttttgacccctttcggtcaattatcgccttttaaacaagtcaattcttgtttaaagaagcgtatt
ttaggtatattcctttttccgcttaattatctcttatttttcacttttcttgtctgtgaattgattcatcgctgatccttccttcctgtgagatcttatttgtt
cttctttttctatataaccgtttttatttgcgtttatttctccctatctaaaaatggggttaaaatggaatcctttctctaagtctaagaagcccaagga
acctcttcctctggaacagtatgaagcggctgatcaaaaaaaaaatagctggcttagtcaacaaacaagaggcaaagctccttatcatcgcgggta
ctggatttttattggatagttacgacttgttcattatcaacctcgtgtctccagtcatttcttatttgtactggggtggtttagaaggccatgaagacta
cccatcgggaattcgtggtctcgtaaatgctgccacaaacattggtaacattatgggacagcttatattcggcttcttgggtgactttttcggccgtc

agttcgtttatggcaaggaaatgatggtcgttattattgccactattcttttaattagtcttcccgaccgccttcctactcctcttgacaaggcgatgt
ggctttttactttccgtgttgtactaggtattggtattggtggtgattatccgatgtctgcttccatcacctctgagcaatctatgattaaccgccgtgg
tactttgctagcttggatttttttccaatcaaggttggggaactcttgccggttgtatcgctactctgattatcctcgcgtgctatagcacatctttagac
aagcatggtgattatcacaaattggatggtgtatggcgtattcaattcggcattgccctattccccgcttttcttgtcttgattcctcgattgagaatg
caagaatcgaaacaatttaaggactcgaagaatgtgcatgctcttaatgaagaagaaaacaattctcagatcgatgatataaagaaagaccca
gaaacgtatactcccatggaccctgcccagcattcggacccaggaaaactaaccgaaaaaacatctggttctgtacaagttgaaccacacgatg
cttcttctgtgacttccaaagatctatcttctggtaagacggagcagaagcaagaagtaaagaccggctctaccttcgttgcctacttttctgagtg
gagacacatgaaattattgcttggttcagctatctcttggttccttttggatatcgcattttatggtgtcaatcttaaccaatctgttcttctcgacgag
atgggctttaacagcggaagaaaccaatataagaaattgcagagaaatgctatcggtaatttgattatttccatcgccggttatgttccaggatac
tgggcatctgtttttttgattgaaaaattgggtcgcaaatggattcaaatgcagggatttgctgtttgctctcttttgttcgctgtacttgctggaaaat
ggaatactatttccactgctggaagatttgtatgtgttgctcttgcccagtttttctttaactttggaccaaacacaacttcatttgtttatccagctga
agtattcccttcaagagttcgtggcttctctcatggaatctgcgcggcttgtggtaaagcgggtgccattctctctggtcttttatttaataagttgac
ggaagtaattggttttgccaacgtgttgtggatcttctttggttgcatggtttttggcatgatattcagtttgcttttgcccgaaactgctggtcgtgat
gcagatttggttgatcgccttgaaataactgcccttcaacaaggaaagcattctattatcgaccggactgagaaatgggcttggtggaaacacgg
attttaattttaccttgtgtgtattacatattttgttgactacaagtcggcaatttgttgattgttgattgtttgttgtttgtttttttattattattttcatatt
tccaaagtttgaagcagtaactattagagatttcgtgacttttgatttgtttaagctgttcttttgactttccaatatttatctttactatgtttatttttat
gcacaaagaagattgatgtacttggcagttttactgtggatatcatctttaaaacattagtaaacagtgggttttcgtagagttagtttgtttctgag
aagtttgtgtttcttttgataatacttcttccaagaccttatattatatatatcgaaaaaaaaaggaaacgaaactaaagcattgttttaattaatat
atatttattaaagttgtatatatatctaataatttcttttctgtatacaggcataagtggtagattccataaattcgtcgttcattatacaattatgttg
gtagactatagtaagtctcttgatctaagaacttgagtactagccctgtaaaagggcgctcttccttaatccatttagtaatgtttgctagcattgta
atgatgatatggaaacagcatggcatacgtctcttcagtttcataaaagtaattccaacgtattgtatttacttttttttgctttgttttacttataacat
taaaatgaaaacttatactcctcttgctgttcagaaatgctgctaaaaaaaagagtatatataaatatcatctcatttcgtaatctttttttatttata
aatataatagtattcactgtcttaatcgttggtcaagatctaagtaaatcaactttcctatttacagttttaaagggcaggaattctgacattcctaa
cattggagcacagtctagcatactaaaaatacctagtaaatcctactgaaagacatgaacgtaaaacgaagaatcctttcgatgttagtatagtt
aattaaagggaaataagttaagattgataaaaagtgaaggaactaaaacaatcattctcgtgtgcaatattttgacccttttcattatgagtgcgg
catggaggaaaaaactaccaattacttataaatcggaatgcttggatgtttacagaggattgttaaaatgctgtaaccaattcgtagagcccgaat
accggtctgttctattagaaatagtgcgaacaaggacccgccattatcagaaaataacaaatgcatataaagccgaatcacttttacaaaatgct
aaaaaggtatgaaaaaagaagcccgaaattgctgacatcgaaatagtatcgtgaacgacttgaacaggccaatcgaggtcaaatgcctgcgaa
acaaataagacaggaaataatgaaaaaatataaatcctacttatatcaaaaaagacaagaagttcccatgaaacgagaacagcgtttagcttc
aaatttaccaaaaccaaaaaaagccagtttctatccaagattatatccgtgatgggaacattctttaccattttgtaatgactaccggaggagatac
ttttttgcggccaaaatattggaagcaaagtgagcgcataagtatgatgttgaaaaaacgaatcgaaaccaaagagcatagacaccgacttatc
caagcaatgaatcatatggtctatcatgcatccttagaggatgagtttttaaaaccatatacgaatgaagacataggatttgcagaatcactcata
gagaatgtccgggagattggaaaaaagcttgaaaaatcttaccaacaggcgtctaaatcctggcatattacaagtcacttatatagaatgattgtt
atggaggcagaacgacaacggctagaaaagcgatattttaaaacctggaaaaacttaggaaggataaggtctaggcatatagatgataactag
cattgttggcgatattcaacataacaaaaaatcaccaacataattcaacatccacataggaatgcatagtagagaagtccatcacagtattttag
attagttattctaaacaaattataaaattcccatttaataacaattttgtctcagttcatcgctatgtcgcttgctatatgcagtagactgattgataa
ttaactagggtctatgatattattttgttttctcgttcgtcttgggtacttggtgcatgttgtgaagttacccagttattttctatgtcctgtagcctttgt
aaatcgtcatcatcaacaaaaaaatcgtctccatcatcatcatcatcatcatcatttaacgtacaagtagtcgcggactcatctggctcctcctcgt
ctaaggtctgttgagtatgaatgttctctgaaactgaatcttgcgaactgcttgactctcggtcggtatccgaagtctcttcctcattagaaacttggt
cattaacatcagcctgaatactttttttgtcttatgtttttctttctctgttcttctcttcgatctattgtagatcgtagtaagcttaacaaattagatacac
gagaaagcttattctttggctccggaatttcgttcgagttcaactccaaagtccttgaacgctttaaagtattccttgagttatctctatcaatagact
tattggttgtacttgaaggcatagacacagaccggtatacgacttgattttgctgcagccgatcccacaagttgacaacaggatcgtttatagctg
gacttggctctcctgaagatttggtaaatagctgtgagatttgctcattagacaacgatttacttttcattggtgacaatttccaagatacacatggc
tgatcactttttggagaatctggcatagcttatatgaaataaagcgatttcctttataagagcaaattaataggcgactaaacgttcgttgaacaac
gatcgacagaagaatctctagctgtacggttcaactcagacaatactcagatctctatccttgtttttttatcttttttttattaaaaaatagtttgaatc
aatcacgaaagaattctgtaaagctcaaaagagtcatgtgtaaaaaacacgttctttttgaaaatttggagaaatcttggtcctttgcaattacca
aagcgctaattcttagttgccaaataaaacagaaaataaataagtagtattttgtaaattaaactaaagaacgcgcacttgaaatatttggttact
gaaatcaagcctgtagggtaacgtgaatctgactgtaaacaaaacctttgtagaacagataaaagacaatatgcacaagagaaattatctttta

aaaccatcaatgcctatataaacctatccaaaaagtcctgctacgtgtaagatgtggtgtctaccaagtatatttattttacatgacatgaatctat
caaaaactacaattggcaaacatttaaccaaaagaacacaagcagttcattcattcacctatactttaaattgcaacaagtgtgatctaatatcat
ttaacagaacaagatgaaggctaaggcagtaaaaacaaaagtgaagaataaagtctcaaacgatggaatagctcgaggagaagaagacatt
gtaatgagaccttgtgaaccatgaaaagaacgagtagaataggtgggagaatagaaagaggtgtagacagtagtagttttctcgcgagccgtg
gtaggcataacagtctgattaatagtaggcaacctaaagtcagacagttttgtgtgagggtataaaaccattggataaccactggttaatgtagc
ggtaatattacctccatgtccaacggtcagagttcggtgcccaaaaacatcggaaacaacttcgttctccgagtattgggtagggactttgtagac
agttccattatccttactaccaaggttattatataaaaggaagacatcacgctttttagcagcaacatgattcagctcagcttctataattgtgtaat
tatggcgtgtaaaatcggaatcctgtttcataacctgttttcggaaattgactatagttttaatcatggtatagtaaggattggtttgatcgtacttag
atgtccaaagagcttcacgattggcaggatccaatccaccatagaaaccttgttcttgtccataataaacagatggaataccatcccacaaaata
gcaaaactataagcggacatgataatcgcttgatcgtcagtataagatgcataacggggcaaatcgtggttgtccaaaaaaaattagcatttgtcc
aagatcagagtttgagcatttgccgctagtcattagctccgtttgatccttaagcgtcccaaaacctttgtttggctgaaatttgtcatgagccataa
aactaaaattcaaacctagacgaacaacgtagttggttacacctatatcatagttcatgtaatcacacaacacatttgggtcatagtcgaaaacct
cacccatagaaaatacaccggcagatttgacaaaatcagggaaaaagctttcgttcatcatcttgacagcatcaatacgaagaccgtcaaaatc
gagttgctgcacagaactgtgaatgtgcttatcaagatattttttacatcggagctttccacattcaagtcggcaagaagcacaccagaatcttgc
cagcagtcttgaatatttgtcttattgtcggatttgttccaatcaatgatacaataaggatgataatatttttccttgttaaaaggataaaccttagaa
taatcaacttgatcataaggaccaaccaatgccatagagttaacaactgtgtccaccattacccatatatcacgcttgtgagcagcgctaattaag
ctaagaagatcatcttgagtaccaaaatgttcattatattcttcataattgatgttccaataaccgtggtaagcctcgccggcgccagcgggatcac
cttcaatattcttgtcgataggagaaatccaaatggcagtaaagccaagatcttcaatgtagtctagcttattttcaatacctttccaagtaccacc
acagtaagcattggaggaaggattgcaaggatggtcggtgcttccgtcttctttagcaaaacgatccgttactatttgatagataattcgagttcgc
cattcgtcagcattacgggccgtggtatatccaaagaatgcaacaaggaagaagaagggaatcaaaagcgatgatagattgaacatgaaaact
gttataaattctgaactgaatgggtataaatagaaaataaaaatgctccggaaaatgagagatatataatgagagatcgtaaatttacagattaa
aaaagagagagatgagaagaaaagaaagcaattaataaaaaagaaaagaaagtaataatgaataaaatatgcaaatcaaaatgcaaaaaa
gagggatgaaatgggtatcaggtatttttcaaaaccaaacggaccttttttcaaaaacgaactttcgctaagctaagcccccgaaaaaagaataa
tgcgaatagcttaaagtattatttgtgaatatataacgataaactcctgttatatcaaactcatatatcttcgtaacaaatttcaaaactctgctccg
ctgaagataaaaaaaataaagtagaaggagggttgaatcagccctccgcttgaatatacgtagcagtcaccagtgctacctagaagtatatata
tttgtaccttgaagagtctccttatctgtacttacggaatagtaccgtaatgacgttatcttgttccttgcaggaatgttgccttttcgtaggaaatga
aatgctttgcttctgctcatttggatgaatctctagttataagatcacccaggacgatctgtaatcctggacaaggttgtgttagcattgccatccag
ttttcgtagggtcaattcgctgtccaaaaaaattcatagaactagttcataaattataacaggagagcaagaaaagttgtttgacgaacttgaacta
cgtgagcacaaaatggtggattttcaggagatcgattcgctaccaaccccccctccctcctacgtatgagaacccaagtatagaactagtgcataa
acaaaagtaagcactggcacgtctccacaaggagatagaaagtctatcgattccattgaacgcttgattagttgatcttttttttaagtatactatag
tagattccatcaggggttgtttgttactctccctgtgggccaggttttaaggagttttaaagaagaaagtattggggtatgtatgggtgatggaaac
aatgaaaacaaagttgcataattcatctatttcagacttttgtgtacattatctagtaggaagggaatgaaatccacaaatcttgttgcccatttag
gctcgtagatgtacagtcttgttttcatgtatattccatgttgtatgcatttcctttctctatcaaaagcctctggtcttcttggatattgtcacggaaat
catactagggaaaattcataaaatagggttacgaatagaataacgactcggcggttatattgtcaagttgaaattcagttggagttctagttgcta
tatattttaaaattcgtgattgttcagataaatacgctgactggtcaactaagtcgtagcattcgaaatcaagttcgctggctctcacaacggatac
ttctaactaaaggttcttatggaaatagccttttgtttgtaattattctgttctagaaaataagcaagtacttctaaactcattgatcactgtattacta
ttgttcttttgttttgtatgtccgtttgtgtttatttttttcttgatcgaggcaacggcataaaacaatttgctctttaccgtcatatctgttgataagagtt
agtaatatttaagcttcctctgacagaatccaaatggcactattggcactggcactactagtactactccattgtaccttcctacaaaaccttcgtat
gtagaaatccaccaacaaagtaacaactcagaggtaacgctttgatcatcagtattcctgtcaagtcggtacaacataacctgcacgtattttcga
cgtgggaatctttttagcgatcgtcccttttttgctaaagaataaataaaccagaaggaaagaagacgttctttttgtggtgctataaaaacagagt
cgacaacgtcacctgactttagactcgaattgaaattgccaagtactctcctttctgcatgctagtttattaagttctattcctatggaagaagcaaa
ggaactgtacgaagttctcggacttcctattgatgtacgtagaaatatcccagttcttttctttactaatttttatgaatgacaggcaacccctgcac
aaattaaagatgcatattataggctttcaaagctgtttcatcccgatagacataatgttgaacaaaagttaaccgcagagcaaaaaattccagagt
gtacaagctgcttatgaagttctgtctgatccgataaagcgacaaatttatgacaaatatggaacgaaaggactaaaagtagagtgggatgtcg
gacctgcaggacaatcaactgaagaattgcaagaagctattcgcgaacaacttcaggctaaagaatcccagaacttagatttattggtacaaaa
tagaactgaaacaaatgttatgattaatgctacgcctttattcgcaaagaatttgcgtgtacgtaatcccttggcgttaggtatggcttctactagag
aacttacattgcttgagcgcttttctttagtacagtgggtatcgtttcatttgaaaaactcttttagtattcctttgagtacttctaaaaactcgaagtt
tcctattccaaacttctttcaaggctcttccaaggatgatgatttttccgatccgctagaagataatgagtctataaatggctcaacacgtctcatttt

tatcaccgaagcatctgcaaaaccaggcgccaggacccaaccttctctgtctgccgttattcgacatcaaccttctgctcgcttaagcacagaaac
aggattatctattttacgtccaggaattgctactgtaaaagctatatatgctttgaacaatgaaaccttcgttattcctatgattcgagtgtcttccta
caagcgaccacctcaagctacagtcgtgatagggcgtcaaatttctcgatacggcacgttatctgtacgctggaaaactggaagttggtctctagg
tagttggggagctgcatatccttcaaacaatgcgtcttccttttctatcatctggcaacaactaaagccctcactagatgatccatttccagtcttga
aatggaacgccgaactcacagctggattgttatatagtggggttggctacaattataacattgacaccttaggtccatatcaactgcaatgtggaa
catccctcagcaccgtcggaggtctacaagtcactgcagacgccaatcgaaaagttttccgtcatagtgccatgggcttaactgttgctatcggtct
tccatccggtgcccttacattgaacattaggtatgtcgtcttcctttgcgtaagtgattacatactaacagaatataagctggtccagattgggtcag
aagttttcatttccaataatgtggagtagtgctcttgacagatcttctgccttttgggggttagctttacccattggatcaattttaggcgttgattatt
tgttcttacgtcctagacgtatatccgaggagaagcgcaaaagagctcttcgtttgcaacaacataaaaaattacatgatactaagaaagaagca
gctgagcaaatcgtctcattaatgagggagcttgtccaaaagaaacagaaaatggaggctgaaaaaggtggtctcattatcgaatatgcagaat
acagaactattcctagaaaatcagccttaactaaacgcaaagagggagttataaaacaagatgttactataccaattgcttccctggtcgacgat
tctcgcttagttatcccttccagtgtctctaaagtaagtaaaaaatggataagctaagtctaaccactgcagtcttccattattgggatttatcctta
ttttctgatgcagataaagaattgttgattgtatataaatttcatcagcagtctcatcatgttttgttacgagataagcaaggggttcttttacctaat
cgaggtatgaatttaaaattaaaaaggtcaattactaattaataattatagagcataagttgttataataaaacttattatttgtttatatttgtattg
ggattctgaggtttgtttgtttacatatgaataagttacatataagtctatcaacgctcattaaattttaaccagaacaattccctgaagcgtaagac
aaactgcagtctttttcaaggctaggttagaaggggttttcgttgaatcctgactgtatctcgccgtatccagttaaatactgtgctttagaaaaaaa
aaagacattctttttttgaaaagttatgtttaattctaaatgtacatatgtcctgcaaagccttttctttttttatagggttctccctagcagaacgaata
acggctgatcagtatggctttgacgaaatcactttaaccgctaatccacttgagcccctcaccgagttcaatcattctttgatgaacctcagtggtgc
cgcttttgagcatggttctttgaactactcaacggatctttccagcgacaccttatggtcttctcgattttcaacgcctctaattgagacccctgaagt
taaatatcccgaattaaatagttcggtagttggtgagcaatttgctgagtatttcaactctttagattttgaaggaaaggcggcattggctgctttaa
atgctttacagcactcttatggctcttcgcatgttcgcaggtttaccccgtatgagctttttggtcaatccaattgggtcgaagaagctaccgaaatc
aatcatgtcgcttggtctattatgtttgataaccttggccgctactttttactggaattgagaggacttcgtgaacttactttcgccttctatattttgtt
tgctcttctccctatcattactggtgtttcgacagtttatatctttaaaagaagatattacgcagtaactttcaatcaacggggtcgatccgataaatc
cgttaaacagcttgagaatacttctgagagtgattttaatgactccccaaaatttgcagcaactcttgcgttaccagaaaacgatgaaccgttgatt
gtgagggaacctcctcaggagcgtttaagtattttatttgctactcttgaatacaacatcccagactggaatattaaaattaagattggtggtttgg
gagttatggctcagctcatgagtaataaccttaaacgctatgatttaatctgggtagtcccttgcgtcggcgatatcgtgtatccagttgctgaaact
gcaccttccatcatggttaaaattgtcaatcaaaattatgaagtgaaagtttactatcactatctggaaaacatcaagtatgtcattcttgattgtcc
tattttcaggaagcagacgtcccgcgatccttacccactccgcatggacgatttttcctctgctatattttatagtatttggaaccaatctatcgcagc
cgtaatgtcatatgagaatcctgatatttatcatatcaatgactatcattgtgctctagctcctttatataatcttccgaaaatagtaccctgtgctgtt
tcattgcataatgcggagtttcagggtctttggcctctccagtctgcacgcgatttacgagaagtttgtggactttttaatctttctaaagcagtatgt
aagggctacgtacaatttggcaatgctttcaatttaatgcacgcagctgcctcatacgttcgcaagcatcaatctggttttggtgtggttggtgtttc
cgataagtatggtcataggtcttgggttcgttatcccgttttctggagcttaaagaaggttggacatttgcctaatccagatccaactgacattgatc
ctgctatgcagtcggaagtacaagagttgcctgatttggttcaatataatgaaaagcggaagactgaaaagcgaaaagctcaggaatgggccgg
gttgattgttgacgatactgccgaccttttggtgtttgttggacgctggtctgtgcagaaaggtgttgacctcgttgcagaccttgcgcctactctatt
agagaagtataacacacaacttatcgtcgtaggtcctgtaattgatttatacggcaaatttgctgctgaaaaattcgcatatattacggagcggtat
cccggacgcgtttttctcgccctgaatttgttcatttaccttcattcatatttgatggtgctgattttgcgttgatcccttctcgcgatgaaccatttgg
tttggttgcagtagaatttggtagaaggggagctatttgtattggtgcatgcgttggtggattaggtgaaatgcctggctggtggtatactgttgaat
cgtcaactactcctcatctcttgaagcaatttgagactgcttgctctagagctttaaaatcttctcctgaaacacgccatagtcttcgtgtggctgca
gttcaacaacgctttcctgttactgagtgggtgaccttatacgacaggttaattaagaaatgcattaagacgaataggaaggtatttaagtcgaat
agctttagtgagcaatctggcaatactcctgccgagttgccaatcattcaaatagacgatacagaccgagttgacatggttgacgaacctaacatt
gatagccaggaaatccctgaaacaacagaatcacccactccggcgcctgaaagcaatagcttaatttctgatcaacttagcccagaaaaacttg
acgaattaatggaagatgataacgattcagtatcatcttccgtaattagtcgacctttatcggaactaacttacatctctcagagttcaatgaatttt
ggcactaaactcgatgaaagatttattgatgcaaatggtgtggcaatcacagaattttcagcagaactaagttccttgaccccagaaaattccaa
atcaaaactgtgtatcgatcactttttgatgaatgtcttgcataaatggtacgacgaagaacaccactattataagaccggtttccgtcctcgctttt
acaagtacttgagaacgagaggcaagaaaccacgagaacttaatgatggctctagcgaaaaccttacggaaggctcctttaaggaatttgacgg
ccccgttaaaggtgtagctagaaatcagctatctacatgtcagcgagtgatgtatgtaaaggtatttggctggccaatgtatgctattttccttgctt
tgggtcagattttatcaatctcttcatttcatttaactcttttatccggattcgaaacaagtcatgttgcctctatgtatgtagttaacgcaatatttata
ctctccactttcttttggtggggcctttacagaaattttgctagcattcattgtctgtcaatcccgtttactatatatggtctagcgtttaccatgactgg

tatctcatccatgcccttcattcaatttacgacgagagggtggttgagttatggagctacttggatctatgctatcgcagcatctagtggacctttgt
attttacattgaatttcgaagacgagcatactaaaggcttaggttcttgggttacgcgagcttgcattctacacggccttcaacagttgtggacagc
tcttctttggctttggggaacggtatatagtcgtttagactggaactatgctatactaatcaaacctttgaatcatgtactcgttgcagctgctgtttg
gccaattgcatttgtgctcatgatgactacgatcatgttgtacaaaggcttaccccccttttatcgacagacgccaggtagtataccagcatttacta
agtctttgttccatcgtcgagtagttgtatgtttcttacttatggttgccaaccaaaacttctggatgtctacactaattggtcgtggatggagattttt
ctggaattctgagctcaccgtgctttggaaagtagttgtcatgatgttctctttccttgttttcttatgggttgcattattaacgttgttgggaagactat
caaatacccatacttggttaattccggtattgggcttaggatttggaggaatcaaatggattcatgtattctggggaacctcgaacattggattcttt
ttgccttgggctggaatttcgggagcatatttgggtcgtgctttgtggctttggctaggagtcttagattctatccaaggaattggatgtggtcttatt
cttcttcaaacgctatctcgaagacatgtgacgaatacattgctgactggtcaagtgattggttgttctattacactactagcatcttcagtatcacct
accaaaaccggcccaggcagtgtattcccagatataacttcttatacgccagtggatggattacagccaatttctaattttcccttttgggcatgtct
tttactaaatgttattttgtgtggtgcctatctaaaatgcttccacaaagaaaatataaacaggccttaatgatttcttatagaaaggtgttaataca
ggtctttttttatgatagtttaatataagatttatatgatttgtacattagcaaatgttttattaaaactttttgtatatactcagtgtaaattggaacgg
tcctttagaaagaaaacttttagatgaaataattctcttcgttttgacagtgctgtatgcatgaaatctttctctctttttttgagggcagttttatatt
ttatttttggtaaactgttaatgaaggaatgtgcttttcaatcacctcctatacatcaagagtacaccaaaagtgctcaggttagaacgtctccata
gaagtatccataaaagtcattgtaacgaatatttaacgaaattacaacgaagatatacaataacatgaactcgaaatctttaaaagatttgttaa
atgaaacaaaccaactgaaagaaacgaaagggattactcaagaaacaattccgcaattggttcatggatatgaatcctgtctaggcttggttcat
aaggcgcgtctattttctatgaatgaagatattgatgaagtaaaaacgagtgagttaaggtacttattattggactttgagctagctaaatgtatgg
aacaatggatgcagaatgatcgattgaaagtcattgaatactcaaaaaaccattacgagagtttccttcgtatctgcgaaggctacgggctaaag
ccattaccggtttcttcgtttaccaaagattcagacgccagaagcattaaaatagcaaggtacaagctacgaaaagatatggaaaaagagatga
gttcgataacgggagaaagtacacaagatgaagaactagagcgtaagaagtggaccaacaaaatacaaatggcattggaagacacagttgac
gatcttggacatgttgagatggagattcagattttaaggaattctgaaaaagtattacagagaaatgaagatgatgaggaaaaaaaagatgatg
aaacacttaagaaggaagagcaaagaaagcgagatcaaaacacttggaaactggatatgcttactaaagacggccttttggatcccaaaaata
gacccatacaaccatttacaattgtttcagatagaaatactaagtcgaaaaatgtttttggacctggttataacttgccgactatgagtgtggatga
ttatttggacgaagaatacaaacgcggaaatatcgtaagccaaaaggataatccaaatcaagaagagtttgaagatgaagaagatgatgaaat
ttctgatgccaaaactatgaaagaacgatactgggatgaatataaagaagccaatcctcgaggcagtggaaacacaatggtaaaccgaggata
ggaaaatagatcatttgaaagaaaaagcttacactcatttctatgaggcttctttctcttctataaaaaaaaggcgaccctcaattatgcgattaac
tgttataataagcatcgcaatacaagttaccttgagaatattacgttgtgaagcagttttaaaaatgtttgtctattctttaactagacttttgaatcg
agttaaaacggtcttgattttctcgattgtcaaacgaataccatcgttcatctgttcattagctcaaataaaatgtttaaatttgaaaatttagtaga
aacgaaaatttactatatcagtacatgaaaattccatactagttgatccttcaaatttcatcaattctctcccattgcacgtatacgctccgtagtta
aaccactcctagaaagaccttgccattttctattgctatagacatttataagtttttttggttgaatactataaaactaaaataaaaacaaaaaagtt
gtgctctagtctttttctgtctttctcttcaccttccttgtatacttaaaatatatatatatatatatatattgaacccgatggttgccgcaagctcttcg
gttagaattggaaacgatgctgagacgtatgtgtttgacatagctcaactaaaagaccatattgctgctagttgttctaatggtacctgggcttgtt
atgataagactactttacgagaggcattcaaagtcccgaaagctcattccaatatcactggtattgtcagttatgagcagcataatggagtgttaa
cgtgcggtactgatggaaagttgcaactttgggatatacgaaattcatcagctaagccagttcgaacttggtcctcagagtcggtaccctttacgtg
tttggcgaccaatcgagcggaacaaatagcagttggttctgaattaacacgttcgttagcttctatcttcctgctagatgctagaagcgagcaaaa
gatcattcgtcaatggaacgatgctcacaacgacgatataactagtttacaatttcatcctaaagataataatttattactttctgggtctgtggatg
gactggttaatcttattgacattactaaagatgaagatacagagaatgccgatgaagatccgttgttgcatgttataaaccatgaagcatctattc
atcttgctcgctttctaggtaaacgaagagttttggcactgtctcatatggaatcttatgctttatacaagttgaagcgtgatttggatgacaaaaca
tggacaagtaaagaagtcgcttctgaccaagacttacgacagtcgctcccatgcagttatgtgattgatgctttccctggtaatgataagcacagc
tacatgctggcttttggtactttttcaagtaatgagacacatttagcttctttggatacggatactggcgaaataaataaagaaaacgtcgtgtttga
aaggatgagtactgagatttgtcgggctatcacttatgattctaaaaatcacgtctactatacaggtggtgaagatggcatgctacagtgtttcgca
gtatgattccaaagtttaaagcttagaaaaagaaacatataaaatagacatcattatttatttatttttatcgctactggtagttttgagtaggaaca
tcacaatatataatgtcatgtactttggcatcattgccttaaagggatcaatggatctgttggagcatacatctgtttaacgctgtattgtcatattag
ccttaatagtaatgaatatgtttatttgtctagggggcaatgaatatatttacaaatatgtttatacctacataatatgcttataacatagttgcttat
ttttgtctttagtaactgcaagcatagtctttacaggcataaagcgttgtaatatggttaccctgtaagcatattctcaaacttcattaacaccatttg
actgagagtaagtaaacaaaactcacatattccgcgtataaatcctgtatatagtaatatcttggttcggttttgggggtctctacttttaaattatg
gctaattctcggtttgagtatgtgaaacaatacgagcgaatggatagactgttgcctgaaacatatattgtgattcgaattgatggaaaaggattc
cacaaatttactaagaaacatgaatttgaaaagcccaacgatcttaggtgcttgagtttgatgaacgcagctgcaagagtcgtaatgtcagaattt

acagacatcgtattggcatatggagacagcgatgagtataggtatgaatatgtctgcactttttggtatctaacaaaccatatatatagttttgtat
ggtcgaagagtactgaattatatgaacggagagaaaacaaattagtttctcatgtgtgctccttgtttacatcggccttcgttttcaattggcctcaa
tattttgagattccactcttgtctcttccatccttcgacggacgtgctgttttatatcctaatcttagcgttttacgggactatctgaactggagacagg
tcgactgtcacataaataatttgtataatactacgttttgggctttaatacaaaaaggcggattaacaaatacaaaggcagaagaatacttaaaa
gtatgttttgattatgctcggaactatgatattaacttatcggtcctttgggaccgtcttagggtactataagttctcaaaagcatgaaattctcttttc
tcaatttcatataaactataataacgaacgtgaaatttataagaaaggttcaatctgggtacgcgaggtaagttcattttcagctacactagttctt
ctgcttactctatccagcctgaagatgtatcagctccttctaaaaatcaaaagtactctactaaacaaaaacgaaaaatgggtttacgattacttc
atgttagtattattgacaatgagttttggaattctcgaccctacctcaccattctgctgaactaagtcttatattttttattaaaataacccaatggtg
acctggcgttttctctagttactacaatagatgtgcaaaattaatttattgaagccatagactttatgatatatcatgcgttgcttctctattatcaatt
aaatatagattaaataattaaagtaaaaaaatattagcggtaaaaactggagtcataacaaaattaatcgaaattcataacgtgaaatcggttg
gaaagagaaaatctttataataacgtaaacaaagacaagcgaataattcatgcataattcgtagtaagggtcatattagctaatccttcagaaac
cttatctgtcacatttggaagacttccttgtagtttactttgcaataagtctaaacgctttgaaatcaacgtagttactatggttgttagctttatagct
ttctcaatatatgaaaagcttacgtttctgaatagtaaaggtattgttaaaatcttcagtgccgttaggagtcgaaaattcaccttcaagtagcgatt
ctaataaattactggcagccttatagcttctcaatgaattaaccaaatcattaccagaaagttcagaagtagcagcgtttcgagactgtcttgttag
taaatgaatattctggagttttcctagtgacttactatgtccaaagccctttggtagataagttgcgatgcaaacgtgggtttagccgtctgttgagt
attaacgaaggtatatgaattaatgacggctttcaaaaattgagttttctcgaggctttctgcgtatctttgttgataccaattcatcgcttctcggca
ctcagacgtatttttctcatcattattagaccatacggttgcgacaaaatcaaacgcaatttgcagaagttcaatatccctttgatataatgtcaatg
attctttcactaaggaaactaatgatgctgatgtgagtcgagcgtctttcattgaatcaaactgtgtatcatcaacttccaaagatggaagtacttgt
attaatttcacctcagccatatggaaaacagcattggatttagcggctagattttccaaaacttccattaacgttctatcaatggtctgagaaataa
taggtgcgttcgtacggttcggtaaggtagcttgtttgggtaatgactgaatagggcttttgcttgtaataccatcaactccatgattgagttttgcat
tagcaacattgagtgccctttccaatgcactttcatggctattaccagcaggcgcatttggtaatgttattggcgtctgttcgaaagctcgacgtagc
tgcgtaacaggtgtatgcgataagtacgcattatgaggcggataggcattagccttcatgtaaggtataggaatgcgacggggatggatatatgt
ttgcgggttggatggcaaactttcaggagagaagctcccatgaaaaacgtcttcgtttggagaagcagagttcccatttagagaaggttcctgatt
ttgaactaggtctaattcatcatcagagtgatgctcaaaggagtttacattatggcgatcgacgacgacataagtactacttttatcggatacatgg
ttattacgatgtttatgctcggaaattggtatgttcgttttagaattgagatgataagggtagttgctggcaggaggagcactttcgggcggaaaaa
tagcgggggcatgcggcagtgctgttccatcttgagcataacgtgtggaaagccgtcgattggatatgtctcctggttgatgtacagatccttgggc
ttgaaaagcctgtcgtagtaaaccgccaggaggattgttcggattgcttttcgggaaaaagtcggtaatatatgcgggactggaagaaggaatgt
ttataccccctgacggcgtcctggatgtcactaccagttaaagtagagtcgtctaccactggagttgttattaccatgctggaaaaaaattggtcgta
gtcgatgcgatctgctgggttttgctttagcaatgcgcaaattaaccccttgatatccgggtgaatataagcctcctctggaaattttataacatcttt
tgcacgctggatacggcgtaatagttcaacatggttaggagctttaaaaggcggttttccaacggccatttcatagagaacagcgcccacagacc
ataaatccgcctttgcatcgtatttctcatatctcaaaatttcaggagccatatataaaggagatccacataacgtttcggccatgctggatgtttga
aggtatcgagcaaaaccaaagtcagctagttttagcattggcagtttcgggttaccaataaattgaggatgttgttttaagaaatcttcggtgggtg
gaggttgtaacaggagattctgtggtttaacatcgcgatgaatcagagaccgggagcgtaggaattggagcgcagaagctaattgcttcaaaaa
atgacgaacgagcgtttcgttcagtccccctttataaacaggaggatggtctacattaatccaagctagagaaggtatgctgctgaacttttctcgt
ttccgaatgaagtagcttaaatcacccaaagagcagtactccatcacaagatggataaacctgcctactttgatacaatctattaattctactacgt
gaacatgtcggatttcctttaaaattgatatttcactttccaagttctccaacagtttcttggttaatttctttgtcagcacgctcttaatggagaccac
gcgctttgtttcctagacacagtgttaattgtctaataaacctacatcaagcacttatttttaacttactgaatgctttcccttgtaaacaatggcaaa
agagccgcgtccaatttctgatcgaataacatagggaccgatactttgattgttgttactatgtgcgttcatacaaaaagaattccacaaaatctga
atgaaaaaagatctcaattcccaaataattcaatccttagcatgggagttggcaaagacatcatgagattaacaatagctttatcatcaatataat
taaagtaacgcgctcgagctccttaaattgcaacaacacggatacgattcaaggtcagtgctgcatgcgtagtctatcaaggcagacaatactaa
gtgattgaaggtgtgtggtttgatagtatgtcagcggtgtgtatcattgttgactgacactggtaaacattcccatactgttattagaccctaattta
ctgcatataatattctttataattatatgtatgttcatgtcgcggcagatatcttacatcgtataatatgtaaatcgagtggaatgaacaactctagc
tgcatcgtaatatgggtatcggaaggttgtactacgtttatagtagctactgttgtaaaggaatctgattagattaactaaatgagacgcttgaaag
acagtctagtctcatttagtcagaccgatcaaatcttccaatctggctgtaaattgatgcgtatatagctcttttgaaatagcgaacgtaatatgctt
ttttttaatagtttcccaatgtgctcaggttccttatctcataaaccatactttccatagcgatatcttaaaatcactgtgtacagtaatgtttataat
gtcaacaaaacacctatcgtgacaatgacaaggacttcctttgaagtgtgttaagagtaaatataattatcattttcaatacgatatagccattgta
agcaggagtagaaaacgttccatccttaagaacaaaaacactcattaataagacatgcagaagaaacgttcacagatggagacgagaaacgt
atattaaaagctagtaaattagatggattgtcaatttcatcgttcctaaatcattctattttaagtattttccaatgcccgatatagaacccacatgta

cataggcaaaatgagattgtcattttccaatgagacaccttctagcatagcagtaaaaacagacatagctaaggtatttttaggctaacatgagg
gcaaatatgcagtcgttgtgcaagctgcagaaccataaagacagacgcggaaaaagcaaaagtaccttccagtgtttttttgttcttcgaatcct
aaaccttccatatttttcccaatgatacttgccatagaatctccacatccaagacaaagtacaccaaccaagagctctgcagaggcgaccggtcc
acgtagtgcatgagaaaaccaaacagggattgcgcatccaattaaaaggtaaatgtgcgaaataattaatggtccacgatgatctctgttatccg
tatactgccacaaaaatttatgtatcatttcaccaaacggctccaaacgcagtacacggatgccttcaataaacaaaaacaaaaagagcacccct
gagaatgcaagatgtacgaaataggggtctgtacaataagtaggaaggaacataaacacaatcaaagcgtgatacgttttccttctaaaattta
aaagttctccttttgtgccaaaatagtaacgaaagaagctgccgttatgagagcaaacaacaaagcctatatagcacgacaggcacaataccca
ccaaatcaaaattgttatccttttcttagaataaggactcaaaaaaatttgagcaaatacccaatattcgggcttttgctggatacgttttgaaacc
caaaatcgagagacgaaaataacaacaaaagtactagctgcataaactagtagagaagatttctcctttgtaagtttgcaacgagaacatgtttt
gagcaggcatgaatttagtaaaactaacgaaggaacagatactaacaaaacaccggctaaaactgataccagagggccagcctcattcagaa
aagtagcgtatgctacgctgctaagtccggtggagatcaagacgacctcaataaatgtaaagctgtttttttgtgatttctataaaggcattcataat
agtaacatgaagttctaaaggaacagacccggtttgcagtcccttcaataaatatgagaagaaagagaattttaagcttaaggcaagcgctgca
ttgtaaaagcaaaccatgtacatttcatgaaaaagcgcagagtcccttccaaaaccaatgcagaggatcgcaaaaggaaacgaggaaagccat
aacagcggcctattgtcaataattagcgttcaactgtctaccgcatacaagccacttactcataaaagcatttttctttgggattggcaaacagata
tacgaaagctggcaaaaaaaaggcaatattagagaagctcggatatgagacaaaggaatcagatatgaatgggaggaagagaacagagaag
atctccaaaataatttcaatttcaaaagaaggaaggtgcatgagtaataatgctaaaaagctttagataccaacaaagagagcaaaaatatctg
tttttatctataagtagattagagtgtactttctttatcgtatatttagaaaataatcattcatgtaattgcttgatcgaccggcgtattatacaaatcg
aaatttttgttaaagtgcgctagaagttgttcaactaggaattagaaatgaaagattgtttgtaaaaaagtactatagcattctatacaggttttca
ttaatagtgtcaactgctttccctaaggtaaagtagaatacaaaggtcggtcggtgaaacgaagaagattctgctgcaagcggaaaacaacaaa
ttcatatcattgtagttttcaccaaataaatcgatggaagtaagagatgattaattacaaaataacacatggattacatctttgaaagcgaaagta
agaagacttggaatgcatactaggaacggttacgattagcttgatactactacacgaaaaaataagacaaaagcgtcaagcgtaaaataaagt
aaagtaaagttgagtaaatgatgaagagctctatggaaagtaggtggagaaaggatggtaaaagaagaaggaaaacagcacataaaaaagg
tatcaatctttttttcaagaacatgtcatagtatgtgcttgcacattacaataaaacaattcaaacgagccaagtaaaaaaggtaggcattcataaa
cggaatttaatcttgacgacccttaacaagtcgttttatgcatttgtcgggtgtaatgacatagacaaggcaaccccagccactaattgcatcacg
atcttgagcatttaaaagagcttgggagatagtttcaaacaaatcttcaggctcgagattgggctcatacacggactcacacataccataaagttg
ttctacggctgtaccactcacaataaaatcatcggcgaaatcaatacatccaatggaatcgaaaccgcaaataaaaggattgtcgtctttagaaa
gaccagctacaactgggaaagaaaagtagggtccgaatctagaaagaaaggttagtttgagaatagctattaaccagagaatcatttaagcaa
caaaaaacaaaaaagtatagaatctatggcaaacgggaaaagccttaccttttttcgtaaagagaggtggacaccagattaccaaaggtttttg
gttcaatttgacgctcctcacggaatttgtacatgtttactttataacggaatagttcataaagcgtctgaacatcactagcaaagcctgtaaggcc
caaatacgtcttgtcagtcatagggaatacttttggaaaattgttcgccaaggatatagattgtacacccaaacgcaagtcagatgcgatagcca
cgcagtttttttcctgccatggctacgcaggatccaccattatattccataatactcattttggcaatgtttcgtaaagagaagaaggattcatacctc
tctaaggatgcaagtaggatgagtgtacataatattcggtagcgcagtgcactcggtataatagatttcgaaggaataaatcaatacagtatgca
ttttaatgaaacacaacaaggtatggattttatttagtgcaaaatgcgttatgagcattcgtcaacatagacgtttaggtcataaaattcctcgtat
gcggtaggtaaagaaaggtaggaagcaagctcatctcgtcttgtttaaaaggatttgtttacttgcatttttttggactgcatagaaagtctattggg
attgactgatttgttctgctcctttttttttttcttttgtggaagaacgcatgtggggttttacgagaaaaagctcagtcattatagaaagacaaaagt
accttgacaaacgcgggcgacagctcagcgattcaatttcattctattctatactataccgttttcattccgtttaaggtctcaccatataactcttttc
cataaactatcttttggaatagaagaatctccaacgaacattttgaacaaaacatttccacaaacgtacattgacatatgtgaacctttgaaataa
attccaaataaaaatagaggaaacaagatagatgattggaatgtcttttctgtgggaattgtttttttctgaatcatataatacccttttaatacgtttt
gttcttcgtacatagtcacgctacacggttatgaattggataacgcgaaattagcacaaattcactataagagttcaactagaaacacgtaaacg
cgcaagacaattccagctcttgtcagatcaacagtcttttttggaaatgcaaaaggattattactccatacttaacgtttcgcaaaaagcaaccca
agaagaaattcggtttgcctataaaagagctgctcttgaaacgcatccagatcgagtgcctcccaatgatcgaacacgggccacacaacaattcc
agttagtgaatgaggctttctatgtattaggagattctactcgtcgtgctcaatatgatcgtgaaagttcttttccatcttcctcttcacgaccgaaag
ccaaacaatcatcttctttcttctctggtggtcgtaaagacgaggatgcttcttctagtcaaagggaaaaacaatcttatggttccagctatggattt
caagattctttttcaaactcccaattcggcaatatttttaacgaaatgatgaacgagtcttccaatgaaggtcttttgcatcatctttggactgttgtg
ggcggtcttgctggagctgcgcttgggttcattacttttgatttcctaggcatccctttgggatcttttgctggtgcgaaattgggtagagtgcgagat
aaacatggaaaaagtgcttacagtgtttttatggatctcccagctactgagaaagctcgtatcctaagcaaactacttgcacacatactgaatacg
tctaaaacttttgctagtaatccgccttagtgctttgcatgtgctggcatccattttgtttttgtttttgtttttttatgtttgttgtactcttcgtccttacg
ttttatgtttcatgtcttatcctcaccttgttataaaatgtttaacatcttctgaaaaagagaataaaaagaaaaatttacgttatgaatatcatattg

atgatcgcctcttcttttccatttcctgacatttcatcctgttttagtaagatattggtagtaattatagcaatattactgtattggaaactccccttttt
gattaaacgaaacgcttgtttctcatgtttcgctattctacgaaattttgatgtcattatactgtgtcaatatgatgtagaatgtttttcttccctttctg
ctgctcgtaagaattctctttccacctatatcgacagtaaaagtaaaggcaataaattaaaagtgattcattcagatccttttctttgtatatatttta
atttcgaggatttttatcgtggcttgtcgtccagtatttgaatctttcccattcatttactgatattgtcctttgtcttaccgttgtgtgagcaaagtgga
aagcccttgttttacaattggtaaatccgcttttcagctttatgattgaaattgctctatcatgacttctttgaataacacatctccaacaacgccctc
gggcgatgaattaaccggtctccattcggtcgaccaacaaactacaagtactacgggtaataaatctcttaaaggtcattatggccctcatcctca
ccaggatcatccttctgcttatgttgccgctaaatctggatacacctcacgaaatttgacacctaattaccgcgataatcaaccttctcggactgca
aatgactattctacggcctccgacggtacagctgctggaacaagacaccaagctcgctcaacaagtgctgtggagagtcctgagtctggtcctgg
ctcgatggatgcttcaggttctttcgactatcagcctcatcctcacgctggcaaggccgctcagtccgctcatgttttcaaatatataaatttatcag
aaggtgaaccgaatgcaaatgctactgattacgaggaacatcgcaaaccattacaatttctcagtggccctgttttcacaggttctgctcaacaac
ctccacctactgatcttaatccacaaccttcgaatgaacatgatgaagaagcttctcgcaaaatccgtctcgcttctcagatggctaaacattctgc
aactttcgctcatcaaacaccagaacctcaacccgctactgaatctgaattaccccatcgtatggctgcttctcaactggttcataatatttctttac
aaggttcctcttccttaaaaccaaaagaatcttcttacgaagtttctccacctgtaaaccttaattctcttgctgctgcatcttatgctgcctctcactc
tcgaccctacgatgaatcgtccgctcctcctactgtagaacagcagaagtcgtcttacgatgatggaagagttcgcaatatgcatatgttttccgcg
tccggtaacaacgctttacgggatcgtacacaacaagatatggtcgcacatggaaatagagatgatgctttcgctaacttttcaaaaagcaatatt
ttaaataatgcgccaggctatcaaggctcacgagcttctgcatctattaacactaaaaatgttagcaatgagcgtcattcaaaacctatttacttca
gtccaggttctggtgcagtcaccagtccgtatcagcaaaaaccaaaagaaccatcatatgatttcagtgctgcgtccgctgctcttcgtcgtcatcg
tactacagatgtaaacacatcccgtgaccagacgtttgaaagccaagaggattcaagggctcgtgcattcatccgacaaatgcgttttccatcaa
gtagcccgattgcaaccagtgggccaggaaatgatccaggttatccaaacatttctggtactgaacgcgagcaaattcaatctgaggcaatccag
cgtgttcgtgatatgaatttggatctctcaaaaatccaaaataggacagactctcaaattcgtgcttaccgtcctagatcttctgccgattataagtc
ggttggccaatctgttcatcctcctcatacaggtgccagtagtagttatacaccccatagtcagagaagattttcgcagacaggccaattccctaat
aatgaaggggaaatccccgagaatggctcagatggtataaatcctgtaggcatgcaagctgctgctcagcgaagcaatagaaactctatggctg
ctgttcctaggcggaccggtgttgcttcaaatgatgtagatgttgaagctctacaaagaagaatttctagagcttacattgatcatgaacgtgaag
aagcttatgcgattgatcttggtgctggtcgtcttatttctccggaggagttagagtcaattgccagacgtaatgtagatcctatggtttctgaacta
gctgaacgtgccacgcaagaaaatcaaagaaagcaggaggcaaacgaagctaaaaatgccaaaaagcttgctaaagaagagaaacgccag
aagaaaagagacgaaaaggcgcgtaaagcagaggaaaaacgacttgctaaggaaagagctaaatttgctaagcagatgtccaaggaggctg
gtaaaactgatgaagcagcccccactgtaattgcagaccaagaacaaggcagcacctcttcagaagtatcttttggtgaacaagagcaagaaga
tcaatcttttgatcctactacccaagaatttttgattcaaaaaactgatatagcaaatgctacgaaacaacaagaaatgtcttcaaccgaaagagg
agcagaagaagaagtacagcagcctacccgcgcggaactttctggcgcaactgaacgtgatttgaatgctaacgttcctgatcagtatcttcatg
atgaagcgaatgaactggatggtatcggaagacctccggtttctaccgttgagagcaatgaaagtgatttacagagacgaaatgaggccgctta
cacaacctcacctaatggccaatttactgattccaatcagtttcctgttgtacaagaaacggtaatcatgtatgatggtcaagaacaggctgtaag
tccgaatcttgatgaggtcacagaggctcctatgtttgatgatgctggcgaatataaaagagacgtatctcagaaagatgatttggacgaaagcg
ctaaagtacttaacaaaaactcaccaaagtcaccggtcgcttggttaaaaaaaaagctcaaaaatcataaagataaggcagctgtcaagcgtat
gttagaagaagattcttctaagagactatctgagaatgaaaaggttcatgtgagtgctcttcagagagatgtgtcgcctattggtgatggtatgac
tccagctgctgaaaatgaacaacgaccgccggttgttgaaacccattacgagcctggttcagttatgaaaaagactgagggagagatgccggct
gttgtggatgctcaacctcatgtaaatgtttcctctgttcctcaaacgaacggtggtgttgaagaagcattggatgaagcagctgtttcaaaaactg
atgagtatccagttgagaacgggaactttgaacagaagaaagaaaatacaggggaagaaaaagaggagagttctaatcaagtggtaggtcatt
tccctatccccggtaaagagcctattggagccgcatttcatgaggatttataagtaagaaaactacggttgtaggactgatttcatttggtgcaatt
atcggctctaattaaatttcttaaaccttgaccgtggcatgggccttttgaacgggttgataagtaagaacatattgtctttttctgataatttaagt
ttaattattatttttgtttattactggaaagtcaatatcctgctgaagctgccacgcatttttttactttggtaaagatagaaacttacgatttttactta
agacaggttgttatttattactgtatgacagtctataagaaggaagacgataacttttgtggtgaacggctgtcagcgaactgatcttttccggatt
atttattgtttacaagtaattagtgttatattaataggaaaagaatgtgactttcatacatattattaattttagtaattgtttatccttgtatgatgga
gttcgtattggaagtgctatcctacgagcgaattggtaaatagtaagtaaatgggatatagtaaatactgactagattgtgtcttcacagtgagat
catcaggcaattagcgaacaggtcattatgaatattatcaccgattgtagttgtgaattagctatgaagaaactgataatcatttgcatgaagaat
tgattgttaacacccaattacatatatatgccttcatcatatcatcaggaaaggaaagcacagagtatgtagtttgtagtgtttaagccttcccttta
atattttagggaccagtctaaaccaaatataatatatgagttattatggaagtcgtacttacaaatcgtgattctcgcagttctaaatagcatgtac
ctctcgtataatagttgtttgcttttactttaccttttctttgtttactcgaaacatggttaggtgacagttgtggtaacgattatgaggacaatattac
aagaggatttacctaacttcgaagatccaacccccccggggtagcctgacaagtttccccggggtagcctgacaagttatgatatcatttacgtaa

agagtttatgaaggatgtttataaacgttactttagccaaacaccaggtggaaagtgcaacaagagggtataccgtaagatttgcgtttttcgcgt
attttaaaaaaattaagaaaaatctattcgaatataagaccaggaactatttaataaatataatacttattattaatcaacggattatcccgagtgt
ttacatacataatatacaaaatacgcgattaaagacacgagctcttaagagcaaagtcagtcttttgatatctctttcattgttagtgtctattctta
caataagatactccttcgttcgattctagtcaggtaaatcctgtcgcatctggaagttttgatcaccaacttttcaagtacatttatttacctggcaat
ccgttacattcttgctacgcaacttgttactctgtgaataaacatcaagcagtgtggaaacggaattttttttactgttaattaatagtatccacattgt
tgtgtgattcccatcttttatactcaagatacattttgattttttgataatttcatctttaatactttaattattcctttatttgtgaacgacaagatggt
tgaacgtattcatactacacctagtcgtccccgctactgggcttatcttttatttttactacccccttgtttgctttttaatgtggacaattgggttaattg
tccttttgggtctttggagtgctcaagacaaatggcatacgcctggtgaacctgatcccgtatatatcagtgatatgggtgcgtataccaaaggatt
tttcattcccatgtgcgtaataacaggtgtttcttatttatttacttttatcgccattcgcctgtgcagacaatggcgatttctatatcctacctctgcca
agatagagaccttccttggttggtttgcagtgctgacgagtgctgcttgtgccgcttgtttaatctctctggcaattcgtgatgatgtacatcacgata
gcgttcactggaaattcaccgctgcttttgtagtattggcttttgtttctgccgctagcaatatttttgaatggctctctgcttatcgccattatcgcttt
agtaagttgttagctttctcgttttatgcaaagtttataattatcgtcacggctattgtttgcgccatcgcgtttgctggtctgcgccattacgaagatc
gttctcgatcttctagatttgagtgggttgttggatttttatgggctctctatatttctcttttgtgtctggacgtgcttccagctatttatcatgaacgtt
atccgaaggattcgggtgacttggagaagggtgttcgtggtaatcaagaaccagagactacttacgctcctcctgtcgagcccccagcggctgcg
acacgagagactgagcatgctgatccctctcagccttaagtcgactttgaagtttcttcaaattcgtctgctaatcaagggtaatctaattcaccaa
ttaaaaaacaaccctcaggaatgcactgcgctgttcctgttgtgacaacagccgtttggagtctggtaaaaaattacttatgacaagacaagttac
ttgtctttccttatccgtatccgtatatcatttaattcaccttaccttcgattggtttgtcacgaatgataattttggtaaaaaaacaacagcactttttc
tttctcatctaatatgttattcatatcaactgtttataacctcaaaatgaaatttgtttctgattattttcatatttcattgatctttatagtaagttacgt
ataacgaaatgaaaagcacttcgttggaataaggcagtacgttagcttgtattaagacagaaatatttaacacacttgaacgcgaaacttccgta
gatatgcagaaagcaactttgtcttttacccatatcgctttatggcaccaattgcagaaaggactcggtaagttgattaaaagcaagaagattact
aatgcaacagatttggtcttaaaaggagcattgagcccatttatagcggtggccctattggcttcgcttctaatgaaaaaatcctagtcaccagttt
aggtgatcggattatgggtagcaatggggagactggtgaaaaaatttttctattaataaggatgaagatgattacgttacttcgatgattatgac
aagtgatggaagcaagttggttgttgcttttagatcgcgcttggttacaatatatgaagtaccttcaggaagacgcttaaagtcgtttaaagctcat
gaaacacaagtaattactatgaccattgacccgacaaacagcgtcttggctacgggtggtgctgaaggccttgtaaaggtgtgggatttagcagg
tggatacacgacacacgttttacgtggtcatggcggcatcatcagtgctcttcgctttggaaagtatcaagaaaaatgggtgctggcaagtggtgc
cgacgatactcgagtacgactttgggatttaaataccagtcggtcaattgctacattcgaaggtcatagcagtgtgattcgaggccttacattcgat
gagtctgggtcgtttttattgagtggctcacgagacaagacagtgcaagtttgggatctgaaaaagcgttctgctattcgcactatccctgtaatgc
atggcgtggaatcccttggatgggtcaatcatcctgggaaaaatgagaaagtactatatacggctggtgaggggaacataattttagcctggaat
tggaaaactggagctcgtttcaatcccgatgttcagattattcctaatgaaaacaatgcgattatccaggttatttctatggatgataattcacttct
gactgtgcattccgatttgagcttgcttgtacagagcttatcaattgaagaaggtttttttggttcaaaagaagcttcatggatctttcgatgaagtca
ttgactgtgcttggattggcgacgatcatttggcaattgcatccaatactgaatatattgatattatttccgctgatggcacacaagtttatggtgttt
tagcaggtcataccgatattgttctcgcaatgcacgcttctgaagatggcgtttggttagcaactggtgctaaagataatactacacgactgtgga
atctagatgttgaaaataataaatttacttgtgtacgtgtgtttacaggtcatacggctagcgtgacagctgtggctttgggtcccttggacgcacat
ggtttcccctcatatcttgtatccgcctctcaagatcgcactttgaagcgttatgatctaggttcttctttgactaattccgaaagtcagaatagagct
atctggaccattaaagcgcatgaccgcgatataaatgctgttcaagtatctgctgatggacgtatcgttgctactgttagtcaggacaaattaatca
agttatgggattcatctcttggtgaagttatcggagttttgcgtggtcatcgtagaggtgtgtgggcctgcgtattcaatccccatagccgtcaatta
gctagtggtagcggtgatcgaacagttcgtgtatggagtattgatgagcaacaatgtcttcatacattagaaggacataccggtgctgtcttaaag
ctggcttatatatcaaggggcacacaacttattagtgctgccgccgacggtcttgtaaaggtttggtccatagcctcgggtgaatgcgtcgcaacat
tggataaccatgaggacagagtttgggctgtagcagttcgtgatgatggtgctcgcatcgtatctggtggtgccgatgctgtcgtaagtgtttggca
agatgtcacagaagagcatgctgctgaggaagctgaggaactcgaacgtcgtgtcgaggccgaacaatcactggctaattacgaaaaggctga
agattggcggaatgctattgcgttggcattgtcattggatagacctctcggtttactacgcctttttgaacgagtcatgaattctccacatcaaaaag
gaagtattacaggtaacgccgacgtggataaggtggtatcagatttgagtgataatcagttaacaattttatttcaacgcatccgtgattggaatgt
taataacaaaactagcatggttgcacagaggctgctacatctacttctacacgcttaccccctaaacatcttttggaaattccgggaataaaagg
aatttttgatagcatgtttccatatacccagcgtcatctttcaaggattaatgatttgttggaagaatcctatattgttgattatgttatgtaaattaaa
accgaacactttaatttgaggttagtactttcggtacttaactatttttcaaaggtcaattttcagctattgattattttctatgtttcaaaatcgggtta
aataaaatttgggtctatatttttggaaaacttgcaaccaatagcaattgtagcttttctggagggtaagggtagtagaaattaaataccttcaaaa
tttaggaacccagacatgagaaggatacggtaatactttttcttagaaatctcgtttgtccaattccaagcgctggttaaccggccgtcaatacaac
ttccagcgtcgtaccaattgtcagcaccacgtccatcgaatgcatgtttgaaaaaatccagtagtaaagtctgctgcagacctctaatgcttccata

ttcttcttcaatgtattgtaccaagaagggatctctcaaatattccagttgttgtaatacctttacaagaggtgttaagatgtgagggacttggcggt
atttgtcgatgaacattaaagcgttaggtacggcacgatcacctaaatgtacaacggagctacctatccaaggcaattcctgctcatgctgtactc
cggacaatattctattaaccgtcgtacggagcaaagggcacgcctgaacgcggttcaaaccttgtccagtatcagaaatgaaatatctcgtatctt
tgtcaacaagggatgcatcgctttgaagccatagcaaaaacatttggtccatcatacaagaccaaaattgcaagctttggtcaacataagaaaat
tgcgttcggtgatcatgtgttagacgggcaccattcataaaaccaattttcaagtttccacctttctttgaatcttgtggtgaaaagaattcatacaa
ataagatcgcatttttcaacaggaacccgattagccaacagaaatgactcatgatctgctaatgaaaccagacagtcttccaaaattctctttttt
ccatcattcccctttgcgtatttttgagcaaaacatcactgcttttttttcgttcctcatttattttggaccgtaaggaggtaccagcatagggatcta
tttgttggagagcagtgtctagaagctgcttttcagaaagaatatctagcaaaccattctcttctaaaacacgaaaaacggtttttacagggatgta
aaagtcaaagcccatttgcatacggacatcaggtatcatggaatcttggcacatgtacatcatagcgccataagtagtacgcatttttcggggttc
atgattttataacgtcgacctatttcaaatatcgatttgaagaaatctgcgttcgtggcatggtcctttccttgcagtaaacttcgaccaagttttacg
tccattgcaacaattaagcctgtcaaaatgctgcacatttccgatagataagtcaccatcaattttgtccttgatgtataggatataatatcgacatg
atcggtatactcaataacatttagcctgaatgttagatgaattcacagaagataaagaaagaaaaggaaatggaacagtaagtctttattgattg
aaaacatatgttagacaatcttttggttaaatcctctgccgaatgcctcaagaagcccatagcaaagcaaagcaaaggaaaatttgagatccaat
agagaatgatatagctatacaagctcgtttgaaattaccaaataatattttatctaaagattttgtggatgtgattcttttcgctcacaacctttctg
tctcttacggtaattttacttacgctgcttctaataaacccagaatacgacgttcatcttcgcttaaacgtacaggaacaaattttgatttttcgaccc
agctttttgtgaataatccggtttttgacatggtggtattaatcgagtagtgcagcgtaagtgcatacacttcaccaaaaacttagaatccacaatt
ctttattttcggtgattaaagttgtggtacaaagttctcttaaacaacgtttgcatatttattttttattactaccgtccatcatcaattctatcgatttg
ctgaagatcgcgcatgttgacttgtgattttcgtaacgctttgaatccaaattgctttctttgaaagaagaaattatttttcttactttggatagagaa
ttggatcctgtaggaggttaatcgactctacacggtttcatatattaaacctttgaaagtcgacaaggatttttcttggtaatctacttcttaatcttct
tttcttagttaagtatttaccgctcttttctgttagacatgctatactttgttatgataagtctctaaatcgttagttctgttaatcgtgtccatacttatg
tcccataacctaaagatttattttctttttgctctttgcagtttttttatttgggtatttttttcggagttttgtcataaaaattgtctacttatgggcgactg
gttattttatttggtttcatttgcattcaaagcatctcggtaccttcacttcaatcaattcggctgagccatttctcgtggatcctacagtttttcggaa
acggagtcaaaatctactaaaatcgagattcctactgaggcaagctttatgtccaggatatccagagatttcagaagaaatagagttcatcctgg
aaattttcatggaaaaatacgtgaaaaaatggtcatctctaattattggcgaacctaccattcctaatagtattgaggatcattttcgaaaatgtat
tcataccctatttttgagtgttgagacggttgactttgcagatgttctcattaagaaaatcttaccactgcttacaaagcacctgcgtctttttatcga
agcagagcaactcgttgttggccacaaggctgtttcttttactgaccattctgagctttcaaaagaaatagctgcgaaatataataatggatcatta
cataaagctattcttttgtcgggaaatccgatggttgctcaaaaaaagtatttgagaaattggaccatccaggttttatcaataatactccctaatt
actaccaaacaagccctctagtaatctcattaattacggaaataataattaatgctgcgcttcttccgttaatatcctacgtttctgaccccgatttct
tcaattacttgattattcaaggtagtggaagtattatagaacgacgcagaaagatacgtcgcttaaagcatgctatacgcaagttgtcttcaaattc
tcaaattggggcacgcaggctaggactcaaagatagccaacatgctttcgaccaatatattagggaaatccgtcgtatttctaatatttctgatgc
aaggcgtttgcgatccgagttgttagtgcaaaggcgacaattagatcaacacacccccctttagttctgagcttcaacaatatcatgaacgtttacg
agttgctataaatacaactgaaaaaagaatatccgttctttctggattccctttttcaaaggaggcagtctgccctcgttgaacaagctcagtctttgc
agcaaatattgtctgattccgcagcagtttcatgcttcttggaatttatggaacgtaggcataggtcccatttacttcatttctggcttgtagtggaag
gattaaaagaatcacaagatgatcctttgaatgcacacataatggctcctttttctgatatttttttcagattatactgattttacagccattgctaaat
cttatataaaaagaaatgagaatccctttgaaataccagattccttggtgaattctgtgtactctttcattagccaacccgaaaataatctctcgtct
gaactttggattgaggccaggaatgctatgctggccgctcaggagcatgtttttaatatcatgcataatgacgattacttggaatttgtaaatagtg
atatatattatcgctttcttgctcaagacgttatttcagaccagaagtcattaacatcatccactgcatacagtcctataaacgatactctagataat
cctccaaattcttccagacaagcttcgataaaaagcatatcttcttcttcaaaatctttcaattcttctgcatcagttccattgtccatgccttccttga
gaaaggatactactttgaacgatgtggatttagaagacacaattagtagtgatgaagaagaggtgctttttgcttccccgggtgatcttcagttatc
tgaggcgattgaagaacttcacgcggacatccaagagtttgaaaaccaacttcacgccttgagcacaatgatgaagaaagccgaattaatctca
gatcaaaaacagtttaaaagtctgtctaaaagctatcaagctatagaaaaggccgtaaatcgaaaaaaaagacaagtggatcaatatttgtcac
aagaagaagacagtaaattgtataatcgttccagggtttccattgattcttataaactttgtaaggatgagaacgaacaagaatatgctgtctata
cgattcgtattgaacgcctggagggagatataataaggtcaggttggatggtcgctagaagatatcgagaatttgctgaattgcataagcaattg
aagcaaatgtttccacaagtacgctcgcttaaatttcccaataaaaccgttatcccttcaataactaagtcgattttggaatatagaaaaacggcc
cttgaagaatactcgcaagctatttttcagatgcctgacgtttgcgatagcaaaattttaagaatgtttctatctcaacagaacgttactgcacctca
aatgttcaatccaaagatagtcggtgaaagatggaagcgctcgttggagacactgggttttgaagtaaacggttcgtttaaattgtcacaagccg
atagtacatcctctttttcagctcctatatgtgaatttttgattgagctcttttcaccaaataatgatataaagcaacagtggatgccgaaaaaagca
tatatttccattctagaacaactattcggaggtgctctggaaaaacgtatacgaacacaaatgcatcagtgggtcaatcaagagcgaatatatca

gaaagtccaccagcttcgacatgaattaagtggaatcaagaaacctggacgatcggcagctgggtctagtcgtgctcgtaaaccagcttatgctg
atcgaaatcaactgaaagctgaggctggtatcatgcttgcatctatgtttcctggatatacgcctgacatagccgttaaaagaattttccgaatctt
acaaaaccaaacacttaatgcacatataatttataccttgctagatgaagcactggaaggtttaaagcagcataaagaggattcagcaacaaaa
ccatagcttacgagctttctcccgactccttccggtttaataatcatcgtactaatttgctacgcttaatatttgctctaatcgtgatcaatttacagat
tttaatattgaaacttatcttcttaacatttatgatgccctatttcaatcggtagcgacgctggattgcaccaggacatagacctgataattatttgta
gattttggcctgaaaataagagccatattaatcttcattcgcctacagtttgtaatctactagcctttttcgtttactggggtgtagaccaacagctat
attagactaggtacagcgcaaaaggaccgtgaagcattcaacatccagtgttccaaaccccgtttggtgccttaacatataaatagtaaatttaca
agcgattaattagggaatgacatgtatgcgaccactcctttactctggcttctgttcaattgacgatgaaaggcagtgaatgcaagactcttgacat
gaagttttaatttgtaatgatatctttatttcttctactctaatgggatcaactttaaacgattgatcttttaggaaattctaaacagaagaattgatct
gggaagttgaattttgaatttttaaaaattcgttaataagtaacacgagtaacactgctaaatagtagtggataacatttcaatcaattgaatcga
attggaatagaatgtaaaacatattaatagtagtattaaaattgtaggtcaaaagatctactgggtcagtaggaggataatgactcaatcttcaa
aaagcattgtaattgagtcgctgaagacaaatgtcgtgaaggatgctaaacggatgtacaattaacaatgatttcgtaacaagctgagaggaaa
atttactctgctccaaaattttcaactcaagcattgagttatacaggagaacctgctcacaggtatggtaggaaaagctccgcgacgatggctgta
ttagatactttgtttgattggcaagtagtcttcaagtaggattgtagttaggaacaatcaacaagacttggtagtcttacaagtagacgggaccac
gagcattgatttgcattgtgagagcttgggtctctctttccgatcgtcttagagtattccgggagccattggatttcctaaggcgttgatttttgcttgt
tataattgccgttccctatcttgcctttccttctcttttcttggtaagcgtgaagaacggttttggttaaacatagaaataaaagatatgaaccatata
acataggaaataaaatgaaacaatgcgacgggaccaaatttcttatggtatttcaaggttaatgacagcactacattaacaaagcatatgaacg
caagacagcaaatatgcataaacaccttagaatgatagtttactatctcttggctgactttctcgtatgtatcacctgaatcatcttcagggcgtcg
ataatcgtacacaatttgaggctgaaacatgttgaatccacaatttttgatgaggattcgctgtgaagtttcaatggaaagcgaaggaggatgata
ttcgacgtcagtcggaagggagaagatggatggcattcaaggcagcctattccattcaaagttaccaccatctttttttttatatctttaagaatga
gtcaacctgaagaggcttttgaggaaactgctcctttagcatctgaaatccctcctcagtatgaagaggaatcaagcaaatgtctttggcaaagtt
tacgaaacagagaaatttcttttcgcgttctattagggactttccttctcacctatacttttgtctttttcattctttttcttgtgtactttattaatattttg
gtagatgataataaaagagaaattagtgaaggtatcccatattactttacatctatttcttgttcgatacttggccttgtctgctttatttttatttgtgt
ttactctcgacctgttgttgtcaaggttgaaatgggttcgattcccatggaagatttgtcacgaaaaaatgatacaacgaaaaggtagctttaccc
acatttgaagtataacccaatcaaaaagttatttcttcctgaatatcagatattctaaggatgcctcctcaagtttccttccagtaggtgaatattaa
caaaaaaggaattctacggaaaactgtgcaaattctatctgacaagcgaagcggcatgcctactgaacacgaatggttataaaaatacaagga
gcttcttagtgaattcaacatttgagttccttgggcgttgagaagctttcattttatcgtctaataacgatgtcgtgtggaatagtctttaggtgaaat
aacaagaccttttccttttctagctcctctaaaaaccgtttcaataatatcaatcatttcctgtttatcttccaaagcccaattgattttgttgttattac
cggtacccagatcaatcatcatatgcttatttcggtagaaaaacataatcgtagtcctatcataaagttcgtacatctttaaaacgttagtatggcc
gtctcaactgttggctgaaccaaatagacaatgcctttgcctaaagtctcataccttgttgaagtctggaacttcgtcaacgtcaaccaagtaaaa
aacagccatatttataaccttttctgcaattctataaaggacttcatcttgtttcatgcactcttcatcctattccagctaaattagtacaccggtttcc
catgatccttagacgtacgtgatcacgaccaaagcgtataattacaagcctttcttgctcttccaagattaattagttaatgaagcttaaaaaaag
aattcaaccaacatgcgtgttcataccagaaagaattgcttggtccacatgcctaatgcacaagtgttagaacggcatatataagtgaagctttcg
taccatccagaatgaagatgaggaagaaaataactcatcgcgatgtttctcaaacttttgaactttgctttttcgtcaagaatagaaattgaattaa
ggacactttggaatgcataattgagcttactgaaggcatgttacaattcgctactttgttaaagtgacatgtatcctagatttatacaaaaaatgaa
taaaggggaataatcattgtgtaaaagaaaggaaaacaacaagcatacttggatgttcatgaaggttactttgttaatgaaattaaggctcttcat
ttaatagttgagaattggaatcgcaatttcttcgttgctttctggaaaacatggagtagtcggtataaacatcagattgtaaaaaaagatgaaaag
aaagaaaagaatctatctctatatgagcggtgatccatatagaattgaggaatgaattgatgtcataaacattacattagaaggaaaataaaaa
aatatattcatatgcgaaattcgttatcattcttcgttcaaagattggctttgctttttgtaaacaagtaaatcacctcgtcgctcttcactttctggaa
tccaagagttaaagtatgttgcgaattctgtgtataacagtcctctagtgttaaaatagttctcgagtaatggtaaacgttcttggaaaaaaatcaa
gtagtctggaaatgtatctagttgatctagataagcaggcatgtccgaataaaatcgatctgtctcatccagagggcgctcaaaaggttcgcaagt
aaggaaccgccaagtatgatctgcgtttggaacgtgaatatgggattgccaaggtgtggtatggcaaggtgcaaggataatgccagttttattctg
ggaagcaagagagtgaatcaacggcatcacgtctgcagctcctaattgatgaaaacggcaaagaacaattgcaacaccaatatgtacagcgct
gatgaaaagaaaaccacggcgtatccatttggaagacacggaagccaatgcattcccaatagcggagagaagtataggagataaagggtaca
caaaacgaaattctttatgggaaataaaggagtagggaagtaaaacaaatccaacggcaagaacagcttgcaagtccaacgacagtaagagg
aaaataagataccctccacaaattagtggtatagcttgtgaaagataataatgcgaggaatttaccccatacagggaagagagtcctgatgtga
cattaaagttaaagaaagaaaaaataggaaatacgaaatggccgtaaacatatcggtctactaagacatttaaaacaagaatcatgcctatgac
cgtaagggtagctggaagatgatacaacacttcgaaaaataagagacgcactcttgaaaatattttgctagggctaagaaaagattctcttggtg

atttcgtccacagcaaaacaggaactacccaaacaagtaaattggtgggtcgaatgagaaaagcaattccaataaaagttaaaaaccgtaaaa
aagatcgaaatcgtagatttggattatcgatggcattggttcgaatttgagatgaatacttcaaatatttctgccattcccaaagagcaagagctgt
cagcggtgtttctaaagaattggcaaaagttcgaacaccgacatacgcattaaagaatgagaataaggaacaagcaagggtccattgtgcagct
cttctcccatagcgatttagggcaaactgataagtagcataatccagaatagcagcaaaggttccttgtaggattcgaggagcacctaccataatt
gtgtaattggaatcccatcctagtaattttaaaaagcggtacaagagcgcaaaaacaaggggatgcagaccagaacgaatgtactttctccattc
ccaagttaaaaatccatatccaaacactaaatgatgagcgacctccagagactggtaaaactcatcgggttgaaaaaacgtctttacccacaac
gcattccaccatcgaaaagcaaataccacaaaccacatccaagtctgcatggcgtttcgaggtacgttgcaatgttttgagtgctttcgagcatcc
aagctgatgccgtacaaaccttcgttcttgtttcggctgattagtgatattgatgagtatgagcggtttgtaccgttctgtcagaagtcaaaagttac
gagcagagatcttacaacaggactgccaaataaagcggatttaacaattggttttgggaatttttgtgagactttttgatagtcgagtggtttgtgat
ccagttgcgagaaccgtcctagcagatgcttctcatcaccagttgtttcaacatctaaaaacaaactgggaaattgtggaggcttctaaaggaag
agtgcgagtagacttggaggtggattttgaatttacaaacaagcttcatagtgtcgtgagcaaactgacaggttccacggtagctacagaaattat
caaaggatttgtgaatcaagcaaaacaaaagtatgaacttgaacaatcaaagaaaccactcgtagatgatgcttccagtgacctatgaaattgg
ctgattaggaatcctatactataagatatcgatactttcatccgaacagaaaatggagttactgattgaacgaacgaaaagctgtagcttataaca
ggtgtatttacaaaaacggattcttcttatcctcaaaatccaactcaacaaatctacgaactgtctacatatttctaagttgaatcggcaacaatttt
gaatgtttttttttttgatggatttgctaaaagttttggatacctctggtgattctatttcatgactttttcatactctccattctgtataaagtggaagc
tttcctgtactacgtgtgaatgttcttacatattgtgactggaaacatgatgctatgattatagagtagcgacaagagtactagcttcaacatacctt
agttaatgaattgctttcaagtctctcttttgatatcaaaacctatgttgatgtttgactaaagggaaattgtggagatgaatccattgataggttac
tttcaatttttttatcttttaataatgtcttagatgaatataattcaatgaccattttccaatttctattcgatggtcattaacaagtctctatgctcagt
aggaacatcaataactttcataatagatatgagcgtctaacagagtacgaaataaaaggaataaaattgttttaatcctttaattgaaaatacac
aatgcagcaggtttgcaaataaaaaccaaccaaaatacagacaccctaaacataaactttcttcatggattatttgagtttgaaaaaatgaaata
tatatttcgctctttttattttattttattatcttgtatcttgtttgtttttttaaaatcaacggtttttttacattcatgaatattgagataaacaaacaaaa
cagtaagcaaacaagtaacaaccgagtcactgtgctccaggatttcagtcgttacgtcaacgtggtggtcccttgtacaaacgaaccccctcatat
cagtagaatagcgatgcgctccattctaccggaatcatttagtgctatccactaatattcgctatctccatatttatagggaatcagagacctgtcg
ataaccaaccaaatatctgttatgaatgtaataaaagaaggaatatataaagaaagatcatcgttagactcacctgaaacttccgaaggattgg
ataggaaggccgatattcagattgcgattgtcagcaccgtgacatcttcagatggatcatatgtggaatacgaaatttcgcaccatgtacgttgtat
aaatgaatttttcaatttgaaaattgaattgcttgaggatgaattgctaatacatgcgattgctatagaagcgatctgtttggagacggtattcaga
tttcgagtctttggtaaagttaatgcatcgacgatatccagcagcaattgtgccaccaattccaggaaagcaatctttatgtaagtgatgaatgag
cagaagctgttcatggactgtttttttccaagttcacttgctaacgaagggtttatgggctctgttgtgagcccaaaagccttaaatacagtaagtta
tgcaaaacatcctagaaaagcaaaaaatgatgccgatttttgaactttcgctcccgtatgcttgagctttttcttcgtcaatgtttgttacatccatt
gcttcgtgtcgactctgactttgacaaatttatcaattccagcatttcgtggcacgttataaatatcctctatgaatttatcaaaagattctggtatcga
tcctcttcgcttgcctccgattggtactgaaccagacccctttgcgcacttacgttcttctatgcctttagtaatgtctaatacagtaagtcccccgtct
tctagaagtagaaaaccagcagaagtttctactcaatcagttcgccctaccagcaacccttccaccacctcttctacggacggacctgtctatccc
gactccgaagacgaatttaaacttccacctacatctagcgagttacttttaacacatgactcagacgataatacttcttctgcttctgtccaggaac
cctcccccctaccttccatcagctattgacggactttccgtaacttcaaatcccaaagacacgacccgccctgaatttaaagacttaacatcatatac
caatgaggtgatattgtgtcgaaagtttttgcatcacaaccttagtccttccattcattctactttaagtggaatttctaaaatggaatcatctctctc
caaattaggatctgcttttcattccctaactgctttaaacgaaatccatttggcaaaccatcttcaggtaattgctaatgcatttgaatttagcggaa
tgtatgcgaaagctttggaacaagagtttaattcctctgtgtacgaaaaactaattcagtcgatgcagctggctcgttcagctgttagggctctcaa
atacaagaatttaaaaatccagcaaagagaatttttacaggatcaactagttcattcaaactctattgatccagtagattcatcgtctccctctgctt
atccatctaatcggttaaactcgattcaacgcgccgtggtatcacaagcaaaaaagggatacacaatttttggaagactacagaatgcaatttat
gactttgttgatggagaaacatcaattagtaaggaaacgttgcaagagcatagaacaactattgaaaaccaattgaccgctgcgaactgggatt
gccaaataataaataaacttatagatgaagagttagaatactataaggtttgtcagagcactcaactaaaagagattgtgaatgcagttcatgct
tccttttcaaaatgggcgcagtcaaatctccatcgatggttacggacaagagaagaactagaagaattgtcaagaggtatgtgacagctcgtcct
ttttgatcgtttgttcatgaatatcaccgcatatatctatttaatgtcagttatgatttacgatgatgactttttttttttttcttcaatctttttaatacct
gtttgccatgtatattatttatagtttattttttccaataagcttttttttttgtttcattaaaaactttgcaattcttcgtagcatctcgcgaaaaaaaat
tggattgtttgaagctggagttctcatgttttcgcctactattattgaatatggtcaaagattacgcataattgcatgtacagaagatgtggaatgaa
tttagtagtaaaggtgaatatactttgggaattttattgatgataaagacatgcatagttcccaaaaaggtaaaaataaggatcaagtagagagt
aaagatggtttatatttttgtgggcgatcattaagcaaaaaagcaaggtaaagctgtatattatcgcatccattgaccaaaaaacgttggaaaga
atgtaaatggtccgccaaacgggactagactagactttactttcttgcaatatcgatttctctcgtattatatttaaaaagtaagtaagtagaaaca

aggtggaaaaccagcatccaagaagataggctgaaaccaattaagaaattagaatcaaatgtgatacaaacaagagagatttcttattttatttt
tgttgttgtcaaaaaaaccaaggtaaaaaaagtacaaagatgaaacaatgcagttcaatttacggtaaaagagatgaaaaaaaggaaattctc
ataacttgtaagaagaaattacacgcaaaccacgaggaattataaacaaaaatcatagtcagtccttcttgctcgtcaaagctttcttggcaaaa
gcccaaacaccagctgcagaagccaccaaggtactactgatggccattgcacgtacaccacgagtggacttgaacagagcaccagttaaggca
ccagcagcaacactattttcccagctatgcttttggcgtttgtaaccgatgaaggaattaattccgttgtacacaatggcgagcacaccaagagag
tttccgacaaagggaccacgacgtgtgacaccgttcaaaacaccgttcaaacgaagtctagaggaagagatgtctctggttttctttaggccttca
ttcaaaccccatacgccaccaaccgctaagccggtcaaatacgatgtaccggttccgtagcaaagatcgtcttgccaaccacgagaagggattgc
catactatcaccaggcaaagcactcaaagcatcttcttcaataagcaaataatccaaaggcttgtctaattccgccaaaggatgaagctttgcag
gatcaaactctgattgagagaggatatccgaggcagaggatcccggggaggcttcattttcagaagattttacctgagtatcttgattttctttttc
tctcttgtaaataaccaagacatgttcagttttgtgatttggacctacaaaaaagatgttggttgtcagtcggtgaataccgcctcttaggcgtgcta
gggacttagggccagtttcatttgatgaataattttgataatttctactcataaaaaccctttttttaagaaaaaaaatttacaattgatattctatat
aatacttccctttttcataccaaagagaataatgatcgaagtaaaaatgtagattttatagcgtctttcgtctgcttgttttgcggttcttttcttcaaa
aacacattttcaagaactgctttcaaataagaaaacgaaaagatggcaaagagcgaagacaaagctcagacactgttctatggtagatacata
ataatagctaatataagtaagagacgaagggagaatgagaccaaagtttacagatgtttaaagacaagcgagtgttataaaataatttaagata
ggtaaagtaaataaaaagatcgagaggatgggccttcgggatcagacccattctgaaagaaaaagaattcgtacagaaaagccatccaaaata
attctaagtggaaggaaaaggggatctagggatattcattgccaaataaacacaaacagatatttacgagaaaggaaaggaagcaaaggtga
ggacaagaacaatgaataacgtatgaaaaaaagaaaatgaagaggtattaccatcaaagacgaaacttcaaaccatcaacttcatccgtagct
ggaaaaaaaattgaggattgttcctatttcttctttttttcttgaggttatgaattgcatttccatgttgcttacttgataaaatacgttcggaaagcaa
tgtaagcaccaatcatagcaacaacaaagatccacattgctggatgactagaaggcaaaaggttagtcaaagcttggcctaacagacttgaatt
tttgtgtctcaaaggcaaagcatcaactatatctttctaaattgactattcgttgctttgcctttggagacctgtgaccacttacataggctgggaag
agtcatagcacttgctgtcagagaacgcaccctttgtgggcaattcgggaccaccttcagtctaaagcaaatgttagttagtaaatgtccaaatag
gtgttctgcaaccaaccttaaccaacttgccgacgtgaaagtttttgagcatttcttgagcatcttccgagtgaccgacatcctcaaaaggaccgct
tgcatcttgacctagaagcaaaagaattagcaaacaagaatgacaaggaatgggtataggtttcagcggaaagagcttcagcaggtacaaata
caaagggttccgcaggatccaagcaaaaatagattggcaattgagtccaaattattaggagaaaacacttttcatgttaccgttttgtatggtata
aacaacctttcgacgcgtcgtcaactcaaaaaacgcgttggtttcagttttctctccttttccctacgcgaaaattaccgaaacagtatcctagtcct
ttcaacttcgcccttgtttagccgctgatgttgaccagacatatagactcttgctttcacctatttcctcttcagcaatcaatttcttaccagcaagatc
aaccaagacttcttcaccaccagggtgggcctcaatgaactttgtaatgtcaaagacattatccttgacgacgatccacaaatcctcgcgagtgtt
gtgtttcaacacttcttcaacagaaacggtcttttcagtcattttctctcaatttctttcgattctttatggtctctttaccgtaaacagtgccttagaag
caattgatgtaggaaatcaacgagtcgcggtgcttccaataagaggagctatatattccaataacggcttttaaggattaacacaaagtggtccg
atttacgaggtcaaaagtagtcacgccttggaatttgcaaggtgcacccagaatacatgatttaacaacaaccaaccttttgccagtactactact
ttttctacttttcttcttttatggtttgagagtttgaacagactgtagtcttaaagaagttaattcacatttgcaagatttttgatcaagatccgtgtatc
tttttgtctttcagttttttggtagatccgctggaattacgcttcttaaaatatgtttggcccttttcgcagtacgcttgcccggtttggaggtttagtta
aaaaagatccctggaggttgtcacacaatcgcaaataccgtctccgtcaacgccttcgtgctgttgatcaagtggtcgatgtgctacgatctgcttt
gtcaaaacaaggtcagtcggtacgtgctatcgaagactttgttgctacaaatccaaaagaatccgaaatgtcgcccaaagacaagtacactgtat
tcaccagaaaaacacaaggtgccggccctggtggatttcgcaaatctattcacaaagttcctaaatggaccaaaattactcatcgcaccaatcct
gagggattttaatcattaaatttttacgtttcttttatttcttctattttgctaatgataactcttcaacctttcttctcctctttgttttgaagtctagtatg
cattgtttaatacctcgtttattaataatatacaagtctttcgtttacaggctttttagaacaggatcttttttttcgaatatccttacctcttattttttttt
cttggtaaaagagcacatatgccgcagatgtgacaatggaatcgtctccaacgctcctaacgtatgcatctatagcacaaacattagtaatctttt
aagtttgtacaaactttcattattcttaccatcgtaatttaaaaatttacccgtcaatgcatccttagcatacgaggtatagtggctattgatcatgta
cccatgatgattatcaacagcgtacaagtcgtatgtatatgatcttttcttagcttctttctcctttaggtttcttaaaggactaatatgtgcttgcatg
tccaagttcattgtaggaaaaagtacataatcttttcttttccgaagttttcgcatccggtttgtgcaactaaaacggttcaaatgtataattaaattt
cttggtaatttccaaatttccatttgtttggtagccgcccggaaatccttacaagatggacaataccatgaatcttccaacgaaagctgttcaggcc
gggaaaactcttcaagacaatcatccaaagtgatggaactcaaatggtattgctggccttcgaaaatccactcatttttttataaatgctttttttctga
ctgtcttccttccatacacacatgataatcgtctggttatttaaaaggacaccttcttcgcttactggggtatattcatagctttcaaactctttcaatc
tattccacacatccacaccaggtggaacattctgaacacaaatttgaaacatatcgggagttacaataatgtcagcatacttatgatatagtttgc
gtactcttctatataactcttctttggagatataattctgggaaaaagaaagttgcagcggatacccgaaagggtcacaacgttgaaatgatccag
gtaattgaggacgacttttgcagtggacaatgggcgtaattatcttctcttttgtagtttcatacatgaacaagcgatcataagaatggattattttg
gagattttctcgtgttcgtttaaacacttatacacctttccatcatagatatctactgccttcaccgactctgtgctgtaaccaaactgttgcaagcgc

attacagcataacacttggcttcctgcacagtgtagtcagaaggaatcttaatgtcgaacttttcgggggaatcatgatctgatataggcacaaaa
tttacagtatgtctccacttgggtaactttggaaggggcagagtaaggtacataaaaggatcaaagaccacggacttcaagcagcaagttgaac
attgcaatgtacttttgtacattccttgaaataattcaacaatgattgaatcattccttttggaataggtctcccagcattcatctgccaccttttgaac
ctgcaccggattttcactgtacaagtccggtctttcaaaatatggtttgttgataactctgttcaaatcttcatgtaaaccgtcaaggaaaaatgcaa
tgaattcttgtgaatcattttgactatagccggcgaaggatgaattgaattcactaacaatatattttaaagtgaaaggagaaagtgagggtccag
aaaccccattattatttgcttgttttaaaaggttagcataagcaagagcaacctttcctttcattcctaaaggattggtggaattaatttccccttat
accgatcttgaataaaatattctgaaagctcgtaagtatgaaataagcattggagagcagaattcatatagcaagtatttccaatattatttatacc
acagtaaccccgctcaaccatactctttgtgtcaactgtctgaggtgaatccatcagccatccttgaaattgagcgtttagagtttcaataataagg
catcctttctccatattcatctcaaacattgtcatgcagtcggcaaaatcatttaagagaatagttcccttaaggcttctggcataagatattggtag
aaaacggttcgatggtaataatcgttcattataccatatacggtattttaaagattttagtatatgaaaattggtacgaacaaattgaaatagatct
ttaagggtatgacttcttgagaaatggaatttataaggaccttcgtagtgcagagttggcggaagaaatgagagttctctgtttatcgaatcggcgt
aaatgaagaccgtagaaaaactaatggggtatacattcacgaatggttgcaaattgtgttctggtccagtgcgtataacttttcgttcaatcgcga
atccatggactccaaacctaaatgaattgttagcaagattaaaaaaatgaagcgtaaacacttcaagtctggctcaaattataactgcacagcac
tacctattatttgtttgtaatttgtgcaaagaatgctatggttataaatagtacaagcatgaaaaatgaaccctttcaaatatacaaagcatttacta
gagaaaagtaatcttttgaaagaccttaccattgtgctagcttttgccaggcagaagaagatataatcgtatagtcaattgattcctcaagcccag
gtaaaagctggtgatttttatccagcaattttctttgctctattggtccagggagatggctttttccactcaagaagtctttatatgcttcgaaccattc
gtactcaatgagatatgctgaatcatcggttttccacttatctagatcgccctctaacgtggaaacaaaatctttaaccaatgtttcacaaaaaata
tctctccaatttgctgaatgttgtaattctccgatatgagcagcagttgaagccattctcaaccatggcttttttttttttcgtggcttccaactctttt
attaaattctaatattttagtgaccttttatgcaaagtattctggtattttgagggttttggaatgattcaagatttcgttgaaatagtaaagtcgcttt
cgatttcaccaacttcttatagttgccgcgttggatttctttctcgtttttcttgcataaacaactataattattaattatttatcttgcaaactagctttt
attatttcagggattcatttcttgcttcagcattccttgacagaaagaaacatctgtgaacgttttaacggcattattatatgagactcacgttttatt
aaaaaaagttgaaaaaaaaacaatttaatatttctttaacttttgcatagtcgagtggacaggaacggggacttaaagaaagaatttaaaataa
aagttattgtcgacacagaatagacttggattgaatttgactattgagttgatttgaattaaagaatggctgccgaacaaagaaaaatcattgaac
agcttatgggaagtaacctttccaattttacaagtcgaggccaagttcactttactgatcgcaaagtatgcagaagtttcttatgtggaatatgtcct
catgatatttttacaaacacaaaaatggatttaggcccatgccccaagatccatagtgacaagctgaagtcagactatgagcgagccagctactc
gcatagttatggttacgaatgggactatttggaagatttggaacgacatgtagatgaatgtaatcgaagaattgatattgctgaggcaagaaagg
aaaaaaccacagaggaaattgaacgaacggatgaactgatgcgtgaaattgctcatacagaccattccatcgaagttttgattgaggaaatgaa
agccttatcgcagaaaggacgtgtcaaccagactatgaaacattatatcgagctacgtcgtctgagaactcatagacaggagttgcatcaagaa
gtgatatctatgaatgaagtccccagtcaagctggtacgacacatcaaaagctgcaagtttgtgatgtttgtagtgcatatctgtctcgattggata
atgatagacgattggcagatcatttctctggaaagatgcatcttggatatgcaatgctacgaaacctggctaaggatcttcgtgaccagttagaat
tacaaacttctgataaaaatgaagaagaaggaggactggaagctgagaatcaagaatatggagaacaacattcaatgctcacgtaacttgatg
attctctcctagacactgctacatctaatagtatcttcacgttcctactagtagggccgtcctagtacctattatacatgatattctcttggtatacttt
atggataatataggcatgcatctttccttcgttttctcctttagacatactccgaagtttattttatttttctaatatttagggttttgatttttttttaaaa
aaaagtagaaatttattcatggcaaatatgaaataggatcaatgatgcattcacaccttgtgaaaactcctaccaaatgtgtacacataaaagttc
agaaatttttagctttcaattcttttagtcattaagtttaattaagggctgtgagaatacaacggtcatatatgaacgatggagtacgggccacatg
gacagtaagacagcatcaaaacaattaatgggataaattgcaaattaggaaaaaaaggtttaaatagtttttctttttcgtaaaatgtattcgatc
gactcagattgatgtactctggaagacagttttaaccctaagcataccacaaaatctcatcaacttgatgaatcaagcttgcaaaaccacatacca
tgctcgacttcgaacggggaatacttctatattacaaaaaaatgttacggtctcaacacggtcacatacctcgtatacagggcgaaattctcttcg
aaatgtaccagtagttaattacgcggcttttgagcaaggtgatgacttcgagcttccttctgagccttcaggaaaggaagaagaagaggaaaatt
tcaaggtagatgaagaaggtttcccaattgaacgtcctaaaggaaaaagaattgttccgaaatttgcaacaaaaacaagacatacatacttcac
agatgaacaactacgctttgctgctgaagaaagggatgtctatgttccaatacgactagatattgaattgacaaatgactttcggttgagggatac
gtttttgtggaacatgaatgaacaggtgatgacaccggatacgtttgctcaaattctatgtatggatttagacattccagcaaattcatttggacctc
aaattagttcaagcattcggacgcagattgaagaatatgcacccgtagctgaagtacctatgcctcaagggcaggaaatgctggtcgtgtttaat
attcaactacagctgtcgcagcatgcatacaatgatcaggttgaatggaatttaacaagtaatttgacaccagaggactttagcacccaaacttgt
agtgaccttggactggcaggtgagttccggcccgaaatggcacatgcaatccatgaaaatttacttaagttgaaaaaagatgcatgtgaaggag
atttaccggattatgataacgattccgtgcctggagcgatggctggacctcgtcaggacatggctacaatgggagccttatggcaacctacgttgg
aaacagtaagtttggaagaagcaaaacgaaatgaaaccaacaaagaaaatatgattcgacaatggagaagagatgcttcgaagtttggcgga
aatgcggcagatgtttcagcggagcgttggagagcagcagctactggaagtatggttgctcagacggctacgacaaatcttcctagcgcctctgtt

tctgatgcgcagactctgttggaccaagagcgaccacgttggcgatgtagatggtgcaatgttttgggaacaggaactttttgcgtgcgtcgtggc
ccggaaggaaacaagtcgctgtgtaatgcctgtggtgttgcttatgctcgtacaggacagttaccatactggagaaaaggattgtatacatagag
tttctataaggtagatcgctatttctgctgctatatctcctccttctcctcttattactactactgctactgctactgctactgctagtctggtataagta
gatcttccaaaaaagaatttaatagataaaaaagtagaaggattataggcaaaagaaaagaagcgaaaggtggataacttaatatgtgatcac
ctgttttactggatgtggatccatgacgaataacttttgttaattaattattggtggaatgaatgaatgaactattagagctgctgtatgtggaaaag
tatatgagatgagattatatacgttattgaatatttcttgagtgtatcattcgttaatttttttgttaaaacgttcacatcaaaaggtcctttcattattcc
atagctaaaaaaggagttcaatcataattcttcataaacataacatcgtagcgcaaagacttacaaagaaaaaattaggcggcagcaatctttat
aattcatcgaccgaaggaggactctttctgtcgttgacggatatattttcagaacgttgatgcttgctcttgatggcagtctctcgctctagttcttcg
atgggcgtattatcccaatcggagcgaacataagcctctgaatcggtagtattatcacgactagcattatcttttggagaaattgcgtcctcttcag
cgggtatgcgatgaagaggctgatcactgcctgatgttgtggaattatttgaataccagcgatttggtaaggcactactttcgaacaatggatttgt
gcgtaagacgttacgacacgatttgctgagaggaaagctcttgaaagtggtaagacgttgagaaaacatgttgtatcacaataaatgctaagaa
acaaccacgtagaggagaaattcacacaaacacaaagcaaagaattgtataatctaatccctagttagtgacttgaagcaagttcctacaactg
aagagcgaagcaaaatctgactacttttgtcaacgtttcattagtttttttcttcatacctgtgaataaattgagtccgctaacgggtgattgagttg
actctatccttgggttcagttcctaagtaatgggaaaagttaaggaaacaaagctggaaaagcgagccttttctatctgcgtaactggctacggaa
gatgacacacattggtgagccttatttccataaaatgattcacagatagtttatttgacgatttcactgtctagggtgatttcatactgagcaaagtt
gctacttgaatcgcgatttagagtagcttagtgaactgaaactgttagatgtggcgatccaaaaagcagcaaatattcttaaaaaagaaataaat
attcatcaaaaaaaaaagaaattcctgtaaaatgccttttgcttttgtccttctctgatgtcatttcccgttatcattcgaaacattgttgattaacga
tacgggaagtgtaagaccgtaggtttcaggagaacgtaatttgattttcataggtcattacaattcgctaaaaatatatgcactgttgcactgtagc
ttggtaattctttttatgggttctatcttccaaacctatcaatcccatatgaaatggatatagattctttttatggaacaaaataaacagaaagtttca
tcttaggcgacttaaagaatgtattattttcaaggacaactgagttggtgaaatggtttccttcaaaaaaacaaggatcaaacgaacctcaaattc
ctcactgcattgcactaataaacaaggtgctggcatatgaaatggaatacaatattaggttgaagacttgagacattcagggtttgaatttcttttt
tttttctgttcctatccttgaaatttgctacttcggttgttcctttatggaccactcttgtaagaaaacgaaaaagtttcaagatatcaagagagaata
cttattgccagttagcgtagttagcgtcaaaaacccaaaaacagtatttcgaaagaatttcatctccaaaataaacagtgaaaaggcttcactga
aagagttttgtgctgcaatattcgattcccatactcttagacttattattgattatcattgacaaccaccaaaaatacacagtagttcaaatcttcttt
ttgttaagaattgaagcaaattattcaaggaatttttcgtttctatagtgagaatgtcatgaaattgttgtcgataaaagtaatgtttttttatattcaa
agaagatacctgcaatcttgatttttctgcagaaatgaaattccacattttcctataaaatggaaaacaatcatacgacagtaagaaataggagt
aaggaatagaaaataagttttgctaaaatgcaatagcgggaacccaaacgcatacatacgctatatacaaggtaaatatacatacttgtaagaa
taaggataactatacaagagtatattgtcatagaagcaaagctagagttttaaaagttctgtgggaatacataaccaaatttgataagtgcctgtt
tgaaagacgacttattaccccaaaactatccatcgcatggacatatagataaacgttacaagcgaatcgtcatacaaaaatcattgattaagatc
tctcttccatttccaagtcctggtcttggttacgatcgccaccccttcactatcaccaatgctatttccatcttcatctgtgtgggttatacgagtaat
tacaattacaccttcagcgaccatgcgatcataaatcgatttcaaaagctcctgaagcccattaggaccagaaatttgatcggatgaaatttgcaa
acgcgaacgcaaagactcaaactctggttttacaattgcatgttgtaaagaagagaaatgcttataaaaatcgcgattttgtgacaattcatctttc
ctagaatcccgtataagctctggaggagcatgagggaacaacttaggagagattcttgggtgtgtaaccacccgatacatctcatcatccattgc
aaaaggttcaaccggagtcgtactattctctacagaaccagataattgttcaagcatgtctaatgtttgagaacgagagtaatcttcattcacatca
aatgcaggtcccgaggtatcctccatgcttgaagctgtttcgccttctcttcgtggttcagtagcagagtcattagtaggttggagttgattcacaga
ttcattcattggcgaaaatcgaggcagaaacccaggtgcttcagcagtaggcgataacacctcccagttgaaatcctgaagagaatcttgactag
tagcaccaaatagctgtaaaaattggtttgcagacgaagtgtcatccatactacgactacggctggatgcattatttctagcattaatgcttctagt
aacattggaaggatcagcaatcaattggtttactaatgaaaccgtttcttcatctttttcatcactatattcttgcatccaataaaaatgaatttgag
atgatgatttaaatctcagagcatatacacgacccgtggtacattgatcaatttttatgaaatcggcctcatccggaaatataataaaatcctaacc
caacaactgttaataattaccatagacacaattaagcaacttgaaccctccagagtgcacattctataaaagttcagttgaaaataattaatactg
gaaatatcattccgtatcgcattcttccttacatcttctatatccgtagagtttctagatctctctttccaataaaaatgaagcaattcatctgtctcta
gctgtaggtataggatactgatggactattagtatcgtacttataaatgacgatgctggcaaaacacaaaaatacacagaacaaagataaattc
atcttcaactgctttttgtcagtgtccttaccctttacgatgatctgcttgcaggatttttgtaccaggtttgcgatttaacttgccagctttcatgctg
ataattccatattttcttgtctctgcttgatcattcctaactcttccaaaaacactttccatctttggttttgatatcaaatatatttttttgtctgtcaata
tttgttttgttgaggtttcgcaggtgttggcttttgggtattccaaaaacattgagcatggaggcatgtattgaccacgaaagtacattcgttgaact
acattgctaaatgtatctaaatatatatttctatggttatatttaactatctatggtattcttcttttcgactctttgatgtctcgagttgggtccaaaag
gaacaggtttcagctataaaaatatagaagccgttaagttattaaaggagattctactggatttgatgctcttaaacagaaggcggatatttgaat
ccaactgttacgaaagtgccttttcatagaggcaaagaagcgatcctaatacaggcttgttggtcaagtcgatgagtcaaagactattgattactt

aatagttaagagcaaattgctttcaagatacattctgtctctgggtttcactctacttactttacttcgcttcacaatgatgctgttcccattattaagg
cttgccacggcattaaggaatactcataaaggactatgtgtactcaaacttttctcagacgcataatgaaactttcactctaacctttcttactctgc
agcttgtcagacttgagtacaactgtctttgtttactaatacataaacattagcagaaagaaagaaagaaattaaaataacagaaaaatttctttt
agtactatttattttatgaattcacgaaaatcctttgaagcttttctattaggtgttcattatgaagcatttgaatagcatttcgaattattcccaagtc
tggaaagaaatgtgttcagcttgaaatagtagttttacttaaattaatacaagaaatttatatctctgagcgttactattccatttgctataacctttt
cttgtatatttcagctaaaacgaataacctgcatgtcaaaaaaatttttttttgaaatgaaatttatgcttgaacaaagcaaagaccattcacacg
tctattaacttcataattgattcttttcatcgcttttgttgacattcctttctatccactaccaacatgtcctcgagccagcaaagcaaccagggcaga
ggagcaacaacccccaaacgagctaatagctcttctagggaagcaatggactcatctccactgtttttccaacttcctcccccggttctactcagc
atacaactcctaaaaccaaaacgcgtacatctcgtaagtctttatttttctgcccggaaacctaacatagctttagtcgcttcctctcctttattattt
gaatctagttcacctgctcctcaggctcctcatagctctcgttctatccctctttctcagcataatgatttatttttagctgggagctcacagcgaactc
ctaaatcttcaaggcgtggtgatattcattctagtatacacttgaaaacgccttccagaagagaagttgatcctcagaaaattggagctgctactc
ctagcagtctgctgttctcgggttcagatgctttaacattcagtcaagcccaacctaccagtgaagccaacgatgataccattcgtgtcatttgggg
aacaaatgtctcaattcaagaaagtattgcttcttttcaggggtttctgcgcgggttcaagaaaaagtaccgacctgaatacagaaacgatcccat
gcctcctcctgatgctgaacaagctgtttatatagaagctcttagaaatatgcgtattataggtttggatattctcaatttggacgtgcaggatttga
gacattatcctcctacgaaaaagctttataatcagttatactcttatcctcaggaaattattccaattatggatcagactattaaagatgtaatgctt
gatttattaggaaccaatcccccctgaagatatattaaatgacattgaactaaggatctacaaaatccgaccgtttaatttggaacgaaccattaat
atgcgcgatttaaatcctggtgacatcgataaattgatatctgttaagggtttggtaatacgatgcacccctgtaattcctgacatgaaacaagctt
ttttccgatgctcggtttgcggacacagtgtcacagtggaaatcgatcgaggtcgcattgcggagccagtgaaatgtccaagagaaatctgtggtg
ttacaaattctatgcagttgattcataaccgctcagaatttgctgataagcaggtcatcaaattacaagagacgcctgatgttgtcccagatggtca
gacaccacattctgttagcctttgcgtctacgatgaactcgtagattctactaaagccggtgaccgtatagaaatcacaggtatatttcgttgtgtcc
ccattcgaatgaatcctcgtcaacgaacaataaagtctcttttttaaaacatatgtagacgtcgtccatatcaaaaagcaagatcggaggcgcttag
gtactgatccttctactttggaaaacgacttagccgaagacacagctgcacaaattgacgacgtccgtagaattaccgatgaagaagttgagaat
attaagaaggtctctgaacgaccggatttgtatgacttactctctcgttcactggccccaagtatttatgaaatggaagatgtaaagaaaggtatat
tactacagctctttggaggtacaaacaaaagttttcaaaagggtgccagtcctagatatcgaggtgatatcaatattttaatgtgtggtgacccttc
aacttccaaatctcaaatccttcagtatgttcataaaattgcacctcggggtgtctatactagcggtaaaggttcttcagctgttggtttgacggctt
atattactcgcgaccaagatacaaaacaactagtcttagaaagcggtgccttggtgctttctgatggcggtatatgttgcattgacgagtttgacaa
aatgtcagattcaacacgttccattcttcacgaggttatggagcaacagacggttaccgtcgctaaagcaggtatcattaccactctcaatgccag
aacgtcaattttggcaagtgcgaatccaataggaagtaaatatagtcctgaacttccggtcacaaaaaatattgacttacctcccacattgctttca
agattcgatttggtttatttgattttagaccgagtggatgaatctttagacagaaaactcgctaatcatattgtctcaatgtatatggaggatactcc
acagaatgctacggaatttgaagtattatctatcgagtttcttacgagttatattacctatgctcgcaattgcgtacatccgattatcacagaagaa
gcatcaaaagaacttgttaacgcctatgttgatatgaggaaacttggagaagacgtacgtgcttcagaaaaacgtattacagctaccactaggca
actggaatccatgatccgtttatctgaagcgcacgccaagatgcatcttcgtgaaacggtcgaggtcgaagatgttcgggaagctactcgtttaat
taagactgctataaaagactatgctaccgatcctgctacaggaaaaatttctcttgaccttatatacgttaatgaaagagaaacacttgtttctgaa
gatatggttcgagaactgagtagtttggtctctacactaactgcaggaggcaaaacgattatggttagtcaattattaactcgatttcgcgagcaa
agtactacacatgttgatgcttctgagtttgaaacctcactggcagcattagaaaagagaggtcaattgaaagtgataacgagcgctggacatcg
tctagtaagaccgattgctcaaattgagtgatcaattaagcatgacatccttaagctggtattatggatgaacccctttatagaatttactgatata
atcaccgtacgatgtggattcttatttatatttattaagaatttgttacaactgaaaaaagaacattggaaactagtatgtaattaattaaaatttat
tttgtaaaagcagcatagaactataagttaatttagataggatagaaaatcattagggatctgttcacaagaaaaaactttaaaccaaaacctgt
cgacaaccaaaaggggacccaagaaaagagaggaatatatacataaataatacaaaggctggtacatgtaaaaattcgtagatcatcacatcc
tcataacgaatgaggtacacaaacacgaaaaggaaggtccaaaggtacctttccaaaacaaagtagaagattcagtctacaaattttcggtaga
ttcacggttataggaattctcagtgatatcaaacttatcatcatgcatgtctttactaaacaccgtactttgttttctagagaaaggtttttgtctttct
cgcacattccatcgcaatttatcttcaagaacctttgtccattgcttatgtgggttttgaatcgtggtgaaaggatattgtgaagtcacgatggacaa
gtaatcaccgggggttatttctgttttgctgtgaccatcgacagagaagaaagcagtagcccgagaatccaagggaacttcaatgtttataactga
ataatcaggaaggatcactggccggaaagatagacagtgaggacaaataggagtaatcaaaatagcgtttaagttaggatggacaagagagc
caccggcattcatagacatattagtcgaaccggttggagtagagacgattaacccatcagcttgaacgatcgtcattaactctgagttgttgtaaa
ggttcaggttactgatgtaaggatgctcaccgcgtgagacctgaacctcatttagaacataactggtagatatgtgagagtactgttttgtttctga
atcatacttgtgatacgaacattccagtcgagaagaaaagcgtaaagaaacactgctgctaagcatgtggtgaatatgctctttatattcggtaac
gggaaaatgagtcaagaatccaggaatatcagagtctgagaaagtcaagacaggaggacaatgtttttgaaataaccaagaggtatacaaaac

agttgtattatcgccaatagtgatgaccatatcaaactttgttggtttcactaggcaaataaaaggcgtccaataaccgattctgtcttcgtgcagct
ccaaatcatgcaggatatcgcttgcggaaaaccgtttggagtttgctaatcgtttgtctacatacacttttgagtcaggagaggttgaagaaagca
agtatttcgtaatatctgcggtgaaatataccaacgaatggttccttggtttggtgacaatgataatattttttggatccccagaaaatataccattg
atagcttcactgtacatagatttcaatctcataacattttctatacgagagtaacaatcttcgttattgatttgttgtgtgaagctgcggaagtcagta
atgtccaggctctgcgtatttccactactaaactcgtccacgggagtatttaatccagacaagtcaaagtttgcaggactttgcaaaccttcattgct
tctctgacgaaaatgtaagtttttcattttaatcttgttcagttcctccaacgcacgatcccctcgaatgtcagaaaattcagacatgttcgtaagat
gtctcgagttccaaaattagacctacttttccaagcccttaatttatctacaaattcagtaagaatttcacaaaaacagccctcaaattttgtctaaa
tatacgcttttcttagatatacgctgtgttgtttttaccatgtgcatacgtatgaatttgctctctctatataatgggtaacattgtaacccggagcatt
aagtcgtagttattattgttgacttttactatatacgaaaggatgtaaatatgaacgaaaaaaataccaagaaaagtttttcattaacatttttggc
atatcttgtgatttaagaacaaaattgtaaaagagagcgtgaagtgtgaagtattgaggaaattctgaaaaacatggattcaattgtaaaaaaat
aaaggtaatgaagtcgaaacgcgttcagccagacggttttatctttttttttctcatttacacgtaaatctgctgaaaaacggtctaagaaaaggta
atttacgtgtattctctattgactatgcattggtatatgttgtatgctggtttcgttagttggaacgattgaagttattgttttcatatactaaaatgcca
attgtcattcaattataagaatttacgccgtaattgcaatttcttgctcaaacagaatttcagcgttgagagagctagctgccatttggtaaagatg
agctcatctttcaatttaaaacaaaatcagcaaaaatacagtcaatggattcactccaggagtaaaattaccttcaaagcttacaatttctgtctaa
ttatcctggatctacggaaactttgacacaagcagaacatggtttaaattttcattggctttaatttcttcttataagatactagattccacttggac
tgaaacttttcattgatggcccttatgacagaaactctcataaaggccaccaaatattgagtgttgaaacaaaaatttaaaaatttaaaaatcttat
ggttcacaaaatgcatcttaataagtaaaaaagaacgggttcttttttttttggcgatttgaatcattttggagaacataaacaaactactatcgctc
ctctactactaaactctttcttacaatatggatccattgaaaacagaagaaaatcatgaaacgcaatcaaagagttttaaaacaaagcaaatacg
aatgaatgaatggaagcatccatcagagaaagtggatggtcgatggttgcagttggatgcaagagttaatcgtactgtaagtcagacacgaaaa
agtgtatcagcaaaggagaagctgagttaaaagttcctatagtttatctctattctcttgagattccagaaaaaaaagtggctaattttattgcgat
ttttgtcgtttgaatgagattgaatgaaattacaaagaatcactaacaaaaattaggttgaagatcttgaaatttcgaactataacttgcaaataat
caatgagggcttggaaaaaaaaattagaagaagctacaacccaatcttcaccggaagaaaagcaggctcccagtagcctattgcaaacaaaagc
agatgaagaaaaggttttgctgcattctgtcaataatgtactagacaatttcatttcaattgttgatgggatggttcaacaagcaaatgatgtcttg
gatgactagtaaagcagggtagatctccattttaccaaacagcatagcctttccaaaatctgaatcccggcttgatttgcttcaggttggatgcttg
agcccctgtacttaatgatacagtgaaaagagacctgttctttagcaggccacacattgagatatctctcaccttaaaacacacaagaaaacatc
atgctgagtttcaaaaattatagcattcaaatattgaatttcatctcttgattaggccatctactttgagcatttacatatttattgtcaaaacaaaag
acagaggaagatttaattgtatttattctttaagttaatatttcagaaaatatttttctcattgtatccgggccttaagagtggaatttcaccttgtcct
tggtggtccctacttctccaaagtgttgccaagttcctacgcgagccatcatccaaaatcgggtgttcttacttcaaatcacccaaaaagagggact
tttagcgtttgatgaaaagaaattgattcttaatgatttttggaaagaatttgcgtctttttcggccacaatgtatgggtttcattccatcgactcgttc
atggtttcataatgccagtgttcaacagagtattttaggaagaatcaaagacatgtttggaaaagaaaaacaaaaaggaaaagaacagattccc
gagaaagatctccctacgttagaggtagtatttccaaaaaaaagaacgcgcgactcgaaatggaaaggacagcaactttttgagaaatttgccg
gtgaaaaacttaataaaactgagagtcttgctgttatcgaggaccttcgttcttccgaaaaattacatgctccttggtctccagaagatcgtttgcgt
gctgcaaagttagcatataaaaagactggacacattgtttatgattctccattacaaggactttctaactgggatgatatgtatcggtactacgatc
ttcatgttaaatatggagataccactgtgcgaggacgcactacttggaaacctacagaagagttgatgcatctaccaaatgttgttttgcatgttga
taaaaatgggtttcccactgaagatcaaacaacaggaaagccagtgaaggcatctgagtccatctaaacaatatcctttttacttctaacattttgt
gtactagcatattatctttacgcttaataatttcttctctcgtctggtttcctttttagcctatttttactgcttataaaagctaatccctcatgtatccctc
atatgttgttaagttttcgtactcccatctccagtgaattcttctattaattaaaataaaattgatactcgagaagcaaaagttcgccattgtattatt
gctagcaatgctttgagatttcgagaatgaggtagctcaaggttaatgtttccattgcaaagtagaaggaaggctcttttaaaatccatgaatata
aaatagtttaataaagaggaagacctctgatgttctgaaatatgacagctcaaagctccagctcaactcaaagctcgtgttctacatcttgtacaa
agtaaggacagaatcaaaaataaacgttttctttttttctgtttaacataaaattaaaaagtttcaatatgattataaaattcattcttttgcaaggga
cttttacctggaaactaacttgacttctctactatactgctttgaatctaccacccacctcactttcagcgaaagatttaaatctgcagccatggaat
acttaaaccaaaaagaacaagaacaattttctcaagtattagaagcaaagcaaattaaagattatacgaatgtaagttgttgatgactagcaga
gattacagaatgatggaagctttcaatcgatgaagaattgacggtagaaacaggaaagccatcatctagatatgtttttctttaaaaaaagggag
tttaactgccaaatgatagcgagtgccgcgggatcctagttgaaagtgaaatttcatatatactgaccagcatatagatgatgtttaaccttagtca
aggatgtttcaatgactgtattcttgatttcaccagcagtaaaattggtgaaaaggaaagtgcatgcttgactagatgcgcagacaagtttttcaa
gcatacccagcgtgtcggtcaaagatttgcagagaataatgctaagcttatgcaataaatgtatcatatctgtcaattgcacagagtccgctcgac
tcctgattccctgtgagtgtctggtgtacagttcattagttttgctatcaggacgtgatgaaaaacctgaatgaagtatccagcattaccacaatttc
ttagtttttgatcaattattagggaaaaatgcagaaaacaggattcgtatttttcaacttcactgtaatgtacaattgtgtcactaatccattcgttt

gatggtcctgtagctaaacaatcgccaacacattcaagctcggatttatgcgagtcttgctcgtctttcacttgtgacttgtcctttcatttgctattca
gagtgagttctgcgtaaatgtcaacactttttatacggtctgattcaaaaagacggaacactcaagcacgtttctgaatagctcaagttgtacaag
aacagtataagtacgtaggacgcattgctcaagaacgagaatgctctctttcgtaggctttgcaaagtttaaaaaaatacaacacggcacatgcg
tggaacacaagaaatcgacattttgaatactgaaatctttcatttgccttttctttttatacgtaaaatcgtgttcagttccttcaaggtagtgctcgct
gtatagcaattggtagcagctagtagctttcgagaaaggaactggctctcttcctaaagtcaaaagggatccaatcaattcttcttcttttatttact
taacatttaacattcgaaaacgagcctgcttcatttgtttatggtctctaatagacgtaacatgttacttgtagttggtgtcggagagcttttaccttc
tgtacagacttcacgtagcatcagcacaactctcgttgttcattcgaacagatctattggcttttctcggcataattagcatcttactcctctagttttt
tctgtagcagtctccatttccgctagaatgtctgccatgaaccaaaaatcgcttttggttgctttgtgctcctttttcttgcttttctcttcggcaatcgg
tgctttggttgcgcctatcggagatgtaaacaatcttcaaggtgttcaacttatgtacagtatgtaaatgcgttctggtcgcttctaccgtgtcatata
ctaacgaattccttagctgagcgtgtactagcaacacccgctcctgattttaaacgggcgaagcgtgatgatgggcctaaaccatcgtattctact
actgaagacaacgttgtcattgttaccgacaagtattggtcgtatttttcgggagccaatagagaagtccattatccctatagtttctctgaagcac
atccttctacgacagtaacaacagcgccttcgggatcaatcggttatggcgcgaagaatacaaaccgcaaattgatggaaggtagctcatccact
cgcatctccttcccttacatttcctttgctttagtagctgttgctagcttatttgctttgagcatataaaaaatctcaaagtgtcattattgttttgagag
ctttcgccttgttggcatatttatcgatattttcatgattgtgatatttgtgtgaaataatctttcttttcttattcactgtctttttattcattcacttgtat
gattttatttcaaacaaggaatcaatattgacattcatattctttttcctagttttaatgttccagaacggttttctgatgttctttcttattttttttgtt
cacactcttttattaaaagcacagtatgctcttttttttcttgatgagtgcttaatatgaattttattgttttatttctatttctatttgttattgaaatattt
ggcttatcgtaaagacaagatgaaatccttttaaatatagctggttattgcgtttctgttgtcactatttgaacttcaaggaaaagtagttactaaat
gttagtgcttttctctaacataattaaataaaattaataaattggatgatgagcggatccatctaagataaaggcaaatgagtaaacttcatttg
gttgttccatcatgtaatttcgtccttcaaatcacaacagcaggaaagaaaaaacagatgcttggacaaaggcggttcaatcatccttcccttcaa
tttacacttgtaaaaccaagttcagagcaataccaaatacaatactatttgtttacactgccctattttgttttgttttcatttacgtcttttctacacta
aaccttgttatttcatattgtgtgctccttaaaactcattaacctacataatgcgcccaaaacgactaccgcaaacgggatatcttaccatacagct
agctgtagaaagcaaaataaactggccacaccaacagtctaccggcgggaaaggagagaacatagagaattgctgaagaaacgcattttcgc
aatctgaaaccatttcagatcaattcctcgaaaaattggtttcgattggtacccaatttataggcatttttttgattctcagctgacttagaaaaaag
tcgccatagctgagcgacgaatttgtactggaaatctattatttttttaaaagtccttttgttttttcttactttttctttaactaatatagaaacccctttc
aattcagtcagttcccactggaaatcacaaggtacttttgctttgtacttaaaatcacttgctttgctccattgcttgtgactcaacatttttttattagc
atacaggcgaagagcgtatctaaagaacctttgtaatagttatatattcgtttgcgtcctttcccttgtttttacgccctgtccttcctttttcctgtacc
cagcgagtatctagttcttcacaaacttttgttcttttgtataatgtcttggaaattgacgaaaagtatgtgatggttttttcttgttgaatttgttttct
aatccttgatttttcagaattaaaagaaacacatcttgcctccgcgatcggattgaactcgagtggctcgataaaaggtttgcgaaataatgcgca
ttcttcaacatcttcccaaaaatctagcgaagaaaaatcttgcagaagctcagaagaccaacaatcccctcaaagtaccgtggttcagccaggttt
gcttcaagtcacaattattgcagccagagacttgcgcttgccttctggatatactccaagtagttatacagcatctttcggtgctactcctagtacca
atggcttgggtcactctcgatctcgaagtcatgcatggtggctaccttacatcgttgtagaatttgacaaaaatgaggttttggtggatgctctgaat
actgccactctagaaaacccaagatgggactatcaagcaacttttgatgtttcccgatattcaaaattatctctaaacatttatcttcggtcttcttcg
agtcgattgagaaacgggatgggcaatgacgtgtttttgggaggaataaagctttctccatcttttattgttaataaacttactgatgaatgggttc
ctttgcagggaggcagcggaaaacttcgtgtccaaatgctgtacaaaccgaaccaaaatacccctcttaccatcgatgcttttgaccttttaaaag
ttgttggaaaaggctcttttggaaaagttatgcaagttcgaaagcgcgacacgtcacgtatctatgctttgaaaactattcgaaaagcacatattgt
ttcaaggagtgaagttgaccatactttggctgaaagaactgtgctggcacaggtcaataatccttttattgttccgcttaaattctctttccaatcccc
tgaaaagttgtatattgttttggcattcgtaaatggtggtgagctattccatcatctacaacgtgaaggttgctttggcacttaccgagcaaagtttt
acacggcagaattgctgatagctcttgagtgccttcatgaattcaacgtcatttatcgtgatttaaaaccagaaaacatccttttagactacacagg
tcatattgctctatgtgattttggtttatgcaaattaaatatggccaaaacagaccgcacaaacagtaagttttattcttagctttttgattctgcata
cattctctaacgtaaatttagctttttgtggcaccccagaataccttgcgccggaattgcttttgggtcacggttatacaaaagtagttgactggtgg
actttgggtgtgttactttatgaaatgattactggacttcctccgttttatgatgaaaatctaaatgaaatgtatagaaaaatattacaagatacgtt
acgcttccctgataatattgataaagacggccgtaacttgctatgcggcttattgactcgctcgccggaaaaacgactaggtagtcgcggtgccca
agagataaaaaaccatccttttttttgcggatattgattggaaaaagctttgcgccaaaaagatccagccgccgtttaaaccaagtgtcgactctgc
tatcgatacgagtaactttgactccgagtttacttcagaaatcccaatggatagtgttgtcgcggattcacatctgtcggaaacagtccaacaaca
gtttgcagattggagctatcaacgccctacaacgatagatgcatctgatgacatttcaacaattggtcctggaagcataactcggtagtgactcgt
atatgctgtacctgttcttttttttgataagtattccactctttattgtttttatatcatctaactatcgaaaacatagctgtcgcttcagcaatcattcgt
ctcagcacagctatttttctttcttaacaacttagaatcatttgtatttaaagattatgaaatctgtaccttgcaattacatggtatttttttttttatata
aggaagaaaccttaaagtagcattgatcttacacaaacgtcgcttagaaagataatcgtgcatgaaatctatatgcattgtgaaagtgaattattt

aacagaccgtagaagaatatagaactaaatatacatgggaagtttttgcatccaaaagaagtttaacaagtgattagttgctcaaaagaacgaa
cagaaaattagaacattagagaaggggaatgtgttcgagttaaggtgcagtgatccaattaaaccgtttaaaaaaatcgtataattgttctacctt
tgtgtgatcgacgtgctttaatagttgaatagcatcgtctttagagaaaggtttttttgaaataagggaagcagaaattaaagcgtattttaaagcc
aaaaagggttttgggaaaatttgcaattgcagacataatgaatgctcctcgtcggagagaagatgacggtcagcagaagtaggaaatgaggga
acaggaataggttgtttggcaaacttttgaatatcccgaatccgaaatggaattggtgggtctatagaagttataggtttattttggcgtattaaac
cactagtcgccctgctaatcaacatctatgaaaaagttagtcaatataatggttgtcaaataaaagaaatcttaccttttgcgttttatcgcgttggt
aacgaagtccttgctcaagggtagtaagtccaacctgcctccattcctgtaattcttgtatgcgttttacaagagcaacttgttcaaataagccctgt
aaaaacgttcgataatcctctcctgaaagtactcgagcaaaaacttttgttttatttagtagcagacgttcttctttagagagacgcttttcattagct
tgcaacctccggtaatctaataagttatgagtgaagatgacttgtttcctgattgcacgacttgttagacgagaattataaatattcatcatagtaa
gctttatatgttcttcttgttgtttggattctgacgcatcttcctcaaagttcatatctttgacagagacttcggcttcattcatgtactcttgatcaaatt
ccaagcgaccaggcatgtatccttgaatttcatggcactgtggggttgatgctaacggttttgggggaatcgctggaggaggttcgaatttttgtat
gcgagcccgcttttgttcagcaaaggctattctgtctatggatccattaaactcactgttggaaaggggatagttttctgtttcaacatatgtgtttat
ataatgatccctacaattttccttcgtacggccgtttcctacatattcggcgatatctgaccagttccccaaacccaacgtctcacatgcatcaatca
gcaaaagctcttcatctgcaccccagtcttctgtaaagataggataggaattcgtctctataatacgatatggatgacttggctggtggttgccgaa
ggaagctccagaagtgtagcagggaatgcacaaatcaaaatcggcacattccgcgcatctaatgtgaatcatccttgttatatcttgtgcacatac
attacaatgatacttttgaggcggcatcctgcaggggagcgaaaattttctgttccactatggtggagcaaaatcccgtggtacggtcaggttggg
taggagagatgaaaaccaaataaactcgataaattgtaagaaccctactaggaaaagtagatagaattactcaaaaagagctgccgaatttttt
gaaaaaaatcatggtggaaaaccagaaattccgatatcgggaattgtcaacaccacaaggcaatcctgatcagacttcgtctttaaaaacggcc
aaaaacaagtgatccctaactctatcgtttttcatgaaaggcaccaatcttcagacgatattttgaggtttaccagaaatctcaaaccaaggttga
ggagcccttcaacttaaaatgagtccgtagtttgcttacgcttccagttcgcatagaagaattcttgatttttagtttttcatttaccgtatagaactg
aaaaaaaaaaaaaaaaaaaaaaaaagaaatgctaaaagaaaatatttccaaaccatacttttacgacgatacaaacctctataggaagatattt
actgaattaaagcacgttgccgttcgttaatgagtttctccgtgggctagcgtaacataaccctaccctatgtggaatggtcagtaacagcagtca
cgtactacaaacacgaaaaatcacagaaacgaaatcatcacaggaacagaaatggtaaggaacagcatgctcgatttcttagaacgttgaagt
gctaactattaaatagggtcgtgtacgtaccaaaactacgaagagagcttctcgcgtagtgatcgagaaatactatcctcgtctcacgttggactt
ccaaaccaacaagagaattgtcgatgaggtcgctattattgcttccaagcgtcttcgcaacaagattgctggttacacgactcaccttatgagacg
tattcaacgtggacctgtccgtggtatctccttcaagcttcaagaagaagaacgcgaacgcaaggatcaatacgttcccgaagtctctgagcttga
aaaggagaagcttaacgttgaccaagacactaaggacatgcttaaggctcttggctacgactctattcccactcttgtcgtcaacccctctgctcgt
cctgagcgcactttccgctacaagcactagaggttttcttagatgttacaaatcaaatttgtttttgaatgtaatcattgattttcaagtgtttttgatt
tgtatttttaaaaagtctgtaaaaaagttgtacctttgtatgctatagaaagtttttttgtaagcattaaaaacatttgtatgcagtagccgttttgttg
ctgctggatgtgtatataattgtatgtagtagtcgatactaatccatggtaattatgaaattcaaacatgcagcgatattattgtctagagcttattc
aacgtctattgacaacccttctagagaattatgagcaatgaaatcttctgtttttaaaggccgcagtcccccgttgtcaatttctgttttgattctatct
aagtcatttccaaaagtagcaaacagttcccgtcgaggaacagacaaagcggaacggcatgcttctttgatgtaagagcccgacaggcctgccg
tagaagcaacaacagcattccagtcaaagttttcatccaactttacacttttgagatataagtccaagatctttcttcgctgactagctgtgggtaat
gggatggagtaaacctttggcattcgacgacgaatcgcttcatcaatgtctccagggcggttcgtagcacctagcaccaggactctagtatcacct
gttaaaagaccatcccacatggacataaactcagccttaatttgagccatggcttcatggtcggtacgctgtcgttgccgtaaaaaggaatcaatt
tcgtcgatgaagattatagagggttctaactttctagcaagtgaaaataatgcatccacaagcttattcgactctccaaaccatttgtcggttagaa
ggcctacactcacgttgatgaatgtagcattgctttgttttgccaaggcctttgccaacatggttttaccacagcctggtggtccgtacaataggag
acccttgggagacgataaaaggtcgccatgtgtattaaaaacttctggatattttagtggaaacagtacgtcttggaaaagatgattcacatgctc
gtccatcccagcaatgtcgtcaaaagctacattaatttcactcggcaagactagctgagacgccaccatttgttcgtactcatccaattcaactgtc
tcaatttctcggacatgttcttctgcccagttatccaatttcttgcgactctttgaaacagtgtcttgtcgtttttgtcggtaaggatctaacgaagaaa
gaagcttacgagctgcataccaagaacagccgaaagccaaagcataaacagcgatttctttgatcgcgcttttcgtactggggttcataggaact
tctggagttaaaattgttctattatattcagaacaggatattataaacgcagagtgatgaattaaaaaccctctttaagatggaatccaaagaaat
gacacagaaatatgtaaataaataagctcaatcttcttgtttttctcttctcttttaaacaaataaaaatacgctaaattgaaaaaaatgaattgt
gttgaaatccagctctctttggttcatcaaaatgctaaccgacactcttcactctgttcgctagcactaagtttatttgaagagctgtggggtttgaa
gacgagtttgaaataacgtatgtagaacataaacacataaacagaagcttgacttcctttgtgaatcaataatacagagtatctgaaaagaaaa
gtgtactagtatggaaggtagtaaaattaataattcttattgtttctagaagattggatttcgtttatggatctacttgctttaattcgtatgttgaaaa
ggtttcatgaacttttggtctcgtttctgcaacgatcgtaatttgaagatgtactttctagtaaaaccaaattcattatgtctataaaaataagcatg
aacaataaatgatggattcgttcctttggataattgaacaacgagaatattccataacgctttaaagtggaactttctgagaggaacgaaaaaaa

gaaaaaagataaaaataatacacgtctgttcaaatgcaaaattcatctcattcatggcaaatgtcatcttcgagcttcaaggacaaaccgattttg
tcatttgatcaaacaaacaaataaacgtacaaatagaagccgccgtatctacttagagccagagatggacgaagatggagtgtccccttcactg
gcaatgccttcttcagaatcggaatcagcattattggatgggtgaagaacagctcgaaattgttggatatcttgaacgatcttttcaaaattctctct
tctgtgattccattgcagctcgtaatctttattttccttctctaattcctgaatagcactttctaattttgcaatggatctattatcaatggttaatggat
atcgtaattggaaagcaacgtaatttggcacatactctaattgttcattcctcgaacgtaatcctcttgccataataggctttgcaagatcatcatat
tcttcttgtttttctgttcctgctgcgcaagaagtaaatcttgctccaagtgtgaaattccttcattctcttgctcataatgttgctctttacctccgtta
gataaatctcagaagaaataagattattacgtacgggtttgtttcaattcatcctgatagagattgacttctttgttgtgagtgtccaattgcatttgt
agacgaagccaaaaggcagacatggcagcaaactctacgtccaaatttttttagacttgcttcatcagacatctcgtttgtgaaacccaagcagcg
gttgatcaggcgccgtaaaggcctttcttcaacatttaaacgcgaacgaattgcagcttctgctcttgttaaccatgaattcgtatgaaaagataag
ttttaccctccatttcaaaaataaactagtaaatttaataaattgaaagatgcaaacgaaaaagaacgtccaaactttggagtcggtgactatttt
cggtgaagtagagactccaccgaagtatatctaggaaaagcaaaagcaaagtctaacgtttcgctagaaacgcctttttttttgcttaataatgtat
ttaccattattccaacaaacacctgtgaacgtatttgtctttgaaaatgttgagaatgcagagaaaatgcgtcaagaactcattggcaaaaatcct
gtatatgattatgccttcattgacgcttctagtgtatgttcgaagattcacacgaaggaaaaagatccgatgaatgattcaattcctaatgttttcag
cggaaactaactttcggtgaacgcagattgtatccgtaaagcagatttatagtgctattatgcgtgctttgcaagctcaattggatggacaaatga
aatcaaagacattacatacagaagtcattttatcactgagtcctaaaacagaggtttgttcttacgatatatcgttcacttcatacaactcggctga
cacaacgaacaatagatttccagcgctttacgtcaatttggcatgagtgtaaagtccacaaaggtcttggttgtgaagatcaacggcaccctttca
gaggtatggcaaaaatgttcatatcaaatcgaataggtcaggctgagagccattctattctggaagactcatagtaggattctaacttcatcaattt
taggctgaagaagaggcacacgtagacgaaattgttcaaggcgctcgtctttcttttagtgatgaaacatttcaaaaactagctgacatgaaggt
gataaaaaaggtgagtcagtgaacctttttgtttggaatctttctaatttatggtttagaactacaagctcgacgccgctgctatgcagcaaccctt
aggtgccatcttgtctgctattgctttacgaggttactcttagttttgtacttgaagctttacctatagaaaggtggattgcaatttaaaatgacctct
aaaacggaaacctttatacttttatttaactatatcttgtgtgatttgtacctgcgctgactgttctttcctcagtaaagcttttgcgcgcaaaatttgg
gagtgttcgttaggagacagaagattggaactaaatgggaaaaacaaaaagaaaaaagaaaggatagcaagaaggtttagctgtctatgcat
aatttgtttgacagtttctttactttatgttattttatacaataaccaccttcaatttttttttcttcttgtaaattagaatttcttaatgaaatctatcta
aactattgtttgtaaagagaaaaagtgagtgctggttatctgaaatttttccaaacacattttccttcacctctaccaaacactcgtttatgttttcaa
aatcttagtttagtgggatttcggctcatttttacatttatttttgattgttatttctccatgaaatatcaaagtggttggctgtgatcctttactctttga
tagtaaaaatctgctttgttattgagtgattagtcgcgcgtatgctgaacctcatagagacttgagaggttaaagcatcgtcaatatttttcgcctgt
tttaatcgcttgcaaaaggttgaattttattgaaattgcgtcgcagtgcgcatcaccgtgtaggtgaagtgcagaaaaaaatatagcatagtaact
ttgctatacctcatacgctctcttttcactcgttttgttcgtttgtcaaatatttgaaagtcatttattatatgtgagaatcgtttgctattctagtcaatg
actgaaagcgaaaagaaagatccttactcggttatcgggcgagattacctctatccagtgggtcatgaactgtttctcggattccatgcgcgagta
attgagaaagctgaagaacaatatgaggacaatgaacatgtagatgattcagaaattataaacggagatgctgattctattaaatctgaagttga
ggatccggatgattcaaacacaatacaagctgtttgtgatgctatcgctctttatgattttgaacctttgcatgaaaatgagcttggtgtcttcgagg
gtcaaacgttatggattctttcggaagctagtcatggctggctagtcgcttatgatgagctttctggaaaaagcggcctagttcctgagtcttacgtt
actttgatccaataatttgtctactttttttttgttgttgttcacttatggatgtctatgtatattagttacttaatgcactaatctggaaaatatatgttt
gtgctatcccgttcgtctttttcctctaaagcaagtcactctttttcggccgttttaacgattcgtatttgtcgctctttttttggtttatccgcggcatctt
gtcatttctgttgttatcaataatattaatagattgtttggaaaaatgatggatatttgcatcagtcaatcaaacacgagaaaaagcaagtttatgtt
ttgttgcttgttttgttgcaaccttctaatagctttacatctcctaatctcaaggagaagggttagaatgtaccgtgttttacgggagttcttttcctgt
agagttacaatggttgctagaataaccgtaagtcatagtacatatcaaggctgaattacccttggtgtgtctccatttgagaacggacataaaaaa
ttctgaaagtgttttgaaaccgtagatggttggattgtattttgggtcccgccataagagtagtaaaactataattgatacatgtgaatcatacagt
aataaagtgggaaattagccttatggctatgagcgttaactaaccaataaaatcataatttactcgtgaacgtttaggagagatcagatctacaa
aggctgaacgctttcttggtgtagacggagtggaccttgctgagcgttctgctgccgctttgtcaatccaagaaagatccttaagttgatgggatcg
gtcttgcacaatagaagttaaggatcccgcagaagaacgagtttcagaggtgagtaagtgatatgtgagtgggaaccaaatcaactttgacaat
gcttctggcggctctagtcggagtgaccaggtccagtcaagctggatgtctttggttactttggcgttgcacgttaaagaaagagattcatcattttt
tgtttcttctagtttaatggtgctttctgacgaaacgttcataagcatctcgccgagtagttcaaccaatgtatcgaaagtttcaaaaggatcaattg
gacatcgttctttctggaattgttagttgcatgtcttaggcaaaaattgagggttggtatggcaatccgtttactaaagaaacacaggttcaaaaaa
aaaaaaagaaaaaaagtaacttacctctgcttggtcgatcatctggtcctttgtataagagcatgtccaaacccttgttagatcagtaacagaaat
tttcataacataatctgaaccttcaggatcaaaactagcctgaatgaaatgatatttatggcctcgtaattgagtccacgtagacattaaaggcaa
tataataacaggacaaataagaaagaaaaaaagaaaaaaacaacagcctattcaggacctgatttgaaatcaatcaatctttggatcctctaat
gatttattacgttaacgcacaagagttaactttttaaagatgtattcaaatttaatcataggttcctttgaaaattttaatatgcgaccaaaaataag

aaaaaaacagaatatttgaacgccaatgctttatatcgtcccaggatagtggccattctaggaatacgtgaaaccaaaggtagtgtaaacaatg
gcttgaagggaaagagaagatttgaaagagacactagagggtttcaagtgatgaaaacaatgaaagatgaagaaaatgagttgccgtggtag
cataagattgaatgaaatcatcaaaatagaaaattacaggtactgttcttttcgtttcaattggtgctttgcattttgctacttagctagctactttag
ttatgtaaacaaactgaggaacgatgttgtttcattgagttaagagaagcatatggaggaagataaaaaaaaagtaaaaacagtaataatcaa
gtcataatgctgaaatatttcgaggttgaaaggtcctctgcgtagtgtttacatgttcatttacgtcttcaactattgtttacagatgtacacaaatgt
ttgactttttatggctaccgaaatgttttgtttaaatgttggaacgttctttttctcatatttaacgtattcttactgcgtttgtttcatgatttactgagc
ttaagctaagcactttcttctgcaaggcaacgtaaacaatactaaaattggagcgggttttcttgtaaagatgtagaaagcgaagcagtctcaaa
ggcttgtgattttttttaaataaacaaataaatacagcagcattacagcaatctaaaaagaacagtaaaaaatagaagcaaaaaggaaaaaa
agaaaaaactatgaccccgccaggagtcgaacctggaatctcctgatccgtagtcaggcgccttaaccattgggccacgaggcctcttgttagta
tacttgaaatgctatgcaacctgaagcagttgtggagaagaggtaagaagttccagaataagcaggttttatgaaaggtttataatgtcattaatg
aacatcgctttcaattgaaagcacaaggattcttctgtgaattgaaatgaattgaaataactatgggcacgtatacggacccattcttccaattttg
gtaaactagtgacaagctgcttcaacaaatgaacttgatcagataatcttcctactaaattctttcgcgatcatcttgtctttattatgaagggcaaa
tttattattatatataagtacgatataatcttgtgtttcaatagcgatgctacttttccatacccacaatacgtacagagaagtacacgaatggtagg
atgctatgaggtaacaatgttgtttgactagtcataagaatatcgaaagtataattatgttaattgctatattacagtctttaatttatgaaacaaat
aatgacatcttttaatgcagaaatagctctaaaagggttcaattcctactgtgctctttttctcgtatttttgttcttaatttgctgtttctacaaccttct
agtctgtattttgcatatcttgtgcttcaattggacgcggtaacaaaatttgttaatcttttggctaattgcaatactgtctttattgtccttcttcataa
gactgagacgaaaacacagttgccaaattcactcttcaatttacagttgtggcccatttcatcaggttttcgtcgcttttaagatttgggtatcgtttt
ttgtaaagccttagcatttcttggaaaaggttaggtttctttatttttaattcttttcatcttttgttgtactctctttacttttgccttggccttgccttttttc
ttgattgtcgtttctaaagacaatatggttgtaccgtcgcttttgagccatgtagaggagatcaatgaccctatggagattgattccatcattgattc
tatcaataaagatcggtctgaaaataaatgggcttcgtctcgttccccatcactatcacaagtcttacccaaattagattcaatatcgtttgcttctc
ccaaagtacaagagatcctacgacctcaaggtttattcgtagtggatacaaatttccttttgtcacatcttgttctctgtcaaaatctcttagaagta
ctttccacgcggtttcctaacatcgtaatactacttccttgggtggtgcttcaagagctggacggtttgaaatcagaaacttcagcagcttgtgggta
cttggccagacaagctcacgattttctgctgcaatgctttcgagccagccttcctaacctacgtggtcaaaaaattcacgaaagatgctctccaaat
gagtcgggagataatgcagtattagattgctgtctgtattttcaagatgaaaaactcatccctgtcaatcttttgtcggatgataaaaacctatgca
ttaaagctgccgttcatcatgtttcatcacaaaattactccaaatccataaatgcgatatctctaatctgtaatgctttccccaccatagatgcgtcg
gttccatccgccatgttggagcatccaactatggaaatcgacgcttctctcgccgcttcaacgcagcaaaaaagtctttcgtttactgaaaacattc
aaccaagcttagctattgacatgcccatggatgcatctcgcgacaggtccacttgggggtctcggtatgcgcactttccggctgaacaaaaccaa
agtacacgaccaacatccccttctcaatacgttccatatacttatacattgctgacaaaggaacaaattttacaagccgctcatccccgagctacta
aattaattgaccaaattacaaaacttctagtggaagaaacagcgtttttattgacaaggattttgaccaaactatgggacgattatcgtctggctat
gaaaaagttactagcttctcccagtttcccaccagaaaatattcatgacgtcgtaaatgaactctatatccactggtacacttgcttcgacgggtac
ctgccatcaagtgagcgaaaattcctaaaagcgaaagcaaccatgtgggacgagtggttcatgtgggcagaaactggtttggggaaaggacct
agatgtcaagaagagttgactgaagccatttttttactggacaaatatctggtcgatcctgagtcgcaaggaaatcttggaagataaagcttccga
atatgttgtttttagagaagaaaatatagaaaaatggattcaacgatctatgcgacccgcaattttatcgtgaggatgaaagtttactaaaactta
agcatttgaattttttttttgctcctcaatcatacttcttacagcacccattaccattgtaaaagcggattggatttctggatcatttggaaactctcct
tgaagtataacgctgcagaacgcttggatctgataacgttttgacagaattctcgacattcttcttgacaagtcgtcgatagatcccactcctacta
aatcggtggtagccgtgtccttgccgacgggaacggcaagtgttagatttccagatggacaagatccactgttgcttgcccaaacgtaaattgag
gatgaaggaagcatttccacaagctgaatccaggtatgaggagctccgtgaatgtttgtttcaatcacttttcgacttacttgaaacatttttgatag
ttaccaattggcttattcttcttgctaaagtgaattttggttacacgtccaatggaccctttgtattttcacctgttttgactttcgttaatcgttccttaa
accaatgtaataatttccttttttaaaataaaaatagtgtatttctcagcacgaatcactttcccagtttcgtatctttaaccaaagtacacccaatat
ggagtaggtttggtaaaggaatacacttaacgaattttaacatatcggttaatttaaattaccgataaataaaaaagcatcactaaaagagatac
agtaaagcacttggaaatgctaaatgtcctttgtttacatcttctaccaagtgtcagtttccttctaccaacaacgtttattgaaaaagacaaatcgt
tgccttttatggtgaggaagatggaatctttaaatatgatgaaactcatttttttgggtttctctttttttctattgtttttggacacggaatccatttacaa
ccccatcaaaagggtatggtggtacatttaatggctgtttagtccagtcgtgctaactttttaagaatgcttttatgaaactttaaggacaaatgata
aaatgtcggttacatttcaaaccaaccttggtggtgatcagcttgtttccttaagtgtctataatcctcttaatcaacttatgcaaaaagaagaacct
agttctatgtcacagtattcgtttactgttaagaacccaggaaagtatacatattgctttgaaaacgtaagtaatgaatgaacaactttgacgaaa
aaaaaaagtggcatggatataaaaaggcgtcttttctttttttcgggagttttgaagtcatttatggcactatgaactaactcttgatttaaggatgct
tttgataatgaagctaaagaagttttatttaatgttcatggtatcatttatgtttctgatgaggatttagaagcgaacaatcctttgattggaaaagt
acgcaagcttcaagatactatttcccaagtaaagtttgaacaagaatatatccttgctcgtgaacgtgttcaccgtaacaccgccgaaagtactaa

tgaccgtgttaaatggtggagtattcttcaaactgttattttactgtctgtttgtggtttccaaattatctacttgaaacgtctttttgaagtgaaacgt
gttgtataagctctgattttacttacgcactaatgcaagctcgtctgttgtgtctaaaaatcaagcaaaaaaatacatttcttgagaaaaggattat
ctattccatctttacgaaaagaaactgaaatattgtgggaggaacagtatgagaacgctggctaaaggaaatgccgaatttctccttcacatggtt
gtataggcgttgataattatgttcatcctattagcatcttgtgttcatttttattcttctttcttctaattatgaaactttgatgtgagtaatacatatat
catttttttttcaatctcatttgctttattctctcacctcattctacgaaacgctagcaacacatatggagcagtattgcagttttcttataatacatccc
gaaattcggttcttggtttttttttttaatttgttatgtgatttgaaccatgtctattgacactcaaactgaatatcaaacaagttgtccttatcgagg
ataaagactgacaacaaaataaaaataaacatagaataccttctttcctatatgattttcttctgctgattccctgcattccaatgccttcttatggg
tattcttgattgttacttggtaactgttactcctgtaaatatagatggcaaaacttttctcgtatgtgaattaatccaattacaatatgcctcaaggag
aaatcaagaaaaaaggtccaaagaatactaagaatgcacacaaaatttctaaacagaagaaaaatgtaaagaaaggaggtaagtgttccgat
gttgctatatagtttttgttggcaaaaactatgtgtagttgtaggtagccattgcttgtttttatgaaaaatgtcgtattgatttctatatacgaaaga
aaagaaaagaggatggctttggtgctgtacagctcaatactacatacattttttctacttcaaaaacctttgctcgttcaaagcttatagaaatcat
attaacataatctagcccgttattatgctcctagaaaagctgctgcagttaaagatcatattgccaatgaggtatgtacttggaagttattcttcggc
tgtctagttgaatgtttctgactttttcaattagggtattagcaagactatcaattctcgcaatgaacgcatggctgcaggacttgccagtcaacag
atgggaaaattaacagttaccaaaagcttgggagatgctgctgttcaagagttaaaagagcagaagcattaatgagctgtttcaacaatgtttac
aaaaggtttttgggactggtatatggtaataaattattgatttcatatttcggtattatttaaacaatctgccaaaggagaaaatacatatatttctt
ggaaggacaactatgtacatgattcaccaaatatataataataataacaaataaaatctttttaatctgtgatatatgccaatgaacctcataaac
gcaaaagacaaaggtataacataatatctcataatttcgatctacgacctcttgttgaaccaggtggcctacctcttgggttcgggtgaggctgatc
agcagagctttgttctgcattattgattgctcgatcgagcattttccattctttttgccaagtttcaaggaaattccgagccttgaataatttttctcgtt
ggactgttactccttcggaatcccctttcctattcagaaagctgagcagctccgtgaattcttgttctgaatcagaagactcatctagtcgttgttctt
ccatggccggttcactttcaatttgctcttcttcagtgctgtcgttcgatacaacattttgcaaatgcaattcttgcaatatgggaggttttgtaacttg
tgaagaaccgtcgtattttggagatcctggagaccttggcgatatatttgttgttgtgataaaagcaatgttttgtaaaacattttgatcggatctga
caggagaggaagatttctttgtaggatttgagggaagtgggttagaaatattttgttggtccaacggattttgtaaaaggaaatcgggatcttgttg
aattccgggaataatttttggaagtgaaaagttttcgggactgagagactggaatcgttctattgatttcctcatagaaatactaaatagatctggt
aatattaaagcccgttgtgtatggataaacgcagctaaaagtagagaattagaccatatctcatacgcatttctgtaagatggatcatagtaaatt
tggcttccatactgaatacaaaggttggcaatgtatttacgaccgcatgttagcttcgagacttggcaaagccaatttgggtcgattgctaataggg
ttgcacgagtgctgtttacgtctaggtcggtttccaaggaatctaataactgaacaactatttggttggaaaagtttttttcgtcaattggaagaact
gttgacttttggtagttgttgagatttatgtaacgacgagtagcagggcgatctttataagcaggtgtaatagtttctataggagtaaaggataact
ctgaatttaatttacaaacgtatccttcattattggcgaagcgtaacgtccgatacggacgtattaagacgggtcgaacatacggttttcggatcgc
cagttcgtgaacacctaaaggaagagcttcatatggtactgaaaaaatgtgttagtaaatactgtgaagaaactagtaagaaacacttccatcct
tggaaatctattcgcaaagacatcaagtaatcgtaaatttttcaaacgaaggacagttcgatctagtgttatagtcaaactatgttaaatgcctatt
tttggaaggatcacaaacaagcaaacatggattacagagggaaaagtaggatgaagcaaacgaattctcatcttttatgaaagaatgacaacg
aaaccattcaatccattcaaataatcgtcagtgatccaaacatgctagacatattggcgaacttttgcaaagaccgctgtacagaatgcacagtat
atgtattacatatctttaattgtacttacccaaggtgatcaagcagcgaaagggccctccttgtccgtcatggtcgaaaggattgcgaacatagga
atccaatacatgtgcctctagaggttggaaagcattttgtaagaaagagaaaaattcttcaatatcgggctttttattatgaacatccaccattccc
tatactatacaagggcctcctaacatggagcatgaagtgataaggctgtctgtttatattccacaagaaaacgaataattcttaattctcggggttt
tcactaatttctaagccaattttcaatataatgttttattaaaagcaatgtttataaatcatagaaacacgtacttcttttgcatagattgttgaatttg
gtaataggtggttcagaggttcttgtctacgtacttgaatatcgtaaatagatattccttttcgacagaaaaatggcttgccttaacaaacaaagct
tcttaaaattttaaaaaattgacaaattgcgatgcacaaagctcaatatctttcctttacaccgaacttcaggcatcataacgatcattctgtaaaa
aattggacgatccatcaagggcaaatggagtaatggaaagtgaagcaaaacagtttagaaagtttttttaaataaatatatctttcagtttcaattg
gtaaatccttcaaaatagctactattttccaattaaatggtttattataatatcatattaaatggtaacttgaaacgaaagatatctggaagagcag
ccatcgttttaatttgtaaattactttgttttaatagaaaataaaacaacattctattttataatacagggaagtagcatctttagcgctgtttctagt
catttgaaaggtttattttaagatatgcgcgtggtactttcaccaacacatttcgtctaaagatttggtttttaaatttacatttttgctttgattatcta
aaaactaaaacatgtagggcctgtttctatttacgaatccggtttacttctttgtttacggtcctgcttgttactcacaaacatattaccgtaccaaag
taaggatcgctacggtagcacaattaacgatatcgtacatatccatatatatttaacgagaatgcgggacagtttgcaatctaaagcgggcttctc
tggaactttctttagatccgcgccaaaacgtaagcacatgtaagtttttccgcacaactaaggtaacagagtcattacggattaaacagaggcga
aaaactgtaatcaattaccgaaatatcccatacttacttgaattgcctttgtttcatttcattttcggaaaacaataaggtacttttgtgttctcatac
gcaaactttctctttatgtaattgcattatgatgaacatatcatcctccataaactctagccactgagcgtctttcggcgacgacgcgttcttttgtttt
ctttttcgtgtttacaataatcttgtggtttgtctatactttatacttttcaaaaaatggcgcttgtcgatcctagtagaagcagctaacatactgtacc

cgaagtcgtcaggggaacgtaagggtggttcggagggagcattgataggtaaaaacgcaatagagtcgttctcttccgaagaagactgtcgaat
gagcggagcacgtctcaaggcatctccttaatatgtgactgtcataaaatttatatataataatgacggacattgacaaagttgtttgctaccaaa
cattagacacaatagtaaatatggctgctcctcgtacatttttcgtcggtggtaacttcaagatgaacggctctcaggagtctatgaagaagattgt
tggcgctttgaacgagaacaagctcaacgttggacaagtcgagactgttatcttccctcaaaacttgtaccttatccctacccgtgagcaagtccgt
aaggatatcggtgttggtgctcaaaacgttcatgacaagaagaacggtgcctacactggtgagaacagcgctgaatctttgattgacgctggtat
caactacaccttgactggtcactctgagcgtcgtactatcttcaaggaatctgacgagctcgtcgctgacaagactcaattcgctattgaacaaggt
cttactgttgtcgcttgcattggtgaaaccttggctgagcgtgaagccaacgataccatcaaggtcgttgttcgtcaattgaacgctttggctgaca
aggtcaacaactggtctaaggttgtcgttgcttatgaacccgtctgggccattggtaccggtaagactgctaccccgcccaagctcaagaagttc
atgctgagatccgcaagtgggctgctcaaagactcggcgctcaagttgctgaaggcttgcgcatcatctatggtggttccgtcaacggtggtaact
gcaaggagttcctccaattccctgatgttgatggctttttggttggtggtgcttctctcaagcccgaattccacaacattgtcaacgttcacactatct
aaacggaatgaatttgttagtacagttgtttaattatttttaagtaagtaaattcgtgctttgtatatttttgaagctttaaatagaaatggtagcata
tatatccgattgatgcacatatccttttgcttacataatgtttatgttgaaatgtaacttcttaaaccttcagagagctgttagttattgtaagtatgcg
tgcattagtcttctaataatttttcatgaagaactgtcctttcatatgtttatttatttgtgaatcctaaaatgattaccgtatcaaatatatcgtaattg
accgtgcacgtaattccattcctactgcaaaccaataattaaacaataagaaaagtgaagaaatcgaccactacagttgttgaccatttgtattgt
ttttcagcactttaaacaatacttgtttttaggctcatttcatcgttcggctgtcgtatgccacggcttgtttgctgagcccaactattacagcaagtcc
caggaatagcttcttgtttcccagtgagccatctatatatgtatggccgccaaaatcgatgtctatattcttaactttgaaagctctagtacatgcca
ggatacgcagcccgtaaacttgtctcacgatggctttttgtcgttcaaccaaagctagcttgccatagattctgtgtaaatagtcgctcattttcaac
gcacaagctcacggatgatggcgcattacgaatcaatccggtggggattcagtacttatcaccagccttacaatcgcaggtatttcctggaaaacc
tcataaaccacctgatttgtatgtggagcttgcaaagcttcatctgcaaaagcatgatctcttaggtagagagactacaaagttaccatcctataat
tttcgtcttcctgctttacaagggaaaaccctaggggaacactttgaagccatcggtcacgaatgtgccgaaccttatctcacaaacgcaaagtca
tttggcctttcttctttgccttccatcccaaaaaactggcttcatcaatctggatggaccagatacggtttcgatgataccgtggaacaagtcccttat
cctaaggaagccatgattgtttttgacgttgaagttttgtataaagtttcagattttgctgtccttgccgttgctgcatctcccgaggcatggtactgc
tggctgtcgccgtggctgttaggcgaaacagagcaggatcgacaccttattcctctacgaccagagggacatctcattgtcggtcatcacatcagt
tttgaccgccaaagaattttgcaggaatacaatttgaaacggtccaacaatgccttcatcgataccatgtcgctacacgtagctacaaatggaatg
tgttctcgacaaaaaccgacttggcttaaggctagaaaagctttcagacaaagtgtgattaatgaagataacggtttggacgaaaactcttctca
gattgagtctaattatcatgattatattgaacaggaaccttggttaagacacagctctgtcaattctctcaaagacgtagcaaagtttcattgtaat
atcgaaatgaataaagacgtgcgtgatatattctctgaacttgataaaggggtaattttacagcatttggacgaggccatctcttattgtgcttctg
atgtccatgccacacataaagtctatcagaaggtctttcctcaatttcttgaagtttgtccacatccagtaacatttgcagctatgcttctcatgacta
ctagctttctgccagtaaataaatcttgggagcgttatatcaaaaatactgaagattgcttccaaagcatgtctaatgcagtccaagaaaaactttc
ttattatgctgaacagacaaagaatttacaggaagatgaaagcgttgtttctaaagatccatggttgcgtcaactagattggagtccttgtaagttg
tatcgtaagccggctaaatcaactattatacctcctgttgtcccaaaatggtacaaagtagtatacgacaaaaatttacagaaagcggttgtttctg
acaagtctcgtcttgcacctatactattgcgtttacagtgggaaggacatcctttagtgtggtcacacgttcatggttgggtttttggagtggaaaag
aagtctcaagaacatattgacaactttgtgcgccgtaaattcttccattgcatatgtgacccacaaaaggacccgaagttggaagctgaaagttat
gtatactttaaggttccacacaaagatggccctgaagctaggtgcggttctcctttatctaaaacataccaaagttattttgaagaagggattctta
cctcaaaatttgaaatagcgaagaaagccttagaaatgagtacgtcgtgctcttattggcgtagtgcacgtgatcgaattcagtctcaaatggtaa
tttgggaaaaagatgctgatcttggacttaagtctcagcccgatggctttgggattattcttccaaggattattcccatgggtactgttactaggcgt
gctgtcgagaatacttggttaacggcctctaatgccaaaaaaaatagacttggttcagagcttaaagctatggttcgtgctcctgaaggatatacg
ttcgttggtgctgatgtagattcagaagaactttggatcgtggctgctattggtgactcacaatttagattacatggtgctactgctcttagttggatg
actttggaaggtaagaaatccgaaggtacagatctacactcaaaaactgctgcaatcttaggtgtgagcagagatgctgcgaaaatctttaatta
tggtcgcctgtatggtgctggattgaaacatacagctttactcttaaaacaatttaattcttcattggacgatacccaggcggctaacttagctaaa
gccttatataagtctaccaaaggtgttcgctctgaagtgagtaagcgtttgcttgctatgggtataccagaacactatttttggtcaaaaggaagtg
aaagcttcgtttttaacaaactcgaatcgatggctcaaatgcctttccccagaacgcccgtgctgaacgcaggaattactcaagccttaagcacaa
agaaccttagtaaaagttcattcatgacttctagggtaaattgggcaatccagtcatcagctgtagattatttgcatttactcgttgtttctatggag
catttgacacgtaagtacaatttggaggctcgtttatccttgacggttcatgatgaagtccgctatttctcttcagttagagacagattccgtgttgcg
ctcgccttacaggttgccaatttgtggactagatccttcttctgtcatcgattagggattgacgaattacctcagtcggttgcatttttctcttcggttg
atgttgatcatgtattaaggaaggatgttaagatggactgtgtgacgccatctaatccgacacctattccacctggtaaagaatataatatagatg
ccttgctttgccagttgactgaaaacaatcaatatttggagccacttgaacgtctagaagtcgatagtatttcagaaggtgaaagtatattaaata
aggaagaggatttaaaggctttggcatacttaaaagcccaagcttttcattaacactgggacctgcaagattatcctcgaaagagataattatga

aattatttgtcattaaaattcattatattaaaacatcttagttaacattcggattggggcttacaccaaaaggtttagtataaattaatagggatcac
cggctttttcagttcataaagtagcaataaaaacgattgtaatgaagaagtgagaataaaaattggaagatggtagaatgcgtcaaggattagtt
attacatatatgtatatacatatatactccagataaaaaagggtaaaccgtaaaggaaaaaaaacagactttaatgagagccaaatactccggt
agttctgtcaagagatgttttcagagctgccacccaaaggattaaagtatcttcatcatccactcggaattccattggcttattgagagtaatcaca
gtaaatatgtactttctggatttctcaaatatattaatttcagcaacgtcttggatttctgtgatagggataagctttacaggcttatactccatgttcg
agttgtaaatcgtcataccattaccgcgaactactgcccaacattttgaccagcgatggatattaccgtgacgccaatatatgggtccctgcattaa
aaccttgttttcatcaagcatcttgtacagattttgtagcgaggaagaatgtttgtgttttcggaggttattattattcagtgtgtggaatggatcctt
gatttcttgttctgaaaagccttgaggcttcaattttcccaattgtgtcaagtcatacaaagaggtacaataaaagctcggaaaaccatttttccgtc
gaagagggaccatggggattattggttcgagaagaagagtagtttccaagatcgttgtcagagacaagtacatccggatcgcttgttgttgcagg
aggggaggaaattaaagcaaagcgtaaaggatgtcgagaaaatggagctcggtaagcagttgttgttcgtaaggcttgtacccagctttcagctt
cgagattcgaaggggcaacgaatcgtataattttttcgtttaaatacaggtacaaagctagaacagttagataaatcttgcatcgttctgaaatca
ataaaaaccatacattttggagatttctctttataatatgctacagccgaaatgtcttgcgttggaaaaataagcttcgttttatactcctaaaccta
gttagaaaacagaagaggaaagcttttaaaaaaacctttttcgtctttgtaaaaggacaacttgtctctgcgaagtacaccccataaatgcttgcct
ttctaagtcaataggttagtgattgttaacactttggaacagttgaagtaaacaataaaatagaatcctcctttcataagaaagcagcatccaaaa
agtatgggattcaactccgtgaacaaggttactttctacctagtcaaaagagcataatagaattttgtagcgaattcgccaatgaaacgtttctaa
ggaaggagaatatttgaacacttttagcgatcccacattaccggatgcgtcttaaattcacagatactgtccaagctgtatctggactgtctacgcg
aacataccgtagtagaatgactttttcttatcaaccaacctgatttcaatactcgctctaattcttggagtcgtcttgcttcatcaaagctggattccc
ggatttcgcatggattcatttcctgttcagcaaaaccgaaactctacgctcatttgccgcttcaattgattcatggacaagtttctttacgatgattttt
ctcaaattaattcctttcaagattgctttgagaatttgaagttttcttactattgcagcgacggtgtatgacacaacatttacagtcctatagggagtt
tcaacgaaggtaatcagaaaaagaaaaaatcagacttaaaaaaaaatctattatttaagaaaacatttgttttccatgtaacgtttgttctgagct
cccaagggtatggaatctatagatgaagctgacaagctctttggcatctatgaaaacagctattttgaagaattacttctcgatgagtatttaaggt
tgaatgctgattctatttttgtttttcttttttttttcaaggattttgtcagaatgctgccgttagtggattcgacgatttacgagagacttgtaaacaa
actattaatttcatgaagcgaatgaaaacggattcaaaattaatatatgtcaacgagtgctgaagaagaccttatcttgtacgtcgattgagcaaa
gaacatctgctatttaattaggagctgttttaaaagtgttcatttcagcccttcacgaaattcctatatataagaaaaggtaagctctataaattcaa
acgtattccttatgcataacgatagttggcaatttttctgctaagcatgactggataaatccgtttgaatatttgaatatatttaaagctcttcttgaa
ggtaaacatcttggaatagcgtaaggaagagattgactggatgcagcatatattgaaaagcattacaatagtaatattcacggtcacacctaggc
gaaaataccagttcccgtccgatcactgcagtcaagcgtctgagggcttcgttagtactatggttggagataacataggaatccggggtgctgtag
gctacttcttttgctgctcttaacttctttattttgtttttctttttttatacgtctacgactattgcttgcgatcgttttctcgaaagaatttttgcaagctgt
tcctttacaagtgacaaaagactaattacaagttcaatgaaaagaaatgaaacgatcgtattcaattgcaattaagaataagaataataggaat
aataataattaagaacctagtatgatgccaatgatctcagattacggttcctttcaatgcgcggattgtatgcatgcctttctattgtccacttttctt
cgccatttccaattcatgttggtacaaggatgagtccgtagtcaaaattaaacacaagaacgcttagcaaagggatataaataaatacttatccg
aacaaattgcttgtaatgttatctttttccacttagtgactttcgatcggaagagttaagactcatgagaaaagaaaggtaaacaaattagcatta
actttgagttgtaggattgctttgcagctctatttcttcgttcgtagttgtcggcggaggagctnnnnnnnnnnnnnnnnnnnnnnnnnnnnn
nnnnnnnnnnnnnnnnnnnnnnnnnnnnnnnnnnnnnnnnnnnnnnnnnnnnnnnnnnnnnnnnnnnnnnnnnnnnnnaatcgttt
tctcgaaagaatttttgcaagctgttcctttacaagtgacaaaagactaattacaagttcaatgaaaagaaatgaaacgatcgtattcaattgcaa
ttaagaataagaataataggaataataataattaagaacctagtatgatgccaatgatctcagattacggttcctttcaatgcgcggattgtatgc
atgcctttctattgtccacttttcttcgccatttccaattcatgttggtacaaggatgagtccgtagtcaaaattaaacacaagaacgcttagcaaag
ggatataaataaatacttatccgaacaaattgcttgtaatgttatctttttccacttagtgactttcgatcggaagagttaagactcatgagaaaag
aaaggtaaacaaattagcattaactttgagttgtaggattgctttgcagctctatttcttcgttcgtagttgtcggcggaggagctagtgcatcatcc
ctttgttgatatctgggaagcgtaggcgaaccggtgttttcagctgagctctcaaatttcattccaaggccaaaaacagtaatgttataaattcccc
aaaagatagcaccaacgaaccatccaagcacttcaccaagaccaaccacgataaagattataactttagcggcaatccgtattgtcaatatgaa
aggccaaataagtgctaggctttattagtaacacacacacattctaatagcgtttgctattaacaacaatgctccacttaccatagaaaagagcaa
agaatattgcccatatcacaagccaaatgcctaaaggtccaagaagggccaaactgtatccattttccatatatacttgtttcagaaaagaaaat
agttcgaaacgataggagattgctaatgtacaaaggaatgataaacccaatatcaaaaagaaaaacatcgacttacttttaacgactacattttc
agggaaataatgatctgtcttcttcctaaagtcggtaaagaaatcattgaagctatcaaattcagtaattatcgtttttttgctttgtttggctttgac
agaagttgaattggaagttttgtttatgtcttttggtgtatacaaaggaagtcccttctcaaggtcaacattgtaaacattagcatggttgtaagcag
gagggtcgcttgaatcgaatttcagctccttttccgacatctaaaacaaataaagagaaatttcaacgaataattagcaaaagagagtcaaataa
aagctatcgatccgtattcgatataaaaatttcgcaaatgttcagaagccactaagaaaggcgaaattgaaaggggtttagctcaaagtagactc

aatttcaatatttcatctaaaatagctcagactttaatcaaaatcagcccagttggcaggaattgaagaaataaacagttggctccagcaggaaa
tctcgcatctcttacgcaacattttagctcaggatagtgatatgttcagttgcgctatctcctataatgacatatagcataaatcgttgaatatacgtt
taagtcacaatgaacaagtaacaagtttcgctaatctcgcgtttgtttacacctctgtaaacaaagcagatcccatttctctatgaacttatgaaca
atttttaccttgaaagtgagtaacagcctacaaccaaaaagcagccctcatgggctcttcaggatcaannnnnnnnnnnnnnnnnnnnn
nnnnnnnnnnnnnnnnnnnnnnnnnnnnnnnnnnnnnnnnnnnnnnnnnnnnnnnnnnnnnnnnnnnnnnnnnnnnnnnnnnnnn
caaaagagagtcaaataaaagctatcgatccgtattcgatataaaaatttcgcaaatgttcagaagccactaagaaaggcgaaattgaaaggg
gtttagctcaaagtagactcaatttcaatatttcatctaaaatagctcagactttaatcaaaatcagcccagttggcaggaattgaagaaataaac
agttggctccagcaggaaatctcgcatctcttacgcaacattttagctcaggatagtgatatgttcagttgcgctatctcctataatgacatatagca
taaatcgttgaatatacgtttaagtcacaatgaacaagtaacaagtttcgctaatctcgcgtttgtttacacctctgtaaacaaagcagatcccatt
tctctatgaacttatgaacaatttttaccttgaaagtgagtaacagcctacaaccaaaaagcagccctcatgggctcttcaggatcaaaaaaacc
tataaattacttctttgaactgaaggaactgtccacggccatacctaggcgaaaccaccagttcccatccgatcactgcaattaagagtctgaggg
tcttgtaagtaatatgctctgagacaacatgggaatccgggatgctgtaggctcctccaatttttatttcatttctctttcactatgtttatttctccgtt
ggtttcaaatttggttcaactcccattctgattaatacttttatactatgcgattcttgtttcattagttagaatggagagggatgatttcaccatacat
tcaggaatatatagctcgctagaaaattatatccgcctattttcgatgattccctaagtgtaacgcgatttaaatatccgtttacagactcgatttttc
tcctatttctacttattaccattgtttatttacgacgcatcatatacctataagtaagggagtatggaaatgatactcatggaaccgaccaatattct
cattatattacgcacgtagtcaagggtccatgattgacgaatccgggagctaacctttaaaaaaattcttttactagaagaaccatccttcttcctc
gatagaaacgatagtataaagaattccttaccgagttacccgtaaagaaacataatgtaacttcttacaaatgacggtgtaacctgaaatccacc
atggaaagtaaacatataggttaaagttcccaaataaaagtctcgatttgtgagaaaaatccattgatgcgcaagattaaaataaattggtttgc
aagcttaaaccaaaaaagatgcctaagaggcgtttgtgatggagtaatatacacctttgactcgtcgaagtcctatcccattcgatcccacgtaaa
aaggtaccactttataagtaaacctctaaattattaatatatgcacccaatgcccaaagttcgtgttcactgaccgttctccatagcttgaaaggcc
aaaaaataaaataataaaataaaaggtacaaaacctcaaccttgagaacgttatttaagatccatgttccaaattaaacatttcataacagtaga
cacaaggcatactttcgattatggttagaaaaagcttttcataatatagtgagaattcacctatttttcttgaatcaattaatttttttggtccttgctc
tttcttcaaatttatcaggcgcctggtccatgcattcaggtgtaagatccttcagctcaattgagctttcatcatggaattcctggtcacgtgtgtcaa
agatttctgtaacgatttgatctaaaagattacaacgctttgcttccagacagcattttaatacaaaaagaagatcataactatactccttgataat
cggaggaccgtattttaatatattatcgataatcgcaacttgaataaagtttttgtgtactgcattcgatgaatgagccatatgaaattggtttcgat
agtaaaatataattccaattaggaataaagaattgttgtaaaaatgtttgtatacgttccatataatccttataatgtttacttttggatattcaataa
aatgaaggataggaaaaaccaaatggacaacaataacaactaatgtttggattataccaataaggagcaccatacgtatttgtttattggagatg
ggacgagcaatccctaagtaaagcgtgacaataagggtaatagaagaataaaatgaagatcgaatagcatgaccacaaaagaaaagaaaaa
ctttcattaaagaggtaaggctagttggattttcaaatatctttgtcacatcaaataacagccaaaatgcattattccagtcatccaagcaagtgaa
tattattaaaaagcaatgagtaagacagcatttgcgcagtgcacacagtataatagatgttatggaacccaataatccgaaaagggtcatcagct
ttgaaaagatttccataaatgataacttggtaggagcagagctaacaaattcagtatccataatgggcctcttgattaaatccatatcttctgaata
atctatgtcttggtatcctagaaagcaaaacaaagtatcataaggtatatgaagttttatagacggttcatcccgatcgattttcataaaatattcct
gatcaacctttggaataagttgttgcaaccttaccaaatctccatcagaatcaatggcacgtttataagatttaaaaggatctgagatttgcttgctt
cgccagtaaacagaccctcgagtattttcctttaagcttatattagcagtggccatgttgttccaatcaccggaagaatgtaagtaagcacacgaa
agaaacccagttttcccataaggttcatttttaaagtctaatagaaaaacacctgaattgatgtgcttaaatgaacgtagacctgaaacattacttt
ctgatgaagcggctaaatatgttataattctataaaccacatacagcttagcacagtttgataagaatctatccatccaagaccaattcatcttgta
aaatttaaattaaaatatgaaatagacagccaagaagcataaggcatgaagcttttgaaacaagaagcgaagagcacaaactgtgaaatttct
caagttatttcagtttttaacgccgatctcttcaaaaaaagcaaaaacaaaaaattcatagggaaaaacgctgtatttataatttgatagattaag
taacaactgtttctcaaaatatcatgcgtgaggggaagcgagcggtgtagcctcggttaaaaatagctgtctatgttagggcagatatctcacatc
gcgtaattatataaatcgagcggaataaacaactcaaggtgtactgttgtatacgtaaattggacgcggacaatcttatagcaattagtagccgg
ccattttgtagtgttatttttggattatcactaatattcttccaccaatgactgtcctatacccgttaggattggaatttattatgacctgattaccata
catatatatctctgaatagaaatccaataaagtcgatcactcctaaacaacatatctcataaatacattataatcgatagtctatcaattcaaaggt
cggatagcacagttggctagtgcgcatgactgttaatcatgaggtcgagggttcgagtccctctctgaccgtttttttgaattacatatttcataaat
atgtcttcttaaaacaagaaaatccataagaacccgtttcattactatttctttctatcgaatcgttacattcacaattaattttagtgaccaaaaatt
tctttaagaaatgccgtactacaaaacatattaaaagttaaaaataagatctaaatctaatgatggaaaagacgcaagggaatttcagtgtaga
ccatgccaaattcatcagcagcatcatgaactgccttgtccataacacttccaccaggggcagcaatgtacttgacaccgctttgggagagtctgt
aaatattatcgggaaaagggaagaacgcgtcgctagcgcaaacgacgtcattgagagaagccaggaattcttggcgttcttggggtgtgagagg
ctcagggatggattcaaaaacagactcccattgttcacgctcactgccttcagaaggaacggcacccaagacatacaaatcgatggcattagact

tctcaggacgcttggcagtcttcttgaattgcatgcctaaaaccttaggatgatgacggaaccaccagttatccgccttgtcaccagccaagcggtt
gcaatgaattctagattgttgaccagcacccaaaccaacgaccataccatttttagcatagcagacagagttgctttgagtaaacttcaaagcggt
agtagcaacaaccaaatcaataagagcagacttgggcaaatccttgttcttactaacgactttgtcgaaaagtgaataatcaatctttgcatgatt
acggtgctgttgtaaactgataccatagacttgacgagtctcaatctcagcaggaacataactgggatccatttgtaaaatacaatatttaccacc
cttctttttcctcaatatttccaaagcttcaggctcaaaatctggagcaatgacaccgtcagaaacctcacgggaaataatacgagcagtgcacac
gtcaaccttgtcggagagagcaatgaaatcaccgaaggaagacatacgatcagcaccacgggctctagcataagcacaggccaacggagtca
actcaccaatatcactgacaaaatagacactcttttcgacatccgataaaggaataccaacagcagcgccggcaggagatacatgcttgaagga
agcagcagcaggaagatcaaggttctcatgaagctccttaacgagcggccatgagtttaaaccatcaagcaaattaatataaccgggagagcca
ccaagaaccttaaagggaaggggaccactttccatgaaagcctgagcaggagattggtgagggttagcaccgtagcgtagagtcatttggctga
caccaacagagtattgcttgcggaagtagtcagtgatggccgaatcataggaagcggtagaagcaaaggccttcaaagcgtattgatttctatcc
tcttgcgtcaatgtatttgtaagcaacttctcagcaaatataggatagtctttgggatcagttaaaatgctgacacgagcatggttcttagcagcag
cacgtaacaaggtgacaccaccaatgtcaatctcctcaactgcttcaggaatggtgacattaggcttagcgatagtttcccggaagggatacaag
ttacaaacaacgacgtcaatcttctcaatatgttgttcaagaaggtccttttcatcagaaggtatgtcacgaccaaggattccaccatgaacagca
ggatgaagagtcttcacacgaccacctagaatttcgggagcattcgtgatggaggagacgtcttggacttccataccggcctcacgaatcattcta
gcagtaccaccactaccgagcaacttaattcccttactcacaagtgccttggcaaggtccaacagacccgttttatcgtaaacactaagtaaagca
tacattttggcgctggtttgaacaaacctggcttttaacagagtattggttacgcccaagttggatgaccatggtaatttttcttttatctcccatttgc
tgatagcgagttcacactatgcgttgttatcagtaaatcaacaaatctgatctatgctacctgtaacgttacttttctgttttgcaaggtaaaaacaa
tgcaaagtgaaagatctaaaaatcgcttttcctccaacgtcatgtattcactataagacttttactaacaagatcatttttttcttaacacagacttct
aatccttaagcttgtcctagcttcaatgtaaattttgtaacgagttctagcttgcttcagtgagtcaatattgaatcagattcaatcatgattagcata
gaatattttggaaaacatcatcagcaagcatcaaacagtcttataatggtttgctcttcatctcttttacatatgctctaaatgaacacaattgacta
ctacgtccatatgaatccctatagcaatccaatttgtatagcgtaaaaacagtaatgattttatttcctattcatagttccgcattatctagcaaatgt
taatataatgaagatgataaagagcaaagatgcaaaatcaaaagtatcagttattgggtttggcttccgtgtcaatcgtcacaaatttcagatag
gggcgacacaatgttgttcatcattgtaaagattttgcatccgttttagatcacttaccttgtgcgttaagtatagatttgagtagtgtactttaggca
ttatatagattttggaattcaaaataccgtcacaaaccctaccctacggatacattaaaataaaaaattatactatatcaaacaagccaacggaa
cgcacttcggtaaacaaatcggaaacgaaggaataaatggtgaatatgtggctggtaatttatcaaacaagtttaaaaaaagtttgctccagcag
tgactcgaacactgggcctcttgcttactaggcaagcgctctaccactgagctacaagagcttctttgattgcgattttcattagaatatccaagaa
catccagagtgtttggtgcaaaatcagtagtaagtgaatgtttatgtctgctgtgtaagttacattacagatatcgaaatgaataaattcttttaaa
attgttgattaccatgtttcaggagccagctacattgacgtttccaatcagaataaggacaactaaaatttatggtgaagcaaaatgtattatatta
taccttttatcaatccgtaaacccacgtaaatacataattagctgtgcacaaatgcatactgcgcatcaaatcaaaaacctgccttaactttttttat
ttggcagggtctggcattcgctcgtagcaagcagtgaatttctttccaccgtttctagcaaccttttttccttcttcagtctcatttgtcataagtgcaa
cacagacgtccgcatcctcttcttctttggagagtcgacggaagagagggtgcttatcaaggtgttctgccatccagagatgaagttcttctacatc
cgtgatagtgtacagtctgccaagaggttgaatgaaataagcatattcagaagccaaggtactggtaactatacggttcttatgcttacgagcttt
gaagtgaggatctggaaagcagaaaaacatttttgtgagctgtcctttctcgaaaaaatttggtaagaacttttgacagttcattctcaaaacaga
aatatttttaaaacctccaacaaccggttcttcatgttctgctcggtaacgaatggcctgaatcttttccttcaaataatcggtgacctgcgttcgaat
ttccattcccagaaccaaggaatcgggaaattgaggaccaagagctacagtaagaccaccgtaaccacagccaatgtccacgatctctacctttt
ttccagagtcttctgggatatcagggtaatatggtgtccaatccatttcagccgggcttctaggactttttttttgttagaaatatgaaacttagaaa
ggaaagaatccaagaattatgattaagcttgattgagtgttgacttgataaaacacacttactattccagttcatgatcagacaaaacattagcat
gagctctttggcgaaataatcttttctaaacaagtaattagacttcaagctgaggaattagaatcaatataccattggtaaacgtccttctacacca
cctcctttcgccagacgtttcatttttttttctggcttcttcctcttcacgttgaatctgtccaggagttttctgttctacagccatattgattattgattatg
taggttactactacaaaaaatggaaaataacaagggcagattgatttttgaaaattttgaagtctactgtggcacgaagagggagtttcaggttc
aacaaagttatctaagcgagagtacttaaatattaagaaggaaaagaaatttacaaaaggaaagtacgtaggatgaatgaacaaaaaaaaga
attgtaaacgtttaggatatttcattaggaaaatactaaattagctactgtaaaaataaaagaaatgcgttacaaagatccagagcccttagcgg
cgcccaaaataagctcgacgatttcgtccattcccttatcgctcttgacctgggcaaacactgtagggccatcttcgcgaaccttcttggcatctcg
atccatcacttgaaggtctgcacccaccaagggagccaagtctgtcttgttgatgatcaacaagtcagattctgtaataccaggaccacctttacg
aggaattttgtcaccgccagagacatcaatgacatagataataaagtctgccagttctcgagaatagttagcagcaagattatcgcctcctgattc
aacgagcaagaggtcagtcttgaattcggcatgaagttcttccagggcgaccaagtttgcagaaacatcttcacgaatcgctgcatgggggcagc
caccagtttcaatggcacggatccgttcttgaggcaaagctttgttgctgatcaaaaattcttggtcttcacgggtgaaaatgtcgttggtaacgac
tccgatactgtattttgaggctaaatgtcggcaaagttgcaaaagaagtgctgttttaccggaacctacaggaccaccaacaccgatggtaaagc

tacgtcttgaaaagtctcggttattgtatgtaggaagctcgcgtttcaaatagcttcctggggaatccatcgcatcgtgagcatggccatgttcaag
aatcatgcgttcgcgttcagacaagttggacaaggcgtgaggaggaggaacaggatgaggatgctcgtggtcgtggttgtgaccgtggtggtcat
ggtcatgatggtgatgatcgtgtccatggccatgatggtcgtgatgatgtccatcatggtggagactcatggaagcattctcatttccatttccttta
cgaacaataggaacgaccatttttaaaaaaaactcgtcgacaaaagaaagggaaaatgaaatttttattaggaaaaatatatatagtcggaatc
tctcggattggtattgcactcaagaaccgatcgttcaatatttcaaacttacagtagcaagtataactattgatactccaaaggtgaaagaatgaa
agaatagaaacgcaatttcaactcaaaagtaagaagaaaaaggaaaaaagtaaaagctatggttaagatatgtgaaggtgaatagaatctga
taacgcttgataatcgacatctcaatatcaagtccgatttcagggtcctgggaggcaaaagaggcaatcaaacctttatggtttgtattttttttta
ttttctttattaattatttcattttctttttctttttggttttttcaaacccaatatagtcgaatatttcgcctgagcaacacttacagtgtccagggaat
agaatgaggacgaggagcttcgtctttgaaagctaggttcgttcttttcaattgttcaatgaacgaaacgatcggttctttgtaatacgcacgaact
cctttatatgttaatatgggaatcatatattggaatatgaatacaactgagtaaattatctaagtaaagtatgtactgaaaagttgatgttgaattg
aatcttaattactaatagttattagtattgtcctctgggtagattggaaagatgtacaaacattttactcgttcggccactgaagatatagccagaa
cagttaggctagtatatatttattcttattttccacatctcaacttttcacaatgttcaattgtttttcttgtcacatggattttctttttcttctgtctgttt
aacaatgtttttgttttgtttactttcgtctacaatctaatactccgtagtattatgggtattatataatagttgatgatactaagcttcctccgataatg
gtatcctgtaaatcataagaacctctttaaaatgtttgttttcatcgaagttccagtcatttttttgagaatggacaagcgaatcatataagctttttat
tgttttttttggtttattgcttttttttttgtaaaggtccacacgttcgtttgatttgcgctgtgtttgaaacaagagtcgaataaaaagctcaagttcaa
ctactgaatggagttgtccttttacgggattcaaattataagaactaggtcgttataaagtaatgtacaaatccatttatttactttttttcttttttcgca
aactgcgtcaccgaaaggggtcctaaaacgaaatcggcaaagaagcacttggaatggtcataaacagttctttcaagaatctacaaggatatcc
tatttttaaagcttccgtatgctaaggaaaacggcctttttttttttcatttgttttctgatcgaacaatttcaacccttgcatcatgaaatttcttatatc
atatcccatgacgtcaactggagtagcgagacttaaataacgtgtcaaaatcgcattgttcaacagcattcacgacacgtaaacaaaagctgcgc
tgtaatatttacgcttgtttaccaactttcattcatttgatttttcctttttatctgattccttaaacttggttcgaatcctatcatcatgagtcctgcacct
tacaaagatctcagcaaagataccgttcatacccatcctgcaattcgtgagtcggaacgtagagaacagcatggatggggcgatttaaacaagg
aacccccttatggaagatcaagctgaacgtgatgctaagaatgacttgtcgaaagaccaaaaagaagtcaaggagcaggccagtcgtcctgccc
ctaacaactaatttctttttttttatgaaacgaatggattcaatacgagcagcaaattgtattcatccttgcgttaaagagaatctttttgccctcatcg
tcatgacttataaatgtgtataatgattggttacgataatatgttttcgtatatatgatttgaatacaaacagaggtttcttgtcccaccgtatcttgtt
tctgtgcagtattggtgtaattggtataatggagcttatgaaacacaaaatcaacctggtttctattcttaaactattgtgatttgtatgaatgtcata
tttcaaacgaacaaactattgtctgtcttgaaaagatatgtttgtccaaaatattcagctatacgatgcgatacattagccatctctctattcaagtt
gattgaaataaaaagacggaaggtgttgactctttccaattaaactattcgttcggagactgatgtttggttactaagtacaatttgcttattccatg
cgagatgaaatgctacatcaattatggaatttacttccaaggagaggtttaccttgtaaaataatatctcataaatatcatcaactgaagagtaat
gcaaatatcagcagagtttcccttgtttcgttccatgtattttttttttctttttacccagcattagcgaatttctttacggaagtatttcctccaagcttc
agcaattggattcggaatcgtttgatcatgaagtgaagtcatgttgtcagactcatcttcccggcttctgtcgtttaagtcgtagctgctattgcttct
gtttgtcgtttgttctactttattatgccactgattttcgctcatatcaggattaagctgtccgactaatgatcctcgagaacgagagcgataccgttc
tccaaaatcttctgacaacgtagacgcacgagatccacgtctttgtaacgtgcagtttctccaacggcgtgttggagaactggacagatctgaagt
atctaactgattatcatcatcaaatgtgatgctttgctcctttgtatcattggcagggcgagtattcataatgcaaaatcataaataagggggtgta
acaattacactggtttaatttttatctgcatttataatgttaatattgaagtttttttcagatgatgatcattctgttctgactttcatacctttaaatgtt
tccaactaaattcaataactttatctaacgttgatgaatcgataataaagaaaagaataataatgtaataggtgtatcgtagtgtgagtgttcaatt
tatcctatatgtccaaaaacatgccttccttgtgtatacgtgcatatatatatatatatataaatatatatcgacataagtttcataagcttctacgtg
tgttccttctcatatgtggacatgtctacgacatctctgtggcctaaaatcttgattgacgaagtgttttcctcagttgcgtggccatcgttgccacat
ccttggggtagggatattgtcttaattagtagtaaccaaaatcatagtataggagaattacaataagttaaaggagggaagcgtataatttgtgtt
tcctcgaagacacaggttcttttatcaagagaatgaacgtttcattcgctctttggcggggcttacagtaaaagattttgtgcgacgaaagtaagat
gaaaacatctttagtattattagggaacttttaaattccagcatgggcaatttccaatggtaattgactagaacgatctttcataaaacgtaggttt
gtgaaagccaccaccgaagaaatcacccgttgacgaagcaaatagtcaggaagtaaggtagactatttagagatgtagatgaacattacagtg
gtggtgtttatgtgcatgaacaatgaaggaaatgcaaaatcatctcaaattatgaagagacaacgttactaagcacagccgcaatggaataact
acaaagataaatttaaagtagactaaaggtaaaatttggtgtaagtgtttacatctagaaaatgtactttatcgttgctcttctttttttgtataacaa
tcctattgtctatatttgttttctaggtaaagtaaaccatttatactaattccaattatttttttagctgctacaacgaccgtaggcattacttatttattt
cagttcaaaagtaaacaaatggcgctactttttaagtaacagcctgtaacatattcatgacttgagtatgagtaattctttagggatcatatcattgt
tttgtcaatttggttcatctcttttcatatggcttttccgttgttttactgttctgctaagctccttgttcttgctaaaattcacaaatcatgttgtcaaaa
aataataataataataaacaaggaaataccgcaacgcaaagtatccggtcaaatcttacctttattcacctaaagccacactatttgttttaacgt
ctttaacagtcatttgctatcctcttgtctttgtcgctaaatgcactttcgtttgccattcttcaacaagcccttttgcttaggaaacgattactgttttcc

ttattgggaagtttctattcaactaactttaccatgtattcaaaaaacccaagctatgacttggcagcatatcccggcgaccctcgtcgtcgtcttcg
taaaaatgttcgatttgaaacgcctgctgaagatgatgagtatcgtgattttgaagacagtgatagtgatgatccaaaatttgaaaaccgcgaccc
taggaaacgcgttgatcccattcaacacatgctactcgtccgccatctcaaccgtgtctcccgatcttctaaaaacattttcctgctaacccttatgt
gcttcattttcttctttttcctcttaatcggttttatgtcgtttcgctcccatgaaggtcatcccttttcttaatctgacgaaatcaactccccttaatatg
tcgaggtaaaaatgtccgaattgttttcttttccttattctcccagccatttctttcaatattcaaaaacgtcctttttgctttttactgtatcgccttccg
tgcggcttgttccgccttgtaaataaagattcctacttactgttgagttactgttttcaatttgtccgatcgtttgttcttctctagattaagccttggta
tgccatcctacttctaacattcacaatactcctacaataatctaaatcaatattcatctccttcactttaatagtcaataataatgtattagcttcagc
gcgcatttctattactacaaattgcagaatcgtaaaccttttcccttaagaactatatacccaagaaccacaatccaacaaaagagagatgctga
atttagacccaacctctctagaaagaaatatttcccaaggctctagagcagcacacccaaacagcctcgaaaaaatatcaacatattcacttata
tgttgtatgcccgtcgtattcgtacattcactctaatcatgctttcttctgtaatcttgaatttttttgtattaatttctccatgtcaatactctggatatctc
gacacttttcaatcgttgacacagtcatggtgagccatagaaacaacatgcatacaaaaacttgtcggatcgttcacataacgcttccacatctgc
aaccaacaaacgcacgctcaagcggatatgtcaattcgatttttattatcatttcatctgttactaacgaatcaatcaaatttccttcgtgcaaaacg
ctcaatcacagtgaaatattattatattcactccctcattgctttctctttttttctctatccattaaatagatatattctatccaaagcatctttatttc
cttttaaacaatacttctgacagaccaaagtttcttgacataagtgaaagaaatttcagctgcattagatcaaaaggatcagtcttgtaatagctta
ccaccatgattgagagctaccactgttataggtgtttccatgatgactgggagcacgatcatagccactactgtaaggactgtagctgctggtggt
gttagtggtacgacggaaatcacgagttccgtggtttccaccgccgccacgtccacgaggagtaaagcgaccgcgtccaccaccacctccaaag
ctgttatcacgggccatagccaaaaggaaactaggacattcttggttggactcttgtaaaagatcaatgaggtccttagcaaggcccttattatta
cggttgaagaaggaaacggcatgaccaatatttccagcacgaccagtacgaccaatacgatggacatagtcatcaatatcagtaggtaagtcat
agttaatgacgtgtgttacgttggggatatccaaaccacgactagcaacagcagtagcaaccatgatggaagtacgacctgaacggaacaaatc
taaagcacgctcacgttcacgttgagtacggtcaccatgaatacttgtagcagggaagttactgttcaacagatagtcagtaagagtatcagccat
gcgcttagtctccacgaaaatcaaagtcaagccttccaggggaagggtatgaaggatatcaagcaagtaactgcgcttttctgaatcttcgacat
ggacaaccttctgagtaatattttcagaagtggatccaacacgaccaacactcaaaaagatgtagtctttcaaaaagtcacgagccaacaattgg
atatcgcgggggaaagtggctgaaaacatgagagtctgacggttctcaacatcagtcatatcagcaccttcgacaatttgtcggatttgaggttca
aaacccatatccaacatgcggtcagcttcatccaataccaagaatttaatattagctaaagagatgcgaccgcggtcaattaaatcgaccaaacg
gccaggagtggcagagagcaagtcacagccctggtccatttgacggatttgaccgcggatatcagcaccaccataaacggcacaggggcgaac
ccaagagcggtagcaaaactttcttgattcctcgtgaatttggcagactaattcacgggtaggagcaagaattaatgtggtaggataagccttacg
aggacgatagcccataccggggtttcgctccatgggaataggagctggtcctttgtcaaacgcttgagaaagaatggggaacaagaaaccagcc
gtcttaccggaaccagtttgagcacatgccatcaaatcgcgaccggaggtgacgatgggaatagaattcttctgaacaggggtaggttgtgtata
tccagatagaaaaatatttgaagcaactgattgttcaaaggaggagaaaggaattcatgaacaggttcaacttcacctccagacacctcaacg
gggatgtcatcatacttttcaaagttgattcctgttgaagctttaatgccatcggcgacagcaccaaataattggcgttccaataaggtatttctggc
accgatgatatgttgaccgtctttccattggccagctccacggttattgcggtatccaccgccataaccttcacgagagccgcggggtcctccttgg
cgaccgtcgccacttgcaaaaccgccttgacgaccttcgccaccacgaggaaaaaactcacggcgtccgccgtagccagaagaggagacctcg
ctaggagtttcagatcgagaagatgcaggctcagagctcacaggagtgactggggcagcatcaggatgatcacgggcaaaaggaggaatatat
ttcctaggtctgttgttgagatttactctttgcaaattttcagttacggaaccaactgaatctacttgttgctgtacattgtcgctcataatattttttat
gttcgttgttgtgtgtggttaaggttaggttcaaatctgcggttgactcccccaaatacagggcgggactgtccttttatagacagcgatcatcttttg
gccacgaaataaatttgttgcttgaaaataaaaatttccgttaaactcgtaatgtcgctttatgtatagttcccgttggtttaaaaaaattttatgga
gtttgaagagttctttctttccatactgaattctctttatattatcccagctagctaccagaaaaagaatttttacttgttttgtagaaaagattaatga
aaagatgctgaattatgttttaatcaccttttggtaattagctcatcctagctcagtcaagttgatgcattaggtactaatcctttaaccaaagcaag
cgtataaattactagagaatttagaatagtttgtttaccatctttgaatttgtgcactgtttcttgatatctttttttgaagaactcgtcatttagcttaa
agcttaacattggcgtagggtacgtttgtccttccagcctcttttctcaaaacatccaattctttctgaaatacgacatttttgagatctcttacccata
aacgtcgagtcagcttttctatttattttataaattacttttggcgattgtcaatagagaagtaggtgctatttgaagttcttcatccttacaacgtcga
gtacataaaaggcaaccaacaattcagaatttttttcattgcttgcagaaaatgctaattttaaactgcttatcatacttgggcgctattttgggattt
gttggattaacccttagcattgcttctgctttgtattatgtatcggagttcatagaagagcatacaaagcttgctaaagcctttttgacacggttggtt
tactttatcatgggtacattagtcctactcactttgcttgacggctttcccttttggctttctgctttctccatcttcagtaactacgtctacaagcttaat
tttgatacattcccattcttttctttccgtcgtctacggtttttattggcttgtgcgttgattattatcaatcacctcttgtgggttcgattctttcaaacac
acgagtttccgggtcgtccacggggcgtcacttacgactttcttggtcaaagagtggcaacataccgtgccaccttttctcaggtagcctcgtttatg
ggaatttgcgtttggtcggttcctattggaatctttgtgtcgttcaccgctgctgataatgctttgcccaccaccatagctaccgattcttctgagacct
cttcctatcctgtttcctcaagccatcatcaagtccgtgcatccaatgtcttgcgtcaaatgtacaagaaaattggtgcctacattgaacggtctctt

gtcgccttaggtttaagtgaccatatgggcgcaaatgaacgtttcgtttgagcaaatatctcttttcttatgaatgtacttgtaattatctatccctttt
atttctttttatttacattctttatcagctcttcactgagtcattgtatcctatccgtgcattccttttcctctttcttgcacgttatgcaaaataaaaata
atttttcttagtttgtaactactagattcatctgtaaataaacgattttccccttttatactcatttaaagtgagaaacacgatgattcattaccactct
tcatttactgctttctacatatcttgattttctaagttgcaaataatagtattattgataagatggtaggaacacataaaaacatccaagaaactttg
ttgttcggagagaaactatgatgctctatcatagtcgctctcatggaagatatattgtaagcaggtaaacctgtagcttctaccgacacattatgtc
gaaaaagttctccataggttatcgaattaactttcttggaagctggagagaaatgggttgtttctaccaattctgatagtttggtacttgtggggacc
tggctgcacatacttgctccaggaacagattcactcaagattgcagtgaagtttgtgctcatattatgataggcctgagcaatggatatagtttttg
ccttttggtggaatacagtatatgcatttcgaaaaaaagccaatcctaacacagcctgcgatgatgatgtcggccggatacctaaaacacatatat
catcaatagttccaacaatcagcattgaagttttaatgaaaattgtcgaattacaaccaaattgaaggttgataccactgttgtcgtcgatgctgtc
acaggaaactgcaaggtagtcatacgcaacaaaacctcccataagatcagttaaagcatacgaaatgtcataaggtagataaataaatagttct
cgagtttggagaagcgcatctaccactagcgaaaagttcgaagcaaacgttgaattcacgtactgcaaggagtacagagtagttgaataatgac
gggttcgtgtctgccggtgtgtaaccaaagctccgctatactttgttccgtctacaccacctaaaataagtgtcccgtttccggttgtatcaaatgtta
aactgaaggacggcgtacttaaaatatcactgtctactaactgatccaaaaacgtaggtggtttgaatctcgaaatagaatccacgaagtctata
ctattaatttcagaagaagcagccaaccccagaacacctttgtatggtaataaagaactgggaacgtactccttcaatgtaatcccaaactgaaa
gttactaagttcatggtaatggttgtacactaaagagtcttgaacgttaactccagcaaccgtgatgttatcatcatatttgattaaatacgtcttgg
gttcattcacaaaggaaaaacccaatgaagaattaccggtatagcaagagtcacttggattggcgtcgcaaaggaagtaccgaagttcctcact
accccattgggaaccatcaacatcgattcctaaaaaagttggattgaatgataacacatcgtagtatgtgagccaagtgtatggcatgtcaaggt
cgacagccacaatatatggaattgcaggatgacctatatacagcgtagtggtgtaatacgcataactttgaagagtcaatggaaaagtggacat
atcagaaccaaaaactgaatagcgtttttgtaagtgaggatttgcggacgaaaattccacactcctcggaacaacatccaaggcaacaatacctt
cgtccacatcgtgataggcaattacggattttatccataaaaacaacagagggacggtaatgcattgaatccaatacatatttaaaacgaatgaa
gagatgaaagaaaagagttagacaaactaagatccacccaagaatagaagatagaaattttatagtcaatgtattacacaaagacgctgaact
gacaagaaaaggaaggaaatgaaagacaaaatccaagaataagttgcaaagcaaaagcataaatcggaaacaacaacaatatcaaggga
agaagagtaaataaaattgaatgctcctggtagtgccaaatgcgaaatgaaatggactagatatttcatttgcttactgaagagagggtgaattg
accttaataacctaaaaagccaagaagtctaagcgtaagaataaaacaaaagtaaggcttttcaagctaataataacagaagaggtcgtcaat
aaaccggtttgatgatcgcacaaataaatggacaacacagtaaaacgaacaaaagacgaaaaaaatgcaataaataaataaattatgggaaa
tcaaataattaagtaaatttacaaaggcccctgtcagctttgttagacaaaatgcttcttattagaagctccccgaaattaaattaggatgcaagg
acaataatttcagaatgagtgcagataaaacgctgaagacaagcgtaatcttccaagttgggattgcgtttgaagttcgtattattgatgctgttca
gtttacgctttcaagccaactatagcgcagaaaaaccctccaattcaatatataattattaagcattcaaagaaattattttaggctcataaatatt
agcagggcttctcacattcaactgaattggtgaaaggtaagttaaatcgttgagaacctaagtttgaccaccaccaggataaagaaaacattctt
ccaatattgtgaaggagtctaaaatcgtttgtctgtcaaaatccttgaatactgttacaaactatttatttgagcatcgagtgatctttgcttttgtat
cttgcatgaaaatttttcataggatgatatggaaggggtaagtttgcctattgaggatttttgtagcattttattcagaaataaatatatactaac
atatattagaagtctgtcgagcttgacgcttcattaaacggaacaccgcaagatttcagttcaaggcagagtactcccaatggagagagaatgca
atccatagattctttatttaagagtgaagagatacctgaaaatgaaaatagatcagacagtgccgtgcttgcgttgcaccggaaatataaaaaac
ttttactactcttgtggttaaatactaaatttccggatggtttaggccagattctaactgatcatgaaatacaaactgattatcttaaagaatggcat
gagttctttgaaagaaatcctttactcaggtatgttgatacatggtcattagaatcattcttaaaataatcattttaataagctgggttcttgcatcga
gtgactttattaatattttatttagtaaatcaagcttggattcatccattctcgattcagttcaacctatacatgcggaacaggttttacctccagcttc
ccctactgctccttctgaatcggtaacggagccacctgttaatgaaggatcacaagaatacgaagacgaagaagttgacgtagcagaaaagccc
gaaattgatctacctagttatatttctgaaaaggagactatccaaccaaccttgcctgagaaagatgcaatacaacacgaggaaggagtggtag
atcaagaaacacaagaaaacttaagggaaacaggaactattgtggaggaagaaaatgcaaataggccttcaacttctccaacgaaaagacctc
agacgccggatagcgaatatgctgccctgcaacttaccaaagagcagaaaagcaatgagcggccgaatttagtttctaatgctgatgttggttta
gaaactatccaaatacaacctgaaacttcagtgactgacaaagtaggagatacagagaccatagaacaaacagtcgtttgcaagtctcccactg
agcctagccatgagatatctcaagatacaactgaagcttctcgtgaagaggagagtgttggagaagtaacaaagcaagttattgaagaaacag
atccaagttctattatcgcgaacaacgaaaagcctgaagctgaagcagttgatcagcgagtacaacaagtagattctttgattccttcaaatgatg
aagcagaacaaagaaattcgccgccaattccactatctactacggagaaaccatttgcccaagaagcaccaaaaagtgagtctcagaagaagt
ctgaaggtgttaaaatgttgcctgaagctttcacttcctcgaaacctctatttcaattatcaatctctcttccattttcattacttcctccctcagaactc
tctgttcagaggaaaccggaagatactttagatgcttggctacttcgcacagaaatgatacccttacatgatgatcttgagaacgcttacaagtat
gtttctactaataactggatgcacgcttatagagagaaaatagttagggagtctatccgcagtgtgttcaatgccaaagaaaaaggaacttggtc
gtttcgccagccgaaacgacaaaaagaaatggtttcaacgaaaacgcatcatgattatatacttgatgaaatgcagtggatgtctattgacttttc

tcaggaaagacgttggaaaattgtattggcaaaacaaatggttgattcagtcatggactatcatacaacatctgataaaagtaccgtatgtaccc
cgtcttctttatctaaaaacaaaaagccatacttatatatggaagcagaaagaacagtatgtcttcagtatcaaattacccttctcctgattccgc
gagtatgatgcaggatcagtataatgctttacagtcgtcgtctccccttcgagatacagacttttgtagcactattttttcggacgatgtcattgctac
taccaatgttgaagaatctcgcaactctattttgaatgttccaacctacaatccacctaacgctactgaaaatcagttgcgcacctcagcaattaca
tatcccatagaaccaatttccagattcgctttagctaaaacgagacttaaatctagttgtatacacactgagcggaaaagattattcagtgaacgc
gatactgttcctcaccgatctggagatcaaagcaagaacgatgaggcttttgaagatatcgacctcaattctgaatttcgagggggaaacacaaa
taaaaaacgagcattcgcaccattcactgtgcgccctccgtatcctcctttggcaacagaaaacacttccgaggttatttggttgccagaagagga
tgaactgcttcttttactactaagacagtattccttcaattgggaatttatagcgagtcgactaactccgtctgggttatacgtgtctaaaacagaaa
gaagaactgcttgggattgctttgagaggtggattcgaattgatcctcaagctgcgaatgttcaatttacaggatctcacgcaagattagcacaac
aaaaattagaggaatcactgcagagatcacatcaatcaatgcaaaatatacatatgcgtgactctactactcctgtacctcacctattaaatcaaa
gttcttactttgtattaccgaccgtatctcgtcaataccgccctatagctatatttgaagctatcaaaaaaatacttaagcaacgagaatttgctcaa
aaaaccgctactaaacggcagaatgctaatgtctctacttcctctgagcgtttacctccggtaccttctccgattgaactttccagattaaaattcga
gcgtgaagcacagattcagcaagttcaatctcaaagaaatgcaaacctatatatgcaacatcaaaatcagaccaagatgaggccaggagcccc
tatatctggaacacctcagcaccaattggcagcatatcaagcagcagtttctcagaattctagtgctacatcctcaacaggcacttctccagtccct
tcaagagctgttcctcgattgccaaatcggttggattatccaaattctccaaggttggcagcggagcagattcagccgtttcaacaaagatagacc
tttatatgactttgctatgataaactgtatattactttaacaaaatgtaatggttaattttttgaatgaattgaatgcattcaagttctttgcgtctcga
aagagcaattgataggtttttctctggtggttagtgcatagtaattttattgaaaaaatcattatgttaaagaaaataaaagtatatacgttgggac
aaatgtaaaagagatgggagatatttcagattaagctattcacgtacagtcagtcattctggataatcaatttctctaacgaatatgtttgattttct
gaagaattgaaacagcagttgggagcacacgattccatcctaatgttcctatcgaactagtactgtaacttcttctttgaaggcggtttagtttcttt
gaggttttggcattggttcttgtgttttgtccatttactggcaatctatattcggtatattagtatgaatagcttctaggaagaccaaaaacttaccca
gcagcatgtcgcaaacctcgataagaacggatattatagtatcgagaaatattggcgtctctttgtcgcttcaagtctccttcaatggtcatattgg
atagcaattgtgagagaccagtttgttgactgtcactgaggtcttttactttcaatgtatcatgaaacgaaagcttggcacatatattctcagcaac
agctcttccaattccatagaaacctaacagagcatactttaccggccgctcatctttaataggcacacccaaaatataaaccatagcggactattt
caaaaatcagaaatacacacaatcggggacttttctgaatatatgagttaaagaaaacgaaatcctttttaggataccaggtttttagaattgaaa
gctgaaaatgagaggtattactagaaatcgtgaattcctgggaatacggagaggcaacgattgaaacacgaaacctgaggaaatggacgtctt
aatagaaggggaccctgttagaggtttactttataaattatcaataatattaaagggaaaaaaatctcaaatatatgaagttgaatcgctttctctt
ccggaaggaaaaacttgtacagacatttgtgtttcgcatgcgctcttgaaaaagctagcaacaactcaaatcttttaggtaagcatagaatgccat
tttggttacctttgatgaaattgataacgactattcttgacaaaatagacatggtcgtcgtctgttttccttcaggtagggtcccctgatttacgaaaa
catttatagagtattctcgcctaatttaattgaattggtttgatgggtttttaacttgggaaagacttgcgcaggttctgaaaagctaaacccataca
tacgttcttgactcctaaagggctttgcttgtcttcctggttaacagaaatgaaactctaagaaaactatctaagcggtaagcacaaagctcatggt
tttccagttacatatttttgttgccttgaaacgactgcgaatgctgtactccactttatgctaaaccataatacttaaaatattatggaaagaaatat
atatcatgaaagatgattttagtcttagggttctcgataagggagatgaatttttccacaccatggattcatatgattgagccttacaaattaaaag
aaggcaatacggaggaaacgacacataggacgaagttacctctttgtgaaaagatattcacatgtcttccgtaaaagcttctgaaaagaacggtt
gagtcaagacagaatatccacattcgaatttgggccgcaattgacaaaaaaattatgactgaacaaatccatgtgcttagtaatcaaacaatact
agcataagaaagtttgctagaattccagcaattcctatacttcaagtacgatgccgtataatttcgtcgataactgtaacttgatggcttgtatttga
ctttaaatcaacagattcattaaggatgtgattttttttttgtttaacctggatgaaatatgctaacttcatacatgcagtttcccgaaagctgttaaat
tccagtcagagtaaatgggctcttatgttgacacaatggataattgagcaatctacaagccttcgatgaacccatttgatcaattcaatactggagt
attataagagatttcttttcaactcacaatctaaagttggctacgtgtttacaaataacgtttaacaatttttcgttgaaaaccgacaaaataaaaa
aaattagagtctctgtattcatttgcataaattataggcaattaccttataaataactactaattagaaaagcattaggttcctcagaggaaattaa
gggcagctaaaattaacattttcactgcaagtatagattgttgctaacaagcatgggtaaaagaaaattcgtctcaataatgcatcaatcttcaaa
atgagcagtaatgttcgtctgtgccgcggtagaattgaagggaacgttcatgacatagaattctaagaaatgatggtaatcctccttgtccttatca
gtctttagaacaggttccttaacttcagagctttcacgcttcttcaagtctggtcggtcagggttctggaaactccaccagttcttatagacacgaag
gaagtcttcataagtcttggcaggaaattcaggaacttcacgcttcttcaagtctggtcggtcagggttttggaagctccaccagttcttataaaca
cgtaggaagtcttcataagtcttggcaggcaattgatcgttagagggtaccacagaattggcatcagagcccccagcaggagtaggtatcggag
cagcagaggctaaagcaaaaagaagagcaacaagagaaaagaatttcatatcggaaagaaaaaatgaaagaatattactcaagaaaaaaa
agtcaagcaaaaaagaaagagtttcagaagaaaagaaattaaaatcaaaaaatagaatgcgtaaagagcgttttacaaggtccaaaagttca
actcgtcgaaaggattaatgcaaaaaaagagatttgcaaatataaacaagttataaagagaagagaaaacttgaatgtgttctggttaattaga
aataaagcgggccctatatatatcatcatgaaatccatgagtttcctttctgggatgattatggacttgccaatttctttgtttacatgaaatcctcat

ttcgtgtttttatgggataaacctaatgacaacacgattggttggatgaaagcacgtttctttgaagcgaggcacgatttcgcggggttatgtactt
attagccattgaaattgtttacatcaaggatgatataagagcaaaccaatattttaatgtaattggttcgtttcagtagagataaaaaagaacaat
agaaccatctctattgaatcagcaacttaagatggtcacataagccaatcataaagcagtcgccccgaccaccaaatagtcggttaaggaatttt
atgaaaacagaaacaaagaattcatagaaaaagaggagtgtgtacttttctgaagaggaatccagctgtttaacaagatttattcttccattcatc
cgctcattcttcatatcgtttgtttatgatcttttcaaatcatagtagtgaaattttctttacagggaaaggaatctttgtgatgtatcagcggtcatct
ccaaccatatctaatttccgccttccgttccttatcaaaataaacaagtaaacaaacaaactaatggataggtccgtttttacttgtaccgtcattgt
ttccatccatattaatagcgctcactcagaaaccagattagcttgtctattaaacatagcaaagggatcagattgaatggttcataaaagacaaa
aatagaatcccttctgttaagcatattaaattacttagaaacttgtacattttgtagtgattttcgtatgaatgctttcctttctgcatttcattctccat
agggattttctgtgaaacaacatcttgcatattgcatttatttacctcttcacgaagccctcttttctacaattgatgaccgacttctcggaatatccg
aggaggtaccccggccgtcccgaagacaatggtctgctggagcagcttccatataccagagtgaaacagcttacaaacagcatacatccaaacg
gcaagtattagacgagagtttggcctttggtttttggtcttccctaggtctgtacatatattctattctgttactttttctggcatccttttctctcattttc
atcttctgtaagaaacgtaagttgtccattaaacgcgcattgagagcggcatctattcgagatttactatgtagctacatgtagaaagcgatgcttg
tttgcaatatttccgtcacgtttaacttgttacgcgttttttttttatacctaagagtggtcataacatgcattattcaaagttgacaacatctggagtt
cgcgcattccgtcggtttcacgtgacttatcctatcttatttgccagctcattatgcctgtgtctgctatgctaactgctacttcagaaacccttatagc
gcatcatcatgtataaagtaatctttgttttgggtggtcctggagctggtaagggaacccaatgtgaccgtttgacgacagagcttggctgtgctca
catctctgctggtgattgccttcgcgaggaacaagcccgtcctgatagtaatgatggcaaactcattaaagaatacattacagagggtaaaatcgt
acctatggaaatcaccttgcgtcttttggaagctaaaatgaagtcttatagccttcagggcatcaataccttttttgattgacggtttcccacgtaaaa
tggaccaattcgaaggattcgaacgcgccatttgccctgctactgtagctcttttcttctactgtgatcaggaaattatgttgggccgcttaatggat
cgcggcaagaccagtggtcgagatgatgataatgccgaatccatcaagaagcgtttcgttacttttaccgagacttccatgcctgttgtcaatgcct
tgaaaaacgacaatcgttgtatcactatcaacgctgagcatgaccctgatactgtctttgaaactacggtcaatgctttgcaaccctacctttagct
gcttaaaacctgttcaattatagaccgtccttcaaaaacttatttttgactgttattcatccgaaataggctcttaacgtataagtagatatggtatgt
acctgtgttataagatttaagacttcactcatcgcttactacttccatatgacaatttacaaactagctctaccaaatttcgaaacttacacaagaa
aattttattattacccaaaatatacgtgcatctttttatttttgacaccaacaacaatattgtgtaagttccaataatgaaacattatagtaattttgt
atgcttcagtcagaaactttgcaatgaaaataagaatcaagtgagcaacgaatcgtgacattacatcctttctaccctttttctatgttcctcattaaa
ctatacctatgtatcacgggcgcagctactcttattcgacccacagaaatgaatcataccagacatacagacaagattagtcggaatcgagttgc
ctgactttcgtcaggtgccgcacattgcactaaaagacatcttctcgggtgcgtttcaggatctcacagaacatgcctatgaaatcgaatttctggt
acacccggcaggactttcgtcctccgctcctcacgagcatgcaacgaagaagttcctccgaccttacccccctcaatgcgctaaaacacttgtaaac
agtgcgtttgaatttgaggaagtgcggcgtttgtccgtacgcccggcatggttcttttctgcaggttccatcttttgatctttctcaactaaatggaga
aataaaatccagtctgtcttacactgacagaagcctcaaattacttatttcaccaatatctttgataactcatcgatcgtacattgcatccaccatat
ttttttcagcttaactcttttgcattctcattcatttcacacaacttctccaattggtaaatgctaagttttaatctgtgcaccctatacttatataatatt
atgtgactgccctgtagacttgggagctggtactctcaagtaccttcaccaactacgcataacataagcaaatggtaagttggataaatttagcga
ctttactcacattttagagttcaattggtacaggttacgatttgggacttttcttttccccggatggacgtctgtttcaggccgaatacgcgtataaag
cagtggagaatggaaggtaagtgatttttgaggtttagaaagtattcgtctaatcattacagtacttgcattggaattaaatgccaagacggtgtg
attcttgctttggagaaaatcgttattagtaagctgttaaaacctcgtgccaatcctcgtataggatctgtcggtcgtcatattggtattgcaactac
gggattcattcctgatggaaagcatatcgtaaaacgtgccagagatgaagctaattcttggcgtgataactacggatctgccgttcctggtccggtt
atggctgatcgacttggcaactacgttcaacttttcacttgctattcaagtgtacgtccttttggtgttatgtcttttgttgctgcatatgatgacctcg
gaccccacttgtacatgattgaacctaacggcgtgtattggggatacaacggcatttcagcaggcaaaggtcgacaaatcgctcgtaatgaactg
gagaagttagacttttctacattgaaaatgaaagatgctgtaaaagaagcagcaaggatgtatgtattaagttccgtgattggttatccttaggtg
attacagctagctaaccatttttgtagtatatacgctacccacgacgaagagcataacaaagagcacgaaatagaaatgacctgggttggtgaa
gaaaccaatggcatccatacggctgtccccgacgatctattacaagaggcagaagcttatgctcgccgtaccgttgaaggcggtgaagatgagg
atgtccccatgaatgagtaatttataattacaaaattctcgtgaatgcagcttgtttcttacatttttatacataatatatctttgccgagtccttggc
tttaaggattgacagtttgaatcccttcaaattctatcactatcttttgtcaaaaagcaaccaagaacgtccttattttggcaaccttattgtcctttct
tttataaccgatttccaacatgatacgaaaaattgtttgaaacaatatcctcacttataaagagcttcacagcttgattaacggtatatgcgttttcc
tatccgtcgttttcttatattccacagttgatgccgataacaccgttcattagcttgaataactcggaatgattcatgattctttatacagcgtccttta
ttttgattcacaaataaggcaaccaaaagctccttgtgctttattgggacttttctatagactattctattcttcttttcatggctcgtaaaaatttatg
aaattattatagctataaatgattatgaaacaaagtcagtataatatatagttacttaaattcgactacttcttatacataacaaagacattcagta
tattgtattttttattcattttcctaaaagttttctaaattttacttaattaattaattaattatgtagcaacagttattgaggtgaattccactagttcga
aaaacatctcacccaaccgtctcaagtttgatctccagtatacatttaacaggtaatttctgtatcatccaatatgttaacagccctgcttcgtaact

gttaaataagcgttgttcattatagaaaagtatgtgatttaatagccttatataactactttttagcagtcacgggttttaccaacaagcgttggaa
actgttgggcttctttaaacggtaaacaaaaggtattgccgtaatagcctgccttcaaagagcataaagaaacataagaaaagcaaggcttttct
gtcgttggctgcgtagttattgaaaatggaataattttttcttttttttttcggtttcttagatcactttcttggtgaaaccaaaagacttttccccttca
gacaagttgtatctttacatgattatttagcccaaaaaataagttctgctatttagaaaatctttctttacaagattcatgaaatatcaagtatacgt
ataaatagtattttgattttaaccgctcaagtatatatcaaaatgctgaaagaattattaactaggtattatgaaagcatatgtacaatgctataga
cagagaccattcctgtaattttctacagtctgttttcataaattagaagggaaaacagtctagttagttgagtaaattaaaatgaagcgtttttgag
agagcctgtaaaaagaatgtgaaccgaaatcgaatagagtagaattgagtggaaagaagaaaacaaccaagtttggaaaactataacaaaaa
aaaataaaaataaaaatattatcactgttgagtcctttttgagctttttgcgcttaattcttggttacgttggactcggcggcgtcaccggaagcag
cctcctgtgtagttaaaggaatatagttcccttgatcaaccggtttttgctcgggctttgtatatagaaacgtggcggcaatgacgaacgctactcc
aacaacaaaggcaaatgagatcttaaaatccaacaagtaaacactcaccaaagaagacaaaatgatactgatactagtggaaaagttcttcat
aatgttatcagcaaaagcaacacagagtgcaacaatgatgccaccgaacgcttgaaggaggatagcgaaccaaacgacaggattgtaaccgta
aaagaaacctttttgagcaatgttggagtagtcgctaacgaggatggtgaatatacaaggcacaatagaaaagaaggaaagttgaacgttacg
gacccacagagagggattcgtacccttcaaaacctttcaaaataaacgccagctagaccactaattaagcaagcagaaagaacagctgtaaat
ccagtcaatggattcatgtacgtagaggccggtgatgtactatcagaagccaagttttgcagctgcacgatagcgataccaccagtcaagatgaa
gagagacacccactttgtaaggccgagcttgcgatggagcaacacgacagagaaaatagcggtagtaaggatttttaactgataagtcacctgg
aaaatggcaaccgataggttaccagctgcaacatactgcaagttgttttgacatgtatagaggaaagcaggaattccaagtttccaactgtctcc
accaaagatttgaggtaaaaaggctcttaagcgagcttcactgcccacatttctgcggaattggtagtagccaactataaaacatacaatcagctt
gatcaactcatttaacaaaacggcggtagatgtaaaatatcgcttatcttcataaccaggcataacacgcgaataatttaaggtctattatgtaaa
gttaatatctacattcccgaacaaaatcaatacaaagatagaaaaccaatgatgcccaacagcattcatccgccagcatcttcaaccattcacaa
tgcataccaaaataagagcgctgttttgaatcgtcaacaatgccagcgctatatacttcataggaatgcctttaaatttcacttcatcccctttataa
gccatgttcgaaagtctcagaaattggtgcgaaagaagatacgcttgaaatacgaatgtaagattccaaaacttgcttaaatatcccagtacaag
aagcaagtcacctttaaacaatgaaacaaggaatgaaggaatgaagggaaaaaaggaactcgaatcctgtacagttggttgagtccaacttga
atttaccaccaatccccctaaagcttactttaagtcgacgtcaacaaggcactacattgctgtacgctcgacaattacgcatcgtggtataataccg
taaatggttgtttccaccaaaaatctatgcagcaagaaaaaaatccaatacaagataaaggtttttattagagtaagagaagctttatatataatat
atattctattattacattttgaaaaggttgactttagttttagatggtagaaacttcatggaaacaaggtttaaatgagggattatcagttttcgtttg
gcttttcatgagttggtcaacgacctaacgaaaacctcgtagttgttggggacgttggtactgcattcgacaatttcaaacctcttgaactctcagg
ctcctggtagttttgtatttccaagtgcgcttaagacatggttgaagtaatgtccatctcccataactgatgaaaaacggccataatatcaattgcat
ttcatgagattatggctaccttcagttcaatccataaggttggaatttattactctttaggcttgcagacacgaaaagcttgcatcgaccttggatag
cagcaggttggtcacagtgttggtacggatcaattcataatttttgaagagtggtagagcatacaactttttgttaccatcaagtacgcagactttg
tgaacatgctttagtccttaaatacgagcgttgtcagcttgacgaattactcgacgtaaacaagggattctgcttctgtaaaaaactcctgtgtcat
cagcgtggattaatagcttttcacagccttatgcctagacggttttggcttgtctttacatgtatatatgcattggctcaataaacttcaaatagctgc
ctaaacttgaaccatcatacagagacattggtaaatcccaacgtgaatcagctggaaacgtgatcttcctatcatgaaatgaaactatgatacttt
ttcgtttttaaacttcttcgtttgtttaattagtgatgacggaggacgtaaacataaaagaaccatctattcgaatatcggttgcattgatttttttta
attttctttttaatttttctttcgttctttctattttgatgcgttcttatgttccaacacaaaaattcatcataaaaagcatgttccctccacgtaaaactt
tgcctataagacgtagtgattgtcctattcttttgcagtgtcaaatctcgtacaaaacacttactttcctaatacgttcgagtaaactttaggcttgaa
tattaaaagtagcgtctaattttcttgatatttatcaatttatccaagaaatttttgctgtctaggaaattggtcaccctcccaaattcctgacattact
gagatgattgagaggttaaagatagctaaaactagattagaagctatgaattcatttcaatactctactgatcgcagagaacgtcttcctttgcttg
gagacgagcgcgatcaatctatggcccgacacaatgctattgtaaccacaaaatccgttcttgcctcaagttacattaatttcctattagtgtttgtc
cccatcggtcttctaagtggatggtttcagtgcaatgcaaagtatgtcttctttctgaatatgctagcaattgtccctctctcttccctccttagctttg
ctaccgaacaattggcgtttgtcagtggtcctactcttggcggattattgaatgctaggtaagttgatttgtttttacgaaaggttcattaccagaat
gctcttttgaaattctatatactttttctctgctaacttctttcagttttggtaatgcaatcgagctcattgtcggtgttttggctttaagacgcggtgaa
acccgtatcgtacaagcttcgttgcttggctccattttgtccaacttgctgctggtatttggtatgtgccttattactgcaggaattcgacaccaaatt
gctagattcaacgttactgttgctcagacaatgattgcaatgcttgccttgtctactgccagcataataattcctgctacctttcactttagtcttcccg
ataataagactagtgaaactgctttacttcaaatttctcgcggtacagctatcattgtcctaattgtttatgttttattattggttttccaattaaaaac
tcataagcatgtctgcagagatgttaatgaatcagaagaagacgaagatcatgaacccgttcttggattatatgcttctatttttgtgctagctttag
ttactgtttttgtatctttatgtgctgattatcttgttggttccatcgatgccttggtggaagaaatcaacatctccaagactttcgtcggtttgattatt
ctccctgtagttgctaacgccgctgaacatgtcactgcagttgttgtatcttaccgtggtcaaatggatttggctcttggcgttgctatcggttctagt
atccaaatttctatgtttttggctcctttcttggtcattgtcggttggttcatttctcagccattaacgctgtactttgagtctctagaaacagtgattct

cttcgtttctgtattattagtgaattacttaatacaagacggagcctctcattggcttgaaggtgtccagctccttgctctttacgccattgttgttttg
gcattcttttactacccgactcaataagctgatgggtgctgagactttctacatttcttacgctttttttattattttcttctaataaagttttgacattcct
ggtctttggagtcgtcaaccttctttcatgacttagagtatcaatgtgtgttttggcttctaatgaaatccagctttcaattgtttctgtcaacttgttta
ttctttagatgatagccgagatttatatataaaacagggcaaaggtttatacaccacttttggccgtttcaactgaaagaatagattagaatcattt
catgctatcgaccaatccaacagaaacttttaaactctttatattttgttatgtacaataacatttacacaatcacataggaaagtgtaatattgag
agttgtttaaagggttccatctttatacattacacatacaaattccattgagacttctgcctgtttttaaccaagaaacaataaattatatgaatcca
aactggccatatttactgctctcagatatgaatccaaacctcatttttccttttacctctccccccaaataagctcatcttataggggcacaatccca
aacaaagaccaaacaaaataaaaagaatataataggtattacagcctaatgtattattcattctttcactaatatatcgggtttcttacacattctc
taatcataattgagaaactgtctatttatgataatcaattgctttctttcctttttcaaatcccatatcgattaatttattaaagactggtcaaaaatca
agcattctacacaatggattcaacatcgaaacaaaaccaagcatctatcgatataatcgagcactcgaaagagaatatcgaaccgagaagaca
aggtcattccgcttcagcaatcagcaaagctttctctaaagatcatgagcaaaaatacagcaaccaactacacgaggaaagagcgaactttgag
aaaaaactccaagtgtcagacgaagaggaggatcctttacaaatttggattgactacattcaatggactttgaactcttatccccaaggcaatacc
actgaaagcgggttgctttctttgttagagcgctgttctcaacattttataaaagtgcctgtttacaaaaacgatatacgctatcttcgtatttggatg
caatatgcaaagtacgttgatgatcctgctgaactgttctctttttttgtctcttcatgaaataggcacgaacttttcactttattatgaggaatttgca
agttactatgaatcaaaaggatcatacaaaaaagccgaagaaatctatcaaaaagggtttcttcgaaaagctaaacctttcgctcggtttcaaga
acgctatcatcaatttgtccatcgtagagtcatatatgctcctgacactatttctacgagcccaactaacgaatatcctcctctacaaactacttacc
aagcgtctagtcttcaaccaaaaagccggagcaatgaagattctattccagaagcgagaaagttttctgtttttttcagacgctgaggctattgaat
ctcagaacggtaaattcagcaatccctcctcctgggaagcttttggaactgtggaacaaaaaagaaaagaaaatacaattccttctcgtgcttgg
gtcggtgaaattttacacacgcagtcctcccgcaaagtcgatccattagataatttttctgtctatcaagatgaaacttcatcccattaatgtgtctc
aatttttaatgaacttctgaaaattatttttttattccctatgcctttatactttgaaaaattatggttaagtatcaaaattctaatttgtcaaaggaat
ccagcgggttatccacatgtcaaaagttaacttccattatataccatttataaacagatctataataaaaatataaataatttattcacagtcataa
aacagaggctaatccgaagagtctttagtaaaaaccatggaggcagctttagaaagacgattcattatagccaatccctcattatcttcagtaac
accttccacaagaacaaaatttacacctagtaaatccaactcacgtaagtgagcaaacaagtttttcgttatttcggaaccttcttgtcctaaatca
agaaaaatcaagttgctgaattcctgaaaagattcctttttccactttctgctacacaaaactccaattttggcttcatctttatccatagtttgttgtc
ttagctgttcatataaacaatccatcgcttcatgttctttgtagtccaagaacagcataacttttgcagtgggggaatagtgtcgatatttcatacct
ggagtttgaggaatataattttttttccatatttattgcggtaggtttgtagacttgagtgtttacccagattcctccacaagcacgaatttgttcaaga
ctaacaccgcctggtcgtagaataacaggcggattacaaagaccatttactactgtactttcaagtccgacaccgcaagatccgccatccaagat
taggggtatcttcccttgcagatcattatatacatgtttagcctgggtgggcgaaggtcgagtagaagcgtttgccgacggagcggccaaaggaa
catcagcaagagctattaaggcaagagcaacaggatgttcaggcatcctaacggcaaaagtaggttgatttgctgtgaccactggtgacactggt
ggatgctcattatttttttacaggtagaagaatagaaagaggtcccggccaaaatcgatctatcaatggatcgtaaacttgcggaatcaaagagtc
atctgaaaaacccttgttttgtgtattcagaggctgatttttcgaaataagtctacgaagctgatctaaagaagcaatgtgcacgatcaaagggtt
atcagctggacgatttttagccttgtaaatagagtaaacagattctgttcttctcgcatcagcacccaaaccgtaaacggtttcggtagggaaagct
acaggctcatttgtatttttttagaagttcggcagcttcttgtaaagcgctcaaagtacttgagggttcaattgaaagccttgcaatattgaaagggt
gctctttaggatcctctggcttgctaaattttattaaagatgaatccaccgttcgaatttttgtttgcgtcgacattctacgatagaaaaggctaataa
aagccaagcgcttcaaaaaacccaatgtgtgcaatttgtaagtaagaaataaagaatcactaacagctaaaaatgggaagccaatagaaaaa
aaaatttaaattgaccttttcaaatatccataaaactcaaataaaacccaaccaacaatgaaagattgattttctgagacttgataatcttaagaa
acaaatagttttccttggaatttgcacttttatccaaattttgatttcttgcaatacttgtttagtaatcagaatggattcccaagaaagaggagaaa
gtgccttacaaccattcagaaatatgagaataataatattttcaatgtaaaaaaaagaaaccaataaaggtggaaatttctaggtttatatattac
tttgtgaattgaaagctgaatttgatcaattttatgagtaacagcataacttaataagttgagttaagaaaaaaaatcattgaaacaaatctatctaa
tgagtcagataatacatgttttctactacatagtcataaagaatagcgcatagaagccctttaggaatatcatgacaggtggcttgaggggattag
tgctggctttagcgaaagtaaaccaagaaataattagatagagaaatgacttggaccatattagtagcacatatgaaaggattttcgttacgcga
ataaaaaaactcataaacccgaattcagaatttttgggacataaacaacgtagttcagagaaaaggtaagagggaagcgaggtatattagaca
attcggtccagagccagcaagagtaagccaaggatagtgaaaagaaaaaaaaaatattctagctgtacgactatttcgtgatttagcgtgaatga
gttgacggtttccaccggtaattgaatcagctacactcatagcatcatcatataacttttccgctatagtagattgagcggaaagttgagtagatat
ttctgtctgcaatctagcaatttcagttaacgattttcctgtctcatgcacttgttgcatcgtatgctcaaattcttgcagcaagagatcattttcttgtt
ctaactccattagctgctcttggctaaaataatttggaagctcagaatcaggaatttctgccactgacatatcatttgcttgctgtggatgcaagata
tcagacatgatcgtctttctttcttgagcacgcctcagtcgcaggtcttgaagtcgatacaaagtggaagagacgtctgatagttcactttgcagat
accataagatagaagaataatgagcaactatggtttcggttttcgtaagtttttcagggctcttaaagctttgcaaccatcccgtccgtttgggaatc

tggtattgtttctctagatgtatcagttaatactatgaatggatcagtaacgctggttcaaatacctttcaacttctgctgaagagagctaattttctg
gacgcaagagttgataattgttgatgcttgatggtcaatttcatccctctgtaaatcattcagctccaacttggaaagctcctcaaaagagcattcc
atcaaaggccttgacaacttgtgaacattggaaacagtacgacctttcaaatatgctccacgaatcttctcaagaaacgtacaaagatgaacaat
cgtattgtgctacaataattagcggtgaataaacacctaatatagaaaaggaattgcctgcttatagaaagagaagggaacacaccctttctatg
ctatataatgtccagggtcaaatacctctctctagtgtgcaaccgtaattcgcttcaaatattcaacttacgattaaataagcttctgcgagaaa
agcatcttgaacgtttgaaagcttgggctttcgcagagaaacgttttccaagtccacgtttcctttttcaataaatccaaaaaactcgttcgtcctgc
ttgtcatgtttcacgacaaatatgctttatttatactcataaatacaatccaaatggaataataggtatgatttccgattctcttaaataaataaccc
aggtgttttcttttcgcaagagctgtaagactacagattctcataatagcacaatctagcgaccaacgctaaccattggaagtagtagtaggatta
aaaaaatgagagctagttgttcttaaccattctttgttatttgtttttctaagcttgattatgtcttagatgctgccgaaagttgtttgtttgcttacact
cataagtttactgaaatcactttacgtagtttgtaaacaaacaatcgaatgcaaaatttagacgaatttgggcgaatttgataggattcgatataa
aatatataattaatgctccaaattgaactaaaaaagcttgaattaatcgattgtaaagggtttggaggaattcgatgtatactgtaacttgcgcga
gtacacatatatctgctcggttccccagagttgtcactttacctttaccatatatcattattgtacagttgtattccttaccataattttttttgtttcgt
actattttcttatcctctatatttatttattaaaggcattaattataattggcattctatctacgtccaggtttcgctttcctttgaactgggaagcaatt
ccttcttcgcttggcaaactcgcattttagtccatccactttactgctaaagctaagttactaagggatcttttgttttctggtttatatccttaccggcg
tagctccctagtgaattgtctttttcgttgattcacatccttaatttacctaccctctttccttctttttgctgtttccattccgtttgtttatttattagtcgt
ttttgaaaatgtccatacactcttttcgagacccttcagaagttgattctagagattatgcttggaatgcccaattctacggagaccgttcggccact
ccttcgacaatgtcactttctgagcagtatgggttaagcaacattctattacaatcagagaatgaatctcgcgaagatatcgcttcgattggtcaag
aatctgacaattctttgcaatatatgtgttcccgaattgtgtatcgtcacaaaattaaagtttttgacaagctttatgcggaagcaactgaaataag
aagaaagtatcctatggacgataggctttttccaaagcctcctgtcgtaaaggaaatacaagatccctcacaaaggctaggaaattatggccccg
atactcctttaaccaaagatgttacgattttagatgaaaagacgtatgccgtcattctgaaaagaattgacttttacattgaaaaggctgtcgaagt
accagtgaatctggaatatttagtggcgttgaatttgcaattacaaatgcagcccgtttggtggattgatgaatttgctgaaagaaatggtgttgaa
agcttgttgacggctttacgaaatttgggccacattcccgaacgtcatgcaaatactgaagcagaggtgttgttgattcaatctttgtttcattcctta
aatagcaacattgcccggaatcacctttatttgtctgccaaatgtgctgttcccggctttaatgccatcggcactttagctgaatcaatgttttccaa
gaacctaaatgctcgccgtatttcaacatttattttgaaagctatttgcagtcgaaaatttgaacatgggcagtcatccgtcgtaaaggccttagag
tggctagttgaaaaaggtgcgtccaaaactcgttttagcagatggatgaccgccttacatgatgttgtatctggcattcagggttacgccgataac
ctaatattaccaacaaatgaacttgccagcctacaaggaacggactgtttgattgattatttggtggttacctttaatctaatacgtggtatatgttc
atgtaccattgatcttcattcaagatgtaacatacgccgtgaattcttgaatgctgggcttggcaaaacgttggagataatgcagcgctggaaaaa
catttctgttcgcacggttgttgttaatcttttaaatgaacacaattctgatgcacagtcttataggaagttcattattgctaattctgcaaacgaaga
gaaatcagaagtcgcatctttaaagcctactaatgttaaccaaggtcagctgcctgaagacacccccagcaggcgcaaatgctaaatgcgtttc
aaaaaatttgttctcgtttaatagatcatgaatctcaaactagtttctacaatctacttcaatccttaggtaacgaaaaggatcttgagaaaattga
taaatccatgcatcttttagtgtttacgttaaatgcaatcgacgaatataaatccgctcgtatcgactcaaacgtcggttttaatattgtatcacaaa
gattacttgaccgcatgggcacagctgatgaattatctgattataagaccaggcagagcgagttactcttggaaaacaaacgtttgcacgatcag
gtggatgcattgttgtcacagcttaatgtcggtcctcgtgatcctatgcaattccttaaaagtcaactaaatgaattgcgaaaagaaatagatcaa
agagagagactcttggcgtctatgcaacgtgaattcgatggccgctataaagctcaattacaagcttatagtaaactacaatctcaactagaagc
cgctcaaactgcaggaaaaacaaatttgtatgatggctatccttctggaagaccattgtataaaaggtcatcctctcaagattctttagacgcaat
gtctaaagaatttgcttatagtgttgttccaaccttagaagattctgaacattatattactccggaaaatgacgaacaagaatcagaaactgagtc
agaaatttcttctttggaagagtcaaacgaagcaactttacaacaaaacgatattcctagccttgcgcaacctgcgttagcccctacaactcaact
cggtccttcccctcctcctcctcctcctcctccggttcctaccatgggcgtttcgaaagtagttgtaccgccagctacagcaggtcctccaccgcc
accagcacctccttccgcagctgggggcttaccacctcctccccccctcctcctcctccaggagttacacctggtggtagattcgtggctacagcccc
agtacagacttctccggctgacaccaaggaaggcactgtgaaaactgaagaagagttgaaagaagaggaagaaaaggctagacaacaaaga
gaggaagcggaaagaagagcaagagaagctgtggaaaagtataccaaaatacctagcttcagagactattacgaacctaaaaatcagctcaa
gcgcgttcattgggaacgtgttgaacctccctctggatccaatattttcgttaagttcccaatagacctcgaaacgattggaagaactctattaaat
agcggctggttacaaattttagatgaaaagtttgacaatactagaagaattgaaacaaaggctatctctaaagaagctactaccacatcacttct
atctccaaatgttaagcaacatttggaaatcgtgttgcgttctgtttcagatttcacacctgaacaattagtacgaatgtttctgactgatcccaactt
tctacctcctgctatccaatttttccataaatctggatttgctactcaagaaggacttttatctccttttgcatcctattggactgattacagcaaacct
gaggacaagcgtacaccgcctaaagatgacgttaatgaactaggatattatgagagattttttgttttatttatcgtaaacttgagacattattttca
acagcgtatgtatggcttacgattacgtgagactttcataccagagcttgaaaatattgaggcccacgttagaaaagtcattgatgtatgcaggtc
aattttagaagacgaatatttttctggatttttccaagttttacttaacctgggaaactatataaatgaccctcttgataaagctcgtgcattctctttg

ccaatggtttatagattggaaacacttcgagactgcacgttttctaacactcttttacattactttgaggatatcattcgtacccaatttccagaatac
gagaagtctgaagcgacttttaaaaaaatacagtctattagctcttacggtattgatggattagtatccgaaatggagtctgcttacgaacattttt
tgcattttaaaaatgaaattgaaaaaggtgctttgtcgaaacgtgaggagcatcatccagaggacaaagtgattgatgctatttacgaatggtac
caaaaggcctcagagaagataaaaggctttatggctttaaaacgtgatttcttgcaattaacagaggatactgttaagtatctatgtgaatttaaa
gacgtggaggttgttcgaaacactttttttgaagaacctaacttcattctatataatatactgtaacgtcagaaatgataatgcaagaaaacgcgag
cgtgaagctcaagctagacgaactgaagaatctaagaggctaatcatggagcgtaagaaaacgtcaatagttgcacagtatagaaaggaactt
ccgggacagaccaacgaacttgtttcgaatcaggatcttaatacagatatcggatcatcaagggccattgatagttcggaaacgttagctgatga
aactcctcagttatcggatgatgcggatcaagaacttcatgatacgcttctcagtgagttaggtaaacaaatctccgaaggcgctggtaaagaatc
aattaacgaagattctgttagtgagcttggttcagcagcaaaggatgaagatgtcattgaattaacttctgaagccgatgatatcgctaccaaggc
aaaatcaatgctgcttaaaatgaaaaatagcgagaaaactggtactaacgtgaaaattgaccctatgaatacggatgaagatttttttgcgagca
cagctaaaagcagccaatcatcctcaaaaaatttctttacctcagaacaagaagaaggaatcggttgaggcgtcgttagcaaaaagcattttagc
aaatttgacaaatccttcagctaaggaattacaatcgtgatgatcctctgcacagtattatataaaaatcaaatccgttctttttgttagattttaag
gatagcaggattgaggagctacttttatattattttacatgcactcattacgcgcaaattgctcagtaactctactctgtgcattaatcagaatgagt
gatatttttacagacatggtcttgacaaattttgatagttttgaatgaggatgagtactaagaaggaaagataaaaagggctttgatagcttccca
aggaaagttgtatggtgactcaagttcatgacgtttaatgtgcatatatggatgggttttctccttcattcgttttaggattatggtttcgcaattaga
atgagaactatattacatactcacgggatatcgtttaattctttcctttttttatttacgacgatgatcaatgatgggacaaaattttatttatttggct
ttagccgttaatgtgtttctaaaaagcaaaggttaaaattggaatgctttctagatgtttaggttttttttttttttttttttttctgttttgcaatcatag
gtttaataatattgtaagatacatgagccttaatggatattatattagccaaaattgataatctgtgaaaatggtataagaaaacgaagaaactta
aaagtatataaagttcaaacaaaacaaaataaagactatgagattggatatgtaatctgtattcatcttaagtaaatcccaatttcatccttctcat
tgccttttatttatgttacctgactcgtaagtaacgagaaaatgttcaactgaagggtttgtacttaaccaagtgtacagcaattctaatagttattt
actgcaatgtttcggtatgaaacacttcatgctggatgtaaccgcatttcagaggcagctacatggacaaacaatttcggattagtttatggcgctg
aaaaactcatagcgattgccgatcctgtaagatttagaataaagaatttattaaggaattaagaagtttaacatataaaaatagcaaaaggaga
tacagtatttattagcggctcatacgggaagagtaaattgtattcaggagctgaatgttgaaaaatcattgattatttcgggatcgtcggacaaga
cacttcgcgtatgggagaaaaatgacagcaatgactcctttgatgttcttcaaacgataaatttggatggtacacctgaaagccttaacatcaatg
gcgatttggttgtagttggttgcacaaacagcaagtgtgtggtctttcagtggaaaggagcagggaagatgctggaagaagcatcacgatttgat
actggagagctaatcccaatggatctaaaaatgataaagtttaaggatggaattgttttggttgtttgtggctctaacaaacgaatttccgtttatg
gtagcgataggacatctcttagttttggtttaaaaaccgttttaacagggcatcttgattgggttcgcagtctatctattcgacaaacttctgagtca
atggctacattagtctctgggagtcaagatcgttatatgcgactttggaaaatttccctttgggaagaaaaagatgaagtcgtttcagatgagtaa
gtttatttactaccttgaaatgtatactaattttatcagatttgctgaaactttattatccaacaagcccattcgtttcatgcttggtggagtggaatat
aagattgtttttgatgctctcttaatgggtcatgaagattggataatgagcgtacaatggcatcctaaagaagagattattttatcttcatctgctga
ttcttcgttaatagtgtgggagccggatgtagctactggtatatggttagccaacgggcgcatgggtgaaatagcgtcctctcacggatctactact
gcaacaggtagtgctggtggattttggggaggattttggagccccgatggtaatgctgtcgcttgctggggaagaacaggaggctggagattatg
gcataaatctggagaagattgggagcaacttccctgtgtctccggacacaccaaacctgtcaaaggtattgcttgggatccccagggtcaattctt
ctttacagtgagtttagaccaaacaactcgtctgtatgcttgttatgaaaaagataatacatggcgggagatggctcgaccacaaattcatggcta
cgacttaacctcaatccaatgcttacccacacggactggatttattagttgtgctgacgaaaaagttacacgcgtttttgagtttccaaaaacgatt
gccagattgttatcgcgtctttgtcgtactcagatatttgaggaaaagcttccggaggccgccaatgttcctctcttgggcctttcgaataaagcaac
cactgctactgagacaggtactgttaatgctgatgaaattcaaactccattaattgacgtgattgactctttgcaccatccaccatttgaagagcat
ttacagcgtttgctgctttttccagaaattgagaaaatttacggtcatggttacgaggtttttgcgtgcgatgtttcgcatagaggtgattatatagc
aacttcctgcaaatcccagtctcctgaacatgcaatgatccgtttgtttgatacaaacgtttggaaacagcaacaattgctatccggtcattcatta
actgtaacaacgattaagttttcatatgatgataaatacttgttgacagcaggacgtgatcgactcgtttgtattcatgaattttctcaggcagaatc
gaagtatctgccgtatgttgttatcaaagcacattcccgtattatatgggatgctgcttgggcaccaaaggacatgggttatttatttgctacagctt
caagagataagatggttaagttttggcaaattttggatgataaaaaggttaaagacgtagtaggtctgcaatttgcagatgctgtaactgccttgg
atattgctccattctttcatgataatcatcttttactggcagtcggtacagaatcaggaaaaacatatgtctggaagtgtcctaaagataatctaac
gaaatggactcctttaaagttgccggatacaatgtctcccaaggaaccagtaaacaaaattgcttggagaccaatttacgaaaaagagggagag
tactctttgttattagctggcgaagacacgtcaattcgcatgcttcgtgtacagctcaattaaaaaaatatataaaagacgcgatagagtcattgg
gtttttgattttggacaacgccagataattaatataagtccggaagagcgatctttctttaacaataaaaatacgtgttcttgaataatgttggttac
ctctgaaatgtatatggaaaaaaaaaattactttgatcatcttgatttttctaatatggagttgcgagatgactagtaactatcgatttggagtttggg
tttttggtttttcagcttatgactaagatagttgtttattaacctaagtttagtaactgaaccaaaaggtactagatttatattatcatgagtacttttaa

caagtgaaggttgtttttcattagtgtttatcatatgaaaatgaagttgaattcggttatttactttcaatgtttttataaagagtcttaagttgcttca
acaatacaaagccgtaaaataaaataaaagtagggtaaagtaaagtggttttaatgtatactttttataggacagtcgacagttggctatttaggt
acatttttgaacttttctcgctgttttctattttgtacccatgttgtacaccaagtatgaaatgctttctcctaacctactccaaaccattactaatttag
agtaaaaagagcgaaccataagactaagaacttgaattttattcttaaaagaaaaggttcgtaaaaaaaaaaaaagatcgccatttaatttgaa
tgaatttctagaagaaagtggtttaactgaattctttcgttttaactcagttactattatacaaccaatacttatgtataaaagtttaattaaattaaa
gcctctacttttctgatatctacgaaaactgcttctcaatcctttatttattgtttcccttacgccagattcatttgaagtgaaccgtgcctttcacttctt
aacttagattattagtcttctttgctttgtttttatctgtaccttcgttcattcattttttaatccataatgagtcaggatcaagaagaagattatacaa
agttacctttggaaacgagaattacacacaaagtttggaaggctcgtctaggtgcttatgaggaattaaataaatctttttctctttctggctctccg
acggatccttgctttgacctttgggcccgtcaacccgatttatggaggtctgtccttgcagatagcaatgtcgctgctcaaggagcaggtgttactg
ctttcgtatcgttctgtaaattttctgacccttcttatatcgttaaatgtagagaacttgcttctgctgccatttcagaaaaatgccttacctctcgagc
ttccacaaaatccgatgctgttgaagccctgatgttaatgattgaagttgatgctgcaggtcctgttattgatgcccttcttccttcgttatctgcgcg
gtctccgaaagttattacggccaacgtatcggctatcgctgacttggtcgaacagtttggtacaaaaatcgttccgccaaaaccaattgtggcaca
attaaagtcactatttggacatgccgataaaaacgtacgtcaagaaacgttccgattaaccgttcatctgtatcgctggattggagagcctttaaa
agcatctatctttggtgatttgaaacctgttcaagtgaaggagcttgaagctctattttcgaaattacctacggatcctcctaaacaactgcgtctat
taaggagtcaaattcccgagcaaacggaagctactgacgaaagtgaaaaagaagatgaccatggaatacaagaggagccaaaccttcaggag
gaagaagatgaatttgatttaatagaagaagtagatgtgattcctaagattgattccaatctggaatccgtaatgtcctcgacaaagtggaaaga
gcgtaaggaagctcttgatgcactcatcccaatcctcagtcagccaaaaataaaagatggagaatttttcgggttggtcactttgttatctaaagct
attgtgaaagatgccaatgttatggtggttatgaatgcagcaaattgcataacggctttagctaaaggacttcgttctaattattctaaatatgcca
ttacttcaatccaagcacttttggaaagatcaaaagaaaaaaagcaaaatgtcgtagaaacattgtcttcagcaatggatgctgcttgtgaaacg
atacctttcgatgaaattgctgaacccatttccgctttttctaacaacaaaaaccctcagattaaaattgcatgttttaatcttcaaactcgatgtttc
cgtaaagtgcttcaatacccttcaaagttttctattgaagtgtgtgctaagtcttgtgttccgggagtttccgacacattcgaacctgtacgttctgcg
gcagctgaggcccttggaacccttatgaagctagtaggagagcgcccattagcagtatacttggattctttggacgaaatacgaaaatcgaaaat
tacgtcctttttcgagtctgctacggttaaggctgtagcaccaccgaaaacaaaaccggcacctacagcttcagctaagaagcctgacactaaat
cagtagtaaccacgaagccaagattatctaaaaacccacctttgcctccatcttctacttcgactgcatcgatttcccctaaaaagaaggcgaata
ctgagaagctttctttaaatgtggagaaaacgaatgcttttgaaaatggacctttaatgccaagaccaacaactagaccagttgcccgtggtcttt
ctcgcaattcaccacttttaaataagtctcccggtttgaaatcgacatcacctctacctgctgggaccataagccaaacggtacaaggaataaaaa
acatggaattagacgatgcggcacatcagccttcaaaactttcaaagacatcctcaaattacttagaattgaaaactaatcataacggctcagtc
gatgaaatcgcttccttaaatgaagagaatgaagagcttaaaatcttattatctcgtgaaagagatgaaaaaactcgtttgcaaagagaattaac
tgaagtcagaaaggaactaaatatgattcgtgatatgcaaccttcatctccattaaatgatcgaagatctgctttttttgcgaagagctaattcaga
aatgttagacacatcggcaggtccaagcaatcgcattgaccttctacaatctccattaggaagagcaagacctttaagctctacaggctttaatca
gcactctccatcagcgttttcaaaatcagcaaataatctttcgtttcaaacagatcctccgaaacagccattttctccacgctcaaacttgacttcag
attggagtaaagcaattgatttgacaacgaggttgaaacaaaagattactgaaatgaagcagacggatataagacatcagggaatgattcattg
aatactcacagaaagaaaagatatgttttatgtttggttttgtataaaggatttgcgtataataatggttaatttggtgaaccttaagaattctcatg
atttatgtttgtttatatcccttgcaatagacctttgttccttgtgaataaaataaaattcaaatcaagtgattaataaactatgggttaatcataata
aacaaatttaaatataagaatgtaaatttcataaaaataagtcatgtactattagatcaaaattaatagaaggcgttaacggaagaacgaaggg
ataattgtagtctttcttagaaaagccaggacaacaataaacaaaaaacaataaacaaaaacacataaataaccttaaccgcttttggataaag
acggattatgagaaatatcagttatttgatttaaagaaatagacgaagtcgttacctcttcgtcgtcttccaaatagaagtaaggatcacgactag
gggaataaggagggagatgggacaattcagaatctaggtttcgatgaaagatgaagacaggcagtttaaattcgaactttttcagcttgtaatgt
aacttttgtgagtttctaggtgaatattgagataaacactttaaagacaactcactagacgattgcggtgtactgttattttccgagtctaaatgcca
atttgaaacaagggatgagctagatctgaaagaagaaaataaagagtaagatttttttcttaagtgaagagttagatgaaaaacctttttttagta
gaacagttgatggttctgaagtatgaacggacacataaaaattatgctttacaagaagatgaggatttttggttacacttccatggatggtgtcttc
aaagggcaccataaattcaactcttgaatgaagccctggtacagtaggaggggtccagcgtccatcctcgtaaaggacggttgttgatactctga
agggaccgttgaaaagtaaaaaagtgactttttgaacaagacagacatgaataaaatacctttcataagtacattgcggagcgggagaagaag
cacggcttttagcatgagatagtttatgttcaccaagttcgagactaagtcgactcaagcgctctggtatagaatcagcagattcatgtaagctttt
cgaagcattagaagaaggtgttcttacaataggagtactgctaagtttttccggatacgcgttgtagatatttccttcctcaggttgcgctagttcga
aatcgataagaaggggacatatgcaacctggacctttgtaattttgttgaggatacttcactacaactttaaggtctttatgtttttttaggtgacaac
acaagtttcgtaaaagagtcctggtttaaaggaagaccaaatcgaatgtgatgcggccctggcatgcggtcgggattcagggtaaaaggtacga
caacaggaacagtagcgatcctaggcattcggcaaatatcgtctgtgcttatattcacagtcaatgtataatgaatagcaccatcaggcgtaaat

attgttctcggcgtgtacctaggaaggggtgctatgatatcaaacgtgtataacgttccttcttggaccaaaaaattccctcctagattccactcaat
gtgattgaagtgagtttccagaggaagtttattttcggtaggaaacgccttgatgcctgtaaaaaaagcccctatgcgcttgcagaaaaatgtatc
tgttgtagtgaagtatgcgcgatactttaaaactgaccttaaatcctcgacattgtcataagtatctgaaactgttcccagtatatggagtttgtctct
tgagtggtccataaatcttcaattctaattctctccttttttgaagtagagattgagctcattccattcgtttttttcctcaatttcccctctattcaaatgt
aatgaaccgattaactcgatttcaatcttcttagttcacggatgattttggcagttttttcttgacgatttggagtttattacgtttagttggtttaaaa
caagcaatgacagtggaaagtatgctatcttagatacgatagataccttataattcgttaataaaccttaaatgctttagcgaatacaaaaacaat
gtagtccagttatacaatactgattttaattaagtaggcatttggtcgatcgtatattaaattcgttacaaacaaaatgaaaaaaatacttggagtt
tggtcgttactttaaacggcacccacacgatggttcattttgatacaactctcattcaaacaaatatggatgcaccgaactgaatgatatagcttct
tcagcacttgtttcttattttttttttaaatttcaataaatacaaatacgaacttttaagtcaatattgaaagatacgacacgaaagacaaaaaaga
gaaaagatacaaacgtattcatccaaaaagtttatttttcttgctcgcaaaagcagttaagtgccattactggcaatcaatgactgtatgcatctgc
gaatatcatcttcgtaatatacctttgattagactccaatctttgatgcaaagcaaaatccaaatagcgtctaagcaatttaagtagaatcacaac
cttcggccaagctttcacagcaaaagcattatcaagaattaagccctttcctaaaggatccactataatgtcatttgtcccaagcaaagccatccc
aaacgtattgataggagtacaattctgcagaaatggtttcgtagtttctacctttcgttcataagctataaatgtagtcttggatggattcggtgaat
tgctgaacatcacgcttgtcggatgaataaacaacctgtttttagattgatcaaaaaatttggttaaacgagcctcttgatcaacctcaattgagcc
agaagatgaagacacgtactttttgtctgggtaagcgcatttcacgatatttggtcgcaatgcaccagcaattatagctgaaagaagcgataaat
cctcactagaattccatgagaattcattgtacttcagattaatcacaagtttcaacatttgtatagcatcgcttaattctgacctgttttgtcttatttg
ttgcatacttaaaacagaaatatgattctatatgttaataaacaattagacgggaaaacacttacatcatgacaccattgagctgctttttcaaac
cgctatttttcataatctcattccactggtcgtatgctttagcatctgttaggacatcaccccaaccattacttagattagatcttatatctcgtgcacc
atcgtcatgcaaaaatggggacttcgaagacaatactgccgagatacttaaagcaggttcagtgaatccgaatatggaggctaaaactaaaagt
tttcctagctttaaatctattggtagagcaacctaattgttagtaatgaatacaaataagtaaacgacttaccaaatactctcccattccagtgagtt
cttcattttcagttaacgcaccaacctgaattaacttctctatggcagtatccactgcttgatcagtaggaggacttaaaaggtactgcataaatttt
ttaacagcaccttgtctgtctgcatctttaccgctgataaaacggcttactaaaggtatgacgcttaaacaaacctgtgacaaggctgttcgaaga
acatccgggggagtctcatcagccattcctttttctcaaatcccctactgtataatttgaagcatattccttcttgaacacgacctgctctacctcttc
gttgtctgcaagcagctctagatgcccatgtctctttgaacgtaactaaatcccgatcgagatcatagtcaatctgtttcacccttccactgtcaata
accgccactacgtcgttgattgtgatcgaagtctctgccacatttgttgaacaaatgacttttcttttcttataagacttaaaaactttcctttgatccg
tagtattcaaagaagcatgaagaggaagaatttcaaaaccatcaccacatttccgtttaatttcttgcatacaacggttaatgtttgaaactcctgg
tagaaatattagaatgctacccttattcaagccgagttcagaattaatatggtttacgagctgggaaataagattatagtccacttcgtagaaacc
atttcttttttcgtaatttcgttgtttgaatcctcatcgcttatgaaattgaaatcttcaatataaagatctctaatgggatacgttctgccttcaatat
gtaaatgcttagactttggaaaatacgacatgaagctttcagcgttcactgttgcactcataagcacaatcttcaagttgggtatgcgaggtataat
aagctttagtaaggcaagaagtatatcgttatcaatggatctttcatggacttcgtcaacaacaatgcatgatagagtacgaagatacgctaagc
catgcaactggattcttctcagtaacaaaccagtagtgcaaaattctaataaggtttcttgcgaaatacatttatctccgtgtacactatagccaac
ttcacttcccaaagcagtagttctttcaaaggaaactctttcagctaaactgattgctgaaattcttctaggttgagtacaaacaacattagcgaca
ttaccgcgaccttccgagagaaaatgatccaaaatgaattgaactacttgcgtagatttaccacttccagtctcaccacttataactatggactgac
agctttgtaaggacgataatatagtctcttttgacttgaaagcaggaagggtttctctatctttcgttcttcgtaaatattctgggctcttttgtctatct
aaccaaatttgatagacctttctatccattttggggtcacgtttaaataatttcggcattttgctttctttttgcatatgggagataacatctttagtctt
cgatacatccgttccaattgtaaccgacgaaagacttaataaaggttcaagattctttagaaaactggtaatattttcctgcaaataatcatataaa
atgtcaagcataggttctccaacataattatgagcataaagaagtagctttctcactatacttagctttatataggatgccattcttttagttgaaga
tataaatattataggtatgttttgaggataaggactagcaggcaatttaaattcaatatatatctgcccaaattcgtcggacggtgaattgagctta
attttacaagaatcctcttgtacagaaactttactttggtatattgattgaagcgcttctgcttcttccttaaagaaagtaattgaatcgctggaaaat
tcaggtttatcaggaaattccttaccaacaagatggaattgtaaatattctgcggcaagtgccaaatcatttctgaatctttggaggacagagaaa
gaaaagtcataattgtagccagcatctccaaggcctatacaaggtgattagcaacagaaaaaataagacaaataatgaagaaaagagtttcat
acgttttgcattgtaatgaatagcaagtgtctcagagttattattttgaatgctaacaccaacattataatcagaaggcagaaatctttcaggaagg
tcatcctctggaacatgaataatcaaccactctagtgcttcctctaaggacgagcaatactgacatgcttcctttgcatgtaagtctcgaaatccca
aacttactaaatcttttactaaaagttctttaagactttctagaacctctttttctcgtatccatcccagatgcttaacgagcgtaagagagtttctat
agactgacgatgattaaggctcatgttgagagtaacagcatgtttgaaatcgtactgggaaaatcctgaaagacgagttccttctgtagaagaga
atgaaggtttgggaagtctttgaacaggtggttttgctgaggcttgtttactcgcagaaatttctgcttcgcgttctttcgcagctttaaacgggtcct
cgtgataaagatactgtctattattttgacatcttctttttctgtttttccaaatttaaccatatatctctatagtttggaggaagtgcatgctgtaaa
gatttatgggacagaacctagaaacaaattttgggttaacttccacaatagcactttttatcacttactctgtgtaatgcgtatacagctccgacatt

tctggcctctaccaacgtttctttttcaggaaaatagcctttttcgtaaaaggccttgggaggagccaggttaacatgatcaaaaacagatgagttt
tttggatcccttttcgataagcgtgcggaagtgaatatatattgtttggattttgtttgtttctgcgttacggatactattagaagctgttcaattaagt
tagtagtatcaacttaccacctgtatatccgacttttcccatttacttttctggcaatgctcatttaataatacaacagggattttaccagtccatgaa
cttccccccaccaattgcttagctgtaggacgagaaggagtattgggctccgttttattttctactgtctctcgagttcctttaccggctggaccacct
ttttttttgtctttaacatttttatcattagaatcctttttatcaactataccatccgatggttttcccttttttcccttttggggccatgctccgtacagtttg
gaagaatgaccaaaaatgtatatacatttcgagggttgatgatagaatatgagttacttttcatgcttattccttgttacttaacttactaaataatt
atcaatttatagtcaatttaaactgtttgtaacagcttatgtccagttttcgacatgagcgaagccatcagttttcaatgtcttgttatgtttcaataa
aagtaaaaatagattcataaataatatcataattaaacaatagacggaatatcctagctacaccaaccatccatgaaactttttgctgaaactgc
tgaaatttcagcctttcagtcatcgtaacagcttgacatgtttttactctgttttggtatggattttcagtaatagtgttaagtattcataataataaaa
aaaagtacctagccctgttttaaattgcttgaattccttggcgtgtgtctttacaatttgtttttcgagcacttagtcattcatataattcaaaattatt
acatctaagtttgtgattgtaaaaagcggcgttcttctgcttatgtagttcgccattatttttttgcctctactagacttactctctacaacaaactac
cctatttctagtatataatttgagagcttcacagctaagctttccttcatttaatatcattctttatactttttaattggggtaacttgccttgtaaaaaa
ttttgggatcaattcttttgatatattgaatttttgaaaaatggctaggggaagacgtgcaaatcaacgcgataccgatttggacttagaaaacaat
caaggtgagattgaagctgtaaaaccagctcctaagccaaaaacgaagaagaacaaaaagtctgccaagaaggcagaagtttttctgatgaa
gaatctgactccaattccgatgttaagcaaattcaagaagaagaagaagatgtttctcctcccaagaaatcttctcgccaaaacaagaagccagt
gaatgcctttgctgcatttgctgatgagcaggaagatgaagaagaatctgaattagaagagtcagttgttgatgaagaccccaaaaagaaaaag
aagaatgacaaaaagacaggtcgcaagaatgctttcgacaatttaaacgaagacatggatgagatgtccttgaatgacgaggagaaagaaga
gtcttctacttccaagaaggaaaagaaaaaatcgaagaagtcggatgaacctaagaaagaccgcaaaacccgcaagaaagaagaaaagact
cgaaaaatgactgctcttatggagcctaaggaggaagacgagaccgccggtaaggggcctcttaatcagaaccaaaatttgaaggatggtttac
tttcaggacgcctcatttttgcctatgcttccgggcaaaaagtctccgctgatggttctgacccttcagccggtattaccgttaccggcaatttgctca
gccctcccaatagccgtgatttgcaagtagaaaaattatctgtatccgcttatggtaaacatcttattaaggattctgaacttagcttaatcaacggt
cgtcgatatggtttaattgctccaaacggaactggtaaatctactttacttcatgcaattgcttgtggacaaatccctgctcctcagtccttagacttt
tatttattggatcgtgaatacatgggtaattcacttacctgcgtggaagcggttttggacataaatgaacaggaacgtaagcaccttgaaaccgta
atggaggacctactcgatgatccagacaagaatgctgttgagttagatactatccaaactcgcttaactgaacttgaaactgataatagtgagcat
attgtttataagatccttaaaggtttgcaatttaccgatgaaatgatagccaagcgaaccgaagatttgagtggtggatggagaatgcgtattgct
ttggctcgtattttgtttattaaacctactcttatgatgcttgatgagcccactaatcatctggatcttgaagcagttgcctttttggaagaatacttga
ctcatgaaatggaaggtcatactttgttgattacctgccatacccaagatactcttaatgaggtttgtactgatattatacatcttcaccatcaaaga
cttgattactatgctggtaactacgatgtatttctcaaggtccgtggagaacgtgatgcgcaattatcaaagaaagctcgtcaacaagaaaaaga
catggctaaacttcaaaataaactaaacatgactggaagtgatcaacaaaagaaggccaaggctaaagtgaaggctatggacaaacgtcttga
aaaagataaacaatctggtaaggttctagacgaagaaatcattcaggaaaaacaactagttattcgctttcaggaatgtggtggtggtatcccgt
ctcctgctattaaattccaagatgtttcctttaactatcctgaaggccctactatcttctctaagttgaactttggtttagatctgaattctagagttgc
tttggtaggccctaatggtgctggtaagactactttgattaaattgatccttgagaaagttcaacctacacttggaagcgttgttcaccatcacggtc
ttcgattggctttattcaatcaacacatgggtgaccaattggacatgaggctatctgcagtagattggcttcgtactaagtttggtaacaaacctga
aggtgagatgagaagaatcattggtcgttatggccttactggtaaaagtcaagtcatccctatgagtcagctttctgacggacaacgtcgtcgtgt
gctgttcgctttccttggtatgactcaaccacacattttacttcttgatgaacctacaaacgctttggatattgataccatcgatgctttggctgatgc
actgaacaacttcgatggtggtgtagttttcattacccacgatttccgtcttattgatcaggtcgctgaagagatttgggttgttaaggatggtactg
tgaaagagtttgatggtgaaattcgtgattacaagatgatgcttaaagagcagattgctaaggatagagaaagcgaaaaacaagcggaattgg
ccaaacaaaaaagtaaggctttgagatgatggtttcagattcatttgataattctcataaagtttttgtttagtcggttggtttataatttaaaaata
aaaaacatctatttatcttgacatgttttaaaagatgataatacaatccgtagttattttgtattttacaaagttccttaccaagtaacctagtactgt
ttaaacaataaatcgatgaacttctaactcttaattaagttgtatatgaaactgaaaaagtaattaaaaaatcaaacagctacgtccttaaaaag
gttatatgaccccaatccctatcatatctcagcgatttacttgctactcaaaagcttctactgccaattcgcagaatgcatctcaaatctcgtaacttt
ctggcggcattgttcggtagtttattgttttcttttgtaaatgccaagctttcaattagaggtatttctgaaagtggtatgttcatcgttggccatgaat
tccatgcatactaattattgaagaattgcatctctataaaccaactgcaaacgggcaatggaaatgtttaggaagcgataaaacgattggttttga
acaaattaatggtaggaacatttgaaaaactcatgatgatgctaacacatatttgaagatgactactgtgattgtccagatggatctgatgaacct
ggtataatatattaatttgaaagctgtactaatgatttttactaagggacttctgcttgctcaaatggaaagtttttctgcaagaatgatggttacatt
agcaagtacattccttcaaattgggttgatgatacgaaatgcgactgctgcgacggttcagatgagtctttaattaagtgtgaaaatacttgcaag
aaagaagccgaagtctataaacagtctttaacgaaacataatcaacttgtaaatgatggcctaaaaattcgggatgactggatagcccaaagtg
ctgtaatgaagaaggaaacaaagagaaagtacgattcagtatcagtgaaaataacagaaatcgagaaattgaaaaaagaactcgaaaacac

agtgcaaacgttaaaacagtctccggaatcagatagtagattcgttctctcaactgatttagaagaaattaagaatgacttattcaatttgcttgat
gagagaaatgagtacaatgaacgtctgagtgctttggagaatcttttagatgaaatctctcttcagtatgaaacagataattttgatggaacattg
aaagaagccttggattcttgggaggatctcaaatctcaaaacataaaaagaaaggtttcctcagaaaaaattcacaatgacttggaatcatcttt
gacacatctttctcggattgctaaagaagaaggtaccaaactttgcgtttaaaatttatagaatgttttgagttattaacaaaatatagctacttatt
ttagcttctttaaaaaaattgagaaagacctaacggaaatttattggaatgtgaagcgctctttaattgatttcggaatactaagtcctagtattcct
gccacattattaaaaacggctgaaggaggtatggaagccttgttaatttttctgacagttgaactttaggttcttacttggagcgtattcattctgac
ttggatgcagcagaagatgaattggaaaatctgcataaagaacaggctactcttcttgaagatttgcaagagcaccataatggttgggataatct
gtatcggttcatgaaagggatgaagacagagcttaaagttggtgactatctgtacaaaatcccttttctttgaaaatgtttatcaggattctattaatt
tagggtatgagtgcttttttccatttttggacataattgctgataccttttaggagcttttcagagcaaagtggaaacacacttaaatatttgaacg
gtcaaagctgttggaacggtcctgatcgatctgccacagtcgtcgttgattgcggaactgaaaacgaaattgttagtgttttagaagctcaaaagt
gtgaatataccataaagatgaaaagtcctgcagcttgttcgccatctcaatttcagacatccttgttgaaggatgacaatgacgttcctgatgagct
ttagattgactacttttgatactatttataaaaatttattaaggtaactcttatgaatttactgtttttatcaatacctatttcgttgatgagcatagata
ataaaggatattgtattttcacttaatgcatacagatgccttacagaaattagacacctattcatgcagtgaattcagtgaaaatgaaaagggaaa
taagtatataaacttaaaaagactatgaacctattagtctatttatactttgttgagactttgtaacgagcgacattataatacatttacgagctccg
ttgtttcgacccatcaacgacttcaaggcatcaatcgattagaaagggcgcgcatacaagacgatatgtaaattaaccaacaacttaaaaatgta
attgaagtaaacataaacgcaagtgagaggatactgaacattaaaagacgatcatagagagtgaaagatgtccagcaacgagtataagagtta
gaatcaacgggcaaagtagggcaaagacttagaatatcaatacactaacgggatgagtaaaatacttggtgttcgatcacccgtacatttgctcg
gtgacttaacaactcctattagatgagagtaaactgagtaaaatggtgattgcggtatttttaacatgactttgggatagacgagcatgaagaag
aattactaaaccaataaatcaatgtttattatgattgccaacaacaggaatcaaaacaccgcaaagaacagcgactaaaatacagacgataag
cacgcaaacaccgagacagatccatttcttccttctagcggctctggcgctcttgatagcacgatccatatgttgtgtaccctcacccatattggttc
gtacgttgacggcgtcggtaactattttatcaacagcgggctcttgctcttggaccatggtagccatgtcttgaaacaattgagcaagttcagcaat
agtacgctcaatctttttgatgtcagcatggcgttcctgaacttctcgaagagcagttctggcttcaccagaacggttagagcgcaacaaagcctg
agcaaagacctgaccaccgttttcttcgctaatggcggtttggaagtcttcttcggtagctcttgggttcgcaatttctagttgacggcgcatgcgtt
gttcatattgtgcgcggtatgtcttctcaatctgcaagaagtgtcgaatttgatccataaacttctttttaacagcttccgtttgagttcttctagtagc
tgtgtcattatcaggaggcagctgcatgttttgtgattccaaagatcggattgccaattgaacactggtttgtaaacgacgggtatcggcagtatat
ccttccaactgacgagtagtagcagcaatgttcgattcatctacttcctgaagagattgctgatgcagcatttctattctgccaacattatcctcgat
ttgtcgaatggcatctcgtatatgatcaatttcctcaaagaattcccccatgctaagtggaatcatttccaccccaagggagtaatcagtctccttat
tggtattcattttcacaccaaataatactacttttggtgcttattagatgcccaaggtcctctagttgttggtgaaagtcaagtttgtttttaatacaga
aggaacaaagcaaatcagttgtcattgcttaaggcatagtaccgcggcgccttaaatcgaatatccgtgccaccaccactatttatttatcagttca
ctatgattcggtaaagtttttgggaagcataaggttatcgttaatagtttaagaacgtttctgttttggtattcttaataaacaattgttctataagtc
aataggtaattcaatttattcatttttacaaattccttcaatactctaccactaaactcctatattacctttgttaaaagatgtcaaataaaagaaat
gtaatgtcttcattgagaaacgatctaattgtgtttgtcatcaagttttcgagtaatatagtttcgataaagtatgaataaatgatttccttttgtttg
ttgtatttctatacagtatatacgcgcaaaagcgattttgctgttcttccctaattattctttatattagtgtacaagatgtagtaatctggatgaaca
actctgccagaccgaaacgttttcgattgcggagatgttggatgtggataaaactggaattgatgatagaaattgagagcttgctcgttcttacca
aagacggtcaaaaagacatacggaatctgtctttgctcagcgatgcgtatcaattgtttcatgagccatgacccacatcctcttcttctcatatcttt
tgtaatctgaagttcatacaagtaaaggcatggaagatcagcttcaactgtttcttcaaaactcataaagccgaccaatttacgccgttcagtact
atataaacaaatataacaaagagaatccatgctcatctctctccatttagcttcatcatcccaactaaggacactgttttcgtaaaggttttgcatat
tttcctgaaccaaagcaaaacattccttcaatgtatatttatcccgaaaagaaataatttccatacataatttattcatttctaaatatgcctcatcat
taatttcccattttagctttattttgtcggtcatttcctaatgggttcagagtacaatgtaggtatggtcagtgtctgctctttgtccctctcagatatca
ttccaatatccttgattacaaaaatcttttagtgtaacatatccttcaaagttgcttttcgagccataatcaattcacagggttgttgcacgtcagttg
tagctcaagaagtaaggaggcccaaactaacaaaagatttaatattctctatttcaatgcttcaataacggtgtaagtgaatccaacccgtaatat
tctatcaacctcccactttataattctatcgtacaatccatgaaaagcaatacattgaaaaaccattattcataacattattctaatagcttaatgct
aattttggaatctatcttccagaaaacctttcttcttcactgtcataaggtgaacggcttctctgtctcctggaatagtaaccagaatctcgtctttca
gaaggtattgatcttccaaattcatcgaaatcttcatgccttctatgactagtccttggacgatgagaagaacggtagctccgtgaactgtgacgat
ctgaatacgggcgctcatagggatcattctcatacctgctgttacgatatcgacgtggtgacctttctctctcataagatgatccttcctgttctcttc
gcgtaaattttcgaggttctcgtctcggccgggttcctggcctttccctaacatcacgatatcgaccttctctggaagtatgctcttcagctggatgca
atgagcgatcaatctctttttagcctcggattccgaaacgtctctttggctctagttgtattagcaattattgatcgttccaatgaacttacccgaag
tttagagagttcttctttcttctcctgaatgaatttttcaggtattccatagggattttgggaagctgataatatcaacatccacagttcttccgtaaat

tcagacgccttgctctccagaaaccctgtaagattgagttgaactttttaggatctagagctacttcattatgcgaatctgtgcttttcgatgcttcg
acagcctcctctagcatgctatacacaaaatcaatcactacctcatcttcaaatcctataagctcgattaaccgattagctatccaaggctttagta
cctcaatattcactttttcatatccacctttcatatgaagttagcttttatatcttaaccttcgatggctcttctaggtttaccatcttcaaccaaaccg
gcgcactcatacctttgcttcaaaactaggtggaaatttcgtgtcccgcatcaatttcctgtcagcactagtatatagcgtttcttgctcagcagcaa
ccccctaaaaacagttagtatatcaactgcaagttcataacttccatgccttataaaagccactcattttgcaacgatagtgtatttctatgtatact
ttcttttacagaatagacttaagatgtttttaatgatgttttaataaacgaaaactgctgaaactcaaatggttaaaataatcgagtttgaaaatgg
cacacggtactttaagacacaaacactattttgttcaattaatgttccgaaggaggacacgccttcattaagcgaatacagtattcacaaactttta
tattacgagttaaacttaatagacttaatacttacacatgtgaaatcgggtttcttactaaataaacattttcgcttcaggtaccgtattataccacc
ggaagtgtcaaatccaattttaagttctacaaagttatgatgactatttaaattatttcttcttagagacaccaacagtcttaccacgacgaccagt
agtcttagtgtgttgaccacggacacgaaggtcaagagcgtgacgaagaccacggtgagtttggatcttcttaagacgttcaaggtcttcacgga
gcttactatcaacgttgttggaaagcaattggtgagacttgccatcgacgacatccttttggcggttaaggaaccaagtagggattttgaattgagt
agggttttgaacgatggtaacaagacgctccaactcttcagtggtaagctcaccagcgcgcttagacatgtctacatcagccttcttgcagacgat
gttggcgtaacgacgaccgacacccttaatttgggtcatggcgtacatgaccttatgcttgccatcaacattggtgttgagaagacggagaatgtg
ttgaaactatacaaattagtataaccctttcgttttatttcattttacttacgttctcgggtggaacaagagacatgttgagataccggctaatgcgct
gtgggtaaagttttgcacagaaggatgtttgtgttgtcaagttgtgactgttctttccgctaagaatagtagagctctaacgttgattttaacgatat
ataacgaaccggatatatgatataaaagagaaaaagtaattgatatacgattgaagtatcgcattcatcaactgtgtgtgtttttcagggctaatc
aatattatccggtttttacatttcttattttctaaatactttaatagcactcatcagatgcacaagggatgtgagtctttaaaaattagctgccttattt
gacctcaaaaaatccaaaaatttctatataaaattgcatttaaacttcatttggttaagatttatttacagaactttttaaggattatataacaaaa
ttattaagttagtctaacttaaaggaagcagatacaactccaatacaaagagcatccgtccagtccggcaagtagtcatcatttaaacttgagcac
atgcgcatactaggacgattttggccttccacaaatttagaaggatagtgccctatagcatcgagacctacaaagtcaaaaaatattggctcatcg
atataactcatattgaaagaatatgaattgatgccataggcacttacttcacgaatatgatgtctgcgcacatgtgcaggtacctccggcaaagac
caagatatcacatttgcgtcaaaagatatgactgtccagatcaatccaggatagtcaacagttaccgtaacattagcaattccattttctatcactt
tatcagagaaagacaaacggggtttgtactcaaagttagatttttcattaagagggatgcgatacgatccaaggaattgactgaaaggatatact
gtatcccaatcagaattccaatcatctatttctgtttgaacaacaggttcctcggagactttactagccacatcatatacaagtttctcaaatcctgg
ggcagaatcagcggtggcaacatgcaaagaaaactcatttgatgtgatattctccatatgttgaatgaaaactcgtcgaggatgtaacgcgtcat
agaaagtttcttgtcctcctaaaacgtaagagctaattccagctattagtatggacaacagaaaaccaagctttaggaaacggggagaacgcag
acgttgaatccaagctgaaaagagagggaagctaatatagacagagcaaacgcaaatacaactaataatatgatcagcaactttttcaattcca
atacgaccagtcagaggtacaaagatatcgagcattgtagtactggcttcaaggccagcagttaaagaaccaattgatccgattacgtagctcaa
cagatgaactgttttctgtttcgccaaaacccagtcattaattaataaagcgagtatcatgaaagagtctataatagtaaaaaggtaagcagagt
gcaaaggtactaaagttatcaaagaatatagtaagaccgtggctttaaaagtttgatactctgatttttttatttatccaagaggaaaagataatgg
aagcaataataacaggcaagtaaataaataagggccaactctcattacggaaccaactcattcctctgtctaagacattgccgaatacgaacgc
ccaaatgttagggaaaagaaaagcgcaaaacaatgtgaaaagctgaaacaagaagggtacaattatggactcagaacctttaattgagagat
aaattcccaacccaccaacagcagtgttcaagatcctggcagtgacatctgaatacatgaagaaaaatcggttcaagattgaaaaataaacggt
gttagattcacccatatttttcatgtcagcctcaggagacgttaagtaacgaagaatagccataatattctcgccaaattgctgggcagtaccggg
agtgatatacggggcgaggtctttcttcgtatgatacagataactgttttggacaacggccatatccagtccagtaaggtttccatattgaacgaat
tggcgaaagtccgtatcacttatgataatgcctgtacggaaaacatcatcagctaaaaccgtgccaaaaggacgaggtacatgagagtatgcttt
aatcatttcattacttgtagcttgaaacaagatttcagcaccagtggttccacaagcttcgagatttacgacgcattttacggtatcctttaagctac
tttgagtgataaacagatgagaagcatcctgcaaggattcttcagcatcattgaagagaaaaattattgaatgggttaaaggaataggttgagag
gaaagaacacgcaaattttctagaagaatacccacggctagagcatcatcggtagcgccgggactaggcagagtagaatcaacgtgagcattg
atcaaaatagcttcctctttgcaagcatcagttccattactgagacgaacagcgatattcttcaaaccgtgatactttttaattacatatttattcata
aagtcgaaaagatgagcaccatcgccagattcgtaaaaaacctcaatttcatgcgtattcgaataaggactattctgtaagttcttttgggtggct
aagacctcgtccataatgtattgacgagcgcggtcttgttcaattgtaccaaggattcgatagcctatgttttcggaaagctccttgacatgtctcat
aaccgccctttcagaaaattgtgaggtaccatcaggtttgtaaagactgtcgactggggtaagcaaggaattatggaagtacgacacgacctgca
ccatgagtataacaactatagacaggaagagtgttgaaattccatttttctttaaagattgactacaacgggatgacgatgtcataataagtgagg
gaaatagtaagcgcaagagaattttcaaataaaaagtaagaaatcaaagtagtaagtcagtgaatcagtggttatagaagaaaagaaaaaaa
aaatgctctataaaataaagaagcgttccaaagaaccttggtatagtttcagctgcaatataagccttcgggtttttaattcaagtatttagcgaaa
tgacaaaattcgaaaagaaagattagagaatttagaataaaagatcaacaaatatagtatttgaaactaagctgtgtactgttatcgcagaaaa
gatcaaacgggaagtgtcagaaactgacgaatcaatgccaaatttgctttttaacaaaaaaacaaacaaaagagcagaagaaaaccaatgg

aagatcagaggcttcttaaatcctcgaacatgcggttgtcgtgttgttttcaagtataggaagttatgagtggtttcggatcagacatgagatatga
cgagtatggccagaggtgagaacaagtagcaaatttggctaaagtcttaaacgtttaagtttaagtaaccgcaaaaaaaagaagagcatggttt
tgaaatttttctttccctggtgggaattcgtagagtttatcctcagtttagtaacttggtatggattacgctttgaagaaaagacagaaggaccagg
gtttgtatttgaaattgactttatataggaacaaaaaaacaacccactgtttttatattcaaggttacagtagaagagtgatcagatagatgaaga
gtcagaacgttactgttacgtctttgtcatttgtagtggaacgacataaaaataagaactagggaattgaaaattgggattcgatacttacaataa
cctgagacatttgtaaacctccgatgccttgctttgccgaacgatgaagaaaaaaggagcacatcatgatcgataaagtagtagatagaaattaa
aattttttatcatttaaaaatcttttaggattgctttattcattgtactatcatcaaatttttgctttccctctaaccgtaagtttatttactaacgtgcg
tttcgctattttacccaataaagtatagtaaaccctagtacgtagaacgataagtttgctttacttttgatgcaaaaaagaaaatgcgctcctttcct
tcacctttgctttccacctaaaggaaaagacaaaccaacatgtcagccattggatccttggtttttgctctgaatgcggaaatcttttagaaagta
ctacggcacaatggaccacatgtgatcaatgtcaatctgtttatccatcttcgcaatttgcaaatttggctgttgaaacgaaaagtggacccaatgc
ttttccttctgctctgaaattaaaacatagtattgtccaagtggaaacacaaaaggaggaagctgcgacggtatggagttttatgttaattcatctt
gattgacttaaaggcacaacgaacatttgtcaagtcttcagtggaattaatgatttctttattttagttcttaattattctaatactaatttcgttctag
attgacgaaaagtgtcctaagtgtggtaacgatcagatgactttccataccttgcaattacgaagtgccgatgaaggttccactgtcttttatgaat
gtcctaggtgtggttacaaattcagcacgaataattaaaagaaaaagcaggtgtcttcaatttgggtctcatgtccggttctttttcctctctccttac
gaaagtgaaaaataaaaaagcgaggaaataggtatatttcttcattttgggttatttttggtttggttcatcggttaagcttgtaatttgtctttcttct
tttttttttggattaggtttctcttctccatatttaataccctggagacgaccattacccttgaacagacggtacattaactatagttttcttgaactata
taactatatcgcatactatctagaaagcttttccatcgtttacagtcgttcacttctttcctttagtggtcctagacatgcatttcataaaaaaacata
catagaataaaaggaatgataaatggctttatgtatctgggatgtctgattacgttatttttatactacacctagacgatttcgtttttgtagtatcgt
atgttatgtattgtcaaaatgtattttataaaacttgattctctgaaaacttttatttatggaaaaggaatgtgtcataaatgaaagcgactacttta
atttggcatattgtttattcatactatgtatggataaatttcattatattataatcgatgtaagctaggggtatcttttagatctaatgaaacatagca
attcgatggtcagaaaaagaccatttcttttatgtttgtttggtacataaacaaataaaattgaattcactttcaacatccctgataataaaatcctc
gtatgaattcatctgcctagtgaacaatagaaaaagtgaaatataatagggtgtcgcaaatggttcaataccatttgcttgcttttaaaaagaaaa
tcaaagcagcttgactatcgaattcttttatttttgtttattatttatcacttatttacagtacgaaaggatttgggtatcataaatagaaaaccatttg
ttaacatatatagaatgaaccaattgcttaaattctataaagataaaaagcgaggtcactaggatacgctatcctgacaaggaaggagccaagc
atgagttgagaggaaattgacgatctgtattcaataaagtaaaaatggcttctgtaaagtcatttaaaaggaaatatttgaaactgagaaaatctt
ttgatgttgtccttcaagaaaccgatgaaattgaacagaaaatagcagacatctctcaagtttatcgacggattttgatggaaaatacgtatgtttt
ttatgctatttcgagaatgaaagaaatagtgaataagatagtctgaataattcctctactaacgctgcttgtagtcatttggtcgatttatttttggat
atgaatgaaacaactttgcccacacctcctgctagtcctcctggatctccatatcggcaagcaacgactgttcctcatgttgattatttagaagaca
catcagttgcttatagtttagaaaagcctattgccagtacccacaagtggttatctggtctaacagcccactccaagtcaaataatccgctcttgtc
agctggaagctccaattctttctctaaacccagcttaaatttacctaaccaatcttctcctgcatcaacctcttcgcccattaatcctagctctttatg
gaaacaaaagcggaaaagagctacagactcgccctcagagcgtcgttctaggaaaagatcatcttatgaagcttaaggttgatatgacgactttc
tgcaatcattttcttttatattgcatacatttatctgttcgacatttctttccgcatactattatttatgattagaacacgggtgtgtaggcataagttta
tatgtattttcagaagaattagtcaagagactgcgtacttttatccaccctctctatgtgtcgtttttttattatggtaataaaaggagtataagtccat
ggggagcttcggtcgaaatcttcgtcgccgtacattatcattatcattatcattattactcttattcttatatctcttttctctgcagtaaaaactttgga
ctttctaacctttcatcttcattttacgtacagaaacctatttaacatcgtctccatccattcctcctccaaagggagaataccaaaatctcttttaaat
ggacagtttcagacagttcggatgaaattgggatcattattatgtacaggatacattatttggctacgggtgctttaaatacatttcagtctacttgc
tccatccttgctagtcaagaatcaaaatcaataaaaaattaaacacctttctcattgaacaagcttttcgtcgacaaaaggttaacgagataattt
aaatgaagccaattagtagtaaaaaaggaaagcaaattcataactgaaaaaaaggaaaaaaagaccagtatggataactttaatgctgaatta
cgcacttgcaaatacgttcaggggtcggacttattcggaagccttttcagcagcaagcttcttatagaattcctcacgctgcttaaatgcaggcttgt
ggtaaccataccaccaggccaagccaccaacggtacctaaaccaagaatcgtagagaaatcaatgattacttgtctgcatcaaacgttagaattc
ccgattttaccaacaagttcgtgctttttttgttctaaacaagatcaaaacataccttttgaaaagtccagtaataggtccaacagccatagtgaaaa
aaaaaacacaaaaactaagcttgaaattgaattgaaagagaagggggaagttggtaagtttccttttagctggtatacgatttgtctccgccagtt
ggagacatagtgaactcgaaggatctccgtatacccaatggaaaacaaaacaaatacgcaacaggtattgcttaatccttgaaaattttgtcttg
gtatcctatttatatcggtttttcaagaaactgtttcaccatatgctttctaattcttcccttccttgagaactctctagctcttttgattcttacggctac
ataatctcaaaatcaaaagaatgacttgtatagaataaactagcaaaaatttcccgcgatggaatgattcaactcaactttagtatgtttgtttact
acattaagctgctgtgcaacttgacataacacggtggcgcacggtaaaaagtttaaaaagttatgctcaaggttgctatttgcaaaaactgaattg
agagacccattcaacgcgaattcaatatatatgaacctgtttctttttcattcttgtgaagaagaatcactttatctttttctcttttaatttatttatttt
gtcgatatatgagattgtaacggaaagccaaatcagtttaagacgcgtacctaggaaagcgacatacatcaaagtacgagacagcaaagtgag

cttcctcatcaagtattctttctgttaagggatagagtgcttcggtctaaaacgagactcgtgttaagtatctgcattcatcgttgttacattttgacgt
attaaacgtatctctttatcttttagttatacaacatggaagctattctatacagtattctacagcagaatgggtttgacggaataacgaaagctgct
gaacgaatgtttttggatgcaatcgataaacgtatgagaaaaatcacaattcattaacatagctaactggttcttcagatcttaagactttatttca
cgaattagcaatccatacacaaatcggaaaacataatgaacccactactgaagacgttgcacattggttcaacttgagcaatatacctgtgtcag
gtcttcgaaaagagttacaaacagtatacaagcctttatctaaggacttcatcaaaaatttggaagaaagaattcacaagtcggctgatataccg
gatcgatttaaatcatctttggattctagcatgatatctcacttgctgggttctttggctgtttctcaaaaccgtcctaattatgtagcaagtcatttgc
cgccttttcctgcttctcatacttatttagctagccccgtgtacccagtgagacccacgtctcctagacggatacgggagcttgctacgaaagaatc
ccgtttggctgaaaatgctttacgaaagatcttagatgccaatcaacctcaggttatgaattcacctcgacaagccgcttttacgaaggcatgcca
agatttgcagttggatagcaaatcatttcacattgtaaattgggaatcacagaaatggaatgctccaagataaatgctcctaatcaacctctagtt
ctaattatccattcaatgatcgcacttcctctagatgaccgcgaatcctgtgttttatgttagaattttccaaagaagaggactcggatttacaagtt
gattttctttaacatactttcgaaaaaaccgatgtaccgttaagcaaaccaccttgttgctatcattttctactttctcgcacatgtactgtttcatgca
tttgtatttgaagctactgggttgttcctcttgtgattcgccatgttttgtctcaagagaagacaccattccattgttggataaatttgccatgaagctt
atcctaaaacattaactttatcgttagcatatttttttgtaatgaatgtttcttttatgttccatatacgagttgctgtccgcagctgctagcatacgaga
actgattaacggtttcttgcttttcatacatcaatcaaacgaagttggtagcgctaggtaaggttttctgagaacgctaagtgccttgctcgagaatt
attacctgaatgtatataaagtatgatatatgcttaaatactttaagataatgtttgctatttactctgccgttcgttcactcttagaatactgtctgtc
gtctatgacattactgctgtgctgcactgtcttcgagttcggtattctgcagaagattcgcgtcgaaacatgtaaatacaacagaatgtgaaaaca
aatacaatattcatggaaaatcaacagtcgacacaacctgccaggtaagaagcttaggggctacacattctaattgaagtgctagtaaagaaca
acatcaagaaaccagcaatgagaatagtatgcaaagaacaccaaaaacagagaaggtaggattctcagtattatcgattgggtctattttagaa
aacccccaaagcccgaaaagtcccgagacgattacttctcatcaaagtgaaattgagtctgctcatacagaagcagtgccaatggcaacttcaa
caccgacgtcgtcagagaaacgaccaataaattcccctacgtccagtagtcattcggaaaaaaagacaaaagtagaaggagagcaaggaaag
cctgaagacgaaaagaaaagcttacagcaattcagtggtactggagaaccaacggcagcttttacctctcaccaaggtgcaccgacaggttcac
cagtaataccaggttccacgccgtggccctattcgagctcagcgtatggagcgcctcagagttacgatggcgtcagtaaattatcttatgcgtattc
ccagattcctgttcgtcaatttgtatcccctccaattgatatcgacaattctgccctcgatactttttttagagacatgcttatcgtctggaaatgaacc
tgttgctcagttcgtctatactccatggtttgagttgccgttactgtacaatgcaattggcaaatttatccgaatagaaattgatgctaaatggttga
atgctgcaatcaatccgcgtcttagtaaacgagaaatatgggggacagatgtatatacagatgactcggatatagttaccattttggcacattgcg
gttatttttctctcttcaaacccctaacgaagattgctgttgtagacttatttattctgccacccttaataggttacaaaggaaccaggaaaaaccaa
attgaaagcagaagctggacttctcgacaagatggtgtgagtttaaaagtaaaaaaccacgaatggaaatctcccgaacctacggtttttgaaac
tgctatgcataatatgacattagaacaacgattacaagcacgacttgagctatcacgctcctctgttttcaagatctaatacaagaatgtactgatt
accttgaaaaaaagttcatgtattttagaaagaaacaacgtgcaagatgactaaaagcaagtatagcaaatacatggtatataggaacattgtt
acaagctctgttatacttgaaagcatgataaaagtctgacttgatttcaaaacccgatttattaataagaaacttcaacttttattcattctatttttt
gtgaatctttctatataatctctaaacgttcgatattaccattcgttgtttgtgccatatttggtaaaaggaaagtttaaacagttagagatttaacag
ttcagcttcatttaacggaaacttatgggaaaaacaaacaaattgatgaacaatgtaaacccaacagataaaacgtaaaagcagctgatttccc
cgacccgctattactccacgtgatactttcaagcgtaaatctggtgtaagcactacggagaagtaaatttgccaaaaactataaattggttgtttcc
cctcttcttgaagtaaggttgtatccgttgctcttttgtttttctattcccgacttatttgttttcacgaaaccctaccctaacttcgtggtggacgacttc
agatcgccggggcaaacttgttttcttttaccaatttattggactgtgaggaaagggaaagagcaagttttacttgtgtctgtactgtgcgatttcga
ttcgttgcattttggggaccattttggtttctagtttggcgataaacggctcaaaagtttcaaagctgatttatttggaaaagtgtttgtgttcgccgc
aaacgtgatttcaccagcctagtttctactttttatagctagaagggatcagtgaaatattcggaccgaacaggagacactgaattaactctcag
catttaaccgctcgcttttgagtaatcgtacttttctctggatattaaactgtgaacgtaataaagaagccatcatgttttctttataacgtatctcgct
ttctttttcttataatttccttataacccgatttcttgtattcgctctgcgtgtgtgtgtgtgtatctccttttctagtccgtaaaaatcgcagtttttttgtc
tacgtgcctgtccttgaccggtcgtgtttattcatttgtcaaaaaaaggataatcgattctttatcctgaaaaataactctatagttgctaactcgctc
agtctcagctgcttttaattgttcattggaatcccttaacgcaaagttcaattttgggttttattttttttactttataataataaagtataaaaatcaag
tacttcagtcttgcatattaatttttctttttcgttgacttactactctatctgtacctttcttgcatacttttcgcgatgacagggttactaagcatcttat
tgcattgcaaaccttctaactttgccgaatctaaagtttctacatcagaacatcactcagtacaattttgtatagagcgttctcatgagcaatcttct
gaaagctctccatatgaaggacgcgttaaattcgccgtcttaccaagtcgagttggtgaaggaaaacctaggaagccttgccgatatgggaaatc
tccacctcctgatattagtaaaaatcagctgcttaaccgtcctcaagaatctaacgtttgcgatggtaaatcaaaaggtgaaaattccaacttacta
cgtaaacccttaggacatttgaacagagaacaacagtatgctaagcagccccctaaaattagttttgctttaggaaatttaacaaatggaagcaa
tcaaaatacgtcgacgtcccatgctgccgcttccaagcaaattgcaactacttgtcgcgatgtattggaagatgtgttgaaaaaggataatgactt
aaagttagagtcttctgatgaagaagaggatgacagcgagtttgcttatcctgtgaaatttcaaaaagatgctagcaatataggtaaaggtaaaa

tgtatcgacctattagatctcctaaaccgcccgcaaaaaagctaaacgtttcttctttcgaagaagatgatgatgacgatgaatcgtccgatgaag
aggctaatagcgatagtaacgatgaaaatgacggggcagtttctgattcttctttatccgatgatatcgaagagggccgatcacctttacagtttcc
ttcaaccccgatacagactgctgctcctccatctgaaaatttaatcccagatgatactgattttgtgccgggtacttttgatgaggatcaacccgcct
gtctagcatttgcttcctccttagctaaccactcccctaagcattttctaatggttcctcaggacattgatcctacctttccagattctgattccgacga
tgaatctgtggaagagaaatcaaccagagcacaacgatcatctgctaaggctaaatggaatcctaagcacagcagtaacaatttagaaaagaa
atcttcatcaggaatgtcaaaaaatagcagatctccccctgttaatataaatcgaaggccatcgatggcttcatgagttgcgacggctctacctatt
gctgtatattaataaaaagaaaactggaaaatgcgtctacaacattgtatatgtgttcgatgtaaatttctgtttcattccgttatcgaatcactcgt
tatgttctaatcatcactgttgttaatagagtctggttattatttttgtataattgttgttattgtaagtcaataagaaaactatagctagtgtaagga
aagatgtagtttgctatcttcgattaattccgttagagatctcaatggcctctaattattaattaagggaagctacctccagattccttttaatttgtt
cataaatgaagctgcgcggtcactgttaatgcgagtactgtacgacaatttcaatacgatctcttcatttttcaactgtgaagggctaggcaatgtg
tgttcccgaacagatcccaagagacaatcataagcatcatcaagcgttgtctttccgtttaatggaggatttggatgagcgaatgataacactgca
ctgccagataactgaccgggtaaactaaaacgattagcagtctttctacaaacattaatgttcacgtacattatagaagaatggcaaagaaaatc
gacgacattttcaactggtccttttccagaaataagctgagttttcggcaaaacacttgctcttgacgaagacaatcctaataaagcatcataggc
attctctacaggatcttgaaataagtctagaatttcggatgaagccaaagaatcagaaacggctgacgatacgatatttgttatcttttctggaagt
ttttctaaaaaggtgagaagtctcgaactaaatcattgtataacttaccattcttttctttcgttgcattagtcatattgattaaattgaccaaagaaa
gactagtctcgacgacattgagacgaggtttttcaggaatagcctctgatggtttctcagtagttgtaggagaagctacaagctgacctttcacag
gtttagcctcaactttagaaaaatcaagcttttcaagcttttgtatagctttctgtacggagcctggtgcatcctttttgatttcaccaataaaagata
aaacatcacctttaagtaatcgaccacgaggacccgtagcagggataactgaaggatcctggatcttgtgtaaatgcaacaagtacgcaactga
agggagcaaaggggtgctttgggaggaagatgaatcagcatgactagatgtttgcaaagaactgggctccgttggtgtcttcaagttttcggactt
ttcagtagtactcgaagcatcaggaagattaattttagataaatcatctccttcttcagcgataatggctatttctttacctacgggaattttggtacc
ggaattaataataatttttgctaagacaccatcgtcttgtgcctcaacgtccattgtagccttgtctatacgtaaaaatgagtattagccattgtcaa
gaaactatacataccagtttctacctctaatatcacatcaccagccttatacgaatcccctaatcattgtaagtattaactcatcttatgtttcacttg
tatcccataccttctttcagactccattttgcaatatttccttcttccattgtaggtgacaatgcaggcatgcataagttgctaatagcatttgagaaa
gctgtattatggaaaactatagttgagtaagtaagcgaattcattgtcccagtcattctcgatcatacattttccatgtctacactgccacaagacct
gccttttaggcaattgaaaaacacttcgtcttagcattatattgctttcaaaatcaggtttaattcacgtaacaagcgacccctttaaattttgttaag
gctgaggcttgctagttgcaagttgggttgatcaagtcaagtcaagaattttaggtgaagaccatggtcagttagtagtttctttgaaatatcggct
aatgtaaattttaattaagcgaccacttatttcgttaaaaacaaaactctggcaatgtttgaataatggtttatgtttctgaatatgatttttcttgca
agaaaacggcaaaacttttaaaaatgaaatggacaagccagaactcgaatctgggacctcttccatgctaaggaagcgtgataaccatctacac
cacatgcccaataattaagcatattgtttagtatgttaccttactccctgttatttatatttttgagtttttggcatgaaatttgagttagcattcagctt
taaagcatattttgtaaaatactataatgtacgcatatgcttatttaacaatatgtataccatttttcgcttcatatggggatttatttttagatattct
acatttattacctaattagtttttgggtttgcataagtttggtaattaggaatattagtactaaaccgaagataaatcgcctataaacataagattta
acggataatcgaatttctgtctcatatatggaattttatacagtggtaagggcgggttcgcaatagtgagatagaagagtatgaattaatgacagc
agactttgcatattgtcacaataccagttgaattacattgaattttattttctacaagttcatgaaactttttgaaaggttcacagtaaaccaaaatt
catgttacttaaatagtttctaaagtaaatggtataacttccgttctttttggtaatgcatcgattgtacagacttagcaatcagttacattatggatt
caactgtacgagagtgataccctcatgtcggaagattacatctctagcaatcgagacatcttgtgatgatacttctgtggccgtgtaagcaactatt
ctgtgagcccaggtgaaattgccgctaacggaaacagtgtccagaaaacttcgagcgagtccttaccaaacattgtttgtctcaatactcatcgaa
cactttcaaaatatgaaatgtatgggggaatacatccatcaattgttatacaagaacatcaaaagaacctggcaagagttgtttacgaaactata
aaacagtctcaatcgttaggtgtttctaactatgatcttataactgtaacccgtggtcctggaatgctaggtcctttagctattggcttgaattttgcc
aagggtttagctgttggccttaataagccccttttagcgatacatcacatggtatgatttaattaaacgcttctcaagaagatgctaattcattatta
tcattagcaagcacatgcgttaacagtgcagttggagtctcaaatcccatttccgtttctttcaattctagtatctgggggccatactatattaatttt
atccagatcggttgttgctcatgaaatcttggcatcgacaactgacatagctgttggtgattacctggataaatgtgccaaatatttagaaattcctt
ggaataatcaaatgccagctgcagctcttgaaaaatttgctgctcccactctcgatgatttgaatgtagattgtaatccaccgaaccccatgagca
ctggtgaaaaggcacataatcctaatttttctttctcgggattagaatcttatgctcgaagacttattacctcaaagtcatggagtttttcagaaaaa
aagatcttggcttataagcttcaggaagctgcctttaagcatatttgccagaagattactaaagccattgagtctactgatctatcggatgtgcgttt
tctagtttgtagtggaggcgttgctagaaactccgttctgaagaaaatgctggaagaattgcttatttcacttcatagtagaggcctaattcctgaa
acaaaacttgtttacccttctttggaactttgtagtgacaatgctgctatggttgcttatacgggaatacgaatgtttgaagaaggattcaaatcctc
gcttgaagtagaacccatcagaaaatggcctataaattcaatattaaatgtaaacggctggatacatcgataatgcaaattatattattaataaca
aagcctaaatactgtagggttcaaactatgccataagataacaataaatcgaagtgcagaaaaacatcctttttatcaataatggcatatcttttc

gtaaacgcacatttattatggtgcgttttctttcgtaagcattcctttgactagtttctttataaaaaaacctgagtattggtagtagcatagtgtttgca
gcaatatactgaaattgctctagagatatccgaaactccttttgattttgtctatgtttattaaagcaagagcgcaaaaaaatcacttattactgttag
cctttcatcatcttcatgattcccttcataacagcaaataagaattaaggattctgataattcaacaatttgatccgtctgtagttcctttccttcattt
agatcatataattgaaagctgaatacttctggagcctcttcggctaacatattcattgtttcaataccaataacaagcttttcaaagtttaacggaa
cgctaagatccaaacttttattcaaccagttatataatcttttaaatagctttcgttcacttctttgataatggatggcctttttgaagaaaatttgccg
tggaatcgactttttataagcttttcaaagccattgtaatcaattttctgttccaatatgcgaccatgacatagatgatcttcaccgataccatcgat
aaaagcatcgtaaagatactccaagtctgtgtttgataaattcgtttgttgatatagaccacgaagatgcgagcgtttagtaaaaagttttaaaga
agtgagaaccgtttctcgaaattctgttctttttgctaatatataactattggtaataaagtcaaaatattcaaaagcaatggcctgaagctgtgaa
aatgtacatgagattggtttgctattagtacttccaatttctgtcgttaaatattcggagttaatattagcaaagcaatcttgaaaaactttcataatt
gatgcatcatcagcggcatttagaattccctgagcattaatcttaaacagagcaagagctgtttgaaataaaacgcaaggtccgttcgttatgaaa
taatcgaggattcgcattgcataatccaatcgtaaatccttgataaaaagtgaaaaaaaccagtttattgtaataagtttcaagtccatcttcattt
cagatatttttgataaagatgaggaagagcttgttgtaagacgtactcaaagacagtcagatcaagtaaaagaccgtgaatcaatttaccatag
tatccaggaatgtacagctcagagagctgagagaacaagaaataagcctgttcttcggtacagtaaaccagtaaggctgcaacaacgatattta
tagcctgacagtagcccatgtcactgtttactccagaataaaatctcaatactttcctcagagcatctatccctgtacttgattgataagcaggata
agacggtaagcttcttgataaatctctctcaatctcatcgctatactcacaagtttctgcaagtactttatgtacattattgtaataaaaacccttcga
tttccaccgccaatataagcttccagaacatgtttcccatatgtccgccctcaaattgtccggaataccgttttgaattagcttaatgaaggaagga
gtttgtacaaaattaaaattggctccgttttcaagaaaatactttttccactgatctatttcttccttttctgccctttctgaattaaattctgggtctatt
agtttagagggaaagccaaattgcattcccaagcctgtagaattatatccatgatctgaagttgcttgattctcattattattttttatggataaccct
gaatgcagaagcttttccgatgccaaagcatgtaagaattctgtaacatcaccagcctcatgcgaagtcaatcgtaattgtcgtattaagcgatca
caaaagagttcggattgttgacgtgtaccgtttatagtaagatgaatagtttgattgtgaacggttattaaatcaaaaacaaacacgttggtgtctg
aagctgttctttctacttgtcttatagtcgacagaggaaacgtaaatcgagtctttaaatccaaactatggaagcaaaggaaagaggtacttatgt
agatgcttccaaaaattagcaaatcattatatttaatttcacaagacgacccaatgataatttcttcttgcttcgtcaaacgaaagaaaacagttgg
tggtagtattccttcataagcttcgtagttattttcaggaaaaacatgcttcaataaattcattgattaatcattctggaccttttaccaataaaaggc
tccagaaacttttctcttaatttaaagctgtttgttgttgtcaaaagtcggtatgaatgaagtcttgttgccgggtaacaacatcacacttgtgccgtt
aggttcaacagtcaatgcaaaatactagctctgtattcccaatatacagatcaattaccaattcatagctattctatttatttagttaatgcgttgtta
ttgaggaagtaagaatacagtgttaataaaaaaaagcattataattcaataactcatagaacattatcatcgtaactcatttctagccagttcatg
gcaaaatgaagtctttttgaacattaataagagtgtttggtaaacatttgtgttgacaaagcaaatcattaagagcaatcgtatgttgcaaaaatt
gctccaattggctttcaggttgataaccgctgaaccctttatatagttagtaagcaaaatacagtaaaaaagacaaaacgtacttagaagcaatt
cgttgatacgactggctatatacttctggaagtcattttccaaaaagaaagataaaggactcgaagatggattatagtaagcgagaagtcccata
cttaacttgatcattttttttagatcatgactttcagctttttcatactcatcaaaaaaattgttgggaataactcatgactgggtccaaaattttcatct
cttcttgttctgagattacgccggagaaagacgctgagtacgagcgaatttgttgcatatttcgcattaattgaagaaattgcaaaaatcggagag
aaagggataggtcttggcaatcttgaattgcttggggatattgtaaagtaatttcctgtaccgcagaggccaagtccccattacacaacaattgat
tgatccctaaaaaggttagtaaggatgtgtaaagggcataaacatactttttcggatttgtacatctgagtgcagtacctttttcgggacagaactct
tgtgccgtttctacatagccatgattaatgaggaaggaagagataaggtctttaaggaaattttttcgttcattttgctcatcaactgaagctatatt
cagagaatcgctcaacaatttttctttctcttcttgaacggcgtggtctatctcataaagaaatgctgtttgaccaaaatttgccctgatgtgttcacc
ttggctttttagtcctatcgtcgggtacaaagaatcgctcacgttcgtgaaggctacccctaaatatgctccatttttttgtgtagaatattgttttgttg
ataaggttcacaccacatcccacaacatcaccagtttgaaattgaggtccatatcgttctccggttttcgatccatggtaaatttcaccattgtgtcc
atgataaccccaactttctgatgtgtttcctggtaaacgtcctgtttgtgtattctttttacaaaaaccaacacatattctaccttctttaccacatgac
aatacttggatttcaaaatagtagactgaacagttatttggtataggaaaatctgatcgtattaaagccgcatcatgttcgcttccgctatccgggc
ctttataatgcaaaattagcaagtgttctactaacatttaacttaccaaaaaaagaagcctccaagttgctttgcatgacctctataaaatcggatt
tttctcttgtgctccacgtttgaggtaaaggattgacattactgcctgtatattcagtacttgatgattcggtatctttcaagtattgttcccattgtag
ccattgaggttctttagtcagcaatttttcaccgtaatttgttccatgaatatattcaggcaattcattataggaatctagcgttttctttgagtttccta
acgtttttgtcatggcggttgtttcctggtacgtaagtagcccggaggtaataaatagatgaatgaagggagtaatacaagtttgacaatgtagct
agcaaaaataacaaacttttacgatgttttggtatattataagaatataaaataaattaccgtttgtatgtttgggtttcttctaatgtgcgtacaag
ttttcgaaactatatagcggtaaattgaggttttacagtactaacttacaatccataagtattagacttacaaatttctttttagtaataatatagca
aggcatactatgaaattctcaccatctaatagcactattgagtataagtattctgtttgataacaaatagatcaagatatctcgtattttaacaaac
agccaacgaaaatacgaggaatcagaaggttcggaggacaataagtatgagctataaatattgctaaaggctttttaagatttaaaatcattata
aagcaacgtagattaacgaaaagctcagttttaccctcaaaagaatccgaaatgttttgaaagaggatccaagtttagtataatgaacaggga

tttcaccaaaagtataccgtatgtaaaacttgaagcagtggataaaactcatttaatcctcatttatagaagttatatctcgtcaatttccttgtagc
gttgcattaaaaggcgtttatactcaacaggatccttactccatagttcagcggctctaatgataaattagcatttatgcaaggtagtaaaatgaa
acatactgagcatttaaaggtgaagcattgttaggttcacctagaaggctttgaagggatagtaagatagtttgtacgttgtagacagcagacca
cttgtccttcaaaatatctaaacaaatgtttccactcatgtcaacattaggatgccacataggagaagtaaaaacgatcgttggggctgagtaagg
atagttggggggaaaggtcatagatatcttgaattttaattcttcgtagtactaaaagataaattagctaggcatagtcagataacttaagtaaac
cattcacatacagtatcaaccggacctgtaatagtgcctgcccaatgtagaagatttgaatcagagtcgggaaaagcactaatccccggggtatt
cgacatctttattgtttggttagaattatatggtttgaacaaatcgcatttcataccattagactcattaattcacttcgtagcctatttccaaaagtta
gtacgaccatttcatcaaatcaaggatataaagctcaccttttgtaacgctatgtccgtctgtagacatatttgaagaagaagtatttttgtatttg
aagccatgctagggctctgattttgcatttctgaatccataatccttgttggaagttgactttagtggatgtgcgctagtcctggtaaaggttaaata
acaacaataaacaatacatcgactatatagtagacaacaaataacttcactaacaaagttttcttatgtttttttgctaaaaataagaaaacaatg
ttaagttactgattttgtttctcacacggaaattaaggaatctcgtataactaaaacattctaccgctcctaggaaagtaaaataacctaaggaatt
aattaaaggatagaaatgacaaagatcatagaaaagtttccgtaccatattggaaaagaaattggaactggcgcttttgcatcgtatgtttcata
gattcaataacagctaatcgctttatagtgttcgtatttgttatgatgacaacgcaaaaatctacgccgtaaagtttataaataaaaaatttgcaac
aaaatgcatgggttccaatgtttgggcacgtagaatggctagtgaagtccaactacatcgtctgtgtaatggtcacaaaaatataattcacttctat
gacacggcagagaactcacattggcgttgggttactcttgaatttgcccaaggcggagacttatttgacaaaattggtaataatatttgaaatgac
caaataacagttcagttctactaatgcgaatacagagcccgaggtagggattgatgaagatgtcgcgcagttttattttgctcagctaatggaagg
aatatcttttatgcactcaaaaggtgttgctcatagagacttaaaacctgaaaatattttgctggattataatggcaacttaaaattatcggattttg
gttttgcatctctctacgcttacaaaggtaaaactagacttttaaatagtccagttggaagcccaccatacgctgcaccagagattacagggcaat
atgatggtgcaaaggtcgatatatggtcttgtggaataatactcttcgctcttttgtctggtaatactccatgggatgaagctattagtaatacagga
gattacttaatttacaggaaacaaggtgaacggccgtcttcttatccgtggaatcgtttgtcaccgggcgcatattgtatgtcttttaaatattggag
catccactaacatgatagctattatcactggaatgttacgcagtgacccatcaaagaggttctctataaggcgtatcttacagcatccatggcttac
ttctaatacttccttccgtactgataatggaaattgttctgatcctgttgctctcgcttcaaggctaatgttaaagctgcacattgatttagataagcc
ccgttcatctttgacagatacttcgacgtgcgttatgatcatgtataaataactgctgacttttacaggtatgggaatatctctatgacacaacctac
aacaaaagtgtcagagtctgacatacataaccatactgctatatatggttatctgtctcagccagttcaggtgaatgaagaggttgatgtcacgga
acttatggagaaggatccttcgctgagtcagttcagcaatacgaacggttttattgaacgacttgtaagttaaatcatcgtagatttttctaactcaa
ataggctggaaaagctaaaaacttttatgaaatttgtcctccggaacgtttaacaaggttttactcgaaggcttcaaaacagtatataatcgatcg
actttatgatactctacgtttgttagcggtttctttgacaacaaagcatacaagtgggcaaagtacgctatatgttaagcttcatgataaaagaaaa
tgtttgttacagggtgaaatagaattgaaaacactcggtcataatttggaattagtgaatttcataaaaagtagcggagacccattagagtggag
aaattttttcaaggtaaagaaattttgatatctaggctaacactgagcagaatgtagtaagttcacttggaaaacctattgtcttgacagacattac
ttcttgaaaacttaatatacttcgaagattgcattatgcatataatttcttttattataatataatatttagagaaatgataactccatttgttcaaaa
ggattaaatattagatgattcggaacaaaacatatccaatattgattattgtaaaccaaaacaaaagtttttaggatttgaacttttgaaattttag
acgttatagtaagctattttgtctaataggttcattacttaattttattacttgactcttaaaacatacatacctttaggtgttcttattgaattgaaatt
catgaagtcctaagtatactcaaagctaattaacgacagtgcatttgaaggctgtataagccactattagtgtactattgtgcgctactaagcagt
acagttaatatattactatatttaataatcttaaaataataataaatgctttattttgatacgtagcctttacaaaacgattcatcatagtacactata
taataaatgagaatccgacgtaagaatagagcgggccctatgtttaactaaaaattgaagatatttaaataactttcgttggttctaatcggattat
ttgttgactgttcttttagtaaacgaaattcatttccgctagttttgcaatccaatttttttaatttcctcagtttgaggttttgagatgaactatcatta
taacatctttttatttgcattcgaaggcagtaataaatgtatgaaaacttctctaccttctccatagaactagcgaattattaagttgataatcttagt
ggaaagtaaatacataatctcaccttcataacgtgttggtttatactcatttatcaattctgttagaggtgagtaagtcgaagcctaagtctaatatg
agttaaaaaaaaaaaaaggttccaaatcttttctttaatgtagcagaaaagtaaatttggaatttggtgcttaagttatatccaatgaattatgttttc
gacctttttttactcataataatttgtctactataaactcaattgcgcaattgtattttctgggctaaaaaaataaattgaaaccagtcgttagtaagt
aaagtttcgttagcaaaactgtctggcttgttactttataattgatatagtagaggaaagcattatttattgacttcagccaccaaaggcgattgttg
atgaaaatgaacaaacttttagttacctttccgtctgcccacaaagtaataccttcaccacctctataaacatagactatatattatttggttattgt
aattattaatttnnnnnnnnnnnnnnnnnnnnnnnnnnnnnnnnnnnnnnnnnnnnnnnnnnnnnnnnnnnnnnnnnnnn
nnnnnnnnnnnnnnnnnnnnnnnnnnnnnnnnnnnnnnnnnnnnnnnnnnnnnnnnnnnnnnnnnnnnnnnnnnnnnnn
nnnnnnnnnnnnnnnnnnnnnnnnnnnnnnnnnnnnnnnnnnnnnnnnnnnnnnnnnnnnnnnnnnnnnnnnnnnnnnnn
nnnnnnnnnnnnnnnnnnnnnnnnnnnnnnnnnnnnnnnnnnnnnnnnnnnnnnnnnnnnnnnnnnnnnnnnnnnnnnnn
nnnnnnnnnnnnnnnnnnnnnnnnnnnnnnnnnnnnnnnnnnnnnnnnnnnnnnnnnnnnnnnnnnnnnnnnnnnnnnnn
nnnnnnnnnnnnnnnnnnnnnnnnnnnnnnnnnnnnnnnnnnnnnnnnnnnnnnnnnnnnnnnnnnnnnnnnnnnnnnnn

nnnnnnnnnnnnnnnnnnnnnnnnnnnnnnnnnnnnnnnnnnnnnnnnnnnnnnnnnnnnnnnnnnnnnnnnnnnnnn
nnnnnnnnnnnnnnnnnnnnnnnnnnnnnnnnnnnnnnnnnnnnnnnnnnnnnnnnnnnnnnnnnnnnnnnnnnnnnn
nnnnnnnnnnnnnnnnnnnnnnnnnnnnnnnnnnnnnnnnnnnnnnnnnnnnnnnnnnnnnnnnnnnnnnnnnnnnnn
nnnnnnnnnnnnnnnnnnnnnnnnnnnnnnnnnnnnnnnnnnnnnnnnnnnnnnnnnnnnnnnnnnnnnnnnnnnnnn
nnnnnnnnnnnnnnnnnnnnnnnnnnnnnnnnnnnnnnnnnnnnnnnnnnnnnnnnnnnnnnnnnnnnnnnnnnnnnn
nnnnnnnnnnnnnnnnnnnnnnnnnnnnnnnnnnnnnnnnnnnnnnnnnnnnnnnnnnnnnnnnnnnnnnnnnnnnnn
nnnnnnnnnnnnnnnnnnnnnnnnnnnnnnnnnnnnnnnnnnnnnnnnnnnnnnnnnnnnnnnnnnnnnnnnnnnnnn
nnnnnnnnnnnnnnnnnnnnnnnnnnnnnnnnnnnnnnnnnnnnnnnnnnnnnnnnnnnnnnnnnnnnnnnnnnnnnn
nnnnnnnnnnnnnnnnnnnnnnnnnnnnnnnnnnnnnnnnnnnnnnnnnnnnnnnnnnnnnnnnnnnnnnnnnnnnnn
nnnnnnnnnnnnnnnnnnnnnnnnnnnnnnnnnnnnnnnnnnnnnnnnnnnnnnnnnnnnnnnnnnnnnnnnnnnnnn
nnnnnnnnnnnnnnnnnnnnnnnnnnnnnnnnnnnnnnnnnnnnnnnnnnnnnnnnnnnnnnnnnnnnnnnnnnnnnn
nnnnnnnnnnnnnnnnnnnnnnnnnnnnnnnnnnnnnnnnnnnnnnnnnnnnnnnnnnnnnnnnnnnnnnnnnnnnnn
nnnnnnnnnnnnnnnnnnnnnnnnnnnnnnnnnnnnnnnnnnnnnnnnnnnnnnnnnnnnnnnnnnnnnnnnnnnnnn
nnnnnnnnnnnnnnnnnnnnnnnnnnnnnnnnnnnnnnnnnnnnnnnnnnnnnnnnnnnnnnnnnnnnnnnnnnnnnn
nnnnnnnnnnnnnnnnnnnnnnnnnnnnnnnnnnnnnnnnnnnnnnnnnnnnnnnnnnnnnnnnnnnnnnnnnnnnnn
nnnnnnnnnnnnnnnnnnnnnnnnnnnnnnnnnnnnnnnnnnnnnnnnnnnnnnnnnnnnnnnnnnnnnnnnnnnnnn
nnnnnnnnnnnnnnnnnnnnnnnnnnnnnnnnnnnnnnnnnnnnnnnnnnnnnnnnnnnnnnnnnnnnnnnnnnnnnn
nnnnnnnnnnnnnnnnnnnnnnnnnnnnnnnnnnnnnnnnnnnnnnnnnnnnnnnnnnnnnnnnnnnnnnnnnnnnnn
nnnnnnnnnnnnnnnnnnnnnnnnnnnnnnnnnnnnnnnnnnnnnnnnnnnnnnnnnnnnnnnnnnnnnnnnnnnnnn
nnnnnnnnnnnnnnnnnnnnnnnnnnnnnnnnnnnnnnnnnnnnnnnnnnnnnnnnnnnnnnnnnnnnnnnnnnnnnn
nnnnnnnnnnnnnnnnnnnnnnnnnnnnnnnnnnnnnnnnnnnnnnnnnnnnnnnnnnnnnnnnnnnnnnnnnnnnnn
nnnnnnnnnnnnnnnnnnnnnnnnnnnnnnnnnnnnnnnnnnnnnnnnnnnnnnnnnnnnnnnnnnnnnnnnnnnnnn
nnnnnnnnnnnnnnnnnnnnnnnnnnnnnnnnnnnnnnnnnnnnnnnnnnnnnnnnnnnnnnnnnnnnnnnnnnnnnn
nnnnnnnnnnnnnnnnnnnnnnnnnnnnnnnnnnnnnnnnnnnnnnnnnnnnnnnnnnnnnnnnnnnnnnnnnnnnnn
nnnnnnnnnnnnnnnnnnnnnnnnnnnnnnnnnnnnnnnnnnnnnnnnnnnnnnnnnnnnnnnnnnnnnnnnnnnnnn
nnnnnnnnnnnnnnnnnnnnnnnnnnnnnnnnnnnnnnnnnnnnnnnnnnnnnnnnnnnnnnnnnnnnnnnnnnnnnn
nnnnnnnnnnnnnnnnnnnnnnnnnnnnnnnnnnnnnnnnnnnnnnnnnnnnnnnnnnnnnnnnnnnnnnnnnnnnnn
nnnnnnnnnnnnnnnnnnnnnnnnnnnnnnnnnnnnnnnnnnnnnnnnnnnnnnnnnnnnnnnnnnnnnnnnnnnnnn
nnnnnnnnnnnnnnnnnnnnnnnnnnnnnnnnnnnnnnnnnnnnnnnnnnnnnnnnnnnnnnnnnnnnnnnnnnnnnn
nnnnnnnnnnnnnnnnnnnnnnnnnnnnnnnnnnnnnnnnnnnnnnnnnnnnnnnnnnnnnnnnnnnnnnnnnnnnnn
nnnnnnnnnnnnnnnnnnnnnnnnnnnnnnnnnnnnnnnnnnnnnnnnnnnnnnnnnnnnnnnnnnnnnnnnnnnnnn
nnnnnnnnnnnnnnnnnnnnnnnnnnnnnnnnnnnnnnnnnnnnnnnnnnnnnnnnnnnnnnnnnnnnnnnnnnnnnn
nnnnnnnnnnnnnnnnnnnnnnnnnnnnnnnnnnnnnnnnnnnnnnnnnnnnnnnnnnnnnnnnnnnnnnnnnnnnnn
nnnnnnnnnnnnnnnnnnnnnnnnnnnnnnnnnnnnnnnnnnnnnnnnnnnnnnnnnnnnnnnnnnnnnnnnnnnnnn
nnnnnnnnnnnnnnnnnnnnnnnnnnnnnnnnnnnnnnnnnnnnnnnnnnnnnnnnnnnnnnnnnnnnnnnnnnnnnn
nnnnnnnnnnnnnnnnnnnnnnnnnnnnnnnnnnnnnnnnnnnnnnnnnnnnnnnnnnnnnnnnnnnnnnnnnnnnnn
nnnnnnnnnnnnnnnnnnnnnnnnnnnnnnnnnnnnnnnnnnnnnnnnnnnnnnnnnnnnnnnnnnnnnnnnnnnnnn
nnnnnnnnnnnnnnnnnnnnnnnnnnnnnnnnnnnnnnnnnnnnnnnnnnnnnnnnnnnnnnnnnnnnnnnnnnnnnn
nnnnnnnnnnnnnnnnnnnnnnnnnnnnnnnnnnnnnnnnnnnnnnnnnnnnnnnnnnnnnnnnnnnnnnnnnnnnnn
nnnnnnnnnnnnnnnnnnnnnnnnnnnnnnnnnnnnnnnnnnnnnnnnnnnnnnnnnnnnnnnnnnnnnnnnnnnnnn
nnnnnnnnnnnnnnnnnnnnnnnnnnnnnnnnnnnnnnnnnnnnnnnnnnnnnnnnnnnnnnnnnnnnnnnnnnnnnn
nnnnnnnnnnnnnnnnnnnnnnnnnnnnnnnnnnnnnnnnnnnnnnnnnnnnnnnnnnnnnnnnnnnnnnnnnnnnnn
nnnnnnnnnnnnnnnnnnnnnnnnnnnnnnnnnnnnnnnnnnnnnnnnnnnnnnnnnnnnnnnnnnnnnnnnnnnnnn
nnnnnnnnnnnnnnnnnnnnnnnnnnnnnnnnnnnnnnnnnnnnnnnnnnnnnnnnnnnnnnnnnnnnnnnnnnnnnn
nnnnnnnnnnnnnnnnnnnnnnnnnnnnnnnnnnnnnnnnnnnnnnnnnnnnnnnnnnnnnnnnnnnnnnnnnnnnnn
nnnnnnnnnnnnnnnnnnnnnnnnnnnnnnnnnnnnnnnnnnnnnnnnnnnnnnnnnnnnnnnnnnnnnnnnnnnnnn
nnnnnnnnnnnnnnnnnnnnnnnnnnnnnnnnnnnnnnnnnnnnnnnnnnnnnnnnnnnnnnnnnnnnnnnnnnnnnn
nnnnnnnnnnnnaaagaaacccaaaaaaaacagaagcgcgattttttcgcctagttgctacgtactagcgatgaaaatgcatagagaaagg

cgtctaccaacaagaggcctcgtggcccaatggttaaggcgcctgactacggatcaggagattccaggttcgactcctggcggggtcaaaaagct
cttgatttttttcctttttcttatttcctttttgcttctattttccattatttggtttattcaatcgctatttttctgccttcattatctttaaagttcataaaaaa
caactatagtttatagcttctcaaagcctcagaattatattttcatatcgccgataaaactgtaaaagacttcaaaggttagcatctttaccttgaga
agctggggtaatccctttatagaatccattgaataatgtctgaaagaggttttccgcgataggtaatggttgtaattgaccacaacgttagtatcag
acaagaaaatgatactcttttcggagagctgcaatacttgcataggcttctttttcccaagtagaccaaattctttgccgatctatgaacattgctga
atggaacgcaataggatgataaattccaccgttctgtttttgagagagtatggttcctgtagcaaaaccagaagcatcagtttctaacctagtccct
ttaaaagtcggataatgcgtgttcctccggtatatagatttctggttctttcacattgatttggtctaagtatgggtttaccctctcccatatatgcatt
gcacactttcaaaaatgtctgtaggttgctcagggacaaagtccttcatcaagatccattcgactcatttttttctcttccattctctccttatgcattaa
gttgatctgatcggacgaaacttttttttacttgtttactcaagtcctgttgtatttcttgcttgattcctctccatctggtgaacgacatctccgttcatg
atgttttcagggtcaatatgcttctatatcttttgacagtcaaattcaaacagttccactcttcagactggaagatttatagtcgcagcaggattctc
acgccgtataatcattgtatttcgagcgaagaaacaatttatatagcatttttgaaatagctacgtaatacgtttctccagttccagtcaatagtttc
tctgtgaattaggttccttaactcataaaatatatttccatgttttgtttactattcgtaaaatttcaagacccacgcttttgatatattaaaaacagtt
gtcaaaactcaatcgatcatccgccaaaccaaattgggttcggtgaggaaagttaacagtatatccttgttactctatgaacaagacaaaaacaa
gtttgttctgaagaaaaatgaattgttatgaagcgaatagccaaaaagtatatatatattcaaactcacactcataggattatctatttttatggca
taaattataaaatattgttcatcaggtccgatgaaatcctaatgatctgtgaatcttcgtaactagtgtaaaatgatatagcgaaactttaagacgc
tacgaagataaaaagtctatagataaactagtagcttctcaaattgttaaaacgaaatgatgctgtatacacataaaccgcattcctattttcttct
aaagtagagtaaagatgtataatgcatcaaaaggcagaaaccacaaaaaagtttaaacgtccatgtaaaataaaataacgcataaaacatttg
gttcatattgccacgataggtgttttgtccatattgtactattacaatacactaccataagaacgtatatctgaatattaacaaagaatgtcgtagg
actaatgaataatatattacaaatggtaaacaaccaatttaaaacgaaatacagaaattaagatactagtgatttgaaaataaagacacgtaaa
cagttgattatctattgatgaaatttattactcaatttgattagcaatactatatatactcttgtgtctacagtcagaatcttcagattgcaatgactc
aactgatcaggctaaataagatcatcgaaatactttctaattagtttaaacggatccctggacaatagtagtgtctatatatgtagtacaaggata
actaacgacatatatacaaatattaaaatcgttagataattactgctttgcttcaacttgataagataataggatattgttgagaaacggtactctt
gaactttgtgtgcgtgtgaaacttaaaacaataggaacaaaaagaaaaaaaaaaaaaaagaaaagaaacgaactnnnnnnnnnnnnnnn
nnnnnnnnnnnnnnnnnnnnnnnnnnnnnnnnnnnnnnnnnnnnnnnnnnnnnnnnnnnnnnnnnnnnnnnnnnnnnn
nnnnnnnnnnnnnnnnnnnnnnnnnnnnnnnnnnnnnnnnnnnnnnnnnnnnnnnnnnnnnnnnnnnnnnnnnnnnnn
nnnnnnnnnnnnnnnnnnnnnnnnnnnnnnnnnnnnnnnnnnnnnnnnnnnnnnnnnnnnnnnnnnnnnnnnnnnnnn
nnnnnnnnnnnnnnnnnnnnnnnnnnnnnnnnnnnnnnnnnnnnnnnnnnnnnnnnnnnnnnnnnnnnnnnnnnnnnn
nnnnnnnnnnnnnnnnnnnnnnnnnnnnnnnnnnnnnnnnnnnnnnnnnnnnnnnnnnnnnnnnnnnnnnnnnnnnnn
nnnnnnnnnnnnnnnnnnnnnnnnnnnnnnnnnnnnnnnnnnnnnnnnnnnnnnnnnnnnnnnnnnnnnnnnnnnnnn
nnnnnnnnnnnnnnnnnnnnnnnnnnnnnnnnnnnnnnnnnnnnnnnnnnnnnnnnnnnnnnnnnnnnnnnnnnnnnn
nnnnnnnnnnnnnnnnnnnnnnnnnnnnnnnnnnnnnnnnnnnnnnnnnnnnnnnnnnnnnnnnnnnnnnnnnnnnnn
nnnnnnnnnnnnnnnnnnnnnnnnnnnnnnnnnnnnnnnnnnnnnnnnnnnnnnnnnnnnnnnnnnnnnnnnnnnnnn
nnnnnnnnnnnnnnnnnnnnnnnnnnnnnnnnnnnnnnnnnnnnnnnnnnnnnnnnnnnnnnnnnnnnnnnnnnnnnn
nnnnnnnnnnnnnnnnnnnnnnnnnnnnnnnnnnnnnnnnnnnnnnnnnnnnnnnnnnnnnnnnnnnnnnnnnnnnnn
nnnnnnnnnnnnnnnnnnnnnnnnnnnnnnnnnnnnnnnnnnnnnnnnnnnnnnnnnnnnnnnnnnnnnnnnnnnnnn
nnnnnnnnnnnnnnnnnnnnnnnnnnnnnnnnnnnnnnnnnnnnnnnnnnnnnnnnnnnnnnnnnnnnnnnnnnnnnn
nnnnnnnnnnnnnnnnnnnnnnnnnnnnnnnnnnnnnnnnnnnnnnnnnnnnnnnnnnnnnnnnnnnnnnnnnnnnnn
nnnnnnnnnnnnnnnnnnnnnnnnnnnnnnnnnnnnnnnnnnnnnnnnnnnnnnnnnnnnnnnnnnnnnnnnnnnnnn
nnnnnnnnnnnnnnnnnnnnnnnntggagattaatgtcaaatgcgcctagtgtatctatttcatggatagcaattgtttaataaagaaag
tagataactgtcgactattattttaattttgtttttctttatacttatataaaaatagaatgagaaaacttgaacaagaaggggtatggtatgaaatg
gaattgtatggttggtagtttgatagtttgatagtaaatacttagtatctaataaatagccaaaacaaaacaaccaatggccagttggccgagcgg
tctatggcgtagcactcaagtgaaaacttggactgtacaagtcttgctatatcgaatgatgcgagggttcgaatccctcactggtcaaaactttttc
aatcctttttttacgttacttaactattacaattatcttatctttgtttttatttttctctcttttttttttattttgttttcatttcagtcttatgctaatatctc
ctctattccgcttttatttgttttccgtttcccattcctcgaaattgtagtcttatatatgcataaaattcttaccatttttttgtttcttcttttttttttgcct
gtttgcatgtatttattcttttctatagatgattatacgcaaatcgttatcgcttccagcagataacataggctctatgtgtctagtgaaatgaaaacg
gcctttatagtattctttctagtaagagcaagaatttgagaaaacccatccgtctgtttcgtttatcctgacatcaaagaacctgttattatgcatcct
gcatacagagaacaccagttagccaaaacgtaaataataaaaactgattatacatattttgtaatttatagcaagaaatccgtcatggtccagtg
gctaggattcatcgctctcaccgatgcggcgggggttcgattccccctgacggagactttttttccgaaagtgtcttttttaacgcagtttcgttctctg

ttttatgacattcgttatagttagtaaaagtaaagtaaaactaaaaatgagagaaaaagaaaaagagaagagaaaaaagaaaagaaaagaa
aaagttattggtactgatgttttaacaacgaaaatctgttagtacactttcatttaaatagatcgatagatcttcgataatgaattgcacaaattgct
ccatttgctatcgtcctttacgatattaaatatttgatttcctgggttataaaataaagttttggtctcatttctcttgtaacagctcctggtgggtgagt
gaatggtttgctacaaatagcaatctttcatcactttgctgaatgaacgaacgctaaatataacttagtatgaacgtcatgcagcgaaaagcataa
gaaaaggaaaagaaaaagaaaaatttgtcacaaaccgggatcgaaccagtgaccaacagatcttcagtctgtcatgctcccaactacaccattg
cgacttggagtgatggatcataaggacaaaacaaacctatcaagtttttagcttacagctttattttgnnnnnnnnnnnnnnnnnnnnnn
nnnnnnnnnnnnnnnnnnnnnnnnnnnnnnnnnnnnnnnnnnnnnnnnnnnnnnnnnnnnnnnnnnnnnnnnnn
nnnnnnnnnnnnnnnnnnnnnnnnnnnnnnnnnnnnnnnnnnnnnnnnnnnnnnnnnnnnnnnnnnnnnnnnnn
nnnnnnnnnnnnnnnnnnnnnnnnnnnnnnnnnnnnnnnnnnnnnnnnnnnnnnnnnnnnnnnnnnnnnnntcccaact
acaccattgcgacttggagtgatggatcataaggaacaaaacaaacctatcaagtttttagcttacagctttattttgaggcttatatcgacgtta
gagtagttcaattattgcccttaccttgtcagttgttatttactaaatggcgatattatatactcagtagagaacgatgggttactcttctggtacgttt
tgaaattatcagaatacgataaccgtctatggtgcttcaccgtagaaaaagaaattaattcaatcagaactaacgaaaagaaggataatgacaa
gcatcagtccttaattattgcctttttttgaaaataaaaatccgctgtcatcatcatttcaagtcgtcttagtttgcgtttttcctttttagcatattcaa
gtgattaaaacccatttctactttcaatgcatatgaagaatggtaacattattgatgcttacctaagtgtctatcaaattttatttacaccttatttaa
gctaccttatagcaacttagataaggctgggaacatttgtgaataaagccagctgattggttagcttagactggatatatgatacattaacacgcc
cttttcgtcatcgtatgacacgacatctgttcgaattgtcgagttcgttttcgcaagtcattgcttgcgtcattttgatcttcaactttagccaaacgtt
cattatataattaagctaagcgaaaataaaatcatatagaataatattatatatatagcttagttatgggcactggcatattcattggtttacgtatc
gttctggacggtgatgaattaattgttcattttatttgggcttttggtaatatgtttcattgaatgataataagctacactcccgattgacgaggctag
aagcttctttaggaaataactagactgattcaactacaagaatctccactttatgattcctctagtatgccgctcaacaactatagttgatactcata
ggacctggagagctttatttatcctctagatcatgcgcattatggaagaaaagaaaagcttgaacgagtgaaatcaatttcacaatttcgaaacta
cactctcatgggaaatcaaactattaaccttttgtagctcacttctatatgcattaaagaaaagagagcaattaataagagattgaaaattattgc
attcagtgggaaagagacgttaaagagaaagcggaagtcgttgtaggttccctattcccctaaaatatatattagccatcctttaatttgatttctt
gaaattcacatctccaaatttttatacgtgtatctttgaaaggtattacgataaataagaccttttcttttcatatacattcgtcacctttggcatgtaa
ggctgaaaatggaatcgttgtgtatggtatctaccgaaacttttaagaggggtaattagttttatattaccgagtaatcactcacgcttgatgaagc
attggattcaatcccaagcgaagtttgtcttcatccatttggtcttctactaattacaagcatcgagtctatatagtaatgcacaaacgatctcgcttt
ccgatgaatcgttatagaaaacaaaagtcaggaaatttatatatttatcgaattgtaatcataagcaatacttttatatgcaaagtttcaaagatg
ggaaagaaataatgatacgaaattatgcgacattacatagccgattctatgaagcctttctgcattctttttataaacttttagtttacagtcggtttc
tattttattctcgcactttttattttttttaatttagtctattctatctttaattttcataagcgttcgttcatgatgtattctcaaaatcactctggtactta
ttctatattgaaagcgcaatataaggtttgtaaactttaataacttaaggatttccttatttagtaatgtagttggctttcattatttctattacgatat
cgtgaaccggttaatgatataaattgatgctcatatatatacaaggaaagttcagtatctaacataaatattagtatttgacgagaagattatgctg
taattactgactttagtttatttatactccaggcttgtcttaaattgtcctaactttataacttttcgctgacttcaactatgctttcattatcgtctaata
atgtcattgcgttaatacaagcagcttaaacggatagtttccatgcttatacaagttgtattatatgtcatcgctaaagcagtgcatattatcttaca
ttaatacgtattatttatatatagaataattttatcatcctatagaaaaaatatcgtttaatgcttaatatattattactcattagcaaccgtaccttca
agtttaaacctgacctttttatcatgataaataacgaccctatggataacattgctaagcatacaattgttacagaaagccatttttttaaagcaaac
taaatttttttaacaggggttcaaacgaatacaaagtgacaaaattgctgctttactatggcacagaatttatgcattattacattctgaaaacttat
tcatattgaacctgaacataacttataaaagaacatacttttagatattaaattaggtatagttggcgttttctaaacattcatcattaagttttttat
ataactgagaatttatttactccattctattatttcatgtcttttatctcttcaccttctttgaatgttagctgacttaaatacacatagtagacatttca
atgataaattattttatattttcttgggaactggtaactgttgcttaggcgttcttttcgaatataactgtatgatgtctatataatatgtataaatcaa
aaatgtccccttcgataatagattttgtttacattagaaacgacagcatcttagaaactttcaccttgcatgcaatgtatatcaagacctatgtatttt
tacctgtaacttttaagaatacttgctttcgttattacaaaattgaacaaaacaatttaatcatcgatgttttatagcgacaaaatcgtatccttgaa
tattcaatacttcttcaggttatttatacagtttatattttactaatttatttccgtgtattattattatttttttttgttcaaaggtaaactagatttggta
gtctcaaagcaattgatagacgaaagtttcatatgactccttcatgtaaataaaccgtgtttctaccagttccataattttgcggttctcccaacttat
aagcatatcttctgtttattactgatcattataaactagatttatttggtatcaatgaataaagtatattatagttttacagaatttgaatcaaatggc
tgactgaccttttaattaattaagaagttacttatagttctatcccttaatcaatttttaccttgagcatactttttattgtcgttaacttacaaaaacg
acatttactttctagagtagaagtacgaagcggtactttcgtgaaatattgtacattgtcagaactaacgttcacattaagctgctttatgcaattga
acacaccgagaaacgatgtaatcttcaatttcaacgccgtttcttaaatcctcgtcagaaagatgcaaccatgcaacagaatcaagtgtgacaaa
cgcaactgaccttctccgagtcgataacaagttagcaatgactagctgatgattatagcggtgtaaagcagtcctagccttatcgatcaaaatgtt
ctcgtcagtctctagtttaaacgaaataatgagagcctctggtgcccaagaatctaccaaatatcgaagaaactttgggacggggcgcatagtga

gttcaagtttatcttttccagccccactttggattttgtgttcactcatatcttgtgaaggtatatggaaatcgctaacagcagccgccaaatagaat
aaagctcttgatcttatacattttaagcattctgctacagttctcagataccatacgtattcagtaagcgtcgtatatggtagacagagtaactgatt
ccgctcattcatgagctccaagttttcaagtgaattattcaacacattcctcagagaatctttaatttcccaagatgaagaagatccgacactctttt
tagtaaagatacctgtccatggtccttcaaagtgtcgaacaaaaggcttcataccataattcctatagaggaaaattacagcataccctgatttac
aaaagtactcagcagatgcagccccccggtttcccgcactaaaattgtctatataccgaaccatgttcttttccagaggaactaaagttcctccgct
ggtgactaaaacgatgtttctatccttaggctgcaaatcaatgaatttattaaccttattttccacatcgactaaagtagaaggggccggattctca
ataaaaaatcgcatcgcgtcttcaccttccatactgtctgatgcttgctgatttaccgtttctttccctttttttatgtatataaacgtaataactctttt
atattatattaaaaatatccaataagtttggatattttactttcaatgactttatatatgtttgctagtcaacatcaagttcaattcgataacgcacca
ttttcacaatttactgccaacatctaatttgacttgaagcttccagagcctagatacgctgccaaccttacataagggtaattcaatcatcctttttgt
tataatcattagctcaactgtataacaacaatttcccaaaattttttgtagtctccgtaaagataattgattatgggcaataaatcagctattcaaga
agaatttgagatacgatcaatatatcaagagaaggtgaaaaagaattgccaaaaggaaatcgaagattttgttcaatgcgcaacaaaccgaac
tattagtgtgatttggaaatgtcgccagcaaagtcatgttatgagagattgtcttaaaaacacgtacgtcttatgttttacctagaactgaatttaac
atgataaaaaaatagaatcgaccaaacatctgaatggggattacgcagccatcaagatgaagaaaagcagaaagcattcaaaaaccaaaccg
agaagtagataaataagttgctaccaactcaacaaattccgctaaagaatttttactgtttgtcggaaagaccttactgttactaaagtcactaaa
gctctccttgtaaatttttaataatagtcaatattacattctattttttacacatatgttctcaagctatcgttgatttattgtttttatatcgggctaaag
aagctaacaaaggttaacaattgataatttcgactctcaaattacttcagcgggtcttttgtattataagttcattaataaaccttttcaatttgctga
cccggtggaatgaataaggcatctctactactcatgaattcgtcatttgaaatcttcgatagttcaaaaacgttcaaacgcacaaaagcgacaatc
tctgctaggatagtcaagtctaagttatgacttccagcaaacaggtttctccaaatcgcggtcgccaaaaccgcatcgttacctaacattgattgat
cgtatccaaaaatggcacctgtttgttgttgaaatagatccttcaaatacattcctgttatacgatcgctattaatcctataatctttatgaatgcgga
attccatatcttcaaaaaaacgagttactaaagcctgtgagagcgcctgtttttcttgtggctgaagggcgcgaatccgtgtttgaagaatccaaa
ggtgcaattgtgtgatttgaaaccaagattgaaatgtcattggaatttcacacctgcgaaaccaaaactgaaatgcatcttgggaatccctttctg
gagttgcttggcgtgaagcttcataatagaaatatctcgcagtactggtagcctcaaaaaacatgttctgttttggcgagataatttttgaaataat
aaactcgctttagctaaaaagctattaggaggggagatgtttcttggatcttttgggggagttaatggtttatactttgaatcattaagggaattcac
ctttgcctttcctgcggtttccttggataaaacagaggcattatgtaaacctcttataggggtaatccgattgctttgaaatttgtagaacttcaatct
ggcttcggagcctaaacgcagctgtgtacaacttttcagcatgattccctcagttcgtacctctaactcaaaatttcaaggtttacgctttttaaagt
atattagagtatgagtcaaatagcttaacgattaactttttccgaaagggcgaacaggaaatctcaaggtataagtgttggttaatgcaggctcc
attaatgtaacattttgacatgctcccacccatttttgaaactttgcattaccaaaggtaacaaaataacggtgtatattacttttaataaacagaat
taatttcaagaggtgttgcttgctatattctaaaattaaccgtaatctgttataatcattactttgcatactttcgggtaattgaataaatcagtgtaa
tattacataaatataaggagaaaagtattaaacttgggtcatccaactcaaatccattatgagatcaaatgctgagtaaacattctccgcccgtaa
tagcttttgtaaaagctcatgcaatatgtggcctttatacattcaaaaaaggaaacagtgcataagggttaatagttattgttccaccatcgaaag
ccaattatcagctatacaaacttgcaaaacttcatttaaatactgcgcaggtcaatttaaagtgcatcttgaagctctgatactttatgatacagca
atggtagactttcactaagccagttattatcgtccaaaagtcttgctttatctgatcctaggggtatacgcttgatgacgctctcataattagattctt
ttttacgaaagtctttcattattcgtttaccatccaaatcgatatagatccttgtctcattcacgcgtactataacattatccaaacgcattaccaatct
tgctagcaatatcaaatgtcctttcactacccgtactcgaacatcaaacattgcttttccattgtcagctaattcatcttcgaataaaattagctcgtt
gtaccataagttagtaccagcttctaaaatcttcctcgcaggtatatgatctcgaatttcttcaaaatgacgctttcgatccaaaagcgtaccttgat
attcggtataataagtccaatcataaggagaaacaactggaaaactttctccagttttacttctcgattttatccaatctcctgcaaacgaaacttg
cattaattcacctggtcctttacccaccgtttctagagcatccttggtaaaaaaaccataaattgggctatctttataataaattgaaatgaaattct
ttccaaaggtcatctcaggaggcgcaaatcctaattctgtctgcaaattatcaacctcaaatgatttcaagatagcacctctttgagctttaattttc
caaaaaccataactaaattcttctattttcggatccatgttcctttttacagcttttatcctttggttctatatgagcttgagttggatgcacttgtgctt
gtgattataatacatgaaactctgtacagttctaacaaggtatattcaatttaccaatattaaaatgtatttatacatataatctaatagttttcaata
aaattaaaatatttattcaaataagtaagacaaaacagtagaccaacttgtattattgaacaagattaaaactcaaaattaatgaacaagcttaa
caaactctttaagccaaggtgtttgagttcctctttccaatatcaaagaccgtgtattcagcgactgaagccattttgtacagtccttctcagattcc
aagtaaaacttaatacgaacacctttatcggtatttagttggattcccctcgtcattcctaagtttaataaatctttgtcacaatctcttatagataaa
ttatttgcatctctcacagccttcaagctataaagcaaatccccacctctgtctgtataaaaactaatcttcttggaatgcaaaacagcaaaacgtc
tgcgaacagatgtaccttccaatttgtaaacataaccctctttgtaaacatagctctgccgaacatgtcggaggtcttgattagcttgatttaaagta
ataggcatttcagattctggaatggaaagctccggaatataaagacaacttattgacaaatttccaataatatcattcaggctcagatcagattta
ccagaaacagcgtgcatcttaatgggaatttctgtagctaaaatcttaccaaaacatttatccttaacacgagataatgccagagtggccattcct
gacactttcacagggttatcgatttttgcttaccaccactcaaagaattagacttggctagttttctctttttgcgtctattaaaaagacgttcaaag

agtttctttgttttcgatttactttccaaattgacggggtctgagtcacttttcgcggaacttgcttgtttcatgtctcggtttttaccgtgtagcatatca
aaaaacatgaaattgaccgtaaatttatcatcggcaggaaaggcaaaatcatggtctaacctatcattacctttattcaactgccacggaagctg
aacgctctgggaaggagtaactacgcttattttcatacctggttgatctccaacaaaaattgaggaaaagtttcgaaaataatttaaatgaatata
gaagcggccacttgctggcatagatttaggtttatcttcaaagatggtttgtttaacaaaacccagcgaagttgatggccgaggagcaacattctc
aactccaacagcaccaggtctcatgctaggcttattcatgcgattagttttcgaacgactgagttccaaagatctgggtcgattaattgatccttgat
ctgaaacgggcgtagactgcctaacttcatcgatttcgtctgacatttcataagtcggctcataaaaagcaacagttcctgtttctgagatattccat
gctcctcctctttgtgattgcggactaattggagcaaagtctgattgacctgcgatatcattctcagtaatattgctaaaggtacgtttggagccagg
aataggagtcatcatcgacttgtgtcgaagttcttctgaacttagtatagcactcaagttgtcgattgaggaactgcttttgatgcgacctcgctgac
tagctatactaaatttaattttggcagcagcaagggaatatcggggaacctctttataggtaacagatccatgtcctgaagataatccagaattat
cattcgtagctatttttttcactttcatccatattcatataatcctctaaaacattgggaatttgctctttattcattatcgattccttcgaacatccatcg
tcctcttgagatgagcttaagtaaaaactaccatcagaagcacgcttagcttccaatttggcatgcactttttttacgaagaccatcagaagatatta
atgatttatctgcatcatcatcaaaaacggacgttgaaatacgttcagtggtatcttccacaggtgcttcctgaatgggtgcaagaggctgttgagg
gtgataatctatgctttcagtcgctgaaggatcatcggatggcacttcataattgaccttagaagaaagcaattttggcgtattcggaagagctcg
aaaatgattctctgcagataaaggtagcggcggcaaaggcttaccggtataagaaactgaataaggtaaaccgtcattaacctctgcttcagtaa
aattatcaccaggcttaagtaaagaaccagctggagtattcttaggtgaaaaaggcgatgagataagattagaatcatataaagaatcatctcgt
gaagcagctccttctgggtaaacattatttggagccatttctatgtagtgatcatcctggctaactggtacaccatcgtctttaatcatcgagggtcc
agaagaaatatttgagctcccggtagtatgtgttaagctaggaattcgttcttcattagaaggatttgagcgtcgacccaaagaaaagccatcag
attttttagtggggctttgggatgaataaaataaagcatcattgtgaccgtatccttgcatatacatgctccgtaaatccaatgattccaaaaggtc
ggtagcaacgttaagttctgacatattaccaaatgaaggttcaccgctctcgttagacaataaccttgatgcattattaggtgaagcatccaagct
aggtccttcaaaaaacaaaggtgtttttttcacccattggagtagcctgagtatccaattcagaattgagttgagtgaccgactgctctcccgtataa
atatctttccttttattgtcttccatcctgtatttattacttttcgaagactttaatgaaactttaaaataataggtccaatttcgttaccaataatataa
taaacgagtgtgagcgtcaaaaagagtgattagtggatataaatacaaataattaactgatacttgttcaaaattatataatttaaatgatagctc
ctatatctttttttatttagaaactttcatgaattaagggaattcagaggggtactggatgtgaagaagtaagtccacgtcgaaggtcaattcctaag
aacaccattatcagggagaatgaagatggacaggatactaaaagaataacttttataaagaagattaagatatcgtaaataaagtataattata
ccaaacgtgaaataaaaactcataattgctaaataatatcacagtggaacttgaaagagggctttacaaaaaattgtgtagtgatcaccaaaaa
gtaaattaataacctaactagcacctttattccccatgtatacttcaaaagtctcttctgaagaaaaaatgcactttgcactctttttttattattgtcc
ctatactcaaaaataatataatcattaaaatccataactctttgatgacgcctttttatttgcgcgttgctgttcaaaataagtttcaaaactttccttt
tggctaacagccattccaaccaaagatgataattgatgccgaccaggtgctacacttttaattgatggtgttactgaagtatcttcatgagcatcca
ctttaggtaactgattcatatcaacgtcgataaatcgaaccttttctttccgccttccttcaataatatccgtttcaggcgacagcaccttttcggtatc
ctgtaccttgttaaacggttgcgaagtggttttatccaaaattttcggagactctgcattaatgcgcgttttctttcgttgaggttttacaaagatggg
ttcataatttggctcgtttactgcggatctttcatacgtacgcttagagggatctgacacattaaaaagtgaaatagaatcgacattcgctttttccg
caccatcatctaaattggcgactgctgaactctcgtcatcatttatttctgtatcatcatgtacagcttcaatttctaagtcggaacgttttgattgttt
tgggagaataggtttttcataatgaggggcgggcagaatttcattaagcgttacaggatccttgatatgttgtgtagttctcttaatcggttttgtatc
atcttcttttaaatcatcatttagatacctcttattatccacataaatctctgattttctttttgttttctcatcatttgccatctctttaggaactcttctcg
catgctgaaacaagttttcattaaacgtcgattttgtaggaatctgttgttcatattcttcatcggaatctgaactatcataagataccaaagacatt
aacgaaaatttatagttttaaggttctgatggtttttttatttcaaagatgaggttggttgggtgaaggcggttcaggtctcagataccttaattaccg
tatatgaaatatgcagctttttttagcagataaataagcagaaaaccagtaagttaagaactctcaagaaattgactgatcataggaagttctaa
gttcagtaattttcttttttttttttctttcaaactggatcgtcccaaaaaaaaacaaaactaataaactttttactctttcttaaaaataaaaaaggtt
accaaaatcaaacattacgagcttcatcaatttgttgttcagcaacttcaaccaagtgttcccaattgacatgaagagcacagcgcgtttcaacgg
cagctattacaaaaacggcaacagttattccaacccacaaaccaaacaaaccccattcaaggtagaagcataaaacaatagcgataggcaaag
ctagaatataataggcgacgaagttaactatgcctccaatacgctgacgtccctggccacgtagaacacctccagctacacactgagtgttatctg
caatgttcattagggcacaaagcggaacgagctgagctaccaactgtaccacatctttgtcagaagtgaaaaggtaggcccagtgatttctaaag
ataaccataatggaagtaatgcaaagaccaatcaatgctgctactgatacagcgacgtaagttgaaagcttggcaagttttgtattacctgcacc
aatcaaatttcctactcgtgtagctgcagcagcagctacaccaaaagcaatagtatatgaaacagacgaagtcgtagataagactgacattgag
gcaagatccgtagtacccaatacacctgcaacaaatgtcaataattcaaaggcccaatattcactacatatcatcactacgccaggaatagcaag
cctgcacagtggaccccagtttccaagagcttccttactaaaacctccccaagcctgacgtccattgactttaacaatataccatataagaattgca
caagccatccaaagcgtaattgtaacagcaataggggcacctatgaacccgaacccgaaccaggggctccatactaagagataatttagaaga
atgttgagaggaacaatgaaggctaagatattggtaacaggttgaaatataccttggacttgtaaaaaccgttttaaagactcaaaaagtgcata

tgcaggactagcaagcaacaaaaccctcatatatctacccgctagatagcaagtggtagggtcttgtttaaaaaacagaagaattggttctatatt
ccaccagatgaaaattattggaatttgaagtaaacataaaacagaaagaatcctttgaaggtggagaccaactaattcatagtttccggatccga
agcactgagtgccaagagtatcaagagcagaaacaatgccttgatagatggcgaatgccgttatagtagcagtcatatttgaaagactagcagc
tgataattctgtttttcctaaatgtcctagtgaaaagaccgttatgattacttccccatattgtagtaaggatgttaaaacaactggtataccaaattt
aaccagtaaatggaattcctttttccaagtgcttttccgaacattatcaatttcttcactcaatgctgaagcagaaggactggtctcccgttctgcttc
ctctaaagagccataagtaggtaccgtggcaggagaagcatgtcctcgcctcgaagctctttggcgtgcttcaataaattcttgtaaggaaggag
gtacgctggttgcatcagaaccaacaaattgaggacgagacaacccataaacaatgctcgatcttcttctattcggaggttgttgtaaatttggcc
ctaaaatgggctcttgttcactttccatattaatataaaagatgaacttaattagaaagcagaacactctcaagtcctctcttacttttgttagtggt
gatggaaagttgtactgtcgctaccgtagaggcactacaaaatttttatggaatcgtttcaaacttggttatacagcttaagttgacaatttattgta
ttatatcctccaaattttaaagaaatcatttattccctaaatcaattatgattgaatgaaaccttttttttagtagtgcacaatagcacccacctatta
ataattaaagatattagtgaacaaatgaatgaatctatgcaccaaaaatatttttaactaagtaatttagatgttatagcttataggctttttacgcc
gcaacgacatggtgtgttgcttctgggcaacttctttggtagggctactcaatctaacctcttcaaattcggatttacttcgatacggccatggaagc
ttataaagataaaagtgacgtgaacatttttggcacattgtaccttgagcccgagtcaaggaatcgataaagacttcttgacaatctgaacaagc
atattcagtattgccatttaaccttctctttttgagctcgtttcgaaatttgtcaaaattgacttgttgatctgattttattgccttttccaaaaacgctg
ccttgattgatcttcgttcacgaggccaaactcttttttatgacgagggatataaagagataattcttcatctaaatcggaatccgatgcattactt
aaatcgctgccggtatcccaatcactgagataaagtctgtcttgttgagatattgagaaaggatgcccttccctttgctgactttgtttagatgatat
actttttaggtttgaagatgttaactggcatttaaagaattttctgtagccatttattccttttcgttcacaagtagaacaaagacattcacagttatc
aggaccaaaatagtttgatgaatagaaagtagtaatttcttgtcctggctgaattggcttaacagatctaagccaaattcttctacctgatgtgtta
aatcggcaattagcatcacagtcatggttcacaaaacgagcaggccctaaaaataggcacattgcgtccaatctacttgaatgaagaatactga
aatctttctcggcccccaaagttcgttcttcttttgcggttaatttcacaatcgttcctgacaggtctaataaatcatctccagcttgtatgagttctcg
ggcgattacacaggcctcgtgtttcgtagaagtaaaatatctgttagtagaacatatttcaaacttacaagaaggaagatacagagataagtaca
aacgaaggtgccttagaaaagcattatatgaagattctgaccatctttggattataggctgcaattcgtgtagttttaacgtttcgtcaattgcacct
tccaaatctttccgtcctattacattttggataacaatattaagaagaagctgcgaattgatccgttcaatagagcgacttacaagttttcgaacttt
gtgtatttcagaccaataatgtattttgtcaataaaaagcgaagtacaaatgtcatcaaaaagtgcaaaattctccaactgagtcttagagttccg
cattttgttaatctaatacacgaccatttaacggaggtctataaattgtaacagtgaacgttgaaaagcaagatccgatgatacttaagcaaattt
gtgacaagacatttctgttacaaaacgtaagatgaagtatttctttgtattcttaatttcctcctatatcagaaacagtaccttttgtcctcagctttct
caacgtttaatagatggtcgtttttctatttctatccttctcgaagacctttaggatttcaaatgcttgctatcagactggaaactgtgctttcaaaccc
gttagtcgccaattgtggtctcaaacgttatttgtagaaattttagatactctataaaggaaacaaggaaaagataacaaattataaaaagaaag
atgaagaaagaaaaataaattcgtgaatagatagtctttcactttttcagggcgctctaggcgtaagccaatgaattgttcagctttagtatatatt
tcggttagtgatgaaattcattttactttgacttttctcctattcatctttaacctactaattataattctatataatttgttcgtttcattttattcattctt
tacctgaaagaagtattttagaaaatgaatagcattcgtttctgtcgatggatcaagggtcctcttaaactcatgcctcatcaaggaggaggccat
ttccagaaaacgttcaaacggttctcatctacaggcggtgaaaacagaataagagatatcttaactgagaagttatccccgagctctcttcgagtt
atcgacgtctctggaggatgtggaagcatgtaccaaattgtgattaaaagtaaaatgtttcagggaaaaaatacactatcacagcaccggatggt
taactccattttaaaggaagagattaaagggatgcacggattaaatctcttaactgaggtggacgaagaaaattctaaggatccaactgtttctg
gaaagcagtagtttctgattatactactgcacatatgacaagttcaatctacggcgaattagtttctcttataaacatgaatgctgtaaaggcaga
aataacggtatattttacattatcttgatttttttcctaagtaaaaaagaaaataataccagaattaagatttttttggaatcctttggctttatttacta
agccatcacagtaaaattcggattcatagaatagtgagacctagccaatgatatctaaagtctttgctaacgatgttgaagataagcagcgtaatt
tagaagcagctcatcgttcatgctattgactgacatttcagttgcaaaccaaacgtatgctgaaaactcacaggttgtattcgcggattatgcaatt
atattgctgatagttagaaagtttggctatttattatttcctttttttgttgatttcttgcagaaatttatatatatttttattttctggataaaattttgttt
tgaagctgcatacatcaattgaatattcgagcacttagttcatttattttttgcatgactttttaagtattgaatcagagattcattacatatttattat
aattaattttgtcgaaatgcacttgaaaagtaagtgaggcttgaaatgcccgtatgcgtttgaaaacaagggttttgtttggatatacaatacagg
gcgtataccgactcttcgaacaagcacaaaagtggaggctaacctggagatagaaatttcaaaaccaccttcaagaaaagatcataaacactca
gaagtgactgtgcctgaccaacaaatagaacaatgggggtatcagatatatcaaatcgacaagtcgattcgaagcatgatagacaacgctaggt
tgttttatgacgcttggcgatgtgtggtttgtcttgcaccatcggttactgcgtcttggatttctttatatagacaacttccgaatgaaaagaaccctg
ctccttctcttggaacactagtagattggaataaagctctagaacggcagagaagagaagttttgctaactttgcaaaactttcaattaaccgtaa
tagcaccttgcaaagaagtaaaggcacatgtgaaaaaggccgttgagatgataaaacgaaggagcaggaaggtgagcgaacttgagaagatc
caaaaggaactgctggttgtttatgagttaccagatccggaaacgaagaaaaacaagattaaagcactacagacccacttagtacgagtcaaca
atgagctaaatgaattgcaaagaagcttgaccatgtcagttcctacactaataaccaaatgccaagtctttttttgggcaattaatgcgacatttttt

ctgcctccaactgcagatgttccggaaaatgcataacattactcgtccatgggattgttttcaagacgatattcctaaaacatggcttgtcgagtttt
cttccgtatgtaaagcagctgagggtcttcctataatttctgttgataataatcgtcctcctgtcgagctcccaaaagcatcagaggctttatctgcg
catgaatgggaagctggtaagctcgatgcaatgaaggctcttttatcacagaaccctcatctttcgccctctcaaagcagcttatctcctttatctac
cgcaagctcctctgttgctgcttctcctattgatacacatgctccttcgactcctgtggtttcaaggcctccttccatgcaaaatttggcttctgcactt
gaaccagaagaagatccgatgtctcctagtcaagttcctattttgagggctcctgccgtaccacatcaccaaaaaataaatagtaatacaacaaa
tactttatatacaactgaatgggcgtttttggcttctggtagtgctaacgttgtttttgaatacgttgggaaaaacccatactttcaagacaaagtac
tccgacttcgtcgacgaggtcaggcttttacgaccgaacaagtttatgactactatcaaaatactgtttatcctttatttgctggtatggagcaattc
ctaatagaaatatatcttcaaccagttactaaagattttctacttggtgctcaaaatgcatcaggaatttttctaaatttaaatgaatcgttttgtatt
ctaatgcaagacttgaaagaaggtatggaaatgaagccgaaatggctcactcagtctccgactgctccaccggactgggttgtctgcaggacttg
tgccatgagccgtatgcgaggcaaacccatcagcttttgtccacttcagcttgattttagtaactggccaaaattccttagcgcgcttcagggcatg
gttactcctgacattgctatgtcattatttcagtcgggcatcctaaagcgactccgcgatttacaagaagagtattctcgtaaagacatcgctttagc
catgactcttcgagatgtgactctatatatcggcaaaaaccacataacgttgcttgacttggatcccaaagatattaatgcaaagctagcaaaatg
ggaacgtgatgagcagaatctttcagagggcggttggtattatgggcgtggaatgaaatctaccgaccaggcatgtcgcagtgcctaacgaatgt
tttctatttatatgattatacgacatgcctcatagagttatgaacgccttgggttcacgttagatatgttgaaatttgtaagctattacaaacttttaa
gttactagaatttaatacgaaaacgttttgatatcaatatttttactgatcaaatccttcaaaacctgccgtgaaacttattaaaaatttaattctaat
tttttaaatgctcattatgttcatttttgggtttgtccgttgaaagacggcgtttagatcttcgcaacctttgtgcagtacgtgttatcccgtatcattca
actaccaacttcgattactcgtttacaatttactaaaaaaaaaaatgctacgtgttttacctcgtttaaacaaaaatttgtaagcaataaagattta
aataactcaaagtttattaatgaatgcagaatttccgtggttcgtgcaaaacccgcaacttcattgttgagaacattccacagctctatacctacta
gaaatgctgaaggaaaaattgataatcttgttgaagaaattagtaaattgtctttattagaaacctctaagcttgtagagcaactcaagcaaaaa
ctgaacatttccgacattgctcctatggctgcccctatggcagttcccggggccgctgctggtgctcctgaagaaggcaatgaagagcccaagcca
gaagaaaagacaacttggaacttaaaattggattcctttgatgcaggttcgaaagcaaaggttatcaaggaagttaagtctttgctgggattgtct
ttggttgatgctaagaaatttgttgaaagtgctcccaaggttcttaaggagaacgtattgaaagaggatgctgaagccattaaatccaagttggaa
caacttacttgcaaggtcaccttggagtaagctaggttaaaatgaaaacataaatacgaaaaagctgtaatgctttttccaatacagttttataag
catggttcaaagtattctttgctacttctcatctctaaaatggaaatcttttttttggaagaagaaaaaatatgtacgcatacatatataaatggctt
tgatcatttgctgtaaaactttctctactctataaatcccctttttgacccacagttggactatgacttacaatatatgctttccttcgactctaattcg
gccttttcatataattaacaaagagattcacgaatgcattcattaaaccttacaaaatcgtcgttcaatgattccaaatcaccttctgtgattggttc
tatagtattccaagcaatctcaggttctatttctggtttcgctattgcccatatgttttcgcgcatgcgcttatgccgttcccgatcttcttctaatcgct
ttaatatgtcggctctagaaattgacgcggtgccttggtcttcacctgctttgcttgcttgttcatgagcgtctaccaagttcattgcctcctgaagcc
gagcgtcatctattatgtttttagacgcagagagtgaagcacctttcttacttcaggagcattggctgctcccgcaattccaattggaaccacgtat
tctaccagttttaaaatatcacgagtaatcatagtaatataaccactagtgattccatttttaaacttaattcacataaggtatccacaaagtacat
gatattaaccctcgtattaaaagatccagattccaactcttcccatatgcaggaatataaatcatcttcaaggtccatgtttttatagcaaactggg
caggcttcagttgcgataactgggaagcacttagcttattcaaaagctgcaaaaaagccattcttccttcaaagggatccatattgaatcaaagcc
ccagtaagtatacgctataatatatacaaacgaattaaatggtttcttttatatatctttttatcaaattataggtttttttattacatggaacatataa
ggactaacaggacgtttatattcacatagtgaattttgatgaaatgcgttattttaggtgtataaacatgaagtttgcaagagagcgtgaatagaa
cttttatatagtaatcctactctaagattacattcgtttagttactttttttctataagtaataaatgaactgtacaaaccagataagccaatagtaa
tgaaagccaaagaatataaaacgacgaaaagtcgtcaatatgttaaacgcatttaaaacatgaaggaatccgattaccactcaggcttaccatg
atgtacacgaacatgacaaatatctaaaaccagcaaattgtgaatcaaaatgtgacaaaaaaaaaacaaaaaaatgtaagaacaacgaaagg
ttgaccaacctaaatactttttaggctgaagaataagcttcaacaaattctaaatagtcgggattgagttccacaccagccttctgtgcatcctgca
aagcggtaaaggcagccttcaaataattatcttgtgattcttcctgatcggttatttgtgctaaatccattttaatatccgccaactaaaaaccagtt
agtaatttataatacaggaataggttgtggtcgaaaaaaatttaagccttttacaatacatacctttaatagtcgttcagcagagttatccaaaga
gtaaatctcactccaagcatcagccgaccgagaaagccattcggaggcctttgaagaccacttatcggcgttttgctcgtcggtttccatcaagtct
atataatactcggcaaaactagtatggctagccaaaaccgcttctttagtagcaaggcgaacaggaatagggcagtcagggtaagaactgcatt
tttcccaaagatttataataagggagtgtacatccaaaagtaagctttcagaatattggaatccaaattggaacatgtatgatgacaaaacataa
aggggacgcaaagagtcaggtgtcaagtcatcgaatggatcaacagaaactaaagggcgaagataattgagtgcaagttctatgtatggattg
acaatttgaattataacggagggatccgaaggcttctgttgatcatcctgcttccacaaactgatacgtactttttccaaataagcacgtccaaaag
cgagataaagtcgacagtgatcacctggagcttctaaaccaagttgtgctctttcgatggcagcatcaataaatgactcctcgtcattagtttcctt
aagacgtcctttggcaggctcatacaattcggaaatagccaaaagagccatagagtaggctgcatgaaaatctttaggaagaccggaataatct
tctgtgttacggagtatgcggtcggcctcatgaatcgtaccaaaaacaaggctaatgctaccttcgatatcaccactttctaatttttcacggctcat

ctcatacaatccccaaagttgatcaatgttgtcatcttcagtatataccaacatagtttcgtcttcgtccccttcaactttgtttacatctccatttcctt
gttctgaagaggcagatccattagcagcggcctcttgacgttgtttttctaattgctgctttttaaagatgctgcagcgcgcaaaccttgaggacgt
ctgccaatcatctttatatgtaaaagttttccttacttgtaaaagaaacgtttgaatcgagtgattttcaggtagaaattacacacggtttagtgata
aatagtgaatctcgttgaaaaatttaatgcaacaaagaagtgcatagtcatatgcgtaatattaaaataactaaaagcatattaaaatataatat
gttttatttaagtgtttgcattaatcatatattatactagtttaaataataatatatattgttactaaacgcaaacaagcatattacttattatttttac
atattacacataaaacgcttataagcatattatttactaggaacttaacttacgtttatctgtcttaatactcatataagcttgttttaaaagtaaaat
cgaaacattaatattccctttttcctatatttcctaacatttgagatcaagtaaaagattatcatacattatttatgagttttagttgatgtctcataaac
cttccgctattgagtataccgacaagtttcgtttatttgttagtatactcaagtgtactcgctaaccgaatagtaaacaaaatgacagatgtataat
gttgacaaaatgatttgctaaaggagtgtgtctaaagaaacttctaatgttaaattccattcactaatcatttgtatgcttaatcggataggaaatg
atgcagaaaagtctgtcacaaataaaaattacacaggctaagcaaacctaatacagttaaaaatccaatttatgaaaagatgcacagttacttg
agaatatcgagctaatgtagttggaaaaatggttcctacctgaattactgaaaaacgtactagctaatatccaatacgactcaaagtagctttcag
gaataagctcataagcgcacatgagtctggatgaacattaattatagtgaacacatgaagcactgaaggggttcatcgaagttcatatagatcaa
aagttacatattattcccttcacctaagctgaaattttctcaatatattcaattaaacatgtctttcttccgattaattctttattatcttttaaacttgtg
actttcaactcagctgactttgagcgttttctttccttccactccaaagtagactgttgggtaaaccagtctcaattttcagtttcttagttttaaacat
gtatccttctatgaaaaatgatttaatcttaagagcagcaaggggtatgctgaaaacaaggctcgcatttataaagataaacgctggatactaac
tgattaatttacaggggaaacggtagaacgaccacctgtttggattatggtacaaaatcttgaaagcattttcaatatagtatattaactctatcga
attttagagacaagcaggacgatatctgccaggtacgcgtacattactcatcaggttttcggggtttcactaacaattttcctttagaataccacaa
actcagatcaaagcagagtttttttgaaatgtgtcaaactccagagacgtcttgtgagttgactcttcaacccattaagcgttttgacggtttgcttg
atgccggcattatttttagcgatatcttagtaattcctcaggcgcttggcatgcaggttgtcatgctcgagcaaaaaggcccgcactttccggagcc
gttggtaacaccagaagatcttgaaaagctggaaaaggtccccaatattccagctaaattaggatatgtaatggatgcgatttctttgacaagag
aaaatttagatgggaaagttcctcttttggggttttcaggagcaccttggactattatggcttacatgattgaaggaggcggttcgaaggcgtttac
aaaagcaaagacttggctcttccgttacccagaggcttctcatcgtcttttaaccatggttactgatgctaccattgtttatctgacgcaacaggttt
acgctggggctcaagtatgttactactttgttgtattgatgtcaactaataatattttagatgctacaaatattcgattcttggccggagagctttca
cctcaggatttttttcgaattttcctacccttacttggttaaaatttgtgaaggggtgaaaaaggaactgaaaaaaagcaaacgaacccccgtgccc
atgattgtatttccaaagggagcctggtatgcccttgacaagctttgtgattctggttacgacgtaattggtcttgattggactatagatcccaggga
ggctgttcgtattcgtggagagcgtcgcgttatatttcaaggtaatttagatcccaatatcctatacggaggtagagaaatcattgaaagtcgtaca
aaagagatgattgaggcttttaatggtggaaagcaaggctatattataaatttgggacatggtataacacctggtgtgaatccagacgatgttcgt
ttctttttagaacagtgtcacaagtatggctctcaaaattgagccacaaataaacttatgttttctttagagtatgctcatttgtctagtctctttgact
tgactttaattctcttaatggcttggttctctactgcctttttattcgctccttcgaagtttaaatgcctttctatttttttggggagctttgcaaagcaattt
aaaatagtatctagctctccagtgtttccacacaagaaaataatttcatttctttttgtttaactttcttgcccatctttaaagggataagcattcactc
gatcactcaatgttgacatttttgtaattactcgtagatagcggttttctacacttagcttattaattgaaaaattatgctactttctttttgactgttat
attttaggtctcgtttggcaatattggggaggatgtacctgtcctttaaggcaaaactattttctataaataatgtatatacataccagttaattagtc
aattagaatttatgaatctgtccatagctaaatagttgtgcttatccaaacatatatacgagctttatttattttttctgtagcacgtataataataat
aattattatttattcttctatgtgtttattctttaatgtatgtatagaaaattttgtgatatattatccgaccaaaaagtacacagtacttcatactacc
ccaaaaaaacaattctccgaaaataaagcaattggtttgctaaataaaaaaaaggaattaaagatttgtagcgtttgatacaagaagttaccac
ttatacttaaagtcgagaattattcacgattgtgtctcaaaggtgagatatattaccaattcatggatattgcggatcgaattttgcaaaacaacgt
tatttcaggaattatatttttttaccttaagatctataacccttagtcttgttttcgtcacaaaattttctttaagtatactagcattcgcttcaataacttt
accaaaatggacctataaagttttaagttactccttaacgattcaatttagtttcaagtcattgtatgaagcatccagtttttttttcaattaaagtgaa
atgctaactcggtagacttttcttatcctttgtttccttttgcgttgccatcttgatcattcgttacaggtacctaaacaaatactctcgacttcctcacg
aagccccaataagagaggctaagcttggtcctgatgctagcgtccaaataagtgaaccaaaaatcgggtttcaaaattatctcgatgaggtatgg
ttcatacatattagggtcacaaaccactaacactatctagtttttaagtgccatcagtatatttgggtacttagaaaaacctgtctttgtagaattag
ctcggcatattcaaactcgacgggttgctgaaggagatacaattgatcttgacgaagaatcttcttttatcctggttgtggatggctgttttcaagttt
atacatctacagaaccttccaatgatgacggttcaagagatccatcatccctgcattctgagtacaggcttcttactgaagtttccaatggtgcccc
gctttctagctttttcactgtcttagaattattcacagaaattgttccagaagtttcaattgtcccaagcaccactcagtccacaggtacgccaacag
atcttgtagacaacccctttccctgtaaacagcattggctctaaaaaatcacagtcgaaagatcaatttaaactaaaggagaaatcttcaattgttg
ctaaagctaaatgcgatactacgatagctgttatacccggaagcgcctttcatcatttggttcgtaaattccctaattccagtgctcagatagttcaa
gttattcttacacgttttcaacgagttacattctctacaggctttgagtatcttggattgtccgatgctattttcagtatagagaaaaacttcaatgct
atcacctcttatgaattgccaaattatattcgtggtgatatcattgaaactttcaaaaatgaagcaaaatctgagaataggaataagaaagttaaa

ggcggtattattttatctcaaaaaaggggccctgcccatcgtactggcaactttaatagaaattttgaggtttttgggtctgaggttgatcgttcaac
agcaaatgcgggtgacttactttcttctacaaatcctcacgctactctttctcaatcagtcgctccaaatatgaagcggtcaccgcttcgtcgagcat
actctagagaagtgggtgattatgatgtatattcctcttttaggaatggtttgttacattgcattctgaattctcttggcatagccaattatggtataa
aatcccagagtactgcttcttcagtggatgatgagttgtcttctggcgagcaagaaagtattaaatctactgatcaatctgaaaagtctagcaaag
ttgacttcttaggtcaacttgctatgatgagtgctaccggcgtcaacgaagatactaaaagccctgcaataagcaaccaaaaacaatctactatcc
tcgagtttgccaaagaaattgaaattattttctataagaaaaagactaccgttgttcagcagggtgatcatgcagatggcgtattttatatcattga
tggttttctggatgcaacttgtccatctaaagtagctacttacggcactcaagaacaattagagcaacattcttttatgatgaaaccaggtggtttg
gtaaattatcaagcttgcgtttccaactatcggtcgtttgtgaacgtaactgctaggacagatgtcttagtaggatatttgccgcgtaccgctcttga
aagaataattgaacaggaacctctaatttctcttatcattgctaaaagacttatatctttagttcctcctttactacttaaacttgattttgcagtcggt
tggattcacctaaatcccgatcaagtattatttcataagggagatccaagcgattcaatatacgtagttcaaaacggacgtttaaggtctattgac
gacagtaatacgaaagcaaattctgattactttgatgaatatggaaatggtgatagtgcaggtgaactggaaatgctaatcaatgccccaagaa
gcagtacattatttgcaattcgagattcagaacttgcaaaaatccctcagacattgtttaatgctctctctcttagccaccctgctgttggtctgcagc
tctcaaaaatcattgcaaaccgaatgcatataatgctaaataacaagttcatttatggaggtattcatcaaactaagagggaaaagcatagtata
cgtacactagctgttgtaccttcttcttcaaccgcatttttaatacttttctctcaaaaattaacattttccttatccgttttgggtatatccgttaaaat
attgagacagtcctcagttttagaacatttaggaaaatacgcttttagtcgcatgggtagattcaaacttgcgagctatctttcggatttggaagac
aaatatgatattttgatttacgttgctgacacaaatgttggtactacctggtttcaaacttgcgtacggcaagctgattgtatatatatccttgcaga
agcagatcggaatcctcgaattggggaatttgaaagttacttgattgcaatgaaatgtactgctcgaaaagagttagtacttatacatcctgagag
attctgtccttcaggattaacaagaacgtggttaaaggagcgtccatgggtatacgctcatcatcacttacagttacgcgctggacgggacaatga
aatggttgatcataacgatggaaaagaatttctcaacataattaggtccaaagtaatgaatcttcattatggatttagaaaatacatcgattggaa
acacctacatcctgtctatcaaacgaaccgagcacaggagagtgactttgcacgtttagctaggaggatttgcggcaaagctatcggactggtttt
aggaggtggaggtgccagagggatctctcagattggtgctctatatgcattagaagaagctggaattccttttgatattattggtggtacgagtata
ggagcattcaatggcggattgtatgcccgcgaagctgatctggtgccaatgtttggtcgggcgaagaagttttgtggaagaatggcaaatttatgg
agatttgtactagatttgacttatcctcaggctgcttacaccactggccatgaatttaatcgaggtatctggaaaacatttggtgaaagtcatattga
ggatttttggctaccattttatgtaaatacgactaatataactcattctcgtatggacgttcactcaagtggttatgcttggagatatattcgagctag
tatgtctttagccggtttggtacctccaatgctctccgataatggggatatgctattagatggaggttatatggataaccttactgtaacacacatgc
agtctcttggagctaacacaatatttgcaattgatgttggaagtgaggattctcgtttggcaatggattatggagatactgtttcgggtgcttgggct
ttattcgctcgttggattccttttatttcgaaaaaagcatatccttcactcgctgatattcagtctagattaacatatgttacgagtgttgctattggcg
agaaagttaaatcgacaccaggttgtttttatatgcgtcctcctgttacagatttcaaaacgctggaatttggaagtttcgagaagatctatggtgtt
ggttatgaatacggcaaagcttttattgataagctcaagacagaaggtaaacttgacgggattcttggaactagtactcagtaccaaaagtatcc
aaagtatttatcgccccgacggaactcattataaacgaatgttatgatttttgatacaaactgtatttacaatatagtcgtattgaaatgtataatga
atattattcatattcgacaacctttttcttgcaaggttcaactactgcacaagtggattttaatactttttgtgatacacaaaggaaggctagttaga
ataatatatatgaaaatatcttttatttattgattgtttgataaaccgtgcagtaatagcgtaaaccaaataaagaaatactctcgccttgagtactg
tgatataataaccttcactaggagttactacccataacgttgcagctcgttaaaccagtcatatatacgaatggagtagtcaacgaaatctttacct
tattgactgcataagcctggccgaaataaagctgtcggctaggagtaatatgtttaaccttttacatccaaggaaatcatcacttgcttatgtacttt
tattcattgtcgtaagtttgagaaagtatgcggtggcatttcctttagctttgcgagaaaaggctgtcaacggttgcttaacagaaccgtctgagtc
atccattattctgaatcagcaatctaatttttcttactattcgaaacttcttattagtgctgttctagtcttgttgggtggcgtgtttgcaggacttacga
tcggtcttatgggatgcgacgaagtgtatttgcaagttttggagcagagtggtgatcgttacgaaagaacatatgcgaaaaaagttttgaagcttt
taaaccgtggtaagcactgggtgctggtcactttgctgttgggcaatgtaattgttaatgaaacattgccgattgtattcgacagtatcattggcggt
ggatggccggcggttttaatctcaacggcggcgattgtgatttttggagaagtaattccacaagctttatgtgtccgttatggattgtctattggtgc
taaattggaaccactggttctgtttatgatgtacttgctcatgccaattgcttatccaacggctttattattggatgcatgcttgggagaagatcatgg
tataatgtataaaaagtctgggttgaaaactctcgtcactttgcatagggatctgggcttagataaactgaatcaggatgaggtgacgattattac
ggctgttttggatttacgggaaaagtcagcgaagagtattatgacacctattgaggatgtttttacccttccaatggaccgcgtcgtggatgaaca
actaattggcgatattatttgtgctggttattcgcggattcccgtgcatagaccaggctttcctcatgattttatcggtatgttactcactaaaacgct
aatcggttatgatccagatgactgttggccggtgagcaaatttgcccttgcaaccttacctcaaacttttccagatacctcctgtttggatctaatga
actattgtcaagagggtaagagtcatatgatccttatctctgatcatcctggcgataaatttggtgctctaggcgtcatcactcttgaagacattatc
gaagagttaattggggaggaaattattgatgagacggatgtctatattgatgttcacaaagggttaccgcgtgtgagttacaacaatgaattttgg
aatagattattccaccgctctcatttgaatacaatggctgctttaacaaagcgccgtcgtacgacagtgaatggagaaagtcaaatggatgagca
acgcccagttttgaaccctaggaatgccgctatgaatcctcgttatgttccgaatgagcgagtaaaggtgaaggcacctgttagcatcaagaacg

agacgggttacggaagtatggaagcatcaaatgaaagtgttgcctctctttctaagaaggttgacaaagttgaaacggaaatagaggctcactc
aaaaagcataccaaatattcaagaatacacttcagaggatatttctcagcgtgacttagaacatcaggcaaatgacggtattcaagagcatgtta
cacatgaccacaaccgtagggaagaagaagacgcttctccaattgattataataagattgctgagcgtggttttgatcagttaaacggtaaggag
actgcaaaaaaaccttctcgatttaatgcagactccccaagaattatgagcaaaaagtcagatagcgtatcttctgattctgagaggacatttaca
ggaaaaagagtaagacttggtaacgtaattgaaagcaatcttgtctctgcggatggtatcgagcgcattgttatcgaaccatatgaaaggaccgt
taacgaaaatccggaagatattgttgaaatggtggaacgaaatggtattcttgtaccgaaagtcagcatcacgactaatgattctcttgaagaga
atgagtctctaaacattggtgatgccaatatgcatagtcccattgaaaatcactcctctgttagttcttcgagaccttcaaaaaacaaaaagaaaa
agagaaggagtaaaaataaaaaataaggtatggggatttttttaagctcactagatgttttcatttaaaactaaattatcgttcatgatgtcgcggt
ttgcttttcatatcgccccattggttattctatttctatttattcctaattattcaaatatgatcgatctagacgctcaatctttatttattctttaacttaa
atttttgatgatttcgatgaatctgagtcctatgtgaaatgcgtgtttcaatatgatgcaccaccatatataccttcctgctagtatgaacagataat
gccaattctatttgtatacattctacatagtactgttagtcaaaataccttagaagtagccagtaatttgtgtaccctaatggatatttaactggcaa
gggtattcgtggttcataaagcaatatacctggaaaatgctttatactaagatgaaatctcggatggttggatgatgccgatgctatagaattatgt
ataaactaaaacaaaaataatatttaaagtcaaacagtgcttatatcaattttggtaaagggaaaggtaattgaaggcatagctcaatgaaaac
acataatattcaagcaatttgtagactcttcaccatcaacactgtaaagaaagggtataatatgtggctttcagaagtacaaagtatgtttaaactt
aaaagtaaacaattactaaagataaaactagaaattggaattggtgtaacaagtttggggcttctattcatggtacttgtaaggctagaagaaga
caagacctacttgactttatttttgctattaagattctaatattacatttagggcgtaataatgctgtttgacggccctctactatctcttggaaacgta
tgttgaaatcagtcggatagctggtgtctgtatagtcaactcaagcaagccagagaacagaagtctatgctttattctaagaaggacctgtaatta
gactaacctcttttagatcttgtctgtatcaggcgtattcatgattataggaccctcgaaatcagcaaactttttttataagaaaagaaagaatgctg
ggaagcataagttttgttggaggattcctcctcacgatcctacgctttcctgttcttggtttcttctttgaatgcctcggattctttaatctcttccgggt
gttttatcccatcattattagctttttaaggtctttgccttatattggacctttcgtggatcgtttgacttcttaccagcaaagtcctgtgtaaagcgtgt
cctgtttctaatgattaaaagattacagcataacatactctctttaagataagtttttaagtaaatgattcgaatggagattagtctctttcttaacgt
tacaacaatctctttataataaactcttattactttcttttcttttctgcttttcaaacaaactactacggttgcaattttgcatggaatcatattcattc
ataaacgttttcttacctttttgaagcaacagaagggttataaccttatttgtagactttttagttacattcatatgtgcattgctgataataaacgag
tttcttaaaagtaggggtcaaaccaagaatctgtatacgggacaaagcatgaacctgaaaacttaattaacaacttggtatatgcatttctaagg
accagtagctaataaggaagaatcttgagtggcacgttgaccgtaaccagtccaagattcatcatacactcgaacatttgtaaacccacataatt
caagagcaacaaaaagtacagaggcagttacaccagaaccacaagaggtgatgatgggagcagatttatctttaagaccatgcttggcaaaga
tatcttcaagttcttctacaggctttggttcggtaattccgggttttacaagatcagtaaaaggaatgttgatggaagttgggatatgtccgcttgcg
atgccagggcgaatttcaggagcacttccagtaaatcgtgtatgtgggcgagcatcaatgatacgagtgttgcttttcgacggatctttaaggacc
tgggcgatatcttcaaaagaggcaaccaagttattgttcatattagcaccttcataattgactttagcgggtgtagaagggctttcagtttctaaccc
gtaaccagaagttttccaatctgttaacgcatttggaaacaagaagacattcttgtgaccgaaaactttaaaggtccaatatacacgggggggagg
aaaaaaatccttttctgtcataaataactacattggtctatatccagttagaaatcacttattcattctcaagcacaattttacgtacatttcgatcaa
ttccaagatttcctacgtatgcagcaaattcatcagctggtggaagcatgtgaggtaagctactacgatgatccttggcttcatcaaccccaaaaa
actgagcgcctggtagtcgattttgtaagaattcctgttttccatctttggattctgtaggtaagtaccaagtagcgtccaaaagaaccgatttttga
gcttgctcctttaaaaggttaaccacgttccttgcatttaataatgtagtcatccttgtgcttgctgaaaacattctttttctgaggactgttgtttttgg
ctattttctcgtttctacttgggaaatttataagtacgaataggatattatgtaatgtcgcttataatattgctaatatatatgcttgataacgaaaat
cataaatacaaaacgcagtgttatactacttactatggcttccccaatatgatcaattgtagaaagcttggatacagaaatttatacgaacaatga
tattgcattcgactaggtagataaagcatgtttggagaaagaaaaaggaaaaaaaaaaaaaaaaaaaaaaatacccccaatagtagcactat
agatgctacctcgtattctagaaaagccatcgcagaaagacttgtttacactaacaagcagacttaatgttctctaagaaagaaaagcttagctat
ccaaggacacgtatttgtattagtatttcaacatatatatagttttaaattgtgtatgaagcaatacatggaacaaaactcgccattattgccatga
caatgaaaatagattaaataaaaacgaacgtaaaatgaatcagacaaaaaacaaaaaggaaacaaacatatagccattcgttgaattttttttta
gaaaaataaataagagttatcatttcggttgcctaagtaaaaatattctttcgaaagttttctctgattgaaaactgtgcttcagagatccttaccag
attttcagacgttaagacagatagtcaaaaactaaagaataaaaaacaaaaaaacaattcaaaattaaagcggtgaacatctgtaatttaacgt
ctttgcttgagtcgcgccagcaaggtactagaattaactggtccggaagccttggaactagtagaagccgaattttggcgagaactgttcatggag
ggaggagcagctctattccgattagcagaaagttgttgtcgtcgagaatttgcaacggcacgcaaagcatcattagccacctttgaagcttctttct
ctacaagtaaagtctcgggttgagaagcctccataatttcgtcatgctttaacgtgctttgaattccggcgcttgcaaagatatcatctagcacgga
ttcatcgccatgtgtatttttagaagaagtaggctttgtgacgccgctatcttctggtggtttaaacttttctatagaagcaatcccaattttatcaaa
aacctttcgattcttgtccttttgaggatttccctcaacatcctctcgagatctctgctttttgctttttcttcatcatgacttgaaggttgctttttgaag
actcgttcagctcccaaaaacatgttaccagtctctgtaccttcttccttatcgttgcctaatgaaaataaatcatgcagatcggtcatcttaaagaa

tcgtcgctgctttggatctttcaaaatcttatttgtcaaaaattgtttgaaaatttgacgatgatatatcttttcttcaattgttccagcagtcattaaa
cggtagaccacaacatctttttttgtcccaatctccaagctcgttcacgagcctgggcatccgttgatggattccagtctgggtcgaataatatgac
acgatctgctccagttaaattaatacccaacccgccaacgcgagttgtcaataaaaatacatcataaatgtcgtttttgttgaaattatcaactaaa
ccttgtcgaagaccaattgatgtccctccatccatacggcagtaacgaatttcaggaatacctttgatgctgttctccaaaatatcaagcatttgtcg
tgtctgggaaaaaagtaatgtacggtgcttttgcttcttccaaagctgtaacagagaattcacaacttccaattttccagactttttaggatctccat
aatgatagtccttcttttgagataaaaattcacgaacaaccaaatctggatgattacatattttcctcaaaatatcaattccataaagcatttgacg
cttgccatttaaaatttttgcatatccgaaccattcaagaaatcctggtaggctttacgttgaagaggagtcagcttgcaaaacaaaacttgttcg
gattttttggcaggtctgccgctacatctagcttcatccttcgtaacaagtatggagatattaagtcgcgaagcatacaggcacacttgtaagcag
tttgtacttgaacgtttgatgcatttgcatacccaccaatattgataggaagagcgaattgattttggaaaacaggcagcgttcctaagcgaccgg
gaaaaacaaaatcaaataagttccacagttcaattaaattattttgaataggggtacctgataaaataattcgatggggggttcttacttgcttac
aatttacagacatttcagaatctggattccgaatcttatgtccctcatctaaaacgcaataaccccattctcttggtaataataaatcgctgtaaag
acgtaacccagcatatgttgtaatcaatacatgtccttcagtaaacacgctatctaccaaagctttggcataactgtgaaatgatttagaagcattt
gggttcacattagcagcttcctctacagtatcctcttcctccagagatcgatattgtctcttttctcgggtagcaagagcaccactacctgtagcatg
caaaacaacaacacgtaaaggaccccaccattgatggaactcatttacccattgtttcattaaggtagccggacaaacaatcaacgctggcttttc
gtacatccttgaatggtgaagtgaagaaagaaaagatacaatttgtattgtttttcctaatcccatttcgtcacctatgataccacctgcttcttggc
tgtaaagttcccataaccactgtacacatgtaacttgatatcgaaaaaggtgaggtcgaatgtcaccaggcacggtgaaaccgccttcaaaagtt
tggccgcgtttggtaggatgtggtctaaaccattcttccgtatctacgcttaatgaagattgattggtagaattttgttcattagacgtctcggattct
tcttgttttactcgtttcgtttttcgcgtaatagattgcgctcggtacaccaggaatgtaaacgcttacgatacactaaatcatcaccatcgtctaca
gttgaagtagccgtttgttcagtcacattctcgacttgcgcataatcttcttcttcttgctttggcacataatcatcatgctcttcggtttgctcaac
actagtgcccttgatattcgtgtcaccagcaaattcatgtttctcgggtatcacttgattacttggttggtagtcatgaaagtccgaacgtttctcaat
tcccgaaaaatttccgaaaggtgtaattttacctgtacgaatcaattcttggcgctcgacttcttcattagcatgaggatcagtaaacgcagtttgct
gaccctctccaacaccttccatacgtaattttatatcattctcatcagattttaagttctgaagcgttgcacgaaatttatccagttgctttcgaagat
ttcctttcacgctaatttgcgtcaaacgactatcaattcgcgcttccaattgacgagtcttttgttgtacggtatcaatctccttctgcaccttttctaac
ctttttttttcaatctctctcgtctcactagcgatatattgatctgctgaatttgtcacatcgcgctccaaactagcttgatcaacagtgaagactccta
attgagtcaattcctgatttgattccatattcctgtatattttccgtactagtagaggatcattgtattacaatatcataaatgatgattgctgctgtat
tgtgcagatggcaaacaccaacaccttgtattatttacttagcgaaacggtgcgctatgctacaatattttgggtaacatcgttcaagtaacatgta
ctgcctcctaaacttgctgttgacgcgcattgttcttgaagtcgcgaatataaagggagtgtagatgggaatggatgctaaactctccttgactaag
aagtttacagatatctattcaactaagaccattccaagctctacagagcaaccatttaataccaagttcagagaattcgtaaaaagtgaaatatg
gtcaaaaatcacagctgaattctacaactgtcttgaatttgaaactgccattcgttttcgttgttatgaaattagtgtaagaaccgcttgaagttgtt
acgctgaggaatgtttgctaacaagaatcacttatagtctaaaaaacatgactttgaggagaacgttaaggagctattttcaatcctagacttact
agttgtcttgaatgaaattgatgcttcctactgtgatgcagtcttcccgtttttaattttagaggagttaatggacattcatagtctaacggagtgccg
aaggctgtatgagtattttgagacaagatcagttcttttggatggtttagtcagtaatcgtggtcgtggacctgttttactgcgtatttcgaacgaatt
gttgcgccgactttcgagacaaattgaccctgcgctttgtggtcgcattcatattttgctcagcaaagcttttgctccagaagaaaggagcggctct
aatgttcgtggtgattataatctagaaaattcagtaattactaccaaggtttcgtcaccaaatatccctgcatccgatggaaatgaagcctcttattc
tcaaaaagtgcagtctctctatcaaatattttgggaattacaagatctgcttgtccagccaccaaaaattgcacagatttctgataatttacaaaag
tttttgaattccacaaatactatcttggaatcttttgagtctattcaaaaaaacacattttttttagataatcccaaacctactattaaccctacgtcg
aatgctttgcttccggaaaagtacacagttataaacgacaatctatgtccaaattacattcgttcccctcctctttttgactttcaactgatagatga
aagttttcgtcttcagtttttgctgcaggctgttattttatgtgatttttttgttagacttttctaaagaacgattggagtcacgccaaaaccgtcctttca
caaataagtctgtagcgcctaatacaagtttatctgacgaagatgtatgtttttgatcgttaaaggaattttgattgactaacatattgtaggagaa
gaacgtgtcaaacctactgaatcggatttttgcacttttaaaagaactaaagaatggtgcttattttgatacagtgacatccgtgatcaacgtggaa
agtaattggaaaaattggaaaggttttggttgtctttcgtttgaaaagcctttacttgataaatccattttagatgaagcggttgctggtctaaaaaa
agttgcaggtacgcctccgaaaatccgttatgccatgggtaatgcagcgcttgctaaaatatggaaaaatgctggagagaattctttagaagattt
aaaaaagaaagaaagatatcagatacctactccgaaaagtttcctttctggtgtaagacacgactctgctgaaattgaagagtcggtaaaggaa
gatgagcgagaatatcatcaacaagcaaaagcttcaaaaacttggcgtgcttttcgttctgcgatcaattcgcatttagaagcctttgaatttaccg
ggttgggtgatctggagctattgtctcaggctattgaaggtcatacgacattggaaaagccgaaaacaaatatgaatcctgctttcgacataaac
atcgttgagaagggtgatgaaaagttacatgagaaagtgatagaagaacaaaaaaagcaggaaattcaagatgctacgccaatggatgaaga
taaagaaccagagtctaaaggagagcagcagattgagcctgtaaccacaaaggaagatttgaaaaagtcgaatgatcagaaggatttatctac
agatcttcagaaggaaaatggaaaagatgaagctgaatatgaaagcaaaaaagaagatggtaccgataaacaaccaaaatcagtgcctaaag

atcaatccgataaagtaagtaatccctctgatgaaaactctacagcagcgcatgaggaaagaacagaggaggccgagaacgtcaaagattcag
aaaataagaagcgatctcgagaaggagatgtaaatgaagaaactattagtttaagtccaaaacgtcaaaaaactgaagagaaagagaacact
gattcttagttggaagtcttaatgtatgtaaggataatgtctacaaagttttttgttggtgtaggaatttttcatcatagttttaattaatttatcttaat
taaagtaaaaagattacgacgtattttaggcctaatttgggtttttcttgttcttgaatgctgtattcggttagtttaaatctaggaaatctaaagata
aaataaaaatcaaatgaaggtttaccagaatactactcgcttttatgcaggctcgtgaattttgatccagagagtacattcagaaatggaaatcct
aaaagcatccataacatagaaaagacagacagtactgaaaggcactaggtttaaagagtaagtaacgttgactggaataaagtacctaccact
aagacattttcgatacgaatgagttgtaattccagtaaaatctctacaattgcaaagatcgcagtacctccataaatcaacgctacaggagcaac
agtaagcattttttttattttatattaaggatatcaaaaaaagcgtcttgtaagaaaaaagcacttgcaaaaatatgagagaaaatccctcctctc
aaaacgtcaatataaacaagacactaacctattgtaaacaaagtttaggaggacataaacaatgcacgaaattggtagcgctcacacgtttcgt
aaatgattttcttaattgctttggcttcgaaagttcgaacaaagcatcttttgctttgtatcgagttggtaaaaataatcctcaagaaagagacaaa
ggagcatagaagccgtacttgtgacagatgaatcaagttatttcttttttttctgaattcatattgatagggcagcttgtaagtggcccatgtctacttt
cccaacactttctaaactcattcatctaatatgccaggtcagatttgtgacttcacaaaaagatccttaatttacctcttttgagcattttctccaatc
atgtttatcccaataacaatcattaagccttaaatgctcttcgacacatcctccttttttcaagagcggtgtcccaagcgtccttctcattatcttccact
gagttggtgttggcgtctggagtcaaactcatttttttatttattcaattgcagaataaaattaaataagtaatatgaaaatagatcggaatttggaa
atatatagtcgtcgagaattgatgagcgttcgagtaaagtaaggatagtgagtaggtgacggttatcatcaggaatacggtcaaaagtatataga
tcctttctaggttcatctatttcacttagagtacttccaacaaaataaaaatatatatatctgctcttttaccgaggatactgtgatgaatattgtggtt
gacttggagaaattggttgagcaacagcaggagtcggagcagaagatgagccagtattatcagctttattaatggattgatctccgtcggaaggt
ttttctaaggtagcatttttttccgtgcataactaaatctgacaattttgcatttctagtctcttgctcagctcctcctaaagaacgaggttttccccatt
gagctttaagacgaaatccgtcaatagaaagatcaccattggcacaagaggcagctgcaatttcagcacttaatctgcttttgaagttgacaaag
gcacattttgcccgatgagaacaaacgacagattttaagggaccaaattgttcaaaatgtcttcgaattttataatccgggagttcgtcctcaaca
ccaataataaaaagagaagccttgataatagttagttttgaatcctggatcctgatgatttacttacaattttttttatctttaggaggctccatgtca
actgaaagcaaaacacgaccgtttgcaactttttttataggaaagagagacttttcagaacttttggacgtatcacttgctttagcatccttcaaga
gctgtttagattcttgttcatcaatcttcttacgcgcgggtttttggtagggctctcgtcgctcggtctttcgaaccagatttctagcagcggcatttga
ctcttgactatcatatggtgtttcttcgtttttcaagagtctttgctggttttgagcaaagaactcccggtttatgtcgtttgttggtccattgtctaccat
ttttaaagcagcatcacgtaaagcaatctattgtttagttagttttataataatccatctctttctatgagcatcacataccggtaacccaaattgca
aatcgagcatacacgcttgacagcaatttttcaatcgagcacaacctacacatatttgagtttttcctctctttctttccctagtagttgaccaacgga
atactgtacacggccgcgaacatattttgcattcttgtcctaaaggttcacgagtcatacgaacatacgaattatctcctagacaacgttcgcaaat
acttggaaattcaacatcttcccattcaattttattgacgtcgccttttggtactaaagacatgattaaaacgacgtttagttaagttcgtggagtta
gataggttgtacttggtaaactcagaaaaccgactaaaaatggtttttacgaataaaagaaatttaagctagtacatttttaagtaattacatgct
aaaattgtatatcttcataaaacttagatcatattcttgaaatagctatggagcaaaaatttacatatacaaggaggggcaaaagacaatattac
ggcggatgaggttttgacattcttccattttagaatacattgaagcatcacctacaacacgagctgcacctcgacattctcgaagaacttcatccaa
ccgaatgattgtacgaacaatggaaccttccaaaatatctgtcaaatcggtgattcgattaaacgacatacctcttgcccattcataacaaacttcc
atcaagccaaagcgtggttgagattcaaaatcatttccctcattaaagtataacacttgatgcttttcctggatgcgattaactcgctcagcaacct
gaagaatcatctcttttcctttttgtagatgggaagaaatattgggttcaacttccgtcttctcatcaaaaacaaaagcagaaagcaaggctatagt
ctcctcacaagaaaactcacccaaactattttccaaaatcagttctgttaacaccagttcgctagttgagtttatctcacacgcaacacgaccctttt
aataaaacggttttattttcatcaatgtattgaagctcttgaagtacccgaattctctgttcataatcaggaagaagctcaaggttttgatcagagat
aaaatcccgcaagtttctgatattattttctaagtcatattcttgaaagcatagtgcatattgggtaacaaaattatcttgtagtaatggttcgtaaa
acataagtttttgtcccacatatgttcgcttctcttgagcctcatggaaatcaaaatctttcactttgctccaatccgcttcatcaaaaacattgtcatt
aaatcccttaactgaagcaaattgatcgctcaaccttctaaaagcacccccctcgacggtcacgtatacctccacaatcaatacgtaaaatggtttt
acatacacgctcaacgcttgaaagactaacaagtccaaatttgaactgattattttcagtaaagagtgaagcatctaatatatccaagaaaggtt
ctgtgaaagccagtaaatctgaagggcgcttcatagcattctgaggtgtcaggtaagcaacttctaatgtacaatctgcagaagtagtacgcattg
ctgttcctaatagcacgccgatgctacgagtactatcaggttgctgatagacaattaatcttccatcacgaaagacgcgtcttcctgtagctgcgga
gatagctttcaaatgcaatttcctagagtatgctttatagtcttcactgtaaactatgcattccttcatctgttgtatatctatttcattgagctctttctt
aagtaaagaaagctgctcttcatttgacttgatttcagcttcatgttgcggcatcagtgtctgatttgcattttcactaaaacttcgtttaatcatgtct
tcgattctcaatgtttcaacacgaagcaaattgaggatcatattatatgttagtctgaactgactgatcaattttgtcgaaggaccgagaatcatgt
gtcgtaaactggctgtatctggaagatcagctcgagataaaataattactgtccctgtaacatctaaaccacgtcttccagctcgacctgaacattg
agtatattcaccaggtaaaagctcacggaaacttcgtccatcatgtttctggataccggagaacacaacagatcgagcaggcatattaacaccca
tcgcaaatgtttcagttgcaaaaaggattttgacaaggcctcgttgaaacaaaagctcaactatctctttgactataggtagtaatccgccatgatg

taccgctagacccctagaaagcatttcacgcatacgaccgatctgaggaagtgttctgtcttccttcttcaggcgagctacggccttttcaatgact
atatgaacttcactttttttcttgatggttattcaaatccctgttcgacaaagtgtcaacgtattcctcacaacgtttttttgaaaagacaaagataatt
actggtaatagtttgtttttctgaaggtgcccagctaaatgtacccaagtatttgcatctcggcgttcagcagacttggcattacctcttccacgcat
caaattaacttgagatccccgaccacgaggtgctccaccacgacctctagcattgttgttgttggacggtggtaatattttatcaggcttcttaagcg
tatcattcgcagctttgtatccgtccattaggaacctgccatgttgatccactaccttatagacgttatttttaacccataaataatgttcaagagga
acaggtcgcttagaagtagatataacgtatatatttttcttcttagtacgtccaacccatgaagcaaattcctttgtgtttggcactgtagcagagag
aaggattacagttatatgaggagggagcatgataattacttcttcccaaacaacacctctttccagatcattaacgtaatgtacttcatcgaaaat
aacaaattctacatcgcgaatcaaatcagcaccacgatataacatgcttcgtaaaatttctgtagtcataatcagacatgatgcctctggattaact
tgtacatcaccagtaaggatacctacatcttcaaatttttccttaaagtctctaaacttttgatttgagagtgctttaatgggcgaagtgtaaatagc
cttggtcatgtgcttctgggcaagcgcaatagcgtattctgccacaacggtttttcctgcactagtgtgggcagccacaaatacggaatcacccatt
tccagatgataaacagcttctttctgaaagctatcgagctcaaaggggaagctaagggccatttcaggtaccaactgatggaagttatttatagta
gctcggctatctacaacatgagcatattcttttcgtttggagacagtgccacttgaggaaggaagggcaacgatggtgcttttgtcgggaagtaag
tcatcaatctcagtgtgttttgagttaatgctcttcagatgctcatttaattcattaagtgtagtaaattggcgggtagaagggcgcaccatttttgtc
tcccatgtatcaggattaaattcatcattcaagttgtttccctcgacggatttgttcataactaagcctctagaaaacccaggaggaacttgataca
aaccatcctctcccttcaacgccatttgtactggcttaggggcagctgttttctggggatcatcaggatcatccgtcaagaagggtatattattggta
ctgcctcgaacaaattcacttttggaagcaggtttcctcaaaaagcttgatgaatttttcgaatttagatcagcaatgtcttcagaaacttctttgta
atctatgatatgaccttctaatccttgccttcgaaattgcaagttagtgcgcgacaagcttttggggaaattgaaaagaacattatggtctacctctt
tttcccattgtttttgcaattttttttacccattcatgagagaacgactttgcaggtttaagaaaggtttgtgattcttgatttatatccaacgatgagac
gaaatcaccttcgctcgtacatttttttgtatttagattatctctaacatgtcttaactgactggaagcatgaatagaacttaaaaggctttcagaca
tggatatttgtcaagtcgtttttgggtgaggttagatggcaacatccactacaggtcttcagagatcaaccagtcttgcttatctttggcaagaattg
tctttatttaattaaatgtttttttaaaggttttatttatttctgagttttataactagtggtggataaattgaagctctgcagtactgggttaattgggtt
attactatttgaccaaacagtttcgtagaaaagtagacagctcagtttcttcaataaataaagcttcttttgagtaaagatacaaaatgacatcaa
gtaacccaagttttcatgaaacacgaaaaatgatgatacatttatatatacttcattagtagaaagattgaggaaatgcattgatggaaccagcct
tgaatttgtttggggcaacttggaacaaagaaaagtaccaagattaaaaaactagagggattatgaacgaatatacaaaaattaaagaagggg
tttggaagaagattcctttctaatggatttgagaaaaggtataaaacgaaaaaaatgggtatagaagcgaaaagcctcggataagtaagataga
ggttggtagagccgatgtaaactaatggcttgttaatctttattaggtcccaactttggcaaagcaatgtcccaaggaccatgaatttcgctagaga
aaatcgttagcatagaaacagcatgagaggaagtcatactttttaaacctttgaagttttataaaccaaatagggcaaacaaaggatatgaaga
atatggatgaaagaaaaacaaacgctataattaccgacgagactaaataaagggacaagtaggcagtcattataaggacaagagtcatgagc
atatcagcataaaacgagtaaaacttgaaatattagtatttatcaacaaaaaaatgcaacggttttgaaaaagaaagcttacgtgaatgtattttc
gaaaaatggggaacagcgcaaaccaatgaacggcagaaagaacgaagaaaaagacatgtatcgatctatccaaacgagaagccaggaccac
ggaagcgcttaaagcggcgtttgtggataaagaattgtgaagtctaggtattaaaagttagcggaggaagactccatgttttataacttaccgtac
gtgtggttgaatactatccacagagtactcatgaaagaatatattagcgagaaacagccatacggcgatggcccaaacggagtcctcagaagta
gattttgtgagagacatgagaatcggggataaaccaacgagggtaaggacaattaagatacaagactttattaggccagcataattgaggaag
gatctactttggcacggtttgctcaacaccaaatcccagagtataaaagcacctacagcgcttacatttgatatcatcccgacgattccgcaagac
agctgttttctgtgaattccaacaaagacggcaataaatatcatcacacttgataaatgctgagaaatcggtaggctgtcagctactagcgacca
aaaatcagttacctgaatgttaacatttcgctgcaaaccacttaaaaaggactgatcaatatagttgtcgggataattttgttttctccaaaggact
tttttccaaggttttctactgtgcaattcttcctcctttctcttgggtttcgcatacggatctaacaacaatgacggaagcactttttttaggtgctggtg
cagcgcagatgtcttccatagattctagcttttattttttttaccatgtaccatttataatttagacaaaattttcgtttagaatgaatcccacccaggc
atagagctgaagcgttagaggactcgtttgaaggctttcgtatatgggaatgggaaaggaggtagggttaccaagaaaagatgaagatgcaaa
cacaaagcaaagattatgttgtttgaatttgtttctttttactatcttttctgtcgttgttattctttttattcttttttgtccttctctagagagtactgaac
cggtttccctttgatgtttctactacccaagtatgctttgtcaagttttaattttgtggatttacatctattttgtttgtttataaaagtaagattgatatc
aaagggatttactgagcgtttacggattgaattgatttcctgtagaaaattaccatgtaaacaaacaagtaaataaataaacagttgggtgttgaa
caaataaagtgattttctttggctttatcttcttattggcaggttctgaacattcagaaaggaggaaattgggattttcttttgccgataatttgtttat
aacaagaaaaagcgacttttagttcagatatagtattacagtcagtagaataacgttcatcagaaatagtaatgttaggggacgaatatatcata
aaaccgttattttcttgttttattcttattgtttttatactattttattttttttttttgatatacattcacttccattaatggaaatatttttgtaaagaggaa
atcatactactttaagtgatttcttgcttttcccttgaacgcgcttatttaatatttttccctttaattttggtagccagaaaaacaaagacagaatttg
taaataatataagctattctggtttctccttgctttttatgaaagaaattttgatattgttagtaccacgttaaaaaaaaaaaaaaagcagagcaaa
attaggtagttcctttgggaacatattgtttcatcctaaactgttaaagtgttatggagggagacacgcggattgaagaaaacgcagaaaagcaa

aagtttgtgctacaatatgtgatgaatcgaactttcggagttgatgaggatgtagtacaaggcctgattcatgatttatttggtgagtcagcatcac
tgagtcaaactatgaatgaaatcaacaaaaacattcacaattacgactttaagcttcaaaaaattcaagaccagctcgatggccgaccgacatg
gcattttcaaaatctatctggtgaccctgtttcgcaaatggccacaccatattcgacgaaccaaattgaactgatgcgaagaattatcgattggatt
atgcaagctgacgagtatcagtactcgataactacactccagattcacaagtatgcacgacgggacatgtcgctggctccctctattattgaaaac
tacttgagcacttttgaaagagatggttggttgaatcaacgcgaagggatatggacattcacaaaccatgcattggcagacatggaggcttacct
acataatgagtatgaaagtagtctctacgagtgtaatgcttgtcgcggtattgtcacagcggtatgtgaatggaaagtacacagagcgaccaaaa
ctgacttacgtttatagggctatgtttgcgaatgtggatattgtttgcatgtgtattgttgcaaacatttggcctatgagtcatgtcccaactgcagca
ctaattggacagaagctacaacgattggaagatggtactaatgggaaagagaatgttctatgcatatcaccttttaaaaacaggatagacgaaa
aaaaaaataaagttcttgtcaaggaagtgacacaaggactatcgtaagctaacttaatactcataaaaatcatgagaatctttgaaaaagacat
agagaggaggaagagagtaaaactgtttcaaaatgaaatcatctcttttatggaatatatttcttttcaaaattatggtatggagaatatgctgcta
aatatttcttgggttccttcctaaggaaatgggatccaggttcaagaattactattttctttcccaaaaaaatataagatttaattcattcaacaaga
aaagaaaccaagatccaaaacatcacattggacaacgaatcatggtttcttattaagcaaaatctgcaatccaaagcttcctttactctttgatga
cagccaacaattcatgatcacgatagaggctatattcctcttcgccaactttaatgttgtttccaccaaaagcaggtaacaagacacggtctcctg
gggtcacactaggaggagagagagttcctgttttgttcaacatgccctttccggtagcgataacgcgaccttccgacaacttttcaaggcttctttc
aggtaagaaaataccagaagcggtctttgggtcagccttgacccgttgaacaaggatacgatccaaaagaggaacgatagactttgcgctcttc
aacttagtagccattgtatgtgtggggatttgcttttcctttaatcttccactttcaagtgtttgaattgctttggttagaacttgttaaaacgaatcga
aaagacactcgaatcttttgtattttcctagttggtttttggcaggttgtatcaatatccgcatcttcctgttctctcctctccttaaaagcattttgcaa
accatctggaaaattcgagcatgaaccatttagcgaatcgctaccccgctttctctttccgtctcaatgctttcatatacaccgatccaatgagtgcc
tcttatttgctttaaaactcccatccttaaacgcatttaccgccttctccgataaaataagtaacgagacagaaggatgcgttatttgggtttcttttc
gatgaagtaagaggaagaggaaaaacaaaagacaaggttcactgcgtcgtcagataaacgttgaataggagaggagctgatcgattacgaca
acttgtggaggagggttgacggctacctaccattacgtatatcaccctgaaagaaataaaagcatgggataggatattagccgaatcgtttaattt
tagtttccaatcaaggagataaactgcccacggttgcagcaaaaacgattcggcaagagccatggggaggttggtggagcagtggaaatcaaa
cacataaatatgaagtagatcggtcgtcttgccttgattaaatgatgattaaggttgaccaattctttatctaagttccccgggaaaagatacgccg
aatatggtataaagaaagcatagagtcgcaatacgaattcattcatcattgtttttgttaaggtttgcctcgtactttaggtcgttcctttgagatcca
ttggtggatccttaacgtttcgtttttattgctttggtgaactctctaatttttactttctaacttgtctttctcttttctctctttacatatcttttgaacga
aacaacatctccataatcatgactatccaatgggaacagattgctgctaaatatagtgctcatcgcgatagcacgattccttcttttatgaagctga
aaaccattccttataaagaagtacttaacgttacgaatgtacctgctacttgtgggcttctctctcctagggaacttgagttgaccgagaaatatga
cgctactgcattggccgatcttattaaagagcgcaaaactacaagtgtagagttggtaacggcgttttgcaaacgtgccgctgtcgcccagcagtt
actgaattgcgtaaacgaattgttgttcgatgaagccctcgaacaagctaccaaactcgacgaacattatgccaagacaggcgaactagttggtc
ctctgcatggcattcctgtttctgtaaaggaacacatatgtgtcaaaggacataccgcaacagctagctttttagccaaagctgaagtgatagccg
aagaggacgcagatattgttgaaacaattcgtaaagctgggggtgttttattctgtcgcactccccaacctcaatcaattatgcaattggaaacctc
tagcaatttaataggcgtcactgtgaacccctttaatcgaaaactcactcctggtggctcttcaggcggagaaggtgcacttttggggatccgagct
tctgtcctaggcattggcagtgatattggaggctcgattcgctctccagccgcaaataatggtctctttggttaccgtccttccacattgcgcttgtcc
cgaaaaggctgtcaaggagctgtattgggacaagaatccattcttggtgtcgttggtcctttggcacacagtgttcgtgacttgaatttatttatgaa
tacttgtatctctagtaagccttggttgaatgatgcttctgtcgtaccaattccttggcgtaatccggacttgccggccaagatgagaataggtgtta
tacgtagcgataaggtggtgactcctcagcctcctgttcaaagagccatgcaaatggtcattgacaaacttgagaaatctcccaagtttgaactcg
tcgatgtcgagccaatggaacataaatattcgtgggaacttatcagtgagctttactttcttgatggcgggaaagtgtactatgatttgtttgcaaa
ggtgggcgagcccatagagccattgacagaatggatcttgaatcagcccagtacgaagaaccacgatatggctggtgtttggaagctcgtaacg
gaacgtgatgcctaccgcaataagtatacgaagtatttacagtcctacggagtcgacttaatattgtgtcctgctggacctggtcccgcacccaaa
ctaggagaagcgaagtattggacctacacttctgtctggaacttgctggatctcccctccgctgttttccctgtgacgaaagtggatccaggtttag
atgtaaaggatgaatcttatgagcccatgaatgatgaagacgccttccactatcaaacgtataatcctgaggactacaaagatgctccagttagtt
tgcaactcgttggtaaacgttgggacgatgagaatttgctagcggctcttgagaaggttgtggagacactttaagtttgttataatgactatcgttg
gagatgtattatatttgatatttcatgattttacttgtatatagaatgattttgtatagaaagtaataatatgtttagtaattatatattcaaacaaatc
tcgaatgtaacgactcattcataagtgatttcttcctttattaacaatatatcgtaggtgaaaagaaaatttggaattcaacaaaaataattagtaa
atgtttggagggtcgcatactcaatcatgtcgaaagtaaggtatgtgactaaaacagcaatttatttattgattctttattttcacaaactaaagag
atatgtacgcttttatagacttctttcttctatttattaatgattgatttcatgaatttgtttgtttacatattatgtaatgcattacagtagggaaatac
agcattggcagttgttttcaagaagcgttactttcttgatactatcatataaggaatggatgaaccgatattctcttcacaaatttaccgtaattcat
gttactgaaattcaactctttgtggcatacaaatcaacgttgcccgtcggccatgatttcgggcaaaatgaattgaccgatcgactcatttgccttta

ttcctaacaggtacagattgcgtaaacttaagcgctcttatggaatgtttcctaaaggtaggatggtttggaaaagagatatatatatatatatagg
tctatgttgaattgattacagcctcaattgaaggttcaagaataaaaagtcagtggaagtttggagttgaaaaaaggtcatatcttgtaattttcgc
tatcaattcttttgtttttccctattgagattatttgttgaacttcataatttgaaaaaaaaccccgttcatttggagactcaattacccgtttgttgttt
gtatctatagttctactaaatattcagtatggctgctcagcgtgtgaaagcaaaagattacagaaaggcagcttctgtcttggtggaagccttttac
gacgacccagtttgtttatatttatgtcattgcagttatgaccagcagtacaggaaagtgctacagggaatgatggagtacatagtgtatgctcat
attatgcgcggcattgttttgcaagtggatgatttctctggtgtttccttatggatggggcctggtaataacatggacgattggtactcaattgtacg
aagcggcatgtggcgcttgaactatcagctagattcagaaggccgccaacgtttcttccgagaatttcttcctattcttcatgatacaaaggccgaa
attcttcagcaacgagatgactcttcctggtacctagtctacgttggaatcaaacgaaatagtcaaggaaagggatgtcttcgcaaacttatccaa
ccaattatggacgtttgcagccagcagaaccttcctatatatttggagtctagtcatcctcacaatcgagctatttatgaacattttggcttcgcgct
caagaaaacgatttatttgaagcgtgacacttctcagattccccttgaaatcatggtacgtgaagcagatactcttttgacttcggacacgaaacc
gaaaaactcgactagcaatcctgctagcatttctgataatgtaaatagtacagcttctattcctgcagcttatgtttgctaagatttccgttcgctttc
gaccatgttttgatttacacttcttatggtctttaactcattgtatttttatatattctttggtactttatcacccgtaaacaaagaaatttttgttctcttc
tacatctttattgcttgtagcactgtacaacatagcatcctttggaagaagattcaataaatgctctctcaaaatatgtcagtttataacttctttact
tcatgatccttgcatgcgattataacactaacgtttgtatttaccgcatatagtcattgtatacttgtacactgtactgttccttaactgaacaaaggt
tcggttcgacaggcgaaaagaaataacgccaacaattgattcatttttattgaaggttgaaatttgatttgacttttcttttggtttatatattaggct
tcccaatttgtacctttatactatttaaaatctattttctctaatcctcgcaaatacgggcccattggtactttatttattaggtaatacactattcgttc
atatattttctccttttttttggcgaaattatgtctgctatacaggctcatttactctccgagaaaaactcctcgcagtccacagtacgatctacaggg
tccgaattgttagatattctcctaaagatatgcctttggattgcgccctggtatgcatgcctgagtacattgtttttttacatggctgattattctctacc
cacggcctacccttgcatgctcagtgtctgcttggcttttctggtttaccagtatgcggaaattgaaagacgaggaatctaggaaagattcttctag
taaggaaagcgaagaaagcaaaaaggcatcggatgaaactggttcggaatcagcgcgttcaaccacagaatccgatgaacaggccgttcacgt
tgattctgattttgaagaagaggagcccagtaagttaattcctggtcttcacttactacaaggtaggtgggctcatggaaaagcggcgaccacgg
gcgcagaagacttgcatacaacaagcgatttgttatctcataaggcgaagaaaaagccacaaagatctgacagtataggtcaggaagtacagg
aaggaaaagctccattaggtgaagattatctgcataaaattggcagattatctatctcaaaaattgtgacggatcacttaattcgaaaagaagatt
accctgttgaaacttctcagaagatcaataaaggaccgatattcgtgaactcatcaactggtcccactttgctggatgcctatcttgagcccgttca
ccatttgtatcagtattcattagaatcaaatactctattctttagttatctgcccttttttaacagctgcctttatttttttgcctccccacccaagccgtgtt
tattttcttttcgacaatagtgcttgcatggcattcccctcctatgaaagccgttttgcttgcgatttctcgaatctccatcttcagctcactttcagaa
aaattcgtatttgggaaaattgaagtcagggaggaagaagatgaaaccttattgaacgagcccacatctgttcctggagacgtttcagacaagg
aaccaaaagatactgctactagtaccttgctgaaagcattgaaaaaaagcgctgtcccttcgaaaaaggagattacttctacagaaagcaatgg
aaatgaaaatagtacggaaaaatatctatacctgattgagcatcagcgatactgggttggtgttggctggttgaatcggacattaccaactgacc
atcctaatttcacaagcttagacagaaatattcctgtagcagaacctaccgctcagctcttacctccagacgggattgcttggatcgacgacaagt
ggtcaattggtccttggacatatacggatactttttggagacaaccttctttaacacagtacaggacggcatttacgagattcagagaatggagaa
gaaggtatcgtatattacccgaggaatctgtgcactctgtgccggagccgttatcatctgagaaaggactagatgatgaacaagttgtcgacttaa
acaacacaaacagtacaagcgcaactacgaaaccccaatcaaagactgaggtaatttaaaacgtataatgaatgagaatctacctgctactaa
gtcacggtcttccccagcttcttctacaagatgataattctctttttgggagtttgaaaacaacaaagaaaatagtgcaaagtcctagttctctaac
gatgaatactaatcaaaattttgatgaattttccagaatcgctaaaagctttctacacagaaacgacaaaaaaagaaaccaagataaaaaaaat
gccgctattctaacttataaaaaaagtttaaaacaagattcacgttataaacaaaaccaactctggatgaaagatgattaacggattcaagtacc
gttcagcgtacttctagtataaaaaggacccataatttttacctttttttattttcacattgttaatttgttctattcacactacaggtataaatatctaat
gtattttccccagacacctaggaatctttaccttaagctttctcccttactaagccaaaaaaaaaagttcacgataacgaagtaaaaaatatgaca
tgatgtaactatgaacgggaaattacttcagtctccgaaaaagtacagcaccttcaagggcaattttagccaactggatcattaaaaagttcgctt
gtgacataaccttgtgacttgccggcgaaatttttcatgggtaaacaaataaaatatgagtgtgcgaattgaaagaaaatcggccaagctgaggc
caattacaagaagcctatcatgtgatttatctttaagtatttgtatttaaaaacaggtcggcttgaagacacttaaatttaccttagagacgaagtc
ctaagaaggctattaaagtattacttgtgcaacttgaaagaatttttataaaggaaatatatatataaagaaaatgcagacgaacgttacaactc
gtcaaaatgaaataattgctgctcgtcgagtgcctgacaataacatcttcattggaggccaatgcgtgcagcctcaaggagctgatccaaagacc
attcccttgataaatcctgctacagaagaggtgattggaacatgcgcagatgcttctgccgaagatgttaacaatgctgtcgaaaacgctcacga
agcattcgaatccggcatctggtccaagcgtagcggaaatgaacgtggagccattcttcgaaagattggtcagcttatgcgcgaacaccgagagt
tgcttgctggtttggatgttttgaactgcggtaagcccacctcatatgccctatttgatgtagattcctccgccgacttactagattattatgccgaaa
ttgctgagaatgatgaggtaactagaattgttcccttaacaaatgctcctggttacgtcggtaaagaaaagcgctttcccagaggtgtcgtcggtgt
tattactccttggaactttccattgaaaatggcccctttggaagtcaattcctgccctggcgtctggtaactgtgttgttcttaagccctcggaagtcgc

tccctggtcctgcctcgagtttgccctattgtgcaaagaaggcggcttgcccgacggcgttttcaacgtggttattggatccggcaaagagaccggt
gttgctcttagccaccatgataagattgcctatctcgcctttacaggttctttgttcacaggcaagaaaattatgcatgcagcctccgaccacgttat
tcccgttactcttgagttgggtggtaaatctcctttaatcatctgtaaagatgccgatctttctctcgcttgtaaatccgctgcctatggtattttcttca
atcaaggtgaagcttgcactgcggcctcacgtctattaattgaggaagatgtgtatgaggaggtcatcaaaggcgtcattgaagaagcaaagaa
aatcgtcactggaaatggtttagaaaacaatgtcaacatgggtcctttagtaagcaaaccccaatatgaaaaggtggttcaattgatccaaacag
gttttgacgaaggtcttaagtgtgtgtttggtggattacctaaaactgatggaaaaggttattttgtccctcctacaatattcaccaatgctgaaacc
gaacatactttatggaaagaggaagtgtttggccccgttttgatcaccaagactttccgaacgaaagaagaagctcttaaattagccaatgctact
gagtatggtcttggatcaggcgtattctccaaggatcgtgctactcttgattactttgccgacaatattgaggcaggaatgtgttccctgaacactta
tcacttttccagtcatgaacttccatggatcggttggaagcattccggtttgggaataggtttggctactcatggttactatgagtatacgcgtttga
agcaagtttccgagtacgtcggcactgcttaggatcgtaaaggccatgaagggtatctgtttaaaaaacttcatggatttgttaaaataaaaaatg
tcaaggttaatgaatggaatgatatttcatgattatatacatatactgcgatagtggtttgaatagatgtaagaactcatgagtatagtaagtaggc
tacaaaagtcgagtcgtaatgaagacgtatctgtcaaaggtcaggattagatttgagtacgtagacgataaacacgcaaaatacaaatgagaa
aaaaactaaatccccaatcccaataagggaattggggataaaaatatgcatctaccagggatcgaacctgggaccgtctgcgaacatatcatgt
aaagcagaaatcataaccactagactacagatgcttcttgttaagtaggagttgtatatatacacatagcgcatgaatgctgaatcgctaaaattt
agttgctggaaaataatcaatatgtaaaacagccaagctttttaacatctaatacatactacattatatagcagtcgcacatgatttcattccttgat
ttacgaaaaaaaggaaacgaatcaagatacttacttctgaaaggtttcgtatgttatcataaaaaaatgtttactttttttgtgcccataaaaaatt
aataatctatttactagtgcagtttcaatggatatgggtattttatataacgaatcgcaacacagagtctaatcgtatagtgaacataacaaacgtc
gtaatgattttaaagcattagatcgtttgttccttaaagcatactcagaaagggaatcaaaaatttgtcatttgcctacaaccacactgaactaaaa
tccgcaaatctaacccttaggcttttgtggtcttggacaaatgcaaaatgtctttggttgagcattttgacgcgacgcttaacgcggacccaaatac
acgtactcgagctgaactgtctttaaagcaattggaaaaagaacctaattttattttggctgttcttcagctgctaagtgatcagagaattgctcttc
ccacccagcaagctggtaagctcaataaagtcaactatgatttttgggacgtaaacgtagctaaccaaagattttagctgtaatttacttaaaaaa
cagaattgctcgttcttggacgacagttgatgatgccccttctttactagatatccctgaagagcaaaaggccctctttcgccaaaaccttcttccgg
ttctgttgcaaagcccaatatttacgaggtctcatttaatggccatattaaacattatcctttccacagatttccctgatcagtggcccggctttagtg
agttcacggccaatcttgttcaatctaatgagcagtgcgaggtatacgcaggcttaatttgcttacatgaactttctaaagtgtacagatggcgttt
agatgaccgccaacgcgatatcgggcctttaatatccgccgtttttccaattattcttcaaatagctcacaatttgataaatatggaagacaacgac
tctgccgaaatgcttcgtctcatcttaaaaacatttaagtctgtcattgcgttagaacttccttccgaattgctcgccaatgacatgatccttgcttgg
attcaacttcttttggctgttgttcaaaagccattgcccgagtccgttatggcccttgattccgaagtgcgccagacccatgtttggcataaatgcaa
gaagtgggcatactattccttgaatcgtattttcacccgatatggagaacctagttcgctttacggagaatctgccaacaaataccgcaattttgcc
aaaaattttatcgcgaatattgtccctagcatcttggagacatatttacaacaaaccacgttatgggcacaaggtcaattatggctttctcctcgca
tattgtatttccttggttgcttctacgaagaatgcgtaaagcccaagagtacctggcagcttttacagcctcatctgcgattgttgattggtgattttg
tttttcctcaactatgcatgaatgaagaggacgaggaattatgggaacttgatcccgtagaatttattcataaatacatcgatatttatgatgacttc
aattccgctgatgttgctgcttctcggtttcttgttcgattggcctctaagaggaaaaagcacaccttcatgggcattttaacttttgctagtgatgctt
tgaaccaatatgctgcttctcctccaaacgagaagaaccctagacaaaaagaaggcgctcttcgtatggttgcggctgtttctaattccatccttag
caagaactctcctgttgctggtatgatgcaagagtttcttgttgctcatgtagtgcctgagtttactagtcctgttggttatttaagatctcgcgcatgt
gaaatgattaaccgctttagcgagattacttggaatgacaaatctcagctcttgatcgtttaccattcagtgcttaactgtctacaagactcggatat
gcctgtacgagtacaagctgccttggccttacaacctcttatgcgtcatgtcgaagttcacgacgttatgacggcccacgtacctactataatgcaa
aatcttttattcttggcaaatgaaattgacattgatgctttatcttcctgcatggaagaattcgttagttccttcagccacgagttaactcctttcgctg
ctcaactggctaagcaacttcgtgatactttccttaagtttatgcaagaaagtattgagcagtcaaactctgccgaagattttgacactttggtgga
tgataaatctattgctgctattggcatcttgaataccctaagtactatgattttgagcctcgaaaacactgttgacgttttgagagaaattgaagtaa
ttcttttacctattattagtttcgttttagacaacaacatttttgacgtttttgctgaaatgtttgaaataattgatggttgtacttttgcctcaaaagaa
atctcccctgtgatgtggagcgtatatgaaaagcttcacggtgttctaaaggagtctggcattgaatttgttgaagatgccactcctgctttaagca
attttattacttatggcggtaaagaatttgctgcacgccctgactatattgctgctatggtggatattattttacaagtcttcaacagtgatcatttgg
ctgttaatgaccgtattgctgcttgcaaactcatagagttgctcatgcttaattatcgtggcttgctagaccaatatgttcctacttttatcgaaattg
ccggtaacctactcctagttagtgaaaaaccaacttcacaaatatatcgcgtctttttatgtgaagttatcattaacgctttgtactataaccctggc
atgactctgggcgttcttcaaatgcaccaatggacggctcctttctttgccatgtggtttaaggatattgcctcctttactcgcgtgcatgacaagaa
gctttgtttagttgctattttgtctatgattccccttcagctgaacaggtcgctttggcattgcaagaaaattggaaccaagtcatgaaagtaattat
ttttttgctgaaaacattacctgatgcactagcagctcgtgctgaattagaaaaagaatacgatggggaaacattcaatctttctagctctggttgg
aatgatggaattgactgggaagctgatgaagatgatggtgttgatgacttcgctgtcgaatatgaaggtcctgatttagaaggggatgcctctggt

gaggttgccgatgaactggatgatttcgaacggtatcaaggtaattatcttctagacgaggatcctttattccatacttctcttgaccaaattgagcc
cttctcccttttccaagaatttatggtacaccttaaggataacagtcctatagtaatgcaggatatgataaaggatcttaatgtcgaggagcaaca
atcactccaaacattggttactgaactaccagctgtgctcgctgcctccgctgcttctaccaaacagtaattcttgaagttctagtgaatttgaacta
aaacaaaaattgggtctttggtatcggaaaaattatttaattattagtgttttcaaagtttcaaatggtttttgcagtcttttgtttgacttgtattaac
gcaaagccttaattaccttttgtttcatctctggtaaatttggtcactgctgatggaagtagaagattaagaaaattctttttcatctaattcaattca
attactctccttgtaatattaaaataatgcaagtaacatgatatgaatatatggagtagatcatcgtaaggagctgtattttctcttttgattttatttt
tatctacaaaaagggaaacagtaattcaaaccaaaacacccattctttagtacaaaaatgctgccgacctttccaccttcaactggttctaatgtt
atgtaaggcacccactaccaccaagcattagtgactcagaacaattcaatccgccatgaatcatggggctaccttccaaaacaattgtattagat
aatggtgcttatactgtgaaagcaggatttgctgatcggaatgttctgtaagtaaaagcaaagttaacgtagttagttgatcgtaagactagtatct
ctaatttgcgtttagagaaatacccaactgccttacacggtcaaaggatggaaaccgcttgtttttaggagacgagttgtcgaattgtaatgatttt
accactttgcaatttcgtagagcgcatgaaaaagtacgtatattaagccttgtattcagagagagcttctgagtaggccaaaaatgtcaatttctat
gaattttactaaacataaagggatatcttgtacactggagtacagagacagcggtttgggatcaagtgatgcgacggttggggattactgagcct
agtatggacggtcattcattaatcctaacacaggctccattcacgatgccatctatagaacttaacacaattcagctaatttttgaagaattcgggt
tcgatgcgtattttacatgtacgccggcagaattaattccatgggatcatgattgctttacaaaaggcgacgaaacttcttacaaaaatacacacg
gcgaatgtgttttagttattgattctggttatagttttacccatataatccctgtcattgattctcgaatacaagaaaatgctgtacaaagaattgatg
ttggaggcaggtttttgaccaactatttaaaagaagttatttcttatcgaaaatataacatgatggaggagacatatttaatgaatgaaataaaag
aatcggtatgctttgtctcccaaaactttgcgcaagacttgtctcttgcacagacaaagccaaaatcgaagcacgacatcctttatgcgctaccag
actacagttctggtaagtttggacatgtagtcaaagacttgaacaaagttatcgaccagcaggtattaaaacttgccaatgagcgattcgctactc
ctgaactcttattttctccgtctgatgttggtttgcaggaagctggcattcctgaagcagttatgcaaagcgtacaaggtttttcagaagaaatcgcg
gccatgcttcttagtaatatcgttaccataggaggaagttgtaaatttccagggtttcatcagcgattggaagctgacttacgctcgctttcaccag
aagatatagagcttcgtgtttttcaaccgtctaagtaagtgtttttgccatatgtactttgtactaaacctgttctagtccatccacatatgcttggaat
cgtggagcccacatgcctattgaatactggaatacaaatagtattgccaagtcagaatatcttgaacatggtcctaatatattatatagaaggaaa
aagttatgaagttacgaaaaggtttctaaaaattcattgcattcaaataagattgccagttgcaattattactactattgttactacttgtttctaacc
actgacctcatactatcacgctttttatttattcatttcaattatcccattttcattgttcgtaaaatctatataatctattgtgtactctacactgtagat
gtattcaacataaatcttcctgtacttcatacttcaaaaaacaattcacctaaaagattagtattaagttcaagatattaattacggtgaagcaaca
tttgatcagtgtgtgaatacgtaataaccatgggattgtcttcaagactaatcgatttttttaacatcatttcatgttactgatggacattataataca
taccgattcaaatattaatatagcaacttgcagaggaatcttcaaaagattcctcgtgtgtgtgtcgtaaatgttgcatatggtaaaaggatgaag
aaaaacgattgttgaaactattgaatttaccagcccaaaagcagtcacggcacatcgtgaagaagaatgtaaacaaacacgacgcgacgcgtc
aatccaccatcgatataagtgtaatgagagtaaaattataatatcgttatatggaacttatagtaattcatgaaatgaattatatatttgtcattcct
tttacatggtttccgtgtatagttcaaagaatgcttaatgcctatgttaagattaccctacaaaataaataacaaaagctaattgaatataacattt
acaattaggacgtctttcaaactttgttacctcatgtgtgctacttgtatgtacgaagggtgttctgtactatacaactctagctctccactaccacca
ccattctaaacataaacaaataagaaagcacctaaacttggcacatataccaggggatatttgtgtcattttataaaagagatcaaaatatcttta
tgactttagaaagatgccctcttgcacctcgatcagcaaatataattccccagaaacatgaaggtgtggtgaagcagaaagccgcttcattggca
gaggatgctcctggtgaacaaaaaaggtctcgtactttagaggagagtgtttcttctaacgaagatgaaataaataatgagaattcggtaactac
gacaccgaagcatcagaaggttactactcccaaatcttaccggaagtcagtaaaacgagttaaacatgatgaatctatatctaaagaagttcctc
atatgaaggcacttgcgcctatcgatgaagagtcaaaaaacactggagctgatactacaaaactagccacagcaaagtcgacctctcaaaaag
ctaagttgctggaatggaagcgacagtataaaaaggcatttccaaactttcgtttttatttagatgggtttgaccctaaccttcagcaacgagtcaa
acgtcaagtagttcaattagggggccacgtggagacctttttttctggtaatgttactcatgtggttacgacgagatctacgcaagacataatggca
aaaaactctaagcaggacgtgcttaccaaggcacgtcaattgaatatgaagatttggagcatggaaaaactgtgtaaccgggttcttaaaacgct
tttggaagacgacccatttggtgctggcccatctcaaaagcaagggaacgatttaagttatttgctttacgtagaaaaggttcaaggtaccaatga
gcgtgatttatctgtaccaaggcaagactttgttcatttcaaaggcccatatctatatgttcatgatatggcaaatgtatataaacctattcttttgag
agaatggccaaagccgtcaagcgataaggatgttccgtggcccacgtttcgtgcaactagtataggcagatgcccatttgtacctgaaaacaagc
agcgcctaagtattaacgcacacacggcagcagcagcagctgcagcaaaggccaaacaagatcagtcacaagaacagcagcagctgcagcgc
atacaaaaacagtgttcccaacaagcacctccaacgctttcacgtgctaattcttttaaactctctgttccattgctatctaacaatactgtttctact
tcgaactcaaacagtgtatatgctaatgccgaaagtgctccattgccaaatgcaaatactggagatattcaacaagagcaaccttcggccaaact
taagccatatcgcatgttagatgatggcatgcaagcttctggtatcgtccagtccaatctaacttcggcggtaatgtccaataattctaatattcgtt
cagggagtgctgcaggtgttcctgttgttgcgatcaacgggcgagacataaatgagcttaagaaaagaataattcagcaaaaatccggagcacc
aaataaagaacagacttacaagaacatacttcagaatgctaaccagagaaaagttaggattgacccaaaacctggatattgtgaaaactgtcg

agaaaagtttgacaatttcgaattgcatattcggagtaggcgccatcggaaatttgctgagaatgatgaaaattttaaagaattggatgaactatt
tgagcttgttcaacggccttttcgtccactctaagaacctcaatattgttttatctaaacatttatgccatttttttagtctgggttagagtttcttcgctt
ttctgtgttttaatgttgtgttgaactttttaattcttgaattgaaacagaatgattgcgttgaagttttacatcggtgctttgtcgattcttcttttttta
catgcattcattctttttcttctaatatatatatatacctatttcctgatctaacagcggttacgattctatctctgtgatttaacaaaccatctatcttt
tcgaagatttaacgaacgatttatatctattttcttttaatgaatatgggattattcataggatgctatgaaggattacaatccttatttggcaatttg
acttttgatggcaaaagtcagcgtacaatttacatgctcattttattgtggcatgttttcatagctgaaaggcaatatacttgtatatctattgcatat
gcaggcaattagggaagagttctcgtgtagagacaactttgatgataagtactttgaagtatcgagagtagtaagttgtatcactttgtatttttgt
agcttcttgtgttttgtaggttacctcagtaaaacagtattttaagaacttttatggacttacaaaagaaaaaccaaatgaatatcgtagtataaga
aggaaacctaggatttatttgttaacgttgccgaaggatgaaatgtgacagaacgctatcagtcgctattccggtacaccagaagtataggaaca
tcaaacagactaaggcttgccctgaacgaatatctttagtcgacgcgactttattcgtagacattatttgcttgaaacaacacgagtaccagtcgtt
aagtttttacttctttctttacttcaacgtatagcgtcttgacgctcaaaagaaagcagtgttaaaagtaaatacagctcataaagaatatttcgac
ataatctcacttggagtcacaataatctgtattttcactctctaaccagtcaatctcttattgaatacactttctggaacctgatttatggaaaatctc
tttacttccgaacaattcaccatgactgcgaacaatcttgaagtcacaaatacctccgccagtcttccaactgacggtaatgaagatataaaggta
caatacgatggattactcgcttccaatgattccgaaatcaatggtgtgaagcatgaaagcttagacaatgcgactacgactgttacttcaccggaa
gaaaccgaaaatcttcctccttctgctttattacaaaagaagcatgaggttgaaggttattctaaggaaaagcctgcttcatcaaagaccgtatcg
caaaaacccattattgatgtccactcagaggctgctttccctaccctttcgcccaaggtcagctctagtagaccaaaaactgcgtcttgggtccgaa
aggcagctggtgttagttcagccgctaatgatggtgccccttcttcagctgactcagctacttcttccaacaggactgcttctgcttcaagagaaac
agattatatgacagacactttgattttgggccctgatcagcagatgtctcgcctctcgttcaccggtaaaccaaactcagttgcagatattgtacgt
accattatgcagcaaacttctactcgtataaacgtctcaaccgcatctaagacaaaaagcactaccttcttaattcaaggtaaagttgcttctgtta
aggctgcccacagacaaataataagggccgtcggtcgtcgaaacaccattattgttccctgccctgtctttgttgtcggcgctgttatcggtaccaa
cggtcagaatctaaagaccattatggatcgtactggcactcgtattcaagtccctaagcgtaactcctctgccactgacgataatgaaacatcttct
aagaatgaaaagtccatgctctcttcctcgacttctttggacgaattagaacctcaatacgagctgacttccgttaccatcgaaggcgattttgaag
gtgttgaactcgcgaaaaaagaggtggaaacaatcattaatgaacgcactagtcatactactgtccgcattaatacaattcccactgatttgtaca
acttgcttcgtggagccgatggttcgaatattgccgaattggaagaaggcaaagatcttaaagttcaaattccatttgcatactccgacaacaattt
gcccgtcaatccagttgtcttatctggtgaaaagtcttgtgttcgtgaatgtgctcttttcttacaaggaaaagccgaggagttagctcgcactacca
ttcctactatgattcctgtccctcgtcgtcaacatcgttttatcaacggagaaaagagtactggtattcaagatatcttaagaagcactggatgttca
gttatacttcctccaattaatagcgattcagatattgtcagtgttcgtggtcctgcaatgaatattagtgatggtattaagatgactatggagcgtgc
taactctacaattgttgatgctgttaatatttccactgtcttcacttccttgaaagatcccttcagccatgctaatttgcttgccagatttttcattcgct
ccaagttcttgtctcaatatgaaaacgctttaaatgttcagtttcatcttccgaagcgcgaagagctccaggaacctgccaataaatctgtcataat
cgagatttccagtaaagccgctgaaagcgttcgcaatgctcgtgctacgttaactaaggttattagtcaattccctcctcataaattccataatgttg
agattgatcctctgttgcaacgttacgttattggttctaaaggtaaaaacttgcaaaaactcagagcggatcatagcgttgagctattaattggcga
aaatggtgaaaatgattccaatgtcattctttgctatactgggttggatgacggaaagtctcctgaacaggtacaagaagaattggcaaatctgg
gtgaacgtatcaaatcctctgccgatgcctccgctaagattgtttctgaaactatccaagtaccttctgtctatcataagcatattgtgggaccgaa
gggtactacactgaaggcaattcttggtaattcagaggaaaatgtaatagttcagcttggtaaggtttcttatcgcactgactctaccgatgacgat
gtctacgttagaggattttccaacgacgttcagcgcatcacaaatgaaataaaacaactcgtaagagatgcaaagaatcatgagatccttcactc
atacgttgctgaatttgattttcccgctcagtattcaaagaatattattggtaaaaatggaagcaatgtgtccactcttcgtgaaaactttggtgttc
agatcaatgtcgaggaaggacatgtacgtatccaaggtatcaaaaagaatgttgaagaaactaaagctcgcattcaagctcaagtccaagctct
catggacgataccattttgcgcgttaccatccccaatgaattccaccgccagcttattggcgccaacggaaaatacgttaaacgcttagaggaaa
aatttagtgtccgtgttcgtttccctcgtgaagatgacttgaacaattccggaaacgaaacgatcaagccctcatctgttgatgaaattatcattcgt
ggtagtaagaaggctgtcgcttctgctaagcaagagttaattgaactttacgagtatgagaagtctattgcttatagtgaagttatagaaattccct
ctaaggctgcttccagagttgtagggcgcaatggcgctactgtagagaagattcgtgctcaatatgatgtcaaaattgacattggagacgaaaga
tctgagggtgcaattaatgtttcagtacgcggttctcaagaaaatgtcgaaggtgccatcaaagaaattaatgctattgctgaagaaattaaaaa
ccaagtcgagaaagttgtcaaaattgatcgcgaataccatcgttttcttattggccctaatggaagcaacttgcaagaaagtattaaacaatgtgg
tggtacaaccgacaaagccgagactgctcgtcttgttagtttctccaatggcaactctgaagaagaacgcaattctgttgtgttacgtggtaacaa
acaactcgttgaagctctagaatctcatttcttacaaactgtagaggaactaaagaatcaagttgaaaaaactattgatgttcctcagcgtagcat
cagttctatcatcggaagaatgggaagtagccgtcgtgatatcgaaaagaaaacattgacttctcttacaattccgaatgttttagatcccgagca
acttgtaaccatcacaatcattggtagtcccgagaattgcgacaaagctatacagatgattcaagagaaagttgctagtcaatacacagaaacca
ttgaggttgccaatactttgtgtgaacgattgacgaatggtccgattatcagaagacttcgcaatgatcttaaggtgtttgtggacgtgcccgacgt

tcgtaatcaattagaaggttcagaaattgtccttgaggataacgaagaaggaccctatccctggaagttagttaccaagcaaaaaaataccgac
ggtgtttcaatattgaccattagaggttacaaagaaaatgttcaaaaggccattattgctttggagaagaacattaagcaggttgaagaagaatg
cactggttacctttctgttcctccaaacttgcatcgtcgtattattggtgctggcggctctgttattaacaagattcgcaaggttgctcaagtgaaaat
tgacgttcctcgtgtattaggtgtcgatattattgtcattcaaggaagtcaatctggagctgctaaagcaaaagatttaattattgaacgcttaagtg
agtctcaagattaaagaaagaaaaatttttttgacatgctattttatactcctttcccttatgggaaatttttcacttcatgcagctagaaacccatat
tattcgtgttatatcaaatcatgttgtcactctttttctactattttgcttagtatgggcgaagttttatagtccttaattacgttgcaaagaattaatttt
gttttcatccagctcaaaagtgaatctgagaagctaatgttcttttaagtgtacttcgcttgaatgagatgaagctttagaacttactatataatgttt
aatttaattttcaactttagataattcactttaaatgagttaaattgaactagaagtaagacccaaaagatacataaaactaaaaaatgcctttatt
tatgggagtatttaaattatttattaaagctcctaaaacagtcaaccatcagtgttcttcgattaggaaaaaccaagaccaaccgacaaacatatt
catagacagaccagtaccagtacaaagctatacaaaccaaaatagttcctttcttttccttgggggtttttttttgctttcataacaagattgtaa
caagacgaaatgataccaagaaattattgcagaagtggatcccgatagtgttgcttcgtgttattcttgtaagcaattttagtaaagaaagcctctt
tctttctacggtcttggaatgttttgatatgagaggcactataattctttcttccgtctcggacaccagcaagactagtctctacagcgcgattacga
gccaatgcttctccactcatagcatgtctggctacattttgatgaactgctgcaccgtcaccaacagcgttagtagtggaaggacgaatattttgac
ggaaataacgagataaagaacgatgaccagcacgagcaccggtagggaggtgaagctcgtactcttcagctataggtactctacccacatctaa
gctatctacgctggaatcagaatcactggaagcgtcttcccagttttcatcgttttctgcgttttctgcatctacaacctcatggggtttctcgtaatc
aggataagaagtggtgaaatcgtagtaatcagcaagttcaagctgttcctcatcggtatcgtaagcaatagagaaatgtcccttttgctgcatatg
agagcgaacagcttccaatgacttaaattggcgattgcatgttaagcaagtgtaacctatagaaaccttttcagctaaatattcgaaaaggcccg
gttcatctacgacatattctcgctcaggaatatacaggctgtgaattgctttcatatgcttcttgttagcttcaaaagaggagaaagaagcattgca
aaacaggcaatcccttaaactcaatttttcgctcacacgtcgagcgagctctttttcaaaatcttcaccggttggttcagcggttgtttttgcttgaga
agtttccgtagacatagctccattctttaaagaagaatcagaaactgagaccgattcagatgccaagcgttttgttaagttttgaagctcttcctctt
cagcactttcatcgacaggctctcccattgaaagagtcgatgaggtaacgctacttgtttcatcatgtctcaattggtctgtacgagacttcatttca
aatttacgaacattttcatggtgcttctttgaagctaaatgggaagaataagcgccttcggaataaaaatttttgctgcaataggtacattcacgat
aaaattcagcctttttctgaacagcttcattatgcttttggatagagagaattttctcagcaaagatatcagcagacaagggggggaaggcttgcca
cttttctctttaaattataatgatgccaatcagatttccaatgtattttctgagagtccgcatttggaaatgctacacggcaagttgtacaggaaaat
gcagtggacatcttgaatccttaaaattgagaattctcgaagctaagctgagaaatcttcgtttatatctttaatacttctttttctttcaaatagaaa
gaataaaaaggtaagttttcagctttctccctgaactttttcacagggaaatatgttggtggagaacaaaagtaatcactagtaaatggaataag
ataccttagcggcgatacgattaatacttcgtttttcgataaaaatattattattttaaaccttgaaagaaataaaaaagttgcactttataatttcg
gcacctactagttctgtaccattagatatcgaaaacagaaaaaaaaaaaaaaaagacaatgtttgattcgtactgtatcatcgactcgtttaggcta
aagcatgatcaacttggttttatttctttaattaaaaacatcctacacacagaaacagtttaacttctcttagaaaccgttctgagggactcaatgtt
tttgttcaaaagatgagctcttctgaacagactagtcttcaaacaaacaaatatcctaaaattaaagctatagaataagtttctttaaaccttgcat
taaatcaaccaaaagcatgggatccctacaatgattaggataccagtcttcaaataaattgttccataagtcctgttccttttcatcttctagcattg
atggctgcactgatctgtttgattctcttgtctttaactgttttgataaatttttaagtgtcataagctgctcttcaatatttaatgttttaatgttatttac
ctctttatcatcaagtcgcgatgtgccatccagaaccgattttcccaagtagacacttaatagtacaatagtggactatgatatgttagttgaaaat
tcaaaaaagtgcgatcttatggacttacaaaaagttttccggtgaaaagctcttctgtacgtttagacaacagttctgatgcttcaacgaccacgct
gaagtaattttccaaaatttcctttgaacgtttttgattcaattcaggaagaatgcataaaaatatagtccttaagcaagaatataagagcagttca
ttttgttcccgcctgcaaatatctaataggtgggagacaagacgcaaggtggtatcgctttttttcagaagaggtacgaatatttcgaataaaacca
caataagatttcaaatgaaataccagtttaccaaccctgttgaatgttagctgtgtattatcagattgcaacttctcgtacttttcatcatgatttgat
gccttccgacctgactggatatatccaaggaattcttgtagttcttccattaaactatctgtcacggaagcgaaataaagttttatatttagttgaatt
tcggtcgctatcaggttcaggttaattatcctgtgaaagaattcaccgcataatagatttatttggaaattatttgatgctttagcagcatactccact
tctgtcaataaagccaacatatctttttttagattctggtaaagcgagagtttgctcttggatataatccaaacttttaataaaatagtagcttgcaac
attgcgttctcgatatttcttcatcaaaagaggtagctcatgtaataaatgtaactgctttgaggtataatcagactcagactaatgttgttagaata
cgtaagaggtaatgccttctaacttactgattttctttttaaaaaagaaggcttttcgatgaagttaagacagaaatacacgtaggaagataagaa
gtcgattattttctcatttggcgcacatgattttaaaatgctattacagcttaaatgattaacatggtcaaatagtaaatagccaagaacttcttccc
aagaacccaatgagttcatttcattatgaatagatagcatactgccctcgtatactttccactcctcagaattttccaaatatttagcttccatgttga
aaccaaagggccaaactgagtttctatcgtctgattgtgaggaattcgcttggacgttttcaatagtccatgtttccagcgcctgcgcaatgaaaga
aaaaatcaagttttccttgaaaagatttttgaaagcagaattggatattttataattctctcgtgaaagaatctttttgagactcgtttctttcgtagc
ttgtttgagaaaaccttccagggtttgatcacagtacctcactagtaacctcataattggcgagcgaatttgagctttttttattaaaaatgcataaac
aaagcaattcaatatcatcctggctgagcaattgatggtccagtaattgaaagcacaattgaagtgctattgcgcgaacactttcaatgtcatacc

tacaaatttctgcaattttggaagaaaatctttctaagaaattaagtgaagtaaccgataactcttgttttcgctccgtaataaaaagctgttgaat
gatttttaaagcaacctttcgaattcttgcatcctaattcaaagttagtaaagttgtgataataaacttatcttacagtatcagaaagtccccatccg
agatacaatagcgttgatctgttttgaagatgagatggaagcagtagacaacactcatgaattgcgagaaaacattccaaccttataatagacct
tgtatcgcgtatcctaattaaaatagcactgttgttggtaagtgttctataatcagatactaatgtttgaaatacctttatagatatcatccaaaag
ataattgagattctcaaataaactttgattttcatccccttcaacaaaatcacgaccatcttcaaatttggaaagggcattcgcgagtgcaacgtag
aacatgcatgaaatggcacttgaagtatgccggatagataaccatggactagttgtcatacacaaaatccatgatttaatagtttccatgaaggat
gagcttgagagcctatccttttctatagacatattaatgagctcattaacaagaaaactaagtttggttctaaatgactttcctgtacccgtggagc
atgcatataaatacggttctgcaaaattggcaatctaataattgaggttaattttcaaagaaaaagagatttgcatacggaattaaaagttgaag
attggatttgagctagggtttcaggtattgattcagtatcctccatgtcaaagtgatttaaaaaaggttggcaattacacgactattgtaattgttag
tatgtgaaagcgcgtaatctcttaccagcagtataagattaagaaatccacgcatgaattcagaagaatccgtttcagagtaggaacgccattttg
tgctcagtaactccatggtattggtgtcattcaacagctcatctagaagattgagttagttatggtatccacacttcgttatgcttcaaagggtagat
gcttactgagcaaagtgaatgagaaatcttgagatgtgttttgagagttgtttgaactggttattgttctttggatagtttcagcagaagcaattggt
tcattttctaactttctttttaaggtagatgtcaacatttcagacttatcgccatggtcgttggaagttgtattaacagcgggatttagctcaatatctt
cttctaaatcaggtaaggaaccatctaaatgactactaagatctgattcaaaatcgtattccatgatatttaaatgctaactagtttcgtgaatagtt
gttctgtataagcgtgtagtaagtgtgttggtgttgtaacttaaatatggatgcgcgacgcgttcactttaattaagtacctttgacgcgcaaccagg
agaacttcaaatgttcgtagaatatatgattacatttaataattgaattagaaattatctaaataaaaaaattacgtgtaaaaaataggtattgta
gccgaattttgtactctactgcaggttgtcatctttcgagtctcaactttacggcactacttggttcttgatttcagtcttttccgtctgatttttttcggta
ttaattataatttggattatttaatctttttcctatttatttatttgttttggtctaccatcgctatcactattgaaactttgaagacttaattttggcgaa
atttgaaacaaaacacggcaggaagtattcctttttgacgtttattttctttcttgctgaaaaagataccaatgttattcttttatacgtctctgtaaa
gttatggaagcttcaagcagactcgatgctcatgaattagctacagatgaaaatcaaacagaatcctttgcattaaacggaaaaggcagaaatg
tttatcgatcgcctctaagagctcctctcaagtcattagaaaacgtttctcatggtcccttgcactctacttacatagacacaggccgtttcataaca
tcacccactagttcagaaacagaaaaatatgttcttccagatgagaaagaactagatagtgttttgttgaactccgtgcctgagggttcattttctg
accagaaaaactcatccctggacatttctgaagagtcctttgatttgaacacctccaaagtcacagagccttcccatgtcgcaatccctttctttac
ggaagaagactttcgcttgttatctcatgcttcgaaatttgtgaaagcgcacccttccaaggaagatgacgatcaagacccatcaatggtttccga
ttatgaaaccgatatattccaatatatgcatgagttagaaatgaacttggcaccatctccttcttatatgtctattcaacaggaaatcgattggcca
actagacatttgctgattgactggataatccaggtccatcattattttcgacttcttccagaaacattgtatctaacggtcaatttagtcgacaggttt
ctttccataaaattggtatcgttccaaaaaatacaactggtaggcctttctgcattgttgattgcttttaagtatgaagagattcatccaccgaccat
ctatgatttaggtaagatcgtagaaggagtctacgaacttgaagaaattatccgcgcagagagatacatgctaatgctgctgaaatttgaattaa
gttggccagggcctatgtctttttaagaagaatcagtagggctgactcctatcaatatgatatccgatctttatcaaagtatctgatggaactgac
attaatggatgaaatgtttgttggaggatatcccagtgtaatagcagcagcttcttactacttgtctatgcaaatgttaggatatactacatggacgt
tgcatcatatttattacagcggactcactgctcgacaactggaaccttgtgcgctaggtatcatggaatgtattttggatgctcctaatcatcaccgc
tctatttttcaaaagtacagtgaaagtaggatgaagcgcatcagtgtctttgcttataaatgggtttctagcattctataattctgttccatctactaa
cgatttcttttttttttttaatttctttttttggaagatttgctttacgaaactatttttgatcatttggttaatgcggcttcccactcccaaacactttttgt
tttctttttgctcgaattaataatatcacttttgtaatcatcattatagctaattaattaattaaataccaattgtatcaggcgcacagtcatatggaat
gaaaagtctcatattcactgaccacctttatcaatttttttctagttgaaaacatagacgttcgtacatcaaagtaggcaatcatactaacttacagc
tttcaggttgagtatactgaataaggatcaattagcatatttgctattctacagggtaaaaagattcttttattctatgaaattcaaccatttaacttc
atgtctaccttcaaacaattcttggtgtcatagcagatcataatgcttcggcctataactgcttctaccattaaaatcttttgataatttcttcttatttg
acaattcgttcaaatttttagaagaagtaaattcaatacttatcatttacattctatgctagtgaactgggaagggattcgtgctttttttcaaactgt
gaaacgtcctaggatactacttcctcatgcaaagtttccttcattcacagatattccgattggtattaacagccatttaagccgcctatacagtggcc
ctgttgacattcgaggtattgtgctagacaaggacgattgcattactttacccgatgataatgaaattcccaaaagaaaccttgaaaaactacgag
agttacaaaaacagtttggaaagagcaatatcttattggtgagcaattcaattggttctaaaaagcaagatccgacaggagaagaagccgccca
gtttgaagctcgctgggaaattcctgtactgcgacactccaaattaaagcctctttgtgaacaagaagtgtggaattattttcagaaatctacaaa
agttgaacgtcccgctcaacttttattcattggtgatcggctactcacggatagcactatggctaatcgaatgggtgcatggagtttatggctacat
gttggagtacaaacatcctcaggttgggtaatatctttgatttacttttatactacgacaatgctaacaagagaattcgtttgtagatgaacaagttt
gaaggaaagctttaccaaacattccaaccgatgagtcccatactaccttcaaccaacaggaagaacgagaaatgataagtgtctttcaaattatt
tttgaattcattcaaaactctattgcagactatagcactgatattgaagtaagtgagatcctctttcgacaacttctatcaagaatgagaagtatctt
cccttcaaggtattttcaaaccacttagaaaaatctttgacagcttgtgactctcgtcgtttcgtaaactatagatgtgcagaaaaaaagaaaagc
gaatcagagcaaacacagcttagaaataaaatcatcatattactttacgcagacagcggatttcacaaaaattcatataaaataaaaaagccac

aatagcaaaacatcattatttgttattaggtaagtagttgattaaaagaaaaaggaaggaaagcacaggaccagtgctcaacgcagaaaaggg
gcataaagcatgaacgaagaaaccaacaaaaagccattgtagggaattaattaataaacgttgcaatcataagaattcgtaaaacaaagctctt
ttaatttttatttttttgtacttttgcattattttatcgtgctgaaagattctccgcaaccacaagtagatttgatattcgggttggtaaaaataaacc
gcgactgtaattcatcatccttgtaatccattacagaaccgatgatttgcagtaaagcccttcgagctactatgatacgaattccgtcttgctctaca
atttcatcaaacttctcaggctgtgcaatgtattctaacgagtaggcctggccagcacaacccttttgctttacaccaattcgcaagagtttatcctt
catagcgggttcattcagcatgtttcgtaaatgattcactgcatctggagtctacaaaaagtgtcagtatatgtaaatatattaaaaaaaagaaat
atcataccaatttaataacatttttacggggcatatgtctagtaggagcaagttctttcttagaagtttcattggagggagttgcaccacgtaaacg
ggtgggtggaggaatttgaatattttgcgattttggcggagatgaatgagggtttttcaggttatgaatttcatataaagagagatttcgaacgaa
attcctaatcacgccctcgcgcaaggaggatcgcgaaatctgccgagtccataaatgtccaagcatccttgagaatttgcccaaatttttcgggttt
cgatgatcaaacctcaaatccttgatatgattattaataacaccaaattctttcaaatttaacgtgtttggagactcaattgttgtctcccatgaatat
ctgtttttggctaacgacaagtactcatcatgtttccagtttttatataattgtcaggctcctgattggttcaaggataaagcaattagcgtttgggaa
tgcaatatatgttaatggagcgtagcgttccagaagatgcaaacggaggattgtgttccaccgccgtatttgttattttactaaggagaataatagt
acgatgctggaataaaacgagacggcgaagcagggttggatttcctttagaagaacgaagtcgggcgtctgccacgacttgaagagcttttttttt
tcgttctctttcatgtctttttaattgaacagaaaggaatgaagccatacaatcgctttcatgagaaagacaagcacggtaaaggcattgaaacag
aaccgaagatcccaattgtacgctataagtccaacaatagccggcaaagaaaggcctgcacattcgagaagcagtcaacaaatatccttctgct
ggactctatttaccactgaggggtaacgcagtatcgttatattatgcatagactttcatttacagacttccagcaacctcgcaaaacgaattcctctc
gacgcgctttcgaatttcctctcaaaactcgaaaacgcgatcgctggaagtcacttgctttactttgtaatttccattcattgtgcaaaatgaaatga
ctcagctcgatgatttgcatagaaaaagagccaggaacgtagaacctagcagctcccatggaacacgattgacggcgtcgcagattattgctga
cgaggaagaaggatcagagggcgaacaatacatcgacgacgacttggagagcccaaatgaagacgccgaatttgaacaaagatccggacgc
gtttcctctgatcgaagtcgtcgaaagaagagcaaacagagtgttggtaccaagccattggacggactagaaaaagacgaaactgactctatga
attttcaaatgcttgtaagaaacatcgtgcgtcttgccatttgctcccaaagctctcaccaagctctttcgcgaaaggaagttatacagcgtgcattt
ttgcaaggaagttcccgaagcttgtttcagcctgcactagaagaggcaaaccaccaacttcagtctgtctttggatttcaactggttcaagtaagcc
ccacaaaccgaagaaaagaaatggctatctcccaattgcgaaaacaacatgcttcgaatgcttcttcttcttccaatattccaaagtattggatttt
aaaatcatctctccgtcctgaatacagtaatgatcaacgacttgttcctgactcattgggtgatacttcgtttcttggatttttgatgcttgttgtttcg
cttgtagcagtttcccatggatttctaggagagatggaactgaaatcttatttagaaaatttgttatcttctgaaataaccccttttcaactagactta
gatcgctccctcaatcttcttgtaagatttggttatttggatcgcgttaaagatgatacccataatcaatcggtttactatgttggttctcgttctgaag
tcgaaattggtaagcagggtttgaaaagcttcatttcggagtttgtccaaggtaccaatcttgatgaagtactacctgacttcacaagtgagagat
cggaaagtctttcgaatgtaacggaaaatgacgattagaacgtcaccgtatttttcacattcctttcttgtttctcttttgtatgtcttagaatctattt
actagagatacctgcatttgttgtacattatgttcataatgaaaattcaattcatcacatctttatgatctatcaatatacaatattctattaacatga
aaaatatcaaccatcttaaattaaagaatgaatgtgtttcgtgtacaaggtggtaactaaaatcatgctcgtgaagacgccattaactcttcttcaa
ggctagcaattctgtctcgaagcgcccgaacatcaattagtgaataacggttttgcttctctaattcggaaatcgacttttgtaactctttttctcgttt
ttcagactggtattgcacagcaaaaatcttagatttttcactgttgatttgatcctttagtgctttcacttcaactgcatgagaaaaggcaacccgct
ctaaagatccgccggtggcttgtggtgttaccatagatttttttaccctcctgcatttgtttctcaagatactgtttctcgaggttgagcctatcataatc
tctctttaaattctcaaatagggtctgatttgcatttcgctgttgtaagtcaaaaattgtcttcctctcaggaagtgagctcttaaaagatgctgggg
gtttattttccttttctgcaggcttgtgttcctttagacgatggagttctgcatctttctttttgtttacctccaatgcggaattcaactcctttctaacttc
ttttaacttcaattctgattccgtcagtttgttcttagagtactctacatcagaagctttgtttctcagtaacgaaacttctgatcgcaaacttcgggtg
gttttcacctcctccaaaagcttctgataattcgtttccaattgccgtttattttgaagctctttctttaatgaacgtagttgttcattatcttgactagct
aaaacagctttctgtttctcttcttcaagctctttaacggtctgtttgaattcttgcttcaatttggaaacttcgttttctaataaaggaattttaggaa
gcttttgcaatgcagaatcgagctgacgagttaggctttccactttttgagttaaatcgctgcgctcctctagtagcttttgatattgatcattagtag
aaaaattgctttttttctaactgttccttgtctcttgttactgcgttatgctgaaccattaattcatgataatcagtttggaccttgcgtaagttcaaaag
ctccttattttgagtctcataatcgaacttatacttttcaagttgccgatttttcctatgtagatcttctgtttgcccaaccagttcttcatactttgatgg
gaaatcctgtaatttcgctaacttgttgttggcatctttaagattgccttctagcttttgaagtgtaagcatatgcgttttcaccgattcttcaagctctt
tattcctagtagattggactttataaatgttgtctttttcggttaaagcaccgagctctttcttcagctgagatacttgcttaacatcagcttcaaggta
gttaactttttgttcaagggtttcaatcgtcatttgttgaacacctttcagtacctcaagctcctggttttgctgcttaagaattgatatatcattagcc
atttctgatttttcaagcttgttacgccctaattcagaagacatattttcaacagatgaattgatatttttcgtaatattcgtttcattcgtcagcttttc
ttctaacgatttactcaattctatgagtttatgaagttcatcctttaaagcagagttttcttgaagcaaagaagataattcgttgtttttcttctcatca
tcttctctcaattttcctatatctcgatcaacggtattttttgttttgttattgaagctatctcaaccttggcagcggataacgcagatttgacttcttc
attatacattcgaaaagtatctagctcttttcgtaacttatcattgtcttcttttcttttctcattttgagatgacatgctttcgagtgcttctctcacctc

ctccatatgcttgctttccacttctttttcattcaaaatatccgctaatcgaacttccatatctttgtaagtggcattcaaactcacatttgaatcttcca
aagaagctaatcttccttgagttcgacgaagaatttcctccttgtcaagtgcaatggctcgttcactggtaagtgcattctccaaagcaatataaga
tttagtggactcgttcaccttggtttcaagcccctctttagcgtttgtgagcttcttaatttcgtatttcagttctataatttctgcatccctccgtttgag
ttgttcatcattatgagtactactaagcatgggtcttaacttaaaatatagctttgtccagggttgctttcgcagctcgtcatacacaagcaagttag
cctgtatttgttttatagactctatatctttcaagcgcttttggtatttccttctttggacaaaaccacggattcgaacttgcaaagtagttacaatctt
ctgaagtgtttgcaatcttctttcttccaaactagctagtacccctgccttgaagaaaattttggaaactccgattctaaactgatcttctgaaaggc
ccatttgttgaagtatcaagagagaagcacgtcgagattcaatataattatctttcggaaggttggccatcatttcataacgaagcctgaagtcatt
aaagggcatcctattggggaatccagcacgagtaatccggatgccctccaaaacaccgttgcatcgcaattggtctaaaaccaaagctctgttaa
aggaatggctcttttttttcttcattaggaacaatacatctaatgaaatgaggttgagttgtgttaaattgattcatcaattgattcaattgctctttatg
cctttgggctacagtccgaaaaagagccttacgagtgcgtccacgaactgtttttgtttcaacctcttggtagtcagaaaaaagctcagatatatgc
ttattcgtagaatgagctaataatttggcaatgttttcgttcaatgggtcgctattcttttctaaccaaccttctgttgaataaggaacatcagccgcg
taatgtgtcaaaataaaaccagaaccagcgaatttgaacggtctatactttggagatttatctttccataaagcatctaacttggaaacaaaggta
gcgtctgtagcttttggcataacacattcttcatcaaggcaagatagaatgccaataggattggacttctcaatcaaatcaatggtgggctgtaaa
tcatggccaaagtcaacaaagtcccatttgatttcttctttcatatattcttcctgttccaaaacaaacatatgatgattaaaaaattgttgcagacg
ttcatttgtgtaatttatacataattgttcgaaactatttgtctcaaaaatttcaaaaccagcaatatccaaaattccaataaatgtgtttgacgaag
tacttcgacttattgcagtatttatacgcttcacgagccagccaaaattccgttcgtatatcgctttagccaacgcctccacagaagaaattacatg
ctcttgggaccgagcactcactacccattcatgaccggcacgaatgcgtggacggataagattttgtgaaaataactccggagatacgcctagaa
gatggcaaagtttatcaacatcatccatgtttgtaaggcgagcgacgccggaacgatcagcgccgatggagatattacctatgtgcaaaataata
gaaattatactgaataaatttaacatttctttttcggaaaaccctaggatttttaaagaagatagtaatcctttaaactcttcggcatcatttatgccg
ggaatagtacgagaagaattcttcaagtattcataatcattaaagtcattagagagaaggagctttttccgtaaactggaatcagcaccaatcag
gagttggtaaaaaatgtgatagttacgttcttgagagctttgatgaacaacacgggacttttcgagcaaataccattcaatggaagcattagcaat
ttcaccactgattgagaattcgactttgatgaattttccaaatcgagaagaattgttgttacgtactgttttagcattaccaaatgattcaaggactg
gattcgtcttcagaatttgctcttctagttgttcgccagatatcacatgcggattgcttgcaacagaagcaagatactgtataatccgtttggtgtttt
cagtcttaccagcaccagactcaccggtgactagaatagattgattttccttattttccaaaaggttgttataagcaaggtcagcaatagcaaata
catgaggaagctttcgttcttgacttttatccttgtacaactgaaccatatctttaccgtagataggtaatcgtcgataaggatttacagctaccaag
aacaggccagaataagtgtaaatctgatcagaaagatatcgttgctcaagattatacgtgaccgacggttcattcaaattcgtcagttcagccata
tcgttgactcggtcaaaccgagcagggttgagtggctgcatttcgtcatggctaacgactttttgatccctggaattgttgtatcgaacgacatattt
gccatcaggaagttcctcttttatccaagcctttgtgaaagctgtttcaggatgtgaaatccaaacccaacgtttgttgtctatatcattcaagctatt
atcagctaaagattcgccaagatttccttgtaagtttctatacataagttcaattcttaatcatatattcctaaagttgaagtaaggataaacaaac
gaacgaataccacaaacagcatgcaattgctaaatgaacgacaagagctactcactaacttatattaaaagggtatcctatccaaaattaaagg
tatcaaaaagttgacaaaaagatcagttgaagccaagtggtgtggacggtaaggtagctcttaaagggaaagcaagtctttcttgctattcagac
agcgtaatttacagagcttcaacaagacttccatttacgatgcctactgtaatgcggaatacccataatgcttatatggatacaaaagtcgatcta
aaattcctaaaatgatatttgcgttagaagagtagcatactatgcttttaaagcatacaaacattatgcatacaatttttaaaataatactatattac
attaaattaatgaatattactttcttttatattacttaataataataccttagtgctaatgttgtttaaccaacaaatcgcattccgatggtagagtgtt
tagagatcattgatacattaatccgcccataataaaccatcattcgacagcagcgaatcacccacagaggatttggcttctggctgtttctgtcacc
attactctccatccaaaaaattagactttacatatggaaggttttccaaaaaaggaaaaaggtaaggggccaaccggatttgaaccagtgacctg
gggagctgcaatccctcactctaccactgagctatgaccccttttactgttacataataaaaataacgtaaaatatttagatttaatttccaaaata
attgtgtattattagaattcgcaagaatctctcagaaatgttgcagtttaattaatgtaaccccaaatattcaactttacttgtcggttgcttaccgtc
gttacagacgtataggtagagctgattcagtgtgacggtatagctatagcccattattagtgctatcggtgttgcaaaaactcaaaagtagccata
taaaaggttcatctatttattatatggataataaaaattttaaataaaaaactaaatttcccaatgcaaaaataccggaatcgatatgccaaatca
taaataaaagtaagatgcgatatattctatatttggtaaacatcacaattcatatataattggataaaatgtatctgttaataccgtatatggagaa
taagtaaaaataataacaatatgatggttcagaaatggtcaattctgtttaacaatcaatccttgtgtaaagtataaaccaaaaccaaccaacga
aagaacaataaaaaaaaaaccgtttcatgaaactttcccaaaaaaataaaataaaaaaataaatagggtgtttggtaaaactaaacgctactt
atacatccataagattttcttccttcacaaggtccagtgcaaggattaaatgacgatcatggaattgaggatgtgtcatactaaacataacatgctt
tccgtcagaaagtaagtcgctgttactagcatgaagtctgatattgtttccttgaatgacttgtacttgcttcatagaacgagagttccatatctctat
aaaatcgtcactaaatgctaacaggtatccttcgtaacatgcaaccgaattcggtctacctatccattgtagccatgcatccttcttaattaggcttc
cctcgccattaacaaataatgctccgttatggtaacagcaagcaaatattgtggaatcgtgaacgcggaaaactcccagaggtttcgaattattta
aacgtgagcgatagaaagggtcggtcagaacagagggactagggaaactgacagccaacttgggatctagacgtacaatatcaattccctttg

acgactgatggctatagaatacttgaagcaattcatagaataacaatccgcaagcactcgaaattcttcgagaatacgaaaagaactgacgttct
tttgtttgaaattataagctttttgattatgatgaccaagcccaacaacaggttcacagcaatggatagaacttgcattacctgatccttgtttgaaa
aaaatgagcgttctttctcgctttatagacgcaccttccgttaatttagaaatcttgaagaaagatatattgtgggaagtaccaacgggtattgcaa
ctttgggctctaaagttccagtttcaatcactcgacttaatttgtgggcatacatcgaccggtcacgcagctctagtaaacagtcaaactcttctaac
aatcctatttgcattactcgtctatgacttgaaattttacgccattcacgaatattgctattacctccaaaagaacccagaaagacgcccgtcgtcgt
agcaatacaaattgatagtttcccatccgcacctccgaactgagcaatacaagttatatctccatagttaatggcttcaggtaagtcatcctcgttg
tgtataggatagcgccaagccgccattgttaacgcacggtccactgcggatccctcaaccaaacttatttcaggcgatcgagaagaagactcaaa
agtatgggataaatcagcaggaggatactggaaagaaacatcgctcacaatttgtaatgcaaatgggctctttttgtgcatcttctgagcgtgccg
ttttctcgcaaaattaattttgtccatccaaagttttcgaagttgtaaagattccgtatacaaagtataggttgaagataaaccacccagatgcctta
ctgtaaaaggataaaaatgttgcttttcaagtgctaatgtattcaaggaaccctcattcattttatcagaattataatcataaagaatatccaagtt
aaggcgactttttctctttgacctaacgttcgtagacccatcgtacggaaaattaccagtcaaacttcctaggaatttgttagagctgttccttgagt
tccattcttctgttaaagggtttagaactaaaagatccatcggtataggtcgtttggacaccaaaaatttcatcgcatttgttctcttgtcccttctcg
gtttggccataatgaaataattgtccaacaaaaatatatgtatctcaatccaatcgagtcgtgattcacttttcctaaaaacaggcccttcgaagaa
gaattcacgttctggatctcttaattctaaatttatactttcatggtttttaaataacaattgatatgaaaggtcgcgaatacggttagcttcaagtac
tgtagcaattaccgcatcgcattcttcacaggttgcccttaattctttctcagcttgacttattagttgataatcccagcttgtaggatctgtatgctta
agaattgtatcccattcaagggtatatctttgtaaacgttgagttggtctttgtagaaagtgctgaaaatcaagtcgacgacaacgaggatctgat
ctgcaggttgctaaccagcgagcaaagtaagtatttcgttggcattctagcttgtaagtttcatcagccagtggaaactgcttggcataatttatgt
aagatactttagccatttgtatccactccagaagaatatcaccgattagagacactacaggaccttgtaatcgctctcttataattaattgtggcaa
tataaattgagaatgtaactgacgaattgattcgacatttccaaagacggactgtataaaatcagccactcgattactaggtacaatggaattctc
ttgaacgatcccatccctaaaaagttcagcaagagtatgaagatcagctacataatctgattctttacatataagttcatgaatattgttttgtcgg
gctacttcctttttggtgagtgacgatggcaaagagtcctgaggtgagaggttccaaaacatattccaagaatcatcacagaagtctccttgcgta
ctagtaagtctatgggcgggaattcttacacacctgcagctgtaacatgtataattagaggaagaggctttagaacgacaagtgtatgaataaca
gccaccgtttgttaaaataggaagcacaccactgcttgaaggaaactgatcatcaacaaaagttatatatagtttttcaagattagtaactttcata
taaccattctgaatccaatcacttatgatatttttttgcaaggacttcagcaattagaaagtccatggatgcaactgacattgtaaatagtatagcca
ttatttgggcaacctcttgccgaggtgcgccatctgtcgttgtagcactaagagcagggaaaacctcttccttgcaccatgagaaaagtctcttcag
agaccaaggttcacgacaactactaaataaagcattcgcttgagtggctcttgaggagcgtgacactaaaggagatgaaatctcgccagaagta
tactgttgaacaggaaccttaggaagttccctggaaatatgtagttcttgagagggtgaaggaaagttcgctggactaggagaagaggtcaaatc
cgtaaatgaaggatcgctccaacacccaaaggaatcaatatgagaaagagagtaattatcagcctcgttcattgcagtacgaaatggacttgta
gatcgagagggagaacgagtaggagaaagaggtaagtacggttcaggagaaaaggaaagaggtagctcattgtaaagggtataaaagctgtt
cgaggaactactactatatggagaagaattgtttgaatgggtgttcaaaagcggtggacgaggtaaaggatgagaagtaggagatgttggagat
ggcgtttctggtaaagggcgatgcacgtctgtatattgaccaccaggggacatgaggtaaggatctgtatctaaggggtcacttggggagaaatt
ggaatggagggcattgggaaatgaagaaacagtcccattcgggtcagcatcgtaggaatagacagaacttgttgttggaagagcaggggatga
tcgcaccgtataagaagcggtacggcgggaaggagataaacgagaactttggggtgtcatggggagttgacgggaatgcgtgctgtcataacgt
tttggagaattaaaaggagatgaaaataaacgaaaagaagaaaaatcggatatagaaagtcgttttggcgatacgggtgagatattaggagtg
gaaacgggagaagccaaaaagtcgtccggatagtctatggacgtgcgtttccttctgcgctcatctcgagaatttttgcggaaaatatcattggag
agcttcatataaaacgagtaaacacaaaataaggagggctgtaagaaacaaaaagcagtatcctatgagaaatccccaagaacttaaaaaaa
ataggcttgcaaaagaacgaaaaggaaccaatgtatatctattaacaagaaaggaaagaaaataaagacacaatgtaccagaaataaacggt
tctactaattaacaaagaagatcagaacgtttaccgtaaaaggtgaaggaatgtcacggtaaaactgaaagaaaagaagaaacttttgcaactt
ataaaagtgtaccaagggtgtgaacttctaagaaacacagaaaaattcaattccgcaaaggcagacaaaaagtgtcgccagttctaagcttacg
aatgcaaagtacaaaagaaaaagtttggaaaagagagggcgttaaaaaaagccttgatataagtatagcggtggtggtggaataatgtaagat
ggctggtctggctgggtaatacgacgataatcgaaccgagaatgcttattattaaacatgaaaaataaggaattagaaaaagaaaattgatata
gctagtcttgaaaattaaatttcagaaaacacgtaataaagaccaaagttggagtgtgttagttgccgacaggtatgatgacagtgaattatgctg
aaacatgacagccataccggtttatattaagaaatcgaggaaacgatctaattatttttggtgatgaaagaattgaattacaaagtttggaaaga
gatgggatgtgcagctaaagtgtgaaagaatgctccatgttttgtaacaaaggaccatttttggagattgcaggaaagtaagcaaacaaaagga
tgctcattcaaacctaaaggaaaattctacactgagaatcaccatgaatggagcaagcaagcaaggtaatgatgaaccaaacttagagaagaa
caagaaacttgggagtatgtgtttacgagttaaaggagacaagattagtttcttgagaagcctctaggggttgagacacttttactatgcacggat
gaagaattgctctgtctatctctcctaggaagttttgagcacaacgaaggttgaagccaaggggcaaagcataggcgtagtacattaggaacta
tgcgactacttctcgggatgtgaaaagaaacaactgtgaccaacgacacggcacgtattggttgatttcattgtggaactcttgcgtatatgagaa

tcaagaaggcgaaaatgagtaaaaccattcttttgttcatcctttataatacatatatctcgaacaagacacatttcttccgaactctcttctttggtc
cgaggctttcattggcaactttggcaatgaacgcgacgttgttggccttgctgcgagttgcttagttacatttatgcttagatctgccttagaacgttc
ttatccatattctctctcatatacaaggctatccatgctctccatataaccttttccaagaaggaatgaagtataaagataaatacccttcatataga
atccctaagcttattcaattctcgtaaaacctatcctggaagctacaaacttccttttttgtgctcaaaatatgcaatttattgcaaactcgtttaac
caatgttaatgttaacttggtagtatgacctcaatgtaacaaatttattaaggattgaatctgtttcgtcgttatacagagtgcatcacagagactac
ttatcgcgttttcctcatgtggtagttacctttcctcaccaccacaaccttacttttgcttccctaaaacattcagagttgtcgattaaatcgtcaagct
ggttgtttttttacacaatggagtatcggcatcctaatggattggatggaaatgaaggttctcgtgtgtatgaggaaatttttggtgctccacgtaaa
cgggatcctatccgagctgttagtagtccagtgctaagcgatgaagctgctacaaggaaatcaaaaaaatctcctacgcgcagaattcctcgaaa
accgatgccgaatcgttctgccagtttgcagacgtccattgcagccgatccatcctacaaccattactcagaggtttctcctattaaaattgtagact
attatgataattcaccttcatttattaatactagtaaccctacaagtccgttgaatgacaaaggagactacgagtttaccccttctcttccttctttcg
atgaatcaacgacccttaatgcaatctcaaaacaccctatcccttcatcttataatcggaatgattcctttacttctacgacaccaagacgtgcttct
tcgcctgaaggacatagccttattgcttctcaacgcgaaaattcattaatcaactccacgatcgaacctgaaccctttgaagaatcatatacgacct
cgaatgcttctcctgtgattgattctaggagtaatagcatgcagtcgagccatatgtcctcgcagaatcctccaaatgcattcaggtctccttcggc
ggctagcatgagttttcgccaagttgcggatttacctacaaagcggccggaatccggctctttgtattctacaaatcgtcaaccatctttttcatcgt
ccagtccagtttcaccatcgactactatgacttctggatttaattttcctatttcaatgtctcccgcagtagaatctcaaatgagccgaacgagtacg
cctgctcatccttcttctccgtcttctccccattcaaaaccatcgatgtcgagacccatttcctctgcatcggttccggtccgtggtccttcattaccga
atgttcctaccacgaacctcagaattgtaccccctcaacaaagcaatctaagaaatcactctttcgatcatgctagtattgcacatttggaggctat
gaaacagaaacgtctacatgctgaggctacctcacgctcttatactttaagctcgtataaatcatccggatctgccactatttctgactcttcgactt
atccatctagtcctactgcctttgttccatccgaaagaagttcccctgttggaaagggtaagaaaaagcgtaaagtttacgctgctttgctttcccgt
gttgcggaagagttgacagcacgaatacaacttaatgacatttccaaagatggcctcatttattcaaatgctttcacaggggactatgcggtcacg
ctccttatggggattatccatacctcggatcgtactttggctttacttgtgggtagatctttagatgctcaaaaattcattcatgatgttacatatgac
catcgacttcgtgattcccatcgagaaatttatcaatttcaaggcattgattaccgtcgcctcattcctcacgatgcagcttctccaagagatcgtta
ttatcgtgatcacaatgaggattcagaatcaagtgctcgaaattcctccgcaacaattgaagacgtacggtttccaaatggtgtttttactttgttaa
ctcactgctattctcctacctgtgccaaagatcacccttgctattctatttcttgtccacggcgcttagagcaacaacaccgcatgtttgctaaaatgc
gtgctaacgctgaacagtcatcttccttgacttttgatgataaagagcaaaagttatggatccatagtgttcctaaagaaatagcaaatagtgtta
gtgaacgcgagcgaaagaggcaggaagtaatttgtgaggttatttatactgaaagagactttgtcaaggatttggaatacttacgcgattactgg
attaaacctttatgggcttctaattgtattaatgaacggaaaaaggaaaagtttattcgcacagttttcttgaatgcactagaggttcaagctgtga
actctaaacttgctgaagctttaacccgaaggcaaaattataaacctattgttgacaacattgccgatattttcttagaacacgtaccaaaatttga
gccctttattcgttatggtgctgggcaattatatggaaagtatgaattcgaaaaggaaaaggctaacaatcctgctttcgccaaatttgtatacgac
gttgaacgaatgaaggagtctcggaaacttgaattaaacgggtatttaaccaaaccaaccactcgattagcgagatatcctttgcttttagaggct
gtattaaaatatactgatgaggaaaattcagataaacaggacattccgaaagtagtaaatgttatcagaggatttctctctcgtcttaacgtagaa
agtggaaaagccgaaaaccaatttaacctcttccatttaaatcaacagctcgtatttaagcccggtgagcattacgatttgcaccttctggatgcca
acaggcaacttatttttaagggaccgttaaaaaagaagagtgcaacatccagtgctctggattccagttcagatgttagcatgttcttgttcgacca
tgcgcttctgatagtgaagcctaaatttataaataaaagggaaatttacaaagtctaccagagaccaatccctttgttgcttttacaactttatctac
ttgatgaagctggaacaagaattccttattctaataaaagtcaacttgcagcggtctcaaaagttgcaaatggaaagccaccaggaagattgcac
ccttttagtttgcaacttttgggtaggcgaggatatgaaatcacactttttgcttcaacagaagtaggtcgtgacaaatggttagagcatatcgatc
accagcaaactttgttgcaacacaggagtcaatggtttgaatctgtaacagtatcatctgatttctttgttaatgataacaaggtcaatgcaatcgg
cgtttatgatagtggtcgtcgattattatatggaactgattatggcgtgtatgtatcgcttcgcaaatctaatagccctcttcagttcaagccagtccg
agctctcaacatttccaatgtaattcaactggaagttattgaagaattcaatttattacttttgttgtctgacaaaacgttgtattcttatccccctgga
aatgatagaagcagatacaagccaagctcccaaaaagcctaggaaagtttgtggtcatactaccttttttccgtgtcggtatatgtctcgggcaagt
tttggtttgtgccgttaaaagttcagttctaagtgctacaatcaaagttttcgaaccagtgccgcatctcgctaaaggaaagaatatgcccagtttc
aagaagctattggcagtaaaccaagaccctttacgaatcattaaggaactttacatccctacagagtcaacttcagtacattttcttaaaaataag
ctttgcgttggatgtacaagaggtttcgaggttgttagtcttgacaatttggaaactcaatcccttctagatcctgcagatacatcgcttgaatttgcc
gaacgcaaagaaaatgtgaagccgattgctatatatcgcatgaatggtggtgaatttttattatgttacacacaattcgcattcttcgtgaatcggg
atggatggcgttctcgatctacttggttcgtcgtctgggaaggcaatcctcaaaactttgctctcagctatccatatattctagcatttgagccgtctt
tcattgaggttagacacgtggaaacgtcagagctagtgcatgttattcctggcaataatatcaggttacttgcggatggacgaggaaaattagga
gaaggcggtgaaatttttttatgcttgtgatcaacgtagagataatgttgacacaagtgtcgtctgttcccttcgacttaccagtgctaatgcagatgt
acatgtaaaagaagctttgactcctagcaaatgagtcttacgatgaaaacgatgtcattcgttctatttatttaggttaatttttttatgggttacatt

taggttcatattttatttaaaaacgtttaagggtgcatgaatttaatgatgaataaaattttttgtgcttgcataaatttatcctaaactgtactaata
attcattgagtatcacaaaaagaatgtagactttaaagagcaaaaaatttgaaaaaagaacgtaaaatctaaagtctcaacttttattcctcgtat
tcaagaggatcgccatatacccacataccaatatggttaaggcgtgcttgctgctggcaatcttgcaaaatgttcaagctttcagaatctttctcaa
atggtgctaactttcttttaggaataacgctagccatgccgagggcaaccaattgagtattaatacagtccgagaattcaaaaccatcactggggt
tgtaaagagttacagaaacaaaattatttgtttttccatcaatcttggcaacgagacccttgttcatagcgagttctctaaagacgagtctggcatc
ttcgtaataatcagaattaggaggaggaagctgtacgaatgtaagacgggaaagttgagcttggggcttcaactttgaaaacttttcaggaaggg
tatacagatttttgaaaggaatttgttccacactgccgtagtcgtataataagacgtcagcattcagattctctcggttacagcgtagaatttttcca
cgataaattgcattgtccagagtagaaacagcagcaacgtttattcctacattatagcgatctaatggttgtgcagatttttgataattagagagct
cggacatcaagctttccaattcttgtatattggggccagcgacttggaatgaaaactttccatcatcggcgatatcactaagggcaacatccaaat
acaaaggttcagaagtagtaggatgagaaggtacatcactttttttcctggacaacttcaggtgcatagttatgccacatgccacttttcttttctttg
gcagaggcttcagcatcatgatattgggtatactgaatgtttgattgattagaataaccttgacaccaggctaaaccttggtttaataaatccaaag
caaaattagagctgcgattgacaattaaatcacccaagaaacaagagttattatcgaccgaaagaacttcaacttcaacttcttgttgtaataatg
atttagcaaagttaaaggagtcagaagcaaaaggttcttctttttcgtgaatattacgagcagttcgaggagtacggattccagcacaagcgaac
ataaaagagcagttttccttgggacagaagcagcgaaaacgggaaccactgataacgttgtcgatgacaacagaaagctttctgtttctctgaag
ggatgaaaggtattgacgagaccttaacggggtttcagaagcattgacaattgcatcaaactgaggcttctttccagaccacatgccctttttagct
tcttgtgcagacttctccgcctgaattaggtaatcatatactggagaacgatcgacatcatccgctctatgtcgcaaaacggaagcataaccatttt
caacagccaacatagcaaggtttgttccatcattaaaagtaacggtgcaggcattaatttcgggtaatccattttgggcaggacgaacaaaatca
agagagacgttaactggcttaccgactgtttttttacggaaaaattcgcgggcatcaaattggtaaggtgcctctttctcattagaaggccttggat
ggcgtatgctgctaagttggagtctagtttctgaaccatcatcacggcgtacttcaagagtatcggtggagattacacgagaaacagtaccagaa
tactcacgaagagaaagcgagccgatatttggtaatttaacatccttccagatccccaagttactcgtctttgctttacgttcgatagaacgtaaat
tctgcatggcttcaggaccaagataaacgctatgattgtcggcaacacgaccaagaccgccagaaagcaacacgtgtgcaatattaccagctgg
atggattacgtttgcaatgaagctggtgtcgtttggtgttacacctaacaactcaataaccacatttctttgtaaaagtcttgcttcaacaaattgcc
tcgcagcatttccgaaaggttcttcttcggtagcgggttgatttccgtatgtgtgggtagctcgaggacaacggacaccagcaagcgatacatttat
gtactgatggcgtctaggctttaggagaaggcgcacccgtaattgatcaccgtcgcgaatattttcaataataccattgagtttcttcttttggttttt
ttttatgaatttcggaatgtcagcaggcatagctttttctgtttgaactgcatcttctaggttactccatacaccaagtttagcttgttgagcagcggt
ctgagcttcttctaagcttttaaaataagggttttcgttattagcttctctgcgaactgcctcaggacgaagcttggctagaccagcgcgtaataaa
gattcagtcaaatcaaactcagtacgaatgcaaccatattcacgcttggtagtgggagcaacgttgaacgtacacgtggaagcaggacgtcctac
aacaagacgccgagaatgttcctgggcttcaaaagcaaatggctcatcaccttcttttcgaaaacgagggcagtcaatgtaagcaagagaaaac
tgcttttctttgatttgtttcaagttatttttaaccaaaatagtaaaactatcaccgctatgagcgtatttaatcatagaagatacatattcactcattg
ttttatggatgaaaagtgttcgttaaaggatggaacgataagatggtggaaactttgcgtgtagcagcatttatagaagtataaagaaacaaagt
agaatatgaaagaaaataagataataatagttgaagtatgcaaaacaaagtctgagtacagattctctttccaactaccaattcatgggaatctg
tttcgccttttgtttagcagtactttttttagcttcttgtttttttcttttaaataatatttctaatcttcttttcgtttctttttttcttgcatgtcttaataaaat
atgtggttggtttatatggagtatacaaagaatgaaaaaaaattgccgatttcaggttaaaatgtatccttcaatgtataaaacagctttttttttttt
tttcttccttttccttcgtcttggcggatgaagagatatttcacagtttgtttacggtgagaaaggaatcgtttatattgacgggttttcgtctgatttg
gctagtttttgcttctttttgttcccttatagagcagcttgttttctctgtttcaaaacgagacttcgctacaaactgttgggaatttgctttgtgaattct
caggtaatatgataaccctcgttattacgttaagagttcttttaagaaaatggactgtgcgatattgcatggaacgaattttctttgcctgaaaaag
caaaagatgaacaataagtaattttaattttattcctttcttttataaaagcagcacttttatatacggaaactcatatacactgctgcacacgacta
aaaaggcgctcaacaaagagactcaacatatgcgtataggtttcgtgttcataagcttcatcatcggatgaaaaagaaaggaaaaacgataata
atttaataaaaatttcaaggaaagctgaatatctgggttttaagtttcaattttgtagtcacatcctgcttttgcgtttcttcgttcaatcattaggtgg
tacattcaagtcttttcttctcacatataaatacttaacattaccactacaaactgtaaaggacaacgtggccgagtggttttaaggcgttcgcctgc
tatttagtagcctagcggattccctttgggagcgcaggttcgaatcctgccgttgtcgtcaacagtttttcaaaattttaatgtttttttcttagaacac
gaacaatttttttctactcgtatcggaattttcttacttttcaattttgttttctaaattcttttttttgtatcccgtttacattccaatatatcctaccttgtc
tgttcgtgtatgttttctgtgcaatttcagagaacatagaattcgttttaaaagtaacaaaaagtaacatagaacggccaaagacaattggtaaat
aaataaataaagaaagaaagaaaccataaaaatgcaacatgaaaatgtaattaaaactaaagactaaagactgaatcagttcaattgacata
agtctcgaggttcatttctactgtccttttgcaaagacacgtcttccttggttaaaaaggattcattgcaagtcgcgtttcgttaggtttgatgaaaga
aatttatttatttttctcaattgaatctttcatagatttcgtatattcgtcctctttcttttagggaatgggtgcctataccctctacttctcctatagaca
acgaactatacttagctcttcgaaagaacagctcgagatcacttctgaattcagaaccaaacagttttcttttggctttatatcctttaaaacactg
taagtatcctttttttttgctttccttgcgcatttcatccgcttttgggattttgcaagacttgattcctcggggttccaaaagctgtccatttgaaaagac

atacacagttaaacttccccttccctctgtctttaaatccacgtaagacatcaagccatgctttgtcttcgtttacctcgtactgctgttcttcaacaat
ctgcttcgtctttcttcgggagaagctgttttggtcgcaagtttcatcgctctcagtttgtacaaaaagaagggacatcagagaagcaaggacaca
acgatgcaactagtcatgaatcttcatctacgtttcatcctaccagctctgtattggctggtacaattctgaaaaacctcaacatcaaagctggagg
cattgacccagtagccaagcaagaccatgaatatcctgattggctttggacgcttttagatactcctgccgaggactctcgtactgctcaaagtcgc
acccggaagaacgcaattcgtacatccaactttttgaaaaagaaataagcttttaagggtttcagggatctcttccaataactattactgcttttgt
actctcttcttctcttctctccctcctttcttttttttgtttcttcctcccttgcacatgctcctttttattcccaaacatttgaacagataataagctatggt
acaatactgattttgtaaaaaataaatgcaaaaagtcattttattctcttcaatcttaaagacagaccctgtagacattaaaactgctaatcagaa
atgaagtttctcttctactcgatttcttgacttctgacttatggatatctgaaaatttaaaagtctcaaacagaatccagtaaacctatagaaggatg
gccagtaaaatcattttaaatgatgtagaaaaaaaggttgtccaacttttaaagaaaactgccgacttcatcgaaaatggccataatacgaacgc
acctatcgaagtacgtttagccggtggttgggtacgcgacaagttgctagggctttctagcgacgatttagatgtaacattaaacaaaatcaatgg
tatagatttcgccaaagctatcttagaatatgtaaataagttggacccaaaagctgccgttccttacaaaaagtcaattggaaagctgactgttaa
tccggaccagagcaaacatctagaaacagcgacgctttcactttatggattggacattgatttcgtcggtttacgagaagaaacttacgatgacaa
gagtcgaattcctactgttcgacagggtaccatagaaacagatacttatcgtcgtgactttactgtgaactctctgtttttttaacattcgtagcgaac
aagtcgaggacataacaaaacgtggatttgctgacttgagaaacaaaaatttggtcacgcctatttcaccagttcgatcctttttagaagaccctc
tccgaattctgcgtggcctaagatttgcttctaaatttagcttttctctagatccttcacttcacgatgccatacaaaatcttggagtccaaaaagcct
ttaaaattaaggtcagcagagagcgtgtcggtgaagaacttgaaaaaatgttacgaggtccaaaccctgacctttcccttcatctgatctattcctc
tcatgtatcttcttttatgtttggccctgtacccgaagaaaagagagaagtccccaagagagccacggaatctgcgacttaccttttttgattattttc
aggaaaatttctctagactgccatccttaagtgatgatgaccgattttctatttggctgtttactgctttgctgccttgggctcatcaatccattattca
aaagaaaaaactaatccatatacccgcagtaatagctaaggataatttgaaactaaacaatcatgctataactcaactcaatatgaactgcttat
acgctgatgtgtttgacactcttgcaaacgacttaaccaaggcttctcgatgcgaactgggtaatataattcgtcaaattggaaaaaattggcctat
tgttattctttcatctgccttatattcgtattgcaaacaacaagatgcgcagttacccagtgtatttcagaggttctctgacctgtacaagttaatata
cgatcaaaacttacaaaagagttacgaattaaaacctttgttagatgtatgtaatttcatttttaaagcctatactaacactaaatcagggaaagc
aaatcattgaacagatggaaattaaaccaggtcctgaattgaaaacaatcatggaacaaatgatttcttggcaatttgaaaatccagaaggaac
agtcaacgatttaatcgcatacttacgctctctaaaaaatataatagaacagaaatgaagtcgtgggaatacacggatttattgcatctgctcata
ttagaagtggtagcaactaaaaagaaatttgatatgttaccaatctcgagcaggctctttgtactcatattctgtagtttccggaataacttctgtac
aatcaggatagcagtaattcaacctcacataacagtgtgttccgtccaaatcatcttcaaagtgtcgagtaccacaagaagcacattgtaagttaa
gctgatcaaattgctctataatttcgttacccgtacgtttgggatagtccccatcgcactcatacatagctgtacaccagtttgctgtatacgaggtg
tatccatgatatatcagggtacgattgtacttcatatacgcataaggacaagcttgattttcgccgcgcaacataggacataacattgagcctttgc
aatcgttaggaaagacagaattgtcataacccaagaaattaaacatgctcttttttttctttcaaacctttggtaggctcatcaataattgctggttca
agctcgtttgcatctttcgaaatcggtgaagcgaatgactgacaagcctgcaataaagaaacaacgaagctcaaaaaaaaattcctggcgcatttt
ttactcctattagaagataaaacttaccggggaatggatagaaaagagttgtttatatacctgcagcgtcttgttccagtaaacaaacataaaga
aaacaaaataaacacaaaaattcgttgtaattgtttacgagtaggataatttaagtgttttcttgaattccccacaaagaactatcgtctataactg
aaccctctaattcttcatcagaaacatttgtttccgattccacaggttcatttcgttttctctgaaaggattcttttattgaggagagcttctgtttataa
tcttgattcgagtcttcaagatagagagcgtcataatcatcaggacgtaaattagtatcctcttcattaaacaaagcatggccatttgaagtttcttt
atccttcaaataagcttcctcgtcatgtttctttagttcttcatcctggagcagagactccttttgtgtggcttcctgtgcgatgctgttgatttcatctt
ggaaagcaacccattccttgtttataagattttcatttcttggtatggattcatcaaaaaagtcatctggtaagctggatttggattgtttttcaatag
tttcatcgtttgatgactctaaccttttttgagaaatgtgttctcggggaattcgaagctgcgaaatttcctcgtaactatcatttgatgaaggctttg
ctttttttcttttcctgaattgagtcacttcgcgtccgttttactccagttctttcttgctttattttttgaagtaaatctcgggcatctcccatttttatacta
aatatgtattttttttcttattaaaattacccttaaattatataataacaaaagtttggcagtaagacgaacttcccaactcgcgtttaggtgtagcta
tgactagcaactactcatcacttaccatttcaccaaaccacagaaaaatctttcaatagtcaacaatatatgattcatttgttatggatttagggat
gcgttaatttatatacgactcacttatattaaacggcttgaatacgaattggtatgaatataagccttcaaaattaggaaactttgctaacgaagct
tcacaggaaatatacagttctattaaaaaaaaaaatgagttctactagaaaatctatgagaaaaagaagggttactacaaaagctgctgaaggag
atcttgcaattggcgatgaaagcgaatcggcagttcgttgcatttgcggttctcaaacagacaatggcgatgtttgggttcaatgtgacggttgtga
ttgctggcaacatgctggttgtgttggactttcggaagacacaattcctgaaagttatttttgtgaaaaatgtcgacgaccagctgaaagtgatcat
gaaattgagcaagaggaatcttcatccatgaaattacaagatccgcaacctcccactaataggccggcatttgactcatacatagaaaaagaac
aagcaaatggaccatctggaaaaatcgccttgaaagacgaagaatcaacgtttgttccacaagaaagagaagaatcgccttcatcaatatcttc
ccaatcttcttctgattctgcgccttcccctgctggaggtacggattctcctaattctacgactggtaagcggaaaacgtcttcttccagcttttcctct
tatactcccaaacgcattaagggttcagaacaaaagttacaaaccgaaacagcttcccaggaggatataaatttaagtttgtcgatcgaggaatt

aaaaaatcctgtccgaaagagtgtagcgaaagcttgggtctcagttttcgaaaccattataaaaaaggcagagaaagaaggtgtcgttggctta
caagggatgaatactacagccttggcactcgaactggaaaatacaatgtttttagagctgtcgtataatgcagatcaaagtacaaatccaaataa
tagatacagagaaaaatttcgagctcttcgctttaatttggttgatgacaagaatcctgcgttccgtgcgcgtgttttaaagcaagaaatccctttttt
ccaaattggttcacatgaccagcgaagaaatggcgaatcccgatctaaaatccttggctgaaactattcgtcaacagagtactgaaaatactgttt
tgaaacaacatacccttggttatcgcattcgtaccatgcagaatggtgaaatgattgtacaagatgatcaaaatatcgatacaaatagctcaacc
atacttctgccaagcaaagtaactattccacctccgcctgaagaaagtattcccccggagacgaatgagtcacctgctatctctgaaagcaaatct
gcgtccaactctgaagaaccttctaatgattatattaagaaagaagatactagtgaaataaaacaaaatgtaaatgtaccttcgattgttgagatt
gatgatccttctgtactagatatagttgagaaggaatcactggttcataatgaacacctgtcttctccgtattcacctaaagaagaagaagaagca
tcagaagtagaaaacgttccgaccaaaaagaaagttctatggaagggaaaggtgaagatggcttccatctcagaattttcggcagaagctatat
tatcgagtggtcatattgactttaaatttttatttgagattttgtcttcaaccgctttgatcgaaggtcgaataagcatctcttcgactcttcaatattt
acgggcattacaaaaatctcagtccaagagcattgtttctgttctattccttccgagtaacaaacgtgaaacagatggatttaacgttttgtataag
tattttttggaaaggaatcggtatggtgtcctacgatccaaatctgcttctgttaaggatgcatacataattcctatcccttcaggtcaggatatacc
agaagtagtaaccctgttgcatgattcaaagttaccgaaaaatcgcgaatctttatctttgttagggatatttgttttaaacaagactaacgcgaaa
ggcgatgctattgagcatatgccctctttgggttcctcctctacattgttatcttctcttccaactacgaagccaattgctactgaaaacgaagatatt
ttggataattccttaaaaagattacttaatgtgctaagtctagaagacatctttttaataaggcatgttgtagagaataatccattaattaaagcca
acccaaatctcgcgattgatccaaagtttatgcagaaggcgatccttgatgaacaaagcaaatttttaaggtagttagtttcattagaggaataca
cttcaacataattatactgtccaattgctccatgaaggtgtaaatggacgacatgcttttggtggatttgtttgcccattcattaatgagctaactatt
catcaaaaatgtatgaaagattgtggattatgacaattaagcaaagcgaaattagcaaaaggattagaaatccttgcatatagcttttattgtaa
aatcatacatggctttattaatgctattctacagtttggattttggtaaatacgatatgacaatgaaattataatttacagtaagaaacgaaaaata
aatttggagaatcatgacctaaagttgctgttattgaaggtatattaagcaaacatgatcaacaactattaacgtaaaaaaaaaaatactcttttc
tctatggtttccacttagacatgatgaaagatttttaaattaaccaaggcgaataacaccactagcaataagcttgtcaatcttgcttttaacgaca
gggctcttcatatgttccattaaggcagcagggttttcacgggcttggccaaggatggcttgcatggcaggatcttgaaggatagccaaaacgtca
ggatcgttttggatacgtgccattgtttcttcctcagtttcattttgtctttgagcagccattgcattcatgcatttttgcaattgagattcaatttcacg
aatgtttttaccagtattgggctctttacgatcaacatctgcagccgcattgcaagcatcaatacactttccatagtccttcaacatgaacaaagctt
gagccttacggacataagcttttgcaaaaagaggatccaactggatggcttgattgcaatcacgaatacactcggcaggtgccatcaccttaagg
taagcagcagcacggttgccgaaaccacgaggatcagtaggagcacgcttggtcatttcggtatattccttgatggcaccggcaaaatcaccgga
cttgaagagatcgttacctttagagcgggcctcatcagccttgtcaggattaatgtaagattcacgatcttgcttttctttcaatttttcaacatccttt
aactttgtaagtgtatcagggctgcggtgttcagtaagagaacgctggtaatattcaattgcggtagcaaggtcaccacgtttttgataagtagtac
ctaaacgaccaagagccttagcgattaatttaaagtcagcacgcaattcacgtccttgctcaatggcttctttgcaagtagattcacagttgtccaa
ctcatcagcttcgtagtaagcggcagcaaggttgtttaggtacgtaatgtctttataagtatcccaagccttcttgtagttctcaatagcaacaggg
aagttgcgcttcttgtaattttcattaccgatttgtttggcctcatcagctttcttacgaagggctccttcgctagcttgttcttcgatttcttcaggaga
tttgtcttcttccataggttgagcggcaggctcgggtttagcttcttccttgggagcactttcaaatttgggagattcagaaggagtagcttcgttatc
atttgcagcggcatcatcctcgcccttcatgttgatatcaatgcccatgagcataccaatcaccttcatcatacgtgggtcattcaattcggacatga
tagagccagggttcttttggatgttttgaagtttggtcataaagcctgcatcacccagcaaagaagatgtttcggggttgcttgccaacttttcaata
aactttgggtcattcaattgagaagtcattttggcgaatggattgaaaccaccatccccgccagcggagctttgggcagcctcaacactctttagg
ccgtttaagagttgggcattcctggggtcaaacttcaaaccttcttcataagcagccttagcagctgacaggtcacctaaaccatgcaaagctgct
cccttacgcgaccagccttttgcccagtcaggcttgaggttagtgcatttggtagcatcttctacagcacttctgtagttcttttctgacgcataacag
gctgatcggttcgagtataaaatatgattgctaccgtctaattgaatagcttgtgtaaagtagtcaatcgcattcttgtaatctttctgtgcaaatgct
gcgttgccttttgctttcaattcttctgccatttttgctttttgaaatcctctttcaattaaaactttctttttagcctaatataacacgagaattcagtga
aaataatcttaaatcgcgtttcggacgtgtttttttagttcctttcccgtcgttttgtcgttgtatactactacgctcaggtcaacttcaatcctgagttcc
tacctagtacgctatataagggaatgacgattccattcagcgaacctttacgaaaaatccagaataagcaagatcgtcttccataacctcgcaatt
agataatgtaacttaagagaaaattttgtattaatatatttaatgtttttctttatcgattcaatcagttattttttaaaacgattcctcaagtctgcaa
aaagtatccttgcactgcgatgcgttcgtaaacaaataaaaatgttcgatgatttcacaagaatggctcctatttagctgctccatggatagttcat
agtcaatcctcctttgtatttgatgtctagggttagaaatcaatggacacaaagatgctcttctctttttacgttgaatttgtcaatacattccattgat
tgctgactctcatcaaaaactcaatacctatctaacttggaaatatacaaagttaacaaatacatacctctatgtaagatgctaccggaataagat
ccatgattctaagactaaatacatatattttattcatatattctggtaaaggtcaccttaatcaatatgagacaagcacacaattctaatggaaaga
agtaatagaaaaataagatataaaataaagcgttcatacaaatcatgtagcattaaatttcgtaattcgaaaataaagattttgtagaatggaac
aattcagttcttcaaagcaaacacttttgtatgtttcacattttgttttctgcactcaaagagatacagatagcaaatgcacaagaaacatcttggtc

ctttcttaatggaagcttccttgacaccaaacgtccatctctgttgaaaacttccaaagaacaacgcgtctctaaagacttttacatacatctaagc
aatgatattgtaaaagtataaaaaaaagacctctgttgagaaataataaaactatccaaacccgaaacatttacttttagcattcctttctccacat
atattacccaaattaattttcctctaattgtgaaagaactccttgatccggcaattgagcatcatgatattcgtccacagatccattttcacttcgata
agtcacgaaactagaagcagggttagatccgtcacgtaggtgatgaggacgattctcattcaatcgcgtccggccgttgtctcttacatgcttatg
aggttcatttccttgagctgcgggttcagatttcgagcctctaccaataaggctacgatagtacgcacgatcgttttcgctaaaaattggtatacga
tcgtccaaactacagagcacttcagcatcagacttttcgtacggtatatccatatcaagtacatattcttcgggtccagtttgtttgaaatattttggt
tgccatttctcgttgttagcttcccgagctctagccttttcgcgctgatgatcttcaatcgcagtctttgactttgttgcaacagattggtctcgggcgt
tcacagcctcacatgtctcatgccataacttctgtgattcataaatagtttgttcttctaggggtcgagccatgacaggatagaccggcagctctgtg
acatccaaaaatgtagaggtcttccctgtgctgacattttccagtacaatttcagaatcccactttccagaaagtttgtacagcacgtcacctgtgct
tatgcgttttaccttccctgcaatggaattgtaggttccactaataaagcctttcactttaaattcgatatcagctgccaagtctgtatctggacaacg
cacagtaacatgatctcctaattctatccgcattttaccaaacaaaatgccacgagcatacatatttggctgggtcaaaagatactcctcattctgt
tccaaaaatttgatgctagcaactccctccataatactagctgcagaattacctaaaaagcgtgacctcggctttaggacaccgtctatgcgaact
ttgtattgaggacagtaataaaaataggcactgatgggtggatgatgggacacctgttcggctatgtataccgcaacaccattcaggttattcagc
cccggactttggatgtcggatggaggaaacttccaaaagcccgtgaacgtctctcccaaaactggattcagcggtttctttacacccggagggcg
aatatgccatcctgaaaggtaaaatcgacacacctcaaggaaccgttcttcggcagaaggaacatgaggaactgggagcagaaaatctggatg
actcataaagctaatcaacttgttagctcacaaggtgtttatcaataaaaaagtgttttcatcgaaacatcttcccatatagtatagcacgtactttg
taattcgttccaacattgacctcggctccaaaacaaacgtagggagtgtcacctttgacaagtccatatttggtctaaaacagagtcagcgatact
gtgtctaaaagcaaatcttttgcctacctcaattgagacacaattccaagaatgatattctaggaaatacgttaacaacggtctactagacgaaaa
gaagaacaaatcaaaatataataaagctgtaaacccactatcgggagcattgtatcgtctacactacaagtttgcttccttccaacgtcaagcact
acaaaccttgccctcatcttcgatttcttccgtatctctatcctgtttgtcatctagagatgtttttgttagccacatccaagaccaagggatttctaat
ctttgcatgagcgtagctatttctttacatacgaggcataatggatcgcctattacaatttccttattttcgctaataaaaaatggcttttcttagtagt
tctcagtgaattcttccttctttgttgttggttatttgcaactcttactcggtcatgttcgacgttggccaaaatatatatgtattgtaccttgacgtcaa
acccaggaaggaaatcaaatgtaaacagaaagtccccaagataagggttgaagagaattacaaaaagaattaaatgaaaaagaactgtttgc
ttctattcctatttttttgtttgctactttctggtcattctgaactctaaaaattttattaccgttcacaaacaaagagtaaggaacacataaaaaaaag
ttatgaaataggaagctgcagctggcttgcaattcgaacaaagcgcagctctttatgcttcaaatattactagtaaatcccaagaaaaaaacacg
aggggaaacagactttcataaatcaaattcttagttccgaaagaaaaaacagattgtgcttttctgcatgaaatccagtcttccataccaaaaaca
atcgcaagatggccagccctatccgtctgaatttttgccaaattttctaagagcaaaataagaccaatgttttggacgggaaaccaaaaaagaaa
aagtaaataccccaaaacttcctaatccatagtaaccctttacccaaagtacgattcatacttaaaattttcgcctgtattactcgtcatcgtctccc
ttctgagcccttgttagtttcaattcttcatagcattgtaaagaatcactatcatcaatacttacccttctttccttcaacttctcgtgtttattctttgctt
ttgaaactttggtttccagacgctcttctcgagattgtgctttcatagctgcttccaacttttcttttctctttctattcacatcttttgtttgtctttttttct
tactaattccattttctcccgagtttccagatcgtagtgcaatggttgcaatttctttagcaactttttcttttacactttgaggcttttcctcagaagga
attgttccaacatcatcccctgtaggatcagatcttctgaaatttctacttcttacggattgcttctttgccatcttgtcaattttcgatattggcaaaa
gacaggtaggttcactgtgatgaacctctggcacgagaatatggaaaattttattattgacgaatcctaatcagtagaattataaaaaaaaaaaa
gcaaagaaaaatttcttctttttttgctctcattaccttaaggtttcgacaaactccataaaatattatctgatttgaattcaatttttatctaaacata
gtaagtcatcaagtgtgttttactagtttctagtatacacgaatcataaacttcatgaaggaatcgtcttgttaggcttatcactcttaacctcatatc
attgaaacgcagaccatccgcagaacatgagagataactgacttggatcccgcgcaatgcgtattaattcaccgaccgaagcttctattgacaaa
gtgttggacagtttttttttacaaccaaaagtgccctttcagcttcgctttccacagactgcgttatacgttttctcttaggatctttgtaaatagatg
aagtggtctcttccatatcctcttcattctctgaacttactatttgcagcttctgtttccgcatccttcgtataggagatattaaccatgaaaataagg
gatcatatcgcagcacctctaatatggagactagcgattccacttcgtgtcggagagtttcaagtgtaaactccatggaacggcgaaatacacctt
ctacacctgttatacccataccgtctacaacatctcgggttaatcggaatggaacacattctggcacaggtaattttttttccctgaaaacgattagc
atttattattccctttccatctgaatcccttagcaatacttacctgctcaaacgttataccaagatcaatatgtaccacctgtcctgacgttacatcta
ctaatatgttattcgcatgtcgatcaccaagtcccaaaacatatcctaaaacggatgcgactgcagtacttcttgaatagtttgtctgatttctaaac
caaatcattggatccggatacttttcaaagaaaaaatgacgaaatacaggatgataatgcctacaaaccaagtcgaataccttcagacgagtgc
ctgtatcttccatctgcttttctactatcaatttccgacagacattcaaagaccaatctttaggataatagcgcttatgggcaggatctaaatattca
gccataggaaccgtattcaatacccactcaataacacccgtttttaatgctaagggaattactttataagttctcatagtcagtttcctttgcattgtt
gatctgtagtttttcaaaaatccgttcacttgctcaaatacttgttccattactgcatcctgtcgtaaatcgtcatttcctcccttcactaattgtttatg
tattcttccatcggagctggtacatttcacgatttttggaacattgataccagaagcaaatccaatagtattagaaaaagaaacgactgtaggcac
atcagaatagttcgccatatcattaatcgccaaatgtgaagttatgggagctaaatctagttgtggaaaatcattcaaaaaccacgcagcacaag

gggagcggctgaatgatgtactagcggttttactgcgagggttccattcagcaagggtaacataattcataaaaacctttaaaagcttatcgatta
atgatttagattccccaaaagtttgcaaagttttcatgatattttgaaccgcagcgaatcgtgactccccatttcgagtttctgcttcggatgggctg
gaaattaaagagtaaagcacatgaagtgaatgataagggtgcaattgagcaacatctaagcatattcttccgagtagtttttgaaacttgctgcc
atcattgagaagccttgcagccaactgataatacacagggataaatttcttacgaggcgtttccaagatatggtcctcaatctcaacattaagtgtt
tccgaactagagtttgaaagccacataatacaaaacctagatattgtaacgtcgttttcatcactctttaacaaactctttaaataatgaataatac
cctttttaacatcatatctttggaagcacaaagtgtttgatattctcgctggtctgaaaggagtgccgccatttctctcaagtaatgatgtttcagaa
ttctcttctctttttcttgcttgcataattttagagagtcctccaattgtaaaatgtcatccttcttttcaagataaagcctttccgcccttctaaactcct
cattaagttcagggcttgaataattcgcatcacaaaatgaagcaaacatataatgaatttttgaacaagtggtgtcatccaggtccgacacgtatg
aaatggccttttcgaagcagttttctaagatgaattcagtttttccgacttagtagaaaacaaccactttcccagataagcatagcataaagatga
aaccgaggcagaacattcagtactatctaaagactgtttaagtaagcaaatggcatccgacgtttcattttttgccataatattcttgatgttaaa
aagtcaatcttttggcgtatattcgtatccttcacgaggtgcaagctatacaatgaatcactggacattgctgtagacaaagccaactgtaactttc
ccgtgagaagatacacttctccattcttaagtaatgctaatatgtgcttcttccgaatctctttcagattagattggttcagatgctctgcacgattttc
taataaagtaaaagcaacgtcttgaaaacttaataaaggttcgatataatcatagcgctcaagtatcagcatcttttcgttaattaatctatcatgg
aggtcttcgttcatgttccacccttctttgaatagaaaagaagtaaattcagagatcagagagtgaaacaataaagtttctttctcacgggagtga
caattaactctcataaatcgacatataaaatcattaacccgtaattccaactcatttgaatccgaatcgtacgcgctttgcaataatccgtagactc
ttccatcaaactctgtctcatcacctacaggaggcagatcccatttttgcattctccaagcagaagcgtaaatttcattattacattcatcgatagaa
ttttgactcttcaagcagtcgacatagctttcattcaaactgtaaaatccgccatttaacaaagtgttagacatagtcatttttgtatcagggattgat
tgtgaactaataaaattcatggcatccagatacgatatagccgaaaaactcttcttttcatgaacagctttcattaacatattacggaagttactttc
tcgatgtattccataataaacatctagccaatctaatcccgttaacacattcgatagatgagacttattaaactcaagggtatccgtttgatgcaaa
tttagaaatagcaagctttgttcgcaaagaccactctgatacgctgcttccgatgcaaccaagtaatctgtcttaagccaaaaatttcttgcaaaag
gagtgctttcatgaacttttggttgtttccgaagatatagtaatactttcatgcataattcccgtaccgatttttctcttgcttgctctataccttcgaaa
atcaatgaattgatataattgaaggcggttttatttcgaccatgaaattcatgatcaagcaaaactatatgcacaatataatggaaaaaatgaag
ggaaactccttttatttccgtattcaataaagatagagcccgtacttcgtttgtcttatccagttgtgctaataattcagcaacggcagtgtctaccca
caagaagagttgatcgttttcagaattaaataggctgctgtcgtttgaaggctttcgaatatctatcatacctttttcttcgtataaaattgagctgtt
accaaaaggtaagcaactctcatactcatgatcaacgtcctcgtgtaaaaaaagccttatgctactttcaatgaaggatccgtaaaaaaaattat
acgaaaataaaagtaatccgacttctctcaagatacgtttaaaaacatttttctcatactggaatatgtagtactttcatagtcaatattatactcg
tcattcattgttgattcattcgtctcatcggttaaggaaaggaaagatgatcccatcatattcgaatagtaagttttcagataatcaggaaatttag
atatatcgcataactcatggtatttacatatttctggtagcgcttttccaaaggcattgaggggacaagagcagagtgattgccaaaaatatagag
aagtattactttcttttagcaatacgggaaggattctttttcctaaaaacaatgattctaaaatgggagtccagttgttccaatctactggcaaagcc
tgaacaaaatttgcatagcagcgaagaaacccaaatccattctcaacatctagcgaaggcgataaattattagaaaagccgctgaaccaaccgt
aaaaataactcaatgtgtctttggtcaaatacgttgaattatgacatgaacctaatatctctgtgcaaacattgacaagatacacagaatcgaatg
tgctttctatttgaaaccgtttcaaataataccttatgattccaatgccgaggaaagccaacgaagatatttgaatcagttccttagctagacttact
attaccataataacgtaattcggcttaacaaacctatcaaagaaagacaagaataggcaaagctggtaaatgcaatgctttaaaagattgctatc
tggttcccaatagacccgtaaaccgattgtcctaattactatgcacattagagagtacacaaatgtagacgactcgaccatgctgctaatatagtt
aagataatatataaccccccattgattgaaagccaaaataatttgtaagatcaaagtccagacattcttcttcaaagataaataattgctcataaac
aacgaaaacagaagggcttattttttttaagtttgctccaatttcattatagtctttaataaaggacaaagaaaaaaattatggaatccactagattt
gggaaatcaagtttacatcgcgtattcaaaggacgaagtagattttcattcgaaacttcaatattgctaaggatttttgcaacatttagccttgaag
caagtctttcttgaattattgtagaagatacagaaattctggagcataatagctgcgctaacctctggtttccagttagaagaaggactaaagaca
tcggttcaatattgtccttcataaatgttaatccattaggataaagagaatgaaagggaaattcaaaaaaatcgtttttttgtaaattgtagatcatt
attcacaaaaacccgaaagagttcaaatctataagttttgaaaaggtttcttaaaccgaacctatagtcttcagttgctgttaaaaggtagttaagt
acattactcccttcttttgaattagaaaattttaagagttgtagtaacagttgcggccgattgaaattcaaagtatagaccagaaaaacaataaaa
cgagtcaatacaaaccttctactttcatgattaggaaccaccaaagactcaaattcttccattacttcctttagactttcacgcttagcattaaatat
aagtcttgaaatgttaggacttaactgaaaaatgactatgggttcggggtcaagaataaactgtttaaatgtcgttttcaaatcatgtacggaaga
gatttcatagatttcttcagaaaaatccagtaaaaacccatataaaataattcttccagtagaagaagagtacccattttcgagcatcacttttcga
atgaacttagtaatcttcagtatcagctttctcaaagaagggccaaaaggtacctgagaatttaataaacaacgaaaaatacttaaaaattgaag
taggtaacactcatctcgttcgaaagagtagtccatcaagattttttgcgcgaatatttggagcgaattcagaactattgactcagaaacacatgc
aacgttgtaaaaatattgataaaatgaagatgagagataacaaaaattccgagaatcacataaataaataatgttacgaagcccattttcattat
agtgagccatgcggtccaaattatgctggatatacactatgctatcaatagttttctgtctttgtgagtcctctatatcatttatagaggtggcagatc

taacaagagtttcgggtctgtacatagaaatatcgtcctgaaaatcttcatcagctatgtctagcggttgagcatcactactttgaaagatcgagca
ctttttaaaatgaacacaagcagcgaggataaagaagaagaaatcaactcaactaccttatcaccaaggcataatagtttatcggaaacg
attaccgttagttgtaaagctctagctaaaattaaagcaataggctcctctgaagtttcaaaatccttcgtataattgtgaattaccgtaaaaacat
cgttctcaaaatgagcgcatgaagtatatggctcactttgatggaatgcattcatcatatacttaagagtaacatgcagaaagaataaagataca
agtaactttaattttccttccaggctttgtatatctttagtcgagtatttatcattcaagccggctaagatcattctcaaattgttttctaagatttcaac
tagcttttgtagttcttctacattattccattcattttgcatattcggattaacgtgttcattagattgtgacccacgaaaatttagttcctgacatttga
gatatgtccaagcatttttaagaaggtaagcgagtggccgtgtgtaagctcattgcaagatcttatggaaagaggtgatactttcttgaaatata
aaaaaatgattatgtttataagctcatcaaaaaaggacgtggagtacataaaattattaatatcgggattaatgtaagatttcgataaatgttgaa
aaatccatcggtgaaaataatgaaagaaccgtactttttcacagaattataaccttgcagcaaaaaatagagtgttttccaatattgtagggcct
cgtgggaaatatccagagcgtgattttcagacattgacataacctgattaaggtaagggaataatttaggaaacgagagaggttgaaaaatatg
aaaataggatagtatatgatagcacactgaagaagtttcactgtataacaaaccccgaattgataaagcaagaatgcttttgagaactggttcag
acaggactatactataagccttattatagcaaacattagataaaagaaaataataccagcaatttgatacactcttttcttcagctataacaagtg
aattcttagtaaggtcacgtactttttcaggactcaacatggatgaaatggacacgattattgaaagaatttgcacagaaaaagaagattcttgta
catccggtgaattaatgcttattaataagcttgaaaaagaaatgttgtttctgcgcttcttaggaaaatttgagatggaacgcatgttaatacaatg
ttccattaaacacacgacttgagccataaataagtaaataaaataaagatccaagtggctcttatccgatacttcataaggaagaaaaaaatctg
ggtggaatagagaagcaaagttccgtttccgttgagtcgcatcgaacataaacatagacaagttgaagtgagatacatcaccgtaaacttgcag
catactattaagaatatcaaaagaagattctaacaaatcaaaactaaagaatgactgagaggaacttaattgccatatagtacagttagaccat
atgggaaacgaagctgctaaacattgcaataacaaactccttagtgtagaccttttccatttctgacttatttctaatatttggaaaataagctccgt
gacttgcaatactgcagatcggtcattagtcacaatataaacgcacattcgatataggcaattcaatgcaggcaagtgagcattagtctcttcatt
aaaagtcctaaaaaagtttcgaagaaaacatagcagctcattttgcaccagatgaatcggtgccgcaaaccaatccattaaaagttttataactg
taatatactccactatttcctgcttaattcggatagtatcatgagaccgccatatagatttatcacttttagaactctgtgaagctaacatttcttcttc
actatcactcacgtaggattcatccacgaaactaaggtcttccacagctttggagcaataaagcaccatattctgccaatcaataaattgcagatg
ttgtacatgaggggagtgagaaaataagagatgtaaagattttccaaaatccaaacagactggatcacatattgtacctttgggagaatatgtga
atctctgaatcaaattcaataattttggtatttgagactcagtaaagactagaataaagctttctacatagagtcgaaacaaactgcctaacttttg
aaaccgatcatttatggaagctatagaaacctgccttttggtgttttgatagttgatttctctctccacttcaaataaacgcttaaggattcgttgaat
aaccgagcaatcctgttcctttaagtcttttctatttctagaataaactataaaagctcgaagttcttcaatggcatcatttctggttttgattttcagt
gatgaaacctcatcaagaatttgagcaaggcgggccatatgcgtctaactatttctttttgaataaataattatatagtcattcttttaattcatatgt
ttaaaatcagatacagcacagttctggtagtggttagtattttaagtagacaaaatgagtgaatgaactggtagtacccaacaaagaccggtaat
aattcactaacgctcatctaatggaaatgaaatttctaaagcaaatataaaggatgtagttataaacaacaaccagatttatattgatatatacta
aatctgctaataactaaaaaagcttgtaactgttttcgtaaaataccacgcgtccagttttgaattaaaattattttcatctattaaacacgtatttc
atggccaaagcttgcaaacgagtattcagcaacatagtctaaagagagcgaacaaaatatgaacttcaatccatcttagtataaataattcaatc
cataccatccgcagttacaaatgacccagcatcactagccgtagatagctttctagtatggttgaaatcttgatctgccccctgaagtttctgctgc
acttgttgccgttcattattcagataagctccgaaatcagccttctctccattgatatttctagattctagattactggaatccgattctttgtaattcg
taaaaagcttctcttgtttttcctcgttcactgtcggagctactgtcggagaagcggaacgaatactgaatccaggacgaacataacttctcatatc
cttaatataatcatttccccctttaatatcccattcactagtaccaattttagaacccatttttctccatctttcaagtccaatttgacgacggattttac
ataattctacaatctgaggccaatacttttcatgaaagtactcgaattttaaagagcctccaaagtttgtgtcaagttgatccataggaacataacg
atctaaaggttcattaaatttgagcttctctcgtgtgattggatctataaacggagaaatcaatttaaaaaagccccagaccgcccagggaatatt
gataactaatgccttaccaagacgctcacaataatgagtttgaagaatattaaggacttccttaccctggccaacgcttggattagaccgagagg
aagaatccctgaagttaattaataaagccaatgtttccacgcctggtggcatcaagtcgatggcgcattcaaggaaatacaccaaatgtcgaatc
tgcaaaggtgatgtctttgtattttgcctgccagggtacaagtacaagcaaggacgaccatctttatcataccccaacaaaacctgcttcccggtg
gcattttcctcctgcacctcatcaggatgcatattattgacaccaaattgacgacgccatacaagtgtatctaaaatgcgctttttggtagcacttgc
gttccactttgtagcacgcaaaaatctcaaaatacattctttagttaggaagtatttttcaacttccgacaagccttctgttttatcggattttgaaga
aacaggaatgtcttttaaatcattacagtaaatgaatacttctttatacatattcctttgtccttcagttaaatctttgtcttggtactgaggcgtcaaa
tgacaatgaggactgggaaatggaagaggagcattccatgacccatcttcttcaagttcatatggtaatgaaaagttttcatccggtttttcctgaa
tgacaccatcaacgtttcgtaatggtttcgcttttgagtcattttcagtacctgcagcaatttgattagattgttctgtatctttgttattgttgactcca
tttattgtagacgagctagaagaacgctcatgcttggattggtctttcgttatactttcattgtttgctttttgattgttatccgtccgtgtctctatagg
tataggtacaacagtttcatgcgatgagaacgatgacctaggttgtttagccgacacggaaacaacacttttaccggattcaggggtaagaggta
cagaacgtgtcgattgctgctctggaagatcagtagatgtcccttctattttcatcgagttcgaagcagaaagttctttattcaatttactagggtttc

ttcctttgcttatcatttcttcaccaagatcattggcattgggagcagggcttaatacctcgctcacagattcctcagcagtagatgccggttttaag
gattcaggcacgtcactaactttcacagtactggagaccaatcgtgactctgtcggcatgaaggaattatgtactttatcatttgaatttgattgaa
gtgtctcggatggcaaaggtgaagaactaacctcagtaacctgcttcattgtgtcgggctgcgtcacagaagtggaagcatcattacttgtgtcca
tagtagctacatgaccggtgagagcggcaggaggctgttgattggctgaggaaccaacgtcggttctaaaaaaggaatgactcttgaattcagg
agtaccatgaggagtcgtgctgtcctgcaattgagagggaaaaacttcactaaaattcgaacgggcaggctgcaaagtcaaatgaggtcgctgt
tcactgttgcttcttggaggcctgacaggagtagtttttttcctttttttccttcttgggggtacagatgccaagtatagaactgcaaattcgacctaga
ccgttcctacgacccgcacgagtgttttgtgacattgtatacttaatataaaaatcactaccaaactgaaatagccaaaagtgaaagcctataaga
gaatggttacgattaacgatagattgcacagataataaggatagaccctggaaaactgaaaagagcctaatttatactggataatctgcaatgc
aaactaatagaacgagaaagggcttaaccttcgaaagctggcaatccaacgtaatttgggttgtttacctaactgtgttcgaaggaaaaaaaccg
aaagaatgaaagcgtgcgagaaaacaggaattaagatgtaaagcaagcaaataagtttacgaattggacattcgaatgacaaccaaagcttca
ctactagttcttaatcaactaaaaaaaaaaaagacaatagttctgtttattttagatctttccagtgctggatttgtaaaaggactccaaattctttg
taatatcctttttccttcgcagaattccttccggttcaatttagccagaatacacaagcgtgtatggtagttggatagagcctactaagtagctattg
tggaaagtattttacgaagaaacggtaaacgctaggctccgtatcccgagactatgcacgttcaccctcgttcgagctccgtaaagatcaatctta
ggtaggtcaacgatgtctaaaaattgtatatttggaccgatagatatatgttttcaatattcgaaaggaccttagagaagttatttcgtaaaaaag
aagacgacgtggcatgaaaattagctttatgacgcgttggacgcgacgaacactggactggagtagaaaaagaccaaaaagatagttataaaa
ttaaggagtttatccttttgagagagaaagtaatttattcgtagaacagcgatttgtacttgttgctaaactgaacaaggtgtttgtatacatccata
tctatgttcgtaccaaaatcaacgcaggtaagaagttgtactgaagtatcaggtttcacagagatgacaaattcgtaaaaatgcataggttccaa
attattatcgaccgtaaggttcatttcaaagggatgatcgttgcattccacttgcgcagcacggtcactcacatggttcacttttccaacaattctga
cagttctaccggcgcattcgccaagcatatcgctagtaacacgcggcgtagggcgttccatcctttataaacgcgttgtttattatatggtaaaggg
aaagaatctcctgagagaggaacactaatcagaaacttacaatattgcaagaaagtacgacagggtaacgaaccattgttacttgtatgtgtaa
acagaccattctttgtcgaaagagaattgatgaaaaatagatttgtcttctataagaagtatgttttaaggtgcaattggaaagattgactttattt
acgagcatatccttcatgggtaggaaagcgtggtgatatacttgaaatctgaaacgaatgcttctctttttttcatcggcggcgatataagctctttt
aaacaaggaagaattagcgaataccatttctttttttatttctattcatttttatttccaaaaaagggcatcgctaataaaaatataagaaacatgtt
acctgtctaaaagaaaaacgatgccaaacttgaatgcaatcaacccattctttttagaggacacacttttctttgcttatgctttctaagccatccttt
tccttacgaaaaggctcttttctggggtttagatcgtttgtatcgccttctctcgggacaatgagtgagcttttgtgcattaagagatatatgcatcag
tggtgtagcaacgctcgactaaaccgttgccaaagaagtattgtatcgataatattaaaaaggaattccttctctacatttgcatttactcggaagc
aggctgatgactcaactaggattgcgagtaataatgcacctaagtctattgatgtcgagcaattgctcctcgatctcgaaaataaaaagatgcctg
tttctataaaggagtttcttcaaaatacctatgataatacgaaaataaaaaacgtcaccttcgatatagatactcccgatacactggaaagccaag
agaaagtgggatgcgcctcggtgccaggccaagatcctttagggccttctcatccatttcgttatggagatttactagtagtgatgaaccctatgac
agggttgcagattggcgtgtatgcgggaacaaatcctaagaattatgctaacttgataacgaccatcaatgaaggaggtgttttgggtctaaatcg
tacaccaagagttttgcttcgatttcctgatttcattcctcaatttaatgattacagttacaacaaaagacttaagggccaggtggttattcctcctgc
tttattggaaatcattaccaagagcctaaaaaatcttagtaaagaggtacaaaaagccggatttttgaattatgaaaaattcaacgagatttatcc
cacgcttcaagcccgtaagcctatgccttttcagactacttttcctgagcttttgaagatagtatacggcgatcaattcaccacaattgctgagaag
atcgcacttttgacttaccttaccccacgcaatgagcgtttcgtagttgtggaccacttgttttcggaaattcagactgttttcatgaacccgttatac
aagaattcaaattattctaatttttttgactaccgtacggatcaaatctcctagttaccttacatttctacgaaaagcacggcatctgatagaaaata
aacgttataagacactccccagcagtactaaaaagataaagccatttgcattgcgttctttcgtatggggagaatatgacaaaatgatgattaatt
ttataaaatttcagacgattctgtgtaatgtgcagagagtgccccatcctcttctttctgaaatatataaagaaataggtctttatgagtgtaagga
agacttggcagttaataattttcatgagttcttgtgtgatattggcgtttggtctccatggacgatatccaggttgttattaccagaagtacgcattgc
ttttcctcagttacatcctacaaatcgaaaacaatacgaacaggatcttcattctttctctgatcctagtcgttccgtttcaagagatgttctcgaaga
agtgagaacagactttacacacctggatgcctttgcattcgatagttcatcaacagttgaagttgatgatgcaatttccattgagaaaaaaaatga
cgggacttggatgcacatccatatagcaaatccttccagcatgattgatctcaatagccctcttatggcttttgctgagaaaaactttcagaccgta
tatttaacgcatattgaaaagaaatttatgttaccattggaattgaccaaattactatggacgttggatggaaacgataggcaaaaagcattaac
gtttagtgctaaattatctgaaaaaggtgctgtgctagactacaaagtccagcctagctggatacgtcgtgcaaagcgctatactttctctcaagtc
tgtgctgccctaaaaaagaaagagaatgtaacaatttggtcttctttacaagtaggggatcttgtgaaggagcctgcaatacctgttgatgaccaa
aagaaagttttggaaattttggaagaaatgaaaaagtttactggctttagaaaaaggaataatgctttcttatctgaccaaccgtctttttccgtgg
atttattaccagaaacaattccagaaacttcaataagtggagttaatccagtttattggagctcttttccttctatatgcttaaaagtaaaccattct
ggtatgaacatcgcagaattattggttgcggagtgtatggttttagctggccatgttagcgcgaaattctttgacgactatcgtattccaggtatatt
ccgcggtcaaaatgtaccctcttcattcgatagctcatttactgaaaactttgattctttactccgcgcgcgagacgaattcggtatggttcctttga

agcaagtgtatcctatctcttcgtatttgacttcttcttacatggcgaccagaaagtctccacatttttctctgggtatcaagtctggttacatgcagtc
tacgtctcctcttcgtcgatttacagatttgataaaccattatcaaatgcaatgcgtactcttgggggacaaatcaaagttgatttctagagagcaa
atagaatcaaagctgcctctttatagtttcaggggaaaagttatgaaatacacgagtcgatatactgatcgcttctgggctttggaatatctggcac
gacttccaaaaaataagctacccatttgccatggcatagtgaacattgacagacattcaagtgtaatactagaagaatttgggcttaaggcacaa
ctcaatgccttaactgggttagaaaaataccatttaactagacaagcattccgcattgattacgttcagccatttcaaaatcagctttttgtttcaat
cgttgaaaattaaaatctgtatactagggatctatggtttattataatatattaataagattaacttttcaattcatattctgtttcattgaaagatcat
cctcattcaaggttgtttgcactaccaacttgcgatgacttgtttactagcgaaaattaacgcgacaaaataaactattcacaaatattttatatata
catatttaagtgcagtagtttcaaaaaggtataagaaaggagcattaaattgtcaaaaaagaaataatttttcttatatttaaatttaatttttgag
atgactagattatagggtcattagcgagaaaacctgaaatagaagaagaatatggatgaatcaacgacgcagcaaagcgaatggaagaatga
attggaacaggatcaaagtactgagaagaatgacccaatttctttatttactgaaaggttggaaaactttcgatattatatgaagtcgcatagtcg
accgcggcagcaaacgcttcgttccttcgtgaacaaagagtcggagatggtagaggagttctctaaaattggaaggaatccaatcaaggaaaga
aaatccaaaatttccgggaataccataacgtctcgggtgcaaaagtcattcaaggagctacatcccagccccaagaaaaacacaagaaaggag
aacattggaaagggaataagatcgccgttgggtttctttggtcacgttatcccaaaaaagaaaccaattatacctttgaaaccttcgtcagggatt
gacttggagcgtgcaataaggacgaaagtaagcaatacattaattgagccacggattgcaattttgtgtgccaagaatcaaaaaattgtcttctct
agggcttcggggaatcaaaaccctgaacgtgaattttccgagagcaaattaccgcaaaaagtagctccatatctatggatctatccgtcccgaat
cctacataatcaagtcgttgatgagcacggatacctcatgtattacggacaccctactatggacgtggaatatcagttgcatgaaaacaagcata
ctttgaaaaggattaaggaatcctatgatttggagaaaagtggtacgtattttggaatgtcggagtttcccacatcagatgcgagtgaagaatctt
gtgactcttttgaagtcttggaagcgcctgccgatgaggttataaatgaagatgatgagttcaatggattgaccactccaaagtattcaagtatact
gaactcttccaatatcactaggtttttcagagtcctaaaggaaagaggatgtatgcacagttttcaaagaaggacactgtaaatgcctcagtttcc
tttctccaacttcataatgatttattaagtcatggagtacctgttgacttttgtacattgaatcactggttggatgaacgaaacatttcttatgtgtagt
tttatcgctttatctttacgatgtttgggttatgtaattagtttctttcgtttaatgatggataatgatattataattttaggaatttctaatctattgaaa
cactttttctactgttgctctatatggtgttgtctggtaaattgtattttctaccttttattgaaaagctggcttactggtgtaatgagggagtcgatgtt
cttttactgagaagtaccacctacactatatgaccctgctcttacttccagtaagcccaaaaacccagaatttattgaataaaggcaagaaaaga
ggaaagattaactgaaaccaagttgttgtataaattttagtcgtggtaaaaaaaaatttttgatttttttaggtgcaattgcatctccttttcagaaaa
gtcactatagtttgaagcacaaagaagcctagtatacatttgtgattctagtattgacacgaaattcgtttgcaattgctttattgggttgaaaatta
cagttcaaaataatgtctgctattcaataagaatggcttatcgagtgcttctactattcatctcgttactactctttttggctcgtttttcaaattgcg
gaaggatcaccaacgttgggacattttcaagattgtctacctagtcataggaagcatgatgtcccttattttaccccgtacgcgctggtaaagcact
tcgacagacaaaataggcattattatattcaagtgatagggaatcttactgaaaagataacagtagttggaggttctggtgaaaacgtccatgct
gcgacgatatactctgaaacgtatgccctaggttcgctagtcacaaaaacatccacgaagctctgtgacgctgtgatggatgttcatagctttggt
gtacccatcaaatgtccctggcctgccggaagtgtatcattcatgatagacattccatttggatcaactgttctcgattactccaaatatgaactcac
gtcacttacaaatagacttataatagtggacgggactcctgagcggaatttgatagcttgcgtcacatttgacttgtcgattgtgcttagctactttt
atcctgtagtcgcatatacctcgttgatttgtcttgctttcgtgtccttttcaatagtttacgccctctttctcaatccatggactggaagtcttgatccc
tttagagcattgttcaactttagtatggatcctgatgctcttcgtctaaccgcgcttgggttttctgattttattggatatatgcaatttgctgtatctac
gtctcaactgaatgttgcatttccgaagttttatattaatcttatggctgctctgtcttggagtaccgttctttttagattccctatcttttctcagtatgc
aacctatcaatttgcagattatacagatttagcaaacgtcgatgatgttgcatacagccaatttgttccggatagctatggtatggcttcgtttcttg
attccttaggcaccaggcaatcttgctggatcccatatttaatagtagtggcagtgtatattggtgcagcgttattgttggctcttttgataattctgtt
aaagtgggttatgtcaaaaatgtttcatgatacaatcgttgaaacgcgctgggatacatggagttttatcggtggctttttttattcgaatttatttctt
agcctattttcctactatatcgtatatgtcttatcaatttgtggttgaaccaagtggctatgaaattataccggtcctcatttttatattctttggtatttt
tttccctggatgtttatacttatttttgtcatttgtcgagccagtttcaaagctctttgaggaccaaacttatttacacctttatgggtctatttataattc
atatcgggagaagaggctgatgttttgggtgtttcctgttattgtgcaatttatacgtggcattactgttggtgtccttggagtttctgggtcagctca
agttacgatatattttcttctagaagtagcgaatctagtcgcatataccgtgtttcgcccacattatcctcagacgaacatgaacatattaagctgct
atttatctatcattcgtctgattatagttgttttaatgatgcctctagtccatgcacgtgatgttctgagtaatagaaataagctattgacttatacaat
tcttttcatacatgcgcttgtctatgtcatatttttcttaatatcaagtcagcgattttttgaagtcaccgcacgactcttaggtgctaatagtgaacgt
aaaggagttcccttggatagaccctttggatgggctcgcgtttttggtattaatgagttaaaacgccgtcgtcgaaaaaatccgtatgaaaatagtt
tgatggcacactcgactcttgacaaaacatcgcattcattcattggtaactccatccctcttggtcttatctctcctacatctaaacacgaatttccta
gcttgacaattcaaacaagcggaattgaaatgctgaatacggataatgaccctcaaaatccgggtcaggctggtactgtaaatatggagtcacca
atttcgccattatctcctttcaatgatacccgagggtattcattctaccgccctccgaaagtcaatacttctgtccgaaagagagacagggatcaat
tacgcacgttgcagttagactttttgaatgataagccaagtctctttgcgcatgacacgaattatgccgttcgcgaagccgatgtgtaccatcctca

ggttgatgctgacgttgagtcgttatctcagttatcgatgcgtatgaatcatagtagacctgacttagccgaccctataccacaaggtaggttagct
agaacttggcacgtagttcaaaatgtgtttcgaactcttcgagatccacttcggcagaaagagaataacaaggaaaaaggattcgttgttgttcg
gtcggcaaggcggatgcgaccacctcaggtctcaaatcctgagcaaccaacaatttttgatgaacaagaaagtgaagaaaacaaaatgacttct
tccacagaatcccaaagttaaaatagacgctaaatttacataaaggacttgaaacatttgtgctcctttctttccttagttaaatacgtttggttcct
ggggacggacgctatttttactattttttttgatgctgtacttatttctttttcttagacatgccagtcattgctatatttttccttcatccattgtttatat
attacctcagctgtattttcttttgcccgaacattcccgagacctcacaagtaatcattttcttttcctttattcggcttttcttgatttaatgcctttacg
gtttaaggttaatatgagtggtccgttctttacaatcgttttaaggatcttttatttatttccttgtgaattatttattttgttttatacttttcttctgttga
gtttttcctgtagatgcaacgcaatgtattgtatatccaccctgggttcctataatcaaaacttctttaaggactttaatttttttttcttcacacctagc
aaatgaaatgaaagttccaatgaaatatgttttgtacgaaagtctaaaagaaaaacgaaaatcatttccaaaacctcctcataagagccgtcaat
ggtgaaagtggtttcaaatgtacaaattttgaatcagcctataaatgctaaaccgtttaatttttttactattcaaggttttcctatttttacatacattc
acaaaggttgtcccctatcgaatatggtcattcttgcaaatcacgtatttgtttacatggctggccatttgtgtataaataaatgtcagtggttgctgt
ggtggagtccttcaacaagagtcataaggtaacaaatgtttctatgccaacggtaccttccagaaccttttttacgagacataaagaatttattgaa
ggatataaacaataacaacagaatctttttattgctgcattaaaattatagacttacattatttatttattattttatttattgcttattttgctggtcta
tgtcacccggaccgtcctcaaatagaatgtcatcgtaacatctgttaccagtgttacctaaccctgatgtcagacaaatgtttatataagtgcagcc
acacataaacttagcctctaccattaccggttcactcacttgttatccatcgttttagaccgttcattaaagaacaaagttagcaaaatggagcttc
cctctcgtcctcttggccgccagcacagcaagtctcaatccgcctttgcttttgacactactgctactagtattgctgctagtactatgaaaaatgag
ttgaatcatcttactggaactgtcaaagatcctgaaactaagaatgctttcaagaaggagatggacaacttctttagcttgttcaaccgctacttgc
aagagaatgctaagggtactgagattgactgggatttggtcaaatctcctaaacctgagcaagtcgttgaatatgacaccttgagtgatactactc
ttactcgtgactacttgaacaagctggctgtcttaaagttaaacggtggtttgggtactacgatgggctgcgttggtcccaagtctatcattgaagtt
cgtgaaggaaacagcttcttggatttgtccgttcgtcaaattgagcacttgaatagaaaatataatgtgaatgtcccttttgtcttgatgaactcttt
caacactgatgaggctaccgctaaactcattaagaaatatgaagcacataagattgatatcttgactttcaaccaatctcgtttccctcgtgttcac
aaagagactttgcttcccgttgctgactcatttgattctcccatcgaccaatggtaccctcctggtcatggtgatgtttttgaagcacttacaaacag
cggtattattgacaagcttcttgctgaaggtaaggaatacttgtttgtcagcaacatcgacaacttaggtgccgtcgtggatttgaacatccttaac
cacatggttgaaacgaaggccgaatatattatggaattgaccaacaaaaccaaggctgacgttaagggtggtacattgattgactatgatggtaa
cgttcgtttgcttgaaattgcccaagttccttctcaacatgtagaggaattcaagtccattaagaagtttaagtacttcaataccaacaatctctggt
tccaccttccttccgtcaagcgtgtcgttcaaaaccatgagctttcaatggaaatcattcctaacaacaagactattaagcacaagggcgaaaata
tcaacattattcaattggaaactgctgctggtgctgccattcgtcacttcaagaacgctcatggtgttaacgtacctagaagacgtttcttgcctgtt
aagacttgctctgacctgctccttgtcaagtctgatctctattctattaaccatggtcaattggacatgaaccctcgtcgtttcggtggctctgctcctc
ttgttaagttgggtactcacttcaagaaggttgctgatttcacctcccatattcctagcattcctaagattttggaattagatcatcttacgatcactg
gtaatgttaacattggccgtaatgttactcttaagggtactgttattattgttgctagtgacgctaatactatcgatattcctaatggttctgtccttga
aaactgtgttattacgggtaacctcaacatccttgaacattaaagttcttttttccttatttgcattaagtcattgcttgcatggattttcatgtttatga
ttagcttttgtcatgaagttttcctttagttctattgtaaattactgttgttcactacgttagcaagaagatgacgatatatgatttctttacgttctttct
acgatttgctcacagaaatacttagaagtgttcaatttattcttatttcatgtaatctaaaaatgtgtctagtcaaatatgtaaaattatctttgtaaa
tagtaattagactaagtagagagatgtactgaatcgtagatatttaaacttgggagatgttttagttttcgctaatacctaaatccttcttcacaagg
ccggcgcaaatcttatggtaaaaaccgctatattttctaaatgtcttcacagccaattcacgatcagcttggtttaacaaacggtaagtagggcta
aaaaagatgttaataaaaaaagtaaaaatcattaatgtttccaataaagaaggaaagaggaaagaacataccggacaaatttcatacgaccaa
ccttgcccacctgctcaacgacaaggtcgtagagagggcggtattcgctcttgaggacaagcttaaagtagcggaaagaaatttcagcatttcta
gaactggagaaggaataagcagaatccatggctgctatataattatgtgggaaggcgtctgcctcaaaaagaacatcaaggaaaagagaaag
ctgaccagctgaccaaccttctatatcatctttcttaaatctatttggatcttgagaagatttagcgctgctaacccacacatcggcaagtttgtaac
agttgtcagctaatgtagtatcaaatttgggcgttacagggggcatgccaggtgcataataccacgcatcccaatcgatggaatcaagagcatca
gagagacccaaaggcttaaagaattcataaacagcgttcttaaactttgtttcgttgacagtagagttggcaaatttcctaaaatagaaagacaa
gaaaggctcaaaagcagtgggaccaccgacaacccgctcgatttcatataggaaattgaaaccttttttcgtacggaatagttgagaaggcatcat
cgggatcctttccgttgaggtctacgataagcttggtaaattcatggtctttaccgaaaagttctacagcttctttcaattcaccccaaccgataatg
gcatcgaactggcgatgttgttctccctgaacgcggccaataatttttctctctaaaaatacagtcataccttcattaagccagaaacattgccaag
actcattggtaacaaggttaccactccaagaatgagcaagttcgtgagcaatgacagagacgttagaacggtctccagcgataagagtgggagt
agcaaatgtcgcattggggttttccatgcctccataaggaaaagaaggaggaaggacaacgaagtcataacgcgtccatgcgtaaggagtagt
aagagattcagcagccttcatgaacttctccatgtcatgttcaaactcaaacttacaagcaacaatgttgtcaggctcggtatacacagttgaacg
agggccaacggaagaagaagcgaggtcaccacttaagatggaaattaagtaagaaggaataccaagaggttgtttaaacatgtatacgagag

aatcgttgcggaaatcacgggtaccacagggaataccagatgcaatgacgggaagtttagaacgtatgtggaaggtacagggaacttttacaga
aggagtgtcttggcaaggtagcatagaacgagcgtgaatggcctgacattgagaaaacatatagggagcatttcctccaatggtctgtttaggct
ccagaaactgaatcgctgtacattccttagtagtagaataattaatacgtaaagttgcaacattcacggctgggattggctctggaggaatgatat
gaagggcacatcctaaaaagtcatggcgttcatcgatacgaaaaggaacaggtgtaccatctaaggatacgtccttaacatccaggtaggatgt
gtctaaaataatatgggacagtggttgagaaattcgtgcagcttgaattttgtactcgacagcaccaaaaagaagttctttgtcaaagtcgatttc
agcatctaacagtaaatgttgaatagcaacatcataataattggattgagtggaaggatccaaagaaatcttcattgtccaaacacgtgttcgcta
taaacgaatctaaaaaccagaaatacaaagagaaagtccttgcaacgtacaatgagctaccgaaaatcaatctatggttttaaaaactcaaaat
ttaactaccctctattcttttagatgaagaataatttgtttatgtttagtggaaagacgaggaggtcacatgccgggtgaagtaaggaattaacgac
ctgttgcaatgtctttattctatataaagaaaacaaagaatatgcggaaaacaacaaagtatgtctataagaaaaagaagtaaaacacatgcat
ttgtctattgaactaaaattctaagctcttgatgaatttcgttgttcttgtagagcaattcatgtatcatgtctttccttctcttttttttaatgcttattaa
accgcagtttgcaaacatgtaaacaaacttaaaagatgaagcagtcatttaaaaaattgctgctgaaaataagtgctgttgggctctaagtaatt
cattcgttttctaatcggatgtcaagtaaattttcatatattgtgctggagacactttttctttatgccttcccttgtttcgtttttactactatgctgtaa
ataaaggtgcatgtttttttggattcctttctgtttgtttaatttctccaatttactataatgatattttattttttttttgttttgatcctgtcacggggataa
tacaaaaaatcattcatgtaattgaaattcgaataaggaataggtgattgtaattgcaattcagaattttcctttgggcttttcgttactatgtatgat
gaaacgaaggcctggttgagtaggaccatgattcttaaggatgaattggctttttcaaaaaaatcttaaggaccgaagagtgtaatggaaatgat
tatcttaattttaatcattctcttctacaatatgaaaagcatcatgaataacgatctttgaaatccattttcatcgttcgtattatttagacccaggtaa
catcattatatcaaatgctttagtcttatatattgaggtaatatttaataatattaaagcttcctctagagaccggtcgtcatggtagaaacctcaag
ccgctcaagaatgtatatcgagcggaataaacaattaaaatgttatcgttttagttttactacgagtatagaagttattaatgttcagggatatggt
taagtgcgtaaaatgctttgaaggcaatcttatttagcctgtacagtggaatattccagtctgaaaatcctaactatagactaatgagtaaatatgg
tttttctaaccttgtaaacgtaatcttcttctttgtttttataattccttatcacgtttttttaatgtttgcctacttcgtagtatgcgacagttctttaagctt
atgaatattatataatattcgttacctataacaatttgtatttttgttcatcttaacgaagtaaccgactgtgacactattagggaccagttcagtttc
tttagaatgttacaatcaacagctcttggtttcgttttctgtttctcatggaaatagatcctgtgctatatcaattaaaacgagcagccagccaagat
cccgccgctgtgaaagatgcagagacgtatcttcaagaatggaagaaagaacctggcttctttgccaaattgtacagtatttttttagataaacaa
aacgatgtcagtttgcggtggcttgccatcattcaaattagaaactctattgacgttatttggagaaaaaaatacaaagtcgtaagttggattttgga
cacaacgttgttgctcttttaaccttgcgtagcaaacactaactattccaacagatctctgttgtctggagaaagggatttctttcgaaataatgctc
tagctgggtctatacactcagaagaccaacttgttgttcaaaatgctttggtcttatccagaatagctcgccaagattacccaacggaatggccttc
gctgtttcaggagctaatttctagccttcatcaaaactttgatgcccaacaaccccatttagcattgcgattacttataaccttacaccatatagtca
aagccatggctggaaaccggttgcttcaagctaggaaaacattttatgaggtaactccccatcttcttttgactttacaaccaattttggattggcat
ctttcccaatggattcattcgcttgaaacttcgtctacccattcgtcggaagcaatgtattactctatgcagatttcacgctatgtcctcaagacatgt
agaagattggttattttcggtgtaccaaaccctagtgatagtgaattcagtagagagatgctttcttttggagcagtttctcaacaaaaagtcttga
acttactttccaatctctacatgtcgccttcatcatcgcagattgatgaccgtatctctgagctattgtttgcccatggttatcttttcaacaagttattt
tttgattttgcttcatattctccatgtttgacagtcttcccgtctaccattgactatatttccgtccatatgtcatggctgttacaggtcgacaagcttttt
ttggcttcttccgatttccctgagacaatgaaagacttagaaaagctttctctaatgagtttgagagtattcattactattttgagagaaattgagga
tacaaaatcttctcatcctgaacaatgcgaaaaaattcgtcaatcattctttttaaatgatagacatttcttgcttctgcaattcattattacaaaatt
gcttgttctttctgaatccgactttgaggaatggtcagaagatcctgaacaatgggtccttgatcaatccacccacgacgccgactataacattcgg
gtaagtcatgcttattaaattggtgaaatttatactaacatatgtagccttgctctgaaaacttattgaaatgcctatttgaccatttcggggagtttt
attcccatcagtttgaaagtatgctagaatcggtttttcattgcccatctacattggaagagtccatccagcaagatgctcttttatgctctattggta
ctggtgcacctcatttgaagggtatgtttccgtttgctgactggttgaaaaaggcagctatccccaatttggcgaaaattaccaatggtggagttgc
tcgcatcttaagaagaaggatatctatctttttgtctcaatggatagactacctaccctctgatgggccattgatggggttcatatatcacatttacg
cgctattactcaatcgaaatgatccctgtaatgactctgttgtacggctcacatctatagattctttgcgagctgtcttggatgattggtcgttttcag
aaagcgattttctgaatgtcaagaatgatctttttttacagtgttttatcatccataaacgagtttcaaactttggaaggaaaaacctcaacgttaact
ttacttggcacgctactttctcgagctggaactaacatagctcctctggaagaaatgactgcttctcttttaggccagttatggaatgaatgggcta
gtgagcctatacttcgaacaagaattttgggagtcttgactcaattcgtgattgtatgtataatgatctgttatttttctaaccttatttaggcaattaa
acataaatcgatacagttttcccgaatgctagctatgattatcaactattgcgtgaatcccgagtctcctgaaatcgttatgtacgaagcagatgcg
atggagctttggagttgttttcttcagtatgttcaagagcttcctgaagagtttacgctactcattccacatttgttacatcagctttctcaagctacat
catcccttcctttttaccttgatgattttgaatagctatctcttacttgaaagcggagtcatcatcgacaattattcatttgaggttctagaaaggttga
cgagccttttgaatgacgtgaaaaacgatacgcttttagctctatgtaaaacacttagtttaatatttgaagtgaatgttaaagattctcttcagga
atccctgaaccgctctactttgcttcctgttttgtcgaaatccatcataagctcagaggtatgcatttgaataggatcaaaattactaacgtcatata

gaaacatgctcaaacacttgtacagtattcattgctgttatctcgaattggcctatcatttccaaactttataataaacacgtgtttggtacacaggc
taagtgtgtatatgctgttgcaaaactggatttcattggtatgaaaaaaggtgggcaaacaaataactaatgaacttactagtttgattatattacg
catgctaaagacaggaagctgacagcgcttgcaattacctccttgctgcgtacgaatgctgaagaagtgttggaggccttgagtccaatcatgcat
ctttggtttagtgttttaagcgaggttgaggaagacgaacagggagagtaagtatatatgttatttcgttatgcaagatttagactgagaaagcta
acaagcagcgcacctatctactataaaaatgaagattattccacagttggattctacttggacgagagctcagaggagatgagccggcgtaaaat
ggtaaggaatatcaaaataatgatggctaacagagtttaggttttggccagagatcctgttcacagtgtgcagtcaaaagcctttttttgtgtccacc
ttcttcgagtgtcaagaaaagaatggaggaccagaattatttgaaaaaagttacttatccatggtggatcctgctcttttggatcaaatcaactcca
tgttataaaagtaaaaacaaatgtgaaatacaaaatattagacaggataggaataaaaaagtggaaagcgttactttttgatttttgatcgtga
actttgacattacattgtaaggaaagaaaaaaaaaaaaaaaaaaaaaaaaagtcagaagtatgtaaaataaacacacaaaggatagggccaa
caacgactttggttttcctgaggattcattaaagtaaagagatttccgaatggaatgaatcattactaaaaaatataacgattggaaccatgcaaa
taaatgaaaagggaaataacctaaattgtatttgtttatagttgcaaaccctgttaaaatacagcattcattatgacaaactatataatagaaata
agattctgtccacataactatattataattcctcttttcgaaggaacactattatgtcttgtttagacatggtggaaattgattcaaacgaaacgaaa
ccaaagaatccatttgggatttgagaaattatttctatggttgcagtaggcgtaaaatcatcagaatttcggaataaaaaactatttttagaataa
aaataaacgtgttaaaatatccactaagcttggggaagatgtcaacgctgacatttttatgaaatcaatcgtcaaagaaataaataaataaaagt
aaagtgacaagaattccaatgcaatcgataaaactgctaactcagaaaggccagcctgaaaaggaatctactagttcgttctactgaatgtaatg
tcgttattgacgttttcttttgcgatccattgcaaaaatcaccgaggccacgatttcatatctctaaaaagtgcaaaatacatttcaaataaataaat
aaaagccgttctaatagtggaagaactaaagaaattcctaaatttacatactgtttccttcaataagccttatcctgatttatttactaacaaacttt
tgttttaatcctgttactagaatgtcccttgtacgtctttttttgttcatttttacgtcaaacgaaatcttaccgaagcttagcgcacaactctattcatta
tgacgtagtatgtgaacgtcaaaagcaaggaagccgcatatttatgttcatctttgcgcgtaactcacgattccctgaaagaaaagaaagaaacg
cagaagaataagaaaaacgctgaatagaagacgaaacagagaatttctggtttgagtttttcgtgattaaatgatatttgggaaagtatgttttag
tagttatatggtaataggaagttgcagtcttggcttgaacatcacgggctttcgagagtgattgcaaaaatcgaaagagcataatgtgggacagt
gtttcgcacatttctttatatcgttacctggctattcaatctgtagcatcttgtattgaacggatgatttgtacttaataggaaacaggatttacacat
gtatgacaatattttaacttctttctggagatgattgattccaatctcgaatgcgttcatttcttgccagactggccatcttgcttctctttctgttatat
atatatatatatatattattttttttttactaactattgacctgtagaacccattttacacatcgggcgactcaggagaaaatttcgattcccaggaa
atgaacactcctgtcgctggtaaaaaaggtagagctttagattccaatgaatatacaaccggtttgccaagcgtcatgaaggattccggtaaacc
agagtcgaagtttgcaacgcgtagggctcattttgatgaaaatattagtccctctttccacgttgaccctaaaaaaaagaaaagaccaatgctga
gtatgagcgcgctatccaagctatggtggccaaggctattgctgacgaatcttctggacagcaattccctatttataaaggtttagaaaactatacc
ttgttatcgaaaacaggagatggtgcatttagcaacgtctataaagccattcacaataccaccggcgaaaaagtagccgtcaaagtcgttcaaag
ggctcatccaattgatcctaataatccgggccgtcgtcaaggcgtcgaatctcataacattttaaaagaaatccagctgatgcgtcgagttcgaca
tccaaacatcattcaacttttggattttttcagactcccgagtactttttccttatacttgaattagctgaaggaggtgaactttttcatcaaattgttc
gtttaacgtacttttcagaagagttggcccgtcatgttatcatacagactgcgcatgctatccgctatcttcatgaagactgtggtatggtacatcgc
gatattaagccggagaacttgcttttcgaccccattgattttatcccttcaaaagtaagaaagttccgtgctggtgatgatcctgataaggcagatg
agggtgaatttcgccccggagttggtggtggaactattggtcgaatccgtttagctgatttcgggctcagtaaggttatctgggattctcaaaccca
gacccccctgcggtacaatgggatatactgctcctgaaattgttcgtgatggaaagtattccaaaggcgtggatatgtgggcgttaggctgtgttcta
tacactattctttgtggattcccacctttttacgatgaaagcatttcttctcttactaacaaagttgcccatggtgaatacacctttttgtctccatggt
gggatgatatttcgaagagtgccaaggacttggttagtcatctgttaactgtaaatccggatacccgctatgatattcatcagtttttagcacatcct
tggattactcaatcagaagagcccacgttccctgccagtgatgctcctcctatgaataatgaaagtccatttgcttacgatgttcccgcgttttctga
tgatgggataccctccgctcgcacaccaggtgtcaattctttacgtgaggtatttaacatctcgtatgccgctcaccgaatggagcaagaaagatt
gcggaagcgtggcttccgtggtaatcaaggaatgatgaacttttacgacaatatgaatgaattaatagaggaagaaggtgagtatgattctagca
cttctaatgttgagcacgccttaaaacgaattaatattgcaaattctccacacacacgatcgccaaatctgtcacctcaacgaaaaccgcgtgaga
atggatttaaaggattccaattaaatctttctaatgctaccctgtataaccgaagacataaaccgagaacttccaaccgccatatataagttttact
gtatatatatcaggatgccgtcctttaatcatttgacgtatgaaacgtgtgcagtaaaataaatttttttccctcgcactatttattctttcgttttttttt
aatgattaaatctttattttaggctggcgtacttgatgtgaaaagttattaatgctgtggtgttcattggtgttctatttgttctttttttgtttttaaatat
gacctcccaaaaaagaagttgcgacgaccattaaataatccgctgatgaccaaaaatccttttttgttaatatatccaatgcaaaaatctatttata
acagtttaattgcaaaagatcataatttataaattctcggctgaagtaagggtttaaatgtatggacacgataagtttaggcgatttcctttaaaaa
atactttacgattcggcataatgattggaaatttaaggcaatgaagttgaatcagttttgctgtgatctaagaggatatgtttatttttttacgaatag
ggaaatatttttctttaacccttttatggtgtactttctagtgtaaaaaaaaaaaaaaaaccaaaagggaaaaaaactacgatattactcaagagttt
gtaccaattgattgtattttcacgtttattttggtctttgtttaggtggcatcgtaaaacagcatactcgttagtgttattatagcaagtaatatttgat

aaaattgatacttctttgggcacgaattttggatcaataaaatccagtaaggccacggagaaaaagcactgtatgttgatcggttaatcagaactc
ttataagcgaatattacacaaaagaatacattacataggccttgttacttttataagcaatacgcttttcaataaacagcttgaaatgagatgaat
gctgaatgaaaaatactagaaagatttaatacaaagcttgttttccttcagagagagatctcagcagctctaagcagtcgaaaaacgtatacattt
gttactgatcgctcagatagatagaaggaaactggcttctttcgtataattattaaattgcattttgaaaggtgaattattatcattaagacatgttg
aattacaaaggcggttaccgagctttctaatgcatataattataacgtttcgtctcttggaaacttgatcgaaccaaacaacaacttggaaaagga
tattgaaaatgtcgaaaggaaatgaagtaaacgatgatgtggaatcattaatggcttccttggatcaattgggagtcgaaaacgctgaatcagag
tcgaaaccagaacagaaagattccacagaaagcaatcatcaggtgcatcctgctgagaacgagaaggagatcttggatttcttagatgaattag
aaaacgacaaacgtgaaacagctataaaagaaccttctaacggtgctacacaggaaccgtcggaaacttccaacactccagctgagccaaaag
agaaactaaagaacgagccttctcataacccaacctccgaggggcccaatatccaaaatataaaagggggtaatgaaaatccaaaagaggat
accaagaacaattggtttggtggattatggtcaagtgcctccgcagccgttcgctctgcagaacagcatgttcgttctatgaagaatcttgaagaa
acaactcattgggataatcatgttcgaaaaatgatggacttgaataagcttggtaaggagctatcatatagagtttgtaggttgtactaactagtct
gtttcaggagacattggaaatgggattcgcagcaaagccttaccgacattgagtaataccttccataacgtaatgaacgttgttgctccccctatcc
aggatcatgaaatcctgcaggttacggtttttcatgatttagctgggttttctcatgttgatcgaattatttacgatagttttgataatgtaaaggaat
tcttattacgctgtatatgatttaacaatactaacttatctctaggttatgtctcaagtagagggtggtgacttgacggttttattggacaaagaggc
caaggtacgaagcaggactagcgatttgtatgagaatttcgcctcatgtaacggtctattggaagcaaaaaagcttgcgaaggaaaatttggca
gaaccactgcagcatgcaaaaaagcttgtccaagaatcgcattccaacgaaaagtcggcagaggaaaacaaccagcatgcagaaggatcctct
gttagggttacgcagctttttttgtcgattcaagcatttacaacgaagttgaaagaaatatcagacacggagcagctttgtttttttgttatacttgaa
ggatgtcagtcatgacctggaatttttttactacttcccaacctattcctctagaatggcatcagtgggctgtggatgatcgctatatcaaattgtttgg
tcccactgctattcttcctaatgaatgggctgttcagtggactgaaaactgtatatctcttgctgcaggtatccttgcacaaatgtatacctccaaac
gcatggctctgggcgatccagcactctttgcaaacctggacaatagacaaggagaagaagaaaaagaacagcaatctgcttactatgacggttt
ggtctacacgtaagcatgaatacgtacgtatgcaaaattttgttttgttaaatcttgaagaaactgatgtttgtatgttgaaatgaagattatcgga
agctcaattttgtttcatcatttgtcttatttatagtcatgattaattggggtgggggatgttttctcccaaacggaaatgaataccatcaacgtttgtt
gtctatttaattttccaaatttaggtcattgttataaagatcattcttgtactctggatcaaagtctatatttatcattttatcctcatcgattcatcaaa
cattctctgtacttggtctataggcaccagagtaaagcttgctgtcctagacttccacctattcacctatcattcaatactgtattaaatacttgttta
aaaaacagaactgatggccactaatttaggtagtatatatacactatggtttttttacaaaatggtaaggtcggatggctctaccacattgtttgcag
agtattttcacaagatacacacgttcttttcctttaatgaaagagaaatcaggacgcttactacttactacaatacccttacttgcgatacagttgac
agaacgaacagtgtcgtgcttattgatgttaagaatacataacattccatagtgccatcctacggcagctggtatggtgtatggcatggttctactt
tagaaagaaaaaaaaaattacaaaaaaatctaaaatttatttaaaaattagattcatctggtgttttatagaagaatcgcattggtttttctgttgtc
gtttatttacatcccggttactgttattaataactggaacctaaaagagtaatatgattggaggggtaaaggggagtaagggggcctaagaacgc
aagctcaagccacctgttacctactgtgcctcacggaacaaacgtgacgtgtattttgtcatcggaaatacatatatatatatcgaaggtaagctg
ctgtatactcgaatcaactacgatttagtgcatcccggtgtatatccttataccgattttcattcatttttttcttttaagacgttcttttgcataatgcttt
tccgtgccggatttttagtttgtcttgccagcgcttttgtcgctaatgctatccaatttaattcccctgaaaagggcactgtttggacgtctggcgaga
caaatacagtttcctggactcctgttaacaccgaccccagtaaaatcaatgttgttttggtaaacatgcataactggcctaacagaaaatataacc
ttggtaccgtcgaaacatccaaggaacacttagatactgatattagtttctcctctgaccttcctacttctggatggcaaatttacctcaacagtgtt
gatgatatgaacactggttcattggcccaatccgaggagttcagtatcaagggtggtgaccagaaggttgcatctaattccatcagtggaggtatc
gtttctagcacttcttccagcacttctgccagctctactcctgcttcttccagcagcggctcttctggcagctcctctggctcttctgctgcctcttcctc
ttctcctagcagcagtgctagctcttccgctcctgtttcttcttcctccactgtcatcgcctccactagtggctcttctaccacctctcttacctctgcag
tcgcaaagaccatgagtggtagcagcagtggtagtggtagtggcagcagcagtggctttgctactagttcttctggttccggttcctccaactcaa
agggtaactctagctcgagcggcagttcctctgactcttccggtgttgctgctgtgggcatgactaagctttctgttgctgcttgctttgcagttgctg
ctcttatgcttttagcttaagctcaaggcagatgatcaactgtcctcgaaaaaaagtccagtcgtaattccgtaggatgactggttaataaggaag
acagcgttttactgttctttgcacattttcatttaacttacattgtcgcatctcattcatctatcatgtttttgatcttttgcatggtgcattgaattaatt
ggtattggtctatcatttgtttttatcttatcatttcagatttttataaagtctcccaatttgcattttttgaactatttggattgttttaatcattcatcgt
ggaagaacctgtcaaggtttctttccttgggtttgcaatatttaatatcatatatattgtcctagttatcattacgctagtagttatatgctagtttaaa
tttatcatactcattgcatattttataataatttcataccggagaacagtaaacttgtaaataaaaaaagctgttaacagtagcatattgtcaagaa
tttcggcaggtagtttggaaaaaatagttaactcccaaggcgagactcgaactcgcaaccttctgataaccttcataagagtcagacgctctaaac
cgattgagctacccgggacgtgttagtattcggttgaacaataatcataatatcatatatttactgatctgaacgatgtccttattttaaggcatacg
tttctacgagtgctggcatgatgagtaggccaaatcggacatggcttttatataactagacggctcccaagtgataatgtattctatgacgaataat
acgactaaacaactgctcattaagataaaaatgaacgaaaacgaatgatatgcctacaatcaaaatggatcaaagagactccataaaaatttat

tgaattttacatttacaaagaaaaaaacaaaaatttaaaattaaacgaataattttagcgaaattttgattcgtttgtaagaattttcactttcgtct
tagtccaaaaaagcagtcacacttttactggaaacactgtcaaactgcaaacacacaaagtactttgctcttcccaacaccaaacaccagcagtt
aattctacacaaaatgtctcgtggacgcggtgcagcttccggtactaagtacagaatgaccttgggtcttcccgttcaagccatcttgaactgtgct
gataactctggtgccaagaacctttacattgtttccgttttcggtactggcgctcgtcttaatcgtctccctgctgcaagctgtggtggtatgtacatt
ctttacgaaaagtaatatactttttgggtacatttcgttattggagatatctccatgaacgaaaattcagaccctggaagctttactatcgttgatag
cttagggaaattgacaactaacgtctttttagatatgattcttgctaccgtcaagaagggtaagcctgacttgcgtaagaagatcatgcctgctatt
gttgttcgtcaacgcaaggcctggcgccgtaaggacggtgttttcctttacttcgaagataatgctggtgtcatcgtcaaccccaagggtgaaatg
aagggctctgccatcactggtcccgtcgctaaggagtgcgctgacctttggccccgtattgcttctaacgccggcactgtcgtttaaacaagttaat
aaaaataccgagcatattttattaaaaaaattttgtctttctcgttggttagtgttaaaaaaactaagcattttgtagctagaattacaagttgtaag
cagaagatcgctctcacatgtacatccaacctcattagcggttcattatgaatattttgtgaattgcaaaaagagaaatagaataataattattcct
atacaaaagtactacaaatacacggaaattaggaaccattattttttggagaaacttcttaaatcagctagcaagcttttgttcaatttttttagca
tgcctttaagtccaattccactagtggaatgataaacaagccaattctccatttctaatcgtacatttttcaataagtctggaatggggagacttcca
tctgatgtttgcaacgtaggatttagtaaaacatcaaaaagccgattccagagttcttggttccaatatctcttcatccgctgagtcaatacctgccg
tctctgaccgccggaatagtcaagttttgtctcaatatattgtccaaaaagcatagtgtgaataatagcggctagtccatgataatcaacttgatac
gtccatggttcaccttcacgcatttctatacaatcttgagagtcagtttcccagtttgccataaattttgttttaggatcaaacaaagacatatcaag
acctctaccaaaatcaattaaaaaaattccttttgaagaccaaccgcaaaaaccctcaggggagtaggaagcgtcccattctatatcattcactg
cttccaagcgtagcaacgcattgtctgccttcaaatccccgtgtataatttggtgtgaatgcaacccttcaacgattctaaaaagttctatcgagaa
aaagaatactagcatctcatcaattcctggtacagaactagaggtgtttcgtaatgaattcaccaagtccaagaccgatccttgtgggcgataatc
cattaacaaatggcttgtattccgatacatacataaatagtagacgggaagtatagaacctacatctctagtaccgctcatccttcttcgagcttcc
agtgtcaaataatattcaagtgaagaagcaggagtctctatttttaatgcatacaacttataatcaacggtatcactttctctcttttcagatagctc
attttctaagggaagtcctttagcaagataaacaggagcaaaagctccttgcccgagtttacttatgatcccaagttttacgtttggaggataagtc
agaggaaaagtggggctatttaataaactaagacgagtgctgttacttcgtcttctaccacgcccactaggcgtagtccttggtttagaaacaaaa
gactcaatagcttctatcatagtaaaattactatcgatctcatgatagttttcatcagatcttaagtaggggcctagtgcatggtataagcagtcgc
gtagcggttgatcgaggggatggataatagtaggtaaaggaggtagaacaggatgctctaaaggtttattcataaccagcgaatcatgttcatga
atattcgtaaaaggttcattttgtaggtaggggttcactgtatgaagttcgtgtttttcatttaaggaaaacatgggctgctcatccaaggaaggaa
tagagccataatggtttctataagtcattgaaggaggagagccggacaatgttcgtctccgttttgcgttactcgaaatcgctggaagagacgact
cattaacaaaggtatctgctggtaatccagaaaataattgggaattttgcggtgaatgggctaattggcttttatacactctgttctcataagggct
atcaacagaatgactaggtactgctgaaggttgggcaaagttgtttgtagcttcctttacggaagaaagctcattaaatgaaatcgtttttgttaca
ttattcggactaccaaagtcggaagagtgatttggtttcggagacttcggaggcgtctgttgagtttcagaacggagaggttggttaaaaagatcc
attatatcagccaaagcagcttttgtatggatagtgggagaagaaggtttcaaagcgggctttggagaaagtgcaacaggtgttaaaacatcaa
acacttctttttgggggttggttgcaaaggttcattttggaaagaggacaaaggacgatcggtaggaacaggaaaggattgggattcaggagct
tgcaatgaagaggaaagcgaagcaaaataacgttcagctctatattcttccattgacttttcttgcccgttctcatacaaaacagggaaatgaaaa
gccgcatactcaattcgctttccagaagtagcgtcataaaatagaggaaggtttggaccttgttcaccaataacattggaaggtggaactgtcaat
gcagtggataaagtatgttcagaacgaggagtaggggcatcagaatatatttggaaaggtttcgctgcatgaggaacaccattattctgtacggt
agataatttttcagaagtggaagttgcatctgtactggcttttgttccaagtatggttctaggcggaggaaatggtggctcataaggaagcgattcc
aaagatatattttcatctgataactttttcaaataagcattgtagacatttgagaagtcggaggcatggcgaagaaggcgatttaatggacgagcc
tctcgagatacaccagtctgcaatactactgaagcttctctccatcgctgcatacgaactaacaaagaggtatactcctcatagtataaggctaatt
ccatcccaatacgtttgcttgctaaatgataaaattcttgagcagcgtcagggaacgtctccggagaagcacataatcgatgaattttggcaagcc
aaattcgtaaatgtcggacgtcgttttgagcaaaccggcatttttccaagtaagcaagagcatcttctacaaccttcggtaatgtgttctcctttaag
tattgtgtagaccctagccattccacgcagcgataccaaacatccacaggatcgtctaattcatcaataacttctagctcttcctgaaagacagcg
aaagtttgtgtctcctctaaacggtttcctaggggtttcaacggttgttggttattcccagaaatcgcttcactcacaatcttatcctccgaagagtgc
cattcgggcatattttaatacgtcgaaagcattgaccccgtatctaaggacgggaaatatgatcgacggtagacgttgtaccggatgttttccttta
actactgaattaatcaactgtggtcaagcaatgtaagatttcttggcgaatacagcaggagtgctgattataccaagaagggtcaactttaacaat
aaatatgcaaagtggaaattgtagtcgtgattttaattctttccttaacttctttttcacctaagaagacaagtagagcattcaggtttgtggtttgaa
aaaatcacagcaatgcttattatattattttatagagggactttcaataatgattctttgggaaaacaaacaaaaaaaaagaagagaggaagag
aggaaagaatagaaggaataataaaacagaacttcgtagactgactggttacatacgatgcagcctaaaaatctacatcaaatatacagctatg
tttctataagaggtttggcatttcttgttcaaaacttaaagttactcaggtataaagagggaactcatatttttagaactgaataaaacaattctcat
actagaagacactgatgacttggtatttcatagttaacatataagatgtatttttctacagcattaggtaggaaacaaaaaccgattattttcttctg

ctgaagaaaaaacacaaaaacgacaccagcaggatttgaacctgcgcgcggagaccgcaatagatttctagtctatccctttaaccactcaggc
atagtgtctattgttgttgtacgatcaggaattgtagtttctattaggtttttaaatttatttaccttcttttgatgagttactcaatcatgtatcaaaca
taaggcatgcaagcatccttcgagtctgttctcaatccctcctatctcatggtcgttgctgaacggacgaattcaatacgtacgtaaataaaaatag
cagatacgatacagtctcttgcatgttgcagttgcattttgggatacaaacaaaaatcaaaagcaatagccttgcaggagaagactatgaattaa
aaagggaattaaaaaaggaaagaaccagaacaaaagaaaagaaaagaaaattagaaaaaaagggaatcagaggaaccgtactcaaaaaa
agattggcaaaaactcaaaacaaacaaaaaataatcctttttttctggagatgcaaaaggaatatggatttaatgggacatgaacttgatggtca
ataagaataacttttgatggaagttcaatagaggaaaacggtaacaaaatgactgggaagcagtttcgtaaagcatttgatacatccaaggata
ctcgtttcatctttgctactctaaactgaattgagatcctgtttagcattccttcatatgaagcgaagctcgggagtttagaaagaatgctgtttgga
atgcttctgaaatgcatatgtttgcctaatcgaaaaggaccaaaactctacctcaatttacacctgttcaggatgtatgtttgtttacacatcaagta
aacaaacatgaaacgaatgtagaagaccatccacaggagaataaaaagattaatctgatttcttctagaggtaaaacaaaaagagaagcctac
agcaccccggattcccatgttgtctccaaccatagtactaacgaggccctcagacgcttaactgcagtgatcggacgggaactggtgttttcgcct
aggtatggccgtagacaatttctttaaaaatttgaataattagtactaaaggcaaggactcttaaatgggtgcgtgttagtggtagtttttgtgatc
atctgaatttaaaacttattcaaatctaacttaatcttactacttgataatcccttaagtgcaagttatggagtctattcaaaatactgtaatccacat
atgatcttctcatatagtctcaccccctgggacaaacttgctgatctatgttctcacaaataatgaattggaaccttcacaatagtaaacattaacatt
tgaaacaaaacaaatgtttgcgtgagatgttgccccggattatgagatactttctaaaaaaggactgcagatagtgctaactatcattctcttgtgt
tatctataaaagtatgtcttatcttggtctgcaggggatgatcaaataacagaatcgcgtattattctcttttaccctctaccaaacagcacctttgta
tatagcttaattggaaacgatcagaacagaatatatgaattgtaaaattgaagaatatgaaggataacatcaattgaaaagaattaactggtaa
agtttaaacattgtgaagagagctatgaaacataaataataatatttacgcgcgttgtttagggaaagaacagttctttgagttaaaattatgcgtt
attcaattgctggatttctcaactttcaattcgtttctatcatagtgaccaatatcctcagaaagacataaattttctttatcgtggtagagcaaaaa
ataaatgtctttgaacttaaacgcttaaagtgtacaattatctacctcgcctacaacttggagacggcgtgtaagttgaaaaagtagaagttcatg
ctttcactcgtgtttttcgttatcctatttacatatgtttattggctttttgtgttattcgtacccttcttgctttttgggtgttgttggtttctttcaactact
caaaagcaatgcaaaatgaactcctgtttcaatttttagagcatgatttatcgcaacctaggttgctcaaagtcgaattgaaattatgggaaaaat
aatacaaaaatccttgctataaaacaaaaaaacaactttttcagaagtaattacatcacggcttcaatgttttcttgctcattctaaatgcagcca
ctcttccgttagtatgatattgttacttacacctcttcatttcgtcttccctaccctaacgggctttatttaccgaaaattacttatgtctaaacgtggat
attccgactaatgacatctagaatctcttacttttcaccactcatattagacatagccaaacacgtgcaatgcttccttactgtctatttagattttga
gttctatataaagtctttggtaagagcgtaaagtttgtttggtacaagtgattctcctataaaaaatcgtccaatattcgtggaacgtgagaaatca
cgacgaactacggttgaaaaaggttataggccgtcgtctaatagcagtttggaattgcatacggtcttatactcttgcaaaaaaaaaaaaaacaa
aaaaacaactataacgtgatccctatttaaagttgatgaaatcaacagaattctaaatatttattacaacgtttcatttaactggcgtcatattcaat
cccccaagtttcaagtcattttataaagattcggctttcataacgttttcttactgagttgtttgtttcccttagttgaatcaatggcagtacaattccc
tctcatttcgttaagaggcatcaagtttctgccgagtcaagcgatatctcgtgcttacccctgttcgtgtactcgaggaatttcatcatctctttctctt
tcactttcacaaaaaaaccgtatattgaacgagggtgtgagaaacttgcaatatgcctcgttttcgactgtatcaaggtctcgcagactaagaggc
gcaatctccaaaggctatttttcaaagcaatacagtgccaaaagcaattccgattcaatctcgtcaggtgtcggttctagagggaacttccccaac
aagtataaagatacggtctattttggttataagcctaatgtctttgccggaaaagaagaacagaaaaagaaagtaattgactgcctccgaaatga
acggaaatcaattcctgatgggtcaattgaagacgaggtatatcgtttttatgatcatatagggttagatgactattactttcaaatggaacctatg
gaagcgatcgctgaacatattgaaatcatttatgctgctaaagtagctgctcgtgcgtctcatgcaagagaagaactcaatattcacgttaaaaat
gagaatgatgactccgctatttatttggatagtgctcctgttacacagatggaattggataaatctcatgaaatagaacaggccatttctaagcgtt
acttggataacccaaaagatgatgccccgtcttttcgccttgagtcattcacctcaaccgccaacattgaccgtgctagtacagaaaattctaacat
tcacacctttctcgtgaccaaatgtaatttcattgacccctctaacagtatatcttcctcacaagagattcccaaaattgcttgggtttcagaccaaa
cgtttttggaaaaggcctccgagcaaactattcaaatgtaccaaaacgtaatggataccgtgctaactcgtttcggtcccgctgttggtgtgtttga
gcatcaacagcgttctgaaatccgtttggttatcggttatcgtcgtggatccattatgagatactttccttcaatctcaaatctgctccgctactacgg
tttgtcttcatttcgaacatatattgagcaatttagtaatggtgtaaccatcatcagttacaactttaaatctgaaagttttaagaatgcagccagttc
ttcctctgttgaggagcttttcgatcaaattacaaaggaagcttctcttttatactgtttaccttatactgatatcgattccatgttcacgtcttccaag
ttatccatccaagaggttgcttacgctcatagtgtccgtgtattttctgagcatgtaatgaataagcttggcagcgaatacacaagtttatcagctat
tcttgaccattcaaatcaccttcatgttgagattttggacacaattaagcgccgcctgtcagttcttgcgtttactcgtactaagatccatgatataat
agttaaatatcctcaactggttcgcatgatgttcgatcaattctacctccaacattctatcggaactggatcttctcgacgtctacaccgtgctaagc
ctgatctttcatcccaatctctagacccacaattcataactgagatatctaccgagaaacttatggaaactattcagaaaacttgtgctaacgatga
agacgtatccgttatggaaatgtttgttaaatttaataatcatctattaaaaacgaactttttcttaactacaaaagtcgctttaagttttcggtttga
cccttcatttttagaccaagctcaatttggagatcctctatacgctatgatcatgtctgtcggtaatgaattcagaggtttccatttgcgctttagaga

cgttgctcgtggtggaatccgtttgatcaaatctgcaaattccgaggctcttaatcttaatgctcgtggactgtttgatgaaaactataatttggcta
atacgcagatgctcaagaataaagacatacctgagggaggtgccaaaggtgttattcttttgaatctcaagtatcaagataagcctgcattggctt
tccaaaaatatattgacagcatcgttgatttgctaattccaaatgaacaagagcaaatagtggacaagtacggtaagccggaaatattattcatg
ggacctgatgagaatactgctgatttggtagactgggcaaccttccatgcacgtaatcgtaaagctccttggtggaaatctttcttcaccggaaaa
agccctagtatgggaggtattccacatgataaatatggaatgacttccctttcagttcgtcgttatgtggaaggcatctatagaaaattgaatctcg
atccaaccaagttaagtaaaatccagactggaggtcccgatggtgatttgggttctaatgaaataaaactttccaatgaaaattatattgctgtga
ttgatggaagtggtgttatctatgatccggctggtctgaataaatcagaacttttgcgcttagccaacgagagaaagatgattgatcactttgatac
cgctaagcttagtgctgaaggctacagggtgttagtcaaagataacaatgttagacttccgaacggggaagttgtaaacaatggaaccactttcc
gaaatactgctcatcttcgttataaggcagatgtgttcgttccttgtggtggacgtcctggtgccataaacatcaacaatgttgatcaactaattaat
gaagctggaaaacctaaaatcaaatacttggtggaaggagctaatctattcgtaactcaagatgccaagactgttttagaaaaagcaggagtag
ttgtgattcgtgatgcttccgccaacaagggtggtgtgacatctagctcattagaagttttagcatccttatcctttgacgatgcctcctttatagaga
atatgtgcgtgcgtgatggaaaagaaccagcattttacacggattatgtgaatgaagtgaaagagataattcaggaaaatgccaacatggagttt
gaatgtctttgggagacccataaaaagcaaaagataacttacaccagcctgagtagtaagctttctgaattgattgtcaagttggataatgagatt
tacaaatatgaaaagttatggtccaataaacaattccggaatcttatcctagccaaagcaattcccaagactctccagaaaaagattggacttag
tcaaataattgaaagagttcccgaagcatacttgcgcgctattttctcaaaatacttagctagtcatttcgtttatcgctataccacttcgggagatc
ccttcgcgttcttcgattttatttctactgaactagctaaagttcatgcttaaattatacctgtgtcgtatctcttcaaaaatctcaaatttcgttgctgt
tactgagtcagtaagaaagcttttttttttcttctatttagtgctttgtttgttttttttgttactcttatattttagatacaaattattacggtaaaggaat
attttcaccattaaacaaaaagaataatgaatatatgattgaagaaatacttttggagtaaccgagtatttgaaagatcatgagaagtaactaga
taaattgtaaaaccaatatatggaaaaggggaaaaatataatagaaaagttaaaactgaatttatcagctcataatcacaaaaatgaaagttga
ttaatgttggtacaatgaattgactgtcagattgcctttgtttcatggtcaaaagtaggtgcaaaagcaaattgcttacagactctagtgtataata
agatataaatcatatacaaaacaggattagtgaaagaaacgagttcattttcggtgctttctttttcagtactaagtaattataatttatttttgtcat
aagctttcctgatttcatattcccctagatacctaaaactaaatcatacaaattacaagaccaaacgtagaccccagtatgccgtaaatcaaatta
ttccttatcctcgcttcctttcgaaaccggttcttcctttttaggaggagatgatgtcgaagtaacttcatctttttctttctttgatttttttgctcggca
acctgttcggaagaggaggaagaggtaggcctgttttgttcttttgtcttctcctctttatcattgagaggagtatcagccttcttttcgacagaaag
ctgtgcaatttcctctgctaaagtatcaatatccgtcgatgttagtttctccaactcttccgaccaaccaagcatttcacaaagtaaacggatatgca
tttcacaatcacctaatacaataatgtctttttttgcgctctccaaagtcaccggccacctcacgattaatcaaaacacgttcacaattgtcggaaac
aacttcaggcaattcagcaaaaggatgaacaagcaaactcgttccaacgacgagtgccatgtcgcagttcatcgtgtcatcttccatttcctcaaa
aaaaccgagaagggagtccttctccataaaacgtaatcattggttttattaagcccttacaatgtacacatttaggtacttgcttttgcttgatgcag
gatcttacatattccgtgtcacactataaaaggttagcctggagcaatctcgttatctcgtaccgtttggtaacattctatacagcggctatactgga
aagaaccatgagcttccactaagtagtcttcgggaacaccagctaatcgttccaaagtatcgatgttttgtgtataacatttttcagcaaccctttg
tcgtgaagcaaacgaataaaataatgggtgtatgtgggtctgaatttttctggcatcaattcgtatgccaattcgtaaaatggacgaggatttttcc
gaaaataagaaaggtctactcagttagagcgattgcagcaaaaccaatggaacataccaaatacagcctccgcataagggaggtcaaatctaa
tagagttagattaaggcttcttcatgaaagtgattataactttttctcttacctctgtaaattattgtagataccagtctcagggctgaaaattgttaa
ccattaccactcgacattgtaatctcaatgtgtataccttcgaaaatcagggattccagcagcagtactaattcctgctcctaccataacacatattt
ggtttacttttccattgttaacatatgttttcaccaatgaaatccacttcagatccttaacaaacctttcttctcttttattaaagacgcaatcttttcca
agtttttgtgagcattttcatgcgtggattttgtaaataatcccatttttctctaatattgccttctaatctgttccttttcaaatcacgcttcaaatccg
tttcaaaaagacgtgtatatcataaactttccctaattttactcaattggacgctatagatccaaaatcgctgtttttcactatatttccagtgtttctg
gtttgtttacgaaagacgtagaaggtagcattttcaaaagatgaatttttaggagcgtttaacgaaaatcattatacttagccacgtcataggagt
ataacttaaaggttttcgttagcattccctagttttcgataaaaatctttacaaattggaaatagagttcgctaaccttttccattttcaacttgtttac
gtcttacacagtaccaactacattgccaaaaatacagaataactaggcggtaaagacacgatattttagatgaaacaaaggttttccgctttaga
tatcaccgcaattggtgcagagttgcgagaggaaattattggatgtcgtttaaataatattcatgacttgaactcgcgtacatttttgttgaagtttg
gaaaacaggtaatatattcatatattgtaagtataaatgtattaacgaaattaaatttaggacaaaaaatactctctggttatcgaaagtggatttc
gtgttcacttgacgaagtttcaacgcgaaaatgcccaactttctggatttgtcacaaaactccgtaaacacatcaagtccaggcggctaaccaatg
tgtcgcaacttgacaccgaccgtgttcttgtgtttacttttggtggaggagaaaatgaccaagatccttcatggacatactatcttgtttgtgagtttt
tcgcagccggaaacatcttgctacttgatggctctcataagattctaagtttattacgcgtggttacgttcgataagaaccaagtctacgctgttggt
caacattataatcttgatcaaagccggttaacgcacaatatgggcaaccagcaaaagatgcctcagatgactgctgaaaaattgactcaactatt
ggaagaagtggctgagacttccaaggcggagagactagctgcttcccaaaagccgaatggcaaacagcctaaacccattacccttcgtaaggc
actaacagttcgcctaggagagtatggtaatgcattagtagagcactgcattcgacaatcaaacttggatcctttacttccagctgatcagttaagt

gccaatgaagacaaaaagaaagagctgctctccgctttccaagccgctgatggtattctatcttcaatggtaaaaccaccagtgaaaggttacat
attttcaactcagcaaagcattcttgctgatctctctgttcctaacaaagaagagaggaaggatgtactcatgtatgaagacttccatcctttcaag
cccctgcaattgatgcaagcaaaccgtgtgtgtagcgaatttcgttcgtacaatgaatgtgttgatgaatttttctcttctttggagacacaaaaaat
ggaaaaaagggctcatgaccgtgttatgaccgctgaacgtcgaatccattcagcaaaagatgaacaagaaaagaaaattaagaatctacaaga
agttcaacatgtctcagccaccaaggctcaggctatcgaaatgaattccgaattggtagaagccattgcttcgtatatcagcaatctcttaaacca
aggaatggattggttagatattgagaaactcatacaggttcaaaaaagaagaagtcccgttgctgcttgcgtagtacttcctcttaaactaaaaaa
taacgcggtaaatataatgcttccaaaccccaatacaccattggacgctgtagacgatgattatgctctttcagacgaagatgagtcagaagctg
aatcttctaatcaggctaagcaagaagcgtcaaagattgttgtcgaaatagacttgtctctaggtgcttatgctaatgctcgcaggcaatatgaga
aacgtcgagaggccatcgtcaaagaaactaaaactttggaagcttcttcaagggctttgaaaaatactcaaaggagaattgatcaagacttgaa
gcgctcaacaaatgctgagaaccaaagaatccttccaaccagaaaagccttttttctttgaaaagtttcattggtttttctcttctgaagggtatatgg
taattggaggacgagacgctcaacagaatgaattactgtttgcaagatattgcaataaaggtgatgtctttgtatgcgctgatttaccgaaatcttc
catagtaattgtgaagaatcgaaacgcaaatgaccctttacctccgaataccttgcaacaagcaggctcattggcgttagcttcttcaaaggcttg
ggactcaaagacggttattagcgcttggtgggttcaaattgaacaagtctcaaaaacaggatatgcaggtgatgttctgccagctggaagttttgc
tattcgaggttctaaaaactatctacccccaagtgttcttactatgggttatggtatattatggttgttagacgaacaaagcacggaacgtcgctgc
aagaaacgtgccgaattagaactggaagatacgcaacaaagaatctctgaacttaaagtggatgtgggttttgaagctgaaatttctcaaccaa
aggaagaagcttttgtcccaagtgtaactgaatcaaaagacagttttgatgaaacaccacctgaaagctccaaattacaaaatgtttttgaccatg
atgttcacattgtgagcgaaaaacgaggtaaaagaggttccaaaccgataacggctaaaaaaccctccgcaaaagagagaagagaagctcgt
aagaaaagacgagaaactgcattggaagaatcatctaagaaaccagtttccattgaagatgcgtcagatcctcaagcaattctggctatcctaa
aacaaaagaaaaagaagaaatcaacggagacaccatcaacacctgaagctgagaagccagaagtccaaagagaagaaagtgaatcaaacc
ctgaggatgctgacaaaaattcagagaatacgtctaaagaggtaaaagagactactcatgataaggatgatcaagcgacattgaagcctcaaa
gatcagagaaaaagcataagcaagcaaaattagaacgtgaagatatgtctcaagagttgtacgaagaaatgttatatgctcgagatgctttaac
accaaccccagatgctacagatactattgtcaatgcagtaccaacatttgccccttatagtgcgatggccaagttcaacctgaaagtcaaagtaac
gccgggatctgcgaaagttggtaaatctgcccgagaatcgattgcatacttcacaaaagcccttgggaaaccttcttccaatgaagtaaagatttt
ggaaaatttgaaagatggagaaattgttgcaccatttagtgttaatagattacgggtggtttttggatctggcttgaattcaaaaaaatccaagaa
ataaatgttacggcttaattagtgagtttagtttctttataatgacgatataataaaagtaaaaatttggtttataatacgaacagaaaaaagtaa
agcataaacaatggattcaaaactataaaagtattaaagtagaactgtagaaactcacgtatagaaaagagtggagagtcattaaaaccattct
gattgctagttatccagcaaaagtgtgctagcgctcaacataaccttgttattactcttggaaagaatatttgcgatttctcgagctgtttccagttta
cgaagctcaacaaagcctggcttatttttaatagcttcaccaatcaattgggcggctcttccttcaccttgcgcacgaacgataaatccttgtttttc
catacgagcatggtcaacgtaaaaggtagctaaaaatgagtgttgttagtcgtcgtacttctcaaaaatttctaaacggattaacacacgtacctc
tttgagcatcctgttgagcaatttgtttagcctcgacagcggctgtaaactcgggggaaaattgaactagaattgaacggttagaatgtaaaataa
atgaagaaaagggatcatgctgatgtaaaacatggaaacttaccatgtgtcaaagaaacgtcgtccaacagaatattaaatctagcagcacgtt
tcatcaagttttcacgaaccaatcgagatacacgttcacgttgggtaatcagttgactggcattaaattgagcaacaacacttttcaggacttcatt
gacaatggatggtaagacccgctcatcgtaatctccgcctagtgtccgataaattttgggtaaagcaagaacgtcgggacgagaaagaacacgg
caattgatattgaccatctgaagatctaaaaggagaaggttagtaatgagtatttaaggagattgatgatgaatggttaaagttttcgctaataaa
ggaagaaaataagggaaatcttttcaacagaagaagcaaccaagattcttcataccttttgttccggtcaaagaagagacatttctgggttttgca
cggacatcataatcaataggagtttcaatccaaggaatgaggaaatgggttccttccgggtaaataaaattcttcacacctccgacacgactgta
cttaatggcacggtgaccaccgtccacgttgaataaagaagtttgaacggcaaatccagccagacctaaagcaactagcaagccagttcccgta
aaagcgtattttcccttaggcattccacgctttatgatctcattcatttgcttaaagggtctttgacgattcatcctgaaattccctgtttggcagttgc
atcggacttgctgctacgttttctttatacagtctccttcggttgtgtacaagttcactatgtgaagctacggtggttggtatttctctatacacttgtta
tgataatgcaaggagcacgtaataccaatcccatcccaagctgtatgttaaaatgacagtaggtatggtgtagatatgaatgagaatcaggatat
ggatgggagagatgaaacgaatcttggtaaatcaatcgactgagtctatatcttattgttacaattgtgtatctgtcgttgttctcttctacaaaaaa
cttgatttgaccattacaaccgtcaagtcgtcgttgaatagcaaatcaaatttggctttgcaagaacagctcttctgcaaatttactaatctgtagtg
tgttggatcatctagccaaggaagcttataaatgaatgaaacgataaatagacgaggataaaatggatcactataagattatgtactcctagagt
tgcagaaaaaatggaatctattctacttctctttcactttccaaacatgcagtgattttgccctattctttattgtctcccctgcctttattgtttatttat
agttgacctaataatttctttagcatgatacactttaggtatctgtgccagtgtcataataggttacctcgttagcattcatcatattacaacctgttat
aagaatacattagcgaatatcagttgctattcataagcctaaagaaatgtgttttataataagtaaacagaaaattgactcttagtcgttttcattt
atttaatcagatccctggacatattactattcttctacgtagtacgaaaacgcccaattttaacatacagtcctgtaattgtacttactacttccactc
gataaacattctcttaacgtgtgagatttctccagtgacaactgacagtaacctgctatgtatacgtaaatattctcttctctgacatttataagata

atatcatacaaaagatgttacttggcagtgttgctcgaaatttatattgtaaacaaatgaattttgggggtttcggtacaattggccagcagaagtg
ataaaagaaagttccaattcgtagacatacactcatcaagagattctttcctaggaagctttccttggttgatttctctggaggatcctgcatgattt
taccactacaaactcgaaaagaaaggtgccatttttgtaaaacactaccaaaaagtcaccattgtgcttgaccttttgaatttcagatactttgcgc
taccaaaagacgaaacgtttttatgactggacgagcacagttcttttacgagaatcaaaatggaaattcatcaatccctttgaaaaaatatgttcg
ttactctaggttattactaaaattattcttacatgatagactccaatttcctacagtgttcttttctgggaccgccaaaggaaacaaaaatatcatta
aaagacagtagggaacgatatgaaagggataggttatgaaaaggttcattgaatattatcagttcaattagaataggtagaatacaaatcaaag
agcagcagaagtttttgtggtaggatcagctgagtttaccttgtttttctcaggttatagtttatcagccagaaaagttttagcggttcctatttctatt
caatctcacgttccatactgacgatcagacaaaagttatgcactgaaaaggatagcccatatgacgtagaataaataccgtggtttcttctaattc
ttggcagtcatttgtttcaatctttgcttaacgatcttttgctcctcgatcaaaaaggcacggacaatgcggtccttcacagcattcgcactcaaatt
gccaccataagcacgctggacagttttcttgttgtgggaaagacgagcaaactcgcgaggacgtaaagcaggaataccttgcaaaggaacacc
agtatcaccacagcgaggaatggtccctaatttttttatatgcaaatagcgcaccttgtttccaggagtcttgataattctggttttgttgctacgagt
gttgtaggccaaacgacgacgataagtaactctttgggccatattctctttcttatttaatttgtttggtaggtgttttgttatttacttaaattcagaa
tttacttgaattgaagatgggtttaaacacttaaatttttcaacaataaaggtgtcctataaatctcaaaaatccccaatcagtccctttttctactatc
tcttagaagaaaaactcaggaaaacttgaaatgaacactccttaacaaatttctgtcgtaaatatccgtcttttccttctgaattgaggcaaaaga
cttaataacaaacttcctctctcgctgagtatgtcattcctcaaagtttctgttgtgactgtttcaactctgtcaaattcgatttcgttggtgttttctgt
cgaaatttaccagactcgtactgatgtctaaagtgggattccaaacaggcttacacttcgctaaacgagtcctctcccatataaaataaatccaat
tcaagatttctctgtttgtttatgtttttttttttttttaagaataaaagaatgaaataaataggaatggaattctacgctgaaagacgcttctgagttc
tcgaaggatttggccccaatagtgattcacttcttgcatcaagtgtgtgcttagctattaccatcaactacctatcgctacaatcgaagttgtccgga
aacaactctattttgggcctaaaagaagagctttataagtaaacatagattttaggaatcttttaagacggattgatctgctttttggctatctttcat
cttttgtacgagattcgtttcctggatcggcaactcccctttaagattaatctcaagaaggaaatagttgatgctttgtttgttttgatagactcaaag
tgacggattcattgcttacggattcgtatacctgataactcgaaggcttttggactggaatcgagaattatttacagttcatcagttgcttgacgata
agactcgactgttgtttttttgcttttttttgtattcttgatttttttgttgttgactttgtgagcgagcgaagaaatgaagtttggtcaactgttaaaagaa
acgctagtacgagagtatcaatactcgtatgtaaactatgacaaactaaaaaaagaaattaaaaagagaaatgaagatgggggttggtctgaa
gaggatgaatctgactttgttgaattactggaaaaagaactggacaaagtgtattcgtttcaaaaagcaaaatccgcagagactctggaaagaa
tccgattatgtgaggaacaagcttccgatgccatcagtcgtctcaatagcgattctcctccaaacgacactgatttgttactttggaaactgaactt
acagatattatggaaacggtgcatgatttggctaaattctgcgaactaaattataccgccttttacaaaatcatcaagaaacatgacaagcatacc
tcttggtctttaaaacctgtctttgctgttagaatgaatgcccgtcctttttacaaagaacaatatgacttgctgatcgtaaagcttagtcgactgtat
gatctcattcgtacccgtggcagccccattaggggtgataatgctgccggaggcacacaacagaattttgttcgacaaactacaaagtattgggtt
catccgtccaacgtaaccgagttaaagctttatattcttaaacatttgcctgttttggttttcaaccccaacaaagagtttactcgtgaagactctgct
atttcaagtatctatttcgataatgacgatcttgaattttatcttggtcgtcttgaaaagcacgaaggcgccgaagctattcgccttcgttggtatgg
cagtatggaaaataataacattttcgttgagcgaaaaactcaccgtgaagattggaccggagaaaaaagtgtaaaggcaagatttcctttaaag
gaaaaacacgttaacgctttttttgcgtggtgattacaccgtggaagaagctttttttaaaaatgcgaaaagaaggtaaaaaaccaatctctgaaat
tgaaaatcttgaacgtcttgctagggaagtccagtatactgttttgagtaaaggaatgaaaccatacgttcgaagcttttatgaacgaacggcatt
tcaattgccaggtgatgcccgcgttcgtatttccttggactcaaatttggccttggtccgagaagatggcacaaaccgtgctggaaacaattggcgt
cgtatggacattggaattgactatccttttaatcaacttcctgatgaagatatagtgcgcttcccttatgctattttagaagtaaagcttcagacgca
gtttggtcaagatcccccggaatgggttaataatttagtaaatagccatcttgttgaagccgtgcctaaattttctaaatttattcatggtgtttcgag
cctcttttatgatcgcgttgacatgcttcccttctggttcccacaaatgtcaatagatattaggaagcctcctgccgatgccattcgtcctgtcaagag
cttgagcgatgttcctagttcatcggcatctcaaaatgtgtctgacagcgaaaatatacaatctggtaatgttttagatgaagaagcgcgcatacc
atctactggtatcagaagaaatgaagactttcaaagtgctcatgaccgccactttgatgacttagatgaacgcagtaccctactaggtgcaggca
aacgaaaaggacgcgactcttttgctgctgcactcactacaaagctaagggatataaaggaaaatctctttttagaaactgttccaaagtttgaag
aatcgaatgaaccttcaatattatatgagcagaaatttactgctcccccggtaaaaggatatatgttcctgttcgtgtggaacccaaaacctattt
tgctctagagcgtacctatttggattatttgcgctactcaattttgatgggttctattggcatcactttgtttaacttctctcacactcgtgcaggtatac
tagcagcatcctgttttactctcgctgccttgtctgctattatttataccacccttttgtatttatggcgagctgttaatattgccaagcacaatgctgt
gcgatacgatgatcgacttggtcccaccgttgtttgcgctgtaactatctttacaatcattgccaacattgtttttaatttaaaagcctaactatgtga
tctgacgacctttaataattttgcaataatgaaatactaattttatagatagatagattacacagacaattgagcataatatttctgacttacaatag
acaggacaaaaactaataattagtattaagattagaattaaagcaaaaaatatgtaggggtaaaggatagaatgccataatatgggtatatatg
atgaagaacagcgagattaaatccatatcgcctcaaactaaatttgatttaaacagtatagtttctccatccttcttgctctaatcggtcggctttttc
aagtactacatcacgcatactgtccatatattcttccaaagcgtgcaacaaatcaggccggaaagatgccaaaatacggcatgccaaaagacca

gcattcatactgttgttgatagcaacggtggcaacggggacacctcgtggcatttgaacaatggaataaagagaatcgacaccatccaaagagc
tacctttaacaggaacaccgataacaggtaaaggagtcatggcagcaaccataccgggcaaatgggcagctcctccggcccctgctatgatagc
acgaaggccacgagaggcagcagaacgagcgtaggagaccattcgttcaggggtgcggtgagcagacacgattgttacttcataagggacttt
gagttgatctaagatgactatagcatccttcatttttgataaatcagagtcagagcccataataattcctaccatcggagattcggaaggaacctg
aaccttttcttcttcctctatcagtaaattgtacctgcgttcgcattcttgagaagagtcagcaataatggttatatgacccatctttcttcctttt ctag
attcaccttttccgtacaaatgcaaagttgcaccgggtactgagagagagcgctttgcgatccgagagacgtagttggggtcatcaccacctagc
aaattcaccataagagcatgagtagtggcagtgcttaaacgagtattggcatcggtgaaaggaagaccagtaatcgcgcgtatgtgtgcttcaaa
ctgagaagtaggacaggcttcgatcgtgtagtgaccggaattatgaggacgaggggcaatttcgtttagcaaaacggtttctccatcctttagaac
aaacatctctacaccatagataccagcacctttgaaggttcgaaccgcatccatggcaagggcttgggcacgcttctgaagataatgagggagac
gggcgggagcgtaaaccagatggcagatgttgtctttttgtatggtttccgtagtagggtaagcgtaaacatttccgtctaaagttcgaaccaccat
tacagcaatctccattgagaaaggaacaaatttttcgacatataatgggcgatctcccaaagctttgatgccgtcaggaatttcttggggttgacg
aacaacatagtttcctcttccgtcgtaagcaagtgtcttgcttttaaggacaaagggataaccgaactgttgtccagcagtctcgactgcagcttga
ttaggagcgtcgcaaaactcgggtaaagcaacatggaacgattgaagatgctgtttttgcgtgtatttgtcttgaatgcatcttaaggtagagtaa
gaaggctccacagagaccgtagacgagacagaagcgagagcatcggtgtttatatgctcaatttcagtcgttactacggtacactttttttgcaagt
tctgaaattgcattttcatcacgaaaagaagcatcgatatgatcacggccaccgtcaatttgcttcgctgggcaattaggagcgtcaacaatgatg
caacgaatattcaaacgatgagcagcctccaacatcattcggcctaattgaccacctcctaaaattcctactactgaagaagaatccattgtatcc
cttcgtcgtgatttcgtcgcagcaatccttgtatgctgtgaataaatggaaaagtccctgatccatggtttcgtaccggcatgatgaaaagggtgat
acagaactctaatgcagtctaacgtacagatctatatctgttttctttttattgtaataacggttaatgtagttctttcagtttcgttttactgataacg
aattttttttacaaagttgtaataaaaatttaacgaaaaccaacatggccctgtaagtaaacaaactgatctggcatggtaaaggtttctttaaata
aaaagacagaaagaaaatccaagtgatgcttgcttgtatcgttagttttataaacacaaaggaaacaaataagccaacttttaaacttcacgaaa
aaaacacacctgaaatttaaaacaaaatagataaggataaaagatgaagcaagttgttcaaatacaaacgtaaggaagaataggatgccacg
cagacgtagcaaaacgcaatttaaaagaaaagcgatacactgccgaaagcgaaaaatgaacagcgcaatatgcaagaaaataaaaagaaat
taggagaaaaatactagcatataaatagcgaaaaaaagaaggaattaaagacattttccaagaacaatcagagtagtatatcgaagccatttt
gcaagattttccgtttgcactaaattgttaaaccatagaagagcaagaaaaacaaaagctaggtccagtataaatgagaaattaaaacaccaaa
taattcgaagtttctcatttttcacttttgtcaatggcctctgaaatcctcgccaacttgaacggacgaacaacccacgagaatactagacggacat
attggatggaaggcatatcctctgtaatagaaatccagttgggttgtccttgacccttgttttcaacatatgatggagcacgaattggacgtaaaca
taaacatcctttttccaaatagtccagatgagacgaatagtcgatatcgctgtattgcggtaaatcactgtcgttgcaatcaaactcccagcaaga
agacttggctggtttgacagcttcgaagtgtttttcttgctctttttcgttcatttttaagagaaaaaaaaacaaacatgttccaacgattactaaaa
agtctgaaatgaaccgcgaaaatgttccaagttggtagcaacgaacaagaactgcgtgggactttctaagaaagagagtgactaactcaactca
taccacgaaaaaatagaaaccccataaaagctccaacagacgcgaattgaaaacgcaatggtaatgacttttggttcgctaaggtgaaactgtc
atcaaccattcaggctttttggtgaacttcgctatatcacttaccgcttgtttttaatcgtcgttataaattttacctcgtacattcaggtagcatgctc
ttcccaagtaacgtgtcattaccatgaagattcgattattttaggcatgtaaagacacttatcaataaaagttataaactttttaatgccttttt aggc
gcttttaatggtgttcaaacaaccaaaaattaatatcgcaaaggcttagttttcagcaatagtcggcgactacacagatgctgtacaagactttcat
tataaagttcacttcgcctgtggatggtcccctaatcttattatgagtttattacttgacgtataaacaaacatacatcctgaaaaggctcgaaaaa
atagataaatcaatggcgcagcatgcattcctttt agcttttaaccatttgaaaagatcatccaatgatgggatttcctctgtttgttatggttggttt
gtttacattctcaaatgtcaacaaacgtacatcctgaaaagatataaattgaggtatagtttctttccttttgacagggaaaacacatgcgtttcaa
atagcgtcttttctaaactcatgagcttcactccttatgaaggaattgctaaacaggatttcaattcagtttatagtaacaaaagcttaagaaatga
gtgtcctcggttgtatcaattgctttacatcactggaaaataattaaatagaggctgctcccagttattctgacacccttttttctctcttcaaattccat
caaaagtttgggctacctattaatgcatcatgtttcatttcccaataatccctattccttttgcatctctagaaaaatattttttgtttgttttgagttttt
tgacatctttttgagtacggtactttgattcccttcctttctaattttattttgttttagttctcccctttcgagatttcctgtttccatttctcattttctccg
ttgctttaatttctctttaactcaaagtcttctcctacaatgctgctttctgatatttgtttgtatcccaaaattcaactgcaacatgcaagatattgtat
ctcacctgctatttttatttacgtacgtgtggaaaaacagtgtttagcaacgagcgtaaggtaatagaatttgagagcagaaatcgatcccctattg
tttcctagccaagcgttagtttacatcgcaagatgcaatttacagcatacgagagaccgcgttccatctacttctgttatccacgtgcgtgttgaata
gaaaccgtttagcaatgagcatgagataaaagggtttgagaccagactcgaaggatgcttgcattccttaatcaatcaattctctcaatagtaatg
ttgaatctgtcaacctcaacaagctttggctctggccaagccaagcttttgggtttatgttccaataacgaattgagtaggattaataaccaaagga
ttaagagctgcagcatctgggtatgaaagacaattcgcttcttaataaaaacaggttgatccttttctcaacaatccagcattgagttttcttcaata
aggagacgtcacaaataattccaaaagtatcctaggacaaatcgagtagctctttgtcgctagtccgcttttctggttgtgtctgtttgtttattcga
ggaactcttaacgttgcgtttcttgaccaaaagcaacttgtttattgttaaaaataaatatattttgattaatatttatccttctaatagttagtaaaat

tgcgatttttaacttgagtgtatgcatttgcttggacaactatccggcgtaaactaagaacaatgggtttagcgttgacttagtctgttatttattggt
aaaagcaactttttttcttaataagcgaaaccgaaattagatttgaacatcctgactgctgttggaaagttaaaaataaagtaacaaaaggtac
ataaacacaaaactggtttattggcataaagtcgcgttcgggagtagtagaagctctcaagttaaaacatgaaaagttatggtcaagcttgttcat
cattaaaacttgtaggcttatgcgaaagtaatcctgaaacataacaaaagctcttcccatcaatagtttggaagacaatctagacctaaccatcac
agataattcaatcaaggcgaatttgtatttataggaaagccactaagataattaccacgatagtagcttgataaatcccttggttaccgattgcat
gcataaacagccttttctttcgtcgctttcaatacaagtttctaaattggattggatagtatccaaaaaaaaaattctcagtcataaaacaagatag
ggaattgatcatctctgaattgcaaagattaaggcaatcatcattgtgagacaacccatttcgttttcgttgtttaggaagattcttgtgaggttttgc
tttcttggaagttcgaatggttctcttttatattcaacgttgaaaacgactgtcttcttgacttatctttcttctctttggtaagtgagaggaaacgtctt
gcgtaaacggaagcacaagtcaggaagaaacaaaattcatacagcgagtgagaaaaacttatactatacgaagatacgcgattttgcataaat
ttcaagattcatgtccatataggaatgcagcaatccttgactgattgatgaacttgcataaatcatttcagctaggaaaacttgagatgtcttcgttg
aaacgtattgtattccttttaatcagtaaaaatcgttcttcgaatgctcctgttcagttgaatgtaatgtttctaaggtaagggcctttacgagtcgtt
catacatacattgtctctaattttaacaatttttaaaacttcttgcctaaggaaagaccagaggatccgtactttctcttctgcatgtttgttgattgag
gtacttttgaagtttcgcttcaccggtgtgcaatcatctgtttttttaagatgaaatgcatatcaatccattttcataaaagtagattgaaccatgttttt
tttttttttggttcgggacatcagcaaaaagcttcatcatcttagctgtattaactgtactgagtataacctctttagtacgattattgaaaatgttcg
aataaaatagtttccgaaaaagaaaactgtgagagttattttttaataaattcatcaattcattccgacgatctttttcacgtttctactatggtattc
caaattgccttttggacggtaatataaaatctacgtcaccttactgtagtagctacttacagtacacaaattgcaactcaacttctcaataccaaaa
tttgaatagcagagtgttggaacaaatatgagtgaaacgaaggaagatttaaagtacattcagctttcaaatgggctgcctttaccaagaattgg
attcggtgtgtttcagttaaagtatgcggattgttatgatcttgtgacagcagcactggatgccggatttcgacatatcgatacttctgaggtttttgg
aaacgaagatgtatgcggaaaagcagtaattgattggtgtgaccgaaatcgagttcccagaaccgatatcattttaagtacaaaattggccaact
gttccgattacacggcgacgcgcgcttcaattcgtagcagtctccaccatttgggcacttacattgatattttttcaatcttttcgccagcagcaggc
agtaaggctcgaatcgaaagctggggagtcatggaagaatttatagagagaggagatatccgatgcttgggcgtttgtaattatggtatcaagca
tttagaagaactatatgcttctaaaccaaagcatcttcctgtaattaaccagattgaactacatcctttccttgctagagacgatatcgtcaactggt
gcaaagaacacaatattgttgtggaagcctattcacctctcacacatggcattcgtcttcaagatccaaagctcgtggagattgccaactccatcg
gcattgatgttcccagactactgcttcgatggagtcttcaaaaaggttacgttcctatcgtccagtgtaccaatcctgatcaaattagggataatttg
gacattttctccttaagaattcccaaagacatagaagataaaattacctcgctcgacgaacattggcacgcagattcctcttatgaccctactattt
gtgaataatttctttgattgaacgactttttcctgcctttgatctcggtttcactcttccaataattgactatcatcggagctctgatgaattttttttttt
tttttaatttaatgactttaaaatgatcgatctcaatttgaacatatatatattcatgggtacattttaggtagtattagttccattttatactttcaact
caaatcatctttcattatcctaaatcattataatttcaaacacatgtagttcattgcattaatacatagtgtatatcgagtcgcaaagaaaacttcgt
ttttttgtttctcttttccctccacctctctaatatacggatatttttccataaataaaatatcatgacttagttggttcagcatttttcgcttttcttcagt
atcagcggtttggtaaagacaaaataattatattgtccattagcaaatggttgacttgctttaaaatgacttcttaaggaatatgtaaccacatctc
attagaaggatcagcaaagtcgcagataaccatattttcgaacatttaaaacaccagtcaccaagacttctgttactcattcacccaacagtaatg
acaacaaaactttacaaaaaaattaaacaattcatcaacttttcggttactttattccaaaggctcctcatccacataattgctcgttgatgaagat
gcaccagcattcggtctagtactgctttcattatgcacgtttccttcagtagccgtagcttcatgagctccacgttggaaaccgttttcatggttgact
gcttctcctacttcttgagcattacttgagccttcgttactgttatctttatttacctgtatggaaggatcaataggagcacggcagatagcacaggtt
ccattcacacgcaaccagggcttcacgcaattttcatgaaaaaagtggctacacggaagctgaataacctgatcagtttctttaaaattctccata
cagatggtacattcattttcttctcctagtaaatcaaccggaggggattgtactttgagtttcgagatgatttcttctgaagcaggagggggtgcgtt
atgaccttgggcttgctccattagctgtgatataatatcatctagccctcttgctccccaagcatagtctcctggatttccagccaaattaaaaacgt
ttgcaaaaatgttagctgggttgaatggggtttcaccttcaccttgttgaccttcattttggtttaaagagccaaaaattctttccaaaatagagcca
aggtcagcgataggtatagcacctgcttgagtctcttgagcagcttcaccagaagtattcacttgatgtaaagttccattaggtcccatggagaag
acaaaactttgagcagtaaatggcaatcctgagtcagatctaagaaaattgggttgaccttcatcagtagactgagactgttcgttaattgaaggt
tgttgatcagaaccagaagttgcattccggggttgttggttgatcgaagaagcataagagggaagcgtctcctgcagtggagtatttacataggct
ccagtaagatgttcagcagggtcggtttctgcagactcggcaccgtgaggagctgtctcatgggtattagcggtggaaggttgattggaggtagg
agatctagacagccgattcaaaccaccggggcctagtagagtctgaaataagttatgaacttgccctacgtttacaattccaggtgtaacaaatg
gggtcccggtagcattaggggccgcgtttcgagggaaggatgaattcgctgtcgaccctgtatcggcaaattcttcggagtgaaactcagaggac
ggattatccgcttcatttccggaagctgtatttggttgggattcagatgcctgcgcattggtgccaaaggcacgaaatatgttctgcaacatggcac
cgggatcagccaattctggtataggaatcatattttggactctaggatcatcttgtggagctgtacttggctcgatctaaatataagagtcgtaagt
cttcaagttcagcttctttaaaatggggctacgaaaaggtttccaaaacggaaaaaatatcactcaataactgtttatttcctttaaacaagtaac
cctgaacataccatttcgacaaagtcactattgcatctggtacaatttccaggttgttgaaattcatttccgcaagcatgacagtaccaaactactg

gtagcgactccattttgctatgaggtggctcaagcaaaagcgatctgaaacacttctcttcaaaaatagaaccagcactcaatggtggtggtgtgg
tgtttttagtgtagttagcaaagatgaagttcatgaacaaatgatttaaagggttcaatttggttgttccagatattgctgtggaaattggggagtct
tttttcttgtacttacaaagactttcatttttatttttaattcttcgtacttgaatgtggattgatggtatgcatcgataacctactgaatgggttaaattt
ggaactacgggtggagatttacttatcgaacaattagtaatatatggtcttttatcgtaaaattctttactaggtaaaagaggtaaagaatgaacc
agacatgcgggcgtattgtgctttatccatcaacaatctggtaactaaataagaatgccacctttatggttgttttcattttgataaaagacgttcaa
aaccccaaatccagtctgaaagctggaagtttcggatgaggtagctgaatgcccgatctttcgaaaactagagacattcagactagttatagaag
aagtcatatggagacgttaaaagttccttaagtgaatccttaagcctttgatttctcgttgacgttgctcgtgtaatgagtggtttaacggtcgtttgt
aactaaccggtgaatccaagaggctaggaggagaccaaaagtcgttgctgtacaagcagctatacaagagcttgcagcaatcacttctctatctc
tcatttcctttaactcggcatggagtttgattgtttcagcgtcgttggggtactcacgcaaaagtgccatggtgcagtatcgagcatcatcgtatcttt
ttagaaaactgtatccaagggctattgaatacatggattctcgatacaaagtaaggtcatactccatacaacgtcggagcatggctatgccttctt
cgacgtcctgagagcagcgcctagaaattaggtaccaaccatattcaaaagttgtaccaacacttggatttggctgctcagactcaaaccggtttc
ggatgtgttggccgtaactgtctgcgctgcaggaactcattgacccggaattggatgctgttactgacccgagagggcactaaaaaaattggataa
aaagcccaaaaagagattcgtctgaggtgcttagtgttttgctctcggaagaaaagcttttggctgacattttgcattttgcagaacgtttgtaaac
aaacattgctttcctataggataaacaaacgaacagagcaaacacttcagaaaaatcgagatagctgctcaataaactgtaataagtaagataa
tgaattgattgaaaattcgttaatgtttcaacaccctttcttaaattctagaaagctctcgtattcttgccataaagtctctcagtaaaccatcatggat
actcatataggtagtatgaatatcgaagttttccactcacgtcgtttgcttcaatttctacttatgctgcatattctagttttcctttttgtttacaggata
gcgatactttaggatgtcttaaatatatggcgaatcccaccgtatcggtaatttgattagcgaatgtgactacgcctcggtaatggtatagacagtc
acagagtcgaaactttgcaaatgagatgcgaaatgaaactgacacaccgccaaaatggctttcgttaaacaagtgaagagcagcccttacttttc
acgcttccaaacgaagtaccgccgtcgtcgtgaaggtaagactgattattatgctcgtaagcgtatgatctctcaagccaagaacaagtacaatg
ctcccaagtaccgtttggtggtccgtttctccaaccgtttcgtgacctgccaagttatctcttctagagtcaacggtgactatgttcttgctgctgctca
ctcttctgagcttcctcgctacggcatcaagtggggtttggctaactggtctgctgcttatgctaccggtcttttggtcgctcgtcgtgctctcatcaag
attggtcttgctgacaagtatgagggtatcactgagcccgaaggtcaatttgagctcaccgaagctatggaagacggcccccgtcccttcaaggtt
ttcttggatgtcggtttgaagcgtacctctaccggttcccgtgtcttcggtgctatgaagggtgcttccgatggcggtttatacgttcctcactctccta
accgtttccccggtttcgacgttgagactaaggaattggatgacgagactgtccgcaagtatatctacggtggtcacgttgctgaatacatggaaa
tgcttattgatgatgatgaagagcgtttccaaaagcaattctctggcttgattgctgacggtattgagagcgaccaacttgaggacatctatgctga
ggctttcgccaagatccgtgaggatccctctgcccaaaagagcagcaaggacgtttctgctatgaaggccgaatctctcaagtacaagcaaaga
aagctttctggtgccgagcgcaaggagcgtttcaatgccaaggttgctgctgctggtcgtgtttaaatgcattgctactaataaaaataaatttctc
gttctgtgctatcatgaatgttgtagatgtagaaatgacaatttatcaagccctatataggaaacgaatttactgaacatgtaataggggtttatga
taccgaagtatagaaaaatcttattttatgaagattatttcttctattgaaaatttttaaagatagagaaaccaaacccagaactcgtttgcaatga
atgaataaggaaacttgttagtagcaaaatattaagaataaggaaaacattgaagggtcaaaatgctttccgctggtatagtagaacaaaataa
gctaaaatgcctaaatgaagtctcgaggagcagatccaagacactaccaatccttacatacgattccttcaaaatatacaaaaagttaaaataa
ggacagtggagacgtacgacacttcatcattaaaagtggctctaaagagttcatcgtaccgcgtgaatatcacgaattgcaagtcgatcaagcaa
aatagatttaagaatatttcacggcaggcgttatcgttttatctacatgtgatggtactttgaaaatgtcgattttcaactaaagcgcttacgataaa
ggaaaaacggacaaaacgactaatttaaaaggaataggaattaagcgacaaatttttttacctccttttttaaaatttactcttggcgttcgctatcg
gctgcaggttccgcgggttgagcaggttcgacagcttcagtagattcagcattgttttgagcaggttcagcattgttttgagcaggttcagcattggt
ttgaacaggctcagcagtggtttgaacaggctcagcaggagaaacttctgcgtcagctgacttttctgattgttgaggtggttgttgttgttgctgtt
caggatgctgatcagaggatttctgctcaacgggctgctggtcggtggaaggagcagcgtagcccttgggctcctcggaaggaacatcttgggca
gggatagaagcttcattggagggttgaggttgagggtagtcactaggggcactagcttctggagacgcagcactacgacgatactcaacttcatc
agggcgggggctacggcgaggagagggtgaacgatagtaaggagactctcggccacgttcataactaggagagcgatcgcggcgaccagggc
ttctactacgagaagggtaacggggactcatagaacgtctgctaggagaaagagagcggtcaccaccgggggaaggagaacgagtacggaga
cggccacgctcaccacgctcaggaccacgcacacggccgccacgttctctccggccaggggtacgacggagagggcctgctggtggaggaggtt
gcttagcccattcgacgcgcaaagtaccaccaccgcgctcaaggcgacggccatggacttcatagtaagcatcttcagcatcccgggagtcttca
tattccacgaatgcaaaactatcgttcataggttagttgagcctcagtatgaggaagtagcgtaggaacaatcaatgaagtgatgaagcggttcc
gattactacgttccaatcttacgtacggtctagaagagcgagttcgaggaataggaatatcgcaacgaactagggggccaaatctgtaaggttag
tatgtaattttttttagatttcacgcaagacatacggctcaaactcaaaagcaagttcacgtgcacggagtccatctcgaaaaccggtaacataaag
cgtacgagggcttctacgactcatttttattgaatcttcagtatggtacccaagatggtggttgaaaaaagaagaaagacacccaagtaatagtg
aacagcaagccgctactaactcatttaccgaggcttgtgaatatcggtaaatttttctagcgctattggatggtaaataaacgaaaatgttctatag
aaatcaattgacttggggattataagagataaatttgaactgtatgctatgatgttgtaggtattctcctttgtcgtgtacagggtggcagaaaacg

caagaatagcaaaatacagaaggtgtgcgacctcgccgacgatgcatcaaactcaaaaattgcattcatactgaaactcctacaactatctatct
acatgcttgagagaaagtagtgatatatatcagcgtagacacagcagcaggaattttatctacgaatgtcgagtggaattagacacacaagatg
atgtaaaaaccaggcaagcaacccaaagaagaaacaagtaggaaagtacacaaagggaaaaggataaaaagtcgaagaaagcattttatac
cttgaaggcacgagactttctgcgaccacgagctctttcgaacttgcgtccctcagaacggacatggggagctttgtgcttgtggggaccgaaacc
aaagtgacggaaggcctcacgagcgtgctttctaccacggagaaggacggtgttactgccagtaggggcacggagggcgagttggtcaagagt
catgacttcaccgccagccttcaagatacgagcacgggcagacttggtgaagcgaagagcagcaacagagagcttggggacagtcaacaaac
gctcgtcatctgtaacggtaccaacagttacgacggtcttaccctcaaggttggaagccttgcgactggtcaaagcagcaatcttggagatagag
ataggaggacggttagtcttggattggaaaagacgctttaagatagtcttgttgaagcgagagtcagtacgacgggccaagaagcggtacaact
tgaccaaaagcttcaagtagacgttctcagaggcaggcttgctgcgttgagactttctgacgtggtgtctctcaatgtcaataccctaaattatgtt
aaaaatcacattccggctacgattttctcttttacataccattgtgacagaaacgctgctggtttgtttcaaggtttgccaaacaaatttggtagtttt
tccgtgactgcttgtctctcttttagggtagggttcttttatcacccgctttcggtatgtctgactttgaaggtgattatagaagagacaagcgaaga
gacacggattcgttattccaattttcctagttaattttttagctgtttctctgattccttttctgcaaacaaaacaaaacaaaacgaattgtttcatat
gtcccacgttcgaattcgttaataaacatattcagaagagcactttttgtattattctaaggactatgcaacaaaatgatagtgggcagaaaatga
aaagcaaaaaaattggttttagggtaattcattgaaatttattttcaaaaaaattataaacattattataaaaggcaagaacgattcatgttcttattt
cttccacaggaacgtatataccatacctaacgataaataagtcctagaacatgatataatattaagttatgatttcaaacaatattaacagctggc
ttcgtggtgtagtggtagcatacttcgtttgggtcgaagtggtcaggggttcgagcccccctcgaagcccactttttttgggcagcaacttttattcta
aagtgtgctgaccgtacgaagtcttgaaaccaatgttggctacctatgccaatgaaagcatacagatatcgcctatctttcattgaaactcattga
agtcactttttcgtcgtttaatcatttccagaaaatgatgtaggcggaacaggatcgtgacatcattagtttttatccatgatgaaattggtcaattg
gcaggattcaatcctatcaagaacatattctttggaatacaacaagtttcaagaattcagtgagtgttggaactatcaccgacttaactttttaggt
tgctattttattattcgtgaacgcttctgtggattctatttagactgatttgactactttactgaataccttgttaatatttttcattactatttacctatt
tgaactgttattgtatcatggcggactctacaaatgccccctagtcacccttcttatgcggatattgtaaaggacaatgcacttcctcagaccgagga
agaaaaggccgctcctcttcctcctcaggttgagcctgttttgagcgaagagccacgtcaaacagaagaaaaaagtggttgtcttggacgagaatg
aggcagattcttatttgcatcccggtgtgtctggtgaaagtttaggaaaggaagacgaagctgctgataagaaggaaacaggaaaacaaaaga
aagatatagagaaggaagcgaaaggtatatgcgataaaatgtccgaagttttccgtaacagcagtaaggtttctgctttcgcggctatttccctcg
accttgccggtatcggtgctctttctgcctacttgctcaaccgccgtcaaacagaaacgttaaccaatcgttccttgtgtacttgcaccgctttagctg
gaattttattggctggcactgccattgtgtataaaaagccaaagattttcaaaagaaaaaccacttgaacgcgatgataaaattcgctgtagaag
aatttcatcgtggatccatccctctacaatcaattcgtctcataatgaatactgtgttatttacgaatgccaatgattcctagtttctactccgttccta
tatgttatttttagcttatgaacttgttgctgtcatttcattttccagtgatttatccaataattagttggtaatatatatgaggtttgtacctttgcttttt
cgtaattatagaatcttgtattgcgtaagttaatgttgaaaaactttgtaccccttggccttaaaaaagcataatgggaacaaagtgcacaatccct
accctatctccttaattttgtttgggttggaatctacctaatgtgcactatatgactgatacttgcgtatatcaatattctctttactccagaacaatag
atgacttgtataacctttaaaaaaaattgacattgaaactaaataaaacgtaaattaaacaaaaccacaatcgcgcttaacacggtgcgcatgtg
tagaaagaaaaatttctataaaacacttgatcgaaaaagatcgacgtgcaactataaagagagtgtataattcaattaaacgaatgcagaaata
aaagaaagaaaaataatattttcgaatagatcgaagatgctcatgaataaccatataagtgtgggcgaatgaagcgaaaccaacatgagtact
agagaagacaattgttgcaacaaatcatagcaaatgagttgcaaggctacaacattctccctcagaaaattttttttcagtagaaagagaagatc
aatagacagattcaatatagggcagctaagaagggctgttcaattcctatacctagatatatacaagaggggaagcaaaaaaaagaaatttctt
tactgtatacagcagcaaaaagttcattcacacatttaaggatggagcaaaccacggcgaggttcatcgaaacgcatacgacggttctctaagtt
cataatttcttcatgcgaccagagatctcttctccttaggccatatgtaggagtggcagtgaccaccggagaagaagccgtagaagcccagttag
cataaagggtgtaagtataagaagcaccaggtttgacggtagacgtggggtaagggttaccaccatatgtcgtaaagacactgtaggaagagg
atggccaaagaggttcaggactcatgtccttagcggttaaggtagaacccggtctcggctgcataggacatgtgcgaaggggacctgattgttgc
agataccaatttctcatagttcctgttggccacaataaaacaaaacttgtaggagccgggggattggaccaaacacccaaagccgcgaggctgtt
aaaaacgttaccgtcaaaggtaccggtgagtccagtgacagtaaagaaatcggaatagtcattgacagtctttccattagtagtagtagactttg
cccaaacaaaccatactctacccgcgcggccccaactcactggcatagaaaatggaccataggaactagttgaagaaggcttaacagtatctgg
aatttcgtagcaatacttaggatctgagccagtattcgtacagatctggaaaactgtctgttgaatatcatctaaagaaactccagagttcgactct
tcccatttgattgtgatggcatcgtatgaaaaaatatctgaagtcaatggctgagtaattttaatttgcgccaaagcgcattgaacgattgaacacc
aaatgagcagattatgaacccaccacatctttcactttgatttccttccttcgggtaggcttggtaaaaggaaaacaattgggaatgaatagaaga
ggaaccattcggttaccatgacgacaaatagttagcgtccaaaaatgagctacgttagtaaataaataatgcgaataaaaaacaagaatggatg
aatgagggaaaatgaaaacagatttggaatagaaaggaatagcaacaatgttttttttgaaaaaggttaaatatttgcgagaaattgggttgtag
tttgtagttgtttttcacagctgcataaaactggaaaactaaaacacagactttctatcatatagagaaacacaatgaatagctgaaactttccttg

taaactattgcaacgaattgctatggattcttggtctgtttttgtttagtaaacaaaccgatgcctgtgttttaataaagtgagagagaacttttattt
ggatatattttcaaaagcgatgccggaaactttcgataaacgcttactacgaaattgcatccttctctctcttcctttcccgctcacctttttctttttt
atagcacccttgtggagtcttgtatcactcactttccaacacatacggttcatagaattatgtcttcctaataaaaatcattgaacatttggatttcta
tcgctgtaaatagaaaaaagaattgtcttggaaaccgcaatacactgtatgtcgaaaagtctaccttttcttcatcatttgaaaagtccgtaccgct
ctatacattactctctttgcttgtttttattaaatccttcattattttcctgacagatggaattggaggaagaaacaactgggttccttatacccaacg
aaaaagaattcttaacaggatttgttagtagcatcgaatcttttcgtgaaagccttgacaccgtcgacgatccctctcgaaaacaatatctatgtga
tgatgtgctaaattatgtatgaatctccaactcatttttgattctcttctgactatttttttttttttacagatacagagctgtcaatttcagcctacctt
gttggataagatccttgctcagtatgtccccatattaagcaggtttctgcattgtaaggacgttctgaacagtcttctcagttccttggttctatacaa
tttttgccgaattcgaggccacaaaattattcgtgtcttgtttcccacagaagtacagcatttagaaacactttatacgtttctacacaatgcacagg
atttgtcatggcaaacgacatatgtgaatctcttgtggctttctcaattgcttaatatcccgtttcccctttcttccgttgagcaaagcaagaatcttca
acttcgtattctggagcttgcactctcttgtttagacaagagtggactggaaagagaagctgcttctttagtcttagcacgattactctctagacaag
attgcatgcatgtattaccaaatattctgcaatcctcaattgattcatgggactcaaaaaatatcttttatagacttggatttctttcatctttggcagt
aattcttaaagtatgtcaaagagaagactttttgaattatagcagttacgttatatcttttctaagatttgtacgggcgtcgaactcctcatataaaa
gtactgcgctacacaaactgctctcaaaatgctatgcaaggtttggattgcttttgattccaatgagcgcatcctctaattggaaatacaatcccttg
atttctgactcttttcactctttaccccaagataccgaaatgcatgttcatagctatttggaggagataatcgattttctactttatcaattgaatata
acgatacttttgtacgatggtctgctgcaaaagggctagccagaattgttgaacgcctcccctggtacttggctgaacaaataattgatgctgctgt
ccttttaactcttgacaacactttttaaatccaatttataatactgttaacatttctattacttgccccgacctttggcatggttctatgttattctttgc
tagattggcaaacatgagacttttaaaatataaaacaattctgcatatcctaccactcgttgaattggtaagttgtacccaaagcagtactctcctt
aaaaatactaacactcattctacagggattatcttatgaaatacgcgttggaacaagagtttctggtcaaagcattcgtgacgcttcatgctatttc
atatggtcattatacagatcttattctcgtaaagatattgaacctgtccaactaaatcttatactcagtttactccaaactctattatttgacagagaa
gtaaatatccgcagggcagcgacagcggctttattcgaaatggtcggaaggcatggttcagtacctgatggcttgaaccttatttcattcctaaatt
attcatctgtttccgatctttctaattgttatggcgactatgccttaaaggtagctaaaaacccacattttcgtacttgcatctaccaaaaattgttgct
aaatctgcatcacttcgactacaagattcagaatttagcaaatgcatccttatataagttgttttcaatataccatgaggaagctaccgtatatttac
cttttctgaaagaccgactgtcaaccggcaatgctgatcatgcatatggaaattttctcgccattggaagcattgtgagatgcctattagattatgat
tcttccatcatcattactgaagtcagttctttattgaattttggcacttatattccgattactaaattttctcgttcacaaaaaaccaaagcattttctg
gaatctgtagaatgatagactcaattttctcttctcgatatgactttgataaaaatctttctgattctgcttttgtatgccttaaaaacgcaatttccct
tcgtgaagagtcaattactgaggatatatcaaatgcattcaactctttggtgcggcacgacccatccggtagctacttaattgaggttatgtctttgt
ataatttttattcctcacctgaagagttttcaacaagcgtttctataattggaaaactacctgacgttgatttatctcaccaagaagatattatcctgt
acttgaaaacagcttactgctctaaggtaagtaaacatgcaatctttaaaacatactaatcgatagaacatatctatagagctccagtcttcgatt
atttcagcttttcaaggcttatgtccaagcgtatatgaaaagtcaaccagcgtcaaactgaatgagcttgtagaattccttgtgtctatttcttataat
tatagaattgataccagaggagatgttggctcatgggtccgaaagcaatcattacaggttttcagaaagatggtaactttggactcaagagctca
gaaacttcagaaagtttgcgttatctcagttctttcctttatggttcgtcaagcattcgaaaaaattgataatcttcgcttattggccttacattgctta
gaagattcaaaggaacatccaattttaaaacatgacgaaggcttaagaaacattattgactcctctctcaaatacgagactaaattagtcgaaga
agacaaaatagaaagtccagctatccggtttctcaactatacagcttttcgtgaagatgctttcaaaggtatagtgactttcacaggaaacggcttt
ggaggtggaaaggtatagtaacagagtaaattttggtcattatgctaatattttagattacttcttattataaaaattaccagcgttatgtgtatcaa
ttgaaggatttcaaaaatgaaagttgttcagactgtggattatccaagcaagatatttttgaactatatatcaaattcttctcttccaaaacagagg
atagccgtctttattaccaaatcgcttcgtcttttacgtccttgtcattgtgtggtgtatttgaaaagtttatttcctcaaaatcgtatgtacttatctca
catcatctattaacagatttctagtgttgtccgtgtagctttcctggctcaaaaacgggctttaaaatgtacttccacaacgggaattataattattct
taatttgttaaggacttttctggtatctaagtatgatgttttagctttatactctttaaagtatgtatccaatttacttgcacattctcgagagaacata
agactacaggctgcagatatcctttttctttgctattcaagctaagattactacttttattcccgaacatataatagaggaatttcttactgtggattgg
tttgcaccttcatatgcaaacattaaatttgttaagaattttagacagctcattcaatcgaaaatagagcagcatgaacaatcattaaaaagcata
aatgtctctattcaatagtttaataacttaatttaatggaacaccttatttattcaattattaagcatcactaaatcccggcattatagtagtatacac
gtagctaaatgaagctaagctgaggaagcacaaatctagcttctgtttaacaccaaaatggtggcatgttatgcacaattaaaaatattagaaga
aaagaaaattaaacaaaaaaacccattacactttagccgaatgaacaaattattaaatatcaggaccgtaaccaaaatcgttaataatgaaaga
caaggactactcaagttttgcccgaagttcctcaaattctttcatatagtccatttcaagaccctctaggctatcttggatgtacagcttaaactgtat
aatttcactgagcatataagcaacttctctgtaaacattatttttgtactccatgcatgaatgagccagttgatctgcctctatagtaatagtctgtat
ccgctgatccaattgtagcatgctgttatgcgatgaaagtttaagctgttgtaaatcatgctctaatttctgagattctgtattcagagaagcctttttc
ttggtttgctgactcgcgcaacatagtacactcctttaccacggaattcaaacgggactcaatggtattcaattcatcttgtaattccgcaaccgtgt

catttaaggcatcaagtacttcttggatttttagagacttattttgttcttcatgaaattctaaagttattgagtgacgtacttgattcaaaaaaggtc
gtaaatagttctttaaatcaaggtttatggatcctgtttctggatctaagattacttcaaaattgccatttccagcacgcggagcagttggaggaatt
atcccaatccggtaagctaggttgctatatttttgagattccttttcaagggtatcaattttcgattgtactaagagatctttgtcgaaaacctcttttc
ttagctcggttatcttattgccaatgaaattgatgtttcgatctaactgttcttgttcagacatcatttttgaaattcggctgtagaaatcgactgcat
atccactcttgattttagggatttctttttctcattaagctcttgcaactgggcttctttttcagtaagactcagctgaatttgcttctttaaagcagtctt
cgactccattcttgattgaattgaggacataaatgattgaagttttacctcatccctttgcattgttttaaatctttcttctagtagttcgattgtagagt
ctagatcacctgctgcttgcttttgctttaaaagctcatcattttcttgttgtagcagctgaattttcgtattaatttctttattttgctcctcaaatgacg
cctttaactcctgttctggttcttcttccatagaagcctgtaaaaatagatgatattttcgaacgaggtaatcaaatagtaccttgttgacaaagtga
tcatttaacgtatcgttttctacggaataagccatatcgacagcctgctccgtacactggattaaacatacaagccagtgcaacattcctaaaatg
gcaggccagctatgaggagttcctatagccactaacctggatcgagatatcgaatctgtaaaaggatacctcaaggactttaaacaatttataac
atcttcttcatagcgagctccgaaccgaaattttgggtccagcttattatacaaaaacttgaatatggaagcaaattcctttgtagtgggtagaaat
ctattattcggggtcaatggctgtggatacccacattctaataaaaaattgactacataagctgcacactcttgttgatagcgtttatctgtcaacgg
acgagggtccttcatgcttgaagtagatgcagcttggcccataaaagatgcactagacatccttcgattcattatactggacctgcgtaccctagta
cagttagtatgtttcatcgctgtctctgctacaagcccaatttccttaccccggtgccaagctgggtctcatagaaggtgttgcaagccgcgaagaa
gacattgtcatgccatccacgtccatagaaggacggattggaacaagtgtacgctgaggtgctagacttgttcgcccgctcactaatccattatcg
gcgaatgttgaatttcgaatacctgaagatgaaggaacttgagaatatcctgaatttaatttattagtcataaaattcgtttagttttacttacttctc
acgtaagacgaagaatcctgcatttgtaaacaaacaaaagttaattgttgttcttctgtttcctagataagcatatactgagtctctaggtcgtaca
caagagttctgtaaatgctggtaaaacaataatggttatttacaggtttcgaataaaggattagaagtaaaatacaatttattatggtagttaatgt
ctctattatttctgtgaatgtcagcactttgaagttattagagttgtcaaccaactagatttcaaaacgataaataaactaaatatcacaaaataaa
caaaaagatggaaagcatgcaagaaaaaaagaacgcacaaggtctgaaaaacatttactgaggattaaacatcaatttgacggacaaaatta
gaagggaattggcctctattatgatcatcgtcaccaacgacgaagccttcccaccagccatcttcataaagtctgattacagcaatggtatcgcctt
tctggaaagaaatctcttcaggaatagcagcattgtagtcgtacagagcatgaacgtatccaaggatttttttgtccgtcagaacttgttggggtttc
tagatctaaagaaggatgttcttcagtatcgtattcaccaggcataggctgtgacaagtttgcgacgccgtatccatttgaagcaagaaagtcatc
cgtaatagaggattcgagttctgattgataatcgtcatcattcaaggatcctcccaggcttccaacaggatctgctttgactccatattcataatccg
gttcgatagcaccaggtgtcagagatcgtggtaacctagacgcacgacgcgatgtagaagacgaaggccttgaagactgggtttttcgcaaact
gagagaagtattgtttttacggttatcagaaatgggtgatacggctctattttccattttcataacactactaaattcatcctgaatctgttgtcgtct
ggaaattggagaaacgctttgataaggtggggggttagctgttcctgattcagtatcaggcttattgctttgcatggataatttctgaaaattttcat
ccataggagtcatatccgaagaaggattcaaaacattaggggtaacaggacgactcgacttcttaaatttactaaaaaatttttttacgtggagac
attcgccctgattgcgacgagttggaagacaatgacgcagctgtctgattgactgcggcagagacgttttgtgaaccagaggatccttccaaagt
agcattccctgttcgagcagctgcggaaagagtgtaaggtcgatttagggtcatgttatcgttttcgacgttggacggagcgcgttcgaaatttgct
tggactagctccacaacgtcttcaggagattcacctttgtaaaagtcataaaatttaggaggctggggaataccagttcccgtagctcgagaatcg
ataaagcccaaaacgtcgttcttgacgttgcacgtctctaaagattttcgaattcgctcgcaagcttcatcatcattaaaagcgcttcgagaaacga
gaagagtaaacgtccaaaggacgcttcttaaaaagtgacgacgttcttcttcgatgtcttgtagcttatcgcaagcaatcttccactcctttatcca
acggttgtaggtatctgagacggtttggacgctagctttgtattcggaagcgcttgatgcaattgaattttgagtcttgttcagtttataattattcctt
tctagttcctttccaactagcatattctgctgggaaacgtaatagttaaccttttttgcataggtaatggtatttgtcttgtgcctgatgaggatacatt
agcaaaacaagtctattttaaggaacaaattttctcataccttttcgaaactttgtttctttgaatctttatccttatggcatttttcaatgttcgctata
aactaaagctgcgttaataagagacattttgagagaaaaaaaaaaattcctacagttttctttttatctttcaaagatatcgcatagcgactaaaag
cgctggaaacatcatcttgtaaaagctgagcgattgcgagatggtttttgcccattgtatccacctcggtcttcatactctgaatggattgttttaaa
gttccggtttcattggcacccacattcgagcgagccaatttggccatccgtttagcataatcctcttcaatgcttgctctatagataacttgttagaat
gggaaaagtaaaagcaaacttgacgtggcactcccatattaggctttctgctcccctttttttcctgaaaaaagaaatagaaactatggaaatgct
ttcatgctgagaatagaaatacctttcgtgaaaaaaagatcgtaggtcttcattgatgtgtttgatttcactgaatctagaaagtaatgcatggtat
ccttcatcattcactccctaaagatgaaaacgaggttagaaaagtaaatttaagcagaaacagcagtgggatgccaggaaattgtcctacccaa
aagtagtttgaaaagctcttcgaaggattggatgactgagcattgaagtttaatgttgtagccattctataaataacaaggcaatgcgagaaggtc
gaattcctctctcgcagtcttttctgtgtttactcaaatttggtagtagacgcctcggtgtaaacgaacagagtaggcaaggtaaataaatggcagc
cgttgctaggtaaaacctgaatgtaaacaagtccgagaaaataaataaaaaaagagtaaaattaaattaaattaataaaatacagcagaagaa
attgtacaatcataagtagaacaaaatgaatgactgattcataaaatggttctaaaaagtaatttgtataaaatttgcctggggttttctgacatgc
ctggtttggtggcttcgtagtcagccatgtatacttttcttgacttgtaccagaagtatgcaattggattcttaaatacaacaatatgatttgtagtaa
aatgaaaaagtcatcaattgtgttttgctcagatgcgaccttgtatggatagtaacgaatgttttaccaatcgaccatgtcaacagacagtagaca

actgctttgctgtgttaggaaattaatccattccaggcttctttcctcaatgtatcctaaaggttagagtagaatattgtgttctttttctgcctttgtta
ttcaaccttcctcttttcctaccgttctgtatgcaaaatgtcacaagcagactaacacgaaacaagcttcaacgaaagaactcatcaacgaaagtt
ttgaattgtgaattcattccatgactgtatccatccatgttctttcaacagaaacattcttttccgtcgaattgtttatttacctatggaacaaggactt
aaatcatgatgacgtaattgaacacgatatgaggcgcgtgagcctaagaggcaattactatatatagagaagggtccgccgctgtgtttgacatt
aactaataggacaaattcaaaacaattcagatttccattgagaaacttcgttttcgtgtcgttgaatttttgaacgcatcactctatttgcaacgtta
caactaacttgaaacaatatgtcagaagaacgcaatgttaagcacgccgtcaaggatacagaccataagatcgtttacaacgttgaacgggttc
gccacggtataaataactttttcaatgatattggcaatgccgtcaagaaagaaagcaacaccggtactaccaagatgagcagtgaagctgaacg
caccgaaagtgctggaaaactttaatgattttataaatcctttataaaaaaaaaaaaaaaaaaaattgtaccttggcgttcagcaggttttcttaca
actcttattatgttgttttcatgtttttgttttgtgttttatgatattataaccttatgtttgtgtagaatctgttgactggaaaatcctaactttcttttca
acaataactatgaatacactatattttaacattagatatttcgtaagtctagtttcacgcgtagtgttgtaaccattaagtattaaaacaaatcctca
gcatgcattcgcatctttcgggacacgaatgttcctttagtaaaactgggtttgaatttggattttacgtcgccagcatcctctggtgtggagggagc
agcaggagtagatgtatttgacggttgactatcccgatgctgatgctcctctctttcaagtaaaagctccaaaacattcacaggactcccattgtat
tcttgatcatcttgctcttcttccatttcagctgcagctgttgcccatctattgtttttagaagcgtataacggccgggcggaagaccgaacggacgc
tgtaatctccttgctcttaaacatttgccatgctgaatctagaacttcttccggaaaagctacccaaactcgccaaatctcaatgacttgtgcaacct
ttttacaaaaaatatccatttttaggcgaccacctaatcgtcgactaaaggcatatagattatcaaagatagatggtacatatggctccaaactac
acttgaattagcatgcttaatgctgaactatctctcgtttcttacccaactcgataccgccaaactagcgacactccagatattccattaaacagca
ggtcattaatcacgtacaatagtcccaatttttttgtcccccttttccaatatagatttatcattcatccttccgtcattaacctcctcattcttcagtaac
tcttgtacaatcgctttagaaacctatattgatatatggttaattatattcttctttcctcgatattcacacacctctttatatttatcaatgtgattaat
agaaaaacataaaacacttgctatagaccccttttcacagctcattcgcgtcaatagccattccaactttgccctatctagggttgacagacgatct
gctgacgcatttttctcttcttcctctttctttttttcgtagctctttctgttgtccagaaaatattgaatccttgtttgcttctccttttccaacagtagctg
atatgtaatttccatccactggctcagtagacaggcgccgtacggcatcctgtgcgagcttttcgtttcgaaatacggaaattcctttggaaatattt
tcttcggaagagaacacattgattgcaatcacagagtcacgtccacctagagcttctttcaacgttgctgcggttgtctgtttagcaatgccatcca
gatgtacaacacagggcttttcagatacttcttcgtattagcaaaaaaaatacttgtaattgtttttttcctaaaagacatacaacttgtagcttggtc
caccgattccaatgcataattttcgcgtcgttcaatttcagcctctagtccagacttattcgtctgtctaccaattcaaattagcttccatgacacaat
tataatacatacggaaacggttgctgttgctttttctctgaaatactgactttttgccgttctccagctagaaacgtatcttcatcttcatccgactatt
gtcttagctttcccttcctttttattccaatttcatgcatacttctgaacttatatcttccttcgcagcaaaatgccgccgtgctccgataattggccttc
tttctgttgataaatttcgtcagcaaactcttcttcaaagctcccaaacgaacttgtttctaaagtttgtaatggaaactgatgatcgtgatccattct
aaaatcatctttctctgcccacttttcttgattagttcgcatttggagtcatcagtagagtactgatcttcaattaccatttacgcgtcctacacaacct
gcttaatatatgatgctctgagagtctaactgctacagtctcataaatgttagagcttttatttgtaaaatttttactaatcatataacaaaatgatta
atacaatcttgcacttcatgatgaaagtagttagttcgtaggaacaactgacatctcctaaaaagctagcaagctagcaagctggtttaaaatgg
aaaaaaacactatccaacgttataatctcaatgcctttcagatacgtctacgttcaataccttacgttgagaaaatcagctttttttatggattctct
gactaaggggatggctgtgtttgctttacacctattcaaaatcaaaaaagcttaaacaaaaactttggttttatctcgatttctgtttgtttacaaac
attggatttgagttctccaatgtttgtttattgccatctttccaaatatacatataaaagaaattccaagacttattatttaaaatatacagttgagaa
aaaacgaacctgagagtcgaagaaggaattagaatggttgacttacaagacccaagagcattacgggaacttcaagtgaatgcaataccctta
gcgatccctggaaagatgatgccatcaatttatcaaaaaagctgagaaatgaatccgatgcgagatgcggccgtgacattgagcctcatgaacg
gaggaatcctttggatcttgatatctgggtagcatataactatctttgtaacgtctatgaagctaaagcgtaagtctttaaaaatggaatataacat
taacaaagaagatggattgaaaaagaaatggaagtggtttatccctgtatagatgactttattacttctcttgcagatggaaaggtgctttacgatt
tatcgatgaagttctatcctaagctggcaaacaactggaaacctcgatgccgctctgctaatcgaaaagtaatgtacatgggtgttttctttgagtt
cttggactttgttggaatgtttagtgtaagagttttcacgtatgttgaattcaacaactaattatataggcttttcgctttgaattaaagcatctaattg
aatattctaatataccgcgcgttatttattccttacatgcgctttcttatctcttgagctttctgcaagtgactgagcatgttcctcatttatttggtaaa
atacacctaaaacgtagtgaggtagaactaacgaatcaccaaatcttacccttgaggaattccaaaaagtttcctaattttaccaaactgaaaaa
acatttttatggtcggctccatcggaacaagcaaagcactagtcaattcaacgagcgcattctggcttcgaaaaagttaccttcttggatcaaccct
ttgcaagccatttgcagaggatatctgttgagaaatactataaacacgcgtgatattatgagagaaaagtcccaacagaatattgttaagcttccg
ttactggaaaagttactgatatcaccgaacgcagatgagagattgacaatggttaaagaaagccagcagctgtaccgaattatggtttgcttaca
ttcgcaaatgtttcaccttccttccccagagctttctctagatttgagtggactcgctcttctgggctcttccgttgaaacttattatcagcttattccgc
caattctgaacaacgtagtttttttatcttcaagaaaatccttccttgtggctatcagtgttgaaaagactcttagctttaggtgttgacagggtggac
atatccaattatggattacttgtgttgaagtttttcgggttttctgttaccaatcgagaccgaaaacttttaaatgaatttatgcttaaaggtctcata
acatccttaagcaatataaaatcatcagaaaacaatgatcgcaattacgggatatctcctatatttttatggatttccaaaatattttcaagcagttt

gttgtgagtactatgaagcatcactctattttactaacgtattagcttggatttggattctttctttgataagcgagcttcttttgtactggatagctttt
atgaatcacaaagcagtgttggatcacttgaaattactgcaatgcatactgttagtgaagttatttacagcttttatttgcataacaaagaactgcc
aaaagaattcttttatttttcaattcactatattattccgaagcggtaagttatttaatcgattttcaaccgttcattttactgattaatagggtcaaa
aaaatcctcgttccttaaaaagctctatgttatggtttttattgatcaagttataagctgtgtccaccggcatcattctcgaaaatactataaaaag
cattcagaaaagttttcgttcatgaaacattatttacgatcgctttttgaaggagtttctaatttagcaactagtgaaatccaatggcacacgcgaa
gttatcgaattgttgaagcgttaataagaaaatgtacgtacacctgtaacttattgatttattaacagaacagtaatacatgctccaatcaagcgcc
cagacatcgagcttgcgggaagatctttgagctgcacacacaaagatatatttagtcttcactcggtattgaaagtctgtaatgacaatggtgattt
tgatgattacccttcgtttaagcgccttatcaccaaacttggttctccgaagttatgcagaaaatatgaaggtcaagtcctgtttctagacacaaga
cgggagtatatgcattctagcataccttgctcgcaggcagccaaatatcaactgtcacttagtttatgtttacctttagtagaaggtcatttgcgctg
ttctttaaaagaaacattatggggatcacccacctcatcagaaaaccaaaagttcaaaggacttttacatcttgatcagaggattagggaccttca
tccttcttctttgacaggtatcactgaatccttttctaactcttcacaacctatacaacaatggcaatcacggcttagaaagttgttgaaaaacttaa
ccaagattgatgatgatttggaaagtattcaatttcttatacgacatggatgcacggaccaagcttgtgtaaaggacattaaagaagcttacaga
aaagtggccccccttagctaaaactttaaagcaaactggaattgaaaacaatgaatttttaaattttctttcaaacataggacatggctctaaaaag
tcgaaggacttcaattataagcatccattgcttgacaaatccatgggtgaattgctacagatggagatcgtttacagttgcccatcagagcttgtga
atcacactttgaaaatcttcgttaacaaaaaccgaactgcacttcatttttttcctttttaaataagaagaatctgatcgatgagtccgtgattttggta
aatgatttgatacaagctgcgtatgttattatttggaaaactggtttttactaatattgaacttaggaacataggaatctacttcattgatgtacttaa
tctccccttccatactaaacaattgcaggtttggttaactccggtttcctcgtatcatagtgaagaaaactatgctgaaatgaaaaaacagaaaag
aaagaaggtcactgggtttgaacatgcaaagaaacataaacttgcaaaatgagcattgtctttcccttaaaataaatattgattcaaatttaactc
aactttaggtgaaataactttattacaaaggcagaaaggagaattaacctgttcaaaatttttagtaaaatatataaaccttgtccttgttaacgta
aaagatatatttttctgtttaaaccaaaaatattaaggaatcccaaaaagatcggcatataggtttttttttcacttaacacactgaacaataaca
atagatccacaaacataatcctggttgcttttactattcgaaggaagaatgttaacgaaaattaagctcaatcgaaggtaattttagtagcagcttt
cataggagcgtcatttgtcacacgattaccataagcattcaacttgggcttaacagctgtaccatatttatataatccctcaggaatatcttgtttag
cttgacgcaaaacattaataagctcacctgcatgggacttgtcttcaggcgtaaaaaatgtatgagcaacacccttggtatttgctctacctgtacg
gccaatacggtggatataatcctcaattgtcaaagggaaagtaacattgattacaagttgcactttggggatatcaagaccacgagcggcaacat
cggtagcaaccagaacaggggatttacctgacttgaaactttcgagggcttgaaggcgagcggcctgagacatatcaccatgaatacccacagc
attaaacctcctagccaaggtattttcaacacgggcagcttcctttttgtacaatacaaacaccaaaatcttctctccttcaccagcagaagcaaga
ttcttggagatgcagttaattagggacctttccttggatctggaatcatccaacagttctattatttgagtaatattttgagaagcagacaactcgtc
gcttccaatagtgatcttaacaggatccttcaaaaaggtagccgcaagtgcacgaacagattctggccaagtagcactataaaatactgtctggc
gggcttctccttttaatggattgggcgtttgacttataatgttacgaatgtcttgctcgaaacctgtatccaacatacgatcggcctcatccaacacc
aaatatcccacgtttgagcaatcgacagcaccgtcgttaattagatcaagtaagcgaccgggggtaccgatgactacactagcattacgagcag
cacgagcttgttcacttttaggggcaccaccgtaaacgacggcaaccttaaaagagacatcggaagttaagcctttgatattttcatatgtttggat
agccaactctctagtaggactaacaaccaaaatacgcgttgttggcttttcatcaggcagattttgaagatattgaagagctggaatgccgaatgc
gactgttttaccggatccggtttcggcaataccgacaacgtctcttccggcaaacaaataaggccatgaagctgactggattggagtaggttcctt
gaagttcttcaaacccaatcttaaattgtctgcgattggaagctctgaaaaattcaatacaggtaaaagaggagtttgactgataggatccacata
attaatttcatgcttctttgcatattctttgcaagcagcttcgtttgccacggattgagagggaccctctgttaaagtattcttctcttcttcatcattgc
tacctccctcttgagatgcagactctgaatcggacttctcctttcttttcttttccttcttgtctttctttttcgtgtcggatttgtccttggatttcttttcct
tcttttcctttttgtgttcatgcttggctttttttctttacgttttatggcctctccttcaacatttttttctaaaccagcatccttaacctccttaattgag
cgttttcccattttcaaaatttttctttagagtaaagagggagggagttgtaaagttctcttcagtccccgattcatataccgcagttggactatggc
atgctaagtcgaaaatttatgatttgataaatggaaaataaaaacaagaaaagacaaacgaaataaacaattgaaagcaagttcattccattac
taacagcaatacttcgttcagctttgtttgagacttttcaccctcataaacaagggtagtaaagtcaatttattttccataattactctgaaaaagaa
aataaaagcaaaaaaaatttcagattactatttctatattattaagtgcatattaataaacaatcgtattttcctatgtgcaactcggacgctcctga
aaagtaaaaacaatttcgaagaaaaccatgtcctagcgtttttttccaaagcaaacagcaatctaactctttttctttttgtcccatttcttcctttcc
tgtcaagacctgccatgaaagaaagataaaaaacgactaaagtcctattaaaacaccataatctatttccatgtctgtttttttttttattatttttct
ttctctttctctcttttcaatatatatgtatgttataataagagcagaaaaaaaaaattcccacaacattttaactgatccaagaacctatagaaccat
tctcaatcacccacagcctatttttatatcaattgagataatcagatattaaggattcaaaaaattttatcctatgcgaaaaaaccagtttatgcaa
agcggaaagaaagattcactatcacagtagtaacaaagattcaaaaacgattctttcacgttattagaaaaaggaaacaaagaagtgtttgcat
tctttcgaaagccagcatgaatactactgccctttccagagttcgttatagtcaataaaaatttagttccccaaaaatttttaagaaaataaccgct
acgttcattgcaactgcttgtttaccaaacattaacgccaccaacagagctagcaggaggagaggaccaaatttgagagcccaatgcaatggag

ggatgatgtgacttcgacaacgaggaagacaagccagacatgtcttgattatttccgctgtactcttgcttgaagtccaaacttagtgtaggaaga
gaggaagaaacagacgaaccagtcgtttgaggagaggctgcgacagcagagaccttgttcacagcggcaataggaggagcattcccgttggta
agagggggattcggtggacgaagcgtgctatccgtcgaagcagagccgttgctggatggaatattaaggtccctcttcgcaggacgcgttccaat
gggcgctatggaagatggacgacggcgtgccgttggaacgggtaacgaatccacattcaccgaatccataaaagaggaagacgggttatagct
attcgaagtggtttctaacaatgaaaatgccggaggctgcttcgccgtccgtaacgccgaaggaggaggcatactagaaagaccaaagttagga
agactatttccacctaccgtcgacgaagaaaacgaagatcttccaggagcggccaaatctgtaaacgacgaaaatgggttatgatgggcactac
ttgacagcagcgacgtaaccgatcgacgagggtgtagaagcgacgatgccgtagttcctcccaaagaagtgctacctggaaacgaggtaccaa
agccggaggaatgattggcggaaccaacaatcgtgctaggctcagcaggcgaagtcccaagcatattcgaggctccattaggggtgctggaaa
gcgcattgcgtaataaactaccacctactccgtgattcattggtatactgctacttaaagcctcattaagtgatgcggtggagaaagggtcattcgt
ccctgcaaagctgcctttccgagctcgtgaggccaacatgccattcttcatcaaattgtcggatgccaattcgatttcctcgtactgactcacaaatg
cagcactgtcatctggttgaaaaacgtttgttggacctagaccatcccaataagaaccggccgagaaccaactgttgactccatctacaaacgac
ataaagacaacttatggtttctagaaattggccactattccaagatcaaaccttcaatatatttttttaaaaagaagttcaggctgtttgtggtttgc
tctgagtaagaatcgcccgctcggatttcttagggataccccacttccccatgattcaatgagagtattatttcgatcctacggtgatatcatacaa
agcaggatcataagataatatttgttttgaatggatagagtgagtggtagcaaggtgaaataggaaaggaaaaaaggctagttccgtaagaata
aaagtaaagatgttgttattaatgtttacaaaaaaatttgaccctatgaagtccatccctattgagccatacgtttgcatcaacttcagtatgctatt
tcattcattcaagcaattatagcaaaaaaacatatatacaaatgctgtattgcgtatttaaatttagatgttcaaaactcattcagcgaatcctgct
gctgatgcccttcctgcggacgaatttgttcatcaattgtgtcaagcattcgatggaccaaagtctcatcctccgattgaagaaactcactttgcatt
gaaggatcagatttcatttctgttgcatatcttagttgtgacagatcggcaccaaaaagacgccggcattgctctgttgccttttccagagcacttgc
atcctgttccgtcgatgaaaatgcacctcgtgtgagtttggcaagaccataaacaacctgccaatccatataagataaacgttcttgcattaaacg
gcttctctgttgttggcgaaacgagtcaatggcgtctaataaacactccaatttgcgttgaagcactgtagtaagtcttcgaagtgtcatcgcatttg
gatactcttctgaagaaggattcttattcatctgttgcagtttatgcgaggcaatgcgaacagctaaagctgccgtctcttggcgaaaacgactagc
tcctgttgtcagaaactctgattccaaaaaagtcgactccttcgtaggccaggccgtccagtatctgcttggtgttttggtatctttttcctcctcatga
ccatcatttccatttcgcgaatgtcctcgatttttagcgtctcgtgcacgaaaaatagctgagttgctaaatcatttcttgtttcttgcttcaagttcg
aaattatctctttgtacgctcctatttcactagtcaagagagttcgcttctctgggacctctgcttcacgctcagtaggagattcctcatattcctctct
ttcttctaaatacggcgtctcagaatcacgcatagcatactgtttatgagcctaaattctaagaaagcaactgtttctgtcaggttgtcgccttcgac
agtttcttttggtgaacgtacttggttagaaaagtgaaacacaagagaccatgtccaccaacctagtaccttcaaggaaataagcttttctttttca
tgcagtataatagacgtttaaaaatatgaacactcgccatttgcaacctcaaaaatcgaaattttccaatggaactgtgcaccttggatactgggc
aatttgttgatccattgagtcaattacaattattttgtttttgtatcctttacaactgcctttttgatgctatttccgtctacaattctctctcagaactttg
gctgaaatggacgaaaatagaaacaatgtcaatggagcagttgtcaaatggaaaattgattaattccatgcatccaaatgtactttggcgcatct
gcattctttgatttcacatactttttaccggctgaacgaaggaaagtgaaaagtagtatttcaaataccatcacgaaaggagcattaatattttcttt
taatatttcttttcctttatatcagtttctttttgctcctttttcagttcaatttgtcttcagagcaaaaatccacatgtttaggtacactctctccaattgc
aaacaactgcaattcatagacgcagtttacattgccctgtatttgtagatacaaaacaggttttcaaatttttttatttttaaattagaagtactggag
gttttatttattttttttggggattcgctctcagttataaaaggattcagccatatactttttttttgacattagacattatgagtgatgtcaaacagcttt
cttttacaccttcgtatgtaattcacgtggatcttgtgtctatttagcaggtttatcctctttgaagactaacgaactgatgtttaggattttcaagaaa
attgctcctgatcaatacttgtctcacttgctagatcaaaatatacgttccgacggaagaacggttactgaatttcgtgaaccgcaaattcatactg
actgcatttctacagctaacggaagtgctattgttcgagccggtgaaaacgttttcgtttgtggcatcaaagcagaaattgctgaacccacagccg
atgcacctgatcgcggttggattgtccctaatgtggaattaggtcccctctgtaattccaagtttaaaccgggcccgccatctgatttggcccaggtt
gtatcccagcaattgtaccaaattttgattcagtaagtaattgtcacaagaattctttcagccttttagacatttgctaacatgtactactttagaacg
aaactaattcatcttcattcactttgcattcacgaaaagaaagcagtttgggtcctctatgccgatatagtatgcttgaattatgatggaaatgctct
tgatcatgcctggacttctttaatgtcagcattagaaacaaccaaacttcctacggctgtttgggatgaagacttggaaaaggtagtctgtgcatcc
aatctcactcgaccggtccaactcagcacaagggtattttcattttcttggtctgttttcaacaatataatgttggcggatccaatcgatgaagagg
aatctttgtcaaacgaattccttaccataatgatggacacttctaaaaacatttcaaaaattacgaagttgggcggttcttttattcaagtcgataa
gttaaaggaatgcgtagaatttgctcgtaaacacataaagtagattttaatggactcttaagttgtctgcttaactaaaaacgtttaatgcctagct
gtatatttttgatccggttgtttagtgaaggtttctatttaattttgacagtttcaagtaaatgaaattttgtaacattaatttattatgtaaacccaaa
acaaaaccctctattttctcacttttaaccattctcttggttatcatcttctatccaaaaaatgtagtagcttttcgctacgtttttccaaaatacatatg
acagattgaatcctaaaacgaaaaataaaaaaaaaaaatctttaaacatagggttcttcatcattcttttctcctttattaccttcaccaacatatcct
ttcgcaattcctttctgcccttcatgaagcttacttggagctgtaccctcaggtacggggctcaaaccagcccatgaccgagattgaaggtacgcgg
cagcaggcgaatatacaccgttaactgtcagatcccggttagaacgcttttgaagggcactagacccatcttcgttcaaatcacctgttttattttca

ttgctgtccaaagaaagttctacatgcctcataggtgtcgaattaacaagcttacccgtaactagactaaaatgaggactttcactttcgtcgctttg
aatttgtttctcagtgttggtttcctgttcttctgttaatgaaagtacacgatcgaatgagagaatgcattcatttaaccaggaggtgtcagatgaaa
gcgctttcattaattcgaatggagtaacaattggacgataaaaatcctagattttgttagtacaaagaacaaaagattcatataaaaattcatacc
ctactatcaatcaagctgttttcaccacatgcgattaaaacgaaacactctatttcctgaaaattggccaactttgcaggattcagttttccgatagc
caatatatacggtttttaccagcatttagaatcatctttcgaagtctatctaaaacgtttagataattcgatacacccaaagtaccaacgataattc
cgataacacctgcatctcgacatttttgcaccaaagcataacgtctccgaagtttggcaccaccgaaagacgattcttcagtaaaagtctcctttac
aggatcgaaagaccagaaagctttaacaacagaataatgagacatcaaaatcgatgttaatgtgggtgagtcgggtccaacaaaaaccaaaga
aacatcacttaacgaaatattttctggtaaccgaaataggcgtcctggaatcgttgtacaggcttccaattctaaatctggttcttctcttctaacta
acgtcgaagcaatgactgttttgtaaccagcatttttcatcttccgaataatcaaatcttggcagtagtaccaacgagtatcactaacaagcaaaa
ttggactgtcaagagggaacttctccgtaaaactggtaatcatcttgtccacattaataggaagccttccaaatacataaagaaggggaagcctt
gatgtactaatcaaatgttagagaataagtttttaaatgtattaacatacggagaaagacatgctcttccatagtgtacaatggcatctgcagaca
tatgttctgcagctacttcatctacgcaacagcttccgtaatttgtatcagcaagtatttgaatatttgcatccacttgtttggatatcagcgaagcta
cacgaccagaatctgcaagatgctcatcgggaaactgtaaagctacctagatcacaagtgttgttagccagactgaatttacgaattatacccttta
aattcaaccaatgcttaaaagtagaaataacttacagatttatagttgttcgagcgaataaagtcaactgttctatcaacctcaaatacatgtgac
aaatcaccctcaacttttagtgattctccaattgtcttggttaatacactttcagagtcatcgcttaagggtacaggagttgaaagctggaaagaca
tactcctgacaatgatttttttatgtttgcccccaagtgtatgtttcggttgaataaattcagcgtcagaattcaacacatgaaagcaatcctaagca
gtcagatataaaaaattgcctgcttttagctagtctaattttattacttctatcttttaataataagaactaccatacctgacacctacaagaaatgc
ataggttgttatccaatttgaatatttacagcaatcaaaactaaagaagcagaataacaccaatgattatattattattaggtcttccactgtaaaa
gcatttaccatcagcttattacaccaacgaagtattaacaaaaatggaagacttcatatgtaatatataaacccctaataaatcaaactaaaaca
ttacacttaaagtaacaatggcgagaagagaattcaatttgcaaatagttaaaatgaaatccttcacaaaacagtaaaaaagacaaaaaccga
cgagcagtaccataaacgaacaatgaatcaagagacggaacgatattcaacagctttatattttgtaacaaaacatcacatgtaagtaagtagt
caagttcaatttgtttgctttccgacaaaaaccccaaaaaaaaacattataacaagaaaacgtacataaacaaccaaaattggcaatgactaaa
aagaaatgcaaagcaatagcagagtcttgattcttccttaaattcagctaagtagaatcttttaactctcccaaccagcaaagaaagcatcagaa
tgtttaaacagatagtaaaaaaaaaaaaaaccccgacttttctctttatctaataaactcgtttgcttaatttcacatctaaatttcaaaatcaaacc
gtttttatgcaacttttgttacacggataaaagatcggtatggcagcttccatatatcccgaatcaccgtaattttatacacagaattaaacaaaat
attagcttaataagcctgacattaattcatcccccttacttattagcttttgaagatcgttgaaactcaattttctgcccaggtaacggagggaaac
caagagcatgtgatttctcggaagccgatgaactagaggctgactgaggaggtgttatgtcagttgtggtgtcggctctagaaatgggcggtaaa
gttacttcaccaaaggaaggaaaagcggagggagattgaggccgactaattgtatgcgaagaaaaatcaactggagacaactgcggggaagt
agtggcatttgtatggaacggtgatgacggagttgtagcagcatgtcgtttgctgtaatatgggaaagcacggtgcttatcgcgagcttgagatct
cggacgcataggtaaagaagaatcatgatttgaaagattagcatgagaaagatctgtagaaggaaaactcatatgtgcttctgacacaggagat
gatacagttgaagcatgaatcgagggggcaagaagatcaagaggcataacctgggagagattgaagtcggagctaggatctgcaacactggc
aggagtagaattatcggaaagagcagaaaaaatggaatgattgtcggtttgggagattaaaggatgcaaaggtgcttgtgcttgaccttgagca
ctagtaacggcgggatctgtaaaatcaagatgaggcaagttttgtaaaccatcaatggcatttaagccaggtaaaatggcactttgctggcgttca
agttcgaattgtcgcatagcggcatcatgggcttcattgtgttcttgaagagcagataactcatgttctggttgaggaatttgagggttagggacta
cgtcgtttaaggcaccaagattcaaatcggtttttttgtttggatcaaaatccatttgggtcaagagattatggataaggttgggatctttggccaga
gcactgagagcgttattgatagccgatccttgaagaacagcgttagcagaagagtttgtagccaacaaatctaaatcgggcaaaagtccttgag
aattcgaacgggatacgccattgctcgtattgttttgagcagaggtacgctccttttctttacgttttgaatggcgacgattgaattcagcctcaatc
cagttcgttttctcgatactcggttcgctaccaaaaagtttttggcctttttgttcacacgacaacgcaaatcgttgtctttattacgctcctcattttg
ttccctccaacgcttcttccgttcccgattttcagagcgcgttttctcagaggaagtcatcttcctcggaggaggaggatgctcaatggcagctacct
gtttgtaaataggtcagaatcatcaaacaaatgcaaattataaaccccagcaaccaagcggctgcaaggttagtgaacccaaacaaaagataa
agtccccgtatcattgcttttgtttaagcttgcttgcaagaagacatgtgttttaacaaactatagtatacgatgtatagcgaacacaaaagaggaa
ttcgagactataaccgtcataacgaaagcaaacttctcaatttccgggtcggtaaaaaatttagcatacctgttgagctatgtaaaatgcagctgc
agacggatcatttggatcaaaggataaggcagaattgttattattacttaaagatcctaagttggataagtccggaatggctgagagaccgagtg
aatccaacagctcctgggtgttcccagacattcctaaaaatgcctccaggttcagctttgtcaatccgtcttcagtcattctgggtttctccacgcat
atatatgtttcctcagtgatctagcggactgaaaacggttccaaattgattctcgttgtttgtgtagttaagaaagcgaaaccacctccaccagtca
ggtggctaaacaatccctaggattatccgcggttcgtttccactttaccgaataccgttttcgttcttttgttcgctaactatttcttattcgaaataac
gatatattccatctacattaagtatgcattacatttccgaacgaagttcgttgtcatatgaagcgctgcataattttctcgttcgactaccattgggtt
tcgtttctaaagtacagtttttgaaggaaagcccaagtatactgtgtataaagaacgtagtcattatcgtaggtcattacatggtagtgctcgaaat

gattacccagtttctacttcaagtcatattgctttacgaggagaattcaagcagacacatatgatcaatgcatgtaataaggatggttgttgattatt
cccgtttatgaccgaatgtagaggaggcgcttttgagtttcatcgtctcattagattgtccctacttcgagttaggttgttacatgtggcatgattata
ctattttctctcttataaacatttccaaaaaaaaaccaatttactatttatactcgatgaaagcaacgtcacaaagcagtctcctttgtgactgattct
tgatagtgtgtgcctctttcttagcttagagtagtgacttggcttccatttacgacgtttgagaatagcgtagaaaaaagattcaagttaaagatac
attacgaaccttaccgctattcttagagacgtttacgcagattcgtggacctcagaatactagattttcgctaagcttgttttatttgttaaattattat
aacgtctatcttagattacgatagtacgagaaaactccaatgtaataatgatgccaatgctaaatagattggacacacaccaatatatctttaatt
gtaataatcattatagaatccagggaaagaaaacttggcataatttatgaaacatgagaacgaaggttagagagtctgtcgaaaaacgatgag
aatttttttacgctttttctcttccattttttttgttttttttgtttttttgggggtttccatctattaagtagtagtagtgctgttgaaaaatagattt
tcttgaaatcaactcatatctctttcatttgatcctacttatcttgaccattcgtttaacaaattataacgcgagtagctctaatgatctaggtacatg
attaataaattgttgttttcaaagatattggacttactttccgtacaaggcagcgatcgagtcttgcttaccagaccaagctatattgtttacttcgtt
ttcgagtttccaagaagcaatgggagaataacggttttcgctggatgaacccgtgctttgagcggttgacatgggcgtagcatactctgatattggt
atcgctgagccagacggcgtaacaggattcgatcctcggcgagctggtgttggtgtagagctcaaaggacgtgacaagtcccataaatgaacca
tgcaatcatctccgcatgaagctagcttgcttcgcgaattaggcatccagctcacagcattcacgtctccacctcgaggactacttagagttgagta
tgctgttcctggcactcgaatatcaatcatttttatatccgatgatttgtgatggaaggtagccatcaaattggcatccgcatcacatgtggctaacc
gcagtaaaggtctacaagtactaacagcgctgtttgtatctccagtacgtttccaataactgctatctccttcatagataatagttgaatggtccag
agaactatgcatatgtcagaacacttggtcaatacaaatcaattttaaacgtaccgtaaatcgaacattcgtacacttccatcagcccccacgctt
acgaatacatgggcattatcttttgtaaacgcaacatcgaagacttctctgtcatgagcaatcaattgggttttcgattgttgagcaaccacgtccc
aaacagtacacgttgtgtctaaactactgacgataattaaagacgggtctaccttgcaccaatcaaaagaagtgaggggagcactactgtgagtt
ttcccgtgctgaacttatgttagtcaaggaccaaaaaatgtgtcgtcatacgtaccgtggataaagcagcttggcatgatagagaggcagtatttc
ctgattctgtaggagcattccaaagtcttaattttgatctgttgatgctaaaaggttcgtagaattgtttgagtttaatgatggagggttccacatca
ctttggtgacaggatactctaaatcgtttgctgttgaaagcgactttttcacagcaggtttatctccttccgaattcgatacttgaagaatttgaatct
gatagttagtagattggaatcataaagtgttgatgtttattacaatctttcaagcgacatactttgttggcgggctcttcaattaaactccctacagc
aatccattgtcttgtatcattgctttgttttaaccaatctaaactgtaaggtgtccaaggaaaatcaaatcccgaccgttcgttttcgctattgaattt
gctggggaaaggccgcctaggagagcttaaaaaccgtccaaaagaatcataagtagattttcctactcctgcagaaatagaatagcggtttcttc
gtaaatccgtcatggttttatagtcgatcccaaatcctggtacctaaggaggatatgatgcgtaggtatcaactgcacccacttttcaacttgaatt
aaacgaacaattaaaatacagaagatatgcaacaagaaggctggagacaaacgtaaatacctaaccttttcgacaagcagcctttcagtcgcg
gcgacagaaggtgtcatcatagtcctttaattatacttacaccaacaaaccgcagtagcacagcacagcacagcaaagcctcaaacatttcaaa
gtattatctttagtagaatataaaacgattgatattatcatactaaatagagaaggatatgacctgtttcaactaaagtcctctgtataattctataa
agtgaatttgttttcaagaatttatgacttctttgcgtgaattaaatggaactaaatggatgcaagtgtgaagatccttttctttactatttattatatt
tttttaatgttagcatgaattttcgacgaagttcaattgtagcgaaaattaatattacgtcgtactgtgcttcatcaatcctaaacgattaggcaattc
aagtactcttttgtacagtatctttcagtaaaatatactaaatgataattctatttcctattagatacaaggacagatgcttcgtaacaggtaccttttt
gttctattgtaaagtacactgtggaaaatgaaactaaaggattccaaatgaattgtataccaccattacaataataagtaaacacttgatatatgg
atttgaaaaacgctgtaaataagatatagattgagttactatataagacttgtcaagatgaaaaagagtagttagacaggaaaaaagcattgga
attgatttccttttaatagaatgaattgcttcatacattcggctgaccaaaattatcgttttaataacattttattacagaaagattccattcattatct
acgacataatacgatattgctgcttgagtactcgatactgttatataaccttaggaatcatcgaaggcaaaagaataatccctacccttactaaaa
tcaaaaccttgcccaaacgcacggacggacctaaacaattatccaacaacgaaataccgcctgctgttgcatttcctcctatattgcaagacagg
atatttttttatttaaaatgactacggtgtatccagaaagcacctctatcgttacatcagcaggtatgttggttgattatttaatgaatgtaaaaaaa
gactaacgattattaggtggtttgatgtccctgctggacgaatcagaaaaagaattacagatacacgcactgttaaagatatatgaatttatagat
cagttatggcctgagatttctgacgatgttacaaaaattgaagttatgtatgaagatcgctcttttcctgaacgggaattggcagcgttagtggtttc
aagggtctattattatttgggtgaatacgatgaagccctccaatttgctttggcttccggtacaaagtttttacaagacaaaaacagcagcttcaaa
gaaaccctaattttcaagtgtattgacacttttattcaacaaagctctgagcgctattccaattatgaacttgagcctatggatgaacgtttgagtgg
agtcgttgagggaatttttcaaaaatgctatgccaacaaggaatggcgacatgttttaggtattgcaatcgaagctcatcgtcttgatttagttgaa
tacattttgaatactgctaaagatgatgatttaaaaccttatgtccttgagttggtgttgactgtcgttcttgatattgaatttcgcaatcgtcttttgc
gtcttcttttgagctcctttttagtagagaatgagccagattatttctctgttggaaagtgcgtagttcacttgaatgacgcttccgttgctgcgaagc
ttttgatgaaattatcttctcaaagtgatgacaagaatttattgactgcctaccaattagcttttgatttagaggattctgctcctcaagaatttttaa
acagtgtcatagacctcttacctgctcctgtctcatcagatcatgaaggcaactccgagaacgtagaggtcgatgaatcttcaccaatctcccgga
tccattatatcctgtctggtgagcaaactgttaaatatcatcgggaattcttatattctcataacaatacggatatgctcattttaaataagacaaag
gattctcttgaatccagaaactcaattttccataatgccgttacttttgcaaatgccttcatgaattttggtacctcttatgataactttttccgcgaaa

atctttcttggttgtctaaagcgaacaactggtccaaatttacagccactgctgcccttggtgttattcatagaggttactacaaccaagctatgaat
attgtacgcccttatttacccgaagaagatgctccgtctagttccgtttattctgagggtggtgcttattatgcgatgggtctcatcaatgccaatcac
ggccgcggtgttacagattatttgcgtgagcaactgaagcgttccgaagatgaaacggtccaatatggtctcatccttggtttaggtcttactggta
tggctagccgtgacgaaggtctttttgatgccgttaaaaccgtgctttttaacgacaatgccgttgctggttctgctgctggtatttccatgggtctta
tcatgctcggtactgcatcatctactgctatcgatgaaatgttgcaatatgctcatgagacacaacacgagaagattatccgtggtttgggtattgg
cattgctttaattgtttatggtcgccaacaagaagctgacagtataattgaacaactaaatgctgactccgatcctgtacttcgttatggtggtatgt
ttgccactgctttagcttactgtggtactggtaatagcaagattattcgcaagcttcttcatgtttctgttagtgatgttaacgatgatgtgcgacggg
ccgctgtttgtgcattaggttttatctgctttaaagactctactgttttaatatctacagttgagctccttgttgatagctataatcctcatgttagatat
ggtagtgcaattgctctcggtattgcttgtgctaactctggatctaatgccgctttggaattgcttggccgattagttgaggatgctactgactttgtt
cgccaaggtgctatgattgctcaagcaatgattcttactcaacataatgaacatttaaatactaaggtttcaggaattcgtaagcattttgagcaag
ttatcggtgagaaacacgaagatgcattggccaagttgggagctaccttagcccaaggtattatcgacgctggtggtcgtaatgttactattgccct
tcagactgctaccggtagtttgaggctttctaccgttgtaggtttggcagttttcttacagtattggtactggttccctttaactcattttatgtcacttt
cattttctccgactgcacttattggcttgaaccaaaacttggatggccctaagtttaattttgtctctaatgctaagcctaaattatttgcttatcctgc
taagactgtccaacctgctgctaaagctgttcaaaaggtcgaaacagctgtactgtcaactacggtcaaggcaaaggcaagagcaaagcgcgct
gagcgtgaaaaacatgctaaggagactgaagacgatatgaaagttgacaagaaacccgatgaacgagaggcagacagcagtgctatggatat
tgatgaagaaaaacctgatgacgctaccaagtctgaatcaactagaaaagaagaacctaagcgtgaagttttagaaaactttactcgtgtggttc
ctgctcaattaccgtacatttcatttaatgctaatggaagatactatccagttcgtaaatttactggtggcgtgttgatgttgattgatcgtgaatctg
acaaagccccagatttgattgagttgagtagggatgctccaaccccacaggctaatgctgaaccttcagagcaagaggcggcaccaccagagg
acttcgaatacccttttgatgatgatgagtagtttaaatctatcatatacaggtgaaaaaaaaaaagtaaatattagaaacggcttgctacgattt
attctagatgattattatcaatatatacaatatatataagcttgtaatataatcaatttgtaggttatgagaacgtaaattgttaacagtgtggccac
taaaggtggaagttaaaagtaagattaaacgaacggacggttgtaaaagcagtagttgctattccatatccccttttttagtttcattaaatgctta
ggtttttttacatcaggcctttactgatgttctgtgctgagctgataatattgcaaagggtggtgaatgctttcttctaaagtacccttctgaggatga
gttaactcaaagacacgtgtacatgcgatatgataatgtcgaccttttacagcatcgactacttcccggcaaccagacgaatctggtgctataccc
attgtacttaaagccgtgacaagattatcgagtgagtaagttctatagggacaaccatgagcatcgccaggtcccaattggggtcctgtcaaaatt
tgctgacagctataacccttgtaatttttttctgttaccctctaaaccataagaatggcgaatattgtaacgatactccttattgaatttgtcatcgtta
gtattagagaagctacttttccagaaaatcagagcctcctcaacagataggccaatgtcctgtaagttagtaaaggaaattgaaaataatatacc
tttaagaaaaggccatactgaagacggctaaaatggcggaggtgtttttctttcctcaaaacattgtgaagatgcctggcacacaagggaaaatg
agggacaagactatcaacctggtctgctgtaacttcaccaggtttagctgcagcgacagatagctctggtgctacaaatcctttactgagatgattt
aaaataggcaaaagacgatcatcttcatctaaccgcggtaaagcttttgctgtattttcaagagatgattttagatttttctcaaattcctctatcac
gatacttatctgttctgaaaaaggtacatatgcaaaacctttgtgaacaaagacagctcttcgttccaccaagtcaggtacttttataaagggtact
ttaaaaaaagattcgttttccacgtttcgttggcacgcggcttggattttactcaaaaactggcttttctcttctttagaaactgcattccattgaaaa
ttaaggctgtcaacaaaatattggcgctctcttgtttcttcattgataaatctatatttaaagagagcagattcagcacgaagaaatcgatatctta
attcctctctattgtcaaaaaacatgttagtatatctgtaatgtacatccaccaaaagaggttcaagttaaaaataaaagagagcgcgtccttttc
cgtcaaaaacccgaacgattagcatttgagcgagcatcttaactcgatacaaataatcgaaaatgttccggctgtaaacttctcaaaaggcaccc
ttcacagatatgaactttgtcttaaatctctctttagtcatataatgtttttttacttacgatctactgaatgcaagtcgcaaaatgaaatgagaataat
ggtccttcctacgttcttcgtctaggcccgctcctttgacgactctagccatattactgctcatgggcaagtgtttatcaacgacgcgtttaataatca
tatccagttcctttttcgtcttattccggaaaagggctgactcaatttcgcctaatactattatccagaaggattagaagccgtttaatttatttgata
aataaaaagtagatataaaagaatatatagcacactgaacttaccataaagtcgatcaattgcccatgtttcaaactcttctatactgatttcctca
tggggtggattcaagtaaaaatttaatcgtgtagggtaactaacgtttgctggtacatctcttgcaaaatttctgtttctttctaccgccctgctttttg
tgcttcgaaacatggtagcctgactggtaaattttcggacttgcgattggggagcccacgcgtcgagtcggccctcacaatattaaattcaggttgc
aaataatggcttaaaatcattcaaatcagaaacttcaaaattaaacctttttctttcatgctgactcactgaatttaatgtaaaaagtatctgaatac
atttaatctcagtcattgacattgacattcacaattacattacgttgttctacgaacgctttttcctagctgatataagtatgtattaaatctgaaaat
gatttatttattagagtatcagtgattcagttcaaacctaataacaaacagaaaatgcattctgcattttaatatttcaaaattccttctccgctcgg
agacaatttattaagccctacatttttatgcaaccctaatgacagtagtaatctactgctgttgttggcgtttgctaggaggaacgtaggctcctcct
ttaggaggtgtcacggattcacgagaagtgtcagcactggcaccggcaccgcgagcacgaggaggaacgtatctttgaccaccaccagcggtag
caccaccacgtttccatgcagttacgggtggaggactttgagaagctaagcccaagttagctttcttggcagcaatcttccgagcagcgtcttcttc
acgcttcatttgcatcactcgttgacgttcaagtttctcgctcatttcctgttgttcacgctccttggcttctttagaagcacgagcgttttcttctacgg
ccttatgttgactttcttcttgttctctgcgctcacgcgtttcttcttcttcagcggctttagtagcttcttcttcagcctcagcaattgcatcagctaact

tctgttcgtaaatcatctcgtattgtttttcacgttcagcttctataacatccttagcacgagcataagcttcttgataggctttttcacgttcatcatca
atgctttgtttgtaatcgtgaatatatgaagcaaacttagaaaacagtttcttgtcttccaaagcttcttcatgcttgcgttcttggatctctctacgct
gcttctctctttcaatgaaggttttacgatcttgttcgacttgtttccttgcatcctcctgcaacaggggaatagcttcacgacgataggcacgttcca
aatgatcaatacgcttaccgataacacgaagtcgctcattcatgcttctattttgtttctcgacctgttcgatttgcatagctctcaatttatcagcatc
caagtgttccaactcttcagcatcgacttccaatccacccttggccttaagttcgtcagccagtttcttagcttcttcaatacgaatagcttccttttca
cggcgaattctgtcagcattacgtttcataacttcatcttgaacacggcgagattcagcctcggcttcttgtttttgcttaagagcacgttgagcggc
gagttcagcttcacgctgagtagccaaggtatcagcttgaccgcgacgtaactcaagcaagctacgctgagcgataacagcctttctctcttgctc
aacaccagctaaagcacggtcataagcagcttcagattgttgcttacgaagaaggcaaaagtcagtatcaaaacgcatcaagacactagtaag
cgatttcgcaatacgagaaagttgatttgtaatcagttctgcaggggtggattgtaaagatgtagtgtccacctgagagcttactggactagtgaa
aagatcagatgcaaaagtaattgagctttcgatgtgattgacacgaacggaaaaagcacccttttttgttgccgttcattataaatttttcaatctga
ccagaggtgcaagagtaaggttcatcaaaagtagccaaatccataacgaatttaagggaaacagcatcataaacttgggagagttgttggaaa
agacgagtaaggataacttgttgtaagggacgaatgtattgagcggtatcagtgttctcagccaatttcttcaaaataggctgcaacttcttgcag
atacttaaagggtggaaatcgacttccaaaattctgtaaagatcgcggatagcgggatcacagtagttcaaaacattacggttaacagcatcctt
aaccaaagcctcacgattgggggctttagacatgttgagcaaaatggccaaacgagcgtttttgtttttagcatcttcggcgtccaggggaggacc
tgaaaatctagtggcatcaataataggaattgacaaagcactgaggataacaaagttagcagtagcaggcttttgaacatttgtaaaggtaaag
tatctgttccaagcagcagcatgcaacaagtagttgtcgcaaaccaagaaaatcttgataagtttgcggtaataatttccgagcatgacaggga
aggggcacgcttggagaatgttaaaagactgtgaatatcctcaattgagcggaaagcctcctgccaaagctccaactcgacagctacgttgagtt
gagagaaacgcatgtctaaatgacgttgcatggtctcagcatcgttcaaattgattgaatgaggcgcattagagaatttggaggcatttccaaggt
gagaacgcaataattcacaaagacgacggaattcagtcttgcgttgatactttaagcagaattggaaggccgagttggcaattagttgatacatg
acttccaagcgagcattattccgcaaaatgtcaaggacagtacgataagcatcccaaaggaacttgagccagggagtgaccaaagcacggtcc
gtacgggtcttggacatttcacctgagacgaaagacatcatgatgctttcgggagtctcagtagcctccaaatcatcgacatattcaagagaaatc
ttgtcagctttagcttgagcttcttgaaccttttcgttggccaactcaatgaagcgcttcacgacattttcaatggcggtcaccgaggtgttttgtaca
gcattcttgtacatgtagagaccctctttggcgactttacctttacgtaaaaagacacaaagttcaataaagcgaatcatgataggttccaaagaa
aagccttgggcatttctggaccttttcaagacgatggtctcgtgaagggactggagtgcagacccatgctgatcaagagcaataagctcatcggc
aagctactgatgttagtctttcaataaaatttacgtgaaaaaacgtactcttagcacattttcgggctttccctgaggaggtgccatgttgtgcggtt
gtgaactaaacttgctctggtaaatttttttgtaaggaaatttgtgcgttggagcgcataggagctgaacgaacctcgatgcagtaatataacttctc
attatataatcgtatgaaataacacatttgtatcacttaacgttagtatgatctctgtacaaatccaagcgagcttcctaaacaacagataaggga
gtacaccgagatatagggatggacttaataccaggtcatagggaagaaaagtcctgaaaatatgctttgaaaggactcttaaacaggctgagct
tttcatgcaagcgataccaaattcactggatggtccaagtttactaaggccggaggatcttgctttccaaccacgggaggagatttcatatcgagt
gttttatcccgatttacgcactgatgagccgctagaagtcatcgaaaatacatatccagaactttccggaattgattcctctctttttcttaccagcc
gacgtcttcgtatgtggtcaagacgcttccggaagcaaaagttgaaaacatcaaagtagtagattatcataccgacacattgcctccaaagatca
aggaatgtggttacaatactactacgaaatgcaacttaccagcagatcgcttccaaaggtttataggtatgtttctcctggtactatgagcgccttc
tatcctgtgtactctttccttgcaaaaatgttattaaccttgtaatttatagctaaagatcctcataatgataaccaatttccggtttcttatgacctgg
atgagctagatgcaatgtggttgatttactacaatgaagcgcgagcctcctcagatcctgcttggatacctctatcccacgaatttcttgaaattatg
cttaccattatcgctcgacattggctcttgctagatgcatggattcccaagattgaacctgttcgtatagaagatgagctcgatggccgttgtgttat
ctgtaatgagtcagattgtgaaacaagtaatgtcattgttttctgtgataattgcaacatggctgttcaccaaaattgctatggcattccctttgtgcc
tgagggtcagtggttttgtaagcggtgtttacttgctgccaacgatgtcatatcctgtgtttgttgccctgatcgtgatggtgctttccgcacaactgt
cgatggccgttggtgtcatactgtttgtgcaatggcagttcctgagatctcttttcatgatgctggtcgcttggatttggttcgggatttttatgctattc
ctaaatctcgctggaagctcatatgctgtgtttgcaaactacgctggggtgcttgtgtacaatgttctgagaaaagctgttacacagcatttcatatt
acctgcgccaggcgtgctggattttattacaaaattcatcctccttttagtaattatagtcaaacagaggtagaatgtttctgcgataaacatacgc
ctccttctcatctcgaaggtgcccttcaacgactttatccacttgctgttcagtattataggagaatggcttatgatgtccctttaaactatgttgcttg
tcgggcaccttctgctgttccagaagaaccttggaactttcgaccattacctcgctacattgtagaaaaagtaacgtccacactcaaagagttgcg
gttgaatgacaataccaatgacttggatgttattattattgacatttgcaagtactatcaaatgaagcgcaagtttcgacgagacgcaccattgtta
cggtctcagagacttgcagatgaactggtaaatcttccagtgaatgccatcaaacgacgctatcactttctagacattgcaaagcagcttcgtgga
caatatgaagacctcttatcccttgttgaattgactgctcgtcgacagcgaataaaaagtgaattatcccagttaagaaaacaatttttgactacta
tttattttcctttacaaagactcatagaagacagctttgaaagaatttgccagttggacacaaaaggtatctttaattctcccttggagcacgggtg
gattggttggatttccttgaaacaactggttgtaagttacgatattgatgatttatttactttggaacaatacttgtctcatatttggaacatagatgg
acttattcaaagtatagatgacatggagcaacttaccgctgctgtgcaactggctcagcagtttgagccgaaagccaagaaaatacttgaagatg

ctcaaagcagctctgaatcattcaagaaagatgaccgtggaaacttatctttcccgtcactaaagttggaaggactcgaatgcgttggttggccgg
actgttttgttgatactgtggagcaagcaaagctggaaactccttccgaagaagctttccatatgctggagagttttgttataaatctcagtctaga
gggaaaatacggtcgttttgcgttgtccgatttttcctcaagtatacaaaatgaacaattagatggtgtaaaagcacctgaagctggcccaaggga
agtgttaaaagaaattaacaccaagaaccaaaattcagataccgagaaggaagaattcgaggctaagaatttcgatggctccgacagtgttccg
aacttagcgagacgaacgcgttcaatgaccaaaaataatgaagcggatgaaccataggtccctcgttgaccggttctcttgttaaaccgtttttaa
gggatgttaataatgtttaaatgctttctttttgcgtaacaatagttttgttattatttggaaataccttactttctttctttctatggaccactgacttg
taatttttgtaatgagttgattgattataaatacaacataaatatattattttaattttaatttttattcgaattgttattcaaccactaaaatttctag
taagtaagtcttagcactttataataaatcttatccttaattgttatgaatttagagatgtcttggtgagcgccattcatagaccaacaaatttgattg
gaatttatcaagttgtttctactttaagtcctttttttcattcttctttttctatttttatcctttttgacaccagattttcatccagcaaacgcatttatactt
agaaaggattcgctatattgattcatagtttaaccataatgacaacctcaattcttcaaagatttagtggattacttgaacctacgaacttatatata
gtttctgtagtagcgtattcgtcgagcaatcggccagaaagtattgggggagtcgcaagagaggccgttgaacatataggcccttccatgtttaca
aaattgcgcgaagccttggtcaagtcagctcctttggttggttttcctagagtaagtttgcattggagaaattgcatgcaagtgtaaagatgttgaa
tgaccttttacattaactatactaactggaatcccaagaccatcaactctttgcgcgtaatggctcagtgtttcccaaacgcatttccaaatgaattt
agtcgatcttcagatgaatatgttaacacttctgagcgaggtaaaagatattttgaaaagacgtatggcaaggtaacagaacgagttctcaattc
aatgcagtcttcttcttcagacttgggtaagtaagctcttgtgtaagagggtttatagtaggttatcaacttctaacgtctttcggttctgcagctcat
attgctatcgactatgcatacggaaaagtactctcctttaacgaaattctctctcctttagaaaccagtttaatgatcatcgcttctctcgtcccgcaa
gatgtgaaccccagctccgaggccacttaaagggtgctctaaatcacggtgctacaaaggagcaggtaatgggcgcacgaaacgtctctatag
cgattgcaaaagaatgtaaaatcatttttcgtggtgaggtagaatcactttagatttcgcactaaactttctatcagtgttccttatgtaacggacgc
agatgaatattcttggtaatgatggttattctcattttacattcctgaataacaaattacagagttaccatcttttccactactaagaaaacaagaaa
aaggcctacatgtgaagcggttaatttcaataagtgatcaatacgatgacttttggaaacagaatcatggcatatttcttcactaaaccctagaag
actgaaacaagaggacaacacatgtggtcagtgaacttactttacatcttacgatttaaaaggcttttacgaatggtacatggattcatgtcacag
accttgcactatgatgaagtctaactaacaagagttgtaaagaacttagtatctactttcgactacaaaacaaattaactctatttcattcattaaa
ttaaaatagacaatcctttttcatgcttcatttctttggcagcgtatatggcttttcggtgaacttcggtgactgaatgtcaacccaccgaagaattt
gaccgatgtctgacaaactatggatagtatcatatcacacttgtaaacaacattgtggccgacactcccaaaatctctgtgaaaatgaagttcata
ctttatgtattggtaagttaatactagaagactgtttcttacaaacaccaggcttccattttgttgtttgggttgtctgttttgttaagtctatacagca
gcgccaacgtgtatgctgcgacagtaatgatttcgcaaagtccgatccatatcacaattgggcttaatgtgtgcttgtgtctgttctttgcgattgcc
aatgtattaaaggcactcctgtttggatccctacagacgtttgaattagaattgctgtatgaacaattttggataacactaacagaagccatgcttt
ctgttaccaccttactcgatcctatttctatttcctttttttgctgttgagcacgctcatgtttgctcgagtttttcattcaatatgcgcatttcggaccg
aaagacaacaggtgcagctgacagaacgtcaattccatctctttaatcgactcacatgtgcttttctcgttttgtctcttttagacatcactctcgttt
atttgtgtagcagcactgattacttcggcgatatgtcgacgcgaatactctttatctgtgagttttccgtattattcattaatttaactgtggaagccg
taaagcttgggctttatgtttatgaaactcgtcatccagagcaggtatggcaagaaaaaagcgtctatctttttcgattagaggtcgttcgagattc
tttacgccttcttgcttacagtaccctccttgtttttcaacatacctatgtgagcttccctgtttatgcaattcgacaactgtatattagcttctattcgct
gttccgtcgccttcgtgaacatgcccgctatcgacaagcaacccgtgatatggaagttatgtatcccaccgcaactgaagaacaactttccaattct
gatcgtacgtgtaccatttgcagagaagaaatgttccatcccgattttcctcctgaaaatattgaagaaatggagcctattcctagaggtttggata
tgactcccaagcgtttaccatgtggtcatattctgcatcttcactgtcttcgaaattggctacaaagacaacagacctgccccatttgtcgaagacct
gtttttggagaacatgcaccttcacctgcagctagtaccccaagtcctttaccagttcctactcctaccgctgccgaaggtacaaaccagaacaca
ccacgatcattttcacctatgccaggcccagggaactctggctttcaatctacacccacaagaatattgggaactcccgttacttctttgggtcaaa
ctccaactattcatggtagaattcctttacctaatggatggagtatgctccctatacccggtgtgcatagatttcccgctaatggccccacaaatag
ggctggtgatatgactgctgaaggacagttgacggcggatccaaacttatccggaataataccatctggatggcgcttggtgtctagaaataacc
atccggttgtttctcctttacaacaagcagtacctggtttgggaatctcttccgtaccgttatcaaatgcagacttccaaatgcaatctcccttacag
gtcgcacccatcgaaaggcaacaattttcttcgccagaaacagactccattcaaagcttacatcgaaaaatagatgaaatgcgtagtatgaccga
tcgcttacattatgacatgcaagaacttcgttcgtcagtaagaaatcttggagtactgataaatgaaaatgctgttgcaaacggatttatggaactt
gatcaacaaagtaggaataatcccttatcttattctcgagtaggagaacaatttagtcagcggccggctgtctcgtcgagttcaacgcacgcaaca
ccgactcatagaaacccaagaaatgtacaattagaaacactagatgaagattcatgaagttttgtgataatgaaaaattgacggatttagaaaa
cttgaaatctaaatgtttatgtaatcttaaatgaatgatcaatatacgcagaaatttacccaatttacttcatgaccgtttaaagtcagaacaaaat
tactattagcatattgaagttataaaataaataaatcattaaattagtcgttttctattatcgtaacgttttatttagcatacgtattcttttcatgattg
taagtagtacaggagttagaatcaaccttttaggcaagttcagaaggagtctcaaatggatttttttgatgacgaagtatagggtttttcgtgagca
aggtcttgctttaggttctcaggctgcgtctggttatagtgaagttcgtgatggtgagaatgatagtgatgcttcccactttcatttttttaagaacggc

gaagaatcatcttggggcttgactagctcattggcaacaggatcactaggaacggaagagggagctagcttggcgtcttcaaattcatcaaattc
ctttaaggacgggggtctgttcctgatagaagggagttgttgttgtatatcaaaaaacacctcatccgttggaaatttgtcatcatcgattgagttg
acagttttgccatattgctgtacaatagattccagacacgtactcaatctatcacgaatgacagagaggtcaggttcaaactcttcgagttcccata
aagattgtgtaggcataggagactttaataaacgtcgcacaagttctaacgtgaaagcagcgatatgaacaccacaatcataaccatttcgctgt
tgttgtacaggcatagactggattgtaaaatctaaattgttaataactaaataaaagcgacaaaattaaattcataccttttggaaaagttttgag
atccgatcaagcgcactacggcagtcctttgttttgccattgctcatagaatcataataaaaacattgtgaatctggtacactgacaaccataagc
gaccaatgagtgccgcctgtcccagtagcatatttgtctatgtcattgatgggcataaagatgtatttgcaggataaaaggtcctgagggagagca
gatttaagctcttctaatgatgcaggagcctcagcaagcagaaagacaagggaaggtctcaatagcagaatccctttcgactgagttgggcgtaa
gggaaaccatagtcgctcaactaatgtatcgacataatcgagtgtcacgtctgtaaaccagttaggttttcgtaggctttctatgtcttcttgtttga
agcagatatcaaacaattccacgattgtatcattttgagtcatttttgtgcgagaaggacaagaatggcaaagaaaagtataaactgtgtaaggg
aatcactaatgtttttttctctgaacggtagaaaactcttcaaatccaagatgaaaatgggcaaggaatacaaactcaaaaatcaaccgacaaaa
tagaaataaatgaaaacttatcagaacctggaatctctggaaaaatacacacacacacacacacacacacacagacttgcaaaatatgctagg
aacgaggcgaatatcgaaggagtatggaatttttctctcaatcaggcctcaagtatattaaataaagaaaaagaaatagtgcgtatatatagaa
acactaagaaaatacaaagaatcgctggaataaacaagcaagggaatatggcgttaaggaaaaatgaaaagttccctgatgacttcagtatttc
taaagaataagaaactagagtgtggtgtgttatcctacaaatggcattcgaaggaatagcgtaattcataaataacgatatcagcttctagtaca
gtaaaaaattcttataacataaagggctagctctgaagaaagagagcggttgaagggggaagaaagtaaggaatatgtaacggaaaacaaaa
aacagagcttaaatcataggacaacatttttgaatcttccaaacaactatacagatcggccatgacaggagaggacagaacaacaaaaagaca
aatgtacaatgggcaggtagaaagaaggaaaacgaaaggtgaaaaggtatatgaaagcgaacaaagttatagaaacgatacaaacaacaac
accaaaaacaatgaattcttttcatggtgatagaaagtttgggaaccgtgacaaaaaatgtttaacctattgaatgggatgacaacagtttatgat
ccacagtaggtcgacgaggagattctgagtatatgggagttgtttcacgttgctgcatttgttgttgtggtgcgttggaagattgagtctggtccgtg
cccttgtatccgggagatgctggaaacgttgatgaaggaggatgtgtcgaggagtctgtctgtgaaggagaaccgacataatgaggactagggg
gagaggggacaggatacacgggattgcctgtggaaggcgacaatgaggatactggggagggataggtacggtttgagtaccagtagaccaac
aaatctgcataggattgaagatcttcaagagtaataaacaagttaaactcgttaaaaaggaaaaaacaaagctccaaatgattcatttcctgcac
ggggattccaccgactttggagtaccggatgttggtataaaagacatcgctaaaaaatttgctgccagcagtgaagccagcgattaaaaaccgat
gaacattgaaactattaatcaaaaaagagaaattaaaagtccgtacaatacggtcaagataaattaggacacttaaaaatacatcattcgttgc
cgggcagtattttaggatgcgttgcaaataagcatgaatcgaaatcgatggaacatttttagctgaaaataccaagcttggattctttaaacttgtc
gctgaaaaaggtacttcattagggtcttttccttcctgtcgtttgtcattcaaacgcgttaaccgcaaaagaaacgtcgacaatatttccagtaaat
actcttggttaaacgtcgatatgtctacatgttcctttatatttgataaatcttgttcagatgttaaaagcgtacatgccagtgtcatagagctgagct
aatagtaatagttggcgaaggacaagaatgtttttttcgtgaaaacacacatgacgcaaaagtgccaaggacaccactagcgtcctataaggatt
cgctggactctccataatcagtaaggaagcggaacacggctcgttaattctgtaaacagacgaagaatgggtgtaaacaaaggaacatttcaaa
gcaaaaaaactagtggagtagtgcgaaaagatgtagaggtcttaaaagcccatttcaaatgaaagaagccttaaattaaagaaatttggttttc
gaaccggtttgactggtggcgcacactggctgtagtcgctgaagagaaaacaaaccaaatgcattttcgaatctcattgctttttcttcaccagttt
cagtaccaagtttgcgaaaatgttgtttgttgatgtaatcgtgtaatcttatgcatgcacttgggtttgtctggttttcaatgtatgtaaccgtaggaa
aataaacaaactgcttaagcgatgctaaaatcagcggatgcgaaatgactgacttttggtcgaattgtttcatttgtttgcaaaagataagcgctc
gtttttatagcttgtaatttattaaaatccgtccatatcttgttattacttgcgttggcctcgaagaaccttagcaacaagtcgatcctaccatcatca
tagctccatacattgccgatgcataagaaaggcttcatcggttgtctatttgggatcacaaatcatttgaaaaaaaaaagggtttccaatttggttt
aaaacaaactataccccgtgccctaccagtttggtttatttacactccataggaacactagaattgaattggagaatacgtcgactgttttcttgtt
cgagttctttttccctcaaatttatctttcttttatttgaaagaaaggaagccaggatgacaacaagtatgggattggtgatttcccttgaacaaaaa
aaaaagagagaaggagatttttttcaccattgacaaacttttgaacgaggattcttgcgtcctaatcattttgcttacgtttcattttgttattgattc
cagcgaacttctccataataataaacatccaatatataaataaacagttagaatccattggtaaggaaacaagaggagtgcagtatttaacgttt
gaattggttacagtaattaattggggaggtttgtttttttttattgttttaatgtttaattttaaagaggaaaatctttcatagcttcctgtgcgtcccata
tcaattattgatttttagaaacgtgttgttttctcctgttttgctttgtaatccaaattgtttcttcaattgtgctttttataaatgggcctttgtttccagc
ataatagaagcaagtatttaaaatcagagttattgctgtaactagccataaatcacataactcttgtccaggcattgaggatcaaaacccttttctg
ttggctttgattcttccatttatttatttgtttatttttttttaaatgtctttttcttgataccagtcttttccacaatgtcttggtttctcaaacaacccata
agaagccagcaaacatcgaaaaaacctgaaaaaacaatgcttttcaaagaagcgacatcttcctatagtccttgtctgtcgggtatatcattcgct
ccctaccttggacgatacgaatgcaaaatttgtttattaacttgttaagttttcatttgacttgaatctcgaaagcaatggacagcacaaataatgct
tgcttgatattttcatccatgtcatggaaagatatcctgcaaataccaaaagggagggatgactgctttctttttttttggtctttatgtctttgttctt
tttattatcagcgaagctaaacatgtttaatacatttggactgaatgtaaagtaaaacgttcttccatacttggatcgctgcttttttttcatttaagtc

ttgcgaagatattcaattttccaactttctttaatagtggctcaatgtttcttcaattaatgaatatatttgaattttattcacagctcactttctttcaat
agtacgaaactccttttcatcaatgcagaatctagagaaagccttctctagattcaggtatacgataagtaagcgtaaggtaaagtaaagattcat
ctatccattgaattcatatttcgtcttctttgtttatgttatacatctggttcaaaattgatggatcaccttcatatactaagcgttcagacaatttttca
ttggaaaacttcaacaaggaaagaaatatttacataaatacttttattcaggcttgcctcctggtatcaactaaagtttcagtcagcatgccattcc
acaacagcgcataatgatattttcgtatgccttaccatgcacattccgtttccatttagaagcatttctgagtgcttttctgttgtaccattgatcgtgt
cactacacacaatgttaatacacgtctttctttccaaaggtgcatacaattcagtaacatttctcaaaacaatgttcacataatcatcaaaaccaac
caaagtgcccacaaattctctctcggtcttcataatcacccataaatttgatccaacgcatttatcaattaactctgcaagattagttgcgttcggac
ttcataacgtaaaaagcttcaagaataaatcttttatcatgattctcttaaaaatccattgaaatttaccgttatttacatacctaaaggtaaaattg
tcatcgacatggtgttaagtagtatggaattgtaccttccaactgccaacttgcctattaagttgaaagaccttcataaacattttgtattttattattt
gatttatttttctttttttatttggcccttcttctaaaatacgataaagcttttcacgcgtatcgactgtgtgaacgaattgacgattattaatgaatgct
cactttcgataaaagccgttacataaattcttattttttgtttggtttcaaggcctcgagattccatgcgcaagtatttatcatttgataattcagaata
ctccatctatatagtttaaaacaagtattatgaattcagaaaagacacaagaaaagaatgcaattgaccagattgtggacttaactagatctttga
acagtaaaaatgactccagttctttgcttacgaagcttcaaaattccgttgggaatcaggacgtcttggagacactattgagtctgttaggtgatat
agtttcttttgacccggatacgccagtcagcgttacttcaataaggtgtttgcaactctctattcacttggtatttctattaggcatttatactcaaatc
cccaaggagttacttgctcctgcaaaggtaaaagccgttcccccgttttcccccaaaacaagtgtccttaccgtatttaagtttctagttcctgctctt
cttaaaccaaatttgcttcaaggtccagtaagccttcattatgcagatctactgatggtatatctttacctaaaaaatatatatggggatgattttag
aaacgaggattttggggttagtaaagattcccttgttcgcatcatgtctccagatttcgttgatcccgcgaaaatgatttctgcatgtttgcaattact
acaaccaaagatccctgcttggatgagaggacaactcaatcagtacctgacaactagcttatgtaaagctaacggcgttgaaggatttctgaaag
tctttatgcaagcaaatcctaattcaattgagcgagctcaacaagccgcccgtctgatctcatcagtaccaaaaaaatatgacagaacaggtaagt
cctttttctttttttcttagagttgttgcagaaagctaatcgaaaggattatttgaacctaatatttccccaaatttggagcttattctttagtttaccac
gacttgcttgccaaattaccatcgcacttttagcatcacataggcatttggctacggagttttttttataaacaactttcttctttaagctctccaacag
ttattgacctaacccctgttgaaaactgcttaaaggttctggatgcttttgcttctattggaaacgaagatatgcttaaaatagcagaaagatttgtt
ccttcattacttcaattacaatctaacaatactcttaaaaagcaagctcaggaaagtctgcttacaattatttcaagagcaggaaccaagcctcta
ctacaatctttagaatccattcatcatttaggttctttttgtcatttgctttccaatactcaacttgctcaatttttaccccaattacttgaattatgggtc
aggcaaccttcagagcatcgattagagttacttgatgttgtacaatatgcccttacaaaagttgacacagatgaaattccgagtgaactcatgatt
tcagtttgcagtgatttgatcagtgaggttacagaaaacttgaacaactctgaatcacttctcgaaatatcttcaaagaaagatgttattgaagaa
agtgaggaaattttgcttattttattacatcttttgtcttccgttattggaagaaaccgagaattgggtcttgaaaactcagttccacttcttctctccc
ctttgagacaactagcagcttaccccaatcaagttatttcaacgttagcgaatgatgtttataaaacattagtaatgggtagtgatgactattcatc
tgctctatcatatatccataatgatcaagttccagttcgtggtcaaggaatctatatgcttcgaaagttgattgaagccaaagataagagaattaat
cctcttcggatactacacactcttcttggacttctgacagacgaagatagctatgtgcatttgaatgttattgctgctttagtatctctttgtgacaagt
atggagatatgatgatacaactcttaactgagtatacaaaggtagaaaagtactcaaccgatgaaacattattgattgggcaagctatatatcaa
agcatggagcgattgggtgaactgacttcaaagtattatggacaaatagaacaaacatgtttagttctgttgaacgctgtggttatagatgtcaaa
atatctgctttaaatatcgctgaattactatgtcgttattcattgagtgatgcttttattgaatcagccacagctattttgaatctagaaaagacaga
ggataagaagtttctgcggagagccgctcttcaagttcttgattcgtcttctcatttgccgaaatctgtaaccataactttggagtatttaagtgttca
tgatgaagatgaatttattcgtgagcaatgctctcgactggttaataaaaacgccgaggaaaataggttcttttttggaatgatacagacatgttac
gattatttatatcatgaacatagaggtctttttttgaaaatttgttgaatttcattagaaacaatcattaaaaataaaaaatagtatatgctttcgtta
acttaatatacctcgaaaaaagcactgtcaaaattatagttttaaagaaactttagcataaacaatcctacaaatttcgttcaatttccattttctca
agtctatccttgagcttctttagttttcgttccatatagtcagtagctactttaatttttccattttctaaatctgtagcgtcaccgtcatacgaaaggtc
tcgctcttgctcaatactgtccattctcgacgaaagccacaacttaaaagtgaagtcgtccatgggtatatggtctatagaattctcttctaaagcat
tatttcgaaaaggtgacgtgggattattaggatgaactaactgtctcatataccaccattgtagcataatcagtaaaaacattagaataaaaagc
agataaataggttgggatagcaaagacaatccgttggggactttatttgcaagactgcctattaaagagcttgctgaagcaaatatagatgattg
cttattcgaaggaagcctttctttagattctgcatccctctgaggacaatttctttttctttttcatgcctgtttttcgtcttcgtttacctttcccagatgac
ttataggactcgagagcattagtgaggctcttaatgtatgacatttgaccctcttgtgctcccttttcaataggacccttcaaccaggaactcttattc
cattcaactgtataacttacatccatctttgtctttgagttatttccccaatagcatgcatactgagtttttgttacaaaagaagaaccactgggtacg
tctggggtagatgctatagataaaacctcaatataatcgttgatgtcgagatgttcaatagtatccgtaatgtaacacgtggtttgtcgatacggag
gagctaccggctttatatactgtacttttcgtgtccatttgttctcatgtttcatccatttaccaatcttaatgtcggaaagcttttcatccttaaagtaa
ttcattagccaagtagtatctgaaccccaaagcaataagaaaaccaacgataacggaagtgagatggtttcctttgagcaaagaatatgagaaa
gagggctacccttccactcggttggagcatgagaagtaacctgtgtgacagattgaaagttatcatcggagatctcattaatacctttttttctaaa

ttatcttcagagccgctttctaacttggatatattaggctcttgttcagaagtctctgaactctctgatcgattgccatcatcttctgaaggatctgtgt
tctcttctgtacctccatccgtatcatcatattggccatcttcgtcttccgagctgtccgaaaaaccaccatcggagcgattgtattcatggccaagg
tgctttcgagaagcatcggatacattatacccattagcggccatgtttaagaaggggtgaatgtttttccaaattgtaatgattaattgaaaggtgg
tatcacgagaaataaacgaagcgaacacatagcgagcatggagagttgttatttgtatggcattaggaaaaacgacagcagtggactttttttct
acggaaacaatctctgagacgggtataatgatactcgtaacccagccaaatatgctagaattaaagcatatgtgttgttccgagagatacaacct
tccatgtaaaaaaatgtctctttgaagagcacagccataatcgtctatgagatggtcatcaggaggaagggttttaaagatgcaatgaaaatcat
gatttcgcttgttgctagcaaaagcatacccagacaagcttccaggatcacctgcagaagaggctatggatactttccgagaagctgaagtgacc
gcagagccttgggttgccgcatgttctaatggcggcaaatttgaagcatgaggttgcgtattaacatctttattacggacagaaggtgagcgatcg
gtagcttctgtttcgttatcaacatccacgtcttcagaactcattgatgtttcatgacctgctacagcgtttccattattcctggcagtatccttttcga
agttttctagttttaattcaccttccccaaaaggaacatcatctttactactttttgtctttccagaatctgtcctagcatgcccgttatcgttcaattta
gaatcatcttccgaaaatttctggtggttactaccatcgccggctcttgtatttcctaatcgaatattatgttttggttgaacagcttcattgaccaca
ccgctcaatcgggtcactgcagaattaagaaagctagaaaattggctctcttccttcatcggttgctgaaaataaacaatctatttccttgacaatg
gaactctcatgaatcaggtgagtattcgtagtagactgaaaaagcaacttaattccggctttctgttccgggaacgagtgtccgatatatgaaatg
gagagaaactaaatatatgcaagtatattcctgacaatatttctgctaatcccagattgtgacgttcaaaaatgattcaacggctaattatagaaa
aaatgatgacatacagtttcaaaactcttcactagtcgtctgtttgcctttcaatgaaaaaagttaaacagattctctctgtataaacggccttcaa
attaaagccttagcacttttcgtacaatctcttggtgaatgggtgattctaattttacatagcacaagtgaatcgattttaattgcgtattatagcgac
gccacgcaaaaaagggataacggtgatgacgaccttttataaaaagatgtatcgtaagcgaaaaagttgatatcgaaatcgatcatagaactcg
gtaaaggaaagtaaataaacaaaagcaaatagaatgcccatttcatgaaggaaggaatcggtcccattgtagaccactcggaagagtctagcg
tttgacatcgttgattataatagttctattgagaggttacttttgggtcgcaaaagcattcggaaattgaattacggatcaaagcgataggcaacct
gacaatcaattttatgcatgctggtactaaagcttcgtagctggacttgttctgcattcttaattaagcgggggtcgctatccacccaatctgtaggg
acttcactgtcgcgatcggcgtaaactagcacattgaatgtacagcgtccttcgagttggggcaaaaaagtcacggttgctgtaatttggcgaatt
agagcctgaacttccttttgaattttggactcatcctgggcatgttctgccactgtcgtagtatctttttgcaggatttcaacgttaaactgccagcgt
tctaaatcttccccagagtctttgtttgtgataactaaagcaactttttgtatctttcctccacaggtccaacctatcattgagttagagacatgcgcg
tctgcaaatattacggataaccatacgatgaagttgtaatataatctttctaatgtatgttttgacttcttcgtccactgtgacaagcatacttatccc
atatttacgcactactttgaaatcttcaggtggatagataccgcgttgaaaaaggatagaattcaccgcgtattctatgaacgttaacattgctgct
ttcatttttcataacattaccaaaaaattcactaattactttagatgagcctcttaaggagagatttgatcgaacaggtgctgccgccataatttgtt
gtgttttggtttacttggtgaataatgatcctcaccttttataggcttggtgtcatacaaaattgaaaattccttataaaaagcgtttttattttattttt
gtttatgatatttaagtgtgtttttattcctttgtcactaacaactggctaaatctagttcttcattatgttgactaccaaaatatattacggactcatc
agtcgagattatattgtactacaccagaaaaaagtcaatgacttttatttagtcgttagtatgcctgtcagaaaggaaaacacgagaatcctgagg
caattgtagataatgtttcattactgtggattccaaaagcgttgaagcaacttcattatgtatacggaaacccttctcgatcatcattaaatagaat
accttagcttcatcaaggacttctctggtctgcagtaacgtgaaaatatacatgtataaatctgaatatatgttagttaatttgtggaagagaaaag
cgccataattcactcaccttttcgaaatggacaatggcttattgcttttcttataccaagaatgcaatccatttcgcttgaatcagagttcgagaaag
ctgtgtgtaggtaaatcttccaaaaacttaaaataaaatcgagtcctcgtattaaaaatactctatccagtgcatctttcattcttgttagagacatc
ttggcctcttccataatgaaaacatatgaaggataaatagccttttcttgataaatttgaatgagttccaatgctttgatttttactcggaactgcaat
tgatgcttagattcgaaccatgaaaatatttgccagagaataatattattaggaaacagctcaacagctttttctaaaattggcctcatcttcaata
cgctgaaaattttatgatcctttgcgtaatgtagggcaatcgcaaggccaactgtaaaaaagtattctaggtcttccttcgtaacaacgtccagact
ctccagttttaatacgcacttctcaatggtataatgaatgggctccatctctgtaaaaaatgaaagcattaaataaataaatgaaacgtttactacc
ttcttcttatcctgctcctcttcaaacagcatagtggcttgttcagttaacccagtcttgtctgatgtactcatacaagactgatcagcatgaaataaa
tgcaacaatacattccaaaatccattttcgtcattagaaaagaccttttgcattgcccaatttttatgaagccatacattctctaaatcagtaaactt
cgaagagtgcataagataggcagttttgtatatttttttcacacagcgataattttccattcataaattcagtttgggcgaaacaattccatacaaaa
atcgaattctcatactttttttaaaatttcttttatcgtagagctgcttagcaattagcatccgttccttgatacatgatatatcatacctaaacttttcat
attcttgctcttcaaaatctagttgacataacttttgtaaaacagttatatacaacccagcaaagtattcatcatccacctttataaaaatttgatgc
aaaactgtaatcaaacgacgaatttctatttcagaaaaaccttttaataccattagaaaataatcgggtaaatcagtcgagcagggaaaatagat
atccaaaaatcctggagatagtactttagaggaagaaacaaaataattgacatttttaggatccttttcgaaatgttcaacaagcgaaatctcaat
gtcaagacttgatgaaaaccatggatctgaaacaaagaaatggtcattatttgctcctggtggaagaattgggatgccatagatgttaagaaaac
agtattttagatcaagaagtgcttttggcgtaaaaaatcgacatataaagtctttgacatcctcaaaaataacatttcgaaaaggatcgtccttgcc
attcaacttccgagcaggcttcgcttcacgccaagctttttgttcttgaaacctttttcattctcggtccattgcaaaaatgaatcgtaaatcgttatt
ggcgattgagattcatcacacccttggttaaaggttgatgatacactggaactattactccaaccaagggcacctggctctccaaatttcggcgtat

cagaatcccagaattttgacagctccagttttatataatctaaatctttgttgttaagccttgatggaagaaaatagttaatttccatattcacctgc
gtaatcgcataagccaactcaaaatatcccgaatcccgcgcaaaaaagcataccctaagcagaacatagatagcagttttctcaagttgttgtat
ttcacattccaatgactcatctttttagaaagttcatcaaccttattgtttattatagccaggcattcagaaaagatttgtaaacacgaatcgacag
aaaacgaatgtacgttagatataagataattgatatatagaatccacaaattttcggataaaccatatcgagaaagcaaacgctcccaatggttt
tgtgtagttttggagtcccattcaagagatcccagtttaaaatactccaagaccaattgctcgacatcaatcgaactgaagtcaatagagaaaata
gctttctctaaaattgatatttgaatgtcacgtaatgaatgctgtaggcttctttcttcacgaatagacatgtttggtcgagcatattcttgaaggagc
aaatctttttgcaacgaacatagacgaagccataatgttgggttcaatccatctttttctaagtctcttcgtaccttgacaatctcagtctgaataact
ttaacattttctgaagttggttgcagtgttgactcgtcgtcagttacattggcaaaactttcaggaaaaggttgataatctttcacatttgaaaatgt
gttaagcgaagaagaagcattgactgttctttcttttactctagtacttggtacacgtgagatatcctttaaataaattccttggctcctcttatgaac
tatttctttctcaggatggctgcctattattacgttagatttcctggtatattttggaatagaatatttatgaatgccaccgtaaaagaaaactttcct
atctccctttgtatcgtattttacattatacaactgttgctcgcgatcagtgagcggtagtgtcgtttcgtacttagaatggtttatttctctttcattgt
atccacgtcctctttttccctatgcttatctttttcacgctttattggtttgaagcttccaaacttgggtacagggacatctttccttggcaacatatta
aacactatacaccagaatatatcttataaaaacctgcaaactatattcataaaatattcattttcttagggaaacctacaagtaagcaaagtgcgt
tgtcttggtggaagtatttgagatacggtgaagaatctcctaaaaaaacaatttttggttatttcttcattgattcttgatatttcgttctactaagtcc
taaactttacgttcatttggtattttatatctttgactagtgattcacaactttttcgtatataaattgctttctatactataatcaaaaagcattagatc
gaacaaacagtttgccttagctgtcttgaaatatcttttaaattaaagcaatatatatattttagtctgttgatgcttaaccaggaagatgcctcctat
tgacaagcgcgaccttcgaaagcggtaccgatcactaattaatcaagttcatggtaatattcatcgttttcctttttcagtttgaaaatcccgctaat
ccgattagaatctcgccttgagttggtggaccaagaaaacaatgccctaattgaaacagtggcctccgctaatgatttattttcatcaggtaagttt
ttctctttaatctgtaaattaaccgataaatagttgaggcacccacagaagctgctttggatgctttattgttgacaaaaactgtggacttggcatct
atgagaaccaagcaacttcatataggaaaaccaaaattcaatattgaattgtatatcaggcagattaaacaatttttgagtcaaaatagttccaa
cgccgtcagctcacaatctaacaatcatgaactcgcatggtcaaggttaggaaagattgcttggaagtatcaaagcaggccagcttctattaattt
aatgtatgtggcattctgattatcctttgctaatatactgaacagggttgggcctttgtcttttcggaaaaaggaaagacaagtgcaaagaaggga
aaagctacagcgagctccaaatacgtcgactcagcctatactggtaagaatttcgtattcttcagttgttgattaaccggatagctcaatcaagat
aatatttctgcccaggaaaacaataccacaaaaaatgttttacagatttcccgtctacttcgaaagcatcaaccaatcgatcttttgaagtttatta
caaacccggaaagttattctcaaaccgttgagaacctgttttatgtcagttttctttttaaagaaggaaaagcggccttaattgaagattcattagg
agcgcttttactaggtatgctttatgtttcctttcttttggttactaattgaatataagagagtaaaacgcctccatcggatgagcaagtaaattctgg
ggaagtacgaaacgttcaactcgttatggacatgactttagatctttacgaagtaagtagtcgattatttattcttttctaacaaaataggatattat
acgaacttttgaaattacggatagcattataccaacgcgacgaccagtagagactgccgctagtagtaatacatggtacagttaattttggaaag
gaaagggaatgtttattagttgtaaattatgaattaatttttattagcccgataaaattttataaattgtaagcaagcgtgcttaatgataagataa
atataatcataaccagatatccatagtaaaaaaaaaaaaaaaaaaaaccaaaacaaaagggaataggagtaaagtcaaatgactaattaaaat
agggtccaaaggtatataccaataaaacaattataattcgtgggctcggagaagttctttttgttgctaaaaccatttttaagagaaataataaga
attgacaaagtcagttctgtttctctgctcctgacgtctttgtcggtcacgaacacggtctttttctcttctttcggcgcctgtaagaccccttttctggt
catcaatgtaattttccgcttcttcttcttcattgtcggattcagaatcactcatagctataccagcaacatacccagaataatccatgaaattacga
agaaaagagcttttcgcaaagtaaacaggaaatgctgaccgtaagatgtacttaagactttgggctcgtagaatctttttccaggtgtcaagctga
actgtcgacttgcctattctcatagaagaccttaagttaccagaagatcgaggattttcaattgtttgaaggacgtttcggaatgtttggtgtaacac
ttttctatgctttttagcgatacttttagaggattcagtggaaaggtcacgaagaatagaaagtaaatgaatgcgatcaggaaaaatatcattatct
atggatccttttccattttcttcttcagaatgcgtgtctgtaatacgctcatacaaagctgccagagcttgcccagctcccatctgtacgtctatactg
gcagcttccaattgctcagcaagtgcttcagcagcagagctgacgaattcactgtaggatgttatgccggttagcaagagtccaagggattgaca
agcaatcccaaccacgtcttcgttatcctctgcattaaccactgcaccatcagactcgatgatacttgcaagttgataaccaaagtcgataatatca
ggctcagagtccaagaggactgtcaaaagcgataaagagagcatagcttcacactttactgctgttgactgggcatcatttacgagagcattgaa
acgaccctctgtagcagtccaaagtccctctactgaaaatactgaagccaaacataaagcacacaacagtctcattgtgataataacttcagcat
cagaacgtgaagagtggaagagctttaagagtatttcttccagaggaccgatttttcggcaactcgttttacataatatcgagtgcacaagttgta
tagtttcctcaaatgtccttcccgctcttttgacccaacttttctcacttctaataattcatcaacaatgctttcgaattgctgattccatgttcttggatt
ctccaccatattcaaatcttctgaagaccctacacttaactgatcaacagaactgctcaggatactaatagtatccatcgtttcatcttccaaaccct
gtgatttatcctttatacccatattataaacactatggctttaggcagaattttaccatgcagagaaatacatatacatatatatatatatgtagata
tatattagttcaaactatgacatagtcgccttcggttgcgttctgtcacattaaaggaattagtgatgcttatcgaacagttgagagctaagaacag
aaaaacaaagctttcctaaaatatggaaatgatgctatttcggaaaagatttcattaaaaatttctctcaagattcctctatgtattttacttgaatt
gtcgtacatgaaccataatcagccgacgttttaagaatgaacctgccatattcgtcgccaccagccttgacatctcattgaaacttttcaaaagtct

cctcatagtttttgataaaacatcctgtcaaaaccagaaagacgtagagaagaaatccattgaaatcagatatcatagttcgttataagataggt
agcaactactctataatatgatgtaaacaggtagcataaataaagggaatttgtatctatatataaaagaagagctataaagtaaaaaagaaaa
atagttgacatttacaaaaaaaaaaaaatatgaccattgggaacaggaaatgcttgatttttcaactcttcttgcatgtgcattattgaacaatatca
ggtgggtataattaatatttaagcaatataggaaagtaatttttagcctttaatttcaaccttgatcctcttagtaacgagatggattatctttcttttc
aaatcttcttttgtttcattttcgatgtttgggtatggtagtttttgggcttctattgagcaattttactattattttatttttttttttaaatagtaaaaatc
aagaaaattgatatgaagactcttactgttgtagtttttctcgtaaacattcacaaaagttgctagtctggcttttgtaaagagatgttctttctttttt
atttttgagttgttttcccatttcagcccttattctttcttaaccgaatagtactagaaaaaaatcttccttaaattatctgcatatgtccaaccacgcg
aatcacttctgctcagtaaaacctcacatttcaagaggaacttcccaactcatagtatatagacttcaaactggctagcaaaatttgaatgcataa
agcggctactgtagacacaactccaaaaatagttacactctataaatgcttaactagtcgatactggacagggaacctggataatgtattttggcg
cgaattcttccttctcagtcctcgccttcaagagttaaagcaaatactgcaagaaacttcgactcaagatctcgcctccaatggcccaaaaatccat
agcatgtatctattcgtggtggatttgcttctatcgaaggatgaacatttacgaatccataatgctttgacaacactgcaaatgtttctctctgctatg
tccaatcagaagtcttcggatgtgaattttactgtatatttgttactgggaaacattgattctatcgatactcagttttcactgtttattcgaaacttgt
gtgatgtaatcaaaacctctgacaatgctcagtgcatacatgactctttgtctttcttcttgtgtttccttgctactctgtacaactcctcgttcatatct
catatttttgacagctatgatgtttttacaactttggtcactgccatcttgcatcaaaagcccggttttgaagttgctatatatgcacttggattactaa
gtgcttgtgacaaatttgaaactgtcaatacattgcgagttcatctgtcccaaatatctgaagaaccattctttgaggctgttttaaagtattctatcg
ctcaactcattgctttaagagatgaatacgttgctataaaacctgatgattctcctgtttcttcttttttttagcttctttactattcggtctttaccttcga
atcctgatgtaaaacaagagcagcaatttgctaccctacctgataaacgcttagtcatgtttttaactatttacgaactttgcacttgcaacaaacg
tttttgcaaatttctttttcagttcaaagaaaatgaccctgactctccttcttcaactctgctttcactagcttcttacataaatgtccatcagaggcaa
tccaaacgcgcctatcatttctctatgcttttcctcattctctttcatttacaaatagatgatcccattacttctgctttgctggtgcaaagcaattcag
atttgcgcgtacgaatgtgtttacagagatatcctttcccttcttatccagcgaaaccatatgttcccatgggatatcttttagatatatgttgccttgg
gattcaacataatgcaaaactgaaaattagtactccaatctatatcttgtaccttacgcttctgcataaatctttcgctttttttagcgcaaaacctgat
acgattggaatatcattggaaggaactttggagatttctattttcttttctagaattctgtaatacgcttataaattcggaattttccaacaacgcacc
cctaattgtagagcctttattaaatttgcttgcgtatacagtcgcaaatgtggacgcctttgttcagaatactgaggaaatggtggatctgttatata
aattaattcgtaatgcacagattcttcaactcattctttcaaaggtaagcctcaaaaacaggcgcgcagctgattaccttcttggagtgatcagtta
tttgacttcaaaagcttctgaatcaccagatagttctccagaggaactaatggttataataaaggaaaatattgaatcaattcccgtggcagaacc
acaggttctttcaaatatacctcctttaagggaatctaattatcgtttatttcataaacgagtcagtagagatatggctgaacttctacgaaacgtcc
aaatttcttagttagctatgttaattaatgacttatgactgttacaaaatatttggctcttagaataacaaaacagagtattttgcttcatgcatcttt
aacctttttttccatatatatacaatatactactctactcattaatacggtagctactattatttagctttattcagtaaaaaacgattcaaaacttatt
tttacctatattaaatgtaccacagaatagaaagagagttaaaactttaagggatacaacacgaactatactagttctctttataaataatgaaag
cagaaagcagatataactgtttcgtggaaaagtgcttcgagtaccggttgaagtaagtcaaatcaagtcataaggatatgactatgtaagcctttc
attcaggtatgtggacaatgcaaaaaacaaaatttctatccaaaaaagccagccccaaactctccttcacaactaactggaaggatataaaaga
gaacccgataaaatccatttaaaggcgatttattcatctttccctttaaaatcttccgcgaccacggaaaccaccacgaggaggaccaccgccac
ggaagccgcctcgaccaccaccacggaaaccaccgcgaggaggaccaccaccacggaagccgccacggccaccaccacggaaaccacctcg
accagcagcagcagtcttaggtctcttagcagcaggacctttaggcttgggcaagaagcgctccaagggaagaagcttatcacctccaatgaata
ctttatcgccaactttgaaggagctagaaacaatgccttctgaaggctttacagtgaagtaaacttgattcatgggacccaaaatctcgtcaatttt
gccgatttgagactaaagcttcgttagcaaagtcttcatttttggtattattaaaagaatgaacctaccttgttctccaagtaaataggagcattga
agtagggaattttagggttggtagattgacaaaccatatctccctcacaatcatgcacaaaacttccaatctctaattatttcaactattagttttcc
tatactagaaaagagaagaccaacaactcataaaacgatcgatgctgtctcattattgagctttcttacctaaaacttgatcaggaggaccagca
ggggcaaaaggacgatatccaggacgtccacctcttgatccacctccgcgaccgccacgaaaactcattctcaatacttaattaggctcgtgagg
tgataaaaccgttacctccaaatataaaggagttttgtaaaatcgaaaatttcactgtcacgtagaattcggtgtagcaaaattataacgaacttc
gttgtaaatgagtaaatattataacgtatcgtcataaatagcgttattagaaattaacttagtgtaggaaggcagcaaaacgagtttatcagagca
aatatcacttgttgtaaactggggagagcaaattgcttgtctgtgttaaacagcgatatacaaaaggtaagtaagctgaaaaggaaaaaaagga
gacattcatgaagatgaagtggttgtttgcgtttcaacacagtcctcggtacagcatatacaggtccgtggaaagggaattgaatcttgctagacc
caagttttaactgtagatcacgtataatatggttttattaccgtatttatttagtacacgagccgagcttagcgcaaatcaaattttaggttccttcag
gaatttaatacgatagaaaatgaaaaattaaccctggttctagtcttaagtagaaaactacacccagataactgtctcattgattgattggtccac
cgaaaagtcattcctgaaagttcacattgagtttcatcgttcgaattaaaggatgagtgcagctactgttcaacaagtacagaagggccttggaca
ggtctctttagaagttccatacattcaacatcgtgttgaactttttgagaaatacaaaaaagaagttgaggaggagattgccaacaagcccagag
aggaaattgacgttacccttcctgatggaaaggttgtcaaagctactagttgggaaaccactcctcttgacattgctacctcgatttccaagggtct

tgctgagcgtaccgtgattgctaaggtggatgacaagctatgggatttgacccgtccttttgaaacttcttgtactttgaagcttttagactttgaag
atcccgaaggaaaacgagtcttttggcattccagtgcccacgtccttggtgaagcttctgagttattctatggctgccacttgtgtgttggtcctccta
cggatgatggcttcttttacgaaatggctatcgagaatgagcgtgttgttacctctgaagactacaagagcctcgaaaacttagccaagcaagca
atcaaacaaaagcagccgtttgaacgtttggtcctttctaaggaaagactccttgagatgtttagccacaacaaatataagcaacagattataca
aaacaagattcccgatggtactagtactactgtctatcgctgtggccctcttattgacttatgtactggtcctcacgtccctcataccggccgtgttaa
atcttttgccgtcatgaaaaactcttcctcttatttttgggtgatgctaaaaacgattcccttcaacgtatttatggtgttagtttccctgacaacaag
caaatgcaggagtataaagcctttttgcaagaagctgccaaacgtgatcaccgtaaaattggccgcgaacaagaacttttcttcttcaacgaagtt
tctcctggtagttgcttctttttgcctcatggtactcgtatttacaacactttacaaaactggatgcgtgaacaatattcccagcgcggttaccaaga
agtcatcacccctaatatgtacaatgtaaacatttggaaaacttctggccactggaataactattctgaaaacatgttttcttttgacgttgaaaag
gagaagtttgctttgaaacctatgaattgtcctggtcactgtgtcattttcaagaaccgcgatcgtagttaccgtgaccttccttggcgcgttgccga
ctttggtgttttgcatagaaacgagttttctggtgcccttagcggtctcacccgtgtccgtcgtttccagcaagatgatgctcatatcttttgcagacc
cgaccaagtgagaagcgagattgaaggatgctttgacttcttgcagcacgtctacggtatctttggttttactttccatttagaattgtctactagac
cagaagagaagttccttggtgacgtcgctacttgggatcgtgctgagactcagttgaaggctgctttggatgcttctggttataattgggagttaaa
tgctggtgatggtgctttctatggtcctaaaatcgatatcaccgtattcgatgccttaaaacgacaacatcaatgtgccactattcaactcgatttcc
aacttcctgagcaattccaacttgaattccatgctcctgttaatgctgaagaagccaaggaaaccggaaataacaatagctatatccgccctgtca
tggttcatcgtgccatccttggcagtttggagcgtatgattgccattcttaccgaacatttcgccggtaagtggccgttttggttgtctcctcgtcaag
tttgcattgttccagtttctgttgctgcgtatacttatgcagagaaagtcaatcgtgctttagtaagcgctggtctctacgctgacgttgacttgtcag
acaacactttacctaaaaaggtacgtaccgcacaactttctcaatacaacttcatctttgtcgttggtgccgaagaagaaaaatccgagagtgtga
atgttcgtaaccgtgatgatcctaagaagcaaagcaagggtgaggttattcccatgaaggacattcttgaaaagttactcaagcttaaggagtcc
aaatctcgcgtcaacgacttgtaattcgagccttatcaactttaggtccatttttcgggaattagggttatttactataaatgcaacattcatttttttc
tttcaatgaaaacaaacttcgcatataggtgctttggattctacaacagaaggtccagcactgcgtataaagtatagataagcaaacttgtatgtt
agtttattacttagtaggaccatgtgattagcgagatgactcttcaatagtcttaaacaattgccaatcattacgattaatgttaacgttacttttctt
acgtatataagtagatgtcttgtaaatgtgataaatattgaatggtgctactacctaccatactgtcacttactcttaccgacttggtaggatctaat
gatttccaatgggtaatttacattgtacgaagagttagatgaattaacatatattagataatttaatagctcatggtttgactcataaattatacgat
aaacggaaatctactgcctatgagttggagcggtatgttattattcattacatgaaatccagttcatttataggaatttattacatgaagaagaaat
attttttagctaactgaatgcttgtattagtgtcgtccgccaatacttagagaaaggtgaaacggacaaagtgcaggaagtaatcaatgagcttgc
agatgactttatttattcaccgacaaaaggatccaatgctgtttttggaggacttataggtcttgccgcagttgctattgccctaggtccggtaagtt
tatatacttttcaattcgtcattctatgtcttaacacgcgaaatagaaaattgatatatatatggaatcaattataatgccagtgctatattgctttaa
tgattccgattccaaaatccgttattatgcatgtgaaagcatgtataatataggcaaagtggctaaaggagaagtgtttcggttctttaatcttatgt
ttgacgtcttgtgcaaactatttgctgacacagaaataactgtaaaaaatggagctgaattgttggatagactagtcaaagacatcgtcatccagc
aagcttccacatatatgtctagtgtagaaaacttggaaacatttaaagacaaaactgctacttcttctatgcaagatgttccagtcttgtcttgtgaa
tcttcccggatgcaaacgttttcgctcgcgaagctcgttcctttgctatccgaaaggatatatgtaataaaccccaataccagaatgtttttggtttct
tggattcgattacttgacagtattccagacctcgaattcatttcgtacttgccttatcttttagatgggcttatgaattatttgagtgatcctaatgaaa
gtattcgaatcgttacgagtaactgtttatacgactttttgagagaaattcaaagaatagccaaggtgaagtaccatatccttcaacgagacgaag
aaagcgaacctgactttttcaactcgatgattcgtcgagtcacatcggattcagaaatgcaggagatttcagagtacgttgagggttctctgaaag
acggatcttttatattagaagcacatattcaaatcgactgcaaaaaactattagaaataattgtcaatcatctgggttcttctgtacctcttattcaa
gaaaaggcattgaagtggctctatgagtttatttacatttcaccgaaagatgctctcgttttgatttctaaggtattagaaaatcttttgcctttaatg
tctaaaggagagaatattcgtcagactgccaaggagcttagtcaaaacttggtcattctagttagtaaggtgatggatattgaaatatctgagctt
gataccagtcaaaatgataacagtttaactgttgattttcacacattgatagaggttttgcaaaaacttctttccaatgacaacgaagaaactcgtc
tatgctctcttgaatggattctactactacaaagaagggcgggtggaaaacttatcagtatgcatgatacgatttttcaaacgtttttgctgcagttg
tcagaccctagcgacttagtcgtttctcgaattttggaacttctggcttatattgccatttcccacaaaagcgaaaatttggttcctttcctgaagtat
cttctacagatgttttctgaagataggaagttcttgaattctcgaggtaatcttattattcgccaactatgtaatcacattgaaggggaacgagttta
tactgcttttgccagtattctagaagtagaagaggtacgtcaattccattaattattatgctaatctatagaatttggaattggcaagcactatggtc
gaagttttgaataacaacttatttacagctcctgaattatttgaccttcggaaaaaactgaagcagagctcattaaaggtatgttatcactataatt
ggaaggttctaacaaatttaatagctacaaaatattttcacaaccctttatcgtgcatggtgtcataattctatcgcggttttcgctttgtgtcttttgt
cgcagaattacgaacacgccgccaacctcctatctgtattgtaagtattgccaaggctagctataacacggaacatcactaatcccaatagtgctg
aaatagagtttaatgttgatatgttgatccaattggacaaattggtacaacttattgagtcgcctgtgttcacatgtatgtgaatagagtggttaaa
gagttactaactagttagacatgcgcttacagctgctggagcctgagaagtacccgtaccttcataaagccttgtacggaatattaatgctgttacc

acaatcttccgcatttaggactttacgagaccgactccaatgtactacggtgtcaaaaagcaatgttttactaccaaacgaacgcatcaatagatc
aagaagggatgatccatactggtcagatttgttggaaaggctgcgtacagtgcaactttctcatcaaaataattatcgagaaccgatacgggcaa
ccaaattagctttagcaggagcgttgccatcgattccgactgccacaacgatctcgacgactacgtcagcctcaggaataacgactactgctagta
attccaaagatttaattgcaaatcggtttccgcaagctgtgacgatgccaccaggaactcgaaagaaatcgaaatgatcaacagaatttttgggt
ataaaacatgctttcttaagatgattatttattcatactatgattaattatggaacgcttagattatattttaatgaacttgaaatgagcagcgcagt
cactgtattatttctttgcgaaaatccgtaataggtatgcagtgttaactagaaaagtaagcggtattaaagaagaatgggagacaaaacatgaa
taaccaaaagtgcaacgaaaaaggtaatagtatagggaaacgatagaattaggtttgaaacaaagaatgtgggtgtatatctttgcggcatgta
ataagtattagtcgtaccaattcatcatccatttttcgatagcataggaaaagacactgaaaccagcaccacctaataccatggcctttggtcctg
atcgtctagctaaaatgctaccggtaaaaaaaccggcgatggttgggttgtagatgtcagttttagcacggaaagcttcgatactgcactcagag
ccagcgaatataagaccaaggacaccaaaacttttagctgacgagtaagagcgggagcccatatctttcaattgaatacgtagctgttcacgga
atttcttttcatagatttgggggtccatactttgcatatccagagaggacatgaaaagaccaaatataccacccaaaccaaaccccatacctccag
cagcgatacttttgaatacacagctttcttgcataaatgtaacgattttagattgtagcatcattgacttttcctctggtgtcatctcatcagaggcag
cattttggcttgataagaaagaataaggattatcaggattgttcatgtttaagtacgaaaaccttttcgttcactataagtaaaggtcggagagatc
tttccaatcaagagatcagtgagggtatagacaaattgaatttaaaaaagagcaatctaaatctttgaaagactttaaggaaagtcgtcaacttt
aagtctaggaatttttcagcctgaacttttgcgactcgaataccaaaacactcagataaaaagggttacctatcacgaattacaagactaatgtga
ttacaagtctcaaaaagatataattgaaagaatcccttttttattgccaagcagatataacattttggtggaatacgagaaaggattaggaaaag
gcagtcacatatgttcgaagtagatcaacaccaaatttggcagccaagaactcgaacttttccaacaacaaaaagtagattataaacgtaaagc
aaaatgtctgtcgaatctcaacccgtcgaacaagtttctgaacttcccgctggttctaccaccgttgtccatgccgaaaaggcccaaaagttgatcc
aaaagttgggtcttaagcgtgttgaaggcatcactcgtgttgctatgcgccgccctaagaatatcctgttgatcgtcaacgaccctatcgtctacaa
gagcagcaacaacgctttcgttgtcctcggtaagatcaccgtcgaagacatggctgctcaagctcgtgccttcagcgagaaccaaaagaagtctg
gtgctgagggtgctgagggtgctgctaccgaagagcctgctcctgccaaggaagagcccgctaagattgaggaagccgaagaacccgttgacg
aaaccggtgttgacgccaaggatgtcgaattggtcgtggctcaagccaatgtcaaccgtgccaaggccgttaaagctttgaaagaaaacaacgt
aagtagcaataaagtgctccataaagggaagagtggaatgtcgaaattgaaggtcttgaaactcttcaaacctttcgtctgccttcttttgaaaat
cttcaacgtagttgatttctttttccaatatactaaccatgcttttgcagagcgatgtcgtgaacgctatcatgtctcttactatgtaaacttgcaatgt
gaaaattgtgtagattttgtattcatggctccgtcacatgaaaaaaatattattatgtttattttatgtcccgtaaaagaacattgtagagaacgag
gtagacggtaggaaatatcgtataggagcttgcaactaagttcatactctagagaacacggtggacaatatgaaaactaatagcaaagctttcta
agaaccttggtaatgttcataaagggaaccctagtcagccacccatccattgatccatgaagtctgtctatgtattcaatctaaattttatatcctat
tcataaagagtgaatagttttcatacaatcggtatcataatttctgtgcgttagagcaataataaaaaaagggaatattgaatatgaagtcatgga
gagttgttaggaatctttagctagttaaccaaagagcgaattataaaacccgttcattcatgacaaagaaaaagaaataaatcaaaaataatcat
acatcaaggatcgacagagtgaaaataggaactgtccagtcattcaccgaggaagcttacttgagcttcaagtagtagagtataaaaccgagaa
acaataagggaaaaagcaacctcataataaaatatacataaaaaggatttgggaagcagcaatgaaaaagagaaaatagatccgtcgactcc
gttgatgggaagttatggtggcattaatagccatagtagattcgggcagcacggcatattttaacgagcgtaaaaggaaaaaggccgtagctaa
gtacatgtaaagaaatataaatcttgtaagccagggcatattagctatccggctgagggaggttaatatgaggccgacaaacttgtacccactga
aagccaagagatccaggacctgactctgatttgaaatatttaataaataacatccaaggcgagtagccaaaaactcgaacaagacgataccac
atgctttgctagcacgtaaacccaaaagctcaggttgaaatttatcctgcaaacctgcaagcgcacaaatcaaaagaagatgcgtaacaaatgc
cattaagggaatgtacatgtctggagagttcaggtcttctcctggagcacagtatccttcagcgctaccattcatttctgaccgtttcagcttacgag
cccaagacctgcggcgccaggggaaaatgatgagcaacagtttcgctacaacatacgagttggttactgagaagtagtgatggagtcgggtggt
ggagagccactttccgaaattctgttccatatactcatggccagcgcttactgcatttcgacctaactgaaatcccatttgcgcagtagccgaattg
ggtaaataggacgcagttgaaggcatgcccatgccagcagccatgttgggattgtcgtatggagaggacatggaggctcctgaaggaaatccag
gttgcggctcgtaccgactacctgctcgcatattgggactagggacggcgtgagtcggttgaggatgatacagttttggaggcatttgtaaagagt
ctctgctctcctttgggtacgtataatggggaaaggtcaaggctggactcgatgaagttcagagagaaaaaagagagcaatatacagaaaagat
ggggaaaaacagaattaaatacttaaatagaaatataagaaataaataaaaatgtaaaatatgtataccaaaatcaagaagaacaagacact
ttccgaaatttttggcatctgaaacgaacccagaaccagaatgcccaagaacaaaggaaagggtggtaagaaccgccgtcgcggaaaaaatg
agaacgagaatgaaaagcgtgaattgacctacgccgaggaaggtcagatttacgctcaagttaccagaatgcttggtaacggtcggattgaggc
agcttgcttcgacggtgtgaagcgcttaggtcacattcgtggtaaattgaggaagaaggtttggattaaccaaggtgacattattttattgtctcttc
gtgaattccaagacgagaaaggtgatgtgatcttgaaatacaccgctgatgaagctcgtactcttaagaaccaaggcgagttgcccgaaactgc
caagattaatgagaccgactttggtgttgaaggagaagacgatcttgactttgaatttgatgttgatgctatttaaattcactgaaaaaaattttat
ggtgcacttggagatgtggtgaaataaagacgatggactaaattttataaaaatgcttgtttttttatttcttccatgttttcttttggtgtatgtggttt

cttggtttttctttttattatttactagaaaaaaagtaaaaatccctcctatgaccgaaattatgtctcccttctttttccgttttggtctccggtatata
atggtatttaacaagattgtactcggatttccgtccatgattttcaattaactattgtcttgttaatttatatcttggttgtttcgttctggctcatgaaa
ataaaatttatttaaacaaaaaatgtcgttatgtagatgcatggtatcatagtttagcagggtatggtagctttttctttttcctttttaagcaaacttgt
attttgattaaagtacaattgtaccaaagattaatactgaacgttgttaaattgtagacgagcaatttctctcaagtatgagtttgaaagaagaga
aaagaatcaaatttaccgcttggtttcttttaacaaacaaacaaaaaaatatgctttttctatgcgacaacatatttgcccaagagataccaaaaa
atcactaaaatggggtatataggcggtttcaaaatgaagaatataacagtagagaccgatttttgagttaaaaaaaagataaatgaagatgtaa
gagaatgaaaataaggctataagacaattagcatgttcatgagtatttggtatgattacagataaggcggtcgtaacttttcaggttcaattgtca
aagttgggcgatgattaaacgccatttgaattcgcttcaaacttcgaatctttagaatccctatagaacttaaaatcgtagccatcgtaaaaatcat
tcccacagtcgaataaaaggccgctggggattcttcgaaaccgtttggcaagttcataccataaatgctagcaattatggcaccaaaccccaatc
ccagcgtgaccgcagaaagctttaacccaaggagcataagggaatttctgttcgcatctaacataatattacagatttcctctgtacttcggatatt
gcccattaaattatctgtttgttgcacaatttcgtctacttgcttgtaatatgtttcaagcaataactctatttcatcatgtttatcatgatctctaggttt
gccggttcgaaggcgctctgtcaagtacatgcctgctaaatcttgatcttgttctagcagttcgtctaaaacatcacgaatcaatgtagctttttca
gaaatccagaaagacgtcttgaaaagatcagaagggtcctaagccgatcttgattaatatcgagttcaaagtcggctaaaagatcagagacaag
attctgcagaacacgcaattcagaatccagagtattcacaacgcttattaatattgtttctaaagccatcatttcatatggcagaaattcaaaatca
ctggaggattgcttcaatctcccttgcagttcatatataaattgagattgcgaatgggagtgctggcttccatatacatcaaagagtaatacggaat
ctgccttgattaaggctcgaaggtgcaagagattaaccaaaattgacccttcacgtacaaggatcaccggaacgatagagttgatactagtgttt
agctttcgtaaatcacgaggtagcaatccattttgtttgcacaaatccatttttttgaaatctccacagattactcttacgtttccctgaaagtcgaat
tcagtacaatttataagtaagcggtttttttgaggcacttcgatgaaaccattctgtccggctttccgcgaggtcacgatggctgccgtttttcttttga
aggttagcaagggaattgtcaagttgtcttgggggaagaagccgagaatggaatcggacaatggatgaacgaaacggatgagcaaacagttgt
ggttttgagggtaacgagccgaaaaacctcgaaattcctataggaaatagcattggaagtagtccgaatgtttacagctgaaaagggtaaaccc
acaaacttgcggatttgatcaaaaaaatccttattcaacgacgaaaattaagtacttcagcttagaaaggaaatctttagaatttcccttttaatcg
aaggtaagaagttatttaaagaaggcgaaaccgcaattttcaagatggtagatttgcaaaccttcaagcttgtaattttcgactgtggttcacaatt
gtcgttgattttgctttcttttcttctttattgacagtgaaaaataactggtagttatctaatcctctaatcgagcataataacgacgacgatttgtaa
agtattgcaagtagtagtgtaggaatggtacagaatacaatcgcagacagtcaaaggccgatctctattaaagaatgtatacttaggattcttaa
agaatataaatttatctattaaaaggatgaaagacgaaaggaatccgtgagactttgtaggtaaaaattgaaccagacacccaaacaacggttt
ttttctttctttccttctttctttattaacaaaaagaaatgaattataagatttttaataatcgcgaatgcaggaagtgatggcaactgttgagtcact
gaactaatgtgtaagcaaggaaaaaactctcgggaatgccagccaagtttattcagcttactttcatataaacattatgtcctttgaatttcaatat
ttcattattatgccttcatgttcatatcttgagacacgcttacaatatgcgaacgaacaccttttcacactttgcatgatctttgttcgttttactgcaa
caactgttgaacataaatgcacaaagttatcctaagctatataccttttctaccttttttatttcatagttgtcatgatttgctacatttgcgatcccag
tgtaaggtttggtaagcgactcgcttaagaacttgattgctttttgctcgcagccctttttatgtatcatcaacagtatacatcgtttgagtcttatttt
catcaattctttttgggggggatttagttatcctttccaactttaggatctctttcttctgctgtatgaacctttcgtttgttgagtttctccaaagccagc
aaaagacaacctggccagagcagaccaggacacaacggtattcttagtaagtaattttttcgtatgtaccacagaaatcgaagtatgtgcttttca
ttgactgagtcattcattttgaatcctcgtattatttatagtagcaagtaacggtgttacttatgaggtttcgcatgcgaacagacaaatgtgaacct
gccatttccccctctctttttcaattacacagttaacagaaatcttgaagatttactatgggtaataaaccatctcggcaagaatttgttttgcacgc
acctacagagttttcggaaggcttggtgcgccatctcaaagagtctccggaaacggacacaagcagatggatggatatggaaggatatatccaa
gaacgcgtacaaacggaattgaaaaaactgcaagaccgtcaaaggaaagtcattgatgtccttatggatgaagaatggaaacaaaacaatga
attggagaagcaaagcgaaggatccttgaactctaaccttttgaaaaacgagtttgccgctcttcgtgataaattagaaaaagaatctactgctca
tcagtcgataaaaagcgagaccttgaaggagattgaaagtactcggtccgacttatttgcatgcatgtcagaaaatgctgatagatcccttctttg
tcgcccgtttgcagaaaagtttgttgctctcacaagtgcttttaaagaaaactcttctgattcttaacacaaatgagggtgcttttctgttctaccttt
atcttgcaactgaaaatccgaactttcgcttactgtttcacatgttcatgtaatcctatctacttgtatatgtgatgcttatacgcttgcaaaacctca
acgcttaaaacttttcctttttgaatcgtctttcacctgtatcctgcgttctttccggataatccttcgtcaacgagttttggtgcgaggcatacatttc
tgttgtcattttgcatccaaccagatattaaatctttcctgtttcccactggtattatgacattcaccgcaagattaatgaaattcatcttgttaatttg
cgattgctacttttatttcaagtacacttaacactgaccaataaatagaaaacctgtatttcattgtaactttctcataaaatttctgactgaactgaa
cgattaattaaatgaaacaccctcctccccaaaaaaaaaaaacattttttattttattttttttagccaacaaacatttttttgtgtgcccatcgttaa
gagttagtagactcttttggttctggaatgtctgatattggctttgcttcagcagtctgtttgttttcatccttgttgatttccttctctacaggtttcgtct
tctcgtcaacttttgagcttcctggctggtaaagctcatttaaattaaaccttcgcagctgctcataatctctcacgacactcattccaattatgttctg
accaatgttaataagaattcgaaaaatgagcgtcaatgaatacatactttaaacacctggaccatgataagaaggtcatagtttttcaagtcaact
ttatgctcacgcccaacacatttcgcaatggatctataaatatcatccttctttagaattgagtgatttcgaatagtagcttggacagcaaactaag

gcaccggtgttaatacatctatatttgaacgaattgcttaccttcaaatcctgttgtccttcatgaaaatgaggtttcaatacttctaaagccaagtct
tccaattcttcaattgaggctcctgttgtcctgactatgggcgtcaacctttgggtaaagcgagtcatctttttcgtcttcccttctttgcaagtgtattc
caccaatcgaacaggatctataggaggtcttatttttataaaaaccacttatttcaatggttactacttcatcatcatataattatagcgccatacca
cattgtaagtcaatcaagattggagtcaataactcctttttactctttttcttctctttcattccttccaattcgcgagaaatggcttcttccaagtcccc
ctcatcatcatcactctcttcaggtcctattgtttcaggccagagggtagcaacatgctggcaactttgttagaatcagtaacaacaggaaattata
ttgacatacttgagagagaacatttataatttcgttggacgcttgtctctctttaccacgaggtaccgtaatcaagattccactttcatttttctcgat
gcctgtacgtttaaacttgagcttttacaggttaataaaagttaaaaatgtaaaatatacccttttttattgaagcaccaccgtgctttgtgtctgaat
gttcgcgttttgccatgataatgtttattactttagttggacctaaagtgaccagatttgacactttacgaacgtcggcatatggaatttttgtaagaa
tgaaattttttttatcttaaaacaagatgattttagtaagcaaaaaaaggaataatgtttcaaccatctattcatttaatctgcttttaacatacttat
ctacacagggaaattttgtattaaaatggagtcagttctcatttcaaaattgttagttaatgtactctacaattcttatattgattccaataatatcac
tgacggaataaaaagagcataataatctacgtgtctaccttcattttgttttttgaaatttaccagtcagccatcgatacagtataacgggagatt
atgtacatttagaaaattaaacatctcaaaacttaaaattgaaaataaataaccttatgctttctcttcttgcacagtagatggcatctcgttggatt
gtgatacttcagcatttttttaagcatttcttcttccgtaaagactgattcaaaaacacggaggatgcaatgcgttttctcccagtcgtgatctttccat
tgggaggtaatcgaggccgtggaggcttgtttcgaggccgaatatcttcatcctcaattaattcatttaggccaaactcatccatccttgaagacag
aaagccttggataaggccaatttcgtccttcagactctctctggtaatcggtgggtatctgggagcaggagggtattcttttgtattttcagtggttct
ataaagtaaaggttaataataaaattcctttcacgaattttacgtacagttctggctgctgactcatattaagtctcagcctactttgcaatagatgc
aaaggtacagacaatgaatccaacccgagttctttctctaaacctagttctcgtaatccaaagaaatcatctccggtatcatatgaaaatcctcctg
ttataaatgaatcactatctcgtccaaaaaatttcctcttcatcattcgtagttgaaagtgcgggccgtaaaagctctacgagatgtctatgcagcctc
tgatgaacttcatccaatttaataccatagcgtttaatgtcatcaaatacgtaagaaaccaatgtttccggaccagatacaccgagctcttgaagg
gtttgattgataatttcctaaggttgttaatacaaaattcgcataccaccaatttgcatacttctttggaatccttttgcttctccgataaatacttcttc
atcaaagtaccagcattcttcatgtaatctgctgctacatccgtcaatgcgtttaaggcctcggaatggaaagctaaatggttaacaatcaatatc
aaagtttaacttacaatcgaatccagcgtgaaatagaattactgaacagagtttctttaaaatcccatgagcaacaggttgaaaatcggaaactt
cttcaaagttttgcggaacgtctaataaaatcgattcctcattggcgaaaggaatgttacttcgataataatcagagtaaaactaaagatgttaat
aatctcctaagaaacattcataccggttgcggaagttgcatctgcctaactgtctgaattttattacacagctttcgtatctgctgcattttcgatata
ttcttgtaaatagaagaggaaagtcctggcgattgatgattcatttcccaaagtaggtaattattgaagactccatcttcagtaggtccgacattgc
tctcggcaaaataatgaacaggaactcctgaagctacatcgtattcgaatagagagtcgtaattagagcttaaattggagacatccaatatacca
cttaaactttctgagcgtgcagaaaatttttcgtaagctttttctctattcataaaagatgtcatggcttcaggagacctacaagacaaagagggat
tttcaacagcattcaaatttccaagcatattcctattccgcataatgcattcaaaacgtctagactttgaattatttttccaaaattcatgtgcaacat
cggagacaactttatcctcaatctcattttgagcacgagcggtattttcttcttccgcagagcgtttctcttcgggaatttcagcaaattcagaggaa
ggaggtaaatgtgaactttcttccttcttttgctgatcttcttcttttcgcccttgtttatcgccctcatcaggactatttgggacgtcagtgtgtacttc
aacagatcgcttgctttgtccacctctcgatgaggttttctttgatgtagtatgctttgaagttttttcgctggattcttctttacggttagtgtaagact
ttcaatagaacgtaccttcgtcactctcaaggtctgcttccatcaaagtttcatcgtaatctttcctagactgaattacgatatctggaatgatgttga
gtagctcttgcgactttttttttcataattaaagcatgagctcgtagcggatgttcctactcagattagagtcttatccattgacacaaacataccgga
tgtgaattatatgttaagcaattagaccaaataagcattaaatcatgaacaatttctttttttactgttatactgcagtgcttttaatttccgaagcatt
gttcccaaatccattggctctttgataactgaaaaccacgttaatacttaatataaaatactaggaagagcgcttatataccattgtagtagtcagg
tgcatcgcgcttgctgacttttgtcaaaaaaggcaaggaatgctcagtgtaattacgaaggtgcataatgactttttcagcgccctcatataattcc
tcttgccctattctttgttcatttgcctaaaaaagttagttctaggtcaaagatgtgttcgtacccattttgatctgctttttcggacatccgcaagcaa
ctgtttgatttcagcgtcagaagcaccaactacgtttcggttgttttcaagatatttcaagagattctgatatgcaaaaggctattagtttttttatgca
agattaacgatgaaacaaaccttgaaattcgcagtaaaatggccaaatttggaatgtagttgctgaaatatagttagaaaactgaatgaatacat
tttgcatacagaaaaattttcagtccatgaagtatcggaagaattttgaggaaatgaagctttcccaggtgaagatggaccatcagtctctaatttc
tcaacttcatcttgatcgagtaaatcatcttctagggtataatacacatagttataggcaatattgctaagaaccttgctagactcaatagtgcttga
tcctgaaacgtctatttataagaatcagctatatgaaagtgatgtaaaataatcacttacccatatcatcaatatcgatggactctaagtctacaaa
cgaacgagatgaagctccactagcttcctgtttttcgtcatttttgggctgattagcatcgctgtcaggttcgtcatagtcgttctcgttaatttctcga
gttgaactcttcttcctcgagtcttgttctatttcattctttttctcttcttcgctttgctcgatattctgagtttcatgaggtatatccttctcattctcctt
agttacttgactggaaggaccttcagcgtttcgtgtagcgaacaatccaccattaatttgagcgtcatataacatacatcggcatcgaaatagagt
tgtcatgtctagctcctcatcttcctcactgcctcctgaagggtctttttcgtcgcatagtgtatcccagtttctcacgtttataaatcgattccacaac
cgtacatcattaatgatatttatatatttccgtttttgttctggagttaaataaagactccaatattcgttctttagaagagcaattgctaagctatga
agaattggctcgtccaacgtcttcgcgtcttcaagattcctgtccatccaatgctagtctttgaatttataacaagacattcaactctattacttattc

gttaaaccagctcgaaaataccctaaattttaaatggctataagttatggacgtcgcgttctaattacttggaacagaataaggatattaagtgag
tagctagcagttatgaagtattcatccatgactttattagataatagagaataataaaagcttaaagaagaaagattgaactattttctataacaa
atgcataagtatataatgagtttttactacaattagctgagaaatcacacagcagcaaacattttccatagtctgaaggatataaggatacgtcgg
aaaaataaaagctttggttttctagtttctatatttcataagggtatcaacgaaaaatatgtagtagatactttcatatgcaaaaacaaacatcaat
tactccttactaatctttaaactgaaaatttatacttaaaaaagaagtgcataaaaagtaaccaaagttttgcaatatattttaattttttcccagtc
ttctcttttgttcaatcatcaacatagcacatcaacgaggcataccacgacctctgccacgcataatcctttgtgcagcaccgggaggcataccacc
ttgtggaccctgaggttgtttggaaggctcattcattatagtttcgtcgacagaaagaactaaagtagctgcttccgctgctgacatgattgcatta
gatttgacagaactcggttcccatacaaacttttcaaaattgttggcaacaccttcggttgccatatcaacaccagcccatttatcacctttagcatg
catcattcggagtttgttcaatatatcagtggagtcaaaaccagcattgtcacaaagttggcgagggacaacttccaaagaacgagcgaaagctc
caatgaagttttgttgttttccagaaatggtaagggagtaatcccgaagatatttagaaagttccatttcacaggcaccaccacctgctacaacaa
gattattcttcaatgcatgcttaacaatcatgatagcatcatgtaaagagcgctcaacttctgcgataaactggtcagcaccaccacgaagaataa
gggtgcaggacttcgacttgggacaaccttcaaataaattgaagcgctcaccaccgatctggcgttcttcaaaagtgctgcaagttccgaggtgat
gttcttcaatattcgaacatgttgattgaatggaaccgccgacagcttcaacaacgcggtttaagtcatcagtacccactctaccagcgcagaaga
tatcacggtcagcgaagtattgagtagccaaatctccgatgggaagcttagacaaaacaaccttggcaccagtggcaacagtagcctctaatttt
gaaaaaataatgcgccattcagcatccacaatgttttggtattcctgaaccttgtcgacacgaacttcagcattgtccttttcagccttcagttccag
ctcgacatccaagcaaagaatcttaggatttttaaagtatttgggttgttgctcaaatcctgcgtatgaaaaagtctttttgaaagcaacaccattt
accagctgagaatcttccatagcaccgcctggaacctttttataccaatcaagttttcatttaaatcttctctgtctaaagtaaggacggcgtctac
aaccattttcgtaaagaaagatgaattggaacgaattagtttggaactcatggcagtagcagcacatttcgtcaacaaatcgcgtaattttctaac
cggttagtatcgaaaaaaaaaaatcataaggggaaaattcaaaggtttcgaattttcacagttcacattgccttgctcatattatacatacgtttca
tctgataaatctaattgaatggctatttctttgattttgttgactgctaattgagcagctttacggtagccgcgaataattgagtgcgagctaacacc
gtcttccacaaaattgcgggcttcccgtaataattcaccagcaaagacgactacagacgtagtaccatcgccaacctcagcatcttgagcgcgag
caacgtccacaagagtcttggctgcaggatgaacaatgtcaagtagcttcatgatagtagcgccgtcattcgaaatgacaacctcacctctgtcat
taaccatcaatttgtcagctcccaaaggtccaagggttgtccgaatggtatcttggacagcgacgcaagcattaatgttcgagagcagttgaccac
gaccctgcgaatcattagtgccttctttcagaactacatcacattcattagaagctattatttgatcatatacataccaataattggaatttgagggg
cccctacagacattctgtaaataactcaaattctagtcgtttggtgcttggaagaaaggacgtcgcgaacaatactactcttccacccatagtcga
aatagtataaggcacgttacaccacggtaaagatattgaaggaaatgtgaaaatttatcagaacattaataaacattaatattttaggaaatata
atcgtgaagtaatataaaaaaaaggtttgtttatgcccatactcactcgtatcagatcgaatacgtcttatgtttttatcctcaataaccatatgtaa
acagaaagtctattgaagtttactctacaaaaaaaaaaaaaaaactcaataaagcgaccggcgcttacattttattatggatccataatccaaata
taaagtacaaaagggatatttaacatcacagcatggcagctattgatcctgctgtcgctcgtagaattgtaaattcgtaaaagtcagcgactgctt
cataaactttcagtgtttcccgaaatttcatcggtgataatcctaatacccattctccgccgcatatgcaacgttctttgagactccattcctttacac
gtttacattttgagcaagttaaatcttggacttggtacaaaacgagctgatggtgaatattgtcaattaaggtttcttcaaatacccatcgatcaaa
aggtgaattgcaaccctgacaagtccaacctgttggaaagcttaaacttccagactcattacgctcaggccataattgctcatgaagacaaagat
caaaatcatgacccataccacactgctgacaggaaatctgctctaatctgagtttttggcttggatactcaaattgagcaatggtagaaaattcac
gtacttgcagcacagataacaaatctttccttaataattgagtctccaagcttaaattttcagctaaaccgtaaacagcacacagcatttttataaa
ctccagtgtgacattggaaaactttttaaatgaacctggaagttttgggaattgagaagtttctataacttcgtcattcaaagcaaactcttggaat
cttctttgtaatgcaacaatttgtctttttaatggatgtatcatcttttcactaaggatcccggctcccagagtttggagttctgcttcggcttcagaaa
cattatcgcgcactcgcatattcgcactttttccattttgtttcagatatttctgcttgtagacttcctcaataaattcaactacccaattctgaaactc
aggttggacaagagcaggtaaatgacttttgatatgccatgacacaaccgtttctggttctcgtagtggactaattgctaaagtttctggcaaaatc
atttttccaccataatttactggatccatccataataaataatcccaatattcttgaatatttaggtccaaaaagtgaaacaagggaagagttttga
gcgactttaagatatacttcgtataagatatagcattctgaaccgtggtcttggacgttcctattaggattctattggtagtagcgtatatgatttga
gctccaagtctttttatttctgcaagtagttgtaagaacgtcttcttcatgacaacttgtatatggctgaccaacaatggactatataagtacgacttt
gatgaataaatccacctggccagatgctgaatcattagatcagctagtgtgtttccacttgcagcttcctgccaccaatttttataagttctttaagt
acaggaagggaatgtgctaagccaactgaatcatacattcctaagcgagcttctaaatcatccgttacgtttgcggtaaagccatcattcaaggc
agccaaatcacttgcaccttctatctcattgattaaggctgagttaaggatactgcaaacagctaaattagagaaggatacatccacgcaagcatt
tgaataggcacccgatcgattaatctcaagagaatcgttgggactagctacatgcaatacatcatccttttctaaaccacccaaatctggcattgg
acccttgttccaccaaagaattatattttgttccttaagttttcttgcatatagcaaatcaatgacatattgtagctcgtcactttcgaaattgcagag
tggaatgtccgcatatcttgaaagttggattcgatgatttatccaagcacccacagctagaaaatgttggatcatttttttggaaacgtattgcttcc
aggatagagattggattgctgcttctcggcgactaattatcgtataaggcaattcctctaattgcttaagattcgctttaagcctctctggagaggta

ctttcaatgactagcaaactgttcttggttttcgcagaaagatactgaagtattctttcgtcaattaacttgtatgctttcttttcgtttttgctatagga
aaccgaaaaggtgatactttcaggatagtctataactgacattttaagcatgtcattatacttttgccgcaattcatgatacagatcttcgatttgag
ggaaagaggtatcatctccagaaagactgtcataaacgataatttcggctttggctaaatgaggtagaaatagacatagtacaaatctttggttc
aatgaaatatggaatgcatatatgtagttcatctcaacaccctcgagataatcagcatcactttcataagggtgcacgaacatttcaggttcgaag
cctgtttcaaatgctcttccgagagcccccgaaagtgtattgttaaatcgtctgtggcatccaacgtgcaggattccacgctcgataggctttatctt
ggtttcgtatgtaccttccactgaagggtgtgcaaaaatcatcgatatagcgttgttttcattcaagtaagttttctccggtatctccaaagtgaata
atttgtcggagacatttccatgaggaaggatagcattactttttcaatcttacagtcttcaatttcaacgttcggcaaattgttgtctttaaaacaaa
tgaaaaattttctctgaatgttaaaacggactgaggttaaccgattgtttataattacccacgcaagcaaaactccaggactgtcagtgtttctcaa
ttgcacaatatgccataaattttggtacatggtttcagcggatgctttaaaaacattcgtaaattgtctagattggaatcctacgagatgtcgtcttc
gagtacgaacttgtttttggtatttccatttctttttcaggtattttaaccatgcactatatccttcagaaatcgatacgggtttttcgttaaaagtaac
ttggatctcatttccggatggttgagctgattttcttttagatacgcgagcgatagtcggcactaagctcttaccttgggatccaaaatcttcgatatc
attaacttgagctgaagaaggtggaggagcaacgctaaatattgcatcaattttttctgcttataacctgaattcagcgatgcaatttgtttaagc
aaccagtcaggtaatggtattcgaggtactggattagagatcttttgtaaagctgcaggaatagtaattaacttttggactactgaaccaagacgt
tcaatataatagtcccaatctaatacatctcgaatatcaatattgtataggctattatctttcaaccaaagttgcagaaaatgtcgcttaactgattc
ttcagctgaaaatatggccactggaatagcccgctcagcaacgggccttcctttaggacttgaagagataatgaacttacaagcgagacctttatc
tttaaccatctgatcgcccaaaaaatccgccaaacgtctggctgtggttatggatgtggatttttgtgacccgtattctgcaagtgacttcgacatgc
ttcgattttctgaaatcagctcaaatagctccccatcagtaacgtcttttccttttgtgaaaagaatttcaagccatgtatctgcaactagtgcgactt
gtttatagcattcttcaagagttcttccgtttaaaaagattttaaagatttgcgattggaaatccttgatgagttttaactcgcctcttcgcttgacttc
aaaacccttaagctcagctagacttccatcaaaattgaaaacagcgtaacgtttcttcaaatttttaccttcttctttagaagccggtaaaatcata
gccctataaggtccatcgacttcaaaaaaaatactattttcactggtagtttcatattccaaggtatccggatttttttagcagtgaatattggtgatt
ggtaaatttttcatgaactaggtgatttaacataacacaaggataggagataaaaacttttccgccagattttcttttaaactcaaaattctctgga
aacgattctggcaagatacaccagattccatcggtatctaactctaagggacgtcctgctctttcaacaatttgacgtgccatttgaataatagtag
cccctgtcaaacaagtaatacctgccatttcaattgagtaccaacgagatccctttctcattacataaccgtagaaagaattcaaaataaccttgtg
tgcaagttgaagagagtcatacaaaatcaccattttttttagcttcttctaattcagcaagactgccggaacttgatctggacgaaagggctttgttc
cactttttttgtaagcctttaaaatcataacgacgatctctgaacgctttaactgtatcaatataaaacgaattttccttctgacatatgatggtctct
cgttcaatgactttattatcatgaagtcggtgatacacctttcgactatagtcagctatacgcttttgaatcttctcagccctttctataggtgagagt
tcgtcaaatgatctggatggagaaaagggtgttaaacctgggaatctttcaacctgtaaggcagagaggatcatatgatactcatcctttttgggc
gggtaaaactcacctctccatgcccataccattcgtctatcacacgttttatctggaacgttgaagtcgcatgtagcacaaaatgattcatccttcac
agaatcaggctgcaaacgatttgtaatcatgatattaggatacatagaagcaacatccagatggtaaattttagggttttcggaccgttttggttta
tcccgtaaatcggacaactttgacagaatttgagacttaacctcctcatagtcttctatatcatccatttcaactttgttttcggttacaattgaaaatt
gtaatgctttatctagctgggaaatcaattcttgatagacagtcggatccatatcaaagggagtaggaatatcactgcgaaaaacaccactttcta
aactttcaacatgaccaccaacgtatgtttcactagtaagcatgtgtccatcaaaaaatttttgaggtgtctccacgtgcttattaggtaaaattata
tttttcgtagacgcctgtacagtaagcagtgtctcacaaagcgtaccggttccttttcgcaaaacttcatctggattgagaggaataatattacaga
gagaaaaaatgaatggatgtacatatttcatatacagataataagtcgcgacagcatctgatactgagtattgtgccaacgtttgagctttctcact
agcatagatagtcatcaactcgggatccaattcgataggattataacccaacttagctacagtgacggatttcaagccttgacttccttgcggcaa
ataactatctctttttacccaacgaaaagcatccatgtgaatacaatatgatgatttgtattcgtcctctgtatctctgctgaaaccgatttcttctgct
aaattcaaaccatgaaaagtagtacgagcatcaacgaagggccaatcgaaaaaatcaccgttataggtaactatgatggtaggcttgacggatc
gtatatgtttaaaaaaacgctgtaacaagctcaactcgtcctgctcattaaatataatgaaaggtccctcaaactcaggcttgggagtataatgaa
aatcttcaatatcctcaccaacaatttcgcgatttgtgatcaaaaaaccttggccgtctatcatataagaaatcatcataattttgtcaaatgagcta
tctggaaattttaaaggaagctttgtagtttcaatatcaaacgccataactgttggctcagcacgctcgatacgagtaggaagatgggctatttgta
cctgtccttcgcgaaaagatactgtgtaccacatacccacacgaatgtcaagatcaatcagagtcctagcatgaaatggcacgtcaaattcgcga
aagtccgttatgtggtccattggatcatcataggcattgtcgagagacaaatcgtttgagtcattaagaggcgcataagtattgaaagcttcttcct
tgacattatttgactttactgcagacataatggcacgtcgaactgattgaaggtccgttaaattgtcaaatgttaattttatatatagtttttttatatcc
aactatgtggtttttcaatttcaaatcatccttgtactcccgtgaggtagacttcacaagtccagcaaaatttttttttcatgcactcttctacacttgct
acttttccttcccgtgctgcaatgtagaagtatggtgcataaggaatggtgacacggaatgtttcaccatcgtcctgaatgaagtaaaagtcgaca
gcacttaatgtagagttgcgcttttggctctcaataacagttggatgaacgtttattaaccaagcttcacgaggaggtcctccttcacaggcatcaa
aacccatgattcgatcgatttcatttttccgattgacatagtacatgggatcacttagaccattttcctccgcaggccttcttacctcgccctggaagg
catttgagtttttatagatatttcgaaacgacttgggagggaggttgggacgcttagcagaaaatttgggaggcattataaataaataattaatta

attaaaacaaaagaagaaaaagaaaatgtatgaaacacagcttaagaaaagaatgtcagagtgtatcaaaaatgacaataaagatggaacg
accgtaaaagagtgtactatttatacgaataactattgatagaattcgccttagtctatacaaaatgcatttgaaaattgtcaagatgccaagtttt
gggacggttgttgtggtaatgaaggaaagggtaacatagtgagcgagaagaacaagaaatgaaaagcgcgtcgcgtcgcgctccctcagaaa
gtaaacagatggattcttgagttctttttctttttttatgttttcaattgatcaatgaattgttatttcaataaatgtagtcttactatggttttggatgtt
agatggaggaattgccctagaaacaataaaaacctatacacgtttgcgacaagtttaggtcagtggatagagaaaaggtttgttagatcttgcttt
acttcaaaaatgatagaatacatagattggattgctttcttggtatgaacagtaaacagccgagctttttgtttttgctctgttcatttggttgctgta
ccgtaatctaatgcaacagattttcttagtttgttgaaacatatgtatttcatgaattcactttgtatctaaactagtatagtaaggaacccttggttg
catattatatgatacctcctggctttcacccgctacaacaacagtttccaccatgagtgaacagaatgagcctccgaaagaaaacattgtccaggc
atttaaggtaatttattgaattgaaatcgattagtagaagatttgttaacgagattttaatagtccttaaagaaagacgactttgtccatatgcttta
tcacccctgctttaaagaatctgttgttactagtgttctttgcgggattggagtaggatgcatccatttgtttatgaaaggtaatggggatggtttgtc
aagtctgatggcgatctttcatctttccttagacgatcagattacttacaatagtctagctcctcctagtaaagctttaaattggggattcggcacat
attttggcatgagtttggtttcatgggaaggatgtaattaccgtaagcaattggaaactcaaaacttcaatatccaaattcaagaagcggcacaag
gagtccctaagcgccggacatctcgacctgctgaaaacgattcgacggatgcctaattatattaaatcatccataaaacttgacataccctacaat
cgaattatcactaattgaattcatttagctcattttaaaagtttctttgctatggaataataatccatctttttggtatttagcttgcttgatgaaagaa
aaagacatagagagtgttccttgtataaaagaaataggggatgactattaagagtattccatatctctatatccttcctttgttttccagggagttt
acaaatagaaaactagatggatttcaattgcttacatccattaagtgtacctcgtacttctttatggactacaaggattaacttttattttttttaaaa
aataaaaataaaaaagatgtatatatacatggaatatatttattgtaatttcaaacatccaatgcaaacaagaatactttgataaatcatgttactt
tgtctttcagtagcaatctctgagaacacaggtaaaattagaactttttgcttaaataacagaaagtataagataacctataaatctaaaatctcat
ttcaagttagaatgtatcgtgaaatccttttgtttaccatgtaaacaaaacctgttcggtaacaaagaagtggtctatgacgaatcttcattgaata
aatggcaagcggatacattgaataaaacaaaagtcatgcctacgtatactcatcatgaaaccaggcataaatcaaatgaagatggatttcaaat
cgaaagcgagaaacaaatctctgaatttcaagcattccaagaatatctcgcaaaatttacttttaaactgctttttaaaacaaattattaaaaaga
aatagtaaagaataggatagtttgaattctttccaatttctttggaggaagataatagttaataaatatattctattatatagtaagttaataacag
gcctcgtggcccaatggttaaggcgcctgactacggatcaggagattccaggttcgactcctggcggggtcaatttttttcaaccattccaaccgta
tggttaagtcgaactaaggtacgccgtagtggaaattgctactgaataattattatcaagttaaaccccagtcatcaaaataacctcaatagtaat
caatgtaaggaaaggatatggtaataatctcgacctaataattaaactaaggtcaaataaagttgtctcaattaatgtcaataaagatccagttta
aaagcactagtttattctctttattctcttctctaaacaattaaagaaaagacttgtacttatattataagaaatgataagtaattgtacttctatcga
atcaataaaaatcaaataaatcataggaaattttaattgaataaaatcattttctacttatggttaaaaaggaggtgccttaatagttcttagtttat
tgtattctatgtgtttttttttattttattttttttttgtatctctgcagtaagtaataattctgatctatatttttaactacaattgcctgtatcgttcc
gtagagtacttaaatgaaaaacagcaattgttagttacgctagtttgttgatttttgtgtctcggtaaccaaacctgtattcttattcttattcggttga
ttcaattttataataaatattgagcctttctgcatccagagtggaatttcaaattacttcgatatgtatacaattcttataaaagagctgagctacttt
catttctggttactatgctcacattagtttctaagactcgattctacaagtgtattactttttcttggattgaacagtttcttttatattgttttcttttact
tagttgagtgaattgaatttactagatggatttctttctcatttttattgtttatttatctacttgtcatcgttatttttttgttaaacttctctatgtcttata
aagaactttagtaaacgtcgctctttaccttagtcattcatacgttaccatagctcgctatctgagtggcttccctcaaacgggtcgttcatcccttcc
atatagaacgtaaacaaacctgatatttcccaagtgttacgtccttccgatcatgtctgcttcagttcacaagaagttacgttctttcaaactatcac
agatggtatgacttctctaatggcggaaaattattaatcactctatctttaggaccttgaacgatcgttatgggacatggtgaacttgtttcgtgcgc
ctcgtcgcgtttatcgatccattagcttacgagtatgtagttttatggaattcttcctgatcaaaaatgaattgaaagctttacctttctctaacacat
gtatagaaacgtatgcagagcattctgtcacgaaagacatgacattaaaagaacactggtttaatttgctaatcgaatgtaatatagaaactatta
accagtatggacgtgaagacctatcatttattattttattttccgggttaattgtctttacatcattctgttgggcattctttttttgaaaatactctcaaa
ggttacatttccctgttatgttccatgctgtttgtcgattttctggctgttggatttattctctcatccatttattattttgttgcaaagaagtttttactta
aagacatttatcgagagtctgtaacccttgggccatttgagagtcagttagaatggaaatattgcttcgatattcattgcaattcttttttttcctgtctt
tgttcttttatacattcttcagctgattcttttgccccttattagccgttctaatttgtatgtccattcattttccatacctctcaacttctaacgaacaaa
aagtatttctttatttttaggaaactcgctctatttggttgctttgtgctactacacctatttaactttcattggatatcaaagtacgtcttattattatca
aaaacaatttgtcaacatactctttctaaccactctttaagctttaccgtttttaaaagatactcatactctcttaaccccaattcccatgtttctcatt
atgtgggcactgtcactcttgggttacaacgtccctaagcatgtcgtttccgtttatttcaaaaatgatgcttaactcgttatctttttggtaataattg
atttcaattctactaattgctttctacggatgcagaaatgcaattacacaatttctttcttcttctttgtatattaacggacttgacaagtttccctaatt
tttttaacgcaaaagacttactggacactggttaaaatcccaaagaatttacaacattatcatatcgttatttttttatctttctttttttaaaatccgtt
gttcgttttttttttttttcttttcagcaaattgctttatgaatcgtaattggttagctacaaaagtttggctaggtaccccagatgtgaatatttcgaa
taaagaagtcccagatcagtattctacgtttctaggtgattcccctgtatttttgaacaatgtttcagtgaacccagatgacattcgctgtggaaact

gtaactcttattgccgttttgtacttcaatgttatgctcccatagaaggggatgaaagcaaagagcgtgtgctgtatatatgggcttgccatgaaca
ggcgtgtagaagatctcccaaatcaatgatttgcttacgtggggttcaaatcatccaacctgcgaaacaagaaactgaactacctaaaaaggcac
taacggaagaaatatttaagccggaaaagattaatccattcacgtcttcctccaatacaacaaaggctgcaaatcctttctctagttccttttcttcg
cccaaccctttcaccttgtcttctcaaccgccatcttctcaccctaatcagcttaatgatgaaaaggatctgatgaaaacgcagacttcatttgcctc
tatggcactttctggtgctaataaaaatccggaatcgaagaactccacatctgataaggtgtatgttgatgctcagaatatgcctattcatcctcag
gttactactacatatgactatcctattatttcaaaatttccaggaatgttcttaacgttcgattcggaatacctaccgaagtcattgagggcaaaag
aaatgaaagaagcttcaagttcttataagttacaggaaaatttggatgcctatggaacctgggataatgaatcatatgagaaatcacctgctgtct
atgaaaaggcttttcgaaagtttactgaacttatatcacacaaccctactcagtgcgtgcgttatgaacgcggcggaaaacctttgttagcttctaa
tagagatacagttggaaatatgtttaccgaagttaatccttacagtaatccttttcccacttgttctctttgcaagtctccaagaatattcgaaatgc
agcttatgccctactcaatatctgtgctgaatgatgaagtggcagaatggtctacaatcttagttgctacttgctctatggattgcagaccgccatta
aataagaatcaagttggttatgcaaaggaatgggttggtatacaatgggagtaagtgggttcatatttaattaataaatgatttatcgatgaaaaa
tgaacactacgtatagctaaaactacgatgctttgtttttaggaaaaaaaaactgtatgacaagaagctctcattaagtcacaagggcacatatc
aaatctatggtaattgttctaagtcagtttaactgtcgattgtcctatatgttcttgttgaacgttcttcacataatatgtaagcttgaatcgaatggg
caattcagtagtatctgtagatacatttgagacgaacatctcctgatgaattccattttgttgtagaggagacaagcgcgtgccatactgaggattt
aattccaaagagtactttttgggtaaagcaataaaagattgtaagttttcgatactttcggaaaaattggaattggaataagtagcaagtataaca
aatcttccgtcagaaacctttttaggcgctaattggaaccttacatgttcattagagaaaacagtagtaggtgaaacggtggtgtttggagaatct
gcttggtgagataataaagacatgtttgaaaagttgtcggagagagagattgcacctacagcaggaggggtttcagttctatcaagggacttggg
tactccagcagacaaatcgaacaaaggattgctcttcttcatggatggtatctgaacatttgaagtgtcagattgttgagacgaaccaacatctaa
gtctattagcaagtccatggcagactgagatgcaggagcagaactgactctggaatttgcatttgtatccttggaaaggttagaggttgactggag
cagagcatgagcgttggtagacgtattctgtgcagagtgaaagtcaccctttaaaagatggtcatactggctgagtaaagcgataattaaagact
taaggtcaataacactattgtaatagtcatccgaagaatccaattcctccaaaatcttatctactttgatgacggcggctttaagggaataagcaa
gttcttcgtacaaatcgcccgaagagagtttatcagcttccgttatttcattaagcatttctccaaaaagagaggcctttcgcttcactttttcgagct
ccactaagacatgttccttgtatcggcgtttatgttcagagtcgaaaccggccattattttcataagtttgttggcttccataaggtcatgaggggta
ccgcggcgaatgtgttcctgtagtttagcagacatagcatcacggtcttccttttcaagttcttcagcagtttttagcacggatttctgacttaataca
gcaaggttatcttttgcaatttccggaaatgtatagcctagaaaagttagaatccctaatcagttttcatattgtgtcatttttctcttacctttatagg
acaagagtctgtgcatgtcgcggataaaacccaggtcttcatggtaccgatcagtgcggcagatggttcgtcgccattcctcaataagagaaagg
atcataacttgaattttgtttaaacgagaaggtggtctctcaggaaaacgacggacaaattcgttgaggaattcttttgtagcgatttgcaaatgaa
atgcataaccacaatttttcacgcaaatatcgagaagctaaaggaaaaaaagagattagtattagggcaacgacgattgaggttcatacattca
gggctaaataagctactgttggattccgatcattcactttccggacaattgcgaaagcagcttcacgcggactagaaaaacgaacatgtcagtca
aattccaaattaagaatacaagctcaatggcttcactcatacatatttccctgtgcttgattgattaagtccgtaatgtccaagttcagagccaagt
cgggctcaaatgtgaatggttccgtagcattttctggcattagcatttattttccttcatcttaacttacgaattaaacggtaaagcttgcgttttgca
ctagacatgaatcctacgtttcgaattcctcttggcgtttggaaactcctctgttcttacatcgttctctcaatatcaagaaggaaaagattacgtag
cgcgtaccatcgttctacaaatgtttacgaattgaaacgggtatcgttaaattaaaaatactgttaaaaaggatggaattatcagaaaataagta
gcgtatttgtagacaaacggagagagaagtgaagtatgaaatctagaaagaagcagttgaaaccatcacgaattcaaggatggaatgactggc
agttctattaccttgtatagttgattctcgacagcacccttagcaatgtaagtatatactttgtacaaaccaatgtgtacttatactacgaatgcgta
gcatctaaaataaacggacactacaaataaaagtttgcttttgtcacgaaactaagcttcataccagagcaaactataccatttgccctttggtact
tcgtttctcttgtaatcactacactcacttttattgaagatcaaaactaaattgatttctctactcctaaagacatttaagcaactcccctaagagcac
gtctgcggtcacgctcttcagtgatgctgagcttgacacccttgcccttgggcaaagagatccagctcttgttggtctcaccaataacgaatacgtt
ggagagacgggtagcgaactcacggtccaaggcatccttaacgtggatgatttcaaaggaaccaaggtggtgttcacggtgaataatggtacca
acacgacccatgttacggccaccagtgaccatgacttgggcagaagtatcgaacttaatgaatccttcgatcttgttggtttccaagttgagcttga
tggtgtcgttgaccttgatcaaaggatcggggtaacgaatggtacggccgtcatgagtgacgaggaagggaatacccttggcacccaattgcac
acgcttgaccttgcaaagcttgtacttggcttcttcagcagtgatacggtgaacggtgaaacgtcccttaacatcatatacaagacggaagtgttc
accggtcttttcaatgctaatgacgtccatgaaaccagtagggaaagtgttgtcagtacggacctttccgtcgaccttgatcaaacgttgcatgag
aatggccttgacctcacgaccgttcaaggcatacttgagacggttgcgcaagaaaacgatcaagggaaggcactcacgagccttatgaggacca
gcagaaggcttgggagcgtaagtaccagaaagcttgtctaaaagccagtggtgaggggcggcgacacgctttaaatgctttttgggtcctcgaac
catttttggtggatgctgtttcagcaagccaaatttttggcctaatgctatatgtgactgctttgctttggtataccgaagtaataagttatgttacat
agtgcgttgcgttttggtaataaataaacttacattttgaaggaattggaagaaatagaatttgtttgtaagagtagaaacatataatatgactttg
aaaacgtgggaatcataaagcttctgaaaccaacacgtttaaagaaatgctataagcaaaaagtgaatgttcatgaagacagaatcaggctttg

aattacacaaaaatgtatatgaatctgatatacgtagggaaacaataaaataatgttatatagtcgtcattcgtaaattttgtaggaaacttttcat
agaaaaatcgggaaagactaatgagagaaagagggatccaaaaagaattagcttacaaattttatattcggacgcgcataattgttaacttgac
gttgcaaatcacttgatacatgcttgataccttcttctccacccttttgggcaatccggaaggatgagtagggcgtggcaaacaagaaaagtatt
gatgttctaaagcctgttgagctgtaggcctgctgtaaggatcataatcaagaaacctcttcaaaaggtctaattcctcagtccctgcagccgaaa
ataatgcttccattcctccgcttggaggtggaatggctttcatgtcaacgtaatttggtagttgtttcatcctctgcaaaacggctggctctgggctac
caagggcgcggaaaatgacattcagttgatccaaatctgtttcacctggcaaataaggagtacgaagcatgagctctgcgaaaatacatccaac
gctccacatatcaacgccagttccatagcttctacagcccatgaataactcaggaggtcgataccatcgagtaatgacctgatgactcatgtgatt
aggcataccgtaatctcttgaaagaccaaaatctgctagttttagaactccatcgctagagatcagaaggttgtttggtttcagatctcgatgcaga
ataaaacgagaatggatatggtgtaaccctcgaagaagcataagcatccaggccttaatatgcgcaggttgaaaaacgataaatttgtccttaat
cagcatttctaaatcactgtcaaggaactcaagaataatattaagattagatttggttgcaaaaacatccattaactcaattacattgacatgtctc
gtttccttcaaaaatttaatttcacgtaaagcgctcaaatcaattccatccttgaactgccccactttgattttttaatggcgacccttcggcctgttt
ccttttggcgacctaagtcatgtctcattagtatttgacagatcaaatttgctaaaatttcggcatcttcatgaaaaatcatataacttacctaaaaa
gaccactgcataagttccttcacctactttacgctccttaacatatgaccatttggttgacttttcaatatccatagcgcttgctcttataagttcgagc
taaagaacaaaaaaccctaaaataatacaaatctggagtgtaaaaactgggtttaaaggtgcaacgtttccattcgttgaatttttgttgcaatg
ttcgctccgcttcttgaaatacaacgtctaggaaatgtgcttctacgtttttactttttcaaggaaacaaaaaaaaataaatggatccctctagtaa
ataagtatgctaattttctgttacagggttttataaaaatttgcttttcagttgtggtaagtttaggtgaatgaatcataagttaccaatccatcgtgt
atgtacagcattttaactattgaattgcttctctatttctctcacatttttattgttttgtaaaaaaagtagcacttactatagttaatatagttgaggt
aatttgctttgaggcctttgtttgtttacacctaagggcctaattatcatagtccatgtcttaaataacaaggaattgtattgtctaacaaatcttta
gaagtatacatttaacgaaactaaagtaattatatgtgtcagttcatccgattgtgtgaacaattgtaatgccaacggtttttcaacctcaaatgaa
actacagatattgccaaaacccaaaagctaagtactatttctccactttttagaatatattttaggtctgttgattgagttcaaattacctgaaaaca
gtctcaattgatgtcaagcatcggtaactcattgagacagtcgattccatcatcataagaacatagcctttcaaaacacttcaaaaacaccgcata
tcctctcaattccttttaagtaccgtcttccaacgaaataaatttttctgataaacattgaaaagggcattcttggtcaatatccaaagacgctgac
tcgatattcgctccacactttaagcgataacccattcattccttctttcattgctttcgaaccctagtggctatcgttgtagcgacacaaatactttta
tatctatcgtctgaagaacctgtataggcaaaatcaaaagttcatgacctccagtttttacgaagacataactacaaaccttgaaaaggcaatgtt
aaaagacacagaaagattaagttcacctgttgacaaaaaccctcagagctttgaacaacttgacattgaccatgaccttgaaaacactgtggac
atcaaaagtaacaatgctgatgaaggccttaaaagacaattaaaacctcgtcacatgcgaatgattgccgtcggaggttgcgttggaacaggac
ttttcgttggttccgggaatgcgctagcagacggcgggccagctagcatcatcatagcgttcgccatcattggactctacgtcttgtttaccacaag
tgctcttgctgaactttcagttctctatcccgtctctggttcattttatacatatttctcccgcttcatcgatccagcatggggttttgcggtaggaatcc
agtattggatatcctttgcagttacagttccgcttgagcttacagtggcaccccttattattaatgtctggcatgcccctgggcgtgcttctgtatgga
ttaccgtcttttatgtaataatcatcggcatcaatatttggggaacagcgggctatggtgaggttgaattcattttgagtattgtcaaacttattgctg
tttttggattcttattccttagtatcattattgctgctggaggggtgccgacagataccagaggtgtgattggcgtctcctattggaaatcgcctttga
cgtttaataacggaattaaaggcttttgtgctgttcttgtcatagcaatcttttcactttccggtacagagttagtaggattagctgctgctgaagca
aaagatcctcataagacaattccaaaagcggtccgtcaaattttctggagaattcttcttttctacgtgttagcattatttatgctaacattggttata
ccttcaaataaccctaacctacgcaccactggagaatcggatgctcttttatcgccgtttgtaatcgccattcaattagcaaatatcaaagcacttc
ccagtattatgaatgcagttattctcagttctactctttcggttggcaactctgcatcatatgccgcctcaagagcacttttcgctttagccaaaaatg
gtttcgcaccaaagttccttatgaaaacaaccaaaagagggcatccaatttatgcgataataattactttattactcggaagcattgcgtattttac
agaggcaggagtgggaggtgctctcttcgggtggcttttgtctatttgtggactctcgactaccttcatttggggaagcatttgtcttagtcacattca
attccgaagagcatggaaagcacaaaatcattctttgaaagaacttccatttctcagtatgtttggaatttatggatctatatatggagtagtcatg
acctttatagcacttgcagcccaactttatgtggctatatttccaattgataggccgccaaatccaatcagtttcttccaagcgtatttagcagctcct
ttggtggtaatcagttatacattttggaaactctggaagaagacaagcattgtcaacgttaaagaactcgaccttttagaaggcatggaaactga
accaaaggatgagcagcaggtaggaggaaagacatcaatggaagaaattcttcccatggtaccaagcaaaaccggaagtgataagagcagtc
aaggtagcatttggaaagagaaagacgagactattgtgacacccgagcctatatggcccaacaagccttagaacatcaaaggaaaattttggtt
ttattcttgatatttttggaaacccgtggattcgttctgcgtgatcattgtctcaaatccttcaggaataaaaatagcatcagcattagattagagttc
agtacatcaatatgcatattcacattcatacacacgagcttaagaggaattttgggaatcttcattagcattaggtgacgctagtttccgtgaatat
aattgatccatcacagtttctgctgcatggttcatgatgtcttcattcgttctggattcagaactgttcagaaagatgctagaagagtttgattcttct
ctggtaggaagttgagtaaaaacatcatttggattattgaaagcattattgattaagttttctaatccgtccatgtccacaacgtactttttgctgtcg
gcgaaatatatcttggctaactgcttaaccaatgctttttttgaacggtattctttttcaaagatgttttcctgttttctttgtttgcgaatttccgcatca
ggatctggcagagggtaagtatcaggagcaattcctggtactcggatatccaccctctcgttttttttgttttactgcggtacgatgtctttgtacaac

atcttttagctttgcggatgcttcttcactgcgcttcatggcagattcggcttcggtggcataatcttttcggatacttttgactgcctccttgtaatcgt
gttgcaaaaagttggcatctttttggcttggagctgtgctttggaatcttttacaaagggaggagacgacggaagaccgagagatacgtgaaaac
tggttgattgagagcatgttgggatttgaagtaccataaacaaaaaaagaaaaattgttttaatgttgcaagagaggttcaaaaaaggttaccaa
aagaatccctgccttggaaaacgcgcgaaacaataatgaaaaactgtttcaagagacttgtagaaagtacacaacaaactcgagaaaatctcta
tataaactctgccacaataagtcgttaatctgtagaataaattggtttagagaacgccgtgcagatggaaaggtgaggttgagccatatatcccta
tagcagttcctctcttccccatttcgtttcagtggacccgattaacgatgtcacgttacggtcccttatatattcataaaagccaatgaagaagtgtg
ggaccatcataaatataaacaaatattgccatgcacgaatgaagaaataaagtaaaaaataaatcgtaattgtaatacaaataaaagaagaag
ttttaaataatgtaaaagttgaataattacaatgatcttctcgtgaacaatgagctatcattttaaaggtttggtgatcctctcaattgaatctataa
acgaaattatgattatttttaaggactaacgaacttctaccatgaaagacgatgcatgattatttgggaattaagtgaaaattactgaaatacata
cctcaatacgatgaatttaagaatacctgagtaatacagtatcagggaataaggagaatacattgacgaaaacttgctatacattatggattaaa
gatgtctgaagccttctcactgtgtgtgtaataactcgaaaataatcaatagactctctgtttgtatcttcaggaagcatgaaaaagtaaataaatc
ttctcgtattgtttactaacgagtactcaagagcatcgttattatatccagtttttgcttgccaatgcactagttactcttggaaaggaaaagaaaaa
aaccattattcctaatccagtatataaataatcaagaaaaaactatttacatttttctgatatatgtcatgtattttgtatcggtctttttcttctcagta
ccgcgtaaaaaattttaaagggttagggttatcagttaagaaagtataaatattgctgttttggcacatatttctttgtgcgtcaaagcattttgcaa
aagctagcgtatctgtttcttgccttttaccagaaacagccgtttgcaaggtgtgctaatttgactgtttagtttttgtaaaccatttctaaagctttaa
gctttaggatatctttaccaatgttggggtatggctctgagataggtaaaggtttcttgaagttgtgaaatgattatcggttattaaaatggggttta
tacgccttgcgcctagtaagtacgtgttattctgtttgcgtcgagcatgtgatgggaagagttgaatggaccattgcttggcgtatggtctgaagaa
gcgataagagttgcccattgtagttggcggacttgtcttgatctattactgaatgatatattgttaggtgtatcatcaacgattatgattcagcatga
acgttcgcataatatgatattatgtcaatttgtttattctttgaaattatatttactcttgtaaacaataacatttgcataaaagattggttgattcaac
agtataagtagcatccataacaaaatcgagattaaaaatttacttacaattgaaacgcctagacaggtaagaaggcctatgttcctttgatgaatt
ttcaacttagatttcgtacgggttagtcattgtttcctccaaaagctagcgttaactttgccttgtttgttttctcctgctaagcgagtatgcatagtttc
gagaaagagaaaacagagagttcacatatctatattgcgacggccgcagggacagtgtccctgttcagtatttgggcggtttggcgatttcggag
gttcgaaacggtccataatatcccgaaagctctctttcctaaaaagctgccggatcgaactcaaacctttcgatcactattcggctatgtgaccaga
gtgggtgatggggataattttcgtttttaccatacgcctggaggttgttggctaggatggcattggttgcgacaagtgccttcctcacggtctgcttt
ggcttccaaaaccatttctgttcgattagctggggtggatgcaccagaatcaggctactttggaaagaaagcccaaccattttccctagatgccaa
acaatttctgtcgaaaacgattctaaacaaaaatgttcggataactcctctaaagattgaccaatactctagactggtagctggtgtacagtactat
ccaatctctcatttttctggaaaagggatattggtcccattatggtccaaaagggattagctgtagtttacgatgggaatgatggtgtcttttatcc
atcaaaaaaagaatacttgcaatctcttgaaaagaaagctaagaagaaagaactgggtctttggtctcaagggaaacagctcatattaccatct
caatataagcgaaacaattaggtatacatacgtcttactcttccttccttaatgcatgtcttttgaatctagtattgcgatttttgagcattttccctag
gaattagagattttcctacgctcactacatattatctatcaactaactcctctatcaaaaaattattttactcttaaatgcattgtaaagcttctgttca
actattgtttacaaaataatgaggaaaccctaaaagccactatccaacattatgcactgccttattcgatactaatggtttacgaacctccgtaaag
gtctgctacttaccataaacaggacgaactcttgaagtgtaaactatagcttttaattaatactttattttttttttattttaaatcaaggaattggtgt
acatagaagggactcatacttgagcagaagagccgaaatgccccccttccaagtggatgtcttcatatgaccgagctgtgaaaagtaagtttcaaa
ctttctataaaacactaataataaatgtttagaggctacgaaaatcaaactggctgctccgaagagtaaacatgtggaattgattttgcaagctac
gtttgaagatcctgacacactagatacagtcattcaggatctttgtgagcggatgaaggattcttcatggactgtcgtttttaaatcactaatagtat
ttcatttaatgctaaaagatggctcccctggtactacgatagtagctctgtcacagcgacctcgtattctagaggtggtgcgagcgtcctccttgac
aactcaaggacaaaatatatataattatagccgatttctaagtgaaagggtaaaacaatatggccgtttagggtttgattatgctcaagcgagcg
acggagccaagaaactcaaacaattgactgtcgacaacggtcttatgcgtcatgtagaaggcatccagtctcagcttcggcgtctcttaaagtgtc
agttccttgtagaagagattgacaacgatattaccgtgacatctttccgtttactcgtgggtgatttattagtgctattcaaagcagtgaacatcggt
gtaatcaaactgttagagcattattttgagatgagtcatcctgatgcagaacaatcccttcgcatatacaaaacgtttgtccggcagacagaggat
gttataaactttttgagcactgctcgttccttggaattcgtcaccaaattcccggttccaaacataaaacatgcccctattagtttgacggcatcact
ggaagaatacttaaatgaccccgattttgaacaaaatcgtaatcaatatatgaacaaaaagacaaatccaaatatgcctcgaaatgtttttaacc
tttccgagtctattcatccacaagcaccagaaggaagtacgaaggagaaactctcgcttcctaaggataatgcttcatcctttgtagatgaaatgt
tgcaagaaggaaacaaccttccattgaatttaaaaaattcctctccttcatttaatcaaacagcgtttgatattccctctattccacagcagtctgtg
ccgaactcattaaatcccttcctaaatgcctctataccgtatcaaccacagcaacctcaagctgctcctatgtcggctccacaaatgacaggaaat
ccatatgctcaaataccatacaactcccctgctaatccttacgtccaaaattcgtctcttgctccgccaatgcaaattgattacactccttcaccagt
tactaatccctacaactctcaaagtagagcacagtctgtaaatctggatattagaaaccctttccaaaactatatccgtcctaacacacttcaacca
cagtcgacaggatttcaatcaccttcaatgactcctgcagcattgcagagatatccgacggattccgcatcctatgttagcaccgaaattccatcat

catttactggagcaccgaaaggcttaacaatggagagaccaatgtctgtcgacttctataatacgggtgcgaattataattcactgcctttgagcc
agcaaaacaccatgaacgtccccaaccattctacctctactccttttaatgtttcaaaaaatcctttcacaattaggaaatccgaagagattgaaa
gaatagcccagaattcaacgatcggcgagtctagaaatccattccgagtataatatggtcttacctttatcctttaccgttagaatatctagctagg
catattcacaaacgcactgtaattatagcaatgttaagttaagtcgctccttgatttcgatttgattatgcttaaagccatgtaattgaaagtattcc
attagcgttcataatctactcatcttgaacattaataatgtaaatttgttcctcgcactatcaagaaacatacttcaggatgatgcagttgtgtaaac
cgctatagagagtctttaagattttggactttctatcgtatatgatttagctcatacatcacttgtgcactagtatatgcgttccagcatccacaatag
taatagaggtacttcatactcttacatgaaaccattttgatcgctcttgctcttaatttgaagggaaccaggtttaaccttcaacaatcgatgtcctgt
cgcctacatgtatttatttctgcaagtttctttccatttaatacttcaggaactcgatttgcaatttagcttcccctaaactttgtttctattcaaactgg
atcaatgaaaaactacttgttccatctattccctgatgcttgctagtctgagatgagatataggtcacaatcttttcaaaggttttcccgttcaatttt
ctagtccgtgagcatttcctttatattggcaaattaacattagaaaaatggaaggtttacaaacattgcactatcccaaattcttattaaaagtaag
cttatactgtaaacgatacgatactgccacatttggcattttcatccattccttacagtaacaacgtcttctatcgagagacattgagagtactgtga
aaatttattactgttatagcttatagatgaattggttactcttgcttccgtttaaagcatttcttataactattctttgcaagcctcaaaatgtatcgatt
gtttattcttccagaatgtattaggctgtgtttattttttccgtaaagaggatgtaagcttattctaaatgggacggttacgttgtatttatttttgcatt
tgccggtgatttgaagttaaggcttctttaatactttcaatggtttgttcagacacatttcccaaaaacttctttatttactcgttcgttgggaggaata
aacagaagtatctattatttatttacttatctttgttgttgtttactttcactaacgaatttgttgtcaaagtcggtattgaagcaagtgtaaacaaacc
gtttccaacttcctttctacaataaccctcaattcaatattcaaaatgagatatgctcccagattttattctaaaaaaggacaaaagtttcgacttat
ggtcactggttccagttgtactggatatcagtcttgtattaatgctctttgcgaaaaacacatcttagacgagcaattggaagttgatcctgaacaa
gctcacttggagagacctttagagattaaggaacatgaagctggtacgctcatagctagccactgttttctaactttttttccagatattcaaggag
aggaattccgtgttgctctgactgtcattgaggcctgcggttttggcgatatgcttgatagatcctctaagtatgagctatgaaaatattttgacatg
tatgctaatgaacgatagctttgatgttgttactgagtatttggaaaatcaactcgaccaagttttagttgaagaatccaagattcgaagaaactct
aaatttgttgataaggttagaaatcttttcttttatttatatctaacttgtaatagagagttgatgctttactctactttattgaaccgtacggacattg
tttatccgaatttgatttggaagcaatgagacgtttttccagaagggtaaatgtcatacctgtcattggagttgccaatgccttcacaaaggaggaa
ttgaatcgcttcaagcagagaatctttcaggaactcgttgatgagaacatccaagtttttgacttcccttttgatgacgatgaagatgaagatgaag
ttatcgaggaaaataagcggctcaagtcgatccttccatttgctgtttctggcgctgccgtgggtgattatcaacaggattccgacaagcttgctaa
acacttcccatggggaactttttatgttgacgatccgtctcattcagactttttagatttgaagactgttctgttcatctctcaccttcaacaccttaag
tttattactcagcagttttattatgaagcctatcgtacagaaaggctttctaatgcttcgttatcctcgaattctttgtatgttgtttactttatattcaa
atgctaattatatttataggcatatgctagttgaccatgagaagccaaagctagggcctgacgaactaagtgaaactacccatcatgaaacgacg
gttgagtctgaaattcatcaaactgtcccagatcaaacttctcattcacagacgagttgtcaatccgcgatcaaaaatgcgcaatcaaattcagat
actaattctgcttctccaagtctcaaatccaaacaaagcttacaaatgagagactctattaaatccgagacatcaactagagttaaaactccttct
attgaaatgagtttacgtgaatttcctcaaaggaactcttctaagaacaacaagcaagttaacgaaataactgaaactaaagaattaaaatcgg
acgatgttgctcacggtcgctttgaaaatgttccctataataagacaaagtaaagaaacagaaatggttattgctccttcctttagtcaccaatatt
cgggctcatgaatacggttcgtttattgttcaattaagtacttagtatacgttatcagaactagataattgatgagattccacaagtccatttttaag
taaatttatagtcatttaagttggatatgtaaacacaagactatttgcttgacaatatttcgcaattccacggcgatttttgcagcaattctctatcta
gctaatattttttaatttctcagacataaagctgctttacccaaaaaaagattaattgcttatctttagttcatttaatagcaaaagaatgaactgtg
ctattgaacaaagtattcttcaaaaaatgatcagcgttctggaactattttagacgactaaaaatcagagaaggtatccaaatttgatacatgtgc
atcagcgatgcaacaggagaataacaagtctagttccataattgtgcgatggttctcttcagaaaaacttatcttagaaaagaaggatatagaag
atgagcaaattattgaaccaagatcttcaactgactatactctgctgcgtcggttaattaaagaacaatcgattgctgctaacgattttgccattact
ctgaattcctctctcaaatatatacttcttaattgcaagtagcaacttcactatgggcatcttgaaggaattgctgaatgatcaagtgcttctatccg
cattgaacagatgaaagacaggaacaatagaagaacgctttgtacgcagccaaggtaatccttaacgcgctcaaaaaaaacatcaataagaac
agtattgcctgctttaaaacagaatgggccattcattctcagcgagacaacataaatatcctcaaagggtacatcccatgtgtaacaacaaacat
aattttaatcggcatatacatttatgcataattcaagagtactgccaacaaggagatttactgaaccaagttataaaacaaagatcaacaactga
agataaatactgattttaaaagcagatcctaagggccattaactacttacacggaaatggaatacctcatcgagatcttaagacaggaaatatct
tgataggttgtgaaaatcagtgacttcgagacaagtgaagcagtaattgagaaaaacggatattcaaaatccaagagaatattaagttggctgtt
gacagattgacatatttagcaccggaagcttttagtgaagagcgtgagataacgcatacgacaatctagtagttataagccattaaataacaaat
ttagcatattgtgggatccgatcagacctttagttgtgcggagtctttatcaaagtcgattttactgggtatattcccttccagttagcaaaactaaa
ggatagaatgtatagttgatacgttcagaatttataggaagattctaaagaaggcactactcagtatcctgggaatcaagatctagcattaagga
gtctgccagctttacaaccacttcggcgaatacatcagggcgcgaaaaagacaattctgttattactaactcctaacccagctcatctatagattca
atccttcattttccttgggtacgtaaaatcgattgctgttgtccaaagtcaaaaccttttttgcagaatcacattcttcttccgatctcaaagagttct

tttcgacgttccaaacctgtttcaaaaataaacaacttaccaagaatccataatcacaaacggttgtttatgaacaatgatcagtgactagttcatc
ctataaggaattcatccatcagccaatagtgattaaaaaaaaaagcagtatacagatgtttattaatttttaagtaatcaaaatacttttacattgc
ttttctataggaaaagggaacatatgagtaggtgcccggaatccctactaccttcgctgtctaatcaaatcacccataataaataagcaaacggtg
aaagcatttctaagggaaaaggcaactgtattggctttttctaatttaatagaattatttatctagcctttttttgaagtcatgcataattttgatcatg
cgtcgagcatagtaagctaattttcgatttctttatagaatgtgtcactgatcaatttagacattgggcgattgtctaggtaaaggagccttcggggc
tgtttatcggggactaaatataaaaaatggagaaactgtagccgtaaaacgcatcaaggtgtctaaaatcatgaaaatggacctttctgtcatca
aagtacgaagctcaactttgtttctggttacgaagtgattcctaaccgtgtattgttagatggagattgatctgctgaaaaacttagatgtatgctct
ctactgttgtcgattgaaaatactaataatacgacagcaccccaacattgtaaaatacagaggctcttatcaatttgatgactcactctgtattgtgt
tagagtatgcataatttacaattctgcgtaccggactaactaagtacattagatattgtgagaatgggtcattacattcaatctgcaaaaattttgg
aaagatacctgaaaatttagttgctatatatacatatcaggttttacagggactacattacttgcataatcaaggtgttattcatcgcgatataaaa
ggtgcaaatatattgacaacaaaagatggaacaatcaagcttgcggatttcggagttgcaacaaaaatgaatgctttagacgagcatagtgtcgt
aggtagtccatactggagtacgtacaaaacattcggtaactatgttttgactaacttatttagtggcacctgaagttattgagttgatcggagcaac
tacttcttctgatatttggagtgttggatgcacagtgatagaattgttagaaggaacacctccctattataatttagatcctactgctgctttgtttca
catggtcaaagatgagcatcctccctttccttctaatatttcattatcagcgaagagtttcttggctcaatgtttccaaaaggatccaaatcttcgag
tcgggacaaaaaaactattaagacatcactgggttactgctcatcaaacgtctaccaaattcacagatgctattgacgaggtccaaaaatacaac
gagagagttaacgaagataatctgaatcttattaatgaaaccacaacgcataacattaatccaacgttgcatgctgacagacagtcctcttatcat
cctcctgagccacctaaaacacctccacctattgaaacagagagtaagatttttttgacgttgattttatactaacttatttcttcagaatgcgatgttt
gggatgacgaatttcaagggactctaaaaatcaataacgatgtactggagaaatctgaacattttgtgaacttttataacgattttaaaggtaaaa
acactacttcaagttctacaaattcgactccttcaaaaagcagaaaatccgttagtgttgatcgggggaatgaagttgtggactatgtacaaaaac
gctcatcaccggaattattgacagtaagtgtttgtaatatgttttcctatgctaatggatatagcctgcgcttaataggtccattgatcttcaacaga
ctcccaagcatcatagatatctttcaactggtaagcaaaatatgatttatcctttgctaatcgagatagaactaaaagaaaatgttcctgatggttt
ggagaaattcatcgaaacccagaaggacagtgagttcactgatgtgtttcctgcttcttcaattaagattcagggcctccggaaagaaaaagggt
tcggaacactagttttaaataaatcatataattcgtggaataatgaggatgaagaaactgaggatgaaaatgagcttttcgattctatcgattctg
aagtcgaaaatttgaacttggagaataatatcctactggataaacgatctcgattaatttcttatttgaaagatattttgaagggattgattgagaa
aaaaccggaagatagacaacctattgtcactcaaattgtatgaaatttacttctaaatttgtaatgctaataattaggcttccattttaactgaggat
ataagtctgaaggacgaattggtgaaggcgcatgggatgctccctcttattgaaacgctgagcgaagttaacgatatgcctttacagctattatta
ttaaaactcattaacacggtatgtacaactgaagttttcgttagctaatcgacaagattatttatgataactattctacgttacacagattctgtttct
caggcggattgcccgtcgtgttaaagttctctcaaagctcctatccatgggattttcgttatgagagcgccgttttcattcagcaaatgtacagagc
atcgtcattatcccttcaaatgtttattggtgtttcagggcttcatgttctgttgctatttctaaaggaagactatacaaaaagtaaagatttagttttc
gtaggtgctgaaggactatggaagttacttagacaacaagactgtataccaaagaatgatgtctgtcgactagtcgtgcgttctgatgttttagag
ccgatgacaaaaagtatgcttaaggctttagctgcggatgatccttcttctaggctttgtttgtcaaggctctgtgaaatattgttagcactgtctca
gtcagaaacttatgttaaggaagccctcctgaatgaaaacattttccgaagaaacttacgtattcttttatatctacctcttgaagaaatggtaaca
atgttgcagtttattaagcaaatttctatggtgccgtcttcaataaacttactcagaaaggtgcatttaatccccttttaacacacatactcggaga
tgcaaaactaaaaaatagtagaaaagagatacggaatgaatctttggctactttattcaatatctgtcgtttagataagaaatcacaggaaaaag
cggttattgctggagctatacctctattacaagaaacagccgtcaaagacaaaattctaaaggaatttgcattgccaatcttattgacactgccac
agtgtagccccatctgtaggcaatacttatggaaaaacaaagggcttgaatttttctctcacttcttcaagatttgaattggcaatcagcttccctg
gatactatatctgtatggcttcagtatgaatttaaagagatacaacatgctcttttggaaaaacgatatgtgcatctagtcctcaaagtgttctgttc
ggctcagtccgcttcattcaatcgcttgttagacggcattagtagaatatgtcagctttctcctaaactagcagcaagctacggtcagcctccaattt
tccaaaaattaagagatcgtttattacacggaacggccaaaccgattacactactgaatgctttccaaatgctcaaatccatgtgcgaaagtaatt
catattgcaagatgtatatcactcattatgatttgcctgatttaatccatcagcaaagccggacaagtgattccgtgttagtacgagagttggccaa
agatctcttaagacaagtgaagtactctaatatcccgagcagtccgtctatatcaaaacctgacatgcccccaccgaaacggtcgtcaaaatcaa
ctgtgaatcgttaatccttacgatcgaaattgcgaaacatggttacatcgcggttactatataatctgaaactttcaagcagtcctaagtcagagat
gatggagacctctacattaattatcacgttttccgtttatgacttcctttataatgtttacttatgttaatgaatctttgtaaatttcatgatgtagtgca
cgttaactttgagtaggtatgcgatctttcattttcagctgaacaaaccgatttcgtactataccattgaaatcaaccttaacatatcccagaggtaa
ccttgaattattatggaaagcccaacttgagtttaagtgaatatcaatgagaaataaagtattacaatttaattgaactttataattattattgtattt
aaaaatagatataatttaggaattaatagtgtttttactctgcttatagaaggtataatatctgattacgcatatcccaacttcattttctcaataag
gccttcgtttctatactagcctcgttttgaggaacatctattgtcctttttattaaattttgatcttaatttttttgtaaaaaaaagctgtacatgttctaa
atttcccgtgaagtaataaccaatgcacaaactcagttcgtgctgaaaatacaaatccaggatcctagcttttgtctaaggctattgtttttttttct

ttgagttaaaatatatttatttgagatgcctcatacagcgtcaaacacaacattgaaaaacacagtcaatgtatttacttcttcactcttaacaagta
caggaacttttccaaagccggttgttggagaatcaatagttcaatctggtaatcacgcttatatatttggtggtcgggatgctgaagattctagcct
cacgaatgatttgtacatcgttgacttagaaacgtcctcctggaaattggttgaaaatcaaggatctataaaacctactccacgatacttccattct
ggtattatttggaacggcaatcttgtgttctttggaggcatcgggtttgacgaagataagaattcattgtgtggtttaaatacgaccgagatttttaa
cctggagaccaaaacgtggagaattattccagcgaaagaaagtgaaaccggaagtgaaagtgatgcaggaacgtctacatgcgagcctctacc
aaggtatggtcatttatactgcgttttgggagattatatgattgtatactgtggacaagatctgtcaaataattacatcgaacagatcaacgtttttg
acctgataactgagaaatgggtccacagttctccatttgcttatcattgtggtgtttacagatcaaactgtacttccttagatcatgacagtgattttt
ctaaattttgcactgctgaaaacaataacaatgatgaagagaacactcgaagacagaaccaatttgttggttcattactgttctacctcaactcaa
acttcgtcaacgttaaacgcgaacttattctattaaagctgtacaaagtatctaataccaaaggaaacggaggaggaacagataaacgtgaagg
atctaactcctgcttttactttgaggaacaagacataagtgaaaaatttgtaggttcctccatgcctccaggacttcgctttccacgtgtttacatggt
cggagagaatttggtactttctggaatttatcttaccagttctcgccaggcgttcgtcttgtgggtgtattcgttagatcgtgcaaactggattcaact
agatactttgggtcttttaaatcatggctcgtggtttaaatgtctaagtatgcaacaaaataaccagcttgttatctttgggaataaaacaaggaaa
ctaatacaagactataatgttcgacaatcaaactacgataacatggtttatgttgagcttgaggcatatggtatatatcgaaaaccgaagattcca
cattactcggaaagaagcgaaaaattaggtaagctgctattaaccggagtaagtgacatggaaattttgactctagaacgaaaccgtattccctg
cctttccaaaattctgtacaagagatggccaacatttcagagactgttagatcaagcagccgaacagaatcaggatgcctttcgaatgagcctttc
cgaacttgggcctcaggtaacggagctaccctttacacctataaacagtactggaagtcgaatgttatatatgccttatagcttcgaaacatgctct
actttcctgtattacttgtattgtggaaagataaacgaaacttacttgtcaacaaaaacactgtgtaatttattaattcttgccaaacatgatgaagg
gatggagacgttattccaatatattgtacatcttttgcacaatacgctgaaccgaaataatgtaaagactatttatgaaatagctgtattgacaggg
gccaaaggactgcagctacgagcattaaggaggatcgcacgcatagaacagaatggtgttgcagtatccaacccgtcatttttgcccaacttgga
aaatcaagaattaccttcgtcctgatttcataaatgctagtatttttgttaatgtttgttgggcgctttgctcttcaaaagctctttctgcctctaattct
ttttgattaattatttcgccttgcagctcttcctgtcgagttacataaccggtccaattatactgttcacgttcttgcgttaataaatctcttgcttcttcc
cacagctcatcttgacgaagaggttgcttttggttcttggactttagagacgtatcattttcatactcctctaaagttgtagcctcccattcttcaacat
ttggaagaggacgagttcgtaacaaggttgtgagaagcggttcttgctcacggactggaaattcttcggaaggaaaaatattagtagttcgtaaa
ctttgaaaatgtatagctagagtattgcttagggaatggatttgggataatagaatattgaaattcttatgaatagtaggcctatcattgattgcatt
aatatgagagttcccaaataaagtcaatcaaaatttgattaagagctttttttcttccttcaaacttaccatggtgcgagttgttctgtttggtgcaga
atcgccaggaaatgactcaaagagtggacgatttgcgtagttctttgccgaattccttctaaagattcaacggctttttcagaggaaatgatatcca
tcggaaacaattgattctaccttccaaggcttaaacgcgtgaatggattccctcttcaagttgagttaaacgacgaattgcgagtccaacggtcac
acttatagcggaaaatagaggatatttgtttaccttttttacaccaaaatacaaaatgtttaaaaacctccaatcactggcagctcgaaatctctttt
ctattatgactcaaaaaagcgaattccagtctttgacttggttttactaagtagaatgaagtacctcgctccaatgcgctatttaattttacaaagct
tcttatagggcactagtaaacatccttggtgtcctttaggaaacgtggtatttccaatgttctatggctattatgaaggagaggattgattttgaggt
ctatcatgaaagattatatgggttatggaacttacatcgataattttgctggctcaactaacgatctcgttatgaaccctcaaccatcgcagtgtgtt
attaccaaacagttactcccgaacggtggtcgaaacatttcaattatctttctaccctttctctttccttatattctacagcttggtggaagtttcatta
taatctgaaattgcactaaaatggcagacagggaaggcggtaatcgaaaatactggctcatgaaagctgaaggtgagtcccgtattgttaaagg
cattgacgttgcattcacatttgagatgctggaaaaaattacaaaagaaggaaagatggagtcttggagcggtgtcagaaattatgaggctcgta
atatgattcgagatcaaatgtctataggtgattatgcgttcctttactgttccaattgtaaattccctcatattagaggtaagtgaacggtttcaatct
ttatagctagaatactgacatgcatttcaaggagtaatggaggtaactcgattttcaatattgaatggatttgaaaaggatcacaaaatacttctta
agctacagtggatgcattacctagagtctatttagatttttatccattcattcaagagtatgacacatagtccttttactaacacaatttttttgtctag
atttgctcagaacctcatccggatggtaagtgcaacctgcttatttcgacagtattttaacctacttgaagattcttgctgggacccgaatgatccct
actatgatcccaaatcaaccaaggaaaagccgcgatggtactcggtaggtgtacaatcacgatatccttttaaaaggcctgtaactcttagggaa
ttaaaaatgcatattaatggaaagctttcacatatgtccttacttaatcgggcacgacttagtatttccaaagtcaataaagatgaatgggatttca
ttcatcaacttgctgaactgcccgaacccagtgaagtacaagaggcaagggagaaggaaaaccaaaaggaaacgaagaagagagaaagaag
ggtagacgacgatgggaaggaggacaacaagcaaaagaaaagcaaaagcccttatatcgcagcagctggagatgatgatacttcattaaacg
ctattaagcaaagtagaattaaagaatgcaatataaaataaacaataagtgtaacaacattagcaagcgaaccttagaaaacgagaatttaatc
tctcataaactatcattcatctactttttcaatagattcagtcaaagctcattttccaagcttaatcgtatttttcataccggacgaatgtttgattccg
ataatttcaagatcatgaattagaaatctttcttcaaacaatactcccgttaattgcttcccttctccgaatgtcccatgcccagcatgtctaaaaaa
cccatactccaaattgtcgtcgattagcatagataccccggggctttcgagttgcagtttattcgtaacatggttccgcctgagcccaaagcatata
cctaaatttttatcaaatacacagcaattggaatcctttcccgtagctcgaaaaacttgatgtcttgagcggagctgaaatagtagagaatctgaa
ttaccgaaacccccagaatgagactgcttccatggagttgttatgtatgcaccaaggacaatcgaagattcttcttcaggttcatgattctccaata

atcctaatatcgtcgcgcatggatggggagcattcggatactttctaggatatgtgttttcaaaagtgaattgtcgagaattccttttcttctcagga
attttatgacctttaagcagtaggatagtaggttcgtaaaaattcaacacatgacgttcaattgcggacattgagtaaccatgacaattagcatta
aaaagacaagttaagtttttgtaattaagtgaaggattaggaataaataagcataattgggcataaagaagggggttcaagacagagaagtttc
tgaaacataaactgttatcctgagctggttgagcatactgtggatgtgaatagaagatatagtcaaacagataccccagatgatcaaggaagaa
cacattttcatctagaaaagttataaagtcgtcgaaaagcagaaattgatttgtcccgtctttgttaatgccagaaattaaagaatcagcaactttc
tctgctttttcataatatgggcttgaaatttcgtttatatggtaaccaatggattcacccggctgaagatcgacgatgcataatactaacacacaaa
ctcggtgaaattttgtaattcgcatacgaggcaaattctttgggtcaagtgactttttccctacatcctgagccgtttttctttcatgaacttcgttgag
tatactcttattttcctgaggaactgagtcacaccaactatgaaaaataagtaatagtcgttcgttgtcgctccatacattctgatgtcgtttcgtaa
aacacgctaagaacctcgaaagagctgaaaacgttaagcagcgatgcttatgatgatggtaaccaaaacagcaggctgctcgataaaaaaacg
aagaaagctgcaaggtatcaggaatcccaaaaaaggtaacgctaatggatcatcgtcagtaactttacggaataacagagaaaataaatgaat
cattcttttcattctcaagcttacaaggcatcttcggtccagactttctccccattttcaaagcttgcgagggcattgaagttttctaataggattagc
aagtcgccgtttgtagaaagtgaagtgtatacccttttaaattgtatcgttctaatggattgaagtttttcaagagagcagatgtttcctgcatgtatg
gtttcacaattttaggagaatcctccgcatttcccagtaaggctttaagatttttcatatttttaacatcatttctcttcacaattgaaagaagttaagt
tgtttgaaaaaagtgtttagagaaaaagcactctagaattgctttatgtataataatcatgatgtaagaaatttttaagttaaattaagcgcaaag
aaaataaacgctggaaagtaaacattgatgcatgaaaggtttgcttcaattattggagaagactctcataaaagtacagacttgaacaaaaaaa
taaaattaacaattaacgatactaatgtacaaaatgctttttctatacacaaaaaacagtaaacagacagaaccatgataaaaccaaggaaatc
aaaaaagtaaaaattcctgaccactacggatgatagtttttcagataaatttttaacaatgcagaaaggacgaaattataatatatagataatgc
tgacaatcaagtgggattcgaaagccctcgatatacatttttgagcagctgttgaagctcttgtaattgttcgtaaatgcccctttcaccacataatg
catttgagaactcgtctaatgaataatagtctacatcatcagaaggaatcgttttatcgatatctagtaaacgcttgagggattcacattctatcaa
aaattcatccaaattctccattttctaaaattgcgttagtaaaaagtacattggaagtagaataaagagtaatttgcgtacttgagaagtatcagat
atttctcgatagcttaatcgcaactcgttttttttattttgaagagcaagtctttcctgccgaacgtcggacagttcctgttgtaaagcaaatttttcttt
agctgcttttctagaagctgcatagagagcttggttcacattcagcaaatcaatgaattctaagagccgtattgagacatgctctccaaaagcattt
ttcacctttaaaagatagggatcttccggttcaatttctgcaagcagttcctgaaataattgttgaattacgtcaaatttgtttagtttcttcgttccat
ttacatttttgatagccttgtcaactcgatgctcactagttcgtccgttgcttgtggcggtgactgttctccagaagccgaagatgcttttgttggtaa
tttgttgatagaggcgtcgttagttgacgtctctttttctatttcttttgcataaatatgatgagcggaagatgatattggcgaggggattcgttcttg
aggagcctctgaactaacggaggccctgacactagagtctgcttgctctacttcaaaattatcttcaaaagtagcaaattcttcttgctgttgtctag
ataagggagatttctcgttttcctgttggatgcttgttgtttttcattggaatggtaaggttgaatgaagggttcaaataatttccgcacagcttcttg
tttgttacgtttcttatcactgcccttctttgtggaaggttgtcgcttgtctgtagctggattatttttaccagcggatgcattcggagtctgattctttcg
aggccgtcctcgtttttctgtctttaacgaagaagttggttcaggccgtttctggggtttcttatgagataaaaaagctttgtctacttgtgcttgttga
gaatcaattactccattcacttcctgggattcgacagtagctgctccattcaatcctctattatttttaccatcattaggagtggaagagagactacg
atgttcagtatttgagggagaggaatcaaactctgttaaaaaatttggatcaatagaaaactcaacatctttaactttgtaccgcctacaaatcatt
agtttctactacaattttcaattttcatactcagcaccacgcattcgcgcagctcttctctgattcacatcatgaacatcttctcccatgatttgaatag
ttaattcagcgaattggggtacccattaccgtcgacaggataatggttgttgacgacgtttctacccagccttctcaacaaatgctaacagttttaa
cattcctagttaagagtattctcaacaaaagagtgcttaatggtttatttttatttttatttatcttgatccttttttttttttttttttgcaatgaaagaa
aaagcgtaagtatgaatcaagtaatatccaaagttcgtttatatatgagcaagtaggtcagtaagaaacgcgtcctttcgcgtaaatatctctata
ttgtaagctttcattaattcgttagatatctaaaggacagaaaatgatgagttgattcaagccctgtaatttaaccatcactacttcaagtaactgta
tacaaatagtcaatttttgaagacagtaataattgatcatctcttcttaccacatcggaggttgattccttcgtattctgcgctaaactagcatagtta
taaatttgcagacttcctccttcgagagattcctactcagtattattctggaaattcaaaatttattgactttacggtccatcaatttggttttcttctta
gctgtgaaaaggacaaacttgggtgaagtcgtccacttatacctagtacgctacatagaaacaatattgaatttctatgactggaatccccccttatt
gaccttttttgatttaaataataattctgtattgctgtaataatggaggatccagaggtcccgaagaaaaaatggtatgttctgaatactatgcgatatt
gtatatactaatacaactgtagacgagaaatgcccgctttgtcgagctgatcgttacttaaaccccaatatgaagttgctcataaacccagaatgc
tatcataaaatgtgtgaatcttgcgtcgatgtatgttatgagggttttcgtgtttgctacacattaatcaagagcgaacagctaatcatggatttcta
gagaatctttactacaggaccagctgcttgtccaactccaggatgcggcaaaattctccgaaaagctaaatttcgtgaacagaccttcgaagatg
cacaaattgagcgggaaattgacgtacgccggagaattgcaaaaatgtatggaaataatttattagaacttactaacgagaaatccagatttaat
aaaggtcagcaagattttggttccttacaagaatacaatgactacttggaagaagttgagaatttaagtatgctgtaacaaaaatgtgcaatttat
caacttgtactaattctatatctagcttttaaccttatctatcaatttgacgtcgaagaaacagaaaaaaaggttcagcaatacgaaaaacaaaac
cgcgagtcgattgcactcaactctgcacgagctgcagcagaatctcgattacttgctcaaaatgaaatcctgttaaagaaacagaaacaagaag
ctagagatgccattatacgagaacatgagttggagcgagaacggcgagaacaagtcgaacagcaaatcattcatgacttggctacgtccggtaa

agatccaaataagattgtgaaacttacagagacgcttaagaagcatcgggaaaaaagggattctccaaagccaatttcttcgcaatcttctgctg
catatcccgatcttcaaaacctggcacaacaaaggctcaaagaagagcagatgcctttcagcccgcttgcaggtgaacgaaatacaagcaaata
ttatgattattctatagagaactataatgacccgtatgtattctatttcttgaaaatctttatgctaatcgtgatcagctttctggataaaattgccaa
ggaacccaatcggtcaattggttttagagttgaagattgccatcttcgatgcttacatgaagcgttcagtggacttgatttcgatcttgcaaaaatta
accctccagtcaaggacgactcgaaggcgccggcagccatgattaatacttcataaccattttttgcaataaaaacattatgactttttaactac
gtttgatcaatcacaaaaactttttccctaaaaccaaaccaaatttcattcataccaaaaccatgcaagtagaagtttatcacaataaaaatgtctt
aataaggcttaaaacgccgattgactttaagatgctgaattcgttgcttgtccaattcaaatttgtgagccaagtcctgcaactttttcttcttttcgt
ctcgtagttggaaacggtaaaagtctttgtggattcctgtctctttcttcttttgtaaaagcttttcagcatcttcaggatttccaacaggttgatgagt
accgcgaacaacagggataaaaccgtcttcgtcgggttggtttctcaacttccgaagtcgattcttttcagtaatttcttgttttgttaatctcttcata
tacgattcaacgctttctcccaagactcgagcatttggaaattgtcgatcatatttttccaaataacctatccaacgaaaagttaacatttataatac
gaatcataagaaaacttacgttttaatcctgatgattgtgtagtattccaaggaatagtggttgatttctttgatgctgtctttagaacctgttttacct
ctgctttttccaataacttcacgcgtaaattcgttcctgctttaagtaatccttgaattgaagggtatacagaaacgaatcgtcctcctaatttcgag
atggcatttcgtaaaaccttttcagtggtagtgaccggcacatttactaaatatacagatttttcttcaggatcttcgctgtcagctcttaaaaatata
ctttgagtcttttcagaatcactgagagtaattctgagcttaacgaatcccttttctttcaatttgtccataattgttgtaaaggaagttgagagcgaa
gactatgttagaagcggaattttagcgatgtccatttataaagttaccactagaaagactaaactgggagaattatatcaatcttttacaaatgtac
gtgtttttacataatattcatgcctttttgttgacaaagtccttccttttctttgaactgtcatcctttttaaaggagacctcacaaaaaataaacgact
tggttcatgcattaacggtaaagtagtcaatacttaaataataaaattaaaagtttcagaaaatttaaactgattaatagtagaatgtacagcggt
tttggtgaattagagcgtcggtgattcccttcgtttgccttctttcataattaattttccaaacgtctgtagcttgctcaattcagctttagggcgcttcc
gactgacttttattttttttaaagaacttcgctcttgaagcacatcttttttttacatcgtctaaacttgaatgtgactccggagactttgaagcgtattt
attggaagaagaagaagttgaaggttccttagaaccgttcgcgaagttaaatccgtttgaatcatcaaatatattaatgtcattcaagagagagg
attgtttctgaacttcaggtatttcaaaatccccattcgcgctagggaccacaaaccggtcttgagtgatatcaaacggggcttttatgggagttgt
aaaagcctctctaggaaatacactttcaaagccattcttctcctttttttgtatcagatgccttgcctatataagggctcttgggatcatcattgtttgta
gccaagctagactccttttttctgttttcctgtggagtcatgaattcgtctgctctttcccttgctttctgaatcaagttttgatttcacactcaatcgact
ggttgtaggagaaaacggttctttttttctgttcgtattttaaaacagtttttttgctttaagagttccgaagcttcttttatgagctttgatttaggcaatg
attgaaacacccgctcaaattttgagtccacagtctgcttcaagtaaggatctttgttagaaattagcatcaacataaaaatcgagaaaccagag
agttcaactgatgattgataatggatatagaactccacctctagattcaaatcaaattcaaattcaaagacgaatagcgtgatttgatagctatga
gttttctcgatgctttaactaaagatccaaatacctctgtatgaaaaaagaaccaaggaagctacgttaattgctacttttccgcaaacctccacgg
tcttttgacgaaagtaaaatgatataaaagattgatgaagccaatttaaaaggaggcctgtgttcgaaaagtggtatagtagaatatgaataact
ttttgcagttactaaaatatctttatttatcttataataagtcttatgatgtctacggccacacctaggcgaaaacaccagttcccgtccgatcactgc
agttaagcgtctgagggcctcgttagtactatggttggagacaacatgggaatccggggtgctgtaggcttttcttttgttgctctttactttttttcatt
ttgcttttcttttttaatacgtctactactgttgcttgcaaccgttttctcgatagaatttttgcaagctgttcctttacaagtgacaaaagactaattac
aagttcaatgaaaagaaatggaacgatcgtattcaattgcaattaagaataagaataataggaataataataattaagaacctagtatgatccc
aatgatctcagattacggttcctttcaatgcgcggattgaatgcatgcctttctattgtccacttttcttcgtcatttccaattcatattgatacaaagc
aaaggcatagaaataagccttcatcggaataaaccgcttgtaatgttcccttttttcatttggtgactttcaatcggaagagttaagactcatggcaa
aagaaaggtaaacaaattagcattaactttgagtggtaggattgctctgcagctctatttcttcgttcgtagttgtcggcggaggagctagtgcatc
atccctttgttgatatctgggcagcgtaggcgaaccggtgttttcagctgggcgctcaaattccattccaagaccaaaaacagtaatgttataaaa
gccacagcacacaagatcaacgataaaaactccagcagctccaacacatccaattaaggctccaattaagactggaacacctccaattacgact
gcaacacctaatataatctgtgcaacagttcttgttattttgacagcgagtgaagtaaagtctactttttgttagcaaatgtaacggcatctttccttt
tttttattaaaaatgcttcacttacaataaaatagaccaaacaagcttgcccatatcacaagcaaagggcctaaaggtggaagaagggcccaact
taatttatcgtccgaatacacttgttccagccaagaaagaagtttgaaactgtatgaaattgctaatgtacacatcaacgaaaagaagaatgttg
aaggtagacgctggctttcatcgataatgacaagattatcagaaaagcgaagggaaagttgtttaaacacttcctcatagcttttctttatcaagg
cgatggaattttgtgatccattcctgggcgttttctggctattgttagaattgaaggaagttggataggacgatttgttgatgttctgttgtgtatcca
aacaaagacccttctctaaatcaacattgtaaacattcgactgactgtacgcaggagggtcgcttgaatcgaattttagctccttttccgacatcct
aaaaataaataaagagaaatttcaatgtaatattagcaaaagagcgttaaataaaagctgttgatccgtattcgatgcaaaaatttcgcaaatgc
aaaactcaacagaagtagcagccaaaactaaagtagctctacctggtcattccaaaagctcaaaataaacgtcaaaatcagcccaattggcagt
aattgaagaaataaaaagttggctccagcaagaaatttcgcatcacttacgcaacatttttgctcaggatagtgatgggttcagttacgctatctct
tatagcgattattgttaataaatcgttgaagatacgtttgagtcacaaattataagtaacaaagttcgctaatttcgcgtttgtttacacctctgtaa
acaaagcaagatcccatttctctatgaactgttgaaaaactttttgcacttgaaagtgagtaacagcctacagccaaaaagtagccctcatgggtt

cttcaggattaaaaaaaacctataaattaccttttgaactgaaggaactgtctacggccatacataggcgaaaaacatcagttcccgtccgatca
ctgcagttaagtgaaaacgtctgaggggcctcgttagtactatggttggagacaacatgggaatccggggcgctgtaggcttctcctttttttcttgc
tgttctcaacaatgttcacgtaaacacaactcggcgcttcctcaaacataatttcatggatatttgatttacactgagactgcaaggaagaaacttg
aaaaacaccaaaacaaagtacagaaatagctttattaaataaacgaagcaacggacaatttaaatttttttcatttggggtagatattctgttgaa
actgattgaacattggaacttgttgttgaccagtctccggtaatttttgctgaatatttgaatatacatcttgggaatataaattttgcggagcacga
tttgattgaatggtatcgggggtagtcgtagccgttgatacaggaggacccatacctgtagagtttccgaccatcattggattcatagtagcaaatt
gatttgatgtgtaagagctgtaagggcttattatatcttgctgcgctcgtaaagtattaccgactgctggggaggcacgttgcccactaggattcat
tccattatcgaaagatttgttagggaaagaagcttccgaattcttagcattcaaatgaccaaacggtgaactttgaggtttggactcagcattttgt
gctggatacgagggcgtattaaattgtggcttttgaaacatttgacgaagcatctcataatggggttctaaataaggcattccttcagggcaagtta
aaggaggtaaaggagtttgtgcttgtcgccccgaggctaaagatgccaagaacgcaaggttgtctttaatgcgccgcatatacctgttagtttgtt
agctagttaaacactttcgaaccttttattacttgtaaatagtatctagtgcttaaatcttaccttgttagcataatagccattttactctgcacttctg
tatcgctagcatacattttcttgatttgatgtaactcagcacatcttcgcaacagatggctattaatttcaagcattttgtttattcgttcatgatcctgt
aaatttgtaccgacattactaccagccgtattcatgactttttttttttttggttgactgcaaatgcaaactttcaaacacaataatacgtctatgtga
gaatgaattatttaaacaaacaaatgataaaaaaaaaacttataactgaaactggttcaacgaaactcaaatgaattcgccaaaatgtagttcaat
ttacctggaatgctttagagccacccagtgctaatattggtttcaatttctttctctgaaacgtagtggtttttaaaacagcaaccaaagtcatgcag
gaaagtttacagttctgtaagccaagcataagtatgcttgctggttactaatagaaattgaaaacaaaggtgaacagcttgttagtttgaattaca
gatgcttaactgaagaaaagcgtgaagaagaatgcaaacaccccgcaggcttatatatacaaaacatgtaatgaaaccgaatttcgttgttttca
tagaaataagaaaagactagttaaaggagaaaaataaaagattcattttgtattatataaaggataggagaacttaagataggttgaagactat
ggatatagcagatcctctatacacacacttaagagtaaacggccaagtttgaaaatgaatagaattatagacatattcgcgaaaaagaaataat
aaatagataatttttaaaagaatgtacatttaacaatgaagtgttcctggctatagtttcattttatcttttcataaaaaagcatataagcttccacct
tagagacctcttcccatgtaaccttcgagacaatattatcactgatgtataaccaccgacgttctttacttcctggttctgtcccagactctggtttttc
agttgtcggagacatagacttatccgctggaacatgcttgtgagacaatacataggcaacgtaatgaccgtaatttaaagtccctgaatgtgcaa
caataccaaatagtctatatttaattccttttgatgatcgtaacgtaggcatgacaaactcattaatatctaactcttggtcaaactgaacgaaccc
agtgatttttttggatcgcaacttccatcagctgaaaaattatgatgaaaccttttcaaatgaatcacaaacacaggtggtaatggggaatcaat
caaaagtcgcttataagcttctcgataagtgaaattcatagaacaatcacatgcactttcatcttcagtgttttcttgcgatacactaccatggttag
agggggacttacaatcacaactacggccacaagattcacaagcaaacatgttgtcgccggttaactcttcaatgcgagtaaagtttcgtaaacaa
tcaacaacggaatttggctcgcttacagacgcaccgttcgcaatcatcgactctgggaaaattagggacgatagattgctgaattgtttttgtgacc
gacgagaattattagatagattactcaacctgcgtaaattttgaaagtatgaaccaccatttgatttttcatcctcagcaggaacaccttcactagc
tgtttcaggcttgttttcgtgggaatcatcagctttatttttttcgttctcgggatcaacatcgtcaataatgattttaacatctggcgttcctgttgacc
tctttggtgatcttccaaagcgagaccgcagcgcgcgatgaaatcgatgacgcttgcttaaagattttgaatttgcatgaacagacaaagagaaa
tcttgggtaggttcgtagcttttgctaacttctttgcaattcttacataaaatgatacttacgaggcgacctgtaaataaagaatcaatcaccgtacg
ggcaggttcatcatctaagataattgaattatcacattcatcagaagagactgcagatttgtccatatttgacaaataatgaatattcgtctcgctg
ggttcatcatatgaggaatgtaatggcaaaggaatggtaaaggcttctctattatccgattttgaatccttatgaaacgtatcagtgactaaattct
gacttgggagtttttcagcaatgttatcaatgttaggctcagaattttctaaagagcggctctccttactcgcataaggagtggaagaaatgatagg
ggaatgtatatgcgaagaggcagaatcagtaccttcatctgaaacagccccttttataggagtaatcaaagatctggcagaaacatggttgctac
ttgattgatgaccattcgaaaagggattccgttttgaatgaccattctcaggagaaatgtttgaatctgactggagcagctgcgacccattattatt
atcggctttggtaacttccttttcctcagtgattaaatcattgagtaagtaccttaaaagttcatgtgcatcctgttgaatcccttcagaaaactgag
gatgacgatatcgaaattcgccgaacagaattcctggacggaaaacgggtacggacccgtctgctttgtagaaggattccataaaccttacgaa
agcggaaagcaaagaggattctaaatgatttccgaaattaaactttggatttctatcaggatggaaaggatgccggctaatgcagtcatgaaggt
attcggatgcagacaaaacttgtaaaacagaattaaagtagcatgttgcacccatgtttttttagccctggttgaatgatgtgcagcttcggagaag
gagaggattttggggagaaatgctttgaaaacgtggatgacttttcctgagatttcatgttgaatgaagcatcttgttgctcagaagtgtaaggtga
aacaagattgatccggatgaactgagaaacgtctcgaattaccatattcttttttttcagacggaaggacttccaagttgcatgcataacaaaaaca
tttcagtgtaatgatgttcacaaaaattgaatgcttgttgtttttcttgtagtgagttagcgaatgattaacatcgtggtgggcacaaaaaagatagc
cgcagttggcgcagagatttaattcaaaatcttcattcgtccttgagccaactgcacaaaggagacacatgtgtttgctagggtcacggagagtcc
gggcattgtttttgatatacgatagtttcacaccttttttcaaatgcaagcatgatttgacctgtgaagtcgtctcctctttgatggacagctgggagg
cctcttcctggatttgcatcgataaggcctcgtgtgtgttcattgtaatcaacagaaacttgttgactgcgcaagttagacaaagtgtaaaaaatcg
aatataagtttagagaatcaaagcagtagcaaccccccagagcctttctatacgtcgtagaaaaaaaaagtacaggtatatatcttttgtagaacc
taatgatcggccaaaagtttctaagaaaagaaaagtagaagtagagaacaaaacgaaatgactgaaacgaaaacacaaacactataaccgat

attcgccaaactcggtcgaatttccgaaaaccagcggatctaaaagaaagaagatataaagaaactatacaccagcaagcaagcaagcaacc
aaaagtatgaacaaaaaaatcagagctctacgagatattcctacaattcagtttataaaatagagcaaaagtatggtatggtacagtatggtga
acgattttaacctgacagcaaaataagcgacttcagtataaatcgtctttcaagaaagtaaaacaagttagccattcaaagctctttcgccaaatt
tgaataacatgcgcgaatcgcgaatcgaataaggaacgacaagttaaaatcgttgctagcaaggacgcttttggacgaaattaacactacaaaa
aaacaaaacttcaaaatcggccacaggaagctccaatttcctcaacaattcactgaaactactcaaagttagtaaacaatgagtgtttgcagtag
tcaaacagattctgctattataaaccttatttagaggtagaggtcgtgtttaaagacgcgtaaggcaccttgcaattgtagattaaggtttttgaat
acagtaagggtagggcgtgtgttttgctagatttgaataaaggcgaagctgcgtagaacaagcatcggtatcaagaaattggaagtaaaaggca
aaagcattgacatccagctattgagattaattatttattgaatggattttagtaatgacaatatgaaaaggtatggagacgatgaagaggcacctc
aagtgaaacgtacaagaaatgaagattaccaagaatatccagcgtatgtgaacggcaggaaggcatcttttggaggaaatcaaggctctggag
ttggtaaattacattatggaccttctcgacctggaaacgtgccttcgtcctcaggcgctgctggaaacacagcagccaccggcgatcgccaagaa
gcagatcaactgcaggatgctttgatatcgtgtggtattcaattaaaagaagaagaattaaatctttctacttccttctatgatccagcttctctaaa
tacatttgcgctttctactgaagatcgatcgaaaaagtgcgactttttgaattcgtataccttgatgcaaacagtcagcagcattgtgagcttacac
cgtttaaagagtatggatactgatatccacgcgctcatctctatggcagtgcgtgactacatggccaatttgttgcaaagaatgattgtcgaaagc
aatcatcgtactgcacgccttgatacgagaaattatacgcaacttgataatgttcgccaaaccttagcgaattccgcttacaaggaatacgagagc
gaagaacgaagacgcgctgtcttgaacgttcgccgagctgagcacgaagcaagactcgccgaattaaacacaaatgcaaacagtgaagaggg
atcttccagtcgtcgtaaaaaagaacaaagctcatcggctgctgcaaaaaatatttcagaagatgctcagaacaggatgacaaatgccactgca
agtattatggcgggaagtgctttaccttccggtggtaagaaatactcttggatggccacagacattaccccggctacccctgccataggaggtggt
tttggaattcgtaaaaaagaaaccaacactcaaaaaatgatggctcgtgatggaacattacctattcaaaccgaagagaagggtgtcattaccg
ctcgcgatgccttagcgatcttggaaatggatcgtgaaggtgctggacgtatctttggtagaggagctcgagcgatgatgcgagcatacattcgtc
taaaggactagggactttcaatcgtgcattgattttttttcttacgggagtgttgtcatgatcgtttttgattattcttatgaagtatggggttctgtatt
tgcaatggctttttgataggtagatgatttcttttaattgttacacgctagttttcgtatatatatgtatatatacttattttttaattatacaatgatat
tttgtttttaattcactttccttgaatggtcggtgcattggtagttagaatccatgacattttattttctagaagatgcattaagaatttcttgttgttttt
tgctgtttaatctatttaaataatgaaatgcactttcctcaacccaaggctgttgatagtattttgagtcccttctttcagcataatttgcttcagatcc
atccaaaataacctctagatccctactctgagattcagtccatcgatgaatgaagagcacaggatcatctgaaaattgtcgcataaaatcatgttt
tgatttggaataatggatagcttcaataaagcttgaaattttctcgtcaaggactttaatttcgttttgattcatcacgttggtcaaaaatgtacgaat
ttgggattgtttaggatcgtctgcattaataaaaacatcgaaaatagcaaacggcttagcggaaacttcattgacgctagaacctcgttaatcatc
tttcatctggcctaaacaacatacttcaattcaaatgacaactttataggatccatcggttccaagaaccggttcatcaactcgggtattctaggaa
agtagagtctatctgtttcaaaaagctacaagtaggttaattaatataataaaactttgtaaaacctacgtctcttaacctgttgtcacagttaatta
aacgcttctcttccaagtcctgaagtcgatggaactatattcaagttagaatgtgtcggctagaaagagaaaagaaaaaaatttttaaacctttaa
cttactttgatgtactgccatagagcagccacaatagccggacgtgttccctcttttatacctaaaagattggcgaatgagctggataatttgtacc
gttcaggctgttcatctggatataaaagtatgcttattttcacatcctgatctccatttctttttattttgagctcattcgccaagttggagccatttgag
agcttattccactctacataattactttctggatataattcttcagagcgatgtacttgaaaagcaatctttttaaaaaaacttgtaaaaggtacttc
gtccaatcgcttcttgtcttcatcgtttccctttgtctgttagtccatttttaatcaaacatgaatatttgaacatacaggaatcaatcttccttccacat
gaattgtccaagaaggaacagaaagagaacttaaatcattaggttgagaatcattgttttcttgaaatgtttgaaaggcttgattttctagggtctt
acgaatgaaaatccgtattgctctggtctttttgattgacgactaagagaatcctgcaagtcgaagcgcttccgtacaattaatgaatctaaacgt
ttctcaatttcctgtaactcaacatattgacttgcttctggaatggtctccaaaagtttttcaggaatttcacgttctatgagccggatctctttttttag
ttcacctgcttctttggcatctcgttcatttattttcccgttaagattagcatttcccgacatgaattataaaatgaaataaaaagtacgaccgaattc
gctggttcgttcaaaattttagaaacttggaaaactgtatcgttggatgatggtatttaggtaggaagaactggtggtgttgagtaggtggatccat
tttcctttccaaagtgttgagaattggcaatgataattatttttatttaattcttcggaatttctactttttttgatacccatttctgtctaattatgatgg
ttaagtatgatcatggctgacatccaaaaagctggtgtctttcagtgtcttgggacgattatcccaaatgccaatgctttctttaaagaaatttacaa
actttatcataaatgtttctactggaatcgcctttcttcccttttaatctcaatttaataaagtaacattgctacttgtaagtttcttccatgaaataag
cagcagaagcatgagcaaacagctaccaacaacatgtcgtcaaacgcaagcattgaactagcaggagctgagttaaaaagcaagaattatgc
aaaagcagaggtcatttataaagagattctgcaaaaagatgtaaagaaagatgagtctctaattaacgagcaagaacaagcgatggtcggctt
gagtgatctctatgttgctgaaaagtatgttataaaaaagcagtgttgtaaagaaagatgctaacaatttagtcgtcatgaagatttagctaaatt
cgtgcaagaagttagacccttgatgtcgaatttttcgaaagccaaagcagcaaaaatcagtaaggctcgaaaattgctttccagaattgttctaac
aaaaaactagttcgtacattgattgataaattttccggtacgaaaaactcacttcccttacaaattgaagtcacgaaaagctgtagagaatgggct
atcgaaggaaagcgaacttttcttcgtcaagccttagacacaaaacttatttctttatattatgaaaacagctcttacaccgatgctatcgacttgg
ccaatacccttctttttgaactcaagaggatggacgataaaatgctcctcaccgaagttcatttactagaatctagagtttatcatgccattcgaaa

catccctaaagcccgtgcttcccttaccgctgccagaacagcggctaatgccatatactgccctgctgtccttcaagccagcttagatatgcaaagt
ggtatcttgcatgctgaagaagcagacttcaagaccgcatattcttatttttatgaagcttacgaaggatataacacattggatgacttgaaaaag
gctctttcttctttcaaatatatgttactctctcagatcatgttaaattctatatctgaagtaaaatctcttttaacaggaaagcacgctgtaaaatac
tctggaaaagacatagacgctatgcgtgctgttgctcaagctcatgaaaatcgttctcttgccgaatttgaaaaggctctacaggactacaagtta
gagttggcctccgatcccataatccgctctcaccttagtactttgtatgataacctttagaacaaaatctacttcgtgtcgtcgaaccatacagccg
catcgaagtttcacacattgccaaactgattgggctttctactgtacaagttgaaggtaaacttagtcaaatgattttagacaaagtgttttacgga
atccttgaccaaggcagtggtgttttaattgttttcgatgagccccaacaagataaaacttatgaggctgctttggaggtcatcaagaatatggga
acagttgtagatctcttgattgaaaacaaagctactgctttactataaaatgtatttttcatttctaatgaacccaaataaataatttttttattcaaaa
ttttttcgtgtaggctttttttagtgatttatgacaaaaaaaaaatatgcgcacgtattgataaagtgtataagtttcaagaagtaacccttcagacctc
cttttctttttaatctgcagcagcagaattggtatctacgtcttcttcaatgaccaaacccattgagggattgtcttcttcacgcccttcatcatattga
acagctcgccaaccattttcggctttaaaatcacgcaccaaatcaagagtaggaaaatagtgttcattcattttgatggttaaaacaactggtactt
tgtcaaatgttactgtgctcactctcacataagaattatcgcgaactttgcgaacttcacgaacaaaactttcaagatctggagtagggataccatt
gatggcagtgatgaattgagcgatacccaagtcatattgatccgcaggtgagcctctgctgcgatttgtgacaaatacattagatggtagaccctc
actttgaaggcgaacagctctatgtggagcttgaattgttgcaccccaacatataattacccggtttgtttcacagttatcggtagggaaagtaggt
accttaattttcatttcctttcctccacgaagaatgtccatctcaatttcttcagtgtcttcagcttcgatatcctggacacgggtcacgggttttccgtt
agcagttaaaattacatctccgtcggttagaatacgaggggtgttcgcttcgacgtgagtaatcataaagagttgatgacgtttttcgtttgcagttt
cgatttgctgaatacgttccgcagataatcccatacttcttgcatgagctaattgaatagcacggaattctacattcagtattctaatatgtggatta
acgtcagtctgtaatcgtcttaaagttgacaagacgacaggcgatgctagaccaagatgatatttcacatcggtaccgcggcttgtctgctcgccg
taatgggtaagccaaagggcaacaacttggccctcgtcatctgttagaacaccggacccacattgagatcccaaaccagattcaatggttatcga
ctcaaagttgacagctctgaatcgtggcatcggattaggaggaataaccatagtaatgacatcagttactgaagttttcgtagcgacgactctaga
cttagaattaaacccgaaaaaatttacgttatctccttgtgaaacataatggttgcgcaacttagcggctttaataggagtttcacccaacagttta
ggatcatatttaataaaagttaaattttgagttgggtggagatgtaaaacctcagctggaatttgtatagaatcagcaaccgtgatggtcaaatct
cccagcgcatacggcacggtgcatcggcttgtcacagctataccattgtttttatccaggactagagcagttccttgttttctgctttttcgataacca
tcaagatgaatgggcatatagtaagtgatttttacgaagcaattattgatatcagcagtaggaccaaagttctcgacattaagttttggaatagaa
gtggtgcatggaatattaggctttgcaggtaacggatcaccaagggtttgaaaatcccaaaaaccggtctcgtcatttctactagctaagcgaaa
agctttagcccaatgacggtcaacttcaatgatatccgtaaccagtgagtgtttatcatggataaaacgatagcccacagcaacacgagaacggt
caggaatggtcttcatgacattgatgaaggtatctaaatcgggcactggtttgtacgcgacgctctcaagcaaatagcagtattctggaccttcca
gacggaaagaaccagcaggctcactaataaaaacgcctttcactggcaaggcatattggcgagctagttggtaggaaagattgtggaattttgc
accacagacttctacatacctgtcaggagcaatatcatgagtactttgtactcggcaggtaacgtcgactgtgctgtttcctcgttgaacggtaaga
acaatgtcttggcctatggattcatctaaaatgctttccaattctatgagatcaatcaagtattttgaattcaaatgaagaagaatatcaccttcctg
caacttgttgtgagaaggtccttccggaagtaccgtttccacgactaacatgctagtggcaccggggaaaaggcctctaacttgttcttcaattgcc
gtatctaatcctaagcgagagcattcatcaaaaggcttgattaaaaactgagtttgaatagtacctctagtaataggtttttttattttgcaggcatcg
taaagcacgcaatggcctatccaatggtaaaaagtagtcagtcgctgcaaccatgtggccacccgcttgtaaagcaacgacattaccgcttctttc
gacgacaggactaccactagaaccaccacttgcattcgcagcagcctgaatatagttggtgttaaaatcacagtacgttagttcaccatagtcgg
gcacattacgatcaacacgggaaatccaaccagcgagaatacttaattttcagcagcatcattaccgataactcgaatttctgttcccacttttgc
caaatcaggacgtaattccaattgttcgacattcatgtaacgaattttttggggtcaaatcgcaaaaatccaaaatcatgaactggatcacgata
aacggggtatacctcgacttcttcatggttatccaaaattgcatgtccgataaagggaccggcacacactacatgtcggttggttaacatataacc
atttttggcatccacgatgaatgcactagcttctccagtgccagattcatctgtatcaaaggaagcaacctgactgaaacgaatagaaacgacgct
acgaactacacgagcaattgattctttccactttccactttctgcaagatgtgaagatggagctgtctccacgagttgattctctagttctgcaggtt
cataagaaccgttcattcttggtactttttcatcattttcttgcgtaatggaatttcctcttgctctttttttttatagccatactaaaaaaaaaaggtaag
agagaactgttgaaacttttcctcgacgcgagaaacgaaccattttgcgtatcgctacgttacataaattatcttagtgaggtacgctgcgatgtgt
tatgtgaactaaaataataataggaggaaagatggaaagaaaaacaagattaagaaaatatgatttccagtgttttattcaatggtttaatttagt
gtacgtttttagtttatcgtccattcataaagttatctcaaaaatagagttgctgtttaataaaaaacacatactcaattccatgtaccaatgttattt
gtagagtcgtcaagagtcggtgttaaaaagctagctttccatgctggaaacaagatccaagaagaaaggggcgtttataatagaatcattgtag
ggaaaaaacagataaatgagaaaatgctataaaaaagtagtttgagtttcagacattcttttgatattctgaaggaaacgaactttggaatcaat
tgaaaacaatctcatacatatcataccgtttagcgtccaaagaaagataaagcatgattggaattccttggaaaaggctcgagtccgatacggat
aaaatgcaaaaattcatctatcgacatgccattatgtcggcataccttccacaaactggcggtttgttctacgattagattgtcaagaccaagacg
gtcgaagttcggatgtcgtcttcgttcaggcaaagtgcgtgaaccgctatcgatttcattctccaaaggttgatccacgtcataacgatcccaatat

gctttttcttccaatcttgtaggtgaaacagtcttctcatcttgttgctcatttttttatcaggcgttggacggtttgctcttgttgtttttgttttcgcttctc
ttcgatatcatacatgccgttgaatttcaaggaatggtcattcttaaagtaataaacttcaaattcatatttgtctgcttcaaagatgtaggaaacag
taccctcatcaggatgttcatttctaacaaacttggcagtttgtggcctaaaatgctttagaaaaccagataactctttcccttcaacttcattgaact
ctttatttaagactaatgcctcgtgaaacctgctaagaggtagcaaaaacttgacccgaagttgtaaaataggtgttaacacaccttccaattgac
ttcggtcgcttaagggaaagatccctaagcaagccaattcctcacgacgattgctgttcattttcgtaaagtaaagagaccctgaacttttctttgtt
ggtgactgagttggaagaattcaacaaggaccttgtactatatatatacataaatatttgcgcggtggagtgacatacgaatgttagtacgtaaaa
gcatgtataaaggctttgttaaaaagcgattgctttttgaataaatggaaaaaaatagaaataaagcaacatcattttaattaccaaaacaaaac
atccagaaaattggatgaacagatgggatttcatgtggtttgtttactaaaagaggagaacgcattgcgttcggtatgaatagcgttagtgacgac
aactcacaagtaaataaactaaaacaatcagatttgcaaagaacaattgaaatatggaaaagaacaaggatgaaaaataaaaagaaagaa
atggaaagctctttttttaactctcgttccaaaggattctctacatttccacttctttctttttatctttcatccttgttcttttcctgttcttttcttttttcttc
cttgttgacgaacgtaactacctcatccgcgttagagtgtcgtactaaaaaataaggattacattcgacatcactattccagaaaccaggcttcaa
tgagaatcaatccttgcagcagcagcagcagcagcagcgacgttataaaccacaaaaaaacgcccagtgaacgagagcaatcaacttcactttt
gctaatttttgtctttgatttttgctgttcttaagacattgcatgcttcgtagcattcgagtctaattttgccattttgtcaaaagaaaggagattcacg
gttcctttaaaaaaagccaagttccctattggattctttaaatagacttgtttcgaaggaagaatcctgtctactttcttgcttaacttaactgttcctg
gatttcattctttatatttgaaagaaaacaaaaacactatcgatttatcttatttctcgtcatgtctacagttcaaagaacgttacgattaattactga
tcaggtacgttattcacgattggttccatcgattgtttgacaggagaaaaagaggtagcaggagagacatcaatacatggagaagagctattctc
tttctttgttgataaaaaacgttttccctacaaggatctcttcgctgtcgttttcaccgttgttgcttgcttgctccctactaacctcattccttagcatat
tttacctggaggggaagctgccgtcttgaatgatcaaagctttgctatgcgagaatgggctatcaaagttgtttgtttagacgctcagcaagaaga
agtagatgcaagttttattgatcgcgtgacttataaattacatcctacttttcaaaatcccactagaagtatgtttttatgaaaccaccattagatctt
gaattttctatgtcagttggttgggatttgcagagagaaagattactaactttcatcatagccgtccgtaagcctccctttatgattcgagagcaag
gttggggtgaatttgagatggaaatcatcatttactattcagacaaaggtggtgaatatcgcttttctcactacttacatttccaacaagagcacta
ccatgaagacattgagctggtaatccatttttctaacctattcttgttttactgactctcttctagacaattactgctggtcggccaggtttactaaaa
gctctatcccttacaggtcctgttcctggttactcacctgaagccgatgatacaagaaaggataagcgaaaaccggaagcagatgctggatctgc
aaagaaaaaaacaaaagcgaaacctgtatgatgcttgtttttatttttttcggttcttccttctctaacccatttttttaggtggacatggaaaaactg
gctgatggtcttcaaaaactacaagaagatgaccttttacaagttgtacagatggtaaatgaaaataagacaccagacatgtacgttcgtaacga
tattgaaggtatgttttcacgaatagtcacttccatttcctttgttaacttatgatttacaggcggcgaatttcacattgatctttatacactccctgat
aatttgcttcttttattatacagcttctgcgcaaagcgcgtgactatgtaatgaaagagaaagaaattctagctcttactcgagtttcactagataa
aactaatgacaaatcatttctttgatattgcattttgtgtttacaaatcttatgtattttatttcctccattaccaacaattcatatccagattcaaatg
acattcagagcattgccaccagcattgttctttaaacagttgatcaaaagaaaaaaatacttatagtaaagtttttatatataagaagctatttgatg
tatttcaaaccaatgtttttcataaaatttttttcctgggacttgcacagtcctataaattcaataatcgatagtctatctgtcaaaaacgtataccct
aaaggtctgaaaatttttcaaagtaaagatgccctttaaacctgttcaactagcatttgaaaaattttcagctgcagctcctaagaagcctccagtt
ttaatccttcatggacttctgggttctagaaaaaaattggcgatctttatcacaaaactttgtctcaaaacttgacagagatgtatacaacatcgatc
agagatgtcacggagattcacctggtgttggtcctctgtcataccagtccttagcttttgatactcaacaatttatgaaaacacatggcattgagaa
ggcttctattatcggtcactctatgtaagtattactctcaaaatccctatatataaattacaatactaactggttttaggggaggaaaaaccgccat
ggcaactgcgttgatgtacccggacatggttgagaaattagttgtcgccgataattcacccatctatactcaccttcctgagaaaactaaagtttac
atcaaggctatgatgcgcatcgatgaagcaaatttcaaaaaacaaagcagcgcagacaagatgttgaaagaggtagagaatgatactttagtc
cgggcattcttactttctaatttaaggaagagcccagaaaatcccagtattttcaaatcccaaatccctcttgaactcattcacaattccctccctga
tttagcgggattctccgtcaatgaaaatggtgctgatcaatataaaaaaccaacattggtcattaaagctaaacacagcacctttgtaccggacg
aagccttgcctgtgtttaaaaaaatgtttccaaattataaattggaaacgttggactgtggtcattgggttcattttgagaaagccaaagagtttac
tgaacttgtcgtaaaattcctatcgtcggactaagctaaattagctatgaaccagctattaatcgttgatgtcaaactagttggcttgataatctact
ccctcctccaaaacaatagactttacgtataaatagaatatagcctactaaataggtacaatggaaaagattataagatacaatcaggaaaatat
tgtaagaaacaaactaaggaaattacaatatactatacaatttcagaactacagacttcaggtgtttatataatagacgctcttctcattagctatt
ctatatatttcaatttgcagccccaataaattcaataggatatgtttatacagactccaaaggagtgacatccttgacaaccttagtaccaacatct
tggccctcaccaccgtgaagctcaagaagcttttgagcatcgtgcttgggagacttaaggatcttgaccttacggacaagaacgttttggagagg
gaagataccgttagtagctttctcaatctcacgaccaacaacctcggggataagcttttggacaagctctctcatggagcaagaggaggtttggtt
ttggatgacttggaacatcttctgacggatagcgcggatttgagaggattgagcataggtggccttcttgacttggttggcacgacgacgagtgaa
gccaataacgaagacacggcagagatagccatcagtagtcttgatggtttggttggcttcgatagtggtttgccacttacgaacaagagaacgca
acttgtcagaagtgatgctcaaaccattgaagctggtaagacagttcttaccttgaatgtcttcaacacggagcttaaccttgcggaatgcgtgttc

ctcgtccttttgcaagtctgcaagggaaacttcaaggatacgtcccttaagggcgtcattggcgttcttaagaccggcagtgcggttgacaagagt
ctttccaacgttcttgacctcgaagaaggaaggagccttgatgtcgtaccagtccttacgagcgaaagggtcaacggcacgcttcttaattcccttc
ttgcctttggagagtctcttgtttttccaacagccattgtggagaagtttggtttcgtaaaatttgggaattgtcaaagtgttctatgtgactgctatg
gtttttctaggttagggttaatttgcgaagctctaaacgttgtcaacttttgtggatgttaccacatatactttgttacttcaggtgatttttgtttgttta
ctatagactgattgtaaaaatacatatttaatagcagtttaagggtgtatccttgagcgtataagatatcgcaattgatatgtttattacgacgactg
gcggtttttttcttagacatacctgaaggattgattccagtattttctgtagatcagcgcatatgttaatgatgatttactaacagtatataaagaag
tagcttcataatgtcattatcgtctcaacggtagtttttaggctacatataaaattatactgccgcgtgaacttgttaattgactagatcctttatgtta
taaacagtaaattagaccataagagtattctctgatgctgtaataatattagtctatcttgcagatagcatgaatcttaattcaggatagcattaaa
attatcttcatgggaacgttctattctttattatcattaaaattatttcacgagaatgaaaatattagggtttttcgtatttttccaatcatttagcgtcg
ttggtcgaacattgtgaatataatatacaaatgttgaagaatttttcaaactgagtgagcaggacttccggaacgatgaattgatgaggaacggc
gggctggcaccaacaaaggttctctttctgcagcccacagtttttgttgatatattttgtaactcttgtagccactattggtaaagactgcagcagtt
aagaagccaaggagagaaatcgcgaaagtcctccagctagcgtctcgttttgtaggaattggagatcgtacaaagaaaacatttccacaactag
agaaaccactttcttcaatatggacccacggcccattatcaactgatagaagagaatcgtggataataaatgactgttttccagtgtcaatgatgt
gtcgatcggcaaaggaaaagtcctttgatttgatatcagagtccgataggggagggactacaacagaaaatccttgttctaacatgtctaatagt
ccctgcttttcgagtaaacttaaaaattctttccttcctcccgcaataatcatatcacgttgaggtatagcaaagggaacaactaatctatccaaga
tatgaacgacaccttcttgaaccaacatatcacgcgttttcacattgtaagaaagtccatttatctttaatatatcgttgttctcattcgtgctttcaac
cattatacttctaccggacaaggttgtatagtttccagaagtccaagaatcctcagttgacccgaagtaatggattccttggaaaatgtactcaag
caaaacattttgcatcatctccaaattatgttctaaagcctttcgtaaaagtcccaactctgcccatgcatcattttcaaccaaaaaccaagtactgt
tgggatcttctccaaggccgagtgaggctaggtatcgaatactcatggaaatttcatccagcaaaataagttgggagagcaaaggcctaggggg
ttcaatgtctttttcaagaacaaataaacttacattaccgatcgtctccttgtcaatggaatgcgagtcgaaattaacgtaaagtgcaccagcagta
tctgtagcaactcttattaaagcactatgttcatcatggcttttggcattagtcctcgataagtaatatttgttgacatctggcatataaaagttttcc
ataatatgataatcgatatcagagctttcacggaaagcccaattcgtaggagcaaaaattcctctgtgagatgatgtatccacagcaatatattct
ctttctttagaaatttttcactaaaccaggttgctcctaagcctatcagatatttagcgggcgtaaacgtaatgacttcaggatttagcagagcag
gtaagacatgtatagctccattgcgggtgactatatcaaaattatatgaaggagtgtcattgaagtataagatttttttgggcttatcgtattgcaag
gttataataggcccgtttcttagagtaaaattcttgggttcaacaacgtcgtcagcatacactcgttcatggatgacagagtgttgattgaaaagag
tttttacatcttcagtggcataaaaagagtaaaaatagcttagttgggtacttgtaaaagtattcacaaacacatccgttgtcggaacaaagagag
tagttgattcatactcactaacccaggcagaagataaacgatagaatatgctgtactcgctttcagaacttagtatttccaacgatgtcggtggaa
ggcgaagcagttgatccacaacttggacaacaccgctatcggcacgccagttgggtttgatgatgtagacatcgtttacgagatcataaggattat
tagactcctctctgtgtgttacttttaaagcgattggtcggccatcggccgctttgaattcagtagcgaaaactgtacgattcaaatcagtcgtattc
aatatgtggtagggccacactggatcaatcgtgtctctacgaaatgcttcattaacaggagcaaacagcgttaatcctttatttcgatttaagtaag
gaatgaagttttcctgctgtaggtgccttattaaacgaaaaaagttgggacttgaagacagtaaatctacaattgacgttgaccgactatgcaatg
cagcctgtgcttgaattgaaaaaaagaataaagaaaagcatatgagattaatccaaagcaccattgcatttcttggtaacaatgatattgtaattg
tgtgtagttgaagtttactaggaatatcccaagggatcaaatttaggacatctaaccaccacaaagggttatctttggctttgttttatttatttatttt
ttctaattttgtttacgaccaaaagctgtacatttgtcacccaacgaaattcacactcaattgcttgtaaagcaatgtctacctagtcagcaatagtt
agatgatctcttgttatacaattcttttctttattattttggtacaggtctatccgacagttatttcaccttggcttaaacaaaatgacgcttgcttattc
tttcatggaagtggcttcaagtttgtttatgcttttggtttttaatctgaatttgcaaagctatggacagtttttgttggcaatgctcctgcaattgcaat
tggttctataaaagctcaactttgaagttttgtttatgtgaaatggatatcgcaatggctttctttctatttttttatatataatttccaagatctttttat
tttccttttctttcattagcaaagaatgatccttctgttttgtacatacttgttttcatttatacattaattatttattagtaaactggaacgtgctgcttt
aaggctttaccaacaccactaaatgctgtttaagaatacgcgttccatttcgcttcgtagatgctttgtcaacaataaaaaaatcttgaaatttacc
attaaaagcatattgggacgcttcttttagaacgctactgcgactttatcattcatcaaaaatggcggcaatgacagatgttacagaccctacctcc
gttgctatggagttgggtagtgaaaagatcacaattgtacgtgactaagattgcgtagaagcattggaggcgcttggatgttctcttctatgtccta
gaagctaagcgtatggtttactaacaaaatttgctagttgcctggtcattctgcagactttacttctgtcacgttccaaattcttaaagaagatcata
ctttgggaaactctcttcgctatatgattatgaaaaagtaagcgaataccattcattatgaatacctgctcaccactcattagccccgaggttgaat
tttgtgggtactccatccctcatccctcagaagccaagatgaatcttcgaatccagacaattcgtaagtaccaaagctataaaaaaaaaacacaa
aaaggtgaaaaagaagatagtgtgttttttcctcacttctcatcgtttagggataccctttttgacttgttcttttgatttttatatgaccagtgactaa
ctttcgttttgtagcaagtacaacagcaattgaagcactaaagaaaggattagatgatttaatggatctttgcgacgttgttacagactctttcaat
aaggaactccaggatagtaatcaatctatgcagattgatgcttgatttggctgtgaacaacaagctcatgcgtttcctttttggatgctgttaatttaa
aatgcatacagcgtttcaagcgtttgtgttatgttccctaattggaacggtcatttacttttcttgacatatataattatgggatttcggttgaaagcg

gctatttagggaatattgtaatttcattttcattgaagtttataaactgtgaatattaaaaattattctcttacacgtatccattctacttctaatagtat
tttaaataattgtatcaaacaataacactataactagataaactacatatcattgttttctacaatcacatctccgtacctcttccttcaactcgacg
accgtcgtaaatatatacaacatactcttcatggtccatccttttcttatttctagctgttatacccttttgtacattgggtctttatacatcttttcagta
ggaagaccaaagcctggtttggttcgtaatgatcccaatgtagttttggcacgatgcaccggagtagtagttgcttcctttctttcgtatttttgacc
aagtggttatgcaacaccaattctcttgtttttctcaatttaaaacagattacaaagtatctgtttcatgtaggaatcctttttttaggaccattatatc
agactttcttcgttgaaaaaatgtacaaggttcctctaaagaaccttcttttcgaaataaaagatccagtgacttggcgaaacctagttattgtatg
tagcttagcatgattgttattgcatccaggtatactgacttgttagggacctctttcagaagagctgatgtttcgttgtgcaatggttccgttatttga
aaaattagattactcttggaaagaaatcattctcttggcaccccttttgttcggctttgctcactttcatcattcctatgagcttttgctgaattatccg
ggagcatacgttattgcaatggtacaattccttgtgcaattcttctatacgacaatatttggatggtatagcacgtatgtattcctacaaacccatag
cttatggcctacttttctgattcactctttttgcaactttatgggattgccagtattttacggaaaaattggaacgaaatcgcagacgactatctatta
tattttactaattaccgggctctttctttttgccattacctggaatttgctatattcgtaaacttcggtatcaaatgggtactatacctcagtattttggg
gttcttttgttaaaataaaaaaaaattataccacaaataactgttgattcatttgtgattttgctagacgacagacaagtgcactcagctgaagcag
tgtctgcactccttctaaaacaatcatatgcgtagttccaatttccttgagcatttccaaacggctgaattccggtatctcattcattgttttcacaac
acgaaacatgttcgttacgatgtccaccgcgctatagccaagaccccatatgtctcgaagctttgccaaagctgtatcgacatctccagaatgaca
tgcttgaaggatcgcatatatcgctactggggacggttggtccgctacccgaaaaacattttccccgttaactaagtcaaatcccgatactgtactt
tgtaaattgttgatcgcttgacgcatatctccttcagaagtcataatgagagcggccataccgtcatccgtatactttacgttttctagtttacatatt
tcaagcaaacgctgcaggacttgttgttctgtcaatctcgagtatctcaaaatagcacaacgagattgaataggctcgataattttgttggactgat
tgcaggctaaagcaaatcttgttgtgttactgtaaatctccattgttcttcgtaaggcttgctgtgcacctgctgtcatactgtctgcttcatccagga
taataattttatgacgacctggaggaaggttcaccttcttctgggcaaaacctttgattttgtttcttacaacgtcaatacctcgctcatcagatgcat
tcaactctagtatcccctctcgacaggctgaacccaggagcatgttggccaagcagataattgaagtagttttaccaatgcctggcattcccttaa
atccattagttgttaccaaaacaaaatttgcataccgaaataataagatgaggcatattgccatccttagctatcacttttaaacgatcgactgttt
cttcatttccaacgacatcatcaagcactactggtctatatttttctaccctatatctcatgtgttagctttttacaaggctataatattcaattcatcgt
cctttctcataaaccaaccgtttgacttgtcatccaccaaagctaaaaaagacacgttcttcctgctcaaacttaccaaggtaactcatatcgtact
gctacattctttgtccctgaagaagacataactgcatcgcccatttttgcgcacggtctacctctgggcaagtattcggtggagggtggacgcttaa
aagtcccagcgtcaagtaatgttacgatacctgcttcatccgtataaaagaataagaaaaaaccaagtaaatcagtgtgtatgaaagagtttttta
cttgaagcgaaaagctatgcatccgggctcatccaagttgagaaatcagagaccattcctttaccataaatcaacccttgaatagattcaagtctg
ttctattcttctttatttcattctatataaaatataacgttccataataagaactactgaaagcccaaagctcttcaattctgtttcatttcgcttagtgt
gagctagaagtaagcgaatctaccccatactttatttctacaaacttggtttcgtaaatcaagacaggttattgaacaatctcatagcatatatcta
aaaaaatatatagactttcctttttctctatacataggaatagtataaggaaagtatggtggtccaattatccgaagtgtttttaaaagacccacaa
tcctttcttcaaaaaatccaagatgcctcgttgtcaaacccaggaaaacctgatctccaaggaagtttaggtattgaggaggcaatttctgatttgt
ttttacataaggatgagttagaaaaagtaagttatgccgttatacctatctttgaatgatggcgctaacactcagatcccaaattttctcatttgtaa
tcccaacgagttaaaaaccggacaattacttcgactacagggaatggtacaagatacaaattttggacatgaatttttttgcaggtgccatcaaaat
gaatgatcaatattggcggggatgtcgctacgttctcaattacagcgaagatgaatcttatgtggatgagagtaaaatagcacttgatgaacggt
attctttatttgtaactaatattcctggcgaacagattccaaaccaatctaattatttggaaacgaaagaagaaaatgaggtatttaaagaactga
ccatgaaatattcaaacagaacgttagagaaaggagattttggcttttgtgtaaaatgttacggcggaatggaaactcgaattcgtgtttgtcagg
ctgttgacatgttaggcatgtacgaagagccgagtgaatattctgatgggcttcctattcttcatttgatcacatttcaagactataagcaacctttc
actacgtatccgtctgaggaagaggctaaacaactacaatcaaagttgcttactcaatttaacaaagtattgggagacaatgtggcatccaagtc
cttgctgctttgcttgctttcaaatgtcgttcatagggcagacagcttcatcgttggatcttttagcctgaatttgaccaattgcacaccagtctgtgc
atttaggataattagcctgttgaaagatttgacaaacagaacagtcgtacaaaaagtaaacgtgcacgaactaaacaaggagccgctatatcct
cgaagtgatggagatactttatcaattggtcgcttgcaattgacgccaggaacgttactgattttggatgaaaccgaactttcgagtggcaccttga
atgatacaggcgttcgtaatgtcgcatttttatcctttctcctttctcaacaagaaattacgtttgcgtatccatttagtagctataacatacagacgg
acgtgcgaatcgttgttttaagccatggaaagtcaatcttgcctgttaatgttgtatatgcaagcaaattcgacgagtcatcaatgtcaagcgaga
catctctaccatcagacgaatcggaagcattgtcaaattatctcaaattatggactcgtgctactgacataccatatgatatgttagagttcattca
atcaaactatgttggttcgaggcgaaatggttctcaaacaaatgaaaaagtactttctattgagatcaattgctctcgcctttatgcaaaaagttta
gccagcaaactggtttcgagggagcattttgaagaagcatctcgcttggtggaacaatggacaattccttgaaaagcaaatgggttagaacaac
gctcttgtttcaaggtgaaggtttcctttgtgaaatataaaaaacctcatttatgtatattaaatctttctataatgatttcagccttgtattatgatta
ctcttaccgtacttccctctcaaaactttagcgagtcgctacgaacgttggtaaaatcttttcactgaatatggcctttctgaattcaccatccagaat
aggtactagtttaagaaattcgatttgaatccgtctttcttactctgaattttgttttaaaatctttttacgtatcgtatcgaattaataatcaaaacgt

tgcatatgcggatggcaaagaggttcaatttcatttcggaaactcaatgaaccaggcagacttcttgcaagagcttccagtaagtatacactaata
atctggataaattattttaactttttttagcatgtatataaacggttagaatctgggcttattatacctcagttaaaagatgttttacgagtatttggac
tacggctgtcaggcacaaaacctgaacttattgcccgaatcaaagagtttgttgaaaaaatagctattaataatgacatgaacacgtgggtttcac
taaaaaaagcggttgatggagacatctctggtgctatttgtacattacaatctaatctttcgcaaatttatcctcagactttagctatcccgcctcct
gtaaataattctggcagtagagcgtatactagaccgttcgctccagtcgtccattcacgaattcgtttccggaaaactccattctacgatatactgg
aaccgttcaatacaccttttctataccggcttgtgttggcgcacgtaatacggtatcattttcttttcaattgacgccaatggctctctcaaaactatt
aagcaatccgaaggaatatagagtgtacttcttctcaaccccgacagatacaattggttttggtgaatgtttaatggagtatcctactcctcaaatg
gagcttcgggccaataatcaagttgcacatgctaactatcgcggcttgaaagggagaagtggtactacaaacccagctgacataacagactaca
taaccaaatacgctggtcctccgggaaataatgttgttatttattatatgaatgctacgaaaaggtattctgctgtcgttgctcttgttaaggcgtatt
ctgttgattatttggttgatgatatcaagaaacgaaaaattgaaacgaaagaaaaaatcgtggagcgtattaagagtgaaaacgaggataccga
tattattgcgacttcgacagatatttctctaaagtgtccgttatcatttaccagaatatctctaccggtaagatctagttactgcaagcacattcaatg
ttttgatgctagagcgttcttagagatgaatcagcaaactccttcgtggatgtgtcctgtttgtaatatacacattcgttatcctgatttggttgtcgat
gggttcatgcaacatattttagagaatagccccagtaattccgaaacaattaccgttgatcccgtaggaaattggagcatgaatacttttgatgaa
gaaagtgaaagttccgaggaagaaaacttgccaaaggaacagattatagaattgagcgacgaagagcgatctgctacgcctgcaaatcctagt
gaacctccaaatgtaccaaaacgaaaggcgtcacctgccgccgccaattcaacaaataaaaagaagagagaatctttggtaattgatcttacta
tttctgacgatgaagaaccctcagctacagcgtctcccccttttatcagcaacgaaggagacagataaatcttattcgaagccagcatctccaagcc
ctattcaaagaaattcccaaccgtcagaatccactccatcagaaaacttttcgttaccaaaaagttctgtgaatggtatttctcaggcatcaacacc
gccaagcaccactacggtcccctctactacaacgacttctcatagatcttacagtacttatgaccctaattacgtatctagaaggagttcatattcc
ggcagacgtccgacagagtcatttgatagctccagagggataagttttcctcctgtcaatactcctcctcctagaggagattttcgtttagggagttt
accagtcgacgacagtcatattatatatcatccttcgatgccgtcgaggtcgtcagtgatggataattccactccaggagcccatatgacgactgc
caatgaccctgcgaatttactaatcagtgacttagtactgcctccagtgggagcgaaccccaataatagctccccatctgttaggtcggatactag
agaatcacaactgtcaaacatttatacacccaatcaaccagtgttatataataatattccaacccggccacctttcaccatccgggagtctgaccg
tgatctgcttagttatgatgattcgagagggaggtctatgttaagcttacctcaaatacaagacacgcgacagaggacaccactgcagacggctg
gaccgctgtggaatgaagaggatgataaccgaatcaattgggattctgaatttcatacctctttcccgtttaatagctctacgtaattacaagagg
gattgtacgagtgatagtgtacgaaaaaaataaaacggtgctctaacaacatgggaatgataatggatttctatttagaatattgatcttaacaaa
aataattattctgttaatttataatataataacaacaatacgaacacattgatggataagcatgaagtcagattttattttctactaacaccacattt
aagcagagttcttcttggggcgttgagaggcaagaatagcagaagagcgagcgatagaaacgttcaaaaggtcaggacggtagccagtcttgc
caacacggccagcaacgctcttgtaggtcttacgggaagacttgttagcaattacgtcgttgcggaaaagcttggcaggcttttgagcgttcttgg
ggttggtcttggtgaccaaaacaacaccacgagggctgttagcgttgataccaacggcacggtcgttggtgagaccagagaaacgttgagagtt
ctttccggtgacgttgaagggctcacggttgaattggatgccaccgaactcagagcgcttaacaaggaagcggttgttatcacggataacttgcca
gatgagatcgttgctaacagacatggtgtgagtgcttgacaagtttcgcctttgacgcatgaaacatttacttttgtgtgactgcctttttgggcattg
gaaatttagggctatgctttgttgtgtgttcgagcgggcagggcctcactaagtatagtattctgagatacaacgcaccgtaagttgtaaacaaag
aagagttggtagaagaagtaagtgaatgggtttatactttattttataaaaatggtttcgagggagaagagtttgaaaaggaaaattgcaatttat
tatcatttttatacttctacttctaacactcttgatctggtcaagggcattgaaaaatcaatcaatgactgggatgctgaagtagagcatgagtaaa
tcagaactcttttccatagcaaacgttagtaataaatgaattttgttcctcaattagcttctctaaagcgaatagcttgcaacttgattgttacgattg
agtcctctggctataactcggaaagaaacaaaatatgtgaatggaagcaaaacttttgaaatattttttaaaagaaaaaaaatatagacatttta
ggaacttcccttactttgcaatctttttacattcagcaacttgacatcgacgtaatctcatcccatacctgtgcatagtctaaaaggtgtattcttcac
atgctacgtaccaacgcttataaacattgagggagtaaattgacctatatctaggaaatagtggatagggagaaggcacgtcttctctaaaacttt
ttaaagcgatagttatttgcaattccttgtcaactaactgttttgattcaatttgttacttttgtttcaataggggtttattcattaattataaaattattt
ctgcaagaaggaacagacggttaatttgctttgtcttttatatgtttagggaatactgacatactatatttgctggtttttatacgagatggctagctt
tcatttgttattttcttatgctactatcgtccttgcaattatagcgggaatcacctatgcaactagaagtatttggggaccacgtttgcgtcaattccg
atactttcctgtgcatgattcgtttgaatccgatattcagaatggattgacctccaattcttttgatcttatccagaatgtgcttgcacaagaccctcg
agctggtctcgatgaggcagcttctatggaaattaagggcataatgaaaaaacaacaatgctccttcgatcaagctcgcctcatttaccttcgaga
tacgatgagtaaaaataacattgatccttcaggccttcccatggatagtaaggctgtaactaagctttgaagcattatcttcattttagaagcttac
gaatgtcgtaacatgattgactgcatttaagccaaaatggatcattatttataacgtggaatgctgatttctttgtctatatgctcttttttttaaaac
aaaaataacgacttgctatcatatcctatgtcttctctgtttagaaaggccttttcgttttgtgtctgctgattctcttgcaatatctgaaataacccaa
gaaaccatatccggcatgataatcgactttagagctataatcaagagcgataactattgctattttttccataatgagaatctgttgattacaatttt
atctttagctttgcctttttttagacttgtttcgttctggctttgtttactaatgcgctgtttttcaaaggctcgttatgaaaactgcgcaatgcttattgtc

ccctattgccaaatacgatgttctagtgtatcgcagttttccaaacgtagtagagtttacaatgtttatgctcttcatcaccaccacctctccataacc
aatcgctcttatagagacgtgttagcaaatttgtttaatttttttatcgtagcatcgttgacattctctattttggggttgcttctttttccaccatttcaat
agggaagctgtaaagcgcgtatttctatttgtctgatccagtgttttcctttcgcagctgctacttgtcgttcttttgccgttgaaacctccatttggag
tggatacatatgtactttttccgactgcggggtgatcggcttacattaaaccgtagttttatgccgaatcgaagatcgtgatttgtttccggaattggt
tactactgcaagctagcaagagagtcagacaactatcaaaatagtaattgtgtgttgagagcgcttttgctcaaacgttagcgatttatgaattaa
tcgctagtgccggacagagacagacggtgttatgagtgttcattcctcgtcattgcgaaatggtgaacgagacataaaattatcgtctcccgtaga
aggaaaagcctcttcttctgctttgcatctaccatttcatcagcttcaacgttcttctgtccggtctcacaccagtgacttcacttctagagcgtcttct
cctcctaaataccagcgtcttcatgtaagcgtcgagcctgcaaacccacttgagcctcttgctgctgcaaaggccagtgttccattgtccagcgaac
gcatgaagatgatcaacgggcggaaagatcaatttggtacatcaaaggctgatcgttacaacaaaaatcataattcacttctcaaatctccttcca
aaatgtcgggttttgtttctaatgctgcagcaaaggcagcctctagattatcgacatcaaaatcccattccgtaccagattctagtatcggaatgcg
tcgtcaaggggtttcctcttcgaattcgaagcggcttcataaagaatttttgacatctggttctttatttgaaatcaaagatccatccgaatgctttaa
tgtcgcaacggaagcttcagcttctatgtcacccgccagccaaaagcccgttcgaataagaaatcgacagagtattgcaagtcccactagtgatt
catcacaagttcgtccttctgattcgcattacatatctgctagtcaaaattcaatttcgaagtataaagactatatcgggaaaaccaagtctccttat
ctggtgtaccagtcttcggatcatccatccagggcaaaacctgcatataagtatccaagtccaaattttgatcgaagttcggaaacatcttatgact
tctgtcctcttgaagtagaaactgctcctcaatcaaaagctgttataaaaaaaaagatgtgacgattcggtttcccattcatctattatcaagaaagc
tgcggaatccagctttacatccgcctactctgaagttgattggaaaaaacgaaaggatgaacaaagttttccgaattatacgaaacctaaaccttt
accatatacaagtttaagcgaggagcctttacctgctgtatccttcgctttaaagaatgaagactcaaatgataagcttagtgatgaaagtaattc
ctattctttacagccatcagtacgaaatgctgctgtggtagcatgtaatcctttttccaatctggaaaaaaatcccggaaatactctcgaacgcagc
actttaccgtcagaggttgctgcttcaggactgtataaaacaggagtatctcatcctgtgccatatgaagcttctaccaaaactcatttctcatcttt
ggcgagaaaagtgcaacctcagcacaatatttccatacctaatgagataccgttagctgctgctcgaagtacagcattattttccaaacatgactc
acagagtttaaaagactctcattctaatggcagcaaagctttactttttgcatttaatgcggctaatcaagtgtctgatttaaaaagtcaatcacctt
tagatgagagtctttcataccatcattctccgaaacttatttcatctgctcacatcgctgctaatcatgccgcgaacgaaaaagtgaacctttcatta
cggcccgatacattgaatgagcccttgaatacaacttctttagtagcccagaataagaacagcgtcgcagttgctagaccatcaatttcttatcatc
ctccctatcgtcagtcaggtccgttacccaatcgaaaaatttcagcaagcgacgacgtttttttatgatgctgtagatgatgaacgtgcattggctcc
tccttcctctttaccaggattgatgtatgattcatctcacaatacagaagaatcttttgaatcgcctgtgagtgctgaagtggataaactaggcagtt
actcggatactgatagttctggggatgaattgtcgttggttgaaaaaggaaggcccatctctagctttaaagattcgattttaaatttttcaaatgat
actgctaaagcattgaagtggcgattggcaggtgttcttcaacctgagaaagcggtcgtacctagtttgctttatttacatcgtgaagctttagattc
ttcatcagcggttcacgcagcagccattgcagataggtcgttcccagttgttcaagaaaaaccaccacctttaaaatcaaaaacactgaaaaagc
catcaccgcgtacgttaagaggacctactaaaaagaagaactttttttcaaaacgaagcaatggtagatataaaaatgcaagggtatcccaaat
atccaagaaagttgatattcctcgtttcgtgattgatgaaaaccggagaaaattatatgaaggcttgtgggcggccaataaagggtatcttttacg
tgataccgatgtagcgaatccggaaaactatgtttgtgacatagtagtccgcgaactatggtcaagatgtggtgctcccacaagtgtcctagcaaa
tatttataaccttgtggatcgaaatcattcacacatgttgaaccgagaagaattcattgttggtttattttgatagatcagtaccttacgggcaaaa
agctgccgcttaaagtacctgattcggtttggatcagcagtaaaaggatgggagagatgctttggcgtcttgaaaagctgcagaagaaaacgag
aagcaaaaagaagcgttttaagaggaaagtgtatgaaggccttaaatgggaattgggttcggaccaggaggacaagggtgaagaagatacag
cgacgttataaacaccaatttagtttgctgattgactatgtaaagctcgatataagatatgttaatgatctttcaaaatcccgatacacctcaagat
cgtcgttctttttttctctttcttctattaatgccctgttggttaattatttatgaatgatgacctgcattcaagcacttgtattttcgtatgaacataaacc
agatgatgaaaaaaaaatctaaattacgccttacgatatctttatcattactagtttaagtatagaagtctttcttttgttttctctttgttaatataactt
aatgtattttgattttcttttttttttttaagcactacatttgttctattttctttaatgtttgacacttctcattaataatagcgttacaatcttttgagaa
aagatgcgagaaaatatttcatattcctaattcttttgcttgtttttaatagaaaaatattaaacacaatatcatcttcattaaaactactgaatttgt
ttaacctttgttctttgacatcgttgttaattaactgtatagcaataatggtttcaacacattcattacaagttgggctacaacttttacaaaaatgttc
ctaactttcttatatttatagacaacagaatatgacggatgaggaatttcaagagtctcttgagaattattacaatgagttaaagcaactttcgtct
agccttaatgttcctttcaatttgacgattttcagaaatcttatcctcaataacttatcggaatactgtttaaatttgaaaacaaagttacaggacat
aggagtaaaatgcaacaaacttacgtctcatttgtatgagcttgataggtgtctagggagaaaacaacatattgaaaatgaattacccttagaaa
cgcattaccagcatttactttctttacaagaaaaaagccaaagggaatatgactctagacttcagctagtaaaaggtattattttctggttctttcag
ttctttgaacaatatcaaattcctcctgctaacagtatcagaaattcaaagcaaagtactgtaccagttgttttgtcttggcgaaaattctgaaggat
tattcttggagaattccccttttgtagacgtttctgactcaaagattgaacagctaaatcatttgaaagagagtattgaaaaaaaatatcaatcacg
aaaagaaattttattaaacaagagaaaggagatttttacctttggaaaagcacctctcgaccaaaatcgagataaataccaataatttgtcgacgg
gctatctggaaagaataaaggaatacaaggaaaaattagactgcttagttcaaaaaaaagaaaattattcatctcgatcgagtgttcattctgat

ttgagtcctaaggtgaactccccaagtgcttatacaagtcccagtagacgagttaatggaaacgtaagggttctatacaatccaaaagattttaac
taactttttttagaaagaaaaagagccttcaagtaactctgtatcgataaagcatcgtaaggagtatgaatcattattgaagcgctatttgcttcttg
cgaagtcgctatttatatcaaagaaagacttgttatttaaggaaatcattgaaaatctgaaaaaggatgacattttggaaattttgcataaactca
aacaggaaattcacaacatggaagtagaaactcatcttcgaggtgaaatcaatgcattgattcgaaaatatttagctttggaggctagattatata
aagaatcgcagaccgttaaaggttcatcaagtccatcctcgaaaccttttaccttgaagagactaaaaaaagattttcaactcacacgtgcaaatt
taatttattctttaagaaattgggaagaagaaaacgaaagaaaaatcatagtttttgggaaacctttgtctgatagactacagttttattcataccc
tttacagcgttcgtctagtaaaaaacttaatgttccaactaatgatcaacgtccaatctctactcaaactaatggatcccagccagaaatgaaaac
ggatgatgttcatgataatgatacaataggcccaacctatcctaacagaaatgcatctgaatcagaaattggatctgttaaagattctgtctctcct
tttaagagacaaggtacggaaaactctgtttcaagagtaagtcatcgaaatgcgtcgccattaattcgttcatccagcaaaaccgatgtgaataat
gcgaatgtcgaaagtcttgaaagccaggttgaagctcaatataatcacttcatgtctgtcaacaatatcaataatcaatcgtctatgccctcaccct
caaggtctctgcgagaaatgattcaaccatatatccctccaaaaggaagtcccactgcattagaaaatcttgcaggctctcatacacaagaaaat
catcttttaatgtcatgcaccaaggatgacaataacaactcctaccttggaaagatgagcagtcatgaagaagagcgcctatgtaaacggctaga
ttctttttcattctcaaaagacatgatttctaaaaagctgaggaataacattcatggattgcacttttccaccccgactcagggtgatccaaatggtc
ctaaggattgactgagactatacttaggaagtttgttatattgtctcgtactttaaagtatttggggtatgtacttgtcagacttatctctaattaatta
atgttctcataatgattccttttttttcttttttgttcccttcacctttaaaacataggttcagaaaaggtctaatattcatttctaaaatcataattctttt
tataattgctattttcaataatatacgccctacttctcataaaccatagtggttattcattattatagatttttgggtagcaacttttgcctcctgtcctt
aaccatcttaccacccctacaggattaaaatgggaaaaactcaaaaaaagtcggcaaagggtattatatttcaacagttttctttttctaacatatt
ataggacgtttggataaatggtataagcttgctaaaggtaagaattgcaaaaaacggtgttgaaccattggaaatttttggattcagtaccgtgta
acaattttacaatattttgatgattgctgacaattaatagaacagggataccgttctcgtgctgcgttcaaattagtccaattgaatcagaagtttaa
cttcttagaaaaagcaaaagtggttattgacctttgcgctgctcctggtggctggttacaggtagcttctagagcatgtaagcccggttctttacttg
tcggtgtggatttgactcctattcgacctgttccaaactgtcaaacgtttgtagaagacattacttctgataaatgtcgctcgcaattacgaggtatc
ttgaagacatggaaggcagatgtagttcttcacgacggtgctccaaacgtaggtgctgcttggttgcaagatgcatatggtcaagcagagttggtg
ttgatgtctatgaagcttgcctgcgaatttttggttcccggcggtacattcgttaccaaggttttccgttctcgtgactataacaatcttttgtgggtgt
tcaaacaactattcaatagggtcgatgctacgaaaccccccttcttcgcgtaacgtgtctgcagaaattttcgtcgtctgtagaggttataaggctcc
acaaaagttagatcctcgctttacagatccacgcactgtgtttgaggaagttacggagccaactccaaacgtagaggcaaaagtttttgaacccg
aaaaaaaaaaaagaaatcgtgaaggttatgccgacaatgactatactcttcataaaacagtacccgcgtctgattttgtcaaaggaaatgatcc
aattcaggtattaggtacctccgcggaaattgtttttcctgaagaagatgaagattctcagcgtctgaagaattcggaagcaacagatgaagatat
tttaattcattgctctgatttacaagttttaggcaagaaggatttccgggaactgcttaaatggcgactcaaggttcgtaatgagttgggtctaggc
aaacgtccggaaaccgaacaaaagaccgtcgtagaagaaattcccgaggttgacgaagaaacaaaaatggatcaagagttacaacatctgaa
tgagactgaacgcactaaggtcaaacgtgagcgtcgtagggccaacaagcgcaagcaaagagaaattattagaatgcaaatggggatggagg
cccctatggagattggtatggaacagagcactatgggagacgacagcatgtttggattatctacagccgagaagcatggcctcaataagttaact
gatgcttcaactccagaagttcctcccacagaagaagaaaatgaagttttggaagaaaatgaagaagaatatgacagtgaagatgaacaggag
cgtcttgaagctgagctggactctatgtattctcagtacaccgaaagaaaagcagaatccgacgtgaagtataaggtcaaacgatctagaggtg
acgctgatgacgaggaatgggaaggaattcaagaacaggctagtgaaaacgaagaagacgagccgaacgaagcttcagtggaagaagacga
gcatcttcttacttcacttgacaaaaaggattcaggcaaaaacggtttaagtaagaaagctagtcttttctttgatcaagacatttttgaaggtatc
ggtaatgacgatgacgctgataaggaaatcgttgccatgaattctgctgccaaggcaaaaagatcatctgcgaaggcaaatgaaaatgaaagtt
ctgatgaagaagaaaaaactcggaaaggagatattgaagttgttccacctgtttctgcccaagaaaaggaggacgattgggattcagattccgg
tgacgatggtgaaaacgttgacatagtgacggcagaagcaatgacattagcccaagaaattgcttcacgcaaaaagtcgacatctgagctcatc
gacgatggatacaaccgctggacatttcaacctaaggatggacttccagattggtttgttgatgaagaaaatcatcataacaaaccaaataagcc
tatcactaaagaagccgtcatggccttgcgagagaaaatgaaggctataaatgctcgtcctattaagaagattcttgaagctcaaggacgcaaa
aagatgcgtactgtcagacgtatgcaaaatgttgctaaaaaggctgagggtattactgaaagtggtgagatgaccgaaggcgaaaaatccaaa
gaaatttcacgccttatgagtcgtgcaatgaaagcgaagcctaaacaaaaggcgacacttgttgttgcgaaaggtgctaacaggggtatccaatc
tcgtccaaagggtgtcaaaggtaaatacaaaatggtggattctcgtatgaagaaagatcttcgtgcgcaaaagcgtcttgagaagaaaaaacgt
aaataaacgatatagtttaggaagtatgctttatggaaaaggttctttattggtataggttcgatataattttgggtaataaaactatggttatatttt
tttttgactaaggtttcaaatttaattttgataaagcttttgcttcatgaattcatcattaatttattcatatgtagcgttacatagaggaaagggtaa
agggctatgaacgatttagagatacgagcgtaggtatatcatttacagcaacgttaatgcaaaggtggagctgcctcaatcctaatttttttgtcgc
acctcattgatgtctcgtgtgagttctttctcccagtagacgtttatcagtgttttagcaactagtccgttacgtacagcccatggaatatatatttttc
ggaataaggctgcctgattacaatttaaacgaaaaggaccaaacaaagcagataaagcaccgacaggtaagcccatgttaacaaattcaagcc

taaaaaagttaatttcattcataaaaaagtcactgttcataataatttcactaaccatttaatagcgagctcgccttcaatgtttgtgggcatatgac
aaagtgcatggtagaaatcatggcactcacgatatctctgcataacgtatgcttcttctgggtcatcaacatatcgcgtaggagaacgtgtgtccg
ggcttacatgctctctatcaatccagtcaacatagatacgtcctagtgtattcagtggaagagtccgtaaaaagggaagatttaaagatgctgaa
gttatgcgtggcttttccattaaaatttgacgaccagtttcatcagaaagcatttgtttaagcaaacgccgtaagaaaaatggctgaccagatgag
tctcctaggatggaaatcatgtctagagtgttcaattagtaccaatccagatgtttataatgtttttaagtaggactataatctcttttgacgtagaa
agcataactttcaagcatatatccagaagagtcaagcgtatatcgtaaccttacctcctcgccaaggtgcttttaaccccattatagcagaacccg
caacaaggaaaattcgttgtaaaggagacaatggaacatggccaggataatttggctttttttgctaatataactatgtgaataatgacattcattt
gtcgtatggcattaagttggcatacccccgatgtaaacagtgaacgactataaggccgggttatggcgatattcaaacgtttaagaaacaaact
catgatcctttaaaataaaaaataaaaaattaagatgccttagcgttatataagtaaaaggccatctaaagaggataagtacaaaaacagacga
ttcgaactgacttctcaagtattcggggtttcccctgtcttccgcggaaaacccgtctaggttatgggttatggtcggtaatataggtcaaaaataa
agtaaatcaaaggggaaataaaaaaagaaaacgatcatttttaaataatcgatcttgaagctccaactgagacaaaaatgaattggcttccct
gtaatatggtttggtatgttcaaattcctgaacactagaggatggacgttttacagttgcctgtacaatggattccaaaagataattttcagatttct
taaaaaaagacgggtccttttcaaagttagtatacactgttaatgaaaataaagagaatgcttaacgtacctctaagattgacggactggatatg
gtttcagaccattttctcgtctttaaaaactctgtaactttcttttccgtaggggaatgaaaataagagataggtagcgaataataggcgtcggcat
gggcaccagcatcggcgattttggaggaaggcagattatcatagagaaagatatcttggtttttgaaaggagaaaatgtgtgtttgttcatattttc
aggatgagtgcgaaaagcaccgatatcgacgtaatgagattccaaggaacgtttaggatctatgactattgcgacgaatgggtcttgaaagcgct
ggttttgcttttgtgtttcaacatcaacccctgaaagccaacaaccgtagtttggatgagaatgataccaaccaacgacgttttcatatcgatgaac
agacttgcacaaggtttggtactggacactatactcatcagcttcttcatgagcatttacgcgagtttccgttcctttcactggaagagcaaaggcg
tccaaaatgattaacttgttgtctttcaccttcccttgtacgtagcccatgacctcgaaaggcattccttgcgacacgtgtcgcagcattttcaaaag
agcaacagcatcaatatggacagatttgaaatagaatggactaggaatatgtcagtaaaacatttcaatcgcaatgttacggggtatatcctagt
ctaatgtagttttagcatactcgtgtttccatggtgcttcgcgcaagacttttctttctttttcttcatcaaatttgtaaatatcttcaaaagaggtatca
ttcatggctagctacgctgttcaaaaaaaaaagtaattattagtacgtttcacttgtttgagtagttgtctaaaggttaagaatcactattaggagc
aaggtttagctaggaaagaaaaaaattctttcctaaaaaaataaatgcttttgaataataaatgtaacgatacagcgggataggaatgagaagt
gagtcgaaagttgtttaaagtggaagggatttttaataaacaaatattttatttatctttttgggtcataaaaatacatattcatttcatttgaagga
aatctagatgtttttcaaaggctcttctgattgcttcaacatagcagaaagtttatgatggttttagaatgaaagaaaaaaaaatcaaaaaaagcg
attataataagattaagagtaaggattgaagaatcttcttcggaagagctagtttactagaaaacaaactaccatgtttgctagcaaatggggag
gatgaatccatcgaaataagaccaatcttatttctttgaaatctttttttgttttcttttttttccgtttaccgatgcaaattatgcaatccaccaagctca
gaaataccttactagcattcttttaatatgccggatgcaataccatatgaccgaaagatagataaatactgaaggattatataaggacggagcag
gaacaaaaaaaaagatataaataaaaaaaacccccaacaaggaaataaaacacaaaaaaaaatcattataaaattatgcaacacattttagc
atcagtgaaggcaaaaatatagaataaaccaacaaaacaaagcagaatggacatttcgtatgcaaaagaaagtggagggatcaccagaattc
caaaacacatcaacgatgtgcaagatctgagtgatgtaaggatgcaaacacagaaaacgcgacaaagtataaagagatacagatactagagc
aaagagcaagaaaaaagactctcaagcaaggtgaatccaaagctgggcgaattcttcgcgggtgactctcccattggtaccagcaatgtccagc
ataagcttaagttgttcttgagtcaaatcttcaccaagcaaagcacatgaccgtctcaagtcctcaaagtcaataaagcccttgtgagttacgtca
aacagatcaaaggcctcttcagcttcttcgtccatgagtacagtcttcttgacaatagacgacgatgacatgatgaattagataagaaatttgata
atacaatggggaacaataaagtcgaggatgatagatataagacaaaaaatttaaacaacgaaaaaatggtacagttaaggaaggattgcttca
agtggctatagcatgcaaagaatgaaacggcttcgtaggattgaaagaaagcaagagaaagaaaaggataaatggaagaaaatcaagaatt
agcaagaatgaacaagtgtaacaatgttgtctgtaaaatcagatggtgcttgtattctaccaaggataggattgtactcaatatttataatgcaaa
agagagaatattaaggaaggccagtagatagaaagaagcagtgtcaataaaaggtgggaaaaaagaaacataaaacaaaaacagttgataa
aagaatcaaaatacaaacaacaaaagaataagaagaaatagctgcaaaagtacgttctcaaaacaaataattcgaatagcgaagcgtatcttg
taatttgcaaacggttttatgcggtagaacaatgtagaaagtagcgctgcaaattgtttacgaatgcgaaacacattgttatgcaagcttaccgaa
tcataacgatttataacacaggtacatattggagaaattatgttatttatgacaaacaagagagacaatggatgctgttagttctcttccttaacaa
aacgaaaatctgttagaaaataaaaaatgctttcaataagcttttgattgtttcatgaacgtcgtcatccgttcccgacctcctccatagtactttgg
gatgaaagtgtcagtgtgaaggaagaagaggagtggtgatatctttgcaagacaaacggttgatttgcaaaagaaaaggcgtttgcagatgatg
taacgattgtggaaaaagcgcaaaagaaaaggaaggatagttcctaacaacgaatgatctgaaagaggtggatcaaggaagattgggattttg
aagtttctcctaaaagttgcatccaaggaaaaagatttaaagaagaatttttttagaaaaacaagaaagagaaatgtaactccaagcacaaact
tcttggtttgtttaccttcttacttttcttttcggtagtgccttcgctaaccgtatgcttattatttatactctttacaaggttgcgtaggcgtcatggact
gcctcttttctacggcttcgtgcttttcaagataagaggagacaacgttacttgagttagcaagtgtgagtaacgacagttgctctccacatattttg
ttacctgctttacacacgttctctccatcctcctctaaccacttctccagcttccttcttccagcgtattccctttgagatccatcttgtatttccgcgttt

tctgtttattttcattggtttctttgtacaaataacacctcaagtctttcttctgaaagctgattctggtttgccgtcgctttaaaaagttcatcgtctgtt
ctcgtgtttctacgtcttttctttcataatgaatcatggtctgcatcgtacgtattttatgcatttctctggtttacggttttgagtcttttccttcatttca
atttactaaccttatgttcttttcagatcctgtggacccattccgaattatcaaggaaaccgctcgtgatggagccactggcgaagtaaagcaactc
tcttatacctctagtaaagtcgttggctctggaagctttggtgtcgtcattcaagttcatctcattgaacacgacagtaaagctgccattaagcgggt
tttacaagataaacgtttcaaggtatgtttgttttcttttgtttttcgtaatattttagccaaattcagaccatcgtaaaaccccattttggtttgagtct
ggtttggcttaacttgcctttttttttttttttttttcttgttttccaacttgatattaaccttaacagaatagagaactccaaattatgcgactcataaag
catcccaacatcatcgatttgatcgcttattactacacaactggtgaaaatctcgacgaggtctacttaaacttggtcttggagttcatgcccgaaa
caatctaccgtgcttctcgactatatgctcgtcaaaaattatctatgcctatgattgaagtcaaactttacatttaccaactcttacgatcattggcct
atatccacgccagcggaatatgccatcgagatatcaaacctcaaaatttgctgatggatcctgaaactggtgctttgaagctctgcgactttggta
gtgcaaagattcttgtagccggtgaaccaaatgtttcctacatctgctcacgttactatcgtgctcccgagctcatttttggtgcgacagactatact
cctgcaattgacatatggtccactggttgtgtcatggccgagctcatgctgggtcaacccctgttccctggagaaagcggcatcgaccaattagta
gaaattataaaattttgggtactcctacccgagaacaaatcaaaacaatgaatcctaattacatggaacatcgatttccacaaatacgccctcat
ccattctccagagtttttcgcgaaacgtaccttcagaagctattgacttactaacaaaattgctgcaatacactcctacccaccgtctaactgctat
agaagctatgtgtcatcctttctttgatgaactccgtgatccaaataccagactcccggattctcgtaatcctcaaaatccacctcgtccactgccag
atttattcaattttagtcatcaggaactctctattcgtcctgatttgaataaaaaattgatacctcctcatgctcgagcttatttacccgttgatttgga
caactttgtccccgttgaaatcaagcgtgcccaaattgactaaagaacaaacatgtgaaagtccaatgtcgctctgcaaatgtgtgagtgcaaac
gatagttttccgatttctgtttggcactttttcgatcaatcttttgctccttggcagctttcttgtttcttttgaatatttatcataatgcgatttatctatct
acaactttcaaaggttatctagttgtttttggttgttctttaatatttttattgagtgcaagaaagagaaatgcaagactaaaatgccaaaagataat
aggacttttaaattgcgtcttaagaatttgagacacgatctacaggatcacgaacaggataatgtttatgttgattggtttccagtaattattttgtt
gatcatttctgctttaattgttcgtggaacttatttatatatacacgtatatacatgcttacaacgttattggaaaaataaggtacttgttttaccggat
gaatgtgtcatcaaaagtagtgcacctgtgaatgtatagagcagcagtactagtgataatgaatctgacatagaagaaggattgaatatttacat
tgataaaaaaaggaattttacatacgatatgggaattatttcaataaatcaagaataaagtcccgtataataaaccatatagtaaaagggttttat
taaacacgcaattttgaaagctaattcattacaaaagttttaggatgccaagtattatgcaactaaaaggagcgaatggaaaaagacactataat
gcaatacaactgaagtttctcccgtatctaaaactgctttggcgagaaaatcatgatccggcttgtccagaaaagtgcatacaaaaagatacacc
gagaacaaaaaagatgctataaactagcaaatggtagtagtcattgaaaagaaatccagtatgcaagaaaagataggataaacctttatttttt
caataaacgaggaaaaaatcaattcaatataagttgtaatttaaggatagtatagtaagcaaaacgacaaaggtaaaacgccattgggagaag
aaagctaaagttaaagaagatggtttacttaatttttttccatcggcagcatagtcacgatcgtcttcgccttctacttcgcggcgaatggcttcat
attccataagctccctcttcaaaacatcatcatctgtgcgaagagatttgtcaaaccaagcctcgtcaggagcacggaatgaatttccaaggcgat
caaagagtgtagtaaattgaccatagttgtagttaaaatatatgtgatgtccgttgtgatgagcggccccattgacaatggggttgttggaaatgt
acttgccatcatgaatcataatggtccagaaattaacaaaagtgaaaagggcaaggtaagtccatttatggagaggaaagaagaaagggaaa
agatgatatggtaaagattggaggaagccgtcggcggacttgaatgcatgagaagcatatggtgtgcaaatgatccatttatggtgcaacttgtg
aagacgaggataaacccagcgatgatggagaaagcgatgagcccaatagatgccaaagtctgagaagagaacaaacaagggaagagagat
gaagtaataagttttcccgtaatcgctaatgtgatcatacaaatagccataaccgtggacttcggccaaaaaccaaggtactgtgagtaatgcca
ttccaggaagatttttttgacgccgtccaaatctccatccatacttggtttttttagaaattttggatgcttcattaggctcttgtcaaaaacatagtggt
aagacaaagaagagaatgttatatacaaaaggaaacctccgacccatgttaaaaagaacaaactaattgcttcacgcactatgtgatcgcgagc
caaaggatacgctttattgtaaacagaatccaagacataatgatcggctatatccagcaaataatccatggagtatgcagagtgctgaaaaaag
agtggttgcgaagcagcgtataagactgtacagaaaatggtctgatttcttaattacgagatggttggcctgtttacattaagctcttactaaacga
atttttagattacgtaccacgttgtattactttcgtaaacaatcagtgattcagaaaagaaacgtgtattcccatgaggagaattcattgatgtttta
gtcttacactgaaataagcaaatcaagtgagaaagacacgaactgtcgatgttactggcatttcgaccaattagcttgctgatggggacagccaa
tttatgtttacgaatgttttgtttccgtatggaagaaagatgtattttatttttttgttgcctttcctcagattttgcgggatcgagctgaaatgagttttc
atacttcagacactgaacgaactacgagatctacgattcaggaccattctttctcatggccaaagaatatgcaatagtagatgaccttttgtaatc
gaaccaaaatacttctaaaacatcttcaatacccatgttagtagtgatcatttgctattctttgtttgtagtatgtgtctttatgaaaaatggacttttc
ttctcaaaaaagtctggaagtatacaaattgttcctcttaggttgtggatgaggacctggaatttcgagtcaccgatcaagatggatgaaaaaaca
acactggaaaatcaaacatgtgaacaaacagagtaatatcaccggctttacgattacaacaagcttaactatacgtgaggatgaaaggatcgta
atattttttttacgctggattatgttttttccttgatttttggaaaacaatccaacctatgaaagccccatacagtggattgtttccttgtcttcattaaca
aagtattgactcggcaaccatattcactttggattttaaatctcaattataaacgatccctctgtataacttaccaaaaggtattatatacaacagg
aagaaaaagaaagcaagtggaataaaccaaatacttttcttttttttagcaacggcaaaggtctaaacgctagccatttcatacatcgctgagtag
ggaaccgatggtctcacagaaaaatcctaaaaaaaaccgacagacaatttttataaaaccctttctaacgcattcgtaaacgtggacaaagttgag

acatcatacaacatcggatttatcagcagtctttttaaccaaaagcagctttccactcatacatactgcaatcgttaatttttacggaaacttataaa
attcaacctaatggataagacaaaattgcaagtcacctaaaagaattggactcagtaaaattgtatatcatcactttcgatcccattcatttttacc
tcaagattgcaagcatttgttcatgcttgcctttctttacaaacataaatacagcaattaaatagtcgcaaagtcattaaagaagaaataaacgtg
gccaattaaggcactaattaaatccaaaagagggaaggttccactacgaaaccatcgtaaaagaaccaaaacccatggaacatatggagctttt
acatcaaataaccctaaaatggaaactctgtacaaagggtgtttccaagaccagatatagagcatactgaatgaaaagtaagtagccgcaaatg
gcataagaaaaaaatatgacgtgactagtaaagatccacagatgatgccaagtgtcttcaagaagttctttgtctgtggaggaggacttgactctt
caagcatactcataaatcgtaatagaaaggataggtacaaaatagactccaaacctagcgggccaatatacaaaaaagtagtaatagccctcc
agtactagactatatcagttttccggtcgctattggctgatatttttacatctcatatcgaatgtttacacatcgatctagcaacatacctctcctttga
ataaaaataatttccaattaaaaaagattttcaattccgaaaggtatccaaaattcttgaacgattagctcgcgttctatgctttactatgttttacg
tactaccgccagagtcgtcaataaagtgccaaaaatgaggtatcttgacattgttaacatttataatttcatacttaatatatttaccttgtaacagg
aggagtctgccttataaactctggaagaatggccattgtaaacgaacttttgatatggtggtttgtcagaacatataggtgagtacagtatttatat
attatgatgaagtacgtatttgactacccagcaaggaagaatctggagatttggatatgtttattctgtatatttaatgatttattttacataactta
gcttcagtatcgaacttttggataccaacaatcctctatgttttacccgtaaatttaagaaaagaaattttgaatctctttccacatattcgtattctct
tctttttgacgtgtcaacttttcaaaatcgtgaatatcttgatatagtactagagtcattagttaatcaactgtatatttgagtgtcttgggattaactt
aatccgttgagtgcaaacaaacttacgttctaactttgttccagaaaagaaagaccaaatgtcatctttactttttttggctaaagtgagtgctgag
ctagccattttctcagcttgctcaatatttcctaaacgtgtacacaactctgtaaatacaatgtactgcaactgagtattcgaaaggtctattgaatg
ctgggtaagcttcaacaagctgtttgtttttttgaacatgagcgttttctcgagatatttttaggatcatccaccacagagtatagattagatttttctgt
tccttgcaagatttttctatccattctaaagagtggctagcttcagtactatcttgtaacaagttgaccaaattgagcgtagacaaaatgaaaatct
ctttgggagtatcaggctttttaataattaatctgtaaatagattcggcttcttcaaattttccttttttatgcttaagcataccgtgtaataaatttcga
aagtcgttttgctcaaccgcgcttgaaaataatattttctcggctttcgaaaattttgaacggctcaaatgtgccgctaccagataacacttaagatt
atcaataatagaatcaaaccattgatccgtttcaaaatatctgttcactgaggtttcatgaggtacaaaatctcctgaaatgcgtaatccttcttga
agaaatcgactggcacgtcccgaactagtttccggtaagtagcagactccagaaattaaataagcgagaatataaatttgattgatggaaagcc
atctcagatatatcgaggtttctccgtctatcgtaaagtcaatgtgagatgatccccattttagagtatcagattccagaagcatatgtacagtattc
aacttttctctagatttcaaaatctttccttcatttatacatttcacaatctgtgttatcaaaacaaggacagctaattgatgtgttcgtgttggaattt
cttccattggtaaatcttgattcaatatcaatgccaaaagagccatttcttgatgattatgatcattggcataattatatatgtttttagaaagttgct
ggacactttgaaaagacgaggaacagagataaaattcatatacccatttatgagcattggcaacaccttgtgtgccatacatttccattacttttgc
atactcttctgcctctttgatacaacgaagtaataaaactttcgcagcccttggtgaggttttacaaaagatacgcgctaacagaagttgtaagctg
aatttctcttcgacgaaattctaaaaaacgttagagacatataatagacacaaagacgtacgtgttgcttcgcaattagcatggctcgatttaatat
agcctcagcatcagcatattcttgtgtatggtgcaacaaaagttcgcatatacgctttctagtaaatagttcaactcgtggttcaacagtgctatcct
atgtaaaataatgtcaataaaatgtactaataaaaattactaataaaaagtacgaaccagtaaaaccgcataaagacaacggagaccacctgt
cagtaaatcactaaaagctcttgtaactccattgtgctgtagagttttttcggcttctcgaaaataaacctcagccaaaggccatagaaatagatg
cctggagcttacaaaatgctctccttcggtgtagtgaacagtcataacattgtttggttgtatacgagagcagttgagtcaggatgatcaaaagtcc
ttaaaagtgcttgaggaaatattcaatttatattatattcgatttgcaaagttatttattattggtataatgatttatagagcaggaaacaagtaata
ttaatcacgatgaatctaattaagttctttttatattattcctatcaagttggcagtctaaaaaaaacaaacctaactatcgttttgagaaagctact
aaagcccacatagcttttaaaaacttaatttttttcctcttagtcctctaagagatcaatattcaccaaacgtaacgcatttacagcccattcgggata
agaagaacgatctccattatgccacatagaaaaaaaatgtcttgacaaactgagggaagcagtcctttataatgctagacctcatgtcacgcataa
gttgcaattgaaaatgagtattatgtattgttacgaggtgagcaccaacggtttcctttgataccaatgagttcagaaaagctcgggtaattcccca
tccacctttttcctttgggacgcaacaaggacataggcatccttcttccaaagaactcatgtctgttttgaatctttggtttcgaagattcaggatgcc
atttctcgttaatgcattcccgaaccttgctgttctcgtaggatagacacaatcaaacatgtccatacctaaagcaacacaaaccactagatcttca
gcatacccgacacccataaggtatctaggcttgttttcaggaagtataccagtacatgtgtggacactaacaatcgttaatatttctcatagagac
atttgcatacttacacttcacaaaaagttgtcttttcctctcctcccgataacccgccaactgcgattcctggggtatccctttttcatcatttccttaca
acaaatctctcgtagcttcttattcaatcctccctgaataatacaaaaaagattttgagtatctgtatttttgtgtgcctctatacatcgatccaacca
ccgtatcgatcgatacatagcttcttccattcgggtatcttcagtcaaggtatgaactacatcgtccaactgcatcataatgtcagacccaattgtat
tttggagctccatagagtgctctggggttaacagcattggtgttccatccctaggagataaaaaggttactccctcttcagtgatggtagcaagctt
caacaatgaaaccatttgaaatccaccactgtcagttaataagtttcttttccacgactgcaatttgtgggctcctccaaggctatgtaaaacctctt
ttcctggctttagccccaaatggtaagtattgttcaacattatttgacagcccattctgtccaactgttctggtagaacaccttttaaagaggcctcta
caatatattagtaaatttttactaaaggtctactggtgaacatacgagttcctacgggcataaaaacaggtccttcgacaggaccatgcggcaact
caagaatgcttgctctagcacgcgttgttgagcattttgcgattactttataattcaaagctggataagaagaaaccatctaattgaaaggaagctt

tgtttacgatggccgtcctgagtctcaacgaaaaattttgggttattagtagcttggttgtatgaagtaggacataacactttttaaaaaaaccttca
acgaccggataaagagagcaatgattgatgaatagctttataatgctttgtttttaaataagaaaataaaccattttgaaaagaagaccaagatt
gtgctaagtgaagctttctttgtagttctagatacattggttctgttaatcccattgaagggatctgaatgaaagtagggtcctcttcctgtttcttttg
taaatattgtgaagtatagtaactgaactgggcaccagatgttaaagaccagattgtgtcagaaacagactcaaaataatccattgattgtagatt
caaatcagaaaagatagcatggataaactctgatttttttgaattatttaataagcattgtatcatttgctgatgtataagcttcatatgttcttgtaa
atctcttgggccttgatttcttaaattttgttctgctgactggatcaatcgaaaactagtctcaatagcataaggaaagcttttcataaagagaaatt
gtatgaatttttgaatagggacaatgtctgaccatacacgaagttttgtatcactcattgaattaaagatggttgtcataatcagggctaaaggag
ggaaaagtttgtcattccaaaaaaatgtttctagtaaatgaaacaaatgcgcgaaaatgtaattcaggagaacacctgccaatcccaacctctgt
ctcgagtttgttttattgacgagacgcaatgtccagataaatatataactcagatggatctcacattctctcaaagatagttttttgtgtatagatta
agttgcttaaaaaactcagcacattcttgctcatccctcgcaacatccaggcaggtcattaagtgacatatagcttcatattccttaaaaggcaag
gaaggcttcattaagtattttcttataaaatcgtaaagaaatggaaaggtattgtttgagtataataaatgtcgatagaaaaaatgaaaaaattca
agtcgttgttcaagagttgcataattaaatagtaacccatgataaagactagcatataaccattccgttagtctgcttattaaagaattttcatttaa
gaggcgatagtacaggaaaaaaacgtagctatgaagaactgctgcaacttggtttggttgtttcactttttcgaacgtagaagagagctgtgaaa
tagtaaatacttcattcaaagaaacattttctattttattttccatagtgagcaattccggaattaaacttggtactcttttgcgcttctcagataacc
gaagcatactttgtgaagaaaccgtcgaagaatgaatcaaatctgaaaccctcgcaacccaaaagacatccatcttccatgacatagcccttctt
tgttgaaacgagcctattatgacatctggataatgctgcttgtaagtgtacaacaattcgaggatatagggatcattggcatttcctggcttttgata
taaggataacaattggtaaattctgaatctctttatataagagtgcttcgtcgtcaaaattagtaaacgcacaagagaaggacggagggtgacat
agtccagaaaatgaaataagactccatagaactttgcaaagcgatctttatcttctaaaaattcatacacatgagctaaccagttcaacaatttct
cttggtatgcatatgcataacggactccataaggatccaaacagcctaaaattttgacggctatggagtgcgatattaactgcttcggatacaagc
tttttataataatcgtcagttggcttttatcgaatttcgtgtgaaaagacaaagctacgttcagcaatgactcaattgcttcgtttgtcaagccttcct
gtgaagccaaaccttggaactgtcgtaaacgactagaaacaactttcttagaacctacaggagcctcagaagccctatttatccattcaatcccat
cttgtaaatcataaaacccatgactttgattttcagtcatgaaggaacagaaaggtagtacacattactatcccggcctgcacaaacccaaatag
accataaatttgctacttttcttccagttaaaaaacaagtaagtgaaaaagagacttcagaaatagaataagtctaataaaagagcggtaaaca
gagtgtacattgaatcacgaatatttagtctctgtgagaacgatctacttcagttggtgctacaactacttatgtatgttcccatatcttctacttttat
accattcataacgttatgtcgctctgtcgttctttaccttaacagaattgaatacaccaaatatatactaatctcgcagtactagacaactatttacc
ttcttgagtatcacatttataattttacataagatataaaaagatgaaagtttttttgattcgccatggtcaaacggagcaaaacaaagtcggaatc
ttggtatgatgaaacatgaaagaactagttgttttctcatctattacatattgggacattttgttaatactctatttagcaaggatctgctgacacaa
gtttgaatgaaactggtcaacttcaagcgcaattattgggccgcagactttcgactctaggaatcgatcaaattttctgcagctcaatgagccgttg
ccgagaggtttgcgaaatgaaaacttgagaaatatctaaactcacatagattatagactattgcccctttacttgaattacggccggaaattcctat
tgaatattcagatttaatcagagaagtaagttggtactatattcatactttactgactgtaatagagaatctatggagaattggaaggtacagtatt
cgtagctttttaatatacctacaaaagtcttggaagtgctaatgagaaaacaataggaatgaacgtggttgaagcaaaacgcctgatgggtact
aagcaccctgatcattacggagaaggtttttcaagtttaatgcaaagacttgttaaattttgggacgagcacgttgttcctttatctgaaaaaaaga
aatttgtggttgtcctttgtcatggaggtgtaatcaatgctcttcgtgctcattttatggaagaaaagggttatacctatgatcgtaccaaattggaa
aagaaaattcttactgtcaacactagcattacggaagtcgaagttacttctgaaggtacaggacatatttatacttttggagatgctgctcatctag
aatcagaggaacacggtcgtctcatcgcataaagttatcatctcttaactttttttattatacctgacgatttaattcactaaggattttatcaaaaat
tagagtcgattcattttgtctagaaattaagcgttcgtaaatcgtttatataaagcagaaaatccgtgaggtgtttagagtcagacgcgtcgtcata
tgatgctaaaaaaaagtgatataaacacatgaaataagaaatattcatagtagtaacatagcagtaacgtagctttgtttctcgtaaacaaacta
gaatccattcatcataaaaatcgtttaaaaatataaaatataaaaaacctttgaatttatatacagaatccaatcgagcaactaaacataaggaa
aacacaatcattcatattcacaaacctctgaaattagtccttcttatcaagccttgtttcctttgagttttcgcttcgtggattttgacgatatcgttca
acagacatgatcaggtgtttaccataagtgtattttttagagttgatatatgagggtatatataagaaattagcaattcacgctccttttcgtttga
agcctccagaagcttttgaaccacataatttgcatacttgtctttcatcatcaacattaaaatgctgacattgtcaactttttcagttagcactttttga
ataatataggcgcgatcgttttcggaaatgtaagatatacatctttcaacaacgtttgatgcgaacttatgacaactcaagtatagcaaattcttag
ttattagtttaaaaacaaagtttttatccttattggtgcctttttcagtatgtgctgaacaacatagttaccatactgaccttgggtcaattgtaaag
aataaggaaggagatcatcaattataatttgacgttcatatgtaaacttttcaataactcgttgaattactcgacaaccataaggatgagccgata
acacatgtatttgaggtcgcaaagcctcaaaaacaaaaccaagcttctttgagtctatacattcaatagacttttgaatgacatggtttccattctg
atcacaaacacactccaatacatgaccatcaagttcactaacaatttcaacttgctgttctgtagcaatatgttctattgccttctgtacaacacgac
aaccatacatgtggacagccaatacaaaagtgtgacccttgattttggaaaccaaaagggttttctgtttgtctgtaccatattctaaaaatttctgt
ataacataatttccaaaaatatccatcataagttgtaagcaattactagaaataatctcttcgaaaacagcacttttctcttcttcagtagcagttgc

taatttctgttgtataaatcttgagccatattgatcactactgaatagaacgacgtttcctaaaatatcagaaagctcgaatcgcttctttttattatt
atggaagacatgcagtagcttagactgagaaaaataattatgtccatgagatcccttaagtaactgatcctcgacgtttaaggtcggatacggatt
tgagataccatcgattggaatacttccaaaaccacctacaggagctaaatcctggttgatatttgcataagaccttgaagaatctaatgcattgtgt
ttaaccgtcggattagcaattggataaacggattggtttgaaacaagaggcatagaagtaaggtcagactttgaagaagacgatatgtcagaag
cgagagttggagatgttgcggaaatagaattccgctcatttaccacattaggaggagatagaaaattcacatctagcttatccttatagtttggca
aagttccggataatggagccgcttgtgtaatagatgccatatcaccgatggaagaatgcgacgtatggcggacagcacgtcgattaacaggcttc
caaaagtcagagtgagctcgagtatggcgagataaaatccgactagaaggtgtttgaggaagaccactagaaagagttcttcctaaatcggcttt
taaaggaggaacagagttttgtaaagactggggaaagcgaacaccagaatcagagccgagctgaccgtataaattaacctttgagggagaact
ggtgtcttggttaagggcgttgcttttattactcgaatcaacaaggcctttagagtgaaatggagactcaatgttagcaggatagtgagtaaaaac
gggatcctgtaaatttgttaaagaagaaagaggcggttttgccagatgtttgtttcgaaaaggatgcgcactgttcagagagacggtatctgcggt
ttcttggttcattgacaaccgatggaaagacggaaaaggattgaggaaagatgaggaagtatcagagtgatcatcacttccagaagattgggag
tatggtgataacgaagaaatcatgttttcacgaggtgaaccacttgaaatcaagcttggacgaccgtcggtatatccggaagaaaagttaaacg
gggcattttcactcatgccatcagaaactttcgcgtttgattttacagtagcatacatggtgaattaaaacgagtaacaaagatgataaaaagata
ataaacaacttgaaaccaacaaaaagtgggttaaaaagaaagaagcgaaacagaaaagatcaaaaaaaaagaataaaataaagagaaatc
aaccaggaagtacaataaaggtcgtaaagagaagataaaaaacaaccagaaaagaaaagaaacaataagaagaagaggttgccttaagtat
aagtaaaattatgcttaataacagggcttaaaggaagtataggaaaactaattatcatatacaactcgagggaaaagaatggaagatttcttca
aaaactttagcgtataatgaacaatcagtcgatgctgccaaaccaccgagcttggaaatgtcacggatatcaaaaaaattgttatcaataattta
atgaaaatacagacattcaaatgtatggaaataaacggttggaatctgcagggcaatgagcaatggaaggacgaatcaattctgcaaaaacaa
cgagaacatccaatgttcaattctcttaatgggatgagatagtcaagaaaaaaaaaaaaaaaaacacacacacacacacacgtagaatga
aatgcaaataagtcaattgaacccttctagctttttcggatcaacagacgagttttagaatcaaataagtcgccagtaaatagtttggtagaattgt
gttcagaataattcctcttgaacgagcgaaaaagcttcttttaattgagatttgcttaacggaaaggaaaaaggcaaaaggaggagtagtgaaa
gtaaaaactgaactaagaaaatatgaggcttcctcactaaaatggttcccgacaagggaggtacctacactccgtttatgtcatgccatttttgga
aacggtagattgaagaataaaccaaaatcaagacttttgtaaaaaaaaatacttgaattttacaattttttagaaaaaaatttcttttccgatgaa
gtcagacaatgtcgttgtttttcatttaataacgagatgatggaacgccccttgaatcgaactaaaagaacgttcgttggataagggagtaacaaa
ataaagcagacagatgttgcaataaaaaaattaacgtaaagagccttcagcatctgcttgttaaatgatcatactgcggtttaagaatcagaaatt
catgttttgtatcagtaaattcgacgagcaacacagttatttgaagtgactttaggaggtggaagcaactttggtttctagaagccaaagacagtt
aaattgttaaaatgaactcatcaacatcaactgcaagtataagatagttatctttgatttcaaaaaggtttgacggaacaattcgttggaacggaa
gactgacgatctgacgatcgactcgacttgggaaaagcaccccgtaaacaaattagattcatttttaaaaaactttgaaacaaagaaacagcc
atggggctcatattttattgcgaattccatgttcataaacaaagacaaagagaagatggaggggatcgagatgaaagtttgctttaagagtgtta
ctgaaagaacattggtgtaaaacgatagcaaaggatgaatttgaaaaagtttgaaaagtttcgacgaccaagtttggattggattgtaaaaata
agtccagcaaaagtcagcaggtttgaagggttcaagagatgtgaaagttaatagtatagtttaagaacgatgagagccattgtagaaatacaaa
atagaatgtattaaaaaaatttttggaataaaggttcatggtcaaaggaattgtacaaacattttcctttccatgatgagacatggttttctgggaa
actagtgaaaaccatccatagaaaaatctgtcataaagttcttactgagggggagcattttgttcgccgattttcttcaagtgctcacctaaaacttt
actagtatcaaagtacttggcaacacaacggtcaatacaaacactttctcctttggtcaaatcggcttccttgtatggtgtagcaatgcactttttat
gacaagaagataccattctgcagttaattgttaacaaaaaaaaattcagaagtatcattgtgaaaatgaattaggaaaagacatgctcaaccgcc
cgcaactccaaaggtttccgatacctgcgtagcattcaatagaaccattctttaaggctgattatgtctttgtcaaccattctttataaaagcgacga
aacatacctgttgaaaatctccacacttaattctacttcctgctcagccaaagcaatgttttgagggttcacagattgatttccccttccgattccga
aaaatgacatgttggctaaatttgaattccaaacaatgaactgggcgttatgattcttccttttgggcaaaagtttggttttctcttgtctatggaagc
tgtccaacttcacaggactaggtaacgttcaggtaacagctggagagtgtaaacaaatttagaaaaaatggggtttcgaggcttttgcagcagat
cagaggcttgctttgctggagaaaagaaagtcctttgaaacctttttttacttgaaaagatggaatgtaaattctggcttgttcggtggtaacccttg
gattttttggtagatacttggcccaggagactgaaactgttcccttcctttctaaatttgaataaaggacatgattcatgaatttgacaattaaacct
tttcttgtatcaatctacaaaacttgtccctgctacaaatcaacgataactctggtcgaaagccttagccctggtattacattgcacggttttgttgca
ttccttttcattcggtatggaattgtcactacaatctcaactccttaattaatttattctctttcattttattttactttacttcttttctttctttgtcaaaa
gcaattcgttagataatgctacgctgccttacctttcttttgtgttgtacaaatcactcgtaatccatatttagcaaagcgtgtttatgaacgcaatcc
tctaagactcatccaccgtaaacaacctacaagaagctttgctatggcttaactttggtaacaaagtcgaagctcaattatcggaacctcttttctg
ttattctttttcaatcgacctcttttccatattcttccccttgacagcagtcattgaatactgctatttggattcaacatgtcagtttctcaacaattggc
agagatcgcaaggtatgtctacatatccatatttctttgagctaaaccttttttattcagtaacgaaaaaactgtcatgtatgccgcggacgccaattt
acaagaaattttgtgctatcccgaagtgtctgataaaccaacacttgttcaattaacagatgcatgtcttcatgctcaagaacttgctaaacatctt

gattttggcagaacagtgtctatcacaaatcactattctcgtggtagctgcgttttacaaaccgtcagagaaaaaaatgatggtacaggaagcgtt
gtttctactaccattgcagctcaggatgtcttgagaaacgcattaaaatccaatcgagcattagacaaagtgatctctcaactttaaagttcatcca
tgaaaaatctactctcgtccattatagtttctttaccggttatgaaaaaacttggatttatacctgggcggaacaaaacgagtcctctttcaaccag
ccgaatttgggatgcgtttcagacacagttcaacattcagcaacacagcacagaagtaaaacaccacttttcttaatctccgatatttatcatcttt
aataaataatagttaattgaataaacatatacaaacacatatgagtgtacctcaaggctagatagacttctcaatcctttcatgtcttgtctgggttt
tctagctctcgtacctccaagctatagagcctatttatacactattttaaaacaatatccgaagaacgtagtttaagatattcttgatcaacacctga
tggtagttcccgaattaagccataaatttctttattgtgataccgtagacgtgtctttggatcagtgtagggagctggcaaacctgtaacatcgcaa
tattttggttgtggaagaacgcttggggggtgcctctatggaactgtaggacaatttagtgggatcattctgaattgggtcactttgcaaagtttgctt
caaatttcgatttcttctggactgagctttatagttcggatttcgaaacggttttggaactaaactgatgtctaaatcttccactgttgctggtttttcc
atggctttttttggcgctatggagtttgtggttcgtgatgttgttagcaaggcgtccctaggtttatagctttatgaattttcttaatgaaaataaatct
tttagaaaataaaatactaattttatgttttcatgaaatggaaatacaattttcgtgcaatgtagtaattgtaaggacaacacttagtactttaaatg
taagcataagagaaagactttttcatttaatcagaaaagaaataattcattttcctacacaaaacctagaaaaaataatgtcaaacaaactttca
acgttcactgctccagtaatacgaccaatttcatcagtcgcaagtttcatttcttctgctattatgaccacgtcggaagcatgattaagggatatcttt
aaatgttttatgcattcttttaatgcttctctttgacgttgattccagccatatccagtgtcaccgttgttctcagtctgcacacgtgaaataattcgga
atgatgatgccagcgcatcgagtgtttgttcaagacccttttcacttttaatagacacctcgtggatatgtgatgatgggatgttaagcgcctgatgc
aggaatttgcgcaccgggcccgctgcttcagacgcttcctgaacgaggtcggttttatttaacagcacatgaacatgttgcttctgagctttaaaat
tttcgataaggtcaaaaatttcgctttctaaaacacatgattctccggtccaggtttcagaaggtaatataagtaaaattacctgggcctgttctgcc
ttttctttcgcaacctcaatcccaatgctttcgatttcttgaacgtctggtccttgtcggaatccagccgtgtcgcttaagaacaccggaaagccatg
aatatccaacaatgtctcaatagcatctcgagtggttcctggctgctcattaacaatggatacttttcgctttgccaaataattcaccaagcttgatt
taccagcatttggtggtcccactacagctactcgaatgccttgtcgaaggatttctgtcggtcttgccttggacagatgacgttccatttcaatagtt
aagctttgaatttcctttaatataggatcgaactctttttgatccaattcatgctcttctgaaaaatcaatggtagcctccaaaagtgctctgctttcg
actaacttattcttccattggaaacatatacgatacaggtcgccatgagcttgttgtgagacagcatagagttgctctgctgtttcagcgtcaatgg
catcagcaagtccttctaactgcgtcaagtcagtttttccattatagaacgctctctctgaaaattctccaggcaatgcatatcgcaaattcggcaa
cttggtttgctcaattgcatccaatgcttcagcaataattgccctaccgccgtgtaaatggaactctgctatgtcttccccggtaaacgacgccggtt
tctgaaaatatagaatcaatgctttatcaacaatttgagaccgtatagggtgttttatggtttgcaaagatgccaagcgcggaggtggtacctttcc
agtcaatttttgaattacctaaaatattcgcaagttagtctatgtttattgaaagtgaactgctcaccttactggcagaaggtcctgaaacacggac
aaccgaaagggctgcacgacctacaggagtcgataaagcataaatcgtcggtacaaattgagagataccttgaaatcgttgcattactatacctc
tttttcaatctttcatacaaagaagtcttcaagacccgactcgtattcccttatgagcttgcgtgtagaactttgataaaagtacagaaaaaaatag
aagcaaactaaatttttaatgaaaatacagtatcaaataagctcagtagagtttataggttataacaaatcaatattcttttcatgctacttcatatg
ttctcacttactacgtgtgaaatatacaataaaaaaaatacaaaaataaaaagatccttgagagaaaaaaaatcccaatttcttcaacaaaaat
atgtgtttttttgaaggtgtattgtagcgttctgttagaaaactcattaactttcgttacaataccctatttcgtacacacacagaatttgcaggaaag
aagagtttgccaaacaccatctgccaaaatgcagcaagacgaagtcatttggcaagttattggtcatgaattctgttcttataggataaagtaagt
gatacggagttagaaggatacaagtgctaacatttgagttagaggtgaagcattaaatttttgcagaaatgagtacaacgttactggactttgtaa
tcgacagtcgtgtccattggcaaattctcgttatgctacagttcgagaggataatggaaagctgtatctgtatatgaaaacaattgaaagagcaca
ctcgcctgcaaagttatggcaacggatcaagctgtccaagaactatacgaaagctttggaacaaattaatcaaaatttattgtactggcctgggcg
tcagattcatcgttgcaagcaaagacttactcgtcttactcaatacttaatgaaagccaggaggcttgctcttaaacatcaacctactttaattccc
ataaagcccaagcaaaagcaccgcgaaacaactagagaacgtaaagctctgattgccgccaaattagaaaggaacatcgagcgagaattggtt
gagcggcttaagagtggtgtttacggtgatcagcctctcaacgtgaacgaggaaatttggcaaaaggttttgtctgctcgtgagggtctggttgac
gaagcgaaggaacaagaagatatcgaaaatgccgaagtcgaatatgttgatgaagacgaggatgaggaggaggaagagatttctgatttaga
agattggttggctgatcaaggttctgaagaatctgcaacttctgatgaaagcgatgaggatgaagaaacttcagacgaagaagatgcgacatct
aagaaacgtaagtctgttgaagataaaaagtctcgcaagagacatggtcctcacatacacgttgaatatgaacgcgaacatgagactgctccgg
ccgttcaacaagagcatgcctggtaaagggtttccaaaaaaatggaattttgggtttcgggttcaggttttatgcctactctttggttttaggaaaa
gggtttttgtttcatactttattttgattactaataattacttgtatttatgccaaaaaccgtttatttgtcgtagctttcatcaactatatatgcttcata
tcaattgtaatttttatcaatccgcgtaaatcccaacaaaccaatcctttcattactttttttcagtcgttctagttcatttcgtaaaagaatgttttct
tctcgtaattgtctgttttcctcaataatcttcaccaaataacgattgtcagttccaatggactgagatgggaacatagcttcaggcttttcaaaactt
acctcagcgacgtgtgagggtagaaggttacattttaaaacctcagacaacatcgggcgtttggatggatcccttgccgtcatccacaaaatcag
actagattcacaaacgtaattttcaatgaatttatctggtaggacaccttgtcgtaaatctcttaattttgaagcccgctccatagccgtatcaaaag
gatgaagaagttcaaagagaaccatacccaaagcaaaaacatctatggcctcggatacaggaacgttaccatgagacgctaatgcatcaagta

gctcaggagcagcataagtcatagtaccaacgtgctgagtagcatccattggagatatatgttgaagatctttcgaaaaagatacagactcgagc
gttttcccttcgttgtcaatcaccaaaccaaaatcccccaactttggtgtatagttatccaagtcattgttttccttgtcccgatagctgtacaaaggt
acagagccacgatctactgtaagagattttgccaaaaagacattactcggctttatatcacgatgaattagatgagcgatttcatgaatataaatg
acaccgtgcaaaatagctcgaaataaatctatgtgaatctgagcatctatcgtggacaagggaaacgaaaccctagaatttcgtcgaaacaaat
atgattccaaatcgtccgtacagagttgcatttgaatgtataaaataaagcaatttaacggagctatagtagaagtgtaacgaacgcttgaatca
cctggaggttcaataatgtcatcataggaagaaccatacttactcgccgaatctcgatcatggtaagagtcactggtttcgccagcttcgggcgat
gctttctgaatatatatattgtccgactgccccgatacttcagctgcgaaaatgatactgtcgttatttgaatagtcatcataataaagagagtcttc
tttgttggctattgtgatggaatcttcgcggctaatgtctagagactccaggccctgtatcattctacttgaagacaaggtaggcttatgtacgtttg
gcactatttcagaagtattttctaaccaactagcatagtaccgacttgaaaagcgttagtataattagtgtaccacaaaagaaggtacttacacta
tattgggatgatccagctttgcaagactttttatttcgcgaaagattttagtgtaaggcatctgttgaaaagtaggaaatattttttcatggcatatt
cagcgccatcgacacggttgcgcacccgatatacggcgccaaagccacctttgccaaggatttgtagctcttcaaaatcattcgcatatcgagaat
gcttcgtatgcagccacggatcagacgtaaagctatttggttgcgaatgttgttgggaactagagggattggaagaagccgagattctttgaaga
gctccttgcaattcctcataagatctctctggaatgtatctttctataggcaatggtgcttgcttaccttgatcttcctcgaacaagaccccccctagct
tgagtgattaagtgatgcagagcactttgataagtcgatcgaattccagcaagttcctcaatatactcttcatcaacaattccaatacgttttaaag
tttggcaaattaacgtaaagagccttctggagcgagacggatctgaatcatataattcttcgaaaagggttagtatatcatatgcttatacgcgtca
gatttggaacatacgacaaaagttttctagtaaagccgtaaataaaagtcttcctaaaaaacaagttgttagtatttgacataagattaccaccga
gcgcatgggtgcaaaagtttttaatgttttggaaaagaacacataccatgcctgttggtagacaattcttttaatttcccatcttctcgtaaggataa
agcctgatcaatagcaagagtatctgtgttagaggacgaagataaactaccggaattgtgatatagcatttgacactttccagcaaactctcaca
agaaaggatcttttaaaagataataaatacagacatcaacccttttcttcgatataacctcactaacctatttataaagaattcccttcaattattgt
ttctggaaggtttcaggcaatgtaaacatacttttgtgtggcagtctttttttgctaccttggcgaagcatggtgtaacctgcattgataaacaaacaa
atagaacccaatagtaaaatatataatgaaggagatgaaaggaaatatgtacaagcatctctatactatatttcaagataaataaatagatcgtt
ctactttatttctttttggttctcgtaatgtactctaccttgcttttcaaactgatccttcagagaaacgtcgattccgtgaaaatagaaatcttaaaa
aagctattcttcagaacaaatttaaaaaactacggatttttacatataatatataatttctttcaataatagagaagtcagtaataacaaaagtcat
atatatacactagcgacacatcaaaacttgtatacatatctctggagtagagaagtagaaagcaaatccgttgtggtccaacggctaggattcgt
cgctttcaccgacgcggccggggttcgactccccgcaacggaagtttttttagatttactaattttttttttgtctttcataaatatcttatccacctttggt
attatatttcttctagtcacgtaaatatgatatataaatatataacattctaagagtacatactggtttaataaatattcatagggtatcatcttaag
attgccaactgaactggagtgaaccgttttctatatttagtcgtcctcttctagatagggatatgcattatccttgaagtagaataaattaagcttca
gtgtttttcataggaattacacgttcgttgacccaccacttgtgccaactatcgtccatagtcaattcgggatgaaacgaagttcccaaaaatggac
cctgttcaacagctacaaccacctctttctcctcatgaacaatggttcccaaaacatgaacatcctcagaattgatagacgaagcaatcggagctc
ggataaaggttgcatcaaaggcttccagacgtttcgtattatgaaatctcataagatcggtgggtcgaatagcggtagtaaacgacatggtttgag
gaccaaagtaatttctagttacatcaatatccattgctcgcaacaagtatgggtcgggaaactttccacctagagaattcttggaaagaagaatca
taccagcacaagtgccccacataggaaccccccttgactaccaagctttctaatttttcccggagatcagggtcaatggtaatgagtttgcccatggt
agtagactcaccgcctggcaggactaggccatcgatgttttcacaatcggcagcagtctttacgggaaatgagaagacatcgtcaaaggagttca
ggatattaatatgttcaatgaaagctccctgcagggccaacacacctatacgaacctttgcgttcgaagggtcatgaacgcgagaagagctagta
aacatagaagccgatgacatatcgatacacaaatgaaagaatccaaaaaaaggaggaaaaagaaagagaataaatccaacaaaaaagaaa
actttctaaaaaaataacttagggcaaaaatcaatccttctgaaacccaagcgaaaaagtttcttcaatatactaaccaaaaggatggccttcaa
aaaaggtcgtttctattaaacacgtccacgaaccagtgttcttttattcgtaaaatttcgccaggaaattttctattttatatactttaaggatccata
gcgttggtggccaaggtgagtttgcccccagtgcataaaattatctgattagatattagcgcgtagcatttttttcctttacataatgattgattggat
aaggttaacgatgtttgcgacaatgcttctcgtcaaggtgtaaacaaaagagaaaaatagaaaataattatacatgtaccaccggtagcagata
agaagttaatttttagctggaccatgcctattacatttgatcaggtttgctcattgtagccatcattactagcaatttctttcgcttgaaacactacatt
ctcaaagaaagtcatgtctcaagctttaattacgtaaactaataactacgaaagattcctcaaaaggtgtaaccattttagtttcaagttgtattga
cagatggctgtgcaagcaacttctatcgatatgcaagaatagtgtatggcgattgaaacaaatcgtattttctatgcttctttacagtaacaaagta
ttctcatgacttggctttcttatttcttgattgaagaattactagctcctggatatgttccttctaacatccaaagtgataaccattaaatgatagtga
aatgcttcaaccctcagtttatcatttcaaaagctgatcagcaaatgaattcagtttcgactgtacgctatacggcgtaataaaagaaggaaagaa
gaaaagccgcaatcatccaccaactaactctttctacaaactgctttcccaaagaactacctagacattccctgaaaatatctgcaatacatatca
tgcactgactcaaccttgctcttatccttcaagacaagctccagatcctcaaacatctcaccattctttcaatcaaaaatactatgtatgtcacattt
gaacgaaccaacatttgttgtcaaatccatgaatcacaaatttgtcaacaaaagatgattcggcgtctcttgccggtagtgcaatagcaaggaca
ccggtgccggaaaacatagcaagccagaggctttccagcatggaccaaaaacaagattaatcggaaacacctcagtgaggcgatttcagcgat

taaactgaaaagaacagatactacacttgatctaagccaaaaggcaaagagagaatactgaacatgaagtattagggccagtatatataccttt
ttgctgcgtagaacccttaccctggaaaatactagaaggtcaatcattggttggttagttgccgctctctatgaatgcttgtttgcaaaagtatagca
agcataaaagtaaatttataaaaatgttttattcaattttattatctgagatttaatatttaaacctttgaaaaattttatcttgtgagtctctaagctg
cattacccttcctcagggcgagtttgcctctttgatagctaccagtatgacctatatttctgcagtattggtagggagtactttgtccttagcataaca
ccacaactggaattaagaagcttcaaagttgctcatatctttgtatttctatatataaatttatccaagtaaagcattcttttgtctttttagaaaaatt
tccatctggagctttttttacaaagtagaagaagaatgtcaggaaattcagagtttgaagggattttagaatccctagaacactctaaaacaggcat
atctggttcgaaaattttaaaattaacgaatttggcaatggaaaatgttggagaaaatgctcagttcattactagtgtgtataagtatgcgaaacg
cgctcccagtacccataagttaggcgccttgtatatcctagattccatcgtacgtagctttcaggatggcgctaaaaagaataatgaatcatttgaa
tctccagtggatgcttcttttagtggtggttggtgcaaagctgctgaagtcactgaaagcttagttgctgatgcaatgcatcatgccccttctgctcat
ttggtatgtgtagcgttggtattggtataaggttctgctatgttggtaatctctggcccctttggtttagaatgtgttgtgttctattttgtatgcttctc
ttcaattgtatatgtttagaagagaaagtacttgtcagtattgctgtattaacccgtttaccaattatctttagctattattgtttttctactgctgtaa
ggatgttactaacgtttcctgtagcctaaaattttaaaactttgtgacatatgggaaaaagcttccactttccatcaagacaaattagaatctcttcg
gtctaaaatcaggcaggctattggctcttctgaccaggctaatacttcaacacctgatactacagcttcatctgcgcaagcgaacgtagaaaatcc
tgcagctgcaaccacctccactacagaaccttctgctccttcgcaacccgcaggtgcttcaacaaatgacccagcttctattctagaagcacttgct
gcttttgcccagaaggcccctgcaccttcatcaacctctactgcttcaggcacgccaactgcccaagactccgcatcccatcctgccccagcagca
gcagcgagcattagccctgttccgtccgctacgacttcccctttaaacccactacctcctcagccggttccatcaatgcctaccccctatgtaccagcc
tatggctggtattcctccctcaatgttaggcgggcctacggtaccaaacccagcagctcctgcctctgatacggaatctcaaaaaataaatcttatt
aatgttttggcttctcaaaacgtcccagctcctcaaattgagactattattaaagctgcttttccttcttataacgctcctttttcaaggaggccctccg
gctgcattgtctgctactgccccttcagcaggaattatggaacctctacataggtctcgtagtcccagtcctcgcagtaaacgagctggtcgtacgc
ctagtccttcacaaatgtctattccttctgctatgcctgctaatggtatgccaaagccaaccccctgatggcatgccccgtaagttagagcgtgatatt
accattcctcctgattccataaaaggtgtgttaaatttttctttgcgttcccaaagtctaaccattgtacagtttttagtcgtactcttttccttggaggc
attactcgagcagttcgtgagccagtgttgcggtctatgtttgaacgtttcggtgcagttcaaagtctaattttgaatcacaattatagacatggtttc
cttaaaatgttccgtcgtgaagctgccgaaaaagcacaagttgctatggagaatgtgccttttgctgatactacaattcgcgtatgtataaacttag
ggtttattcctctgatcgtttgctaactatattatagacaaaatggggtgttggttttggtcccagagaatgttctgattttagctctggagtgagtat
aattcctatacgcttattgactgatgccgatcgtacgtggcttgtatcagctgaatacggtggtactgggggtcttcctatcactcctggtgttgctgt
tgacgaaccagacattgaaatcgggttaggtattagttccaaagctataagtaaacgtggtaaagacctttctgtacgacgtgatgacagattac
ggggacgtaagcctttccgtggaggtttacctcatcaaggagaaagacattttgatccttctgctggttggaacaatggcggtgctatgcctagta
atgtatataacccgggttattcttcttactatgatcctaattattcttctgcatcaggttatgcaacacagcctccctggcaaccacaataatgtaaa
ggttcgcggttaattaattaatggaaaaataaatgggttaataatcggttttacggttcaattgttcaagcgttgttaataagaaataagtacttatt
gtcttgtattacaaccttagagcttaataattacgttcaatttagtagcagataccagagtaattatatatttaaccgaattaatattatatatacaa
tattgaaaaaaataacttgggtacaaaaatgctgtcaactaatgcatatttccaaaaaattaagtttactgctcagcatttaatatattgtacagttc
ccaaagccgcttcaagttgttcctttcgtggacaaataccagccatctacccatttgcgccaaagcttctcttctgtgtattgtttcttctcaaattct
gggccgacatcataaatttcaccggcgtcagaaaaaagagagttcatagacttgttaatactgtcattactgttgtccgctgattgcgaaacagaa
tcttcattgtctaagtcatcaagcccagcaaaagggtctggttcttcatctatagcttcagcgttattaatccatcgaaatctagtgggatcaaaaa
ccatgccgcccaccacttgaatgcctttcctagctgaaatgtttgaaataagtgccggctttgaaggagatataggagcatcaaaatcttttatagc
gtgatcattaccttcccaacattgttttgcagggttatatcgcattttaccaaccaacttaggagtcttcggagagctcaagtttttaattaaagtag
gctctctctttttgtggaaagatttatagttttagcgtttagttggtcattagatagagcttccaaagtcgtttcttttgagggagtaaccgcatttttc
cgattgcccttatagttataggaatcatttttctgaatgttagtgatattagataacaagttatcgtttctcttgctacgagtagagcttgggcgaata
gaagaattatgtgagtgtttcggcacagaagaagtaagtttaccttggtatccattttcgtagtcataattcacaggtaagtcctccaatgcgtcta
attctgtcccgtcaccatacttgcgcacatgtctaggacgagttctgtatttactcgtttttgctgccgactttaaatcatctatgtctaagtaaaagtt
atgggcattctgagcttcctgtttcaaactggaaggtgtccatgtagcaaaatttgatggattttctttctttgaagaagatgagatgtgagaagctc
gaattgtgtcatgagaataagggaagccgttctgagtatacttcgatggggaatttgggtgttgttttgagggacttgggggattagaagatggcg
cttgttcatttgaaagagaaggaaaaatctccttgcataaaggataagctctggtctcctggggaacctttggatggaaagcaatggtggaggat
atatacggatgctttggattaacagtgttaagttcgggagctttcttacgaaaaatagtttctggttcttctgtcggatcaagctcaaaatcgtccca
aaaatcatcttcttctctagcagaagaggggggttcgaagagatgattgttcgacactggaaaaggaggattttggatcgatatttctattcttccttt
gtagtatctcagcaagatcggtatcgggatcgatttctaaatcgttctcaaaagacgagaaatcatcaaaatcatcatacaaagcggaattagaa
ctattaaaagatctggcagcagtatctgcgtgcggttcatagtcactttcgggtgcaagggtatgtatactaggtcgaatggtagttggatgtgaa
gcacggatggtttgattttggtctgcagaagacaaatgacttggtcgaacagtctgaaactctgagttagtttccccttcggaaaaatcaaagtcgt

cttcatattcgtcgacacgtcctttatggttcgagtcaccgtgaagctgacaaacattggaattcatcgacttgtcaagtaaaggagaattggaag
aagggttgagggaaagagagttgagcctaccgagagagttttcgaaggaaaagtcatcatcaggagtagtgtcagcgcttgtggtggtggtagc
tatggactgagcaaacaggctaacgttgtcagcgtctgatgcaaaatccggatcatcgtcccagctctcaatttcagtcattcaaatttgtctttga
gtaggatagagagaagactagtttctaaaaggtggcttgaatttaatcgccagagaaaggttaatgaaacatagaatgccaactgttcgtaaac
aaactctaggaagatctataggatttaaggacaaaacgaaaagatttgcagaatagaaaaagtagagaattcaataaactaacgaataaaag
aaagcgacgagaaagcagtcgtcaaattggaaagatgagaggaaagcactaggtgaaaaacaacaagcaaatccttaccaaaagtcttcctt
ataactagaaagaaattgtaatttttaacaataaacagaaagtagtctcttctttttttttctttttctgcttctcttacttcaagtgttaagaaggtg
tggaaaagggtatgtcgtaggaatccaaagcagggattcaaacaaggtaaccaaagcactagactgatgttgtcaacatggaaggaatacatc
ctttgaaactaaattaatgattcaacttctttctcgaaatttgaaaccataactttttgagatgctacttggacgattttcattttctaggaacccatct
aaaagacgttgggttaggaaggagaattaggaatttatatgaaaaagataattagataattccgtaagctatgtaactttgaaaccgaaacgac
caaggccatcatcgttggacgatttcataaaactacactggacccattaatgtcttgtcaaagcttaaagtacgagcgacaagttatgatcgcctc
ttcctgattctccttatgtagaaatcggggtttttaacgagagaagccttgccttgcatgtttcttaaatcatctgcaagagaaggaaaacaagatt
cctctatgtttaaaagacctggtctctttcaagaaaccttcaaaagaatcctttagactacgctctgcaaatccatagatacaacgtttgtgattaa
aaccattcagacttttgtttttttcctttggaataccttgtgtttgtttattaacgaacgcgtaggaactctaaggagacctgaccaccaaagacaa
ggaaaaatagctttagggaagctgggtgtactccatcctaaggtgctacgaagggaaaaagtcgtccgattttatataaaaaaggtgatttttca
gatcttctttttcgtatataaagcgtcccaatccagtgcatagaacaagactgttcacgtttacattattcagaaacaacttgactgtcacaaagtta
aggacttgtttttctaatacttgcatcaatatcccgtctataaacctacgaagaaacaagagaaattaaaatactgctcttctatagattcactgca
ttttcctgtcaacaacatgtcatgccgtattcaggtcgaacctacgggtaaacttatggaagagttgattctcgatgtccaacaaaaaattgttgct
ggactcgaagccattgatggccaaaaattctaccaagacagatggaccaaaggcgaaggcggttacggtatttcttgtgtgatccaggaaggaa
atgtctttgaaaagggtggtgttaacaccagtattgtccaaggggttttaagtcaagatgctgtccaaagaatgagagccaaccatgaaggtatt
gatcgcagtgcgaaagaattgcccttctttgctgcgggtattagcatggtgatccacccaagcaaccctatggcccctacgactcatcttaattacc
gctattttgaactagttaacagcgatgggagaaagatttggtggttcggcggaggtgcagacttgactcccagtgttctatttgaggacgatgcta
ggcatttccataagttgcacaaagatgcctgtgaccgccatgatccaaccttctatcctcgtttcaaaaagtgggcagacgattatttcctcattaa
gcatagaaaggaaacccgcggcatcggtggtatcttctttgatgacttgtccgaaaaggaccctaaagatctgttcgcttttgtcaaagattgtgct
tacacttttcttccttcctatatacctatcatggaaaaaagaaaggacatggaatttaccgaagccgataaagagttccagctcatccgccgtgga
tattacacggaattcaacgttatgtatgatcgtggtacttggtttggtcttcaagctcctaatccccgcgtcgaatccatccttatgacacttcctcta
cacgcttcttggcgctacaattatgagcccaaggaagagcgccataaagctcttttggaggcaacccacaatcccgttgaatgggcttaagaatc
gctttctcctgtgctacttactctttgtccatagttttatttttcttcatacctttcccatacttcttcggcattagcatgggcattatgttcttctcgcttgt
ttaatattctgtcctaatatatcttcctgtattttggataattctacttttgcgtttgttttaatgaatttgattaatcaactcggccttcctggtttattttt
cgtttgtaaaagtattgatcctgctatttctggttttcctaaacattactttattccaacaaaaataaccctgtaacccttcaatggcagtcggtttaa
acacgtttcaaagaagaaaaaaaaacaaaggcgtaagacactcatacatattatttatcgaatgctatcgaataaatcgtaaggattgaaaagt
tataagtcgtaaactttgctaattccaactgagagatgggagatacgagacgaaacatactgtaagaatcatttcttcttaacagttgcaaacaga
cggtttaatccataaatttttacattcaaagctaggcggccgatttgggtccgccgataatgtgcatatatacctgaatattatgttagtgctcaag
tgtgaaacggctattctatagcaagtaccctaaagattgtaatgttgaaaataactgggtctctaaacccccaagttcaacgcaactcaactggtt
tgtctcattcaaccgacagaccttttttttgtcgttttccagcccttcaacaaaaactcgttctccttctttggaatttcctaaaaattctcgtaatgtat
tgtccattttgcgtttctttttcaaacagtgtcacaagagcttacgtggaaaaggtaagtcaataggcttaattacgtggtaactcacttcccatcca
ggcaagccacgttgacgttttcttacaagataaatcaaccaaaactttcgtttttcatactgttctttatctgttcttacattacgaacaatagcaga
aagttactatctacgttcattcaaagcgatacttgaatcactcactacgtttaatattactagcacgctactaaaccagaatcctctgaaaacaagt
atcatttttgaatactacatatatcagagaaaaaagcattataactgtctgctgctgatgagaagaggaaggatagtaaaaagtaaaatcaaagt
cagatatctgttaaaaagatagaatgtcatcagacgttagttaattaatttgatcaagaaggacatatcacgatatgcctagttgttaaatgagcg
tagacataagcataataaacatacaagtggaacacttttaaaatttattccacgtttgtttactccttaagctcttgggcaatcttctcagccttctta
atggcatcgtcaatggagccaaccatatagaaagcagattcgggcaaggagtcgtgcttaccctcaacaatttcagtgaaggaacgaatcgtat
ccttcaaagaaaccaagcgtccctcgataccagtaaagacttcagcgacagcgaagggttgagacatgaaacgctgaattttacgagcacgttc
aacagtcaacttgtcagcttcagacaattcgtccataccaagaatagcaatgatatcctgcaaagacttgtattcttgaagcatttgttggacatca
ccagcaagcttgtagtgtccctcaccaataacacggggatccatcatacgagacttggagtcaagaggatcaacggcagggtagataccaagct
cagaaataccacgggacaagacagtagtagcatccaagtgagcgaaggtagtagcaggagcaggatcggtcaagtcatcagctgggacataa
acagcttggacggaagtaatggaacctttcttggtagtggtaatacgctcttgcatagcacccatatcagtggacaatgtgggttggtaaccgaca
gcagaggggatacgaccaagcaaggcagatacctcagaaccggcttgagtgaaacggaaaatgttatcaatgaaaaacaagacatcttggcct

tcaacgtcacggaaatattcggcgacagtcaaaccagtcaaagcaacacgggcacgagcacctgggggttcattcatttgaccgaagacaaga
gcagccttggagtcgcctcccaatttgatgacaccggtttcctgcatttcgtggtacaagtcattaccctcacgggtacgttcaccaacaccgcaga
aaacagagtaaccaccatgagccttagcaatgttgttgatcaactcttgaataaaaacagtcttaccgacaccggcaccaccgaaaagaccaat
tttaccaccacgggcataaggggcaagcaaatcgacgactttaataccggtttccaaaacttcgggagtagtagattgatctgcgaaagaagga
gcatcagcatgaataggggagtatttgacagcatcgatggggccacgttcatcgacaggctcaccaatgacgttcatgatacgacctaaagtacc
aggaccgacgggaacactaatgggagcaccggtatcaataacatcggtaccacgtaccaaaccctcagtaccatccatagcaatagtacggac
ggtgttttcaccgacgtgttgggcaacttccaatacaacacgcttgtcggccatattcacctccaaagcggtcaaaatactaggtaaagattcaga
attttcaaaatgacagtcaacaacggcaccgataacttgtttaatggtacccttgttcttcctagcggcttccgtagaagcacatctaaggcttgca
aattttgcggcatttagccatgatttttttagagttttgtaatctgttcgtaacaaaggggggattttgttgctaaagaaaactggcgaagtccgctaag
cgcctgtttcttcagcatcttgtaagcggtaggtttcttccgatcgtgtgcgaagggtgagtttataatccatgtaacaagccatatggtcctatgtcc
gtaaccaacacttacataaaaaatacaatagatatattaaataaaatcttttatattttatatcaaattctttttgtatattttcgttttattcctttgtt
accgatgttactttggaaattgctgaaccaaggtcaagctcggatgtaaaaagtcataaaaacgcgtttccgctgtatttaaaaacaatgattta
tttaacaaatctttctttcaatctttggattataatatgaatgggtttttttaccattaagtcttggactaccgcggtgggttccacgaacaggcgcgg
tacagtgcaacggatatctaagctggcggtccttgaaaccaaagtcgatgtgaagaaccctcaaggtttcagtattatgctttcatttttatgaatt
acgaatttgtttatgcttacctatttctaaatttttcaataatatttgtgctactttttatctcttcttcaacttgtcaatagaatagagaaatcgagtttt
attatatttcacaaacgtatatttctgatataatcaatcgtcaacaagtttatctctcagagttcgagttcgtaacaaactttgatcatgaataatgg
aaattaaaaaattaaatataggtacttgaaagaatatgtcattcagaaaccctcacaaaactcctttcctcggcatcataattgtagagtttacat
acagtagaatagcgatctgattcaatacaataaagagtatgtaagggaagtcctacatcaataatttcctaaagagggttagcttttctaaaagtg
gacatgcagcaattatacacatacttcaggactataattgagaaaataggtcatcaggatattcgacaccactcgatgtgcgatgaccaaaacat
ttgcagagctcctttccaattctacaatgatcggttgcaagcgatgaacaacatccaaataactttctccgccaagcccagaaaatcggtaagaa
acgtctttttgctgaatgacccggtattcttcaggaaaattttcagcaaattctttcagtgttaagccttcgtgggaaccaaaatttaaatcattcag
cattcgcattttcttgacatcgtataccgcaggattaaagaactgtgatgtttccacagcacgctgtcgaacggaagaccaaacattaaagccttct
tcctcaaagctcagtttcttggagttcaatctttcggtcgtttcacgtaattgccataacacacgttgtcggtcgacaaattttgccaaatcttcggca
tattgcacgccaaggtcggttaatggagcatctcctcctactctgccagcaatggtatcattggattctccaggccgagtcagccatatttgacgttt
cctgatattcaaattgagcatgaagtagacggcttgaccagtcaaaaacccttcaatgttatgtgtaatgactttaattcctacgttgataactttaa
cgtattgcaaaaagggaaggccttcttcgcgttcatcaataggctcataatatttccgatataattcaacacgcttttgtaaatctactaatgcttctt
ctgcatcaaacccttcgtaatcagggcctctagcttttaaaaccatgttttgttgaaaaagctacaggaagggtcagtaaagtaaaggtaactata
aaaataaagttatgacttacggcttcatcagtacacacgctttccaaaaacattatacctaacttttcctcttggctaattcggtcatatattaatttt
cttctttgaaacgtagagttggtggcatctaatacaccgattacaccattttcgttcagtaaccaacgcaataggttttcaagagtttccatggctag
atattctcgaaaatttgctaattcctgattttcaaagtcgaagaatggagcagacatggaatatttgctcgctccttcttcacgacgtttatcaccaa
cactgaagaatcgacaattatacctaagccaattataatagcgaacaagcctaaatgttacaaatgtattagcacctgaggaataattttctaga
gacatacttggaaccgatatacgactttcctgttgcgggaattcccacaagcacaatcaccaacttttctctaatgccgtaggatccacaattcatt
tcattttcagctttccgggatacggtcagaccaggaatatcgattgttgactcttggcggatctggtgtttggcaatctttctggttttcactcctaatg
gagtagatccaccatcttcttctcgtatggaaggaaataactcactagtatttgctttttttcttgacatcatataacagaggaggcttcggacttaaa
tcttcgagctttatgtcttctttttttaggtatttgatttataccggaatggatctctctagtcgccgcaattgatggccttcgaggcttccgttccgggtt
ccttggctccggttgtacaatttcaggagaaaactgtaccttttttgattctttcgcagaattgctttcggaattggaactatctgacaaattgccatt
gccatgcttatttaaagaacattgcttcatatttgtttacttctttattgtttttccttctaactagcccaaatcaattgactttccggtttcaattcacgt
tcgacttctttcagaagttcaataagcgaccaaaagcgacgagcgccaaactgtttagtaattgttatatgttcttgacacagaattggtcctttctt
tcatatatagtcttaatgttagtgattggacaggtgatcactcatccactttctcttacctagtatgtatggacaggtaacattgactggtcctagtat
attgtattccgtagtatgatgtctgtttatttatatcagtgaatacaaacatacaaggaacaaaaacataaagcagaatggttacatgatgatattt
aaatttaaaatggattcagttacaaaaattaagctctttattatatggatgaatgtctatttacagatatctattcatcaatagttattttatttacttg
ctcgtgttccaaatttgaaccgttttgtattttgctgtctgtcgattagaatgaatttgatagtaaaattgaatagtagttgaggttgatttattttcat
agtatcattttattctgcttgatgaatgttctttggatggatagaaatggatttcttactatacgtacagcatagtcatacttatagaattgagcatac
aaaccatagaatcacaatcatactgcagggacaaacaaaaggatattttttttttctatctgtaatactttaacaacatgtatattaactcatgaac
gaaaatcatgtttgagcataaaagtcaaataaaatacgttacatatatcttacttcattcaacaaaacaatatcgcgcaggcctagcagaaaaag
ggtccacacaaaacacttaggaaagagcggctttctctagttcaccgtctatgctcgttttttctgaggaattttctcttgaaaagctactttctcgtt
gcatatttgtgcgatcttcataggaatctgtaaccttatcagtaacataatccaccacaccggcagctgctcgattggcaataactctagctggccc
tatcattcctgtcgcataaagaatatacaataaggaccctcctaacactagaagcataaaaaagaaggggtttcgaagaacggccattaattcgt

tccatccaagtaccaaaagcaatgcccaaaagtatggtggaatccttgtttgtgtactcacgagagatcgcttacaatccaaatatatcaaatccg
ctgttctcttaaggtttacagaagtacttgagactcgctttcgatttaaaattgtcaaaaattcagtaggagttgaaaatgaggaatcaatctctttc
acgtcaattccaagttcaggaattcttccatccgaatacttgatacaggaaagaacttgtatcaattccaacgtcttggtaacactatctctgtaatc
gcgatcaagcgtgcctgactttttgaagagctttggcatgccatcagaatcatatcgaaatgtttcttcaaaaaacatgcgcaatcgctgctgaag
aagaacatcagacatttcaagcgcaaccttcttccgaaatgcaatccatgagtctttttaaagtcatgaatgtagctttggtttaattccggttctg
ttgaatccataaagagaggaaatagacgagtaatctcatccgtagcctgcttttgatttgtcaaaagttcatcgagcaaaccatcccaaactttaa
gattaggcttttgaagtgcaatttcaagtgattccgcaacccgagtttccagggtagtacgcatttctgttattttagaagacaatttttgcttgcaa
acagaattacaagagctttctaactcttccgataatttaagcaagaattgctttgagtcccaatcaatattagcaatcattagagattgagcctctt
cttgcatacgagttttagatttttcgatttctttagcagctaattccttaaatggtctgttacacgggaaattttcggccgttttcataaatgaactgac
taactctttttgcaaagcattcaattgagcctggaaaaagacatacaaagatgtatccacgctccgtaataattcttgtttctttttctcataaatgg
cgttattatacctcttagcttgtcgatcgtaatctttcataacttgatcaaacatttcctgcatacgaattggcaagttttcacaaatgtttcctggtaa
gaattcactttgtaaaatgttacaagctttcgtaaaaggttccatagcttcggctatgatttcatcacagcgatattgtgccagaagctgctgttgtg
tgggcaagtcaagatctttattattttcaatggtttcccatatttctcgtgtatacagagaaaatccgtctgcaggaatacgcttgtgatagctagga
tcaaatacataatcgggagcatcgttgttcgcaaatcttgtgcgaagtgagtcaaccgctgtatcaaatgcttctgaacaaagtattttatgggga
agcccagtaaatccaaggtcaaagaagtcggtaatcttagaactttccaagccagggggtttcgctagcgaagaccagacgttcatcaaatctgt
ctttatcgtttcagacaaattttccatagacgtagtgcctatgtaatcacgaattacaaattgaagtaagcttctttctttctttgcgtggaacagttg
aagatggacttctaacacagttttcagtaaaaccatattagagccttgataaagccctacttgatgttcccacatgttcacgatgattacttcacta
gtagcgatcgaaaatagagctgactttcgttcaaaatcctttaaagatcattagtaaaacatattccatcaatgcttatacaatagccacttataaa
aaatctaacagctttttatcaaatcatacatacctgatcatcacctcgttcacggccatcagttccctctacatccataacaaggatgggagattctt
gagcctttgacagccagataccttacatgttaactaccaacatcctttccctacacataaaactgacaatacctttttgttgtttgctgtcttttaccag
catccataacatcgaacgttgtaccgaacaattggttcaatagcgtacctttcattgttagtatcaactttaaaaagaagcaaaaaaacacaaga
aaaccatacttttgcccgtagactgagaacccaagacagcaactacatgataattaaacccagcatctgccaagtttacctgtcgcatgaattga
ggtatatccttattaaatgatttattctcatcaacaatttgcgtacagatcctttgagggaccattttaagtgatggtgttggtggaagaaaagtaat
tccaaaattagctagctcactacaacaatgttctttgaggttttagttgtttcgaaaaaggaattaatgctgtttaaggtttcatttttttaatgaacca
aactcgaagaaaacaaaacaaaccaaaagctaaaccataaccttcggcgacttgtacttggacttcagcattctaacctaaagaaatataaggt
gctaacttaagttactggcttttgtttgcatttgtattacctttttcattaaaaaactacggcgttttatgagatccaaatcctttgacttgggttactcc
tcaagtggtttcttccaccgaaaacaggtaataggagaagagaatactttcactttctagaattaagctataagaatcgactgctatttacctttgc
tacgaatcggacttgttagaccttgcttctgaacataattcgagtgatccctgtgtacgtaccggattccttccaacaagaagcactgaaacgaaa
ctcgtcatttacttctcgcaattcatctgcaatggattctctacatgacacatgtgaatttctttcttgaacaatatattctttcaatccttatttccttat
tttgaccggctccttctttgagtatagcaatgctcaagctcaaagttagttacaaaagacttctatttaaaacatgaagcgttctgatttctttttgttt
attagaacatatcttttctttatttctttgtttttattttttcgttcatatatcgttatatatatatatcttccgctgacacggtactcatctttaacgtt
cacctatataaaaatggcaaaagcaaaagaggtgaaacccaaaagtgttaaaggagaagagggtaacaaaggaatacataaagcaaaaaag
aaaaccgtctcacacgcgtttagctgaaaaactgatccatgactatttacgcagaacaaatcgtcccttctcagctagtaagcacgcttacttttat
tcgttaaccattattaacaacttcgaagccgatattagtgcaaatttgaaaaacgctgttactaaacaggtagcgcaaaagattttagagcaattg
cgcgacaatggccaaatacacggaaaatcatatggtaaacatagtatatttactataaaactaattcaattcaaggaaaacaaagcgtttacgta
tcccttcaagattgccttggagaggcatcttctgaccaactaatggatttggatcgccaaatccaaattttaaaagaggatcttgaaggcgtaaag
gcggactacaaaatgaagtcaacaggtattaaattcgcaagctctggattctttcgctgtaataatctaaacttggcttgccttgaaaactaagtta
attggagttattaactgaacgatagaatttcaaattttaaacaattctcctagtcctgctgaaatacataaaaaaattcttactttggataaagatat
tgaagaaacgagtacaaagttggagtgtctgcaatctggttccattaaaaatgttacggaggaggagatgaaagatacattatcaaatcatgact
ttgttaaaaaaggttacttggaacgaagaaaaatgtttttcaatttatggaacctcattacagattgtcttgaaaaccccaaggaattatgggaaa
agttggggtttgaaaaagatgagattactgaggttaaataaatagctgaaacccttctttattttaatggatttacacctttaaacatgttccttaaa
ctttacgatttacgcatctagaaaagtcaattcatcgatttatatatcattatttagttcataataataatgaaaaccataagcctattttgaagaat
aatgttagaaattcaatttgttggaaacgtcaatagcttgagacaataggaaatataacaagatggtttaatgaatcatgtaggtcgctattggga
acttttcattgcgtatacttttctgcaattgcttgtggaagagttttcattcctaaattacattatcataaacggttgcaacgtcttactcgattgacca
aagtatttcaggctttggtctgagtcgtagactttttatatggcgatttcgagaaatacatagacttcatttcctttacactctcaaggatttttgcgct
ctcagaaagtttgtcggagagctccttacattgtataaaggttatgggttcaagcgggtcgaattcaacaaagtattgaccatcctcgctctggata
agtttggatttgtcatcatactttctaacaatttcccatgccttttcttctgatagagaaagcatgtcagcaacgtagcttattggtaaagaccgaaa
atttcttcttaatttatgtatctgatgctttggaaattgttccagagaaaaaagtataaaagggatagcattatcctaaagaaatgttaataagtga

ttcccaacttgtttttacttcttaaataaagcttaccgtctcaaatcttaaccagttctcttttaaaactgctcgaagaggatgaatgcctttttggta
ggcatctatgtagctttcgtagggagccataaaagtctttaagggtaaaagattaccacaagtagtggaatttataggatatctctgtcataaaag
ttaaaacacaaagcaaagtaaacgttgtttttgcatacctttccattcagtccaaggtgaagcaacaaaaagctaaataaagccctttcatgaaa
gacagtcaaagccgtaacgggaagatatacaatctgcagaatcgattaaaggttagcaagggaagctggaagattagctagcataccgaaaac
agccatccttgagcttcatataactcattattaccaagacaatgataggcagccaataaacagtactcgagtagtaaattggtcggtagatactat
gaaaagttagaagcctaggaatccacgaacttgcttcaaagacacgacatgctaatctatatgatcatcacaatctttatgatggacatactttgc
tgatggtgtcccaacgtaaagttgatgcagctacagcatactgcttttgacgcaaaagttctttgagaaaacagacatgaatcacagatatggta
ggctgttgagcggcatgagaaatagcttcaaggacgagattgtatttctaaatagagctattaataaaagatcagaaatcataagaatgctaacg
tccgttatacctctgagtccgtaaaataatattctttcactaaatgatccattgcagcagttactttcatacgattattagacatcttcattacaaaaa
gagagcaattcattccttattccatacccataattccatgtgaattacattcttcaagagaactactacgaacaaagtgtataaaatcaagaagca
atgtctcaggatcttctaaagggttaaaaggaatttgatgatccatattcaatatacaataaattgcatcgtttaattacagacaaaggttcagaat
ggcaatctctcgttttgttctaatgtgtactgtaatagttagcatacgaatatcgagacaaagtgaacatcattaagcgaggtttattcctaaaactt
ttgatatgaaagaactttacagaaaaaatctatgtaagttcttgtcctttcaattaagtgtttgttatatatatagagattttatttattattgaattat
acgccgatcaaaaaaaggctttctaatcgacgtagaagctctaacaattaaaataattaaacgcgacaagattcatctttattagtcctacattta
ataagataacaagatttcaaacccgacgcaataggtaggagccttcttgccatagccctccataaaatatagactcaaatcaaataccggcaaa
actgaaaattttagtccaccagtttttataatttcagacaaggtcttttgggtatactgataaaaaagtaacaggaaaaaggaaaaaaaaaaaca
aaacgaacatagggaacatctgcaaacacaatatgcaaataaaattaacgggaaagagaagcaaaacaattgtaagacattttttcgaagaa
tagaagcatgtcttattaaccagcacaaacatcaggatcattttcgactgagactatttaaatatacgagaaactttctgaaagagcctcttgaaa
agacctcttttctttttttgaggtgttgcttgtcctggctgtgttacaggcataggccgcttagccggagttgtagtttcatatggtaaggcttgaaag
ctgttattgttgttatttttatatgcagcttttcataataatgacttgcaggtctttggttattctgcaaatttatattggcctgagaagcccttggcgt
aatttgactggcggtatttgataaagtactatagcgatctgtcttgggaacacgaggctttgataaatattgatctcgcactcgccggtaactgcgt
tcgctgtccgaatcattcatataaggatcatccgcatttgtatgcacgcctgaagcctgcatattaccacctggaaaatccacatccgtgaaaaag
ggacgtcgacgatcctgcattgtcgcgttcgtgtatccaatattctgctcgaacgcaggagcattagcatatttggacggactaagtatcgcagaa
gtaggttgatcctcgaaagcgtattcattggatgcatatgatgtactgtttccagaaaacctggcagttagcgggttatgattagcttcttgcccaa
gaacatttgaaaacgaggattctggttctgttatttgggaaacacgcatgggttgtccattcttggataccagtttcgagtccattggtttcattcgg
gcttgtaaatttattgaattccttcttcctgccatttccgaagtctctgaaaaccttgatgtcctaccagactcaaaatcaggatccgtaaaccgatt
gttaaagtcattgtttgtaggaacatcattttcatctgcaaaacgagcaggatctgtagtatttatttcgctcgccctactgaatctgctagaaggtg
agcctgaccgacgaaattccagtcgttgagctgttcgattcgaataataccttcgtagcgatttttggggtactaaagggaaattcgtgtttccactt
gttcggttttcagactccaatttagcgccaactggaacacctacatcttgatgactccttctacttaagcgacgctcgtctaatgatgggagtgttgc
ttttagctcagttgaagcaacagttaagttatttcttgctgaacgtcctttattgaatgaagctagtgcctgctccgttgaatatcccgacttgggcgg
ctccatgtcgccttcaccatgatatgcatttaagccattttgaaaatcatcatagtgactccaccgacgacgatgtccattatccgactctactttgg
ctggttgacgacgaaccagtgattgagcatggcggagtttttgagatattccattcgacgaaccctccacaaaatggggcttattattatacgcctg
aactgttgcattcatcttttaggctccaacatccagtcaatgcccaccaattcgtatccttgacaattatctctcgacgagaatctgtttcttcacaca
aaaccgttcgctgtcgaattgtctctaatttctttgttaaaattacttaaaataatgctaattatttactttttttgaaattattgctccaaaagcggct
cacgctcagttcacgatgttttacatgctccgtaccgtatgctacaataaagttaaggtaacataagacaaaaacatcatcatacgcaaggtgac
atcacgttgaacaaaagcaaggtgtaataccaagcaaaagccgtttctctaccaaacgctctccgtctaccttatcgttaaatcattttgggtcata
taacatcggtcgtaatttccagggaggagggctaacagtgtatcattgcaaaacgagttggaatcagaaggataaatcactaagtgtttctttttt
ctgtttttttttttttaaaattcgaatcgaccatatatatatacatattctttcgtaactcttcattgcaactgtgtcaacagaggacaaaagcaataa
taactgacaaggatgtaatcaggaaatatggttttgttgttttctgataacttgcccgccttttcattttcagctgtcgaatgaagagcaagcatgat
taaagaggataatcagtagagtaaatcaaccatagtgatatcatattgattatttgcgaacttctgaagggcttatactgaaatttatatctcgatg
aagcgattactgtcaatagacatcaataaacactttggatgaaatgacaataaaaatcgaatcaaggattttatagcctgaaacattcaaatacc
atgttttctttattgttggctcaaagttcacctatttgatcgtgggcagcttatcatgacaatttgtttatgggcatagaaaaaaaagcatgttaaaa
aatggtgaaacgacaaacacaactgggaagaagggtttaatcaggtataagaagattcttaagtagttaggaaatatgaagtaaagctagctc
aactcgagaaaagatggactttttcataaaccataaattaaccaaacttaaagcgacttgtgaaaggaatatatatcaaaacagaaaacggttct
tcctttcattcgataaaaccaaaaagtaatgagttaggtattttttatcttggcagctgtatctccataatttttggatttttcaaaacaatcactcaa
tccaattaaatgaattaataataagatacagtatagttgatatgcaaaaaaaaaataataataataaaaaaaaatgaacaataagagcaga
aaaaagttttcaaagcatacgtataggtcaccaatttatcaaaataagtaggttatgctaacaacatggatgccctaataaattaacttttgaaag
caggaacaggttgaaggatcggttgtttatgaagatgaacgggagaaagaacttctgaagtcgtagaggtatgtgacgggatattatcactatcc

ttcgatgataaagggagagcctcagcagcagaattaaatgaatcgccttgattccttttccttgacttgtgatttcgagcatttctacggaaatgatt
tgagtgtttcccgtcgcgacggttccagtctgctttattttcataaaagcgattatgattagaataacccattcggccatataatccataaagatcaa
ggtaagacggtggtaagtagttagagttattcatataatttgcataggcatacggatccacaaatgtacaaggtattatagggagagatttgtcaa
acaaggcactattgtctacgttcggtaaagctgtagtaacgaaaggagggggcataaaagtaagagaatcataaacccaattattttgagaaag
gtctggaggagagtttgagaaacttgcatcggaacccagcgatgcactattagtttcagcaagtaattcatgaacatagctctccatatacaaggt
attttctaaagggggaaatacaaactgtgaacaagcatcgtttaagttataattctgacttaggtaacgaaaagctcggcgaaattcaagctgta
gacccttgacggtaatatcatctgccgtattagccaagtttcgagagtttcgaaagggctcctcaacacataaacagttattcagttggaattgcca
gcccttggcacgctttgaaagaattgtaccatgacgcacggataatacggagtgatcataatcaaaaagataaccaaaaaaacgaaaaaattc
aataaataaagtaccaagagactccctgtttctatcaccgaatcccgaaattagctcaggatactcacaaaaagaaacatctaagtcttcattga
gagttaagttcgtttgtagatgaggaatcatttgtaagctaggaagaattggagggttccttttctgtaaaaagtttataaccatgcagctgattgt
ataagaagtgagagtaccgttttccgcggcatcatttagacaccttttttagcccagtatttgatcatcactattagagggcggacacgaggatca
gaaagtacgtaagtccgcatcaacttggtatttaaagtagaaattgttttgttaatgttacaatcgcatgacagacttaattctcgatcccaaacttt
gacgattggaactttagcagtagaaatacaaacgaccttttccataccaatttcagcaaaagcagaagatatttggcaggtggtaggggccggtt
gagacggatcagtcattatacataaatcgatatctgcgtgcttatcagcaagtaaagactcggtggatccaaacagaactgtctttatattcttttc
aggccatttttctggagtatggcctccaacttgcgaacaaattgatatcgtttttgtacaccatcttcgtccaagacaatcgaatgaaagtactcgt
ttaactgagatgtcaagtgctctgtatccacatccgataacggttgtaaagataaaggatttaaaggaagtaaaggatctgttcctaaactatacg
gaatacgtcgacgatattgaatacaacgggtttcgtacttacaaccgccgctaacggaagacaagttcatttgatacaaactttctcagtcctcaa
ggatacgtaactgtctatcttcagcttatttcgtaaatacctactagaaaagctttagtcttacaccaaaaccgttcgaatcagttctcggctttcaa
atgcgttccaacaagacaaggcgagctaaaacaacagttcaatccttctagcaggtaacctacaacgagactcttcttcttgatagcagagcgaa
tttctatgcttttatttacgtaaaagttgtcaatctccaaaatttccaccagtgttggttatgagcaagctgacttttgtcttcattcaatggataggca
atatggaggaccataacaaagaaactctatgtagtgatattaggcacacagtgccaaaagtatgtgacattggcccttgatgtcaaggatgatag
taacgaggtacctattcgtatcgttacattatgtaattactataatcatcagtctctacttggcagactttcacaatgaatgaagtgatcaccgaatc
attattacctctaatgtattgaagcatactttagtattctctagctctaccttcagagtttaaagtgataaaaattggacaagccagaactcgaatct
gggacctcttccatgctaaggaagcgtgataaccatctacaccacatgcccttctattaaaaatactgaccatagtcctatatatacgtttatagcc
tttgagttcaaacaattaactgagtaccctagccactactcacaattatgattgtagtaaaaatcaaatccagcctcaaaacaatacattcaacag
caacttccatctaactttttctatattagaatactcccattaattatagatcaagcatttttaacacaacatctataattaaaagaagtacacgtcag
aaaacgccaactaccctacatatcatgaccaaaaaggaacatgacatgcaagagatcatcgccttgaacagatgctataatgcagctttatcctt
taaggatccaactactaagcatccattcagtaagaaagtattatctcactcttcgggcaatggcgctaccaatcatcatccgtgttgagttctgtgg
ctaggttaggaaacccaagccggacgtatactatagaaatgatcctatggagtatcgtcgatggttttcaaatgagcgaaagcagtggtatttata
ctattctgttcacccaaaagcagtcacacaccgacaaaaagcagtcacgaaaacagtcatcatcagcgaatcaaatctgatacacgtcaccgtat
agaaaatgtttgggaagtgcaacaagatgcgcagtactacagacaaaatagtttacgatggttactagagctcggtatttaagagaaaaaagtt
tgcatttttatgctggaaaaagcttgtgaattttgtttttctttaataaaaagttaataaaataaaagaatcggtttttctttatgtgtaggtgcttttc
aaaagttgttacttttgtcactttaggtgctttgaccttccctgtctactacttttagtactctacaccaactaaccaacgaggaaaattgggaatcat
gtcagaaacgacatctgtgaacatgtatgttgaaaatctagagggacattatgctaatagaatagcccagagttggaatctccccctgtcgagag
ggttgagttggaatcaatgcttgtgatgaatataataaagcattgtcgtgattccatcacaaatgtaggcactataggtaaggtttttttggattttttt
agttgtctcagtgaaagcatgaaccttgttgtgaatattgaagactacggaaaaaaatattaccttttttttcctgtagttcaactttggcaattgattt
gttattatagcgagtccacgctaatactttctaggtcaacttgtgggcattgacatcgacggtgttttgcaagttagtagtagttttgaatccccttct
gtccttgaaaacgaggaatcggctgttaataagtctgtttcaggaagagcacgtcaagctcataccgaagctactttgaatcgtatgcaatacttg
ggtgctgttactggtcacgttggttggtaccaaggaacctatgttggagctttccttaacagttctttcttcgttgagacccaatatgcttatcaaaag
gagagccctaatagcattgctctcgtttatgatttatcacagtcttgcaatggatctttgattctccgtgcattccaactcactcctgagtttatggctg
catatgaagaaaagacatggactgttacttctctaaacaagcaccatgtttcacccgctaacattgtgcgtgaattacctgttgcaatccacaactc
gcacttggcaacttgtcttcttcactctcttagtgagccttctaaaccctcgtctttgctttcatccgaagctgctcttgactcaatggagcgcgaattg
cctttgactgaaacttttagcaatttcgatctcaaccttggagttggtcacaaaaagaatctcgaacatctcttagagtctgtcgatgaataccatta
tgagcaaggtaatgttggtttccaccaacgtcaacttgccagagagcaggccaagattcagcaatggttaaccaagagaaaggctgaaaatgcc
aatcgtgctgcagaaaaccgtcctctcttgcctttagacgaatggaagcgcatctttaagcttcctgctgaacctcgtcttctcgactctttgttaata
agtgctcaaattcgtatgtcgataaaacaataacgtttgtgcttttactaatcattatttaggtcaacactgctctcaagttgatgagcaaagcttgt
cgtttgtatctaaattatctggtgtgcgtaacgcttatacttcttagatgaatttaataaaagaacggtggctttgattttgtgtagacctgctgaatg
tattttttcgtaatctgttagtgcttttgtcattaactggtaggtggttgaaaagcatttattttttcttttgctttcaggctagaaagttttgtctaagca

agggttagttgagtagtacagtaaaataacaaatgccaattaagattttcttatccacaattgccagacctttactgaatcggatagttcacattta
ttctaaagacctgagtataatcacatgaatacgaatccgttcacaaaagttctaactacaaaaattatcgtataacttgatttgatttacttttccat
gctataatagcttagtcaatatagtaatgtttgtttttattttgtaaaaacgttaacataaacaagttaatgtgttcgtactacaactactgaaacttg
tgtttacttctttgtttactttaaggggttacgcatttgtctaccataccacacacccatccatagcaagaatcttaacattctcaaccacgtttgaa
tgatggaaaatccacaatctgttcagttaaataattatgttggtaagcaattcgagattcaatgaacatatagtactaaactattaggcttcgattc
cattacttcccaaatcaaccgtaaattagtccgccgtggatttcaatttaacgttatggtagtgggtatgctttttcctatagcattctattataatgta
taagctgctaattgaatttcaaaggtccttctggaagtggaaagagtacacttgttaataccttattctctgctcatttaatgaacagcaaaggccg
agttgatattcagactccatatcgccagactactgagattaatgttaccagtcatggtaggtattcttggatctcgagtttcatgtactttcttgctat
aactacgcgttgaagttcgagttctccccttctttacaatccaatttataattactaactaataacttaattagcggtacatgaaaatcgtgttcaatt
gcagttaaatctcattgatacacctggttacggcgaccaaataaacaatgacaaatggtaagtgagacgaggactagtatgaatatctaacaaa
atttctaaagttgggaacctattgtcaagtacattcgtgatcaacattcttcctatttgcgacgtgaattaaactctcatcgtgaaaagcgtcttcaa
gacaccagagtgcattgctgtctattctttattcgtcccactggtcattctttacgccctattgatattgctgtcttgaaacgccttactgagctcgtaa
acgtcgttcctgtaatcgccaagtcagactctttaactctcgaagagcgcggtgctttcaagcaacagattagagaagagattgcaaagcatgat
attaaattgtatccttatgatagcgatgatattgatgatgaagagctaagccttaatgcagccgttcgtaatcttattccgtttgctgttgtcggctca
gaaaagaatattattgtcgatggaaagcctattcgtggacgacagaacagatggggtgtcgttaatgtcgatgatgaaaaccactgtgaatttgtt
ttccttcgtaactttttaatgcgcactcatttgcatgatcttatagaaactacttccttatatcactatgaaagattccgttccaaacaactcagcagc
ttaaaggaacaatcttccatggcctctagaatggcaagtcctactcctactttccctactgaccatcttcacccatctgctactatcgcaaattagtc
tttttatttcaacttttcgacaagcattaatattcaataatatattagtttattctatctcaaatttattctaaggtaatgtggacaaaatctttagtaa
ccgaattttcttgttcatccaacctaattgtcaaaaaaaacatctaaatgtacagatttattacttagtaaatacctattgtacaatctcactgttgtt
aacattcgaatcaaaagatttgctaatgctaagtgtcggcctatcaggttcggtacattccataaagcaattaaacagaagacggctgtaatagc
cctggacttggaacagcacttgggcttctttgcttatgcgaggaaagtgattttgcaacgacatagagcactggtaaacagaataagtgatagcg
gagttagagcaatcctcttgagctagaatttgaagcgcttcaagtaaatcattactgtttcttgtcaaagcgtgaccaatacaatctaaagccttga
atgtcactgttgaagaattcgaatatagaggaacgtggaattcgtccctccaagaaacgatacgttcaagatgaacatttccacgagccaaaggt
tgtgataatatctttattccttctctaaaaagagtgtctgaaattgccgcattgtttcgaacgttaaatacttcagacatgcatagaccgtcaaaccc
ccatacattttgacgcggtaacaaagcaagaactgactcacgaattaccattgctagtaacgtatttacaaaattagatcgaacagcagaagacc
aactcaacttcctgaaagcattattaacaaaattaaaatcgcagtttgggaggcttaattggctcacagctagcaaggcatacgctaacaatgatt
caaaggaatttgggtttggtataggagtacgtaaagccttaacaatacaacaataagcgttacagttgtccaaacgcttagctgcagtaaatgca
agtaatgagtaattgatacacggtatagattgagctaactggggcacagtatttaaccaaaaatgcaaagcaatcgcatcttcaaattcaaatac
aacaatagatggtacaacataatgaaaataaaaaggagaaacttccaaaattggagagggcatcgtgactaaattagaagcgatggaaccag
cagagcgtaaagatgatgaaggataagtagagttcatgatattagacgatgtaggatttatagttgtgtagttatatggattcgatgaagcagaa
gatacgggcgcaggagtttgcatttggtggttcgattgaggataaccggatggaattggatttgagtacccagtcattggaaatgattgaggatgc
tgctttggtggttgattaaagtgcgtaaggttcggagttattgaagaatgatttacttcattagggagaaaatcttgatgggaggtattgaaggtag
gatccgcattcgcagaggaatggattccagatgctataggtgcagcattcatattggtgatagaagaagatgcttgctgctgtggtagcaaagcg
gcgtttatatttacattaggaggagataagctttcagttgcttgaggagtggtcgaaggatttggataaccttcacatttgcgattcgatttagaac
agttaacacagaatggctttgtctcgtcgcatttaatcctcctccgacggcaagtaaggcagccagtcttggttcttcgcttaacaggtttgagcga
ctgctcttgttgctgcttagctagtataggaatcgcagaagaattagatagaacaggtgttgttcgaaatcgttttacagaagcgtctgggtatgca
acgtaaggtttcataataaaaacgtataaactgacaacgatgaaacccaagtattatcaatagacttcagagggaaaaaaaaaaaatcaacaaa
aaagaaacaaaatcccctgagaaaaaagattgaaacacagtggatagcaaaatataaagcttactcaacatgaatattgtaaacgagctaaaa
acttcgaaattttcagctaaaacataaagaaagaatgattaaaccaaagaaaacacaactcaaaagaaagaaaaagaaaaactcaaagttca
cgaaagaggaaccttttaactaacacaaaaaaataataccccttcagctaacagcaaaaaagaaggaccacaatcaaaagaactagctctcaa
aacaaaaaatgagatacagtcaataatagaaactaaatgaggaatgagatgatggtatattaggctgtttcaggcttcaattgcaaaagcttcca
attcagctgtaactatgtccttacaatataattaattagtacaatcaggcattagtaaaattataacttatagatatgaccgttttccttctgtctattt
cttttcaaataattatttcaaaaaaaaaagaaaagataaacaatgttcgttgtggtatggctgcacttggtagacgaaagaaaaaaaaaaaaaa
aaaaaaaaaacaggatgtaacacttttcaagtaccatggctattttcaccataaaactgataattatcaacaattaaaaaataagtctaagcttta
tggaaagaattatcatgtagtccgcttttttttgccttccaaataggaagcataaaagtaaataatacaggctttttaatatgtttacagaaacttata
cagaagtgtgtaattatagaataaattcatgtattgtcaatttcttgaaacttcagtttgtaccatacttattccataattggatgaattcacaggaa
ccaaatcagctcatcctacttaagtagttttatatttaaatttgctgattggagaagctaaaaccctcagtacaggatacacgcatttacaaaatat
ataaacatgaccgagaaaaaccatcaacgtctcgtctacacaggatgaagtaataacatacaactgttctatttttcgcactttcaattacttccca

tcaacacaatacaatgtgtctcaatgtcgtactactccttaccaatctaattttcaactataccatggatacagtgttcagttaaagaccattaagta
acatattagtattaatcataagaatttatcaacgtcattaatgcattaggcaaataaatagtcagctcattaagaggattggtaaccgaaaggaca
aggtcgatcattgaacgtttgtaagtatattttgtacatcaccggatagtcatattggaaggtccagctttagcaaatattccagtcctcttctttgtt
tcactgtcatttactcgccaaaacttcaaattttcatcactagctgctgtagacaaaattcgtccatcgggtgagagagctgagtataatactctgct
atcatgtgcgggtatatccacttgcttagtgacacctgccgtgccataagaccagaccgacaaattattatcaggaaaaccgtgggtggatataa
cttctcgaagttgagggctccatgttagcgacgttacttgactaccagcatccactgtgttgactctcgatccggtagcggcattccagaaatgaat
cttcttatccatcgtacctccaccggttgctaatagattgctttgccaaggacaccatgctacagccttaacagccgcagtgtgattggtttttgtaa
acttaggaatagacgatctagcatcccagatttgaaccaaattatcattaccaccagaagctaactgaagaccatccgaacgccatgccagtcca
cacacttcactctcatgtcccaataattcgcccactttgtgctgagcaatacggacatcatgatggtgaatagcacctgagcgactgccactggac
agaatgtgacgatcccaagccaaaacacctaccctcgcctgatgaccggccatcgttcgaagctttttttgagactcaacgtcatagatctcaacc
aatccattaccaagtcctacagacaagaatgtaccgtcttgagaccatttcaacccagctacataagtagattcatccgtttctgctaaaccacga
acatcaccgctgtcggcattccaaacgtaaacgtttctctcgagtgcgatagctacatcattcaaattagaccagtctaataagttcaagtaataat
catcaacgatgccaggcgcatccaagactcgctctggcgtggtagcaaacttgcgctttgtctgtgaaacaacgggtttttgaggtcggttatattg
agcacgcaaatctacaggcttctttgattcaggtgcatctggtttaaaagcaaggatgcgtgtatttaaatccaaaccacaagcttcggctactgat
tcattgtactcagccgacaattcatgtttttgattactaacaaatgcgttagctgaattttgtcttgaagggatgaatctgtcactgtgattcaagca
acggtgtttagcacgaggagaattcactgcagtcggcaatgaccagtcagatgcaacgacatcaagttttggtgagcctcggacaaaattgaag
gaagtctttgttgggctttttttcgctgagcggctaactttacccagaagagcctgctggtgcagaggcgtaattggactatttggatataccagag
gtctctttttcgtaggggtagaaaatttagaaacataagacaaattgctgctaacgtccatattttataggaatactaattttaagtataacgacaa
aaatgcttaagaggataaagagagactaaagaaaaagaacgacgaagaaatatatgttgggtgtaatccaagaatctattgacaaggtctcag
tattttgtcaatgtcttcttttactttttacttttcaagtgaccagcacctgtgtgtaaagaaaggaatataaaaagggaaaatcaaaacaaagaag
gagaccagctcagagaacaaaaaatggcaattaattggaaagttccagctttcctttaccacattcaatataggaccagcctaggagaaataaa
atgtaacagagaatttaattctgcaaaatcgggatttttgagtctgttagtggtttgtttacaccaagttccaaccacctcgacttgtttataatccaa
ccgcgaaacccttacccttcgttaccctcgcgaccattgtaaataaacaagcatgtaaacagtgagcggtgacagggggatacaatttgcaaag
attgaaagaaacgtcagttgctatgattgcagttgagtcttcctttgtttttcttactccattcgtaacaatgcaattttgggcattttaaagaacaga
tcgatgaaatctttagtggattctgatcgcttaaaaaacctatagtaaacaatggacacatttcaaaagaaacgacatatagcattccttcaatcc
gctacccatagataaaaaagttcatttgtttaatttcaatttcgattttactgtttcatatcgattcttttctcttctttcccagtgaaacagtctgtagtt
ttgcactacattgtaacattatatcatactgaggttttctctcttatatgaatcaagaaattctggacattattatacttaccaaaattctttcttttga
aagagaagacatttttttccttttacataattctgcttaggcttctgatggctggtagttggactcaccaccaaatgtcaactttttccatttcacatact
cgtctaatatggctggtatagcgctgaatatcgtatgaaaaaaaacccctgagttataatccaatttactataaagccgaggaaatctgcagttat
gttcgccagtcagttcacatttgtatattacttaggctttttggtggttctccaaaggtcggttgtaatattggttaactgctaattgaatctttttaata
cctcagtattacaatcggtattttcctcataagttgaaccattgattataataaccttgcggtggttattcttagatatattcttaattggtctataattt
ctccgttgttcttttgtatatataattactcttggaccgtattataatagacaatgggattctcatctcgatccctcgcttatttcttagtgggctgcctt
caacttgttacggcttctccattgtatgatggtccagaagttatggaagcatcatctcaaaaactcgaaaaacgttcaactgatgtcttcaatcaag
tcattgatacatctgatccatcaaccctatttggctccgcaagccatcctttgaccccagccgggctttccgaaagtggaaagcctaccgaaacaa
acaagttctatgataaccttttgatcggctcaagaacctcttttctctatgctgacccataccgctattggtggcagtcgtccattactcattctggta
tatgcattgctcatactgatgaaaatcaacgtgtcttcgactggggtagcaccgttcccaatttctattataatccaattggtctttgttctttagcttt
tggtggctctggattgactaacgccattgctccccaggtcgacgaaatcgatcagttgtccgctcgcgtaaatttcaactggggttcttcttcaatga
aaatctctttggtcgaaggtttggcctttaccactgctgaatacagcaatgccgttcccgaaatcttcacatctacattctttatgaatgctgtggtat
cggcttctggatcctccgctactcaaaagtacaaagtcacactgtccgatggtcatatctggctcatttatgtgtttggtgatcaccttactttgaac
gccaacggaaaccaagttcttgttggctcaaactccttcaatggctacattcaaatagcaaagattccctttggcgatactaaagctgagtctattt
atgaccaacatgcaggcagttatgtaactggtatttctgtttccggtcatgttgatggcgacacaggttactataccttcaacctgaagacttccggt
gatagttctgttacgcctttgcatttcttgttgccccaccaaattgaatcactgtcctctggtgcatctactaccgatatttctttggtttcacttgttag
tggctacgcgacagctgttgccggcagttcaattacttttgctgatactgttcctaaagatatccatttcttgccctggtcacctgagggtaagaagg
ttggttattcaaccgatgcactcaatatgatcaccactgttgctgatcaagaggtgaacggtgatatggctgctgaaagtaatttggactctatgta
ttatagcggtaaggttcttgccaagtatgctatgctttgccttacagttaacgatatcgttaataatactcagacgagcaatacctgtgtgcaaaag
ttagaaagtgccatggctcgcttcgtcgataacaagcaaattaatcctttggtttatgattctacctggaagggcgtagtcagcaaagcaggacttt
ctggctatgcaatggctgattttggaaatacttactacaatgatcaccattttcactatggttactttgttttgacagctgccgttattgctcatattga
ccctaattggttaactgttggtaacaacaagagattcatcaattcgcttattcgtgatgtttctaatcccactagcgatgatccttatttccctgttca

aagaatgtttgatttttaccatggtcattcctgggcaggaggtctctttgaggctaatgatggcaaggatgaagaatccacttcagaagactataa
tttcttctacggtatgaagttgtggggtgaggttaccggtgatcatgctatggtggatcgtgcaaatattattctatctgttttaaagcgtgcactaaa
caagtatattctttattcggatggaaatgttcaacctaaatctatgcaacctaactatgttgctggtattaccttcatgaacaagattactcacgtta
cctacttcggaatgaatcctgaatatgtcgaaggtattcagatgcttccaattacccccatttccgcctacattcgagagcaatctttcattagaaac
gaatggaacaatatgcttatcaacgctcttgcaacagctagtccccttggagaaccttgttgtatgctgattatgctattgcagaaccatggacca
gctacaactattttgctacacccgccttccaagctggctggttagatattggtgcttctcgtgcttggtatttagcattttccgctggtttggacggtag
aaatgctgtttactaccctactgacgtttacgcttctgattccgattcttcttcctcttcatctggctctagctcttcttcagcttctgttactaccacaac
aagccaggttcatcgaactacttccgtcacgagctcggttagcctcccaaccgacggtatcgataccagctccactgctgttactataatttccgta
tccagtttcgcacctgtgcccacttctacttcaacttcaagcttgaagatttgtaacaaggctgtgtacgatccatctctatatgtctgcgatgatgat
gtactgtgccctatagtgaatggtgtttcatacgcgaattgcaacggtgcttgctataacccagttcattacgggtgctacaacaacgctttaggctt
gcctacaggcacttcttcttacactatatcgtcttctatccgtacatctgcacccaccgcaggcacttcctccggtaccccttcaacagatactaattt
atctgaatcacctagacccacttctggaatgcatacatgcggtggtaccatgtatgatgctagtgctattgtttgctataaaaatgatgttctttgcc
ctgttattgatggtgatgtctatcaagaatgcaacggttcttgctacaattcctccaagtttacctgcaaaaatggttcagtatttcacaaaaatggt
tcagaatcttccactgctagtgccactactgccacgagtgctactcctacttcttcgtctattacagcaacttctccttctgccacagcatcttctactc
cgattatcgctcaatgtggtttggcttggtataattctatgagctatgtatgctatggtaacttactctgtcccattgtcggtaataaaccaatgcaag
cctgcggtagcgcgtgctatgattctgatgcttataattgccttggtggtagacttgtttccaactaaattgagtgggcgctatttgaaagcagaga
caagcaatgataaaatgcttccgcttacatttgttatttttatttttgttaatgaactgtttctttacataagaacacgtctggtacgtgttacaatatg
ggattaacataatgatgcttctgcatttgcttgctgcctttttttgtgaatgaaaagtaaggggctaatcagttaatcgtatattttgtgcatatttgttt
gggtctttttgataaagacgtttgatggactattgttcaatttttttgagagaattgaatctcttgtttgggtgtatatagatatagacatacatataga
ctgtatagacacatacttataaatattatttttaatatcagtgtcacggtaataatctcacacgttatgagaatgtttatcgagcggatatagtaagt
acaattacaggattgtatgtttcaatatggacaacttggaactacgtagaagaatagtaataacgtccaaggatctggttaactaaatgaaatat
atgaaaggtctctgttcccatagctgtccaatttgtttttgtcgtattttctaagcttcgtttactatttgtaatattttatccttagccttatggcatttgt
taatattcagaaatacgctttgataatttttttgtaatacttcaaaatatttataaagcatctatcgtaacatttggtatgtaattattcggtttcagcta
ggtatcgttgaagcattaagaaaagtagcaacttttgtgaatttttccatatatttcttgcagaaaatatcaatgaatttgaaaaatgagaataaga
ataatagtaatgataaaagaaaagtaatttcttaatgacttttgaaaaggattaacgactggttctcaaacgcgtcaattcctcgatattacttcat
atgttcatctgatatgatttctacatctagctcagtgatatggaaaaggtgatatcgttttcgaattcaaactaaaaataaatcgttcttcatacttta
acatgaaatatagatcaaatttactgggaatgtttcatgatagattttatctgatcatgtcagtccacttccttcgttgaagtcaatggcaccaggat
ctatcacatcaattccataaagaatacttcaaatgaacagaaaaccaatcgctcatccttcatcctaaaaggtctctcaatcatttcttgggaatgt
agaagcaaagcgagcatacaagtcggaaactgagcatcagccagctctattcaccataaaatccggtattatgtaagtcccaaatttagatcac
gagttaccttttcataaataaatctctaaactaggtaaaacaactgtataaatacaagcatgcgttactaactaccttgttactaaacgtcttcttttt
acaaacgagtcttgtgaaaaatattttcatcattcaaattcaatcatacattaaacctcaacacctaacgaatcatcaccataaaggaccttttggc
gtaaaagatccatcatttaacatcgtgaaacagatatccaaaaataatttggaaatcaagtaaatggaataatgggataaacaaccaattttaa
acagcaacaccaatgacaccgcaagcattgcgaccaccggcgttaccggtcttcaaagactcctcgcttgtacccttgccgaggtcatcttctcca
gcatgaatgacaatggtacggccaacaatgttgtgaggaccaaacaaagtgatgacagcgtcttcaaatttggtcttgacatcaccattggcatc
agtggtgacattacccaagtcaccgacgtgacggacagaggcaacacggtcaccatgagtagtgccagtgggattgaagtgagggccagcaga
ggtacagccattggtgttgtcaccgaattggtggatatggaatccacgcttgacattagcatcgttgcccttaaggtcgacagaaacagtgacagg
ggacttttcgttttcttgttcgaaagtcacaacgccgtttactttggtatcaccacggagaactgctacagctttcattttggaataattcaaggggga
atattttacacaggatgtttcacttgagagtggaagttgttgaattccaagctgacgtggtcacccttatatataatctcaggtaataccactttata
catccaaaattgacctacatgcccttttttttataaaattagagattgttgtttttttaaataccatgcacttgaaaattcaaatgtatccacacttgcttc
ccttagcttggtatgacaaatcactccaaaaacatcagtttggtttcgagaaaatcagcgtcagtgcctgccaatcatccaaagcatcaccaagtc
agctaaccctttcgtatcgtccttgaaggaatttgctttccacatcggtaatctagtttacaaaatggaacgtagcggtgcgtccttccatggaacat
tcatatttgcaaagtttccgtattcatatttacggatgcatagcaatttttcttcgaatacggcaaatagactcactcatccaaccgagcttacctcg
gaaagcagcaatttgagatcaaatgggggtcaatcgccgaacgcttaaagaaacgagaggaagtgtagtctcataaaaaacagcttcacacca
tgttccacgcgggggggttttgttgaaggagcatattcgctccatcttcctttgaaatttatttgcaaggaaaacaataagtaagtgactataaattta
agacaaggaagacatcgaacccttaatttttttatattcattaaaaataataaaaacccagatgcttgctgcaagtttacattgtgcttgtctgacga
aaactctggagaccaatcttcatacttaacgacccactatagcgcaggcaaaatgggaaaggatagtaggtgaatgcttgatttatagttaacaa
aaaagattgacacattcgcaattcgaattccgtgtttttatttgatgaataaatggtaaaaaggctcacgggcacgtaattgagagaattaaaaa
gaccgatgaattgctgatgcatttttgctattcccgatacaacaaaagcctcacgagcgacttttgcgagttctttttgaaaggtgacttaacttgtt

actttgtagatcattcacgtagaataaaattgcttattaaacatttccttctctcctatatatatatatatatataataaaaaagaaatcggagattc
ataaaaagagaaaaaaagggactaaagtgtaggctttgataaatactaggccgattataattacaatattatatgcgtatagggaactccccac
aagtaacagtaacgaaatagatcggcgtagatctggtagatttgcggaagtgaaaatctgtaaacaatgggtaaaacaaaatgtaagattacg
gaagctgaaatcaatgaataatgaaacccctgtttttaacgttcgtaatcaagtctttttatgtctcgaatttactttctaagtccttatatttattgac
atcggtactctttgcaggtggctttgctggaaatcgacgaattgtattagagaaggaaaacaagagaaagattcattggcaactagagttaatgt
ctagaaagtatcagtaaacgacgtagacgtttcaactacatactgaatccttgttgtcttacgtttcttttcatccaagagagcgctaatggtctaca
ttacttatgctaaaaaaagtttttttattcttttattcttttcgtttattcatgatttttttcatttctttttctgtgcgttcagaatagtagaaacgatacc
ctacaggtaacagagtataccctgttactttacctccactgcatcttcagagggtccaccttcagcaaaattttagtctgttatttttggacataattt
ctttattttttctcatttcattatagacggtttcatccctttttgttaagagatgtctttggaagcaaacgtaaagacggctagagactccttcattactc
ttcaaacactctcagtcaaggaacgagatgaagcactagacataattgtagaagagctaatatcgaaaaaggacactatactaaaagctaatgc
tgaagacatggagtctgcaaagaaacagaatgaagctggaaaattgagctcatctatgttaaagcgtcttgacttatctacttctgctaaatatga
atcaatggtccaaggtgtctcagacgttaaaaacctcgacgatcctctaggccgtgttacttatggctgtcgccttgatgacaacttggatctgtatc
gtgtttcttgccccgttggtgtgcttttggtgatttttgaagctcgcccagaagtaattatcaacatcaccgctctcgccatcaagtctggcaatgcag
tcgttttgaaaggaggtacagagtctacaaagtcttttgctgctctgtcggaagttgtacgctcggctctctctaggagcaaagttcctcaaggtgc
agtacaactagttcaaagccgtgaagaagttgctcaacttcttcaatttgatcgctatatcgatcttgtcattcctcgtggcagcgataaattagttc
gctacatcaaagaaaacaccaaaatccctgtccttggtcatgcagatggtttatgctctatgtacgttcatgatgatgccgacaaagacctcgcgg
ttaaagttgttttcgacgctaaaactgattatcctgctggctgtaatgctgttgaaactctcttggttcacgaaaatgcgctcaccacccaattccctt
taattgccgaaaaattggcccaagcctccgtggttcttaaatgtgatgaccgctcctttgaggttttagaaagggttccttatgccgcttcattgctc
cagcacgctacggaaaaggattatgatactgaatttttggattatactctagctgttaaaacagtttcctcattagaagaagcaatggaacatatt
aatgcccatggttcaaaacacactgactgcatcttgacctcgaatgaatctgttgcaaataaatttatgtctggtattgatgcttcaggtgtttattg
gaatgcttctacccgctttgcggatggcttccgttatggctttggtactgaggtaggtatcagtacaaacaaaattcacgcccgtggtcctatggga
ttagaaggcttaactatatataagtatcaacttcgtggaaaaggacatgttgcttccgcctacggtgtcggtcctaataagatcccctttaagcatg
agccccttgatgtccatcatatttctgaggttaccaaattggaagattgagcttcaatatgtctctagtttctgttttatgaagttcacttctgttttgg
aaaagtcccctcttcctttcgttttaataatttgttcgttccatataataaattcaacttctgttgtcttgctgggtaatttcgtataaatgaaaactatg
tcaaacgtcccgctgtaagtatagtatataacataaacaaaaaaaaagaaaaaggaaacatctcgacttatttataaaatatcttcaataaatcct
ctgttggaaataagtaacaatgatacctgtaatctgtccttcaaggtgaccgaaaagatttacttttcaactacttataccattgagaatccatatc
cctttattcgcattatttctatagccatagatattctattaattttatcgtatatgtacccctttttatctggactgaaggtaaattgattctccaatcct
gaagatacttgcatgatcaacacaatggaaaacaatggatatgctcaatttgagagaaacccttcccttttgccgggaacgacattagaaaaa
cgcaaaaaactaaaaaatatatagagcactattacaaggtagcagtggaacatgctgtagaaagaaatcaacggtaagtggaatgctacaatt
ggagaattcgaatctacgtttgctccaaaatataaaaaaaatcaatctatttcaacattgaattacgtaaagtgtccgcgatttagctatatctcgt
tagaggccaattctgtcttgatccataagaatcgcagcttctgcagataattcaaataagtgatggaattatcatattcatatacgattagtgaaaa
ctcttatattaactatgttgtagaagaattgagcttgaacgaaagttaacgacggaacgtggttctgatgaaagaaaaaacagacagctaagag
cgttgggagagagggaatcacagtttttgcgttttcgaaggacgaggctctcgctggaagactttgcgactattaaagtcattggaaaaggtgctt
ttggtgaggtatgtttttaattgtccttctgtattactaacaaaaataggtgcgattggtacaaaagcgggatactggaaaaatttatgctatgaag
tctttgttgaaaactgaaatgtttaaaagggatcaattatctcatgtcaaggcggaacgggatttgcttgtagaatcagattctccctgggtggtttc
cctttatttttcgtttcaagactctctctacctttacctcattatggaatttttacccggaggggacttaatgactatgctgattaattatgataccttttt
cagaagatgtaactcgcttttacatggctgaatgtgttttagctattgcagatgttcatcgaatgggatacattcatcgtgatattaaaccagacaa
tattctcattgatcgtgacggccatatcaaattatctgattttggtttgtccactggattctataaacaggatcaaagtgcttcttatatgaaacctcc
caaaagcggcaataccttaaagagaggacaaatggtagactctatttggctaaccatgtcttcaaaagataaaatggctacttggaaaatgaat
cgccgtgttatggcgtatagtactgtcggtactcccgattacattgcaccagagatctttttacagcacggttatggtcaggattgtgactggtgga
gtttgggtaccattatgtttgaatgcttgataggatggcctccgttttgcagtgaaaactcacatgaaacctatcgtaaaatcattaattggagaga
aaccttaaattttcccaacgatatccaccttagtgctgaagccctagacttaatgaattgtcttatgactgattcagaacatagactgggtcgtggt
ggtgccatcgaaattatgcagcatccctatttcagtggaattgattgggatcaccttcgtgaaagccccgctccatttattccttctttaagatcgat
cactgacactcattatttcccggtggatgacttgtgtcgagttcccgatcagcctgttgcaaccccagaagcaaatgtggatccaaggactctcga
acaaacaaatatggccttcctgggatatacttataaaaaatttaattacctcactatgaagggagctctttaaaaagaaaatacccaagatttgtt
attctgttatctcattgcatataaggattggaaggacaagactgaaagaaagacttattcttacttccacatttgatgaattaatcgacgtttttgct
attataataaacattgtattaaggtatgtactaagaaatagaaacttttgtaaaaaataagactacattgaagtgaaacccgacaatttgaaaac
taccaactgtgaaacataaaagataatcgcatgtgaaaacaaagaacgatatatgcattaaaaaatgtaaaacgtaactaataacaatattata

accaactcgaaaaacagataatgaaggaaaaaaaaaaaaaagaagagataaaaacattgccaaaaaaatgggtcgattttctttcaaaaacca
gacaacttaacaaaattgaagcaaaatataaataaaaacaaaattgtaccaaatctgcactttttttaatagatataataaattaaaattaatcat
cactcgcgagctttaaggtactgttcaatttcatcgttttcattggaattggaattgaaaaactccttactctcctgctcgaaccgagagtgtatgaa
cgcatcgctttccaattccacaaaaatgtcagtatctgtcggtttcatcattcggcggatcatttgtaaaattatatccaacattaaagcagcaaca
gtaaccaaaaacactgtaagccaccacccagcatttttaccgaagtgatgaaagaattgtgatttcgcaaggtaaatattttcatcgggataatgg
tcactgataaacatattgaaaaaaaaccatcccaaaagtgtaagaataattacaataaaagaaataatgtttacatactgcatctcaataaaaat
gagcttaaagttcatcacaacaactgcagcagtaaaaatgagctgcccaaaagcaaacaagttattatcggtgagctgttttacgccatataacg
catatgtaacgtaaaatactaaaaaggcttgcataaatgccatagatgaccagcttatataaacacgccaattaaaagcttcgttgttaattccttt
ttgatatagttctggaacagcaatgacggtactaacatttaaatctttgtcgaatataccaagaccaataacacataaagacgaaaagagagtat
taaagcatgtaagcccccaattctcatataaactttgaccggtatatccaacaaaaggctgcataatagcttgcattaggaagaaaaactgctctt
tgtaaaatgtacctaaaatatatttagatagacgtacataattccaccgaccgtgacaaaaaagtagtttaattaagaatttgaatctggcgattg
agaaatcagatgatcgagctgcttgcagaccttcaagaccagcgataccaatcccaacgtgggcttcttggatcatagcaatatcatttgcgccgt
ctccaatcgctagcgtcacagcagaattaacattctcgcgtacgtttttttaccatgagcgctttttgcataggactagctctacagcatataacaga
gtcaactttcacagaggtgttaataaaaagtgtaaaaacttcacggtcattttcaatactgtcgagagtagcaccatctatgacaaggactaaatg
tgctacagagttgctttgcattgctttcatacacgacaccaattggtgaattacgaggtttgttgtatcaatctcttggggttgctctaaagaaagtct
ctgtccgccctttatagtttcgtcgcttccagatttttcatccatagatcctaaaaccacgacagtcgagaacccttttataactccacaagaatgac
caatattaattgcagtttctttttttatctccagttaacacccaaaattttatgccagctctgaatagtgaattaatagactcaggaacgcctgtctgg
agtttatcttctatcgcagtagcacctgaaaattccaagttcctttcaattaattcagcattatcgtctaatagctgttgtctattagtcaagctgctca
atgcttcatcattcaatgctttccactcttgatattcttcctctccgagaaatcgatgtgcataaataagtgttcttaaaccatctgaagcaaatgcat
taacgtgcttgaaagtgttttcaaacatcgcagcatcattgtgacttaaactttcaaacatcgatccctctgcttcttctttcttagaacgagatttctt
gtcggagagtcgttctcctactgcaaaagagtgtcttccaggagtaggacggcgcctagtttctaatatactggtgctagctcgtggccgaatcga
gcccatttttttgtctacgttcatctaaccaggaagtgacattattgatataatctgcacgtcttctggtcaaactgggtcgggatgctgtcaaacttg
gtctagccgtactcatactgtttcttataatagctttatctatttcaatactttttcgagccttttctgctttagtaacagtatgacttttcttttttgcaag
caccgaaaggcgaagtctttcggttactgtagaatcggcaccttttgtaaataagcaaatccgaccatttggcattcgtacaattaccgacatacg
cttccttttactactgaattcgataacattcaggatcttataggtttttgtaatgggctgggaacgaggatctaaaggataatgtaaactgatggac
acagtgtccatatcacgatccacaacaatatatcccagttgttgagcggcatgtacgagagctagttcatcaggagaagtggattggtaacgata
aatattttccttttcatcgtaagatggaagacatgtgtgacacacagccatatttaaaagaaatattctgactttttcgagaaagtagtatgtggat
tgtcatgcacgtatttgagcaattgaatagtagtcccttcaatgttttctgacagttcaggaatttttatttcatcattatcttctttcttcttatttgtcg
gaagtaaccttggattttcggcccctacatgttgccaagcaaaacctcccacggataaattccgaaatagcattacgttgtctgtaagggttcctgt
tttgtctgaaaacacatgagtcacctgaccaagctcctcaaggatagatgaacttcggacttcacagcgcgtattggtttcagggtaatacatatc
aatgtcactctgaactaagaaagtctgacagacacgaataatttccattgatacgtacaaagaaattggaaccattgtgttgtataaaatgataa
atgatacaagaatagggacaaaagcaaggcttccatctgataagtaccacaacttggtttcaaccttttctgccatataaaatatgcaacggtac
agtaaattgccatactgacgactaaagcaaaaatgaaaattacaattttgttagtatccttttgcatggcaggcgccttaactcgaagattacgag
aagcgttcatccgaatcttcgtttcttctcctgtaaaaataaccaacccaaacaattccggagtgttccttaaaattgatcctctgtaaatgacatct
ttattagttataggaatctctttctcttctgaatttaattgcatccttccattaaattcatataagtttccattaggatcttcaactatacattcaccaga
aagacggctaaggtcattgatgtctttgcaacaagagcgtaaggtagagttaaccaacttggtttttagagatgtctctccatctaaagccgctgtc
tcgacataaacagcgccattggggtgcggactcgataaagcaattatatcagcaggaacagactcgtcagaggttagtcttactatatcaccaat
acgcacatctttccaatcacagttagcccaaaagaatgtaggcttgagtgtggtttcaaaaggagttattttttgtggatctaaatttgtagaaaca
tcctgcgattgaacgaacgtaggtgattgtggaggaggaaggtcaggtatttcttggagggagttggagtaggtagtacttctagatccagtatcc
tgggaccttcgttttctccactttcttctacgaaaaaaagaaggctgttcgtctacaataggaggtgcattatcaacgtgtcttaggacttgaacttg
aatccgattttccacacgatcttgacgataacgacgataattatcaaagccttcacgtaagatagcaatagagataaaaatcaacaacggtatta
tagtagtgtaagtgcctgtagtggaccatcctgggatcatttgcataatcgcaattaataaaaagtaacaattagcagtctttgaaaattgagcaa
tgatctgcaaaggaacgaagttatatttattgtaacgagacgatacaatgttgttcttaatgaagggcttccccatacgttcatcaatttgagaaga
gtcatcatatggatcaacgataatatgtcttccatcttttgatttcaactggcggaagtccctctgaaacaaggatgaaatctttgtactgaggtatg
tcaaggggtttggaaccgagaatttcttgcgatttttctgcaaaaattctttaggaatatttttacggccataaatttctggtgcaatctcaacttcat
tcgaagcatgcgacggctttcccatatcatccaagggaatttcttcaatttctggaaaagagacatgaactcttttctttgcctttgagggtgacttg
tcagttttaactgacggagttaaagcggacttcatagaatttgctgtaagtccaaacactgactttaagttacttctctttttcccttcattctcagtcc
ctgttccggagacgtcagagctggcattgtcttttgttttagactcctcctcatcaatgttaacatctcgaaattcatcttctataatgcttgacggcg

gcgacaagtcctggaaccgcacatgaacgcttccccgacgttctacgattcgattatttacatttccactttcatcgttagcatcaatattcgctaca
acgtcttgatcgttcgatgattgtctttttagtccagtaaaatcgttatcgttgccattggacttcataagtgcatttgtatgaattgaacttcggtgtt
ggaatgggcaagcaagaaaagtgctctccgaagtctcctccaatgacttttgatctaacgaaaatcgttaggatacactatttaaaacataaaca
aatgttgtttttatcatttaatattcttttcttactgtctgctgcattttatgattgaccattggaaaaaaagaaatgctaaatctcagctttgaaatac
tatgttcgtataaattcgtgcaaatcatataaaggaagcagtttgaaatatactaagcacaacaaaatcttcgtgtaagtcgggaagtagcatta
aacaaacatctaaaaaagttttctaaatgtgatgatcttacggtttgtacgaaacaacttatatataactagcagcctaaaaccaagagtattagg
agactttaaagactgtaaatccttgttgtcttaaaccaaacccaaagacatgagccagtcattagtaaggtatagtttgctggcaacaagttttcca
atggcactagtattttcggcttccttaacgccctccataaaagcagcccaaaaacccaaaaatctggctataaagacttttgcatctgtgacgtag
gggaattcctcctttatctgtttaaaagtattctcaacagcacctgctttgttcgtatccttatcacttccagacagagtctgcagaagcttattcata
attatacctgacagtaggctacaaaaatttcggcgaaaaggaaatctaaaacaaaattagtaaacgattaatcgggtagagtaacaggcatacg
tttttcttatcgagtatgggtttacaagatttctgttggcattagcgttcataataacagatgctattaaaatagcggccaactcatgaaggttggca
ccaaagcttgtttgcatcaaataaaattgtaacaaatcgcggtcaggcgcagataactgataaatttttttcttttcagttggaaggaatgtagcaa
gttttgataagaactctataacagaggggatagaaagagtggaagaagttgcagaatcaaatttaggctctttaatttgtcgtaagcgcagtaatt
caaacagaacacacatggaactttgatactgaacgtcacatttttctagagccatcaacaaaggagagccccaagaagtaagttgatgggtcgt
agatataaacgatcggatctgcaggaatcgataaaaggagctcaatactatttctggagtggtagaaagcaaacgcttgggtgggtttttttatac
ctataccaccatccggacagaagctcttttcaacaaatttttcatctcttaaacaattaagcatactatgtatacttatacgattattctcacccaca
gcttccttaatattcaggggtatgcattcatcgtaaatcatccaggctctagtgtcgttgtatagcggaggatcatagctgtgcataaccttcaaag
atgcttgctggttatgtccgtgaagggaaattacatctcgagcttgataaaatcggtgaagaggttgagttaatagattgagaccttgagaccaaa
tctcctctagatcatcaacaaacctccgctgattaactgctgccagagcagcagcatttttacgggaagtgaggtctggcttttcgttgacttcctgg
cttgaggaatccccaacatgctgctctagtatggagaccggatcactgaaacaaccagacactagggctccaatcatcctagaagggagcaagc
cacgagaaatataatagtatagttcgttaggaagacgcgtacccatgtacgcgctaatatcattaggaacggaatcttctggcacaggtgggata
gtaagtcccttttcgttcatgataacacagtaatttaagtaacaaaaggcacgtctgtattgctgaactaattcttgagcagtaccggatggcaatg
tgtcggcaaatccttccacaacgcgataagcggaacctagcatcattaccaattctatagcagattttaataagaaagttccttcaaattgaggaa
gtgtgggtgaaatggcagtaccacaaagtagacacgcttcaacaaattgactccaggatgtgcatctgctatcttgatagatagattctccatcta
accattgaattgtggaagactgtttctcctctttaattaagtgaggaacggagagagaagaacgaaaggactccgactgcaaaaatggaatagt
aatagacacgatagggcgtttcacgttgaaaagaagaagatcttctggaccatatacagcatcaacataacgttttgaagcacctctctggaagt
aggaaagctgtgccatagctaaatagggagcaacaaaatagtcaattttcttcttgtccaaaatgtttttcaaaaaatcacataatctatctaaag
aatctcgttgactactgtagagaaaggtgcgtcgattttcaaaattgttacgatgactgccatttgggtccattcgatacaaatttgtgtgaatgtta
gcatcgtacggatcaaagcttgacaatgaatattgtatctggtataactttttttgtttatgtaaagcttcactttgactgctagcttcaaaggttaag
ggaatgccattaaaaacgaataaaggagtaacattattttttttcaaggcttcaagaaaagaattaacataagcgtaaaggatttcggattctcca
tccacagaaattgcttgaagctcttccgggcttagtctttttaaaagttctattagaaaataactggcatcaactcccagtttcctgttggctatttga
gatatgctacatgtaaattcatgcttattttttattaagaataattccaaagagtgtactttgtgaaaagatgttagttgcaacagttaaaaaacgtt
tggacttactagtcatttttgaatatgctgaatcaagaaatgcgacttttaattgactttttaattccttattaataagttatgcgtccttttgaagtcct
tcagttaatgcatatattgtcgacacttatgtgagaaggttaaaaaggctaatgggcggcattacttagtacttgaaatggtctgtttagcttggaa
accccactataaatggattttatcttgaaaacattcagaataataaaaaaaagaaaagaattaaattatggtatataaaattagcataaaatatg
gaagttagataaatccgtaaaagtttcgacactgcttaacacatttagttgtttttgatgtagcgattttgtcaccaaggaatatataaaaaaaaaa
taaaaaaaacgttgtaaatattagatacaataaataaaacaactcttattaaaattaaagtaagctgtatctaggggagtaaaccataggatga
aaaagaagcaatcgctttagtaaatacgaagcaaaaaagaagaaacaaaatgcagacatatgtaaagttcttgattatggttcaacgtaagag
aatagagaagagaagctaatctctagcgaatttcaaaaagaatttattagtttgctcggcaaaagtaagcgtgaaaatggtatgaggagcaatt
ctagctaaatgagcgccaaatcctttatacaaagcgaaaaatccctctgcacggactgtcttgaggaaacaatcaagaggatttttataaagttcc
ttattttttgattatacatacgagtcatgatcgtatcaaagacttgcatgcagcagcatacaccaaaaccggaaattgaactagcagttaagtgtt
taagagttccctccttcataaggttgtagtattcaatattgcgtttagcccaattatagataggaagctgaactgatgaacctgacactgtacggat
aatagcagcatcagcgccagcaaagattccccgaatcccactttcactaacaactcgcttgaatccgtcaaaaatgtgcttatacccatactgttg
accaatagggaaagcgggagagtacgactgcattcgggttttaatcaaaaagaaaggagaaccaaacagagcaccaaaaaaaccagaacca
gcaccgctaagaacattgatagccatgttattaccccttggatcctgaaggaaggcacgatttagatacgaacgaatggggtcataaaatccaag
acgacatccatttaggcaaagctgatatatgtaagcagttccaagtcctcgttggagtccttgaattccttcatgcttggcaaccaatttaaaagctt
gcccgacgcttttataaatttttttcgattcgtccacctttgtcaactggccctgaagttgaaaacgagttttaatgatttcaaaaggattagttagag
taaccgcaccacatgcagcaatgcctccagaaacgaaccccatcaccggtcctagtctctttggttgtactggaggttttgctgcaacattcgccg

actcgcttacagcggattgtaatttttgatccattggtataaacgatctaaacggttatattgcctgtggtaaagctggtctttcaccaatttggggt
gttctgaatgctcactaataaaagaaaaaatctcatttctttcttatctgattttgtaattataaacatcctcggtaagacgatatatcggcgtaaatt
ttcaatgactttcgtcggtactaaagtaagtatatttatttacgtttacatttttaatatctttatacaatttaatcactgaaaatataggattcaggta
taaacatgtaggatatatataaaaaaaatataaaatagttgatctgcagaataatttaatgaagaaattccatgaatgaaaaactttggtaaatg
gaatgacattcgctatgtaacttttctttatcaaaaataggcggtttaaaagttcctgtacttataacatggcaaagtaaatagctttttgtcttttcc
taatattcatggtatcttatatgcttataacataaacgagaatttatttatttttgtcaaaatagcaggaacattgatcagtgctttgtgattcattga
gtttttgagaaccttgtttgtcaaccccttttctcttggcttacctttatttctcgttttatgaccattatacatatcaattactaaatcttgtgaaaacc
caccgatgaacttttcagcgcattgctgggttaactggtccaatatttaattcaattgccaaacataaaaagctggatagtctcttgaaacaccgat
ataacaaaacaaaaagacaccccattgctttgtttatgaaaacctttacttacatttaaacgtcattcaaagtcgccttcctttgcatttttaggggg
ttcggtcatttaaagtatacctttagcgaagcgggttgtgctcaaggtatgatgggtgaatacgtttttttgccattcccaagccaaatacgaggaat
ttgtcaaaagtaactagggtccttactattttttatgctttttttgtacaaaaatatcaagtgaggatacagtgaaaaaaaaaaacctgagattttcag
tgtcttcattatccgataggtttattccttggccaacgcttctttttctttatttttttgtgtttttgctttagatttttgggtgtgaaaactatcttgctggt
tttatgttttttttctctactttaacctggttatgcagcattagacatggaatcgtctaaagtcactcccaaaaaaaatgggtcaaactacgcgtcgtgct
atccattcttgcttggcatgtagaaaaaagaaactgaaatgcgatcatggaagaccttgttcaaactgtaagaaaagagcaacaacatattcgtg
tatatacttgcaacaaggacaaccatcaacaaataactttgatggaggaaacgaaaccgattctgtgataaatgagcttttgagtaggatttctca
tcttgagcatatgctcggagaactaaacccccgtggctcttcccaaggtaaaaatcgattatcacctaatgttgtattaaccttttgttattagatag
tgcttcagcaccgttgaccccagttaaagaagattctgctgcttttgatgagactgaacgaaacataggtactctatcattgcgagattcaacaaa
aactacctatgttaattatccttcatcttatcatttggcctggcatttacgaaaaacgaattctctgaacttagaccatgacttttctgaagatggaa
aggaagttacgcaagaatcgtttttgtttcgccttcctgttcgggaacacgctcgaaactttcttcctccgaagcacatttcagatcttctcatcgag
gcttactttcttcgttgtcatttatttataccggtcttacataagtctacgtttatggaaaggtacaaagtattttgggaggaacctgctgagagaaa
gccagcaatgacatctcttctttttgctatgtttgctagtgccctacttgctaccccaatagaaactcgacttagttggaatttaggagaagaagaa
aaatatttgtccgttgattatcatttggcatttcgttacgcgttagcatcatccaactttttatttgatcctgatctcaatgctttaagagctttaattat
ctgtcaggtaaatactctattccatttttttgcttatactaacttctcaggtcgcttttgattctgatactacccttgctcctccctcgatggtgggcttaa
ttacacaaactgcttactttatgggactacatcgagatggatctttgtatggattaaatagtgtttatgctgaaattagacgacgagtttggttctct
gtattggttttagaccttagagtatctgaaatgacaggaatacctccacgaatccatgaggacagttatgacacgagttttcctagtcttttaccag
aagaaaactatcaagcggatccatcaatttttactgctggacgctttgtttgtatatctggtagagcatcacgtgaagtctcaaagtacatgcggat
gctgttaggcttaaagtctccaaattatagcaaaattttggaaatggatcagaaactgactgattattataaagaaattaagcaagagctacttcc
tgaacctacaactgttcatgaaagattattacaccttttttacgacttacatctttcagcggtttcctattttgtttcattttccttttctgttaaagaaag
gtgcttcagctttttcatacagtcgtatccgttctgttgaatgtgctttagatgctttatcctgtttgtatgagcttggcacgactccggagttttctatg
tattcatggttcttttggagatatccccctttttcatccgtgcattgttcttttttttagatattttacattctgaacaaactatatattcggatgataaaag
agtggtcatgattaaccaaatattttcattatttcctaaaataacggatatacaacgctttcatcaggcatggtcactactaaaaacttcgcgaacc
catgtctgggaaaaaattaggactaaaatcatctaataatttagcagtctcggacgaaaacatacctgaattggggtttctagatccgtcgaaattg
gatattagctgggtaagttttctcaatagttgaaactttttaactaacttttgtaaaggatgattgggatgcgatttttcaacattgcccattttaactt
tgagctgcacaattactatacatgtgttatacaatagaaagatggtttatttatttgttttgtaggtaatagttaataccatatcatatacgaaggat
actttacttcactttttcttttttaaaggcgctgaattgtttaattcttttcaaactcagctactttacaaaataaaaatcaattgtctgtcgtaaaact
agtattctttaaatcaactaaagatcgagtacctctccatttgacctccttgctttggtttttcaaatctttccatttccatgctttttgccattctggtga
taatgtaccacatacttttttgcgtaatgtacttacctatgtttggtaaaaacttaaaagcatgaccactatccccattagcaatcaggacattctctg
ttccaggcaacttgtctattaagaattctgaatcggaagtatcggaaatccagcacattttgcacttttgcaaaggacggtccgccaaattaggaa
gatacatttgcagaaattgacgaatgtttttcttagcgtaatctgggatttcagtatataaagatggagggtttggtatggaggtaatgatggattg
atcgtaatgagtatgtgtattgtagacatattcatagccggtactggagaattttaataacttggacttgtgatccggtggaaaaacgtatgcgaca
tcaggatcaaatataattggcattttcgtatattgtttgtattcctcttccgttaattggaggtgagcaactggtagtcctttggctataacacggcga
tctgtatgaacatacgcattcatataagcaccaatggccagtaaaatggtatccgcttgtatgattgagccatcatcagtctcgacagcggccaag
gtattgggaccagaggaagtatttggaaaaacaaatcgcttaaaagagccacgttttccatatcgaaactcaactccatattttttagcttcggact
ccatcgtttttaatgcttcggacgcattagcataaccagcatgttcatttaagactgctgaggcattaacttttaaaggagcatttgataatagaatt
ggaaatctttgacggatttcgtccggagtgttgagattaattaatgcagaatcacctagtagttttctcaaattcgcttgagaaacagcatcaaact
gagatctattagggtcacttgaagtgatacttaaccggccgcattcatggtaagaggaagaaaccggatcattcgttctccatagatccattgcttc
taatgccaacttcatgtactttatatctgcgtactctggacgtacgattttgtttaagtcatttgcagctgacatgctagatggtacagcttctgcatc
aatggcgatgattctatcaaagacatgttgttttgctaattcgacagcagcagaaaggccgaaaacgccacaaccaacgatcaaaaaagtttttg

tcataaatgcaaaaaagtgttgtgttttgagataagacgaggactaacgaccggactagaaaactaactggcaatctgatagagatatccacca
atacgccaggcaactttatttccctccacctagcattgtacacgtgtgttcaaggaggataatggagaaagtgtttcatagacgaagaaggatatt
aatgataacagggggtatgctcaactttattaatatgcgaagagttaagctaaatcacggtttcaagatgcaatctttgaaaacaaagcacaaaa
aaaaggatcagataaaaagagacgaaataagaataaaagagtagaaaaagataagtagttgaaagataatttctagtgcttgagatccctaa
atttttggtaaatacatccatcgtcgacaactgggaagtttcccttaaggtgttattataaaagaaaacaattaacctatttttaattttattctgtctt
tctgtttgtttactattttctattagctactcttccgatctttacctgagcatcccagattgcgctagtcgtgcctttatccttaccttctttggtaagtag
gggctacgtaatgtgaaatgagacagctttaactagttatttttagtgacgtttgggtgagcgagttacacttgcttattaaatgaggaaagattac
ccgcccgaatcaaccagcgtttgtaactttttatagctatgtaagtcgatcctgtttcacctaatgtctcccttcggaatgcataaaaaaaaaaaaa
aactcccagaagatatccggggatttcgcttattttcagctttaattgagtgacgcaagaagtccgtttcgcttaaattttcagcctatattccatca
atagatgtgtaagaaaaaaaaaaaataataaaataaaaagcttttccttcgaattcttgctattcataaatgtcacctagtttgtatctttcttttta
gttcgattccgttcattacaccatcttcctgttttgcttacaactttgacctgttgattataagaaatatgattcattgtactcatttcggaagacattct
tggctcttgttactcgtaatcttacgattacaaattacttagcttcttattagcctcaggtttgaactttggttttccaggaaaatactttctcatatagc
tcccgagatttccgtctaccgaacatgatgtaactaagattttctttattgtttactacattatatcatcgccctttgtaaattataaaatgctcgaga
aattcaagaatgcatcaattgtctgaggtttaatttattgtttgattcaaagttggattccttattccctttacctttcttttgatattgtgatatttattc
ccgtttaatctttcatctatttcatttatttatctttttcttttttcttttttttgtttattttatatattatacaggtaaattatttattagcatcatgtcatcga
ttgcttctatctttcatcgtcctgaattgtttggttttgatcaacccgttgctcctacaacagtccatggaaataggtttacttctaatagcaacaaatg
gttctctgttgacaatcccgctaccgctcaagaaatcggtaaagtaaccgatttgaccgtggaggataccaagaaggcagtagatgctgcttcca
acgctttcaagtccttcaaaaacaccactcatgtgcagcgaagtgaactattgagtcgctgggctagcctgctagaggaaaataaagaagatttg
ataaagatggtcactttagaaaatggtaaacctgtttcgcacgctgatatggaggtctctacttccattggttacctcaaatggtatgcaggagaa
gctgttcgtatttatggtgacgtagcgccctctagcttacagatgaacaactttattttgtcggtgaaacaacccatcggtgttgccggattaattac
cccatggaatttcccttctgctatgattactcgaaagggaggagctgcattggccgctggttgcactttagttgctatgcctgcaacacgaactcctt
acacttgcattgctctcgccaaactggcactggaagctggtttccctgaaggtgtttttaatgtgatcactacttctaatgttagtgaaaatggcaaa
gagctgactaccaatcctttgattcgtaaaatctctttcacaggaagtaccgatgtcggaaaaatcatcatgagccaaagttctagcacagtaaag
aagatttctatggaattgggtggaaatgctcccttttattgttttccccgactttcctgttgatcaagctgttgaatcactttacaacattaaattcttta
gtgctggcgaagtttgcgtctgtcctaatcgtgtatacgttcacaaggacatctacgaagacttcagtaaaaagcttgcagagaaagttaaaagt
atcaaggtaggcaacggacttgatccttctaccgccttgggtcccttgatttctaaggaaggatgtgataaaattacaaagcatattgatgattctg
tctctaagggtgccaaagtactagcaggtggtaagcgaattaccgataaaggtggatactttttttgagcccactgttcttacaggaatggaccaga
atatggtcgttgcttgcgaagaaacctttggtccattggcttctctgttcaaatttgacaataccgatcaagttttagagtgggctaatgacacaga
ggtcggtttggcaggctatgtttttaccaagaacctcacaactttagtccatgttgctaaggaactcgaagttggtcttgttggtgcaaatattgaat
tagtagacgaacctttcatgtcctttggtggtatcaaacagagtggatttggaaaagaagcaggtcgtcatggtgttgaagagttcatggtcatca
aagaaatcaaccttaaagctctttaaaaggcagaaatgattcttcctagcaaacagtttgatgattctatgatttttttgtctatcgttatagtctattt
attatagtacactatgatttcacggacaaccgttatgaatttatgaatatataaagttttccatcaaattcattaacagtatgcttctaaagtcatcc
gatgtaggcagcagttgtttgtttattttcacgcatgacttcaacatgcgaaagctaccaaaagggtaacgatgtaacctcaaaggtcaccaccaa
aattctaccaaaacataccaatcgaaagcagttggcttgttttcatcctttaaaatgagcaatattgacgagtgggttcataatgcgaatgaatgt
atatcaattgttcaaagtatgctatagagttatgatacagttgctgacgaaatctttcatagaaaatgagaagggtgatgtcgactgtgaattccat
cctacttttacttacagtatttttggagactcggaagttctatacggctacaaaaaccttaacatcgaggtatgtatttcttcatctacgatgtagcg
acttttcaattcttagtagtggcatttatgttaacgaatgaaagattcaatatatatctccttcaatgaagccacagctaaaagttacgtgttctgat
cgacttgctccagaatcaggagtggaaccaacaaacgtcgataaaacgttgtcaaaatacttgagggatgatcgggttccagaaagcttggagc
cggttgatgttcgtaatatcccggtcgctgagacttattcttttgatgaacgaaaattctgtatattaaaggttggcattgtttcctagctttttatttat
gatctactaacttgcatagagtaaacctagtgacctagctgcaataatgcagtatttgcaaattttttctctattctttatcgagggtggctcttttatt
gatatatttgatcctaaatggactatttatttattgtatgtgtcatgatgaatatcgacagttgaagactaacaagctgtttttagatatgaaataaa
agaggaacagtattgtttacgagggtattgtaccgtttatcactattacaaatgggataagacgagaacggatggtgagcgtcctcgcatttctca
gtttgtgatatttcctccttttcaaagagaaggccatggttctagactttacaataccattgtaaatatattcctagaggatccaaaaattcttgagtt
cgcagtggaggactcatctgaggcatttgacgaccttcgtgatcgttgtgatttccagcgactgaaatccatgaatatatttactagtccagagttc
cgtttgcctctatcaaaggaatggttgcactcgcaaagatcagccactaaactaccaccggtatattgaatgattatagtgaagttatgttctaacc
tcttatgtagcgccagtttcttcgttgttgcgagattgctcttaccatgaaaatgaaggatttatccctgttagaaagaaaagcgcttcgtctcttgat
caaggaaagaatctttcgccagaaccttgacgttttgcttcagctagaccgttcggatcgtcttgacaaaattcaaggagcctaccaaaatcaact
tgatgaatacaaaaagattgcaaaaaagcttccaaaattattgaaagaaggatctcggaagcgacaaaaaactaaataggaaactcaataata

gtgaaatggtttaaatgtgtaaatttactctattcaattgttttcagaatctttcattttaccactttaagtcataagtcataagtcatatagttgattt
attttcagtagttaccttttattagtctagctatcagcttgattcttaaaacttttaaaacaaatttaatacaatttgtgctttttagtctttcaaaaaca
ctttctatacatctatgaaatattgaactcttagtaataatcatcatagttagtccgttcgagtttgatttgttcaaaccaactagttaatagacaaag
gaatccttttccttctcttttcttttttttttcatttgttttttttgtttttgcatacctgaaaagaaatcttctcttcgttaatacagttcgttaaaccaat
ttcccaccgccagaaccagcgtccttcccaattccggccaaaaaatattttggctataatttttttgaataatttaaaatttgaagtaagattttgct
ttaatttctacaatgagtggaagattcgtgagagctagcaaatatcgtatgccttttgtttcaagggtgtaaattaacttattaaggtcacattttttg
gacaaacatgcaagaaagagctttgctatgataatttaaaggttcgttattaagtagaagtgtatacggttactgaacgttgaattagatcagtaa
taatgcatgggattctaatttaataagcgcgaatcctttctatttaagcgtcaattggaactcaggagctggcggtgctattgccgtcattcctttag
aaaatcgaggaaaactccccgaccaaaccaacctattccgtggtcacactgctgctgttttggataccgattggaatccattccatgatcaagtat
tagcttcaggaggtgatgacgccaaggttcgtgactcattaatttatgaatatttacttacaatgatagattatgctatggaaagttcctgaagact
acacagttttggagccctacgaagatgtgcatcctgttgctcacctaacaggacatagtcggtatgtttagatgaagaaggctatttgactaactgt
taaaaagcaaagtaggacatgtacaatttcatcctgttgccgcgaatgtgctggcttcttcgagcgctgataaaactattaaattatgggattgcg
agaaaggaattgctcatgtatcacttcccatgaatgaaatctgtcaatctatttcttttaacgctgatggaaatttgcttgttagcactggtcgcgat
aagaaaattcgagtctgggatcctcgtactgataaaccagtttcagtcaccaatgggcatgctggagccaaaaattcgagaagcgtttggcttgg
aagcttaaaccgcattgccacgaccggttttagcaagatgagtgatcgacagctcgctctctgggatcctgctaatatgtctgaaccaattggagg
ttttactaccttggatgctggttcaggtattttaatgcctttttgggatgacggtactaatgtcatttaccttgccggaaaaggtgatggtaatattcg
ttattatgagctggaaaatgatgttttccattatctttctgaatttaaatctgttgatccccagcgtggtatagctttcgttccaaaaagaggcgtaaa
cgttgctgaaaacgaggttatgcgtgcctataaatccgttaacgatacgatcatcgaacctattagcttcatcgttcctcgacgttctgaagcctac
cagagtgatatttatcctcctgccccttctggaaaaccaagcttatcggcagatgaatggttttcaggcaaggatgctctggttgatagaattgata
tgtctttactctatgaggccaaaggtccagtatctaaaacaacagctgtgaagattcctacacaagaggtccaaaaggatgctgataaaaatcct
atcgaggtaaagccttcgaagaaactagaaccagctggggctactcccgtacaagagaaggccgctgaagatattccatcgaacaagaaagaa
gaaaaggagcttcctaaagaagagaagaaagaaattgaacaaaaacatgtacagaaggagaaaaaaccagaagaagacaaggttttacctg
agcatgctataaagtcagagaaaaaagctcccgaacaggaagaagttcctgttcctataaaagctttagaaacaaagaagggtgaaattaaag
aagatgtgaatgataaattgattaaacgcgagaaagagttatccatgtctcgtatgcccacaatttcttcccctgaacaagaaggtagtcttagtc
aaaagttagaagctatcgagaaacgatataatgaggagatgcctattcgagactggaaaattgctcagttagaggagaaaataacaaaactct
ctgagactatagataagcaattctctcgttaaggtttatcatttttctttctttattaattgataaaattcttctattaacatatctacttacttttatata
tgtgctttgagcttctcctgagtcaccaccaccatcacataacacaggaacacatgttccataaatgtggaaatgtcttccgggatgagaacgtat
ggtaccggtcggcgctatgtgagtaaagataatgccatatgggcctcattagacgaatcacctcaagcattacacattcttgacactaatgtaaca
aacaacgaacttaataagcatgcctcttctttttcaattgaaatcggtaaaaaagcttcgtgtgttaaaaaaagtgaagatgaatctgttaaattac
ttcctaaagctgccgataaaacagaagaaagacataaaaaaagtaattcacatttggtgcctacgattattgttccaagtcatggtaggaatatt
gctaaaacaagtgatagtgatgttaatacatccattactgaatcgaagatgactgagaatttaaatgcagggcttcaaaatcgtcctcaaaggaa
gtcaacgcaactttcgtacctactaggtttagtagatcaacaaattccaatctcttttagagattatataaaaacgtttaaatcttctatttataagat
cggggaagctagttatagtgaagtctttaaagtagagaaggatgaaccgatcgttttgaagattatgccttttggaaaggaaaatcagattcgct
gctcagatattataaatgagctaaagattactaaaaggcttagtagtctagaaggttacgttgatcttcaagaagcagttattgtcaagggaacgt
atccatcaattttgatcaaagaatgggacagatacgataggcaaaaggaatctgagaatgagcgtccagatttctttcattcatctcagttattttg
tatactatgcttaagtcatggtggtactgacttggaacatttggaactgtctgattggtttgaaacttggaatatattcctaaaagcagcgaaggct
cttgctacgggtgaaaggaaattcgaatttgaacacagggacatgcattgggggaatattttaataaagagacgaaacgatgaggacctttcgt
atttattggatgaagtgtcctttgatgaattcgaagactctaatacttcatttccggtgtctgaacgaagctttacagccgactcacttgaaattacc
ttaattgattttacattagctcgaatgagacatacaggttcgtcgtctcccatatttaacgagtttgatgatcctgaattattcagtgggcgaggtat
gttttaacattattccaagtaaaagcatgaacgaaactaatccataattttaggtgattatcagtttgatatttaccgactgatggccaaggtcact
aaagggcaatggggtgaatactttccaaagacaaacattctctggttacattatttattgtatcaattattgtataaaaaaaccattcctaaacctc
gtgatcttcgcgagcaacaactaaaggaacgcctaacaaaattgttcaagttactagaaccagtacggcgtcctcacaaaggatcttgccctttta
cagaattgtgcatttccatccgccgcctcccttgatgaatggatcaatcagcaatatgatgactgaacctacaagttgtcttgttctcattgcttgta
acttagattctttttgtgcattgcttatttaactatcgtttgtataccgtccattattttgaaaactactttttttgaaactgtcctttcgaaaacattgaa
atttttttgttcctaattcatgttgtttaatgactattttatctgtttattttttttatgcataattccattgtaacttatccagtaaattcattcatccttgat
atacatacctctggaaacccatgtggtgaactcaacatcagttgacgttttgataatcagtcaatatgaatccgtttttttggcggaaggtcttcagc
gttaatgttagcactgactttgggagtttgttcgtctgtatgtaattaattataaacgttttatagttctacggtcttaggaaatgaatttatgaatctg
tagatttttgcttaaaaaggagagaaatctaacttggtaatattagttgtatataatgcaacccgtattgaaggaacaaaggtaagaaaacaaag

tattcagtttggattattaacgattcgcattcgtagggaatatcaaaattcacgagacttaactctagaaaatgagaaaaaagaagaaaatatgc
gcaattgattctttaatcggccttctgaatgtcaattcatcttcaaacgacagttacttgcagatgtattttctggctgcacatgctagagcgaatac
gattgaaaatgcacatatttttgtgtttatatacgtcgttatcgaccttgatattaaatttataactaatcaagccctatcctttttaaaaaagtaaat
ataaatacatagatgcttataagcaagccgcaacgctttgcatggattgatttcaatgaacaaagacataaaaacgaaaaagaattgacaaaaa
ccgacaggcaacatagaaatagaattgtaaccacaaccaaaaaaaccaaagctatttaaaaccttttaagccttctagggagatagaatataag
ctcccttcaaataattcgctaaagctgttttccagtgtttcagtcgtcgtataaaggaggggccttgaaggtagtagtgaagatgaggaagtaaac
gctcaaatgctttttgtttattaaaaggaacagggactagctcaccaaaagattccagttcttctttcaactcatcttcggtccaactggattgcttg
gagatggcaggatgagaaaacaaagaatcattcagaatttcaagttgtgcaggagtccattcggaaacagcatccatataaaataactttgcag
tacgaagaaggtcatgacgagagttgtacgacacgcttgaaattaagctatcttctttacgataggttaccaaccacgtcaaaagatcttcggag
gaccatccatctaaaacatttgaaggagagaacgtccttggggtgaagcgctcattatatacctctctcaataaatcctcacgaggagatagaac
agaaacgggaataccccttctcttaaaaaatcaataaggcgaccatcggaccatagatgaatgatttctttcatataagaagcgggcagttcttt
cgtttcatcccttgagcttagcggattttctctcccttctggcatcgcagaacagtagcgtttagcaaggatagtcaagtcctcacgtgatgcaccca
aaggaatacgtacattcttttcatccaacaagctaagcagtaaatgagtagaccaagattcaaattcaccgttggtaagttcggtgctttccaaaa
atgcctgaaaaaccgtttcaagcaattgctccctggtaccaggttcaggaacttcgaaattatgttttattagccattcggagagctcagaattcga
ccaagcatcaaagatccatacacccaattcaggaattttactcaatgattcgcgcaattcgtctaaattactctcatggggatagtatttttctttca
aattagaaatgaattgcaaaggagacttccaaggtcctaacgttgactctggggcttgcgcgtccaaggactttactccatggcttttcagaaaat
catcgagcttgcgttgaggccaggaatcagatgtaggcttgtagggcgtttcaacgtaaatggttctctgaacaacttctgttttcgtattatggac
actaggcattgttgtttgtctcaagtcccttatttcaccaaaaccttcctgtattagtatgaatgattcaacaaaagtacatactctgtactacaaaa
aacctaaaagacaccaccaagactgccaatttaaacaagaattgaatatacatatttaaacagttgttggtacttcttaaaattgccaggatgtaa
ttgaataggtaagctggcagagtcaaactaagcgtgacgtaaccataaacaagtcaaggaaaagaaatcaaataaatgtatatacaaagaaa
aaagtaaaaagaaggaaaaaggagagaaacaaatctcattctatttagtatctggcaaaatctaagtaagatggtacaaacgactctagaaag
taaaaataatatgtgtaatcactaagttggtatccaaaattgtttaagatgccgtcaaattcgtgcaatccctgtttataaaggttaagctcagtgg
tactttgtacccttctatctgcttgagcgttctaaaatgcgatggaggctcccaaacatcactttagtagataactgttatttttaactaaggaatac
agaatgatagagccgagtacagtttcgtgctccagcgaacaccactgtagaagatggtccatgaaggctttctcattttcatggcgtttttgtagg
aaataaatagtttggtttgcttgcacatcaaacatttcagagccgtttccaatgccgaaaaactagactcgttgtacataacgtcggacatgagaa
tataatcccatttttttgaaggctaggagttatctgggatgaccatagaaacacatcagtagatatattattgtgtaggagatgtttatttttagca
atgtttggttctaagacaccgagggcttcttccaagtcagtcatacagatagatgcatttggatactttgatgctacagagatgcctagaattccac
atccagctccaagttccaacacctgtaaatttttgtttttggaaaggtccattctggttcttgtccaatcatcttttcagataaaacaatccctgcgtc
ccatatgtgttttgcgatgctctctcccatttgttccataaagcataaacccaatggctcccactggttatgctctaaatatcgccaaacatacttcg
gtccttcatttatatgtaatgagaaggagtcactccaaacaggcagtattctatgagattcatgtgaagaaacattaggagtaatcgtctgcggaa
gaatgattaactgaagtttgtttgaggtagctggaattgtaagggaaatagtcgatgcaacttcgtagcccttccagatgatacattcacttattaa
aggagtaggtttgcctttggagacatctacaaccacggcctgcagtcgacaatgttgtggataaaaatattctccaaggtctgtagttagagtgaa
gaccgccttaatagttcccgttttcgatccttttgcatatacaggctgaacctttaagagtcgtatatgataataatctaccattaagaaatatttcat
agccttattgtttcaaagaacaatggaatataataaagatcaagtttacaagttttactcttctttcaatacatgagtgtgctttctagttgagcgag
tagtacgtactgaagaagtagcaacggcagaattctcatttaatcaccaattcattatttctttttttttattatacattcaagtatttgttcattttgcat
agatttttcatctacacaatgctgcatatagggaatttttttctttgtgtgttatatgagaggtacgcgttggtaatccacaatcgtcgcgtctcaaga
atcgttcttctgcaattcctattcaaccaccatggtaaagtcaatgccaaacattctggtggactctatacaagaatgggagtcctacatggaaaa
cttggatgatgaatgtgctacctttgggagggttgctagaatgcacccagaaagcaatatggaggaaccagaaaactttgcaaaggatgcatttc
attattttgatgaagaaaacgaacaccttagtcgacgaaaaattattttgctccgtgctaaacggagactttgtcaacaggatctccgttggaagc
attctaaagattcggagaaccagtgtttggagaaactaacgggagtattaaaaaatcatgttgagcggatgcttaaattctgtaggatgatctgcg
aaaaactgtccgagacgtttgaaattgccatgaaagtcgcaaaagagtttcaagactgcaagccttctcaagcatggttctatgtatttcaattaa
agtatctaagaaaacagaaaaaccatcaaatcctaccttttgtctctgaatggataatcatgatacaacagtacattgacttgttaaacgaatttg
ccttggttcaacaaagagtattgaaactttaactttacgataaaactacatcttacttacgaattgtttttagtttgtacatatgtacgagcaactcc
atttgaaactagtaattacctataaacattgcttaagaaagtctctcatctatttgtaaatagatagttgagctatagagcttttttttataaatagaa
ttcttttctagaaggaaaaacgaagctgataaacgccgaaattatcatttcttaattcatcatttttcatttttttgcttataacgtacgaacttcatat
tgtaaaaccatacaatgaggtagcaacctgacttcacaatgttatttttaagaaaacacactaagtggggcaatccaagaattccatgtggggtc
acaggaactacacttcaaccaaggggttcttgcgtagtagtgtcttcagttgtataagtttcatcctaaaccaaactcttttgttcaattaattaaag
cataaaaagcttttaatcacgaaaaaccaaagaaagaaagaaaacaacaagcatgttcagttgctttggacgcaagaagagatcgatttacc

gaaaaattgtgattctgggtgatggtaagagcatcggtgcatattgaaccaccgcacgagaaacgtgaagatggctaatctctctaggtgctgct
ggaaaaactagtcttttaaatgtgtttacgaagggctacttccctcaagtatatgaacctactatgtaagtttcgttgtaagaaggaaattccaaag
gaaacccatccattgattcctccccaggttgaatttgcttttctttgttttaaagatcactctttcttcggcgagtatccagatttgtcgtggtgtgttca
gcaatcaatcagaaattcccttgcgatgctacttcaaccttaccattcatgctcttgatccttgcttctaacaaagtattagatttgaaaactatattc
acgatatatttgtggacggaaatagcatagaactttccttatgggatacagctggtcaagaagagtatgatcaattacgctcgttatcatactctga
cacgcatgtcattatgatttgttttgctgttgactcgccggactctttggaaaatgtcatctcaaaatggttgccggaagttgctagtaattgtccgtc
agtaaagcttgttttagttgctttaaaatgtgatttgcgcggtgcagatgacgaccaaccaaactccaccagaattataggctacgaagagggtct
tgcagcagcaaaaaagatcaatgctgttcgctacctcgaatgtagcgcaaaactgaacagaggcgtcaacgaggcattcacagaagcagcacg
ggtagctttggctgctcaaccacgaagtgcaaaagacaatgctgataattttcataacactggttgtattatctgctaagtttggtcactggaaggc
atttttctaatgtactatattataattcgtccctccctttacatacttgttttaaataactgtatagaatccttttcgttccttttacaggctagaattttt
agcttcccctgttagttgccatatttccctttcttgtcatctccaaaaaaagtttttttataggacattcgacttgctgctatccgcctttcactcttta
ttattttcaatatcttatattgacacagtacatggctttttctgcgttttggccattctattgcattttccttgactgaagtaggcatatatgctctgtca
ccaaaccaacaagctatagtatatagcgttgcctcgctgttatcatacttccgggtttttatgaacttcactccttttctacgattcgtgtttatttctt
tttttattgtcgatatgggatttatttaattaaagttgttaatttcttcatatcattgtaaaatacatctcattccttcttcgataattttacattattttcc
aggttctcgataagtttgtaaatagcttgaagttcaacaaatgaggaaccttcaaaatatcttagtatagttatatataaccactgcatactgtgtg
taactaaaaattatggtttctttttccttctcctattccgattaaagatcccaatcgtcttcatcatcaacagcacggcgactatactgcaatcaaac
atgttaatattcagcaagagggtttcaaaacggtgatttgcataaagcatcatccaatgaaaacgtgagtacatacaacaatcttagtttctctttg
ttttgataattgaccagagatcttttctagaagctcgattaactcggattcagaaatcttctgtaggacttgtccacttctagccattcgaattaatag
ttcctcaacggctaagaaagaagttagaatcttcaagattaaagaaaacaaaatagagaaaaagtatgcttgacgtaccttgggcacgatcagg
ttttaccagagcaattcgacggagtctgtctctagcagagtgttccaagatttgggtaagctgtaaggtcattaataatcaatgcttcattaagtga
tattaagcataccatattctgacgaagctcatcttgagcatctttttgttgattttgatctgtaggagcgtttccgggtctaattgcatgttaatcaac
agtgacacaagcaaagtcaaaaaatgaaaaaaagcactgaaaggactaagccttttataaagtatactttgactaacgaatgcatcggcttaga
aagttgacattcattttagtcagtacttcataacctcttttcactgaaatagtttacttacccggaaggttgttgttggccctttagttgagcaagacg
agcttgacggatagcttgtaattcttcatccatattaaagaactaagatacggttcttgaaatttcaaaatttttcttgatccttggtggaacaaatc
gaaagacgagacgcgtaaattccctaccaactgtacgcttttgaattctttaaaagcaacttaaaaataagaaaggactaaggaacttacaatgc
ttcttcaaagctcaataaaacagcttgttacaagacgcgagaactatataaacgaacaaataacgccttgcaacattgcacattacgttgaaatt
gcaaatgctatcgaatcatcactaaatgagaacttgaaataggaggctcatttggttaagttattaaattaaccttggtaaatttctctatagacga
gattctttctattcgtgatgcgtataaagctttccttgggaatcggtttatattttttgaatccatcgtcttaagtgtttttaaacgaaatatttagcac
ttcccctttaagtttcgctaagcttacttatggcataatttttcattgtgataatctctcgttaattactttacagaggtgcagcataggttattaagaa
atggattcaaaggacagacgatgacacatggaaatttctcatttccttgtgacagactttatgcaacgttgagtcagtaagtgtaaacaattgaaa
agaataaaagttccgcatgggatattagaaatttaggtgctgctaaacgccaaagacaagtgattaggcgattttatcacaacgtaaaagcctag
tagtctatatgcctgatgtgtcggttgagtcgactgatcctttcagctcactggaaacgaatatatggaatgataatgagaaagtaataaaggacg
aagaaagagccatgtctcaagaagaaatactgtcacacaaactgctggttgcaattgaaaaacaagcagcattagacgaacgatgcgctgatg
aaatccataaaaacgaagtgttagaagaacgaattcgagagttagaagggaagcttttgcaccaggaaaaaactagacagcccttgaatgaat
ttgaaggtgtagaagagaaactacggcaaacggaggcaaaataccaaagcgcagagtcagaacgagatcgcgtgcaaagtgagattgaaga
gttgactagctctttatttgaagaagcgaatcgaatggtcgctgatgctaggaaagaaaccctcgtttatcagagacgcgcttctcaattccaaaa
acagctggctgataccgaaacactcttaataaatgctcaatcccagctggtggagctaaaatctgtcatgcagaatatgtccgattcctatgaaca
gacgattcattctcccatgcggcccgggtcttctgctcatgactcggcttctgttgttagtgtaggttcttctacagctgctttagaagaaaccttgaa
tcctcctcgaaatagccctggacatgtaaaatcggcttcttttgaaagccctaggacaccaggtaattccaattatgcgtatccggctgctcgccgg
catttcgagccggtgcaagatcaaagagctgctactagtctaggtcttcataacccttctcatgttcatcgtgtacggacgcttgtcgaggataggt
ctagcagtgcctctcctcctgctaccccagtcgttgtcacagattcaccgcctccttctgctaacagtgtgacgcctatgaatcctgctttatggacta
tcagtcagtcaaacttatctcaagcaatgaattccagcaatccatcattttccgaattttatgctttattaacgtattcctcgaaatcggtccaatcaa
gaactactttaaaccgtacatcttcctcctcgtcattgaaaaataccgttgtactacctcgttctgtgactacgccagctaatgtaaaaccgttgtctt
cgtcaccgtctcagtcaactttcctgccacccgcttcacgatctcttcgagaatttgctttttttaaacgatgcgttgaagaggacattgatccgacct
tgcgcttagatcacgcagtaggcctttcctggctggcacgccgctcgatatatgctgctattatggatgcttctttaattattaatcctgtgccttcatc
gttatcacaggctttctctccatgttccctttgcggagataatcgggaaagcagccttcgctcttttgaatttcgatctcgtccagacggtacgtccta
tgcctgctgtaattattgtgtcgctagactacgtagcgtttgtggatttatatgctttttataccaggttgccaaaggcgtatgggattcatgctctgtt
gagaaaacttggacagagtgtattcataagcgtgaagctatgttttattcacgggccggattagcaaaagaacttggaggttgattccttttgtcct

ttgctgttatttcaaacgctcccttttgaatctaaggctttagtaaatacggacattattatatttattgatactttagagagtgccgttaatctaacgt
cctatgaagttacgcttgacattaacacgagaaaagaaaaaagtaaaaaaaagaaggggagcgttcaatgatactgtaaatatggacctattta
tgagctattcttctgttctatttaattttaaacaatactaatatatagttatcttacagcaaataagtatcatcatttttttcttaatctctcaataaaa
aaagagttcagcatttgcaagtcttaataaacgaaaacgtacaaatgtatttgatatgttaattgtattatactgtgattgatcttaggaccgaata
tccttgcatgctatgggagcaaagaatggatgtcaataaagaagaataaaattaaaagtaaagccagaatataaaggaaatattgtaaagtttc
taaaacatagaacttggacaaggctatccttcataggaagtatcgtctatatactcccgaattggttccactcatctatcctctactcccccctctt
cacttataaagtattgttgaacaaacatggcattctttggactacgagcttatcctacacctattttaaagccaatgtatcctttctttttaggaggta
agttaaagaagcagggacgaagtgaattgggttggagactaacaattttaggtgctgtcatttttttacggaacatacaaacttcgtaatactcttct
tgccggtaagaatatttgttaaaaatagaagaaaacgttcagaaatgctgactttaagcagcggaagaaaagaaggatgagaaacctgcttctg
gtcattaatgaatgcacctttctctctgtgctcttttgtctccgacaatgaagagaaatcatcgttgaattgattaacgataactataagttgaaata
acaacccaatgtatttctctttctttaccatattcgtatacgcgcttgctccataaaggattcctgaatctgtgtttcgttctgtgtaagtgtgtaatgg
tatgtagaaagtaataatgatttcattctatacgaagaaaagaaaccgctacaagctatcaaagcgacttttgacgagagagtaaaccggaattc
gataaaaagcttccgttcgcatacacaaataccaccaaatctttcagctaaacacttccattagacttatacacatgtgagcctcatctccttgaag
aaacaaaaagcttctggggaagctttttgtttctgcaacgttggttcatctggtgcataaacaaaaatttgtcaacgaccacacaatcaaccaact
tttattacaaataaacccacagcatccgagaagttttctcgcagatttagttcatcaagcaaaacccatttggaacttttggttgttcataccatacc
ataccataccataccagtataccctgagtttcatgaagtacaaccagaaacacctttagtaaagaagtacaattcagacaataaatatatgtcttt
tattccattcttacatgcaaaaattttagaaagcttcttctagtttactgatttgtgatctacttttgttaccttttttaaggattgtcttgggttaaaggg
ctcccatctcgtttcgaaataccaaaagtcatgaaaaaaaaaaaagaatctactagactatgcaacccatattaaagtatagataaatattgcaaa
catactgtattggacccttgtttttttttttcaaacaggattcttgcagaaacgatttcttctactacgacatctgagacaaacttttatatactgtac
ttttttgctatgtaccctatgtaaataatttatttctgatgatatgaatgtaagtaaagtaagtaatcttcgcataggtcgcttatctattaatgctatg
cacttccatacacgcaaattgcaaagccacgctggaattcggtattatgattattatttgaattctttatgatgcgattttacctactaccgtctacgg
atgaagaaaacaaagcaagaaaaaatgtcagtagttgatataaaaattggaaataaatatagaattggaaggaaaattgggtcagggtctttt
gggcaaatttacttgggactgaatgttgttaacggagagcaagttgcagtaaagcttgagcctttaaagtcccggcatcatcaattggaatatgag
taagtgtataaaattaaaaagaaattccaaattaatggctttcttttttttcctttttttctgcttcctcaatagagtaaacatatattcactggtttcccc
tttagagtataggatggggttcagtgaaatatctttattttttgttaagcttttcatatatgacatgaaatttcaggatggttttattaacatatcgaa
agatttcgagtatatagtatcctgaaaggcaacattggaattcccatgattcgctggttcggtatagtaaacaattataacgctatggttatggact
tgttagggccttctctagaagacttgttttgctattgtggaagaaaattttccttaaaaacggttgttctattagcagaccagttaattagccgtatcg
aatatgtgcatcaaaagtcatttctccatagagacatcaaaccagataattttctcctgaaaaaacacagcaacgttataacgatgattgatttcg
gattggccaagaaatacagagatttcaaaacttacgttcatattccctaccgagataataaaaaccttacgggtacagccagatatgcaagtatt
aatacacatatagggattgaacagtcgaggcgcgacgatttggaatctttaggatatgtgttgctatatttttgtcggggaagcttaccctggcagg
gcttgcaagcggatacgaaagaacaaaagtatcagaagattcgcgatacaaaaattaatactcctttggaagtattatgtaaaggccttcctgaa
gagtttatttcttatatgtactacacgagacaactttcttttaccgaaaaacctgactataattatttaagaaggttatttcgaaatctgcttatacga
aagggatatcagtatgattatgtttttgactggatgatactaaaataccaaaagcgtgccgcagctgcagccgcggcctctgccactgctaataag
actcctaacacatatggaaaccttagtgccttaccgtctccagcagtgtctcctcctcctgctgccattgcacctcaattgacacctagcctgaaga
attcacctggccgtatacccactgatcgattgccaaaaacgcctcaatcttattcaaccaatgtcgtccaatgtacttctccctctccccttccttgtc
agctccgtccaacctcccaaagtaatgatttcaacatcatccaaccctcttttcaggctaattacactccacacataagaaacccccttgaatgatga
ccatgcttcttaattttatacctgccagtcatctctcttgaagaaaaaaaaaaaaagaaaatggattattctctttttcatcctttgtttcttttccataa
cttcaaatatttttttttttntttttgcgctaacgaccgcccctcctactaatttatcgcttcttgatctacataaagtagaactggcataatactttcca
taggttttatctgaccgatatgaagttttgttgaaagaatcgccgcatgtggcttttctcacttttcatcttttctttttctttgttacgagaaacatcctt
ttttattcatcagcacttgcttggtttcgcatttcgtttaatttctgcttctgtattaaccacagacactgtttagcacttttgtgctttaattttgtcgtttt
ttgcgttccattccatatctttgaaattaatatgcatcatctaatttggcggcgtatttaagcattcgttttcctttcgcaacttttcatattgcacgagc
ctggccagaccctatcaataagcgtgtaggagttacattcttgttgggtatattgaaaccttgatggaagataattattcaatcctagtaaaatggt
ttcaatcatatttgaataattaactagttgctctatttaattcactttaagtaacaggtagacgcttttatctctatgctaacatgtatcgataactgtt
tttcttgtaatggattctattttacatgcttcttttgtttttgtttttttgtttatttgttgatgcctttttttcagtagatgtatagaaacattttctcttaacca
aattagtcgtcacttgcctgttacttgacatctgtaggtcaactagctctcttggcatgctcgtatgttgtgaataaaccaattttcttaggcaagagt
aacctgattaaaccatatagttttgttgaaagaaagtgattggaaaaaaattcaatatataggatccaatggctgcaaggtatgtggaataaaggt
gaaacaagcttacataaagatttggtagaagaaaagatcgcaatggggaagttttgcccttgtatgaaacacgatcttcggcgcatatggacgat
ttggaaggcatcaatgaagatcgaattgcaaacttaacgggaaaagtacagtctctgaaatcggtgagtagaactgaataaaggaacaacgaa

aattcataatgaatatgaagctaacagaataagttgacgatgaatattggtacagagattacttcgtcaacgaaactaatggatactatgaatga
atcatactttacgacaaaaaacgtactgaaaggcacaatgggccgattgaataaggtttccaagagtagtggtatttccatatggacttggctagt
tttcttttgtatagtggcattggtgttcgtcttggttcgatattagggctgtggccgtggtatcggtaccgtaaacgtattcaatggaaatatttggct
acttttattgaatgaagggctggtcccttatacacttgttattctcggatacattattattatattaaattgaagcatttcgttttcttgaagatctacg
cttcccaccaggttggtatggatatacttatgagtgattgagcagcatcgtgaacaataattgatgacatcttattttaagactgtaaatattctcaa
ttttattcttcgtttttctttctgaagtcccaaagactggatatcgattttgtcggtgcttatattttcttgtcactgtctagtgttaatttaaaactagaa
agcaaggttctgaatatgtactcattgggctcattttacaatcatttggaaataaataggtcggcttttatgaatgaaatgaaatccatctttctgat
aactagctcagcttaacatcatatacaagcataaatattcgatcacatttgaaatttatttcttaatagtatcttatatttttatgaaaactcctaaca
ttttcacggtaataaaaacattaccacttagcgtttatatgaccttcgtttcctgtcactactgtttgccttgtagccatactaagaccttgaaaggag
ggtgtctttgtcaaataactttacgaaaggaagcttttgcttgatttcgtattttaattgaaaatatatagcgttttcgttcatgctttattcaataggc
acattaatttattaagatgctagctattcgaatcctgctctttctgtccgcagtcatctttatcaattctaggagatctgctaaaagcaaacgaattat
tattctcgttttgggaagaattgatcaaagtccgcgtatgcaatatcatgcgctgagctttgcaaaacaaggatggaaagtcgatttagttgggtac
caaggtaggtaaatagttgatatatgctttattaagcaaaggaaaactacatcatgtttcttaacaacgattgattgcatatttctctataaaattat
tcgtacattctctaacctatttcgtgtagacaagaaaggaccaggcttgtttcagaatcatccaaatatacaaagctatgctattccaactcttcctg
gaagccttacttttcgatcgcgcttatccttcttactggttgcgccggtaaaaatacttcaccaattattgtcgatcctttttatcttatttaaaataaa
gcaggcatcctatttccttgtccaaaatcctccctcaatccctacatttttattgggacacgtcctttatctgttacgcgggacccgctttgtcattgac
tggcataattttggatataccattcttgccctcaaattaggaaaagagcacccctttgtaaagcttttgcgcatatatgagcgtgtacttgccaaag
gtgcctttcttcatataactgtttcctatagcatgcagcaagtattgttggaatggggaattcggtaagggttaaatatatataggcgaaattcgcat
agatttaactaacagttagaccttgtcatgtttgttacgaccgtccaccagctcactttcagcctgtacaagatgcaactcgaaagtcaaatttgca
tgtaattcctgaagagtttaaccccgaaaaggaaaagcttttagttacttccacatcttggacccctgatgagaatatttatgccctctgggatgctc
ttaaaatgtacaacgaaacacgaaacgccgtacctcttctcgttctcgttactggtaaagggccaggaaaacccgattttgtaaagcacctgcaa
aagaatcctcttggcaaagttcgattttgtttgccttggtttagttccgaagactatcctcgagccatcgcatgtgcagattttggagtttctcttcac
caaagttctagcggtcatgacttacctatgaaagtggtagacttatttggctgtggtgtccccgtggttgcgtggaattttcctgctatttctgagctg
gttcatgatcacaaaaacggatttattgctaatgattccaacgagctattcaaaaaaattgaggtatgtgaagagaactgagcagtaaattttag
gaaagttacttgtagctgaatcatcttcgttttattcaatgtttctaaccttcataatagttgctttgttctgacgagcatgtacttcaatctgtacgac
aaggtgcttttgaagaatctaagaatcgttgggatgatgaatggaacaaaaagattcctcgtttgttcactatagactcctaatagttctccatcta
ctaatgatacaaaaaagcttgtcccaattcttactatacaaatttatttaatgtaagcttccttgtttatattttcggtgaagttcggtgacttaaacct
acatttaccgagttcgccgaagaacttccaccaaattttcttgaggtgggtaccatcttcgcgccttaattgccttgtcggtacctccaattaccctttt
ccttctttaatcggctactaaggggaatcaagcattgtaaatttacaaattgattaaagattaaagttgttctaaggatgtcggcggacgaaagaa
acattgtcaaagtttttcgagcttggaaaacagctcatcagttggttcatgatagggtaagatactatagaaaaatgccttattatgtagcttgcta
actttagtaaaagggatacggcgtctctcaagcagaacttgatctcacattagaccaattcaaggcaatgcactgtggcatgggccgcaacttag
agtaagtatcttcctgctctcttatatagaaaaggactatgtagtcatttctttaagaagtgatgcttgaattccgataatatcctaacacttgtgttt
agtcgttctactttaagtttttatgccaaacctccggctgattctgacaaaggcaccatttatgttgaatttgcgaaggagccatcggtaggcattaa
ggaaatgcgtactttcgtccatacactgggggatcataatcacaagaccggcattttaatctacgctaattctatgacaccaagcgctgccaaaat
tatctcgactgttactggacaatttacgattgaaacattccaagagagtgacctgattgttaacatcacccatcatgagctcgtaccgaaacacatt
ctacttactccggatgagaaaaaagagcttttagaacgttataagcttagagaaactcagttgcctcgtatccaattagctgatcccgtggcaaga
tatcttggcttgaagcgtggtcaggttgtaaagattgtgcgaaagagtgaaactagtggaagatacaacagttaccgtatatgtgcctaatctaaa
ggattgtgtttaatagattcttaatatctctatttccctcgctatgctaacgttctggtttgagggcaaagaatgtcattaacagggaattcttagtca
ctattttcgttccttgattaaaaaaagaaaaagaaagaaaataagttataaagtcaattgactacaattttttgcttttttcttaaaaacaaaaaag
aaggtattatcgaattttgaccattgtcagaaagaggttattttctatatctatatatatttgaaattttagattaggaggttctgtttatattctctctt
ttgacttcgtctttatgaggtttaaattttaataaaaggttaaatgggtttataatcttactgtgtaagtcgtaatttttcgactgtttctagttcgtgat
catgaagtataaaaatgtacacgcatctatgttggagagcatgagtactaaatgatatattcgattatagtatccaagagagaatttaattggaag
aattaagacatgacttttcataactagcattaagatataggggcattaaaagcagctgttatcttcatttttcatcatcaggaaataaccaaaggg
cgcagtccacacaatttggtaagtgagtgcaagtaggtacctcatcataactatcaatcacatcgttcaagacactttgcatacggtggtgcgtta
aggaaatatgccaacctttatctttggcaacatcgtatatacattcctggcccgtatgacaaacagactccatagcatatccgccagcccaacata
aagaagtgggtccggtgcatttacccaagtaaattggatcagcagtatgaccaatatgccaaataagactttcctcatcaggaagcccaggagg
cataggaagatggagacgacgagcagccatacgttcacctggagcttcgaacgtgacggtaggaataccaaaagataagcccatcaatgaagc
ggtggcacctcctaaagaatgaccagttaaccaaacttgtgcattgggatatagttccttcactttatagtatatatcgagagaagcggagtagta

ccgattgccggattgcacttcgtcctctaagcacgtttgactacaagtgtaggtgcttctgtaacagtcgcacacagttgtccatgcccaacttactc
gcgcacagcagcaagaaaatagcaagttatcattgacacgatccttttgagacgttccccgaccaataccgtatatagaagtacccttcatggca
ataattatagtagaattatcgttattggaaaatacatggccacggagcccatcttcatcccaaccaaatccttctggaggtgttatattccagtccg
gaccaatgtcaagccaatcaccatcaaatggaattttttgatatgcattgaagctcatctttccgagagagtaaatcgttgccttatccgtagtatct
ggtaaaacaattttgcgctccgcccataaatccacatgggattgttcagtatgcaaaccgcgcatggagctttctcgatagtgattcaatgcttcac
gagactgatttttcagagtcattgatttggaattcttcattttcagccgaaaaacatcattgctattttcataaacatctttcttgtgtatatctaaacg
atactgttgattatactgttcgtttaatccatgatgaaatacgtgtcgcaattgcacattaacaaaatcgtctgaaggcagcagtctttgtacccatt
tgtctgaaatccgaaatgagttacaaacttggaaaattatggatatgatagtaatccacagaacaaaagataaatgcggcattgattaccgttttg
aggtaagataagaaaaaaaaagtaaatacgaattctcttttttcttaaattaaaattactatctttaaattatttaactaatggcacaatagctttg
tttgaattcgcatacttggcaaagagagtgttgtgttttcggtaccttttagtaaatagggagtacgctctacaactttaagaatcgttatgtaacttt
tcgtataccattccattatataaaatatttttacaaataaatacagcgcttccctaaagggtatatcaaaacgaaacaacgcttcactagagcggag
ttctactactagtataggttacttcaggatttggaaaatacttcaagaatgggaaaaaaaaaataataaggtaaagtgatgtttacttacattttat
taacaggaatagcccacgcaaggaaagcgtgcttataacgataaaagtgagattgtgctaaagtaagtagtttcatcctatacagtcctatatttt
gtcaaaagcatttaagaccagcctttgaatttcattgagttagaatttttcttaagaattgactgtattaacgatttggtatgtagaaacgaaaaatt
tgagggatattaccgtaaacaaaacatctttggtagcaaaagcgattccgaaaatgagtttcaaacattgatagaattcctaaaaaagcctttac
ctacgacttttcgaatctgcggttatttgcaacatgcttttgaactaaagaagcatttcgaaacacactatatccccgtctccagaatgtcgagttt
gaagggaaaacgattccagctcctcaaattatcccttggtatccaaaccaactggcttatagcattgacgctcaaaaagatattattcgtaagagt
ccacctttcaagcgcctccaacgcttccttgtaagtgaaaacgaagccggaaacatcaatcgtcaggaggctgttagcatgctaccaccgttattt
ttagacttgtctccgcatcatctggttttggatatgtgcgctgctcctggatctaaaactgcccaattaattgaagcaatctacaaccaagcacatgt
gaaagacgccgtccaggacagtgctaatctcagttctcttcaagggcttatcatcgctaatgacgcagatcctaaacgtgctcaaatgcttgtaca
ccaaataaatcgtttgaactccccgaatgtattagtagtaaatcatgatgcgtcaactatgccgaatatattcgtcccttcttcctcgtcgaataaac
tcaacgagaaagaaatcttgaaatatgaccgcgttttggctgatgtaccctgttctggagacggcacatttcgtaaaaatctttctctatggaggga
ttggactgcaactagtggtctgagcctccatccccttcagttgagaatccttatccgtggtcttcagcttttgaaggttggtggttatatggtttattct
acttgttctatgaaccctatcgaaaatgaagcagtagtttccgctgctttgaaggctacaggtgaatctatatctcttgtagacatcacggagaagc
ttccggaactgaagcgcgaatccggtctatttacttggaaggtgatggatgacactcttcaggagtacaatactcctgaagagtgtcattccgaaa
acatcacccttacttctacaatgtggcctttgtcaatggatgaaatgaaggcacaacacattgaacgatggtatattgtttttatagtaaccttctgt
ttctaacataatctagtgcacgtatctatcctcaaaatcaaaacacgggagggttctttatcgctatgttgaaaaaggaaagtcccttacaggcga
gtaaagttgatagtttaaagagagatgaagcaccggaagctaaacgccaaaaactcgatatagaagtttctgtaccatctcccgctcctcatcct
actaccaagactggtaataactattttgatgaagaaccatttgtctatattaagccagatgatgaaagcatttcttctgtttcgtaagtatcgaaaa
catttcaacgaactctattctaacccttaagtaatttttatggtttagatcccaagtttcctcgtgaccaatttttcgttcgtaatcaaagcggtacac
ctacaagatctgtatattttgcatgctccttgttcaagaaaataatagaaaataacatcaaccgtgtcaaatttgttcacggcggagtccgtttttt
gtcaagcaagatatcagccctttactaaaaagtaacccgtcgttggaaattaacaaggatgtttgtaattttcggattcatagtgatggtgttaata
tcattagcccatatctaaacgagaaattcttctgcgatgccaatcttgaggacctaaagatactaatgaagaatgattatcctcgtttagagcagtt
tcccgaaaatgggaaattgaaggccggatccgagaaaatggtttttgggtgtaacatagtcaatgcacatgccaagattgaggatggatcatac
actgatatgcgcattctgctgcctgtctggcgaagcccgaatagctgtaatttaatgcttgccagaaaagaaaaacagtaagtttcctaatttgcat
tacgtctataataagtttctaacaatatactaggaatctagaattggaattgtttggaatagattaactatacaggttttgaataaaataatagtttt
ggttaaaaaaaaaaaaaaaaaaaaaaaaaaaaaggaaacagtaaatttatataactttgtaataaattattggtcagctttacctcggtcgtaaa
ttacgtttttccttgcttattaaaatagaggtattgtcttgagaagaaaactcattaatcaatggaaaggaatcattaaagtttgaagtcagctgag
catatatttttttgtttttctctctgtgcgtccggatcaacgtatgagacactttcttgttcttgcttatttaatgcatcttcactcagatcattgttcaaac
gagcgttagaatcagtggttggttccgttacggacctaattttctttgttggagatccctttaaactcgcatcagtatcattaaaagttgtcttggacc
tgtgcggctgctttgccgaatttctataatgctcaaatataaaattcggcgcaggtgtagactgagcatttggtagaatagcaaacggagcatcat
ctttaaggacggatttctcacttgactgagcgaccttacgtaaatgctcaaaccgtttgagtttttgattttcctttttgtcaatcgcccaaggggata
tgtcctctgtaaatttgccggttcggtatatgtgccatagccatgagattcccaacacgcgaacattccaaccagtggcatgttggattttatatctt
gttttcatgctaaagggttcgtttgtgtttataagcaacacgcttctttgcacgcctaaaaaattatcaacagtagctccgcaaagtaccaatgcctt
ttttaggtgtgagctttctacacctttaaacccagaaaggtgaatacttataccgacaaattcatggttaaaataaggacgataaaatggcttcga
taatacatgctcatcaacagggagtactttttgaaaaacaaacatctttcaacccaccattcagttatggtaggaacaggaaaagtaggaatttc
ttcgattggcagatgatgagggacgacaacaaaattggttgtagcttctaaggtcgacgatgcatgccctccttctctattcaggcactttttcaga
cgattgtatttatcatcctcaaatctatgtagaaagaagtgaagtttgtcaaatagccgaggttctgtattcatttctatagttgtagacagttctttc

tcctcaggtgcctggaaaacttcaggtgtgaattcatctaaaagggaaacattttgagctgtaaatgaattttctaagtcttttgtgttcagctcact
ccagaggatactactttcccttttcttgcctcgtttcataacgactggttctagctctgtatcttcggaaatatgcttatttttgatatgatttaggtcag
aagataagttttcatcgtatatcttttgactgtttttgttttcctccggagccaatttaatgtacgcaccacgtccaatgcgttctggaggcatatccat
ttgaaagtactctggctctaggacagcaccccttttttaaagattcccatagccattgtacacagacaactctaatcttccaccgaagagcatattca
tacttccgaccagcggtactcccagcaattaagtgtgtgacatcgcgagttaaatcaggacaaaatgtacctccgttttcacaaacagtattttcaa
gttgaaatcgctctggctgctctatatttgtaagacatacccgacatttaaaaagagctggcaaagtaaacttgctgatcagtaaatccgactcta
agtcttcaccttcgacccatctatcgtaaagccttgggatccattcttgagagacaattttcatatctggtctagattttgcagcaaactaagtagac
atgtaagcaggaagcacatgggagctatcattataccttatacttgggggtatcaaaatctccagcaattaagtgagttacatcttttgtcaaatca
gatttataagaggcgccaagttttattgctttcgttgaaatttccgtctatttgtaagcgtttagtcttcaacacttcaagcttatcatttacagaggcc
tgtatgtttacttacacgtttatttaattcaatattagtacaacatataacgaaacccttgagcggttttctagagctcattctaccgtttcaaaacat
atgagtacagtagatggcaagctgtcgaaaacttgtaaaaataaatacgtcgttgaactcaaatccacttctaatagtaattttttttcttttataatc
agagttgaagagtttctttcttttcttttttttggtttgtcctaattatttaagtataagtcaaagggcgtcaaggtccaacaaggaagacgactgaga
atggaagtaaagaaaggaaatctatatcggtaatttttttttttttttcttctttaaaaccttctagtagatgaattgagggctacttgtcattatagtat
gcgagagcattttcaatcttgtgaaaattaggtccttaactattaggctcctgatggctcttggggaattgaatcagttccagaaagataaacaaat
agccagctggtttataagagccttactatcgtgattacagcaaaatactacagtcttgtgggagcatccaaagtaccataaaccacagttccatct
tcgttttatacatttatatttactttacaaaccacaataaaagactttatctataacgagttttgatttcattttactactgtttatgcttttactgtttgtt
gttagcatccgctacttctgcagagggacagtgttgcctacaggtaagtgacaacgactacaggcttgacttcttctaataccacgatacagcggc
aaatcttgaaattgggtataaaaggccttaattttcgctggatatatatattttctaatataaactggagggatttaattcaagtcccggataatacg
tcgcaatggacggtaatgtaggaaatgctgatttaggggaagaaagatatcctccaggaggaactgttcatgaagaatctactgagcagacgtc
tccaaatgcatctcggagaaatgatgataccaatgattcattagttgattctaaagagcaagagaatgtgtcggacgatgaagatgaacaagga
caaaaagaagatgccgttcaaggagatcgcaatggcgaaaaaaatgaggaagagaacgacgcagttgatgaggaagcagaagaagacgaa
gaagaagaagatgaggaagaattagaagaagaagaagaagaagaagaagccggcggtggtaggagaaaacgcgcacgtcatgaacg
ccggaatcaattcctcgatattgaagcagaggtagatgaagatgaagaggaattagatgacgaggaagatgaaattggaagagaagatggttt
catcgaggaagaagttggcggagctgactatgtgtccgatgatagacgacaccgagaactcgaccgccaaagacaagagttacaatctgtcgat
gcagaacgtttggcagaggaatatagagaaaaatatggaaggagccaaactgtcattagtgatactagcaatgttcctcaaagacttcttcttcc
ttctgtcaacgaccctaatgtttgggctgttagatgtaaagtaggaaaagaaaaagatattgtttttaccattatgagaaaagctatggatcttcag
tatacgagcatgccattggaaattatctctgcatttcaacgtgattcactagtcggttacgtttacgttgaggctaggaaacaatcgcatgtattagc
tgccttaaatggcattttgaacgtttatacgaataacatgattttggtaccaatcaaagaaatgccggatcttctgaaggttcaaaaacagaccgt
cgagcttcttcctggtgcatatgtacgtatcaagcgtggtaaatatgcgggtgatcttgcacaagtggataacttatctgaaaatggcttaaccgct
cgtgttcgtattgttcctcgtgtcgactactcgaactcactcaagagaagtaaatcaatcactcgtcctcaatctcgactgtttaatgaaagtgagg
cttacaaaagtaatcctacaaaattttatagaaggggacccagaacatttgtttttaacaatgaggaatttgaagatggtttccttgtaaaagatat
tcggatttcttcgcttgtcacagagggtgtaaatccaacccttgacgaagtatcaaaattcagcccgagtaatgaagatttggatcttagctcactt
gcattgagtgttaagaatgatcatgcagaatttcagtttggtgatgtcgttgaagtattcgttggtgaacaagctggcgttggtggtgtcgttgaaa
ctgttcacggaaccgtagtcactattatttcgaatgacgggttaagattagatgtcccttcccgcggtttaagaaaacgttttcgtcatggtgattat
gtgaaggtcattgccgggaagtacaaggatgataccggtatggttgttcgcatctcaaaagatgaggttacattcttgagcgataccatgatgac
agaactaactgtgttttctcgtgaccttggtgaagctggcagcgctcaggctattaactctgcctacgatttacatgaccttgttcaactagacgtaa
atacagtcggttgtgttttctcggttgaaagagacacttatcgtgtcgttgatcaacatggcggagtaaggactgctaaagcatcacaaattacaa
tgcgacactcaaatcgccgtggtgtagctaccgacagaaatggtactgaaattcgtgtaggtgataaggtcaaagaagttggaggtgaaaacaa
agaaggaacaatattgcacatttatcgtgcgtttgttttccttcataatagagagattatggaaaataatggtgtatttgttgccagaagccgtaat
gttgctacagtggctgcaaagggtgctcgtatttcgacagacttgaccaagatgaatcccgcattaaacaacggtccgtccttacctccagttgca
aacattaaacgtactattggtcgtgacaaagctattggtgctactgtacgtatacgtaaaggacctatgaaaggattgttgggagtgattaaggat
accacggatgcaaatgctcgtgtcgaattacatactggcaataaaatagtaccagttcccaaagaaaatttgttgtacactacgaagaatggaga
cttaatcacttataccgagtttattgaacgcagccgtggtattcgtcctggcagtatcggtaacgtggacggtccaagcgttccaagttgggcacag
ggtgcacgtacaccagcttatgccaatggtggtcaaactcaaagttggtattctggtagtaagacgcctgcatggagttctggagccaaaacacc
ggcttggaatgctggaggaaaaaccccagcctggaactctggtaataagactccagcatggaatgctggcagtaaaaccccagcctggaactct
ggaaataaaacccctgcttggaatgctggtgctaagactcctgcttggaattctggtggtaaaactcccgcctggaatgcgggcacaaaaacgcc
ggcttggaatgctggagggaaaacaccatcttggaattcttctgctgcttctaatgttgtaggacaaggtccagcttatgggggcttttctgaaacg
acatgggatactgatgataataatggttcatggacagcacctactcctggtggttgggcagcacccacacctggtggatggaatgaagacgaag

ggaattcccctaactacgtgccccccatcaccgtaaaaaaaaaaatgaaagaaaattcttccgtatactagtcaagaaaaaaaaaaaacaaaaaaa
atggttatatcctaatttaatctatgatttttttggtggctgtttatattggaggataataatttagcaatgattcatgaaatagaaaactttcgaatttt
gttgtgtatattattgattaggtactgcattgaataaaatatggcgtgtaaaatcatttcttattaaataataaagttagtaatcagtcttcttcaacc
gcgtcctgtactttgaccaactgcgatttatttttcggcaatttcctttggtaaaccgctcttaaatttgggttgatcttgaggacgaaaaacagga
cgtgctagttttctaggtatacgagtgacatttgaggatttttgtttagtggaagcctcaatatcttttgtttccataaatttgcgttgctcatcatctg
gcatctcttcatctgatgattcgtctttctggtccactactactttttaccactcaagctaattgaaagaaccttttcttcttcttttcctttgagcga
gtgcctgctgcaccttgactaagtttaaagctttttcagcaggagatgcccaggtgtcacttgctagcattgtcggatcaaagtcagcagcctcatc
aataattttagttcgttgagcagctgtacgatcgaaatttaacaagacatttttcctttgttcagcttcaatagctttctgctccaactgttttgggtca
atgaatatagaatgtatagaggagttattaagtttctgaaactgatgtttggaagacatatgcttagattttgtcttttgatttgaagcttgttttaaa
tcgctccctgctttcttaagttcttgtagaagttccaactgctgcgttttactgatcacaggagtcttgcagaatgtactataagaagattcgttagaa
tgatatttatatagaaacttaccatggtcccatgccttccaacatgcatattatcttaccgcaattgaggcaattgggagcagcttcattgagagga
tgtttacggccttggcaatcgcaagatcttctattcgaatttgaagatctttgagaaatttccacctgatgaatagctctttcgatgtctgctaaaccc
tcaatcttactaacctgaggagctgcttttggtttagaggctttttttagaccccattaggtcagacgtcaaagtgcctggacctgtagacttttggga
aaacttttgtattttatatagttcatcgtctgtttttgtgcttttgtttttgtctccatcagaagatgataaacgtgcttgattagtttgttttcgttggctg
atgttcgaggaagccatagtttctgcagaataagagcttggcctattgctctctagctttttagaaattgcgtctttttgtgaaaatgatgccgcaaa
cctcctccggttaaattcactcacaaactcaatcgtttcttgagattctcctaacagagatatccagtgagttttcgcgtcatcagcattttctgctgc
taatgctgtctgtgccacaagcacagcactttcttcatcaagtggtaaaagcttaaggacattatctttagtccactgctccaaagctgaagaagg
cattatgtatatatatatatgtacctttaaatttttttaataaataaatgttacaaaacggcacttaaaaaggacgtaggaatcgaatcaccagtcaa
aaacgaaactttcaggtgaatgtcctataactctattaccaaaaagaaaccaagttcaaatatttataacactcaatttctatagatcaaaaagct
aagtttatgcgattgaaaggaagcgtttgtttctgatggtgttgaatgtgtagtaaggattcctcataccactattttgcttttcgccaagtgtcttctt
tcctatgagcaaaacgaattcagcagcgaaactaaaacacgtctttgatgacgatatattccatttagtggcttatggagaaaaaaaataatttatt
agttggattctcaaatggtcgtgttgcaaattacagccatgatatcgaaaacaactctttagtccaagaatggagcaccaaacgccataaaatca
gttgtcgaaatttatcactaaccgaagacaactctgaatttatatctgttggttccgactgtgtattaaaattagctgacactgcttctggaagagtc
acctcgaaatggattcaagatacagcaagcacggaaccttccccttacagcgtggttgagtgggtggaaggaaatcatatatttgctacgggtga
cgatgatggatgcgttactatttgggatagaagaaaaaagtggtggagtccttcattcacacagggatcagattgactatatttcttctatatgtcca
ttcgaagatcgctatatggttgctaccagtggtgatggaactttgtcgattctcgatgccagaaacttcaaaacagctgtcatgagtgaagaacaa
gatgatgatttaacctgtggagcctttacccgggataataatcccaagaagaaattcgcggtaggcacagcttccggcgtggtcactttgttcaca
aagggtgattggggcgatcatacggaccgcattatgccacctgtccgttcaaacgactattcggtggaaaccatgaaccgtgctgacttaagtac
cctttatattggtggaagtgatggatgccttagacaattgagcatccttccaaatcattacgaaagagttatcggacagcacagttctaaagcagg
tgttgacactgctgacgtttccgaaaatggcgagtttcttgtttcggccagtggttccgaattgatattttgggatacaaaagatggttctgctgagt
caaacgaaaccgaccaggacagtgaggaagatagtgatgcgatggactcggatgaatctgaagacaatactgatagcgattcagatgaaccta
cacagaaatcaaggaacaacaaaaagcctgccaaggcagccccaaaaagaaaggttcctaaaacagatttttttgatggactttaatttatcgt
gttcgagaaaaactactcttgtgtaaataaagaaatacttgtttaatgatacggttggaacaggaagaaacgttattgttgtacattatacgtacat
atattattttatataaatagatatatctagaagtatttatttaaatatttaacctaccttggtaggtactacaaaccacaaatataaatatgtcggat
aaccacaatactattcaataagaatatgaatcgcatctagctttacttaacaagcaaggtttatgcgattagaaaaacaaaaggaacaagcgat
aaaaggatgctagagagtcctaaacttacggtacgagaagcagaagatgaatctttggtagaaaggattgtaggattatagtcagtagctttgat
tgaagcgacactaccatcccagcagtattgttgaaaacaggtcttacatctttcaggagtagacatatttttacgctccaaagatcgttgaaccac
agcgcatgcaagacaagtagcaaagtcatcgctgttgttttgagtggcagccacacgagcattgttaattacagcttccgtttttttcgttcgataag
tcaaatgtaaaggttgaaatattggagtcgaagtcccaagggtagttgggcatataaacgactagaggaggagtcttgttatcaacatagttttgc
tttaaggtcgtgtttttgccgtcgcagccaaagaaggtagggcgtgtgttcaagcccttagaaacaaatgtattttcgtcaggaacataagggaag
ccacggacatcaatgctcttattgccattaacaccagtaactctttcgtatgtgttaacaagcgatgagcccgttggccagctattatcgtcagcgg
tagaatcaacggcaaagatgacatcgacgaaacgctgaggatgaagcaatggccaaagaggaatattctgattatcttcaccaccatcaacaa
ggtcaacagtcgggtaattcttaaaattattggcaacagtagaattcttagcggtgtagttttggaaaggattagggtagggggcaatatcattttg
attttcagagatatgttgtaaaatgtcttcggcaagatggcctaaggtactgtttgaattcaatctcaacaaagcctcgttgaaaagagtagcaga
acttcccataacaaagccggcatcgtcgaacccacgaacacattgattgtttgggggaacgccattatttaaatgagtaccgacgtattccatggg
aataaaagcgttaataccgttatcccaggagccaaattcataaggggtaaattcaaagatagttgaattttcgggaataatgtgctcatcttctct
acgactgtcggaaatgataatgggataaggatactcggcgttttcgaaccatgactgattacgtatgctcgaccaagttataccagggccacctct
tggggcatcgacgagctttctagctaaagaacgaccccaaagatcagtgatagaagtgtcaaacccggcatcttgcttagcatcaatttcttctcg

aagatgattataatagagacctaactttattgggttcgaatgaggggcgaaaacggaatgttccatgtcccaaacattgtctttcaagtaagtgat
gttggagaagttgttaaccgcgatggaaccgactagccaagagccaccagagagaccggaaatatacatggaactttgcataagcccagtaaa
ctttgaagaattttggctgcggctgtcaaaggcatcgaaaaacccggcaccgttcaacatagcacggaatccaccaccagagaaagcaatacca
aggcgagggccatctttctcggtaataacatcattgacactgatgttaagaccagcattttgaacaaactcacgagttaatttgttgactgaatcaa
tacgcttgttcaagtaagattgctccccatcactaatgcccttagagccatcgcgcaagagatcgttggggcacgttacattgtaaggagcatattt
agaacttgtatcatcacgcttttgcatccgcgtggcttccacatagcgagtggccagttcaacggcctcggggctcaaatcggggacctgttcatat
gaagaatcaagagcacgctccaaaaaaggttcctggggtaaagcagtgataatttgagatacgacaaagactagaatagacgaaaagagcag
gttcatgttcgcaagtaaaacagagagccagttatatcaccaaaaagaaaattagacaattacgacaaagatagatcctctcaattaattgagat
atttcaagacggaataaaaacaattttactgcaacacgattgttagcatttgcatatgacaaattagaagctcagagagaaaaatgtagctggtt
ggatagctgtagctgttgagacaagtggataatagactcatgataccgttcaggaaagaagaagaaaaaaatacaggacatgatccgtctatgc
atactgaaattctgacactcgaaatcagtaactggaaactagtatcaagatgaagggaaatagagaaataaagataattactgtgcaattgata
tataatctttgatcgaatttccgatttctcacaaatcacaaatacatatatacatacgatgaccacaactcatcgtctagcgttcgacaacttgtttc
tccttttgcaagtcttccttcgtaacgtttctccatttcggttgatcctgtttacatttcctcaattttttttctctgagtttctttgcatacctcttgtttttt
atttttacaatctttaatagattgattataccaaaacacccagtttcctatatattacacctacaaataatgcctgaaaacgaaaatcaaacacaa
aataaaaaaggattagaaatggtgtgtaggctctgggttagttctcaatagtgtggaagatttatttctcatgttctgcgtattacgtaacctctctc
tcatttgaattgcatgtttacagagatcgttctcgcttcggaaccttttcaaattccgagttctaattttagtaattgacctcaaaattatgattacctc
tgtgtctaattggatcaacttttgttcaatttatattccttcttccccttccttcatgtctattgcttgagggattttgttttcttttgtaaaatttactccgt
agcacatttctaactgtaaaaaaattataattcgctattatattactgtaacgaacgcattggttttctttcatttagttataaaattgtagaggttttt
atacaaccttttgttttgctgtgattgaagagtactggaataaacaggtaatgttgaattcgctgttcaacttcctagttaattggcctctttcaatct
gtaaaagaatactacgtctgcctcaatcattgattctttctctgctttacaaaaaaaaagcctatgaaactcatactttcttaccatccgttctttcca
ttcaaattacatatatacattacatttgcttcattttattcttttatatttaaaaaaaaaaaacaaacgagttttaacctatctataaacataaacttg
tcacgaacttccatacagaaattgagcccttagaagacaatcaacttttatcaacggaagacgagtaaaaacaactaaaaaaaatgaaagtctg
caaattgacaaaagctctcgccataccttcgtcctcatatgagataactattaaaattttaaataaaagagaatgcttctccgatcaaatgagcga
ttcacaatttcgaacattgaaccataagcttcctagtaacgacaaacattacttgccgttacaaataatgcatccatttttaaaaagtaatagatac
agagtgctttgaaacacatactttgtgttttacctataattatatagataaatcctatcccaaaaatctcaccaagtgcgcttacaaacgaggagta
gggtcatagacgagatcatcgttggcagaggtgtcatcatagataccactccagcagtagtcattataacattgctcgcatgcagggctagcagt
aatgttttgacgttccaaggtacgcttgacaacagcacaagcaacacactgaccaaagtaggtttcattgtccatggtagaagaaatcaaaccat
tcaaaacaaggttgttttgttggtcctcggtatagtaagtacggtcagtggaaatatttgtatagtaattccaaggaacattaggaaggtagataa
tcagaggaggagtatcacgagtgacaggacggtcaccagcagtagtgttacgaccgttacagccaaagaagacggggcggtcattgtaatgaa
ggcttacaaaagattgagtgctgggaacataagggaaacccttaacgttggcgttgcccttcttttcagcatccaaaacacgctcataagtcttaa
ctagagagttaccgttgggccaaacgtattcagagtcagcgctgttgtcaacagcaaagatgacgtcgacatcacgacccttttgcaacaaaggc
cacaagggaaggttttctccggttaaggaagtatccacaatgttcaagttatcatcatcacccaagttgttggcaaggctggtacgagcactagaa
acaccacggaaggggttgggaatgggtgcaacttgttcaccatgctcggaaaagttattcttcaagaattcttgaataatacgaccattcttggta
ctggagtcgttaaagttgcgcatttgagtgttgaaataggtggaagtaagggccatcatgaaaccgaaattgtcgtagttcacgacacaagaacc
attagcactagagccattatccaaggttgtacccaagtactcagtagggataaatgcatcaataccagcatcaaaagtaccaaagacgtcagga
gaagcttccaacataatcgtattgaggttatcaatttgattagaatagtcctggttggtagcaaaagtgatgatgggataggggaagtctccatttc
tataccaatcttggtcagcaatgctagagaaggagacattggcgaaaccataagtagtgtcaccaaaaggacgacccaaaacacgaccccagt
agtcagagagcgagacgttgaagccggcatcttgcttggccttaacttggttcattagggacttgtaaaagttggcattgaagacaatagcactct
tagggtacatgacgggtttagccaattgccagacactattttggagatcatcgacagcagagaagttgttaacagccatagaaccaagagtccag
gcaccaccgtcggtgccaacgatgtaatcagaggcttgaagaagaccagagaaaacagtatgattgttatggcgagcgtccatagcagcgaca
gcaccaccatccaaaagcatggcacggaaaccacctccggacaaagcaataccaatagaaggtccattttgcccggaaaggttatcggcagcc
atattaggtagttgggcattcttaaggaaatccttcaaggcagtgttggtgatggatgaacgggtgctcatatactgagactcagcttgaggaatg
ctaccattggaaagagactcaatgatgttttgggaagggcaatcagcatagaaaggagcgtaactgggttccttgctgccgctactcttccgctttt
gtgcaaaagcaacagagggctcttcgtttgcgggaaacgcaaatgtattaaggcacgaagccaaaaataaggtccaaagactcaatccacgaa
gcaacattgtggatctatggaagatttttgactggttggcaaccttcctgtttttaagtttcagaagaaatttgtaagtggtgggagatgactatata
aaagtgaaaatttcaagggcacgtcacttggtacgagattaccccgaataaacgacgtaaatcaatcagaggccggtgtgaacgattccgaaaa
ggtaacaagttctctcagtaagagaagtaacaggagatgatgaagggatcggagaaggtggaggaaaatacacgaaacactcctatttaataa
taaatccacaaagtactacggaagaaatatgtagtcgaccgtattaagtagctgtgctgtataagaaagaagattggaaagattgtaattgaca

gttgactgacttttaggaagtaaacgatgagatgagagaaaacaaaaaaaagaagagaagagataaaacggaacaaattcagaaaatcctgt
cttatacaaaacaagaagcgcagcgtactttgtaaatagaggattagaaaacgagtgagagccagttgaggaaataagtactacggatgaatg
tgtcgtcaaattacgttaatgctaaacaaaacttgttgaatagatgtgacggcatttttacgaaaatatatgctaaataaacacttttaaatgcata
cacaattagtagaaagaaatagaattgaatcaattgatttcagaagataatttatttttatttgtatcgtattcaggttctgcgctataagttggtgt
gcttatttactgaatgccccacattgatgtttcaagcttgctgtatccatacttttataatgtggagaaactctcgacactaggagacaagggaact
gtgttccactttttgcagacgtccgacccgtcccgtttctcttaaatgtaattggttattccatgtagtggagaaaaagaagccccttgagagcagc
gttcatgcgagaccacaagtcaagaaactggaaaaatagtctgaaaattagttataaaaacctagagtaaaaatgaaaacaaaaccaattcta
tttgggaagtacatgcataataaaaaaatttttttttcgtattttatcatggtgtcacttctttcactagaatacatcttctctttgttgtttcgtatttcc
cgtatgatggactccattggatgttctttagcggagaaagaaaactttcggtatgtctgcgtcaagaaaagtgacagttaatcttatcatcgtcact
acaatttgattggcttaggacatgggtttacccacagtggaggtaccctttccccagaaaacatttcgatctactgatcgttgtccagaagttagga
cattattccgtcgtcgttatttgacggccaatcatatgccaggagccttttgttatgttcccgtacgaagaaggtattatcaatcactacatgaaaag
cgttatcatattagtaacagcgcgatggaatttcgaaatacttattaaggaaactattttccccttcattcgggtacgaaaacctttgctaacattct
tatcatgcaaatcattataccaaagatacgttgagtttttttcgtattcaaagtatgaggtatgaagaaaattaggtaaataaatagttcagtataaa
gaagcatgcaaaattggaagaggaaggatgattgtatactgtcatttcctcaggtccactgaaaaggggagcatggaaaagtacatatatatat
atatatatatgcaaaccctttaattccattcttgttgcagatttcccaaaaatttttttctgtgtttgtgtttttcttttcttttacttcgctcgtgtgtttaga
tgagtaatgttcgatcgataaggaatcatatcaatttcacgttttgtttttccttcagcttgtagtttttttgacgatccttgcgtacgcaatttcaagaa
acatttcaatagcgcagtgattccgagtcactgggatcctcttccatgttccgcgttttcctcttgccatagcgtcgagcaaaaatgaaggtgttggg
ttttgaatcactttttttaacttgtcgataattcagttttggaagaattgtaatgtcaccagagtatttctgcgtaataacagactgcagcttggtaaac
agagtagagcaaatccccatttccgataaaagccgaaacgtaagactcacctcttgcgctgcaagatcaattaccttttttcgtacatcgttccaac
caactatgagaagcggggtccatgataaagggtacgatatgaaagttcacttgcgatacaataaagtgattcacatgaaacaattcggtgagctt
tgagtgtggaatatcattgtcgaccgaaccatccatccacagaggggcatcagttacgcaaaaagccctgacttctcgtgtcaatggatctctttcc
cacagtgttgcgggttgaaataaaaccggcactgaacacgtcgctacaaccgcactccaaatgagaacgttcggagtagttatgtagttcaacaa
actcggcatctcatacgagctcccacaagagactgtaacattcaaaatataaccggatcgattatatgcttcttggaaggtaaagtcgccaatca
aaagcttcatgaccctttccaaatgacaaatgtccaagacacatccctttgtaaaaaagcgttttacacttgtccacaaaggaagcggaggaacg
tttggatctgtaaatacattgagatcacctgtgtgaaactgttttaacagtatcttttgttcttcactgtttcgtacacctgcagcgcatgcaacaatg
gctccggcagaagaaccacaaatgattttgggtatgagaccttgttcatacaaatctttcaaaacacctatatgattcattccaaaagttcctccac
cactcaagattaaagctgtatttcctacagtaaacttcagttttgagaactcaaccagcttttcctccagtgatagcaccggggtctcttccagaaa
ctcgagacagtaaagaacttcctgaataaactcatcaataagtttctttgttccagaatgagcataattgtacaattgactgttatccaggtttcca
aaatctctcgcaactgaattttgtaatactccaaacatgagccgtacgtctccagcaagccgaagtttctttaattcatgaagcctgctttcaacta
acttgtaattatactcatctgattcgggatatttccgccattcgtattttccttctaccatatccaatttttcggcggcctgaagccagtcttcgtatgtg
gaagcatattccatttgaaatttcaattcatctttagacatttacatttatttaacctgatcttgctgtcttcgtagtaatggtcgattatacttttcaat
gagattggtttatagttactttataatgttggagtatgagaggcgacaaatgcataaaatcagtatatacaaacaatcacctcagattagggcga
aataaaaaaaaaaaaaaaaaaactttccaagaaaataatagtagtaggttggactttaccacagaaactgacaactatgtggcactagtaaaca
aaacagctgcttctaatcgatgtagctattaccatagattgaaaaaggaggcggatacctattattctttatttttctttttctttttttattttcttactc
catttctttataggaatcgtgccactttaccgtttggtatgatatcgtattaattgatgaaaattgacggttgcatcaccatagtaaataaaaacaaa
agcgtcgagctttccccacgaaaaaacaattgctttccttagggattcctggatacttggtacattggtaaagcccaaatcattataggtcaatatt
ttggaaataagaagaggcgaaagaaataagaagccctgtgtttttttttaaaaagaattgagttatataaaaaatactatctggtaacttttattta
tgcgctgcgcagacgaaaaacgaacaatttataaagttataaagtgaattaaaaaaaaataatagaaaccacgacagatatttgtactatgtat
tgtatttatacttttcttgccgatgagaatatgtgcacgaaaaaaaatattggtcattgtaagcgtccatagatacatggaggaatagaaaaactg
gtgctgaaaaaaaacatatcaataaaagaaagaatgagccaatattctagaacagcaagggaaaccatatgagcctttcaagaaaaataaga
cgctgaacgaaaaattgtttacaattgaataggcttttcaggaagctgaaagccgttatggaaagcaacttcttgttccatgtcatcagaaatatat
tggattcccatgtcttcagtagaatggattttaatgacattattgaagactaataagcaacaacccaattggaaaaagctatagatgttggatgca
gcaacgataaccataaatactttttgcatattggagctgcaaaccattatgttgtcactactaaagacttcgcatagtatatagggattcatggaaa
agtaaataaggacgcaagatctcaaacacatgctaaaaaaggcatcagcagtagaccacaaaagcaaaagatgagaccaagagattttttttct
aaagaattagcatgtcaattcatcgtctgtttcgtgaaagcatttcttccggtgaacaaaccttgtaagccaaaaaagtaccaatgaatccagaaa
taagagtgccaatgctccaaagtccaacatgcttccatcctcgcatgaggcaatacagcagcgagagattcatgccgacaatggctgcgaacat
aaaaaggcaaggaatcttaacatattcgatagacttaagaaaagtacaaactttagagaaagaaggcattatggttttctgcttttcaaaaaaaa
aagatgcttgttgtttgtaagtattcataatccttctcccataaataaacaaggtcttttgtttttttttgtacacacgtaaacataaacatttgctgca

taacacagccatatactttatgtttgggaaacgcttgcttatgttatgtcatacgtatcgtacaagagagtcaattttagaagattgatactcttttct
gcatgactccaaggcaagaggactctaatttttacttaaaaacccattgactaatgaggtccgtatgggttttggagtattgcctttattttttcatt
ctttttattttatttaatagagggtacgaagaagaataaaaacagagttgtacctgagtacatgtacattcgaacataactagcctttctcagcaac
tataataggaagccagtcatttcatattcaatcttttagtatcagatttcttcaaaatttttttttcttttttttaacactttgtctgctttgcgaattttc
acttttatttttcttttattttctatccttttaattatttactttattcggctatttttgttttcacatcgtcattattcttacgtatgcaatttatcttttaagc
gaagtgtctgaaaacaatagtcgtcatcccacaacttatcctctgccaacatctaatcgtgtttaaaccgtttagacacgacattttagatcatgag
caatggtccaaaaaattacaatgcgcagttgactgctgcagcagcttcaacagcgcttcatcgaccgacgacatacaataatgatgaaaactata
aaaatgtaaactcgtttcttgggaaccgcacatctactttctcaagttctacttattctcctcagtatgactatttacgatcaaaggcaatgaacgta
acgtataatccaagaacaagtggtgcaaccaatctgcgtagtcaaaactcggtctatcttccttcctcccctgttgctagtaattatacaccctcaa
gtactgggaaacgatacactctgacttccgcttctgcaaaatatctcacaaaatctgaaagacagagaaatcctactagttatgcgcacgattta
ctccttcttttccttctcctatagagcgtcaacgtgccccgtctatctccagtaacgtttcggaaactccgagtctcagaacttatgaggaacctaga
tcacaaataaagacacgtcaatacgctcaagaaggccctcatcaacccagtaatgtcgttcaaacaacacaaggaaatgcgacctcaacgaaa
cgcatccctctctccaatcgaaacagctattttggtcctcctatctccactattaattccggaaaagttggtgcgagtagtgtaccgtcttcgccagtt
gatataaaaacttcatactcgagtgcatctcctttacaacgttccactaccttaggtcgtccttcaccagcgataacccagaagtcagctttaaaaa
agcattctcctgcactttcggttgacggacaattgcccgagtctatccctaagaaacacgtaagtatctacgaggataaggaagatgaaaggatg
aataactcatcatccgagtctctttatgaggatgtttctgaagattttgaagctgcgccaatcgaaatgaatacaacacctgctttttatgttccatc
aacaaagcgcatttctgttatcccttctaatggtactcgtagtccttcccgctccgtcaagtctgattctatgcaatttacatctttagaagatgctgc
aaggaaaagggttgaggaaatgctaaaacaaatggacgatactaaagttcatggaatgagtcctacatcaaacaacagtcgtccgttagagac
gattcctgatgataataagtcatttgtttcagaaagcagctttgatcgcgatgaaaacaggggccgtgctcataacctatttgctaaattaaaaccc
aaaagatctagctcaaaggcatcacgtgatttaccttatggtcattttggtgcttcttataattctttggaaggcaatggcggtggccgttatgcaat
gcgtgggtcctacctaggaaaccaacaacgtgaacctatttactctcttcgacagccatctcaatatgataataatcgccaaggaagatcttacac
ttttagtgatgctgcccctatggcagtggataatacaacgtatgacaataggtttgccaacgatagtgacagtatgctggatggtgagcctgttca
aaagcccaagaaaaagggaaataagctgaaagccttaaaaaaactctttaaaatcagattttagaaaaagcgcctagtactgacaggttctaa
agtccatcaatgtgtcttttggtatgtctcactatctttatttgcttggcgggttcgtctatttatcatttactgctgcggaatctttttaatctataattt
attattactgcacagtcattgcactgttgtttatgttatttacctctgctaatcttatatattgactatacgttgtgatgttaatttacatcctttttctttt
tggtggtatttttggtgaataatacacattctatatactaaacactatcaacatagtaaatttcaggaaaaaaagaacggtttgataccactacgat
ggtcacgactggcaaggtcctctaagtattttctcgataaaatttccgtaattgtaggttctctttcttcccagttcattatagtttctttgagtaaaaa
cctgtcggtgtattcttggtaataaccaatctgttccttcaaatcatcattaggattgtagcgaaccggttcgacgtcaacgaaggacccttggcca
tcaactggataaccgttttccatgcttgataaaccatccatcagtgaaactctgcaactctctgcatcactcataatttcaggggagtggacggtta
acttttcatcttctgattcaagctcaatgatttccacggagccaatgttttctttctcttctacgtctgatggcttcgagtttttttaccaggtgttgatga
tctgacttaacacgcggtactttatcagatactgacctaaccggtatacgattaatagcattagtatgagtatcgtttaaattcaaggaatccttatg
cttgtcattggatttcatatacgataaacgagcgcaaccagttgaaaaagaatatctagtatgttttggttcatctttataaacgaccagtgcgtcg
caatttgaatcattgtcttgattatcattatccgaactttctatcgcattttcagaatagttttcacggacagctgatatccatgagactaaaggatcg
taattagaagtttggttgcctgggaattccattgccaaacgagcagcaaacgacgagactcgtttattatttaaattagaagctttgttctctttaaa
tcctttcatacttaattactgaaatgatcaggttgttgcttgcgttacaaagataccacttgctaaatatgctcaacgtaaacatacatacataaac
aaacaaaagtaatgttgatcttgaacaatcaggtaaaactttctttcaggttaactaacataacattgaacaaaatgctaacatatcggctgacaa
tcttttaaatgaaaatgaacgaaaaactaacttttctaattaatgttcaataaaaacgggtaccacttaaaaagtttgtctgcataaataaacagc
gttacatagatgactatcaggactttttttatcggttgatcaaagacagctcataacattcagcattcacaggtattggaggttactcctgaaatcctt
tcagccgtttttttgagtaatttgcattttggaaccctcatgattaggttaaattataataaagaaatcaaaaatcaaacttgctattaattattgctt
tttagaatcttcatctggctcttcttcatcgtctgatcgactatccacagcaccagcgccagagaaaacacgatacgcaactttaacaatcatgcca
aaccatatgtatattaagagctgtaaagaaaaaagcaaaaaaacaaaagcgtaatatatttgttcattaaagatataaccttttgaaaagtccac
attaaatccgcctatgatattaggagcagaggttatcacgactttaagaattctgtagtaaaggtaatgtcttgaatatatccagcttaccataaag
agaccgaataaataatcacagattttaccaaagccgaggtactttatcattttagacccagacagaatatagtctgataaatcgaaaatgtacaa
gatggcgttgccaactcgaagaaagttaaagctatagctcaagaaaattaacgaacatgtgacgatgtggtgtgcaaacatttgccaatggtca
gcacgacgttgttccacatgaaggacatatatctgctgtaaccaaaaaccaaattgagcaaggtaataaaatttaaaaagggtagacatataat
attgaggataatgttcgaaaagttgttcttcgtgatcccaatatccactatttcgataaatgtaaaatcctagcagccaaaagacgaaataataca
agaagcaatatccttgctcacagaatcgaacaacgacttttcgtttccgaataccccaagacaggatgaaaggacggaaacaatagtccattac
aatgcttctaatagcagtgaaaattactaaccaataaaaggaaaaaagaaagtctttaggtcctttaccataaacatctgatgattctttatacga

taaaaacataaaaggttcagcataaggccttgtcggcggtaaaagataaagaaagatgcataatgaaataagagctaaggaaatttctttttgc
cataaaacaagaaacagaggagcttgtaccaattttcttccgtcttttgtaacgaagtttctcgggtcttcaagagagcaactgaaaaaattattg
ctggtccctgtatgagaaatatctttgatagactcatgaccaatgggacttggacgaatacgcttattttcctttcctttaaaggcttttttactcata
gtgaagattcgcacaaactatcaagattcaattaatgccaaataaaagtctgaagcgactttaaactcaaataaatgtgcttattagcaatttcaa
actattaaaattaacaaagaaaagagaaagagaatatgaaacgcgaaaagaaggtaaggaagactgggacgctggtatatcgaagtaacac
aaaacaaatgagaaaacaaagactagaagcaaaaagatgttaataaatgataaatgaacagaaaaatgtgcaaactcgcttgtaaaagtcaa
gctatataaaggtttcaagatatcgatccgcaaccaaagaaacgtcctcgtaaatttccaacaaaaatactggtaataggaagcaatgttttggtt
ccataacttgcaaaagtagcaaaagcggtaatcatgtaatatacgacttttccaattcacgcaaatccactcagctgcaaaccgaacaagctcac
cgtatgtatgaaccttccagaataacatcaatttacctttcaaagcaatctgtttctcgaaagcttctgacgcagatccaagttgaatagtttaaac
gcaaaacagagacattcacaagcgttatgcagccatttctcctacgaaaatgaaatttcttcaattacttaccgaatataacttttaattgcaaca
atcctatagtgttaaaatcgtgcgtgtagacggtagaatgtttagcactatcgatgactacgtggtcatacttcaagagcagaatacgaaagaatg
ttactttcgttcgctactcggtatgttctcatatgaagtgccttgttacttaatattgctacttaaaatttgcaaaattaaaactcggcaggaatacttt
gtttatagttgttttcgaatgtttaaatgacaagaatacttctttcatagtaggaaggaaggcccaagtaaaccaagtatcgggtcaaggcaaatc
actcttcaaattacaaacataccccctggaacactactagttcaatagtagcttctatttcagaattgcaattcaactttaccatactacttggtagga
atagcgctaggtcaattaacactagttactttcatcgatactctcaataccctcaataccctcaatataatagaaggagcatttcagaattaagaa
gtgatttatatgaaatgaataaaaaaatagatgtaaagtacaccacaagacaatatcactgttaataagcgttctcagatacatttaaacggcaa
ttgaacgattttcttaagccacctagtaaaccgtactttttggccttatcattcctattttttttatatttattcatttgtttttaattgaaagcagttcgttt
tgtgtcaaagacaggtttttgtccatgttacttacaacgtcaatcggtctaaacaaagatgctattcctcgtcgtagactaacttggaagaatgtttc
tatatatattttttttttaaaaagaaaatttcggtaatttcagtcatgtgaacaagggtcaactctgagtttactcaagttggtgtctcgagacatagtt
catcaccctatttcatccactggccattcagtgatgtttcaaacttctatatcctaacaaaagtatttgaaaagtattctgcctcttaaaattcaatta
aagcaatacttacatggaaattgattgtgattcataagtagcatgtcactcagaaatccagtatgctgttacaaagaaattactacttgcactggg
aattttgcacaaaaccagaatggcaggttggaagcttccatcttgggcgatgtccaaatcattttggattgctgtatcttcttcagtgactacagctg
ctgttattcttggtacattggagtctcggaagtatcgctcaatccaacacttgcaatcgatgattgtaagcgattcaggcaaaagtattcagctctct
ccctctggtattcctattgattatgaagaaaatactgatgagaatcaaacagaaaaagatgtgaagattccatatgacgaagaattaattcgtga
gcaacttgcccgtaactatgcattctttggtgaagaaggtatggcaaacgttcgtaatgcctttgttatagttgtaggttgtggtggtgttggtagtt
gggcaaccgtaatgttggctcgctcaggtgtacaaaagattcgaattattgatttcgaccaagtttctcttagtagtcttaatcgtcatgctattgct
aatcttgatgatgtgggtacaccaaagaccgtggctttaaaacgcgccattaagcgctttgctccatggattgaaatcgaggcttataatacactg
tataaccctgattccgctgatgaacttctttccggaaatccatcatttgtcatcgatgccattgacaatattcaaacaaaggtggatttattggctta
ctgctattatcacaagatccaagttattgcttctaccggctcagcttgcaaatccgatcctactcgtatgaacattgccgacatttcagctactgccg
aggatccgctgtctcgcgctacccgtcgtagactaaggcttttgggagttatggaaggaatccctgttgccttctcttccgaaaaacccgatcctcg
taaagctaatctgctaccccctatcagaagaggaatttgataaaggagatgttgatcaattaagtgctttacctgaattccgctctcgtatcctccctg
ttattggacctatgcctggtatctttggtcttactcttgctactcatgttttaacacgcatcgccaaatatcccatggatcccatttctactcaggttcg
tcctcgtctttacgaagaagccgtaaagcgtttacatgctgaggcgagaaatgccaatgttcgtttagacaaaacttttaatgcttctgaaatgtcc
tatcttattgaagaagtttactgtggtcgaagtgctctttttccccatgaatcccaaaaagtaagtgtggttagatggaaccctcacgtgcctttga
gcataccaatctcgtagctatgacaagagatgaagcacgtaagcatgaagctactgttttagctcctcagctcgatccctctgccgtctatcctaag
gaagtcatcgacgtcgtcaataagtttttgcatcgtttgcgcatctggaagatgctttattaagctaattgagtacttactttgtatttgtttactatat
ttttttggtatcattaggtagttataggtgcattcactaaattggagaaaatgaacagagggaaatcgattcctctcaatttgggatgtttgattgaa
tataattattatttttcttttgtttcaaatccatttgagtacatttttggagtttttggaaacatgtctatttcgtttattacattgttactttttgctaccta
caaacatagtcttttcaaaatcaataatttttgacgaaacttaacatatagcatcttcagtcttataaatttcctttatgtcgagctcaaggcatcgtg
gttatcaaaatgaaggcttttctgaaagaagatctcgcttttcccgagatttacctgcgaacgaacgtcccagagagtctcggacttaccgtgcag
ataataggtctgtaagcccttctgcttctagaaaacgttattcttatgatgatgaaggtgacagatacacccaaaaaccacctctggatccggtgg
aggcagccgctgctgccgccagacgagttgcagagtcattacaatctggtagacccgccgatgaaggcaaagacattatcaaagaacagaaca
tcgagcgctctgttcctcttcctaatgcacctgctccaccgcttcctcctaactctcaatcaaatctaaatgatttggtaccaccctctatagctgata
ttactgggaaagttatagaaggggagggagtttttacaatcaatgttgagattaataatgttaggaatcgttatatactagttaaatcttctacatt
atccgagattgaggataagagtggagtccagttgtattcaaaaggccgctattatcctaacaaagctctagcttctgatcgagatcctcctttatac
cttcatatagtagccagttcaacgacggacttatcaaaggccctaggtgaaatagaatcttggatacacaaagacattggtcctcttgtagacgag
agaaggtttcgaagacgtgatgaagtggaccggaacccgccttctcaaggtgcatcccctcagggtatctcaagtccaatagaatctcaaggaat
gcgcaaacgatggaatgaagaaaaggtttatatcaatttgacacctactaagggtttccatctccgtcaagcagtggttggtcctcaaggtgctta

tgtaaaacatattcaacaagaaacgcgtactcgagtgcaaataaaaggacaaggttctgcttttattgaaccgtccactaatcgagaatctgacg
aaccattacatttgtgtataatgagccatgatatgaacaatattcaaaaagcaaaatcactatgcgaggatttaattgcgaatgtacatcaacaat
accagacatggaaaagccagtacaaagaatataatcaaggatcgcaagcatataaacagactaatcgtaaccattactctaatcgctcgtccaa
accagaaacacctagcagtcatggaaccccaccaatatccaatgtctcacctgttcctggagttagtaccgctacttcgtcagcaccaattgttcca
cctggcacctcaatgccagcagccatgtatcctcctggtatgtctcctacggttcccatggttccaccaggtatgtcctatgtatatggagctcctgg
attgtctggtcctccaggacttgatgcgcctggcgttggtgtcccaactatgtctgcacccggtgccgatccatatgcagcttttggaggctataatg
cgtatgtccagtactatcaacaacaaatttatgctcaaagtcgtggagccaatccacctcaataaatgaatgtgatcaattggtaattggacaatg
aacgaacgttcatagaaaaaggcatattttacatgaacctgaaaagtacactcgtttttttttgaatttagtgttgatttctatttgcttaaggtctga
gtttatttataataattcagctgattttatgtgtggattaacttattgcgttttttttctaggaaaaaaatgtagtaaaaagtaataggactagtaacgac
aatgataataaattagggatacagaagggaaaggagctctatttttacggtgcattttctttggcttgtgcgcggacacgccgttcgtactctatctt
gtcttttctagattcattaagattagcaactagtaaaaacaaaagtaaaaataaaaaaagaaacaagcaaaaggaattgctatatgaaacatac
ttaaacatggtataagcttctgtttgagctggagaagcgatattggggtcattcaataaatcttgaattcctagcaaaatctaaaaaaaaagaaatt
agtaaccactacacaagtctagaaaaaccgaaagaatatacctgtttaatggtaatagctggcttccaaccttcttcttcattcaaaatagagaga
caaacagtaccagagggataaacatttggatggaacaaaggtggagtaaatcggcctagaatcgtgttaggaagctatttggaatggggagttt
aaaaaaaaaaaaaaaaaaactaaaaccatacactttggaggacgtgttggatattctagagagaaatagttagcagggagaaaaacaataaaa
aaaaacaaggaaaaagtcattcaattatgtttgtcgaatactgaattgctttgatgcttggcaaattttgatggtgaattgcaaggacaggtatta
agactagcactttgaatatggagacttgaagacataccctcagggaaagtcatagtgagtttataaagaccaccttcccaagaagtttttggttta
ccagggattccgaccgtccagttcattaagtcaagaccgccatcgggagatttgctaggctttgcgaaaaatccctgtataaggatgttagaattc
aagtccaaatgagtgaaaagaaaaaaagtttttatcccagataccacttacaaatgggtggtctctcctccattgtttgcgttcttcttgaagtcgag
ttttacaaagagacgacattcttatataagtttttagaatatattcaactgaggacgtgtttatcaacaaggagtaagatttgaatgccaatgctttg
aaagctcgaattttgtgggtagggtggattggaaaaaatatggcaaacctcgtatagaaaatctctgttgtgattcatgaatcctttgaacactac
gggaaaagcaatacaaaaatagatgttttctaatttaaaattaacaaataattacatagattattttactagtatttattttctttctgtgctgtgtta
attctagtttggaaagaaaaagctgccttgctttatgagtaaatacaaagcgcattaccttagtatcgcattcgacccaatatgtattgatcaagct
tgttattttcctctgtttctgcctgcaaagaaaagccctggaatgcgtctttgcaggattggtttgtttcatgctttgtagactgcaaacgaaactagc
tttcgggttgatgagccaaatactgaaaatgaaagtaaaaaaaaagaaagaatctctaaatttcgtatagaaaagcaaagaaatgctattctttt
tcagttaccccattgtttactagtgaatctgaaaaaagggtacgttagtaaacaaatccaacatctatctttgaaagcagaaagcaaataacatat
taatctaatattttgtttctctcgtatgtatacaacgaagaaaagaaaagagaaattaagacatgcaacagaatccatcaagtaccttttcgtttag
gatttccaattgatgctagaaagcgaaatacataagggaacgaaatccgtttccataaatttcccaacctaccttatttttactcttgcgttattttt
ctacaaaagtattataaaatgagcatgcctttgcataaaattgatgcccgactattccaaaaatcagaaaatgcaagctaaataaataaacatgc
taaccatgttttatcaatgtatttcctccaagagtcgcttttacatatattattccttagttttctaattctttcacagtagtcagtatgtccgagtggtt
aaggagttagactcgaattcttcattcgaggcatctaatgggctctgcccgcgcaggttcaaatcctgctgctgacggtaataaatgtgcgcggta
ggagagtggaactccgacgggctcataacccgtaggtcccaggatcgaaacctggccgcgcaagtctttttttttcgtttcagaccactgcatacg
aacgaagatccatcgtatggacggattccttttatctccccatcgtcgctttctttttggttggtttttctgtgtgcgtatgtaattcgatttgtcatttat
gcaaaggtaaagttcgctaaactagagtgaaattgaaaaccagtcattattgtacccataattctacagcgcgcaccttcacaaggcccccagta
tggtggtaacccgctctgacttccatgatgaatttgcctacgatcacaaacagttaaataatttaaaaccatcttccggcaattgattctacaaaac
aaaaccttttcaactacgttatggccacattacttgaactactttttctacaaattcttccctctcctaactgtttgcctgttcttaaccggtacttcatt
gattgtttctaagccttcttgccaacttcggcaacggtgagagctagttcagttcgttagtaattctaagtcaaaataaaaaaaaataaaaaaagcg
agaacggacaaacaaaatactttgaatggatccactccatttttcgtaattttcacataccaggaggaagggattgacgaagcttctcggccttct
tggcatcagcgacaaccaaagtataaagatacttggagcagcgaagcttgaacttaacatccttgttttggttcttcttcacacgggcagaagtcg
catcacctctgtgagcaagctgtaaaaagagcttaatatcagtaacctggcgaggctataacacgattcattagcttttcatccaagttatccaata
aaatttacgtaccattttgtcgacttgcttggtaagttgctgtgcacagaatcaagttgagttattgtgttgtgtcctatacattatgtgactgcttttt
ccatcgagtccaacactttggcattttctaggctggaaatatgcaaattcggttgtttttcggtttaaaaatcttattttatcatagaaaagttaaat
gtgctgctaatcgattttgtccacttggtacgttactcaaatgcacaattcattctgatagacttaggatggtatttacaaatttgcaaaaaatttcat
agaatccatttcattgttcttgaagaattaaaataaaaactacttgtaaagttacataaactacttcgaccaacaaaaacgggaatcgttttcgat
gaattctcgttgatgttaaaatagattgattatgtacaaaacgtaagttatcgaatattaaaacaacaaccaaaagaacaaaaaaaaaaataaaa
gaattagaaattaaaagaaaaaagtgagaaaagaaaatgagtcgatattaggtcctttcgtattatgattcatgtacggtcccgatccggtcccct
agtcatcattgagctttttgcaataaatattacaccaggtcacaggcaaatccttggtgataggtttcaaatgtcttaataccataatagtggaatc
ctataccaataataatagcaatatgccataattgatgactatttcccacgatgtcaaatacgcctggatagagcttctctggcacatgcaaagcat

aaaaaaggactcctataatatagctgaaaatagacagaaaaacaggtttcaaataagaaaatgtttctacggcgcctcggatataacacattgt
aagtaacggtaagaatccgctgcaggctaaaccaataaagaaaaagattttaaaggaacggaacctgtaatcgttaaaccactttttccagggc
atatatattcctatacatccaagtgtcccggtaatggctatataaagcacacgaaataaggtatgacatttaaaagcatggtattcaaccgagata
atagaagcagcaatgagcgcggaaatacccacataatccatgcatgccgcacgtcgcatatgctcatacttggacaaactggaaaatgtatgcc
aggtaaccgaacacgctaagcacttcattgcggatactaaaaagaaaatgcggacgattcgattcgaaaacgaagacgaaatccaagacgtag
atgatggataaaagtatgatataactgtgaaaaatccaatgaatgcgattaaatgggtccagatattgaacgtttcattatggaaggcaaggacg
gagcgaatgcatcgacgcttactggaataaaaccgataacctcgtaaaatgaaggggttgtttttccactccaaaggaatttcatctagctcaag
aagccgtctttcaccagcacggatagctctctgaagtttttctttggtatcttcaaatatttcaccaactgtcgttcgactttcaacactggcgatatc
ttctaaatcagacagcaaagtctccaggaaatttaaagcttctagtactttcgaagggaatgaatcataagcagccaaggcgtcgtgatacttttc
ctcaatcatccgaagaaagtacataacctggtttttccctaaaatggtgctaccaataaatgaattccgtaaccttactaaaatttcataggttgta
tttggttcttctatttttggaagtcctacttcttcaattcgtttcattcgatcccccaggcgagtgagaaatagatcaaatttataaacaacaacatct
tccgggatatcgccctttttattctcaacttcgtatgaagaatgtcgacgaccatgaacccttttccttaatccttctgccctttctgattcacacaatt
gcggttgagccatcttcgacaacgatcaatttgattccacttctcaccgttcttccttcgtttttggctgtcaattttacgtctgtacacttttcaaaag
gatgcaattctgggattctcgtcgacgtttgaattggttctgcgtttggtttggggcaacttaaaatcacatccgtgggaaactactattttgtccatg
aaactgttgcttgttggaccttgttatattcttatgcttaattatctgatataatgataaagaggaacacttaccgcgctaatgtcagcactttaccat
atagaaagataattaccaaatgggatcgactttgtttatacaagtgtaaacaaaccaaagaaggaaacacaaaacgcgaatttgaggttttcat
ggggaagattaatcgataaagatatttaaaacctacatttcagtcttctattttctcttttctttatcctaaatataattcatggcaaaaaggaggaa
ttaaatattatctgagacgacttatttacataatcaaaatacctaaacttaaacatcataaaaatcaaacaaacgagaatagacttaggagaaat
agcattaaatgatttcatacaaacgaataaaaagtgtattcccgggtccttatcagcctgttttaggtttttatcaattcctgggtcgtcttcaggcc
gctttgaattagtttccattccagaaatcgaagttcccttgtttgtttccattgaattttgacgagaaaggctggtttcttgaagagatcgctttgtgcc
agatgatttttgctgatcgaatgaatcactaggcggtacgccaatttttccattcggtcgactttggctttttttccacaggctgtacaactgacctatg
aaccggaacagattttgaatgatctactacggatgactttcgtatagatggactatccaatcgggggttgaaatttgctgaattaggagcagaaac
aactccgtctgcagctgcagcagcacgagcgctaatgaattttgctgaagaagaggttccatttagattcaagccatcgctttttgaaggtatactt
gggacggcagatttcgttctctcgtttataggctgaggaatacttgtcataggatacgtattgtttcgttgaattgttttcgtagtgggcttttgcattt
ctggtttgggtttcgcgtccgactcctcaaaggatttattagcaggagatccagtatcatttaccgctaggtgagcaatcatgtcatcttctacaaat
atattatctaactcctcatcagaatacatttctagatctctgttttcctcccttggtttaacagtagatggatgaaagccttcctcatttttgatgactg
gtctgttgttaagcctgttttgagcatttttctttctgataaattctcctctggaaccagattccgacttacggaataaatcgccgctatcaaactgat
actgtaaaacatcctattagtagctgtactaactttgctttttcatttcacatcacatacagctggaggtttcatttttccaatttctcgtagataatac
ttgtcatacatgcagtttccaagactgtttccgaagtttcgaagggcacgttttaaggcatcggtggttccctctttttgcactaacaatgagcatta
atagaaaacactcttcgaccattaagtctttgtgattctttgcataccttttcaaatgctgacccctttcctctgcaattatctattgatccataaccaa
tgtcctaaatttattagcctttcttcttccaaagcaactagtattttaacggttacggtatgaacgctacctcatgatatgctccatctttaatagttac
tcgaaccacgacgcttattcctaaactcactcgaccgttttctttgttttcgtcaacaaaatcaatatttatacttcttatagaggatgaccatccatt
aaatccaaaaatttcattagcaagctcaattgccttccaagattcgatatagctcactgaaaatccacctggaccagatctccgcgaaacatactc
tgggcccaattttctagtgagtgatgcctgtaagtattggtactcatccgctgaatatggatctccaaatccatttcctttttcttcttctattgtatgtt
gtttttcatgaaaggtcatgtttcttttacactttaagactcctaaatgcagttccaattggtaaacttcttggtaggtttggtgttgagtttagactaa
caccaaaactactgaagcagtttgatagttccaagttatatgtatatatatatttatgtatttatacatttcagtagtctaaagtttctgtaaagatca
taataaacttctattcaaaagtattcactataaaaatattttttagttagtctataagtttccatccttgagtctaatttaagttgctattgttagtatt
ttctctctaataaaacttaaaagctgcaatatatttggaaaaaatgagaaaactcatttccgtcctgcttgctccgctcttcgtcaaggatttaagat
atttgaaacgtaggtacgaacgaccgattgtatatataaattaagattacttggaacaatttcgttcttgtttatgagttttccaagcttggttctcaa
ctaatttttattcaactactatacttccttgtaatgttattgttttatgttatcaaagaaaaaacatgtcattaatattagtgaccataaaggataaat
gaggataaaactatcggatgactgttgaagccaatgcatttatagagaaggaattcataaaacgatcagttaaattgttgaaaccaaaaccatcc
cgaataaacaaaaactgttgaaagagagtgtggggttattaccttacattaatttttgagcatgaagcccgatacaaacaaaacacaaacaggt
gaagaatagaagacaagattagcaacacctttttatattagatggccaccccgttataaagctcaggatggatatcctccaagctttttattttga
aaaaacgtcaaacagagattggccacgttcaaagttattgtcaacaataacacaaagcatccagctagacatcatcgataccaacagcttgtga
gtcgcagagttagggtcatgggctgaagcccaagccataccaaggaaatacgcttcgtccttccaagccaaaaaagaggtagattcgacgatgg
ttggttgaattacctcgcgattaggaaatacaccccacgtaactgcattaggaacttcatatgcggcattggtatccagtttcccatctacagttata
aaaaagtaggaggtatcacccatttggtgaagagaagatttcaattcctcaattaaagaagggtgtacaaaaaactcaacgtaaggcttttggta
tacatatccgttcgtaggaccccatccatgaacaggatcagagcttttcacgccattcaaagcaggctgagaattaattgttaaataagccctttta

ttaatgttcaccaactgactttgaatcattttagcctcatctgagataggcacatcgctccagggtaagctgctttgtttttcatgcaataatccgca
aagagttcaccgagctctttgtaagactttggactgccccaaagagaaagtatttcacctgtcgactgacggaggccatgacgagaaggatcaa
attcaccaaaagctggactacgcgaatcaccccatcgaccatgaggaaactcatcccactgatccgtccttgaaatataactttctaaacgagtcc
tccaaaatataggacgtacatcttcatttgctcgtttcgttccgtcaggaaaaatttctgtacggtttgtcgtcagttgattttctgatttttctgtcacg
tcctttacttcttcatctagcaatccaagtcgctcaatgatcattttcacggctttttccaaattcatagtgtagaaatgaagacgagtaataccgttt
tcgattaatgttcgacaaaactgtatcattaagtcggtgccgcgttcacgtacatcatcatcattatctttgactggcaaaagggtatccataaaat
gttgtggaatttttactccactccatttggctcgtcgaatgaatgaatcccaagcttggatgggcatgatgcctgggaaaatgggaaccgttatgcc
cattttccgcactttcttaacccattcaacgaaattatcggcatcataaaacatttgcgttacaataaaagatgcaccagcatctactttttcttttaa
atgctttaaacttctctctaaatcgtcatctggtgaatacccttcaggataaccagcgacaccaatgcagatttcgtctccataattctttttatata
acgaaccaaatctgatgcatagcgaaagccaccctccacttgggtccattcagtttcatttttgggtggatcccctcttagtgccaaaatattacga
caaccagtctcgtaagctttcttcaacgccgtttcaatcatttcttccgacatatttgtacatgtgaggtgcatacaagtatcaagttgaaattccttt
tgcgcaacatttacaatcacaggagtcaaatctgaagtactcccaccggcaccccaggtcacatcaatgaacattggatggccccatttttcatg
cgatctatacgactgtacaagttttggatacctgtgcttgtttttggaggaaaaaactcatagctccatgtaattctgtctttccagttcggatgttga
agcttgtcacttattttcattcttaccacccaattctgaaggaccttgccctgagtagatagttggctttggttaagttatggaattccaatctcctata
aaccgctgcaggagacggtacttagatgtggactataacgttggattcgttttaaagtactgcgcaacggttcattaacttattaaagaagcttaat
tacggaagtttcagcgtactgtaacaatgtgcgatttagtttttggagagcgcagaaatgtaattaaatagagaaatgcttgcagtgaataataa
aaacaaaaaaatagcacgaatatgaatgagcaaaagctgacaggtaaatagtttcagttctattattaagtaacaaatgcttctatattataaag
aataaaatgaaaatttcaacaatcattccaaagacccattatacagtacagacatatcggcataccaaaaatctaacaaaaaaaagctcacc
ggcgcttatagatgcaagcattaaggattgggggaagagtctgaggttcttgacgcaaaggctgtttaacaaactcaaaaagcgtaaacattta
ttccccaaaacaagggatgtattctcgaagcccaatttaggaagttcttcagcaaagatcttaccacttttgtctgaaaagcggcttttgatctcag
ctacccaaagctgtcctgtattcggcttcaaaacacgatgtgcttcccgcaaaaacgattgccaattcgttcccatcaaactcagacaaaagatag
caatgtcaacactagcatcctccaagggaagatctgagatatcagaagctacaataaacttattcgccgaaacgaggtcaaaactttgaacctgt
atatacttggatgcttcatatgtttcggcaattttagcttctccacatccaagatctaccacactgacgggtcgtcgcttcttgtcgtatctttccttca
aaaagcgaataaaaacgtcaacgggattttccggccagctttcgatctgatgtcgaaatccggcgtggtaaacatcgaacatgtctgggctatctt
ggaaaagtttcacagcctcctcgctttctgtagtatacaatctttcattaatccaacgaaatttagccccgtctaatttccctttcattttcttctgaag
cgccgtcaagggaccttcatttgctagctttttttcaccctcgtgaacaagagatggttgctttttttcatcaccagtcgtttcatttcctttcctttcttg
tttttgctttttcttagacggttcctgacctttgggatccttgttctttaccggcacaggttttgtcttttcaggttgattcctccttttcttgttttccttcg
gctcttgctttttggatggagcacttcctaaatcccagtttacttgaaacatactttatgggtgtagatttctgaagtttgcccaaaaattgagtttgg
tgaagagtagacgggcttgtggctttcatgctgaatcaggcaaggcgtcgaaactgtgtgctttctattcgttgattacatatctaagagattcttat
ttgtaactttttgtcttctgaaaataaatttcattacttggtatggtactacttacagaaattgtaaaaacgatactttaggaaaaaaataacgagg
aattcatttacatatcataaaaaagttgaatttttctaataattgaaatcataaataaagaaaaattcccaaggtgtgcatacgtctatcgtggct
gtttctatcagtcataattaagccattataaaaggaaagaagccgcaacatcaatatctccattagcagcttccaaagcctgagcagcttctaacg
gatcaaagcccatgccagtaagttgagcaatcttcgactcaactgtgggatctcctgatgtgttaacgataggattactagaggaagttgaagctg
ccggaacgcttggagatggtgcagtatcaccttttgtacctaaacgggatccaacagaggcagctgaggcagaagcgactgaaggatcagcctc
aatgccgagtccctgggaagcagcagaaggttccacagatgcttgcatttttggaatttcagattctcctaagaaaggaacctcctgaccatggat
tcgaagaacattattttccaagtcgatgcatgcttggtatctgcgtaacatgtctaaaccaagaagcatatcaacgtcacgaccctcaattacagt
aaaacgacaaggaaggaataagtccccaatcttcaaaggagcactgtgtacggagcccaaaatttttgccatgcccacacccttcgcgacacct
gaaaatctcgtatccaacaatcgagtcaacccacatcgtgaagcacaatcagcagacaaaatggtggcttgagctcctgagtctacgaaagcctt
tactttatgaccattaatttcaacattgacgaagagcatgtatacttgcccaaaaacctctgggtgattttcgatggcgctttgcatattttctgcaat
atttgattgacgaatttgctcttcaattcgtcgttgcacttctacatcaaacaattcatcgtcctagaatgtttctgttagtattacaagcaaagatta
tgttatggcaaccgtacagaaaccgtcggcatggaaaatggagtaggagcagaagcagatgatctgggcatcgaaaggagttgtgaaaaattt
aatgtttgccaagtagatgcaaatcgatcaggatcttctaagacttctgctagttgtggaaattcacttcgtacgcgatttaagagatttggtgtcg
ataaaatcgtttgacgaatttcttcaacagataattgattcattttctataaaaaatagtcttacttaaactttttttgggtagtggaagaggtggca
gaagactgggatgctgcaacggtgcaggctcttattaatcgcttgcacatgtgaaccgacatcaacaaaggaatcaccaagtaagggaggtaaa
aagagataagaatccattttttgttgagcatacaattcaaagatacagatccaagtcgtcgtggcagcatgtattccatacatcaacattgaaagcc
ataatttaggactctgtgtgcctttccagaggttacaagaggcccataagaaatagggaagctgaataaatgcttcagaagtgaataaagcattc
tgccatggagcatgatttttcagatttggatcatcatatttgtccaaataaaagcgtcgaacagcagaaagagagggatatttgtcttgcacccac
tcgaagactggaagatctacaaagattgcagcaaaaaagtgtgtcagaaaataaaccaggtaaaagatatccattaagtttcttgtagtcggtg

agtttcaaagccagacgcaatccaagaaacaaacaattggtatcgcttgcaaagtttttcccattttttttactttttgtcttgcctttaagaaataac
atcttagccatttctctatgctttagcaaacaagattgccagacaagcaactccaactataggcttgagttacattggattggaggttctttagcca
actgtttttcaaaaaaaaattttcctgaccgtaaacgaattcctcgggatttcatgaaacgaatcctctgtggttatgttttgctaatcctgtcagcgc
tggaatggatatgtgtctaaaaacacaaacgtttactttttggaacgaacattgcgaagaaaatttcgatttctatcaaacgaattgaacctattcc
ctactactatccttttgtgatatgtaatactttatggaatcgtaaaccaacaatccagtgataattttcacatcaggtgaagggttcaaaagatcag
atccatcaaattgaattaccattttgtacttgtctttacatcaatcgctgaaatggtatttcgatagaacgatcaaattgtttgtgtttatgaatggaa
atttggaaaaggtaaagttctaataatgtatgaacaacgattatgatgagcctgaaaagttgcttatctagaactatttggcaactagaaaagctt
cgtagtgtttgtttactgacactctttaaaacctttgtcgaagaccaaggcgtatcagcaagcttataaaaaaacaactaacttctgaaaaggatct
ttgaacagtaattcaacctcaaactttataatttgctgtttcttgtttacgagcattcaaagttgagatattcacagacacccaaaagggttgtcaag
tttcaaatgggtccaaaacttcatcctttaaaagggtttattttttaatctatctcagaatatgagctcggtaccgcatcaattgaaggtgtacactt
gtataacataaagaaatacctcttgtgtaccttttgaaacaaaatctaacaaagatactgctacaatcagaaaaaaattgaatgaattcttgaaaa
aattcaaaaccttagtgactctctcgaaactccattgtaatccataatctagctacaggaaattgacttggaattctcattactgactttcttttcgttt
ctgggaaaaatcaatgatatctttattaatttagttctttcttccatcttaattttcctaaacttaatatgagcataaaaaaaaggccgtctggtaaaat
cattatatggagccggtacaggttcaaatatcgccgtattgtggatcctgacaagcctcctatcatttttcttggtaaaccgttatgtcaatattcctt
atatggatgaaatatttcatgtttctcaaacccaaagatattgcaatcaggattgggaatgggatccggccatcactactcctccaggtttatactt
actgtcttatttttgaaacctgttctagggtgtcatttggttgctcttcgctctctaaattttgtcttgggggtcgtttgtttgcccgcagttttgtttgac
atcaacaaacaattgagcggtaaggctttcagttctccacttgttctttcttctttgcctccactatggtttactttcttactttattatactgatattgct
agtactgtaatagttctgctggcttatgattttgcactaagaaacgccgccatcctcagttctgctttttgcttcttcagtttgttctttcgacaaacaa
acattctttgggttggttatatataatggtcactttaattacgaacgacttgtcgttttatgatcctgtattgtctgaagcgacctttgtggattttttttg
acgattacttcatttattggaagtgttttaagaaatttaggaaaaaatatggaaaaaaactacttccatttgttttcgtctttgctttttcaatgggtttttt
agtatggaatggaggtattgttttaggggacaaatcaaatcataatgcggttatccatctttcccagatgaattactttactacatttttttttcttttt
tcttttccttcttgcttttctccattgttcaaaagtgcatcgtggaaacctaaaatacattctgttatattctcgcgcacaactctgctacttcttccctt
attgctattaattgtaaaatataatacgtaagttctttttatgaattatatctaacaaaactagaatcatacacccgtttctcctcgctgataatcgac
attatgtgttctacatatttaatcgaattcgcagatattggtggttaaaattccttggtgcacctatatacgcattttgttacattttcttcatcaaaaa
tatttctcaatacgtatctgtcttggattttactattataactctagctactttgctcactttagtgccggctcccttggtcgaatttcgttacttcattct
ccctttcttattttggcgaatgaaagtcccgtggaaatcaaaaaatgcttggttagcagaatttttctttcatatgtctcttaatactatcagcgtgta
tatatttatatatcgcccatttttctggcattctgagccaggaagtttacaacgatttatgtggtaaattacgacagtctttttttttttttctcttacaa
gacgaaacctttcattagatgggttgaatgaaatgagtatggatttaggaaatgtctcagtatatcgcttaaacgatattatagaaaacacagtag
gtaaatgctactacaagaacaaatttgcgaaaaactgaatacatcaaggaaaaaaggattgagtatacaccccttaaacaattattagtatgatc
accaatttaaaccgttgtacttgctacgtataatgtttaatatctatctttaataatgaaactgcattccgaagaagattgtaatacaaaggtgaaa
gctcttcttccctttcataaatcttaactcaataaggacttatcattacaatatctcttacttttactaaaaaataaaaaaataaaaaaggttatcttaa
ggcggttaaacatttcttttcctgtttaaaaaatgctcatctactacttagcaaacactcacccagtcgggtttttgtctttctcgcatatcaaggaaa
ttccgctcatttgtttttttatttttatattcgtctttttttgttccattttagcaaagttgtccatttggaattagtgaaaataatatttattctgggaaag
attgttgcgcaaacgattagaagtaaaggcctttgtatttcacaaagcttcatccttaccaatttagtttattttttcaatcttttagaagaacaggaa
tatgttgaaaacagccccaagaattcatcatcctttcattcctacacaatgccaatggactaatattttgttggttaacaagcataatacattctctg
tctctctgcggcagacctttgggtatagctctttaagtgagtcgaagcaaaacaaaactcaacctataccagctgttaaaaggtcttggttttcacg
tttacctgaaagatggactgcatatgctcacttgtcgcgtgcttacaatccgactggaacattcttgctttacagcccatgtacgtggtctattttaat
gggcgcttattccattaatagcagccttttcgatgtatcaaaaatgttggcgctttttggagttggctcctttttaatgcgaagtgctggatgcgtggt
gaatgatctttgggaccgaaaattggatgctaaagtagagcgcagcaaatctagaccgttagccagtaggttggttagtgttcgtgagggcgtat
cagttttaggagttcagttaactgcaagtcttggtatacttttacagctcaatccttatacaatcaaattgggagttgcatctcttcttcctgtttgcat
atatcctgccatgaagcgaataacttattatccacaagtcgtactaggcttaacatttggatacggtgctgtcatgggatggccggctttagtaggc
gaagcttgtatgaattggtcagttgttgctccccttttatttaagcgcagtatcctggattgtcttatacgatactatttatgctcatcaagacaaacgg
gacgatgtgaaagctaatgtttattctatggctcttcgctatggtgacaataccaaaccagtcctgactggcctagctctttttcagctgagcatgct
tacaactgctgggatttgcaacagtcaaggacctttattctatactattggtgttgcaggagctgcttaccgcatattttccatgatatataaagtaa
atttggactctccaaaagattgcttcagctggtttaaaaagaactctaatacaggctttctagtaactgctgctattgccttggactggctcgctaaa
gcttatacctcctgaaagcacactgtaatttcattaagaatttgttcatttgagcaggttacgagtacataaatgtaaggcataataaatccttagc
aagtaaagcctcgaactgaaatacttgaggattattcctttataacattttgtaacaagaatagaaaactttattaaaattgcgaagtaatatttca
gtacgctttggctaattaattgctaattcaattactcttgtaattaaatttccgcaacatccaacataaattcatatttaatccaaaacctacataag

cagattaaataataccaaaaaccttttttaagttgaatgatcaactgccattcatctctgtgaacctcattgcggaaggcaggatccttcaacgcatt
aatactttcacgagtgatgtgtgaatacaaggtatcactttccgtaacatgacgaccgaacaagatctgtgaaccaccaccagcttctagtttcat
ggcaacaccaccaccagcttggcccttcttaaccctttcaacgggtttgtggttcatttcaagactgactacacgtcccaattcaattatttgaggtt
cctctccgggaacatttttgacagacacaataggcgtacctttacgcaaaacaccatcgacaacatcaacaccgagaatgataggatcccgctta
ttgaaagcagcaatggtcttcaaaacacaagggaaaaccgcaacatcagagttttcctctcgcttctgttccataattttgttttgatgggcagtga
aagcatcgaaaagatgatagattacgttagcggagaaaacctttatacctaactgatcagctaaggcttcggcatcatgatccaccttaacatcg
aagcaaagaagcaaggcatattccttggctttttctaacatggttgcacatttcatcacatcctttttgtaaacaggtccaatgttgacagaagcta
cgggaattttcatttgtttcaaaaattcaagcaaagcctccaaagaacccaaagtagaagcctgaaccgaaacaccaactcccgaagtgtctata
cttcccaataaatcttcaagatctgccataagttcatcagctaattcttcttcatcatcttcagggccaacaacgagtaaacgagagccagcgaca
gccttctcgaggtcattggcacaaacctttacacccattgcagctctaatttccttatgatgaacatatgccgatttcacacgcatctctttcataggt
tgtggcgttaacagagcacgaacagttgtaataatagggccacccataccacagataacaatccgatcaccttctcgcaacacaccgttcgaaa
gaataacgtcgatagtagcacccagaccttcaacaacttttacttccaatactgtacattcaagggtcgaaatatacttgatgcgatcagacatac
gagtttgcgttaatgcaaccaaaagggccactaaatcaggaacaccttctcctgattgagcagaagtaggaacaagagaaacataacgacccat
ttgtttattttcgaaatataatgcagcattgagcccttgctcattgagctgcaaaataatgctgtcgactcgatctctaaattcgcgttggatggcttt
cttttgctttaataagctgtcttggaatccattttccttaatcgaatgccagccgtaaagacgatcaaccttgttcaaagcgaccacaaaaggtgttt
tattgtcacgaagaagacggatggattccattgtttgaggctccaaaccatgcataatatcaataacaaggatggcaatattacacagagaagta
ccacgcgaacgaagattggtgaaagattcgtgtccaggcgtatcaataatcaacaaaccgggtatattgaactctaattttcccttcttattaacg
ggtttagtcttttgcttaatcgattcaatagggaagtaggtagcaccaatttgttgagtgatacctccagcttctccttcttgtacatggctacgacgc
aaattatccaaaagtttggtcttaccggtatcgacgtgtccgagaatacagcaaataggagaacgaagattgtttacaggttcagattcaacggg
agtagcttcaggaaattctgagctgtcggtttgatgcttttcttctttaacctcctcatgggcaggctctggctctggcttttgctcaggttcaggttca
ggttctgattgttccaaagcggcctcccagtcgtcaaaaacggcatcatcctccttcttttcttctttaatctcttcaacaggtttagattcttgagga
ggagcaggagactctacgccggagacgggagcagaagtagcaggagagggggattccattataccagaagaagataaagaagaggatccac
tcttgctagatttcttcttattagtgtaaacaggtttttgtttctttgctttttctgaaagaccagagacttgaacgcctgactccaacatttgttgcaa
gcggcgttgagcttgggcttgttgctccttttgctttttcgaaacgtactttccttgagccttaagttcttccttcttcttcctatctttttctttcttctttttc
tttagcctcttcctttcggcgctcttcctcttcagccaagcgcttttcttcctctgctaaccgctcttcctcctctatttgtctacgttcttcttcttctttag
ccttcttttcttccagcatcttctgaagagcagcaacattaggacctttacgtttaccttgtttgcccttggcagacgcatagtcagattcagaaaca
gtctcttcttcaacctcgggcgttgataccccttctctctcttgatttgtggaaggctgcttcttaccctgttgtttcttcttttgcttctcacgttctttctt
ttcacgttccttttctttttttagactttacatggacagtggaagcaggagcagcagcaggtatttcttcctcttcaacttgctgcttcttattttttttccc
tttcttgggcttttcgtaagcaaattcatcatcgtctaattcagtaacagctgtcagttcaacaggaccgctaggagcttccttttcgtcaactatttct
tcttcatcaagtggcgtttcttgatccgccttcttgcccttctttttcttcgacttcttatctgaagaagtgtccaaaccttcgacttctttcaattcatct
gtttcgttggcttcctcaacattttgaggtataaactcattttggccgcttatatcttctcctaaatcatcttcccagttggcataagaagctttttttcc
tttttttacccattttaccacctttcacaacctcaatgcagagtggttctggtttactaaattccgaattctttttcattgtttattgagatgaaccatttc
actcagcctcagtaataagtatacggtgcggtgtactcagataccctatagtgctttttacgataaatatacctttcgtaatttactaaactgttatat
ctactatatttcactacaccacttagtaattggtataaaaacaagtcagtcttctctctgatatgcttataaaatgcgactgtcaataagcttaaaga
aaaatgtaattaagatagtatagaatgaagtaagaaaaatgaaacgaaacttcggagccatgggcttcttctgtaaaatactatgctatatgcaa
cgtaaatgatagaaaaaagagtgaaagaaaaaggacgtacatagataccattaatggtgtcgataaaaaaatgtgtataaatatacattacatgc
aaaacttaaaaaaaaaccaaaacctcaaaagacgttaaaacgtaatcataaacgcgattttgcagtacagaaaaatgcaactttcgaccaacg
cataagaatcttttaaaaactcaaatgagtacaataagtatcatcaaaaaactcgttgagttgccgtacttctcgaactaatggaaggtgttgcac
tagtatttgaactaaaataagggatgtatgtttcttgtgacttatcagcagaacccctaatataaaatatttgtgttgtgcgtaatcttctagtaatcc
atgaagcgagaatcttacctcgttgtttctcacgcagacgagatcgagggaagtttcctcgagatgtaaaagcgacagtgtcaacgatgatttggt
gctcatccaaaatactattgataatcaccggaacaattgcagataaatagtcataccctattacttcaacaacaatgaccacatttcctccagattg
aaagacggcagaaccacctcgcgttatacggggatgagacctttcaattgtatcttcaatatcaggagggaagtggcttaatccgttaacctcaa
atgtctctcctacgggacccaaaacgtataatagctgcatatcaacaatcgggttaccaggtcccattgattgggatgtaatttggaggaatccca
aatctccggtacgcacgtattgcccattaccaatgaattcattgttacaagaagcattaaacctcatcatatcgactggatcactggattggaaaa
aggaaatggcattggcactcgaacgtacccaaatttcgccatattcacctagacggcatagctccctagtatctggattaacaatggcaacttgag
tagatattggaaccattcccgagtccaaaagaggtaaggagtacgttttattttcagagtcacaaggctgtactaacccatgacgcagcgcacga
agatcaacacagtatttagtaggagtagctcccatataagaccttgtagtaacaaaggaattaaggcagccgctgtataaaatactaaaagcaa
acggacttacgccatatctcataaagaacttttgcaattcagaaatgtaaaaagcattgactcgtccgtcatgaggtatcattaaattttctaagca

agatagatttggccatttggtgggttgcatattctgaacataatttagtgtttgaaacgtagcatacgtatctttgattttatacttggagattatttg
gaaaagcaaaagcggattattcgcaaaatccaaaggggacaacaagtatgtagttgttcctagaaatacacccatgagacaggtgtgcaaaaa
gcctataccactcatgctccgtacatgacctagtacaggccttttgtgcgtcatttggcatgtttccttctgaatttggcatgaagagagcaacgtttc
atgattataagccgttaaagttattttgtgatctggagaccagtacgccgataccaatgcaggaaaagcagggtccaagcatacaggatccaacg
ttacgcctaattctttaatactcttcgtttgtttgctcaacttagaagtgactataaacgaaggaagaaccactttagcagccatagctgactgcttc
aggtgttgcgctgcattcttacctcggattgccatatcagcttccgaatttagcaaaatagcactgacataataccgctttatgagaaacaagaac
gccggaacgtcttcagagagccgagtattatccaaaggaggaacaggtataggaatcaaacctaagtaaaagcaagcgtataaagcataaac
gtaatcttcagaatgcgtatacattagtacgacaactcttccagtctttaggtacttcttcttttgcaaatatcgaatcacggttgcaactctttgatc
gaattttctccaagttatgtttttccctcacgtccacgtccatctatagttacatatgcgatatcatctgcattttcttttacacgaagctgaagtatgt
gcaatatacttctgtatgatgatatattgaccaatgtttttccatccaactgaattgaacgctcgatgtaaccactatattgtagttcttgatcaaaa
aggaaattttgtcgaatattactagcatagtttgaccagataccatcaatggcatcttcacctactggaagattaaggacggcacgttcaactgca
aactttacaaaaacaaatggaagtgtaccatgctcaaaagcccttcggcacatcatatttccgatttcctgtctaccgttcttagtcactcttggtaa
cgtgaatggagcgcaaatcaacacacaatagacacgaacctgatgatattccaataggcattcgatacattctgtggttatttgattcagcatatc
atagtttagtaaaaactgagtaccacctgcttcagctggcacgcttgctgctggactctccagtaaaacaacgggcaaatgctcactgtttacgtat
atgtcaaaagttgagcaatcaaaaactttagttactgtacgcatgatagtagtaaccaaatgagatgtataatgatgaaaaaatatagtgtcctg
cttcccgttatcgacccattcaactttttgttgaaggcgatcctcatataaacctaatacatagattttacctttatggataaatcctagcaacccagt
tcgcaaaaattcctgattcgaagcaattggttcaaaagtatcagaagatataaagtaagtttttgcatgaaatatagcttcagtttgtctaggtaaa
ccgaagaacccaccagacaatgaaggagaatctacccaaatctcaccaactacattaggtatgcataatacttgagtctcaggatcgacaattgc
taaggtagcatcaacaaaaggataccagaatgctccaagtcgaatagtactagggtgtttcgattgacgacgagctttctcatcttcagccaaaa
caacgacttcattaagcttcaacgattctttatccaacagcacttcagcaatttcattttccggagtacgaggaacttggaatcctctaggagacat
aaactcttctccacctacccaatccttcatagagataaccataccaccatgttctgggagacatagaagaggtaccaatgttgcacgagaattatg
gttacctaatggttttaaccaccgatcggatacaatttcctggaactcacaatcgacgttaaggcaatcaaccatacacatcctcaatgctgaaag
atcgactggctgctttttactaaatccgagagtagccttcggatcgttttggtaattataagctactgccttcaaaccagcgtagtcagtcaccatgt
agtttgcacgatatcgtgtagcgatatttgcccataaaccaggcacaaaaacagcactttgtgggcaccatacagttgtgtaacccgaaaacacc
gtatgcaaaacaccgagaattaagccaatagcttgacgtaaatctaagtaagtcaaaaataaaccagccttactaacaccttcaaaatattcacg
atccacggataaataatccaatttatcagaatcgtaggaaggagcagtagaaagagtggccgcaaaacagttcatttgatgtaaaatagtccga
tgagatatgactacaccatgtagttcgccaagtggagacctagaaaattcaatataagcgagatctggtacctgtaagggcggtaagtcaccttt
ctttttaaagtggaacccaccaaactcatttgttttccaccactctacgtttctcggccaatgtaactttttagcattaagatcccgttgaaagctcttc
aggttagcatcagtggtcaaagctaatcgagtgccggtagtcgcgaagatggatgttagctcgttgaaatcatcgaagtggtttactggaactgct
actacaccggcaataaaacacccaaaaagagaaacaatgaaatcgatagcttcactatcacgatacataagggcaactcggtcgcttctaaata
aaccactcttgtcgcgtatgacttgagcaaccttttccgctcgggaagcaatcttttcccatgtgctagacgcaaactctttccccttactatccaag
attacaaaggccgtttttttagcactatggactccacgatagcgaagaacagcagcgatattcgcaaactgcttcatttcaattttcggattcaggg
gatcagtaacaggaaaaggaatttcacggggttccaaaggatgctgttgtttaccagccaaatgtgcccattgagcatcagcgaaagaatcatcc
atttctagtcgactcccaaagtttttaccatcaaacccgtaggcatctgcatgatacttggaattctccattaagtcatctgccatcattatattatcg
tctttagaaggataggatggtaaataacccgaattttcaatagaaggatactgaaaatcctgggaattcattgtcgaatacgaaggacgggcatc
atactcgtttccagaagcaaaattcgaattgtaatctatataatcattatagccattgttcccatactcccttgcggtgttttccgcataggatttact
agcattgctgtatgtcctttcaaaatctccggcatctcccggcgatgaattcatatcataaagaaatccttaaagttaataaatatacatgcaggac
aatgttatttcggcttttgagaattcagtaacgctgccaatttttggtgtttgctggcgattggaacggtccttcataaacgtgcataattattcttcc
ctcatatagtaatggtattacctaaacataaaagttagtaatacagtattccaaattcgcagaattcctatttacagcgtatgtcttcatattttata
aacatttttaacaaatatagaacattatattggcttattttcgagacaacttgtttcaatcccaacgaggtttaggagaaagaagtacaatatcga
tgtgattataatatgaacgctagaaccgaggtttcaatcgttttacaaaggtttgcgtctaggttttactggctctattttatgatatttggtattgaat
tacatgattcaaataaatcaaccgaaggtttccttcatcattaaatccttgttttgtttctcttaattttgcgaatatattgtttcgacaaagatatagg
taatcgtgcttccttctcgcttgcaaaaggatttatttccttaaaagctaaataaatacaaagcgagagaacttagtttacgcagttatgctaaaca
acgcaaaaaaaacatacggatgcttcaaaattgcacaccaggtcgttatttattttcacttgatggcaaacgtgcaatgtactaagagccaagtg
cgaattgctcctacttattccagccttttgcaatgaaggtacaggacgattttattttaactattgatgactcagaggatgatattcaatatgatgac
aacgatgtcgatgctgcagaagagggagacaaagttccagtaaagacacctaaaaagaaatcgaaaaagcagaaaggaaaggaggaaagt
gaatttagcaacgattttatgtttgaaacggacgtaaatcaaggtctcgttacagaggaagattcgaattgggattttgacctttccgcaaaatctg
gttaccgtgatgattcgaatgtagtaaatttggacgcgattatcgagcgtaatcgcatcaaggatgtctcaagtgaagatgctgaaagggaacaa

aactcaacagcaaaggaagatcctgaggaagaagatattaccctgcattcagatgacgaagatttagccgtcgatggttttggtactggagcga
aaacattggaggcttctgatcaggaagaggaggaaaacaacgacgatgatgatgatgacattaccgaacctgttccctcatttacacccttacag
aacggtgtagaaggattaaacgaagagtcatctgcttcaagtgacgaggaagacgaagaggaggttgctaaaaagaatgcttatttctccgaag
gagacaaagacagcgcagatataaaaatgcaatcgtcttttcaatctatgaatctatctcggcctattctgaagggtctttccaaaatgggttttga
acagcctacccaaattcaatcaaaaacaattccagttgctcttcttggaaaagacattgttggtgctgccatcactggtagtggtaagactgctgc
attcattgtccctattttagagcgtttactctatcgtcctaaaaaaatacctacttccagagtattggtcttggttcccactcgtgagcttggtattcag
tgtcacagcgtagctacaaagattgctgcttttactgatatcgtttcatgcttatgtgtcggtggtttgtccttaaagcagcaagaacaagaacttcg
caagcgacctgatatcattattgctacccccggtcgttttattgatcatatgcgaaattcgcaaggctttacggttgaacatattgaaattatggtta
tggacgaagccgaccgaatgcttgatgatggtttcgctgatgaattaaatgaaattattaacagctgtcctaaatctcggcaaacaatgctgttctc
cgctacaatgactgataaagttgacgatttaattcgtctttctttaaatcgaccagtccgtctcttcgtagatgccaaaaaatcaactgctaagctac
tcactcaggaatttattcgtgttcgtcctcaacgtgaagtccttcgtccagcaatgcttatttacttgtgtaagacactttttcataaaagaaccatta
tttttttccgaagcaaggcctttgcacataaaatgaggattatctttggacttttatccttaagcgctaccgaaatccatggttctttgtctcaggaac
aacgtattcgtgccttggaggacttccgggatggtaaatgtaattatctattggccaccgatgttgcttctcgtggtattgatattaagggaatcgag
gttgttattaattatgaagctccttcatcgcatgaaatttatcttcatcgtgttggacgtacggctcgtgctggtctaagcgggcgggcagtaactct
ggcaggcgaaggtgatcggaaagttattaaagatgtattcaaaacttccgcagcccaaaatacaaaggtggttagtcgaaatcttgagttcgata
aggtagaatcatacgggaaacaaattaaagacttggaagacgtagtcaataaagttttgaatgaagagaagcaagagagagaggtatgatttt
atgatgaatttctttttcttttttttacaagatattaataccgttagattaaaattgctgaacgtgatttgaaaaagggagagaacattattaagcat
agtgatgaaattcgctccagacccgctcgtacttggttccaaagtgaaaaggaaaagaaggcttctaaaggtatgttttataaaataatgtcttttt
gctgacacttattagctcaaacttctgaaaccctgataaaacgtctctagctaagaggaaaaaacagcttgaaaaagaggaaatgccccgttc
ctataagaaaactaaaaaggatagaatgacaaaaactcgagctgcagggaagaaataaagaatggaatatgcattggtattaggtttaaaata
ggttttgggtagtaacagttgttaattattcttttatgtttttaaattgcttagacaattctgtaaataaaaaatctataactttatttctctatcctagt
tacccatgtcattcaaatatttaataaaaattcatttttagcgttttcttctgctgcgaggaactgcaatcttccttccagcaggaggattcggctgct
cattagttgatcctaaattaaattgtataccgctttgagtttgaggggctggagactgagtagtacttgaaccagcactaaaagcaggtgtactgg
ctccactaaatgagaatcctgcattgggcggattggacggctgagatgcaccgaaactaaaagcaggcgttgtattaccaagattggcggtaaat
ccactaggactagatgctccaaaaccagaaggattcgtgttatttgaagctccaaaggtaaatcctgctgaaggcggttggcctgtgttactaaaa
gtatttgctgtacttgtagaaccactaggatccgcatttggcttggaagagaaaggagaattggtacttgcaaaagttgacgatccaaagatacc
agtaggttgggaacttggttgtgagaaaccacttccaaaaccagatggggcagaagcgttggcggtgtctggctgttgggtacttgatgtacccg
ataatccgaatcctggtgcaccagtaggaccgttggctgagaactcagaactaggcgccttcatattcccgctagtagccgtgttgaatggggaa
gagccgaaaataggagcaggaggattagtattggacccaaatgtaaatggcgctgttgtttgagcggtatcggattctgtaggtttagaaaaaga
aaaaggcttatcagtggaagtaaaactcaatgggtttttcttctcctggtcatttaattctatttttccagtaggacctttgttctcatcgtttgctgtc
aaggtgtttgaagtagaagcatttataccagtggaaaaagcagttggtttttccgttttgttgaatgtatttgtagctccactagatccaaaagaaa
atacaggagtagctggtttcacatccgcgggtctttcgttttgacgatcagtattatctttcacattgacatcagagggtttagagaatgaaaacat
agaagtagtgggtttttcatttttagctacttcatccttcgattgggaggttccaaaactaaaggtgggcttggtagcagtcgacgttcctaagctcg
tgggctgaccaaaagtgaaagtaggcttagcagcattattatcggcagcgggaattgtcgattccttattttcattcttggcgaatgattgtccaaa
gctaaatgaaggtttagccggagcaatctgctcatcacctttaagagtctgtccaaatgaaaaagtcggtttcgagacttcctcctttttgtcttcgg
tagtctttgaagcttcagtaacctctctttcagcattttggccgaatgtgaacttcggggtttctgtattagctgagggtacaatattggttgatggtt
cagtagcaatgttagcactcttttgtcctgtactgggttttccgaaagtaaacgtcggttgaggtgattgcgatgtggcggttgtagcagcttgatca
aaagaaaacgctggtttcaaggcagtagattccttgttctcggacgaccgaccaaaattgaagtttaacttgggaacgttcaagttgtttttgtcac
taggtgtcgaatcagcaacttccactttttgttcttcggatttcttgataggagctaactggccaaaattaaatgaaggtttagtaggaatttctgat
atcttgcgatcagtaggttgaccaaacgtaaacgatggagtaataggtttagacggctgttgctgagaaggaagaccaaaggtgaaagaaggct
tactactattttcgggttctttttcttcgagtttttttgccgaaattgaacgaaagcgcgggagcctgctcgtctttatttggtgaagcaggcttgaagtt
gaaagcaggtttcggagcctcagttttctctacggaaaggtctttttcagaaggctgtccaaaagtaaaaggtttttgagtggctgcaggtttttca
gttgtaaaagttgtttgatttgaagtctcattggctttatcggatgatttaccaaaagaaaagataggtgttttattctgattctccgtaccttcggttt
cagcaatacttttaggagcactagtagtagacttaaactcgaattttggaatattgtcggacaaactttctggaagtttttcggtatctttcttttcgg
gtatcgctgcaaaactctttgatgtgcttgtggtttgagattcagccggagtgaatgtagtgtcaattgtagattcagctgtcttcgctgggatatctt
ccttctcttgggttttaggataactgctcggtacgattgctgactcttctgtaccagaacggttacgctttggtgacaatgaggactgaagttgttca
aaggtaggacctgagcgaggaagcaaagttttaggagcctttttatcaggacccgaaatgagtggctcatgatgctttgtcatagaacgggaagc
agcatcggtcatagcggtgtcttcatctttatcaaaaagctcatctaaatcttcatctaatgcggaaaaggcagctctcacacgtttttttgttgcccc

ctgcggcaggtgggaaagaatattcatattcagagacgaagtcctgtttgtttacggaactagaatttccaggttgctcttcggtttggacttcagc
attcaccagtttctgtttctttaaatcaatgcgttcaagagtctcgtaaacaataggaatcaactcttgacgtaattttccttcctagcttcctttgaa
gctgaagtcacacttttcaatcctggctcttccctacggatctttttgggatgtaactcgatactactggtacttttagggtgctgcgacttctgatca
gattggaaatgttcatctgaagaatagctgggagctatttgaccatctgtccttcctgtatcagtctttctagtttctgaaatgggtctctgggggag
atccctctttgtcgaagaacggcgtcctatacgctgtgaaggcacattctttttgctagattgactagtggataattgagatacaggagtaatatga
ggtaagctacccgcattggggtttgttcgaatggagcgcaaaatgtcgagcaaggccgcagaagaggaagttaatggtggggtctgaagatgag
gctcagaagaaagctgttggtatttttctggcggtaagccggattgcatgctacctctgcgggaagcggttttattttctacaaaatcctgggaaaa
ttgcttagaagacaaatcatcttcatttgactttccttcaattccatctagtgtgcgaatgtgactacggttatttatgcgaacaatatgtctatatga
aattagcaaaatgttctgaataaaagaatcctccattctttattactgtacttacggagttcgtcccaagtagtgaggactcaattctggatccaat
agacttcttgaagctctcttatgattaggcgtttctttttcagcattgctcgaccaactattgctgaatggtcgcaaaacagaagcgactgtagcctt
caaagccgaaaaaaagggtcggtgatttcccgattcatgtcggtcataaggagttctttgtaatttcttagtcgcacgttgatttcttgaagacgga
ggcattaaaataagcacatcgacgatgagaaaggaactgtggttaaggcgaatttgtcaagcatttcctcgtggcaagtggttcttaagccgaat
agtgagctctaaggaataagtaactttccgttaactaaggatattgtacgtgtaacatttcgttatatgtttaaaataagtaactaatgctactaatg
cgaggaggtaaaagtacgagttggtgtatacctttagacattgggttttctgtatgaaagataatcctaagcagggactctttaaagcgatgagca
aaacaagtcagctagtatatgtttctaagaatgactttgtacatggagggattacgatgccaattatgatcgactgaatctaatgaaaaaaattca
ttcatatactatacatgtagataaaacagtgtatatatatatatattatttatgcaaagaaatagagactttcaggcacgcgcatttctgcttttc
ggttagtaaagctcaaaaatagagttgtcataaccgaaacaagaccacatgtattttccacctaggtaagcttgtctttactggtaccaacaagta
gtgaagggcaggcagtctcttggacatgatttacgcaatttttatcttcaataataaagggaaacctagactgacaaagttttatactccaattgaa
gaatcggatcaacaaaggttacttggcgaaatttatcattcagtatccaaccgtacggctgcatcgtgcaattttcttgagagcagcttgattgctg
gaaaaaacaggattatttatcgtcaatatgccactttatattttgtgtttgtggtggatgaagcagaaagcgaacttgggattttagatctcattcag
gtttgtttcccttccccttaattcggtaaagctaacaatcaggtttttgtggaagctttagaccgctgctttaataacgtctgtgaactagatttggta
agtctcgagtgcatcgttccaataagaacggattccaaagtccttgcctctttctgttgaccgaatcatgatattttttttggtccttttaaaatattttt
tgattaatgctgtatcataattgaatgagctttaacagattctgactcattcgacttcaagtggataaagacgtccatcagaagatcatcaaaccg
ggtctatgacgattcgcagaaagagaaaagaagatgtagagaaatgctttatattatacaggaatcataaaaaacgaactattactaactcgttc
atttccaggtgtttaagtttcaggaaattcatgcaatccttgctgaaatcgtttccggtggattggtcttagaaaccaatttgaacgaaatagttgcg
gcttgtaaaaaccaaatgccaaaaacaaagcgacctgggggactaaacttcggtaatacactcagttctttcgctacgagattctaatgaacctg
taatcatgtttctgaacgatttcttcttataggagcggcttctgaaatagtaggaattggaagtactggatagttataatcaccaaaaggcatactc
tttgcctgagtataaagcggttctatctcgtcatctatttcaaaatatattcctccttcataatgctgagattcgctgtacaaaccggggatagagaa
gtaagttttacttgacgctggggagttgttgttgttaattaaacgtacgcgtgattgatcgtatcgggaaacccagtatttgccctggccaccttgac
tgtaacgcaaaccgcggattgttcgatttaatcgtgcatcatgtcttgatgggacttgacattcagcaggttcaaaattctcatagacataccgatc
gccctgtaacgtttgaatgctgtcgcaccaaacgtctttgaacccagcaacatctttccaggcttttataatcggcatccttgaacacgatatccatg
atggcgttatataatctttaatacgaaaggcatgccaatgagataacgcaaagaaaggcatttctaaacacataaggacattctggtctaataag
ataaatatcccatgggtctttcgcaaaatgccaagccagttggctacggacaaaagggtttgttgccaataggaggcgaaaataatagcttttac
agaaagaaatttcgggacgggtcgaaacggagccaactcctcgtgcaagcaaacccaaaatgttgttaaagaatagaaagataaagtaatcga
aatattgtacaacaacccaatccataaagcagctgtaacaaagaccggctttccctcttgttcatatactcctaaagctttgctaagaagtgtagcc
ataatgagaaatggtttaagccatgtgtactgtaagatacctcgtttaatggaaagaaatgtgtaaggatcgcttaagtcaatatcactttgaagg
tgattcaatggccataaacgaggacgaggtaaatgtccatgcagaatgatcacagcagctctttcgccgccaaggtagtcaataagcaaacaaa
agaaacaataaagtacaaatgcctataaggacatgttagatgtgattctcaaaaaattgttcatatatagcaagaaaatagattacacaggcttc
ggaacaaatcaaaaagccctcttgtttttgacacccatttgtagactcaaggatgcagtttatcaatcataactcttacctcgtagatttccctgaaa
ggttcaaaataggctccaattctttcattatatacagccaaaaaagagacgatagaataagtaacaatcctgtgaataaagattagcaaaacatt
catgaacgaacaatttccaaagaaaaaaataaaatgaaaaaagaaaacgaaaaaggagaaaaacgaacattaacaaaatccttactacaga
gcgttgtaaaacaggctttctataatttttaaatgagaagcgattgaaataaacgataaaaccagggaaatagaaacaaaaaagccgcataaa
gcgacaatttcgttttccatggacaagtgcgtgaaaaatagatgtcaaaagtgacaattataaataaaagtttcgtccttgattgcttttctcttttt
aaacttccagatatttttgctttccctcagctacatgcaatttcagttactacttaaaactcttgggtatagttcctgtatacgtcttttcaaccttcaa
ttcttttgaattgctcttgctagttcaactcatcgattcgcctgaaagtaacatcgtaacttgagaaatactgagtgtcaacaagcatccaaaggta
gaaatggaagaatttttctcatcaaataacgaagaaaactcaatttgactcattctttgaagaattccttttcatatttcaaaccctttcttgttttgc
gagcgaattgagcaataccgaatacatgaatcccatattgtttactagttgtcttgtccctttgggagaagatgatgggagaatgctttagcaaca
agaaagaaggtttcaaattcacataaatcgtattcaatcgtgctcttcatgatctaggttattcagaatacctagcctatagaaagttttgttactac

tacttttactactggagctatctggtcttttggagttcaacctaacattcccaaattacttcctctcgtcttgctattatttcaagaatttcctttttttttt
tctttttgcatgggcgtacatttgaaaccaatagccaggttccagtaatcaacaaatgggttatattctctttcttttagcagccttcaaccaaaga
cactatgcctgagtggttaaagggatagactagaaatctattgcggtctccgcgcgcaggttcaaatcctgctggtgtcgtttttttctctccttcca
tatcgaaaggaaaacccaaaatttacaaggattcaagaatggtgataataacagctgacttcctaaaggcggtcgactttgaaagtggtttctat
cagtgatttcaaactaccctgttcattcatttttactttctttatttgtttttttacgagcacgtttttccttaatgcagtaaaaatttttgcaaattctac
tgttctactctaactatttccttctataagctggtttctgtataatgggtttcaaggggaatggaaagcttaaaaagctctcaaacgatgaatgaaa
atttcccagataactgggagactgtttacaatgagattcgagagatgaaaaaaaaatgtagttgctcccgtggacgtacaaggttcacatacact
aggtgaaaaaacagaccctaaaatctttcgatttcaaactcttgtagctttgatgctgtcgagtcaaacgaaggatacagtccttggaccaaccat
gaagaacttgaaggaaaatttgccgcaaggactatgtctacgggcaataagagaaattgacgaggattccttaaacaagcttatcgaaaaagtt
ggattccataaccgtaaaacaatttatttaaaacaaatggctcaaattctggaagagaaatatgatggtgatatccctaaaacggttgaagaatt
gatgtctatacctggtgtaggcccaaagatgggatatctttgcatgggtattgcttgggacaaaattgtcggcattggcgtcgacgtacatgtacac
cggatttcaaatatcttgggctggtgccacacaaaaacagaagagcagactcgattggctctgcaaagttttctgccaaaacatctatggcatga
agtgaattatatgctcgttggttttgggcaaactatttgtcttccaagaggaaggcgctgtgatatttgcacactcgcttccaagggactatgtccaa
gtgcatatcaggaaaagagtctggtgagtgcaaagcgctcgaaaactacgaaaaaggttaagaaacgttctcctacagcttccccagaggttca
ggtaactcaagagcagaagacggaatacgacgtaatccctactggtctgaatgaacttccttctgatgctgtcccggaaaagaaagttaaacttg
agtcttctgtaggtgacgaaagtcagaaagaaagccttaaggaacaaaattacacagaaaatgcagatcaacctttacgacggtatcgaagaa
cggctgctgtggcctctgaatatttttctcgccagcatcttcaagatatagaagacttgttttaaaattactatttttctgtcatttttttcctctaaact
gttatttaattatgattgattcatctcttgtttattatccgttctctcgtttttttaaaaaaaaaaaagaaaaaaacaattttcctattcacaaaactttt
attgctttgtatttctatttagtttctttaaattagattctataaaattcatctaatagtcatttcattcattttgcaaagcaattccattattttttttttgg
atttgtatgcgatgcaatcgtcccagaatatcacaatgcaaataatgattaaagtacagcagagtcgttgaaaacataatgagtacagaaagatt
catctaattcgtctgtatttggcgtgtacaacataaaatcgataggtattggtccagttacagaaacatcattacgaatggaaggtttttgtctatcg
aatgctcgatccaattcgtaagagacaccagtggcatcccggagaacacgcccctttcgtaccagccgattcagcgttctgattatatctgaattat
ttaaatgtttatcaccataattgctatttaatcgttagtaattcctgatgaaacaagagcttgagcaacaaacctttgaatataactaattatagaat
caagagaagcaacggctggaaacttctctaatacttcaagcatcacctcgtcggcgttactgtgagtttgcatatactcatcgatagatggaacat
gctctttttctgagaattgaaatatatgatcttgagaaacaaccgaatcagtgacagaatccaagttaaagcttgaggacgttagtccttgaaggt
ttgcttgagaaacctcatgggcatcggatgaaatatcaccaaatcctggttgggacataacttccgaagatacaacaggagtttttttcttgcttttt
attttttataaatgagactgcattcggacttaaagtcggtaattctggatgagcttgttcgcacataagtagtatgtcaaccaaatgtgaggttggt
agactaaacaaataagacctttttttcttccagggacaagctcaattcttttgcagtagaccctacaattaaaggcttttgatgttttcgttgcgagca
agcgttacaaaaccattcgccattaggagaatctaaaagtgaatcaggtatatttggttcatgacagagctgatgaaatggggcattgcaaccat
cacaaaaaacgatgcgatttgtaggtggtgattgtaagcgctgacatatacaacatttcatcgtcaaagcaatagtaggccgccgaccaggacc
acgttttctggttggtaatttcaccaaaggatcgaaatgattaggtcgatgaactttacgtcccgacttcgttttcacaccttccatattaaattgttc
ttctttaccttcttctcttgaaaagattgcatcagcggtattatcacgttctaaagataatcagttagaaaaaaaaatactgtatacgtcctaaattta
cttaccaattgctaaacttgcaggccaagcttttttttctggcaaaattcatggcaatggacattggaggaagaggtgacgctccaggaggagcca
aaggattatgccttttgctatgagactcgttactttttggagaaccactctccggatgaaacggacttgtcgaagagggaatccttgtcttgcctttc
agcccatcgtcattttcggttaacagcaactcatccttagagccattattagataaggagccaaaatcttgtgcttttcctgttgactggaattgact
gaattcagtttccatagggttagccgcgttcacatttgttttcggtatatcgtgaaatggttggtcaattaaagcagaaccacccgcgtcgttacgtc
caggatcatcctcatgaaaaaatcgcttgatgtaaggaattctgaaatcgacgatcatcatcgaatggaaaaggatatggtccagaattcagtgat
ccgccgtcatcttcataatacggtgctccagacattagttttatacaaggcaataatttagcacaatttaataaagtacatccactgaaatagaac
acttacgaagattctacaaattattgccttgttacgattgtattataaggaatctggaagactagtgaattgcgagtggaaacgtagtctgtaactt
tagaataaagtagtatatcatgactcgctaacatgcttggtggtgaggcttctatgtgactttacaatatcatacacataaatcatactaagagtttt
tttgatttcttttcttttattcgcttgctgtacattttcaaactgtttttttttgttttgtctttttttctttacttttgagttaaagaacccctttttttttgagagg
attcctgcctgagggattcataaagcatgattcttctcaacgccttactcaaagacagaaaaggcttgataaaggatagttatggatccaatccct
ttacaatgagctccttttgtctttgccactaacaaaaaaacattaggatgctgtctatcaccctttgttggccttttatttacttagaaatgccaacgc
aatttcgttaagccctgaaatggattcgacaaccaaaccgatataaaccggctgccttcgctgccaaattggcgaattcgtaaagaaatattgga
ctaaatgtttccaaatgaacatttagataggaagaaggatgagagaggagcatcttctcggtatgatggacatgatcgtgaatatcgccgtagac
ggcgtcatgaacaggatgaacgtggcttgagaagaagagaacgacgtgaacggtcacatagtcctcaaaggaagaggtcttatgagggtcaag
aatcttataaacgaaatgcggagcaagagttttcgcggaaaaatgattattttcgttctcgttttactggagctacagatcaggatacctcacaaa
ggaacgattctaggcttggaaatttggagggaatggactttggagtgtatggaacacgtatgtcgaaagaacctgcttatgaagaacctaaaca

aactcctgggcggtttatcaaagctactcaatcagatcgtccttctggggacacaactagggaggtgatagatgaacagcctgcaaaaagtcagc
gtttagagtacataattggtgacaatggttcacgctggcgaatgatgaagcttcggcgtgttttcgacatggcaaaggatagccatcgacctgttg
aagatgtggctcttgaaaggtatggttctttagaagagtttgatgatgctttagaagaaaaagaagagttagatcatcgaaaagcaacgggtaa
aataaaaaaagatgcaccgacaggcgaaaagtatcggaatcgcttagagaatgagcgaaagagtaaccccttaccaccagaatcttcttcttct
ttttcaaagcctactcaggatgatttgaataaagtaaaagcgcaggttttacgagcgcaggctaaaaactcttcagactatgaagctttgaaaca
gcaatatgataccatgtataaaaggtttgaggataccacagacggaaatgatcagtcccgcttggaaccatttttaaataaaggcgacccctcta
tcgaagaaatggttcaagaggagaagcttttgaacaaattggacaagcatggattgaataaggactatgcaaaacgtattaccagagatagca
gattctcgaatgatttcgattatctcgatgaaaacgcaaacagattagccgatcgtatgaatcgaagagaaattgatatgaaccatgtttttcaag
gaggaaatttatcaaaaatgaaccgcgtactcgattcttgcccgttgtgtatgagtattaattcacagccgctagcacctgtcattgcactaagcca
tcgtgtgtatctttctttaccaacgaaacctgaactagcaaagtatcattgcttaattgttcctactaatcaccgtgtaaacatgctctcctgtgatga
agatgaatgggatgaggttagggtatgtctatatgtttgttcttgaaaacatttgcggaaattaaccttgaaatagaattttatgaaatgcatcgct
ctaatgtttgattctatggggatgggtgttattttctacgaaaacgctccttcacctcaaagatttctacatactgcaatagaatgcgtacctgtacct
aaacgcacactctctttggcaccagcttattttcgtgaagctattttaacttctgatgacgaatggtcgcagcatcgcaaaatcattgatacaatga
aaggtgctgaaaaatatggaaaattggctttccgaaaaatgatggttaaaggtgaggaaacatgagaattattatttgaattcctctggatgctcg
aacaagactaaccatttttagagttgggctattttcatgtttggttcacggtagacgggggttttggacatgttgtcgaaaatgaaaaaatatgggg
tcaacatgatcaagttccaaggcaagtattcgcaagtatgcttaaccttccaccggaagttatacgaagaagaggcaaatggactggcataagg
gatccagctgaagatgctttccgagctcgttttcgtccattcgattggactcaaagcctattggaatgatttcttacttaagactttttatttattttta
acatagtaatgttgtatactagttgttaagaaaaaaggaccattggaagatagcaccagggaataatgttgaaataaaaaaaaagctttcttatc
tcaagaaaccttaaaagttttacagtacacaaacattgttcatatcatatcataaaaatgcacgttaaaattcaagtttacctgtaaaagtaaagc
ctttcgataaaagcaactatatgcatttaccgagtattatagcagtctaaagcaagcacatctacaacagcaatagtgtatgaaaatacttactga
aaaacaatctagagcattcaatatataatcaacatgttattagatactacctcaaatcgtatcagtgctttttttcagtaagctctcttctatgttgatt
cttttatgtgcaatcatcactctccagaatcttgttgaaaaccccagtgctgaaaaaatccttccagcaactgttcattatgctaaatagtaagtgtg
atcttgaatgaaaagttactacgctttcctctcgaaactatagccaattttgtctttgctactttgttcaagaaactcgagtgcgagaatgagaggat
gagaggaattggtaaagtataccagtttgttattgttttgatgatgaattttgatcgtatttgcttggatttcttgaacactggattactaacgtttga
gaattagcaaaccagccagattttatcatgccttccacaataaacggcagaattatgctcaaatcaaatttgacttggatgccgatttttcaaatct
atggacttggaacaccaagcacattgtcgtctatttggtagcctcatacccaacggaaaaacatgtatgttgctcaagtgcatctacccggaattt
gcattgctaatcttgcaggatgtgaataatattgttgtctgggatcgcattctgtcaacttcagaagaatcccatttgcaacttgagaatacgctttc
aaatttacatgctcatccatttaacgagtatagcaatgattttagtaacttgaaagcgaactatacaatgcattggacagtgtctcctaaactagga
tttttgacttggggctccggaactccgtctctggctgtaccttttacgaaggtttaagagtttccaatatatcttaagcaaatacgttcattttactgtt
ctttttcaatggtacatccacttcagaatgcgtacgcaggggacgagtttaaaagcgagcgaattcaagaaacttacagctatgaggaattgata
agaaaaccgagttcaaatcaaaggagttatttatttgactattgcagtttacaacatcctgaagtcatcgtacctttgcagatagaattaagaccta
gaaaagtaaatttgcgtcagcagttggtaatgtttgctaaaactatttaaatcgaataagtaaaagatggttttatattgatcgttcgttttagaga
actaaacagaaagtataatatactctttttatctttgggcaaaaggaagcaaggcacgaaatcatcacaaaggaagacaaaagtagacaatac
aagaacttttgataggtttattaaaggttttgcttagaaaaacaagagaatacgctgtgatgaatgctactcaactagaaacctttgaacgcttttt
ctatcaagtgtatggaaatggtacagttggcaatggaacgcattgctatttgtggagacaacataactggccaagcataacagaagaaggaacc
gttttggatggagtgaattgcgatagtccagtgaagaaattggcagggcatggggctgttgggatcgtgtccgcatgcttgggtttcctgttaatcc
catttcttttaatgaacataggaaaatttaatgtccccggagcacaaaagggtttccagagacgagccgaattctactggatgattttattgatcct
ggtgttggcggtctcagggtttgcatacatagatgtggatcgaaatccggttcaaggtttgtcgatgtgcatattctcattttgctttcactcgaatgt
accgattctagtgttggtgtattggcactatgtgagtacgtttggatttatacgctggcgagagctgacggtagtcggacgagaagcagatggcag
tgaacgaagaaaaaggatagagacaatggaatacggaatgccaattgtgatgtatatactgtcgatgctgggattcttactggccgggtttcatt
cttgggcaagaatagcgcggggagaggcagcagtcgcgacggacgcacgatttaaggcatcagcggtgatcaatttatttggattaggatgctg
tgtgggagcattttgtgtatatttgaaagagtatggacgtgaccgaagaaaacgatggatgagcgtttatatgggaggcctgatggtgctcctacg
aggtgtgtatggatgtatttccagctggaaatacaactggaatgcgagcaatccaaaagtgaacccagggatagcattcggacttggatatgtgc
cggaacttattgtgggaacagtgatttgtttgtatggagtattatcgccaggaatcgctgaagtggagcgacttgcagtggaggaagaagagcaa
gagagaattacacaaacaacgatggatcgtatgcaagtagcatcgacggattcgacctggggaagacggcttcctgcggcggcgcccgcggcc
gcggcctcgacggtatcaatgccacctcctccgtatcaagaagtattcgcaaatgatagtgaggaagctgaaaaagaagagcaaatggaggtg
acgattaatagaatgaactagaggataattgcataaacaagaacgttcatttctccataaaagcagcaaagcagactagtgaatctacattattt
tattttccaaataacaaagaagaaattgtaacaagaaatttaaaagaaagaatacataatggcaaacctagtattgaaacccaatcgatgtaaa

catattttcatggtagaacattggaaatgaagaaggaagaaaaaacagaaaacagaaaacagaaacaatccaacctctcagtgaaacaatgt
agctagaagggactattttttgttttatttctatttgtttattcttcgataaattgagaataaagatgtacacgtgaaaacgaaaacgaaaaatgtc
agaccctaagtgtgggaaagtagacgacaaacgcaaggaggcgcaatgcatatggtcatagttgaaaaggtggacccgagaaagattcattcg
caaggtttgtcattcaggggaaatgagtttttaatttgaggagatacttcgcaaaatctttggcacggaaactcaaaagtttttgcaaaagtaaac
aaaagatgaaaacaaaaatgaagagttgggtttcatttttactttttttttcatcttttttttattttttttctttttgtttttctttttttttttcgtttacag
atcattttcctatcgcaaaactccatcaacagttattttttcatttttgttttcatcttttgttggttggtttgtttactttccgtgtaatctttttccctctcc
atctcgtatctccaacgagtgctctttatctcgcgaaaatacttccttcggatctccgctttttccacctgattacgtcgctagtcagatatgacataa
aaattctactcttatccctgtttcattccgcacaagcatctctctctgccgatcttctttcttctcttctatttatcccattttataaaaagatctctcttcc
ccaccacactactccatccaccgaccgctgttccgatcttccatcatcagcaaaccatatctttcctttaccttttttgttatcaacgctgcaataagc
accgccaccaatacaagagtcatagtaaagtacctcccatccgcaattaaaaatcaaagagaaaaagctccttgattattttttgttgtcgtcgttc
ttattttcgcggggttgttttcttttctatcccaattcatttaattttgcctttattacttttaggttctcttgttttgtagtcatttcttgtcttggttctttat
cccatctttcattatttttttttttaagatccctctatacatttctttgacttgctcttttatgttgttctttcgtcagtattgatcaagcgacatataacgat
ttctctctcttctcttcttttttttttttttgtttttttgcgcgttcaatacatatcgatcttctgtgtattacttcctttggttgattctctcgttttcttcttgtct
ttgtatttctgagtgtgtctctatctttccttctttgtttcttatttggttgttgttgttttagaattccctctttctatttgttccatcatcattatttgtagtc
atgtctaatgcttattctcttccacatttgcattctatcaccggcccaggctcaggtccagctcctccaccacctctttcttcctcctctcaataccttgc
ctcggctgattcttcctactcgttttcttcttctaagcctcgccttcctcccattaactgtatcgccaaaccgtttcaacagcacacatcccagtcctct
tggccttctctctcttcttccgcttcgtccccttcctcttctgtatcttcttccgccatgaaggactctccctcctcctcttcttcctccatgccttactctta
cggcgttcctcaacttccttcctccaatcagaatctcccaagttcctttccaacaccctctttgaactccaagaataacaacttttccacctctaataa
atcacccgaatctgttcactcttctccttataccaattctaacaactatataccccttacctctcagcagcccatgtcttccagtcatacatacgccaa
ttccgaacctactatccgaaatgatgcttttaccacttatgttcattccgtacataatcctccccttcagcctttatatttgactacccagaatttacct
ccttcttccatttcttcctttccaagcaaaccatccactccttccgcctctacaccagttcaagtttcttaccctgagtatcagccctattacaaccatc
agaagcagcagcccgatgttacttcatctaccttggattcaaccttgccgaaaatttatccgcctgctgagccttatcctctcaacgcgaacgttcct
tatggccgtaataattatttacgtcgcgtaacctccatggtccccgttcataccgagtatatgaattcttatgtgcgcaacccagagcttcgcacaa
atcataaacttgccgaacgaaagcgtcgtaaggaaattaaggaactcttcgatgatttgaggaatgctcttcctcttgataagagtaccaagtctt
caaaatgggagttgttaacccgtgccattgaatacattgaacaattaaaatcagagcaaacaaccttggaagcccatataaggactcttgagga
aaaacgtgcaagagaaaaataaaactgttattggataaccctgtttcatgctctcaggtttcaattcgtcatttttttgggtatgttttaaagattctg
ttttcatattctgctatgttataaaactttttattttatgtttttatttattctcaaaacataacggtttctttttacgatgttctctgcctttctttctcatat
gatacttctcatatgatactatactcttgttttctttggacagcttgatcgtagcacatctgttgaactgcacttttatttcttattgaagccttcaaccg
tgctaccaccattgcggttccttttatgaatgttcagacttatgttttcgttatttcagtccttttttattcttttttttttcttattgtggtgtcttgttttc
ttaaaatttacatatataattgccttgtcgatttgagtaatcaacgtttttgggtaattacttttctactttcttaattcttgtatagtttctaatgaatgt
cattataatcagattaatagacctttagtttaatagttcataagtataagaagcacatatatgaaatataattttctttgttttggtaaaagtcttcta
gtaataacgcgagttatgcagtcattgaatatagagaggaactgaacaatgtatgatttgtttccatcaacagtttacttactagttattctgaata
caataagtatcctttctggtttttaaatagtatattttctttattagaaaccttaccttttgtaaaactttttatctatttcatacaatatgctagatgcta
tatcttcattcctctaatgtgtaaccctatgtaaaccgatgtataataattttatcctctagaaatgttaccatttaatatcgtccaaagaaagcaag
ccttgtttcttgcggcgccatgcccaccggtctagccaaatttgcatttttttctcgagatcgctcgctacccatattgaccgtaggaatcgtggagag
gatattcatactgagacaaaacatagtcaagtcataaaaactagaatgttcactgtagggagctacaaatgcagcaactttgtcacttgctcctct
cattgcacgcaagtcatgagacacaaactgtggtggccgagaaataatagagtcaaggttactgctgagatcagctcgtttttcgaggggttgaa
acgtccatccagtgacccggtatccaattactcgctcaaatgaattatgttgctcgagatattccaaaagagagctgggttgtataccacccatagt
taccatatgaactgatgcctggctagggtcatctgttaacaaatccagcaattcttggttaccaagctgttgaataataagtcgttttctaggtacg
acaaaaattttggaggaaagtgctttcgctatggctatggcaaccttttccttgccaatactatatgtgctaacaacaactagcaatctcgagttgg
cagcattctttacatctagtgctttgcgagcacatgcatcaactacgtctttctgtgaaggaaaggtaaacttcgggtttaggtaagtagtatccaa
atacaatttatgaatggttttatcttgcaaagccgggtgggtaacgtgtgaccgagaagctcgaaaatcaccacaatgaaggactctctttgtctt
attctcatgaagagtttcaaacaaaaacattgtcgaacccggacaatgatttgcgtcaattaggtaaacagtaacaccaaggacgttgtggggct
cgtttaacgaaagcttgtgaatatacttctcgtccacgtgaaggacattgataagtaagtttccggtaacttcggagcaatatataggtccatgttc
ccatttcgaggacaatccaccataatgatcagagtgaaagtgagataaaaagtaagcttcaacaccgtcaatagctccatagctgaaagcatca
acggcgaacggcatactatatggcatgagcttataaaaaggaacaaggctggatttttttcgcgagtttaccagcctccaaggaaggcgtgtttct
aggatcaacaacgattgtatccggttcttgtgtgttcgcggaggataaagatagtttcgttttagcatttttagcaaaagtttcgggcgttcggccat
cgagacaggcattcacatgctcattgggttcaacagaaagagccgaaaaattaatttcgcaaacagggcaaagcattacctctggcatactgac

cttttcattttcgtaactagaggactttaaaggttccagaaaaccagcttcactttgaggcgaccattgaggaggttccacattttccagttttagcg
aaggatcattaaatcgttctaaagcttcgggagaaatttcttcaatttgaaaactggaagtctttgctttaccctgttgttgggatgcctcaattgct
gcagcctcattcccccaaatgttgcgaccggtgttatttttctgaaaaaaatccaagatggtggaagtctttcttttgctcatgataccgtatagaga
ttggaccgtgagatttatttatttttcttacgacaccttaccaaaacaaattcaaggataccagttcaaaaaagaaggattacttctgggccagtta
acgctaaaccaatgcgataaatgagatttcgctttgtttgcttcaggaaataaagctttacaaatagtagtccttacaaaaactgggaatgcgaca
aatggctcttttgggataagatttccaaatgcttttaattaattatttaagaaagtaaaacaaaaaggaaggaacagctctaattcggtagtaaaa
acctaacttcctactagttatgttgtagaaaggacagctaggtctgagaggaaaactgctttatgagtcgttaaaagcagtaaaaacctcgaaaa
aggttttcttataataaacagaaacaatccctcccaaaaagagtatgtttaaaggtcaaaaataaacttcaaagagtatttagggacaaattgaa
ttgcgcgtcatcttgggagtcgctaatttcgatttaggagtttgaacaatttcgctattagctgaatgaatagtaaacatggctattgaatcgaaatt
gaaagatccggaaagtgatttattgtaaatattataggaaaatccagttaataccttgctcttttgtctttgagaagcaatgctcgagatcttttatg
ctttgctttcgaagtgtctctttcgaaagtaaagggcaattcttgaggttcaatagagcaaaacacttgtgagtcggagacgcctggggttttcttat
ttccaaaaaaatctttcattagttaaagcaggaaagcgttcggtagtgactgcacctacagattgcttattacgagcctgggaagcaacgaaggag
aagggaggaggagccttgcaaatggaaagaaatccatcgacaatgtgctcttcaaacttaaggcaaggattaaaataatatggatctgcttcatt
ttttcgaattagctcaaaatcctccatttgctttgcgttttgtgtaagtgcagtagacaagttcatgtaaacaaacaaatgatgacccgtccgacttg
gaacaaacgaggtcaaagaaagtctaggaaaaaacattgataaggacaatcttttcatatttgataaggaggattttatatacacaggaaacga
ataaacaagcagtagtagaatataaattgaactttctcctaacctctagagttgaaccaaaccgctacaggcgttgtgtattagcatatcgagaaa
taaaacctatccgtattgattcaatatctgtgaaatattttcaatgtaattaggacgactcaacatttttttcggaatcaggaagaaaggtaatatag
ccagcaaattgatcaatggatgcccgaattcgttgctgaaaaatcatatttgccaaaacggcatccaattccttcaccgttatgtggaagtaatttg
ctagattctccaaagatgcaacttcatagaactcggatacaaaaagaagatttgtttccagaaaatgcttagggttagcgattagcgtatgcatat
actgaacttggtgaggaagtaagaaaggtaaaatcgagtttgcatcctccagggtgataaactttctttgaagatatttttttgataaaattaaata
gcggcaattccgtcacatttggaagcagggaaagttcttgtaaaaactgtaaaacaatcatagaaggcgttgctaaaatcagacattgtatagca
aggcttaagttttcgatgaagacgatctcattcccaggcaccttgtattgaagggctttatagtagcttctagcggcgtccaaaaactggaacgttt
catctaaagcccttgcattgcacagctgaagttccatcaagagttgatgattattcgttttgtgtgcgataggcatacttgttcgcattaatagaatc
gccttctctggcttaccaagctttatgtagttttctcctagacgaatccttaagagtaacagctgttccaagtcactttcaaggtttgcatgcttttcta
gcgtctctaaagcggcgcaaaggttttcgtaatcgttttcggcatcaaaaacagcagcaagctgcaagcagcatttggctaatagttcacttctaa
ttccttctaacggcgacaattgcgcatacatatattctaacaggttttttcgaagtgcgaggcaagatataccagctgccatgtctaataaatgatg
aacagcttcatacgtccgctcatctcgaccagattggatacatatttgaatgcaattccttaactccacgtgcagttgttgctcgcttgtgtaatgta
aagcgaacatttcctgaaattgctccttcgaaagcttcccttctaggaaagaatgcaagacttcatccatcgcttttaattagtattgtttttctccttt
taactataatgaaaagttccctgtttctccagatgaaatggattttgtgttgttcatgtacagtttgtttatagagaacttgatcgcattagcagaatg
gtcagtctatcgtgggatctattctacgagaaaccacagctactacagcaaatcttttattgaaaattcgttgatttatgaaaagtagattgggcaa
aacaagacggatccaaaatctactctgtagaacaagaacctacaaagaagccccaaaattctctggtgttccattcagtaatagtctgtctaggc
taggaaatggtacatcttcaatttcacagcaatacttgctttcattttcaacgtccagcttcattcccactactcacagtaagtaaagttcaccaaaa
gctacatgttcatactacatgatttccctacatgatatctacaaccaaaggagaaaacaaactgcaaacaaaattatgaagactagattgtcaaa
ggctttttatttgcaaagaacggcgactaaatctagtagaagaaaccgaggatcttaccaccagtgtccttggcacgagcttcgttgtgagacatg
ataccacgggaggtagtcaagacaatgataccaacttgacgagagggcaagagattgttgacccacttttcaatatccttgatcttgatgttgtag
cggggagagataacaccacacttgttgatacggccgttcaattggataacaatcttgccagaacgatggtcatcaatctcctcgaattcgtcaatg
taaccatgcttttgcatgacggtcaagaacttgacaatgaccttggaggaaggacgaataagcacttgacgacgtccacgacgctcagcgttaac
aatgttgttcaaacagtcggagaggacagattgacgaaccatttctgcaaattgcttggtatggagaaaggaagttctacggcaagaagtcaata
atttcacacctaaaaaacagtcacattgttggagtctgactttgcgcagtcgaatgatccatggtagccaacttactgcacgttgacagccccagt
atagtaatataccgaacagcaacgtgaaagattagtaaacaaaaacaaaggaacaagagttttgtgaaaggctgcaggcataaacggagtgtc
tttggaacaacgaagtttcataaatggggaaaaaacaaaaagaaagagaacgaaggtaaaaggacttaccgttcttttagataagttgatggtt
tcacatctctcgtgaagccaacataacggagcgatccattcgttttgaaagacgattccataacaatgactcaatttcattccttaaacctttctgtt
tctttgatgatgaagcgaaaccattggaaaaccgcttgcttacacgtttaacgcgtatttggtaaacagacgctatgactcactatgtgcaattttct
tttatttggcaggaatcatggattttccatagtacatggccttagaagaggcgaaaccttgaaacgttcagaaaatagagacgacgataatttag
aaagaggaatttgagtaggaactgttcacgagaaagaagcctgaaaatagtatgcaattttgcccaacttgcggaaatcatcttgttatagccatt
gatgaggaagggcgtaatgcttttgattgtaaaacatgtccttatcaattccctatttcagctctgctttatagcagacacgagtttccacaaaaga
aaattgacgatgtgctaggaggtgatgaagcatttgaatcgaaccaacaaacagaagtggcctgtgacaatagcaaatgtgacaataatcgtgc
ttacttcttccagcttcaaattcgaagtgctgatgagccaatgagtactttctaccgttgtaccaagtgcaaatttcaatggcgtgaaaactaaaaa

tggttttggtatttacctaaagttaataatggttatcggtgctgatccttggatataaaaatgtacttgaaaggtggaattggccttttccttctttatt
tcctttttcttgtttacatgggttattttctctgtataatatacaatatgaatgtataatatttatgagtccttttgagttgataaaggaaaaacattact
gcctatagacgaggtaggaggatttagtctatatcaaggtatatgtgtatttcgtcaaacaatattctaataattacaaaaatcaaagtcaaacaa
caatccaacggaatcggacgaaaaaaaaaaaagatttgggactgaggaggtcttttctaaagatctaataatacatgaatagaacatttcaaaga
atgcaacgtatcaagcacggttcataaagtgctgattataagcgttcccacatttgcagcttcatcgtcacgggttcgccggattcatcagtactca
aatgctttaaacggctttgtccagtccactgacgtatctgagacgatttggcctgtaaccattctttgctatactccaaaaatggtacagtgtgactg
acggtgtttaccgaaggatccatctgaaacttgtctttggcgcccgttacggctatgatgcgcttcgtgcaacgacacatcaagcccaaagtcaac
aacctttttatcaccaataacatagatacggccaatcgaatcctctctagccttcaatttgttaagagcttgtttgaacgtagaaacatagtgaacgg
attcatcaaaggacttgggctcttgaccgtctgtaatcacgagtgaagacactttgtttttcaggatatcttcatattcattaaacatttgaagattgc
ttagaacgatattgactccattttggaggtccgggacacgatttttgaaataagttctttctccagatgattattgttgctacccagatgatgcatac
tagtgtgctgggagacagtaagggaattgggttttggtgtaggaataattagagatatatttttcgaaacgagtggccaatcttcgcggccaaaa
aagtctataaagtcagctgcggcttctacataagcaataggagagggtatatgatgggcaccagggtggaataacacttgtgaatcctcacagat
tcgaatcaaggtagtagagctttcggtggacaacacgttatcaaactttcctagaaaatggagtgtgggcatttcgattttcattatatgagtttctc
catcgatagccaaggcacgataatatccactaaagaacatgccaaatcgaaacggcttttgaccgaaataattttggtatttcggaagggtgaca
aaggtcgcaagattggctgccaaatttgttccctgagaaaaccccataatcccgtcaaaagggccatgctcgcgaatgtaggaagccaagtgctc
gtatgaggtctttggttctaaagttttggaatctccatactcatcaattctccaccatccccaagtgatagaggacccgtctaatgagtcttcatcag
agtaatcactttgacctttgtcattcgaaagttcaatgggaccagtggggaaatagaactcgacatcgtcgactaaacgttcgcgcagtgcacgc
atttttaccgacaaaaggttacccgattcgccataaccatgcaggcaaaggactttaatttttgttgaaaaatttgaggccattgaaagggtatcgt
ctgtaaagtgaaagaaaagagcaaaaaaaacacaggtcaggattcaagcgtaagtttcagcttttgcaaagtgggagaaaagtaagaaagcc
cttttatagcatttagcacaaggcaaatgaacgcatggatggttcgatcttgcccaacaagcttcgtgtctacacgagcccaaacttgggttcgtgt
acttggtgttttggaaaattttgtattgttacttggaaactgaaggtaacattatgcagtctgtaaacaaacaaattcttacgaaaattcaaaaggt
agagatgtctcaatgaaattataaactgaatatatcgatttcgactataaaagtgcaaatcatgattcgcttactacggtaatcaaaagtccgttct
cttggttataattctcagcatgctttatcaagtaatgcaaagaaagcttttgataatatttcatcgttttctgatcgataattacttacaaagtaaaca
acgaggttgcttcattttgaagtatgagcacaacattattcactttttgattcccaattcccacgagcgagtcaggttatttcccaagtatccaagtg
gatcgatcgatacgaatcaagcaatagtaaatgtatacttataggtatttcgtaatgttatggttattaaatttcgataaaaaggaactttctcaaa
aaaaaaagaagaaaagattcgtgtgagtctcaagcataaaagttgctcaaatgattcttgtagatgtacaatgtaaattacatacataatttatat
cacaacatttgcattcatttcaaaatattagtgaatttatgagtcatcgttatacagtttcagtttagttttagaggtttttaagttatagcgtatcctt
cacataaagaaagaaaatagtattacatctacaaacagatataaatgtgtcaagtcaaaatcgataagaataaacaacctcatcctgctaccta
tagatagacagtccgtttgtttgcaaaagatcgccaaattctacattaatagttataagagaagttttccagtaattactttgatttgtaagataaat
aagaacagtactttgcaatagtaccaatcgtaaatccagattttgagcaattcgtaactgattagttacttcaaaatttctttttactcaaaggtcaa
ttattattatatcgttatgagcttagccgcccacttaatctgatggctcactgcgtgaagtctcgtcagaagacatagaagcggcttcagctgatgct
gcttcttttgcttccatagcagccttttcttcagctgcaatcttgtaacccatctcaacttgagcatcccaagctggataatatactgcaaggttctctt
ttagaggaacgtctgagtgagaattttcagctggaccattatacacaaatggatggggagcagcttcctcatagaaaattggatttactataccag
aattgtattgggataaggattccatgtagctattgcatctgaaattccaagaacctttcaattcaagggactggcaggacgattgagttaaagggg
tcatgaaggtaaaaggactataaaggtaataaacaccaaacatcaatgtaatgaaggctgctgtaacgaaagaaaaggctggacggtgctcaa
aaatcacatttccaacgaatgcccagcctacaccaagtgccataattccgaagtacaaggctggaaggtaatgatataaaaatacacgatccgtt
tcaagaatataaggagcccagtgaataaaccaggcaagagcaaatttgccaatgttatacgaatgatgataaaacttaggaccacaatcgttgt
atcccatattccatcttgcagcgaaaaacaacattaccaacgcaaagaaaacaattagggacactacgctgtaccaaacaactggattacccat
gaagtaaacttggaggttctgatccgacataaatctcaaaggtcctaacaagaaaggccatgtcaaagcatggctttcaaattcatgtccatctcc
catttttcggtccttcaaccacatcaacttgtttgcttctatgaatttttcaaagaaacttggcttcctgtaattcgtttttctagcatcagacggcatg
gcatcgtggaaatttgactcaatgacccaagttgtacttcgctcaatacagtttcgacagcatgtaacctcacgatggtcttggccccagtcagga
agacggcggtgagaagtcattaattcacagttagtaaatacgtgaatcagtctaaaataagtattaaatgcttcaacggtgccgttggaagaagc
atgtcgagaaggacggtcaagtatttgtatacggaataaatcatttccatctccttcgaaaccaaagccaccgtaaccagaaacttcaagctgcc
aatcatgattgctcacaagaggtctgtgatcatgagaatgtaaagctctccctgtcataatatggcgaagacgaacgatactaccatccttcaata
attcaatttttttgcggtcattatcagcagcattctcatgctcaatgatccaaagagtattctgattaggatgatccaccaatgatattatttgttgttt
ggtgccttcgggatacacgagttcgtttgaatgcaagtaaccatgttcaggaattgcattgcggattgttaccaaagaaccatatgctacatctgc
atattcttctccaaaatcgttaccctccaaagaatacttatactcaggggtcaaaaatgaatcataaatattagacttataggctaattttgaatga
actgcgaatatacccaagtacacaaccataggaactcctataagatagaatgtgtagttaaaaccataacgaataatacgagtaataggaacag

taaggtcaccaatcaaattccaaagatggaaaagtgccaaaaccaatatccaaaatagtgtaaacagtcctgaaagttttgtgctaattgtaagg
ccaagactgatacccgtatatagaaggctgatatgccattttagggcaaaaggatatttttgttggttttcatagtacatccagaaaaaagaagtt
aagcccataaaaaataaagtagggatttcaatcattacataacggctcattgtaatcaatccattgtcaaagatagcaaaaacagaagccatga
aggcagccatttttgagtttgaggaaagatacacattaccatacattaaagggatcaatagagaaccaaatgcagcgatgatgaggcgaagag
atctcgcagtttcgaaagaagggaattcctgtcctggatgacttactttcgtaagatcaggattggagttcataaatgttagaccaaagctcaata
atagctttccaagaggaggataaacatccattacaaatttatttgatgcatagtcaacggcataattcacgtatcttaactcttcaaataccacctt
agaaggattcattaaacgttgaactcgaacagcgaaagcaacaactgagagtaacaaaacaattccaaaatccctagatgtaaagaactttcta
ggccgcgaaagctccacatcacgaatatttcttttctgcttttcgctctgcaaagcagacggtgtcgacttgcccatttttatgcaaatacgctccaa
tcaacgctgttatttacgcaatattccttttgatatttatgctaagagagagaacaaaggtgtcatataaatatactatgacaaagactaaaaagt
aagtgtgtgtagtataatactcatagtctgagcaaattccatgtaccaagtaacattaagttgctacttctagttgatcttagaaaaatacaaagaa
tgttacaaaattctatgattgttcattatattcatttgtcaaggcattattaaaaataaaatagtcattcggtatcatatcaaatatctggttcgttttt
atctaagccaatgcaatgtttttatcttcaattttagtagatacaaaaaattccaccttcctataccctctatttactcaatttttttgaattgcaaca
gctatttcttcgctgaagtcaaaatacgaaatgtgttgcgactaaaaatataggatgcaccggatgcaattgttgtacatcctaccagataactaa
acatatgtgaataattagattcctcataactctcagtcagacatattaatacttacaatagaggggatttggcaaaatcaatgtcaatcggtagca
ttggctcagtaattaaaagaccaagtaacatcaactgaaaagcagtattccactaaattcgttagcaacaagaaactgactctgaaataccttgc
tggtttttgtgggccgtaattcggtggtgggaagactaaaatcaaaatagcgccctaaagtttttggagacgggagagctagatagcgaagaaa
gctgaccatggacacaagtaaaacatctcttccaattattaaaccagctaatagtgctgcaaatgtcaataaaaagtcaacgaagctaagaaca
taccaggcattgagttttggtatgctaaacaaagagttaaacatgtcattaatgttttgtcagcaagtggatccaaaatagtaccagcaaaactcc
gcaactgaaattttctagcaagatacccatccaccaaatcggtaaacccagcatacaaaaacaaacaagcagcagctttggcgtagttattcac
ataaagaaacgcaatcagtggagctgagaaaagccttgacaaggtcagcatgttaggcaatgtaaatacattctcacgaatgggcaactgaaa
gccctttagttcattacgtttttctgctttggttggtggcttggacgaattatggaagccacgaatacctcccattgtcttatccaattttggaccagg
cacgtgccgggatagagcccatcgcatgccttcccaaacgtttttagcaacatgtttagcagggtatttcgaactgttatcggcgccctggaaaat
acctgtacgaaccaagatgcttgtccatccataggcgttggctccacgaatatcactttcaggattatcgccaaccataaaaatttccttgggga
gttgggaatacttaaagcttttcgttttgcttcaaggatgttggttgcataatcataagtaaccttatgtggttttccatactttgtcaatttcatttgct
ttcctgtaagctcattataaatcttttcacaacaagaagcaaatgcgccttgtcccaatcgaggaagtgggtattcatttggccaaaccagatccg
agctaaaaggttaatatattagaagtaaaaaactgaaatcaccatgttgggtcttttgggagcttactttgaataataaatgtcaggtccttcgtcg
cccttttcagatacagtacctagaattccatttttcgattgagccaattccataacaagttgcaaatcaagtccaaatctcactggatcgacataag
taaatacagcctcaataggttggttcaggtcgaaatcgcgagcctgtttcaaatcgttttctgtaaaagaagtaaagggccaaaagggagtacca
cgcttagcgattacatcgagttcatttataacgcttggaaagccatacatctcagctgtctttcgtacgctgtcatcacttcctccgagaacgagtat
atgcttatacttatctttaagattcttgaacggagtatgcgactgacaaaagtccacttccttcaagttcacttgcaaaaggttggacaagcgttca
gcccgtgtcgtttcatgcattccacctccattcgtgagcaaaatgaacggaatgttatgcttttgaagataacgtaaactaacaggtaagcaaaaa
aataaataatattggcattaggaaggtaagcatacgtttttgttccttctgtcaagccctttcctcctcgaataaggacaccatcaatactttacggc
gttagctttcaattctttaagacacaaaaaaactatttactcaaatgcgaaagctacatcagcgactcgacctccagcaaccggcacttctgttgtt
aaacgacgaactgcataatgtttcgcttgatgagcagcagcctgccagtatctatgattaatggcacgaaacatgaagaagttggttaaaatata
tccagaaatagatttaatttcccagatttttactcgtaaatctctcttcagtatggtttttagtgtgtagtgaaacggggccgcttcacgggtatacat
aatttcacatatgataattagtgaactcattcatatttgctagtcagaaatttacagacaaaaaggattggaagctcgaaagaagagaattaaaa
tgaattatcttaaaatatctttgttttatgaaaacattgaaagccggtctttgctacttctatcaaaagttcgctattcatggatacaacatgttctgtt
ataaccaaataatctttcatggtaaaccaaaatagtctaagcagaatgtatactatgagcaggatacaaagtgagattgataaaaaggagtcgg
tagtattatacaagtaatagctcagccgtcataaactataaatacattttgtaattaatttttagaagactcagcgacgtttatttggagggagagt
agcaccaaaggaactttgtgtagctgcacgcttccttcgttcaacttcttcgggagtgatctcgatgccgttgtgtacgtcaaaaatatcagttcggt
tactgactaatcgtttcatatttgctgcaacgtcaacgttttccaaattaagtgtagatcttcgtgactcttcaattttccgctgttcacgccagcggg
ggtctaacaagtgaacgcgcatatgttctcccatttcatttgctggaatcatctcacccgtaaacggactgcgctccaaaggtacatggcgctgga
atgccttgggtacagatttggcgggttttgaagcacggatgggcgctggagtagatgcaggtgcacccataggtgaaggttgcactgcaggtgag
ggagaggtttctgcagacgtcctttgtgaaggtacagacgaaggcattgcaacgccaaactctgctttggcaggtgcattcaaaggttcccatgg
agctgaagtagggggagcttcttcaattgtgtaattcatatcaggcatagtgaacattgcagacttttgttctaatgtagcggtttgcaaatcagcc
agatttgtaggtttggaaagtttggctgtttcatctgcctcagtgaattgaatgacttctactaccgcgaaatcgttccagtcaatctgggcatattc
aatcttttccctctctaattcttctttgcgtttttccctttgggtctcttgatacttctgccactctaccctaggctgaattcgcgtaagaacgctgtagg
gatcctggatgttttgacgaagcaaaggttcaaaaggtggaagggtattctttaccaaagaggaatattgttgtactatgcgcataaagtaagaa

taaagtgcatgattttggtgcaagaaatcaaactgagtatttccccattctttttgtgaaagtttagcaatgaatgagggccctcgaattgctgcgt
agcgagctgtgagtcgaagaacgtttaaatcctgtgctgatatagaaggcaagggttctgaaaacaaatacggaggaggagcgggaggggcag
tttgaatggattttgtgataggacgagccaagttagcagctccctgtccgggtccagaagtagcttttagttttccttgtctggcttcctccagcttgt
gctgatagtacttgtaataaggatcgtccacttgtagaaaagcaaattttggattcctctgctcattttgtctgatcttttcttcaaacgccgggccat
ttctagcaacatagccagcggatttgtcaatgatttctcgaatttctggcggaggaagaattacattggtaggaagacccaagacaccatctttctt
tgtatcattttgattagatggagacaatccaggtgtggaactcattgccacgacaaaaatggtaaattgctagatttatgcaatctaagacagaa
taccaaacccaagcccaaagaaatgttctattaattttatataactaaacacaagccaaataaatgataagtttcgcgaacaatatcgtctttaag
aagcaagtggacgcgtggtaatggctctgttagttgaatatcagaggtcaaatactttacgacgaagttagatgaattttgcttttagagagtttta
ccaataaaataaaaaacaaagtttatttaaagcatcccttcggacaaaaagaaggaagtacggtttttgtactttttcgagaagtaacgaaacc
ttttcatttttacaaaacacccgttctttcacataaacacccgaagagcgtgtttttaacatgcatttaaatagacgtcaattaaagaacctccataa
ctatcaatatatcggcgttgataattctttattgtctcggcatgtgttgaagccatactggtggagccagttgctcaaaataattccattgtccatggc
gtaagttgaattagtaaggaatgaaaaactaacatcttcgtgaatagtccgaatttgattaccttgacgggattgggttttgttgccgtgaatgtca
taaccatgctcgtatacaaatatggatacggattggacgcgtttcctggctgggtgtacgcctcttgggctattggtctatttttataccaaagttttg
atgccattgatggttctcaggcacgccgtaccaatagttcctctccacttggccaactgtttgatcatggtgtcgatgccatcaacacgagcttgga
agtttggttaaccacagaattacttcaactaaatcttccaatgtccatccttactcagtgtgctagtctgttttatttttatacttctacttgggaagaa
tatcacacgggcatcctgtatttgtcttacgtatctggtccagtcgaaggaattttgttggtttgtggattgtatgctattactgcctatactggtgatg
gtttttggacaaggcttcgtcctaccccagagtctcttggtttccttcgttctttagctccttactatacgtatggctcttgtctttttaagcttttaggcg
ttggtcttttttcgaatgtttttcagagcatttggaatgcattgcgtgctttgaaaaaacaacagaagtgcgcttgcaaggccttgcttggactttctc
cttatcttgtacaatggactgccgtctttttgctttatgtgaaatacccctactttttgaagagcaatttgtcactatttatttattaaacgcgatcatc
tttgcctattctgtcggtgtcattattgtctctcatgtcacagaatcatccttcccttactggaatgttttgattcttcctttttgtggtaaatgccttggac
gcctatacgttcggaattttaaaaaactatcaaaaggaatacatatttgcgtacttcggttttggactaggtatctatggcaactttgttgcccatat
tgtatcatctatcacggaggagtatgggatttattgcttacgacttcgtcctaagccagaagaaaagaaaaaataatgtatcctttataattcccct
aagctggaatttttttatttcggctattttacctttatttttttctcgttcgttgtaggtgacttaccaacgtaaaaagatttataataacctattaaattt
acgtctttatcttgaaatcattcttcatcttcatccatacatccatctaggctagaactaacaaacaaatcaatgaatatgaacaaactcttgtaccg
attgtcttccaacttgatatcctgtgagttatgtccttgtaaggcttgggttttgtagaaggactacttattgacctttttgtttacattacatttacgtgc
aaaagaaaccaaaattagagttcatagttagggcttgcgttcaatgaagttgcaggggttcaagagaggggtggtcctctaaagtttattttgtgg
agaaaatctgagcctctgagaacattctcctttttcttgttttaagattaaagcttgacaaatcaaagtcgcaaaggttttaaaagtttgttttaacg
ggcgcttctactggggactctgctatttgcgtttatcaaaagcttacaataagtttttgaaagcatactttgcattggttatgtcaccctccataattt
aacgctattcgcgtcgagccgttattcgcacttgtgcttgttttacttcataaaaaaaattgtttttattcgctataaatattattaaacggttgcatcat
ccattccgttacaaaataagcttttggtagggctaataaggacattacataattgatgggtcaatgaggttgttcatctatataaacagacccgact
ctcacttttaagtattcacacaacaaccactttagtactacagcgcgctttatcctatctacacaaaaaaaaatttaccaaaaacaccccaaaaaaa
tcctacattcctttttctcttatttctctctatctttttggattaatatcaatcttttagtatctcttcactttatttattttggaaaaataatttcattttaaa
aatggacaaacactttattaacgaccctgaaactttggttgtcgaggccctcaaatccttgacttctttgaacaagacacttagtgttcacgaagaa
ggaaaattcgtttattaccacgattataataagaaaaacgtgagtgttatttctggcgggggtgcggggcacgaacccacccactcgtcgtttgtt
ggcaaggggatgctcacggccgctatttctggctctatttttgcctccccttcgtctaaacaaattaatgccggtatcaaacaagtgcagtcggaag
ccggtactttggtaatttgcaaaaattataccggtgacgttctccactttgcgatggccgtggaaaaagaaagagctgctggaagaaaggctgaa
ctaattgccgtgggtgatgatgtctctgtaggccgtgccaagagtggtaaggtcggtcgtcgtggactctctggtactgttcttgttcacaagttggc
tggtgccgctgcctcccgcggtgttcctttagaaactcttgttaagcttgctcgtactgctaacgataatatggtcagtattggtgcttcattggccca
tgtccatgttcctggacaaagtcctcgttctgaagatgaaagcatggcccacaatgaaatggaacttggtatgggtatccacaatgaacctggttg
ccgtcgtatgtctcctatacccctgtattgatgatcttgtttctgaaatgttaaaacaacttttggaccaatctgataaggatcgtgcttacgtcaaaat
tgacggtaaagacgaggtggttttacttatgaataaccttggtggcttgtccttacttgaattcagtgctatttctagtaaagtgcaggaggctcttg
ccaagcaatattctattcatccagtccgtgtttatgctgggatttttaccactagtttgaacggtcttggttttggtatcaccttgttccgtacgactga
tcgcgtaaacattggtggccaagaattttccttcttagatctgctcgatcaacccgtcgaggctcctggctggcctttctgtcaacctgctagccttg
aatctaagaacaagattggcaacatcagcatagaggaagctcatagtgatattcaatctcctgtgaaaatcgacaaggaagcagttcgcaagat
tatccttacagccatgcagaatttgattgatgctgaaccccagatcaccagattcgatacaattgcaggcgatggcgattgcggcaccacattgaa
gcggggcgccgaaggtgtcgccaaatttgtcaaatctgacaaattttgtgacgacccaattcgtctcgttcgtgatatcgcagacgtcattgaaga
caatatggatggtacctctggcgccttgtatgccatatttttccatggctttgccaagggagtgaaggaccacctacaggaaaagaaggatgtttc
cacgaagatgtggtccagagctctgaaggctgctatggacacactattcaagtatactcctgctcgtcctggtgaccgtaccatgtgcgatgctctt

gttccatttgttgaaaattttgttgaaaccgacaatgtcaagactgctgctaagactgctcgtgagggcgctgacaataccgcaaatttacaggcc
aagcttggtcgtgctgtttatgttggcggtgatgtgaaggttcccgatgctggtgctcttggtgttgccgcaatcgtagaaggcttcgccaaataag
caagcaatatttgtagccgttatttgttatattattttattattatttagatgttcatatatatagtttagacaaattctagttttatgtagataagatat
aatactttcaatgaaatacttatagatagaattggtttctacagtctattgtcttttgagcgaatttaaaacaaaaaattattctttaatgtaggtaa
gtaggacatttggaaaacatgagaattgaaacaattgtattcatcaccatttatgttactgtacaatgaacgctcttttcggaatctagtgagaggt
taaagatttttaccatagactttactaactgaacccgttcatcatcgtccaaaaattcagactttttaatgcaattggtaatcaaaatagtagtcgg
gattgtttcagagtgaacccacacacgtccattcattccaacagcaatttcaaaaggaacataggtaccgagagtttgcaaaagtgcgtttttagg
ggttaagaggtttctacaatgagataaagataacccagtgatcatatagccatccttcaattctccaaagccaccagcttttccagttgttgcatct
acgcattccagctctggttccatatcgcggtcagccaaagatacgcgagcataaactaggcttccgactgctaggtttggcttgcttttcctagtaa
cgttctcaaaagccaatgcgttcaattgagcactgtgtgctgatccaatatccacccggtaaccctcagcaaatcgggaagtaacttggccaatga
cctgatcattcatagcaggaacatactaacattgagttagcatggaaatcaaaaaatttaaagtaaaataagtacatttggcgcccgctttgcttt
gtcttgccttgtctttcatgaacgttcacagccataatgttcgcctcgtctggtataaaattttttttacttactcttttcattctagtgtccactaaaac
agtatttttggctccctgatgcagaaagccagcgcgagataccttaatttcatcttcctggtcgtgtttttgatgaactaagccagggcctagtcgga
cagttccttgctctgcgactgtagacagctttacattgtctcctggaaagtagtatacaggacccgactcttccacttctgacatgttgcaatttcca
gattctttcctgcagtgtttaccttggactcaacataccgttgtacgcagcactatcatttccaaacgtaatcattatactgaaagctaagcaacatc
ttcctaaaaggttctaatgcatcccatcgcactgtttttatttcgtaaagtcttactagcacgctatctttgactacgttatttggatactatggcacat
ctcctattgacatgaaccattataaacatatgtctgaatggcggggaatcagtactcttcgccatcagaaggacttcacatgactgtggatgatga
ttttaatgacagtcgctcaaatacagtgttcgatccaccattctatggacatcccatgctgattccgccgtctccttcattgactacaatgctacgtgc
acggtctacgacacctgaaggagacgaaattgacgtctctgaaatggatcagcaagattgggacattatggttaaggtccctacttatgagtact
atgggtttgttttgtacttggtgtcaatggttgcttttggcctcttcgtcttttggtcgatccttccatcttctgtgttaaattacctggaaatccattcct
atctttcccgctggtgggcactcgccattccaagttggatattggtgctggtcatatatatccatgttgctttgagtgcttacaacaccgaggtgctg
acaagaccgcttaatagcttagaatgtttagtagatcagtatgccctagttggagaagaggatggagcggcttgtggtcgtgttgtcgaccttcgc
ctatgcgatgtaaatcgtcaacgatacgaagcggcaaacgcgaagtaaagattcctacaggaaaccaaattgcatttttttcggtaatttcttttttt
tctactatcccctaatgaccaattatttcatgaaatatttacaaaggtgctggacgaatcgtgggctgtcaactcaataatttattcgtaggaagct
aggttttcatttttaatattcgtactatttcctttatacatatattcatcagatggacaagtagaaactaatggtcttgctccaaagtggataattcct
ttgacaaacttcatcaaaggcattcattaatagatatagatagtccagaacgttggaagataagagtttagtaacaacgaaaaaaaagaaaaa
agtaattatatcacttctagtaaacaacgaatctactcccaatcgtcgtcatcatcatcttcaggattattcttattaggcttcgtgttctttttgtccgt
gttcggagtctcttggtcttctttttcactaggatgctcagaagaagggtccaactcagtgctcttctttgcctttccctcggaaacaagctcgtttttg
gtatcttctgaaacatccccatgtctttcgcttgtatgcttagtatttttgtcttcttcaccatcactttttttcatcatcccaactaaagatttcttcttctt
cattatgaatagaactcatagatttcgttggtcggatttcttctttgtaccaaaaaaaccgtttccaaaatgcatcgtaagaaacttgagacggcac
taatgtttccatctgattacgcaaacttgaatacgtttgaagaagatctgaaatctcatctgtcttatcgtcaatggaaacacctttttcccagtcttc
gtaggcttcatcagacacaggctttcgaaaaaggtctgtgttttcaataagttgcaggatttgtttttcctggcggcttaggctaagaaaggttagc
actactgtatttgaataaattaatgtaataattgagatttccatccccattatgaaagatggtttttgagccacagaccagcaaacgccgagattaa
ttaacttactataaatttgcacgagaagacttggatctggttagtcttgattcttcttcctcctttggcgtaatgtacaacgctttgtccaaaaatcca
ttgactgttgttccaaggctactccagtaatgacccgctgactgttccactttgcgaatattctccagggtagcatttgaagaatttccatctccaaa
ttgtgctttcagattactcactcctgtattcgcgcgctctcttgtattatctattaaaccttcgctcttcatatgttaagttagccagttcccctcaattat
ctcgcataccttttctttaaatttactccaaaagggaccgaattttcctccagttaaggactgttcattgtcagttcgaaacgttgacgttttgcaata
tgaagacatacgtccaatgcagagcccatttcttcttttaatttcacatatccttcctcattttggtcttctgttataacattaggactcatataatcgt
aaagatccatcctctctttgtttagtaatgtggttcagttttcgtgcgttctctgcataaagtagcattcaatgcaattgctgtcagggggcggctgta
gggtcgatacaagttgcttaaatgtagaattaaaaagaacaatcgttttcaacataaatgaacccaattaaactcatatttcctactttctaaggta
cctacctgcttttcacaaaatactgctttcatgaatatataatgaaaatacaggctcacatatttataaaagataaaagaatgtattattttaattag
agactaaatactcttcatgctaataacaaaggaatgaattgttatacacgatttccgacttagactgggttctagacgattcacaactcaaaagac
gttagctaaattatacgtactattcgcaaagataacttcagcaccagttaacttggcaaaaagtacatcaaccaagctaacggctttccaagggtg
atcgttcagaaaaggaataacatgcactgatattaattacggacggacagctaattcagttatccagctttcaaaaaaaataagagaatgaatgc
tgtagttaaactcattaaaaaataactgatttaatcataaatcatttctagagaaacattaataaaattattcgaaaccttaatattctttaagattt
agtgtcatattagtgcatgaaaatttttgaacgatgaatccacatcaaatattttaaagttcatcagcatcatcaaagtcctcatcttcctcgccagc
gccagctccttcagagtaaagcttttgagtgataggatgaacgacggtgtcaaccttttggcgttgatcatcaatttcgtccttagatgcatcagca
ccttcaacctcaagccattcaataacatcctcaacggcgtccaaaacagcttgcttgtcctcatcgttaaccttgccacccaactgttctttatcagt

gaactgggtcttcaagccgtaaacgtagttttcaagaccattacgagcttcaatacgctccttcaacatcttgtcctcttcggaatattgctcggctt
ctttaaccatacgctcgatgtcttcctcggacaaacgacccttatcattcttgataacaagtttctcagccttgcccttaccagacttgtcgacggca
gaaacggtcaaaacaccgttagcatcaacttcaaaggtaacttcaatttggggaacgccacgtggagcgggagggataccacgcaagtcaaatt
taccaagtaggttgttgtccttggtcatcgtacgttcaccttcaaatacttgaatcaaaacggtattctggttgtcacttgcggtagagaaaatttga
gacttacgagtgggaatgggagagttacgacggataagttgagtcataacaccgccggtagtttcgatacccaaagttaaggggataacatcca
aaagtacaatgttatcacttccctcttcgccagacaaaacaccagcttgaacagcggcaccgtaagccacagcctcatcggggttaatgccctta
gaagccttcttagtgaagaagttctccaaaagttcttgaaccttaggaatacgagtagaaccgccgacgagtacaatgtcatcaacttcagatttc
ttgagtccagaatccttcaaaacttgttcaacgggcttcaaggttctcttgaagaggtccatgttcaattcttcaaacttggcacgagacaaagtttc
agagaagtctttaccatcgtaaaaggaatcgatttcaatacgagccgacttttgagaagacaaagtacgcttagccttctcgacctcgcgcttaag
tttacccatggacttggcgtccttagtaacgtcaacattgttcttacggttatacaagcgagccaagtagttgatgaccttgttgtcaaagtcttcac
caccaaggtgagtgtcaccagaagtggccaaaacttcaaatacaccgttgtcaatggacaaaagagaaacatcaaaggtaccaccacctaagt
cataaacaataatatgtttttcaccatcagttctgtccaaaccgtaggcaatagctgcggcagtaggttcgttgacaatacggataacattaagac
cagcaatagtaccggcatccttagtggcttgacgttgagcgtcattgaagtaggcgggaacggttacaacagcgtgggtgacgggttgaccaag
gtaagtttcagcggtttgcttcatcttaccaagaatcatggcagaaatttcttcaggagtgaaatccttcttcttaccaccgtaggtaacttcaacga
gaggacggttattcttattgataatgtggaaaggaagagatttgacatctctagtcatagtcttctcgtcaaacttacgaccaataaggcgcttaat
gtcgaaaatcgtgttttcgggattggcaggagcctggttcttagcggcctcaccaaccaaacgttcttcatcagtaaaaccaacataagaaggag
taatacggctaccttgatcattggcaataatttcgacacgaccattcttcataacagccacacaggagtaagtagtaccaagatcaataccgatg
acagttccatagttttcggtttcatttttctcagaattggcaaaaaccaaaggcaaaaaaacaaacagagctatgaaatagctgaaaatgctgaa
gagctgaaacttcctcattttgaaagaatgattcagcctataaattgaagaaattctttatttctctaacaagaccaataataagacttgattttttt
aaaaaagtgaacagttaattagtgagttaattactgcgcaacctttaagcttaatggtatatataatctatctggagaaatgcgtgttggtttgtgg
taagtgtttatagactagttaaaacaccgctaagtaacatatgagagcagtcacattgtcgtttactagtatcttccagaacaatttctttctataga
gaaataaacataaagcagtgcaacgaattgatttcgcttcttaaactttattgaaaagaggtaaacaaaacaatgctctgcagcaaaaaagaag
taagtcataatcacggaagaggctcctgttcatgatttactttccaaagggaagccatatgtatcataattggtttacagcatacagtgtcacgaat
gcaataatattcacatagaaatgcattggagtaatttttaagaacgataccctctttcatgctgtagaatactttttagtacatttgatattctttca
tgatatttttatttgttctagttcaaacatttcaatccttgtgatgtttccatgttggtttgtgcgggtgaatcagataaaaatcaacaactcataaaat
gtacaaggtagagacgaaaatcaaataagcatagttctatcttctacgatagttgaggtgagcgcctttttcaattccactattatgaatgtgtcat
ttcatttcgaccatcaaatttgtcatatatactaaacaaacaaacaaacaaacaaacaaacgagcaaacgagcggtcataacttctctagtctat
atctttcatacatttttttaatgtgatagatcaattggtttttggttaggatttgataattcaaagggaaccgaaaacatccttgtacaaacaatttg
tccaaagaagaactcgtaattttcatctttttttttctgtttatttggatgaagaaagaaagaaattcttcttttacgttatcctacacgtagtataa
agtaatattggggaaatcaacgaaacacctcgtatttccgtcttaaatactcttaatgcttcttactgctgaccagttccataatgtcggcaaaatcc
attagagaggtaaaagagcagcagagacatcgcatttggacgaattgtatttatttactaacgaattttagtttgatggtaaggccgttttggctta
ttggttgaacaagtctcctagcatttcgaaggaagaatacaagactccttctactaatcccgatactcgccttgctcatattcaatttccagttccta
atgatgaaattcctgcgaattcgtctacctataagcaagttgttgataatgtctttgccgaagtgactcgtcaacatccctgggtccttgaaacgaa
acttgttgcaaagccagaccaattaattaaacgtcgtggtaaaactggacttttgaaattgaatgctacttgggatgaggccaaagaatggattcg
tgaacgtgctggccaaccccaacaagtccaacatgctgttggactcttaagtacctttttggtagaacctttcgtccctcatccacctgaaacagaa
tattacataaacattaattctgtgcgtgaaggagattggatattgttttgcaatgaaggtggtgtcgatgttggtgatgtggatgcaaaggctaaaa
agcttttgattcctgtacttctcgagaattttccttctcgtagtgaaattgctacttctcttctttcaaatgttccccaggaacaacatgcttcccttgtt
gatttcattatccgtctatactcagtttatgttgactgtcaatttacatacttggagatcaatcctttggttgttattcctactggttccggtaccgatgt
ttactatttagatcttgctgccaagcttgaccaaactgccgagtttgaatgcggtgctaagtgggcagttgctcgcacagctgaagcattaggcatc
aaaactgaaaaggaagattctgaaccgattacagctgattatggtcctcccatggcttttcctgctccatttggtcgtgacttgagcaaagaagaa
gctcatgttcaagagcttgatgccaagacaggtgcttccctcaagttgaccataatgaaccccaatggtcgtgtttggaaccttgttgctggaggtg
gtgcctcagtggtatatgctgatgcagttgctgtgaacggtgctgcagatgaattagctaactacggtgaatacagtggagctcctacggatggac
aaacatatgagtatgccaagacagtcttggacttgatgactcgtggcaaacctcatcccgagggcaaagtccttttcattggtggtggtattgcta
acttcacttcgcctgctgtaacattccgtgcaatttctcgagctttaggcgaattcaaagacaagttgcatgaacataaagtctctatatgggttcgt
cgtgctggccccaattatcaggaaggtctccgtgtaattcgtgaggccggccaaaagcacaaccttaacttgaaagtgtatggaccagaatgcca
cattagtggtattgtccctatgggattgggcaaggtacctgtggagccagaatggcagatggcctaaaaaccttgaaacgtatgatttcgcaatgt
atggctagtatgagactgtttgttcaaattttctatgtatgtttttggtataaaatctgtttcctcttaagtaattgatgctaaattagaaaggatatag
tagatattttctaaatgttgtaaaataaataagaaattgagtatttactgtttttcaacttaaacggcatataaaataataataattaagattatcaa

ttaagagatgctatccataagaagtgcactttttctgaataatcttcaggttttgatagttattatcgtcatattcatcatcaataataaataatcgcc
tgcatgttgaacaaaggaatcaaaatgttaacggaacaaagatggaaagatgggttggtaggtaataacctataaacgttcttttcttggtctcgt
gttttaaatggcagtgtaaccaccgtcgacgataaggtccaagccagacacataactggcagcatcactagccaaaaatacataggcaccggtc
aattctttagggtaaccgatacggcggaagggaacgtagggaacccattcatcgtgcattccaccggtcatgtcagttgaaatatagccaggaga
aacacagttaacacgggcgaaaggagcccactctacagccaaactcttacaaagatgaatcacaccagccttggaagcgttgtaagcgcattgc
ttctgagggacgttggcaatggtacctgacatagaagcagtagcaataaagctaccaaaaccttgcttctcaaagacaggaccagttacgtgtcc
aatgttgaagataccgttaagattgatgttaatttccttagcaaactcatcaggagtcatctcagcggctgatttgttggtacaaacaccggcattt
gcgacgacgatgtcaatatgaccaaactttttcagagcctggtcaaagacactcttaatatcgttggaatcgctaacgttgcatttaagtgcaactg
cctcaacaccatgtctttgagcaatcttcttggcttggtcttcggcattagttctattgtaggtaataactacactagccccagcttcggcataagctt
cagcaacgtccaagccgatacccttagaacatccgtaaagaagggcctttttttccttttaagctcaaaagatcaacgacagttctctcgttaatgtt
cttagcaggaggactcattttggtttctaatgtgggatgaaagttttcaaaaaaaaattaaaaatactgccgatttatgagcaaatgggcgactat
ttatatctttctttttcatcgaaatattcatgatgatgatgatgatgatgatgttgagaatgatgttgaaggtaatgatgttatgcgacaatgatcatt
caaacgatgatttcacttttggatttccagttttattcatagttttcgtggatctactacagtgtacttcttacgataatctcatatcaaagtcggttttt
ctcgaaattctgttgaggagtacaaacacttacagcaaacattttgcagtgctacctagttaaggcgtaacgaagaaataataaatcgaagaatt
aaatcaaagaaaaaacaggcaaagaattatgactggtggtgacttatagatgtaattcttttgcattgtttactacatttgcgacctgtaaagtaa
ccgatgctactatgagtcgctttttttataacatcgttaactcgagtagaaaaatggccaatttaaaaatttccgaatttgaagcgattttcttctac
atcaaacaaaatgttcggttgcctatttgaggttgaatcatcgcgtatcatgatgtaagtaaacacaagtaatatgaataactacccaagcaatac
agtttcttatagaagtacctttctgagaatatctttgtttcgttatcgttccaaattgacttttccttgctactgctctctattcattgctgccttccatttt
tcccttcgtttatgttaccacatatctaccatgggaatatcactcccaaagtatgattataacttaagtagaataaatccactttttttgaattcaatac
taagccagatatactttggaattacttacttacgataagattaaggtatgtatagtaatgtccaagcctggtaaactagctaggagatacaaagac
tggcaaatgtgggctctatttttttcttttgatgaaaaataccccacttaaccacttcatcctggtacccagttgtctgcttcaaaaactccaaagtttg
tcttgatcataactccccatatattttttttattcacttctctgggtttttcttgcatagcactagtgatattaccgaaattcaatacacaggatgtccctg
tttcagcaaatattcacgcatcccgatcatttgttatggctggagagcgaaagaaagcagcataaggagcgattcgtgtaacaggatgctctgttg
aattacaaccatttgggctttaggaagataatttacccaattattctaattcttttgggcgtaacaacgaagataattttccaaaatatgttttttttt
caactactgttagagtaaggtgcaacgaatgatcgcattcatattgtctccaatcgtcggcggactgaggtttatgtgatgatcttacgtattctttc
catttggtcggaaacaaagacacgttctctatctgaatatatgtaaacagcctttttttttgaatatattaattaattatttcacttcaaggctgatgcg
ttgcaagcaataaacaaggccatcttagtagaacattccacgacggtcaaaataacctccttgccttgaaaatatggtaatcctatgatgaaactc
ataaagaaattcttccaaggtttatctggtactgatgatatttcaaaaatctcagaccatagatcttttcctgtattatgtcgttggcaaatatcgca
attttttttcataactttgaatgtcgaccgtcatgccattccaataaaacttttcatttattcaattcagtgagctttgtgccaaaatgtcttcctgaagg
ggtcggtcatgatacatctgcactaactgctgaatttttgagatgagaatctgtcctcgattgttgtttatgagaagcccgtctcctactgagtattg
ggaattagtacttgctccatctatagtatctcgaaattcctgtgttgtcgattttcaattctcaaataatccacgatgctctctacatgatcaacttctg
ttgctagatgatttcgagaaatggtgtcggttgtctcgtttaggtcgacaataggagtcaaaaatataacaatccagctctgaagtccacctgttca
tttttaaacgagcatctgtgcggattctagatattaagtcctcatggttagtcaaaatatgaaaaggtttatctaggtctgaaagcaagtggggcca
ttttctaagagctgttataattgcataggcttccttttcccaagttggccaagttcgttgttgtcctgtaagtgatgctgaatggaatacaaccggatg
atatattccatcgtcttgtttttgagacacgacagctcctatagcatggtcaaaaacatctgtttctaatctagtttctttagttcggtcgtgatgtctg
agaatagaagaattttcaaatgcttgcttgatctgcataaaagcttgttgttgccgatctgtccattcatatttagcatcctttttttaataagtcatac
aaaggtctagataattttgataagttcggcacaaattgcctagaataattaacaaaacctaagaatgatcttagctttttaaaagtgtcaggtacc
ggccagtctataatggcctgaattctttcgggatcgacttccattcctttttctgaaattagatggcctaagaatttaaccttgttctgaaaaaaaca
acatttctcttgatccagatataagtcagcttcccttaatcttcttaatatttctcgcacatgtttctggtgttctttctttgaagaagaataaattaag
atggtgtccaaataaacgataataaaaacgccaagaaacggcaataacgctttgttgatacatctctgaaaatgtgccggagcattggttaatcc
caacggtatgaccatatattcgtaaactccaaatggggttttaaaagcagtcttgtgctcgtcacctttctttattctgagcagttgataggcatcctt
tataagtaacgttgtaaagactctagctccgcctaaatgtctaataaattgttctaaattattcaatggataattatctcgctttgttattacattcaa
aggcttataattgattaccattctgtactcatctgttcccttttttggaataattagaggagtgcacataaacttggaggaactttcacggatttttcc
ttgatttaataattcttgcatctcttctttcatgcatgatagttcccttttattataatgtaataattttgggttgtactttggtaattcccttgtcaattta
atctctaagtcattttctgagtgtttaggtactttcggaggttcctcgaatagtttggaaaatggttttaatagattccttgttttctgttgttgttcttca
tttttcttatccatctgcatcattcgactaaccttctttctagacatctcactcatctgtgtttaattttggtgaacgtagctagcaatctgtggtcgaag
tctatattcgaataaactcaagtcctgtttctatttcaaatcagctatcccactccgtcaggaagatatttatcccaggaataaaaccaagaattttg
aagaaactgcgaatggagtaagcgacggttaattttcctattaagtaagaattcaattgatgctctagcttcactttagtaggaatgttgcaatccg

cttagttggcgagatttatagtacaatttttatagctcaatgccacggtagaaatctcacaccatataataatgtaatatcaaccgaataagcaatt
gtatcgttttattaatgaatggaattaggttgtactttgtgtatagaagctattaatgtccagtcgaatcttccaatttgctgagttcaagtgtgtaag
ctgaaaaggtttatatacctttgcagatatggtcaacgctatgtagctctttgtggctgatagtttctttgtctgaaaagtctccgttccgaaaggtgt
ctattacgtttaaatcgttcatgaatatctcatcctttactctgatggatttgctataactcggcaatatccttatatcataatgtactgtaatatatat
aatattaacaaaacacctctcatgacaatgacaggttccaatttatgcaactaattttatcgtactttgttcctgctcactatctggaataacaggcg
gtcccttagagtaagctaaatgtcgctaaacattctgaaagtcaattcattctcccagtgggtcaaaaaattgctgctaatataggtgtttacctaa
catttgaagctttttttttttcttcttttccaaaaattaaccacacttaattcgttgtcatactctctttattggaactttgaaattggaccgcgtttcgtt
cgtgttgatcttcgtagtttaagtccatttcaaaataaaggccctctgtttactttttctttttgttcttactactcgatttccttacactcgcattgtattt
tctccattcgcaactatttgagtattatcataacatgtctgctcaaaaccgcatgtataaagcttgcttgtttgatatggatgtatgtcgttttttttccc
ttaaatatcgcttgatggtaatttgtttttttcaggagttaaccgttactacccatagggccttcttgtcgatagtgaaaccatcttaaccaaaactact
agtatggattttgtcaatatggtattcaaactaacatctcctgcagatcatttactggaacgttatggcaagggtccgctgcctctctccattaaggc
tcaaatgatgggtaagcagtttcaagcgatggctttcgctggtcatggtcgttagtcctattattctttgcttaccctgttccaggtcgtcccggggaa
aaagctgctgaagttgtaatcaactggtccaagatccccatgacaaaccaagaatttgttgacgagcagcaagtcattcgtgtacgcatcttgtttt
cttttctaccttcttaaactaatccccatttacaggccaaattttggccttccatgaccttgttaccaggagctgaagctcttctccagtttttaaacac
aaagaacatcgatatggcagtaagttactcttttttttttttcttcgaacgagcctttcaaccttccatgactaacctatttctttattttagttagctacc
tcttccaataccgcaaactatcaaatgaaaaccaaccatcttcgtcatgcctttgacaaattcggcgacaatgttattactggtgatgaccctcgca
ttcctcctaaccgtggaaagccccaccctgacatctggcttcaagctctaaacttaattaatcaaaaccgtagaaagagaaatctccccgcattgt
ctcctagcgattgcattgctttcgaagacagcgttcctggcgtaaagagtgcaaaggccgccggtatgcatgtcatttgggttcccgatactgcgat
tctcaatctttttcgtgatcaattaaaagaacttgttgatgatcagtgtgaatttttaatgagtttagaagattttcagccagagaaattccttttggg
tgcttgaacaaaatatttacccataatctatatctatacatgtgaaaaaagtgaaaggtcgcaagacttgacgcacctattttggattttagaaga
aaaaatttgtttaaaaggtttccttgtttagcgttactttataattaatttgtgtttcatgttatatacaaatgtctgtttcattgttcatggaagataat
tagataagtataaaaaacaggcgaaactattaaagtgtacataatcataacatttttctatgtaatggaaagaaaaacaaaatacatagaaaat
aaaatcagtaaaaaagagaaaggtcctttttttttttaggaaaaagagaaaaataaaaaaggctttggggtggaaggaatttcctatcgggttca
gacaatcatgaaaagccagagttttcctgaaaatatcggtgaaaggaaacctaaagcaaaaaaacgaaggtacttgaccagaaattgggatgt
agacaaaggttaggttggtgaatatttaaataaaaaccatcaaagtcattgaaaagaaggcagggttttctcatcgtatcgtacattaaatatctg
tgcgctgacaaaataatgttgaaagcaatgaaaagattgtcaaagccaaccttaaaatgagatatgtaaaaatgtaataaaatccattaaacac
attcatgaaacatttttgacgtatttgggcgtagaggcgagagggaaaataaaaaaaaaggtcaaaaatttcacttaacaactacatccgctttcg
ttttcgtttggttgaatacttacatcaattaattgggtactttgtgtctcaacattctccattccaggaagcatttgagcaatttttcggaaaagttgtt
tcacattataaccagctttggcactagtctccatatgcatgatttttagttcatttgctttttttttctccttcttcttggctaacttgcctacaaagtatta
gcaaaaaaaagagggtccataagaaaagggagttctatgcttcttttcaacatgacatacctttttatctgacaaatctgttttattgccgacaagcac
aataattacatcgtcacctcgttcagcacgaacatcttcaatccatttttcagtattcacaaaggaattatggtctgtaaggaagattagcaaacgt
tcatcattaaacatgtcactcctcttttttttttaaatccttcaacaaacaccatagcaagtatgtttgcttgtctgccaaaaggttgcagagatgcttt
gctttctttggtcacatacttgtaatatcgtaaacaatgatagcaacagaagaatctcgaatatatgaaggaatcaaagaccgaaatctttcttgg
ccagcggtatcccaaagttgtagacgaacagtgcggtcttccaagtacatcgttttacttaaaaagtcaatcccaattgtagcctaagtgtgatga
aattagataatcacttcaatttatttcattcattccatacctgatatgtattgtcaaattggtcatacataaaccgggttattaaagaagttttgccga
cggattgttctcccaaaaagacaagcttgtatttacgcaacgaaaacgaataattgtctgacatttagaatatctattaagtcaaaagagcaaggt
tcgcgtagtcaatcctctgaaactaaaatataaccgttaaatgagaaggaaagaagtcgtctgcctcgagctttacttcctgttgtggttgaaagc
aagtttgaaataaggtggtgcaacgataccgttgcggaatggggaaacaagctaaatatttgacctagcttaaagataagcttaaatttgacgag
atgcttcttgtctgtgaaacaattaggatcaagggtatggtaaacagaactaaaaatagtagaagaacgtccaacgccttgatatagagaactga
caaagtcaaagtgtgtgtatggaaaaaggaatactgaaacgtgatgagaataagcactcaaaatatatgtatatttaatattgtatttgtagaac
cagtagacgaagctaagaatggataggttcctgcttaatttttatatactgttcctctattttcccagcaaaaagaaataatccataataaaaacaa
tggatcaaatggcctttgaaaaaggaaaatggacacactggggattgaacccaggccaactcgcatgcaaagcgagcatgctaccactgcatc
atatgcccgaaacttagaaggatgcattgttataactgttatataagaaaaaatgcgggaggtgactgtgaatggaaggatggaggaaaaatga
gaaattactcgaaaacaaagcgtccaaccatgaaatggtctcatgggattcattcactattgtgatcggaatgctatttagtctaatttggttcttga
acgctactcagaaacaaagtgaattttgttttttttcgttgatggtaggtgtacgggtttacgaagaatacagttactaaataaagtaagaagaaa
agatagtttaaaacttgcttgcgtgttcatttaacacaaaggaatttttttttttgacagagaaacaaaaaaaattggcaatgtttcttcttcacactt
tttcgttgtcactgacgagtagactctttcatataaaaattcattaaatgaaaaagaaatgatcatatgtcaataaagttcacaattgtacatatctt
cgttcaatttttttttcatttgtttatttacactttaatgacgaattcttataccaaaagacaatttcgtgcaaaagaaatcggctgtttttttttataaga

cgattcctttcatttttttctttatacctatcttctcatattcttccttttcatttcattcattgccccgcgtcaaagtcgcttaattaggacttaaacttac
agattcttccaaccaccatttaccgtccaccaaacgttgcctgctttcaattcacactcaggtacaaattggtttttaaaaagagtagttcatttaa
caatagtttttttacacgtgataaacagcatttattcgattcctgtaccgcccttttgaagctcgtaccgtgatctgttagatttgttcgattcccagct
ggattgtctatctattccttaccttagagatagtagacaaaaaattgtctcgctaactggtgtattaacattcgaatcgtgggccgtatatgcgtacg
atgtcttggagcttcattctcgatgatgagcagccacagtgccctttggctactgtaacaagttttgtatcagccatagttatcattattggcctcgtc
ctttcctatttgcttcaggtataccgaattgtaaagcttggaacttccaagggtcttagcttttcctaccttggcttaggctacataggtgtattgaat
gctcttgccaacgtcatcactttacaagtcattccattaacaagatgctgccatgaattctactccaagcgccagtgctttgccaattctaccggttt
gatgcaagtcggttctcaggttatggcaatgggagtcgttttgctatccttttggatgtaccttccccgccctatccatttcgtctccgatgatgatga
aaacggtgagcctattcctgaacctttaagtatcacgaaaagcaaaacttggcgtagaacttcttacggcttactagccattttttgcctggggtcttt
gcatttgcttgttatctgctaccgttttaacttataacttagcttgggcagcattccttggctttagtgcttccttctgcgcaatcgtccaatacatccct
caatttatttcaacagtccgccataggtcccatggcgctttaagtattcctatgatggtggtacaaactcctggtggcttcttgatcggttacctttttg
tcgagacttcctggtacaaactggactacatatatgatgtatgttgtcagcgcaactttacaagcagttctgctttctttatgtttctactaccaccta
caaaacaaaaagaccaaacaagaagaagaggcttttgccgcgcaacgtgaagaggagtgtcagcgtgctgtaagagtgttggaagaagaaac
tggtccttaaagacgtctatgaatcgtctacttttcatcaccgacaattatatgtaattatccgacacctttgtgaatatgatttctttcttagaattct
caaaagtccaatcgtttttttcatttccttacacgctcctttttattgttttataatgctttattatttataaatgcttacctcctataaatgctaaactaac
aattcttttatcatcagttgaggaattgcaaaatcttatatagtcgtgaaattataaatgtttttaataatatgcttcatttttttttagaaaattatca
aatatgctggttagaaaacgtagcaatttctgggtcatttgatgccacatacgactctacttgaaagctattgcaaaggaagacaagagcttgttg
agaccaaacgtctcatatgaactgaaccgggtgaacatggaaaaaaagggcatgaattctgaagaaattgctttatatgatagacaaattagac
tttggggcttcaatgcgcaacatgcgtaagtgttttgctgatctatgagaactcctaaccaacgttatttagactaaggcaatcacgagttttgttaa
ttacagcttctcctttatcgaatgaaattgcaaagaatttagttttggcaggcattggagagttatgcttgcaggacactcgttctgttgaaaaaca
agacgtgtttgaccagttctttgttgaacagtccgacataggaaagtccaagatatcttgccttatagaaaaattaaaagaactcaaccctctggt
aaaactttcctctttggatacaccagttgcggagctatctgaaagcacagtctcttcttttcagatggttattgcaactcaacttcagtacgaagagt
tctgcaaagtaaattctctatgtcgctcttgtcagcgtccgttttatgcttcgagctgctacggactttacggattcgctttcgcagatttggttcatca
tgaatttgtaatgtaagtcaatggcctatcaacgtgtgcttaactgagaatataaactaacctttatcagtgagaaaacggttgaaaataaaaaa
gttgagcaagcattgtcagctactcaaaaacccatccaagaagctctcgatatccctcttggttcgaagctaaaaccacgtttggcaaaaaaagt
acctgccgcttatccggcaatactttgtaagtttattagctacagaggagaatggttcattcaagcaagctaacatttggatagcaattttgaagta
caacaagtcagacccggaatccatcaagaaggtgtgtcatgaacagcaattagactcaaatgcaattgtgaatgaaggatttttaaggtaagat
ctagttcctttttttgtatcgctatgttattaactataataagcaacttttctgataactccagttttcaatggatgccagtgatttcaatcattggaggg
gttgttgcccaagacgccttgaattcaataagtaagcgtcagtatcccatagataatctttggatctatgatgctgaaaccagtgctggacctatat
acatgctgtaatatatgagttggttatttatactaagaaaggatttttttttaacattacttcctaaaatttagatctctatttatgtaaatcctatatat
agacgctgtctaaccactttctttcgctactcatttttgacatttgtttgtttagattatagtgagtgctgctacaccgcgttctcaacaacgtacagta
acaacctcgtcagttaaatttcgtaagaaacggtaaatggactacgtaaccaaaaaaagcagtcacgtaaacaataaataaggacaaacgcgc
tccctttttttacacacatacgtattagttactattgtttttaccgaactttagttaataattggtaagtatataatgaacacaattaaagatataaag
tgatgacaaaacgacaaatatagcagaataactcgtgttgaattttgttgtatatatttaagtttcagccttgtctgattagtgcttttttttttttcta
tttgcaacgctaacccttcatagacttcattgaaatttatcagtttaaatagctgtcgttactaaatgtagtgtgatgtgtaagttaatccttccaacg
ttatgcgttaagaggatgagtttccaagacataacgggcggactaaattggtcacatttaaggcttcaactgtacattgaagtcgtggccgctttgt
tgatttcgagtcttactgatttctcgcattactgttgggttccattttagtattcaaattatcactaacaatttttagagaattgtattgtagcgttatat
aaatttttttaagggaatgatggctttaaaatatgacctgttgcagtgatgacaaaattcacactgtctgaatctgcccatgatttctttaatatactc
tgagcaacgctgtcatattttcttagttatcttctataaataaaacagtaatagaaaagttcatgttatcttgatatattgtcacaggaagataaatg
aagtgtgaatataatgaacgttcatctactaatggttgttataatgtcctttggataaactagctaaagatgtgtaaggtaaaggtcgacaggcaa
tttaacagtttacgtttggactatatatgctcctattatttttcttttgattgtttactttgtataatgcgatgattttttaagcttatgaatattccctaat
tttcaagtactattagcatttctaatattataagtttaatgaagggaccattgtaacacttccaaagcaaagtgaacattcttaggccacgcctacc
cctaaaatgaaatggaagtagacctgtaaacaagccatgcgaactgtcaagtactcaatactcactatcattctagtatatcaatgaaataaggt
ttcaaaagtgttatgaatttaaaagtattaatgttcatattcagcggtatgaagaatccgaccaaatagctttttatataaatgtattcagtgataac
gaatgcatttgatacacaaaacaagtaatttaattgttattttaatttttaccaagtattaaactcacatattagatcctttttttatgttactaacgcat
gaaaacccgtcatgaaatgacatattaaaacgcttcgggagaaacaccttcatgaacagcgccgtttgcaccgccatctttaacttcagtaacaa
ctccggcgcggataacccaatcaccaaaaggttccccatcctcgcgttctaaagcataacgcttgataagaggacgtaaaagatccaaaatttca
cgctcttgaactgaagcgcgataaagcttattaagtctttggccaaagtgaccaccacctaacattaaattgtaggtgtttggtgctttacccacac

aggcaatctcagctacccaaggacgagagcaaccattaccacaaccagtcatacgcataacaatgctatctttctgaagtccagcttcatttatga
tgtcttcaaccttggtaataagtttgggaaggtatcgttcactttcagccaatgcaagtccacaagtggggaaagcaacacatgccgctgaagaa
agacgtaagccagtaaaggcagtattgtcaagcttataattagccaaaagctcagaaacccatcctaactcttcagaagaaacattagacaaca
taaggtgttggttagctgttaagcggaattcggccttggagttattcttgtaaagcttctcagcgagctcacgcaaacctgtcttaaaaagaagatc
tccactgtcttcaacacgaccattctcaatgaaaatggtacgccagtagtggcccttttcggtttggtgccaaccatcaaaatcatcagtatttttga
caaatggagcatgttcacgagcaggctcaaaggtaaagccacctacttcttcagttttctgtttaaacgtctcaacgctcagagtatccacagtata
cttgagacgggcgtgtttacggttttcacgatcaccattatcacgttgaactatgagtacggctttaacagcatcaacaactttatcattaggaatgt
aaccaataaccgaaccgagacggggatatgttttcttgttgttgtgagtcgttcccataccaccaccaacagctaaattgtatccaactactttagt
accttccgtaatagcgaccagaccgacatcatttgtatatatgtcgacatcgttatgtggaggagcaacaataccaaccttaaattttcttggaag
ataagtaggtccatataaaggctcgacatcttgagcgacttcaccagcgatcttcttcttttgaaccgtctccggatcttcaatccaaatttcatgat
aagatgaggttgtagggagcaaagaattgctaagttccttagaagcttcaaaaagttgagcatgaatttcctcatttccgggaacggatgaacaa
gtaacgttacgagcaacatcaccacaggcacctaaagtagttaaaaatactttagaaattgagcgaatagcatttctcaggtttcctttcagaacg
ccgtgccattggaaggtttgacgagtagtcattttcaaagtcttattaccccatttcgcggaaatatcatccatggctaaccattgctcgggagtgc
aaacaccagctggtaaacgagcgcgaatcataaacccgtaagcaggttcaaggccttgtttcttgcgttcttcacggatatcacggtcatcctgca
tgtaaataccatggaacttggtcaattgacaatcgtgctctgaaagtgcaccagtagtagtgtcagccaaaccctccatgatagtaccacgtaaa
aagttcgaagccgtcttaatatcttcgttagtaatcggtttgggctcatcaatatcaatatcaaccttgtctaagcccattgctgcccaaagttcagg
ttcccaagcattgtaagcagtctcccaaccatcaggatcttggtcattacccaaccctaatggaagaaggggcttggctccaaattcttggaacttt
gcatccaattgtttaccgggacgattgtaataaagtttatcctccttacgaggccaatattcgtaatcaccaaaaccaaatacaccataatttagat
tattcaaaaccaaatcagcattcttaaatgcttcccagaattcaaaaccattttgaggaaaatcaccttgaccagcagtactaaccagtaaaaca
acgttcgtttcttttggtaattcttcaattggataatcatccataaccatgattttgcatttggagccacgagcacttgctctactgtgaaggcgttta
gcgacattttcagcagtaccaccatctgacgcatacaaaattgtcaaaggaggacccgatagaccttcaataagtttgttaagagcattacgaga
atcgacattttgcttatgacggacatcagcaccaaaggattgggtaagagtttgagataaagcaggtttacgtaaagtcaactgtgtcaaatagtt
atccctttcaaggaaagcgcgaagctcgttacgaatgcgttcagaatccaactgaaaatcggagctttcaccatcttcaagagcaggagtccaac
gatacaagggccagtaaccaacgtcaacagctttcttggtttcttgtaaaacagtaatagcatcatcattgggagaatggtaaggaagataagcc
aaaacaacggaaggacccttaaagttgtcagcttccaacaaagaagaaatgacttgagtgtaagaactgtaaagagcagtagaagcgacataa
gcattgccaaaattcattgcgtaaagaccgatatcctttttgcgtgaagaagcacgagcgacttcacgggtggaatacggttgggtgtcgataatg
agtaaattaacgttagattcgaggcaaagagcttgatgcaaagccgagttgcccaaatcgtaagcccaagaatcagaaccgatgatccaatgag
aaaaattggtaaaaaagtgagaacgatcaagcaaagacttgataccagcggacgaatctttttttgagttctgcgacaacgagctcaggatcgac
aggtgactccaaattgtcgacagagacaatccaattagaaagaacctgcaaagattctgctgaaacatcagcagattcagtttttaaaatagaag
caacttcatcacaaaaggtgcgtcgttgtttttcatattccaatacagtacccaaagcgaactgaggtgaaataataagacgcggggatgcaaca
ggcttggaggtattggggtcatttacgacattaagacgagccttaaaaacgcgctgaagcaacttgtgataggcatcctcaatggcgggcggagt
gggaacaaagacctgctggccaccctcagtgaaattagcaacaatagaaggcttagccgagttgttaatgttttcgatagcggcagaaactatgc
ctgtaacgtgctcagaagattgcaaaccatacttaacagccaataattgaacattggatttagcagagacaacagatgccaaaatatccaagtac
aaagggtcccatttacgaggaaggttgctgaggggttctaatacagcaaggcgcttcacggtggatggtaagatggataataggtcgtcaggta
accatggacgcagaacgcgaatgacgacgatgccaacagagccttgttgaagggcagatttagaaagcaaaggggaagcactaccgaaagcc
aaaagaacgtgggcggcatctgagggaccataatattcaaatggcttgaaagaatgtgaagaaagaaaagcataagatgcggcatcacgagc
tggttgatcataaacagggggggacatatgacgaagacttttcgacttcttctgtttttgggattcagagggaagatcggcaacccgagcagcctc
taaatcttccttggaaggaagcgaattcaggtcttccaaagcaacagatgacttataaacggggttgtaaaagtgcaaaacaccggtattctgtct
gacagcgagattgtgtgcaagagtagcagtttgaaggacgtcgtttagggcatcttgttcggtaccgttggggcagtttccggaaagcaaaataa
caaatcctgtgtcacgtacggaggcaaaatcaaaagcttcggtaggatccagagcgatgtgggctacaacaggctgatgagagagtttgtacag
atggggggtagcaaagaagagagaatcagcagaagctacgactgaaataatatgtttaggagtagggactaaggattcttgctcagcaaagaa
gaaatcagtaatatgtgcaaaaggatctgcatgagatttcaaggaaacaatagaagtggttttaccggaaatattaagggcctcaagtttccgta
aatcttgtacagcctcttggaaggttgtatcgaggttgtcattcgctggatgaacaacatagagggagtcagcacatcggaaagcgactcgagtc
actgcttcctgcgtgtttgcgatggaggcttttaagctcatagcgaacagataccgtttaacaatacaacttggtcaaaaatcgtgaactagaata
gaactgtttgcttgaatggcgatgacagtagcaaaattatatcgaaataaagaaaaaaaattagaactttacaaatttataaccaaaagaattca
acgaatttaaataattcctatgaaaatgatagaataaatcataagactctaatccaaaatgcacagttaagagaatttctagtttgttccgttttat
gaaccgaaagaatcagcaattcctacggttttcccttctgattcaactttgcttccaaatattttaggaaagcgacgaacaaacaaggaaagcaa
aactcacaaagaaagattgcggtagagggcaagtttatatttcgttgatttgtgttcttgagaaattgtaccgtatattgcgtgattgccgtggtcgt

aatttcgggtctatcgcgtaatagacttgttacttggtcaagaagcctaaagggatatagtcaaaagctcgataaaggaaacggtttttacatcttt
gtttttttggaacaaattttcggatttcagttttgtgaacaaaaaaaattttaattgttttccctttcttgatacgttcattggtttgttcgtcaagggttc
aaatgcttttcatcgcacaatcatattctttcctccattgcgctccttatttaaaagcgtttcattgatcgttagcgacacggccaccaatcatcacta
aggaaaaaaaaatagcgataaggagttgaaactttggtattgaagatatggaggtggggcaagtaattgtttgtttacatttttggatgcgaaa
gtccttcatttgtatgtttatatgcttgttgtatcttttatttttctttatttttgaatacatcttttttttttattgtttttatggaaggaatcgtgtaccatt
tgctggaactttcttgtactagcaacctcctactacggtttcaataagaagcaagtgcctatccagaaaaagtaaataaacaaaaaatttctgaat
ttggaaaagtcaatagtttcttttccaaaggaaacgttttactattgttttctaacttttcttttcttcgtttctttgtgtcccatgtcttcttttgcatcat
gaatttccctaattctgtagtggtgttattttgtttttttctggagagcacaagcggagcgcaaaaagatacatcgcagagtcatgggaaaaaaaga
aaaaaggaattcaaaaggactgttgggtcaatcagacacaatctaatacctaacatttcactacccatgttttaatgttaattacgatatgtaaac
aattcatcccaacaaagttctttcgcaccatgaattttttttgtatacttttttcctaaaaatttttattttgctctagattcacaaattcatccactgattct
tacattcttataaatacctttgaattctaacccaaaatccatgactgtagagacctccaaaataaaaaatatttctactttccctttcatcaaacgct
tcaaaatacctaaatacgataaagtaatattttcccatagcattaccttcctctttttttgctttgagagtctccattcatctgctttttcaaaccgatta
aatagcggcacaactttttgtcccttttttcgacttggatatttggttcggattcgcaaatttgaaaaacgtcggtgccacctccacaagccatcgtgg
atgaatcgtactgaccgtatgcatatactccttcgtcgtctgaattaactcatgataaattacccactcagctggtttaccaaacaaaacactactg
ggatgcatatacacttgtgcattctccactgtcgttctatagcatccttcatgcgagtctctcttggctacattcgtaaagtgtccagaacacaacgc
gcgcagaatcaattcccgatttcgaccacacgatattaccggctgacgataacggtccatcaatctcagtatttgtttacgtacatcttctactctcc
gaagccctcgcgcttgaatgtaatgttcataacaccaattgtctgagcatctgttagttttccatgccgtatatacattcaatagcgtcaaatgatca
ctttctggattcgaaaactgcgcacgatgtttatccgcttcttgctgcttctcccttggccggctccagatattgggaacacttaacatggctataatt
gtcaaaagctcttcagaacatcccagttccacactcgtaatcaaaacctttgataattgtggctccataggaaagtcagccattttgcgacccaac
ggtgttaacaatccttcgtcatccagagccgataatgcatacaagttctgcaaagccgcaaccatcgtctgcgctggcggtggatccataaagtca
aagttcagcagatcattaattcccatggcttttagcataaggattgtataagaaagattttgacgttgaatctcaggaaccggactaggtagcatct
cattctggaaagcattttcagtgtataaacgataacactttccgggtcctgtacgccctgcacgtccgcttctttgtctagcttgtgcctgcgaaattg
gagtaactacaagagaatccatacctaactttgggtcaaaacaactttgctttacaaaaccaggatcgaccacatagtaaattccatcaattgtca
aacttgtctctgcgatatttgtagcaataactacctttcttcctcctggaggagcaggttcgaaaatacgactttgtgtttcagaaggaagcgcaga
atacacgggtagaataaccaattcaggtacagagtcccctagcatttttgttctttcatggagaatttcacagctggtatcgatttcctcttgtcctgt
caaaaataccaaaatatcacctgggccttcagtcaaatgtatttgcattacggttaatagtgctgcatcgagatagtcactttctggttgcttagcat
ataaaatttcaacaggataagagcggccaggaattgtaaaaataggacatttatgaaaataagaggagaaccgttctgcgtctaatgtggcaga
tgtaacaattagcttcagatcaggtcttttcgttaccgtatgttttagcaatccaaacaaaacatcagtagcaaccgttctttcatgtgcttcatcaa
gaattataacagaatattttttcaagagtggatcaaccaaacattcacgctgaagcataccatctgtcatgtatttaatttgagtcattctacttgtc
ttatcctcgaaacgaatggtataaccaacttcttctccaactcgacatccaacttcttcagcaacacgcttcgcaactgacatagcagcaacacgt
cttggctgagtacaaccgactattttcctattatccgtatacccctcttcagctaggtattgggagatttgagtagttttaccggagcctgtctcaccc
aacaaaactaaaacttggtttttgaaaacggcttctaaaaacgccttccgaagtttaaagacagggaggttttctctttgttctttcatacttaaag
acgtcttctttccgtatgatatatcaggaccctttgcagattgtcgccagctgggagcctgagtagtcatttgtttagcagctgtattgcgtatatcct
gagcaaatttcttatcttccggacgggccattgtatcattccagaccaaagataagtcttgtttttctagttctttttcgctttgtaagcgcgtttctttc
tgacgcatttctcgacggtcactagctagtacttgcccttgcattgcagctcgagataaagatccgtcaggcgcttttaaaacttttataggagaaa
gcttaagggagagctttgtttgaccagctaggaatctaggttcttcatcacgtaattcaatttcaacatcttcatcatcctcaggcttcaactcaggg
ttattaaaggtagtaaaatcctcctgaaacccaggaatactcgataatgagattgttcctgaagcagccaactgttgtagttcccaaatctcagga
gatgtcagtctctttcttccattgttcctaccgggatcaaagaaatcagatccctgtccatttttctgatccccgtccccgtttaaacccactgagtta
gccccagtaccacgataaacggcattgtcatcagttttcggctttaaatcctctccagtaacctgatcaacctctttcatggagaggcctatcctttt
actagcttcgtctacccgggtaaccttaactaaaacaggttgtccatatgatacaacatcagagggatgctcaacacgtcggccacccattaaatt
agatacatgcaccaaaccatctgttctttgggaaaagccgtctagtgttacaaaagcaccaaaatcctttatactagaaacaataccagggtatat
ttcatgtagtttggggaacccgctataagcattcgtttttcgacgtcgagaagcagaaccaccatcgcgagggcgattaggtatataccggtcagt
gggagatcgatcccgaccacggtaagagggaggagggcttcttgatctttctctgtatgaactgttcctagagttatccagccttcttgaacgaga
agagctggccagctgatttaattcgttcatcgtatcattcattaaatcactgtcttcattagacttttcaagattactatgatcgggaatacttagacc
aggaaacaactcccgctttcgacccacatcatccctcttagacgttaaattaggctctgtaattttattttgatcaacgatatcatcttgagctttcgc
cttcagctttggatgtaattcccgaattaatctagcgatgttttgtaaaaatgactctgcaaactctcctcctgatgctaggacgttttccttaaattc
atcataagtcttggaatgctcgtacaagtttattacaaattcagaaagagttttatcatcaatccctaattatattagcataattctttaaaaatgtc
ccaattctttaattgccaatggcaaaaaacataccagtatgatttttttatttcagaggcaacctttgaaaccagagacaggtactccaattcctcta

agtcatccatttcctcaattaaatatataaagatgaaaattggaattaataaaacgaaggaaaatggtataacatttaggcgttactaatgtgtaa
atagaagagtacaaatgtatcgaattccaataggagacacgattagatgtactcgttatatcaattaatattaaaataacgatactgagggtgaa
aaatgcaatcattggaatcgatgcaaaacaagttgaaaggaagcattacagcttagttatttaaatatttacttttgaaaatgaaagtatttaaac
ttgaactcataacatctttatatccaaaataaaaaatgaacacaaaaacagatgtcaaaggttttacaatattttaatgctctataaaacttacaa
taactctaattttgaaataaatagtattgtgtgtccactcaatgcaacacatcattatccagttgaaagttaaaatcaagtgtattaaaacaaccct
aagtgttaaccaagtaccaaaaccaaaaccttaagtaatcataaaatataaagtgagagacaatattaatgtatgagccaatccatccccaattt
tagagaagcattgaagtatacggtttaaactacttaatcggtagaaacaatagtagcttggctggtaacctctacagattcggatgaatcagcaa
gagccgaatcagcattaaagtgagaaacatcactccattccacagattcaccagaactaatgaactgatcaagtaaaccagcgctagcggcaac
caacccgaaaatgggagtagatggtttgagaggcttggaaagatattcagggtgatattgcacacctacgaagtaaggatgatcgcgcttctcaa
taatttccatacgctcagctttttcatctttaccgacaaaagtgagacccctctcttccatgagttcgacatacttggggttaacctcataacgatga
cgatgacgctccaatacttcttccgaattcttatgaagcttacggagtttactccattcactattaggctggaagatagtaggacgcaaacctaatc
gcattgtaccacccaacttgtctttattgatttcaggcatgtaaacaataacatgcttatcggcttgaggatcaaactcttcagaggtagcatctaa
aaaatgttagttaaaagaacccaggaaaataaatcccatttatttccccatttattgtctttctaactttaatatagcacctgtctacagttaaaatc
aatggggaaatgataataacctctagtaggtgcatgttcactgaatacagctaaaaagacagtaacgagggacctgggataccaatatttgtaa
cttacgctcaatttcacaaacatgacgggcaaactcaattacggcaacttgaagaccgagacagatacccaaataaggagtgttattttcacgtg
cccactgagcagcagcaatcattccctcaataccacgagaaccaaacccaccgggaaccaagataccgtttgcagagcacaccaaatgccaag
ccttgtggtaactaaggggatcagtgtgattcatactagcctctaaatgggaagcctcaacccattgtaaatcgagcttacgaccacaacgcatag
cagagtgctccaaagccttaatgacggaaatataggaatctttaagatgtgtgtattttccaacgaggacaatgctaaccttttgaaaaagacggt
catgaccagaagtcagaagcttccaagagttccacataaccgaaccggcgtcagcatttacagaagagataggaatctgatccgacttgaagcg
aattttaagaaaatctaacaacttattttcaagaagttgaggaacatgataggtactagaaacatcatggacagccaaaacctgttcagggccaa
catgacagaaaagagaaatcttttcaataacattttcctcaagaggtagcttgcagcggcaggcaattaaatcaggcataatacctaaactacga
agatctctgatcgcttgttgagtaggcttcgtttttttgttcaccgttaataacaggaacaagggatacgtgaatagacacgaaattatcccgtccaa
cacggaactgaaattgacgcatagcttcaacaaaagcagcagattcaatatcgccaaccgtaccaccgagttcgacaatacaaacatctggttct
tcaccactttgatcaactggtacacgagcaacacgttcaacccaatcctggatttcattcgtgacatgggggacgatttgtacagttttgccaagat
agtcaccacggcgttctttctcaatgacttttgaataaaccttaccggtagtaatattgttgtccttagtcaaagtaacgttcaaataacgctcatag
tttcccaaatccaaatcaacttcaccaccatcatttaaaacaaatacttctccatgttccagtggtgacatggtcccagcgtcaatgttcatatatgg
atcgattttaatactggttacttttaaacccaaggtttttagcagtaaaccagtggaagaggcaatcacaccttttccaataccgctaataacacct
ccggatactagtacatatttcatcttgatatccccttttgtttgctccaaagttggtggtacgtggaggagagaaaaagaatgtcagtcatggtcctt
atgagggcagggtgaaaatttttttctttattttttttaaccccagcaatattggtattattttgttccgttttctttattatttaagatttttataataca
aaattgacagcgttgctcaatcttatagaaagtaacagtactttggattgggtgtccacaccaacataccactaaagttgggtaaatggaccgaat
aaagataaattatgatgttatactctctaggatctaatggaaattatcagctaggattaaagcatgaggaagatatatcaaaaccagagaaggtg
aattttccggaacaaccaaagagaattgcatgcggaggaaatcacacactgattctggatcaaaatggtcacctttgggcttgtggagataacag
aaagttacagtgttgtcaaacgtttgacgacgttattgtagatgaattaatcaacaacctgaaacctttgaagtcagattatattcatgagtttcaa
atcgcgaacagaaaacaatggtccatggtggctgctggatgggaatttagcgtcttggtgggaaaagacagaagaactgtttggacatgcggtg
ctggtcctcatggagagctaggccagggagaggttggggggtcgtctttaacgaaaattatgatcccttaccttaatcaaaatgaaattatactag
acatatcttcaagtgtgaaacattggatctgtgttacgaaccagggtaacgtttatggctgtggagacggacgtaagggtcaattaggaccagtg
gttccaaagatgaccaacaaagttcagtttttggccaaagtagagcatgctcataaggtatgttgcggacttcagttttctgccttcctccaagaaa
cgggtcgagtaagtatactaggcggtgatcgttggaatctttctgaggaatgcatgcgatggcaggataataatccgaagctttcttctcttattgtt
gatatatcttcgaattggtctacactgtctttactagattccaatggaagaatttatgcattcggcagaggtgatcgtgctcaaaaagcacatactg
tacaaaataacgcgcaaacgattgctggaggtagcgaacacaatattgtgcagacaaaatcaatggaagcatatatttatggatggaacgaac
atggaaacgcatttaatgaagatcgtcaagatgtttaccaggctaaaaacttgagattgccaggagcggtcacttatgtagcagcaggctatgca
acgacatggatagcaacagaaaacgagtaggaagtctaaaataaaacaaagtcgaggatgcattgataaatttaaaggctagatgaaagtata
gcaggacagcacataaaatgaaaacgaaatcctttattaatgatagaacgtcaaacattttatagcgtgtattacattgttatatagatatttatga
aaaagcagagaaacgaaatctatttgaatataaaatggaagacgaaaagatagaattaagggttaacatcttatgacaatgaaagctcggata
acggcaagaataatttaagcgtcagttccaaggaaacgagctaatgctagtgcgagaaaaaccacaacggcaatgccccaacgacttttgaac
cattttcgttcttgacttcttgatgcattcgaatctgcctccgatgcactaggatttttattctgagttgctgattcggcatttacctttgcctttgcggc
agcttgttccattatgtctcgccgattctgctctaaaagttccggaaatacggcaacaaacttgggattatccattaaattaaaccgcttagtatcct
ttgcataggctcgtcttgtggattcgggcgtgacaatgccacctgtagttatttcatcggaggtcataaacgagacgaggccaacaagaatcgtcg

aaaccatccatgcaggattccacgacttgggatgaaagtcagaaaacgacagacataaacgagtatttgtttgaaaacgtccacttggcgtaatc
atccgtattgctggaggcttaaaaggatactctgaagggaatataagagttcctgtataacattatgttagcgcaaacaagaaagacaaagcatt
tgcttttatgcacgaaccatgatattgaccaccctcatatggcgtgtctggaggaccagtaacgatataatgccattctaaaatgttttcaggtgct
ggtctggcgtcaatatactctacaggatttctttgaagcgttaaatattctttcattaactaaaaatgttagtagaattactttcttgatcctataccct
cttataagcagcctttgatgccatttttgactcaatgagttaggaattggaaagaagccaccgccaaaattattaccatcaaaaatacaaacatga
agattgtttcattcttggtagtaacttaaagagaaaaattgtttattttacttttttttttcattattttttttacatctttattctgtttccaagaacaatga
tgagcatccaacgtttctgttcatccttctagactcggctgaaattcggtgcgggagagctttacttttactttcttagttcgttattcgttgatttcatg
agaccagagtcaatttctatgtttgatgtatttcatgtactccatcctgacaaacctgcccaaaagttcaccccgtaaacaatcccaagttccaaag
aaagaaatacgtattgttactaaaaataaagctatgaaactggctgcttcataaagcatggtgttcactttaactatctgtcttgatgaaatggaat
gaaagttcatactgttcaaagatacctatccggtaagcaacacttcaatttacaataccaatgattccttcttaggacaacaagaagtatgaatat
taaaaccctccagctcccttgtttcatggcatattcatttgttagcttgtttaatatcataattagcatgttctgatattactgtcatccttataaagcat
attactataatccttaagattatagatgaataaaacataattaagtcatacaattatataagaaagtctttatgacgtatttatttcaagcttataat
ggcgacctcatgtgtagtcagcctttaccaactaagtatttcgaatagtacgcaatgagtaatcaggttattgttaagaatatttctttagacgttac
tgagaagcaaatctccgatttcttttccttctgcggaaaggtttccagcatctcaactgccaaagaaggagatactcaaactgccaatatccaattt
gaacgccctagcgccacgaagactgcattgcttttgcaagatgccctcttaggacaaaacaaaatccaaataagcagtagcgacgccccagctg
ccgctggtaacgtcttcgacaagggtggtgctggtggagatcaagctactagccaagaagataagcctcgttctgcgattatttctgaacttttaag
tcgtggataccatctgagcgatgttactttggagaaaggtatacaattagatcaaagccttggtgtttcgtccagattcaaaggtgttcttgaaagc
gcgttgtctagtgttcgttccatgaatgagcgctaccacgtcagtgagaaagctaatgaagttgataacaagctctctatcagtgatacctttaacc
gtactacttctctagtcggcaactatttcaataaagcttatgaaactgctgccggcacatcagctggtcaaaaggtttctagtgcttacggctctggt
aaaagccagttgtttggaattcacagtcaagctcgcaaacttgctgactccaagagccatgctcaaaatgctactaccgggcatactgaagctac
acctgccgctcctaccgatccctccgtagacactaagccttccgagtaagctgtagaaacaagatcaaaccaaaatatcttgttcttctaattatct
tggctttttgatctttacgttatgggatacgtggcttcacgttcagacatattgagaatatggtatttcgatcttggtgaaggcctttcgaaattaata
tttaatgattatgaatacctgtgatatattgaaaagagaatttaatatataaacttgatgttctgttagtacaaagaagatttcttttacatgttaag
atgaattaatttgttaaatttagtctcaaagcataggttttgcgtaaacatataatcatagtcgaactcaaaatctacagtaaatatttattgcttttc
caaccgattaacgattccagggtcctcaacaacctcagatttaatgactcagatgccttctttagaaaaccgaattgaatgatcagaggttgtaca
ttttacatgcaacttaatgtcttactcaaaggaaactttataaaaaaaaacaaagattgttaatcctccttttcgttgatccgaccgttcctggcttg
atcatcaatcaacacccattgagctttcatatcaggataagtaagaaaataaacctttctatacgttttcttcctatctaactgcatctttcaggttta
ccgtcgaagcgattcagttattaaaattcaggtattttggtgagctattagatccaacctgttaccttcttatatatctctgtcctagccatataattc
actttgtcacatattattgctcgtactttattaagccttcattattgaaggtgtaatgcctaaatgaaaagtcattattctattcatcaacgtttatgtc
atgtttgtttcttgagaaagtaaaacaaacaagtattcagaggatatacggcactcccaaaaaccagtctgaatagaggatcatagcactaaatt
attttttcacctgtcatcgtcgcttttcactgaacagtctctgacaaaacgttgcaactctatataagaaaacgttcaactttgattttgtatatttcat
gttcttaggatcctatgaatagcctatttcaagccttccaagtactttaataggaaggttttgctagaaaaactcaaaatcgataaaaagaaaaag
aacggaattcaccctcataaatatgcttaacaaaaagaaaattattaaagtaaaaattagtgaaaattattttagacacactttgaacaaagttc
ggtaaaacatttgattcttaagatgagataatcagccgagagtaacctgtaagttcaaattgtattttttaacgaaatagaaagtaagaagctact
tttctaccttcggtgaagggttgacgactgtaaatccaccggtacgcacaacttctggagcattcgtcgctatgggttcatcacttattccttgattg
ggttgactatattcggtaacggcaggctcatcaacaccaacttcttcttttacttctggttttggggttgctcttgttgtctttttttcgtttaggttcttttt
tagcctttgcttcagcggcaccatcaaagtatttttttcttttcttcatctgtcaaagcattccaagaatcttcaagcatttttttttaatcctgttcccttc
aatgcactaccttcctcttttaatttcactttctcttgagaggaaaaataggtaaaagccatataatttgtaactctgggtatcgcagcattggctttc
tgtgtggtgttttgagcatctgcatttattattggaccttcctctcgttcactacttttcagcgcacccaatgatgcagcatccttctctgtgtttgtacc
agctccgatgctcgttgtttcctctgacttctcatttgcaatttgttggtcctcttggatttcagcaacgtttttttggattcttcgaagtctctgatttagt
ggaaggggactttcgtttcttgctttgtggttgagtacctttggtggatatttttatctttattccttcaggaggtggctacagaagttagtaaaaatt
agcttaatcaaatagtttttttattaccggaggagattgtcgagattgctgaccaagctgatcagccttttcttgagattgcttttccaactgttccatc
aagaaagctcgctctagtcttgcgcgtcgaaccgctcgtctaatataatcatattttttttaatagatactcatttcccgtctgtacttgtaatattctag
cctttaaatccttgcatttttgtcggtacttaatctctaaattctgttagaaatactgcctctacattttcatgagtctttcctcaccttcagactttgata
cagctgctggatagggactatttgtgctcattagtccacaattcttgtttacttgtacagaaggtaagtcgctggccaccttaactgagagtgactta
gtgactttgactttggaagattccacaaaaaaaggagttaagaaaatataaaacttatattcatagatattagaaatctactatctatgaataattt
atttgaattccagttccagtttgaaaatgttcaagcttagtacttaaaaaaatgaggatctacagacgatatatcttataaaccatattataaaaag
taaattaaactcatatatacgtattaccttaaataaatcatttatattaatagtttaaagtaggagacccaactcttgcaaatgaaaattactcaag

agtaaacattagagcgtcatcaatttatgtataaaataagttaagttaacaagttaacggagcataataacaatcctttatccttcaacaaacttc
atagcttcatctttggaaattgtttcgactgttgaaaccttatgattgaacataacaccataaaacgtgtcagcagctttaaccatttcaggtcgga
aagttgtgcatataaattggcttgttttagacatttcctttaccatagacgccaatgcagaccggtattgagcatctaaatttgcatcacattcgtcg
agaatattaaaaggtgcaggatcacatcgctgaatcgcaaaaattaaagctaaagcacacagagacttttgacctccactgagttgatgaatgct
tagctgttcatcatctttactattaaaggatacacggataccaacgcctgtataattatcgattgaacttttctgagctgcttggttatcgatgtccat
tgtattttcttgttcagaactctgactgagttcgcttcgtttattcataaccaattcaccgcgtccagcaggaacgagcttttcaaaaatttcagaaa
agttctttgctacctgcttgaaagtgcgctctatggcttcatcttttcgctgatctagtaccattgtaagctcattaatagaatcctgagaccttcttaa
ctcttctcttctagcaattagggaatcacgctgttttgtaaagttgttgtattgctcatatgccttcttattaacgcttccatattctttcaaagcctcat
taaccttgtgtaattttttactatagcatttgaactagtggaaacgtatttaacgaaagcttcttcgggtaaaacaccaagtgacttaatattttcgt
tacattccttcttacgagcaagtaaaaggctccttttagcagcatttctctcatttactttcgactcgtgttctatttttgctgctgcttcatgctgtaatt
cttccaatgtctcctgttccttctcctcggcttgcaagtcattaaccaggttttggatttgattttcaagtttttgagatgcatcttcaagtttatgaagc
tttgcctcgtgtttttcgagagttttcttaacactggagagttcggattcatcaacaagattatccataccgagttgtaccttcaggggatcacgtctt
aaataaagattcgtattcaactgatattcaagattaaccttttgggactcaatttcgactcgtttagtatttatatcattgtaatcaacagacaattgc
tcaatttgagtttccaaatgtttaagtcttttcatatccctaggatccatttctaccatgtctgactgaagttcttcttcaaaatctttcaattgctgctc
tgaggtttggagttctaattttgccaaatgtagatcatgaacctttctacgatgagagccttgaacttctgcttcctcttgcgttatagacacttgttct
aaattaaggggagcatgatcacgttccagctgcttgaattcaaactctgctttttgcaactcatttaagcaaatagttatttcttgatcaactgaatc
gatttgagtagtgactctttcaagattcgtttgaatatcaacaagtcgtttctgatatttttttgacatttttaagggcatctaaacgagagtttttataa
tcacgataaccaccagataaggcacctttcttgtcagaacggtcaccacttaaagttattccgttcagttgatgtgatctagcatactgtgatgcaa
cctcaatagaaggacatacaatagtcttagagaatacttgttgaaaagcgggttgatattttgtgtcgtactcaagaaaattaagcaaaggtaaa
gcatcactggcgtcaggataacttactggtcttggatgaagcctatttaaaggcatgaatgtgactcttccagcattttgtttatacattacctgtaa
aatctgatcggctgtagagtccgaatcaacgacaacatggaacaaactatttcctgcggttgcttccacagctgttttaaaccggttatcaacgcta
aaaagatcgcataagggaccatagcaaccagtcagttttaagcgttctgcaatttccttaacagactttaaaccattactagtgtttctatccatgg
tcgtactcatgctttttttcagattgtgaaagatcgtctttgatactttcagtcatggattttagcttagcctcttccctccagagttcttttcgtctatca
attagattctctttttgttcgttaattgaagaaaccttagccattgctccagcggtaagttcgcctctagacgaaagtgctgattgaatctcttgttta
cgaaaagctttcgcatccaatgctgcagaaacattctctaattcaatatttaattcttgaatccgttgattttgggtcttgatacttgaagaaatctct
tctatttgggcctttatccaaatatcgcgttcttctcttgtagcaaactgggatattcttgattgtttgtctagaatggttcttcgctggttttgtaaaga
tagtatctgttgatttaagagatcaacttgagaaataatttcattgtactgagggataatgttcgtcaattctgattctttatcatggatttcagattc
caacgactgaagcatttcaacctttgaagtctcatcctctttagaaaattcaatttgacgcagcaattgctgaaattgcaaatccacagctgcttta
gacttgataaggtttgtgtaatcttcgtcattctgttgtttttcgagccgaagcaaattgacagaattctttagttcggtgatttcacctttgatttgctc
gatacgcgtctctctctcaataaacgctccagagtctatctcatttttttgaagcgtgctagtgcgatcttgttctagagcatccaagacggcattga
tttcatcatgttcccgagaataaatcgcatattccaaacacctccgttcattgtctttttttatgataaacagcgagttcattcttttcttcttcaagttca
tgtaatctttcttcaatatactctaataattcgtcaattttctcagatttgaggaccgtctctgtcataatcttgttggattcggcgcgtctactttcgta
aatctgcgttcctgcaacttctttaagaagttccaaacgctcagaatctttggcgttcgtaagtgaagttactcgaccttggggaacaatatagtat
gggttcgaacgcgaaaatccagcagactctaaaagattaattacttcatttttgttcactgttttcctatccaaggagtattcatcctttttaagacca
atggttcgacgtaaaacaacttcacctttcccagtgggaaagcgattatcagaattttcgaatgtaacctcaacatacgcgctcattacagctgaa
ccaggaccttcatgtaagagagcttgcctttcttcacgtgataaatgcgtgtaatcatcgcccaggacaaagcgaatagcagcaaagaagttact
ttttcctgaaccattccttcccacgatcacattgtgatgaggactgactggttctataacagtataatctttgtaagacttaaacccttgaataataa
gctaaaattcgttaactatgatcaatccgtttcaatactaacctttttttatatgcatagattttgttgttggctttggacaaatgaagcataccagact
cgcgtcgcgtatgtaccaataagtagcaaccaaagaacaagatcaactaacattgttctatgtaccattttcgtttgcaattatacatattatatatt
aaagaattaaagaaagactttattgatagttactactatgacatttacgaaatataccaaacagcgaacaaaccgtgggtactcaaatagttcta
aacaatcagcatagcaccaaaggtgatttctattttattcaacttaaaaatagaatgtttctaaaaaagttactcaaatattgagaagtaaaaagt
aaaacaaataaaagtaaacactttgtcgcccaagggaaaataaacgttcgcttaacgaattctgtcgctaaaagtaatagtttaataggattttat
tctagtaagagcaattcaagcttttctacagcaaattagtgtaaaatcataattcctctccttaatactcatatacttctagtagttagctggaaaaa
taatgacagaaaaaggagaagcatcctatctagattctgcaaaggatgaagagcaatatgaggattatagtgagggttcagagaacgaacagt
atgaaaatctcgacgagattgactggacattagcttcctccggtatgcacttttctgttttatgtgaagaaacaagttttactttgcgtcggtcaactt
tgatctacagcttactttgtcatacttaaagaatatttgctaactcatagactatgcttcctttgaggctgctggttctgcttcaacaggattacaagc
gtctacacagccaaatagtagagccaaaatagcaaagggacaggatcgtgtttcaaccctaagccgttacatggatcacatgaatgtaaaggac
caatttgacggaagtggtaagttaaaggacgatcggcatgagtttattcggatttttttactaacatgaaaaagaaaaagctagcacaaaagata

aaagtgacagagctacatctgagcaagttttagatcctcgaacacgaatgattctgttaaagttgattaataacggaacaatttcggaaattaatg
gctgtattagtactggtaaagaggcaaatgtttatcatgctatgaatgaagaaggaaaacatcttgctatcaaaattttcaaaacatcgattcttgt
atttaaagacagggatcgttatgtatctggtgaatttcgttttcgccatggctataataagaggaatccaagaaaaatggttagggtttgggcaga
aaaagaaatcagaaatctgaagcgagtgttttcggcaggtatcccttctcctgagccattattattaaagcaccatgtcttggtcatgtccttttag
gtgacaagaaaggttggccttatccaaaattgaaagatgtggatgtcacagcagaggaggcaacaaaactctatggaatggttgctagatatat
gcgtacgatgtaccaagtttgccatcttgtccatgcggatttgtcagaatataatatgctttatcacaaaggaaaaatctatttaattgatgtttctc
aatccgttgagcatgatcaccctcgaagtttagaatttttgagaatggatatactaaacattaccacattcttcaagcgatttgacgtaaactgtttg
tccttaccaaggctatttagttttgtcacggaaaaaggtagcgttgagaaacaagatatgaaaaaccgtttggaagaacttcgagagggagacc
acgatcctgatgatgaccacgaagaagaatttctgaaaacgtacgtaccacgaacgctggaagaagtttacgatattgatcgtgatactcaaatg
gtggaggagggaaaagcaggaagcttagtataccgacacttactgaatactggttcaaaggaaagcgatgcggaaaatgtgaatgaccctcat
cccagcaatgactctgtttcggtgacagaggatgaagagtcagacgcctctgatttggatgatgacaatgattctgtcaggaaagataagagtgg
ccgtgaaaaagaaagtgcagaggaaaaacgcgctcgtaaacaaaaagtaaaagaagagaaagctgagaaaaggaaaacgaaggtctcaaa
gtatgagaaaaagcgtaaaataaaacagggaggtcgtaaaaaataagccagatgtttattctttttattttttttacgtttattttttggagggttt
attttggataatggatagaggatataataatacataaaaatttcatttgggtatttgttcattgaatttctcataagaatagaattatagtaatcatc
gtaacttatctatatagctttgacataaaaaggaaatcaaaagtgaagaattgtagaagggatcatcattatttctcgtttcattatctctttgttga
gttttgttgctggcctttatctctttttgcttttctgtcgacaagagattttacatttggtagctcagtatactgatgctctataatctcaaaatgcaaag
gtgggtctgtaaccgcattccataatgtttgcggcaaataatcaccgtactgtgtacaaaagtcgatacgagccttcgtttcagtctgctgctcttgt
tctagcttatataccagactgtcaactacagagcgtaccctcttttcgtaagcttgtcgtctatgtatttcttgaaggagagcataataggattttgc
aaattgcgtatagtgtctttctagttgtatcagctcttgggctaactggggtatctccaacccggtgtgagcactcataagctcatgcgctgcttcat
gacgccgattggtaagttgatttttatttaactcttctgcaagttgttcaagtttcttcgtgctagcatccacgttttgcttgtatctaagaacggtaga
atgataagtctttgtttcttcaaggccagttcgaagatccctcagcacagaaggaacatgatttgaatcatatcgcaaaatctcaatcaattccttc
ttctcaacatctgaaagaccggaataaattgtcaaagcctcagaacattggtcgtaatgttgagctacctgaagaagcaattcagccaaagtttcc
gctatatcattgttcacaggaggtaagaagtctaaagagatagctccaagccggatgtggtattgttcacatatgtcttcttccaacaatccagag
agcttttcatacgcattccatacaccgtctgtgactcttcgaagtcttgtcttcaaatcttccagggatgcgtcatcgacaaacgaataaagattgg
gatactcacttcctaaagcgggatccaaaacgcattctcgaagccttttcaacgatagttctaagcggtcatgaagatcatttaattgtccaaatgt
tggatgattttgaacaagatttaaaagttgttttattaaggaagtggaagagtccagtctggcacattgagaagctgttagtttagctagttcgctt
agttgttgatgctgtttaacagagtttttcaggcagtttttttgcatcttcattgtattcatgagcgtctccacaaagttgacgagcttctgtaagggcc
accttagcttgctcggtccattgcttcaacaagtccatcaccgcacttaagcggcttttgtatactccttctaagaaatcactcaattgcccttttaaa
cgataaaactggtatatagaatcttaaatgattaatatttattgttatatgacctcaaaattcgtcaattacaataacttgaagcacaatgtttgag
agtgtggaaatggtagaatgaattcgttgtaatatttatactatgatcacgaatttaaaagtgattttacgacaataacaattctctttgcaagatat
gtttttattttgttaaccaaaggaattagattgaaaaggatataaagagagagaaatatatagccttagagctactgaagatgatcaaggaaac
ctaactaattttaagataggaacggtagaaaaaatataataaaataaaagactatttataactgtgaattattccataaatattctatttttaaaaa
gctcgtgttagcacatggcaaaagaattcactcactgaactcgacaacctaaaatggggtaaaagaaggactagataaacactattaaggaac
aaaagggatcccagaaggagtgtttttgcgatagtccttgtagtcttcatcgaaaaatttgaccaaatacatctcttctgcaacaattcgtttgctg
aaaaagtcccacagtactatagtgaaagcaaccagagagaaaacattctgtaaaaggagctgcgttcctaaagcccaagcaaaaaatccaaca
tatgacggatgacgaacataatgatagacacctgatttaacaagactgtgctcctgtctcttctcactagcaacgtgatgcgaaaatgatttacca
gcgtgtaccattgcaaatgatcgaagtaactgtcccgaaacaatcaagagaattgctaaacttgtgtatacccagtgaacgcacaacaggaccc
atggagggaaatggagccaggatagaatttttgagctgctcgatccatcagtgaagtggtattctaatgcacctattgacatggcaatccaatacg
ctaccccattgttcaaaacaaaagagtcccaagacaactgaggtccttgaaactgtgctgttatataaaattctaattcatgaaatagccccaata
tcgcgacaaaagcactggctctagacttcgtaaatatgctcaaaatgcacccagatccaatgaccagccctaaaagggtacttgtagcgcttatg
aaagccacttgatttgatgtgtgaatattcggcatttttagaaaagatttatcaaaaaatctagtcacgttcggtcaaaaaatattatccacaaaa
atcctactagaatagttaaaatttcgcagtacgtattaaaaaataaaacctatagtagaagataaatattgatttaactttcaaaaacaagaaga
ccaaaacagattactatgcctgtacagacttggaagggtgtacatcctattgctccatttcttaacttctattgtctattgctaacaatagacaaaga
acttcattattctatgtcaaccgaagaattggaaaaaaaaatgaacacgaatgaaaaaaagtattaaatgagtcattagaaagatattgagattct
ggtagcctgtatttccacttggcaggaataaataaagtatattactatatatgtattacaaaatagagcatggtttctgtctttcagaccacccgttt
gtcgtcaagtctttcatcttttatcatatcctttttatcttcatcttttgtttttattaatttatcataacatgttaataaaagagcaataaaaggtgggc
gatcaattcacttcacgaacagcgtgatcaaaagccttggcaacatggtcaacattagaactgtttaggccagccatagagatacgaccattggc
actaaagtataggtgatacttttcttggcagaactggacttgagcaggggtcaaaccagtgtaggaaaacatcccgatttgctcagtaatatgatc

ccaagtaccaggagtcttcaaagcaacgagagagtcacgtaaatgacgacgcatttcattgatacgcgaagacatagtccttaagtctcccttcc
attgttcaaaaagttcagggttatttaatatttcagcagcaatacgggcaccataagccggagggttcgaaatggtgctgcgttgaatcaagcaa
agcacagattgcatcttacttttcacatcgttattcttagctacatagtgaagacaaccagtacgctcaccgtacaaacccatgtttttggcaaaag
attgacagacaaagaaatctctgtcaaacttggcaaactcattcaaagcccaagaatcacggtccaaatcaccactagcaaatccttgataagc
gatatcaaacacaaccaaatgcttgcgggcaagaagaacctcgaacatagacttccattgctctctagtagggtccattcctgtaggattgtgagc
acaagcgtgaaggaggaaaatgctgccttcggaagcagatttcataacgtccatagttccttcaaaatcaaatcttcggtttttagcatcccaata
aggataagtctctaccttgacattgacactttcccagatagtacgatgaacaggccaagttgggttagaaatgtaaacggcagctccactctttcg
aacatagtgttcagctaaaaatctagcagccgtgaaattggcaccagtaccagaaacggattggatagagcaaatacggtcttccttcaataatt
gctcgttgggcttgaacagaatttcggcggcaccctttgtaaaccgaccataaccagcaatgggcatgtattcatgattgaagttaggatctttttc
tacaatttcagtagccttctttacaactggcaaaatccatggcctaccggtatcatcacggtaagcaccaatgctcatgttcaccttcgaagggtct
tcatcttggctgtataaactgttgagtctaaaaatggcatcaggctgagcttcaatcaattcagtaaatccaaaatccgacatttttttaacttctcgt
gctggtaaagtccaagtttagaaaacggtttccgctgctaaataagtctttccatcttgatttttttctgatattaaatcacctcgtttagataatctttt
tcttaaaatggaatgattctcagtaaatgatatctacaattttccaaaaaatgaaatgattagaaataattaaaaggatttaaaaaatataaagtt
ttcaatgaagcttggttcatcactcagtcaaatgtagaatgaattgcattgttgattttgtgtaatcttctaatgttttagtgtcttgttgaaattcaatt
gtattattcatatattatcctaattagagaattgctctcttcatagtttgcaaaggacaagataaagtgtttcgtagaaataagtttcaatttcctatg
ctttcggacagtcaatgaacagatatcatcttgtactcttaatcaggaatctccgggacccgaatgtcaccagggaacattatgtgttgaaaaaac
caatatatgggtgaaagtagcttttggtaccatcggggtgaaggtttttcgtctacatatcggctagcaacgtttgccaatattgatagactattcac
cgggtacccaaacatttgattgttcttttggatgagcatgagacagcgaccaagcattagtaattcttgaggtaaacgatgacagtcttgtaaaaa
gctcacaaaccttgatttctgagtaagctgctcttcaaataactgctcttgagacttcagaactgactcgtctttaaagtcaccatcaataggaatat
tcaacattcgatttgcaaatagatcgggctccctaattccccaggaatgcatgactttcataatagtggctcgatcaagcgtataagcagcactaa
atagaagagcaaaactctttcgaaaagattccgattcatggacatacaatccatgatctaaaatcacgacctgaggctgtcgacctactttccgca
caactatattacctggatgaggatcgcaatgcacttgctttgtattaaacatttgaaaagaaaggaaattaaacaagctactgaggatttgcttttt
actgaaaccagcatctgacaatgcttgttggttgtaaaggggaatcccatcaatccactctgtaatcataatcttttctcctgagagtttgtcgtaaa
cctttggaacgtagacttttcctctcaaataatctgtttgatttatggcttcacgacttgatcgagtcgcttcggcttcaacgatgaaatttacttccg
accgaagtcgttctgaaacatagtccaccgtgaagtataagggaatatggaatatccatttgtcgtaaacatacatcatacatctatagaccaata
aatcccagttcatttgcttggaaacggatggtttttgcaccttgacggctactttctctcctgaaggaagaatagcctcgtgtacttgagctatgcta
gcactcgcagaagcgtttttttcaattgtagcaaaaacttcttctgctggcatgttgtattgagttttgatgatctcagatacttcatcccatcctatct
gtggagcgtcatcaaacattcccttgaatactctcccgtatacaggaggaagattagaagattgcattgcgacgatttgccccattttaatgtaaa
gaccgccatttttggataccatattcaccaagcgctgagcaactcgctcatgtaaggcctcaattcgatcagctctctgctcattgaagtttagcttg
taatccaatgcaacaatggttgctgtatacacggtccgaatactccgagtcaaagcttgggcgttatagtatctgtcccaaacgtaaattcctgttc
caatactccccatccagactagtctcttggtccaacttgaacgtttgcttaaagcttgcttggtagggggctctttgctgtacaaacgacgagagga
tcctcgacgcataacattcaaaagctttacattatgagatgctaacggtgaacgaattcctatccgagtaaacattcccaaacttcgtgacccaag
ctaatgactatagcttttgaaaattattcactttcgtcaatgggaatgggaattcacgataatatatagctcgtaaatggaatcaaaaatatccttt
acttttcctttctgttgctacccagcttttggacccagaatatgcgaatccggtaagtttgttaatgcaatttaactctttcactggcttatttttacggg
tataaattcttcactccttacattcgcagtgtaatttctcttgttctgaagttgtatgaagacgtttctactcaaccttgcattttcccaatgaacatctc
ccatgatagaacgtaaccaattcgagtagcatgttactcttattcaaaagcataaaaattagacataatgtgatataggaagcaaaaatctttgtt
tgctagaattatgagtcagttaaaaaatgaataaaatttgatgaacaaactcatacttttcaatcaaagtccttgtgtgtttacaaggcgaaaaac
aaagtcaggcgacatataaaagtgtcgtactttacaaagtttcttaggtgcgaagccaacgagatttttttattctctaaatgttcatgtattttctta
ttaatatattaatgtacctagtggattttattcttcatattacttctgaatatattgtttgacactgtacgagatggtagttgtatcaatttttttaaaaga
gggagcaaacttatcgaataacgagcaaataaaacgaaaaaagcagaaaaaaaaagaaatagaatggtattttaataaaaaaaaatcgaccgc
ctttgtcgattactttctattcgattaacattatacgatatcacagaacatgcattatcgttgtaatagggtagggcaaatccaaagacagtcacga
aaaggtctatatatttgttggatttccgaactacagctatgcaaagacacctttgagctcgaacgatcgttgttgaagcaatctaacgaaacacta
cgtttcttttgcagctttagggaaagtccccggactcttgcgtcttggtttcttttgaaaccaggacaagcggaaacgcgctgacctaaagaaccag
cggtttcaatggaatgtttgtcccgtggtaggcgctgtcttgatttttcttgtaacctttctatgtgcttttcgcttccggaacggagtagacaaagat
ataagtctatgaaatgtgcataggaaagtgtttgaggaagagcaaaacggatgctttgcgggacttacggtttcattgaactgtgtttttttcgtcgt
aggcgatagttatacgatcacatccaccagtagatgtaactcaatggggcttagtcatgggctcagaggatttttgccgtctttttttgaaaggtttg
agtggtttctcttgtttaagagagcttgttcgattcaagccaaacctgcataagatagttaagcttagaaggtatggatccaaaatatgatgatcaa
caaatacaaagagtcaagcactgaagtacgaaacgtactgtgttgagttattcggtctgattttggaatatattccataggtctgtgcttggtagat

ataaagtacatagatgaatgtatatgtttatttacttagcattcttttgttccatatggagcttaagaaggagggtttattagcagagctataaaatg
tttttaaattgtaagagtcgcttattacagtctatgggcacttggaatatatcactaaaacccgaatgttggttggctttggctaactgtttacattttt
gcattcctggtgtttagtttatgcgttgtttgttgacttttggagggacgcccataaagggcttttgatgtttatggcaatgtagcggtcgcatctcttc
ctttatcgacttgctcactttaagtctgattttttttgtttttgctttttatttttaaatttattccttaaaaattgtacacagttaggatcaggcttaaagc
ttagatgccctatttatcacacatatcttgcatgagattttcctctagtgcattgccctgttttctgagtgttcttgtcattttctctcggttctgaaagct
gttctaatacagtcatatgcaaccaacttttacgtatcagagaaatgttagtcaattctgatcaccctgaagaacacatgcaaacgctttatcaaa
gaattgttaacgcctttttggatgttttgttatgttgtgtatcctgtgacagttgcgatctaggctgtccagattgcgactgtgaagatccaacagttg
aaattgcatatgctgatattgacataccaaaagaaactcatttttttactcctgaactctctaccctggatccctagtatctgcgaagtgttaggatta
ggtgagtttgagcttccggattattatgatattccaaatacgtttacgcaaggttctctcagtagtgttttccatgcattcagtggtacctacttaaaa
agtagctatgaattggatgagtctggtttttttatgccagacaaaatatatcactttacacaacgcaatacatgcgctcaggaaaatgtgaaattat
tacttaaaagtcatcgtagcttgtttaatcatctctgtgaatatcttcgtgccggcccgattccaatctcggttttgattcatcatgtgatgctatttca
gttttacccaccagaaattcaagaatctctttggattgctgtcgagcattatctctcaaattatgactttgctcatactaaactactaaagcttgcttt
acaaaacagactaggatgtatacgcatgtatcttattaacccgaatgatatttatgctctcaaagcaaagaataattgggttgccgttacacgttc
aaatttcctaagctatctccagcttcggttaccgttatgcgagagcagtcttccatatgatttatgcggttttgaagcaatgctgcattctgcttcctct
gaagctgacattggctggttatctatgatgcattgtataggaaataacggtcatgcttttcctattcatatgtatatgaacacaaaggatgcagtttt
tgaaggaaaggtcccagaatccgctctttttctgtataatactgatgctgttctatttaaaaaagattcttttattcgagggttttacgacttttgcaa
ccaattactcttggacttgtgcaaatgtcgacaatactacacagaaaaagatctggatgattatatctcaattcatcctctgcgtaaccaaaaaac
aatcgcgtaccctgagtctattttcgacggttccaacagtagcactttgaaacaaagcaggacgcatttaacttcttcagattctaccaaggactca
tctgaggatcatactccaaaattaaaatcgttaacataccatgcgtctcatagtagttaactttttattcatcaaacgtgtttgcggcactttttatat
gaaattaatattagacttacactatgaataaaacacaaaaagtaaaactctgttgaaattttttaatactaaatacatatgaaatcggtgaatttc
atatcgatgaaatctcagctaaacatataggtaactgaaaatgtctagcaaactgtgatataagtcataagactccaaaaatttgctaatgcgaa
aagaaattaacagaatttgcaaacacaaagctcaactcgtaattacttgttcatctaattcttaattcatgcagtatatagaagtcgttattctcaca
caaaaatattatcgtatcgaggaaatataattatcgcgatcactgggtcccatgacattgatgtagtcctcattgttggcgacaaccaaactgcttt
gaaggtatgacatcgggtaatccagagaaaacggtggatatggcggtatgaagacactggcagagagcaaaaaattgactaaaaacctttaga
agctcagtcccgaggagcaaaagttgttccgtctctatggaagacttcctcagcgtaaagatcttttagttcagaaattgcaggtatgtttgaatag
ttttagaaaagatacgtaattagggttgaatattgacttggtattttggtagcaaggaagaaagtacttcgattctggtgactatgcattgaataaa
gctggtaaggcgtctgatgccggcataacctgcatagggaaagaaattccatccccggagacaattcctcacagggtcgcagtttctggctctccc
tcaaaagaaccatcgattcacatgaagcgcccaacggattcagcacctagtgaagtcagtagtcgcagagaatccattgtccagcatttcgataa
cattgatgatgaaaaattaaataatgataagaagcaagatgagtaagaacgctgatgctacgttacgtataagcagcttgcgcgtgctgctcata
atgaatcttcgcgattattaattataaaggctgaaagactcacctgcaggtgtcgctttttgaaaatcactttgttgttcgaaactatataatagtttt
ggctcatctgacaatgtacaccggccttgcttatccatcactagatggtatatacgatattttctccttttactacttacttcgctactcttttttttttttt
ttgcgtaaaaaatgcttttacctgttccattgaattttttttctttgaattatatatttttagtttaattggttatgtgaatgcttttgaacttttggaatgg
acagtgagttaaagacgagcagtttgtagatatagtgaccgtattactatatcttgctatgagactttactcttatgttacacaatagaatttgtgttt
atgcaacgtaaaatactatcacctcaggtattgttgaaatgttcatagaattagaataaatacaatatagctgataatgctaccaattcagaaacc
gtacttgggtcataggtcgtgtcaactgttcatagtcacattaaatatatatatacatgtgtaggatgaaagatactttttttcatcttcttagttcatgt
tggtagtaattaaggtcattccttgcttgtaattttcaaatcattccctacaaaccgtttaagttctttcattttcttcaaagaaaatcaccgttactgt
aagatcgtctcgatatctcctggagagtggataggtcaaagtaaggagagcagaaaccatttcatccttccgacccaaagcgttgcgaataaggt
gagtagcagcatttccatctacaaccggtaacgttttgtgaaaccactttaatggattaaacgtacttggtgatgtattctcagaagcatgattttct
gcgaattctgaatcaatccattggccaactaactcgactgctcgctcactagacattgtgtcccataagccatcggaagccataataagaaattta
tgttttttagaatcgacctttattgtttccacttcaggaacagctgttacgtacggcggcgattttacaggaatagggacagcggaaaagtactcttt
atgcaaacgttcacttacttgtcgggaccatttaaatcttgcatcaccaaactataaagttagttagaatcataatataaagctcatgctgaacaac
ttacggctcgagaaggcattaatcgcccaagtatacggttgttgcgtaaaacaatttcgccgggatgttcaacttccaaacgagcagcttcatctg
gattcgcgcctgtttggtcacgacttaaaggaacagcgttccaaattccaccggaagttcgttctcccaatacagctcgcgagtcaccggtgcagg
caacttgaagagtttttgactttgcagaatatgcagtcagaagggcgcaagatccagaaatagctggtaacagtaagctagctgcattctgtaat
gatttggggtttttaaatacacttgaaacatgctcatgtacgatgcgatgatccaacttctggaatgcgtcgcacaaagatcgctcgtaacttaatg
gtgaaggacacgccttactttccctcactttcaatgctgtttcccttagctcatcaactacggctggaactaaatgttgacgcaagtacatagaagt
attccagccagagtgaccatcaaaaacaccccaaaagtaccaattcccatcatcgatctcctttgaaaccgactctacatgatcatcctcacaagg
atcattacttggcacttgattaaagtcgtatctggcgatgccatctcgattcacataaacagtcttttggaattctcgcaactgcttgttaatgctttc

actatcctttggcggcaagcgaacctttgaaggatatgggtaaaccgtattgggttgatctagttgaatggcattctgttcacgtagctggtatgttg
cataacccgctgaagaaataattgcagcagtaattgagatgtgctttagagagcagatgcttctagccgactttgaggtaaaatatcccattttct
ggatgaatatctccttagttcaactggttagaggactgaccattatatcagtcagtattatataagaaattttacgagatttactaaggaacgcagt
agtaaacaaacaatagatgaagttaatggatacactgttgaatgactcgtttttcatagagcgaataaaaagaaacgaattattcgttagaaatg
aaatcattcatttcgatatccgtaaatgctctacctaaggaatggaaacgtcatgttaattggatatttgcagtgagcatagaagcaaatatcaaa
atgaatccctcattatgtattaggagacaaaaaaaacgaaattcagactgtagtatacggcatgaggtggtaagcactcctgtgggatttgaaag
tgagacccaagtcagccgcgcacacatagaaaagagcggtagcaggaagctagtagagttgatttatttagtcagtacatcgcttaaaaccttct
gaaaagcagaatattcaggattaggaaatggctttaactttgtattgaatagagcctcaacaccttgagcaaggtctttgtaagaacaagttatca
gatattgtaacgacgccagcttgtttgaaagctcaagctgatggttgtccattagagattcatttgggacgacaatgagaggtttctgggcacgca
aggattgcaggatactacctgcgccggcatgcgaaataacaatagaagacgactcaatatatgttccaatttcaggtgcatagtcaaatccagat
atagagattccttcgatagggcttggagtcccaaaagcctctcttccatttccaaactgaacaatcagttgagtgattccatgttgtaacagctggt
attgtacattgacgtgtaaaacaccagaaacaagttcatcaaatctggtagaaccgacagtaacaaaggctttcatgtcttatttctcattcactac
taaatatgtataaatctttacagtaagtcaagtcaaacgcagtcgctgactatttacggtaaatgaaaacgactcgagaaggagaaccgaaattt
aaaaaatttgatttcttttgaaaggaaaaaacccgttttacacagaatgttttcgtcgttttgctttccgttctatgtggtgaaaagatgcgacaact
ctgatggtgagtgaacaaagtaagcatttcttttcataggaaagctcaaatgaatatgtggataaaaaaaaaaaaagttttcgtcagtctgttcca
gaagaattgctgttagcaaaagctcgaagaagtgggtatctttcattaaaaactataactaatagctagcgtagcttacaaacagaacaaacttc
aaaagttatttcatcataccaccacggcaagtacctgtctgttcacaagttctaagcgctcatgaagacaattcgttttacagtgtcattttcagtat
aattccaaaaccacaagacgcagaaacagatgcaagaaagaattgcaaacaaaagataacctcttgcaaatttttcgttagtctgctttttttcaa
agtcggtctagtctgatttggtctgaccttatacatgcaatgctattctcgacatgctgtatcttaaaagcatacctaaccaagtcgctggaatacat
cctataatggggtttcatgtaatatgacccattcatcctttacaacaccctttccttggatccttgttatatactcattccttgtaatgaacaaagcga
attggtaacttaactgcaatcgtatcccaaccgaaaagaagaaagaagagaggatatctcgaatgttcaacgaagaatgccccttctcatccca
aattttagatgctaattccaacacaactaagagctagatcttccttactgtatagcacagaaagaattcgtttcgtctccctagttgattttcttcagg
gaatgcttgaaatccgtaattgtatgaatgtacaagaaattatacttacacgtctacagtacgtccgatctaaaaaaatattcagtggaaaaactg
gaattttgtttgaatattgctccaacttagaaatatagctacagcactctccttactttatagtatcagaaaaggagtcaactgttgatagtcagtcc
ttcgatattataatttatcgttggtgcatctgcagcggttaaaacctagtacctgcaccgaagtagaaaatttctaaccataaccaacacccaaaa
aagttggtgtgaatcgcagttctcagaagtaaaagaaagacaaaggccatgtcaattcaacctaaagatttgtctcatcatctttcggtggaaagt
gcttctcgtaggccatctccactaaaggcaatcgcaggctctgtgagtaaaaagggaatcaatgtgatttctttggctggaggacttcctaatcctc
aatatttccctttccgtaagatgggctctgaaattccttctatcggttcctgggttcgtggtgctgaaaacgagggcaaattgtcagatttctctgtct
caatggattccgaagatcaagatgacttacccctttctgtcgctttgcaatacggaaaaggctctggttgtgctcaattgacaagctttttgaagga
acatacccaaatgattcactcccctccttatgccaactgggacattttgcttactaccggtaatacccaaggcttggacatgtgccttcgtatgctta
tgaaccgtggagacagtattcttgtggagaagtacaccttcccctccgctcttcaggctatgcgtcctatgggcttgaacgctgtctcagtggacat
ggataagaacggaatgttgcctgaatccttagaagaaattttaaataattggaagggtggaaacaaacctcgtgttttatacactatccctagtgg
tcaaaatcccactggtagcacattgtcagtagaacgaaaagagaaaatatatgcccttgctcagcaacatgactttataatcattgaagacgaac
cttattattatcttcaaatggacgagtatcagggaaaacctgaggctgttgatcccaagtttacaaatgaagagttcgtcaataatctgatcacatc
tttcttacacattgatgtcgacggtcgtgtcattcgcatggattctctttcaaaggttgttgctcctggttctcgtgttggttggatcaccgctcaacctt
tgttcatcgagcgtagtcttcgctataccgagactgcttctcaaaatgcttccggtatatctcaagccatgttgcatgccttgttcaagacctgggga
catgatggatacctcgcatggttgaaacatattcgttattcttacactgaacgtcgtaatgctctactctatgctatggataagcaccttcccaagtc
tatctgttcttacattgcccctgaagccggcatgttcatttggttcgaggtggacaagtcggcgtacaagtttgctgaccaaggcaagagcatcgcc
gaaattgagtcagatattcatgaggaagcaattcaagatggtgctaatgttgcctgcggtaattggtttgtggttgattcttcaatcaatgacaaga
tctttttccgtgtcacctttgcttatgctgagcttcccgagtttgagactgctatagaacgttttgctaatgttttgaagaagtttttaagtcttaaatg
tatattaaatatctgctgcagttttcagtatctgaggatgatagttactcattataatttagatgatccgccatttttgtttttctttttctttttaataca
aagctactttctattaataaaattcatatgtttattgatgtatatcaaatacatccaagctataaactacaactcattgtaaattcaataaacttcatt
tacagtaagatcttaacagtacatagatgatcagtaaagtatgttcatattcatctcataagattaaaattatttcattgatagaaaagcactatct
atcctcaaaggatgatttgttcctaaacgaacggcgtatatgcaacaaattgatcaaatcttcgatttgtcataatttatcaaaaataatgaaaagt
aaattcgcatctcctatattcagggtataagtacattatgtaggtttacaaggtgaaatttccaccggggcgcttgtaatccatataacagactaac
ggaagaaagcctggacgaaataaatacaagacattatcctcagagttcataagtttattctcagttcctagcagcaagttttgcagctaattccttt
ttctctgcttcatcaagttcggttaaaaccaaagcaaggctatcaatcatgctttgcttatcagcacgaatcaattcagcaatcctattgctataata
cttgctcttcaactctagggtgtgcttaattaggctttcgctgttttcttcaatccatgtaataacggatcggtcagcagtatcccaatcttgcttaccc

ctccattcctcataccatcgtttaaggcaacattgtgattcttcgtggcttaatccaggaataagctgcatcaatctctgttgggagtaatgctcatc
caaacgtcttcttagacgccagtagaagaagcgtctagcattggtccactttaagggttgacgaataactttcttagcaaccatacgaccaacgcg
atcatgcaaatcagcaaagtgaatgctaatttgttgataaataggcattatcttttgctcacgttccatcaattgcaccttgactgaagaaagctca
tctgggttcaaattcttttggttaagtttatcctgtaaagtagcatattgctcatcgcatctgcgcattaatgaaagaagcttttcacgtctaaacttg
atactgaccattccttcaggttccaatacaccagctctgctttcctcatcagcatacatctccatttgcttgttattaatggtagggtcaacaaccacc
caagagccaccacgaagttcactgaaagggggtatgtatacaaatacaggttgcttgtatcccgcgagagcgtcaacaatataagagccatattt
aaggacttcattgaacatatcccgttgaccaccggagaaaccacgccaattagcgagtataaataatggtaattgttcaccatggttaaaatcatt
aatggcttgagcagtcttaaaagcagaattaggataccagacttgtccagcttccattaggttctgctctgtagagcttgggttggctggatcagca
ggtaaagtgttttcgatagtacgagtttcaacggcaataacaccagttggtattccacctaaacgtgcccgaccgacgacaaccgtcttagcccat
ccatccaaagtttcttggaatgaacctttgtcaaataaaccatacaggaacaaatcttcatcctcttttccatcaattaaccagcgaggatcataac
cgctttggctgggataatactcgacatcacgatcccagggatcatttgagggcgatatagggacagggttattacgtttgtcagggatataagaa
agccagttgacgatcttagaaattccgtcgaaatcatcttgggcagtcaagtgagaaataccgtttctatgcataacttgagtaccacccagttgta
agttagaattatagacttcacgtcctaataccttattgagagcaggagcacccgtaagaatgataggctgaccttccacttgaacagctctttgtcc
aaggcgaacgagatatgcaccaataccaacggcacggcaagttacaagtgtgcaagtgaagatatcaccataagctcgagatgtaacaccagc
aattaaaccagatcctctcaaacattcaacaccgagtccttcaccagaaccaatgatggtggtgatcttgtaacgaacttcaccagattcagtttc
aactttttcagtttcaatagtcttagactcttgcttgtccattcgctcttgaacttcaggagttatatataaatattcgaatcctttctcgggtgaatcg
gcgtcgttccaagctatattgaataagggaatcaattcatctgcaacaccaatacgggcaccagaattggcagccaagtagagtctgggaacac
cacgttgacgggaaagttcagttaccttaaaaaagaagttgtcctcacgaggtccgaacgagccaatttggaatgtaatatcattggccacaatg
atcatttttctaccattaggatactcaggagtcttggcggttactgaccaagcaaccataccacaagagttagtccctggttcacgactaacttcttg
taaatcaccctttcatcctcaaccaactccttacaatcaaacatgtcttgtggaataggattctcggaatcaccctttgtccaaatttctccagcaat
cgctaaaagcacgacgaaataactcagggaaatcatatacgaacgttgtacccatcaattgtgccttgtagcgacgaggttgaagccattctttat
ttggataaggcgtgttaatacgtctaagatgcatggagccaggttggccaatgcttttgaatatccaagagttattttcagtcttcacttctgcataa
agttcaatatttacgacaaatccagacacgttagatattattacacgcaagggaaaagagtattagtggtaggatcggtgcaaataatacgaat
ttcagcagcggttacacgcaagcgccataaacgacgtccgaatcgctctaaaaatccgcctaaagcagcttcgacctgtttgggggcaagtccaa
aagcaggaaggaaattgataaagatatgattaagatcagtttcttcagggctaattacctcaagagcatcaagaatgtcatttatcaagcggtga
gtttcagatataagatactcagcggttgggatctcgtcacgaagccttccaggacgtacaagagctctagtaaagaatcgcttatctgtattcatgt
tcttagctgttgcacggtaaacatgaatattgtgattgtcaataaaaacaggctcgatatcgaaattagagagtctacctaattccaattggaaag
ccaaagcgggttctatgtgacgaattcgttcatcttcaacgtaacgaaccgactgatgctcaaaactatctgaggaagcgcgataagtgaagtat
gatggatgggccgacttattatgtcttccaccaatcactgtaattcttcggacgttatgttctaacaaatcatcctggaattctcttagaattgagcta
agcctgtccacgatagcggaatcttcaaggtcattagtagatgtcagagcaacatttaaaacattagtgagttcttgaagaggagcagtagaagg
gtcatcactgttcaacgtcaatccggcattgaaataattacgcgaaagaggcaaacgctcgatggccagagcaaaattggtttccaactgatcaa
agttctcagccgcaacaacagcaccaaaacggaaaggttccgaatcattcttgtttacataccaagaaagatcactataagattgaagacgtttg
ttttcattttccaaagaaggagagccactgctgggaagaagaccatttgcaccactggcaccttggcctggtgtactactagcacgaagctggaat
ttccaagaaagcacgaaaggtgtaccactctcaacatgatagtttaattctaaaacggcatatgctcgataagctctacgcacatacacttcaag
agcagctaaagaaacccatggatcgggatgacagaagaaagcaggtaaaacatcgaaaacggtatacttagagtcaatgagctcctttaaaat
atcaagattaggaggacgatgagaaaacttagaatcaccataatgactctcgacaactgaggttttcaagatatgttccatttgggaaaaccgct
ccgaaagcgaaggcatagcgcacgaaatcagtaattcacgagccttaagagatactttggcagtagctctggaatcgagatctgtcaattttctta
aaatatcattgaagtataatgaaacaaaagtagagggttccgattttataagatcaagcacagttataagaaggttattcttagaaccgatacga
gaatgagacaaggcaagagcaatgactttatcaacgttatccttgttatcgtcccgaaggcgaagggtaacatcttcttctcttttgttaggacctg
aaaacaagcgctcaacgttataatactcctccaagatacttttgacaacattatattcgtgcacctttagaccgtctttataatcattgataacatca
atcaagggagcaatttgctgttttaactgttgggtcaaagatagtttctgaccctcacagtacgtctgaagagccatttctagctgtttggcaggaa
attcagttctgcgggctcttgatttttcaataatggaaacaaataacttatctaacacgggcggcatgcgactaaccaatgcagaataatgagagc
tccactcactaaatggcagttcaggatcacgcagaacttcaataatctccttatatgtactgttaagaattatttgattgtcataacccttcaaaata
tcaagcaagatggataacaattgatgataacgttggcaaggcttgttaccagcaatttgaggatcaccaaagttaggtagctgaccgttaaaggg
aagtgcatgtttaacacggctagggtcatccaaagtaaggataccaaggatgtcacctgaaccaacagaggcaccgggctgttttattaattgca
caataccatcctcagtggctactaatggcataatcattttcataacctcgacttcagcataagcttcacccgccttaatatgttcgccggtttcaacc
aagaaacgaaccaacttacccggagaaggagtacgcagttgagttggatcattttcccgttctagcatacatgacatgttatctatagagattcgt
gtaccggtaacctcatccctataatagacagtataagactgaccatttagcaacacaagtaacccaccatcagttaatgagcgaactcctgcggt

acaacgggagccatttaagaatagatgataggaacctggagaagagcgagaggctgtgaacctgtaacgattaccgtcgtaaataaactctata
tcataaacatttttagaaaatcacgagaaggaacttggccacgttccaaataagatttaaaggcacgatattgttcttcggcaattgcatgagca
cgaaccagggcaccgcaaacaactgtaagcatcttatcaggacgggcagaagttactttttgagcgataagtttgtctaaccaagcggtggtaaa
ttcattatgagcaaactcaccagtctcaagtagacgaaccaaatattcaacggttgtcctaaaatcaccacgaatagacaattccttcaaggctac
aatcatagacttacgcgttgcttcacggttatctgcaaaagcgaagatgtggccaaattgggaatcggcgaattcatgaataccacctgcggtac
cgacggagaaataaccccagacatcgctggaagaacggaaatttaagtctctaatcataccgctggaaggcttaaaccttcaccgggatcctct
gaggtaatacgacaagcgatacaatgaccctttggagtgggcatttttggattttatgggattctggtttttcaaaagcaaaatcaatctcggtct
cgctatcacgggatagaccgtaaagttcacgaatgtggggaatgcgatttaaaggaatacccatagcaatttgaagttgagcggcgggtaagttt
acgccggaaaccatttcggtggtagggtgttcaacttgtaaacgagggttcaattcaaggaaataaaatttatcattttcaggttcataaagatac
tcaatggtaccagcagacgcataaccaacaagttcacccaaacgaacggcagcgcgctccatctcacggaaagtactaggaggagcgacggtg
acaggggcttcttcaataatcttttgatgacgacgctgaactgagcaatcacggccaaatacggaaatgttattgccgtactgatcggctaggatt
tggacttccaagtgacgggcttgaccagcaagcttcatcacaaatacaggagagccagggagttcggctaaaacctgttgataagcgtgtgtga
actgctgagaggaagagacttgacgaatacccttaccaccaccaccttcggacgccttaatcatgacgggatatccaattttctcggcgatagca
acaccatcctcagcggtattgatacaggccttttgataaatgtcgtcatcgactgttacaatgttggtttcgggttcgatgcggactttttcgaggcc
caaacctgaccagggcatacaagggaccttcgcactttgagccacaatggttgagctaattttgtcacccaaactacgcatagcactaccagggg
gaccgataaaaacaatttttgtttgctagcggccaacatttctggaagtttggggttttcggaagcatgaccccaaccagcccaaacggcatgga
ccttcatgcgctctgcaacatcaacgatgagttccacgtttgcatagttgttattatttgaaccgccaggaacttccacgtactggtcggccatacga
atatagtcggcgtttaccttcaaatcatcgggagtagccatgacggtgaatctgatagctctttcattattaaacgtttcataagcccattttcggat
gcttcgaatttctttcacagcagcgataccattattagcaattaaaatagacgtgattacggtatgtccaccacgtgaagcaatataatctttcacc
ttgctggaaggtgcaaaatctagggaattgcctcctacagacaaggttagtactgaaaaaatgaaaaattacttttcgaaagaatctatgatagtt
tgaaataaacgccaggaaactcaatgtcaatgctatgcttttacgataaaacaaaatttagtcatattctgaaaagtgcttctgacaacgcgaaa
atattagcagcactcccacatatgtgtggagaatacaaatacgcacgttaaatttcgaaaagcaagctgaaaaaaacagataatgagattgtttg
ttttaatagaaaaaaaataacgacatttggttcctggccttcattctcgtacaagaatggatcaaactgacagatttcgcattcttgaaaggagaa
aaaaaaagaatagaacatgaaaagtaattctttacatacccagaaaatgggaggctatacggggagccatagttctaagtctacgggaaaaga
gacgtggaagggaaatggttgcttttattgcgcttggaaggagcaaacccgcagtaagaaatctcctctccctacacgaaaggttcggagttacc
gttcccacgagtagcgcttgacatttgaatacaaggaagcttttaaaaaaaaactaagaaataatataatgtatttaaatatgtatcttatggaat
agaacgaatcatacaatttttaatgtatatctcaccagatgcggccggcatagacgagttatccgtgcactgggacgcgaaacgatagtcaatg
attggaggaggaaaaagggggttttaccttagataactttgtctcaaggagaatggacgcatcatgaagattgtaatttgtaaacaaacaaaa
aaagaaaagttaaagcaaaacaggaatcaattcctgtcttctagtgaacagccttaaaatggaactttctctagttctttcaaatacgtttcctaat
gtgattttatagttctggatgccctttatttgttaggttaccattcaatctgtctatggccgactgagtgcccaccacatgaactacctttcccacattc
tcttcttttgtgaaggattccaccagtgatggaacttacttacgaaccagacgtgcctaccacttcccgactggttagtaggaaagcatgtgacttc
acgttgttaccctaaaccgagcaaatgaaaattcaaaggtcgtactcggaaatatgaagtgcataattgggcataaagcttttgctttatttctcga
aaaaaaagtaggttcggagtgagaaattaagcgattcattatggcttttttttcgaaggaactcgcgaaactgaacaccgaggttttaagaagta
ggcgtattcgttgacaaagttgccatgacaaaggggaatccatggagaagaaggattctttgaaaaaaagcgccctagacgggatctatgaagg
cgataacattttctttgtttacgtcttttggctttataaagtactatctgataaaacagcctcgttcacttgttttggatttcgtatcgatgctacaataa
tactctgccagacgtatatcatctgctagtttcatagatatcgtaaaatagactcatagaataaacacgactttgagtttttggttcgaacagttgac
ttcattgctaatacgattggcgaaataatgtaaaccaacatgtataccaatttacctcctctgtttacgaactggcctttttttatattagccagtcag
ccaacaagactcagaatggaattgctttagatcatcggaaccttcaggtttttttttttggttttgatatattccatattattcttaatttttgcttgatc
gtgtctttgtttacttggtttttaatatcgtattatagtaatacgatttcaatcttcctgttagctcccttcctttcactaaaaagggtctacttgaattg
aaagtattaaatagcaacattgctaaaaaaccgtagcctaccgtaagaaactataattctttgcgaaaagtgtgtaatatatcgtccttaaagtag
cgactctgacaactggactttgcacttatctttccatttggcacttctttttattttatcttatccataaaattacaaaatggcaaacgctgctcaaaa
aaaactagcattgcaaaacaagcgaattctaatgttcatgctcgtggccgatgtcactgtcaacttcttattcgtcttgcttcgctttttttcttcgtagt
cctctttccaagttctccaaactcttatatatctttgctgcgggctcctcgggtttcctacattaccagctttaccgtgcggccgctcccaaatacgatt
ctaaaggcgctctgctgtacgtgggccaggatctcttacagcagggtgttacatcgtacatgattgattatatgtacttttcttggattgtaattttcc
tttctgccctgacttctaccaagtttttttgccttgtatttgctagtacgcaacaatgactctccgtcctattttgcttttactaacacactttttaggttcc
catctttgttggatataaagcctttcctttgttacgtatgggacttgagcaattacaaaaattcaggaaccaaggctctaatgaccctgcctcctcgg
tacaggaagagcctactgctagtaggcctctttcaaaacgtcaagaaaagctccgcaaaaagtcgaacaagtaccattctggttgattttcaatg
ctctagttcttttaagaacctttctgtaatttacatagacggtctaggtcaacacatcaaagagattctgtaagttacatttcttacaaagctaatcg

atacaagttttctcttcttcttatatcttcttttcgcaaattaaatttataaacaaaaaaaataaaaaataaattaatcatttaatcaaatcaccttgg
aatcattacaaatcatcaatatgcagcagcttgtccgtactttcgaggatcactcataccatatattatatccttgtgctccataacagcttgaacag
cactcaatggccactgtctaggatttaatcgaagcacattatgtctgtactttttgattctcgatgcaatcattttcgaaaatccgttttccaagatca
acacattaggtcgcagttgatggtgagagcgtgcgctagcaaccatagactcgatgtcgtaatgcatatctacacgctttacaatagcatccaga
acagcagtaactattcgactaccaccgcttgctccaatcaccaattcgaaatcaccttgtttgttgctaataatcgtaggagcggcagaagagacg
ggacgcttgtacggttcaatatagttccatggagatggagtcaaattaaatgcatttgaagaccctggaatagagaaatcatccatttcatcatta
aaaaccattccagtaacaggatccatcaactgcgacccaaagtacaaattcacggtggatgtaagagaaaccgcataaccttcatcatccttgac
agcaatgtgtgttgttccgtggttgacaggaatatcgtatgagggattataatgctcccacgaatatgtttttgtcatagagatatttctacgtacgt
ccgatgcaaactttttcgacatcattgttgaaataagaggcaaattttcttgaataaagtccgggtcaccaaactgggtgcgtccagaagacaacc
atttcatcgcctcaacctgcaagtgtaatctttttgtaaaagataaagaagaagaatccatggggaatccatctaatatatttagtccttcaattaa
agctggcccactggtcggaagaggacatgtgagaacatcatggcccctataggttgtctcaatggcatcagataccactggaaaatattgttcaa
aatcttccttcattataatgcctccacgttcctggataaaggcaaccatcaaatcagctaaagagcctttataaaacggttcaacaccttccttggc
gataatctccaaggtttttgaaaaggcaggacgacgcattatatcaccagcctttaataaccttccataaggagcaataacagaagcccaaactg
ggtcattaatcaggtctttagtagtatcatccagcaagcagtgttccattgccggtgttactgtaaatccttcccttgcaagcttgatacttggttcga
caattttgagccactcaagctgtccataatgatcccatgcatgatataaaccggctaattcgccgggaacgccaatggatagagccccaacccgg
gctagcatagggtttccgtcaaacatatgcttggaggcattagcgggagccatttcacgaaaattaatggagcgagctgtatcatttggcagtcga
ataagcataaaacctcctccacctagaccgctagaacaaaaatttacagtaccaatacaaagagtagaagccaccgccgcatccaccgcattcc
cgcccattgctaggatgtctatgccaatgttagagcatccaacgacttctgtcgcgacagcgcctgactttcctttaactttatgtcctgtaaattga
cccgggggatgaagaggagggttaaatgcataaaggaatacgagcagagctaagatcccagttccgaatattagcagtgcgacatgacgtatc
ttaaatcgaaaatatcgattttgacgagcgcgatgtatttcttcccagtcagagtcatgttggtttgtcaaatacgaactccagtcattcagcaacg
gagttgtaattgacaaattagacatattaagtagagaatgatccgttgtttactaaagatctgaacgaagaataaagacaatatgaaatgaaata
aacaaagatattaggaagtagaaagattgtcaagcggcaagaaaaggtttcaatgactttccgaacactgatttggctcagagagagattgtcg
cgcgtttaggatttcagattgagttttctactgaatcctacaacgttgaaggaccccgaaaaatgaatcacgagaaacgagaccttttcctttatgg
gaacctaacttgctattctattgtgaccgattagatcctgagcaattcaggtctaacaaaaaattcacgaagcctttcaccgtccaaacgctcaaa
gactaacaaaacaatttgaagaatttatataagaaaagactgtttaaacacttataagtctcagcaaatcaatggaatgattaggaagagctttg
cgagaagagtgaggtctgtacttaacactgtaatgcgtacggatgcactttgttcattcctcctgctacacgcgacaacagcaaaagaggagata
ccattctatctccaaacagtagtgagtattctcaaggagtaatatagactagttcttttggaagctgtaatattagttattggctttttggctatcattt
tggtttcttttgtgaaataaaagtttgaaggagggaacgcgtccttgttacactgaaagacccgtggttgaaagaaaaagctacttttatagacta
acaaactcttgattgtgtgttgttatttggagttctttacaaataagcaaaatatagaaaactggatcttttacccagttctctttcgtcgtcgagcta
catcatttattttttttttttagaaaaagtgggataccttattgttgattattgaaatgtcagaagctcatttggtagatggtgaagccttgcaattccg
caacttgaaatatgcagaccttggaccagttacggctctgaaccttcgtgcttttcaggacagtgacacatggcaatggagatttcgcggtgtctcc
ccttcaggtttggtagcgattacaaagcattttggcagaaaagcttatttcgatcctcatgtaaaatgcaaggtagcatatacttcgtcccgtcctta
ccttggctttttggcttatgagcagtttcctcatacttcttcgcagctctcttattatcaaaaattgatgaattgggtgaattctctttatgagcgatttc
tttattggtggtacgatgctggcgctgtcaaacgtcgcatgcagtacaatgaggtccaatatggaactgcgctttacaaaaccggtttatttaatca
tcctggtggctttttacacgttcacttggttgccgttgatccggaaatccagaaaagggggattggaaaggctttactgaacattgtccataatgat
gccgatatgcatcgtcttccttccttcttggttgctagtacgatcggttttggtatgtatgaacatttaggctatcgtacagctgttactactgaacttc
gtgatgacgaaactggtgaaatcgttaaaatcagttcatgcatgataagagatgccaagaaggaatagacatgtaacctactggtaataatactt
tccatatcatgctgcatcaccttaatcttcctttagatacttgatgctttgatagttccctttcttctggtccctaaataccatttccgcatacgaaaaa
tgttccttataaattgctgatgtttctgtctcattattctaaaatataaaaatactacactactaatacctaatggttatctggaaaaataacaaaac
aaaaaatcacttctcaacaaaatactacaacgtattgtttatgactgttaataattaaaaaaacataccgagcattttacttaagtgagatatggg
tgcatttactatttctttgtggttggaacaaactcttgcatatctctaacaaaagataataatcagatatcaccaaaaataaaaaaaaaagcttact
aatttttcttttgttttcttgcaagaattcgaaacatatctatttatttatatgagtagtatattacaagtgcatgcgccaagtattaccttttcacactc
tcctttttttctttttcaaataacaagctttccaataagatatataattataattaatatacatacgtttccagttcgaaacattattaaacgttattaa
cagaaatgatagtaatgttaatgaggaactcgcaatgatcaaatactcttcacaaataagagttcccgaaatttcagtaatccatgttcaaaactc
gcgttcaaggctaagttttctttacttttaccaaacggagcaatcattcgaaaatgctttttcaacaacttctttgttacgagcatcttcatcttcagat
tgattcgctttataatgccttcgaaggactgcgctgctcaaagatgctttctgcctccgctcccataagaggtctctccttaaactctccgaaatttcc
gagtttagtagttttgcccggttagcgtttttatgagaaatttcataaacccttggttctgatgactgggaagccgtatcacttaaataactagagtg
tgacatgtcaaacttggcttcgtggacatcgatattgtctgcgttaaaattcaaaactggtgaaggtgacggcaggcctactttcggctcgtcgtta

tgcccactttgtggtctatcctctttcgacaaaagcaatgataaagcactatttcgataagaagaagtatgatgaggtgtaggagagcgcttaaaa
aagaaaggaggagtattggcttcttcatcttccgacacccatgcatcttcatcgtcactctttgcagtaatatttccactttcgtcagtacaccttgtt
ttatacttagaagcaccagaatatcttgtagaaatagcttcatcatccgttgtagaaaccatagaagaagactgtgagtggtttttgtcagaatag
gcacctctaagataaaaaaagggatgatgtttctttacatcaggtatttgctttaaaaatccggcattatgatcttgaagtgcaggctttgtcttgtt
ggatgatgaaggagttgtttttgaaatgcttgggttaaatccatgaataacgctagttgatttactgggtgtcacagttactttagcaatttttttctcg
ttcagcttccttggctctagcagccgcgacgacagaatcgctaattgatttagcctttgacttgactctggaagagggaacaggttttgaaatgctt
tcttttgttgtatattcgttttgcaaaatagaagcagaaggaatcattttaccagaacatgcatcgttttgacgttctggcatggaagaagtagaat
acggttcactaacagttatagtcaaagtcggtcgcttttgacggtttgaagattcattcggtggttcttggtggtaatcggacgtttggtaagcagac
gaagaatctttttctgaacgtttctcaaacgacttctcaaggggcttctctaaaggagaaaatgatgagataagttgattaaatgtatccgaagaa
agtagacgggaactgggacgtctggtgtgtgggcgtatgcaagaagttgagttcgatgaatcttttggattagctcgcacggaagattcctggctt
ccggaattagtagttgaatttagacggatagtgtcagatttgctattaggggagtaaatagaggcaccagcatcggaatctgcagatgaagctat
agaatcacaactgctagaaagatctgggagagaatattccatgcattcattggagtttgcgccatggtcagtcatgacggcttcacgataccaaa
gccgccatgaaagattttcaagacgacgaccattttcgagattttcggcgcatttggtgaagactaaaaacagatataagttaatatagagcatct
gttaaaaacattaccattccacatgcagctcaaattgtctgcggtcagagttcctaatttcgcaatattttctgtctccaaggatagcattggattcg
aacagagtactttttttcgtcatattgtatagtaaaggaggagaataatcttatgtgaagcaataaagggaagaatactagtaaaggtgaaaagat
ataagtaagaataggccaatgaacgaaaaatgattaaaaatgaaaagacgtaaaaatggtacagaaaagagagcgttctaattttaaataaa
actgtggtgaaactcagataggaaacttgacgattgcttgatgattaccaactgagttattattcggtatccaaactttcgagattaataaaaatat
gggtttccaatgtagtcagccaaagtcacaaaaaatactaaaagcgatgaagcatataaagaaaaatcacggcataggttcgcttttctcgcatg
cgcgtgtacaaaaagctccacggccgattcaaaagtgccgatttacaaaatgaatgtgcaacgtgctaatgattttaaaaaagctttagaacgca
caataaaagaaaagctcgttctaaaagatgatgtcccaaaaaatgcaatggtaaattgaagaatacaatcgttctgacagcaaaaagatcgtta
aattcaacttttacgtcgaacttaacgacaaacagcctacttagctcaaaaaataaatgcttttagcaaattctctgaaatttagcgcttggttcttt
gtccgttgtcggcatcctgagggtatatctataaaacactaaacaaacatgtttacttcatatcgttagattgcgttatcccttcggctaagaaaac
attcccgttccagcaaggtgtagaaattatattgattaaaaacggcattttagagaaagtgtcttagaaggaattttctgaatatgtttcgcggaga
tttttttcaattaaaaacacaaagaagcccggcgttgaaggtaatgattcattaaaatcgatgatgaaaaactgttcaactgattcgctttgtttgtt
tacataataatgtgtcatagacaaagggctgaaattttgtatttgtattagctgggtgaatgcctttctttctaatattttttaataaagctttaaaaa
ataacaattaaaatagaaatggaagatatgtagaaacggtatgtgagagctgattgcttccggctattttcttggttagcgaagctcatgtgaaat
gaatatattctttgtttctagaaaaaaaaaggtaggaatttttttgttactttattgaattttatagataaaatcaattgctccttataactagaatgg
aatcctttgattttaaaggaaatatattcgttagcatacagtgcgtaggctcagaaccattgaatctggttgactaatgtgtttctaccgctatacct
aaacgaaaacatcagtttctgtccgaccagtgtagttacatctgagggccttgttggggatccaggccgctgtagacttttctctgattatatctttg
tttttcacaatagtctccttattattgtctacggcaactgatatactttatttatacattttgcagctttagtttcaatgttattcatttccaaactatcaa
agagtttttttttgagtcaaccgaacacctcatttaagccatccacgtatcattcttaattgtactcatcgtctttagaaccggcaacaattatgtcgt
ttaccttcaaaaccattctgcaaagttgagtcgccaaaagtaattgctgcttcttgccaataagggggtcaataacgaattgctccctcatgttattg
tttcctgtatgaagacaatcgatacccaaatgagagttgttttctttaacatgacgtgctttaaccgcggtcaaggcttcgatagaggataaaccac
tattttcagccagggccaaaggaacagtatccaaagcatctgcaaaagcacgcatagaatattgatcaatgccaggaatttttttctgcttcctgat
tgactgccaaggaacatgagacttctgcagcaccacctccatacacaacgcggttatcacgaatcaagttacgtacaacgcacaaagcatcatg
aagagcgcgtttcgcttcgtcaacaatcatcttattactaccacgcacaaaaatggtcacagcacgagtattcgcacactgttcgattactaaaat
cttatcacgagtagtgccaaaagcaacttcacgaaccaaaccggccttaccgagcttttcagtagaaagatcttcgaagcgaggaacaatacgtc
cattagtagcaatggcaattaactcaatctctggtccacccacccaacgtacggcaggaagatcattctgcaaaagtaaatgattagcttcatcat
cgaatccccactgacaaataacaagatttgctccagcatctttcacgtgtttgatcatatcttcgaacttttctttctcatatttctgtagagcttcaaa
ctcactgaccgatgtgatgtccaacttatgtttggtcttgggcttgggaggctcaaaaggacatgttaaaatagcaagttttgcatcctctatacgat
ggggcatttgagggtgagacatatccttatcgacgacaacacctttgacaagtgtagtatcatccacagaagcgccaacctttccgtctactttaat
caactcaaaatccacatctttacgctcaaaatcagcaacagaaaggaccgcatcgacggcaattctggcaaagtgatcgtgggctttcgatatca
ctttacttcctaaactagtctttgcagcacggaacaaatttgtggtatccaatttatcaaagttaacaacgtcggatattgaatccaaatgcttaac
agaatgttggcaagctctttcgaatccatcagcaattcgaataggatgaatacctttgtcaataagaacctcagcttgctctaacaaggcaccagc
caacactacaacaccagtggtaccgtcaccgatttcgttatcctgagattttgataactgaaccaataacttggcgatttggtgctcaacttccattt
ggtccaaaatagtagcaccatcgttcgtgactgtaatatctccatccggggaaatcaaaattttatccaaaccacgaggtcccaaagatgtacgg
acaatattggcaacagttttggtagcaaggatgtgacttttgacggcatcaatcccgtgcaatcttttcttcttctcctgatcgcgaactaaaataaa
gggatttccctggtcatcgcggaccataacagcattgtctaagtttgacatgattctttaatatacaaacaatcctttttagcggttctttgagtccac

tttaggatacacgtttcctttcactttctgctttgctttgactctccacagaaggtgctcaaattaaaccacgctaaaattaacgggattttctatttca
aggatttaataaacgtccttaagatgataattgaattcgcagaaagcaaatgtggtagtggtgaagtttgaacttcgtcttcatcgtcccagttact
atgtgagcagaactcagtaaatagcgtatatgattgtacttcgctaaaccaatggttgagggacggaatcctatccaaaaagtgaaagtgaattc
cttaaaaaatgaaaaataaaaattagtgatctaacgtaaaaatgtgtgctgaagccgctaaggaatccgcccttcgtatcttagttatacaataat
atcatgcttgaggagaaatgtgatttcaaatgtgcaatgtaagtaaggactttaaaaagtaattagatatcattaactacctacaaagtctgaagc
cacatcctcttgatatttcatctatacaagttaatatggtttttttttcttaatgcgtacacactgttgtttacttgtgtgatgtaacgtcgttgcgtaaa
caagagtttgtatagaatacaatagtatgttaaatgcgtaacacagtgcaacacaaactagcgagcgtcatggaaagctagacatggcatctaa
gacgtttccaacaaccaaaacttaggcaatggaagtaaaaccagaaatcgattattccaacaagtttgacgaattaaaaaaggatctaggtgcc
gttagcactgtagatattgtaagggattgaagagaatattgctttttttcttgtttattaacagaatgtttaggacagttccttggaaaagcttctaatc
tttgaaaaacaagtgcgtcaagcttcggacgcaatcactaatactaaagtattatgtttcattgcggatcttttataccatgctcgcgatttccatgg
cctgaatgagcagctggtaaacctgtctaaaaagcatggccagctaaagcagtcaatgacgagtcttgttcaacatgtaatgaattatttgcccac
tattgaagacctaaagattaaaatagacttgattgagactttacgaacaatcacagatggaaaaatttatgtagaagtcgaaagagctcgtctca
ctcagcttttgtcacagatcaaagaagagcatggtgatatcaaggctgcgcaggaaattttatgtaatgaacccgtcgaaacatacggctctctag
accagaaagaaaaggttggttttatactcgaccaagtgcgactgttcctattacgttctgattattatatggcatctactttcactaaaaagatcaa
cataaaatttttcgaaaaagaagaagttcaatcactgaaactcaaatactatgaacaaaaaattcgtattgggttgcacgacgatgcttatcttga
tgtttgcaaatactaccgtgccgtctatgatactgctgttgttcaagaggacccagaaaaatggaaggagattttagagaacgtcgtctgttttgtc
ttattgagcccctatgacaacgagcaagccgacttgcttcatcgtattaacgccgatcataaactgaattctcttcctttgcttcaacaattggtgaa
gtgttttacagttaatgaattaatgcgttggcctaaaatctccgaaatttacggttctgttttgagaaacactcctgttttcgccgaaaatgacgaaa
agggcgagaaacgctggtctgaattgcgcaagcgtgttattgaacacaatatacgtacaattgccaaatactacgcttgtatcgaatgtgatcgtt
taggtgttcttttggacatgagtcccgaggaaaccgaattatttttgtctgatctcatcaccaaacatcaattttatgctcgtattgatcgtcctgccc
aagtaatcacgtttaagaaacctaagaatgttcatgagcaacttaacgaatggggttccaatatttctgctctccttcaaaaactagagaagacta
ggcagttgatcttaaaagaggaaatgatgcattcgatacagcaggctgttgccaaataagcgtttaaacagaaaagacgaaatgaagtttggct
cgtgcctttatgtataatgacaaaatttccataattgtttaacgtgcgtagaatgatttcaataaaatattgttggatatcaactttgatgttaacgat
agtattattgtaattatttatttctgacattatctaaagagcttgttaatttggcttagttagcagatttccattattatcgtattttgcttcaaagaacg
tgtaactaagtaatatatcatctacatatttcatatttggatcatcagcaaactcgggatccaaaaagaaaaatacaggcaagtcaactttttcgc
gggcatccagcttttgctcttcgaaacaaaaacaggcaactttggaaaaatacacagcggcttgacctggtacaatgttataagtagcaacgcca
actatgtcttcgtcagaacaattttcagctgtgtaaaaagcaagagctgtttctcctggttgaacccaaatttctcgttgttgaggatacaacttcca
gtgaagatttcctgcaatatctccattaaatgtcactcttatccgtttagattccctacgtggaaccattctatcagaattcatactctcttgatcggt
attgacggttccgccgtatcctgttttggaacaaaacaagcggtaaagaggaaccgatgcatatgttataccaagtactaagatagagagagca
atcaaataattaatagttgtttggcgttgatattttaacttcggattaactctataccctttgggaatagtatcgcttgttaattaatgaacctttcagta
gtcctgaagaagaattgaatgcattttgattcaaacaagatcgcgtactgtaagatcgtctaccggaaaaagcatagcttggtattggctcttcag
atagctgcgaaggatccttggaaggggaatttagagaagcaggttgttccttagcttctaagtgcttatggaaatgatgtgttgattccacactttg
ggttttaccttttatggggaaacggtctcccaaacttgattttctggctatttggtaatatagtttaccttgagacttaggcacgatgtgtcgacgaag
ttgagcatcgtgatcgcacacatcaacaattacatggccgtgacgctttaaaggtgatcgaattatacggggccagtttatagggctaggattaag
ctcaaaatcggtccgcaataaatcctgttcattttgaatagctttatatgtatctaattgacgagctactcccttttgtaagacgacatagctaaaat
ttgatagagaatgtgatcttcttctagcttgtggaacgtacatgcgagaaaaggaaggaaaaaagaaatgttgtttgaagctacaaacgtttgctc
tcacaccgccctctatttgaacgggacaacgatcttcgtggggacaaggagctagtattcgggcatctaaccgattgttttccttgtctttttctctgt
ttagcaaaaacgacctagcccttttgatggcattaaagccgtgttttgagccttgctcagtaagaattacgatacctcccttgtttaacaaagacca
aagcgcacgtaaatatccaaaaaattcctcttctgaaccacattcaaggagacgattagaagctataattaaatcgtactttcctttttgggaaag
cggcaatcgacctaaaatttcaccattcatgtccccagaaccctgtgaggcaattatattttgattgacttgttttatgatttcacgaaggaatctgtt
ttcttcaacgacagctggtggagttgttgatggaaagaaagatcgactcaaaatggcgcccaagcttggtccttttccacagtccaaaactttcaa
ttggtcgctcggtttacccttcaaaagacctttcacttcgtaaaagatgtgatataaggaagcatactggtatggcatcgtttctttcaaatacgcca
aaatttgataatctgtcattgtctttgtagactcttctttccaagggagagatattggggttcttacttcttcttccctacgtttgtgcgttttattagtta
ggtttttgattcgacctttgtcagcattggctaatagttcatccatatagagattgaaagcagtatgaagatgcaaatcacccgatttttttgctctt
tcaaaagaaagttaaataaagtagaagattcacgtttcgcagcatcactgtccaattgattcacttgttcaacctgcgtggaatagcttcttgatcg
agagttaccccaattcgctcctgtcagctgcacaaggcgctgatttttactgcaatttttcaatttgataattgaatttttagaatgacatcgagtaa
accatcttaaatgatacatatgagtgtttcataaataattgagcaaaaaataattaatgctatggagtatagggatgagcgcgtgttcgcatacta
aatcgcattgttacttcactataaggtaacgcttttaaaaaaaatttggaaagaaaatcattttttgccttctgtttggttcttccttgtttttcaataa

aaaaataaaagaaattgacacgtggaagggatattcgctttgtttaaccaagtgttacactttggctcaaaaatgacaagtcgcgtaaacaaaa
ggcggtgtcaaaaattcaaatatgaatatttgtcattttctgttattcttttattgaaaactgcaagttttgtatgggtaaattacttttaaacactacc
gcgttgcccatatctgttaaatttttttattttttttggcttgtttgtttacgccatagcattggttcacccttgcttcaagctgtacaattgacacaata
tgttttattattttataattctataccgcagaagtattcagatgaatcacagcgtaaataaaagtcattaattcacttcattaaatattacagtaccc
aaatcaatgaatgtaaacaataaacaaaccggaggaaaaacgcgtttctattgtttttgcttagaaaaaattggcaggaaagaaaaagtatgaa
aaatggattcctttcatattgcttgaaccaattttctgcgctcgaatacgcgatcgagtattttactaaaaccaaccacatggagagtatttcttact
agttaccttacacatactccactttgaaaactttgcagaaattctacaaacgtacgtccaatatggcaatgaaaaggaagcgcgttttccataagt
ctcaggcaacggcaccattagacgatgaaacaaatcagcgatgcgcatttccagaaataattgagaatcaagttgattcacaagacattggaat
tcctctcgatggtttagactatcttgccgccgtaaggtaataactggcccttaaaaaagtcaatcaagaaggaaggccttccagtaaccttgattc
actggaatatgaaaagtaaccttgctaatccaactagaaaagaagctcagtctctgaattcctttaaaagtgcaggcataaaattggagaaacc
agagtccataccgcataagcctttgtacacagatccaacacactctgtttgctctcaagaacctctactgaaggatctctatcacattgtggagga
aaaaaacaaaaaattaactacttaccctttggatttgttgttagacaattccacattaccttctactctacagcaatggaggcatactatagctaac
aagatcccttcgtggcctattatagcgagcaccgacttagctactattctggatatttttgagaactgtgcttattggttggaaaatgattctcttgat
gtacaggtaagcaagtttcagttatttattctttcaaatacgcaaattattctataggagttctttgttaacactcttatagtctcaatggttattttgtt
tttgttgtaaacttccggatattttgaacggtgaagatatatccacattgaggtctgctttacgatcattacgaagtacacatactaccttcccgtcc
attcaaacatcagcatctactttgaacgctgttttggtatttcgttatggtcagaatgatctgctatagtttttcctccctgccattattgctgaaggag
tcgataacaaaaccatatgtaacgtttagtatattatagatcgtaactttaattattagttagtcttttagtcgcacttaatcacgttttattactatgg
gtttatttttttatggaatcatttaacctttttgtccagcataggcgatttttcaaataaaaatcaaaggaaaaagataattaaacttaatctttctat
aacagctcgataatatttaaaaacgtaattttcacatgaacaatattttcatatatcactttacctcagtcgtcgaaaaataaaaaatccatggaa
agcccgtctttgctagaggttcttcaagtgcaagaaattgatgaattggtcttaccatctataaaatatatttttgcagtaagattagttaaaaattt
cttatactaagaagtcgtagtatttcacacatagacatcctagacagttgttacgtatttacaactccttagacgacattcattgtgttttgaaagtt
atttttgaatatcgaagcctaaaaaaatggaatgctacatcagttgaaaggaggtttcagttaaagcgagtgaaatctgtgaaagacgcaggag
gtcttgtcaatacatttccggaagaagttaaatcggctgttcagctaagtcaaataaacattttagcaaaaatctttttgagcgtaagtattctgtat
tcgaatgtgctccattcatattaactttttttttttagtactgtctgccatacttattttccaaaataaaaacattgtcacatagtacaaggctgctaga
gttgagagcaagttacaatgtcgaacataataattcagaaaatgaaggtcatcaagtccatccaagtccttctctaaactttcgttcgaagattcta
ctaaagctgaaaaggcttttgtccatactaaaacttatgtttcaatttggatcaaagttttacaacagtattcgatgggcttattacgttttatttgctt
tgggtacgataccattctcaaatcctgttgatcatttattaaagcaacgtcttatttacgatactaagattcccaatcgaacttccgagaaagcactt
cgtttttatacgcttccttcatttctggacaattcgatgagcatatttttatctctagtccaagtcttagactggtggcagacaaatgactatgccact
caaatcaagaaaggaagagttgcatttactgaacttgagccaccagagctttcagccgacaatacatcttccacaaagaatatttgtaaaatctg
tggtcatagtattaaaaatccagcagttttatctactggcttcgtgttttgctatccatgcattcagagctggctaaaagataatcccttcatatgtcc
ggtttccaaattgaaattggtcagaaaagaaaaatctttttggaggataatggtataatataatgccgaaaggcccatcacgtaacgaaataaaa
ggtacctgatgtattcaagttactaattctttgcaatatcggaccaaagtttttgagtatttttttgttaattcatcacttaatatagttaacgggctttg
ttttacataaatttcatgaatcatgtccagttttccttgaattcttctatccataataagtccaataagtgattgttcaacaatttccgtagttacagca
agcttctcagctagcaagcttaattgaacagtcgaatacggttgaatcatttccaacaaaacctgacttctgatactatacaggattttatcaacgt
actggcgaatgaattcatcttccatgatatcctgccggtgctcatgtagtaccttttccacctctttaatatcacgtttttggtatgcttccactaattta
gtcatagcaaagacgtgcggattttctttgtacggctgagtctcgggggaatcataaggattgatttcagattcgcatagcatattggataaaaca
agatattttagcacccgaatacggtccatagagccagcttcatcgtaacacttgaaggattcaaaaaaatttgtttgagcttcagaccattgttctt
cctgcatgtgcatcttaccaccacattcgcgaataatgcccataattttagggtgagggattgcggttttaattctaagagagctttggtataactct
tttaatcgtttattatcctcgagactagagtacatttgtatttctagcgaataaagttccaacaaatgtgtctctctattttggtctgtaacggcggtgt
tttcgttcgaaagaaggagatgcatttgtcgtaaaagagacttaaatttgacataattcttttgcgaaaggaaaaatcgggctacgtgcattaacg
ttttcagtgttagtcgttcattatttaacttgtgaagagcgtgagttgtaatttcataaaacctttccaagaattgagcatttgtagaagacgatgcat
aatccacgatgttatatatcgatttttctgcataatttttcgtaatactcggccattctgcaaactcaagcagtttctgatagtgctttagcatctatga
taaaaaataagttagcaaagagccgctttcatatgcaatgatgcatacctcatcaagctgatcagtacgaaacacaatctttaaaacctgctttaa
agacttaaacgccctataaggttatttagcaaaaagagcgtagagatatcatagacatttaccattcatccttttcggatttgcatttgtcgacgat
ctccaaaaatgatctaacagccgcaccagcatcatcctcttttaaggctaggttgaatttgtaagagcttgttcaacaaaataattatcaccaaaat
aatctaccttttgaattgtagtaagagttctcaatatccacgtccggttcgaccatctcatcatcgtcatcctcaaattcgaactcataatcttcatca
tcccttcttttgttagtccgattatttcaacactgaacagaatccacttactctaacataaaatcgtccgccattctatagttactactttggaattggt
tggtttcggtattcctctactattgatatatcatactagtaaagaagccatagaatgataagtaaattcatgcatattagtaaagacccaagagaa

acaatcctcagttatatgtgtatgaatggatatttagtaaagtaaagtaatatgcatatcgaaaagcgtcatttagcatatgtacccttcaatgtttg
caacaaagaggcaaggttgagcgaattaacgagagattgtatgtgagcatatagaatcaaagaaaatttctatttcattgttcaaaaatccaaag
gatctgtactatgcgaaaggtaaattgcaaacaaaactcattccatttcgctcgactacagatgtttattaacgatacagataaacgtaattatttt
taccgatgcacgctgctacatcctaaaagttaacaacgaactgtatggttaagggaaatcgtgactgtctttttacaccaacgaaacgtgaggctc
tagtatgtaaaatatttctcttcagccaaaggccctgacattttcaagaagcagcagcaaaaatgactaagggtacatcttcgatgggtaaaaga
ggcaataagtctcataccctctgccgtcgttgcggaaagcgttctttccacaagcaaaagagcacttgtgcttcctgtggttatccttccgctaaga
cccgcagcttcaactggggtgctaaggccaagagaagaaagactaccggtactggtcgtatgcaacatatgaagaaggttcaccgtagctttac
caacggtttccgtaagtgatattcgtttagatggtcgaccgtgttcttttactaatcaactatctaggtactggcaacaagcccgctactggatccaa
ggctaatcccgctgcttcttctgcttaaatctagtgcattcaggacatttttcggaagagggatccctgttgcgatgtacaatttgtattctttgactg
gatccgtttttattggcagtttttgggagagaaatctaatctgtgttcttttcattttttaatgtactgtttttaagtgtgtagatacatctgagtagcg
gaagtcccgatgataaagtaaacaacacattggtgaaagcatgaactgaggaaaagtaaaaagcaaccccattgctgagcacttacaggatgt
agtttattcctttattgtatgttatgtatttgagcagatctccattagaatcacattaataatgtcgggtttgtcattgaaaatctgataattgcttcat
ctttttctttatctgcttgtttttttatcgtttactgttagctttcttgtttattcgtgttttaactattgaaaaccaagtctagcatagattacttgttattt
acagtcgctctcatagtatttattgtttacacctcttgcctatctgaaaggtggattcatcatttcaatagtttgtatacacttatgacctcaagtctttt
ccttgtagaataaataagtaagaatcactctttccaatccggtttctcaatgccacgataaaatttcaaacatgtcggaacattctcgtatggactc
ggagcaagagtccttctctaatcaagaaaaattaattgactcgtttttggcaaaagggccccaagcgttggctgaatcagcttcgtccagtgtaaa
tgagcaatcaggtttatcgactgaacaagagctcaaaaagtacaaggaattcttctctcaactcaagttttcatatatcgaacaaggaacaaaag
aaagatatttacgtgcaatccttgatgaccctcctctgctagtcgagccagaagataatgaaaagctggagtctacgatatcagagctgaaatttc
gtttgaaacaacgaaaagacgaggttgaatcattaaagaaagaaattgtttctgagtgtaatgaaattgctcaaaagtacgatgcaacattaaa
agaatccaaagaagcaaaaacactattagccgaattgagaacattggaaaaagagctacaggacttggaccaagataacctaattaagacgcc
cattcttccagacttagaggaaaagattcctgtcttagaacaagagcttttggttactgatgattcgataaagtttaccaactcacagttggaaaga
gatgaagttcgcttagaccagctgaggaagaactgttctttacttgaaaaagaatatagtcatctgcaccaacgttctaaccatctacgtgaatct
atggaaatgcgttctcctggagacgatgcaaagagaaaggttcaagcatggtatacatctatgcttaatatttacgatcaaatacttccttaacgc
acaaagaacgaatctttttgatatctattcacaaatcttaactttttttctcttttgttatcattaatccctgtttctgtcaggaacccagcggcgcaac
gtcacgcattgaatagaataattactgaggattaattttgaccattcccatggatccatattcttttgccgttgccagaggtctttaaacttttccggt
gctggacactgaacatagcataatacaagagtctttttcacttggccaattgtgcggttaaggcaatgcagttcatgccatttcagattctgtttttg
ataatctcctatgcaaggaagtaataaaattgccaattctgcatgactgaacacagggcctctcttatagagaactaaaaaaggttagaaaattc
tattcattcacaaacaacgtacgataatccactgagaattttgttcctccttttaccacccatccctgttggcgaaagtaataatatgcagcaagctc
tacaaggaatgtgttgtctggacgaatgtaattgctaggatcaagttgactgaaagaagtaccagggaattcgttccaaatttccgtgatgaatcc
tggggatttggcagtaagctcggcaaagacgtgaagcaaaggagtagatgaaaaattatctgtcccattaacaactagagttcctagagaagat
agaaaaaatgcctcaggaaatgtcaattgtaaatgctcaagttcgggcacaaaagtaacttcagagggattttgagcagacaaagatgaagaa
agaggttcggttaattcttttgggagctcggcatcttctttgagataatctgggacttcttctccacgttcaatacacaattgacgttgccgcgcacat
tgttccctatatgctcgttgcgctttaaatcgttttctttgcaaccttcttcttgcagttatctcttcggccaccaagtcttcatcgaaacccaaaagac
ctagcgatcgctttgttcgaacgtgccacgttggctcacttctggaaagattcccttttccaaagaatccgcttgtccagagtctctttatactttcttt
gtctgttaccagacagcttaaagtaccgacgtccactgaacattgtacagggatttgtttaggtgtcctcgaaaataagtaataataaatataggg
aatccaagtgataggatttgtgggaattataggaggcagcggtggagctaatgaaaatggaagaggagctttatatatttcatgcacctttgaca
tctcgataaaattttagattactgatggtattttgtaatttgacccagagtaccttaccctaccatagaattatatgaattcttttctataatcctttaa
ttaaagacttttcagttaattaagcattagtttgacacagaatacctaaaaacaggattcgggtctccatcaattgcgttggtaaaatgctccacta
tgcttgcttctaatttcagccaatatcgaaggtctgaatgaagcggtcaattttctttttaattaagtctatatcttgaattttgatcatgaattcatttg
ctgatctctaaaataccgatttttcccaaatttaataagaattgaataagtgatgctatgatagttatatgcactatattcagttcaaaaaaaatata
caggatttcttgagtttttaaatcctatcacttactattacgaagcgtttgattattttatcgaaagcaatcgtgtccaatatacttttctgcgcattga
aaacatagacaatttatgagtaatttcgaatctgatagatcttctttatgatttttttgtttgaaattaaaaaaaaataatatacatatatatatatata
tatatatagattatgaggcggtatcttggttccgtggtttcaaatcctcgtcctgcacaagctcttggtataaatcatccttttatagtcttttgctttat
tgctaccaggacattatcgctagctcctgctgtatactggtgcttgaaatgtttacaccttttctttttttggtaatggacgtgcatggctggcattgact
tctgcactctggacaattgtatgttggattgtgagtattcactccgattgtaagcttacatcgatggtttaggtttcagggtatttatctttcgtactaa
ctaccgggtttctattaaaatggtatgtgacattatctgaaaatagtagctaagctactatgcgggcgttggtgagttgtgctaatgcttaggcttat
tcattattctatcggaccaactattatacgtttattatcccttaatgtgattaatttctcgtttgtgtctttgagtgttaattttatcactcatggcgaca
attcacttttgcttcccgcttggattgcaatttcctgctttcagacagcctcttatattgttcaagattggatcacctcccccattacccgtactgtccc

atttccgtcttcgtcatccgctaatacttctctttcaagcagacacaatttagatttcttagaaattacagtatttgctgtcgttcctgttggaattgct
agtttttttacaatgttaatgctgttatggcagatctacaatgatccaatttattttttaggtacctgatgtgcctacaaaaatttgctttaattttataa
agtttaacgttttatctttttaatactttctttaataggttaatatacttatttttattatctggtgcaaacaattcaagagggaaaatgaatagaagta
acgtaaataatcattcaagtcaattgctttagtatttaattctcgtaaaaaaataataacaaagtgctccggaaaaagcggtaattcgttttgtttgt
ttatttttgtttatgtcgaaagaagttctgataccactccaactgaacgaccataattccttacgctttggaaaagaaaaatggaagatatcattctt
aaggatcgaacagatatagttaatatatcctcatctgttgcgccaaatgaagagtaccgccctagctcgaattcaaactatgcgataaagaaaca
aacaaggaacaaaagttcagcagatacagaatatttttcggacgctgaaacatcaaagcaagaacttagcgaatcggatgttccttttatctata
tgaatgaaaacttcagcacggcaagagatacaaataccagcagtactatcttgactgaaaagaaatgtagaacagacagtttcggagggaacg
aaaaggacttgctcagtgaaattcaacgcttaaaagaggcgaacgagcgtcttcagatgacattattacagatggtaaaagaaactctgattata
tgtatatattttaacacaaaataggctatttcagacacagtttctgaaaaggagcaagaagagatgcaaatcattcgaaaggagctaattcgtga
agtaatgggaagcaacaaatcacgcatacttcagatggaagctgaaaataaagatttaaaacagtcaattcgagacatgcaggagtatataac
aaaattagtagaccgctgtttacagaacaagtttgctcagcaaatcctttctatagatgaagcatgaattaagaatcataacctatatcccgtctcc
aatatgccaagaatataagtgatagagttagacgttgagtaactctatcggtggtgttgatattttcccggaacataaataaacaactgagttctc
cttttcaaatacacctttcatattatgtttagtaactttggaaaagctcatcgtcaaatcttccagtaagctaggaagatttggaccatccacgacttt
acaaagaaatgtccctgatggtttcaagccacgtaacgaaaaaattaaggcagattgtgctagaaacaaagagttcatgtgatctttaacagtta
agagctcacgatttcgcatggccaaataaggtgtttttgaaatacaaaattcaaaaccctgaaccattggaaacggacgatttaaatcacttata
acaacatctgcacgtaattcctttagcaaggattcctggttttccatggccgcttcatgctcaatagccaactgaagataaggcacggaggaggga
tcaccgtactgtgcagatgcttcttgtagttttctagcgcgcagtcctgcttttacaacttcatcttgaatagccatactcaggaaattcccctgtatg
gtgctacagttggcgggagatcttgagggaataatatcaacagaaacaactcttccttcagagcctatgtgttgagaagcaacattattccatatt
ccaggagaagatcccttgccatgttagtaatgatgatcctttccaaaataaggtcttaccaaattaataacgagttgacctggtgcaaacatttttt
ccttctcttgtatctaaaaccatttggttagctgtttatcaaattaagctctcaattgtgactaggttgaatatatttccctcaattttcgaaagaaaat
aataaaagaataaagtattcagtattcagtagtgcaacaaacaaattcagagcgaacgtatttattcaatcagtaattttcgaaaacttacacttt
ccaaaaatttaaaatattcattttccaaagaagctctaaaagagtacagagtcttttttttccctattctttgcttcgtcgaatagaagcatttttcatt
caaagtagtggcacaaaattgccatttcgtacttcctaccaagtatttttttccagctatccagaaaggctgttctctgaaagatattcatttttcatga
agcatttttacttggtttctagatgtttgcttctgatcattgacagatttctaaatacggtgactcgctcatagtaactttacgactatacaaagtatg
ctcagtcaagacaagcgaaagtaatcgaatcggagtcaagtttgcatatagtttcttttattaattgatttatataactgaagaacaaaattcatat
tggaaggacatattgttgaagaagagcattggattgcactcatcataaccatacttcttacatgagtctatcataaagatatcaagcaattctattt
cctaaatctttatcattctttttttaaaataatttaactcttcgctttttttctctattcattcccgacattacttagaatgtttcatttatcgttaattggttta
tttattaacgcacaaatactgtatatcggtatacgacgccgagtaagatcaccgacccggtaacctttcggtgacagctactccaaaatcaccgaa
tacgcaacaagtgctgatggtagtgtacgcgcacgtacgcagagccaatatgaagtttggcaaaattcttacttatttaacccttttttgcggttgca
tgccttgcaaaaccagatttgaactctcgtgctgatgacaatgggattatcaatattgctggtagacttttccagcgaattgtcactggtaaacaag
accatactacagttgcactcttttcagtggatgctggaaccatgaactgtgaaatttgtcggtatgtcttcttccttattctttacactttcatttttgct
gacggttcagccttattgagcccgaatttaaagctttagcttattcttacaaattacaacacggccttgacagcggtatccggtttgtatatgccgat
tttggtaaaaataggaatttgtttgaacaggtacgggtctttacacttaaaataattcaatttgttggattttaacatgtttagttcgctatcgaaagc
gttcccaatttttggatttttaagcccaatactatagttcctatttcaggggatttgagcagtgggtaagcaatctgagtcaattcgacgttatgacg
tcgttaaccattgtttagtgtttctgctgaaaagttggctgccttggtcgagagagaaactggcaaaggcgccccgatagtttatcgtcaagatcct
gctaaaaaatatgctactctggcttcatctattctatgcggtgctgctcttttctttactagaaaaacgcttttgaaaatattcaccagcagaaaagt
gtgggctgctcttagcattatctctgttattacgttgagcagtggattcatgttcactcgcatccggtttactccttacagtcaacgtggagcaaatgg
tgaaaacctttggttagccggatcccaacaatatcagttcggcgcagaagttcaggttgtatcattgatctgtaagtatcgcaagccacagtggtt
gatgagtttactgacctagtttagacactgctttaactataagcaccatctttcttgccgtgatagcaccaaaagtcgagggtgcaaaacgccaaa
cccttttttgtctttttctggctaattgtattatggcttggctacagtttccttgttgacgttttccaaagaaagttgcagatgtatcctttcaaacttttac
tttaaacgttcattatcgatttgagatgaaggagatgaaaaaagattaatctcatgatttattcttaacgtacattcattaatcaagacttatttcta
aaaaatcatttacacttataatcgttaaacctaagaatcactgcgtatggcataagcattatgtctttaaatagttactccatcgtttaccttttccta
cacgtttgaaaacactttactcactgtttcaaatgtataccaagtaacgctcatgacggggataattttcaataaattactgaagtatccccggaag
aaaccaaaaaggccttccttgctgtaaatttgcgctattgcatctgatatacttgagtagtgatagccaatgccaggtatccggtttacttgaaatct
tcgtcgaagaacatcggctggaaatgcaaatgtttgagctatgaacccgctaaacccgcctagagaaagtttctctaagctgttgagatctctacc
agaaaacatatactctttgcaccattcataagtgaaaaatacaatagatacatagggtaccacattaagtagtgtcgctggaagaccactgtaaa
atgcggaaatccctccttcattgcgataaatagtccttaaagttgacataaggtttaatgactgagcattgcaaccattggtcatattatctttgatt

aaacccgccgtctgaattgagagtcttgtcctagcaatgtccagtggataagtgctaataacacttgcagctcctgaaatggctccgaaaattagt
cttttataattactcaaatcttttctatccgatttctgcaatgtgagttgctttaatccactgtaagttgcaaactgaactgcgccgtaaggaaatgcc
cttaagcaattcgttccatttccacgaaaaaaaccaagaatgccttcccgtttccatattttggcaactgtagcaaacatgctattatattctttatta
ctttgaacctgaaaaatgatcttcatccgttccagaggactggttacagttctactaagagatgctgctgtaccaccagacagtacagatacgata
acgcttttaagaaaacttccagattcattagccgtatgtaatttaagatctttttccattacaattatctaaaatttctcagtatcatttactaaagcat
cgaccttcacaattcagccctaccctactatacgaaaacagtcacaattcaatacacaacttttgagtgagaaatgatacctacaccgagccaag
atggtaagtttaaaatgatattacgcttgtaaaggtacaaaagactaattcaatttttagggtatcgacattgagagacaccacgtgagaaagtct
caacgcagcaagcctgcctctgagaacgtgtacttgaagcttttggttaaattataccgcttcttggcccgccgtactgactcacgtttcaacaaga
ctatcttaaagcgtctctttcaatctaagactaaccgccctcctatctctatctccaagattgctgctttgactagccgcaaggcctccaaccttgag
ggcaagactattgttactgttggtaccgttaccgatgacgagcgtatgttgaccgtccccaagctctctgtcgctgctcttcgtttcactaagtctgcc
cgtgctcgtatcttgaaggctggtggtgaagtcatgactcttgatcaactcgctctccgtgcccccactggcagcaacactgttcttcttcgtggtag
aaagcacgctcgtgaggccttccgtcactttggtttcggtcctcacaagcacaaggctcctcacgttcgctctgagggccgtaagtttgaaagagct
cgtggtcgtagaaagtctcgtgctttcaaggtataagcttgttagtcccaaaatcaatgcaccaaaaagggtttctttatgtaatgttatgtgttttgt
agaaggattttgattcttagtagctcctgtgatgtatcagcgaataacgaatgctgttgtatatccagtcatgctggcaaagtagtagagtcttcga
gtaaactcttattttgaacatttgaagagattttaagttaagtggcattcaaatcatttattaatataaatactatgatcagtttacgaaacagtaaa
cagaacactctgaaataaaaaaacgagactatgaatgacaaaaatacaaatcaaaatcctgcatttttttgaacaaaaggttaaaaaattattctt
tttcttgtttatttcttcataaacatcattagacaccaatgtcagtttagaaattttatatttctttgtcagacatggaagggtacacgggtgaaagtg
cgagtctgcgggtcataattatattgataaaaatttgtgccgtacggctgcggctcgatacaataaacagtgtgaagaggcacatttaaatgtgca
atatcttctcgacggcatccaagaaaataagcgattatgattcttgtaatgactctatgtccaataactaaaacatgatgcttcatgcgttctatttc
aacaataacgctttgaagccgataaataacatctaggtaactttcaccaccagaaccgggatatctgtaatttagtttatccaatgctcgagcttcg
tactcgtcggggtagttattttttatctcctcgtatgtatgttgatcgcaaatacccgaacaaatctcgttgagcattcgcatagccttgatatcatat
tgttcatcgtcaaaatatgctgcggtttccatacttctcttcatcattgaagtccacacactaaacggtttaattatcttttcttcatcttcgggatcca
attcaataggtgttgatacggcgtgttcagtcgaatatagattattaaagcttgaattatatgtcttcccttgaaaagacaaattaccatggttcata
agattggattcccttgtcacatcaaaaaataatcgatcttgaaattcggcacgttttgtttcaataaacattgctaaatcttctccgtattgtttacca
agtttggttaatgaagcattacctcctatacgacccattgtgttatccaaggactctccgtgacgagtaacccatatttgtctaggcgaaagattaa
agttaagcaagaagaaaactgcctgaccggcaaggaatcctcgaatattaaaggcaactactttttttacctacatcgattaccttgatgtagcgta
gatttagatcttcctcgaattttccaagcggttcatacttttgttcgtacaatcgaacacgctcctgaaagtctctaagggacttctcaggatccatat
ttttatagtctggaccaaagagtttcattcgcatgttggcagcaattaactgttcgtcattgcaaacagactcaatgaaaagggtagttacgtatgg
aacattctttaagtgctcgacaatagccttccttcggctagcagtactattggtagcgtcgaaaataccaacaacaccattctcattttcaaaccaa
tctagcaaagcactcagtgtattcattgccatgtcctcgcgaattttagaagtttcagggtttgaaggatcaaaaaaggaagcatcatcatgccca
tgaaaagcttgttttctcctatgatttccgacattaaacgctttacaattatattggagccagttatagtatcgcactaatttgttcacaatatacgac
ttgccccgagcaggaagaccgaccattattataactagtttctcctctgggccagcaccttcaccttctgaatttggattagaataaacactcgatg
tttggcccggtatactcattgtggaaccttttctcttcatactttgagcaagcataaggggagaaggtggagtactatcaccgggtgaatggaaact
ggggacatgatgaagagaggcaggacgatactctgtgcgtaaagaagagacttgctttggcattgcaaagtatccttccgaggccattcgttgag
gcttatgctgaggatttaattgaaaaggaacaggagacgaatcgctagacgttaacgaggatgcattgggagaagtagatgttgatggcgggg
aggggtcgtgttttcttttttagtggaaacgtttgtctcacgggaaggggcagcttgcatcgagtctgtgtcattttgtgttaattgagatggtaatct
tggaccgtttaaatccttcaagttttgaaaactaggacttaaagaaggcgaagatgtgggagactcatgcaaggaaggtgagctgactggggatt
gaacaagctgatcatccttgaagaagctcgtttcccgttgactatttggtgttgacataatctacaataaaaggtatagtgacaaaaaaggatacg
agtatgtaaaacaagcaccaaattcccgaaagagaaaatatgtaccgagcggattgttataatgcacctaaaaaatagaataaaataaagaga
aagtggaatgagaagagaaaaaaagttttccaggccacaggctcggttttggaaatgaattccaagagattgacaaaaatctattcaaaatgaa
gcaaaaacaaaacaaaaagttgcgattctttaaagcgtcatgaaatagaatgattctttccttgtacaaaccaaggaattccgaaaagaaatca
gagcaattccaaaacactaaaagaaatgaacctagcgcaaacgaacaatatctataaatagcttgttcttacttggtatgttggttgttgatgaag
ggcatatatcttacgttgacatagtaaaaagacttgaggtaacaagaaggaacaagtctccaagaagaaggaaatgacagcgcatattttcaac
gtaaaaggaaggttggattactaaatagggaatttctgcaaaataaatcttcgttaagagaatggtaataaaaatccaagctggaatccaatgct
ccaagaaaaagaaaaagttcgttttgtaatcaaataagcgaggaaactttaattactttattgtatttaacgaattagccaccaacaaaagaaaa
atttcattcgatagtagtaatgttttcgaaacctctgaaattccaaccgaaaactcgtgtataatggcttccacaccatgaaaacgaaaataaaaa
acaatgcctcttttgttttatttaatagctatggacccggttctcgcatgaagagaaggctgaaatgaactgatcctaaaggaaagaaaaacacca
aagtggtgtgtgttttctacttatttcaattcatttctttggtttgtttacttgtttattttattttattattttttttttgatcagacttcttacaacaacaat

agtaaaaataaaaaattcccttctttttttattactatatcctttgtttttaacttgcccttttcatgctttcgcctgtttcccttttctctttattcggtttta
ttctgggacacgggcataatactataaagtagtaatacaactatgagtgaaaaagtaaaggtcaactatttggttaaataaagtacattgaagtg
atagactattatttttatatagtaaagattcaattgcacctgcttgacttgacaatcagattaattgatgaaacaaatcaatcagctcatctgcgaa
ttcttcccaagttgaaatttcactggaaacgcttagattatctgtagatagcgattccattctacgctagtaaataccaaaaaaaaaaaagaaaaa
tttaatgactcgtgaacttatgaacgcacatgaaccaaagactttccgttgtttgttttggtccagtaagtcgtaaaataacatatagtaatgttagg
attgctctaacattttgctgaaatgactcaaactaatacattccctacccaccggaagctgaaatcacctgccccgcgagccattgaccgctatctt
cggcaaggaaaaccaacaactatcgctaaatcttgaggctgtccatagtgtttttcagaaggagtcagttctgagaactgcttgattagactttga
ctttttcttaccatatccgttacaacaactcctcgattgacttgattcacggtatggccctatgggcctaactcagcaatccatcattttgtgaaacttt
caagagccgacttgctttcgctgttcagatattgttgtggaaagccttcccttccgttaatacttccaatattaatgatcttttccgttttgggaaacat
tcagagccatgctatctgtcatgatgcaggcggcacgaatgtttgcatcaaaagtttggttataatcttccaatatcgccatctctataggttttggc
tcagttaaatatcctgcgttattgacaaggatatgaatggtggaaccaaaagcttgaacagtggtatccataatatatttcactctagaaacatca
cgcaaatatgcttgaatcttaattgcatctgccgaagtaccaaagtcttttatttatttatttatttaccaactgttcagttgcgtcattgctgctgtca
gagatgaagataatagctactttaacaccgcgacgtgcaaatttctcagctattcctgctccaacacctcataaggcacctgtaacgattgccactt
taccttggagggagagattgtttttagccctttccagatccacggtaatattagattaaaagttatgtaatattcatctaattataaaaaaaaccttt
gtgaacttttgtcgtcgaacaacaagtgtattctgtgtcatttttggcgtccgcagtaaattacttaatgattaagtatcagggaatattgtccagctt
gtgtatttatgcctcgattctttaagcttatggcggcattcgctaatattcaacaagtctagtatcaccggtactgtaatatatataatgttaacaaa
acacctatcgtaacattgacagtgacactctgacattagcagtgctacttcatgtttctatataacgataacgtaaaggtaccgtatatatactgaa
cgaagttgtgtacgcttcgttaaaccttgacttcaaaggttagtttccaccatacaaattcgatatcgaaaaagccaaatgataatggttggttcaa
cgatagtctaagcatcatgatttggcacctaaaggaatttcggaagcgttttcacgaaggtagctctcgggcttacttgaaaccctctgaaatgat
acaagttatcctttccgacacactcactgactttttttacaaacattagttattataggaattgaaaatatccagttcatttacactgaaatatgaag
cttgctaccaaagaagaaacgcaagcttttaatcaaaccatttactcggctctgttacgaggcggaatcgttggtactggtcttggcatccttggat
gtatcgtgggaggtaggtattctcctggctttcgtcgtctgtcttttcctcgaaaatcttggctggtcattggtagcggttctgccgtgagtgtaattta
cgctgataaagccactgtgaagtttgaggctgaacgctatggtaaatttgaccaaattgaatattccacaaagcatctcccttggaaatatagggc
gttgtactattacaaccaacacaagtggcccatcattttgggtacatgggcaagcactcttggcttaagtatgtacgctgcatctcgcaatcgttac
gataccctacctcaaaaacttgtacaggcaagaatgtacgcacagggtgttaccgtgctcgtccttttaggaagtgtttatctgactgccgttgcca
atcgtttggaacctccaacccaagagaaacttatttctgaccctaccaatcctacccagctgattcctttccagtccaagtcaaaagaacgttatcc
tggtgaatttcaatgggaagttttagttgccaaagaagaagagcgtcttcgtcaattgaaccttcccctt cgtgccggccagtcgtcgcctccgccc
aacgcttccgaatctgcaaatgtttcttcaccgtaaaacaatatgctgtttctcgttccactcccttttgagattttctttttccgacagctcttatttct
gatgaatttatttcgtttcatcggattgtttctctttttacgatcccatttcacttagctgccctcgatctccttttcctatttcacctatcgtttatccagt
tctcgtaagtttctatggatttctatgcgcgagatttatgttttgcttacgaaaaaataatttaataattttttgtttttgttattgtctgttaataataatc
atcattttcgattgtttaccctttcttttacgtaattgattgtcaaattttcttgattgtaagttaatcctatagtttcgcttttgtctatgattgtaaaatt
gtaaatatattaaaatcacgtttttcataacaaaacaaattaaactttaaaataaaataaaataaatagtatgaatatgttagtaaaagaaagaa
tctaacgttcaatattaacagtatacgtcccaattctctgagactataaacaaatccttccagtgagcgatagattgttgtaaaaaccggaaataa
ggattggatcctttttctttcatgactagcaaggatgttatatcgtttgactgaagaaaggatggcccaacgcaaatcaccaggttttgagagtcca
tcaaatttttgtcttgatgctgatttacttcggataagagcgagaaaataaatctagcagccctttgtgaattctcattcaagttttcttggacataat
cttgcaaggacgcaagagaatcattcgtttcttgaagatacatatcttgggatttcgataagttttctatgaactcactggaaaacagaggtatgct
tgtcgaacgaaagatgtgcttaattaatgcacaagcagaaaagggtccgaagctctcaagatccactgtgcaatgattctcgtaaagctcaatga
gaatatccaggtgttttttgaacatgaacgacgaaacaatccttgcacgtctaaatactttcgaattacacgacatgcgtctatgaaagcatccg
gaggacgagaccaattttgaatgggggtactaaaagaaaaagaagaagtatttgtccgaaaagaatcctgcgggacgcttgcttctaatgaaat
ccgcggctcgactgtccgatcgaattcaaatactaccactggtaacttcaattgccgaaaaggaagatattttgcgagatgagaaagcgaagaa
atatagcggaatttttggtaaaacttggggcttacaaaacttagcagtgtacggatagctacacgagacaaccaatctgaatgaaccacataaac
agcctttgcgtgtttgtgtaaataatagtccatatgcatatatgcttttacacaaaaactcaggtaaggaaggatattgctttcatgggcaaaaaag
acggcagtgtaattttcgtcggcggaaacgagttcatgcgttctatgatacaatttttcaataattcctgctgcacccgagtctaaaagggggttgta
ggaatgctgttacaatcacaatgactttgtcatctgtacatgaattagagttgtgttacgaaaccaattgcgacagggtaattcttcacaaaatcct
tgcttcatgcataattgtcttgtgaatccttatcccaattttggttaattaagttttcttacgctcattatcgaaccccgagacaaaacagaagcgttt
caaatcatcttccattccaaaatcctacagaattttttttattaatgctttctgtatgaggtaaaagaccttcgttcgtctctcgattcaggtgaaaag
gaacaattcgttgatgagagaagttagtaattatactccttcagaacaggtatcatgagcagcattttgtcgcttcaacgagttgcaccactctgag
taaggaagtcagtagcaatgccattgtcttaaggacagcctaaagattgcatccctaaatcccactttgtcttatgtggtgggcaatgctatcctta

gtgggggttatatagccttgggaagtggagtctcaaacccaaagtggatgaaggatggctgattatgtatgcataggagaccgaaaaaggaaa
gtcatgctaagcagtaggaagaaagcataaataacccaaaagagtttctttgttggcacctgaaatatgaatttatttaagcaaaatgggcctcgt
tgaattgaccattttcttctgcaatgcctgcggtgtctatatctgaattaccaagtcggttctcctggtcaagcgactcgtctctctctacggaaaatg
actctttagattttggtagtttgacctttagtgaatttgaatcttccttccatagaatcctaggacataccttgaaccaatctgttaaatcgtcaacttc
cacaccttcgtgcgatttaagtgtttccccgaacgagttgagtgagcgacttagctttgattctagtagcactgttacaaagttaaatgtcgaaagt
ccaggtcaattgagtgattgctcatcatcatgtcgaaaaggttctttattgaaacacgctgatactcgtagagccaactcaaattctacaaagagt
attaaacagagaaagaaagaacatgggctcaaacttctaccgtcaaagtcactttccataaactcgaatgtttcgctcctctggtttcatccaaga
gtgcagtttttcttcaacatttcgctgcccttggtcgtccttcttccgcttcatatccttcttcttcctcggtaaaccaaacatggaattcttgtacc
atttgttaaatcaagcagcctgcgattcttcgccaggtctgtttggattctcagtttccccgaataaccttagattcgttcattcccatttccactacat
attttctcttttatgttttcttatcacttcctttcgcatagacttagatttctttttatgtttcgcctcagtctcactttcttctccttatgacctttgcatgta
tatagtaaaattgatatatctctgttaattaaaaatctgcattaaaagtatatctagtgaggaattattataagtaaaatcttaatgttaatagaaa
aaaaaaattttaatttctttatgctttcttcacattgaaagtcatacttcttatactttctttccaagaagaaagagatacaaaaaaaaaaggacaa
aacaaaagagaacaatcataccaaaagttcaatgattacgcttttcgagaaagtataaaggaaggcgattttccatgcttttgtaaatttctaaat
aactggagaggctttacatccgtagaaatcggatgttcttttccatcatagctcagcaataattcatagctaagccaggtgctaggatcaatgtgat
acttttgtaaggtagacgttaatacctcatggtaggaagagtttatggtcattcttggaacctttttcttttcgctagcattaaggggtgcttggtcat
ttacttccaaatcaaagcttcctgccacagttggagaagattgcgagggctgaaaaatggaactgctctcagagcaggtatctgagttgatcaatt
tcgtttctttaaaattcaatgcaagctttctcaaaagaggaaggaactcagaattcagtgaattgattgttttcacaagcttggcattttccgcttcc
aaatagtcgatccgtttctctaaagccaacagcgataatttcactctttcttgagtgttggtatcttcatgacaatcagttttctttaaggataaagga
ttttctttttttcgcttgttctttatggctttctttcaagtcctgtatagctgacaaaatatccaaacggtggcctatactcaaaattcccatgtcgcgtag
atcattcgaattcatcaatacaagactctttccaagaatacaattttctagagcagttagttcaatgatctccgatgcatcttttcttcacaaaaatct
ttgcaggaaacaggatgcgttttctttttttaaaaaacccaacccatcagtttttccttaaaaacacatataagaaataaaaataagatataacc
atcaagcatgccatgcttcctgacttttcttgagaatcataccttgaaaaatagacttgtacggaaaccccaactgctcaatccaagagcatacag
cttcgttattccaatcgcaataatttccttctgtcatccgtttagtattgtttacgcacgaaggttgatgatgccagcaaaatccttccttcccaaatct
ccctttttcatataggtccaaacaaagaaagaatacgaggtagtaaaacagtacgggttggaataataaaaatgtaatatgaagattattcaatat
ggaacgagtttaaagagacccaatatgttatatttcccgctagaaatgtccgcgaaacctcacttgttgtttttgttgtcattgtagtccaacgtaaa
taaacaaatattacgtctccatattccaaaggtaagagatctggcaataaccggttactgttgggacccaaataaactttttcaattcttttctttat
agtaaattaaggaatcacttttcggtttccgtttcatactctttcattgctgcaaaataaaaccatttcagccatatttgacttataatggctttagcg
gaaaaggaaaaaaagaacaatatataagccatacgaaaaaatacattatattagctttatcattcagctttccttgtttctttttattcattgcaat
acttgtatctttccttattgacatgaattgactgaataacgtattttgatcaaatccagtatgcaaaataagatattaataacgacatgcaacgaaa
ccttctcttaccaattctgaagagtgggaaaaaaggagtttattttctgtaattttttttctcggatccttttaataccttattgaataagtacaaattga
aatactttttttccccgaaagaggttgtacttctgcagcattggaatttgtgttctgcatacgccttagtctttaacttttaccaccaccaaatccttgta
aaatcgaaaagtccacaaaacacagccattaggctttttcttagataatttttagaacagtgttttgtttttaaaggacaacatggcttcttttggtg
aattgtctttattgggtgagatatctgtctcctcaaacgctgaaaaaaaggatgaatatcgttccagtggtactgtggctttagagaacggacaag
aatcttcatcgttcattgcagtacaaattgagaaacatgggattgaaatctataacttgaaggaagagcgtctgtttgcatcttgtccgctgcctga
aaaaactgttttctcttgtaagcctatgtacttacacgagggaaattcccattacatctgggcctgtacatcttctgcacgaagtaagggagaatgg
aagttgttaagctggaaatataatgaccttaaagagcatggtgaggtacgctttcgggatttattaaataaacctgtttttttctatacactttattcc
atcatctggacagctcgttctggtttttgttaatggagaaatttcttttcttgatccagaagatgatttgattcaaacttccgcttctattaaagggaat
gctactttggtgaaaacgggcagcgtctcaaaccacgttgagaaggatattataagtaattcgacctcagaggataagtctgcagtcgtggatgg
aactggtaaagatcagtccggagaagatttgtcgccttcttccctcagttctttgtcgactactacaaatatttatctcctgtatgcacttagcgttga
aaagattaaacagttttatatgagtgtcttctctacgtcagaaaaacgcatactttttacgaagccaattgaactagactcttctacttctcctgccc
atgttctgcttactaaaaactgtgaaaaggtattcggtttcttttccaacagtgttttcgtttatgcttccaatgaatccaccagttcttatcaacgtga
aaaatcgtttcagttggagaatattccggctttatctaatgtacatttaattcttgataaattttgtctggtacaaacaaaaagccaggtttccttatg
ggatttgacatatggtacaattcaggacgttcttgacactggtaatgatactactttcataacagtgaccataaataagaacccttctaaaagaag
cagccttaagttaaattctactgtgtctggaaatatcatactattacagaagaagactattgcatctgttccatttacgatgccgtctgttatgtctct
tgcggacgccattggaaagagaaaatcaaagatcggtaaaattttaacaaaacccgaaactatagctgacggcgcccttacaaaatcaaaaag
cagtgtcactttgtgcgaacaattattaaagaatgtgcaacttcaagatcattctctaagagatgagttgcatcaactccatacatatgtccaaaat
caagacggtgaatcctttgatgttaagtttttgaagtttgttgaaggatttcaagtccaaaactctactaaccgaaagctgttaaaaacaaatagtg
ttcttcctattccgttcgtccatgctatcgaaagtattctcttttcatccaacgaagagcaagacctagatctcttgtgtccggctaaagctactttaa

attatctcttaagaaaccgcttgttcacttcttctattttaaggcttcatccttcgagatctcttttcaattgtatctacaaatttcaaaaggaatcggc
attattgcttttagagcgaactcttgatcttcctgcttttgaggttggttgtgccataaagaaagctttagccaccatgaagacaaaacttttaaaaa
tcggtttattaagactctctcagtttgactgtgtggaagcccaggaagcattgctgcttactttaggtcaggaagattttgatttactattcaagcta
atttgcgcttctattactggtaaaaaaacatcagttcctttgaatcttgatgttgaaatccttatttatgttgcttccattgtcttggatgctatgggcgt
tggtggtattgctgctagtacggataatttaactactgctcaagaattatattcaggcttacaatcgaaaatcacttcactcacagcaatgtctcttg
ctctccctgctgtctctgaactcataaagcataagaagaaggctatagctgaaaaaacttttgttcaccctaaccctcaaccaaaggctgttgttga
tgataaagccgacttagcaacacttttgaagaaagatggcttggctgagcaaagaaagaacaagagtcaaagagctcgtggtagagagcttga
tataactataggaagatatacaattgagcgcctagaaatctaactagcaagctagtaaaaaaaaaatatagtgtttaataattagggtgtatttg
gaataaaattttgactgtttaatgggagaaacgtgggtgactggaaaataaaatcaatagagttacaaaacaatgaacttttattagttttgactg
tagggttagacaatcgattattgcacatattggtttttttaaattggctgagaagttaaatgatataaagatcataagacaaaaacgaatgagtat
aaataattgtaaggtataagatgtcaaaatataaacataaaaagagtccaaaaaagaacgttaatgctctacgtactccaaaattgggtctcca
atattcttaatttctggaatcgtacatttcagatcaaatactttggcaaggacaattttaaaaatctagatgttagatgaattttttttatcgggcgt
actttttgaacattaatagaatgagatgtcgaacagaagattaaacttgctttcatagcttttgcatatcgtcgagcctaatcagtcgttaattttgt
aaccaaacttagaatcacgtacctgctttgttatttcttcttggtcctcacgaggaaatttcataaaatgatcgtatttcgtgcctataaggataggt
acagcagtcttcaaaagttagttacagtcaattaaggaatcatgataagtccattaggtatcacagaaacagatatagttgcgaaataaaccttt
gtatttattatcattgaatacgagttgtgacttaccttattgaagccacgagcttgacggtaccattctttaattgaattcagggtggattttcgggac
aaatcaaacataaacagaatagcaacagcatcattgcagaccataggaagcatatttacaaactctctttgaccacccaaatcccaaatggaaa
aggtaatctctgtattccgaatgctaattgttttctccataaaattaacacctataattagcttgttagtgttatgccttatgaaagcttgattcgttta
cctaacgtttgagtactttcttcatcgaaagatccttgaacgtatgtaaccatcaacgaggtttttccaattgaactatccccgatcattccaactttt
atcgtaacattatttttccttgcttcagccatttctgaaaagcagaaagcccttgtgcctaagtcttgcaaaaagtaaatacagatccacgttcggg
tcgttccaaaaatctacctaaataaggagcagttttaaatctcgcaatttcacttggtaagttgtattttatgttaattaatattcctacagtttagct
atatatggattgtctaaattaaattataaatataaatcatctcgatttaattcatcttcattttgtaatatcattaatctcatataaccatcaagactat
tcacaatgtaacagtacaagatacactgtataagactagatttttcttgaacttgctagttagtttatacaattttcaatccttttaattcattttcaac
atttaagtactctagagcataaggcccttttcgagtctcattgtctaaagccagaacttccggtttgttgcattttgctttcaaggaaatcactactct
caaagcagcgcgaactaaatcaaggatattttccgaatcagtttttagtgacttctcggtcaactgctttttcagaatatttttcaacggctctagta
cgaggttcagtcgttgaaatatatctgatggactgacttctatcaatctttctaaaatggagatagaaagtaggttaatatattgttcatcctcaagt
cctgttgaaataacctgcgtaaatatatctattttatttttggactcctggaactccagcaaagaataaaagatttcaaaaacgagttgtctttgtag
aagacaatcgtcaactttatgttggaaaggtcccatctgtacagttcgtatgctattcgggtccaccacagatttggagagtaaagcagatacaag
atcattaaatacttcttctatatacttaggcttgttccttattgcagtcaatattagctgcagcgcttcttcacgaacgtggacatctgaatcatttaat
aatttatagatattcaagaaagttgttttctcagcattgtaccatttctgatgttgataagataggctaaaacgaagaatgcttaaggcttgcatttt
taactcagaagtgtttttcgttgaaagctccagcaattttgggtataaattaatagaatcttgcaataaaagcaatccaagacattctgagacatg
cagcctagcaacacttccgtctcccaaaaatcgacatacagtagtccaaatgttttcggcagtatctgctttcaattgatttctgagaacattaata
agagaagttactaaaagttcacgagaataagcatctgaatcatataatgtacataaggtctgaacgaagaattcatcctttgttgccagttttcca
actccaattgctgcagcttgagttatttcttcattaggtgaatttaattgattcgttaaagcttcaaagtagctttgtatattaagagaaggatcataa
aattccatggaagcaacaaaacagatacagaatctcttttggagtttctctgactttgggttcactaaatcgttaaaaattttcttagaaatgctaa
catctgcagataacattgcgagtttgtttgctaataatccaatcgtaaaagattcttccagggatttttcaagagtacttagcttattaatgaattcg
ggaagagattctttgtcgagaattttggctatcaatttcgcaacagattcaatgaaacctggatcattgggaacttctttattttgtaaaaatgaaa
aggaatccgataaaatagcctcccgatatttcatcaaatattcaatcgggatctccaataggcaactatattttgcaacgataacatcaggttcga
caaaatccaagccaaacaaaaattcagtcaatttatttattttatcttctgttaatatgtaaggctcaattggaagtattactcgcaataaacgtaa
actgttgataacaagagacaagtcaactttcttggcgtataggacacaacaggcaaaaatcttttgtaatagcatttttttgattttcttttagcagat
caaggcttcgagactgtgaaaatatatcagtcagggcattcgcagcttggaggcgagttggttcctcatcaaatttatttgctaaaactgaaatca
cggcatacagaaattcagtagaaatgtttccttgatggtaaataactgttttcgatagaaatttgattaggagtaccctgatacgctgatccaagtt
tcctgatgaaagaagttcaatattcgtgttagccatttccattatttcattgcgaagagtgttgggagcaacgcgtgcataatattcacattgaagt
atttctgcttgaacggctttaaaaactaattcaagatatttatcttcagaagcagaaatgagaacggatgttattaagggttggagcttttccaaac
attcaatttttacattggaggagacgatcgcagaaatcaagttcacaatatcaagcttaactcttagttccaaggtcgcataattttggaaggaatt
caaaattgacattattccaataaaatgattatcaagcttatctgagaccgtccgaaaaatgcgagatgctaaattaaccaaaccaattttaatcga
caatgaagatttttttgagaatttctgataataagcacataaagaaggaagagaatttgcgaaaattacatcaggtgtactagtcgaaagcgatg
aaagctctgaaaattttctctttcctttaggctttaaattttctgatttccaaacagaaatttgctcgcaaaataagtcgagaaatccaattgcagaa

agcttgacgttatcttctctatcagacaaagctttgatgatatactctccaaaatcttcccataagacacttagcaattcaacgtgaaaagttattaa
tgaggaggcaactgagatagcttctcgcctaattaaccatgcattgtcttcttcatcttcgtacatacttccaaaatcatcgtcttcatcaaagtcttg
gatatcttcttcatcttcagcatctgaaacaatattagggtcaaattgaactttttctttcagaacattcagaacttctttgcatgtattttcaacaatt
gggttttgatattttaccaaaagtgaaacggtattcagaagtaattgcactgtgtcgaaagaattatgatcatcctttaataaattcaagatgaccg
taagataacctggtgctgatgcagaaaacttaatagataattctgtagtactacttgcagatgcaagtagtaactggttaagtaaaaggacggac
gtatgaagattggaagtggatggtttaaccatcagttgctccttcagaatggaaagaagtgtagttaaacactgctcaggtccatatatagataaa
atacaaatacaattgatagactttttttgagatcgaggcacgcttggagttcatactatagttttgaaataccttgaaagacgcagatgcaacatca
gttgacaaaactgaaagtgacgaagaatataattccaatgaatcatttgcaattgataaaagacttaaagactcctctgattcgttactttgagttt
ctagtatattaataattttaggtaaaacttttgaacagtaaaaaaaagtcaaactagattgtgcatcagattgagacagaatcatccacaatgca
gcaatataattcttttcataggaccttgaaaactcaattaacagtaggtcaattaggtgttccaatgtctgatcagaaaccttaagagacaataga
gaaacacatcgcgcagcttcttgttgcacctcaggagcaggatcttttaacgctttaatcaacgtttctgtaaaattttgactagagaatgattctgc
aatacttgatcgtctttctttattgatttcatttcccaagtcgctcaatgccatgtagcgtaagtccttatcagaaccttgtctttgttagcaagaaagt
aaataaatagaagataactagatataacttactttgtgagtatttttgaagcagtctatcctctgccatattgctttggtataaactttagaaaaag
gccagtgggtacgtacttgtcttgtgctgagctgcttaatatcagcatttctagttgggcattcaaaaaagaaataggaaaaatataaaatataaa
atgaataagtaaagaactattgaaacattaatgctcctgtattcttaccttattaattttggatttcaacatatttattctgatatctatatgcttaatt
aatatgctttagattaaaaagtaagcatattataatgcttttttaaggatatatttaagaatgaggaatgtgttctccgctcatagaaaaaataaata
gaataagttatttattcacgttattgagcattgtaatctagtatttctcataattagaggaagaaggtgctttattatgcaaaattggaaatatttatc
tttatatgctattcgtagaatacgaagttgcattctctatagaacataaactgagtccttccaaggaaactcgaactgtcatttagttagaagtttta
attcaataattcaggagcttttacctctggcctacctttttaaaggaggaaaatctgatcaatatcgtttttggcagctacatcttttgtttatttactaa
atagggagccttgttacattatcgtattttctttccggatcattctcgggttcctaaacgtaagtaggaattgatgtcgtaagcatcatggaaagggt
tggaatcagaaataacttttaaggtagacgagaagaagaagtgaggtttatacatggaaaattacataagttaataaattagttcaaaaaacaa
tagttaataaattttagatgtataaactgatatatttggttttatatataagaagtgctcgtatgaagtataagacgaaccatctaccatgaacgat
aagaacccagaagtggaacgtcttcagggcacaaaaccctcagaagagtaagtgtattattatttttgaggaaattcaattgcgattgttattaac
tgtaaattagtgaccaaaaagcagaagatactgcgactgcaatccttcgcaaaaagaaaaaacccaattccttagtggtggatgatgccactaa
cgatgacaattcagtgattacactttctcctaatactatggaaactttgcagcttttcagaggagataccgttgtcgttaaaggaaagagacggaa
agacaccgttttgattgttctaactgacgatgatatggaagacggtgttgcccgccttaaccgtgttgtacgtagtaatttgcgtattcgtttgggag
atgttgtaacagttaatccctgcccggatatcaagtatgctgaacgcatttccgtattgcccttggccgacactgtcgaaggtcttaccggttcactt
ttcgacgtatacttaaaaccttatttcgttgaagcttatcgtcctatccgaaaaggcgacttgtttgttgtccggggcagcatgcgccaagttgaatt
caaggttgtagatgtcgctccagacgagtttggcattgtcagtcaagataccattattcactgggagggtgaacccatcaatcgtgaagatgaag
agagtagtttggcagaggttggttatgatgatattggtggatgccgccgtcaaatggctcaaatccgtgagctcgttgaacttccccttcgtcatcct
caattattcaaaagtataggtattaagccacctcgcggaattcttatgtatggacctcctggtactggtaaaactttaatggcccgtgctgttgctaa
tgaaaccggtgccttcttctttttaattaatggtcctgaaatcatgtccaaaatggctggtgaatccgagtccaatttacgaaaggcttttgaagaa
gcagagaagaattcgcctgccattattttcattgacgaaatagattctatcgctcccaagcgtgaaaaaaccaacggagaagtagagcgccgtgt
cgtgtctcaattgcttactcttatggatggtatgaaagctcgtagcaatgtcgttgttatggctgctaccaatcgtcccaattctatcgaccctgcttt
gcgacgtttcggtcgtttcgatcgtgaagtagacgttggtattcctgatcccactggtcgtttagaagttctccgtatccatacaaagaacatgaag
ctggcagatgacgttgaccttgaacaaattgctgccgagacacatggttacgttggttcagatttggcttctttgtgttcggaggccgctatgcaac
agatccgcgaaaagatggacttgattgacttagatgaggatgaaatagatgccgaggttttgaattctcttggtgttactatggaaaacttcagat
ttgcccttggatctggcaacccatctgctttgcgcgagactgttgtggaagttccaaatgttcgctgggaggacattggcggtttggaagaagtcaa
acgcgaattgcgtgaaactgtacaaatgcccgtcatgtatgccgaaaagttcttacgcttcggtgttaccccaagtaagggtgtccttttctttgga
cctcctggtactggtaaaactttattggcgaaggctattgccaatgagtgctctgccaacttcatttcagttaagggacctgaacttcttagtatgtg
gttcggtgaatccgaatccaatgttcgtgacatttttgataaagcacgtgcagctgctccctgtgtagtctttctggatgaattagactctattgctaa
gtctcgtggtgctactgctggtgactctggtggtggtgaccgtgtggtgaaccaattgcttactgaaatggatggtgttaatagcaaaaagaatgtt
tttgtcattggtgccacaaacagaccggaccagattgatcctgctcttatgcgtcctggtcgtttggatcaattgatttatgttcctcttcctgacgag
gaagctcgttccggtattcttaagacccaattccgtcatactcctatggcagaagatgtggatctgggagctgttgcaaaggctactcatggatttt
ctggtgctgatttggagtttgtcgttcagcggactgtcaagttggccatcaaggaaagcattgaggaagatattaagaaagaaaatgaagcaaa
agctgctggagcagatgacgttgttatggaagaagatgctactgcttctcaagtacaaagacgccatgttgaagaagctatgaagatggctcgtc
gttcagtcagtgacggtgaagttcgccgatatgaagcttatgctcatcaactccttacctctcgtggcttaacgggattccaatttgattcttccaac
aataatgctaatggacccagctttggaaacgatggcgctgatgatttgtacgcatagaaaaactgattgtatgaatatatatcctgatgacctttg

atttcgttttgactatgaggatgatatttgagaaggtgtaatgatttactgtctactaagagagcgctagatttttgtgatgtagtgtaaagaaaga
aaaattaaatattatttgtttcacgatgagtaagaacggaaaagcagtatatagacagtatgatagatatttccgagttagaaagtaaatcaaac
acaaacaaaacagtcgataataacgaaactccttttaaggagctaggcagcgtaaagaacagtcttacggacatcgggttccgtcagaaaaccg
ttgccgagctatgaaacttaacaaagataggaaaaaacaacagagcttgaaacaaaggataaatattcaattaataatggtgataatgtacaa
ggatgatattcgagaacctcaaagtaatctaattgataaaaaaacttccatacgaggctcaacaagaaatctacaaaagtaaagtaaaaaaaa
atgcaatacagatgtaaacagaaaaatattagataagaaataaaagaaatcaactaaggggattctctatgtaaagtatgtctagtactgcttac
ccccaataagcgacggcttataagcagtaagtatgatcaagtctatcaatagacacaactaagaaagacgctccacacaggttaaacaacgtgg
tcttgatatccttaaaatcttcgctacctgtcgtgtgctttttcttggaatgttacttgtttggaagtaactacataaacggtggtagaacgaattaac
aacccaaattaaacgagatacgcctaaaagtcgagagcataggctttatataccaaaagaaactgaaaacgacgggatttgaattgaatctgct
acaatttagcactaggaaagagtttgggtagttcaaagtgaaaaacgaagcgatttgccactcataaaatctgtttgtagatcgtttgctgcacttt
gcaatttcaagacctatttttcattggaaacgtgttttattatcgcgcaaattataaaggttgcgtttgttgagcaggggttttctaaaaacaggcaa
tatatccaaaaataattaatcgtttggagcagtataaccattgttgtatgcgttcagtctgacgtgcataacttaatacagtcataccataccgtatt
gctgggcttggtcgcgtttttatatctctactcctttgtgtagattaattatttgtcgtttgcttgtcggttgtatgggttgcgttgaatctaggctaac
gtaaaggatttgtattttctcattattgtctagtctttttaatcattatttatttgcggttggtgctgattgttgtttgttttctaattttctttccttgttga
ccttcaataaacatcgttctctttttgggttaaaacccgacgctcctttaaagattgtgatttagttcattcattgaatgactctgtctctttgggtgttt
tgacgccaatttatcatctaattctgtatcttcatcatacttagatttgtttgtttaggaagcgtttctcctccttccttcatacataaactccttctttac
ctatttcttcacttttatctacaccataagtcacttctttttttattcttcccctttttttgttgtcttcttaccatctctccatttctcgatatctcgcattccga
cctatccctactaatctttaaaatcttcattcaatctctgcagccttttagtgtcgttttagcggcctttttttttcgaagatttaatttcttggacatataa
tactcgtacgtgttttccttttcttttcccttatttacccccttttacttttaatacaaaaaaaaaaggagtatttcttataaatctataattaatcctaatt
ggttactgatctctaattccctttttaattatacctctctacaattagcgatgaaccaggctactaatacagctccttctaattctcgtcatcaacattct
tcttccagtgttaatgctggaacagcaaatgttatgatttctactcctacaggagtttcaaattgtcagacaaaggaaacagtacctttaacaaaca
attctaataatcaatcaaaattgttgacagatcaatcaaagagctctaattttatgagtaccactcagaaaaccaagaaggatgccaatggatct
acccaaaaggttaagcagtatgtttgcactatttgcacccgcgcttttgcgcgtttggaacacttgaagaggcatattcgctctcataccaatgaaa
agccttttgtctgtcaagatattgatggtttgccgattggttgcggtcgtttgttttccagacgtgatttgcttcttcgtcatcaacagaaaattcataa
aaatcctcaaccaagaagacgaagacgttcgacaaactctatccccaatatgactgtcgcggacgcccagtcttttattgcaaatatgaatgctcc
taacaaacgcccgctacctcaaccatccacaaatgccgattcccttagattctccaatcttcaatcttcgaatcaacaaccctcggtttcttctcctt
ctgcatcttccctctattctaatgccggttggcttgaatctcaaaagcaacgtttagtatctgcagttaatgttggtgatgctgtagcctctagtttac
cctctacttcatttccttcttccgcaggttatcctataccaaatccctctgttggtttgcaagacaattctttgtttttccgtcgcgcaacaatcgcccat
gaactatttcctcctgttcaatatcaaccaatgtcaggtttgtacgatcagaaaagtcctgactgtaattattcatcttttgatgatgacccttctttat
tatcatatccttcattttccaaaaatgcaggttcaagtacttactggaacaaattgactgttcctgaaactattcctgagggtcacgaagttgataa
atctgattggggtcatttttctgatcctttgtctgttccagtgtctcagggtatgaattcaaatgacattcttgcattctttcagccatctcctcatccat
cacccaaagaagccagcaatgcaaataccccaagtaactaccgtttagaaacatctaataattctaatacgagttcagttgattatgttcgacca
gatcatgttgagaacgctgcccctaatactatgtctccaatggggaatatttttctcctagttattatagtcctgactcgatgatgcaaccttctatg
gcggctagtcccacaactttgccaccaaccatgcgaatgtcgtcgtttgggagtgtggatgcgaacttggcttcagatgtttatgacacagatgcat
ttcctccaagtgggtatgtaggcatggatggagttaaaggctttgaaaaccccgaaaggattgcatcgactccaagtgcagctgcaactaattatc
ttggtccggttagtgaggaatatactgatattatcaacggtgggttagcacaggagatgctgtacgcggataataatgattatcgacaagacagg
tttaatcctgctatgttaggcaatttggaatcttacggtgctcgtcctccttttcaccgtaacagtacttctacttctaagtggtcatctgatttggatta
tatctcctctagcataccgaactttagtactggtttagaacaaccttcagagcatgctaccttttattgatacattttatttcttatattccatttcttaa
aattcgttgatgtttaatttttttttttaaaaaaaaaaggaaagacgtgtttcggttcatatttttttgacaacaaattactttggtgagagttacattca
taaaaaattaatctcaatctagtgtttgtacaatcagcagttcatgtgtatagaaagtatcgttagaattcaggatctgaaacatgtaatgtgatttt
gagatgtccatcgagttgctgaaaatcaatttgtatttcaagtacagacatgcggaattcaaaagttagattatattcaaaatgcacagaaaacta
gaattcatttagacataagtaaacaacgagaaagatttatgaggatctaatattacagaacgtaaacttgcttatgaaaattaacaaacatatgg
tagaagagggagcggggcgtaatgaagacattaaataaaacaaaccaaaatgagcaataaagaaagaaaaagaatgatacagcaacgtgg
atattgctttgctttgcgagtcacttttctcaagcgatttctagccaagtttagccgtttgtctacccgatctacatcgtcatccatatggtctattaact
gattatgttcttcgagttcatcgttcattaagtagcccaattgcttttgctttcgaacactagcctcgattccacctaatgactcttcttgctcgttgag
catctgctggtgcatctgcgtcagtaagttgacatcaatgggattgggatcatttccttcgaattggtagatggattccatttcaatatctgatttgtt
ggacgttgcatctgaagtcaatggagcagcggcagcctgcttggatgagtaggaagattgaaaacttggtccttgggaagcagcatttcgctttct
cagcagtgtttcctcggcagactcaactcgtcgctgcatacgaattaaatatgcttcagattctcgaagcctgtattcaggaatttgagcattctgt

ctaaccttgtttgttcttgacttagactttgtaattgtctacgaatatcttgtaaagaggaatcgatctctgcatctggtgagtttccaaactcgatca
atttctcataatcgttgattttcttcgtcgttgaatccactaacaacagtaactttgacatgatcgttggtgttttgattactcttattgcagattgagtc
actctacgaagaagctactacgaccatgaacgacagctgtgcggtgctactatttacggttgtaggacttgacgaatgaaaatctcgattaccgc
actcgaacggcgtagtgttatttttacaaagagaaaacttaggagagattgacaaagttgtagtagaagaactataaaaaaacgaaatgtgcga
atcctctaactaaaaaaaggtataaagcaaaacatacaaatggtggattgaaagaaatttccaaaactcgatttctcgaagtgaaaagtaaaat
taaaaaagggcctaaccaggattcgaaccagggatcacctctaaactcataaactcagaagtgggtcttacccgaaagaggtatgttaaccact
acactattaggccgttgttgtcatcttatgatttaagaaaaatatatataaaaatcttaaggtagaatagttagcttttgagtgttgaaaggttcatt
tttttatccagttttaattcggatttctactttttttttattttttcctaaaattagaaggattcaatcgtaacttatgtatttggtgctaaaaaattactgt
acataagggtcgcaaacttatttggaagtcgccagcaacaacgacgaagtatgtttatcgtcacagaaatccataactagcaaatccatatctag
cgaaataggtgactaaagagggtttccggaatttctctgttaagtctctggtctggtatgtctggaatgtgaattcattattgacgtatcgattgtca
agcgaagcgcctcctctgtgatggattggaaagtgatactgcttcgatgtaagcaaaattgcaaatcgcccttagtttgaaaggagatttgaagg
gacaatttgaaataccaacattctagttacatatgtctaaattattaccttactaacagttctcctgatggtgtagttggttatcacacccggctgta
atgtctacgataccggttggtcgctagttcaaatctggctcaggagattttttttttcatttgaatccaaaaaatagggttcttcgcgtattgttttgca
atgctaattggattcttacatgattttcttttaagtgcgagtctgatgttgtcaaaaaaaaaaactaggtaacagttacctaacctgaatcaccactgc
gtaaggcttggattgcagtttccaagaccaacaatcaagacaagcaacgggtttttacattgaaccgattgccagttgagcaaggattactaaatt
cagtgctgtaagcgtgtctttctttttctgaaaaaaaaaaaaactcttcatttgagcatcgtagtcacggagtttataaaaaatggacaagcctaaa
atctttagcgccttgaacttgggagtgggtgcttttcttgggctttcgggtcttatcaaggttttctctttcagcttggcgaatgttcttttaggtttattc
atcatcgcctttggcctcggtaccatcgcattagaacgtgaagttccctctgtagcagtcaaatatgcctcctttatgttctccttcttgggtagaggc
tttttctatctatttatgggttctttgctttggtcctataatggattcacatctttccttggctttttagttcttcttgttggtgctggcttctgtgctgctaa
ctacgttgccggactacaagattatgctcctcgaagcatgcgcgatacagactgggatgatacggtagatgaggtttaagcacctaacggcgatc
ttcttttcatgtttagcttccagctgtaagtatgttgtatgtttttcatctcgtcttttttttcctaatagtgccattctttttattttattttatttgttttctcg
catttcatggtagtaatttctctctaccttgttaaaaatgtttactagtaagcgaataaaatccgcttcatataatttaagacttacctacggccatta
cttgaatggagcgtttccattttcattgtatgggctaaaaactgtagaagaaacattgagatgagtagaggctaacttgatcctgaaaaggttcctt
gtagatatgaagcagaatcccttctagccgtccaaatccctaagatcacaaaatatctgcgaatttttgaattatggaaaggttccctctaaaacc
atggaattcgtttgacatcttgttgaatacatgcaggcaggtgattgctagaaattgaatcttgatagaacagtttgtacgcatgagaatttcgttc
aaaataaggaatacttttctggtcaagctaataagctcattccttgtaaacagcttcgtttgtcaatacaaatgtgtgaacatcatattactctcgta
aataaacactaatcaacttattaagcaactgcagtttccattccttacaacaatataatacaaatactgagataatatttccgacagccgtgacgg
gttgaaaatttcctcaggtcaaaggacattgttacctaagatggtgaaaggcgacaaactggacaaagtcgaaattactatatatattgccggga
agcactacttcttcctacaaccaaacactgtagcttacaattaccagcttggttagtgaactgttgggtatatcaaggaggaaaaaaatgacatgt
caagctcaacaatatgaacatgtgtcatgatttgctttatttcttcactttctgaggtgtatctttcagttcacaaagcttgttggaagggtaaatgcg
tggtgagtgggttccgtttgaatacggatgtataagtaaataatcaacataaattaaatttccttctacctattgtttagtcaaattagtaaagataa
agaattcatgagagaaaatcgtaaatattatgacaaaatgtagacttaactgctgctttcgtttcttagtcagtaggatctggcgcttccattgtcca
tatgacaggatcttgtgtgagcacagtaatttgaggtttgcagatgatattgcaggaacttattagtccaccaaaactacagacctgattttcgaga
ttccatttcaaaccatcggttcttaaaacaaaagcgtcacgacctacaggaagaatgccacaatgcttatttacaatggtgccacagtttatagaa
ctttctcctgaaggtagtagccaagtcacgtttgcttcggacagtatgtaaagctgagtttccttatgctttgcatacgaccaataaagatgatttag
gttccccaaagcatggtcaactcgtccatccatgccacacaaagtgaaaatgacagatattccattttcttgaatcaccttctgagccttcataaaa
tctgtagagtattggtcaggatcgtggagtatttgaacacccattcgcttgtagtgctcgcgtgtctcattcgtcaaagaatcaaaatctcccacaa
gaatgtcaggcttcaatgtaggatcgtagtttttttaaacgattcgatccgccatccgcgcatatccgaaggcttgcattatcccaaagatgatggaa
acgattatcagaaatactaattgagcggttcaaaattaaaatggcatatttatagttgtaatcacgttcattgagtctttcagaccaggtagcacat
gggtcaaaatgctgagactctgttaattcgttgggaggaggattcacgctcgtgctgactgctgatgcatcatgatttttgcttttcttatacttgcca
attggatgaggaagatttctgtgtatccgctgaaatgtttcgtaatattgaggatgttctggacttaaaatgccatgtcgcattaagaaatcaagaa
tcacaagagcacaatttggcttgaaattccccgcggctaattcttggataacttgatccaaggtccaaagatgaaactctgcaacctctccatcatt
tggccgcaactttgtattctcgtcgatcggaacatcgaatacgtattgtaactccggctggtaccatttcttctgggaaaacttgatataggagaca
ataccacgggaacgtaaactagaatgcgagacactcaagtttgcttcttccagacattcttttaccatagtccccacaattgagtcaccatggctg
attcctcctgcaacgctgttatcgagatagccaggccaagtttgcttggtggacgaacgcgtgggaacccatagacacataggagttgttggtgta
ggaggaacataaatgttacaatggactccatagctaacaaaaccaaaaagccagtatccacctcgttccacggcaaaagcaacttcaccattgg
gaccaaagacgctatataattcatttcgccattgatcagccacgccaaaaagattcgattgtttccaaactttgagaatcttttctaaaatggaaga
tcgcttctcaaaatctgttgcgttcagttgtatcgatttcccgggcttatatatccaatcgtaaacgaagctatcgaattttactaactcttgaataac

ctctgggataatgtagccgatgacagcatcattcaggtaaaatggaattgttccggagatgaaattgttatggaaagagtcacagcggtccagta
gctttaccagatcgagctgttcgattgtcgaaattgaagtcattctacttatttgtgaaaggaatcttctatgaaaagtaaacctaattcccttcccc
gaagtatgccaggtatgctagcaaatgtaaataaccctgaatgccaaatgcagaatagcacaatctaaaaatccaaaaatttaccaaataaaga
aaaaaaaaatatcaggagatagaggtaaattccttctgtaagtgagtgaaggtatcgtagagagtatggagtgtatagttgaattatcccttgta
gcaaggatagcttaaatcaaccttaaactttagccctagcagttgcgaaggaaaagtaaataaagaatcaaacaaatttcaacaataattcaat
agaattaaataaaatgaacttactttagaagtcaattccattcaattaaagtaagtaaataaataaaagggctgacactaattgcttcaagcata
aagcaaaagacttgcgaaatgaaattatcataaagaaattgaccgcagggtgtcctctcataagttaaagaaagtaaatatacagcatttcttca
tttggatggttttctttgcattgttagcttcaaaactctcacagagttataaaaactagaaaagatcatgacgaaaagtcatttatcatggaaactt
gagagtaagtttcctgtttttcaatgcataaacagaatgaatatttgcattgatttcaagcaaactggtttctttcttcaaatgctgttaattttggat
acatcgcagaaaaaaaaaaaatacgtaaaagaacttcttacaaagatgaacaactaaggaatttttttatttaaatttttctctattaaaacattac
tttatgataaaaaaaagaaaaaaaaaaacacatgaagatcaggaaaatattggagcgagttccttttaacatcaaagtagcaagctactctttg
ttccctctgtaaaggaacaagagagtgtgatctaatgaaagctacctgaaacgatcttctgcaaaacggaaccacaacaaagaattaatagaga
aaacagttgttcgaagacagtgatagaattgatttggttcactgatttctatagcaagttttaataatttttggatatcacattttacaaggaaaact
caacttacataaagagacatcatgacttctcaagagaaacaggctggaaatggaactaatgaacttgacgatgaatttgtggacaacagtgttcc
cgtttctttgggtgaaaagaagactttgaatgaatacgtacatatggatgccgaggacccctctttaaccaaatggaaagagtctcttggtatttct
aacaagggatactctcctcctaatgaccctagaacagtggtgattgaacaactcagcttgcttgtcaatggtcgtgatcccatccatgtcgatatgc
aagatgaaggcagtgtggaacaaattcaaaagaagggtttcgtcattaaggaggatagtgaatatagaattgctgtccgcttccgtgtccaacat
gaactcatttccggcttacgctatgtccaagttgttcgtcgagtaggaatgacagttgacaagtcgtctactatgattggctcttatagccctaatga
aactccatatgaatttactagtaagtattttgtttttgttgattgttctcggaatgaatgtattgctaattagagtgtctagcggaacccgaagaagct
cctagcggattccttgcccgtggtcattacgaagctattggtaaatttatcgatgatgaccaagttgtccatcatgtagttcaatgggtatgtttctta
tattcttgataatttgcctactaactgtggtgtagtcatttgatgtcacaaaaaattggaagtaagtcgaatctgcgtgactgacaggcattattctg
ctgaaacaacaggacccaaacttattatcccaactttgtctaattttgccttctttttctattgttgctctatcactgtttgattcttgatcacttgcttg
ctttgacagatgctacgcttttacttttgctaaaaatgcttacgaaaacaactagcacttcctattcatagtatatcgtgctttgatgaattatattcc
aatttattccaagatttctttgaagtttgtttttgttcagagcctttactaccaaaaccctatcattcaagttgctagctttccacgtcatcttgtgctga
ttatcataccctcttctgaaagctgtaagaattaatcaaacggtataaaccggcttgtattactttctttttcattcatcctttttcagttctagcttaag
ctcatattataatacaaagtcggaattccctaagtaataaagggctttaaactagagaaatcagtaatatttgttacttcaccgaaaagtaacaat
atcacggcctctcccttactaccgtcttttggtatcctcctatatcctacaaagtaggttaattcttgttgctagtccgagatatctatcttgcagtattt
ttttgagaaaataataaactaaactttatttttttaaatctcctttcaattttcttttagggatctctactttcacatttttaacatgaggaattctgtca
ttatcggttctcttctcgcaactgccgccgttggtaagtaggaatgcttggtcccgcaatcgtttgaaggaaggaataataattaggcttctacggct
gaacttgaatgatccgggaggaatcatttcaagtgtattttgcacgatgtctatggagaaagggagaaagaatagtacattttatgactggtgctc
tcttttgaaaggattttaaagaaatgaaacaaaaataactgattttgcgtgcatggaaagctgatcgatcgaatgcgggtttccgagctggcttcc
tttcttgtgcatgaacatgtttgctaatatctctcaggttatggcatttattttgactacaagcgtcgcaacgaccctactttccgcaaaacattgagt
atgtccacaatatataataatgtcatccgctgtatattctttctgttcgctaacgaaacggtttttagagcgtcattacaaaaaggtgcacgaagct
aagaagcaagaagaacagattgctgtcaagaaatttgatgaagttatcgaagatgctctcgagatcgtcaagtctacacctacccctagtagcgc
tgaagaaaaagagctcttctttatgcagcaagttgcccgtggtgaacaactcttccaacaacgtatgaattattgcttctatgatctttttctaaca
attatagaacccgacaatatcaaggaatctgctgcttgcttttatgctgctcttaaagtataccctcaaccagttgaattgttttccatttacgagcgt
accgttcctgaacccatcatgaatcttttaagagctatgcaagctaaggaaagcacccctactgtggagtaaactcttaattgtcaataaaactat
attctcattatatcaataaaagataggatgcatgtaaatagtagccatgccatttataattttatttacaaaagtcgctgaagaattggggttatctc
cagcttcatgttctttaattctgtactatacatcataacttttgataccgtttcagcatatgatttatcacgaggaaatcctttgtactggttttcaatg
aagaaaaccaaactttcaatctgacggtacaaccagtttcgataaagtgaatctggtttcttgacctcaaacatttgagtatacaaacgcgtaatg
tgagccaaataccgaaaggcaataaaagagctggtggtaactaagtttgatatatccttttcccattgtacattataatatttctgtttgagctgttc
ttcacgagaatcaaagaactgtttccgcgcatcattttcaaaaccaatggatcgtaaatatctaactaactgttttccttgcgtaacgctaatcccg
gggaatccaatttgatgtaagaatagctctttcagcctttccgtcttatactctagtttatccaataaaaaatccgttagtaattgttggatcctttcg
ctttgtaagcggtttctcagtaaatttagtatgcgtatagcatccgaatactgacgcagagaaataaaattatctaattgattaaattgttcaaaaa
gatttagcaaaacttctcttcttagagcataagactgtagaagtaacggagaatcaatagttttacaaagcaactgaaaagacgtgtatgacgat
aagcttgcctgctcacttctgacgaaatgttcaagctccattcttaactgtgtatcacctacacgtttttgagcatgttcgagcagctgttgagactg
gtattgtctagattgacggataaagaagtctcgttcttcaagattactatttcccagtacaaaaatgcttctatcacataatactgaagcgtaggat
aaattcgagtctttctctaagttggatatcgcatcatcatccgcttgttcaggagtttcaattggaagaattgtaaaatcaataaggttccattgata

aagcgtctgttctttgattctcttttctttatccaaagaatgcgaagaggtaacaaggatgcagtcattaaataagtaaagattgacaatcaaacg
aactctttgatttctagtattaatgagtgtccaacgatcttgttttgtaattaggcttctattcggactcttccaaaaatttctgtttacaccatcaatg
gtattttccaatcggcgcattgatcgttttagattatcgtcagaagtgttttgtgaaagctcgactaaatttagtccttcgatgttttcctccaagaca
cctaatagcgatttgaaccggtccaaatcttggcttaaattgtttacttcactggaaataaagacgtactcgttgagatttttataaaagttcttctcc
aaattctttcggactcccatgtggatacgtaacagtttgttatacaaggaaacaaattcctcttctgtggcatcatgcaatatatcgtttaaatctga
tggagtagactgttaataatcggaagttagatgaaaacgtacaagtctgagggtcataatcttcattatcgaaatatttcggatttagaaatacttc
atttttgacataatatgttttgtttgtcgcttttgtcgataaatatcatctgttttagaagtgccaactcgaagccgagtaggagactaaaatgtgta
agtactttgaatcgtctttcacaaacctggcgtaccaatctttcttcagaggcttcgtcggctgctatatttaagtatttgagtttcctggtccattagt
ctaaagcaatgaaaacgtatcgacatacgaaagctcatcattagcagttaaatgttcgtttgacattatagcaaggatacttatgaattataaccg
gaacttccttaattcacgagaaagaagaacaagttctctctatttcgtgatttcgtattgctggacttgaatgttcgaatatcaaatcataaaagcc
gtttaaattgcttttagtaaataaaaatgaatatattttgctctattaatggattaccagtaaatattacaagatcaacgattagttctgttgttggta
tcattatattaaaggtatctatttaagtaacaatgtacttgcgctacctgttcatgtcacttggtctgtttgtttacattgaaaaagcatgaaatatac
tgcattgttgatatcgtctgaagaaagagatacataggaaaatctagatataaaatagtggcattcaagctagaccaaacagagtcccgggttat
tagatttggttccttgttcttgtttccacatcataggatataaagaacgtgcataaggctttctataaaagtattctttttcattgcaattacactcctt
catccttgtaaatgaatgtttctttgctgcaagcagtgcaattaaacatatttctacttatgagacgtaaacaagcgacaaaaagtagttcagtaac
agttatagattacattgtaaattcacacgaagtcaagcttaaattatagaagaacgtgaaatataaagtacacagaccgaaaattaattgattct
attcaaagtgcttgctcaactagtgagctaaaaccatttacagagaagtagtcgatggttgccttattatctaatttttgttgtaatgttcattctcc
aagcgaattctataaacgatgtatatcttcttaaaggacatctgtaaaataaaacttaaaacgtagtcgtagccttcccacctccaacgtttagtat
agtatgccttgcagatgattgatattcaataccgttttggagcaaagtttatcaaaatctgtatccattagaggttttaatgaacctgtttgatattat
gctgaaattatggatacaacactacgtgactttataggtactgaagtaaaaacgtattacagagaatctccatgggaacgtttaaaggttcctttt
aaacaagcaatttctccgataatagcgtcccaattaccaagatgtcgttcatcttcttcgggaggaagacgtcccaatcgtagtaatgtctggaga
ttttgggatagtttaacacttacggtatttgacattccttcagaatggatagaagaaaatggtaacaaactatataacctgtggaagcctttgcata
aacatggcaacatatcattcatgaaattcatagagcccccgaataataacttgactacgactgccatcgttcgcttctttcctcccccgagagatcc
tttttggcagcctttcaacaaaattcgtgttaatggcgtggacttgaacgttcgtatagacccttacacccaaaattccttgaccaaacagtcgtctt
ttactacggttggtaatcaatataataagattgtccaacttcccctgtcttccttaacaattggtcaaagttaccatgaaaagatggttcctctgtttg
gacacgaatgtggaacgccattacatgacatctccctttcggttgatttcaataataggcggtttatgataagctttagaataattgctggagaaat
aactgatgattatcgtttggatatcaattttcggagtgtagtcgatgatataggtatcgatgttgagaaggacagcatcaccttgtcattcagatac
agattaccacctgtattatttcgtaaagaacttttggaatcagaaaccaaattgagaaaagtatggagttcttcaaatctttggcgtcgtcaagttg
atattttgccttcttcaaatcgtgcagttcccaattctcctctacagttattaaattcaaacaatgttccattaggaagatccaacatggttatgttttc
atatgacataaggaatagtcgttccgaggaagctaatgctttttttatagaggaattagaaaagtataacctgaagagtcgcatgtaccgagtctc
tacttaccgaattgatggttattccctgagatgtgaattactatatcgcaatacggatctgtctcacaccgttttatatctactggaaacgtgtctttc
tcagcagttgctttccgaggttgacttacctgttctccttgacaacttgaaccagcttcaagaaaaacgagctactgtttttttaaaaaatatccttg
cttctcaaaaacctttacacaaccctacgcgggaaatatttacacaacaaatggcgttcttcgatctaattaatacttctagtatacgtataagaaa
gctctttgtgactccaactaccattcgaattgtagaggattccatcgaagcaggaaaccgcgttatcaggcattatagacaattcgcggaccgatt
tatgcgggtacaagtcgctgatgaatttaccaaacgaaaagtttacgctgataattcgaactgcgaggctgtgtttgcgcgtgtacttcaattattg
gatcagggcgtaaaagttggaaatcgcatctacgagtttctggcctttggcaattcccaacttagggaacatggtgccttctttttgcttcaggacc
agatttgaacgtggaagacattcgtaaatggatgggtgatttcagtgaaattaattcagtgtcgaaatatgcagcgaggatgggtcaatgtttttc
caccaccaaagacatagatcgtttttctgttaatattgcacctagaaatgatatctataaaaataataattgcttcactgatggtataggtatggctt
ctctctctgttttgcgtcgcctttcttttgagattgatgataatgaactttttccttcagcttttcagtttcgtatgggaggctataaaggggtattaagc
ttagctcctccgactaaactagaatatcatcaaggtgacttggtttttcctcgcccaagtcaagataaatttaagtctcctcatgctgtgttggaggt
aattaaagtgtctcgctttttaaatgctcgtttaaacatgcaaatcattacattattggaaggtttaggggttcaacaagatgttttccttgaaatgg
caaaaaaccaactatccgaattgaataatgctatgactaataagcaaaagtgcatccttatgttgagggataacgtcgatgaaaaccgatcaac
gcttgttatggccgacttaatacagtcaggatttttagagaaaaatgacatttttacaaaaaatctccttaatctttattatgaatgggttcttaagtc
gataaaagaaaaacagaaacttaacgtgtctaaaggagcttatttattcggcgtagccgatgaaacaggaactcttaaaggtcattgcgatgatg
ctgttctttcgattcctgagatttttgttcaagttacaaatcatcattcggaagctgaagcttcctctactggccggataaaaacaacagtaatatta
ggtctttgtgttgtagctagaaatccctctttgcaccctggagacatacgtgtctgtcgtgctattagaagtgacgatctaatgcatcttaaaaatgt
cgttgtcttcccaacaaaaggtgatagaagtgttcccgcaatgtgttccggtggtgacttagatggagacgaatacactgtaatatgggatccaag
tcttttacctaaaattgtgaattatcctcctcttttagaaacgacacggaaacatgcagcaaaatttctggatggaaaacctctaattgactcagtg

aaagcatttttatttgttatataaaaaacgataatttaggattaatatcaaatgcttggaaagcatgggcccacgataaagacaataaccctgac
ggaatttatggtcaaacctgtttaaaactagcagccttacactcgcaagctgttgattttcctaaatccggcatcgcttgtactatgcctaaagaatt
aaggcctaagcaatatccggattttatgcaaaagcagcgatccagaacattcaaagcagaaacggctgttggacggatctttcaatatgctgcta
aatttcaaaaggaaaatgaaggtattcggaagtttgatccgaacatggaaatggtttacgatgatcgaatgaagttaccaaaatacaaattaaat
tatttacacatcgcggaggaagtcaaagagcgctatgattttgatatgaaagctttaatgaatcgatatgagatagcttcggaatatgaagtttac
actgcgtttatactattaaatgatgatatgaaaacatctatcaacgaatatggagtacgtgaagaaattatgttacaatttagcattataaagaaa
gcctatgttaacgaatactttgaaaaatgcggactttccaccagctgctctaaccaagacaataaggcacgaattgacagtgccgtagctgctgc
gtacgatgtaacgtacaaacaaagactacattctttgcgaaatggaaaaaggctacctttaatttcttttccttacatttttgtcaacatactttgtcg
cattgctcattctgcagctataagcaactgaacattgtctaaatccaacgtcgacatacaacatgatataaaatccttcttaaggtacttttattaat
ataaaattaattatatattatccaaaaattgttttgaactttcaaacttattaaagttttcgttaggacgaattaattagcaaaataaactcagaatc
tcactgatgcaattaagtatgaaatagcgttaagattaagtagctaaaatttagcccacccatttacgggcggattggaacaatcggatccatggt
gcatgaatgccccatttttcggattctgatttgggatagtaactattagccacctttaagacaacacgttctggatgtggcatcatagctaagaaac
gaccgcaaggtgagcgaatgccagcaattgcatccgtacttccattggggttgaaaggatattggtttgttctgttaccataattatcaacatagta
caggatgtccaaaccttcttgcttgaatctttcataatcagacgcagaatcgaacatagcacgaccttcaccatgggcaactgcaattggaatacg
cgagcctcccatttccttagcgaaaatagagttatccgaattcttagagtctagttgaagcattactgtacgtccttcatactgcgaagactcatttg
aaaggaaaagtggccaacattgagcaccaggaattagtgtctttaaacgagtgaacaactgacaaccattgcaaaggccgatagcaaaagtat
ctttacgctcattaaagaacttgtaaaattcttgacgagcggtatcgtgtaaaaggattgaagtagcccaaccattagcagatcctaaaacatcgc
cgtacgagaagccgccgcatgcggctatgccaacaaacatatctaatgtatgtttaccgctaatgatgtcagtcatgtgaacatccacaggagta
aagccggcagcatggaatgcataagccatttccaagtgcccattaacaccttgttcacgcaaaacagcaaccttaggacgatttgtcaaaatcaa
atctgacttgggagaatgttcaaaggtcaaatggaaaccaacgccaggatcggaattgtccaaaatgttttcattctcctgttgtgcacatttagg
attgtcccgaatgctttgcattttgtaagaagtttcatgccagagtgattgaagtgtagcacgtgtagacttatatactatctcatcagatcgaacaa
acacaatctcttgagcctcactagagatgactttaccaaacttcttgacagcatccacaagtccatgaactctgaaaatatcatatatactatcaag
atcactatcccgaacttgtattacggcacccagttcttcattgaacaaagtagcaatattgtcgttggagaacgaatcaagatttgaaatcacacc
aacacgtccagcaaaggccatttctgaaacagtaacaaataaaccaccatcagagttgtcatgataagcttgtatataatctgtattgtgaagctc
agttataacagcgaaaaaagatttgagtagttctacgtcttcaacatcaggagcctcatctcctaactggttgtaacactgagcaataattgaacc
gcctagacgttgtttacctttagccaaatctatatatagcaacgaggatggtccaatttcagtctcacgttttagctgaggagtccagatagattca
acattattcacagaagaaaagcctgtaataataagcgacaaaggagcggtgacagattgctcgcgggagtcttccatccatttcatactcattga
catagagtctttaccgactgggatactgatgcccaattgagggcaaaagtctaaaccaatagcctgaacagcttcatataacttggcaccctcacc
agaatgagatggggaagacatccagttagcactcaaacgaatatgatttaattcagggagcgaggcagcagccatattcattatacattcagcg
acagccatacgagctgaagcagctgatgagataagagcggtgatgggcttctcaccaatggcaaatgcttcacctgtagtggtacccttatcgta
agaagtgacggtgacagcaacatcagctacaggaacttgccaaggtccaaccatttgctcgcgagcaacaagaccggtaatgctacgatcgcca
attgtaattaagaatgattttgatgctactgcgggcatatgcaaaacacgctgaacagcatcagcgattggatgactggcaacaggtatgtaaga
aagtaaggaggaatcaaactcctttaagacaattggatcagaattatcaacacgagacatttttggaggcttaccaaataaaacttccataggca
agtcaattggagtattcttataaaggcgatctgtcaaaattaaccgttgctcctcagtagcatagccaacaacaccgtaaggacatctttcacgttc
acaaataccccttaaacaagtcgagatcctcgggtttcactgatagaacataacgttcttgagattcacaacaccagatttgcataggtgacatact
aggttcgatacaaggaatatcacggagctcaaatctggcacccaatccagcatcatggactaattcaggaagtgcattggaaacacctccagca
ccaacatcatgaatggattgaataatgttgtcttccatcgcagtgcaagtatcaatcaccatttgtgcccgacgttgcatctctggatttcctcgttg
cactgatgcaaaatccaaatcttcagaaccttcaccggagctcatgctggacgcagcaccaccacctaaacctactaggagagcaggaccaccg
agcacaataataggacttccaggagaaatgggtttttttgaaagcgtgctgtcttcgaatgcaaccaacaccgcctgcagccataattggtttatga
tatccacgatattctactgttccatcagctctaggcacctccatggtaagtgtgcggaaatagccattgatacaaggacgaccaaactcattattga
acgaagagcttccaattggggcagataacataatatcaagggcagaggcaatatgaaaaggcttgccaacatctatttcccaaggctgtttaaa
accaggaatatgtaaattagaaacgttgtatccagctaaaccagcttttgattttgacccttgaccaacggctccttcatcgcgaatttcaccacca
gagccagtagcagcacccggaaaaggcgaaactgcagtaggatggttatgggtctcgaccttcccgagaaaatcgatcttttcatctttcattgtc
cattcaccattgatgggaaagaaattagtaccaggatttccttcaaaaacagcggcattatccgaatatgcagagatggtataatcgggatttagt
ttatgggtattacgaatcattttgaataatgagttgtccattttctttccgtcaatagtccagtcagcattaaaaattttatgtctgcaatgttcagaat
tgacttgaccaaacataaaaagttcaacatcggttggttcgcgatgttttaatgatggctcgttgctatagcactcgactaggtaatcaatctcgtc
cgaagcaagagctaaaccaaattttacattggcttccgctagtcgacgtttggcagcttccttatcggcttctttgctatagccaagctcaattgtta
caagaggcgcaggttcatgagtgttaaaaacttcatcttccttgggaggctcataacggagtgtttcggtcatacgatcgaacaaattagtgaaag

caatttggtaaatgttctcagacacaccttgtttcagtgttaccgaatatgctataccacgttcaatacgattcactcccttcagattgcatacatttg
caatattagtagctttcgaagaccatggagagatagtaccagtacgaggaaaaacataggcaatcgtaggacctgatggtatataatcatctgat
ctcaagtcagataaaattggtttcaggtcattagacgacacagaaactccctcttttgtttgaattaaataaaaatatacagcttctatacgttcaa
aggctgaaaccgtagcttctaaaagcttcttctggttatgagatgtcaaggcagaacctccgtaaagaactaacattttagcggattactccaatt
actttttaataagcactgaggagttatagacccgactcgccaatttgtttagctccgtctaaattgattctacaagaaaaaattcagttaatagctat
atgtaagccgccacgcaagattgcggtatctcctagatttggcaaagataatctcataaaagctttcatgcaatcaaaatcattgaacgaagaga
aaaaaaaaatgacaatgattgtataacataagtaaacaatgctcaatagtgcataaatatgaaaggcttagaccaaaaataaataaaactttct
tagtgtgtggaagaattttagtagacaactaagtatttattcgccaaatatatcttcatagtcttcgtcaaaatcgtataaatcatcaaaaccttcat
gttcttggctccgctccatcaaattcttcataagttcatttacccgggtatcagaagatgtatactcctcaggctccattgcaatgtctttcgccgaag
catctttatcatcgtcattcgtctctttgtcagaaacatccacttcttcagcattcataatagcatcaagcgtaacagctgaaggttgtttgccctcag
ttaaagcgtatagtttttcacgcattaaatccctaactttgtagaatctaccactcttataccaaccagtctcacggtgacattcaaaccgtaatgga
gaatcccttagcagggaagatatttgaggatcggttatatcacagatttggtatatacggttcgagggataaagtgtttttccatcaaaaacatgat
ttttaggtcctttttttattttcattcaactttaatttaaagaagagggattggtatgcagctgctttaggatcctttctagggtcgtagccaaaaatagt
atatgtatcgcgaaaaggacccgaggaccataaataagcaatgtgaggcaatgcaaactttagataatgtacataggaagaatcaatgtggttc
aataaagctcggcgcgtccaaatagggcgatctcgaaataaagggcttattgcatcaattacttcttggacatttttactcggagattccgaaagc
ttttgatgcttcgtagtggggacgtggtcaatattcatggtggctgtaatgatccatacacgacattgacccttgaggttcattatccttgttgaacc
atcaggtaaccgaacgcgccctacaagaggattttgctggtagttgtaaaatgttggaaggggaacttgtgagaaaagagggggtggaggcatg
tctagatgatcgcgctgcactggtgataagtctatattgaatttgctaattgcatcaaagtttaacgtgcgaagagaagtgtctaacttcttagcaa
atggggaattttccgtgctgtattggaaatcggccatatctcgaaaaacaaaagcatgacgaatcctaccggcactatcgatttccccatttttctt
gactcgtactaaaatatcatttctttgtcttagctttgcttgtactgggtgatggtaatgatcattaggacgtagctttagctccaagagtgcatgttt
gggattgcgaaacgacgttgaaacagcctacagcataagtcagtaccttagaactgcatttcatacttcaattcactagtcagaatatctaaaaca
gtcgatatattaagcctcatacacaaacaacgagtctgtacatagatctataaagtgtatctgctaatgctttaattacaattctatacgcacctttt
gtatcgcaggaaccccacctaaggtttcataaaatgatagctcatgtttcataagcccaggatgttcaatcgaggtgtattctttttctgatattttg
ttttcacggacatggatgttagtgaaagttactggcgataaatatacaatcaaaatctattcttcggctagtgacacaacaagtaacctttcaaac
aacgaatatttaggtaaagtaactctgcctcctagtagctatatctaagaaaattaaatgttgttttgttttgctttatttgtggtgtagtttctatgttt
gctagtaagtggtgcaaacagtttcgctcggtatagtgtagttcaacgaggtggatttgttcttgataaaccattctatttacaattaacttcaacat
tcctcttttagacattcactaatttcttagctgtcctttccaattggattattgcttcattaaattgggtttctttagaggaaaaaagcgttcactcgaa
gactcgaacagcgtttaagggaagaatatctttccgacgaattgtacatacggaacggaacagctctcagaatagagcatataaaagtaagaaa
gacggtaaaacaactgtttattactagataacttctttcattctcttctggtttatcgcataaagaattccgttctagcttctttactgaagcatatgag
gaagctttggaggctcgtttaattgtttttttagaagcaattaatcttcacttgaaattttgggactgcatattagaaaaaaagaagataaaaaaact
gttgaattctgtatatacgacatcatagtaaagttagttcggcaccgtttacttttcttaggttcctgttcatgaaaggattaagaaataagtttgtga
aagcattgtcgttcaaagatgaagccgggtcagataaggatgttagttattccaagaaagactataccaaaaatacttcccccaaggaaaatca
accctcctcggaactgaaatatagtcgctcaaagaaggaattgaaaaaatcgaaaagctcaagctcgttgtccaaagttaataaatcctttctgg
ctagggaagctggtgatgaaaagcttgagagtggcaaaaataagcaagacgaaaatagtgttattgcagaatcaaagagctctcctaccttttc
aaaattgcctcgatttgacgtagatgaacaaagcttgggttcgttaaagccagcttctatatcgcttcctgatgtatgtataaaacataagtattgtt
tactattatagttgcaagacaatataaagcaatgattgcgactctttttttgaatttggttacgctaacaaagtttcagctgaatttggcaacggaa
gtacatggaaccttagatccctccggtacaagttcttttacatcatttcctcaaaattcttatttttctcctgcagcatcggcatcttctaataagctct
tgcctccggtatcggttagtgatgttgatcttaccaacaatgtacgccggatctcctttcatcgtcaacattcttctcagttccaagtttctgaaaaaa
gaaatctcacccgatttccctcttttgatggtaagcactctcagtggtatatcttcgccttactaacagagaaaatagaagtccacccttccgagtgt
gaacagctatttattaaaaaattggatcagtgtaatgtaatttttgattttaatgatccttcatccgatctagaaagcaaagaaataaaacatgag
gccctggttcagatgatcgattttgttacgtttaatcgaggagtagttaatatttctcttttctctcatgttttacatactgtatgtttgtttacacttacg
ttattcaatcctaatatatgtagttttccgtcaatttgtttcgtcctataccttcgtttgcttttaatgactatagaaatgttttattatcgctcgaagaa
gagccatatctcgagccagcttggccacatatgcagcaagtttatttacttcttatccgttttcttgagtcaccggactttagagtcaccaaatcgaa
gtctttagtggatcaccacttctttaataatttattaatgttatttaattctgaggatcctagagagcgtgaacttttaaaaaccgccttgcatcgcat
atatggaaagtttctgaatcttcgctcatatattagaaaggccatgagtaatgtttttttacaattcacgtatgaaagagaatcatttcatggtatag
ctgaactactagaaattcttggaaggtatgtttgctcatttttagttgtatgactaactcgtcggctagtattatcaatggatttgctgtgccattaaa
ggaagagcataaaaccttcttgacgagagtactcattcctttgcatcaaaccagatccttatttttatatcaccctcagttgacatattgtattgtgc
agtttattgataaagatccgtccttgacaaggttagtcgtgactggtatattgaagtattggcctcgcgtagatagcttcaaggaagtaatgttctta

aacgaaatagaggacatttttgaggtattagaaccttcggagttcgttcatattcaagtacctctttttcagcagctttcccgatcgattgcgagtcc
gcattttcaagttgcagagcgagctttgtgtttttggagtaatgaatactttaccagcctggtaagcgaaaatctaagtacacttttgccaataatct
atccttatgtgtacaacaccactaaagatcattggaatataactatacaagctattgcttgcaacgtaatgcagattttcgttgatatgaatcttgac
tttttcaatgctgttgccgagcgctgccacagcaaaatggctattgagaaagaggtgatggaatgttccaagcaaagatgggctgcactcgaaga
aatcgctgcaataaataaagcatctgtggattccgtaaagcccttgaattcaaaataatacttacgtttgataacacatcaagaaaaaactgaaa
ggattatgattattgagcgctagtatttactgatatacttaattataataaatcctaattgtcagatgtgctatgaatattcccgtgctctattattact
gaatatatcatccattgtatgaattaggagccgtaatgatctaaaagccaagtttttaaggtgtaatttttgtagataagatatgggtatcttattaa
ttaaattctagaacatagaggtatgattacttgtttctcttgtaaatcttgcccaaaaaggttaagaagacagacttctttaaaccaccggactcat
caacagactcaatacgcttcttaatttcagaaacttcggcttcttcataatttgcaaactcgttgcgtataccaagttcttcaaaaacaatcttgacc
ttagcctcacattcggagtctttaacaccataattcttatccaagatcacacgttgctcagaagatgcctttgaaagggcgacattaattaaccagg
aacatttgttgtccaatatatctgtaccgactttaccggtgatgttgggatcgccataacaatcgagaaaatcatcttgaacctggaaatactttcc
aaggggaataagaatatctcttgcaatttgcaagttttctctagatgcagtaccggaaaggtacatggctaaggcaacaggaagataaaaggag
tagaaagcagtcttgtagataacaataaatgagtgtttttcaagagaaaatctggaaatatcgacatggtcttcgggggcagtaagaagatcgag
atgttggcctaattcagtttggaaggtaacatcatggaagagttcaagaaggtcaatgtaataagtatcctgacggaaatatttcttaagaagga
aataaatagcggattcaagcatgaaagcatcgttaatggcgatgtttccaacatttggcatcaagtaccaacaaggttgaccacgtctcgtctggg
aagcatccatcatatcatcagcgacgaggaagaaagcttgaagtaattcgatggaccatccaagaacagacgccttcttataactttcttcatca
aggggcttaccaagcaagatttcgtatgtatcgatgacagacaatccacggttatactttcctccaagggtattatggtacagattctaaaatttcg
ttagagttccttgccatttattaaaatgcgaagccgatcggagggaattgcagaaattaagattattatcattcatgaaaccaaagtttattcaagt
tcacttaaacatctacaattttatataaaagcgcacatcccacatatcagacgtgtcaaaatccagcgtatgaaagaagacctacactacgaggt
tgatacatacctttttcgtaccactgagcaacgtccttgggaatattgatggacttcaagtagtccataatatcattgacgaaaactgggtagatgg
actcaaacttttgtctttttttcggcagcagaattctaaaaacaagttagtctctttaaaaagcaaaaaataaaaatattttgtaagaaaaaggaga
caatgaattgtgatttcgcgtctattttcaaagtattttgacaagaaatgtaaaaataaaactacagaaataaagctctagcttttatgtaatcaac
atacctgagaagacatatttggaaaagatcgtatactctttggataaagtcaagtagcgcaccggttgtaagcgtcagccttttcagcggggagtt
cttagatgaggttaggttttagctcatatttagcgacaaataagtatcactaaaaaagctttttcttgtttttagaaaatattctcttaaggaaaaaa
atgtattctaaacttgcttgggactctttgattttagtttcatttcatatcatttaaattattcctttcttcttttgatgtaaattctgctttttagcttgcag
acgttgagcaagtttgacttcaagcttttattttttcaaattactagagaatgagaatgtttttagctttcttaacacatatacagatccttctttccttt
tttttctttctaataaaattcgtaaatgaatataacttctctcgccattcgttctacagtaaatacgaaaccattctacctaaaacagtttttattcaac
ctatttccttttgctcctatgtctcttgatattcttttattcttttttttgctatgcttcgtgtttatatggctccctatatctttaccttcttttttttcctatagc
atttttttgttggatttatttacctttctacataaacaaacacattttgtaaggcgctctacgccaacgttatacttccttaaacggagtctgacacctc
aagatgtaaacaaacttggtaagcacaaacagtcggattgatttccttttgtgctctaccaaatgctattgaataccatacactaaaatcagaaag
gaaaattagaaaaattgaaagtttgcaatatcgaaaaaactctgttattcgtcttatttcttggatcagaggaggattgaccgtttttgagttattct
ctcagatagtttgtcaaaaggaaaagtatcgccatcatgatgctggaagagggactctatacccagcaaaaaatagtggaaactcggagcggct
tatggaactgcatcaaggaatgctcagaaagaggtttggtctacagtgttcgatggtaagtcacttgtaaagtgaaagctgatgaagactgtgttt
tatcttggtttttatgatatatttcatggatggcaaatgacaaatgtctggtggacgtttatgatgatagtctgcttaaactaaaaagaccttggttg
gaaacttgcagcaatagaattatgttttaagctctaaatctaacataaatcatattttttgttgaaatactgacctcctttagggctgctgaaatgctt
gccggtttgaaatctccaaatacccttgatgctcctttctcttctactcctaccgctgaacttgaagaagatacaactacgacaaatgagcgtatttt
agaatttaaggaagaaactacttacactctggccaaagcctactttgactgtaaagaatttgaaagagctgcttatacacttcaacactgtaaatc
ttcaaaatccctgtttctgcgactctatagtaagtatttatcaggagagaagaagatagaagaggaggcagatactgttatgagctctatctatcc
agcgtcttcagctagtcgtgagctttactccatatcggaaaccttggatagtgtgcaaaaacaagggaaccaggatccctatctgttatatcttagt
ggaattattaataggagaaggaaacagtatgcgaaggctatcgattttcttctttcttgtgtccgtaaaactccttacttttggtcagcatggcaag
aactgtcattatgcttggatagcccagaggtgttggccagtgtcattcgtcgactttcaaatgaccatgtaatgactaaagtgtttattgtttacgcg
tctcatgagcttcaccaaatcaatagctcggtctacgaaaagctagccgaagtagattcccttttttcctacaagccgtcacttaagaatacagcgt
gctttactagcttatgatacaagaggtatggatactgttattgatatttcctaatttatttttctagattttgatgacgccgaagaattattcgaagtta
ttctaaaaagcgatccataccgtttagacgatatggatacttattccaacattctttttgttttagaaagaaaatcaaaacttggattcttggctcaa
gtagcctcaactatagacaaatttcgcccagagacttgctcaattatcggaaattattacagtttattatcagaacacgaaaaggcagttacatat
tttaagagagcaatccgactaaatcgaaattatttagctgcttggactcttatggggcatgaatatgttgagctcaaaaacacacatgccgctata
gagtcttaccgcttagcagtcgatgtcaatagaaaagattacagggcgtggtacggtctgggtcaaacttacgaagttttagatatgcatttttacg
ccctttactatttccaacgtgcaactgcccttcgtccatatgaccagagaatgtggcaggctctgggaaactgttacgaaaaaattcaacggccgc

aagaagccataaaagcatacaaaagagctttactgggctctcctatcaatagttcaatcttaatgcgtttaggtaacctttacgaagaattacagg
acttagacactgcttctgcaatgtacaaggagtgcattcgaagtgaagaagagggagatatgacgccggaaactattaaagcgaggatgtggtt
agcaaagagagaacttaaccaaaaaaactatagacaagcagagatgtatgtcagtgagattttgaacggagatctggaacttgaagaggcaaa
agctttgatgcgagagttaagaagccgaatggaacatactcctaattgaaaaaggagctgttagccaacctatgccatcttctaaagaaggattt
tttatgatgaatattttaaggtgtaatgtaaaacaaatttttctttattaatcatgaaattcattaaaattagtcatgcacattctatcacgtatttgct
ttaccccattgttattgctctgaaagatcattatccataacatccatcatcatttccgagcgtgtatccatatataagagagagttgctatttaatgg
gtcaccaaagcgaggtgaatgattttccgcagtgttgccattacttgcgttaatagtcgattgctgcatatactcgctgacagcatcttcatatacca
aatcatcagtgatagcaggaatcagggaattttcttcaagggcgcgtttcaagagatcagacgccacaatctcgttctgagagatcgccaggctc
taaaataggttagtaaagttatattcatgaatctgcttgtaaagcaacaacatacttcatgtaaatgggtaattgcaagatcaggcaatttcttatg
caaatagactaaagctatggcagtatgtatattcgcatcttgagtagatagcataagcccttgttcaagggcatcgattgcttcatcaaacattcta
aaggttagatgtaagttattatcttaaacgtaccttaactttctgtaggcatgaccaagatttgcccaagtagcggcccaaggtttttcattgctttg
agttttcttaactagttgcaacgcagaattaaaaaactttattgattgctgcatatccgccttattaaatgccacaactcccatttcattgagtaaga
gcggatcgtactggaataaagaataggaacttcgcaagtattcgtcagctaatgaaatatttccgagttgcatatgttgcattcctaaaaatagat
agggaaggtgagttccttgaaacaatcgagcagcagtcgtgtaagctgaaatggcttggtcatgttcgccttcgagagcaaaagaatgagcaaa
tccaatccaagcagggccaaactgaggatcaattgttgacgatttggaaaagtatctacgcgcttcttgcactttattaacagataaataatatatt
ccaacggcaagccaggtgacggctttttcaggatggcgatccactaaatcatgagaaatcaaaaatagtttattcttttcacccatttcatgaaga
gaagccaaatgtatgggataaactgccatgttgtaaggatcaccctctaagatctttgaggttatatttaaaacatcaacaaaacgagatcggac
gattaaagactcagcctttgatagcaataaatcagcgttattttccaaaccaggtatggaagtcaagtaatcttcggctgctcttaaatcagtttcg
tgggaagtcttgtttaagcgtaacatatacagagatttcaaaaaattcgcatcttcttttgaatatgtagagtagtcaagattcgctattaattccca
ttcttcggaagctgtaaggagatggtttgatactaactgatcaaatgcctcaaaacattttgtgtcaaccattaaagcttctttatagcattcctttgc
acggtcaaaatcacttaaatttgtgtaaacttggccacgtagataacacattgaagcttctagtttaattccgccatcttgcatacgcaatttgtcgc
tgtttccatgtgctctgctaaaaggattggtttcaccaagcaactctaaggctccttgccagtcatacagtttgacagaacagaatgcggctagata
tctacaagctgagctctgattacataaatcttctttcgttaataagctctttgctctagcataatcttcagtacaacagtagacttgagcaagccaga
atgcatcgttcgggtccttagtcatatcaaggaccttttcaccaacaaatgcagcacatttgtattgttgctgcattaatgcatcatgtctccacattc
gcaaatattcttcgcgcgataaagattcaagaccagcagtcgacaaattttttcccttataaaagataattctgttgatgtatttgcaacatatgcc
ataggactaatcgtcaaggtagacatctgagaattggagttgtttacatttagtgctcccattgatggaggtgtcatataagcattcgggggacgt
gaatctccttgactaaaggaagaaggtgtagcctgtaatttaagtgaggcttgagaccgtttccccattatcgttgttgataaaggtatatatttact
ttttcaattgttagtattcttaggcgtagtggatgttagacagtatagcgagtagtagggagtagaatatatagcaagacttggatgaccattatat
gtaaacgaagaatttgaaatacagatagttactttttttctattctactattttaaacaaagaattattctctcttacttctttcctagcgaacaaatt
aacccaaaaccgaatattttattctttttacctttagacaaataaaaaaatcacagtaattccaaaaaaatcaatctaaatctttttcattacaaat
acaatgaagcgctgaaaacaatatcgcgatgtatacagttaatcgtgtcttccatcttcttttgtaaattggaattaccaataacttttgcagcttca
ctcatttgtcgaagaagctcttccaaacgcctaaacatacgaatcaaggagccttcatatacatcagtcatcttgcaaatctgtgcaaagctggct
ccatgagcccaggcatacacaacctccatcaaggatggcttgaaactgttgacataatcctcctcattaacatcaagcttgctttcctgagaaactt
tagcaattcgacgagccatttcttgtaaaattcgaagggggccaccaagttcttccttcatgcgctgattttctgcttcacttttctcttggaaaacta
cgcaactaagaagagcagcacattgttctggactcaagtcattaaaaataccgttaaaaacaagctccgtcagtagcaatccatctccagtacta
atttcacaagcaacacgacccttcatttctataacatcgtccgaagaagtaaatcctaaacggcggaggactcttttacgagcacttaattcatcc
aactgcataatagcttttgcttttgaaagttgcttcttgagatcttttatttgatcaagcagcgagagcttattaagatattgatcatatttttctttaa
gatcttcaaagttatgaaggggattagacagcagtctagcttctaaaacatccaccttcttcattaacttcaagaaagctggttccttaatgttcata
ttttcaacaggatccaaaagactaattccatcaggaaaacgacgcttaacttcactaagagctttaccaacagtgtccttttgagcttgagctttca
agtcattgggtaaaaatatacgaatatgagcaattccgttcaaagacgaaagaggaacaggaaccacttcatatcttccattgtcatctcctttag
gcgtatgaatctcgggtaagcgaccattcttcatccttggacgttcgctttccgttggaaccttcaaaataacatctacaacgtacgactcctgagg
aagatactccttagtctcacccttgggaagtggtctctttgatacgcttacaacagcaccccaaccaaaatctttctcattctgctttacggctacaa
gtcggccactttgcaaaaatgaaagagagaaattaggatggttaacaactgttcgaacgtcatctcggtacttattcaattgaagctgaattgtat
ggtaatcctccaacgcgcgctcatcaggaatggatgtattgtcatattgttgttgaagtttttccaattcttcttccaacttaggaacttccatactgtt
ttgaaactggaaaaagcaacgttcaagcataaactcgggagaaatgccttcaacacgtagcaagttcaaaatcatgttataacttaagtgaaaa
gcagagtctagacggtctgcttcacctttgagcattgtcatagcaacaggaggttccatttttttcatcaatcattagaattacaacaccacggtcat
ccaaaccacgtcgtccagctctaccactcatttgaatatattcaccacctgaaatccaacggaagctctttccatcaaactttctaacgttagtgaa
aacgacagtcttcgcgggcatattgagaccaattgagaatgtttcggtagcaaatagaacttttaataaaccttcttggaacaaaatttcaataac

ctctttcaaaattggaagcaaaccagagtgatgaataccgataccgcgacgtagcagaggcaggatatgctcaatttgaggtaaatcacgatctt
tttcggacaattgattgatggcattattaaaaatagtagtgacgaggtcgcgctcagtttcatcgttcatatctagttttgacatttgcaaggctaaa
gcttcgcaatcacgcttgctgaaactgaagacaatgacaggattatagttctttaacataatcattttcacgatcttgtaaatgtcagaaggacctt
taacacctccttttccagtttttcctttttagcatttttcttggacactataccagctgggtcatcgccctgtttttctaccaaagcagtcattgctcttt
ggaaattttcctcacggaaaacactcttttcatcgacaaccaaatgtattccatcgctaccacttgggaacagataatgctgtaatggtgttggtcg
aaaatccgtataaacaacgtgacagggttgacggtggattttggtaatccattcggcaaactgcatagcattgggaatggttgcagaaaggaaa
acgaagtgagccttatcaggcaataaaataatagtttcttcccaaacaacaccacgttccttgtcacgcatatagtgaatttcatcgaaaataacc
caagcaacttcacgcattacctcggaaccacggtaaagcattgagcgaagaatttcggtagtcattaccaagcaggtagcgtctggattaatagt
aacatcaccggtcattagaccgacatcaccaaattctgctaatagttctcggtatttttgattgcttagagctttaataggactggtataaattactc
tttgcttatcccgaagcgactgagcaacggcatattcggcaacgacagttttacccgcagatgtatgagcactaaccaatacactctcttggcgttc
gatcgaagcaatggacactgcttgaaaaggatcaagggtgaaagggtatgttcgagcagctggatcggggcttttatgctgagatataggtacat
aattgtagttaggaggcaaagatacttggtgacggacttgatgacgaagttcaacaacatgatcagacgtcgatttggcttcagccatgccaactt
ctccagcaatcttcaaagacgcttcctgttcaaaagaatcggttaccaaagcatcagcaggagctttgccattatccaagccattctcgggagaaa
tggggacttctcctgaggcatctacaatttcatttacgacaccattacctttatttgcacctgcaacgggctcattcgatctggtccaaggagcagca
acagattcctcaattttttacgcttaacatcagactgtttctcttcagaagaatcctgtcgcttattgttttctttttcaatatctacaggagaaccaa
ctttagaggatgactgtctgccattttcttccttcggactgtctaagtctttctttaagttctttggaacttttccctcaaaaacgtcaaaagcatcatc
taactctccttcaaacattgcaatgatcagttacaatcctgcaatgatgaattggtgaagtgcttgagaaggtcaagttagtacaccttgaaaattg
gaagttttttcctacaatattcagttataacgacctcttagaagctcataatttgataggtacaaagtaaaacgcagtacgttagattttttaacgga
attcatttctgttccttattaggcacagtaccaccaaagcgaacggcatatgtgtattcgtgcctacctattcataatccttgattctatcaattttgtc
ggattgtataggactgctttgtgctaagacggagaaacttagctatagtccatataaagactcacgagaaaagacggactagactgtctactaag
attcgatcacaagcgttacctaaaaatagctagataatggaagcaggagataatgtggacccaagcactataagggagtcatctactaatacag
aattagaatccacgccaaaaataccggcagaagatgttgccgattctacaacagcaaccagtggagtaaaacggaaacgcctaccgaatgaca
gagttggtatgttaaaagatgagattcaggaaaatcctcataatctttccgcttggtatgcgttaattgaggagtatggatcgaaaggaaaacatg
aggaacttcgagaaacatatgaacaaatgttgcgtccatttccttacgttccgcgcgtatgggtagattacataaatgcagaacttgctttcaatga
tttcagagctgttgaagttttgtttagccgttgcttggtgaaggttttatctgtagatttgtggacactgtatttaagctatgttagacgcatcaatccc
gaaggagaaggtcaatcacgatcaacaattacacaggcatacgaattttctattaatacaattgggatggacttacagagtggtccaatttggtc
agactttattgattttcttcgctctggacctgcggcctcaacgtgggagcaacaacaaaaattagatgcggtccgtcgtacttatcaacgtgcagtt
tctactcctatttataacattgagaaattatggagagattatgatgcatttgaaaatagcgttaaccgggccacagctagaaagtttgtagctgag
aaatctcctgcctatatgtctgctagagctgccatgcgcgagctttctaatatgaccaacggattgcgtgttttagattatacttttgaacgaaagca
taataatgctgaaaagacagcctatgctcgttggatgaaatggattgaatgggaaaaaaatgatcctttagatttacaccatggtacaactttgca
aggaagaattgcatatgcttatgaacaagccatgctctatatccctctttgtcctcaaatttggctggatgctttctcttactttttaaatattcaggat
gagcaacgtgccttacagataatgaagcgtggtatgcgttattgtccctcaagctttgtgctacatgttcgttatgcagaacatgaagaagcgaac
ggtcgaacttccgacatccgtgtatcctacgaagctcttgtaactgcgttggccagagaaatcactgcggtggaaaaacatgtggtatctgatacc
tcccaaagtaaccaagaagcgaaaccgactgctatcttatccagatcggaaaagcataagaaaaagttggtgaagcagtatagcctagcatggt
gttgtttgatgaatgctattcgaagaacagaaggaatcaaagcggcgagatctgtgtttacaaaagcaagaaaagcgccgcaccaatctcatga
aatttacattgctagtgccttgatggaacatcattgttctaaggatagtacaatcgcctcaaaaatatttgaacttggtttacggcactttagcgacg
taccagattatatatataaatatttatgttacttgatttctattaacgatgagaccaatgctcgcgcacttttcgagaaagctatttcaaaaattgat
gctagtgatgcgaagcctatatatcaaagatggcttgattatgagagtaggtatggtgatctcaatgccgcgatcaccctaagccaaagaatggc
tctagtatatcctcaggagtctactcagagcatattcttgtcccgctacggtttggaggaagagcgtgaggaagatgaatttgatgcagatacata
tggcgtccgccctacaactgaatcaattaatcagaacaaagatgatgatgctgcttccgaaacatcagactcttcgaaaagcaatgtcgaaatgg
aggatactcgattgttacctgcttcattagagactggagcagctctagctcctcctatttctgcacaagctcctgtggcacctgttccattacccca
atcattacagaactactagaagagctaccatctgctcaaatgattaccggtatgttaaaattgactaatattatgttattaacaaaaatagaagcc
cccataaatcctgccaaactcatggattacgtagtgaaatccgatattcccttcattcgccttagaaatgcgaacacgcatttaaaaaaagcacgc
tacaattgataaaaaacttgattgaaaacgttattttggagcttaatggttataattaatgaaaattattttggttctttctagatttataagcataaa
aatgtgatttttaattctgaggaatctggtcaatactaaaactcatcgcattagaatacatattttgtcttgcttatagttcttcttggctttttttaatgc
agagctttgacagcttatattaaatactgattctggcacctgaggaccgtaaacatacttatgtatttcttgcaaacgatattggcgcaggagaga
ataaatcacattattcatatcagagaagtaaaaaaagaatgttctttattaaatcatattctaaacgttttgtatattcatccattatttacttgtcaa
gcaaagaagctaatttacgcttgctgatataacgtaaccttcctttttcaaatgtattatacagtaaggttaaaccaagttaattttgtacaaaccg

ataaagctcaaaatttagaatccaaagagcgtgtgtagaatcacctgaatacttttgcctaacaaagagctttgtccggttttgttatcagttacac
ttgccaatttttcttcctcctcaatagtatcgttcgaggtatttccaaaaagattttgaacaaaccagtttttagacgggtccttatgggatgatttaa
aaatatcctcattatgttggacgactatccttttcccgattgtcgcttcgaattgtgcacttgcttctaattgagtcgtcagaggcggaattcctttggt
atcagcttcagtcatttcaatatctaaagcgtctaattttgtagccgcctgtgaatgactctgcttatcatcaggactatagatacatccaaccataa
aagaatcatgaataatcattccgtttgactgaactgctgtagcagcagaagaaggttcagcataagtgagttgcagccaatttccatgagcatttt
tcaagcccggcaaggcactagtagaagcggtaagagagtgttgattgacaatctttccgaaccgagagaattcgtttagaacttgagtagttaac
tctggagggaacccgaaaactatgacagatctgctaacctcctttaatggctccatgggactatttaatgttctagcggtaaaaggggaagaaag
aggcgaagcagtgatttgatcgacatttgaggtgctaatttggcgagaagaaggaaaatcataaatagattgagtaggaggagcgtcttcaata
atcgaacttcctcccaaacggttggctcgcaaaaatggttttggtgtaccaaagctaggtccaccaaatttggaagaaggagatgtagaggaacg
gattccagaactaattgtcggttagtattaaaattaaacatgatcctaaggagttcttaataatccatctcgaaaatttatttcaatgatagagatta
agaaagagtaaaataatgataaacttactgtgagtcgaagaactcatttagcggagagctaacatctggagcgaattttcgttgacttgctgtca
aatgcatcaaatgactaggaacgaatttcttttctactgaagacggagcaaaagatcctctatttccacgtaattccctttgaggcgaaggagaac
gcaagtcatccactgagagaggtttcaaagggcttttattatttggtgaactactagctccaaaagacgacgaaggattcatctttgaacaaaatt
aattaatttataagccttatgttctcagactattattagatgaattgaattagaataacaagacaaatcttacttttggttgttttgagtagtggttaa
atctgatcgcctaaagcaagaccagccagtatgttgttaaaaagggtagaatatgactaaagagattctcaaaaattctttttttgtatagaaaat
gcttattgatttacgtgaatgaaaattggacggtgtgaaatgaatctaaagaacctgatatgtactcatataaacaagcagtggcggagaaagcc
actaaacgtacaacgaaacgtggatgtatagaatgaatcaaatacatatcagtcgactagactttagcttgtagtaagttttccagttcaaactgt
tcgttcattactgtagtgagaattctcttttacgctcagtatgctgaacgccgtacaaaatagcaaatcattctagtacacagtcgctttattctatct
catttattaatacagatacttcaaattcattaaaatactgttgatttgccgtcaatttgtttattactgtaacgttgcgcagcatataagtagcattttg
tttatatagaacagaaacgttacccaacgttgtttacatccaagagacaaacaagtaacggtgaagaaatactgaggaaggctgtttgaacttgc
actcttcacacaaaaacaacattgtttcgtaagtcgaaagtttttggaaataaaaagacttatatttcattggttttgtcttttgaagacgtcttggat
cgccttgtgcaggtttttatgcactcttccttactatggccgaaaagtacaaaattctagaatgcattggtcatggaagctttggcaggatttacaag
gttcaaaggctttctgacgggctactactagctcaaaaagagattccttttggaaatataagtagacaagagaagcagcacattgcagacgaagt
taatatccttcgacatttgaagcacccaaatatcgtccagtactgtggcgaggaaataaacagacttactcaagtaatcaatatttatatggaata
ttgtgaccgtggagatctagcaaagcacattgaaacctataaagaagaaaaaaagagatttccagagcatcaagtacttcgctttttttacacaac
ttttactcgccttgtatcgatgtcattatggtagagatgctcctgttacggagagccaatggatgaacgaaagccacattcctaaccagtctgtcttg
catcgagatataaaacctgccaatgtattccttgatgaagatcattcggtaaagttaggagattttggcttatctaaattattggataatacccgtat
gttcacccatagttatgtaggaaccccgtattatatgtcacccgaaataattcgaaataatccttacagtgcgaaatcggatatatgggcgcttgg
atgcgtcatgtttgaagtatgtatgcttagccatccatttgaaggaagatcttatctggagttgcaaaagaacatttgccaaggaaacctctcccac
tgggatcgttattacagtgatgatctctatcaattgattcggcattgtttggaaattgactgtgatgtacgccccactacatttcaactattgtcttctc
ctgcgttaatggacattagaaagcaactagagacagaacgtttgatgaaggatcaattcaaacttctccggaaaaagcaacaatccttgaacca
acgagaaagcgagattactcttcgagagcaacagctggctgcccgcgaaagcgaactcggtaattacattgctgccaatttggcgcaacaagaa
gaggctctgcgtcgtgacatggaaaaacaattaagggaggtagacaatcgctaccaacgatatgtacagcaaatggtggggtctatgcagcaca
tgcagatatcaaatcctcaaaccgatggtctggctgaagcagccaatgcatctgcgatggagatggttatggacacagccattgaaacaacggc
cattcaagaaccgaaattgagcatgagtcaagtagtatgtacagagctagcgccctcttttgcaccaaagaaaccgatgttaccgatcactgaag
cacctagtgtgaaaaagaaagacatgtcatcacgggcactgcagacgactgcaacacttatgaagcacgcgtataaaccgcgaagccctatcg
agaaaaagtctgcatcgcttcgtgatcgacatgatgtcaaccatgtgatggattcaatggcgaaattacttgatagttctttacaatgcaaaagca
aaactcaggaacaaaagaatgatgatttacatgagattttgcgaacgcgacgaaaaacaagcaagggaatcttcgacaaggagcaatcaacg
acggcggtacttcccgggtcacttccggggcatgtttttgaagaaaataggcagaaaacgaagcatgactctttggattttcgatatattgaaaag
ttagaaaagttgaatttgcagtatccgagcattagcaagcagccatccaaagttgccaaaactctgcatggcaagtctggttactcaacggagca
agagccgatagtcaatgaaaacagaaaaagtagaagagacacgcaaaatgtgtatgatatggaggaaccatccataaacggagaagaatac
acgattcaaatgcccaaaaaactgcaattgctagaaggacaaaagcggtcaccagtcaagcaattggcacgattgaactacaacaaattgaga
cgttcaggaatggatagacctacgtataattgggaaggagaggatttgtcaggacaactaatgaaaaagacttggaattctcgaatgatgcaag
catgaaatggaagagggtttgaattttgaaagcaacagaaggacagaactaccaagaaaaacaaaaaggtttcgactctttgtcttgtattactc
tgttgtttttttttatttttttattttaccttttaatttttaatttttttgagagaaaagccagacactggaattgttttgctggtcaaaacagagtctggata
tcatgaatgaataaaatgattgtatgtctagctagatttaaagaggggaagaggaaagcgtaatgaatcggttttataggcaagacccttaggta
gggagatgggaaagaggagcaagatgatgaaccttaagttgaatgaaaaagggagaaaaaaggttagaaacatgatgaaaaaaaaatttgttc
ttttctaacttatttcctacctttttttttttttttttttttttttttttttttttttttttttattttagtagtaagtaagtaagtagtagtacgtacgtacgtact

actactactactactacgtactaataataacgtaagtaactacttaccttaccttaccttaccttacttaacgttnnnnnnnnnnnnnnnnnn
nnnnnnnnnnnnnnnnnnnnnnnnnnnnnnnnnnnnnnnnnnnnnnnnnnnnnnnnnnnnnnnnnnnnnnnnnnnnnnnn
nnnttagtagacgacgactaggtaggagagaggaaggagagancannnnnactatananagaagagaagananananaaattttctatattc
taacttttttttttttttttttatgactaagaatggtaagatagcgcaagattcacaactgttccagaattacaagaatgatcattcctccattcctata
gaggaatcaaacagtcaatcaaatagaaaagtcttagagaatcatagacaaagagtttgcctttacggtactgaattgatgaaattacgattggc
ttggaaacaaggttacaagaacaaaaaatcaatatctagatgatcagagtttcaaaaagtacatgaattgcattatcaaaaatgaaaacaaaca
gcaacgaaaagcatcaattaaacaatggaaccatactttaatggagagggaattcttgtttgatttataaacaaaggtcgtagccacggggggtt
ttttgagaattattggaatcaaatttttttctttttttgttatcaaattcttctatctttactttattaatttttacttctgtatagttttcaatgtttatttacac
gaaattatcatgccggcggagttgtgcagaaggaaataattcccttgtcatttgatccgtcacaaggttttggcaattccccgcgaaaaacgcaac
cgtgaaatcctgcaaatccatcgaccgacactggcgttatcatggtcaactaagcaaacgataaaaaaagacaagcggtcaaaagagatggtc
agcagaaaaacaaacaagaatcgggttacgaaagaattgttaaaaaacgatggtctatgaaaacattgtttttaattgaaattagttcactcgat
cgcttcttaaaaaatcatttgataattaacaaggtgttctctccaatcccccacacccatcttgcatcgaaacatgctcctcctctaattctcctccct
tgcctccgcccattgttacttctttccatctttctaccctagtgggggagctcgggatacatattggttgatattttatctcatcacaatcataatcata
atcataattaagtccatctatcacatcacctttaccgagttttggaattgcatatttaaaggcttgttgaagcagcttattcctaactttttcttcatat
ctcttcaccttttttccactttcaattcacttgcttttaccagcacttgtttattctgtcattacttccttttcctctcttatcctctgcttaagtcatatatat
atatacacaccttttcgtccgacctttttctatatcttctcgttcaacattcactaattatacttgtccttcatattattatattataatatttttatttctat
atactttcaaatttttctttactttttgttcttcttggtgaattgagttgctttctccttgtctttctatccgtctgcatttttctattgttttcgggctgtttcc
ttattagtgtacctcttcacttttttttttttttttgttttgtcttcggcaattcgggttttctttctcggtgtgaatttttaccttcattccgctattggcatat
acgtttcatattcccgttggtggtgtggtgatttgaatttgtttgtctcttcttcctcttgttagaaagcgacaacacatctttttactaagatcgaata
aacatcacatttggtgtaaaaggattcattggtctcctttttttatctttttttatttttccttcttcattttgtataaaagaaggtccagtttatccttatct
tcttatttactttctttcatttcatttcacttctttttctacttcttttttttttttcgtcttcttcctttgtcttattctgttcaaaggaatttaaaaaaggctt
tcattggttcttcaactaaactttgaaaactcacttctttgtctccacttttttttttgttgcctctttgctccttttttatttatttgtttcttatatttattattt
ctattcctacttaatttcttacattcgtttgtttcgcattttcattcctctttctttttctttttcttttcctttttttgtttctattctttctctgttggcctatact
tgttctttttttgtgtaagcatctctctggtcgcttctggattgatacatacctcaaaggttttgtagtcgtcttttcttactttcccacacatttcccttgtt
tctttgccaaaaaatctaggttttatttttcctttttttcatcagctcccttattttgacttatcgtgtatgttcattcgaatctccgtcatggttctcttctaa
acttttccccgatttctgtttcaggagtgtgctatactcttacacttgacgtctcttttgttagttattttcgaacgcaactacgattctccataaactct
gttctcctttaaaaccacgtggctttttgcattcttaataagtttctcgatattttgttcaagagttagtattggttcaaaagggatacattttcttttttt
tttgtctattctaattcgctttgtttcctcttattttcacggcatctttttttttaataaacttctagtttcttggtttctttttttttttcgcaaaatgtctaaaa
tcaatattcgagctttgcttgatgctttttctactgttatcctgtctatctatccgcttgctcaaaaggcgaaccagccttttcattcacacacattgcct
ttgaatcagttcttatacgaaaccattcgtcgtagtcataccgattacaccactctgatacttgctttatactactttataagctttcgtgaacgggct
tttgttgaacccgaaaagtacatccctttactttgcggtcgtcgtctgtttttggtttgcttgatagcagcaaccaaatttctacatgaccgtagcttta
gcaaccgcgcctggtctagactttctcaattacccgttgataaactgcttatccttgagcgcttattttaccaaggcattgattatcgccttcttgttgc
taaacatgtgtttgcacgctggtctttattgattggtgaatgttgcatgcaagcaacttcttgcttgtcaaactctgctgaccctcctttggattttcgc
aagttttcgccttattggatatctttgttttccaatattcactcttctttaaacgattttctctccattgcttatatcacaaagattgcccagcaaaggat
gaacaaccggatggcagccatgcatcgcatgaatacgtgcttttctcaacaagtcccaacttcgccggagagctctacctccacaagtccctcatc
aactcccattatggaccagttcagtccaagtaccaactttattgctcctaactatggcctacagacgtctgctattcaacctcctatccctgctaact
atcccacttgcccaagatggttgagtcgaccgaatgtaaatgcgcccactcagtttgccatgcctgctcaaacaccttttgtcagacaaccaaattt
accacccaccccgtcctctccccagcctatgtcatacagaccagtgcaacctgaaagctttctgatagaaactcccctttcgtcagctcaaccaatg
gcaccccttccattcaacacatctcatgtacatcctcttccttctacgtttcataatgatagaaatggccctttttctaaaataatgccagagcctgtg
accgcccactcagcagttccagttaaaaaagtgagcattcctatgttaatatcgcagcagaaaaggcctttcaacgatgtcattactatttctccaa
gtgttccatctgtaaagcgcaatcgtctcgtttgatgaagaattgcttggtttgttacatgtataagttttcaaaattcatacgtcttcctttttaattat
agaaaacctttaatcttctctccttattaggttatttcatatactttttactgaactctatgtcgaaatacgtttgtatatgtgtatatatttatgtgctta
gacagcatataacttggaatagggactttgttattttcttttctttttttcgtttttatctttatttttttagccatgtgttttttttcaaaacaaggactatttt
atgattttattacattttctttctttttttgtttccctttttccatagtacaatagaagaatagtaagcctttatcaggttgcccaacattaacaactatcgtc
attacgaatgaaacttactcatttaagctgaaaaaaaaaaaaaaaacctcttgagtactaatggtaagagggtccaatatcccattctggcgtattgt
tggttttcgaactgtaaacaaacaaaagtcgtacccctaatgacacagtagcttgaacaacaggatacggtagcaacaaacatgttagtagcaa
actttataaggctctagattttgcttaaaatgcaaatgtacgaacttcaaaagaagaccttttcttccaagtcaaaaagtaagggcaatttctatac
aagaatgtgttgattgatttgattaaatcaataaaatataaaatacatctttttcgttttttttttttttcatatggattatttagggagcagcacggag

acctctattaataggagatacttcacgtatccttgcaaagttacctttccccaatacccccctgcaccaacttgatccccagttttcctgtttttggcttg
taactctattgtatgaacacagtttctgaagttggaccttgggagcttggtctttccttgggatcagaaaggcaaaacagtactcgcttggcgaagc
acaagcagactgggcagcttgcggtcgtcaaaccattattgggatggtctcaatatacgccaagccagcagacaagcatagaaagtgagcttgt
cttgctgcgattgattgaacatcccaatgtcttacagcttatcgatgttattacccaacaagaccaactctatgttgttgttgagtacatgccaggag
gagagctttttgactgtatgctccgaaaaggaagcttctctgaagaaactgcagcaaagtttttgtggcagattctttacggtttagaatattgtcat
aaacttcatatctgtcatcgggatcttaaaccagaaaatttatttttagacgcccatggttctattaagattgcagaattcgggatggcttccgttca
acaacctggtaaattgttgcaaacatcttgtggctctccacattacgcctcccctgaaatcatcatgggacgcccatataatggttgcgcaagtgac
atttggtcttgtggtgtgatactgtttgctttgttaactggtcgattaccgtttgacgatgataatatacgctctttactcttgaaggtttgtgatggcc
attttgaaatgccgagtgacatttcccctcaagcccaacacttactatatcgtatgcttgacgtgaatccttctactcgaatgactatggaacagatt
cgtgaacatcccttcgtcagctatttagccaagcccaatatttctatacctatcgtatcagcacctattcaacctgttgaccctctcattgtacaacat
ctttctttactttttgttgttcagaaaatcctatgtctttatatgaaagtttggcttcacaatcacctcttctcgagaagaccttatacacgctgctga
gccggcacttgcatccccccttctaccgctgctattgatcgcaatcgtgctgtcgtcaatgacttacttggtacagaggccgcaaaaaattatatcgtt
gatgaggaagacaagaacagaaatgaaactgaaaaagtagttcaaggtccatcatcaaccctatttcccgctacttacgttgccgatcccatatc
acgtccagcaacttctgcctccccttttttccgtccatggctagtgctggagttaaatatccttatactctcaataatcagtcttctttctttcaccgcc
ccgctacctctacttccgctatgccgcaaattcctaaatctgtaacacctggtctggctactcaatattctcacattcctccagtaataccaagtttgc
gttcgactacgaacgtgcagcatccctttgccgttccaaatagtgaccccgagagttccggtaatcgccgagctgtctctctagacatgtctaatga
ctttccaggaaacgaatctgaattacaattaaagtctggcaccccctttttcatatgttaatggttcaggtgacatgggtcctcctaagagacgtgtc
gtttccaggttgtcggagtataacaataaaacacccatgggttattcagctgctaactctggcaattcgaaaacagctcgcttccaagttcttccag
actcaactccatctaacgaaaattatgctcataatcaaccactaactaatccccattacgcagcgtctcgcagagttcgtacacccagcggtgaac
gcatggttcctgtggacatggtttcctctatgggaagttatgactatatcaatgttcctaagcaacgccgccagtcactatttactcaatcagcttct
caaaagaagctttctgggtctccatttcaacctaagagatcattcttaagaaggcttttcagtagcgagccgtcttgcaagtgcatttatgccagcc
ttagtgcagctgaacttgagtacgaaatccttgaagttcttagacgctggcagttgctgggagttggtattgctgatatttttatgatgcagtgagc
gtttcgatcagtgctcgtataaaacgtcagaactcccttaacctcaagggattaagatttcgagtttctgtccttactgaatattttggttctcaagcc
gtatttgttttggaaagtggttcttcttccacatttgaccacttagccactgaatttcaactaattttcgaagataagggatttatagataatctagag
ccttcttattttcaatctgttgcccctcgtccatcttcgagatttagtgtaacgaacagtccttttgctgcttttcgtcaacgccagtctattcaaagttg
aaaattcctttttttatcatactacagaatttacgatttgactcctccatacgtttgtcgttcttttaatctcttagttttcgcttataatttctatggtacg
agttgacgtgaaccaggtattccctttataaccctttttttgttgctttttgggaaccaactacaataatttttttgcaatgtaattttggatcggattaac
actcgccgtttgttggctgttaggttttgaatcgttttcttcacttcattctccgttttcgacacagttgaatacaatataagaaccttctttttttattata
atgtaatcgatgaagaaccttgagaatactctcgctttttttaagattcacaatggagctctcttttactttttgctttgcagtctttttgtttattttttttt
ttttttttttttaccttttttaccctctaaccactatttccttttcgtcttttatttcttgctaccgatggttttaagaataacttctcgttcatcactaagg
atttatttactgttataatttttaataaaacggatgttaaactatataaatgtttgaaataagaattttaccttcccttatatactaaatcttacatata
catgctcaagaaggtagggtttgagtatatgctggtgtctgtcaaagggtaacaaaaagctagtagcttcagaatttgtaggtgtatagtaatgat
ctacaagtaaagaattactgaaacattacgaaaagaagaaaaaagctatgtcttttattctagtttattaccaaagtttcactataagtgcgtaca
cccacacttactatttcatctcaagtctaaaaacacttgataaaatccatttcaagtcttagtgagtaaacttgtaaagatgactcaagtagaatta
caaacaagtttagtaagatcaatacattgatcgaacttgtctaacatttctgtagggaaaaattttgtttgagctttacactgagcacgctccaaaa
gtatgaatgatggaaattagatactttttatgatttgtatttagggaactaactttggaaagacttgtcaaaacttttatactttagcaaaagaagg
atattatgatggtgttcttgtacgtatgcaagcgaaggatttcgtttatagggatgatcatggagaatacaaacatgggttatatagcctgtcagtt
ctttctttctcttgtgtgaatgatcttttatgaagtagctaacgtggactcagttccatagaataattcctgattttatgattcaaggcggagatccta
ctggcactggtcgaggcggtacttctatttatgggtaagttttttttaaatgttttatttgctatgtattttttttctcacagattagagattcatttgagga
tgagattcatcctgaattgcaccatacgggagcgggtattttatccatggcaaatgctggaccaaatacgaatggatctcaagtatgcttatttttttt
aatatcactctacttcgtactaaccagcagtttttcattacattggcacccacaccttggttgaatggtaaacacactatattcgggcgagttcaaa
gtggactcggtgtttgcaaaagaatgggtcttgttcgaacggattcctctgaccgtcctattgaacaagtacgaattttaaaagccatcgctctttg
aagtaagagaagcgctttctgtgtttacggttccatttttctgacttttaaacccaataaatggaaatatgataactttctcaatatttgtcccatctc
aggttgtatctaaaaattcgaaaaagtcagaatgccatcttttttttaatttgcattaccgggaatgcagggatatggatatttctgaaattcattcaa
aagctgaataaatttgatttggtgtttcaaccttttttttcccttggtatttagtctaaaaccgatttctatcacacagatgctcaagcatgttgacctg
cccttttttcagtaggagctggaaggaagaattagaaagtaaaaacttgaaattactctcaaggtctctctttgttcttgcaattctgccgtttccttct
atgtttactacagctgcttttttttatcgaaagtttcgaccaatgatctcaaaggtcctcgtttccggaggccatctcgctttaaagaatagaaatggcc
gctgatattcaagaccctatccatgccaatcttctaatttctcacatttttgtaagaattccaggaatctgtatttaccgtattcgaaggaacctcctt

aaatagtaatgtttggcagctgctgagaatgtttctgcgtttctatttacatgcaagatggcatttttggaaggaatccaccttttttcgctcgttgaca
cgaggaaaatccttccaaaaagaaataaaaaataataatatacagaaagatactggttctcatgcttttattgaagcgaacagtgtaaaaacac
cttacacagaatatgcgagtattttaaaagaattgactagcacagagttaaaatatgtattagaactaaagaagatattaaacgtaattaagaga
gatttttgcttgcagctgacgttaaacatacttactaacattgtatttttgtttaggaaatcatacttccaattgagagccaaagcttgagccaagaa
gcaataggaatctgcacatgtgtaaaaacgttgactcggtttcatggtattttacatggcgaaatgctaattactagatcaccagtcttgcttcttct
aaaatatttaccagtgtttcgtgatgtgtatcagatgtacacgatcaacttggctggaatatatgaagtagagaaagctgcttctccatataccatt
tgcggaaatcggttgcttattccactgcaacatttgttgcgataccccatgcatctagctcgaatttgtaaattacttcaaaagcacccttggttgga
aaaaaatttgtatatgttcgtgcaagcattagaagggtttaaacagttatgcacatttatagacgaagaaaaaggccgtgatgaaggtaaccatc
ttctaaaggatctttgcagaaaccaaggcatcgcacttgatattcctcgtcatattcgatttaaaggactgatagtcgtcgctaaacctaaagtttat
gactttaatgcatgcgtcttttgcgatgatggattaattgtttggacaaagctcttagacacgaaagagatgacgcccatctctattgagcagtgttt
atacgtgtcacaaatatccttgtctgtagtagtggttacccttacattgaagggccagctgttaaaacgacatttagccttgcataccttgggcgcat
ctgtttttctgaaatggtatcaacagagaagtaaacttgatagcccaaaatcaaataacttcttctaaaacctaatgaaccgatgaaacatttttca
ggaacatttcctttttcctttttttatttttatttcttttatttttgaagttttccttatctgaagtgaaagtaagaactgactgttgctgatttttacagct
tttgaacctatttattgttacttacttcttggctgtattattacttttcttttcttttctttttcctctaaaagaaaggatgaaacaacttacaaatcatta
tatccatcaaagctatgttagccgtttcaaatttacctttctttcgtttcctttttccttaacgtctctcaatcctttgctagttgcttccttaataaaaat
ctgtcgtttcctaatatctcataaatttttttctctgttactaatttaaactaactagctcgtcggaacaataaacattcatacatgacttttcactggc
gtcttcgaaaggtggttcgaaataagcagtaaaatcatgaatcctgactattctaatttatttttgtttccccgttctcgttcttttttcttgacaaaaa
cggccataccccttcatcgccctacaaaccccattattatcataaaaaaattatttaatcctcgtcattagaattatctccatcaagaagactagcata
agatgtagctgatgaaagtgaccgacctttacacttttccatatgtgctgcatatcgggaagctgctatttcacgccggcaagaaagacattgata
atatggtatgctagagggtgtttggatattgtttgcataaatatcgaaaccaggtttggtacaatgttgtcggcaataagtattacatctctggcact
tttgtaatgcaagcttactcattctatggatcctttgagttaattcatgagtataatctcgaagtaagtcttcaaatatagaacaagactaaatttag
aaatgttagagacatcaaggatataaactataatgagaataaaatcaattccatctagcgataacttacgattgctgcaagagagacaggttgc
atagtcatagtgatcctcaatcgcttaaggaagatttcagtcggtggtttttattgtaaatagatgaatcgagcaagatcgttgcgaaaaacaaac
cataaaagaaccaaaaatgagccaggtatatgtatttaatcgttttcttttaatttattgtctttttctttagtttagtttagtataatttcagagtcgg
ataaatagtgatttcagtttcttgaaaaaataacgattggtaagaaatactttataattgaaattttgtatcagatcaagacttttcaaaaattcgtt
tcaaagtaaacagcaataaaaatcaaaaagaaaaactcaaaagtcgaaaaacggtgctataatcaagcattcaaataaaccaaacttctctag
aaacgagctaccgaagatggttttggtatttttctcttacaaaaagaaaacttgctgaagtctcaagacttaattttcatttgtatatttattataagc
accttacagtgtttcttttataggtctaaatttctatgattttattaaaaaaagcaattaaaatacagactcgtgcttaccaaaacagataaaaagg
aaaatgaaataaaataaaattataatgacaaacaacataatcgactcttccaaataaataatagaatgttgaagagtcaaccaacataaagtat
agacagcattaaaatggcaaaagcaagtaaaacaattatgtctaaagcaacaaatgaacgacattatatagaatagattaggaacataatcatt
tgggaatgaaatgtttgcaaattatagatcacacacgaagcaacaaaagaaagtaaacatataccataacatatgatctttttttccataagctca
gccttctggatattcaaattaatcattcacgctaaaaaccaacaacatcagcgggaacttcagatattgagtaaccattatgctcatcaggcacag
tctggattattgctgtgggactacgcacaatggaatcattggcattattaatagtggatggacatattttaaaactgttagcgtcatcgatcaagtta
ttaaataaccatagatgaatctttccatcctctgagccacttattacctcgcaatctcccaaagccacagaagtcacaggtccagaattacttctca
gcgtctgaacacattgacaagcttcccagactttgactgctccatcatgtgcaccagagacaagacgtagttgatccgaagcaatctcccatacgc
cctctatatgaccaaacaaagtgtgaacacatttccacttttcaatatcccattgtttaattgtaccatctaaagcacaagagaacaaccgattgcc
tgtcaacgctaagctttggacaggtcctatgtgagcattgaaagtgtataaacacatatttgtttctagagaccacactttaattgaacagtcatcc
gatgcggagaaaacgagacctaactcgcgatggatacgcacagaattcactggccccgtatgacctcttaaagtaatacgttttcctccggtaaa
atgccatagctttacggtatgatctgcactgcccgacactaaaagtgtggaatcaaaagatagggagagaacggtgtcggtgtgcccgtgtaaa
gtagatatacattcgccggtacgatagttccaaattcgaatggttttatccattgacccactaattagcttacatttatcgaattgtaagcaggtaac
gccaccgctgtgaccttctaaaagaccgatttggcgaaatgtagtaaggtcccaaaggcgaacagtcgcatcatagcttccactagctaaaacat
tcctttgaagctgcaaacacatgattccatcattgtgtccatttattataacctgacgacatcgaccatggcgccaattacactcaattcgacagcg
ctctgcatatacttccttccacggacgtgtttttaaaggaggcgaggaagctttctctgaaaactcgggcaaagaattatgcttttcgagggctgct
gacgaatcaattaagggagcgggttcggtgcgcagtcgctttgtggaaataccggatgtattgctatctggatgacatcgtttagtaagagtatca
tagcgtttttggatagatgccttggtagcgtagaggagctttcgttctaacaaaggtaaaccccagccgcatttttcacattttcgattgatatgttg
ctcgcacatacgatgccatataatatcgtcatcagcaagctccttccattgcctacaaacctgagcagcctgacataggctgcgagcatctaaaaa
ggataaaatacggaaagagatttcaggagggagaattgaaaggaagtcaagacggacaagggtatctagggtactggaagcaaaggatagc
aaagaaggacagcagcgatttagtatattttctaaggcaacttttcgttcttgtatagaggcgtctgaaaatgtagagcaaattgtattcactcgac

gacggcctgtatcatcgagctttaccagatcctcgttgactttttccataaagagaattaatggtaatatttcttcttttgttcgtaaaccgttaattcc
aacaactcggaggctatcggatgtttggagaagataccactacaaggatacttgataattgggatccgtaaaaaattgatatgatagaataaaat
aaaagaaactttttttaagtatctctatcaatcccaatcattcacaatacaaagaaggcaagattccaaggatcagtttatttgcctgcaacaaaac
aaaatagaaacagaattcgataattattagtatttcacaggttcaaagtgattagtcgaaatcaaattccttccacaaagaaaaactcaacgaaa
aaaaaactgaattggggaatacaagcttggactttggcagacttctttttaaacagatacgtcttgcctattacagagcaaaaccgtaaatgcaa
gatttaataaatcttttcaagtaagatgaaggaagttttcaggaataatccacaaatcaattaatttgtataacccgatagtccaaaatgctgaaa
gtggatgatggatcaagagaatttgagccgaactcgagttgtatataatctcgttgttgcctgacatgataggtaacaatcaagcaatcggcattc
cccaacactgagtgatcccagtaacattgttgcctcctgggactcttttaaaagggtaaggtaacataaatgctgtcaaattttgtcggtcacttca
aagcgtcacctcgttttgaaaaaagtcaaataattgcacaatcttccgtggatttcattgcagaaattcctcttagaagtaacaaacgtcgcaatta
agcacgattaaattattgataggtgcgaatatgattgaacgaaaggcatctaatgatagcgaattccatgattggtaatttagcaatcgttgatag
aagctcatcaacatagtacacctaaaattcgcagacgtgcattcgaattacgtaaagtgctaaattaagtttttaaatgagcacttaattcaagttg
agtctgagattcaaaaaaaagaagtcgcaatgggtgtgggcgttatttcgattgccgcatgtgttaatcatacagcataagttttctatcacgcga
aggggcaatgcgtgcttctgtatgagatgagcgtttcgccccccttcggcaaaagtacggatagaaaaagctaaatgcttatgtgagagaaactaa
gcgttcatacttggagaggggcttcttggaatacacgaacgaatagagtggagagcataatcttgactgcaattcaaaccgtcgttggttttgtttc
agaagcgaaagaaaataccaaattaaaatacaatataaactaaaagaaagtatgataggataaagtgtaaaggactagtcataaaaaatata
tatatatatatatatacttttatttctttacgataaacaagctggctatcagctatttactcgcctctttgaatctcccctatctggcacatttgctct
tcatttcttattcctgaagataatacatgggagaagaagggcaagagtttgttttagggaaaggtatctaggaaaaattgagacagcatttactaa
gcatatacccatatataaatacatagtgattgtttattttatcaatttttttatttttttctatataaagcctctcgttccgtttccatagaaatgagtttca
ttcgaatccttattatttgtacctatttatatacgcatggacgcgttgtttcgtttttttttaatgaattgaaaagggatacaaatgatgagcttgatatt
atcgtgaataagtttcatcagttagccaaaaaacgacagatgaatagcatcatccatttgttgcaactacaagatactttgcttttgaaagcatccc
aaacacgacgcgtaaatgaagatccttcagagattttggttaatcttcggcaagaagagaaaatccacttattagaatagaataggatgaaacg
cgtaattcttaatcattatgcatggtttgtttacctggttttaaaattcaggtgttaccgaaagttgtgttgttacttcaccatcatcctttaccctttca
cctagtggtggtgtgcactggcaaaagcccactgtagggaacacttaaagcaatagcacttgaaaagtcaacagcaaaggttggtcttccacca
aatccttatgttgaatcgacgattaactatccaccttccgttctatgcaaatcaaaatccttctccatcgtggatttggcgttacttttctcctaaatcc
cgaattggcttatttttttatgtatcgctaattatacttgggactttaattatgacatttaaaactatgaaattgaacacctcgcaatgcttcactcctg
ttccattggtgggagctgatcccttaagcaatttaaatcacttaattattattgctggacacgctgtctggttgggggggatcttctcagggacaaga
ggatagcgaatggattttagagccataccaaaaaggagagggaaaagtatttgcagaacatgcccgtaaaggcttgaacttgcttgagcaagac
gaatcttcacttttagtcttctcaggtggacagactcgtccaaatgctggtccatattctgaagcccagtcctattacttactttccaagtcgatgaat
gatgatgcctttttattatcgcggaggaccaccgaagagtttgctcgagatagcctagaaaacgtcctcttttcaattgccagatttcatgaagtaa
ccgggcattatcctgagaaaataacagttgtctcttttgagttcaaacgagagaggttcctaaatttacatagggaggctattcgttttccaagtca
acacttttacttcgttggtatagacccagaaggcggcgtccctcaagctagctatgatgcagaaaaaaataatgcccttctccctttcacagaaga
tccttacgcttgtgtacattctttgttggtgcgaaaaagaagagaacgtaacccttatcgaagacaacattcttatcttattacatgccccgaacta
attcctttaattgaatattgcccttcaaaaagttctaaatactactcaggaaaattaccttggtaatttattacaaccacgtctcaattatttcactttt
ttttttttttttctttcaaaggctggtggtatatgttcaaacacttattgcttttatgtagatacattttcttttcatcttttgtttacgtacaaaaataa
gaccattctttttgtccaatcttattatatataaaaaactagaaataaaaaaaaagacaaaacggcataattttggattaatatgtgttcatttcag
cataaagtacgatttcaatagagctcgaatttgtgtttgtcatatctagtaaactaactagaaagtcgtaaaaagttatggattttctggtagatcta
ttatcgttagaatatgtcacacaatatagcaaattatctctttacctcgtatggtttttccattagcaaacaatgtcctgcttcaacaacttgaaaag
aaaagtctccttggatgtcttgatatagttcctttttctgatgaaggagagagagagtcctgatgatgtgaaactttattagtaaatctattttttttaaa
aaaaaatacatacctcattgcctgttataattaaagtaggtaccgaaggaaaagtaaaggaagatgcgagaacgctttcaatacccatcagcat
catcttccatacataagtatcactttcacgattccatatccaaagttgtgcacgttgagcctgagtagctttactagagaaacatcggttcaccgat
ggacttttgagaccctgaaaacggtcaaaaagtcggtacaaactgaaaataggtgaagggattttcttgattctttgaactaactcaagttcacttt
tgttaaaacagtttttaggagcaagcaaaacaagagcctcacattttttttcccaaccttttggctaatcgagcagcaatgctcgtacccatggaatg
cccgacaagtacaatggagtcagaaggaaaaatatgcttttcaatgactttttcaacaattgtagtcaatccctccgtggacaattcacgaggtga
tcgtcctaaatgggaattaaaggcacgttttgacagtccgcatccacagtaatccagtgaaaacacggtatattcaggcggaattagattgattaa
cccttcaaactgagccatttggccgcccaaaccatgaaggaagacaaatgtctttgaagagcgaccagtattcttgtcgaaacttgtgtcagctgc
cgtgcgcaagtaatacgccacttgatactgataatctaatcggcaattctcttctacgttctttgataaatctgtagcaaagaagaatgagttggga
tcggcttgcttgtttaaatgagaacgcatttgttctttctgctgatccacatacatttgaaacttgacagctagataggcatcgtgtaaataaaggga
aagaaggaacaatatagctattccgagtaggaagaacaaaagggcaatcatgctcgaatgtttgaagtgagtaaacaaagctttctttctctaac

aaataaaaaaacgagtaagaatttaccaattcgactccgcacttgagaaattggtaaattctccaaaaaatcgttcaggaacagaatttgtattg
caattaactaggatcctcaactttcaattcgaaatcaagagataaagtgtttataattagtatagtataataagaacctccttttccaatgatatctg
cttgccttaggggtatgtactatcgaaagtggaaacctgagtgtttcttggtattattttcagttcgttatgtggctagtggatttctcgacaaaaggc
cgcaagttggatttgctaaacaagtttatttcaatcgatataaatcagtccttttgtttgcatcacagttagagaatcgatgaatgaacttgaaagc
ctttaaggaaacctctgtaaacggacaaaggacgtgtttaaaacataaaaaaaaaaaagaaaagaagaaaaattatatacatataaaacaga
tgaataaaataaggatgtgctgtaaaagatcgtagcacacattcaaaatctgtttatgcttaaacttttgtttttccattatttgacctaatttatac
agtacttatctatttattgcttattgtatccttcagtttaataactcaaaaagaataaattttacttatcctattttttaagcaagtgtatgtttacatg
agcaaaatagggtagggttagcattgggaagagaaagaatgaagttgctaaagctattctctgccatattcaattgcccagtgctactatagtaa
tcatgtctgacgaacaaaaagaacaagttcaaaccgctgctgtagagaatcaagcacctcaagaaggtgttcctaagactgaacaaaccacag
agtctacaaagccagctgaaaccgtttctggaaaagctgaatcttttgatgaaaatgaagttttaaagcaagtgggtaagttattactcaaaaatc
atttgataggaattcgctagaaaatgggcacggatttcggactaacacacgatgtagaattttacttttctgactccaacttgcctcgtgacaagtt
cttgtggactgtttcccagaaaaaccaaggttgggttcctatccaaacgattgcttcctttaagcgtatgcgtcgctatcagcccttagaagccgttg
tggccgctttgcgcaagagcccagagttacttgaagtggatgaagccggcgaaaaggttcgtcgcaaggttcctatcgttcgtcctgatccttctgc
tatacaacgttccatggaaagtagtgtgtatgtcaagggctttggtgaggaacaagaagatactcaatttgtattggaaagatatttcgaaaaag
aaggtggtgaacttgcttctcttcgtttgcgccgtgatgacgataacaagttcaagggcagtgtttttgtagaattcaagactcccgaaattgccaa
aggctttttggaaaaggtcaagggatctccattgaagttcaatgaaaatgagttaactatcatgtccaagaaggaatacgtggatatgaaggctg
aacagcatcgcaatgatcctcctaagcctggcaacaaacgcagacgttttgacgcctttaaaatgatggatcgtgagcgatccagcaagcgtgct
cccaagagaggaaaaactgcttataaggaaaccgaaaagcaagaaaaaccaaaggaacccgagactgttgaagaacccaagaaggctgaa
gattctaatgaggctgaaaagtctactgaacaacccaaggccgagggagaggctgcagctgccaccaacgaaaaagaagaagccgctcctgct
tcttctaactaaaataaagataaaactggttttattatctttttggaaaagtttttattttaaggaataaaatggttagtatgggatgaaactcagga
aaatgaaaagtaagataagtaaggtttcgagaggaacttgacttgaaactgtattgcgtctttgtatggaacgacttgaaacatatttcatcacttt
ggtttaggctcaaatttcaggttaaatttagtattcaccttgtatataaattttggcaatgaattgccaaatttgctccttacaaaaaataacagggc
tatgaagagtgattgacaacattaagagattcattaagcaagacgataaacagactttcgcaaaatttcatttgaaaagtgattaattccttcaaa
aacttgaaagttgacaaaagaaaaagggcaccatattcatttctggcatcgctatactcaaccattttcctagaaaaaggaaagaaaataatga
aaagaacaccatatgctatagttacaactctgcaagccatttcttcaattttattgcatactagatttatttatttgttagtgaagtggaacgatgtta
atggcaaactcctgaggaaactatttacgttgttatgtaaaagaacatatctcacccggaaaaattggtacacgtaaattttaaaaaaagtatatt
acactacgaaaaaagaatggaagctcgtttttcgggagaacaattccatggttgcttacctgtggtgaaacccccatactatatccaagtggggc
gagtccttgatggaatttgtgtggggtagtggggtattgtagcggacattaatgtagattacaaaatgcaatgatatcgtaatcagtttaatttgttt
acgtcaatgtgtggggaaagaaagaacttacatttgtaaaggtgatgctacgtgctgtttacactcaactattactacacaattttcatcctcccaa
gcagaaatacgcgtgaattacaatgccaagagcacctaaatctagcaaaaaaaaggatcctaatgcaccaaagagaaacatgtcggcgttcat
gttcttttcgattactaacagagaaaaagtaaagaacgaaaaccctgatgcttcgtttggtacgcgtcttatttgaagaacagagcgtcttctcatc
aattctttttattgatattgaaaaattcgtgtttatttgtcaaactaacgcgttgcagggggcattggttctctcttaggaaagaagtggaaagaatt
gccagcagataagcgcagggtatgtaaaatgaaagaattctttacaaagggatttcaccagtgaataaaagtctacgccactagaggatttgaa
tagtggaatcttgcaattcgtcaaatcgtttgagagcgatccttgaaatgagaatccttttgatatttttcaagtttttttagctaacgttttactatta
gccttacgaagaaagagcacgcgaagacaaagatcgctatgaacgggaccgcagcgattatgacaataaaatcatcaacgaagcaaagaaa
aacgccaagaaggaacaaagcgacgaaaaagaggattaatattatattcattaccggttataatttattgtataattttcccttgctttttaatga
actctataatcaattcatcttagactttttttatttcctattggtcctacatggcactttagagcatattttacttgctttgtttgtttatatttcatcttctt
cgcgtttctttgtgccgagtactcaatattgtccggaaaatgtttttaccctaaatgaatgaatgaccgacattctgcaatttttcagattatttcgat
ttgttgctgttttgttttctttttttcccatgagtttgaataattcatttcactcgttaactttttacgatttgcttacagattttatgtgaatattgctccct
gaatgtttatattaatttagtttttatgcttctaatgagtaatgtttccttcattttctcctactataaagaatcttcaatgaaaataaccattacaatat
acgtactgcaaacttataggttgattgctcaagctaatggtcaacggtctgaatctgttgacacttagcttgtaaaacaaagcgcaatcggtcattc
attaccataagtcgtactctgtaaagttggctatttttagcgtctcgttttcctaaatggcaagaaaagaaaatactgtttattaaagtttatagtca
cctatcccttatgcaaagctaatactaaagcttcatgtattcaacactatgaaaatacataatgatcgtatcgctgtttttttcttcaaaactcattgt
atataataccacaccaacaaggcgtatataaactcttttcgtgaacttaggaatcatccttagctggacttgtgtttgaagtagaaatttccttgact
tcatcgttactaacaggtttatcattcgaagaagctgcatcctttgcaggaacttcgctgctttctccttcttctttttcagtagtcaaatctttgtaga
catcaggatgctgttggaaacaggtttgcatagcctgaaatttttcaagacattccatgcctttaggctcactttttgagtagacaaagcaactaaa
ggcggccttgaactcttccccacagggaccatgagccattcccccaaacaagggcaatcccagttaatttctccggtttcaggatcaaaggcgg
attcttcactgccttcttcttcaacttcttctagctgcttggttatgttaccttgctcagtcgaagtctcctgctcaggaacgtcatcatcggagtagac

gctggcatcagagtcaagctgttttggatcattattgctttgttcttctggtgttttaggtttccatgctttttctaccatctctttcttcttttcgtctaatt
ttgcttcaattgaatcttcaatgcgatgttgttgcatatacggttcattgtaagctattattttgttaataaagaaataataaatcatataaacctcct
taccctgagtttctgttcggacatgctctccaacataggctccgttatctgagtcgggttttgaatcattaaacaccttttttattgaggtaaaatccaa
aaaacaatgatagaccggctgcccctgataatatggatgctagttggctccatgaaggagcagcttttgctcggaaaactgaactcgtttgattga
accgttttggaaagctgctacttaaacataatgactttttctccaaagcagaatttctttggatcgcagttcttaggaggaaaggagcttttccacg
aaacatggtctttctgtaatttgctatggtagagacttggttagtaacgtcaacgatatacctcaagagaagaatataagctatcacttactctgat
tagttgaacaaataaaacgaagcttttattactttgactctagtttaattcagttatcaaacaataatccataacttgtacgtataaacgattgtctc
acaggcattaaatcaaatcctaatattgaattcaaacaaaatactaagttctattttaaatctaagaattatttaatttattatgttacactgaaagc
agcaatcggatatgcctttttctatattcaggaaaccaaaaatatatacacatgtatgattctatcgtgctaaattatacaatttttgtttgagcactc
agtaacgcttaattctccttaccaacaatatcaacccaacgctttccattcaattcggcaagaatttcttgcttaattttatgggcgacaacaggtcc
atcgtaaccaagagcagtgtatacttgaaccatagaagcaccagcgcgagcatattcaatggcatctttaccattagaaataccaccacagccga
taataggaatgtcagatgacaagtgtttccgtaatgtacggagggtgttcagagtaatgggctttaagggaggaccagacaaaccacctgtttctt
gaacgtgcttcgtggattttaaggtatcaggacgttgaatggtagtgttgccgacgattacaccgtcaaccttgcattttttcaagacagcagcaat
gtcggcaagctctgtttcggacaagtcgggagcaatcttaaccacaagcggaggattaggagcattcagcttattgcgttcggcaatggtggctg
acaatagttcagttaaggcagactttctttggagactacggagaccaggagtatttggagaagaaacattgatgaccaaaacatcggcataattg
ccaaagttgcggacacccgttacataatcttcaatctcatttccgtttttgttctttcccaagttaatacctaagtacttgttgggaagtaagctacgc
gggacgccaagaacagagggatcagtgtaagcagcagggtttgcatcgaaatctttcaaaagccctggcttgtttttagcaatatacttccgaat
acgcttttgaaccttttcgagaatagcttcgtgaccttgtgagttaaagccataacggttcaccactgctaaatcgggatccaagcgaaagtatcta
ggtttgggattaccaggttgaggcaagggagtgacagaaccaacttccaaatatgaaaacccgaaattcaataaaccggaaatagcatcagcct
gcttgtcgaaaccagcagccaaaccgacgggattagaaaatttctttccccaaacctctacagctaaagcttggtcgtcaagagtaaagtcacga
ggagtgatgccccaagaggcagcaagaacagctacgctgtgagaaaactctggcgtagtaaaagcatggaacaatggcatttcaatgcgacca
tggataaagctgcgtacatcagacatgtcatatatcatccaaccggcagagaaactaccaagtaagctccaaagcttccaattgcctcgaaaaa
aagaatttggcttggaagaagcagagtgtaaattagactgaaaacttttaccaccgtgtttgaatacgtttctggaaagaagacgataagcattg
cgggacaacatattcgctgaatttaatgaacgacagaaattaatgtcaaatatgaggatcgaatctaagaacccaaaaaaaattaagaataaaa
acaaaaaggtacaaattatacggaaatattaaattaaaagcaagagagaattagcaaagaatgaggtttcgatcggtcgtaattaatcggagg
ataagaaaacgaagatgaacgaactgctaaagaaacgatgaattatagttaaatttcaaggcaggttcaaacttcaagtgcaagcagaggtat
acgctctgagagagaatgcattttgcagtcgtaccataatttttgttgttactggattcaaggtaacagtcagagatttgaaggattggattttcata
taaacaatgtagacgagaaataataacagttcaatgaggaacaaatggatccattatgggtattcaagatctcaaattagtgtgttgctaattgat
ttcataccctatcggctcagttttcaatagtaaacaaataaaatccaacagttttagactcgagaggcgtacagaagcacatcgacgaaaatgga
gtgatatttgtatgattttccacctataatttgcatttacattacagaaaacactttgttttctatttcgtatcttcatgaataacgctggcacttaattt
tttccgtaaacaaacattccaactctgtttccaaagcagatatcattcctttcataatttttccaaaagtacacaggattaaatatggtcgtcaatgc
ttctacttatatcttctattattcattcattttctctcttttgtttacttgtattacgcaatgcaccgctctaccctaaacggttgcccatcttccagcatg
cgactcttcctgggataaatccgacatgggagctgtcttgagtccccttcaatacttaagacgcgccgtatggttgagacctatgtctggaaggca
atcaaatatcaggtaagtacaactattagaggagctcgttgaccagtattagcaagttcttcactttgggcccagagcaagcgcgtaaaaaacga
actccagctgctccagcgaattcagagaatgaggacggttctcgagtaataaagaaagaagagaatgatgagaaaactagtggaaatgatgga
atagcagttaatggagagaagagaacagcagacgaacttgagagtctgaaagaggaacatggagaagcgacaaattctatggaaaccaagc
gttctaaggttgatggcttgtcattagcttcatcgtcatcttcctcttcaaatcttgcttctgaaggtgaaaccactccttctgagattcctcctcctatt
gctgaaccaccgctagagtctacgagaaaacagcaaacaaaaaaggctcacgcaacgtttgcagacatggtatctacttttacgaaaattgaga
agactacaaagcgtctggaaatcatcgatatcatgggtacttatttttttaagatgctgcgtgattatcccaatgatttacttatctctgtatatttga
gcataaataaggtatagtacaggtccattttccgtcgaatgtatactaatgagagaaatagctaggtccagattactcgggaattgaacttggtat
tggtgaaagtatcatcatgaaagccgttgccgaatccaccggccaaactcttcaacaaatcaagcaagcattccacaaagttggggatttgggtc
tcgttgcacaaacatcccgccaaaatcagcctactatgtttcaacctgctgctttgacgctaccttttctgtttgattctttaaagtcaatcgcacaaa
tgtccggaaatcagtctcagaatagaaaaatcggcttaattaaacgattactaagctcttgtcaaggtgctgagccgaaataccttatccgagctt
tagaaggaaaactacgtttacagttagcagagaagaccatccttgtttccctagcaaatgcctgtgcacaatatcacgctgacaaaaaacaagtt
aagctgtctcagcaggaccgtatccatgctgaacaaactcttcgcgatgtttattctcaactaccttcgtacgacattattgtgcctcggttgattga
acatggaatcgaaaatctgcgagaaacttgtaagctaactcctggcgtccctacaaagccaatgcttgccaaacccacaaaacagttctccgaa
gttttagatacgtttgatcaagctacttttacttgcgaatacaagtatgacggagagcgtgctcaagtccattttaccgaagacgggaagttttacg
tgttttctcgaaattccgaggacatgtccgttcgctatcccgatattatggcgtccgtacccagatggagaaagccagaagctacaagctttatcct

tgattgtgaagctactgcttgggataaagaggaaaacagaattcttccctttcaaaaactggctacccgtaaaaggaaagacgtaaagttggaa
gaaatcaaggtccgagcttgtctctttgcttttgatattctctacttgaacggcaaatcattgctagatattccattttcagagcgtcgaaagcactta
tatacaatgtttcaacccatcacaggtgagtttgattttgctaagcatagtgaccaacggtctgttgagtctattcaagagttcctcgaggaagccg
ttaaagagtcttgtgaaggtcttatggtcaaaatgcttgaaggaccggaatctcattatgaaccttcgaaaagatctcgacattggttaaagataa
agaaagattacctttcaggcgttggtgactcacttgatttgattgttgttggtgcatatcatggaagaggtaagagaacttctgtgtatggcgctttt
cttttgggttgctatgaccctgatacagagactgtccaaacagtttgtaaattaggtactggcttttcagaagagcaattggagcaatttcatcgcc
aattaagtcaaatagtcattccaaaaaagaaagatttctattctcatagtgaggctccccctcatcaacctgatgtttggttcgagccaaaatattt
atgggaagttctgactgcagatttatcgctctcccctgtctataaagctgccattggttatgttcaagaggataaaggaattagtcttcggttccctc
gatttgttcgagtgcgtgaagacaaaaattgggaggacgctacaactagtgagcaggtttctgaattttatcgctctcaaattgtgcacacccagg
cttcagcgcgtgcatcgtcacctaatgcggaagaatactaaagaaaaattgctagctttaccaactacctaaaataagcccaacattaaaacttg
gacatgttactgttttttgctctacaaagacgctaattatgtatgatggaattagattattatatagttttcccttctaattaatattccttttaattgct
ttggttatgatctaacaatggaatttcaaagggttaaatcgagaccaatgccgtattttaaatgtcaataataagttgattctattttatattttgcaa
ttttcattaacataatcctttctactctcagaaatttcaatcataattagagattggaattttcctagttcagcttagacctcactcctacattgtcatt
cttacctaaagtcaatgtgtcccgttaacgaaacaataattgagactaaagctggggtaaaataccagtaaaaaagatggagaatagaacaga
gtggccattaaagcatgtacagcctcctagagtttcatttcgacatcaattgtcaaagtccagtgctgatgcaccttcccttcgtgttcaatggtttta
tgcgatagatcgacctgtaagaaaatccaaaaaaggaagtctagaagctaaaaaatcaaaaagttttaaacctttttccgtcgaagactctgacc
gaatagaagctgcctaccaaaacacagaagacacggatggtcaatcggaaacaataagggtaaacgaggattatctttatgctgtgaatgtaat
atcgagggaagtatttccaatctattgggatgggcctgtctttcgtattttaagaggaacctggttctatgctagaggcgacaaattatatccgtgtg
aggagaatttggccattcaagttgaggaaggatacttaaatatatgtccttatcgtgaaaaagttgaagaaaaagatgatcacagtatgtcagct
caaggcaaaacctgggctttacttggaaaatatactggagcatttattgaatattcgggagatagaagcgcaagaatgcactttggaggattcta
tcgaaatgtgtcaacaaaattatttaccaaaatgtcttattctagtaatcatcgaagtgataagcttatccgaggatacgatatttcatcctctacaa
taacagatgctaaatctactacaagctcaacagtggatgatttaagctcctctagtacattggtaaacgattcttctgaaccgaatgaattactttc
gccttctctgactgaaccagcatcggaaataccaacctcgacagatgcttcgcacttaaagtcttcacctgatcgggaaattaatcaccttattcta
tgttgccatggtattggtcagaaaatgggcgagcgcgtggaaacggtaaattttgtacgagacatcaatgaatttcggaaaacactaaagaaaa
ctttcaatgcttctccagatttgcaagctttatatcctaacttcaaaggtggcggaaatgggattcaatgccttccattattatggcgccaggatatc
cactttggcatggcacgagacaataaatctgacatttcagaggaagaagaatcagacagttttgatgtttcaaaagatcttgcccttgacgatttg
agcgaggatcctaatcccactttagagagcattaatataccgaccgttgtaggattgagaagcataatttctgatgttttactagacgtgttactct
actgtcaaccaaaatatagagagaggattttagttgctgttgttaaacgtcttaatcgtctttatcgtttatatgtgaaacataatccctcgtttcagg
gtcaggtttctttgttaggtcattccttaggcgccttgattttattcgacatagtccgtttccaaggaaacataaggtatccgcggcttcagctcgatt
ttcctgtaaataattttttaccctcggcagtcctcttggcctttttcagatgttaaatggaaagcggattgcaggccagatacctcggtctacgatta
cgagaagtgttagctattcagaaagcaactttgactcatcagtttctgttctcagctgcaagaatttttacaatatatttcatccaactgatccgatt
agctatcgcgttgaacctcttatagtgaaaaaaatggcacatttaaagcctcagagtattccgcattttagacccttaagtcatgacagtagctctg
gtgttggtcacagacttgcggatggtgcattaaatgtgttatctggattacgttccggtatcgcaaatactttgattttgaaatctttgagctatgcat
cagtgtttaatgaagccacttccaatcaaaaagtccacgttgttgaagaaggcaatcaagcatatcaaattgatgaaagaatgtatcgtttaaac
aaaactggccgcgttgactttatgttacaagaaggatccttagacacatcctatagttacgtttctgcaatgaatgcgcattcgaattactggaaaa
acgctgatttggcccattttatattaactcaattactttaatttattcacctgctatgttaacttgtcgatgctgttaatgatcgagcttcattatatata
tggtatttttggttaagtttatttatgaagaagcattgctcgatggctacgagttaagctactgaacgaaatatttttttagttctaaaatttttaatct
atttattttaatcctttatttataaagccttttatacattttccactgtaaggatacgtctaatcaaatgtaacgtaaactttgctgattacttatcttg
agtttcgcttataacgtttggtatatctatgaaaagatcacaatctttcttatcctctcccaagtgctttaaatgtcaggatttaataactttgtaaatt
aaacaaggattacgaaataagggaccttaatgaagcacttacatatcaaagcctcatcaacatagacactgtcgtaattgaacaagaccacata
acttagagattcattaacacactctttcttatatattacaattcttttacattttctaatggacataaaaggcattccgcttacgacatttgcgagttca
ttcgatagcccgaaagaggaccttaataagccgttagtcagttatccaccttcaaacaagaagtctagtgatggccgggaaagcttagaagaggt
gcctatccgagctactgacaccaacgtggtgttatccttagacccagacaacccagtagacgaaacttctcctaaagaatttcatgaagaaaaga
ttagtagttggatttggatactctcaacggttgctggcatttctggtttgctatttggatatgacaccggtgtaatttctggtgctttagcagttatagg
atcggatctgggaagtgaattaagttcgaaacaacgagaactcattacgtctgcaactccattcgcggctttattgtcgggtattacttctggttgg
cttgcagatttactaggtaggaagcgtttacttatgtatgctgacggtatattcgtattggggtctattgtaatggcagtagcaaggaatatagcta
ctctagtagctggtcgttttattgtcggttggggcattggattagcttccttaattgttcctatgtatattacagaattggctcccgctcacatgcgtgg
ccgacttgtaattatttatgttgtttttattacaggagggcaattggttgcctattcattgaatgctgcatttgaaaatgtaaggcaaggttggcgtat

tatgatgggattgggagctttaccagctctttgccaactcattccgctcttttggacaccggagtctcctcggtatcttctaaaacaaaaccatgttg
aaaaggtatataaaattatgtcgcgaatccatccgaatgctaggccaggtgagattgcttacaaggtctctttgatccaagaaggcatgaattta
gttccttagaaggtaatcaatttcagagagtaaaacactcgtttaatcgtctttggtcagttccttccaaccgacgatcactgttcattgcatgctttc
ttcaagcttttcaacagttctctggtacaaatgctattcaatatttttctgcgatacttttcgaaagcgttggcttcaaaaattcaatccaaatcagta
ttgtcgttggtgcaaccaacttcctattcactatccttgcgtttatgtttattgatcgaattggtagaaggcggatattattgtatagcgctactactat
gataattgccctttcagtttgtgcggtttcatatcattttctcgagctcaagaaaactgaatggaaatacgttacactagtttccattattgcatttttg
gctagttatgcttttggtattggtaacataccttggcagcaagctgaattatttcctatggaagttcgtgctttgggaacaggtttctctacggctgta
aattggtctggaaatttgatcgtcagtgctacgtttttgacgatgatggagtctatcacttcagttggcactttcggattgtatgctggattttgtttcg
tcggtcttatcacttgtttttttacatatccggaattagcaggaatgtctattgaacatatttccatacttttggagaaaggattctggaatgccgtaa
aagaacaagataataaaagtaagaacgagatcaaagatacttactagtttttttattgcatgtttatttgttatcattagaaaggcgtatttggtaca
gattgtattaagttgagcgttatgaactaattatacataatattataacatatatttcttttataaagcaacaaagcaaatagtaaagggttaagaa
agtaaatctcaagtcttctggaatcacttatgtcaaagtttgctctgatagaagttagttaggaaagtaatttgtttaaaaacttacttggaccatca
ccgccttggggcgttttccaatcaatgccttgaagaggatctttgatatcacaagtctatttcatcattagtaaacttgaacctataggtaaaatga
aagacttactttacaatggacacaattttgagaattgataacgaaacgttttccagtaggaagtgtctcatcatcgacgtactcatatacaccagc
aggacaaaaacgagattcaacgcccttgtattttcgataggcttcttgacgatcatttgggcgacgatcaaagagatgacaaggctgattctcagc
atgcatagtgcctgacagagctacagacgtaggaatatcgaatgaaagaacattgtcaggcttcgggtagttgataggtttatatgaagaggcac
ttgcagtaacgacatcgtctcctttcttatggctaaaagtccaaggtactttaccttttaatacgtatgattctagggcagaatatatcataccaccg
tagttccttaagaagggaaatgcgtgaaatgatggccgaacgtttcgtacagaatataactctttaaaaacaaaagaatttttttaaattttcttcat
aagtattaatcgtaagtggttttccagtatcaccagtattaaaagcttcaacaattgcatcagcagcgaccatgccggatttcatagctgtgtgagt
accttttattttgggaacattaagaaatcctgcactacagccgatcaaggcaccacctgggaatactaacttcggaatggattggtaacctccctc
attcaaactacgagcggcgtactctaaacgagttcctcctttaagaactttagcgtaaaagggattttgcttcattctttgaaattcagcaactgga
gacacatatggattaggataatctaagccaacgaccaatcctacagacacatagttatctccgaattggtacatgaaaccaccgccataggtatc
acttcgtaatggccaaccaagagtgtgggcagtctcgccatacctaaagttttcatcagggacacgccaaacttcctttatacctaagccgtatgtt
tgtggctcacaatctttttgaaggttaaagcgttgaatcacagtttttgataaagaaccgcgggcaccttcagcaaatagcgtaatgggagcatgg
aaggccattcctctctcaaaattatcctttttgtaatcccttgctatcaacaccaaagtcgttcgtagctacgcctcgcacggatccatcttgattgtat
aggacctcagaagcagcaaatgagggatacagttcgacatcgagttcttcagctttagcagctaaccacttggttaattctgccaagctcataatg
taatttccatggtttttcataattggaggaacaggcatagggagttgcataccaggaactaaaaacctcatgatatctgatgaaacaggcgtgca
atggcgaggtggttcgtttttccaatctggaaggagctcatcaagggcagtcggctggataactgcgcctgaaacgctgtgattaccaggttcggc
agccttttccaacacaattacccggatttctctaccagacttggcagcttgttgtttaatgcgaattgccgcacctaaaccagctggtccggcaccg
acgatgcaaacatccacctcctcgacttctcgttctaaagactgtatacgcttcaagttatctaacagttcattaccttgaacaattcgactatgaca
gaagtttcgggcttggattcttttcatgggcggtacatattgcgttttaaccttgggaagagcaacgagaagcgaacatccttttaaactctgtttac
ggaaagcatactttggagtttttaggggcccagctaacgttcgaaccaggttgctcatgttgtatcacgcctttttcctagaccaaatatcttcaagg
gatgtcaagaatccactaacgctataaagatggagcaataatgcttctgaaaatataaatagtgttacttcaaagtatctaagcttataaagtcaa
agagttgaattaccaagaaatttcgagtacccttcttgataaatcaacaattacaaagttagaagaggcttatacgcagcaaaacaatcgattttt
cctttttgaatatttggcttatacagctaggaataggcaaaagttaaagtaccttcatgtaaataaattgatacgacacgcttaaagatatcgcatc
ttttattacctaaaccaattaaagtctatacattatatcaaataaacgtggataattttattaaggatattctattttctaaaggaaaatagaaaact
cgtttgtaagatgacatattgtattaaggaatgaaaaactcaatgcgtaaacatttaaatgaatgggatactgtacttgctctattgaattgtatta
acgctttctcacactgtaatttctttttctgatttctcagaaggaagaagcgatggtgtttcatttgcttcctataacgagcatgggtattgtttctcttt
taagtgctaccaaaccaatttttttatttttttaaatctagctactcttactctttgtgttcgactaaaaggaaaatacgctcgcctgagatgctatcgttt
cctccgttcttaccttacctgtaatttacatgttaactgattatttaccatagacacattatattcaaagttttccatctaaaaggcaacctaaattat
aactgtattcttctcctaataaatggttaattaaatctctttatatgatggtacataggataatcgccaggatgagccaatttcaagaatcgtaataa
aagagaatgaaatgagacaaaattacaatcatatatcaaaatgcttcataacaacatcacctcttaccttgagatgataccaaaccgcgcaacat
aattatacaaagacgtagccgccttacgtcttccttattagacagtctgcacaaaattagatgggaataatcctctcttaggattcataatattgat
aacttcaccatcccaccatccatcttcctgcgtacggagtaccatcaaggtatctcctttttggaaactaatttcctctggaatttgggctttatagtc
atacagagctataacataacctaaaataggttctccttcggctgtgaaagaagctctaggttcagctttcgaatagccgcttcttattgaggaagc
aggcgatcgggagcgacctgtcggcgtacgctccaacaattgccttgatgaccttcctcctaaagaactatatctcatatccattcccgaggacgg
ccttgagtgtgaaaaaattcgcgctcgaccgatttacaggattcggggaagtagagcgctgcatattcataatcgaggaagattttcggtgatgtcc
taagggattcggagacacagaccgttgcagcgaaagctgagatggaggtctattagaaagcgaagacgcaggtcgggaagtttgtccagacag

cgattgtccacggcttctactgacagggtttacttcctgttgtctcatacttaaagatgaagaagagcgttctggtacaccacttcgtatagcagag
cttcttctagatagcatgggagatggtgatctacttaaattgcttctgctatggcgaagcgagtccttcagaggaacatcaccgcgcttttcaccaa
aaacacgctttgtttgatttgcaaaactgttgctcatacgttgcatctgagcagaagtatgagcagcaggaggggctcccaaggagcttcttttag
ctaagccatccacttcagtagaagattgattgtgcgaaccagaaccaagcatacttaaacgattactagatgaagcaggtttagcagttgcagtg
ttgtcaccagcagtagaagtagactgacgtcttcttacagacatgctcagatgagccattgcatccgcgattggatcgctacttttgtcctcagtac
ccttgggttgttcaggaacatcgccaaccttcaacatgtttggtccaacgtttaacactacgtttgccctggggtcaatctcatccatctcatccgaa
tgacttgagccaattgctggagaaactggctccttcaatgagtctttaattggagaattactacggcttcgcatactagaaaatgtagatccgaca
aaaggagaagtagggcgacccatgcgttccataattgatttccgcttaatagatcccatactgctttgagagccttgtccatgacgggaaaacga
cggtcttgatactctactattaaaagaaggaggagggaagtcttgcccaaggattgcatcatccaggtcatttttgaagatctatcttctacaact
ggttcaattggcttagtaggaacaattaaaggcgaagatggttcaccaagttcaaacttgttcgtctctacttcagtggtttgtgttgggacagtag
ttaaagacggtaccgaaggagattccttaaactcggagtttctctgggaatcatttcttagttcaggaacgctaaatgtcatttcgggagataagg
actgttcaaaactcatgggttgcattttaccagaaaaaggagaagtaggaggggaagcaagttcggatgaaaaggagtcggatttttgattcta
ggagatgacatatgcggagatgcagacagcaaagcagaagaaccagcccttgaactagcacgataaaggctgggagctttggaagcaatttga
ttaagatcgacctgtattccattctccttaaagtagttgtgttcttcaggaagagatggtacgtttgtaccggttgactccttttgaataagcaaagt
aatgtcattggctacattagttttctcgagagtaagtcgaatacgttcacaagcttcgtcatctctgacacaggcggtcgaaataatgtttgcatatg
accacatatttgttttcagaaactcaagtcgatactcttcaatatgctgaaaagcatcgcaaatttcagtccatctatcaacccagtcttgggtgact
gtccgtaagagactattagtttcacggtactctttttcagtcctttggactgttaacgcggcttgacgcaccttaagattatgcttgtccaattctcgg
ccagagtatttcgacgctaactttaaatatgagttaagcttattagcgctgtactcataggcatcatacttttcagaaagatcaatttccaaagctgt
cttttgttgataaagttcttcaatggcatctgtggctagggatttctgttgttgagtttgagaaatataggtatttactttttcgagaagctcttttcaa
tttgaccagaaacttcattatgagcttgagacatattacttgtctctgtttttaaagattccatattctgatatgtagtgccatattctgccagctttgg
agtttccgagactagttcgtttaattttaaagcatattctctctcaatagcagccctatgcaggttagtaagtattaccaacacacattaaaatttgg
aaaatgttgtaaactgttcccgcttaaaaaaccaatgcagctcagatacgcagccgaaatcactaaacaatccatacttgattacagtaatgccc
gctggatgtcaaaccggaataaatcaacaatgaagtatcaccataccttctggaatagaacctgttaatcacctctaatgtggaaagagaatttct
tgtccgagacataagagcatccatgcccgcatcttcatatccctgattataccagtcgttagagctgtccttccggtgaatgatttttaaaacgtacc
caaaaattctcacgaaacttcgttggagatttcgttatgtaaggagcgacttcattctcatttacagcaatatctgccattctattcaacaaaatggc
ccaatgtaccaatttcgtgtagttggaaagtatggaggtggataacagagaataaacaaatttgtttacgctacctctgcatagcaaccaaacac
agcaaccgttaccttacttgatttattagtaaacaaaggagaatccatgataacgttagaacgcgttacatattttaacgacactcacagtatttca
gcgcattgagatcccgtcgttaactactagctttctcagcttgcggaccgactttaatatctacatccttcttcaaaacaccactacactcgcacatc
gaaaattttaaagatatttcgccttggttttgattttgtatatattcagaggtttacctgttgaaacaagagaatatagagttcagaagccttaggga
aaggcatgaaagattattattgaagaacctttatacaataaaagcagaaatgtcggatatgagagataaatcaagttcaatgtcggattcaaatc
attcattaccattagaaaacaagactacagctcctaacgactccaaaattgagagtattgtgaaagctcaaattctcttcttgttgtccactttacg
ggaagaccagtatgacactaaacttgagcagattaggcagcttattaataagaatgcgccaggcgtatatcatcatttcttgcgtcgtttagtgca
aggtaattcctatcgtatctttggtactgggaaatcatctgatggtttagctacatacaaattgttgctagacgagcttaagtcattaacaaaatcat
gggctttagcgaggcggttcagcgatgctatctcggggtctgattccgaggtctttgaagatttcgatattgagacgtttctaaaccactttcaattt
tctcctttagaacgctcaaatatattaattggtttgactacatctgtaaaaccatctctttctaagaaggcttcggctttattaaaggaagagtttcag
cccttattaaaccagctcgctaatccaaatcaacaaattaacatggaaaaagctgatttggatatgttgagagatggtttatttacatcagattatg
accgcataccсctgattcaagttcctaaattcgattcggtggttgagaagcgtttagctaattgtcgccctcttactgctctgtttggcaatttaaagc
attcgaaggcaactttaaaaaccatgaaagaagccatcacaaagaaatacсctcccgaatccgtttccgaatcagatgtagctttccttattaatt
ctttagttgctattcatgactatggtgcctgggacacagtcttgattggaaaggcagttgtctcggccttcgaacatttagattgggaatcaatgctt
ccgatgtttgataatcctaagttcatggttactggaagccctagtctcattctcttcatgacaatttttctaatgcttttaagctaaggcaacgaact
ttcactttagattttctttggaatctttggaagaacсccttatctcagctatctattatttcgcatattattttgacgcctacttcgctatttgatatcaag
gagcataatataactcccattgtttctgttaatgatttggatgactatagtgaagaacttattaactatgctaaaggttatgaaaaaagtaaccttа
attgcttggaactcgttcgtgtgcttcttcgattattaactgaagttgttacttacgaaacttccttattcctcaattttctggatgaaaaaatttctgcc
gaacttttgcttttgggtactcttcagttacctatgccatggaaccaagttcaagagtcattagccttccaatggtgttcagatttttcactaacttttt
cccaacataagtttgttttccatagaattgagcacatgaacccgcaattcctctttgcatttttgcgaagcctatggcttaaagacagcatgtcgatc
aatcaaattgtcaattttatcgttgaaaataattttgtcgagcatgttaataatgtccaacctaatcgctttgctctagaaattgctgctcttgcttccg
ctcgtaaggcactaaattttcaaacttggcttgatggaaggttatctgagtttcaagatgctgaaaacctgaatatttttctggttgaggttttagac
tttttgatggtccatgctgccttacagaaaacaccaactgaaaacgaagatgaacttataccattatcgattgacgtagtcaatattcttttaacta

cgctaatggataacgttagcatttcggaagaggtcagcgagcacattaaggatgtacaaatactttgccttcaagtttatcctcgcctttttgctctt
ggtcatgatcgtgatgatcttattatgaatgctaatggtacgaactctttctcccccgatgtggaagctgaagtagaatctttttccaagctttatat
gagcgcaagctatccattggtaaaattgtacttactctgcaaactttcaaaaaatctgataatcctcgagatatggatctctttgcttgtttgcaaca
cagtctgtttgacgaatatagattcttccctgactatccctttggaggctttggctttgaccgccgttttgtttggcagtttgattcagttcgatcttcttt
cttttgtgccgctaggtgttgcgttgcgttatgtgtaccaagctcttttaatgcctacggactctaaaatgtttaggtttggtatgcaagctttagtaca
atttcaagataagcttccaaagtatataaactactgtaatttgattttgggaatcccttctttacaattaactcgacctgacatttataactccattaa
agagatgattgcttccgcagaaacttcaacgatggaggcagaaaaggcgcttatgataccggagcccactgctcctgctagtccaaagttacctt
cctattcattcgtttccatcagagtaccagccttatcttttcctagtgtcagcggagaaattcaggaaacagtttgcgataacattcttttcgctttga
ataatttatctcaacttaattttggcgaaaagttgcagattgtcaaagaaagcctcaaaccgccttatttagcatggttttctcactatatagtcacc
caacgtgttagtcgggaagccaatttccttgctttatatagtaggtttttggaagagttaaaaaatagcgatttatataagatcgtatttagagaca
cgttgcaagcggctttggacatcttgaacttagatactgagacattgccttccattgaaaagtcaagtttgaaaaaccttggatcgtggttaggaa
gtataactttggctaggaataagtccatcactacactaaatgtctcatttaaagacctattgctagaaggtattgatagtgagagaattgaccggg
tgcttccttttgtctgtaaagttctggaaaaggttaccttgtcagacgtatttaaaccaccaaacccgtggttgatggggattttgaggttgttggtcg
aattatatcatttcgcagatttccgtttgaatatgaaatttgaaattgagttattattacagagtataaatgtcaaaatggatgacattgagccgtcc
gaattatataggaaccatttaatccataaagccgatttagagaatgatctcccagaggatgttgtgagccaggacttgcgaaacgacgcaaatat
tataatgcaatatattgcgacagcatcgcctcaaataattgtttctgatgccgttgctcaaatatttggaaaatctaaagccgcaattaagaacagt
atgcagttaattatacagcaatcggttttggaaatcatatctgctgttgttaggaggtctgtaggtatatcagccataactactaaaagcttaatcc
aaaaagactttgcagccgaatcaaatccctcaagtttattattggctgctagacagatggctaaaacattagctggaaacttggctatggttacat
gccgggaaccgttacaaatgctgatgattaataatttacgtacaatgattttgcaggatgtggaaaccgtacatgcagctgttgctcaagccatag
atgagcttgtgcttcagaatttgtttattgccagttctatcgttgaaactgtggcatccgaaaccgcgattgctgagattgatgcagaaattgaacct
atgatagttgaacgtgttcgccatagaaaatccacccccaacgttccgttcgttgatcctgctggagcagcaaatctgcatcttaatctgccaggaa
tattgaagctttcttccgagttagccccccaacaatttcaactatatgaaaatttcgatcgactaagtctgtttaccattatgagtaactctggttatg
gcaatatgaacggggtacgaccagaagttgctgagggttcagaaactccaaatacaagcatgaatgttcctatggaaaacgaagtaaatcagg
ctgctttgaattatgcccgaaatcttttgttggtaatagcacaaattttacaattgtctagtcaagtaccatatactagtatgcaagaagtacccgct
gagcatgaatttcatgatttagtaaatcaattcctaacgggggtcgcttcaattaaccatcctattgctgaccacgtctttcttttgtgtgcacagga
atgttgtcgaattcttttaactgaaagcgcttctccatttgtattagaagtttttgcgcaatattagaatacatatgccaagcttctaaaaagactgc
gattagtgtttcattatattggaacttttctagtgatattgagaagttgaatcttcctgttattcttagtctaattcgttttggaattttgacgactgctg
aggtagactaccatgttgctcgaggtatagatgctgaacagggtaaaggtcctgttactacattcgcgattgggcttcttaagactgctgttagtgg
agaaagtcctatggcattgccgggtaactttgtgaattccattgatagtctttataaacacagttctatgtttactgatgaagttaaggaagcttatg
atcaattatttgagtcaatgggtaaaattgcctctccagccaaggaaatattggcggtgcttgacaataaacagcaactaaatgaccaaatgata
attatttttgtttcttgggttcatcttttgcgtaattctgcaactgatgatgttctgaaggccgctttcgtttatcaacttcataagcaaggtatcctagg
tgacttggagttaacgctacagtttttccgctgcaatctagaagctgtgctagttgctttcctagaggctgcatcagcaaatgctccggattactgta
atgttgatgcttttgcatcactcatagtcaatgttgtcaaatactcggaagctaaaggagaagagaatacgagctcagttagaagtgtatttttccg
aaaaattatggctctcataactggtatttttgccgaattacataatgctatggacgagttcgttcatcaaaagaccttcttcaggttgttttcgagcat
tttatctgaactggatgaaagcaaagaatttgaatcgtcttttacggatatattcatcatatttctccgtagtcttcttgctattcaaccgaattatttc
cctgcctttacatttgcgtggttaagtttgatatcgcatgcatatcttttgcccaagatacttatggccaataatgagcatatcaacactctttatagt
gagattttagctgcatttttaaagtttctgggtttaaaggatggatatgataaggcgcagtttaaattgctgtacaatggatttttgcgtatcgtcctt
gtcttgctccatgattttccgggctttttagcaactcattgctatcatttaattccccatattcctttggaatgtgtgcagcttcgaaatatgattctgag
tgcttttcctaacgacatgcatttaccagatcccttttgcacaaggcttgaaggtaggaagattaccggaagtggcttgtgcacctttgatatctgatt
ccatcctttcttctttggatgatttcggttctcaattgagtttagatgcttgtttggcttccaataaaccagctgatgtagccaaattattccttgaggc
tttctcgaaccttgaatctgctgaacagcttaatgaatgggctaattggtttgttttatggcttattcgatatgccacacgggatttgcaatccaaac
aggcacctcaattccagccgaaatctcctgagtgcgtcattatcagtacatttaataagcattgcaactccaaatgccgttactttttattaacagct
attgcaaatcagctacgttatccaagtagttatacatattatgctagctgtttcttttttatacctattcaagagttcctcaaatatcacccaagaactg
ctgatcaaggagcagatgacgatggtactattagagagaattatctgcaatcgtcctcatccatggggacttttaattacttttactgaacttttgaa
gaatgaagattataatttttggaaacacccatatatccgacgaaacgacgaaattcgaagattgtttgatagtcttcatgaacatattatggcacc
ttcagcgaacataatgaacgcaagagatgaaggaacagagattatgggtcaggtataagaaaaaaaaaaaaccccctaagatgttattacaatt
tagtgatttttaatacggtgaatatattaaaaaggaaaatatctattctctactacaagagataacgcataagaaggtggtattttgaaaaagaaa
tgaaaagaaaaaccgctcgttgatccaaatgacggattatttattgtaagatacgaaagcatatagataatgataattgttgaaatgcattaccca

gtttttgagtatgatagtggtatatatgagtaaacaagaaaaaaaaaagatgaaaagcaaaagatcggatgaaatgattcaaaagttcataaact
tataaatggatagaaaaaataagttttattctatacagaaagctaaactctagatggttttgtgtaaaagaatgaagaggatggatgcttaaaac
ttgcctaaaaggaagctctttatcattcgcttgacagtaaagtagatggtcttataaagagtagggaagtcaagagttgaaaagagcgtttcaatt
aatccaaaagggagcatgctagggatccagccttgagccaactcaagggcaaaacaaggttgaccagaaggcatactgtattgagcgctttggc
cccatttatggacgtgttgatcaccaggaaggtactcacgagcttgcaagtcgttagcagaagcagcaagctggcgaccaaccagaatggtgaa
gtaatcatcggccatgtgtaagccggtgtgaccttccgttccaatgggagtaccaaagaacatgaggtattcggtgaaagaagcgtgaagaata
aacacgttacccatagctccaccagcattgttttggacccattcgtcgtcgttcatggggtggatcaaatcaccatactcagcaatcagccgcttat
ttaaatcgtacaataatccacgggtatcatttccataaagttcaatagattgacgagacaactcttgtagtttggcaggatcaaatctgtaaaagg
aagatatgtttacctgaatatagtatatgacggctgcaatagctggcagtaaaagcccgaataattttaagcacctcatgatgcaatgaaaggaa
aaacgaacgataaaaaaaaaagtggttttctgtaaatcttttctgttttccaacgcgaagcttatagcagcagtttatttgttacttccggttgctacct
gctgatcgtacggtttttttaatttgcttttacttgataaaggggtaaaattaataatattgcacaaccattatataaaaaataattataaggaatat
atgattgtacagaatatcaagcttgagtaagcatataagaaaatctcactaaggaagatgggaatggacggttcaacagaacgggagagacag
gcatacctttggagtagactaaagtaggacagtataagcatacaatacattgttcttccagcgaactcttttcaagaagattctctgtaatcgaat
ccataaactcaaatacgaaatggcttctatgtgtgtcggcagcatagaaactattacgttacggttgaatttactctctttataatcgccatcattaa
aggatgagcaaacatgtttgttattgaaatcgaaaccataaataaactattaagataaactatgaattagatcttcagtaatttccttctgaatccg
ttcggattctgtagtaagactcgactcgagtgtcagcttggtagctgctagcttgcgtatattttcctgcagttgttagcatccttatatcaaaaagta
aacactcacctcaatgctattttccgtaatcagacgataaacgtgaacaggccgagtttgtccaactcgatgtgcacgatcttctgcctgcaaatca
tcaaaaggattgaaagaacaatcatacatgacgacaacattggcacaagtcagattgattccaaaccctcctgattttgttgaaagcagaaaaa
ctttatactcaggattttcgtgaaactcgtctagtagactttgccttgtatctactggagtagaaccatctagtcgtaaaaatttaataccgatggttg
tcaggacgagctcaagaatatctaatacttgagtaaactgactaaaaattaaagttcgatcttctgtgttaacttcatgtaacaatgaaactagctt
gtttacttttcccgacttcatccaaggatctccttgcaactggaactgatgcaaagacttgaattgcatacagaggtgatgaagctcaaaatccga
catcacttccatatcttcataaatataactttcgttcgcatctaaatattgatcttcccgtaagattctttttgacatcgtcttcaacgtctccggagag
tagatagaacgaaacaaaagttggtgattgacaacttttcgaagttgcattataatattttcacgactgctagatggatcattccgcagttgaatca
atttttcgtaaagacccttttgactaggttccatagaacaatactcaacgtgctggattttctttggaagatcagacaaaacatcagccttttttgcga
cgaagaatgaagggattcataatcgatttgctctagatattctttgttgagaaaggtaagctctttcaaaatttccatctgaagaaggcttaatttt
ataaattatatccaatccttgcatattactatcaaacatcctaggtaacataaaggcaagtaaagactatatatatatatatatattagttcagtttca
aaacgtataaaactgtgcacatacgattagctccttcaaattattttgcaatggcgttccagtaatcaaaaggcgaaacttggctgggatattcatc
aagtgcttgtatctttctgacatgcgattttttaaatagtgcccttcgtcaaaaacacaaatctatacgttagtacatactttgagtatggatggattc
gtacattaaatttttgtcttcgtaagaatgctctgtcatccctagaacccgaagccatatgatagtttgttaccaaaacatcgtactcgttatccatc
agataatatctttttctaccctctcagtttgatttcccgagtattcttcaactcgtatagaaggacaaaatttagtaaactcccgaatccaattttca
agagtagatgcaggaactacgaccaaatgaggacctttaatgtttttctctttcattgaagctaaaaatgctataacctggcatgtttttccaagcc
ccatttcgtccgctaaaatccctgataaatcagcatgatacattaaacgtaaccagttaacacccatcatttggtagctctttaatttcattccaggt
gccagcaagcccggttgctcttccatgtactccatcggtatgcattcttcgccgctttcacttttaggattgtcattatttttgctttgttgtaagtaatg
ccaccccttttaatgtgtttgctatgaggcgtccgtatttttcgcaacgattaatcaaggaatcaacggcatcgaaaccttgtaaaacttcatatgtag
cattcacaactttctttccaactcgatgggcgtccgattttgaacgcttttgtttttcagtaattttttttcctatgaacgacttcagccttactcagactt
gggtatggcctcttggaaataaaatattcaacaatatcgtgaggacaaccagacacatccagtaattcttgtgaagtacagctgttcaaaaaatc
aagtactcgcttatcatagtcagtttcttcatcactctcattttggacttggtgttcatcaggcttctcctctgaagtgacttctctttcatcctcagtatt
ggctttaccagtaactgatgatgtactaatcccttcttcttcttcttcttcttctttctcttcatcagaaagttgaattacacgtgaagtctttttccgag
atttagctgattcaagaggctttcggctcttgatgtctctttttctcggtatattggttttggaaaacaaaggtttcacagacttatcgtctttttgttct
tgtgaagttttgttcgaaacatgcccacttggctctttaagtagcctttgaaaggtgagtttatgaagaaccatgttttctttccaattaaattttttca
acttatccactatgtcattttcgtctaattcaggaaatcgacgtttaaccatttcaattttggagtttatcatttcagtatttacagtatcatcgcttccg
gaaacagtcttccaatctgaatccttttcaaatccttgcttcgactcaatttgcttggcgttcttttcttgaaggtttctgtttatagttttgggaccgtc
aatatccccagatgtcaccgattccaacgctaaaggattcagtctttctcgaaaatcttttcttacgccaaaatctatattattctgatcattttcact
cccagcgttatgtacaggagactcttctatcggagaactgtttatatagaattggttattctcgcacactggagtaccaggaggagatgaacttgg
gataactatttctttattttctttactcaacatttacgcttctagcatacttaggaagatagtgtgaaagaaaaaaagtataagtttttgtttacaatcg
aatagtttgcgctactgctagcttcataaatttagacatagcgaaaaaccctgatcattaaataaaatagggagatataagaagtaatggcttata
gttttttgttggaaaaaccagaatttagtaaattcatttggaaatatccaaggaattgcatcgcacttacaaatgtaaatgaactttagacaatagc
taatactcatctctttggcttacaaattcactattcactattaaacataatgttattgaatgatagacagttccataacccaaaaccacaaaaaccc

caaaaaaattctaattaattctttgatttctttttagaagacttctcctttgaagattgcttcggtttgtttgcttcttcaacttcattttttctcttttgac
ctttcacactaaccacggtagatcccttacctttagttttattgatttgttgctcagccttcttccaatcttcttcatttccgccaatagcatacttgtcc
aaatccatagcatcaattaaggctttttgtttctcacgaagggatagcatagcttcgtcagcaccttcttgtaattcttcttcaagtgattgctgaag
aggagcaaattcgggaactctgttatttggcttctcaattttctcattggcttccaaatcttgttcgatattcttgctctcaatttcatctaagcaagtg
gtaatcttcttcaataacttaaccaacatagccaataattggttcgaaggtaagttaaattcccgttccaagtcatcaatcgtcttgaattgtaaacc
aagggcaagcaaaacagattgttgaacaggtgaaagcttaacagtctcagggaatttatcactaaaatagagaccagctaccttaggaagcaa
atccataactacatgatagtcaagcaaattgttggcatatgcctccaatcgctttaaatcgaaagattcaaagaattggtgaatttcttcatcagat
agcttggggacttcagacttgatgtatttggtaccattgttacaggcgtctaaaatactcaatgaagtaatagcggtaaagcttctgaattggtaac
caagtaaagtcaagaatctcttatagaagttttgtgcgaaagcacccaaccactcggtatcacgttctgccaaaggtcgaagcataacacatgtg
tgttcaccagtcaaatcgttggcagtctgtctgaggtataatggacaataaccctctctcttccaaaacttttgcaatgttggggtcaaaccatagg
aaacacccatataatgaagatattctggctgaagttctgataatttcaagagtaagggtggcatagttctagcatctcggacattaagcttctcagt
ttgtagtgcgttttcttgaagttcttcatcagaaactcgcttcatattttcaagaggaggacgataatcttcagaaacgctaacgaatttaccttcaa
agtagtcatgcaataactctagagctctagtaccgtatcccatctttgtatgctcgggagaaaccgcaatacggacaatacgagcaccacccaat
gtagcaaaattttcatcttggtattgttgagaaacgagccaaggaattaagtcaccacctgctctttgacctctacttaatccatttaaaatggagtc
cctcgaaatagaaccttctaaagctagttgaataacacagataggatcaggtaatttgggattcttcgggtcaacaggaggcagtaatacaaata
attgatgagcaggagcatcagacattagttgtaaatcatttggagaattcttgtaatgacttgcaacgtaaagagccatcatgcgttgtagaaacg
cttccgaaacaggatgataagaaaacaatgtgtctctactaactcggaataatgaacactcagaaggatggggaaatccatgagctgcagactt
ggaaatataattagtagcgtccaaacagagcaatttatttaaccatccctcaatacgatctccagctgcatatctaataggttcatccaaactgatt
tctttcaaagttcttccgctaatcgaagaaccgcttgcagaactatttgcatttgctgaaatgatgcgagattgctctctaagttgttgcaatagcttc
aaagagagagaacgaccagtgccttcgtagccgttgatggtggaagccataaatactaagtaaggtccaatcaatttacgaaccaaaggcaaa
gggatcgctgctgcttcatcaataactactaattcagcttgacctaaaacattggaatcttctgggcggatatattgaatcgtctggcgatggtcgc
gaaaaatattaacacgcacaatggcgttattgaaagctggatttgttgactggacaatatcataatcggtatgctcatcataatttaacgcatcaa
atcccttgaaaatgaattcaaaaagagtttttaagttttcaggactgggagaagtgataaaaatattagaataaccgtaagcaactgcagctgca
atagcaagacctagcgcagcagatttaccacgaccacggctagcagttagcgaaacagtgcctctcaaacttttctcaacaatagattccacgaa
acttaagacagcatgggattggtcaagggtacgagtaatcccaacaagttcactggctaagttgtcttcccctaaggattcctggagatctttaatc
ttgttttctgtcgtcctatcctcctctacaacgggaggcaaaggtttcacatttttaccgccagatattggaagaacgttaagttcatcatcaatgac
gagacagttttcgcaattacctaaagacaatataaatctctcattgaatctagcggtcacgtcactgtgcgcttcagttctgtaacgagagtgaata
tccatagacatggtataaagctgcttcaacgaattaattttatgaagcaagagaacaacaacaccacctccttcaatggtctcaattgtgcgtgca
aggatattaggagtcaatgcctcaaaatcttgcaaaaccaacattccataagtagtacccaaaatttttttcagattccttgtagtaacaataacgg
atattggtgatactgcagaaaagctcaaaaggatcctccgaattaggatcacggattcccctcttgacttctttcttgattttattctctcgtttcttac
ggtgactttaaaggttagattacaatagggaacatggagtcataaaagccaacatacctagtaaatcccaataaatctttttttatacatccaaagt
acattgggacgagctgcaaccttcgactgagatagtaaccaatgaagatttacaacctgatctcgagctttgtcaccgacaacaacgaaaaaact
tcgttgtttttcctacaattgtcagttaattttctaaactcaattgcatacctgacagccattctttataagtgctggaatacgagaatcgagagctttt
tttggcattcctggtagtttaacgtttctggctgctgccaagttctattttgcctttctgaagataaaaaacgaaagtgtcgttatggttcgttaatag
gaatagtgaaaagactttattcaaaaataaataaaaaaggaatcattaatttaatataaaaaaaataaatcatttagtgataaaaaggtcattat
tgaattccttgatttgggatgagtatctttaaagctaattcattaagattaacgggagccatggtaatgggaactatgagggaaaataactcgattt
aaaagttggtatgcttttcctgtgaagaaacataggcttctaaaaatttgatattcgtcaaaacaaattttaaagcccgagtccaattttcgtcctg
attgaaggcaagtcgaatacacttgcttccaatacgatcgttttctataggataaggtaagatggcagaaggtgtattggcagcacaaaagtcgc
ccagttggtgtaaatagtccaagaaggctaccattccttgatcaaaacgacggttcataaatattttgagttcaccagtagaaaataaatctaacc
ttacaggctttgtctgtccagtgctaggatcctgatcaaaacgaagaacgtatgactgactaccgaagggtttcagctggtataaaggagaatga
aaatctagtctttcagctagtacgtccaagagcaaaaccgtcatcccccatgcggcattaatttcactccagcttacctaaaatacgagttgtcagc
taaaaccatatctcaatttgacaaagcatgatagcttaccttttggctaggcaacctgcccaaccgcaagccattgatggtagcaatagatccttct
ccatttggctcagagtaatgagatatgtaaaaaatgtcgctaaagacattcattttctggagcttttcaaatttttttcgaattatgttcaaattctagt
tttgcgcaatcatactgcttttccaaagcactttctttcatctgaagttgattaaatgcctttttatattcatcttcttctatatcaaccttttcgcaatca
attgttatggcacgaaactgactttgcaattcttgttcctgggattccaaatcaattaattgttgttctttttccttgatcgttccagaaatctaacactt
tgttagtaacagcctgttagttaaggcgcatacctcttgaacatcggcttccaatcgggagatatcttcatcctcataatcttgattagccaacatgt
tactgtaataggaatataaatttttttcagcttttgttgagttcagtgttttcgtcatttcctctaccagtaattcagcacattcttcacacaatggatg
attcacttttgtttttgcaaacagtaagtcaaaaattcgattgtagttctctattttccagctaaacaagtcgttaacttccgtagaaggattccgttc

gtcttcattctcattgtatccatcatgtgacgctggtagaaccacaaacgagtcaagagttggtgttttcaattctgtttgaggcgtttgagaaaaat
catattctccctctttaagcgcttttcgatacaactgtagccgctccacaggataatcctccactgaggaagcagtttcatcatcgctttcactttctt
cattcgtttctgttaaagatggcgtccacaaacccggatttttggaaataatttcaatttcactagttcatcgtcataattatctttcaatttaagca
gcgaatggcatttttggcatataaattgcatgttgtggagtcgaaaagatacgttaactctttcatatcagtactacgtgcacactcataccatatat
gcattaaaggagtaagggatcgaaagtataaacaaacagttgaagaaattgagaatagaataaatacaaagatatggaattgaatagaattga
ctttaaagaagttacatatggcaccttcaccaaactaacagatggtttcgtaaatacttcttctatcacaatagtcattctgtaaaccaagacagga
aacaacagactctcgaactgttcaatgtgccctctttgcagaacaagcttctcattcatcaaatttcaactctaatcctttttagtcaagtattactca
atatagtacgattatcgtattggggtcatagctcagtggtagagtgagggattgcagctccccaggtcactggttcaaatccggttggccccttacc
ttttttataaaaagaaggattgcattaggtagaaattaataaatccatcgtcagtggtaaccgttccgtctactcaaaaacctattttctatggctcc
tttgtctgcatatgctatgtgatcaggtcttcattttagacattctactgacaaaaagtgccgacagaacgaagaacactcaatgtaaaaataaac
aacgaacccttgcaccactcaacttctgcgaattctttgctatgattctgttactttattaaatatgcttaggttcgcaaacaacctgagtgagtatat
aaatcttcgatgaggtttatgccaaacgttgtctacagacaaacaaacgctttctataattccttgaaggattccgtgaatattttcttaccaaaag
attcgattttagtatcgaacatttttttatatcctcatgcatgaggacttctcttagctttatataaattaattattctattctctattctcaataatccgtt
gtggtccaacggctaggattcgtcgctttcaccgacgcggccggggttcgactccccgcaacggaatttttttgagattcactgaaacagttattttt
ctaatttaaacgaatcattacgatttccagcattcttgtgtttctcccaaaatgtcccctagcttcgtggtgtcctccacaagaagctgtgtttaaaat
gactattaaagagatggtttgctaatttgaagagaaaacttatcctcggttaagaggtgaatcctttatacttttataagttccagaactttgtatttt
tcaaggtgatgtgcttttaatggttttggtcgcataaagtacttggttgaaataaaggtctttttttcgtttaagctcgacttactttagacggtgcctc
gtctattatgttacaaaagaataatcctgtttatgcagctactagtataattgccgcccgaatgtcttgtttaacggcatgcttaattcaacaatagc
atttgctttattgtcaaagcataaccttatcgtcagacttattgtctccttgaataatgttcgttgctatggtgttcaaaatcttcatatggatgcaacg
attcaatggtctagcattgtacgaaggagggtatccattttcgatgggtaagcttcgctgacaccaagaacaatgcgatgatcgttcaatcgagttt
ctgagctgtggttaaccaaacattgacaatatctcttaagtccttttggactttttttttgatacaatttgtcatcaatccaattaaaggaattaagatg
atagtcatgtgcacctctttagggatttaggaacaacattgctactagaattaactttaagagtatgccggaattactctgttgatttttttaagacat
cattcatgaattttcgtaatcgtcgatacaagatcgaaggactccaggatttgagacctatctatgtatgtgatattaaaaaaaaaaaaaaaaa
aaaaaaaaaaaaaaaaaaggaattactggaataacagtatatagaattttatcggtttattccgcaaggtattttattcagcctttagaaatggat
tctaaaaaaacctttgctttgttttaaagtatgtcatgatttatatccttctggatgtttcatgaatcattcaggattacaatctttccttccaaatattg
ttgatcggtaattttctcgaaatgcatcctatgcattccagtagcaacactcactgagctgtttctaattgaaacgttatcatcgatttcgccgttttat
attagagaatgtgtgggtttctgtaaaatgcatattggatttcatgctagctcttcaccagctactaactataattctttttctttattctttaatttcttg
attctttttttattatttttttatacatctcgtctttgaagattaaatcatgaattcaacctcgaatgcatgtctacgcaattattgagcagcgtagtgaa
attgtttcgagcatactcccgtatcgttcgtattttctcatttccaacgatttctattgaaggacatttgactatggtatcgtgaaagcatcaaaaaac
agaagcatgaatcctttcaatagatatcaaatagatgcgagacaacgttctgtcaagtatccaatgtaactgcttattttaaaacacttaactaag
ctttgaagaaaatgactatcatgatcgttctaattaactggaattttacatttccttcttcacgacgttacgtattacttgctccaaagccggctactc
tgaactgattactgcttgtattatatttttaattttatgctaattatccttaaaactttctcctagagaaaaaaagacagttatcttataaacgatatat
ttcagtataaagcgatatattccattaatcaagctgtaaagtgcaaaagctcatagtactatcgcctcgcttcttcaacctaaaaagtacttaaaac
tttttcactcttataactgatttcttacaatcttttcctttttattcaacgtttcatttttttgctatatttctttttttataaaagtattgcttttcatgtcttctg
cacgtgcaataaccaactttctgacaaaatataagggaagacttcatacaccggattttggtgcctattgttttaacaaagaaaaacctgtgtcttt
cctccatgatatacctttgtcttcgcaagaaggaacattgaatctggtatgcgtttttggacagcttgaatgaatgaattgaatagctaatccgtga
gtaaacaggttgtggaaattcctcgctggacgcaagcgaaatgcgagatttcaaactcagtacccttaaatccgattatgcatgatataaaaaat
aaaaaaattcgatacgttcaaaactgttttccatatcacggttacatatggaattatggcgcgtttcctcagacattcgaggatccagtcaagttgg
atgcacgaaccaatttaaaaggggatggtgacccaatagacgtgtgtgaaattggaggttctattggatacattggccaaatcaagcaagtcaa
ggttttgggtgcattagctcttttagaccaaaacgaaactgactggaagatcctaaccatcgatatcaacgacccgatggctaattctcttaatggt
aagtattttcataaaggaatttttttgacccagctttagacatcgacgacgtaaaaaagatcatgcctcgtttgttaacctgcactcgtgattggttt
gcagtgtataaaattcctgatggaaaatcaaaaaaatacatttggtctttcaggagagtttcttccaaaaagtgttgctaaggaaatcattaacgaa
tgccatacaagctggaaaatgagttctcaaagagctgatcatattcattcattcctcacgaattcagaaaaggctacccaaattacccaacattta
gaagaaaggaaggaaagctcattcccaggaacttcttcttttccctctattgggtattattatcaactacgtgaataggcctttttgatactcaccta
atttcctcctttattatccgaaagaattcaatttctttttttttttccttttaattttcttttcctgtgcatatccttggatcctccataaatactttttttgtttt
tttgttttttttttgtttttgttttcggagtacattaccatacttgttttgatgcctgcgacaatataaggtgatctgatccatttatactgcgctgttcata
aaaaaaggcattctgctatcacgaaaagatagaactggatttacaaaaggagcagcataattatcctcaagttgggtgtttacaggttcatgtgct
aacacatccatgttggaagaactagctgaataatttttcgacttataatgctttctgctatagtcttcattacagagagggcaagatgctttcgaagt

agtcatccaagtatctatacattgagcatgaaaaagatgcccacaaggaaggacacgtaaataatcagtataatcataatttgcatagcagattg
tacaaaagtcatcttcttcttcctggtttgcatcttccgcgttcatagtgatgttcgcattgtcttctctcgaacaacttccctttggttgaccataatta
tccatttcatcggaaacttttgattcaaaggaggatttttccgaaaattttgttgtcgatgaattcattgacattcctaagagaaaggggtccttgtc
gaaagtatgtgaaagtttatcaattggaagattatcactctgcacattttgtttattttttttctgctcttcttttataaagccagtctctgtaggttacta
ctggagataaatagcctagctcattttgggataatgtaggaggctgttgcctatggcgaggaatgttataacctaacatgttgtgaagtctttctgtt
gggaatgaagtaggaggtcttgaagtgttcatcctgttcgtttgaattaaaggaagaacatcgtcatcttcttcatcaaaatcgagaaaatggaag
catgttaagcagtagcgacaaatatatattaatattaggttaaagaaaacaactgcaaatatgattgcaaatgcgaatggcaagacatccttgttt
ccctccgaagaagtatcttcatccactttcatggaattatgtccctccattgcagtcccgtgtatctactgcaaaagtagcaggctaattatctgtaa
tatttttgatggaatcctcagattcaaaatctcgtaagcatgtatgataaggtactcattgaatttgattcccattcaaacctttctacctagactgta
aacaaacaagcgatcacaagatacactataaagtaaggaacaaagttatttgactaaggaacaatatataaacataaataaaatttgaaaaat
aaaaaagaaggaaagtactttttattcaaacagctcaatctataatcaaataattatattatagcatcttattttgcagtatacgcattttttttaaac
caaaaaataaagtaattaattaaacaaacaatcaaagcttagaaagtttctaaatctttttatagcagaagcggatataggcttataattagcaa
acagacaatgtacgtctaaatcaacatttcccaaaaccagcaataaataggtattattttacttaagcagccgtcttcaatcgcttgttcgatgtag
catctccttcatccacaagcttaacagcagagcgataagtatctcttcttgtttctgcatggagtataacagagcggcagaatccgccaatagcat
gcctagcttgatcttgcttcatatcccaaaaagtagctaagtttataacatcaaaaccagcacgacattctaatgggctcctggtttcactagatat
caatataccgcgtcctcttgtagcacgaaccaaactagaagcgttcgtaaccaaattccttctgctagaagcttcccgtaaacctgaagcgtatga
aatttcaattccaattcctcgagaaaccgcaagtcccataatagtatgctttagataaaagggtagtctctgtgaaaagtcaaaggacaagatatc
aaattcgaggtcactggcagtttgctgcagcaacctttctcctacaggccttaaagccaaaatatcgaattcttttgttgcattatgctatgagagtc
agctatacgaatatttgaagcgcataccaaaactttgttctgaggtgttgattctatcgttatggtaattcgtgaatatatttttatcttttgccctgga
tatagtgttgcagggatggggtttttctgttttgttaatatgggcccaaactagaaatgtataaacgttctatatacaacgtacaataatattttgta
actttccatcgtactggtaatttaatgcaaccgctgcataacccactgttcaagttagtatgaaaataattcttgcctattcgtacatttctcaagcat
ttttacagtgttcactattgtcatgtcctttacacctaacgaaggccatattacatttaaatctacaaacatttttgcctgctagtataggtaatgaag
gacgctaaacggtccaagttccaatggtgggtgtggatggaaacccatgtatgtataagacttctatcgtctgaagagccgaaagctcaataggc
ttgaatactagcagatttagcaatctattcttttttttttttttttttttttttttgtgatagcaaattaatataagatacgctcacttgaatagcatagtct
gaacgtttatgcagtactcaccattcagatttttcctctggttaagttaaagtagaataggccatattttgcatccgttttacaaatgaagttctaaat
aatatgatatattcatgtaaaatacagttcttaatcagcagcgaacttaaagattatactacctgtttgcaacacagaaattccaagttagagaac
tttcaaaactaaatttataatacaactaggcaatcaaatggctataaacagtacaatagagtttatatcttaaagctgtagataatagcatgaata
tcaacatgcaaaagttcaaggtcctatcaaaaaaacataataatgatttaacgtttttattggtatcaagcttcagacaccttcaagaaataatca
tctaaagctttaatttaattttcaaggtcggtcctttaggcttattattattatcattaacaacgggacttggtgcaggtatctgaggtggtgactttc
tttgtttttcccaaactgttttaatggcgcttttcttataacaattttaccctgataagatgttagtttaaagacattcacaaagactatgacactcact
tttccaaggtggatggcacgcaagcgtggtataagacttggtatcttcaatgtaacaatcaatgaagatttagccttatataatactcgacatatca
tcaataagtttcttttagtcattaaatctattttaggatcactataagacattgttagttaccttttcaacaaagaaataccataaaaacttacttaat
agctttccacatttcctcagataatactctgtttttccaacgtcgtttcgcagtgcctcaatggctccagatatacttgcacgagatgtaatatctat
acgtttttcgatagccttggtaatatcctcttcaacaatcaaatcatctgatgcaaattctgttttggctccaccccgcgctaagcaaccaaatccttt
ttggatagaatacattaaagaacgacgcacagcaggcgaaagatcgtttataagtatgtaaaacatgtatttaattaaagcatcgttttttaaagc
tccggattgaagcattaaatcataagcgactgagcggaccgaattgtaattaccttcctgcgtatatggaaatagctccctcgggccaaaatctaa
atgaaaggttctggctaaacgattcttagcttccagggctttgattgtgataatgtttttatatgaaggcatgcaggcatcaatacgcatgtgtcgat
caatttcgttaattgcctggtgaacatattccatgtgctcaggatcactaaatgcatactgaactgtttccccttcttggaataagagcatcaactag
agaatcaattaacagacagataaaatgagagtcagaaaactcattattagcattgtcattaaaacaaagtaaatccaaaagaagcttcttcaca
gaagatgtagtgacacccctttcgatcacggacattagcaaaagcctcaaccatagcgcacttaacgtaatactcagaaatattagagaaatcatt
gctttttgggatggttgaattttcgaaacaaaaactttccaaaaatgccatgcgcaaatggtaatacccaatccaatccagttcaggatttgcaca
gttagccagagcacgagcggcttcttggcgcactccatagtaataacgggtatctagaagagttctaagtaaaaccgtggatacctgacgggaa
attgaaaagctgttacccgtaaaatgtctgattgtctctaattgcgcaacaacgtcacgatcttgttgaatttgggaaacaaacatatgctcgggtt
gacgaactcgtaaatcacatatccactcgaaatcagcgtcaacacggatccactcaaaagcttcagtagccatcgtatcttcatcttccttggtata
atcataaagcctccatttttcaatatcttcatcactttgaagaacgtctcccaagcttctaataacatagtcagaatcatcaccgccgtgatcaccgt
tatctttgcgaacgtttttagtcgatctgttacgactgattcttttgtacttggtattatactgaatatcaagtttcgtaaacgaatctttcaattcaac
aacatgttcgtaaggagtaccgtcagcttcgtgaattcgaatagtcattggtccactaaatactggaagaccggaagcaactggttcattgtttaa
gtgccgtatagcgtcagaaacaaagtttccttctgatgcgggtatagtcggagtttctttagtttgaacctgatcaatacccatttcaattatcatctt

ttttctgttaaatctttgaacaacgcggaatataggatatccataaccatatatccactgttgagcaaaaatatctaaacgcatatgactagccttc
tcacacgttttgagaaaatgagaagtactcaggcatccgttcaacatatcaccactcataacttgaagcaatagcttaggtattacccttgacattc
ccaaagaaccaccagtcttcgttagacgacgctcaagtatgtgaattacaagcggagcttttaacgctataaatttaaagtgttcggatcaattg
gaagaaccaaattaggattagctataggaggtcgaccaatatctaattctaatagctttaaaacatctttttttaatcgaaaacgatgatcattatt
ccccattaatttttcaaaaataaaccagttataaaataggagagaccatatataagccataaatcgttccaagctttaggaattaagtatacccc
tatccattgactagccagagcccaggtaagtgtcttcgtagactcataaatatcatccaaattagctttaggaaataaaagggaattacttgcgat
gacaagagaaggagttgaaataataggataattcgtttcgtcaacaaagcaaagtttgaacgcattaaacgggtaagagccatattccctcacg
aaaaagtccatagctttatacataaatacgcaactattttcaacttcttcagcatatttgggaaggtaatagcctgcaatttcgatagcgcttgagc
ccatcgcgtcatcatcttctggttctcgaaagtcagtcaagtttatatgtttaaatggaccagctgcaaaagcaatataatttggagcgaccggcgt
agaaacggtggaataaacagtttttttatgggtggctttcggatgcgtaacctgatccagtaagtcaccacagccaataacctccatgtcatggtc
ctcagaaaaatcgggttctgctacagaagtacgtgggtcgtgatcgagctcgctttgcatatcatcatcttcatgaggctcttgagaagaagactt
gtccgtaaatccatttagttggctattggaagacttagagagagttccaagggttttagggatagtgatttcaaactcccaggtggaacgttcccat
ataccatcgacacaagggagccaagacgatgtgctatacggaacaatagaattgttagtgtaagcatgaggatagcgatgatcaatggggtcaa
cgccaacaaacgttatgcccgaagtaggctggtgaacagaaaactcgattgacaccaataaagttgcaggatcctcacccggcgattttggtaa
aaaaattgataagccacctaaatcattcacagtagattcatactgagaattcagctgatggtgatggcttacgttactatttggttcctgtatgcgc
agccgccgtaaagcatcatcataaacaaaattagctggttcattatttatatagcatgaatgaacatctgtttcagtaattagcataagaattggta
ggaatcaaagaaatgaaagcctctgtactgtattcgttctttcactggaaacgtcgctacttaccagcttgatagcaatccaggacgaccttttttta
aatcaggattaatgggcatgatatttatatccgttctcccatttatggtttgagaagcaaagtcaatgtcaattgcaactttctgatgacttgtacaa
acattagcataatcctacacaatttatacatatattataaacccccgcgcggggacatccaccgattgtgacatcttacgctctataaatctaacat
atggtgaattttccaagcattcagtacatcttgcggatggtagctttctcgtttctgacaacaagcgtgaataacgaggtgcagtgagttaaaaggt
agctatgcttgttttggagtgagagttcggattgagaaagagagcaaagaggttttatttctagttcggtcagttagcaattgatgttagatagcta
cttaattactcatgaagttgaatctatcatgaaaaactatgtagaaaaattgattctaaatttttaatcaaaaagaaaactactactatacaatatc
gggagcttcataaaccgcaatcggtatagcacttcattcgtttaatgtatcacgattggaataaccgaaagaaagctcaagaaatattcattaaac
tcatacgatggttcctatatcatagctgggcgagagaagtaaccggttctgtatcatcttatacttttaggtttactcatccttttcctggagcagctc
cattaccacactaattgaggtacctgcgaaatataagataaacaacatcgctcctagctgacgatccattctgtaatggtttattggaacataaca
taagagtaaaaccaggcaagctagcaagaaatatgctgttattgatagagaatgaggaatttcaatccggctcaccgccccatggtttgagatgc
tgttatacaaacaagcaaagccaagacctatcaggatattcaaagttggtcccccaaaaactccgccgatggccatttcaggaaatccggaacg
agaaaccattacatcagcaataaaatcaccaaggctatttccggcagcaaaaacggtcaaccctaggatagattcattcaaatccagtataatac
ccagtgtcttcaagatgcccacaatttcattggctattgttgagatccaaagaatgcctaggataaaaccggaaaagctaacccatggtaaaaaa
tgaggggccttatctggattagtgaacttatacagtatcacaatggccaaaaaagatgcgacaccactacacatcaacgcataataaaattgcct
gccgttgaatgttaaaattgaaaaagctagaggaacgaagatgcactgaagaactcgtagaggtctgcaccagcttgtcgttgtttgggtatatc
cttccgttgggcctaggggatccactgggtgccgagaacactggtacacgggtaaagtcaacgtgaaagccaaaactgaagggacaaccacaa
tatctaatgcctgttcgtaccaagcctttttttgaaaaattgcgcaaagtcggaaaaagcaattgaagaagggttggacgagattctagtatatctg
tctcgtcaaagtagtttaataaacgactattttccgataaaaaagactgaacacttaaattgttgttacaaacgtgatacggtctacactcagtacc
acttaaaggagtattcattccagaaggagtatagctacttacggcttgacgagcatgcaaatgatgcaaggataaatcgccatcagctacggcat
ttgcatccatagctcgagtaggtggatgattctctaatgcgtaacgcaattccagagcaccaataaaagatggacgaatagtaggtccaggcgtg
ggagggcatacaagttccattgaagctgtgggttttgacaaaaaggaaacatcatcgtcactcgacaatgacgactttcggaagttggtatcgtct
acgagaaaaggggatggactaaagcttggatgtgatgtttgtttagtaggggttttcaaatgtgaagcgacagtaggcaaatcatcatttaacga
attaaaggtagggcaacgactaataacaggggaataggtaggtaatgccaaatgaggagcgtctgtacctgtgtaatgctgacttgaaggattg
ctagaatagaacacatataacacataaaataaatagtagcttatcatgacgaagctttgccataagctcagggtaccatcacgcatcaagaaga
aaatcaataaaataatagtgcccgtaaagaagctaacatctctaatgaaatggtgtttacatatctgaaaagggctaatcaaacaaatactacca
gcgacaatggcaacaatgaaaaaagcagaacctaaaagctcgccaagtgcgagaccgccgctttggacgcggacggcagcgaatgtgcttag
cacatcgggcgaaccatttccaagagctaaaaaggtaacgccaacaacggcgtccggtaactgtagaagccaggaaatggtgacgaggtttgt
ggtaaaaaagtcactagcggaaacaccaatgtttgtaaacaaaaatgctagccaagctagcatgattgccaaaccgactccatgaaacccgcta
aatgaacagtaatatagttctggataatttacatagccactatattcatccccacaatatcgttttgcaaaatgacattgtacttgaggttcaatag
aacccacatcactgcaagtgaaattgtcttcactctgtggactttcgaaacgtgcaaataaatgtggaatagagagatcagaattagacgggagt
tggttagattcacttatgcgatgtatccatgtgtagacacataccgcgctgagaaaacaccaaatgagcagcggtttatagcgttttacaagttgtg
aggtactaacagggagtttccattcgtactgctcctttaagtcatgtttagtaaccatcacaattcaggatattttaagttcataagtgggagtgag

gatatataatacggtatttaatggcgagaaaactcaagaacacgtaggctttctgaagtaatatgtaaggcttagcctgttacgtagagtagtaat
agagatatactggaccaagtactttatcttataacatagtaaacaaacacgcgttactgcactgcggtattccttatagcgtaaacagtacaaggc
catgaaaggaaggaggatttgtttggaaaagtaaaggagagattcaaaagaatgtaaaaaaaaagaagtgtttagatatgttggctttatgatt
ttgccagatcataatatacggaaccatctaaaccattggcacctttgggatatgcacctttcaagggctttactttgttggaaacataaggaagaa
gcttgaattttgtcagtacagttacaatttattagcatacatacttcgcaattgccagcagggggacaaaagtacggacacgcgcccattcaaca
gagaaaccacctttgcgcgtaatttgacccattttaccggtccttgtacctcgatagtattgatgtcccattttagtagtcatttgaaggcgtctagcg
cctgaacaaatcgttgtagggtgcattctctcaaatcgcttgggttaaggaaaaggctaaggcaacgggacccgtgccggacttgggggagtagt
gaagaaaatgtaccttgaacaaaggtaacagaaagtaaacaaaagaggtttcaaattaaattctttttacgtggcttcgaattcgataaataaa
atgtatagaaatacgaagaggtacacgaactaatagttttgcataccaagtgaaacaattttgctgggtatgtttacgcttttataattaccacgcg
taacgctttgactattagtatcaagagtgtgtgtgaatgagagagaaaaagagagttgaaaaagctaataagcgaatgtaaaaatttccttctga
ggttgacaatacgtttcgcttttcaacgattacgaaaattgaagtctccttcactctcataacatcaattcgccaagagagaaatcagtaaacaaa
ccgaaattttataaaatcttttcagctatccatttaacaaaaaaattcatattcactctgtattgtcttttcttgccaattcttcttatataaacaaaca
ctataaataaatagatagactggtttagcgatgtacattctcctacactaatcgatatttgcacttttggttagtgcgctagaaggaactccaacga
aagcttgctcagcggacgaaaacaacagtcttctttggcgactaccaacatctttgttttgaaaacattgggcttttctgttaaggatgaatcaacc
tgagcgttatgagctaattgagctcatgggattgcccaagtaagcttaagcatggatcgagaatgagaagtttactaacaattgctcagagtcact
tatgagttggacaataaatcccctaatgctgccgttataactattgagaaagaagatcataccttggcaaacatgttggcacagtaagttgtataa
aacccctcggttgattgtaccttgactagcagcttaccatccactagacaattattgacagacgagcgggtactttttgctggatacaaagttcccc
atcctttgaaccataattttattttgagagtgcaaaccactgaagattgcacgcctaagcaggcaattgttgacgcagccaaggcacttatcaccc
atctcgaagaaattaaagtgaactttatgagggaatgggaactcaagatgatatctgtagaaggagtagaaatggaattttcgtgaagtagtaa
aaatgttgtttttgtttatgagctcgttaccctatataaagtccctggaggatgtgttgccaaatctgcgtaacgaaggatggtctgaatattgttgc
aatcaaggaaaagttggttgaatcccggaaaagaaaaccaaacgtaccagaagcctcatggcgaatgaccatgcgctcattcatgtttcttcctc
tttgaagctgtttaccatatcctttacatcgattcaacgatgatttttaatgccatttattcctttctgtaattttgtttctatcgtttcaggcttgcttgg
caactcgagtgtttaattttgctgatcttgtgtttgagaatgagaatttttttgattgcttggtttggttaatatatagatacatgaaagatagaatttt
tagtggaacaattaaaaattttatgcttgttgcgggagctatgctttttaccaaagacgattctgaaaactcgagattttgaaatgtcaataaaaaa
taaaaaaagtgacataaattgatggtatctttttcataagttgactatgaaactttcaataaaaaacaattgtataagcaaagtacaagcaccttc
ttcatgtacgaatgtcagttacaacataccaactcattacatatgccaaactagctgtacggtaaaatctagattgatgtgtttttaaacaagaatc
aaatttgatgtttttcttaggtctaagtaataaaacctttcttactgccacggctggtggactgtctgctggtgcctttttatcatggttcttgtctagtg
atgcttacttggcacatcgtaggttgcctgcgagtgccaaaaagatgttaagcctcacagattcttcaacggctgttcaaatggtggaaaacttgc
agcgtgaacgagaacgtcaaattcgtttacaagagcacccttctctatttcaatctcattactcatctaactttttatcgtttaaggatggtcttttgc
ctctattcaaaacttactatgattctaagaaccacgagtggatcagtattgggattatgggaaaggcattaactggttaccaaaaattgcagcatg
gtggagctattgcgaccgtgctcattgaatgcctcgaaactgttaaaaatctaaattctgatggaaaaggtctaatccgaggcgaaccccaagat
cctgttttgatggaaacctatgatgttcatactccctcctatactataaattacagaagaccagtccctgcaggtgattggataatgatccgcgtga
atgaaagggctgcgaagctttacaattctaagaatcagttattagctgaagcatatgatttacaaacgtgagtattagttgaagtctgttgatcttg
ataatggatcaaatgttttgatgaagtgatcttttaatgaatttttccctttaatttgtcttaaacttacggatttgttgattttgttagtccaagggctc
ttcatagaaatcaagaattgtatgccgtttacattttaccttccgataccagttaattgatctgtagaaacatattacatcgttgtaagcctgaatac
ttgaagcaatttaattacttgcaaatgcacccattgatttgaaagcatcactgtcgtaaaatgattaggtgtttagaatattagcattatttacataa
gggtattcgttcgtcatgaatgaaaatcttgtatggttgtatgaagtgacaaaaggaggcattatgaaggctacacatgtgcaaaatctaatacca
gaaagcgcgaagggcttcacgatgaatctcaggtgcaaaatctccgaataggtgaagaagaaattctcgagctttttctgtagaataggatgcaa
acttaatgatagtgactgagcgaagctcgcaggctgtccaaaaagtattattttgctcaagtaagctagtagttttccccattcggctttccttatttc
tgtagtggtttaaaattcctttctttgcttcttcaaactttggccctacaaggtaaagactgccggcgcattcaaaattgttcatacgatcagctttac
tgccaatatgaagagttggctcatttattagctgtaatgtgtcgcgcaaaagaacagatagatttccttcctcatcaaatcctcttttaatgatatttt
tagagatatacgacttcattgtccagcgttctcccatatgacttcgaccctgaatagtccaatccaatagtgccaacgaagaagtggtgtcttcaag
gacaaaattttgtgtttgcatgtaattactagatccaaatggttgaacaggatcttgtagcaacagacatgtggcattcgctgaaacatgcacatc
catgagttgacgagtaattctgcctactcgcgtctccttgtaaactttggtgcttccttgagtttgaatgcataacgtagcatcttttcctaaatggatt
ttcaaagcaacctgatccccagatacaagtccacctccatagctcagaacataaagaattgcaaaatccagcttcgtgtttagagagagtagcttt
aaaggatatttacagaacaagtgggtaacatagtgaacattattgacgcattccacccgaaattcaccttctttagcagccatctctggttcagga
cgaattttcaaggaaaccacataccaaagaattcgttatcaaagacaatatcaaagatgttatctatattttataaagcttaaatcaattttcgcag
aaagttgactaaaacaaattatttcatccgtcataggcatgatgaattgatgttgctttggcggctttttcttaccaatttcaatttagttttattcagt

gtaaataaacaaattaacagtgaagagtaatttacatggaggtaataagagttactttttaaacagaatgaacaataatttggaaaagaaaaa
aaatttttaagagcaatgcatggttcaagaagagaaatgcgacttatgaaaatgaccgcaataaagactagataatcccagtagaagggtacat
ttgggcggtccagtcaaggattataaagagagctaaatgctactgctgctttgaatcatatataatcttgctaccaaggttaagaggtacacaggc
aaacgtgcttaagtaggataattactatatgaaatgatagcggttttgacagctcacaattgacacgtataaagtaataaaagcgtaaaatcacc
cgtaccttaataagaaaaggaagtcgcttttattctcattaatttcttccttgctagcgaaacatagttacttgaattgttctttgttattgttacttgcc
agatagctgtcattccttcaagccaatatggatgaacacagctcaagaaaaaaacaacattccaagtttgaaatcctacaaaccatggataactg
tcaactgcattagtacggcttagtgctgaagttcggaaattgtagtccaaacttttccaatgaccctttagagaaccatacaaaagtcatcaaaag
tctccttaatctactacaaaatctctttatttatcttataataacttcgagaatgtctacggccacacctaggcgaaaacaccagttcccgtccgatc
actgcagttaagcgtctgagggcctcgttagtactatggttggagacaacatgggaatccggggtgctgtaggcttctccttttttgttgctcgttgc
tctcttgttctttttctttttatacgtcgactattgcctgtaatcgttttctcgatagaatttttgcaagttgttcctttacaagtgacaaaagactaat
tacaagttcaatgaaaagaaatggaacgatcgtattcaattgcaattaagaataagaataatagtaataataatcattaagaacgtaggatgat
gccaatggtctcagattacggttcctttcaatgcgcggattgtatgcatgcctttctattgtccacttttcttcgtcgcttccaattcatattgatacaa
agcaaaggcatagaaataaacctttatcggaataaattacttgtaatgttctcttttttccacttattgactttcgatcggaagagttaaggctcgtga
gaaaagaaacgcaaataaggcattaaccttgagtggtaggattgctctacagctctatttcttcgttcgtagtcgtcggcggaggagctagtgcat
catccctttgctgatatctgggcaacgtaggcgaaccggtggtttcagttgtatttgcaaagtctaaaccaaggaaatacatgaaaactatataaa
atccttgggcaacagcataaataatatcaccaagcaatactccaacattatatatggcatcaccaagcaatactccaatattaccaatggcaata
cacacaaagattatggagtagggcaaggctcttattgccccgatggcaattggatgaaagactgccttttgttagcaaacagttgcatttaaaaa
aaatgattcacttacaatagaaaaaagcatagaatattgcccatatcacaatcaaaagaactaaaaaaccaagaagggtccaatcgtatttaat
ttcattaaaggcatttttagccaagaaaagggttcgaaaggataggaaattgctagcgtacacaacaacgaaaagattgctgttgatagtaaac
gatagtttctgtgtttgaggaccacattttcgggaaaaccatagtttactatttctgatgttttgcagtgaagctcctttatggactcaggaaagctgc
tggattcagtcactatattttttttctttccttggttttgagaggagttgagacggaagatttgcttacgttctcttgcgtacacaaacaaagaccctt
ttcgagatcaacattgtaaacatcagcatggctgtatgcaggaggatcgcttgaaccaaattttagctccttttccgacatcctaaaaataaataa
agaaaaatttcaataaataattagcaaaagagcgtcacataaaagctgtcgatccgtattcgatataaaaatttcgcaaacgcaaaactcaaca
gaaatagcagccaaaaactaaagcagctctacctggtcattccaaacattcacatgaaagtcaaaatcagcccaattggcaggaattgaagaa
ataaaaagttggcttcagcaagaaatttcgcatctcttacgcaacattttcgctcaggatagcgatgggttctgttgcgctatctcctataatgacat
atagcataaatcgttgaatatacgtttgagtcacagtgaacaagtaacaagtttcgctaacttcgcgtttgtttacacctctgtaaacaaagcagat
cccatttctctatgaacttttgaacgactttttactttgaaagtgagttactaatttctcgttgcgcacagaagaagtaacaaacaaaacacggaaa
ctgcagacacattagctaactgcttttcattagacaactgagtaacagactacaaccaaaaagcagctttcacgggttcttcaggatcaaagaaa
cctataaattacttctttgaactgaaggaactgtccacggccatacctaggcaaaaacaccagttcccatccgaccaccgcagttaagtgaaaat
gtctgaggatttagccttgtttgtactatggttagagacaatatggaatccggggtgctgtaggctcctcctttttttgtttcttttttttaagtatttcact
ttctattcatttctatttatagtaatcttttatgcaaacaatagcgaatccaataggaagggatccggttgacctttccaaatgtgaattttgctaccc
gatttgttttgttgtacaattttgatggttatataacgcgttacagccaagtacggtatatacagcaaggcactgtaccgtatatacaataacatttc
tgtttcaccaaaaaagcagtcacaaacattgggtaagaagattcaaacttggcagcacaaaacttgtccatagccagagtcagcaagaatggca
ctgaaggagtatcaagttgttggccgcaaggttccaactgaacaggaacctgtacccaagctgttccgcatgcgtttgttcgcccctaatgaatct
gttgccaagtcccgttactggtacttcttaaagatgatcaacaaggtcaagaaggccactggtgaaattgttgccattaaccaaatccacgagcct
aagccccttcaacccaaggttttcggtatctggatccgttacgactctcgctccggtactcacaacatgtacaaggagttccgcgacaccacccgt
gtcggcgctgtcgagggtatgtaccaagacatggctgctcgtcaccgtgctcgtttccgcagcatccgtatcctcaaggttgtcactgttgagaaca
aggaggatgtccgccgcagctacgttaagcaacttcttgatcctaagttgaagttccctctccctcaccgtcgtactggtactctcggcttggctggc
aagaaggtttttgctcctcaccgtcccaccactttctactaaggtttatggtctgtgtcgctagtaaaaaaattctttctcgattcccttcttgtctata
gcaattgtggcatcgtagattcgtagattcattatggtagaaaacaagattgttgtaatgaagttatgcaatagtaggtctgtaaaattggaagaa
caaaaaaaaaaacacgtagttagctactagagtcgatgatccaaaaaaaacacatggagagttttagacatagcgaccaacaactgaaaaga
aaaacaaataaaagtgaaacatttattatacaaaaaagaaataaagtcattaatcttcatcgctatcatatccaacaaccagtgctggactctttt
gttgcgagggctgggatactctgcgaagatcgttcaaaacgtcaggatcaaggaatttgttggacgaagtggaactttgctctacttgatcagcat
tctcctgggaagcctctttctgttggttatgctgagtggatgcttcaattgatttttgatttcggatggaagaagatccgtcagcataaagtaaatca
tcagaaggtacataaccctcaagtccaaattctagctccaatttccgctttttttgatttccttttccggtctttgattcctgcaaatcctctaaaaact
ccttgggtaataacttcttcacgacgtcggctgctcgttcttctaaacgtctttgatttcgtgcttgtgatgctgggttaggatctaatgttgcccatcg
acaattaagacactcgtcattgtcaagagattgatgggccattgcttctttagcgaattgggcattggcttcatttacatatgtgacgaaagcgatt
cctcgagaattcaaaactcgtatgcgctcgatgtcccccattcggcaaaatgccgggccacaacctcttcaatatcgtctgttggttgaatgccac

caatgtaaagagtgtgattttggcgtaaaaaagaaccaacaccacccatgtcatctcgatagtcggcatgcttttctcttccaaaacaatctacgtt
agcattgaagaaatcagtttcattgggcaaacgatgcaaatattcacatttagatctatacgcagttagtttgtttcagtccccatcaattattttgtt
tccgacatgaaatatcgacctacccttccgaacacattcctctggcaaaataaagacaaaaataagacccaggaattttgtccgcctttgtgtatc
cagaatcctgctcaatgttacatcttgtgtcggatttcacttggcttttcaaagggtcttcacgatttccaccactccatttattataccaactaaaga
tagtcagtaattatatcctatccagcatttcatccatcaagtcacttacatattatacacttgaccttgttgaggtgcttccatttcaaattctggtctc
tcttcaatttgtttacgcgccggtcgtttcttacgcttcacgatttttttcactttttaatgattttaccagattcctctattgtttcagtaatttcttcttt
aaccacatcatttttgttcgtttcttgcgcattatttccatgcttctcctctacttccgccatattcgattttgtggtagagaactggaaacatccagctt
gtctctaacaccttacctccacttggtgttgagctttttcgcgttatggggcggtataacgctttaattaaaataagccaaacgaacagcaagttatt
taacgcagcggagcataacggtaaacaaaatcaagcatcgcattcgagcgagtagtctgtctcctttgtttatacaccgaaaacgcacaagttgg
aattttgatccacttttgattgtttcttttctgttttggattgaaagtattgaacccctttgttgttttattttttcagataaatttccatttcctcgcatct
cttcttatagcagctgatcttttcatttcgtaaagagttctcattgacaatatatatctagatttctatataaaaaaggtatttccttgttttcgtggtaa
ccgttgcttgttccaaaaactagaaaaaaaacctgatactaagcgtctgtgatgatttattcaagattcgtaagcgcactaggatttcgaaatgctt
gcaggacaaacaattttcaacgttgttttctccgctgttacagtcaagaatttcaagggagtgctggcaataacgtatacgagagatatcaagag
aaactaaatcaaaaggctaaacaacaaaatacgccagtggaaaaacttgttcaaaatgcacgcaaaagttccgctcctaaagaagcaatttctg
aatttcaagagttagcgaaaaaatcatccgagaaaccgcaggtgaagaaggtgaaaaaatttcccgaattaagcgacttcattgacattggcaa
atttcaagaccttccagcatcggcgattgaaaagctatggcgagctagaaaccttcaggagaacattttgagtggttgcgttgaaaagtccacat
atgaaaggatgatttctcgtgcaaagaaatatccactttttgtgcttcctctacctagagaaaatcaaggcatggaaacccatttaatacaatggg
gcttccccggaaataacactgcccatctgataatcactactctgttagaatataaactcaaaaatgcttatgctaccccacatactgtcctcatcca
ctttgcagatctgcttgagtcaaatggcattgctcttatgcgatgccaatttgaacatgacaagtctttaagttcttctgacgtacagctattattgct
tacggttcaaaagttctataatgcctcgccagatagcgctttaggcaaggaacggctctcgttgcttgaaaacttcgcaagtggaaaggattttaa
tctttacaaagtggctgggcatatggacatgcttgaataactgaaatgagatatgtttacgaattgtacattgaacgataaaataattgtttatgcc
ttctcccttacaagataccccatgttcattgaatttcaagctttactagtaactagtgactaggaactagaaataagggaaacaaccaagccaaaa
cgaaagaataaaaatccatcttgaaagatgtaacatttacttcacagtctacataaaatcctcaaagtcgtctgcatagtcatcgtattcgtcttcg
acataatcttcgtagacggattcatcaacggctcctcttttagaattcgtgttcaccgtaggtttggcagtctttttagcagcattcgtagtaggagc
aggagcaggagcgggagtaacggtagcaccgcgcttcgattgagccttttcttgttgctctttttgagtgacaattctattgacagacttctgaata
gacttgacattagcagcagttaaactagtaagcaatagaggaattaactcttgaacaaagtcattgtattcagaggaagtttgagagtgtttaatt
ttgcgaaggatttcagcctgaaaggcggcatagtctgctttggttctcagctcgcgttgttgttcagcttgtctagcacggttaccactggcggtattt
tgatcaacaatgtcaaaaaggtccatagcattggctaagtctgagtcaatttccgcttggcgcacttgttctttggaggcactagcagattttgcgg
cttcctcactggccttgatagctttttcaatgttgcgttcttggatacgttgtttcatacgatcagttttagtgagctttggttcgctagaatcgggaga
cgtattttggttgtttttgttattgttggtttgaggttcttcttcttcttcgtcttcttcatcatcgaatctctttttgggcttttgagtacttcccaatggaa
gttcaacttcagtagccttactaggctcctcaaccaaaaaatcctctattttaagatcagtttttgactttctacatttctacataccccaactatccat
agtacaacgtatcttttctaagtcttaaggactgcaatttttggtggtaagggaagaaaaactttgtcacaaaatttataaaaacaactttgcgaa
gaagtaaagcttatgtatgagtttataattcgttttctttttcttttctacaaaatcaaaaacaaaaaggaaaaataaataaatgaatagaagaaa
gaaaatgaaagaaccaaagaaggattcgcttacaaaagatgtaaagattcttgtattgctcggacttgctaacctttcaagaagtattttgacata
ttacaaacttaaactgcaatttctatctgctattgtttcattcttaccttcctctcacacttaatatattcgtttgtgtacatcatattaacctctcaata
aaatatttgaaaaataaccaagcagagaaaagaggaactaaaaagacttgtaaccttagtcatgttaatcaaaagtaaattttttttattcgtttca
aaaaacgtagaagcctgtagaagttagggcttggtcagtgacatgcagacttaaggcaatcagcttctatgatattatcgtctttttagttatacca
aaaacatggcgattcaacttgttttcattcctaaaaaagtttatgaaaacaatcaagaataagtgactatgtgattacatagcaatttttgctttac
aggttttgtattgagagggaagacaaaacaagacactcaaataaatatccaaaacaggtaagatcgactttcaatttgctcattttaatttcattat
aggtattctagcaaggaatttcttctaaatattatgctgaatcatttcaaagctgtttcataatactatttgtaagccatttcatagccagattgtttg
atcttaacgaaaacagcagtggaaaaaagaggacgatggaaatagtttcgttttcgaagaagctgaatgattgattcattatttattagcggattt
atatgtttattagtgtacgattgtcgttacgctactcaactttagatactttattgaaactctgtaaagtctgacatcacccgctttgttctctcaaagg
aaagatctaagtgacaacctttcctattggaaattgaaacgtaaccagagcaaaacagtaaaggtcttaccaaaggacgcaaaaataaagaag
tacttcatagaaataaatagaatggtatggatatttttaacatatttattctgggatggaaattaacataccgctgagtagggatcttcagatggat
tctgaagatatccttgttcggcaaacgtttagactacacgcaatcttacagatgaacaaatcgccgaatttcgtgaggcattttctttgtttgatcgg
gatcaggacggaaatattacctcgaacgagttgggtgtcgtaatgcggtctcttgggcagtctcctacagcagctgaattacaggatatgattaat
gaagtagatgctgacggaaacggaaccattgattttacagaatttttgacaatgatggctcgtaaaatgaaggataccgataatgaggaggaag
tccgtgaggctttcaaagtattcgacaaggacggcaatgggtatatcacagttgaagagcttactcatgtattgaccagtcttggtgagcgtctctc

tcaagacgaagttactgatatgattcgtgaagccgatacggatggtgatggtgtcattaactatgaagaattctctcgagtaatatcttcgaagta
attatcctcactttctttaaaacattcacaccgtgatcaaaaatatttcaatcaacatctgtgtccttcataatttttattttcaatcagagtaaactc
ttaacatcgcatattcccaattttcctaaacttccgtttactattatcatgttccttcttacagcttatggaaaactactttcctttttcatttatgagaa
agaaatggattcaaaaagttgatttccctcttacctgctaactcttaggaatgatttttgtaatgaataaagcatttttctgtcagtcagttatgtga
aggtgaaaaagatgatttggaagcttaccaaacaactttcaattcctccttgtcctcattaaactaattctccattactgatctttttcttttcaaaaa
acaaggcttcataggtggtcgtttgcaaacaaggttgtattgtctctctttgtaacgactttgttccaaatgacttgtcaacaggtgttcatcacagct
aaggaatcttatcgtttttttgtatttttaaagatagaactataaggttgccacttacaaaaaaaaacctttcaattcttttgtttctcgatgttacgtag
ctcggctttacggcctttatattacgtccgtgatcacaaccatggatagcagaattacaagaatcctacagatactttaacccttacttgttgatgtg
cttctctgtatagaatgctttcattaacaagcgtaaactacaatcattgcatacttatgacacaacaataagatgagtatttcatatacttttgtgca
tttaaatttatgtgaattcatattttcattttgtgtatcctgtcggtattcattcctagcagaagaattgcaaattcacatttaactcttcatgtcctcct
agccagtaatttgcacgaacaagtgccttcggaaaatctattgaatgcaaaactataattcatcgaatacaagctcatgaaattcacttctttttca
gtgcctctggaaagctcacacttatccttgttcctaccatcttccaagaaccgcttcattacagccgaaaccaaatctttagaaattagaaagaga
acctccaatttttttagatcatatcaacttgaagttactttgtataaaagatactggagcattttagaaatcagcttcataatatccaccaaggcattt
ctcatagaagtttctcaaggcgcgtccgctattaactagatgtcgaacgaacaagaggaagttttgattctgaacaatttaggaaaagatacattc
attcatgcagattctcttcgaagtttgcttccttcaacaacttctatagatcaattgaaatctgttcgaggtaatttactgaaacaattaaagcaatc
acaggataatttaggttctctaagaaatggatatgaagaatccaataaaaacttgatcgttcagaaaaatgaattcaataccgctgctggagaag
tcgttgggaagcttcgatatttacactcgacggctgaagatactcagttatcaattgtgcaacttacctcagaaatcaagagcttagatcttgccaa
aaagaatctcacatcatcgatgactttgctaaaacgtctgcagatgcttgtgacagcttacgaaaagctgcggaatctcagacaaaataggcaaa
tcggagaagctacttctttaatgctggctactttgcagttgctacaatattttaagaattacagaagcgtggagaggatagcttcgttgagccaaaa
catcacagaatttcaacgatcattttatgaacaagtgtttgaaaattttcagttacaatttaagaaggcttctagcatacgagggggatttaatgct
gcttccatagaaactctacacgaactctgcggcttcattgatgttaacggcccgaattgttcaaatgcattgaaaaattggtattgccatcaccagt
tggatgggattttcaaagtgtttcgtgaaaatgaagaagctggtttattagaaaatttctcaagaagatataactggtttttttaaactgcttcagact
tatgacgatcaacacgttcaaattttcccgcctcattggagaatggattttcagctttgtatgttgttttgcaatgaaacgaaagcagatttatctaa
aatcttgaaagaaaaagaaatatcctatgaagtattttttacttccattcgccatacgttggattttcaaaacgcgctaaaaaagcgattttctcgg
cttttaaaagagggtgaattgaacgatcaaaaattaaaacttcatactactcaaatgtttgagtctctttcaaacgaattcaatccttattacaaag
catatatcaaccacgaggctaaacaattggacactctctttaattcttttcattcgaaaaccgaaatttcatccgaagaaaccaatcaaattctttc
gtcttccatgcaactttttcaactttatcggaagattatggggcattttcttcaatttactagaggaccacctcttataggattaaaggatttatttagt
gattggttacggaaatatgctgaaatagaactcctcgccgacttgtcatctcttccttttaaacaatatgcaattcgtttgaatactgctgagtatatt
catcgaacaacattggaacttgaaaagcgatttcaagaaatatgccatgctgacttagcggaggaaatgtccttccagaaggtaattgactttat
gttatattcaaaaagcttgttaatcaagtcatgcacgaaaaagtacgaagattcggttaagacaagtttagagcctttcggaaaattggatcttag
aaacctggatagtgtgggtgaccagagtgcttatgtatttactgctatacacgaaatgaaattgaatgccgatgaattcctgggaacaattgaaca
gaatacactggcgcgcaatttttgtgatcgggtttgtgaatcatttaccaaacaattttttttattatatatatacgattaaacctgtgtctgagattgg
cgcagaacaaatattgctcgatttatgttcattcaagaatttcttgctgaaaatgcccagtagcaagccagattatacgattacagatagctatatt
aaccatctaacgatttttatgggatacatagaaacactgctcaaaactttgctgactccttcccttccaaaagaagggattatccaaagttacctttt
tttgatcaaagatcgctcggtgtcaaactttaccatagtgttagatttgaaaggagtctctaagagtgagcaatctagctatcttcaattattctcttc
tttcgtcgccaaaaagaacgatctttccgacagttctcctattttttttgtatttaagcccatcaacgtacacggatatgaacacgcggtctcgcttatc
acttgagttaactcctgatgcaagcagtcgtaccttgcagaatcttggacgattgtttacttctaagaaaaagtatggctaaattttctttgctgtctt
ctgtctctcttttctatagttaggtcttttatgtttttatgttattgttttgatgagaagccatttacagattcatgcgatagttattacagtatgctctttt
cttttagacggttccatgcgatgattttcattatcctaaacagatatgtttcctctgccaagtaaatgaggacaaaaattgccaaaaagatcgggct
gttctgggaatcgaacccaggaccaccatcaccctaagatggtatcataccactagaccaaacggccgctgtgttataataaattgttttaataac
ttattagagtagtaaatgcaattagttttgaaggttatgaaattgtatcgtcgaaatgtcaaaattgaaaagtagttcttgaaattagatagtaagg
ttcagcttataaacatacgtgcagcatctatataacgaaaaatatagcaggtcgaagggagagtcttctgatctttacttacaacacaaactcaat
tgtcctatttaatagctaaaggtagaaaaaacaaaaagaatccatcatattgtttgttgacaaggtttcaatttgaaagaaagcaagaatgggaa
agatgactatcctaaatggttcatacagccaatatcaatagccttggagactccagaagaatttttagaatattattttattaaacatagaaatgtc
taatgtgaaggtaaggtaaattacatgtcagcggaaaggtatacaaggtaaaagaagatgatggtaaaccaaaacgagccttcatttgccaga
acaaaacaatgcagaacttgataatagctttcctttttttaaaaaacatgaagacacaaattataggttattcaaagacagagcaatgtgacaag
gtaaagctatcaaaattttcttcacgaagacaaagaatcgcagccagcgaagaggtaaaagttgtgtagttaaaaaaaattaacggcaaaaag
tttgccagtcaaggtttcgctgtgagcgaaagagaaagaaaggaaaagagggcaaatggaaaagccaaataatcaaaatgcagaattaggat

aatatttaaattttcgtctgccatggtaataaacgaagtgataaaaaaaataatgcccatgattaaatagcagcaccagcgggagcagaagaag
acgaggaagcgggttctgggaggacaggagcaatgggacgatttgcctctgcttgcatacggttcaaagcaatacctaaaccttcaaaaacagc
caaaatacaagcacatccaatagcaccattacgggtggctctccaaccacctcgaatagctaaagcaccaccagtgaagaagccggcgataat
ggcattgtaaggatcttccttcctacgaactccttttacggcacagtcaaatgtactgaacaaaccaccccaaacaccaaagttaccacccaacac
tggtgctctagttttagcagctgtaatagcagaaatgcgcttttctccctattaaacaaaatgcattagccttcgaacgaacaaacatttcttatgca
tgatgcgatcctgtcaccatgcaaccaatgttaaaaaacacgtggcaattcataccgctggactatttctccaccctttaatagagtgccaaattgc
accaccaattgtacccatcgaaaaggcagcaccaaagtcgtttagaacaacataaggacagggatctctagtatgatcagcgctcgccattttgc
aatttttgtcttcaattccaaggaagctttttaaaaggaaaaacaggtctacaacaccaaaaaaaaacgacgcctacaaacagaatgcgaaag
cggattcgtctcagaccttttcgttgtctgctaaggacaggtcccaaatcggaacggtttgttcctttctagataatgagacatagcgtcaacgttg
gctatatatatcgacctcgctccgctacagtatagtgtaaacaatagagaaaggtaaatggaaagcacaggtatatgtatagttttataggaattt
ggactacaaacgatagatgggaatggtagatggaaattcaggagagaaagaggtcagagagtgaagatatgaaaatgaaaacattgatttcta
gtgtacagttctttctcttttttatagagtagaatgaaatgactttcaacaaggtgtcagaaggaaaagacaaaaataattggaagcaaagaata
ttgtgaatgagaagctcgatcaaacacagattttgagtaacagggataagttccaaaaagtgaatatcaaaaagaaaaccttaaatgcaggag
aaaacaaggagcagatctttgtgagtcgaatgtatatgaagaattagggattgacaaattctagagaagaggggagagatggatccagctttct
ctttctcaaaaattccgggttgtaaagcttagagaaatggcgatttccaggatcacagagtaatgtgacaattcgatgaccaggaccaagttgctt
ggcaagacgaactgcagcaacgcaattaatagcggaagaactaccaacaaagaagccatcattgacaatcaaatatcgagacatagccacag
cctgttcgtctgtgactctatacgcataatcaatgtgaggcctagctatagaaaaattgcgagtcatgcggtttatacccacaccctcaacaatagt
gtcgacttgatgtcgacgacgagttccttcccgttctgctaagtcaaacataacgccatggaggactttgtgatagagaccacttcctggagggtct
gccaggcagattttatagaaggtttttccgatttaagatactttgcaatacctcccatggttcctccagttccggaaccagctacaaatgcatctaa
ctttcctccacactgacgccaaatttctgggccggtagttttatagtgtgcttgccaatttgcggggttttcaaactggttggaaaagtatccttgtcc
tggaatagatttgtcctttgtatgtcgaagagcatggtcttgagcagagttcacgaaatgattacgatcaacgatgggagcagggggcacacgct
gaacatgggctcccagcaactccaagacatcactttttttccttggcttgatcgctcggcataaagatatgacatttataaccaagagaacaacata
gcatagcaagagaaatcccagtagatcctgcggtcccttcatagacaacgtcgttttggtgagggacgagctgacccctgtcttgggcactttgga
tcatttcaagggcaacacgatccttgggagagtttccaggatttaaaaactcggccttggctaatatttcacagccagttgcttcagacaacgactt
gattttaacgagcttggtatttccaataagaccttccacaccctcaacaatttcttccttcaagtccgtctcttcttggttggtttggtttcgtgatccta
tccatttctgcaaaatatagcagacgacagcgccggcaataacgccgccaaaaatgtcttgatgtcgttttgacatgagaatagcgtgaattttca
gttgaatatcaaaagaaataaatataaaaaaattatcgcaaagatgtttgcctcaatactctatcaaaaaccaggaagacttgttccaaaagtac
caaaattccaagtagtagagatgacttacgaactgcgtttcaacaaacaaacgaaaaataaaatagagaaaccgtttgattctcgtaaatacca
agaatgtacctgaaaatcgacgatggttcgtaatttgttgttatatatgtaatggtgtacacataatctcccaaggaagtaggtgacagtcagcaa
tctcaaaaaggtcaccagaagaacggatcaaaactggagctttttcctagatttctcgagtttctttcttgctttttctctcagatgcaatcaaaaa
accttgtagtctcttttcctacggcattttattatcgagaatatcatacaaggcgtctcactggatggcaagagtaaaaaaagatgcgatggcgtgg
aaagagaaagaaacaaaaaagagccggaaaggccaaagggagtattagctgacctggtactgtattgattaatatcaataaacaaaaacata
attcctttgcatcagccgggagtcgaacccggagcgaatgcttggaaggcatccatgttaccgctacaccactgatgccacgttagtagttatggt
aaagaaaagcatatataacaataataatatctagaagagattgcaatcttggaattgggttgagcatcttatcatttcttcaatgtagtgtctcgttt
cttcagcaattttttttcttttaatggatctgacttggatccctttttcatgattttttttgttttatttttagtagcgaaagcttaaacttattttcagagt
ttaagcatcaaagttgaagttagtcattcgcgaggcccaggtttctttcagatattcgtgtaacgaggtttctttcactccttgaattcatggattcct
gtagaacaaacgttaccctactcggatgaaacaaaaatatatagataaataagtaaataattgagtaatttataataaaagtcaaaagaattga
aaaaaagttgcttcatgaaagagaaaagtcgaaatacccatctcggacgagggacgaggaatggttcggacgaattcattcgtatcgggtagtc
ccaggctctggacttagaacattgcggatcgtttcagatcaaatgtcagagcctcatctcgtccgttcgttagaaaccggattgtcccaattgctta
ccaaggctaaatcggtacagtaacgtcttggagagcagctttcgttctcttaaattcagtttttttttaatttctattttggttcattccttcatgcttg
atttgttttctttctctttccttttgcttccttttgttcctactacgaccatcccttttgctttattacataggaatacaggaacgaattatcaaaatgaga
actctccttataaaaatatttattggtacaatattgattgttggttaatctagggtggtcccgtaaattacaatagcgagaacggatttgtcatcgga
tcacttgcgtcatcagatcatgcccaatggatcgacatttgacgaatatttcgaaggagcccttgatatattaagtcggtctgagcggactgtttgc
acttaccatctgccattcattaggttaatccatctttctctctttcatcaaactctcttcttatagaataattgtaaagatgagttcttctattcagtaag
gatttttattttatttttatttctagtctttggatgtgcttactaacggaatcgtttttatagacctatcaagcaaaacgcttctatggtcgtttttgg
cgcctctggagacttggctaaaaagaagacggtatgttcctgattgtttctagttgaaacacgcgtcataagggtttttgcaagctcgaatctataa
atgccagtttcatggaaatactatgctgtgctttgcgagtccatcgttccatgtatttcagtttgtgctggattttcaactcaagctgcttcttgaacac
gagttgcatgaatttcgactcttgcttgcaaaaaaaatctttgcgttcgtttttattagttgctaactatgtggaatagttccctgctcttttctccttgt

ataaagatggccgtcttccccaggatttggtaatcgtgggttatgcgcgctctaaaattgaacctaatgacttcatcgaacgtatcacccaatacat
caagattgacgataatgactcggaaaccaagcaaaagctcgaagaattcaagaaaatgtgcacttactaccgtggctcttatgatgaagttgaat
cttttcaaggtctaaataagcatctcttagaacgcgagggtgaccgtactgagcgcaaccgcgttttctatcttgctcttcctcccgacgtctttgtac
ccgtcgctacccatttgaagcaacaatgctatcctgaaaagggagctgcccgtctcattatcgaaaagccctttggattcgaccttgactctgctcg
tgagcttcaatccaagttgggtcctcttttccatgagaacgaggtttaccgtatcgatcactacttgggcaaagagatggtacaaaatctcgttcat
ctccgtttctgtaaccctattttctctcaattatggaacaaggattctatttccaacgtccaaatcactttcaaagagcccatcggcacagaaggccg
tggtggttactttgactctagcaccatcgttcgtgacatcgttcaaaatcaccttgttcaagttttgtctttgattgctatggaacccccctacaactctt
tctgctgatgacctgcgtgatgccaaggttcaagctttacgccgtacccgccttggtgaactacaagatgtcgtccttggccaatatattaagagca
aggatggcaagcatccaggttacttggatgacgaaacagtacctaagggtagtaagtgtccaacttattccgccattcccatgtttgttgacaatg
atcgttgggatggtgtacccttcctcttgaaggccggtaaagccatgaacgctggtaaggttgaagtacgtgttcagtttaagcctgttgccaatgg
cttgtttaacgatgcttatcataacgaattggtcattcgaatccaacctaacgaggccgtttatctcaagatgaacatcaagcagcccggtattagc
tccaagcctttactcaccgacttggatttgacttacaagaaccgctttaccaacatgaagttgcacgaggcttatgaagccttgttcttcgacgcttt
tgctggtgaccaatcccgtttcgctcgcatcgatgaactcgaatgtgcctggactttggtaggccctcttttgcaatatatggagaaggagaagcct
gagcccaagccttacgagtatggttctgaaggaccagactgtttgaatgaattcttgcaaaagtttggttactcatacgataccccctgattactatg
aataccctgtgacaaatttgcctgaagacaagtaaatgagcttgctccattcctttaaaaagagagtatttttttattccctttttaaactgtacctagt
tttattatttggacaacatttaacttccgtttttggtctatgtattagtttggtcattttagatgttgtatgagctcttcgatcccaaggcccgttgagtg
atttagaactatttagaagggtttgtttctgttttgtttcattgtgtgttatgacaatttattacttcaggctaaagtacttgatgctatagctttcaata
aactgcttttaatgaaaaacgaaatttattcaaaatcataacaagttggaaactcgtagatagttctttaggactggagttgtaggaagaacaga
attgtattcgcatgtactacaaattaaatttcataaaccatgtcgtaccaagtatcacctccgaatttgcaagcactttcaccaatgagtttgaaacc
gacttcctcgtacagagaaatcaaaggcttatgagccagcaaagtcatgatccttggcttggggctaacggttgtttcacaacggaattgtaattc
cttaatcaattgtttggcgtaacctcgaccacgatgagcaggatgtacagaaagggagtggatggcaacgttctttcccttcggttcatgcttggac
attgaatcaactgtaacactttctgaagatgttctggtagccattaagtgccctattaacgtggtagtatgttccgtgggagcaaaaaggccaagct
ggagctcaggtgcttgtgaaaccctgtaagcgatctgttgaaaatgtcagtattgaataccatccggaataaccaggaagaaagaaattatcact
ttttcaaagaaatgtatggtcctttatgcccacaacagacaatagtgttttgttcggcttttccttatttcgtgtgaggtatgaaaatgcctccatagc
aagataaatcacattctctttatcttctttaaaaaagttgagtgtattcacattctcttcttttcctgcctgcttcgacataccgttttaagagacgcac
gctcattttcaggaaaacacatgtcctctaaatgagctacctgttttgtttcggatttttcaatatttctaatgtttaccatctttttaatgaatcaggat
caagtgtatggaaaactacaaaaaatcctctatttgaaacgctctggaaagaagtattctcgcgttatatgacacatgcgtaatacatcgtcactc
acatcccataacaaaagttcactaaaaaaatgaattctgaaaatatagtttagtcatagttggatgaatttgaaaagttcgtaaggaatttcgcac
tgtccttcttcattatggaaaagtttggattacagtagttgttttatgtatttgcatcaaaatactaaataattgaaacaactgtttctttgcgtttttta
agcattaaatgtagttgtaattggttgaatcctttcaagcactgctgttttatgatttttccttgataccaacccactccctgctattcaactctttgctg
ctgtttaaacccttttttcattgttcattacgtccaagggaacaagcgaaatcttgtacgtttcatttcaatcctgagaatgcgaggcactcaagttgtt
cggtcctcgttttcgcgagatcatgtcttcaaaacgttaaaataggaaatcaagcttgtttactttttataacattaagcaagaaactaggcgaatt
gttaattagctatcggcagttattcattgtcaaaaaaccaatctattcattatttacaaaaccgaaaagattttatcctcattataaataaattccctt
aaactcatctattaaggccaagacaaaagtcataacccgatccaaatccttcctgctttctcttcgacaaaatccaaaacagaaagcactcattaa
tttttttcgtgtataaagaattgcaagataacatccttgggccagcaaatattctgaatcaaagttattaagcgttaaatattatttattcttctgcat
cttccatttcttcatccaaaggttccagaacaccacaagaaaattctttctcgtaatcaactcccatataagagtcacagaagcaactgagtttgaa
tgacttgttaccggcttcaggtggtacgaactctagttttgtcgttaaggatcgacccaaagtaaccttcttaatcgccagaagattttggtcatcgg
aaaccaccaaccaccaatgttctgtctttttcatagggaaataaggagcaaacacagtgatgtccacttcttcctcttcttctacttctcggtccagc
tgtattaataacaacgtaggagagtttgcatgtacattctctggatcgtgaacttcaaaattgatgtcaatgtctggatatttattaataaactctgc
acatttggcaagatgaacattatccatttgcagtaatttcatgcgttcatcatcgtccatgtcaattatatcgaatacatcctgcactcccttctcatt
gcagcgtttaattaatgcatcatcaaagtagggaatctgtttcaaaggactgtcacgatcccatacagcttgtgtcaccatttgtgacatttccatg
ggtcgtatgcaagcggtgagatggccgtccgaagagagggtatcaacacatgcactgagcagattcagaactttaccgagtacgaacttttggtc
taacgctagttcagccggtaattcaaagcgcgagtaatgggcagctaacagaataaacgattttgtatgaggatcctcataatccggactgctta
agcgaacaggcagctgtgaatgaatacgggctaatataacatcttcatacttccttataggtaactgttcatattctgcagcggatgttacaatctc
taacaaacctttcatcttagtacgctcggttaaactgagagcaaatgtttgcatagtaacatatgtaattccgtaaaacgatgctatcattgccaaa
ttgaggggtacacaactgtcatcttcttcatcaacagtaatcagacgggcttcatttagatcattcattgtagtttctactagatctgataggaattc
cgaaacagactcatgggttatgtcctgaagaccgtaatacgtgggatttgcaaccaatcgtctgtacatataagtccatgttaaccaatccacagc
atcctgtttgttttcgacagtttgtgttgagatctccgaaacaaaagcatcatgaagccacacttgtaaatggctttccatgggtaatggttcattta

agaatctcttgtaatagtcctttttagtattcaccgttagtaaaacagcctggctgataacgttcgttctcatattagccgtgaaacctagcatttgca
gtaaatcactaatgggataatcaatgtatcgatgctcttttccatcatattgctgagttcccattacaaccaccacattggatttgacattcaacgaa
tagataacatcatgcgtagcaatcaaaacttttattaatccatgtaaaaacatggcttcaacgatgcttttgtcatgtgcagatgtaatttcagaga
caaaagctattccatgttgaagagactgacgcaaactaacgtcctggaccttttcgattgcccctgtttctgctacagagaactgatattcatcttca
tcagccaaactgaatgtgacaatgtcaagtgcaagttgtcttgcaactttccgatcaggagtgaatacaagactagattttctttgagatatacttt
gcacaagaacacgatatacgggcttagacatagccaacataagggaaggaaaatggttaatgctaaacgattgcaaatgtatcgttaatgggtt
aggtctatccttgggactaaagttgaatatattttgaggactagctcctaaccattcgccaaggtctcgagcattagctaaggaaacagaaaggcc
aacaattcttatatgtttttctaattgcaaagacatgtaacggatccttgaaagcacaacttcataaagaggaccatgggcaccacccaataattg
aagattgtcacatatatacatgtcaaccttgtgtatactacgcattgttctccatcttttcgataatgaatcccattgagaaggagtagcaaatataa
ggtcagccacttggataagcttcaaatcttcagatcggtcgccggttaacttaaataacacctttccttcaccaatattaccaagcctttctttccact
cttcaaacttttgatcgataatttcttgtattggagcgagataaacagcactaccagcatcaggatttttccagtggttcaacaatgcaagctcagc
acatactgtttttccactaccattaggagcaccaataaataaggtatcattagttttatataaggcactaaagacctgcgtttgtaatttgttaaaat
ggctaaattgagaactgtacaatgaaatatattccggattccccaaggtactaacaggtacattttgcagatcaagaagaggagtcggtgccgga
aatttttcaggcatgattagccgtttaaatgacaatgggaccttgaagttgcaatgcaaccaccgatcagagataatactgatgaaataattagga
ggcagaggctctgttaaagtcacagtaaaattgacaatgtgttcatcttcctgatatttcttaagtaaaaagaattgttcgtaatgtagaagcttttc
accattgacatcttcaaccagtatccagaaagcttcagaattgccacttaaagaatcatcccaagtgaactgggaggatatcgacaattcaaccc
caagtaacgatcgagtaataggctggatatgagcttcgatgttaactctggggaatgactgcaccatattataaacgcgtcgaccttccttgggaa
caccaattaattcacctaattcagcagggtccaaatcaaaataccgataccaagagaaatctttcttttctactcggcgaattacttcggcagggc
aatttgcaaactgtctcagaggactcatggtcggccaaaggcgtttttcaatcattttgcaggtgtctaaagccatctttgcaacagctgaccatcc
acgacgtaaggctatttcaaaaattgccctcataattcttcctgcagactgggttacataaaccatgtctactactaaagcaaacccatcaagctg
ctgcctcgaaatatatgattgcaataacgcattaatctttgcggcagaatcatcaaggccctcacgtataggaatcggtacacgttctaaaagtctt
gcaagttcaactttttcttcctcccgtaccggaatatacttaaattcgtctgataaactaaagactctgaacagttctattaaagatatagactggtt
aagtaacctgttatacgtagccattgagttatgagacacataatagctggctgcaactttaccaagttcagttgccgttaatgtaccactctctttgc
tataaacaagaagtctgcatttttcaagaagtaaagcagcagagtgaactaaatcagctctttttttgtataagatacttgtcattttcatattctgga
cctacactgtataaaccaggagacctcagcattctaacgtataggtaagtaaaacccaaccattcaacaccatcctcaatgcttcttactgagccc
atagctatctcagcatttaagcaatcagctaagcgggtcataagttgtgattcaattggcaattgttgattcattaaagatagataatactgtaatt
cagagtgtgcggtgatgataataccctcaccatatgtgtcaaactgtggccgaccagctctaccaagcatttgaagaatatcttggggtgaaagtt
cagtccaaatacctttctcaggtgagtatatttgagtacctttgataattacagtatgagcaggtaaattgacaccccaagccaaagtagcagtac
ttactaaaacctgaattgtaccttcagcaaacagatcttctgccgttcggcgatcctcacgtcgcatacctgcatgatgtatagcaaatccataagg
aagaagatctttcaaatgctcattcttggtggtatcagcttctgtttgcaaaatttcacgcgaagcagcatcggaacgtaaaatatgacctattgttt
cttcttctaaagctttgtctcgaataaaacgagcagttttataagtttcttttctagaatgcacaaatataagaacctgatttttgccagcttgctgca
ttgtcttctcataacaagcttcattagtaacttgtaaacgcttgataggtttcttttcagttactccgataaattcttgttttaatgggcaagggcgata
tgttgcatcaaaatagaacagacccttattaggatttactcgcagaaaagttgagacatccttataattaggcaacgtagctgaaagaccaacta
aacgaatcctttccaaagactcttcttgatgacgaagagtacgtgcaacaatcgattccaagactggtcctctttcatcatgcagtaagtgaacttc
atcaataattactaagcggacaagattgacatacgatagatcgttggattttctggtaataatgtcccatttctcaggtgttgtgacaataatttgtg
tttcgctaatctgttgcttagttaactggctatcacctgtcagctcagagacttgaattccgtatgggtttagacgctttgaaaaattattgaccattt
cctggaccaacgctttcaacggcgcaatatatacaatcttgaaatcatttttgcgaaatgttaaagtttcagcattaaggtgcttttgtaattcgttta
aaatacataacattgccacattagttttaccagcaccggtaggagcacatagtaaaatattctcgtctgtcccataagctattgggaataagtgac
tctggattcggttcaatgaaggcgtattagggaaagctccgtgcgcccactcaggaagttcattcacagacactaatcgttcatctgagctcatcgt
agccttagttggagcaggaacatgaatttcttcatacccttttaccagtgcgtcggaatgagccctcaggtagctttactgctttatttgacatgagat
gagcaccttcagagaagacatagttttccaagttaacttgttgcttaggaatcacgttattggttatttttttgagcactaggctttgggaccacatcc
tcagcaggtacaaacccttggccatctaggacgggtttcgcttcttgaggggtgacttcagcaggaataggagcattatgttccaaagcttgcaat
atccaggactggccagcctcttgcatagcttccactatggtagaacgctccttatcatcagcagctcgcgtaagtcgagtgcagaaaactatcatc
catcggtttttagtcaaaagttgtaccaaagaaaaatgctcataatcaaaaatattcattagctcgttctcaagctcaccaagatcaacattcgatg
aaagagcatcgaatgcaagatttgtttttttcctggcatacatgagcgtcttggtagtattttgcaatttcccgttgaagccagaatgcatctatgtct
cttgggtgaagtttcgacacttcggatactttttttagtatcgctcgaaataatggctacctcagtatcctgctcttctgtgggaatttcttcaccccttta
catcctcggtaggggctttctcttcttcttcttcttcttccatcgcttccacggcttcttcttcctcatctgcttcattaaacaatactggaacgcctg
aatcatacaaatctctttcatccatggctgtctcttcttctctggtatagtctgtcaagcgattaccaaggttaattaactgtgaaaatcgatcgtcag

gaatcttgactcctaaaatttcgttcacttgttgtttttttgcgagtatctgctagggtatcatcttttatggtttcgatcaccaaatctgatgcagaccg
taaaacttctggtgcctgatcacccaagtattgttgcacaaatgctaagatatagtcataaacttgtcgtgtctcatttgtcaaaggctggtatctta
aggtttctaatgtatcggaagcacctagcaatgtcgagccagtggtgccctttacgcctaggtaatctcttttcagcctttacgggatatccctcagta
gtaggaaccatagtttcctgtacttcttgagtgaatccctgaggaagagcctcagctttactagtccgtgctcgagtacccatttcggacacattaa
cacgattagcaagtgactctggctctcctgtaggttctgtatcgcgtcttgacacgaatcttcgatcaggttgcgtaaccaaatttgacattgcattg
taactatactgcgacgtgtcaattctaggtttatccttgtcgcttccatgtttggaggaatctttcgatcctcctttggaatttacactcgacatgattg
tgagtggcggacggtaagttccttctgctattgtatttggatattagctactaggttgaaaaagcaagtggtatatgggtttctcaggaataaagtg
ctacagcgcacgataacgtgtgccttaagaaaatgttaacttttgttgcgttcttttacgtaatttaaagcctctcgttatttgattctttcggttttctt
agtaaatgaaacaacacatttgtgaataaacagtatttgtttattatatctggtttttttaccgtttatcaccttgtttgcttatatggatgttagatgga
caaaaattacttacgattaagcttgtaccctgctaagtagcatagtgaatggtgattaaaggatagtttggatgctactgaaaaagtatttgtctct
aaactaagagttaggaaaagaagcccttgagaggatgactgatgacgagtcgtttgaaggagacgattgggatagcctggatgtgcaagcggt
agatgaaattgagcataaattacaaaacaacaccattggattacaagggtattcagtcgatgaatatttagaggtgaatgatcaggaaccacat
cgattacaacacgaacttgatcgagatgctgctcaacaatggatctatccaatcaacgtgtcatttcgggattatcaattcaacatagtcaaaaaa
gcactctttcaaaacgttttggttgctttgccgacaggcctcggaaaaacgtttatagctgcgacggtgatgatgaattactttcgatggttccccaa
aagttatgttatatttatggctcctacaaaaccattagtgactcagcaaatggaagcgtgttataaaataacaggaattccacgaaagaaaacgg
cagaattgagtggtcaagtgcaggttggtatccgaaatcagtactatcaagaaagaaatgtattttttgtaactcctcagacacttttaaacgatat
aaagcatggaatttgtgatcgcacaaaagtatcatgtttggttattgatgaggcgcatcgcagtactggaaactatgcttatgtagaggttgttcgt
ctgctctactttggaaataaaaactttcgaatcttagcgttgagcgcaaccccgggaagtaagattgatactatccaaaacgttatcgactccctgc
acatttcttgcatagaaatccgtaatgaaactagcatcgatatcgctcagtatgtacataaaaaggaagtggatagaattctagtggacttatctc
cggacataatagacttaagggatcgctttgcgtctcttttagaaccaatgctgttaaagttgaaccaaggaaataattaccgagtacaaaatgcg
agagacattaattcgtttactctagtacaagcaaaacaagctttttttagctacttccgggcaaaactttgcaaataaccaacgttgggacattttaa
atacatttgacgcacttgctacctttgcgtatcctcttaatttactaatgaatcatggtgtcaggcccttttgcgaaaagcttcaagaaattgaagaa
gaatgttccacagggcgagcaggttataaaagaaggattttgtcgaatcccgaatttaaatctttaatagtcgatactaaaaatttgcttcagaat
aatgatttttatgggcatccgaagcttgaacacctgcaagaaattgtagtaaatcactttgacgtgggaagtaaagacaatggaaacaccagaat
tatgatttttgtcgagattcgatccagtgccgaagaaatatttcgaatactaagtcggcactatcctattgtaaggccggcaattttcgttggtcagt
cttcagttcgaaagaccacaggaatgtctcaaaaaacacaaaatgaaacagtgaaacaattccagaaaggtgattttaatacactgatagctac
ttctataggtgaagaaggtttggatattggagaagtcgacatgattatttgctacgatgcctcttcgtcaccaattcgaatgctgcagcggatgggt
agaactggtagaaagcgtaatggttatatttacatgctcttaacacgaggaaaagaagaagcgaaatgggagcgtgccaaagatgctcataaa
aaaattcaagataatattattaatggaagggggctcgttttgtccgataaaaatcatagaattcttcccaaagagattcagcctgtgtgcgaaaaa
aaggttgtggagattccaaaagaaaatgttgctcctcttgtgagttcaaatgaaaaacaaaaagcaaaagcgaaaaagaagttttttatgccag
aaaatgcattgcacggttttgtacaagtatcaagtcttacaaagcctaaacgttcgtctactgttgaagaagcttttgaaatttctgaagatgatctt
tctcccaaacatgatgaacaattattatattatagacaagttccaaagaggacgatagatatacataaaggacgagattttaaaaacatgaatac
gacgggccgggtcccccattccttggctttgaaatgcatacaatcatctttgaataatatgagtcggcacaaagattcctcgataacttataaatgg
aaagatatatatcaacagagtctgaaagaagaaaccatgaatgattcttctcataagaaaccgaagatgaatgatataaagcaacatttgaatg
ctagcttgtaagtattatatttctaacttccttttctaatatatagagcaagagaatctagacttttgtcaaaagaaagattagaacaccgaaaatt
acaccaattggataacgatgttaacgcggcaactacagacgaaaacctagagaaagatcttccaaatttaaaccttacatcctcccaaaattata
gaaatagagataccggttcttatacggatagacaacatcgccttcagcagttagtggaaaggcgtaagcgatccaaaggaatgtgggtatagaa
gggaacagaatcattcatttattttatttatttttactttaattacaaatttttaattttataatccaattcaaagagatagtcatagcattccatttgtg
ttttcgacttttcccatgtggatccccaacttattgctccataaccgcgaataagaattgcattggtttctggatattcttcaagcgtttctttaatactt
tcaaaaattccttcaagggtgtcattgtgtattactagtttagaacaatcgttagtatcgatacttcagcggcacattaacaaacttacttggaactt
tcagagagtcaaaacaacaaagataaccattgctaggttgtcccttgggaattcctttaatcatctctttcgattgagtatgaaagtcttttccgttg
taaagagaagcaccaagggcagcaagagaagtggataaaatacaagccacagcgttccttttcctgttttatttcttagaaatatattcataacat
taacttactctaagatgtaggcaaaaaccttagaccacacaacatcggtattcttggaagaatcgctaagcgattgttagtcaaatacagccctta
agcaatttaaaggataatagtttttaacctatagttgaaacaaaccttaaagagaattgggcaaggtctccttcctgtagcatgtctctttgaatac
ggtctttagcacagactacttgatcgctggaaacgagtcagtaagtaaaaatttataaaattgtgatattaccaatttcttagacaaaagtttccgc
tatgtacagttagttacgagtattatagttttaacaaaggcgcttacctcccaaacctcatccatccttgtctataaaaatgaggaatcagttccaa
aaccaaatctttttctttttgttggatcattgcaatggtattcagatctattccttacaacggaaatcaatccctttttctataagtgaaagaggtggt
ccactaccctggaagcaactataaatttggttcgcaaaaagtagtttcaaggataaaccattttacattggagtatgtgtgaatagcatatattgct

tgaatagaacttaaaatatatatacacaaggataatagttaaaagatgtttgaaatacattaaaccctatataaaaaaacaccaccagttgtgaa
taataacatgttgaaaatttttgtgccaaacaaaaacgctttttcttttacttaaatatacgactcaagaaatgataaaggggagaacaggaa
aaggtaaactgaaaaagacagaggaagaagtcgaaaatgtgagatagtccggttttactttttgacaattgtaacgacatcttcgtccaacaaa
acatgagcaagaccaacacgcatgccgggataaggaacactcttaccccaaacgtaagcgtgcttgaattggctcttaatactagagtgaatgtt
gttacagaaatcttcaacactagagtgaccagaacgaagaatgacaggttccgaatagtcaggatcaaggcccctaggacgagtgtaaatacg
aaccaagtttagatagtcccacattgttgtcttaagttcgtcaatgttccaacccttgtgaccgcagataggaacagcgttaggaatacggtcgatc
aagtccaattcttccaatgaaatttcatcgatcttgttgaggatgtaaattgctggaatatagcaacgatttccttctaagacgtcaatcaagtcatc
aatagtggcatcgcaacggaaggcaatatcagctgaattgacacggtattcgctgcatacggcacggatttcgtccagatcaatgtgattgaggg
ggacagtgttggtaatgttaataccaccacgttccttcttcttgaaaacaatattcggaggatccttgttcaagcgaatgccaaaaccttctaattct
tcctcaataatgcgcttatgagacatgggcttcaggacatccaaaacaatgaaaatgaggttgcatgtacgagcaaccgtaataacttgcttacc
acgaccacggccgtccttggcaccctcgataataccaggcaagtcaagaatttggattttagcaccgttatactgcatgacaccagggactgtcgt
taatgtagtaaactcataagcagccgcttcactacgagtaccggtcatctggttcatcaatgtacttttaccgacggagggaaatccaataaagcc
aacggtaccaataccggtacgagaaacatcgaaaccgactagaagagttagctttaaaccaatgtaaagtcaaatattttagtaaacaaaatac
ataccaccaccgccaccaccgccgctaccggtcggtgtaatgagctcgcgcttcagttttgcaagcttggctttaagcatgcccaaatgcttagcc
gtagctttgtttttttgggtcctgctcatttcatcttcgatgtccttgatcctttgagctgtggtcgacatttttggttcccttccaagatgtgatattgga
aatggtcaggtttgaaaaaaaaagacttttgcctacgtaagatattttcggttgttccgcggccaataaattcatattatcgagttatccgcggttcg
ttaatggttaaattcgctattgtgttttgcgtattttactgagctgcttaaaaaagttcgttgctgcgtactctcatattttacttcaccgaatattcttct
gtctccctaacttgatttttgtatgtgttgttatacgatatgaacctgggaagagaatcgcaaaacgtttcgtcaattttcgaatagctttattaaaa
aataaataaataaatatctacaaatttacagagtagtcagacccgcgttgccaagttgtaagtagtcactataattccaaatgaaaaagggtagg
gcaatcgtagaaaccgtcatttacaaacgaatatgccttgctaacgagaccaaagtacgtacttccataattgttaagaatcggtaaaagcaact
agactttttagttccaagattttgggatggatggatgggagtacatctcttttgcttttgccttggtagttgcagtcttgcaattgcgagcctttatgct
tgtttttccagtttcaagttcaccaaaagaagaaaccatgaaaacaggtttggcattccctcatagtgcaaaaagttcaaaatcctagaactccac
gaatgaaatttggagaaaaaaatatgaaagcaaactaaaaagtcatcacgacaattttaagaataggtacgaatattataaagcatcaagata
acagttgagaatgattaattaaaatgactttttctcccatacctaatcaattgtcttgagcaataaagttacaaaacaacacgagttttatcaagca
ggagaatcttagaaaagataagggataacggcatggcgatcttctggataccgcgagaaatgggaacggtaccaagaatgaatacggtaggcg
cgtggaagcatcaacagtacctccgctaaaaagaaccaccagatgggttctgcttgcattccagcggccaaatagcaaccaaaccattcaatcc
attcacagaaatagtttggacaagagacataggtgaaaagaccaccgtaaggaattttgtaatgagattgaaaggacgaaataacctcttcgtc
actggaaggggaggaagtctgggagggttcgttgttttgttgctttgcaggttgtcttcggcggaggtcgtacagtatattgtcatgatacaaattg
ccaatccaaccaaaaagccacattagtcctcctacaatgaaccaaggcaaggagactgggtgttgttgcaccgtggcctgcagcaagtacaagc
ctatggacattccgttaaggaagttgaataaaatggcggaaataaacacagtccaatgcataggagagacgtgaggagcgaagacgagtggac
ccacgatagcacggttggcatagtggatcataaacataaaggccgacgctttacgaaatagatccaagtcacgaaagcatacgtcccccacaaa
cccaggagccagagaatggtttccttgctgcaatgcatataaaaaggtaacaggagctactagttccataagtacccagccgagacgacctggt
atgtagccccttgaaaaccaaactgtcggctgtgctagttttccatgaggagcattcacaaacagcacaaatgggaacacacccacaacgtatgt
tttaaataaattcaaataatcggtatacagcatttgatacttagctaatgattacttgtcttcttttaaacaaaattttaacaatcacgcatgcttttta
gaaatgctgcatccgtaaacaatcaagtgcgtcctaatagcaactaagctcacaccctatcaagatggactattgatatagccgatcctgatcca
atcctttctcctttcgatagtagaagtgactggcttttagagtagtagtgattgtgggggtcgggattttcggcgtctatatatatatatagagtgcac
cccaaacatacttgtcttgtacgacacgcattaaaggtaaagcaaattctcttcgttccctcaggaagaagtttcttttcctttttttttctttttataac
agtataattttctcgtcttgaagaaaatatactcaaatctattgatatctagattagtgttcttttatagactcattaatcgttccatactttttatacct
atcttcgttcccgtggatcttcataatggtacaatccgctgtcttgggtttccctcgtatcggtaaaaaccgtgaattaaaaaaggctaccgaggca
tactggagtggtaagatttctgccgatgagttacaaactgctgccaagtccttgcgtttggagcactggaagcttcaaaaggcccaaggtgttgac
attgtcccttctaatgacttttcctttttacgatcaaatcatggatcactcttttactttcaacgcgattcccgagcgctaccttaagtatgacttgactc
ctttagatacctattttgccatgggtcgtggtatgcaacgtcccgctgtcgacggtaagcctgctgtcgatgttccttctcaagaaatggtcaaatgg
tttgattctaactatcactttttgagacccgaagtctctgacaagaccgatttcaagttgaactccaccaaggctgttgacgagttcctcgaggcca
agcaagctggtattcaaacccgtcccgtcttattgggccctgttactttcttgttcctcgcaaaggccaccaaggaatcatccgccaaacccatcga
ccttttgcctaagcttacttctgtctatgttgagcttttgaagaagcttgtagaagccggtgccgaatccgttcaaattgatgagcccattcttgctct
cgacttggccgatgaggtccttaacagctacaagtctacttacgagacccttgctcaagccaatgctaagctcatcttgactacctactttggtcctt
tgaaggacaacgctcaagttttgaagggtctccctgtcgccggtatccatgtcgatgtttgccgtgctcctgaaaacttggatgctgctctttctgtc
cttggtgacaaccaagtgctctctgtcggtattgtctctggtcgtaacatctggaagactgatttccaaaaggccgttgccgttattgaaaaggcca

ttgctgccattggtagcgaccgtgttatcgtcgcttcttcttctagcattctccacatccctcactctctttctggtgaagaccaattgaaacctgaaat
taagcgctggttcgcttttgccgtcgagaagtgtgctgaattagccatcttgaccaaagctgtcaatgaggggcctgcttctgtccgtgctgaactc
gaagctaatgcggttgacgccaaggctcgtgttgagtcccccatcaccaacgttaaggctgtccgtgaccgccaagctgctgtcactcctgaaatg
cacacccgtgatactccctttgaaaagcgttactcccttcaacaagaatcccttaaactccctcaattccctactaccactattggttctttccctcaa
accaaggagattcgtgtcactagaaacaagtttgttaagggcttaatttccgctgaagaatatgatgctttcattcgcaaggaaattaccgatgttg
tcaagttccaagagaacattggtctcgatgtgctggtccatggtgagcctgaacgtaatgatatggttcaatactttggtgaacaaatggaaggct
tcgttttcacccttaacggttgggtgcaatcttatggatctcgctgtgtccgtcctcccatcatcgttggtgatgtttcccgtcctggtcctatgactgtc
aaggagtctgcttttgctcaatctatcacctccaaacccatgaagggtatgcttaccgctcccatcaccattttgagatggtccttccctcgtgatga
tgttgaagaatctattcaagctcaacaaatcgctcttgccttaagagatgaagttttggaccttgaaaaggcaggcgttcgcgttatccaatgcgat
gaacccgctttgcgtgagggtcttcctctccgtcgtactgactgggctcagtatgtcgactatgctgtcaaggctttccgtttggctactggtggtgtt
aaggatgacactcaaatccactctcatttctgctattctgacttcaatgatatctttgatgctattcaaggattggatgctgatgttgtttctattgag
aactccaagtctgatatgaagttaatgtctgttcttagtaactacaagtcttgtattggacctggtcttttcgacattcactctcctcgtgttcccccag
ttgaggaattcaagcaacgcattgatgctctcgttcagtctgtccctaagaatcgtctttggatcaaccctgattgtggtttgaagactcgaggctgg
cccgagaccactgctgacttggaaaacatgattgtggctacccgtgctgttcgtgagcaatatgcttaaggttagcctaatggtatttacatccaaa
taggcatttcatgtataattaaggaaagaaaaaaagaaaataaaggttggttgttaaccttcaacatggttaaccggtaatgaaacgatttatgct
atggtaatgtttctagaaccgcatagctgttttctcatcaactgagggtaacaagttgataaatttttgctatgcgatggcatctttgtctaattctttt
taataatagtttaaaaattgcattattctttctcaagttattctaaaaaggaaggaaaggttgtaaactagtaagaataaagtgtatattgcattgg
cattgtaagtttgcaagctgtctaagattttatcaataaaaaaatttatctgcaattcaatgttttgtttcctttttttttcatttttggatagttgaata
tgctaggaatgactagaagccgcaatacctaaaaaagatgaccaagagatacaaataaaaggctgacaaagttcttagtagactttcattaaat
gtctttttggtttatcttttaaacttcttttcctcagagttttgcatgggtatcaaacttgccgagtgttctgtttatatgctagagattgatttttttcctgt
tgtttgatagaaattgataatttttttttactgtttgtgatttttataaagcctctttaggaataactgaagacaaggttccaaagtcttttcttaattta
aaggagcaagtaattattgctgagattgagaaccattcacttacatctacggtacgtaagctcaatagaaatgtgaacttgttatgtaaaagttag
atgggtttcttattttattctacagttgcataactaaataaaggtggatgcgaattgcttacgaattgtattggcaaggaataatgtttataaaaaac
caatctctttttgcattattagcattcgttaatcgacgataataaactccagccagttaatgatgatgagatttttcgtgcagatctacaacatttgag
gaatttcactatcataaaacagtttttgagactttaattgtggtgattttaaatgcgagtaaaaattccctttataaatttgtcttttagcaaatcactt
gcaagagactttgttttgtaaatggtgtgcattgtttcggtgagatgatacatatttctttacatatgtacttggagacctgaggattatctttgaaag
ttcgtttgaatttaaattctgcatttgcttggctatttgacgttcttttttttctaatatcctcaatgaacgctgatgtttcatttgaaatgagtctaattt
catcacataaaattcggttcacccaatttgaataggagtattaaacattgcaaaacatttggataaataagtaaatacaggaaaagtaacaatat
aatgaaatctttctgtatgatttataaacacagagaatacagcagtaacagcttagtatgataagatgataatttggtatgctacttgacatttgttt
acaggatgtacagtttgacaatttttgaattgagcgagctttcatttgtaactaagtgtttgactcgtagtcctaacaaatttctccacgaaattcttt
attttcttattcaggtaaactagcaatgtgggtcttttcattaaacagaaaaggagaaaaaaaaataagagagagagagagagtgagagtgagag
agtgagagagagagtgaaagagatgaattccgtgagaaaatcaacaaatcatttcttaacagagactttgtaaactttcagccctacaaaagtc
cctggcgaacatcgtttggttggaacgaaatgcaaaccgaaggaatgtatctcttcaaatttgcttcttgaatagcaatttcctaactagtcgaggt
acgttacgatgcccccttcgacgaatcaataaaaccaatctccagaaatttccctaaaatgcggttttcaaaacttttgaacttgggacacgagaca
gctatcatccacaggaatttttttgattttaataataacattcaatggtaatcatctataaaagctatcttgtatggaatacgaatttatgttcctaatc
aagcttcatctattgaagaacgcaaaacgggagattcatatgtatgagctcctaatttgaaaaataaacgtaaattaagattaaaaactgttttct
tactgatttatttcgaataatttacaagaataccatggcaatttttattacttggtcatatttcaagaagaaaggaaacaacattgttacttggcggg
ctcactaagatgatatagaacggcgataaggaagactgctatgtagccatcgcatgcaggtatccgtgaagcttgatatatatactaagcggcta
aatggcatgaaaactgcgtataaacagtccaattaatttggcagtgtttgtctgtttaagagaatcttttttccccatatataacacgcttggctgtt
cccatagcgccttcgtttcgtaagggtcgactcgtaaggagtttgaaaaaaggcagctaaatcctctttttattttttgattttacgaaaaggatac
ttggtttttgaacttgtcgtaccaggaattgcaaattatggcgagcgttttaacggatgaacgatctacagatggagacttgtttgctcagcaaaga
tcaaacacctcgggatctacgacttcttcaaccggtaatgaaaaagtgaaagtgttccatgaaatggcgcatggtaaaaatgaacagtcagatg
gggtttcctcagatgccgacagtgatgatgcattgatcagaagtatgggctacaagccggtacttcaccggacctttaagttctttgaaagctttgc
ggctagctttgctgccatggatgttgtcagcggagttcgtcttacatttagttatggtgtcacatttggtggaccggctgcttactgggtgagtatgct
ggtcacaggtgtgtgctctacaattaccgctgcctctttggctgaattctgttctgccttacccacgtctggatccgtttatctgtgggctgctgaagc
aggtggaccccaacttggacgctttttcagctttttggttgcttggtggtccactactgcgtggacaacttttgttgcctcgactgctcagtcttgtgta
aactttatttttggcgaagttagcactttcggaaacagttggtcgacagacgattctaacgttaaatttcgtgcagtccaatggatcgttgctgaagt
tgttttagtatttatcattcttgtcaatcagatcccaccacggtactacagatgggtctttaaactttccatgattctgatctttgttgattatatactaa

atttgatttggcttcccatcgccacctctagaaaaagccatggattccaatccgccaaatgggtctttactgagaccatctacgatcaagccggcta
cagcaaagaagtgtatgattcaagtggaaacgtcgataccaacttgtccaagcttgttcccagaggctggcagtggtgtcttgcttattttgcgtct
gctggtgtaatcgtcggctatgatgcttcgggacacatcgctgaagagaccaaagatgcctctaataaagctgctcgaggaatcttcttttccgcct
ttacatcctcgattgtagcttttagtcttacgattctctttttattctgttgtcctccactagatacctttaacgatattatgtacaatacggcttcacctc
aaccgtttgtcaatttttattcctatgtgttgggacgcggaggtcatgttatcatgaatgtgatcattattcttgagttatttttgaatgccgtgattgg
tgtgttagcctgtagtcgcttggtattcggcgtctctcgcgatggtgtcttgcctttctctggctggatcagtcaggtaagtaaagaaggacaaccaa
aaaatgctattacggtaatttacattgtttccgctttgcttctttgtaccatattgcccagttcagttgccttctcttcccttgtttccgcagctggtgca
cccagttttgcttgctatgctcttcttgcttttggccgtttgttcattacccgtgataaattccctaaaggtaaatggtctctgggaatctttagcaaac
ccttcatggttgtttctcttgtttacaactcattcgtatttattatcaacatttctccttattcttatcctgttactggttcgaatttcaattatgccattgtc
attacaggagctgtctctatatttgcagtcatatgcaccttagtgatcccacgttctcgatggattgctaatcgctatcgatatgaccaaagcatatc
ttccaacgccaagtatgaaaaataatcacctacgtttaaaaaagtacaaaaatacatactagcatttaattcttcccctaataatttcctgtgcttta
caaatgaaagcataagcgatccaggttcgtgaatttcgtttgatattgacacagtgcttttcttatcttttctttacgcccattattgagcgactataa
tctttttggaaatacggtaatttttttgttttctattaataaaatttggtttattggaacgagtaacttaaagcagtaatataggaatcagcaaccca
tgcgtttttgaaagaaaataaacatctgtaactgtatgttctttagcatgatcatgcgatcaaaattccttttctttctgaagaagacaaaaagaa
gattttgcttctggtgaggctcgaactcacgaccttcccctttcatcttatgagagggacgctaccaccaactgagctacagaagcttcgttggta
agaggataatttcaagctatatattccatattgcactaatgtgtttataaaaggtgaacaaggatctaatagcacaagaatattgtttttggtaatt
cattgaattttggatctagaaaataggttctcgcttgtagcattcaatgcaatttcgtccttactgtgtcttttaatctctagaactttttattgttgtaa
gaaagcaaggcaaaagcgcagaaacgacagcatcttctacgtgcgctgaaatgatcttccatgtattaatggattctaagttgctttcatttgact
gtatcaatgttatactctacaatacaaaactcatgttttgtttttgatagcagcattcccacaaattttagactagaaagaaattttgatccatcagg
tcaaacaaaaataaattctcattttccatgttccttttgtatattgaacagaataaaacttgtatagtttttctcatatgtcaacatcttgtttcatttta
ctagtacaattgttattttttttgggagagtctgattcctacacttccttgcttgtttatttactaagaacccccaagacaaatccttatttgcagttatc
tgcgaaatcctatatattgatattctactgtaaacaaacaaatttgtacaaaaactaacatcttttccttaataacataaatcctttatccttctttat
aactgttaatttctactcgttttgattcttcctttttttagttcatttttttggctgcttctcttttcgttgatttcacttcttgcgtaaaatttgctaattgct
tatattctgtgtaaaatgtctttcccttttgtctctgattttcccagaggtcctttaggtaggttacgatttgctcgtggtaaaagttactaatgcagta
atccactagaacacgattgtatagaggaagatatgcccatggggtcctcaacaccaaccaaattggaaccgggaaatggctcctcaagaggttt
cgatggctctcatcaagaacgtcttcaggaggcgctcttcgaattgaaccaagaatctacgcattcgattcatttaagcatagacgagtctatttct
gataataacgatctgttaaatgatcaaggcttacagtaccctgcttcttcctctttaccacaaaaacatgcgaccgcagaaagcccttcaaaaaca
aaaactcccatgagcacctgctttgaggaatggctaccatcgtcaaaatacaaatactctagtcctttgaaaagctcttctagatttcctgcttcca
catcacctaccagacgaggtattcctctcatggcaattaatgcaaatcaaataactcctaggaaccataatcttcatggaaaagcaaataacgaa
gactcttccttacctcgacctgtagagccaacatataactcctttcaaactccttctcgccctggattgcgaatagactcctattcgccttcaaagag
tatcaacgaaaaagaactattgaaaaggaaagatgacaatcatttgaaaaacttaacatcaacacctatttctattcaaagagatcccatggga
agagagaatgccacaccagaaacccgacaatcatttaatgcctctccctttagaagtttcatggatactactttggaggaaagtttgacgcatgac
gatgtaacatgccctcatcttgtaagattagaaaaacttcgggaattactttcttttgttcaattggagttgcaatgttatgttgatccatacaaaaa
agagcttcgagaagccaaagaacagctagccttgaaagaccaagagctggaaatcttacgaaccaagctaaaaaatgctggtttatcttgattc
ttataaatatgttgttatgcggactcagtggtcgctgataaattttgtactttgttcccttacgttgatttttttattctgtatttactcctctttaatattct
ctattaatcctgaataagatatcttttagaatacgtttatggacagtgatttgtcttactagcatacttcattatgtaaaaaagccaagatttacctca
aaatcataatcaaattcggaatgagagataaggtgtcatatcatttagtttaaggtgtttgcaaactgaaagactagtgcagaacttgaaactttttt
gtctcaaatcatgatgatccatatcatgagctacatttcgttaataagaagtcaaacaaaaaaaaagtgcgaaaaggcttaccatcttctaaagat
ccccatttattcgtttggaaacaaagctccaagttagttagctcaacaacttcttcggaagacaaacttccctgaaggagaagcgttgacaagata
aaacttttacttgaagcaacacaacgctcaaaggcagccaactgccaaggatttaatgagtttagccatttttgaatagtctcatgagtttctattg
attgcttattcgatatgatcccgacatcgccatctaagtgtttcagttgaacattgaacatttcttcaaaggcctttttcaaaggcaaccatttcttttg
ttgttctttgagaagctttccttcatactcttcagcgggtgcgtataccagaacggtatccgtgtcgagaaactttatcacttggtcgaaaactctttt
cttttcattctccgcatctgttaattccatcgccctcgaaaatagcgatgtaacaggaagcatgtgtggacgaatcgaacttgttggcaaacggtcc
cattctagagcaaccaagtgagctaaaggctgcatagtagtaggaatttctagtttttttgccagaaggtgttttcaaggaatgtccgtccagcttta
caaacacttttccattctcaccggagacggatgtgtttttccaaaaacggcgaaagccatcagattttacattgtttttgattgcattagcgcaaaa
atttctagaaaacagctgttttcttgccaaactaattccacgtagcatcatcgctcaaagggaaggaagttattttctttattcaattacagatagat
agtactgaaatttaccaggtcattcctttttaatataaaacaatcgccttgtcagaataacttctcagaaaggaacgacagagttatcttcagttgc
tgctgtaacactttcgagcttgtttaatgttaatagaaaggtaaccgagaatagtgagaatccgtctttcggtttagtggacgtggttacattttata

ttaatattagttaaagaaaaatattatatgaaattgttttaagcaactgtgcaggcttgatctcgtttgaaaactaggtagcatgaatacataatttt
gaatcaaggtacgcggattaaatcttttcataaagcaaacgaaagatatgtttaactagcactagcagaagggctctacattgatacactagtca
agctgttgtaacttcgactatttatcactctcgaagcatactcataacgaataaaccactcgattaagtttaacgacaacaaaaagaattagacat
atagattatcattatcaaaatttgcaagactacatttcctgaaccggcagagaaatttcacataacagtaaacaaggaagagggataaaaaggc
catggacaagctaacctgcaagaaatcggaaaagtaaaaaggtaaaagaaagagagagcttataaacaaaaaaagagatattacaaggaaa
gaaccatgttaaaggacaactttcctttttccactttccgcctgtcagttcttttaggcgcttgaaattttggtagtggagacgctgttaattcagag
gaaacaagttcgactgctttcggcaattcattttgttgatcagtagaaactctgcgaactagttctccatcttgagaagtaaataaagactgtgctt
ctttttcaatttccttttcttcctgctctcgacgtaaacgctcgtctttgtccatcgagccatatgattcatcgtgcaacaatgttatagcatcatcaac
atttaccctggatcgctttgtgctttttctcttaaatcatcaagagcatcacttatttgcatttgcctcttggtatccatcgtcctctgttctaatttttcc
atttcatcttcttcaggtatggctccactttctgctaattcagtctcctcgtacgcttgtaatttttcctccctccaaggttcaaaatttctagacgcacc
tctttcagcggcataatcggcatgcttgggatctgtaacaaaggtaacttctgcagcacacctagtacaccgaatataaaacctcaaaatatcaat
tgaaaagtacttctccccagtcttttcttttcgagcgttaaatttctttcccttgtatatatattcaccacaagtatgacatttcatagagaacggtaac
attagacgtacagtcgttcttcctccattcggcccttgacttttctttttcttcggaggaggtaccgaaggatcataatcaggtggaatgtacttgttg
agaacctttctttccgacattttgtgtacgtttaatatataaattccaagaaaattgacttttcccttcaacgactcaccaatgctttcagattttaca
aaacctctgtaaacgcaagcaagttctacctactaagaatgttaaattcctacacgcgtccaaataaatgatggtaaaggacacgttcttgtttgc
ctttcgacattcattcatttcgaaaataccgagctagaacccaccgccctataaggtaaacaaagttacaaagtcaatcatttctacgatttttttac
agcaatccgcaagcattgtctacataaagcaaagtttatgcatgaatagtacttttaacgagtcacgagaaagttagcaattttgaagacttgtta
acagattgctattaacgctgtaggaagctccaggtaataaaaaaaaaaaaaaaaaaaaagcaaagaatgaatattacagaaaaacgtatcggc
tatatgaaggagtggaacttatacccatatatattatatatcgttagagtaatttggaacttttcaaaaaagaccacataatccagtcgactactca
ttgacgtaaataagcaaatgagaaaaagagagataaaacttttgtcaaaggaataggtgaacatgaaacgaaacgaagtgcagaattcaatg
accgcatgaaaaaaagagtaggataaagggggccatatattaaattccaaagtcattgaattaggttataaacaatgcaaaagaaaagtcga
catttgatgggaagtcaaacccgttgattgcatcgaactcaaagcataagtttcataggacaaacctcactaaaaatatcacattttcaatgttac
atagaagttagaaaaacaacaagaaaaaaaggatcggattttaaagcaaggagtccagatgagtgaatacctgtaatacagcagcaacgacc
tcgatggataacatttgatcctcagataatggagccaattcttccccaacagcttcacccaagttcgctccaacagttgaaggatggataaggaag
ggccattgagaaaattcagataaaagatcatcgacttgagcgagggctctgtcgcgtctctcaaagtctaaaactaaaagagcattttctaaaac
attagctgcataggcagaacaaacaagagctattgtacgcaagtagcggaattgcaagttagcaggaaaagctggtgtgctttccaactcgacg
acacggtccaccaagatagacaaaacgcgtcgctttagaccctctttgatagaggtatctgcgacaggatgggtgggcatatcgaaaagcagaa
aatttttcttttcagttctaagaatgcctttatcgaccaaaccttttgcgagacgttcacggacttgacgaagttgatacccaattttcatgacattcc
acgtttcgccgctcatcaagtcaatccaagaagtaaccgacattggttcactcgagtgaatcatttttatggcttcatcaagcaatacttcgcccgt
caatcgatcatccacgagttccaccaagcgatcagccagtgggaaacgctttcgattcacatctttctgcatacgcaacttgccgcgaaaagcaa
gctcgattaatatgcacccgcgaagagaatacgagatactatcattccaaaaagacagataaccctgtttgtcttttagacccagcaacagtactt
cctccataagagtcactttcggttgtttctcactttcagcaccttgttcaagatctttgggatcaaatgcaattttatgatcttcatcacgattctgcgc
tttgcttttcattaatgcgacatttttggtttcttgttcattttcgtccaagttgcctgaagcgactcttctccgagataatccgcttgtcataatttaatt
gagagtctcaatgtatataaatgatgttgctgatcggttgcttaacagccgcagaagaatatatacaacctactcaagtttgagggactgaatctt
gaaaccagttccgtcaaagctctattaaaagcagatctttatttcgtttttaagaacaaatgagcacactgaatgttttaggtaaaacagtgttgc
tccaaggggtttcaaagtccacaaaatcccacaacaatcgtaaggacaagcacctataaacaatacaatcctaccgaaactattcccttcaagta
ttaccacagattgaaaacttctccttgcacactaaagatgtaaactatgcaatgaatatgttaaagtcatactacctcagttaaggtcctggaagtt
gcttttgacgagccataacaaactgcagtattaccaaatgctgcagacactgaagctcgttatttatttatttattcaccaagaaggaaatttacga
gaaagatgcaagaataaacttatagaaatgaatggttaaaatcagaaatctgaaggctcaataaatagtaagacgaaaaacgatatgtataga
cactcgcaacatgtaagaggaaaaaacggtgccctaaaccgtgctaggattttattaagaatttttttgaaaagaatggaaagaaacactagat
atggaatggattcaagtatacatgcaagagaaggggaattacaaaatgagctcggtatgcaaaaatttctgtaatttttgcaaatgttgggcaga
ggtagaaggaagggcatgagcaggccatgtggaaatctgttgatggatggcgagaaagacttgtagagtttcggaatcaaaggggagggcatt
tgcctccatttcggcaagaagccgaagaaaggagaagggatcttgagaagtttgccagagagccaaaagagcttggttatagacttgagtggta
caagcttggacgtatgcatagacgctagcatgcttgacatgggagaaaaaggaatgtgcgagaaggggatctagaaaattaagacgagcgaca
agcatggagtcgcgaagagcgcgggaaagaggaaagtaagatgacacgttggaagaagcggaggcggaagatgtagggtgtttggaaagtc
gcgggacaaaatgggcatggaaagtttgatgcaactggaagctagactgacaggtgaggagtcgatgacgatagacaattaggtcggtagcct
cttgttcagaatgagcaagagactctcggagagtgacaaaaaacttctccgggtgttttggaggagggtgaactccaagagtagttggagcacct
gcagaaaggatttgctgacttacatcatcagaaggagaagggggagggtgacgagaaagtgcatgttcaaaagacgatttgacgttacgcgga

agaggtatatgaacctgaaaaggaggttccatggtatccaagaggcgagtagaagcggtgggataataggttgggacaacgggcttcgcttctt
ggtcttgaatgaaaaagtcaaaaagctcgtccaaaggctcatttgtaaaggcgcgatgaggaagaaaagaagaggacgatgaggaagcggtg
tgtgagacacggcaattggtggaaaaagcagttgactgaggagtagcaaaacgccagcattgacgacgaacagcagcaagaaaacgcgaaa
gatgagaaggagaatgagcaaaacgagatattaagcacatattaggagcattatcgagcgaaaataaaaaataaaaagcaaaaaccaaaac
tagaccaaaaagaaggtaactaaagcaagatagtagaaaatacaaaaaaaagaaaccaagaaaaagaatcacagaaaagagacccaaata
tgattttttctaccaaggggaacacaatgagaagggctttagcaatagcaattcgtaaaacgagcttgggatattaaaacaattgaaactagcaa
attcttctacgaagtagtacgcaagagaagaatgtgtcttgtaagggaatagacgaagttggaaaataaggaaaaaccgtactacaacgagtg
gtagctaatcttctttacgaaacacatcattatcgttaaaagggataacgaactgtagaaaaaatagtatcaatagaaataattaatagaaaaga
aagagtaaaaatttatttcctgtttttattttttatctacgtggtttccatccttcatccatacttttatcttctgtctgcttttcctttgttttaggcggttt
aggaccatttgcatcacgaaagctaaaaatgcaatgcccttttctattggatatcgtttataactccgcagaggcaaatcttaccccatcgttaaaa
acaatgatcgcatcgtttcgattgtttccccgtactattcgttcattttcggactatttacattttcttttgttgacatttctttgaatatcatggatggtt
aaaatctcttacttctcaaactgataatagatggaaaacagatattacagcttgtataataaattaaaaaacttacttttagttttagatgtccgttt
atgctttatttttttatacattatttcattttcagttcgtttgtttattttttcctatgatatcaacgtcaaatcattggatatatcaatagctccacccgatt
caatgttcagtattatctcataatttaactttatttaacataaaatcatttttagccattcatggaattcagaggagaacgttaaggcctatatgagg
ctgtcactttggcaacttaagtaaacccacagaaaatccgcatcattaaactttctctttagggtggggttcaatagcttaaatctagatcggctctc
gtatttggaatttgtttgcccccctacttcacactttatatacggcttgtttccctgccgggttgggttttcatttccttagccttgacttttcttttttttttt
atatttttcttttctcctctaccacctcctctgcattttaaggtttctataccaaagtcatcatacgttggtaaaagaaaaaaccaaaaactgagcttt
gtattttgaaaaggagcgaatcctatactttcttattgatccaaccagttttacttgtgtttgtctgtgttgtgtttatcgtttttgctcgctacgtatatt
atctcctattctctttttttacaccaaaaaaaaaggaattttcctctggtaatctcttgttgcttttggaaagatttttgacctatttctggctgtctctctgc
atcttgtgtgtttcacctttcttgttaagttgctttgattgaatttatttcacatacttgcttctctcaccatgtcgcctactagtccttctaacccaccttc
catcagtggtactcatcagcatgcccataacccatcttatcctactggcgctgcctcttttaccactggtactactggcgctgacggtagccatgcta
tgaatacggaaggttcacaaccaacccatcctacttttcttgacacgaatcaccaaggcctccacaatgctatgccaagtggtgcggttactccta
atactgttgatgtgccctaccatatccccaaacactctgaagctcggaagcttaaacacttttacgccagtcgtgccgaacccatttatgaccaaac
agaaaaactagctactcaagttgccgagctccagcgtcaacaaaacaaactctttggtccctcaggtgccgatttcgaacccggtatccaccgtct
tcgtttcaacaagaatgctaaccctggtcctttcggtcttttctctttcgccttcactacgtttcttctttcgcttattaatctcgctgccggccatgtcac
caattctcgtatcatcgtagctcctgccatgttttatggtggccttgctcaaattcttgtttctatgtgggaaatggcttctggtaacactttcggtggt
gctgtctttggcagttatggctgtttctggctctcgtatggtgctatatttatccccctggttccacattgctaattcgtatactgatcttgacgagtttaa
ttatgctgttggtctcttccttatatgttggtttctattttccatgcttattaccatatgtacagtacgctccacgctcgctttcttttgtttgtttgctactc
tcgatgtaacgttcttggtattggctgccggctatttcaagactgccggtggtgctcctaatacagctctgatccgcgtgggtggtggttttggtattt
cgtgtgccgcttttgccttttacaatgcccttgccggtatcgctaccgtcgaaaactcattcttcagtgtgcctcgtgccgtctttccctggagtttgga
gggtcttagtcccgctgaggctaatcgtagacgtatgtatgatgacgatgacagcgataaggtcgcttaatttctttcgaattattacctttccccat
aacttgtgttccttaattttcaaaccagaggagtcacccaaacctccgtatccctctctcctcttgacttcaatccatttaatctattttctattatgag
attatggtttgcgttttcttctttagcggtatgttacggtgaccaagaacccttaattgaatttcaatgatcatagtttctaatgatgacactttaccct
tactttagcggctgttctttgtgtcatactaaatccctcttacattctttttttctgtatattttttttaaacggtcggtttccaatgtccttacctttcttcct
tacggagtctttagctattgacatttcatgagtacgaacggagtgaatgacgacgacgacaatctatatgtcttatatatattgaattgtcctatcta
tgtgtcttctacatctctctatttttcatgactttgcttcacgagctcttcttctcccaaatgaccaaagaaaaggatatctaccttgtacctttgagct
ccaatcttgcttataggcacgtacatgtctcgtcttcaaaagcgctaattttctttacggttatattctgctttagctagttccattctttcgtacactat
gcattcataataaaagtaattgtgtatatttttttatcatttgtactttcaactatggataaacatcatgtttgtagtacgtactacttacggcttgaagt
agcttttctttataatgtaatcaaaacgtcctatgaatttttcatcataaagtcttctttttctatttctttgaatgcctcgtgtgtgtttcattggaagtt
tttagcttgttactttgttgcacagtaacttgtttaaatttccttagatctcttttctctcttctctttgtttcttttttttgggaatttccggtctcctgcagct
cagctgcattgaataagttttaagttgtttgttttttgttttctgtttttttgtatttgttgatttttggtttgtttacccagaggttgttttgaatctttctctt
ctccctataaacaagaggacttgtcaacgtctctcgcacccaatatctttccttccattttccaaagacgtcgagtttcttgaagagaagtctaacg
acgttgtctgtgattttgtgatatctttttctatcctgaagacgacatgctgaatttggacaacaaggaaaatgaggttcctttttttcgctaaaaagg
aaccagaatcccctcctgctctatacagggtacagcgtccgttgttgcgtcgtcctttacaggaactgagcattgaaatggtccctcctccttcttct
acttctggaaaaggagtcgtcgagtctcctaaaaaggcggcgaaaaaggtatttcaatttttgattcaagaattctccccaacaatgttgctaactt
acaaaattctagcccttggctccctcgcatggtttccacccttataagatctctaccaaggtgttaccccattcctctcatactcacaagcagcctctt
aaacaaaagagttcgaataggttgatgtctatgcgctgaagcggacttgtcttttctttacccctctgcccctctgtggttctcgctggaactaaata
catgcttcttatacttattcattaccctctgtcattcccattctatatcccgtcctgtttacagtatcgctttatcaagtattatataccatgaagcaatg

taatatcatttgcttccgttgaatggaatttgaactgtctggatggtatcacaaagtgacttgtgatgcccctctaaaactattgctatgaaatcattt
ctcttgatctttcttttattttacttgaaccgaaccgaatgtgtgaactctatgatcgtagttattttgattcgttatatactggatttatcagagtaccg
ttcactaatatacagattcaccgaatcaataaggtcttgatctttggaaaccattctcatggtttggagctcttcttcttttaatcacaatgaggccct
caccctcatacctatgcgatgaatctctaagaatgaacacattcgcacaattattgctattcttttctcttcgcttattttaatccaattctattcgtaa
ttaatctgagcaagtaactgtttgttctgtatttattctcattaggcactttgttcaattcatgaccatagattctactattaggaacctataaaacaa
actgaaacatagatgacttaaattcataaatggtccatagttcgagcatcaaatggtctctagaaaacgtatatattttaagttagcccgaaagag
aattgagcgttttcaacacatctatccttcataagtgatggatttttcttttcttttgtttctattatttttttcaaaagtgaaaaatttccttaataattt
aattcttagaaacgacatttgaacatttcaaccaaacgtgtatgtaccacatatgcaattcaaccaactggaagtacccaattagtaacaaggtta
tatgtttttttatcaatttctttaaagtatttatgtttaatattcagaattgggtttccaactataggaagactctaataagcgtggaatgcaatgtcga
agctctctgctcttctcgatcctatttttttcaaagattttagataaatacgtgtattcctggtactatgtgatcagcaaagatacgctttttccctcaca
atgtgaacaggttggcaactctgtcatttatgaattggagaagcgcttgtcgcgccaagatttgacgaaccttttatatcatgagattcccttttttgtt
aactgaacacgtaaaaaatgtggaagaggcacagcaacgaacggtgatcccacagggacaatttttgaatgttgacaggatttatcataatttg
catcctcatatcgctttagaccgagaggaaaacgagcttatttattgtcgcctaataatggaagacatttgtcgacatcttctacctgccgtcaatg
ctaagagtgaaatcgagtatgttatattgagggaagctttggctactcaagtgtataaagcaattcaaaatttttcagatcatgaaaaaatgtaca
agggaattctcttactttcaaacgctttacagcgtccctctacagattcttgggtgctttctgttcggcgttttggttattggactttacgagcaattaa
aataatttccactactagaacccttccgtattattctacctcttggtttcgcttttattttcagttatttacaaagcaagtcagtcaagttccctttgacg
aaacaaaacggttggtctttaccactttgttctacccatggatcgtattgttatccactttttttttcggtatcctaacttgctttttattgcaaatgaa
ttttatgacaagggggcttccgaacgctggtccaatttcgtattaaaccgaatttctgaagctgaaaagagcagcacttcttcgcagtatacatcta
gtacttcaactaaagagtaccgaaggcaaagcaagtcttcttctacaaaagcttcacaaaggcagcgcaaacaatcggttgcttcgaaaagtctt
catgaactgtctatgaaatatatccgttctgaaggcactaacgattcgccatcaacaatatcttctgaaagaaggaaatctttgctatttacagatt
ccacttttcctgataacaatgcttccatagctctatccacggtaacgtctaccccggtttttcgacatctccaagccaatcttccaatacaactcga
gacaatttactttcctttgttccttcaaaattacagagtgtttcaaaggaaaataagtcatcggaaacgaaacataatcgaactgcttcgactccta
ctgacacacacaaaccggacccatctccaatgacggatgtgatggtcacgaagcaagctgcgcttgaggcttatgcaaaacttcctgtacttcctt
cgttgattgcacctgaaaagcttgcaagtttggtggatagtgattatcgcaataagcacatttttttactccctcttgaatacgttaactgttgtgatgt
tccctgaaacaagacatgaagaattatgatggatctaagcataaatttctatgaatataacgatatgccttatttattatataacccttttgttttggt
ctactataatactatatacgaatgagtggaaaatacagaatctatcgcatctctatgattttacattaatgttacataagtacagaagaaataagtt
ttgcataatttttttggttactaagatgtttgtattaagtgcctacctaagcagggtataatctcatagttaatacaataaaaaattgtttttgaagagc
aattgattttgtaaaatagatttcatcgttcaaatttcaagtacatttcatatatttcctttcttaaactcctctccaacctattcttatggattaagagt
ctcataaacaaatcgggtttaaccgctctttttcgtactttaactattattatttattcattaattaagcaagcagaaaaaatcagctatgctcaaa
acgttatttctattaggcgtatctgcattcgcaagcgtgtttctgatacttgtaggaagagaatggtactgttcttcgaagaaaaatggtctcgcaa
atcctcatctgctagtatattttggttctggaggccatacaggggaaatgttgaatttacttggcgctattaaagatcatcagtatcctgtacgatca
tatgtagtaggtgccgacgacaaaatgagttactcaaaagcaaaggctttaatatctgataatcctcataacaagtcttcaattttcacaattcctc
gggctcggtatgtaaagcaatcgtggtcaacgacacctttaacggctatctggagccttctattttcactgaaagtcatttttttggaataaaaacgg
aatcccagatgtgattctttgcaatggtccagggacatgcatatttctatgtttggaaggatacgtaactaaggtaatcaacctacgatcgttcact
atagctaaccatacagtttttttggtaaacgtatccggatagtttatgtggaatcgtttgcaagggtgaacaatctttcactaagcggaaagatacta
ttaccgttcgttgatcgctttttagtacaatggcctgacctcgcactcaaatacaaaggtgctgaatatgttggcattgttacttaattggatgtgtca
tgatatgtctttattaacgctgccaaatatgatgatttttctaaaagacttagatagaaaccaaatctggatcgactaggatttaggtgattattgaa
acatgcgttctatagagtacaactttcatttcattctttttcattcctggtttttttgtttgagccattcaattcctttatgttagtttagatccaaggctaa
agcatgactacaagtcaaacagagcatcctttaatactttacattcggacatttttttagttcacaaacactaattagcaaactttttacatgttccaa
gttcgcttcagcttgtaccattgccttttggttgtttgcgtccgttacatgggtgtaagcatgcaaaacccatgcttgatcttctgctaatgggtggaa
tggagggtcaatcgaatgtagttgtgaaggaattaatgtttggaataacctgatagtacagttgctggaagcatattctaaaccttgtactatgtat
tcatgagaaaatctagagaaaaggtgagctagaagtttgcagtttgtaagcaaaataatcaaaattcttactcgtatccaaggttttctacaaatt
ccataggggagccgttcgtaatttttgcccgactaattgggcgaattccgcaccttccttctctcttaggttctgatgtaccttctaaatgaatagccc
ttatttatattagctcctttttcattttataggtttgcttaccattgtgccttttgtatccagtcagactcactatttcctattgtgtctaattgaccgcata
atcgaagccaataatctgcctgtcgaggtaaattgattggaacatctacaaacatatattagtactctaaaatcgacgtaggtcatcatggaggtt
cttgaatttgaaccaaatgaggtattttcccaaagcattgctaaacctacctttcggtcgaaaaaccatcatatactcaaaaattattttcggttcat
tgaagattcttaagagtgaattggcgacaatgtctgatcgtctagacggtataactccaattaagtatagctcctgcatttcatcgtatgtggaact
agtttgagcggactaaccaagaatttgaaaatgagtaaaatttccagtgaactaatttcttattacagtcgctatagacaaactgtaattctttgttc

actttgaggaagtcaatatcgacgaaaacaggtgtgatattttcttaatggtcagtagaataattctgcaaatattatgagatgcgtcaatgaaca
aataaatcaggtttatattaaaagataatctatttagaaaagtttatgtaagatgatagtctttatccactatgctttggatttctttctttgcaggca
cgtctcttgcagaacacatccaaatcatcaataaaataagttagcatctcaaacgcaaattacaggaagctcaggtaaaataatgtttcacttca
aggaggcagttaaagccattccttccataaagtgtagattagtattgcttatcattctaaaattgcaacatatgaaccaagtttagaaaaggcatt
aaataaacataaagaattaaacatcaatattatctagtttgaaaacatgagtattaaaggagtgccaaattgcacaagaatagcggttggaaag
caaaacaagtttagccatactggattctatcagaatggggatcaagatttttctcatataaaagaatcgaaattctaaggatttataactttggaac
gataaaggggcattcttcaaaagggagatacaatttggctctttactttgtccaatcaacaaccaaaagactggcaaaagcaagaagcaaagag
ttgggaggtgtgaaaatagcataggaccaccaaaggaccttggcgcattctctctcgaaaaccccattcaattgacaaatttgtgtgagttgaatt
gcagtagggcttcctaccaaaaggaaaataacgacaacgaaaactggatcatctacggtgctaatttcactaaaataagacagtaaagaaaaa
gcgggcaaaataaccataggaacaaccaccattcgaccgagaagactaacgataagaacgcgtttgtcatttactttgcccggctgttctggacc
ttctgtcttcccaatatctgttgcaaggctagcacctagaaccactaaaatcaaaggtacagcaacatcacctaccattcgaataccactggtgat
actggcctcaacaaaagagccttcttcaaaaaagaatctttgcaatggaggaacaacggcaatgaaaagagcaataaacaaagagtacagag
gagggctgaaaaaatctagtaagaggataattgccttttttgagcttggagtttttaaactcaatgccctgagtagtttcttgagtaaccgcattttcg
gaataattgtttttgttattagtggggcttatgcccccgggatgtgacaacccactagaagaaggcaagtctaattgactgatatcttcacgttcatt
aggagtcagcaaactctcaatttcctcctcactaggattaccgtggacccgaacacgatcgtggtcttcactgtcatcaggttgaataggtgaaag
gagaattctataaccatacgaccaacgaagagcctgacccagctgagaaaaaattaaaagatacatgataccgcgagaagcaactttttcagc
agtgtcgtcgggaattttatcccattttaggttttccaccgtattcgccaaggaagtaactaaggctagaggaagagaattggactagaaaagttt
cgttagcgactgaatgaaacagttttgttttcgtacattttggaaagtgatgcatgcagtagcaaagttacgttgtcgtcgactaagtcgaaaaata
cgagccatagcataagaaactattatagaactaaagacagtattagcaaagagggaaacacaaggttgaagaggtaaaattttcatacaccgc
tgtaataataacataaaaaacaggcagaagagagagatcgataagcatttttagatttaaaccattgccgactttttcaaaaactagacatgggg
taaagaagtaaacgtttaaattggaaatagcctaacaaattagtaattcgatcaagtgaagaatgcaaaaaatagtaccttttgagattctctcg
acaggaatcccttttttcgccagaataaatcctccaagggcgatgacaattaccccaagatcgctctccaaaatcggcttcaaaagaccccaaaca
tcaattcggcctaacaaggaagacatagcaaaagaaaagaacaactaatgtcgctaaggaaaccgaaaataaaagacacaagtcaatggga
gctgacacaaggagaaaagtcaaagagccaaaagagttttaatttttaaaacttgatatgaaaggtcaattaaagtgaactgacgcgatttgta
aacgaaactaaaccagctacgtggcaagtactatacagaaaagcgctaatctttgttcaatggagaacaaaagtcaaaactgtacgcttcaacg
acggatttgatatttccttgaaaataacaaacgattctcttttcctgcaaaaaatcgggctaatgatataagaaaaagccaccaaattggctgaat
tgatgatgaaaacggtagatggagaaggaaaggagactgctaccgcattcatataccgacatcctcaagtagcattgtgacttgtcagggtacg
aaggcataaacaaagagtgcaaaagtgggattcttttctatgaaatgtgagacatgaaaaaggaatgaacatctgaattactttttatgacaata
cttgaatacttggggcttcttttttttggtgcattgtgaagaagatggtttatttacattcatggcaataaaatacaagctggaataagggagtccg
aaaaaaggtatggaagataattagtaaactttatggtagtaattggatttaataatcttcgtagcacagtacgtttgaatattgggtatatgtttat
gcctttggatatacatgttcatatagtcaaacgctttcagcgaaaatgattcatagactatgagctattttatacaaaaaactgctagttatccctac
aacctgaagggtacagaaagcgtttgtccatatatcatattcataagtgtattgagaaaatatcatactattcatgagatggattacgaaacttgc
atgaaaatgcattttaaaattaatagattggattggcgattactgaattcagagaggaaaacagtaaaaacaatactttccattgaagaatggtc
gatttgtgcgactgcataccaacgaaaaaaaaaatgtcaagattgtctactttcgactcttgtttgattccatagctcactgggattttctataccaa
gctataaaaaagagagatccgtttttgttactaactcgtactttccagattgccttagcttagcttaaatgaccattgctgtggttcatgaaatcatc
atgaaaggtcagttctacctcatgcatttagtttataaagaaggatacagggatttgaaaccgtctgcaaattttttttttcgcttctttaatctccat
ccttatttctttgtagacttgaccatatctgcgtcacctttttttattcgatggcttttttttgtcgccaattccatcatccctctacaaattcaccaaacat
ttttactttcgacaacttttaattttctagtatttctttttatttcttacagttataaacattcttattatcgtcaccttttttgtttttattgatttattcaat
aataaagcaacccacttatccatccaaagacgttgttgtctatttatttattaggatctacgaaccattcttatagcttgcctctccacactggaattt
ggggtgtcgaaattcgtttccggcaatttaccttcggaagccgtttcttgctcatttttttcatttaaaaattcttaaaggacgtgcccaggctggtac
cgtttccatttacctcactttcttctttttttacataaaaatttctcttgtttttcttatagcaaactacggtcgcttgctttgtataaggtaacgaacttt
agctttgcttcgctattaatttttgcacagaacgtaaataaacaaacgtacctccaaccgagatttacttttcaagatttctcttgtttcttattttttcc
taaatctatttatttattaaatttaagagggttttcttcctttaaagaatatttcgctcgttttcgatttattggctttgattgttaaaacaggattttctt
gtttgtatactttgtcatcgattcaatccaattatttggttctctcaagtcttctttgtcttaataataattatctgtgtttgtttctgtcacgactcatca
gttatcgtaagtcattgttttcttgacggaaaatctgttttgttttgtttttctttttttgcaagaaggctttcatccaactcaaaggtcaagttgaacttt
gatatttttcagtttcatccgactcgtatgcatgaaacagatcttttaggttttatttgtgcctaaattaacacatccttactttcctgattcctattctct
acaaggttgaacgcgttgtaggttttgcatatagccattgtcctctcaaatattttcttctatgtctattgaaaatccgggatttcaatatcctgacac
tcctcctcttcgcttttcttcacaagagctttctggctcgacaattcgcgctcccttaacctctaaacctcaaagtcataaaggattgtatcagacgct

aattgctaattccactccttcttcttctacccagaataatgtatcggcttctttatcctcatcctcttctcgcgcagcaagttatcctcgtcatacaaag
cgagcaaggcggacgaaaaaaatctaaagcagatcctccttccatggtgaaaaaggctcgcatgcatcgaagcaaaacaaccagtgatttatca
gctcttgaaaataagactatagctgtatcatctaagtctacggatactttatcaaatttattttcttcagagaatataccaacaatcgcccaactga
aaacccgcaaagctactcttgaatctcgcttccatcgcttatccaatgcactaggtgaatattatttaagtcatattgaaacagcagaacatcttat
gcatcaaaataagcacagtgtcattaatcgggagcgacagtttttcgctgccaattacgaaaatcggatacgactctctacaaactctacaaggct
gatccaacgtcagttagaacattacctttcctacaggctccaaacaatctcaaaaaactaccagagatacgctatgcttatacggcaacatttgct
atacaagacgatgcaacgctatcaaaacttatgtggacgtcgtctgtatcataacttggttcctatacttaacccttctacttttcagcaagaacata
agccagctcaaccaatttcagaatctacatcaccacaagtgacccccatatacttacctcgagtcccaagtgtcctttctgatcaacaagccctag
atgacttggctctttgtaatgtctaactatgactatgtatcaatgcgacgcactttacggtctgcgttcattgctaccccttgatatccccatataca
atacatatatatatatatttatgtaaaagcaaataaaattgaattttcggtacatacaacaatatatagaaccaaccctatgagaatcagaagtca
tactcaggttgctccttgtgcaattatatatggatcaacaaagaaaagtcatatctgaaatcaaaaatgccatcctacatacttaaattttacaaaa
cccattatattcacatattattatacatagtcaatgaaaagctcatgtctcatgagattcaataacgttaccagaacggtttttttaaatcgcacaaa
cataactttctacaaccagattcaattaaaatgataataaacatcagcatctattctttttctggtttgagatttcaatttaatagaagaaaccaagg
attttaccaccagtgtccttggcacgagcttcattgtgagacatgataccacgggaggtggtcaagacaatgataccaacttgacgagagggcaa
caggttgttgacccacttctcaatatccttgatcttgatgttgtagcgaggggaaataacaccacacttgttgatacgaccgttcaattggataaca
atcttgccagaacgatggtcatcaatctcctcaaattcgtcaatgtaaccatgcttttgcataactgtcaagaatttgacaataaccttagaggacg
gacgaataagcacttgacgacgtccgcgacgttcagcgttaacgatgttgttcaagcagtcagaaagaacggattgacggaccattttgtttttct
aatgaaagcgttagatttctcttgagtgtcttgtttaaagattcaagaaaaggttctagttattgagtacattagctcagtgaagaactaatgatgtt
caataaccatacgctttcatctttcctctattaaaatcatacctattgtttaagattttatttttttcagatgtttcagaaagccggtgatggcagaaa
acttacaccctgtcgaatagagtggtttcctttttcaagaacagtcactaaaccctaccctaaagccatgaattcgtactttcatatttacaatactg
gatatttgccattgacaaatccaagaaaaatattaatgaaataaacaaaaaataatataatattcaaatgaataacattaatctgtaggtttatca
aagtacaatgatctagcacgggtttttactcgatcaacaggtgtcatgtttatttttactttagcatttttagtgcctaaaccaacacctggcaaata
ggctgtaggctcaaatgattcatgggagtgtgtttcactggaagtgtttcctagggatcgattccttgagtatgggtcaggcaatggagtgggctct
tttgaaaagtcggtattatcggtgatatgaatggctcttctttctgttgctcggtctctgtaatcaggacgttcgatttctcgaatgcgattcatgaac
ccctcagcagcagtaactaaggagctattctttaagttcttttgatgtaaaaaagattgagttaaatgcttcagtacatggccccagctattaaattt
tctctcgcacaagacacagataagagatgatttccaaatatactgtagaagaaatttctcttcttcttcagtaagctcgggcaaatcttctgttaatt
ccctttgggcgccttgccaacgtgccagagtaagagcaatctacaaaagttaatcaataaattcatatgtatatatatcataccttttttgtttgtagg
tggctctactaagtttgttcttaatcgcttttctttcttttgtttttgagtctttgcaattggtttgaactgttcttggtcaacgttatcagggacaaacgt
agagcagtataagttttcatcccaataagcacgatacccgtctttggtaggaaacttccaagtatcaatttgtccctgtgctggaacaaagacgcc
ttcgtgaatgtaatcaacaagcacttttcgagaagcaattgtgaacgcctttcctgaaaaggttcgcgccattgttaaagttttagcagcctcatca
acattcggcaattcaataatggcataaccacaaaacgaatcatctttcttttgccgaatcatgaataacctttggacattgcattctagttttgctat
gccttttagtagcgtaaggtcatttaaacaacgatccaagtttcttaataagaggtaaaaagtaggtatatcgcccacatcgttacttccatttcttt
ctgtattatcgacattagatcgtaaagttttacacttgaagcaggcatctcgaaaggaatagttcaatgaatcacactacatttgttagtcaatacc
taaaataattaaaatagttgtaatacattttcacatttccatccgtcaatctcctctcgagcttctcgtgagtagcggacactaacgtctctatcatcg
atatgaattgacggaaagtatttctccatccactgtttcgaagcagttaaggatacaaattttacaaagctaaagcacttcgattcacgagtaactt
tatcacgtattagaacgatgctgtcaatatctccgccagtctcactcattacttctgaaaccttcaaatagagggtaagcgtatgaaatacgaaaa
ttctagtgttcgtacatgttgctcatttgttgtgggagccagtccttgcaaaattatttcttgatttggcactcccgcaaaccttggagtaaagttgga
tttggaatctgtttcgtctctgtttctttcattatcatcgtttttatcgtaattatgctcctgaaaggtatttctctctgtgttgatgaatatatcccttgta
ttcgaaacagtctgtttcatcgtgcctattttttctttcttatgctcatctttttttaccaaaatccatataatagaatgaaatgaaaaataaatactaat
ttgtgaataaaaatgggttgctattctcttgttataacacttctaagttttctatgcggtttggcaatatatacttttggcaagcgtgtcgtccataagt
atactactattactcataagttataattaagcactaatagtaaacctgaataatcttttaattaattgcggatggaaaacttgaaatttaatgcaact
accaactacaaccacttggttaaaccacgtcaacgcaacgctataaatccattgtctgtaaaatatccaaaaaagtagaaacatatttacccata
ccatctactattcagacctttcaccttttttcttatagtgatgtaaagtagtataaaagagatccaaacgtgtgcatcacatagaaacaatacactag
taaatttagggaatcatatattatagaattactagacgtttttaccaaccaaaaaccataattttatgcagtagatccacgaacgctagaacccttta
ccatccttgggacgagcagtagcggtaaggtttccttcaacatctaattgaagaaccaattcaacctttgagttggctgggacatcaggaatgtta
atggtagcaagtaaggtcttggggacagcaatgcgttccttgacttcctcaggctcctcctcttcttcgtcagaaagtgcctcctcttcatctaaagg
ttcggattcaaccttagtaaccttgactgagcgctcagcctcaaaaacagggatataagcaccaccttccttaggggtaacaacctcaacaatttt
acgggtgggcaaggcagtctcaatgtcaaagacggtaacaaagttttcaccctcgttaataccaataggagcagtaagatgaggagcttcaacg

atggcgggctccatagaagaagcgatatcagaaggttcaaagtgaccaaccaaagaagcttgaacaccagaaccaatagcagtgagttcaga
agcttcaaaaggcaaaggggtaacagagctagtggagcggatagcagtctgttcagggaagatcccttccattaaagaagtcaacttgggggta
ttggaagcaccaccagccaagatcacttcagtaacatcgaaaggctccaaattagctttcttaacagcttcctcaacaagctgagacatacggttc
aaaggagcagcggcggcgagttcaaaacgaaggcggttaagggtagcatggaaatcaacgccttcagcaagagaatcaaccgcagctgaggc
attggtattgttgctgagaacacgcttggtaagttcacattgttccctgagctttgcaacagaacgagcatttttgaggggatcaattccatttttctt
ttcaaactccttagcaaaatatttgaccaaaacatcagtcaattgttcaccgccaagattttcatcgtgagcagtagataggatggtcatcatacca
cccttaatagaaacaacggaaacttccgagcgattagcaccaaagttggcaacgacaacagtcttatcgacaagaacggactcagaatacatg
agtgcaagaagaatagcagcagggtcatgaattaattgaaggatggggaggccagcttgggcagcagcagcttccaaagcctttctttgattttc
agtgaagtaaacagggacggacataacacaaccatcgaccttaacacccaaaaagtcttgggcagattcacgaagacgttgcaagtggcgagc
actggcttcttgagcagtaacgatcttttcctttggtttagattcctcatcctcttggacggtgaccttaaagcccacttgtccctctacgttaatagg
agtagctgaataatggcagtggtggtttgtaacttcttgatgagacttgccaagcaaatcacggaagttagttacggaattgtcggcattgcgaac
aagttggccacgagcttgaacaccatggtattcttggtcaccttggtaagacaaaatggaaggaatttggcggtttccttcttcatttgccataaca
tcagtctttccatcctaaaagcataagttagtactctattctatgccaagaaaagaagggtctgtcaacatcagaaaaagtaatgtctgtaaagtt
gtaacaggccagtttttcctgtaaataatttacatactctgttaaaagagatagaactattttggtttccaaaagaaataccaatgacagtcttatat
tcttcggattctgccattttgaaaaatttggtggatggaagaaggatagttcaatacccaaatttatcacaccaactcagtggtacttaccactaaa
aatttgtgcttactattactccttaaaattttagtagtttactttaacaaattagtatacttttatttattttgcagctactaaatgtttccatttggaaat
aatttacaaagaaacgattgtttgcaaggattcctgtgacagtcggtaatgaaacttgtatttatcatgaataaatagatgtttgtatgcatgtaaa
tggaaaaaacatgcaaagtattgctaaaactgaaccatcaattcatgtttctgctctgcttataagatgttgcatagcaacatcatggctaaaaac
atatttattactaaatgaaaatacagaagtgctatactgattgttcttttggatgcctaataagaaactattgcaaaaaaatcggtaagctagattt
tgcggttgtaacaaaactttcatttattattatatgaagatacaaaaaaagacttaaataaaatgtccagttataaaaaatttttcccggaaaacta
gaaacgagggttgtaaacagacaattcttgcttaagtactattagaaaccgggtggtgttattagtagtattcattaacagcattggtaattagttc
gtttttattacaagagtaaaagttaatccgattgtccgtactgtcaccacatgccaacattaaatgatgaggatggaatttcaggcaacttagtcca
ccaagtttcgattgcgtcaaaaatcgagagctttcacggaacgtattgatatttttaccatcagtactccataccttcacatattgattcgacgatcc
agtggcaaacacaggcgcatggctatgtacgcttagagacgtcaaatcatttgaatgagcaggaattgtcaataagctatgattagatcgaatat
cccatagttttacttcaccatcaacactagctgaaatcaattctctcattcccgaagattgcatttccaaattcacaattcttgaaccatgctctttcc
aaacgtctgtcatagattctcttgctggtaatcgtttatcataaacccttaatgcaccatcagtaaagcctgcaactataacattacatccaacaag
atcactagtaagactagtaatgccattactacttcttgctggtagactagaatgacaaagttctttggatgcgtcccaaattcgtataacgcgaacg
tctccagctacgaggagatgaccacaattttgttgccaatttagtaataaaccggcatcacgatctccaaataataagtctccaagtgcattccaa
gaagttaccagttcgacatcttcggtatcatagttgcgataaagacgaacgacaccctcagaattaccggtcattaacagagcaatgtcatcctcg
tttaataactgcatatccataacatcacttctcaaattcggatcagttttgaaagcattaagacacttagaacgcccccagtcccagacttgaatta
agttgttgttacttaacgatactaattgatcctcaaattgatgaaaaattagtttctttggagtgaagacattgtttatggacgaaacttgctggttcc
aatggttattcccagatagttcagcgagaggtcgtgtttgataaattagcttttcattcctatttcttctccaaagacgttgattgtaacagatgctac
caggttcgtcgtcttcattaggtctcatctggggctctgtaaaatatttacgagaccagtcaaagaaatgacttgttaatggcatatcgagttcaga
ctgatattccatactattaaaagaaggagggacaggaactttagcatctgtcaaccgagaagctccataatcgctcgctttgctttctccagtaag
cgaaagtatttcagaggctttatcattgccagctaagcttaaggccagtccctttaatgatttcgttaaagagaatgatctggtcagcattttccttt
ctgcgggatatcgagaagcagaaaaagatcgcgagtcaccgtcaagcgaactattttcagtaattgtatcctgagccgaaacagggttccgaag
aaccggcaaacttttagaaacgaaagcagaaaattcgtctttaagtgatgaattgagaatactttggtacacataatttacaactgcttccgcagc
catagctacttccacgcttggatcagctgacaaactcaaaatagcctgccaaacagtctcaaatatagttgaaacactaataggggtatgttgttt
ctttcttgggtttaggaaaatgctgctttcataggcaacgacaatgaatctcttcttatagcaaactacaaaacgcgacaaaaatatcgtaagttct
cgacgtaccataggagacgcatctgacattgccacaagcgcagataaggcgatgtacgtttcgatagctataacttcttcagtttttttcggggaaa
cccaagaacgtagtgaaagctactaaagctgaagcacgaacttccggaacaggatccgaaacaatctctgcaagcttcatgtgagcattatcgc
gtatagctgaccattttgcttcagaataattatcccacagctgtgcgatacaaagacaagcccactgtctaagcaatgaatcggaactattcagat
gactcaaacaatgtgataagacctgaggcccaagacaagccaattgtccctgagggaatcctcgacaaaaaatgcttaaaatgaatgcacacat
agcacggtgttcggaaatattactagaaggaaaaattgaagaatttgggttcaaaatttgtacgaaataaccatagccattatccttcagtaagtc
tgcttggcatgagtcatcaacggctaagattcttgcccaaataaatactaaaacaggttttagttctatagcaggtgactgcagtaatttcaaaac
gtaaggaaaaattccaatggatagagctaaatcgacggcccaaaccccaaggtcaagaaatttagataacagaattagagctcgaagacgatg
tacctgcgaaagtaaaacttgcaatactagtggtaactgatctggtggcttgcgagttattaagccttgagatagccaaacttcgaaagctgtcaa
ttgttcgctaaagaacatggaatgtttatattcgtaggaaactcctttactctcagcttcaagcatatctggaagctgggataaacagttgtcaatg

gcaaggtcccaagaattccacattgggtgatcatgagttgagggcaatggaggactagactgtggatgacatgaatgcaccaacatgatacgtt
cggctaaaagaaagttacggaaaagggcagctaccatgagatcctgacggaaaagtcttcgaaaaaggtgtttggggaatacgttccaggctat
tgtatctgtgatagctgtgaaaatccaattcaattcaccaagcggagtgcggcgatcttgtaagcgaccaggtatcttcaaaagcatattcaaattt
aatttgctgggaaacggattttggagtacataccatcgcacagaaatttcaataggggaagtcaaacaggaggtgaaaagatcagcaggcaga
tcaggattcataggaagcgtttcctttggaccacatgccgcaagctgtatactggtagtatgggatggcatagccagtatattgggattttgtttag
caagacgaaaagcctctctatctctctgttcagcaaatcgattaaagttagcaacgatatttccagcagcactacaatcatagacataaatacag
ggtgcacccagccatgtctgtaggtcatacaaagacactggaatatattgagtatagtttttattaaacacccatatttcgccagaagaagtaggc
ataggaactccatggccattataatgaaacaagatgcgctcttctttagcatttcggcgttgtgcaatacaaagctttttttacttcttcaattgccgg
atcaagatagtgacggtatctggtacgcatactgagcgtttcatactgctgttgcaaattctttcctatagcttctaatgctttagaagcaggtaaag
aaaatggatcaacccaacactcgtatttagcacaagggttaggtttaattatatcaggaggatctacccctatgtttaagcacactaacaaagctg
cagatattgttttcagcctctcgcgcatcctccagtcatgtatagtttccgtggcttctgcagtctttttactgatcaccccgcgatgccgtttatcagt
ataatacatataaaacacttgttctagcatattaatatattctgcattattgtattcttcttcaaacccatgcctgggttgaggaaatgcaactccaa
aaccatcattagctgaacttgttgtatccccaccattctctgtcatataattactagtctctgtataaccatcactccctgtctcattcgtattatagct
taaaacagacctatgggtcctagagctcgtggaatgctcgctgtttcggtcattcatatcgtgcaaaccagattttttaattcatacaatcatttctaa
tctgttcaaagactcctatttccaatcttttaaagtgttttaaggaaaaatacgttcgtaattagtaaatggttataatggattctgctaaagcaact
ggtgtttagccgatatcattgtaggctataaaattatttagtgaactctttggtaatattaaataggccgcttgtttttgttgctgcttttagtgtggtg
caaggcccgatctttatttcgagctggatcacgacgttgtaggacagttactatttaccctcaacaatacacagcttaggcaaaattagaagggaa
gtaatcttgctactttgggtagacgagagggaaccttcgtaaaaatgagttattccaaaaaagatgaagataacggtaagtacaaggggagata
cgatgtgtgctaattgtcgtactagatgagtcaatattctccaatataaatcaggtttctgtaacgcaagatgcgagagcatttcaccaatcttcaa
tctcacctcggaaaagccgaatgttgctttgcaagattgcatatctactgtatactggagatcgatttcctgagaagcaagcaactgagctctttttt
ggcatcacgaagctttttcagcacaaggatcctagtcttagacaatttgtgtatattataattaaggaattgtcttcggttgcgcaggatgtgattat
gattactagtagtattatgaaagacactgctactggaagtgaagtggtttatcgtccgaatgctattcgttcactcgttcgagtgattgacgcgaac
actgtacctgctatcgagcgtatccttacaactgccgtcgttgacccagtttccgctgttgctagtgctgcgttggtttccgcttaccacttgtatccg
gtagccaaagatgtggtgtcccgttggagtaacaaggctcaagatgctacgacttcccatattgttggtagaaaggttgcaaattcgccctttttttg
ctacttctctaggctatacgccttcttccagtggtatttctcaatatcatgcacttgctcttttgtatagaatcagacagtcagatcatatcgctgtctt
aaaaatatttcgtgatattgcttcgaaagttgctggactttcaaattctcatgctttcgtcatgtacgtccgctatgtcgctagtcttatggatcaatat
cctgatatgcgtaaagatatggttcctctgttccatagctggttgaaaggtaaaggtgatatggtcaatttggaaattgcccggaacatggttcgct
taaagacccttgacgatgaggacatacgtcccgtagtcgctgtattaaccatatttcttacctctcatcgctctgccgctcgtttttctgcagtccgta
ctttaaatgagttagccatgactcgtcctcatcttctctactcttgcaatcctatgattgaaagtctaatcactgatgcgaaccgctccatagcaactt
atgctattaccactttacttaagactggaaatgaagaatctgttgaccgtcttatgttgcaaatagctaactttatgtctgaaattcccgataacttt
aagattatcgttgttgatgccattcgttccttatgcctgaaattccctcgtaaacaggaggcaatgttatctttccttagcaacattcttagtgacgag
ggtggttacgagtttaaacgtgctgctattgatgctattttcgatatgatcaaatatattcctgaatctaaggagaaggcacttgcagaactctgtg
agtttattgaagactgtgagtatcctaagattgcagttcgcatcctgtgtatattaggtgaagaaggacctagggctgcagaaccgactagatttat
tcggtacatatataatagaatcatgcttgaaaatgcaattgtccgttctgcagctgttagtgcgctcaccaagtttggtgttaatgccaaggacgac
tttgttaagcatagtgtaagagtcattctttccagatgcttggatgatgctgatgacgaagtgcgcgatcgtgctgctttcagcgttaaagttttaga
tgacatggattccttcttaccttttgtcaaatctaataaagttccttctatcccagcattagaacgtagtcttgttctttacgttactggtgacaaattt
gatcaacctttcgacattgaggccgttcctatgttgactcaggaagaaattgatgctgagaatcttaaaatcaagaaggcgtctactgagattgct
catattgcaccggctcctactgttgatgagagtgccgaagaggttgtgaaagccgaaaacgaatttaaatctgttttgaattcaattcctgagttca
gttctttcggtccggttttgaaatcgtcttccttgattgaggttacggaacgcgaaactgagtatgtggttaaggccattaagcatatattcgaaaag
catattgtcattcaggtaagttgattgatctcttaatgtgaaatatgttttactgacagattatagtaccaactccataatacacttacggaggtttta
ttggagaatgtcacggttggctgcacaccatctaatgaaaatctaatcgaggaatttgtgattcctgcaccagttgctgccaacgaaccagtctca
atttacgttagtttctcgtatgaaggttctgaattgttcagtactcctacaattattgaaaatgtattacgatttacttctaaagaaattgatcctcata
cgggcgaagcagaagaagaaggctacgatgatgaatacaatatcgataatttgtggcttaagtatggggacttttgcgtttcttccacagtttcca
actttgatgaaatctgggagcaacttggccatgaaagttccgcggtttattctcttagtgaaattgagtctttcagtaacgcatgttctcacttggtg
gaacttttgcaaatgcaacctgtggacgaaacggatcatgtaacagacgaagaacctttgcatgtactgaaattatgcggtgagtttgtttccgga
ggaaaggtgcttgtagttataaaaatgattcgtgcgaatggcacagaaggtgtcaccattaaattaattacaagggctgaaaacgagaaagctg
taaaaagtttgccaaccgcttttgaaggttaaacaatggaaacaagttacttcatccaacgagaaacgaatttcatgaaagtagatttaattcaa
agatggaaccgacaatgctatgtataagtggaagataaatagaaacaaaaatcttaattatattaggcagacgagaatgaatgaattttgaaag

agtatcatattagtagcttaattcgacaaggaattcactgttagacactgtatcaagtattaaaaattagttgaattattttaaccaaacaaaacga
acaagctgtaatgaatttatgtgtaaaaaaagtaaataaaaggagtagcatggatgatactcgtggatttgaaagcattaaaataaaataagttt
caacatcaaacataacggaatatttcaaagaaaaagaaaataacttaactcatgtaatgcaatgctatgcgaatgaactaaacgaaaaggaaa
ttaggaacgaatgaatatcgggtgctaggacataagcaagagtgctcactcggaaggggaaccagcgaaaggcacgatagcattacaagtag
ggcaggtatgtctgtaatccacaacccatttagaaatgcaattacggtggaattcatgcttacaaggcaaagcaataactttatcgccgcggttaa
aagcttcaaggcaaatgacgcattcgacgccataggcagcacgccgagaagattcatccattagaggaaccgattctctatctgggatatcatttt
caatgtcttcctcgtcctcgtagaagccttctcgagaaatcgtccttgacggtaagttttggataaattctttggttttagaacgacttttgtaagtac
ggatgacacgacgaatagcaagtgcttggacagttgcaagcattataatggaaggagaaaaacatagaaggaagggccaaaacatgtcactg
agttcctccggtttggcatagactacgagagagcggttttcggaatagagtatatcagaatacagaatgttaaaggaagcggtagaaataaata
gcgatggaattgagacctttgagtcttcctttcctggtcctaccatgtattgtagatgtgaagaagattgttttctgttatcacctactacaattccttt
atatcctaaacgttgagcatgaagggctttttcaaagaatgtacacttccctcgttgaaccataagaaagcagagcgtgtcgtcatcagtatcatc
ctgttcctgtttttttcacgaagtcagaccagtcttcttgcatttttctaatagctttcctttcttcatcaaccgataaagacatacttaattgctcatgttt
catctgtacgtcagagcagcctgtcatatcttcttggaaattggagggtaaggagagttgaatcttcgtcgtgtaagagctattagttaacctctta
ccgaaactagcctgccttgcccaaagtgtcacatttcgtgctggaagttgcagagcaacatctgtgctagcagcaaatcgtttgggagagaagat
gttgtctaaaatcccaaaatcaagagcaatgacttcaagcaatatgacataacttatggaagctaatgtacataatataatggaataattagaca
tatcgtaaattgctagagaatgcgttgcaaagtcgaagagacaggcagcggtggagttgaattatgaagcaaacatcatcttaaagaaaaaata
cctctttactatgattcttcgttttaaatggaatatgataaagacgatgatggttgaactgtcactatcaaaagagaaaaacaagagcagaaagg
cgctttaaaaaacaactttttttatagaaactccttaatttagtaggatgcagcctttgtttaaacggacttccatcaccattcaagcatgacgtata
aaataaacaaatcaatacgggcacaaaatctcgctgtttcatcaagctggaagcgtttcttcagtagagcatgatttagaacaggaaaaaggtg
atccatcaaattgtactcttgtaccagaacatatagatattagcgagacgtcctataaacaattgcattaacgacctttagtgaaagctttcgctgt
cgttatacaaaaaataaaagtgaaaaagttattgacgaaggtggggttcgaacccacgcccacaaacgtagactagcagttgctttcacctgaa
gctagcgccatagaccactcggccacttcgccttgagttgatagtaaccaaagtaagactcttatgactagttccaaaagcaaggagagaaaca
gtttatcttagcttatttttcccttgcttttctaataaacaaaggaaaaaaaatgcatgcgaagaaatctgttgcaagcttccatttgtaaaatttatct
atacacaagaagaaaatatcttattttttttggtgaaatagttctttgcgtttcacatcatgttgttatggtacaatttttgaatgaatgacattttgtag
ttattgaaccatttacatttttcaaggattgcttttcttaaattctgttggttttagcgcctttgttattattatttggttttttttttttttttttttttgt
ttgaagttttgattatttatgtcggtgaatcgtcttccgtaaattaaagtccatcccattgattcaaggaaaatttgaaaagtactgactaaatctttt
ctatgtgaaagagtttcatcaattgattctattttagtgctttctgcatgaattggaattaactgtatcacaatgtgacgatttgagaacgatggcgt
ccattgccttgtccaatctcaactcagtttttttcagtcagccttcagatcttttgaatttatgttcaaatatattgactacctagtctattctgattttttt
ttctctctcaaaaaaaagacgaaagatcttaataaaggaactgagcattacaaattggttgggtttttcgttactaccaacagttctctcgatggtgt
agttggttatcacacccggctgtaatgttcaagataccggttggtcgctagttcgaatctggctcgagagatnnnnnnnnnnnnnnnnnnn
nnnnnnnnnnnnnnnnnnnnnnnnnnnnnnnnnnnnnnnnnnnnnnnnnnnnnnnnnnnnnnnnnnnnnnnnnnnn
nngttttttttttttgtttgaagttttgattatttatgtcggtgaatcgtcttccgtaaattaaagtccatcccattgattcaaggaaaatttgaaaagt
actgactaaatcttttctatgtgaaagagtttcatcaattgattctattttagtgctttctgcatgaattggaattaactgtatcacaatgtgacgattt
gagaacgatggcgtccattgccttgtccaatctcaactcagtttttttcagtcagccttcagatcttttgaatttatgttcaaatatattgactacctagt
ctattctgatttttttttttctctctcaaaaaaaagacgaaagatcttaataaaggaactgagcattacaaattggttgggtttttcgttactaccaacag
ttctctcgatggtgtagttggttatcacacccggctgtaatgttcaagataccggttggtcgctagttcgaatctggctcgagagatatttttttgtact
ttttacttacgaagtactcgtttaactttaattgttttaaaaagcaaaatgatcttctacgggcttttataaaagctacatctttcttcttgatgcctttt
tcatgactcctttaccgaattacatttaattaatttcatttcatcatgtaattcccccaggacagggaacaatacttcatagcaatcagagttaacta
taacttattttacaataaacttgaaaaataagaaaatcaaaaagatagctagacaatgagaaataacagtttttataaataaattcgtttttcaaa
gatctatcagtgaaccattgagggaatgcagagaaaaaagtgtcttcttgatgaaaccgttacattgacagctcctattatcgaagtcgcaattaa
tttccattaaaataaaaaaagagaaaagaataagaaatgatcctcaggaaaaagagttaccatacgaagtgcaaatgtatactcctaagaatg
aataaatctctcttccaagggacaaaatttataataatgcatgacaagaaataactcagacactttgcgtaacctgctcagcaggaggtgattggt
aaacagccatactatacaaaaggttagtgccatgattctactaataattatgaaacccataataaaatttacatacccactgcgtttactctttttca
attcccgcctaatggcagcctgatccaatggtttgataagttcgaaagagcaaagaagctcagaattaacactcatgacagcaccccaattatcg
aattctccacaatagttgggggcagaaaaaaccgtacataacgtcctctcattaaaaaattcataaccatcttcaactaccatatgcgcacgaca
aattaaatcgaaatcatgccttgccaaaaattgccgaattacatttttgttaaaaacaaacgaaacgcctcgttcattatcttcccaatcattttcta
ggtctgcagggtcagaccatagcaagtcattcaataaaccataatcaggaacatcagtgggccgagtgatctccttaatgtcatccatagaactt
aacgcaggagacaggcctccatggacacaaaaaatcttgccagcgactacggaagcaataggtaggcaattgaaggtgttaataaacgtcttcc

agattttaatattgcaacgtcgtttacattcatcgtagaatccgtaaacacgagtaatattagcacattcatgatttccacgaaggagaaagaaat
tttcgggatatcgaatcttatataaaaaaaggagtaaaatcgtttccagactttgtttaccacgatcaacgtagtcaccaaggaataaatagttag
agctcggaggaaatccacacatttcaaataatcgaattaagtctgaatactgtccatggacatcaccaacgattttaacaggtggcatgagttcta
gaaaagtgggttgtgaaaggaaaatctccctcacagccgtgcaaatactaacgatttcagcgtttttaaggcagacacttttgctactctttcgag
agtacccggcatgaatgagccgctgaatcatttcgtccacatttaaactcaccaatgcatcaggatcatctgggttcatcgccgataaagcagaat
tcggtattacaaccgggccctgtggatcgtagtgaaggtaaccaggagtgggtgccgatgggtccgtaattgtatcatgattcgagtcataggacc
caggagaagccattgaagaaggcggtggtggtgaaagagaccgattgcttgacatgcccctggggctaagattacttgaaggcgaagtacctgt
aggcgattgagtagagctcattccagcagcggccaattcttcttggtcttgtcgatgtaagttggagctcgaaccagaagtagctagaatgcctgg
cgatggcggaggaggaagactaggcggtgtttctggaaacttatggcggctgttggtggtatcagtagacgttcttcgatcatgatttgaatcttta
tctttccgcaaagctgtctttagagatttgaaggatcgaattgaaccttgcgtatctgatcgactaaaggaagggtaggaatccaatcggtgatca
ctatgtttactactcccttgtcccattgcgtctcaatccgttcgtttcctaaagtaagttttcaataattctttttaaaacctttaattcacgttattta
attgctttcttgcatttagtgccaaaaaatccgcttttcctcttctctatatgtcttccaattctatttcgtatctgttcgacagcccttcaatacttgttc
acttcttgtatattttgtactaaaaatcgtttctttcgtttgtttatatcccgataaatctgcgaattgtatatattcgtaggtttgtggtaaaaagcga
gtttccatggaagggaatttgcagccaggtgttacagacgcaatcgcatgagaaaaaaatggaaccagcagaattcgaaccggtaattagttta
acgatgtggaagcacaggtcggaaaacgatataagaatatttgaaaaaatattgcaaaaacattcttgctaaataatcaatatagatagaaaag
taacttcatttatcaacattaacaacataatcttaaccatcttgctcccattcctcacttgtgctccaaggcttagtttcctgtgcttattcctttcttttt
ttattccattctgattttgatctataacactcgaaatgcttcttctagatgcaggttctacttccatcttgtccaagattcccgtggaaaaactagaga
cctcaagactgtggacttgtgaagcccttgtgcaatttccaaacgaagtattgcaacaccataaacaatacctccaagtatgtgatatcataagca
cttttacctatcagcttgatgaatcaatttacgacgaacaagtggaaaagatccctctcttagaagcttactcgcgcagtatgactcttgcgctgca
agctagacaagaaactcatctacctaataagtatatcgctcttacattaggtagccatgccgcaactatcccgggtgccatggaataccgcatggt
ttacgataaacctgatgactttgatatgctttacaactttcataaaaaccgtattgagctgttccaggcttccaatcctgaagcttttaagcaaatcg
actttttggcattcgaaagtcttccgcatattaccgaagctctagccttgctcaagcttttggcagattacaagagctggaacaagcgctgctggat
tacttgcacttgccctagtatagaaagtcttgaccgcgtgaaacagattctcactgctttgttagatgctaattctcaatatatttggggtgttggcgt
taattgctgccacatcagtttacttcccaccatcacgaatgccctcactgaactttttaaatcgcatgctgataagactatcatgctttatcctgacg
gccgtggtttccctaatccttatactacttcccccaattctgaaacgaagcctgctccatctccccaagaatgggctgagtcctcttttgagtactcta
ccctgaacagtggcaacattgtaataggtggttgctgtgaaactgatatccgccatgttgatcaattaaggtctagatataattagattactcatat
tttgtttaccatgcctccaatttctctctactgatttcttaaaagaacctttgaatgcaatatgcttaccaaaacatttttattaaattcctaaaaccct
tttatccctaattttaattgttataatacatctatttcaattccttatatgactttaatattgtcaatacggaaaagtccaaaaattttagagaccatac
aatatgactgtattcaaaaatagtttattttggtgctttcagtggttgaataaccttaccacgctttgtgacagtcctgggagcaacagccttgtgat
gagaggctctcgtagtccattcaggacccattggtagattaagagctctttcatattgttctcgagattcaaatggaaatggaacggcatcagcag
taagcttagctgatttcttattgcgtttttcgttaataataacatgctgtaattttgcatcttttcgcttggagggatccagccctgcgattttcttaaca
gctttcggtttattcttcttctgcttgactccggctcctgcccaagaaccccaaccgggtaatgtttggtcttcctcttttggagcatcctcgttaatcc
aatcctctttatccttttcaaattccgcaactacatcgtctcccgcaaaagccctagcaaccaattccctttgttgaatagccactctgcctttcgtag
acttcaatgtaggtacctcttgatctgactcttggtctgattcactttgaattgacgtctcgttgaaaattttgtctggctggatttcagggtcatgagt
tagttcttgtaatgattttaccttatcactgttcattctgtgagcaagtttatcagcttttgagctatccttattaacttctgctaaggtagatttccgttg
acgtggagcttgcaaccatggattctttgaatcatctgatacaccaacttccacctctttattatctttttgttattattcttctttccttgtttgctgtct
ttcccagcatcatttatttgttctttttgggtgttggacaagaaaggattcttacgttccgatcggatttctttcaatgcttcctttgatggaacaaatg
atcgacgacctgcattatctcctactagaactccagaactttcgtcttctaccgcatcagaagcattgccatcaggtatattagccatttcgtcttca
aaggctttcatatcgttttctacttggcgagcttttactttagaagcatcttgcataaatttcatacccagaacacccttcatttgtggctctttcatct
ctcttacgttgtcaaaggcacgtgaaacatcgtcaggtgtttttaaatcgtcatcagaataatactcagcagcaacaccgtcagcatccgtttgacc
actaattcgtttcgttaattcttccccttttctgacttgctccaaaactgcttcccttgttccttcaccatgatttgctcgttcaagcatttttcgagtcca
agcactactatttttgtggcgctgcgtcattctttctttagcacgagcttcttcggctcttagacgctcagtctccaaatcctcttccgtccttggcaac
aaagattgttccttttctcttcgtttcttcaaatgtttgcgatatgctttacttttaatttttgagacacgtttagctttctgttcctctcggaacatgagtt
ctctcataaaacgcaaccgtcgagttctttcagctacttcttcaactgagagtttatttaaagccaattcttcctgctttttcattgactcttcatcttta
aggccagcgtcaacgagagctgtatccagcttcttctcggttttggtttcaggagtataactggaagccaatccagtgctacttggtgcaggacgt
acactctcgttcatggggaacacaagctgatccgcctttctattttctgaaacaattggcttccacctttcaaggtcttctttggattgttcataggca
gcttgtctctctaaacgatcatgaataggttttgccaatggagcatttaatttcgacgctgctacagcggatgactcactaactaaaggcttcaaag
aagatttcaacctattatcaacttgaacagattccaaaagatcagaaagtccgatttttcatcatcatttccaaccaaattgaattcagattcacg

attggattcagtaggattatgcttcaaacgttttttctcagtggcttttcctttatcttcatcttcttcggaagacgcttcagcctttcgcttatcatttaa
ggaatggatataattcttcaagttatccaacttagcatcattttcttcctcgtcgcttgactcttcatcttcagagctgaggatatcacttccttctaaa
ttttcatcttcattttcctcctcttctgattcatcttctgaagaaaaggaatcatcgctttcaataattccatcagtatgttgtaaatgatgcttctttag
attatctttggaatgattttcctcctcttcttcttcactagagcctgactggtctaaaaccgcatctaggtcgactaattcgttttcatcaatactagct
gcttcatcatcttcgtcttccgactgctcattcagatcctcattttcgtccaaggaaattttctgttcctttttgtttggagccgattgaataattttttc
ttaggaacagatccagcctaggtgtttgttagtaactacacaacaagatgattagaaatttaaatatacataccttagacgcgctgaaagaccaa
tcagcaaactttttgtcatcttcctcgtcaaatgcttcgtcactgtcaatttcttcgtcgtcgtctgaattcacaccaacatggtatccggagtcgtttt
cgtcgtccaattcatcttcaatgaatgaatcctcatccttttcatcgtaggagtgcaaaattttattttctgaagcttttcttctttttctattgtcaaaa
ataggctgagtattacccgttttcgattttcctctcctcgccatggtgttggtagtggaagaatttcaacaacttctgaatgtaacataaagtagaaa
catcaattcttttgtatgacagtaaataataatttaattttaaatattcattatggataaataagttactgtaattgtactttttagagttgcacaaact
actaccgccacggtataaatatttatatacctaaatttttcaggaattcatagatcttttatagtaaacaatcttagaagagtaatcgattttatata
atctagccctgtaagtaataattagattttgtcacaaagtcaatttcatctgcttgataaacgaaggaaaaaatgatagctattataacgctcttcc
aaaaagaaaaaaacttttgatatgtactcataaaccatccatcaataaatgaagctataacaagatataacttttaagttgaagttgaaattcata
attaaaaattttaatcaaatcatatcgtgcattcgtcgttgttgttcagcaacttcaggctctttttttataaaagccgtattcatcctccattaagaaa
ggccattcatccatgtcataaatttcaccgcataaggatttcgtaataaacataattcgatcaatctgattaacaattgcatatgcaatcgtacaac
cgacagcaaaattaaggtaactcatattatttaacatctcataacttattctcgtatgaccttgagattgggtatcgctgaaaacgggaggcaaga
atacatcgccgcttttagtaaaaaagcggtctactagaggggaaggaacgatttgaggtaaagcattaccagtttgaagtgcgtttgaagacata
tagagaacggccaattgatcagcaacaaacttatgatcaaaccaaccgactcttcttaataatgcatacatccattcttcatcgagggttgtaaag
gttgtcatcaatgaattaagtaaatccaacaacgataactcggtttcacagagagactgatatttatccataggccattttcccactagggaaggtt
catagcttaccataggtaatctctctttcagagcttgaactgtctgagttagatttaatatcttactttggatttctgtgtcaatatgctcatgatcaac
tcttcgtgaaaaatttgaaatttcgcaatgaagtgctccgagttcaataagggtatttccgatgctttttcgaatagcatatctagctgtaaggggtt
tcggcaagaaggaaaaaataaaagccgccgtaattccagccataacgacaagaaatctgcgataagcaacatcccaaccgactccgaacgtta
caattccaggctcatgaaaagctttccaagagtagctaacagtaagagcaactgtcactgagaaaataactgacggcattggggtcaaaattat
aagatgaaccctaacgaacatgacaaaagggagaacaactgccaaaaccacagctaacccgtaagcgtttcctaggccatgtccatcacctgta
taccatatgaccataccaacaacagtgccgaaaaatgtgccgataactctcatgaggtatccataaacagtatcagcagtataacgttgtaagct
catcgtagccagaatcacagcccataaacctctattgtaataataccaacgagcactacttctaacaaatgcagggatcgtacaaatgacagcca
gcgctccaacttttaaaacaaaaatattcatcggcctgaatatagatttgaaaaaacgcattgaatgtaaacctatgacatgatacaaattggatg
gacggtccgcatctggatttctggtttgtgtgctcgactcgttcatatccttctctacgtcttccacatcatcatggaaatcatcatgaccagaaagtt
gagagtacaaattggctcgtaaagtcctcaaaggccaccacaaacgtcggcgaggacgtctttcttccagatcgataactgtatttaaaatctgca
aaacgccccgaacggcctgcatcaaatgagactcgtaataatataccacgaacagtgatcttaaagaatgcatatagaaaccttctgtatctgca
ttttgaaataatggctcgtaaggttttcggacttcaaaacacttctccttttcaaaatcggaaagtacctcggataaatgatcatagtgtaacttcat
aagccgaagacgaccatcactggcttctttctgattttttttggaaaaaatacgcttgaaccgatcgtgattaacttcgtttagccaagcacttatag
catctaaagcataaatcgtttgatcaattaaatctgcggatgatgtctgtaaaacagacaggattgagtcaagaaactggggccgattatatggc
aacctattctccaaagctaggtaatgttgggcttctagcattcccaccggtttatatgactttttggtttctctgtataactttccaattgctcctcgca
aacgactccgacttcggtgacttttagagcctggtgaactttgacgacccctatgatgaccggggatattaccggctgctatagttaaggcagaat
gcgaccgtttatggtgctggagaaaattaccatcgtttaaggaagttgacgacaagttgtttggaccgaaagatcgaatagtccgtagattatcat
caacaggaacatttaactgacttgctgctgcatttcggcctctctcgtattccgtttgatttgaagttgtacggtggtgggttggggacaaaaaccta
tcactttcataagcagcccgatttggcgaggcagatcgtatagaaggcaccggtgtcccagctctagaatgcaaagttgctttttcagcttgcatca
tagtttcttcgtcataaatttgcatattgtgatcaataagtttgcagaaataagaaaaagaagataaacgaagaatagttttcctaaaaagtctttg
caaagataccaagtcctcacccttcaaacgaccataagaaaattcagtatccaaaaaaacctcattcgacttcattttcgttaataaagttttcaa
atcagcattatccttttctaaagtagaagcgaaaaacttccagccttcggaagtaggagaatgtgacaacatttccacctgcttttgcataaatttt
ctcgtattttctgtaacagtacacaacatactattgtacgaaaaattgcatgtttcgggaattataaagagtgtgcaaccgacagtaatggcggttt
gaattgcattggggataataaaaaatagaccaacacgaggataaaagtaggggtacattggtccataatttgcactaactgcagcacaaatgat
tgcaaagacgcaagggaagtttaaaggtttatacttcattttgatgcgaataaaaacggcaacagcggaaaactcgaaaagagcccatataatt
gatggcaagggacggacgaaataaccctggaaaattgcttcagtcaaacatccctgcattccttggctagggttactacagtgaaatccatccgc
aatggcattctgtaaacattgttccttcgaaggggagccacgagacacagcagcaagtttggatgccaacacagtccaagcgtaagaactaaat
agcagtaaaaattggaaggcgcacttccataacataggagctatcggttccatagcaggtaccagcatggaagtaattacagcaaagaaacta
gccatacctaaaacccggttggtatggttgcatatgagaagaagaaaggctatccaagtgttcagagatgctcgaattacaggctttatcccttt

ctatttaggtttttggaaatccattgcatatacgaagggaccttccaggtggaagggtgtttgcaacggcgccaaaattgttttaaacgctttttaga
caatgttggtgattgttttggaagatctggggaagtttctttttctgactgtaatactccatgtccagatacagactgaggatgaagtgggcgagaa
gaattagaggaagaagaccggtcgcgagagtattcggaaggccgctgaacaaatggttcacgaaaagacaatttttgttcctgttgcgggtttga
gggcggtttggggaatacaaaacgagcatgttttggagaatcaggagactgcaagtgagaagatgaaggaagattaaaagaagaatggatag
aagtcctaaaagaattgaaggatgaaccttgagactcgtgatcatgatcatgaccttcggaagtgttcgacttggaggaactccgagatgaccgg
gaggatggattatactgcgagaggatactgggggaggattttgagtgtcgaataggagtagaaggatcatggggatgacttgaatgttgagaat
gtctagaatcacgagcaggcgcttggtaagcggtggaaaggttggagtaagatttagatggaggaggaggaggaggaggaggaggaacgtga
gggagggtggcatgagagagaggagaacgtacaaatggttcataacgagccggagaaggttcactagacaactgtattttggggataggagta
gagggacggggatgaggggaatctatgtaaaaagaggcttttgaatttcgcatgggattgattaaagagaaataagacgcatacagaacgaaa
aatgaaaagaaaagaagttgagaagggaacaagagcaaaatttgggaacacaaacgaggtaataaaaaccgtcaagaagtttgaaggatta
aaaataccaaggaaccgtttcaaaaagaaggtagaaagaagtttttcaagaaaccaagacttgtagatttatgcaatcaaacaacgaaaagt
attttaacaaaaaaagcactatgaaaaacaaaaatttgtgtaaagggatacattccttttatctgttttggagaagaatttacccagcgtgagata
cgaatgaggcgattgtaaacaaacaaacagagtgaggatttgtttggttttattgagcagcttaccaacaagcaattgaatatacgatgaatcaa
gaacaacaatttggcaacataaagtaaacaaaaagagtaaacgaacaagaaaagaaaaagaaaaaaaaaaaagagaatcgtaaaaagtgt
agattgatgtcgtagtggggtaaaaggaatggacgtatgtttatacacaacgtgagcggcagcgtgcgtatagaggggagcagtaagtagagat
gaaggcggatctgacaaatcaaatatataagagaaaagagtaaaatattatataagcgaggcagtgaatccaagacgaaaaggactgagaaa
cgaaaggttctgctgagataaatcgaaaaagaaaagaatctttttagaagctcctctaagagccgaatatgaagaaaaaaattgaacagatag
gcgaacgaaagtattatactggtttgtgcaaatcaatatgattaatatcatacaaaggaggaggattccatggtataagaaaagagtgtgtgtgt
gggagatgccatgttaggtttcataaaaaaaaaaagaatctattttattcaatgaatgggaaaaataggatgaaaagaaatatgggtaagaagtg
agagggcggcgatgtccgtacaattactatgagaaaaggtatatatggggaaaagaacaaacactaggtatcaacagaaacagaaaataattg
taataataaaaaacaaaagctcgtatcatgaataggtcatccaagacccatgggtgaaatcatcctatggttgagaggaacactattctttttctt
ggcagagacaatcctcactcgagaaatccattcctagaaaagagagaaaaaagtaatcatatcaatcgacgaatgaatcaacagcgaaggtaa
gacaactgttgtagcagccatttcctttccatacattgagatttaatatatgattattattattaataacacttctatttctatattacttgataaatact
gttgtttctattttaggaaatcatgtgttggatgaatcatgtcagtcgaaatggaaacaagaatccacgaccgaaagaaacaatttaaacgaatg
catgttggacacactacgaacaatccatccctattgtgggttgaaaaagcaatcgacccaaaagtgtcttctagtattggcaaagatgggtgttgt
ttcatatctctaattttcacaagatacacattgttatattcgtttttcattttctttgttgtttcattttctgtttttatcacgtaaagaaacgagttcgttc
gattcagttctgtgatgaaaaaaatttgtatcggatcgtctgtcatgcgattttcttgaagctaaatttctttttatttttattgtaaaatttcgtttcatt
gttttatttactttttttttctttttctttttctttttcttatttctttttttttttttttttatttttttcctgtcatagtcgttcgttagtagtagttacttcgtcgtt
tccttttaccttagctactatacgtaagtagnnnnnnnnnnnnnnnnnnnnnnnnnnnnnnnnnnnnnnnnnnnnnnnnnnnnnnnnnnnnnnnnnnnnnnnnn
nnnnnnnnnnnnnnnnnnnnnnnnnnnnnnnnnnnnnnnnnnnnnnnnnncttttttttaagtttttaaagttttaaacgtttttacgttttac
ttttatttttatttttatttttttacttttttttttcttttttctttttctttttctttattcttatttatttttttttttttttttgcctgtgcaatgccgttgtagtgaat
ttctcggctttcccttttttaacagtccaatcgtataacggagtttagtgttgcatttaaattttcgcccaccacatatttcttccttcgcaaaccacaaa
tcctataatataaaaaaatttggaaaaatacaataaaaaccaagttaaaatattgaaaacgccatggctcttacggatctgaataagcaagctg
atgtcgctactgagagcgaagttaagcccactacctctgttgagactcccaaccaacaggaatctggtgctggcgaagctgaaacccaaaagcc
cgccgaagaatctgcttctgctgatgtttccggtgctgctaccccagctaccactaccagctctttagctaacccctctactactgctccctctagcg
cttccttgtatgttggtgaacttgaccccagtgtgactgaagctatgcttttcgagctcttcaacagtattggacccgtagcaagtattcgtgtttgcc
gtgacgccgttactcgtcgctctttgggctatgcttatgtcaacttccacaacatggaagatggctccaaggctttggacgaacttaactacactttg
attaagggccgtccttgccgtatcatgtggtctcagcgtgacccttctttgcgtaagatgggtactggcaacgttttcattaagaacttggaccctgc
tatcgacaacaaggctttgcacgacactttctctgctttcggcaagattttgtcctgcaaggttgctgtcgatgagttgagcaactccaagggttac
ggatttgtccattttgacactgtcgaatccgctaacgctgctattgagcacgtcaatggcatgcttttgaacgacaagaaggtttacgttggtcatc
acgttagccgtcgtgaacgtcaatccaagtttgagcaaatgaaggccaacttcaccaatgtttacgtcaagaaccttgataccgaagttaccgaa
caagagtttagtgatctatttacccccttttggtgaaatcacttctctttctttggtcaaggaccaaaatgacaagcctcgtggttttggcttcgtcaac
ttctcttcccacgaagccgctgaaaaggctgtcgatgaactcaacgacaaggaaattaatggtaaaaagctttatgttggccgcgcacaaaaga
agcatgaacgtgaagaagaactccgtagacgttatgagcaaattaaaatggagaagatgaacaagtaccaaggcgtcaatcttttcgtcaaga
acttgcaagacgaagttgatgacgagcgtcttaagactgaattttctgctttcggtaccatcacttccgccaaggtaatgactgatgagcaaggca
agtctaagggttttggtttcgtttgctacactactcctgaggaggctaacaaagccgtcactgaaatgaatcagcgtatgctcgccggcaagccttt
gtacgttgcccttgctcaacgtaaggaggtccgtcgtacccaattagaagctcaaattcaagctcgcaatcaattccgtcttcaacagcaagttgct
gctgccgctggtattcctgccgttcaatatggcggtggtcctcttatctatggacctggcggttaccccatccctgctgctgttaatggacgtggtatg

cccatggttcctggtcacaatggccctatgcctatgtatcccggaatgcctcctcaattccctgccggtgtcgctcctggctaccctggcatgagccc
tcgtggacctgctcccgttcccgctcaaggtcgtcccatgatgatgcctactggtgttcctcagcccggccctgatgagtctgccgctgttcctggta
tgcctgaaagatttactgctgctgacttagctgctgttcccgaggaaaacagaaagcaagttcttggtgagctcctctaccccaagatcttcatgcg
tgaagagaaactcgctggtaaaatcactggtatgcttttagaaatgcaagattctgagttattggaacttcttgatgaagatgccgctcttaacga
gcgtattgatgaggctctcaatgttttacgcgaatttgccgatcaagagcctgctgctgatgagcaataaagtagtttggttaatggatcttatactt
tgtaagaagtgatcttcaggttagaaacctataggaatttagtataatctttatcttaacgttgggtgttttattcgttaacagggacattgttttgca
ttttacatatgtgcggtgaatgatttgagattcccgtgaaagcttttttaaatgcagaatccggtttgataatacaataaacttttataaaaattagtt
atggtatggtcatgtagttggatgataaactttttctattcattacgtatattatagttgatcgagttgatatgcctattcattgccaaaatacgctga
aagctttgtttctttttatgcttcaaaatcatgatttcagttcatcacaagctttaatcacatttaaaagtgtttaaatcaagtacagcttaataagtg
actagatgatctgacagctatttctggcaacctgaaaagtaaaattaagcgttacatctcatcaaggacatcgaagatgtttcataatgcttatgtc
gtagtatcagtacgtggaaaaggacggacgactcattctgtcttgcctcatttcatcaacacagattttaaagtcttcagggacatgctgcctcgaa
atttgacagtcctttcacctaagaagtttatgactggagcataatatgtaaggaaaatccatagattcaatcaaaagaagctgttaagtcagaaa
cagttgagttgctattataatgccagcttcatgaatagtactttgcaaacgatcgaggtaagaaattagctttctaattttctgaacaaatactccat
tttcttttccagaatggtatagaaaagtctcagtagggaaggcaagtattcttacagggcaaagcagaatgcttcatgaaggagttaatgaaaaa
ggtggctttcgatattttcaaatagattacaatactaaatttcgttttgtttgttttatatattttcagatatcattagtaagctgacttttgcatcgttta
aaaataaacgtttacttcgacagaccaagtattaatatttccgcctcacataccaaaaaaccagaaaacctccacatatttcaggttcaaacggtt
tttgcagtaaaagcattgagtgtataatttatttggactataaacaaaaaagatttagtggttattctgaaaatagcctacttgattgtatatttggt
gccttttcttagaaattgaaacagaatcattaagtctacactttttcaatgccattgagttttgaaggagcattcaaagccaaaagaaacgtcaat
ttaggcggaaaacgcacgacgaatgatcgtggtgagttggttagaaaagctgcgattgaacgaaaaaatcgagaggaaagacgaaaaacaga
gtttgtgtctattcagttgcaatctctttctcgtggagttctttgcagagaacgaataaaaaacgaatttcgctcacaatggtacgaaaattatttac
cggttggtgctcatcagcttaacgtatctgactgggacaaattgagtgaatgtatacagaagtttgttatttttgcagatcctcaactggaatttgga
gctttacagaacgtaatcagcgcattgcttgatttttgcatagtgaattttcccctaaagatttgacgtcttccaaaagtaaatcaggcgggtcag
atgaaaagatcaaggaagcagaattatcaaatttctattcaattaaatctgaactttcttctctcagctttccttgggtgtggaagcggtttactacc
ctttgtttaaagtcatttagcaaggcgcttccaattattaaggaagaaaggcagtatgcagatttatacccgtttttagaagctttaggctatttctct
gtctttcttgaggtttatgaaattcctatttactatgattgtttaatgaccttttacgaatattctcaccttcaacaagaattttccatgtttaggagtgc
attttccttttctcttttaagtcctcttgacgatacaaaagataccttcaatgaagttcagcaactttttatttttcatgtttttacgcgtgattcctatga
gtctttgcttgatatcatttcatccaacttggttgtttcgaaaatatttccttctttgtctttcgccaaagaagcggatatcatgtcttctctaacaagtt
tagaaacctcaaaattattctgtctggcaggcgcatgtctccatttattgcatgtagaagttcaatcttcaacgttatggtgtttttgctcgttagcttt
ggatgccctatatactcattctgaaaactctgtcatcaatcgctttaatgctcccgtagacttggaagaggatgaggatgacgaattttcgtttgttc
aagactactatgctcacgtacaagttgttgctaagcaatttaacaaattactaacctctcaaaatttgagtactcaacgtgcttttgctgaagcttta
tcatccaactttataaccaaggtctttactgagattacgagttctacaatattgaacgtttcccatttttctcgtctatgttaaagctatttccttcaa
atcgcacttccacattaatgtacgtatccttaattgaccatgtacgtgtagaaccgaacagctcatttgtacaatattcctgggatatctttaccgag
tctcccatatatcacttattctcgaggaaagtcgatgtcaacagtattatgcaaactaactccggttattggtatcaacttcaattgctacttgacgtt
tactctcgcatgttgtttacaatggttgacgaagagtttcatagcgtccagcataatccattgtataatacacgggacaaattatgtatccttctaaa
aaatcttgttttgggattgtattgggatgtttacgcatccaaagaagtaaacgacaattcactggttgatataactcagcttagggtttcttcaacat
ctcttcttcaacgattgttccgaattaattatcgaaaaccatatctacatgaagatttctttttgatggaagaatatttcaacttgaatgaatttgaaa
cgggagcactgcaagaggcacagtctgcttccactatggatattgacttatctacaggtatgaaagtcgataattataatgaatcaagaccaaaa
ctgaacattttgaataactgctcctttttcttaccatttcgttttcgtattcatctacttcagcaacttattacaatggataagcaagcaaatggtttta
tacaaccatttggtcatttaaaacatgcggttattcgtcgaaatcgaatttttgatgatggtttcgatgcattttacaatctaggaaaaatgttcaaa
ggatccatccgaatcacgtttgtggatgagcatggtgtggtagaggaaggaattgacggcggtggtttgacaaaagaatttttgacaaggtgagt
aaactatacctacgtttgaagtttccggtactaatggttctagtatatgcaaaactgtttttgatataaattatggtttatttagcgaaacgaaggca
catctactctatcctaacactcatgcatatgcacaagatagtaagttagcgttatgaggttgcatgaaatactaaccgtctattgtagttgagcgat
tacgttgttatgaatttttaggaatgctgatcggcaagtgtatatatgaaggaatacaaatagatgccgcgtttgctcctttctttgtatcaaaatgg
ctaggacatcctagttattttgatgatttgacaagtttggataacaatctgtatgagggattgatatttcttaaacattatgatggcgatgtagaaaa
tgacttggctctcaactttacagttgtccatgaagaatttggtgttcgcaatgtcattgaattaattcccaatgggggaaatgtggccgtgacaaat
gacaaccgattgcaatatattcatctaatctctaattattatttgaatgcacgcttatctaaacaatgccgggcatttacaaatggttttactcaaat
cattgatcctcactggctggccatgttccatgagaacgagattcaagttttagtaggcggagatccagtacccatcgacgtgagtgatttgaaaaa
acacacagtttatgctggtggttatgaattaaactctcctactattcaactgttttgggaagtgcttcgagaacttgatgaagaagataagcgtaat

tttgtaaagtttgtcacgagcgttgcaaggccaccaattttaggtttcaaagcacttatgccatctttttgtatccggacgaatggcgaagatgaatc
tagactgcctactgcaaggtaagtttttagcttttccactattctgattcttactgtttgctaacattgttttagcacgtgtgttaacttgttgaagttac
ctgtgtatacatctaaaaagacattaaaagagtaagtaatttaatattttgtaaaggggctcttactgacacgttttcaggaaactaatagtagctg
ttcgttctggaggtggtttcggattcagctaaatgccattttgtttgttattattattaatgaactttttttcgtctccatctatggttaactaggtaagac
atgatgttgatgaagtttatatttatgaaattttttttttcgtttcgtcttcattgtaccaatataaaagtaaacaggaaactctttaatcaatactcttt
caaggaaagatacaatgtaattgacatacagttagttatccagttatacttctgataattgcgaagaaaaaaatactcgcttaaataaagcttaa
aataagcgagtaaaagtcataatgacgttaattataatataaaggaactgttcataaagactgctttgtctgcaaactacctatactcaaaataca
ggaaggaagattttcttgctaaactcttgctaactaatatgtttaagagagctattcttccgatggtaaaagcgtcttatagatttccagtgcaatca
tctgtttttgcttctagaaatagggacatagccattcgtgcattcacctcaaaggtgaagaagtcttcgagagatcaagagtttgcggtcccttctgt
cgatgaggaagaagatatgctaaataacttgatgggaaacgttaaggtcaaatctgccgcagaggagggtgttcccgttgaagaggtaataatg
aaacacccagagttgctaaaggatgcacctgctcctataagaaaggctgtggaacaaatgatggcctccagaaatcttaaatcgggaaataaag
actccggatcaccgtaagaagaattaaggatttaaatgcttccttttgccttgctacttcttcaactatttatgattcccacttactatttaaacacatt
tatgatttccaatattacaagtgtttacctgcgtacattctttataatgattaacgatgatgactatgttcgaatagctatggcctattttatgaataa
cttagttcattaaatataatattttatttgacattacgaatttcaattcctttccttgctcatagtaacttcttcaataaagctgaaatttacaaaagga
atattttacctgctctcataagtacgggaaacacccatttcctacaacacctatataaattgataagggtatcaaaatggtatcgtgaagctatact
aatcctgatataaagaaagataaatagaaaagacgaattccgcaaactgagaagcatgaagttacaattacaacaaacgaagatcaaaacga
aaactaaagtcaacgaccactttactctaaaacatgaaggcaaagaaacgatacaccgttaaaaataagataaaaattaaagaagccttatat
gaaacataatcattcaactattagctaactgcctccgttcgagtatcataaaacgataaattccaaacaaacgacaattcacagatatatgcgtat
gttcccagcaaaatttcattcatcagtacttcaataaagtaataggtatatatatgcagaaaaccattcataaaaatcgttgctttaactaaaaca
gcattatgtaatcccgaagcatcaaataacataaatatccataacctttgcaaaaactagcgaccgacttcatctacaatagcatcagcaagatt
attggcaaggcggtctgaacgttcaaaccgatgcttatttttctcagtcgtttacgagcaaccattaccggcacactcgactaaaatttgttagtac
cattctagttttctaaaaaatattccgtaccttgttaaccaagtagttactgaaagaaccaagcaacacaccttttaaatgactgcgtccacgactg
cccataacaaccaaagtcggctcgacatagtcaatctataatcagttaatatgacaaaaaaaagtgacgaaaaagcaataaacataccatttcc
acaattaagtgtttggctttctcgtgatgaataacttcaatgttcacttctatttcgagaacagttttactaagcaattttaaaatgtactttgtaattt
tttctaaagtttccatctgtgaagattccatatgctccatctctaatttcggatcatcacattcaatcacatcgactataataagagtatcaccgtttc
ttaagagaacacctgcggcccattcagcagcatataaactttctgatgataaatctaatgtaagaaagtaggtactatttctttttgagttactggc
agcgctttggaaatttcctctacgaatccgtcggattgttcgactggcattaggaatccatcgtgttggaccgacattcacatctagacttttggcag
tacgaaggtcttgttcaatttcttcttggtcagaaccataccaattatcgacatgtgtagatacaaaattcgtatttggacgaacaggttcggacttg
acctcatttgaagaagaaaggccaggattggaacgagatcgtcgtaggacattactatcacctttcatattcgaatcatcatcaaaaagtgatttc
cttttagataggggagcaccaaccagtttcttgaggcgacttcttttactggctttaccacttttgttaattaaaggttgagaaactttcggagagtc
gtcaaaagatataaaaggtttgtttggacttggtagcccttcaacatccgaatgcgatgaaaagccatcttcgtcttcgtcttctgaagatgaaga
ggaggaaggtagagagcgagttaaatggagaggttcgtcattttcatcaagtgcatcatctgatttcgccggagaatggatcccatccttggcggt
cttggatgtgccagaagttgaagagacaggaggaggagaaacaacaaagtggggaatatccggtttcgccggagatgtttcatctggtgacgtg
aaaggtttcgttactggagagcttggagtagtcctggatttcccagatgctttctgtgcctccttttgagaagatttcgttattgttgtacttatagaa
ggtctctgtacagaagaagaagttgaaggtatactgccaaaagaagctcttcgctgatgtgtcggttcggagggtttctttaaatgcgattcacca
taaggatgcagagcagcaaatgtctgttctggtgaagatgacttggaggtaatactagacaaagaatgtcgtttatgagaagtactcggaggag
actcagtagaaaataatgaagatgccaacacatcagacactttgttacttgacatggcgagcggattcgaagtctgaaaagaaatatttctttttt
tctcatcttctttttctttttctccttttctcctttttcttcttgtattcgtctgatataggaggattcttataatgaggtaaagaagtcatatctgctgac
tctattcggtatcctttctcgccaggttttacaaaacaataatcacggacgataggatcgtaatcgagagccagctcacgatacttcttctcctccct
ttctttcatccatcttagaacttccagtcgttcttcatctagcgctgatgccagtgaattaccaccatatgccttcgaggtcattaataacaatgcaat
tgtacactaaaattagttaagaattgtcataaaacagcatgtaaacttttgtaaatggtaaatattaaactcggtagatttgctcacttatgtaagg
agattgtgttgtattttaacgcagtgcactcagtatcattatttaagaaatgccagaatcaagccataatggaaagaaagcattaggtttaaagaa
acgagtttgatatatttgattagagaattatcattcgtctttcagctggaagcttttctgtgacgattagtaaatactttactattcatacgttccgtga
atcgattcaatcgaacgagaaacgacttacagacgacgcttcttgaatagtatcatcgcttttcttttcttacttgtagactttttttctttgattagac
agcaaagaaggattcgttataggttggtttgtttcaacactttggttactgaaataaaacacttggcctgaggatataagaaacatagaaagtatg
tttcacgtgcagtaaaacctttgctgtgtcgataacagttccttggagcaacaggctgtaaaccatcaagttatacaaaatgcaagcagcccatgt
ttattattttcagttttaggtcttatttcttgtcgtaacttcgctcaagcaagtttcatccttaacataatttagttcatgttatgtgtttgaaattttaaa
aacgtaaatgatggcgtagggaagagtgaaatacttgatggttaatcccactcttatttgcaaactcgcaccggaatcgatgtcttttgtattcatc

ataaaaagaaaagataaaataccaaaaatattccattcatcaaataaaataagtagcatgtagcactgcacacctcaaagtcagatgtgccgag
ctgtcttgataagatgggagaattaaaggaaactttgtcgagattccacatatctacatagaattactttcgtggagtagtgcgcaagaaggaa
cgttggatgtatgctttctggtcacgaagccaacggcgataatcctgatattgttagtgagttcatttatagataaaggaatcatctcgtctttttata
cataccgacaacgagaaaatcttttctccataataatctgtcaacatatttcgttagccaaatacatctcgtttcttatcaccgagagcacagcaca
tacctgggatagggttggaatagctttccattgaaggaattacacgattcttcttcaagtgcttgtagaattcctcttcacgctataataaaacgacc
gttagcaaaggccctctgtttatctcaaaatcttttcacatacgcatgcaatctgatctgcttgtttccgattgatcaatgtccaaacctgtcgtccta
tagaaggtcctatcaaccagccaagagcaccagaagcgatcgtagctaagacataaatcgtaaacatatccaatcccatcattgttacagtagg
atcaacggcgcgcgtcataaaatatgaggaccccactccaaggccaagcgcagctgtaggaatggaagtaaaagtttcaaagcgacgtcgactt
tttcttagtttgaggaatgtaggccaattcaatgtcggcgtctttggaagttccctttctgaagtgtacattcgaacattcttcacacagcggaaaaa
ggaaggaaggactttggcatttcccatacccaatcgatttaatccgcttcctagaaccgaatacataattcttcaacttatttggtagtaaacaggg
aaaacgcgctcttttgcttgttcatctctgctcccatttaccgaactgcataatcgtaaaaagggtatgcgttcaacctcactatttcgtctatgtcag
aaaggaagctatgtataaaagcttcctgcaaatgcaacattagtagccattgttggtgatacttgtgtatttcttggattttattcaatttttagaacg
atctagagaatataattgacttattttgttgttattggtgattgataatactttgcacgtttaaaaaaaaacatgtctactaatgttggagcaacgga
ttcaatgtccatagaagagacgaataaacttcgtgtttcactaggtttaaaacccttggacgtatcaggtgattctcgaccgagtgaaccggataa
aaaggaacaagccggctcttcgcttgaccaagaggccatagctcttcagaattggaaggaagaagaagagaaaaagagcaatgaaagaaaa
caacaggaatttacatctagaattcaaagtatacgagaaaagaatgatcagcgacgtcgcgtgcaaggaaaatctttggctgatattctcgctac
agaagaagaagaagaagcacaatcagacgatactcgctcttgggtcctaaagatgagagcaaaagcattagagaagcgcacgagcgaaagct
ctggacccaaagaaacaccgaggccagtcaaaaagaaacccacaaaggaactccccaacggaccagacacttcattagaaggaatgcgcata
gctcatgaacttcagaaattgaattctgagaacggtttaacattaacgcttcgcgattccgacattttggaaagcaatgatggggatttattagaa
agcaatgagattgcccaacaagaggccctttccaaaaagttagaagaacaaaaggcgaatgccgggtataagccttatgaagacgaagaaga
agtttataacgattctgcaccaaagaaagatggaacagaaggaataactattatcggagagaacaatgctttatttcaaggaaaagatgcaaga
gaggaggaaagttcggtcccaaatcgaggcattcgaatttcgctttcagaaactacttcttcaattcctgaagtttcagactatcgggatatcaag
ataaaaaaaagtaagaaaaaccgatctagagaagatagaaggaagcggaaatttgacgaccttaatgatgatgaatttcagtcaaccaccaca
acagaagacaatgtatctgaagcgcaaaattcaaccttcactgctcaagaaaaacaggagaatattaagaaaaagattcaaggattgaatagt
cagtcagtcgttgaagatgatgatttacaagaagttttggctttacaaagaagaactgctcaaaaaagggcaaggagcttacgacctgaacaaa
ttgcagagcatgttcaaaaccaaaacaatgtagaagaggaggaaaaggaagatgagaatgatactagaggaatgataattgatgatactcgct
ctttcgttgattctcttcagcaagtaaatgatacagagtcttctgtctcacttaaagagaaggaaagcgaacaaatttctgagaatccatatactgt
cacgatcccaacaaatgttgaatccagcaatgatatccctgtacaagaggaacccattgtacaaaatactgaaccccagtcaacaacggataag
gtaactgacgttctcgaagatgaaccgcttgtttctggtggtgttggcacggtgttgaatatcctcaaaaacaaaggtgtcgttaaggtttctgcag
aagctcaagaaaagattcgcaaagaagatgaatataacagatggtttgcgaaaaaacagagtgcacgacggaagcttgaagaacaacgaag
gcttcagaaagagcaagatcgtgctagtggaaaattgaaccatatgactcaaaaagagcgtgaacagtatgccaaacgagagaatgaaaaat
gggatcgtctaattgccaaggttgaaatggagcagttccaagactacaaacctcaggtgaacattcgctacgttgacgaatttggagttgaattgg
gaccaaaggaggcatacaaatacttattatctcatcagttccatggaaaaggatctggaaaagcaaagactgaaaagcggttacgaaagattg
aagacatgaaaaaacaagagagaaagccgatatttgaatgatgaaggacatggttttatgtatatgtaatttaattatatgtagtaatcaataaa
tgtcatcatcagaggttgtatttttcgtcacttctagcggtaggcaaaatcgtgcgtctactggtttcaaatctgggtattgtaataaggacaaggc
ggttaaggaaaagcatgtgtgatacacatctggaacgtcttctctacgatcagaaaacccacctgtctcttgatcttgagcacttagaataaaacg
tattaagtcatttctgtctatccactcaagctttttaatgatcgccaatggggataacacccaccatccataacaagtctaaaagttaacgagtgaa
aagtaaaagtgaaagctttcacacttacatctgggagcttttcaggacgaccattcagccctccatttttcgtttgtcgttcactgagccaccagccc
agtaagtcttggtcaattctgtctagttgctccaatatagcaagagaagctacacatgtaaaaactgaagagaagttagaatatgacaagaaact
tcaggtaatttgaagataatgattataccataagcagcatgtgtttcagctccagggcatagaccaaaaccaccgtcaaagttgcggcacttgag
aatccaatcgactgcggctgactggttcaacatatctagtcgacctagaagtgccaaacaattaatgccagcgtaaagaaaacgagcatcggttt
cgccccatcggtcacctttcatagatccgtcttcattttgcaagctaacaatatcttttaaaaagcaggttagatatctaccaaaaaagattatccgt
acaagaagctactttttccttattgattctgtctagtgaatccagcatcaacaaaacctgaatagcataaacagtattggtaatatgatcatcatgt
ccaggatagcaagcaaaccctcctgaaaattctcaattagtacacgatcactataaaaatgctaaaaaaagtgatcaacgaacctgactctgta
agacatgattgcaggaacaaaacaatttgctctttgtttaattcttctttcttattcaacatccagtagctcatgcagctccaataaatagcactaac
gtgtaaatgttctttcaaccaaaagtctagttcatcctggcgctacgtcgtgaaacgtgtaagcacgatacattttcttcattttactttcgtacctgg
cccaattcattcaaataatttaaatgcttgactcgttccaaatccatactcatggcgaatgatggttataagggtgagtgttgaacccaacataaaa
gaagcgtgtgtgctctgtttcgacattttaatattggtgcctttatgaatttcatcttataaagcttcttaaaggaaacacctgttcattaaatctttt

attttccatttccagagagcatcaaggagaatcgatcatttcaatgaagcatcaaacagcaagaatctactaactttttcctatttgtccaggattct
ccacacttcatcttagcttacatagaattgatttgattcaaagaattgttaaatgtcgactgctttttctttctttctttttataactttattgacttgcct
tttcggtctcactttaggaaacactgaaattattaattttaaatcaatacatgtgtacgacaggaatggaagttgtcaacttaggattccagataca
gtgtcgcaatcattaatattacaaccatatcctatagaccccgaaaaaggcatttccgaatctgcgtccagagttgcattgcatgcctgcggcttaa
aagagaatacctggtatcagctgagagcctcttggccagcggtagtacgttgaacgcacttttctattttcttctaacattttagtatccttctgaaat
agaccttgcttggaatggtacccatgcacttgtgcgtctttacgcttcattctattctgtcgatgattctctcatgaatcatccacctcctgttccgatc
caaattggtaagcggcttctgtttcttagtcttaaggcaccatactcatcatcttatttagatctggagccattaacattgggatttttacccagcagt
cttaaacccatcgttgctgtaatagcagtaataattatctctagcatccctttttgctttttttaattttacgaaaacagaagctggattaatatacatgc
tcgattttaacatcagcttacatcttcctcaaaactatacctataacacctaaacgaattttcaaagtctattaattctaaaagttctaaaaacatta
tgtctaaattattatgattacttcctttccatagcacaacaaaaaaataaaaacaaatgagaaccagacaaacaaaaattgtcagtctcccggca
tgaccgaactagcattgaaaataacgtttaaattgcggttcatcaattatctctgctttaattacatgaaaacaatgcaaaagtaacgacggaaac
ttttccgtaaagtatgcagtaaaaccatcaggaacgcttcccaatatagcacgcacattctctggtaaatcctgatagtgatgcctctatattttcag
ttagaatccatatcctgtcgcttgtaatgttcatactcaccttatttcttaaaactcgaagtatatctaaaaccttagaaccatcgtattttcgatacct
tccaagattgtctattagtgagctgtgtaaatcggccatccaattttcaccaacaactaattgtgcattattctctaaacactgaagcaagggcgaa
gggggatcacgctcttccacttcgaacctatcagatacgtcgattaagaaatcgagtttttttggcgtaatcccagaaaaacggatggttaattata
ctctctatggatggacggtccttagggctaaaactaatcatctcagatattaagtcaatagccaaagctccatattcacccatcaagtcaagttcct
caagattaaattccccttttagtatattagattcgcattcatattgattcccaaacggatgtttgccaccggaaagagtgaaataaaaaatacatcc
taaagcgaaaatatcagttgcatggcttgcttgacgaatcttcccttcttgactcgtacagcgaaattcattggcttcttttgatattaaacctcgaa
gaatctcctgagaacgccatccataagagcctgcagtctcaaaagtggaagtatgaaatgtactttgattgatattcactttcttacttaatccaaa
atcagatattaaaggtttgataatgaaattggagttgtttgcaacctttcgggagagtaaaatattctgcggcttcaaatcacgatgaactaaatct
aggctgtgaaggtgtctcaatcccagtgcgatttggtacaacaaaccaatcctgtctattccctgtaccatatcatggtaaatcagaggatgctcta
tcaaatcagctaaagtgcattcacctaattcgacaacaacataaaggaatctatcactttgttgtctgcaatagtagcgaacaatgtttggatgatc
gtcactttttttgaagaagctgaatctctcttgtagccaactcatagaaatccaaaagaatacgtttcacagcgacatctctatcttcgtattttcctcg
atatacaatagtaccatggctcccatacccaagtatttcggggaaaatagttaaagaattcatagtcaaagagccgtcagcattttcactcgtagt
gttctggataacaggccttatgttaccgactgtgtcagtttccttgtcattatcagtttgtaccattactgtttcttggtcgagtgcatctggcacgtttc
cacgttcatgttcatcactaatccttttctttttttcggtttctgcgacgtcgtttagctataccagcctttgtcacatctattataccattattagaagttt
ccgctgcggcagccttagttccaaaattttgcttccgtgaaacaaaaaattaagaggctagctgtcagagaaatgccaagaaaaaatgaaccagag
caatttagatttggaagccgaatcactgtcactcgcatccaacaaaggaattttactttgagagggaggaaccaatttatcagatcctgctccctct
aaatggtattcagggaaacaatcagttggtaaagaaagtaaaggaatttccgtagttttaagtggatgtaatcccatgatactgctaatgggtac
atgtaatccctggtacaaataacacgcggcatttggaactgcagaaacaagaggaaattgctttgttgacatagcgtaccaattgtaattagaaa
gagtgttaagataaacgctttcagaatcgtttgaaaataaatttggaaacatattatgaataaatacatcaattggttgatgaagcaacaatgaat
taggagaacgatcgcatttatctccgtatgcttctggaaatgatacaacatcgaagacggaagtagcgggaagagcgagattaacagtttgaat
aacacgttttcttttcaaatctaaaacgattaaattattatcggcattaggaaacgccaatttggaatctaaaggggctgagtaagaagaactgtg
aattaagtcactcttgctaggactccattcagaaaaaacaatatccaaaagaagagaagaatcctgataaatgctaacggtatattcagttctgg
caatatctataattgctttgcctgaccgtggcgcttctcgctgattttcatctaacatgtccaaatcatcagctgctttattattggtgttcgtctgaaa
aaattcagcgtctcctttgcctcctaagtcttctaatttgagcaaacgatttggaatagatgcatcatatgaagatgcaacggtagaagtagcgga
atcagtagtcagggcacttcccctggaatcatattgagaaagaacctttccacttaaagcatctatcgtaaacaatgttgttttcttacttccaacaa
agatgttgttatccagtaggcgtacgggagatgcactgaccagattacgaagactatgaggaagtcgaaccaaaccagtcttggtgttaaaagc
atataagattcccccctaatttaggttccacaaaccatagtgtatcattactagagacatacgaagctgtttgtggagctcttaaagacttatcgcca
gataatgatttaagagatgatggagccagttgccttatctcgtttcttggaagaaccggaaagccaaacttaatggcttcatcttcttgaagaccgg
aaatattttcagtctctccgagtgaccataacctatggccagatgaccgatcgtagccgtgcaaaaggccatcgatggtagaaacaaggaagac
gttatgtaattttgcctccgcaaagcttttagagacatctaaagacggtgaaagggattgtctattgtcataatcttgcggtaagctcggggaagat
aacagtattaaatcattagcagaattatgaggcttgggattcgacaatgaatgacagatctgaatgctagaaataaaaaaaagcacaataaaag
gtatagaaaagaagaaacctgatctcataagtagccaatgtaagctgatgaaagtacttctttttgaatctgaaaattcgcttctttcttgaaacga
agctatagacaattcctttaaattacaaataactcattgctttttcagctaacatatatttatacaggatagaggcaattctactcggaatgttaaag
cgtggttgttgtcgaaagccaagtttggataggaaatatactgggagacaaactacgtaccagcctcgaatgttcgcgatccttttgacatttaca
aaaatgaccattcatctttagaacataacaataaaaccacacataggacttccaaaattggctgtaggatgaaagaaaggtgctttaaccagcgt
atacaattgcaaacgacaaatcgttcgaggatgatgagattcgaaatctgaattcttttcttcttacttttaagtttcgtttattatttttattgtttaat

tatagataatcattatttttctcatacatgttcaacttcacaattgccatcgtttgtttactaatgaatgatagaaatcctcactcattttcgcaaaaa
attattccgtaactttctttcgaactttgacaatgcactcaacttcattattgtgctttaacaccgtaaacaaacacaaaactcaccaccatatattc
cgagaaacttgtcgtctgaatgactttaaagtactcaatcaatttttttttccttcactatttgtaagatgtctcgtaagtaacattgaaaatataaaa
aacaacccggtaagaaatccatgccgaagccttctttgtcccaggttccttccttcataaccaacataacatgatacatcgcttcttttattaatact
atcatgtttctttgcttttaattcatccactttgctgcattttgctatagaacgactttagcgtttcaggttgaaaggacgtaatgtcttccttttctgtg
agggaagtgagggcgaaagggtgaaggtttgattggattcaaggagttggttggttttacccttttagttttgtatcatctgctaatgcttctttttct
ctctacaggtggtaaccaacgtgaagtcgatcgtgttcgtaatgcaaaaaagcaacaagctgctaaaaagaagcaagacgggaatcccgctaa
gcgtctggaagcgtaagtgttggttatctaccgggttttatgtggaattcttcgtcaatgaattgcataaaattaacacttgttagtcaggctgaaat
catgagagcaaaacaacgtgctgctgatgagcggaaggcagcttcttcttctaaaaaggtacgtttcgaagtactcaattttagttcttttcctaac
cttagcgttatagtaaattcctagaacaaacgttcctagattaaatgaattttgtgaaattaatagatcgtttcaacctatgaaatctacctcttttac
ttgaagtatgtcttatattttcataggttaattcgtaaaaaatttgttgcttcgtatttctcgtgtacattaatattataaacactgaactctccgtcac
cgaaaacagatgtcaaatagtaaattctaaagagcttacgtaaatagtattgactatacaaaaacagtaaacatttttttcaaaagatacattgct
ttcagcatcaatgtaaaacagctagttaactaaaaatcatctataatatgacaataaacaagaaaagcctgtctcttaaacacacaagtaaacta
accaaacattaaaaatttatatccaccattaacaaatcacgctagagggtatatatgcaacaaaaacagaccaaaggaaatcggagtatgacct
aaacaactatgtaccaatttcatgggatataaccttcatgatatccccagaaccttgcctattccatagacaagccaatttacctaaggaagtgac
ggacttgtgacacctgaatagctaaataaccaacacttttgcttgcatacaaataataaatattaaatcccttcgccactacgaagggcaattaat
gcaaacgctggataaaccatcatattttaaggaatattaaaatgaattcacttacaagctgagagcagcaccgaggagggtaacgataccaacg
ataccagcagagaaggcaacagtggaagcaccagaatcggaagaagcggactttccatgagagtcggaagagccggaatagctcatagaag
aggaagtggcagaagcagaggagtcactagatccagtggcagtggcggagacttcagaagatttggccatgctagaaacttcgcccttgcaagt
ccagtcattacccttaataacgccgtcgctcttgctgaaggggcaggtgaagttgctggacttagattcaagaagcaaactaccacgaacatcag
taagcttgggaaggtcaacataggagtagttaccgagcatggtaagaccaccaccgacttcgttaagcttagggaaaccagagatcttagccaa
gtctgtgttgttgatggtcaaactaccaccaacagtagtcaagttgggaagactcaagctattaagatcttgggaatccataaagtagagagattc
gctaacggaggtgaggtagggaagagagacattgttaagggtggtgtcggtgaaaccaagactgcccttagcagacttcaagttaccgacgaa
aacgttgctgattccattaaagttagtatcgccgctaacgttggcgagcttgctaaagttaacgtcaacctttctagcgttggaagcgatgttgatg
gactgggcagtttccaattggggcatatcaatagattcaatatacttgttgttattgatgttgaaggatttaacactggtgacatcaataccctggat
atcctcgaggttagtgtcgacgatttggacatcaccggccttcttgataccactattaaattggagatgttgcaaagcgggcaagaccatgagatc
caaggagccaacgtcagtgagtttaggagcgggaagcgagttcaaacgagtattatcgtgaatgctgaacttaccagaaacggtctcaaggtcc
tggaagttgacggtttgcaagtattggctgtgggaaacttcgacatctccagtgatcaccttaacaccatcaatgttaaggacagtgacaccactg
ttttgcaagttgttaacgtacaagctgccatccaacttgctgcagctgctgatgttatcgaggtcactttgagcagtaacgtttttatcgccagagca
cttgttgtcgtcggctaggacgcgagaagcgaggaaaagaagagaaagagaaagagacttgaaaaacaacatgatgaatcagatgcttaaga
taatgaaaagaaagaaaaactcaaaaataatatcgaaaattaagaaaaatgcaaaagacttggtcttaaatgaacgatgcaaaaaagaacaa
tgaatataaaagatgagaaggtcttcaaaagaatgtccaactgaaaaaaatataaaagaaatgaaagacctaagggtcaaagaagcgatgaa
agcaaataaaagagcttaaaattcaagaagaacttgaatatagtgagaagtggttcgctttggtctgatctcttagtataggaaatcgtgccttat
atagtagaaaaagccgggttttccgtgactttcctcaagccacgtgaacaagcacaaccgttgcaatgtttacagttaacaagatacaaccagcc
aactcgttgcccgagcgatgaatgaaagtaggtacgagaacgtgagcaacgcggctcctgattggcttaacccctactgatagcaaagcaaacg
ggcccttaaggtctgggttgctagggtaacgagatattgtattcatgtaaacaaacgtgctattaaacaaacaaaaggcttgtttataaagctggc
acagaaatgcaatagacaattgcgtttgtctagtcataatatgcaagccattttagaggtttgtggacaattgtagatggctgaaccgttggtttcc
cgtgaaaaaagtcatgaacgctgataaaatgaggctgacttttcacatccgtcgtctcctaccagcactgaaacccctgtacccatctatgtgttca
gttattcctgcctgatatgatgtgcaagagtcaaaaacaaaaaaaaacaaaaaaatcaaacaacagtctgagttctgtttacagaaagtagcaa
ttggacattggtagattgtcttctaaactcgagaaagtctggtttcgtttttgctcgttctacgcggtataaacataaacaaataaatcaagcagga
cggacgggacgggacgagactgaactgggtggtgcttcgggaacatgctgataaagtttgagttatctcgaagagattgacgattggcctttttaa
atttctttgcgacatattactagattagacatggaagttttctatattattatacataataaggattattatagagtctggaaacaagtaacccaagc
aatataaacaaaagcaagaagcataactaaaagaagaaacaaaaagacaaaaggaaagcattcatagaagggattgttcatagaagggatt
gatttagaaatgataccgaagaaaagaacaccaaaaatagaatctaacagactcatctgacgttctttccattgtttctcagcagccgatttagga
ttcctggttttatcgaggactagccagtagaataattcctgctaaaaaagtcgtcttttgaatgtgatttagttattagttgtcaaaaagagtgttgtg
gcaatgttttctacagattaacagaatgaaaagggaaacaagaattttcgatactttgtatttaatttttgtcgctatgttttaaatttgaattttgttt
atgctttcctttccgtaactggattcaattcaataggaaaagtccctttcctctacccatctcgctaaccacttaaggttagaaaaggtaaattaagc
gatatgaaagatcaacactacactgactgccagtattgagttttcttgaaaaaaaaaaaaacactccatttgtatcaaaggcggtacaccatgtc

agaaaatatgcagattgatcatccagaggatatgtcgcaaaacgagactccccgttttgatataaaaaaggtatgtggcttacgattgattttgta
gttttatgtccaaggcttgttattcactagcttactctgtgaaatgttttgtgatactctctttttctttaatgttttgctttattttcgtttgtgaggaatct
ataacttactttttcgttagtggaacgcagttgctttatggcagtgggacattgtcgtagataactgtgctatttgcagaaatcacattatggaccttt
gcatcgaatgtcaagcaaatcctgattcggctgctgctcaagaatgtaccgttgcctggggtacttgtaatgtacggcacaaaacagtgtttattta
ttctcttcaattactgacccatccttttcagcatgctttccatttccattgtatttcccggtggctgaatacaagaaacgtttgtcctcttgacaatcgt
gaatgggaatttcagcgttatggtatggactacaaaccctgaaaagggagtttatctttctttttacttacatcctactaaccgttctttttcgtagg
ccattaatcgtaagcttataaaataaaagattccgtatttttattctctttctcattgtctaactgcttcctcttgttcaatctttccgtgaacggcagt
ttaacatcttacattggagaacggttttttattatgaatttcaattgatgaatgtacggtaaagacgatagtcatgatactttttctcctttgttatac
tcgaagcagcatcaccgcttttacgactcgctgtgtagaagcataagtttatcagaggaactatcttaaagtagtgatactctctaattttgctttca
ttttcattcgaatctaaaagctagtgtatagccaattcaaatttatatgttatgtctcacacattttcgtacagcaaaaatgttacaaaagaagcatc
tcttcaaagagttagctaatctcgtcatccgcttttaatttcaagctcacaaatttggccggtagcgttcatcctcttatgaaaagaatgaaccattc
atgaaatcagataagtaggctctcattttccattcctttacaacccggccagttaaataagcatataataaaaaatcgaactcacatttgcggtccg
tattttaaccagtgctatagattactttatgcaggcagccagacttgcaccttttgcaaagttatttgacatccactgaacaaacagaatttaaattt
ctttaaactattatttatcagagctcattaacaaggatccttttagttccctttttccaggtaaagtatgagattcctagtgaagaaaaactggatac
ttgatttggctttgctgtttctgcatgaatattcaaggattgacaaaagactaccaagtagtaggatttatatcctccggaacttacggaaaagttta
caaggctatttcaacaaataaaaatgaaaagcgctattttgctataaaaaaattcaaggctgaagcaaggaatgcggctccaaatgcgcaacaa
acgggcatctctcaaagcaccatacgagaaatgatgctttgtagagaaatccaacatgaaaatgttgtgaagttgattcaaatattactgaaaga
cgggaccattagtatggttttcgaatacgctgagcatgatctattgcaattaatccactttcatggccgaacaagaacgagaaaaataccccatt
tataataaaaagtatcttgtggcagttgctcaatggtcttgcctaccttcatgaaaactgggttatacatcgcgatttaaagcctgccaacatcatg
ataacggcagctggaaaggttaaaattggtgacttagggcttggtagactaatccgtgaccctttacttccattctactctagtgatcgtgtcgtcgt
tactatatggtatcgtgcgcctgaattattattaggggctcgagactatacgccagctatagacatatgggcaattggctgtatttatggagagata
ctcgctttaactcctttgtttaaaggtgaagtgaaaatggaatccaaaaaagtgattccgtttcaacgcacacaaatgctacggataatggagata
ttaggtacgcctactgtagaaaggtggccagatttaaaagattatcctgactatgatcaattgtcgacatttgaagtaaagtattggaataatctgc
ttccccagtggtatcgcacagacaaaactagagatccaaaaggtctcgatttgcttatgaagctacttgtttacgatcctaaaaaacgaataactg
ctaaagaggcaattcagcatcctttttttacagaggactccaactggagtaccacgtaagtattttcgttaccgagatttttctctatatggttttattt
tttctcttttctatttttcaactaacatatataggcctttctccgatcatggcgtccagtatccagaacgaaggatattgccggatgattcggatcttc
cacctggtaaacgaccgctgacttctaccatgcgtcaagaaccgaaacgctttaaaggaaattaaaactatacaatcttcgttaaaggtttcatac
acggcgaactagcgaactgacataatttcaagtgtctgaattgtcggacgctcagaatgaatcaggcctaattgaagattaaacaaatagaagct
caaagagtttttttaattacaaagagatgaggcttcaaatccctttttgacgtttttgaaacagcttctcgatctctccttctaactctgtatttgaacta
tttgcttggctaccagcgttccatagacatgttcccagtagaatggaaggcgaaatcgtttctggaaattggtctctattatcaaaaaaaagaattt
gatttgaatacacagtttcaaaatgctctacagctgatggattttgcagatcacactgatgaggtaaaaaatcattgaaacgaaattttgtctaatc
tgttagcctgtcttattagttattgcagactatttaatttggttcatctatcaaaatatccctttgaaagaaaatccatttctggcccatttttttcatac
ttgggcgcatacatcttgccttggccgacaatacctcttagctaacatattatccggcagaatccagcaaactgtaagtatgattcctttggctatat
cttcatatgactcttaagtggtagtcattcttttactctactcatgatcttaactgctggttattaaccacttttaaagagctctgatttggtgagcattc
tcacaatctctccattagaacttgtttgttcaacgacaaaagaagatgttttagcagaaaactcctttattgatcagaatgatttacgacagtggtt
ggaacaacaagaattgttgcctgaaaaagcatcctctaattcaaatactacaatatggttaaccggaacagaacgtgccctaactacagaggaa
gtggtaagattgatgaaaccgactgcttattaacttttaatcagcgaagtttttttggaaaatcaaccacgtttttctgaaaaggatgtacctacagtt
aaacaaatagagacgtttatcttactaaatttgccttttgctcctgaaatcttttctgatttgcttaatcattctgaagcaaattttaatcaaagatttg
tcaagaaccttacatctctttcgataacagtatccaacatagaagtcttgattctgatgttgctacacgatccttctttggtatcatatatgactggtt
ccggtacttttatgtacgaacttttgtcgtcattcacttctcagatctccaactctaatctattcgaaaaagatcgaatggctcatataggaacaagt
tttttcataaaagtccttgatgtacctttcataaaaaatatactggtgtatgacttgtattttgacttacaatcattctgcatggccgctgtacctcagt
cggctatattatatcagaagctaaaagtcattaggtctacataaaagttacgaatttatattggcaaaatcgtttacgaaatttaaatagagatat
atccatatagcattaatttaatctattgtctaaccctaaccaaatttaaaccaaaaaaattgagtgcttttatccttgctttccatttatatgctataa
gcacactgagtggataacaagggaaccaaaattaaatttactgaagactatcctcgttaagagtgagcaaacgagatcgtataggatacatccg
cttgtacaaagacatagcaatcgtatcttgaacggtaagcttgccattgaatgtagccttaaagtatccatgggtacccaatggctccttgataaa
ccctgtacggccttgtttagtaaacagttgaataggcttgaaccacataacatcttcagggttgaaaaacatgtaacgaacagtgataagtttttttg
tggattttgaatggatggccagttagaacagcacgcttaactatcacagaagaagtatcactcttaaggaaagaaccagtagcagctaggcgta
atgaaccatcaggcataggtttaaaaagtgttacaggcagacccccgaaaactataggaccgataatagtacctaatgcaggttgcttagcttgc

aaatatttgtaatatttttgaaggttatttgcggcaccagaagctggagcatcggaatacagtggattcacgtacaagcgacgaggtccaacttgc
ataattaattcttctttagaagctataggatcttcatattctgaatgcttcatgactgtaaagtgggaaacagtcgttctattctcataggccattaac
gaatatagcacaagcatattgttaggctgactataataattgtaaacagaaacaggcacgtctcggagctctacaacaactgacattccaggttt
agcttcaccgatgaatggctgtttgtaaagcttgttctttaggttgcgataattttcaagcttgtacaattgtcgccattctggaggctcacttgggtc
atactcctcagcatcccaagggctagtatagaaactacggagtccgcggtatttttttcaagcgttctcttgctaattctttagggtctaattcgagttc
gtcagggaattcaagatcttcttgttgttctttcatccttcgacgataatcttcatactgccgagtttcttcgtcttcatttaattcaacaaacatttcag
acttagcatcatcctcaacttcgctttcatcctgttcgctagccggctgttcctcttcaggaccgccctcattttcgacaagtaattctgtgtcttcaac
atcactatattgttcttcttcttcatccacaccagggatccaaacggcctgatagctggaagtgcccttaggaacgcgtttagtgaccggtataggc
tcttcctcgttatccaaatagtaaaagtcatcaagacgaacacctcgtggaggttcagattccatttccatctccatactaacgtcatttggagcaa
gggtctctaattgttcctgattttcagttggagcagcaagttcaactaaaccagcctcattcacgtttttgtcttcttccatggtatcagaaggttttgt
ctttaaatcagaagcatcatatatgcaattaacttgaaaatcaccgaaaccttgaatatggatcaaggagtctggatctaatcctttaccacgtact
atacctttaacagcaagctttccttccttataatcaatggactgagccaaaagataggcccgagaatcacgccaaaagataccacggggatgag
aagtgcacagagcacgcataacgttaagagcatcctgtgaaacgtcagaagcaaaaactcgatcctgatcagagaagaagaagttcatgaagc
tttgcaaagatttcttaacttctccgcgaatttttatgttgtcaataactgacatatcgttgactaccgatatgacactagatattccttggcactgag
ccgttctgacgactaattccccaaattcatcgacttcctgagtagcagaaagaccaaatattacatagtctgacgccttgcaagcatcaacaagac
tataaaagttctttccaggcatcaaaaattgtaacttttgtttgaaccgagtgattgtcgtgttatactgataggaattttcttcaaacaggggaaga
tcagcctcaggatccaccgacactaacaagtttctaaggatactccacggatcaacgttcgcgcaaagaggaacgatagtaattacctttggagc
accatttttgccgccaaaaatccgattattcatagcagcctcatttcttttgttcaactgtatttgcttagcagtatttcgtcgatctgctttagtagag
gtatgttgaatacttttaggaccagtcctttttggctcaacgttattactgtaacgctcttttaacgatgactttgaagcatgtttagacttaaacggc
tttttaggcttaacattcgatctgtgatgatgagccatcttcgattttggaccttaagtcaaatataagattccctgtcgacaaaattaaggattagg
ataaaaatatatttttttatccaaggcaattaagctttacaagcttctttttgtacaattacaaatttttcttaacaatttccccaatgccgtttttagca
aagaaaaagaaattaaaaccacttaattcctttaattatgatttcttttatactaaaaatcagttgtctctgctaaaactagtgttttggacaaatgg
agttttgttgagggcaaagtgaaaactgaaatatccttggagttagttatacttaattcttccttctttattgtagaagatagaaatcactattaaga
tttaattttaaaatatcataaaactctattaataaataatcacctaaagattaatatgcttcgtagggaagatttctttgggccaaatatttctttgca
caaccatagtgtacgacagaaccactaaattgttaattataatgaaaaaggaataaacttactctggaaatatagaaatcacgctttttcctaatc
gcttgtggcaatgtaaacaagtcttctctcttgttataaccaccttttctgaacgaatgcgggccagctctgcatttaggtcattaatcctcttttgat
acagccttgattgtaattccttattattttcttcctcgtagagttgccgaaattgggaagacagaaatgtccggtatgttttcatgctggaatttttgg
gtagcaacggaaagattctttttatcttcaaccgagagctatactttgttataaatgagattatgtcttctggccgttcattcacaagatctgtaagg
tacatatagtaaggctcggtctctccagtatccatatagacagaatcacaatagccgttcgctcccttccaatccttcattttatctagatatacattc
aatgcatctctatgttttgataaacgtcgataaaggattaccgacgcttcaaacagatgggagttatcagagtcgatttcgttaagaattgcattcg
catcatatgactttgattcatttaagtgttgaataagatcttttaatactgcatcaaagaccactgatgattgttcatagttgctctctctttctaactc
ataaatccgacgcaaatatagttgaccaaggcgggtccagaaatattcaagagttgttttcgcagaagcaattagttcctgtagatatatgatgga
tacttcagaagatacggtttcaaggtagttcaaaacttcatcatgagctatggtgctgcttccacttgtatcattaaggaataactgcaccgaggct
ttgggaacgctgcctaagggaattctggcatactcaaagataattttttttttcatcgggacgtaacttttgaaggtaattaagaatatcatcatactg
agaggtttcttcttgagctttaggagcggataaagaagttccctcttcgtttaacttcgtcaacagtttcaaagcatcgtcatgatacccttttactaa
aataataatctattaactcgcgaaacataccagccgaagtcaaatttgcttctacaactgaagtttcacaatgattaggtaaacgcaacaaagac
cctacaagggccggcgtagaaaccatatacgttagaaaaagagtagtgtcaacttcaatagcaatattctccaaatcctttttcgatattatactg
ccatcttcgttacgaaagagatctttttgatatgaatggtaagtttttttcatcccaacttaagaagagattggctttccttctagaatcagttaaataa
gtacttaaactacgtaaccgtttattgttaaacactagagattttccatcatcctgttcctgagagtcggcttttgaggttggctgtatgcttctttggg
gagatccaaccatggacggatctgaaggagaaggtccggaagacaggatactgatagcatcagaataatcattttccacgagcttgggaaaaa
gcctcaatacaaaagagggagattcacatatttcagtaaatagtttcatagcggtttcatagtcaccggaggaaaaggaacgagaagctttttct
cgtttcgtcatacgaaggtagtaatcacgttttggaaatttcgcatcgttaatttggtctaagacacttatcgcttcatcaagttgatttgactgcaac
aatgattctacttgacagctaaaatcttctggaataaggatccaaacataagaacttgaggtaaaaaaggtattttcttcgcaagaaaaagtaga
ggatatgttactaataccaatttgttgtatcagagcatgagtgtccttattccaaacgtaaatgtattgagaatggagagatattaaataaggtga
attgtatataacagcttgaggactagtaggccagactaatggctttcttgcaacatgtaaagatttaagattaacaaggaagccttgcgtatctctt
gatagtaaaacttcttcgtccgaaattcttgttatgtgtaagcgatttagcttcatagacattcctatataactcatgccgagtcccaaagaagagg
atggctgccaaggaagatttaaagacgaggactgagatgtttcgacgttcactgcaacaaactcatttgaaaaagacacaaggatcatagttga
tgacatccatgttagagaacgggctcgatcattaagggagattgatttgtcatgaacaagatgatcggatccttgccatttatatatattcagctgc

tttctctctgccaaaataatttgatcatttccgtataaacacatgttttggcaatgactaccaacacttagaagcttctgaaacgttgatattttatac
acatgtatggaggaatctaaagttgaggtaccggattagcatttaactcgtatacaataaagaatcttcctaccagatagcgcgtagaaaatgtct
ttcgaggcatcacggatcacctttgttattggtttttcgtaaaggtaggataaaactcaacatgctccataacaaagtcatcttacagaagttagtt
caacaaatagaaaagaaaaaaaataaaaaaactaagtaccgtttaaattatcatttccatggtggatcttgtacctatccaaatcgccattgctgt
ttgaaacataaatatattcacctttcctcgttagcaggaacgaattcaagagaaacagatcgtacccaattcaaagatgcattcaatttttgtttttg
aaagctccaaaacgcgaaaaagagaaaaagatcgatgcatagtcaaaaatcataagtagttatcaaaaaacgatgcagacgatgctgttcag
atttctcattaaattaaccaactttataaatgtaaatgacagcctctttcttgttgaaaatactagccgagcaagtctctgtattgatgcatgactcta
acatgacgctttactgagcatggtcgaaggataagcgtgtcgcgttaacattccttaataaacaaagaaagaagaacaaacatttggcaaatttc
gtagaggtattcttgcttagattttaggagttgatttgttgaagtgttggaaagtttctatgaacataaagcgtcaaaagatttagcaaaggtgga
caattgaaattagaataaaggtattaacgttatcgagatgaaataggcagagcaatcaagctccaaagcattctcatttttaagtttagaaccat
ttgtttacttggaagtagcaggccaaaatccttcttcaacccagccggcttttaaaacgcccacctagtaacaattagtttttgaaaacaaagcaa
gatgtttgaacacgttccataaatacgctttttcatacaattgagcttagtttcacgggtcgtttcagtttttgttcttgatgcacaagcattttataga
cgttcaaataaacatctttagaaattaccacttcatttgaagaatgctcattcgtgagtttatcatgatccaacaaccatgcagaacgccttttgtac
cattcattaacaccaacttcccaatcttgcatatttcgtgtttcttgcatggttctggttaaacataaggctattgccccttgtacactaatccatagg
gaatggaatgagaggatcttattgtatcgtattagttcactaaaagtgatatagaaatcgtagtatgttctttagagcgatataaacaaaagtcga
tgggtgcagaggaaaaaatataaagaaaaggattgtgtaggatgtcttataatgcaagtttctcctaatggataactcttattatgtttgtcattac
cttgtttgctgaaaatgcttctcatgaaaaagctttgtttgctgcttggtgtaaacaatcaatcagtcatgtcgagatgtttttctaagaaggattaat
aactttttttcttttggtgtttacaaaaaaaggaaacacttgaaacgaaatagcaacggtttcagttcctaccatatgtctgtagatcaataccaat
gaatgaacctttttttcattcctgtgcaattttttttctgaatttttcatacaaagaacagctttctttaagttatacaatttaaattattcctgccttaat
agccctatttgcagacttgcttccttcattctttagtattccaagtacgttggttcattgtttgaatcgtttccagagtaagacgtattcttttgtcacct
tttttcatagtttaaagattgttcaaattataaacgacgagcttttcatgtcatgtcggtaactacacattctgcaaatgcagtatcgaaatcaccac
cactgccttcatcgtttgctcccgctcatgcttttaaagaagaagtgaatccctcttctactgtgctctctttttgcttattcctctcatacctaggctct
gtaactgggatatcgagttatgcactcttaagggtataggcagacaactttttttcacaaagtccttatttattaacattcaatttagtattacgtacg
ccctagatggcttcaattgctcgctgtacggcatgcctatgtgaaatccaccttgacaaagctagaacatgtttcagcaaaagcaaaagagatag
aaaagaaaacagctactttacaatcaaaccacgacttgtgggttagaacaaaggttgctaaaagtcggatatctacactaaaaattgttgcaca
aaagaaagaagaacaaataccagcagaagcatctctaaacctaagtctacaggggttcaaagatattttgaatgctgaagttggcaactttcttc
ctttgtataaccctaaaagcattccagctgatcctatatcacaattccatgatgatctttgcaattacaaaaggcttcatttgtctatctcatgagata
atcatttttccaaaaaataactatgcaattcattaaacgccaattaaggatcgatagttcggggaagcatcttgatacttattaataaaatactcat
ccttatgctgtacgagatatctagattaattgtcagtcagtaaaataaaaaaacacttgttgtacttactgtaaaaattcctcgatatatcctagtaa
acagtcaatcgactgtgagtccatattcgtatggtcgattaattcaggtaagctaacgaaaagccgtattaggtgttctgcgccataaatatcgca
catctcaactccaggatactctctccgaacttctaagtattgctgcctttcaaaacgataaagtaacatgtttccaaggcacttattgaaataaata
gctagtccagccatagcctgctcaaacacatcaacgtcgacttcattcgttaaagaagaaatcttagattccctgaaagcagcaatggcagctctc
acggtcggctttcgaggcagtgaaattaactgaagatgttttgtgatatgttcccaatcatctactaaccaaagctttaaaacatcaggaactcga
accttgtgtttaggcaatggtggttcttcatcaacggattcttcttctacggctttcactgctgcatctacagcggcttcagaaaccgcctcgtttttta
tcacatgaggaacaggagaggctgacttttgggctttcgctctttgagacgtttcacgggattcaccctccatggtggtagcgctagattcccgaga
ccgttttccgataggtttagttgcttgaccagacgcttcaccaaccttgttagtagaagaagctgcctttttcgtagcagcagacttttgtttagctgc
caaggctgtactctttaattccttttgtgttttcaaattttcgtcattccactttaatatacgatcgggtccgacccactcatcccagctatttttccatc
ctctatagtgaatcaagtaggttgacgaatcttcctttttctctgtgtctataactttagcttcataaagtaaaggtccgtgaaagcacaacacccgt
tcattcactttgtacgacatggaaagagtaatgtatggaagcggctcgtggtggtgaggtcttctacagtaccgccaagaaacagaagagaaag
acagcagtatcctgaaaaagaaacttttaggcacttaacggaaaccgttgtggtggtgaacaatttgttgtccagttctgagtccacttctgaggg
aagccaagttgtcgaagcgggttaattgctgtagaaggtttactattctgttcttgaaaaggctaaaagtgctagaaaaaggaagtttttattgta
aaataaataattcaaggtaagaaatataacaaatctactaagcaagtgaggagtatttttttgcatcaaataaaatcgaatttattctggaaaggg
caaaggaaggactattataccttttcatataaacactatgatatgaaagctgaaatattttaaatgtagaaacgcgaatgctgataattacgacga
ccatattcatatggctgacaaaaacttggctgttgatttccttggttcccttgtttcgctttcttgataaacttcacttttcaagtgaaagaacggttaa
acaagacatccattcgtattcctacagctgagctaacaagatagcaggggtttgttttcatggaacaaaaaattctaagcgattatttgctttccca
aggaactttctcttctatcattcatctccaagaatggaaagaactatttcctcgtcgttttcgcgacgaacctttgctcgttgaactttatgacagatg
taccaagcaacaacaaaaccgattaaagaaaatcaaggccaacattgagattgagagtcaaatactcggaaagaatgaaagagatcaaatgc
tttctgcaaatatacgaaagttctttcaaagcgctgctccccaaggttccgatcaagacgaattttatacaacgtatgctgctaaaccgctcgacat

aacggctatcaaccaacgtctggaaaacgcattgcaaagtgtcaaagcggaagttgatatagagcgtaaaaaatgttctacgttgtcctctcaat
tggacgcgtacgtaaagttcttttttttcttcacttatctaacttgattagacatattgattcgctaaacgatttgaaatggtcacaggaacctaatct
agaggaacctgtggctcttcttgatgatttgattgaacaattggagagacgctgcaacgaagtaccttttgtcagtaataatgacgaactagactg
agttttctatacaactgttacaatatttattgatgaacttgaagttccatgcgtaaaacattttattttcgtccactaagacttttacgataaccttttc
caaggaatttcaattgctgaaagcgagttgttacattctttcttttcaaagaagtattctttctctatttttttgagttttctaaacgttgtttcaactattc
agctgtttcaggctaagcttgcatcacaaaagttctattttcatggcgaaagcgataccataactctgagtctacttttacaattcatttcaaggaaa
tgctttattttttttcctttttttttcattattgagtttcttcatttccaattatttcccactaatatagcagatatagcaggtatccgagatctcttttggaa
ttcgtgggtgttttcttttatccgaagccaggttcgattaaagaaattttgttctgaaaaatcaattgaaattcaattgaaatttgttcagcttaaaat
cagccttgtttgcctatatagaagcaggaaacgaggtgtttcaatctagactttcggttgtgcaagataccatcgattctatattctttttgaaaag
taatctctccgttttattacttttatctgaaaaatgaagcctacagtcaattactctttgtatctagtaacaagctctacacaaatagcaccaggatc
cagcttggaaaagcaagtagaggaaggaatcattggcggagttactttggtacagcttcgtgaaaaagatatttccacaaaactgtttttggaac
gggcgaaaagtgttttaaatatttgtcgaaaacatggcgtaccattgttgatcaacgatcgaatcgacattgcattagccattggagctgaaggag
ttcacattggacaagacgatatggactgtaaaaccgctcgcaaaattctaggagaggattccattatcggtgtttccacaaacaacattcacgaa
gtcgactgtgctgctgctgatggtgccgattatgttggaatcggctctatttatgataccaacaccaagaatgttaaagaacgtattatcggtatta
caggtctgcgtgaaattttggaacatatgtccaaaatgccccgtcagctgaattcagtggccattgctggtttaaattctagtaatatccagagagt
tatatatttgagcgaagttaacggaaaacgcatcgatggtattgccttagtttctgctattatgtcctccacgacgcccctcgaaactgccaagcac
ctccgtcacttgatcgtctcccctccttgtttcgctcaaatccattctaagatttcatctagctcggagttggttgccaaggtgggtcctttgtttgcca
aaacaaaggattatacccctttaatccatcatttgaccaacggtgttgctaaaaatttttctgctaatgttaccttggctgtcaatgcttccccgacc
atgggagaaagtaaagaagaagttgctgactttgccaaatttgctggctcacttgttttgaatataggaactctacaggttcgtgagacccttattt
atgcgtctcaaatctataattcattgaatcgacctgttgttttagatccagtaggtgtcggtgctacaaaatttcgtaccgatgctatttcaacattac
taaaccatgcttatttcgatataatcaaaggtaacgaaggtgaaattatgagcatcgccggcgaagaaggtattatgagaggtgttgatagtgttt
ctcagcatactcttacttcaagagtttccgctgttcaacgtttggctgtagaacggcgctgtgtcgttgccatgtctggtcagacagatattgtcagt
gatgggaaaaccacttatatcattaacaatggaacttccttactaggtaaaataactgcgtccggttgttctttgggaagtgtgatgggtgttgctg
tttcccttatcccagaagataaactacttgctgcggtctctgcaacattgctttataacattgccggtgaattggctgttctagaggaaaagcctgca
acatctcatgctgtccagggtcctggaactttttgcctgtttttatcgacaaattgtatttccttgttaatcagtgcattaatggtgactataattggc
ttaatcgcgctcttattcaaaaagtctaagaaagaaagattacagttgccatgagagggacgataattttcgtataaaataaatgaattactaaat
agcatttacgataaacactcacaaagaatggaaaaaataataaaggaagttagtaggatgtaatcccagtagcgcactatcgaagaaaatgag
gagttattttatttttaacatgagtaaaaacatatcataaagaaactttttcagtggtttggcaagggatgctctacatcagcagagaccgaattttt
ttatgatacaattaaaaaagtatcaaaactttactaagtcctgatcatatttgttaagcagcttgaccttcgccagtagtcttctttttattgttcttttt
cttcttgctagcgctgctagtcttcaaaggagtctcgagaagggagatgatttccttgtcctcaatcttcttgtccgactggatgttgttgcgaggctc
agaatcactaaagacaatggtaccgttcttggtcaaagcaatagtagagtaaaactcagcaatatagccaccctccttgtcggtcaaaacatcat
aaggatacaataggtcgtgctgagtgcattcgctgaaacccatgtttgttctgctatcatgagtaatgttacgggtagaaaaggggtaagcacca
aacttattttgaatctcagaatagaccttgcgactggcttgaagcttcaacatgtatttggtatcagtcttcttgtaaatacgggtagcaatgtcaga
acgcttgatttttccactaggagaagaagagatcaggatatcaacaccatagacttcaccttcttggaaggtaaaagtgtcaaattgagcgcgttg
ggtctcagaaggattcaaaatgatttgctttttaccatcaatcacgtctctttcttgttggtgagtgagcataccagaaacaggcttgcaaccataa
gtagtagcaattttgtcgacaatatcagtgacttgccagttattatttccaggcttgatagtacgttgggcagccttcaaggcagcagaagcagca
gcaatcacgtctgccttttcaccaatgacagcttcctcagaaacgacaacagtagtagcaactaaagatgcgaaaccatcaatgtgagcacctaa
gaataccttaacaatatcacccgcctttaaaataatgttggcttcagggtcattagacaagggagataagtgtgcagcaactccattaggagaaa
cggcagtagggaaagcaataccccttataagagtccttaccgcgtgcaaccttgctaactgcttcttccaagagttggtcaccacggacgcagatgt
cgaagaccttagcaccgtctttacataagtcgacgaccttgtttaaaacattttgggaaagttcaccagcaagcttgtacttgttgatagtttcaga
attgttcaagctatagtcaacagcggtctcttgcttaacttctttggtagccatcttgaaaggattatacgcaaagatggacctaactttccaggaat
ttctgaagacagaatgagataaattttgcaagtcacagaaaacatcatctcttaaataaagttagctttagcgaatcgcttgtttacatggattgaa
atacaatggggagtggaaaacggcaggaaaacaagaaagaattcatgccgaagagtaatttgtgtattacgaatgtaaaaaagttgaacaag
gccaagggaacttgatgaatctcgaaaatatccgttcacccatgcaaccgcattccattcattgtctaattacttatttatccatataaaccgtctgc
ataattttcgtcagacgcaaaatcatcttgaaaactatgttcactggatcctccccattcatcttcatctggatagctgttttgatagaagctttctgc
gtttgaatcctcgtcaatctcatcccgcaaagggtcctcttcttctgcatcatgactttccgatacaagctcattttccagcgattcacgaaaagcat
caggaacactaagagcatcgataactccgtaaccagtcggaggcttagatggctctttatttgacgcttcataaatatcgtaaacatattcttcacg
ggcttgttgaagaggtaagtcgccatttgaaaggtactcatggatcatagaatctaattgaggatgcgtatgatacatcccttgactaaaaatgca

gtgttagttaagcattcaagaaaaaaatagaaaaaaaatatatctatatttgccgacagaatcaaaaagctgattcattgcaacgtattcaactg
acgaaaaagattaacggagaaaagcctacctttttggagacgaatagcatcaaaaactttttcatcttttgtagatggtaaattttggatcgcaa
atcttggagagtccaaagtaggaggaatggaaggaactgatccttttacatccaaatccatatgagaatgaacttttggaattgcaggttcagag
ggttggataattggaatcccataagaattggcctttggcaagggtggtttgctaggaacattcgttgattctagcaagtctgacgcagaagaagaa
cggacggcgttcacattcgacacatattgcttatcgtttttaacgtctgagagagtctaaaaacttcgttcccttattagtttttactcttcacagaa
tttaattgcactcttccacacagatgtgatgaccgtggaagagtaaatctattcagtatatacttacaatatcggcctgaagatttcctttttttagca
attgttcgttcccctatttcatgcccaagctccaaatctactcggtgttagccacaatttcattatggttgtttttttcttacatagagcatttacggga
tcatcagttgcttttcttttgacacgcaagactggtagaggcatagcttttctaatttaatttacaatgatacaatcctcattgaccataaaaagact
cgcaaacaaatgcaattctattttgttgttggtagctttttcaccactgggtaaaatccaaaacaaaaggtcaagtgaccatttccatccaagtat
agatataaagtgtacagaattctcttgaactcgtagaaattattttttgtaaatgctgtttgtaggaattgaagatacgcttctgaaaatctattttc
atttaattttgacctggttgctcaaaggaaactagcgtagaagtacgtgaataaaatttgctatgatttttctttctattattcttcttcgctttcctca
attgaaagagtttcgaaaagaagctcctgaatccaaaaaccaaaggcaaatcaggttcacagcaactatgaggatagcactttaagaatttatg
acaaaagaaaaggccattctagctctcatctaacagacttcattattcgaatcatcaatcagcagtctacacgatacaaaggaattttatacatgg
gaatacctgcaaaacaccaactgaccatatcacaatgcttgaaaattggtggacaatacttgttgcgcttgcaacgattcccttagctacatttatt
gtttttcccttttctttgcatcgaaaaaaaccattcagaaaaagacaccggccatctttttgtgggaccaactgaatctggaaaaacgaccttgtt
ttgtcaggtaggattatgaaaattgaatcattctattaactgtttccctttagcttgtttacgaaaagaccaagtctactgtgcctagtattgagcca
aacgaggctttatggaaagatgatattgttcttgtagacttacccggccatgctagaacaaagcattggacagaaaaaaaaattaaatgaaagtta
cgatatacggcttgtggtttttgttttgaatagtgcaaccatggaccgagatttgcatcaaattggactctctttatttgatgtactactgaaatgtag
aaagcacaagatatcgaaagttttagtagcgtgcaataagtttgatttgtttactgctcaacctgcaaaacagatccaaactgctgttttgaaaga
gttggacaatattttcgaggaaaaggaagctcaactcgacgctattgtgacagaggacgtcgattgggatcaactgcgccctcagcttcaagata
tccctttggagttcctttctggcagcgctcttaaaggcaccaacttggatcaatggattcaatggatggaatcctctctctagaggcatatcagtcgt
tactcctttttaattttaattttaatttttctttaatttagaatcttgatgttttcttgttgaatggaatttaggacttggcgaattccttttcaatgcgaaa
tgtttactagaagcaactatatcttctgctcgttgacaaaacatcaaaaggaaaaagacttaaaattgtaactaaattcctcacatactggtgtaa
ataatttctaattatgactttccatctcttctttcttactaatatcctaacatagcatacaagtttcttacgacacaaagtagtttcataatctttaaca
aataaatgacattgaaaattaaaaggaaaagacaaaaaaggaacaaggaatatatgtatacgtatgtatgtatgtaagtatgtatggaaatata
tacgtttctacatggaagcaaaaaagaaagcgcatcgggtgaaatttgacaggatataggctgtcaacgtgaccagcacgcaaaaaaaggagc
attcgcctgtttaaaattcaaacatggtttcagtaataataccagtgacaagataagggtcgatagaggaagcaggacgacgatcctcaaagtac
ccacaacctttcatagcaacagaacgaggaatacggacagaggcaccacggtcagcaataccatagttgaattgttctatagtaccggtttcatg
cttaccggtaagacgcaaatcattatcatcaccataaactttaatgtgatccatgtgacgctttgcgaatttttcaagatactgctcaatggccttca
tgccaccttcggcacgactatccttggtggaaacattagtgtggcagccagcaccattccagtcacccaaaataggcttgggatggaaagagatc
ttgacaccaaaatcttcggcgatacggtggaggaggaaacgagacatccagagttcgtcacccatttgaataccacggcatggaccaacttggt
attcccattgagaaggcatgacctcggcattgacaccagaaatgttgatgccggcataaagacaggccttgtagtgggcctcgacgatgtcacga
gcaaaaacattgccggtaccgacaccacaatagaagggaccttgaggtcctgggaagccacccttaggccagccaaatgggcggtcataatag
tctaacattgtgtattcttgttcaataccaaaccaaatatcttggctggcatgcttttccataagcttgacgcaaccatcacggtggttgaagtggtt
agcagagccgtcggcggtataacaagcagcgagaacgagaatgttgtcaccgcggcgaatgggatctctatacatagcaacgggttttaaaag
ggtatctgagttataaccgggggcttgaccagtggaggaaccatcaaagttccaaacgcgcaagtcttccaaagattttggcttttggtcgagggt
catggttttgctgcggatgtgattgaaaccatcaatccaaatatactcagccattaccttgccatctaaaggttcaaggttggcgtacttggccata
attgaggccttggacaaaagaggatcaacttcgtattggcacatgttggagaatctgtgaataaaaggaaaacaagtgagaaccagaaccttct
aaatctattcaaaaaggaaagaaaactcggtgaaaaacaagaaaattaagatgcaaataaaaatttaacacacccttttctataaaataggac
aaataatttccgaggaatgataaattataaaccaaaacaatataagaacaaggattaaaattataaaattaaaataaaaaggggaaggatttg
tgtgtctttttttacctaatcctggtaggaaaagcggtgtttggagaaggtagttggcagggccgccgagtattatatatacgtgtaaaataacagt
gtcaaattttgttaccgacaaggtcgcctacttctgctcatcccattgataaatatagggtaagggtttgcaaatcaaaaatgaagcaatttgattg
gaagatagagagaaaataagagagcttggtgcaagctggaagataaagtccgccaatgattcgacgactttttaggggaaataaaaaatgaca
gacgattggggaatgggttcataagataagataaccagcacgttcactacgctaggaggcttcggaacattcttttaacgaagaagaaaggaat
gccttattcagtagaaagatttacatgagtgtttgtgtttattggtaaagcgaggagcaagatgttgtttcagcctcattttcaggtattattttttttt
attctggattgtttgagagtgaccctttgtcttcatgattggtttgtaagaatcactcagggttgtgtgtgttgatttcatgtttagcagcagagtagg
cagtgatgtggggctttcataattcactgaataagattcaccggatcctcgtgttctggaagcgaggtatttcccgcacaaacttattgtgtcgtcta
gaagttcatttgtttatattcctttttcataatttttccttttgcatctttgttagcaagctctaaaacttgttcagtcgtcaataaacagaaacgcatc

aaactaaaactcttgccgcctttttttagtaccttgcgtgtgattcatattatattatcttcctgaccctcctttacgaactcgcacattctaccggcga
atacttttgaaataggttagagaaaaacgaaaaatcgaaagaatcatcagcaagaatcattgagttatcgaatagacgcactgtcccactcatc
gcttccattctcgtttcaatctgaccccatgttactgtacagctttcgtcaagcggcggcttaccatctatcctttttcacgtatcgaaatccatagtgg
aataaactaggtggggattggtaattaattccaggtgggggttgagaacagattgtttcgcactgaggtaggtagtaaacagatcattttatcata
gcaaataaatgtctataagacttttcaaaatatagctatcttaggattcttcttaaaaaatcttatggctcgtctgctgcaatggatcctcctcccaa
acacaactcatcattgtacattgccaaaacttgaccaggcgttaatgcccgttgaggattcaaaaagtcaaccactaataaaccatctccttgaaa
taaagcctttgcgggctcgagtgtttgctgatgtcgtacacgcacagaacaatcaatgggccctttcgatttgctcgcttcttgtcggatccactcag
tcgaccatgtccaatgattgactcgaattcgttttcgaaatagtaatgggttcatgcttccatggcaaatgaaaatcgtgtttgtagaagcttctttct
tccagatataccatcgcccttgaaattccggatcgccctgcggaagactcaagccacagcgctctccaattgtataagaccagcaacctgtagat
ggagggtagaagccaaccactttcccatttgctacgacgcgtataggttcattcgatgaagctagatatcgacccaaaaagcttcgaaagtggct
tcctacatttggtgatacaaaacatagtccttggctttcgagtcgatcggcactctcatttagcccggcatgagcggccatcctcttaacgtctttttt
ttctagattatgtagcggaaatattgttcgctgaagagctttttcttggaccgtgcacaaaaataatgtttggtctttccatcggtctttgggtaagca
aagtgcaacttgattattacttgcatttttcattgttttcgcatagtgtccagtggctagccaccattgttgagtagaacaagagccattctcccactc
ccgagacatttcgttttctaatgcctcatacaaggctccaaatttgacatagcgattacaagggacatctgggttgggcgtacatccttgctcgtat
agctccagactcggttcaaatacccgagtccaataatccttttcaaaattaaacctccgcgaaggaatatctagttgtttgcatactttctgaacaa
gggaccagtcttgttccgcggggcatccgcctggtgcatcatcttcatcaagccaatttcgcataaaaacaccttccacattaaacccctaattaaa
aaatttcattagtttctacctttacagcttaaaaaccatcaaggaaataccaacctgtttttttaataaataagccgaaaagctcgagtccacgcct
ccagacatggccacaaaaactagattaaaaaataaaaattagcttccataccatccaattccacaagttagtattaaaacttacctttatcgtgttt
tcgaggccattgcacttgaaagggctgcgctttccacctctttacaaatgaatttgcattttgaaagaagaattttgttattttcataaacaattcctt
aaatcgttattgtagcaaacgtttctttagtagttgggaatttctagttaatccgttaaaataaatatttctataatataaaggagaaaaacaaaa
tagtgaactaaaacaaaagactggatttgccaaagtagggtttctgaaggaaacgttgaaacagtccagtactaaggtgttttttttttagtgctc
aatgactgaggtagatggtagcaaaagcaaaagaaattctactcgattcatggtttcttattccaatttaaaacttatttctcaatagaatcgttcat
ttactttcttttttttaatcttcagttttttttttcgtttataaagcatccattttggcgaaacaaataaatcgtcaagattcgtaaacgtaagtgagcagt
ttccagaaattttttttccaattaagttataagcattcatctgaaaaagtccattctttatctctattttctgcttatttcaaatttattctttctcatattct
gtttatccatggtttgtttactaataaataaccgtatacaaccgtataccgtttactataaggtagcgtggtatcgttattctccaaaacacagaaat
tcagcagtgtgtcaatcatttcagttaccaaaaacaaagggataacgtggatgcaaagtctgtaggaggattgaaggcttcacttaaagtatttta
ctgacaaagaaagaaaccatgttccttttcaaaccgttgatcttacttttctcacttattgtgtgctccgttcaggctgttcactttgacattccagcc
gagtcgaatccccaagctttttgcttacgtgaatatgctggagaaaaaacactaatgattgtttcagtgagaacaagcggtaaccgtggagatgg
gcaaaagcttagcatggaaatcaaagactcccagggcactattcattcaagtgttcaaaatttaattgacgaagagcgtattgtttccgaagtatc
tggacctactgtaattgatatttgtttcatgaatactttgacaccgggtatgtttttgctgaactgaacgcgtaggaaacccccaaaaaaaagaag
ctatacgttggacctttgtcctatgtttgggggctaggctgttcgtgagggtaaataatgaatggatgaattaacaaaattataggtgctatggaat
ctaaccacaaaaaacgatctgttaagttagacttcaatattggagctgatgctcaagattactctgctttacaaaaagcctataacctggagccag
ttgaagctgatcttcagcgcgctaaggactttattaacgaggtaaaaggaaagatgtactacttacaaacacgtgaaactaaatttcgcaatacg
aatgaaagtacgaacgaacgtgtcaaaaactttgcttactttacattcattaccttgatcgtattggttacttggcagattctttatcttcgttctttct
tccaaagaaagcatcttatcccctaaaaagcctgctcttgaatcatatatgtaaattgtggtgctggaatggtatgtatacattcttaaaaccatgc
ttatgctccctctattattttatttcaatgcctagagatggattgatttgtcatgacactgtgtattacagggattcaaatatacaacgattcttttttt
ctctcgtatttctattataaaatatactacactacatataggataagcaaagtatgattcaaggcatgtaaaattactattcataaattaaagatta
aaataagttaaaaacaaaaaagaaaaagagaaaacaaaatcttccagcagcaagtgaaagatccatttgcttcaacaatgataaaacttcaaa
gcaaaacaaaattacacgtgtaagcataacaaaaataaaagtgtaatcattaaagtcattagaacaatggctaaatgatttttcgaagaaactg
cttaacaacttcaaaacagggcatgggactattgaccgggttattgattgccgaagctttgttgccagaaaagcgaatgtcaataatattagccaa
cgaaccaagagaaaatatttcgaacgtcaaataaactataaagctgtccgtgttgattttagcatctacacgatatttattttcacgtaaaaagtct
tctgtgctataagggcgaaaagaagctccgaggagctgcaagctctggcacaattgcaataaaatttctcgcggagatttattggtctgtatgcca
taatgccaccgaaattttcgtaacctggttgaaatggtcgcgtggaacccatgaatattatacattttggacatgtaagtcgcatgttcgctgggaa
gggatgtaggtagaatattgacatgcgattcggaagacgcctttgaaggaggcgtgactaaaccagagggatctttcttttcagaatatgacaat
gagtttggatgtgaatcagaacaaggaaagagctgaaaggacaaattatcggcggatgaactaaggtcggtgtttgaagacgcatagacagga
aggtatgataaatctagagattgaagctgtgaattgttctgactaactgaagaagaggttaatgatgaattaaaaaggtgtgccaattcagctgg
gtaaaagttgacgatatgatctttctcggagaagaaggtactatgcgaagacaggcgaactgatggaagattatcggctacaaaccaagggtgc
tgaataatatcagatattttgattctatcagttgggatgacggtgagcatttgtcggattagatcggtagcacctgaagaaacgtgagaaggtact

gtatagattcctttacatgcacggctcaaaacattggatatggtgacgtcttcaaaaggaaattcaccgacgagcatgagatagagcaggattcc
acaaccccaaatatctacatcacatccatcataataccgaccttgaatgacttcaggagcagcgtaatgaggagtgccacaagaagttctcaaa
aaactaccatcgtacataaagttggaaaggctaaaatcactgagcttaaccgttaagctattggagacaaggatgttttcgagctttaagtctcga
tgagacacattgttttggtgacagtattcgatagcaaagacaacttgttggaagattctacgggctacagcctcgttcagacgtttacatctagcaa
gcatgctatgaaggtcggtctccataaattccaatgcaagcaccgtgtactttgggacacgaagaacttggtaaagggaaataatgtttggatgc
cgaaatgatcttaaaatcattacttctcgtaaaacatggttccaagtagtctcatcttccagctccgcatttcgtatcattttcaatgcaactttttctt
ccgtctcagaatgaattcctactatacaacaaaactgtaagttcctcaaaccacacaaataattgtccacgaaagaactctcaagaattcaaggc
caggagtcaaagatgcctcatgcacaaagctttctatataatccattgagagtgatctttctcattcaacttacactttactttggcaagattaccct
cgccaagagtcctccccaacttccaaggaccaatgaattgagatacatcaacattttcaaattcaatcgatgaaaaagtagcggtttgcatatgg
cagagcaaccacctaggatttgtagatatttttatgttcaaaaagagagaaacagcatgtatttgaagacaagaaattgtttgttgttagtagctg
ggaatacctagtttacttaagaagtaatatagcaccatttttgatgatatcaaattagagttcgctacgacaagcgatccgtaaacaaagaaacg
gtgaataaaacgagagaaacaaataaagaaaaacaaaataattggttttcattattaaaaacaaactttttttaagacctatacatcccttaaaaa
atttgctaaaattaaggttatctaagcgagaaggtgaaatattacatggacaacggtaacagtatacagtttatatacaaagttacaaacgtgttt
ggacatctcacagagcccaagacaaatgaagtttcaaggatagaaatagatttatagtccgcttcactgtatatgaaaattatacctgttttctggt
tttacgcttgtgagcaggagtaaacgtttcttaaacgaacagtgaaaggtaaaactagaaaacaaataatgtgctttttttatttaaaaatgagatc
atagacaatacaacttcagtatctgttttaaaaccgcttagaataagtgctagtaccataccgttttaagttccttctcataaacagagaccgattc
ccggaatttcgtattcaaggaaagggcccaatagcgtttcaatacaaagaaaaaaggctttcgttatttctcttgagtaaaagcggcttcggttctt
ccgatccatcctaaaaaaatttttatatcggccgtcgagatatagaaatacattggttcaaacaggagccgcgactcgatagataagaaagatg
ccctaataaggactcaacaattttctatattttgcttatttctcttggcaaatccgagatatccagtacttttcgctctaggtcgctttgtttacatttta
tggaatcatagttcggtctcgctaaatagtaagcaacggctagtccgagcggacgacgggctccgtacggctccgagtaaattatgtttggattac
ttgttaccaaaacaaagaacgtttttataaacatagcataattcatcctttttccattacctttttttatcaattccccttcgctcaaggggctcttttg
gatttattactttgtcagtaattgggaaaaagaaatatgatggaaaccgtggaagtacgaagtaagagtctattgattcaatggctaaatgtgaa
accaaacagtcttcttagctggcaaatctacgtgaaacggaaaagtatcaaatacgatatttactataaacgaaccaaagaaaagggaagtcca
gaggaagactataagtcgccggccgttttggatgccaggcaacagcaacaaacccatgaaactgaaattcggaagctgaatgccgctggacttg
agctttttttatcacggcgaaaggtgcatggctgaaaagcccaacgacggttcggtgttcattgagaaaggaggattattcgcctttgttttcgacaa
cacattctctaaaactactccaaaaatcattacttttctactgagcactcaatcgttctcgggaccagttcagcgaaacttaaacagtaagaatgcc
cctaaacagctggtgtctggaacccttctcaaaaagcgacgcaaaaaaggccaaggatatgctcgtcgctactttactttggacatgctagaagg
aacactttcatattatgccaacccccattcttccattatgcgcggaaagctacctttgagtatcgccgttgtcagtgtttcttctgaaagtcatgaaat
taatgttgattctggtatagaggtttggaaccttcgcgctcatacccatcatgattggctgcgatggtgcaatgctttagaacgtgctaaaaacacc
caggctttttccagattgaccgttggtgaacgatcccagcagtcatcatcgaaacagcttgattcgatatactctcggcttcgcgaatgcttagata
tagctcaaacctaccgaataggtcgctctcggtctgctaataacctcaacattcccaatatccgtattcaactgcctgatgaacatatgacaaatag
tggaagtgcaagagaatctatcgatggtttgccctctgaaaattcttcagccgtgattacacttcgcaaagttactcgccagcttggctctctattgc
acgaactagaatgtttcattcagcatcacgagtttacaaaagagcaaacatccgtttctgcaccttcctcccgtgtatccatggattctactgtatct
caatcatggtatgatgctgaagatgatccaacaatcagtcatgtgacagatgctttggaagaccttgagcattcagacggtttgttaagcagtgat
cgcaataccaaaaattctgatcctgatattacttttgtcgaagattctcttcacagagcgagtagctatgaagaagattcagtctccgattcctctgt
ttctgacatttcttctgaaattacggaaccagaagagcctgcgcagtcgtccgccagcactgattccgttatatcaagcgataccacagaggaagt
ctcagaagaaagtgaggtcaatggaataaaacgtattgaaagcctcaaagatattaaggaacaaaaaccaatcccatttaaagagaagtctga
tcaaaattctgctcttggttccataaacgtgaagagttataatgattctggcgaaccgaatatttccaatgaagagaccgaattaatttcctcgaat
tctactaaaaataaagatgtatctagcgattccaaccggccggctaatgaactgtatcctcttccccacaaagctgttcctcgaagacaaaatata
ccagctattgtagaacctcctcctagtattctttcagtcttgagaaagaatattggaaaagatattagctctattcgtattcccgttgtttccaacgaa
ccttgtagtttacttcaaagatatgcagaagacttggaatattctgaccttttagacacagctaacgaaagcaatccggaattgaagatattttatg
ttgcggcttttgcggtatcaaatttctcaaacatgaggcacaaggaaagaagtgtgcgaaaggtatttagtccgttactgggagaaacgtatgagt
tggttcgtgaagataagaattatcgatttttagcagaaaaagtctctcaccgccctttggttgttgcatgtcaagctgacagtcctaaatggaaatg
gcatcactctccaagacctatccagaagttctggggcaaatctatagaactcaatactcacggggatgtgaatatacaattgaactgtgggcata
agtttgtctttactaaacctgcttgtttttttgaaaaatgtcgctataggagaaaagtacgtcgaaccttatgatcatatggtagttactgactctacc
actggagacaaagctatcattcagtttaaggtcggaggaatgttttctggacgttctgaagaagtatttgtcaaagtcataaggtctgacggaagc
caagatcctcgttgtttaacaggcaaatggacgacttcactagagttggtgcatgttgaaactaatcaagtggaaaagactatatggcaagtcgg
aagtttagtaaaagaccctcatatgcattgcggtatgacactgtttgcagcacaaatgaacgaaatcacacccattgaagatggcttgattcctcc

tactgacacccgtttacgtcccgatcagagattacgcgaacaagattacttagaggaagcggaaccgttgaaattagatctcgagcagaaacag
cgagagttacgacaagaaatggagaaaaaccaaatttcttggcttcccagatggttttcccaagtgaggacaaacgataaagtggatgattctg
acccaccatggctgatgaacgaagagccaaacaattattgggcctgtcgacaggagaaaaattggtcaaagtgccctcagttatggtaaattga
aaaagttttgtgtattctcattttcatgtcgttctaatcgcatgacttatctttcgttacttcttcctttgcattgtattaatggtcatattaatcctaata
ctcatatttaatattgagattctaatatacatattttttcaaactgtatgaatgttttggttatacaatagtaagaaaatgagtccaaagtcccatttt
caaaccgggcatatgctataagaataccatcatagctacctcgtaaccgaaacagcagtaaaaaggtaggaagttgttaaaaaattcatttggta
gaaaatatatgattcatatatattagtcttttgtttttgaatgatgagaattcactacctatggagacagaggactttctggcaattgatgactcat
agaagagcatcaacgtattacaagaaaccattttggagacgcccatggcctgcaacactgctaggatgcgcagtagcaggtactgcaattatag
tattctccaaaccgtcgccgattgatgcaaactatcctacttctgttgccaggcatattcatgaagcactatacagacaaacaggatcgagcatac
aagactttcagcaggcctggaaggcataccaatctgctatagaacaagcagaaaaggaaaatatggatatggaatctgctcccgttcagggtat
tcgactgcagatggccaatcttcaggaatcagcaggaggatatcaactggcttggaagttattttgggacatttttaagcgaacacaaaacctac
aagacttcactgaacagagaatcttaattgcgagtaaaattattgagttgtccgaacctcttggcttgcaagtggaagcgacgaaagccgcagat
tatattatacagtgtttgcttcgtaatgaaattaatgatgaaccagaaaaaaagagtcgtttatttgaacagaccgctactttatattttcagcttgg
aaagccttcctatgcggttcctttatatcaccaagcacttgaattaacaatgaataatccgacttgccatggattgatattgatgaataaccttgcta
ctagcttacttgctcagactgattatatcgacaaaagatatcacgaaaagctacttcaacagtctagtgactggagccaaaaagccgttgactcat
attacttggcttatcctaaggaccgtacagtggaatgtcattctgggtgcgctgctgccttctttaccttaggacaaattgcagagcgaagtggtcat
atcgaaacggctattaagaattatcgcaatgctgaatctttacgggcccacgacccatataatgatgggtctatgatggctcacattgctttggacc
gtttagacaaaaactccttttttgcgacataacgaataatattattttgtgatctctactatttgtatatatctattttaaaatttcgtctttctcataggt
aaagcttctaatgaagcaagcaccgactactattatttcattacttttcaatccattttcaagctattttagaatcagttatgtttagccaaaatgttc
ataactttgtaaattgtgtatggggttctctaggctttcatactgccgccggcttcacatgtgggtttataaaatataatttaatactatggaatatct
ttatttttatttaattagtttttactgaagtttcgcttttagatctttcgctcaaagtttgacactactctacggattctcatacttttactaagcagaaa
aagtaaattttgtctcttggttactttcgaagctagaacaacctgaagagtagaagatggctaccaaagttacaaagaaacacattttaaactgtc
aattttctaattggtattcgttatttcgtactttgtcgccaaaagcaagagtcataaagccaattcctgcgtctatactaagataccttaatgaagat
ggtatttatataaaagacgtttctaatacaattgaagatttaacagatgaagaggatgggaacggggaagaacaagaagacatacatgtttcttc
ttactatccagacaaaccgaccattcaactcatagacaggatcatttcggagctcgatggagccgtggtgcccaaattaaattggtcaagtccca
aagacgctttatggatgatgcccacgggtaccatgaaatgcacctcagctgaagacgttcttttgttactaaaatcatcggactttatagctcatga
ccttgattatgcctttgatgattgtatagaagaagatgctcaagaagcagatcttacgaatcaaaagtttccatctgaatttagtcacgagctaatt
ttaaaagaatggtttcccattcattcttcaacggagttccgttgctttataaaaggaaaaaatcttgttgctatatgtcaaagagacgacaattatta
cgatttcttagaggatgacatgaaggaattcctgcctcttttgcaggatttggcatccaaactttcagagtttcctgatcccaattgtatgtatatttt
ctatttataactgtgaccatctaacgatatttttagttgtttttgacgcttacgttcaaaaaaatcgcgcttggctaattgatattaatccattctgtgc
aagaacagacgggttgctctttgcctggacagagttatacgcaatggactctgaagcagaaggtccagagattcgattaattcctaagggtagca
tgccttcttctggaagtgccaacttttacaccaatcgtgttccattcgatttaatcgctgccagtcaaggagaaaatatactagaatttgcagaaaa
gatgcaaagcctggcttcagaaagtgctagagatgaagcataagcaaaataaaaataggatatatatccaagggaatcgggttgtttacaatta
atctcataacagttatctgtttttctatttatgataaatctaagttagtctatgttacttttactttaaaccatagcttgcttcgctctagtagttattat
acatgtacattcctttccaattttgggactttttttttccatccactgaacgctagataccgatggtttagttcagccctccaatgaattccatacaaa
gactcaattcctccggttagtgattgctgttgacaataaactaaacgatgtatgcgagaatgcagtaaagccatactacacataacgcatggttca
tgagtcattaaaaccgttaagccacgacataagtatctttcttcagatgttttatgcggaagtcttcgacgttcccgttcacctgctgctaccaagtt
aatcgccttcatggcacaatggtcgactgggttctttgatacatttctttcatctatagaaattgaaagaactttacgtgtacttagatcataaactg
cagcagcacagccaatttgttccttggattgcgactcaaaagccattttaattagctcgtccataactccatcaattacttccaaatctcgatctgta
aaagtatcctgccgttcggtatgccttctgtaaaaaacgggccatatgggattccattccttgtactgttcctcagttaatggagcgtaagaaggaa
ccggaaggatataaacctttgcttcaattcctaattcttgtaggtctctttctatctcattttgagacattgcagattcaggacatactataatttctag
ttccaagggctcattgtccttttgtattcttcgaagtctagcgcaatgtaatagagcttctttctgtttatttcttatttttttgacatattcagttgttttt
gaggcaaactgcggcttcaaacgagcaatccaaacattttctattacattagtcaataccgtaactagaacctcatacctgtttctaaaggcctta
attcaagatgtgattttactaatttaaaaggccagtcctgttcttgaatttttatctcccgttgttgacttcttgatgaatttgacttgtccattatatttt
acaaaaaaatgttgttatatgaatatacaaaaatcacctatgaaaaagaagatgcattgactaaaattgtttctgaagaatattcttacattttga
aagtaccttctgttaaatttactttaaaaaactgcttttaaagcattacccaccaaatgttatgaatatagttccagaaagcttccctcaactgaaac
actgattataaaaccgagaacaattatttatttcatatttcagttcctacatacctttaataaaagtcatcgtcacaatgaatgccgtcgttaatttca
caaattttcatcaaaccagtcagtgagtaagtcaagtcaagaaatgaactcaaaaatcgactcaaaaaatccattccattaaaagaatagtagtt

atttttttaaagccattagtatgctcagcttgctccccattgtagactcaggcttaagtcgatttcgaacatgagaagcagcatcccctaacccatga
tcagaagccatcgtgtggatatcatatcctaacgaagtaacgcttctaaattcactggtttttgtatcaaagacaaagggaagtggttcttccgata
agcttgagtagtcgaaattgactaatccacctacattacctaaatttgctagttaataactttatgaaataatgcacatactttcttcattagagatg
tccttgtcgacctgaaatccgatattttgatccgaatggaattctttcactcgttcaaccgcttcatcttcaaataaattatgacaacccaggtgaag
gaaaacttgtaattgatgatcagaaagaagcttggaaatcgagtcttttgcgtggggcaatttcattccttttggaatcctagttattaaattaagc
gcattttcatcggatccaatattctccaaaagagtacatataaactcaggtttatctaaagaatatgaaattaaatcttcccataaatccttatctcc
ttgctctttgacatagcgaatggctctaccgatgtcagacaattcattgattataagcatcaaggcatctttgttatttcccatacgaccaagaacat
aaacaagctcatcaagataacggtattttacacaaacgttatatgcgtgtcccaaagaatagcactgtgtgttcattaagaactcaaacagccttg
accggtcaaattcagcaaaaacgtccactttcaaatcaccatattccatgagcgaattcggaaacataacttcataagagcaaaaataggcatat
aaaaatcttgtaatcgactggatctgtgttataacttcattaggtggaaaagaatgtacgtgctgaacaagcatgtttatgacttttggattaacatt
ttcaggacgtccatcggctgaaataagtaatagttgtaagatcgattgccttgcttcaccatataggttgtgttgaaaaacaagatcaatgcattct
ttcttgtttaatttaaggtatagccagaatgcttccacaggcatactgtcgtttacataaagaaaggctaagctattggctagctctctgctttcttgg
ttttggtcgtactttttctgtgctgctttgcgaattgtcggcactgaatataagtttgtcggccaggtatgtaacttctcatcaaatttttttctcgttaaa
ggccatgtaatatgccaaagtcatttcgtatataacaggacttaaatgatcatcaccaagtggaagatactcagcaatgtcttctaatcgattatg
gtccgcaaaaacaaaaatccattgctcccagccactaggattgttactgtataaagtagggattaccaaagccgcttttcgatattgctcattatca
attaaatgtgctacatatttttttgccaagttcagggacttgcaattctggaggcacgtctttcattgttttcactgcagcaatcgcctctgcgtattctt
cccgagaaacaagatacatgacgtgatcaatctcattcctctctctagcacatataagatcattaggaaaaaggacatatgaataatctttcttcg
ctggatctggaatcaaatgataatcggaaggctggagacgagaatagttagtcagtccgatgacatcgccagcgagctcttgaaatgaagagtc
tataaggcgaagctccggtcgtactgcgtctactcttttgggtctagaagaagaggtaaagtcttctacctcggcgacgtaagcaagaacaagaa
tgttgaagccaaacatcatgataccactgacaatagagtcgatttctaaaacagcctgtactgaaacttttggtaattcatttggctgacttgagag
ctgaatggagactatagatatttggtccgcccaaccaattatcagtctagactcagattgccaatacagttggtaagggaatacttcttggttagg
aagctttttttgggtgttccaagtgtctaaagatttttccatattcattactatatagataaacaccggaatcagtagcccaagcaacgtaagatttgt
accaatctattttgtataccgttccgcaatcaacctgaagaataatgtctctggagcccagccatcctaggatttattagcatacagtatctggagt
agtacgcgtcgcaaatcttataccttttttcggacaatatgactttgcctgctcgtcctccacatataatttgacgtgaagatctggtgctatagtagg
gatcaatcgctaccgataatagtggccgtttaaagtcatagacatttgtttctttagagctgatgttatagacaattagtttccctgtgtaagtgtca
attaaatgttcaaccttattaaacaaataaaacgaaccatccattgaacacgatgctaagttttcactttcaagatctacagaaagaccagtgacc
gtagcaacatgtaaaatcatttttcgaagtaaaactccttgtcgcgaataaatataaacagcaccattatgtgatccaaaaaaactaaaagaagaa
actgttaatccctatacattgttgaaagaaccacctacaaagtgttcctttgaaattgcacaggcggatatagaatcgttcacaaaacaaccttga
aatctgtctgtaatcttttcatagttcaatttaggctcttcgtcttcatccgacgaagttaaagtgtctacttcagactcggaactggtttcactaactg
acatgaagtatacacaacccgagggcaccaaaaataattaatgtagtgcttgtataaggtcttgtttttcaagttccagtactttaattaatttaca
aacgactgaaaatcttaaattgtaattatataatccaaaaatttgattgtaaatagtagaggtggagagcaagatatttgcgttatgagtcttggt
aacacctaatgcttttaccccgagctttcatatattcatttaaattataaaataaaggaattgaaccttcacttacttgcgaggatagcaaaataatt
aaaagcatatatttgatctattagaacatgtgcatgcaactcatcaaagaaaagaatgtgtttttttccaacattaggtaaaaataaagagtataga
aagtcaaaacttcgtatatgagatgaggtgggcatacaaccttactttcgtaaggagtatttgttcctgttcctcagcaattaattagaggaaaag
aaatcgggaaaaaaaaacttcttgataaacagataaaatgaaaaactgacacagggagtctcttgatatgggaaagaagtaaaagaaaaacac
aaggatatttaggggaatcgtattgcatacctcaacaagtttttccgtatggaggctgataggtattttattttcttcttcattagtatttttttgacattt
ccaactagtttttgtatcgtatcctgctgtgcatccacctctgattcagcgtctccggctaatacagcagataacacatttgttttactggattcttga
agctcaccagctagtaatagttcttctaaaacatagtaggcctttttcgaaattgaaaattaagtccaactcgcaaacctgttaaacaaaaaggtta
gtaatggaaagactccgagatttagaatgagcacaattaacatacattgccgaagtatttgtcaaggatctcaacatatttatgtatgacttctaa
aataatcaattcattatcatcctgctcgattccacaaataaagaataaagatgcatatcgcctgtaaacgattttttctcctatacaagcgttagtca
actcaattcaatacatcgggacataaattgcttcttttacctttatactctacaaagttgcacatcttaggcttgcggacaattactaaagaagaaa
catcacggataattttcaacctctccttcgtcggcaaagcgtaaaaccatttgccaaacgaacttttccttggcgacttattaatagaaaaaactt
gatgcccatagttgaggtttatagtttgcacgttcctttgcccgataatttaacaaacgctgattattgaagggaatcgaattgcgagtacaaatac
ttaagtacttaaaagattaatttctgtttttagccacgatttattagaagaatgaaaagtttttggaaaagtgtttgctacataaccagtgtagtctt
gctgtatcagaccctctttaagataatattctaatgggttatttccaggttatttccataatgtgaatatcatattcataattatatgacaagataata
tctatataacatttttgtgctcaaaactttagtttattattaaaaatgaaatcttttagcctaccatagtccgcttacgaccacgcaggcgaacctctt
cgtagcgtcgaagggtaggtttgcattgcttccacttttgagcatatgcttctaaaacataatgaaactgagattgttagctatcacaatgaaagaa
tagaatataacttactagagctgcactatctgggagggtactagctatagcacgctctaaaacgtataaatcaacggccttgtcttcctcagactcc

gaaacacttcctagtccaaaatcaataaatacaggttctagagtttcaatagagcggaacatcatgttactggtggtaagatcaccatggacaat
gtcattcatatgcatctttgcgacttcagatccaatgattttcattaagggcgccagcgttgtctcatattcttcctcagagacaagttctttaatgta
atctcgaactgaaggaccactgatgtattccatgaaaatctgtccacgattgggctcaagaaaatagagtaacgggcaattcacacctacagcat
ggcacctagccaaaagtctagcttcaactaaacatctttttcttgtaagtttctgatcaagtactggatgcctccatcgtttctcaggacgggatttta
gtatgcatggttcaccaggatagaattcaattatagttataatggcttctgcaccttgttttactactttccaccctttttctcgaatttcatgatataa
ctgtccacattgtttccgtaaatcctgtttgtccgtcatattttgacgttcttgtcgatttcacaaaagtcgaatagaaagagggtgtatcagaaagc
agaaaagatgttaccagctatacagaatttcctatcatttgtaaataaaagattcaaaaatatttttctattaccttttctaatttattagactgtaa
agtttatgaaggttaaagtagcataatttgattttattaactgacatatagtacaacactagacgatccaattaatgaaccgtccattatcatcata
ttctgtttgaaggctcttagattcaaaaaattcaactttgtcgccgcacgctcgagctatgagtttaaaacgacggtataaaaatatttcttctagtt
gacagatttcagattcgttcatttcattcacttcttctggagagtagcccaaaaggaattccaaaagatttgaatgaaacttgggatgttctaagca
accgctttcatcaataatggtttcaacaaaattattaaacttattaattccagcttcgtcctgtataagaagactttttgaatttaaaagtttttcatt
ttacttttcacaattataacatttacgactccaacgacttgaagacccgatgaaactgtttgctcttcaagtgccttcagcgtccattgcttaccagc
atcttgtagtaatatactaaatttcttagaaattgaacaggaaaattcttgaattcttttatcctgaatcaagcaacggacttggacttcggcatctt
caatgtttaattcagggagaccatgttcatcggcaaacacatttgcatgcgtaatatacagtactaaaaattagtgaatattttcaaaatcaatgtt
actttttacctgtctgcgcgttccatttttcgcttgcacatattgatggctcatcgtgtaaaactagaatcgcagagtaagagccatcgcttacctca
attagccttttactcttttcattcctactccacattgatagtgttcaacatcatgcttccactgaagaacaacaagagcttgaactgctgtagccgtc
tgcatatcttttagtaaacgaagaggagatacattcgaaaggtccagaaagagaaactctttttttagattgactatagaaataatttcaattttac
tttcagcatcctctgcacataaagaaagacgccattcattcaaagatgttcgacgcgttacgacgttgctgtacacctgaacaacatttcctaattt
taattgctgcaaagttttataacacggagaagaggattttaagttgtatttaatctttaagacacccgtttcgtccatgactttcagctggtattgaa
ggtttcgttgcttcgtgtccattctcgaaatgtttacaatttcgccttgtacgcgaaatttcgaagactggtattctaaattacatattttgagattcgc
ataggaattccaattgaaaaaatcttcgtaataaacgttcatggcaagcattcatttattatttgttaatcctctctaaagaaatattgatggcggtg
gaattgtagtatgcatggatatattcattcatggaccaagaaagaaattaaaataaaaacttactcccaggtataaagatatttaaatattagaa
atcaactgttattaaaaagtcgtggagtatctcaaattaattgaaacagtgtgtagcagttcacagagaagggaatatacaaaaaaaacaccta
cttcggttctattagtctccttccataattcatacaataatctttaaaatacttttctgtaatttacaggccattccttaattcctgtgtgaacgaaacc
aagggttggtcaaattttgacccccaatctttctggctataaacgaaaaactataaaaagctttttttcgtttataacagaaagaaaaaaattgaga
aacggtgggtggacgggtgatcaaaagcggaattgcaattagttttaaaaagagagaaaaactgttagcaacataactgcaaggataaataaa
gaaagaaatgataataagaggggaattaatatctgtcgggtatatctaataaatgtcccatttttttcaattttggttcgaagataggcgcccacttc
agggctgtcaaaacctccctcatcacctcgccaagactttggaatcatggacaacctttccaccacttgaacttctttgtttggaccttcaatattgc
taaccttttccgggttgtttgtcagtaaacggatgtttctttgaccaagatccaagagcatcgcagttgcaatcgcgtaagaacgaccgtcagctgg
atgttgtaagagaaggttagcttggacagtatcgttaccaagatcttgcagattatatgctttcaatttttcagacaatccaatgcctcgaccctctt
gtctcagataaataattataccatccccttcttcactaatgagacgagcagcctcatccaattgttctccacaatcgcagcgagcggaccaagctg
tttcaccagtgtagcactctgaatggatccgtactaatggcgtaaaatttttctttctaacctcctccttcttttcttcactagcaactaaagcgtttgc
atcgacgtatgagctttttcttcctggatataatgttcccacataagcaccacgaatcatgcgctccatctcagtttcaccttcctgaaccctatcga
ggctttctgatcgaatttgctttccaaaaacaattgctaaatgctctttgttgtcttggtcgttcttatataaatttaggaaaatttccgttcctgtcgta
gtaggtattctagcacgaacaatacatcgtacgttcggcagcttttcagctttgctagttatttctcctttggtctctaactccttccctgggcttaaga
ttggtgagttcacacaaggtgcatcaataaatctctcgtttttggacttttcgttggctttttttgatgctttctcttaacatagctttaattgtgttttgttt
tggctaaattttttttatctaatttacaaaggatttcatgcaaatgaatcgttgattacttcaaggcttctaataagttctgtgctcgtaccatgctttgt
cagatcggtcagcatctaaagataccgaaagaaatttcataaaaagaataaatcaatcattgaaatgtaatagatgttttattaaatctaaaaga
aatgtattatgaaagaaaatccacgtatcgcagcaagtatataattttatgaacgcccccatgaaagcgtagaaaatattcacttttacaagttat
ttaataattgaataatactcattatctgtataattggattgttacgagcactaggttgttagtttctcattatgatttcccacggctttactcttttcgaa
catagacggtatcagaaaattacgccttttagaagaagtcctaatcacattatcatctttcgctttcaaagttacaaggatataataggtacggtta
gttgtcgatatatccataaaccattcatttgttcccttaaataaatcttttctctttatctttttctgtatcaaagttgaaaacctaagtctatacaaaa
acagatctataaaaacaatacattccttccgctaagactctatatgagctcgtaatttatcaagctgcatacgaatcaattttgcaaactgggttttt
gtttccttagaagcttcagcatcctctttcggtaatccgataacctctatggcatactcttgttcatataaggtagcaggctctgccaagctggatag
gaagttagggtgagatattaggtagtcaataccatttaacatttcggccttcaactccgacactcgaatctcttgtagcaaagcgggattaaagta
ctgtaaaaggtccattaataaaaccaacataaaagattcatctatagacgccaatgaaactgaagaaggaggttgagaccaatttaagaagga
gaattgaatttgaggtaaaaccaaaagtataaattctctagcatcattcattgtgcgattaccaaggtatttcacgatattaaacagcaaaggaag
aattcctcgaatccacagtttatattgtacagacgaaacgtcggcaccaagccctcctgtttttaataaactactcaatggactttcagcaattgca

aagaacactccctgggatgctacctgttccgctcctgctggtatggaagaacaagttaccaaaaatgagagcgctgcatcagcaaaataaggct
gaccgtctattaacaaagcgtgagaccatgaaaacaagcgcaaacagttttcaactgatgagtgatccataatatgactaatataaacagattgc
agtcctttaattaattgggaattaactataacgttctgaatggcatttaataaaacaatatcactatgcacatctggcgttggttccgtaatggcatg
ctgaaataactgagataatttacgatgacacatcgataggattcccgaaacaaatcccacttcaatattcactttctgattcgcgttcgataatagc
ttattcagaccattgtacaaaacttgaagtaaaggacggtaataattcatttgaagcccattatcaacctcaaacaaagagaacttatcggtactt
agcgcttgccatgttaataccattagtgattctaagcgttctttaaaaccatccgtaagaggagctgacattgcatgttgtgaaacatgaaaaacc
aaagctgctcgttctatagataaatccctaataataaatgaatcaacagtatcatttccaagggactccaaaatccaattcataatgtcgaagtct
aagaaggataacgtactatcctctttaacaaaatcaataaatgcggacaagaatatactccatgaccgaaggagtgcagtttgagcatctagcgt
agagagagcatcattagcactccccatactgctaatcattggctcaatgttaccataaacggatagcatggctttagcaaggtgtgtattgtaaaa
atagtttttccgtaaacaggaggagtaattcctgtgtttttaaagacattgcgtttagttcgctttgaaatctttcaaaattattatttagcgtttca
aagactgaaggatcataggtcgttattgtaaatgcagattggataaggtcatgaagacaatcttttaacggatctataacaagtttctttgcacta
acacatttattaaattttgcaatatgcaactgaatagctgcaatccttaccgcatgagcggcgagaacataaagatccgctttggatatttccaata
attcagggctcgaaactgtaactggaagcttaatagcatcaataacctttttccagaaatctgtctcttgttgtgccgttccgtaagacgcagacca
aaaatttcttgataaaaatataaattcaataataggcacctgtgtggagacatcattaatgaaaaagttatccattcgtttttcagccaaattaata
aggcttcttgacgttatttgggtttgctcattgtgtcttaaactcttcatgttttccaaaggaaatttcttgccagagaaaagaagcactgccagccct
tgttgctgtcctttcatgattgatgacaaaaatttccaaacacatccctcgatataacttgaatgtataggattttccaataaacgtacgcatacacg
accaacgacgtcggtaaaattgaccatataagctaaaagagaaggagtttcagaaggccaaggggcaagaattaacgaagtaagtagtctga
gaattgaacctacacatggataaatctgaggtaaagcataaaaaagttgaggtgaacgagagaataagtccctttctaattcggaaggtttaag
gtttaaaaatcctctcagatcaatcaaaatttgcatcgtttctaaagacgaaacaagccagttcttagcaacctccgctaacttattatctgtagaa
gaaaatatttctgtgtacaaaggatctatgcgatccaaaattgaaagtaaaggatgcaaatagcgtttggcatcttgctgaaccaaaaatctttgt
acaacgtaatttcctagttgctgtaacggtaatattcttttatttgggactttagaaccaaagtactcgataccatatatgacattaagcaattttgta
attagacttgtgaatcgacaaccaatttcatactgttggatgatcttagagtatctccaatcaagaaagttggcaaatatttcgcagaaacactga
atagattgtttagcgaactcactttttaaatttatggaaaagtcatcaggaataacggacgttgatatgcaattatcgagtagtaattcgtaaagct
caaagaaagccaaagtaaaatcatatacaccgcttagcgattcgtgttcaaaaataacattacctaaagataaaaccttttgtaaagagcgactc
gcatatgcccaaatgctagttggttcaatatgaacaaatgcacaaagaagctgacaggatagaacacatatttgatattcagtatcgctcagact
ggaatttgataaatagtggtctaaaacactgcaaataagatcgataaagtcatattctccttccaatgaaatagaaactaatcgagtcagttcacg
aatttcagtagtgttggatttgtacaaatcaaccaaagcgtgcaagaatgtcaccaaaaactcatcatgcagatgataaagattatttgttacgat
atgctgtaaagatataccaatacaaggccaacgtggataaaccacatcccacatgacaatgggagggaatgtatctacagatacaattcttcctc
tagtacctttaggaatagccttctcaggattaggaaagaaaaagccagtttgttccaaaggtaatgagtcctgcagttcaataagaacgttcgttg
ttacgttcggctcaggtataatttcataagatttgaatcccttcggtaaaacttgagtaaaggtggtatttaaattaaagtcaggattaactggacg
ttgaaggtttaacgaagaaaggacaggagtgagcaagagcgggaactgagataattcaaaagggaatctagctcctgcataagagaatagat
gagctacactttcgatatcggaaaacatctcaaagatttgtgggtttttgaaaagagtggtagcacaaaaaatgacagaagatgagaactttaca
taggaaatagcttccgcaaataaaacagccaaaataattaaataaccatcataattaacatcatcttgtaacccatttaaaatagaatgcataag
agagaaagggtcaagtctaacagccgagataatcatactctgataagaagtctgcgggtttttgaaaagagtatgtgagaccacgcttgagtttt
gaacggtgcttagattttcgggactattatgaaggttatgaagagcaataccccaagtcaatgctactactgaaccaatcgggtcgccaagaaaa
cttgacataatgtcttgaattttcaaaatacaatctccagatgtcatgaagaaggcatcattcgcttggaacggcaaaacctgtttgtctaaagaa
atgattgttactgaaatgaccgttgcgagataagtaagcttattgcacaaagaaaatcctgtctccatgtctaaatgcatgaactcttggtcatgca
aaaaccttgtttttttcatgcaagaaaaccaagcaatagctacttccgatgtgcatgggaggagggaatagattaaaaggaacaaaactcgaag
taattgcagttgtaggtggaaatagaagaaataccaatgagctagagtcgcttctcctagtaaagtgtactgaattggagcagcctgatcggaga
gttcattaaagtaggcgattaagtcaatacaataggcggacggttctcgaggatttgatttcttggattgttccaaagttgcaacaaccatgtatttt
gaagctttataccagaaatgatttggatctgcgcaagcacgaagcaaaaagacaaataccttccaacaagaaaatagctcagcataatatcgct
tttggaattcctgaacaaggaatgcatcatcatttgagtatgaagagtatttaagcataaattcatcgttgtaggagagaatcacatacaggactt
cggcttcatcaaatcctgttaagtccgataacctccttgcaaattccttttgagactttttcactgggtatgaatgtttatatacagacaaaacgcaa
tcttcttcttctttttcagtagcttttgaagcttgcacgcttccccgcaaaggatatcgaatgttttggaatgcagaaaccctttcagctaccaaactc
tcaaacgtttcctttgaaatgctttcattttgagcttgaaaactgcttaagatccaagcccaagagctacacatttagtcagtaaattgaacacaaa
aacaaatcttgctaaattcaagacaaccacatacattagggagtcacccatggaaaaaagactgacgaaccgacaaactacaaatcaaaaaa
gaatgatttcaggacgaaagttgattccgttctaactcttataacgtctttcaattgtttcttttgtatgggttttgcaacgagtactactactaagtcg
cttaccataccacggaatagtgtcccacaatgtaagccaacgccgcgttgttaagattcactaataaacaaactatctctaaaaattcgataattg

aaaaaaacgaatttacttgcaaacatgggcttcaagctgccacattttaatggacttcttattgaaaatgagagattgcttcttactaggttactttc
tgtttccttttagttgtatatttacaggtaaagctccaagttttacaaaacatatttgaagagtatgaaagacaagctttatagttctttaacaaact
aaatattggtacaacaagtgaattgacttgaatgaaaaaacgaaatcactcgttttaatgaaggatcattgtttagtagcaagaaaaacgccgtc
tagttattatttaggaataacgaataatataattattatggcaatataaactatattaataaggtaataaacgtgtcacgggagaaatctcacacg
ttatgagaatgtaaatcgagtggaagtaataagtacaattacaaaattgtctattggaattggacgtgttagtactacgtagagtaatagtaatat
gtccagggatctgattaactaaatgaaaacgactaagtgtcaatcttatttagcctgatcagtcaaatcttccgatctaaagatttcgactgtcgac
acacatatatatattattctgtatatttgccatcgtgactggcttctctaattaatagcaataatcacgaattgtaaattttaatcggagtttctgcaa
tcaaagagaaatttgcattttcaaatttttgtttacttattatcaagcacaattctttaagcttatgaatattaactgatattagctaatattccattctt
ataacgaattgttatattatgaatgctaacgagggacccaattatgacaaaacgttcgttttgtattagaaattgtattagaaattgaatgaatacg
ttcatcttattacaacaaatagccccagtatactaaaaaaatcttaaccacaaccgttaactgaattgacgactcgtctttgagatattcgcaaagt
aattttaaaagggtatattgtttatatgaataaatggaaatgctaaagagatcgcgttctaaccaatatacttagagcttaaggtttggcaaacag
aaaaggctctaaaacaaacgagcctttcgtcacgtattcattctgactataaaaatcatccattaggagttttcttctactgctcaagaataggagc
tcaattgcttttttctttttctttaagggtgattgattttcctcatagggattctaatctgataatcacttcctatgcaataacccttcgtctcatggcca
gttagaaggtaaaaaaaaaagagatttgttattattgttcttattttcgccagacagaagaaggttctggtctgaagagaatccaactcaaatcc
ctttcctaaaatttctcttgtttcactgttataatataccttacttttgacattccagttatccccaatccattcgttacgctttttacgactaccttttttt
tttttttttttaagcttttggtctttgtcttttcaactgatcatatcttcgtccttcaccattcccatagtccatgggcagtcagcaatcactactccaa
ttaaagaatgcttccttacgattaactgaggataagaacatccccaggacggataatatatgggaaagcttttggtccttaccggaaagcttgga
agaggtgctagaacaaatttctgttgttgatgttcgaaatgcgctaagtaaagcaccggaaaacatacatatgctggttgatatactattcaagaa
gctggtttctttgcagacggatagtgatttttctatacattctaatgaccttgcgacaaaacatgtcctgaatagcatgcgtgtattgaccagattaa
taccccttttgtttgagtctccagaatgcgcttcttggtttaacaagtacatgtggttaccgcaggaaagcgggataccccgaggtgttaatattatg
gacactatcataaactatctatttctgactaattttacaatccctcccattgacgaattcacaaaaggagttagatactgtatttgggaaactggtat
tgcatgccagtctcccttgaataaagaagtaaactttatggcgaattctgtcgaggttcttcgtcttttgttagcgctcttttccgaaagtctatatgt
aaatgatgacagatctagtctttgctgtttttacattgcagcccatccaaacagacggttaacattatgtttgatatgttctttactaaacacaacaa
tgcgtttcaacacattatgttggaaacccgagtttgttccatccaatcaattttccctacacatgtcgttaattgaaaactctctatctacattgcttgt
gattctctctgaatgtagatttcagtcgttacctgactttaatagctattttcgaggaaataatgtgaagcctttcaaccaattttcttcgttgttgtca
agcttgcactccatgtcagatttccaagttattatagacggaataagtcgattactatatcctccaatgcaatctgacattccaaaaaaggaatctt
tggttatgtatgattattacccaatggtattatcactgtgtatgttaatagttcagtataacaaaaatttctgtttgtttctgatagaaactgattgtgc
cactgatttatttattttcttagtttatttgagcttcgagtacgttgaaaatcccggtacaatcaatcatctgagattgtgtgcgatgttatttaaactg
ttattaagtgagcccgtcttttgtcataagctgaatagaagatttcagcaacatgcaggtttacctacaaatatgagagttcctttccacgatggttc
atttgctgattttactgttattgcattttcaatattgctgcagtcaatgagagattgccagtgggatgtgatggagattttatcagcgtctctttgtcat
ctttccctttatataaaagagataaatcatagcgctagcaagttacttttaaacgtttttcagggagcctctcaacctggatttctcgttgcaaacga
aaataattacattattctttgcaatatagttggtactatcagcaacatgctacaaattcatttttctgtgaatccgttccttacatatacccttgtcatc
tattctcactatgtggaaacgcttaacttgtacgcatttggggatataatgagagtgagacagtcaagtcccaaccttgaatcgttgtattggagtt
gtaatggtaaaacctattccagtagagcgtttgatgctatactatttgcagcttttcgtgttatcaaagttcaaaaagaactttacaaacccgacttt
gctgagaaggtagctggtgttaaattcccttccgctgtatctgattcaaaattgaaagacgaaaaagatcaactacgaaaaaacattaaacctgg
aattacgggtgtgaatattatggatagtcccgccagattacggccaaagctgcatcaaaaattggctgaggatctagactccttaaagtcccaag
aagcaaaatctgataataagaagccgaaaaggaatgtgttggctcatgcggtcgactatagctttaagcctacgcaacgatggtgggatagttg
gtcgtcaaagatgaatcttcaaccgctgcttaatgtggttaagaccttacgtccaacactctctgaaatgaagcaacgcgactgttccacaattga
aatattatctacattaaggaatcaaaagttttatcgaacaacaaaacccatcgttcccatgtatcacgatacgatatgggagaaaagacaatgtg
agtctactcaagcctttgcgtggaatctgatatacgatttggatagcccggaacgacaagggcaaggtatatggacgaagacgaatgtgaacgc
tatggcatcaaccgcaacaaaagtgacttaatgttatttctcatatctggttttaccgcttggggaatttactctatggtataatactattctttattga
ctatgtaacaaaacaaaaaattatatttacttattctttgttaaactttgcataattcattcatgtattacagtttatgtttatctagactggtaaatgt
agtgttttcgagagaatattacagagcacagtaatgcatcctttgcctttatataacaagtctaccttaagtttcaaatgcatcctaatggatattca
tctcttctgacccaatagcaccaaaaaaaaattctattaatttaccgcaaattgtacaatcatctttgtcaaagctttagaaagatctggcagcttac
agtggtgttagccttccgggaaagaactaggcggtgttctttaacataccaacttcataaggaaggacatggaagcgctttttcaaagtataacgc
tgacgagaatacttgtcatcaggagaaaatcttgcaggatgggaagagacagtggctttaccatttggagcccttttttttaacgtataaatacgct
ttccctcttcgttcaaataatacatcaaatgcattttgaacaatggcttttgcttttccgtggaaggatgctggtaaaaggtgaattgaagttcaaat
ttttacagacggtgactaccttgcttgcgttaccgaaaaagaataaggcaatggtaggtagctttgtaattacagcgctgctagttttaggattacg

tacattcaaatgcgttatacatagttaagccaacgaaattaaagagtgcaaaaaaattcatgattcgaaaacaaagtagcgtagtgagctaatg
gtaaaatactcgaagaaaaacaaacaagatctatttagaagcagcgaatttacaaagatcttccttctaatgtgttaataaatacaattttttgct
taaatgctaattattaaaaaaaaataatttatatcttgtttacaaaatgaaacgtgtgttttcatggaaatgatactaaaaggtatattatattgga
gaacgcaatgagtcatgggtaatgtatactgccatgctggaatccattgactcttcaatattatattacttattagtatacaactaaaagaaagatt
ccagcgtgtctggttggcgaagtaagctgcaatagtaaacaagttggttagcaaaaccttcctaatccgatactttgtttggaaaatgtctcatcat
tacatagctgttctatgtagtaaaaaataatattcattaataactatatttctcttgttcttcgaatctgtttttccaatttcctctttaacatctttaaac
accatattctttgttactttctgtagaagtatatcatattatacataaacttcctcatgcagtcataaacgatactatgatatcgctacctctatggat
atgttcgactactttagtaaataaataaaaaaagtccaaaagtaatgattgaaagcactattcttaaactcaactgaaggtgaaagctctttagaca
ggaaatttccctagggtttacattagaaagcaaagtaaattaatctctgtttgcggtctgttaccatttaataaaccaacacaaagttgctgaaatt
tctctcagatatttgtttaatttattggtttcttttcaaacccgaaaaaaataaagaggatattacccggattcctataagtaatgaagaaacccaa
aaatatgtgtttcatcgcattatcgattcttgcccaatgtagacgattatgattctttggttggtataggaaaaagagcacaacgtctaagaatttcg
taagctcaatggttaactgaaaagaatggtaaagagctacttcgctatcagtaatcgatgcgtattctagatggaaaattggatcaaattctaacc
aggattggcatactaatatcttttgtatgcaaacaaggaactttacaagttttcaaaaccttctgctattgtcttgcttgcaattttatttgccattcga
ccattgtagcaaagaaagaaaggtatctcaactatcgctgaattatacaaaccaggtatacacggcttagaagaaacaaaatgacgaatagag
gattgagaaatcggtataagcctttcatgctacgtttgtcaagttgactcttctgagggtgttataatgtaagcaggcaacgattaggtaacttttac
agttaacgcggttgaaagcaacatctcagaatcctattttccatcgaaaatgcctgcttccttcatatgtctctataatggcgcttttcagcgactat
gcttcaaaagcaacaacgtgtcaaagttttgggatcggttactgtataaccctgcgttgtagtcagatgagtcctggtttcttttatcatgaaaagtg
ttagaagtgtcaccctcggtacagattatgcatttctgaacactgcggcaaattgacaagccattgaagaagttaacacttcagaagaactgccat
tttttaaacaaggtggaaagcagtatcaagcattgttaaatgaagcaaggagacaacataaagaatcaaataaaaattcagtaggcttcctagg
gaccgtaggagggtttcaatttatactcttgtttctaatccgatccattccttttaaaattccaattataaagcaatcattctcgctatatccaaaaaa
caaataggatgattcgtttatgcactattccaaaaagaaggaggaaagttgaccgtaaattttctctcgtttacaaacagcctaaatagaaaattc
tttttacttttcatagttgaagggagtaatcatattgtatacaaagtgaagataatgaaaaagcaaactttttactatttacttcaaaactgcacaca
gaagaaagactttaatactttcatacaacagaggttgttggtcaaattcatcgtttaagcaaaacacaatagaaaactgaattagcgaatttcatt
ttgaattactttactattgaaaattgattttatcgtcacttgcaatatttgtattgacgttggaataaaggatgaaaaaagaaatttgggagtttgag
tattaaagcaaacaaagctgaatctttatcgagtaataaattatggcgcggaaagagcatcctttttattgttgctatttgatccaatctaagaaaa
aggcttccagtagatctctctacattggttcaaccccaaatccaattcgagtatgtagagaaatttcaaattcaatgacgattcaagtcagttatta
actttgaatttagcgacttcgacaacataacggggaaatacaaggaggagcctggaaaacgagaaatggacgaccatggatcgtactttgtctt
gttcatgggtttcctaataaaatttcagcattacaattcgagtggatctggcagcatcccaatatttctagacatactaaagacaaggaagcaaaa
ataaataaaacaccgagtttaagcaactctctagttgctcttcagcaaattgtttcttgtaacggttggaatcgatggcctcttgaaataacattttt
ctctcagcatgcatttgaaaaatggaaggcaattagcaaagggaatacctctgtaaagttcaccatgaaagaagaggaattaatggacttttacc
ataaggcttctgaaatggaatcgacaaagcctcagtcggaaaataacatgactcgaaaatccatttgtgatatatgtctttgtgaagtccatgaaa
gggactcattgcttcattgtctatatgatgattgtgatatgaccagccatacaacatgcttggcggttcattttctagagaacgaaaatcaaatctta
ccaatcgtcggtcgatgcattcaatgtttacgctttcttcaatggagtaaactcattcaaagcattcaaatgttggaaatgaatgtcaacgatcacg
agaacgacatgactgatcaagaataaagaactataatgctttcccaaccttgaaatttataatttataagaatggattaaggataaaaaggtttta
aaaattgttcacaacggataaaccaacatccgtaggacccacttgcataggtacaatgttagaggaattccataagtcagtaactttttcaattac
atgtttttttattttgtagaatgcagaattgggcgtcaaatccaccagcgccaggaaccccagctccaattaccccttcaatagtttccaattgatcaa
gtaccttagattgagcttcaggttcaatgtctactttttgcttctgctgttagatattgaaggagttttctaatacattgaaaatggtatcgcaattcaa
ctgaggatccagtcgcagaaaggaaaagttcttgcaatgcggaaacatgtccatagagatcgtcaaagagcttatcggaaacttctctagcttcg
gttttccatttttgaaccttcttcaccattcctggtgttccactccccccggcaacgtctcccattagcaaacgatactgagaaggtagggtaaaagg
ctgaatcggagaccattgttctgagacgacttgtcttaagtcatctgagaagcttttactcgccaactgatgagtagggtccacaaggagatgttg
aatgagttgaggatcaaatcggcggtaaatacaactaccccaaacagctgcacccacgtcaaacccgctaccaactttaccttgagcagcacaa
tgagcaatctgactcaagttgtgaatgattgttttggaaaacgaggtcagttgtggttcggatgccggacctttaacacaaatttgattcagtgaca
aaaataaagaacttataaagctcgtaatcattgcagcggagcttccaagccctgtttttttaacttcatgcagcttgcaattaaggaaattaaattt
gggatatgaatccagtccttctggtaactttggttgatggtaatagctgttatctacttgaatgatgatgctcaagtcttgccattcctcaggttgctc
tccggaattgaaaaagtagttcaagacataaaatagagcaagctggacaaatgggttaggactttgtttctcgtcctcgtcttgttgtaagactcg
gattggggaacaagaccaatcgactttgtatgaccattcagcatcgataaattgagggcttctcacataaataagtccttggcggcttttcagtga
agtcgtcgtagcaaaaccttttggcgtctaaaccgacaacaagcccgctatactgaggatcaaggacaatgtatccacccgctaatagcactttttc
caggagatgagcaagtactgattactgaattagtaaagattcaccgaagattttacttggaaacgcacatattcatgagaaaaccgagtcaacgt

aaacagattcgacacgtacgctaataaatgaacgaaaacaaaaagaataatgaaatctatggagtatagaaaaaccaaaagaatgaaattttt
ataatgagcaaaagagacctttgtgtttcttactaaaggtttccttggataacctcgttacgattatataacaaaatattcaaggacacaagaatg
gaaatggaatgcaatttcgtttcagaagcagtatgctctctctcgtccgttactaatcctttttggcttgtcaactaaattgtttttgataaagctctt
gtggaaccatcacaaaaaatttacgacccaccattgagtttgtttacattcccttttgcgagcatgcctttcatttgacactccaaaccaaccaaat
ctggctcttccatttgataaaacaagaatagatacaagctgcgaattttcaagcgacggtgaatctctcccactcctttctttcgtctagcttagagg
aaacctatcggtatcccacaatactactatgtgggatacacataggcatgcttaaagacgtgtatataacgagatattttgaagctcgtaatgtatt
caacgtgttctgtaacatttcgtaatttggcaatgtagcgtccgtcaaagcaagtctgggatatgtctcataagagcaagttgaaggaattgttaac
agaacggagttgtttgtgaaaaaaaccaacatattagggatcaagtacaggaagaaaatcgtatagaatttatgaattgtttaagaggaacacc
gagtatgaacataggtattctatcgtttcaaagacggtttctgcatactttgcggagaccatttatctttcctaagccattgaaaggacccaaatgt
aaaaatgtggttgcagaatacgagaaaaagtaccagaatagcgtggatgttaatggagagcgtttccagatgtttcaccggtcgcaaaaggatc
gtgtgagaccaggagctgtcttattagtggaaagctattctaaatacccgttgaaagaaagtataaacagatttgcaggatacttattgcgtgtac
gccatcgcggaccgaaaagctcgtttttactcagaaacaatgtgatgggtgtcggtgtggaagtcttatttcctatatactctccacagatcaaaag
cataactgtactgaaagataacggaatttcaaagcgtccaagaagagcatatttaagttacttacgccaacctcgattccgactagcacctgtcg
agccgcttgtacgcaagtatatggaagcaaaacgaaagtagtgtttctcttctttctttactttgtgtactcctgttgctagcaaaggtttcatttgcg
atggttattctcattattcattaaaaattgtatatctttctatatttgtccgttttctggatctctcttctttttttacctatcctatcctttcactatgtgatt
catacccttgacttgactctttggtatgagcttttaatctttttgagtccccaaaacctggcattagtttgttgagttttcttcttgaaaaagaagatgta
cagtttttttacgtataaaaatgaaaactactactctacttttcactgtttttgcaaaactgtcgcaaacttaaagctcggaacctattccattgaattt
cctaggagagcaattactctcgggatttgtagtttttcttagtttaaatcacacatactacttgtaaccttcatacgcttgatagaataagccaaaga
attcattcgatagagctttttgttgacattcgtcttttccaacttttatttactgttttatgtcctacctacgaatccgttatttggcattctgaaaaaca
atgaagcgacccaggctttgctctaggtaacgactttctactcctaccacatagaaaacaatgaaagtaaagatcaaaagtaggagtaaacaat
aggaaatggagctaacgaagtcgtaggataccatgcagttgctaattggacctggaagacacctaaagatgatgtttgtggaatttgtcgagtcc
cttttgatgggtgctgccctcagtgcttaacgcccggtgatgactgtcccataggtaagtaccgaatgcttttattagtcaatgttctttaaatttccg
ttttcgtttcttctaccaattcgtctatgaaagtttgcttattgtatctggcttgatgatttttgatacgtttcattgcttgggatcatggctcgtttttgaa
ggaaaatccttgaaagaaaagaaaagaaatgactaaaaaagagtctttctatgtttagcatgctgtttaggcactaacgtgtgagtttagtatgg
ggaaagtgtacacacatctttcatgctcattgcatcgaaaattggttgtcaacaacaggttctcaagggcaatgcccaatggatagacagactttt
gttgttgctgaatccagtatcacaccttagacacctgcacctaaattattgattatcgttaaaaatctttcatttcaaagttgctctcgttgacaacaa
catggctggctataatatttattaacctgggttttactttgtttatgaaaattatctctactaaatgatgaatgttacggaacctagtttatgatgact
atacatacttttatgacaattcttttttttttttttttttaccaaattagacaaaacattttattcattcaatcatatataatggagacattcaaaagata
ataatcattcacgttcataacgaaatgaagaactttttttctttttcataaaaatgcacttccttattgacggtctttgtttgaccatatacgaatagtg
ctatcatcactagctgaagcaaattgataggggtccacaggattgtaagcaatgtagttaacgcatttcgagtgcccgggtaaagtagtaagtaa
ttttccggagtcacgatgccatattcgaataaactcgtcttggctaccacttaaaacaaacgtattgtcttgaccaccaaaacaagagccgatcaa
ccagttccccagtttatgacccatgtattgccgaataatacgatattcttgtatatcccacagcaatattgtgtgaggctcaatatttgtcagtgcat
aacgagagtcttttgatagcgagattgaagtcacctttgattcgagatagattttttttcaggcattctcttgtagatatggaataaacggcaatatgt
ttatcttcactttcaagttgcttttggtacccaactgcatacaacttcgacccgtcgttactaattgccatatcgtaaatattaacatctttccatttgt
gcaagacctctccggttagcgaccaatgaataattgtcatgtcaggagaccctgtaatgaaagataacccgtcaggtagccagcaacaacagct
cacaaagtaggtatgctcttttagatgccggatcttttcaccagtaaacgcatcccaaagaattaccgagtagtcgttagagcaagtaagtaaata
acggtcatcgggagaccatttcacataagcgacttctttgctatgtccaattaatctgtgtagcatcttcatattaactaagtcaaagattatagctg
atttatcttgcgatgcagacgccaagtatttgccgctatttgagtaggaaatctgccaaacttcactcgtgtggtcatcgaaacaatggatttccttt
gttggaatttcactaggttcagcatggtaatctgacagaaaggtgaagtttttcaaaacattatgatagcattgagaagaaacctgacaactttttt
gcttgatttaaaagatctaagagccgattctttggcatcataatgtcggggctgatgtagaaagatagctcatttaataaagcaggacgattgtctt
taatgccagatgcaggctctgcaatactttttcttgaaaacatgatttgagagatcaactcctctttgctttgattaaaatacgtattatcggttgttt
gtagaacaaagactgcctcacaaagcgatcccttctcagctagttctaagcatttttgtttttgcaaaagaaaaagggcttcctttttgctttgtcag
tattgcatctccacgaaaaacaaatcaagtaacttacgtttcttttgctttcgtctctgagcttcatagaagcaaagcaactctcagcagtttgccag
tcaccggaagcaacggctttttgaaacgatttaacgttttccgcctcgtaaacaagcccactttcgttttccaggcattttaaagtatctctaaaaat
aggtcagctagcaatccaaaattgaaagaaaccgtgcaggtggactgaatgtagtgactgtacccataaccgtaatcatttaagaattgaagga
taagacgaacaatttcttgctcccatgtttttgtcaactccatcttacgacgtgttgagcgattttctggctcttgatcaaaggagcgtttcctatttgg
ctgcgtctgaaagctcagattgtttttcgttgtcggcttcactttaacaaaaaaattgacgacggccttcaattgaaaatgacgttaactgaaaatc
ttagaaaaccgaattattatgaactcggatttcctttaagtccttatttttttaaacaaaggaattgttaatgaggtaaaggtcaagaacggtagggt

gtagcacaaagcgtttgcagataacaaagtaacacaagcagcatttaagcatcggttatgaagttgaatgtttacttactaaattgaggtgtcttt
gtattcttcccaatttatattttaagtacattggagaccatcgaggaacctgccgaacacaatgacaaaggacatctgagtcgtatatggggaata
ctggatctaagctattagtttttcccttttacttgcttagagctgcaactgcagtgaagatggaagaaagacttgcaagttagctaaatatcatatta
atttgaatgatgcggactgggacgagcacatactgggaggaaacaacgatttctgttaaagccaatgtaaattaccatattgagggtatatggta
atctacggccacacctaggcgaaaacaccagttcccgtccgatcactgcagttaagcgtctgagggcctcgttagtactatggttggagacaaca
tgggaatccggggtgctgtaggttcaaaaatttttatttatatatgccgatttcctttgattgtaaatgctttataaaagcgttctcgataaactctttg
caagttgttcttttacaagtgacagtaaacaaaaatcactaaataaatatgaatcaaatggatttacgatttcaagaaagaaaacgatagtaatt
gattgtaactcattatagtacattagacctgcgcatacaattctataacaatattgatattttcgcaaaaaccgatttgctcttcttacgttgtaacaa
taacgacatcaacttatactattgaagtgacagagaatcatacacaggcctttttattgtccgctttaataactgctttttgcacgtcaatgtcattcc
atattgttagaaggatcatttcttagtcaggatcggctaatagatggtatgtaaaaataattattcatttaagaaaaaaccttcttttcgctcacttt
agtccatgttatagtagaatgttttgacgagaattaaaccactgaaacaaattccacccttaaccagcaaagactaagattcatgaagaagaaa
aaatttaaatgaagggagtttattttgagtaaaaggattactctacaactctaattcttcattcgtagtcatcggcaggagagagaatgcatcatcc
cttcgttggtatctgggaagcgcaggagaactggtgtgttcagttgagcttacgaattctagcaatagataactttttttaaatccttaacaagtag
aaaatattagcaccaacttgctgtccaacaaaaaccaatggaaatacgtacgaaaaattatagaacagcagaagaatcttgttaccatgacaat
aataggaaaagcgactacacttgttaacaaagaattgcattttgtgacaaacaatgattctccactcacaaataaagccaaccaaacgaaacat
gtaagcctaaagcaccaataaactttcaattgacccatctcgtccaaaatctaaataacttcatacagtggttgtaagcaatatctgtatcattata
tctatctagttcttaacagaagctttggtggtttagtggtataatgcttcgttgccatcgaagcgacccgggttcgattcccggccgaagcaagaatt
tttctttacagacttcaatttacttttcatgatttaaacggtttacagttggtttatacttaaaactttaaaaaatgcaaacatgctgatgtatgtattc
tctgatgctgctaaatcgcatgggaaaatatcttagtattgatttcagtagatggattcataaaatcatagattttctatggatggaatataaggat
aaacttgtagtggcttatttgaattactacgaaggcacttaaaagaattttcgttactacattttcttgtttttgtctgcttcaaatccatcgtcttggtt
aagtaacaaatagagacaagcataaaacataatggttagatcgctcattcagcaatagagaagcgtttaaaaagaaaaaaaagcgctcgtcat
aacaaaagaagactgaaggtaaatacttttcattgtttacgcgtcacgaatattacgttaaacaacgagaagcttggtaaacatgacgatttagc
agtgtatcgataaatatactttgtacaaattggttttagaaatttaaagagattataaaaggtaaataagtcgcttcagtttataagctcacttaaa
aacgtgaagattataatttgcttcggccgggaatcgaacccgggtcgcttcgatggcaacgaagcattataccactaaaccaccaaagcctttatt
ataaatgatagctatgaataactattttatcaacagtgacatgtatataacaaaataaatcagttaacaaacgttgaagctagctagtatgcagat
gtttctgcattgctgaaacaagtttttaaaatactacgggaagccatagcaaaacgacgggttcattgtttgcggtttacacttcatctgtgttgata
aatttacaaatatattactctgtaaaaactgtgaaatcataatatttaattctctttcggtcgctttattctcgttaacgtacatattactagcgctcgt
tgttttcagataagacaaaacaagctcttcagggcagtagcttttgagattcaaccttcacctacattcatacttcatagaattattactcaatggta
agttactaagtggaacttcgacgatacattagtaggatgaaactaaacaagtaggctggagggattgaaattgccaaaaaggcaattagaaacc
taaaaaattacgatgaacatgaaaaccgttttggtaccgtttttagtgtttcaggaccaggtacggcatcttgaatttctatgaatactcctttttgg
aacagtatattgactaaaactagtcgtcgttgcctccaacatgcttggatgctccatgtacgagcttgtccgcgtaggtcatgatgaattggttggt
gaagtcattcgaattcatcaagacaaatgtacaattcaagtctacgaagaaacatcgggccttaccgtcggtgatcccgtccaacgtactggaaa
gcctttgtctgtcgagttaggccctggtctggctgaaaccatttatgacggtattcaacgtccgttgaaacaaatccacgacaaagcccaaagtatt
tacattcctagaggtattagtacagaagctttagaccgtactcgaaaatgggatttcacacctaataaaaaattacgcgtcggagatcacgtttct
ggcggcgatgtgtttggttctgtattcgaaaactctctctttaatgaccataaaattatgcttccacctagagctcgtggtaccgtaacttatctcgcc
gaagctggctcgtacacggttgacgaaaagcttttggaggtagaatttcacggcaaaaaacaatcttttggtatgttacaaacttggcccgtacgt
gctccgcgtcctgttgctgataaccttaccgccaaccaaccattgcttactggccaacgtgtcctcgattccttgttcccttgtgttcagggtggtacc
actgctattcctggtgctttcggttgtggtaaaactgtaatttcacagtctctctccaaatattccaactcagacatgatcgtttacattggttgtggtg
aacgtggtaatgaaatggccgaagtcttgatggactttcctgcattaaccatcgatgtcaacggaacccccgagcctatcatgaagcgaactact
cttgttgcaaatacttcaaacatgcctgttgctgctcgtgaggcttctatctatactggtattactttggccgagtattatcgtgatcaaggtaagaat
gtttccatgatggctgattctacttctcgctgggctgaagctcttcgtgaaatttctggtcgtcttgctgaaatgcctgccgattccggttatcctgctt
atctcggtgctaagctagcttctttctatgaacgagctggtagtgtccgttgcttgggtagtcctgaacgtgaaggtactgtttccatcgttggcgct
gtttccccacctggtggtgacttttctgatccagtcactagtgctactttgggtattgtccaagtcttttggggtctggacaagaaattggctcagcgc
aaacactttccctcaattaacacctctctttcttattccaaatatgttaatgctttgactccctggtatgaagaccgcgtttctggtttcaacgcattac
gtgatcaaatcaagcaaatcattcaacaagaggattcgatgcttgaaattatccagttggttggtaagtctgcactttcggaaagtgacaaggtta
ctttagacatggccggtcttatcaagaacgatttcttacaacaaaacggttattccaactatgatcgcagctgccctctttataaaacttaccacat
gatgagaaacatgatttcatactacgggaaagcgataggtaccgtcgaacaaggcaatgttcaatggtcgaaagtccgtgaaagcacctcagat
attttctttgaactttcgtctatgaaatttgaaaatcctgatgatggcgaaaaagaactcgttgagcgttatgagcaactttacaagaagattgacg

acaaatttcaagcccttgctgaataatactttaatttttgcagctaaacattagagtgtgatactcaattaatttacaatttagtcttccattttacttt
tatcaatcttacaagtatctaagtttttttttccctttattaaaaaatatgcctacaacttccatagaggtccgatgaatgaatgcaattaatttttat
atattttgactaactaggcacacagtcttaccaaaaaaatcaaacttaatcaaatttatctatatatatatatatataaactcatatcttttaggcat
tattatattcataattatcataacatacaacagcaagtggctcttcaataccaaaatgccatcttcaatttaccatctgtcttccgcatccttatcaaa
aacgtcccaaaaatcaccgggatgatccaagtcagcatcggaaagaaaaggagacttttgttccggcatggaattttcaattgacgaatgtcttg
gattttgttcatcttttaatatctcctgagcagataaatcttccgattggtaatcattttctggagaagacgaaccgaacataatgggaaaagtaag
gccagccgtaggactattcgtacggctgaaatagtcgaaatcaatagtcacagcagacccaagcataacagctcgttcttccaaactcatttcgc
gaggacaaacttgactagtatcaccttcagcagcctgaagtaattgattattttcaacagtacctaaattggcatctttggacgtaaaccgcaaga
cataattacctgtatccgtaaagaattctctcggcaaccccataaagttacgagtaacgcttccaaggatttggttgtcggcatttcgtaacaagaa
atcccagctcaaaactcgctcatcaaccgaagcaaattgatctaacatatcagactgcgctaaaaacaattgatatttacgacgccatgggtgcc
acttttgaataatttcgccaactaatgatggctgatctgaagaataattgaatgcactcacgtttagatgggaattgatccatgaaaaaggtctttg
taactgtaatatgggattaccatgcacatccatcacatccgctacgaacggacgatgtgtacggaatagttgccgagacatactagaaagaaaa
gaagtatgtcccttttcagctatatagccaagatgttgaccctgatggtttaatattacatatcgattggcctgttcatatccaagaaatacgttcat
catctccaattgccgctcaacaatcagtacgtcctgtgatagcaaaggagccgcaggtgagttgtgattgatgactcctgtgctactaacagcagc
gggtcctcgggacacaacttgggcagaagcatcaatatacctaagcgccggcgatttatgataaaagcgacatttcagagatcgtaagccagcg
gcagctggaggaatacaatgtcgaagaaaagaatgacttaatagagaatttgataatgtaagatttaacataaatagcaaaattcggctgcaat
ggtaatcctgaatgaagcttgctaaaggtaagattctctggtaggaagcaagccctgaatatacgatagcatcttcgtgcgtggtatctgatttac
gaaaaaaaactataataaacgttacactcaggtaaagaagtataacgacaatgtttgaataaatggtatttatgataatgacacgcaacacttca
ttatcctgtatatttgtttacattcaattgaattctacttttatcaaccaaccagtcttatagcaatgaaagaagaaaagagagtgcaaggcataaa
caagaggcagaaaaatagagatctatccgtttacattggcaatttgccatatggttgccaagtattgaatgtcgtcaatctttgccagcaatatggt
aagctttctcattgaaatcaagtccaataaactaataaaacaaaggaaatgtaattgaagcacatgtcttaaatagaagaaagaaaaagagtat
caatatcaagggttcagcactgtttgcatttgtttacctggaaacaccggcttgcgtagaagctatcgttcaggcattgcatggcttcgaatatgaa
ggaaataggttgtaagtaattcttccatcgagcgaaagtaaaaggtgattatgctaatgtttcataggatcgttgagaagcgtcatccagccaaat
cagccatatttaaattcactcaaactcaagagaaggatgaacccagttcagcgaagctcgactttaaatataaagaagcattatgtctcaaggaa
atgcaaatgaagcaatcagacgcggaattagaaaaacacatgaagtatgacaattaccaaaatcaaggcagtgaacaagaaataataaaaac
ggacgaaaatggcttaaattatccccgtctagacagagaggtacgtaagccgcaaatagggcgtgcttcaagcgaagaagattatttacgctca
ctaaaaaagagcccactaagtattccgaaccttcttacacaagaaaattcatctgctgtgcaggatgaaattaaaacaaagcaagccgatccag
aacatacgttcctcaacaattatcaatttctgccaagcatacctttagataaactgttttactccgaaactgctgagaacccaattctggtaattggc
attcttaacattccaatgaaaacttcacccgtagatctttatgacgacttttctcagtatggaaaaattttaggagcggccatcaatcaaacaccga
atccagatcaaacgttatatgctgagttggcggtcaactctcatcaagattgttctaaggtaaagttggttatagagtctctcgaattctttctgata
attgctaaccaattcagattatcgctgcctttcaaaatttcttatataaaggtatccaacttcagttctttgtgaagcagccaatgcaaatgcaggcg
cctccttcttccccaatgtcttcgacgcaaccaccgatagtcccaattcctttgctaccatttgaaccagtagcaacgatggaaccgcctgtagcaa
tgattcctcctcctccttccttcgtaccgcctcgcgttccaatgccgagtgctattaacacgattgatatgaatactccccaagcttccgacccgtgta
atttgtttgttaaaaatattgacgataatgttgtaggaacctcagaacaatttgaggaatttttctctcagtttggtcgtgtgaaatcgtgtgttttag
caagttacccaaacactggaatttctaaaggatacggattcgtttcttttaatcatccatccgaagcgatgcaagcaatatctttttttaaacaatac
gtatgttggcaaaaaaagactatttgttagttttgcggaaaagaaagaagaaagaaagaaaagattgaatgctctatttaaccaagaattggca
ccagcacctccagcaccggttgctgttcaattatctcctgaacccattccatttgacatgtattctcaatatatggtcgttcccacactaccgtcagc
accaatggcaaaaccaccggtcttaatggctgaaatggagcaaccaaaaccgtttcataccgatgaaaatgagtatattgtaaaatccgggatg
aaaccatcaaagagaagttccaaacgatcaaatgcactttcaactttaacaaatatggttaactaaaacaagcacaaacagtgagcatttacttg
tttatatatatatataaaaaaaaaggaaacataaaccgacatgaatgattatttattccttcgagatattatctaaaatattaaacatttaaggagat
tgaaatgctcatcaataccctgtaccgagagtaaaataatactcgatgaaatgttaaccgagatggtttaacctgttgtgttagtcgaagaaagcg
cggtttacatacttatcagactttttaaggaaggtataacgaaggactgttggttctaaacgcagaatccgctggagttcttgctggacagtggga
cttgagtaaaaagacattgaccaccagtatccaaaattatgcttattgttcagctttttcacgggcttggcaagttccttaagccccatggactcgat
atcaacaactacgcctttgctatccaagactgcttttccacaattccgagcaacattaattgccgatgtctagaagaaactgttagtgttctagaaa
aagagtatattggtgaaatgtatctatgtatacagcaatccacgactttcaaaatttacatggatctgcgatttcacatacaggagcagacgtgcg
agtggcatttatagcaggtctggtgatacaaaaaagttcataaagaaccattatacaaatgtttagtttgagtagccttgaatgggtagtcccagtt
aagagcagtatttggtgtagcccaaaggtagtaagagggtagaccaccaaagaaagacagaatgtatggtaggatcagtgaaacaagttcact
aagaagttactatataagtaagaaatcaaaatataaatatgtttgtattttattatgttcaaatgtactctaaccttcgatgaatgttgtctttcataa

cttagatgctaataaaaacctgacaaataccaaccacaaaacgattcagtaattgttgtatttaagctgaaaaagggtattttatcagtgtatttat
tttctattactttaatttttatgtaaagaaagactttgcttgcaaagaggaatatttgtgattgtttataaaagtttgattaagtattgaaggataaa
gcttataatcatgaatcatgaagatctattaactcagttttgtaatgagttttatgttgaaagcacgcaggcacgatttttcttggagtctactaattg
gaactatgaattggctgctgcattgctaagggaagtttttcctcctgagcaagaggaaggatttgaaggctcggatcgtgcaactacacctgatac
gaatgctgctttgccaagtgcttctgaacgaccaaatccgccttcgaatcccgctgtaaattctaaagggaaaaaagtttctcaaaagaagtttgc
tactttaaaagatctacaagaagatgaagaggatccagacgaaaagtcgaatctatttactggaggagaaaaaagtgggttgtcggtacaagg
acctgatcccaaaaaacagcttgtccgagatatcattgagaaggctcaccagcagacagcatcgactggtaccgaacgcgaaacatcgccacca
gcccctgtgcctgcctttagtgggtctggacaacggttaggaacggaagatgaacttgctaggcccaccaaacaatcggaaggaactcctttcac
tcaagcaaccgtgccgcaaccaaggaatgaaccagagagaaatctgggaacccctgttcgtcgtaccttgtatttctggcgaaatggttttagcgt
ggatgacggactgctttactcttatgatgatccagcgaatcaagaaacacttcgtctaatcaataatggccgtgccccacttcacctgttgggcgtt
actatgaatcagcccattgatgtagtggtacaacatcgtcttgatgaagattatcgtgcaccaacgaagcccttttctggaaaaggacaacgtctt
ggaagtacatatgctcctcagcaagcgcaaattcctggcgcatttcatgatcaagcttcctctcaaagcagtgaacctgcgaaaaaagttcaagt
agacgaaagtcaaccgtctacgcgaattcaagtccgcttaacgaacggttcaaggaccgtaataaccctgaatttgtctcatacggtgcatgattt
atacgaagctgttcgtgtctcttcaccaggatcatttattctgtgtgtgcctttccctgctaaaactctagaagatgatccatctgtcaccattgagca
ggcatctttgcagaacgcatctcttgtccaaaagcaacaataagcaaggagaaaggaggtattccgtgttatatatatatatatataaaagtaaa
taaaatttgaattagacaagacttgtttttcggtttatgatgaaggtgcgtttccatgcatacagaagattagtgcatggaacccttttcaacgaat
ccatcgttggacgtttgaaaagctatcttccatatgtcgaatgtggtttgggatgaagggggttccatgttggcatcaggtaaaataagctacaaat
tcgaaacaaaaaaatagaaaaaacttagaaagaaagaaagagggagactatcgcgccaaataattaatagtcagtttctacttaataagtcca
cctcgaatcgttatggaagaagcgctttataatgcttatactgccttacatttcaatcaagtcgcggtggtgtagttgggagcatgacagactgaag
atctgttggtcactggttcgatcccggttcgtgacactttttatttttctctgaatttgttgcttcgattcgtttcattcttgttctgttctcgtcttgaatg
gaaaaaaggctttgtttacagttcccttctccctacgtcccacacaagattccctagaaaaggtcataaatatgcttaagaaatttctttttttattca
ttagaaatgcactataaatgaaattttcttatatgcgagactttgcttcgcttcttgtttcctgattttgtctatttattacttgccaaacaaactcaaa
ctatgtcgaaatcataagctcagttaagccaaataaaggtaaagtaaatcaagcattctagatccaaggaaaagataaacaaaaaatctttcta
tagattgattgatctcaattgatgtgttccaactcgttaaacaattttgaagacattacatttcgttcctccctttagtcctaagtgcaagctgcaaga
aaggtaaagtgacttgaagacctgtgtgagacattggcaaagacaaaaagaacagaacgagtggacaattatgaaaatagtggacttaattca
tgggcttccgaaaggaagcacgttttattcttttgagtattttcctccaaagacaacagtgggtttggaaaacttgatgagccgaataacaagaat
gtcgaataatatgcttcctttgttttctagcgttacatggggaacggctggaagtacagcaagtgtttcagtgctattggccaaattgctggaaaat
caacaccgtgtgccggcttgtttacatctgacttgcacgaacgtggaccggaaagtaattgacgagaccctagagacagccaagaatgcaggga
ttcgtaatatcttggctttgcgtggagacccacctttgaatagcgatcattgggagggaaagctgagtgagtttgagcatgcagttgatttagtacg
[truncated: 1,658,723 more chars]
