# Supplementary material for: Assembly of Schizosaccharomyces cryophilus chromosomes and their comparative genomic analyses revealed principles of genome evolution of the haploid fission yeasts
Source: Sci Rep. 2018 Oct 2;8:14629. doi: 10.1038/s41598-018-32525-9 (PMC6168568; doi:10.1038/s41598-018-32525-9)
Supplement: Supplementary file 3 — Supplementary file 4 [file 41598_2018_32525_MOESM3_ESM.pdf]

Supplementary information

Assembly of *Schizosaccharomyces cryophilus* chromosomes and their comparative genomic analyses revealed principles of genome evolution of the haploid fission yeasts

Lajos Ács-Szabó, László Attila Papp, Zsuzsa Antunovics, Matthias Sipiczki and Ida Miklós

Department of Genetics and Applied Microbiology, Faculty of Science and Technology, University of Debrecen, Hungary

Correspondence: Ida Miklós, Department of Genetics and Applied Microbiology, Faculty of Science and Technology, University of Debrecen, 4032 Debrecen, Egyetem tér 1., Hungary

E-mail: miklos.ida@science.unideb.hu

Tel: +36-52-518-600/62056

>Chromosome III of *Schizosaccharomyces cryophilus*

atgaatcctttcaaggaaaagactgattataaaaatttcagtaaagtacagtgttatatatactttgacttcttgatagattggagctctgtagggct
agagtaaactataataaatgcctcgattgtgaataatgaattgagaagaaaacagccataaattacaattagccaagtttcgtaatctttattaga
tccttcaaataataaaaatacggacaggataatcaatgagaggaattccgagagtaaatcttcataaaaaaatattacgatcggccactaacac
cgtgagaaatacattagagcttacagctataacttggtgaagtaaattgattaatcacgtaaatccaaagaaaaaaaatatgcgttttaattcag
ggactatacatacattcatgactccttgccatgacaggtttttttcaataccccttgacaactaaaaaaacctaaaatacttaagggaatcacag
actttcattgtgaagctgataacttcttcaagtatttaattatgaaaaatagagcattgggcttcctaagtgtaactcagaatcgtaattgtagaaa
atctcgtttcatacagagaaattcctcatttatcataagggttttgagtcgattgtttttattactatctgtttatatatattaaatgtcataaattaaa
aaaaaaaattaatcataacttattctattatgcgtaattaagatgagaacgaaaattgctgcgcaggaaaattcatatgatgattttctcaaaaac
tttccccaaacaattaatacatatacattccacctgaagctgaaattacctgcccagtaatccatcgactactatcttcagcaagaaaagcgacaa
tttcagcaatgtcagaaggtaatccaagacgattttcaaaaggggtcatttttttttgtaatttctaatgttacatctgaaagtccctttagcatatccg
tttcaacggctcccggattaacttgattaactgtatgccctcgagggcccaactcagcagcccaacatcttgtgaaaccttcaattgctgccttgcta
gcacaatagagaggcattttaaggaatgcctttcttccacaaatgcttccaacgttgacaatcctgcccccactcccgtcttcttgtttaggaagatt
aggtgccatggcttctgtcataaaaaagacggcgcgaacgtttacatcaaaagtaaggttatagtcctccgcagttgcacttcctagaggttttgg
ttgacctgcgtaccctgcattgttaacaaggatatggatggtaggaccaaaagcttgtactgtggcatcaacaatatgtttagcacttgaaacatc
acgtaaatctgcctgaattttgatcgcatctgctgaggtgccaaaaccctttattttttcaccaactgttcagtcgcatcaatgctgctgtcagaggt
aaaagtgatggctactttagcaccacggcgtgccaaagtttcagctattgctgctccaattcctcttgaagctcctgtaacaattgctactttgttttg
aagagaaagattgtattctgacattttacacagttatggggtgagttaaaaaaatttgggacgtagttataataaggggtcacaagttgtaccttg
ttacttttacggatctactcctatctcggtttaatgatgattttcttcaacaaagaaaaacaactagctcaatgcttcttattatgtcggttctgacca
atttatcagtattttaatacttaagttcatagctgtttcaattcggctctctcagcacatgtctttcaaagaacttcgctgtcgttaccatgctgatttat
acaaatatacatggataatgtgcctagtgatgcaccagcaaaacaaattgctatttccatgaggcagctaatgcaagttatgagcgttttaaaca
gtatgtggaggcttgtagatagatataccattgtaatccataacgagttcaccggggatcattgcagatgatggttaattgcctatgtggatgcaat
aaccattaaaccacgcacatctattatcacagtcgtttcttacctagcagggttaagttacgtaggcgcacagaggacctaacatccatcatttgg
acgaaggtagcgaggaagacgaacagatgctcgaatagcggggtaataaagtgttttattaagtctcttatgatcaatgacaactacttagagat
aatagtttctgtaataagtttaagaatgaacacagttgtaaggatctgtcagagcaatccttaatattaatataaaccattttgcaatgattatgct
actgagtatattactgcatattaatgttaataaacaaacaaactgaggattagttaaagtttattgtgtctatttcctagttttagattactatttata
aacgccgttctcctcagacaaagtcttcagttaggaagattttgctggcagacctaataaacttgttcgttagcaaattcattagtatcaccaggga
cgttactaaagatatattactcgagatatttagtaacttaacttaattaagagtattcaacttgacggttcaagcattaacaactgtgttccacttac
actctcattactaaaggagtattttccgtgacacgaatattagcggataccttaagactaaacgatgagatattataaatagtaaacaaagcatg
gaaactttttcaaaatgaatataatccacggaattaataaaataaagaaacagtttacgctagctacttccaaaaagctatatgaagaggtaact
atcttaaccaaaaaaactattgtaggatagcttccataaaaattattacaatacggacaatttgaggtgtttcgtcaattcaatcatcacttatcctt
cttttatttttgtcacatatcgtcttttagttatcaattgttgtcgcaatacaaccgtagttccaaataccaaaaggtcagagtcgatgagtcgagtg
acaagttatttacatggagtccttcttatacaaatcctttttgggtctgctattgcattcttaatgttttatattctctacgtgttttgagtaacaaacat
tttacccgcttcagtcttcaaatgaaaactactcccactaaaatctgcatcagaggctgttcaaccttcatagcttctatttaacccttaatttttaat
agagaatcgatcctgcgacgcttccttaatatatcaatttacaatctggtaattgtcccttcataggtaacagtaaataaaagtaagatgaataca
acttcacggagaaagcgtttcaaaaggctgaatctgcaactttagcatattttttttcttttaaccctagattttgcttctttattcgttgtatgtccaa
tgcagtagcttcctccgatacatactctccgtcatttatattttgactattcgtataaccttcaacaaatcatattactgtataactgtactatatactc
acgaatacgatgtggttttcttagatgaaatttgacagagtattgtaaaaagacactgcgttgaccatagctgtgtgacagcaacgttaattacctt
gacatacatgaatggaccttttcgaggattcgggtaacagagagacatcattggaaatcttccattcctcataatatatactcaaataagcctttt
ctagctttgactaacgcctgcccataaaccggcagttatcaataccatgacgaacgaccttaaaaacttccgtaaactttccaatgacgagctggc
aaggctgcacgaaggttcaggtaaaggtaataaaagtatttccaattggacaagattccttatttgattgtaaaagacctcaggcacgatccctca
atactaacgatggatgcattcagagagctcaatttacccttcagcatgtcagatgaaaccatcgttgcatgtcgcagaatcattataggaagcttc
aacgaattcgcatttgattcaaaccagttattttccttcaagcgtacatacatttattggtcagcatagcacaatggacattttgtggttagcttcag
acatgtatttccttttgaaggcctgctaaaacgaattacttactagcaaagagctggcatccagaaacctttcccgccagcaaccttatcccctgtt
agatgactcttataatttagactccaaagctgatcatcaaattgcaaaataaagtgtattatatacctatcgtcacttatagcaacttgacaaaacc
aaaacttcccttaatacctcctcttccttcaaatatcctggccttatttcaccttccattcctcttctttctaaaaactcaaaactctgaaagcacttgc
tacgaaacgtgttacaatttttaggatacatttccatggacgacgctctcagctccttgatctaagaatcagttgtactttttgcttgctcgcttacat

cccatagctgagccaacacaaaattgcccacgcctcggtatgcggcgatacttggatattccaaagacataccaaaggcttgtacaaaaatgagt
tatcatacgtatgtgcacgtgaaaacctgcttgaggagtcgttgagtcttgtcgcatcacagcttaatttgacggtaaacccgaaaacatcgaact
cagagtacaacaccagtggtatcgctactttgcgtagtcgcagcccggaaaaaatttcatctcctttgaacaaagatggaaatatcaaatggaac
caccctcaatccgtgggcgaaaagattgttatgaacttgttttccaaccttggattgggcaagcctgaggctgtacggagactacgctttgtactgc
tataaagggctggtatatttcatgccaaagaggcaggatagcgtgaatgcgatcgatttgtttgtgagaggatgatatggaaaggctgaaggcgg
atatgaaatttataattatggaaaagcaagttagatagattatggacgataagaaagcaatgtgaatggttagattaactttagaagagcatcgc
gattatagggagccttatattatattatttccaacaacttgattgctcatacgggaaatgacatagcggaacgagttgatttcgtcaattcagttatt
ctattaatgttgttgtcatcaacatacgaattaatggccactgctttagcgaaaacaaaaaccatgaaactatgtttttgttctcaacgcttgtaagt
ttgtacaatttttaaggatctagttcttatcaaatctgtctttaagcattcatgcttacgaaaatcgactgagacttctgcagattactgctcaagtttc
tggataaccgaaattagcgggaaagcttcctatcttaagtttcaagtaatctttgtcctcttccttgtgataaaggatccatttgtttttgaaggatg
ccagatatagcagggaattagaattttagttacataacgaaacgtccaacagataaagctatctgacagaaaagttttcctaaatatttcaacata
accaaaatatgattttgcaaaagttaaaatcagaaaaagcgcaacacaatagtaaagagattgctttgcgactcgaagagattcttttgattttat
gtatggcgtagatgttaaagttcggtttaaatatagagttgttaatccaaccaaactaaagctagagtcatttaaaaatgtatcaatggaatccaa
agctgtttcacttagcttagtcttccaatgaagcatcttcaaaatactatgatatgcaaaaggaaaaagtaaaacaagtgtcattcctaaaatgag
ctttcggtgaattggacgccatacaccaaagccaaaggaaacaaaaaggagtaacagccgttcaatgaaagaatcgaaagcttgtttgatatct
aaaacatgccaagggacaaaaaaacctaaataattgtaattgcaagatttttttccaccaaaagaaaaatgggagcccaaagacagaacttaaa
atgagcaccaaaagtccgtattgaatgggaaggatttttgatctctgagaatacatcgcacatatccaataaagagaaaacaaaagagacccaa
aggtaaatatcagaggagcgtagtaaagagcgtctttagtatatacctcagtgaataagggttcctggctgctattgtaacccatttgaacatatg
gtatacttggggatcctttctttgaaagtggcttcgttgcaaggcaaaacgttcctctgtgtttttcttcaacctcaaagaattgatttgggtgagtaa
tattggatggaaagctgaatgtacgaatggaatcactgctgtaggaaaatgggccagtccaaatatctctttttttcagggcatatgtattcaaata
aaaaaaaatcccaatttttgtgataaagctcgtttaaacaatctttttcaagtccgctattaccaagtactaactcccattcttccattggaaaataa
gctactgccattcctccacctatcctttcataggtttgtatggagctatatttaggatgattttcatctgcattcacccgtttgcaataaacagccaatt
ggaaactttgataatcaataagatcaggatagactcggggttggtacaaaaaaagtgagttatctcgagcttgaattaatgaagaaaacgtgca
taaaagcaaagaaattaatcctttcttgctaaataaaaacattttgcatgtctgaggtattgaaaagaagttggtgaaagacaagcacttccttag
gaaagtcaagacgaactccccgtttaacgtaacaaagtcaatgtttgagaaggttacgcttcatccccttagtttaatttcttgcctatcctgtttttt
tctatatataacatcgcatcatcatttcacagaaacagagttagcaaacatatttttttgcttaacgttgacagtcgtaaggaagcttgtggaagaa
gtaattcttgcgaaatccgagatggaagaaaagtagtttggaattggctctgacatattcaaatttaaggtaataacaaaaatgagtttgcacgct
ttacgggtgccttaacattgtaagcgaaacagttgctgatttcaagtagaagagcaaggatgtagtctagatagcaaggtgaactttaaaattaa
aactcggcgattaagtagttcattcgtatctttatatagttggctttagacaataaaaatcgtccgataaatttttctttactaagacgggctgatga
atgtccattcgaggatgacagaagactactttattaaatagcctgacagacgtatatttgttaacccaaaagaaagaaactaaaggtgtggttat
ataagatcatcaaaaaactttcaatttagttaagcagatccctggacattactagcgtgtatatacgtagtacaacagaatccaattcaagtatac
atttcttcaatcgtagatattatttccattcgatattaattctcatgaaccgtgagaattactaacgtgacatggcgcatatggatagtatttgttgttt
tattattcaaaatgaagaatttaaatgaaatctttaactgtctacggccacacctaggcgaaaacaccagttcccgtccgatcactgcagttaagc
gtctgagggcctcgttagtactatggttggagacaacatgggaatccggggtgctgtaggctacttgtttttgcttttgatattcttaaaatttgataa
actgggaagttggtttttcaccattcataaatataagccacatcagcaacggttcattaataaaaataagaataagacgaactaaactgattgac
catgatgcaagcaaataaagaaactaaaattagaaaagaaaattaaaatacgcagtacattaactgtcaggaaaacgaaaatatatcggagtt
gtatgattataaagatatgtattagtcacccaccagtcaacaccaataaagcacaaaactgccgtaaagtacaatggcccagccagtccggggt
catactgcttctttttaccagcaatcctaaggaattgagtataggtaggcatacatacaatctcaaacaagttggttcttgttatttgttttattattc
tgaagtatttcattttattatttgttatacatttagtgaattgattaatacatctgcaaacaaacatcctgtatacaaaaacgaaattttacaaaagg
ttaagtgctcctgctaaaacaaccacagtttagctgagataaagatatgtcataaagcatccacgatcttcctcaagatcataattaacactcaca
tccctcaagaacctcgcagagcgaaaaatagcactagggaccggcaccatgctcatgaaaagaactactactactggtcgggctttatgaagcc
aaccatgagtcattctggcaattacagtttatatatatgaagacagagagttaggatattataaaaataggtaattaccaattgattcaaatgaac
ctcattcatagtactgaatgccgtaaaagtcatcacaacaaataatgtttccgtaaatttttcgtccactcaagaagttgcacattttaattcaaact
tagaaatgaaaagtgctttccaaccaattcattgttgtagagcttgttttcgttccgctatggtctcaacgacggcaggacgctcaataagacgctt
gtgccaagcatgtgtcttagggaactccttttcgaagtccagctgaggaagctcttccttaaattcgtgtcttcctgggccaaaaattgctgttagca
aatcgttccaggctacaaaagacaaatcggcaatcgtaaacttgccagcaacgaggtagtccttgtcttggagaatactttctaaaacacagagc
acacgctttatttcattcctatatctagtaacggcagatgcaacaggttcttgatgaaaaaaattaaaccagcctgcttgtccccaaatgacaccct
gacctgaagactggaaaaaaaggtattgaattaaatgatggtactctggattatcatgaggcaatgaaatatttctttctttgtcgtacctgtctgc

caagtagaccaaaatagcatcggactcccagattgcataatcattgttttgatgatctattaatgtaggaactctaccattgggattgtaaaaaag
gtgttgttcgttcttttgctcatttttttcaaaatcaaagaagacgggtttgtaagaaagatttaattcttaagcgataactactttccaaggatttgg
gcctcctttatgagaaaagagcataaaattaaaattaaccattgttaagagaaagatggatgtaagtattaggagttgactgcttttatattatcg
acatacttttacgttatcttgggtgcttgatggctaaaaactgaagtattatttaagatagtagcacgatactgcttgcaaagcaaaaatacaattt
ttagaatgagttcgaatgtattcttttctagctttttcgaggattgaaactaatcagctcattgcgaaagcgattgtttgttcaacgtatgaattctctt
tgtcgtcatagtaaagattacaatagttctttgattatgtctgtattgttagatcgtacttcgcctttatgaaatatcgatagaatcggattattaata
atcattagaatagttctttgcgattcattaatagcaaagggatgagagtcaggactagtttaggagtttgctgtggtatgggtcaacgatcagaaa
acattgagagtattctatatatatatttttcttttatttccaagaggaagatcattacaatctccgttgtagccttaaatctatttgaagccttaagtga
ctacagtttcaaatctatgacttttagctttttcattttcaaaaaataacaaaagaaaaaatcgttaagagtccagtccatctttacgtcgtactaca
ccttgagttgcatttttttcctttttttttctttgattcagtttaagaaaaactctgtaaaaagtatttgcaattgatccatacaaaacaaatagaaacta
atcttaaaatgtgacaattcatacggcctacttaaactattaccaatttcgtttttttctgcttttgttataagcatgtcataaaatcaacagctataa
aagtatgactctatggagtctattgtttgtcagggcagaaatcctacaccatattataacgtatctcgactctagttaacaactttagttttaaatag
gatctaattgtactacgtgttaaaaggtgagtgtgatatccaaggatttagtaactgaattgataaaagctagctagaatgatcaagaaagatttt
catattcataactttgtctttgctaattaaacctaagtttttttatgtatcaaaagattttctgattccgtctagtcaatgaattacctgcttatctctgta
atgcatcttttctattcaaatatcaaatagtatgagattttcagaatcaaaaaattcgactttttttcgggtatcataaacaaaaagctcgaataatta
tcaaattgtatttgtgtgagtcgtatttttttattgtctaataaataatttcaaatcttttatttcgagtactcatgattgtctattaataaaataatactt
gactgtacctaaattacttgaagatttttgtcaaggaagcacatttttttcctggtctgtatttgtaaaactttgaaagataatatcagatctttagagg
aaatcaaacttaaaataacacatttgacactttcgcattttactgccgtgcaaattaatattacctatttataaaatataaaaagttaactaaggtt
gtatgaaaaacagaagagattttatatattctttctacattttatttactttcaatttcgtcaaactgtatgtttattagcaccgtagttggtttgcgca
aaatcttttaacgagtcagtttctatttcgtcagattctaaaacatcgtatatatttcaagattagtatccgttattcttctttcaacaaagtatttgta
gctaaataaagaaagcaaaatgagcactgaacagggatattacgcaaaaggattcgatagcagcatttccaaaactcatgcttggagaactgtt
gaaaatagctgcgcttacttaataaggtatataaaagataatgacaaagtactagatgtcggatgcggtcccgggaccattactatggacttggc
aagacgatatgttccgaaaggagaagttatcggtgttgagccaattcaagagtttgttgatgcaggcaacttgaaaagatctgaaatgaatctta
aaaattgtcattttcaatttgctagcggagataaacttccgtttgaggataatacattcgacattgtacatgctcatcaagtcttggtgcatgtcgaa
aatcaggttgatgtgctcaaagaaatgaaacgtgtaacaaaacctggaggaattgtatgtagtaaagatgctgaccttgaaacctctcaggttta
tccaacaaaacttgatgaaaccttaaagtcccagtttttggcgaaaacgaagtgctcatctacacatccacaggcaggaaggcatcttcgaaagt
gggctattgattccgggtttagtcctatgagtatctcatccagcgcgtctctttggtttatcgcaaatgaagaagatcgtaaatggtttggaaagatg
ttccgagatcgtgctctacattctactgaaacattgattccttttgacgccgaaggggacctgagaaaaagacaagaagtggcacaagcttggga
tgattttattagggactcatctagttgctactatttaatgcatggtgaagttgtttgtaagaaataaacattcttttctttgctattgttgtcgttatcag
ttttattggtaagaaattcttaagaattcaattgaagatcaatgaacgtacttaaggattgaaaatggcttttgtcatggaatagtacatgaagtaa
taatatagtgggaagggaatgagtaatcgatatttttattactaggttgtgtttaattgaaattctagttactagatttgcaaaaatagtagccatgt
cactatacttctggttaactaactaacatgtatatataagcgatttgattagctccatcagcaaaactctcagtaagtgatttgtataatgtaagccg
ttttacttttaagctttcgtatattatcgtatttacaattccactaatatagggaacttgggttactccattgttttatgatattcatggttctcaaggtg
tcagggaaaactgttacatcgtataataatgtatttcaagtggaattaaaaaagacgactttatcatacttgaattgtacctggtatataagctagt
aatgtttaggaatttttgtaactgaacagaatcttgacggctagtattatttagccagacaagcctgaaggtttcgactatggactcaaagtcatac
aattttctgataatctcccctatcactttataagttccgttccaatctcgcatccacattttctgtattaaatttttttatctttatactttaatataacacg
atgactcttaggccaatgaatattcgccaatggatcaatcattaaaacagtttgcaatgtattaacagttttaacgatgtagccgattgtggaaatt
tatgccaagaatttcctactctagaattgactggtcgtttataattaaagtaaattacgaaataatagaaagaccatagaatacaagtggcatgat
aattgcaatctctgacataaggaaataaataaatttagaaagattatatatttatccaaatattatccgtggatatatttgtttggttgattaacttc
gcttatacaactcggtactacaatgggatttcttttagacttgattttccataacaagataaaattcactaaatgattataatcactaaatgggtaa
acgacttaacaacacaaggagaaagtattagaattacatttcttttagttaagttatattaaaccacgtaagatagttttaaataagaacaagaaa
ctgagcagaggtacagatattttattattatgatatccatttagtaataatttaactactttctgaatgcattgttcatctatagttttgagaaaatcc
attactggcttatattcataagtcaataattctaggcttaaatacattctaaaaggaatccctgtaatgttttgatcaaaatatatttattttattatcg
tttgcgactgataaatataaaacatggtattagtacaggaggtctctttcccgtactatatatagtattaattaagcacgtctctgatttacttttgtt
cttgctttaaccaaaagagttactgtttcgtttacagtagctctataacctatctccttagccaacttgacaatccatccatttaatgaatcaatttct
gtttctctcttagcaagtgtgtcgattctcatggaagtactgtttttcgcattttctacgaagccaaggtacaaaattaaatctaccaacttcggaac
gtttaaaacttccctcgcttttccattgtctgctaagaattgattggtcttaaataacacatcaacacattcggttataatagattgaaaaagtgttct
aacctcggcgatgttggatagctcatagtttatacaatcaataatggtggttgtagagttcatacaagcattaaccataaatttttgacattgataa

attagtaaatcatcataggttaaataagtaccattcaacattttggactgtaataaggcttccaccatagcacagggttctgttggaacgtcaaca
gacaagtctccatccattctggagactttggcaattttcataacaccaaagcagctgtgacaatagtaaaaatcttgttttggtaacaccgagtga
gaagtgactccctggtaaagattcggccgtacgttggcattcctccaaacggtcttaactaaggtttcgatcacaccaaaaccgttatgaacgaac
agaatattagagtctcttttaataagggaagataatcgcgtaaagccgcttctgtctgagttgtcttggtagtgacaattaagttgtcaatagtatc
cacattaagttctgataaacatgtggccttaaatttacgtctaatcaccacctttttatcatctagtgttctttcgagtatcaggtatgatccatttctt
ctgaattggtcaagtctgtctttgtttcttagcaacagtacaacgttgtgttcatagcatgataaatttgccagctcacaagctaggaaatttccaat
acttccagctcccaaaatgtaaatagcatcatccataagacaaaaacaaaacaccgagatggtttattgccttggacctaaattctaacgctcgtc
ccgatcgtcaaactgataaggtacggatgtgactgtcatgccgtatttacgcgtaactttatctgctcttatctaattttcgttattctttttatcttgtt
tgttttgttaagaatctccgaaaactaaaatacatgactaaaacatccgtatcaggtttatactagcgagaattagtttttttgattctggttctttgt
ctgatataagtagaatttcttctactttgaacccacaattaattaaaattatttgactttgttaaattataaaattaaaaaaatataatcataatttga
atatagtcagcggcatactgacgattacaacgcaaaatattgggtatttaaattcgctaagtattagtctaattaacaaagttaaaagtaataaga
atgaatgaatgtaagatgcaaagaactaaaacatgcttttcttttgttaattccttactagttttttttttttttgttttggaagcttaagcatcccata
gtaagtcaggttgaaaaggcccggctacttcgataatttgatatttaatttattttacgctttacccctaacattacttctttacaaatttgttacatgg
ctcacttatttatgttcaagaaagttttaaatcagtctataaagttgatctgctaactttagattatggttgtacgatttactgttccatccagcctata
tctttgtctctgaagcattgcgtccaacgcgatagtaagtttcttttaaatataaacaatatactcacaataaaatacttataacttccactaattaa
gatgataaatacagttaactgagcagatgatttatgctacgacaattgagtttctataaaatgtattcattttatttgattttgtgacgtaaacttaa
aacaaaacttcagattaactgactaactgactaactgacgtatatatttacgtatgtcaacaaaaaacgcttttcgatcgttgcgtaaaagaaagg
agaattgaggatgctactcttaagaaaccaaaaataaactaatactaaaataaccacagactaggaaagacttttcaacgttttacgtaacatca
tagcagttttacacaaacgctttctcgatattataatgaatgccttttataatgacgcaattcgctttatttacgtaactataaaagtcaaatggaca
gtcagtatgtattattgtttctaacaaatatctatctttcaattttggatatgagcgttcaaaggaaagtaacttcatgttcattaaaaggcactgac
cttctccgaaatgctcgctataacaagggtacagcctttacaaaggatgaacgtagtaaatttggcttaacttctagacttcctccagttgttgaga
ctttggaacagcaacttcagcgctcctacgatcagtacaaacgtttaggagattccttacaaaaacatgtttacttatccgatgtctttgatacaaa
tcagactctttttacgcattagtcactcgtcatttgccagaaatgtatccaattatctacacacccaccgaaagtcaagctataaaacaatactcta
aaatataccgatatccgaatggatgctacttagatattgaacataatgatccagaatatatgaaggaacaattcgctgaatttggaaaacccgga
gatatccaatacatcgttataactgactcagagggcatcttaggtattggagatcagggaattggtggtatcttaatttcagtggcaaaaggacat
ttaatgactttatgctcagggttagatcccaaccgctttctacctatcgttttggatgttggtacaaacaataaggatcatcgagaggatcccaaat
atcttggattgagaaaggatcgtgtacacggagaagaatatggtcgctttttggaaaatgtcatccatagcgtaaaggagacctttccaaatgcat
tcattcattttgaagattttggtcgctcagatgccaaacctgttcttgaccgctatagtcccaaattaccttgcttcaacgatgatatacaaggtaca
ggagttgtaactctagctgcaatattttctgctatccgtcttacgaagagtaaattgaatgagcaaaagtttgtcattgtcggagctggcacagcag
gagtcggtatagctgatcgaatcgtttctagtatggttaaggaagggataaccagagaagaagctcgagataaaatctatctattggataagcaa
ggtttaatattggataagcatgcggacagggcaacagaaggacaaaaaccttatttgaaaaaggggtcagtttttgctgatcaatcatcctctgat
gaagttgatctagaaacaactgtttctacggttcatccgacggcccttataggtgtctctggtcaagctggcatattttccgaaaatattgtacggg
aaatgagtaaacatacaaagcaaccaattatatttcctctttccaaccctacagacttaatggaagcgaaaccgagtgatctgaatgaatggact
gatggaaaaggattaattgcctcgggatccccagtgccacctgtgaagcgtaatggaaaggattattatatttctcaatgtaataatgcttttatat
atccttcagtcggcgttgctgctgttttatccgagtgtaaaacattgactgaagaaatgctttcatccgccgcagacggcttggctagtacaccgag
atctttatttaagtctgatgattctttactgccagatcttactaatgtaagagaggtgtctcgtcatgtgaagtttgccattttaaagcaagcgctagc
tgaaaataattgtgctgattatgttcctcgtgaagatgaagctctgaaaaaatggattaaacaaacagaatggaatgctgaatatgctgaatatg
aatagcttctttcgtgatttaaaagagataatctaacatctccatatacaaaaggctcaaagcgccttaccctaaataatgagagaataaacgaa
agaaacctaccgagagtggatcaaaattcagtttatgtggaatttaccagaaaagttcctgatatttggtatcgtaaacatgagaaatgtaattat
actgaaagcaacatatttaaggatagaaagagtagaataagagcaacgtatagatatttaaaaaaatagagtatttcattttgttgaatgaaaa
tgtattagtcaattggcattacggttaccgttgaacctatgaaggtatttatatgctgctagtttatgaacttacaattgtcctaattttttccccaca
aataatctcagcaagggtttgcaaatatatgtgtccttttattagttgatgactcggtctatgtcggctcttccatgttgttactacaatctatagtttg
gtttttcaaataacgccgaactcataatttccgcgaagtacatgtgtgtgcaatggctgtaagcaatattttacatcaaattcaaaatgtagccagt
atttactatccaagggtatggtaattctgttcatagttaactccatctaaagggtagccctacttgaaaagaattgaaaaccttttgtattagaagc
gttgattgttccctgtagtaattggcaaatagtctgtcagacgaagatgtgttgttgatagataataatgcaattatgctgtacttggtaaacaacc
gtattcccattactttggattccacataaaaaaatttttttgaattaaagagtcttagtcaaagcagagctacaaaataatattggacttcttggata
ctgtcgtatgtattaatggtactatggcaaaatgactattagcattcaaagttttcgcttaagtcgaaaatattggctgttttaactataagtagata
cactacgcaagcttcagtgagtagtagttcgttaaaagtgcagctgaatactcaatagatcgagagaatggagaatatttatgaaatcattcgctt

aggatgttctaataacgaattgaataaagaatttacgaacttcttttgttttagtgtttgaattaatctggcgcattgagattcgtcatttcgcttata
aaatctacgtataacctaaaatcaagtcgaattgtatttttctgtttctttttccctgatataaacaaatggtatgaggacatggttgagtaaataac
ttaaagggtcgcttatattttttgatatgcaatattacatggtaaaggatgttagcataatattctcagattacaattggagattgttttattaaaat
cagtattcaacagtatcggtcaaataattcattttcaagaatgaattatttattatgacaggcatttttacaatatcatgtattgtcaatttttcagtgt
atcatgtattatttacaagtaaactctttgtatgcgattgactacctattttcgtcttaagtatatttattatgtttttgcggcagcaattagaaataaa
tcatggtaaacaatattaagaattgtacattgtccttgctcttagtgcatgtcataagaattaatcttgttgcgattcggacgtaaatttattctgaca
tttagattcgactcataacattgaatgatacctaactatatttgtttcctacctttcgtataaacacgattagcagtatctacaatgatgagtagattt
ataaagcaaacactttcccataaaactcctaacggaataacttcatacccaaatttttaacttactgcaatggaattttctgttaatactatagatca
taaaatccaatcgaatttcagcctaaaagtacaagtacccattaatgattccgctcacgataccattcttctcgtcaagtaatattgtgagactaat
gcaaggccttaacggaaagtgcaacatgttacttagctaccttacataaaaagataaaagtgagtgataattgctttgatatttctaagaagcgat
aaaatacagttaaataaaaggtaggagcaggatctcctcgaacatctagaagaccgtcatgatgaaagtagcataaatcgcaacgataaaaaa
aagtagcttatttcagttcaactttaaccttgtgttaagggcttcatagctactcgattagggtaattgctgaaatttctatacttccatctctagtcct
tactgagaaggtaaaataacaaagcaagctacacattgaaatgagattcgtctccttcttttataagaaatttttttagctactaaactcttattcat
agctttactcaacaagtaacattagttactgaggcgcattataatttcgatgcacgtaacttgacaaccttaaaaatagatagccaaaaagactca
agatgagcctaatcgtttcagtatgaaaaatgttaacaatggctgtaatttgcccactgacagatcgaaggctaagtgaaaatgaatgaaccact
tcaacgaggcaaagcaagtagtgcatgagcagaaagattacggaactctatgcttagctaaggttcaacatcgggctggttttctctaattgctga
taatgtacgaaagtggcgtcccctgaggctatgctattaatacggatataaacaacctacgtacgttcaaggagacttctttaggtaattcaaaat
atattataaatttatttttctgtaatctatctttccttacaatttgcttactctctaaacttccgccttgctattcttcgattctttttcgataccgataatc
atatttgaaatatgaagttgctactttttataacgattgttgcttgtttttttgaatagaacaatttcttcaacaacgaataaagccgactatccgtttta
cattcaagtttttccttacgactccgaatgcagcggaaacctttctttcatagcgaatgctcctgtcgttgatggaaagggtgcttactcgtaccttg
aaaacgtgtcatcggtgttggctgttccagcagaagatggcagtgtaacatgccttgctttaaataaaacacttccaacatgcttctctggaagtg
aaccaagatgctttcagatgtcaagtgatattaatcgatttactttgagtatcaatgactcttcccatccggtgaaaggtacatctaatgattatact
acaggatttcattatttgcttaccccccaatacaacttttgagtatgtacataatacttcagaaagcaaaaaaaaataattcatctgtgcttgaggaaa
cctttttgtttgaatgccatcgcaataaaagctgtgtatttaatctacaactcagtaaccaaaccctcagcaattgcagtgacgctaaaacctgtct
acaaagcggtaatggtggagcctttgggagtgttgatgtctttttaagtaccattttcaatcaattagggaagacgtcccggcgtacccatgattca
tttatatcacctaacttgaaaacaatttcaagctccaatgctgtaaatgtaacgaaaattgatgtttcagcatcaccttccccttcgagtagctcatc
ttcaggctcctcttctgcgtcctccacgtcccctacgttggcagcaaacaaattacttataacagtctttatttttcttacgtttgctctattcttcgcat
cctagcttccatatgaagacaacaaaaaaatggggtttattttgaagcacataatactaccatcatctctctttaatcctcaatcatccaatccattgt
gctctcggttagaaagggtctatattaatcagaagccatatgtcacctgaaaccttttttgtcaaaattctagcgaaagcctctgctgtgataaaac
aaataagaagggggtagacgaatgatgcaacatgattaaacagtggatgctaatttatgaattcttagaattgatagtgtaaatataaacgaggt
ctaagaagaaattctcgtagcaaaagtatttaataactaaagcgcaaatagatgatttcagtttagtatcgtatatactcatatttgttccattcac
aaaaatcaacttgaaaggtaaaatccttatatactttacccaacttactgggagtctttttgaatcgattagttttgttaatacatttttaagagaaa
tgacattttgtatccataaagattttagtgaaaagattaggttatgaaagtttcataaagattcgagtacttcatttggattcataaatttgaaaggt
aattgaaaaggacacatactaaagcataactccaattagaatcttgtatagtctaaaaaaaatgctagcacgcagtcaacaaaagatgtcctgcc
catactgaatgtaattgttatacagtcttaaccggctttacatagatataaagattgtgagaagcaactaaatcctgctcccttatgttgatcagttc
atcccaggaccattcaagtaagcaaatctttcagtaaaatactaataagtcattgtaaaaatagattttaccttacaaccataattgaagtatttta
tcatagagcagcagcatcgcattctacactctcactattaattgattatatcaaatgtgaatagaattatagtatttgaataacaaaagggaatatt
gcagaaaatatacaagcctatttcatcggttgtttgtaatgccttcatgtgcaaaaaaaataggttggagaagccatacgaaagttacagtcaatt
ttctacttaaaaccttaaaacaaagaaatgagtttgattttaccaggtgatctttcaaatcaaacaacgagatgaaagagcacatagaattgccg
aaggtatcgcaagagtggtggttatcttgtaatattgccaattttctagaatgtcgtttgtatacttaatccgttttaacgaagttccttatagattaa
ttaatctttacttgtacttgaaaagtctcaaaaagttttactgccatattttatgaccttgaatgcaaacaactaagatctttcattaaagtttttaaa
aagctgccactgatgatacagggaggaaatttattttaaaattaaataggagtttataacagtataacagttaaactcaaaaagcatatgccact
atccttttgattctctctataaattagtaggcattataaaattaaaattcgagtcaaaaatatcggaaaacatgtcaaaaaaagtttcgtcattttgt
tcaaaagcattatcttccaattctggatgtgatgaaactccatcagctaatgcttgcaaaagattatgcaacttcatccaaagtcgcccagagtatt
gtttagttttgataattccttgtatagttgatactgagctaaggaaatcatcttgtaaactaaatcctttatctgagcataaatacaagagaaaaaa
ggtatgaatcacttgaattaacatacaattaagccatccgtactcttcgaaaatttttatagagttggttataatacggctcagttgtttcaatgtttg
tgtcaaatttcgaatgagtttctcataattagaaagtgacacaggagatatagcaataaaatactggtataagtttttacggaccacccagaaaat
aactttcaaaaatgagtctcgaaagatggcaccggaagcagcagaggaagaattggactcaatgtccatcaaaattaatgataattttgtctcaa

agtgaggaatggtatcgataaattcaacagcagttgatgaatgtattgatttatatacctccaaaccacatttcaaaagtttgccaagtatacaat
gaaattcgtaaatactatcgtcagagtaattacttttcagaggtgttaatattccatagatatcgtgttggatccaaggtggacaagattttagtac
acaatctaatgcatcaatctcgcacaccgctaaccaagtttttgcttctgatccagtatccgaatccatcttttgaaaaataggatttacaaaacag
gcgatgtgaacagcattacacagatcattatttagcttccgaaattctgtcctttcattacgctgagctcttaatgcgtgaagtcggatcctgtcggt
gtctctccaaagacgatcaacttcttcatcttgtaagagctcatttattttcagttcgacttctaaacagaaattagacctatttgtacctatagacgt
aagataatttacaacttctataggtgcttcaccgaatgataatactgtagatatgatagccgaaaacatgacaagtatcgtttccggaattaccgc
cgaattttccatatcagaaaggtactgttgaatttgtgcttcgtcgaaaatgctgctcacttcctcgagagtatcaaaatatcgctgaattaagcatc
tacatatctctagaggaggaagaagttttcttaaatctcgtaaaatctttatcgtagaaggatttttttttaaatattcatttaaagaatcgacatctt
tgaggaaagagccttttagtttatgacccgttaactccacgaggtttctccaaccaacaaagccccatttcctacgctctatttcaagatgattcggt
aatgatgaggcatcttttagcttccttgttgcttctctatcttccgtacctaagtcgtctttctggctatccacaacgttttctccataaatgcaagattc
attgctttctttacagcgggaacaaggaatttgtcgatcgcatttaaccttacgaagtctgcacttaacacaggcttttatttgggactttttgataaa
cggttttgaaacttttgtaggcattacagactcaatagtttcttcttcggtttagtttagtgtatttatctaaagaacatgttgcatttgaacagattca
aaaaaaatcattccttttattcattccagcgtttccccactggtttttagagtaaaaagcaaacatttgctgttgaaaatttttctgagctaatacact
gctagattacgaaatctaaagataaatttgtaaaacactttatcgtatatactgaaataataaatcaaaactgtaagtttgttgcgctttgttagta
atcaaatctattcaaatgtaagaaaattggaaaagtaaataaaaattgcattgattataatctctgcttttaatatcatttcgatattgacggctata
cgaaacaagaagaattacacttcattttacacaggaaagtcttaatgggtgaaatccatggcactaccggacatattgttctacatttaactaagt
acttaatgcagtaagtcacaaaagtgaaatatatataatgagtaaaagagaaaagaaagtcatcgttgaaataaaatatgttttacttataacct
acttcttaaagatcttcccaacccttagatttttccggttgaaatgttatgatgctactcaatttaactgcattattagatcctcatcattagaatttat
aacgaagagaaaacaattgatgagaatataatcatgctatttttttgaatgctaatcctttttaatatagtttgtacgttaccaacgttaaaaattgat
gtgcgcaaaagcgaattgaatgccattagaaagtccaaaaacaagcctcaagcaagctgaaatgcgttcatttatagagaacctagtatctaat
aaatacaacaagaaactatggaagtaatagaagttttcgtaagaaaccctcccgttagccgttaggggatactataacgtctatatgtgtatactg
ccataaaaaattgcgaaaatgggtataaatgagaaaaaagggactttattactcgaatcacttagttgcactattaagtgctttttacaatactcct
gtagtttattcatgaaaaaaatatacagtcccaatcattaatcgtaacgacaaatataaaaaatcagtaatttgtaaaatggtagccagaatgaa
ggcatactgaaagtaaaattacatttaaagaaattaatattcaatttatcaagtacactatagctttttctttgcttccaaatctgaatttattcacttt
tagcacctagtcgaactttatgtagcttacatatgaccccagaattaaattgtaccgatcatcaagtaaattgtttataaagatgcaaatccttaaa
actacgtttacagaaagagaagaaattctttactagtatatctaaagttttgcatgtaatgctacaaataaaacctgataaaaactgtacttacgtt
tgactgacacagtgcgaacacttccacattctacgataccggagaaatgagtatggtattcttatctacagcgagctccaatattataaaaagatt
gtagtaaaggagaatgatgagaatttgcaataaatataatcctgattacaatgcaaataaggaaatcatcttaagacaaattaaggtgtaatgct
tgagcggtatagtttgacgttcctagtgcaagttgtttaagttattcaaagattctattattgtaagcaagatgaagaaccatcttcaatcattactc
agtaaaggagcaacttaatacagcgcaaaatacgtttacacaaaagaaaacctcgctaactttgtgctttaagaaatgaaagccttcgtttcgttt
cattggtttaagattaatatattcattgcactgactctgtattacgcagataacacatgacaacttggattcgactatattaagtgttgactaaagta
catacacatgaaaaggctggaatcctagtttcattatatctaaagaactactaacataaaaccaaggttaatagatgttgttttcggtgaagctat
aacatcattgaaatgatgttacttctatctataccaatgttgaaaacaaagtttgcttataatcatctttgataacaaaaataacctgtttttttcatcc
acactggccgagctccaccatgggttattgtatctattaaattttctactttgataagattaaggaaaaggggcaattttcatataaacaccataca
aattaattgtttttttggtcttttgaaaacactaaggtcagatttgcatggtcggtgcattattttatgtaagaatccagggatttataaatgtcttctcc
tacatctatggaagtaaatattatggatccagatttcaaaaagtcttcacttagtaacgaagagaagaaatctggctttgccggctctataattgat
accaagatcaccagggtgacttcagaggataatgttgatgatcccgaagaacttgctgatttgggatataaacaagaatttagacgtcagctatc
ccttttttggtgtcttttcaattgctttttctgtattggggttgcttccttctgttgcttctacgttatcgttcagtctttcgtacatcggtacacctggcttat
tatgggcttggctaattgctattttctttcttatttgtattgctttaagcatggccgaaatatgctctgcgttgcctacttccggaggcttatattatgct
gccgctgtttttgcgcctgagggttggggtcctttagcttcatggattaccggatggtctaactatattggaaatgtaattggacagccgtccgtaaa
ctctagtgctgctagtatgattttaggcgctgtcagtattaatagaccctcttattcacctaccaattaccaagtttttttgcttagtcttgctattcaat
gtttacattgcttcttagcttctctccctactaaatacatctcgaggattaaccgaattaacacttttataaacattctatttctttttatagccgcattc
gctattatcggttatgccgcgaaaaacaatggattcgctcatggtacacaacgttggcgtgaaattgacaatactacaaattggcccaacggattt
gctgtgctattatcttttaatggtgctatctggactatgtccggttatgatgctccatttcatctgagcgaagaatgcgcaaaggccagtataaatgc
tccaaaagcaattgtacttactgccgttattggtggtatagttggatggcttatgcaaatccttgtttcctatacaattgttgacgttcatgccctcat
gactaaaagtggtaacatgtggagtaattatttaagtcaagtcatgccgaactctgctgcaattgccattttaagtctgactatattttccactttga
tgatgggtcaaagttctttgattgcatcttcaaggattgcttattcttatgctagagatggcattctccccttttccggatggataggaaaggttaata
atcaaacaaaaacacctgtaaatgcagtactatgtaatggattaataagtttttgtattttattcttattgtttgctggtgaagaaacatcaagcgcg

gtttttccgtaggagccgttgctgcattttcagcctttacgattcctatctttattcgcgtttttgtatgaaagacgcggactttcctcgaggtccctg
gaacctgggtaaatactcaagacctattggtgctcttgcttgtttttttgtggctttaatgattccagtcttgtgctttccaaatgacaaaaatccgtc
accaaaagacatgaactggacttgcttggtttacggagggccaattttcctgacacttatttggtatgccatgtcagccagaaaatggttcaaagg
acctaaggctagtcaggtatttaaacgagatgcatataatcctccgtctataccaaagaccattgaagggaaagacttataatccttttcattgcgt
acgtataattttaaaatttgtagctttagttttgtagatattcattaatggctagatatctttttaatcacgtttgaggaatttaataataaactagtcg
ttaatgatggaatttttatctggacaaactgtaacattcattttataaaacttggacaatggaaattgaagaaagtagtaattcttattttgtttttt
aaactgtgtaagttgtagaatatgagtgggcttgactgattaatatttcagctttgttatagcgaagtataaataggatgtaaacatttttgggca
aataataactgcccaagcaacatatatctcgcaccgatagtgtaatatttgctgaaggacaatattcaaaccacccaaaaacatgtacatgtata
tatatttcaaaaaactaaactatcaacctggttttcataatcagcagaaattgaaacgcaaagtataaaccgactaatcacaagacttgcattcta
aatgtaacaagaaatctgacgttatgagaagcattcatgttagacgttttcattctacatacctagctctataaagggagataaataagaaggaa
agaaagaaattgcttatgaaggagataaagtcaccggattcagtcaccgtcgttttttttttctctctttggttttaaagaaattgagttggaaggttt
gtttatctctgtggtcaatacaatggaagtcccttctaaagatcaacattgaaaacattagtatggttgtacgcagaaggacctatcgagtctttca
tttctttttcttacatcaccaaagtaaacaatctacagagtcggaaaagcgaggtcttatcctgaatacacaactcacacatcgcgcggaaacgcg
aaaaaaatgcagccatcctaaagcaaatcttccagatcgttccaaagagttcaagttataatcaacccaactggcaggaattgaggaaaaaaag
aattggctgcactaatttcgcatctcttacgcaacatatcgttcagcattgaatctccacaaaaagtaagaaacaatcttcaaaattcgcgtttgttt
acacttttatataaaacaacggaaaacttttcctatgacttgatcgatttttattttgaaagtgagtaaataactcttatagcatttagaaagctaga
gttattcacttttcttattattttttatataccatacagcattaatatacattaatatacattaattggaaactgcagacacaatagctaactgctttt
cttgaaacttttaatgttagattatctaatatagcctacaaattatattttgaatcaaaataaatgtctacggccacacctaggcgaaaacaccagt
tcccgtccgatcactgcagttaagcgtctgagggcctcgttagtactatggttggagacaacatgggaatccggggtgctgtaggcttcttcattaa
ttttctttttaaaactttatagtaacagccggttactgcaccaacgtcagtaatataatgcatccttatagttattgaattttagcgaatataagctga
tattcataagcataaggaattgtcgcaaagtacgcaagtaaataaacaaaaaaaaaaggttttacagaaattgtgaacacgaagtcggaacaga
ttacgattggtagattagagaaaaggatatagaccgagagtcaacagtcagaaacttccgactggaaggttcgaactggtctggctaaataaga
ctgctttcaaacgtttcattttgttaaccgtgtccctggacagtactagcgtttatacacatagtactacttagtccaatctacgtaataaagagata
caattttacgctcgaaatacattataccctggcaccgtgaaagctacaaagcttcgtaattctatatctccgtaatagtattctattcatattttatttt
tgacatactaagggatgcttagagatatcactggtcttagctcatgataatagctaacggtattgaatgtgtaaaaaagtaaaccaaaatagaaa
atgcctttcgtgttacttcctgtaagcgtatatacaggttcctctggtatagttcaatcaactgaggatctttagttcgttcgtgcattaaatacgcaa
gtactcagtgagtagcaaaaaatatgaccgtgccaagactcgaacttgggaccgtctgcgaactcttagatgttaagcagaaattataaccatct
aaaccacacggcctaatacttacatattatgcacgtagtttttatatataccagtgcatttattactatcataagctaatgccaacattcgtaaacta
actcctaaaaatacccaaagtatataagtttatttctgacaatggaaaagttttgggctgttgcaatgacgacagcttgaaactgagtaatctccg
gaattatttatagtcacacaaatcaccatgaaattgtccagttcgacgaaaaagcaccattcatggtattattttctaggttgctgataataagctc
cttagtagcatttagataggtaaaagtgtgaatttcagtgttctgcatcagtactctactaggtgcctgggataaacccagtaccgtaacgttagcc
aagggaggctgaagatgataatctcctaccgaattaatagacaattgatttgatttacaaagtaatttgattgtcaaagtactgttttgcgtgatag
actcgccatcatcaacgtacatcgttccttccgagtattcattatcatcgagggccaccaaaagattaaacgggttttgacgtgtttcaaatgtgtta
agtgcgggcttttggaggggaacaatctttccacctcgaatcgcgacattaatatgctctaagggagcatatagtgtaacattttcgccatactgtc
gatcaatgactgtatgattgtaccaatcataccacttagtactagtatttccagggaaaacaccttggacgtgactaacgtttggctcaagtactgg
tgtaatcaaaagtgcatcacctaccataaattgtctatcaaccccggctaaattttgttcattgggaaattcaaaaaagagtggccgaatcattgg
gaacccctcagtatgagcatctttcatcaaagtgtaccagtaaggtagaagagaataacgaatgctcatcgccctcctggaagattcagcaacag
atccccatctatatggttcttgagaaatagaaccgaggctattgtgatttctatagaatggagaaaatgctgccatagccatccatcgagaacata
attcctcatctgtattgcccataaaaccgcaggcgtcagctccaaccattggaaggccaaccaagttgaatgtgagcatacccggaatagaaaag
aacatgtttgaccactgagaataattgtcgcctaaccaatgagcggcatattttccggacgacaaaaatgtcgatcgacttaaaacaaaaggccg
tttattctcgtatatagaagtcaacgcgttgtaggttgcttttgccattccatatccatatatattgaaaagattatatcttaaagtaccgttataata
agttgcatttgcactaactccttcgtcagctaggtcgccattaccctgttcaatatctagcgcataaggtggattattaacattataatccggcttaa
cactcaaaggtgtaggtgttgctttaactttttctaaagttgaggtagcagtagcatgaaattgagaaagagaagacatatatacggatgaatatt
cagtagcatttgtaatattaaagccttctggatatgaatagacaacgttattggtttcaccctctaaaacgaaggcgacgtgcacaggattttcatc
caagtgagctgaaccacatgatccaacacaaaaagaagtcggctcattcatatcaatccataagccagtaaacgtaggaaacccactagaatta
gctccaaaatcgtgagtgagattaactaggcaatctttccagtattcgctacttgctggattagtaaaatcggggaataccgtatatccgggccaa
acatttcctacataaattgacccgtcgggttttttataaagacgtcattctcaattccgttataatatgggtaataggaatcatcacttctattatta
ggattagcagcatacactgcggcatcaatgagaggaacatagtgttggtgagtggactcgatttctttaaaaaactccaacatattatctttggaa

taagaaatcggatcaactgtaaaatccctgtacatatacatgtaatcgagatcattccaaaatgtatcgacaggtatttcaaaatcggaaaagctt
ttacgcacatctaaaatgtcagagacgttagaatatccccacctgctgatatggaatccaagggtccaatactgctgcatggacggtgttccgata
gactttacaaattgagaaacggtttttttaggatcatctgaacctccaacgtaaatatacagatcgatcactcccccaatcatacggtattgtaaat
aatttggacgtagtaagacttccatgccatttgcggtaagcatgagcacgccgtgagaagaagaatggtatccggtttgattaactggagaatca
ttagctgcagggcggtatctttgctccaaataaaatggatgagtaccataactattcccatcaagaggagcattatcaccattcgcccaaaaggtc
ctagttatattattaccaagtcgaaggccatggactgtctctgcgagcccatataggttgtaatcatcaaccatatttgtggtcagctctacatattg
gtcttggaaaatgagatgatgaccacgagtgtcaaataaaagttcgttatctgtatttctgactacccagaactcaaagggatctgaattataaaa
gaaggtgtaagagcggtttgaaggatacgtcaaatcatggtagagaggagcatcccaaacgtcggaatggttagtaaagcggaattgtttacctt
caatatcagaaatagaaacgtggatacggtctactgtgtcataagagacatttaatagtaagtgggagtaatctttgccataagaatagcaagga
gatccacgaagttcgagagcagcaattaacccatttgctgattctgagacatttaaagcacgataaccaggacaggtactaccacgataaggctc
attaactgaaggctgttgtggaattccaagagtagcaacagaaggtgtcggcaaagccctataggttgttgagctagtaggcaaagtgctcgag
gtaattatggttccagttttggaagcatcgagtccgaaaacaaaagaagctttgctaaaacatagcaagaaagtaaagaaagacacaataaag
gaaaaaggtcgaggaagcattaaattaaaatagatgaattaaagaaggaaagtagaacaaatttaaatacataaacgagcgaaatttcagag
tgagattctcactaaattagaatgattgtcaactgtcactaaacaaagtgtggatgttaaaagaaagttttaaaagaagcgtaaaataagaatct
ttcaagacaaaacgagggatgccagaataaaagcacaaaagcctacgaagagcttgggatgttaaaatcaaacgaagtttttaagcaatctgc
aggattccaactgttcattaatatattcctggtaaacttgatttcgtttaatctcggtcataattgagtcacgttcagcgaactcacatcgccaatagt
aagaggtatgccaacctaaaacgagtggtaaaagcgaatcatatctatgtcatttcggcggaaagcaatacaatgtttactttaagcttactgtta
aaggtaaaacttcgatggtaaagttcaaatgaatggaaaagtccaaagtaaggttaaagaatataggtcgaaaacactaaaatttaatacataa
aaaggttaacaaatagaatggtgaatgcttgcttttaagtgacattgggaacaaacaaatttcgaaaacaggaagtttaatttacaatttggggtt
caccccttcaaaataatgagtaagtgaatatctctttataattatataaatatatgtatcgaaggctaggagaaatcgattagaagcgtaaaagcat
atacatgatagataatagcatattactggtgaatgaatgattcagtcaagtccaaagaaagaaaggaagcgttgctgctgtaattgtaaaagaa
gataaaatccaagaaggattagaattatagaatagggattttgagtgttaattccaatgtgctagatcaaactataaacaactagttacaattatg
agtttaggaaaccgacttaatttactgtattgccagtgctatgaaccacttttcaatgcactaagtaatttattgaaattgtatttttcacagctgtt
accatttactttacttactttgctttattttaatttatcattattcatttgtagtacggattccacaaagcggcgatgtttaaagaagaatcatcctatct
atttgtagctttcatcccacaaggaaaacctagaaagagtgaatcatgtactcagttaatgagtccccaagtccaagttccgtaatattgatatca
aaacaaccacattgtctacaacaaggaaggactcttttatttctctaatttactttcgtacctttaaaatcttaagagttacccaaattaacccaggt
tattatgctcgtcatacattttatattttgagatgggtttagcattttccgtttgctcataactgactagaccataaatatttgattagtccatagatag
tttaaagtccgataagtaggttggccgaaaattagtttgccggagtgagtcattattcaagtttaactattggtgaaaagatggcgtcaggatcgtt
acatccgtccttaacttacaccttggatcaatatctaagataccgagttcttgagcaatcggaataggtaacctaagtagtagataataatgggat
aaacaaagaaaaagagagaagtaattaaagaagataaagaagccgccttctggactcaatgcttcttcattcatgtcccttcctttcccttcgtca
cttgcttttcattcataatagtaaaacgacaacaagaaaatcatatccagcgacggcattagtttgcttctttagcattggaaaaagacatttcata
cgattttgatttggtttgtaagaacgtcttttaagacagggaaataagtttcggttattcatggcaatgaaataaacgaatcgcataatgaaattta
atagaaccctaaactgttttatcctattcttttgttttgtattgtatttcattctattttattctattctattcatgaatcttagtattcgctaacatctttct
ttgaaacttgaagagagtaactattagtataggctgaattgaccagcgtttgaatgaatttaaacctgaatcgtaattttagattcggtatattctta
aaagcaattctcgtaatctcatctattcatctattctcagtcatataaaattttccgataaactgataaacatagtatgatccaacgctatctatatc
atgtatataccaagacgtaaaagacacataaattatgatttgcggcttgccaaataaacgctcactagtggtttagggaaggtgacaagatcact
gatatctatacgttatagaatagataaacagaacaatgtaatttaataaaataaaaggaatatgaagagctctattcaatgaaaatcgttcctttt
ctgaacaatcctttcaagatgactgaaaaagagtcagaaaaagcaactgtgaagtcagactctgaagagcccattcaggctgaagtgcttccca
tctcgaatgttcaatttggagaaacgaaaagagctttaaaatctagacatgtacaattaattgctctaggaggctgcatcggtacaggtctattcgt
tggatcaggccaagcattgtctcagtcaggaccggcatctttgtttctttcctatgtcattatgtcttttgtaatttggtctgtgatgaatgttttggga
gaaatgaacacttatcttcctattgctggagcctctccacctttatatgttgctcgctgggtggataaatcattagcatttgctgctggctggaattac
tggtacgcttacgtgttcttagtggcatcagaaatcacagcagctgcaatcatcatagaatattggacagaatcggttcctgtagccgcttggattg
cgatcgtcctctttgtgttaatgctacttaactcgttcctagtaaaatggtacggtgagaccgaattttggtttgcaattgtcaaagtcattaccatta
ttggtttagttatccttagcattgttatctttttggtggtaccccgaatcatgatcgcctaggttttcgttactggaaaaacgggttggcttttcgtga
gtacttagtaaaaggaagctcaggtcgcttttgcggtttttggagcgctgtcatcaaaagcggtttctcttttattctctctcccgaattaatcactat
agcttctggcgagacagaagctcctcgtcgaaacatccctaaagcgacaagtagattcatctatcgcttggctttcttttatattttgggtaccttat
ccatcggtgttattgtctccagtcaggatcatcgtttgttgagcgccgtctctaaaggctctagcaatgccgccgcttcaccatttgttattggaattg
aaaacgccggaattcatgtgttaaaccacattattaatgcggttattcttacttcctctatctcttctggtaattcattttgtttgccggttctcgttca

ttatattcacttgctgtcgagggacaagccccaaaaatattcaaacggtgcaatagatggggtgttccatatgtttctgttttatttaccattgcaatt
gcctctcttgccttttgaattcatcctcctcttcagctatggtctttacatggttcacaaatctctctactatttctggcttccttgcttggatatgcatc
cttatcacttatctgcgattcagaaaggctatggttaaagaaaatttattaaatctgatgccttttagaacttcacttcaaccatatgccacctacat
tactcttttccttgtttccttaatcaccataaccaatggtttcagtgtctttgttggccattcgtttactgcagcaaattttattgcggcttacgttacctt
acctattttcttagcattgtatctgggacataaactttggacgaaaaactggaaatggttcaaaagaattgatgagttggatgttaccagtggtttg
gctgaagctgaggaagttgaaagtagatacatactcggaaaaccaagaaacttcattgaaaaagtttggatgtggattgcttaaacccaactccc
tccttttctcaaggtcatgcaccaaaatctactattgttgtcatgtttttcttttttttacttgcaaactaatgataattatttaatgatataacccccctcc
tcttttgtttccaacttttaatcagaagtcgctatcgtatggttttgctcaccaccgttttcttttgtcttttctctatttttaaaattcgaaggaaattac
ttagtttttcgaattatttaaacttgattattttttttcggccgctcattaatgaactttagaaatacatttttgaataaatggcttaacctactttcatta
aattcaagtgatactaaagccatttcgcaatatacaacatgttattgtagtatttcagaaaagaataaataaattaaaggtaagcttcgctaaaag
tcaatcagaatcatgatatataatttttggttcattctgtcagatcagatcatatgtgatttcaattataattgcattacttcaagcattagcctcgtca
tcaatttcgtctaatgattttcgagcggcttctctcccgcttttagtgtttcttttatttttcatggcagctttgtatccaccacgtacgtttccaaggtttt
tggcttgggcttcatcattttcaaacaggtttgaactctgaaatccttcctttctcaattcggcttcatcatctcggtcagctttgtaaccacgcgtggt
tccagtagtataatgagcttcgtcaccgtgactatctaaccattcttgagctcttgctttggcctcttcggaaacactaggattatgcaaggcagcct
tatggccggcaatttgatgtgctaaatctggcatattggttgaaaaaaaagttgattgtactaaatagcgatgcaaatttcatttatatatgattctt
tgttcaacatattctgaaacgcttaataatgactttgcttttcgtaatttattacgtctattataatgctttacctcaactggatttcggaggcaaagat
cgattttatggatggcaaagctcgcgacaataaatatgactaaatacttgatttcctaaattaatactatagaagattacaagattgggcaactat
aggaaacgaaatgataaatcccgtcatacattgctttcgagaaatcgttccgctgcaaccggtttttctagggacggactatttggtatgaatgaa
tttgagcaaataaaactaaaacttgacctaaatgtcgtaatccctaatcactcaacacgagttcatgtttacgatagtaattcttcagttaaaaagg
aacagtttgtggtactatgagaagaggcttttgaccttctaagccagtcttctttctaatgcttttgacataagtcgaaagctatgaaaatatcttac
acttcagtagcaatacgactgcctatctgtttactgtatggtagaaacaaatttcatttggtagaatatgctaatggttacgaaaattacgatgtttt
tgtatatttactataaaaagaccattaaattcagttcatactttggttggaacataaataatattcatttcaaaaaacataaatgctcgctcaaaat
atatagtacatgatattatcgtatcaaaatatttggttatccatgaaaatcattatgctttacttttagccagaaaattaacacctggctaacatgca
attggtttttattccaattcctcaaggacttccttggagtgttccttcgtttcttcagaaacattggggttgtgcaaagcagctttgtaacctccagct
acgtttccagggttcttgggatgagtagggcttctagagaagtcttcgtgttcaagaacatccttagagtgttgcttggcctcctcagagacattgg
ggttgtgcatagcagctttgtagcctccagcgacgtttccagggttaggcatcttgaatcaaataatcaaagtcaaaaaaaataaaaaccgactt
aagttttcaataaaatttggtagcacaaactgtgttttccctgtcttatataattttccattgcagaattatagagcggtgaaatcatacttgaataa
gaaaattcctaaacttctgtcattctgacttgaatagtatttcacttttggtgaaattattgaattgcgaaataccaacgtaattctattgtaggcgcc
actttggagattcatttgatttcagaaagttttcctaaagtcctcgaatcatgcccataaaacctgtctatagactcagtacacaatatagggatca
ctattcctttctgcaatgtattgcaaagaatgtatattgctcattttgacaaagaatattatctgattttgctaatcggcgtattacccacataattctt
cgtgaacgagcgaatcaggaagattattctattgagaatcagaattagaacttcaatatatcatagtgtataagctttgtaacagttaatataggt
aatagtcataaacttgtttcctagattctgttagcatcaaatgtattataaggtaagaacttcatatcatatgtgcttgtttgtttgccaaagggaagt
aaaaccaatatatacaaagtgctttgcactaagtgggcaattgtttttcgttaattgaaataaatgctcatctggtaaattatagaattgtcaatca
atcactgatatgtattgcgctcggctttctgtccttctagaaagtctcatccatagaatcttagcttgctctaattctacctctttgtaactgacaatgc
cattctcgtcttgttatcagtttttgaggccttacagaaatgtaggatccgtgtcggatgaggctataatatgatagacgttttcagaaatttagatg
aggatataatttcacaaaacttcttgatacgatcctcacgttcattttcttatggaaagttttgacggaaatcgaatccttgaattatttcaaaaaac
cgactaaaaacctattttccagtaaaattcaagtgatatttttagtcacgtccctaaacattcagaaatgaacgttatagacatgtgtttcttagca
tatttgtatatttcataatttagcaatctcccaggagttacatctaagtaagcttgtcaagcactatagtttcacgattatttaaaactaaagcttgtt
acccaggtttacatgaacagtttttagatgtgtatacgtttcaatgcctggagctcctaactcactgccaattccgctcattttaacacctccaaaag
gagcctggatatcactattttctgtggaattgataaataccataccagtatctaattcctcactaacgtgatgagctctctcgagatctttggtaaag
cacatggctgccaatccgtatatgctatcgtttgctttttctaaaacttcatcttcagtcgtgaatttggatacagcaacaacgggtccaaaaatttc
ttctttcacaatagtcatgtcttgatgacagtcggcgaaaatggttggatgaatatagtatcctttttttcgaggtgacctgttcatttcccatcaccat
tctagctccttcctttttaccaacatcgatataatcaaggactctattatattgagctttacttacaacaggtccgacaaccgtcgatttctcaaatgg
agatccgagcttataatctgccaaaacgtgctctttgaatttctctaaaaacttttcgtatacttgttcttgcacatatattctagagtttgctgtgcaa
atttggccgctgttgaacatgacgcctaccgcagcccattttacagcctgatcaatatcagcatcctcgaaaacaataaaggagactttccacca
cattcaagtgttatagtctttaaattagaagaagccagtttttgaattgtactgccaacctttgtagatccagtaaatgcaattttgtcgattcctgag
tgagaagcaatgtgagatccagcgaccgtacccaatccactaatatattcactacacccttaggaaatccaacttcatcaactaattgagcaaa
gtacaataaagataaaggggtagtttcggcggacttaatgatgatacagttaccagcagctaaagccggagcaatcttccagcctgccatattta

agggataattccatggaacaatttgaccacaaacaccaactgggactcgtttagcgtatgcaagtttttcggggcctgttgggatgacagatccat
agattttatcagcccatccagcacaatatcgaagtaaagcaatcgttccttccacgtcgccacgagcattcgaatcctttggcttgcctgaatctatt
gcttcaatggcagatagtgtatcaatgtgtttttcggtggcatctgctaatttcatcaacagtgaaccccgttcggaaccgtgcaccatcttccattc
tttaaaagcatttcttgctgattttactgcgagatccacttcctcggtcccggctaaatagacttttgtaataatttcttcggtagcaggattaactgtt
tcaagtacttctttggattttatccactgaccatcaatgtataatccaattggttgtctaactgtattaccatctggaagttgtagttcttggaaaagtt
cttctgacattacaaaggagcaaaggagcgagctgggtatactgagcaagggaaatttaaatagaaaaggacagggaatcctattgattccaat
atatactaaaaccttttataatccatgatgtaattttcagtaatctttgagatacttttatatagagctaaagctagcgcccttaatggctggagtttc
gcaatttttgtgctttccactcgaggctacagatagcgttcatccagtgaaactaggttgtatctttctttgtataccctttttttattatagtataattaa
atagctatgataatcttaaaccatagtggaagttattcgtacttttgtgttgcacctgaaagtcgttagtttgctaaaggaagttcccatatattaca
gcttatttttcaagtatttctatttatcaacttctttatatatcatcctaactcagtaaaaatttgcggctgtaaagtagcgtaaacattgatatcgcct
gtttattttaaatatgttaaggacttttgtaagcgtaaagctccctcccgataaaggttttgtatgcctagttccttggagggggttaaataaactcc
ggtaaaatagacctaagagggaattcaaagatgcaatgtcttgtttacaaataaattaggcgatatttaccactctgtgaattagatcatcgtcaa
atattattctggatgacaatgtgattcgaaacgatcccaaggaagatgatatgtatttactataacaatacttaattctcaactaacctttactcttt
atcgtctcttccaaaagcataaaataaaggtttcattttgatatcacaaactatttggatactttcctagttacaaagtaaagtaatgtgtgttgaga
agcgaaaaagaagcctcaaaaataaagcaaaatccatggcattcatcgtattaccggaagaacaactttattatcatttgctaatcagttgacttc
actacgaaagaaaatataattgttaccacagtttgcgcggtagcttagcataactctcttttctatccatgtaaagcattaagaatcgaaaggcaa
cattgactaccatactattcaaaccgatatttattagcctttcaacattcgtcataaatcgaatttcagatcaaagaaacaggacgtctcttttcttct
tttttgggatcaaattgcaattccttgccataatttgcgaaagtgttataaccatagttgttagtagcatagaacaaagaacgaacgtaaggagta
agctcaagacccttctttaatctatcaattaaatctggatttgatgtaaaatatctcgagaatccaatcatagtacggttgtcaccattgacatcagc
cttcaataacttaaactcaggactgtcataagtataattaccactctgcaaaatggctcctttccataattttttataaagtcgttggaaccaacaa
tgtcagatgtgttgacatccaagtttccttggaccctaggttccacaagggaaagataagcaagctgtttaccattgtttgcacgtttttgaagctca
ttcaccacataactaaatgtggtaataggatgcacagtatcttgttcagctttcattccttggaatttggcccaaggagacaatctgattgcaagctt
ttcagcaccaattgtatcgcttaaaaggtcgataatctcaagcacgattcttgcacgcttctcaatagaaccaccatagttatctgttctttgattcgt
agcgggctgtaagaattgatcaagcatgtatccatgtgcactatgaatttccacataatcaaatccagcttccatagcattgattgctgcattcttgt
aatcttcataaataatgcccttgatttccttttcagtcaatgctctcacaggattgcctgcagcttctgcagccttcttagactcttcgctttcatataa
agcagagggagatatcaaatccaagccatgctttttcaaaagctctggggatccaactcttcctaagaaccaaaattgacacgccatatagcttc
ctttagcatgtactgcatcagtaatctttttccatccttgaacatgtttctcattccaaatgccaggaatattagggtataagccagcgcgaggaga
aatgaaagtggcttccgtaatgagcaacgaaccaggagcttgtgctctatcagaatagtattgtagcatcaagtcgcttggagtatgatcatctgc
agcacggaatcttgttgtaggagcgaaagctaccctttgatccagtgtatttttaccaatagtaatcggtttaaataaattcgattgttccaatccga
cagtcatcttttataaaaaatgtgtgaaagcgtttgacctatcttgtcgagcttgcaagtaagaaaatgagcgcttactttctttgggatagtgttgc
ttatatttatatattttcggtaacgaagagcggagcggaacaaaaatctttttgattgctagcttactaatattatttgtcagcggaaacgattagta
agtcgtacattaataatgaataaacgataaggttgaaataagattttattaagcgaagttagctaccctttggaacgattattgaaaagtgctttat
cggttctaatacatagtggatttttaaccgacaaatactatacgatttaattacttccatcagacaaagttcatacatactttattttttttattgaaa
agttaatataaactgtgccatatctattttcttgcatgtggataattccaaaaaatccaggataaaccaaaagttgaactttacaagaggataata
tatattcttcatttacatacacaaatcaacaggtatatgctgaaacagctaaaaaattagaattgaaacgtagacttgccagtacatataaaatac
agaatcaccagctctagcataatgaattaagcaagagtatcaaggtaatgtttttcaccatcaagtccttgcagtcgcacagcggcttgttcacta
gtcaaatctctttgggcttcgcctagcatttcgaaaccgacgcaaaatttctttactgaggctgacctcagcaatggagggtaaaagtggatatgg
aaccaagaattgttgcattcatcttcggtaccatggagaggtgcttggtgaactcccattgaataggggaagcttgtacaaaataaattatcgtac
tttgtggttattttttttaagattgaagccaaatcttgaacatgcttttcttggaagtcaattaacgaacacaagtgctctttcgcgatcaaaagagtt
tcaaaaggccataaagcccaataaggaacaagtactatgaaggaatcattctcaaggacaatcctttgcttttcttcgacctcgagctcgatgtag
tctcccaacaagtgagtaccgtttgaattgaaatatttagtcatattcttaatttcttcaccaacaattgtgggaatggtatctaagcaccaggcctg
accatgaggatgaggattcgagcagcccatggcggaacctttgttttcaaaaaattcaatatatttatagtagacaccttcagaagactcattcttc
aagttaaaatagagctgtttccaagtttccacgacatgcataatcgcatctacttccatgcgaggaagcgttaaactatgattggggctaaagcaa
atgacaaagcaattgccttttacgctttgagttttaagcaaacgagactttaaggagtctcttttctctttagtatctagttcttccacataatcaggc
tggtcaattttgacagctggatagtcgttgcgaaagacataggttgtcatataatttggattctttattcccattgccctttcatttccaggacacaaa
tagcactttggatcatactccagtaactcgttcttcttaatttcttcttgttcaccttgccaaggtcgttttgcacgatgaggagaacacagtacccat
gtatcatttaaaggattatatcggcgatgagaatgatctgtaaagtcgaacttcatactcatccttgagattgtagtattatataattcagcaggaa
gaaggtaccttgaggatcagacatttcttcatggtggtttcatgatgtaggttacataattctttaccccaccgttcactccgcatacacaagcctca

ttataagcgaaacagcaaatttaacctaaaagtagtcggagagtttgcggagtgaagcccttattagatcaggggctttaagtcattgattcgtttt
ctaaggaataatacatcttggcttcgatatctatgtcctttcacaccttagtaccgccatgcattgtcttatcctttcacctccttcttccagtcgggcg
ttagtcctgagtattttctgacgttttacggagatatatcgatatttgttttgagctttactaattacattattagctgtgcacaagagaattaagtcg
acaaaagcacgggtgaacgtttagggataagggtatacggtggagtatatttcgtaaacaaagtacagaaaggacgaatttaattatttttttttc
tttcttggttcataagctattaattttgtaatcaacacgtgttcatttattgcagggctaaagctccgactgtgcgaatttttttgttaaagccattaaag
gtatctaatatgaccgtttgcgatggttacgagcagcatgttttggtaacaggcggggcaggattcattggatcccattgcctagacttccttgtgtt
taaatatcctcaatttcattttacatgtatggatacattaaactacgcaagcgagaacagcacgaaatttctccagaatgttttgggaaggccaaa
tttcaactttattaagataaatttggtttcagaatatgctgccctccaaaaatttatcatcgatgagtttccaaacacgcggatcacacagatcatca
atttcgctgcagaaacatgtgtcgatcgctccttctcagatcctatcttctttactaaaaacaacattctctgcactcaacatctgctagagtgctctc
gacttttgctcgatcgctatccccaccttcgtagtgtgctcacatttatgcacatatctactgatgaagtctacggcgaacaggaagatcatgaaaa
tgttgatgagctttgcaaattgaacccgagtaatccgtacgctgcctccaaagccgctgttgacttaatcatcggatcctatgagttgtcgtataatc
ttcctgtgacaattattcgagccaataatatttatggatcacgacaagatggagaaaaattaattcctatgacaatttctaaactacaccggtttctt
caacagaaaagtcacgaagtcatgtttgacaaaattaccattcacggtgatggctcacaaaagagaaaatatttgcatgtgtatgactttttaacc
gctcttgatttaatttggtcgaaagaacgaatgaatgctgcctcttataatcgtcatactaatcatcaactttttaatgttggctctgaagacgaaat
tgacaattattctctcgtgaagcgtatatgcgattacttcctccagaaaaaatttccttcgaacaactttgactattcgaagtttatttcatttttttaaa
gacagaaattataatgacacaagatattctttaaattatgaaagactgaagcaatttggttggaaacctcaaatttctcttgatcaaggaattaaa
gaactggttgacgaagccttctaatctgtgtccttatgtcctttttttatacattgctaacgttcatcatgctttcataaaccgaattcctaaaatatcgg
tcacttttttactacgtattcttgtttgtctagaacaaacaaaggttatttatgagttatctccttaacatttcattcccgtttttgcgacatatcaactga
acgtactaaaattatgcatatttaaaaattcattaatcttcatttccatatttaacatgtgaaagaaaacattaatttagagccataaaactctccct
tgtgaagaagaacttgtttggaccagggcaaataattgattgcacttaaatagcgacctgcttcaacgcagaaaccggctctagctctaaaagga
tcaatatctacgttgtcccctgtgtatagttgaaaagccggttctgtagatgccacggagattttaattgatgtggaagggtgacttgcttcagcaat
tgccttgagctctctgcttcttgtatcaagagcaaaatcgctttcaccgactataaaacaattatcaaaagaaacatccggtacaagttcaattgac
ttggtcaatggctgggttgattgagtgatctcaccgcttggtaaaagagtttttggatctaccttaaggtatttatcagaagccaatttcaatatagt
accatcaatggtttcaccatcagaaagattccaataagagtgattagttaaacttatagctgttgcatcttttaacgagtcttcacaaatggaagat
tgatactctatttcaatcgagttattgtctaccgtatacttgactacggtctctacatcagcgggaaatccattgttaccatctttatccagtagaata
aactccaaggtattagaatctttatattgttttgagattggacccaaaaagtattgcctgttaaatgcattccttccaccgtgcaaggtagacccgtt
ttgatttttgtcaaggttgtatgttgtcccttctatttgaatagatccatttgcaatgcgattagcataccgaccaactgtcgagccgaaaagaggat
tgtcatcggatgtataaggcttaaattcttggaaaccaacgaccaagcctcgatctttgtatcggacatcttgtattaatgcgccgtaattagcaaa
ggacacctgcaagtcaggaaatttcacagtatgcaatcggttcttataattgggtgtatcggattcattaaataatttccactgataattatcaactc
gaaagccaaaagggttttcggttgtccacttccaaaaatctctacatgcatcttcaacattcagggctgcattccatttcaattcttgcttggcacgt
gttgggttagctgttaaattaaggacatcaccatctcgtctgccgacgacttcgtattgtagttcagtacctactgttttgcagaacgctttataaata
tcaaagacagtggatcctttaccagttccaagattccattcacgatacaagccaatatcgtgcttcaagttattcatatagtctaaagccgcaatat
gacctttggccaaatcaacaacatggatataatcacgaataggcgtaccatcatgactttcataatcatttccgaaaacataaagcttttcacgtct
accaattgctacttgcgcgagatatggtaataaattattagggatgtccaatggatcttcaccgatcaaaccagagggatgggcgccaatgggat
taaaatagcgtagaatggcaccacgccatgtccgatcgctggtatgaaggtctttaatgagattctcgatggtgtattttgttttaccgtagggattt
gtgggatcattcgggcaggtttcgggaatagggatcatgttttcaaatcgtgttgcatctccatagactgtggcagaagagctaaagacaatcgtt
ttaacattgtgtttgcccatgactcgaagtaaggtaatggatccattgatgttgttgtcataataatcaagaggaattttggttgattctccgacggc
tttaagagcagcaaaatggataacgccttggatgttgtgagatgagaagacatgactcaattgcttttcgtcgcgcaggtcaatatcataaaaca
aaatggattttcgaacgagaaactcaatgcgggccactgcttcgtagctagagttacataaattgtctatgatgataacattatagccatggtgga
tcagttctacgactgtatgggagccaatgtatccggcaccaccagtaacaataatgtaagaattagatcccatggatatgaattacaaaaaaata
aaaaaattaaggagttttcgagaagacgaatgatatggttttgagtaaaaagataacaattgagttcttcccagataagaatctagctttttactc
gcaaatgatttctcaattgcaaaggttattatgttcaagcttgagggacttcctaacttgctttgatttttaagggttttctacgtccatgcgcccaat
atttatacttggttcgtctgatggtacgatgatgtgtgacaggagggttacgatgtgatccccgatatcagtactcgaagaaggttttcgcgacggc
ctctctccagtttctccaatttaagctttgcttcccccttttcccccaatcaatcggctgtatgctaggcttaggttttcggctgaatgggtaacaat
ttgaggatgagattgtcaatgtttcttgccgatcatttcagtgatgacacaacatcatccggcttaggttttccaattacaagcaatgaacattcctt
aatttatacatatatttatgcttagaggcaaagaaaagctatacttaggtctgatttctgcgaatctaagaagagagaagaataagaaaaactag
cgaagaaaggtcgacaaagagaacaccaaatggctacaatacctatatatcataatttatcgttttatactgatcctgaaggaaacaaagctcgt
tatgaaagcttgctctcaaaattcaaggaggaatataaaaggggccccagtttttttgagtcgttcgcctggtcgagtcaacattatcggagagcac

atcgactacaattatttttcggtactccctatggctatggaggtagatgtggtggctgcggttgcggtaacggatgatggtaagatggttctgacga
acacggatgaagaatttcagaaagaacagtttgatttaccgtcagacggttctgtcatttccattaacaaggaccagcacagttggggaaattatt
tccgttgttccttaatagtggctcataagtttattttagaaaaatatccaaatctggtgcaaggtggctccaaacctctcaagggcatgagactaac
ctttgacggaaacgtccctacaggcggaggtttgtcttcttctgctgctttttgtgtggcatctatcatggccgttttgaaggccaacggaatcaaca
cagttaccaaggaagacttgactaggattagcgttgtttcagagcattatgtgggggtaaataccggaggcatggatcagtgtgcgtctatctatg
gagagagagaaaaggctttgctaatccagttccgtccaaagctgatcggtattcccttccagatcccttcgaccaaaccagaggaaatggtctttc
ttgtaactaatacgcttgtgcaggctaacaaacacaagaccgctgccaccaattacaacttgcgagtagtcgaaatgaccgtagctgctgaaata
ctagccaagaagtacaatttaacacttcctaaagactctaaccttaaaacagcagggacattacgaagcttcatggacacgtactatgaacaata
tttaaaacagcctccttgggatggtaacgacatcgccgttggccttgagcgcacacaagaaatgcttaagattacagaaactttttttcagaaga
agaaaaagtgggctttactactgaacagatggccaagaagttggagatgtcggtggaacattttaccgaaacgtttctaaccgcaatgcctgttc
gttatgatttaatgaaaatatatcaaagagccattcatgtattctcggaatttttacgtgtattacagactctcaagctctttcacgaacataagccc
tcggataactcagaaaacttcttaagggcgtttggaaaacttttgaatgattcacaaagatctgctgacataaataataattcttcaagtccagaa
ttgcgagaagtatgtgcgatttctactgccaatggcgcatatggtgctcgaacaaccggtgctggttggggaggctcagcagtacacttaaccact
gttaaaagactatccgaagtgattcgtgctttaaaaaagcaatactataacaaacattttcccaacttgagtcaggaaggtttggacgccgccatc
gtcgtatctaagcccgctatgggagcttgtctggtggaattgaacggtcccgttttgtaatacaaccgaataaattgataaatcacttctaatagtg
cctatgataacaacagaaggattgttgcaacttactaagggattctttaaaatttaatcaaaaaggttaaaggtaagcaataaagtcagaatatg
caaggaaactttataaaaaaataaggttatatgttttatggtattttctttctcacaatccaaattctcaagtcttcattcaagcagccagtttcctta
tgacagtctgctctgttatcaatatattcttttaaataaaaatttaagcaagggaatatcaatttgtgaagtctaatatctcaaccagtgagttttggt
tagtaaggaatgccaaccgccttttgctaacggtaaacggtttacgattatggtcaccgtgtcttggaagtttaatacatctgattattggcggtgtc
cagtaatttacttgtttcttcatttacgtcgccgttggtgacgaaatactgtttcgaccagttgaaattttgactcttattgtccctgcttattgtaatct
aagaacagcagtatatatgatttcgggcgtttttttatcaccgttttattcatttttttgggtctcattctgctactatttccacttttaaaatttgggatga
gacttttgattatttcattcccttagtagttaactccattgctctcttaatatggctatgtctttgggtttaaaaactagcagtggttatcttcctcgttg
attatgaaaagaaaggtcttacttggaatcataaaccttacatgctacatcttcatgatatcggctagtgttcttataaagtaaggcgcctcataat
tctcgcggactccatggaacgaattcatatttttcagtaaagacactcaagcgttctttcaatgaaagactgtgaaaacctatcttcatctgaagag
tgtttctcgtagtagacaatatatagcaaattatcttgaagagcacaatgatgttgagtttacggaaaagattcacggggtcttctcctgtatgatct
ccttatgaatacttaaattgctaagagtgggataacttagttttacgagattagggttcctctttttgtttttttttttactaaaaaaaaagtataatc
ttcctaattcgctacaggaggaatgtgaaaaagggttttccgtttgttttatctaagtttaagtaagaacacgaaaataattgtgtcggttataaat
gttctttcccgtataatctttgatccttggtattaccataaagatcattggcgatttctaataatatcataacttgggccaaatcttttgtatgaatggc
gaacattgtagcgaaggactcaattcgtaaacattcggtcaacagaatgcaccacatattaacaataaagatcagtatcaaaattaaataaatc
gaaaaatgggtctcccataaccaattcagaagatccatgcaagttaaaatcgtcgaataaatcctttttttgtttttcaatggttctctataatcta
ataattttgcttcgtttattttcctattccttaatgcatcgcacaacgctgaagcaacctgaaaagcatctctcgaagcaggagtattgaaaatttct
ataacagtgttcagagcttcaaatgcctttggaattagctgcaagctttctttggtatcaatcgacaagaggacacaaataccatgaaatggtatg
tccaaaatactccaccatgctagtctttttttggcataaagtcagacatttatccaatgcttcatcagtcattttaactatgataccgacgatcgtttca
tcaggatgagagtttgttaaaaaaaggcttcggtacaaatggaaacaaacggttgctctcataagggtaagttggacgggttgtaatttcgaaag
atgagatatcctctggattgctgcgagatatgccatttggggttcctttgtcaactcgtcactttcgggtaaaatacttgctaaacgtacaagttgat
gagactttgatttttctggaaaatccactcttttaatattcaactgagatagttttataggagttgtcccgtattctgaggctaatattctgttaaaag
cccatccaattgaaaaaattttgttctttgaagaaaaataagtgtatttttgctcttcaggctcagataaaactgcctcttcctcattggtatcatgat
gaaggcaaaaaatttcaatgcattgcatataaacacaagacgctaaccaagcgttatgcggatgagcagcggttcgtaagtagacgcttcttaa
aatccaggtaataatttggctgattgatggaatactgttaattgagaatgcgctattatccagcatgctttcggcgtgttgcattagttgaagctcag
ttgacaaggaattttgatgcgaaaagtaagagccaagtactacgacagtgctaagtaaaacctcaaactcagcggtcatttctgcagcccaaag
cgtatttagtttttcctcaaaagcaatttgttccaacaattcaaaaatggggttcacattctgaaaatatatagagacataatagtggcattgttctt
tagataaatatgtgtgtatttttggatcttcattccgcttttgacagaaacgagctccgacattccaagccaatggtttatatcttgttgacggagaa
atttcaagcttctcactcacgatagcaggaaaacaaagggcatttgtttttttggttatctttcgtttttttcttttcttgaattatatggtcgtcggattc
gtaattatgagaagtccatacctcattcgagttatcagattcagatggagaaaatttggctttttcaaatttgtttgactctaaaacttgtctattact
actttccgtctctgaccaactgtcttttctgtgtaaatcattgctttgaacggaatcctgggcatattgacattcatagcaatatttttgacaatacga
acaaggaaaagtgccactgcattttcgtttttttcttatgacagacaaggcaggcttttttttgttctaatacgtttcggctccacgctttccattaatccc
aagaacttcgtcttttattaattgaaaattagcgacatttgcacggcagataattactacttgctttaaatttgagtattgcaaaagccgaagtctgt
gcatattagcatagtgagctacctttatacgcacttattacacataattataaagaggagtccactatattttcgaaatcttgaaattacgactttaa

gaattattctacttttattaaattaagacccctttcgttgcacgattaagaaggaaggaatgtaacggttttcctctgctgacaagctgaaaaataa
aaataaacgcgtttatttgctcgaataaataattctaactaagtatgtaagctttttacaaatccaccaagtattcattttgtagtaatagaagaggt
cgttcaccttgtcacgcatactaaaagtactaaaaggatttttttacttttatggtacttaaaaatgattggagtaaatgaattggcatgatatttata
taaaaactatgaataccaactggcaagatataattattcatgccctagttcattccgggagaaatcaacgggtatctttcaatgattaattattaac
taaccattattccacactcacacgaaagcgactacagccttaacacttgtgtttacgaaacgaccattcttttacaagtgaaaatctttcaagaatc
tgagggtcggtaaattttaagaattgtcatgtaaacaaatcttatttgcgatattgtttatggtgttattgtaagctttatactcaaagttgagacgat
tgttacctatatgctgtttcggtaatgcaatataaagggagaacatgatatggcgcaagcggagtacgcaaaacctacggttattgcggatcgcg
cggcaacggttattgcggaatcgctgaccgcaagctagaattggctcttgtatttctatatatagtggaaaaaatcaaacatgattttgatattgttt
ctttaaatattttcttgattttgaatccttagacagctccggttatactagccaggccggactttggctgacgttatctcagatatttatttacgcatac
ataaataaacttttaggtatataggtctgttacttgtgccgaagatggacttaatttccctagctacaggaaaaaagtttgaagagcattatgcga
aactccatcggcaaaatgtagaaaacttgtcaattgatcaccgaagctttgatccttctatatcaaagacttcttcagatgaacttgacaaagagc
aatggttcttggatgtcatgcaaataaataaaagcgataatgtaattgaagagaagatgcatgaaaaagaaaacaaagattaccttgttacttgg
gatggccctcaggatgcactaaatccaaaaaaattggtccaacatgaataaatggtggataattattcagatttgtattataactatgcctgttacat
tttcttctagcgtctactccacgggaatttctgaggtggtgaaagtttttcatgcatccatccctgtcgcatctctaggcacgtgtacttatttggtagg
cttctccatgggttctttaccctttgcacctttaagcggcatttatggaagatttattgtttaccttattactcttatagtatttactttacttcaaatagg
tggtggtgttgctcaaaatgtctggacattagctataattcgatattttcaaggtttttttggtagcactcccctagccaactgcggaggaaccgtta
gcgatatatatagtccagttgagtatacttttactgttcccattctttgtgcttttcccttccttggaccagtcataggtcctattataggtgactttattt
cgcaaagctacctcggatggagatggacattttggataaccatgatattttcaggttctgtagctttgctcgttatttttactcttcctgaaacgcatg
ccgacacaattctttactacaaagctcgttaccttcgtaaagtgacaggaaacccttttatggtatacagagaatgaaaagcaacgtgatcccaaa
agagcaattgtacgagcgttcacaaattctatttcattgctaatatcggaaccgatcgttgtttgtttcaccatttatgtaactatacttttcgctattg
tttacatcaactttgagagctatcccattgtgtattcccagtatggtttcaatcagggtgaacagggtctttcttttattggtatcggtattggtattctt
ttggccacttcctccactcccattatatattattattattccaaaaactacaaaaagatgaatggaaatgtgcccccggagtatcgattatatccact
ttttattgggtgctttcttttacccattggcttgttttggtttgcctggacgtgttaccccccataaaatacactggatagttcctttaatttctagtatctt
ttttggggcatcactgctacttatattttcagtcatgtttacctactttatcgatacttatcgctctctggcaccgagtgcgcttgccgctgcgacgctt
gtacgctactcggctagtggaggaatgactttagtcgcacgacccatgtaccataatttgggtgatcattgggctacgacactgcttggctttatat
cagctgctatgattcccattccttttgtgtttttttaggtatggaaagaccatacgttcgcacagtaaatatgcctacaaaggataatttggaagaag
aacagaaaagtgttatatgaaaaggttagagttcgctcgtttgttaaatagatctcgctaattattaactctacagaaaatttaaagttcgcatgtc
agaccttcgacatggttaatttgtgttttcgttttggagataatggatcgaaactggaattggattaaaaaattgaatgaacgtgaatgcctttgca
gaataatacaaacgtttaataaactaggtatattactttggactaggattctccttttaagttaatgaatatgaataataattgcactgttcttacga
ataaacaatactgaatatggagcttcaatatattcagattttttttttagcgcattaagcatttctcggttactagagatagcaattgtatatttttctt
acactctactgaatattactcaactaaagtttcaagggaacgagccattttcgtattttaagtattaacataaaaagacagataaacaagttaagt
cgttaaaattgaaacacttattagttcatgcatatattgttaacaagaagcattcttgtcatattcaaagatacatgtaaatcttgttcagcaagtaa
gtagacataagaagcttcttaaactacttaggatatttgtgataattactctattaaaaaaagataaacaaataaagtaatgaacaaaaatatttt
tttaagggtttagcatctgtattacacaacaatagtttctccaagatacattaccagtggaataaacctttagcatattgaagctctgttttcactaa
tcgttatatttgattgtattcctgaagcacaagttgtaaacttaaattgagtgtaatgactgaccgaatttttagaattgaaaccgtgtttagttagtt
ctatttctcaactcagctcaatcgaaacattgtaatcttcgaagtaatgctatacgcagaaatgctaaatcatatatagtgacaaatatgctcaaa
aagacagcgaaaatgtttagacgtttagatggtttatatattaaggcattgaataaaattagacttgtaaaaacccacccacttctataatgatag
tatcacgccgttcatcaattgtaacaaaattaagtttaaataaacttatgccaccctttagttggtagcataacttgatcgtaaccgaagtcttagtc
catatttatacaacacaaaaggaatagggaacattatacaagatatgatacccaaaatggtgcaaccccaaccaacaggagcataagaaggac
cagtatttttaaacatagcgcgcgcaaacagaggaaagctagaagccatcaaagagcgacataggttgtttcccgcatagacggaagcaatata
gttggggtatgaagaagcaaggtattggtagcaagtttgaaaaataaggaaagagccagcagcataaaacagagtgcttacgagagaagaaa
agcagttgattggacgctgagccgtccatccaaaccaaaaaagacacacaggaaaaaagaatgcggctgggaaagatactcgtaagacttctt
ccgggacaatgcgtccagagttattcgtcagctttggaatcagcactttgtgtaaataataaaaataaaacggagttggtaagagtgttcctatga
gaattcctaggtaagttaatccgctctctattaaagtgaaatggtaaagttctgtatagacgataggaaatgcttcaaaccacagatacatgagag
agtaaaccaagccaaggtataggtttaaagataggacaattggctcatttatacttaaaatgatcggtcgaagaaaaatctccataagtagcttc
gagggattcaattttgaaaattgcaactcatctggtgtttggagatgagaattgttcgtcaattttcgtaatcggttagctttgcgatgcaaaatatt
agaagctgaagtttcagggacaaagagaaagaaaagaatgaacacggctgcagaaagcatcataagaaaccaaaattgccatcgccaagatt
tggcaaccaccataatgctaccgaatagaggacctaacgcgggaccacctacgtaagaggagtggttagtagaaaaaaaaacaaacaacata

tgttacatactaaaagcgcagagtgaccatgcagtgacgaagtactgtgcgtatttactgtcccacatatcagttatagttgcacctccggtagca
agtgctggagaaccaaaaaacccagctaaaaaacgtaaaggaagcaaagcgccaatccctgagcaaagtgctgtaggtatttgtaagcagatg
tagataaccaacgttaccaaatatactttttgccttccaatctgcgggacttcagtcaatggcgcgaagaagagcggaccaacggagtatccaac
gatgaacagagaaaggccaagcatggcagtaacattccccacatgcatgcttttcttgatttcatccagacctggggtaattattgaggcgcccat
atacgtagcaattgttagcaaacaaacacaaattgtaacataaacttttagtggtctcgaccagtttttggatgatctgatatatcaaaatcttccc
atgtaattaagttcgttgactcgtttggttcatcggattcttctaacttaattttcgttaaatttgcaaagcactctctcttttcgtcgacaacgtggaa
ttcatctggcaatgacgccgatgacttttctttatttccagtagcaaagccacgaacataacgaaagagttcaaaagcttctgagcctttgataatc
tggtccatatactctcaaatgacaacacaaaatacaataaagcacaccctaattcaatatctaagtttttagcactcactgctttctatgcctaattt
ttttcctgcctttgtagaatttcacaattttattgactattcatggtcggaccggaaaccgggtatccgaagagaggtatgaaattttttttgttcgtt
tttctttttacaaactcgaaacaagcatttgccactttaactggcagtcgtttaattaccaaatctggatgcgtaatccataattttcagactccatat
ttttgttatagcttctaatcaataaacatctaattttgtaaacatacagttttagctgtaaatgaaaaaaaaattcttaaagttacatagttccttttc
atttgataagaagtgaaagagttacgaccgtaatattaaatatgatttcaattagaaatgcttgttttaattgttgaaccgctcttaataaaaaaga
gcagaagaaaaaaacgacaccagcaggatttgaacctgcgcgcggagaccgcaatagatttctagtctatccctttaaccactcaggcatagtg
tcgaaggttaatagcatagtaatgcttacgtatatattattctaaatctaattcttcagagtacaggttgagagtcacaaagaaatgatagcattct
taaaagcgtctatcctgttagagatgacatatgattccaattcgttaaagattatactctatttttgtttgtaaattatgaaaaacatattgaataaat
ggaatcaaggttaagtagaaagatagtcaacattgtttttttttttttttttttttttgttttattccttctaaagaacacatgcggcgatggcaatgt
tagggtttccttctgtaattagtaggaaccattacgaatcttggtattacacaaaagtaagctacggctataacccgaatacgaaaaatttattttc
tgagaagctgccatttgccaagttttttttcttccctgttttataatctgtacataacacttgaatgcgacgtgcatacaatttttttcctttcatttagaa
ggtctattactgatagaaaaaaatgaagattcgatttaaaaaataattcaaaaatttgcttcggccgggaatcgaacccgggtcgcttcgatggc
aacgaagcattataccactaaaccaccaaagcagcatcataatagtactttgtgttaactcaagtttatttatgtgtaatatggaatggttcaatgg
taaacatgcattcagacaggtaaattgtgagaaaaaccaaagcgatcttttcttttctttttaaagtctacagtaggttcaaacttctatgatgtcttt
cagctagacattcaaataaactagtatcaacttgatttgccctcaaagtcaaaacctttcaatgcaaaagctacgttccggtcttactactactaca
caaaaatgacagatattgttaaaagcatatcagtatatttatactaaagccaggaagagctgaagctagacggcacagcgattcccatcaaagg
atttttctttgctacccatggtaattctaatgatggacttagcaggtgaatacccaagagccggaaacggagacatccaagttgttgcagccaga
ttggatctcttgtctcaagataggatcaccagagcaattatcagtagcgtagatggaggcagtgcaaacgccacgatcgacatatgatttgatgg
aagcaatatcacggaacttttggcagttgttcaaagaaatcgcataacttttactacccttacagttgatgtcatcgtaagcttgcattctcaaggg
ggaggcaaaagcaataccaatcaaggccataatggcaaaaaatgcgacactgaaaaacttcatttttccaaaaaactaaagaagaaccgata
aacagctatatatgtttatgtgaaaggaataaatttgattattcaacgttgtctcaccaaaattgaaaaaggaggaatgggggaaggagaaaga
atcaacccatatttaaatttatatacgtgttgctatgtcggtacttatccgtggatgacgaaactataaaagggtttgtcgtacgagattgtaacttc
gcctaaaagtataaactgtattaaatagactcttccttaccgaatttgttgcaaagttacactactataaatgaatgctcaatggtatttattgatta
caattgcaaaattcatatgccgttttgcaaagtgactttcgtctaatattataaaaaaataaagataaatcatctttcaagaaatgaatggataaat
catattcaaacatactgaagttttactactgcattcgtactcagtataaacataaggtttgaattaattcctaaaaatatcttacataagaaaacttg
gattattttagttaagcctatatatatgcgtctgttaatcttttaagatacttatgactgattttatagttggttactttattttaacggtagaataaga
attagagtcaaaatcaatattctcaataaaaaagaattttgcacatgctcaatttaccattcttaacgtgtaatatactaataatatagttttccgag
catttcattcacgaatatccaaaaccatcattttaaagtcaactaatgtttaacattggtaatgaattatttgtttcagttaggggaatttagtatact
acgatacttgatttgcctaccttatatatttattttgcataaaataaactcatttaaactggtcgttttaccttggaaaaaagaaaaaaaaaaaatc
aaaaaaattagtatgttaattcttcctttgacgactttggagagcttgtaaacgagacttcaagtacaaaattatttcaaataaacattggccaaat
ctaagcgaaacaccctaatgcttattttattatcgacatataacacgacttcatatattaccgactataaaccgaagcgtatgtttgactgctcaca
atttacagtccagtgtttagcaatgtacctttgaagaagttgtttatgattatatttactgagaatagaattgtaaatttcttaataaattccaaatag
gaaccgaagcaagtatgacttctcaagtattgccatccagcaagggcgggacgtgttgaagtattcgttctgacgaggctgttaccgaaaaggga
taatactaaggagtgctgtaatattgttacttcatgaaagtgttgatttttctgcgtcagttgttgttgtttacaaagaaaatgatactctagatttga
atagaagatgccgttttaactgcaaagagtcttgtgatcaatccaaagccaaccatatacaagagaggcttgaaggaaaccttgattgaaatgta
tttctgctcgagccaatccaacatgagacgttggagtgtgcatgatttctttagtgtattatttttttttttcaaattaatagtaaatcactacgctttc
attgtaaggcaaaaaaaaaaacaagtttctatgttttacggactaccgttctatatgtttatagcattcaatctttaatttggtcagccctctagatgc
aatgcttattgtgcttgctcgtttgttccccccggggtccgtcatcgaattttccactactccatactgtatatgagtctgttacctggggcgttaccttt
gcttgtctcagggcaaacaggagatgagacgacttgcaaagaacctaacacaaactcaagagcacgtcataaaacatgagcatcaactgggtt
acatataaagaagggtctttcaaagagccattgcttcttgaaaatgaatgcgtcttttctcactacaatggaattgcgctttctctcttatgcaatcct
ccttctgccaaatcttggacctccacaaaaggatgcctgtatcttacaaatcaacgggtacgctttatgaagaaatatatcattgaagactccttgc

tgatccttttccccagataatatacatcactgacgaagagaataacacctttgccgatttccaagtaccaatcagcaatataaaggataccaagct
gaatcaacgtatgatgatcaaaccacaatcttctttccttttgtgatgctaactgccttttgattagcatttttttggtgctaattactattcaggcgtag
taatgccagtcaaaaatggcggaattccttctgaagcagaggtaggtacaggttataaagttgtactatttattaacagtactgtttttgataggtt
aagttccagtttaacgaaggcggaatctttgatttcgtagaatgttggaatcggctattacaaaggtatcaggaagttgatagtgcttctcgcgttc
aacatttggatcctttaccaccttaccatcgtccaagttccagccaagaccagcctccccaatatgatgaagcaattaagcaggtataaaatacaa
ttcccctttgcattaactcgtttcatccttattaaatgaagctttaaatcttattgagcttctcgcatgcggtaggcagagagtgctcaaacgtttttgt
ttgtgctccgtttttaaggacctatggcttttgtgagaaaaaagccaataactttttttttcctatcaaccagaaaacaaaaaacaaattgttttgtaa
cattatagtttaatcgtgtttgcttttctcctttttttaccgcggtgtcatacagatagctatactcgattttatatgaacagacgcaagttcgtcttaat
ggtgattttgccaacttctttttgttccttttcctagatttcaagtgactctcatccctacttaccgttttctaccctaaaattgtgaatcgttcttccttcc
tgtgtttatggcctactctatactaatttaaccagccgttagcttcccctgttaatgtttcgtaatcatgatgcatattattgtttatatgctaaaatag
attatttttttctttccctttccagattccttattgtatgtgcgtacgaagaaagtttttgattgttgttatctgcgaaccgaaacatgtcgacatttttat
aatcatttttatatatagccgactgaatgctttgatatcaaatcgtattttattccaatgtcttacaatgattgaataaaagaaaggtgctatcactgt
tctaatttaccaaaagaaagcttgaataatattaagtttacgactcgctttttacagattttctctgatgaaaattaatcaccctctattacccttttttg
ttttttttgccctttttttgttgttttcatttactcgaaagcaaatcaatgagaatatactgcaaccgcttcgtcttagtgacacatctttcagaaatgac
ggatcatacattgtgcttcaatttatgcaatatatcgatgaattgtcctccgtttgcgttaactctaatgttggatatataccccaagttgtatagtga
ctgttgtaaacaatcttaaacgtctcaaattccaaatcaagcaggttgtaaaggggttcaccgttttgtaaattatagaaaaacggcatcacttca
acaacagagaaagtataccaaaagagtgctcagtttgtatcttaaaaagaagcggtcaccattatttcggtcaaaaatgagttttgctgtaaaac
gatcttccagaaaataagcaatttcgctttttttatgatgctatgagtctaatgtctctttgagggatgacaactttaatgttagttccatgtttaaatg
aattaatatgtatatatttcaaaaccgtactgtaatcaaaaaagagttttacataacaaacatgaccgttaaatgcctagcaagacaatggtattg
gtcttacgaattaaatgactttgtcacagttttgaacatgaaaaacagagaagcatgagataatttgaaagaagatttccgagaataagaaaatg
tttattagtttgagaacagtttgatccagatggtttacctaccttcctttgtctatcctcttcctatatttctattaatcttacacaataagacactagtt
cattccatgtttgacacaaagttattttttcattctttaaaagaagcttcatagatctttattcaccaattaagtgcagttttcagacctgaacctgtat
tcttaaaaacctcccaattctgagcgttcgtaaaaattgaagaacaaggatccaaaaaaggacattttgagaaattgagttgaaattcagcattg
tgagagataattcacttacatcggtctgcgtttttggcttataaaggaaattggctctaagttgtaagtaaccacaatttaaaccatttgcttgcgat
ataccttctcaaatggaattatcttcttctatttacgacaaagagttggagtatacaacgaaaagcctgacagctctttctatacagaaacatgagg
gatctgttgagccgggccaaagtatctccaaaagatttctcactaatcgtgatatttggtctctcattgttcactaccttgacgtttttgatctgtaca
gacttatgcatgtcaatcgacacttctatcattggcttcaaaaatgtcgtctagaggaatgtcgatttacagatgcgagccctacggagcttcccta
ccaaaaaaccatttctgctgcaaatgatgaactatgggcttatgaaaagcagttatatggaagaccctccatcttacggccgcatatccaagatcc
atttgtatgctctatgcttgaaaaattccagggactaagtactatcgtgctggatggtaccggcatcaaaagttccacagttgcttttattttatcaa
acatacctactcttcggacgcttagtatacgctggtgtcaaggggtttcagtattaagccttcttgagcttttgcaagctgtagcaaaaaataaagtt
ttctcgcttgaaaggttttatgttatgggtatcgatggcttagaactacaaaaacctttactacccgacggtacagaagacgaccgattaactagc
aattggcatagtcgattagttgtatttcagagcgctttggatcaaatgacaacgacacatggacgtcccatagaaactgacgtacaccgatgtcct
ttgaacgcatgcaaaatagccgatcaagaaagtgaacttgcagattcttacatgcttaaagtacttccccttgtgtatattgctcacaaaaatggt
caaaaccactttgtcgttattgcattgacctaagacggtgtatggtttgcggtgcctttatttgtccttcatgcttgtctttggattttgatttacaaatg
caagcatttgctcgtcaacatcgtgtcatttctactttaacagatatttaccctgaacgtgaggatacctgctatcataaaaccagagccataaaat
ggcaccagttaagtccgagatcccttgtttttcaatttcaggaacaaaatcatattcatcaccgtcgaattcgtcgaaaccttcttctgagtgggtgg
tcttggccttctgcggtgaaagtaatccttgctcaagaacattcaagtcggtaattttttttatttcttttgaataaaaattaaggtgccctaagccatc
acaaatttggtatttcattcatggtaatgactattgattctttttgttctttctatttatgtctaagtggacttggatacgcgtgtagtattgccgagaa
gtgtcctctttatttatattcgaaaatctagactttcctcaactctgcacctggtttactcaaattactgctagatttgactcgtcggatttcttctgtta
gctgcattctcacaagtgaattcgagcttcaagtgccacgaaatcgaagaagtacttgaacagttttcaagactatttaatttaaaaaattatacg
aaatatgttaaaattactgctttagcagttttctcaattaatttctataatcgtaaaattaatataatatttgttttaagtatatttaacgtagttcggt
atgcttcattaaagtcacgctgaagaacagcaagtaacagttaccagaacctgtatcgttttggtttttctccaatttgtatgcgtttcaagaacgat
ttagttaagacttaataatcacagtaatattcaataattcagtagcaattcgagaatattggaatgcttcctttctgctaattgggtatatgtctaac
cgtgatccacttgtgaccaatattgcggacttagtttcagattcctcgtttgacgaagagtcactctcgttccttgaggattcagaggatcaggacct
atcattaaaaaatgacacattctcgtcaaaaagtgcacattctgatgggacagtcatcagcgataatttgcgtagaaggtccagtgatgcgaaca
cagcagaaaaaacctttcaggctcgtataaaagagccagaactgtttaacttaacttcatacggaacagctcctaatagagaggtgaagtctcct
gctttccaaccgattcgttctcatgagcacatagttaccccctatgccagctcagaactcgatgcgcatgcaatctatggagccacaagaaaatgag
tctatgtcaaaaacacataacaagactcctgggtcactgtcggtctctcgttggcgtcgaattggccggataggtctgggtccaccaaaacgagct

gaatatacgttatcctcagatgtcaaggttccatcagaattgaaacctatgaaccatgccatcgacgatgataatgataacgatgttgaaatgaa
atcttctgaaaactcaactacatctccatttgtgcttcctactcaagcgtccaatttcccatctgatatccaagcaggttcattgcctaatgcacatga
tgttcctatgcgtgctacggttaatcctgcccaatttgaagaatcgttatttgtaaagccgcatttgaaagaacttgcttccgataaaagttcagcaa
tgaaggcaccttctgtacctttgcagcatcgttttgatcagcaagaacaattacatgggccacctccgaaaatgaactctttggtggatccttctca
caacaatggcatacccaagtttcttcaggatgtagcaacggttgctaacctaccgtttatcaaattagggattgtgggtaaaggtggcagcagtac
agtgtttcgaatttattcacctgagaatggtcgattgtatgctctcaaagaagttagtttttacaagctgattattcgacgattcagggctataaaa
acgagattgctcttttaagaaaattatcggggaacgatcgtattgtaaaattatatgctgccgaagcaaacaattcaattgggcaattaaatatgg
taatggaatatggggagactgacttggcaaaactcctaaataaatatgcaactaaaccaattaacctcaactttatacgaatgtattgggagcaa
atgttgcaagcagttcaaatcgtccatgagcaaaacattgttcattcagatctaaaacctgctaactttcttttggtagaaggaagtcttaagcttat
cgattttggcattgcaaaagcgattgggaatgatactaccaatattcatcgagactcgcatgtgggaactatcaattacatggcaccagaagcatt
gactgatatgaatgcaaatacaaacttaggcgtgaagctagttaagctaggaaggccgagtgatgtttggagtttaggctgtattctataccaaat
ggtttatggaagagcaccttttgctcatttgaagatgatacaagcaatagctgcaattcctgatgagcggcatcaaatacaatttcctgaaattgcc
gttcctccttccgcaaactttgaaccgtcccctaatgtgcatgcacatggtgtggtcgttggacccgacttaatggatgttatgaaacgatgtctgca
acgagaccagaaaaaaaggctaactattccggagctattaatgcatccctttttaaaccccgtcccttctttcccttcacataaccctcaacggaat
tccctttcctcgccagattccaatactcactcgaagctaatgacagcaatgtcggcctctcagttgtccatgattattgaaaggtctatagaactca
gtaaacgaaagtctttacccaaggaacttattgacagcatggcgtacgattgtataagtaacttgcaaaagatgcctcgctagaagaatgacaaa
actatgtatctatttatgaaaggtttcggctcactatctttaaatttcgttgaacgtgatttaattaaggctttttttgagatttaatgaacgatttagc
atacatttctttcttttcttaatgaattttccccttttaaacagagaaaatacatatatatttttcgttaaccgttgtttcagtaatttgaaaagctactt
ggcaaagttggtttccttgactatgttttcgcttttgaagaaaaaaaaaaaaaagtatacaaatcatcaatatgactaaaatacaaccgtcctgtc
gatttcctttcagaccgctattttaacattgtaagtagctaagtatctttgcgatcttgtttgtctcatctaataaagatgattttattatacattacaa
gaatgtttccataaataattctaaagcataaacaaaaatttgtaacaagacaaaataaaacaaggtacaaaggtcaaaacaacttaaaaaatg
aggattttcagcaagaaattagggttttataatttgggtctccatcttagcgaaaaaaaggattgataaatgtcaacaaaaacaaatattacttcc
ctttcacttcctttagttatctaaatacttattcgcagaggcacccaagtacaagcgcaaggtattaggagaacagtttaccaatttacagcgaacc
ttttgctttccagactcgtcatgagcgcgtagacgatggcatccaccaaatgcaaagtaataagttcgaccacttttcttaaaagtcaatacatcaa
caggaggcaattcaccttttgtaaggtctcctggattaatattgcaagtagcaggaggcacatttccatccattgtatctttcatactgttgactttttt
catagtccaaaacaggaggaatcgggcgaatgatttcagacatgtctaggtagacaatgttgttattgctagctgtatgaatcgaactcatggtta
ttgttaatctgcggtttcgaatatgatgatataagaaagtcaaaaagaaagcaaggtatcctttgtttacttgtgaagtgtagcgaagtaacgatc
actaatcagataacctttagctgtggtgtagttcatgtggggaagaaatccgaagcctttaaatttactctagcaaggacaatgacgtcagtcatt
ctttgtcagataacgtggccagacaaattctgtgtcagcaaacgaaccatattacctaattttatagcgtcccataagacgttgggatgcagaaaa
taaattacggtattgatgttatccctcacaatttaggtttttttatgtaaacaataggtagaattcaatgtaaacaaaaaacgaaaagataatggaa
atcagcaaaggagaatgatggttttgttgagttttctgaacagtcaagtcataggagcgatgagaccaataccaactatggaattgggtggaaat
tcaagtgaagtgatgatcaagtagcaaagaaaccgaagagcttgcttcgttgtattattccatagctcatccaaagctcatttaaaaaaccgtttc
aatccagggtgctgaatatgaaatttggtctgatggatgtatttgagctttgagctggtaaacggttccatctgtaaacaagaagatttttttaagta
cgaagaggttattataaagaaaaagtttgcttttctttgcctctcgttggtatcttcaggacactgtagatttgtcgcatcagggaatgtgttggata
gctgggtagaacaaaaaagacaggacatagacgtcggttcttgaaaaatataataaaggtacttttgttacagctatgcttgtttctggtggaacc
tgccaatatgaaaagaccggtcgccacggtcaacttccgatgcttctagtgagtgagtgaaattgtaagaactgacatactttttcatgttagactt
catttttgtaaactcatacttcttctcatctcaagtttctttcttttaagcagaaaagcttctcatggctgacctgaagcataaaagagaatcattaat
aaaaccaaataaagatcatttcgaattgtaaaaatcagtgtaataaggctaatcacatatccaccttgactcattcagttccttctggttttgatatc
attttactaattcccactcccaactctcgtgaacagtaccaaattggagtaagcgtttgacgatctgttagtttagtcctctttattatctaaagtaag
ctctcttgtttttttattcatcacttcttatctgttttcttcgatttaccgcatatttgaatttgaagtcaattgacgttctgtattaccgcatccaccaca
gcgattatagttttcaaagtttttataactttttaagccttttgcattgattttgatagcaaatttttgacttattttgcttattttctgacaatgaatcct
gaacgttgggcggctgatttgtttggcaatgttaaagactttagtctacatgtgctagactttgtccatgaaaaaagtacttcttcaaacccacctgc
tgctcctcctgtcaaacctctgggcagtcaatttgtttccttcatgaaccgtaatagagtctccgttggtttgggttctgctgctatatttcttgctggtc
tctcctattacaagcgctatgactacgtgaagaaacgtcgagctcctcgagctcctaaccaccagaaaacccaggtgatggtgctttcggcctgga
attccttagcttccattattgcacacgatttggatcgccggggcttcatcgtcgtagttttggttcgtgatgcttcggaggctgctaccgtctctatgc
aacagcgtccttacattcaatcgttgattgtgactcaaaacgaagcagtagaaagatttttatcttctttttcgaaccaaaggggtcccaaagaata
tgctcttcgtcttcgtggtcttgtgcttgttccaactggagattctttgtacacgccaattgaaaatattggaaaagatgcttggttccatgcctttcaa
caactcggcgaatcttttgccaatgtttctcgtctaattccagtgctaaaagatcaaaagtccagaattattggcttgtcccatggaattctctccgc

atatgaacctcccaactatgccattccttctatcctttcttcttccattgaaacctttttacataccttgaaaagagaaagtggccttcgtgttgtttgt
ataaaacttggtaatctcagctttttgaactacgatcattctagttcccctgcttctggcgtctatccaccggctgtttgcacagagcgcaaagtact
aaacaaaatttttgattgcttacttgatttttggagcactcctaatcgtcacgtcggaagccgtaccttttttcttaattcatatttctcattttcttccta
cttgtgttttggatgtaatgttttcctttttcataaaaccaaacatttgtcctgcgcattagtaaaacgagtttaatacgcctatgcctaggttgtatt
aaggtatgaattttgaaagctttcttcttatgtctttttagtgatgatgacttagggctcatatactttttttagtattgaatcaattattaattttctcttc
aatgtacattcatgtgctcaagtctgtcctgatatcgcagatgcattagcttagaccctacctatttcgctgtgtgctgtttcccgtcgttacaaagca
attctatcacaactatagcgtaacattaagttttatctgaaaccctttatgaaaagacattcacctctcgtaaattgttcttcattacaattcattcat
catgctctatagatactcgaatatagtaagcttgtgatagaacatggcgaatttcaggtgctctacccactatttcattgtcagtcaattcacttact
cttataaataatctcacaccattaatgttactacacacataataaaaaaatgtcgcctcttaccttttccttatttatttacttctttcttacttctaaag
tctctttgcagtatgcacaacgtcggtataatatttaaagatatccttccatttctttaaatattgaaggcattgatacatacagcttgcaacaaattt
cttggtttctaccaaaaggtaaagttatcttttagtggtagaatttaccttttataaaagctgtttatcttttttttaagcaaggaaaagattcaatga
tcccacaatttaaccaggacgaaaaccagaatgttgacacagctcaacaacaagccttgaagcattatatagccaaaccaagagcgctagagg
atgaagacgagggtgatttaaacccaggaatttctagtctagatactccgtattttgattatgcctttgagcgttctttaagacaggtatgatatatt
tcgtccgggagctgtgagcaatatttaccaataaagctattaagtgtatcttaaagaaagaaaaggtgctttttttagttcccggatgtagatggtat
agaaagcagtaatctgacaagcttttatttagcaagatgccaagtacttggctatgaatagctctcaagggaaccggggaagtttctcaagttccc
aaccttctcgtagacagacaaacgagctggatcccgctcaagtcaaccaggatataaaaaattcgaagactggtcctgatagctcaaaattacta
aaccccaagctgcaggaaaaagtcaataatattcatatgccccatgtccaacaaggaaacgatgcttttgtttccaataccggaggtcctgagca
agaccccggcaacatggaaacaaatgatcccttattttcagacgaactggctgaaatatacatgtccattcacaaatgcatggacatgcgtcaca
aatacattcgcattagtttacaaggagagttagataacccaagagacgacgagtcttggaatatatatccctctgcaaaagaaggtgaagagga
agaagaattttgttttgaaaaatgcgagatcccaggaatagccaacgagatgatgtttcatctcgaccaccagggtatttatcaggtttacgaaaa
tgaaagtgcctatttggccagtaccccttcctttcatgtgccaaccattcgcgattattacatggatttagatttccttctttcagcatcctctgatggt
cccagcaaatctttctcgtttcgaagattacaatacttagaggggcgttggaatatgtacattctcctgaatgagtaccaggaacttgcggactcta
agaaggtgcctcatcgtgatttttacaatgttcgtaaggtggacacacacgttcatcactctgcccttgccaatcaaaagcacttgttgcgtttcatc
aaggctaaacttcgcaaatcccccgacgaaaaggttatctggcgtgatggaaagttcttaaccttacaggaggtatttaactcgctaaaattaact
tcctatgatttgagtattgacactcttgatatgcatgctcacgcagatacctttcatcgatttgacaaattcaatttaaaatacaatccaattggcga
atcgcgtttgcgtactattttcttaaaaaccgataatgatattgaaggtcgttatcttgctgaactgacaaaggaagtttttactgatctttgtactca
gaaataccaaatggcagaataccgtatttccatttatggacgcaacaagaatgaatgggataggcttgcggcttggatcatcgataataaactct
acagcgctaatgtgagatggttaatccaagttcctcgcttatacgatatttacaaaaaatctggtattgtacacaactttgaggaggttgtcagaaa
cattttcgagcctctttttgaagttaccaaagacccatctagtcatcctaagctgcatgttttcctgcagcgagtagtcgggttcgattctgtggacg
atgaaagtaaacctgaacgtagaacttaccgcaaattcccattcccagctcactggaatatcgggttgaatcctccttacagctactggttatatta
tatgtacgcaaatatgacaagtttaaacgtttggagaaaaatgaggggcttcaatacatttgttttacgtcctcactgcggtgaagcaggagatac
cgaccatttggcaagtgcatttttgcttagccaaggtatcgctcatgggattttgttacgaaaagttccatttttgcagtacctttggtatttggatca
gattccaattgctatgtctcctctttccaataatgctctatttttggcgtatgataaaaaccctttttatctcttatttcaaacgcggtttaaacgttagtc
tttcgacagatgatccacttcaattcgcctttacgagagagcccttgattgaggagtatgcggtagcagctcaaatttacaagctttctaacgtcga
catgtgcgaattagcaagaaatagtgttttgcaatccggatttgagcgtcaaattaaaagtcgatggttaggtcctgataagcaggacattgatca
cacaaatgttcctataattcgtctctcatatcgcgctttgacgctgagtcaagagattaatcttattaataagcatgttcaacctactcctgttacatc
tcaccatgacttgggggatctaattagaaaatacgataagcttactgcctctgaacaactttcgagttccccaaacacaaacgaagcaacgatcg
ccagtaaaaagtctgcacatgaaggaccggaccatggagctttctttcctgggttgagcgttatttcggagcgtcgtcgaaggaaggaaagtacg
acgagtgctagtccagacatgaaagattaaaagtggaatttaatggtgtaattatgtgaattatatataatttttttgtggactcttaatacagttact
aataataaataaaggtttcaacatgaaaatggttattgggtttgtttttttaaaaaccgcatcttttgtgaaacatgttttgactttgtagtttctttctc
ttttgaagagatatgatgacatttacaaaagtgaaattgattcagtcatggcactactgtctgcttgacggacttttgtatgagttactatttccttct
tccaaaagtttacgtccatttaagcgcctttttcaagtcgagctaaataggcttccaagttgtctgcatccaaagtcaatgacggactctctaacaa
tcctgattagtatattaattcacgtaaataacaatgagccatggaaaagcacaataaaatgaatcaacaaaatagacagattttccatttctttga
tagatcgcgtcgtctggcttatcttccttccttcatacctgttggaggttgcaagggctccttgctacgggcataagctgccttagcatcctcgattaa
aacttgttgttgatgatatttatgctcaattgcatgctgcttttcattcttgacatggcttcgatactcaaaccaacctatgcctaaaccggcagccaa
agctgaccagcgcaatgtctgcttaccattaacatcgtttctaaagttacaacggatgaatcacttttcctacctttaaattaagagaacctatcatt
ccgaatggattacaaaggagagagggggacggagaatccgtaatactgggagatactcaactggatataaactatacggatgtgaaacgcaat
tgcggtatttatatttgtatgataaatggttttagcatgatattatattataagtatcagaaaacatttaatccataacaataacgagtatatcgtttt

acgaagttatctgaattaagacgataagtacaaatggaaatgcagattgaaagagtgccattagtctcttaataaacagtatagaagaatgttac
aaaaaaaatctctatggttaggaagatttagttggttgaacgattcacctttgttatttttttcatttcaattcgttaactagacatgtggaccattgta
tgcatgtagtaaataaacgttcaatgaaatcccaattcagcaatacagagtctcattctttatgcttcgggcaattacatttgtactttatgcctaac
acatgtactgtgtttgtttatgtgcttgctactcattacctaagtgccagagcaagccttaatattactataaactatttggcaataattatactatgg
agtatatgacttaatatcaatgttaataaacaaacaaattgagaaaacactgaaagattaaaaaatcatcaaggatttagttaaagtttattgttt
gtttatttcccagttttagattactatataaacgccgttcttctcacacaaaatcttcagttacgaagattttgctggcagaggataatagacgtgttt
aatcaccagagacgttactaaagatatatcactccagatatttagtaacttaacttaattaagagtattcaacttgacgattcaagcatttacaact
gtgttccacttacactctcattactatacggagtattttcccgtgacaattacctaacaacctaccttctaccagcgtgaaagaaacttgaaataatt
agcatgtctatcacaagcaatgaactcaatgaaatattttccaacaattttaacgttccaatccccactgagccctcatcaaaggagggaaaaag
attcttgaaggattatgatatcgccagcagaaaaagaaaacttgaagacgacattgaaattaacgagaaacaaaaaggtttccaaaaaatagt
cgatgaatggacaaggtcggaaatgtcagtcgaaagtcaagaacagcctccactatggtatcaaagggaaaaggaggaacagacgagaaatt
ttaatctactgttaaagggcgttaatcaactgcaaaggaatacggaaaccttacaaaggagcatggctactatgcagtcaagcatggttactatg
cagacaagcgtcgatactttaacgggcaaggtcaataggatcgatatccgttccgtacgcatggagaacaaggagttgagacgagcaggttatc
caatcatggaagtgccatttctggagggcaataatccagattcagaattacctaggattacttgtactcaagacatcgatcgcttgtctaaagatg
aatgtattaggtacttaaatggctatggaatatcatttaacgcaaatgaaactattgctctgaaaaagaagcttgctaatgctgttggtttggtatt
ggacttcgataaagactattctttctcagggttttcaaatctttaattataatttttcagttaaaatgtacgtttaaagcatatatttattataagcaat
gaaatgatcgcttttcagtcgctctgcttatgacatgaaatcaagaccaagaccaaggaagaacatataaaggtaatagttatgaaaattctgat
acgatcaaacactatgttgcaatgtgggttacaaattaatatggcaagttatgactgtttgtgctttctgtgcttccaatgttgttagtgatacttaat
gagagaagtacttaaagagtaattctagtgtgaattcacagagctgactagcaattgtacaaggattatcatctcgcctgcctactctaggatgaa
acgtcgtcacatagaactttggcttttacgtagagatttttgtagatgaaaacgtgtctttttgttttctttaagagaaacatgtgaagcattttaacc
ttattgaagtacaacaatcataacatacatttttagctttcccattcatgaatttgctgtactggcactatattaactccaagatagaaagaaaacg
aggacacttaaacgagggtgatatcaaaactgtatattattcaaatactagtcctttgctcctgagcttatccgtctaaaccaaaaccgtattataa
tgcaggattaaacaagattattctgcaaaaataccgaaaaaccacattctatattttatttgattatatgacccttttttttttagttaagagtataaat
taagtgaaacgtttggagaaagataacaaagattcatcatcaccatattgagcaattaattgcattcccattggtaagccaccgcataccttgcca
aaaggaatactaatagatggtaaacctgctaaacttgatggtaccaacatgctgtctgacaaaaagtcagtaggagtacttgctccaatcggctg
acttggttttaaaaaggagggtgtaacaatgaagtcaacatcaccagatggatctgatgcttgtaacgaaggaattctaaaggatctattaaattc
catttgaacagctcgtctcataagctgagctttttgatttatgcggttgtcctttctaaacgaagaatgttagcatgaatcgtgatcttcacagagaa
cgtacccaaaagataaagaatatgcaccaattagtaagcgtttttggacctcttgtcccataagatgggttcgaggatctaaaccgtcaggatatt
taccttgttgtaactgagatccaaaaaatgggattgctaaatcgagccaaattagcaacaccctcagcataagccaaagctgcgtaaatgcttaaa
ccatatagggaattaggaagttgaatttcacgaatctcacaaccttgtgacttcaacaaggaaataaattcattccatttgcataagacctctggat
gggtttcagcaatattccaattcgttggtactccaattactatagctttttctgttgacttaaatttttttacaatgctcatcgatcgttttacgccattgt
aatgacaaacacgtagcatctttggaatccggttgattcagcacattgtacactgtttgtgtactttcaacatccttagtaacaattccgacagtatc
catgctatgcgcaaatgggataacgccataacgagaaattcgtccaaaagaaggtttgaaaccgacgcatccgacatatgcagcaggtaggcgt
actgatcctcctgtgtctgtaccgatggatgcaaaagataaatcagcagcgacggatgcggcacatccaccagagcttccaccaacctggtataa
ttcatctttaacctgtgggaatgctgttctcccatgtaaattgttatgggtacttgttcccattgcaaattcatccatatttgtcttgccaataattactc
caccgtttttttcgagattctccactaaagtagcttcaaaaggagatacataatctgattgttagtcttttattaccttcaaggtcctcattccaggca
aactcataccactcaaaatctcagaactggctgaagtagtccaatattttgtacacatattttcttttacagtaatcagctttccgcctaagggaag
attccgtttgacagccaagtcttttattacatctttgatggattcagctgtatgaaaagaagttaacgaattgatctcgacatcttttgacgctaaag
atgcgcaattgtctaataaatattgatccattcgatacaaaatgcgtcggtttctaacaaaaaggcgttggatacgaccttcaatgttgtaaaattt
tcttttccaaagctgcttgtagaataaatcatgttacctagcaacttgtcacctgtgagtcattcctgaaatgtttatttacaaaagtagattctctcg
atgaaccgatgttaaaggaataaaagaagaatctgtaaagaaatctattacggacgaagaaagaaaagtaaaagtgctcacccttgttgagtgt
gtacatttacaccttcgaaatcctttcagtcattcatctagtaattcaatagtatgctaaattttgtattcggactctacttatcatccttgacttgtaat
taagtctttccaaaagcaccaaatataagcaatatctcaagaaagcagttgatcgatggaatcgtctccttgaaggattatctctataagaacagt
agttgtcacagaaataagacactgccaatagtatgcgacgaatggttccatttcattgaaagaaaagtcaaatttctgagtagaactcttccaggt
cttcaagatgaattttcctatattcaggaaaatggtaaagagagatgaaaagataaaccgcataagcatccaagtgatattctgttccaactctag
ttagatgtacacaattcgaacctagaaattagctagccctatgtttaaacttgagaactgtattgatatctacattgtagctgtatatagatgtcacc
tagaagcagcaacttagagttacaaaattcaagacaaatatacaaacagagactgtacttctcgtctatacatagcaaaaaggaacccggtagc
cctaccctgtaaacaatagtttcatccctttaagagagtattctcatcgtatcgtaaaaaagacgtataagtataaatattagtatgatgattagac

tcaacaaaattcataagcctcctttcaaaaataacctattccgctataataaagaaatttgttccgtttatttcaatgttaacatctatattaattcaa
atattgttccaataatcccaatttttcctgaccgtaaatgtccaaccatttttcaaattccatagtacccctttctagtgcagccgtaccttgccagt
aacttcaatcaataatcccattaaagcaataaatgacacttcaagccttattgattaagccatacgagaaacagaattttttcagccttagtggcc
aaccaattaactaaggtttctgaggttatgggagaccctttttagaatacgaagacaaaacaagctcagctaactgctttggggaaattgaataa
aatccgcctctttctctttgctcgttgaaaatagttgacagattagtgacaacgaaagatgctaaagaccttaaagcatcattgattatatccgaag
aaaagttcttctgagagactatattattccagacagaagtataataggaatagccctcaggatagaataaaagtgcgcgagcttccggtttaggc
aacatatagggtttagacgttagcgttgcataaagaacgtcaagaagaggcttcatggaagaattcagtttttcaacatatgcctagattgcatta
gcttaaatttttttgaaatctcatatatataccttagataagttgatgggtggtatatccgtctcaggaacataaagatgaccctcaggaactagttt
ctcataaaggaaaaacgtttctccagctttatcgtcaaaccctcttacttgagtacgcaaagggttactattttcggtgggtgaaaactgacgacgt
acttcaaattcaatggcagtcatcacactttgcacagaagataaattctccagctctatcttaaaaggttttataccagaaataatattcttttggtg
cgatgttcctgaaggactagagatattcacactacaattcattccatcgagctccgttttattattcaaaatccctgcatgacaaagaaccattcgt
aacttacgtaaaagggcaccagtagtttgctcttcgtgtagacatggaggtacagtaatttttatcactggtgatccagcacgattaaaatccagg
aaacgcttggtaggatgactcgcctggattgactctatagcatcctatgttgaaaggttgttagtctcaaaagaaatatatatatggctaaatcata
cctgctccaactgtatggactgaataggaatacgcatatcttcaacctcatccaaatcttgagagaggtctatgaatccattttctcccaatggcat
ctcatgttgggtgatttgataaccagctagcgaatcaggatgaaagactaactttcgatcaaattgacaaattggcgcaatatcgcagttgagtgc
cagcgctccttgaatagcaagccttaaagcttctgggtttagagactgttggtttcctagttagcaaagagcacatatttagactaaaattttgtgat
tctgtaaaaattcataccggcatagacccaggaagggccgcgtcaaagaaagccacagaagaatttggcgtttcatgctttacacttgcagaact
aaacaacttcttggctgtcgccaattggaaacgaacttcaaggctgatgtagatattccaattcatgatgctccaagttttaggctatatatctgatt
tcttaaatcgattaattcatggattactctgctccaacttgtttacactgtaaagctaagagagcaaaagtttttgaccatcctgcaacatcaagctt
gaaagaaattgaagaaccgtatattagatagtgcaacggtacgtaggtttaaaaaaatttggtaaatactataaggatctctacagtattactttag
taatttatagtgaactcttaagattcgttaatgtatgtttacaaaatctttttttaattcactattatgcagctcagaccttctttaaagaaacagctca
attctgcctttcctaaacgaacaacaatgtcgaaagctgcaacgtctcaagtggcatttcaggaccgagaaaagcctcaggaagttcgcttgtcg
aacattactgctgcgcgctccgttgcagacgcgattcgcacgagtttaggacctagaggaatggataaaatgattcaaaccggaaaaggcgagg
ttgtgttaacgaacgatggtgctactattttgaagcatttgtcagtccttcatcctgctgctaagatgcttgtagacttgagtgcagctcaagatgta
gaggcaggagatggcacgacaagtgttgtagttttagcaggatctatgcttagttgtgcagagaaacttttgaaaaagggtattcatcctactgtg
attgctgagtctttccagcgtgccgctgaagttactgttgaatgcatgaaaaagggagctctcgatattgaattatcagacagagaatcgctcttac
gtgccgctaccacgtctttgaactctaaaattgtctcccaatatagctctttgctcgcccctattgccgtggatgctgtattgaaggtgattgatcccc
gtgtcgctacaaacgttgatttgaaggacattcgtattgtaaagaagcttggtggtattattgatgacaccgaattgattcctggtcttgcattaacg
cagactgctgtgaaaacggctggtggaccgtctcggatggaaaaggcaaacatcgctcttgttcaattccaactttctcctcccaagcccgatatg
gaaaatcaaatagtcgtgaatgattatcgtcaaatggataagattttgaaagaagaacgtcaatatttactcaacatgtgcaagaaaattaagaa
gtctggtgctaatgtaatcctcattcagaaatccattcttcgtgatgccgtcaacgatttggctcttcatttcttggccaagcttaaaattatggtcat
caaggacattgaacgtgatgaagtcgagtttatttgtaaatccacaggctgcaagcctattgccgacattgaatcctttactgaagataaattggg
ccatgctgacttggtagaagaagcctcttcatctggtgaaaaaattgtcaagtttatgggcgtaaagaacgctggtaaaactgtgtcaattctttgt
agaggagcaaaccttcttacattagaagaagccgagcgttctcttcatgatgcgttgtgcgttattcgttgtcttgttaagcaaagagctttgattcc
tggtggtggctctccagaaattgaggctgctcagcaccttttggatgtggctcgtacgttggaagggcgagaggctctttgcttccgtgcattttcag
aagctttagaaatcattcccgttacccttgccgaaaacgctggtttaagtgctattcaagtcgtcacagaacttcgcagccgtcatgcaaacggag
agaaaactgctggtatcaatgttcgtaaaggaattgttacagacattttggaggagaaggtcttacagcccgttcttgttaatattagctctattca
gctggctgctgagacgacaaagatgatcatgaagattgatgatataacattggctcgttagcatgtatttgtcattgttgcttatttttaatataaaa
atggtgttttggtaagaagcttcacacaatatatggtgtttgtagtttttgttccattcaaatagaaaatatgtttttattgtacagaaccgtaatata
aaaagttagaaacaagattacctgtatggtgagctgggaaaacatttgttggattgtttcattacgatcaagaggaggataacgaaaaaagata
cattcttattaaaactcatgctgttcttatttttttcttctggaaatcgtaaaacaaggaaaacaatttatgtagaagtatggttcaatttatcgtaat
cgcatatttctgccccggaatcgctttactagaggaggaacaattgcagctgtgatgggtaagcgagctacaatcaaagccttatgaatcccata
agcaacgaccatttctgtccatatgctagaatgatacgtttgaggctcatctggggagggaacttcgttgggttcccaattgaagaagcgacggag
gcttcccactaccgattgttcaatgtaaccaatcttctctgtgcctaatgtacgaacaaaagcaaaggatattccaaaatcgatggcagagatgcc
taagtatactcccagagcccaccagccatactttttttgtaagatccttcacacgttgactgaggcttaaacgacccgtaggagcagatgagcccat
tgaacgacgcacaggaaaattatataaaggaaggattggatttttagtccatgagaaccgggatagacggaaatttcgccaaaaactgttgctg
ctcatagagatgcttaactataggagggaaactccagcgtaaatttaaaggcaggtaaaaaagactcaatttctttttttattattttttaaatagat
gttagttttattctcttttcttttccaagaattgcttttcttattcgtataagataatagagccatcttaggttatatccttctgaataccgatgagaaac

gatgtctctatcaaagatagcgaatttttgcgtataagagagtgtgtctacatgatgtttatctgtttattgacagttacaaaaaaaattggctgtat
gaatgcttgttaaaaaacatgttttatattacatttaaaacctcctaacctatagggtagctgaggtggtgtaaaatgatattcgtgagtacatattg
ttcagtttatgccggtgtcatgtgttttgatattaaacaactgatcagatataaagcggaggaagtatttcgttcgtggtccttttcagtaggtatttc
ttgtcatatgaaagattaattttctctaagaaatcgagaacatgtaatgatcattcaagatggtagttggatatttgaatactgggtaacaaagag
agaaaaacattaacaaaattggagacaagaaatcaggaaaagatgaatcagtgtaggacgagtatggtatatgttggttttcttgaatacttcac
gtttcgttggtgcaagctgtaagaaagaaagtgtttattaaggaagaggaggagtcagcgaaggtatcgtttggttcatgcgcaagaattgtcttg
tctacccagtttgatttttgtttccatgaacatcgtatagttgttttcgagaaataggttcagttctcatgaaaggtttgcaaaaaaaatttaatataca
gaaaaaaggagcttggccaaaattccaatgaaatatctgtttctttagacaattagagacaactaactaatttattgagtttgttactcttcatgaa
aacaggtaaacggaagacgaaagaaaataagaacagcggaatcaattcttttttaagcgatcattttaacaaactctacgatacaactgaaag
aaataacgaaacgatcgacgaacaattaactttcagcaagcattttcataaaatcaagtttcatcaagaccacatggtttatttactaacataacg
tactagttatccttatcgtaaacttagtttaaggtgatacagtgtgttatctagcatcctgttgcagaaataaacaggaactatgtgaaggaggtttc
tatcgacaacgaacttgtaatttacagtaaaagtgaaaataaacgaagtataaaaaaaaattagtgaaacaaaaaggatcgaaacaggttctaa
ttgtaaacaaaggaccagttacgacaacaacgtggtttgttgtttgtttacattttccaaagacgattcctccaccacttaaccgactccaaactcct
attgtgaaggaaatgtgtagattttagagactaaactggataaattttgatgaagaatgctggaactaaaaacagatattatcgacgaatctacg
tctagcacaatacttgatttacaagaaacagaacgaatcgaaatccaaggaagctcagtaaactattatgtaaacgatcacttgaatcgcacatt
tgattatagtcttgacggtcaaaacgtgcgagcggccctcattacaacgttcgatgaaaacaaaaaagcaatcgtagttattctcgatgatattgg
gtacatatattatgtgaatgatcaaaataatgacacgtatattgtgaatatcccgttttctaccaaaagcgcttgggcttctccaaagggtatttatt
tagaacgtcttcatcttgatagcgaagacgcacaagatggatggcctcgcatcttttgtttgaatgggccattggacgagttgagtttgatccgtat
tgaagggaaaaaaaagctttctgctcatgattccattgtttacattgtcaatgacatcgttgtgacccataatgagaaagaaaagaggctatcgtt
ctggagaagcagattttcagatacaagtacacacggtattagtcaaaggcctcgaggaaagcggagatctagtgtcgcctcaaaagacagactt
tccgaaatcactcggtcagaatctttgtattactcggccactgattccatattcaacaagtttgaggaagagggatttgtatattctgctgtcttttct
cacattgaatcgttccccatgagttccgtaacttcattcgattccgtcatcgttaatggggtacttgttatcttctctcttgtcaaagaacttcaaaaa
gcttatatgatgatttttcgtcttgtacaaggaaaaccaccctattttctggattctttacagctacatgctaccaacatcgcggcattgaaatcaaa
gcatctccagaaaatcgttgttctttctgctaaaggaaaggttactttggaatctcccatgtcgccttcacttaccttagaagggtcatttcgtaattt
ctctgtgcacggtgcttccctttatttggaggatacgcaaggaaataaaaggtatttatcggttgatcaaaacgtctccaacagtcttgttaaatgg
ttgtttagagtactacggcatattttacctctccgtgagtatgaaatcttttatacaggatatttattttctctgtttacttataaacttaatgatgatga
agcattttttcagcagttttagcatgcttttttcttcttttctcctacccgagcgcttccaaagaaaacagatacactggaggaagcagtttatctttc
atctctaataaactttaagaaagaaatcgagatcgcctttttcctttcctcagaatttgattattcttccttcaaatcgtttcttccacttattagtctat
ctctacatttcatagcagaggagctgaggttggacattactgcaaaaccgagaaaggattatttagtatcctttttgtatcaattaagtcgtctgac
atgctggaagcgtttcaatgaatattatgagcttgatgcctcagatatttgtaaaattgaaccgctattattagaaacgcgaattcaagaaccaaa
tgaagttccttctatatataagtggatgattgaatgtttttatctccaaaaattttaagctcttacagtcctgaaatttatggtctaccatcttcatgc
tatgccctatttcctcaaagctatgccattttccaactctttgattgcttgtttaatcatcaaatgtcattgcataactttgttgaagagatggtgcgtc
taggcataacaagaaaacgtattgaaagatatccctcgggcatttcatcgatttttttacaccatattggatatatgtgcttatgattttccaggatat
tggggaagcgaggaactgcagatcgtcaatcgcttagatgttgattgttttctacatcctaaaaagtcaaattggtttctctctactccgacagaag
aaaataagaatatacgagttttaacgaattctgtaaccgactttacacttaatgatctcaaaactacccatgctaaacagccttatactgacatgat
tttccgcgaggaccgtcgtttagcagaggttgaacagttgctatgttattccaagcaaatcagcgttatgactgatcaatttgatattgatctgagtt
cagtcacgatgcaacaaaaaattagctcagtcaatttgtattaggagtctagcaatacctatcggcactggtatgcttacgtatggtaccaaacacc
ctcttccaactgaaagaataaatccccttcctttttaagttcaccgttcatctgcatcctggtactttgctaattaatcccgataaagatttcataaccc
cttcgttaactgaatggcctgagttccatgcaggtgtctctcagggtctcacagtctccaaactatctacagaagttaatactagttggataatgttt
aatagacctggtacccttttcccctatcatgctggattcttgctcggtttaggcctcaatggccatttgaaagaacttgctacatggcattccttcatt
tacttaactagcaagcacgatgctactagtattgggttgctgttaggattatctgtatcctacctcggtacaatggatgcaaaagttaccaaactgt
taagtgtgcatgtattggcactattacctgtgggatccagtgagctgaatatctcgcctttaacacagactgcaggaatcctgggaattgggttgct
gttttacgactcctgtcatagacggatgtcggaagtcaccatggaggaaatattaacgcctgaacaaaatcaatataaaaacgaaggttacaag
cttgctgctggtttcgcgttaggattaattaatatcggaaaaggctcgaatcttcctggaatggcggatttgaagcttgtatcaagactacagatgg
ggatatccagtcaagctgcctttcaatctttggaaaccggatctcctggagcaataatggcattagtattaatatatttaaaaacaaatgatgtcga
aatcgcaagtaaaatagatattccgaaatctagatatttacttgacttttatcgaccggatattattcttctacgagttttggggaaaaatcttgttat
gtgggatgaagttaaagcagactttgaatgggtgaaaagtcaaattcctgatattatgatctcacaatttgatttgcgcgaaaaaaaatatactatc
ttcagatgatctacttttgtataatgttataagtggtatctgtttttcacttggacttcgttttgtcggcacaggaaacttaaaagcgaaagaaattct

tatcaactttttggacaattttatccgcctttgtcgattaccagcaaagacttatgatgaaagagtcacttatgttaccgtcatccgttgcacacatgt
agtagctctttcagtagcatgtgtgatggctggatactgtgatttggaagtgctccaaaggcttcggttgctgcacgggagaattgaacccgtaaat
tatggtgctcaaatggctacgcatatggcacttggtattctaacgttaggagaaggtcgatattcgttgtctcatagtaatttggcgattgcagcatt
acttatttctttatatcctcagtttgcgaagaatacacaggataaccatttacatttgcaagctttaaggaacctttgggcactcgctgttgaggaaa
gatgtttcattcctagggatcaagatacgaagaagccatgcgttattccaatgaaaatatgtcataagaatggtacagtacaaaattttgaagca
ccaggccttttaccttctcttgatagtatacactccattacgacattgagttcaaagcattggaaattgaatttggatttagatgcaaatagtacttat
cgtaaactgttgcagcaatcacagattctaacattgatgccttatgacgatacagaccctaaaagcgaatctccaaacttattgagtcagatagac
aaatcccctaatccactttgggatttgattaaaacaagcagtttattccaaggaccgaagagtgccttaagtcaggcgactcagtcaaaatcacgt
acgaatagctcgctttcgacaaaattgtcgcttactatgtcgttgaataagctttcaaatgatcagcttacgagcgtccaaattttgttacaattttttt
gaatcttgctggagtggtatcttgaaggacaaattccaaaattgtcaatattcgtttctatctcgtgaatttgttgaagatctgagtcttcgagtttgg
gagcttatacatgccgttcctaatacaaataatgaataatgacctttatgaatgaaaaataccgggtttaatgatccgttatttgggatagcaatag
tattccaattatagaatgatacttcattatgtactttccatattaaataaaatgttgctatacttttacaaagcataaggatcgctgttatctaaaaga
acagagcatattttctatgcagccatggataacttgagcataaaaaccgaggaattcaaacctaatacagatcatgtacgtgaattcagttagcat
gaacatgatacatactgtaaacgtcttaatccactgaagatgaaaaaaaaaacttcaatacgttctgcattttatgattgtaagtctcaaccaaaa
acacatttctaccttacaggactctcaaagcaacttcataaacttactggctttttatatttcgtttccttgattttcatttctactcgctactttctttgt
atctattgaatgtcttgtaatgcttattttaccttttagtgtttatattttaacgagctcggaagaatcgtttaatggaatagccgtcgatacgcaagt
gttcaatggcatattttgcgcgggtgatgggctacgttattcctcctcaaattatgacttcaaacatattggggaatgacaacaccaagcttcactt
gccctctgcaaaacacaagttctaagaaagcattcttaaaaacgaagagagactatttgtggaggagttaatttctgcaatattgcaggggaaat
agaaagcacgaagcaaataaggtagaataaagattacagtcagtagtatcgagttatcttgtttgtcttccaatcaacgggcttctttgaggataa
agtacgttgaatttgctgttttattacagaataaaaaaagggattcatctcttcagcattcggttactttcaaaacaattggaaggaaaaaagtgg
atcgtttggactttaaactatcatcggaaaattactgagtttatcgataaactttggttcttcattgtcttgtaaatttcaagacttttactgtgtttgtg
tcttatagtaacacctatcatcttatcttattttataaattttccttttcattaggaaatatattcagtgtgttttaaaatgcagactgcagcatatcctg
catcatctaccgatcaaactagcaaagtggatggctacatggatcggcaacccgacggtacccccaaatctccgcctcaagagcaaaaaaataa
tggttcagagcaagaagagcaaaagtctgtggaaaaaacaaattcttcgtctaatgtctattcatctggtattcctgccgctttagacgaagcaac
tatgagccagccagcgccatcagcttcagagggacctcaagtagagtccatgcaatctatgcaagaagccggatctgcggaacaacaacagcct
tcattttccacggataagccagccccttcgtctagtgaagccagttctcggagtactgagactcagcaatacagaaataccggaagaagcgccga
caactttcgagatcgccaacttcgcaaggcacgccttgtacagttacttgatctacagagaaaacgaatccgtcctgcggaccatacaaccaagg
gtcgctatggaatccaagattttgattgtctgcaaaccttaggtacaggatcgttcggcagagtacatttggttcaatccaaacacaatcggttgtt
ctacgctattaaagttttggaaaaacgaaaagtggttgacatgaagcaagttgaacatacttgcgacgagcgccatattctttctagagttcagca
tccttttatcacaattttgtggggaacgttccaagacgctaagaatttattcatggtcatggattttgcggaaggtggtgagttgttttcccttcttag
aaaatgtcatcgttttccggagaaggttgcgaagttttatgcagccgaagttattttggctttagattatttgcatcataaccaaatcgtatatcgtg
acttaaagcctgaaaatcttttactcgatcgatttggacatttaaaaattgtcgactttggctttgcgaaacgtgtttctaccaataattgctttacgc
tgtgtggaaccccggattacttggctcctgaaattatttctttgaaaccctataacaaagctgctgactggtggtccttgggtattttcatattcgaaa
tgcttgctggttaccctcccttttattcggagaatccaatgaaactttatgagaatatccttgaaggtaatatctcctatccccaatatttttcctcgtc
tagcattgaccttatgaatcatcttcttcaaagagatattactaatcggtatggaaaccttaaagatggaagtatggatataattatgcatccttgg
ttccgagacatcagttgggacaagattttgactcgtaaaattgaagttccttatgtacctccaatccgtgctggcatgggtgattcgagtcaatttga
ggcttatgctactggtcccacgaattatggtggcactgagaatccagaattcacggatattttccaagatttttaaactgttgtgaaagactcagaa
aaccgttggtatttattcctatctttgacaaccgtacataggataaatcgtgaattattatgtatgattttgtatgattttgttatgttattttggaatcc
ataactttttgatttttcttactgctttttttgagttctattattgctgagttttccactaattcgtcctcatcttaaaatatttattaattgtggtatcgtgtg
aatataccttgtcatatgatctattttggacgagatctttgaaaaagaaaatgctgatgttatgcgtttttagttcaagaaataaaaagtgttagtct
ttttttcctatatttttcttttacagttttgatttctatgatgcggtttagcttgctctgtatcaatcctaatgaaagaatcccagctttttatttgcaagta
atacctacaattcagcaaaatttatatgctatttctattgtgctttgactaaagtagtaattcacgttttaatttaatttcattattcatgtcacatcccg
taatgaaatggttattataggttaattttacatttgtgtgtagagtgtcatgatttaaaatgatcttttataaagcgtaataaaaaacaaaacaaaa
ctgtgttatgaagcatcagcagtcccttgatatagaaactttgtaaaacgaagatattccattgttttagcatgctgaaataataaatagaaaaca
agcaaaaaggttagaggtgggccatctttttcaaccaaaataccaaaaaattcacttaaatgatttttttcccaaccaggaatgacttccgatcgtaa
acaatcggggtcattttccccttgaattttgattccattttcccgcaaaaactgaacagatgagcgagtatttatttttacagcctcataaggatcag
cctttacttttccctttaaaattttgagaaccatgcttggtaaaaggataggcaagtcactttgatttgcatggactgtcccattcaagacgaatacg
tgcgaagtcttctcatttgaaacaatatcaagtttgtctgcagcactttcctgaccaatttgagactccgactcgcgatccagaagccacgttttcaa

ggcctcgtaattatcggtccagcgatgaaattcataggagataatcatcaatgtagggtatcggattttctgtatattcagctgacgaacggggaa
catccaaatatcatacaaaatcgcacatttgtattcttcacgatgcaggtaattgtgattgagcgaaggagttgaagaatttgttatataagcaca
ggtggcagctccaaaagagtgacctgaaagtaaaatctctcctttggtagtattcaagcgaccctttagtgattcaaaaagacgtgtatataattt
ggggtcagcagagcaagagtaggctgtgtctagactaggcacaccgtgttcgttgattgagcggatcattttcatagcaagttggatttctctttca
cgaaaaaaaaacctgtccttatgcaaagcaacagtactatcgctataaaaatctttgatttcatggtattgaagaagataacctttatcatccggg
tcggcaggatcacgaacagtcgaaataatggcagtctggtcgcggtgttctaatgccaaaacaacgatcccatacgaagaaatatttccacataa
agaagaataaacgtttctcgaacctaaaagtccatggctaaacaataataccggcagcttagcattttgactgtcctgatatatatccccatcgta
aacaggaagcgacgttttcgaaaaccatgatgcacatgcgcgaatcatccaccatttaaaccctttagcaatctgtggaactccctcattgaaag
gaagccaaagctcgtctttccggcgtctcacatccttgttaggaagaaggggataaaacattcgaagtttaactgttttcatctcatgttcaatgct
aggaagctggcaccgaagttcttcaggaacactcgtttcaatgactaccgaaccgacacgcaatggaccttcatactctggcaaggaagtagaa
gtgaattggaacatgataacagaaatataattcagaatatcaataaaagtgacagtaaatcactcggaagatgacgcagaaaacccagaatct
caaatgaaaacaactgaatgaaactatgaaaggatcaatgagttagctaaaaaaacatgcatagaaggaaaaattaatagtttttgacgatat
attgatgcaaattggtcggagttgattgctacaatggctgttttgccaggtcgacaaattgtggaaatggtgtgttttgtatggatgtatggggaag
taggacgacaaaatagttcatactagaggtacaatcgtctacagtctgttagttttaaaaaagaaataaattcattaagacctacttttctctgattt
ggtgaagtacaagcttcaaacgctatgaattgtcaagctgcatatcctctgtattctgaacagataccgaaacgaagcctttgttttctgtttctata
ggttttgtattatcaggagattcctttggcatgtgttcacaatttgttggtgtttggtaagaagctgcataagcatccagttctcgatcgaggtcctca
tgagaaatgggatggacatgtgctatttctttttcattgcctggatcccttaaatccttgaatgaggctttttatgagaactcctagcattaacggg
agaagaaattcgatccagaagggaagtaggaggctttaaaaacagttttatcggttgattattaacaaaatgaccatccaaagattcaacagca
tgttctgcttcttcttttgaggagaaaaaagcgcgacaagttccttcagaacgaccggcacgatcataatccatgagaatagaactaggatgaaa
ttctgataggaatgactaataaaccttgtcaataataagatagcttaaaatattttgagaagatagattgattgaaggtactgcatcgagaaagct
cttggtttcaaaggatgacttgtaatgcgtttagaagaccaatcttttctctgagccttttaactagaatgatatttgaggctgcgcgttcgaaagcc
gctgattgtatgtaaagttgttttcctaaatacaacctgcaaggtaaaatgccaataaccatggtcactatggagcgagaccttaagggaaaagc
cttcgttgctacgcattcatgaaccggctttttttgcacacaatgttcggtttcttccagtatatctattatcaccagtagcaacttaccctgacatttt
cttcagatagctcataatgtaaattttcaattaaaacaatatacaaagattcggtggaagaatttctgaatccaatgttagcttgcatgacggtcca
atacatctcaaattgctcatctcggcataacagactgtacatctctgagcatagaagcatacctgtctcttccctttctctgtatagcttcttgctctg
aggtaaatcatcttcttggttcaaatcatgctgccatggttcattgtcaatagaaggaatctgtgatgtaaagcgtttcactggtctctgaatgcaga
aggaatcatgttagccttatatcaagctaaagattgtcactctgtcctaaagtatatacctttcctcgctttttgtcaatcctattctgacgatcagatt
ttcttggttttcgaaaaacactacttttctatattgtaaatgattttgtttattttttagaacttactttttttgtccttaataatttcatctaaggatctatc
cagcgacatattcatcgttatggttcttgacgtgaacaaatccccagtcgtttgctttgcatcctctaccaatactaaagtaggtcgttcattgtaaat
atctcctttcataaagaaaatcctattctgctgaacctttcaaccattgcttctgcttttgtcttgtggtattgctttttttcctgttgtttttttaaagtct
attgttcctgcttcatttgagtgatattgctctttcttagctttataaattcgctcttacagataatctttttaattcttttctggtatagactttactgtga
atatctcttgcaggttttatacaatcataatgagtttctccccggagcaacaatttatgctggaaaagcccggaatcttgctgtcttttgagcaattg
agattaaattttaagcaagtattacggcaattggaacatgagtctcatgtaatgacctctacgcttacagctttgcttcaagagtcaatccccatcg
aaaacaaggtcaaacagatcgatggcttggttttaaggacacgaaatttgaaacaaaaggtaaaaatccttgttgcttattgctttactgacgttg
gtttagatgacgcagctcaaggaatccgaaaaaatacatattcaacgaacacgattacgtttacagtttataaatactctcagtagaattgaagat
ttggattctccagaatttttggactggtcgaggactcgtttgaatcgattaattgcggattatatgttagcaaatggatatcatgactctgccataaa
gttgtgcaagaactctgacctgtttgaaatggtagacattgatgtctacaagagatatcagaaaatacttgattccattcttcggcatgagttgaaa
gaggttcttgcatggtgtgccgagcatcgatccattctgaaaaagattaacagcactctggagcttgagattcggatacaacgatttgttgaacta
ataaaatcgaataaaaaagttcaggcaattaactttgccaagacttacttcggtaattggaccaaagttcatgcagtccgtcttcagcaggtggca
gctctcttggcgattccatataccacgaaagcttctcgatacaatgtaagtgtactaatttttttatgggcatcattttggtctaacttagttcttagga
gtatctttctgataatcgatgggattttctagcgtctttattttccaaaacattcactcaagtccatagtctttcttcggtttccattttgcatattgcaat
tgccgcgggtctttcttctttaaaaaccccttcttgttgtagttacgaagctgcacatacttctcactcctttcaatcctctactattcggcagtgcccc
gtatgcactccaaacctaagcgtgctcgctcaatctttgccttatgctcatgttacgcaatccgtcattgttgattcattaacgggtgcacgactgga
tagtgataatcgtcctgtggcatttccaaatgggcatgtttacggaattaagagtttagttgcatggaatgaagcaaacggaatacaagaaggat
atttaagggatcctttttcaggaaaagagtttgcttctgaattactcagaaaggtatatgtggtgtaactccatccctatcttcagtaaagcgttcct
aaacaaacatgtaatgatcgtaatgagtctaatgctaattaatgatatattgatttttttgcttaaggtacatcacaaagaagctttatgcaattgct
gtattataatgcaataatattaattgtcctaatccctaaaccgattcctttctataaaacaattaccaaaaaaaactcaccagagatatcttaaatca
ttgacaccttttcagactaatcttcctttactttatacgtttttttctcgcgatcgatatgatcacgcaaagatttccgcttctgcataaggcttcttccag

cctttcctttgactttatgtttaatcatcataaagtttttgttcttttgctttctcttatttgaaagactcgtaggattaaattgattactcttggatccaa
acttctcgcgaccttcacgaccttccatgacacgtgccaaacggccttcacgatcgtctttcatccttttctttggaccttcaatatcacctgcatcaa
caacggcgtcgccagcatgtacacgttttggtccatggaccaagcggtcaacgttagctttttcacgcagttcttccagcttctttaaatcagctgga
gtcaaaattgatctagcagccaagttagaagcttcatttttatgctcgctgtcctcaagcttagctttatcttccgatgttcctccggtatctttttgatt
attattttccccttcactgtcactaatattgatatcatcatcacttcctaggttaacccaaccttcgctttcacctgaatcactttcattgtccacagctt
cccagttttcccatttgctatcgtcgtcctcgtttccaccatcttcattttcatttttatatttttcaagcagttctaagccttcaataccgtgaacaacgt
ttagttcttcaccaaacttgattggagcacgatccttcaactccatcgatgcaagctttcctcggtcctttttcttcaacatgtcaggagcaacttcac
gatacaaagtaatgagaccacggctagccatcataacacctttatccttgctagacttatattcggttaaatcttgtaatagatctgccgtcatagc
aaatgggcaacgtgcacaaacttctcgaatggcgttaataccggcgcacacaacttcattagcgacacctgtagtgacaaattcgtcagcgatttt
tcggatcaaaggttctaaaacatctggaggaataaattcatggctagcttgagctaagcaagctagaaactgagtaacttcacgctgatggggag
tcagatatttcatcatgtacgagtaaagacctaaaacagtcaacttgtgagtaccaatcaatctcgtaagaagacgaagaactacaagtttatga
tccatagagagagaacgatttgaattcactaattgcttttcaaaaagttcttctgcaaatccttgaggatcatataaaagttgaagagcaggaaag
ttaacatttgcagggggtgccatcttccccctttctttttcttcacaagagcacgaatacggagtatctgagcttcacgagattttgttttcttattaa
tattcgattgatgcattaatttggagacatccaaaccatcgtcttcttcttcttcttcttcattttcttgggtatcagcacctaagaaaaaatatat
accgctaagcataactttggtgttgtttgataaagcggcagtcttcataatgttgaccgctcgagcatcaccagaccaaatatttcgcttccataag
tcttggaccatttttacagcccacataccggaaagctcgtttggggaatattcatgcttcttgttagaataatccgtatgtgttatagaggccgtatc
agaagtttccactaaggtaaataaagcagcttgaacagctttgttaagcttatcattcttggctttcttgtttgcattacgtactgtaagagaaacct
gctcatataggagttcacggaaggctcgagaaggattactgctgaagagagggaaaaagcactgtagaagtgtgataggcgacagagcacttt
tgtttttagtaaaacaagagcctgtacgatttttcagatagttcaaaaggaaactggtcatgattttttgaagtaagtccattaattctttcgaaa
agtccttagtaatgtccgtataatggttacatgtttgagcaataaagtcaacaagggaactgaattcagtaacatcattggatggttgaataagga
aaatttctctaactgtctcataatggttccattgctgtaaaaattcgtcgcggtaggatttggggtcctttttcaccaaatgctgtaaatgaggaaga
ttgatgggaagaactgcggccgtacgacgctttaccatgactcgtttttttcttataaatttatgaaaaataaaaagcgcgagaacaaaagataca
gaaactgaattatgatgtggcacctactgaaatgcagataagtgagaaataagatttcaattgagatttatatcaaaaaaaaatcccaaatgaa
aagaaaaaagaaggaaatcttaataatgtctttctttggtaaattctgaattcgctaggaaaaagcgaaattttgcagaagcaaacgggcttaga
cacttattgattaaagttttaattaaaaaagaataaaagaaacaatcaaccaagaaaacggagtgtttggaaacaattccaagttttctcgtagtt
ggttgttggtgggtggtacgaaggagatgccagaagttctcttacccttcaccaacattgacgatgagcgtcacacagcagcaaatgctctatgat
gaagagcaattgagattgatggaagaagtctgtattgtagtagacgaaaatgataagcctgtacgatctggcacgaaaaaagaatgtatggaac
tttgatatgagagaaaacagttaaactatgcgaactcacaactaatggataaactaggccacttaatggaaaatattcataaaggattattacat
agagccttttccatgtttctttttgataatgaaaaccgtcttttgttgcagcaacgggcagctgaaaaaatcacttttccttccttatggaccaacacg
tgctgctcacatccattagatgttgcctctgagcgcggatctaaattggatgaagcggttatgggcgtgaagtctgccgcacaaagaaagttgtttc
atgaactaggaatcaaggctgaatatattcctaaagaaaggtaccagtacttaaccaggattcattaccttgcacctagcgttgacaaatggggc
gagcatgagagtaagcacaagaaatcctttgaggctcacttttgctaattatcaatgtttagttgactatattttgttctataagggtcatgttgagtt
ggaaatcaatcctaatgaggtacaagcctataagtatgttactatggatgagttaaaggctatgtttgaagatcctgaatacaagtttactccctg
gtttaagttgatttgccagcactttttgttcaaatggtggacagatgtggataattcgagcaaattcgaagatcaggaaattcatcggatggtatag
aagggtttgactttcatgatgtgctatctctgaaggcgttctttttttactttctttctttttggggcaagcttctatggttttgtctttgaattcggaaatt
aatatagatttgaggatataagtaatcttttgttgtatttacttgtcatatataccgggcacgtcttttgaagagtctatggaaaagctcggtctaatt
ggcactattagaagtgtttcgcagatttaccattttgcaataatagataataaatacagggtgtaatgatataatattcgatttggcggtatcccga
gttgaaggggaatgcatagtttgaatggaacataaacttttttttgtggtaaaagaaggaagaaagaacatgaaacattttgaaagctggaagc
aagatacaaagtgagttcagctcgttacgatccaaaatggatcagcttttctattgttaagacgcaccatttaagaaggcatccgaaggcatttac
ctctttatgcaggaatttaacatacttataaagctcttgtggccctccctcttagtaggtttcttatagcgatttctttagcttgctactataatactgc
gtttacgggaaatcgtttcctggtgattattctctaaaaacacaagaggttttctgtcataattcagtaatttgatgaaactaataccaaagctctgg
agcgcttatggatttttgcccttggtttttttgtttatgttacaagggtgttgatgacatcaaccgttttgcccaatcagaaagtgacaagatatcgct
tggtaatagcttgctgtactctatttttaacgtgcggggtatttgaagctttttattggtgccgttaattaggtgaaggcacatgcgccatacatttcg
gtttccccaggaaaaagcgaaggcaaaatgagcaaaagaggacaaggtttgtttacttatactttcattataattagaaaatggcttacaaaatg
aggggaaggcaggagaaattaggtggaaagcattacgaaagattgattttatctagtaaagatggcttaaataggagcttcattctcctggttttc
cattcttcgcaatactagctttctgaatttttggctctgcaacccccttcttggacactagagatactagtcttatataaaaggttatgaaggagtctac
tgcttcctatcatggcgatagccaaccgaaagatcaaagtgtggtcgagcacattgtaccctccgagactatccaaacaacagaattggaagtca
gtgatgatatacttggcgagaaggaagaagataccactcctgagttagagagtaacttcttgaaagaattaggttgtcaacacttggataataaa

caggaagaaaacttcaagtcatttgattcactctctgcttctacttcttttgactctcctaacgatattgatttttcagaggtgcctggcgtctcaatta
atcttttgaaagagaaggaaggactgcttgacccaatagaacatgaacttgctcagtcagacacattccaagaacctcaactgcaatcggctgaa
aataaattaaaagactcattttctcctgctccaaattttgctcctgcttccgatctatctgcgaattctgttcattctttacctaatggcgcatcttctgg
aaatatgtattatggttctaacccgttttacccagttcaaatcattcctattgtcgataatccttactcagctcatcctgttcaatgtactccttttcaac
cgatttcttcttcatctcctccatttgcttcacaacctccggatccaaaagatacttcaaatttaccctatcaagaaccaagttatttggccgacttta
gaattttaatattgagtaatttaccctctgagtataagataaccgatcttctctcgcatattcatgcgggccctttggaaagggttcaacaaaagca
agaaaaaaacaaggtaatattgtctttcttatttgcagaagatgctatcaagttccatgaatgttgtaatcgtcggagattccaatttcatggtcgct
ctttgaaagttactttcgccagaccgagccgtctctcagaatcagtatacaatgtttgcaataatatggcggcgagtagaaatgtctttattgggaa
ctttcctgctacttttggggaacatgagttggttgatcattttggcaaattcggtgaaattgataatgtgaaaatcctttccaaaaagaacattgcat
tcattcattacctaaatatcaaagatgcaattcgtgtcgtacagacgttgtcttgtgatccaacttatcatgcttggagaattttctatggtatagatc
gctgcgagcatcgtggtgcaagtacgtgctcctctccatacggttctcctaaattttcttcgtcttccgtgtctattaactccgatcctgaactaaaag
aatctacttctgagatatctcataaacctgacatagaaaaccggaccgttttcctaggaaatttggatgcaaaagtaaaagattccgaaatatgtg
acctcatttactgcggacctctacaagactttcattatattcctgagaaacatatatgtttcgtcacgtttgtcgacgttgctgatgcaaaagccttcc
acgcatacatttccaacgaaatggtcgtccttcatgggcgtcgtataaagtctggatggggaaaagcaagtggccccttaccacgtgtattagaa
acttctatctctatgggtgcttctcgaaatgtttactttagcaaagtcagtgactctcttaccaagaaggagttggaattgattttacggcaatatgg
tgaaattgaaagcattaagtacttaaagcaaccaaatacgggatttgtcagttatacgaatatctcaaatggtatgaaagcattccatgggttacc
tttacaaccagctttccagaagagtaaaatacagtatgctcgcgaccgatgtgataaatctgactcttatgtttcgtctactccaagtaactctcctt
cattgaacgcggggcaaacacaacagacacttttacctcctgcaatcccgtttaccattcctatgtatcctcctatgtttacggcaccttcgaatatc
aagtcggacgcatatccttctactccatatgtaccccttttgctcccttttctaatgtatatgcgtctattccttcttataacatccagcaatcaactc
atatgcaaaatcctaaatgattgttttcactgtctatggtcttatccttccttttccgagagagttaccccttttgtgacatcctaatcccttttgacgta
cctgtaattatttttgtaaagtgtttttcttactcctagaattcatgacttggctgattctctatttttttcttgttggcctttgacttatctgtttgcatat
caaatttgaaagcaaatttcttttattactgcttaaggctttggccaaattattcatttttctctttttgcttatttttctccgtttttgtctgtttctctttt
ttgctctctttttagattgtgtttctttgtttacacctcttctctataacttgttcaggttaaaaatttataaatctaccaaaaaaagctagaaaacaa
taaaataaattaaaaaggacaagaaagaaaattaatgacttattgtaaattctactaatccaacgtatcgacttttctgacaatacctttgcattgc
aactgcatcgaatttttatagaacttcatgcttacagagttttagcaattatactaacaataaaaaaaggatcatttacgagtaaaaattggacca
aatctcaaacctcaaaaaaaaaaaaatttttttataatcatgttcctttgtttatttacactttcgtgaaaaaaaccaatttccatgcgttcaagtacg
ttattccagatatgacctaacaacacaatccaccatactgtttaccgtaacgctaaagcgaacacttagccaaaagttgtatgagaccaaaccaa
cctacttggataataccaactacgaaccatgtcaggatacgatcgtgcgctatcagttttctcaccagatggtcgcttgcttcaagtagaatacggt
caagaagctgttcgccgtggtactactgcggtatgtgaaaacaagaaacgaggttgggtgaagctgtgagactaacattttggtagattgctcttc
gcggaaaagactgtatcgttatcggtgtggaaaagaaaaatgttcccaaattacaaaatcctggaagcttccaaaaaattgcgatgatcgacaa
tcacgtttgtttggcttttgctggtttaaatgctgatgcacgagttctgattgataaagcccgtgtagaggctcagtcacacaaattgaacttagaag
accccgtttctgtagagtatttgacccgttacgtagctggtgttcagcagaagtatacacaagcaggcggtgtccgaccttttggtgtgtcgacact
agttgccggctttgatgttggcagtaacattcccagagtttaccaaacagagccagcaggtgtttacaatgcatggtatgtgatattatttttgggtt
cgaagtattttcaattcccgagtatggtcagagagtgtactaacactttgtaatttaggaaggcttctgctatcggtcgttctagtaaggctgcccgt
gaatttttggaaaaaaattggaaggaagacttgcctcgtgatgaatctattcgtcttacaatcaacagtctcttagaagttgttcaaacttctgccg
gtaacattgagctggcaattttggaccctggtaaggacgttgaatttcttccttctgaacaaattgattccatcgttaaagtacttcagcaagagaa
agaggctgaagccgctcgtaagaaaaacggtcgtactgctgcttctgcttcttctacaactcaatcatgatttccttttctttcgttctgcagctttgtt
gaaagactttcctttcctgtattttttttacgatgcttgtctcctaactaacgcctacaaatcttcgtcttatataacctttctcataactgatcttatga
aattagagttttcaatgatacttttgcaattgatgtggttcttaaatatggttcccattgctaaaattatggcctatttattatccttaatccttaattaa
acacgccgtatattccatctatataatcttttagtatatatactttttttttctattaaatattccatgatcgatatcaggatttttttcgtataattattcg
cttttccgtgaagaaaacgtacatgaatagaaccaagaaaagtccgctcaagcatagtaactcataacgaaacccaaaacctcttttaggaagg
aagttctacggaaacccatacaaacatatccaattaattaataaaagtcccaacaatcgtatggaataagagcacgcaaactggttttcgtaatg
cgaaataaataccatatcaatcatgtccgtaattattttcttctgtcaaggaatataaactttagctcttttcaagaaagttcttcagcttgcttccga
ccaagtcaaagtctttacggtaaatcaaaaacgtatcgtggccatagtaactttcgtcagcattcaattcatggtatatgactttctcatttccggcc
ttcttcaaacagcgagcggtttcacgctggcaagcgacaggcatcagattatcactctcaacacccattatcatggttggaatatttcgtagagga
attaaacctgcagttaagtctgaatcaatatcaggttcgggagccctatcttcattggatatttcttctattggggtatcgggcaggacggcaatgtt
cgaattcgcttttatcacatctttttgtgagacgtcgggatgctcagccaaatacttttctagtttgtgttgatttttctttctgttttcagcaagaatctt
ctgatttgaagcagtcatgtcatgcatatccatagccttggaaatatacaaaagggaattggcgtcatattgaagacagaacttttctccggcatg

atctaaatacgtctctattaaaaaatcagggcatagggcaggtggtacagcagggttggcgcgttgagtggaaaagcgttgttcccactctggtcc
ggaacgataggaaatggtagcaacttcccgagcaagtttcatacctgtgtgaggaggaatgccatcataataaaaacctcggttccactgagggt
cattcattaatatttggcgctgagtaaaacggagtgcgattgaataaggatgagatctagcaccaccagagatactagcgatacgagcagttctg
ttaggtgcaagagcaccaaatgctaaagattgcataccacccaaagagctaccaatgctagcatggagtttatcgatttttaagtggtccaataa
caaaatttgggtacgaaccatatcgtttacagtaacgatcggaaaacgggttgcataatgttttccgtcaccagggtcgatggaagaaggtccag
tgctaccgtaacagctacctaaaatgttacaacagataacatagaacttgttggtatctaaatcttttcctggtccaataaattgttcccaccaacc
aggttgtgggttatctggatgagaacgagcatgtgaagaggcagaaagacctgtatgtaaaagaatggcattcgagcggtctttgttcaacgttc
cccaagtttcgtaagccaattcgaatttaggcaaaataccgccgtgatcacaaaggaatactttatcatacttaaacttcttaaatccggatacaat
ttttccgtagataggctcaggacctgaagcaagtgaaggagaagtaacgcatacacttggatcctcgttgccagaacattgatgctgctgaatgct
ttctgcgtatctgcgattgcgttcctgtgcttggtcaacgcaaggaaatgacatttgagggttggagctttgaaaccggcgaaaaaccaatctactg
ggccccagccgcttgctaatcaaccgaaacatacttcccatcatgagaataatgccttaattgaatatgtgaagaattcaaatttttttgaaaggg
gatatagtggctttttaaacccttgaaaaaaaatatttcgatctttaacgagacaacatatatcacgattgcaacatttcatcttctgcttacaccat
cgcatggatgctcactacacttgtggtaaacgtcgttaagcgaaaatttcaatttatcacggtattccgcaatttgctgattcagcatattaactttt
gcttaacgaggtgtcagttcgttcttttaatgcttctttttgttacgacagggcaacttacatttttgtgcatcattttaggaatactgtacttgtttg
cgatctgttatcacctttatcaaaatcgaacgaaccatctttatccatagacgcaaacacgctgctaattaatcccaattgtttcaatttaaagaag
aatgaagtttgaacaagataagaaaggagaaatctttttctttttatagtttgatattggaactcatgtctcgtagcaaaatatccttttatttttgat
ttcttgcccaatggagatattggtagaaaacaagagtgcaatccagaaagtataaacatatctcggatattattccatacaatattttaaacttcat
aacgaagcctatagatatcgtatcccagctttgcatgaaaagacacgtaaggaaatttaatgaagcatagcgtctgtatacggtaatatcaaatg
ctcttttaagaaaccatatgagcgtttttcgctaactgtcccaatgatagtcctgttttctccttatacctcaaaaacgcatttaaccacttgttttaat
agggtagggttggactgctttttgggctcaagaagagcaaacttttgtgattcacttgctacaagcaacaaccaactcttagttgacaggacatc
atgagcgttgctaaagctaaaggagcacagaagaccgttcaaaagggagtccacaacaaggttgccaagaaggttcgtacttctacaaccttcc
gtcggtatgtgaaataatttgagcttagctctgtgaatatgggagaaggtttttttctgaacccagtttcttaaagcacagtagatagctcgtgcatat
ctggtttccaatttactaatttttttaatagccccaagactcttcaacttcctcgtaagcctaagtatgctcgtaagagtgttgctcatgcccctcgtct
tgacgaattcaagatcatcctcaatcctctcaacagtgaatctgctatgaagaagattgaggacgacaacactctcgtcttccttgtgcacatgag
atccaacaagcacaccatcaaggacgctgttcgtaagctttacaacgttgaagccatcaagattaacactttaatccgccccaacggtaccaaga
aggctttcgtcaagctctccgctgatgctgatgctcttgacgttgctaaccgcattggtttcctttaaaccatgtattttacttgagcattaaggataa
ataaaatcttggtattcatagatccaatcctttttttagtagtattgaaaagacgtgtactcggggttttcacgttttaaattgtatagtaggcatgcat
gtgcaacaaatgtattaaagtgaacgctatcattttacattcatttaaacatactctaaggcagttattggtgacctacttgtttctctagtaaacgt
gaaaccttttcacagcaatagtttgttagattttttttgtctaatcttggcagtgtaaatgtcgttaaaaatatgattctattcacttaaacaacggat
cagaaatgtaaccttggatttttggatcaattttggcaattctttagactgtaagcctgaaatgttttgtggatttgtcattgtattaatctatgaactt
tcggagttgttcgtctttctttttttatgccaatatccttttgctaggcatgaacaaaaggtgattgactatatattaatttcttcgaatttagttatcatg
tccatttcccataatcgttgttttcctacactgtgggggggggggttttcttttttttttgatgcatcactaaaggaattgggtttgctaacataggtaac
tttgtaatggtataagtatcgctcattatatattattcaactgtttcagcaagagtgttttaaaagctaccgatgcgaaatccacaaaagcgtttctt
caatttaattactggccttcgttgctcgtatggggactttggttaatggaatccttttaattctgaatgcccaagaaaaaagactcaacattggtata
acctctgtttgccaacaaccattttcgctgtaaatctagaggttttgctcacaactatgtcaaggtctgaagcttaatgaatacttgaatacaactcc
tttgatcattgcaaaaaaatggtttcaatttattcttggccatcccgatgctcataaacataccatgctacaaatatatcctgaatctcagtattggg
ttgaattttcatattatttagaactcaatactacttcattgcatatctcaattttttaattgagcttataatctgaaatttaaatcattcacggtatagc
acttataaggtaaagcatccacaaaggttgagttggaaacagaaacgagctatttgactcttgtcaatgtaacgttagttctattttgtaaaaata
aattaagtcataacaattcagatttgacgtgcttggaaactactttgagaatacttaaacaaagttgctttgctagtaaccaaggaaggacaaacc
tttcgtattgaagaaatgttcattaatcagtttcctgtttattttttatacaaatatatcaaaacaaagtccattgcttctacgacttttgtttttgtcttt
attgtattgagggtttattttgtaaacaaacatatttaccagagtgacattgttaccctcatgtaaacactctcagaacaaagtggtaactcactga
aagtgtattcacacccaagagcataaaaaatacttagaaaaaacagttcaaaacgcgagttttctttttgcaacattaaagaaaacttcaatgaa
attttggttgatatcttaagctctgttgacaacactgaatgtggattatctgaaaatagtaaagtggaattcagatgtacgcgcgatgtttatgaga
atatgtaacagaaatgtattttccaatgctctttttgcttcaaaagtgctttgtaggaatgtacatatgcatccccgtaccttagaaagccgggctac
cttgaccacgttggagaacctgaaaacgcatgctgaacggatgaagtttttctcttctattataaatgacaaaaatgcttcacgaaatatcgatatt
ggagatctgattacctcaaaaagcagtgaggacgaagtgcacgatgttttaaatcaatacggtaatgataatagcttattgactagtaacttttta
aaaaaagcgagctcaatcgctgaaaaaacccaatccggagaacttgcctcacgtttgcgcgagattgctgctaatagcatgatggtactgagtcc
tgaagtcttaaccaatcttctttgggtatttgtaagggaaaaaaattcttatgatcttatggcaattgtctctttttttccctaatgaattatcagtgtac

ctacatgcctttcgcagacttttacttacgtctgctcgtaccggaaccaatttggatacttgttattttctagcagagtatttgttaccaaacttgatgg
aaagaagatctacttactctattgcccaacagcgggaacttcaaaaaatttattttgaattgttgctctgctttgcctattcctatcatactaaggga
ataaagcttttgctccctattctttatcaaggaaaatttcaaagtatcaatatgacttcgatggatatggtaatcatggctctttgtgcaaaccaaga
gcttgaagctgctatccaacttacgctttatttggccaaacgtaatttccctgtcaattacatgctatatcaagaaataacagacaggctatgttttg
aaaacaaacccctactagcgctcaagttcttccagaatttttacaaaggcacccataccaagtcgaccgctgaatttctgatgcctatagctgagt
ctttttacaaaaacttcaagtcatcaaccgaaattgagaaaagcttatccgcttttctcgagaatgcccctaaaccaactcatgctataatttccag
actaataatgctttgttcactgtttagccaagatttatcacttatattacgttactttgctaatttacggtcaatgaaacttttgacgctggttgactac
aagattgttttccaacttgcatttgatcttcgctctattgaattaggagatgctataaataaagaaagtaaacacctcagccgctctgatcaactaa
aattacgagaaaactatattgctttacaaattttagtcagaaaatacgcggatgctattggtaatatagaacttcttcacagggaaggaaggacta
tatctaaacagttgttgctatttgccttatactccatgaagacctatggaacggaagaggacttcaaattacttcagtcgattttgaaaagactagg
ttatggtttttttgtcgagttctctggattctcccagacccgttacttatcgttgcttatattattaagataaggccatgatatgatctattttgctttcga
agctcagtcattttaaaatacagactctcacctatgcgaatatcaacttcctcaatcttcgttttactatgaactacataagaattctactgaccagt
gggttttcctaaactataatagaaatgtatagcaccctgaaagatcctctttattttaattatcccagatttagtttaattttggtaatttttgacagtt
cacatctcctctttcagtacaaaattagaaactgcttttaataacatgaatattctcatttttttaaaacagtaatcaattgaaaatttaagagacatt
aatgaagtccaacgtttttaatttgccattaattactttgtggttttattgatgccttcagcagttttcaattgctacttgactcacagttgtacttctcg
aaccaacttcttttctagatttttttttcaattataactgatttttttaaatgttttacaatgtaagaagctaaatacaattagtttttaacaaatatgtag
gccaagggatatatttgatgaaaataaaggtagaacccattgattacgatattagcagtgtactaactcacttccagcgggcttggagaatttcca
aaggagccttcgttctgtcgcaaaagatattgcagacgtgtcagacatacttatgtctatggataaacgcttgggtgtattagaagaatccacaag
cgtaatccgcggagatgttacgaattataatcgtgtatcaagcagtatgtattgaataagtgattggacttactgaaaatccattagacatttatga
cactttaaaagaaatggaggttctacaagtcattcactctaatcttcccaagattaaagcagagttagatactttggataaaatggatatacctgtt
acagagaaaattaaggttgtgatgagaatgctaagagttcttgctcggtattatataagcttgcagtcgagtccgcttcatttttcggcgaaaacgc
agcgtaaggccgagattgggctaagtgaaacatgtagccgactttctagtttgtgtcagaagattctgcaaactcatgctgcatcttcattgaaaa
aggcagcgaccatattagatttagaccaaacatggtcttttcctacagaaactctacagcagtttatggggttaatccaacagttcgataatgaag
ttttgtatcctatttcctattcctcagatatcagctctaactacgtcaaaatcaaaagtgagtgtttggtccagttactaagatcactatcttcacaga
ctgatgcagggcaattgaatgaagcttcaaaagaatttattcaaggaaaagaagaagttgctatttatttggttgctttagcaagaattctttctgc
cactgctacagagctactccatttatatgacgcagaaacatcacaagccttgtaccagcgaattggaaagaatgtgatttcaactgtaacaaaca
taatacaccaacgggaagctgtttatgagaaacaatctgctcttgataatcatgttttatttccattgatacactgtgtaaaggttaccgaggattac
atgcaaatgaatatgccttttgaagatgttgccttagtaggcctagtttcaggtgttggaaaggaagcagaaaaaattattatagcttccttatcga
gtctgtacaatggctattgtaacagttccaagcctatagtactcagtaccaatggtgttagtgatattactcaaggaattatactgtttttgtcaaatt
tctgtcaatataaggactcattttatttaatcgaaagtatcggtgattggggttggcgacatgaactgtcaccggatttaaaaccatcttcaacaac
acgagaattggttcaaaattatgttatggattgtctcttatctcatctgaattccgtggaggagagtgcgcaaaccgtagatacaattaattggaaa
aagggtgtaattctcttgaacattagtgtttattttgagtctgcatgtgttgattccgagattgcaacctatttggatgaagaacattttaacaacttt
tcggaattatcacagaaatactccagtatgtatatggaaatttggcgccattgttcacaaaacatgttggacgctacttatacgaaagcgaaaga
aaaaccaaacatgtctacgaaagatagagaggcaacaaaggaaaagttccgaaatttcaatgaacagatcaccagtatagttgagaaccaca
aagactcagtacggtttgaacctacagttgccagtttcttgttgcaagaagtgcggaaaacggttttgcctctatatcaaagattttatgataagta
tgtacaactccatatctttcccataagcattgctaaccaggcagatatattacgagtgattttacgaaatataaagaaaaagtatgtatctaaaca
aactttgcttttctttaacgacagaagtactaacttttgcttagtatatacgatttacaaaagctcagttggattcatacattacggaagcatttagc
ccttcgctatcttgattcttaaataaagcttcatacatgattttatatactttatggaaaccattgcacgaatgaaatcatcactcatttccttttacaa
tctatagctcaaactcgatcattaaattctcatatcaattgttcgattgtaaagaaatttcaacgtctgcatgaaggcttaaagcgatacaaccgga
aggggacctttggtcgtataattagcagcgtctctcttctaaatattgatacttctgtgaaacatttgtttgtttatatcgctttaattgtatgaccctta
cccttttgtatgatgtattgggttaccccgacaacccttgtttacatctcctttctacaccaggaaccaaacttgtctttaaccaacgtcctgccaaat
ctccttcattacacctattacgctcatttattcaaacaaaaacaaaaagtcggtttagactgttgctactgtttggagagctggaggcgaaacaag
aaacggaactttgttggttaagtagacagttctgtataccttcataaactcgtttaaatactttgtaagtgtaggaaataagcttatacatttacata
atgtaaaaaaagccggaagacttgggttttggaaaggttctgagaagatatggtagaaaattttcttccttggacggtttcaaggaatgaagata
gattgcaactagtaaacgatgagttaactagtttcgacatactcttttaagtagttccttaaaaaaagtgaaagaagaaaccaaatccagcgata
tattcctggttcaaccgtcattttatgtttcagagttcgatattaacagcttttagtgaccgagttcttttgtcaggttcttattataaacgcctttgtgg
attcccggtcgacaatcgtttactgtccaacgttgagactcccgttcttgaaagattgttgataccttttgtgattttgcgctccccttttgtctttcttttt
ctgcataatattatattctacacagtcgtgacgtgcacttgtttacaaagttgaggtgattataatatttgttgttttcaaaaaagataacgtgcgttt

tctttataaatatcctgcttcattcttttctttgatttgtattgatactctcgaatttttgtaatgtctactcgacgccttacacgtcagcacattttggct
aatgccttgagcaataacgatgaaaacaatccatcaaagcaccttttgcagtcatccgagaatgctataataccaggaaagaaacccctgtcca
cctctacagctaataaaaaacgtcacgctttagacgatgtctcaaacttccattccaaggaaaccaatatgccactcgcttcgaagagttcgaatg
ttcgacattctaatgctgcttacccaatccatcgtcctcaggaaagaaaggatactattaaatcagttgatgacgaaccggtttccaaaaagcgcc
gtcaaccttctgccctaagctctttatcctcattttccgctgcccatcctgtcgatatcgtttcgactcagcctgtaggggagtataacacagacgcct
atgttaaagaaaacgaagccctttcgaagaaactgaagaaggatactgaagatcgcgttccacttcaagagattctgcaccaaaattctgccctt
gaaaagccagaggtacgggactgggatgatttggatgctgaagactgggctgatccccttatggtttctgaatatgttgttgatattttccaatatc
ttaatcaactggaaatcgaaacaatgccctcacccacatatatggatcgtcaaagagaacttgcttggaaaatgcgcggcattttaacagactgg
atcattgaagtgcattcgcgcttccgtttgcttcctgagactttgtttttggctgtaaacattatcgataggtttttgtctttacgtgtttgttctctaggt
aaactgcaacttgtgggaattgctgctttgtttattgcgagtaaatatgaagaggtgatgtgtccctccgtgcaaaattttgtttatatggctgatgg
tagttatgacgaggaagacatacttcaagctgaaaggtatatattacgcgttttggagtttaatcttgcatatcctaatcctatgaactttttacgac
gtatttctaaagctgattactacgacattcaaacaagaacggttgctaagtatttggtcgaaatagctcttctggatcatagactgttgccttatcct
ccttctcaacagtgtgcggcagctatgtatttggctcgagaaatgcttggacgcggtgtatggaatggtaatcttgtacattattctggatacgagg
aacccgagctaattcctgttgtgaagaaaatgataaattatctgcagaagcctgtccgacacgaagcttttttaagaagtatgcatcgaagaaat
tcatgaaggccagtttgttcgttcgcgattggataaaaaagaattcgatgcctttggaagacgatattgatgaagactatgcgtatcatcaacagc
aacgtatacatcatgatatgagggacgagaactggtaaatacattctttttctgctttgagagcccctgaccgtaatgttgtccgagtttaactcag
ggaactggttaatttaaataatcctttttagcgtgcatttttattaagtttttttgttttaatcattattatcttgcatttgcacgactatcgcccgtaatg
tttcgttttgtttagttctacctgcctatcattaaaggactccctagttacgacagcaaaagcgtattatctaggctataataatttatttcttttgcttt
cggagatgttatggtttgataatgctttaaatatagtggtttcatcgctcttaccggctttcctttctcaaaacacttgaatccttgtttgaagatttaa
tgatttctggacggcattaggtacctatttagtttcacagcttatttaaattataacgttgagctataaggatggagtttattttcttcctcagaacga
ccagaagagtatgctcttatcgtattgttcaagataatagtaataataatgattattttctttaaatcgtgaacaagataatgtgaactagtgtcata
aaaatgctagaataagtagccaaggtgatactgctaacttgcaaaatcaataaattgaggaggacaaagatatgagaagactaaatcatagag
tcgctaatagaattgtccgcttcaaatatattctgatcgccgtcgaaatgatttgcgagggaagccacttgtaaagttatagattgagaatgataa
atgaatgcctcaatcccttttggaaagaacgtttgaagggacggctcgtaagtaggagagcttccagataatctgtgttgattttcgacaaaaagg
cgatcccaattgggatcccaaaatgataagccatagcgaagtatagaagtgtcctggcctaattgatacgtttggattccaggttgcctatcggaa
tcaatatatttgctttgtcccattgaatgtccctcgaagcgagttaaatgaaggtgataaccagaggttatatggtaaaatttatgttttaatttaat
gattatagtttgaacaaaaatttgagaagtattcgaaaagtaaatccgggcgttagagtccataacaattgaagacgcgaagtcgctatatgcttt
cgaatcacgggaaggcttcccatacttaatataaaaattccaagaaagcgtgtttaaattacagtgtgtaatgcttagggcatccttcatactactc
tcaaggtaccatctgttaggccgtccaactggtttatcccagatcgcagttaaagtctttttacaggactgagtatcgattaatgtaaaatgaacga
cagtaagatgatccgtcctaaaagcttcaaatacttgatttcctcgttcatctttgattgactccattgatgccaaaatcttctcagatacatcattag
gagccaaatacctaagaaaggaggactgtgtttgtttttcaagcagaattttagacgcatcttctaccgtattagtgtttgtctcgttttcactaattt
ttagcctctcaaacaatggaacaccaattttaactcttacgtaaacaatcccgatatataaatagacattaaacagagaggctaaaatgtaatttt
tcatgatttgaacattgccagtacgaatcctataaattcaaagtaagcaatcaatctttgggtaccttggatttttgaagcatacttttcaaatgttgt
tccatttgtacaagaagaatgagaagcaattgctcgtaaatgaatacggcgaggaagaaattttctcgaaaggatgaattcccgatcataaaat
actttggtatttgaataggaacaatccacgttgagaatatgtagaataagacgcgacctctctaatcgttgatatgaggcctgctcaaaataaaac
aatcttacgtgtcgtaacgcctctttgagttttatataatctgcacaacgaatatgaattagctataaaggttagcaagtacaataaggaaactatt
catacggataatttcggcgaatattcgataaagcaatgaagcttgatgcgaattgggttcaaaagtgctagttgtgctcccccaaaatattcgtag
acatcgagttcgtcagatggaataacaagagatccctaatttagcagcaaaattagtacagaaaatccatattgaaagaatattttacctcacat
ggaaaagtcaacgccggatccaacttttgtgaatatcgttttaacagagtttttttcacgtttttccttgtttgagctcaatttttcttgaaaagtatagt
tctttttcttgggttctttcgaacgtttcggcgctttcttgctgaaatggctccgtctattggtgtctaccaacgattgtagagaattcaaagcaatag
aagctcttaagttatcttcattacttaaaaaaagtttcttttagaaagtagtcagtattacacaagtaagacactgaaggaatcattgataaatcca
tacatacaccagacgtttgatctatttcaaatttggagagagtacgccgttcaactttcctaatttcaaagctgaatttctgaaaacaacgatttag
atctgatatgtcctcaaaagaaggcagtatgcattcccctatataccccaacattaattaccttcagtcactttataaaaacatacggcgaaaacg
aaccgaatttacactcatctgcgcatctctagtgtcttccgaatctggttcagaagcttcagaaatagccatagtgcttcgctgtttttttaaatgag
attcaatatcacgtaaggttgttcccttttctcattcaattgatcaatttttcctttgactctgaattgataaaccaacttcaaatatcctttaaaaagt
ctttgcttcactaaatggggaacgaagtagttaagcgttatttgtaatgtccaagatgcattggagggcatgttgtcaagcatgttcagcatttttag
agctgacagtacaatttgttgaaatatatgaattcaattaaaagaagaagagaacagataaatctggaaacatctgattattggtctattttgaag
acgtactgaggtttacgcgggcacaatcattgtatctaaacgcagtactttcaaaaaaaaagcgtatataaataactttttattaaacaacttaaa

aaaaaaacagattattcattatccatcttagttttcaaattgcgtccagataccttcattaagaaaactcactagcaactttctctgaaacattcaat
tcctgtctcaatatcgttctcataagccaatcgtaattctgtagaaatgtgttgggataactttcgatggaggaccgaatgttttcccatatatgcct
atcttcttgacaggtaataataattatatttccgttttccaaatgctttatcaggcgcttgttaaaactatttattgtagaacaagctccggcattcga
tttcactatattcataacagcggaaacattttctggagcaactgttttagaagtcaaataaattacataatcttttagaagggttggtcctttttctct
ggctcgttgcaaagattcttgtaaagaacagccgatctctttttctttttcctcatctttcaggagatattcactttccaaagaaggatgtcgatgctt
gatgctatcttgaagccattccatggtcaccacttgaggtccttttggaatagcacataaaaactttgccgtacgcaaaatcctaggagcaactaa
atgggtacacttacttggattcgatatgatagttatattaatctttttcaggtcatcagaactaggcttcgtttcgtatccagtaataactacatatcg
tcgttcttttaaatttgtattcggtatgttttgatttgcttgcttttctgggactgtactgtcaacgtgtttttttagcctttctctcgttcgaatgccacc
acatctggcattacaacattctgcagcatcgaaagtgccatagaagccgattttcgcttacttcttgaccgttcttgcgttgaaggaacattgccgtc
agtctcttcaatgtgcatcaaatcaggtatttgggaaattggaggtgaggagtgccttgaaaatacttgtgattcttcctcggaattagaaaagcct
tttaacggtggaatgatgacggcgggagtgagtaaaggtgttgatgccggttctttctgattctgggtactagaaatcgtacttttgtttgttggtgc
ttgattaacatccacttctttatactcctgtggcaaatccgttgttatattatttaaaaacggtctcgaaatgtcctcttcgttatttgtctggatattat
gaattgatgctttattgttatcagatggtgatcctgccatgatggggccattaatactgggtaagtcatcatccaattgaacatcttttgaaattaga
ggatttgctaatggtagactagaatctttgttacatgagttacgctcttcgggcttatcgtccgtttccttctgtaaaccctctttctctcgctcaaata
atctttcctctaaatcttttgacatttctgatatcaaagttgtttctgttccactttggtttaccaaatcttcgttattatcatggggcaagtcattagca
acctttacttccgtataaatcttagtgtccagtttacatggaagcaaaaaatccatatatgattcatcaagaggaacaggaaaatcagagtatggc
tgagacgaaagattcttaaacgacgagaatagccaggtatgatgaaccgatgaaatattccaaactttggcagcaccatatttttgtccatatga
ggaagcagctattagtgcagaattcgtaggttttaggtttttcgtgtaagatgctcccgaagccacaattaacttctctagataagtccgagcttttc
ctgtataatttgtcaaagcaatcgtttgtcctttaagaaatttctttgaaggtaaaggaaagtgtagtgcgtccaataggggatccttccaagaccc
taatacaaaactattcagtagccaattgatagtagctatagtagattttttttcgactgcaagcttgaactcatttgtttccctttgaaatcctataaa
tacatcacagttttgaaccgactcagctatgccagctcctaatttaacagtaaatttgcggatagcctgtttggctgcagtaggaagattgagatca
ccagagaaaaagacctttttgttatggaatacgtttcgaaataaattgttatcaatattatcctcaggcttctgctgaaaaacgggattggggaata
gataatatttttggtcaatgcacttcccttgaatcaaacaatcaaaaatccatctcggatgaatgattttgacatcaagattttgcttttcttttacctt
tttacactttggaccatctccatcaaaggaaaatacatgcgttatggactttatcaatactggtgtgaacttccccccctagaacttccaaagagtca
tcaatgaaggaagccaagaatggatctatttcacatgtggtagcgcaaattcccttatagatcagatatgggttgctgctataaggcatataatcc
aataaagcattcttctcaatgcaatcctcaatccaatgagtctggtcaatgcaattgttagaatcaactcattagttatattattacataccttaaca
agtcgaagcgatcttcgagcgccttcttgaataactggagaaccaaaattgtttcctataacgtgcgtagctaatttccagtcgtacggatagttta
aaatattcccgccatattgaaggaacaatttttctgtaaaaaccattcagttagcgatccatcaagaagcagctcattcatacaatattttgatca
tctgcatccaaaacggctactaccgacttaaataaaccatttaaagtatcattttctgcggcttgcattcgtatctaccgttaaagttgagtattttgc
gaaagccaattttatgataactccaaggttgtttactccaggttggaggcctccaacagcatgtttagatggcataaaatgattatgcgaataaaa
atgtagttttatcagaaatccttaaattgcttttataaaaaaaataatcgatttgaaaaaaaaaaagaacaactaaagacttgctaatgcttgaaa
tccgcgagtcattctccctgagaggtatcagacggcagtgagatttgagtgaatgcttgattttgagattcctgttttgatttaatgaacggttctgtc
gtataaccatgcggaagctgatgagtacaacttaaggaaaatgtgtaagatggcggagagcgattttgaaggagattttccagcttcaagagtct
ttgttttgttttactagagtccgtttttaatttttctcgtaaagaatcacaaatatgccggaaagaattatgttgggtttccaaatctgaaaatgactct
tgtagtctgagaattcgggatcgtgtttctgcggaccttggagaaaatgtgtttgcttggttgttgttgtcacgggattctacatgttccctttctcttt
ctctctttctttctctttgagagttttcacataaattgacatcatgatatttgttacgctggtaacctggggatctttttgctttgatttttttccttgaactg
ttctttccaatccttggaaggttctatttcagcgtgtagcccctgtttggcttccctagtgtgattgtatagtaaagtttttaaactagaattttcatgc
accaaattccggatttgcatagacgatgctttatagttttcttcaattgttttaagatcttgttttagtagttcatgtctcgtttcattctctttttttggca
gtaagtaattcttcattttggtctaggagaagctggttttcatttttcagctgtccaattgcatcttggagtttcattaccgatgcctgtttgaattgttc
atttatccttcgttcctcttccaactgtaattttaagttcttgatttccaatgaattttgtaatgaatcgcgttcaaacgcgtcgataattccctttgcat
tcatctgtagtttcaaaaatcgaatagtcttttcagataagtctacatctccattgctagcaacattttggacgtgataagtatctaaattctcgtctt
ttgcgtaaaaagttggcgacgatggtacacacgttgtgtcaggagaacttgaagcttgcagtaaatcttcataattcatgttttattaaaaaggttg
atctgacaaacttgaatatggaaatttccctattgcttagtagtaggttgaaggcagctagagtacccagatcgcgtttcatatttctacatacaga
gaaagtaaaaaccgcgttcatgaattaacattataaattactatatcattcaagtaaaataaaaataaaaaccagaatcgttacacgtgatgtttt
ctatgtcgtcttacaaaaaccgattacgctaacaaagttgggtgaaaacgcttacaaacataataagaaaaacgtgaaaaaagatttattcttac
aaaccttttggggacagtcatatgcagaacagttttcattacatgagcatcaacaaacggagtttccatttatgaaaccgacttgactttttttaatta
gaaacgagaaatccccaatatttagactttaaaggcaccgcgcataacagtggcttggtatttttgaagcttattgagcaagacatcaccttgctt
aacaacttcttgatattggcggttcttttcatcaggacgatttgtttcaacgacgccatggacgcgatcaataacacagttcaatttattgtcggga

taaaggaagcaacatcgcgatcaatatattcgacagagacgccaaaggcagcagccatagagtcgatactaagagcgcgataagattccaaca
actgagagtaggcacgtcttctcatttcacgaacatagtagcggtaatgagcgacaaggaattgatcgcacttcaaatggttttgttcgacatcag
caagagtacggaagaagccagcatagtcacagagataaaggctattgatacaagcttctaaactagccatagactcgtttcgagggagcacgg
caaggacctctggggaatctataatttttgactttacatgaacacggtctaaagaaatggcaccagaaatgacagcataacgaacaatatcgtaa
taaggaagtagttctgtggaagaaaatgtagacatgcagtcaagcagcaaatcggctgcgccagaaaagtcacgaatggacatcaaataaatt
ccttggtaagcctttaagcgattcttgcgttcccaatctcctcccttttcaataagtggtttcatcttctctagcaaatgactgactgtcatcatatcag
cgtagatataggctaggcgaatctttaggaacaacacatcaattttcatgcctgtcaaagtagctcgatcataaatttcatcacaaagttgaacac
ctcgtactctatcacaaatatcgacaaaatagtatgccttgtttctcatagcttctagaatttcatgttcaccgttcagttcttgggcgtctgcaaggg
ccttgtcatattcttcaatccgtttattgttctcttccatcatttgattgtaaaaggcttcatcatgatcgattttcgacatcgtaccctcatattcgcag
acgtattggtaaagaggtgccagatggttttctcgaatcgctgaaaacagggtttctagagctttttcatgctttctttccagaaaaccgtttgataa
ctcatatatggactgagaaatcgtcaaatcagatatcactcttgctttttcactcattgtctttgaaggtggtagtagatgacacctttcctcccatcc
acctctggattcgccctacattgatccaggtaacaataccaatctgttacttttatttacatcggaatggttttgtgaaaagatgacagtagcattcg
aatgtatgttatttatatgaaaagtagacgtttctaaagggaggtctttcatcgggaaactttcaagaggaaaacaagtgaacagaaaatggacg
atcgtacaaatacctactgagaaggaaacaagagaagcgagcttttaattgaataagacataaaaaaatgatgaagatggattcatcttttagc
cgttcttttgatggcatgctttcgttgaatctggtaagcatgctatgaaatcaagaaccaggagtagccaagaaggtcatatgaatatcattcctac
cctatttatccactctttttcaaatatttgatagcaaactccagttttatatgaaacgtcccttcgatcaagattcattctttcctaactacgtattctga
gagtttgaaatttacccattgttcaacacctttcagctcctttttttatttaaattactttacataaaatgctgcatttacattcattattaaaacggga
aaacgctaaaaaccaattttgtctaatatcgtattaccggaaaacagtttttgtaaaaagatacgggttttttaatacgatatttggatcgataag
cagtaggaaagatatgccaaaagtcaacaatggtttgctatcaaaaagatgagtgtcttttggacgatctttataacgaggattaataataaaatt
ttctttatcaggtaaacagctttgagatttccatggtttgggtcggtttgggataacaggtccttttatcagtcgaaatggtttgtcctttccaaagca
ttcatgaaatgatgaattccaacaattaccgtcctaacgctttcgcacatatttccactttgaatcaacaagttatcaaagccaattatgcagtacgt
ggtcctttaacagttcttgccgagaaaatccaaaatgacctgttggaagattctaacgattatccatttggagatataatttacgcaaatatcggta
acccgcagcagtttgggcaacttcccattacatttaatcgtcaggttttgtccatttgccagtatccagctttgctagaccatgccaatgaaccatgg
ttcaagagcatctttcctagagacgttgttgagagaacagaattactattgaaagagactggaagcgtaggagcttatagcgcatctcagggaat
cccaattgttcgcaagcacgtagcagaatttattcaggaacgtgataactgcgcttgtcatccagacgacatttacttgacgagtggggcgacaca
aggagccagaattatcatgacattgctcatctctggaccgtctgagggagccatgctccccattccccagtaccctctttacggagctctaatggat
ctttttcatggaaaatccgtatcgtactatctatctgaaaaggacgactggaatgtggatgttaagcaattcaaacaagcctatgaagaggaatcg
aagaagggaacgaacattcgcctttgcgtttttatcaaccctggaaatcctacaggtgcttgcatgacagaagaatctatcgaagagatgcttcgc
tttgcgaaagcaaaaggtctcgtcatcatctcggacgaagtctaccaaaacaatattttcaggaaggaattcacttcgtttcgtaaagtattatgtg
agcttgagagacgcgagccgaactcgaattgggacaaggtctctctcatctgcattaactctgtcagtaaaggacagtttggtgaatgtggacaa
cgaggaggctacatcgataccatgaacattccgcccatcaccaaggaccagatcttaaagttggcaactatagacatatgcccgccggtgacag
gccaactgcttgttgatttgcttgtacgacctcccagggaaggagaacccagttacgggttatattctcaagaaatagagaagattcacagagac
atggagaaacaatgccaatttctctatgaggctaccaaacgtatgaaatttgtcacttgccaaaagcctcatggtgccatgtatttgcatcctaaa
atttgtttacccaaaaagttggttcgagaagcaaacgagattaacattgagcctgatgagttatactgcttacggattttggaacgtactggcgtat
gcttagttccaggaagtggacttggccaaagagaaaatgagtatcatgttcgaataacatttttggcaaaaggagatgaatatgttgaacgcttta
ttgaagcccataatacaataatggatgagtatgaagattaatcttgcatatatggttcatccatactttctttttgggagttactcatccttcctgcga
ctttttattcaactgtcatttctcgtagtctaatgtcttcttaatacatagctttaatgaaattggaactaataaaatcccattaaaattaacttccta
gtaacttttaactcggtttatttacattgatcatgataaattgtcattcatcctgtttccacagttagtaggttttccgattcctgtcttgtttttatttca
aataaatatcttatggatcattttataacaaataatactaaatacttacgagtttttatcattatcatataagcttagaatatcatattttcttcaacg
ctctatattttgcggattgcatacaacagcaaattcaggcgactttacttaattctttgtatcacgactcaattcattagaaaaaatggcggttatat
ttgagacagctcaatatgcacatgttttgcgcgtatgaaactatcaaatatagaaacttcagtaaaggtactaactatgccgttcagatgctgaatt
ctatgtctgctagagacaaggtaatgattattcaattgtatagaaaaaggacacttcacttcgtttggctatgctttgctgtatacagcaaaaccaa
gagtattacattttggatacaaatatgtccctctatatccaatcgactttctcggattaaagaaaaaataatttgctataatggctgagcgatccta
ctatccaacaacaaattcgacattctcttttgcaccaaattcttgctaacattgctgtctagtcttttagaaccttgcagtttgttgcaaagcttttggc
ttggcgtctgtactataacggaagctctttggaaagcgtgaacaagtgggatactttggaatctcaaatttcctttgctagaaaactcttcagtattg
gcaaggtatttgattatctttcaaagtcttattttggcagccttcaaatacaaaatccactttcatcttctgaagcagcacttccttccatccaactga
ctagagatattgcttttactggttattccgcaatggaattggtcggttggttgcaaaaggcaagattaatcgactgtcccaagtctaagcgcgtctct
accttgagcaagcaatttttagccgctgctttactttcctcatgcttcgcaggggtatataattaccaaaagatttccgaacaattgaaatcaattca

ggcatccgaatccggtaaaactaaaaatgaagcatcaattgaatctttaattaggtatggtttattcaccttttcctttccactaacacttatctagg
gaaaaaaacgagattgtctatttcgccatccaaaatgctttggatgctgtaattcctttggctgagttggatgtcttaaagttggatgatggttttgt
agctatgaccggcattaccacaagcataatgggtctctataagacttggtcatccaattagaaaaacttcactacttcctggttgctttttgacgat
ataacatatgattcatttacaatttatgatgttataaatattgctctttcaattcgtaacccaatccgttcttttccgggtaatatatttaattaacagt
catagcaaaaataaagcttcaccttcgtcaattcaagcatcattagtcatatgtcagtattccgagctaattttaactagagtccagcttcagcttgt
ttatggaaaagatacaaaagatcgctaatcgttaattttcctgattttacttttagtttgctgtctcccaaagcgccttttgcaatcagtcgcttttctt
cttgcaaagcgacgattctctcttcgatgctgttagctacaaccagtttgtatacgaatacatcctttgtttgcccaattctatgaacccgatcgata
gcctgatcctcaatcgctggattccaccatacatctgagagaattacacgactagcgcaagttaaattcaagccaagtgctccacactttaaagag
cacaataaaacgcgcacgtcgttgtcatctcttagtattcgcaacgattcctcacgagcagggttagccatttttccatcgtaacgaacaaacttgg
ttttcgcagcaactaagtgcggttcaaggatatcaaggaaggttgtaaattgagagaaaatgatagtctttccggttggtatagatttcccttctttt
tcatgttgtccttctcccttcaaaatacaaagaatctttttttactttggacgacaggtacagttcccgatccattgctacttctgatggatcagcagaa
agctttcgtatgttagataggcagtttttgcaaattgaagtactcgaactctgtggaagtaccgtataacatatttcgcatcgaactttacttttgtga
ccaatttcaatcccttcaagcatagcggcaacatcgtccacggcttgatcaggaagagggtccttaccattcattttcatagtggaaatagaaaga
gcatcggcatcattctgtaaatgtgtgctaataagctttggatgattgcaagcttgtcgtaaacggagaagtaaacagagaatatttgtataattcc
tgttaacttcgtccatctttatgaattctgacattgtagtttcacttttcttttccagagagtaataaaactcctttttcacgttcgctgaattggcaactc
actttctcaacaatccgttttggtaaattcattagcccaccgtctcctaactttccagctgattgttggagtatgctttttgtccttcgcaacataatga
cggaaagaaaggcacgtaagcgttcaacagccaattgccctttcccttggctaaccggacgagtgatttgatctttccatgtagctgcgtcattca
aaggttgtattctcagaaacttaattaacgagaaaagctcatctaaattgttttgtaagggtgttccagtaagacaccaacggttaatactcattaa
ggaacaacacgatacagcagtttttgatgccttattcttgattgtatgagcttcgtcaaggataactctccaccaaaagaaagaaaataagctcaa
tgtttctttggagttctcttctttttttatcgggtgcatattctgaaacgagggtctggtaagttgtgatgaccacatcataatcatgtacatgatttaat
tctttctttctgtttgcaccgtgaaaaaccaaagcttttaaattgcttttactatgaacctcgccttcccattgttttatcaaacttaaaggagcaatca
ccaaagttgaatgacatggaataagagacttattaacttcatccttggtctctaaagatttaccttgatatggatgagatataattaacgaaattgt
ctgtatagtttttcctagacccatctttatgattagcatttaataaacaaagttcttcatacatcatctgcaaggataccaccatatggtttaaattctt
cctttatttttttctccttaaaaaattagcatcttccaataatacaatataacttacttttgaagcagtgttttgcacctcatcattgtcagtgttctccc
ttcgctgtagccagcgaaggccttgaacctggtgaggtagtaaagacagcttcaatcctttgatagaaaactccccgttgtgtccagattccgaat
caatggtcgccttatctacgaatgatcctgcaaaaagatctttcaaagtttcctgagtctttataggatcaacaaactcgtgtgaagaaagcttggg
tgagctataatttttacgcggagacgtagacgctgaagaactaggagtccttggttgaactggcgtatttaaccatgtaggtgcgtcatttttttggt
gtcttaatgacgaaagcatcactagtaggtttgtttaaaggtggcttgcttattttagtgatattctgtttgtaaggagacttccatgcggaagatttc
actggaataggagcaaaagggtccaaatcccattgttctttgccagtgtttggagtttttggtcgaggaggctccttcattggtgaatttattgctttc
cctttagttgttttcggtttcggagagaagatggcatctattttctctggtgatattgtaggtaaatcatcctttgttgaatccacatctatttgttgttt
ccctaaactttgtaaggcgcgaaaattagaggaatgcttagtttttgaaggacttgatggtttcgatgtcaaattcggtaaatttcttgcaaggtcat
tgagatcctctagtccagttgaatgctgactcttcgtttccctgtgtgatttgctaatgcctttttcattaatggtaccttgtttttttagattcctttgttt
actatctttttcttccatagttggcattatataggtctttacaatgttaatgtttgttttgtgtagggacgattaagagccgggaaatttgaattcctttc
aatgtcaagactttttttttctgaacgtttatatggctttgatgaaattctgtacctaatagaagcaattttacagctgataatttcttttatgagtaac
gatttctggtattttgccagaaattcatttctacatagtcgccaatgcttaaagtccaagacaggaaaatgtaacaaattcctttctatgttgttactt
atctaggagcttgaatatttttttgtttagtagacctagggcttttgatgatggtgctagtaatacaacacaataatgacgagaatgaattgaagaat
agtaatggcagttagagtaaaagaaaaggcttatgtagctgcaacttcaaatgaaactacttctctaggagattctcgaaaatgattcactaaaa
cccacaaaacagatcttacaaaaaagggtttaaacaaaaggatatattttcgaattttgcttttgtaaagacttaaatgctgaaaattccctcagg
agaagtaagactgtattctcctacaatacccaaaccagctgcatactcttcaattcttccactttcaagaagacagtgatagagctttaaatcctct
cccgcaacctcatttgcgagagcaaagcaaccattataatcttcattatgaaaatagacattgtgaagatttaagataatttcagggatatatatg
ttccgaatgcgatacaattcattagaaagttctttttcctcgtcttcatttatctccatagtcgatggatgaaggaatctcgcttgaagaagctcatta
aatagttttatacatctgcgtgaagtaagtgtaagagactttttccattctttgtatgcaccactagcttttgtcatagaggtatcattcaaaattggt
tcattcattaatttttttccattcttcatgcacttgaaaggctcggcacagtgcgcgataattcatgaattcataggctgttcgaacttttgaatctttat
cagcagatatttcacctgaggctaaaacgtcgagagtttctgctggaaggcgtgtgtacaataaatatgcagaattcaattctcccctagacaaa
aaaaatcggaaagcgtaattcgcgtaaattaacaaatcgtcggattggcatgttactatcaaccattctagggtggatataaacttcgtataaacc
aatggtaccggatcttctagtgatttcaaatcaatttcacgtaattgttcaacaccggtagcgaattcgttgtaagcaagtgaaacactcattaaag
ctgctctatcgatgtctaacttatactttttcgcgagtttaagctgctcaagacgcgacctctcatctcctataaaactcaaaaagcgagcgtaggtt
tcatactgaacatgatcggaaaggaaaggaatgtacaggggaaccaaggaattctttttttgcactggctagcagctcaatataagattggattat

agaatcggaaatgtaagcgtcgacatgaatgccagcaacttgcagaaaaagaagctgatgaaccagaattcgaagactagaggggctagtaa
attccaaaatgccttctacgtttccagctcgcacagcctctagctgttcgtgagccaatgtatatagtctatcagtttggttcaaaatagtatacgcc
tgtagtcgccttagaggatgagccgctgattttcggatttcttctgatggatattgatccaaagcttcaaaaatactttctgggcttaaacctattccg
ctatcaataggtgggaggtgagtatctggttggggaactttatcggaaagatacacgtctaaatgatactgattcatgctgttataatatgcccata
gagcgtcctcccatgatgtgcaaacattcagaacggattctaaatcaccacagagagcgccatacagagcacgttcataagcgtcggtacgctta
ttttttgttaaagcgaagcatgtacgtctccaaagctctttccttttattacctaatgtttcggattgcagagcatcatctattacactgttacgatatt
cgaaagcaccttgtaaggaagccgagcgccagaaatttccgctttttctacacaaatccaaaagtccttcaaaatccccagcccgataaagcgag
aaggcaacgcgaataaattgacgatcatagctgtcgtctctttcgtcaaggcgtcgctcatcacgaatgtcagcatctggatccaaatttggaaca
tttgactcatctttcttttcgatagagaatttggaatttgttcgtgtatttttcttaatattttcacgagtataaaaccaacgattaccacggatttcaat
gtcaggctcttctacaggactgtcacgtaaccaattgaataccaactgtaattattagataccattcgaagttattattttacatacatgattttcga
atgcagcggggttcttggagtagaactcttcttccaaaacagctattgaagaaaacatgtgagaagaaatagtttcgtctcgctttgactttcgaa
accttcattagagtattagtaatctcgaaagaacgttactgatatgatgaaaaacatacgaatagagtctctgtatcaaatcccaagttcgtagttc
caaaccccaatgactgatcaattcctcatcttcttctttgcaagcttttatatatcttcgaaattgttagactgaacagcgtgacgaagattgaacat
acttttcttcacaaaatttatagaagcttgtcaacaatccatcactggagaataaatcgtccctttttgtaatgaataaatttgcgaatgattcaaat
tcatctccaagttcaagaatgactggtgtctcttcagttgttagtttttggtaagctgctcaattcacccataccatttggtcccaaaaacacgacat
cattgttcatctatacacacgttaaaaaaaaagagctttgagatttaacaaacctctggatcatctttctccatttgcatcagttcttgaaaactgga
acgctactcaagttagtaactcttcgttttctatccggagcgaaataggagttttcaatttcatacgcttaaatcattgtttccacgactaatagaaa
acgtagaaacatcgtttgatacatgttgagacatgttcaatttgagccaaactcttttctaatgtgatgctggtagaaagtacattgtgaccaggat
gggttagtaatggaatcaagttccaaggatatttttacaatatagggtacaagtaaaagcaaatatgaacttgtcttttcgataaaaacttattcac
ttgtctattattcaaatttgggctggaagttttccatttgaacgctattgaaaagatcgaaaacagctaaaatagaatagtaataataaaggaatt
aaatgtatagttgctaatgctacagcaaacttgtagctaaatataactatcttttacaacattatctaaatccatgcttccataattcttattatgcta
cagcacgcacacaaatacgaaatgcaatctattcagactttttcttttcttaaaaaaactataaaacaagagttaaataatactatgtcgatatca
atgaatgaggtcttatcatgtctttatcgacccaaaatgaattcaggaataccttaacaacatggttgcctataaaacagaaaacagggttaatca
aacaggggatggattacagagcttcttagttggtggcaggaatactccaagggtgattagggaccttgccatattggatgtcaaggatccagttgc
tgacttgggttgtgatggggccggcttcttgaccttcaggcaaaggcacatttatttcggtacccttgtagttgatggccttgacaggagaaaccaa
agcagcggtaccagcaccaaagacttccaaaagacgaccctccttagaagcttcagcaacctccttcatggaatacttgccttcagtgaccttcca
gccttggggaacaaggcgctcacgggcaatttccaaaatggaatcacgggtgacaccaggcaaaatcataccgtcaagaggagcggtaataag
ttccttttcaccattcttgttgatccagacggtgaagcagttcatagtaccgacttcagtgacatagtcctcctcaccgtataaccagaggatttgag
cgaaacccttcctggcagcttcagcagcaggcaaaacgctaggagcatagttaccgccaagcttgtactgaccagtaccaccgggccaagcgcg
aacagcgccttcagagcagcaaagacggatggccttaaagccggaactgtagtaaggaccgacgggagaagcaatgacatagagcatagcgt
tttcgcagtcgtgaacgccaagggcagcgtcggtaccaataaaggtgggacgaaggtaaagtgagaagccgcgttcgttagggacccagcgac
tctcggtagcaacgaatttacgaatagcctcgctcaactcgttgggatcgaaagcgggaagagagattctagtaccagtagacaacatacgctca
gcgttcttgttgggacggaaaagtcgggcgacacccttctcgtccttgtaagccttcatgccctcgaagcattcgaaaccataatggaagacagag
gtagcgggatggaagctaagatcctgcataggaacaatttcaggagtaccccatccagtttgtttgttccacttcatgatcatcatatggtcagtaa
attccttgccgaacttcaatgttttccagtcgggcaaaggcttgagctgttcagcctttttgaaaataatattggaagcatccaacggtttaggaga
agaaccggcagcagaagaagagctcatcttgcggatttgagaagaggtgctcaatttcgataattgtgtggtgcgtggtagacgcaagtgaaaa
tttctagggaaaaccatccggacatttcctgcgcactttaaagtccggctggcaagaaaagaagcgttcattttatataaaaaaaaaaggaagga
tatttctatttgagctgtgtctataaggctcgtagtaaaatgacaactagaaatagatttctacctgctttttggtcgttgagtttagtttagttgagct
gaagttgaagttggtagatggagggagatggaacgagggcgtaaagctcttttcttattcttttgttgtaggctttttttatcttcggtgtagatggtg
aaaaaagcaaagagcagtattggaatcatcataccagatgtgataaagaggattccctgtaatgtcttatatgggtgtaccaaatgattgaaagc
gctcactactttaataaataaatataggaaaaaagaacgtatcacgaggaatccatgttatataacgaaaatcttagctcgaagaagtatcccat
atgaaagcaataatgagactggcgacttggatggtttgcgtccaattccttgtatcttgatgattaccaggtttatcgattactggtatcgttcatctt
tatgtcgttgatggtttttttggtgtacatcgtcggcatatatatgacaatcgtttatatatttctgaacgtttgttgcggtcatacctttccagtagaa
cttttcgcatatgcgatttgttagttgactttttttcgtgaattggacttggttgcactatgtgtatagatgctagtaatgtccagggacacgattaatt
ggatgaaacgcttgaaagacagtctattttaggcagaccagtctagtcttccagtctgaagatcgtaactgtaaaatgatggtctatatcctctctc
taaaccagcattcgtaatctgttccgacctcgtattcagaatttcagtatctcgctttttaatgtttgtttacttgcatatttagcgacgaccccttatg
cttatgaatatcagcttcgctaaaacacaattactgaattgaattgtagtattgattctattatcgaagtatcctcttatgacggtaattgctgggttt
gtcacgattccctgcacaagtcggtgatatgcatcccgaatgcagttacagaaacaggtaatgaggtaagtataactcttgccttttcaattgtatg

caaaagattgtcccaataccttgtcacaatcgcaaattgtgtgccctcgtatctcatctgatctcatctgcttattgaggcagttctccgtcctcttct
gtggacctagagttaatttggcgttactgaaattcccatatgtgttcacatttagctctctactgggctcatccatttggtagtattcagtgtacttgat
gtgaactgaaagcattcttcatgtgatctaccaatggcttggaatgaaggtattaattatacacatcctcaagctgattcttcgtcacgttctgccct
atcaaagttaataagtgggtgtttgttgacactgcagagaccagcgtctgaataaatatcatccatatcacatccccattatcatattgactatttat
ttgatgtattatgttctttgtcgctagataggttaatagttaaaggataaactcttttacctgtcttattgcggtctataactaaccacacacgacattt
aggtgccaatcaaaatatttagcaaaattatctgtgatttctaactcttcgaatagatccctgatacgttgtcgagaggacacattcctttcgatgca
agaggcttccattttcaaagtactttcttttcacattcgagatcaacagaaagagaccataaacattatcctgacaatgacggatggaaatgtcac
ttttgaagtaaccatcctttgttatcaaggaaaagaaaataaaggaataaataaagaaaagcagtgaaaatatcgaaaatggattcaataaata
gaaaagaatcctcaaagatgagcttgtctatgttaagaacaacttgattgctcattaccaaaaaaaaaaaaaaaaaaaaaaaaagaattatagg
atctttcagataggatagcctttacgactgcaaggtataaaaacttaatggaatggaagtatctcgttttcatccaatttataaaaagacattcagc
agcaattcgtatttatgaaacacattccagacgaaaggagtagacctcgaatggaagacgagaaaaggataatgagccatccgaaaaagcaat
ctaccataagatgaagtctcttctagtacaccctttccattttcagtgattgattcttttgcttggtaaaaagatattttctttttatttgtttatcttcat
ccaagggctagcagtaacgatatgcttaggtaacacatgtaaacacttttctttcgatctaatttgaaggatttttcgccgtttgagtcagttttattt
gtcattttataaggaaaattctctccttatgaatctttttcacctgatgtgtcatgaattcggtaagtgaaaacaggattgggtgtttaagcccaattc
aagtcataataattccatgcagaaagtatgagagaattatctaccttgatagaagagcccttgttgcgtgtttcgattgagctaagaactacttcag
ctagccgggtttgctgaaatgttgtttcccatctgataacaaattagagcgtttcaaatctgtgtagacaccccaataacaagttgaaagtatcaac
caagcaaaaacgaactgggtccaccgtatcaccccgtctcgaaaacaccggccaactccaatattgaacgcaagcctgaagacttcggttacttc
agttactctagagaatcctttgacctcatgaaagtacaattggaagataaccaataaatttttttttcagtcttatcggaaaaagaatatccaaca
gggaaagttttctcatgagcaagttttacgaagaacacagaagagatgtcagaagtatgaaagttgcaagtgaaattcagcttacaaatcttggg
tatagtcaagtagagttcaacgttaccaatgtatgcagatgttgtttctctagatgtccagcagagctttcttgagtaccgttttctagaacctctgat
ctcctaagatttgtttttgagcaactaccagatgtttaaagatagcaaaaggaaaagctttgttaacacagacaaaaaacgaaaatagtaggaa
agcagctttctttattgcttttgagcaaaaaatcctatgctacgactctttgaaagaatcgtttagtctgtttattatgagcgaatcaaagctttgac
ttgagacaacatcaataccatctcaatgaagattttcattgacttgtttggatccttcgttaacacagattggaattaagagcgacaagccttggaa
aagcttacaggtgaaatgaacaatccttatgtttacaatcaagtcttgcacttgtctaagcaaaatagataacacttagcatagcttacctctagtg
gaatgcttttgcttttgcttgaatgaaacacatgcaaaaagatattttcgttctgtgtcattatctccttaaattaatagaaaaaacgtaaaagaaa
gattacgatattcttttatgtggttttttgcccttaacttgtgcttgaaaggatagtattcatagttaggtaccctaaatactgagacaaaacagctt
tacgaaaaggtattcgtcttgaagaatgtaatctaattctattgaactgtcaattccttcacaattataaattccaataatgctgtttgttttcacttca
ttagtacttgatcgtacctcaaaacatacctccactgaagactcaggagcagcttgattattctgaatttcttcgtctaaaatcatttcttcctttcatt
ttgaaactaaaaatgtctctaactcaagcattttggggacttattgccaagtatatgattgactatggtttgaccgccgaaccagatcaagtattgc
aaaaagtagagcaggtcggtaagaatattgagcaatatgaatcttcatttttcaagtctctgtacagaaggaaacaacctttttcgcttccatctgt
aatcaatattttaacactcattctcattctatacatgtccttggtcatagttaacagagctactcgaattgcattagcacttttcaaaacattagctac
tatctcctttttgttgctaatggtttgtctcggtgtctactggttcctgaatagtcaataattcttcaacactgcctatgagacttattgttacgaacagt
cttacaggcagctcatcgatattattttcttctttgcatccttaaaaaattttcgattgtaagtataattgtcaggttctgttcggcgtcatacagcccg
gaagccttgaaattaatgaaccaaaaaaagaataaaaagaatcccacccttctccaaccgaaagcgcctaggctcaaagttgtctttaacaatg
ttcccttagtttaaaatatctatattgtatcgtctttgcttttacatgttttatactggttaagatcatctacaaaatacatgaatttcgtttacgcaaaa
acagtaattctattcggtattaaaactacgaataattatatagaaaaacgtttagagccagtacttgccttcatcagagttgcgtaagcgaataga
ttcgatttgaatgaaaccagaagtccaggtaggatcaaaaccaccaagttcatccatagaagcaagcttctcgtcgtaaagacgagcggtatca
gacttacgtccacggacgctgacattacccttgtagacggcaagcttaacaacaccattaacaacttcttgagaaacgtcattggcagctaaaag
catacgagtgcagggagagaagtattgaccgttgtagagaatcttggaaaggttgaaagtaacgaattggtcacgcaaagcacggacttcacgt
tccatggtcaacccttccaaatccatgtgggcggaacgaagaatggtcaagccaggggtttcataacagccacggctcttcaagccagaaaaac
ggttttcgacgatgtcgatacggccaacgccattgcggcgagcaatggtatttaattgatggaagattttaacaacgtcggaaaagacaccgtcct
tgcattctaacttacagggattacccttttcgaaatgaacagcgagttcctcctcctcatcaggagcgtccttaggatcaacagtgagggtccacat
gtcagcgggaggagcaagactgggatcttcaagaataccagcttcataggagcaatgaacgatgttttcgtccattgaccatggtttcttagtagt
ttgagtaacagggatgcccttctcagcagcatagttcaacaaatcagtacgtccagcaaaacgatcaaagaaaacggggagacgccagggagc
gataacttcaatatcgggcttcaaagcgtagtaggcaagctcaaaacgaacttgatcgttgcctttaccagtgcagccgtgagagacagcaatgc
agttttctttctcagcaatctggatttgacgacgggcaatgatagggcgtgcaagagatgtgcccaaaaggtagacgttttcatagatagcgttgc
actttgcggcaggaaggacagtgtcatggataaattcatcacgtaagtcttcaatgtaaaccttcttggctcccacacctaaagccttttgacgggc
tgtttcccaatcttcttcttgaccaacgttagccatgtagcaaatcacctcccatccttgttcaataagccaggcaagaatacaagaggtatccaag

ccgccagagtaggcaagaacgcaacgtttaacgtcttcaggcatttttgcttcaatagtattgtgtagaaagaaaaacaaaaaaaataagatcta
gtgtctgagtaggttaaataggtgacagtcaaactgatttgacttggtgattaagaagatgcaataaggaatataaggatgctcagcgatagagt
gtaaagattggtagtagctggaattaactcgcttgattaaataggactggttataagggctgcaaagtgaataaaaaacccgtttaaacgagagt
aaaaagtttcaaagaattaaaggaacaagggtccaatggaaaccattttatacaaagtcagaggtcaataagcttctgatgatcacacgcttgat
caatctttcactgaatctatcaccgcagaaatctatatcttagatctttataaagtaacaaacaacctggtgtcggtttttcgcgaaataattcgtag
ccactgggcagtgaagtagcagtccatatcttggtgaaatttttaatgaaaagaggacaaataaggacgatgatctccggattcagaaagttgtt
tgacgacggccttcttgcaatagccctttaggaaaataaaaaagtataatctcatcacagcatatattaataaacattacgagaacggcattttgg
cagcgtaaatgacctttgcttcatggccaactaagcttatatatatattcatttgtttccactagataatttccttcatattttcatcataagggaaaa
aaatgcacgctgaaacttgagggtttattaattaagacttttgagtgattcatagtagaccagattctatcattcagagtagcggtttcatcataatt
taagtatacttgcgtgttttatgaaaagaatttcctagtccggtgattttcatttcctctaatccccaaatcaacaaaaaaatatataaacagcttgt
tttaagatagaaaacataagaactcgataatccattaaatgagacaattaatgtacaaaaaaggtatacatataaaaaaggcactgtttcctgta
atcagaaatacattatagaagcaacaataacatctttttggtgaaaattattcgttttttttgagcatctttgtcctcatgattcaccataccatcataa
ttttctccttggcaataagtcaaaactttgtctttaaagcgaatcgcgcgtagtaaatccatccgtacatttgaccaacaccgaatgaattccctgct
tttttcaagagctttctgtacagtcccgctaagctctttacattgctccggagtgatcccaaacacctcatgaggcgcttgtgtgagagatgggtcgt
cacccagcaaaactgacagtaactgctgaagttgagcagcatacttatgttctgaagaaatactttcatgcgctgaagcagctaagatgttgaga
taattctgttcaaatatgtatgcaatgccaggatgcgtaaatatgttttgtggattggtcaaaggagccatggtttctatgcattacggttagaatac
actttgatacaaagtctttgtccttaccgttcatatttacatcgtggtaaggattatcgtggtctaaccattctggttcagcgcgtcgtcgataatggg
aaataacgccacgctttgtatttccaatgctgtagtacttcatcagagaaaaaggttagttgttttgaagctgcaaatcctgtactgtcttacatcta
cgtcgacagaagtaccgaccgaattgcccatagacccataatagacggacctggaattactttttcttttcaatccacgatttgttaatgactctcct
atagaagaatctgaatcagtggctaaagaagagcgtgttagtcaatctttcaaagtcaattggcaattaccatcagaatcctcaaaaagagcttc
tttaaacctcctaatgtttcttgttaatcatttgcccatagaacgcgtgcattcatactcaatttgctcttctaattgtttcatggattttggcatgagtt
ataccagtcaaagaacgtaaatacttcgctctttttctgttacttgtgcgtgttacttcttctatggtgaaataaaagcaaatgagtgtgctaaaatg
cctctctttctcacgattgcttatttatttttcaattctttttgtatttttaggataaaagtgtaattttaagactgacttgttttgatgcaaacgaaact
gtcatagtccgacacaaaagcaatatccttcacaaaatcatttggaaagtgtcgagttagagacttaaagttgagaatgaaaacgaaataaatt
agatattactgatacttggaagatttccatacaaaggtctttgaattagcttacgaagtgaggattagggaccatacaaaatacgccaatttccat
aaaatttaagaatagatatttgtcttttaaactgttttattagtttccatagaacgcgatgcagtctcctttttcatagaaacattcccttggattttt
tttgtttatgtttacaaatcataaagatatattaccttttctatacgcgcgaccttattgtagtttattatgcattattatgagctctgtatgtagcggta
aatagtaaacaaacaataccagattggtagaaacttcaaataagagaacataaacaactgtttcgtaatcggactttatttatgcaagtagaatt
gtcaaacaagaagaaaaacccgtatattctaacaaaatgaaggatatcaagagatacataagaaactttaaaaagcaatgaaatctggagaa
gcaaaatggaagctatacaacctataacattgcttatccatcaagtattatcttgagcgccaaagctgacaattcacagacatgtttttcttgaaca
aatgcagtatgtaacaaaaagcgaagttctactgaccgaatgcaatgaagaaaaactcgtttacactatcaaatcaagtataaataaataagag
ggctcaaaaaggaatgtttcagttccttgctttcatcacgtaaaaaaagcatatttcaatttcagcatgttccttttcaatggtaactaagcgtctac
ctttcgtgtcttctttatagaaagaattttatttaaatacatttgattcgtacaaaaaaaaaagggagggatatattagtaaaatgcaagagttcac
catcacataatggacgcacatgaaacgaaagaaaaccaaaaccaactgtaaacatgatagtaagaaagaagatagtaactcctatgtttataa
aaccaagacggctttaccccagcgatccatcttgtaaggaaaaagcctacatatatggggctgcatcccaaatggattgaaagatgaaagaaac
gagaattaaaaagcacagagattattaattttaaataagctgtttatacacctttgctttatgtttcttcgaaaatttgtttgtttttgtttgtttatttct
ttgtccccttgtttgtttgtttttgtacccgcacattgcatgcacaaactgaggatgaggggtggtttcatcctttctttaaccaacactaactttttca
attcgcccaagattgagtcgttgaacgctttgtttgattctgtagcatgtctactcaaagccctgaagcacgacctgaacaggttgatcagccaagt
actgaaagtttgcagcaacattctgaaatcgattcaggatttgctcaggccaaggagattccatcggaggtaactgaaccttccgcttctactgag
caagcagcaaacgaacctgaaccaacaactgaacaagcaaaggaagaggttgaggaggaaaacaataagcctaaagaaactcccgccgaag
aaggaaataagaaggcaaagaatcctgtggtcattgagacaacgcccagcgagtacctctccggaggctacttgtactgccgttcgaatggcctt
attccgtgagtcataaaaaaaaaatccacaagaaaaattacctaacacatatagtcatgttattaaaagatactttttcattaaggactcccctatg
cctttagaatatcttaattattactacaaaaagcacttccaaggttctctcaacaagcaacctgaagatgatccttctttgagttctgagcaagcta
atgagtcatcttcattaaattcgcaaagtttatatgttgatagaattgccaaagcagttgaaggatgcaaggggttacttttctactcaaagtctca
gtgcactagcgtcccaagcggtattatttctctggtaagttccttttaccaattaaatgaaaccattaacaaaatttctagttggatgtcgaaaaagt
ggaagccggtaatggtaacaagtttttgctatttttcgaaaatggaaaaaccgaaacattggaggccttaaacactcaaagtcgagatgcttgga
tcaaagatctaaacgtcattctttcccagagacaagagataaaggaggaacttgaaaattcgagtacctatggtgaaactttgactaagctgaac
aaaattgcaaacaattctggagccagccctttggaaactttacgctctcttttcccatccttcaaaggaaaatctactaaagaggaacagactcctt

ctgaagctgcggaaaagcccgaacctacgagccctaaagacaacgtatttgatttcttccattatcttttgaaaggtaaagaccatgaaaagagc
caagaagaaaagcccgaagaaacagttaacaacgatgctgccgatggtgtgacacaagaggaagcaacagaggctccctcagatgtccttact
caacaacctgaaactacggagcaagctgttccagaaagcaatgaagctgttcaagaaagccctaacaagaagcggggatatggcttgcttgcat
actctaagtcatggaagactcctaaagccccgaaaagtgctaagtctgaaaaatcagtacagaccgaaaaaggaagcaaggcaactcaaccct
tcgaaaatacaaaggcaaaacttgagcacttacctacttcactttcccgcaagcttactgaattcgtgcacaaaaagaagcatgataagcccgct
gatgcagaagtggtatctcttggtactgatagtgctgctgtgaaaccggaagagccaccaactccgacaagcactcctgcagtgaagacagttaa
cgaagctaactccacgacagagaaccacaatggatcagtccctgctgaggaaagtactcaaagcatcatagcaccggaacccagccgtgaacc
tgtagtccctgaaattgctgcctaaaccattttaataacacgataatttcgtaacataattttatggcagcggccatcattctctcctgtcgcttttatt
cgtacaaatagtacaatctatttgttttcaacaaggaatctatataattattgactaacttgataacgtgatgcaaactataatagaaatgtctata
cctgaaagtattcgttataactttgggaaagtaaaagacgtaaaaatctcaagaccttatccaaatagccatccacgacaattggtagctccacat
ttgcacttccttcgtattttagaagacctcatttgctgagattgttgtgaagactgtgaagagttatctcgcactcctgcataatcaaaagtaagttc
ctctagaggttttatatccttaatggcgaagaaagcaaggtcgtatatagttctaaatccatgattacgtattacagaataaatagctaaattaggc
gaacaagaatgattaaaaaaccgggagacatcaccgtaagtttgtgcatcgaccgtataaacactcgaatcttcaaacatatccaagtcaaaca
aataagtaataccatcctgatcataatttttgtcacgtttggcagcctcttcagaggttatgacttcccccagataacaagtaatgaacgtcccaga
gggaataaatcgaagagaacgaacaccccaaccttttcccttggttttgaaaatttctaaaggaagagtacgaccacgttgtactacacgattag
ggcagctcagtggacaacagcagaaggagttgcattcataaataacagccggagcatcgcgccgaactcttccttgtgcatcataagcaaattgt
tttggctcttctaaatcatccaagcattcgcaccgcgaaggatcgtttaggttacaaccatcagcagaacaggtgcaaccggattggaaattcgga
tctggagggacaacgccttcagtaagccgatatgttgtgataaactgaaaatacagagaagggcaaggttcatcatctacttcattcaccaaagt
gatctccggtccttctaagtttcttaaacgttctcgaaacagttcctttttttcgtagaaagaaagataactataagcattttgtctcacagggccaa
cttgattagttactgtgaagttcgacgtggtatgcaagggttgagtaaaaggctcattcaatgtatccgagatatctttttttggtatctgatggttcgc
tagttgtttccttttccttgtctgagttatcatttttgatatagcgaagattagtctcgggacgatgccgcttttcttgatttgatgaaagagaggaag
cgcgtttacgagcggtgcttacagaaggttttgagagcctcttacttttgaggtcagaagttgtattgtcagagcgagcctcttttcgtaaaggtaat
ggatgttgtttgagtttaggacgaggtggcgacttaaaacccttttttatgtgttgcggtttttttccaatttttctaattggatcttccgctttttctggtcc
cattcagcaagaatttctgcacagccagaaaggttttccggaggctcccaagtgtcacttcgagcagagtaatttaaccaacgtattctataaagt
ttcaccttaccatgccggtctaatttttcgtcaacgactttttccacctcatattctttcgacggcatctaccgaacaagagcagccctcaagaaaaa
ctgtaaagaaattcaaacagaaagctattgaaccaaaacaagaccaaataaaaataaaaaaattggaagaactgtagaaacgagcgtgaaa
ataactttaaactcccaagattttgaaaatgactgctcccttaagaagcaccactataatacatgaaatagagacatacagaagaagatgagaa
taaaataatgaaggaaaaggcctttccaacagctgtcggtagctactaatgcaataacttgcaggtggttagagtaagaaagcaaataacgaaa
acgatcgatatcacaaaattcattaatgaaacgtaaaaatcttcctggtaaataggaattctaaaagctgagaaaactaaacaagagtattctta
tatggatattgaagatccatttcttgttttattgaatcaatttgcagtcacttttcagcatacattcctttctctacttgtatgttgcgatgtaccttgac
cgctatgatttgagactaaactagtttatggttacataagacatagtttgaattagtataacaagctcacatggtccagtggttaagactcatcgtt
gtggccgatgcgacccaggttcgattcctggtgtgggcaaaatttttttgtagcgggtttgtatactttatccacgaaaagaatcgatttgattttttttt
taagtcaacaattggtgctttattctactttattttcttttgttttaagagctattcactcttaacgtacaggtaagattaccaatttgaacaagtactt
gtctaaatcaagtctagaaattgttttaaatattgtgagggtatgagcctaatgtccaccggctacacctaggcgaaaacaccagttcccgtccga
tcactgcaggggttcatgggatctcaaagaaagcaccaaaaatagtttgaatcaagcaaaagttgctggtaatgtggtttcctggatttctctagt
aagtttcaggtattccagaggattataatggtatatgtactgtacacttttcattgctagtgaagccccgtcttgctattctgaactagtcatcaatag
aaatcaaaacaaatgaatagaataatattaaatttttatgaaatatgagatcattcgtgaaataaaaaacgaatgcatattatgtctatcaattat
ggaatcttttccatagacgcattattaagtatgcttgaaattagcaaaagaaagctgatttggactttaaagttccacactttctttgtaagcaatct
gctcattttgagagggataccagatatagcaaggaataatgatttcaataaaataacgaataacccaatatataatatcagtctctaaggagctt
ctcctagagtagcagaaaaatttatcaaaggagcttgtacaataaaaaaaagtaaaaacagcgacgcagtaaattagtgaatgcttacaaacct
tcgcagagcgttcagatttattattgaaatgatcctgttctttataaagcatataaattgaagacaaccaaataacattggaaacggaattatttat
ccatatttcgagaaattgcagaggttcaaaatcaatcacattggaaacaatatggaagaaaagtaaaaaagacaacccagcgacaaccctcaa
aagaaatttaggttctacaggccgccagaccccaataccaaaagaggcaaggataaggagtaaacgacgaacaaaaacttcgatatggtattt
caaatgacaataggcatattcatcacagatattaataatgataccaatcgctatgtttacaacatggattaataacccatattgaataggcagtat
ttttgctggttgggacagcagtgcgtagatccaaaaacaagctggaattaataagagaattgtaattaatgtaacaattattgggaaaaagtcga
actctggatggggttcatacccgggatctccagtgatatcttcataaaaatatccaacagaaacacaagcacgagaacccaagcttgtcaatggc
cttgtagcaatacaataaacatccgagtcatttatctgaaagtgtctatttataagagtttcattgatcgaaataatgtagttcatcaccgacgtttt
gttacactcaaatggacctgaccacacttctcgcttttccggaggaatatgaatagaagtatcaagttggtttacctggagctgtgaaaaaaatgc

agaaaaaggttccctctcgtctaaggaacccaaagcacactcccaatcctttgcattgaaaaaggtaatcgcctttggtccaccatcttcatcatcc
caccaactactaaatgcggcatacaacaacgagtctgattgcactttaaagccttgacaaaataaagtcagttggaacgcttcatcttcatcttca
ggattggattgataactgtttatttgtaagaagtaagagaaagaaagcagccataacagccagcttttacctcgcataagacccatttgcaatatc
agtagtttatgtttggtagttggtagaagaaaaagaaattagagaagtacgacagacagctaactacgtgcggggccccgaacgtaattaatttc
atgtaatgagagtgaccaagacacagagactgtaaggacctagttgttgaattttttgcttttttttttcttatgaagagcctaacgaaatcaatc
cataactaagcaaatgtacagaaagcgattgatcattattgtagagctagtgctatttgctagcgctagtgctagtaatagatttagtagagatac
agttacagtctacggaaaaggaatcgctaacatttgagacgacatgaggatttgaaagaaacgacaagttcccattaccggttgaaattgtaccg
gaaaagcttgaatacaagatgggaaattaggtgttctctcagttgcgttatttataactacaattataattataactaaaattattatgttctaaaga
cataggataggatcataagcgacccgtgcccttcgaaagtgaaaagcagttccaaggcgagtatgcaaattaaagaattacgttttccaaaaaa
atcaaacagcacaaacgtgattgtcgtaaaatgttaagtaatctaaaaagactatacagatgtctacggccatacctaggcgaaaacaccagttc
ccgtccgatcactgcagttaagcgtctgagggcctcgttagtactatggttggagacaacatgggaatccggggtgctgtaggcttcctcctcttttt
gttctttttcaaaaaggttctttccatggttgagctctttactgccataccaatggatgaagtgctatgttaatacaatacaaaggaaaagggcaca
catctaaaattacagtacaaacaaataagaagaaaagtgtaacgatgttatttcttaaaagcacagaggagaatggagcgtcacgcagatgca
agatgtaatcaaaaagaaaaatgatactcgttaagggacattgaaaagtcaattcatagttgtcgtcccggtctaaagtacagatcacacagcg
aaaggattgccagtgaaacaatgacaatctattcacgtaccatgttatgtaaacgttcacttaacgttcacttatctcaaagtcaatacgatttcat
cgtagcatcaagaatgaccacgcatctaacgacatcagcaatgcaaagtacaaaataaataatttatgcaaaaaacgaacgtaaaaaagtgtg
ttaaaatgaagataacgtaacataaaaatactgaatatgaaacagagaatgaaaagaaccctctcaatttctcagttcaccaattaaaaatatc
gaaagaagatagagtccgaaaccaaggaatcagctgctttccaaattcccagtgttttcaacatccctagattgacttggttttcgctgtctttccg
gaaattgttcaacgcattcagaagatggttgttcatctgtaagagtattagggtcacttgataggttttgagactcggatgggattgtattcagtgg
gatgggattatcacccgccaagttgtttctttccaacgtcgtgtcgaaaccgaagattgaagcaatcaagggattaataattccaattaactcagt
gaacccatttagtaacaggatgggaacatttagtgacagaatgaaaagatccaacaagtctataattgatccattagtaaaagtctcaaaacaa
atatctataaatcaactcacatgaaacaacaaatacgcaaatccagaaagccaaaatggcaagaagacccgctagactccactgaatccatgc
attttccattgacttctttataaaaggaaaaagaagaaagtccgaaaatacaaaatccagcacaaatgcaaaaatgtagcaccagacaagtcga
gcagtttcatttaccactttgacagtagcttgtggaatcctagcaacctttgctggacagataattaacaataatacttccatgttggaattttcaga
agcattcatggacgcttcttgactcttcttttcagaacatggtggaccgcgaattggacgtaaacataaataacctttttccaaatcatccacatga
gacgaatagtcgatttcgctgtatgacggtaagcagctgtctgaactggtaggagaaacccagaaaggagattcggttggtttagcaacttccgg
ctgcatttctttctctttttcattcatttttattagaagaaactgaaattttcagctgtctttgaaacggtcacaatgaatcgcgaaaactacccaaag
taggtagtagaaaaaccaaaaatgcatcaaagcccacaaaaaagcacctaaatcaatcaattcacattacaaagaaatgaaaacagtttaaaa
tcgtcaagaaatgagaaatgaaactgcaatggcaatggctagagtttgctgaagtgaaacgggcatcaatcaatttaagtttcttttggtgccttt
cattattctatttaccgtcgtctcgaaactcttcgctgtatcagatttaatcgttgttacttcttttgctagggtaacatgttatccttttggctggtaaa
taaaaaagcggtatggtggaaacttgaacctttagatcggctagcggttagtattcaacaaagtttcattatgtgaacatctacggccatacctag
gcgaaaacaccagttcccgtccgatcactgcagttaagcgtctgagggcctcgttagtactatggttggagacaacatgggaatccggggtgctg
taaacttctttactttttctactttttgaaagttattagatttatcttttagttacttttgcaaatgtcatggaagagatcgaactgttgtatacttgctgc
agatcgttacttgccaggtgacaatacataaaaaaaggtctattttgaaagtaaattcattagtcaccttttgctattaaagactaaaccatcatat
ccaaatgcaattattactacagtatataaccttgtatatgaatctaaagtgataccagcaattctcagttttactgttcgttctagttatgttattaca
ataaaattaacccattcatcgcctctcaaccggcggaaaacgcaagcatgccttgtccaatgttttccgtgaatttccaacactgaaggttcatgtt
gtagccaaatcagctaatagataatgtataaaaatgctcaataaagacacaaaaataaatattcggtcgaaatagaccgcttgtaatgttctcttt
tttcacttatttaactctcgatcggaagagttaaagctcatcatgaaagaaagttaaacaaattggcattaaatttgagtggtaggatttcactgca
gctctatttcagcggaggagttagtacatcatctctttgttgatatctcgaaagtgtaggcgaactgctgcttttagttgagcatttgatctcaaatcc
aatacaatataaaaactttataaaaaccttgacaagaagcaataataccaccaaagaatagtccaatactgccaattattcgcccaagtaatgct
ccagtactaccaatggcaatatacacgaaagttatatagttgagcaaggctcttgttgccatgacagcaattcgataaaagactgccttttgttag
caaacagctgttgatgataaacgatagcttagtatttctgatgctttgcagtgatgctcctttataaacttgagaaagccactgattcaattactatc
gttattttactctcttttactttgagggaatttgaattcgatgtaaaaatttcgcaaacgcaaaactcaacagaagtgcagccaaaactaaagcag
ctcagcctgattattccaaacatttagaatcaaagtcgaaatcagccctattggcaggaattataggaaaaaaaaagttttggcaccagcaggaa
atttcgcttctcttactcaacatttttgtcaggataacattgttgttcaaaatcatcatcttatcgtatacaaaaggaacaacaataagtaacaagtt
gctatttcgcgtttgtttacatctttttcttactgcttttcatgaaaatcaccatgttcatttcttgatgaattgattagtcgaaacgtttttttatgttcctt
cagtagctttccgcattgccgttggctcacctgatctacagtcaaaattttatgaatgcgcgttcataagacgaaaagatataatgacgtttacaaa
ttccttgttgattcaacgagtttcaaacgcttactcaaagagaaaatttttgttggatattttatattagtttgctaaattctcgataaaaaccaatgt

atagcagccgggtatcattaaaatatgctcagggttgatatcaaatattcacgcagacaattgaacgctatgggaattgccactaaaagttttaaa
ttaaattctgattcatcgcttagtaactgggtaaaatcaggcatttagacttcgtatcttggttcatttggactctatatattcttcctcattgtaacag
accaattcattatgtgaagggcgccatatatagcaaggaattaaaacttctgctccatagcggattatccaatatataaagtcctctatgcctgga
gctatgcaagcaggacgccacgatgagttggtagtaaaatatgacaatacaaaaggacgcaacgtaatcataccgacacaataaaaaagaga
atgccttaccaaccttacggactgcttcgatttcatgcaagatccagatgtaaatgcttggtataaatgataaattgcatacatccaaattaaattg
ttgctcgagtcattcaaaaactcatcaatcaaataaaaggatgtttcatgtatccaaaacttccaattgattaatttatggacggtatggaacacta
cagggataagtaacaagatcgtacctcccgaaactaaattgcctcgagttggacgccatactccaaaaccgaaagcaataaaatatagcaccag
acgttcagtaaatatatcaaacgatttcttgatttcttcaactttcgaaagagtgcaaaaacttggatatgggttttcacaagatcttgtccatcaaa
agaaaaatggagctccaaaagcagagctaatggtgagtagcaaaagtccatattgcgctgggaaaacctttgatcgttgagaaaccatcgcata
aatccagcaaatagcgagcaaaaggaatccaaaggtaaatattaaagggctgtaataagaagcatcttttggagcagaattatcatcaagacta
gagccccaactaattctatgactccaaactgaatcaaaatgaagatatactggttcactattagatccattctttgagactggtctcgaagcaaag
caaaatgtgtttgggaccttttcttcaaattctatatagcggtatgggtgggtagcgttggttgcaaaggtgaaactatagacagaaccgccgcta
gaaggaaaaggaccagtcaaagtatctctctcctcagggcggatgtattgggatatagaaaactttccgcgttccttaataacctgggaaaaagg
gtctttcatcgcttcgctatcaccaagcgctactttcgattcttcggctggaaagtaagtgacagcaatttcttcacctgcaaaataattatactttgt
acgtttttcgtccgcatttacccattggcaatcaattctgaactgaaagttttcatcgtctttgacattatcagaaggaatttcagaaattgaaatac
caagctcaagatctactttaatcaatgtaaacagcgtataccaaatgaaaaaaataagcctcgtttaccaaactgaaaatctcgcattttgagaat
gttatttcctaaaagttggcggaaggcatgtaagtactgacgaacgtggaatgtatttccctcgctttcgcttaactttagtgcttaaatgagatttc
atcccttatgctttattccctttctgtcccgtttttcctgcattctataaagtcatttttttctttctttgctattatttaataagggaaacaatggtaaaga
attggtttgtcaggatccaatggaagggaagcgagccaagccaacaagtacttagttacactatggctcgtttgatgttgccgaaagcagtccaa
atcgtacccaaataaaagggttgctagcgaacagaaaggtatcataactcttagggcgaaccgaaaacactggtattctttcgagaggttagctg
acaaattgataagattagaatagtcaacaagtaggataaatgcaacgccaaaaaaagcagcttattccatccaaatggtaaacgcaaataaga
aattacgggaaagacatatgaaaacaccatccttaagtaattggccttgaaggtattactattctacattctccatacataccgtgtgagagaagt
caaataattcgtgtacatatgtcatctaaaggccattgtaggaaagatatagaaaatacttttgcgacgaattgtaaacaacagaattttgatgga
aattttttgaattcgcgaaacgatgaagacttccaaaatagtttatacaatcacagttataatatgtcccgtaaattggcattaaagagaaaatctt
tataggaaggcttttctgcccttttgtaccattgaacttgttctaaaaaagaacccagctatttcattaggcgcattcattgtaactgttcacttatac
agtagagtactcaaaatcttaacagtaaagtaagacatccattctacttttaggatatcaagcatgtcgctataaccatgaagatgaaataagga
aaactttatccgcgatcgttttaaaattatcttttagaactcttcggtaaaagcttagacttccacggccacaatattcatcaagtacttagagaaac
gagagcaaatcgcgtaaaacgtcgttgacgcaaatttaatctgtaaagagcagttaatcaaaatatatctttcttaatggttatattaaaaataaa
catatgtgttttacacgcatcaatgtctattgtagtacaaggagtttctttagaagactttttagcttgggcttgaagaaaattcataaattcggtggt
tctgttgtttatgctccacgaactgattcttactattacggaacatcaaattttgatactgttagcttagcttgagttaagatctctctagtaaagagt
aaagagtaaaaaaatataaagatagggattagagaaaattaaaagactttttttaaggagaccatgaactatgatagattaaggtaactaattt
agtcttgagtaaacagaaagttccacattattatctagtcgataaaagtaattaattactgtacactgacaggaggaaaaataccctcctctcgag
ggtgcttaaatttatttacttaaagcgtgaaattagattatctcactcagatctttttagtaggttttgctttgattatattgactgattcagaacttata
taatcacgcttttgttaatcatcatttgtgtttactatacaactaccatgattgatcgtgtggatcatttcgtattgacagttcgcaacctcgaaagaa
gtagagatttttacgtaagaggattaggaatgggtcttgaatcttttgggaatggacgtacagctcttacttttggaaatcaaaagattaatcttca
cattgcagagggatccccaattttacctcgtgctagttttccaactcctggaagcgcagacatttgttttatttgtagcatttcattacaaaatgtcat
tgaaattttgagagaacgtaacttacctattgagttaggtcctgtcgaacgtacaggtgctaagggacgcattgagtccatttatcttcgagatcca
gatatgaatttaatagaactctccgagtataaatagttggtacctatattagttctccaaagggtcggtgcctttctccttgggatacgcaggaagt
gtttgcatgcagctttgcaggttgcttagcttaacaagacgacttgggtaagttcgcacaaaaagttcctacttgcttcttgtgggagcatgaatatt
attataaagtatgagcggtattttgaatggaataaggactgggtgtttattatgtactgctaagctacgttgaaattaataattggacttttgactcg
tctggttgacgaactaagatgtagtatcattaacacgttcattaattcgaccagcaaaatcttcctaactggaggttttgtagaagagctgtcttgtt
atgtatatttatgatattatagcatataaacaaaatgtgactgatgagtatatatagttttgttaatcaaatcaattaggtcaatcaggaaattttttt
attcgaacatattttgtatacgagtctttattctccgatcactcgtatcttgatttctgtatttcgtttttaattgtttgtttaccatttgtaatatatcatc
ctttagtcttatgacgtttgctaatgtccaacaatattcttatgatatttatatagtaatatgtatgatagtaacaagacacctatcatgacagtgac
agtgatagtgacagcgacagttaggtcctgaaacaaaaaaagcacattactttatcttcgttacttcgcttagtgaacatgatggatgggagacta
agagaaaagcaagatgaaaatgtcctagtagagaggaggtagaaactaactagtttttatacaaggcataaaggcatacagaaaaacacatct
cgttttcttctgtgttgatcaccgacaactcatgtttccattgatctgataacagtagataaatgatataatggtcgtctcatgtcatctcgtattgtcg
caatggtatcaggaaagatcataaagaacaggtttttaaatatctgacaaataaagacgaaggtaggaaaacaacttttactttgtatttgacag

tcctttccttaaaggttaagttcccttagtctcgaaagacggtctattcgcatgggtttactattgcatcagccaaaggcaacaatatccgtgtaaat
gtgatgttgttctctaatcacaacagttactcgctagcggattcgttgctagcggattcgttgttgaaattacgacaaatacaaatgttgaaattga
gaaggaatctgagctaatgtttttgtctataaatatcgaacctttctggtcatgcagggtttcaatcatttctgtcggggtttttcttggacgattttga
tacgtggatcattttcataaacattatatagaataaatatagactatatatatatatatattccctcttttcgaatatttggattgttttggtgactact
aaaatggtgttcgacagtgatcatttcgaaactcttcagttgcatgctggccaagaaccagataacacgaccacctcccgtgctgtgcctatttatg
ccacgacatcctatgtgtttcgagactgtgagcatggtggtcgtttgtttggcttacaagagccaggatacatctactcccgaatgatgaatcctac
ctgtgatgtctttgaaaaacgaattgctgctttagagcatggagttgcagcattgtgtacgagttctggtacctccgctgcttatatggctttgacga
ccttggccggaaatggcgataatatcatttctacctcttacctttatggagggacctttaatttatttaaagtcacttttccacgcattggtatcacta
caaaaatcattgagggtgatgatgttgataaactggtggcgcaaattgatgagaaaacaaaagccatttatgtcgagtctattggcaaccccaag
tataatgtgcccgactttgaacggattgccgaggttgctcatacagccggtgtccctctcgtagtcgacaacaccttcggcggtggtggatatttga
ttcgtcctatcgactacggggcagacattgttgtgcattcggccacgaagtggattggcggtcatggcacgacgatgggaggtgtcatcgttgata
gcggtaaatttgactggaagaaaaatgcgaaacgctttcctgaactggtggagcctcatcccggttaccatggattacgtttcgtcgaagcttttgg
gaacgcagcctatgcttttgctgtccgaactcaagctcttcgtgatgtcggtggtaatgccagtccttttgctgcttttcttttgattcaaggactcga
gaccctctctttacgtatggaacgccacgtccaaaacgcttttgcccttgcacaatacttggaaaaccatccaaacgttaaatgggtttcctaccct
ggcctaacttcacacccttcgcacgatctcgccaaaaaatacttaaagcatggctacggtgccgtccttagctttggtgccaagggaggccctgat
cagagtcgcaaagtcgtagactctttaaaactagctagtcaacttgctaatgtcggggatgccaagactttagttattgctcctgcctataccactc
acctacagttgaatgactccgagcaactaagtgctggcgtcacaaaagaccttattcgtgtttctgtgggcattgagcatatcgatgatattattgc
tgatttttctcaagcgctaaattttgagtcagactagctaaaacgttcgcgccctattttgcaaacaagggtctgaatctgtacttgctattatacttg
cgtctttcaatgattacaaacatacaaaagtgatgaatcaaaaagcattctaatcaacatctaattagcacttgcaactacgactttctacaaactt
ccgtctttataatatcatagtgaatagatttacatatgaaccaagagattaattctacaatcttccaacatgcaagcacaagagcacatacatggg
ggataataacacagaaaaggaaggaaccagttattgcgaaagtcacacatcgtggacatatgaattatagcaaataattggtttccaaatattcc
caaaacgtcttacgacgagttggagcggtcagggtaatagttttcaactagcaataacgccatctaggaaaagttcctcactgataagcgtaata
cccgtgcacaaagatggagaataagagagtccaagttcacccttatataacttacagaaagcagtcacggtttagaccctaccctaagtataggg
atgtcgtgatggagtgcggaatatatacttaagaaggacttgctttctcgctaaagtaatagattcaggaaagcaaagagtaaataatgggaaat
cgatggaaatacatatattatagagtaaaggaagcatttattggatagggtaaagtaaagtcatgtatgatactggaaaaaaaagaaacgttaa
tcgctgggttcaatcccattcgacttaaagaacggaatgagtgaggattttacataatgaggttcagaaaatgcatataatcattctaagagttgg
acacagacggaggcgtaacggacgaactaatcatagctacaaaagaccgttagatacaaagaataaatcaagaagagaaagaaatacctga
atcaggaacgggggtttcttcggtagataatgcatagtttgccggtgaaactcttatcacacgatcgtacaagacggaatagttcatgttggctat
agcctcttttgcatcctcgggactttcaaactctagaaatgcaaaggagcgatccgctggtatatatcagctgacgctgtactattgtgtctctttcg
taccttttttctttattatgcagtgctaccgttataatttcaccaaatgctatgaaagcgttatagaggacattttctgtcacgcccggagcaagattgc
ctaaatgtattgaagttagcaatcccgttaagaatatttctgtaaagtataacaaaacgatacacttttgttcattagaagaatgaaaaagtgtgc
aaaacacactcttgtctcaatttaacaaaacttacctacgtgaatcgttgctttacgacggtccgccatgtttcttttgtccttacttgaggaaaaga
ctacactgtaaaatagatgcagaataacaaggcttttcaaaggaagaaataataaatactgtatatgtttgtaataccagccggttaagaaaata
acgtaatacgttttaatgcgttagtcaacaaagagattgtatgaaaacaaaagacaaagagggttttgggaaaagggagagataagttgagtct
ttgttccatcataattaacgtgtaattcaagcaatccattgaattgatgtaacgtatgaaaaagtctgacgaatatagaggaaacaagtggatatt
ttcgaggtgtattaatcagaaagagcagttgttctatccacggtagaatttaaatcatgctttcgactaaatcctccataggagcatattccttgcca
tctaaaagactagattaagttagtggaaaggaaaaacaaaagcagacccatactcgaattctttgcaaaaaaacacaaaatgtttgaatgatgt
attcaaataactttccagttctaaggcaaccatgacatgaaaatgggagcaataaatatgggcataaattcgaaaaagtcggcgaaatacttgtt
ggacaattttcctgaattcgcgcggaaactcgacaccaatttctgttgggaacaaccgtttgttgtcaagcttttcttgggcccattccaagagtgta
ttaatataatctggggcattcattttcgttggtttcacgtataccttctcatcttgccaatagtactcaaagttgggtcccgcattcatttgtggacaac
ttttttctgtacaaaactcggtaatcgttccataaagcatattaatctgggtataaaagtccatagctgaatagttagccccgtcagtctccatatca
tcgtattgaacgtggttacaagcgtttttcagaagctctaatttacttactattcatagcaatccattcatttacatcttctcctttgggaagcttcact
gcttccatcagggatcccgatcccaaagttgcttccgcatattgccgcagttggtaatgcttggttccagtctctggtttgcgaatacgaaatgttttt
gccgctgtactattattaaacccaaacatgactctgtattcacgatgttttgaaaagacgtggttcttgagtccaattataaagaccttgctaataca
atatagtatccgtttgtggaatataatagcacttcggaagaattctatttaataatatgtaaacaaaagaattgtagaatttcttatggaaaatgtg
cagctaatttgaaccgatgtatacaacgactgaatttgaaagaaaataaaactaaataaaactaaaaaccaaagtcgaaacctgaaccagaga
gaggctagaatcgtatatgcattgtcgtcttattattgaaccctctgtattatcaccaaccatccaccaaccagtcgttattcaaagattggtgaaat
acaaggatctttgagccatagtgaaataatatcaaattgtttttttcttcaccttgtcgctcgttttgtctaaagcattaatttatcacttggttaattc

atcgcgtttcttacttcgttaagcatattcttccactcaaaaggatttctttacagtattctttggtaaagaacacctttaaagaagaattgttgctga
aacttgcctagttcgttttaattaagctcagggggtgaattattatatttccactattataacttccgttcgtggccactgttattaaaccggaaaagc
tacttcaagaacaatactccctattgcctttggtttttatttataaaaaaaactaggagcttcacttactatttttggaaattttcactgtcactacata
gactcattgtttcattcatttacatacttattcgtatctccatatggcattaaaaacgtctattagaaagtatctgttcatcctctgcttcgccgttggt
acaatcagcatttatatttcagaggtgattggaatacccctcaattttattaatacgcacttatacaataagtatgtttctttcacaaaaggacttgc
aggtatcttgtttatcttccttattcaatcttttagtcctactcccgtaaccttgacatatgactccgacctgcggaacctcttttacttagatcggaat
ggttgcttggaaacaattgcttcggaacgcaatattgtaatggctaaccatcaactttactcggattggatgtacatctggtggttagcgtacactg
caaaactacatggtcatatttatattgtcttaaaggactccttaaagtggatccccatcataggttggggtatgcaattgtaccggtttatcttccttt
ccaggaaatgggataaggatgcttctacgatggagcgtaaattcaactttattcatcatgttcctgagcctgccagcttgttaatgtttcctgaggg
aaccaaccttgttcaagggacttgggaacgcagcaaggtttttgctgataaagccggcgttgacatgcctaaaaatctaatgcttccacgtgttcg
tggtttgttctttagtattagtcaattacgcgattccatgacttacttatatgattacacatttgctttttctgatccttctcctaagaagtttgctgcgg
atgcattctctcttaagcaattatttttgaaggtgctcgccttcgtcgcatgcatgttcatgctcgtagattccgtatttccgacatcccttccgagga
aaaactttttacaaagtggttataccagcgttggtatgaaaaagatcagctcatcgaggattttcgtaaaaatggaagctttcctggttcgagaca
gttgaataccactgttcgccttaaatcacgtcttgaaattctctatcttttctcagccctttttacatgtttcgctatatccttcgtcatttctctatttctt
tagattatgaccattgtttatagagcacaaatatctaataaatagacattcgatatcgtttacgtttacttatactaagtgggcaaacagagtctttc
aagcgtcgtgtttatctaattacttactatttagcatccttttatttttgcctcattctgttttgctactcgtgctaaaacaaattttcccttagaagatct
tttcaagaagcagaagtgaaaggaattggtctgcagtattttcctttatgtactaaattgtttcttatttatgttaaatataaaaaaaaagattttaat
cattgaagtgttcttaaaattaactccagctatagttgacttctgagtatatccttctactactgcattaagggcgaaaattgaataagagccataa
accgccaaagtactccaaaaaataatcaaacatttttgctggcttctatttataatgggaagagatattttaattggttttgttggtaaaccgtcca
gtggaaagtcaacaatgttgaatgcattgactgatgccactgcaaaaacaggaaactttactttcacgactattgagccaaatcgggctattggtt
atgcgcaagtaaactgtgcttgttctcgtttcggattagtagatcaatgtagacctatttatggcggctgtaaaaacggcaaacgttcaattcccatt
cagctgctggatgttgctggtttaatccctggagcacatactggaaaaggtttaggaaataagtttttggatgatctaagacatgcaaatgcgttg
gtgcatgtagtggatgtttcaggaacaactgatgcagaaggtaaggcttgtcgtgggtatgatccttccgtagatattgagtggctgtacaacgaa
atgactgcttggattggcacgaacctcaaggaaaaatggccaaatactcgtcgtcgtcatatagcctcaaaagccaacccagttcataccttaca
gtctcagttttcgggttatggaagtaacccgactattacagcaaaagtactagattctcttcatttgaatgctccattagaaagctgggatgaagca
acattagatcgtattgtcggccgttttgttgacatcaaatttccaaccgttattgctttaaacaaaattgatcatcctgattcagatgctaatatttca
aaaattgcaagacgtgaggaccccaatcggctagtcttggcctctgctgtttctgaggtgtttttaagaaggcttgctaaacaaggttttgtaaatt
acgaagagggctcagagtttgttgatacaatagaagagttcccagattctgggttgaaacccttgagtgagagtcttcgtacaaaaattacagac
attcaagacatggtcttatttcgacatggatcgactggtgtatgtaatgtattggctagagccattgaattattagacttggtaccagtttatcctgtt
aagaatattcataacttttccaataatcctaatgatggtgtctttaaggattgtgttttggtaaaacaagggactactgctgggcaagtggctcaaa
tagttataggaggtgaggccatgtctattattggaatgaatggaaacgtgggagaatctgatcccattattgctggaaaaaatgatattttgagttt
taaactgcgaaaggatacttaagggtaccgttatgtttagagaaaagaaacggaatgaacgactttatattattctttctcaaagattgaaatgtc
actgtatctaagagtggatagaagaagttgaaagaaaaaaacaatgtagtgatattggaattaaaaaaagcaagtaggagcttttacgaatca
atatatatatatatgctattggttattaagaatagaatcattatcaaattcatatatgttaaagagaaccagtatccaacattaagcaataacaca
accctttccgtctccaggaggggaaggattggcctgcttttcgatttcgctaataataagctcaaaagcacgagcaacgttttcattatggcgagca
gaggcttctgtccaagcacatttccattcgctggctaactgcttgccttcttcagcggaaacggtacgttgcatgtgcaaatcgcttttgttaccgac
aactacgataggaacccattctgtgccctatttcaataaaaaattagtaacttggaaacatgtaatgaaaatgaggaggacgtacagtatggttt
aatattttatcgcggacaattttgaccatctcaaaagaagacttgctggttatgctatagacaagaacgtaaccatgtattccgattgaatgtttcg
aattcaatatgctatattcgtcttgtccagcggtgtcaattatctcagtagcgaactcctgtcccttatacttaatgtttttactgaacgtattttcgat
agtgggatagtaggattccacgaaatggttctccacatattgaacagtaagactttttataaaagttaattttgaatagatatgggtgaatcgacc
acaatgacttaccttgatttaccaaccgaacggcttcccagaacggcgattcttctcgattttacaggagccatagtaaatgagaaaattatttga
aaaacttataaaaaattcgtgaattagagctgaaatgaatgtgttatacgagagaatcgatcaactgaaaactaaacacgtagtagttaagtctg
aactctatccaagggcatcggttacgttaaggtcttctcgagagactctaaggtatcatcaactatagaaactatagcaagaccttgtgtaccaaa
taacattaactcttcttttaaatttatatgaaactgtttgaattcgatgagagttgtttttaactgaggtgccatgggtacgatttgatcaaatgctcg
tggatcagcaacaatacaatgaattaaaagcatagcagcatatccgcttaattcaacttttggcttctataagcgataataattagaactctcagc
cgttaaaatttgtcagctgtttagcttacctgttctttgtaaaactgaagcaagacgtctaacatagtttgagttgtattcactaatataaaagttaa
caaacaattagaacattcatgcgtattaccttggaaatttggaagagttttgacaaactgttcagattcttcacttatttctagcaaaagacctaaa
ctcatcataacaagttcttcggtggacctgctcttgacaaagacggaattgtttaatacctcaaagagcttctttggaagctcggagttacaaaga

gtggaaacagaagagctgtctgaattgactgaacgaatcaaggttcgaagtagtttctcgcaacaagcaatcataagaaaatgcattgaatcgc
ctgtttccatgttcctcatttgttcactgtctttaagtaaatttttcaagaggaaggacaaaagatcataaagtttttctaaaacatcatcatcccaag
tataagtctcagctttatcaagggtagccgcgatagaaaagagcaaaaacgtttgttgagttccgttttccaaaagaaatgttatagcatgttctac
ttctagcttcaataagtagagaaaaaggtcatgaccgctaaaaggaccatcacaagccttcatcaatgcgatagcaaccaacgataataaatta
caggaaagtaaaggctcattactgaattcttttgtcagtggggatggataattaaataattcttgaaatgatgattgaatagtcttggaataagttc
caattagcgctcgaattggccgttgatcgtgttcggttatggctgaacggaacagtttggagatgtctttggagcagttttccaagagagattgaac
aaaatcattttcatctgccatggaggaaaaaatatatatttgtaagattgtttgtaacttatcttcgggtaaaacttctgaaaatatgcgctgttgca
aatcctgtgaagactttaaacaggttcgaaacatgcgctggtatatttgttcacaaatttccataaagctgttgtatcttgagtttgccgagttgcttt
tagaagatattccatctattaagtattctatttcgtccatgattctctgtttttcacctgcaaaccgaagttcatgaatgcttttcatttgagtatcttca
tcctcggattcatctacatccgtagcttcattatggtaatctgctgagtatataccctgttcatctcctgtactgttggtttctgtgacatacgaacgtt
gttcgcgataggtaatactcgtagcagaaccgccagagtccattccgagggttccactttgagatcggcttaaaccattcgatgctttaggggtttc
tgaaatttgtattgcttttggtaattctggtcgaaggacatgattggtttccgcattctcatttcttctcggaaaatcatcaaattccttcttcctcttgt
gcaattgtctagtaactttaccatccatcaaaggacgcttttccctgaaatgtcgatccacagtcgcgtctaaaaagtcccaaactgtggattctga
aaaacttctttgtaatccgtctttagcattggtatgtggtactctcttttccatatctaattgcctttataagaaaattaatataacaattggaagctgc
gtcaaatgaagttagtttcagcgtgaacgcgtaatttttgatccttacgcgtaatattcagctctattcatacgtccggtgggtaacacatttagttct
ttccagaaataaagcctcctgaagtttctatcacaattcctgaatcaaattgtagtgcttttgctttctgttgaatgtggacaacttgcttgccaagg
aatgtatgtttaggtgacgatgttgtttgtctgttgacatgtgaatgaaaagggaatattcgttttcaaaggatatagagctagaataatgaatatt
gtagagtatttctttataattgaatttatgcagctatgcagctttttctgtgcaaagataataaaataaaaagattggtgaaagctttcaccagtgc
gaatgacttggatttcatttttctgtcagcagatgatatgccaattataaacagatcatatcaaggttttgtttccgtaaaaaatgtagggtcaactt
tggccaaggatgacttctatccattccatacgtacgcattccaatcgtcaatcgatacaagaatagctcttcgggaggttaaaacacgatgtacgt
tcgtcctgcatcatcgatgaaatttattcaaggaacaggtaacccttagcatgtttctaggagatccgtttatttatttactatttactaactagtcgt
cagccatcgtcctaacattgttactttgtacattggtttaaacagtcaatgctcgatggagagttcaaaaattcaaaaaaaatataggaagctaag
ggaatggatattctcgtctctcttctatgaggtcgaaagcgcttgaccggtatgattgattttctcaagtcattaaccatctatccacatgaatattat
aatgatctgattcaaactaatttaaatttatctaaacaaataaataaatgattatccttcagtcccattcctgtcataaccgattccctgtttttcgttt
ttattataaggaaaagaaaaagaaatcttttgatcaaatttccagctgttctatctcaagaccacattcacatcgctgaacaataattcctggttga
tcaggtctttcaaaaacttcgtcataagaatgtttatgctttttctttcacggatgctcattctactataattgctggttttcgttttctttcgtagttca
agtaattgcttttcaaatttttttccttttgttcttgtttttggacttctcttcgtccaaattcttgatccagttcttctaaagacccccatttatttttttgc
aaattcttccacctgtaaacggagataaagcatcatgttcgaccatccctgttggtgtggatttgcctttaacagatgcggtaatacttctgcgtctc
gcaattccggatccgtcaataaataatcttgtttacattcagttttcgtgagtaaggaatacttatctggatatttttcacgacaaacatgacagact
ttacacccaaatacatccaaataacgtgagtccagctcaatagaggcacattcgtcacatttaggtgctgtttccggatctaaatttaaagggctt
ttcgtaaagctttctgtcgttcctgttgttcacgaagctctcgctccgctggtttttcacgaaggtctgtttcttgtttcttctcttctactaagtaaccac
ccttggtgtcttctatcttggagaaatcatattcaatgtaatctttggtgatttccgactggtcgtagaattggccgcgctttcgtttattagctaatga
tccttcaaccttaacttcttcggcgccagtcaattgtttcaaccgttctatttcttgacgttgttcttcaactgtcgacgtggatggcgcaccttcggttt
gctccatttctatgtttctgggtacaaaaaatagcaacttgaacggaaaagagccttttctcttttcaattagcaagaaccgaaggaaaatcaggg
taatgatccacaaagatgaacgaaagggtgctacaagttgttgatttctttttttttaataacctttactttaagattatataatccaaggtttaattaa
ataagcaattgaatccactgaagaaaggaattgctttactagaatatatatcttcacaacgaaaagaaataaatagaagaaagaatggaagaa
aatggatcgtagtctcatttcaatttctgctcactacccagaaccagtaattcaagaaatatcttattatcgttattattatatcctaataggaattaa
atatcttcttgtctgccaagaaatcagactaaaaactatggagtaaattagtggatggaattagagctagattattattctgttgagtaggttttctt
tctaatttcatttactttcatagaaacgtttccattcgcctgtttgaggatttgagatgtatccatcagcaagttgtttcgctcaccaacgaaatctact
ggaaatttttctttataaagagctaactaggaagtattaatttattttattcgaggatgagcaggcattttcgattcaactgctattctattcctgtta
attttaaatctgtacttcctctcgatacttttcgtgcctgaatgcgaataacgacgtaaacttgtcataattgggtccctcgttagcattcataatata
acaatccgttataagaatggaatattagctaatatcagttaatattcataagcttaaagaattgtgcttgataataagtaaacaaaaatttcaaaa
tgcaaatttctctttgaatgcagaaactccgattaaaatttacaattcgtgattattgctattaattagagaagccagtcacgatggcaaatataca
gaataatatatatatgtgtgtcgacagtcgaaatctttagattggaagatttgactgatcaggctaaataagattgacacttagtcgttttcatttag
ttaatcagatccctggacatattactattactctacgtagtactaacacgtccaattccaatagacaattttgtaattgtacttattacttccactcga
tttacattctcataacgnnnnnnnnnnnnnnnnnnnnnnnnnnnnnnnnnnnnnnnnnnnnnnnnnnnnnnnnnnnnnnnnnnnnn
nnnnnnnnnnnnnnnnnnnnnnnnnnnnnnnnnnncctgaatgcgaataacgacgtaaacttgtcataattgggtccctcgttagcatt
cataatataacaatccgttataagaatggaatattagctaatatcagttaatattcataagcttaaagaattgtgcttgataataagtaaacaaaa

atttcaaaatgcaaatttctctttgaatgcagaaactccgattaaaatttacaattcgtgattattgctattaattagagaagccagtcacgatggc
aaatatacagaataatatatatatgtgtgtcgacagtcgaaatctttagattggaagatttgactgatcaggctaaataagattgacacttagtcgt
tttcatttagttaatcagatccctggacatattactattactctacgtagtactaacacgtccaattccaatagacaattttgtaattgtacttattact
tccactcgatttacattctcataacgtgtgagatttctcccgtgacaaaactaatatccactttagtataatacattacaggtaatattttgtaatatt
ccataattatacttgcttttagacaaaacgttctaactccctaaacctcctgttccttgcaatttacttactttctgcattcatgcaaaggaattagaa
caaaggttatctagcctaaacattattttcgcgtgtgtctgttggtgtattttcaattgtatttgttcgttcttcgttgttcgggtttcattcgatgctttg
atcttttacaccttcttatcttgttcgaaaaaacacttgctttctctctttttctctggaacttcgtctgttgacttgacaaatttttgagaaaggtgcg
ctaattaatttccatattctggcttgtttaggatgtccaaccaaactgaaaagtcctcctcaatggtcaatttggaaatagaaaatatcattaggga
agccgtggaagcaggtcctgaatatgctttgactttggatttgtcacatttaaatttgcgagaaattccttatacacagctagaaaagattcaagac
cgaatttctcgccttgctctaggtcacaatttactacgaagcatcggtccagagatattgaaattcacacggttacgttacttgaatatacgagcaa
atgcgtttcgtgaatttcctgaggtgctttgccgacttgaatctctcgaaattctggatatatccagaaacaaaattcgagaaatgcctcagtcattc
ggcaatctgatgaatctcagagtcttatcgatttccaaaaaccgattgatagaattaccctcttacttttcttatatgccaaatttggaaattttgaa
actggaaaacaattacatcgtctaccccccctcctcacatcgttaacaatgagctccaggaacatgacatgcccttgtttatagcaaacataaaagg
ttacctccttagaaatgaatcattgctaagaagtcaacaccatgattttccacacaacaaatctactcccaatttaatttctcattactatgctgacg
aaacagatagctcttcaccatcttctgcccctatgactcgttcactgactaccgctactgctgatcctcgtcattcatctgtttcttcttatcatttccct
caaaacgagatttcctcgcatccttcctatagtactttaagagcattacccaatcctaggcttagtcctaaagatcagtatccacctcaacctatcc
atagtaacaacagtgagcttctttctaataatggaaatgagcagaacataccgtccacgtcaccttttttcaacaataaactagaagggaacagg
attttggttcgtcccaaacgatctgtttctctcgcaacaggtctaaactcgtcatctctcagtaaaccggttcccatgctaagtgagcatacccgcca
ttcccatcaaaattcaaattctaccaatgagtcaggagagaaaacccccactgttgagtctcctaatgagcttggtagctcagcttctatcccaaa
acccgttcctaaccagtcggtgccagttgctcagtcaacaccagctggctcccctgaaagaaaaaagaaagaacgcgttcgtagcaatagtatca
atgatgaactgcacaatgtcaggttgccgtcttcagtattgcaaagaattgagggtttgaaggaacatcgcaatttcaccgaccatccacttactc
gaaatctatctcatgactctcatttgccaggcatgaatcgtctcaagaaagaagaattgcaggaaggtactgaaaatgttaatgcttattttagca
aaatggctcttgctaaagaacaaattgcacgaccttcttatccagaaaaagcattggaaacatgtcgcggaattctttttgccctctcccaagttca
acaaagcattcgtcaacaattgcttttctgtaataaccctactattattgataccatgcggcacgttagttttacaacaaatacgcatattaaacgat
tgattcgaagctttgaagaaattactttcatgaacgatggagtatacaatgctactccagttatccacgcctgccttgcttgtattggatcttttcgca
aactgatcgatgtgaccaagaaattttctagtgattttgtgaattctgcagatttacgatatacaagattgtttttactggtactttttggtgctacta
aagagctccaaaattcttatcaacaactttgtcataaagggccattaactaatggaatgcaagaccctctcagacaatctcctacttatactggta
gatctatccctatgtctgcgattaatactcgatttaatatagaaaagccgatgtcggcattgaatcctgaattcgcaaaacaacttctggaacaaat
tgagctagcaactagtagtgcaacgtcggttttgagcttactggttgagtctatagaaaaaagcactttatcatcagacgtttctaatgaatctact
ggtttcaataccgttgctaaattgaaagagcttgccatgttgtcaacttccgctagtgaagttactagaaagcttaaaagccactccaaagcatttc
aggcaaatgtgaatgatgaaaatgagtggcaaaagcttttagaagatacacagatgtttgttaagtcaattatagctgttgccaatttagctaaga
cggtatctagcgagtatacttttcaaaagagtatcatttctggtctttccgccgttactaggtctacgaaggatttgaccattttaatatcatcatctt
cttcgcatcacccataccatggtgcagactctgcctctgcttcggcggcggcaatggcagcgttgtcgccaattatgcgagtaccggctacacctct
atctgcagctttaggatcggctgcgcaatcaattactcctttaattatgtctccatctgtacttcctgtaactggacacaaaacggaatactttactg
atggagacctggaagccttgcaacgttaaatgttcatatctatttattgtggatgagcccagggtaaaatttagttacgagatataattttatatgat
ttactatttattctataacttatacaagataaaagatttttaggaatttggatagcgaaaaaattgtttatttaaagagaaattgtattaaattttttg
atctaaaattacaaaagatgttttaaataatcatcaaagaaagcaaaaatatcccaaagctatgtatgtcctgagtgtccaaatgagttgttttcct
ttgttggttttatgcctcaaggtttttgttgcttttcttcgcaacgacgaacaatatctttcaactcatgcacttcaccagttgttagtcgatggtattct
attgacttattagccggctcttgcatggcctgtaaagcgaaagtttttgctagctgataattgcgtagataatattgttcttcagcagcaaacttgca
ctgttctaggatatattccgggtctcctaaaacaacaccatctttactcaaatgctaaattgggtcaataaatgaatccataaaatgaaacgttcga
gtctaatcacatacaccaaaatcttgaatccgaggcgtccaatcctttgggtcaaacgttactttagcttctaatcgtttttccctttttcgttttcactt
cactcggctcaaaatccaaatcaatggcgaacaagtccatggtacaagggaaggccacagaatttttcaaaagactgttaccaccagtttgcgcc
aactaaaacagcaaaaaggtagtttttttatcaaaacttcacgaatgaaatgctgtttcgcggaagtcgagtggtagtagcggtattcagactagt
ttgaatttattggtcgttttccaagacttaggggacagtattaccatagaaggagttattgaagagcaccaagttatacaagctttagaaatccag
gattgaaaagaggatattttgaatcgcatgcaagtatttcatacagttctgggagccaaagaagccagttttgtatgattcttcatacgcaactcat
tcgcgactacactcaagatacgtgtgccaatgggctctctgtccttattaacgaggacacaagcattatcatcaaagcgaacataacgtccatcgg
cgcgtcgtagaggaaactttgtccgaacgacgacggcatggtgaatatcacctcgttttactttgtttgcagtagcgatattggggccagttcttga
ctttttaacgacgacaacaacttcatctcctatgcaaatatgtgttaatccagaaacctgcaagctttacataccaactgatgcgaactgtccagca

cgtacaacgcgaatacattccgcgagagttgctcccttttcaacgttaacactccgttaatcccatccaacttcaaatttcttaccgaattgtcaatt
acttttaaaatacccttcaaaccgatcattgttgtaagcaaaagatacacccaagtctgtgtctgatggttccatggatagcgtatcactgaaactc
actagtaaacataaaagtacttgaggattgaaatattgattaacgacataaaattatactatgaattcttttttttgactttagtaatcatataagg
tacaatttctgtggtgatggacacccttgtgaacaaaaactctgttttgcttagcatagcatccactacatatagtttgtattcaatagcttcatttac
atataaacaattgatactcgtaagaaaaagaaatgaaaatagaaacacatcatacattactccttaacctagacatccattataaccgatattatt
aattatacatcatggtggaaaagaagatatctttaaaaatttgctggttgattcgtcatggtagcattttctccttgtagtcgactttgaatcacgga
tagatgcttttcaatcagagggtattttgtatcatcgctatttgacctttgagcttcaaagtgaaggagcaactgaatagcttggaatgctttttcgtg
gggtataggagatagagtttgttcttcttcatctgtctcaaaatctttatcatctaaaaactctgcagcagtagcgttatccgatatgttttgcatgtc
gtctgctagactttcttcaaacggattcaggtaattgtccaacttaaattcaatttttccgagtactgcttcgatcgattcaacgatttcttttacttca
tttgacatgaaaggtgtctggagcgcagctccatcctcatcggctatgtaaagcactccagaccggcgaaaagcatggtcaatcacacttacaga
caaagaggtttcccacgatgaagcaatccatttcatggccaaaaagagatttatctttgtttgtggagactgtacatttgataaaagggtcctcca
ataacgagtccaccgcagtctatacaagaccttaaacgcatagaaaacacccaattcaaatggttggtaaacattggttgaattagaaggaaga
aaggcgattgtggtgtttcgaaaggagtgactagaacaacgaatgctgtcaacggcgagtcggtgaacatcgtaattgtcgagaatcaataata
cctttcgtccttccatttttgcatcaaaccaacgaagccattcctccataatcgtaatcgtcatagatgctttcccagaacagcgccaatttatacca
atattttcaggatatgtaccagtcaaatgaaaagctcttggccgctttgcatatccaatgacccataaaggaagtttttcgcttccatttgcatttgc
acacattatgatgcttattttagctttataacgctttgcacgtccagtctcttcaatagaaggggcctggttaggcaacaatttccaaaatagcccag
tttcgtccatattgaatatgttttctttcgaaaaaaagctagcaacatcttgtatccgtaccatgctttcgccaagctgctcaacattgacatgatatt
ccctcccggaaggctcgtccacacgctccaggtatcgtcttcgaaagccttcaacccagccattactgaattctggcataggcgattctttatactc
gggaatggtgctccataatttgttgccgctgcttttaatatatcgcctgtagctttcgtccttgtaaggtgagaatcttgaatccagtcatacagtgc
cttctctagagccggaaactttggcgagcgatttcgttttgcatgcaggttttggtctgtatcgtctaagtatgcaaatctgtccgagagaattctcg
acaaagttgactgagcaattggctttccaaattcacttttaaaccaatccgctagttcaacttgtgtaggttttctcgcttgattataatagaatgctc
gtaatgccttcttttctctcccagaaaccgtgattcttgtccttatagctgtcatttttcatgctatatggaatgcgttgtggtattaggatattcttaaa
cgttttgaatatgccttgtctataaacattctgaccgcagaagcatattctaattattaaccctttataagcatataatcttagcatcattcattccaa
aaaggaagcatatgaaaattcagagattttctagttgtagccttgtttttcctttttgctctattaagagcatttataccacggaatgattcacgga
tgatttgaagcgttttgttagatcatcctggatattcgcattctcgtaaactctgcgatccgtcttggaatagcaaggaaaaaagattcacttaatgt
agaaaagtggaataaagtaaaaccgtagttccattaggtagagcatttacgaataagattgcatacatttctgtatacgtgtaacatacttctaca
ataatatagttcgccttggcttatggattcctttgtgattaagcaaattccttcttttgaaatctttcaatctaagctgttttgaatatctgaactcaag
tttatgagcatcgtctttcctcatcctgtgttacatatcctgtagtaacaatactcgatacctttctttgatcttccataaagacgtgtcaatacatcttt
cagcaacaggtcaatccacagtgtctactttattatcctttcaactgcttttcttttgcatccaagacaagtacaaaagattagcggcagtatacata
aacgaaaagcttgtatctttatccttttagatacaaacttaaaggtgtcgtcgcaatacccgagattcgtttgataaatatcaatcaagcaaagatg
atccttaattaaatgggcacgattgaatggcttttattcctaagctgatgaacaaaagggagtaactattgacatttatagctctctttttgaataaa
aaggcttgcatcctctttccgatcattctatccattggtaataagcggtcgcttttccttggctgctgaggatccaaaatggaagcaattttagttcgt
tatcctcatatagaacaaagaatctcacaagagaaaatcgatatcaagtgtaataatggccttcaatcccttgcgttccattcaagtgtcatgcag
aaaagtgaacaagttaacagaaatcgtacttaaagtagggaagcattgaaaccagtttataaaaaaagcatacaaaaaaaattgattgtagcc
atgaacccttcgtgttgcgatgagtagagcgcaagtgaatagagcgacagaatcacttaccatcagcggcaatatagtttatcggtttcttttcaat
gtatttaattgttttttttttacaagaaaatacaagcaggggtttttcttgtcccgctttgtttctcattttgattgccctcaacaacagcggccttctc
ctaaactctacggaggtattcaaatttgaaattgagcgttcaaacccgtttcttttttgtagtcagctgtttcttcttgtaaaataacgatttgcaaat
ggataaactacgtagacctgaaattcgtcaacaaacgacccaacaaatgtatggtgttcctgaaaacatcctggagatcgatgtatgctgtttgat
tttacatctactaggttgaattaaccagttttccaactaggtcgtaaatcctcaaaatcatggtcttggaagaggcatgtttactacctatgaaattc
aatgtcgggtatggttctttaaatgagtcttgtagtatttggttttcttaatcttacagatgaatgtagacgaatatgccttattttcgtttgagatatt
ctagcgttcgtcgccgttatagtgaatttgaaaagtttcgcgacatgttggaacacgaaattggacgtgtttccattcctcccttgccaggaaaggt
tttcacaaaccgcttccgcgaagatgtgattgaggaacgcagacaaggccttgaaacatttctaagagcgtgagttccatacaaagtaaaacaa
cggtctaacggtatactaacaaacactttctttagtgttgctggccatccgcttattcaaacacattctcgtatactttcttcatttcttcaaaaccaa
gaattccaccctactatttaaattaccaacgaacctgttcttggattttccttgctacgagcacgatagaccgcaatatcataaacgaatctcatcgt
ctattattctcttgatttatccatgttattatattttacgttggaagaatagaaagaaaattacaaataaaaacaaaaatgaagtatgtttgttggtt
ggatcatgaaagacgttgcaagaaaaagtcgatgctgttttgatttccgattcttgctatatattgtttattgaaaaagtaagtttcttaaggattga
aatactgaccttttagttccattcgacaaattgagcgcttaaacgagctggagatggataaaccattgagcagttcatggcacatggattacaaag
acagtgcctatatttatatcggaaatttggattatgatttaaacgaagaagatataattaccgtttttcagaatttggcgaacctacagacataaa

cttggttcgcgataaagaaacaaagaaatcgaaagggtattgctttctgaagtacgctgatcagcgttcaacgatattagccgtagataacatga
caaatgtcaagttactcgatcgactcgttcgagtcgatcatgttgctagttaccgagttcccaaaaaaggatccgaagatggagccgccccttctg
aaataggcagccaaaatttagatgctctttatcctccttcccttccacgaaccgaactagagtctgctgaacggtcacctgctcctgatgagcatgc
tgagcccttagatgagaaggatatgttggatcccatgcacgaatacattaaaaatgaaaagcggagaaaagtggatgacgcgcatcattcttca
cactcttctcgcacccgtagacaccgtcactcggacagacaccgacatacccatcgaagacatcgtgacaagtctccacgagaaaactaggatc
ctttgtaattccaaaaaaaaaaagagaaaaacaatcttttacggcaagtaatcatagtaaatccatggttcagtcgttaaatgtgtgcggcggct
gccgcttcatctatacattatactaaacgaaaaccaagtctttatgttaacaatacactccttctctcccgatactgattagttgcttccatcatccgt
tcaattatttagcttaatattgaacattttattaaaaacacttcaataaattgagttacaaacctcttttaaagatttcttttttaaaaactattgtct
atttgatttcatcatattaaagatttatcatactactttcaagtaacgaaagaaagggaacatgcagcgtcagtcagaagacattaaattacgaag
gaataaacctggatttaactgcttttaagccagtttagaagatataaaaatgacctcctacgtcctttcgaaagaactagtaggacacgaacaag
acgtaagggcgatgaatctagtgcaaagagtatggaatgtacgcttattaacaacagttcaggttcgagcggtttgttctatttcagatgaattgat
aggctctgcttcaagagatgggaagtacggtctttggaaaaagattggtaatgattggactcctcaattctatgaatttcacgaaggatttgtgaat
tccgtatgctatgttcctccaagcgatcctactacgaaaggtatggatatattcaaagaagtagagaattgctaatagttcagggtttctcttttctg
gaggacaagattgttcaggtatcctgcaagaagtagatagcgaatctcctgcttactatttattcggacatgaatccaatatttgctccgttcacgc
acttaacagtgctaccatcatcactggaagttgggactcaactgctcgtgtgtggtcatatgggcaatgcagacttgttttacgcggacatgaatct
tcggtctgggctgtcttagctttaggggaggattcttttgtcactggttctgctgacaagacgatcaagatttggaagggggataaattggtaaaat
cgtttgaagcgcatgtggattgcgtaagatctctttgcaaaattccccaaggctttgcttcttgctcaaatgatggaaccgttaagttgtggtcctta
gatggacaccttttgcaagaattacatggacatacctcatttgtttattcgcttacgtatgttcctacgttgaatcttttggcatcctgtggtgaagac
aagtctgtacgcatttggaaggaatctgattgcattcaacgtatcgccttaccaactactagtatatggtctgtttgtgctcttccagatggggatat
tgcttgtgggtcgagcgacggctatgtccgtgtcttcaccatggagaaaattcgaatggcatctgaagaagaattgaagaatttcgaaagacgag
tgagtgaatacgcaattgctgctcaagaagtaggtgatgtgaaaaagggttctttgccaggccctgaaattctgacaacccctggaaaaaatgaa
ggagacgtggtaatggtcagagtaaataatgatgtcgaggcttatcaatggtctcaagtggagaaagactggaaaaaggttggtcaagtagttg
acgccgtaggaagcaaccggaaacaactttataaaggccaagaatatgattatgtcttcgatgtagacgtagccgaaggtcaggctcctttaaaa
ttaccttataatgtgactgagaatccttttcaagctgctattcggtttttggaaacgaaccaattacccatgtcgtatacggatgaagtggtccaatt
cattcagaaaaatatccaaggacatacgtttgatacgtcgtcaacgcctcaagcttcaaatccatcaactagcaaagaagttactattttccctgt
aacttaccttttatttaccaccgcaaacatccccgctatgtgtcaaagattacttagtctaaacactacacatgctaatgctcttccccaagaagact
tggaaaaattacaacgtatactttcaacgaatcctataactgatactgatcgtcaagttgccattgagatttgtctcaatgtgttggaccgttttagt
ttttctgaacgatttccggctttagatgctttacgattattggcaatccagtgtcctaagcacctttgtgcagttctcttggaagtgtttgcacaacttg
ctcaatcaattacagtcactggaaagttcgagtcgattaacagtatgttgacgcttcgaggcctagccaatcttgcgccaaatctttcatctgaaga
agacattgtaaagatagctgattgtttattcaaggctactccgcaagcggaatatccaaaagattacaaagttgcttttgcgactctcgctatgaat
ctttctatcttgcatattaagaaaaactccgaggaagcagctattgaacttttaccactcttgacaaacttcttagctacagactcttctgagagtga
atcttgctatcgtgccctcatggcattgggtagtctgtgcacgcttcctaacgctgcagtagcagcagctcaagtttatgatgcccgcaaggtagta
gacaatgtttcaaaggcatttgctaaagagtctcgttttctagaagcaaaagaccaattctttgctctcttggataagacgacttaaaagtgatgct
tttcatctatactactaatgatatttatgaaaactgtaaacctagttccgtagttgctctcgtgctcaatagatccttcttgacaaagtctaccaattt
gcttcgctatctatatttgcgaatcgcagttgcagtgtacgtttacagacgactgattatttgcatttggttctttttacatttcgcactttagctctaaa
ctattcaaataatttactacgttttgaaattttctatatcctttttaccattttcttcatttaacaacggtacggtagccatcgctatttattaagtagcc
aaattctgtgttcaacttgaagttgtctttctctaagcacgaagaaatggcgccaaaaaaaggcaccaaatcatccaagtcgaaggtggcttcttc
taaaataaaaaacataaaggagagctcgatccaaaaaaagaatgcgtcagaaaagaagcaattggtatggtgaatgaaagttaaaagaaag
actgtgctaacgtgacattagaaaagaggaactaagcaaaatgagaaaggaaaagagaataaagggaaaaagggagtgccggcgcctgaag
tccctgaacgagaattaacagaggaggattttgcgatgttggatgaaaatccaacgtcactgcaatttcttactaatgtaaagccagaagaattg
acaaagaaggtagagaaaggtcctcgtccagaaatttacgatcagaaactttctaaagatgatgactttgacagtgaagaggagttcgattcgta
tggctctgatgaagacattcctaattactcaaatgattctgatcaagaacaagattatgagcttcgtccaagagctattagctcatggaacatgact
gactcaaatcgtttgcccattaagaccaaagaggggcttttgcatgcagtgaaggaagatgcaacccaagccgaaggtaatgggaaagaagaa
gaagaagaagaaaaggaagattctcccgagggagaaggtcaaggagaagaaataaaagaggaagacccttcaaaagatacttcggtcgaag
agcctcagctttcacctaagcagcaaatccagttggataaagaagtattggcttctcttgctcaaaaactattggaagaaccagaagattctttgc
atgtcttcaaaagtatattcgaaaagtttcaatcaccattaattactatcaaaaaattgagtcttctgactgctcttgctgttttccaggacttaatcc
ctggttaccgtatccgtcctttgtcagaggaagaacaaagcacaaaattatccaaagaagttgcacaacgctggtcatatgaacagacttttctga
aatattatatgcgatttatcgaaattttggaatctatccttaaatcatacgcttctctagaaaacgatgtagaaaagtctctctatgaagtcgcagtg

cgttgtgtatctcgattaattgaacatgtaccacactttaattatcatgataaactattgactttggctgttcagcaaatcagtcacaagtctaagag
aaatagctttgatttgtttataaacagtctaactgctatccttaaggaagataatcttggacgtgtttctttggattgtgttaaattgttgtcaaagat
gtttaaacatcgcaattatgatgttcttccagaggtttacgatttgttcctgcatattcaagttctttccgatatggaattaaaggatagctctgaaga
gcgaagtaaaataaaaaagagaaagcaggatcgtcctcatcttaataagaaagcgaggaaaacgctgaaggaaacaaaggagattgaaaaa
gaaatgaaggaagcagaagcggtaatatccgctgaagatcgtgaacgataccaaaatgaagtgttgaaaactgttttccttacttattttaagac
attgcaggcaagaagtaaattattgggtaatgctttagaaggtttggctagattatctcatctcataaatgtcgagttcttcggggatttattgcagg
tcctgcgtgaacttgtttcagatgataatatgttgttagcagaaggcaaaccggctgttcaaagtacgagggaagctctcttgactgttagtacggc
ttttgaaatcgcatcagcaccaggagtcaataagttaaatttggatctagatttaagcatttttattcaacgactttacagattaattcttccattttc
attgaacccagatgccgatttgtcatacaaggtgaatcgacttcgggatccagatgctccttcaaagaagaacgttgttaacgcttctacccaaat
ggagatgttattaaagtgctatcagtccttcttttttaagagcaagagtgtcagttccactcgtcttgccgcattttcaaagagattggctatttctag
tttgcagctcccagagcattcctcgacggctgcgatcgcactccttgctcgactggtgtcgcggtacactaagttgcatagaatgtttacttctgaag
agcaaatcggcgatggcatttatcagccgttcgttgaggacccggaactatgtaagtcatcaactgccatgctttatgaaacttttcttctgaagaa
tcattattctcctgctatttcgcgatcagcaaatagccttttttaaattgtccacggaatgaagtaaaaaaaatttttgggtctaggttggttataaatta
attacgtacaagttttggatattatcggtaacagggaataacataatggttttctacatataagagttttggtactcgtatcatctcgtatctgtttgt
cgagcttgagaaaaataacatgctttcatgacttctcaaagatatagcttgttctaagatatcctttataccatgttagcttgttcaaacaaaaagta
acaaagtagagcagggacttaccagttttgtgattataccatgcagttttccacgaactaacaaagctacaacggttattcccatttctctaaacat
cttaagtatcagtactggtggagtactatgattcagggtaaaagtagctggatacaaatgatcagagagcgaaaacgtcagatcaatcgaagat
aaactttgttcttcaggttgcaaagtggaactgacttgtgtaaatagaccttcatttaaaggtgaaatatgtagcagctcccctaatttctctctaga
aataaggccgacaaaaaaattctgtttggtcttgcacgacagctatataaggatgagtactatgtttaaatgcacattccatttgatgaatagtacat
ctgtccaaaggtaacttgtaaattttttcactcggagtcattggaagattcaaattaaacgaagagatactatcggcgtctgagataggaaagtaa
gggatacttttaaagtgtaaccaagaatcataaatggaaaatggattgacaaagtttgagatccatctgctgagaagggttgcaagcatgagag
gtaatacaacagtaaaggcacccgttaattcatagatgataaccaccagcgacacggtaagtctagttgttgagcttaggaaagcagttgctccg
acgatagcatatacggtagatcctgccaaggaagaaaaattgcttcttagaatggtgccgatgcatctacctagacaagcaccaattgccaacga
aggtacaaggattccacctggtatttgcattccataagtaaggcctgtcaaaagcaaccctacagtcgcggaagcaaaaaggactaaagaggta
atccatggcggagcttcacataatatagaagcagacgaaggagagcaggaattcgaaacttgaaaaaacaaatccgtcgggttgtataagata
tcgtaatcaaacaaagctgggattgatacaaaacaagtaatcaggcttagtaaagcaactaaacatatactgaaccattttggaaatgaatcgta
ctttttaaaaactttgatatttaagcgaactaaaactgacccaagagatccgcaaagaacgctaataaaaatgaaaggaagtaaatctagataa
gaccaatacctttccaacgaaatgtatgaaagaaaaccagcaccgggttgccaagatctaagcagttgtaaagtaacaaccgaaaacgcagag
cacaagaattcataccatatggagcctgttaaagaagatggaaatgaactagtggtgacttgctccaaagcgaataaaactccgccgacaggag
cgttgaatgaagcagctataccagacgatattgcggcaatcagaatctgacgagaaaaaagatttgatgttcttatttcaggaaatagtgaagca
ataaggatagctatattgctggctatatgaacaaaagggccttctttaccaacccacagaccagaggcaacacaaagacatattgacaatgactt
tgagataagcgttttaaaagaaaaaaaaatcttcgcatcagggagtgtatagccgcttaaggttgctttaatctctggaattccacttgcggatac
ggagggaaagaagaatgcctttaacaaagatgccagtaaagcaaatccaaaagagaaagaagaataaattaaaacagctgtgaaatatgaat
gctcatttctccaataatgaaaggcctgacattcatattcgtcggtaagaatcgcacaacaagctgttttgtctagtaaaatattctttttacaatat
ccctcacgaaaactgttgagtaagaaggttaagccattcagcaaagctgtcgataggcccaccaaaatgcctataagtgttaatataaaaccag
aaccaagaacattgagtatggaatttttttatcaaaccaaatattaccagaaagaatggaagtgattaagtactttgtcgttgtattcaagaagtt
aaacctgccatttttttatagaatcccgtcgttgttttttataccgctcataaacccagtcaatgagaggctcattgtccatttcaaagaataaattgtt
taaaaataaataaatataaatttgcagctttcagcaattgtaccgtgtaagctaatatgagggaacttgagtgtaaacaaaagacgacgaagac
aaaagctttagcaatttttctcaagtacggagacttcaaagcaaaagaagtattaaaatttataaagaatagagttttaacaaagtaaaaaagtc
gaactgaattcaagcaaaatgaattcttttgtccataacgtataggtagaaagtgtatgtagatattgtaagtattggttttacgattgctgctaca
aataaaagaaatgaaacataaagatattgaataaaagactatttagacgacacattaaaagtttaggtattagtgataaaagtagaaggaaac
gtattaagaaaagggtgagaaaaggattgggatccatcgtgtaaacgagaggaaaagaaaagaaaagaagatgacagtttatctgttttaaac
tattttttataaaatagacttatgtaggaaggctattcaagcttatcgagagtttcaattcgctcaagcaaagcagttttgttggactcaccaatttc
acctcctaggagaaattcatccaaaatagcgctaactttgtagaaattaaatatgagatctaactcgcatacgtttccaaagaaagaatctaaaa
tctcgacgaaaaaaatggatcatttctaaaactgccaaatcattatccgtagtgtcaacacaaaaacaaaaatagagtccggcataacgacgata
gacaagtttgctgttcctccattctaagaaatttgcttggtatttttgcgttctttcggatatgaggcgatgaatctggctttttaactgtgtttttcaa
catcctaaaaagaagggttagaaataaaattcgtacagggcagaatagaaaaggtaggttagtctataagagaaagacctgttaatcgtacatc
aaaagggacgtaatattttgaaagtcggtttttaccatggcgattttgaacaaggacaaattgaatcatagtcaattcaaagagcaacgaaacgt

cgtatatacgtctactcgtaaataaccttacagcgagaatagatagatgaaaatagcaatggcaataacaactgcaagaacgaattcctcttaaa
cagtgagaacagtgaaaaggaacttggtttgcagagacatgagcaggatgggctttgagtgcttccaataagacagaaagaatgtgtttccgaa
atactagagtctttcaaagaaaaaaatgatctgaagaaggaaacaaaaagctacaaattcgccaagagatgatgagagtcctttagtgataca
aaaactcgacctgtaggagcgcgtatatttacaaaagaaaatcgatacgattctttaagaaagaagaaaatttaacaaatccttcacagagagg
gagagcgtcaaattggctacttcgttgaaatgactcttaaccgttgaggagtttacgaaaagaccaagtgctgaaaaccggataaggtacgtaac
aagaaaaatccaatcggctagcaatcgaaaaagcaaaaaagaaacaaaaagaattggttcttgtgagaaaatccaactgtttcgcggtagatg
catcgagtttggttgtactaacaaaaaatgatcccgccaggagtcgaacctggaatctcctgatccgtagtcaggcgccttaaccattgggccacg
agacctctttgttataaacagtgagtatcgttctatccattatagtagtaagataatattatacaatttgaagaaatcaagctgaaggaattcggtg
cgaatatttcttttcgttgttttcattcatcttttcttatgaaaaaggttggattaggtctccattctaaatcatttcctttgcacatgctactctgctctg
atgctgattctctcattttgttgttgagagcatgggacatgggttgctaaaatgctgaaaaagaatacacggtttttgttgaaataattcgtgcctga
aaaatcttgcttaaaatagttttcacttcactatggaacacttggtaacccaaataatatggcatttgttttcattcggtgaatccagtctaaacaaa
ttgcttataagacttttaagaaaagcatcgatttagttataattttaaagatttgtttttgtttctttcttctttaattatttttatgctttcctttccttctc
ataaaaaaagcgattgccatggtttttgcttctgtttcattgtttacttatgatctcattcggtaacgtttttaggaacgataaaccatcacccgcata
cttttatttctatttcctttttttacttttttattcttttgacgggtttctttccaactcgcttccttttttgtttattttctcttgtctcgcttttgtttacaaacat
gaccggaaatgccgcggagacttccgatactttttatgaacaccgttctccttggcaatgactgcatactcagttttacagccaacaaataaaaaa
gcgcttgcttggcttctccagccattctattcaaaccatgctcaccgtgaagaggtatccaggagccactgaattcatcgttcacacaagcaaagg
ttatggcacgcaaatgtttgcagtttgctttttgtctttcagtgttggcgtcacgatactagcttttgagccatcgccgagattgttgttgtggatttct
ggtgcaatatttattttgtcccttttccaaattataagcggcgtaaatcgtaagtcttgtggttttaatgtccgaattcctttgaaaccagaaacatag
aaaatccctacagaattggtttctgtttctctctcgcttcttcttgagtacaaaaaaactaactttgaaagatgaatccttacttgttgtgcgcgatct
aggagtccaaaccaattgtcattctattgttccctggaagtcttcctccaaattggttcccttggattccgtcagggaaatatttattcacgaaggat
tccgaaagttcgacgtctgttattacatgggcattgccatcgaccatgaaccccaaatccatgttgtatttcctacattgctacctcgaagagatgtt
ttgcagaaaatatattctgaaactttgcaattgctcaaacaagaaacataactgaaagcttattactatagaatgcatctttgttcatccttgttgct
tgacttttccatgcctcgctttcaccagcattcatacgaagaacaaatccttccctttttcgtttagctcgtccttacaagcctctacacatgttcttttt
ttgtttcgtcttataccgttagatatcgaaatatccgtccttgaaagccctctgctctttgtttttctcgtactccatttgtttacacatagacttactccc
gcattcctttctccttgcattctcttccaaatggtcatttaacaaactctctttgcaatgtgctatggttatataccgagagctaggcgattttgtaatg
gatgggaaaggcagtcacatttttggtgtgcaatttcttttgaaatagtgtgttggatatagcatttctccatctttagcaatggcacagtccggacg
cccttctatactgaatgctactgtaagtaaaacaagctaaattatgctcttgcgtgtcaaagatattaatccaatgtgttaggatgaggacatcaag
cagctccttgctgctagctgccatattggctccaagaacttagaggtccgtatggacccttatgtctggaagcgccgtagcgatggcgtccacatcc
tcaatgttggtaagacttgggaaaagttggttttggccgctcgtatcatcgctacagttgaaaaccctgccgatgtttgcgtcgtgtctacccgtgct
tatggtcaccgtgctgttctcaagttcgctgctcataccggagctactgccattgctggccgtttcacccctggtaacttcaccaactacatcactcgt
acttaccgcgaaccccgtttgattattgtcactgatcctcgtgctgatgcccaagccatcagagaagcttctttcgtcaacattcccgtcattgctctt
accgacactgactcttgcttgaatcacattgatgttgctatccctaccaacaacaagggccgtaagtccattggtttgatctggtacttgttggctcg
tgaagtccttcgtcttcgtggcaccctttctcgttctactgcttgggaaatcatgcccgatctttatttctaccgtgatcccgaagaggttgagcgtga
agaggaggccaagaaggctgctgccattgcttctgaggaagcccaagttgaagaagccgccgcagctgccgaatttaatgttaacgacggtgct
gctggtgctgttgaccccactgttcttgccgctgctactgctggtcaagttggtgagaacacttgggaagagactggtgagtggaataccaccggt
gctgctgctcaaacttctgattgggctcaataaaatctttcctttaatctttaattaaaataaaatggtatccatggttggtgatccatagagttgttg
tattgtaatcctagaggagtgtaattgatttctctacacgtaaaataaaaacgaagcagagctataagacgtatatgctttgttattcgggtggtag
acatggtaaagttcacagcaaaatggttttttttgataatttattatttgtgtggtgtattttaggtcctataaaaggatagcaatcgtctatatgaaa
aaaaaaatgaagtccagctctatatcgatgaatccaaactcgatcccatccctacacggtacgaaaaagaattggtcccttcatattctacacgta
cagctacaccaacagttataagtttctaaattttatatttagaaattaagattcatatttttccaaacactacctcacaattagaatcttaaaggagt
aaagaaccaagcagacttgccggtttggtacttctcttcaaaagtcttcttgacggtcttcttggcagcagagcgttgggaaggctccttgaaagtc
tcagcattgacaaggttcttgaggttgtcaagctcgagagcataacgagtgggcatcaagtggttgtagttaacaactctgacgaaaggcttcac
acgagaacgcttagcgatgcgcttagcacccatagacttggtgaccttcaaggggtagcgctcaacaccagcaacgatggcatgtccgaaggga
tgggacttggtgccttggtcgacgttttgaagaattacaatcttcttaccggcaaagcgacctctggtgataagagcaacctaaaattcattgttaa
tacacttattcagaccctatcgcgaattcagtacctttcctggcttgagaatcttagccattgttttgtcgtgagtcaaacttgctcttcctcgaattcc
caagacacgaaactttgtgactgttttctttgtattttccgaggggtgtcccacctccctaaatggttgaaagaataggaacgaagcgcaacatag
gctgcggaaccactgtggacttctttattcaataaacaaagccatgttggtgtaattttcatcaaaacgtcaagcctgtcgactgaatctgctttttc
ttttccctatttttcttgttctttctagtaaatcattgacacaaaatcccttgttgagtttacagtttgttttgcatttctacaccgcgagttcgtcttcttc

acgcattcctacaactagggaatccttttcacgttttgagggtaaaagcctagcctagaaaggaagaaataaaacaaacaaagaatggtaccct
tgaaaatccttttgttgaaagctaatgaaaatacgattgtaggttcttgtcaagtttaagcgtcaaaaaacgagtatttttctggaagtggcaaaca
aagagacattgaaaaatgctgtggatacattgcgacatattttaaaaaccagtgatgatcaagcgattaaaatcggcgctttgcgtaatggagac
tatgttgctttggagccaagcacttggtccaacactgttcttactgagaatacagactatgcatttgcgtatgagaaggaagattttgaagtccatc
aaccgtctgacgaagatgtcatggagcctgagggctagctttccagaagaaggaaaggatatagcattgtctgaatatgaagcttgttcatctcc
aaagcaaaaggccctctctttaaattatacctgttttcttacaacaacaatggatgaaatataaattctacaggtggaattgttcgaaagtttatttc
gttgcaaagctacaaaactctactaattcatcatccatgtatgttataaagatcgcttcctttgtatattcgattcatattctcttataattgattcgca
catagttactaagtctctatctttcgtaaattctcagtctttagaaacctttcactttttgtttttgttttcctcaatccatattcaccatgtcaacgaaa
ctcacgaacaggattatcgaacctgaaccgctgctttttttactttacttttctcctaaaaacctactctcgagtctttgttgcggatttgcagaaata
agtgacaaaaagtattgaccggtgagggattcgaaccctcgcatcattcgatatagcaagacttgtacagtccaagtttccacttgagtgctacgc
catagaccgctcggccaaccggccagttgttaatatactacatcaatgaaaatatatatagcccttaaaacatgaaatacggtattaaccgtattg
aagagaatgcaaattttggttctttcgaccaagtgtttttcaagaaacatgaaacgaaaatatatgagtaacagccattcgtctttgtctttcgcg
atacagaaagacctttagtctgctcgaatagggaaggaaaaaagaagctggaaaattcgaacccgttatatcacactaccacatattagtaaag
taagaagacatcgttgagctcctttaaacaaccacagtaccgtactttcgctatgcagtttgattttctaatttatgtaaacaaaccagatggtgttg
ggtcacggtatcatagataccgtattagacaacacacggtaaatgattttgtcttgctactcagaagggagcctgagcaacttgagaacaacaaa
acacgtttgttcggcagcaatgagctatggatagatgaaatgtccgagaatattccgtctacaccgaaaaagagctctgcagatagcttctggca
agttccttcgaacccagtgactcctagaacgcctggacatcgtgttttacgacaagcgggacccaataccaaggagcgctctgctaaacggcggc
ggggccgcttggaaattcagcaacattttcttggagaagttgatgaaaatttggcttctgacgctcttttggacacgcctcgattccagtcgcgtgtt
cctccctcaccttcttttaacgtgtcgtctccatcaaaggggcggcttctagatcatagagagattgaagatactgatggaaatgcctttgaactga
cttcttctccatcggcgaatcgaatatctttagcttcatttcaaccaagctcgtccatttcagaaggagaagttgatgaaagccaaaaattacaggg
tttagaggcattggaggaagaagaaaatgaaaaagattcttctcctctgaacgaggatattggccatggccccggtttcgatgaatattttgacaa
gttttcccagaggaaaacgtccagcaatactttatcgcagcttcctcttgtagacaaccagctatatctggataccatccagaaaacagtcctaga
aaacgaccgtttcactcaaactctcgttcactttcaatctcggaactttcaacagtggttttgtgaggctgtatttggatacaatttgctattttatgg
ctttggatcaaaagagcactttctttctacatttgttgaacaaaaatttcacgggtttccagtctttgttgtcaagggttattttccacatttgcaattg
aaatcagtgttgactgcatttctcgagtttttagaagttgttccctctagtgcctctgtccccgatatgttgcaacaggccctctctgtattagaatcc
ggcgaaagagcatttgataaagctgtaatcttagtccacaacattgacggagaggatttagtagatgaacgattccaatcagctttggcaaacat
tgcagcttctcccaatgtttatttttcgcctctgtcgatcatgttaactttcctttactatgggattcttctcttgaatcgttattcaactttgttatgcac
gatgctaccacgtttgcccgttactataatgagactacatacgaaaactctcttggcattggacgcgccaatgtttccaataaagaaaaggctatt
aagcatgttttatattctttacctgccaactctagagaaatctttaagcttttgcttatccatcagcttgaacgccttgctgattttgcagattctacta
gtatacgacctagcgagcgaatcggaatcgagtataaggcgttttatcaaaagtgcagctccgagtttttgtgtagtaatgagcttaattttcgttct
caactcacagagttctttgatcaccatattattgaaatgaaacgagactccacaaatatggagataatatggatccctcacccggcagatttatta
gagaatttattacaggacatgatggaagatatttgaaatttacaacgcatataaatcgattaaaccgtgtcgccctcttatcatatacactcctcat
atatttattttcacttatttatagctagaaagcaaatcgattaataaagtgtaagataatttcattatcatagatcatagaataaacaattgttacta
aagacaaaattatgcgaatataaataaggtgtgcgacttgaataaaaaaattagtaacataataaaaattggaaaacataaatcacccatagtg
ctgtacagtttaaactaacaatatacaaaaaaggcgtgatatcgtgagcctttaaattgtagaatgatccatatagtactctttgcataataatttct
gctctcttagctcggcaacaaattccaagagaccattgatttcccatcttccaaataataggccaatacagaaaccattaacgacagctgcaatgg
cgataactaatgtgaagaaaaggattattaccttgagccaggcaggatcacaccatgctatcgcaacatgtacaccaacaataataggtataga
aataaaaagctctgcgatacggaggcgcatatgcaatctttctcttttcatatattgataagttatgccaagggtgtcgtcaataattgtgtgacca
acgcgagtttcaacaggtactgaaggttgatcgagaatcttcaaattggtttcaatggcaaaatggtctgaatagctacagcccaattgaggaac
acgatcagtaaaaacaactttactttgagtagcattacacgtgctaggtgaataataaacataatccaaacgtttagcataaaaatcatccatatc
cttcgaagagatattttcgcgccagctattgaggcgagagtcgcaagtagctcccgcaatgtctatcaactcctgatcagacatggcataacggtt
aggaggatgcgtaacttgatccggatgtaccgttagccaagcatcttggaccaatccataagaagtgataatttcatgaggtacacttaaaggttg
aatattaaaatctccactggcaattacaatatgacctcgttggactgctgaacggagtaacttggaaatataccaggcctgagaaagtcgatgac
acaaataagtatctggaccttttccgtaaggagcatgcaaatgagtgttgaaaagagaaattgtttttccggatggatgctgcagactggcagttg
ctacacctttacccacgtaccagtcacctcgccaaaaggcttgaggacgaccgttgaggggatacttgcacatagcagattcaatgatggggaat
cgggaaagcattgctaatccagcccccattgcagcgctgtgaaaaaaccttgaataaacgaggttcccgcttacttggctgcgaatttcattgaaa
tcattaatggaccatacttcttgtaatagcacgatgtcgtaatccgaattagcaagcttcttaccaatctccttcaaacgctcggtgcgatatttcga
cacaaaccgcaatccccaacaattgaaggaaaaaacacgaaggtccatatcttctttttttcttgatgcattctgctagttactttgcagaaaaga

gattctgaatacaactataaaaaaaaaaaacaaagaaagaaacacagcactcaaaatgatctgtttatacgagatttgtgattcggagatttcct
taacaacctactacaaaaacatgttaataaaagccattgaatctttaatgaaacaaaaagaaaaagagttgggaaaaagcgatagccaagaca
gttcttcacgatttgcatttctttctttttaggagcgaaatcttttccagccgaaaacagtaaccaatgtaccgatagacattcaacaatcgttctttt
ggagactctaataaattcatttctgagccgcaagagcacatgaaaccaaatttggtgatcttctccttccaactttctattaacaaaacttactcag
cctttgtgtttctgcaagaggaaagaatgcaaagcacctaaacttcaatgaatttgaggcaattgataaattgaaaaagtggtgtgttaatggatg
gttatttccgcacacgtacttcattatacaattgtacgatattgtaatggcagaaaaaaatgtatgtgaaaaaatacctgaacgaggaaaattaag
caagatgagagaaaaaaacataagaaagaagatagattcaatgcatttatgactaccgttcaacatagtcggcgatgaaatttctcatagtcaa
aaaagtttgaatagcaatgacgaattcgtctacaagagtcgaaatccagactataagtatttaaatgaattattctgagaaaaaaaagagtccat
tatgataatttcgatgtccaattgctaaaaacagccggcgaaatattggaagcaagtttcagcagaaaaaccaatcttacaagatgaaatctgat
agttttcctagcaaagaaaaaagaagagttattaagaaagcgataatatttcggcaagagttggacctttctagtcctatttttgtgtaaaaacatt
tgtttgtttacacaacaaacttcttccaaggacaaaagttcatccattaaacacgtagaaatagaaaatcaaaaaggaaagatgaaagtcctcgc
ctcgcatgcagaacatagcgctgagtggaaagtatagtatgccttgcagaatagttcaatctcgtctttaaacaaagaacaaattgcgacagtca
tcttgaatgaacgaaaagggattcatgagaatggtctactttcaatagagtatgaaatgaaatgggccaattgcaagatcattgggttatatagct
gaaaagggtcgttcattagcaagtttttaacatcattcatttttcttgtcttttcctccttcctccaaaagaatgcatcaaaatataaaccgtggatgc
catttcttctttcatttccaatatggcttcgcttctatgagcttgcccagagctacacaaactgcacctattggattcaactcttttaaaatagtaactc
cgtgtactaatagacttccttttaattttgtaacaagaaattattgaattgaaaggttttccgcaaccatgagaagagaatatttagttagtcatatc
caagaaaacgctcacgaagctgatatttattcattatgtgtcatttcaggacgattgtggtcagcctctggagattcaaaaattaaacaatggaat
gtggaaagcgcagaacacgagcttgtcgaagaaatcgacactccacacaaaattggtattcataacatggcagcatcattggaagaacagttac
ttgtttcctgtggtttcggacaagatgtctatctgtgggatccaagcacgaattcatctcgtttgctgggtaggtccttttgttctttttttgtctgtatct
aacatttgtgtttaggtgattcggttgagcatcccggagaatgctggtctgcttgtataagtccagatgcacaaaccatttcctttacttctgtagat
ggacgtgtttgtgtttgggacacacttggcaatacaaaggtagccgacttggacaccaaagggaaatttggactgtgcacggcttattcgcctaat
ggaagatttattgcgtcaggacatcaaacaggtcagctatttttaatcagtactgaaacaggtcggttgttccacactttgtcaggacatacgctcc
ctttgcgaagtctagcattttcacctggttcttccctgttggctgcagctggggactccaaaatgattacagtatacgatgtaatatcgggagatca
ggttggccaacttcgtggtcatagtgcttggatctttgctttggcattcaatcctgttggagacttgcttttatcagctgacattgaaggttgtatcaa
agtttgggatatggatacaatggaatgtatcagtacaaaaaatgaagtggacggtgccatctgggctgttgcttggtataacaatggatttgtagt
agctggtgctgacaggagcatcctttggtacagagctgccgcttctgaataattcctttatgatttgataatggagaagagttttgtctttgatgcac
aagaaaaatttatgctattgatattctaatacatatacccattgtttccatattcatggatcgatgtagtatgaattgtatattaaaaaaaaggataa
agtaataaatagagactatatatgtcttctttggtagtattcttcaagcatacattcattacttcaacaacaaacaacagtctacaacaaatacaaa
agtaaaggttgaaaagagtagaataaatttcactgaattcaaatacttgctttttcatgattctaacgtttcagcttccatccaaaaaggatttttgt
tttagctgatcacattgttttgaaggattcctttccctttcatacactacctcaaacttaaaaagtatatcattacaaacggattctgcttagctgtga
atattctgttttgacttttcaaattctttaaagtcattgtcgatgaatacagtaggaaagtaatgattgagaaaaaataagcaatttggttacttgttt
agagatgagtgaattcagtgaaaatactttggcgttccaacagagaatgagaacactaaaattttatcaaaagatggcttcctccacacccgcca
tcgttgtttacctgcacgaccaaaaggaagtacaggcagagtttggcggtatcgacagctcagaatcaaaagttgctgtctcccatcttcataccg
attggggtacagtagacaaagctgtccttcgatctggcgacattgtaaacatcgaatatgtacttcagcaggaagaaggagaaatctaattcattt
tgattccctttctacacccgattgaacgaaaaggagaatgggaaaggggcggtttcatagaaggagctaaagaaggatattttatttttattatt
atctttcaatgaatactttacgaccactgtgtttaaacttccaatttcagtattcgcgtttgagagaacgagaaaaagtaaaaaatggcaagttttc
aagtataataagaatttgaaagaaagaaacatccaataacataaaggtgaagcgaaataaatatgaaaactaacagtttctttctatactatta
aactcatccggaaagaaaagtcaagattaggcaaattctcggctattcatagagtgattagaatagaaggaatcatcaataaagaatattaaaa
aaaaacaaaggacaccgtctaaagaatactttcaatatcgttttcgatatgctcaggcttactaatactagagtagcgctcgataacttgaccttg
gcggtttacaaggaacttttcaaagttccacttgattctttcgagaccaagctgcttcttcgcgctctttagatactgaaaaacgggatcaacatcat
cgccattgacattgattttggacaaaacggggaatgtgacaccatagttcttttgacagaattcaccaacctctgttttcatattcgttagttaagct
tgacttttacctacgtgaagtcggttacagttttatggcaaaccgtaaccacaagaattcattgcataccttcattgctaccgggttcttgtccacca
aattggttgcaagggaagccgatgacaataaagccacggtccttgaacttttggtatagattctccaaacctttaaattggggagtaaagccaca
cttactggcagtgttgacaaccaaaacaactttacccttcaactcactaaaaggaaagggtttaccagttctatctagaggcgcaaagtcataaa
agtgggaaaccatcttggtaatttttattcttcttatcctttatgtgtggtagaagacaagtttaacgttggcgatcccagcctttttaatgtctttgct
tatgacgtcattgtggcccaggaagattggagaccgatgatttgtgttttccattcgaatttcgttaaccgagaatatctgttcagtcaactactgac
tttcctatttacacttttagtgattacattagttagcgagacaaactttaccttagcgattgcttcatctgtgtctgttggattcgtcaaagattgtagg
gtagggggtggtggattcccaaggctgagtgaggaaagctttgcaaaaagagcccgtatgccaattgtcagtcattggcctgggcgtggtcagta

tcgaaaacaaaacaaaatcataaataaacagagaaatgggaaaggaaaagcatgagagagcgcaaaaatagacagtgttgttgacacaggg
taaaaatatatgctccagcagtgactcgaacactgggcctcttgcttactaggcaagcgctctaccactgagctacaagagccttgtttgattttgg
aaaggtattgattaatatataacctatattatgggaaggacattgctcaaaacaggacaaggaatttatttgtttggtaacgaatgtaggacttga
ttgtgcatgtataatgaattcattttgaagatttctgttgattcttggattccaagagaaataaggttcttgtatgtttaaaataagtatgagcaaga
aaagcctcataaaaagggttactagttgggtaccaggcttgtttatgaaagtgtaaacaaacgcgtaatcatcaaaaaattcaaatgattcgcga
ttttgcgagacgattaagtgaaacagaagaagccgctcaaaattaacctccatcttggtttcaaaattggtttaaatgatccaaatgctatctatgc
ctttcagattaaagacgaagtttgagagattagagacaagatttgacatgttggaaacgaaacttttaaggtccgaaaaccttcagcgacgatcc
atgggttatccagacgatgttattccatttttacattctgatgacgaacctgatgccgattttgcctcatatcacttcggttgaagatatagatcgctt
gactagggatcaatgcgctcgatatcctgctggatacggaactccttgcaactaaaatgaaacccattcggatgaaagaaaggctatgatatggc
gtttcgtttttcaggattacattaaagctgtgcaaattttgtatacatatgtaagagacggtagactcccacacgtatttttatatatataatcagatc
tgatgatattctttaagatttttaatgcttacatgaacacattatttcatacttgctactgtgagtgggtacccttggtcaattaaattgaagttacaa
attggaagtgtcgaaccaaataagcatagcatattccttcattcttgaattaatattcaaatttggttgatgagaacacgtctgtttacttttatttgt
gtgttcataagctgcttcatgaccaaaaaggtaagaaaatatgatatggaaattgattgaaaaggaaggaaaaagagaagatactgagaaagc
ctacagcacgccggatctccatgttgtctccaactatagtacgaacttagacgttttcaattaactgcagtgatcggacgggaactggtgttttcgc
ctaggtgtagccgtaggcatctcatcagatagaagaatagatgtacgataattcacctctttgcttgtccttctcttttacttacatatgacattcgtt
atatgatattactcacaatgactactttctattaatgataataattagatagaaaatgagaccaatgaaaatagcatttacactttcttaattcgcat
taatcgacttactgttcatctaaagctttgtttacattggcccagagcttttgcttagcaatgacatacaaagaatagcaactttgggataccgcagt
acaaggcttctattcataattgtaaccactacgctacataaaacaatccaacgatgtacgttattcttgaattcgtagtcaacaaacgcgacagaa
agaaacctgtcgtttgccaaaacataaaggagtcgtggtttttgaggtttttcgtagaattgaattgggcttggaagatgtcaagtctaaagacat
ttataaaagccgtccgggcgtcgaaaacgacagcggaagaaaacagtgcaattcgtaaagaatcggcacaaatacgtaaaaacattcgccagg
gatcagcggatacgcgaatgcgaaggagaaatgttgcaaaattattgtatctctatttgttaggagagccgacgcattttggacaaatcgaatgtt
taaagttgctttcaagtgcccaattcatggataaacgattgggatacctgggtgctatgctgttgctagacgaaaatcaagaagtcttgaccctgct
cacgaattctctccagcaagatttacaatcacccaacaaattcatcgttagtcttgcattgtcggcttttggcaacatcgccagtcctgaattagccc
gtgatctttccaatattatctccaagctttgttccagttcacataactatgtcagcaaaaaagcccttttatgtgctttacgtgttgtacagaaagagc
cagagttggctgatctgtatattcataacactgaagaccttcttgattccaaaagtcatggtgttttaatgtccgctatttcattcgtgatggggttgt
gtaaaattaatccaaacctgattccccgttttgcccagcataccgatggccttattcaccgtctccgtcaactttctaccaacacttattcatcggaa
ctcaatatcggcaatgtttcagatcctttttgcaaattaaaatcttacagatcctatccgttttcgcacaacacgatccttccatcaatgaccgcatc
agtgatattttggctcaagtttgtagtaatactgactcttctcgaaatgcaggaaatgcaatcctttaccaagcggttcgtaccattctagatgttaa
ttctgatagcagtcttcgtgttttgggtgtcaacatccttgcaaaatttttaggaaatcgtgataataatactcgctacgtcgctctcaatatgttgaa
gcgcgtcgtcaatacagaagagaatgctgttcaacgtcatcgttctactattctttcgtgtctttacgacgttgacatctccattcaagctcgtgcttt
agaactttctactttccttgtgaacgaaaccaacgtccgatttatggtacgcgaacttctaacttatttggataccgtatacgacgatatacgctcaa
aaactgcccaatatattacggaggtcacgaacgcttttgctccaaacaaacgatggcattttgacacccttttacgtgtattcaaaagtgccggta
atttcctttccgagtctgccctttcaacttttctacgactgatcgctagtgctccagatcttcatgagtatgctctttttaaactttatgctgctttgaag
agtgacatgtcccaggaagctttaacccttgccgcgttatgggtcattggagagtatggccagtatctgttatctccctccatgaattttgacgacg
atactttgccaaaaagcatatctgaagacgatgttttggaccttattgaggaagtattgatagggacgaacttcgcacaagctactagcattcaat
atgggcttaatactttaattaaactaagcactcgttttcaatcatcttcttcgttatccagacttcaaaggcttatcgaattttacaataagcatagaa
acacggaagttcagcagcgctcagtcgagtaccaatatgtcattcaagatccttcattaaaccgcaccgtgatgcaacccatgcccgcaccttctc
caccagcacgaattaccccttaccaaaacgctgaacataaattaaacgcatctaaaaaaccaatagaggaaatggaagaaacgaatgaccttttt
ggaccttatcggtttaactagtacttcccaaccccaaacccttgtcgatgaaactccgtctgttaatgtcggtgcaacgtctgatctcgcagaagta
gctccagcaccgaaaaagagtcaagttgacgacattttggggttatttagtgctccagcaccagtaccaacacctgcgaatgcaactgatactttg
gcaagcagtatcgcttcattggaagtgggtggcaataacatgttggacttgtcacagacaagttctgttcctcaaaacaaacaatacgagcctctt
cttgtatatgataaacacgatgtaacgttgacattgtttccttcgaaagacgaagttaccaaaaccgcaattttagaagccagatttaagaataca
agcacaatatcgagagtcgaaaaagtgcatcttgaaattgctgttccaaaatcacaaaaactacgacttcaacctcttcggtcaacgagtttgga
accaggaatggagacttcccagactatgcgaatccaaggacctaaaggtactcaagtcaagttgaggttgcgaatttctataactcgacaaggc
aatgctggtattctagatcaagtcgattttggcaacttaccttccgatcttttaagttaaactcgctatttatggtttgcattgtacaatcttttgtattt
tgcggtttcaataatcatgaactttataatggctaacgaatttctcaacctgttgataatggtaaccgcatgagtcaatggctggaatttataaaaa
attttcttttagatatgcttacgaaaaggcttagaaaatgcaaatgaaggtatgaactttcgtattgatcattattttagaagcataaaatgaacat
aatgaaatgagactggaagactacactaaaaaaagataatcagtaaagcttgagttgtatcgaggacaacaggttctctacgccgtatgttcatg

ttcttgttcttgttcctgttgtttttcttttctctatcagcaatcgtttgtcgaatataatatgctcgagcattttggtttctgggctccaagactgccatc
ctctcttttaagtctctttccaaatcccaagtaggcttctttggccttaatgttgttaagtctaattcttcctcaggtatgctttcttgctcttgaatctttc
gtttcgtttcgttttctatgtccctcgaaatagtttcaactgtaacgtctgccgtcgggggttcaacgaatcccatacggggagcttgtgtctctggat
cataattgcgatgcgttatcagctcttcttttctctccttcaggagcttctttgatggtttgtgattctttgatctttcgcaattcgcgcaatcgttgtt
ttctcaattctgccgattcatccaacgaagacatttttattaattctaatgtgatcaaaaacgtagttattatttaagaataaatcatatatgtcata
aacgcccgatcagagtttttagaagaacgtcaaggttttacctagaaaagaaagagtgctgacttcttcacttatatgagtagcatcaatagaaa
ataactatataacaagccttctcttctattttcacgaaccaactgtaatattcaaaaaagactactctcctttggtgtttgtagcaagtggtttacgg
atacgcagtacgctcctaagcagctatactggtatcgtacgtaatacaacacgacggtaagtacttcgcaaaatctgtgacagaacgagtagtgg
caaccctggtagaaaaaaacctgtcttctaccaagtggaataagaagatgcctcaaaacgagtatattgaggaatcaatccgtaaacatggacg
tcgctttgatcatgaagagagaaggtaagctggaaaagagataattactaaagaattgttggatctaacgcttgatgtttagacgtaaaaaagta
gcacgtgaagctcacgatgcaagcctttattctcagaaggttcgtggtcttaaggccaaattacaccagaacaagaggcgcaaggaaaagattc
agatgaagaagacgtatgtaatgacttttacacgaaggatagatactgttgatcagatgggtgatgaaaatgtgccatctcagcgaatctgttgtc
atgaagacttaaaaatggtgctaacaattggtgcttagtatgaaggagcatgaagaacgtaatactactcaacgcggtacggaagctcctgcccc
cggtgctgttcctatttatttgcttgatcgtgaacaggagtcacaggcgaagatgctctctagtgccgttaaacaaaagcgtaaggaaaaggctgc
gaagttttctgttcctttgccccaagtccgtggtgttcctgaagacgaaatgttcaaggttatcaaaactggtaaaagcaagagtaaatcttggaa
gcgtatgattactaaagccactttcgttggtgatggctttacccgacgtcccgttaagtatgagcgtttcattcgtcctatggcgcttcgtcagaaga
aggcaaatattactcataaggagctcggcgttacaatgcaattacccattattggtgtgaaaaaaaatcctcaaaaccctacatacacccaattg
ggtgtactcacaaagggtaccatcatcgaggtcaatgtcagtgaacttggattggtaacttctggtggtaaagttgtctggggaaaatatgctcaa
attaccaacaatcctgaaatggatggttgcgtaaatggtcttctcttaacttaaatgcattctttccggagtgatacactctctgcatggaaagtgtt
ttatgtcattgccgcttctactagaatttttgaaagtttgtataatatttttgggaattaacgttattttatttgcagtccttgaaattttattagagctg
gtacttgatactagctagtaattggtgtatggtatggttaataatatacatccatcttttggtctatgactttttgaagactgagtcagtcatgtctaa
aacttgaaattatgaattaaagatgaaagtgaatgggaagtgatgatgaaaacgactgaattaaatgatatatccaatatagtgaaactaaagc
ccgtgaaagctgaaaatgaactacgttttggtgacaggaaagatatttaacaagagtactacaggagatcaaacgaacataggaaaactcagt
atggctaaaaaagaaaagtgcttaaattaatacaagactaaaaaaatcatatattttgcgtttagtcgaacaaaccgaagcccatgtcttcgtca
gattcttcctcaaccttgggttcctccttcttttcttcagcaggagcttcgccagcggcagcaggggcaccagcagcaacagggccagcagcggca
ccagagccaatgttcaaaaggagttccttaaggtccttgccttcaagagccttagcaaagatgctagcccagatgggttcaacttcaacattacca
gccttggtcaaagccaaaagcttgtcagactaaggatcattagtatactatttctatatatggcaagctctaagcttcctccagtttcaaataggaa
gtgtttctacaagaatgcgctggaggtaagtttcactgatttgtctgacatacggtgatttcaattccctcgtcggccaaaatgagagcggcgtaac
tggtagcgagttcagaggcagacattttaattgccttttcgtgttttgttgtgaagaagaatggttgcaagagcttgtcaatctcacgcaaaattggt
ttggcccaaattttgtgtgactgttgaattatactttttcgttgcctatcgttcgagtgtgttatatggtaatgtaaacaaaaatgaatatttgttttga
aggaaggcctcattaggaattggatatactacggaatagtttttttaactactattgacaataaactgaggttggttctttaggaccatgacttaaca
agtaccggaagaaaaaagttgaagatttatatcatcagaagtgaaagtattgaagaatgaagaaatgaaaaagtatcaaatctaaagaatgaac
gacaagatggactggttctggtcagacgaaaggaaaaggttcaatccgtgttgagacggccagacgcaacagctgtatgaaaaattgactaccg
gaatctattgttcttcttatgtatttcctttttttattttaaattcaaatatcgatcctttatgaactttgctttggagtgatagtcaatcaattcgacata
gaaacaatccttttgaaaacttaccaattaccaatatggcgacaatcagccctaatatttggattgctgcttctgatggcaacactgaaattgtatt
ggtaagatttttgcttaattgttggagaaatgaagtttgttgctgaagctgtctcactgacataacagaaacatttaaacagcggtgtgtctccaaa
tcaaccagatgaaaatgggtatactgctatgcatgctgcggtatcttacggtacgttttgtactaaattaacattatactaatagctcaggtcatcg
cgatttgctaaaaatccttgtcgagcatgggggagatgttaacgttcgtgatcaggacggagagacacccttattcgtttgtgaaaagatagaagt
agctcaagatttaatcgaaaaatataaagcagacggttgtgctaaaaataatgatggcttgattgccgcgcaagttgttgaagagaatggggaat
tccctgagcttgcctcgtacctttacacgttcagtgaaatagaacctaataatggcaatatgcccaaggatacaaagttagaatatgctcgtttcgt
aactccccaggagatggatgaggctgctggagagcctttgcttgatcaaaaccggaaggaagaaatagagagaattatggctcttagagaaagt
ggcgtcaatattgatgaccagttacgcgaactacttcacggagctctttctgagcatttcgaaaggaatgttcgttctcgcgaacaatagatgagct
atgaataaatgctatggctgatctttttaaagaaagaaacttcgattatctcaatgcttttgtgtattttttaacgtggtccgtcttaaattttcgtct
ttctgctttttttattttttttatttttcaaatgtatcatcataaataggagaaaccaagcaaatgcttattagctgtaaagctcttcttttcaactaacgg
attataatttggtatctacgtctttaagagcaacttggagtaaaaagcagattcgctttgcacagatgtgccatacttaagtttcttcgtgtcataaa
attacaaatactttttatttacttaaataaaaaatatggtactaaatttgtgagatcctatccgagctaacttaggagcgatgttcaggtcggcaaa
tttcctcctctaccaacacctgcctttttcgtctttttgaaagcatatcgaaatcatggctattggaaagagaaaagcagttaatggattgaaaaat
ggaagcaaatttgataataaacgatccaagcctgctgattcagttgaattgaaagttaagtccaaaaaacaagaagttgcttctccggaagaaa

ccttgtcgcaatccgattctgaagcagaggaagaaaacaatgcatcttctgttgaagaaagtgaaaatgaagaagccgagtttaccggttttaaa
gacgaaaacgaacaagataatgaagaacaagaggaggactctgaggaaagcaagaaaaaattctaaggaggctcacgctaatcagaagaaga
tacaaaaagaccgtcgagcatcaaaaccgtttgctgatgttagcataaaagccaaatccttgtgggataaacttcgtcaaaaaggaacactgaa
ggctgatgagcgtaagaagttagtatcagaactatttgatatgattaaatcgcatgtgaaacagctagtgttcaaacatgatatgtctcgtgttatc
cagacatgtgtcaaatatggttctaaacagcaacgcgaaagtattgctgacgaactttctggtagctttgttgaactatgcaagagcccttatgga
aaatatcttgcagttaagcttttcaaatatggaactcccaaaatgaaagaggcagctctaggagaattacatggacatgttacaaaattgattcg
acaccgagaggctgcttatgtcgtagaagatgtattccgcgagtataccaatttgcagcaacaaaatgcgttaatttgcgagttttacggtccaga
atatcaagtctttaaggaaagaacgcaggatatgcatatagacaaaattttgcaagaacaccccgaaaagcgagcaagcgtcatgaataattta
tggaaaactatcgatggatcgattgccaaaggctcaatcggatttacaatggttcaccgagcgatgttagagtttatccagcatgctagctcgaat
gaagcaaaagatcttttgcaagctacaaaggaacaaatttacgagtttgtgcatacaagagacggttcgcaagttgctatgaagttgtttgctatt
gcaaatgctaaagaccgtaaggctatgttgaaaggtttacgtccatattttgtagaaactgctaaagactcatatggtcatttggttttagtcactg
ctttagactgtactgatgatactattatgactggaaaattgctgcaagcggaatttgagaacgaactaatcaaaatatccacgcataagtttgcgc
gcagagttttgttgtacatactggttggttgggaagacgcccgatatttcagtaaagaaaaccgcaatatgttggcttctttagaatcgcttaaagc
tgccacttcaaaaaaggatccagttgttcgtagaaatgagttgaagaccactgttggacctatgttgttgaatgtgatcactaatgctgctggtgag
ttgttggctgaatccttgagctctcaagtggttgtcgatactttattgagcgtgccaggtgataaaaaggaggccgtgaatgccgtagtgaaagtgt
ttgaaggaaatcccgctgatgaaaagcatttgatccaccaaattcactgttctcgtgccttgaaaacacttgttcagaatggacattggaacggag
cagaacaacgtatcgtgaagtctgaagaagaattaggggtagcaggaaccttaaaagatatgattggtgaatatttggtagagtgggctgctgg
agacggtgctttcgttgttcttgctgttttggaagcattggatgaaaaagaaaggaagaagtttttagaccagctgaagaaacatcaaaagacatt
aaagaagtcggaatttcgtggaacacaaaaacttttggaaatgctttagtactataaaattttagttatgggattagccgggtaaaaatattatct
attggatttaaaggaattatggattgcattcgctgttgacgaaggtgaatagtttaaaacataacataaccaagtaccttaagagaggacttggtg
gatgaaacggaaagaatgaaaaacaaagagggtaaagatatcaaattaattaaatctgattgctataatgctttttttagtatcaatcaaactaaa
atggagaagaggaacaaactatcgaacacgggttataaggaacacaatgggattatctacaaactttgaaaccaaaacgaaagtttgtggtttt
ttgggatccagtgattgaaaatgtagggagtagtgaagtctaatcaaacagtagttcaaattcaattcctgttcaaaggtgtaaagaaggttaag
agggatataattcaaactcgtaaaattaatttcttccttaaataagtagaaaaaaggaactgttctttttagccttgactggaaactctgaaggcaa
gagggatacctagcgactaccagctacctccaatgatggctgcatccttagtggtattgatttctggttcgggatccaatcttcagtcaataattgat
gctacgcaaaatggaattttaaaaggagaagctattgtcactcatgtgttgtcaaatcgtaaaaatgcgtatggactagagcgagcatcgaaagc
cggaattccaacatcagtacatcctttgcttccatataaaaaggaatatggaaacgaggttggtcggaagaagtacgacacggatttatcagaga
aaattattgctttaaagccttcattggtcgtctgtgcagggtggatgcacattttgtctcctgaggttttggctccattagaaaatcgaaacattgga
attatcaatcttcatcctgctcttcctggtgcctttaatgggattcacgcgattgagcgagcttttgaagctgctcaacaaggaaaaataacaaaaa
caggagccatggttcactgggttattacagaagtagatggaggaaaaccaatcttggttcaagaagtttctatagaacctcaagacacggttgaa
actttagaaaacaagattcataacattgaacacaagattcttgtcgaagccattcataatatcgtttctgctactactgacgcttaattaactctaca
attacttggactcgcttacgagataaaagattaatggtttaagaaagcgtctttataaatatagtcactaaactaattggacttaaacctaatcata
caaattctatgaaaacaaaaataaaacgagagacataaaaaaaaggctactcttatacagatatgtttgaaccttcatgtcatcaaactttatgtt
ttttggtagcagaagaatcagtctcaagatgattactaaagatggtacatgtaaatttaactttatatctttacgaaagaggatatcataccaaga
atgcaatggagaaatgtcaaatgtgaaaagttaatatctacgtcacacgaaactgagaccttgcctctttcgtgaatatgccaagaagaatttgc
ggattaagacgtagatgcgctaacaaacaatgagtcgatgacatttggacactgacgcaaggcggcaaataagttgcctggatttcatgacgttt
cagatgagatgaaagtataagtatatataatatactacaaaggtagagcgcgagttaaggtctataacaaagtatcttaaattaactattatcag
cgaaaatgtcaagagagcatttgggaaatgagaacgagttactaaaatctggaaaagaaactccttctagtccaggagcatctaagagagttaa
tcgccgattgtcagtggcccaggaggataaaggatttttggacagagagttcaataatctgactccagaagtggattttaatcaggaaaaatggg
agcaaagaactacacatcctaaacctgatactccgttgcattttcgcgaatccaatgaagaatcaggagcagataagacaacggacatcaacttt
taattgatgtctgctgtcttaaatcatttcctggataacagaaaaagaaagttgttttgtctgaagaaaaacaaaactggtctaataatttatttac
gaaactatgttccacgattctgaagtgatcgggaagtaaaaaggatatttctttcatttcactcgttttatgattgtaaaagcaaacgttagattgat
taaataaataattttgaattatatttcaaagtcaatatcgtaagcttagagatgtcctggattaatagatgtaaatatttgagattataagttctctc
accttttaatgtttgataaatacgtacaaggtcatatgaaggttgaattttcctcgttccttgcttacacagtctgataattcagctgacacaggag
taatatcaattgtgttcctgaatgttttagacctttatattgtatctaaagaattcattctcatttgattataaaaggtgcggaggtaattaatatcttg
agaggaaactgttatattcttcaatattattaattttcggttttcgcagaatatagttttggaaatcatcttttgcctttcagagattgggtagttgaag
agctatggagcatgccattaaaggatataagaaataaatagacggttctttgtaataatattggtgttcttatataacacctggagtagaacaata
aataggtctcatcgtcggaaatagaaagggaccttggaatattaatgagcataagtcatgtacggccgtaatccacaatatgtttgcagaaaatc

tatttaaagataattaatatctgaaatatgtcaaacttacgtgaagtctcttacatcgatgtgttttatagagaagtcggtttcgactcaactcttctg
cttccaatatttcgacatatgaaacaagtagacccccctacataaacagataactgttagtacgtacagcatggtgtgagagacagactcgcttac
aaaggattgctagatataagccaagtaatacaatgccgcaagaaaaaaactcaaatcgtccggatgttatataacggataaaaagtttttcttta
aatcgaaacatgtgataaatatcagcgaaggcgacaatcatcaaagtgaatattccccctgttacgaaaaagtgaatccagggcaagatggact
tattggcaaaagtggccgttggttgaatttcctgtccagttattgctgaagatgaactttgactctcgtttgtttcttcctgcttttcttgaggtacaga
ctctgcattcggcgaaactactggaaatccgaaggtactttccataacatctgcaaggtcgatccaatcaagctcatcagttgagcttcgtaatga
agggctggacgactttctattcataacaagcagttcgaaaaacgcaaaacgtgaaagtttggatgcagaaatccgcaaaacgtaaagcaattta
aatcgctgaatcaattcagtctcaatcagatagtaatgtttttttctaatgacaaagcgaagaattggtatttaacatatcgacgtttgtcagtttttc
cagggacgaatacgatcactttcagtgaacgtacatatgacttcagcaagaaaagacaagtccttttggccatcacaaaattaagttatttttgat
tccaatgataccatcaggataagactatcaatgttgttatgcttgaaagtgtatcgttatgaatttagtcgaacgtaagaggagctgaattgtttttg
cctgcagaaaatgtaaagattcctcaaaagaaataataagaaaggatataaaaagatatttgaaaactgaaagtttttttcagctaatatttcata
tagcagatcttaggatgaatttgatggatagcagcatattaaaacgaaagaaagaaaccaagacaaaaagtttaatgaatgtatcatcatagtt
agtattgtttctctcttggttgtaggtgacataaaacgctatgtcaaatgggaattttgcaaggatttgaaaacgtgaaatgatgggatgaataata
acataagagaaagaatattggctttgatctagtacctgtaccctgtacctgagtcatggaaatttcataagcaagtatggtgcgttaagtctgaact
tgttttcttttactatttttacttccaaaggaccttaggtattgaaagattgaaatctgatgtatgcgttctctgtagcagacacaggaaagtaaattc
attgtaaaaacaacccatttttagataaagttaagtcaaaaaaagcctctatagcagtctataccaaaatccttgccattggaaaagggaaaaat
cgggatctcaaaataccctttagatttgtgattgcagaaagtcgcggagcttcatagcagcaaggaaacgatggctacaagcatttctttcatcat
catccatttctgcataagtcttttcatgaccattgggctaaaacaacatgttagctgaagctttttacccttgaatacgtacctggaaaatggagttc
caaccgaaacccttatcaccacgggggaaacaacttcaccatgtaaaatgccatcaaacatatggataggctttcctggaccttcggtatagcc
aaaagtacagccagctttggcatctttggtgggaaagccatccaacatatgatataaacctgtcaaagatgatgttagaatacacttaagaacac
aaaaattttcgcatagtaacctaccatcacaaccgatgctgttcaaaaaccattttctgtaatcctgttagcacaaagcgagaagcgtgttttcgta
ttttttttctcttcccggtacgtacatataaggaccaggaaggccgttcattgcatcgtaagaaagccatgtatcttctgtcaatacaggaccttgaa
cctaagcaaaagatgttaaatccaaactatattggcatgagttactgaatatccgccgtaaaacaataagaagatagaaaaaggaaaaagaga
tattgtctatacattctggagcccagcggagtcaatttctacttacaatttcagcagcagccctgcatttttcagtaacaatatcttgaaggcttccct
ggatttcgggtactaacacaaatgggttagagttcatctaatggatatatgaacaggacatacaatcatagctatgattcttgatttcaaactcgtt
gccaagaatgctggtaacgtcagcaagcttgtgtttatttccagttacaaataaaatagatctagggactgaattgcaaaatattgttagaagaaa
gtcggaatcctgaaatacagtgacgctcaaggttcttccatccaaaagttccttctaaggaaaagacatcactaaaaaatttttaaaacttactag
acatgatgataaaagtagaatttcaatagaaactttagagattaattggaaaccttgaaaaaggagggtaaagtaaatgaagcagatttgggta
tggaaggaagaaaaccgaaataacgagaccatgaatacgcaactagagaataggaaactatcattccaatgaactatctataaaacacaaata
tccaaacaacatcagttgtggatttttcaaacgatatcattccctctttgcaagaaccatgctccttgagtagagcacttgactccaaaatttgttta
cagcccttattgtttttttgtaacgtctttcatatacataaaagaaaagaaacaaatcaatggtacgctttaaatctcgatatttgttgtttgaggtgc
tgtatccacaagacaaacaatttcatgaacattcgacaattccttcagacgaaagtatcacttcctctagtttatccaaactgttaagaaatacaat
agcagaaaactttggagacgttggaataggcaaggtagcatcgagtttgtcagtgaaatatttttcaccttcgacatctacaggaattctgcgggt
ttcaaggcaacatattcgactagcctgggctgccttatgctttctgagagaattgaatggcaagccaattgttgttcgtgtcgtacgtgtgtctggga
cgatcaaaaaggctgaattagctgctatcgacagaaatactacagatatccgactactttctaatctaaatgacattgttcaaaatgcctaactac
acaatccatgcttgctggcatatcttgtttctttcggtttttagcaaacagtatttattttttttttcctaatcaaaactagcgattctaggaaagcatcg
ttgttttaaaataaaaatagaaaataaaaagaatcatataatacaaagagtcgaatttcatgttcttctctacttttgaagaactatgagaccaa
gaattctcgtttgtttatttgtttacatgtaaaaaaattggatccagccaaagagcagtcacaaatgtttcaagaattactatatatattggaaacta
ccctccgactaccaataaggcacttttggtcatgttatatagacatgaaagttgctttgtttatagaaggtaagaactattgaaaactgcgaaacat
gaaaatagcttcagaggatgcaagaaatatacccaaattttatagcaaggcatacggggtagccgttttggttgccttcttgcaatgataacagtt
ttgtcatagttacactgatgaagcacatatcgcgtttctttttttttttattaaatggtcactaacctatatagattgcaatgattgtacagtaaaataat
actagttttcaaggaaaatgaagtagaaaaataatgattatcttttattttaattatacaggaggaaagaaggttgtaaaatcgtggacattaaat
tgatgtcagatcgtccctatgtaggaaaaactaagaacgaaagtatcactcattgtgtttttttacatatagcttcaaaaggggaacgtttgtcaaa
aagaattaacgagttatgaatagtagagaaagagtaatggctatgctgtgtaaagaacaatgtatacatgataaaggaaggaacgtgcttttgc
agaaaccattttcataagagataacgcacaacatgaaggaccctatatttgtggatgaattaaatatctccatcctgtaaaacttggttcattttgc
agtaagttttttcttttactaatctatatatgctttctttttcgtacgtttacgttttctaaatcatgcagcttgattctgccttgtttgtttatcgttacttc
ttgggtaacgaacttgacaatcaatcaatgaaggggtctgataattcacttatttaacagtgttctatcattgtttttggaatacaagactgctctc
gagaggatgaaataaaggactgatttctttttggaatgtgatggcttgggaattgtcatcgtggatacccatagacgaagtagctctgaaagaga

aggctttcatatttgtttttaactgtcaccaaaaaatgctttaggagagctttaaataaacaaaagcgcaaacatgctaaagtatagtcaattcag
tcgcggtagagaaacatgcttgtcttcagactcgatttcctaaagtagttatgtataatggatttgcaagttggtgatatcacaagctaaagtctcc
atgtttttaatggtgaaactgatttgcctgtctgtattttgtaagcttttactcttagttcggatatttctaatcctgcttataaaaaaagaatgaaata
tttgaatcgaaaggtctaagcactttctctatttttctttaatatatatctataatctcgccctttgccaagaatcaatcaagtaatcattcctgcttga
aaatacatggagaacgttaattatatgataaatagacttaaatttcaaaataattaattcagggcaaagggatcttggattagtagaagaacctg
gtttatactaatgttatatagctcacaactgctgcttatgtttaccaatttgtttacaggcaaaaagttgctacattcctcgcattgtttcttgcaaaca
gagtcaactaacgaattttaagataaaaaaaaataaaaaggttatatcacaaacctcatcaatagtttttcatgaaaaaggcattttatttttgttt
tataaggactgtaaataaaagcgagtgttttccaaaataatgaaatataacgataaaaactaattgctacagaggaatcttttacgaagtctaca
agagtactgtaagtatttcatcatgacgttattccgcgtaagtagtaaggatccttcttaaaggaaacttttaacatttaccaactacttttattaaa
gaaatcgtttggtttctcaaggaaaataccgagtttttttgctaaataaaaggaaatatttcttataaaccatcaaaatgtctatgcaagttcgcatt
aagcaacaaatgcaggatttggaccaaagagtaagtggcttgtatgctttgggatcaagagacgcggatcgattttgaaaagaggactattttga
ataagattcatccagtagaaacttgatttcgtgtactttctatgaagaaaccattgccaggttcttttgtgtcaaggtggttggagaaactaacacg
gatttgtagttggcgtcttttcctcagctcaatcagctcgaaaagaccgttggtgttagtaagctttatgtgtttctaggcgttgctggcgtttactttt
tgtttttgatttttaatatgggtggccctattctgacaaacctcttggcttttgttatgccggcctttttttagtattcatgccatcgagactacaaacaa
gtctgacgatactcaatggctcacctactatttgatcacttctttcctcagcgttgccgaatactggtccaagttgattctttactatgtccgtatgtaa
cataactcagttgattgatattgaatcattcctaaactaacaaacataactagctttctactggctcattaaggctgttttcatcttatggttggcctt
gcccaagttcaatggtgctaccgtgatttacaattacgttattcgtccttacattactcctcatgttcttcgtatgtgcaagcaagcttctcaaagcaa
ttcctctaacccaactggctctacttttgccaagtccacttctaccgacgtccctcaccacttgtaatgactttcgtcaaagatgcaagtcgtgcaga
aatcggttgcacaaaaacgttgatgaattctgtttatggaaaagatcaataagttgatttccttattaatcttcttctacgaacatattcttaactctg
tttgcttcataatgtacgctgaatgaaggagtatggatcattagaatgcggacgacataagctcaaaagtcgtccttatatcctatgcataaaattt
aaatcaggaaatacgaaaccttgaatgataattttcatttctagtcttatgaaacgtttcattgtatattttggtgcgatgtaaactgttgcaacttaa
ctttcccattaccgtagatttcatttcaaacaattcattaaaagaatttctttttatttaaataaacgtttgaaaccccagaatgccattcgaaaactt
gaaatttcaattggaggaaattatagtggaaaagatgcttaatttttttgtataacatatcgcaacattctacggaagttactttagtgcataatacc
actgccttaatctacgttacggtttgcttctatggtatggatgtttctcgctattactcgaaacttctagcatttcgtcactttctttaaattgggtacaa
aaatagtgccgtctgccattgctcttgtcctttgcatactacagaacgaccattaaaacgttaaaaaccgagatcttcgatacaatttctaaattact
tgacctaatacacctcctttcctttttccccgttgaatttcgatttgttttgattgattgtttattatattctttacaatttcgaatttttttttataatctct
atttggctttaattcttgttggtttcgttttaatatagtctttttggcagagttgattgtcgattacttgacgaaatggtagcagatacaaaattgtacg
atgttttaaaagttagtccggatgtcaatgactcagatctgaagaaggcataccggaagcttgccttgaagtaccatccggacaaaaacccaaac
ggtgaacagcagttcaaagaaatttccctggcgtatgaaattttgagcgatcctcaacgcagaaagatgtacgatcaatttggtattactgaagg
aaatgctccacctccaggagcagacgctggcgctggccctggtgctggatttggtggcttcccaggaggaggtggtggtgctcgtaccttccacttt
aatatgggtggtcctggaggctctcaattcttcaatgccagtgaccctaatgatatttttgaacgtgtcttcggaagtgcctttggtggaggcggaat
ggatgaggacatggatatggatggtggattcggcggcggcatgcctggcggattttcaagcatgtttggcggcggcggtcctcgtcccagatcca
gtgctcgtccttcctttggtggtgctagacctcgtggtcctcctcctcaaaacgaagttgttacccgtccccttaacgtttcactcgaagatcttttcac
tggttgtgcaaagaaacttaaaattactaggcgcattatggatgcttccggttcttcagtaaaggcagatcgaattttggaagttaacattaagcct
ggttggaaaccaggcaccaaggttaagtttaccggtgaaggagatgaaaaacccgatggttctgttcaggatatccagtttgttctaggtgagaa
gccacatccagtttttactcgccagggtgatgatcttcggatgtctatagaactaagcctgaaagaagctttgcttggattctctaagcaaataagt
actattgacggtaaaagactaaaggtttcatcctctcttcctacacaacctggttacgagattaaatacccaggatttggcatgcctttaccaaaaa
cccctagtcaacgtggtaacatgattatcgaatgcaaggtaaaacttccttcgaatttgtctaccgaacagaaggctgccgctgaagccttttaag
gatgctaagcgcttcttctttctctattacttattatgttgtttttcctccgtcttcgtaaatacgttacccattgttttacacttttgttagattattttttt
gatgaaccaagttccctgtgggattttgttattaaaaatttactctctttctctctctttctctgtctctgtctctctctctctcacaatctccaataca
taaaggctgcgtcatatagaatgcatcagatattccaatattagcagtcgcttttgtttttgtttgacttactaagtttgcaatcttatactgctcaatg
aagactcaattttatgagcattgtctaatgatataatgaaaaatttattaatgggtttcatggcttctaagcgcatgatcaaataatgattcctaagg
taaaaaagcaataataacatttcacataaatactttttactgttccgttatacctgagtttttttttagagaacgtagcgatcataaacagtgtaaac
aacttcgccaatcccaatcaaagttatgatagaagcatgaacaatatttgacgcaacaggttcaaggatgccgtaaaagaaacagttatacgtgt
aacaaagaacaagaaggtggttttactttcttgctgacacagaaataaaaacgcagctagtgcaaaaataacttaaaagaagcatttagaaata
aagtgagaaataaaagatgcatgcattttggctaaagagggtataaaggaatttagaaatattaggagaataaatcgaatgcagtagaattga
attcaatgaaaagaggaaaaaaaaaagaaactccaaaaattaaagagtttatgcaacaaaccatggaaataagcgaataacaaagaaaaaa
ggttaaaaagagcttagcgtcatattgctggtgacattggagccacttacaacttgtttaaactcttcaaaactgatttttccatcacaatccctatc

gacttccatgattgttttatcgacaatctagtaaagcaattagcaatctgaagaaagaataggcaagtaatacgaacctgctgaagctggtcttcg
cgaagattggtcccaaccatcatctttaagaccaaaaaaagctctccatttgaaatgtagccatcacggtcaatatcatagatcttaaaagcaact
gaaaaaggaggttagtgataggaggaacttttgaggaaacataaaacgtacatcgaagcttttcctctttattaccatgaattgaaaacactgag
aggctattaatgaattcttgaaaatcaacatctcctccgccgtcttcatcaacgacagaaaataatcgagaagctagaggattgtttgcgatacta
gggatggataaaaactcattcctgtcaattgaaccagaatggttggcatcaattttcttaaaacgcttccgaattctagcaatttcctcgtttgtgac
tacaacatatcaaaattagtggaaagtccaaacaataagcaggaaaacgcgaaagcttcatccttcggccatggcaaaggaacctaaactaag
cgtctattgcaaaaatctcaatgcaaggtagataaaaattcacagtagttttatataaaccctttcattttccataaatcctcttatacatacacgta
gaattggagatcaaatcttcaaagatttgagactgattttggcccattttgggaatgaaaagcaagtaagaatgatattgaaacagaaagaggtt
gctttaatataaaaactgaaaaaattcgccgttttgtgatgcgtactagggaagttgggaaagtgcgtcgaagcagatgtcagacctgtcatttac
caaatactcacgaattgacgaatggtgtcgacgaaggaaggaagttgaaattaattcaaaaaaagaagaataaaaaaagatttcatatgaatt
acgggaagctgatagcttaaacgcattcgatttgcaatgatgccttctggaacgctaggaaaaaaattgtgctacacgctgtttctattcgccgaa
gatgcaatgctttgaagaaacaagtaagagccacttaatttccaggatcgtgagcttccaaatgacattcgctagggaatgaagaaaaagaaat
tttgacagagaatgggctttaaaagaaacacaatttgttataatatgatacgaatggcttgtatgaaaagtttgttagcctttttcgagccttttca
taaaatatgttttgcgattttatgatttctaccagaggtgacgaatgaatagcagaatgagatgcccgatatgcttgaaggtttacctttgagaaaa
aaagttatctggttcctttcatggtattcttcttggttcatcagaggcctgagaagctgaaagtctgagaaacacaaccatgattctttcaaactatc
gtactccgaaatggtcttctgctaaagagatttctttagaggtcgctgagtacagaaaaatgaagggtttgtagccctgatgttagaaaccttttct
ttcaacgagatcagacaaataattgtaagtcattctgagaaggatagacgttccttgtctaacttgaacatatctttctacagatattcctttttcttt
tcaagttgagaacttatccttctatatcgttggttcataaaggtgtagcttgtccaaagaagaatgatggttgccttttgtactgattcactcgatgcc
ttgtatactatcgcaattccaaattgaaaatcgaatatactctagaatgatttcgcaatcttggtatagcaacctttctcttttggaatgctgtctgttt
atttgtttacatactcgttgaaattcactaatatttttatatgatattgtgagcttttatgaggcataccgcgctttaaagcaatgcggtacgaggct
gccaacaccacaggcggtaaaaccatagacgacaaaatttttagcaggcggatggtggagtgttttgcctctaggaaaacacaatacggacgac
tgcagtcaacaacgccaagatttagagcggtttttctgcgaaaatctcagtggtattgacgatattgaaccataccattgaatagatctcttcgaga
ggaatcgaacggtgccctataatcatgagttccgttgctaaaaatgcgagggcataccgtcaacgaaaagttggtatcaagacggtgatgcctat
atgtttagaaaaagatatacctgattttgatgaagaagtctcccagcagcgaacagtgccgcaggtggagacaggcgtcgaaaaagaagagga
agaggtaggttgtgtgatttaggtcgtagacacattttgaagctatatattttatatgggcagaatttgtcctatttcttctgtgatctgtttgtggttg
tattgcaacttgagggttgtgaaggatgttcgatatttgcctataaaccttgagattaaaaaatccctacgctttgcaaagaagttttaagtctgtct
tctctctactctgttcgtttttttctacaatcctcaaatgcagccgtaaatgtgctctatgacaattcatgtggtgatttctattttcatttatctaacta
acatgcaggaaaaacacttacaacaagtcattaacgctgcacatgaagccattatacgaggagaggcaaagaaggtggttatccctactcgtga
agtaaaatctatcggtgtaatagaccattattataaacggaattttatttgcccaaaaaccttactgcgcttttcattaacagtagaagaatgtatta
gccccgtttattgcatggatgagcaagatcagaaatttttttgatgaattaaaagagtcccaatcttcttttactaagttttccgaaacggattttgaa
tgggtaatccagattttagaagacgagatcgatgaaaagcaaccttttttaaacatggacacctctcaaatattgtctctttcagagttattgcctg
catttgaggagcgtgatctgatgaatttaaaaccccttgttactcatgtttatccctattggcgcgagcggcgcattcaatcagaagggaaacgtat
tctacttactgcacaggttggtgacgacaaggaagacgatgatccttatgtctgttttcgccgacgggaggttcgacaagctcgtaaaactcgccg
ctcagatacacaatctatggatcgtttacgtaaactacgttttagcatggaaactagcttccaattacttgaccaagtatggaaccgtgaaaagctt
aaactacatggcattcagcaagacttctctattttccagcatcgttgcttggtcaaacggctaaagcgctctttgaatataaacgatggcgacgag
gacctaattaatcccaagcgacgccccattgaaccaaaaccggtagctccgattgcttctccttctcctgcaccaccggctcgtcctgctgctcata
ctccggctaatcgaccagctgctccacgtacccttgaggtcaaaccattgcttatgttagatgacatgattgcgtcgcaaatcactcaatttcaaat
tcgcctacaagagcgactcaacaaaaaagaacaaacagatcggaattgggttgatgtttttgaaaatcctagtacagttgttccgattgactacc
ccgactccttttatagaaacatacattcacaaatcgtcacaggctcaccaccacaactctctaatccctctaataatgctgtcctttcttcaactcctt
cctccccttcatctgaaactggtcctacttggtcacctcctacatcattaacttccaactctcttcttgcaagaccacaatcagtatctgtcaaagatt
ccttacaccaatataatcaccttgtatccgcacgaaatcctctttgcgtaagacaacgacgtggtcgaggaggacgtctcatgcttgatcgaactc
gagctttgccaattcacaaacaacgtttaccaagtaaccgtttcgaagatcgctggtttttcgatattccctttgatcctgatgacacagtaatatta
gacgacgattcagatgcctgtatttcgtttcgtgcgaacttgcttgccgagcattccactgcttaattattattattatttttattcattttgggtgaaa
taattaatcatggaaaaatgattccgttgtggcctgtgaatattcttcgcaaagggttacaagttctcttttattaaaggccttttcacatgctgcatt
taattttattacgcttctattatcattattatttatccctaaaaaaaaaacaaaggcctttaagtgcgatattaaaaacgcttcagcttattcaagga
atggataatgttttaaatccatgaattttgttgagtcgtgatttttttttttggcgttctttttcttaagcaaatatgctttgtatgcaaaatgttgttt
atgactctttaatgacaatttttgttttctatatttttcgtacctggtagtaatgtgtatttatatgtaattcttgtttaactttttatcttactaggttctg
ttattagctgtgtttttaatgaggaaaaactttttggagctgcttgtttcattttattgatatgaaaatatttgttacgagtataagactgtaaatcct

cattgaattgtggccctattcaaatgttaattaaaataaaacactattcctccagcgtatcaagttatcacacatatcttggagagaccaaaaaaa
agtatggtttgacgattaagtattagaaatataagtaaaatccttttcaaggaatttagaagttactagaaatgctcatctttttttatttgcaaacc
aaaccataatatttaattttatgcccattaatatataaagccttattcccaaacacgcatataattttttttttttcttttaactagaaaatatcaaact
tcaaacctacaaaagcctttgcttcgatataaaagacgtaaaaattatgaagcatcatgcaagcaatgcccaaaaatggatctgaatgaactgta
tgtatgctgactaaaaaaatcccgcaaattatatctgtaagcaacttttgtatcaaaagtaacacgacaagacgatttttgtaatccataaccaac
ctaccccttttttcttttcagcattgaagaatagtcatcataaacgaaataaacatactttgccatatacaattaataatcaattaacccgttagacca
ccacgacggacaatgttgcgtctaatacgtcttcgctgagagcctgctgcaattgaagcaaaatttctctcgaggtatggtaactcagggagaac
aaccaaacgtctgtcaagcatagtagaaagcggcaattcaagacgacctcgttcgagaacaattttcattccttttgaatacgcgtctgctagtaa
gtccaaattaattggtcctacaaagtgaaattccttttttgactaagtcaatactgcgaagtaaaactaatccaaagcactgggcattggctggatc
caacggaagaccatcgttaacgagcacaggtattccctcaggcgtttcgacaatctgattatcgttccattctttttcatttgatacttcgtatgcgc
aaagtgctagaagagtaccattaatggctttcgaaatatccatcaagtttaatggttctccaattatgttaaccgcatctataccatgttgtgggcc
gctaaaagacgttgccaaaggctggcgcactgccaggctccttgtaaaatcccaaacattatgctctatatcaaagtgtaaataactcaaaagtc
ctaatttccgattgtcagcagaggaaatgcgatgcgcattaggatcaggcaaatactgccaagatgattccaactgagaaaacttgaaatgcgtt
gcctgtagtaggaattccgggataccttctttatgctggtagatactctttttatcaacctgattagatctcgaataacttctactcaaataaactact
tcagtaggtgaaatttgattaacaaggccaaaaagaagttccgcacccccctcctttcatccatccagggcaattaataattaacgaaacgggcgg
ttttccctcaagctctctttcaaagttaaatttttgatattcggagaaaagtctgaatgtagaagcaagataatgagtcggatccctttgaggtgtac
tttcgccgatatgaattcgaaaagatcgtacattagaaagcaagcgacagaaagcaggaccctgaagaggagaattcaaaaaatataatgaaa
tgtggccaaatggaaccacttctggttgaccaggatcaatgtctagaaacgcaacatagtcggtgcaatttactaggcgattcgtaagatatcgac
aacaagtacttttgcctgcacctttaggtccacaaatcaacaacttgggaggggtatgcatggatgaagcatatgtggaaagcatagagtcagca
aaggttttccaagaatctggtataatttcaaaactcgacgatagagaagatgcattttccaagagagaaggcatattaaaaagccgcttagcaaa
aggagaaacacgtggaattttagaaatacctatatcattcattttgaacaccagtacggtctcaaaagagggaagtaaagagtccacgtttacgc
ctaaggcgttctcaaaatcggatatgctagtactttctacagattccgttgcgggatggcctgtcgatgaggaagaatcggttgattcgactgagtt
gaaagtaacggacgtattagaaatattctccgaaacaaaaatcggagtgggatacatcaaggggacatttaaagaatgccaaatttggcctcttt
cattctttggtcgacaagcatacgaggcgccaaataaactaaaacctccacgggcgcaggcaaataagtaggttccgaccatagccagtttttcc
ccagagtgcaatccgacaaacaaagcatccttgacgcgaacagagttcgtttcagttaagtgtgtatatggatacctaaggtcctctttttgcaatg
atgtgttaaacgaagaagatggtgtattcgacctcgactcagcgtcagcctccactaaagaaggagaggcagacaagggagaaggcgaagatt
catctgcaactgaaggtttgtttaaagtaacctgtacggaagatggcatggtcggtacgtcaacaccaccttcatggggaggcggctgatcaata
ccggttttaccagaacgatctatcccgggaactaggacctgaaaagaggcttgtttggaagaagaatgatcttctaaaggagcagatgttagttc
catggtttctagaggttgctcggtaccttcgtttggtaattcatttttatttcccctcttcttcttctctttggtagtattctgttctagatttagttgacga
gcggcgaaagcactgagaggagctactgcatgttagtttccccagcttcttgtgaatgcgaatgtttttcgcatcaataacctatcgacataccttt
cgacgaggattctgcacgtttccgttttgccatggtgatctgatagaaagcagagtgttccatttcctctctttaattatataaatccaagaatgacc
aaatcgtaccaagaggaatgtgagaaagcagtttgtccagcaaacacgatataacgaatgaatgaagctctatagatggaaatagaaagccgt
caatagatttcccctaaaaacaaaccaaaaaaacttttaagagcacgaaacagccttgcttgaacagaattgaagcatgcacaatgtaaataaa
agacgcgtagcggtggaggaaaggactactaaatgactcgttcccctttcttcttagtactgatttacaaatactaccattgtatctaataatacga
agcagcatggtaaagctcactatgtgagattgcaaagtagagtgtgattcgagaaatatagacagagccaaacgcgttggttgcagaaagaaa
gaaactgtgagaattttgaaaggattctaccgcgaaaatgagctttttttagattgaagttacaaagatcttccattggtctttcaaaaggaatccgg
gatgttattcaatctttgggactaggaaagcgccattcgattgcgtattgtgaagccaatcctgtgtctgctggtaaaatattcaaggtaaaggaat
tggtgagtgtaaatgaagtggaaaacaagatgtccaagtacgaagaacggaaagccagagctcctcctagtggatttacggttgtgaatcgacg
atttcctacggcctaaatgaaagtagagcaaaaacgagtcttatggattaattctatacataaatcatatatatcaccttaacaagtcatctagcc
aggactggtctttggaagcggttgtaggaagggacgatgaggacaaaggcgacaaggacgtagatggatcctttgtatgcaatgtggagtcatt
atgagaaggaccttgttcggagacttcagtctcttttaaaaagtcatcaatatcttcctcttcccctatgtgccctgaggcattggctacacgaaact
tgggcttttcgacattatatcgtttccgaaaatcttgaatcgatttctcatgttcggctgcctgtaaaacatcagccacttcctctcgtttgattgtcttc
accttcgtcttgggtcgctgacggccaattcccattccagtaagaggacgagcttgtagtttcgagtaatcgacctcttcctcttgtggctcttcttcc
aactcgttaccagcttgttctctctctttcaagctttgtaaacttggaaattcctcgtccctttgctcttcatcaaaaaggtctgatacctcttgaaagc
gccattcattcgtccccaaatcctttttacgtggttttgttggtacatctcgaatgttatgtttttttcttgtaagctattgtcgcctttttttttccatttatcc
tgaactaccattttcgcctacttggcctacgtatgggttttccatagactcgtagctcctaatgtacctgaggtccggttctgtctttttgaagaaaac
ttaattgagggaagttagaaacttcataggaaatacgataataaaaaagaaaataggtcaactcgttagatattgtgtaggatggatttgcgcac
attgactttaatttctgctttgcattcttttactctgtgttcgcggaaacctaaaacttgactccaaattctatactattctacaaaaaatgcctacctt

gattttcttgttctcagaatcgatgctgttttactcttcctttgctgatcagcaacaaagaattagaaaggaaagctcttgaaatccttggtatctaga
attctcccctcagcacttttctttagcttattttttctaattttttttttgctttattgttctatttttaatatttatctgctcttttctctagcaaaaatcatt
atcacagtttggttccttatagattcgaaccccgggtttttgtgcaaaagattcactttctgtattatcttaaaatatgctacaatgtgctatatctcg
ttcgttgctataacgagaaataacgcataataacgtacagcatgagtcaatgatttttcatgcgaaattattcttccctttaccatcatggctttacg
tgctcaatttgagaataacaacgagtatgtggtttaattataccagaaaatagattactaatgaatttgtttaggattggtgtcttttctacgttaac
caactcgtatgctcttgtcgccttaggcggatctgaaaacttttacaggtatggaataagcccaatgctggagccgtgtcaagtggaataagaga
aaggaaagctatgcgcttgttggttatcttgatttcaatttcattcgagatgtaatgctttttgtctgtcgtaacccaaataatattttcttgaaaaag
aattaacggcgaaagcactgaggtttcattagcagatcttaagctctgcatgcatattataacccgctagcctctagcctttatacttttctttgatgc
gctccttgttcggtgtctttttttttttttttttctcttaatgctttttgaccgtatcactaacttgtatatagtgtttttgaggctgagttaggcgatgtgat
ccctgtcgtgcacactactattggtggtactcgtattatcggtcgtttaacttgtggaaacaaaaatggtcttttggtaccttcttcaaccacagata
atgaacttcaacacattcgcaattcccttcctgattccgtaaagattcaacgtgttgatgagcgtctctctgcgcttggtaacattgttgcttgtaatg
attatgttgctctcgttcacccagacattgaacgtgaaactgaagaaattatcgctgatgtcttgggtgtcgaagttttccgtcaaacagtcgctggt
aatgtccttaccggatcttactgttctttaagcaaccaaggtgctttggttcatcctcgtactagtattcaggagcaagacgaactttcttccttgctt
caagttcctcttgttgctggtaccgtcaatcgtggttcggatgtcattggtgctggtcttgttgttaatgactggtgtgcttttgctggtttggatacca
cagcaaccgaattggctgtttgtgaatctatcttcaaacttcaagatgcacaaccttccgccattatcagcaatagagatacccttgtagaatccta
cacttaaaaggttatgtaaaagttctataaaaaaagaaaaataattatagaatcatttagtctattgttcttttttttttcgtcctgatctttattttct
tatgtattctaagtgtaatttctttatcattgcgtttctaaatcattattgagagtttcagtttactactactactaacgacgtaaaatattataataga
gataataaaagtaggtcattattaaggatgtgcttaaaattaaaatgaaggataacccgagtctatcatagttccttttcttcatcaacgtcctcga
aaggatacattatatatagttacgtatattcagtttttagagtgcctttataccattaccaatatcacgtaattcattctcttgattttcgaaccgattc
gtttcaaccgaattatcatgtaaagttaataatgcttggtcaaaactctcctcggcactagcaagatcaataattcgttggatatacgtaacattac
attcaacgggaatggtaacttgctcaagagaaggctcttcttcaaatgtgtcgttcagctcgtatatcactttatcgtacgaaatcgtctcaggccg
atcaagtccttctgctcgagccatattagataattcatccataactttttgggtactttcgattcataagtctcagcaacatacttgtccagcagtttt
ctcaattgttgcatattcaaaatccatagcaaggaaacattatccagaatcttctgtttattcatacaggtctgcaaaaatttagaagcctggctga
tttcttctaattgaagtgtagcttctacaactcctcgttgtgtacaccactctcttaaacgagaaaagttatacgacatgtttacaccacgcttccagc
taccttttttccttagtcatcagagcattaaacgcttcgacatctataaatcggtacagactggagaccacagatgcaaatatcgctaaactcaccc
gataagtttgacacgaatcgtgaacctcattcagaatatgcagtatatgttctattgacttcacatctttatcaaaaacccttggaataagcctcttt
tctcctttgtcgatttgattaccactcacaattattccatcaacaatcaaaggcctcaaagcagtattgatttgatcaatccatgtagaaacgattct
tgataggtgtgaatccaacatgtcgaatattgtttggatcgcatcataggattcactaaaggatagcgtcaacgaagacgaacgcttaagtccat
acaacttggtgcgaactagatagagcaaagtatgcgtattggcaatccaaaaacccatatcagcgcgcacttctctcctgatttgtgttctctgag
agatatactccaatatatggacacaataacgttcaattaaagcaatactttcttgagatagattggttttccacatttgggaaataatgataaaca
atgccttagccagaaacaaatgcggtttactttcattttcttcggaactattaagcaattcaacagttttctcccaaacgtacgtctttccggcaaac
tctgttaatttatccaaatcatgttttagtccatcattcattagtaaaatcagcaactccttttgagtgcgcatttccattagtgagctatcccgtttgg
acttttcttctgatgtacgagtttgttcagagccaaaactagaattaaggacttgagaggccttcgttagtttcgctattattttattttttccttaatc
tcagtagcctgagcagatataacttctgcatctttgcccaattgtgattttaaagtagtattcatacgaatcaattcctgattatcgtccttgaattga
ttaaaggaatttttcaaatcagacagctccttttctttttcagatatggcggacttaaattcttgatcttgagagtgatctgaaagcagtacatgtgtc
tgttctaattcacgctcctttgacaaagtagcttcggcataattcgacaaatgtgactccagtttaaatattctatctttaagtttcgcattttcttgttc
agaattatctagtttctttgtcacatcgaataatttactttctaacttgtaagacacctgcttgaagtgactagctttttggattctatacgaagttca
acaagctgtcgtttggcatatttcatacgaatcaatgattgtattcgaatggtaaaatgtttcagcttcgtaaaaaattgtcgttcacgataagtttt
ccaaatactttttatggtagcaacggcctgttccattttatcaacaagttgattccgacgaaacaagaaaccacgaacggtgcgttgaagtaaaa
gaatagacgctttagcctctaaaaactttttctctcaatgtgtgttttccagaatagtgaaatgactgaaatactctttgaaattaattcagcttgag
tagtacgacgacgcagaaaaccgtgagccattgcttggaaataggaaacacgtttgcgcaaattaaggaatcgtgtacggtaatagtttgtagca
aatgcatcgtaaagaagttgagcagcatgtttcaacgcccgttcttttgcagcctccagttgtggggtaacaccagaacggaaaaaaattttagat
ttaccaacttgatatttcgtagagtcaatagatttatccaaaatggcagttgaaaaagccaaaggatcactagaccgatcagacgaaggaacaa
gcatataatattgggagacaaagtcgccgaaagtccaccgtgaaggaaaaccagcgcacgatatctttatggtttctaagacaccacaggctctc
aattgagatgttaccattacattatcaaatttccaagcttctttttgttcattaggctttatgcatcgaatgtaatgagcgttagtctcgtttatagtac
ccataagactaatgagggaatttttaaacattgaacccaaagtgttgctctttggctttgcttttgatctcttattattcgatgcactagagtcctgcg
aagcagtttgcttcaaacggaaatgcaccagatctttaataaaagggactgaagactcgctgaaaagatcgatcagttcgtctgatatggtatcct
tatttttctcaataaatccttcagcattataagtaacgtcgagagcataatgcttaacggtgaaagaatcatttccaaacctagccttttataactg

ttcttaaactctggtttatcaaaagcatcgtttaattttgtgacccaattttcgtcactatttgtaggcattcggcactcttcgtctagaagcgagagt
attcccattctattttcaatcatacttatgcattgttggttgtcttggtaatcaatataagaccagttaagaccttcattagcatactcgtcctgttcaa
gtttgaaaacatgtttgtaaaattcttgttgaagtttttcattggcataattgatgcaaaattgttcaaaggaattttttcgaaatgctcaaatccat
aaatatctaatacgccgataaaactttcagaagtttggttttgtttttcagcagagtccattagagcagtattaatcgtagcgacaagccaatcgaa
caaggacgcatataggaatttggagaccgagtcgcgaactacaaaaccttggtaatcagttaaaggctttatgataccctcagaagccatcttaa
tttttcgtttagtcaaccattttataagcaagcttgggtcaacacccaacaacttcgaagcattcaataaatgctcacttttttgaatcaatataagaa
tcgtttctagaacccttgatctctatgttaccaacatgtaacagcgccgctaacaggttgaaaatacttttacaggtatcatcaccaatggctactgt
tttgagtgcgttaactgtttcttgaaactccttagcatcatcgacaccatcgattgtatgcgattcaccttgacgtagataatgatagtcatcgacatt
ggctgaaagacgccattcttctaactgttgaggggaagcgcctgccagtagctgataaaatatgtgataatttcgctctgactttggttggaatact
aaacgggacctttctaaaagataagtttgaattttagcgccgatgataatagaatttttgtcaaacatgatttgaatgtattttccaaatcgggaag
agttatcatttcgagaagttttagaattaccaaaggcttccataatgggattggtagcaagaatctcattttctaccgccgtcaattgtaccgaatct
gaatttagagcaccattgctactaggatcactcaatgtttgcacagaggcaaaataacgcattatgtaacgagctgaaaccgttttgccagcacc
agattcaccggaaataataatcgtttgattcttttttttctcgattcatgcacttgaaactgtcttcagctatagcatacaaatgcggatcgagctggt
ctcgagatttttcagagtaaacacgtacaacctcaggagtgtaaagcttcggtaaagactgaaagggatttaaagcaatcaaaacaattccgga
gtatgtataaatttgtaattgattatatctggtcaacaatgcatcaagaacagaaggttcatttaagtaagccaaattcgtcaaatcatctgggtca
gaaagattggaccgcaagaaaggcaggccatttttgccttcctcctccaaatcctctggttttgcatgaaacgttacttcctattttcaacagttatt
acttcattcacaagatggaatcatgaaagtcattgatggttacatacattgccatcttcatcttcaacgatcaatgttgctttatttgattcacttttac
agcttttgactgttccagataaccattgccgttcattatcaggaatccaacattgactaaattgcaggtcgtcaatacaaaagtccaaacatcaaa
actaaacgtacagtcctttatagtaaattccagaggacataactggaaggcttgcaaaggttttcctattacctctactataactatataggaatgt
ttgtttacgatcctactgattggaagccttcctcctcagttcctaaaagatactaaaaatagtaaaagaagaaatatttcctttaagtaaccttaaat
caaagaacgcgaaagaagcacgttctttaagggaatatatatatatagtacgtcgatgttgcacttggcatactccaaaatgaccctactattgac
taaaatagcgacccttaagcgacttatttaaacaagcattacgtattgcaagacaacattgctaataaacaatttctttacatataaaacatctca
atgatttatactatgtttttctgttcttgatgtgtttggatattggaatccagtaagcttattagaaaacctttcatcaaccttttatatgtggtaagcta
gtggtgaaattgggtagaagaatagtaaacaagtcttttcgttagttgatagttgaaaggccatttgttagtaatttgtatcagaaatactggatgg
gaatatcgtttaatgagatcaggttagggtgttctataggatgactcccggatggaggaataggcttctctgtaaaaggataatcagagaaaaca
atagctttttgtcttctacaaagcaagtagcaaattagaatacattttctatgttcacaaatataaatcacggatataatctcgagtagaagaacga
aagcgtagcctgcattcccattcagacagctttgactggccattgttatttttgtataaacgaatttttacaatcatcttttatttgtgtatatttagaa
ataattattttatggtttctaacattccttgaaacattgcaagatgcactttgattcctctctgatagaaatcaataacattgataccaacttcctgga
ttcctaatacgacatagtacaggataaactattataactgtagtttgactagacgtacaaagccattccaaatgagcggcgagatgtataataaa
catcggtcgtatagaatgtaaacaaacaaacccattccactatataaatcgccttcaatgctagttaagttataactaacttaataaccagacccc
aaaagaattagttgatagtggaattaccaagtgctaatggaattaacagattcccatagagcatagaaaagttcaagcagtagaattgaaaaaa
tggttacaaagttcttttcgtcatggaggtatgagtcatagagtacaaatgactggctaacctttgaagcacagaaagagtatgtgaaaattcgcc
tgcttgtttttaatttttataactgaagggggacgaagccttctacactcagacagagaaaaaaaataataaagcaaacgctaactaatttgtagc
ttccgactgatcagagaattcaacttcctgatagctttttgcaacttgaagaagaaatagcaattcgagaagaaggtaacgacaacatttagcttg
taataaagctaataaataggaataacgaagctatatcaagcaggaaatatctggattgattcattaggtgagtagtataacctcagatagtgtct
aataaataaacaggaaaaaaagttgatggagaagagaaagagaaatgtttccctattgaaaaaatgggacaggttatggtaagccacggaaa
agctcttcctaatgactccccttatggtaagtaacttaagaaaagcgcggactaaccataggtaccgcgctggttagttatggtcaagtacagttg
aaaatcggagaactgttaggctttttttactaacagagccaatgtctgctaccttgatttcttaaagcgctacctggtccaaatgaaggattatcaca
cagccaggaaaaagcttgaaagtcgtcgtcaaacctatgagtcgcttctagtaaaatcctataaggctaaaaaggaggattcccgtttagaaga
agacattcgtctagcatcttacaaattcgaagagtcaacagaaaatgttagaagccgtatggtttccttgaaggaagcggagattgatcagcagc
atcaaatgacccaactattgacacaccaacttgactttttcaaggaagctaccaagacgctaaccaatgtctttgacttgtggaattctccagcctc
gcaaggcatacaggacagtcaagcggaaaaaaagagtgccgaggaaagcgaagtttacttacataaagtttcttcaaatccagttacacctcag
aagcctctgagttcacagccttcgtccatcatttttcaattaagcaacagccaaaataacagttcaagagaggttgtacaacaacaaacggttgtt
caagcactttacacatttactggaagaaacaacaaggaactcgatttgaacgcaggcgacctagtttctgtgagccataaactaagcgctgattg
gtatgttggggaaaaaatgaacagtgaaatggaaatgctaggaaaatcaggaatgtttccagcaaattactgtcgccgtattcgtatctcggaca
gaaaactccaatgcaagacacctaccaactctatagagagggcaaagagccttcgacgtatcgtcagtgacgaacccaaggaattaaactctcc
tataaggcaaagccatggtaatattggtaaactgctcgaaagtgacaacgggaccttcgaacctgctagtaactcatcttcttcttgtattaaggtt
aaaaaacccttgcctaacatccctaacaaaggtgcttttatgacataaaacggtacttcgtttttttttacgttgctgaacgagtcagtttatagcaga

aggaatcattattgaaattcatttcattactaaatcatgtcctctttttttttatgacattgtaaacgacgtagacactttcaacaaaccagtgcttgct
ttcgttcctactgaccaagcaggcaaatgctggaggaaattttcgtaagcgggaatggtaccatcattaatatcatcataaaagcatgtaatctca
gtagatgttggttcagtgatagtaagattaagagtgtaaggctttagaaaatctctttgacccatacggatagaagaatataacttattgacacga
ttttcatttaaatcttcaacaaacaccgagtcgttaaaattgattagacaccatttactttcagcaaggattgtgagatccacttgatgctttccttga
ctaatatcgtagacaataggattctctgcttcaaaatatggcttaacagattcaaaagaacaataggtatcattacattggccaccatttgctgtat
agtatggatttaatttcaaaaccttatcagtgaaaggagcaatgttcttaacgtaaaatgtatggttaccattattcagatcatacgtttgaaaagtt
gccagctttaatggatgagtttcgtcgaaaggacttaagaataatgcttgaacaatgagagttatccaaagcatcgtcaaaactgctgtcgacttt
ttggacaatgcacggaaaataaacggtccactattcaccattgaaaagaaggctattattattgttaatttatacagcctgttagagaatggagcg
tcagtcagagtttgacttcctgctggtaaaactacttctgttaaaatgtcgtaacacaaaaataagggaaggggtacaatgaagatgaaataaag
gacagtccagatattatctttaagcttcgcatgcgctttttttgaccgataaatttgactgtctatgaaacagagattgctgagggacagccccatac
tgggagtgatttgaagaaggagcaccaagaagtgatcgttcttcgtgagtgaaatctatatcttcaacgttatcaacaaaataacggattcgaact
agagtcaatatacagcagaaaaaagtacttagatacgaaagcgtcacccaatatgtagaactcaattcaaagtaaacggaaaagataacaga
gataagaaatactacataccatagcatttgaacataccaaataaggacaatttcgatgcagctcaagtcttcggatgaaagtttgctatgtgtagc
ggcagtgcgaaacgagaaagcaagagcaatcagatttgttaaataaaacagggcagaaacgagcccactgtgagcattaatgaggtacggatt
ataccaacttaaaaaaagtacagttccagtgtcgaaaacaaaaacgaaaacacaagataagataaagcatgctgtatatctaattttctttaatt
gagcatataacgaaggaaaccgaagcaaaattgaaagtaaaagaatcggacaaacgacgactaaggtgatatttatcatttgatataggggat
atttaaattgccagtactttccaaataacaagaaaaatgtcggttttgaaaatctcgtcaaatctttcttggattcgtgaagcaaattatttacagaa
tgaaatgctgtatacatcatatgtctcaaagaccttggcatgatatgggatatatcgtctttagcagtatgatagatattgcgattctcataaaaag
caatgtcaaggccagcggtcccattgtgcatattctcatatataacataatctgtttcactgcgaattatcttacgcttgaaaaagtctgaacctaa
gattgaggctaaagggtacttgttcttccgaaaataagacttagtaacgtagtgattagtagatcggaacagcattgctttccccccagaccctgct
ccttccagattaacaaaagctgttacatttttagaaagctcatgagcagcaaaagcccgagctccgtataaaaagtcctcttcagcattattgatgt
ttacaattaagtctcgctctggttgatactttgcatagtatctgcacaactccaacgtagtagcaacacctatgccatcgtcagtggtacctttaccg
gtagaaactgagtcgaaatgagaagataacagtataggaagtgcttgccgttcctttccttgaaaatgtaccaggatgttattcccctcgaaataa
atcagactggctttatcttctaactcaaatgttatgtttgtatagaggtcgttgacgacagttacgtgcgactgatttttttcttctagtaatctcaact
catccagtatataatcacgaaccacctcattttgctttgaattgtagggatgagggagtagcgagatgaatttcaggtcgtcccaagcacgctcaa
catttagtccctggagtgacttttgcggaggaaacctagcaattcgagaccaatcaagcaaataaaaaatgaaaaggaacgtaaaaccaaaaa
tggagcatgtaaggctggacttgaacgttttcgcgcatatttgctcaattaacgcctttttggaaatagccatgaaaacaccaacaatggtttcttt
atctctatcagtttaattttcaaaaagtaattagatacaatatattcaaaaactttacgcaaatgactactttttctagttagaggttggagactgta
agtggagagaaggtgatatgtaagaaatcgtaataagtaaatgatgtgaatgctagggaacttttagtctatagaatcatcggaactatctactg
aatcacttttctctttaaaggtttttctttgattataaggaacaacgttgcttcctgggagaacgaaacagtaatgtcgagaatctcgcagttcacct
ttccaatttgatttttcgctaattccctgtttccaaccgattgtacgtcgatgagagtattcaggataaaaagaggaatcaaatccttcagtcattaa
actaacgaaacctgcctctttggctaatcctttcagattccacaaattgtatggtttcgtttccgcaagagtaatgacaatcactcctttttccgaaa
gaaattcttttgattgtttgaaaaactctaatagcatttctaaagtagttagtaaggggcccatatatgttttcaaagactgaaaaaagtagtggca
gattccaattccattccttaattcccgaaagctagtagagaaaggtaacttactttgattatctaaaatattccgatcctgatcctttatacccctttcc
tgagtgtgggaaattccataggattgtatcaaacttttgatttctaaactttttattttgcttcagcttagtagcatctacatcgtatagaatttcacct
ccgttttgtaaaatttgttcaatgtactctgaagcgtcaggatacttcagttctacgtctgctttagtgtcgtaacaggtagctattaaaactgcttca
tcgtctaaatgatgcttctgtaaagaagctgcaaatgagaaatttccttctccaattaataagagccgatggttacgctcaaatggagcgacatat
cgaaaattcgtggatttttttaggggtcttcttaactttgtcagtttgcagagagtttctctttttttaaagcagaaaacgagccattctccgtttcttctc
tttgtgatggtttcctttgctttttagatctctccttttttgcccatagtccgtaatttcaaatactctgttttggttaacttactaacatttcgcagaaaaa
agttaaaaataatattgctggtaaatactttcaggatacgtagccaatttatatttattcaagaataaaaatattagatttattaaaatattactcaa
acttatgaggatatatccttaagcctaactcccatatacttggattgacaaggattaggagccatgtcatcccaaaaaaggctttgtaagaaacaa
atcatctcaaacgatcaaacgaacattcctcttttagctcgaaaattacaaaacaaactattgaaacaaaataaaccatgtgcacaaaattctat
gattccaagatttaagaaaacaaaatcaaacgataacccatattaaattaccaaaaacaataaagccaaatattaagacaagaaaccaaaaat
aatatctctttaaaccaattgacccacaacctcaattccatcttcaaaaacaacgtctctctcttgctttggtggaggagcatttctctcttgctcagc
ttcataatttgctataatttgtcgctgctcagctgtctccacagcacgttttctttcttcttcgaaagcgcttcttatatgagccagttctttctcgacttc
ttccacaggacgaaaaacttttccagactctttgctgtattcttccatatctgtttcaatgttagtttaattaagagggaggatagcaccttggaaaa
cttaccaactggctttttcgtccttaatgcctcatttatccgagcagggttgcgcttttcttcctgcttaaatttaagctttatttgtcgacgattgcgaa
ttggaaacatgttagaaataagcgcaaaatctgtgccccattgagaaagtgcctatttgttagctaaacggatccaattggataaaatataccttg

taaaacttctcagtctcatcagccttccacttttctgacttttgacggtttccccaagttgcactcgtaacccttcgagacagtgcattttcttccacga
attcacgatcttcttcaggtacgtaatctcgttgatggcgatctatttccaaagaagcttcatccaaaacaatctggccatctacgactcgagtccta
ggagcagaagcgcctgagctatttagttgtccttccgaatccatcttatcggcaagttggtccaatatagacttggaagctatattagactcttcttg
tgtttctgaatccttttcttccggttcagtttccaaagtcccaaccacctcttcttcctcttcttcgggctcttgtatacttgtttgtgaagctgtattattt
gctgtcttcaaatctctttgtggagactcctccttgccaggtgtaacggatccggtacgtttttttccttttggccatcttttcttccaaaaggagtttttc
aatttcaacaaagcgctggcttttcctgccaataccaggatcatcacataacgccgacattttcgtctcgtttatattaatactctcttgctcagttcc
gacgttatcgacgatgagcttacgcttccttctacggatagaggctaattaattgtcagccataactgcaacaatgaaagacttaccatttgtcatt
cgttttgactgagatccactaacatcctcaaaattagcggcaacagaacttatttgcgaaaggggaatattttcatccacaggagtctctcttttaa
cttgcccagataactgatagacttcaaaaggagtaagatcggattcatcattgttagacagggcagaagctggctcgactgtgggcttaaataca
cgtgaagtctcattgttttccaataataaatcttcatcattgttatttcccagcttttgcgcttcttgggcggcttcgactagtatgttgaatgaatcac
gcccctcctgagaattgacaacgggctcctcattcacgccattcaaatccgttaaaatttcttcttgaggtacttctaaacttccattttcagaagtct
tctcaacagagcgctctttacgagcagtaaactttggggcgaatttagaatatggattttcttgaacttgtgttaacaaaggcctactttgagtaaa
acttacttaaaggagatgaaattaatttagcactcatatcattgacagaaaaccctccaattgacttcaaaaagtttcaattgggttgtggcagttt
ccaagtgtaaacaacattccctcaacaatttcgtggtctaagtgccaaccaccaaatttttgtaactaccgatgaactggtagacacaatctaaaa
agaagtttatttcattatttttataaaggatttaataaagtctgaataactacagaaaaggcgtagagattatgaatgtagtgaacgaaccgccaa
gaaaacgacgtttagtgcatgatgagcaaaccaggcttcttaattttgaggaatctagcgatttagaggtatgaattacacttttgaaaagtcgta
gtactaacagcttgctagttatttcaaaaagaagcaatatggagacgagctcgcgaatatgacattctccttttagaagacgaaaaggcacaaca
aatgtggaaccatttgttagagaaattcaatctatatgtatcagaagcttacaatcatcatcaagaacttatgagtcatctgcaaaaggtagcgtc
tccattgcttgaaaaagaaaatacaaacatagtaccacgagaactttctttagataaagctctgtttcttgaaaaaaatgatttagtgtttgaggat
gtgtggacgtacttctcagattacgataagaatttgaaagttattatttcaaatggcattctatcgaaattagatgaagcaaatgtttcagaatgga
atgattggattgaggaaaaggaaaagcaatttttaatgacttttgaatattattttgagcgactgaaactggctttttgaagaaagagcttaccttt
acgtctgacgctcagcagtctgcggagcttgaatggaaacaactacagcagcaaaagattacgcaagaattggaacaaagtatagagcgaaaa
aaagaacatacgcttcaattagcaaatgcaaatggtaaatcagaatcgctcaaaagtgatgtttctctggatgatgacaatgtggttaagctaaa
aaacaattttgagtcacagtctgacttgatgaaaacaaatgttagtcaaaagttaaatcagcttttagaaaaacaaaatgaagcggcgcatttgt
atatggacttactcagtgtgactgataaagccttagaaaagacacctgaatatcaagctttacaagagagactacttgaaaatgaaaagaatgt
ggagagtataaacagcaagcttgtttctgtctttagtgaattacaaacattgatatcccaacggacgaaaatacaattagaggatgctaatcaaa
aaatgcacgaaataaaacaaaagcaagaacgaattgaagaacttgaaaaatctttgactacaatacgaacagaacgtgacggacttgtcgtac
aacaacagatgcaaaataccaatgatgtatactttcaagagattttacaaatatggacacttcttggcgattcaaagaagtcggttttggaagctc
ataagactaaacttgcatttttagaatgggtagaaggttctgacttacagactgatcagagtgatgacgaactggaaaagatttccaagaacaat
gcttgcttagaagcagaacttcccggaatgcatctagctttcaataaggcttattcgaagcttcaaaatagtttaggtttgcttcaaaataatgaag
agaacttaagaaaagcccattacgataaatccagagctacacagaaatacttttctgcgatgaagtcccgcgatctgctgattacagaaagtaaa
actctgaagctagtggacagcaaaggtcaagatttcatcagcaaactcgttgagcgtgagcatgtactggccagatataactcatcccttaaaga
aatggaagatgtatataagcagattaaagaaatttttggaaggcattctgctgaagttcttgaagaggataaacggttgcagacaaaaaacact
gatcttgaacagtcattaaaaaactcacaagcattaattgatcgtataaatgaagagactcatgctgcatatcagaagcatcattctttacaaata
aaggaagctcaacttcgtttacagaatgattataatcagaaggagtcagcccttatagaaaaagcttatgaaaccgaggaagctcaggtctata
aagggatgctaaagtgccaagtttgtgagtattcaaactggaaaagcaggcttttaccttttgtggtcatgcattctgctcatcatgtttagaatcc
agactaaagcaaaaggatcgcacctgtcctatctgtgaagcaacatacaactcagcggacatcgtgaccattcatctttaatgtccttactcttttg
tatcctaatgtatctatttatggtttttttggactttttaataacaccttctttttgtttttagtacatttatttggtagcctttacaaaaacatatgtata
aaattaattgtaatagtttgattgaataaatctggctttactgacagtgagccattcaaactgtacttgccaacatctcactcctgtatttctatcatt
ttaattcgactggtataaggggtataactgactgttaaaatctacgtttttatttttttctttttgtttaacgaattaaactattgtacattcttcattagg
accggtaatttccaaaattatttatacactgcccatttatgaaagatgagtatagcagaagattgggtagatcctgcgtcttctgagaatacagatt
tacgcaaagatttttattatgaagggaatgaactaaacaatgaacgaaggggcttattctctatcgaaaaagttcaactgcaatttcctgtttcaat
acgttgtttggctgtcgagaataatattttagtcatggctcttacaagcgacaagctaatgattgttgatttagagaaaccagaagaaattgtgggt
acgtaaatctttcttatgtttttttttctatggcaaatttttaaccttttttcagacatcgagcttccgaagaaagctttggctcttggactgacctataa
gatattcttagatccttctggacagtatatttttgtcacaacaacggctggtgataattgcctttttactccttcccgacaaggccgagttttgacgaa
gctgaaaggccatacgatcgaatctatgatgtggagatttggcgaaaatggtaatctagagcttttacttgcttctaggtctggagctatacttgat
cttatgttgagtttggatgcaacaaatgtgaaacgccttgaaaagtctatgaataccgtttattcttttccatatccagaaacaccgttagatatgtt
attggcaaatcaagaaaacatgggagaaggctaccttgttgttgctactaatcaacgtcttttgaagttttcgaaagggcatatttccagtatggac

cagctctattttttttcttttttcaaggggatatcgatggtatgggtgcagttgattacttgcttatgactaacatgcagaattatatcgtatattcga
caatgaatctgttgaatgtgtttcacattctccatatccgcaaatctctgctgagccctacacaattgctttaaaaacgaataaaaggattgtgtatt
atgatttgataaatgtaaccttcgatgtgatccaggaatatcagtttgttgaatcaccgcgcctttcggtgccaactatggagatgaatttgctgctc
acgccgttccatacagctgtctttgatttggatactttatatatattcaacagagttagtcaaaaggaagtttatcaacaaaaaattaccctttcccc
tcatgaagaaatattgggactctcttgtgatcatgaaaaaaatacatattggttatacacaacagatagtcttcacgaactggtggttaataatga
attggatgatgtcgctatagtttttttacaaaaggcggaatatctaaaggctttggcatgtgctaatactccgaaagtgcgaaattctgttttagatg
gttatgctgatcatttgatgaaaataggcgattacgaaaaggccgctactttttttcgctgaaacttacagatctgtggaagaagttgctttgagctt
catagaaattggacaaaaagatgttctgaggcagtatttatggaaaaaggtaaagtcttataagccatctatgaagtcccagcgagaaataata
gtaaactggcttttggaatctttattaggaagattaaatgatctagatgaagaagaaaggcttgagttgtttcccgatgctataattgaaaagcgtc
aacaggttctgcttgaattttcaaggctcttatcgcagtacaaagatggaattaatagagaacttgcatataaccttgccaataattatggaaaag
aagagcagttgctgcaaattgcaacagtgatgaaagatcagtcctatatcatgcattattgggtgcaacgagaaaactatgataaagccctaga
aacattaaatgaaggtgtggatcaaggaacccttattcagcatgcaaccgctttattaacccatcggccgatggaaactgtcagtatatgggagc
ggcaggcagatatggacgttcatgctttgataccgtctttgctaagttataatcaacgtgctcgtgtctctgttgaagaaaatgcagcaattcgttac
ttaaaattcgtaactggggttttaggttgcgcggagccatccatacataatacactgttttgcatttatgcctgtcattctagctcaaatgagtcttac
ctcatgaattatatagaacagcaaggtgatcatcctttatatgacatggatcttgggattcgactttgtttgcagttcaattgtcgcagaagtgctgt
taaaattctggtgttattgaaactgtacagtcagggtgttgaactggctttggaagaaaaggattatgaactagctgcttccgtcgcaaatatgcct
gaagatgacttggtgttgaaaaaaagtctttggcaagccattacgaaggcgatgtttgctactacggaaaatataaaggagtcaataaagttttt
agaacaatccaacgttcttccacttacggaactaattcgacttttaccagaaaaaatcaccctggacgatttacgagataatatatgtgacgaact
ggaaagttgtcgagagcaaatcgagcaacttggcgttgagattgaacaagccacggaagttgcagaagctgtacgtacgaattccgatgaccta
aaaaacagatatattgtgcttgaccataacgagccatgctggcattgtcatcagctgctattttccgagtcttttgttttattcccatgtcaacatacg
ttccatagacaatgtatgcttaagcatagacttaccagtgttcctgaaaaggatattttaaaagagtgtcaactatgcggcccttcatataccgtcc
ggctattagatgaaccattccctgacacattttgattgctattctctataaaaaatggttagcgatgctgtatttatttaaagcatctttttttgtttctt
atttgaaatgtattctctggtttcggtaaaggtcctggaaccacagaagaaacattctcctcaacgttcttttgatgagtacaataataatgaatag
tcatgcgttagttacttttagatgtgtcttaatgaattatcttattaaaaaaatatattaactataatactctacgactcttctccgttcatctgcctga
agtaaaatggtaatagcagacccgtactgtattggatcaaaattgaaaagtaacctatatctatttaaaacatagattatgttaaattaaattcttt
aattggaagcaacgttaacaataatagtaagatttggaacatcatgcagaagctagaaaaagcgtactgctgttccaagctattctgctctatac
atgatacccattcctcttatagatgtctttaattaatatatgaaagatagaagaaggtaaagaaattataaaatgattatcaaagttttagagcgct
ttcgggatttcgttcagaatctaccattatggacgaaaactgtaacatggacagcgttgtttattcaacttgtttcctttatgggtctccggacgagt
aagttggcattatcatggagcaacgtttttcaaaaacaccttggtaagcacccataaaaaggaacgatttgctgatgcttttcttcagtcttcgaag
tctatacatatgtatttttacatatctctcttttacacataatatttaatcttgtttcgttactcccagtaatggctaagtttgaaaaggagcatggcac
ctttgcatgtgtatttataacaatgttgccttataccctctttcctgccttcatgcatcttattttttatcgctttatttttgcaaaagagtcaatttcgatt
gctggattcagcggatgggcctttgcatttatttctgcaacatgtattcataatcctcaaggcccgtaagtatactctactgtatgtcagcactaatc
attacttgtaggattcagaattacattcccaagcactggattccgcttatttacttgtttataactacaatttttgtaccttacgccagttttattgggc
atgcctccggggctgcagctggctacgcaacgccctacatttttgagcatattcctttaaaattatgggcagaaaaggttgattcaatagggagtc
gtttcaaaggttaccagtcctacgatgagttggcatatgcatcactgccaattgcagagccaagtactggtaatcctaagttccctggaacgggga
cccgtttgggaacttgagagttataaaaagcttaactgtaagcgattctattaaaggtgatagtagtaatagaaaactcacagaacaaaaactag
ctatcctattcataatatgcactgagtctaatgatttcgaattcaaaacaactgtttttcctgtattcctaaaaaataaatactgtttcctcggtttga
gctttttgcttaaaatcaaaacacattttgatagtattcaaccgcttaacccctatagttataggcatcttttcatccgtgcattcaaatcaagctgca
aaaaacaaacttctgtctactaaaaccaaagaatgtgtaaattgactcaagatttggtttgctgtctgcaaagcagcttcatcagcaggagacac
atctgctaaccttttgggaaaggataacgtttgggacaaagggatatttacagagcatacaacatctgtttcaaactcttgtagtgttattacatgc
acaagtacagctacgtttgaagcgacgtctgtcatctttttttccctttcgaatactttttggcatccaaatgtgcatgttccgattgctcgctcgttat
ttaatccagcgatatcttcttgacttagatctgtatctttccaaatgatagattcactagcatcattatcgtaagcaatgctatcaaaatgaaacttg
gcgaccgtcgcatcttgaggtttctctacctgttctagcagttccaaaattagagttaagttttctttggagtcttgaagaaaaacttcttgattatca
ggaatttgtctcaaagcgcttgcattcaaaaagtttccaggtaattcagcacgaaaggctccatcaaataactgaaccattttataaataaaaact
atacaatcttttaagaaagaaaaattctatcagttataaaaactttcctgtctccagccctcaattgcttttgtttactagtcatctgaagatgaaga
agttcttggtgttgtggtacttgattcgctttagtaatattttttttacaagcatattagatgataatgtccagttgctgtgaaagttgacggcaaagtg
caagatacgagcttaacgtcttctttaactgttctatgaaaaattagagtgaaaacaaatacaggtaagcgtcgagaaaaagaaacttaagttaa
gtctgaaaaacgcttcggattaaaactgtaaggattctcgttatagagatattcaaaatataatactatagaggtatgggaggtttgaaagaagt

atagtatattgtcgcgggttatgaaaatgtttatagagtggaagtaataatttcaattgtaggattgtgtactttaattgggctctgttaaactacgc
gtatagacgctagtaatctccagggatctggctaattaaactgaaagtgtgaagacgatctcatttgaccttgccagtcgattctgactgtaggca
catgagtacatatagttttgctgaaacagctttttacgagtaaacgtccgtcaatcacttcaagttgcaccctgcgaattcaattttaagcagatac
ctttttccttgatgataaagatattcgttcacctttgctttgatcggaaacttatgaaccaagaaatcacaattcagttgaagtattcagccttacag
gtcgggtagcacttgagatggttctaatagattctttgataaagcttttatatgaaaaatgaaaaacgctgtgtccctagcattcatcatgttcgacc
taaagcattctgtacgtgtgagattgttttatccaaccttagtttgagaaaagtcgatttttaagacacggatgagctagcagttctcatactcagtc
ctttcgtgcttgctaaattcattctgatcatgaataacctctggcaaagggcggaatgtcctctacttctttatatatctaattcatgctggttagactc
tatatccaaataaagttttcttcgtcaataatgacgctcctatcgttaagggtttactccttttctttctaatctttttgattttcaatatccagtatgag
agatggcggattcatcattgtgaagtcttttccttcataagcttcatgcagcttacaagaagaaactgaattcaattcatacttcctatatttgtggat
cttaatcagtgcgcagtggaaaccctaatgttaatatctggatcatttgctcgaaatgacaagaaatattacactggaggacccctttcacatgctt
tcgtattatacatggtaaacaaccaagccataatcggaaatccctcgtgtttgttattatcttctcgaagttgattcattcttaatttaaggaatattg
aaaatctttgggtgatcaaacagattattcgatatccactttgaggataccaaaccaagaatggtaaaacttatattctttcatccttattaaaagg
acctttaattattaccagccactgcaattgacaacggccgtttgttctaaggcaggtttcattcaggaaaacataaagactgttgtttctctacaca
gaaactacttaaagccaaagtaaagcaaatcactagtaatcttattcaattctatagataaaaggaaaaaacatgcatagaaagtatttttaatg
cgtaaaatatcaggcctctcgaaaaaaaaggtctgcgaccgcacaaaaaaggttcatctatttccatattgaagaaataagaaacgagaaaga
aaagggaataaaacgaaaaagaaacgatgaacaactttttgaaaattaacaacccatcctcttgtctttagcatgataattaacaaaaggaagc
tttgcaccatatgcaataaaatagtaaataacatatgccagtaaaatatatataatattatatatagtaaaaaatttcaaatttggaataaatcaa
ccatatttcaaatatgaaaacaccattgttttcgatgttacattactttcatgcttctagcataaaaaaggaaatggtatacaacacatttcatcgta
cataaagaaattttagtcaggcttaatatgaagtattgaagacgaagttcattcccgcatagttttgagagtagttgagcgtccaattaacatctct
tggttgtctcaaacctccgccaatactaagttgagtaaggtttttgactgttccattaggataaagaggactgattttagaaattgcatcaacactct
tgagtcctgaccatatttcactaccagcaaccaaaggaacaatcagtaaaatgataagaaaaatcaaaatggagaaatacaaagcagaataac
gacgcacaatacgcttacgtagtctgctctgcttcgtcgaaaagatgggaggtcggatttgacgactaggtctcaaccaaaatagcaaaaccgag
tgccagcgatctacatatgggatgcagattacaggaagcatgaaataaagcaacaagtgacctaagaaaaagtctgttgcaaagaggttgagc
tcaatggatttgcacataaactcacgtgcaggtaaagtaactgcatgataaccgtaacctttaccataccatttgcctgaccaccatgaaatattg
gagtcgtcttgcttggtttcacgagtgagagtcagcaaactcaaaagtttaaagataaatctgtgaactgcagtaacggcacaaaaagctaagac
agtcttcggtaatgaccagccgtctaaaaaccaacaaacctcaaagacagcgatgaaaattattatttgaatagcgtgagcaattgcagcaagt
actgcagggaatttcttgcaacacaatcctagcaaaggacccatacagcaggccatacctccgaaaaatccgaccataccgaaactaacagca
atcggtacaagagtgataatggcaagtcgtaagatagggttagtacctgtctttggatcagttgtgtagtacatcttatcatgatccatccaattgg
cataggagggattaccaggcatagagttcataaagaagtagggaataacggtaccagctgctaatagagcaggaactaggacttcactgaaga
atacactgctaagcttggcacgaggagtatccatagaaatcttatcactaggttgtcccaataccttgcgtttgtaacccgtaatcctggtacgaga
caaccgacaatagccaatccaggaattagcgtgagtacgggaattacctctggacaaccagcgaataaattctctatagtctacaaagaaatca
ttccagtcaaactgatggggattaaacaagaatggggctacacaaagagacacaatggatacccaccagtagatcaaatgggcgacccaaaca
gtcattgtaccaaagagcagcatcatcaaaaagcgagctccagcataaatagaaggttgagcaaatcgagagtatagtacagagaagggtaag
cggacagtagcgaatccacgaccagtacctatatatcgagcaccgccaaaagaaagatcagcaacaattgcttgacaattaacttgacaagtga
aaacttcaaacaagggagaaaacgaaccaatttgcttcataaatctcttaaaagctctccacacaccacgttcaattaactcatgtactgtcaaag
gaatgaaggaaatccaaaagacgatgaagatagaaataacgcatcgttttagccaggataaaactggagcaagttggtaacaaccttgaggaa
cgaatgtagtatcataactgagtttctgagacttgttatagtaacaaacggtaaccacatggtagaaagcacctaagttcacaaggacaaccatg
aagagttgaactgaaagcataataaacatattgttcaagtggaaaccagggtgtgcgaaataaaacgaaaggaatctatctaaggggagttgc
gtaccaagatagtaatattcacgtgaaagcatttgttcgcccataccagtaccaaccttcgtattgaaattcagaatagatccaaaacctaaatca
cgacccttaccacattggaaatactcacaatgtttaatacgaccaccacgaagtaaagcagtcataccggcgtagatatcttcgtttacgtgcaga
cctttctgagctttggaaacaccaccacgtgtagtcatgaaaacaccgttcaaaaaatctggatgaccgtaatgcaatttaccaccaatctgtgca
agcgtacgagagaaaagagtaccaaaggtttgttctttaccagctgcaacatcaccaagaataccgatattttcggagaaaatgtattcacgagc
accaaggattgcaacagggtggttgttcctttctttggctgtttctgaataaggattcacattatctgtttccatttcctcaaattcagaaagaacact
gcggatcttcaaacattcttccaagtagttgtcctgatttgcgtcaatcaattgaagatattcaccacgatagaatggtaaggccatattttgattat
cagatttaccatcaccaaggattgggttgccggataagcgaatacgatacttgggacgacgacgttcattttccataatttctgagtgaccatctat
aagagcagcaaacagttgtggttctccaccttcttcaagtggaggatcttcgtcaaggtaggctatttgaagatcggggtatgcacgaagtaaga
attctgcattttcatattcttccttgctgaacttagcatagcgttgcatagaaacaacaatcttaaatttacgacgagccatacgatccaactcatgt
tccaaacgatcagtgttaccaccaaacatttgcacaatttcaggattttcaacacgatatagtaatttaatagcacgactgtaattcataaaaccg

ctgatagtacgatataatgtttggcttcgtagagatgcccagatacgtgtacgaagagtgtattcgggtacggcagacttgaaaccgatgcaata
gaaagggaggtcatcgatcttgcttcggatgacactttccttttcggaaacagaatcatcctcgtaaggagcattctcttccaccagaatcttggta
tcttttacaaaacaatcccattcaacaggatgcaattgtttaagatattcaagcaatgtaacacgagaaagctgatcctcttctctgataatttctcg
taaagagagtaagatcttctctgcatagtgaggaatgaagacagtgaaggtaggcatgttatcaacaggaacaggctcaggaataggagtggc
cattgactgggcgaagaatgacagacgacgctctgcctcagaatcggcaggaaagaaatcagtatggactgtgtgatcgtcttgagaaatgaaa
aagttgggagtacgtaacgtacgtcttccttcttcagcaggaacctgatgataaagcaaacgctgaacatgatcgatagatagtaagtgttcacg
atacatagaaataacaactgcattccagatttgagaaatcagaatcttcggcttatattttatttccatatcattggtagcaagaattttgctataga
ttctctgtggcagtcgactataaatgttcctccagggggtcaaaattgaaataccaagaataaaggatcttaaaacagagaagattgtgttaaaa
ataacataccacaaataagagtccaagaaaaacaaaattaaatcagtaaagtacataataccaagtaaaattctaggatgtacaaagcacaat
ctacttcccacccagtaaattgagcagaggtaagggcgcattgtactaagcacaataattggatccctaattgacaatgtcaagtagaagtaaga
ctcggcaaatttacaggtaaagatacaaacccagataagaatggaaacagcctgattatcaaacttcaacggagcaaagttagcagtgaagta
cttgttagccaaatacttccggtcgctcttaacagcccactttccaaaagcagattggagtggaacgatagcaaaatagaagtaggttgcgatag
acatcaaaaacgctattacaccaacagcaattcttcttccacttctctgcttttcctcagatgagaataccaatatataaccgatcggaataagatt
gattccgcaaaccgtaaggattagcatgatccttttccaaattggtttagatcccgggaagcgtcgaggtacatatagatattcacacaaagatgc
gaccaacatgatgatacaggcaacaatggaagacgttgccgtagcggcccattcagatcctccagtaggccttgtgccttcttttctatggaatttc
ttgttgtacaaagttggagaattgtaagcagtaaaaaaccagaacatgcaaaagtggataacccaaatacgattgaagttgacaacgagatga
agccaagaacgagtttcatagaacgttttgtaaaaagcgcgattccattgaacttgaggcagtctatggaaacgctcagaagcaggaatatcaat
taaacgagttccatcctcgaaaatgagtcgagcgataccttctgcatgccaaaataactgattgatatcatcatatccaatgacttggtcgtgagt
gcgttcacggcgaacatatttaccatctataatttcaaattgttggtcatgcatgtactggtaaagaggagtaacaatactgtccaagtaatatccc
tcctcaacaggctctgtaacattttgagcttctggagaagagatataatcataagcacatttgaacaaaaatgccattagctcaggcatgaaccg
aacgttgttagcttcaccccacaaaagtaaccaaagagcaacttgttgggcacgttcaaattgggtcatttgacgcatatgggatcgccaacgat
attccgctgattccaaggagtcatctgtattgacatcaatatactcatcctcttgctcggcaggctcatccttcttcttattcttcttgaagcgggactt
tttggggggtttggctactttaggagctttcttcttgcgggagaaaagctttcccttttctttgacagaggtagctttaccaccatacacgccaaggtt
ggcaagacctatagcgtcttcacggtccatttcagcagcaaagtaccatttttttataattggcaaggtcactgccaatatagtcagcatgcaaggt
caacaaagcttgatcgggcgtcatacgcgatgcacgtgaatctaacaaagtcatgaaaaagtcgtacatattgcgcatagaatcacgctgaaac
ccaagcttgttagtcaaatcaataaagatatcctcaatttcctctttagtcaaaggaagttcattttcaggagtccaggcaggatagggttctctgg
gagcaaaattagcgctatcagcataaaaggaagcgctgtccacatccataggagtgccagagccagaattgccaacaaaagaagaattactaa
attcataaggacttgcaccataaggaacgctattgtagacattggcaagcgaatcagagctaggatcgtagctgttgtagcggccatcaccacgc
aagttgtaaaagtttgcctccgcaggacgagcattgtaatccgaatactctgaaccatactcgtctgaaggatggatatccttggacttgtctgcaa
catactgataaggagagtcacctgaatcaacaggcgtatattcgtcaccgtagctttccgttacgccagtaggataatcatagccttcggcaaaat
cctcctcatatactgagccattatcatagctttcacgggggtatgaggaggcagaaggccgttggtatgcgttttcatgataatcttcagattcata
agcaagcccaccgttggtaggatctgaagaaccttgtggatgattatgattgtcgctcataacagcaaaaggtaagtaaaaaaatctttaaaagt
aagaaaaaaaaagaacgggatttaagggtaaatcccaggaacaaggttaaaagaaagaaagaaaaagaaagaatattcaaaatcagcgaa
agacagacaaatttacaagataacaattatagaaataatcaccttgcaattttgaagtgggtatgattaaaaatccttatattaataattctgaaa
agaaagatgggaaaccgtcctgtcgttccaaatttacgggtataagaggaggtaaagtaaagacggaatatgcttgaatgcaaagaaggaaat
ccgggaataaggaaagaaaagaaaaaagaatgcaagtttaaaaaggaaaaattccgagatacaacttttagcacgttgctaaattaagattac
agacctggagcaagctacgaaaaggaaaacggagcaagctaattggagtgcaaaatgcaaacgcggaaatggggttgaacgcgaaaactact
acgagttcaagcgattactagaccccttgaaagagaagaaaaggtaaagaaagaaaataataataatcctgtgtagacacgttcaagtcta
aacagagtaaaaaatgcaacggaaaaaaccgaaccaaacaaacgggatattaaaggaaatccctaaacggtaggctatatcgggtctatggt
atgtcaatcaaagagaaagataaatacaagagaccaacccaaccggttataaagttggtgaaaagacagagatctcaaattacaaaaactaac
gcgtctattaagcgaagagtaattcttgtaaggagatacctagttttggaaagaaaaaactttggaaacaggttaaattcctcgaaaaaggggga
ttcgaagaaaagaacctagtgagaattcaggaagttctttccaaagcgtctatccaaaaaagtttgttttacaccaaactaaaagactacgcgaa
cacgaatgctttattagcggtttatagggtgtgtttggttcgtggaagacggcaggctggtgtgagggggtggatttatacaggttaggggaaact
gataaaaaacaataaaaaatctctaaaagctaatagggaggagaaatcaataacttgcttttacgaatcccaaggacagccacgtaagcagat
tgaacgaggccgaaaaaaaaaaaagattatgttgtggtcgttctatgaagatgtaaacaaacattttgtgaaaataaaaaaatgcgtttctgtaa
ggaagaataacaaatcatattataatcaagaagtttatagatatatttatctggtaaatgatgaaaaactcgtaaaaaagctttttcaagagtagc
aagctggttttgtaatgccaaaatttgacaagctatcaccagttatatgcttgaaagtaattcaaaaaaaaaaaaaaaaaaaaaagaaaaaa
agtataaaataggattaaactagaacaaacagatgttacaaaggtcttaaaaacttaacattttatgaccttgctttggttcataacgctataaaa

tgccttgattactaccaaagtttctttcagacagcatatgcaatccgtaattttcaataatttaggaataataaaaataaaataaaataaaattaaa
ttaattttataaattaaccagcaagaactgagttcttatcatatattgttgcttcaatgcttattgttcatcaccctagtacacacacactgtaacttc
gcgtgacacatagactagccgtgtatttgttataaagtcttaagaaagaaataaaagtaattcaacctaaagtatattaagatataaacctataa
aaatttctcgcgaggatcctaaagcctttttatttgtaaacaaaacacacttttccttaacaaactacgcaacgtctggttgtgtggtacgatagaa
agctctgaaagtttcaaagttgccaaacgtatgacgttttttggttctcagccacgctgatgtcaaaagttagcgaggtatacagcgactgtttatt
gaagtctggggctagcggagcatcgttattggtatataagtttccgcgggaccgagcacaagcttttggaggatgcaagtaagcggatagatctc
taatgaggtacttaaaaaaccaaatatcatgaaagtatataaacacgttttcctatttacatgaaattaccaagaatgttgtttgtatttccattaaa
tcttgatcaaaaatgtaaaattacatcacttttaaagagataaccccggatctttgacctgccggaaaccctttttttagaagctcagacgatgtct
gtgtgctacggtaatctttgataacaacagtttgaaaacttctattatattaaactttttaatataatgacatgggtttcgttgggccatccttatag
atatttaccacgaagttggaatgtgtttgtgtagtttgctatcgtttattacaagtctcgtaaaagtacgttagttttttcgatttcctaaaagaacct
caggagcatacagtttcttcttttgcatccgcccttggattccatttctgtggaaatactaggcgttctcttttgtacccttatatatttgaatctcatcc
tcttttacccaataacattcgttttgcatcgtagttcgcgtcttgttcaccaaggaagtctacgatttgagtattcttgttgaaatctctccgtaccgtt
gagattcctttgtctttattatctactttttttcttcttctgtttattttagaatcctcgcttaccattggattgtttttcttcgtactagagctgtatcttcat
catgtcttctagtgttgctccagaagttctttcatccttgctggtccttgttcaaggactagcttctcctgacaataccatccgtactgaagccgaaaa
atctttaacggctgattggattgccccacgagctgatttgttgctgaacggtttggctgctttggctgctcaaagtgaagacatctctgtatgttgaa
cgttaataatagcaaggcataaaaaaagggttaaagataacactaataaagttcaccttttgtcctttctttattgttaggaatatgtatatctatta
aatttccctttttcttttttgtctattgttattttatctgttccctccatcttctcttcatgatcatcttactaacttaatcttctctttagattcgtgcatttt
ctcttgtcctcattcgaagaatatctttccgtactctgccaagcgatagcgaacttgaagtgttttcgagtctttctaacgatgccaggcaattgttac
agaaccagcttttgacttgctttgtccaagaggctgttcccaccgttcgtaacaagctttgtgataccattgccgaaatcgcccgttctgtctatgac
tgtcaaaatgaatggcctgaacttaccaccatccttttggtactgttaactctccggaggagtcgtttcgcgactctgtctaccgtacaatagctgc
cttacctctcttgttatcaggccaggaggctgccattgctcctttgttcagcgctggccttgctgatcccagtatccgtgttcgtgtagctgtagttcgt
gcttatgcttctgttgtattggaaagcaaacagtcagttcgcaatcaagtcatccctatgttgcctattctcctcaatattctccctcctttgcagcaa
gaacatgactcagacaatcttaccgaatgcctcatggccatcactgaaattgctgaggtgttccccaagattttccgcgacatttttgaaacttacc
ttcaatttaatcttggcattatcaaagataaggagcttgaaaattctactcgtcaagctgctctggaagcccttgtttgtttctcagaaggagctccc
gccatgtgtagaaaatcaccagaatatgctaatcagctcgttttgcaatgtttaatgcttatgactgacgttgctggtgacgaggaagatgaggct
gaaaatcttcaggaatggttagagactgatgatcttgaccaggatgaaagcgatgccaaccacattgtcgctgaacaagcaatggatcgcctttc
taggaaattgggtggtaagactattcttcctccttcttttacttggttgcccggcttgatctcttctcaaaaatggtctgaacgccatgccgcccttat
ggcaatttcttccattgctgaaggtgctgagaaaattatgaagcgtgagttaagtcgagtattagacatggttctgcctttgttggctgatcctcatc
cacgagttcgctgggccgcttgtaatgccgttggtcaaatgtctactgacttcgccccagatatgcaagtcaagtatccttcccgcatacttgaggc
ccttattcctgttttagagtcacccgagtctcgtgttcgtgctcatgctgctgctgccatggtaaacttctcggaagaagcagacaacaaggtccttg
agccatatcttgacgacgtccttcaacgtcttcttactctgttacaaagccctaaacgttatgttcaagaacaggccataaccacaatcgctactgtt
gccgacgctgctgctaagaagttcgacaagtactttgatgctattatgccccttttaatcagtgtcttacgccaagctgaaggcaaggaatttagga
cgctccgtggtaaagctatggaatgtgctactttgattgctttagctgtaggaaaggctcggtttttaccagtttctcaggaaatcattcaggttcttg
gtacaatccaaatgagcatcactgacagcgatgatcctcaggctagctatttgatttctgcatggggacgcatctgtcgcgtccttggaagtgactt
tgttcctttccttggatctgtgatgccacctttgcttgctgctgctacttccaaaccagatttcacaattgttgatgaggacactgatgatagtaagttt
tcagagcaagatggttgggagttcattcctgttcacggacaacaagttggtattagaactagcactttagaggataagtgcaccgccacggaaat
gcttgtatgttatgctgctgaactgaaggctgacttcgacccgtatgtaaatgaggttttgacttctgttgtattacctggtttgaagttcttcttccat
gatggtgtacgttctgcatgttgcaaatgcattcctcagttgctgaatagcaggatcctcgcaagcaaccgtgatcccgtcaaggttgctgaaattt
gggatcctatccttcgtaaattattagattatatccaaagcgaaccaagctcggaaatgctatctgactattttcagtgtttttacgaaagcttagaa
atcattggtccctgtcttgatgctgcttctatggagaagttggtttcggttgttgatctacagttgaaggctttcattcaacgtgtgcaagagcgtgaa
gagaattcccgcaatggtgacgctgatgtggaagaagatgaagacattattttggccattgaaaatgaccaaaatatgctaaacgagattaata
gaacatttagcgtagttttgaaacaacataaagcaacgttttgtccttattgggaacgtttgttaccttatatgaatggtttttgaatggctctgatg
ttatcgctaagcaatgggctttgtgcatgatggatgatttaattgagtttactggtcctgattcttgggcgtacaaagaccatttccttccctatttgg
cagaaggcattcaaagccaagaaccagaaattcgccaagctgccgcttatggtatcggtgttgctgctcaacatggtggagaaatgtattctgag
atttgctctagcgctttacctgctttatttaacatgcttgagaatccagatgctcgagatgaagaacaaatttttgctacagaaaatataactgttgc
aattagcaaaatttgtcgcttctgtagccagcgtatccaggatcttgataaagtcattactttttgggtaaacactcttccagttacacatgatgaag
atgatgcaccttacgcttatatgtttttggccgaactgatggaacagaaccacgtagctgttgtatcccaaatgcaggttgtcatccctgtcctcgca
gatacctttgcttctggagttttacgcggtcgtactttaattcgtctacttgaagcctctaaggcatatttgtcccaattccctgttgatcaagtcaata

atgtcatctctactttgagtgttgacaaccaaagggctttgtctgcttatttttaaacgactaagtcattgaaaagtttttcttttattagtctattcaa
aaaaattagattaggttaggttaattgctaggtttaattaatgttgttgaagttcagtttagttcaactactttctatgtggataagaatatctgaatg
tataagagaataaccttcaattgctgtaacattatatacctcgaataaatgagtaagttgaagaggtaagaatgaaggcaagcagtggtgtatttt
cctattgctaatgaatgatctgacattaacatcaaaataaacaatttttatttcttgtctgaatctatattaattgtttaattataacaactgaagaat
attgtatgcttactagtagctatgtactgtagaaggcgcaaccagttgattctgtttcgccccagcaaaactttttcttttcaatccacacagcgaat
tcaatatgaattacggctataacggttataatggcaactcagaagcgaattatggtccttacgggcctcaatatcctgtcaactctcattcaaatcg
tggatatccaaatgtaggctatgatcctccatctgtgccacctcctcgcggtaatagacctaatatagatggctacacggaccaagtgcctacaca
aaatgtaggacatattgatatcggtgctaatcagcactatggatatgcacctccttcaggaccacctcccgcaactggaaattacgcttataacaa
cccaggctatcacggaccgcagttacctaattcacaatcacaatcttatggattttctggtggaggctcatttagctatcagtactctacttgtcaag
gaaagcgaaaagccttgttgattggtatcaattatgtgcatacccctaatgaattacaaggatgtattaatggtacgaacctcgtcaatgttaggtt
ttgaattatgtctaatgaaaactagatgtcctatccacaggtgaacttttaaaggagagatatggatataaacctgaagacatgggtatgaatttt
ctgtggtcttcgttacgagtttttctaactgttaagtaattatgaccgacaccgctcaacatccaagagctattccgactcgtcaaaatatgattgat
gccatgcgatggctcgtaagcggcgctcaaccaaatgtaagataatgaaaagagcatagtcagtaccagttttcgaaattcttttagaaaatatg
aacgagactagattactgttatcgtatcttttctttactttatctctatattcggtgatagctaacatagtctaggactctttgtttttttcattatagtgta
tgtttaaaagagtttcttgattatactaatcattctcagggacacggtggacaagcaaaagacttggatggcgatgaagctgatggatacgacga
aacgatttatcccttagaccatgaagtctcaggacaaattattgatgatgaaatgcatgaagttatggttcgacctttacctgctggatgccgttta
acggctatttttgactgtatgtttatcagctacaaacgaagtacaactttgactaacaaatgatagcctgtcattctggaggtgctttagacctgcct
ttcacctattctactaaaggtgtacttaaagaaccgaatatgctgaaagagtcgggtatgaatgttctaaacgctggcatgtcgtatatgtctggcg
atattggaggtattttcaacaatgtgaaaagtgtattccaaaaggcttctggcgggtttaacaatagcgctgttcaatattcccgacagtataagtt
ttcacctgcagatgttatttctatttctggttgtaaggatagtcaaactagtgccgatacaaacgttaatgggttttcaaccggcgctctttcgtatgc
tttccgtgaagttataactcaaaatcctcaaattagctacctggatatgcttcgaggcgttcgacatatcttacaaagcaagtactcacaacttcct
caattaagttgtagccatcctttagacatgaacttagttatgcttctttaattacaaaagacttttcaatttttatttttattatttgaaatagccaattt
acaaattaccactaaattagcttccttaatttagatgtatcttaatgttgatatttattaaatttggaaaattgtatcgaacgtttccatttatcaaag
aaacttttctcatcaaatagcatgaaagggacattttcgcgtagcataaagtcttaaggctcgcccaaaccattttgttcgggcttgaaccattcat
tttttatgtccatacaaacccctgttgcgttcttttgggcctcttctggtgaactacctctggcacaaatgtagtacttgagtttaggctcggtaccgct
ggtacggatagtcataacgacttggccattttcgagttcgaaagttacattatctgaacttttagaagttggtaatttagctttcttatccactgtagt
ggaatcataatcagcagttaaatcacgaacgttggttatgttatagtttcctagtttagtagggtatccattagtggtgcttttcttgcgaagggcat
ccaccaaaccacgaagtttttgtgtatcgcgagagaaaaaatacgagtttcgggttacatagtaaccgtacttttgagagatttcaataaagactt
gagtaacggaaagttgttgaagttttaggcgtttaagaaggtgtaggaaagttaataaagcattcacaccatctttgtcttttacaataccacctac
catatagccaagggcttcttcataagctagacccacgaatttgccttggtcttctaattcagcagccgcattaccaagccatttgaaaccggttaat
gtctctacatgatgaaaaccttcgatctttgccatagatttcataagagcgctggaaacggtagtagaaagcacataaaaattattaacaggttta
cccgcgttcttatactcttggaaaatgaaataagccaaaatacagccaatttcatctccggtaaaacgtctccactcattgtttatcttttcggcaaa
tgcaaaccgatcagcgtcagggtcggtagccataacataaggaatattatgggtgtcggcttgcttaaaagcaaggtctagggcaccctcttcttc
aggattgggaaatttgacagttgggaagtcaggatttggtgagtcttgaagaggaacggaggtcatgtcagcttgctcaccaaaaagggttaaa
gcagacgtaacgaaagggagacctacaccatgaataggggtatatacaaattcaagtggttgcatatctaatgaaaaagtattctccgcgtgga
attcatgtagttgggaccaatactgcttcagtaaatccgttgcaaatgtacgatttgccaacatgtggtgatcgactaggtctttgtcccaaactaa
aggctttaagttcttctctatacaggaagctatgcctttatcatgaggaggaatgattgcggatccattgctccaataaaccttataaccattatag
acagctgggttatgagaagcagtaatcataacaccagcagcagcctttaggtttttatagagaagggaataagtggagtatggatgaggaaac
cataagcatatactttaaaccccttgttcaagaaagcagcggctgttaaacgagcaaattgctgtgagttatgtctatgatcgtgacctacgacga
cgcccatctcttgagatccaggcacggtttggattaaatattcagcaaatccctgagatgcttgtataacagttaattcgttcatgcgagcaaaccc
ggcaccaatttcagcacgcaaacctaggcgaaatgttagaatttacatagtttacattacagttacaaagaggatatctttctgagcacattcccc
aagaacgctcaactcatagagaaagagagagattgtaatttcaaaagctgattggaagcagggacaaactgttcgacccacttagaaaataaa
ggtcgctttttgaaagggtaatcaaacttgccagaaagagttccttgtaacatctcgaatgaaaatgatcgtttaacttacctgaagtaccaaatcc
gattcttggatgcatgatttgttccaaagttgcataatcgccaacgttaactagatttattacttcgttccttgtagtttcattctattttcaataaatac
cattagcatctcatcaagctcatttcatcagaaaggttgcgtacttgatcaattgcaagccattcatttaccaaattttgcaaagaagcatccatttt
gctaaaaaaccaacttcttagatctgcttcttagggattgaccgtacagagttgctatatcaaacagtaggtggtagatcacagtaggcaagaag
tatatgagtcagtacattcgattaagaagtatgaggtagaggtgattgtaaaataggaaatgaataattcaaacaaagtttaaaaattattgtttg
tttgatacttgtttatgtgaaatcggcctacctttgttaataccaaagaataaagtgtaagaacagaatcaaagacacaacgtgcgttctacatgg

gattttagcatgaaatttagggttgattgaaactacgaatacgttaagtctttctatgtatgaacgaatattggaatgaaattccaagcttttcatac
agagtcaacagctttgtagttcagtggaaagatgaacgagcttaagtcataaaacaaatcttccttcaatgatttactatataaaatgctatgatct
gagtggttatcttgtttgttccaaagaaaaatttgtattcaatataagatcaaataacaaccgctagaaagccatattcggcatgctatagctttac
ggatatggcactgcatttgaaacaaacataagcacatattcagcaaacccaagaatgccactcaaccgaatgaacgatcgattataagtggaca
ctgcgagaataaagaaagaagataattaagtaaatttcagacctgtcaagatgaggagaggagcgtaggaaattctgcgttgacaaatgtcca
gtaagcatgacaagtaaacatgctcaaaagaaagattgtagaaacgtttaaatggcatctatataattagcttcacgtcgttggtaaaataaata
cctacctgtcatcagtttgttcaaatggttgacacgagaagaaatactcacgtcaaggatgttgtctccgatttccacaataagaccgccgagaat
ggaaggagaaaccttgttgctaactaacagtttacccttgccatacttggactttgcgatcttggattccaagcgggacaaagtcttagagtccaa
aggagcagcggaagtgatagttacctcgacttcaccacgctggacacgcataatcttttggaattgattttcaatacgatcaagcattgacaaacg
attgtttacggacaagatgttgaaaaagttactcaacaaggcatcaccgccagtcatcttgcttaaggaagatacaatcaatttcttgtcctcataa
ttaatggagggattagcaataaactgggcaaactctgggcgttccttcaaagtagctcccaaggtagtcaaagctttttcaacacggtccaacttg
gattcttttacagcagcagtgtaaagagaagtagcataaacacctatccacgttaataaacgtgaacgtcatttcaagtaatggaacccactatat
tctccgtttcctatgcacaaggaacttaccatctagaccatgcaattgaataggagggcgaacagaagcagtagctgtagttgcattacgaaacc
cgaggcgggagaatcgtagagaactcgattgtacttggcgtaacatggatcctcgcataaaattcatcattttgagtatatcgctgttggcaggta
ctcgggttcttctctatgtctttatacggattgtgaagcttgtaattctttactgcgaaacttcttatcctttgttacctatgatgaagtaacataaaca
gtataatggaaaaaggtaaaagaaaaaattgcgagtaaataagagtatgacaaattttattaggcatataattattagtatgcaattagagaac
gtaatcaattcatcagctaatagaaagtaaagcgggaggttccttttcaaaagtgacatacataaataaggaagagagtgccaggtggtaaattt
ttacatatagaagagaacaataagtggtgaataaaagctgggtgtttttcttagtaaatgcccaaagtgaaagcgcaggctatagtttaacaaca
cggtctctgtaaatggcttccacggctgtcatttcgtcatttcgaaggattcgcaagaccgcacgttcccctgaatacgcagtagacaagcccgtat
cgattaagatgacttggccattgcaacgactacgaattccttcaaattgaggtgtgtgccctatgaccaaacgatcaacattcaagctttttgtaac
attagatgccacttcgcatgcaagttctagaggaagttgagcataacctctgtaccacatagggccttttatcccccaaaacgccttcagctcatct
ggtaactcttcagctttcataaacatctccagaagttcatgtcccatcggattaacgaagttctccataagactccattctggggacagtccggcat
gcataaaatgagatctactggtttcatctttaaatgtcacgttgtatttagttatgagtaaatttccaatctctccttcaggtccccaatcgtatttacg
gttttcaatagagggttcagggtacgaagctaggtctccataatgaacataacgccagtcgccattcgcattcataaactcatggtttccaagtaa
acgaatgactcgtcctccttgccgctctgcctgagtgcttaattcatcaaaccatctgaaaagaaatctggtatccgggccacgatcaacaatgtct
ccctgtatataattgtcagctttttctcttttctctaattcatgcttaccgtttggaccaaggttgcgtttcctgcagtccattcaaaaggatgcactgg
agacacaactccagcggcagaaagagcgtccagagcgttatggaaatctccatgaatatctccaattgcataaactttcacaggaaaatcctctg
tgtttctgtttctattgtcaaaaaggggaatcacgtctggtagtctaatgttcaagactttatcgatattcaagttggccaaagcatatttaatgaaa
aaagcgaaaaacaagccaaaagtaaatatacgaaacaaagataacaatggatattgcctaatttctggtattctcgacatttgcgttgaactgta
gtttgaagaaagacttcgaatgtaaaatacaaatcgtaaaagaattcggattaattaaacactacagaaaaaaaggcttttataatacctactat
tcgtctacagacacatagcgtgatgacaaaaaaaataagaaatgttatacatataaagcgactgggttgggttacttatataatacacattagga
aaccccatcgtaaataaccatttgaacagttcattaaataaaccagacgaaaatacaaggaaacttatggaagcaccgatgttcagagaagttg
aattgttatattagatactgtgtagaaaaatagtttttcagatgcttttccttaggtattgcggcgaacgttgctttagatatgtaattagcgatcga
ttggctcgatacagaagacttttaacggaagaccaataaagtacaaaaataaaacagccagcttgaagccatatatatttctggtaagaagcta
agaattctttgacttgaacaagccaaaagtactcgtgacttactgttaaattatagggaggaggttgtcccttaattgacaaaacactcaaagttgt
attgatgatgcgtgcaggttgatctaacttgagtaatgtaccatcttcagactgatcccaatattttaatcccgatggatgaactaaaacgaatcgt
gagtagggtacagactttatggaaaaggtatcgctaatccatgatttccactgatgggcattcaaccagacgaacgttatcttattattctcgggta
gaggtaaagcatgtttggcggcacgaaaaggaacatcttcgtagcctttcgcttttaagtagttcacatagtcccaccattctttacgagcattgag
taattggtttcgttcaatagatcttatagtctgcacccactgttttcctatactttggatattgtgcaaatcttggctctcaacgtgacgagaaatagg
attcactaaagcaatagctagataagaatcatcatctaatgttttcgcaaattcaggggttagctctggtacaagagggtatttaagcttggcagc
ccaggaacaaagacggataggatcgcgcatagaagaaacagactctgccggataagaaaaggagactccatttcgtactgccattaagtgagg
gagtcgagtgatattataatagttagccagatttttatcatctgaccgaaagatgggagcatagccagctaaagagcgagaaaacatccgcaaa
taattaaaatcttcagatgttgacccatagtcatgtatatacaagaaaaaggtggggtattgactttctaaagactctaattcttttaaacgaacca
cactgtcgatatgtggttttccatccaagttatttaaaggacgtatagtttctgcaaatttaatcaggacttcaaatggagtatggctggtgaaaaa
ggtgaattcacccattaggttatagtaggcaaatgtaggatatgtatgaattcccaactcttgttgacagtaccaagggttagagctgcagtcaaa
tgaataaaaagggatccctgatttatattcctttaataattttacaaacgcagaagcagcattgtgggcataatagttgcaagtagtgcatcgtttg
tccttcgacgtaaacaaaataaatccattttgtcgaaaagaactatatacattgttatgtagctggtcataatttatctcttcgaaatagctaactaa
ctgagactcaagttggttagaagtctcagcttctgtgttgattagccgtttttcctttgttgtaacaacatctaaaagagaggctcctattaaactatg

gtttacgtttttccttcttcaaaggctttcaagctagtattattcgtcagtattaccaaagaggaacctcgcaaaacaatggaatataaacttaagc
aaaggaccagagtctttatgaaccccatcgataagcccatatttagagaaaaaactgaaacccgtcaacttcaacgcctttcctaaaatttacctc
cagcaccctatttctgtacgaaattgagttcgtagtccattgggataaatatgtagcaaggtacagtacgttccatattacatttaaggatggtttgc
gaagttgaaggaaacaagaaataaacaatgacaaaaatacaaaggaatatcatttttaaatgtaaatttattattgcttgtaatataaaatgtat
ttgtcaatataagtaaaaaaaagacgaaatcaaactcttcgactcttatgggctatatcatgagttttgttcaggaatttattattgtttacgacgat
tcccaagcctaaggcttggttaaatcaaaaagaagacgaattggaggaagaagatgctcgattcgccaagtctttgatttcttgtattcccaatgg
accagattttgaaggatcaagaatagcttcaagagcagcttttgcgacatctgaagtcggtaaaggctcagcagaagcagctccaagaaaatca
aaaaatccagagcaaaaatggttgaatccagaagaaagtttcagaagaggagcgaagactccagatataggtcgattataggtatcatacata
aaggtaggacgtacaaaaatggatcgcatatttggtattttagaaatagcttgctcggcctctcgcttcgtacttatatatcgtggatcgagcccag
gaacagatgcatgagcggaaatataacagtatactgggacatgggcttcagccgctatattggctgtgttaatagctaagtctcggttgatagttt
caaatcgtagatttggcttagcaggaggaacagatggagaagggggtttttcaatgggttttcagagccttggaaaacattgctcctcaagttctt
gacgagttgaagaatcgagcctcttgggttttcaagagcttgcttatagttggattccataaggattccgaccgtattaactactgcagaagcatct
tgaacaataggaagaagggaatggggttccttcattgcatccactttttcccaatggactttttccatccatggttctttgtattgaattccaccacgt
cctcttcgagacacagagacaacttgatgccctcttgcaatcgcaagtttacatatgttatgaccaaggaacccagatccacctaagacaaccag
cttcattgtaacaactatagagcactattaaaaatttaacaaattgtcgaattctaggaaaaagaagaaaatgttaaaagttgaaaacaaatatt
aaataactctggatcgattttcaaagatgcctattcctcgaatgtatcttgctttatgtcaactgaaagaaagctttataaaggaggctaataaaac
cggaatgtgagaaaaaggaagtaggaagttgcaaacaaataaaaggaaaattgagaatcttctttttgaaccttactagtggaatcttggtacg
atgtcaaattatgatagaattgttaactcatctgttactccaaaggcatgatgtttgtttacatataagagggaaaaaaaagacaagagtaaatac
attaatacggatggaagagaggaatgaatatcttgcctacatattgaggttatattcgttgagacagatccgtactatgatcattcaagtaaggaa
atgagttattaaccaagaaaagtttatgaaccttttctttgtgtgacaataacttttcgctaaattataatcaaagtcattttgtgtaaataactgga
aaaccaaagagtcttacaaatacaaagattgacatgtgtaaaaaaaaccaaatggtattttgatatcaaataaaaaggtcaattgagatattaa
cctttacgtccgcttataagacatagtttagcgatgttttaaaggaatttgtctgtcgcagtacaattcaaatctagaaacctttgcaactcaatagg
acattcgtcacctttcgtgttaagttttgttgaaacataaaattgaacccatttcttttccttcgatatgaagtgtccattttcaaaaagtctcaacac
atactcatccctattgtaaacaaagcatgagacgcattctaaatggtattaatgattatgctccttgtcaaatagcttaaagattttcgtactcaatt
aatagagcttaattaaactttatacaatcgaattgaaataggttcatatcattttatttctatcaaaaatctcttattaattgtaactagaatatattc
tgtataattatattacgattaatattgttttaagctgttgcgttgtaccacaggatcttgtgcattgcccctaccatatttataaggtagtaccggcct
ggcccagcttgaacttagtagccaacgacttcattcacattcaattaatatgggaaatgttcagtctcaggaaggtaaatttacttcttttcattcat
ttgcagagaattaagttggttattaactattttcttcaggtgactccgctttgtcacgtaacgcccagggtgtaaacaaatccgttacgaaagaaaa
acctgactcgcttcctcatagaacccaaagcatgattagtattgctccggatgatcttaaccaggatgttgaccttatggatgacaattctagtatc
acgggtgctaccgatggttctcaaaatgccgcaggtgcttctgggagatataaacgtcgctctcattcttctaccaaaagagaaccatatacaggt
atgccgttcatgctcaatggaagatatgctctttgtcgagtggatgggatggaactcaaccaattcggaattctgttctccaacttgactctatttca
aagagacctatcattcacctttcttttatttatatgtccaagacttttgttaactgatggttaggtccttatgtccctacagtccttcgatggagaggcg
gaggtgaaatcgtttacgttacaggttctttttcaaaatggaaaaagaaaattcagctgcttaaagggtaagaaagattctttttaaatactaaca
atgttcattagcgaagattatacactcgtcttgcagttacgccctggaactcaaaggtttaaatttcttgtcgatggtatttggtgctgctcttctgatt
ttccaactgctacagatgctgatggaaacctgtacaattatcttgaggttgaagccaacgaaggtgtcgatactacccttgatcagagactcagtc
aagttcacaccaatttccctcctagtgaagagccggaatccgagaaatatgtttctgaaatcccgtcttttctctccagtagctctttagccgaaact
aaactccctaaacctccttctttgcctcctcatcttgagaaatgtatcttaaactctaacaccacttacaaagaagatcaatctgttcttccaaatcc
aaaccatgtcgttttgaatcacttggctgctgctaatacccaactgggtgttttggctttgagtgcaactactcgctatcatcgaaaggtaggctttt
gtaaactccttcttcttgactttatctaaccatacagtatgttactaccgccatgtttaagaattttgatgtgtaatgactttattgcgttcttctttccgt
acttattattttcttctttaagtaactatcatatattaatttcgtaattataaaaaaatattaaaaagagaatacttagaagggctcattattattcct
gatagcaccaatgacgaattagagtatacatgttcttgtaaactgctttcatgactgtttgtagtgtcccttcacttttcgtataaggaaaatagttat
atagaaaaacattccaagaatcactgtgtttgttatccatcgaacggaatgttcatgttcaatgttggcagttggaatcattatatttaaggtggaa
ggtccaattaatgttaagggatgcacaaagctattgagaagcaaatttgaaatatactcttcttttataggttccctattcctagaacagtcccagt
aaaaactaggtgaataaaagcttgtgtctacataaacagagccttcagcaggtaactggtaacgagtgtgcaagggaagctccagtcggagcgc
atgattatgagtatttgtttcatttaaaaacacttggaaccataaaacgcttcctgtacctgcaagggtataagttggggcctctaagtctgtttcgc
cgtaaatcctaataatatgatccaaatttccagcgttcatttcagccattgaagataattcgtattgatcaaagaataatgtagaaggtaaatgaa
ggatgcctcgtaaagaacaatgcttgcttggcggcttcaagtttttaagttgacttgtaatttgggatgcaagcctgtcggttgtaccaattctgca
gcatatacatttttttgatctttgttttgaatactgatatcttttaccttccagaatttggtttttcatcccaaaacccgcttgtatttatattagcataa

tcacttgattcatcaatggcttctgtcaattcataatatcggatcttttcttctttaaaatacatccacttttcctaaatatttcaagtgagttagtaag
cattctagtttcaattccatgaaaactatgtacctcttgattttctatcaagctatcatttttttacaaaagggattgatttaaaagcatctctatatatt
tagaaaattagcagtttactcatattcttcgacttacgagttaaaagtatctttgcctagaaaaacccaaaaaccttttgggaatacccgttttcat
gtaaaagagcaaatctaccaggaagtttagcgtcagatgcggaattgaaagaactaaatggattgtttttatcaagaaaggacttcaaattatca
aagaactgcattgtttgtcctttaagtgctcgtagaatagctctgtgaacaccaaagtttatttacttgtcaaagtatgagcatattgttaaaccgtg
tttacaatgtagttatgtggtttcagttataaaattttaaagaattcaacgtatatacttatttacatatatatacacaacaaaaacccattagaacc
cagtacctatgtctgtagtactatagtccatttacccaaaaattcgttttatgttttatttattctttgtataaacattcaggaaaataaaataaaagt
tttcctcgctgagggaataacaacgacactgaagccgtcggagagtctatagtcaattccaaaagaaaatagaaaagcaggtataggaaaagtt
gcattacatttgtttcagaagtaagtaaatgaaattacgttaatatatatatatatatacatacatacatatacttatgcagacaaaaccatcatct
cgctcgttcgttcatgaagccttaatgatgattcgttctatgtcacaaaaggatccttcaataattcttgagtattcatccttctttgaggatccttttc
gagtaatttcgttaatacatttcgaagggtatcactagcataatttggaatctcaagcctgaaaatccacgttaacgataaatactttggtatgaga
aaagcgaacttactctttctctgttatgttggtaatcatttcagaaatcgaggttccagaaaaaggaagctttgcaaaagctataacataaagggt
aactcccatagaccaaatatcagtctttctgccatctaccttttgataagggctgctaagtagttcctaaaaattaaaagttagagacaaaaaaga
agaagaattataataaacctacaggcgccataaaagctggcgttgaagacacaacattgaccaatccatcagccggaagaaactctgcttgtcc
aaaatcaatcatacataaatttcgagcagcatccaacaaaagattgtcaggctttatatctctatgaataacgttttctctatgtacttgagttgga
atcgttagtaaggtgtgcttagatttagatgtaagttcaaattaaaaacataccgtagtctacacagaagacgagctgacgaaaataaaactgac
aacgctcttctggaaatgcagaaaaagatattccatgatcgacagaggccaattttccattaggacagtaatttaaaactaagcaatagacttca
ttagcacagtaattagcgaaaggacgccatttgacgactagttcacataccaacgagtaacatatccaaatattcattattaatgacatccaatac
tttaacgatgttcggatgatttagtgactgaacgatgtcaagctcgtttcgtatataacaaaacggatctgaccgctcatcttgaagcatattttttc
taaagatatacgatctataggaacccctgcatttcgccggttggcattttgaagcaattcaactcttttctgacggcgtaaaacgcttttacgaaatt
ctttgatcgcctagaacagaagaatgtgttagttgctttaatgatgaaaactcagcaagtctgcatgctttcatgcttacatattcagcattggtaga
gcttttcgtagccaaccatacggtactagaagaaccgcgtccaattattcggtgagcaaagtattcatttgcagcgaagccaaagtcttcctcatc
gcacggtccctccgttaaaagactattttcagaaattgctggtgataaagattcttcgacgttgttcttttgtttctctgtttccatactaacattttta
atttacaataaataaataaataaaagcttgacgtggtttttaatcagacagcttaattttgttgcatttagcagaaacggctgtttggcttcaatat
taataaataaagttattcattcttttgtttactttcttatatattaaaagaaaagccgaaaacgctcatgtgatttcaatcaattttcttctgttgtatg
gtctgagtgaaaactcgcttagctcgtccatctcccagaaaatagccaaattcagttaattaggacaagtaaaattgacaggatatagagcggta
aatttactttttaaaagaaagaaaaaaaagctcccttgatactttaagcatcctttggtagactaaacgtactttctcttgaaaacggtgctttcctt
atcaaaagcgcgacttctacattcatttaggaaaagcaaatacaaaaataattaggtagtaaaagagagttctgtgaattattaaaatggctacg
gaacaaagaaagttacccaatatcattctttgcggcactcctgggtatgttcattgaaagggacattgaaaaagaatagaattacaactaacaac
aaacgtagtacgggaaaaactacccttgctgaacagcttgcagatgcttctgaattagaacatctgtgcatgggcacaatagtaaaggaacatc
accttcattttggattcgatgagaaatggcaaacgtatgatgttgacgaagaaaaagtcttggactatctagaaccaagactattgaaaggaggt
tgcatagtggactggcacacatgcagcatttttcctgaagaattgattgacttggcgattgtcttaagaactgatcattccaaactatgggaaaga
cttgaggctagaggatactcattacacaaaatccaagaaaacaacgaagccgagattatgcaaatttgcttagaggaggtgagagaaagttttg
accctaaattaatagtagagctcgaaagcaattcgacagacgacatggaatccaatctgtctaggattctccagtggattgatgcttggaaagag
aatcatgcaaactgatgaaaaagaagaaaagccgggtactttgggataaaatttctggagtatatggattgaactttttgtaaaataaagccttta
ttaacaactagcatgttcaagtgaaccattgtgttttgacgttactatctatttgattttaaactagcttatcgggatggagcgattggatataaaaa
aaagagaaaatgaaaaagtgaagtacttttcgctattaattttatttcaaaagacggaatgtattttcattgagttgacttgcataccatgcgcata
aggtaccagaaaaacacttctataccatgtccgtgtactttgtaaattgccaatttatcaaattgcttgcatgagaaaaagctcaagatgactccat
ttgtaaagcatacacaggtccgtaactcgacaacacatattgaatggaaattaacgaccatttttactgactctatgttggtagtacacgaaggtt
agtatgataaaatgggaaattcctagtagagcttcccgtaacggttcatcatcgggaaacatagaaaatataaacaaaaattctaattcatcaat
acaagcaattgaattgctcccaacgacgaaaactcaaacataatctgtaaataataacctttgtttatcatgctacgtttgtttgcgtttgtgagttt
taaggaattggtagcctttttttcggttcactgcgtaaaatgtggagttacttgattagataatacctatcagggtatagcgatctttattatattatga
cgcgagtaataaacaaaacaagtagaattcgatcgattttataccaggtagtgaaatgaaaaaaaaagcttatataaaaatgaattcaagagatt
tgactttgtatttaggacatgattaaataaagtagtttctttatatccatcaaatcaatagaacctgtaactttgcaagaatgtgcataataatgcga
tacagaattgtcggtttcgctttaaaaatatgaacattataaattataattaattagagatatactaggtactgcaactttgtcaatatccacaattt
aatcaatcatcttaagggtagtgattgcttctgttaattgtgttgaacaagatgctccatatattttcataatcattcgtcttcaacaggcaggtcgct
tcgcttgtcagatagaacttgctccaatttccgtagaacctctactctacttgtaaatattcaattcctgaaaggaaactcaaacaggcatcttaag
aaaacatttgaatttctgcaagagccagtcgattgggtcatacttcgaacgtttgtaacgctaccttttcgtcaaatttccttttcttgacaagttttcc

atcggaattgaattgacttcaaccttgacaacaacacgtcgaaggtaaattaaaatcaaaaccaagcaggcaagaccaaatatcaagcaagga
ataagtgtaagaaaatgagcaatgtcttcaatgatttttctttcatttatacctaaggtatatgtatagccgtatatagatgtccctgtttcgccatat
ttggcgtatgcagttgacaaaaagttacaaaggtaaacacaaaacaaaatgcaaaaatatatagaatacggcacaatgaaaaacaatgaaca
gacttttaaatcactttttttctttctagttgttgccaaagacacctgcttgattcctcttcaggattaagagcaacgtcagacgttaaaggagcagt
ttcctcaaaattctcttcgggttgactcattcttaagattctgaaaaaaaaaggtggtgaatttgaatgggagtagctggtttcaataccatccatct
tctcccttccgactgacgtcgaataccatcctctttccttcccttccaaattcacagtgaatcgtcctcaaagaaccatcctcaattttaaagcagcg
gattcaacatgtttcaaccaagagttgtgtacgattatcggcaacctgaagatgattcttacgagaaagtcaaccaagaaataacaagttggaat
tttaaaatgtttctgcatatttgctgtctttttctcatatttttctcagcggagtgctgtcagtaaccttgaattaccataacaaaccagatctagtcg
catggttccattctgttagctatattgtatggttgatatcttttattaatttgcaaatccaagacgtttcttcagacttagtaccaaggaaagaaaag
gaaaagcactatagaaaacagcaattatcaggcgcaggaaccaatggcccttcaaatcctcaacctcaagaaggccagtctttaccggaataca
ctgagacgatagggcaaagcgactcacagtcccactatgcgactcaaacgtaattaccctttcttctacttgtaaggctagcaaccgcgtcttctgt
ttgtttccaaatacggtccactgtacattaatcaggttctcccgtatagtacagatgtttggttggaaaccactggaccaaagggagttcccgttag
tttgtaatggatcttctacctgttttgcatgagtgcaccttcacaacatttgtcctcaacactcaataagaactcatccgaagattagtatttatcctg
ctatcaaatccacaaaccttagacttataatttttctactaatattaatatatttccaattttgttgaatctattgaaatgctatccactactatttatca
gcgttacccgtgttacccactttcgacctttctaaaattcaacttcccagatctattcttactacattgtgtttcctaaaaagaacaatcatttcaagtt
gttgatctcaaccgagtagaagagctgtttcggaagataaaagatattgttttattttaaaattctatgtcaagggtcttgtattcactgccattattc
gtaagttgaacagaagccagaatacacgaatggtatcttaaaacatgccattcctcaatttttcctttgttaattaactagttttgtgttagggcacc
aaacgatttttgaacactggatgtcgaaggcttcaatgggttccttttacacaattcgtttggcgaaaccgcgggtactctatagctgctggtatac
atcccagtaaacgaaaaaggctggctgattacgaacagttagcaaatgaaaatttgtctgacccatattttcaggccaagctgtacaaggaatta
tcagatttataccccgaagcaattcagcatcgctacgaaactttaggagtagctagaaattcgatatgtgatcgattttaccaagaagctttacaa
aggttgtctatcaaacaaaacttcccgtcatctttcgcccctaatttaaaacacgagttgagttcttacccaaaaaactcgaacgaattcatgaaac
ccctcgaaagtacaccaaatctatattcttcgacttatgcacatgcaccgcaaccaccacaacctcctacgcaagcaatgtatccgacgcctccgc
tggcatctactctgggttcaagcagtaaaactcctatgtacgtcgtggtcgatgagcctcgctttacaaagtttttcgtatttttaagtttattgctgg
tctcagtgtagcttcctacttcgtcttgctgggtgttagtatctttgctgaaacatccgggctcaataacatcatgtccaacgctgctgaacaagaac
cgattgaggaacgttctatcgatgtgcgcttttcagatgttcagggtgttgacgaagcaaaagaagagcttgaggaaattgtcgcatttttaaagg
accctacccattttacgagactcggagggaaacttcctcgaggtgttttactaactggtccaccaggaacaggaaaaactatgcttgcccgtgctg
tggctggtgaagcaaatgtccctttctttttcatgagcggtagccagtttgacgaaatgtacgttggtgttggtgctaagagagttcgtgaactgttc
atagccgctcgaaaacatgcacctgcgatcattttcattgatgaactagatgctattggacagaaaaggaatgcccgagatgcagcccatatgcg
ccaaacgcttaaccaactactagttgatttggacggattttccaaaagcgaagatgtagctcatcctgttgtattcattggcgctaccaactttcccg
aaagccttgatccagctttaacgcgccctggacgatttgaccgccatattcatgttcctcttcctgatgttcggggtcgtatggcaattttacgccatc
acacccaaagcgttcctttagactacactgtagatctaagtatcattgcacgaggaacttctggatttgcaggagcagatttagcaaacctgatta
atcaagcggccgtacgtgctagtaagaatcatagctcgaatgtcggcatgcaagatctagagtggtctaaggacagaatactcatgggtgctga
aagaagaagcgccgtgattactactgagagtaaactaatgactgcataccatgagggtggtcatgctctagtagctttgctaactagaaatgcta
tgcggccatataaggcaacgattatgcctagaggaagtagccttggtatgaccatttctcttccggatatggataaggatagctggactcatgatg
aatatcttgctatgatggatgtggctatgggaggtcgtgctgctgaagagttactttacggaaaaaacaacattacctctggtgcccataatgaca
tagacaaagctacccaagttgcccggagaatggtcaccgaatttggcatgtcagaacaaataggacctgttagtcttgaggcagatttggattcc
ctctctcccacaacaaaagcgatagtagaagccgaaatcaaatctttgttggaagcagcatatgaaagatctctcacacttttgaggtcacacaa
aaaagaactggatgctattgctaatgctctggtcgattatgagtttttgactgctgatgaaatgagacttgttatgaatggcgaaaaagatttgacg
agagccaaactggcctgaaattacctgtttgtatggtaagtttaataacggaatttggctttatgcttgtttattcctcttatcagataacgaactgtc
caaggtatatactttcgtgtatttttattttgtttctgctaaaaattttgtttaacttattggtcagtccgaaaagtttggttttgagattacaaaagtta
cttgttttataaaaaaattctgttaatgaaaacctgtaatggaataacgatatcaacaccacttcttctagtttgtatgcataattgtatattagatgt
aaatagtcatgtcgctcatcaaatttaacagacaactataactggcatcaagatgggctaaaagaaagatcaacacaaaagtattattgtgcaa
aaggcaagaacaccaaaatacgacgttttattagattacttggtggagttagtaacatgtttcataataaacgagaaacccttcaggtaattttctt
acaaggtagtttatacaaaactaaataggagtattacagaaagcattgaacaatgaactgtgtataaccaatctgggttaaagacgagaaaaaa
aaattcacccgtctaaaaaatgacatcttcgtccgaatccgctttctcatcaccagatacgttagtagaggttgaaggaagtgagtaagaatcttc
cttatcggaatttttctctacttctgaaacaaattgctgtccaacttcagtagaaggaggcttaactgtttcaccttcggaagtagcctggattacatt
ggcaccagtttctgtagtatctttaacagcctcattctcttctccttgttccttggtcttttgtactttcttcaatcgtgtaaaatcttcacgctcaagctc
ttccaactcggattcgatatattttatagtattttccagtcttggaattataatatgttcaattgagtttacacgtcggtttgttacctgtagaacatca

cctagcagaataaatgcagattggtaggaagctaattgaacaagagtttctacggctttcccgtatacttgtcgagctttgtgaatttgttgtcctcc
tctacctaagccagtcaattgaaagtcatctatattttcgttaagctttgtttcaaacgcaggaagaaaaactccgctaatattttcttgcttggagt
gaactttgagtcgcgcttgcttcacagactgctgcacttcaaaattgatgttacttcccgttgcaaagcccacttcagccatcgaaaaagcggctat
ctgcatgacacgtcccatttttgtttagcctaagtagaaatgttagttgctccttcataaacttcattcaaatgtataatgcgactggttttaagaaa
taaaagaagcttcacgagagtttttcctggtagacaatgatagaagggtttaaatcgataagttcgcatcttattgaaggtgtcttacctgatcaat
attcattacaatttcgcgaaatctttttttcagagcctctgattttcgttttaatagtgaatgtcccgtttgagctcccttgagacgagtttttatggctg
taagtgtcctatgtctattagtgctttttcagatgacacttatcgtacattctagtaggaaacacattttctctttgtccagacgacatattttaatatg
aaacttgtccagaattggcgtcctggaggtaaattgaaattaaagtatagctgggtagtggtcaagtttgtctgatatcaagagtcactagttcaa
atttgatccaacaaaggattccttactcaagtttctatcgttttataatactgatgtaaattcatatataggatactcctaattactgtctcgtattctt
gcgttgataccaactgacagcagacaagaaacgtattcgtaaaagcatcaaaagatgcaaattacttacattcaattcctttgagttgaccaactc
aatttatactactatatcggcgaaatttatgtggaaaggtttgtccgaattcaatctatggaataaacaaagtacatataaaagttttgctctggaa
gctttaaacgagagaagcactgtagcaatttaacatccattatagttctaccaaacatatccatattattaaacctgataccattgaaaatatatcc
atgtgtacattatagtaaacaaaaacgtgaagtaagtaattttgaaagtaaaggagaagactgaataagaaaataggcgttaatatcccaaaa
ggaggaggatgatttcgttttaatagtcccgacgatagtatgactcagatccgaccgaaccattctatcaaggaatcttggtaacatcacctttatt
catccaaaataggctttgcgcatttcctttgttcggtaaatccggatcctgtctgcatatgttgataatcaactgggaacatctgttcaccggtttcac
tcgattgggcaaatacaacgcccaagtcattgcgcgctgtggtcaagaaataagctgatccgtctcccagagaaagctattgtataaggtcagtc
ttctatatattttaaaaataaattttgggtgcgtccaaatcatttatttacgtaccaccaaagctcgaacaatatcacctggtcgaaaagagttttg
gactttcactttatttttctcagtagcacggatatcctgaacatgaatgactccttgaaattcttcttttacgcagatgtcatccacaacacttatact
aaccgttgcttgtcgaacattaatccttgtgacccgggccagaacaagagacccaactttcggtaaaattcctgtttcttctatcttctctgggaacc
taacagatattgtcttgggatcaaaaatgcctgttctagtcgagataacaaaatcgccttttgtggtcgtgccttccccatttgcaactcctctagca
acagtttgtccaggcaagacgagactcattattccagatatcaaaaacctaaaacaacaagataactacgtttccaaaggtgtgttttcttttcctg
cgcttcttgttggagaaaattaaacctactcaggtaacattgtatcttcaaatcagcatgcagaatgacgaaggtttttttgtccaaggaagaagca
ttgcaataatgtttataagaaaagagcttcttttggaaaagacgatgaaatttacgaacgaacgacataatttcgatccattagttttgcccatcct
atgcaaccatgtttctacctgcagaaaacaaaacttgagaagcaagtacggggaaagaataccgaaaaaggagtaagaagaaagtgttctatc
gatgagcctgaataaagtatattcagctatgggaaaacattaccaacaacaaatttggaaaagacatttcgagcaagacagagtagagtgcgta
aaactaatctgatgaagaatttcagaaatctcatgctcttttatgtttcgtaagctgacaactgagtgaaaagaccatgttttatttttcagaagagc
aggaaaaaaaaaactttccaatgaaccgagtcatggccatgcttccattctgaacgaattcgagatccactgtttactgctttcttgttgaagttta
cgaaaccaagagacatttttttcttatttggctctattggaaagcactttctctttaatgaagaatatcaggaattgactcaccccgaatcttttccca
atctatgtctgcatccaaactttcattcaccagtcccactatggctctatagtaagttagcacctcccatttgctccttaaaagagacaggttgttac
gtacacattttcaccgcgcagcaaataaacacccaaaggaatcgtttctgtgtcttggtctaaactaatgattctttctacagaatctgaaagaatt
aagtttgttgtgtgatcaaatcctcgtaacagacctaaaacaacacgcccatcattcgtaatgacttgaactttctctgtcgagtttccagtgtcaat
actttccttcaaacaacatttacttacgttcaacaaagtcagccaaactcataatgacaagattttgcgctaagattcgattcttgttgtatagttga
cttggaattgttgtactctgttgaacttggatataaagtaacacgttactcgtctgctaaagggaaagaaatgggaaaagtatgaaaatcgtggtt
agttttgatgtaaagtagaagaaaagaacatctttagtaaaggacgagcatcaatctcgcgcaatcgagaaaattgaaggctttcaatgctttac
ggaaaaatgtagtgtccatactccatccatcatcaaaatcaacatttttagttttcgaaaaaaaatacaaggtataataaaaaaattatgctttcat
gaatcgtgcaaaacactcaaaaagagttgttttgctagttcatgctacctacgaaatatcttactaaaagcgttaatacgatgaaaagtaaggcta
ggaaacattttgatttccataggcatttccaaaggtacgaacattatagagatactctgatctgtaaaagactgaaaccaataaatttgtacttgc
aagtgacagtcgatggctcaaagagtctatgcgacaaaacatatgtaaacaatattaatgcaagcgaaagaatgagaacgttgagtaaactctt
ttcaaatcatttggaagccgtaaaggtaaacatagctttgtcttcaaatttaaaagtttgagtgtttactaggtaagtaaattcttggcataaagttg
aaagacaaagattcatttttatgaaaactgaagttgtattctaactttttcctttctcaactgcactttgactgcagaatggcttgagcgtaacaaga
tgaaatcatacgtaggtagtttccgatacgggtgatttacgaacaatcgtattctttggcaaacttttcttccatgtaccttatttttccagtagacat
gccaacgagtgttttcgattctcttgtctttcggggaatttttggagaagccaaaattagccaaatttggtcagacgaaaatcgcacccaacagtat
ctgaattgggagtcagctttagcgaaagcagaatcttcattgggactcattccacaggaagccgcagaagaaattggtcgtgtatgtcaagtgaa
gaatattgatttccagagactagaagatgaaacattgacgattggatatccagtattgggcgttgtacatcaagttgcaaagctttgtaaaggaga
tgcaggtaaatactgccattggggtgccaccacacaggacgttacggatacagctacgattcaacaaatactggagagccttgaagttattcggg
gttacttggacaaggcgattgagcttacagaagccttggctcgaaaatataaggacacaccaatggtaggaagaagtaacttgcagcaagcagt
tcccatcacgtttggatttaaaatggctcgtcttctttgtacgttccaccgacatatcaagcgcttggatgagttggtcccacgtctctctgttgttga
atttggcggtgcttgcggcactcttgcctctttagggaataagggtttaaaagtccaggaattacttgctaaagaacttggactttctcctcctgaga

ttgcttggcatacagaacgagaccggattgcagaagcaggatgctttttgggtcttgtcacaggaactttaggtaagtttgccttggatgtgaaatt
gatgatgcagaatgaaattggggaagtaaatgagccttacattgccaaccgaggatcatcttccactatgccccaaaaaagaaacccaatttcct
gcgtatatattacagcttgcgtgtcttgtgtgcggcaaaaggttgcggctttgctggatgccatggtagaagatcatgaacgctcaactgggacat
gggaaattgaatggattgtgttgccagagatattttccctgacagctggagcattaaagcaaaccgtatttgtgcttcagggtcttgaggtgaatcc
tgagagaatgaaagccaatttaagcttaaccaaaggtctcatttcctctgaagcagtgatgatgggattggcatcattccttgggcgtgatgaagc
acatgatttgatttattcattgtgtaaaaaagccaacgaggaaaatcaatccttggaagaagtattgttacaagaaaagaaaatcactgatcactt
gacaagggaacaagtgctaaatctcaccaatccagcaaattatttaggacagactttggaaatgattgatcttgtcttgaaatgaatacacttgaa
tgtttatgaatgggcgatattttataatttggaaatgggatatatttttaataaagataattagactctttaaagacatgggggatattcatttgtaa
aattccaaaagtaatcattcagcatacctatttgcttttcgtgacaaaacagaatttccaaatatgtccgcctaatggaatccatagcgttagaaat
taggaatggttttactggggtccacgaaggccatgattacccttataattgataaattgaggagggttccaaccttttagagtgtctttctcgtcttgt
aattgatatacttcaatacccaagggacgacagcaatcaaccatcttgctttcttccttgttttcctcgctttcaccccaaccccattgggacaagtc
gcccccagggcatgcgctaatagcaacaaggagatcctgttcagcaaagcattcaaaataatccaaggatgtcgcaggacaggcttccatgaaa
tattgatcgcgttcgttcaagccggtgacttgaaagacattcaaaacatcatggacatcaaattcggtgaggccataaggcatgatagctcttgtt
agattggaatggcaatgtaaatcattgtccatgccggaaataagtttgtctacatagggatcacatcttgtgcccaaggtgtcatggactcggcct
ccccattcgtcatggcgagcctccatagaatcggcagtaatggtaactaggggacgcaggtagggcaaagtagaccagagtctgtcaaacgtgg
aaacatgagcggaatgcagctggcgtgttctggcagcccagaaacgctctctgggattatgaaggttccaaatattaagatcacaaacttgagga
ccttttggtgtagtgatacgaaacaccgactttgcaggaattgtaacagcaatgcctgaacgaggggcacaattatgagtttttactaatttttag
gggactttgcaatacgggtatacaattctttgttagcatatataggagaaccaggatgtgggtaataagcagggacgggttttggacaattgtatt
ttgtttttccagtcgttgggacgatgtagctaaaggagtcgtttcgttgttagtcatattgtatgtcttgtttcgagtgcttttcagtgaaagatcttgg
catacatccgcaaacgagggaagcgggtactgtggagtatacttggatttttgtaatactttaggtgtgaatagatgatatttaaatggccagttat
atctttcctatcgccggtttttgtttctggcaccggctttcttgtaatatcttaggttattataagcgaatttcattttcctgtgataagatgggatctat
tatggtgcttatgacatttttattcaataaaatacaaaaacaaaaagaaaaatgaatgaaaatgtgcaaggtaaacaaaaagcttcaaggtga
gaaacaattcacacaggaaacgttccatttttattaggaactagggttttgtattgaaatatgtcaattagaactgatttttacttcgatttggaaag
tacatttttcccctttgacttatgtcgtcaattaactttgatttttctatttacgaaacacagtgacaatgcgattaaagcgtcaagataactcatcgc
tttagtaattagttagaaggactaggaatacattgtttttctggtgtaaatctttcatgaaaaagaaaagaaaaggaatgaatactctctctgctttt
tttactcaaaaaacaattctctatcgtttctgtttataaactataatatatataataacatggagttccccccttggcgttttataactatattatcaagt
ccctagtatgcgtcacatggagtttgtttataccgtaccgagttcatagtgacaacttgaagtcatgaatcaattcgttttagagaaatttcaggcta
tttcacctggttttcgacaaaaggctttacaatggacggtaaactttacgaaaattctgtctggctttctatatattgcgttcgtcgttctgtggagca
tcactgctgctctctttcgacgcgtcctcttgcccggattctttatttttcgaacagtttcttttttttatcattaaagctgcctccatgctacttttgatcat
tgctgatcctgccattctgatgattcaatctgtatattggtactttatacgtgcgccagctcgctttattctcatggtgggtgtgaccctttacccattg
tatgttttgctatcgtgggctgtctttcttggaatccttactggttttctattgcatacgatatttacggccctggattcctacgttgctcccagcacag
aagaagattctcaaatgcaggaccttcctattatatctccttttacctctagtttgtttaacttgcgctccaatctcgaacctccatgtaaacctgcgg
actctaccaagtcaaaagttacagcctcgaatctgaaaaaaaccgaatcttccgtatcattgaatggaaaagatttcaagaaccaaagttcgcct
caatcatcagagaaatatgacaaagaagattcaaaggtacctccagtcgaaacaagaattatcgccgaatttccaattcctcagcctgctagaa
aacgtaagcaccgttcaaaaaaatccactggaagtgcatcgataaatccataaagcactgaagaatggttgccttctgcttgatttacgaaaacc
aaattttgttatgatgtgtataatatatcaatgaccttcgtttttttatgctttcatgataagaatgcttttatttatcaataactgctaaaaattattgaa
ctataaatctatacgttggtttgcctgccacttaatccccctctcttcctccaagttgctttaaatagagttggcaaattggaattgtgtttactcttaa
aatagacagtacaaaactcaatggaattatcaagctgttatcaacatactaagataagtatgctgtccaagtctcaaccagctgtatagttattga
agattgcgtgaaaggattgatggttgacatctttttactttttcgtttttatttttaaatgaaataaagaataaaacgcacatattattcttcttcatag
ttacttggattttgccgagctagtctcggacttttctaatagacgacggacatacgcaatggaacataggctttctttgatttataaatattctctaaa
gtttcctcctgagattcgtttaaagcaaatgtaatcattcagtcctcaagtgggccttagagaagttcgtatctatgaccactcaaactaccaccat
atagcaagcaagccattgtcatggcaaaacccgcgttaaaagctgcaagagaggctttaaatacaaaggattacgaagaaactataactcaat
gtgaaaaagctctttcttttgaccctgaaagttataacgcttacgtatttttaggtgtcgcaaacttttcattaaataaggaacttgaggcgaagaa
agcataccaaaatgcaatagaattagatgataagcccttgcttgcttggcaaggtttgtggaacttacaggaaaaattcaaagacgttcaaggac
ttcatcactcaacagaagtcttggcctataagtttatgactgccaatgaaagcgaaaagtgtttgaactttgtaaacaaatatttagaactcattcg
aggggaaggttctaaagctgatcaaatgaaagcattgtctttgctgacgcctagtcagggagatttatatcaattccttgaaggacgaattgagcc
tgttcaaagtttgtatatgcaaatggcagatctgcaagagtcccttgatcatgactactaccaaagagaagttaataaacgaaagagcagacttg
gcgctcgtatagatcaagtaattcgcgatgttgaaaaagaaattattaataattctcctattgagaacatctacatggaaattattaattggtctca

ggatgatgaaattcgtcgcttgactgaaacaaaacttctgtacatgtatttgaaacgactctacgcatcagatagcaaagaaaaagcaatttggc
gaaataaagtttgggaactggtgcaaggtatgattacgttacatattccagaacagacggcatggactatttatttggaatggcaggatcattcgc
atgttgatcaattgaatcccattgaaatcaaagagttctgtaatctttttcagaatcacctttatcgaaagcgttggtcgcaattcaaagttctgatt
actggatggaaacacactcacccaaagaaaaggaatctgaagattctgcgtccaaaggagatgaaattgataatgaaaatcagtccaatggca
attttctgccacaagatcaagtattagccctattaacgcaagcataccaggaatgtcctcactccttgctgattgctgaagtgtatacccaatactgt
gtatatttaaaagaatatactgatgtagtccagcttagttcatcagcattggatctgttgaaaaaattggagtctgacacaggtcttcattatcaag
aactgccatatcttttctatctattcctagctattggatattctcattacgaggtgccgagatatttattgcaggcagaagaactttataatagagta
ataacccatgaacctgagaactatagcgctcttattggtttagctgatgtgcaagtggaaaatgagcagcatgaagatgccgctcaaactctaga
gagggtgcttgaaaaaaaccctcaagatccatgtttatctcaactttcatggtgttactatatgatgggagatcttgataaagccatagagttagtt
caattttgcttacaaattttgcaaggaaacttaaaaaataagccagctttagcagaagcttactatcgttatggtatttatttgacagaaagaaaa
gtggggaattacgaggaggagtctttttcagcttttgtttcttctctgagatgcgagccaaactttgcttccacttacactcaattaggatactactat
aaaaatgtagatgatacttctcgcgctatgaaatgttttcaaaaagctttcgaattagaagcatcacaaactgatgctgctgaagaattagcaaa
aatgtttgccgaagttgaagaatgggagttggtggaggtaatttctaaacgtgtactaagtactagtcaaaacgatttaaagcgcaaaagaaagt
tcaattggcatcataagtctttgggtgtatttcaattgcattttaagaattttcatactgccattgtccattttcaagcagccttacgtatcttccccaa
agacgcacattgttggagtggcctaggtgaaggttatgctcgatctggtcgctacatatctgctttgaaagcatttaacagagccagtgctttagat
ccaaatgattggtacacgaaatactttattggctctattcaaaaagatatgggagactttgagaaggcaattgaaacgttgtcattagtattacag
gagcgtccctctgagttatgtatactggtatgtttagccgaatccagcctacgactttcaaaatattatatccgtcacggttattaccaaagatctgc
gttggcttcacaaaagacaattgatatttgctgcatgattttgaatatggattcaaatacatattctgcctggaaaatgcttggggatgcgtgtatg
gtatattcgcaattaggaaagtaccaggttgacttccctgtaagcagtgtttcagatttactattatccacggaatcaatgaaccttgcaaacaatg
gtcgacagattgatagcatgatttattttcctgatttagagaaaaatgatcctgcttatatgattgcttgtggatgtacctgttttactattttattgtc
cctttcagccgataataaactttgctatcggcttcttggtacaacacgggtgtttcctactttactctctttaatttcacccgaaacaagtcatatctg
ggagtcgctattgattgtcttaaacaagctattaagttagagccaaagaataataatttctggtgtatgcttggtgtcttgttatcccatagatcggc
tattcgtgctgcgcaacactgctttattcaagctttgcagataaacgagagagacgctgatgtgtgggtaaactatggcgccctttgtttacaaatc
ttggatattgaatgcgcagataccgcctttacgcgcgctatttccattgaccctgacaattcgcgcgcatggcttggatacgcatattgctcgatttc
tactggataccacgagaaaacagttcaagctttacagcatgcatttgaaatctccggcggtaacgtacgagacgtaaattattggtatgcaagca
ctatgcttaccttggcaaacacagaacaagaaatagatgtcatgaagttatggaacgctgtgtatgctattaaacgctacctttctgaagtaccta
atgatgtctacgccaatttcatcaaaagtagccttttggaacatttgggagaatcaaatgacagtaccgaaactgcgcataccctttgtcaattact
agaaaaagaatacgatgtcactgaatcggttggtgcactcgagaaatacgttgatgccaaggggcaattggcgagattgtgtttacgtttgaaca
agtttgaggagtcttccgagcatgcttccgttgctttggacttagtcaatgaagaggatgaaaacgccaagcaaatttcgttaggattgaatcttac
ttctggattggcggcatactttttgggtgatttggaaaaggcactacagcactttgaaaaatctctcgagttaggggaatctcacccggatgttgtg
gtcctcgttgcgaaggtactttgggcattaggcagcgaatctggtaagcaggctgccagagaacagctgttttctgtgacagaacagtttcctgaa
catgttgactcgcttgtatgcttgggtgccattggtatagctgatcaggataattctatgattgcggcaattcaggaggccctgttgaatttaaacaa
ggacaaaacctcaaatccggaaaccttacgtgtcgtccagagtttgcttacgatgttaactacagcttgcgatcccaaacaattacaggctctact
cttgcagcaactttttatatatcccagtgcagcatccacttggtctttgctcatgcagcaaagatacagttcgccttccgtagccacggcttttgctcg
cactctgtatacgcatccttctcagcctgatgatttggcacaggcatatcgacatcagagcacagttagccaagcgcaggcgtcagttcatttagtt
ccctgggaggcagaaaactggaactcgttggaaaaaacaattcagtcttcataaaaatagtttctctttcgagaaattttcaatttcctttctcctga
agacgaaaaagaacgacaaaaaaaagttagggacaagagctgtaatcaaacagatgttctttttcttaaccaaaaagaaagcacattctcatg
accctggtttatgttgagctgtagttgtacactgtactaaatagttttatgaagtttaatgaatcaacaaaaatggcgttcatagaaaaaaagattg
aaaacggaaggaggatattcaatctggcaaatttgttttaaagtcgtctctgctttcagcaaaatatttttcccatagtgtggtctttctttctatgct
aaatcatgtacatggacgggtttttatcattgaaaatccacagaccaagtaaagattagcgtatcaagaaatggccaaagtataaatgaaacga
caaagcttagtctttatgaaacgtcgtaaaataaagatgtcattttcatgaatttttgaatgtttcattgtttcagtttcaataaagaagaaatatttt
agatgcaaaagttcaagaatattcaaagaaagataaatatgctttttcgttataatatatatgtttattattccttgaagaacattcaaaatgtaatt
ctttgttttactaagagaaggcctgaaatgaacggctgctgtgtggtccaatttatacttacagaaacccctcattgtttgttatcttgatgacgcag
aattgccaagtgcatatactgtttggacaaatggtagtaagtgaatgttccgaaatgacatactaacagtatatgcatttgaaacgggaacatcgc
acgtattgatttttcgttttgaaaccttgtacccaagattttggttctctaaaagtaataaatgctaaccaatgtctcatagagtagaactctttctac
acattgttttgcgttatggaaagtgatgaaattaaaaaatcaatcatagcaattatcatattaggaggaatccatttcataagtcatatatgtcaag
gaatcaaactaagttcgattgcatagcaaagattcctatgatttatttttccagatatcggttcgtttagatttgtcaattgatggtcatgaactgga
atgcttcaaaaacaaaatcatattggactgttactttattgcccttgtttactgttacttgaaagcagtcggtttctgaaattgtagaagaaaaaact

aaaggacagtaagcagtagtcttactaatgaatctataagtcggttttgaacctcgtagaattgaccttcataattgtttgatgtattctgtaattaa
caacagcttcattatcgtaagttagcgcagacgctctctcggctaaaggattattttggaactgttattgcggtttatttttctttcctttttgagaact
ctgagatcattttcaatctcatatttcttcaagttgaacagtctttttcgctttcagtcactgtacattgagcaagttaaaaaacaataaaactgaatc
cacgatcttgctatttattaacaacaaaattcaaattaaaaataaatccctctgttcatgaatgccatgctattagtttcttttgtttactgctttccttc
aaggtacttccttttgtctccttatttgaaactcaccaccagaaaatcagattcgacagcaaaggagaaccttgttcaccacattactagagcttta
tttatgctgaaagacagatagttaggatgtcactagcaatctcacacgaaacctcatctttgagagaaactcatatacgtatgttaaattgaattac
tgcgcctaaacgtcaataaagttgagggacgtaaactaacctgcgagataggatctttagaaaagcttttaaatttgaatcaggatgttgaagatt
tagaaaaggatccaaactacaaatcagaattcccaatctggaaagttctgatctttgataagtctggttcagaaactatatcttctgtactgcgcat
tagtgacttgagaaaacatggtgtcactgtccatatgaatatcacttcttcccgtcaacctatagctgacgttcctgcgatttattttgttcagcctac
tgaggaaaatgtcgagtatattattgatgtaagtgcttttgtttcttcatgagagcgttgtcattttatacgacttctaaaaactctaattatttcttctt
atcaaaccgaagcaatctatgtaactaaccggcttttaggacctctctaaaggtttatatgaatctgcttatgtttgtttctcttccagcattccacgc
agtcttttggaaaaattcgcggagcttgcttccaaaaccaatacaagccacatgataaaccaggtttacgatcagtacttgaattacgtagttttag
agtctgactttttttcgcttcagcttccaagagtcttccatactattcacagcccttcctcggacgatgtattgattgaacatacaattcaaagcacag
ttaaccgcctgttttccgtcattgttactcttggaactattcccattattcgttgccctcaaggatctgctgctgagttggtagctaaaaagttgaacc
aacgtttgaaggatcatttgatgaatactaaagatgtttttggctctttaaatcctaaagtacgtcctctactcatcctcttggatcgaacagtcgac
cttgttcccatgattaatcactcttggacctatcaagcattgattcatgacactcttaacatgcaacttaacagagtaaccattgaatctgccgatga
aggaaaggtctctaaacaatcttatgacttaaacggaagcgattatttttgggaagcgaatgccaataaaccatttccagaagtggctgagaaca
ttgacgaagaattaactcgctataaaaatgatgcaaatgaaattacaaagcgttctggcgtttcgtctttagaggaatttaatgccgatgcttttac
ggattccgcttatttaaagactgcagttagccttcttccagaactcacagcccgtaaacagcttttagatatgcacatgagtattgcaactgcaattt
tgaaagccattcaagaacgtcacctcgatgatttttccagatggaagataacgttacaggacttaacaaaaccgcaatccttgcttgtataaatg
ataaaagcaaaggtacaccggaggacaagcttcgtttttcatcatttggtacttaagcgttgacgccgtccctagcactgacttgcaagcatatg
aagaggcattgcttaatatcggctgtactttggaggctcttaattttgtcaaaaggtaatattaaactgaacagttatgtttatatatgctaatcatg
gcagagttcgtgaaattactaaaatgaccatgttggcttcttccacggcgcgccccgctgctaatcaatcaggagataatattttccgtggtttttca
agtctttctagtaaagtatgtgaaatgaatttattaagagaaatactaacctatagtttaccgatcgattcaaagaggctggaatcggtggtttaga
aaacatcatctctggtgtgaggaatttaatccctttttagaaaagatgggacaattacctctatcgttcaaagcttgatggatcctggatcttcttcgg
tatcaaagcaaacagagtcatatcttgtattcgaccccaaagcttctcaagcttctagtgatctcaggggcataaataaacgccaaatattcaatg
aggccattgtatgtgtcttgggtggtggtaattacttagaatacggaaatttgatggactgggcaaaagaacaaaacccacctaagcatataacc
tatggcagcaccgacgttcagtcgcctagtcagtttatgaacgagatggcatctctatcatgattttaaaagtagcatataataaccctttagaaat
gattattgatatactttatgaaaaatgtaagtttttgtgtatatatgccgtaagaaagaaaaaaatatatatcttattcaattaatgaatagctcttt
aagagctaaaaaattaaattacaaaaaaattgagaaatgaatacttctataattgatctaatagtccgtacattaaaaagaacaattttaaacac
cgaattatttgagataaaccaaccatccttaattagataaaagctgaagttcataattggacatttcaagaggtatttgcattatcaggtatcatga
gggacgaattttgaagatgttgggaagatgcagatgacgataggttggaaagtatagaactcagatgagtatttgagttgttctcaagtaccttat
catctgcgcggacattagttgaatcaccactcttttcatgaagaagggagtcttcaacttccttagggagtggtattgacacgggagacacagcgg
catcgaatattttcaggggatcggagacatttgaaagaacagacgtacttgaagagatatttacattgagctgaccctctctttcttcctcgtcttta
gattccaaagttggcttcgtatcagcaccaccaccacgcttataacctcgtggatgtaattcttcctttgatattacaaaatgaataggctttggtct
gtaccaaattatttcgtcttcaacgtcgtcttcaacctgattaccaaaagcaacgcgggcctcctgtctatctaaatctcttgcactcccatatatatg
aggtgtttttacagctaattcttcttcatttgcatgctcaatgaccttaatttgaacaagctcatcatcaggtttccaagtaacggatttcttccggcgt
ttcttccttagagagctttcttcactggatgcttcagatttctcagatccttcttcggatttggcagaacgttttctgaaactgggcaattcgtcacgtg
aattggactgagattctgtccgtaatgactgagacttttgcaactcggcatccttccctttcaatccagctactatagaggaaagaggcgttgatgt
agatgtttgcttagcactcacagtggagggttttggcgcagatgtagcgggtccggttttaggattagacaagtttttgaaaaaggaagtaccagg
aacgcttgtaaccttttggttgacgggttcttgctatttgtagatgatacattggttggcttcgcaacggtttcgggtctagttggttctttctcgaga
tctttcagctgatccttgcctagttcaactgcaatagatttctcggcaagatcggagagcttttcagcatgctgtcgaatgacagaatttgctgactt
ctttttacaatcaaaattggtttaccaaacttgacatctgcaagcctttcagcagatatgggtaatttacataataaaagcaaaattgaatgaata
actttatctgatttagaacgaatagcagaaacaaaccaattacgtagtttcggaagaaataattttcgaccaccatctactagctctgaaaagatc
gttgcttcattattgtttttcaaaatttcaagaaatagtagtttctgatcatcctgagttacggtgctcatcatgttcacgaggatagcaacacattcc
ttaggagcttggctagtgtttaatggatcaaggcattcatttagtgtttgtcgcaaagatcccaaagaagtttccgttggtttttggtttgcttttgact
ttaatttttcaatcacttgcttgtctgaggaaggcgacctagagaatggacttgggttgatggtgcttgagggggtattattgcctacgaacgagga
aatagagccagaggtgttattcgtgatagtatctttcaaatcagcatgaccacttggatatcctaatacaggaatttggaaagatgaagatggact

ggagttaagaccagcattgggctgcaaagcagaaaaagacgaaggatccagttgtcctgaaggtatattcgaagcaatcgaaggtagttgagg
aatttgccaactgttatcagtatcagagtaaggaggaaattgggtaggaaaggaagatgcgctgacaggaggggatgctagagatggaaaatg
tggttcattgggataggatgcttcgaaaggaaaataatctaagaattgagatgtcagtataattcatatatgactgcttcaaaacttacttgatggg
taatggctttcaaaagaagcaggcatctcaaacggatgcggtggtggctgagaatgctgaaagacatgagactctaaccctaaggaatagttaa
ttttatcatttctatcaaagaggaagacatgaaaagctacttcttctcaagccctacatcactacttgtcctaaaaatagtgtcaatacataccagg
aaacgcgttcatcaacttttcatctaaattcttttctaaatcctcgcctccactgttcaaagttactggaaattgagaggttccaggagagttactgg
gctgattaccaaatgaggcaccgaaaggagttccattgaccccattttgagtatgattataattctccataaatggaataaaatagtgctattaaa
ccaacagttcttaccagtttactttgaactttctaaattgttcgtatgtcgcgttaaaggattgaagaggccttgctacttgattaatgtctccaccag
ctaacatttgttaccttgcgagccttcgcatattcaccaagtcgccagagtatatgtggtaaaatggtgagaagtatcaagatgaagagtttttgat
tgaaagttttgagatatatgtatatatgtgcatgtaaaagtcaatagaaacatgttgcaagaattggccaatatcattacggttagatgtgcatatg
caagtaagatacaaagaaaacagatatcaatataagctttcaattgattactagtaaactcagatagagtattggtagaaaacacattacccatt
ttcttctcagcaattgataaagggagatatgtcaactttgaatatgctcatagcgtaatggtcaatgttagctccatcgatttcgatcaggaaaccc
ttttcaaagtttcataacccataagctgatctattaattcccgagaagctattgcattttacttgtcctactttggtttaattgcctttatgtatttatat
actttctagacactgcacaataatcgaaagtattaattgacattaaataaaacataaaaatttgatactaatacttttatgcattttcttataattttt
gaaaaggttgtgtacacggcaaaaaacagcgggatatgttctgaaaaacaataccataaaggtgcattattaggttagtttatgtgcttgaacaa
actaaccttttcaagcacttcgtcctaatgatagtcttaagtttaataaacattatggattttattacagaataaacaccgcccaaatgcaagtttaa
gcacgttataatctgcataaccatgtgcaatacatagttcatccgccataggaatttgctcgaaatgtgaattcattaacataattcaataagagtt
tcgtaaaaaaaaaaggaaaagagaagaaggagatcgaataggaatatacaggaattttgaattattaagcagaaaacaatttgcaaacgaga
gtagcacataaatccaaaaaggggaacgaggacttcaaatcatctacttcatgatccgtaacaactgatccatgtcgtgaaaaatctttataacc
atttgccttgacatccaacatataacaatctggcataacttcataaagctgtaactcaatatacgcatagacttttcttccttctttcttgcaatgagg
aatctcccaaatactcttgatcgtatacatgtcggatttgtacttaccattatgctgcactggcactgtaaattgagcaccagcacgctgtagagca
cgatatacagccagtaaaatctcgggagcatctcctcggcagcggacgccaaagtgccaacgatttctcttgctttttttctttcctgtctgtgacaa
agcagcttcattattggcatccaacaaggttttcactatcaactggttattctcgcctggaacaaggatacggatatgactgtcgacagtctgttctt
gattagccggaggctccgaatgtgaccatctcgatccctcaatcatgggatcaagatattccggctctttagaattgtttagatcatccgcatattcc
gtaaaggcaggaggagaaacagaaagaaacgaatccactttctttgaatttgagaaatgactcttttcttgaataatttggttttcatgtagtaaat
catacgcctcctttaactcattattactctcggaacgtaaggcttcgtaaatgtaatcttcagtatatcccatcgcttcactcagcttattaacaatat
gaggatcagcataagaatcctgaaagccttccataggacgtagataatcaggcaaattgatattaaaccatggatcatgacgaatttcttgaatg
gtaatacgctgcatgggatctgcaacaatcattcgtcgaataagagactgagcgcctggcgacaagaaatccggcatcacgtaaatacagtcta
aacaaacattaggatcaacatcaatcgagataagagacagtaacaacacatactatttactttttaaacaaattaggaataaactcatcatcaa
aaggaaggcggcccaccaacatgacatacaaaacgataccgcaactccaaacatcaacctcggggccagcatagagttttccattaattacttc
aggagcggcataattgggacttccacaggaagtttttaaaaagtttccatcagtcataatattcgataaaccaaagtcagcaattttaacattcag
tttgtcatccaaaagcaagttttctggttttaaatctcggtgaacaatcttgtgtcgatgacaatattctatagcacagatgatctgctggaaaaatc
gacgaccttcatcctccgtcatctaatatatgttagctgaatgacccatgaaaatcgaatagaaaaaaaattattagggaaaggtgctcttgctga
agtcgttttcacaatctgcccattcagttcgtccttctctcctaaaaacagaacctacccttttcttttcaacgatataatcaaataattcacctccag
catactcaatgaccattacaatatctgtaggcgtggtgataacatcgtataatttgatgatatgaggatgtcgaagaagcttcaagtaagatatct
cgcgttcaacacgcatatacatatcgctttttttcaataattgccgcgagataaactttaaagccactttttgatgcgtcttgtaatgagtagcaact
gtcaaagctggttaatcatacacggctcgttcctccttgaaaacttacgcttcacttttccaaatgatccctctcctaacgtttccttcatcacatacg
gaccaatgtgtcgcttagagatggcttcaggaggtaggatcctggcaccgttcctcatcgttgagttctccatctcacgtaaaacggttcctttaaa
agccgtataaatcttttgaataaaaatatataattctctcttctattcactcaacttccgtgtaaattctagctcgattataatgaacttggtggtaaa
ggttaggacggcagccaagcgtaacctggagtgccgagtatggttgatgtccataatatgaataacgaaggtcaatgtttcgttataggataata
ctgctttttgatgcggaattaattatgtaacgttaataaaggaatacacactgaaattgaacgctggtttggtagaaatgagaagaaaggtaagg
tcaaagtagaattccatatttctgattctagtaaggttcatagattaataaaaagtcattgcaaagattgagcccaaaaagaaaaagttattgatt
ttattatccaacccacaaagttgtctttatcatcagcaaacattccgatatctatttatcatttgtattaaagcaatattgcttaaacgtttaattaatt
tgcaccgactgcaagtattcaaggacttgtggaggcaaccgatgctcaatcatagtactatacataatcaagttcgaagctataaattgtaagttt
gatattaaacctttctcagcttcttcaggatttttatgcaaagtattaaacaactctttgatactagacatggccataaacagtaagaaagaaacga
cattagtgatttccggcggtaatgtcaagagattaattgctttttgacgcaaaaaaagagaatttaaagattcaggtacatgttccatatttccatc
aagaggtaaaatatccattttcgcaatttcactcaaaactcccttccattctcccttatcatacagggtccttgcatccataacccgaagcagtaact
tgcacatgtctctattggtcttacttactttcgctgatttctcgagattagcttcatagatttccaaaagactccttgccagaacgtctggtgctccatt

agctacaacgttgggtgaggacttctgttgactctggtcttgagaatctgccatccacagaccgcgcgagagtatactctgacttaggtttttcaca
gcaacgccgatcacggtatcataatctcctaggaggtgatataagagaatggaatctccgtaacgcccatcatcatctgcgtttctagcgccttctg
catacaacgcttgtaatgagtcttgatcaaatggtactaacggcagatacaaatcaagtaaaccagttgttcgctcggtatcctcccgaatatctcc
caaaaattttgaaaagtcatgcgattgaagaagcaatattttaaccaagttcatgcaacacgttttatatgatgagacaagagatatacaggcga
tatagtcaacataaaccaatgtatctacggagccaaggtaaccaatataggctacgataagttgcgggtaattaatgcgcacgctatctgcttcat
gaataagaatattttcgtttttttcataattgcaggtatgaagaaggccatagtaggacatggcgatagcataatgggtagcttcaacggggtaaa
aggtatacagataatttacagcacgttcaaactcaccacagattaaaagggcaaggaaataatttacaggagtgctattcttaggattgaaatat
ctcggtccgaaagctacaatcttcttctgaagttgttgcagatcaaagttatttaccgagcgctgattcaaatcagaggtgagagaattgttagcat
tagctcgacaaaacattaattgcatccacatatagtcttcggaagtaatgcatacttcaggaagtgaaatacgatgaggttcgcattgaccaagg
agtttgtaaatagcatgcttaaatggatcttccggagcatatttcatgagttgggcatactcagcttgcaaacgatcacgaagttgcttgggaaga
ggttgatgaggatttctagcataagcattgaaatatgaaggaaaagaacgaccaaaactttcaaatacatgtgcattagtgctaacaaactctga
tgcgagatccagcagaccagcacgcataaggtaaaataagactgcccacacaggaatgtcatcagcaattgagatagttggcttaacccaaga
attattcttgaagaaccgtaaccgactgaaagcaatcattttatccaatggttttgtcgaagaaacatggccgttatcagataattgcgcatcaaca
aacgctaaaaactgaacttctaaaaagcgcaaggatccatggatgatattatactgaaatgaaggattcacattagtcttgtcactgctatttgcc
gtctttaattcgaaagcatgtacattatttgatttctcatccaccatataggctaataaagaccatgcatctaaaagctgaggatattctcgatcgat
ttttctgctagcagaagcaaataacgaccatacccggacaatgctaccatgaatgcgagcatcgtttaatttgtgaatagcagcagcaaagaacc
caaccttttctggaattccagacaggcttgaaagttgactacgggctgcttcattccctaacattgaggaagtttggtcagcgacatgcagtacgct
tgaggctggtgttctcaaagaatgcgctggatgtgattgagcaagtaacgaccccttaagagtaggcttggcaaagctagattgcagagacatgg
taacggatgcacccaaaacagtattatccttggagcgcaacatctttcctgtatcttgcaaaaaactcatttttcggttttcccaagcaacaccgct
atctttcattaattttatttggaaatcgttggcgcttgcttgcaggccccccttcaatactagcaattacattctgctctttatatattttggcgtgaggg
cgttcgtacaacgtacgcttttctatctcaagggaatcccattgagaatgtatgtgaacagagtaaatcttttggcgggtctgttctacattcatacc
actctctgcgagtaaataatgtctaatgtgagttagcttctcatatatgacagaacttccaaggcagacaatgaatacaggtttttttaatacaaag
aagaaaacttctttattcattagctttttttttggtcctctgtttctcggctttccaaataaaacctcttccttcggaaatctatacttacgctttggtat
cgccatcacgtgtggagacaacctttaattctaattttttgctcgctcctctaattgatttagattgtattgaatgacaggtacttgaggctttagca
aagagccaaacaaatgctgagatttttccgacaaatctgacatgcagctggagacttgcaatttatttgcatttttgtattttgagactctttttctg
gctccttcaacgccattaaaaggatttgtttacaaaaaatggtaaaggtcaagtatagtaataataaggcctacaactaccatacccaatcacttt
tcctatagtgaagtgtaacatttctgtaagtaatttaacgatataaaatgttatgcagtaataaataaatacagtaaaaggaagaaaactaagag
ataatccgcttttctttcagatctttgaatcattttggagaccaacctgtctttcctttgcatttgccgactcatattcatgaaattcatggtcgatgaa
acgagcctagttataagtatacccatatcttctccactgatttcaattgagcctttataattcaatgtcaattatgctttttttcagtgacctgatgtttg
gctatctccggttgtgcagcaccaatatcaagcgcttcaatccctccaagagcggccagtttagctattttttttattagcaataaatagctgcatac
tcttttcttctccgtcccttctacttacctaagtttggatgctcctttgctgtttcctcccaaaattcgtcttgtagtttctctacataccgttaattatact
ttcgtttggaatcaaacacgcacggtattgacaataaaaacgagcaattccaaacaacaaagaactaatgaagaataataaaactaggccttta
gaacactgcatatactgtagaactaaactcacaatgccaatttgagctggggtatacagagataacaagaatgctttaagtaatagcttctttcga
tttaggtttcgatgtcttcgtaagcgaaaatatgtttctagttctgaatttcttggactctgttcatgctttctctcatcaaaatgtaaggattgatgtct
tctatcacttaatcgatctgcgatttcagcacccgaattctgcctggcgataggggattcaacagtgtcgctttcaagggtagccttattcatatcct
gtctttttgatttatcttgcatgcccaaaaaccagtctcgttccaatggtgcggaagctgaaaatcccatagctggactttctattcttcccgctttgtt
cgaaaattcagccataattaaaattggaattgtttctattctatagcatacgttcgctcaccatttccgcttctataaagagaaccgcatttccgttg
cgtaactcttacttcaatttccggatcactattccatatccattaattaccaatttatttcccattactctttactttgcaatcagttacctttacacttcc
accaaaaaaatacttaaaaaggattcatgagtaattccccttgtaaaacaatttattcaattttaaacaagtgatcgttaatcatgtctattatatat
taaacatcgtataacaaaatttacaaataaaaaaacaaaaaccgcccattaacatattaattttagaaagcttaatcaacttcctcaacttcagg
accgttctcgcctccaggggcggcaccttcaggagcggcgccaggagcggcaccaggagcaccaccaggaacaccaccagcttggtaaacctt
ggccatgatggggttagcaacaccctccaattccttctgcttagactcgtattcgtccttggcagcagtggtgttgttgtcaagccattcaatggtct
ccttaacagccttatcaatggcttccttgtcagaggcctcaatcttgtccttaaggttggggtcatccatagagttgcgcaaagagtaggcatagga
ttccaagtggttcttggattggatacgagcagcttcagcttcatcctcagccttgtacttctcggcatcagcaaccatacggtcaatttcttccttgga
aagacggcccttgtcgttggtgatggtaatcttttgagtcttaccagtacccttctcgagggcagagacgttcaagataccgttagcatcaacatcg
aaggtaacttcgatttgaggaacaccacgaggagcaggaggaataccggaaagttcaaacttaccgagcaagttgcagtccttagtacgagca
cgctcaccctcgtagacttgaatcaagacaccaggttggttgtcagagtaggtagagaagacttcagacttcttggtaggaatggtagtgttacgc
ttgataaggggagtcataacaccaccagcggtctcgatacccatagaaagaggggcaacatccaacaacaagagatcttgagtcttttcagagg

tatcaccagtcaagacggcagcttgaacagcagcaccgtaggcaacggcctcatcggggttgatggacttgcaaggctctttaccgttgaagaag
tccgagacaagcttttggatacgggggatacgagtagaaccaccaaccaaaacaatctcgtgaacagaggacttgtcacacttagcatcacgga
gaacacgctcaacgggttccatagtgttacggaaaaggtcagcgcaaagttcctcgaaacgagcacgagtgatagaagtgtagaagtcgatac
cctcgtacatggagtcaatttcaatgctagcttgagcagaagaagaaagggtacgcttagcacgttcgcaagcggtacggagacgacggacagc
acgagcattaccagtgatgtccttcttgttcttacgcttgaattcttggatgaagtggttcaccaaacgagagtcgaaatcttcaccacccaagtga
gtgtcaccagcagtagacttgacttcgaaaataccctcttcaatagtcaagagggaaacatcgaaagtaccaccaccaagatcgaaaatcaaga
cattagattcaccagcggtgttacggtccaaaccataagcaatggcagcagcagtgggttcgttaatgatacgcaagacgttcataccagcaatg
gtaccagcgtccttggtggcttgacgttgagagtcgttgaaataggcagggacggtaacgacagcgtcagaaaccttaccacccaagtaagcctc
ggcggtttcacgcatcttcatcaataccatggaggagatttcttcaggagtgaaaaccttcttttcacccttgaattcaacttccaagacgggcttgc
cattcttaggaataaccttgaaaggccagtgcttcatgtccgattggacctcagcatcatcaaagttacgaccgatcaaacgcttagcgtcgaaga
cggtgttgtgggggttcatggcgacttggttcttagcggcgtcaccaatgagacgctcagtgtcagtgaaaccaacataagaaggagtggtacgg
ttaccttggtcgttggcaataatttcaacacggttgttggagaattgaccaacgcaggagtaggtagtacccaaatcgataccgatagctttgctc
attttactgtaatttaaagatcgttaaaaagaaaagacaaaaaatcaataaaaggatagatgaggatattaaattatatagagtatccttaatgc
aaaaaaaatatcgttacttagtaaaggggattaacgacaaaattcgttatatttttataatgcaacacaaaggactacagatgaagtataagcg
gggaatagcaaggtatttatttctaggctctaattctggaagtttcgaataacccttaccctaaagaaaaaaataagtacagaagagtatggtag
aagcgagtggaatttttcaaggcttgacgtcagcatctgtagaaagagaaagcaaatttgagaaagctcacgaacaaagacggataagaagca
acggcgtaacatctgtattttgtatagtacagtcgaacaaattggcttaagtaaccataaagaaaaacaaaacagtgcactatgtacaataaagt
agcttaagctgcattcttactcattaaatagcaatcataggacatgggaaagcatagagcatatagcagagaaagtactagtagtgtatgactat
ggtggtagtaagggcgaaaagaaggtgactcttctttttctagtggagtaaatctccaagtcctgtcattatagtaatgaagagtataataacaat
gctggcaggggctaaaagaatttttgtccttgagagtagcattcaagtttcctggtttctggaaaccactaaagagacgaatacaaacaccatgg
gttacgactgctagattttttgaggcaccttggcaaagtattcagtcaactgctgagtctttagtacactggtctcaaagtctgctttgtggattcct
tccttcttgagatataccttgtccttgtaacaagcagtagagtcgtgtctaggaaatttgctgtttcaattctgcagttggtgttccgacgttacattcc
tagtctccgcagtcttacaaagtaggaaggatctttaaggggacatgatcatctttcatataatcctggaatgcaatttgcatggtttcaatagccc
ggcgcaaaatgaagaacaaaaaatgatttccacaggaagttctgcagctcttaacttgcattgaaccttttcagcttgtttgatgccttcctcattta
acatatgatcgtgatctcgatgaacgccctgttcatttgtcttgtgattaactaatccatggcgtgaaaaagttaatagttttcatattgtcttgccaa
ggagcaaaagccatgtttccaaagctttgttcctccttactttgagatttggcggatgcccttttgaataacatcgctgttatttatattttacctatga
aatcaaggtctacaacacttagctatttcatgacaatacacttatgtgcaccatacgtgatgtgaatatgcagtaattacattttccatctcgtggat
ggaacactagaatggtgaaaatggatctcgaatcaaccgtttcattctgtcacttataaaaagcatttttatttgcttattatttgatttttattttcat
gaagaaagcatagaaacacgaatttatcgggatcatactaaagagttcttattgtttactaacaaagctctttgtaatacactcttctactgtacta
aacaaaagcgagaaggaattatggaaatagtagaaatattcataaaagaaatggtaagcattctataaaccataaccaagaaaaggaaaggc
aaatctaaatatctactcatcagcaatcttggcaagagccttaccaaagttctttccctgaaggacaccaagcaagccttcaggaatcgaaccca
agcccttataaacgtcgtacttatacttgagctttccttccgcaatcagcttaggcatttcacggaagtattcctcttggtaaagtgggatcaaattc
atgacaatgaagccttcaatcgtaatactcttcaaaacgatttcagtgtaattcttcaacctgtaaggattgggattgttatactgactgatggctcc
gcagaaaatgatgcgagcgtaaaagttgacgttcaagagaacggcatccaatgtttcacctccgacgttatcaaagtaaatgtcaatgcccttag
gacagagacgaggaagagcatcatgaacactttcatttttatagttgaaaactgcatcatagcctatttccttcaagtaggcgattttctcgtcaga
tcccgcacttcctacaacgtataagcccatagatttcgccaattggccggcgacttgaccgacagcaccagaagccgcagaaatgtagatagtct
cgcctgccttgggttttccaacatgtttgagtccaacgtatgccgtctgactgggcattcctaaagcacccacaaaggtcatcaaaggcaaattgta
aggattggaaataacttgcaccactttcatagcgtctttgggcacaaaagtatattcttcccaaccgctcataaaaatcacgtgggagccagtctt
gtattctggaactgcagacttgacgacttcagcaatagtcaagttaggaatcacctgtcctaaatccaaaggtggcagataactcttatgcttagg
attccgcatgcgattgcgaatgtaagggtcaatagaagtgtaaatgtttctcaaaagcaaaggatgctcttcatccaccttagctgcttctaaatta
aaaggacgttcctctaaaccaaagtgttctccaactttgggatatccttcgacaggatcaatatgctttttcaagataataccgttattcgaactcat
atttctaacaataattcaaagactgtattctcgtatacaggattttactagagagtaatacctcctatttatattcttctgttttctgcgtcattactatt
acatctctcggttttgcacaccagtcccaggccgatcggaagtttctttagaaaggggttagcgaagcttattgcactttaaacaagcatgatgtta
gtaagcaaaaaacaaaggaacaaaacaaggaaaaacagaaaattgaagtgtttccacttgatatttttaatcaagttcttactcctaaaactaa
agaacatgcctttattgtttccgttgaaaagcatgggcgttcgaaataatctttggttcaaaacttacagatgcagatttccacagattgcttactat
taaagtcgtaaagatcgtttcattccaaaattagctaattatatttttctttgctttaaacgctactcaaaaaagcaaaaaagcaagtatgacacaa
aaataacaaaagcaaactttctagcttgtttttaggtttcttgtccaaatatttttctaattttgcaatattgagccggcgaaaaagaaatttcccat
ctaagttcttggttcttcaaactttcttctcttccaatgagtacgctcaactcgtttctgcaggaattgactaaggagtcgatgaatcggtatgcttcg

aataatctcatatgtaactatggcaaaacccaaattaaaggcagcatagcctataaggataccaaaatttctccaccgatgatggtaataatacc
caaaaccatccaacagttcatctgcaattttgtacggacaaaactcacaacttgagacagcttccggattataaataatgccagcaccagaggat
tgtaaataatcaaaaaagtatgctccacaggtataccccataggaggatctaccacgtacatttcagtacgttcgcatataattgggacgttatgt
agattatttccaatcattccttcaaggaaatatttatatgggctaagatcattcatccatttccaaaatcctactagttggctaggcttttgaaccata
ccattgaaatattgcacaaacacgaaagcagtaccgcttagcattgaagcaatacccgaactaggagaaaccgtagcaatgcttataccgaggg
tagtataatacaaagcgaaaaccatcatcatcaaccaggcgtaaccaccataaaaaagggtattgtgaccattcaaataatagccacttggata
gtaccaacaaagaaagaatattgttccaaaacaacaattaatcactgcttccgtgatgaagaatgccaatataaaagcaggacggttataggtg
gcagagttctcttcatgatactcataaatgtcacgtaaatgaataaattttggtattaatccgttaataagagaagtagaagcaattgttaacatat
aggcagagaacattttattctgtgttctctgtattccattgccttgtttataaaacgtaaatccgataagcaacccagccaacgtattaaatgccaa
ccgattaatcagagctgcagaatctctccagtaaacggtaaagttccgcttcgctatcttaaacgtttggtaaaccgtactactcagatattcctgtt
cggagcgtacttcttgatcactcaacctgcgactcagtaatctgtctaactctggtataagacgtttatgcgtgtctgtggattcccatacagctgca
tagtccactttttttacttttgtttgaatagtatatacactttagtacatagtctgctggatctttgatactattttcatcgtttccatgaattttcttgaaa
tactttatcaacttcaaagcatagctgccaacttcatcgaggtagacagtccgaccataaccatcaagaatcattatagaatgaaataaattaag
aagtctcgagctaggttgcgaaactgaacacataatggataaacctgtatttgctaattttcgaagcgtagcaacaagcatccaagcggactgac
tatcaagaccagagacgggttcatccagaagaagaacagatcgtcttgcaactaactcgatcccaatcgctgtaagctttttgtgatacaatgata
aacctccacctgcatcgcccacaacgaatctagcaacatctaatagacctagaaaatcgataacctcgtctacatactcataccgctcggacata
gatatgtcagcactttgacgaagtgaagcgtgttgtcgaagagtctctcgcacagtatactggggaacaaacattagtttgttcgaaatataacca
atccttttccgtaaatatttggaggtttttatcccctccaaagacacttgtccttcaacaattcctgatacaccaagttgagaaaggatttgaataag
tatggatttacctgatttattttcaccagccaatgccattaaacatccaggttttacgtaaccattgagcttttgtaatacacgtttctgcactccatct
tccaaaatgtaaaaggacaaatctttccaacacagtgacaattcatccgtcgtcgttctaaaagacgtggacatgtccattgccctatgagctttgt
cttctttttctgctttaacgccgctttgtttaaattattttcccatgaactctgtacagttgcgtatagtttttcatttgatattcaggaatagccatac
acgttctatcatatctgatatagttggctactgccagtgatagtaacaagataaagattgcaaacccaaaaagaataccgcaatttttccacaaaa
agtcggttgtatatctgaaaacgacattcaaataattaatgcctatgatataaggaactcctggcacgccggaaggagaaggacatatattgttc
cgtagaggaaaacttaaatttggtataatcgccgaacaattgaatattctatccttgaactcgttcaacatgatggattcatacccatactgaacag
ggttcgcataggcaagccatcgaaaccaccatcctatataattgattggcataacagaaccgcaaaacgaaatgaaatagagagcagcgatac
cccctataaaagcagcaatctcagcggtgctgcaaagtaaagcaattaatcgaaagattgatgccatacaaaaagtgattaagaaggtgaaga
gaaaaaaggtaaaaaaagcaccagcattgactcttaaaccgctcaagaaataaacagctatatcaaaaattacaagagataccaaacgaata
ggaaattccactaaaattgagccaaacaaagctgccgaaggttgatataaggactgaatccgatgctcagcgaatagggactttttgtaaatat
aatacctacctcagacattgtttgaatggcagtaaatatgattgatgttgaaagtacactacctcgagaaaaaagggttgcagaggtgttttctat
gaggtaaaagatagagccaagcataaagccttgaaatagataggaaaaaacaaacgttaaatgaaaagaatagtcgtaaagatagtggaagt
acgttattttagcaaaggcccagacttgtaatctataaggaacgggacagggaatccgattcaagaagcgtttccactttacaatgggcgtattgg
atgagtcctttgtatgaaatgcagaaacgtcactatgatattccatagacgcattaattgaagacattaagtgtgtataatccttactttcaagcca
gcattgacggaattcagcaggagtagatggtataaaccctttatggcttttattaatggttcgaagcccggggtataacaaggaagtgacgaattc
accaacggtactgtgactttgtggaatgtaaccaagatttaaaaagtaatcttttacacgagaagtcggaccgtaaaagatttgctcaccttcata
taaaactgttaccatatcaaacatacgaaggattttgtcaggaatttgattggaaatggcaatcagggtcgatccgaagacgcgagatataatttt
ggttcttgaaagaatgtgggtagcggacacagaatcgaattcacgcagtgcgttttcccaacattgcaataacggccgtgcacacattctctctgc
gataccgatgcgtttctgatcacttccattataaattttcaaaattcgacgattaaaaatgctattcaattcaaatgcatcgcatataatctcacaaa
tccttcgaatcgtcttgtcacgagagactccgggctttttcgtttcagggaggcggcaagcacagataaactctaataaacggcgcacagaaatg
gttgcaaatgacgagtggtcgtcgtcgttgtgataacttaaatcagtcttgtattgactatgcattgtgcggtaatccaacccatcatagtttagctc
tccttctaacgtttgattctcttcaactattccactcaaaactttgagtaaattatgacaacctgatccttcatgtccaattactaataaaaaggcacc
tggatccaataaagcatcaaattcctttaccaaggggatcgcattacgttttggtcttctagaaaacagcacgttcaagggatacatcagttgacc
cagaaaagaagtaatcatgagccgttcattaaattttccattgatcctgaatcttcgcgcgcaaaaccccgtgactcggtcacgaattccaaaatc
ctccaacgtttttgcacactctcgtatccttgcttcaaattcttttgctgattctacatcttcactgacaggccctgtggttcctaccttttttgattaat
cttcttcatgaaacttttccaagttttccgtcagatcaaactcaaaatactctctcacctttttgcctttatgaacatgcagatggcaaaagtcttgaa
attgaaaagtaagatgacgttaactttgctgctctaaaagagtgctgcgccctgtcggcataataaactaccgagactcatcttttgacaggtattt
tacactattctattttgttttttttttttcaaactcggaattgttaatgacaaatagaactcacaatatgcatttttattagacaaaagctttgttcatgg
ttttggtggttagagaaataagggtttcgacattgctttagcaaactactgcggactacacgctatctatcttcctattgaaattggattgtataata
ggtgaagtttaagagggaaatgtgaacgaaatctataacaaaaaaaaaaatgaaatattacttaattatgcaatcaaaatataagtagatattgt

aaagggtggaaatcatagtatgtaaaatgattgatatttaccaggttatagtcctgtagcttgcaaaagttctgttctaatctacgtcgatgtcgaa
gaaacaaagtttgaattgattatgcgttcctgatttcgcaagtgcactcaatcttttgatccttacataacgttagaagaagcagcctttattttctcc
acacgcatatatgctcacactaaaaatccggataggctcttagcgaagtatcacggctttttatttcgaaataattcagtcaaaagtacatattttt
ccctttcttcagtaaaattcaagatactaataaaaagaaaataaaaaagcagggacgatgagagtttcgaatcttttgtcgactgctcatagttta
ttttatccgctgttatttctttctttcatataagtaactgaatccaacatggttgtggattttactttttacaaaacatttcgtcgaaaacgaaaggtg
attgaatttttttttttgaacggtctcaaagttcgttaatatttgtattattattataaaacaagcacacaattatttagactgtcaattgtacgtcca
acaatggccaaggaaagatgacttttaattccattgaacataattaaattaagcaaagtataattaattccgatatttagaaattttaaatttagag
tatttgtattcttaggtatttgacgttgacaaaaagaaacttcaagtctatttacatgatgatgtcgggccttagtctaatcaactgctatagcatat
agaatgttcaaatggtacataaaaaagtatattgatattaaggtactattttaccttgacgaaatcaacaggattttctccattaaagttactaacg
aaaagcgatcagtgaatgtcagatttaccaaaccttggattcataaatcgcgtgttttgtaacacaacaccaataggaagaaaggaagaataat
aaacatatgtgcagtctcattacaaggcagattaaaggacaaaacacaatttcaaaaaatatccatatggttgtcatttgacgataaatcatgtat
caaatactgaaatctattaaaaagtaactttttcttggaatttcctttcacatgaaaccatcaacgcttcatctacacttagcttcgtcactagaatc
atagttgagctataacttctatattcattaatcgttgtagaaattcaatttcatatccaggtgtcgaacttttcacgcctgcggatcgagacaatcaa
acaaccgtctcttatcgctcaaaatctggagcattaaggtctttatttttcatagtagtgacttgactttttccccagatccgacataaagcttagttt
cgagcacatacaagatcaacacatggttggttgaacataatctaaattccttatgttcctttccaattggcaacatgttagaatgaaatagcaaat
cctattataacactgacaagaaagtattgcacaataaagtcggtataaaccctaatccttcaagtaaaagcaacaagtaatccaaacacaagga
ccaaaacaacagatgatgacaacattattcagcacttaaatcacatttatttaagcgtgctttgttttcacgggggtttttttttcaagaaaatagcc
attttaggtaaaaactttctcgagctcgttttggttgcttttagtaaaaagcattgcatttttgcaatttaaaaatctatgatcaatagacttactttat
tccataattgtatcattcatacaatgtctcaaaagcaagatacagctttcctaatcactgcccttaaaggtactatccattggaaccacctcatcatc
gactggttgtcaaaatcaaattcaaaactcttttgggttcctgataagccttctgaaacatttttttatattaccttagaaaaacttcccggttttgccc
acgcaaatatatgtatcgatatatatcaacagaaatttcattgagcaaaaagtacatagaatagttgaggaaaatgctatcgttttttttttaaatc
aaagaaaagacagacaaagatgcttagacattcttaaaattagacgccttttagattataacaacgacggtagattaatgggggcagaaggac
aagcataagagaagagtagaaagaaaaaaaaaagaagcggaggaaatgaatatgagaaattaaccctataggaaaatagatgaaatgatg
atcaaccatattaaattggaaaaacaacctatacatcgcaagaaacccaaagcactgtagctattctaaatgaagtattcacttcttttccgactt
gggggaacagttctgtcttcatccggtacagcagaaaatatgcgaaaatcaggttctaaagaatcatctactttcataacactcgcaacattaccg
caccgatagcaatagttcggagcactccaaacagtaaccagattttttttcatcaaaatgatacttgtatccttcttgaactagctggtgtgccctag
caatcagctttaaatcattgatgtgactaaattcgctagtgacttttgaaccaaacagccagccagcaccacggggcgatacagtccaagactca
atgtcttcgggatccgaccacataaggtcacaaaacgagccttcatgaggaatttcttgagcacgagccagtatacgaatctggtctaatgtacg
aacttcaggggataagccaccgtgcacacatagaattttgttgtcaatgattgcagccagagttagaaaatcaaatacttggcagcagtatttcca
gacatttgcattaccatacttcgtttgacactcgtcataaaatccatacacctgtgtaatttgtctactttcgtgatttccacggagaagtgtaatttt
atcaggatatctggctttcaataacataaacaatgtaaatgtttccaaactaaaataaccgcggtctacaaaatcgcccataaagatgtaattagt
tgacggtagttctccactgacacggaataattctaaaagatcgaaaaattgaccgtgaatatctccgcagacggtaacgggagttcttacgggtt
gaatattcgattcttccatcagtatttccttcaccatctcacataagcgttttaactgatgttctggtaaatatttacattttcgaaccgttgcaatcca
ttcatctaaatccaccatattgaaacgaccttttttcttttttcagaaatttattattcttcgttttatcgcttcttttggcaatactttgttttcttttaatgct
tgttgtaggcaaaggatacgtaagttgtaggatttcactgtcagtctagataggttaacgaagtcaagtgagatctgacgagtttttaaataaata
cttatatcaactcgttcgaaagtctacttattcatataagaaaaaacaattatcaaatcgaattttaaaaagcttattactaaatgtatagagattc
aattatcactcagatgatgcagagaacgtaaagtcagcaagtctagaaatgcctcttagtaatatcttttgaaactttttttttcgttcaagccaaca
acgacagttgtttcaaggaacttcatttatatccctgagaaagaatacttttattctagttctagaaatctttttttattttttatttttgctcttgacaat
gtttggtgtaaaaatgtttgctaacggattgtaccgttgccataaaattttaacgatacatttatcgttcgctatacgaatgaacgagagcgacgat
tctattttttctgtcgataaaaatgtcaataaaaaataattatatcttttcagcaataacaaatgatcctttcattctagagcttaaagttaatcttttc
tttctttattatattttgtaaaaaatgaaagaaaatgaaaaagcagttgctgctcaaaaccttaggctcattgagattgatctatttacataggatg
aaaagaccctcgctaaacttggcagcttcaccttgagatttaatttacctttcaatttcaaagaacctttatttatagcttaaaaccggaaatgctat
gcccatttttagctgtttctttattcatttacctttgaatatttctacagatgaaaaagtgtcgatatcatattaagcagtaggattactgctactagta
gaataccaggtaacgtagagcatgttctccttcggtaaaaaaaaataaacaaaacaagagagaagaaaaatcgcacgcgtataaggagaaac
acaggaaatataatatgttggtaacaaaaagatgtttctgctgtaaaaaatcaaccaatttcccgtaatgtcttaaccctcccgcaacgaaagaa
gaagaaaagtaatatgtcgctcattattcaagatacctcattgcataaaagttacctttcaaatttttgtacttccaacaacagtacagtctgctcac
acccagggtttgggatgaaaataaaaaacaaagaaaatcctatgcagttgttcaaaacacttcgtattttgagtaatacatcttcgaaacagata
aattgaaacattatagaatttgactccaatgctttcatacattgttaatttctttctataaactccaaccagtttgtttataaacatacaagtttcccc

agatgcaaggaataaacaactctcgttaacatacgatatgctttatagtgaaagagaagaagtatcaatataatacacagaagcacgttatgttc
tagcagccaaaagggatgccaaccaaacgtaaacaaacacgaaacggcgtaaagaagacatgagagtagcataaatgaatatggagcagct
gtggcttgaacatgatctaagcgaagaatggattccaaagtcgacttccgaagatactgacagcaaggaggaatttcagtcttctactagtgcta
cgaatacaaactcatctaagaaggtaactaccgctccttccagtgagccaggaacctttaaaaagggatcagaatcctctttagatgttccgcaat
ggaaacagattaacgcgaaaaacccagtggctcgtgacatttttgcaaaactagatctagaaaatctgtttgatggaggcggtagttcacgaaat
cattccagttcatcccaaaaacgaccagactcctcttctaacccaaatcccccgagttcccccttgcatccgtctcctctgacccttcaagaagcta
taatatcaagtcttttcctaagcattctttttcttcacaaacaaagcattctcctagtttttttagaaagctctcctccgaccacaagctcccaaccccc
tattgtctcaggttctacttcacctcattctcagacgtcttcttcatctgctaagcaagaggcttctccgcattcctcccgacagcggcaatcttccttg
gtaggttcctcaaaatcttcttccccttcttccctagcttcttccccttccgtccagaaaggtaactcttctcccttaaagctttttcaaggtgccagcg
atccttttacccgtgaacatctaaatcaacttgcacaggatgttaattcaaattctttcgaagcacctgcagaggattcatttcctttgccgagctcc
agacgagtttccaaacctgcgcatactaccgagcgaaaagccgggcttaatactaaagatttataccaagaagtagaagaagtcatggcaaag
ctccgtggacggataccagctacaagtcgtgaatcaactgtttttctgcctcgtaaattaagcggtcttcaagaggaagaggaacaaaacgaggc
tagtgaatttagcaaagatgatagctctaacgctttcccttctcttagtgatcagctgcacttgcgtgagttagaaaaaaagcaaaagcttttggat
ttacaccgaaaccagccagaaagcatctcgaattactccccgccttctcttgttcaatcatcgtcaggaacaaaaggtgaaaagcaatcttccttt
ccttcttctgatagccgcatgaaagactctcttaagtcaaacaaccgaaaaatacgaaatgataatcatactcaaccagactataaaccacattc
agaaaatccaaacatggttgttatcactccagcagacttacccgacggcatcaacacgacccaagggtcaatggagtatgatcgtgtacacaatc
gctggcgccgtcgcggtcatgactctgatatcgcttttgattttggttctgaagaagaggaagatgatttttcatcaaatctcactgtttcatataag
gctgctcaaggttctagtcctccttcaaataagaaccctaatgttcacaaaaagcagccgaactcttatcctatgaggaaagagaataataaaga
tcctaaatattcaacctattctcttcgtgatgtttccaataccccttctcacatcgctttttccttccaggattttctaccacaagatgatagtcatgat
gatttcaatcatttacttgccaacaattcatccaatattcctgctgatggtccaacctccacctttacttcccctgagaaaacccaagatgatcaagc
gctggatgaggattacagcttttcaatctcaaagcaatctatcactcgtttacttgaagatgtagagccgtatcgtccattctggaaacggataatt
caactagacataagtaggaggggattagattcccttattggactttctgagcaatgcccttgtctcgaagacctcaatatagagggtaatgagtta
tcttacttgactggaagtccttcttcggtgcgaaatctaaatgctgtcggcaatcatttatctagtcttactactttttctcacttgataaatcttcaat
atttggatttatcagctaatcagctagaagatctgactgctctctctcctttgattcatttacgtgaattaaacgtggataataatcaattatggaac
atagatggtatattgcgtttagacggacttctcaaattgagcgcacgcggaaatcgattcaagcaactatcgttcacaaactcaaatttaaacaga
ttggaagagttgctcctggggaataatgcattggaaaacattgaagaaatcagctctcttcaaagtttaatggtgctacagctggatattaatcaa
ctcacaactttgcaggctagtcaaccgatgattcatctgcgtgttttgcgcctaaacaacaatgctatacagcatttagaagtggatcaattcccta
atttaaggacattgtatatggattataactgctttacccgtattcctgatatttatcggcttaagcgattgactaatttctcttttcgatcccaagaatt
caaatctggtacacacatgttgacacctcctttggacataaggaacctatatttatcgaacaatacttctatagtactccaaacccataatatgtta
ttgggaattaaatatttggaattagcgaatatacagataacaggattgcctcctgactttgcaaagagcttaccgaaccttcgagttttggatctgt
cacacaattatataagtgaaatttctgctctgaaagggttttctttattacaccgcttgtaccttgtcggtaacagaatcgagaagatgaaaaacgt
atgcgaaactttgtctcatctgaaaaagttaactcaccttgacttacggatgaatcctttaacgttcaacatttatcctatgattgatgaccctgtgta
tgaattatctgctgcttctaaataccatcagagttttgaccaatcgtctcaaagaacattcagacatccaaaacgaaaatgggctgacaaagacc
atgcatttattgaaaccatgtcagagtcgggaaaaaatcgtcggaaaatgtatacagaagcagttactctatcctgctctcgcctagaatggctag
atggattggaacttgaacttattggaaatgataattctatattgcattctgtgcttcatgaaatttaatagctttcaaagaaactttttggttagtgaa
cgtgttggttaatatattacgatcattctttttgtgaagatacgaatttttgtaattgtaaaataaattgaatgtaaacgacataactgaatagtttt
acaaatagtcaagtaatgtcattgaatgataaacatacataggcaaaaaagaaatcataaaaaagagattaatgaaaatcaacaaccaatcac
aaataagagcacactttgtttctaaaaatagatagagaaaaaccacttatgtatataaataacaaaccacaaaataataaaatgattacttaaa
agtagaagataacaatagaaaaatacgaaataaaaaaacagtggtgaaactcgaaaagtgatatacgcacccaaccagcatgttcaaaaatg
accctgttaggaacgattactttggaggcttaatatcatcgatagaccaaggatttggaggtggatccttgaaagggttttcaggatctctaaatgg
attttcgggagcattgttttgcttgttgctacgcataaaaggaaacctaaatccagaagattgaggagcaggttcaggagcataagaggacatcg
agcgactgtcaccacttaacgtaggcttcaatttcttgatagaatcaccacgtttgtgagaaagtccgtcgtattcaggaagggtgtcataaacgg
gtttttccatactataatcagcaaacttgggaaggtcgccagtacgggaggaagtagagaaaagattgctgttgttttgaagagaataggggttg
taagccatgctatcagcatgagagcggcgaatatacttagattgagccttgacggcttgtctgcgagcaattaacaacaataagaggacgataa
attgaataccagcgttagcaagcacaagccatgaaagccattggatgcgagggacaaaggttattacacagatgacgaatgcgatagcagttat
aaaaacattaaagaaaagaatactagccacaaaattgaaaaagccgggggaacgggaaatacgaccaatgtaagcaaacaaaagcaaaatc
gtggcaagtgcacaaatgcaggcagagaccggaatcagcactaagccatatgaggctttttgacgaacagtggtcgtcgtgcggaaaccttgag
aggcatggttgttcaagtaatccagttcatatccgaccatagtattactgcaatttgagcccttacagtatccgaaggttccaaagataacatcatc

gttcttggaaagaccaattttttggacaatggagcggtacagattgccaaaacgaaaaacacgatggtcacaagcagcagaataagggaagt
ggtggcgctacggaacttcatatttggcagaaaactttaggggataaccgggaaaagaatgaaacgtagtggagagaatagaaacgaaggatt
ttttttttataaaattaagattaaaatttctaaatgaatcagttgtgatatgcaagaatgaccttcaaaaaccggtcaacgctcttgaaaaccgcac
ttcacaaccgtagactgtcggaggcgattgaaaaattcaaaaagccacctttgtaatgaagaagagaaattttcaatgtacgaagcaaaattcgt
tctaattcaaaattatacaagaatcgaccgaatttagccttggaaacgaaaaattcaaaggttaaattaagaaggaaaatccaaatattatggat
tttgttgaggctcgtggtaggttttggtcaagaatatttttgggtggttggtcgtggacaaaggaagtagcacgtcagttcatgaactatccttaccg
aaagtttattgtaaatggttgtggagatgcatcacggataagccacggatggatatagggaaccgcggaaaccacaaagagagagagagaga
gcggacaggtgaagagggagtaaacaacccccatctccttgaaatttcttgaaaagtagcaaaagttgactcaattcgatcatgaatcgctccgt
tggacgcatttgccaaatctctcgtggattgtttaccaagaaggctgtttctccggtttccagagcctacagcagtgaagccaaattgagttggttg
gaagaagtcgaagctgagcaagaacacgccaaacgctccagcggtaggttcttttgatttgttaagatgaaaagaggctaacataaatgcagaa
ttctggaagaaggtgacctactagtaagttcttcgtccacgatttctataaattcgttcccgtttattaatctgtgtttgattagtgtcggcggtcctgc
tttggttttatcaagcttgaatgcttattacatttgtaagtgtttctatcggctaagagaagagggcatcttttggtttacccgtctttttcgttaacgcc
aaagctttgcatttctgttttcaactatttcttcattttctatctatgcatgcttaaaaagttactaacaacatgggttagacaagaaacacaaagaa
cacgccgagcatcatcatgatcctgaaatcgatgttccttatacgtaagtagagcagcagtgctgtcctgggaaggatggttgattgtcaatagca
gcactctaagaaggccaatcactaacactgagtttttagatttgaaaacttgcgtttcaaaaagtacccttggggtgacggaaccaagactctctt
ctggtaagtattaacgtagcatagccagtatcgttagcagcctgcatagcatagaatagcatagcacatcctagctagtataagcgggtcaattat
cctaagtgggtgatggtgtgcttatcaacgcagctttctttctaactggatgataggaatgacaacgtgaatcaaatcaagggcaccgactaaaa
agaaagggaaaacttttctttcttgtttgacaagctgtccttcttttcagttttcttttcatatctgtgtttcgttgtgagcctttgtaatatgtttcattgt
gaagcgtattctatgaaaggtaatataaagtttgattgcatgctttctttgaactttttttttcaattaggtagttcagaaataatacttttactgaat
tccgcgtgattcttttctcgtctaatcaagtattattatttcattgtttttatgttaagaagaggaaggacacaccaatactaaaccagaactagaac
cagaaccagaaccagaaccagaccagaccagagtagatagaccattttcggtatcttgaggggaaattccatgtcccgtgagatcgaatgaaat
gaaaagggtcagttcatttcaacagcactttaaaccctttgttttgatttttgtttattcacgttctttattgaaaccaatttgtatctactcaaccttat
tctatcttactgaacctttcctttaaatgtcatagaatgtcgtaaacagatttgcgaattggttaaacaacaccacgagagtgacgtgtctatatga
aaattttcgcagttttatcataaggagatatgcgtggaaggaagaattttgaatggagactttaatttagggtttattatcgaattcagctctcctttt
ccgctttttttttttctttttttaaaaaaaaaaaactaatttctgagaatgaatctttttttcatttgatactctcaaaagagacgtaattggttacgtacc
cacaacagacccgcaacgcatacgtattcttatggaaataagaatttgcggtccaaccttatatcataatcagaagacataaatagttcgctgaat
cctctacattgtcgcaagcattaatcaagcttttatctacacttgagttacagaaaatatcgaaatgacttttcaagtaaacgaagtacccacagac
cttcagaacagacataccttagacctctttagcctgaaaggaaagaatgcagttgttttcggcggaagcaaaggtatcggtttaaatgttgctaca
gcctttgttcaagctggtgcgaatattgtgatgacttatcacagtactgattgtcaagacattgctacgaaattggccaaagaaaatggagtccgt
gtggatgttgtccaatgtaacgttggctgcgaggaatctgtagaaaaggcttttgaagaaatcaagcgccttcacgagaaaatcgacattgtcgtt
gctaatgctggcattgcttacagaagagaggccactgaaatcactaccgaagagttcgaacatgtcatgaatgtgaatgtaacgtcgattcaccg
cattgcatctagatcaggtcaaattttcaaaaagcaaggatttggtaattttattgctacttcctccatgtcgggatccattgtcaacactcctcaaa
ctgtctcgacctattgcacttctaaggctgccgtcaggcattactgcaaggcattggccgtggaatgggctggctttgctcgtgtgaacactgtgtct
cctggatacattgctaccgacatgcccggatatcaatttagaaaggaatgggcgccttacgttcctatgcaacgtattggtttgagcacggaactg
cgtggtacatatctattccttgctagtgatgcttctacttttgttactggtttagacttgattgtggatggtggctatactgtcatttaaggcggattac
catgccaccgcgcgccattcatggactgtaatgaaataaatgttatgaaggaaacgtaactcttcaaatattgacaaatgctatgaatttgttcca
actttttatattttacggatactctttattttttttgatcgtggagattgcaacccgaaccttcattaacgcttgattaatgaagatccaaacatgatg
ttagtatgagcaaaaaacccctagaattcattctttttttttagaactttttgagatgatatttagactgatgcaaatattttagcaagatgtgacttg
caacgagtgagaaaagcaacatatagaatgttgctgaaaactgacatagaggcgcgacatccgaactggcacatcgcgatttacgataggcctt
gggtttacagaacccttttatttgtatccaaaaaatgctgaaagtctgcatattctctcatgaacgaggaggctgtttcccgtttctgcttctgttaaa
aaaaatcatgagtactctgaaataatggccgttattagaagatttcgaggccaagcttcctgaaactatagttataaacaattgatgtcaaataac
cgtttcttttttcctttgttatacatatagagatagattacatataacaatgatatgagattcgtcgccataaaatacaggaacgtaagcaaaacta
atgaaccacaattcctaagtaaaacgcaaatgtttcaagtatgatgcttgataatccgagaagcagcttcttttgctactttaggacgctggtcatt
cttctcttctgaattaggctggacttgcttttcagcgtgttttatttttccaacgtattgcttgactcgctgtaactcagacatgattgaagcttcatttg
tagaagcaccatgaagcttgtaataagaatacagagcagaattaatggcatagctaagacttacatactgctttgcttgttctaaatcatctttttc
ctcgacaagatcgaaaatagattcagagtcttttaaaggctgcaatatctcttccacctgattcaattgttcatttaacctctcaaataagtgagaa
tactcggaattcattgtatttttttgagatttgacaacgccaaagtagtatagaaaatttgtgtataggatgaatagcgaaacacttgtagagtccat
aaaaggaacttaaaagaggttgtcaaggtttgttagcaaaaagtaatgtcgttcaacactcttcgttacagcacaataccgaatttgtattattttt

attactttgtttaaactaccctatcccagtacgtaatctactatctgattgaaaacacccaatgacacccaagctgagaatcaattgcttacagtca
aagaaaacggaagaacttctgcatcaaaagcgaaattatattagtcatcagcagattagaatgccctttttacgtcaggcagctaaccagattcc
tggacattattaatatatatattcacgaagtagaactggagtcgttctcatatttgttggcctaaatttttgaatcattcaacttaaccaaatttaaat
tttggttggcctagcttttatattttacaagttttgaacgaattcagaaatactgaaaatgccagctataaatggatttcctgttggacctattggtttt
gggttgatggggttcacttggagaccccagcagacccctgatgagcaagcgtttgaagctatggactatgctgtagaacatggagccaccttttg
gaacggaggtgaattctacggaatgaatcctcctacagctaatttggatttgcttgctcgctactttgaaaagaggccagaaaatgctaacaaagt
ttttttgtctatcaaaggaggtttagatgaaaccttgagtccacggggtgatgaagagtctgtttgtaaaagtgttaggaacgtccttactcatctcc
gcggcaccaagaagcttgacctttttgagtgtgctcgtgtcgaccccaacgtgcctattgaggatacaatgaagactctcaaaaagttcgtcgatg
caggcgagattggtagtgtcggtctaagtgaagcatctgcggaaagtattcgtcgtgctcatgccattcttcccatagctgctgtcgaagcggaat
attccatgtggtcacgtgagattgaggagaacggtgtcctttcaacttgcaagagtttgaatattcctattattgcctacagcccctttgctcgtgga
ttacttacgggtgccgtcaagacaaaagatgatttagaaaatattgcaaaatcttttcctttcttacaattcttagatcgttttcagcctgagatattt
gagaaaaacctcatttgtcttcaagctttggaagcgcaggccaagtcttacaaccaaacgctttctgaaatggctttgagctttttgatcacagtag
gtgaaggcaacattatccccattccgggagccacgaatgcacaacgcattgagcaaaacttgaaggctgcttccaaacctttatctgcaacccaa
gtcaaggaaatctatgctactttggacaagtttccagttgcgggctatcgttataatgaacagatggaacctacactttctgtctaaaattttcacg
actaccattgtacgatacaagctttactaatatattgaatctttacattttattaaagatgtatttgtttaacttgtacaaatcgtagactgtacatg
gctcccttactaagagtctcttatatcgtacaagtttttcagtaattgtataagtaaacaaacaaagaaaagaccaaaaatgaaatcaatcccata
ctccagagtttcggtaatggaaacgggaaaataaaatatgtttttcactcatttttatgagttaagatgccgcaaaaaaattcgcatttttgcatc
agccgggagtcgaacccggagctgatgcttggaaggcatccatgttaccgctacaccactgatgcttacttggaaggttaaaatttttgatatata
tatatagagtatatgtaagattcaagcgttggcttatcccatacttatatgttctgattggccaggatcaatagaaacttaggatatcatgttacgac
gttataagtgatgatattcaaagaccaggcttctccagaggaataccttcgctttatatctttttttatttattaccaagtgtctatttactactgatt
attacagaccccattgatcttttgttagtacatacaacctcaaactgtctgatacaatcattgggtaaatttgcaccattagcgcttgtgttttcgacg
tgttaaattctcgtgtatcccaagcagtttcctatttaccctacttggaatacgttgtagttcattaaatatattacgagaatcgttggaaagtcaaa
aagggttgggttacttaacgaactgtgttggtacatacaatttggtttacggtgttgtaaaaatttctcagtatccgaaaagcatcttacaaaacaa
gttcatagaggagattgggaaaacgctgaaactggaacgtaaagaatactgtctctattttatttgtgtgacttagaggagacgctttttgcgtgtt
tttttttggaacaatttgagtgcataccggttaagtacgctcaaaggtaataaattcaagaattggaaattggaaaataatttttttggacgacctt
atcaaattctgcacgattttacttgtgtttttatacataggtatttgacgagaattaaagtctaagcatatcagagtgttggttttcgtaccatttgttt
cgaaagaaaaatgcttatttctacctcattggagaatgccgctgctattgtaattggctctgggaaatcagcatgcaaacgagtagacctttgcttg
tcaaagggtgtctcaaaggttgtttggttctgtggatcaacctctactggaggatttgatttgggcccatttcaagagaataaattaactattgataa
ggtacatctagtacccatttgttctttcgacccttctacttctttgttcacgttaggcaatgctctcactagctatattattgacatagtcttcattaccg
atacgaaatttccgttaaacgagaaaatatatgacctctgtaaaagaaaccgcattcccgttaatatcgtagattgtccttccctatgttcttttact
cttccggcatgttggtcagaaccacctttacagatagctcttagtactacttcaaacggttgtcgaattgcgcaaaggcttttgcgacatgttgttag
tagtctgcctcccggaatcggtgaagccgtacgtcgttttggtgttgcccgttcctctaccaagaatccacaaaagagacaatggattaatcacgtt
agtgatttctggcccctcgaaaaactaattcgtatgtctgaagaagatattctttctgttatctcagatgattatatgctacctctcacctccagtgag
ccctctttcactggtagctcacggacaagtatgtcctctcaagaagaccttacaaaggaaaaagtatctcttcctttagaccccaatgaatttccta
ccaacaaaaaaggctctatcgtgcttgtgggtagtggaccaggagatcctgagcttttgaccattgctgcccgaaatgctattcaaaaggccgact
atctcttagccgataagttagtacctgagtctgttttaaaactcatccctcgtcatacacctttatttattgcgcggaaatttccaggaaatgctgata
aagcccaagatgagcttcatgaaatcgctttggatgcattaaagcgaggtgactatgtagtgcgattgaagcaaggtgatccttatatatatgga
aggggtggtgaagaagtattgtttttccataagcatgggtatgttccaaaagttattccgggtatcagcagtgcgctgatggctcccgtttccgcgg
gaatacccataactcaacgaggagtagcagatcagtttctcgtttgtactggtacaagtcaaaaaggtgctatgccggtaatacctccatttgttgc
ttcccagacagccgtctttttgatggcgctacatcgattggatattctcgtcgatgctctgttacagaatggatggcctaaagatcttcctgtctgtat
agcagagcgagtctcttgtcctgatcaacgatttattttctcaactttggaaatggtagtcgaggtgtacaatacatacgaatcacttcctcctggct
tattaattactggatacagctgccaagcgttaagtgaagttccactagtgagcaggttttctcactaatatgctgttaataatgcttatggtttaaat
acaaaaaaaaaaaaaaaaaaaaatagaaagaaaagaaaagaaaagtgatattgacgtttttgtctgatactcgcaactcaagttcaagtgctc
ggttcctctttttgttaagtactctattgatggtattccctcctttttgtacagattagcttatctacttacttggctatttaatgaaattcgggtttttagg
ggaagaaaattctttagtctaaagtatcaatgtcttttatgatttactataagaaaaaaaaaaaagttgccttctttgccttttgatacgaacgacatc
aacttacctaccattcgatataagagatcctaaagtaatggtgtttttttaattcacaaacacccacaggagttgaaataaattgattctttatggct
atctaacaaacgctacgtatgacatataaaaaatcacaggcttcattatttacagtaggtggttccagtatgttgcagcagtaaatttgcttagatg
taagtccaattgttcatccaactttccctcttgggttcaccttcactcactgcttttgtcaagatgaatatcaagtcggatacctcgttaacataatta

atataacagaatgttaaggaagtatgaataatggtcgtaggctcacaggggtcatcccataatacaaaataaacaaacaacaactcatatgcaa
tggattttcaactgataaggacaatcaaaagcgaaattatattactcatcagcagataaagatgctccttttacataagggtagataatcagatcc
ctgaacatttttgtatatatattcacgtagtacaactagggttgtttactttagtcggcttacaattatcgaatcactcaaatcaaccaaataaagag
tttggttggaacgaatttcagaagtactgacaacatttcttgacacattacagtttggaattgactttataaacatgctagctataaatggatttcca
gttggacctattggttttgggttgatgggattcacttggagatatcagcagatccctgatgaacaagcatttgaagctatggactatgccgtggagc
atggagccaccttttggaactcaggcgaattctacggattggatcctcctacagctaatttggatttgcttgctcgctactttgaaaagaggccaga
aaatgctaacaaagttgttttgtctgtcaaaggaggtatggatgaaaccatgagtccacggggtgatgaagagtctgtttgtaaaagtgttagga
acgtccttactcatctccgcggcaccaagaaacttgaccttttgagtgtgctcgtgtcgaccccaacgtgcctattgaggatacaatgaagactct
caaaaagttcgtcgatgcaggcgagattggtagtgtcggtctaagtgaagcatctgcagaaaccattcgtcgtgctcatgctgttcttcccatagct
gctgtcgaagcggaatattccatgtggtcacgtgagattgaggagaacggtgttctttcaacttgcaagagtttgaatattcccattgctgcttaca
gccccccttgctcgtggattacttacgggtgccgtcaagacaaaagacgatttagaaaagattgtaagttctcttcctttttatgaacacttcgatcgt
tttcagcccgaaatatttgagaagaacctcgcttgtgttgaagctttggaagcgcaggccaagtcttacaaccaaacgctttctgaaatggctttga
gctttttgatcacagtaggtgaaggcaacattatccccattccgggagccacgaatgcacaacgcattgagcaaaacttgaaggctgcttccaaa
cctttatctgcaacccaagtcaaggaaatttatgctactttggacaagttaccaattgtgggtcatcgttataatcaacactttgaacctacactttc
tgtctaagattttcacgactaccattgtacgatacaagctttactaatatattgaatctttacatttttattaagatgtacaaatcgtagactgtacat
ggctcccttactaagagtcgcttatatcgtacaagtttttcagtaattgtataagtaaacaaacaaagaaaagaccaaaaatgaaatcaatccca
tgctccagagtttcgatgatggaaacgggaaaataaaatatgtttttttcacgcatttttttatgttttaagatgccgcaaaaaaaatttgcatttttgcat
cagccgggagtcgaacccggagctgatgcttggaaggcatccatgttaccgctacaccactgatgcttacttgaaagtaatgtagagtaaaaaat
atataattgtaaattacttgtttactcattatatcattcaatatcaacttgcctctgaattcaatgtcggtacaagaaagcagaaataattctttatat
atagtaagttattaagattcactgaaatttctatatcctgtgctatcatacgactaaactaaatgagaaaatggaatctgttagtttttctgcgaatg
aagaaatttttataattagtctctgagtaaactctgtcttatactttagaagcttgtaactttcatataaatgcaattgaccctgtattttaattgact
ctcaacatcaaacactattcaaacgtactatagaaaaatatctttgcatattttttatttatttttttgtttatttttttgtattctctttgaaaaacttgg
ctacctgggaatgtattacttttatagtgcttgcttgacaatgctgttcataataaaaaaataatgtgatagactagccgaaaacaaaaattgtcat
tgattaagaaaagagaacttgaaatggatgagtatattactacacatagagatttttttacaaaatctattagtatgaactacgataatagtcatat
attgtttaagcatggagctgatctatatagtcgaagaggagggagtaaattttgacatctaaagaaattgataatagtcagtagttggttttaaaa
agattatattatgtacataaatgattcatctttgtatttgcaaccttaggtatcatggcatgggttgcttcattgtaaacatagggacgtaaggcctc
aggtacaaaaagatattaagatatcttctttggcgaatttgatcaaacctaaaacaatttaatagcattatttacaattcctactcgcttgggtaat
aatatattaggatgagcttgtggtaattcatttcaattacagcagtgataaacgtttctatcatggcgcacacagttggattgcataagttaaagat
atctgaaaatatatcatgctcaccttggtttttaaaatagcattgacttaatctaaacttacgtcgacttttcttgtatcgttaaatcaaaggagaaat
agcatgtgtgtacttgcattagtaatcaaacaatgatagcagccgcattcatttcaatgcactttacactacaactttgtcgctttgatatagacatg
attaagaggttaaaaaaattgggctgttctgggaatcgaacccaggaccaccatcaccctaagatggtatcataccactagaccaaacggccaa
tttgttttaagactgttaaaatgaaatacaagttgaatatggtcaaaaggaaaatggttaagttaaaaatttaggccaaggagttcagtcgatgtt
gggataaacaaaataaagccttcaataacggaaatctttttactaaaacttatccatcttacttataatgtctaaaaatgccactttcacttatttga
cttgtactttttttttggccttactgtaatcgcagtgttgttccagaggtaacaactgttttcattaaatggaaatatgaatttcagtaatttggtcctt
cattgacttctttagttgaaaactctgtagagcattaaaaagtatctttttttcagaaaaataatttcgtttttgttgtccattaaagtactaaatacatt
tatctttagagaagagatcaatgtcatgagtgcaagtagtcctagataaatctgaatccggactaataccattcaaaaatcacgcttccataattg
gataacattctcgtctcggctccttgttctccaggcttacggaattgatatcaattcaattgacttgcccgttaaagtatccatgcttgcctgtgtata
ggccatactttatagcatagaatcgagacttcgctgtaatcgatcaattactataagaagtatagtaaagttagtcgcctgttccacccaagtatct
tttctgctaaagaggatgatgttcttggtttttttaatgacattcccgatttcgaacattcatccaattttttttggtttttttcattcatttcaaatattttct
tcgagtcttccttttctgctggctatatcataatcctttaagaatatctctccttcctttgatgaggaatcagttagtactggaatgttaaacccattgg
aaaatgtttcatttatttcaccccttgtcttaggaatgttatatatttcatcttatgcttggaagaaaaattgggtaatgatagcaaacacataaaca
aacatatgtgtaatgtagaccgtgaaaaaagcgtatttttgaatgagcacctaatgtggtatagtacatttatttttatggtaacttcttacaaacc
ttcaatatatgtcttaggactattctgtccattatatctaaatttctttgcttcagtatccaaagcatcatcaccaccaacatggctgacagttgtttgc
tatcagtaactcaaccgctatatttggttaagaaagctcatgcttctattgccagattgacatctaaaggcattaatgcttcaaatttaaagacgctc
taccatctttactcgcttgataccataattataccccttttgcaataattgtttatctttcaaaatcgacgaaagacgtatatttaagctttcttctacc
gaaagtaaatgcttcgtttcgttattgagcttatccagagagtagcgtaattaccaatggtcttaggaacgcataaactcatcactcggtcctatct
gcatttgtttatttctagacgatgtcaaaatttgcatacaaatcgaaaaaaaaaaaaatcaaacagttgaagagctgtttttgaatgagccctgcctt
acattttccctacctttcagtgaacactcaaaagcaaacagtttgtaaactatgtaggtttgtctctacacatataatcaaaatcaccttttgttaag

ataggatagtaaattacagtctaacaacttcattttcttgcaattttatgttattcttacgtttgttgctacgactgaaaggtatgtcattttagctttta
cagctgcttcactgagttagaagtttttgattttgcatgttcaatgatgctcccttaaaagtaggccaactagtcaaccaggcgcgttggaaattcta
tcaaactttcattgaactccagcttacgagtataatttatttgtcaatgtttgattctcatgggaatattattgcaaatatctccaaaaataaatgata
tcatattgaaattcgtctaagacgatttgcgtcttttcaaaacacagttgctgtccaaaagtaaacactgatgagaaaccttgcaccagcaggtat
gaaccttcttcatttaaattgaggatctactgataatagtatcaatttaggtagaaattgttagtcaatttccaccaaccgaattcctacgaattttc
aactgttgacatatccttgcaagacttaattattggaaaatggaattgaaggacatgcctcacatcttacagctgtactcagggaatgaacgaatg
ttgaaacacatttggtgtttggtaaaacgcagacaccaagttttgaagactggcttcatactaacgacaattgtgctaacatcttttagtcgcattgt
attatttaatttcttaatggaccaccataatcaaaacttacgtctcttcatcgtatttgatacaattataatgttggtactttgcttttatatctttgttt
cctttcgctgttcaaccgaacgttcttgattgactatgatagcttgcctaatctatcagatcagactagttaaaggcattaatgatatagcaaattca
tacctagtcatgttcaagacgagtatttacgtagaaccctctgtcaacgagtttctatcacattagttgaaagcaaatcagggatttgtatgattgct
cgccaagccgatgatccccaacttcatccttgattgtcaagatgcataaaaagacatagaccatttgtgctttcgttcaacatttcactgttttccga
tgctgagagagattgactggagtggtaacagctttttcttcaatggcttgatatgaataaaaatactctgtttatcccttcgctataaaccagaaat
catgcattaattcctaatgaacgctcgaatagtgctccttcctctactctgttggcgttggaattctaaataaatgaataacataaacactgataca
aatagaaaagacactcaataaagtccataaaagatttgttccttctgtgatactgctttcgttatacatatatgctagtaacgcataatctagagca
ataagtccaatcaatacttttaaacataattttatttttccccaattatcatttgtttgcaaaagagcttcacgatacgtaactggcatgctaaggga
ccgccaaaggtagaagatccatgctggaagtcaagaggggaataccttgatcctggttgaaaaatgtcaaacgaagaagaggagagcgtgcac
tcgtgtaccttgtctacttttcatcttttgttatcttcagttctatgatgcatactacccaacggtccaatattgtacaaagaaattttccaccgatgaa
tttcactttcagttcaaactttgtctgagttggtttttacttgttttgcatattgctccactaacaattcatacctttttctgtgttttcatatgacatactgg
atcgtttacaggaataatatcatcaaccccttgactctgtctcctttaattgcaccattgaaactacacgatgagctgcagaatgcctattgtctaaa
atggtatttcactgttcaatttttaccaaattctcattcatttactttgaaaagtgtttttctaattcgcttcatttgtaatataagcaaatacgatccc
tacgtaaagactatatgccatcttcatatgtagtatttcgggctcaataatatgctgttgcttgacttttggaaggggccaaataaaaatagattta
tctcaatggcaatattctttcaccgtctctatgtttggtcaacctttttttctaggcaactaaactatgtcttattcctctggattctacacatacatgga
cggttaatttttaccttaaataattagacaaaacatagtagctgtctacggccatacctaggcgaaaacaccagttcccgtccgatcactgcagtt
aagcgtctgagggcctcgttagtactatggttggagacaacatgggaatccggggtgctgtaggctttcctttttgttgctctttactttttttatttgtg
gtttggtttttttgtttattccttttcggaaggttgattctcttccaggtaaggtaaggcattctttctgctatgctgattcacgtaacagcaagctataa
acttgaacgttcatcaatacttcattagccaattagaataaaaagaaagctaaacatataacctaacacgaaaattaataatgttacacagattc
aaatgcaactgcaaatgcgaataaaaaaatggcaagttcaacggaaagagagcaagactaacacagtatgtacgatggtaaaacgtcagtgc
acttcatggccaaatatgcagaaagaaagagaacaaaacataagcaacacttttgaaaaagacaagaaaacctaatatgcaagcagagaaga
gaaagggagaaacaagaaacctttccttagagcagagaacccggaaagtgtaaatctacaaaatgtataaaaaattaaggggagaaagaatt
agctgctgctttgcagatttttaccgctttccgtatccctagattggtgatgttgtcgtcttacaaactgttcgaaacgttcagaagatggttcctcat
ctctaagactattaggattatttagctcctctagattctcaagaggtattgcatgaattggaagttgattttctcccgtcgactcggttccttcaaatg
tcatttcgtagcctataacataggcaacccaacccaacagaaaaaataacgaatccaataaaactattcattattaattagcacaactttcaaca
agtatcatccgtctactgacatacctgaaaccaagtttagagagaaccaaaaggccaagacgataagaatagctgccacactatactgaaccttt
atggaagtatctttcaacatattattctttaaaaaaggaaagagaacgtaatatgaaaatgaaaaatcaagtatcagtgagaataaataacacc
aaaaaactcgaagtccttcatttgtcgttctttttgcagcaggtggaattctcgcaaatgccgctgggctagaaatatacaagaatccgctcttaac
actaccaaaaaacgcaccatagtagtctaaacattttgcagcagaagcgtcgtttgacaattcgtcgctcttgttttcaggaacgagatttggatgt
aaacataagcaacctttttccaaattatccagatgtgagtatgggagctcgctgtactgcggtaaatcactgtcagaactcacaggagaaactcg
acaggaagattcggctgctttggcaagtcctagcagcttttctttttctttttcgttcattgtttatcgttttaatatgaagaatcaaaacacaccatca
attgccatttacaagattcagaagcgagctgcgaaaactatcgaaatttgatagtaaaagagcaaggagcgcacaaaagtttaaagtaaaagc
aacttaatttaagtccgctacaaaatcaaataaaacatttcattacagctctgagagacgaaaaatacaatggcaatggcaaaggcaagagtttt
gctgaggtgaagttatcatcaaaccaaataagctttcttttggtgcctttcgctatttcgtttactacttattcctaggtatcggtatacatatgttact
tcaacatttgagtagcacgttactttctgaagagaccgttactcaaaatgcattctaggaaaacatatcttccaaacaaccagtggtgaactacat
tcgataaacaaaataaaaaataaaaatacatcatcttttcaagtttcagagtctacggccatacctaggtgaaaacaccagttcccgtccgatca
ctgcagttaagtgaaaacgtctgaaggtctcgttcatactatggttggagacaacatgggaatcccgagtgccgtaggctcctctactttttttgctg
ctctttttatttttttatacgttgactatttcccgtaatcgttttctcgatagaatctttgcaagttgttcctttacaagtgacaaaagactaattacaag
ttcaatgaatacaaataaaacgatcgtattcaattgcaattaagaataatggtaataataaataatatacgcctgcgtataaggatctagaatgat
gccaacgattctcagattacggttcctttcaatgcgcggattatatgcatgcctttttattgtccacttttcttcgtcatctccaattcatattgataca
aggatgatttcgtagtcaaatccacttataattaaatgcaagaacgttaaacaaaagcatagaagtaagtgcttgtgattcgttctcttttcgctca

acccacaataaaatagatttccttgacgaaaattcaaaaatacttccagtctttatcggcataaaacaaaaattcaaaaagagacaaaacaaac
gaaaaactaactttgtgtagtagagttgttctgaagctctattgcttcattcatagtcgttgatcgtggtaatagtacatcatcccttatttgatatctg
gaaagcccaggcaaattagtgttttcatttaagcgttcgacttccagactacagaattcgataaaaaggtcaaacaacaaaccgcagatggatgc
gatgatgatataagcaaagtttgagccactgctaaaaagtttcctgacgaaggctccagcatagaatccaaacgattcctgaacgtcttgaacgt
tacaatattctaagtacttaaaaaagttcctgacgaacctaccgatggcgggaatgagtcctataatggtacaaaacagccccaaggcaatactt
gatataaaatacacgccgataatcaccaatatagaaaaaagtcctactccataaacgtaaaaccgaggattacctatttcttgttaaaatgcacg
ttcgagctattgaatgggaaatcccaaatactcaacttacagcacaataaaaccatcaagatgaaggatgggatgagaacaacggaaaaaaa
gaatcactcgaaacccaagagtaaaaaaaaacaaaatatactacaaaaatacccactagtaccataaaaaaattcgcaagtatatatataggga
aagcatttaacgactacatttcgtggtaaataataatttcttagctccataaatctattgctgtactcggttgagaaagcatcgaaatagcccgatt
ctgccattcccgactttcttctctcactgattttatgagaagtcgggttgggaggtttctttattacattcggtttattcaaaggacgtccactctcgag
atcaaccttgtcaacattagcttggttgtacgcaggaggatcttgtaagcctcttgattcttttccagacatttttgaaataaataaaaagatcaat
tagtaaaaaaccgtcaaatgaagacgttcaatccgtatttgacgtaaatatttcacaaatgctcacaaactgcaacgaaatgcaagtttcgagag
aaatagcaataaaacttaaagtaattctaccaccttattgaaagagctcgtattccaatcaaaagcagtccaattggcagaaattgagggaaaa
gaagttggcttcaatggaaaatttcgcatcgcttacgcagctttgagtcaaaaggcaagtaacaagtttcataaaatcataaagcgaccttcattc
tcatagtttatttacgatcatctgttgatagctgctttggtggtttagtggtataatgcttcgttgccatcgaagcgacccgggttcgattcccggccg
aagcaactttttcctatccatttacaggatgtattcttgcttaattgtgaaatggacgttacatcaaaatgcagttttctatagtttaatgataataaa
caaaagcttttgtaagagaagcgattgattgatcgattgttttgctttggcttaaaggaagtaaactattttttaaaacattgactaaacattcttc
gctattataactttttacaacgctattttaatcctcaaaggtgatacaccaaattcatttccattaagcatttccaccaaaataataaaaccaataa
aagctttcttcacacgtaaaattaaatgctcctattttttccaaaaaaaatccagtgcctaataatatgcaccaattaagatatcaaaagaaggtct
agtcactatcactatagacgtcctgttccatttcacaaccccatagtgagtcttccggatcttgaaagtcgtctacgggaacaagtgacatgggccg
tacaccttccgccaaagaatgtagctccggttgtttcacatattttgcaatggactcgtaccgagacgaatctacaccttcgcaaagcacgagagc
ttcaagacctctttcagcaaatatggaggctcttttaaagtcgccgtataccacgcatgttcgaaaagcatcgaggtatgaaataacatccatccg
cccaaaaaggttttcttgatgagcaatcaagattctgtgtcgtaaaacacgaagcgcacctctaggattcataatgcacattttcgcaattgtacg
atcataatatgctagttgctttcttcgaatatcacttatcttagcttcctttttctcctttactgaacatgcactacagctacaggggaatccaaagtgt
tccaataacacctttctccgtttatcccgacttttatgtaaatcgatgtaggtagtcaaaatttcttctccaccatggatatcacgaactgagtgaac
ggtaacctgatccaaaactggattccacgtatgttttacatttggactgcaatcatgattcatcctcgaacccaaaagaaacattccacctttacttt
catcaatcgtaagagcattggaataaaagggtcccaagaacggacccatggtatcaggatgcgcattgaatagattatgaaaagcctcttgttcc
tcttttgttttcgatgaaagggcttcttctatctcctttgcatcgctttttgttcgaatcaagggtgtttctgaatatatccttgtccctgcaggaatgtc
aattttcgcaaccatacccattccttttccaggaatgggagttactttgtataaatctgtttcatacgggttcataaataatggtctcgcacctgttct
aatagggactaaatatttttttgttcagtagtcgttatagggaaacaacagaataaacaaaaactatgcgaaatcaatcttctttttttaataaaac
gccaagaaattatagaattaaaatcaatcttttaataacttgcaaaaaagagaaatagtattctcttcttcttctgctaagtatagttaacttgtatg
ctcaaatcaatggtttcatcgaataaaatacgttctttcattatgggtagtttgagtctttatctttgtttatttttttcaaattacctgccgcctgaaacg
gtctccatccagccattacttttaccaattgacacttcctaacgaagaagggtggtagtctttaatatattagtaaaaaaaactttgagacttaattg
ccctaatgctatatgattcgacagaatacctggtaattcatatcgaaaaggaatataccaagaagtattaaaggtaaacaaagatgctctcttggt
ccttgctcgctaattaaagatttatataaaaaaaatatgatcatcaaatagcatatgaaaaggctcataagtaaacaaacttaatgttatttttttttt
ttttaaatcttcaaaacaatccgtccagcgatagctccttgggacatttgtttgtaaatcccaggaagcgtagagaaaggctggaccttgtaatga
ggtttgacaagtcctcgagacacgtactcaagggcttcaatggaatctagacgattaccaacatgagagccacagattttgagcattttcacagtc
aaccaaaaaatgtctgcgtttagctgggcatcagccggcatggatacggtgacgatggtcgatcctgggcgagcaaattgagtagcttgttgata
agatttggcactcgtggaaagcacaagaacaccatgagggcctccgtttgtaaggtcctttactttaccaatgagatccgactccttttgaaagtc
aacaaatgcgtcagccccaagagacttacaaagttcttccttatcgctaccggtatcaatagcaaggactctcatgctcatggctctagcatactgt
actgcaagatgacccagaccgcctcctgcaccaggaagggcaacccagccgccaggattgacgcctgcttctttcaaagctcgatacaccgtaa
ctccagcacacataatgggagcagccacttcaagagggacattttgaggaataggcgtaacatgggcggcatttccgattgcataggattgaaat
gttccgtctactgagtagccagataagctgatgtcaggacagatgctttcgcgagatgaaaggcagtattcacattgaccacaagtgccattcatc
cacttaattcccacacggtcacctactttgacgttatgaacggccgatccagttttaactacaatgccagcgccttcgtgtccacctacaagaggca
ttttgacactcaagggccagtctccctgaagagcatgcagatctgtatgacatacacctgagtatttaatattcacaagaacttcgtctgactgggg
ctcaggaacggggatttctttaaattgaatttgctcagggccgccgtgattgtgaagcacagcaccgatctgttgttttgggacctcttttagtgaag
ggatagcatttgagctaaaggcccttgtcattaaagagtccctcgtttcatgatgcaaactgttggatggacccttgttataacttcgaacaagaat
tatactcgccagagagcggttggaaagcgactgcttaataggtgtagagcaaagggcatttgaggcaaggggcaaggccttttgaacagatcgt

aagtatcgaagcatattacttgctcgacttaatttttccaagtttttctcaattaaagatataactttgagtgaaacaactaaaaaatgaaaaaatg
aagcatcaaaaatgtcaactcagcagctactattttattttaagattttaggtagctcgtgaaagttctgtgtaatttcccagaggactcaaaacat
atattttatagcaagtctatatgacatgattccaattccagcttaagtaatggctaaaacaaagaaaagcatgtagtgtttcttcaatgtcttttcctt
cgatttataaaagaatgaaataggaatggaagttatttttccaaaaaaaacaatccgttgaattttgatctgtttcccatttctcaatgcgattatct
tttcctaagaaaaatagaggcctaccatcgttttataacaattgagaaaagttttcattgtatgttattttccctatgccatcttgttctcgttcgcaat
tttgaaaaaggcttttccttcaaaaggttctatgaaacaacgtgaagaaaagaaatctttagcagccattacaaaatcgataattaagtgaaaac
cattctttttattttcaaatttgaatgaactccacaaaaggctcttttgtttttttgttcgggatcgtcttatcgtgaaacaacattttcttaagcttcatc
aacaaaaaattgctgtagtattgacccaaacgttcatgttcgcttttctggtttttgaattgaatactcagagaaagaaaaaaagacggtgaacaccg
tgagttttgggcttttcttgagaggaaatgagaatgaagaactcgctacagacaatgaagacattccaccttcacgctgcatgttgaacttttcttg
atcatttatacttatttatcatttcaatttccacttccatcgactgttttcttttgtttattacttactcttacaatgatttcctggtaacaatgtcctctcg
ttcaagtcactgctacataaccaagtaaacactacaaagacaactctttccattcaaatagcctttttttgtgtgtttttttgttttaaaaatccttttgaa
atttctgtttagaatgaaatactagaaatttttgtaactctgactttcgcagtgactgtatcgtgcaagcgtttcctttgaactttttttcttcttgatatt
gatattttgctaaagcatcctttttttttctaaacaaaatataacaaccagaggatagctgtctcatcccgccttgttttctcccagcattcatttcaa
atcgcggcctttaaaaacagatgttgaattctcaaaaaaatggaacacggaaaaatgtttgatcaaatattaaagctctcacatgaaaaggtttgt
agtctccatgttgatattccattatatctaactgcttttcgaaaagatttctgtccccgaactgattaccctgttagacattcattacaacgacatgtt
ccatgtaaatccatggatgaaaaaggaaataaaaaaactagcctatgaatttgtgaaaaatgacccaaatcagcagctttcaaaacatgatgct
tgcgatcttgtagaggcgtttattaatgtgcccgtaacatcgcttgagagacagtcgatttcatcgagttttcctttgtattccacaagtgtcgaaga
tatatcttctgatttttctgcagagtatgttgtattgtctttcaaatgctaacgcacgaaaagttccagcctcttacctcagtctgttacattgtcctat
agtttatctcgatccgcgactcctgaacatctttctgatgattcatttgtcgcagcattccagaaaactcggtctcattttgcatattcagactcccta
ctagaatcgactttctcaaatatccaaaattgtattgcatccatccaaaacgtcaagaaagaggtatgtcactttattttatatacagtttctaatga
ctccttgtaggatgtttcatctcaactcgtcgagcttcatgtgggaccggaaacttcacttccgtcctcaacaactcttccttcttcttccaaaaaagc
agttccggaacctaaaaggtctaaaccctcgcaacaagctaaaaattattacccgatttccaacgtttgggaacttcttaattcgtcctactatata
aaggcctgcatagcccttctgatttccatcattctctttttcgtcctccatacttcgtctaaatctaatccttctaaaactcgtccctcataaccaaaca
tgtttaaaaaattatttattattagtttaggaaattcatttcatcgtttgtaatcttctgtcgttcgagttcactgttattaagtatcttttctttttttttttt
cattttttttttcgatatagcattaactcaatttgaagtttagaactcggcgtaagcacaagtcgttagtatacttttcaacctcaaaatcccacatttg
gcataatactagcttcagcttttcgtctttcgactgcctgatttttatattataactactagtttgatggtatcagtaaacgtttatgttctttaacttca
tgttaagttccaacaggtcattcggatctagaaaacattagtaattgaaaccataaatcctctttcacgtaccattgatatacactgcttccttttcg
atttttggttccaaaggaagatagtctcctagtcctggaagttcctcatcctgggttaaggtattaatcacgtcccaccaaaggggatttgcagttg
ctaccatttcagcatttccaaatatatataatcctcctaaatcaattgttaattgaagaaaaagaacagaattcttccttacgtctggcacgcgaaa
gagcgacacaaacccgataactcgaaaccaaaaaacccactgtttgttgatcatggttacgaactaaagaaagaagaatcacatcattttcttcg
ccttgatagccgtcaacagtggcaacccggatgttttctttgttaattggtaaatgtcttgctaacaaatcgtctataacctttttctgtgcggcataa
aatgtcaagcaagttatctgaagtggattcacgccatgattaattaaataagaagcaaactgaactagcatttctgcttcaaatacattaaattta
gaagcaaaatccgtttcttcaacgccagcatgagtaaaaaaaaatcttcgtaattgtcccattcctggaaccgcagacctgccctttgctataatat
gatcagataatccaatatagactgaatctattaatttacgaatttgaggatgcatacctgttaagttagtttcaaaccttcgctgttacacacaaact
tacgtctttgtacgcttaacatatcgcataccatattgttttctaccagacgctcaaacattgaaacagatagattaaagggtgctttggcaagcgc
aaatgtagagcagctcggacgcaattgtttatggtccccgattaagatcaactgttctaaagacggaaaaacagcaggtacaattggaccttcaa
gaatattggcggcttcttcaataaagcaaatttttggacgaatgttttccaatatacttcgatacttatttaaacccgttgtagtcattccaataacat
tggctccacgaaggagagcagcgacacgcttgtcggtacaatttattctttcttcatctgcttttcgataaaggttatgcaaacgtttcaaccggaat
tccgcatcttcataagctaattgaagccaatgcttataaactatgcctctactaaattctggaatatcccaaatgttttctgtagctagaaaactttc
agctctttcaaatgaagcttaatgctttcacaaaaatcatacttccgcttgaaatctctaaagatgccatgcaactcgtcatcctcgaaagcaggat
cttcctttgcttcttgttccaattcttctaattgttcagcctcaatctgtaattcttcttcataaccatcaacaatctctgtgggcgttattaaatccagtt
tcgtttcacctaaccaacactcgatctgttcttcttctgttgcctcatcgctttcaacagcagcgtttacccaaccggagttttctaccaatgattgga
gttgagccgtggtaatgattcctcgactgtgtaaatatgtaagttttacgaattcaccagaaaagttcgacataatgttcagcatttgtttcatgagc
cgcccttttttttttcgaatatcattatatgttacccggaatgtatttcttgtagacttaatcctttcaaaaacagatacagctttaatttcaggaactt
gagtacgagaaccaagtcgtacaactgaagcaccttcgtggaggagctgaaggagaatttgatcaacagcgtgattggtttgacaagcaactaa
aatgggaagcacattcgaatttgaattttctaaaagaatctgaatcgccttcaaagcaacaaatgtctttcctgttccaggaggaccttgaacaag
tgccaatttttttgtgagcatggattgaaatgcttttaactgagacttgtctaataaataatcatcataagaaggaaaaggttccaggatgtcaatt
ggcggctgagagctccctaaaatggagttgattcgcatcaagggattatgttggacatacctggcaatttcagaattgcgcttacagctaacaaa

ataatccttcatagggaaatggtgcacagacaacttttgaagacttgataatacatgcttgtaagcttcccaatagccggaagtagcttcaatcatt
acgaattccttccttgaatccaatgagagatttaagtcattgaaaaaaacatcaacttcatgaggatattttttaaaccgcttaagggacgagcg
caaaccgttcccacacgaaaatgctcaaagttatcttcacttaaaagaacaatacttcctgaaattaaacgtcgactcgtggcccaaggtatccgt
ttttgagctctaacagaaaatgagagtttggagacaactgaaccagtagcaattatgtgtccaacaacgcgaacgtgatcgtaaacagcaaatg
aaggagaggagatggaagttggatccttcttgtatcttaaaacactttctcgaagaggagaaatagcatcttctcgaagcaaggtataatgtaca
cttagataatgatcgaagtctttatatctaccaaatacatcgttttctggcaaatcaacagatgcagatgagagttcttcaaaggtaggaatagaa
ggaagagaacgccaatgactaccttgttcttctttgcccatactattaggacgatgcggtactttgtgttgatatgcatctgcattaaccttagaatc
caaagatactggatcattcattgattttccttttgttcttccttcaacaaaaccatttttctttggcgaaatgctgcaattctcaatttttacgtcaactt
cgtcaatggtagattttcggtagcttgccccgaaccgtttttcctgatgcgttttcatttgatggatcaaagtttcatcaggaatatcatcaaagaca
acctcttcagcatcagtagcgtttttttgttgatcccacatttccaacgtactggcttcaacactttgccagggattatggacttcttccgaaaaagtt
atctcttccatccttcaattcaattcaatcgtacagttaacagagaccctttataatgaatacaattccgcctctctttgttcaaatgctcacgaatag
taactacttatttcaatagcctaaattcacaatagataaataccaatcacctactactgttattgttcatttgcaataaaaaaccgccatgaattttt
aaatttgactgtatacgcgtaaggaacggtttgtttacaatgaaaggaaaccccgtcataaggagtaccgaatattacgaactgttacatggttcg
tttgtaaagctttatatgctttaacagtatttagcgacaacaaaaaagcagtattatattgcctagaactgatttacgaggcagttcgtactagctta
ttcttttctctgctacctttgagcaggactctggcttttctcacatttcattaaggggacgcgattctttgagatttgaagtttgtttatgttgtaaacaa
agtatttctttttacttattgtttctatttgttttatttttgcgtgaaattttgggagcgcttcagaatgagtatgtggacttcttcgatccatttacctaa
atttagcaaccgttttatgggtattttagggccccatggaaattgctcattgcgttcttctggaattcttgctttccgtcctattcgatgtttctctcaga
cccctagtccatggaaccaagactccgatttattagagaccggagagcgaactgggtctagtatttttttcttccctaccaaatgaatttcaggcacc
ttctcgtgaaccttctcagcaaaaatctcgcccatggaaacggatatctacgaagcgtcttttaaccatggatccttcttacacacatcaatcgaat
attctccgctcatccttaaaaaaagctggcaacctttgtcgtcaaatcgccagaaagcctttctatcatgcacttttacagatgaagttctccgaaa
agcgcgtttcccgatctatcgcttctgctctcgtaaatgcacgcaagaatgccgttagcaaagccggtcttgatgaaaagaccctttacatcgacc
aggcatgggtgggaaaggcaacttatattaaaaaaattattactcgaggtcgcggtggtcatgctgttgagagacgtcctcgagttcgtgttaccg
tccttcttaaagatgaacgtgcacttctacgtgatctacaacgtcgaaatgatagaacaaacagccgtcgtgtttggtcccctctacccaaccgccc
tatttacaataaacttaatgcttttacctgctaaaccctttcctaaacaacatctaagcttgatcaattctaatttatatctctttctcttttacgtatag
ctgtttccctcctttcattatggtgattaccaatagcacaaccaaacaaataacaaattaattacagcgaatttctctagcactgttcattgtttttta
tccatttttaaatttatacattcattaatctttataaaaaagtactgctaaacgaatacatgttcttttccgtgcgaacaatttatcttaatgcgtttttt
aatttttcgtttaaaatcgttttcatcaaaaccattaatttgataacaaatcagaggccaaagggcaatgaatcctaaaatcaagcatctcagttca
attcaacaagcataacaataattttcacatttgtaccgaagttagaacctttcccaacgatttttcttttataggtattcagccgcttctcattttataa
tatatttgacgatagcctccttttttagtataaattgaaaatattggacgtatccaaacgaactaacttttttttttgttttgttatcttataaatcaggcc
cataaatccaaatacagctgcccttcatggattacaagaataattgtaacagttgcccttgaaaaagaaaaaaaaaaattaatgggaaaccataa
caaaatgttacaaatgattttgttgctagaaatcatcaatcagcaaatttttaaggtaaaaggctctttcacctgttttattgaagtatacgttgttat
acttcagtcatccatctgaaaagtcgtttcttcttctagtgttgagttccaggcagggaaggtattagcaaaggataccсttgcggatccattgtttc
caggactcaacgtatagtcattctttgtatcattaattccacctgtccctacaggtgatacggacccgcctctaaatattggttcactcttgagccttg
cgagatgctgaggtctagttatttcattagttggagaactgaatacggattcttcacgaacaccaaatgaagagggcattttcattggagcacccg
caacgctagattgttgaggggtagttttagtagagccggtattactttcagcatatttagattttgaaaagtaagcaccaaatctactattctgcag
ggtgtttgctgttgagcgaggtgttaaggttggactcatcatggacaaaggagcagataattgttttaagctggaactcattttaccaataggaga
agaacccaaaaaggattcttctagactacccattggtgtatttaagtacctttgttcatcgtcactcatgtccatctgatgattcaaatccacgcgat
tcggtaaactagtggagactactgaagggaatgcatcggcataagaattactgcgaactgcgtatcggcgagtagccccagaagaaagttgaat
cccggtaagagaatgattaacggaaggacgacgcttagataaactgcttccaagcttagtactcagttgagggctctgtaaaggtacattaggttt
cactgattctgcaggtgtcaatttcgaaagagaaggggttctttcagaagaaggccgatttgcataagtggctaagagagatggatgacgagtaa
agtaatctcggttactttcattactcatctgctggtgcaatagtcctttagcaccacctacatttgtagaggatgaactagatgcagcactgaatga
agatggtgaggtagccaaggaggatggagcaatggaaatgccattcatagaagaagttaaacctgtattaatttcagaagaatagtcatccaac
tgtttgccggaatttattgagcctaacatcgattgagtagacgtaatgttattcatataagaagacgcggaagagcgtcctggagagaagggagc
aaaagacggaggggacgcagacaaagctggattgagaaggttgctttttatagtcacggaacccttcaagtccacttgatcaaaggcggttcttt
gtatttttgagggaacgaagaggaaatcgaggagctcggcatgtgtttcatggtaagaccaaatggatgatttgtagcaggattcatgtctgaag
acaaaccagcaggatttgtagcgtcatttactggtagcgagtgagaaagtgcacattttgagccgaatttgcaatttccttttaaatggtatttaca
aaccggacgttctgtctcaagggaatgactaaaagggcaattctcgccggcagtacaagtaccgtgacgaaagaacttgcagggaacatgctgc
aaatctacacgtacgagaggttagtaaggaggtgcattttaagaacagtaaaacatacttttaacaggagcgacgtgatcctgtgctagctttgg

caatggtgattttaccgttctgcggttatcgagaggacgaggctccatacgttttgctgcggcagaggaagcagtagacattgtgatcattccaaa
aacaagagaggagaattcactataaatcgaaaaacaaaaaattcaaagagatttacaaataacaggggaaaaagataaatatgtgtattcaa
aaggaaacaaaagaatatgcaaaagaaaaggaaaactgttgacgaaaggagctttttgtatagaatacttcacaatggcgaagaaatttagga
aacgttcgagagaaaatcaaatatcacagaaattattgcagaaaggacttgctaaaagagcttcttgcttcaaggaatagacaaaaggacaaa
ataaaaagggtttgggcaaaaggaatccaagaataccaaagagtgccgttgaataaggtaaaaagaaaggaaagaaggaaaggaaaccaat
gtctaaaccttgttgctgaatttttttaaaaactaactaaggaaaacggctagatgtacacaaaaagagcgcagaaatcaaaacagtcaagctc
aattacttcaacacaagcaattaccggaaagaaacacttccaaaaagggagaggaaaaaagaagggaatcgattacagacgagcaattgcac
caagagacacaattcaatttagggtaaaggcgtgtattgcacccgaagagaagaacggatatacaggatttgcagtaagggagaacgagtgct
gcaattgaacaaggaattcaattgcaaccagaagaaacaagtaaagtgtttcccaaaacgttattccgtccacaaactccagaatgattggtaac
gtttgtaagttgaggaaaacaggatactcgttaggagagcttgaatggagaagctgctgtttttttcagctctcttcaactgtctcaaaagaatctt
atccacacaacgaaatcacttctagtagttggttcacggcaagtttttgaatgattagcagcaggacaaagagctgataactcccttgtgatgtct
aaaagacgtgcctttttttatatacctaagttatataaaaaaatcaattcttttcttttgtaaatgaaaatctagctatatgcgtttctaaatgccatttt
tctctctttttttctaatctatccttttcttggttagggaagggtcggacatctttaagataatcttgataccgtgtaccaattcaagcttgaataccca
agtgtacaaagccctctttacgctctctaaacgtgcaccttttttaaacaccttgggaaagttaggtatcttgctggcttgtattacgcaatcttgatat
caaatgctacctagatctgggtcacacaattaacgagatagagataagcttaaggaggtaagaagtagacaatttggtaaatcctgaaaaggag
tgtattaaggagtgtgaggaataggaaaagacagaaaataataaaaaattcatctattttttcattatttcgttaccattagttactttccgtaaata
tttgcatgtatacgaatacaatgtccgtactttttgaaaagatttgctcttttttttctttctttctagaccggagatcagtatcctgatgatgcagctat
tgtttacttgtgatcttctcaataatattacaagagccctctaagaaaacagatatatctccaaaagtagatagaaaacagggcagggcttttggc
gccttccttcacaggaatccatttggttacggaatgctgaatgcattgcgcatatgcttgaaaacatcgcatcgcgtcgtgtgattaaagatggttt
cagtaaacaagttgatttgatttgttcattcaaaaagagtcagaaaagcacattgcaacactccgcggaggttccttatatcctttacatcgtttctt
taatctgatcatatcaattcgtccactggtagcttgtactactggtctactggttgataaattgcagtgggtttcttttcatatctctgtctcgctaagc
aagtgtcgatctcaactgatattgaattaaaaccatgtgacgaaaatcacaagtttccgataaaaaatattacaatctctcgcaagtcgaaccctt
gtcttgtaatatcgcttactcatttagagttttgaagacgaggcaagcatcttggttgattcatttgcattccaaaaaacgatcctaggaactattca
tagaatatctcttccataatggcaatgaatacccaagttgctttttcttacctttgaaatcatccttttcagcccgtcatcagatgcaattcaacagaa
gcaaatggaaaagagaaaaaaagtcccctacaatacctgttgttttggaatttgttttctatttttgtttctcaaatcctcttggctttctttctcttacc
tctcctcatttaaacaaggaaacgagccgttataaacgatactatgatatacatcatagtattggacaatatactatgatactttaagtaaaattct
tttcagtgcaaatcaggcatccaaaaagtcgttatttaggtacattattagctttccacaataagaagttcttcttgaaatgatccacttgtttgatgt
gatatgccatgtcatttcttgaggttttggaagagaagaaaggacttctcaatcctaaactggttctgatggagaaccctctttcttttttttttccttt
cacattctttattcttacatatcacaactctttgcatcttcgttttccgttgtttctttagcttactgctcttctctccttttatttatcaaatcccagtgata
cgctatttcctgtaggaggaacgcgagaagggaagctgacttgacttgccaaaaacgtatttcctgccatggctcaacatgtgggtccactagca
gcaggccttgtctggcaactactgagtgcaaaagaagaagcttggaaaagtttagcacccgcggctgtcgaattggctactgcagagatggacc
aaatttggcaccaaacattaagtcataatcgccgtgggagcgagcaggaggaaattcatttactctatcttttacatcgttgcttgaagacccaaa
agttacatcccacaactattttgcaagcctgcgcgtgctttctaacctcgagcgacacagtcaagaaccttgcttggtcgtttttcaaggactgtga
gtcggacctaatggctttagagtttacaaaatttttttcaaagctttgaaaaaatcttttccgttgaactttgccgcctgctaattgtttggacagcggt
gcattgcatgtcctcttcggtatactatcccgccttacttgacctcttgcctattctgtccaaaaccattcaacaaaatttaaacgatccgaataatgt
tgcggaaatggaaaaggagttttggctgaagtgtctcgttcaacattgcctctaccatgcttctgattcatctttgttgttttacttggaaatgtttttta
gattctcatgagcttcaacaatgtctcattgtaatcttagaggataagaagcttttgaatcgtgtaaaggatcatggcaatcctgaaatccgcatga
tgttagacaatgccctcaagcattacaggaaattacaagctcaacaagcttctgtcgtctctcgtgattcctctaatacttttggtgttgattccaatg
acattcaaaatctaatagaacttttccctcaactttctatagaagaagccggtgattatttagcccagagcaatggtcagatggagttagcttgtga
agcggtattaacaaatctaacttccaatataccgtctacttcatctcttcctcctcccaatcgtccttctgctcccctgtcactcaatgatactgtaaa
agcagtcatccccaaccgtaatacatcgacctctgatttgcagtcctcacaagagcaagatcgtttagatttattaaacgtcgctccggaaaatatt
tatttaaataaaaaaccagaagagatcgacttttggaagtcttctgcttccgatagtcaaaaatcgaaaactctcgacttgttgaccttggctgaa
gatgatgagtatgatgacacatatgataacgcggatgcagttgtgcctgtagatcctggtgttcaggatgataacccagaagcaacgtcgaaaga
tgcccatgagtaccaacgatatattaatcaagtcctcttgcgcgcttatgaaaaagaccccaaacttttcgatcaaagcgccagaaaaacaaaag
aacgtgctgatctcattaagcaagctgataatgctttgacgcatgagcaaattgagggatggcgacgtatgtttaccacagataccaagtttgcgt
cccttatcaagcgtgaagtccaatttgaggttggaaaccaaaacgttgggtctttaaatcgctcaaaatatcgaaaatcaaatgaaaagatagaa
gaggacgtcaaaaaaccatccacacctagaccttcgagacctcctccaaacccagctttggcgaaaaagaagtatgttcgacgaaaaccaaata
aagcgtctaatgaaaaaaagtaatatttttgggattgtctttttgttggatatttatgcatgaattatactaccggtaatggatgaatttagataata

ctcgatagactaaaatttctatccttgttctttacttataccttaacaagtaaataaaataaataaaatcttaggatttattgaaatgattatcttttc
acagcttctgttgcgatatcggctaacccaatacaacatagtcggctgcattactactaggaaaaaaatcacaaagaaaaccaaatcatagaga
aaaagctcttcagacttgccaaataaactgcctttctaacgtttcaagttaaaaacgaataaatgtacatatgaactgtttcattctattgcaacaa
gagaatcaaaaagacagaaacctgcatcctttactaactcgataaaagaagaagagatagacaaaaagagtgtgattttagaagaataaaaa
aggcaatgaaagaaaaagagtagtattaggtacaaagatggtaaagaataaacggaaagagggaagttaatgtaaaagtaaaaaaaaagga
tcctatacataagcaaatttacataacgatagtaccggggacttcaatgttggcgctaatactcttgtgaggcaagatgctaccgccgttgacata
gatttcatcattgacgacaacgtcgtcacccaaaacagagacattttccaaacggctccatgatccgagggtagagttccatccgacgatggtgct
cttcacccaggcatggtcacgtacacgggaagacttgaggatagcgcatcgttgaagacgaacaccatcaccaatggtcacattaggaccgata
acaacgttagggccaatcttgcagtttttgccaatggttgcagaaggatcaaccaagacattgccaataatggaagagccagcaggggacaaca
tctcaggcttgtgctttctcaaagaggtcaagtaaaggcaagtgccagtaaggtagtctttgggttgtccaacgtccatccagtagctctctaaatc
aaaggagtgcagctgattgtctttaaccatagcagggaaaacttccttttcgatagaagtaggacgaggttggatgcggtcaagaactgaagggt
taaggatgtagataccagcattaattctattggaaacaaattcagagggtttctcaacaaaacgctcgatcaaggacttagagttagggtagtga
acgacaacaccgtacttcgaggggtcctcaaccttggtaacaacgatggtaccttcagcgccatgggctttatggaacttggccaaatcagcaaa
aggatactcgcagataacatcgctgtttaaaacaaagaagggagagctgtccttgccaaggatgtcacgagcaagagcaagaggaccagcagt
gcctaagggctcgttttccacagaaaacgtgatcttaaccttgtattcttcttcatacttctttaaagcttgaaccataatttcagggcggtagttaac
agccaagacgatgtcagtaacaccagcagcagccaaggcttcgatttgatgaagaatcatgggacgattgccaaattcaaccaaaggtttaggc
aaagtcaaggtcaaaggacggagacgagtaccaaaaccgccgacgagaatcagtgccttcattgttgcagaaataagcaaagactagagaca
ggaaaaccttaaaaagaagttagtatatggatctataattgaaaccaaagtaaaaacacataggtggcttagctagttgatccgattcaagccca
aactgagtcatcagtcttttgaaggatacagcagccagagaagattatcggtgccaataactaagggattataattgagtggtttataaactaag
ctatccaaaaccctacgattttaggacttactcaagtgctcaagtaaagaacttgaaataaataaaaaagatggtccttcctaaaacaaaggtttt
ctcaatcaaaacctcttaggaaagttgatttaaggtggttggtaaagaacaggtggacagaaggtcccccacaactatcattgtcccaatagggg
aagggtaaggactgaaaatatgtcacgaaaatatcaacaatagggcaatcgtcagtcatagtaagtactcacaaatagtgacaaaatttgttgg
tattgtttattcacttttgcgtaagcatagcttttgcttgatattagcgactcaaagaactcttcaccttagaagcatttccttccttacttagggtaat
cattcgcaagaaacaaatctgcgacataagtatagcaataacataataatatcgtaatatcaatataatatgtatctcacgagaataatgatcctt
tcaatagtatcaatggccgtatcgtagcaacatcagggctcagtaataaaattctaggttgtttggactcgttgccaggaggttatgaaagaggct
tgtagggagagtgccaatgttgagacgttgctacgagtgaagaggcgcatacgagactgatgtccacagcttagcgagagaggacagaacgag
cgagctcgagaagttggtaacaagaagccacaaaaatagaaaccggaaaatcaagccaggaaaagttgcttaacgtttaatgcagataataaa
ggaaagcgagtggcttctgtcgtttttccgcattgtctcatggaaagacgcgactgcattttgcataaacagaacgcagttgctgtttttcgttttac
aagtctcttcctttattgggatttcaaaagaaaacaaaagaaaacatgataccatcaataaattggtagcacctaattaaatagcacctaattaa
atcctcctataatgcgcgttcaagaaggaaataagtcatcgtcgcacacaaacatacatcctttttttatttttcaagttcaaagctttctgtgaattt
agattgaataaaaatcgcattttccagagaaactatccataaacaacatgtattgaggcaaaagactttggtaattgtgaaaaacgcgtaaacgc
gtttttcacagtaaacaaatgtttttttgcttgcgataaacaattcacctgtaaataaaacagtcaatctgttaattagttttatatctttctttcaat
gaatctatttacgtgcagatgcgtagtatatgcatccagagtatatcaaaatctgaatataaaggtcaaccttcttctaccttttcgaccttttttttg
gttactttgcaaatgaagaatttgaattgactttccctgttggactgcaatggaatttataatctcttccattttagaaattagtccttaaaataatgt
cgcgtatagcattggccaaaaagtcatcccaggtttaatggttcagttttattgtgtttattctttggtgttgagtatcaaacaatatgagtaaaaatt
aagagtttttttttaattatgaattatgaatgaataaccaaatgatcatgtttgatatggtatgcgttcctaatcagcacgtttttggggattgcgc
attcgtggataatacgtgttcaagacagcaaaaacccttgtatttgttccgaatgtaacctctgttttctttcaacagatatgatgtgatttttcttgg
ctctagccttgaaatttggaaaaagctaatcgcatgattttgcaagtgtcaatattgagttcgttgtcaaacaggaaacacgcgattttggcaaata
gaagccataaaatatgaacctagtaagcgaaaatgggaaatttttctttgttttctgaattctcaaaagaacaaaaggaaaaaagtaataatact
gcaacaaactacttatttttgtttgttgagttggctgtttcttacaagtattgaatcgtgaaatagttttgttgctgcttttcgataagtttagtcattga
aaaagcaattgcgttttcaaaaacgagtttttaaaaatccttatcccatgagaggcacaaagtaaatttaacaaatccaaatcttgtgttacatac
gcgttctacaatatgacaccaagccgcttgaaagctagtctcttttttgtgtttccatttattccttctcgtattcctcgtcgtttggtttcgtaacgcaat
tttcatgttcatggatgactttcaaacagacaccattccttctgctgaacttcaatacctgagagaagaaattcatcttgcttcaaaaaacatgtctc
tagataatttaaaggccgcggaatggctcccaaatgtctctggacacgaacccttgattcccaaaatcattcaccaaacatggaaaacagataca
gttcctgaaaagtggaaagctgcccaagagagctgtttagctttgcatccagattataaatatattttgtggactgacgaaatgtctagaaacttca
ttgctgaacattacccttggtttctcaagtactttgatgcttatccctacaacgttcaacgggcagatgcaatccgttattttgtccttttgcattatgg
tgggatctacatcgaccttgatgatggctgccaacggcgccttgatagccttttgtattatcctgctgtcttccgtagaactgatcctgtgggcgtctc
caatgatgttatgggctccgttcctggccatcctttcttttccttcattataagcaaattagcgagcaatgcaaagtcttatatcttcccatatccgac

cattatgttcagtacgggccctttatccatcagctttctttgggaaaactataaaaaccatttaccaaaagacagttccgcatctgcaagaatacgt
attatgttagaagaagactacaaattgtccaacgaatcttacttcacttattttgaaggctcttcttggcacgagaaagatgcttcctttattctgtgg
gcaaacaaacatatcttgggtcttatagtctttggcttttccacttattttgttctttcatacttcgttttctctaaattccttgacagtcgtttatgtgaa
agatgctttttctcccgtcaacacaaagttacactccctctctcagaacaagaagatgtgtaacttcatccgagctgtctcaactcataaaacttgtt
acactttttattgctactcatctcaattgaattcaatccaaagaagcaaatacgagcttgacgactaaactgaccactgccgagggaatgtttttc
taaattgtcttggattcctttggcatgtgatgtagtgtctcgtacgatttgctaattttccaataccgcggcacaccgagatgaaccgcgaaggtga
ggtgatttaatttaatctttttctcaagagcaaactctacgcaaagtcagctgagtgagataaacatttggtaaagaaatccgctttagactcattg
ttcagtttaaaaatttctttaaaaggtttgtttattcattggaaagtttcggcttgtttcctgccttcgtcgtctttttgtttataagtcttatggaaggac
attccagccagcatgaagttgcagagtatgcttcccagcttggtacagttttggatgaaatctttttaaatgttggatatttgttgcgctccttgcttg
ccaatgttcctaatgcattatatcatacccaactacaaggagaatgcaataagtttcaagattattgcgacttaattgaaattcgtcttttagaggct
aaaaaagtgattgagagagatttacgtactttggaagctgctcaaagacttgaaaatgttcagactacaaccaccgcaacagcgaagccggcgc
tgccaatgggtatggatacgagctatactccgtcaaacggaaacacgagcaatttagactcaggattgcatcctgatgcatttcaaaataggaat
aatgcaaacaactttttcaacctttccccttcaattctcagcccatggcaaatgaaagcgctatgcctaggtcttcggaagctaaattctttgaagg
catagatccgaagcaaaaaatgtcggcttcacccaaattgaaggcgacttccagtggcgtagaatctatgggtaacgaaacacgttctgaaccct
attcgaatatgccgtccggtattcacactggtggttctcgtgaacctgatgcctttcaatttggcatgccaccggaagcttcctcggatcaagccttg
caggcgaatgattttctattgccaagtgttttaggaaacaccactacttctccgcaagataacctgtcaaatatatctttccctagcttggatacggc
gaatgggtctataccgagaaattttgatttttacaacaacagcagcaacagatttctccatcctcaaatcgccctaagtccgtcaacgcatcttat
cctccttcttcttcctctactttagacaccaagacacaggaaaactatagtgctgctttcggcaatgataactccttcgcagcccatttgaatagcac
ctttggcaataattctcttaatttgtctacggagatgcccaccacagacagcattaatgatttgtttggtgaatcgtttgatttcaccatgtcgggga
ataaccagtaaaccgaaatttattatagattggaatggacttgctttttcgtttgtttctctttcgcattttgttagatagttttcgttttgattatacac
tcttttattaggtagtctattgtttatatggcatgaatgttatgaattgtttgtttaacttcaatttccttccttttgaggctatagtcttgtaaagttgta
attaggcaattatataacgggtgaaaagcaagagattagcctgtttggtatgttcaaacatactactgtgaaacttttgtatttttttgcaacggcaa
ttatgaaccatgaataaaagaggaaaggccatgatggaaaattttaacattgtcatttatatatagagtataaaaaatgagtatgtaaaataaac
catactaagttcatgagaagagggtatgatatgtttgaacaacgtatgaaggcttatttcaaaaacaaaattaaaacatctaagacggtcattat
gtcacatcaatatagataaaacaaagaacatgagaagagaaaatccgaaaataaaaaataaacataatatggtagcctgtaaggacgtataa
cctgagtaagggattcgatattagaaacaacctcgaagaagagtcgtaatccacatctcattgctatttctattgtttcatccaaatccaattgaatt
tctcaaaaaaaggaagccccaaaagattatgtatatggtttagaaaaaaaagctaactgcattaataataatcattactataatcattattggaat
caagcatgactccgggctcatcaacatcgccaggggttcgtgttaaatttacgacaatgcaggtaatattgtctgtacttaatctttttaatgcgtgg
tcgaccagtcttagagcggcttcgcgacagctagtgaatttccgaacaaaatcaacagcttcttgatccgaaatcacatcccaaagtccatcgca
ggctacaatgaaaaactcatcgtggcctggccaaattcgtgtctctgtcgtgaaagggtgagcgctcacgagctctttcaagtaagtgtcacctaa
agctcttgtaacggcaaggacaccattgatccggttctgaaccatcattccacctaattgagtgacccgacgagactcgttttcatcagatcccttg
tgatcataagacaggcgaatagctttaccatcgcgacaaagtacaacacgagcatcaccgacgttggccgtatacagtactcttcgagaccgatt
attttcatagcggaagaatgcaactgcggaagtgcaaccacagacatcattattggtttcttttacaacggtatcatttacttcaacgaaggtctca
tcaagcaaatctgtcacagggcggtcaggaaattttctcaatttttccaacaatatcttgtgaaggactcttttgcagtagtcagcggcttgggccc
cggcatgtccgtcatacacgcctataaagccatcattttgatttcctccaaaatcataaaaacatgtatgtgcatcttccatggctctcctccaacgt
tggtttttgtcttctaccataccagctaaccagttggatgtcttactaggtctcgtactacctattcgcattgtacgtccttcgttatcttttacatcaac
aatagaacttcgtccttctccttcatcgtaagagatacttgccctacggccatgattcgaaggtcccgaatctttaaatggattcatttttttgtaatgg
ctccagtaatgcattagacactgggttatttcctttcatactcaacaacttgcaaatttaggcctttttggccgcaaaaaaatgaaatccaaggtaa
ttatacgacaggatttcaactttcaaagagaaattttaattttctctttatttttgatgttttagtggacgaacgttttaattgaatgtacgtgttcagg
tcaaaattcactctcatcatattttgaacggtttacagcgaacgaatgtgttgcgtcatctatggagttttcaatgctctcttagactacgttacatttt
atcgttcctcgtattttgttttacaacgcttgtagactgcgctatcgtagtaaacataagatctgagaaaacggaattatgtcaaagcgttcaaaatt
cataatcgaaactgcatggtcttggtagttgatacgtgtctttatgttcttaaaaaagataaatataaatggatttgatatagtaaaaggcgtgtatt
ggtgaaacaaaccggacgaaatcttccatagtctacgcaagattccaatgaatatactttcagacctgagtccgtgagagataaaggcaaaaat
gtttgagtccttctttctatgaatattccttacatcctcttcaactttcaaaagtctcacaatgttacttggttactgtcagaatgcatttcatgaaaga
ggttgacagactgggctcctcccattgttttatttaatttcttccttcttgatacaatcgtttttttttttttgtttgacttgcaaactttggctatacgctttt
ctccatactagaatggatgattgatccaaagaataaattattaaattattagcaaagttattaacttaggtcgtagcacaagtgaatatagttttat
acgaagtttaagaattatacaaacaaacctacaaaaacttaagaacgatggtttgcttgtatcatatcacgaggtccaaacataaaagtgaatgg
tcattcaaaccaataaccataagcagcatactctggtcagatacagcaaagcttttcaacttttcagctgtatttgagcgtaattctcttttgctaat

agaaagaggggactataaatagagagaactcaaaaggactgattaaaatccgatagtgatattcaatttgtcaaaaaaaactattatcatgttc
attagccagaatttgttatacaaaattgaattagccagcgcgaacatttggattaaaagataaagaattaaaagggaaacccacatcagcaatc
agcggaaggaaagtaaaatcataagaacgttaaggtaaaggaaagacacaacgaacggacgtgcaaaatccaaaaataaaagaaaattcga
aagataaagccctaattgaatcgggtagcaagtgcaagtcgacaaaacactagcaaccttaagagataaatctatcaattggagccattggggg
actgcttggcattgccaaatctgtcgttaacgaaatcgttctcctctcatctgcgtcagccaaaggcgactgcatgaactctgggaatagctcaaca
ctttctgtattttcaggtaaatgaggtgaattttccttattgtcggcttttacagctgctgccaatgctttggatagatcaaatttgggcttaatttcag
gtgtcagcgtaatctgatccagatcagacaaaaacagttctgaccgccaaggtgatggactttgttttgctttttgggtttcggaggtgttttaaat
aaactttccagttggtcatccacatccttatccaaatcacaaacgggaagagtaggttctgcatcttcgtctggctttcttattacttgccttaaatgt
tgttttggtagccgattttcattttccttatttggattactgctcattaatttctcagaattcgatctttggatattatcattggccgctgacggtaatac
aagccaactgctgtttgtaccgatttcagatagtggctctcttaacgactctcgtacattctcatttgtagttttagtaattggagaagactgaatcct
tcgcaagtcattattaagtaaactagcaggcggtctttcttcaaggccgacgtttgtagacgaagtccttgattcggtcgcagaaccagaagacttt
gggttcacttttggaagtggagaatggcggcctgctgtttctagctgtcgaaaaactggaatcaagggctttttctgttgagacagtttgtacacag
cgatttccagctgagcaaaagaatccaattcagaaggcttttccacctccaggtttttatatgtgctccaaaggctttttggtcgcatcgagtgtcgt
gtggtccaataaattccacaagcattgcacaacatgatggttgtattcataaaattagcattacgccaatttgcagttttgatagcaccacaatttt
gacaataaggaggaatttttccttcacgaaggctatttgatagttgctcttcaattcgatcttttcctcgaatagttggcaagcgcttttctatacgag
cacgggcaatccgaacttgctcctttataaaatcttgaacatgctctcgttcagacggaacgcttaaatttgatgcgttaaaaggaacagcacttg
cttcacgtttacgcttctttttagacgcagcattggatatggattctgcgattgaagtggatggacatacaggatctgtcgcaggatgaatattgga
cgaaggtggctccatactatatggttctgaaaaaggcgcagcagggtagtcattctgagtattatcttgagggtaaatggaatcaaactctaaact
ttctgaaggaggatccgtaaatcgagatgatagagggacaggactaggatacagatcagattcgggatttggaagtgtagccaagtttgcggac
gagtacgcagagtcaaagaagcttgtggaatcggattggtcagaagaagagttgtcaaggatgagagaaggagggagagcttcattgtgaact
ttagaagaagttgaggaaggaacagcttgtaagcgaagacgaatttctagagagcgttgcaaaccagtctctactactctgccattcagccagat
agtttctgttggaaaacttttagaaggccctgggatagattgagaagacagggatgaaaggaagcctaacccgacaaaaagctcgtcagcttcc
aaatgatctaaggcgtaaacactgaattctacgtctgatttgtaattgagttctgggctaagaaggttagtcaacaaggtaatcaacacagtataa
aagcgacggtaacaacatttaacctaacaaaaggatcaaaaaatcacgcatttcctaaaagtatatagtttttatccgttatttttcttccttaattg
ttaggtactaaattcagcatcatacctgcaattcaaaatagcctctacagcatatcgagcatctacctgacaaatgacttttcctttatcaaatgta
actaagacttgcacaggcacagcatgtttagattttgctaagcaacggatggacgatgcacgatcaatgctatagataatacgtactaaaagcga
aatgaagaagttagcaagttttagataaagaagtcagagtcgaccacgttgtattctgcagcaggaacaacggtaactttacatacacggtacta
ccaatacagaacccaatttttcttccatagctttgaccaagagagatatcaacctctggcatcagctctttccaaaccaattaatttacctaaaaac
gaaaaagccgatagatagtgctaatcgggcgatgtgctgttggtaagtttgaacgactagagacacgtacacgacgtaaacacaaggctcgagt
cgcgtcaaaatacatatgtgctcttatctatatgtacatgttttcttttaaatttatacatagaaaaatctattaaatgagattcataataaaaaacg
cgtctaaagtcgtgtttttgttatgagagcaaactcataacaaaggcatgtaagaaagataagaacatatgcattttaaagtacaagcatatgact
ttactgagatttttcctggtactaatgagtttggtatttatccatcatgcatgagtaaaaagagatattctgataaatctttggctttcgaagcacag
aaaataagtaaagctagttcaagagaaatggaaagctatacaactcaaaacgcatatagatgcctttgtttacacttttgtacttaaaattgggat
tgctgtactgcgataaacgttttccaaaattacgctttggcgcgttagtaaacaaatagaagaaaatgtagttctaagagggatttaaggaaaag
aaggttaaagaatatggtttttattattcattaatcagaccccattgacttagaaaaaaagatatggtggacttttgagtgaatggcgtcttagtca
atggtttacaaaccaaacaaagcggacgctggaaagaattctttatcatgtcttcgtggcttcaagaaaagcatagaacgattttcttaagtaaag
gttgttctacccttctaaatgtcttttcctttgctgccgtctactacctttttctcgctgagttcagctgggttttaacgtacttagatggaaactttcttt
tcccttgataccaacgcagatacaaaattgacttcaattcgttgtttttgattcgaaaacagacgttttacctgatttcagttcgagactggattttct
ttttccttttgtaattttatttttagttgaggtatttttttaattatcgtacttcttgttgatccttttggacttagcatacattacttctctgtccataca
cctcgtctaggatacccacatttccccaagtctcttctcacctcaacaaaactcgaaatttcctttttccatttaaggcatacacttgcaggtttgtttt
aaaaaccattaaaactctgcaccatggatgtctgggagcttcagcacttttccgctctcttttacctcttgcagtgcatcgaaagcaatcccagcaa
tcccaatgtctcttcgcgcttgttagtgcagtgtttggattcttataccaaggattttcaaaaatttctttctatagatccccctaatgcttcatctcga
aataaattggaatctggggaagcagaactgaatggaatggtctgcaagctgaatgagacttttatcaagcagtctcttacaatttcttcacagcttt
ctatggacgaaatccagtctgcttgtctcttgcaaaagggcatagaagcctctcaagccttggaccgtacacctattcaagctgccttgtacatgtt
tttcttgtctcgtgagcagctccttgaatccttagaattgcttacaaggattgcatcattcaaagatttggattctgacattgcaaaagctttgcattc
ttatttgaagggtctgtgtaaagcggaaaatgacgttgtgaagacttgttgtgattttgttactgttttggataaaaaaattacagaaacacttcga
aatgaagctagtggtcaagtattgggtattgctgatgttgttgactttcaggaatacatcaagttgtctcatgaagctcatattgccgaattggaga
ccgttgctatcattctccatcaactatcagaactaggattgttgcagcaatctcattttgagtctctcctgtccacgctcagcaagagcgatcctact

aataaaattgcagtacttatattaccttccctgttaagctttttgaaaaagcttctccgggtcgacttccttctagagggagagggtaagataaatgt
agcacccccaccttacagcatattcatcatctgcttataaaaacagctgcttcgagctggcgttgtcctcagttcaagcagatcttaggaatatgg
tggcttacttatcttaatgaagcttgtaaacaagctacgaatattccctctttcattgactatgagtcgacaattaaagatcctgccaatgattttatc
catgctggcgtgttttccgatattataacctttgttatacttccctttcgacaatcggacgatgaaggaatggaatggagctttgcgtttaagcctcg
atcacgtctttcaatcagctggccaactgtccgtcctttgttggtaaacatcatttttccaagtttcgttcattctgtcaatcatttataagttatatgc
ctgagattttgaaaatacttaggctccttgaagaggacagatacttaacaaacgcagcacaccccgatttagctactgaggatcttcaacaagttt
tctttccttttgaagagttttatcagctattgtcttccgtatatacctatgataagtcgtggtcattggatttttgggctgacatcgaaagcgatatgta
tggtttcttaacttggtctatggaatcgcaaactccaggaattgttacttcatttacacttttactggtttctatttgtactaatgaatttactgccaca
aaggttcatgaattaatgtccgaaccggccgcagaaattagtcatttcgataattccgcaatagccggaacgaattggtcatatatattcaatgttt
tccattactacatttcccaattgaagcccatccagaccgttgtaacttcttcgggcgttgctcgtgttcatactgaacctggtgaaattgacattaatt
cttccttgatcttacaggggtatatattattatttgccagcgttacccgtaacagctctcaaatcttagctgccctctgtgaagatcaaacacttaatc
ctatcggtactttttttgaactgttagaatgcagattaatggaaagcgtacgctgcacaatagtaatggcacttgaatcaatgtcttgccaatcttct
ggttttctaaataacgccttatggaactatttggataactggtttattacttccgttctatttgacattgacggcgggcttgctcctatatcatttgcca
ttaactcaaaaaacgtattccctaaatcacccagtgccttcggtcctctattgaataatataagaagattgacagctacgtcaagtatcagaatag
ctttcattaaatttttgacgtctttggttcgttcgaagagtgaactcaaggttacccttacttttcctgaaaatttgggatcttcctatcgtgcacctgg
tataaaaccttatgtggattttgtaattgaaactttggttttatcctccactcaatggagaactgtgcataatgcagaagcgttaaaagtcacatatt
catgtcttcaatttatctctgctgttttcgatgacctcaatttgaatctcttaatttatttccgactctttggtaatagaatcaaagataaagttcagag
caacagcttgtatgtttatttgacaaggcatccagcaatttatttgctggaagcggtttttgttgaaaatgtctattcgggacttttcgaccttgttga
gttcggctatgatcaaatagaagacgttgcagttcctcaacttttggtttccgctgttctacagtctctttttatccttcgtgatgtccttgctctccaga
gggagttattcaaaaatgtaattccatatattacggaattggggataagtaagcatattttagatttgacaatttccaggagagcttatcgggaag
tatttatgactagaatatcttctattgttcaccttgctttattggtagggtcccggcatcaatgtttcgtacaaccagcaattgaaattcttagtttctt
agtggattccgaaggctttatgaataagggtagtactgacgaaaataagctgctttgttcaattgttaggacagcgaatgagtcgaagcgcattat
atttggttttatacgggcatttgaatttagatcccgtatgttgctgtcctctggtagtgagtcgtcttttatattgcgttttcttctgaataatttaaaac
aaagcaaaggcatcttttcacttgctttattgattttaggattcgatataacttccacaaatgcagtaatcgtcagagatgagcctggttatgttggc
tcacagatttctctactgaactcattattaaatttcatagaattgagaacgcaggacgatgtcattctggaaactcctttagttatggtcgaagcact
cgaaattgtagcccatctttgttcttctccattgacctctgaagctaccttggcagttataagagaccgccctcactttcttgtaaatcttattaatgtt
gagcccatattatctcaaaatagcatacaaaatctcgaaaatatctctacagaagaaataaatgttttctcgagaagtttgcatgctcgggcgcaa
gtgatgacaatgttactgacggaaattcacttcacctcttcattaggaaaaaagaagcagtcaaaagaatatgttatttcccttattggaaccgca
gagaggttaaggacttcgcagttatctcaaaaatcgtctggctttaagatactagatttcatggatattctgaggatcgacaatcgaaaaatagca
tcggaattacccaaagttccaggtttcaatttaagcatgttcgtgacgaagtttgataatggagtcaacgattttcagtttgatactgacagagttgt
taaaatgtataaacttctctttgatactgaagcatcgtctatggaagatacaaatgaagaaaaagaagtttggttcaatcaaaaaaaggaacaat
tagaggacctttctagtcgcttagatcttttcaactcacgggtcgttttccttcagcatgttcatgattgtttggaggcatgggctagactaaccggta
tattaatagaagactgtcattctgcaatccctgaggtacagtttgatgaatttatttgggaattattgcgtttgttacttcctggtgcatcaaattata
gcttagaaaaccagaatgcggtttcagtcaccactgcagtcattgaaaccattgttccttatgctttaaaaaaaatgaatgctttaaaaccagaag
aatacggaaagctaacttactttgttgaaggagtacatggaataatcgttagcttaatcaaaggcattcaatgccagtcatcagacgaaactattc
gtgaaaatttatatggctctcttttaagcattttgactacatttcaaaaatgcactagtatgttgagcagggaacatggggattcagaattcttgaa
aactttagaaaagtgtatgaatccaagtctattaaactcttcctttttggatgttttaacgaaagatgcattgtacacaagtggatcatgctgggaa
ttgtcggttattacgttaaatttcctgcataggctatcaccagacatatcaacccatttatacaaatattacttgcgtcgtaatttcattgggtcgtttg
tcgatgccttttcaaaaatattctcagatatacttgcttctgggaaagagattattgaagtcataagtggtttagaagcaggacaatgcttcttaatt
acgcttgcgcaaaacaaaattgctgttcattctgttttaacgatggattatatacgattagtggttgatttgttattgcaactatgtaaacaaggtat
gttgttttgtttaagttttaaagaatagctactaactgaatataggtggtattcaacaccttcgacctcaagttcaacgattgatgcttcaacttttgc
aaatctttattttggcattaatgaaggtacctttaaaggtaatgtctaataaagacaaagcactcttacaatctgtgttctcgctttcgaggaagtta
caagattctgttcaaattaatacggaacaaacgtggagtacagaagctgttaccgtaaagaaatatgttgaagtaataagtcaacttttggatctt
catagagaataaaaggctagtaatgaatacccggctaagggtgaagaaaattacactacattttaatgcttactttacaaatagaggattaaata
aaattattaacgggtaacttggttattaaatactattattccattttcgaaaaaacataggtaaaaacaacagatatggaaaaaagtatggactta
ttaatagaaaaaccgttaatataagcaaaataaaggataaatcggatcgaaacgatcgtactattaaacttgtttcgtttatatttttattaagctg
ttttccagctaatatttttgacccatgagcaccgccaaaacccagaaccaatccaaaaacccaaacattagaaatgaattttaaacctttttgaata
tcatacaatattaatttcaagagtagagacaaagataaacattagttcaaccatttgtcgagatattcgcaagtgaccggtgatacccgacttcca

acagcgcaaatatctgccacggggcaagcgccgcaaggtacttcggtgaaagcatttgtggtatctggagaaggccggacagaacggtaggac
acaccatcagatcttttctcaaccttgccatcatatattaaaacatcaactaaggaaataacatttccaagagaaagatctacttcagtaatatttg
tgttgcgaagccatgtatgtatttgtaaagctgtaggatacccattgtactcaggggaccatatcatatcagggccagtagcgtttttctttggtggg
aagctttttgagtggataaacctgtatacgaccttttttagattctcaatgaattctacatcgagttcttggtcggtaaaccaaggaccacctgtaag
ctcactactgggaaccaggtcatataacatgtaaattttacgggtaggatttttcacactctttattggctttaccagatttttagattcgagtgattt
aaggcaccgattgaccacgtgaacgtgcaagttggtccgcaaggttagagtctttgtccatataccttcattgccagcattcttgataaagtcgtaa
actatttgttcatctccttccatagtagaaacactaagcatactgttaatataagtccatagcgcgaaaaacggactacatggtaatccagtaaca
gaaaagtgtaatattacatacgtttttgcctcttctaaaggaacagcgcgataaactaaggtattcccttgctgaagggcttctaaaagccgccta
ccgagcagcaaattgagagcactgctcaaattgctcacagagatagaacttccaattccatcgatgagttcttgttgtgacaatgcatcaggaatc
tttttagaacaaaaatcatataactgacgagccgtgcgaggtaaggacttcagttggtcggccattacagaaaatattagaggtatgggtgaagg
aaaggtgagagccacaaatgtacaacataacccagaaagttgaactgagtcagagtgaattctcctgttattcaatcttatttcatttaaattaaa
attggtaaaataaacgatagatgctgaagacaatttctcgttcagtttccatgcgacgaaaattttttaaaggatacggcgaaatcggttcattttg
aatcatagcatgggactttgcttttttcttctattgtgacgaattcatcctatactgactcgatacgttgagctacctgcttttcgcttggtttccttgtg
tctacatgaaatgtaatatatggctcatacaagaaagacccaataatttcattctatgctgctgccagatgtattgaaatcaatagaaaaatcttta
ataaaaaaagaggaaaggaaagtgcttgttggtaaacttggtaggaatcgtcaaactttatgaacaagttcaatatccttttggacttacgctgca
atatctcttgaaatttctacaacccttattattcccattgaaaattcgaaacccatccaaaggtatactactagacgttcatattgtttacccttttact
atgaatacaccagacgctgtaggctcacaatccaaagtatctctgaaccgatggagctttttcagtaagtctgaaatcatcaatggatgctttaca
tcctcttatttacaggttatttttgaatccgataacccattttctatgaacatctgtatttggatagctacgatttcttctattcctaccaggggatatgt
cgaaaggtttaaggtcatctagagtatttttttacctttttttgatagtcattttctagagatattttctcataagtatcatctcttcatgtttttgattttg
tcttacatttggtgtttgtttacaaataccaattacgtatgcctgtaaacaagagtgtttgccgaaacactaacgtgcgccccgcaaaattgcacctt
cactataatttttaacaagttacatttcatttccttcaggtttcttttatctcgctatgtcatatactgcttcttaccgttccgtcctttttccctgttactg
gaaacaaagtgacaaatccgacgaagtctcaagcggttaagtcatacgtgaagacagccttcccattacaaaatcccactctctttctgtttgcaa
cattcggtgcaatttcacgagcttttatgtcaaattattgaatctacggatcaaatttcctagtttttgtctgttccggatcaaaaacactcttttctcc
acctttccttcgttccttaaggtaagaccgtgtcaacaaaaccctacaagatattctatttggttttctaacaatagaaaaatagtagtttattttata
caattttctaaacatacataatttttaactttatttgattttactcgttcctttgataggttgaaatagcaaactgatcagatcgatccgcaatctgtt
gtcgtcttttctttcttattttatcgtttaaatacggcttatttgactgaatttcccatgtccatcgctacttctttcagcccagcaaaagctgctttagct
tctggttccgctctctcttgtaagcgacttcttcaatcgaccttgaatgtaagactttttattagcatctcataatctttcgcttttgttggaagggatg
gttgcgagacaatatatgttgctacgacaaaaattccatggttaacgcggtgaatattttaacaagtttggtgttctccttttcaacggttgatttggt
ttcaatgtccagtgttaatttagctcgaattccaatcattacgagttttggaatgtaaattttcgctttttcctattcacaggcgttgttgaaatacccc
tatgcagcatagcaaacatctatactattcgtctagctttcctacccttcctctgaagcctttgcaagcaaatgttgaagattttgagtgctcattatg
tttggatttcgtatactaaccttactattctagtattcttcggagcaaaagagaatgaactctacttcttcctccgagcctttgcgccccaagggtgtc
cagcccggtgaatttcgtcaagaatcggacacctttggccctgtccaagtgcctgccgagaaatactggggtgcccaaactcaacgttcccttcaa
aacttccgcattggaggccccggcgagcgtcttcccctccctttgatccaggcctttggtatcttgaagtttgctgccgcttccgtcaataagactttt
ggcttggatcccaagcttgctggtgctattgaacatgctgctcaagaagttattgacggccgcttggacgacaatttccccttggtagtttaccaaa
ctggctcaggtacccaatccaacatgaacagcaatgaggttatcgccaaccgtgctattgatttgcttggcggcaccttgggctccaaaaagcctg
tccatcctaacgatcatgtcaatatgtctcaatcctctaatgatactttccccactgttatgcatatcgcttctgttttgcaaattcgcagacatctcct
tcctgcaatggagcttcttcgtgatgctttaaagatcaaacaagacgaatttcaggatattatcaaaatcggtcgcactcacatgcaagatgctac
tcctctctcattaggtcaggaattttctggttatgttactcaaatggaatacggtattgctcgcgtccataatgcccttcctcgtctctcacttcttgctc
aaggcggtactgctgtaggtactggtcttaacactttcaaaggattcgacgttaagattgcggagaaaatctccgagattactggaattgaattca
agactgctcccaacaagtttgaggctttggctgctcacgatgcacttgtagaaatgagcggtgctttgaacacagttgcttgctctctcatgaagat
cgccaacgacatccgtcaacttggttctggtccccgttgtggtttgggcgaattggttcttcctgcaaacgaacctggtagctctatcatgcccggta
aagtaaatcccactcaatgtgaagctttgacgatggtctgtgctcaagttatgggtaaccataccaccattactttctctggagcttctggtcactgt
gaattgaacgtctttaaacccgttatggctaagaatcttttaagcagtattcgcttgctcggtgatgcttgcgagtcatttactgatcactgcgttaa
gggcattgaggccagccgtgaaggtattgctcgtcatttgaatgattccttgatgcttgtcactgctcttaatccccacattggttatgataactgtgc
caagattgctaagactgctcacaagaaccaaactactcttaagaaagagtttgttgatttaggttatggctctgccgaacaatttgaagaatgggt
tcgtcccgagttaatgatttcccctaaaaagtaaacattggtttgtttattaacatagcattgtttaggctatgtttagaaatttttcaagcctatgcc
aagaaaagtgagttcattgcgatgatagcatgatattttactacgttcttgtcaataatggcttgacctcagcaataaacacccttctcaaatcgta
tagctatcataaatttttttgaatgtctcttcatgcttctacatggttgtaaactaggagttgtacagtttgcacatgactgattgcaaatagaaatgg

taatgaacctatgctgtttgataattcaataaccttatataagtaagtgcgtgattcccataaggacgtgttcagattatctatatttcgttctttttgt
taaaagcacacgttgaacgaaaccatcgaaagtcaatttataagtaaactaaagtgaaatcaacagagaaagaacacaatcataacgaatga
gttctgtaagcctcctagaaatcgattttgtatacaccggaagatcgaattccagattcaaatcataaacaaatctttctgccacgacaataacttg
atagaaatatatttaaacaatagtttgcctgacctttggcggcaaaatttaactacacccaagcgagcaataccaacaccgaccttgcacattgcc
ttcaccaaactatccatggaagaaataaaacttactcctggtctttctgaagaaggaagacgtttgttgagactgttgaaagatattcctttagacc
ctgttgagatacttgaagaaattccgaatactttatatgcttttcgcgattctattacagcagagaatcgtccggcgtgccttggccttttagaaaca
ttattaggatgctttgagttaccattaccagatgaacctattgttaaagcccttgaaaagctgttggagccgatgagttggaacgatttgaaacaat
ttggcattgaagcatatttggtaacaggtttagaacagccttcccctgaagtgcaggatttttgtcttaaattaattcgaagagcaaattggcaaga
aaaagatgtttctgagaacctttttgacttagtcctcacttgcttgaattctcagagtattggagtttctgaagaagcgtttcagcttcttttagacat
gtctgattactgtttttactttgatcagattgtgcaagagtttacagatatggattatgagatcttggataccactctaaaagtacgctggctattgct
tttcgctaaacttgcaatcaaatccccggaatatatgaataaactagtaaagaaaggaagattgatatttgatattgacgaaaatgatgatatttt
tttacgactttgttatgttgatgtgttgtccatattattggatacccaatttacattcgagtacatttcatcggataatccaaaaccattagaccaaat
cataagatgctttcagaaagaaacgaactcttttacggatcatattggtctcacatttctcccaatcctctgccagaaatttcctggcatatatcgag
caatcgatgaacagtatgatctctttaatattgttcgtcaaagaatttttaagaaagactccacttccgttcttttatatggccattttctagctagtg
agatcattactaaactcttaatccagaaatacgaagtggagtcatctgaaggatacattccttatcctttgctacgtatgttgttaatggatgatac
aggctttaccgctttagccacggccttacagcagccaaattcgggtttatgggctaagctttgggagaatctacctggcgatagtatatcgagtatt
atgaaggcttcatcttcacctcttcgtcgaactcgccagttgggatatcaatgtctacttcatatcgcacaaagatcgcctatccaaattgcgtcccg
agcgtttgccatcgaatatttgttggcaacagatggtgatcatgaaacctgtgtgctccgttatgaggttttacgaactctgtgggaatcatcaaat
gaaggaaatgaattgccattaggaagatatcaagatgaaatccaaagacgtcttcagcggggtgtttttcctggtccctcatctactttgaaaatt
gctgcagagacagcttaattttagagatatggttaaaaatcaattcatagtttattgaatgtgtttatagttaaaatgcaatgcaaaggtacgagtg
atgcttaatcagattatagaatcggcttatggaattgtagatgaattatattccactcatgtacccagcctaattagaagatgggattgtttttcttg
aatttgttaaatacctaatgactttagggaagaaagttctttgactccatgattcgtctatgcttcctttttatgtctatcgttagcagagagtattaa
gttagaagacgtggagccatgaagatcctaagaattatttttctgttattggcttaaaaggcatcaggtagctaaattatagttaacatattttattt
tcagcttagtctatgttcaccattttgtataaattctatatagaaatctaactattaaggaacctttggatatattaatatttaaataataagggttta
attaaagagtttatttccttctatccgtcaaagttgagttctcttagcataggttggttgaacgagaaaactgcacttaactgaagagaatcctagtt
ttatacaaagtgggacatattagcctaaagatgacggctgagttagagaatcaacgaccagaaattgttggaagtgatcaagttgaagatccgt
atcctattcgttttgaagggaaagttgttcatggatttggcagaggatccaaagatctagggattcctactgcaaacatatctgaagatgcaattca
ggagcttttgaagtataaagactcaggtgtctattatggattcgctatggtatccaagcaaatattcccaatggttatgagcgttggatggaacccc
tactataacaataagctaaggagtgcagaggttcatttgataaatagaaaaggagcagatttctatgatgaatggacgcgcgttatcgtacttgg
ctatatccgtcccgagttgaattatgaagggttgcaaaagttaattgaagatattcaaacggacattagagtggctttgagaagcctagagcgtcc
tgattatgcaaaatataaagctgatgcctttttcaaggcttaaaagctatgttgaaactagaaatttctaaacttgtatatgtactctttctgaaaca
tcgtgattaaaaggaataaatattttacagaataaccgtttagaagataaagtagacaaggaatctatttagaaatacccaggtttttctcttttttt
acctacatgtacagtgattttctgtgcttcctatgagatatataagttcttgagaaatcagacaaagatttaatactaacaatgaattgaattgaat
gaataaaccggttttggggtatcacacttgtttatagataaaacgaatgtactagttattttaaaaaaagcggccgacaggaattggatgaatag
aacaatcataaaggtaatcacaaatcataagtatcaagacaggaaaatcaacagcttattctataggataatcccaacacatgcacataaaaag
ggatttaacaaaagggctgagacaacaccggtttttttcttctttttatataatcatacggaccggagcatatagatttttcataaaacgaaaaca
aagaaccaaacgataataattaaaacacacatatacaccacgtatataaaacttggcttaaacggacgggtggtttacgtcttcccaaagacaa
acacgtttatcgtaaaaagagcaagtagcaaccaagttatctctacggcgccaatcaccaccataacacatgctctgatggtcaccaaagatgcc
gctactagcatctatagaagaaaagtccgtcgcgatgcgaagaatttgggcaccgcgatgcatcaagacacctaaaacttgatgataatcatcgt
tagaatacatgtgttctaaacgccaaacgccgccaccaagattggcattggtaagaggtcgaccaatacgtcttgtgtctaaggtatgcatggaat
ctccatactcacccgtggctatatacggaccgaagggaggacgagaaagaactgagacaacccctgactggtgggttttaatatccttccacaca
caggaagaagggattctttgatcatagcagttaagcactgcatcgtctccaccagaatagagcatgttgttatcatcgcgggaataatgaaccgtc
caagcttcatattggtgttctttccatttattcttgatcgctcctgaagaaatatcgattgtaaggacagcgccgttggaaagtgaaatcgaaaggt
ctctgccatcgtctgaaaaatccaacgcaagcataagtacactggaatcggccaccttgatgcctcgcaaaaacataagttcagcacgaaattga
tggcgataaaacgagatatgacccgtagaatgggcaacagctaaaatggaaggatcgagaggtgacctattgaattgttagacagccaaagac
aagaaaagtgagttctatgcagctataatagaactgttcatgcaataacagacacttgataaaccactacgctaaacaaaacacacaaaaagta
cgattttacgtccttttcagcaacgaatttgatccaaccagatgaccgcatattactctacctgcgtgcgatatacacttaccatttgaaatccaaaa
tagcatcacagtgcatcacaaagactcggtttctgagcgtgttagaatccgtaagattagaaatcctctattccttacagttgcttctcccgtgtatc

gtacaaaacgagttttccatgtcgtaacctagttgcttcatccaacatgtacgtgccgacaaccagaatgtcgtcaaaaacgacagagtacttgca
tacgtctgcaggccagtctgtataatccgtggcaattcctttaataccttgatctgccatactcgccaagtttggaattttcaagttgccttttgtcaat
tgtcaaaaataaattggatacctcagtaaactgcgttatcatttataacggacctaacagtgccaaggataaaataaggcagcacggaattgttg
ctcagtatcgctttacgcacttcgctataccttccaatctttcttactgtaaggtacgaaacactcggctatggactgtgggactgtggacaggccca
aaaagacagtcacacatttgttatagaggggagcaacaaaatttggcagctgccttttcttcataaaccaacaaaacaaattaaacaccacgaa
aatggttgtcggcaaggaaaacaagactaaatacttcgagaagctccatcaactcttccaaaagtaccactctgtctttgttgttaacattgacaa
tgtgtcttctcaacaaatgcacactatccgtaaggacttgcgtggtgctgctgagttgattatgggtaagaacaccatgatgcgtcgtgccatgcgc
ggtttcatcagtgaaatccctgagcttgagcgtcttatgcccaacgttcgtggtaatgttggtttcgttttcaccaacgatgatttgaaggaagtccg
taagaccatcattgctaatgtcgttgccgctcctgcccgtcctaacgctgttgctcctttggacgttttccttcctgctggtaacactggtatggaacc
cggtaagacctctttcttccaagcccttggtatccctaccaagattactcgtggtaccattgaaattaccagtgaagtccatatcgttgcgaaggat
acaaaggtcggtccttccgaagctgtcttgctcaacatgttggacatctctcccttcacctatggtatggatgttctttctatctatgaccaaggtaac
cttttcaaccctgccatccttgacgtctctgatgatgatctcgttgccagtcttttgaatgctgctaaggttgttgcttccgtttctttgactgccaacta
ccctaccatcgtgtctgctttgcactccattgtcaactcttacaagaacttgatcggtatctccttggctaccgagtacaccttcgagggtactgaac
aggtatgtattttacgtaggtttcaagagtcgtgtttttttcgttctaggtattatcagctggtttaaagctgcagttctttcttgctttttaagtagagcg
aagcagtgtggtttaaaaaccgctgatgatccttgtttcgagaaaatggtactattcactgatgttgattgatgaggcttgaaaactcgctatttcta
tgagcgtatgttaaacactctaatttttttgctgaaacatcaagttgtactaatcttttgaaaccttatttattaatttctaactttaacatttactaaca
catctgtaggctaaggctttccttgctgacccctctgctttcgctgctgttgctgcccctgctgctgctcccgctgctgaagctcctgctgagcagaag
gctgaggaacctgaggagtccgatgaagacatgggtttcggtctctttgactaaactcctctcttggtagcttctgaataaactggttatttttttcca
aaaaattcaattactgtagtcgtatggtgcttttaacagatagaagttttcaaacattctagttatgctatgtttatgctacattataatgaaaacta
gtgatttcgtaaaacaatccaaccatatgattctcgttgtctataaactattaaactcactcttttggctgctgggaagactctgcttttaaaaacttt
gagtgatttttttctcattgtgtgaagctgtgccaaaggacagaattttgtgtctgtctttattaaaccttcgttctcaatgcgttctaaagtaggattct
ctacgcactgaacaaatccttcatttgtgaagccgatcgtctggaagcaaggaccgaaggtaacactagagtctaaagaagcaatgatatagtc
cgttgttttgtgcaagaaaatggcatgaagcacgggtcctgcaagaggtaaagctgaatgataagctaaacgcttttcagaagaaacttttgcga
aaattagaccagcgataccctcacatgcaaaagctattaaatttctggaaggaataggaataatggaagaaatcaccattggcttgtcagcagat
aaatagttggcgaatgctgagcggacgtcaaaagcgtctaagcattggcaggttttcagatcccataaaaagacatggttgtctcctccgccgga
tattaaggtagagttatcgtataaagagatacgagagacaaagctaaaaaagttgttaattttcaactgcaaaatactggatttacgaaacttact
cttcatggcccatgcaaaatccctcgattacataagctgaagggaaccgagaaattcgaatatgctcatctctatcagagctaacgataatttctt
gatcaggcttacgtgggtcatttgtaagacaaagctgagttaaaatcgaaacatgtcccataataggttctaatttccagtcttctttagattcctttt
tttcttctttattttctgaagctgtattttcttggttttcagaggatgcatttttgaaccactgctcattcacacggtaaatatcgccgaatttatcacca
aatacaaggtcaccagaatcgtgaaatttcatatctgcacagcgtttaggaattgtcttttcgtagcacaaacgagctttagcagcctcatcgatct
cccaaagtcgtaagcatttatcttcgcttacggtagccatacgggattcatctttggaaaacgcaacttggcgaaatgcttgtccgtaagctgctgc
ctctttatttttatgctgtaagtcatcttggtagcaaaactcaaaggtttttttctccagtagtcaaagaaaagcccaataaatagggaccagaacaa
catacgagtaactctcttttagggaggtaagaaatgaactgacaaggatggttaaaaatacctgcttttggtgttgtttctgtcatttttacttcaatc
tataggataagtaaacaaagacgcagaccaagctaatcaaaccggattgtttccgtttttatataaagactgtacagaccctgaaaagacgatgc
tgtaaataattgtcgtaaatcaaattcttcaaagtccttcggccacaaggtatcttggatgcacaaagagttgagagctttgtttatattgtataga
atcaaattcctttaattatgctctatcaaaaagataggtcgtacaaaatttcaaataaatcacagtaacagaaggctatcgaaaacttctccaaaa
tagacgttcaggattcaaaaacttcaagtttttttggatcttttcgctttaaataaaaatatttgttgataaaaagttgtaattctggaatctggaagg
gttggttgaggacatttcaaatttgaaaacttttctgtgtgaagctactctctgcccatcaggctcaaagtctaccaaacgttggattctgctgtattc
gtgactttttaggaaaatattattgaaattgctttcttttttagaaaaattgggcaaaatccgtgttcttttgaaagtatcattgaacatttccagtctg
ttcacttgacgaatcttttggttttctcgtggaaaaaatgagacagaaacgcgcaaagacttacagaaagttgatgcacacgtatcaacttgttttt
ggttttcgagaaccatatcaagttctaggtaagcacttttctgtagtttttcaaatgtccttatgattcggtttggcataaacgcatttggtcttcttaa
atatgatcgtattggatgaaaaagctaattattacttagttgatgctgattttatctcagatttaacgaaatcaaagatggatattagggctgctatt
tctagaaccgtgcaaggagatataaaaccaagtatgtacaacacagtagaaaatttcaaaaggtgaaaggcttgctctggttttgaaggtgtttt
gttcaccttgtaacgaaaccaagaatgacagaaccttgtgcatgcttttcctttttattcgtttttttattgattgaattactaactctgtctttagtggt
tactcaatgctgtatacgacaactgtacagtcggtcccaggaagcaaaacaagaaattcaaatcgctaaatcgttcgaaagaagacgttgtggc
catattgatgaagcaaagtctcctttagaatgtatgcagagctgtatcgatgttaacggaagaaataaacaccgatatgttgttgcaacacagaat
actgaacttcgtgatttacttcgctctgtacccggagtaccacttatttatatgaagcgctctgtagtagttctagagcctgcttccagagctacaat
cttggaaaagcacgccaaggaatcttcccaaatgggtttatcaaaggaagaaaagttaatattatctggcaagagaccacatagtgggtctgaa

agggaagaaacggaagaaacgggcaaagaagatgataacgaagcttctgaaaacgttgaggagcaaccaaagaagaaaaagcgtaaaggc
cctaagggtcctaatcctttgagtgtaagaaagaaaaagaagtcagcatcacaatctgctccatctgaaggtccccttccagtaaatatcattgga
gacgtgggtcaacgcagaaaacatcgtcggaagagagattaaggaacttagcaataaaaaatttagaatcgggtcatgcatatatccttttgttt
agtacgttataaggtaatttggtattgtattaaagaagctctatagaagatggtgtagggaaaagtaaaacaccattgttgttgaattcaacttgg
aaatgattagacacaaacacaagatgaagtatgtgaagtatttgcctttcataggtactcctgcttgtttcattgacagcatgaatccgtattttacc
tagaacaatctttcacttgaatgatataattaaacagagaaatcacttccgattccttttagctgtgttttcatcctgatgtatctgagcactccacgt
agcgcatagctgaaagctagcattacaaatggacgtggaatacaatgctaaggtttttttctaattactgataatgattggaatttattttgtttttct
ctccactttgtatggatattttctgttgttacttaacactgaaaacagtaaataactaggtaagaaacgaagccaaaaatgcaagcatccattgtc
ctctgccttgcaacatataaattttcagacttattgtgaatacgagaaagccagttgccaaagggtcaagctcgatggacatgccgcgcaaagaa
ccatttttgagttgtgtggaatatttatttataatttagtttatgaattttactctcaaatatgagaagattttagtcgcactgttgcattatactgggt
attatgatcgactgcatttcacattatcatattaaagggatttgttacttgtatttgtcaccaaaggagaattataatatgatgggtcgataaaaagc
gatggaaactgaatgtaataatccattacaaaaggacaatacaaatcatttcttttgacattcaagctaatatattgtcaggggcgttccgtaagc
gattctgtctcatcagtttaacgatttccacagctaggctcatcaatagcattcgaaggataattacttaacttttaagctcgttcaaacaacaaaa
aattcttttaaaaggaagggttccactcatttcccaacctaaccaaggaaatcttccgtataggctataacatgtctggctacgaacctatcaagct
agagtaggttcctaaaagtagtaagcaaaaactattatgtaactaaagaagctcttttaagactgactgactattagtcatcgtcattcaccttgac
ttgacagaagtttatgcttcttcaccaagaaccttttacgtcggttttctacgaagaaataaaagacatttttactatttttcagttacttgttcttcc
agctctttcgaagaggtaaaaagagcaaaaagacctcccaaggcgagactcgaactcgcaaccttctgatgaccttcttaagagtcagacgctct
aaaccgattgagctacccgggacttgcttgtttaattatcagctaagacttctgtattagctattattaattttgttattgtaaaagtctaagtgtattc
ggtcttatgataatattaatttaactgacacatttatcttatttacttttctaacaagactcaacttccttacaactgattgatgtagtaggcaaatga
aattacaagaataatgtaccaccaaagcacaacttacataaaggagcaaatacaagcttcatgaataagaaagcacggaccaattgtttttatt
accgtttcgcccaaagattctacttgacatggtgaattcagaatggactgaactggcatgaaaagacgatagggtttggcaatgaaaatagcact
cgtcattctagattctctatttttatatataattgtattaaatgactttttagatcacatatccaaaccaatccactcttcaggtgcataaatattttta
ctcgaaacaagatcatccctagattggatgagtcgaagttcgcagacattgcttagtaaaaactgatctttttcaatgctagggtctcgctgcaatt
gttgaagggtatattcctttgttctcacaagtacagagtgcatacaagttaaaacttgtccaatcacctccgtgaagaaacccaaactcgattcttc
ttcgtttacatctttgaatgaacttcgtttttgtggagatctctcatccatgtacttcgccaaggtaaggctcgaaaataaatctcctgttcctgtaaa
cgttcctgaaagggaaggatacgagtacaggaaagaatgaaaggtattatttttgaaacaatagcctaagcaaaacaacaaatttgattcattc
atatcttgaaacgaggtaatcacagcttgaggaaccttgtataattggtagatacgctttagcccgtataatgcggtcgacttgttcacaacagaa
actcctgttaataatctttacagataatcagtcaataaaaatcctgctaataaaataaggtgtttcattgcaaatcaattgcatagaacttactcga
cctcaaaagcattaggtgttattataaatgcatgggacaaaacccttttatatgtatttaaaacatctttctccacataagcctccccctggtctccc
atgacagggtccgctatccattttacattagggtgtcgacctttgtattcgattaaaagattcgaaactacttggaggatctctccagttgggataa
aaccagtcaaaattgcatcgtaaacaaagtcgttttttttccaataaggttgccaatagttcataaacctcagatgctttgcatacacgacctctctta
ttaccatagcctgaaacatatattagaccaaaaaatccacttatagcaaggtacgggctagaaggctttagaagaatttagattgaaattgatga
ttcaaacaaaagtccaactccaagttgtctcagaactccttcgtatcaagaaaatcttctttcttctgaggaccaaacataccaagatggttggaa
aagtggactgtaggtacaacatccacttcccactcatgcaattgcaatgggaaggtagcagatctatttcctacataaccgtggctgacagaaga
ctatacaaggattgttagttgcaagatcatattcaacaacatacttgaatggataaaacacgctttgaggcttcaggatctggcatttgaatgaatt
ttagaagaaactaatttatgtatatgaacaggtgaaagtaactaaacaatgttatgggtataactcgtcaatggaatacatgaaagctaagaaa
aggtagtggtacatttagtaagcttgtcatttagcgtcaacattctatgggatttgatgttgctactttctatttcttttcttttctttttctttttttagtttt
agttttatttttattttccaaaaggaatgaaataaacttcatcctttattcaatgtttcagcaacgctaaatgaaatgtacagtcatttccaatagggt
tgaaatgaatacgaaaaagacattcttttacatttgaaaagaacagattaggaagatatgagtggaatacaagcttctaattcaattgcttgactt
acgcttcttaatggataagttggataaaacaagaatgaacaagctggcattctaaagtattttaaataaaagctttgtacacaattaatgtggcta
ctggatccttgtttcttctaccctttgtaaagagacaaagctcttttgaagaataaaggatctcacacttgaaattcacgacatttttaaataacttta
atgtaaaacatgaattgatgaaaaagaacaagttcatgatggctggaaatgcacagtaattttggtttcccccattatcttcagcatgcttatttct
gaatcattgaaggttttcacagtagcaaaacctgaggaaaatttggcaaacaatgtgacatattgaaactgaataacaaaataattgcggtagc
gctatcccattagcttggtcattctttcttataccaacaccttcgtacacctgactgacctgtgactgacagccactaagatataaacccacaagag
gttttctcagataatttatttcaattcctctcaggaattcgtttctactcaacccctattcgcatgttgtaaaactcgatacttccttcataccatttgat
cattaccaacccaattaataattcttctgaagtttagatcatattcatcgaagttaaaagagatatttcttattaaatacgaaaaaaatcctttttttg
gaacatttatagggactcttattttctgcagtgctttcattatgtcaaactctgcagacgcatgtatggtttgtctgaaattgggatgactgggagatt
atatcgaaaattctataattcgttcaaactcttttcattgaattcaaatattctgcttcatccctgtcaaagcttcttaagacaacaatgactcatttc

gaaaatcttagtgcttcccggaagttataattgaatcaaagtatactaactatagttcctagttaaaatcctaacgagttctcaaaaaaatgacaa
gccgttcgtgaaaaggaatcctcaacggctttcttttcgagataacctccgagtgtacattgaacatcctcaggcgcacagtaatgtattatattat
gatgaagattttgtttttattagggataaatatccaaaatcaaagatgcatttgctcttgatgccacgggatccctcgttaacattggttcaccctct
ggagattttagcaaagcatagggaattggttgaaaagttggtttctatggtgtttcgaaaactcccggatttaattatgagagaagctagaaatag
tcttttctcgaactctgatctgtcgtcaaaagtttcgtcgaaaacgctttgggatttcatcaaaataggatttcatgcaggaccatccatgaataatc
ttcacttgcatgttatgactcgggaccacgtttctccgagtttgaagcataatgctcattacatatcattcaactccccttttttgttaatattgacac
tcctatcgctcaccttcccgttcgaggatcgctttcctacttatttcaacaagacgtttgttgctaccggtgtggtaaatcttttggaaggcagttttcc
aagttaaaagttcatctgtcagaagagttagattcatgggtacaagatactttagcatctgaagcattacatgtgtgaaatattgctttcactttgtt
atagtctattacgttatttatttattgattaatttattatgaatcaatgagaacggtattaattaaatatatcgtaaagatccaaacggtagtacttcg
tttatccttttctgtccagtcaaaagcatcactaaacgatcgacacccataccccatccagttgttgggggaagtccgtattctaacgcacgtacaa
aatcagcatctggaataggtgtttcttggtcccctaactcggtacggtcataatgttgagattcgaacttcttcttctgagacatgggatcattctctt
cttcgtaagcattacaaatctcactttttccaagatacaattcaaagcgcttagatacgcgatattctgtttccccatatttaattgtttcagactttgc
tagcggagacatggtttctggatggtgtataataaaagtaggaccatcgctaagatgttctaaaacgcaatagtcaaaaagtttatcaagtaaat
gagagactgtaggtgtttttggtagcttaaagttcctctgttcatatatgtctaacaaaaattcgcgatactcttcagagttattaatagtgcctaaa
gattgattaagactttttcaagagatgggataaactcaagagtttggaaggaatcgctttccaaagaaactccaagatggggtaagctcatgttt
ccatgtacattcatgcagatttctcttagcaaacctttggtcatctcaatgaatgtgttcaagttaataaaagcacaataagcttcgcagctggtaa
actcgggattatgcgtggcgtctaaaccttcatttcgaaaattctttcctaactcaaaaattcggtgcatgcctcctatgactaattgttttagccata
gttcaggagcacaacgtagcgagagtggctgtttttgagcatcgtttgtaataaatggacgagccatagctcctccaaaatggtgtgaaagcattg
gggtttctacctctgtaaactcattcttttggaaaaaatttcgaatacattctataatctgaaagcgctttttttagtacacaaagggtatcgggatttg
caactatatcgacgtaacgttgctgaaatcttttttcttgattcaaaaggttggtaggcatttggtgaagacaaggagacagcagaattggaagct
cggttgcataaatggatagttctcctttgctggtcttgccaatgaaccctgaacattgtatataatctcctcgtcgaaaagccttgatgatgtgggag
aaagcgagggattgactggtattgtcagtaatattgtttttactgatagtcaattgtattttaaacagcccatcacaaacgtctataaaagcaagct
ttgaacttgcgtatctcagattactgattctaccacagactgtgtaggcttcattatattgtctggcgtctggcaaaagaatattatcccaggaccgt
agctttgaaattgcagtgatttttttcgtgtctgatatggatggatacattggcatcttcaagctttcaaatacctcttttcgagtgtcgaatttctcag
acgaacctggtttttggaaaataaccttctccatcctaaacgaccaaaattaatatactttgttgaagaacattttaaaatatgagcccttcgtaat
agcattagatgaagaagaaggaatagaagtatatgggttattaaatgatgttccttctgggtttttggttcctcaatgaattgaaaataccataaa
agacacttttactatttaaaataagttgggtttttttacgtttgttaaagagtgcaaagttctaatacaacatgcttttgttaagggcagaattgtatag
acaaccaagaaggtatgtattgagaggtaagtaaatctgcggtggaagccccccctcccaaataaaggaaaaagaacaaaatgattgacaatat
actacacacatggggagttttaacagagttattgagattgattcacaactggttagtcaacaaactttacgcttcatcacatttggcaaatcactct
gttaactttttcattgctatatctattgtacattttataaaagcatcttgcaaagattatgtagcttatatatcaattctataaaagattcaaattcttct
aatggaagcatgaatgtaagtcgattcacgctatcttaaattatcgcaaaaacctttataaccaagaaaaaaccccagtttcattatcaaaccatt
tcatagacattcagcacgatggctcttcttcaatttttaaaaattaggactgaccaccattcttcgttgaaatgtaccaagattccacatacgctttg
attcataatcaagtgctgcaacttcttgaagaaatagacactctgaaccggactgaactataaagtcgacaccacccatgtccataacagtttttc
cagactggtgaactcgcattacgccaatttgaccagcaggaggttgaaagccaggaagtggtaaagaagtcaaaccgtcccgataagtttccttt
ttcttagcttcactatcacctgtggtttgaggaaccttttgctgagtatccggttcctctttgactttagctgattcttcaggccatatgggatcaatgt
ctgcttgaccaaggaactctggaagtttgtcagggaactgaagcaaaacgatctgatcgttaccttcggtttcttctccttgctgtgacaaacccaa
atcttgagcaagtgtttgtaaatcaaatgcgagcttctcttgctcttcctcttctttatccacgacaacagcttgaattgcactttcagcatgatgcttc
aaaacctcatcagctgattcagggagcgtccgatctgagataacaggaacaagcatatcagatccttctaaattgatagcaccctcttcttgaccc
gacagcgctctgagtttttgtatgttgaccatgtctcgagtatgaccatcctcatcatctccagcagcggcagccagctttgacaaagtatcttcttt
actttccaattttacacctgcggcagagggtggagggatgtatgcagagccggctggacgacccccagaagaagaagcgttgggaccaagcgc
aaaaggaccagaggcagttaagtttagctcaggctttggtctctgttttttccattgttgctgggattccacattgtattcaaccttgacccgactatc
gacttccttttcctccgtactgggaccagcttttcttcgaggaacaaacttgggcgtgaattttctcataattgaaaatgtgagagaaatcttttatg
acctaattatattatacaaattttgcgctcaaccaaactgaagggacgtgatatgaatgatgcagaaggcaaaacaaatatatatgtatatataa
gcaaaagtgaatccttcgtttaagccaattccacttttatatttgtaagatgataaaattaataaagatatttcaaagaaacagaaacttcgagtta
tttataaatctacctagcactttttcaatgtgtgttggagttttactttcgatcctcgagcattcctcaaatgcactttacaattctcaaatggtttaatt
ttccaatcaagaaattaaagttatttttttctcttcaatgcttcttccttaatgatggcttttggtaactttttttctggtgtaagcgtggatcatcgatc
gcggtagcattgctacccagtgagaagcatgtttgtttacaaacaattacattaaaaatggatgtagaagaactccttgaggaacctagatatgc
cgtagtttctaatactttataattttattctccttttttttccttttattttcttcttaaatccaaaaacctgtacaaagaaagatcaaaattattttttttag

cgaacgttttccttcataagaggttcaagacaactcaaatttcggtttcgtccattgtttcctgaactgtaaaaaaaaaaaaaaaaagtgaatgctt
aattatgcgaaaaagatccacgcaaaaagtcaagaaaccaaacaagagactgaccttttttcagtccttctttcgatcctgggaacaaaccctttct
gtacaagattttcgtaataaaataaaaatagaaaccaaacctccttgaggaggcaaaaaaatttaattgacgacaacggcaggattcgaacctg
cgctcccaaagggaatccgctgggctacttaatagcaggcgaacgccttaaaaccactcggccacgttgtcttgttacataccgctaacaattata
cagatttatagggttatttgcatacagaaacgaatattctctgcatagaaacttgaatgaaagttagttcccttgtgaacgctcaatatttagaaaa
gatcttcttgaagcagccaggaagttcgtttcgcttacagcgttggggattaatgatcgtctgcatattttgtaagtaactgtataaccgctctaagt
ccttcatattttaatagaaaccatcaaaaagaactcaatgtgtcttttcaaacgaaaaaaggctcttctaatctttttttaggtggtttgtttattgaat
agtgtttttccttctgtctcgttgaagttgcttctccttccttaaaacaacagatgtttatttgttacttaagtgttcactctcactttgatgcgaaaaac
aacaccaagcaacaaccgaaattcataaaactcctggatttgtctatatctttataaaattcagtagaaaaaaaatggatactgggaatgcatcg
tatcctcctttaggaatgaactccgtggcttcgcaaatcgcaccaattcgagacgtatgggcaacgaacctggaacaagaaatgactttaatcatg
aatttggtcacccgttatccggtaatatctatggatacagaatttccaggagtcgtagcccggcctttgggaatttttaagagcagcggagattacc
attatcaaacgctgcgggcgaacgtggacactctaaaaatcattcaaatcggactagccgtgagtgatgaggatggaaatgttcccccagaagc
attcacttggcaattcaactttaaatttaatctacaggaagatatgtatgctccagaaagtatcgagcttctcacgaagagtggaattgatttcaaa
aagcaccaagaagcaggaattaaccctgctgattttgctgagttactcattggaagtggtcttgtccttcaggaagatatcacttgggttacctttc
acagtggttatgactttgcatatttgttgaaggccatgacccagttatctttacctagtgagtatgaagatttttacaaaattttatgtatatactttcc
taaaacctatgacattaaatatattttgaagtctcttctcaatagttccaaaggacttcaggatattgctgatgatttacaaatccatcgcataggcc
ctcagcaccaagccggtagcgatgctctgcttacctctagaattttcttcgaaattcgctcccggtattttgatggcaatatcgacagtcgaatgca
aaatcagctgtatggtttaggcacatctggcagttctttgtggatgaataccccctaaacctcaatttcgcgatttgccaggagctcagcccagtcct
aagcccacagctcctagtatctcttctgcatttgccaatccacctgcctcggtttctcttaattctactttccgctttcctcctcgggtcatttagaattc
tacctcctttcatctttctgccattgttttaccctttatccatgaactatttaattcttttgctttttttgaatttcaaaaagtatattgttagcgttccgtatt
catgctttcaattcccttgccttctattttagtccttttctatttttttttcgcttactacctgctcgcctcacacttcttctatttcccttctttaccgtttcca
acgtttggctcttttgtcccaactattattttcctctttgtttgcctcatgatactttgtagattaggtcatagatcgtttattctttgtgaatttgaaagg
aacaagtggtactctactttgaacagcatattctatgaaataattttgtttaatatccgctattaatataatcctgcatgtattgacttgtgcaaatag
atcctaaaaatcttttcctttgctataattttctatcatccttacgaattgtagttctaattgccaaactttccgtttgtacatttaattattttattgtac
aaacaagaatttacaacagaacacgtctagtaaccaactttcttattttcgcttgtagtaagtttaaaagtctatttttatcaatcgcgctattcattc
gtcaatcaaataaagctcgcttcttggttgctcaaatgtttgaattctctaaaaaaaaacctgttaccacagccaaatttcattttgcttagaaaggct
gtaaaatggcgactttactccctcctaaaggaaaaaaacaaaaacgggaagaacaaaaccctactccttttgaaattcctgaaaagtttccgctt
gtaaatgttcaatttcaagcttcagatggaagtcatgaacttccttctcttttggtccctgggaacagttcagctcgtcagttagatatccttttaaat
caattacttggctctgtatgtaatttaattagtttcaacttaattactaactgtgtttaatttcagcctgaggatcctgttccttacacttttgctttaaa
acacgacgatgatactgtggagattcctgataatctttatactaccgtgttccatagcgggcttatgaaaaccgaagataatctaaccttgttatac
acccctcaagcagttttccgagttcgcgcaattactcgatgtgcagcttcaatgaacggacatgatggcaccatcatttctgcacagttttctccaa
gctctagctctcgtttggttactggaagcggtgattgcacagccagactatgggattgtgatactcaaaccccaatttctactctgaaaggacatca
taattgggtttcttgtatttcatgggctcctgatgcttccgttattgctactggaagcatggataatacagtatgtgtgattttcgtagtcaattaagc
gctttttttctgccagtctactaatgtcttagattcgtctttgggaacccaagaaaggtgcttctcttggtgatgcacttcgtcgtcataccaagccaa
ttatggctctttgttggcaacctcttcacttaagtcctgactctggaccatatttactcgcctctgggtctaaagacaataccatccgtgtttggaaca
ccaaacttagaaccctagtttatacactctctggtcatactgcccccatcacgtgtgtaaagtggggtggacaaaactggatttattcttcttcgtat
gacaaaaccattcgtatatgggatgcaaaggacgtaagtaactattctttatcttcttttctcttactgatatactaacatattcagggaaaatgttt
gcacattttgaagggacatgctgctcgtgtaaatcatctttctttgtctacagaacatgttttgcgctcgggcgcttatgatcatactgatttcaagcc
aaaaaacttccgcgaggaacgtgataaagcaaaagaaagatatgatatttgtgtaaaacagtccggagaacgtcttgtttctgcgagtgatgact
tacaattaatgttatgggatccttataagactactaagcctgttgctaaaatgcacggtcaccagaaagttgtaaattatgcatcgttttctcccgac
ggtcgtcatatagcaactgccagtttcgacagctcggtacgattgtgggatgctaaaacagggaaatttcttgctacactacgtggacatgttgctt
ctgtgtaccagtgtgcttggtctaccgattccagattattagttagctcctcccaagacacgacgttgaaggtttgggacatccgtacgaaaaagtt
gaagtttgatttaccaggtcatcttgaccaggtttttgccgttgattggtctcctgatggacaacgggtagcctctggaggtgctgacaaagctgta
cggatatggactcactaaaaaaataaatagggtacaaagtgtcaatctttagtttcgctaattaattaagggatttttggacatagtttttagaaaa
gtaatgataaaggttattatcattggcttaaactgttttaatactacatttatgcagactggcttaccatctcatgttcgcttttgaaagggagacca
agaaatatttaaaacaagttcacattatatataaattatgcatagatatagtactataacatataattaatctcataaacaatcgttactgaaagta
ctagttaccaaaagtcataaattcaaatcacataatgcagcaagccaatccatgaatcaccttgtcaacttgatactttatgctaagccaatcaatt
cggcaatgaatcatttcgtaaacttcttctgtgtggaatgcagtagaaaaacgagtattatcagtagccagttttccaatttcttcagcattcttacc

gtcgataatgaatagatgtgcaatttcttttaatgctttgaaataaggcaatatggtacgctgtttcagtgaaactacaaattcgtaatacatgttta
gatcacttgtatcatgttagcagatcttgtcaaaaataaataagaataccgacttacctcagtagcatcaagcctccttcaacagtaaatttacgtt
gctttatgtaacgggtcaatgaattgaatagacgcattcctacttctttgtagaagacgtccaagatctcatgatccgtacagcccactaaaagcct
catatgcgaacgcaaaaattgagtgatgctttttgctgccaccgaaggttcaaccatcgtatttgtgataccgacagactgaggacaaaaataatt
gataggggtcttcgattccagcaaaaagtctacatgttcaataatgacatcgattcccttatgtagaccatatgcagctctttcatccaaaagttgtt
cgaatttttcttttcggcaattgctggatcaaacatgtcatctcgaacgcatatagaactcatctcttcattatagaaagtgtccatcatttgttgaa
tcaaatccccgacgtttatgagttcgaggaattttacaataggttcaacagtattattagcaaagtcttttctaggatcaaataatgacaagtgctc
aatagctcggtcaaacccatgtttaagatgcccttgacccaattgcctcagtagttgaacgaagagatccttctgcaagccttgaatatcttcgtctt
ggctgtttgaaatcccgagaaaaattttggttcgagcgaggctaattttacataagtgcacaattttcaaagcaagttctaaactgaacatgctact
tataccttccaattttgaatccaaaacagcggcttgagccgccaattccgtagtaggaagatgggataattgatcttgcgaaacataaacatttgc
tggcacaaatcttgaggcatccaaattctctaaaccatcgtatgaatcggcatcatcagaaagtcgtactcgatgagttaagtttgcttgctcatta
ttctcggaaggaaaagaaaataaagaaacaggaagcaacatgacgttcttgaatgtttccaagaaattattctttgcagtattttgaccaacgttt
ctaaaaaagaaagattcagtagcaccttccttttcttgaatggacatgtcccactgatcaaccacagactgtgcgacaaccttgaaattatgcagc
tcctgggaaatataaatatcgacttgagattgaaagatagtgtctacaagcagaggaccattatgaatagtaaacaagcgcttacataaacgat
aaagaccagaaataaagatcaaatataactcaacttcctcatcacctatgtgttcaacagccgatgaaacataattcttaaggcaattcccaacg
ctttctcttgcatacgggatctcaacgagctcagggactggaaaagatgctgaggtaaaggtatattcagacgttagtaacttgattgtttcgtcta
ttacattttcgaggattccccattgaatgtgtgaaccctcattgtcggaattagtaacgaagagactgtatgggtcaaactgaaagtatgattgtaa
atattctgtctgcttcgacaagtacaattgaatatatacattgggcgctgaaaatttatgcaatacatgagaaaattggctaacgagctctaaattt
cgcgaatcaataccgagcgccaattcatctaagcatgcttgctcaaacaaaacggccatatccaaaattgcattctgcatagatgagtcaaacca
tccgtacggttttgagttaccgaatcgcagtagaagctttaaaacaacagcttgttcttccggcgtttggaaggcagaaaatgcgataggatctag
tgtattacttgcataggccaaatttcgatatattggagccagcgttctccagacacgaatataatctagacgcgctagttcgatcctgtgacttactt
tgtcaaatacatgtatcacatcctctctaccttgttctgaataataatctatcgccgacggtaaagatgtttcgctttccggtagtgtttctagcaaag
aatgtggtaatgttccaatcgatctttcgatagcaatgtaactagaagtactttctccttgtgagctcttattcttttcattgacgctcgatactccttc
actggacactccttcactccgagattctaaatataacgatttcgataatgcggtatcccatgcccccatcaataataactttttgatatagatggtgt
tcgaacgacacaattcgtaaaatttcttacatactaaatgacaattacacaggtctatagtaccaacaagcgagaaaattcgcaaataaatgtta
attgttagctttccgtacagggcacctgtttggtgtatttggcgacttaaaagctttttacgtaatttccttaacgcattttcacgagcttcagaaccct
tcattcatgtattttgctaccggtcaacatttatgaatttggtggatttctacctcttttctttagattctgaaacgttacatttccttaaccgacttaaa
acagtctactggggttcactatcctcttacaactgtcacttagactcctccaatgtcccttcattgcaatggtctattcattaaaggtcagtagtgtag
aaaagaatgaaagccaagttatacacacaaaacgaaacagaaatgtcgagtatttcgattgtttaacttcaatttcgacataaaatgacttcaat
tcaaacactaagcagactataccgtttaacctcttaaataatacatgaaaaattaccactagcaataaaataacccttctttatctgacatttttcct
ctaaaacacccacatgtctagataattcatctaatcattaagtatacctttaaagtagtccaaacattttccactccccaaaaaggtgtttctcagtt
cgcttgataaactaatcaataaatagcaaagcttagcaacagccaactaagcttaattaacatctttttctaaagctgcaaaaggattcatagcac
tcaaagattgagagggtgtcagctttaggaacgccttcttctttgaacggattcgtggcatgcgaaccttactaacgacattctcttcattaacttga
caaacatcacaacgatctgcaatatttgcagtcaacaatcttcgatttaccaatggccgtaatgcaattaatcgtgaggatatactagtcaagtctt
gtatatctatcggcttcataaccacaccattttctacatggaaaatggaccaagtaaagctttgcaagtcacctatatcagccaaagcacttcttat
ttcttcctccaaattttcagatcccttttgaaaatcagtaacaagaagagcattatattctgcatcatgagatgactcaggaatctcttcctgcaccc
cagacaccgatttgctcgtagcagatgacgactcaccatccaaagcactgtcactttgcaatgtcatcgttggatgctgcaaataataatctaacg
cttccgctagaagcacgtttggcatcttcatgatacctttattatagacaacaacattacgcttgggttcagaatcaaatgattcactatctgaacca
tacaatttcgctaaagtatgaataaacagccgttgttcacgttgcattgggggaaagctggtactgggttttccgctttcacatagttcacgaagag
cagactggatctcctgcccaaactctctattgtttcgataataactcaataaacccttcgtatattgtgaaacttctttagctctgcgatctgggttga
tattcaacgcctctgatagaacacgtcgacgttcttctctcgcacaactaactgtacagggtattttatgctcaggagaaggattatcaaaacttgc
accacacggcgctcggcttttaatcaacccgcaagagcatacagcatccaccgttgctctgcatggaaccttttgatcacatggagtttcggaatg
gcaaggtaattcacatgtgtgttcacagtataacctacgcttaccgcaaggctttttgcaaacagtttcgcactcaccttcgggatggcacgtcttct
cacacctgtgaattccgcaacccaacaatttcccacaaaccgaaccgcaacgaacgtttcccttcgagcacggttgattttccattagacgtttacc
acaaaggcagtactttttcaaaagataaggacataaggggcagggttccgaaggtggatgacagttatgttttacctgaggatgtccacatggttt
cggtaaagtacaaagataaggacaactaggtataggtgtgccacaagggataggaggatacaactttgtacgtccacaagcacaggtaagttct
tcaaaagaagcttccaagcatcgaggacaagggccacgatggcacatatgttggcaaaaatgatttccacatcccaactttttattgcaagaacg
caaacatatatgttcttcttcaaattcttcggtaagcaagtgaaaacgaagcaaagcacctttcggacgtaaagccaaccgagcttgcgctttggg

ataaccgctacaacacttcttattacattgatgtcgtccacaactaagtaatacattacaaagccgctcgcaagttggcaaatatccattttgaagt
tcttcgcagggaacttgcacaggattgaacttacacctgcatggaacagtaacaacttcttgacaagagctacaaggacctggatgacaattctg
tttacattgatgcccacaaggtaattgcttcaaacaaatgttgccacatgttggaataggatcttcacatgtagcacgctgatggcctttcaataga
tcagcaatatttttctttccacagaaacaccttgttacgacatttggagataaaggacatcgttcaagagcttttgatcgaggatgacagaccttgg
aacatctatgaacaccacaattaaaaaattgcttacaaagttcaccgcagctataataacccaaaaattcggtaactgcatcatccattaccttag
tgcaaagaataggctctggtaagacagaacacgtgtaagtttttgaatgagtaccacaatagcagcttccaagaataggctcgtgacatgcacca
cataaacctgcatgacaacgctgtttacaagtatgcttaccacacggtaaaacttcatcacaaatattatcacatctgaaacactcctggggagct
ctagactttcctgaacaacgagtgattttactttccttaccgcacagacaaaactgttcaacagtggcagtgcaaggaggacaaggtccaggatg
gcaaagtaatggacacggatgtacacaatcttgacctcgagttttaccacagggttccccacaagaatgaggtgtcaagcctttaacaaattcag
gttcatcttgcttaccgcaccagcatgtatattgaagagatttttctttttgtgaagtttgacaggaaggacagcgccaaacaggctcattcctttgtt
caatagaatttttcatccatttcttgtacacttcaaatgaaacacatggaaacaggttgaacaggtccaaacaggagtgcttggtctcacaatatc
agtacaaacactgcattcgtaaagcatttttttcatctcgataatcattctcgaagtcatatcaagcttagagagatcgatattctcttttaactttttt
gacccttctttctttttttcctttagaagctcttgatgcctctttagacttttcggcttcattttgcttttctgcattcttactgttagattctttcctgtctttc
ttggtagacttattatcggtagtatgtttggtggaaacgtcgtcgaccttcttccttggtgaaggcttcccttgatttctatcgtgattcgtcttattctt
atttctatccttgttctttttggatttatgttcctctttctctgaattcaagggcttcgcgtttgcctcattggtagttaaacggtttccagatgaagattc
cgttaccagaccgatgcccggaacgaaactaggggcatcggctgacaaagaggctgctaaagataccttttgtgagctaggaggatcactactt
aaggaagcagaagggtttcttgtgctgtttttgtttacatcatttgagctagaagatgccaattgagtcgatggatttttattctttctaggaaacctt
cggctacccttctttatttgatgtgcttcagaggagttcttagatgtttccatccagagttgagtattgtataaggacagatctactcgtggcaagct
gtgttgtgttttacagataagtaacaatcaagtaacacaaaacaaccatatctaagtaatatatttatagttatctgaaaagaaaaaaagattaat
tattttgggcccgaatcccaatttttttaattaaaattatttttaaattaataggaccatgcacctgcattaaaacacaaatgacacagagcttttc
aaagtgcttgaaacctcgtatcataaaaaatgtcaaaaagatacttctcaaatcagagctccgaatttaaactgtaatataccacattaggagta
gcatataattaagccgaactatttcattttggatctgagtaggttggttgttccctagacgtaacaaaaccaactgcaaaacaataataaataaac
gcgtaaattccgttcacgtaaccgaatcctactcaatttttgatttttagcaaagccatagattgcggcctcaaaatttttgaaagcaaactcattga
aatccaatttttataagcataagtcaatgatttccactttaatggtaattaagtacaaaaattatcaaaaagaacgaatattaagcagactcttca
gtctcatcacggtcactaaaacaaaccttggctactatgctggcactgaaggatagtattgacaggtatgtagtcgaagcaataaagccataacg
atcgcgaaagaatccagagacaataggaccgataatcattccaagggagtagacaatgttgtaagcactaaagagctgtgtataagggccatta
tatccaaagaaggtagggtgttctaattcatattcagcaacataacgagaagactgtacaaatcctggagatgatgtgaaagataagacaaccc
cattcaaagccaaaaagattccaaacaagtatacgttgaaggtagaatatgttctagggatgcacaaaaggagtaatgtcgcacccaggacag
cgtaggaaaacataccaatggtccgagagcctctacgatctaccatagaaccggcccaagctccacagaaaaagtatggggtactcaaaacac
caaacattaaaccacattgaagggaattgaagccaaaaatcttctgcaactccaatggtacggtagcatcaaaagaagaaaacaaagatgaat
tcacgaagctcgtccagaatggaccaatgagttgacgatgagatataagtttatatataggcagaaagaaaattttaacgactggattcggcgaa
ctgctgttggtagccataggatcatctctaggacgtaaaattggctgactttcaggatcatcctgcaaacctttctttgcttcccttggttcaaccata
agaactcggaggactatatcaatgaacaagatgatgaagcacataccaaacgcagcataatacccaagagattcatagataatccctccgaaa
acaggggcgacaatctcaccaagcgaaataaaaccaaaaattccacccattgtcgacccaacatgctctggtccaaccacatcgactaacaaag
ccaaacctactgtccaaactaccgcagcagacaatccttgaagaacacgagctataataagcataggcacatttgtacctacagtcaatagacc
agttgcagatgccaaaaacaaaagcccaatcaacataggcatttttctacttaagaacttgtcagcaagatacccaataggggagcttgctgtaa
tgtttgcaactgcatagacagaaagaagcgtactgatgacagcttgaactttttctggcggaacaccaactcggtccaccaacgaaaaaggaag
aataggagtaataacaccgtaaagaaaaagatccgtaaacaacgcaattgcgattgtccctataataaagggaagactacttcttcgcttcacac
taagtttcaagaattccatgctctttcctgactaaatctttataaaacaatatattttcaatcgtttattactgagcacttaaattatcttcaccagttc
aaaaattattcaatcaatgagacaattttcctaaatcaagtccaactgaaaaagaattcacccaagaaccaaaagaaaagccagttgtaagtttt
gggagaggagaatcggaaacgatacagagaaaaaaaaaacagaactacgggcgctttttttgctttttctatatttttgcttaggttagaagcact
gttcattgcatttcttttaaaggtaacattgttctttcatcatagtaaatattgcttaatacgatgtaaacaaaagaagttcataggtaaagaaacac
tccttgaacctctctcgttttgctagaagaattaagtcaaggctccattataggcctaacttctctaaagctctattgcgttgtgtttacaatcagtac
ctatttagacatgagaatccagtttgaacactttgtttcttctttcaatggcatgatttaatgcgttttagatacaaaattactttaactagtaccgttt
ttctttaaaagctcacagtgctgctgttttggaaaactgatggatctcacagactgaaagaacataatcacctaaatcacatcgcatggtattctca
tcctacactaaatttacttaaatcttttggtttatttcctccttttacaagtactttactgtctgtcttacttataccggccgttggaagcattttcggttc
tgcaaacaagcatagttgtgatacttcaaatttgaaagcagataatatgtctgtttttttcccttaaaatttctaatattatataaataaccgagata
cggtcaagttttgcagtccttatgcaataaactgattattatttacttaccaaccacctcaacgattttcgtggttctcgtactcatcatgtaacctcgt

gtatcaaatttgtgggtttttgaattgtttaaatatggaaggctttattagtaaacttccttgtcgtaggacagtctgcgttttttgtttttttattttttt
gctgggagatctagggtccaaaaaattgtttcgttggcttactgttgtgttatttaattatttttcttttgttatatagatttagaccttattcgttcgttc
atgctatacaagtacaaaaacctacttttgcttctcttgacttggacggaatttaaagatgtaagtgctttcccccataaccatggacaaactggtc
tccatcatcgccatcaccatgcaaatcttaagcgtgataccgtgcagtgtgcttttccacatgccgagaaaatgatcgctgtaacaccagaagagg
ggaatgctggctgggctatgtctcccaatcagtactgtacttcagattcttggtgcccatatgcctgtgagccaggatatttaatggctcaatggag
ccctcatgcaacttcttacacttatcctgagtcgatgcatggagggctctactgtaattccaatggtagtgccgtaaaacctttcgaaaacaaggat
tactgctatcccggatctggtaacgtatttgcagttgatgaatctgggaacggtgtttctttttgccagactgttctgcctggtaacgaggctatgctt
ataccaacttgggtctctgctagttcaaagcaagtattagcagtcccagatgccacctactgggacggtactgctgcccattattatatcaatcccc
ctggtgtttctacaagcgagggctgtgtatggggttcttccgataaaccctatggaaactgggccccctacgtagctggtgccaacatggatcaaa
ataacattacttatgtgaagttgggtgcaaatcccatatatttggatgatccttactggtctacaatcaagccaaccttcggattaaaggttgaatgt
gagggcgatacctgttctggattaccatgctatatggatcctcgtgaaaaaggtgtccaaggatgtcctgatgggagccctaatggggcaggga
tgcttgtttttgtgttgttggttttcaaccaggaacaaccgctcgaatcgtcgtggtcgactacagtgatcaattgagctcctcgtcggtttttgcttcc
agtactgcaactgcaactactccttctatatctacatcagcaacggcgtcaagcaccttgactagtggtagcacttcctcttcgtctatcccaacttc
tgatacccaaacgacttctcaaagttcgtcttcgattttctcaaccgctcttagaagcactgtcaagcctaatgccagtatagagagaggtgactat
gataaagggagtcaatcttatcttaaaggaaacgacgctttttcttcggctggcgtcctttctgatactggcttcgtaaccgttgatgcgcctagttc
tggaaacggtcgaacgatcgatcagaatagcggatccgttacatcaggaagcaattcttttgttaccaactctatagtagctggtacgagttcttct
aaaagcattcaatctgatgcccgcaacagtgagttgtctacttctaccaacgaccctgtagtcacaaaaatgactacaagtatactcgagggaag
ttcatccaaagatcccaacaaagagggaaaaactgtttccggatctgagatgtctactaacagtgcagaatcgaacattttccttactacggtaac
ttcttattcgaattctgcatccccagtagtccatacagtcaatggacaggaaacgacctctactacgacggtagttgttccacatatgcatgcagag
atacaaaccgtctttgatataacagtggcacctaatgtggcacatgagacagcttttgctactgtttttgagacagcaacgcaatatgcttaacaat
accaattttacagatcaacagcatatttacaatcatgatgattgtttaattgttctttggtattctaaaaattctgaataaaaacacaatgcttattgt
gttttcattgactttttaaatggctacagttattgaatgaagtcaagctctcacactccttaatgattcctttttttgttggttttgctttttgttaccattc
ttttcccagaaaatgtttactaatcaattggtcctcaaaatatagatacttgtacttttataatctaaaatttaaggatagtaatgaatgtttggtttt
gaaatacgattaactcaatacttgcaatcaagacgaaaaaggtgtttatactaacgttatttaattgttgtctttttaccacaaagtcaagggaaga
atcaagttgactccagacgttttgggatgccatcaaggatttaaaggtaaataatgacagattttacaactaagtcttaaagataaacacatttca
tataataacttgtctccatttcaagtattatgctacatttcatctaggcttgtttacgaaaaaagcagtcacatttgttggtagcgtttaaacaaaag
catttgtgataagttcactcatttatataaacgcaaacaacatggctgaagttactaccagaccttccgttttgaatgctgcagaagaagacattaa
gcagctcctggctgctaacgcccatattggttccaaaaacttagaggttcgtatggacccctacgtctggaagcgccgtagcgatggcgtccacat
cctcaacgttggtaagacttgggaaaagttggttttggccgctcgtatcatcgccactgttgaaaaccctgccgacgtttgtgtcgtttctactcgtg
cttacggacaccgtgctgtcttgaaatttgcagctcatactggcgcaaccgccattgctggtcgtttcactcccggtaatttcactaactatattactc
gtacttaccgcgaaccccgtttgattattgtcactgatcctcgtgctgatgctcaagcaatcagagaggcatctttcgtcaacattcccgtaattgct
cttgctgatactgactcttgcttgaatcacattgatgttgctatccctaccaataacaagggccgtaagtctattggtttgatttggtacttgttggcc
cgtgaagtccttcgtcttcgcggtactctatcccgtaacaccccttgggaagttatgcccgatctctacttctatcgtgatcctgaggaggttgaacg
tgaagaggaagctaagaaggccgttgctgctgaagctgaagccaatgctgttgctgaggctgctactgctgctgaattcgatgttactgatgctgc
ccaaaccgctgttgacccctccgttttggatgctgccacagccggtcaagttgctgaaaacacctgggacgattctgctaactggaacactactgg
aacagctcctagtgattgggctgcctctgctcaaatggcttaaattgtttgcttgtgtttttatatgtagcatatttagatttgaagtgtaatgaaagtt
tggttgaaacattaatgaatgctcatcgatgtattgtctgtgcaactagtattaccaaaatctttaacgttttgctcatcaattgtaagcctaactatt
tcaagcctgcatgaaaggctattgccgaagatcaagagctttgtatgaataataactaaccataacaaatagttgcataagtaatgaataattta
atcagaccgacattgcctaagtctgcttctccaaaaaaagttaacaacattccatttatctttatagtgtccaaaaaccccaagtcataagtcatga
aagggaaaaatccgagatactagtatttctttttgttgcgttatgtcaagtaagcatataagatttctaacatttttcctttcgaatgggcttggtt
atgtatctcgtaatacccatatcctcgtttccctcctcaatagagggctgaatatcagctgttaatagaataattggtactgatgacctgatattttca
cgttgctcgagctcccgcattttcttactcacttctataccatcataataaggcatatggtagtcaataaacgcaaggttataatatccacaaggat
gagatgcgattaactcaatcgcttcttttccatctttagcactctcaatttttaggcccaaatgctctaattgtttaaccagcgttagtcgtacaacac
tgttgtcttcagcaactaaaattctaattccttcaaattgccagctttgcctgttggattctgtttgttcatgaccaagcggcaagtaggaagatgttt
cgcgcagcttatcttgttcgggatggacaatcctcaaagttgcatctacgaactgttctttggtatatggtttcgcaatcatggttctccttttacctat
gtattcagaaaattccggcgatattgacttttggtcgtaagttttgttcagttgaggaatacaggagacaaaaattacattcaagtcaggaaaagt
cttaagttcagtattcactttttccaaatgttcaagggagtctacatctagaatcaaaaggcagcgagcaatgtttaaatactgccgaccttgatca
aggaaacttgcaaaagcttcagggcagctggttacatgaaccagcttttccgcttcagtcaaaaaagtaaggatattatcagaatcgagaggtac

attcgttgcacgtataacaagcttatttttaatatactcgttgaacttaactttcgcttcaaccaaatttgcagtaacttctctgtagcgctcttcccgt
aatttatggaccatcaagtcataacaaattctgaacgtaggaggagttttcgaaactgtcacaaaaacagaacctcccataattttacataactgc
aaacatgttgacattccaagggctgacccacttaagtccataggcaaattatcattacagtatttgttcaacgagtttataagattagcttggaaca
ctgcatttggttctggtctactatcttcaaccataaaagtaagccttaaatcatcattttccccattctcaaatatataggctttcacgcttataaagc
cactttcaaccatttttacagaattatcaagtatacttttaagcatttgaccaatttttgaagcatcgccagcaatgaaataaggaatatcattcgaa
agttgataggagagagttacatttttgctgacagcaacagaataaacagaggcgatgcagtcaacaattactctttccaatgaaaactctaagtc
tgattccaatgttactttgccggcttccaacttgctaaaattcaataagtcattaatgacttgtaataacatttcacagctaagccgtgctgcatttac
aatgtctctttgttcataatccaaattagcctcgtctaacaaagagagcattccataaaagccagagaaaggtgtccgtaattcatgtgatatgttg
gccaaaaaattggatcttaatcgcgttgcctctaaagcagcggctctcgcttctttctgatcccctatatcaatagtggcacaaatatatgtcggctc
tgactcattcaaaataggcgtgcagcgtgttaagttacatctgtaaactccatcatttcttcttatacgtacttctttctcaaatggagcattaacgtt
gctttgagaaataatatgttgttctaaaatatcataatcctgcggatgtataaattctttacagatattaggctttcttaaatcagtatcaccgcaat
attccttccaaaaagagccaaatagttcaatctttaatccgctgtcatatttaagctttgaaagtaaacaaggcatagtatctacgaaacttctata
tttcgcttctgatttccgaaccaaactgagtgcctcgcgttgggattctataatttcattattagcatttgttgcttctttcactttatgaaaaagggaa
atattgacaatcgcgaagctgacctgagaccctaaaatagaaagaactgaaacatgtcgacagtgaaacgaaaaaggaggtccttgcagatac
aaaacagcaaatactaccttttttggatgaggggaattacaatcacatttttcctttattgtttagttcgttccattcttcgcagtgtaaatcaaagt
ctttatgaatgttatgtgaaaataacactttttgagtgtggacgacatagtttattacctttgaaggaacgaacggctgtctttccgaaaccggaat
cggaggagtaaagacttcgttgacgttttctgtacgatatgctgcaacggtaaaatgattgtcttctcttataacaattgaggctgcatctgccgaa
gaatcttcaagcattcgaagcgtcattgttgatagtaagccgtctaattcagtttcactagcaatagtttgacaggacttcataactgaggttaaat
caattatatctaacgaaatgaggagttcttctaaagagacagttccaacattcgccatagtacatgcctgatcttcgtaatcttcattaggaaaaga
tattcgataatttgactcgactagatcatcatcttctgctttgtcactagtatcggtagaataaggtctttgttcgtatgcggtatccgtttggacaatc
tcatcaacagttaatttcagtaaattagggggcattttatcaatgatttggttttgtaacgtagtatattttctcttcaaccctaaggatttgtatactt
tcacagactgttcgatgtaatgaattcccgaggtagttaaatttattttgcagaataattttccgacagcttcaagagcaaaagcctcaaccaaata
taaattcaatttagtcgctaaagcaataactttctcgtatagcactgatgctttcgtcaaatcaccgtctaaagtaaccttacttgcctgtaaaaaat
agaaatacaaggttatcggactgcaactttcagatgaagtacttatatactcgaagaatttcagcttttctaagaaaaaggttttctcgttatcaga
aagcataccattagtcaacccccttgttagtaatagccaggcaaagtaaaaaatagccaaagcaacctgtagatgtcaaaaaaaaacccatcaa
agtaccttgagacactatttaagccgtagcaaataacatcttcccagtcctctgtgagaacaaggtttaacaaatgccaagaaaaataccaacgc
ccaaaggtctctgataaattcttttcaggagaggctggtaactgccgctctttgttatacaaagactcgcaagggcccaattcagtttctcctaaaa
ggcattgtattagggttcgtaaattaggaaagtatacactatcggcaacccaaaggcgttgttttaaggcttcaccgtaaacttcgtcaaactcgat
caaaaaatcgccataattggaagatagaagaaaactgtcccagagtgaaaagaccgaaagagagttcccaaggaatctgctagatggttgaca
gtgtttctggagtgttcggatggaaaccatccttttctgtaaagtttccgtcacatatccaatcgaaagcaaataaaaaaattctgtgtaagtctgta
ttgtgtaattttggatctcttgattgagttttctggcaaggttacacattttaaacaaaggagcggaaggacctacgatggtcatcgcagtaacgga
gaagaaaagtaaagtataaccagaaaaatcggtaaatccatactctaaaaaagcatttgcaaaagcaattgaaaagtccacaacaatgggag
agcattgataatatctagatgcggcgctaaagccctgaaacaagaaatagaatatttcagtgctgcggtcgtctaaaatggttgcattatcaatct
caatgctttttcctcgtccgcatacttcaacacttttgggattaatgacttcaaaatttccttcgaatcttcgaagttatgtttttcgatagaaaaacc
tagttgagttaaataatttagaacaagagaaagaagctcttcagagcgcaggctttcaaataatactcggcagcgtaaaaataacgacttgaaa
gcctcagttaaattagcggattcattttcagcaagctttgtcattttcatggccgcctctgcattaccgttaaccaagttggcttcagcacaaccaac
atatatttggatcatttgtttcctctcgtcatcgttttctctattgtaatatttataaggcaatatttcgatgcatctattatacaaattaagtgctttctt
atttgctcctattttaagagcttcttcagcagccaggtagagataagtaaaattcagtgttgaattatcttcattctttatgtattcaaaagctgcatc
aacatgagaagcaatatcgaatagactgtattgatctcgattgttaatacacaactcaaatagacgtgcatgcatacgttgtaacttctcttgacta
tagtaattcttgatggttttcttgtagcttccacgagaaattgagtatgagtttgtcgaagtggcatatatgatgccctttctaagtaataaatccaa
ggactcattatcgaggtcaaacccttcgacttcttgacaaagcttttgaacagtttcaaacgaaaacgatgaacccatcatactaccccaagccaa
gaagcaacgacagggctcaggtaagctagaaagctttgtgattgagtaagaatcgacattgatattataactttcagggctaaacaaatttacct
catcgtaatctattgaccactgacgggtttcccagtcaaagtatatgaccttttttgttgtaaagttctgacagtaaatctctcatcaataaggggttc
cctttacaaacatgataaacataggaagtgaaaggatacaagtcttccccaacacggtgaagagttgtctgaacgtaagcatttaccgcatcaaa
tgctagcggtttaagttgcacttcctgaacaaatcgagagaaggaaaggaaatttttaagtaaattagcataagaatcatcacatgttaaaattat
acttataggaacattgcaacgaattaaacattctagaatattgattgataattcatcagcataatgaacgtttttgatgacaatggttgaccgctta
agcgatgaaatcattgtcagtagagtaattaaggaactagagagagagatctgatgaccaaattgattccgctggtcagaattgtattgagtttct
tggctattagcacttcgacctagggacgacgaagacttaacagattcattcgatgaagttgaaagaaaacttataatattgccaacatatggactc

ttatcaagaaacggctttacttcgggaactatatcaaaaaccgagtttaaaagatacaaatctccttccaaagcatgtttcaatttttgaaaaaatt
catgtactaaatgctgactctcagcgagtacttgaagaattaactgagcgattccacgaatcaatgcagaattcgagaaagactctgatggttca
cattttgtgatagccatgtacccttccttcctatcacaaacgtcttggataaatgtagctttaccaacaccttcaggcccagtaattaatataacaac
gatatgatcactatgctcgtttccttttaaaaagggtgagtcgtgaaaagaagagtctaaaggtagggaagttgattcggtgtgacgcaaaagag
attttaccttttcaatctcacgttcacggccatatgtagaagaaggaagcataaataaggatgtaaaacctgtgttatcaaagattccttctccact
cgtaattccgtttactaaattgtcataacagatttctaagtcagctaagatacttgagcaagaagtgtatctgtcgttagggtttttttttgtcattttt
tctattattttaaagatagtcttgggaatttgcggacaggcatcggcaatacttggtagatgttctgttaggatggagcggatcgtttggataaagg
aatccagatggggataaccttcagctagacaataatagaaaaatattcctaagctataaatatccgttctgtgatcaacagggtacgatgttcgcc
ctgtttgctcgggactgatataagtaattctcgacatggattgagagtcttcatagaaccgctgccagttatacgcttgcaagtaattgtgaaacga
gcttgcaccactcccaatatttaacataattacattcgagtaatcttttatagggcggaaggattcaaatcggatttccccatgaacgatatctttag
agtgcagaaattcaacacatcgacagacagaaatgcagtaacgaatatagtcaataggatggtttcgaaagtaatcatagaaagtggataaaa
tatcatgatgtccagcgtcgaaaagaacgtgtgcagataagtcgatatgtgaaaactcaataaattcaatgggaacagaaacaaaccgacggc
agcgctcatcttttgataaattgcaaaacagctgatattctgcttccatgcgagctatctgagttgagaatttagcaaccactttacgattaccagct
aactcaaacccagcgtatagagcgtaagtattatcgactgcgtatacttcttcaaatggttctgtttctagtgtgtattttaacatgctagaaagagc
taaagctacacaatttcggaactcatttttttgaatgcattttaacacacacaatggctctcttcttacatgatttccgtgtgagatctcttatttaaag
aaaaatagctcaacaatctatcaataaatacttcagaacccaatggcttaccgtttacgagattggtagaagttgcgttcggcgagacgtagaga
acaggaaacatttcttcactacgttgaatgaagtacagaataaaagacgataattgtacagtttactcattgtattatttggaattataaactctttg
gaaaaacataattcaacgccgatttcgactacgggtttaatgattgattctgtttacaaacaattcttggtaatacaaagtaacagagactaaaca
acttcattttagtaaatgatcactacgatgcgaatatgacacagcatacgacaatctccaaccacgtatagcaaaaccttgactacttggcgtatg
cctacgattatatgatggtatagcatgtacagccatctaaactatagaagaactgcattatcgactgagaactacgcgtcgaaagtttccagttga
atatttaaacaaataataaagcccagaccctctattctcatcactaaaagggacttgctttaaaaattatataagaaaaaagaaatctaaatttaa
cactcaaaaatttcacaagtccacgagtaacaagaattgcctctaaaagagattgtattcatagcaacaagttttaaacctaaggcctaatgcaa
aattaacaaaaagaaacttttgctaacgtttttagaagcgcagaggagtgaagaaccaggcagacttgccggtttggtacttctcttcaaaagtct
tcttgacaaccttcttggcggcagagcgttgggaaggctccttgaaggtctcagcagaaacaagtcccttaaggttatcaagctcaagggcgtaa
cgagtaggcatcaagtggttgtagttaacaacccggatgaaagtcttcacgcgagatcgcttggcaatacgcttggcacccatagacttggtgac
cttcaaaggataacgctcaacaccagcaacaatagcatgactgaaagggtggctcttggtgccttggtcgaggttttgaagaatgacgaccttctt
accggcaaaacgaccacgagtgactacggcaacctaaaacctgttagtttcactttccaaacaatcgtaattttgtcaataaattcttacctttcct
ggtttaaaaatcttcgccatcttgttactatgtgtttgtggttgggtgtacgcaccccaaaaaaattgtgaggattttggatccaagatcgtgactgc
tttttttgtaaacatagcttaggtggaaaacgcacgcgtagtagaatgaatgtgaaagttaaaatcattagaaagtagacgatgataaaaagcat
gatgattttggtgttacatgaaccaaagcaaggttgttttatacgtttttaaattcatttctcctaggtaaagggtaagaaaacttatttatgagaaa
taataatggaacattaaaatgaagaaatatctatagataaaaaagagaagcgatatgtagatttgtctgacacacgcagagaaagggaaaaca
ggatattatattcattaacaaaaaatgggtacatacaaaagccatgggaacagtaagtaaaatgtaaataaaaagaaatgtaatgataggcgc
catcaacaatcagcaacagcaacagaaataaatattttatttaggaaacgaaaagcttgcccgagcaaagaaaaccattgaaggaaagaaggt
tggatagatgaggagggcgaaattactttttggtagaaatggacgaggaagatgcatgaataggttcgacaccttccatgatacaagggtcactg
gtacctgtacgaggacccttgaaccattttctagcagaaagaaaataccaaaatatgattaacatcattggaccaccgaacactagacaagtcca
attcatttcagcaggagcaggatgtttaacggtagggaagcataatacgggaatcataagggcgacaaaaaaacacgcaaagaaaccaatagt
gcgggagaacttgcctaagttccaagggccgggacggaatttggtatctttggaaaagacacgcatgcaaatgggggttgtgaatgcgacgaag
gcgccaatggcagtaacggaaaagacagcatcgatagtgatttcaccagcaaaaatcaataataagacacaagtatggacagtgaagtccaaa
aggacagcattgactggtgttttggttgtagggttgatcttggcaacccagccagagaatggcaatactccatcacgggcgtaagagtaagctat
acgagaacaggcgatagaatttccctggcccatgatgaaggaagaaacgacagtaagagagatgattgcgagggcggccttttgcgacatgac
ttgtgacaagaattcagcccacatggaagaatccgtatggacaacggcatcctgatcgaccattgtgtaggcaataactaattgcattacccagc
ccataagaccaccaatggtagcagtaaggacgataccgcggggagcattaacactggcatttgaagtctcttcactcatatgaaaaggagcgtc
atagccagacatggtccaaatggcaccacaaaaggacataaggacagcaaatccgtcgggccattcggtatagttggttatttcactccagactt
tatgattatcgttgaatccatggtttttgccagcaaatgccagaatgacaatgatggtaacaaagagaaagacggtgtttaggtaggtgctagcg
gaattgacacgggcaatgagcttggtgggtaaagaagcaatgaacgcgttgattgcagtaatggttaaagtgacgagaaacagctggtagttgc
ttggagtgtacgagcgatgggctatggaaacggcaccaagaagcatggaagccgtagagtaattgatggaggggcccccgatcaattgaccaa
gatagttggaccagccagtgacccaagcagcgaaaggaccccagccctcaggagcgaggacggcagcggcatagtaaagtccaccggaggta
ggcatggacgagcaaagttcagccatactcaaggcgacgcattcgactaaagcgattgcgattatccaagcccatacaagaccgggagaaccg

acgtaaccaaggttataggtcatagtagtagcgacagacggtaataagcctagcaaagagaatgatacggagaagacactgaacaaggataa
gttacgatggaattcttgtttgtagcctaatttacccaaatcctcagggttgtcagagtcatttccatggccgccaacttctatatcggagctagcgc
tcgctttttctgggatttgtaggatagaagatgctttcgaggtttgagaattagggaaagacatatgcgagttttgactagccatacttttggaagg
agtatggactcgttcagaaagaaggttaaaattacaacacacaatcaatagtcaataaacaagaaacaaatcataaacgaaaataaatgaatg
tgctacctactctaatactcgcaagatcccttgtttttaaaaaaagaaaaataataatgataataatgatatagtatcaacaactttataaagaag
aaaccaaacaatacaaaaccaagtcagtgactgaaagcagcgttcggcttccattgtgtattgttttatactgaaatcataagaatggatcatcca
tcattcagatatctgagaatgtaccttgaccaaacctatctggcatcaaggtgtggtggcattcttatcagtaggcaagaacaatgggggtgaggc
aactatagtagttggaatcgattaaggaaacaaacgaaaattgtttagggtgaagcttttcttttttccaactttgaatagtaaatttatggtgcaa
ccatctcgtcgaaggaggccatacttttcgtcttgaacactcccttttacagtttgggatagataactttttgtttaccaacgtacccacactacagct
ccgtaagcaacaaaactaacaaagagaaataaggaaagaaaactctataagtagaaacaaatgagtattatatatgagttcatcgtaaatgaa
agcgtacaacgatcggtgaaataagatgagatacaagtccaatccccatcgaattgtgaccgattggtcgtatcgtccacttactctcttggatgc
caacgttactttggttggcggaagattttgtcacttgtcatgttactatcagtcgcacttgaaacatcttaactcaacttatgtttgcggcaagcgaa
gatagtgcttaccaattacattcgttcaagataacgcttatcttctgatagttgctttttgcttgacccacgtatattgtacgtgatataaacggtgga
tacaatacccatgtttgcaaggggttttctttttcagtgcgaccaaaaagtgtagaaatagtcagttttatttctttagaattccttaactgttttttta
tattttgctgcgcatcgctttttgaaagaataacgtagtgaaaagaatcatcaggaaacgcttccttcttgaagaaaaattaggatctgttgggaa
tacattgtccttgccttggtttcatcagcacgaattgtatcttctctgttcacttgtacggaaccgtataccttgatctttaggaatcactagattttgtg
cttactgtgtatctgtgtttgtgagttatataatttcatttctacttgaaagctatttttggattcattttcatcttcatttgccgttagattttaagattca
tttttgtgatctttgacccattgtgtctgttgtatccatcctcatacccatcataatgtcttctactacaagtgctacagtttcaccaagcggggtgaat
gggggtgactctaggaatgtcgatttgaaccagttttacaatggtggtgacgtagcttgggtcctcgctagtactgcccttgtttttatcatgattccc
ggtgtaggattttttactctggtttagcccgtcgcaggagcgcaatttccatgctcttcctttccatgatgtctgttgccgttgtcgcattccagtggtt
cttttggggctattcgcttactttttcccacaacggtggcccttacataggttctttggccaactttggtttgcgtcaaactttaggcagaccctcgagt
gtagcttctgggcttcctgaccttttgttttgtatcttccagggtatgtttgctgccattactcctgctttagccattggtgctgctgccgatcgtggtcg
tatgttgccttgccttgtctttgtcttcctctggagctctatcgtttatgaccctattgctttctggacttggaatcccaagggttggctcagtgttctag
gatcctatgactttgctggcggttctccagtccacatcgcttctggtatgactgctctggcttactccattgtagttggaaagagacatgatcatggc
actgtcaaataccgtcctcacaatgttcctcatgttgtcttgggtactgtactgctttggtttggttggttcggattcaatggtggttcggctggttctg
ccaatttaagagccattatggcttgcgttgtcacccatatttctgcttctgtaggcggtattgtctggtgtttttttagagtatattaagactcgtcactg
gtctgccgtcggtttttgttgtggtgcagttgccggtttagtagccattacccctggatctggttttgtgcctccttgggctgcagttgtcattggtgct
gtcggcgcgtctgcatgctatggagctacttatttgaaaaaattaattcacgtggatgatgcattggacatttttgccgaacatggtgtcggcggta
tggttggtaacatactaacagctctttttctgctgactacattcaatccttagatggctccactggaaacagtggtggttggttgtctcatcactata
tccaattgggttaccaactggccgatactgttgcttgtgccgcgtattcatttgtcgtttcctgtgcattgttattcgccatgaattacattcctggtctt
tctcttcgtgtttctcgtgatgatgaggttttgggtttggacaatgttgaaattggtgaaacttcttacaaattctcagaagagccaccagccttatcg
catggtattgaagtcaatcctcgccaaaatggcaacgagacctcaaaagagaatgctcatcaagaggaaaaagagacgaatcgtgaaatccct
tctgacgttgtatgagcatttttttcttctccgtcccatcgagtatatgttgcatattcagtaaataaacaaaccgtctgaatcgtttatcgtttttta
atgtttggctttcctaaaatatatatgtatataaccctttagctacattggaaattccttcataattttcctacccaaatctcgttcgtatcatacttca
gagttggtgtcataggctttctttctatctttggaagtctgttcttattcaacataaacatttttaatacccaactttttattttgactattctaatttc
tgttttatgtttttatatctaatаatatatatatatatatatatatatatatatatatattatattatttattacttacgtttacgtacgtcgtcgtgt
actacttaacctttacgttatagtcgtgttaggtaagttaagtagacacgactaaactaataagtaannnnnnnnnnnnnnnnnnnnnnnn
nnnnnnnnnnnnnnnnnnnnnnnnnnnnnnnnnnnnnnnnnnnnnnnnnnnnnnnnnnnnnnnnnnnnnnnnnnnnnnnnnnttc
gtttctttacttactaactaactaaacttaatttttaactttaacccttaaccttttaactttttagtttacttattacttaatttactttgtttatttattt
attatatataatatatatatatatatatatatatatatatatatatattcatattttatctacgtttcagcgtctgcataacctttcgtatagctgtgg
aatttaagagcagctaaacatagaacttatcacaaaaacaaaacgaaaccctattgtaatactataaatataacttatctcaattgtaaagaagc
acattgtcaatgttcaacgttccataatgcaggcgatgactgtacaacacaattatcactttattgcagattccattcaaataaattttcgtaatatt
atacattataagcttaatagcagaagaagacaacgtggccgagtggttttaaggcgttcgcctgctattaagtagcccagcggattccctctggga
gcgcaggttcgaatcctgccgttgtcgtcaattgatctttttcaagaagttatttttgttttcatccacaactttattaaaaaaaagaaaaaaaaga
attcgtctttgttcattttcaatgattccagttacagttgcatggcaaaataattaaaagtctcattcatagcaaaagaaattctagtgcaagttgta
tattcagcatttgttcgtttttttttttttgtttaagatgtgtttactcgttttgtttattatctttagaaagaagatatcagattcaccggtcaattcgtga
tactttgtacaatcgatgactttgtttttatttgacaaataaacagaaaaattattgtttttatttcatatataagtacttgagaatcgcttgtttttct
accgtccttttactgatatttatggatgtttatcatctttcctttaccgtctactgaaacgaacgaacgattaaggattgctccataataacaattgat

ttactttctttacagaataaccgaaattgaatctttcattactactactttctttatgcaaccagatttaactgtagaagtgggttccctacgacgctct
ttgtgtgcaaggcaaatccaaatgcttgcctttggaggaaccattggcacaggattatttttaggaattggttcatctcttgctgaatcgggccctgc
ggctctccttttgtcgttttgcatcatcggcgtaagcgtctactgtaccatgctagcattaggcgaaatggctgtctatatgcctgtagcaggatcgtt
ttgtacctatgttggcaaatatgtagatgaatctctcagctttgcccttacttggaattactggttgaacgatacaattgctttagccagccatgtatt
ggcttctagattgctggttgacttttggcttcctgatacaacaccgtcaattgctaatacctctttttctgagaatgtcggaattcgttgtacaagaga
aattgttcgcactgctacacctatcatctcactcctagtcaacattggattgaatatgcttccagtggacgggtttggtgaaattgaatactggcttg
cggctatcaaagtcttcacagtcgcactgttttgtgttatcggagttattacgaatatgggagttaacaaagaaaacgagtttattggattccgcta
ctggaaagaccctggtgcttttcacaacggtgttggtggatttataagcagttttgtaaacgcggcatttgcttttgcaggtacagaatcgatcgct
attgctgctggggaagccctgaatcctgtcaaaagcttaccaaaggctatacggtctatgtcatatcgtgttttgaccttgtacattcttggtgttatt
gtcgttggtatcaacattccgtataacacacccggtttgaatggagattccatcagaatgtcaccctttacactcgttttccaaaagtttggggtacc
tggagcagctagtttaatgaatgcggttgtcttatcgtcagctctttccgcaggcaaccattcgctgtttgcaggaactcgccttttgtactctttggc
acaagctggacatgcaccaaagatattttccaaatgcaatcgtcatggcattccatggcttgcagtcctagctacaagcattcttgcgatttttctt
tgttatcatcgcacgccgggaagacatggtcgtttttgctcaacgttatcgctgtatctaaccaattatcttggacctttattggatttactagcttgc
gattcaggaaagcattacgtgtgcaagcaaaaactcatcgacttctttttcccaattggacttatcccataggaccacatctggtgattgttttgaac
gtagttttccttatcttacaaggatggaagagtttttttcctttccgatttctctctttctaagttactatgtagagattcccctcgtgttaggattgtat
cttgtttggaagttctaccatcgcactaaacttttgagacccactgaaatggacttagatacccattggcatgatcctgctctaccagctgaagaat
ccgtgcaagcataattgttgacgaactcttgttatgattgaatgaatattagccaacaactatatttcctaattctatgcaatctttttatgacttaag
atctcttaatatcacacatgacttgcgaaagtccggaattctttgaatcaatttatatgacaattataaacatataaagcaatataatgaagataca
aaagaaaaaacttgaatatatcagaaaaaaatatatataatattatgcaggtcgtactgggagtcgaacccagatctcgagaatcaaaatctca
tgtgctagccgctacaccatacaaccgacttgataaatccaaataaaataaattacaagacgtctaaagaagcgaatgaactatagaaattcgg
aaaaatgtatcatgaaagaaggaataaatggtgcaatccacaaagataagtattcgccgagttgatatttctttttattccctgaatacgaaaag
aaaccgtcagaggttgcaaggttcttatgctttgcaaaaacaaagaagaaaacccataaaagatatcaaggttttttttttcttgattgtttttattc
cttcctactttaatttctctctctttctttttttctttcgcaatttattctttcattcttttatttttggctgtttctagtactttgtttacactatgaaatctcct
atgacaaagaaaaggcgaattcgtcaaacgtggctttatcaatggaagaggtaaaatgagacacgcaaggtttgtgatgtaaacagaagcaaa
caaagctgtttaccaaaaatcaaaagaaaagaaagaaagaaagggaaaaaaaaaaggttactcagaaggaatgacaataaaatttctcaaaa
atgaacggatgtccacgaagaatactgggagttgtgtttttttcttcttttttttttcgtttttctctttcttctgatttttgttgtttgcttttcgatagagt
tatctttgtcgactgacgatttgtaattgcgtgctcccccgaaactactttggtgtttataggaggatatttacgacataccttgttacacctggcaaa
tattcggtgattcttagacaggatgtcgttatcaacggtgataataagaagcctctatcgatggaaatcctttctatatatgtaggagtcattgcgc
ggtcatttgtctaggcttcgataaggtcaattcttaaaggttgtaggaatagaaaaggacaaggcgagtccactcacaaatagtaaacaaataaa
agccgcttttgtctaggggtatttatctcgcttcgtggaagcactattctttaaagttgggatctgctgttattttgttgcccttgtgtcattatcggatt
gtagagggaaatctattaggaagagaagtttgcaggaaccaattgaacgaaaaaaaggaaacttagtttcttaatttggcttggcgtacgaaaa
tgagcgaaatcttcagaaatccccccctctatccatcaaaatgatgttgatgattcttttgatgttgaaaaggacgcaaatgttagcatggaaaagg
aaaagaatattgagcaattcacggttctttccgatgtctccaaggattcgcattctagcaccgaaagaagtatcttgaatcccctttttgatgaaac
tttcaagtatatgcaagtagcaaaggacctagagcctttaagtcctaaacaggataaaaaaattacgctggaagctttactttagcgttcttgcgat
ggttatggtcttagacatgatgttgtatatcgacaaagctaccctcagttactctagtatccttggcctatttgaggatacacatatcacaaaagac
caatacaataatctaaacacactcttctatgttggatatataattggtcaaattcctggccatttattactgcaacgatttcctgtgggaaaatttgtc
gctggctctacagctctttggacgatcataatatttctacattgcacggcttataattacagcggactcatggcgcttcgtttttttcctcggtctcgta
gaatccactttgcttcctacgatggaagcaactctcggcatgtttttcccacacaatgaactaacttttcttcaacctgtcttttacatttcttgctatg
gatgtaacattcctgctggctttattgcgtacggcgtgcaatacgctactaataccgtctctccttggaagctctttatgatcatcgttggaggtctca
cattctttatgaccctttggctcttcttttactatcctgataatcctgccaaggcaagatttttgacaaaagctgaacggttgtatacagtagaccgcg
tacgcagaaacaataacagcggtatcgaatccaagaggttcaaaaagcatcaagtaattgaggctttaaaagaaccggttacttggctgtttgct
cttcatgttttaacaaatcaactttctaataatctggcttatcaacaaaacttgctttttactagtttgggcgtgtcgaatttgaattctacgttggtca
gtgtcgccaattctggatacaattgtgtgagctccatgctcgctacttacctgatgagtctaatcgtgaatcaaagtgcatttcacggagccttttgg
tacattcccagcatcgctggaggaattgcatccgttgccatgtcctggaaccataaaatcggagaattagctgccattattatagcatccaatttcg
gaattccttttattatttcccttggatggatgtcggccacatgcgctggatacaccaaaaagcttactagaggcgttttgtttatgattggatatgca
gtagccaacatcattggccctcaaatgtggcagagccacgatgctcctagatattatcccgcctggattgcacagattgttgttgcttggtcggtttc
tcccatgattcttctaatcattcgctggatccttgtccgtaaaaataatgaacgccgtcgtttacgtgaacaaaacagcgaagaaaagctttccaa
gacttgggtagagtttgtagactcctctggaaaccctacttcaggatttatcgataattccatgttggacctgactgaccaagaaaacttgatgtttg

tttatccattgtaatgaccctttttgccatttcctttcttttgttttgtatcttttcatattttatgtttttatcaattgctttcttgctggattttcgtttgt
ttggatgtctattaaagttgaatgcgtaatggatttcactaatatagagaataattattgttatttataattctttaattcttactcttgtaattgaaaa
gtaatggtagtaacgttaactttcatacacacaaaaaaaagttaaagggaaactgtttacgacgaaaatgtgattgttacagactaagaaataa
agcgtacaaggtaaaccagttaattcattaagaaaaatttgttatacattagataaaaaaactcatgactgcgtaaacatgaaaagtggtgaatg
aaaagaaagaaaaccatatgaagcgatgttgctggtagaaaacgaaaagacgtatcgtatcataagcaatgaatgaaaatgcagcaaggtcg
accagccttctcggaacttgagttaagcaatattggataagaggttgaacataactatatggaagcaatttcttcagcggtaggaccgacaggag
gatgagaagcagctaagttgcattgatggaggatgcgtgtttggctgtcatcataggcacctgtaatgcaatgaatgaggccgcgattatggaag
attacgaaatcgccctccttccagtcgtgacagtaaacacgttcggggttgatcccagggcgttgataatcgtagataatttcgcggacctttgcga
gatcatcaatgatcgtagtggagccgtctggattgtcgatgtaaatcttttcggcacagcagccatgagtttgaagagcaagcttaccggtgacgg
ggttcttccaaagaagagggaaggtcttgatcttgtcctcttgccaatccggaagtttgtccttggggagttctaagccctctgagtacatgctcaa
gccattgggcaaggcacgagccctatgaataactacatatgcgtgcggaaagtattgaacgcgagtgcgagcggcgatcttcttttgttcatcggt
aagcaaatcaaacattttgtagccagaggcaaagacagtcgaaccgagagggacgggcatttgatggcctgatttgtcatcgtaccgtagagttt
gggtgccctgagggatcgagatggcggaaagagtggtgacgacgggaggattatagtcgtaaagagcagcatcgatatgccagcgataaaag
cgggtaatttgcttttccttctcttcttcagcagagatgggatctcgatggaaggtgcgatgatggggatgcttcagacgagcctcgggcaagccat
aatgattttgaattaagccgtgaccgatcaattggacctgaggaacagcgggaatggtgtgcagatctggatggagaatgctattcttttgcttgtt
ggagctatttccatggccataagtggaactctcgggatcaaacctatgggtgatgtcgtactgagtttcaggtggcagatcgtgttggttgggaaa
gcagacgacctggtgttcaaagagagccttgcgaatctcttgaaactgttcatcggtcaccttgcggacgtcaatgccttcaatgacggcaccgaa
agagacatcggtgctgtgagagggcaaaggagcaggagaaatgcgcaagccattggccgtcgtataaggctcgaaagcattggaagacgtcg
cttggacaggcaatgtcatagggaggacgttggtagtggtagtatttgtgctaatcttggtcatgctgatataaacaaacaaaataaggtcggcgt
ccagcacaaaagaagagtccaagtggaaagcaacagtggcagtagtagcagtagcagtaaagttgcaatgggtcaaggcgaacaatcagtcc
gtcaacaaacaaacaaacaacttatctaaaggagtatatggatttattcaagttgagaggagggtatcaggaagaattcatctcctttataaatgt
ccatccaaggcctctacggctacgttgtttgtagctttatcatggaccctgaccctcctcctttcttttgccattgataaggaaagatgacatcgtgtg
gacgaaaggctgttcttccttggaggtccgtatttccacttttcgagaaaccggactcaacgagagattgttttggtggagtgtttcctctcgattttt
ttcttcttttatttatttcttttttttttgttgttaccattactttgagacgtggttttcctaagagaagctccaaatcgttcctttcgctttacttttgatct
ttgtgtttcttttcctttggattctgtaaggtggtttgtctcggtctctgattattgtgcttctcaagctatcggttgcggttacttgtcgtggtagggccg
agccgtatcaataggttctcccagaaatcggttgtactcgtttatctgttgttggatattttttcgctaaacaaaggaaaagtctcttggtcagttgc
tcacttttccaaggcgcgcttcccattgtttcgctgttttcgcttcgccgattctctctcactcttcttgctcttctctctcttttcttgctgatttcgtccaa
taatgcccaccattctgatcatcaatcccaactcttcccgcttcattaccgattcccttcaggagaaattgccaaaactggttgcccccgatgtccaa
ttgcgcttctttacctgcccagctccaggagctgctgtcattgactccatcacagaagccacccttactgccgccctcgtctttcagtcccttataccc
caggtcttagaggggatcgatgccgttgtcgtggcttgctactcacctacgcctctcgtcgacatgatccgcgagtcgtaccagctgccttgcatgg
gcatcgtccaagcctcggtcttggctgctctcaccgttggccaaaaggttggcatccttactagtacttaccgatctgaatgtttactctacgaactc
ttacaatcctttggaattcaacgttctcgcattgctgctattgcctccacaggacgcaccgtcctgcaactttccaacatggatcctaaagaacgca
agacgctcttggttcagcgtgcgcaagaactcgttcgtattcacggagccgattccatttgcttaggtggagccgctttggctgcgattcgtgattcc
attcaagaagccgtaggtccagaaatccccatcatagatggtgtccacgctgccattgaacagctgtcgggtcttgtacgccaaaagctaactact
tccaaatccggcatttatcaatatccttagacatgtgctactacctatgactaacataccagtgctctttcaagaactcggttttcctttctacaccat
taattcacctagactagacaaaacggtactgatgcacctgaacatgtactatcctgaaatgagcgagttcttgattgtttcattgatgtatttccatc
tccatactgtgacaagacaaataatgaataatagactttctttcctttcaaagaacccaataatgaattgcatttttagattcacttgaaccataatg
acacagacaaaatcttccatagcagcaccactttctactgttttaccattaaattcctcaatgctacaaactactgccatccaccgaactaactagc
ttcctactgtttctgtttcccaacaaagttttcaataaaacaaaacttgattcaatctaacataaagctggcaaaatgaaaagctttgagaaggac
atatgtgtccttctgtccatttaagctaaatttctcatttattgcttgccaaagtacttagagggctccttggtgtcaatggagtcggcacccttttgcc
agttagcggggcaaacttcaccgtgctcttcgacgaattggaaagcatccaaaagacggagagactcatcaacggaacgaccaacagggaggt
cgttgatggtgatttgacgcaaaacaccgttagggtcaatcaagaagagaccacggaaagcgacgccagcatcttcaatcaagacaccatagtc
acgagagatcttgtggctagcatcagacaaaaggggaatcttgataccacccaaaccaccttccttacgaggagtgttgatgaaggcaaggtga
gagtattcagagtcagtggaggtcaaaataacttcagcaccacgctcagcaaacttggcagcagcctcagagaaagcgacaatctcagtagggc
agacgaaggtgaagtcgagagggtaaaagcccaagaagacccacttacccttgtattgggacaaggaaatttcttcgaaagcaccgttaacga
cggcagtacccttgaagtcaggagcaggttgtccaatgttcaataccatgattcttgtaatttcgttagtttcgtttgttcgatggtgaaaatggaag
gaggagagtgtccattatatagtctggggataacccggctgatttcaaatcttgcgaattagacgaggtcgtagatccttcgatggcgttctagatt
gctgatgcggcccccgatagcctaatcaggttagctaaggtttagtcatgacaaggttatgttaaacaaagccttaaaataacgcaaccgaagat

tgtcgatccttctggtctggacatgctctacttattcatattgtgctataaatcgttaaattggataaagacacgcgttggagaatggtaaaggattc
cagacaaaggtttcttagtaagggctgttcgcactggcctggtggaaaagaagaagatgatgagacggtgaaaggagcaggaaaaaggcagtt
gttaaaaggaggaaagcgcaaacagatgcactaaagaagatagaggcagagagagacaatgtgtgtgtcggtgtatcaatggcagtgaaaaa
tccaaataaagcgtttttaattaagaaaatatacagaaagaattaattccagcattcttttccataccaaaataccaaaacaaaggacctacctc
tttcaccaaaccatgatgtagctgattttcgcttataagtaggcttattataagcgcgacagaagaaaacaaaaaaccccaaaatatcgtcagcg
aaaaaaagaaaaaaaagaaacaaaaaaataaaaaaactttggataaataaggctcctagaagatcttagaaatagaagaacaagttcagtgt
tcttttccatctcatcaccctttgacttaccttgcaagaaccagaatgtcgatggaatctggagaagagcaacttgggcgttggctcaatcggcaag
ttcgcgtagaagtttccgatcaacgaaagttttatggaagatttttgtgtaccgatcatgagggaacagcaatcttatccgacaccacagaagatt
ccaacggtaatgcgatactccgtaggggcctgattttaacagtttttgtaggtttaactcgagctttgggtttggtcgtgatccctggtaagcatat
caaatccttttctattcgactttagttttgtctgcaactattgatatccaacggctctcagttgggcagaattaaaatttatcgaaaaacaaggaaaa
tgtcctctaattccctaaaggtttgagccaagcttcttttcccaagctcagtcggttccctatgaacttttggtagactctcagaaggacttcgatgca
atacttgtatgcaatgattatataaagaaaagaatctttctcctttttcccagaatattccaatgcattcccatattccctactaaccctgccttttca
aaagaaaaaagggtgtttcttgcctctacctagctacattgcctggaccccaattttattatccaaaaagagactgctcgtctttttagatatacacc
atgaatgaatagctcctaattgaaccagcaccgtctataatcaagcatcaagtcttcaggattccaggtctgcgcactcttacttcttgtgacggaa
tatttggtgatcaaaagtgtttatgcttctttctaataattctaattctctctctttctttccataatttacttatttcttccaacattcctatttaacgat
gttcatttttattctcgttaaatcatacatcgcctgaatatgcgtagacgagtttatttcatgccaaagaaacgcagcctacctataacgatttctttc
tagtttttttttcatttctattttttaacttttactttttcaacatgtttttgttttgacgtcacaaatgagagacactcctcccccctatatgcgttggtctc
cagcatacttcttccatccactttcttaagtgcgcgtgtttacaagatcgataaaaatcttcaagttctttagttattttgcttctttttgcattaattag
agtgcttagtacatccacttatttgctaggactttgcttttcgtcatgagcaagaattaccaaagtgttcctcaagaggaacccatggagggtggca
caggccctactcaggctcctccagcctacacagaagtaaatgactcggctactccaaatcctaccccggatcaattcaaggatacaagaacggtg
gctgaatgtggccgaacggttcgaatggcattcttgagaaaggtatatgcaattctgagtgctcaactctttgtaactgcattatttggtggtatctt
ttatcttgaaccgacattgtccctatgggtaaaaagtcacccttggtttttactcgtcaatttctttgtgtctttgggtgtccttttcggtcttattttgaa
gccttatacgtatcctagaaactacattctgttatttctatttacggctttagaaggccttacattaggcactgccatctcctttttctcatctcgcatc
attttggaagctgtatttattactcttggaatctttatcgcccttacggctttcacctttcaatccaagtatgactttagtcgtctaggaggctttctata
catctcgttatggatgctcgtcttgaccccccctgctttttatcttcatgccttccatgccattagctgaattggcttttgctggtttcggcacgcttgtatt
ctgcggctacatcttatatgatacttacaatatccttcatcgatattctgctgaagaatttattatgtccagcttgatgttataccttgactttatcaac
ttgtttgttcgtgttttgcagatcgttggcatgctccaagatgatgatcactaaaaaaaaaaaaaaaaaaacactactatttaaccgaccgtttctt
ttgtaccctatcaatccatcatatttcatcagtttcgtttgattgaagctccatggagcctacctctccaagcttttcgatagcatataagcaggggtt
catagcgaacaaagaggcctcatcaaagaaacaagatatataaccaatgattataacgaaagcaaaagacacttgttatgaatcttcgtttgac
ttgtatgcatgaatctactaaacaactttaacaagagttctttttaccggcatcaatttcactctatcctatttattggcaatgattatttgcttacttg
ctttcctatcctatataagtttatgtcttaaaaatacttgatattcacaaaaaaataaaataaaggcgataatgagagaagtcttttagttaaattc
atagtttattctgtcgcaataatcaatgcttcttttcaaatacggtaaaatctcatttttcattctattgtttaccgtttagcgcatttccgttgacaatc
atatctcttgatttcatcctgcgctagtcctacttaacgtatctgatatatccacacacactttgatacaacaaacttcgtaaaacttaccaactacc
aacgcctggtaaacaatccattgttcatggaaaaaccagatgaaagcggattcagtcaattgaatggaccgtttacgcgtgagaaaaaaaggag
gagtttttctcattctcagaaaggatcgaaaaacgatgtgtttcctgtagaggaagatgaaagtacgacgccagaggatgtagatgcacaagaa
acacgccaaaagcggtttacttcatccctacaagggaatcgttttgaagaattacgaagattacgcgataaggaacgtgaggatgccatacaga
aaggattaattgacgatccttctaaaccaaggcaactggatgaagcggtgaccttcgtaggtacctgtccagacatgtgtcccgagtatgagcgt
gaacagcgtgaatatcaaaataacctagaacgatgggaaattaatcccgatagtggccgcgtcgacaagaacttggccgtcaaagcttttcatc
gccctgctgcaggaaacgaacaggcacttccttccgatgtccgtcctcctcctgttttaaaggtttgattattttttccaaatttccttcaagtgccgt
cttttgacatatggccgctttatgaaggattcctctttttgttattcattgtgcattttattcacacgtgccttagaaatcacttgattatttaattgata
aaatcgtttgcggaccttatcctttggaaacgactcactttttttgtgcgtgatcgaacgcgctctattcgccaagactttaccttacagaacaataga
agtttagaagccgtagcttgccatgaacggattgccagatatcatattctttgtctacatcagttgtgtgagaaaaaaaactttagctcacaacaa
gaggtcgagcagcttcgtaaaggtaagtatcggttttcgcatcttttcaattactttttgttatactcatctaactgttagttttacaatctttgtgtga
attctatgacgaccttcgaaaagaaaaaatatcatgtcctaacgaagccgaatttcgatcttattccatccttacgcatctacgcgatcaggatgtt
gttcggcagtctcaacttttgcctaccgatattttcgatgatccccaagtacagctgtctttacggctgtcagctcttgctcagaagaacaatgagcg
aatcggtcatctgcttccactaaacacggaagctactccaaacttgtatactagattcttcaaacttgttgcatcttcctctgtaacttatttgatggc
ttgcttattagagtctcatttcacctccattcgaaaaggtgccatcaaggccatgcgtaaagcctatatgtcagtacatgcgaaatttccgtgtgtcg
atttgcagcgtatactccagtttgatgccttggacaatgttttcgagttctgcgagtattacgctttggaggtttatgaagaaaacggagaaccagtt

gtaaacctaagcaagaccggctttttgatgactctaagcctgatacaactcaacgcttttcccatgctttagttgagaaaaaattagataatcgtt
cttttgctgttataataaatggaggaacgacttcgataaaagcagcgaaatcttctgtattcaagaaggacgcaagaccgatattccctgttaata
agccaatctctaatgttgaaactcggtctgtcaccaatggatctcataaaaactcagtcctgtctagaggaacactgaacgctaatgctcccgtttt
taaacctggttcttctccttctgttcttagaacaacgttaaatactcaacttccccgatctgatagccagtcattactttctattccttctgaagaaac
aataacatctttgccgcctccggtcataaaaaagccttctctcgttaggcaaaaaccaatgttttcttctgacgatttaaatttgatttttcaatccat
agtaaagcatacgttaacttcgttatgtggtcggatatgcaaagaagtttacgaaggatttattgacgaaaccgctaaatcaatttattattcaatg
gtaaagaatataatacgcttttcgagcttaaaaactctagcaattgaaatctgcaacagcaccttaaaaaaaagagtatttagtcgaataacact
gaaagctcgagagtgctggcttaaaaagcgagagcgcgaagtacaacagttaagagaacaacagcagcaagaacactataaagttgttttgag
ttctctgttaggagcttcatgtttaaataaccgccgcacacatggttctcgtgttttcaatggagattataatgcgaaaaatgtattagcatctgaac
aaatccgactggaacatgatcgtatccagaatttttggaaggaagaggatatatattctttcttaaagttgttaaacaaccaggttttaagttcgac
atggcatctattgatttttagtgcgtccaacaaaccatctattcatcactggtttagatcaaaattaggtcttaagtgttcatctaatccctctatttgg
agctcaacacataatatcctgaatcgggattataccttactaatgccagagactttagagagtttacctacaaagcggatgtgttacggagcttgt
atttatgacttaggattggttgaggagaatgcttataattctccatcgatgtgtaatgggttgaattctgaaaagtcagcaatgaatgacccattaa
gagatgatcttttctctttcttacgagttgtttccaatcggacgacaacgaagtttcctcttttgttggttatgtggacaaaaggtgactatgcaacag
aaaattacgttgaacgattaaaattcatggacataatcacctcctcatggtcggcagtcagcagcattcatgtgttaactattaaaactattgaag
acgcaaaattgggtgagagcctaaaattattgctgtccaatgttagttctgaacaatcaccttcatttcaagttgagcaagtctgtcaaattaatag
aaagagagaagctgaaatacaacttaacgctacacataaaaaacgaccggttcgaagaacaatacctttgtttcaagaggaggtaatgaaggg
tcgtgacgacttctcttatgcattatctaatccaggcaacgagtcagattcattaacgttattgcgtgaaaaaattaaaaaagcgcaagaacttcta
aacaaggtgcaaacaacgaagtaacgtttgaaggtgaatataaatcatacattaatgatgataacctttttaatgatgtatattagaatgaaattt
tgagttcctgatactaatgtccgtgatatttaattttgatagaaaaaagatgaccaagaaggagtttagttagttgtagtaaaagttacatgaaga
gacagattaagtaaagtccaaaatcaaggcacggcgtcctccgattcgaagaactacaagatcgttacgtacgagaatcgcattaggatcaagt
attgtttcatgtgcaagggagattgcacctgatcggatatgttcagcagcttcttttctcgatgcatagatgcccagctctcgaagtaatcgagaca
aaggcatcggacttaaggatggatcaactttataataacaattagatgctttaaaaataccctttaattggtccactgctggtaatgtggaattgac
ggaagcaacaaaaaagccagagggagcgggttgagaaccgtgcaagagttctgtttgcaatcgtgcaagtttagcattttcttcgccatgtacca
tatggataatattaaacgcaagtgtatgctgaattaaacgagaagaacaatcttgggaatggctcgatttgatgctctctagattttcgctagaaa
gcagagtcaacatatcaagatactttgaagccgtatcatctggtagagaaataaaggactaaaaaaattgagcgttagaatcagaaacgaaact
caaaatgtataggtaaatgatatacctgataaagactataagagtccgtcaaagacgggtctaaccatatagcatttcctgcagattttccaaact
tctgaccattcgcagtcgtcaataagggagtagttagagcatatactggagactttgacgatactgctttcggaaagagacgtcgaactaggtctg
tgccagcggtgatgtttccccattgatcactaccgcctatttgtagatttataccctgatttttataaagatgtaaaaagtcatatgcttgtaacaact
gataagtaagctccgaaaaagatataccagaggaactgtgtaaacgagaggttacactgtcccgagcgagcatttgattgacacgcacatgccg
acctacttcatttagaaaattcagtagattcatattgttataccaatccagattatgtacaatgttccaagtacgaagagaagcaggtgcaacttca
ggcttatactgacaatcttcggcataggccaagactttttcagataagctgaaaacttgcttctcaattgcttgactattccgatccctaataacttg
atccagctggtgccgacctgttgttcttccagagggatctccgagttgcacggttgcatcgccaattaggctgtatacgggaatcccatgtagatac
aaatgtatcaacggcatcaaagctactaaattacctacatgaagtgaaccagctgtcggatctactccgctgtacgctccttgtactttccagccag
aatgaaatctacagaaaacgtgttaacgtaccgaacaaaaaggcaattcatttttactgcttacggagtggaattagaaaccaaagaacgcgca
gtcaattgtttccaacatgctaaaaagcgcgacatgaatcaagggagaaagtagattgaatgggagtcctttgcacttgtattgtttactttgttat
ccaaggaacgaccgcggtttcccctcggaaagtaacagatgccaaggtaacgaacacagtctctggcagatcgtaaacttcccaagaaccagc
aactaacagaaagaatgaatttatctctttacgctgttaatgcgtttttgattttggacagtacgggaaagcgaatattcgccaaatactatagtcc
tcctcacacaaaggaaggagaaggttccatattccagtcagtcaaagaagaaaagactttcgagaaaggattattcgagaaaacatggaagac
tcaaaatgatatttcaacttatgatggcaagcttgtcgtaatgctgacggtgatggatgtggcattttatattgtaggcggaatggaagaaaacga
aattatgctttacgaatgtctacggtcaattcgtgacgctttagaactgatttacaaatatattcccgataaaagaacgatccttgacaattatgatc
aactcttgattgttgtagacgaaacaattgacgatggtatcattttagagtccgagcctgctattattgcttctcgtgttaccaagggccctgtttccg
aggctcaggccattgtttctgacttcaaagaaatgggattcatgagtggattgcagaaagctcgcgacaagtttgccgagactattctgaaaggtt
ctttttgaagcatttctatgtttcgtttcttcgactatgcatgataaaaaattcctatcatttcattttatttataatattataaaatgatggctgaacat
gggaggattttgtgtatgatttgcgcttcattcttttgtttactttttaattctcatcgatcccggaggaactctagcatatgtcatcgctccatttttctg
tcgagtgttttgtaatgccttaatattattttcatcgcttcctgactgttgaccagctcgttaaccttcataagaagcgtattgacatctttaccggttc
cttatcctaactagcttatatttcatgcggtttcctcgtctttccttaatttgtcttacttctttcgcatatatctagacatgtcgaaattgaagaccata
aacgtttacaagctacatttcgcttgtgtatcagttcttggtttgatacgaatataataaaacgactgtacagtacatattttaacgaggcaaagta

atggaaaatagaacgcaaaattcctccatatccacaatgggataaacctcgtctgcagggttttagtattacaactttcgtactactttccactaca
ggacttaatataggcaaatcaagacagcaatagttacccgctaacattccagcatctctactacttacggttacttactagctcattacgaaaccct
caaatttttccagtagaatgtttccattcattttattactccaatttacaagaatacaaaagacgaagtttagtacttcttttcagtttgcatcaaga
tatcagcatactcctcaatgggggtttgaccgaaaggacgctcagcccacaagttgggagtaaggataccgtaggtaagggaggcagcagcga
aagcagccttgacgaagttacccaaagtcttagtggagccacgagattgggtgtagcagtcatggataccagcgagttgaaggaaacgcttggt
aacaggggcggcaacgagaccagcaccacgaggagcagggacaagacggacagtaacggaaccacacttgccagtgaccttgatggggaca
gtgtgagggtcacccaaagcggtaccccagtaaccacgacgaacgggcatgatggaaagcttacccatgatgatggcaccacggatggcagta
gcaacttccttagcacacttgatacccaaaccaacgtgaccatcgctgtcaccaatgacgacgaaagccttgaaacgagtacgttgaccggcacg
agtttgcttttggacgggaacaaccttcataacctcatcctgcaaacggggcaagaagtagtcaacaatttggaactccttgatggggagagagt
acaagtagatctcctcaatagacttgatcttaccggccttcaccaaacggcccaacttggtaacgggcatccattccttttcctcgtcacgcttggct
ccacgacggccacggccacggccacggccgcgaccacctcttccaaatcctctggcttgggtttctgccattttatcttatattccaagtttccttttt
tgtttagggaacgaaggagggtgaaatctttcgttgtgcattttcatgtatatacattgtgactgtttcttgctagtagggtagggcacgaattcgtt
ggcaaagagtccgggtttttttgcacatggaatgcagaagttgaatattcgataaagatttttgttataaaacttaaaaaaaaatgttaagcaatt
cttttctcatcattcaacttaaccacttctgagatgatcataaagtgacgttgttacttgtatgtaaaagaataactgtactgaaacaggtggtgatt
ccagtaacggagtttgtcattccattctcgattgtactcttaattattataatgtttacccaagtcatacgcttcttaccttcgtctgtttttagggccat
tgagtcacagtttgtttagggtagggtacagcatgacagtcacaatatactcacaacacaaatgttaccgagaactttcttccttcacaaaccctct
cgtggtcaccgctgtctacaaaatgagtcaagtcgccaaagatcaaaaggaacaacagttcacctctaccgttcaccgcattcgcattacccttac
ttctcgtcacgttcgtaacttggagaaggtttgcgctgacttggtcaaccgtgccaaggacaagcaattgcgtgttaagggtcccgttcgtcttccc
accaagaccttgaagatcaccacccgtaagacccctaacggtgaaggttccaagacctgggaaacctatgagatgcgtatccacaagcgtctca
tcgatcttcactctccctctgagattgttaagcaaatcacttccatccacatcgaacccggtgttgaggttgaggttaccattgcccagtaaaaatgt
tgttccattttgagcagggtttttggtagtcgataaattgtcgactagtgcttcaataaaatcctactttgtgtttattctaagcgaatagtcaagaaa
gcggctttgtagcgtagtagaattgtatgttcgaaagtatgattacaaaattgtagggtgtgttcaatatgagttctgggtggatgaagtgtatttca
gagttttcatgtttgaacgtgtttattgaaaccttgctctttccttcgacagttaagtgataacatatattggactgagtacgttcgttttgacatgcta
gttcgttgcataggcgatagagtacaaaaaccgaatgccaacctttaacaattaagtaatgaaatagtaaaggtttactaaaaaggtatgctcgt
gttcattgttactccttttttataagggtcgacctatttttgagatgcactttcactcaactattaagttgcgaacataaatgtagctggaatgcatagc
aaaggatcttacgggaagtttcatacagcaaatgtgagatctgtgaaaacaacgtaacaccgattgtaaacttactgtctgtaaatccggatatgt
atgcaataaatatatacggtctcaatccacaatggtaatggtagaatgttatgtatatgttacgttagaaagcttaataacttatcagaaaccaag
cactactactcttcaatactttgtcgtgatgaagacatatcatatagtattttacagatacataaatcgtctcaatgagaatacgttcctgcgggctg
cttggtctaaaagaacatacaaaatgttgctgaaaaaaaaaagagtagcgtaaaaatttatttttgtgtcatctcaattcaagtaatgttgtgaga
aaaagatcaacataatccatcatgtgtcgggaaaggtagaatgtttcatcgagacattcttctatgggtataaggagaaagccaaagcggacga
ggaaaaacatacggaaattaccataaaaaagggaagaaaaagaccatattacttgtagaaagcaaattgttccatgtcagtaggcttaacagc
acgtgcataggcctcttcaatatcgctttgaagaataacaccacggcttttacggacagctaataacccggcatcctgcatgatagctgaaatttg
agcaccactagaagcatctggacgcataataaaagtatccaaatccacctcaggagaaagagacattctagcagtgattgtttgaaagacaaga
cggcgctggcgacggtcacggtaagagggaaattcaattttacggtctagacggccaggacgaaggagggcaggatctaatgtatcggcacga
ttagtggccatcatgaccttgacattagcgccttgatcaaaaccatccatttgagttaacaactcgattaaaatacgctgtacttcacggtcggcac
cagtctgagcatcgaaacgtttcgtagcaatagcatcaatctcatcaataaaaataacagcaggggcattttctcttgccattcgaaaaacatcac
gtaccatacgaggaccttcacccaaatatttttgaacaaactcagaaccaacgactcgaataaagttggcgttgactgagttggcgacagccttta
ccaacatggttttaccagtaccaggaggtccgtaaagcaagacaccacgagggggtctataccaatttgacgataaagatcaccttgcgttaac
ggtagctcgattgcttcacgaacttcttgtttttgaacgtctaaaccaccaacatcattgtaagtaacatcaggacgctcatcaggacggagcata
gaaatagaggagtcagcctcaggaggtaagatgtcgacaagggcatttgagtgacgttgaagagcaacactagcagaaggcttcagcaactca
cgatctagagtacttaatatccgcaccacataattagagccggtggtactgccgacaatggctgtattttgatcaatagcttctaaaaattggcca
atcactaaaggcactgactgaatccttttcacttcttcctgagcacgtattaattcacgctttaacgacttgcagtcttctttgataaagccttcctgt
aaatccaataactctagctgtctttgtaacgctttccagcgattatagagctctgtttcttcagttccattcattttccaagaatatgtgttgttggcag
cttagtagattccaagaagtaagagaaagaccaggaataacgtctaattctatagttcgctatattgtatagcggtttgtaacatctgtaaactagt
aattaacgagctgcagttcacttactaaaaatctcttacatttcacgctacattcccttactattcgcttacgtataagatcgtgttcagaacttgaat
aaaaaaatagaatatctaaatgagtaggtgaaagaaatttaaattgagaaatctaaattaaaaaaccgaagatattaattgctgttcttacctca
aagttattatcatttctattccatttatcgagtagagcacctctagggtagggttcatgtatgtgactgtttttgttgagtaggtcaacacaatgaaac
tttttgactcaaacggaaccaccaccaccgccaaataacaaaatgggtgcctacaagtacttggaagagctttctaagaagaagcaatctgatgt

gaacttgttcttgtctcgagttcgtgcttgggaatatcgtcagatgaatgttattcatcgcgctagccgcccttctcgtcctgacaaggctcgtcgttt
gggttacaaggccaagcaaggttacgtcgtctaccgcatccgtgttcgtcgtggtggtcgtaagcgtcctgttcctaagggtcaagtctacggtaa
gcccgtccatgaaggtgttaaccacctcaagtaccaacgctctctccgttccactgctgaagagcgtgtcggccgtcgttgtgctaacttgcgtgtt
cttaactcctactgggttaaccaagacgctacctataagttctacgaagttatctgcgtcgatccctctcacaaggctgttcgccgcgatcctcgtat
taactggatctgcaacgctgttcacaagcaccgtgaggctcgtggtcttacctctattggtaagaagtctcgtggtattgccaagggacaccgtttc
aacaacactcccaagcatgccacctggctccgtcacaacaccttgtctcttcgcagataccgttagaggagtattgggtatattttgtaatattcaa
aaacttttgttcatttcagtcccttaaaaacacatgacatgcgtgaagaaacgaagtagcagttgttagtagatgcaaaaaatacaagtaaaacc
gtttttccctgtgaaaatgtatctaaaggatcttagaatggaatcaagtaagagattctcagttagtccctagaatttttattgagtaattcaagatg
ttaggaagtatctgtccagtaaaatccaacgaggagctattaatttgacccaatgtacaagaacttattttcatatattggatttaaaatttaaaaa
ttaagtgcaaataatgtaaaagtttgaaaggcgaaaaagatgaccactaagtgacttgaacacttgacttcaagatttggaatctcacactctac
caactgagttaagcggccgtattaattgtcttggtcttataagtcataataaagtagatgctatttactggaagaatcgaaaataaacattcctcca
tagaatttagtaagaataaacaaaatctggaaccgcttataatagagtttcagtattttcattaagatacaaacgtattgagggatcccagcccaa
ttctttccagttcaagcgatgattcatagactgtagagttttccattataatcatgtagtcatcttgaaactaacattaagcaacgaaggatatggat
atggatttgaaatacagaatacttgggtttactttggtggtcatatgaaggaactgctatataggattgagatatttaatttggaccatatgcttcta
tgaatttttctcaagaggaacgttatgatagaagtcgatcacgatttgcttctttttttgtattacgataagccttatatatatatttcttttctgctttc
actagtcgtttaggtataagtgagagtaaactgccattgctgactatgatgttggcaaaaaagatgactggccatcattttcacaaaatgaataaa
gccctatagtggttagaattacaaaatatttcccttagtaattatattgttaacagtagactgaatagcgacctttggatacaacgtcacgataact
gttattcttttacatactctcaagcctgacgacggcttataaatatattttggacgttttttttttcgttggtattgtaaacatcgataaagattttatca
caatggaactacttcaacaaaaataaaaaataaataaaaaatgaacgaataatataataatgtgaatatgataactggttgtatttttttttttata
ctccaataacctagcaaacaaaaacaaatgtgctgacggcttgtttacatatggaaagttcatgcttttgcacggataagatttgtaaactattttt
gtttatcttgcaaaagaaaattgtttgtttatacaaagtatagtttgattagctttagctgattttgacaagtcatctagggagacaatataactctct
gctcccttcatatatgaatgacaatttcccttacacgtgacatatttattcaatttatcttttaaaaataaaataaaataaaataaaaaagtatgcta
cagcttcaataaataaatctgcattgaatatctcttatgaaaacctttggtagagaataaagtaacagatcatattaatattaagacgttacctcgt
tatatcatctagtacgatgccatacaaaaaataaatatgatttaagtattctagttcatacaaaagctccaaaaatcgacctttctaattaattgtg
ataagttttaccatatgttttcgtaattatagattataaacaggcaagataaacgaaatgagcacctgttactctttggtttggaagatgatcgatc
attacaagtttttgattaaaaggtatttagaaaagtgcaaaacccatgtgtcatgcaatcattaaatttcagaaatattaaaagttttagtacctttc
ctggtatagtcctttttttttttattttatttttttttttcagcatctcatgtatgtcagtatgaaatttcctatttacttaggctgaaaagtcgttttgagtttt
gtaataatgtcgacttttctctgggatacaattgactacctatcatgataattaagtaggttggtacagtaaaaattatattttccctaatatacatc
gtttgcagtcatgaatttcaaggtttgtaaaaagaaagactctcaaagaatcactgtcttctaaatgggtaatagatttgtttctttttatatatttttt
ctaaaggtttcagctacgctatatatagcaataaactttcagaaaaaaacaataatgattcatcaactttagaaatgcatgaagggaatttgattg
tcgctttacctttctgcttgttcttcgtcatatatctttataaagtaacgtaaacactttattgttttgaaatgctatcccaactgaattatgcttcaatt
gaagatgctttcgttctgctattcaaaacttgaccataatagaatactactagcctcatataaacaagaagccttttcaagaattagaacgcttctt
tctgattcataagatattcctatgagccaggacataaaagtacaagaattcatttttagcatccatttgcccataagctcttataatatttacgtgta
ctaggatcaaatgggaaatacggttatgccaactgaacttgactcgtgcctcattcattaaatttttattgattcaacttgatgcattttctaaaaga
gaaaacaacgacaatgtcatagtttgacgaatatgaatctcaaaaagtggttcgctgacagtcgacatttaagaactccttttctttttttgggtcat
gaaagcagagactttccaggtagtgattatattccgtcacaaaaactgaaaacaggtcattatcatcacaaaaaaatgtccatatcatcatttgat
atgaattctatatcaatatgtatgaaagcaaaaggattagttgtaacaaaacgcaactcatccaaaaaccattttggcatctgtctacaatttcaat
taatcagcagcatttatagggtaatatacaactctaaaacatttttttctgtcagattctagtcataaaatagtgttgtcattcctaactcccagtgac
tttcatttgagatcgatttgcgaccaggaattagaacgaaataaatagaaaattccataaagaatatacaaaagtcagtaaatgtaaaaacccc
aaatgctgtctttaattgaaaaatgactaaattcaaaaccctttgaccaaacattcctaagagagaagaatccaagagagcgaagtagattatgg
ataaaaaaaagacatcctctcttgacatcttgtcattatatgatggatctcgtatatgtatttatattagtatcaacatactaaaatcaaggacagc
aacgagcacagtaccattcttgaaaaccaaataagccagaatatcatatgaaagaatttcctattatttacaaaaggtagagatgctttccctgct
tcatattcattcaactaaagtccaaagctagttgagccgcaatcccattttccaagtcctccagttcaaggattatatcatcctgatcatttagagtg
attgaagagcgtttcttttcctcatacgcccggctagttttcttgttagaagtgccacccctaatgaaaagcgaaatcagtatcttaccaaacagctt
ctttggtataaattcgaatgtattgtgtaataagctccaaagcactcggcgtaatttttgtacccttttcagaattgaaatatagggtcaaaatacga
gccaaagtgttgagggacaagcgttcgttttcctgctccattcaagtacgatgaccgagaaattgattcaaatcgatacagaactagaagaagaa
gatacggaatttattccacaggatgcttcaagttcagaatcgtcgttttcagacgcagacagcggcgaagagaaggtggaagaagagaaagaa
ggagaagcagagcaaagcaagactttagaagatcccaacccagaagaacatgaatcttccaaagaaaaagagaatttaaaagaaaagaaga

aagatgttcatgaggagaaaactgtagagaagccgtctgaggctgtttcagcgcctgcggaatctgccacaaaggcaaacgaggaaggctcttc
aacggagcagttgaaacaagtgtcttctaaggagcatgaaaagcctcccgtaaagaaaccattggcgagaaagcgacgtgaatcacctctggc
caaggttcaggccaagcaagctgcgaaaaagtcaaaaccaaatactttggagcaggctcagcaaaattggtcggattatgtcaaggagcagga
cattcaggatgagcttcgtctggccaacaaagatggatttgtcgagcgccaagaattcttggccaagacacgctctgcttatgaagagaaaattc
gttccatgaaaaaaattccctgaaatactgaaataacagatatctcttttactccccagtgctctttctattctttttaccgggaatattgcttatttg
taaatctggattttcctaccaagagccattcactcttcctaacttgggcctttagaattttcggtgaagcgagttaaatccgctaccattttaacaat
gttttacttaatcggattgggattgtatgatgaaaatgacattactctgcgaggattacaaactgtaaaaaaatgtcagcgcatctacctagaggc
atatacatctattttgctagttaataaagagcgtctcgagtctctttacgggaaagaaatcactcttgctgatcgtgaaatggtagaaagcaacag
cgacgaaattctgaaagatgctgatacttgcgatgtcgctatgctagttgtgggcgatcccatggggtaagtacttttaattatcttcttatatttccc
ttaactttccttaaagatcatttacgattatggatgttgaaaagagatttgagctatgaattgttaaatgctctcccaatcgcctcgacaggatatct
ctttcaatccataactgcttcttcagggaatcgttatatcttttgcggaatgattgacaaggggtttagaactaactgatgaattagtgctaccaccc
atgctgacttggtgttgcgcgctcgcgaacttaatattcctgtgcgaatgattcataatgcatccattatgaacggtattggtgccagcggcttgcaa
ctatataaattcggacaagccgttagtcttgtgttctttgaaggaagctatcgtcctcaaagcttctacgaccgcatcaaagaaaacgttagcttag
gtctgcacaccttggtacttcttggtacgttaaactttatggttgcatattctaactattgcttagatatcaaagtgaaggaacaaagctgggaaaa
ccttgcccgggatcgtaaaatttatgagcctccacgctatatgtcagctagtttggctgcacaacaaatgatagaagttgaggaggaccgtaaag
aaaatagtatgtatacttctaatccaatgcttacgatattaacaaataaaacatagtctgtttacctgaatctttggctgttgctatcggtagacttg
gttcagatgatcaagtaattgcttctggtactttaagtgaaattgctgaatatgacattggacctcctcttcacagcgttctccttgttggcaaagat
gttcatgatatggaactggattatctgaagatgtttgcgattaacaaagaaaattttgaacggattgcaaagctttaccttcagaaataaagcaag
gaaaataactggaaaatgttagtgtgttagattgttagattcagagatggtttgaattaaagaattatttttagttaaactaatttcttgcaatttca
gtattgactaacgtaagcaccatgaatgataacaacacaaaaaatattcacaagcaaacattattattcaaaaattcagcagaagtaatcttaaa
cagaacttcatcctggaaaacttcaaaagagtgactataaaatattttaatggatccagattataaaatatgaacgtctacgtaacaataaaaag
gtaaaagaaaaacaacaatgctacaaaagaatgacaacataaagtagaagatatcaacaaaacaggtagaaaagacgactttatcacgaag
gtttagaggaatcgtaagaaaaagaaacaaagcaaaaaaacagtctaagaaacacttctcgcaatgctccttgaaaaactagtaacagaatga
ttttcaaaagaagtagtgactgaagttgctttgttaattaaatctatccatgtcgaagccgcttcacgaggatcctcgaatgaccatgacttcgtag
gtgtttccataatccacgtgtgattggagttcttaacactgcggactcttaatccaacgcttttaataggaagctcttcttttatcgtagctttgcctcc
gttatccatcgcaaccaggagacatcttccgaatgtcgttaaaataaacgtgcgaggtcttttcttccggaataaggaggaaaagaaccgtacag
cttcatgagatccactccctggcatacttgaaacgtttaaaatggtaattttggagattttctcatcttccagaagtacgctatgccagcgcttattca
tttcttctaattcaggaggactggataaggtaaatgcggtatttataggtgtattgggggtaggataagcggaagaaacagatagagtagaggg
acgagtttggtcatttgggtcgccagttgatttgggaacaaagctagggacggacaagctgagagtgcttgtgtgtccagcaggtttgagacgag
gagggggatttcccataaagttttatcgaatctaactgcagaaaagaacggatgattacggatttcttttgttgttaaccgttcactcggatctag
gattaaaattttcttaattaaatcagaagccgtctcagagatatcgacagggacttcgtaactaagatgtagaatttttgaaagactagatattcg
tttcccgcaataaacgggggttttcctgtaagcatttgaaacagaatgcagccaaatgcccatacatctgaagcagtgcttgcattcttgtcgttca
atacttcaggagaaacgtatcgagccgttccaacaaacgatctagaatgattgttgttgattggaaggtcgtctggtagtgattcatcctttccaac
agtcttttgtatttttgctgaaccaaaatcaataatctttgttttcatgttgtcatccaaaagaatgttttcgggttttaagtcgcgatgaataactcct
tgcttgtgcataaaatcgatagagtccaggagcaacgcaccatagtattgaacacaagcttcattaaatctgccaagctagttcactgttagaaa
attcttcagaaagagacatatatctctctgtacactgaacataccttattaatataatccaaaagttcaccatttctcgccaaactaaatacaaaat
acagattctgagtatcttgaaaggtgtaatacagtcgaataaacccagggtgctttgaaataaggtacaaggcttctttctctatgttgacatatttt
actttcttttcttttatgatatgtcgtttgtccaacaccttaatggcgtactctctctttgtattattttctgtggccgacaatactgtgctgtatgagccc
tctcctataatttcaccaaaactgaaatctgaaacacgtttgatattgtttggggaagtcgctctgcttgacggacttgaagtcccagtagcttttgg
caaaagattgtttttttgtagaattagaattagaagaaattttaagttcattctgactactgggatcaggtaaagaaggagttgttttggggtcacta
gaacctagcgcatacggtgaattcaaccttctgttcaattcttctactgaaaaaaaactagaatcgtcttgaagttctgtttctgtatcctcagaatt
ggaattacctttgatattttgacttttccgcatacccacgatccaaggagtgtatggtaatagaaaagtcttggttgaggatgaatagatcaagacc
aatccgtcctcgatatgtaaaaaaagtttttttatttataaaacaaatctaattgaagaaaaacagttgaaatcgactcaaaatgaattgaactgta
gctttagcgtttttgttttcacttgagaggtacaattcagttttcaatacgaaggagacgattttaagcgagaatcaacaaaatgtgaaaaatattg
gaactaattaagaacagaatggtatacaatgtctgtgaaatggatcattccatgtcgcatccagcgactgtaaagtaccttcacaggaagagaag
ggtcaatggctggaaaagaataaaaatcgaatttgttataaaaagatctttatctttgctctatgaaaggattgctcttcaacgaagtaaacgaga
tctgactagatgagtcacttttcgactctaggatttaccataccatgcaaagcgaattcgaaagctcagttagctttaggatgcgagagctttttttac
ttgtttaaggaggattgctacatggacatttttctactagaaagaaaaatataaaattcatgaaccaattgctaccattcctctttcagatgcttaaa

aataaacgaaagcaacatattcttccaaagtatgatcagaattcatactgaagaacctttctcacaaaaatcgctgtaaagatgaaacccaagc
actccctttcttgaaaagacaaagctgacattggtccaaccaaaggaaaaaatcgcatttgatttatggttctcaggcttagagtttcaatcagaat
acagggatttgtttgtgctttgtcaacgacgctttcagttattaaattacgagaaatagcagatttcagactttactttgtacaaacaagttacaaaa
ctaatcaaccttaagaagaaaattaaccacaaaatactcgctgttcacaaacgtccgtcataacgcccttagatcttttgtttacatcccatttcact
atgaaatctgacgagaaaaaagtggcttccctgaacttcagccccctggaacacaaaaccaaaagattaattttgtcatagtagaaatctcacac
attatgagaatgtttatccagtggaagttgtaagtacaacaatttacaggattatttgtgacagatatctattggattattaggagcacgggcttgc
aattaattagaagtctctgaattataagcttctacagcatcatggagatgctttatcttaaactgattaaattcttccatatctttcaagtctagatca
ctggcagcagcttctttaatggcactttccacatcccatcctctacagacatccacagggttaccgctacagtttgaaaagttcgtcacaccttgttt
cgtaccccagatcttcccggtaccatctatgtggtataaggtgtcaccgacagggtagatacagctggcacctacaccataggaagcttgttctag
cgtcaattctggatccatttccacacctattgttacatattcagctagttttggatcaatttgagatagttttaaattggcgacatctgcatactttgcc
ataaccatattgatgaaaggtattaaaagaaagattatcataaaaaattacgtttaattgatatgttttaataaagccaacatttattcctgaattg
gtttagcattgctcattgcgaattcaagtcacagctttggctgttacttctctaagacactgggatttagcatacatcgcgtttcttcgtttttccaaaa
agcttcataaattcgaatttaaaactcctctataatgttattttattcctttttaagactttaattgatacggaggttactgtcgagctcaaggtgtgg
gtttgcaagttttgatggtgagactaactgtttgcagaatgatatgagtattcggggaatcttgaagtccgtagaccaattcttgaatgttaaacta
gaaaatatttctgtcgtggaatctgagaaatatcctcacatggtaggttttggatcgaatatttggaatgatgaatgccgtacaagagtatggatct
aaaaagtatcatcgtaaaagagttcaatgtacctgattggtttcttcgaacgagcgtcgtcattcctcaagtcttcgtcttttagcatttgctaatgg
agttttataggctgcagtcaaggatttatttattagaggaagtgttgttcgctatgtgcacatgtctagtgcttatgtagacacaattttactagcag
atgcatgtcgtaggggtacgtggagaggggaagacctgaaattaccatgttttgttctttgactaactctatagatttggtgaacaacaagagaca
ataagtttaaacaatttcccttaaatgcgtgttcttaacaaatctatcgtttggctttatcttctgtttctttcttagcgaaaagcgaaatgcaagatat
atttgttaatagcaagaaaaaaaaaaacttttaccgatatagttgcatcgtttgaggcttggatctatccctatttagaaaaaaagggcgtttttcc
gtacataatattcttcagaaaggcaaaaatagaaaagatgcgatgcaagtgcttgtaaacattttctaacctgtttctcttgtgcaggggtcttttt
tttgtattatttttatgatgagtttcatctttatgaatgaaaactagatttcaataataatatctggaatttggaatgtgcacatgttggtagaattac
tttctcaattctttaacgatccttctcaacttgaatcctgtatccatgttctacaatagtgtattcttaaaatattgtgtgaaaagatcaatcttttgca
aagtcctttgtccatttttgagtcgtactttgtaaattaaatggggtttataaactccgtttcgtatgccaagtttttacagctaaatgtatattata
atttgaaacaatagaaaaaagatctttagtggaaggaacttctcattcttgtaaatttcgacttcatgcatttcaccttctccatacactcacacaat
ggtgcttgtgtacccaatctattttgacaaaaagcgttcgaaggaatttcgacgagtttcaaaaaaagaagctattgaaaatccattagcgaaga
acattgcagacgtcgttcgtgatttgggctataaatgtaaacttgaacctctcaaaactcacccagccgattgggctaatccaggccgtgtagaat
taattttgcctgaaaagatatcaagtacgttataaagcttttgtttttgaatatctgtcactaattccatgctctcaatagaacattatttgttcaaac
aaattgctaaagtcttgcaacatcgtcccactaaaccagaagatccgctttccctacccatacaaaatgttcctgctaggcttcctgaacgtccacc
tacttaccccaaaggtgtagctggaaataccattgtacctttgcattctccagcactttctggaggtggtgtctccgaaaatatgttccaagatatga
tgcaggagatgcaaggtcaaccagaaatggcaaacggcatgaatcctttgaacgcccttgctggtatgggtatgcctcctgctccaaagcaaaat
cctagcccttcgaggccaaacactgccagaatagaacaagaatatgacctggacttggaatgaaaaattcgctatgcatgatgttttgtcttttaa
agctacaacgttcgcttgtgccaaatttggtattaatatgaagcaaatgagcatgtttcatttacaataaatttagactatggacaattatttttcag
ccaattatgagaaatgtaccttctatattcttttcaccacgtttttaataataaaacaagaggtggttgcttatttttggttttctagtttctttcccag
ttcaagcatcccagccatattcgtgcaatatctgctgaaatattcaaattattaaaacaacgaagataaagtttgttccaacaaatatctctttcttg
aattttacatccatatacgttaacgcttcagctggaatccttttctgaaaaccaatcgggatcttcccgttctgaaaaagttataaatcgcttcaaag
cttgtaaacactgtggtggttaatctagtagttcttccttatgaaacataggaatatttcatccattgtattaacgtaatcacttacctttcgtttcttct
catcctttaagatatttgtattaggatctcttttcagcctttggtgagcactacgctcatgaatgcctaccatcgtaacacctatgggccatggtgcat
ttccaattgtaagttgcagatagacgtcgtttgctttcacgaattctttacgctggcatcggtagcatatttcagcaattttggaaaatatatcatcat
tcaaagcgccgtcaataagtagtcgtataagggtctccaggtcttgtttggcttgccggaaaattttcaattggccctgtccttcaggattattctgc
tcagtaataggctttgatattaaaattttgtcccacacgtatattccacgtcgtagaaatgtaactacctgcttcgaaatccgtggtttctcaatacta
gcacgttgatactctatgagctcaacacctctactcaaaagatcttgttctaattgctcaacttgtttttctttttgaattttgaagaatctttttaccag
tgcttcttccgattcgccaaatagcttgatcggctccttcatttctcgaagtttgcgaatgacttctgaatgtgggaggtctgagtgttcacctctagt
agaaggagatgtggactttgtagaaatttgggattcactcactctaggactttcagccgtacctgttacgactggagaagaaggactcaatgcttc
atctactttcgcgatttttgagatagactgaagtctttctttttcgtaactttctttttcttcatctaatttccgtttcttctcttctttttttcagttccatttc
ccgttgttcttccaaatatttcctttctctctccttctcccaatctccccgtcgaaacgcttttttttgctggaatatcatttttaccttctaattgccgacg
ttttttttcaatttcttcttttaaaaagtccattttcatcaatccaatgagttttactccgagtcggtctcaatcgccggtttcaagtgaaagtgatcctg
ccgttggtccttgtccattcctcattcaagtctatcatcaatttcatactagaaatcacattttagacgtattagacgatgttgtaccatcaattgaaa

tttatggatggtaagcaaagttgtgaagcttttcattgatatctgtacatcgacactagaacctgaaatgctaagggaagagaaaagtatagaat
actttttatgaaatctatgatgatgatgatgaaaagaattcctatttagaattgttgcatacagaataatactgacagtgtccaggatgaatatgag
tttatacgagctgggagttttcatcgccgaaaagatattgtttcaacaagaagataccaagcatccagagtggatgctgcaatttcgtttcgttttcc
atgataggtttaaaggaagaccctcctcgagagatatgggtacgatatgcttgcataatccaaagctatttcaaggaagcaagctgctgaaggcg
actgggatgaaatgtggagatcgtatagacgtgtgtattaagccaaataaaatacgtctccgaaaatgaaataaaaaagcgatattatctgaag
aaagaagcttaaaacacaaatgctttcaatgacggaatggtcctttcctatctaatgaatcatgttatattaaaaggaatataaattttggacaag
gcaatcaagtttcaatgtgtatatattacccaatgtttctcttaaaaaaagtaaaataaagaaccatggaaaggcttgctgaatatctgctctaga
atatcctcagattccatctattgctctaacaagatctgtttagatcatctaaacatcaatctttttcacaaagctacaggcattctaattaccctacgg
cttcaactgatttcacattttgcacaagcgccgcccactgaacaagccttggctgagcttatggaaggcacatttcgtaacagtctgacaattactc
atcttaccaagtgctgtcaaaaatgaagcttcaagattatagtctcttgatttttttttgttcatttcaatatctttttactaatttttagtaaaagctca
tttataaacgtctattactgtaaggtactataagtcttccttagtataggggtagtacaccagcctgtcacgctggcagcccgggttcgaatcccgg
agggagagaatacttttattgtagtaatcgtaactttttatttcgaatcaaaaaagcagcatgcactgcggacaaaaacagatagtaaactctca
aagcgctaagttacaatatggagatgattgttccctacatcaccgttaaatgcaagtcaatgtcattcctacatgttttcaaagctacatacataca
aacaggcattgacggcactgttaaatgagcttgtatggaaggcttatcattcaaagctcttctggttgaatcgtcaaaaaagtcgtcagaattttt
ttcgtcctttcattgtatcttgaaaagatctgtcctacattacagtgagagggtctcggtcttcaacagaaattaatatttctgcgtcggcgtctgatt
ctcgaaaagctttggcaagcttttcagccatgacatccttgagataaccacgctcagtattcgaatggccgcctattttggttagtcgaatgttaaa
cgctaaaagggcatacataagataacagagattcctttggcaatgagggcaaggacctgatgatgagacaattctccagtaaagtataaatcgg
cttctgttccagcgagcacggaaccaccagagccggcacatacggaaacggtggaaattggagcatcaaggccctttggagcggccacttggac
taaaaaaaaataggttaaagaaaaaatcttttcatactaaaaaggtctgcataccataagcaaggccagttagtttctttgctcggtcaatgactt
cacgcaagctggcgggcttggaaagttcgcaaagacggccatatccttcagtatcagacatggaagcattttgctgaattggttttgaagatttca
gttggtcttttccaccagcaattccccaagccagccaatcgttgacgccttcatgagcagcgtcaacgcaagtatgaggggagtaaacatggattc
cctcagcagctaaacgcagaagactcctttgttgcgaatcttccatcgtgatagctttgagtccacggaagataatagggtctatacggcgagttt
aatggtaagtaaataatgtagctacgaggaaaactagtacatacgataagttataatggaggaaacgagtttgttagaaatggcttcatcagca
actttttctgtcaaatcgatcgtcagcaatacggacttggcgcctgttctggaaaaaggcgcttgcaacaacaatccggtattgtcccaagaatca
gccaatttgggattgtacagtgactcaactacttgcttaagcttcgccgcaacacaagtttgtcttaacatgatcctttttcgtttttctattgaatttt
ccaagtcgtcgtttacgagattgggaaatgtcaacagtcttcttgttcagtgattttctgtttacattcaaatagggtactgcatttcattgctgttttt
ggaattatgaaatgtagcatatttcacagcatcgtttcgttcatccttagtaaatacaacaaacatagttgagaagaaccattcaagattttggtag
aaaccaatgttttaggagcaaaaaaagtaatcaaggtaagcagaaataagtacctctatctcgtttatcaattgttcattttctagtctggtggtttc
acatgcagtacccttcctataagtgctacctactgcagaagagtaacatctgctaaatacctactacgcgtccttaaataattaacgtggtcgggat
ggcaaagagaggaaaaagtagaaagaattctgatggtaatgtcttttatgaatgatctcagaagggtttattaacactgggccaaaagctcctct
ttctttaggaagacacacgatcggcggacgtgttggcaagaagaacgacaaaaagactgtaagtagctcgggatagacgaaagttggcgtatta
atcgttaattcataggactctggaacaactgaaagtaaaactagcttcagcaagcgcgttgcaaaagagctaaaaaataatgaattatttcaaag
actgaacagtgctggaaaacaagaaacaaatgctagtcctcaagcatcccaaaagaatctcttcaatcgtgttcaggctccgttaaaggcctctt
catctcctccttcttcagttcttgccaatggctcaatttctattcgtggtacggcaggacccaccaccgttgcaattgaaaacttggctccaggaacc
tcttcagaagacgtctctgcaactcttcaaagatttggtgaaatctctcaatgccaggtttttgatacgcagggcaaagttcgggctagcgtaaaat
ttactacgttctcgaatgctcagcaggtggtacagcaacttgatggtgttacagcagatggtaagatgacccttttttgcctttttctgtactattgc
taagatgctaatgctgttcgtataggtcgcaaattatcttgttacataaaaaagaacaaacctaaaaaaggaaaaaataaacttaaaaaattttc
ctgttcttgcgagataatttcctctctcaatttaagcaggtgctttaaccagatgaaagtcatttgtctatatgccagttcatgaatcgatcatttcttc
gttcacaactcttggtgtaactgatgttgttatccggtaagaagtttaccggcgtttttttccccaatttttatttcatttcctctttccttatagactta
caatgtgtaatattgtttttctaaacataaaatgaatcccaaacttggtctatcatattccgtatatattattcgttcttttgttaaagtaatcactcttc
ttcaaaaaaggaattgcttagcgtccatctaagaataagtcggcatagctgtgtctataacgatttctttgtgtgtggatttcaggtgaacataattt
ccctatagtaaggtaactgcattggaagatgtttaggcaagagttgaggccaaagtgttgtgattttagaaacaatctgttggaaaaggatgttgt
atcccattgctgagagatggacgccatcactaaataaaagctcgggaaccaagttatatttcgtagcactgtgcatgtcaatccaagttgtatctc
ctatttcactatccatttccacagccgctttgtacgtagcttccacatacggttcttggttccgctcatagcgaagtacttttgtagttattgacggtga
aacgaaaatcttcttcgaggtttttggaaactttcgtgctaattcttttatattttccttgtattcctcaaccggacaaagacattttccatccactattt
gactgtcattggtacccaaataaataatgacgattcgagtatggtcaatgtcaattgggagtttcgaaaaatatcttaaaaaatgccttgaggtat
atcccgataatccccatggctcaactataagtcgtctttgatagaaatttgccaattctgcgcaatagcctccaggtgtatatccatactgagttacg
gaatctcctaaaatgactaacttttcaaaataattaacagattcattattattgcgaggtaagtaaggtgagaacatagatgatgcgtgttaaaag

ccagaaaaagagttgtaccaaagcttagtgttttacgaagtcacctttaaatcttgcagaaaagtaataaattgattcatgaagatgtatcttatg
atacttatgattaaaacaaaccagttaaaagaaaactaaataactatttactctataagtagctgctcatgtcgaagccaacttataaactgtcag
cctaatggtgaaaagcagacgagagattactttttctcctacatattgtttgtttacagacgaatttaatttgtaaggtattgaattcctcaaggcaa
aagcatacgagctaaataaagttgatcttttatagatctcaaataaatagaatggatttcctctgttgacagtagcaaagtattagacatttaatag
attatttaagcatacagaggtgttaaaaaacaaaaagaaaacaaacggaaaggaatcggtaggacaaagaacaatacaaagatctttccagtt
taaaaaattaatgttataacctgaactgcaaaacccaagaattttatgagacgtgaacttgatagttaaaattctcaaaatccaaaagctttaaga
tcagtccatctagtgaatgaatacatatcattctttgcttcgtaacctgaaagttaagagaacttagaaaactggtaatcatattcatcaagtcacc
catgatcgtataaatatgaaatacaaaaaacaaagtgagttctataaaggagcgctcgattgcgacgaaaaaaatggggaaaagaaggtcag
cagttcttgaagaagacgggagaatgcttttacccttcgtggaacacttccaaacagagtgttcccaaaattccaaagatgaaacataaaacgga
atgcctcttgttttgcgaatgatcatgtcgtaaagcctctggaaaaatgacacgaagggacaaaattagcatcattcccgcggtactagcaaaaa
tgaatccgtagacaaaatttagcaagtcaaaacttccagaagccccaatacgaactaaaagaaaagcgatcaatgctcctaacggaagactca
aactactcaaactagctgcagtcaaaaaagcttttcttcgggatttccaagcaaggtaaagaggataagcaactgtaaatccttctaccatgttat
ggatggtcatagcaacaaacaccataaaccctgtatcgacggttgaagccaaatacgtaataaaaccctcaggtattttatgaaggcaaataag
gaaggccgtttgaatgcccattgtataaacagaatgaagatgctcatcctcctcagacccaaatacagtggtcataagatcaaaatgattatgac
actcgcattcacaagtaggtgcttttggattggattctccggtaggaggagtgccattttcagggcaaccgtcatccggcaacagcatggatctctc
atgaagatgtgagccactagggcaatggctaccatggatggaagaagaaagttgaagagatgctgagtctgagtgagatgatgataacgatcg
ggacgaattctgagcagtaggatatctagattctctatacattgaacttgaatcgttggttttacggagccatttatgaagaaagtgagtaaatcca
tagaagaatatacttcctaagaaaaacgaagtaagttggaagattctagcaaggaacatgtcaatcatggggacgtctaagaaaaaatgcaat
gattcttccataacacttgaccaagagctgaacagtaatattccagcactggtagcaagtgccgttaccaaggaacgctcactgtttaccaagtct
attgctgggcgaccgagtaatttgttagcgacaacatctatccaaactccgctagcaccaaggatgcaaaaacaattagagattaaactgagaat
cctatcatagagttagtcatcgtgttcaaaaagctaaattttagcgtaaaagttgttgaaataactaattggcaaattcttgtagatttcatcggcca
tattgaaatattcgggcatcagaaggttgctaatgtcgaatagaaggggaaatgtcatgtattgaagcgatagaagtgcaaagcaagaaaagat
cagatagattataatggcagaaaatccgtcctaagaatccagagtacaggcatccaaaactacagagccaacgatccaattccaacatcatctt
acagaaaaaaaaaaaaccaacttaccagccttttgaattatccatcagcaaggtaaatagaaggatttcgcttcctaggacttgctatagaactg
agcactatgccacgttacgatacgtaagagctcattagtaaacaagagatgttgttttcttgtattttgaagcagatcggctgtgtgtatgttaattc
tttacaattttccttaaagcaaaaagggaacaatgaaactgaaagtttatacttgtgaatacacgaaactgtgcactcattataaccagctgacttt
atcaaacaatagttcatcttcatagaggagaaaagtagatgtttctgtttcgtaaacagcgtttgcagttttagaacacctggaatatataacactt
tgttaggagtgtgtaattgccaaactcattctgcaaaagccttctctttgtttttggtttattgttctttaatttttcgtatttatttgcgtacgtcaaaa
tcactaggtagaatcaaatatgtctatggtcaatagtaccttcgtaagcctttcttaatcgagcactgtgagagcatgcttgcttgaaaagaagatc
taaatcgaaccaagtaaatagatcctaatcccttatttttacatttcatttacttccagctctactgtttacaataccaagtaacaagtgaccccaaa
agacatcccattgttttagcacattagtcctgctaacaacaaaccacagtccgggaggtaagacaaggagtattctttcgagagccgtaaacatg
attgcttctatgagaatttttagaataatctgcttctttgcgcttgctctaggcgttattgcccagtcaaatgagttggagagactttcaatcaagcca
ccttacattaatcatggaatgagaagcttatggtgggaatatggtggaagcactattatcgataaaaaaaatggtatctttttgactcaagacctg
caacatcaaagcggatggctttcgacacgtttacccactcctactagtagttttgaagtattatttcaatttcgtgtctcaagtgagtccagaaatct
ctttggcgatggtatgggattattcttgactgaagagcgcgctaagacgggtcctgtttttggttttactgataaattcaatggttacggtattttcttt
gatacatacaacaatgatcgccctggaactgttttccctcgtgttgttgtcatgaagggagacggaaagacatcttatgatattgatcatgatgga
aaggccaacgagattgaaggatgcagtgctttacatttgcatagcacggattacaccttgggcaagttgaaattcgacaagaacgctaagaagc
ttcgtttggaactcgcatttgatgctagctccgattttatcaagtgttttgaattggatgatgtcgatatgcccgctaccgctttcctcagcttttctgct
cgtactggtgaattaagtgaaaaccatgaaattcatagcattaccagccgaaccatcaccaatattgatgacgagttcactccggaattttcgaaa
gaagaattgaagggatctactaccaagcgcagcccttggaagcgccgcttcatattcgtccttatttccttgattgtcgtcttttctgtcttttctttac
gttcttatcaacaaaaacaggaaaagattcgtcgttctcctgtcctttaaaacaatctcattagattttctgtcctaaaaaatacagttatctaagca
taggctttgttccatcttatgttccttacttattctttgtttctctccttactgctttcttttttttctattccattatccgtttgtgtaaggaaaccggtactg
gtggtttcggtatttgtatattagaagagctccgacttcttatatatgaagccttaaactagcctatcgtacaccatgttcatcattcataccataccg
cctttaacccttgttttcttttgcttttgattgtttaaatcaagaggaacggctctatttagttataaggacctcgaaagtattcgctctatatcggtcg
ttatcgttatatgctaaagggttgcaataattttcactgatgtatctttcctttcaccatcaacccgctatctataatataacccccacataataagga
aattgacccacataatattatggctgtggaacaaaaccaagaaaattcagaggagaatggggaggaatgcatcatttgtttatcggaactgccc
aattgtcctttagacaagtgggacgttagcgcattacctgcatcactctctacatctcctaatgggatttacgtggctacgcttccatgtcggcatta
ttttcacaatcagtgtttggaatcctggtgccaggttgcaaatacgtgtcctctttgtcgtatagagtttttcaaagttgatgtatacgaatatgtagg

tggtccttggttgcgagcctacccagtcgaggaaaaagtacaggcagtagcgaacgcagaggcaccacccgaatttatagagacggatgatatg
actcgttgtgttatttgtggtggatcacaacaacaagacgttttacttttatgcgatggatgtgatgatgcatatcacacattctgtttagaattggac
gaagttccggaaaacgagttttattgccccaattgtgttttcctaaacaacgataatgttaataccttggcttctcgtatttcgctttctcatcgtcgg
cgaacgaatggtcgtgctgccagagcttcaagagtctcgcaacgacaacaacgtgcatggactagagcgtggaatgcaattcgcaatcgagcat
gggaaactttaaattctgatttatcctattattcgcttgatggtgatattcctaggcgagatcatgcttccgacttgctaaggagaagaattgaaaa
cgctcggttgcggtccgattcgaatgtgaatgatgaacaacagcagcaaccaccatttgtacaaacgcctgttacaaatgattcagaaagaaaa
gcttggaatgattttgatgtaatgctgcataacaatgaagatcactctaatactggtgataggtcgaatcataaggcttcgacgcctgctgaacgct
cgtcagcagcagacacaaaccaaaggaagaagctaaaaaaaccagtccatagaaatcgaagcatttcaatttctacggctctaggttcttcttct
aaccagtcaacgactaatgacgaagcgcataaaccatctctacttgcgaatttaatagataacatcaaccgtccatctccttcttctcagactgatt
ctcaatttctatcaaaagtacaatccattttaaaacgatctcaagaaggcacctcgtcaaataactctaatgagcctcatttaactcctctttttcta
atctttctcctcaaagctctcctccaccccgtctggaatcctcgtctccaatttccagtcactttgatggtccttcacagcctaccgaacctggtgaaa
ttcttgataatgattattcacagacgagtattgatcggcggactgttcctcggtcatctgggcgacgagaattaacaaaatctacgaagttgccgta
tgaagtgaagtatcgaatagaacggttggttaatgctgcattaaagccgtactacagagatgctaaaatcacaaaggagcagtttgctaaattca
acaaaaatatttctcgatcactgtatactttatttttagatggctcactttcatttgaaaggtcaggtcagtatcaaattgcaaacgaaattcgagag
cgggttgattcttgtatgcgttccaatggtgaaagttgttaatgtcttttacatcgcatattatgatggaaattgcacgtttgtatttaaggatgcaag
cgggataagatatgaaacggttatgagtaatgaataattaatttttgggtgagtattttgattcgtactagcagtggtattgaaggagtctataatt
aaatagtaaatcttttagggactgtaggaattgtaaagttaaaatacaaatgaactactgtaaggaaagcattcaaaatgatatttattccaaag
aagggattcatgacttgagcaaatatgtatcgtatcgaggtaaacaatgatgggagatctatttttagcgtctacgaggaggacggggtctagaa
aagccagatcttgtattagactctttagaagcatcaataagacctaaagcttggagaatttccttttcggtgcagtcagcagaatcattcgattcca
actaggtacgttagtgtctgcgagagtaaaaaaaaattaaacctttacccttccgtcaatggtaacatcaagattattctggaccttgtccagaaa
ggcaagcttgcaagacgagtaaaccattctatttttaaccgtggactgcatgggacaaatataaatgaacactgatgagttgtaagtaagttatca
tagaggaattgcatacaagtttttggtttttttatcctttgaaagagaaaaaaacccaaacctcggttccgaagggtgaaagaatgactttacatca
gaggcagcaatgttttctttttcgtcttctatgcaaattacttcatcccgtacagactacaaactcgttagtaacgttaaaagaagtggaatcttacg
aaaagaagaagggtattttgaggaacagagcgaagcttctctaaggctttcaaagcgttgtcgtctagttccatggctacacttccagtcactaaa
gaacgcttttgggaggttgaagattgtagagactaattgagatttttgttagtaatgaaatgaactattgccggtcttaagctggaaaaagggata
cctcattataatgttgtttgtccttttcgtcttgacgaagaggtgcagagtcattttcttttgtaagcttgtcgaaaaaagatttatagttcagctcttc
aatagtactagcaaaaaaggatttatcgatttctgacatgctgataatgcgaattaaaccagcacgactggaggcatagagcatcttgtttcgaa
cattggctgattcgggcacataagaaataaattgtaaaagatcattacgggtttggtcgtgaatcaaagcaaaagcaggctcagaagaagcaag
taatacctgacgaagcttttcaaaatccgactctaaagaagagctttttcaatagtatcagaaacttcaaaggagctctctgaaaatacgttagt
cctctgtaaaagcatacaatcaagctagaaaagcaagggttaccataccgttagaaatccgaataacagcagctcgaataggtttggaggagta
attttcgagatactggttcaaactttcggcagcttttacatggtgagcttcatacttagcgatttcttacgttttaaatcaacagacgaagacattgtg
gaaattgaagagacagtaagaatgattcatgagtatgattacttactctgatgtctcatgtacaggtgacaacgcaggataacgcaattacgtttt
ggtttgttaatagctatatcgtattataccacaaaatgaagggcagccaaattgagaccgtatttatacacgtttcgtaaacaaacgacaaaaga
gaagaaaaaccgttatttttgggaaattaatgaagtatttttaactaaagctagggatttaatatgattcgttttttttcgcgtcaaatttcgacaa
atgcgcgtttttacatgcaacttgcaaagaatgttcagcatagacatatttctacaggtatggagtcaatttaagcaagaattgaatcattttcgac
gagcttttattccataggagttatctatacttaaaaagtatatctttacctaacgctgctaggttgctcggtgggaacgattctaatccgttcacctgt
attaacacgggcgccgtccgaatttgaaagaagtatatataagtataattcagaactatggaatgaactgagcgcccctttgcccaaggaatttta
cttcaaaaagggatcagttggtgaaaaagactggaaaacgagacaaaaggcactggaaggaaaagagtctccctttcaaagcatttttgaaga
agaacataagcagatggaagaaaatgcccttcctgatcccagcacccatttacaaccaagaacgacagaggcagatcaagctggtgatgtaca
cagcactcaacgttgtttagaaaagtcccttttctattaataaaaccaacaaaagatgcagaatggcaattccctaatgttccaatcgagagtga
agaaaaggctttgcatacggtaagcaatgcatgaattaggaagagctggtcgttggaatgatgattcagtgtcttctgcataaaggatttacgag
ccacattccaaatttttatgtaaaatatttctaacgaaccgtttagctttgtcaagaacaattacaaaacatactaggcgagcaatccttaacctgg
tttgtcgcccatcacccaataggtttaattaaacaagatgaaaagaaggtaagtatgactttctcgtgaaaggagaagcggctttattcatgttttc
tggttgtgtaagattgattaactaacctgaatagttgtttcttttaagagcccggctggtgcacggtttgcgagttccaaaggtagaaaatgtttttg
attgggcatggactagttatgaagagctacctaacagactatctccaattgcatggaactcagtgaaagatattttgcatgaacgattgtgagtca
tacattaatatatgaaaaatagcattctcactttaaaggcacttcatcgatgaagctttctatcgtttgaaaactgccaatactgacctagttctaca
tatataccaaaagataatttgagtttatacagctacagttgatatccctttaataaccaatacaagaatgaagtccttaattctttatttctatatcca
catatttcatgcatgttaaacattctgaatggcataaacaaacacctacttacaaataaaaaaaaaataaactcactttcatatgaattatacggct

gaaaatcaaccgcttctatttgaattaatctagaatgattgaaagactaacacattgagatagaccacttctatttttatttattattttttttatacac
gacaaagatttatattatgtttttttagatcttttttttttttcagttgcaacagtctcttttttataccgagattgaaccattttgggtccaccaccaaggt
agaagcctgctcaagacggttttgtttagcatacaaaaggcagctggatacatgaagatgttaaccgagcagcaaactttagagctttttcctgaa
gaatgggagcggaacgaagagattttgttgaagtatcccaacgggttgccaaaggatgcaggtgtagatccgaacacttctaacggtttagatg
atggattagaaagcaaaatagtgcataaagaaagctctcaagtgccgcttcctgcagctgcttttcctcgttttggggtcgagcctaataatacag
atcttgattatgtactttgtcccacatgcgagagaccgtttttgtcaaattatattgaggagcataacgagaaatgtcttgaaaaaaagtcactaaa
gcgccaggcagacaacgaggtttcctctacggtgcactcaaaggaatcaaatactccatctactgcttctataaagaatggtagtaaagcaacgc
atcagaagttgagtacttcttctgaaagtgcttccaaccagagtaataacaacacagaggttgctcctcctaaacgacggaaagtagaagaaag
taagaagtctaccaaagcaaatgcttcttccaaaaaggacgtaggaaaaaagaaaagcacaaagcaaagaggtatgtcactaaatctttcaac
ttactaattgcataggacccgtggatgtggacaaacaatgtggtgtccttttaccgaacggcctgatgtgtgcacgttctttgacatgcaaaactca
ttccatgtcaagcaaacgtgctgttcctggtcgctctcagccctatgatgttttgcttgctgcttgtcaaaagaaaaatcaagccaagattcaacgt
atgtttgctatgcacttgaaactgatcgttactaattttctcaggacaaattctcgaaacagcaaaagaatcagaagaacaacagcatgaaacac
ctgttgattcggatgaggaagtaaattttgttatgaacgccctacaacaatccggcaataagcctttagaacataaaacagttttacctgttaaac
ggagacatacctattttcgtacgagggaattaattgcgaccgcctttcgacatggagagcaaggaatgcaagttactggtactattcttggaagag
ttatcccatttagcgcacgttaattctttatgtatcttctttgcatgattgttttgtctgcttttttttttttgttttgctatgatgagcttatgagtgatgatg
ggttttactttgcacataataatcattgattccttactatttcacctttattttgattgttatctacgcattgaatacctgtgtatatatgtgatatgtatt
ttctaatgtaacccaagtattataattgccttgctttttttgtcaacattataatatcccaattaatcaaccttcactatctattattaaccaaaaatag
attcccaaaatctctgagataaccgtttaccaaaaatcgataactggggcagtagctgaaacgcgtttacccttccaattcatctttattagttttag
cgtcatcctcgtcttcactagataattcatccggtaggccattttcagctaccttttcttcttcagcggtaatgccaatatctgaaagatactgaaagt
caagctccttatagctgtctatgaaggttaaaaagcaatggaatctaaatgagttagcataagaagccacaaatatatttacgtacacttgttttc
gattttcctcttcgaaaatcttatcgtaaatatgagcgtgtttaaacttttcaatactttcagagtacttagaaaagtcaataggatctcgatcatttt
ctggagccttatgctcattataacctttgaaaaatggagattcaagtaaaagtccaaatccggggcctttaggaatatttacttttgttttcttgaat
gcatcttgaatacggctcaaattgcacccagtacgaaccattaatacggccaaagctatcatctttcgaatctgatgcaacataaatgactgcccg
tgtactttcaaagaaatccattcagtgttgccgatcattatcgggtcggaagcctaataaatttgttaataaggatactattatatttgttttagaaa
aaaaaagaaaaacatacgttgaaagagcgtatcaaacggttactattttttttggtgaaaggcttgcccaacggtaaagttatgaaagttgtgattt
ccaacataagtattcagagtttctctgataacacgtaatctcttttcagagattcgatattcctttaatagctgcatttctgcattcttgagagcacga
tcaagtctcatagccggatcagttttgggagaagaggtagcagaagcagattgattttcgtctattgttgaagtaggagcttctacatcttttgaat
cgattgtctggtgttcttttgtgttagattcattctcagttggattagcaggagtagaaccttgaacattctcttcagaaattggtgcaggtagttcct
cttctttggggctctcattttctgcagtagaagaggcggccgcggcagctttctttgaataaggattgacaaagttctcgggatctgcatgataggc
ctctaagattacagcttccatttccttatatttatcccagaattcttttccctcttcatagaaaatcttgcggctcatttggttgatatcattttcgtcga
caggctcactcggcataggagaatttttttgcgatgcaacggcccaagacagaactaggctttgggggaacgaaagcataggtaggaatcaaata
ctcatagatacgggattcgcagtacgttctaggattaaatgaattgattgtacgaacaacgtcccaaacacggatggaaggagggagatgttgat
tgactttttcgatcaattgctcatcttccataatcaattttagagtaatcacatttccagcagcatgaacacccttatcggtccgtgcagctcgagcta
aagcgactttcttaggatcatccgcgttgtaagatgacacggcctcagctttgacaaacgcgtcgaaaaggtctccctcgatcgttttacttggagg
atttaattgcattccatgataaccagtgccacagtatcccaccaggcaagcaactttacgttttggtttcttttctccaacgaccaaatgttcaggcc
gtttttcaccgtattgagcagaatttacacggttcctttttgcttctctacgatcaccattataccaactgcgcttacctccacgtcccataacaaaaa
cgctcgatgtaggttgtttttttttttggaaattttatttctggcagctactcgctttggttgggaaataccgaggacccttgggtatactatcaaagat
acggtattttagtgtatatcgttagtaaataatgtatgtatgtatggaggaagtatggaaagagtataagatttttttgtcgtttatgcactaataggc
atggaagaattgttttaatagtgatatacaaaaatacggatagtaagctcaagttaaactcttaggctgacttggttcgagcgtgacgtttccaact
tttgtgctgtttcgaaggaatgctctttcctttacaaggaagattggagaagtattatatttaaatcttttatgaaagcatatccccaatttagatagg
atgaatgaccaatcctttcaggtttgcatgattcatgaatcccaaaaaaaaaaacatccttgattcagaataattaccaaaaactttttttataaaatc
ctttcatactttgcaataattcattgagtcaaaacagttcatctccactctctgctttgtttatggatggtcactttcctaccaaaaaagaacggcaa
agctatggttctttaccaaccataaatgttctttttccgacttatttgtcttcacattattgactccagcgagcaatgaagatagcagattccttttcct
ttattgtcctgaaataaaaaaatccattttccattgtttacatcttacaggagcaattttacgcgacccagtgaatcagtatccatttaagaaagca
acgataaaaatcagtatttaagatccaaacattgtcttttgtctttgaaatagtatgaaaggtattttccctgttaaataccaagcttagctttggatt
ctataattctgttatagatagaaattgccgtctcttctgttgcttttttttttctttttatggaggtcgaccagccagccaaattcgtcttgccgtgtgtga
ttcttaaaacgtgtactttggtctcttttactttttttgggatttagaaagctttcattgatggatccagaagatccttcgtatgtagaagactttgagg
tgcgaagtaatttctttcagaggtgatggtgctcgttgacaatttgactaacagccgagacagagtgtttacactggtcgaaatgccattctttttgt

agtagaggtttcgccgagtatgctagagcctgtggatgagtttactccttgctcactgcagatggctctcatttgtgcatatcaattggcagctcaaa
gggtgattaccaacccaaacgatgtgatgggggttctgttatatggtactgtaagtttttgttgctgaataattgcaattcttgattcaatcacccat
acttacgaaatccattaggaatcctctactggcaggtttgaaaaccaacaaatccttttggacttagatcctccagacgccgagcgccttaaaagc
ttacagacatatgaaaaaggtaatgaatttaaaaggaattcgcaaagaggcaagaccctgctgaacaatcatcaaccgaaaggcattcgctaa
ctcttgtgtaagaattcgactcctacaaaaaaaattttaagccttgctcttcccaaatatctctgtctagtgttctttaccatgcaagtgttcttttcac
caccaaagctcagaaatatgaaaagcgccttttcctaatcaccgataatgatcaccctgcttggaacgaaacggaaaaaggaatcattcttcaga
gagcccaggatttgaaggatttggatgtgcaagtgaatccgatttttttggaccctcctactcattctttccgaatgaatatttttttattcagactttct
atacatcgtttataatcatcaagatgtttccaatttggtcagtcgtggtcaatcacagctacaaaacatgcttaatatgataactgctctgcaaaaa
ccaaaacgagcgcacttccatttggaaatggatttgggaaagaacgtgagaattgaaattcaaggatatattttattaaagagattagaatccgt
gaaaactagttgggtttatgccaaaggagagcgcttggcaattgctattcctcaaagtaaacaggtaagctttgacacaaagaaggaactgaaa
aggtcggacatcaaaagatcctttacttatggtgggtcttccgtagtatttgatccagaagatttggatgaaattcgatccttcggacctcctgtttt
acggattataggatttcgcgatgcttccaccctaaaaccttggcattctctaaaacctgccgtttttattagaccgatagagaacaaaattcgagga
ggaggagccgtatttgcagcattacataagaaacttcttcattctaacaaaattgggattgcttggtttattgcacgaccaaattcaaatccttgttt
cgtggctatgattccaaccggtacatcaacacatattcgaaaagactttgagctaccagttgggatttttctacttcaattgccatctgccgacgac
attcggagccttcctggtaatcccaatcctaattctcttccaccaaattcaattgagactatgcaaaatgttttacgagggatggaacttagggctta
cgagccaggaaggtacaataacccttctttgcaatggcactacaaggtgttacaagcattagcccttgatgatgagattccaaccgactttgttga
tacgactttacccaaatacagagctatacagaaggtaagttcttaatgaagatttttttttgtctttaaccttttagagagttggagatcacattcgag
aatttaatgagattattagctcgagcaataatatcgttccgataaaacatgacgaagatgaaatggattcagtcaccccactaaaacgaaaacgc
gaagaaagtatagagcagtcatttgcgttggatgattctcagttaaaaagcatgcatgaaactggatcattggaaaaagatctaaaggtataata
tacaaagcgcttgagcctcatcaaacaagactaacatttactagactctgaaggttcctcaattaaaaagtattttacgagacagaagtttacggt
ctactggaaaaaaggcagatttgattacgaatcttgttgcttacattgaaagattataatttgtatcgactctacacatgcttttcctaatgtataat
aattattcgatcaatgattttaatgatataaaatcttatggaatttttctacttttcattgacaacagaataactctacatgatcaatcgaccaatctc
agtccattgtagcgcttcattagcgcaagacttattttggcgctttttgaaccaaagaaagagaatctatagagaacatacatttccctacttcgta
agtaaaacaaacaatgtctacgctgtataaaccgaggtcggcttctttttttaagtccttatataaaaattaatacacctgtgagcaaccaaaggtc
ggatagcacagttggttagtgcgcatgactgttaatcatgaggtcgagggttcgagtccctctctgaccgatttttatcgattattttgttgcttcctg
ttttgagctttcacagtaagtaatattctattctactctctttattcagaatgggttgtgtgtcctctattgctctgcttggtagggacgaagtatttttg
tttctatgttagccatggtaaggtaaggaacattattaacagccataaatgtaagccggcttttccaggatttcttcttaaaattgaagctctggaa
accaaaaaaaaaatttaatttgatgtactaaagcttcagaaaggaaaaaatcctactttattaacactaaaacgatactatgataaagtatcatc
gtatcgagagttgtaaaacgaactacgaggaaaacgttctttttaaacattcgagtctctggagaaacaattctcactcgttacatttgtttttcttc
ctttgcttaaaataatctttagaatagccaagtttcataatgcaaactagtaatgcaacgtatctatttcagacggaaaatgcgattcgaaacgaa
gaacgaaaacttgacaaatccaaaaatgaactgggaaatccaaggaatctgaaatcgaaaatcctaaacctcgaacttgatcgtaccacccca
aagaaataccagctccctagaatattcacatgtgaggcctctcatgtatgcagactgtatgatttgaatgatgggaaaacttgcaaagtttacaaa
ggtcatacagggccggttacctgttgccaggtacagagtcttatggacgacaatcaggaccgctttctgtatacaggttcatgggacaaaacgatc
aaacgatggaacgttcgctctggcgaatgtgttgagactttggtaggtcatggcgattatgtgaagtcactgttgctttttgaggaagagggtttac
ttatttcgggttctgtggatacaacacttggagtatgggacacaactgctcaaccaaaccgtctactatatcaactggcaggacattcaaggggaa
ttgagaaaattgttcgacagccgggaacaaatgtgttttggacgtgtggaagcgagagtagcattcgaagatgggaaatccgacgagacgggg
gacaatctcttgatgaagaaggattttggggacatcaatccaatgtttacagccttatgtttgaacctgaagattcagaatctttgtggactgcatc
agcagataaaactgtccgtcagtggtcgatttacgaagggttacatgaagagactagattcgagcatcccgacttttgtacagacgtccttgtgct
ctctgacggtaatatctgcacggcttgtcgagatgaaaatgtgaggttatggaatgtgaatcttgggaatttagcgcacatatattcaggccattat
gagtctgtcacaaagttaattcagtggaggtcttttctagttagtgcatcattggatgggacgttacgagtttggaatctcgatccatcctctttttca
ggcgaggaaaacatggttactgaagactctttactgaaacaaaagacaaatcagcctcagctaacggaagaagaactcgccgaattggaagag
ttgatgagtgactaacgcgagggaaaatggaccttgctgtttgcgtctaatatattcatagtagagtttcaatgtcgattcaggaaggttttatttat
tattggaagaggaaacaaaatgtttgttatccaataagcattacgacaatacaaaaattcactttgaatcctcgaggattactaactactttgaat
cagagcaaatcgttttcaaaaaaaaaaggtgatttaccactatactatcacaattagcccaacttctgtcgcaacaaatcgaatacccggataca
ctcgacaaggatgaattaaacgaaacttgtaaattcataattttgcttttattaaacataacaaacgaaccacactaaacaacaagtaaatttctc
aataaacaaccaagttcatcatagcattgttagtacgatatattctcttaaagtaaacaggcattcaacggtttttttggtaaaacatataaatgtaa
atgcgaatttctgactttgtaatgtggttttctcgatatttctttttatttttttagataaaggtagctctcacattattatctacggatttttgcaccca
tcatgcatcctcaattcatctcgtttcggatattcgggatacatagatatcggaaagaaggaaagccagccaattaccagccaaattaattttaac

gaaagatgatgacgacacagcacccttaccccgtatgtttatataaaggatggatatccggtatacaattggcattatttaccccagccgcttagc
cggtttgtttgcgttttggtaaatactatccagtaaaacattaagagctacatagaaaagccgtctggtttgccatagaaatcattgattttgactat
attaattgttttattttaatacacaatttagtatgtcttttgatcaatccactttacctaaaaaggtatatcagaaaagagcatacaaaaggaatttg
cttagtatcgtcaaatgcctaaagcttttcaattttatgccattctatctcttagaccgaaacactaagcaaattctaggatgattccctttgtgagtc
aagagatgcaatatcgattccctccgtcattaggcattctatcacaaagtcagatgaaattgatttatacgattttcaggatacttctgattatgcac
agcaattagaaaatatcactatcaatcactcagctcaccctcagaattggactacttggaagaaagcattgttgatttttttatccacctccttacag
gcgtatgttttctggactccaaacttttatagcggtgtacaatcaaaagttatggaagaatggaccctttcctcacagacctctttgctgggtcaaa
gtatgttttgtctaggtgttgctttaggcccacttgtgctggggccactatcggacttggcaggaagaaaaattgtttacgtgggttcattgacaatt
tatttatgctttaatgtttcttgcgcgttggcagtcaactatccccaacttgtcatttccatgttcattctcggcgtcgttggctcaactgcattaggca
acgtggcaggaggtatcgctgatctcttaaaagatgagcattcaaactggggtatgtacatgttcattttcatgtgttcaatttcaagtatcggttct
ccaatgggtactgctgtttctgagaatccaaaattgggttggcgatggctttattggatcaatgtcattgtaggaggatttttcttactcattttagcg
ttcgctccagaaacccttcctttgatagtagtcgaacgttacgaagctcgtcaaaaaaacaaacctgtatccatgattcccaaacttactttcaaac
actttttcaaaagcacgtactttattttcttcatggctttaaagatctttttcacggagcccattgtcttgtccttgggtatttacaatggttttgtaaat
ggacttttgtatttttttcttgcaggccatttggcccgtctactcagacatctacaaaatgtcagatatggctgcttcttccacttatttagcagcaattc
ctgcctgtgtgatactcttgtggcttgagcccattcagtgttggctatatcgtcgtgataaacgcaagaacaggagcttaaggccagaagcaaggt
tcataatgacaattgccttggtatgggggtttccaatcggccttttcatgtttgcattttgttccaaaccaggcattcatttcatcgtgtctttgattgct
cttaccatttttgacatggccgattatcatatctggcaggcgatgcttttgtatatcacagattcatatccaagcgtttctgcttccgctgttgccgcct
ttgagcttccttcgaacttgggttctgttgtctttattcatcttagttcacttatgtttagccgaatgaatgttcactgggctgcagcagtggttggcttc
gcttccttgcctttaattgccttgatatacattttatatttttttcggacatcgcattcgctctcgaagcaaacttgctactcgatatcttatccgtctcaa
ttcttaaccttttggtcgtacttcagtgaaagtccttcatgcaagcaaattctttcaactctaagcggagttagaacaactaattttaataacctctca
tatcctttgctaaccattccattcctttgctaatcgcttagctgtttgccttatttttgtcgcattagtacctagataatttactcgtttaatccaatacta
tatgatgcgcttaattggctgtaagaaatccgcatgagcaaagttgttgcacaagatacaaaatatttttcgtttttttatcattaagaacagctctgt
ttattaaggtataccgtctgatctattatttcacctcggagctgtttccatactctgcggtaggtgactttcgtaattcctcgtaagaaaactctctcca
tgatgaaacatcttaaagtattcaaccaagtgatatttataacaagaattagactaattcaattgtaattggtatttggttatcggttttcacccatg
aattgtttgttttctcgagtttttcctcttcttgtaaagcaattcagttttcgcttgtaggccgttctcagtgcaacttcttgaggaatccactgaaaga
tttaccgaacaaaaggcttgctcattcaggattgtataacgtcgacactggtaaaagtatgcgtttcctagcatgtagaatagtattaacattcttct
agcaatcaggatccttcaagaccaggttccactattgccccaaaaagcgcttccagatgaaatattaagtcaaaatattgagctgactttgtttcc
ctatcatttgcatcttccgaaaattcaaagtttggtgatttacaaattcgttttcaaaaccgctcgaatacttctagcttctgcatacataaatggcat
taaaaatccaattgacatagaaagtcagattcatacacccctttcccctaagactactaggtacacgatgaaatgccattggactgcatctcagac
agacgagtacgattgcatttttcattttgatataaactcgaagggactgatatatagacacattatcgaaaatttagaaagaaaaaaagcggggc
agattgaaagaattccagctttagaaccaagtacttttgaatagattgaaacgacaacacagttagtctcaatcaattttaacgaagacttttcag
cagtatattttccggcactccctctagcatttctaaaatatgactcccaaatataccttcttgaaatagtacctgatcgttatggatcgccggagatt
ctgtgccccatatcttataaaattttactgtctgatcattgttagctacaacaatgaactcaccttcttcccagtcatttttggagcgtttacggtgcgt
gtgaatagccatgatagttctaagatcctggtgaaaatcctcagcagaacaaaagggtacctggaatacttgcgttaactgtggccatcggcaaa
caataacggatgcaatgtttccttcgtacgcataaccaaggctgaaacaaaactctttatatcttcgtgaccaattgatggaagtgatttgggctcc
gcagaagagcctagaaatcagatttcctcgaaaggtatcataaaaataaatatgctggtcatttgatccggtcccaattgctaaaagagacttttg
ccaagggcaaaacgcaagtgccttaaccgctgctgagtggctccagaaatgcttcggcgtatctagtgcattcatgtcaaaaacgcataccaaat
tatcatttcccccgctagcgaattgagtgccatcgtaattccactcgagtccgcaaacctgctcttgatgtatgtgtttgattgtgtacagtcggcac
atcatactttttgttttatgatagatatatatgctgcccatgcttccaccgaccagtaaagttgaatttgtggggctccaagccatgcagcttatatcg
ccctcatgctgaaagcagtattcaggtgtttcagttaaatattgtcgatcccaaatttcaacagatccgttaacgcgagcaattgcaaggtaagctc
cgtctgcggaataggctaggctgcttacaacataatctgtatgccgcatttccagtatagtcgtaccgtacaattcatcccaaacatatacgtgttg
tctcagagcaacggctagttctccgtggcttgaccaggccaacggcgaaatgtaaaaatcgtcgagtaaccctggtgcctccaatatgcgaacag
gatattcatggattgcaccttgataaggttgtgttgaagaatgaatatgttgttcctgaaactgaaactgatttaatgggccttgatttgtacgagac
ccttggagttgtctctcagaaaaatcaataagcccttttgatgtgcgattttctcttttatctaatatcttataatttaagacaccactaaggtttaaat
ttaacatctttggcaagacatcctccttatgtttatgctttcgccaatgctgcgaattatcaagaatccgaaatgcctctgcattggcattagaagga
ataaatctgtctaaacatttccttatactgaaattggttttattggaaggtttaatatcgctttcttttcgtttttttcacgcgtgttttatttaaagtcgtg
tttcttcttattttcgtgattctccagagccttccaggagattttaaagcttgtttctttttcaattccataagatatttattttatttaaacgagttctgtt
tctatgaaaagtgaatcttggggtgtggtgtgtttacgatggatttgtcaagacgcgtttatatatcatatcggtaccctcaaaagagttcaagttac

acgtaactactcaattaattatataattattctgagactatattactgttgttgttgtttgagaccgctgaaagcttgttatgaaatatctttcgaaga
aatccttcgatcgctttcattatcctaggaaaggggctactactgattatttaaaaagaatcagtaatcttctcctgttctcgagtgtctagtctttcg
agcgttctaggtgtatacaaaattgatggaacagacggttacaatccataaaagcaaggaggaattgaattatatgatgagcatagtgctaaatt
attgctgatatctgaaaggattatattcatttgctcacgggattagatttcacgcgataatttcatttgcaagatttgattcaacggttatgaatgtat
cacctttgagatctgaaaaaaattagttagatatttaattcttttttttaaattatttctattctgggtttgcttggtagtaaggatttgccttgttgac
aggtttttcagatttgcttggatgcaattcctttgcaattcaaattgaacaagatagaaatgaataaagaacaataaaaatcttctcatttattctg
aagcattcgacgttatttagtaggttatatgcaatctttgaaaacgatgtctcttctagtattcatgtcaagggtaagaattatcttgatagaaaggt
caattttctgtccatgtcttatactagaacctttctcttaatactctttggctaccatttggttttgccaattttatacagaatctatttggatctagtata
acagtatataatcagacaagatttgagagaatgaaatattaaaatttggattgaattgttacatgtacactaaaggaaaactcggtatacttca
gcttcattgttacgcttttgctctgttcgtacaggaagcatccatacagttcaatttcatcagcaaaggtacaatggtatgaaaatgctaaaaacat
gagaaactgactagagatagcatggaattttaagagtaaagttaaatgaggagcaaaaacgcttgaaactcgagaaggaaagaagtaaaatt
caagaatacaaaggcctaattgagcaatttcaaagagcggtatgttgacttctgacttaaatttaaaaaatattaataatatcaagagacaacaa
aaagactattcttctgaaaagctcaatttgaccaccgaacttttggattggaatcctgaaacatatagcgtgtggaattaccgaagagaaatatac
ctatgtaccgtctttccaaatgtgtatgatacgacatttatcgcaactgtttatacggttgtatcagctactaacggttgtactagatctgtcaatgag
aagcaagatttgttggacaaagagctacagtatgtcctatcaaaaatgaaggtgttccctaaagtttactggatattcaaccatagacgttggtgt
cttgaaaatgctccgtatcccaattggaattacgaaatgatgataactgaaaagcttttaagcgctgatgctcgaaattgtaagtggttttgcttttt
ttatagttagctacttaactaacttggtatagttcatggatggcattatagacgatacgttgtatctcaaatagaaaaagcaggaaattatgatttg
gcggaaaaagagttacagtatacttcaagcgcgattgctactaatttcagtaatttctcagcatggcataaccgtacaaaattgctcgagtttatta
tcaagaaagaaactgactttgagtctcgaaagcaactagcacagaaaatcctgcaagaaggtaaagttattttcccttaaattaatttaattccct
cattgtactatgggataaatttctaacggtttatcagaattggatacgatccatcaagctgcctttacagatccagatgattccagcatatggatct
atcatcgttggctcatgggtcattgcaatcaagttggtgaacctcccatcctgtctgttttatcaaaagaagagcgtacccattacctcagacaaga
aatccagcttcttgaggaattacatgaacttgaacctgaaaacaaatgtaagtacagcattagcatcccttcaggacaggtagtcagttatcctgg
tcgattgcaaccgtctttccttctactaacctgtgatcttgtagggtgttgcgaacttcttattgagtacggactcttacttcgaacattattgaatcac
caacctacgaaagaagaagaagctacatggatctccttgacaaatacccttcagcaagtagacccccagcgacggggacgatatcaaaatcttc
gaaagaaaatcgaaaccgttattgatgcttgagtttgaagactagactgtattaagatgataggataagaatagcataactattcttttttttttttt
ttttttaattgtatttcataaacattactacgattcaaaaaacaccgaatatttctaaatttttccatcgtcaaatactgcttttcgacaaggaaatag
aaaaaaggggactgatcaaatgatatgtaatttttttaaaatagacataaagaagttagattccaaaagcagaccatactaacacaacaaaaac
gaataaaggtgaacgctaacttagaagaaaggaaagtaaggataggttaattgaggagaaaggaggaagttactgagtcagtaagggaattt
atgcaggagcaacgctaaacagaacagaaacgctttctccgtgatgaatccggcggatgcattcggctaaaacaccagagatatcgatagtttc
aatcttattgcaaattttgcgcttgtcatcgtgagggatggtgtttgtgcaaataaccttctctaaagcactctcgttgatgacacggatagctttacc
actcaaaataccgtgagtgacgatggcataaacagcctttgcaccattgtcttttaatgttttggcagctaaaccaagagtaccgcaagtgtcggc
catgtcatcgaccaaaatggccagcttgtcacgaacatcaccaaccaacaccatacgagaaacttcattggccttttgtctttctttgtggatcaat
gcaaaatccaaatctaagcggtcggccaaagcagtcgcgcgcttagcaccaccggcatcaggagagacaataactgcaggattgacacttgtat
cgatgttttcacggatgtaacgcaatacggatggttcggcgtaaaggttatccacagggacattgaagaagccttgaatttgagaagcatgaagg
tccatggtaattacatggttgcaaccggcggtttgcaacatgttggcaacaaggcgagcggtaataggagcacgacttttgtctttcttgtcttgac
gagcgtaaggaaagcaggggataattgcagtaatacgacgagcactagcagaacggcaagcattaatcataatgagcagctccatcaggtgat
cgttaatcgagccacaaccagtctgaaggataaagacatcttcatctcgcacggattctccaatggtgacggaagtttcacgattcgaatactgga
cgacagcagcttttcccaagctaagtccaattctaaacaaaagatttcgttagtcattcttctttcccgtcgctttgacaatatcttcctccaactctc
gttctcgcattctttctccatcaaccgtcatcacgaaagcttcacaaaaaatgctcaacataccgttttgcaaccttctcagcaagttcaggatgag
aatttcccgcgaaaattttgatagaattggatgccatggtggtcttggacacgacaagggtttgtgaaacgacaagttctcaattaaatttctgcca
aagaactgaggtttgaaatatgagtattttgcggcaatgaaacagttaaatgatagaatcaattctaaaactatattcaatagctttttaaggtgg
gtttctgtatttttcaatgaacatacagtttcactgtggtataccagcaaacaatctttaccctgtctacgccttaaatattgaccactaattagggtg
gtaataccgaggcaacgccttttacttaccgcggctaaaaacagaaaagaagtgacaaaacaagtaacagaaagaaaatggaaaaagtctac
aggagaaagatgaagagtgagatacaggcttttatacgatgagtagatacgccttatttgtctaattcaaaagaatatacagaagggaatcccc
atcccttagctgggtaaactcgaagggaaactcgatgaagtggatttacccacaaggtaactagcaaaacaagatagtaatagcagggaatga
aaccatggaagatttttgcatagaaaagaattgtacctttgtagaagaaaagaatgcaaaaggaaaagattaatattttcttatacaaggaatca
acttgtgtgctatctaagctccattgtaccccttttggttacctttagacaaaaatttaaagaaaattcgtgcgtctagtgaagaataaaatagtaat
gtattggtattttgtttgtaaatgaaaaaggtgcataggtcattttcagtcctttgattcgtattattcccgacataaagctatcgaacatttgccgac

gataaaaagtcgaacacaagcaggaagctgatgatatttcaaagtaagggcatgtgaattcggggaaagaccggtttttgaagtaggcttatct
acagaagcaactgcgcttttccacggatacaaagagacgggaatttatagagtaactacttgatgtcgtagcgttaagtgcgaaaaagaaccaa
tagatattataaacacaatcataaaaggtacgtttctatggacgtgtcgaaaaagtgaaaagtagaaggatcacaaacaaaaagttagataa
aggagtatataaaaaagaccatcatatattgcacgataaagtccgtcataaaattatcagcatagttcattcatcgattttgttcttgtatagggct
aagtacgtcaggcgcatcattggctactgtatgaaaggaatcataaatactatcaacagagacatccggatagtccgtatccgcagcacccatag
cagctgctgacgttggaggagtagcaggcacaagactcttccggtaagaaaatgcagaaaaagggataggtgttggagaaacatctgacgtta
aaagaggtaagtcaccaggatgctgcttcgtctcgggtacaggggttgtttctacagggagagactcggataatctactcgacgatgaagtagat
ttagatctccttggagtcaaggaactcaacgaggaacgatccgagtctaaggaaggaccattccatgctttgcagtacaaacagacataacgaa
caaactttgctcgttcaccaacagaagcaagaccattgtgacggaaacaatgctgacatattagtgcttctcggttttgctcttctctctcatctgct
cccacgagaccttccaacactttgtcgtaccaatgctgagactgcgcatcgttgacagaagaatttggaagaatacctacaggcagctctcctggt
tcaccggatcctccctttcctttacgtttcccaaaatcgacgacttgttttctggatcgaggggattgctcaccatatttttccaacaaagcctgcgtc
tcaaaatactcctttcggcttttcaaagcatctaataattggcgtttctcttcttggaacttacgtcgacgcgagttagccctccgcagccgtcgctca
taaatccaagctatgccgtgtcgaaggagatagagtctattattgttagttaaaaaggttaaactgcaaggacggcttccacacagggaagtctg
atttgaaacaagatcattgtcccacccaaggggattcctttttccacttacaatccaacacccaataaatacaagacgatccatagagacgtcga
aagcacacgatggtcaacaagaagagaagaccgataggcccgtaaagacgcggctactgtgtaaatcagaaaagaaaggatggagtatcgaa
actggatgtttttcttcttttctttcaaggtttcaatcttgtattgcacttcatctaacctttcttccaatcgatctaactcccccccaaagtcaacttgtt
tcttctacacagattctcgttagtatggaattaaagcattgaggcagtacatctcctaacaatttcttactttttaaatagccatgacatgattccta
ccaagaaatcaaaaaaagagattttctatatcaatctcttgttgctattcacgtttgaattgcgttttggaagcgtttctcttcctttttgtttttgtgtt
ctcgcgttccattgctgtagtaactagagataagagagtttgttggaatatggtatctttggcttcttgtgatattgtacaaagcaaaaaccagagtt
ttctatccatcgaaatatgctaaagggaaagtggttgacaattcgtatcagaaaattggagaaatttaggcaaggaaacggattctgtgcgcttct
gcaattcatgaggtgtctgtgtatgacgacgctctaaaaaaaaaagttagttcccctgggacactggatataggtccataaaaaaaagcttcatt
ggcatgctcctggctactttcattcatgtttcattctttactatttcgcgtatctcgatatgatagtattgggatttgtataataaagtgtattattatac
tagatgtatactataataaatatatggcaatatcaaaatagtacgttaatctatcgaagatgggactttttgcatttttttatagcaagagtaagaa
gattggattcatccataaagaaaatgaaacccttgatatcatttgtgcatcctcgaacgatgactcgacttcgacttcttaagggagagcaagaag
cgatgactcatgacattcgaaatgtcagaatgaatataaaaatacactttccttttctactcattgcttaaatttgcgtttcaactaggcggaagctc
ccaacgaagactgaagaatcgaatcattttacagcgtccctactaggtagattaatatgttaccagtaatttgcaaaaaggaaaagacgatagct
ttcaccttcctattctttcaattctttcattttctttctttcttcctatttgtctttatgtctagccttgctatttcgtcgtttccctttttgtacaaggattggat
tgtaatcaaagagatattccccgtaactgttcacttcataatccttttcttttcttttccatctctttcaatccctttttgtttcctttcaacgctagttcctt
tgtttactacttaccgagcggaatggaatcctcgttgggcatacagtgttacttgggaagttaaacaactctgagcaggcttcagccagctaacaa
ttaccaagaagtctttagtttttggggaacgtctccagcttcttggaatatctattggatttagttacgtgtttctcgtatatccttcagttcactctttg
taattttactgaatttggctttgatttaagaaatttgtcgtgcaatatgttgtcaagaagtattttaagaaaaacggcacgtacttttactcctcatgt
gcagaagcgcaacttggctcttcatgagtatctttcacacgacgttcttagaaagttcgacgttgatgttccccgtggtggtgtcgccagaacgggg
gaagaagcggagaaagttgctcgtaatcttggtgttagtgatttggttgtgaaagcccaggtcttggctggtggtcgtggtaaaggtcagtttgaa
agtggtctccggggcggtgttcgtcccgtctatgatcctactgaagcaagaatgttttccgagcaaatgattggccataagctcattaccaagcaa
actggtcctgctggcaagccttgtaacagtgtttacgtttgtgaacgcaaatacgttcgccgtgaatactactttgccatcttgatgaaccgtgatga
acagtgtcccatgattgttgcttccgatcaaggtggtgtcgatatcgagggtgtcgctgctgaaaaccccagtgccattatcaaacgctcccttcct
aatactcctactttggatcgctctatcgcagatgaagtcgttaccaagcttggtttcactcctgcttgtaaagaacaagcaatcgatgctatcacca
aactatacaaggtgtttaatgactgcgatgctacgcaagttgaaattaaccctctcgccgaaactaccgatcaaaaggttctttgtatggacgcca
agcttagcttcgatgacaatgcagaattccgtcatcctgatatcttcgctcagcgtgatattacccaagaagatcctgatgaggctcaagctggca
aggttggcttgaactttatcaaattggatggtaacattggttgccttgttaatggtgctggtcttgccatggccactatggacattattaagcttcatg
gtggtgaacctgctaactttttggatgtcggtggtaatgccaatgccgaggctatccgtaaggccttctccctcattaccaaggacaacaagacta
ctgccatttttgttaacatcttcggtggtatcgttcgctgcgatgtcattgcccagggtcttatctctgtcgtttctggtttgaatctcaacattcctatc
atttgccgtttacaaggtactaaccaggatgctgccaaggatgttatcaacaactctggtcttcgtattttctctttcgacgatcttgatgaagccgc
ccaaaaagcttgccgttacagccgcgtcgttgaaatggctcgtgaagccaatatcaacgttagttttgagcttcctctttgattttcattagtattatc
ttcaacttaacaacccccctctctaatgatttctacttagcggttactaattttacagcgaaagcataaaccttctttacgtaatcttaattcatttttat
tatgcagtcatatatcgttacaaaaccagtaaattcgtaaacttattatttatcgtaaatgaagtgttcttggtcattcctataaataatttcttttcat
tccatactctcataacttagtgtccctttcctaacaaccaaaaatgtacgtattttttttatctcctttttcgacaaaatgtacgtgttcttgacgaagac
tgatttctgcaaccattataatagcatcacaaggccattacacgaactatctcagttctatttcaaaaccaacaacttgttttcaaatctggcaactt

ttcgaataaccttctgaatgaccgtacgatttctggtgtctgctttaaacttggcttgaaatagatgaatcaaatctttaatagtgataggtccattcc
tgagagcttctatgacttcggcatcagttatgagattggaatcgtctgtaggtacattcgctgctccctctgctccaacagttgcatcagacgcggg
agcagtagttattttaggacgaagaggaggaaagtcagaagcaaaccgggaaagtttttcacttgagattcgtaaaacaagatatccgggtcctt
taacgataggctctgaagtccttgattgtacaaaagactgagaaggcgaactgagtcgagagactggacgttgaggacttaaattggtactagg
ggtacccaacggtttgggggtagtagcttttgaagctgaaaaacgagatttaactttttcttcacgcttctgtaactcttcttcttctttaagtacctct
tcttcgctgctagcctcgttgggtgaagcataagggttctcatcttcctcgtcactttcataagccagatttttctcaagtaatttgagataacgttga
agtttttaccttcatgagatatctggcgttcttgctccgcttcaacgttaggttcttcagcttcaccaaataaattggcagtaagcatttctttttcat
cttgtcttccaactgtttattgtcctcctcattgccttccataatcggcgcttcctcgtcgtctgcaaactcttcatcaaaatctaattcatcatcacttc
ccgtagccctaggtccagaaacggtacgagcattgggaactgttttgaggtggtacataggcgaagcagtgccctccgaagatccttcttgctcat
tatctttttcataaaccatcgaggaattggagtctttttattcatctaaaaaattgttaataaaataaggtaataacccataaacttaccttggcctc
ggcttcatcaatagagagagtttgaaaattattacgctgattgaatcgatagaaacgatcggctggaataaccttgaatccattctcctaaacttgt
tagcaaaaaaaccctttcattttacacaggaatggcttgttggagaaatttctccaatgaaaaaacctacagaaaacataaacaagacataggc
agaggattgaccgccttccatagtgctaacccaagtgtttttccttcaaaatcctccattatccaaggcgataattcttcttcttggagtctgcggac
ttgtggatccaccttgacaacttggcgggtctttcgtttaaaggcgttttgcttcatgttttgagctccaccataaggagcaatagaagatacgtcta
cattgccagagcttccaggttcagcagactgctgctcgttgtttgatgaagaggaggcatggggatctcttctttgcatcttaatgggtggcacaaa
tgttttcgtagggtcaatggttttattggaatgaaattttaataaatggtggtttacagtttttctttcattcttagggcaccaagatagttgaaaatct
aggaacttttgttggttttgcgggggaggtgtattttgagcaggtttctcttctactttcggttctttcacatctgcaggttcttgcttaacattcgaga
agggtttcgtatctttaatatccatcttgtaacaagaaaagcttaaactacgatctccctgaggaactggaagatggtaaagggattgtgcataag
tgtgatatatctacactaaacaactatgtcgaacgcagagtatattgcaaggaaatatttgaaaaaagacactggaaagaaaagaaaaaagtct
aaatcctcagattttgtagaaattcaagatgaagatgttgctggatggagaaatgaggatgaactagctggctattcagaagaggctactttatta
caggatcaacctacgattgttaaagacgaaaatataaaagatttagatgaaattgcgattgttcaaaaagcacaggaagcgactgttccaacaa
catcacgttggaaacctgtaggaagcactgaggggagccaaggaagagagccggtaaacgaatcaatggagaatggtcctgggtatggattag
tgactggtaaacaagtttcggaaaaagcacggcggcagcgtgaacgggaaaaccaggagatgttcgctatgacggacgaggaagtacgcaaa
agcaaagaaacagtgtaccgggatgctacaggtaggcgtatagatattactcttgcacgccaggaagcacgaaagaaattagccgaacaggaa
ctggaaaaaaggcgtcagaaggaaaaccaacttggtgaagtgcaagttcgccaacaagaagaataccttgaagaattagaacgacagaaaaa
cgtgcctctttcaaggtatgaagatgatccagaatataaccaggagcttaaagaaagggttcgttggaatgaccctgccgcagcgtttttgacga
aacggaatacagccgctacaaaggcatatcaaggatatgctcctccaaacaggtttaacattcgccctgggcatcgatgggatggtatcattcgg
agcaacggatttgaaaatcgttggtttcagcgacagaatgagcgaaaagctcgcgaacacgaagctcaaatgtgggcagtggaagagtaagat
ttaaatgatttttattcatgcactaacgaacattttagtatgtaatgaatatggttgtactgtttttttgttatgagagaatcacttgtcctttcaggagg
taagaaggtaaagtcaagagtaatagactagctgactcttctattcttaaaagtctttttgtatttcctacgatagttgtggggtaacttgtaacgta
ttcacttatataaagtctatactgtcttatcggtttctcaggtgtgattttattcacatttctcttccttcttattgtttttattaaaaatgatttcaaactt
ttcttcaatctactagaaagtatctaaggaaagcagttgctcctgtttctatgactgtattgtattttaccttaggaagaagacatatttcgagttgac
agcagatctcttcctttttatttctttttcttctttttatttacaatttcttctgttttttttacaaattaccaaacgtgaaaactcggtgctttctcggtcac
ctttgatccaaatcaccgagttttccgaatggatttgaagtcgcaaggaagcaagttgttgtttactacttgaaccgtgtcatgactgcagaaccgg
agaggtttcaaaaagcgacttgttcgctgcctttttttagatacaaagtctcggaagcttgtaggtgagctgttacagccgtctctgccttttattcagt
tacatttgggtcaacttgagcaacgcgcgattcagtcacataaccacgttccaccaagcaaagatggaaatacgaaagcgtatgaatagaaaaa
tacgtttacgagatgattactaaccgcatttcacagacattacttgctcgctggaagtggaattaatgcagagagaacttggaagaagattgagtc
gctgtcgctacatgcgaagcctcctaccactctcgagctttcttttactgacgtagacatgtttttaaagtatcaaagagagaaaaatgtgctacatt
cacttgaagcgcttattcaggatgcgcagtctcagtttgacaagtatctggaaacagagtggcagggaaagaagccaggagtaaggaaatctcc
cgatacttttacccctcaagagaaaaagctagtgaccgttacccaaaacaatgctcgcagtcaggcatttgcagcatcattaaagtcttgtttctcc
gatgtttcccagccttctcctggctcgaattatatttcctctttttgccaggttgcgaaagggctatcacaagacacgaaatctcttctcttgtatgaa
acttggaatctattgcgttcgttcatttctcatgatggttcttccgatccttcattcggacccaaatcacagttattttccttctatgaagatagcccttc
taaagttcgtctaaatcaaagaataatcaactgctctcgacgtttttttggaaaaacaattcttcgacgttgttaataaagaaatcgcaaacaaccc
tcaggctgcttccattggaggtgtaccttctgtcgtaaataaaattcgtgcatatattaacattcgtttctcgaagaatggtgtttgggcgaattcga
atattgagtatttaggggacttgcctatttgggcgttcctcttctatctgctgcgtgcgggctttttggatgaagccgtcacgtatgctgagcagaatt
acaccttttttgaagaaagacaagccgccgatttccctacttatttgaaggcttatttgaaagctccaaattcagtaattcctaaagagttgcgaaa
caaactcttttctgaatttaatcagacgctccgtttccaagaagattttgatcctttcaaatatgccatgtataaaattatcggccgttgtgagctttc
taagacatcttgtccctcggtttgcttcgtgactgaagattacatttggttgcagctcacccttgcaagggaatttactgaaaagaatgttgccgccc

acgaatgtttttctctgcaagatgttcaaaaattggtattgtcttacggccctgattattttactaaacaaggctcttatcctgtgatgtattttttcctt
ttaatgttatgcggtctttttgaacgcgctattgaatttttatatccttattatcctacagatgctgttcattttgctatttcatgtgcgcatcacggcttg
ctgcatacagctcctccgaaatcttcgcctcctcttgatactttacttgtgaatgatacttcagaacaaccaagcctacgatatgaagcaatgttgat
gagctacgtaaaaggtatgcaagaatttgatgcagttacttctgcttgttatttaatgagtatgtgtgaagttgcagaatatgcttttgtctgtcatg
aagctctgagtaatctaatccttattacccgtgattatgttaatttggttggtgacattcgtgctgatggagaacgtattgctggatttttggaaaatc
gctactgggtattaaaactttcctctcaggaagagtttctggcaaaaattactttacactctgctaaggttgctgatgatcaaggattatggtcaga
ctcaatcttactatatcatttagctgaggcttatgatactgtcatttccgttgttaatcgaagtttgggttctgcattattaggatttatggaacaaggt
gtattcccagacaaacttatttcgttagtcaaaaatatgatgaatgtttatggaaagaatccttccatctacagcaaaataaattacaagaataga
gagacaacggatttgttactactcactgtggaagctttccatgcttttgttaacaaagattatgaacaagtcttgtctgctttacaacaactggaaat
tttgcctctggatactgagtgtgattcgcatgtggtgcgtaagcttgcagctgaatttcgttttctcaatgacagcctgctacacaatattcccggaa
ttattttggtcgctatgaaaagtattaatgagctttataaaaagcagaagttgtctgcttacaacaacgattcaatagcgaacacaaagttacagtt
ctatcgtatgaaggcacgaaaaattgtaatgtacagctcattaatagattttcgtatgccttctcacgtgttagaacaattgaatcgctgtgaaatt
gaaatgacctagatcgctctttctcttacgtttttgtttttggtttgataaattcagcgtctatagtaaatcaggttatctaaggaagtgtatttatgca
atgcatcaatgtataatataattataataaaaattatttcttgaacatattagaaacactatcccatctggcctgcaattgagagtgtttatcttcta
gatcatgaagttgtcgtaaagtttttctaactcttcattaaggtaagtgatttgttgtatagaagaggcgtttgattcttcgaactttcgatttcggtt
cttcaagactattatttcttgttgcagggattcttgtaattgctcgggtagatgttttagttgttcttccaatgtagcgatagaatttcgtaagtactca
gttcgttcctcactttcacttcttaaaacacgatgacttgatagttcggcggaaagcttggtgttttctctatttaaaagtaccatcctgctctccagtt
cagtaatgtgttcagccaaatccttgttttgaatttttagttcgtttaacatattactgcggtacatttgtcgagacattacagtttcatggtaagata
aataagaaggaaaacgatcaatgcaaagctgaagattgaaagcatcttgaatccatgaatcaacgtcgacaggtgtgcgagtataaaatgtat
acttttgagttgtaggaaggcgataagaaccgacaaaatcagagtttaagtatcgattccattgatctaaattcatcagatttaacagcttactttc
atctttcaggaaaataactacaatcagacgaacaaacatttccaaaccatagcagaaaacaagatcaaaaatgcgtaaggcattctgaagtggt
aaaaagttagcaaaacagcaagccaatgacgggtacaaaggtctcaaagattgcgctttaatcctctgcaatgataaagcaattttcggtgaaa
gatcactgacgcatccccaaacaagggcatatagctgctcttcagagtagctcaagttagctacatacaattcatttaaatttgattttccaagtaa
acaagcaaggcttcggaatgcgtttccctgtggcatgtatgataaaagagatgcagtaggccacagtaaagcatctaaagcatattcaacagta
gtatcaaacaatgtaaacgcatggattacgtgaagtaatccagtcgttgatacctttgcacgagttggagatgcatatttgaatggttctagcgga
ccccggaatgcatgatttcgaatagtcctttttattataggatccaatgagtttatcgaggtactcaatgtagcatacaacgttaaaagctcattac
gctctgcagttacaagagactgccagacagattttctacagggtgaaggaacgctccagtggttctttaaaacgagatttcgaattgaggcgattg
aggcacctgtctctgaatttgcagagtactcattagcagacaatggaagacaagcccactctagccagtcaatctgactcaccgggatggattca
ctttcacattgctgattcaaatcaaaaataagggtttgaatacgagaatcatttatcgcttttgaaggatcatcatgactgcggaataaagtccaca
gttgctcaaattcggattgaggagctctagaggatgaagaagttttatttgatccagtggatagaaaagaggatgcaggagaaggattaggacg
aaaatgaccaaacgcaggattttctgcttgtgttgaatgtgcgccggtctcgctgcgaacaccaggtgaagtgggacgaagcatacttgtgttagt
aaactgcggatttgcttcttctggtatagaagcttgcacttggagaccaggagatgagtaatccaagctaatttcccttaaggaagattcagatga
ttttgaggacctccccgattccattgacattgcttgtaaagtattcaaattctcctttttcttcagtaaattcatgctgtttttctaagggaactatagtg
gttgaagaatacgattcagatgctgagtcctataccaaagctgatttagacaatagttcaataattatgctcaaggaacaatacttacgttcatta
aaggagagttttctatagtaagaatgtagctttcgttaaattaaacgatacaatgttgaagaaagatgttttcagccttccttgtcgttgagtgtaat
gcaatgcaattgaagtagaattaccccaatgtaaacaaataaacaaagagagaaagaaaaacacctaatacgcctccaacaatgtgaaaattt
ggtttttcagtttcactgtaagtatagtgtaaaagggtatagcaaaacacttttaataaacaaaccatttgaaggatgaaattctaccagtatggtc
atattcgtcattttatttcttttaaaaactgttaaatttggtttttcaatacatgaatgtttcgttatgaatatacatggaaattctgtagagtacaatg
cgtcgtgcaggtgaacaatcgaaagcaaaactttaggatcagaatgaaacgagctatttgcttgatagatgttaagaacaagatgtcttttccttt
ggtttatttccttgtaggcaggcgaaagatgaagatacaaacaaccccataaacagtaacgagaacagcgagtaacccaactaagtctttgagt
tgaaaaaatgaatttcaatcattgaaaaaaaaacctttgaagaagctagaaatgtaatttccagaaaaccatgcatacaatctctaaagtcaatg
tcaatcttttaagagggaatggacggtctcgaaaatagaagccttttctaataaaagtaaaggctttggtgcgatttcatggtcgaaagtgccagc
tctgttgaaaacttgaaaacagactagactatcaaaagcatcggaggtgtagatgtgcttggactcctttccttaatatttattatcttcttttttttat
gatagattctgggattaaaaaagtatagcagcaccgttgaaaagatggggaatataagaaagaaaaccaatactcaatctcgaaaaccaaggt
agttcaagtaaccggctgtattttgatttaaaacttctttgtgagtttctcacctagccaagaaaacataggttgcctcgttagctggtattatggaa
acatccatgctgatggaatgtgtttgaaaaacggatcaagctcacactttctctaagtgatcatgaatcaattataaacttaagtaattgtgattta
gcttaataagacttgtgaacttctggtttacgaactggacaattcacaagggaaacagttctttcattcaaagcacccctcacagaatggaataac
aggaatgtatcttataaagctctagctcttgaaaacttctgatgctatttctcatgattagcagcatggacacagtccttctttttttaggtatcatcct

gactttagcacaaaatctataaaatctcccgtttaaatgaatatcgaaaagcactcagtttaaactacttattttttggaagatagcaatccttagg
taatgaaattcaaagcaagccttaggtacgtgttttagaacatttggagagaatctggttgtgtcagatttagctgaaatacgttagtgtctcaagt
taatatattccgttaagaaagacgaatttaattttctatcaaatcccaattcgaatacaacaccggcatcgtttatataagcacttacgcctttgtga
agagagtttcatgagcctcagaaaaaccagtaacagtaaaggagaaaggatatggaatctttagaaagtagcagccctatacaaagtccctttg
attatgctgaagaagaaccactagctgaggtatccttgcgcaggcaatctgaatcttggcggttaataagtcctataagaaatgtttcttcgaaaa
ctagcagagatacatcaaacgcaaattcaaaaaaattcacatgcttattagtaaggcatgcggaatctgagcacaacgttcgtggaataagggc
tgggagcattgacagtgaattgaccatacacggatataatcaagcaaagaagcttgctaagtctctaaagggaaagaatgttcgctgcatttatt
cttctcctcagaagcgtgctcagagaacagctcaggagattgccaaagtaatgaattgtcccatctacacttccagtctactccgtgagaaagatt
taggatcattggaaggaacatcttttagattcacttctaattatagaccgaatgaaccgcccatgaagattcatggtttagaaaatcgagactcac
tactacacagagcacgggaatttgccgaaagtttattagtagaagcagaagaaacgggacttgatacttcagatatagtcgttatcgtttcacatg
gtatttttctgccttttcttttgcgagctgtcttggcagctgttagaacccctcttccttctaagattattccctggagtaatgcttcctatgcctcgattg
gtatcgatttagatggtaatacgactgtagaaatgaatttgaattcccatcttcgaggagttcgaagaatacgaaacttaggcagtacaacttacg
actcaaagcaaaaacctattacggaattctgcaaaaaaataaattaagcttgtaaaataaaagaaaacttatcctggctttcattattactagtct
ctgaacatttttgatgatcattaaactgttcaaaacattaataaactattaaaacaaactctaagttgaactaaaatacacattttctcagcgattg
gcgcgtattagagacaaatcttaagaacgcaatcttatagtaaaccacttttcgtattatgcttcattatcatcgtccggaaatattaagattcgag
cataaagggaatcgagtctattagataaacaagaaaattactcttcagaagttagctcatcaaaatcctgatccatttcaaactccgtctcgttag
caacaatatccatattggattcttccatttctataggatctttttcaaaatcatctttaggctcaactttctctttagctaactcttccttttcgtaattct
caagaatgttctcttcttcaattttttttcatcaagctcgtaagagttcgctccatgtatttggcgtcctcatatacaggagaattctcttcattgtaggt
gtaggcattgctaaacataagcatgaaatccgaaaccaaaccctgcaaatctgtgtatttattattctttatatttcgtccaatcttaccaagggca
attggatgtttgatgataacataataatctgggtatagcttacgactgggcgggtacaagaataatccattaatcatacgaccgctttcgtcttgaa
gataaaagaggctttcgtagatctctttcaaacacctttgaagagccttgtacttgtgcgtagaagggacttcacttgttcttttgttcgaacgcgac
tcgtaaaccaaaggtccagaataattaagattatccaaagcaactttcttcctcggtctaccacgacgacggggcatatcattcctgtttccttcttc
aaatacatcttcgaaacctggttcagcataactgaccgaagctctatgccttttctgacgtccagcgtctaaaggcatatctgttgatgcctcctgc
acaacatgatcaacttcggctctataaatttcaggtagttcattgacagaaagtaagcgatcgaagggctttccctttccatagatgtctttaatag
cacgttcttggtctatcgtacgaaacagttcaagttcgtcatctgttcttgaaataagttcatttaactcatcattttgcagctcgctgtaactcaagt
cattatcctcatcaccatcatgttctaacaaagaacgaaggaaagcctcgcgttcttcaggtgttgatttattatcgaatttgccggcttgaataacc
ttaccatcgagatctaatttaaactgtgctctagacaagatgttttcttctatagatttttcgctgataagccgcaaaattctaacttctttggtttgac
caattcgatgagctcgatcctgagcttgcaaatcttggtgcgggttccaatcagtatcgaaaatgatgacggtatcagcggcttgcaagtttaatcc
taaaccacctgctctggtgctaagcataaacacatagacatcagaggacggatcgttaaattgtgataaaagcgaagagcgatcctcagattta
gtagaaccatctaaccgcaaatacttccaattacgataacgtaagtaatcttccattatagtcataatttgagtcatctgaaagaacatcaaagttt
tatgaccagtctgaaataacttcgggaagattcgatctaaaagctcaaacttaccggcagatctccatagtaagtcataattggtacccagggggt
caatggctttttcaacttcttcaaatatgaaaggatgattacaaatctttttaactgcataacagtgttctgaagtcctttcatcccggtttttcccct
atcaccagctacaaacagcataccatgccttttcatttgttggtaaagctttaactgcaaaccggaaagagggcattttataacttttcaactttat
cgggaagttcactttcaacgtctttctttagtctccgaaacaagaacggtcttagtactttatgaagacgtttgataaccaacaaagattcttcttca
gtaagctcaaatttgtcttgaccaccggtattagcaaaaggggtattaaaccattcatcaaaactcttgacagaattaaaaatcttcggcaataca
aagtttagaagagcccaaagttcaggaaggttgttttgtaaaggcgtaccagtcaaaatcaagcgatactgtgaatgataataataacacagag
tactagtcaactttgattgagtattcttcattcgatggccttcatcaataatcatatgtaaccatttaacttttgagagtaacggtctgtctttaataat
atagtcaaaggtggttagcaaaacatgaaaattattacttttgatttgggcttgtaatgattttcttaactgcgggggtcctctgtaagtaatttttttg
aacgctcggtgcccatttttcaaactcaagattccagttagttaaagttgaaagaggaacaatgattaggaaagggcctggttgattctttttctca
agcaggtaagtgatgaaagcaattgtctgtacggttttacctaaacccatctcgtcggctaaaataccgtttaaattgttattatataaagatagca
tccactctaagcctttaagctgataatcctttaaattacctccaacaaatatattgggttgttcagcttcctcatgaatccggtgagcaacattatag
tagtccacactgattttatcttcatcttcatttgatgcagtaaaaggttcttttaatccttttccagaagtgtcaccactaaaaatagttgattgttgta
gtctcaccgcttcagttaaagattcaagataagaatcggtttgtcgaagtaaatgagtaattctactatccttagctttatcaaccaattctaggtaa
gcggcctcatcgtcagcacgcaatgcttgcaacctttcccgggcacttctttcgatacgtttcttctcctctttttctaagtgagcatgtaaggacata
acatctttgcctatatgagtagacttttgctttcgtacagacgcttttttttgctattgactgcccgtgttgcaatattgacctaagctgggaaaaaagc
ttttccttttgttttaaaacatgggcattattttgctgttctgcaacagctaaagttgcatttgcttcccgtaaactttgatgtttgactgttcgaaacat
actccttagattaccagccgccaatgtttccgactgttgaactgtcgaagaaacagcattgcgtaaatttctttgcttttgtaataatcgtagacatc
gtaattctatcatctgctttgtatctaaactttctttttcggaaaagttggtctcccctttagttacagaaagatcttccagagaattaattctattacc

aactgagcaggcaatagccacctctgaattacgaaaaataaaatcccaaggtagtccagaagaaagtaaagagggaattaactttaagttatc
accggaagaaaatttctcatacgaaataggagcttgaatgtagctattaggattgatacaagacgaataatcaactgaaggaactactggtttca
cagtatcttcacttttgggtatatgaggtgaaaaagagcctctctcagcctttggatttaaccttgcagtctccaactggggctgggaattgggcttt
cgagcaaagtgcatatctgcagatgttggttgtttttgtaacagtgaagaagtcaaaggattcgctactttcgtttgctttgactgggtttgctggttt
accaatggattttccggaacctcaggaagggttttaccaaatacagctttctgaataggctgtggaatttggccgaatggagaattcaaggaatta
taagccattatttgattctttagcaaggaaagctgagcggggctgaaaggaacgggagatggttttggcatagagggtgtatatggatttcttgcg
ttcatagccggtgatctaccttgattcagttgcaaacggttggtaacagactcttttttgaggtggaatctaaagaagacatatttggagaaggag
aagacgctggggccacttgaggaccaccagaagctaccgaagcaccagtttttagtgtattttctgagggaacatatggatttccattcattgagt
caccagggtagtttgaagaaattgatggacgcccataagaatagtgaggaggtactgaagcgctgtccatactcacattaccaggggtgggaaa
gtttggttgagaaggaaaagaaggagaataagtgataggatgcggtggaagactttctcctttatagtttgtatacggtggagttggcatagatg
acccaggagtcgaagaacgtcgaggatttacggataaagaggttcgtctgtccacgatgggctgcataggaactgtactacttaacctttacca
gtatatgaattcatgtttaaattctgatcaaaggaaggagtttggtttctaggcactgatggaggacgatttccatttcctaatgaagtagaatcaa
tgggattatcgtatatagaacccgaagggttacctctgaaggatgaatctaatgactgggggccaacatattggttgggcatcatattactaacat
ttgcattcatgggcgaatccgtattatgaagattggagcgataataattaagtggattcatagcattaccacttgaaacgtttgcgggataactatt
aggaaaaagcgagctagaagtagtagtgttaccttgaagagggtaggatgaatcgggctgagacacctcatcacctgtgtttatgagatttgcag
aagcatctggggggactgaggaaggtgcgacagtattggaagcatgattatgatgtatgttaggtagagccggagacgcacgtgaaagtatcgg
attttggtctctactaatattttcattgatagctgaagacccaatgtttgcagtctgagaaagttggttctgctgagagatctgctgtaaacgagccg
tatattgtaccttttgaattgtccttaaaatagaagtcagatgcgaaaattctgggttcgactcttccgtagcaccctgacttctcaagaattgccat
cgtcggtatatggtccgtacattttctttggtcagccctcgtaaaaagcttggattcacataaggtgtcgacatgaaagaagtttcgtacaatgaaa
gatatgatgatgacttacccaaaaagaagtaaagctaggtgttatagaaatataacaagagataacaatacaaataaaggagaaagtatgtag
tttttaagaaaaacagaatgaagtaaaaacagatcttttggaaacctgttgcgcgtagaattcgaataaccagccaaatcttcaaaggaaggcct
atattgaagagatctttttaaaaaaccacgtatgaataaaaatgtagctctttgtttatatgtacatgaaaaaatatgcaatatctcttgaattcgcc
cttttgaatgtaaattttaatcgttgtagaaaaaggtgagggaagaatcaagtaacaagctgaagtcacaaaatgctggtgttgaaagtaggtag
cagaggtgacaataaattgcaatggcaaaacatggcctatacttatagtaaagtattcgatttcgagcttaatatatattagtacttaattttgtact
ttgttcttcattattttttgtaaaaatatcaacaaacatgtggtgtatagatcggtcgctcgttaagcacctttgaataatgcggtattaaatgcattc
ttcaacatgaggtaaccctagtaattgatatctcatcataacctagagtatcaaataatcacttctgaaatgaaatagaagagcatgaataaatgc
ttggctcaagagcaatcttatttcagttcgataaaattcataatttccagtatgccatggtatctcttatttcttgaccagttgaaacgatggctacaa
aaatgaaagatcactcatcctgctggtgttgattcattaactcgctaaatgatcagtgaatcgcttccgataataaaacatcatgaaaatggttac
atttgtgaacgaaaaagctcattcattataaagattctgctcgaacaaaagctgcacagctattgtttgttttgtttccgtttgctttaacaaattatt
ttgttttgcttaattcattatgactcgtcgaacgcgcgaacccctaaatcttatataaactataaaacaagcgaataagcagataaaaagttacat
catatcagatgaaaaggcccgaaaaagaaagcaacaagaaaaccgatactttttgaatcagatgaaagtgtatgtttttttgtttataaatttcgtt
aaaagagtaaaaagattcgtgcgaaattcctgttctgctctgcatttattttcattgcctttaatgacatatatcctcagacacagatatattcttcat
tacttaaatacttatgcgcagaatacaaaaggaaaagtggaaagaatttgtaatcgaatggctcctgtcctcttcgtttcgtttaatatcgaagtac
tttcgttcagtcgtacttagtgtgttttttttttttttttccctctctcttttgagttttacatattttgtaattttaatgggatatttataacagaaataac
tgatttaagaatattgtgatcttatgcgaccttctgtgggttatagacaaatatggctataactcttccatcattacctaaccatataaaacggacca
aaacatagaatcgattgaaattgaacgaaaacagtgagtttttttgttcatattttatttctttttttagagaagaaataactgtattttcaaaaaatgg
ttttgagatacgatagtcctcatttgaatattgccgaggtagcctgcttgtacgcaggttttctgggtcttgggactttgaaggccatcaagcaacaa
cggcggggctctgaaactttgtcaaggacaagtcgctttggtaaagaacctttgaaaatcgtctcttggtggattctcggtgttgctttgacttatgt
atggtaactgtgaataaaggacattcagccctttgtccgttgaatacttctattagaaatttatagacagttttctctatacagtatcacaaattgaa
actgaagggctcgtctttacatgtaccacttattaacccattttctgtaggtaattgatatcgccaatttagtcatctatgctttgaggactcctaatt
ggtggccccataagtcttccgtggtgtgtcttgttttgtctctactcttttggggtatcgtattaatcagctgcacagacacatctgaactaccaagat
atgccgattcctttttctctgcttatagggtcaatgtcttatatgcttggttaacggaagcagtctttgaatccgtcttccttgcattgacacctatccct
ggtgatacctttcagggcattcttatggctgatcacctggctcgtcttcttttatgtctttttgcttttgccatttatgtaacttatcgtcgcaagaggcc
ttctcgtgacccgatggattacgaagaacgaagaatgtttgaaagcagtaatcaattggatccagaattcgctcaagagcaatcctcccagtcgc
ttcccaatgccaagaaaaacggctctaaaaaaccaaaaataaatacttcttggattgcttattttaaatctttcaccatacttttcccttacttatgg
cccgttaaaaactttagactgcagtttcatgtctttgtttgtgttgctctgttagttgtcggaagaatagctaatattcttgctcctcgacaacttggtg
tagttacagagaaactttctcaaaacgataacggtgtcccctggcttgaagtttttcttttcgtttttttaccggtttctccaaggaaatatgggtttaa
ttggttctttgcgttcttttctgtggattccagtttcgcagtatgcttatcgtgcactttctacgaaagctttacagcatgttcttaatctgtcctatgatt

ttcatctgaataaacgaggaggtgaagtcttggctgcattaaccaagggaagctccgtgaatacctttgttgagcaacttttgtttcagattggacc
tgttcttacagatctgggtgtagcgatcatctatttttcatcaaatttgatgtttatttctctcttatagtacttgttatgacgttgtgctactgttacgtt
actgtaaaaatcacttcctggcgtaccgaagccagaagaagaatggttaatagctggcgggatagttatggtgttcaaaacgacgccattatgaa
ttatgaaactgttaaaaacttcgatgctgatgatttcgaaaatcatcgtttcggtgatgccgtagatgcgtaccttcgtcaagaaaaaactgttcttt
ctagtctcagcttcttaaacattatacagggtggcattttcactactagtttatgtgttgcttgtttgttaagtgcttacaaagtttcttatggatataat
acagtcggtgattttgtggtactcctaacctatatgatgcaacttcaacagcctcttaactttttggaaccttataccgttctctacaaaatagtatt
attgatgcagagcgattacttgaaatatttcaagaacagcctactgtggttgaaaagtgtgatgctactcctttgaaggttactgaaggaagagtt
gtattttcagacgtttcttttgcttatgatccccgtaaacctgttttgtcaaacatcaatttcgttgccgagcctggtaaagcaatagcccttgtagga
gagtcaggcggtggaaagtctactatcatgagaattctattgagatttttgatgtaaataatggatctataactatcgacggtcaagatgtgcga
gacgtcacgttatctagcttacgttcgtcaattggtgttgtccctcaagatagcattttattcaatgatactgtcatgtacaacataaaatatgctaa
gccagatgcttccaatgaagaagtgtacgaagccgctaaggcagctcagattcatgaccggattcttgaattccctgacggatataattcccgtgt
aggagagcggggtttgaaactcagcggcggtgagaaacaacgaatggctgtagctagagctattcttaaaaacccgactataattctgttggatg
aagctacttccgcacttgacacgaacactgaacgccacatacaggcatctttaaacaggcttgccgctggtcgcaccgctattgtgatcgcccatc
gattgtctactatcacaaatgctgatttaattctttgtattagcaacggaaaaattgttgaaaccggtacgcacgaggatttgcttaagcatgagaa
tggtgtatacaagaaaatgtggttgcaacaggcaaacaaagatccaagttcaaaaaagttttcagttgagtagtttttgttttcaaaataattattg
atgataaagttctctaataatgtaacttgactacgtctctagtattttataatgatcaaatatgattatttattaaattaatttataatttccaaaattt
ccaaaccaacccaaaaatcctttttaatataggtttatgtttttcttagtatatattttaacaaaaaaataaaaaagaaatggaagtgattatgtatt
agacatttgttggaaccattgtcgtaaagcagaagataacaattgaggaagttcttctatgttcctgactaccaagtagtactcgaaagcaaattc
atcaatgtacggttgaatattcagttccatagttccatcatcttttgtaatgtagtgaactttgttgatatccagcacggatgatttttcatatcgtta
acattgtcaaggataacaaaaacgatcatcactttttcttcttcagcacgtcgaagaatccttcgaatagtttcatggtcttcacagataccatcag
aaataataatttcaagctgtctttgatcggtcttattgcgcacctgcaagttagtactggcgtaattaaacaggcgcatggatgcatcagttaaaga
taaaacatttgttccagattgctcaaaagagaagtgtgagaacatttcagcaccgacttcagaagagaaaggcttgtcaaaaggatggagtaatt
ccggctgatcgccgaacttcattactgcaatttgtccaacttccagcaaagacaaagattttgtcactaacgctaaagtttccaaagcaagcttcgt
actcttagattcactcattgatttagaatcgtcgattgcaatcataacttgataagttcgcttactaggttttacacggcgcatccagatcttgtctttc
ttgaactgagatgcaatatatgggataatacgtttcatgttcagacgcttacccgtgcggaaatcaccttgcattttagttgctagagttggttctaa
gatcaggcgaagttgttcacaaagttcgatagaaagttgtctggtagattcttcatgctgctgccaaagcacgcgagcctcatctattgacatgga
aggaggaagtttggttgtcgtgataataccttcatttactaaggcatcttctgtatcatcgtattcttccaagccttgatcaaaattaggttgatgaat
aacatttcgagtatttaattcatcaaattttgatttatctgtactttcggatacctgttttgactctacgcccgcctcctgttcttctttgagctgagtat
cgttcaaatctagttgagagtcaatctccataggttcaggagggctgatatcttcttgattctcatttacattgaggcttttgacttggtctttatcagc
gttgcctaaagcttgagtatcttcttcttcttcctcagctatgtgggcaaattctgtgctttcgtcaatattcttttgctggtcagccgcttctttctcagc
attctccggttggcgctcatgtatttgacgagtttgttgccactcgcgcaactgatcgccaagactttgatactgtctatcagcagagtccgcttcgt
ctgataatttattttcctgatcgtttgtttcttgaacatcagcttgcatttctccagcagtagcacccgctccttgttcaccattctcggcagcaccttcg
gtaacatcctcatcagcaccttcaactgtctcatcagtatccatttgagcagatgctgacttcgattgatcgtcctgcccagtcgtactatctttaaa
atcgtccgcaacatcttcgttatgactttcttccggaacttcctcatcaggttcactttcctgatgccctttatcgttttcgagggattctttatcttccg
cttcttccacttcttgcgattcttgcgattcttgctgttcttctccgttcacagcatcatcttgagaagtaaagccatcttcttcttttatatcttcctcga
tattgtcttgtgcatcatattcttcaaactggtcaacctgctctcctgtatcttctttttcagcttcctccgattctttagtatcttcaacgttctccggca
tatcttcgtcaccatcgctttcttcttcatttccttcatttccttcatcaagtttcagatcttccggaagctcaagaacttcattgttttctggttggttttg
attatcttcaggagcctcttgctgaacttcatcctcaacccctggagcttcattgtcactttcttcctctttattctcgttttccgaggtttccagctcatt
ggaatcaccattctcttcttctttagaaacaagttcgccagtattgttctgaacagattgttcgttcgacttttcttcattcgcacctaattcctcttcat
tgggttcatcccacatcttttcgtttaagtcttcggataacccctcattgtctttattaatctcctcatcgagttcttcatcttcctctgaattgctctcttc
agcgtcagatgcaggcgcatctttagtggctccactcatttctgtatcaagttcacgagcctcggagttttcttctagttcatcctgttcattagtgtcc
tcttcatttgcaagttcttcaagatcgtcatcttcattcaatgtttctgagatatcttttgcaccagaaccagaaccaaggccagtaccagattctaa
actaccgccctcctgaacattatcgtcttgaggaatgtcaggagtacagtaaccattggtggccaccatcaaaaacaaggatgcaatcttatgca
aactttgcgactctttcacatgtttaaccaagaaatgtgtaagagctttctcagctgagaagatgtactgctcacatacagggaggaagtgtacga
aaagacgcgtactatcctcatctaaatatttatcttcaaaatgtttttaagatcggacaatttagatgcgaataaatcaagcttaaatgatgctag
aagcttgctaactaaagcactttcatcaaatagagtactgtcagtgttttcgtttcctagggaatcaacgcattttgaaacctcctctgccgatgaca
gtaattgatcaaataaagcagttaacttctcaaatgtagagtaacgaatatctaagtctgtaaccttcttgcccaaaaatgcagactctggcttcac
agaagatagcatggattttgaccaatacaaggcttcaggcatagaagaagcatgattttgcaataacaaactaaaccatttactgccatcttcat

ggattctaaacaatcatactgttcactagatataaacagagggtttggtgaaccaatcgtatcttctgtgaactgcgtactgtactctgacaaacaa
cttgacacagaagcataagtatcttttgacattatttgatgatgttttgataaaaccacagaataaatcgaacataaacttgatagctccttcaaat
aatctttgaagtctttataattaaaactttcggagtgtttaaataatgatgccgtaaagttaagcttcaaagatccatcaactccaacaccttgatat
gcaagcttaatacgattcaaagattcggcaaacttgacaagggtcaatcgttcttcaagcaaccagcttagggcactatcaaaaagtccaatagc
ccttgtaacttctggagttgtaaggtcatcatgctgattaccagggagtcgatgcactttgggaatcaagtctagaacagtcgaaagagacgtgtt
tgaatcatcgatcaaatcgaaagatattgggaaagaaggtatattcgaaaacagattttggagagatttcatcgtttcttctactcctgccttaact
ctgtactggaagccaaaagcatgcaaggtcttataagcgtttaccatcagtagatgctttcgagttttcaagtgtttgatctctgatatattctcatc
attagcagaagatggggtaagcttaaacaattcatgggcgctactaactatttcatcaattgcttgcgtaaacaaggtattagcagggtgagaaa
atttcaaaagtgatttcatcactcgtacagtcgactctgtattgtaaaatctggcaggcaagtttgcgtagacagcattatcaagagtcaaaccgg
aataatccaccttcacctcatccttaaaattgtgaatagataagctagttggtttgagtaacatttcccaattaagcttttgagacagatgaggagt
cacaggttggcgcaaaacctctctgtatttatgcaaggttttataaagctccatatgactctttctggcgttctccttgagagcatagacattagtat
ctttccagctcataagtaatattctttcctttatgctattctctaaatctcgacgctgcacagtaataaagtgggttacattattttcaaacaacttaa
gataggaataaatttgttgaagagtagcacacaatcctcccgattcgttcaaggcttccacgtgttttgcgaaagtaagcaaattactgaggcaag
gacgatattctccaacactcaagccattaaaaaattgcaatattaccgaagcaatttcgttcaaagatgacctgtccaaactatacttctggaata
gaggctgcaatacaacgtaatccaaaatagtgtaaagtcgaggatacgttttctccacaagtttatactcttcccactgtaataaatttttccaattc
gatagctcaaattgtctccactcaataatcttgcttttaactagatccatatcactttccagagaaaactgccggctggcaagcttctcccattctga
caataaatgatacaaacgctcaaattttgctaacatttgtgctacaggaataaaaactgacatgcctaatgtcaagtcggtagcctcttttaaactt
ctcaatacaaaattgtctggccataatttcaataacgaattcgtatgtaagtgtaatccaacaagtatctcacgaacctcaattaatttgtaaggaa
gcggatcggagtaaaagttgtaaacaggaggtgaccaatagttatctacgtcctttgaagatttaacacctaaacagaggatggcagccactata
tcgaaatcggcaggaaccgatttttcaaaatcagaaagtcccagtttcctaaccaagtaagagccgatgtctagaagttggtcggtagagcatgc
ctcttcagtcccgataaaaaaacttttatacagagaccagaatgcatattgaagctgagaaaatctagtcctctctagcttctcttctggttgtggtt
cgacttcttcatcataatctataaaaatctgcatgtactcattattatcaaggttcaagcctctttgtcggaaaagtctgtttttttcttcctcttcttctt
gctctttctgaactaatttggcatgataggtaaaaaactcgtttgctaaaatagtgaatgaattagccgcagacgactgaggaaggtctttcttttc
cttgcatagtttcgctgttgcaaataataaaacttcaaattttaaggcggcattcaattgtaagtccgaagtcaattgtataatatcttgcacatctt
catacataagaagttttgaaagtttctgaggactaactaaatgagacatcaaagaagaggatgcattcacccgattatctaggaaccgcttagcc
gctgccacgagtatatcgatgccgtagaccatataactaatcagagaaataacaatttcaaatatatcagtgtaataaggatatgcttcacgcaa
ccgttcaacaaaagcagtccacttatatttaaatatttttgcctcctccaatgacgagtcatctggattttgcataagctttaacgataactcaaggg
catatgaattattcacaacgttgtccattaagaattgcaactcgtcaaacaaagaagtgctattactttccagggaacggaaaacactagtagtag
tgggagtagactcatttatttcctccatgcacgaggaaattgatttagtgagtaggttttctttttttaccagatttattatcttccatcatctccacaac
ctttagctctccagaaagcgccttcaagacgtctttgcagtttgtggaagataacaaaggaagaagagctggatcataaggcttatcaggaacat
aagctagtaacaacccgtatgcaaaatgaatgtaagcagcagcttgattcttacaagccgtctcatgattagcagctgtcaaagacaactgcaga
tattctaatgcattcagaaaatgtttttttgtatgaggcattaatatctgtagttctttctgcataagacaatatattatcgataatggaaagggactg
ctctttcaactctccatatggtaatgctaatggccttaaaatcgtatcctcatcagcagatggtttaccacttttgattccagaaatcatgaacattg
gaattcgctgtagcagtaaaatgctagctctttctaaaggattttcagaaagttgctcggaacgttcagctatatgcctcattaatgtctggcgtaca
tcttcaaatcttttaacagaagctatattcaggttttcatcaattgaagcaaaagcacgtgtgactaaatcaaacgtggttccgttaaccagagaa
aagaagtcataatcaaaaaggacgacaaatgggatttgagatgcattgaagttgagactcgtgatgatagataatggacttgttgaccctctcgt
atacagagcagcacgtatatagtttgtaaatgcttctgacgaaacattaaaattattgtaatcggcggtatatatagtcaacagatctaatgccgc
taaacaattgaacgctgaataaaacgcttcaggaagctggtcttgcttggactttttttagtgctaatacaccttcttccaaaatttgcatgtacgact
gtaggtcctgagactcgtgctgatttgttacattcataagcaatgatagagttgtgctaactacatgagaagcagtagcctccatacctaaagagg
gagtatttgcacgcaaatattcctttagcaatgtgtcgattctcaaccataatgaccaatgatccatcgtcttaggtacaagaggatggcactcgga
ccagatgttttgcatatattcaccagtagataactgcaaggcttcagagaatttattgattaaattgagaggagcatccaaatatcctgaattatgg
tcggcaataaacttagatgaccattccttaagcattaacgaatagcaatgaaacctgctgaaatcaaactcagcggttccagtccattcatacatg
ctcttccacaacgcgatcaaactaatgagattttgaagatccgcactactcgctaacgagctgtctttacgtaacttgtcgattgagttgaagatat
aaacgctaagatcattcaagagtttccacaccgacgacaaagcacccctataataagaaattccatgcaaaaatgcgtatgcactttttagaaca
taagtatcaatagaaccagactgcacagaagaataaacgtcttgattaatttgttgtaaatcaaactgagcagcataaagaatgttgagtaaata
agctttccagaatacctccgactcacgattagaagatgttataaatgagaaatcattcataagggagacaagactcattgatgctggtaataacg
gatagccatacgtcttaatataggtaagcacctcgttagggatctgtccactggaaaaattggcccgatccaacgcatttacataaggtgttctgtc
tattgcgagcattttctgatcatcacaaaggtttcttagacaatccaactgatcgctcgagaaatagctaaagacaacagctgctacaacatatgg

tgaaacgagacccgctggtaaatcccttagacgcttatgaacatcaataacaaaagacaagctagatgatcgagcatctaccgcagagcatatg
ggggaaggtaataacttggacagttgacgatgatcaaaagaagttagaccgttgttcaacatataaatttctacaccacgattcctcattgctcta
gaaagttctccatagtaaggattcacagttaaaaatagtcgaaaattaagatgcggcttaatgacttttggcatgccagattccgttggcctttcgt
ttacggttagagtacctccatactctaataaagaattaagacgatcaagcacagcgggactacaaagattggcgttatcgagaaccaaccagtg
accattttcaacagctcttaataaataaccatctatccactcaaactttccggtagcaggcacaaaagcttctttaattagcttaccagccagcgta
aagtaggggcgcaaatgcttaaagagggcggtatgatgtcgaatagatcgaatctcggctactacccactctaagatatgtagaccttcatcaag
tgtgagaaagttggattgtaaaactctcagaagagccgttcttttgagaatttcctcaaagtctctgtactgtatcagaagatttaacacttcatgg
gacaagtccaacaaaaaggcagaaatctttctagaaatatcaacctgttcatattcgcccaacaaatccatggtgtcaatttcagaattaattgcc
atttccttcaactctgcaccgcaagcggcggctagaagtcgaatagtacacgttttaccagaagccgaaggacctaccaagatacaaggccaatt
tttgtttatgcaagtaatcatggactcaagcacgtcatattggcattgtagcaacttggtattgtctaatgctgtcctttgagtaaccgaattcctcgt
gaaaaatgaatggccaactttcaagatatttacatctatctcagaactaattgtgcgtggttcgtacttaacaccgaacacttgctcaaaaagctc
gataacgcgttgacgatcgttatctgttcgcattcgatgtagcactacaacctctaaatattcagcaggagaaacgcacgtatacttttcggcttct
gacaaaagctgtaaccaacgcatggcatcacgcaaattgaactcccatggtctaccactaataccaaacgaagcatcattttccacatactcctg
aagcttgaacataaactgaatgatgtggagtcgcaagttttcgtcaacttgacgataattttgaccagctatttcaagcatatcgctttgtaacaaa
ggctcaacatagactacagtaaatcggttaacaaaggatttaggaagtccttttctgccaccaccttgattctgtggattttgcgccgcgaaaactc
taaaattagggtgcgctttgaatacacggtcgagttctggaatataggcttcactacgatggtcaagacaagcatttaagccttctaatacagtttg
agaagctaagttcaattcatcaagaagaacccagtgtccaagacgcattgcggataaaaatggagcgtcacgccatgtgaactggccaccttca
cccccattaacaggcgcatcagagccgaaaagatccattaaatcagtttgatcggatagattaattcgaaccaatggataaccacactctcgagc
cagagccgatatcaagcttgtcttacccacaccgggactaccttccaaaaggattggttttctaacctgcatagcacgcaccactttcgcagcgttg
attttggtagtgtgggtcttaagattataagtagaatcaggatttacgctaggtcctaattctataccaaaatcaccaatgtagaaagaggattcgg
tgaatcttatcattaagtcttctgtaacagaagatgaaaaatcacaatcacaaagttctcctaatttagaggcgcatttttttacgttcatcagaaag
catatttctgttattaccaaaagcaaacgttgataccgcgccaagcgcatcaacgaataccattgaaacagcatggaataacgtaagcttatcgt
cgctaagctcagataaattaataaaatcaactgcagataaaacatctctaattgatatgttggtgccattctggtagactttactatgccagtgtgc
atattgtactaatggttcactaaggtgtttttggcaggaattaacttcccttgcacaatttgaaaaatatcatcaaccgaagtcattggagggacc
caaatttcagtaaatcgattcctcaaagcaggagaaagttccttcttaccgtagtcacccccaggattcatagtcgcaaaaaatgcaaatccgtct
ttggcagttaaaacagtagcggcatcactatgttcgacgagagttagagttcttgaaatttcaagaacactgttaagtcgttcaagtacagaatca
tcagcaagactgatttcatcaagcaagaaaaaatcaccacctttcatagccgaaacaagggcgccatcatgccactcaaataaggagttagattt
ttttatatgtttttcaatgacaacagataagtcatcattcttttccaggggagaaaggctttcaaattttgaaatcaactcttcaactgagtattgctc
ttgagcaacattaaacttctcagatagttgtatatgaagagtattgcttatgagagatcgattgcgcaatggtctttgagctccaataatatcacca
ttctccgtatcttgatgagcattgacgatatgtaattgtttggagaaacattcagccaaaacctgacagacagtggttttaccgcaaccagtatcac
caactagcaacacaggctccctggcttgaaggcaatgccaaactaaggaaaataaccgaatcattggcgcggtccacacgacatttttgagggg
accaactgtggcatcagatctaattgagtctataaagtccaaattatataagtgctcagtattaattttagttttcatcactttctcaattacctcctgt
acagcaaacttgtctttaggatcgcgtgctcgttctgccaaaagcatatatccgttttcagcgagttgttgataacccactgcctcacgaaaagccc
agcgaaacaagtctctaagggtagcaaaggaattcttctgttcaaaaattctagtactttgacgacgaagagaaagttcgcgaaatacttgaaca
atttttgcagcataagaaggagctattttacaacgcatatgcaaaatggtttcaagctcattttcagggatatcgtcaaaatggatttccaaaaatc
tattgcgaaatgcacgagacaagtgcttacgaccagcgtaaacacccggtgggttttgggttgcaaaaagcataaaatccggatgtggtttcaca
agcgcttcggtttcggggatgaataactcacgattatcatcaagaagacggtttaaggcttccaagacatcagtaggggctaggttaagctcgtc
aagtacaatccaatagccgtttctaagagcctcgacgaggataccttctctaaataccaaagatcccttgtcatcagttacatatgagccaagata
ctcctgaagatcggtatgttcatgattgttaattctcacaaatctatggcctgttttaccagcaatgtactcaatcatactagttttaccactggatgt
agggccctgtattaaaatagggaacattctagttgagcaagcacgagtgatatttaagaggtttttctgtacgaaaggtgtaacaatgtaatgttc
ctgatctctgacaggaaatgaacccttactgagccaatagtggcagaacgctatataatcaccaacttccggtcgctttggaatttgtttgagaac
agcatttttctgttggaccgttaattcccctaatgtgtagcgttcgatatatttgtacagtaaattttcactattttgatcaagtaaggttaagaaaga
catacaaaaaccctcatataaggaacgtctaagaccgtaaataggcacaatttccgcaacatagcaaagtgttctgctcaatgtacgaattgtat
aatgaggcttctgtcctgcaccatcgactaatgaaccatcaatagaaagccgttttgcagtttgatataactcagcgatgtcacgaataacgagat
catcaccaatgcaaagggttcctatgtacttacttatgattgataacaaatccgaaaggttcttatcaggggactcaacataaatttctgtaaatcg
tgatcgaagcgaagtttctaactccctcttgcctacatcagttgaaggattcatacaaccaaatagcctaaaattgggatgaggtgcaacgggatt
aacgtcaccacgatcgcttagtaaaatgccaggttcataagaatttaacaactgtccaataggttctaatgtttccatacatgctaaatttatttcat
ccaacaaaacccagtgtcctgaacgtatagctttaaccaaggcaccttcaacgaaagaaaacataaaacttttagaaccaccttcaagcatttta

ccaaactgctcgacttcttctctgaagtttttccaacgagcttccagagctggctgatttgctaatctcttttgacgcttcataggattttcctgcgacc
gagtcttgaagaacagtgatttcgaaagggataaagcctcattccatatttttaaacaggttttccatttaaatcttctagcagcagtagatgctgct
gctaaaaacttcatattcttcttaacactaaaggtcgcctcaaataaacttataaattcttcatgtaaaggaagccccaaggtagtcacgtttatgg
gcttataaccaccaagcatgtctgaagattccgtttgctgagacatgttgataacagtaaccctttgacctaacaaattagccaaaagttggacgg
tggttgttttaccagtacccgtctcgccaacaagcagcaaaggctcatttgcttgaacagaaacagatactttttctaataacgacaaagaggaag
aagtaaaagcatatgaattacttgttgagaaaacgttagacgagattttgggcaacttcgatcgaccaatcttaaccgtagtctcggaatcatgga
aagagggaatggtctcaaagaaatgctcagctttttcaggagacacatttaattcaatagcaatattacggacgacatgatctacgccctgtttat
ttggtataaaagcactgaacacatcaatagcttctttaaaaatagcatcctgaagttcttgagggattgttgcgtgatcaacagacacattgaaat
gtctataaagaaaagcgacacgcttcgtaaatttgtaaaaatcacgaaggcataaccgccgatacaccctagaaactgcaaggaatgttctattt
aaaaagagttcataaatatcattataaacagaaaaaaggatacgagcaatagaagaaagctcaggatagatagttgacacaatttcgatgcat
tcatggggagcagaaaccatttcaacagtcttccaaagcctttgtcctaatatagtggacttcgtagaagtcgttgcaaacatttggaacgaattc
gatgcataaatagtttcccctcgggagggaactacgagctgtcgtctttctagcaaaggcaataaaatacttaaaacctcggcaggtgctttttca
atattgcttagcagaagccatttgccatgcctcacagcttgtgttaaaacacccggctgccattcaaattcacctggtcgggggcaagtataagta
cctattagcattttaggatcagtagagtccgtaagatggatacgaacaatcctttgtccaagcaaggcagcaatttgagtaatgaaatacgtttttc
ccatgccctctgggccagccagaagcaaaggctgctttcttacaaggaaaggacttacacgatttagattttctttggcgctttctgtttgcacaag
agtggtgggcagaggttcatgaaactggaaaaaaaggttaccacctaactcgatgagtggaacagaattaatatttgcatttgataacaattgtc
gcaaaaagtctgcattctcattagaactacgagcttcagctagctcgtttaatctggcaattctttgatcctcaagtacgagaaattcatcatatga
gcacaattgcccattataatcaatcttgcagctcttgcgtttctcatcggacgcgtacaaaaaccacaaatcgtagtcaacttctgaccaagagcg
gtgcaagcgaattcgctgcaaaagagttgttggaacatattgaatatgctctaaaatatcgtatatttgagggtatatagcaacccactcaatcaa
ctcgtccatccccttgagcgttggtagagtgcaaaagctgaattttgcttgctaatattcactggcgtttaattgaaaaattgaattttcttccagttc
gttatttgtggtctccaccacttcaccacaaacttgtataaaaaaaaaaaaattgcttaaaaacgaaaagtttggatgattaaacttaataacaaga
agaaataattgggaaatattggttgatattttttttttcacctagaactttcgaactactaaaaaatagaagaaagcagcatgtcagaggaagctt
gggtaaaattattacatactactttgtttattaacaaataatgaaagattgaagaatattccgaaaaagaactcaaatttatcgaggaaatccaaa
agattgaaattgaggaagctgaagccgttttgaaaaaatacccattacccactctacaaaagaacgggattgctttagttaatcttcgtttgagttc
tatacgaactggttttggtggaaaaacgtaagacaatgcagtttatgaaagttggtgtgactaaatgtacgacaactatagggtcattgaattaga
gagagacccggcgttcagtaactcagatactttgccggcgaactctttctctcctggagatgtagtgagactgaaccagggaggagggaagtcag
atattcgagcttcaaaatccgttggtaaaaattctaaacaaacagacccttctacggaaggtgtcattacacgcgttcaagagaaaagcgtttcg
attgctcttaatttggaagaggatgctccaccgatggctgagggtttgtcaatgtatgctttttttttcgccgttgcagaccaatactgatcaatcaatt
tatagagtaaaacttgcgaatcgcataacgtacgaacgaatgaaagatacgatgattgagtggaggaaaaatctacctcaatatcggatgccat
tattctacacgttaacaggaaagaaagaagctaataccaaccttgcagaagatatgattaacgagttgactttttataataacgagctgaatgat
agtcaaaaaaaagcagttcgtttttctttaggtgtacaagaactagcacttatccatgggcctcctggaactggtaaaactcatacactggtagaa
gttattcgtcagcttgtagcaaaaggaaaaagagtgttggtttgtggtgcttcgaatttatctgttgacaatattgtggaccgaatcagtcattatgg
aataccgatggtgcgtttaggccatcctgcccgtctcttgcccagcgttttaaaccattctttggatgtcatatctagaagcggtgattttggagaag
ttgttaatggtattagcgaagacattgacacagagcttaacaaaatcagaaagactaagagcgggaaagaaagaagagaaatttataagcacg
tgagagaacttcgaaaagattatcgacgttatgaggcgcaaaacgtttcagggtgtttaagaaaaagtagtattgtcttttgcacattacatggtg
ccgggtctcgtcagttaagaaatgaaacgtttgatgtagtgatcattgatgaagcctcacaggctttagaagcacaatgttggataccactcttag
gagcgaaaaaagcgatcttggcaggagaccatatgcaactatcaccgaatgtgcaaacaaaaaaaccatatttatccatgtttgagcgactatt
gaagattcaaggagacaaagtaaaatgtttcttgaacgtccaatatcgaatgcatgagctcattaacaagtttccttcagatacattctatgatgg
caagcttattccagcacctgaggtagcgaagcgagtactaatggatttaccttatgttgaagaaactgaactaacggatgctcctatatatttttat
gatacggatggtaattatcaggaggatgatcctaaagatatgggtacctcattgtaccgtgatagtaaaagcaacaaatgggaagcacaaattg
tgtgccatcatgttactgaactactgggtgctggactaaaggaagaggacatggcgattgtgacgccgtacaatgctcaggttgctttaattcgaa
gcatgttaaagcttcgaggcatcgaaatcgagatgggcagtgtggatcgggtgcaagggcgagaaaaggaagccattattttctcactggtcag
aagcaatgacttccgtgaagttggttttctggccgaaaagaaacgattaaatgttgcaatcactcgaccaaagagacacttgtgcgtagttggag
atggaaatactgtgaaatggacgagtgactttttttcaaagatggatgaatttcctcgagggggaagcaatcgtattggaagtagaaccttccattc
ttgagtagataaacataaaattatctgagctacctttggtttcattaaatatcttttagcagattctactttggaacgatgggattcatatttttcattc
tttgaatctctcgcttgtccattccttctttatttatcatcttttaattaaaaaaatgacattatgaactaaaaatgatatagatgaatacaaaagcta
ctcaagttcatactccaagtagtagagtcaatgtaaatatataaaaaaaaaaaaaaaaaaaacaatattacatatttatatatattaaagaacat
tcatatagatgagcaattttattgacagaaccattttgtttatgtaagcaaacatgctccttttattcagataactgagcatttcccgtattcagcttc

cgtacttcattactgtgaatttctctacattccacttcacagaatattttcgattgtttttggattttttgaattcctttcaagattttgtggatttggaca
ctacgttactgttttcaaaaaacgcttgaatccgtggtctattggaaatttcgttgagcaagaagagacgcgatatttatagcagtttcagttttgat
ttattcttttcttttactttacatttagaaactgttgtacttttaacaggaaaaatgggatgtgcagatgtcaagcaattgctcttatgtactggagat
gtgaccaagaatttaaagtcagggatttggaatacttctcgttcagcatccaatttagaggtaagatgaaatttgaccttttgccaagaactaatgc
aaaagatgcctctacaacattttgacatttaaaggggtactaacatatttactagttaattaaggcattagaaaatggcaactttcaagaattaaa
ggatactggagacgtcgcagactctcacactaaaatatttgtcgccccaaatgatccccttcaaaacctagatggtaaagctgacgactatgaaa
taattggtatgtttatcattaaagactacgagcgttggatgtaatctaataactttactagcgaagctgttctttttggatggaaaaaatatcaatgt
tgagaataggcgtgagacgaccgatcaagtattccagtatctgaaccaattgttgggtatccaggaagtttccacacttgtggtctctttccccaac
gtggcattttcaaaaaaaaacttaaaggatactccagattcgcaggtatattcttccatagaccaaattcctaaagaagagatccagtcttggatc
gatacctggaaaatttttgaagaaaaagttcgccaggggaaaatagggactttaggaatcagtgaatttggtgttcctgagctggagcaattaat
tcccaatgtagagatttctcctgaatctactcaaatcaacactggaggtctgtgtgagcttcccttagacttgacaaagtatactgagcagaaacgt
ctaaaagttttcttacattcagaccctgccgcgttaattgatgaaaaggaaatcacggaagcaattcgtcacgcatgtcctaatttcccaatacga
gctcgtgcggattgggttgtgcgatatacgattctttctagaaaaacatctgtcattcacagaaaagggtatgtttgattattgttgcaaacactaat
ttcgattctcttttagttacttcgtgaaaggaagtttaatttgaatgaacgcacgtttttgagagtgcgtataatagctttatgtggtcgacatggata
ttttatgattgttaacttctataatatagctgattattaatgtacaaatttatcctggttttataattcctcttttgtttgtgaagtgtatttgattcagtct
agcgtttttcaatggactacaactattggcattcgtgttgataacgtaaaaaaaatcatgaattcattaaactacaatacaatccaatatcaaact
gaaccttctccataagcatattatatgaaatcaaaccgtaacaacgagaagattggtgtactgtcaggtgtctcattggtgcccaaaaaaattaaa
ctagtatacattttcttttaagagatacactagcatgtgtatatctcggttggctgcctcgttctttatgtatgaacggacgaggtacgcttgagcttg
acgggttgtgggttccagtaaacaaaccgacgaaattcgtttggagaatgaaacgtccaggaactatcaggggcccaactataattagcaggag
gatcaatgagttccaaagaaggtaccagtggaagtctggtagaatttggggaaggagaataaaaagtcattgtttggccttctttttcctctacat
gtctccaaacttgagcgacttcgtttcgaagctgcaatgaaggaagtctgtctcttttcgtcgaataccattcgtagaaaaagtttcgaaactgagg
atgtaaggcaattaggacaatccacagtccaatcggtaaataacggtcgatggaaattctcaaaaaagccaataaagagaccatgaccaaaaa
tgtaagaagcacggaggagatcgtagacggcgaaatatagtgataataattttgacgaaaatggaccaattggtcataagatgaactgtacgaa
gccatgaaattctgtagatcgcgcaaatttgtggtcacatcatgagtaaggcttatgggcatcattgtaacaaaaatcaagatcagaatggcact
aaatgatacgggatgcaagtataatagagtaatcgttaatcctaaacacacagatttcacaggatgtttccaggtaattgcttccacaagttcgtc
acggatgatgaagaaaatagcgactctagtagcgaaccgaatcgagttctgagataataaaagcggatttagctcattctgcatggtttaaacta
ttattctttctcaagcaaggaagaaatctcgatccttacaaggattgcaatttcctgtcaaatgcgtgagtttgtacgctagaactcgcttgtaattc
aggatagcaaacttgcttgaatagcgttagtaaacaacccaatttctatatgtatgacataaagcaagaaattttgagtatacaacattttcatcc
agaagattaaaggaataagaaaagaacatggaaacaagagggtaggaaccttaccctggtgcttgcatacggcacttttactctcaactccact
tgcagtatgcgtccatatgcacaagaccctccataagacaaggcaatagcaagtttgctaacgaaaagtttcctcatctttgttgtcattgtcattct
ctttacaagagaatgacaatagacgtatttcctacatggacgcttaccatgcagttttcgagagcgctctttttttaacgagatttggttatgttttg
taaacgatctgagagctcgtttagcgatacacagggacttttatatgaattagcaaagtaaaaggtaattcggtaattgcttcattaacaaaaac
gcgacgcgttaaaaacaatcgtgtcttagaaccaaatatttcctagttagagagaattgaaaaagaatgcactgaatttgtttaaaggtatcataa
ttcatcaaacaacatggaaaaaacaacttgaaaaaacagcatggatgagtatatagaatgtaaaaaaacttaaaacgaacggacgctataact
gcgggttaggtgtcttttgaaatacggcttttctaaaaaaaagctcttaaaataaactttttcttcattctccatttcaattcctttcatagaactaatc
cacagaagcattgtttttggaacagcattattttgtcatgataaacgagaaaataaacgagatctgagtttaagtgagaattcaaccattctttggc
cgcattttaatcgcacgattttttttgttgagttatttcgctatagcaaatttcgtatctatcgttctttcaattattcctcaggggtacaactttacaa
aagcataaataaaagggggggttatcagccgatatatttttgtttatgtcctataattctctttttctttttcttttaaagcgaaataaaactgcgtgg
gaacatgaagaccattgaaagcgagttaggtgatgaaagaatccagtaatgatcaattcttttttccagcggaactgaacaacttcttgaaccta
gcgaatattttttcttctttgtgtacttaccaccaaagaaaacgccttctctgccctataatcatcgacccataatttatctagctcgtttatcaatgc
tgcttaacccacggtagagccctacaataaatgactgtttacgtacgtcttagataaacgagatcccctgaattatgtttcgatttattgacggaatt
tttttttaaaaaagattttttttttataaaaaaaagaaaagaaaaagtgtagagtgaattgttttcacctatttattgcagattgattgttttactctct
tagacttcgtttgctcgttgagtaattacttacagaaagattcacatctgacatttgccacgagaagactagttatcgtaaccttcagtttgcttact
gcttttgcctaactcttctatctttaacaggtatttccgaagtaacagccattactgttaaaaaagtaaacaactccttgtcaaaaagacatttaag
gcatcttatttgtctatttttttaaacaaatttattggcttttcgtgatcgttcccctcctccctttacataaaagtctttttttttttttttttcgctaaactc
ttttgaattcggtttcattcttttcttgtcttgtcttgctttgcttgcttatcgctttcaattctgtccgtaattgaaaggttgaataaatgacaatcatgg
tggatttcaattctaaatactcagtcgaaaccgaattggttcgcgtcgaaggtaaccaagatcagtacaatgcttcttctgttcctatttaccaatct
gctacttttcgacagaataggttggacgaaatgggcaaattcgattacactcgttctggtaaccctacacgtaccgccttacaagttcacttggcca

aattaatgaaggctaagcattgctttgtcactagcaatggcatgtcggccttggacatgattctcaaaacctgcaaggctggatctcacatcgtcg
ctggtgacgatctttacggtggctctgatcgtttactgaacctcaacattgctacgtacggatttcatgtttcgaatgtggatacttgtgatctcgctg
cttttgaggccgttttgcgacctgatactgctctggttttaatcgaaagtccaacaaatcctctcattaaaatctgcgatgttcgtgctatcgtcaaga
ttgttcgcgaaaaggcgaaaaatgccgtgttggttatggataatactatgctttctcccatcctttgtaatcctttagatttcggctttgatattgttta
tgagtctgctaccaagttcctttctggtcatcatgatatgatgggaggtgctattgcaatgagaagtgatgaattagctaaggcagtcttctttaaca
tcaatgcttgtggtgctggcttggctccatttgaaagcttccttttacttcgtggtatcaaaaccatgggtatcagagtcgaacgcgcccagcaaaa
tgccattgagcttgcaaaatttttgaaatctaaggggttttctgtcaactttccgggattagatcctaactgtaagtctaccgtcatcttcaactctttt
gctcgtggcccaggtgctgtcatgtctattcatactggtagcgtagagctttccaagacccttgttgagagtacggagctttttgaaatatccgtttc
attcggttctgttgaatctttaattagtatgcctgcttacatgagtcatgcttccattaaaaaggaggttcgtgatcaacgtggtttacctgaagattt
gatccgtattagcgtcggtattgaagatattgaggatttaaaagctgattttgagaatgcaataaccattgccaagttcaaaaagttgtgaatcaa
tcacgtctagaccgtatactttgtaaaacaagtttcctgtcgtgtttgtttataattttcactcttaatttaattactttagaacatgtcgttaacattat
gttgtatatattatgtatatgactcctattatttttcctttttcctcttctagtttatcaaaatccttttcaaaatttagtgtaacattcaaacattttttaa
attaataatgtttatataccatctatgataaactaaaaagtgcatgatacttatggatatatttgcttcatcgcctcattttaaaagcaaattgcaag
cagaaagggagtattaaaataaataaaaagaaagttgcagaacagaactctgaaaaggtgcaataatgtaaacgtaaaggaacactcttccct
ttgtttttcctattcatgggtttcgtcagaccagaaggggagtggctaacgaaggatgaatctgaatagttaggaaatgtatcattttgttcatcag
gtagaatccattttgtaggctcactatgaagttctatatcacgaacattgccaaagaagtctgtaatcaaaatttcgtccaagcctttttgcgtatat
gtggattggacaccaggcgtagatcgaatttctttcggatcgtaagagtcaataagatcttgaatggtcaatttacttttgatatccacacgattttc
caaaaactctaaatttgaaaatgtaaagcggtcaatcactgccacgccaatgtctgagtctgcatgatgggaataagaagaagtgccaatttcac
tgctgccaattgctacaatatttggcgagtaaatcttcttatacaaagagttcgcctggcaagtatcgaccataaacagaatttcattatatcgctta
tgttggtgcatctgctccagggcatcagcaaaatcttcggagctgagctcatctgcatcttgaaatttgataaatccgtctcctccatgtcctgtcat
gtaaaccaaaatattagaatgctcatttgttagcaatcgtttgctagctggtgtattttcagatacacgttcagttaataatcgaacaaatgcctcca
cagtcacctcgtaacccttgtaatctatttctatatcttctccgtacaagtccatatcacgatcagcattatcgaaaacggttcccggaaaaaggttt
cgagaattgcaggcataatcatctgcaaccatgagaataatctggctgtctggaatccccagccgtttcacggaacgataaatacccaaaacatt
agctgcatgtctataattaaaccaaaatcgcgatgtagatattagtactgcccaattattcgtgtgcgagctttcggctcctgttgctacaaacaag
agacataggaatagaagaaagagtaaacgatacatgaatgcagagatattagaaaaacaacctttaaaaaaaaaataagcttaatttacacatg
agtttcgggacttcaactatagaaagccctgctgattcactatgcacaacaataacaatgcaagtcaatccaaacaaacctccttagtaagacga
aaggaagatgaaatgtatcaggacgttatgtcaacaaaaaggttcttttcctacccataggattttattgagtaagtgttgtgtgttcattacgaac
ttacacattgcccattaaaaccactttcagttattcactagaaagatcgtatcctattcataatcattgagatctattttgttaaataagagatggat
catttatctgaatttattgagtcctctgacgcgtcgaaaactaatcaacaagtatgaagttcaaattccttataggaaagaagaaacgaaaattct
aattcatattttagaagccttgtgataatgtattatccaatgaaccaacaaatatcattgaaactgaatattctgttgaagaatgcatgcgggctat
ccaaaaagctagtaaaccaaagcctatgtcagtgtgcaagcctgatgagctgacgtcgtgtgtaaatgaagactatgatgtttctgaagagccgt
attatatgcaacttactcggattcggcgcgaattggaggatttatgggatgaagcgaaatctaccagagaaaagcaagaagtagattctctatca
aacatactagataaattatggctggaaaagctgtctagatttcgaaagagctattctgtagatccagagtctgcttctatcccaaattccggcaca
gaaaatcctgaactttataaacagcctcctgtggagtctcttccggaaaataaacggaatgactataatgaaacttttattccgttaaaagtggca
gattttgaatctcgaatctcaatgctggagaaacgcgtaggtaatgcagaagagcccttaagcatgacaattgaaagttgtttgcagagattaca
agcatttgaaaagaatccattgatttttgaagaaaaaatttctacttgggaaacgattcgaaaccgtttgccaaggaccggtgacaatgttaatga
gcaagctggctataaaagtaatccttctacaccagcaaacttggctcctggtgagttattagatgaaaaagagtacagattactttcggaaattct
ctttgtccatctgcaggatatggaaaactatcaaactctaatgcctgctctgctaaaacgattaaaatctttgcattgcttgcatttggaaacagctg
aaacaatgaaccgctggcaaacccttgaaagcatgttagaatctacatctaattcgttaaatcaatggaatcagctactgaaacgcttggaaaca
tcttcatttcccgaacagagcatgaacgaaattcaaacgctttataaacgcatctctaaccttgaggaactttgcaaataaatggataagactata
attagctaacgataatgaactacagatctataaatgttgtatccaaaaatgaaagcagttgaatcgaaagctgatttgaaaaccctgcattgatct
tacaaaataccaggttattgtttaattcattctccttttcttccttttcgttttctaaaactgactgtcgaatccgaataaattactcagtgcaatgttat
aaattccttgttaatttcaaaaaaaaaaaaaacgacaagcataatctttagaaactaataattttcatctagtacctattaagaatcaaaggtatcg
tctttctttctgtttcactttgtcgcctaccggtggtagcctccttctacctactacataattattatccattttcaagctgtgttatggggaaacaatgc
gaatattaagatttctttgtacaaattcaagatttgaagcattatcccctttaactttggtccgacgtttacatgtccgctcttttgaggaactgttgta
caatgcagaagatcgcaagttcaagtcagccgttacaggtcccattcagaccttcctccgacctctgactccttcattctatctcttgaattacttaa
aacttcgtaataataacagtttaagcaagttgaaacctaccgagtttcgagtgattcttcgttctttcaagctgctcaacagtggaaatagattgac
tgagagagaaaaggaaacgactgtaaagatattagaattgattcttcatgatgctaatgaattgggaatacgactaaacattggagattttacac

atattctccgaattgctatgtatattggacacgagcccgttgctgataaagtgatcaaaatagtgaagaaggacgaattacgaccaaccgtggat
tatttaaatagtgttctaggaaattatggagggtgtaggtggtcaatgaataaattcattagtcctgcctggaaaggaactcccacacaagagccc
gtatcaactgcaatgctggaggcaatagacgttgattttccaagaaatgggttagcaccaaattcgacaacctatgactatcttattatcggacta
gcaagagatgggaaaattgaggaaatccgcgatctacttcaaagggtttggggtataagtcaaggaaaacctggagaacgtttggtcaataga
aataattctttgtatcctacttcacatactcttctttcggttgccagcgctttcggattccttggcgacataccaactgctgttgatttgtgcaaagagc
tagcaaaaacctacgagattcgcttttctgaattagcatggtgttacataatttactggtctactgttgctccaaaatttaagtctgccgctggaatt
accaataacagctatccagatatatattctcgaatgtattatctattgaacaaagatcgaatcccttatcctaaaatatatcaaaaggtaattgcat
tttataaaaaaattaagaagccaggtttactagaaagagtttgtcagagatggatgaagcacttgatgcattcacaaaaattactatcaccaaaa
gaaaaagaaagacagaaggagttgctttttcgagagatttctttcattataaataacaacaaaaaaggtaacgcttccagccagaatcggatac
gtgttaaatggatgaatttgttacatcggttacaggtctttttagaatcttaacgataaattttcatacagcgtttcaaaaagttatataaaaacatc
aagacaagtcaataatgggtatattctcatacattttcctccgtccgcttgtccctaaatcttccttccaagtcccattaaggctttctctaatgtatat
tctttttagtaattgatcttttgataaacttttcatggatttgatagtcgaatctttactatgtcgactgccaagttgatcttgtttgctgcttcctgctttt
ttagggcttttaggaaggacctcatgaacttttggaagatcatcatccgtaaaagaatcatcggttgcttgcagtaatgttcgtgaagaaggtaatt
ccttatgtgattttgaaaacaagtttgttgaaagaagagttggtggctttctagtaactgtagtttttttctctcccaacgtacatggatttaccactttc
ttctggatccaaagacagtcgcttggttttctgaactaaacgtcctaaatctgaatttgttcttccaaaaaaactgggaaatgtttgcatatgaatcgg
tttttatgaagcaggcggggacccatgctgtactctctttcaaaagtaacctccattgattcattagtgaagtattggcctcattatcatttacgttcg
tttcggtgcaccattcacaattcaaaaacttgtttggatagaaagtaatttgtttttctaatatcatgccggtagatttctgtcttcgtgtcttatgaga
cgccttatagtattttgagtctttagcagtagaatggagaacttcaaaaatgagtaaaggataagctatattatttacaaacttaatattcccatgt
aggtttatccattgaaatcgcctttgggcataagtatacaaagcttgaatatcaggtttttgagaccactcgacttgggaaaagtgagcttttaact
tatgttcggaagagatttctggatttgtatagtaatgctttactaaagggtctgggaacccttgctcgatagtgaagtttggccgtttatagccaaaa
gtgttgttcttatttgttcggatgcagttttgtaattgactcctccagctattcagactgtctgaagaaactaaagaatcaccaaagcctagcttagc
agctcctatcgctgtttgaagaccacaatgctctatacctttaaatggtagtctcctcctctcatcaagccgatcaatataataccagtatgattga
cgctgagttcttcagttatcttctccatttcgtaaatcctgactttaaaaaatggtttctttgcgtcagaaccatttcttataactttagtagctccaaa
agcgaaagcatcaacatcttccgtcaaaacataatcaatcaatcccagtctttgaagtttcgcacactcagcttcagcttctcctggagcattccat
acctgcattccaaaaagctgaatgagatgcttcattgaccggtaattccctgagtcatttgagacatctgatcgaatatggcgattccttttccaag
gaggtttgtttggaccatcgaatacaaatacaacgttaataccccattcaaggaaatgacaaagtctataaaatagcgtccttaatgctggattgg
taccaccttgactagcctgaatctgtaccgaccatatagctgcatcaacagcaatttttaggcagcgtttttcggtattccagcaagtcgtagagag
ctctaaaagatctataaacaagttagcgattaaaaattacttcgtataaatttgacatactcgtttcacgatagcaatctcgaaattcgtcccataa
cccaggaatgcccatgatttttatattttcgttttgtttattatatctgttctcttccttggcttactgcatttagggaatacgatatcctttttacatgga
tgtatatttagctatatattaggattaaggacctaaggaaaaatatctttggaaaaggattatgatgagtctaaaaaaacattgaaaataagtata
aggtaaaatataagcaatataggatcaaattagcatacattatattagaaagtaaacaaaaaataaaatagctagatactactcaattccttttg
attgacaattacaaccagttaaagggtgttttatgagaagtcaaaatgtatctaatctacatgcctataaaaggcagcattaaagaatgatgtatc
caaagattattgaaagggaaaaagaattaaataaataaacatatatgccaaattggtagaaagaggtgggaaagaaatgaaaccctaattta
gatgacagagtaaagaacatattaaggcaaaactataatgaaagaagttgatagcgagcggcaaaattaagcatacagtaggaacaaattaa
ggtaaaggagatcttggtgttcgctctcgactaaaattgaaagccaaatgatcagcaagaatatgactggaagtagtgcgaataggagcagctc
tcgattttgaaaagctagtcatttctagatcatcgtcaaaatctgcgatgtcttcgtaatcgccatgagcgttcacaatatatcgacccttcataccg
cgaactcgaagcaaaaaatttttgaaccttgtccttctcttaggattaagagccaacataaaggataaaaagccaaagatgaatattaaaagaa
gaacactcagagcagaaatatacccattaatattatcttggaaggaatctttaaaaggaattaatttcccaaatgcgtccaattgatagtttgcat
atttgggactttggagaaactgctgattggatgaatacaaaagaacttgaccaagtgtccaactaatttcggtccgatcaactttttcaaggctagt
aaagtgagaatggtatgccggcatcaagtcaagtccatccgtttgttggtcatcagcaccacttgtgtttgttttaggaaaaccgaatccttcatgc
agaaaatttaaaacccaagaagctttaaaacaaagcttctccagcttactttcgtctgtagatggagacaactggtgattataaagtcgtgaaag
catggattcccaatcagaagcacagtaatcaccaaccttttggtagaaattggggaaatgatagctgccacccatatcaaatacgtcgtttgtcgt
ataccagaattcagaaacgccgacaaactcgtgattaacaaaatcaatcggcggaatagatataccgtcaaaaccacatggatcctgtgaacaa
ggcgcctctttattaagaagggtatacgtatgttttaagcattgttctaaatctcccgtaccagtaaaagagtgtccatttttggaatactgtcttccc
cgaagggagcaaggttcatcgatagtctcagaaggagagttacgattcccctcaaccaaagcgttcagataccgattgtatgcttgacttgtccca
aaacctaaccaagtataaacaaagagaggatattctatttgttcaccattatccaagcctagatgaaccgtgctaacgctatcgccgtatgctttct
gcacttcaggagaaacctcaaaggcaatttgagcactggcaccgcccatgtccaaaaagcccgtagaagttgcagaagatttatcaagatttcc
aagcaaataattcgttgctaaccaaccgtacataccttcagctctcccatcaataacacgaaccattgatgcacaatcagggagcgaaaaattat

attctttttttatataatggcaagcgctatctaatattttcctttgtgttttgtgcggcaaaagtcgcataccagccgtagcacttaaaaaaacagga
gtttcttcgtgcttgtcatcaggaacgaccttttcagcataatcaagcagttttttcaaatgtttcttcccaacatgcttgggctgctcggcaaatgag
gaaatgcccggatgtacctttatggaccagtctccttcatctccatttccaggttcaatcagtggaagattagcctctctctttacaccatttctatct
ccaggaaacgcccatgagtagatttggagtcgtgatccgctagagccagcgtcaatgaaaatgccatagcttcgtgtcatctaaaatttttttcagt
gaattctctgtcaacccttaatcaattaatatatagtcttcttatttgataaaaatgaaggatcttttgatgattttaatcaattttccaggttggttgc
ttgtccgatggaaggtacctggtaagtcaacaatcatcgtaataaaatgtctttactatgaagtaaggtggaaactgacatggtaagatgggata
attttcgtatgagtgtgtaagtacataatagagttttcatgaatctttaataatcttgaactgaattttccactctaacagatcctggttgtctaacga
aagtatagttaaacacaagttgagctttttcatctcagaaattgttctatgaaggcttacctcgaatacgcttctataattgaactgtttattgacttg
acagaaagacgatcattctgaatacgagccagtagtagtttgtcaccgtttttgaacacaatatttatttacctctgccatcagtgaagtaacgggt
tgtattatattctttagtattatgaacttctacatcatttgggtaacagttcagtaatattatacccaatttggcataaacaaaaatgaccaaagaat
caattcttctgaaaaaaaataaacaataatgaaataaaccgctttagcatgcttactctagctgatagtgatgtcttgtaatacatgggttagtcct
tcgagaacaacacaacagtaactgcagtaggctgtaccagttcgtgaagacgtgctaagggaatagtttatgaagaaattggttaactgctttag
caagatctcgaaatctattattttattttcacaataccaaaatcaaaatcatactcgtacaaaaaatagaaaccaaaaacctttcctcattccttaa
cgtataaacatataacaaaaaacagaaagtcattgatgccaacccaacttcaagtagccaaagcccgaggggtgtgatttatgcgacaaaaat
caaaagtcaatggattttagagatggctgagacttttcattactgcttggtttggcttgaggctgttcttcgtctagtggaggaagaacccatggttc
aatagtaaatcgtctcttggacaccgtaaatgacaaatcaccagcctcgctggaattgtctgtatttgtaaaaggcaacggaagaggaagcttat
gaattcgaaacagtccttgaggttgagtagccaacccattcacagtctcagttatagggaagggatgcaaagagaagacaccagaggcaagag
actctagccaatagacttgttcacaatctctagaaaacaaagttgacgggacagagcacatacaaatgagatgcacagacgtagtacatttgat
gagagacgaaagcgagtggataaagcgaataaaatgctgtggatttgtagcacgaatagaataaaatgcaggggaaagcaaagatggtaaaa
cgagtcgacaaacggttcctggcttcagcgaggctaggaacttggtaagactttccaagacagggatgaaaggatttgagcctgtttcaaggga
gaaggtgaaacagacttcatcattttagagcttattgttaacctctttgtgaggtcgaaagcatgcgtgaagcctggagggattgcgtcgggggca
ggaactttcgttttcgaggtattctcataacgccatgcaattttcattcggttttgttctcctttattagaagaagaagatgagccggatgaaaagct
ttcacttttcgcggcacgctcattttcagcaggaagttgtcgaaaccatggttcgcccatcgaaggccccagccaaacggcatgatcttgtaaaag
cccttctgcagcaaaaaatttcaataaaactgaagaataatcagttgaatcttcttccacaaccagcagactccttaaaggaattcctcctaatag
ataatagtcaaaagaagtactaccagagctagttatccaccgtccgtccttaggagacaaacgaacgccttttggcaattctacaggagccgtttg
tacggcggtctttctcttaaatgacattttcgaatataacacccatgtaggggaagtctatcaactttacaaaggaacggtggaagagcacctgtg
ctagaaaaaaattataaatgaattctagattatttatcttattaaaaaaacaaaaaaacatagtaataaatttaccaaacttcattaaccggacac
atcagacaagttcaattgtcgaaacgaatttggcttgaatagcgtatgtacatgtttactatgaaaaaaaaccctcactcgtcgcttttgcaattatt
tgtcagtaaaagaccttacaaagggcatataaattccatccaccttttaagaccagtatctatccaaaaagaacccaaacagattaataaacatg
aaatgcgaatttacttttctttaaaggtacagtttaatgagttcttcactgacaccactctttgagtctatgattcctaaatcagtgattaatccagag
acaaactccgggggagtaacgtccagcgtagggttatttctaatttgctcttcattcataattaattcattgtgaatagcatctgatggcgtcggcac
tggatcgcgaatggggcccagtgtttctggagatggatcatcgaattcaagaatagatcgactgaaagggatatcatattgagaaagtggaaac
atacgaacaaatttatggctaaagaaaaggttagcgtaaagtgacattggcaactttttttgactaaaaacttacctttcagcaacggcataaaac
ggcttatgagcatgtttggcaaatactgcgagctggaaggtaccaatttgattaattaatccgccgttctctaccacgccttcggcacccacaagca
ccagatcaacgcgattcatagtgaaactcacagcagagtcaaggaccatgcaagttggaatacaagctttacggagtgtgtccgccatgaggca
accagatccgctgggtcgagattcggtcacaaaaaccttgaatcgaacatgacgcttagcagcagctaagaggatggctgctacaccacgagag
aatccgtgagttaaaatcacgtttccatcacgaattagaggataacccaggtttgcaatggtttgacggcaagcccgggcacgttgaataaacag
cttcccgttttccaccaagtgacgcttacattgttcaaaatcctacagagaatattgttaatacactatgaacccttctttgcgtcgtaaacatcaat
cgtaccccaacatcatgcaaagaacgtgtgacgaaacgttggaatatgtcgcaaccagcagacaaggaaatgttgttttctacgccggcctttaa
cgcattggatccattttgtaaaatgtccataaactctgaaattgtttttgctaataatgtatcagctttgctaccctatacagttctgttttaacaaacc
ttgtgaacgcgacagtaactgtacaagagcctcgatggcagctactggcatggtaatctcaggatcatcttctaagaacttcctataaatcttaac
gatgtcaaactctccttgtgaatgccgaacaatccctgtagatttcgattccatgttttgtaaatgttaacctgcttctacaccctattttcctttaaga
taaaacatgacagtgcagcgatgttgctattttttttagtgcttctgtaacaccactaccgaataatgtttaacagacgtgaaagagagttcatgata
tccgtgttgaagtacttaacaattcgttatttaagggaaatcaaaacaaacaaacaaaaaaaagtaaaggcagaaattaaaaataaatagatact
cttaagagagaaacaatgagcttttttttagtgactctaaaatttttggttatttaattacagtatgagtagcaatgttattaaggtgatgacaagact
taatgaaagtgtgagatagctaaaatagaaaactttcgcaaccagattgtttatagtttcgaagctacctcagaagatttttagcacaagcctatc
ttcccctacacttacttaatactttagaaactattaaaagaacctggttcccatcatatggcaaaccacagaaatccggtatcatccattagaacaa
taatcatcatattatagaggattcatcgtttttagaacaaaaatgctactaatttaaaagactcagaaataaaataaacatcacaactatgttgttt

gcatgcccgataagagttccagttaacgcctcaaagaaatagttgattcgttgaacaagaacatccctactgaagaaagcatatactgtagatcc
gggttactattttctttaacttgaagtatgaaaatcgagactttagattagtctagtcaagttattacttacatgctactttttttaaaattaaatttaa
tatctattcccctttatatttgttatacttgaaattgtcagccattttaatgtttgtttatatttacgagaatataggacaaacaaaacagaatgagaa
gactgtaaacataaatatattattttatatgcaacatagtcatcatagatagaaccaatatttaccaattcagagttcagaaatctgatagtctgtg
agtcatagcctccgagtatcatacctcttccttgtctgcctcccttaaggcttatttcaaatttcaactattaagcagcaacgcttaattcaccttttgg
acgctcctgtataattttccgttggggtatgcaatggcttgttgcttctatcagaaacaagtttttataaaagaaaaggaatacaacgacttatgga
gaaaacaagtaatatatcagctgcttactgatcgttttgccagggatgaagatgatttcgaacatccatctcaaggaagaaaatacttgggagga
acttggaggggtataataaaaaagcttgactatattcaatcacttggttgtacagcggtatggattagtcccatcgtcaaaaatatcgaaagtcaa
acaggttatggagaagcttaccatggctattgggcgcaggactttacagaattgaataatcattttggtagttctcaagaccttaaagatttagtca
acagccttcataaacgtggaatgctatgtatgattgatatcgttgtcaaccatatgggacatgctggaacgcaggcgattgactacacgcaattca
agccattcaaccaagctaacgatttccacccatggaagtacattcaggattataataatcaagaggaggtcgttaaaggatggcttggtgataat
tgtgtgaacttacctgacattaaaacagaaaagcccagtgtacgcaaattctttaaaaaatggattagtgatttgatcaacacttataagtttgac
ggtataagaatagacactgctaaacatgttgagaaatccttctatccctcattcattgaaacagcgaatgtcttcacaacaggcgagattttccat
ggtaatccaaaatacgcagggatttatcaaaattatatgccttcgatcataaactttcctttttactatcaattgcgcgaagccttttcatcgcccag
aaaacccatgcttgagtactatcaaaaaggtgtcttggatatcagacatagctttagagatactaccatactatgcaactttcttgaaaaccacga
tgtgcccagattcttttctcaaacgcaagataatgctgtaagtcatcagttaacctagtatttactcgcacaaaatatttaatatagtatgattaaca
ctctttagttggcgttaaatgctcttgctgccaccatcttttcggatggtatacctgtgatatactatggccaagaacagagattcagcggtggtaac
gatcctgataatcgagagggactttgggtaagtaagtacgacacttcaaactttgtgtttgagtgtatctcactatttattcgtcttcgacagcgctt
ggttgccaaatatccaaaattttgcactcaactctctcaaatggtatatgtggacaacagggtctacgcattttacaggccaggaattcttcttgttc
tatgtaacggcggaagaaaaatgagaaaaacaatacaaatagacatatcttctttctcttcacatacaaatcaagtattcgtggatgccttaaccc
aatctcgtatacaagccactgaaaagatattaaatttaaccgttaaatatgggcttccattggtatgcatcttcatgttctcacaaaaggttttctaa
cataaatagttgctagtacccgatgaaagtaaatgattaactcatgtcacaaatattcattgctggtttttttacctaagataggttttcgacgcaaa
tttctcaatcattgattcgcgtttatgcattaataaacaggaattatcgtccttcatcataactaccgtaggctcaatttcacttatctagaatagcaa
attaagactgaaattatatcatattcatgacaaaagctgtacttcatccagcagcattcgtactttctcatgagaattaagatgttcaacatgttcca
aaagtcgacacaatcgaattatctgggttgaagaccagtttcttgtgtcagtaatgatatctccatttacaaacggaatctcattggattcttcctct
tcaatactttcaaaatcggccgtcggcaagtatgagagttcaagctgtcgtatctttttttgcaagttcgtcaaattcatcaatattggaatttaagga
aaatatacttccaaaggcaccttttattgtaccaaaatataaacgggactctagtgttttaaccggataaaaagtttgaggcatcgtattcaaaca
gaatctggttaccctatcgtgaagtcggtattgtgattctgcaataagtcttgaatgaccatttcgtcgttccgagtgtgatatattatccaatcttaa
aatgtgtaatatcccgctatcggatgcaaagatgtatttatttttctcaaagcaaagagcattaattacacgttcgccaaggtagtatcttgtgtaca
ttgcgaaatttctgttttccagtcttaaaactgtaataaaaccaacaacgtccccgactagaatattttttttcttcgtcaataattatttgggatatca
aattgccaacacaaaattttttttcagttccatttttccatttataatatcaaaaaaaccaacgaactgtccaaaagaagcaatgaaacctctttc
atacactgctagacagctaacagcagcagcaacctggaaagtattaatacagttgagtatagtcagtttaggtataaaccggtacagcaacaac
tttccattcaacggaaataaatcatgtggttgatagttattaatagcaacaattaccgtttccttgtctatcctcagtatttcttcacatcggttttgca
ggggaaaatcatacttttcaagttgtttaccgctaaaaaaatctagtaatgacaacgaatacttttcggtaaaaccatccgtaccttcagagtgtg
gatggagatttatggttatcagtatttcgtctaacattactgcccgtaaaggaacctgactcaagggaaacttactcgcatacaaatgaggacttg
aatccagttctaaaaaggaaattccttttgaagagaccgcaacgatcccgttagtacctaattttatagaaaactggcacacatcgtaagaaaca
ggcatatttaacggcatatatccaagagtcccattaaatccgtaaaccatatgaggctgatcattattacaaattatgtaagttccaacatgcaca
ctcaaccgagagaatataattggggtagttcccagtttctttacctcagtaaaaatcaagtcatttttagataggacatatgaaactaaaaatccat
cgccgtaggaaacaaggaagagactcatttcagggtctaaatgttccaatataacatttctaggcaaggaggaaattttcgacttgaaaacgcgt
ccggatttagaatttatcataaagctataaattagaacctctttagaccaaaaaccaatagcaatttttgattcactggccatttctaggcagctaa
tttcactatcaaatttagttcgagatatttcagtgtctttctgaaaaataacaagttccaatccgcctatgacaattgcagagtaatcttgataacaa
gaggcatttgtaattttctccaaatgtattgcttttggtaagttcgtagaatcataaatacgtaaagatcttgaggttacctggataaattgattgtc
ggttcctgatagcgcgatcgttctttcctttattgacatctcaggaacgaattctaaatgtagaccttcttttagatcaaagacaaaagtttcatcga
caaagacaatgaacaatttatcaaccggatgcgaagagagaaccatgatgcggacgataccagaacattcataaaatccgagaactttcatag
acaaagcgtgtttatagtacaacaaagacgcattataaaatgtcccagcacatacaagcataccttgactctgatcgtttgagtatatatttatatc
atgaataggtcctaaacttggtaagacaaagatatcactaagatctcgaagagaaataagtttcgaacaatttccatgactaccaataaataaaa
gggaatcattgagcgagaccaaactcgttatgcaagtaacgggtgatatttccagcttttcaaaggtcaaattccatagtttcccggccaaaatgc
tcgaataaattgcataaaaattaccatttatatcggacacaatgaaatggttttcataaccttcaagagaaacccaatgattatacactgtgggcg

gtgactcaatggtaactattccaccacgataaaagcaatggatggatcttttatctaatactagtaatcctctttttccaaaagggagaattttgga
aggtaaaacagactcaaagaggcgaattccattctctaaatttttgactccacgtcgatgggaaaagctgttatagcagattcaataccttcact
atgtaaaacaactaaagtcggaatctttggatagtttaacagacaaatgtcatatataaggtgaactggaaaagagatggggaagccatcatca
atggaattaccagattttgtctctcttcagcggacaagacatttatgcatagcatgaaggcttcatctatacttaggaaaatcatgcgcattctctt
atccaccagacaatagcttttggcgaaacgacaccaaggaaatgatacccgatagcgtagaggaggatgatctacatttactttctgaaagtctt
cgtcccagtaaagagtaaaatacgtatcatcctgtaaaacaacaaaaagataatcccgactaagaccgggaggtcgataggtctgtaaatgcct
tactgcataccataagggaatagtagtaaaaatatctaagtcatttttatctgttatgcgataaatattaatgttcagattttgaacaaggatcaaa
gatggttcagaagaaaagaactggcttgatatcgctgtatcaattgttgtcgacctaaaagagtgaacgcaaagcgccatgcacaatttccatta
aatggagaaagaataatattcaaaaagattaacaacgatggaatatgaatccagatatgaatcgtttatgttcgaaaggacaattcacttctttc
aaaaaggataatgtgttacaggcatatagttattcagcgaaaatgctttaattacttaattctatgtacatgcaaggctttttcctttatttttggaaa
aattttcttattccctgttatacgatgaaaaaaaaagcaaatacaagggtttaggaaagaaaccataccaggtaggtgcatacaataaataaaa
atgaaatatacagttttttcggctaaaatattcaataaatatatgaaataaactaaaaaaaggaacaagagtttttttcttattgagggggggg
ggggacaaaaaacgctatatggacgtttggtaatacttttaattatggaattaatattgcgaaaggaggattttagtttaatgtttatttgcttacttt
ctgaatcctacctgcaaaagcgggaaaaaaagagtaataacactaaattcgaggaaaaaaagaatttgcttttcagagcagcctttttccatctcc
agtcattctctcacaattaaagaaaaaataaaaatcacataaatatcatctatgaaattcacgtatttccctgcaatatagaatgtactgttacttg
taagggtacactgattttgtgtcaaaaaaaattagaatctattgacgttttcattgaattttagaatatttttcgttttcattactaaatttaaataga
tattaaaaaaattggcaatttattttgcagtaatggaatttacagaaatgtgtattccttctgtcaatgactccaagaagcttcagtaaataggaaa
cttattacaaacaaatttttcaaaaaatcaggcaaaacaaacaaaagaaaattctatacacttagaaatccattccatattcgtcgtaaacatttt
cagagacttaggatctacaacttatggaggaacaaccaatgtactcagtacttttatattcattttgataacccattctcccgtccttcattgatgag
tatccgccgcatggtcatttttttttactcataacaattgggaagggaattgaaaataaaatttacaaaaaagtcaaaaactgcccacaccagga
atcgaacctgggtcgcatcggccacaacgatgagtcttaaccactggaccatgtgagcttttgttgataacatgatttatgttaaattaatatacac
gttacgtaataagacataggaatgtttagccaaaagatccatcgtaattttttttacagctttattctgtcaaatgagtgtcgtttctattgcttggtt
gcttttctgatgagttcgtttattggagataaaattgctcttaccggtataggaattaacgaatcgtaccgtacatcccaattgttctgcgctttcttat
tcttcgttactgcaggcttccatcatgtaaataccaactaaggaagaacgaaatagactttcaaaaaccaaggaaataggaagagattacgtga
aactgcaaattgattctgtcatctcaaatagcctgttttgggaccacatcctgaaggcttcttcatcttgaattttaagcttcactttcaagctccaag
gaaactaacctcgatttttcccaattactttataaaatgaagggaattaaaagcaaaatggtgagtaatttcacaagggaatttatgttgacaaag
tttaatagctctctaggggtaaaacgcaggactcacaaaagtctagcaaaaagaaagaaagtaaaaagtcctcatctcatgaatctttaaaatc
atcacctaaagaggtatgctttttttgtgctagatgccttagtagacctagcatttttattaagctaaagctaattaacacttatactgttatagtcacc
atcagttgaaccttccaacgctgtcatgggtgcacagaatgattatcttttggtccccaagcattcaagtaaaaaggtcccaattgatacgactccc
actccgagagacgagattttactggaaaatgttcgaactgtacgaaaacagcgatcttctttatatcacatatctgagaatcgaatgctagttcgtc
ttccttctttttcaggtatgttgttttactaaaaatcgtctatgagtgtttttacatactgcaatcgtatcggagcgataatactgaaaccctagaaac
ttgagatgttctcttctttcctttttctttttatcgaagaatatcgtagccgaaaacactacaacgactcttcttcttatggtccttgtttttactaactttt
cagatgtacccgtcactgcatggcattctttggctttggaaaaattagaacaatgctgtgttttgtttgattttaatgatccctccaccgatctttatg
gtaaggaagttaagcgcgaggctctacaagacttgatggatttggtttctgttcggaaagaagcaatagatgagtccttgtatccttccattgtaca
tatgttctccgttaatgtttttcgccctttaccgcctccttccaatcctcttggtgaagttgtggatttagaagaagacgaaccagcgctagaagtgg
catggcctcatttacatcttgtttatgactttttttttgaggtttttttgagtctccatctctcaacacttctattgccaaagtatatattaaccaaaaattt
attcgtaaactacttgttttgtttgatagtgaagatcctcgtgaaagggatttttttgaaaactattcttcataggatatacggaaagtttctctctttgc
gtgcttttattcgaaggtctattagtaatttgtttttacaatacatttacgaaaatgagcaatttagtggaattgcggaacttctcgaaattctgggca
gtattattaatggattcgcacttccactaaaagaagaacacaaggtattcttatcccgtgttctaattcctatgcacaaggcaaaatctcttgccctt
tattatccacagattgcatatggtattgtacaatttgtggaaaaagattcctccgtgaccgaagaggtaggtttttttgaaacatttatacttttcattt
actaaacaatgtgtaggtcatccttggtcttcttcgatactggcccaaggtgaatagcgggaaggaagttttatttttgaatgaattggaagatatc
attgaagtgatggagccttccgaattcctaaaaatacaaatgccgctatttcagaaattagcttcttgcatatctagtcagaactttcaggttgccg
agcgcgctttatattttttttaacaatgattattttgtgcatctggttgaagaaaatgttgatgttattttgcccattatatatccagccctctttgaaata
tcaaaaagccattggaacaggtaagaaaaattgtttctcgtcactgttcctcaaataactaacttttgcagggtaatcaattccatggtttgtaacg
ttttgaaattgtttatggatattaatccttctttatttgacgaagttgattcagaatattctgaattagtactcaaaagggaaaaggaagaacaaga
aaggaaagaaagatggcttattcttgagtctattgcgactgaaaatgcttctaaaatgaaagcatcaaaaaatacatctgtacagtcaactacgg
aacaacttaaaaaaatctctattgaaactccaggcgattaggccttttgcattattaataatgattctctatatgctacaacttatttcttttctcttct
atatctaattaatttgctgcaatctagcatctacttattattgccatatttttaaccttaatcagctctatttgtgttattattattttttctttttttcagtta

aaacgtattttagcttgtagcctttagtttctgtttacttcaagcagacggtagaatataataacctcccttaaacgatcgttagattcatatgtttac
atccaaggtgatagaaacctcaatatcaccaacattgagtcaggctgttcagcagctcctctagcttccgttgcattatatatattaagtagagggt
ccatgaattgaagatgtctgctttacaaatcattgcatccagtcaaaattccgtttctagtcacagaaaactgtgtaataaattattcgctttacgac
tgcaagaagcgtttttgcaggatgtttttcaatccataaatattatattaacaataaaaaagggaaatagcaatgcagatcgcgtccttcgatttat
tgtcacgttcattaattatcttcagcaaaaggatccgaagactgacattgctcaaccagttttaaagcacgctttaagaggtttggatgcgaaaga
taaaacagtgcgctttcgcagctgtcaggttgtggccagagtcatcaattgcgtgaaagaaatcgatgatgacatatacagtattcttcgtgaaaa
attactcgatcgtgtagtagacagagaatcgtttgtacgcctggaagccgtcattgccctctctcgtcttcaagaggatactgaagatgaggagaa
tgatgttcgtaacatgctattatttttattacagaatgatcctagtcctgatgttcgaagagctgttttgctgaatattgaagtgagcaaagttactct
accgtttattcttgaacgtgccagagacgtggatgcgaccaatcgaaaatgtgtctattctaaagtacttcctaaaatcggtgactttcgatacctc
agtatcaaaaagcgagttcgtattctgaaatggggtatgaatgatagagatgatagtgtccaacaagcagcgtccaatatgttagcatatcaatg
gatagagagcgcagataataatttgttagaattgctggagcgtttggacgtcgtccaaaattcggaagttgctaatttagcaataaaatgctttttt
acaactcgcctcgagacactcactgaaattgaattccccgatcagttttggttggagttgacggcagagtctgctctccttgctagatcatttagcg
aagtttgtgctgagaagaattttggagaacttgcagaaaaaatacctgaagtaagtcaattgacttatttcattgaaaaacaatacgtcgctctac
gtgatagatctacttatgaggaggcttcttttatcattgaacaacttttgctaattgccttaaaccaagatatggctgatgaaattggaagacgtaa
actattaaagagtttaactaacagtctttccacttcgttgctttctgaaaccttgatatctcttcatgtggaattattgaaaaaattatgttctactgaa
accgatttttgctctttgctggtagaaattattacggacatattcgaacaaggacatgctgaaaccgcgaaggaatctgaactccaacaggctcca
ccaaagtctccccaggaaaaagaacctgaagctgcttcatctcccgttccttcaaacatggaagaagatgaagctaacgaagaaaccgaagaat
ataaggaagcatttagtgaactaagatgcctttcttatgtccaagccttgtttgaaaatatatcttccccactaaatgaaaacttatatgtcattgat
ttactaaaatctttaattatacctgctgtaaaatctcatgatttgccaattcgcgaaaaaggtttagaatgtttgagtcttgtctgccttttaaatagc
gatttagcttatgaaaatgttcctttatatttacattgcttggaaaaaggaacagataatttaaagattattgcccttcgaaccttgaccgatatcttt
cttcaacacgggaaggatagattctccacatacgatgagcaattctcgtcgttgttgcttgaaactcttttcaacatcgatgaggctgagttgcaaa
cccttagtgctgaagcaattgcgaaacttttagtaattgttcattatcgggatgaactagtacgtgaaagtagtttccataataataatactaactat
gaacagtttcttaagcctttaataatccaatattttgagccaaatactgttgagaactttgctttacgtcaggtgttaggttatttctttccagtttatg
tatatggatctcctgaaaaccagtggaaagtcgcagaggtattagattctataatgcttattctaacaattaaataggtattttgcgacacgttatt
gaatttaatggaagtataccgcgatatagatgaggatgatcttcaattatcgacttcccaaatttctttgcaaatgcttgattggactgataacgaa
aagcttttcgagaagaaagtcaatagtgagtcttacacattaaataataagattcacttacacttggcgaataatatctttgaatgcttgcctcgtg
cacctgaaggtaagtagttggtatttttggcttttactaaacgtctgtaggaaaagataggaaactcatggtttctttgctcggaaaacttaatatcc
ctgacacattgccttctgacgactttagcaaatcgattaggaaattacaaacatttgagaatgtaagtaaaacttttttattttacaagaccagatat
taatttcttagcatggcttaacgttagactctactagtatgaatattctaaaaaagtttgaaacaactttaagaaataacgaagcagcagcacttg
aaagggaagaacaggaagcaaagagtgacgcagaacacccagaggtggacgaaagtaaagaaagtgagaacaaagaagaggagcctcttg
cttataacgatttaaattctgaaacaacaaatcaagatcactcaatgacagttgattctgatgaagaggtgtacgtcaaagaagaagaggacta
atgcgcggtcactcaattattgttggaacattcctatagtgtttgcataaactaaaatacgctcgctctttctatttattgccaggttagatttttggtt
actgcttataaaatgcactgctaatattgtttatgtatagaattagaaagtaaaagtatagatattcttaggtctaagaaaagaaacatagtttacc
gttgatattcatttaattaaaacaagtaaagatgccaaaagagtaaattactaaagtacaacagaaacataattagcgtctccgaggtggctttgt
tgcgccttgcaattgattcatcatgcctttaagatcaaatcctccgggttcaccaccacttcccatcaacatgttcgccattttagatatgtcaccaa
agttcatcgaaccagggttaaagtcgggcataccagcaccacccatgcccattgattgtagacgcttttgcatagctgccatttggcgggggtcctt
cttgagaccggcagccggatttccaccgagcttgcccaaaataccatcttttcctcccatttttttttgccatttgagcaaaaatacgaacttgagag
attgtttcttcgacttcaaggacgctggtaccactaccctttgctactcgcagaacacgagagggctgctctataaaagcagaaccatcagaatca
agttcttgttcggtcattgaatcaacgatataaagcattcttttcatacgtatagctccttcttcatcattcatgccttgcatcatattgctcataccgg
gaatcatactggccattttactcaatggtccgagtttcataatattaccaagttggtctcgaaaatcgcgaacagtaaattttccttgctctagatttt
tgaccatattctttttatctaaatttaaagattggacgtgttccatgagaccttccatatcacccattcctaaaagcttggaaataaaggatcggggc
gagaatttctccaagtcattaatgtgttcaccagtaccaataaagacaataggcgtattggtagctgccaccgcagacaaagcaccaccaccttt
agcatggccgtctaattttgtaatgataacagccccaaagttggcactttccttaaaagctttagcctgaggttcggcagcctgaccaatactagca
tcaagaatcataatagtctgatccggttgaatagcttcggagatctcaaccatttcagcaaagagctctttttcttgttgatgcctaccggaagtatc
gacgatgataacgtcaaatcgatcttttgtaaatttctctacaccttcttgagcaataacaacggggtctgtctcagtgtagctaccataaaagggt
acacgagccttaatggcattttgttttaattgatcaaaagcaccagcacggaatgtatccgcagcaactaagcaagacttcaagcctcttctttgat
aatgtaaagctagcttggtacatgtagtagtcttaccactaccttgaagaccaaccatcataattaccgatggctttcctttcttaggtgtgaaagct
tgcacttggggatcaacgagactgcaaagctcgtcaaatacagtttttttgaactatccgctttccattgacgccctgaggaaggttgctaatattaa

ttttctttttatgttggtccttaacgtttggacgagtctaacattgacgtctgtttccaataaagcagtgcagatatttttaagagagtatcaacaa
gctaatttagttagttattacaaggaaataaagaatgataaagagcagtgctcaactgatgattaatgtcaaaaaagcaattaaatgagatactg
aaaagtagtcaaatcaagataattgtatgctgcaaagcatatattctaacaagtcgttttgttcacaagagaagtatactccctgacattttgtttc
caacaagctcaacacagtcttcccacttgtacaagacaacattcgccaaaaaaaagaaaaacaacaatgagaaagtttaacaataatcctaga
gcttgtctactccactccaaccaaactacttacctgttcattgacagatgaagccttggagaaatcccccaaggcagaatttaaacgacgccctaa
atctgcgaataccattttgtcaatttaaatctaacttcggtgatttaaagagacaaaaaggagctgaattgtcgttttgagtttttggtagttggcaa
gttacgctaggccttgacatcgtattgttgacatcgtacaaagaacgtcgcatatgaattttcaaaccacaggcacctccataagaacattctaca
ccgtaaaacaactatacatgaatccatagaaccatacttctaatttttcttttcatcctgtttttttatatagtgagattgacaaagagcacgataat
aagcttaacgggactaagtgtaacgattaatcctacagtatctttttgttgcgcaactggacatacgatccttagtaaacatgatgtccgtatgagg
aaaactccacagacagcggtacgtaggaaaaccttgaaagggaaattgcagaccacactacaagaaaggcatactagtccctcgctcatttcaa
atgacagagtactgagtttgatctagcaaaccctttataagtcggaaatctttcttttttttccactttacagagttagaagaagacggccttttgat
tgaaacattctccatatggatttgtccattgctacacgaaaaggagcaagatattgtttgtttactctttagtgaaaccgaaagagctgaagcaaa
caaagctattagcatgcagctttacgttttcttattaggtggttaacaaatgcgcaccatggtgaagaatacgatttcacgactatggtaaaacac
ggtaagttccaaacaaacgctttttcaacgcgtttttacctaaacaacatcggtttcttaagtgccatagattgaatgcatagaaggtgcgtttca
aggattgacgtctattgattgttcctgaaaagtagagctcgcgaatcgaagaaccactgcattgaatcgcaccattttggaacgatgagtgagtat
caggaactgatggattctacgattgtcaatgaaagtgattccaaatttgagatatttattccatccaatggctccaaaaaatggatttccaaagag
gaatgtcctggatttttggaagatgcttggcttttgaaggcagcaagtcataaacgcaagctagaccactcgctcgacgctactttggatgcagag
caacctcctcttaaacaacacgaggtaaattttcttcatcaaggatttgacccagcagaaaatgactttctgacgcaagatgtgggcgagtatcca
tcaacgcagcaactgttccctattaacgaatcgtccaactcggattcgctgtcattacctttcccaggattttccaaaacaaactcttccgtttttgat
gtcggactccctatgtcttccatccaacgcagaatgatgcatcgccttgtccagtattttgctttctgcatcgaccacttttgcacgggtccaagtgat
acacgtatacaggaaaagattcgcctgtttttgcaatcggctcataaaatcgccaagcatccttggctttacgaaaaagaggttcgaggtgattca
gacatgttgtctgctaaaccagatgagggtaagcctatttccgtgccgaacatgtcagaccatgccgaagcctcctctaagttcatttttatcggga
aattattagaatcgatttcatcaaagctttggatacctctattctgctttgtttcaaacgatgttcttgctactttactcactacatggctgtcttcaac
atctctttcggtaaaatcttggtcaccagagacacaaatgcaagataataaagatgtttcattatgggtatgctcttcaagacacgctccctccaca
gctactctgtttaagtctgatgtccgttttggtgctgtaattttctacgatatggatgcctatatgggtgtcactagcactctctcttcatttccttgtcc
aattcttcgacttcttcatatcaattctgtggaacatgtcatcagaagcttccaatcttcttataatgcaagctttcttgtcaacattgtcggcgtattg
gctgctctcaattcttctactgaagaaaacgcccaagtcgctgacctcaatgtgttttcgaaccgtgccgaaagtagcggcgctattgttgacgcca
gtgcaaatcattcacaccttttcgacgttgcaaatgaagccgcagataaggtagctgagtggattgttaatgatagcgcacctaactcgtggccgt
tacagcctttagtagacctcgcgaacctagctgttgccgaacccaaaccttctcaagggtctccgtcccgtatcgatgatactatcttaaagtcttcg
gacgataaccttttcacccccaaatatctcaaacgccttctaaaggatctcaggaagtagcttcgtcaatctcgaaaccgacgattcaaacatct
caatcacagcaagatccagacgcctctgattttggaaacaattacgatttacagggagccgctgttcaatatctgcagagaagattacgaatggtt
gaagatgagttacatgaagccgtcaatgtgaaaaacataaagcaaagtcatgctgatgaattggaacaacaaatagctaaacttacagataatt
tacaagaatatcgagaacaagtgcgaggacttaaaattgaacttgaaaaatcccagaaaaatctcgaagcaggaaagaagaatgataccgaa
aggttaaaagaagttgccgatttaaagcaacaaattacaaatttgtctaagcagttgaattttggtttcaagtttattcaagatatgcaagaagac
gaacttcgtgctcgactcctagaatccaatgaacagaaatacaaacttggccagcttaataaaacccaaaaagaagatttggattttattacgca
gcaatatcaaaatgcctccgtctctgctagcgaacaaactaaggaagttgctagactacaagcggaaaacgaacgacttaaaatgattaatgga
aaagctatggaagaagtcaaatcctacagtgacgccagagctgatgctttacttgctaaagtttcatcccttgaagagtctctcgctattttaaaga
aaaagaccttaccacctctttaaaaaacgattaaaagattgagttgatcaattaagatcctcagctatttagttaatgtaagttttaatgattatcat
ctagacgattattgttagagaagtctaacccattaataatatacgtttcacctatgtatatctcaacatagtgaatatggatatctgctttgaagaca
tttggacgtagagtgaatgtgaagtaaaaatcttgattgcaaagaataaacattattattgtcatactttagcctttcatttgtatcgcccgatctag
aattgtaggctttctaatttctttttagctctatccagagccaaggtaccgttacactgttgaagaatctcgaaaacaacatcgtcatcatttaaat
acacaacagagtgcatatcaaaatcaatgatagcgaactttattacagagaaaatattgtccgggtacatggttcgaaaacgcagccgctctcta
gtaatagcaaatctctctcccggctttatcacaaatttgaaaggaatgctatgcattcgagcgatatctttatggaaatgatagacaactattactc
tctcatcgtcctgtaaattaatttcttcttcgggtataacctctccaataatcctagcaaaagggttcaatgatcttatcattgttcgcatattaatgtc
ccgataccatttgtattccaaaacttcataaaagcgtaccttctggttaggcaattctagttctggatacttttcacagacagcattaaatacatcgg
aaacattcccaattttataaatgtaaacatcaattagctcggcatttgcaagtccttcaaaaagccaagtaacacgtaaaagtcgctttttatctaa
atcagaaagactaacatccataatctcgtaaaaaagaatattagatacggtatcttcgtcagaactatgaattatgtcgtaaagagtgatgtttgg
attgtttgggataaccatattagctgagtatgtaagaggactgcatgtcgtaagccgaatatggtccgcatgtgtacctaatttttcacctaattccg

cagaaagctctactagtttcatgcgtcgatcaagcacaagttcaaactcaagaatgctctcttgatcaataaaccttggtcgaaaagtaataagta
cacggtttgccatgaaatcatataactgaacggccgttggaaatcttttgtctttccaatccttcgtttccgaaactgctttctcgaaacaaataata
tcacctgttccgagttcaccctcactaaaggttttattcaaccttaagtattcaatataacctggtttaatttcctcatataagtacaacggggtatct
ttcaataagccaacgcgctcacacaattccgggcaaatggaatcaatcctgtcatatttattgacaaaaatcgttccactaccaattatttcttgac
tttctctgtcaaaatatttaataaataagaatgcatcatgagaatcaaaggaaacattaattagagtatcgactgtctgattttcaggtaatatttcc
aagtataaacgaaggatctcgccttgcgagttccatttatccttagcctcttcaattgaaagggtcaactcatttacaggttcttccacacgtacagt
aaaattttgtcggcgaactacataccaaaggcgtaagcgcttcgcagggatatcaattttttcggatatcattggtacaagatccgaaaacttcaa
ttcttttttattctaaagtgaagcatagcttcctcatcaactgtattaccgaaatttacaagatcaaattcgtgatgattagcgaaatagtctggagt
aataatttcaactttggtatagagatggctttctaaacgttcctttcgctgttgatcgaccaatcgtatattttcatcgagtacctcttttaagtggtta
ggaatttcatccttttgaacaggagccattaactcatcaattttgtcctttcgcaaatacaaaagcatataagcactcataaatcttttaagttttac
agcagatcgaaaaggtggatgaacaatataatcaccaccgtaattttcttctaaaacttccttcaaggtcgcaggcgttactctagtatcatcaaa
tttgtaccacccaccgtctttttggggttttaggagagcataataatgaccattatcgagatcgccagaatgaacaagtactccatgtagaacgtac
tcacaattttgatcttgatcagcttcaggagttaagaaggcttttgcattaaattcaagtggaaattcgtaacgatcattaatctttatcatcatatct
ctttcaaagtcatactcaaatctttttagttgaagatgtaagatgggaggaaatgattcaaaaataacacccttttttgcttcttggaaaccatacgt
atcagcgtaatagcagttatctccatctaaagtctcaacttggatgtagtcacggaacgaagcctctagatttttcattcctttaacgtttaactgga
tatcccagaactcttcggtgcgggcagactcataattaacgttcatgcaagcaacgtaacttttcatctttccagaaaaaagattctttagtgcattt
tccatggtgccttccatgcttcgctccaaattatcttgtaaaactcggttaaattcctgaacatcatgctgcatgaaagaatctaaagaatcccaac
caaatgattttgtcaaatcagttgtagggacaggttcattcataaattgtaaattataaaaacagcgttgaagagcatatgcaataccgtttttga
ttcctctctttctgtggggacttggtaaacgattctacggaaagcaccaataatgtacagagactgtaacaaagaattcatataacaagtagcgcc
ttggtttttcaagccaacgtaacctgtcattgattttgagtcataatgattaaaagagtgccatagtacgccggtgggatcatcgagaattcgaaca
taagcgtttatgcgtaatgctccatcttcgacaatggggggtgcaacgggagattcgcgcgaaagaagccgttttgtttcagtaaactgggtaaat
ccccaatcggcaatttccggagagtagcgacaatgcgcagcacttgctacgcgtaaagaaggattgttcaggttggagataatgagtgcgaact
ggcagcacacgccatagtcttgtttctccttttcttctttaaagggtatatattctaagaaaagggacgtgtatccagaagactgcgaaatgccttgt
gggaagtaaacgatgcgaaattggcgtccaccaacttcaaatatacgacttgaagcgcgactatcaagttcatgccaatttttatttcccaagta
taacttccataagcaacatcatcataatccggaagagcaagcgtcagttcttctttcacggtttcttcttcttcttgagttagatgatataaacgaaa
acacgggttaaaaaacattctcatacctatctgcgagtgccgatttaaactagagacggatccaattgtctttgtatctaaatcgacaagaggagt
ctcctgtgatttcaattcaatttcaccattggcctgttttcatcaactcgttagtaaacattcagtcattgcaaacaaagcaacataccatgagaccg
tttatagttctcctttttttcaattaaatcttctcataaaatagcaggtaaggctaattcgttacgaatggcctccgagaaaaatggctctggtcacgg
cttttagagctagacaaaccgctcttcagatggaatgggtggtcgaggccaccgtaataattcgactagttgtgcagtatactactttattcaacta
tagagaggaacaacgtatgcgtgctaaagtatataatgaagcagatatggcttataatacaaaatgattgtttacatgcaaaatcatactgaaaa
taagagtatccatggtgttcattgcatgaaatagttttattgaacttccttacacaaatgtagtctcaattatcttcaacaagagtgggagggcgga
ctctgaagacgcgatgtatttcttaccagagcctatatcaaccagcaactacgaaattgttcaatttgagcaaaatgtcactgttatcgagtgaatt
ggctaggtaaatgatattattactttctgtacgtttgttaacatttgtaggattttacctaatcctgagtccgacgaggaagaggaagatatatatgt
ccctgaagaatcaccaaaaaaagaaagccaggatgtcattcgtaaatccgttcctccttacttacatagaaagggatggattccatcctctttaga
agacttcggcgacggtggagcgtatgtgtgaatatgtggtaattccaggactaatacagaaattaggtatccagagattaatgtagctcaatatcc
attagatatgggaaggaaaccgatggtacgtatattgtgaggagaactgtaccaaaatcgtttgctaattatgtatatagagtacatcttcagggc
gctcacttgctcttcaagtcacctcttcaggaggtgttgattacggtgctattgcgcgtcaaggacatgaacatggcgagcttgtacagtcgagtttt
caggacctaattcctttgagagctcgtatcggtgtgggtgaaatctcagtcgaaaaacccccgaagaagaaaagcaactggttgcaaagaaaa
ctaaagctgctcttgaaaagattgtaactggtaaaatctcgcagtctcaacctaaaagtgctgtcgtgcagaaacccgatgatcctgtttatattcg
ttatacgccaagtaatcagatgggccaagctcctagcaagcagaggatcattaaacttgttacagaagagcaagatcccatggagccaccaaag
ttccgccacaaaaaagttccacgtggtcctccttctcctcctgtgccagtattgcaatctccgcctcgtaaagtgactgcaaaagaacaacaggaa
tggcagattcctccgagtatatccaactggaagaaccccaaaggctataccattcctttggataaacgtctggctgctgatggacgtggtttaaac
gatgtggaaatcagcgatggttttgcaaagttttctgaagctctgtttacagcggaacgtcaagctcgtgaagaagtacgttatcgagctataatg
cgtcaaaaaatggcagaaaaagagaaacaagagaaagaacagcggttatttatgcttgcgcagaaggctagagaagagcggacgggtcatgt
tgctgaatatcagtcccgccctcgctctcctagcgcgtctggcgaaagatatccatccgagtctccggcaaacagtgaatcagaaatccctgaaa
acgaggaagaggatgaaggactgcgcagacgtgaggaaattcgtcatgaaagaagacgccaatatgagaaagagcttcgacttaatagaatg
ggtgctgagaaaagagcccagcttaccggtgctgaccgtcctcgtgatgtatctgaaagagtagcacttggtttatcaaaaccttctatgtctagtg
aaacaatgattgatagtagattattcaaccaagcttcaggattagggtcaggatttcaggacgaagactcttacaatgtgtacgacaagccttgg

agggctgctccagcatccactctttatagacctggtgctagcttgggccggcatgttgatgcttctgcagaattagaacgaataacttcagaaaatc
gttatgatgtgctaggatcagttcacaaaaagtttaaaggaatggatgaaacatctcaggtatgtataaatagatgcttttagatgatacttgtttg
ctaacattacagaatgcatccggtcctgtagtttttgaaaaggatgttgatccttttggcgtaaataactttttggataccgtctcctcttcgaaaaa
gtgaagcttgctattgaagcttgttaatgatttctgtataatatgctatgactagtggactaatgggctttatcgagcatttcatgataaatcgtggtc
tttttgattaatttacagagaattatttaaagaaataaaattaaaaggattacaagggtatacagatattctaaggctgtgaaaaatttcaagtcat
tctagactgattactttctcggtaggatgtaaagtatcagaaacgactaaagataataagttttgcacttttccatttattttatttcacttatgggat
agtgacctagagcagttggttcccttaacttatggaatgggacacccgcttgtttgtttacatatgcttgaaatttactgtttacagagaactgaact
tagccattgccagcaatcatcaaccaaaatccaactgaaaaaggattcattattatttcgtttactgataaaatttcataagtaaaatgccacttgt
agaaaaacctcaattgctctacaatgaaacgagtttacctccaaacaggtaagtcaaacttgtaaccaaaagatcattttactaagtcattacaa
cacgttagagagattttcataaaaaagaaagaatgctctttcactgttatgctctgcggtagctcaggaaccggcaaaactactttctgcaatact
ctgttttctagcttggtaaagcctcccaaaacagacgataacttcggaaaggagcacaagcagaaggaagttcaaatagagaagacgaaagct
ataatcgaagaagatgactttcgactcaacctgacggttcttgacaccgtgggcttcggggacttcataaacaataaggattgttgggatgtagta
actgaatacctagatgaccagcacgagaaatacctattgcatgatcagcagtcattaagaatccagagaaaggacagcagggttcatgtttgtct
gtatttcattaaaccagtttcttatggtatgcttccattagatgtcgtagcgatgaaaaaactatcgaagcatgtaaacattatccccgtcatcgcca
aggctgactctttaaccaaaaatgaattggacagccttaaaggaaaaataaacaggatcttacaggctcaaaaaattgatttattcttcccttcgg
ctgtgtactctgctgatgaaatagccagagatctttcacaagcctgcccattcgcaattgttgcaaccacggaagctgcaaaccgtaaaaacgaa
gggaagattggtcgaacttatccttggggaatgtcagaaattgagaataatgatcactgcgacttcttaaagctgaaggacctaattataaacaa
tcatatgcttgaactggtacaatccacggaaaaagtgatttatgaacgctatcgccaggagcaattaacaaaccgaaaaaatggtatcccaaag
cttaataaagctcactatgaacgcttaaacgaggaaaaagcatttatccagaagaaagttgaagaaatgtcgatggagctcgagaatactttta
aggagagagaagaaaaacttacggttgttcgtgatgacttgaatgaggagcttgctgagtaccatgacaaagtgcgcgttcttgaaactcaacta
gatgcacttcgaaattataagggatcgaacctaaagaaataagtgagagtttttagaaaattcctaaggatattatcagtcaaatatcctatcctt
ctggttagacacctctgatgtttttacagaatccgacgctctaagagcacaatcacaagagtatagcagtaaatttgagtttttatcaagtaattctt
tcagtttgtatttcatattaattttcgcactattaagtttccgagaagtacctaaacatacaactctatcatgtcgaacaaaagaaacggtgtgaaa
tatatagctctctttttcaatctaaaattttcttcttcttttatctctatttcttccttgtggataactttataatccgagcacccttgagaactaagtag
attatgtactttgactaggaccagatttttcttttgcaaatctatcggatctccgaggacattaagtaataacggtaataaaaatcgaaggctttca
aacaagaatccagaatccaaataaatagcacatatcagtgattcaaaaatatccgctacaaacttgggagggtcaatatcaagccaaaagtag
gggacgttatttaatttggcttcttcaatcatctcctggtactcataaatcaaaccaaacatttcggaattgtcatattgaagatatttgtgcaagcc
aagtgttaaagccaagtacgcaagactatggttacatacgaagaaggatttatagttggttagttcaccagcagttgaattaggatatctatgaa
aaagataccgaacaactataaactccagaactgcatcgcctaaaaattccaattgttgatatgttcctgatacctcctgtgaagctaacatggttg
ggtgtataaaggccaaatgtagaaatgctttattggtgaagctgtaacccgtagtatcctttatatatttatcatagggaaacactgttcgcgagac
taagctcgtataaaaatcgatgtctaaaaacttattccaatcagaccatttcttcacgtccagtaacccaaggtccaaacctctacagtaagtaag
tcccgtgtccaatccaccgtccaaggtacaagccccaatagtagattcaaccaagtctgccatccttttttttggtaactgctgaaacaatggatat
gtactttcgtgcttgttttctctcaaaaagccgaacggacaccacttgcgaataaccataggataggataaagcatagttcggtatgtgtatatctt
tcgcacgcatatataagttgtagttgctaataatttgctttcgatggacatgtaactggtattcttgatcataagtatactttaggaaaacagtagaa
gatgcacaaacctttaaaaaacaatcccccaaaaattctagacggtcgtaattttcacccattgaagcattctcagttgctaaagcttgcaccata
gttttcaatgaacagttcaagttatgttcgttcataaattcagacgttagaagaagagcttccatgtgatagagtatagaaggaagaagtaaggct
gattcttttaaacactttgacaaatcagtaagatataattgatgtaaacgtagttcttttattgaatttggctttgcagttatcatagattgtttttatc
caatgaataattaagtctgaatggcatccttttgcaaatcgcagtatgttcttgcctatcctggctaactagagcttcaaaggaatagagctcgaaa
ctgttttctggatcgtgcattaatagaatctggggtttaagcatagtagcgtccttcacaacctgatggttacaagaaagatttcgaattgctgacc
aatcgataaggtttttcagaataccattgtctgagcattccatgtttaaaggagctatccaatatgatcgagtggaatcggttgtctcaagtgtaga
agacaaaccaagctctaatagccgtttggtagagtattcaaggacattttgcagctcatcgttcatgatataagaggtttctaataattcaggctta
actgtgacttcacaaaaattagagcgaagtcgaagaatatcgaactgaggcattcgttgtgactggacgaacaacagaggatgaaatgttctac
actcgcacgtaggaaatagtatacaggcaaaaacttccttaattttatcgtttgaccaacattgcgggattttacgttcaaaagattcatcctgttcg
acatctcgtaaagctttctcatctctttcttcaatatccgctaatttatttcggtaatctaggggttgcaaatgcttatttattaacccacgagcaaca
agctccaaacacatcaaaaaggcggctgagcgtttcgcctttttttgcattagcagctggagatcctcgcttagcaggtatattgcatatagctggta
agtcgaccttgtatatgaatgactgaacttctgagatacaagtaaaaacaggataatgaaaagtcacataatcagcgggcaaagtattacaata
ctgatacaaaagacctattgccaaaaactcaattaacaaagcaccagtttcctctatcctgtgcttaacaatatcattcggagttatagtgttataa
tcagggacgtgaaagtctactctacgagtagattcaagattctctaatgctgttgttaataagcactcctcggtttgaagcttttgatatttaacctgt

tctacaccattcactaaaagcacaaacttcgaatctttagctcttgctcgaccttttgattgcacatattgtgtcacagattgacaaacattaaaacg
gattacaagattacacgtcggaatgtcaattccttcttctgctatggaggttgcaacgagaacattatagataccttgcttgaagtttcgaatgttgt
ttctttgttcacgaaagtgcatagacaacgctgccacatccgaatctccatgtccgatgaatggaaaaggccgaacatttctaagtcttcctgtctg
tttttttaacgttcgtataaatctcgttaacgaataagccgtcacttttctttcaacaaaaattatagtcttaaaagtatcattgtctttatagaagttt
tcaagaaattccaaaagtgttaaaaccttattgctcgtgtcattgccattcaatataggggcccttaatccagaatacagcttcttttgtttttgcgac
catctctgtacctcatgtatgaaattgttaatgcattggttttcctcagccgacaattttccgtcacaattctttcttgattgcttattaacaagttcgtc
aattaagaattgccaaacttgttcaccaaaccagacacctgattctacaatttcgtattgtgcagctttagataccctgcttatatacttgcagtttg
aaaaaacagtttcgcacatccttattattggattttgagatgcatcaataggtgaatttatgtaagggacaactagttcttttgtaacaggcatatg
atgaaagagacgctggtttgatattacataagctttggcatcgagtaactcttcccattcgaaaatcctctttagcggatttgtagccttcccagcgt
agggtgaagctgtcataccaaagatgcttgggagctgctttataggtgttgctaatggttttgcgacatgataaaagtcaatcatgatacgagcgt
atgcgtggtttttgatggcatgatggcattcatcgaaaacaagaatatcaaaatcaaacatagatagaaaacctcgtgccaagcagaggtagaa
cagatcggctgtaataacaattatctcgtaattctgctttatatcatgaaacagcttttcacacatttctattgagacttccccataacatactcctat
acggaacggaagctgtgatcgtaaataatcagcttgctgtataaccaatggaaccgtattaacaagaaacaccgacaaacgcttttgtcgtccat
ttccttctcgtaattgttcctcttgtttttctctaattagctttacagctagcaatgttttgccggccccagttgccataattaacaaagtattctgcttcc
ttgcaacgtgaaatacatctcgctggtactcccttaaaatttgggaagatgttatttctttcataattaattagttgattaagggagaccatatatctc
gttgcttatatactgatcacgaaattcagatattctatcacttttggggtactttcgtttttacttttgtagtttatagtaccaaatgataagccctaac
ttacatgaactggattctctaacccgtaaactgaaaagaaagtcttagcatgtgaagtttgaagtgtgcactttactagtagagaaagaacagaa
caatgtgcacaaacacgttagtgaaagaatgaatcaaaaacctctaaacttcaagtcccttcatggttattacttctttggatatgttggtggtatc
aattttggtggagtatcttattgaggttgtaatttcacgatactatgattgaaaacttgttaaaggttgcttttggatttctcgtagttatttcttttttat
caattgttgttgagaatttggaattcttttacttatatattaagaataatattaaccccgattgttctttgttcttgtagcaataaagcatatctcgtac
tattctcttaagcgcttaatactactttttgcgtgtatctaaaaaaaaaactaaaatgggacagaccggttcggctttatatgaacaacgtcgtgagt
gtcgcgcctattaagtacagtactatgtctagaacacaattgctagacctattacaagtaaacaattattcaagaagacttggtgatcggtttgata
tcatcgtgaacagtgatttcgctggaatcactgctgatgatggtacatcacctattcactttaacacaataaatcatgtccttgatgaagtgaacgg
cagagcgggttaccacggatcttttcaatatggttaaggcttcatatgatccatatataggagctaatttctatgtaacttcaagtgatccagaaag
taatggcgattcggcataaaggctacatggaatatcagagctttaatagacgcgcttcctcatcacaagaatcgcaccgaaagtaagattatccc
tatttaacgaaagaaccttaggaatgatgtaggatttctaccatgacattaagaaacttatgaatttttcaaatgatacttaaaaattgaaaaact
aaagacatatttacaagtttacttgaatcgaaacgagtaaacaagtcattaatatacgagtgctgaaagagtagtttttgatagtccaagttaaaa
agatctagctggttgaactaattaatttgtttcaccaattcaataacctttacttattttcactcatatatacttgaaaactcattaagtattactgcgt
gacaaagaagagatctgtcttctaaaatttaagatgatgaaaaatttagctcttaggctgccatctctaatatcagaagatttgtaaataagcgca
gttatctagtgaagctgaatataaaatgaatcatggagtagaaacaggttagaataatacacttactgtacagtctatacatgttttaaattcgctt
tctttatggaccttttcattaattaggcataaatagaatagagcagattgcatataatgtaagagacaatttgcaattataaaggtttctaacaaat
gaatcttattacttatagcacagttgaattcttaatccaatgttttaaacgggtgaatgaagaaaccaaatgcgcaaaccagaaagagaaataac
ctttagaaataatacatctatgaatacaaatttggtgtttcaaatctcgataaagttggggacagaaaaaatccaacctagttaaacatatataaa
aaatataattcagatataattcagaaccagaagtttttatttttaacaccgattatcctgtcgaaaaaaaaatgaatgtactttaattaatccaaac
ataaaccataacttcaagtgcgaatgtacacaggaatggaaatatgcagaaacgtgaccctatcgtaaatatgaacgagtaataaggaattatg
cactgtaaatagaatgactttcactgtcaaaaggaagaaaagcattgataattttttttaaaaaaattggtacaataaaccactgtttaagacgag
acttcatgaattgaccagaatgcaccagaacagtaaaaaagagaataaagcttttttttaaacatcacctttaagagataaatgtggtttcattaa
acagagtagctttttcctcaatctcttcaccgttaaccttcagtctgaatgtaacaggagtcatgtactggtaaataaaaggttttgtgccagaaac
attgtgaacaaatccacggagccatgtggggatttccgagagaacgtcgatacgctgcaaacgtctacccaaaggtgcatcgactgacatatgg
acaggcttttcataattttcagcatccttaccgtcccactcataggcaatacgagtaggttcaggccattctgtttctgggtccattgttacacctaca
tgctcaattgtattgtcaacagtagcggccaaaaccccatcctttgatacgataccaccgacgttaacgatcgtggatccataactcggaggggtc
ttaaactgcatcataacaccggtatatctgggattttgcaatagagcaaattcccaagaagaagccagatgttgaggcttcccgttttgtaaagcg
tgaacaaacaaaccagtacctgttacatcaactactttaccttttgagacaatattgccttcaacacgggtacggggccagaaaacatgcttcatg
cttgcccaaggtttggaggggtcggtaccgtagttactagtaccattctcaccaatcttaaaagggggactttcttgatgaataacaagatcaata
atagaatccatattaacggatgagcggattctatatgcagactttccttcaaggtcaatagaaatattagcggctctaaaacaagtgtggtcattct
caaagctgaattgatccaaattggtagaagtccataaatcattttcaggattgtctagatcaaaaattcgacatgtagactgagctgtagttgtcca
tgaactaaaaaataaggttaatattagctttttataataatttaagtccataccccaagttagaatgaatcaattgaacaaagcacataaggttg
ccattattaggacgaatataaaagacttgggtttcaacatggctagaggcaggagtggaccaccggtagtcatcatcgtccaatgtatggtaagg

attgataccttgtacagtagcggtgacgggttgaattgcttcagcgccgtaaacaggttctgcagtaccagttaaatatgatatactaaaccgtgg
gttagcaagctatttcaaaatatttcagctgtctctgaatgatatgaaatatgagaaatcacaaatagaaacggcattgaatcttcttataaggac
attgacaataaaacctctggacttgtggaaattgaaggtagataagaaacaagattcatattcccgataaaaatgagaatttgtttctaagatttt
catttctcaccaagtttcatacattgctcggcaattcaggtttttttcagcctgtttcttacatacttggattgaagccaggctctcattattaaaaaaa
ccgtggaattttacaacagaggtaaaaagtcaataacacgacaggatcaagagcacctggaagcaggacgtagaatagccaaaataaagtgg
tgaatagttggtaaaggccaagtctatttttgcattaaagaaagccaaacatgagagatgtaaggagtcgtgctgcggaatgctaaactcgtttcc
gagaaggaagtgaaatgcctaattgaagtatagtatatcgttcttattttccaatagtaaacattaacacagaagaattgcaaatttgatgaagag
atagaaatacggaataaaacaagggagggagggaggaagaaaaagaaaaagagtaagatgaacccaaggctatgctattatagatgaaaat
agaaaaatggtgttttgttggttccaatggatgagatcaggagatgttgggaaaaatcagcttgacagtgaaactttaggattgatgaagaatcg
tttgatctactttgaatggaaatcatgatatgaaaagttttacatcagaaaaaaaatgaaaatgagtataatggaacgtaacataaactgtttgaa
tcaacaatatggatgaatgattcgaagttgatgatttctcttttgaggttatttttctataagagaatcactttttttttatatactaaaattaattgcct
atgttaattgcctatggaggaagagttgaagaacagacaacaaaatctgatttgtttgtttacaaacaaatcaatacacaatcatataaaaagaa
ccatttaggatggatccacatctacaacgacccccacttgatacgggatgagtttcgcttttcgttcttttcgaatatcgaggccgacacggtatca
gtatcgagggtttacgtattctaaagatatttactgtatgcatataaaggaaaatagagaaaagcgtggaggtatgggcgaagagatagaaaca
gatatcgcctatactcccgattctttagacgatactttttcgttcgtgtcatacaagatttaatattatgcaatattgtctgacatttataaggctaaa
gattagaatatttacaatgctgtacacaagccagtaataaaacaaattcaaagagcgaaacaagcatgatgattcatttttatgcaacaactaaa
actttcattccattccatcttttgtacaattccccttttttgattctatcctcacttgtttactatcatattctatggtatgtggagcttatcctttgtgtcat
aaacaaacgatttccactcgtgtgtcttatgaaggaaggcagtttttattttgactcttacaatgaagcgaatcgacgatgatcgcttcaaagaaa
caaaagaatgaatttcatttcttctttctttcatttcttctcgtctacgcatgtcgaactgctttcaattgactcatagatcccctcgctaagtaccaag
catccccactgtttctttctttgtttattcccaattaagaacggacaatttgtctgcttcttgcttactcatacttttatttatttatctatctatctatcca
tttatcagttctttacgtatatagatcgtctgtaagtcatacataaactataataaagactttgcaatgaaatgaaaaggttctttcttcagaaaaat
gtagccattgaattagtcttatagaattttttttatttattctttctttatctttctactttctaattcacttttaaattgattcatgatcatggacacaag
acgcatcagtttttttactccgaaaaaggccttgctttggccgaagaatgataagataaatctttgccaactgacgcttcctccacgctaggtactttt
agttgaacgaacgttgactctttgatttgcccttcgttcatgcttttatagcctagtcttgccttgtcttggccgtaggaaggcttcaccgaggaagat
tctttaataggcaagactaacttgcttatcacaagtgataatcaaaccagagaaaaggcaaatataaaaaaagagaaatgatgcacttttataa
aggccgatccaaaggtatttctcgttcactgtttcttttcctttttcccttatcttcatccttcgctgggttgaaggctttgtgcattgatctgatttagta
tatctaggagaaatcaagaaatagattgattaataattgagaaagaaataaataagtaatctaaagagattctccaagaaccgtttcaaagaga
accggaaaaattggttttctttttttacgtatttttctttctattcgttttcatttttcatttttttattgtttttcttattgtttcttttccatatcttttttttattttt
tgattgtttacatctttataccatgtctcgatccgacaaaatcgatttagaaggtctaaatgcagacgctgcagagctcgctgccttgggctataag
caagaattcaaacgtgaattctctgcttttagctcattctgcgtctcctttttccgttcttggcttattaccctcgttcgcttctaccatgtattatacaac
aggttacgccggcactcctgcaatggtgtggggctggctgattgcaatgtttttttgttcaatgtgttgcctgtggtatggcagaactgtgttcttctat
gcctacttcgggtggtttatattatgccgcggctagtctggctcctaaacgctggggccctttggctgcttggcttaccggttggtccaactatttcgt
tcaagtcacagcagctcctagtgttgcctattccttttccggaatgattctttctctaatccaaatcaagaatcccaactataccccccgaaaactatc
aaacttttttattagcatgcgctgctatgatcgccatgacattgatttcgtgccttccaacaaaagttatcgcccattttaatacttggggtacggcca
tcaacatgctcttccttagcattgtcatgattacccttttagctgtcgctggcaaaaaccatacttttaatagcaatcatagagtttggacgagtttcg
ataatcagaccgattggagtaacggaatggcccttttgatgtcttttgccggtgtcatttggaccatgtctggttacgactctcctttccatttaagtg
aggaatgctccaatgccagtgtcgctgctccgagagctatagtcatgacctctgcttttggcggtctcgtagggtggcttttgaacctcatcattgca
tataccatcgttgatgtatctcaagtcatgaacgacagtctaagtcaaccttatgtcgtctatcttcagcaagtctgtgatcacaaaacaacagttg
cgttaactgccatcaccatcgtttgcagttttatgatgggtcaaggatgtatggttgctgcttctcgcgttacttattcttatgcaagagacggcgtct
ttcccttttccaacatccttgctcatgtcgatcttcgtacaaagacaccaaaccgatgtacttggatgaatactgtggttggtatcctgttgctactct
taattttcgctggtgatgtagccatcaatgctatttttttctgtcggtgctatcgctgcttttgttgcttttacaattcctattttcattaggatcacatgtg
tatctgacgctgaattccaaagaggcccttttcacttgggccgcttttcaaagcccataggttatgcctcctgtgcatttgtcgtgctcatgcttccca
tcttatgttttccacaaaccaagggaaagaatttggatgcagaaggcatgaactggacctgtgtggtgtacggaggtcctatgcttgctattatcat
ttggtggattctctcagcccacaaatggtttaaaggtccttgccttacagaaacgtcttccgcaagcccagaagttattttggaaggtgtatccaatt
atgacgatgaagccagtctttccactaccactaccaagaaaaagtagcaaatttacgttccccagtaaccacattcctggaatttccctaaattgtt
ttttttcatctctgtctttttatctttaaaaaaaatttgatgcctaatctaacccctttccattcgatataatatgatgtctttcgtaatcccatctgtttatg
cttccttttcgtccgtttttttataccattggttctttctcattctagtgttgttatgaatgttttaatgtatgatttccttcctatctttcgcttctttttatgg
acctttattctcttctcctctcaattgatcgctattatattacttgctttgtttgtgtccttttttcttggttttgtaatttagtaatatatctcattcgcttct

tattatgaacgaaggttttgtactcttatccgtgacaatatcataaggcatatagtcgcttacaaaaacaagtttgtccacatctattccatattact
atagtgaacttgtttattatattaaaatttttaaaacatcacttatgcgataaggcaaatacaatactcactatttctacaaagaacgtatattttgt
gaattattgtttgtacggtactgtaaaaatttgagtttttaaagtgtaagtcatcggaattattgttgtgggtcgcaacaatttttttggcatattgaa
caaactgtatcatccataaaaaacgcttctcaaatttattgatcttttcgtttacaatactccaacggataactattgatttttcagattaaactaacg
tgtctgtacatagaaacttcttgtctatgtcttcatgaataatcatcaattggagtggtttaatattttctataattgcaatgaggacccaaagcatca
attgaaaaccgtaaggaaccgcttggcctcgggaaaacatatccaacaagctgcgggggccgcaggtttgttcacagaatttcttcctgcgcagc
tcgaagcttctgtatcccaacaaccatacagtcctctttcttggcaaatacttgcaaattctgttgctggtgattcacccttagaggatgaggcatgg
agctatcttaccaaatttcctcccaagctgcctttgccagtcacaagcgctcaatgttctgcatttgaaatttcggtttggtatcttatactgaacagt
aaacagaggttgcaccaattatgcacctctcctgctggactataccttctcgatatcttggttgatcaggatcgtcatccagattggcagagagag
agtattctttttcgtattgttagtcatattttaagtaatggctatgcagaagttcttatcaaacgtgcagctttattgggatttcgatccatctacttcgt
tgtagatgccatgactcgtgaaccgtttggtgcagtggatctatccctgtttcccactctttacctaattattcgcttaatagctaagcaacttcatga
gcgtatttcttttatggataaatctaatatttcagaagctgatctaaccatcatcacttacttatctgaaagctataaactagctgttagtcttcttttc
actcaatatgagagactcaccctctcacagaatgacccaactcaacgaattgatatcccttacgcattcaagcggtatgctcatgactcttcgttgg
ccaccaaggaagtacttcttttactttgcatccttgatcaacaagttccacgcaaaacgcttgttcaaaaaacacctattccttccggaagcttctcc
gatttttgtgaagctgtaggaggagttaaacgcgaatgtgtccatttcatcacctttgcctcgcacatgtatccagctactcaggacattgttagaa
atgccgacggtctgtcagtgatattatctcagtgtaacatcgatgatttgaatccgtaccttcgtgaggtttctgtttttttgtatccgccacttgttgca
aaacaatcctgagaatcaagcctatgttgctaacctaaatgctgtaggtgttagtaaatctgaggttttggaaaaagccggctatgactctcaaat
tgacaatgatggaaaggtgaaattagtatctaaaaaatcgtcctaaataatttcatcaggatatcagtatgagaaatacacattcgttataatgga
ctttattttattatatacatctaggtacttttacatttttgtgactcaaattagattaacgaataatgattattttttttattaatgtattgtttatgcagtc
attagatatttcatttataaaaggagatgccgaagggatttttcttttttttcttttttttctgttattctttgttggtaaccatttgtaattatagggatt
tatcatatatccctaaagttggactttctagttcttagttcttttgttggtatatattaagcacatgaatgtttactaaaaccaaacaaaatcacaata
aaaaaagaaatttcctctcaaaagtgacaaacaaaataagtagttgaagttttcttatatcttagctaagatatagcaagtcagtgccatgtactg
tacaactagggaacggataaacaacgaacaacgaaagaacgttaatgaagcccgtgatgagtaaggttacttgaggacttactttttattactgt
aaccgagcgtggtgaacttctattaggagtttctatcggtgagacacgcacagtaggcgtaggcgtcgcaacaacagtcgaagcttgactgccat
aaggagaaggggtaggacttgatggccttgtagcctgttcataatgaggaagcccgtcaagaggaagcacattcgagaaagctgaatgtgcttc
atagtaaggtggtaatttacccaattcctgtaaacttaacgtcgaaacgctacgcacgggtagagcacgattaggagatgttgaacgcaaaaaa
gggttttcaggctgtaaaccttgaatttgagaaccaccaaacgatgatgaagtactgctggctctagagcgagcagctctacgacatgcaccaga
aaacggtgctattggagaggttataggagaaagaaattcggttctatcagttggggactgagtggaattctcaatataaaagcttggttgagatc
ctaaagaaggcgaagatacggacgggttggaagcaaatggcttcgtatttatactaaggatgggactcgttggtccaaaatcggaagagtttcg
acggcttgtaataggagtagtgcgttcactgagtacgggagtgctttcggtaggaacaccgtcccaaaggacatcaaatatgtggcgatcgtaac
taggcagaacattttcgtttatataagaagcgtgatttcctgaaaagacaccctcaacaggtctagctccaaacataactggagaaaccactaat
gacaaaggtaaggagtttctaagttcagatatatgaccatcaggatttaacaaagagatggaaagacgaagcttatgatgaacgcgtatacaat
tcaaatcacaatcttgagtacactcacccaaagagtcagggatcggaatttttatttgatcttgccaacagtcgtcgatcatcgcaaggccaggaa
cagtctttttagccaaaacacgcttgaagtcttttcgactagtagaggaaaaggctttcgaagttataaaaagtgaacaatattctttcaaaacaa
tggtcaccttcccgatgccgagtccttttaggagaggatataatttcaagttaattgggatctcagagccaatcgcgtataccttggagggtatact
cgtttcatagttgacttttgatggccatatatcacttacactcatagagtgcatgaggtcaagactgttaggaggaatggttcggatcagccgaagt
tgcaaggcagactccaaatttgaagaagggatcgttgctctttctaagcaggctgtcaaagtgtagacaatatggcaccccggaactccttcaatt
gactcaggaacatcttgtggaagttggtaatcaaaaggatattcgtaattaccgtcttgaattgtcttgttaccgtctcccgacagaaatgaccatt
gcctatccaagatttggtttacttgccgccggattttagtaccaccattggagctattccgagattcatctgaccagctagaaaatggtcagtaagt
gcgacagatgcatacaagcaaaatgtagcttaccaaacaaaagaccgaccagtaagacgaagctttatatttcgaacacggattggttgggag
attgaaaggactacgatgcctgaaagcctagcggctgagtctagtgaattaccatacagcacgattacattcgcttccgcattgtaaagcctaaac
aagaatgaaaggttagtattccataaatctaaaaggaaaaaaatcacaagaaataaagatattttggtatgaatcaagcaggcatgttagagg
aagatttgttactattaggtcgaattcatcgaagcagtttttgattatcctgagccatacgaaaccaatgctctacagagaagcttattctccaaag
aaaaattgatctaactaataattgcagagcaggctgaaacagcggcaaatgctagttcaaaattacagtctagccgtgatacgtcaattgagcta
aactcgggatttttttgtttcaaaagttcaaaaaataactttgaaacaccattcttctgtcctcatacccttctttttcctaaaatttgtagttttacttac
cgaacttcaaataatttcacgggactcttatgacgattacttagactattcgtattcattcataataatgtctggcttgtaccagaggcagattttaa
cttaattattcttttcaatgtcagtttttgtcgtattcactcagtagcggttcttgttgtaaacaattggcaatgaagaacacccgtcataaggaaatc
tgcattatagtttggtaagggtgataccgcatacgaatgtagatatgtgaaagtatcggatgaaaatgagtgaacaaaaagattcaattacagaa

gaaaaacaagaaaaaacaaaaacaaacgagacgtatggattcagtcaattaaacggttattctttaatttttacttctagattagagtcttctcat
taaatattaaaccagaaagagtgagttgctttcagaaaattttaagaatcgagatttctttatcgtaacaaaaggaacaaataaaaaaaaaaga
atattaaacaaggaacagagatatcaacaagaggatgctttgtacaaaatgggagaaagtcctaaagaaccatggatgtcatgacaggaggaa
agtggtaataacaaaagaatgactcaaggtacaagagagggatgaagtatatttaggttgtatattttcacatagagagccatggcaaaaatgg
cggcttcaagataacggaattttatgaagcgtgtagtaaattcgaatatttgagggttaaatttaatttcaagtccgtttagaaaagccacggaca
ttctttgcttttgcaatggtaaaaccaaatctgctttccaagcatttccttccttcgactttcggtcctgtggtttccaaacaatttgtttgacttccatt
aaattggtagtattgcaatcaaataccacccttccttcagtggacgtgattgtccaatacatatagccatcagcaaagctttttgtagttttcattag
aagttttcgagatctaaatcgttttgtaaaaaaccaagcgaaaattctcttgtcatcccttatggcatctgtaaaaatgagacgataagaaaacga
taaaggattccatctttggtacaaattaaataccgcttcttctgtttctgctcgttgttttatttgagtaatgcgacgatttatgcgaggccgtaaaca
gcctaaaggctggcttgctggaaaggcaatccagaaggtaggtaaagccggggctaagttctttaaatcgcctgtgacaactggagaggtcttct
ttgatggtgaaacctgcataataaaaagtaaaacacctattgatgagaagtgtcaactccttttttgctatttcaaatgagtattggatatcaaatga
tatagtatgaaaatcctcgctttttttgatcaaaagtatgcgagtgtaaatactctcatacaaaaaaataataatgagtttgtggaatcgattaata
atttgcaatggtatcctttacaccattaattcgcactgtcttcaacaaaaaaattgaagatatctaataagctaatttcacaaaaaagaagatgcct
aagcaataatccaaatatatacgaaaacagttaaagaatgttcgcacgcggtgttctattgaaatattttaatcggacgagacagaggtatgata
ttatacgaaggcaacagcacaatatcctattggcgccacaagtaggttaaaaaagaaaataaaaaaaaataaaaagatttggtttatgtttgat
gatagttggaaacacttagaagttgtttactgtttttaaaatttaaggagggaactcatgacggggtacaggttccttttttattttgagtttatccctt
acccatcactttctattcggggtaggtagaaaagccgaatccactgaagtccgaggcatggaagagtataaccaaaaaaattggaggttcgggt
taatcagaagtttgaatgaggaaaacagttaagataattgcttcggtttccaatggaagttcggaaatcggatctcttaggaaatttttgagctttc
gcattctgctgagtatagtttaccgaagtttatatagtaagaggcgagcgcataagcaaggactcgcacttaaagaaccttttaaatatttcatagt
gtgaggtaagaactgctcggagtgctacgtcgtcctttagattgtttgaattagaagaaaagaattcctgtaaatgtaattcatgagacatattgg
attgatcaatcactctcaaaagcgtaagagaccccaattttaatttcggttcggcttccttttatattccgtccctcgttcaaaggtattttattttgat
taagttaaggtatcgcttgtttaagtttccttataaccgagattttcaaactattataaacaaaatttatgtactctaagttttaaggaattgcttcaa
taaacattgattcactttatgcaataaaattgcatggactgtttcaataaacccttttacaggttttgcaattatgcggaaatttagagtaaaaaag
aataaccatttataataacactgcaaaatcctttcttttaataaacttcaatgacgaagcgacgttcctccttccattgctctatgaccctgcgagcg
tcaattggctcagaagcagaactttgaatcacctcttctaatacaccggtaatttcgggcaatggccaagtaggaccacacaagtaaaaagaacc
tccttcatctatcaaagtcttcttcaaaagttccttagaagaacgcataacatcttgaatatatatcttgtaaggttgatcacgactgaaagcttgtc
caatgaatgtcagtaaattggctgcatgataagcttcccagtcttcaccatataagtattcttcacgttgtgtacgtgaacccaagtaaagcacaat
gtctccagagggaatgccctgtagtcgctgccactctttgaattgcaaaaatgctcggaaaggtgccaatccggtaccaagtcctgccataacgat
gggcttttctggagacttgggtaacttcatcacagaagacttcaccgcgactgttacaggatcaccagctcgcaatctagacaaataatgagaac
attgaccataacggtcacgtcccataccatctttccagtcaacaacgacgattagcaaatgcacactgtccttgtgctttctttgggaagaagagat
tgaatattcacgacgtttaataagaggtacaacaactgccaactcagatgcgctcaattttgcatgcttgaattcctttaaaacatcggcgtatgta
agcatgtccacctcaacacgacgcttaaagtcaggtgcaccgacagggctgactaaagtctcgagatcactgcgttccttctcgtcggtttcatatt
gagccaattgttcataaaacttcttcgtcggctttccaaagatatcaatattttgagacaatgcttgataaacagtgcgagtttcccaatgacccttg
taaaaagtagaagggacttgaatgagttcgtcggcatctagtccgtactcttcaataaaatcaagaacgtgtttttttattgtttacaccatatacac
caagagcctctccaatgtcataagtaaggcccgaatttccaaggtcaaattcgatatggaaaatgttacggctgtatgaggcgggcgtaagacgc
ttattggatttcactcgcacagtgtaaacttgttcagaaacatccggacgtagaacatcttctttttttgtaagcttcggggaacatcatctgctgggc
aacagtctcccaactagaagtgcgtggtgcaacgtcttcctccagagatttgatagggttagaaccgaaagatgtctcagcggccacagacggta
actgtctgtcactaggttcacgttcttttaatgaggcttcttcgacattaacaaaagataaggcctctttcaaagaatcaatggcactgttgcgaata
gtttcattaaatcccttctccttacaaagaacgttgaaagcttccgtagcacctaatccaggctcgaagagttggacataagcactcaaaataggc
aaattagaaccgtttatagcaaatttctttggattaacgagaactaatttaatttctttatttgtaatgtccgtcaaaaactcataaggaagcttgga
agtgatttcttcatgagaaatagaagtctgcagcaaacaaatagactgcttagcagcattggctaaaactctataatatgaaggtattttatcattc
gctaccacagagacgtcagcttccaaatcatgagcaacgtcagtaagaagaacgtctcgaccagaaagttgaatagtggtaaatcggacacctg
caagcaccaaattgtcgtacagtgtacgaaaagcgacagaccttgatttgtcggttctatgagcagacaccaagtagggaatgtaatattcagaa
gagtcaagttcccagaaactaatactcttttcaaccttagaagctacaggagaagcataatttagacccaaagagtcggaaagagctttgtaaag
agagttagtagtcaatgattctttgacggcataacgttgctcttctacagagaattgaaggttttcagtgtccaacaagcttgcaaggatagagga
atagtaaaaagaaggggagctaacagaagttgagcggacttgagaaaggacaacaacctttctgcaagcagaagggaggctacggtgtaact
cctctacggagaaagggaaagtacggatagaaagtaatcccaaatcctggttggcagcaagtagattacgagccgctgtggtttccgtcttacca
aaagtaacaataaccttagaagcagatgaagaaccttcgtattcaactggcttgtatttagtggagaaatggttattgatatgggtcagggcattg

gcggcattttgaatcggatcttgatctgattcaaacgaattgaaacaatcaattacggcttcggtatcgagagtaggaagactaggagccaaggc
tgagacaattgcatctggttcatagacgtggacagtaggacttttcttcgcagcgatagtgcttgcaagagccatgtaggcagcttccacgttagat
tgagaagtgaacacagtaaaattgttcaagtaggcaaagtccaacacgctcgtattgtctgtgcgataggcttgctcgacatagtccaaagctgc
aacgtgcaaaacaagaggtttgctagtaccataaaaaagaccaatgttcgagatcaagggattcattaaagaaaggcagctggagggggtaat
cattgcatacacgccaggagaggaaatggcagcggtataggatcccaacaataaaccagcggcgccagaacgagcctctaagttaaacaattc
cacattatttgagaatccgtttttacgaccactgtcattccactgggttaagtagcggttcaaaggaacatgctcagcatatgaaaaaatatggttc
gatagcgaatatacaatatcctctatgacttgatgagatgtatcaaacttcaccgaagtttctggggtattagtcatgataaatgttgcagaagttg
atactaaacaagtaattaaaatcgtctctataaaaggtttgcggtaacccttatcttatttgatgcaggtgatttttaaaacagtaaacagcggacg
cgtacaatttccaccaaaacgttcaaaatttgagtacaaaaaactcctttttttttattgctttttccacgccacgaatttgtttatgttggaaagg
gttaatattagaaaacgcggccaaatttttactagtctgcgatcacctgataatcgaaattcttgcgtgatcactacttttgtttagcttaaccctctg
agaatttggtgtagccgttaataacggtgctcgtaggctatcattattttgatcaggatacctctttgtaattccttatcgtgtactgtaataaaagtt
gggtatccttatttacgagttaacaaatcatatcaactatccttataataatcagcaaacaaatatcacaagcctaaaagggtcgagcaataaag
aatatggttgcacaattttatccttactttcatagattatcaggatgtaatgggattgttatagtttgtatttagataaatataattctataggtatcaa
taatattttctattctatcctatcctatagttataaaggacggaattttatgttatactcttcaatggaaagcaccgttaaaacatgcgtttctcacatt
cgatagggaaactaggggtgaagacagtcttcaacttccttgtaagacgatataaaaaattcgaggagagattagtttgcgaaaattcattgcgc
tgaaattatcgattcattcattccatgtattataccatcgttaataatcgtaaccttttgtacttttgtattaggagggtattttattatgagtaagact
cacagtatccatagaacatcatcgtactgaaagtcgaaaccatcgttccacaaggcttttagttatccctaaacgtaaaactcttcactattcttta
aacttaaacgacgtaggaggggaaaagtacaaactcaaacttctcaagattccgctcaataataattatgaatgatcttgcaggaaaaaaaatc
ttcttcgttgctaattacactattatagatcatacataaacattaaagtataccgaagtttttgtcggggcctcctcttaggtttcggattgtttcctttt
ctcaggaccttttcgttaattttatcatttttagttcttctctttggaatctcgtaccctctttcgtatttgttgcagtatcttgaatttttttttcagaa
gcaaacaactctttctttgaggatggcatctgatctatctccgggaacaaatctttcctagatcgaagaaacataggagaatatgcacctatcata
tcttctcgccttgaaggttcctttatttttctattcatcaaagggctagcatagcccgacgttgtttctcgttcgtctaatattttgtttctcgatacgtgc
ttcaaaggagaagaaggttcttgttttacccaaggagaaaggaaagttgaactagcgcgcacgccagaacgtcttgacgacgttgcagaagttg
gggaaacacgagcgtcaccagtgttctctaaagcatgatatgaacctggatcgcgaggtcgctcacgtccttggtcatctcgtaagtacagagag
atttgctcatcgggtagttgaagaacagattcttgagcgttcgcctccaaggcacacaaacgaagaagatattctaataggaccagatttgtgtct
tctttctgatatgctttcatcttctttcttcttgttacaagatccttttgtgtaagtccaagcagttgcatttctgccatatgttcatcactgaattcttca
gtcgtattgtcagaatcctggctttcattatcatcttcctcactctcaaattcttcttcaggtagcagctccattgctatccatttgtatgcttcctgttct
tcagaagtctctgaaggattatccttgtatttgagcagaatgacaggtgtcgtgacttcgtcaagtcgaagaagttttcccgagacactatagaat
agagtttgaatagtacttaaaagaatatctttggtcattggaacttcatcttcttcgtgataacgattatcatcgaatgattgaacaagagagagct
tataagagaggagggaatttttgttatgagattgttgaggtggcacccgaaaaaccttggctacacacacagagcaatcttcttgccaaatgattg
agtcatgatttaggaacttggattgagcatggctctcaaagattttgtaagtgcgaagtcgaaacgtcggaccaacctgtaaaggcttttcccctgt
aaaggaaaggtttgaatgtaaaacaaaagagtttgctttaaaaagctgagaaaaattggtagatttcggtagacttgacgagatagaaagccct
gaagtggtgatagacaaattgagtagagggatggcatcttgaattcgtaaaagtaaagtctttatttcctgcaaaacctttcccttcgtttcttcggc
gcgtttcgggttcgatgcttcttctcccgagtccgaacttgcaacacctaagatgtgcacactgaacttttcaatgtctgagcgcaacgcatcgaca
agttggcgagtgctctgcaaactttgatttccgctggaagacataatatcaatcaactctatggctggtgtgaccacacgaatcatctcaagcaac
tttgctttcatgaattccaattcattgcgatcagcatctggtatgcgtttaatatagctacttacttgctggacagcaaattttccagcaaatgaaata
ctgtgcttgatggcaaagctagtagcaaacgttgctgcatgagatgcgacgtggaacaattcatccattataattcctttagttcgaattcttcaaa
acttacttttcagaaagaatattttctaagaaaatctcaaaggtttctttaaattatagaaagcagttaacgcgtcttaacatatttctacttgagaa
aaggtgtctgaggttgtcgagtgtttcgaaataattattgatgtcaattctaaatgagtaaacgaaaatgtaagaattgaatatggaaagcaattc
tgtctaaaaaaagtctttcgtacttactttttagtcaatgtctttccttttgagttgcttcataaaatgccttacttatgggattcaaatatatattaaa
attgattctctgcaagcttttggcacgtttgaactgagagaaaagcgtacatgtggacttccttgaaagagtctatagaacttgcctaaatatcaac
ccaaatacatccaaacttagaggagcattttcagttctagcacaattaaattaatgaaaatttattgatgtttatattctttttcttttcaatccaagc
cagagcataacaaaccaaaatctacctttggatagtaacccactattttgccacttcaatgttttctttttcatttcatttacgaataaagactagaa
taaagaaagagatgaaaaagtctctttttctataattcgactttcttaaagacattcaacgatttggattgctactgattgttacctgaatataataa
ttttctaccaccaacacagcgttcctttaccacacctcttcctacatataggtaaaaatggctagcaatcagccaaagaatggtgaacaggatgag
catcttgccaaacaaacttcgaaattgtcgatgtcttctttaaaagctccttcgttcacgcctacagcccccgcctttgtgccttctttccaacgtaag
tcaatacctaatcatcgatacttttctcacgatattatcgcgactcaatataaggttgttctaacgcatatttaggccctagctttgctcctcaacaac
aacagcgacaacaatccaatgatgcagcaagctctccatctgatcaatttatgggagctcccaatatgagctaccaacaatattaccaaaagcct

gcttccccagcagcgcaagatcaaactgatgataagaacagagttcccgatttttctaagaagaaatcatttgtccctcctaaaactgctttaccc
aagggtaaggtattgtctcttggtggttcttctggtgctcagaagccgaacaagcctgtttccatcagcttaggtggtgctaaacctgctgctcagc
cagctgcccaaacttccgctcccgctgcttcctctactaaagaggatgctcctgtaaagtctgctgaagataattccactcctgcccctgcgaaagc
tgaagtgagaaagcctgccgaggctccagcaccagcagcggtcaagcaggacaaaggatctactcctgctaaacaagcttctggtaacaagga
gactaccgctactactgagtctcctactgaattagagaccactgttgacagagaagtcttaaaagaaatgtatggcaaggagcatgttaacattgt
cttcatcggtcacgttgatgctggtaaatctactctgggtggtaacattttggtcctaactggaatggttgataagcgtaccatggaaaagattgag
aaggatgccaaggaagctggtaaggaatcatgggctttgtcttgggccctggatagcacttctgaagaacgtgagaagggtaagactgtcgaag
tcggacgagcctattttgaaactgagcatcgtcgattcagccttttggacgctcccggccataaaggttatgtaaccaacatgattaacggcgcttc
tcaagccgatattggtgttttggttatttctgctcgccgtggtgaatttgaagctggttttgaacgtggtggtcaaactcgtgagcacgctgtcttggc
ccgtactcaaggtatcaaccatcttgttgtagttattaataagatggatgaacccagtgtagaatggtctgaggagcgttacaaggagtgtgtcga
taaattgagtgttttcttgcgccgtgttgctggttacaactccaagacagatgtcaagtacatgcctatttctgcctatacaggccagaacatcagg
gatcgtgttgaaaaatctatctgtccatggtatagtggaccttcgttgctcgaatatctggataccatgtcccgtttagaacgtaaagttaatgcgcc
gttcatcatgcccattgccagcaaatacaaggatttgggtaccattttagaaggaaagattgaggctggtaccgtcaagaagaaccaaaatgttc
tagttatgcccattaatcaaacacttgaggtcactggtatctacgacgaagcagatgaagaaattccttcttctatctgtggtgatcaagttcgtctc
aaggttcgtggtgatgactctgatgtccaaagtggttacgttttgacctctaccaaaaatcctgttcatgctaccactcgttttgttgctcagattgct
attttggagttaccttctatttttgactaccggttatacttgtgttatgcatcttcatactgctgtcgaggaaatttcctttgctaaacttcttcacaagct
ggacaaagctaatcgtaagagtaagaaacctcctatgtttgctaccaggggtatgaagattatcgctgaattggagactcaaacgcctgtttgtat
ggaacgatttgaggactatcaatacatgggccgttttaccttgcgtgatcaaggtgctacggttgctgttggtaaagtcattaaaatcttggagtaa
attggtttaaatttaattgtatagttttcgttattaaaaatggttaatgttatcattgctagataatgtacggtatcttgtttttcattgagtatgtggac
tttgaacataagtatgcccttctgaatataagtccgagcgtgaataaccttctcctctaataatcttttgatcgcttcgtaacagaaaccttagaatc
catataaaatacgttaatattatacaagtcaataaataagcattcaaaaattcgattatgaatccatttctaagaacctactattcaatcttagtac
cctctacatatactgaattcggtaaaccctcggaatttccgcgatgatgtaaataataccaccaaagctgaatttaccttatacatgtcagattggt
tttattgtttgtggaaattgcagttttctaatgactttttgtggatttaattagcaaaataacgtctgaatatagtcatcctatttcaattacaccaggt
gaaaatatagcaaatggttttgttagagacgcaaagagaaggtatgttacggttgcaaatcagaagtgctaacatcttgcctagtctttttttaaaa
aaataaatgctatagaatgtgcaacaaaggtaagcaagctttagtcaggaaacgcgaaacgaaactttcaatcccatcccactgtctaccatttg
gcatatatattgggttcatgaactattgaaactatttttggcaagaatagtttgagactcctgccataatttccatagtcagattgagaacgctttg
ctaactctaatcagtggaaggtgttagtagtagacaggaaaactgcagacattatcaatcgatttacctctgtccatagacttttggaagaaaaga
ttgcagcggtggagattttagaaaatgcaaggactccaaactcatcttttgaggttttatatatccttcactgtcaagataaactagtcgactgtatt
tacaaagaccaagagtatccaaacagctattctggtattcatatcatgtttattgatcgaattaagcctcaagttgttgatcgcttaaaaaattcta
gtatcacttcaaaacttcgttcagtacagattggatatttggattattttgctcttgagtccagatattttcaagtgcacgacgagtattcagggttgc
gtctttatcatccttcttctggtcctattattcggcaagaattatcaaatattgcacatggaattctttccgtttgtgtaagtcttggaattgccccaaa
catccgttgttattatcccaaaacagccaaacatacctcaaagacgatgtcttttattttagcgtcacaacttcgtgacatagttgatgaatactgta
acactcatcccgggttccaagaatcttctcagaaatctacttgcttaattgttgaccgttccttagatgttgtatgtccttttgtgcacgaattcaccta
tcaggctatgattcatgatttactccaaattaaagatgagtcttatccttttacgataatgggtcctcacggtttagaaacccggactggcaagttat
ctgatgatgattctgtgtacgttaagatacggcatatgcatatgcgcgatgcgattgaacggttaatggctgattttaataagttttgcgaagaaaa
tactatttttcttgataaaggacaagcctcctctcttaatgacatgcgaagcatgcttgctggtctgtccgattttcaagaactccgtaacgagtattc
tcttcatttagccatggcacaggagtgcatgaatctttttgaaaaggaaaatttactttccattggttctgttgaacaggatttatcgacaggctcca
accctgaggggaaaactccgcgaaacgtgctttatgatttacttcctttactagaagatgattcaacatctgaggcagataagcttcgtcttctcat
cctttacatcacttatagagatggtattattcttcaggatctgatgagactatttcgacatagcaaattgcctagtcatcgtcaagagattttgcaga
atctagaacagcttggggtaagaaatgtaaagcgattatcggattccagtacgaaacgaaaggaggttgcaaaatcgttacctgcaggcgagga
aacctatgagctttcaagatttgttcctactttaaaagcagttatggaatctctggtacaggatgatttggataaggaattgtttccttttatccgtga
tctcaacccggagacagagttggaggtagagcaaacctctttgcgaagctcaagaccatcgtggactcgttcacgatccatagcgacacgcttac
ccaaagaaaagtttttagttttcgtagcaggtggtactacttatagcgaacttcgatcgtgctatgaaatttctgaaaaactcaataaaaatatcct
cattggaagcacatcctgttattctcctaatgaatggcttgagttcttctcgaaattccgaaagtctagggaagaactgaaatttccggaagataat
tctgagcctattccatgtttacaagctcaaccgagtaaactcttaaaacagccgaccgtagatgtaccatcatctccattcgaaagtacgtcggctc
ctactgcattgcctagtaaagaagctgataaaaagaaaggaaaaactagtaagctcggaaaactcatgtctacatcgaagaaggataaagaca
aggataaagttttgacggaatcgaatgataaaaagaagaagaagaagtttggtgtgttctaagttttcttcgcgaattgagaagtaactttttttga
ccttgtctcatgatatatatagaaacaagaggtttttttacgattttaactttttgatttataatgagttttacagttaatgactttgaattgatatatat

gaacaaagctttgaaagtgtcaatttttgtaaggggaatgtttattactcaagttttaaaaaataaaaaatcccatcaaggaaagcctaggtatat
acaaaattcgagaattaattagatagaacatgacaaggtgaacacaaaaccaaaaggaccgaatctaggatcataaagccattgattataagtt
aggaaaagagagcattcattggtcttgcaggacgctcttaaactaaatagaatgttagcaaatggttgtaggaaagagggaaaacagcatacat
ggttttcgtcaattgtagtgtaaatgatgccttcttgttgcaaaaaatctgtgacagcagtaagatctataccaggaccaacgctttgggcaagttg
tcgaacatgtacgccctcattcgtgtcaggagcattatgtatagcttgcatgacgatcatttgtgtgggagttaaagagtactctgccagtttttgtt
ccaaagagttggaagcaggagcagaaccagcattggggacaggattaccgaaagcagaaggcgctgtaccagtcaattggggcttctgggtga
agtgtacatgaacgctgatggcttccaaaaagtgatagtgaatttcgttgtgatcctttatattccgaatatactgggaagcgatatatattttcccg
gagaagactttaatatttccatatacacgaacatatgtatcagtagccaattctgaaagagcatcaatgtgctcccaatgtcggacttcaatcata
cccgtgccatcctcaatctgatacgttgtgtttgtcgtttgttggtgaatgtttctcaaaactcctacaaaggttacttgaccaacttcgaccccgtct
attttgaactctgcatcagcatggacttgggtagcatccaatatctgcttgatagttacaggacgtagcttattgccaattccttgttgctatatttgt
cagtcacttgtttcaatgaaatcacttgagcaaactaggccgtatagaataagcatctcaaacataccctatcaacactaggctgactggacgga
tcataatcattgaatccacctcctcccattcctggagaaaaggttccattgaaacctgaaccataacctcctttattatagggatcgaaagccatga
tttcgatgttgctaacttcagaaaaggttaatttcagataacacggaaatttgtcaccacaacctggatctatttgttcttttctttattttttggtaaa
cagagatccaaccaaacaagtcgttcaattaagtctggtaatcgctggtagttagtgtgctgtttgtaagtttcacgacacgctttttcatattatca
tactttacgtgacgcgttaatatgatattttatgtatgcttataaatagaagcaatttacaatggcatataataaaatttatatgctcttatattccatt
tgcatatctatgtttcaatatagaaagatatttattgctatacgttatgatgaatataaattatcttttatagtaatactaaatgaatctaaaaaaaa
ttcattcgtcgctgactatgttactctttcttttgttcggcatgcttgcatgtaaataacttactaaaatagttatcattagagattaaattgttctaact
tttgcttttctttttttcagacttacttctcaattgatcatatgctaatatcctttttttctatagcctactaaatatatttattacaaaagtagattttcag
aagccaatacatttctcgattagatttaactaaaattgcaagttaagatctgattgcattatgtttaatggaaaccatgttactgttaagtattttgcg
attgcaataattcaccaggttaaacgaatgaagcggaggttacgtataacaaacggtgttattatgaactagaaatattaaagctgcaatgttgc
agtcgtaaacggagaaatttttttagtcattagatcgtctttttttcatgcattaaactgttcttcccaaaaaaaattttttttttatgaaattcgtgactagt
agatatttctttttcttgagaacaacgtaaaagccttgataacaagcaaacagtaaatcacaaagggtcagaattatagcaacaaaattgtcaca
aaacacagctagataagcaaatcccttacagtaaccaacgcattcactgtgaatgtttgctatagtgatgatataacgaacctgagggaggaacc
acattaagaagaaaacagcgggatacgcaaacagaaatttggattgacgcataatacgactcctttgttttgttaacggatcttcatgcaggtctt
tatcgtaaatagccattgcactatcgattgactcttcttcattatgtattctgttttcatttctttcgttaagttctgtttgagactgggaatttgagtctg
gagaacgtcggttttctaaattcccaggtataaaggattttgatggattggggcgattgtacaagctaggaaaacggtaggattgttgatgagaat
cggacggggacgatagggactgaggattaggcaaagaggatgcttcatgagcacaactatacgtatgacttgaaaaagaatttcgtggggaat
gtagagaggaggaagttgaggaggaggaagagtaattttgagggagaggtggcagcgaaatttgagaagggttttgaggaccgacctcaagg
tcatatatgggaaacgaatcaggattatcaggagagattttttgaaactgcttaattttttttctaatggtaataaacataccgagatgaagtgcaa
aaatgattcctgtaagaacataatcaaaggtccaattgagagcccactggaatcgaacgggtcgaaacggaagaaaacatcgagtttggaaatt
cacaaacgtattatctgtattcgtaaaagcaagagcaccaactgtaagagggaagatgaacgacaaggcatatacggtatgtcgaaagcgata
aagtcctccgcgatctgcatcgacagatctagggtgaagtacgataagggagttatgtaatacgttgatcaaaatccaataatcagacatccgaa
gcatgaccaaaagaaaaaagcctaaggtaaaacatcgtctcgctgggatccaaaaataacctgaatatatagataaacttgggtgaagaagct
gaattatggaacgaattaaaaggactataaagagggtaatatgaatatgatcacggaaagtttttttttttttgacgaaaaaatcgtaaacaaact
ataaatatacaaaaggcgatcgaaacggaacaagcaccaataaccattaatcttaggtgtttcaactctatgcctgtcaagacgatgatcgttga
ggtgccggtggtgttagttcgatggagtcgatgatggtgtcgcgcatcggctacaccagtagaattcgtaacatcgctccgacaagtaaattgcat
aagaagaaaggcttatcaatgttctcaatccaacataaggtgataataagcgcacatcgaaacacgagagaaaatcacaaaaagaaaagtttg
gagtccccaacgagaaattaaatattgatgcaaattttagaaacgtagggagaaaaaaaagtgaattgagcacaactaaaggaaagagacag
aaagaggtttgggataggcggaagaaaagaagtggttaatataagcaaggaatagtaacgaagaaaaaaagccagagacgtcagagaatga
ggcgaaagaaatgaaaagaactcaaaatcaaataagacccgaaaatcaattttcttaaagagttctgcaaaaaggtaaaaggaaaaggaaac
agattagtccaaacagtagaataaaattttattatatttgaaagaaagaaaaaaagtctgatacaaacaatcaggaaagaattgaaagtaagata
aaaaaaagaaaaaattaaggataagtcaactttggagaacagaaaaggatggctgatctaggcaggaagaaggtccttttataaaacaaaag
aaccctggccaaacccttacgcaaaaaagagggcaatagtgaatgggaacaacttggttgaaaatccacacaaacagtatacagctctgtaag
ctttggagaagaaggttgatccgaagcttgaaatcactggctttgaggtattaaacgaagtaggaatggtcattcacgacggaattacatcactc
atagacaagagaggggtgaaacacaagcggggagggagagaaatacaacgtgaaaataaagatggaagtgaaaaaataaacggattttttttc
aaagaaaatataacaaatgtttatggtttttcttaaaaagaatcctacctaaagtaagtcacattgttatgcggtttttttaaacgaaaattaaccta
ttcgagaaatgatcttgttctcggcaaaagaataagataacctcgttgtaagagttattattgccgttgtcgccttcccttttcctcttgaaaaaagg
tttgcaccctaactctaacagagataaagacacagagcgggatgaccaaacgcctcctcttttgtacaggaaaaaatgaaccagagagataata

aactctcaattgaaaggcgaacctgataaccgaaatcaatggaaggtgagagtctgttgacctaacactagacacaacagtctactattctgcaa
ataagaagaaaacgggccagcgtgttagctttcattaaacgttgcttaacggtttacgttgggattctgtatatgttttcacttcggcaagtaagaa
acaagaaaggatcatattatatcaataggagcatacttgggttgtggaaattttcctggaagaagttacctaaaagaaggaatatggtttctggtg
tctgtttggtttcttcttttgacgacatcacggattcaaatttgtacgcattctaccataccatcgtcaccaaaaaccgctctttttcggccgtagact
tgagctttgtttacatggtatggcagggcatggcgcgtgtcatagaaaacattacatcgcacgacattcaggaaagcagttcgttttatgtgatag
aacgataatacgatgaaacgatgaaacgatgaaacgaacaggaaaaaaaaacaagaaagaaaaaaaaacccccaaggatgaaatgaatcc
tttaccaacggacagggtctcctttcattctttctttgctttatgaggtttccttatgtttcctgtttgtttgtttactgatttgctttacgtatgttggatc
gtagcccatacacgatccaagctttggcatagttgaagaaagctgttctgctggaggtaaaatagaatcttatctcgcaagagtttctcttcttccg
tcaagttgggaggaagtacggatggtgacgatgttgtcggaggcgatggcaacaaattcatcaaagaagaaacctgattattattgtcctgtctc
ggcgttactgcagatcccataggtaatccttgattttgcgatccaagaggcgtagctgctacaacagaatactgggatgggtgaaacggcgatga
cgaaatagccacaggatcttcccaaagtggttgcgacatgtgtatgctttccatattcgtttctcgtcgtccggccgtcccgggattctgagacggc
cgaatagaagccaacacacctgaagatgatgatgatgatggcgttgttgctggcacttgtgaatagcttaacgatgctacgtcgtcgaccctagtg
cgcttgaaaggagtatgggcagctatgtcggtagcaccgttgtttaatgtcgtaccttgtgatgaggaagatggtggtattacaccaccacctcctc
ctgctgcaattggcggtgtccctggaattgtctgggacataatagaaggtgcgtgaagcgaaattgccggttgagatgaagaattaactgttggat
tcataggtggtaccatatacatctcttgtgaattcccaatctggctttgcgagtttgacatttgcttcttctccgaagatgaccttgcatctttgaagta
taatccgaggttctctatttcaagctttgctttcatgtaaaacacatctgacggaaactttacttgaaaccgtttggtaagctaaggacagtttgtta
gtagactgaaaggaaccatgtcaatcccatttctttggttcatttttgaaagaatattctttttacgtcttacattgttgctatatacgcgaaagttc
atgcaaggtgccttagacaataggaccaagctccgttctttcgtccctgggatgaattggtgccatgaatctagtaaatttatactttgctacgtag
aataacatgttagtagaaagttttatgtgcgataccatcaccatacaattagttcttcgcgcctacgaatttgcaataatcctgatgtcagtgacatt
gtaaatccaccatctgagtaatgagaccagcttgtagatgttgaatcatatccataagagtcttgcgcaacagagtattttgcaattggataaaag
ttcatggttgtgtttatgttgacttcttgggtaacgcgtaagtctatttattgtttcactttgttactctataaaaagaaagaaacgcgtcctactcttct
aatgaattcttatataccttttcttttcatttaataggaacccctgtattgttttcgttcaggaaagccatggcaagtattttctcagtgagtgcgcgtt
acacgcgtttcccggcgtttttgaattttaaatgattggaatgttgaacatccatgctactttcttagtcttttcctattcctcttgaacgttttccagga
gcatatctcttcttgtatgaatctcttaaattcctttttagtttccatcagttgcactgaatttttctaaaaaccaagctatttgaagaaagaaggtt
tatttacttggatatttatttggattcgttcactcaagtcatagttcgtctacagcaaaatctttaacgcatttgctttctcgtatcctagacgcaagtg
gttcctaaatttttggtaacttataaaaacaaatatgaattttcaatttgaaaaagaaattgctattgtcgctgtccgtagggctagctacttgaccg
aaaaagtattcaaccaacttataaaagagaagagcgccgcaggtgctcactcaaaggaagataagtcacctgtaaccatcggtgattttggtgc
tcaagcagttgtcatttcattgcttaaagatgcttttcccaaagaccccatcgttggtgaggaagatgccgaccatttgagaagcaatggagaaac
ctgctctcgtgtctggtcattggttcaagaatcgattcagcaatcgaaagaatataaagagttgggtcaaattaaagacgccgagcaaatgctgg
ccgttatcgatcaaggaaattacaacggcggtcgtgaaggccgtatgtggacactcgatcccattgatggtactaaaggtttccttcgtggtgccc
aatatgctatttgcctttctttaatcgaaaatggaaagccaatggtgagtgccatcagctgtcccaacctccctttgacttcaagcagcccgaaac
aagtcctaagggtatcatcatgtctgctgttcgtggtcaaggatgctttcaatactcccttcataatttgactgtccaaccctcccaagtctatatga
aggatgtccaagatccaaaggattctaaattttgtgaaggagtcgaagcgggtcactctatgcaaggaacccaagaagaaatcgctaaaaagct
tgggattgttcatggacctacaaaaatggactctcaagccaagtatgtcagtctcgctcgaggtgatggagatatatatcttcgtcttcctaccagc
atgacctttgaggagaaaatttgggtatgttgaagatgaataacaatttaaaaagcacgtaactaacttagtttaggatcacgctggaggctccct
cttggtcgaagaagctggcggtatcgtatcggatatgtttggtaaacctttggattttggcgttggtcgtacattgaagagtaacaacggtgttattg
ctgcttacaaaggcatattccaaaatgtcattgatgctacgcgtgccgtcacttctcaagatccccatttcaataaggtctagataaactgaagaa
ggaagacataaaagcagattaactcttctttttgttatataagatcttatacgactgaatttttatttgtttatccaggaaaagaagtgtaaatttgtt
atcgtaatgttctccattctctgtccctttcctgaacgaatttttatccagattagatttaaatagtaaaaaagaaaatccattcaatggtagtaaag
ctttcaagctatcgttttcaaaaatagttcaaatcggcatggtaatacgataatatcttattaaatggtaagcaaatctcgataaaatgattttttct
agctggcagagagatctcacataataaacattgaatacagaacgacatttaataaagaaaatttttggtaaaactcgttgtttttcgtgtgaattat
actatctctgattggaaaagttgcaactttaaacttgcttttgttcgtttgacaaccgttttcatttttcttttgttgggatgtgtcccttaaacccatct
gtatataaacactaatatttgctttactttagaaaatggcaattccgtcggattgggaattaaatgaagtcttggttcaactagataaagttgatga
gcaaactgaacatgttttagtcaatacagtacaaaagtcttcttccaaatacaaaccatggagtctaaaaaactttttggttcgtttaaaaacttac
cgaaatcggtggtctttgacttatgatcctcagctaggtgaagtcaattgctgtaaacatggatggctttgtgactcctatgataagttagcatgcg
atctatgcaataaacaaattgatttaagctttctacagaatataattgagtcaaacgagctttcttgggagttacctcaaaacatcagagatcgtat
tgagaagtctctgcttgatgaacatcaggacggttgtctgtggaagcacatgattttcccagaaaacatatacgagcttgaaattgctactgagctt
ctgaataccaaaagaaggataaaaagtacatcaccttgctcagttcaagtaagtcttccggaagaaatgacccaagaacgattgatgaaagttg

ctgaaagattaaattatgatacaagtaatgatcatggtttctcgttaattgggatcgcactgactggttggacggagcaattgtcaggtcaattgta
tgagtgcgagtattgtcatcggagagttggtgtttggaatttaagtcaagagaatcaattatttgatattattgaccaacataaggtgaactgccct
tggagaacgcccgttatgaatagccaattacatgggtggcagatcatcttgcaactgttcacgggagaaataatattccaggaagtggataaaat
cacagaatattctcattggaatgatttaactttgaatcttcttcaagaacttcggtaaagcatgaagactgtaataatggagcaagaaatgaattt
acctcttgatattcagcaaataggataggtaaagaaaagaaaagaaaagaaaagataagttattattaaattattaaataatttttaactaaatt
aaaaataaataagtacacacaagatacttttagccaaaatttttcagtatgagagaaatagttaggaccgagtcgaattataacaaagagaaga
tatgaaggagtgacttttatttccataattccaaaattttattctccactgtttctggttcctctccttttcgaactttgggaacgtgaatggattttcta
tgtgtattagctaaaggatacgacggctgagagggatgaagtatctcggtaggatttacagagtgagatgtcgaagaaaagccaaccgcattttc
cgagtaaagacccggggttgaagaactcggagcatgcggacttgaggtaaaggggttggaagacgaagagaaatcttcaatttctgaagaaat
tacaaaaggacgaagtttaatcaaaaattcatatactatttcgcggttccttctaacattgcggaaatttgttcgcactgagtctgtaatttgctttgc
ttgaggatgtatatagcgtttggcgggatttgtattggcatcatcttccacggtatcaaatcgggaagaagctcgctgaattaaaagcttctgttgt
gttcgactagttgaagcacctatgtcgtacaattgagctggtgatttggtgacatttgaagaaaatttgcggttcgtaagtatatctcctggaagag
aagtagtgacactttgtgcgtctaaatcgacggttttcgaaggatctacatggttgattagattttaccgaaattaagaaaagtttcagaacatacc
agtattaataagtgtcttagggggggaccggttcctgaccttgattgtgaaatgcggcttgataattagaagtaaggggtccatgctcaattgaaga
ctttggtagtaaagcttctttatgaacaggacttgattgtgttgtactaggagttttcggagcagcaagtgggtaccgtggatacatctctgtgggtg
caaggggacgatcagaaaagtcaggaatgactgcatgctgatctgaaggaggtaaattggagatagcatttggatgaatcgtttcctctttgata
gacgattcacctgtggcagcttgcgaaggtgaaatagctaccgaaggaaagttttgatgttcgacttcgtaagaactctcactgtatgctcgttttg
cttctggataaaaagaaggtttttgttcatgcagtttgtatggttgttcttgtttcaactgcgcagaatcggatggagggggattatgagattctggtt
tgggtggatcagtgcgaatgctttcggtgtcaatatttccagctgtagagtcattatcattcgtaacttcttcccaaacaagtttaggcttcctttgag
tcaatggaattaagccagtcctcgctgcgaccaccctcgacccatgcaagtcattaaggtcattaagtgatctttgtctcggtatagcggcatgcgt
atggtgatgacgatcgtaattaggattatgggaggaagtccttgttaaaacattaggacgagtatttgcggcggctatttttccaggtcgtccaagt
cgaaggtgtccaccacctctttttattctgctattcacgataaatcctttcttattatccctagaagatttcaaagaagctccatctcctccctcagtgc
tgactgtcgaggtagaacctttgcgaacgtgaccgggacccgaatccatggtcaattgcaggttccaatttaataatcctaaaaatgaactactaa
tgaaagtaatttttttccgttcaatatggaattatgccaggaagagctgaaaatttgatagaataatagagcagagggtaggtaggggagtatagt
gcactggttctgcggcgaaaagaaaaaaatacaaaaatgaaagcgaagaatggaagaagagattaaaagtatgaaagttataagaaacttgag
cacatagaaggaatgaaaatgaagaatggtctcgagttagcaaaccaccaaatttaccagatttaggagttcataacagggatatataaacaat
agaaaattctccttttctgttaaattaatttatttttttcttgggttacacgagagacgaacaagggcattcaggatcatagtattatatgaacgaac
agcaccgatttgtctatccttcatgatgaaaaaccaacctgaattgaaatcattagatggaaagatttaaatatttgcgtctctgtaacgtgttctac
ttttcttcttctttcttcttttttttttttttttttttagagcgacttgtttttgtttacgtaaacaaacgattcggattgtcgagaatttatccttgtttgcatt
gtttgaagaaaggaaacagaaatttgattaaaactatgataaaagcttctgaagaaagtctgaaacgtctatgtgtcaagacattatgctttccgt
ccaccttttttgcctaggaaaaaattagattcgcagacataagaaaccacgttccttactgcagacgattcatacaaaagtaaaaaaaaacaatta
tacgatgctttccatgctacaattgctcgttttcttacttccggatatgccgcggcacaagacatggactcgagtaacagaagttctgttacttcttgt
aaaaagttgcgatagtcatttgtttactaaccacaacaccttccaaacacgcgtttaaaaagaaatactgttctcgattaatttctttcgtttttcttt
attcattccatttccgttttcgtttgggtgcgttgagtaggtgaatttcggactaaaggtatactttctttttttaagactttttgaagagaaaggttcat
gattgtttcgtttttatcttgtttttggagagaaatttgccattaaacgcgaggatatttcctttctgttcgaaacacctgtcctctattgctcgtacacc
attatatatctatatatctatatatagatttttttatttttttattctttttattcttttgtgtgctatttttttaaaaatttctttcccttctttttgtttaaaacac
cattaaaatggctcaagcaaatgaggacaaatctatgaaaaacctgtacgagcctccttggagacgtatacgattcatagggattgcaggccctt
ctggaagtgggaaaacttcagtagctcgattaattgtgaaagcattgaatttacccaacgtggtgattttatccttggattccttttacaagcctttg
accatcgagcaaaaacaacaggctttccgaaacgaatatgattttgacgccccagaagccattgactggagtttgttgtacagtgtgttactggag
ctgaaacgaggtcgtaaagttgacattcctatatacagtttccacgaacatgatcgtcttcctgaaacttccaccttgtttggtgcaagtattatagt
tttggagggaatctttgcattatacgatgaaagagttcgctctcttcttgacgtctctgtcttccttgagactgattcagatgtttgcttgtctcgtaga
ttgaatagagatatcaattaccgtggtcgtgatattcgaggtgtccttcatcaatatgatcgcttcgtcaaaccgagctatgataattttgtacggaa
gcaatcactatatacagatataattgttcctcgtggtcgtgataacaaagtggccattggtaagtagaaataaaccccataccttttattgttttcta
tctctttttactaacttatttgatctctttagaaatggtaatcaaatttattaaaagaactttgtctgtccaaagtcaaagccaccgagaaaacatcg
actctttacagcagatcgttcctaccattccaaatttacctttaaatcttgttcagctccggcaaactcctgagattgaagctataaagaccatactg
gtaaacaaagaaacggatgcagataatatgcaattttatttaagccgcataggaacaatgctgatgaatttggccggcgactctttaatttatgaa
gaaaaagttattacccttcataatggaaatacatggaaaggattgcatttagcaaaagaggtattactcaacattgacattatttcgtagctacttt
tttttcgttttcaaggagctaaccattatagctttgcggtgtttctgttttggggtctggtggtactctagaaacagctctctgtcgccaatatcctaac

gtacgattagggaaagtccttgttcgcgttaatcaaatcaccaatgaaccaagcctacaatatcataaacttccccgaggtattgctggaaccaat
gtaattctcatagcttcccaattgactaccagcacggacgtattgatggcaactcaaatcttagttgactttggtgtaccagaagaaaacattatta
ttgtagtttacgtgtcatttgttgaaagcatcaagacactggcttttatatttcccaaagtcacaattgttacaggatttttggagactgctgaggaa
gcagtaatgggtaagttaaatttagaaaagacgttttatggatgttaggttttatacaatgaatatggttggaggtaaatatatcatgtattatcgc
atatagtagaatgaaagtttgtggattatggcgttgtaacaagaaaaaaaattcattaagctctataatacttggcagtaaattttatcataacatg
agacgagatttaaaaattaatgtagcaagacgaacatgaacgatctataaagtcatgatttccttagctaaggaaattttcattataacggaggc
attaaaacacaaaaaccagaaatcaaaaggatgcctctaattctaaaatagggtcggcgtatagcttgccagatttcggtctaaatttgggcgcc
cactttccttgagaaatagtttcgaagcaaacagacgagaagaaagatacatttggaatgtctttttcttgatgtccatatcgatttttatggatgg
ctctaaaaatccgagaatagcggcatgtgcttctttggataaggtatttagttgtttgttgttacgaatttcgagtgaagagcacattgtttccagaa
gagatgatttttctttttcggaggatcgaatccaattgaccaccatcgtgctagaaaacggtctatttgtaaaagacattcgtacatggtgattccc
aaagaccaccagtctgattctctgtaacctatggaatgcacgagcgtttctggggacatgtaggctggagtgccaactcgacctttcacataacttt
ttccatcttgggtttgatgggaaaggcgggctgccacgttaaaatcgacaagtcgacaatggccatattcatcaagaaggatgttatctggctttat
atccctatgtaaaatacctttggagtggatatacatcagcgcagaagctaactcagcaatccatacgcacacgaccgattcttgaaagcgattctg
cgctatatgatatcgaagatcaccgcccaacataagatctgagacaaggtatattttgttatcgtcttcaaactcagaatagaatctacacaaaag
aggatgatctaaagaacgcaaaagcgagacttcagtgagaagttgttttagctttcccggattgtgtttttcttgttgatacatttcaaagcaaact
tttgcttgctgcgtcgatcttttaccacaaaaacagtactccaactcccttttccaacaacgttgcagagcttaaattgagagatgctgcaaaagtg
tttttgtccctcctcaatccatggctgctcggcaggtttcaccttcgacgattgattccccataaaatgtctaatgcaaatgagctttaacctaaaaa
ctagaaagaaaaatttatggaataaaagaaacacggctacttcttttgtagatttcgagttaacgagttttctggtaatcaaaatcaaaatcctcta
gaaatttgttttgaagcaagacagagccaatttttttgatgttgtagaatccaaatattatagttttacacaagtcaaaaaaaggaacacaattttac
ctctagatggaaaaattttcaattagatttcgcaactggtttcgatatctgaaggcgtccgtgttctttatttgtttttgattctaaaacttcaatgaaa
gcgacatccataggatagatcaattcctgaaaggaaggaaagaaaagcagctcagctctcgttcttattataaacagagaacaaagaaatattt
ttaccgttgtctaaagcagtgcatgagataaatggcatatctcctttcgctacaaaagaccttaagtaatagcgtatgaaaatgctgaaagattac
ttgggcagagataattggagaagaatggaagactggtaaatttaatggactgtaaacaaaaagaatcatttgtttgtccattactagagaaaaa
ggcttaatctcacttatagaaataaaataaccttctaattttgtcattatagatcataaaatgcttgctttttgaccgtagtttttaaaaaaaaatgga
gaaacgacgtcttcaccgtctttttttacgaaactctttgaacgactagtgatcttttattactgttttcattctatttgatggaatcgaaataaaagtcc
attgaagataactttgttaaaatgttgaacttttttttgaccacaaaattcgtgtttatgactttcaaaattctcctccgttcaccttgccttgtcctacc
ccaaaacctttaggtcccacgcataccatagaaaagaagcagggctcgtctcgtgtaattttattttactctttacagtgactgggtttctattctta
aaggatttttttaacgaaggacttgatcattcactcgttttgatgcacatattacttcaactggaagatattctgatccctaaaaactgatcactata
gtttgtttattattaattttattcccctccttcttatttttgtatttacctgcttcctaaggcttagcttcttgcttctctttttcggtcgtacataaataagt
ttgatcgggttctctgctgaccctggtttttacttgtcttttctttcccgtaatagatttgtgaaacacattcgatacctccttaaagagctgttttcagt
cttacagaatacccgcacttctttactggaatcgtattcaagtaactgaaacttttctatcgcgtgtataaagaaaaacaaagcaaaattggctttt
agtactgtcttcaagcttgactctctctgtctggtgaaaggttattttctaaaaatttcttcttttgaaaggaatctcttctatcccttttccattcttttcgc
tgtttgtttattctttttatttttgcatcatttagacaatcttttgtccatcgtttttcgaatattataccgacgattcaccaattgccttttcctagtttga
acgatataactcatttacaaaatgatttatcgaatcgcagctaggtatcccattcaagtgatcgcagtagtgtgcatccttgtttcaatggcatatta
ctctttccttgagactctaacacaagaagatttacccgtcttgcttcgtgcattgaagcgatctggtatattagatggtggtcttggtgtttccagtcc
ttctggattaattttgaagctctcaaaggatgaatcttctatcggtaatgctcctgtatgggagcctatcccatctgaccatgctcaagacaatctta
acgtcgactttgacatcacccaatggtatcaccaaacttcagccagtgtaaatgtcgagcagcttgcggaaccctaccttcatgattgtctcttgctt
gattcctcgtcaggttcttgtaaaatattctcaaaagaagtcggtaactggacagtttcaagtatcgccattcctgctaatttggccaatcctcccat
tgactactttttggatgactccagtaccgtcgttcaaagaatccttcctgcaatccgtgaacatggcatttcctggtcttggcttttaaagctaatcgc
ccgcacttggatgaataccttaaaaattacgagccaagcttctaaaatggaacttctcatcgttggtaccgcttatgcctgtatgttcatttctataa
tctctgtttatctaaaaatgagaaggctaggctctaagttctggttatttttcagtgtgtgcatctctactctattctctgttcaattttccctatctttaa
cacgtttttctggcgttcgaatcagccttgtatccttgatcgaaagcatgcctttcttactaaacgtggttgcattggataaggcagtaaaccttacg
agagtggttatttctcgttgtgcttcttctgattctcgttcagccacacatgaagatgttgctgaagcttgccgtgatgctgctcctccaattctccgtc
atttttcctttggaattgtggttttagctattttctcgtattgcaactttggtttaaaacagttctttttatttgccgccgtcatggtttatgatttgcttct
acttttcactttttcgtctctatcttgactctcaatttagaaatgtgcaggtacaatgccaacgaaaacgtgcgacgagtcttaatggaagaaggct
tgtctgagcctgccgctcgtcttgttgctgaaaccaatcaatttgctaagacaaataagaaggatacctctcgacctcttagctggctttacgaacc
tcgtttcctttttagtcatagcatttgttgtttttaatcttttcgagctttgttctattcctttcaagcattatgcagtgacttctgctgcggccgctagact
gattcctttggttcgctcggagtatccagaaattaaccccaagaccttttggatgaccatgtctttgatgaaattctctcttatttgtttgactctacc

tataacaaggacactgtttctgttagagttttgccagccgtttttatggcactgatttagcccaatcctctatcttcaaggcaattcattcttttatac
ataattggactaagtcaattggtggatcttttctatcaaagtgggttattttcggcttgtctttaagcatcggtgcaaacattttccttttgaatgctgc
ccggctaaattctgtaaaaaacgtggatgataaaaaagttgttgagaaagttgtcgaggtagtgaagtatcttcctccttctgagagtccttcttct
gaatctactactgaaaataatgttagcacaataactcgttctttggaagaatgtatttccatatataatgatggaaaagttttgagtttgaatgatg
aagaggtgattcagcttgtccttgctcgtaaaatccccttgtatgctctagaacgtgttttgaaggacattgttagagcagtcgtcattcgccgtgcc
gctgtttctcgcttatcaagaacacaaaccctagaaagctcaaactgtcccgtttatcactatgactattcaagagtactcaacgcttgctgtgaaa
atgtaattggctatataccttttacctcttggtgtcgctggaccactagttattgacggtaagccatactacattcctatggctactacggaaggtgct
ttggttgcttcagctatgcgtggttgtaaagccattaatgctggcggtggcgctgttactgttttaactcgtgatgaaatgtctcgtggtccttgtatc
gctttccctaatttaacgcgcgcaggtcgtgctaagctttggttggatagtcctgaaggaacagaagttataaccaaggccttcaactctactagta
gatttgctcgtctcaagcagataaaaactgctatggctggaactcgcttatttattcggttttgcacttctacaggtgatgctatgggtatgaatatg
atatcaaagggtgttgagcatgcattgactgttatgagttctgaagctggattcgaggatatgagtgttatcagtgtatccggaaactattgcactg
ataagaaaccggccgctataaattggattgatggacgcggaaagagtgtcattgcagaagctattattcccggagaggccgtgaaatccatccta
aaaacaagcgttgaagatttagtcaagttaaacgttgacaaaaatcttataggtagtgctatggctggaagcgtcggtggctttaatgcgcatgc
gggaaatattgtaactgctgtctttctcgcaacgggacaagaccctgctcaaaacgttgagagcagcaattgcatcactcttatggataatgttga
cggtaatctacagcttagtgtttccatgccttccattgaggtcggaaccattggtggtggtaccgttttagaacctcaacaagcaatgcttgacctac
taggtgtcaaaggagcacatttgacatctcctggtgacaatgctcgtttacttgcaaaaattgttgctgctgccgttatggctggtgaattatctttat
gttctgcacttgcaagtggccacttggtaaagtctcacattggcctcaaccgcagtgcaataaacactccagctgattccaaagcttctcaatcctc
aaatacatccaaacagaatgctcctcatttaagtgttcgttaaatatccctccttaaaattgtcatattataatcaactatacgtttgcgtcaatact
gatattagtcactatctcgacattaatgactctaggttctataccccttaaagatattacatttagtaaaaaaacaaactttttttttttatgatttcctg
tagcacttgaagtaagattcagtaagtagcaatcaaagcgttaacagttttagcccacaagaaacgaataaaacgtctaatcaactaaatttaat
ctaataattatatttttttattgaagtggatatttgcagttgcaagacaagtaactatgaatgtattgtaaagatcaaggtcatcgcgacacttaacc
accacagcaataggtaagaacgtgtttcaaacaaagttgaggcttccaacacagaaaaattagcaggaagctctctgtttgtcgtcatgcacgat
tcttcctgggatacagcagatatccatggagtttcagagtttcttgatgtatcgcgaactaccatcgatagtttactacatcaggacttgttttcatcc
atcttttcgtctctaataaaacgatacaaagaatacggaaatttaaaaaataatgaattaattgctgaagtgaactttgaacagcaatctcgagtt
ttagaaaagcggttattggagtctgagaaacaactggacgcgtcaaaatttgataacgaaaatgttcgtaagtcacttctttcatctgaagagaat
tctattaagaactataatctttatcaaaatgcacagaaagaagtagacgcattgaagaaggaacgcgatgaaacaaaaagtgattacgaactgc
tacacaaagaaaatgtacggcttcgagagtcccttgttcaaaattcgaaagatttacaagagaaagttactgtcattgatggctataaaagccag
cttttgacgtacaatcgaaaggttatagaggtacaacatttacaacaggtggtacaatcatcgaaatcttcttacgaattcgagctgcaaaagtta
tcacaaaagaactcaatattggataacaacaatacatggctcaatcaagttattgaagatctaaatacgaagttattgaaactccatcaatctttt
tcgttggaaaaatcacagctgaattcgcaattgacggatgcaaatttaaacaaggatgtattgcaacgtaaagtggattctttaagtaaacaatgt
actgaaagtcaactgaagcacgaaacttgtcttaatgaactttctgagctgaagaaacagcatgagaatacagttttctcacttcagaaagactta
gaatctcaaagacaacttatcgatttatggaccaataaatacaagacgcttcaaggtcattctgaacatcttcaaaatgcgaactctgaattggat
acactacttagagaggcacaacagtcccttgaggatcaacattctgttttcgaaaaaagtttatcttctagccagtcaaatgtagagtttctaaaga
aggaaatagcaagcttggaatcgcagctttccttagcgaatgaaaggcttgctaattctgaagattctggtttttctttttctttaaaacctagagag
acgtcattatctttatccgatttatatacagaaagactatactttaagcaaaagtatgagcatagcaaacagcaagtggatcgtttacaacgaaac
ttgaacgaaactatgaacgaggtaaatcttcaacaccctctcattaaagaacagtttgaacgttttaacgaaaagcagaaggaaatcgcggcaa
tgagcgagcaataccaaaagtctttggcagatttcagaaaaacgcaatcgggcttcaaggctctggaagtacagcttaaagataaatctaaaga
aaacaaacattttgaacaacaaattcgtgatttggctcgccaggttcaagtattgctacatgaaatagaccttcgtgattccggaacagtgtttacc
aataaagagcgtcaagtttttcgaagtttggtagagcagtcggtagaaactcctgaaagcgatactaatcagcttatatctgattgtcttgtttcctt
cagaaatatagctgaattacagcaacaaaaccaaaaattactttcagttattcgcgatctcgcccaagagctagaaaaaaatgaaagcatttcta
acgcttccaatgtccaaaatgatactttaatgaatgctaatcgcattattgatgaactctcccagcaactcgagtttatgaatgaacaatttaacgg
ttgcataaaggaacgggatttttttccgaacgttagttcaagaaaacgaatctttttttaaaaattgacgacgattcgttggatatccacaaaggaat
gaaacttaaggaaaggataggcaatcaacgatcttcgatacaaattgacttactaactgaagagctggaaagctttaaatccgttgccagtgaa
aaagaaaccaaattattggagaagattagatccttagaaactgttaactttgacctacaaaatcttaattcggctgtaaaaatagatctggctaaa
accctcgagaaatgcggttactatgatcatttattgacaacaaaccaaaaagaacacagtgaactatatgaaaaatataagttattacaggaac
agcacagtaacagtttacaacaatttcaggcaatctctggtcaattgagcgtttattataagcaagtggaaacttatcgatttgatttggagaattt
gtacaaagagaagaatctctggaacagtgtcgaatcgaaattaactttcgaatgctcgcaactaaattcctctttattatctattcagcaagaaaa
caatcaactgaaagcacagttacaatcccacgagcaagatcatgatggagttgttaaattattgaataaccgtctttctgaatcttccattgatttg

agaaatgctaatgctaaaatttctgaatacgctgatgacataaaaaggattactctacagaattcttatgatattaaagactatcagctgcaattg
aataccatgggagacaaagtttccgaattaaaacaagagctcacttcccaagctactcgatgcgcgagccttgttactgaaaaccaggagcttaa
ggataacttaaggttgactcaagaaagatgcaataacttacaaacaagtattctatcttcaagatcatccgagggttcaattactgctgaaacag
aaaatattatgaaagatttagagacaactaaaggaaagctacaaagcctaggggatgagtatcaaaaggtccaacaaaagtgtttagcatcgg
agaaaacattagaaatgatgaatgaaacccatgatcagtttaaaaatattatggaatcagaattgagggagagagaagaaaagataaaaagg
ctttatgcggaagcatctcaattaaaggaagatgttaacatggcccgacaagacaaggatttaaaatatcaggagcaaatgacccaaattgaga
atttaacccgagaaagagatgaaatgctgagttacaaggaggaaaaccagtttattcgtgatgaattcgagaaaatgattctttctctcaaaaaa
gacttggagaaacaaatacagctaactaaggaagcacattgtaattatgagaccgaaatcataaatcatggttccactactcagaaactttatga
catgcgcggtgattacgaagaaataaaaggaaaataccttgaactaaaatctaaaagcgaggaatggacgagcacgcacgcaaactcagaag
agaattggaggaaacaaaaggacaatctagagtcggaggtatcttcattgaaagaacagttggagggtctagagtcgcaaaataggatactcc
attctcaatttgactcagtcagtcatcaagttgaatttttacaaaataattcttggcaaaatgaagcgcctgtatccagactggattctactggagat
tctgagttgcgtgagcttgttaattatcttcgacatgaaaaggaaatcaccgctaacaaacatgaactggcttccttggataataagcggttaagc
cagcaagtgaaaagtcttcaagggaccatagattctttaagttttgaggttgctagactaaagggacccacaaatgccgatggctttggaaaacc
agaggtttttagtggatctgacgaactccgtctattatatgagagcaatgaagttcttcgcaaagataatgaattaaagcgctcaaagatcgatag
tatggaaaacagagtcactgacgttgaaagcaagtttgagccgttgaatatcgaaattaacgaccttaaaattgagttggatgccaagaatgag
agtattaagctactccatgaagataatgagagatggaaatcacgtttccaatctgtattgactagatacgaaagaatggatccatcccaatatga
agaacttaaagccaagtatgacaatcttgatgctgaaaagaaagatctagagtcagcttttgaaaagctgaaggaagaaaatgaaaaattaaa
aggagattcagagcaaatatcggcacttaaaacagagattgagcaacttaatgcaaaaaatgtcaaattggctagcgcttggaatgaacgttat
gaaagtcttatcaattcaagtaaagccaggttctctcaattgagacaggagatcgcaactagagataaagaattaaattctaagaaagatgaaa
ataaaagcctgtttaaagagctcgaaaatgttcgtgaagaagcgaaaacaaacactgtcgaagcgacggaaatggtttctaaagataaactac
aagaaatagagcaacgaaaaaattcactagaacaagagctctctattgctaaggaaaacttacaatcattgacgaaagaaaaagaaactgagt
tgtcttctaatacaagtcgttttcagcaattagaaaagcagttagaacagaaaatacaggagaacgaaacgctctctcaaaagttgaatactcttt
ctcaagaactgacttctatcaaagacgcacagcttcagcaaataccgtcagatcatcctgaagcctttgatttccagcaaaagttagagacattaa
agcacgacttcagtaaagaaaaggatgcgactttagaggatacgcgctcgaagcttttagaagaaaaagctaaagagcacgaaagtcttcaaa
aggagctggatgaaagtcagacaacaatacaaaggcttgaatcagatttacaatcgctaaaaagtgcaatggagaataaatctgagccggaac
ttgaaggtctagtaaacgaacgacttgaaaaagccttacatgaaaaagacgaggcctttagcaatcggcttaatagagctgtcgctgagacgga
gattcgtaacaaagcgaaaatatcaatctatgagaaaaaaactagagacttacaatcaaagattgcagaacttgaaggaacacttagcggtcta
aatcagcagttggaatcacagcagagcgtagaaagctcaaaaaaacctaacgttgcgaagggcaaacaatctgttcaaacgtccaatcaaggt
gatcagtctactaagtctctaagctccaggttacaaacaactggcagtaaacagaagccaaatgttgctagacccgctgtagctcgacctgtacc
aatgaaaccagaaggtggaaaattatctatcacgggtgcttccaaacgtttatcaactggtaaaaatgcaggtcaggccaagcgccaacgtgat
gattctaacaaacaggataatataaaatgaagagctataaaatttgatcgtttatgaagtgatggggtttaaagatcttaatatatttaacattgct
tacgtttaatttaggactgaccttaagatttttgaaaataatataatattcagtactataacttttatacaatccaaaagtaaagacacagcttgtaa
tgaagtgagtgaaagaaataaccattccgcttatatagcactacaatataacgaaagcagcagtaaacaaggaagtataacaaacgcgaaatt
aaccattaaaacatcttctatactgagttatgtcttaaaaatcataaaaaaaacgggcggaagatagcacgtgttgtaatgacatagcttgaaac
aagataaatcgatagcaaactttcataaaatttataaacaaaaagagaaggaccatcattaaaaacgttcattaacaaatgaaaaggacctaa
aagggagggattaaaattataaatcaatgagcgaaccaacttgaggttgcatagattgttgctgcatattttgttgcgagtttccattaatcatatt
ctgtggatacgaatacataggttgctgagccccagtaggttgagtatggaaaggattgagtgtatttccagtagcttgcgactggatcgtttgacc
ggcactgttcgtgaatcttgagccagtgtgctgtgtaggtaatcgagcaagaccaacattaccaaaactatcaatacccataggattaccagatg
ctatggcttgagcaatattggcatgtttatcatctccagttcgtacaggttttacgggtttagcattcacaggagcagttggttgggaaggcaatttg
gcgaattgcgcgaatggattattggatccagttttcgtggtttccaattgaggttgtgattgcggttgaggctgtgttacttgtggtttcataaagccc
gtaggctgagtttgcagaggacgagagtagggattatttgcattaaatcccgtattctgcggacgaaaccctgtgctctgaggctgaatataacca
gtatgattaggtggcataggttgaacataaccagtatgattaggtggcataggttgaacataaccagtgtgattgggcggcataggttgaacggc
ctgaggaggcgtaaaacccgtacgttgaggttgcataaatgggaagggattattagaatcaaatggctgagcaaacccggtgcgttgtggttgc
ataaatccggtagattgcggttgcatgaagccagtgttttgaggacgcatcatgccaggattttgagggaaacccccggtgcgctgaggctgaac
gtacccagtattttgtggataaacaaacccagtaggttgtggctgaacaaaaccggtactttgcggacgtataaaaccggtcggttgctgttgtaa
aaagccagtgggttgaggttgaacagggttgccaaaaaaatcaacaggctgaaaagcaggcgtgacggacgattgctgctcacgttcgcgttcg
tgtctttgtctagcttcttcctcttcctttgaaagttggattgctttttgaagatcagcttcagaattgttattcactgaacgcctacgagcatcttcctc
ggcctgtcgcttggattcttcgatcactcggcgaagctcggaatcatcctcttgatcgctactaggtgaaggtctacgactacgacgggagcgtga

gcgagcagaaggacgacgtgatcgactacgatgtctggaacgagaacgatcttcatcgtcatcatcaaagcgactgggagcgcgcgaataacg
agattcttcaaaaggaggaggactcacgctgtcattacggagacgagattgcatattatcacgatttcgacgtgcttcacgtaaagcgtcgtcatc
ggctaagagtgcgctaatttccttcgccttgctgcggacattttgaccctggtcatgtccatgttcatcgacatagacgaactcccgcaacgttgtaa
taacatatatattgtccttggcccagcgaacgacattttcggatccactgtgcagacaatactccagaagagacaaggatttaaagacgtgtctcc
agttttttcctttatcattcaaccgacggtcgatgatgtccattacctctaacatttcattttggtcaaatgtcaaagcagcaatttctgccatggcac
taccactcggtccccatgcatcatttgtggtggcatttcgaaccttgacctgtgtgtctgtgtaaccttttgagaaatttttcctaaaacagcggttag
ttaggtctaactctcagcaaaaccctaatttttcatttgcattttaaacaaaggaaagaaaagtgaagtcagtgttgaacattgttcttgccatgca
gcaatgaagcagcatacatacaaaatgaagtcgaccaattacgacactctgctacatcgtacttttcgcaaacctctcatgtcaacactttctttcc
ttccactctatttgagtctttgataattcattacatacacggatcttacagcggctttcatgctcgatgtaattcaagaagagacaaatcacggatca
aaaccgaacagtcgtctctcaaactcaaattgttcaattcttcgtttatttattagagcaagtagctcaaacctcctaaattcagtttcctttacagtt
ataggtgatggtaaaagttttgtggtgttttatgtgcgtttaccaagttgtcctaaagacttcggtaatttcgtcaatggtacggttataccgcggtta
taacgacttttaatgaactaacggtaaaaatctttaccgcggcatattataaacgctatatctaaaataaggaatgaataaactgtgcttaatgca
cactaaagtcttataaataacaagctattgcactataaagtaaataaataaaaggtgattttggtttttagatgtcaaatgagataaacggaattt
gcaatttttctcaagcggtttatactttactcttctaagtcctgaaaacaaaatcaaatacttcctggccaaatgatatctcaaaaaacacttggcta
tgataaaagtcaatgtgtatacaaatgatctgtgccatagctaaagaatttcataacatatgaatgagtataaaatgaacacttgaaagccatgg
attggtaaattgtaggaagagaccaaaaaaaaggtttgactaccaatctttttttcagtgctgaaatcgtacgttacaactatgtaacaaacttctcc
ctccttttagaaaacctttctcgaaagtaatgagggtaaaaaaccaaactaagtacttaaaaaatctatgccaaccgaccaagcattcaaaacgt
tcgtacagcctaaagtatcaccttcgtgtagcaaacgaagcaacctggaagaaaatgatttgtttactgttttgtgaaccaaaacgaaatctagca
taccataatgcagttcattatcttggaaacaaaatcgggtttaccataggaatagacctgggatttgtattgcggattcatttcattatttttcggtaa
gttactaatgagatcgatagaaaggaattcttaaaggaatggtacttttgattcaggtatcctaccgaaaggacgaaaacttgcgtgtaactgcta
gcttgggtttgctagaaagcgcattgttcatcgggctctttccttttataattcctttttttagctttcatcggattgacaatggatacttgaaaagcctt
tgccaagtggaattcttacttgttccaacgttatgaattgacctttgctaatacatcatggagaatagacttgctttgagatgcacaggttatttgga
agaaagccagcagctggaacgagcccttcattaacagatgcaattgactcggtaagagcttaaagttggaaaaaaactgcttacaaggtgtata
gctcgataaacgaagcgattcacttgaggtgaagatctcaaaattagactcccaattgtctgtatttcaacaaaagattgcaaatactcggcctgg
gccaggacaaactactttgaaacaacgcgctatgggtgtattacgacaaaagaaaatgtaacaattattagcaaaaaaggagctttcactaaca
cgtttctaggtatgaggctcaattacaacaattgcaacaacagtcttttaatatggagcaagcggccatgacaaccgagtctatgaagaatacca
tgaccactgttcaaacgatgcaggatacagctcgacagatgaaggcgcaaagtaaaagtctgtccatagaaaaaattgaaaagctgcaggatc
aaattcaagattacatggatacggcagatgaattcaacgacgttcttgggcaaaacatggcgaatgtggatgtagatgaagaagaactggatgc
ggaattagaagctttacaacaggaagcttcgttaaacggagaccaaataacgcaagatcaacctagttatttacagccatcgaaggagattccg
aatttcgtcgacgaagaacaattggaagctccaaatactgctcagtgaaaagcagtggaaaagtgttatgatattctattccttcatttaagcgcg
gactcattttcaattcttacgacacaaaaatgataatttaataaaaaatacgataaagagagaaacaaacgtatctttaatatggtatgaataag
aatgtagttatggcttttgtgcagatagcatgtagttgcataaagggggcaagcaactatcacccggctttgcaaggatccatttggactgaagag
gatgatacattaccccacggacatcatttactttccaatggtgttcacggaagaattctgccagctcttctgggcgaatatatttttgataatcatga
gttccgtcggggacaattccgagtactttttcagctaaaaagatcgtcaaaacccgagccatgaaggtccgcgaaatggtagatagcatcaaata
gccattgggcttgaccaggttcattaaagattctaaaaagtctcggggattcgtaacatgctctactacttccatgcaagtaacaatattaaactgc
ttatcgaaattctgtttttcaatgggtccatggatgtacttgagacgatcgcggatcttagggtccttttccgaatgatcttttgctacatctaatgctt
tttttgacgcatctacacctgtaacattcgcacccagacgagccatactttcgctaaggatccctcctccacatccaatatcaagaacctgttttcca
gcaaaaggggttttctcgcgtatgatttgagacataaagtcgatgcgagtgggattcattaaatgcaacagtcgactctcaccatcccaatcccac
caagttttcgccaatgcatcaaaatgttggatttcttgattcgacactgtatggttcctctgttgcgacaaggagcccagcagaccccgctcaaagc
ttttgtatcctgatcgtaaaagattatacttcatcatcatcgtatacttacttcatttccttttagcaatttacctactgattgttgcactttgttgtatca
ttttccttctccttagaagaatcagaatagcgtagactgccgagtcaaaattataagcttatagagagacaatacgtatcctaaaacgaattgcac
tctatagtgcggcgtaccgcatttagaaacccatgtattttataataagaggaaatattaaagtctatttctacgatagggtgcgaagcatataga
agaaaatatttcttgtataatctataggattcgattcattagaggggatttacttggttcctacacattgctttattgagcgcttcattgcacaagaa
aaaccgaatctttcctacagaattcgaaaaaaaaaaaaagagtcgctaaacataacattatgccgcttccctttggtatggaatataattttcatac
attatacaattgcttgtttaaaacattggaaaaaattgagtcactttcggtcgaatcgttaccgaacagcaatctcgattcgtcgatcttttatggttt
gtttacactttaacacaacgtagcgcaatctagaaagtaacgagttgcgcagaacattgctctatgcttgcatacctgcttattcatcctttactgat
gtgttctagctcgtaatttcttactctttaactggggaaatccaatacgtagagttgaccattcatataagtttgtcctttgcccaacaacatacaaa
cacaactgcttgtttcaagagcaaaaagaataaaactctgaaattgaggtttcggaacccaaagaaacgattgtgccggtattgaaaaccagtat

agggtaaaaactcaagttgaatacgtcgttttagtgcaggaaattcaaggttcgcggaatctgattacacaaagcccgttaacttgggtatattgc
atagaagaaaaaatcagcagttggcttgatctcaagtgaacaaaaacaaaaaatgttttggagactaggccaagggtttggatttcaaagttcg
actgccattgaggcgatattgaataaaccacaagaagagatcgatttaccagaattattagaagagaatggccttttagatgaatgcaaaagtc
ataatccaaagctcttagactacctttgtaaaccagaggttttgagccaactgatcgacttaattctcgaggtaagcgaagaagatttccttcctgt
tgagggtggattcgaagagttggaacaaagcagactttcttttgttgctagtgaaatccttagtagtgatgtttggtctatatgtgatgcttgcgtcg
agaataaggagcttatgacaaaattatggtcttttttggacaatgaaggccccttgaatcctttacaagcttcctatttcgccaaagtgaatgagca
ttttcttgataagaaaactgaagaaactgtagctttcatacagtcaatcgagaactttgtcgacaaaattctccgccatgctgaaacgagcgctatt
atggatcttttgttaaaatttatttctatggatcgttgcaatacagccgttggaatcgccgactggttgtattctcagggtttaatacaatctttgcttc
gtttactcaatccttatgttgatcctgatgtacaatccacagtagcagatgtcataaaagctattatcgctatttctgctaattctaatgagcctggcg
ttattggccctaattctttatcccgtgaattggtgtcacgccgaaccattaccactcttactgactatatgattgatcccaaagctccttattcatctac
ttccttgattaatggcgttagcatcgtcattgaacttattcgcaaaaacaatagcgactacgatgtgactcctgttctacagatgcctcttgatactc
aaccccctacaacacgtgaccctatttacttgggtacaatgcttcgtctttttgccgaaaaaattccaatttttcaacagattttatttaagccttcttc
tactcctgagggtgtggctaccagttttggttccattaagcctttaggatttgagcgttttcgaatttgcgagttatatgctgagcttcttcattgtagt
aatatgtcacttttgagtgatattaacgcagaggcccttgttatgcaaagagatcacttacgggactatcttttccgtcataataattgcgctcgtga
tcttagtatatctgatgaagaagatgctgattctagctatacggataagcatgtgagtgattctatgcatggacaaatccctcaaatttcaaaggac
tataaagacttacctactgaggttgctggggattcgccgatggaagacgccgaacccgttgcggatgaggagtacaaagacgttcgtgaaacttc
aaaatatattcagccagatttagaggaaagtgcgtcaaacgactcgtatgagccattacctgaaagtgttcttgatgagtttagaaaagaacctgt
aattggagattttcttaaaatcaagtttactgagaataatatcattcctacagtacttgatcatttctttgaatatccctggaacaactttcttcacaa
cgttgtgtatgacgttgtccaacaagtcttgaacggccctatggagaaggaacaaaactgtgcattagcatttgacatgtttttgcgtggaagaatt
acagaaaaaatagttcatggtcaagaaatgagtgacaaggccacttctgaacctaatggttttagacctggttatatgggacatcttactataatt
gcggatgaagttgtgaagttcattgagcattactctcatttgttcagccctgaaattaaagaattaattgatgacgaaaaatggcaacactttgtca
ataatactctagcagagactcgtgctcgcgataatcaacttcttggtggacttgagccttcaatggttggttatttggaagacatggatgatggaga
gatgttagatgccaataatctgccggaaatgcaatttgctttagaacaagaatacagtaacagcagtgatgatgatgtggttgaagtccatcgag
aattgtctaataactcttcatctaatgaagaggatgctgatggtaatgaagaagatcctttatcacgtgaaatgtctcgtcgacttagcttcgattca
aataatggtgcacaagataaccgcgaccactttgctcaatatatgtctcagcatatttctgataatccccctgatgagttcagtagcagttcggatg
aagatgacgaggaagacgatgaggttgtcgaatgggtctcccgtggtaaagacaaaaaatatcctcgctctaatttttttataagtagttcggatc
gggatgaatctgatagcgatgaagatgacgagaatgatagttcagatggtgaacgtgattttgccgaggatgaatattctgaaggacttgtactg
gtaagtggaaaaaggaaattaaaagaatggacggctaacatatgttttagaatcgtaaatagtattgtttactttatctatcagttgatagttttct
gcatgtttacaaactttttgtccagatagatgttcgacatcagcattggttctttttgaatttggccattgcatgttaagaggaaacttgcgttagta
ttcggttcgactttttaattttcggggtgtaattttttttctttgaactacttattgttatagtcttcttatattagatcgagaatgtactaatgttttgcaat
gctttcatccgtttgatcttgtcatgtacatttttgacgcatgttttctttggaaggatatttatgtgaaaacacttgttatcaattcggatgttgtgaag
atgagcagtgttgagtaaaatagcttgctttttaggatcctcttccatctaaaatatctaaggtttcctaagattgatttagtttttgttttaaaattca
cgattcatatgaaggctaatttgtgagatatattaatcacataaaacttacgaattaatcaaaaacttaccatacatagttgaatttttttttattatc
gtactttttttctttaaaaggtgtaaattccgggtctccaaaggcaaatgactgtatacacatgatctgatgcttaagaaggttattttgtactttttttat
tagcaacaaaagaacgactaagttaaaatgactacatatcctgagagaacaaaatctgtaaggcattttggttaaaacaacgaacttgatttaat
ttttaagacatgtactaggaccccctataaaaaggaaaacaaaagcaaaacctaaatccattatcttcatactcattaaaacctaaaagactagtt
acatgaaaaatccttaacaattagcatcctaagtttctcgtctcacaggaagcactgtttcaccaaacaatggttctccaggtcgacgtacgacga
aatttgcatcaagccaatccataacagcagcaaaactgtctttgggaatagtcattcgctcacgtttcgtgatcaatttattcatcatggtaaccag
atgatggtaaaacagctgaaaattcaaagaataagcagtggaataagaaacaaatcgttctacacatccccaaggtgtatcttcgaatccagca
aaaagtgctggattttctaataatcccctagctgacatgataccatccgtatttgtgagattagcgatttcgtagccttggcgaagagaagcaacgt
ctccattggtaactacaggaatattaacacaagaacggacctcccgaatagcatctaaattgacaggaaatgaagaacgatcttgtctggttcgg
ccatgaacagttataaagtcagcaccagcatgttcaatggtttgcattagatgacgggttttatccagatttttttgatatgcgtatctttgcctcaac
acagaaatcttcacctaatgtgtttttaactgctctgatcaattgatatactttttcgggttccttcaaaagcgcggatccgatcccttcgtgaattgc
ccaactttgaggacaaccacaattgattccaattccatccacatacggacctgcgagttctgcagctttcgacaggatgacgggatcctcaacacc
gaattgaagaatcaaacttgcgtctttttcgtttgtagagaaatcaaaatatcttcctttaggatgtaaaaactcgtttgctaaaatcataggagtat
atacgatgtctgtttgatagtctcgtaccagctgtcgaaacggcaatttagagtacctgaccattggagctgctatatagactggccttttgccttttt
gggcttcaaaaattctcactggatgcttattaggatcgcgagtcgctaattctgccatttttatgctatgtagcaacttttagatgttggtagttctca
gtagatgagacaggtcttatgagagaatactacaggaatcaatggtattagtatgaagccaattgagcacaaacgcacgtttttatagcttttgttt

tcatttatttgaacctcttaggcgtaaatacggagtaatgtattaaattacatgaggattcaaagcttcaagacaagtctaattttcaaaaaaattc
attctaaattcatagtacaaaaaggttttggattaggggatccatggtaggatgcaattttaccgaaaaggttgattatttctaaaaaatggatgct
tcaacgcttgtttaggcgtgattctcttctcgggattgagttccaagcattgttccaacaaaagtatgaagtctttttgaatcacaacttcttcattcg
tggaagcaagcacatcctttaatcgggactttatatctcgaacaggcttgctaaaattcaaaactttctgtgtttgttgtccagtaacaggatcaatt
tcattattaataaagttgaaatcttcatcaaaattatcaaaagtaaattgactacgtttgagcatcttatggctaaactttcctttgcattccatcata
agccgaagcatttgattattgcttcttccgggaaatagaatctgtccagcatatagttcaaaaagggagcatccaacactccaagtatcgattgaa
caagaatagggatatcccaaaactaaaatcgttagttcaaatcgaagcatttgacttactaatttccggggctcggtagaaacgactcaccaaat
aaggtgtaatctcgttttcagaagcatcagaagcagaaccaagatcacatattttcaagatatttctcttttcattcaccaacatattatccggttta
atatcagcgtgaattacgttacattcttccaaaaggcaaagcgctgtgaacatttgataagcatacacttgaatcgcctttatgttgagtccaacat
ttcttccaaatttttcagaatatctcgaaggttcagactaagcatttcaaataccatacataaatgatttttgtgcataaagtatcgatcataacga
ataatgtgtctcctcccttcggggtctgcagcctgaaggcgttctaggatggacacctctttcatgccggccttatacattacctcattgtttcttatg
atcttgatagcaaacgatgcttttgtcttttggtccattacattgaccacgctagaaaacatacctttgccaaggttggactgaacaacataacgtg
aatcaagttcttccataagaataaccttatagtatccttcaatatcgtcccaagtgtcttgcatcccagcaagacttcttttgacagtagaaggcttt
ttctccgtcttccgtttttttaaagagggagatggagtatctgcaaacatatcgtcttcctcatcatcttcattctcgttaaccgtcttttcagaaaatg
gctcatttgaaactggtcttgctaaacttgagtcgttcaagccattgggttgctgaccatccaaactgcgtcgttcatcttctgaccgacttgtctccg
gtttggaaaaaggattcgtcacttctgaagtaagagaatcttccgttgaaactcgcgatggtgcattggtggaactttcaacttgatgctttttcaa
aatctccaaccgtcttttccgtctctgttctatgatttcatcttcagtcaatggattatcattcataattaaattagtacaaacttttcagatgcgtcaa
gtctgacgatcgctatatgtttagtatttcagctttatttgccctagagccaaaaactgaatacaaatgtccttcaaattacgttaatttagtacaatt
gatagaaggattataaaacttcctcgagaaacggcgttttctactataaatgagtgtgtgggtaaaattgtctttctctcaagtagttccaaaacgt
tagagacgtaaaactcatcaggtatgtttttcatgctcctttaaggtaatagagtaggcctttggttttatacttcatagaataaataattttaagttg
tatatttgctgtaatcataagcttgtctaaggtgaaatgaaacacaacatagtttcggtaataaaacggtagctcatacgatttaatctagtaataa
actattgaaaaatactagcccatcgttcatcttcagagcgcttttctgctagattggttgttctctccaaatggtttctcatgctatcaactgagaaac
cacccgctgtttcccaaatggaactagcataagcattgggatcattagcaataagatcatttggggaggaagtaagcaatgatcgcatgttttgct
cagtcaggtcgattaaaccgccaatatgtttggcaaggatagaatcgcaacgttgatcatagtagtaaaaatatcgttccaagatattaataaac
aattttaaactggtaatctaattctgtgttagcaataaaagttcatttatcattaactcttacctgatccatgcaggaatctgcaatttttagactcttt
tgaaggcattctaaaactcgtttgggatccctgaataactttttctgtgttagacaacatatggaaaacaataacgtctcattggacataccactgt
ttcttcattcgtttcgttctgccaccatagatgacttgcatgaagaattcccctgggtaaatgttagtaaacaataattattactggtagatcatacct
gcattgatctggtttcttaagcaattttgacgcataaagtgtacatttcgtaatcaacgtatcataatcatcaacagaaaagtttgtggttttctgaa
gctttcctgtaatcatcaatagctgctggtattgtaatcgagaatccaaagcggactcctcataaatagtaaaagcttgggtaaagaattcataag
caaaatccgggtagttttcaacatcggccatttctgcggcaaaaaggtataggataagggacagctctgcagaatcaccgtgattataaagaatg
acgatgcaagcattaacaaaatcccataggacacgaactttttcagcccaatcaacacactataagttgttagccactaatttttgcttttctttaat
ttaccttaaacaatctaaattcacgggcaagaaaaatacatttgttcacaaccacaggaagtaagtacttaacattctcaccagcttgcacaaagt
tatccttcaagcaacgtaaaatctatagtattagtactcaaaactgaaaattagacacatacctcaatctggatttgaggatcgtcattatgtaggt
aatgaaccagggtagctattgttttcaggtcttctaaagtatcattgccattattctcgattatcactgaaataaccccaaggagctcctggacatcc
ttcaattcgctagtaatctgttctttttcaatgatttttttccagtactgttttggctatatcatatcgtatattgttcggctgtgctttgaagacagggag
aaagtttggtaagtttaagcagtacgaaaatgaaggaaaggcgacaataggaagcaacaatattgaacacagagatttctgaagagaccgctc
attcagttcattttgaagagacggttgttgaagaatgcgttcatttataaactgaaatactctatcggcatattgggttttgtaagggtagcattgca
agaaaaaatttaggatacttgacagtatttgtacaatactgcccatagaaataccactacgggcgttaaaaacatcaacaatgtaagtccatagc
acctcttgaatggctaacttgggagaaataactctatctccggggcacactttattttcagaatcggaaggaccattctcaagtaccatgttctcca
atgtttgatttagtgagccttctccatcagaagagtctgcttcgcattcgcgctgaacataggcagtaagtctatccagcatagaaataaccaaag
gcgtaatatctacgcttttgtttaggttcaagatagtattaaagtatatgtctaaagtttccaaatgcatttcatctgggaaaacttggcaaatcacc
tcggataagtattcttgggctaaaggatctctgcattctactacctgttcaacaatagctggaagaatgacatttttgtacgcatcaaggtcgaaac
tgagctgggagagccgtacgagattaagaccgacaagaaccttaagctcgtttcgctcttgagttcgcctcatgtagtctttcatgggccctaaatg
ctggatcctaacccataacttgttcatttccgtgaaatttaagagcaaaaatttaacagaatccataagagtccctttattcgggtcggcattatcat
cgtcgggatcctgcggtagcccttttcgagtttgtgtcaataaataatgtctaaggaataggccacgtaaaggatgctgtacacctgcacacatat
caatcaggtcattcataatttctttaacgagagcttttggagtctccaggtatgcacttcctactgttatcatcaaataaaggcgaggaagaacact
tcctgcatactgtacgagttcataaagatccatcaaatgatgcgtaccgttcagatgtgtctcaagcaatgcatcaccaagtcgccgcagggtttct
gtagtaaacatgtataactcataatattgctttggagaaagagcattgttcctcatctctaccagactgatactgcagtttcgaaatgcgtccatta

atttaccagtctgcaaattcttctgcatcagtcggcttgattgcttgcatattgccaatgtttcttccaaaggtctcgatatttcttcatttaccggcac
agtgttattcattagactgaatttctgtttctaacacccaagaaaactatcgttgcaattgcaaaatgaattcaattcaatctgaatgtagtgtcaac
tcgaatctttttcaatctattaaaagaaaatcctttctgtttatagacttaaaggaaatattggtaacgacaagctaaatgagaagtttgggattat
ggcgctgatataatacaatgtagtaccataagttctctttagacaattaatcatctctggatacagttatcccaaaattggtagtttgaaagaaaaa
atacataaaataaattaaactttaccaatatatctgaatttacattttcttgttggacttggaagacgcgtgttcaatttactgttgagttacaaagg
aattgaagggatccttgtgctgtttgctagaaggaacattcttttctttatcttctttttgtatttctatttttagtgataaataggaaagaaccgatctt
gagacgcattcattatgagcagactatcctctgtttttctcggccttttatgctgaagcagctggtttgcgataacttgtcggttcaaaaggtacaa
aattatctttttctggatttttgctaatcctcagcggtactatcatcggaaatcaaaaccttttttggtgcagctcttgagcaatgatactccaaaact
tggtaaactgggtatgtctttcataaacaaaggaatcaaggaaagaaaaatattataatggatggtccaatgtcctttgcgttgtgacaagtcatc
ctcaaaatcccctctgtctttcaactgttctcccatggtttcttaaaattcactaacacgttgcactaggagatgtaattccaatttctagagggtatt
atcgaaatgtgttgtttctcaaaaagcaagcacttgcagtcagtgctgtggatctcaaaggtgaaacagagcaagagtctgcctcaacaaaagg
acaaaaaaggacttaaaaggttgattgatatcctctttgctcttctctgcatctttgtgacaggacccttacgatcctctccttactttcattgatttca
agtatttatttttgattaagggtagtaacagtttatgctttaagacggtatttttcttgaccataacgttcttctgttgaaataaggaacttgaagttg
attcgtcctgttacattataaattcatcgttggtaacacgatattcattcgagtccattctacccatgtctagtgtagacgttttgccaattatgttttgt
aaatcattcctgactttcaaagtctttaattaattaatttcactgtctattgcttatggtcatatagttatttttgcattgtagtgtacttcaattaaatg
accaactttatagaattgagctagttcaacagaaggtatgcatgttttattaaggtgtcagagtcgttgattttgaaaactaaggagtatcatagaa
tcgagatattcttttcgaagccttgatgaagaaaaacctaacactacggcgaacagtttctatgtacagaaagattcttcatatccctttttatagta
ttttccattgagtataccaaaaagagaatgtttttctgaaaaaactacttggatcctgtctctttctcgggagcttgatgcaagcagtgcagtccgt
cagttgccattccgatgagacagtttctattcaagcattagaagcttccggtgttgcttggaaagaacttgagctaacacctagttgcgctttggatt
catccttggtaagcaatttgactgacaaaaactgggatgagtcgatcaagtctgatgaatggttggttcaacttactttgaatccttgtgatttttgt
cttaaggaagactatattttcaatgtaagtatgaacgtattctttttaacaattgtttcgctactgcctccatgtttatatgcaaggaggacgaagaa
aatatgcggaaagagtaaaaatgtttataaattataaaagatactaacctacaggatctatcccatcacatgaaagaaacataccccaatatcca
atttggccgagtatatgtaaatgacaacccagaactggctgttcgtctattggtggaaaagctccctatttactattttattaatggagcaaattatt
atactgttccagcatcggagctaccagccaagaaggcattaacggcttattcaaagcataaattctcggaatttgagagagtcaagggattctttt
cttttgtgggtccttttatgatttttacaagttccttggtaagatccttgctttgtatagtcgttttagcagtccgtatacgcctcttttgattaatatttc
tatgtttgttatggcgatgctctttattaacaggtcaaacaagagaagaagacaaagaaagcaaataaaaaaagaaaagtaagaaagcaaca
acgattcgtcccgtatatgtctcgtttgaaagttttgcacgttgcaaaagacaacgagagatgtgattctcaatgcgtgacaagaaatctctaacg
aagagcataggtattgactcgtagattaacttttaacttcgaaaaaaatgaaaaagtttcatcatttgaattttgtagccgcttcagctgtgaattgt
cctcctcaagcaaacaatttaattcccagagggtttatcaagagaagtagtgggaaaagacgttttggaaacgaagtagggaggttcggacattt
tggaattttgaaatacattccatttcgtttgatcttcttcagtcgggcaacgtaattcttccacgtcaactggaaaactcaatgtgcgaaacggatga
tcgacgtgagaagtatcgacctgttccatagcatgcattcttccaattccttcatttagtttattaatcatttcctttgtctctatttccgaggaaggtg
catcaatagcgaccagttgtaacaaatgttgaaaagcctctatcgacaaatcaccgggtggttttgcggttaacagttttgctaatgaaaaggaa
gatggttttaaaaagttcattgcgattggttcgtgcgcaagtgtataatcgaagggatatccttgatgggcaatttgggaattaagaagaaaagta
aagacaatgaatagagtgttccaaaaaaaaaaacttcagctttaattaagaatttagcgacaaaaaaggctatttctgttattccttcaaaatggtt
tgtttagcactgttcaagtttagcacaaaactcgttatatgacttttcgtgcaaattttttttttttttgaaatatggcaattgtaggttgacagaaca
cgaagaattagtaaataaaaagatttctaacaaagtaaaataaaaatagaaaaagccgaaagcattgtccctcctgtactagtagtgtaccgaa
gagttaaatacccttttcatgaggatttcttacatctccatttgggaatcttggaaacagaaaacaaggagagactgtttttcatgtgaattcacta
gtagagaagagattgattctgcggccttggaaaaggaacgtgtcatcctatgtaaaatagttattcagttctttctacagctttttacttgttttttttat
cattgtatttcttttctttcgaatgccatttgactactttctagggcccacttctattcctaccttgcattgctttttttagttgtaattcaaataaacaaa
caaaaatgtttccatttctactcataggaaataattgtaagagccacttcttagaaacattttatgtatctgtttcttttcatattttatttgaatgcaa
caaaaaaagcaactcggatatcagatgtaaacagacagacacccagatagggaaaaatctaatttagcgcgttccaccaacctcaaaaaagta
tttagtgagataaaataaaaaacgtatctaatttagcgtactttattccgacttcataaatacgttaaaagaacgcaaacgagtacggtaaacgcc
tttctgtattgtttacatcaagacatgttgtcactgctattctctacataaaacaagaaacgcgtgaacaatgaggaaactgtgggaattgaagaa
aaaagaggctgaaaaagagaagagttcttcaggaattagccctgctcagattcgcgtacaaaaaggtatgcttgctacgcgtttttttatgaaac
gcgaacgagtcagaaatctaaccatcatcttcatatagacatttcagaactagatgttccctctacaatgtcgactagctggccggatgtaaccaa
acttcatgaattgcttctggaaattcgtccagatgagggtaggattgtcaattttagaaaggcagtagctgctgagccgtgccttctcaagtcaata
attttcaaataaattaacatattttcaggatactatcaaggtgggaaatttcagttttcgattcatattggtagcaattacccgcatgatcctccaaa
ggtgcgatgtcttaacaaggtaagttcatgaaaatgagtgatgcagaaaacctcactgtttttttgctctctcttaacacactttcttaatgagata

tggacctgaattctaattcatcaataagatctatcatcccaacatcgatacgcaaggaaatgtttgtttgaacatattgagacaagaatggaatcc
tgttttaaatctaaattctattttagtaggactccaatttttgttccttgctcctaatgctgaagatccactcaataaaggtatgtccaagtggtttctc
gtaacccgaaaagaaaaataccaagcaagtcttccaaactgaatgatgtcttcatttattgattaatttgagcataatatcttctggaagattatct
ggacgagggttaagcattgttattgtcaccaccttttatgtttttgatgtcccttcggctgtactaacaaacatgaaactagaagctgctgctgattt
acggaatgaccctagaggctttggaatgagggtgaaatcttccatgcaaggaggaaccgtgcaaagtgatgtttttgacaatgttcttctttcatg
aaaattaatcaccttttccattccagtgtaatccatttttttttttacctttattaaaaaaaaatatatatagtcgaattccttttccaagtttatcaaaa
tcatatcttgcttccattggcattagcgagatggatttacgtctccgtatctgtactatattaagttggcgcttcgcaaacaccagttttgtacagaac
acctcaaatttatagaaaaaatgctttgcaaacgatctttccaaggcctccgccaacctggctttcaaaccctttctcgtcgtttcttgagtgtaagtt
gaatgcgttgttcacaaaggttttggcgaatttgactgtcttaaagaccgaccggttgaatgaaggcaacaattaatcttgaagtgtgaaaacctg
ctaaaatgtcagtgaaaaatatatgaaaagtctttgttttcacacataatggattctgatggaaaaaatgcgatatatgaaggatggccaaacta
cgagcatcggcataatgtctcgaaatttgtaatgctaacaaaaaggtagattactcacgaagatcctaagcctggtaccacttcagctaacatcgt
tgaccaagagctgggtaacattgtcggcgtctatgctcgctatcccctcgtcatgagccacggtcagggctctttcctctttgacaaggaaggccgt
aaatttattgactttacctctggtgttgccgttacttctttgggtcacagccaccctgaagtggcccgccttgctgccgatcaatgtaccaaactggtc
cacagcagtaacttatactttaatgaacctgccattgagcttgctcataaaataaacacttctttgtctcaaaacagcaagatttcctcacctagca
agattttcttctcgaacagtggtactgaagcaaatgagactgctttgaagtttgcacgcaaagctgcttacgagacgcatggtgaaaaaaagaca
caaattgtttacttcaacaactctttccatggccgtagtcttggttccctaagtattactgccaaccccaaatatcgccgtggtttcgagcctctactt
cctgatgttgttcaagctacttacaacgatatcgagtctatcaagcaggtcattaatgacaagactgcggctgttattgttgaacctgttcaaggtg
aaggtggtatctgccctgccaatgctgagtttttggtggcattgcgcaaggcctgtgataaattcaacgcgtctttaatttatgatgagatccagtgt
ggtcttggtcgttctggtgacttctgggcccattctgtcgtcaaggatcaggccagccccgatattatcactgttgccaaacctttggctaatggctt
gcctatcagtgctaccatcgtttcttccaaagtagctgaaactctccatgccggtgagcatggaagcacttttggtggtaatcccgtcgcttgccga
gttggttctttctgcgtcgatgaattgagttcatcaaagatcttgaacaacgtccgcaagcagcacaacactcttacttctcgttttgctgagtttact
actaaataccctagcttaattcgtggttacgctggtcgtggtcttcttttgggtttgcaattcaagcaatctcctgctgatttcattaacattgctcgtc
aacagggtcttttgcttttgccaggcggtaacaataatacccgtgttcttccctccttgaatgttgaagactctgttattgctgatggtttggacatca
tggatgatgcattgaagcacctttccaagtaatttataatgtgcttatataataactatgggtatgcgaatgcagttgcttgggctttaaatttaaat
atatatagacttaattgaaggaaatagagattttggattttaagtaagaattttactataccataccaatgaaaacctgtaactattggtttagatc
agtaatgagtttgtcttgcctcgtcttattccgtttggtggtttttttgatataaattctggttttaaacatttggcaaaacttaaccaagtttgagtgg
attgtaccggttattagtttttttgggagattatttggtcatggaaataatatgtgtacaatgtaatctttcatttgtatgtagtttggtttagtagtattg
ttggcgcatatatgttggtaaaacttgcacacatcctatacccagagtctttgaaagtctcctgtcaaattggaatttatattcaaaggtatgagctt
atagcaatcttatcctaaagaagagaaagtaaaagttacaatttactgttaaatgattcgcaatgtcgaactagtttggagtatctgcttcttttcgc
gttaaaaagttctttccaagtcaatttgatatccaactgcatatcttaaatcctttctttattaaataatctggctttaataagaacaaaaccgagtat
gccagatagaacatccgtttcatccacttctagtacatcctctaattggactccaggtgcaaggaaaagcagtctggaaagtccaacaaatatatt
cccttttttcaatcaagatttagaagacacaaagagaaaggaaacagaaaataccggtactgacaccaatactttaatcacaaacagaccggaa
actcctgaacaacaaaaaaatcaacagtttccatttaatggacaatggccttttagtcgaagatctagtcaaagcagctctatgcttactatggaa
gacaagcattgggtaaaaccttccaagaggtcaggaaaaacgaaaccctcatcgtcagtaagtcaattttccaataatgtggaccagctgggtg
aaaaaaatagcaaaaaccagacatctttattgaatatttccccccataagaatcgaaaagccaaagaaggattggacatttcggctttgcagaa
atcaatttatgggcctcgtagttttctaaggagtcgaaaagattcgtttggaatatttgggtcatccattcctcagtcgctggttaataaccagatga
taaatggctttggagcagcgtcctttgcctttgctaaattaagcaaagtacggtccccccttagattctaaatttaatgctaattcgatgtctgcggat
gatacttggtctgtcctggaatcagaagtttttacactgtacaatttggaaaaattgcattatactgttgaagatttgaacggaattttagtcctgca
tcttcagtcttgtgttcgggaaagaacgatggatctttttaccagtcatttagattctttgtttccgaaagctaccagtaagttgtcggaatcgctgtc
tcctatcccagaagaagtttttctatcaaaattacttgaaatctggctatttttcctctcttctattctaccatatatagagggcatatttttgcctatca
aaacaaaactttttgacatccaggaaaaagcattattgccttacgagattaatgagttttgtagtacaaatcgagaaaaactaaacgttcaaaga
cttatgctgactgcatttcgtgattacatgattctacctgccactgatcgcatccaaagaatcattaacaaacagagcgagacttcggcacgctca
gatcaactcacgagtgcttatgctcggttgtttcaagtattgagtatgctagcaagtatacattcaaatgaccagtacgaaaaagaattggtaaac
cttgcgtccaaagttcgttctctgctgactgtaagttaatatgactgatccatcttttgcagtttaggatggtttatttcttatcaatcccttttttttttt
tggagagttcacctacactctacataattaccaaatcagtgttgctggagaattgtttccactatgctaagggctgtctaattttcttaatttaatgaa
atgaatacttataaaaagtcatgatgaaagaaactgtaaaagatagtgtctaaaccttgctttcaagtttgtggtccggagcacgcatattgtaat
accatctgggataacaaaattcatactcaagttcaacagtattggcaggatcgcaaatgcatctaccattagcatggagtaggtaatccgaaggg
caatgactccaaggcagatgatagtatccgaaatcttcaaaatagtgaatttgtgatttattaaggaataaagaagcagcaatgctatgaatagg

agcatcgccccaacgctcgtaaaagaacccaaaatgctgatctaagtactcgaagtaatcggtgtaggcatcgcttctaaagaagtttaagtcag
caatttcaaaattgctccaaaaatggcaaccgttatattcaccatctaaaccttttcatcatctacgataaagtctaaagaattatctggggcaag
gtaatgcggatttttttgacaaagtctctggttgtattccatagagtacgaatggtttccgggtattcaggtaaagtaactacaaatccataagctt
tctttttgtcacgaagcaaacgaaaaggatcactgtgcagttagcaactattcaatgtttttaaaagatggacatactaaggaacatcgcaagaat
aaataacgcctggttctacgcgccaataatagtcgtaatctttaagagcagaatttcggtaaaagaagccagaattgaaacgacacataattcgg
taactcaacgaaaagccatagatgatgcgttgtttcgtcatgtcgcggattgtttgtaatgcacgattcatgttaagattcttaggtagaccccattc
atgttgttcaagttgtacatatgtagtgttgctgctagccatttcggtagtagcattgataaattccttggaaaatggtgcatcattcatgaatgtcca
gggataatgatatcgtttattgaatcgatcctcaacgttttggatagaactaagagcagcgataagatctttattgcggcagagcataaacaaggt
agcattcatgagggccggttctatgtcgtttaaagtagaatctaccgttggcaggtcatgctttaccaaatgtaaaggctcatagtcaacagtaag
agtaaggggtgatttgtagaaatacttataggcaataggagtccaaacgatgaaaaaccagagaagcatatacctgtaagaaaacgttaaaga
agccatggtatgataagaatcagaaagacatttctaacacttcaaatggaaattgttgaacgaaaaatatataagaggacaagataccttaaat
tcgattcactaggttgagaaaaaaaggagaaattcaacgattcaagcgaggatgatgatattccactggacaaagaatcaaggtgagaaaaat
ctttgttcaatatggttctagttttcaatatgttagatacttctatatttttttcattatgtaaatgaaaagcagcatacctataatggcacacactatt
ttttctttttaagatgttacttaaaatactataaatctagacatagaactgtaacataagtcttaattaagctctgaaaaggaagattttgaattttt
gtcttggtagtgcttgtcaagcttgctacctaaatattttgttacttacttgtagtcaattgttgaaagtgtttagttgtttaatttttatgtatggtggtg
ctttgaagtctatttcaataagatgctgtagttttttttatgggtttcatatccccatactttggtcctaaagtgtttggttatgtcaaccatcaaaacat
tcacagcacagggtggaaggagaaggctcgttgcagaaaacttctttacaaactttttcaactatagagctcaattattaataagttattaatgatt
atacgggtaaacaacccatttcgtgattcttctgttattgaactagagatcacagtcgattccgattaaaataaatattcaattcattaaacagaga
agacttacgataagtcatagctagaaaaccaaaaattgatttgtttgcttgtctagcatacttgcacatccttccaataaatgcactatttgatcaa
gacacggtctaggaaatacatcaaaggaaacttataatcctttttgaattttattttacaatgaaaacattcattttctttcctaaaaccatacaat
gctaatattctgcacgtttgtttactgccagatgtgtataataaaatatcgttttagcgtgttacttttgctttgtttacactgccagaaaatccaaaa
actgaaacgagtggagtcgaatagggctaatgaagtagttatttttttattgatcgcttatgtacgagtaggaggatgagagtacgaaagggtac
aaaagtattttcgcatcaagttttgaaagaaattttgagaaaacgacaagcttcaacttggctgctcattgagaggtcggtttttcgatgaaacggt
actatttgtggtaacgtaacagaatgaattatcttctaatgtttgaattttcactcatgtttagattggagatttcgtcctctcataaatctcaattcta
tatcccatcgtcacattcaacagtagtaaacttacaaaaagtgagtttctcttcgtttcatgctccattatcccttttttcttttacacactttgctacaa
ggattggcttttcctctcataaataaaaccaagttaagctccccagtcttcgtagctatcggtttcaattgcgcttacagcaatgaatgcttcttttta
cactataaataaacaaagacaaacgaacaggaaagccagaagatttttttatttatgatacctacgtttttcaggtaacaggcagtgaacaaagta
taccgaaagcaaagaaagtaatataacgaactgtacaccacgtcaatattcataatagtgtgcacgtctgaatacgcataaaagacactgttact
ctggacgaaagaaagtcaatttctggcgtaagatgcctttgccttccacgttaagcaagaagcggtcgtttcttcattacttgctgtacgaggatat
tgtcagaagtatggtaactaagatcaataaagaaaatgcaaggattctatttttaggaactggaacgtccaatggcatccctaatgtgtgttgtttg
aacgtcccaaagcctacatgtcgtgtgtgttcagccagtctgacgccagaaggacgtaaaaacaagagaaataatacaggagcaattgttcaag
ttgattccgagggctggagtcgcccgaaaacgatcttaattgattgcggaaagaccttttatatggcagcaatagaattcatggtaaagcataaa
attcgaaacatcgatgccgttctcttgacacatgctcacgctgatgccattaatggattggatgatttaagaggtatgctattttacatttgaataag
gagatagaaagagtctcatggagtttaattaagagcggggaaacgtttgaagcaaaattttttgtggatattgttaaaaagtattgatactaacga
ataatttatagcttggacatctgggggaattgttcaaagcactatgaaaatctacttaacgaaagagacatatgattctatagcaaattcgtttcct
tatatggtcgactgttcacgagccaccgggggtggtagtgtgcctacttttgactatcgaattttcaacccagaagagccatttgaattagaggggc
ttggactctcaaccgtaccattacctgtgcatcatggtttttatttttctcctggtaaggagagtgttccattttataatatgggttttcgaattggtgat
ttgtcatacatttctgactgtaactatataccaccgaaaactaaagacttgatgagtggatcccaggtcgtagtattagacgcacttcgacgagaa
ccttatccatcccattttacttttgaacaggctcatgcatttgcctgttctcttctaccagccccaagacgggtgttgtacaccgggttcaatcattca
gtcgaacatggtgaaatggaagaagaattttcaaaactgagcattcctaccgagccggcgtatgatggccaaattgtagagtttagtgtatgaat
acggtcggagaaggaaggacaaatctgtcagtctttacttgttattctttcttatttttattttttgtctaaatcgtttacgttgtaaaattatgttgttc
acgctttttatgttttgggtttaaaaagttatttctttttcaatattatgctcaagttctaactatttcggttgcttcgtaatataaacacatagattaaa
attcgtggtttttattaaagccacggagccaaaacattattagagattatagaatttaaacaagaacatgatgtacaaaacgaagaattaaaaga
aaacattccaacatatataagacataatcggaaacagacagaaggtagtcctagctagagtaaggcatagattctcatcatttagaagaaaaga
gagattgaatggctgactgtaggcattgcacattgtattcaatgagaccagatttttttgaaattatcggcacggatgatgcaaaccaaagccaaat
actgattcatttcacgcaaaaccaagaccaagtcgttggataatcggatgatagaagaagtttcgtccaaagcatcagggtgtcgctcagaatgt
ttctggcgtgcacctccatatatagagccaatatcaagaataacgtcgatgaaatcggagcagatttcataactttgaacgtcgactggagaact
atcagtagcgatgtagattttggagagcacatcaaaaacgtaagccttttctactaaggaattagcacaaaagatattgagcaggttctcaagcg

taggaagctgaggaatcagtttttggatgacacgactaaaggcttcaaagatggaatggtcaaagatggaagtaagatgaaacgaaataggga
cattctcaagtccaatgtcagcaagttcatcctgggtccgctgctgaatgtcacgctgagtgtctatcttgaattcatcgctaaggccgtccactttg
tgaataaatacctcaatgcagatattgggattaatggcaacgacgcgagcaacagtcacatgcagacgagccaacgcatccagataatcatctt
gtgcatctataacaaaaataagtgccccaacttgaatgaagatgctttcaaaatcaaatgctgcatcaaagacatccacttgaccaggaaaatcc
cagaccgaaaagtcgataaacgagctatcaagaattgtgttagtaaaacattggaatctaaaaaacaaaaatggatgactgcatacgaaatgt
gttcttgtgtgagacgagctgttgattccagaaataaagtttcgttcggcggcattttgtaaaacacaactttttgaattgaagacttgccgcttcgt
cgaagacccatcaaaatgattcttttgggcttcatttttgcttacaacttttggtaagagagaacgttattttgagttctccatcgagaagcactcac
tcaaaataacgagctacttcaccctcgcaaaaagacgaaagtgcggttgaggtctaaaggtactaactcgtttaaggaggtttccaaatttacatt
catagcactgcaaatcatgaagattcacaaacttgtttattttcctttttataattattctttttgcttgctttacgtatcatttctctcgacgttggatcg
aaatcgtgattcttgccattgaggatctacagggttttctctcagcagatcaccttcaaagatctccgctgatccccggataaagatcgactcatgc
ccagtcggccaatcaacatccatcgcgatgttctagaccgctttatcacgttaatcaactcataatcgacgatcatgtatccggatgattttcccta
taaatagaggatctacctcgatagtttcacatccatcttgtatgaaaatcacacacgctttacgaataacctaagatctgcatcattccccttaact
gtatcaactaaggattagcatttttttgatctacatcaaaaacaattccctactatctctactatgtcttcgttcaaggtattcacaacatctaaatttc
attttctataacaaaaacattcatgcaagagttcagagtcagttcttttcctaaaccaatttgaacttcctgacgactagtattttaaattaacgcca
gataagaccattcaatacgaaaacatttgttgataacgtgaatgagaagattagcttcttttaaacgacttggagcaatccttactgagctcttga
acatctcatctacaagattatcagatgagtttacttggagagatccaaaacaatacgtccagcaatcttaccctcgtgcatgaggtggtaaacctc
agggagagtagagaagggttggaccttgtagaaaggcttaacgagaccgcgagccacgtagtccaaagcctcaacagagtcaaggcggttacc
gacgtgagaaccgcaaaccttaagcatcttaacggtcaaccagaaaatgtcagcaccgagcttggcaccagcaggcatggcaacagtgaccat
ggtagatccgggacgagcgaaagcagcagcttgctcataagacttgggagaggtggacaagaccaaggtaccgtgagcaccaccgccagtctt
ctccttaaccatctcgacaacatcgtcagtcttgaagtcaacgaagacatcagcgttgaaagatttgacaagttccttcttgtcttcaccagagtca
atagcaacaacacgcatagccatagccttggcatattggacagccaagtgaccaagaccaccaccagcaccgggaatgcaaatccactcaccg
ggttggacaccagattccttcaaggcacggtagcaagtgataccagcgcacatgacaggagcagcaacctcgaggggaacagactcgggaata
agagtagcatgagtagcattagcaatgcagtagtgttggaaagaaccatcaacggtgtaaccggagagttgaatgttagggcagatggtttcttg
agacttcatgcagtattcgcagttaccgcaagaagagttcatccacttaacaccgacgcggtcaccaatcttgagacggttgacaccaggaccag
ccttaacaacaacaccagcaccttcgtgaccaccaatcaaaggaagcttaacatcgagaggccagtcaccttggagggcgtgcaagtcagtgtg
gcagacaccagtgtacttgatgttaaccaagacttcgttctcaccgggttcagcgacaggaacctcctcgaacttaacgttatcgggatttccgtgg
ttgtggaagacagcggccaattgcttatcggggatagtcatgttgatatactattgatgaacaattgacttcgtagggagaccctctatatatagtc
tctggaagaggctaaacgaaaatttctcatgtctaaatgcttcggagcgactgcaaccctggcgaaaaaagtcggagctttccgccaatcatctg
ccgaaaacctcatcttcaagtcatacgcttcttacaccatagtatttccgacgtaatcgtggaatgataggtgacatcgactaccatttaagattttc
cgacaatccaatggtccgaaatgcttctagataggtttacggataaagcttttcatcgaaaacaattgacggtataatgaaactaaacatccctca
tttcacgcctattgcggggataagaggagattctggaatatgcaagtttggccaaaccttacttaccgcttaaattctttttattgtcgttttctttcct
ttcatgagccttaacgtttaagattggaccttctttgattacttggccatatttttgtttaacaaagacaaagacaaagtggctcggtagctttttgtg
gaatattttaatatttttgccatctacatttttccttatcgttcggttagagtatagaacattgtaactccatgatgtcgacaatgatggaagtttaaa
gaacttcttcggtagaaacaaagggaaggtgagagagtatttaccaaaaaaaagaacattgtacagtttcaggttcagtttttttttgtatttcat
tcatctttattctttattatagttatattcatttcaaatattctcacagtaaccaagtaggtttttaggttgttgaaaaaacatttcagcttcaaatgca
ttgcaaggctttgcgttcgggatattctttccataacatctaagacaaaaagaaggtataaataaatgaagagtatatttttcttattcgggtttcct
tgtattctttccaccgtaaactacactaaagtttctttcttgacagccctgcaaaatttttttgaacctcacccttcctcgtttattagacttcggttat
attagtttcagactttatggcgcggaacgttatgtaaattcggccgtcccgcctcggtaaataacattttgaacggaatcccctcgtaaacaaacg
agttttgaaaaagaggtcaatcacgttctaatggacgattgaatcctccaggtcgattcatgtattgacgatatttagcctttttctgcttgaatactt
gcccaacatcaccatgttttttcctgtagtcgttccaaaccctgagaagcccatcatactctgcatatctgccatcgggtcttgttcgatgaaaatct
catctttattgtccttgtcggtttttgtctcaccagcccgcatttccgatgccggaggcgaaggttgatcttccgaataattttctgaagaatgattca
tatcttgtttccctttttcttttgagactcttcgggactaatgaggtgcctttctttttgttgctgttgatctccagttggcgatccgggggaccgcata
cgcatttcccgttcacgtcgaatcgatcgctcttgctctggccttcgatcacgtccccaattccctccgtactcgttttcactacggtcttcccttcgttt
ttcctcataactgtatcggtctcgtcgatacgtacgctctcgatctcgagatcgcacgtcctcatcatcacctctatacgaatcccgtgatcgcacat
atctgtctcttattctttcagggctcctttctcgccgaagccgtccttcgcgtcgtctcccttcttctcttgaatccctgtacgttcgcgattgactctcat
tccctcttttgtccttttctacgcgacttgaacgaaaatcacgcgagaaatcatctctttctttttcgtaatctcttcgattgacggacatttcaatgca
tatatgctttaattttccaaaatctttacacaactaaatacaaatttagggagaaacgcatgctccctttccgtttccttctaatttttttaacaatga
attttctacgattcgaacaagactagtaattgctattttacagcgccagagattggtttttgctactcttcactctttttacgttcagtgtcgcatcctg

tttgaagtaacgtatgttgttactttgatataataactattgaatttaataagctaaatactaacgattttttttttctcttttcttttcatattaaaggc
atttcttggctcaaaaaatttaaacattaagctgttcaagtccattcttgaaagcttccaaactaaggagatacttctgtttcgttccacttgtaccat
cgttcaatagtccactttgaattgcaaattctgtcaaatgtctcaaggcaaacaagggatgactctccttttcagcttctaaacatttgtctacctcct
gcaaccaaatagacaatggaactttttgcatcttcactcctcgtttctcttcacatgctgaaaccatcgcattaccgtcaacatcatcatagcccata
acacgtccgttcttatcttcaggaaaagactgttgatgctcgattccttcgtgcaaacaacgggcgatctcctcagtggaggaaatacggaatggg
aaagaagcgccattttcctgagtaggccatttttgcatacgtatacatgtttgtacgaatctccagaaaaagtcacgagcaagaatttctccgtcat
tagccaaaatgaatcctggaagaataacatgaatattaggatttcccttttctttgactttgtagcaaagcaattcacccgcatgttttgaaagcgt
atagcccatagcttgtcctttggcttcacggtttgctgttgccaactcttgttgtccacctccggatacaaaaacaaaactcttcggtcctaccatgct
taagtcaagtagtaccttcgtactcatcacattggctttccataagtcatgatatgtctttgtccaatgcaccattgcaccattatggtaaatcttgga
aacgtgcttctccaaaaagtcccatttttcagacgatagcgaaagactgtcatcacttaaatcggaagcccatacaatcaaagatttcgcatactt
ttctatgtttggtaacgattcgtacattcttctctcggcgtccgaatcattcaatccgcgaataagacaaacaaccttcgctccttgttttaagaaata
tgccagtaatcgcttcccaaggaaaccagtagcacctgttaatagtatccaagaatcttgaatgcgctccaatggtttaacgctacctaatttttca
gccagctgattggcatcgttttctacatccacgatagaaacagccaattgatttgagtttccttctttcacagcagcagcaatgctacttggtgagct
cttctggtgcatcagcaagctaacaggtatgtcacaattcaactgctttctcaacaaagcagaaaattgtactgctttaatagaatccaatccatgt
gcctgaaacagaacatcctttccttccagtgtgttattacccaagatagcggtagagattttactgatttcaaaggcgagaggctctgtaactttag
gtaatttgttatcctcttcgaacatacgtgcttctgcataggattgtaaagattttaagtcaacttttcctgtagcattaaacaacaaggtcttaatgg
gaatgcagtgcgaaaaccagacagttgatttcaatgttttattcagtatttcgagtgccttttcagaagaaagggaggtttctttaggaatatagca
caagacgaatcgattatttataaggaaggagaaagcttcattaaaaatatccaaggaattagcaattttgtcgagatgattcagatcgtaaaaca
caccatttcgctttacaatacgaccaacacgaccctttatattgattccccattttccatctttaatttcccaagtggcgaggtctgcaaatcgaattg
cgggttggtgattaactgtaatatatgtctcaagatttgagttacgtgtttcttcatcttcacccacatagggttcggattgagcatcactaacgaga
caaacaaatcctttatttccaacttcgcgtggaaccggttgcatttggtcgtccaaaagcatcacttctttaattgtgcttccaggaagataaattaa
gtccttatcttcaagatcggtgtagaaaccacagaatcctgtttctgtggatccgtacgcggcaccaattttagcgtttggaaaaacactttggcaa
gattgaactagatttgaaggaaccatttcgccggcaacaacgactttttacaattaggaaatgtttcgttttctctaatagtggtctgaagcatcaa
catgagtagtttaggcaaaattaagagacgttcgcttccatatcgtaaagcataatgcacattccacgccaactgctcggctttggagctaactgg
aacaggcgttcgtgtgtgcatagagggacacatgatgcgtccccctggagccaatagagcagtcatccaaaccatggtggtaagagcaaagtta
ggagccataatcaaaggggtcgttagattatgcgacaactctggtataccccagtcagccgcaggaacatacttttccatacgatagggaataga
cttaggaattcccgttgatccactactgtgattgatcaatgcaatctctggttcatgtccaacctgaatacgaggcgcagtgggatctgtttcgaga
gtcgttaaatgaagcgttttcacaccgggaaggtgatacttctcagtaaggtcgctgaatactaggaaagatatatgcaggcgatcactaataac
agccgcaacatcttcatcccaatcctcattgaagatgacacccgtatgtcctgttgcccacaaagcgagaatggaacataccaagtcaacttcttg
tctcatgaatacaccgaccgtggctcctgaaggtaaattcttttgatagaatctagcaattgtgttgacacgatgaaggacatccaaatgagagta
ggtttttgaatcccattctccgtatagaaatccacaaatggttcgtcaggttcattagttgccttttccaaaaatctagagacgggggttggtacga
gatcttttgtcattttaaaaaattagtaaacgtaatgtcagagtgtttggttctctagttatttatcgaatctgttttctttgtcgggaggaaacaattc
gtaatcaaaagttcttcatatactgctttggcttggaaactatggaaacactgctctatatataacaaatatccagcagcctttatccgcgtttacat
gtgtttgtgtgttccgctcttcgtacgaagaaaaatttttcagtgaagtgagaaatacagaccctataagcacgttcaatgatagaaaaatagggt
atagactattatttgagataaattttgattttattcatttattctccatccaaataccttaaccttgcatatactgttttatcggcataagcacatccttt
aaactacttgactatgttctttgtttatataaatcctctgatattgttcttttctcgcctttttggtgtttgatttttaagttatcctgttgacgaatttact
acttcttggaggcaacatagcataccttcagtaattgtaaagagaaacgaagttaactattttccttctgttgctctgtttttcgcttgttacaaatta
aagccttctcaaaatactagctaaatcgtatatctgctgacaagaccgattttgtaacaggatgtttgaatcggtcataaataagggcaacgtcat
tgctttttcaaaaactcgatataaaatttcagtagagcttacgcctttgagaggtgtaactgaaaataaggaaatcactgattagtgaaatcacaa
attgataaacaatgctggttgtggactcgcacgaaagttcgatcagatgcaaatatttgccaactttagggaagctgttcattagaggcgacaag
agattcaacacatgagcgagaacggccgtaggggttgaatttttcagatttcgtcgggtaaggggttatagcttgtcatcttattaaagcatataca
atgcgagcaaatctacgctcgagggcttttcacaaactgtccagtgagaacttgaccgcaagtggaacagtggcatcatgtttgtgccaatttgga
ggcatgcaaacgaatgaagctccaacatgcaatcccatatttaaagctatcatcctcaatttactagaagagctttttgggaaactgactatagca
ccaaagagaccgacccaatgaaggctgcacacgactgatctatagagacgcagcgcaatcgtctcatctaatttgttaacttatactcggtcatg
actttatagagttgattcgaaaacagcacaaaactctgcaagaggaaatggagagaaatgcatctaagtatgagtgtgaccaaaaaggatttta
ttgtcgccgatgctgaagccttaaaaaggaacataaacaagttcgcatcataaaatgaattcgattgcatgcatctttcgctcttgacacatcttat
tttataatactgcgactgtatccaattaattctttagtagaaccgtgatcctctttatacacctttcttggattatagaaatcaacttgtttgtttttgaa
agaacaagtaaattaggaacattttatgaaacgtcgtacacattcaatgctattcctgaatgagcactttgttctttctattctatgattgtttacgtg

tttcagttactacaagacttttcgcctcaatcagatagaataaaagcgtcgtcttccttagtaagataacttaaaatgaagactagcgacaagatt
ggactttgttaaccccgcggttttcggggatatacgatttcattctatcgctttccttcactatgataacccaagatgaagacagcagacctcggact
tgttaaccccgtggctttagggatttccctaaatacataaacactgtaaattcgccgctttattagtaaacttggcaaaaacgtttttcttttcttcctt
ttttatactaaaaagaaataggaacgagtcatttgcgaaaacctgtggacacaattcctgtcattgaaaggattgccaatctccatcaggatttttt
gatagtttattcttcttatcaaggtcacttttatacatttacctttcaaattggtaagttttttttcggacaatattgtttctcctgtttacaatatcgtgct
ttttcagtggtttcaatattggtggaattccttgtttaattattgaattggaaggctgtttacaggatcattcaattaaagacaacctgttgctaacttt
tggtttaagaagaggacttaagtgtatctattgacttcccatacctattatcttggggaaagacgttggtgacgtctcctctggtctgatcaatttgtt
gggtgttctatctatttattttacgaatttattttatacacttgttcggttgattttgtttacctaatcactcatttgtcttggatttctttaatattttctaa
ttctcccttcttccctaattgctttacattgcaaatctataattataattctttaatttacccacaatgacactttcgagaagctcagactctaaaaaat
cggatcttgaaaaaagtaatgagaactccgttcttgtcgataagctatctaccagtagttttgagaacccagaaaaaatcgtcgatgtagtaaaa
gatccctatgaacaatataacccggaagagcgggaaattttgtatcgtcaggttaatgataccaaggctaaggtagccgggtatatgcgaattat
tacatgcggtgataaatgggactatctacttcagctggcaggcagcattactgctattggttctggacttggtatgcctctgatgtctctcgtcgctg
gtaaattagccaaagcctttaccgaccttgccagcggccgtggcgctgatgcctttcaacacgatgttaaccatttcgtattgtattttgtttatattg
ctgtcggtgtcttcggctgttcttatatctatacaatttcgtttattatatcgggtgaacgtatctcgcgtcgcatccgtcagcaatatctgcatgccgt
cctaagtcagaatattgggtactttgatcgtcttggggcaggcgaaattaccacacgtataactagtgacacaaactacatccaagatggtttggg
tgaaaaggttggtttggtgctttttgctgttgcaacctttgtgtctggatttgtcgtcgcttttattcgacattggaagttcaccctcattataagttcca
tgcttttagctatccttggaggtatagccgtcggtgttagtttcgtaacaaagtatacgaaagcccaaattgcccaactatccagttctagtactttt
gttgaagaggtattttccaacattcgtaatgcgtttgcatttggtactcagggcttattagccgactcttatactaagtttttgaatcctgccaagcgc
tatggcattaacaaagccatggttatgggcttaatgatcggatggatgtttttttgtggcttatggtgcatatggtcttgctttttgggaaggtggtcgt
cttttgcatgctggagagttgacagtagacaagatgatcagttgcttctttgctgtcctcattgcatcgtatagtttggcaaatatctctccaaaaat
gcaagcttttgtcagttgctcgtcagcggccaaaaagatctttgacacgatcgaccggccaagcccaattaacgccttttctcctgatggtgagatc
gtacaacaggtccatggggaaattgaattaaaaaacatacgtttcgtataccctacaaggcctgaggttatcgttttggacaacttttctttgttgtg
cccagccggtaaaattactgcccttgttggtgctagtggtagcggaaaatccacaatcgtaggccttgtagaacgattctatgatcccattgcagg
acaggttatgttagatggcaaagatctccgtgatttgaacactgcatctttgcgcggtcacatttctctagtacagcaggagccagttctgtttggcg
caaccgtgtatgaaaatattgcttttggattgactgacgaggtgcaatcaacacttacagaggatgaattgcaagaacgcgttttcgatgctgcca
aaattgcaaacgcttatgactttattatgactttacctgaaaaattcgatacaaatgtaggtcagcgaggctttttaatgtctggtgggcagaaaca
gagaattgcaattgcaagggctattattagtgatcctaaaatcctactgttggatgaagcaacaagtgctttagactcaaaaagtgaagttattgt
tcaaaaggctttagataatgcttctagatctcgcacaacgattgtcatcgcccatcgtttgtcaacaatccgcaatgctgataacattgtagtagta
aatagcggaaagatcattgagcagggtacacataatgaattattggccttaaatggagcttattctaaattagtggaagtgcagcaacttacaga
aacgaataatgatgctgtcggtgacataggagaagaggatattagtggatatgttgatattactgcacaggataaaaatgacggttcttctacac
agaattctttttcttttagtcatgaaaaggaaaaggatccaatctcgcttcgtaataatccacttgccaagttaccaattgatcccgttccagatttg
ccgaataaagatctggacgatctaggtgatgaaagcccgggtacaaaacttgattctacatcgaaacttgagagtaatgtaactaacattagctc
ctggaaagcattgatttttattcattcttttattgaaggtgctttagaaattatctatatgatagttggtatcttagcgtccatggtttgtggtgctgcat
accccgtacaggcagttgtgtatgcacggttcttaaatatcttcacaaatttggcttcgccggattactttcataaaattaatcttttcggtgtctatt
ggttaattcttgctattgcggaatttttggttatgctatgagtcaatatgctttatcttatatattggaggcgattttgcaacgtattcgttggcatctc
ttccgcacaattcttcgtcaggatgttgaattctttgaccgtactgagaatactgtaggtgctttgactacttcgctttcaacttccattcagaatttgg
aagggttgacaggatctactcttggaacattcgtgcaaattcttacaaacgtagtttccgtatcaattctttcactggccactggttggaagctggct
ttggttacattagccacttctcctgttattatccttgctggctattatagggttgctgcattggaccaaattcaggataaattgtcagaatcatacaaa
ctgtcagctgcatatgcttgtgaatctacatcggccattaggacagttgccagtctcacgagagaatccaaggtatatgaacattattgtgcgtcgt
tagttgaacccggtagagacacggctttatcatctttaaaatctggcttattcttttctgcgtcgcaagccgtaacatttttggttaatgcgctggcttt
ttggtatggtgctacgttgatgaaaaaaggtgaatataatctggtccaattttatacctgttttatttctattgtttttggtattcagcaagctggaca
atttctgggatatgcggctgacgtttcgaaggcaaaatcgagcgctggatcgattaagtacttgtgtgatagcagaccaaagattgatacgtggtc
tgaagaaggacgcaaaatcgagtctttggaagactcctccattatatttaatgatgtagagttccggtatcccacgagaaagcacatccaggtact
tcgcggtcttaacttgacagtcaaacctggtcaatttgttgcctttgttggtgcttccggatgtggtaagtcaacaaccattggcttaattgaacgctt
ttatgactgtgaaaaaggatctgtaatagttggtggtgttgatattcgtgattataacattaatgactaccgtaagcagatagcacttgtatcgcaa
gagccaactttatatcagggaactatccgagataatattgttcttggtgcttcaagagacgttacggataaagagattattgattgttgtaagatgg
caaacattcatgattttatcatgggtttgcctaacggttatgaaactctttcaggacagaaagggtcttccttgtctggtggacagaagcagagaat
tgccattgctcgtgccttaatccgcaatcccaaaatcttactgctagatgaggcaaccagtgctttggacagtcatagtgaaaaggtggtacaaga

agctctgaacaaagcttctgaaggtcgtaccacagtagccatcgctcatcgcttatcttccattcagcatgctgactgtatttttgtgtttgatggcg
gtgtaattgcggaggctggtacgcataatgaacttgtaaagaaacgtggtcgttattatgagcttgtagtcgagcaaggtctcggaatagatgagt
aaactttttagcataaagaaatccctttcttaatgctgttgatttttgtatatttttatattttgtgggcatgatgaatgttaagtgcttatgaatagat
actagataccgaatgagtcctttttaatattattttttttttgttttctatagagtgaagttttctaatggtttagacggaattaatatgtttcagctaact
gtcgggctggctattctttcattctgtacttggtaaatacgacgtacaagtctgcagttagttgattttttttttttttctttacggttcattcgcatgcaa
aacacgaaagatagactgctataaattcgtttgtttacgtctgatgtgtttacatgatttaacgtaacctttagcgatggctacctatacgttgtggg
acacccaaaaaacagtcacacattttttggacagtcacaaaaggttttgtagccagagtgtgcattctacttttcacaaaatgccttccaacaaga
ctttccgtaccaagcaaaagcttgctaaggctcagcgtcaaaaccgccccatccctcagtggatccgccttcgtactggaaacactatccactaca
acatgaagagaagacactggagacgtactaagttgaacctctaaaaatggtaaggaaaatttggtgtattggacagacccatgatttctattagt
gcatggataggaacaaggattctaactaggaattaggtcggttgtacttctatttcggataatgtgtttttttttcatatagtgttccgttcgtgcaaaa
tggtgaaatggatcgcattttattgagtcaacctcattttctcttttactattcatatttcgatgtaggccgataattgtaggtggatttattgtatgtat
tctgtaggtgcgagatttttgtcaaattgtttgacagtggtatatactagtcaatctataactcaaaagttcatctaaagtggtagagaagacaaaa
tgaagtccaaagtaagatttacgaatgctcatgagtttcgacatagtacgctatttttttagatttgcaaggtatctcggccacgttcctgtttaataa
caaacaagtcgataaggcattcgtgatcttgagttctcgtgcttcaaagtggtactttcaatgattgtttttaataaatcataaacaaatattgttcg
agagtgctggcattgcaaggaaatcaataccgatttccgatttacattctgttacaagttccacctgttaaggatgaaaataaaaagtagcattag
attaggtgcattctagaatctgaaaaagacaagtaattagaccgcaacataacaagcagtgataatctttttcgatgggtcatgttctcctttttaa
tgaaacgaaattcaattaaaataaacataaaacaagactaagattacagaacagagggaaaggaaagatgggtgggtccaatcgaaatggaa
gagcgacaggaagaataagaatacctgaaagagaacggaaaaagggaaaaatgggaaagaaaaagcgcaagtaataggatgctcgataac
ggaaacgaaccccttacctacgtcgactgtaagaacgttgaagaattgatccattcgatcgggacaaaaggaaaagaaataatcgttttaaagtt
cagaaaatccaattttcgcgtgaacatatccatagaggaaaacgacaaattgaaaagtgaccaagggagaaaaaagaatgaacgcaaaaaac
ttccggtacaatattacgccaccgtcatcgattaccagatatgtagacaacgggttttggtttcgtaaacttcattcagagaataggacggatgga
aacgcgaatttacgtcagacgtagcgtagtattggtttcgtgaacgtagcattatctttttttataaagaagtccccacttgaaacacaattcgtaat
caatttatcacttttgggggactgtctggcgattttgaaaagccatcatgctaccaatatactggcacagatactcgctctctctgctgatgttttccg
attcctgtttgttttgatcaatttcgtccaacagtgctatatagattagtcatggttcacgatttcgctattggtcacaaggattcaagcctgcaaacg
ttcgtaccttgcaattgctgctgaagctccattgcttttctgctttcttagtacaatccttaacatgcacttactgctccattaaattacgtggtaagga
atccgtactcatcgaggaatgcgctttttcgtttttttttcttgagtatgagttgaggcgtgttgccaaatcatggtcagtgtgttcacgtctccatacg
tggattgttacttgccagttgtaactttagtttacgaaaggtgatgtagcacactatcccaaaaaaaaaaacaataacaaaaaagaataggcccaa
ggaatgaatcggtctttcatggacggaccggaccatgtaaacaaaaagtataaataaacaaaccagccgagaaagccaaacctttgtttgtaca
ttccattgcattgcactgcaataaaagaaataaaacgatggtattgttggtaacctgaaaaagaaaggggctcgcaaagaccaaaaaaggaat
gcatgaagataaaagaaagcatctgtgaagggagaagcctgagtcaaggttgagaaccgattccaaccagtcaaaatgacgaacagaggatg
ttgaattgatattcattggaaaaaaatacgaacttattgtttctcggtagtttcggttgctttcgattctttttcggaagcctgagcacgcgagttgga
agcctcttcttcttcttcttcaatgtcgatttcttcttcctcctcctcctcaccagcatcttcttcttcttcgtcttcttcttcttctggttccccttcctcttcg
agttcttcgtcggcctcatcctgctcttcctcttcttcctcttcatccgcatccggctgaacaggtggtgcatcatcgaaatcaggattatcccattga
atgttgaaccgtgtcactcttggtttttcggccaaaatgtttctccatactttcgaaatgtcgaccttgattttcttgacctcggtttcttccatttcttgc
aaattaactccctccatctcattattgacatagtacccaacgcgaacaaactcattttcctcataagcacaagacagcaaaatgactgtcactcct
aaaacatcgctgagttctggaagcaagtcgatgttgggtggatctgcttcaaaaacgaatttattgattccaataggaataggtcccaccaaaag
tgtatccaaaatttgatcatagctctgagatgtagcactacccacgtatgttaacttccattctaggtcgcttttcaatggttctaagcactcaaacg
tgatttcaaatttatatgagtctgaaaacttggcagggttgtttaaaacattcacacttaggatatttacgatagacatccttctttcgacgcgtcta
ggatggtgcaaggtctgaaactcatgaatcttactgagtcttttgttgatggtcacaatagtgagctttaacatatttcccaatcctcgcattgcgcg
tatcctgatattccttcgcgtatgttatcttctattcttgaacacattcttcaagcaggcttatagacaaaacactgggagaaacaaacggcaagtc
caataaagagaagtgaagcatgagaaaattcgctggaatacataggcttcatataggcacaattcgatagcttcgagtagtgtatggaatgcag
ctagatttaaagaaaatcaacgaaaagaattcttattatgtttagttcagaagacaatactctacggtatattatctttttttaaattctatttctcaac
gagtcccgtttagctcaatcggcttagagcgtccggcttttaaccggaaggttgcgagttcgagtctcgcagtgggagttcttttttttcgtatcccttt
ggtttgtttatcctttttattctttcttttaacggttcttgtttttttaactgtattcagagattatatttttctacaaataaaaggaaaaggatctatccta
ctcccaagtgaaattttcgttctgtgaagcttcaacaaacgttggtaggaggaacggaaaaattttctttttcaattcgaagtaaaaacaaaaata
caactgtattttctgatttaataaagacaccaaccgcctgtagccgtttcaatagtggaaaccatggcgtttttttttttgtatatttttttgtttatttatt
tattccttttctagcggatgatgaaagacttttcttattcactctaacaatgtcttgagactccgtattcggcataacgataccgttattgttcgtaaa
cttctcatttcatgcaacaccaagataaagcaccttccattaacaaaatcatagatttgtctgctacaattagaaagcaattactttaaaaaatgg

tatggtacaagaagaaaatagagatgctaattcccgttaggttgttttagcagcaagtattgtgaatcgaggtggtaaaggttcgtggtaatagga
tttgttggttgcttgctgaggatagaaagatagttaacaggttgaatagccattatttcccgtcaattccgtgaaatgtctagagctcgtgtagaga
gtctgttgtcatcatttcctgcattggtttctggaaaatcccaaaatacgacagtcgagacagaaaacgttcgttttgtatatcaaccttttgatgaa
ctgtacatggttttaacgacaaacttgcaatccaatattttacaagacattgatactcttcatttgctgtcccaagcattaacctctatatgcccgtct
tctgatgaaagagagattttggaatatgcttttgagattttcattgcatttgatgaagttacgagccttggttatcgggataatgttacgctccctca
agtcaaaatgtatttggaaatggaaagtcacgaagaaaagattcaagaaattgtctcaagggtacgtactacaaataatttgcatgctccgtgga
tcgatcgatcaatggtttggagaaaggattccgtttatttgcaaatgcacacatccttccacgaattgtctctccttttgccaaaattctgtgaccact
aacacacatgtttagaacaaagaattagaagcaactgaagaaagaaaacgacggatcaaacaattggaaatgcaaaagaaagatgctaggc
gtgctgcttctcaccctgcatctgatatttatgaatcggctggatatcaaaccgtcaacactactatggctgctaacgcagaagaagaatctgctat
ggagtcatatcattctgctgcaaaggctgcatctgcccctcgggtgaagggtatgcagttgggtaagaaaaaaacaaccacttcaatgttttaaac
tatacgccttagcctcctttgatataattccttgcttttgcataacattgttgttctcttctaatgcttgcctatacttataatattcattctttttttttct
cctcaagctacttgaagaatgagcaggaaaccttttttgaacttattcaaaatattgactttttcagcttgatcaacaacaactcaagtctactgctt
atacgtatgtttcataagtaaagaagggaccccttttggccgtataaacatgtcgtgtatgtaatgttgaaatgcgcttatagatttattaaatgta
atatcatgaagaggtttccgtttcgcaactatagtaaccatcgttttcagactataaatagttatgcttgaatccaaaaagataacccatacaaatt
gaaatcgaattagtaaacatcgagcttttattaacagggatgttcccttatgaccaaaattccaatcgctttagtgtataaaaattctaatgatgga
ggtaagtgatgccaaatttttgtattataagaaaaaaggaaacagaaaaaaaaagagtaagtgcaacggaaagtaattctaaaaacaatgaa
attcgaatgattataaccatacacattgcatactgcagactgaatataaaatctcaaacaacgaccgtaaaaccattgttaggaaaatctataga
actgtcctcgttgcacgatttttaaatgtaacagatagggaaaattgcttacttaaagcttcaagctgctccatgatgacggctgcaggatctattgt
ctgaagaggatgagacttcaaatgagttattagcacagtaatattatccatattcccacctaaaccgataacgagagactgaagggagcataact
cttccactaagtgccgaggacaaaaaatggccgaacaataatggatcaggtatatcaggagtagaacattcaattaatatatgagttaacttctttt
tagcaataagattagcgcacattttccaaatataaatattcagtggatctgacgctactttgtctggttctacgtccccaaaggctaggaaataccg
gtccgttaaaccgtttcgaatgaagaatgccgatgacttcaactcatggccaaatgacgaaccatgattcacgggaaaaggtaatatagacaag
gaagaggttatgttggtatacatggtttcatcgattctaaatattgttgttagtaactgcaaaattgcgacaaaaggcagtttcttcttccccttcatc
ttccttccatatccatatatggcaagacacatcacgaaaagcttccttcatccttacatcttgaaatttataaaaccggctttatctaacgcaggcca
cacttggttattgaaaacatgctttttaagggtattgattacatagtccaatcccacgatcgtccttgggttttggggaccaaacatagcgctgttga
taacacaaccgtaaatatgatcaaaatggcagtgtgagattaaaaatgttttgatacgctgttcagaaaacaaccaagctttcgcatatacattgt
cttgcgaaatacccttcgttgcaacctgagaatctctctcttcaacgtcgactgtcagttgctttctttctatgagttccgctaacgcagaaagatgtc
ctcctccatcaatcgaaattatctcctggaaacgcccatcggaaagaagatgcgcactacaatctgactctaatggtcctccctacactatgtaag
tccttctgtctactccatttttcgtgtcttacatttgtgccctaaccttcgttaacataacttttcgatcaattttaaaatcataccaatgaaaaaaatg
tgaaagattcttgagatgtctcagaatatcgctttttcggtggccctttcagcttttcctccacttgtaggatgcatggctctggctaagaaagcacctt
aaaagagcctaagctttcattcttatttccctttgttatgtcatggtgatgtctatatgttaaggtatagctagcgaatctaaaagatgctccagttat
acgatatagtgcactggcttgtgcgttctaaaaatggtaaacaaaggcaatatgcaagagagaaaacaaacaaataacaatttacggaatagct
agtatgtacctacttgtgaataaatttctgtatttctttctttattacgatactgctttttacgaatggttggtggctatggataagagtgagttagtttc
attgactagttcagtctactagtccatatggaagaaaagatgcgaacttaagaaatggtcgatttcttgaaaaagctgtagctaataataaataat
atgcttctttaatcacactgcattttaaagtctttcatacaagtcttgcaacatcatttttattaggaaaaaggaacaagaaaaaccatttcgacgat
ctgctgtttgttttcgctcattttaaaattgagctttggagaagaatcttggctcaaattattaaccatatcattatgtttaaaaaaaataaataaaat
tattcgatctttcataaggatgagttgaaatcgcatgcatgaatttcacgaaccttccaaccggacatcgttcaacagtgcaaaggaaaccatcac
actatttcgaaaaaaaatcaagtatgcattgtatagcagaaaagggagtccgatgaaaaatatatatcgaaagccatcctttattgctgctcacc
atcagatttggaacgtgcagacattgaatgcatggggtacgattgagcataagaggaagaaggaaacccttgaaacgctgtattatacgaggaa
agggattcactttttgtagtgtgcgaattcgaatttgattgcggaatttgcggtggatttacatctgcttgtgtaaccgtctgaacagatgattcactg
accgtgtcccaatttacggtcggttgcggtgtaaaataagaaggaggaggtgccaagcttcccgccggaggagctaatctccactttttgatattt
gcttcgataaacacgtatccagcttggccaaggttgacatggacaacacatggacctgttgcacctactgtcggatacacgtttcgatatgtacac
ggcaattcagcacagcgacggccatttctcgtaaagaaaattctgttcactttcggcttgtaagcaacaccaatgacgtctccttttttgataaaatg
atgcatatggtttatttgtaaaaggcgaattgcatctccgagttccatcggaaatataagctagcgaccagaaattccaaccgggcatgcgaaag
gggggatacggtttcgtagttagtcctatagatattgtagtattttcatcaacttccaacaacttggcttcccaataatacacttcgttaatccgttgc
aatgggagatttgtttgaagacaacattcttccttttgagtaaaggaaagttccgtgcggttagatgcataagcaccatagtccaaattaggcata
aaatgccatgcaaacaccccttttttcttgaacttgcaaaaaatcaattaatttcgcatccgtgttgatgcgagattgagcagcactcatctcaaact
cacgagcctgcaagaatcgttcttttccaggttcacttaggtgtgcaaatccttcttcatcaatgttttcattctcaattgcttcatcatccagttcacc

aggcgtattgtttcgtagcaaaatcctggcatggccagagtaccgaagcaaaagcaaaagtaaagtaataaacagaagtgcacccaatgcaga
aaagaaggtgataaataggaaaagtacaaatttcagaaaggcctcatcagactcatcttgagtatataaccattgcataatagagaaagtcata
atcacaagaattttaatgaggaagaggtacgcaattctttttgtttgtaggtcaatctttgacgtttcaaatttgtacccttgattcaactttggtttag
caatccatgccctcttccaggaggagcttgaatctatgaagtgaaatatcaaaaaatcgaaatataaattaataaaaaatcctgttgattctttctc
aaagagtccaaaaccagtaacttccgattacttttcagatccactgaaaaagcctaaaacaaatcgtctcttctttttcaaaaaataattaaattat
gcaaacgggcctatatttactttggctccacaccgagacaaacgaactcaattctattctgcgatttggcaatttggcgagaagcgatgtctgcaa
atggaataagttgtaattcttaaatttaagatataaagcaaacaaaatcaagtagaattgatgctattgttcttgatcctgcatatagtaaggtgtt
ggtgaacgtacacttgcatgtatgatgttggactcccactatagggagagtatccgtttaccaatatcgacgatatgtcaagatccggtgaacagg
aagctattcaagctttacaaagctctttagatgttcctgtagatactgcccaatctgttttagagtcatttaattggaatgtacaggtacgtaagtta
aacgctcgagaaggataggtcagacaaatgacggagccgttgcacttcataagtcgattcttgtaatacaaagaagatcaattgtgtttttttcctt
ctctaagcgacttgtcgtaaggttcgcctcgaatatcgctaacggttgtactttaggaggcagtggaatttttaacgggtgaatcttcccaggaatc
cagaaagaagaagccgcttcctggatttgggtttttccattccatttttcgtttattttcagcggcttctacaagttttggatgtttttgtcacgcgtac
cattgctgccaacattccttcctatccttggtgctaggaaacgctacttgtcaccagccgatgcagcaaataagttgcttcaaaatttggaagaaca
gtatggtacaaatcatattgactttttcactgaaggtgggtatatggaagcattggccagaacgaaacgtagcttcggggtggctctattgtttttta
caagtacaaagagtgatgacatggaggacttttccaaaaaggttctcatggacgaagaagtcaaagggttcttatcccgtcgcaacattctttgtt
gggttggtgacgtttgcgaagacgaagcttttcaaggatctagacaattcaattgcacaaagtttccttctgcagttttgattatgtattctcctcaa
ctttcagaacttgtagtcgcggctcatttgcatggacctatggattcaactctgcttcaaaatactttaacaaacgctttggcacgtcatttaccttct
ttggaaaggttccgtaccgaaagagagtctagagaatctacgagagaattaagacgtcaacaggatgatgcttatcaagcatcgcttgctcgcg
atcgtgagaggcaaaagcaagccaaggaagaacgtgaacgtaaacaaaaagaacaagaggaaaaagaaagaatgctaaaattgtctcttca
gtatcgttcttggttggctgttagcattccctctgaaccacaacccaatacgcaagcagctcgtctttctattcgcttacctgatgggactcgtgttat
tcgaagatttgagaaagattgtttggttaaaagcatatatgaattcgtggacagcttgctttttgctaaagaagaacctgaagtttatgaacaagct
atgtcttcttctgctgtccttgctcctcctgagaattatcagcacaaatttcattttagtctctattcttctcttccaagagaaattttggaaatggagtc
gacaatttgcgatcttccttccgtgtttcctagtggcaccgttgttgtcgaactagaagattaaaactctagtcgttgatctatctcattatgagctctt
tttttattctaattttcatttcattaatttttgttgttgacgtcgctgcggttcttccatttctatgagcaactttacatgaattgctttctccggtttccatt
cttcctttattttcttttccttgtgtttttatatgctcaatacgttttatatataaataactaatgccatttcttttaccaaggaaactatggttaacattc
aatctaagaacgaacatttgtatcatatggaactagcatgcctttatcatccctttattacatttttaaaggctacataatatttattttgattattttg
tttttaggactatagacttgtcaatagctaaatgaattgctcacgagtaacgatgatagaaataactaccatggagctaagcacaaaatcttgatc
atttcatttctataaatctttatttgattttatttcaaatggaattgtcctaccaaaattgaaagatgcttcaagacttagtatcttgtacaactattta
cgactatgtatttagcgagcgcgccttcgactttccaattctccatgggttcaagaaatcctacctgtacgaagtttggaatttcccaaatcttagct
ttttgtttgagactaagtttaagtgagagaaaaggatttatttaatatgtctaccgtgcagaagcgcccaggacccgccaattctagtcagccaac
gtaaggaatatcacaaagaaagcaccgttactaacaactgtaatatagcgaaaaacccaggaaagaatccattttggatttgtccagatatcaa
gatcaaagaattcaagctacttttacgggaggacgtcaaattactggtgtgctcaagggttatgatcaacttatgaatcttgttttagacgatgtgg
atgaacatttgaaaaaccccgaagatggtaaacttttagataaaactaggaagcttggcttgattgttgtacgtggtactactttggtactccttgc
tccaatggatggttctgaggaaatccctaatccattcttgcaacaagagtagttcaatgaataaaagaaaagaaaagaaagagaaagacgaaa
tactacttatttatgtttacatattccttcactttaatcgaagtatctttccattttacataaattgatatagattgtgtacatggacgttgaaactttttt
gaaattatcgtcacctgttaccctttttcatcatattgttcccaatttatactcgtaagagctattccaaaattaatgactgatctcagtatatttcga
taaatttgcgaaacagacatctcatcatcgttccaaatgaatcaactcgacaaactcaagtcttttaaaatcattatctcaataatctttttctcca
aagttgaaaaagaaaatgacggaacttcgtcggtagcagatagtgccttctcatttacagaaccaccaggaatgtctttcttaggtaaaacggta
aatgagaactgtggcaggctcgtttcttccacgtgttctttttccattacaggagtagcggaagaaaaggcgttgtttctagtactgccaaaggtaa
aaacaggtgtctcttttggcttctcatccacttttggggatgaaggaaaggaaaatggtttaggctccttattggtttgattttctccagcattgccaa
aggtaaaaacaggtgtctctttcggcttctcatccacttttggggatgaagcaaaggaaaatggtttaggttctttagtggtttgagaaacatccgc
ctcgttgccagccgctgaaacagcggagctaataggtggaattgaggaaatgcctggctttccatctttcttttgctcaagtgaagaaacatcagt
aaacgctgaatgattcgttaaggaaccaaaagaaaatcctggagtttcaacagctttaggtgtttctgtcgaagctgtaaaaggcttaggctttga
aaggtttgcaaaagaaaaccccgaaaatgactgtttttccgaggtggtttccggtttttgaatagaagannnnnnnnnnnnnnnnnnnnn
nnnnnnnnnnnnnnnnnnnnnnnnnnnnnnnnnnnnnnnnnnnnnnnnnnnnnnnnnnnnnnnnnnnnnnnn
ggctctttattggtttgattttctccagcattgccaaaggtaaaaacaggtgtctctttcggcttctcatccacttttggggatgaagcaaaggaaaa
tggtttaggttctttagtggtttgagaaacatccgcctcgttgccagccgctgaaacagcggagctaataggtggaattgaggaaatgcctggcttt
ccatctttcttttgctcaagtgaagaaacatcagtaaacgctgaatgattcgttaaggaaccaaaagaaaatcctggagtttcaacagctttaggt

gtttctgtcgaagctgtaaaaggcttaggctttgaaaggtttgcaaaagaaaaccccgaaaatgactgtttttccgaggtggtttccggtttttgaa
tagaagaaacgttcgttttctcttctttaggttcttctttcaattcctccatatcaacatcaattgcttcgtttttgtcagcagctggagtaggcttttctt
ctttcacgttttcaaaaggctttgttgactggggtgaaaagctagtagatgaactagtagctggagcagtgaatttaaaaagtggagtggcttttgc
agtgccagtgtcggttttctgagaagctggttctgtttctttttttgtttcagaaagagagggttctttcaagaattgaggagtaaagtcaggtttgta
tgtctgtaaatttgacgatttttcaagagtctcgacagctggtgtttcctgtttctcttcaattgtcttcaatggacgaacttcgtccttcgaaagcgta
tgacgacgacgggaagtagtaacgctaggtctcgcatagggactaatgtaagaagttttggcagtacttgcggagttttctgctgatggactggta
cttagtttttccttactttccagaatactcattaagtttgcagccgtacgactgggcgttttctgttgaggcgtgctttgtgcaggaggaaaaggtga
agaagcggggacagtgctgaaagaatcctcattcgcagtaaagcttccattaaaagactcgtgcttactacgctttggagcaaagtcaggagtag
tagaagaaatcaaagaagttaatttcgaagtaccgacaggagtagtggaatgacgcttgcgttgtaacttcttgcggaaaggggtagaaattgta
ggtccatagtaacttgttttctgagctgatagtagcgaatttcctgacgtattaggagattttgaattctcgagaatgggagagaatgttgaacgga
aggcagaccattgcctaggagaattagggctagcatgaaaggagcttgcggtttggatacgagactcactcgaaacagcacgatgaggattgac
atatttgggagtgctgaattgctgagaagggatggagggagaaggatgtgttgtcgaagcaggagaattatctttagataaaagactcaacact
ccttctctttcaacctcgttcaaaggctgttttcccttgtttgaaaaaaatgtagccaaaagttggtttggtgattcttgacttgaagctggaggcaat
aagctgccatgagggacgatactaggagtctttggagagtcgacagcacgaggctgcttttgaaatgacggttcttctgactttatttcttcgctgc
tatcagtaattaagctttgatcttcgtcttcggactcttcagaaggtatcttactcaggaaggcaattgtctttttccaagaagaaggcgtaagcca
atcttttgcgcgctgtaaaatcgatttcgaaggtgtctgtggtgacttcaatggcgaagattccgtaattctatcataaggcttgttcttacgcgttga
cttatcctttcgatttgttcgaacgggacctgaagacattttcacggttcctattcagtccaatattatttagatacttttattacagttcaacaatggt
tgtttccttctttttatttctgtcctgcttggtggtagaatcaacgaaagtaacccaaaaaaacgtagttgaaaattagtgtaactccgtgcagtact
gtctagtgcgcaaacaaacagtatcctacaaatttatttttctctattttattgagttcaattcaaccgtaatactatcatctcttacaagcctgcatg
aatgtaaatgtgagcaaatccagtttatttttcgcacaagtttggaatgtttaccttccacgcttgatataactatgaatgcctttcaccccaaaaa
ttgctataatgtttttttaacaatgcggtaaagattttaatgcagcgtagcatccaactggtataacctttctcgctcactttggctgtcaacaaata
ataagaaacgccattgaggaaataaaaaggagtgctttcctgtttttatcttggctaatggttaagacaaagccttctattcagtgtaagtcaaga
ttcaatgaaaggacgcgtttacttaccgaaatttaagactatgatccatttggcgtgtggtcttcaatagcaaccgacgtaaaaagtaaaactcctt
tacgaaatctacaatgggttgaatcatttcaatatagaaaacattttatcacgagcttggagcttgaatttgttccatgggtggaacattacacttct
cttgataattctaaagatgcaacattgctaaacactccgcttgtaaatatgctgattttgccagaagaggtattgtttcgatttcaatggtcatattct
tattaacgttcaattaggattcggaaacatacaaagtgcagcatcgtcctatagcacttgagtggtctaaaaaagtctctcagaatgactctcaat
cctggatgatagctctagtagcgcgaggccaacccgttaagcgatcatctggttcactgggcaggacacattcaaggacgagctcctatttcatag
taaagtccacgatcgaacgccttagaaatgactttaacacatctaacacaaatcgatgtgtcaggctagattatgtccgatttggaactcctgatg
cggaatgctggaatacatttttaggttgcttacagaagggagttttggaggccttggactatcggtttcttcaactttccgaacaagcaaagtttata
aacttacaagacacgtctatttttctgatttttagtttacttcttacagaaggaacgactaggagctagttacttggagttgtctttgtacaatgaa
tctcttgagcaatttagtgagatgttcgaaacttttaatgaagttttgaggattgcaaatggaaaaaaggatttggttttgaaatatttgggtgaag
gatcagatgtggcaccggctccctcagatttaaatgctaaagctttgcttttagatacgacttacataaacctccatctattattggaatctgaaaa
gttctctttattccatatagggttctacttattctcaaagctgattcagttgactttacagataaaagactacgataaatcgtattcgctacttcagca
atcatttcatttttaaattatcctgtttttgatcaatttattcaagaaaatacccttttgctgatacggtttaaatatgactgctgcaatcttttgaaga
gtctcataagtcaaaacgcaaaagataatgaggttaaggtacccgcttgtaatgcaaggatagctttatttagtcgctctatccttcttaagatggc
ttatttgaaaaaactatttcctaaagacacactactactttggcatgaagaaggtactctacaaaagttggaacatgtcggtgatatacaggaag
aatttcgagatatagaggaaatttctacattgcaatcctttttactagagtatttgaggctttctgaagaggccttgattatctttcaagagaggaaa
aacctcttatccttcaataatactgcatctgaagcagctgtggcactttatatgcttcaagattttgaaaaagcatatgaaatgttaaaaatctcga
acctaccccttttctggaaaagcgacgtctttaaagagaaatggattcaattttatctcggtgtactaataaaggttagtaaagctcacgaggttat
tgaatttttagtcagcctgctcaaaaaatacaaaaatcctgattgctttaaagcagctggtcgaattgttgacgatattcctcaaacttatgatctaa
tattagatgattacttttgtgttgatatatcgaacctggctaatatatccgaggattgcaccctagagcttcatgctaaaattacttcttccatttttga
tttggatatgttagaaactgtgaactgtactttggtttctgattcccggcctttcaagttacaatttggtttaagaagcatacagaacgattcagatg
ctgttttgtgttgtaacgtatgagttgaattatatattatgtactaacaatttaggatatttttcccggaaattacttcgtaagtgaattaagcataaa
accaaaaggaatacccttgttatttaaaaagacaacattcgactcccaacaaggaataagggtttgtcaaccatccttaagagggcggcatcttt
ccatagttcaagcatctctacctcagcgttatgtgaatgatcaacttcacactacttttcggatatgctttggggaaaatttcgaggaaaatcaaaa
agtcggtttttcttttgaaactaacaacaaaaaagagagcaataaattaagtcgatacttccttgatgaatgttctgattgctcgttgtctatacatg
acgaggtcgcttatttggaaaatatcaagcgtctagggaaggctattttgcacgttcaaatgtcaagaaatgatggtgatgaaagtaatgatgtac
gtgcaaatctattgttttcatttctatttctaacctaaagttgcagtctaagattaggctaacttataaaactcaagatggtgaggttattaacttata

tcttccggtaatgataaaggaaatcaattttgcagagctttcctgtgagcttgacgagaatgaacaagattctcagacactttttcttcgactcttgc
ccaaggagccaattttatttttcggatggagaataaaaactacaaacctggatagtcaagaaaccagttgttatgagataccttataagttaccaa
ttttggatacaatggattgtttcaaaacgattccatcaaagggttggaggcggatgcaacataccgtcactgtagattgtttacgaattactgacct
tcttttcgaatatatatacaatgaatgtggacgattcttaagccggatgaacgtaaacgttataacaaattggattgagtcgatttgtcgtaaagct
gattttaccaaagttccgtcgaatggccaaatttcattgcctgaatttcataatatggttgaaactaaacaaacaaaggttttaataaaaaatgatg
ggcttttcttggaaaaattctctggtctgttagagtatctgaaagtttttttgacgataaaggacgttctgtcaacactacaagtatcggacagaaat
agagaaacacaatgtttcgtaacgccgatgaacagcttgcatacgctaggacctatcacagtaaaaaataaaaaggatgtaccccaaatcgact
gtgtttggaaactaccgacaagcatatttttgaatgtccctactgaaattggcctcgagtttacggtaacccatgtgccgagtaataaaaatgtaa
aagatgagaaacactgttttgaactattgtacgaagtctcagcttatacaaattatattctctttgcaggtcctatacggaaaaaggtctctttaatt
gatgtgggtgctacactacaagaaacatttacagtcatcttcctaacagcaggaagagttttgctaccagaggtacatgttcattcaaaggatgat
aatattttgacagtgaaaaatccaaagtacgctgtggttggtcgttctccttagaaccatcccgaccatgattatgtactttgaaataaaatcaaat
aaacattggcgatctagaaccgcaaaacaatatcccgtgtgttttcaaattttttaacgtaagcgtctagcttcacgttcagattctaacaagacaa
ggatgtcgtcttcgcgaacaggacccttaacgttacgaataatggaacgagaagtgtcatccatgaattcgacacggacttgggtgacaccacca
ctatacaaacgttagacatttattgatgagaatttcacaaccaacttaccgagatccggtacgtccaaggactttaataactttagccaatttgact
gggacctttgaggaatccatgtttgttgaaaactacgcgttcagtgtagaagttttgatatgaatagaaataacagtcacgaaactaaatagaga
tattggtatatgtcgttatgggggttaccgataattcttaagcttcgttaataaactaatattcgacgtagaaaaggctggtcttattctattaatttg
tcatttcgattaagttatttatcaacctttactaattcaatacttaaaagatgaagaatttaatagactttaaagaatgcttcgttgaatgagtgtgtt
ttttttatcgtattcatgtgcggtaatatcccaaaggcataacaaagtaactgaatggtttttttattttcctcatgaatctcatgagttgatgattaata
agaacaatcatatttgaatcaactgtaagtttactcatccatctattgcatatttgcgggtcttgattatacagtaaacaatataaaagtactacga
aatgaaaaaatggaaacaacagttatatactaaagaaaatatatctgaatcaattaaaagtacaaatttcctaaaaagaaaatgggttaaaag
cttttgattgtagagaagtttgcataaactctgatctttactttctttctgtttcgttaaatgatattggaactccaatatggtcttaatttcgttctattc
tcgtaatctcttcatagctaagcttcatgctatatttatgtttaattcatatctagaaaaaaactaacaattttgtatgacattttgcaagttttcccag
tttaccttctacaatcgcatgatcatattaacgagaaaatgttacctaaatatcatagaaatgaaaagcaacagcttgtcactccagtaaacaaat
tccagtaagaatagttgaaagcctatttaaggatatattctatacatttataatattaaaccaatatttattgaaaggtaaatctgtcttgaatgaat
catagctttatcacctcgcatattcttaggtatagcactatgtatggcaaatctggactgacataatttcttcacagcaatgtggatgcaagaaagt
atcgtaaaaaagatttactataagttcataataacataaattaaatctctgtgtacgatttgagcaaagtaaaacgaatatcatccccaagtttga
tactaaacacttattacttattttcgagacatcacttcgtggaataaaactttctggtcaacagattttaagcatcaataaaggtaacccctatttta
caaattcctacggtataagcttcaagattaatataaaaaaaacaaatactattattcattaaatatcttaaaaaccaggtaaaaaagaatgtaag
tactttcatccaacttttgatttggccagcaaaccaataaatacaatcttctccctacacctaatatattcataaatgtaaatttatagcagtagctta
taaaatcatagaatgctactttttaatcgcctcctgctatacagtcgagcatcaaaggaaggtttgccatcgtcggtggctcctttccttcactaata
cgcattaattgaaagaaatcactagccaaagcgatgtcatctgttgctttatggaagctttttgtcgttgcttccatatcttcagccttttgttctgctg
catcgattaccgactccggcaatccagccatattggcaacattcataccataagacttaggacaaataccatcctctagcttatagaggaacgtaa
cacgtcgatgctcctcatcgacagctgcagccatctgcatcagtcgtacttgttgatatttaataaagtctatacaaagactttgataatgtgtcgag
aaaaatccaagacaaccgaggtttgatactaaatgatgcaaaacagcatatgctattgcatgtccatcatgggtactagtaccacgtccaagttc
atctaaaataaccagactctttggtcctgtttcatcgataattttcttggtttctgacaactccaccataaaggtacttctagcggacattatatcgtc
attagcacctaagcgtgtatgaatactatccatgggagtcaattttgcaaatttcgcaggtacccaacaaccaagctgagccatgataaccgctat
gcatacttgacgaagtagggtggatttgccagccatattaggaccagtcaaaactaccatgttcgctgaatcacctcccagtataacatcatttgg
tacaaacatagcagcagctgtagcattaatgcaaggatgtcttaactcttcaaatactaactcaccttcatcaccctcaataatctctggacgaca
ataaggttcacccaacgcagcagcagcctgagaaaggctgaaaaagcaatctatacaagccgtacatttgattaatcctagccatttttcataatt
actgtcgaatcttgtgtaaaatttgtcttgcattcgcgacattatcgacaaatggatttcttcagcttcaagcaacttcctaattcttttcctcaaatca
tcattataatagcgattcgcctttttttgtacccgacatcttacaccaatttactggcacttttacatcagaaggtacctctatttgatacacttctttac
cgacatgtttgaatgtaagagaggagcaattcagctgctttttatactgccccaaaagctcatgtaagtcagaaatcaactcagtttggtatttttg
agactgatcatattcggcttcaaaaccaggttctggaacgaatacgccctcttccgatgcttttcgccaattaaaagcacgtgtccaagactgaag
ttcgtcttgcatattaggagcgctgtctatcaactgggctaataaagtgcccttagcaatgtcctggaattcagtctgaagttgctgaaacgttaac
gagattttttgaaatccctccaatacacgaacaaaatcacttggcttgcttcttccagcatggactcgtgatatgagtcgctcaaggtcaggaagct
tatgcatgaatccccatatagtatctctaatggaaggattatctgcaattaattcgatcacatctaacctagcattaatagcaacaggcgatcttaa
aggatggcataaccatgtatggaataatcgctttccaaaaggtgttacacaacggcataaaagattgaacaacgtgcctttatcaccaccgtcaa
acgagttagaaaaaacttccagatttttcaaggtttgaccattcataagaagagaagtagaatgttgggatgtatcatattccatgaaattaccca

tggagcacatatctttatctagtttaagttgacgcaaataccaaactaaagcaccaaacgcactagaagcgagttctttcgtttctaggacccttga
aagtactttcggagctccttctaatccattttcaaaatagtgggaagaaatgatctctcgaataactctatctttatcccaaaattcagtagatggtt
taataaagttccaaatcgctgctgaagaaacacagtattttatgactttcattgatttctgagatacttcagacttttctaagataagctcccttgga
cgcgtttgtgttaacaaagtatcaagtttcgttctatgaatatcgtccgtaaattcgcaaagatggaatccaccagtagaagtatcaatgaaacaa
atgccaaatgacggctcatcattatcggatcgaatatattctttgattgccatacaaaaagttgaaagatcggacgtaagcatagcttcatcgact
agagtaccgctagtaaggacttgagttaaaccccgttgtacaaccttttcttctttttgtgttcgttgtcgatccttcatttctttacccaaagcagtct
cgagctgatcaacacgagcaatacgatagcctttggcaacgaactgagaagcccaatagtcaaaactagcttctggaatgccaaccatcttcatg
ttaacacggtccgttaattttagagaaaatatttgatgaccaactgcagcatcattttcatataattcataaaactttccctttttgaaaaaaaaaccac
agtatccataagattcttcttaatttcccagtattgtttctcaaatggtttgaaggatgaccaagccgaaggaggaatatagagtgttcgaggatca
taattgggatctccgggacgatttttatcagcatcacgaacgtccaaaagccattcatagcgctccaaattatctttcttgaaagcttccatgcgca
atcttcgtttctcatctctattcataacgccgcctgccgctgccttggagggggaaaccgatctaggagaaggtagatccgcagaggctaagtttg
accccaggcgataattttcatacatcgatttcgaggaacgtgatgtactcaccggtttcggagctaccaatggtgggttttggacgtcggtatccac
atcttcttcgttgtcctcatccataagatcatcttcagggatttcttctttacttggtatagactcatcagaagagatatcattttcctgttttggctcta
catattcatcataatcatcatcatcatcaaggacgattttttgcggcgattgactctcgatactctcgcatctgccaatgctgtatcctcgtcatcac
tttcttggtacgaaacagactaaaacattagaaaaaaacactggaagcattaaattaaagctacatacctttctggaacgccttcccctattctgc
ggagtagactttttttctggatcctcatgtgctgaggacgaagataataagggatgtttagaaggtagaaactgtgggtccgactgtaatgaaga
attaagttcaaagttttgtgatttatttttggtaggggaatccaaggaaacgtcagagtcgttgtcatgtggagaagaatggacagcagtatccgt
atgttctgaagttcgaggtaattttccgttcaaatacttaggactatttccgtcctcatacgaatccaaaggtctcttaggcgaggatcgctggatac
cgcttgacaatggagatgagaagggatcgctagatggatgaggcgaaagagtgttatgaagagaatcttcaggaacacgaacaggtaattcgtt
ctcttgactctgcaatggcttacgtttagcgtcgctgttaccgccagcagctgacgacgcggaagacttttgcaaaggggtagaggaagactgag
gctctttcttaggagtcggaattctcgagaaaaacccaaaaagagtcttttgttttgattttattgcctcattttgctcatttggttgtgattttcccact
cttcccttgctcatgattctagggtcagaagatggttttgggcaagttgtttacaccgaccgcgacacaatctaataaacaaaaacgcgctcagaa
tatatcccaagaacatatatacatgtttttacaatgaaatcttgaaaatctaacaaatttatttatatatattttcggcgcgttttccattattcttagg
atattcttatgtcgacatcaatattatatgaagtcaattttcgtgagaatccgtattctctaggatagcggccttgaagtactactaccaccaccact
tactacactataccttgaccaacttataataaggttatagtaacactgtaatatatagtaatagaaggatggaccaatataggaacgatataaag
gatattcttggtacagtaaatcgtcaaactgtatcagctaaacaagttcgacaattactggaagaaaaaaagaaggttgatttaagctcgaacaa
aaaagacttaaatgcattaatttgcaatgttttgatgaaacagcaagccctcaaggcggcgacggcgcaggtaagcaaaaaaaagttaaaata
aagctttgtttcaaaaaaatgttgctgacaactggattttctagaaacgaaacctgccaagaaatctcgtggctccaagagcaccgaggaaggtg
gtgccgaaggcggtaagccagccaagaagactagaaaacgtaaaggtgctgaggaaggcgaagaaggtcagaagaagaagcgcaatgtgga
tcctgccaataaccctttgaacaagcccatgcaattggaccccaaacttgccgagtttcttggtgttgaacaggtaagtaggtttattatcggcagg
tgtctctgtttttattgaggctgaaggaggaataaataagtttcttcgaacggcgtttcctgccgcctgactgtaaggatctttactaacttaacag
atgtctcgtcctcaaaccgtcaaaaaactttgggagtacattaaagccaacaatctccaggaccccaatgacaagcgtaccattttgtgtgacgat
aagatgaagcatgtctttgctgtagacagtcttcacatgttcactatgaacaaggtaagacacaaacgatttagttaccatttctgtatttgctaatg
agtttagtacttgacctcgttaatgaccaagattcctgacgacaaactacctaagcaaccttctccagcagaggctgcaggtgaaaaggccgaag
aaactgaaaagactgaacacactgaacagcccaaggaagaaagcaagcctgaagcttcccctgaggaggaagaagaaaaggacgagtaaaa
agatgtaagggtgatttcattcttcttttgtagttttactgtttattcctttgtcagatattctggcaaataggacttttggtaaatcgcaactcaaaag
tcattatattttgattgtttgaattcaaaaggccttcaaccgcaagacccgtatttcttttgtttttgtatcatactctgagatgcttctttttatgcctta
ataaaatcgctatttctatgaatatatgagaccttttagtcttgaacacgctttgacttatttaagctttattttttttgtcaacatacttgtatgcgtttt
gaatcttcaataattgaatgctgtttcagcaaattcttcattatgaatttcaagtgatacgtgaatactctctttgctcttcattgccaggtcgatctat
tttagttttataggcagttgcagattctggaggacgtttgatttgtttctattaataaagcaaactgggtactttcatagtaaaatgcatagagatac
gttcttactagttgttgactattgattttcctaattcgctgccgccttacttatttcgtatcgaataaagcgatggagacgattacctagcttaaaactt
ggtgagatccctttgcctttagaagtataccgtttattccaaatttttaatttatttacgttttagagtttttggttaggagcaatttgactttatgctgta
tgtttgtataaacatttgatcactatgtgagacgcttttcgaaggggttttcagcaaccatatgtctttaaaacaatttttacgtagataatctttcatt
aattagatatgttatgttcgccaagtttgaggatttctaaatttactaaaaccaacggcatcattgctcactggataggacatggagacattcttgc
ataatttgcagtatatcttcttctattaaacatttacgaataatttagatttgttcttcaagagaacgaaacccagaggtgagatacacacggcgca
gatatattctcattccctacatagatgactcgggggagacaacgcataatttacggctcagtaaattcatattattctctactttcttcttggtactaa
aagttggggcgagatgacaactttttataataaggattgtcaggctgaatggaacttttcccttggactttgagagcaacccctaagtattttaccg
gtatagtgtaacattttgagactgtaaccgaactgaaattcataacttcttttcgattgacgtgtaagtttgtctcttgctacttgaaatttttagaac

agaaggactttaagatttcatgtaattacagattaataaaagaataatgaatgtaaaccataaatagtcaaattaccaacttttcaatttattttat
tactgtacttcttgtctctactctgttctttcggtagtatatagtttaagtacgaaagtacattttcttacccagaaccaattttgatattcacaataaa
caaaacaaaatgccgtccccggacactgtttctaacaataataaacaaaaaagcaatcaaaaaaagtctcccgtaaaaaacatccaaaggaaa
catgcaaattctggtatgaaaataaaattttacaaaggtcaaatagatgataaccaatccttttagaggaggcagggagaaagcaaaatcagaa
ggaaacagggaaaaattctgttaagcttcagcctgctggaattgagggttcagatcatgaagaggaagatgaaaaagcgaccactgtaaccaa
aaacatagatgacggagatgaagaagaagagcaaatttgctttatttgtgcagaaccaatttcgtacacgtgtattcttccctgtaatcatcgtaa
gtaatacgacaagggcatactagctgcgtcaatgtgcatttttactaaccgttggtcaacgttaggtatgtgtcatatttgtgcgatacgtttacgcg
ctttgtataaaactaaagaatgtacattctgtaaggtatcatgggacgtgatttcaattacccaagaccataatatggatatccgggacgttgaga
tggataatttcccatttcaagacaacaagcatggagttgtttatgacgatcagaaagtacaagatgaatgtaatcttttgcttcaattcaattgtcc
ggaggaagcttgcgacattacttgcaagggatggtttgatttgaagcttcactgcaaagtgactcatcataagtacttttgtgatttgtgcattaatc
acaagaaagtgtttacccacgagcatactttattttctaaaaaaggattgactaagcattatgaagtaggcgatcagggcactgaacttgatgtta
ccggatttaagggccatcccaagtgcgggttttgtaatacacatttttacgatgatgatgaactttataagcattgtcgtgagaggcatgaacgctg
ttatatttgtgatcaactagcaggtcgtcctacacaccaatactacagaaattatgatgccttggaacgccattttgaaaaagatcattttgtttgtt
ctgagagagaatgtgtagagagaaagtttgtcgtttttggtagtgaaattgacttaaaagctcatcagttagaagagcatcctcagcgacttagtg
ctcgagaattacgggaagcacgcagaattattccagaatttgagtatgaacctccaggaagcggttcggggagaagtcgccgaaaccgtaaac
aaccttctaacgctttaaccgaagaggcgacttcttcattggcgaacttgcatctatctcgtgaagagattgctcatataagacaagaagaataca
tgcatgagcaacaagctcgaaatagagcctttggatttgcattatcttcgccagcaccatctgccgcaacagcttccagaccaatttctgcaacag
cttccagaaccgctgctgtgggaaagcaaagggctttgagaagagaagaattcccttcgcttaatgaacttacaaaggctcaatcgcaagcgaa
tcgaccttctcccagtcctgttcgcacaacagctgcttccactagccgtacctcatctcctgtcatttctcgtatatcatcttcccaaaatgtcagtgct
gccgcgacaatgaagaaaggctctcccatggcttcgaacgtacaggcacaacatcaacaagttattgatcgaatgatgaagctaacaggctttg
acgagaacaaggttaatgagtttaagtttgctgtttcgtctttccgtggaaacgtaatgtcggcacgagaagctgtggatcgtatttccaaactggt
atccaaccctcaagagcaactctcgggagtaattaatcatattgcagatttattagaagataagggaaaaagcaaacagcttcttgagggatggc
aagaatggaagattatgagttctcaggaacaaccacatattggtactaataatgccaatttattgcgtttaaaaaaatcaaatcgaacagctgctc
aaactgcgagtgtgtggaatcgtattgaaacagctgctaaacatagtggaccggcgttgtcagctcctgcgtctaggaataaacacggctctgcg
gccacttcatcagtcccttcgaagggtgtgaacgctggaccacgccctacttggggtatacccacgaaaacaagatcgtcagctgtaaatacgag
ctctgaagccgactttccctcgcttcctccatccacctcaaagaggattcaagtcccaattggtaaaaagccagctcgtggggtagatccttgggct
gctgaagttcaagcagcatcatcttcccagggttccgcgaatcgacctacagcttcaaaaagaaaaaatggaaaaaaacaaactgttttgtttca
tcttggttaatgggcttttgtacataatgagtgtttaattagttgaagtacgtgaatatataatagacatatgacagaaggttacactatcatgtcttt
ttttaaataatatgatcttaatgggagtcactcactataactacattataataaactcctcagatactaaagaaacctttccgcaaattgactgcctt
ttacatttgataaatttgtatcttttcactagtgtgttcaaattcgttgcgccgtattagaagaaatagtgtaaactagcattataaaaagcagtcat
acacaagcatatggagttccaaggctagaatgaacttgcgaagaaccattatggagaagtttaatatagagtatgttttatgttatttcaaggaaa
tgggaatctgctatgctatgttttaagactcgctattattaaaagaaagacctaaaactgcaaatgaaacaaaagtgtgcgataaaacctgaacc
cattatgaattattttttttaggcctactcgaagctttttttagttgcaatttaccagctagaaaagtcaaaaataggccaataggaaatggtgacaac
cagtgactaaacaacttgtatcgggaagcagatgaacggatgcaagaaatcgatgaaagattaattagttgcacatatacgattatatacagatt
agcattgtaatattcgataatatatatatttcaaaattgatttaaacatctaagaagtctcaagaaattgatccagtgaatggtgatggataattag
ttaactgattaagcagctggagggaatggaatgtaggatcgtgtatgcatgagtgaggtgcagtataagatgaatagagacgtatgcttgaagct
cattgtttagtaacaatagcagattgagatgtggatgtgacttagttacaacattggaaagcaacaaacttcagcaaaacagatttaaatttattg
gaagccatcattgtcttgttctgcatcaaggggcaatattctagtacctagaaggctagagacgacttttgttttagcagtgatccgattccaatac
gtgtcgaaacctacttctgtactttcgacttcattatgaaagggcaaagcgtccaatagtgcttttaactgttccagatgtttctttgcctttttaatttt
tggctgctcaacgtactcgtcttggagccatacacaaacacgtccataaatttcaccgatcaagagaaacttttggtacgcgtgttccaagccaaa
taaaaacgcctcctcgtgtccttgctgatgtcctacagcagttccctcgtcatatcctttctgatattggatttgctcgaaatccgtcacttcctcccac
tccattcagcaaggtggtgtttttctcttttttttgccccttgctcttcttgcctgttgtccaggacttactaaatacgctccattcccttcgtatctggttt
aacgcaatacgttaaaactgcttaaacgagaaagcatccaacaattcaactcgtaagtcaagttgagccaggtcaggtcaggtcaggtttgttcc
ttgtagagggtagggtctttgtgacattagatagacaaggaaagggtaagtagttaaagaattttcttgctgctcttgaaggtgtttaataaagaa
gctaatacgtttggatccagtggaaaccatcaacgatacattctacttgtgcttgctaccgtttcgggacattttcattgttgttgacttaaagaaga
aaaggatgtatgatatttttggttacaaaagactttatttaccttggcggccaaagtcgaggagattcgacaaggttttagcgagtttcgctttccat
gctacaagaggcagtcaaacaggcggttggaagcgtacaaaagagaatgatgctaaacaggatgaaaaagaaacctctgacaatagaaagc
atagcatgtatggtggaaaaatatcaaaaaaaaagagaaagtttttattcattcctagtgagcatacaaagcaagcaagtaagcaagtaagcag

cgggcaaaacagaagagaaaacggaaaaaaaggaaggcaaggaatgagagaaaaaaaaaaaggatgaagggaaatcagaaatggttgtttt
ccatcaaaaaaagggaaaacaaaaaacaatcatctttttctggaaatgactttcctttttcctgtgcatatcgaatcaaaaatccagttgggcaaa
gtaaaacaaaccaatgtgaatgaatggatttcaaatttccacggttaagacgaattatcgagactacatattcagtctaccgaactcgtaaacag
acaacaaacataagaaaaacggaagaaaaagtgaatgagtggagatgagattgacatttgaataagaaaagtaaagaaattgaggattgga
gatgatagaaatgaacgaatgcgataaatacaatcaatcatccatccatccatcaactaatcaactaatagatggatcaatcaatcaatgaaata
actgatccaacttaatcagaggtagcgttcttcaagtaagtaacaacattgttacggtcagcgggcttcttgaaaccagcgaatgccatcttggta
ccgggaatgtatttcttggggttttcgaggtaatcaaacaaagtgttttcatcccaagtaatacccttgtcgcggttggcttcggtataagcgtagcc
ctcagcttgaccagtcttacggccaaagacgccatgcaagttgggaccaaccttgtgagcaccacccttctcgatagtatggcattgggcgcaac
gcgtcttaaacaaggaagcacctttcttttcgtcaccgggagcgtaaggcattgtgagagtttatggaaaacacaaatgaaacagaaaaaaaat
gaatgagaagcagaaaaatgaaggaaattccaatgtttcagttgaaatttcgcaaaattgagtttttttggagtttgctgaagaagaatttcaggc
gctttctatttcccatttataataattgcatgaacccttattggatgctttcctccgaaaaactgacgtgtcgaccaatcctggtacttacaataggtg
gggtacactatcgttatcttttattgcgtagtggaatgacggtatgttaaaagaactactgatggaaaaggaaatggagcgatttgatgtttgaaa
gggacacacgaggtgtgacctgccgagtggaggaaatgggagacgaggtgtttcgaaaccatttgtttatgtgacctgcgtcgggtcacaaagc
caaacaaacaaaaactaatttcctgaaagttgactttgaaggaagcttgggtgagtcatacactgcctctttgaatgcatcatcaaggatgcatca
tcaaaacttgtttttactagttcgaacgaatttggtttggtctgatttggtgaagatttttttgcaaataacaaaaggcttaacgttggaagttccaac
cacggtttaatgacgatgatgacggtctttgatattccttttccactcatttggatgactccatgcatctggatctgtttgtaaacaaaccccgccgtg
atcgtatcggggatcccgtaaacaagttacaggagacaatcccaacaaagaacgatccttcccctcgggagaaaaagttcctctttgagacgaa
aaaaaaataaataaaaagataaaagaagttttgagccgttttggactgatgactaaactccacattggtggtcttactctttccttagcaggccgg
gtaacatcttatcatattaactgtgagaggctccactgaaacggactgggcggaaagatacatcatgctcagagtaaagaaaggctgctatcaa
acacgatacaaaacagtgtagtacagcacagctggaattcgccaccacagggtgatttattatcaattagacagaaatatgttccccctgaaaaa
gttccatcgaaacgaaacaaactgaatatctccccaaagcaaaacaaggaaaaaggggcttactgcggtcacgcttgatatgatcccatccttat
agaaaaaggacatgaatgcaaacacagcacagacaatgttgaaaccaaaagtctacttggcaggagatttggtgtttcggcccaatgcctatgc
catttttgaagaactgaaagcgatctgtcgcgaagtcggcgttgtcgggatggccccatttgatggccaagaaggtgtggaagagatgcaaccag
ggattgcgacaagcttaatgattgctactctggacaaaaagttgatggacgtctgtgatgccggcatttttttgcttagatccttttcgcagagcacc
agatatggacccagggaccgctgttgagctgggttacatgtgcgcacaaggaaagccgcttgctgggttcaccgtagatagaagaacgtatcca
caaaaagtacagacctatcgtagagacgcttgggctgatacattgactccccgaaccatcaaaggaggaagtggttctatggaagatgccgatg
ggctgatcgttcattcagaaggcatggtgcaaaatgtcatggtagagggatttattcaaatgtcaggaggtatcgttggcatcgatgcttccttgca
taaggccttccgaattgccatcactgccttggccctagaacttgcaaagcggtccatgcaaacatgatcctaccacaacgagcaatcttgatgaa
atgacaagttttcacgaaattgttctccagaaatatcattcaacaaatgaagacggaagggaagggtccatgcgcgcaatgaccttactctcatg
tcacattctttcatctaaaagaatgctttttttctctcaaagttttgactttttagtaaatatgtatgatttctactacctttgttgtccaaggtacagatt
cttcccttggacaagtttctctagctcctccatttattttttaaaccaactttcattaactggaaaagaaaactacttctattatctctttccactatga
ggctttcaaggatcgtcgctttcctgaatcctcatgactactgaccttttacgaccataacaatatgaaaacttggattcttgaaattctttttcttagt
tactttatttttttttatttttttatttttttccttctttgtagctaattgttttcaactgcatatacccacctccctctccgtatacgctaaagaactcacgc
atctttaccttaccaactaccacaactttgttggggatttgaaaccaacaactcgttgcactttttttttcttctttttacgcgttttaacttgaattttctt
caaagttaaacgataaaaaccacctgcttcttttttttttgagtctcattgatagattccccgaatcgcgcgtcgttcctacttgttccgctattcgtttg
cgttttcgcattcaaactccttttatcatttgcgtggtagcagactttgagctttaacctttgcctgattttcacgaatccattgaatttcacaaaaaaa
gcacagagtacatataaccaatttacgacgatatagatttctctttgagatttatttagttcttttctttcgttttgtgaccaaccttctttcttattctt
tggagagaacaaagttacagacaacgaacataatattgaatccgcagtctattttatagtgtgattttgtggattgaatcatttttcgtattctttcc
ctgtcccataccccaatatcctctgatatccaatttttctttctaatttgttggtataggtatgcacatttttaatagatgaaaaacaaagggcaggtc
ttttgttctattgccttcgctggtaactggtttctgtaaagaggactagcaatttgagggtgttatgctttacgtagtttttatttgactaaccgataca
tttgtagtctttcctttccttttttggtcaaggccccctgatttgattccttctttcttatttttttggtttcaaatcgtttattcattatttgtcgaaagccttt
gcgtttttcgttcaattcctcttcattcgttcattttctttgaccgaccattttcggttttctgagaaactatcgacagatccttcgctaagtttctgcgta
tacatattttttttaacaagatgactcaacacgctgccgattactctgttgttttagaaccacctgctcgccttcacaaggatgcctctgttcccaagc
ctaacttatccagcatggaggaatataaaagcatgtatgaacaatcaatacgtgatcctactaccttctggggaaacatggctcgtgaaatgata
cactggcaaaagccttttgaaactgtcaagcaaggctccattgaaaacgctgatgctgcttggtttactgacggtttaatttcaccctgctataatct
tgtcgaccgtcatgccatagaacgccctaatgacattgctttgatatatgaagctgatgaacttaatgaaggccgttacatcacttatcgtcaacttt
tggctgatgtatcccaatttgctggtgcattggctgcccttggcgttactaaaggtgatcgtgttgccatttatatgcctatgattccggaaactgtta
ttgccatgttagccgttattcgtttaggtgctattcactctatcatctttgctggtttttcagccgaatctgtgactgatcgtatcaatgattccacctgt

aaactcatcattacctgtgacgagagtcatcgtggtggtaagcgtatccctcttaagtctgtcattaacagagcgtttcccaattgcccctctttaac
caacgtccttgttttccaacgttctcccgaggcctccacggctatggaagaaggtcgtgacatttggtggcacgatatagttcctaaatttcccaaa
tattatcctcccgccgtcgtcaaccctgagcaccccttattccttctctacacttctggtagcactggtaagcccaagggtgttgttcatacgactggt
ggatacttgcttggtgctgctgccacttgtaaatacattttcgatctccatcctactgatcgtatgggctgtgctggtgacgttggttggattactggt
cacacctacattgtctacggccctctcatgataggttctgccacccttatctttgaaggtactcctgcatatcccgactattctcgctattggagcatt
gttgaacgtcataagttaactcagtggtacattgctcctactgccattcgtctccttcaacgcgctggtgatgactatgtaaagtacgaccgttcatc
ccttcgtgtgttgggtagtgtaggtgaaccaatcgctcctgaatcattcatgtggtattataacgtggttggccaaggccgttgtgctgtggctgaca
cttactggcaaactgagactggatctcatattgtatcttctatgggtcctgttactcccatgaagcctggctctgctacactcccattctttggtattg
acgctgtaattattgaccctatcagcggtcaaataattgaaggcaatggagttgaaggtgttcttgctgtgcgtaatagctggcctagcgccgctcg
cactgtatggaagggacatgatcgattcatcgatacttatttgaagccttatcctggtgtctacttcaccggagatggtgcttaccgtgacaaagat
ggttacatctggattcgtggtcgtgttgatgacgttgtaaatatttctggtcaccgtctgagtaccgctgaaattgaggctgctttactttctcatgatt
ctgttgccgaatctgctgttgttggtatcccggacgaacttactggacaggctgtcaatgcctttattctgcttaagcccaacgttgtcgccaacgct
gatctcgaaaagcaattaattatgagcgttcgttctaccattgggccattcgctgctcctcgtaagctcattttcagtgatcttcccaagaccagaag
tggaaaaataatgcgtcgtattcttcgtaagatccttgctggtgagactgatcaaattggtgatgtgagtactcttgccgaacccaaggtggtgga
gcacatcattcacgctgttcatcatgctcatgaaaagaatgcttaaaatagatgattagtttgccgtttacaaaattaagcatacatcagggaggt
gttgagctgcggtgtcgtaaattccttcagttcgatgaacgttaagattagccatgatttttgatttcacgaaaagatgtccaaatctcatgagctt
cttatggtgataaagctgtacattttaattgtgttgataaagaattgatttcaacttctccattgtaatctaaaacctttattaacggaaattattatg
actagtaagataagttgtaagtttttgtaaaaatgaatatagaagtattgaaacgttcagaactgttgtatgatgttaaaaactacgaacggatct
cctacaaaggacttgatgatacaaaaacgagataaaagttataataaagaattaatttaaaagaattaagagaaataatataagaaataaaag
aaaccaaaaattaaatatagaattgacataaccatcatgaaacaagttgcagcatactcaatatagcgctggaaggttagggaatctggcgcat
gataaaagagctagtaacaaatgcaagaagcaagaaaaataattgatttcataaagcaaaaatcttagcagccagactaaaagaaagaacaa
cagggaaggtgtattcaaagtatacggagaaagagtaaactttgatgatgagtaacagaaagaaattgagagaagaggaagaaaacggacat
gtgtttaagtgtccattcatttttccgaaaaaagtaaaataaaataaaaaactgaagaaagtgaattcaaccattatttattatcaaggacacat
acgagaacaaagggtgatgacataactaagaagcaagtattatagtaatataaaagtgaaaacaggaaacgaaaagggttactcattagcag
agtcactggacatgacagcctcgttctcctccgaagtaggagattcaaggatggtgtcttgacctttattatcgccttttggagtgggggtgggcat
gtcgtaaccgagaacattacaaaattgtctccatagatgatagcgggaagggtcgacgcgaacagaactggtgggtggagggcagtcgttagtg
gtattggggtcagcctttaggtcaacctcttctgggagacaaagcagaagcctttgagtcgtaataaggaaatcagcgtcggaaacatccgaacg
tgaagcttccaaccaaagatgggagagtttctggtatttttttcgcctacagtatcctgaatagagcgaaagaactgttcgttacaatcaacaag
ggccttcatttccttggggcgatcattgaagggtaaaaggatgccctggcggatgggctgaaggtcaatgcgagcattagtactaagacgacgag
cagccatgatgataaagcaaatttagattcgaaaaaaactcaaagaaacagatgaaatagaggaagagaaatatctataatgacaaaactctc
gtctgctgaagcgggtgtagtgtagtagccacaagagaagtagaagcacagagaaatcagtaatggaacacgagaggaagaaccacaaaag
aaacgtaacagcaattgaatcaatgaaaatcagactgagtcaaagcaactgaaaggtacaagcaaaaacagagaataaggcctcgaaagcg
acgattgtttaaaaagcacagagcgatattgacaaatcaaagagaaaagagaagggaaaatgagacgaaagtaagaagcgttcacagaaga
atgactcgaatgagttcctgtttggcaaatgacaagttgagcggtgcacattggccaaatcatgatgatacaaaggttgttcctttgcaggtaaca
ttgacagatgaaatgaaagaaaggggaagaagaccaactcgtagagagaagagagatagcaagaaaggggaaaagaaaaaaagatccctt
ggaacgaaaaagagacgttgagtgaaactcgggtgaaaagaaacctaaagccaaacaaaaacaaacaaaagcgaaacagaaagaggaag
agaaagaaaccaaaaccaaaaccaatttccatgaaaagctggttgtttttagccactttcaaatggatcttataacttcttaccgcgaaaaaagct
ttccagtcgttgctttctctttttcatgttcttgtacttgaacccacttgtacctctgtaagagcatatcctcaaatgttatctaccaatatgattatatt
gccaagcatctacgtcatgtgtggagagataaggttaggtcgaagaatgtggagaaacgagcccttggaaatcggctgtagaaacaaacattgt
attttattatagagaaagaaagcttacaaaaagaaaaaaactatttcctttttcttttgtatttttctctcattttcaatccgcgttttcggtatatcctc
ctttctgattggacgatacaacaatattaagccagttcattggcctcctccacaccattaaatgaaggtatccggtattgataaaaggttcttcaac
gtcattggcaaatccttcctcctccccttattatttgttcgttacttccaatcgctgcacttacgtatttcggtttgtaccttgaaacactgtctatacca
atcaataccaacatccatatttccattggtcctttggtttttcgtttggaatttcgtttccgcctttaggtgggacatgatctctttttttaatatataaat
ctgcatgagctcctcgtttcctctctactctttaccaacctttacaaatttcctctttctttcggagctttatctgtaaattcgattcagttcatttccttg
aatttacatttttctttttctattctttctgggtaatagacggttattgtctctcttgctcagtctcctaatccttttgtgttttatttgaatttttcttttcttc
gttggaactcattatcatggctttgcgtatgccaaacttccgtaaaatccccatggaattaatgcctttgggggctgctgttctcgccgcttgtggctt
tgctaccttcagtattactaaaaagttggtagctgatcctgaagtgtttgttcatcctaccactcgtcgtactacttaagaagttgttggtttttcgtat
aattaattttgtgatgtgattgatgtccataaaactctctttttttgagagaaacccaacgaacatgtagggtttcttgtttcgatggtttcatctttga

tttgtttgtgttttaatttttattcattgttctttttctcttgcttcgtgttcgttgcaatagaaaccttttcttttttttatattcaagatgggaaaccatgcc
ttgcttttttttttgcatgttcacgtctttgtgtactaatactatatttatggttaataaaaatatatttatcaaatagtttgtacttttcacgagatgaat
gttggattgtagttgtgtgtaagaggtagtttgaatagtaaaagctttttggtttgagtgaagcaaggttcgatgtccaaagggacggttgtaacta
cagaatggaaagcctcacaacatctatctatctacagatacaactcaattataggaatataaggatgaaatttataaacccacgttttcatgttctt
tcacttttggtctgtagtcgtttagtaaggaattcagtaaggttccattactcacgtctgtgtttttttgagttgcttttaagtctagaaatttaaatcca
cgacgaacttttcgaagtaataaaaataaaaataaaaaaaaaagaattgcgcggccaggtttcgatcctgggacctacgggttatgagcccgtc
ggagttccactctcctaccgcgcacattcgtcagcagcaggatttgaacctgcgagcgcagtgcgcattagatagctcgaatggtgtattcaagtc
taactccttaaccactcggacatactgactattgttgatatgcttaccataaaaagcattttatacggatttttagaaaacttttgtcagccataatg
attgttcacaaaaaccaatttttcaagagaacaatctccaccataataaacaaattgctacaaacattccgtcaattcattctaaaaaataaaagc
aatttttattctctttggacaaccaaaaaggatgaataggataggttcgtttctaaggaatgaagttgtgtccagaaaagagttgagcataccgt
aaataaatcattcatggcgttgtcagtgcttcctatccattatctaaaaaagaatttttcaaataaacattttcatcttcgtaccgtccattcttaaac
gtttcttccaacttttttgtttatttatttatttctttttcttgggacatattttccactttaagtgtttactttgtagcttgtcacccatgttacttgctaaa
aattctacaccaaattaccagccaaatccgaaatcttctttacctttcttttttcctttcttctcgaatggcgtacgtgtttattgttttatttatttacta
attgtgtagagatctagtcaatcgacctcgaagtgagctcactgaattagaactcgctcgccttgaggaatacgagttcaccgctgggcctttgag
cgttttacaacagggtatgcttctatagtctgtcttttaagctcttctttagtttcctttcatcctaacacgctcatagctgtcaaaaaccatgaccagg
tcttaatcaactgtcgcaacaataaaaagcttctagccagagtaaaggcttttgatcgtcattccaatcttgttttagaaaacgtcaaagaggtaat
agcattttgcttcgtaccacctggcttttgttactaaccgatatagatgtggaccgagcgtaaacgaaatgcttctggaaaaaagggtaaagctgt
caacaaagaccgctttataagtaaaatgtttttacgtggagatggtgtcgtccttgtcgtacgtgtcccttctgcctaaaacatctctgcctttcttgc
tgctcttcccttcctcttctttggtttctttggatagactatgatgaccatttttcttatatctgttactttttctagtttattctagtttttttttttatgaatc
gataaagccttcattcattagaaaacgtaatgtattatcttaaataaacatttttgccttcacttgcctttacttgctttctcttacttcttatgtattatt
gaccaactcattcaacatgtcttcgtttgatatcttcttgttattattggaatactattatcactagatatgtatatgtttctacatatggctgcattga
atacatccgtcttctcacgtgtatataaatatttttttctcatagtaaaacagacaatttcaggtctcatcattgcttcaatgtatcttcaacaccacga
gatataatgtcttcaaccgcccaacgaggaggagaaaaccagccttgtttcttcttgatattttcaatattggttttaccgacatcttcaccggcagc
tccagatgatagtttcagctgaagcttttctccaatttcatcacatgctctcaagatataatccaaatcggctttattgtgcgaagcagaaaagcaa
aaccttactcttgaagtcaataacgggcatgccggataaccgactactacgacggaaattccccgtctcaacatttcgtgcgaaaatgcgttgatt
ttaccaggattgtacaaaagtaaagggacaactggactgtcgtcgtttccgtaaataataaaacccaaacgcttcaagcccaagcgaatgtaac
gactattaaacgccaaacgctcaatcctttctagaccaattgagctaggagattcatccataatctccagaaaactgctcttaatttgagccaaaa
cggctggactcatggactctgcataaacgtagcctgggttggtcacccgaagcttattcacgatatccgtagaagatgcgacgtaaccaccagca
gcaccgaaagacttggtgaaagtacccatcaaaatatcgacatcagtagtgggaattccaaaataatcacatataccaccaccacgaggacca
attgcgccaatggaatgagcttcatcaatgaacagataaaacttgtagcgcttcttcagctccataatatgaggcaaatcagcgaaattaccttcc
atggaatacaaaccctcgatgacaactaagatttttgtataagggcggtgagtacgtggctgaccttgggaaataacctcacgcaaaacgcgctc
taaatcagctatatcattatgcttgaatacacgaatattagcacctgactgacgagaaccaaaacgaatggaagtatggttcaactcatctgaaa
taataagagagccaggtcccataagagcggaaaagacggtagcattggttccgaaaccctgagagaaaatcacggcagcgggttttccaacaa
acttggcagtcaaatcttcaacctctaaatgaattccataattaccgcaagtcgtactggcactacaagtagaaagtccataattacgaatagcct
tttcaacagcctcagcacaaggtccatgactttgggcaaatcctagataattgtaagaggacacgtttaaactcatcgaagtgtctcctgtaagttt
aaaggtattattatgatcagtagaatagcgattcatgagtttaatcatacgaccaggaacacccatagtaggtcgactaaagcagtcgttgatac
gaaacttcaatcgtcgaacatagaagttatcaaagtgattataaaggggtgcataaccatcactatcctttaaatacttataatcatctttatggaa
tcgctttccaaaaaagtctcgaacatgaccgatgataataagaaccaaatatgagatataggttgtaactacaaagtagtaagaaggttcatcct
cgaattgaaataattgtccagcagcggtatttttagacacccgtcgatgagtgggatccgtaatgggagcaaattctgtatcaatgagttgtttgtt
taatttttctgcagcaacaaactcgccatttacatcaggttgcttcattttctttggttctgtttcagctgcagcgtccttggatatgtaggcttcttgtc
caaatgatttagataatggagcttcaacctgaaccattatctctgaaattgagtatcagtgtataaataagaacgcaaaaaacagcctttactaaa
ctgaagaaatagacttctgtctatacgggtttcaagataaagtagaaaatgtgtgaagaaaaaccgaaactcgtcaatagataataacttaaata
gacaaatttcgtcgcaataaaagagagaaagagttaaaacagaacgctccaaaagtttgcacgcaaaaacgaaccccgttatcaaaggaacc
gattgttacgggaatatcttctaatcatcaacttcctttaggtgcaacaggaaacgaccttttgtaagtagaaaggcctttgttttactcccaaaatt
agaagaatgagagcgaaatggcgttttcttaattacggcggtaagggaattgtaaaggataagttaactcgggtgttcctagtagcgaattataa
agttttacgagctcttacgaaatccagcgtgtaaaaatagaaaaagtatgaaaataaacgaaagaaatgttttgtagcagtagattgatacgatc
ttaattaatgtttatattataattgagttcaaattagtttgaatttttccagttcactgtagtttggttattcatctagttgctggttatcatttacacata
cagcaaaagataatattttcagatgactttgtttcaaaaaaagatgtacaggagctttaaaagagatcgatcgtaaataagagaggctccacaat

ttcgttcatccgttgcccgagtaattttgagattagtgaacaatttgtatcaagattcatttgatggattcgttgatttgtaagacgtaagaaagaaa
agagaaacactaaggttactctaggaataaagggttcagggttcttaattttcagttttcgtgtccgaaggttgtaattgttcattccatatttcattc
atgattcgaataggaaccccattacacataagtggttgatttaaatattccagtcttaccattgcaagtccatatataccttgtgtagccaagacctt
tccaggtgatcgactgctcacccgaccagcagaagaagctctaaggcctagcttggaggacgtctgaatatcggcgattctgctgctttcttctatc
ataaaaggaagaattcgctttcgtgtaactcctgtatgataagttcgtacagtaagttcttgacctagataacatcctttataaaaatcgatgccctt
catccaatccaaattgctttccataggaaaagcagatccaggagctatttccatggagccttcagcaattccatgtcgatatcggaatgccatata
ctcttccaaggctacatccgatgaacatgcatacgacgaaacaactcttcgactcagcataggcccttggaatcgtggatcccacatagatgggt
aactatgtttatcgctcatgaccttctctgcgtcttgttcccatcccacttgtacgctcagctcctcgggatcaacagctgtaacattgcatttatatcg
aagtttgtactttttcacatgtttcaagaattcctcaattctaaacttttctacttcaattaatagtttttgattaggcgtgctaacttctgtatttgtaag
gctttcaggatagcaaaacgcatcaaataaaacacgtccctgtttttttattagttctcaggtagctactaggcgttaacgtacgcttgtattcaaaa
atcctgtataaaacggtttctttaaagtaacattttgtgtcgtgagtccttgaagaaacttttgagtgtcaggtccttcaaatcgcaccaactgtttaa
taggttttaaacagacgcttaaaaaccttttttgctgaacttcaaaatgaaagctcttaatgcttttcaaatatttcaacgacatctctcagttcaatg
aactggcaatgtgttcatcgtctgtaattgaacagaatattataggtccctgagtactgtcgccatccgatatctttagagcattctggatgaatga
aattcgctactaatgagtataaaacaaaattcatacggtttatatacaagtttaccaaatggaaaccaacagttccaaatacctcaaaatcatatc
taatttacatatagaaacctagccaaacaaaaattaactgtgtactttccatcaacgaagccatcttgcatagcttgcgcttataaaaatattactt
ctaaactgaatccataagaagtgtaactttggaagacccatatagtttttttcgttcgagtcatccatataagatccaaaaaatttcctaaatattac
caacataattataatgaaagacttcatgcacgatgagttctttactaaatgccaaagtacagcgtaaaccgtttcaaaacgaaactctttcgagtt
gcaatgattacttgaaagacaatccataccttttcacttttcataggaaagtaataaaaatatctcggttgtttttttctttttttttaagcataaact
tgtttaaactcattaatgattgccggaacagatgttgagggtaggagacccatttcacagacacaaacagtgattaaacgagggggggttacatc
gtacttgagactaagaagtttcaaattcttgacggaatgccagtcagaaagttcgcttagcctttcttggttctcatcgactgaagtattgatgagtt
gatcgccaggagcaagctcattgtaaactaaagaatctaattgaatgcgttcagaaaatttataagattcacaacaagcgatgacaggaacgttt
gcttccttgccaagcaaggcaattaaagaagtacccgcacgggaataaagagctccgttggagagcatggcatgaccgcccaaaaagatctttg
ttacttcctgcatggtatatgctaaagctgaaatcattacataggtacaaggaattccatggcttgataacagctttaagcaggttctgccttcaaa
ctcgggacgagaatcaactacgattacacgaaattgttttccaatatttttgcgtacactaagacatcgtttaccgtagatgagtgcaaataagtc
aaaataacatctccatcctggatcttttcggctgcagcttgaacgatggctttaccagcaataacgatacgatcacgaataaagttatcaatctttt
ctaataatacttctttgccttcttcttccgtcaaatcaatatctaacacagaaatttccagtttaagaaatctaatggcactacccatgctgatacta
aggggacgagtagagaccaaataagctatttgcgaattgatgtgagtcgtcaagtgtcggctcaaagtcgttccatatggagtttggtaatctcgg
atgacctgtttaaaagttttaagcagctcaatgcatctttggttagagccaaagaccttgtagtttgctaattttaatcctaaacgaatgactgcagg
atggatgtccttgggcatgttctccgttgtcctacggcgtcgccagtccagatgtgagaaaatcgaaacttgtttttcttcaaaaaaattttgttcca
aaacagcgggatcttctttttggctggagaggagcctttggcattttgttttgaagacttcttaggctggcttcctcccttcgaaacatgctcttgttt
tttctcctccgcggactttccttttcctccatcaccgttttgttgatctgctcttgcttttctagaagcctgtttttgagcctttttcaaagcttttaagtctt
ttcctgacgcttttggttctgcggcagtgtttgttgttttagtgttcgtttctgctccagctgcaccaacattcgtttcttttacagcagaagattcattt
gctaatcctcgtccttggtctgttggggtggccattaatatttgtatttcacttgttttcccaagtatacgctgaagccaagtacaataataactacca
gtgtcagaactttggcttcttccttacccttacttttagtccacgcgcacagtttactttgggacgattattttaagacctattcaggtatttttttatttat
ttttaacaatattatttgttttacctggaagaacgtgattcggaacgagaatggggaaaaacaccgataaattgtatgtgactcagacagagcatt
ctggtgtccatggatggcacggcggcatgagtgggatcacaggccagcagcctaaaggaacgtttcgtcagcttccttttaactactgttctatttc
gttacagccattcacgcacccttgttgtttggtggattccaccaatcaagctatcatttttgatttcaagtaagtacttcaaatccttgtacacattct
aattgcatcgcagattcattgttccgtggcttcggaaacacaagacaaatccagtgaatggtgagaaagcaaccttgtccgatcttgtaaaactga
atttttccaaaaatgcggcgggagagtattgtgatccagtaactttgaagggatttacccaattttcacacattgtagccatcaagacaacgggca
attgcttttcttgggacactctggaaggattgaatataaaagcgaaaaattggaatgatctagtcgatgaaaaaccattcactcgttcggatattat
tacaatccaagatccccataacgttgagaagcgggatttttctgcgctacaaacacaaaaggaaatatctagagaagaaaaaatccagaaagc
acgtcttgcagttgaaaagtcaagaaaagaaacaatatctccatcttccaagtcaacttcctccgtgcaacctagtcgcatagaaacagcaccttc
aggacttcctgtttaccgtgccgcacatacgacgggtcttgcttcagcgtcccttacatcaacttcgttttctccggttacaaaaaatgaaagagca
attatccctcaggaagattacatgcttaaacatgttcgcattaaacacaaaggctacgctagaatgataacaaattttggggaaatcaatttggaa
ctccataccgattatgctccgcgtgcagtttacaattttatccagttgtctaagaaaggctattaccgcgataccattttccatagaagcatcccaag
gttcatgatacagggtggtgatcccacaggaaccggaaaaggaggtgaaagttgctggggaaagccatttcaggatgaatttggtaatcctttgc
atcatgatcagcgcggaatcctttcgatggctaatcgagggaaaaacaccaatggcagtcaattgtacgtttagcttttcctttgttcttttcatgct
aacactttagctttcttctgttcaatcctgccaaacacttagataataagcatactgtttttggacatattgttggaggaatggcagtgcttgatgcgt

tagaaaaaattccaacaaatgattatgatcatcctaaagtgcctattcggctagaggatattgttgtattcgtagatccatttgaggaatgggaaa
aggaagagcaagagaagttaaaaaaggagagggagttgttggagccaagcaaatccgaggcagattatatttcatggtcaggaagaaacctg
atgcattcatcctccaatgcttccacaacctcacctgttggtaagtatctaagtaatgacacgaaacgctcgtctaatggagcctctttggatgtctt
tcctcaaccgaagaagaagaaaacacgaacgggatttggaaattttgatgcttggtaggaggatacagaaagaaaaatggaaaaaggtattca
ggttttcgctttttagttcatgaaggaaggttaatgataatactatattatatgtaaatctaataaaaaaagacataaacatctctataatccaaaa
taatgaatcgaatagatttgaattaggatgtagctcctgtttcagcttcttcgttcaagggaggaaattcatattgatagatatatgcgtcaacaag
cttgggatatccttttaaacgagcagcattacgaactactcctacgcgagtgaaaccaaggctgtcccacaacttataactggctacattggttgc
gaatacgagattgaagacactagacttgtagcccatgcgaggcgcaaagtacaagaacgcatatgcaagtactctccctataccccgaccacga
gtctcgggagctactaaaaatccgccattacatacgtgactacagcgagaaggatagtttggttttatgataaaaaacccgaggatcattcttttc
caatctattgtagacgtttgtaggttaggaagtggactatggccgtcccattctaagcaaatgactgtgcaatatttaaagaagtattcctcagctt
cttccaggctcaaagggctttctataggatacgtgtctcccttttcaatttcataattaaggatctcccttaggcgagagagcaaaccaggtttatca
tgaatgactgtggagccatcaatagccatacaacaagcctttgcattcgcgcatttccagagaggaatgatattaggatttttcaattgaatatcca
tgacaaatcaattaacaaaaaatacttgaaatagggaaaacttggcaaaaaaaactaaagtcaatgaggtcgtagaaataaaaaagagaaaa
gcttttgaactaattgtctagtgcttatataagcgattgtgaattccaacgaagtttgggaatataataaaccgttgataagacgcataaacagtgt
aatgtgtagttgtgttttctacaaatgcaaaagaaattagaaaggataaaaatcgtttgtaaatgccgtatacattgggactgaaatctggagga
gtgtggtgtttaaactgtcgacaaactgtatctataaatcatgatttattttttacttatttacttgtgcattaatcatttaggcaatgttgaaattcat
attcttcattctgaggactagcttatggtcagttttgttactacgaaacataaccaacatgctaaagaaaatatgatgtgaagaaaatgagctttga
gattgtcgatgaaggaatgacaaagatatattgtagaagtgtcgtctttttgatatatttatggataaaaatacaaatattaagccataggatcatt
tcactttcgattctgcctcatcattgagttttggtaaatactattgtaagaaagaattcataaaaacaatcatatgctcattataatggaatccgtgt
acagatataagctcgtaagaaatgcatagattatcatagaaataaaataaactttcaactcttttatagtaaatgtgttttccatatgaccgtgcta
tatcatattagttgagttaccaggtcacggatcctctctaagaacattattgctagttgaaaagctagtttattttaacaaacactggtaaataatac
attcgaaaccaagaaaactagggaaatctttatgatatagaaagcgaaaataaaaaacaaaagatttgaatttacatagtacgtttttcatgatt
tcgaacttaaaagtccacttcaactgtcttatattctactcataagtatatcaggttctttctatttccatctactttcattatcactttatgttgagtttt
tattatcttcaagagtattttcagttaattcagtagatgcgtttacaaataccaatctttttgtcgcctctggtttctgctagaacgattctaaaggga
aagtgatcgaaaattatgtccttcgttttttaagtataatttggcgacgttctacaggtttatggtttatattttcctttgaaaacaaagcatcaagct
tgtataaaagcttacaacatatatgaatgtatgacatactgcatgagaataaaatttaaaagcaagaaaaaattgagaatggaatacgaaaacg
gtaaactcgatagcatatcctgaagttacaatttttttttctttttttttttgtgaatatctgactacatataaattcaagttcaatgaaaatccattag
cctttcatcattggtaaacatacgctttataataataaatataatacagcttcatagatactattgggtgttctctgaaaccaaaagaagtat
ttggtccaaatggaaaatgacacagcaaagtttttaggaaggcttgtgtatcctaatttgggttaaattaaataattaaaaacaactccgtcggttc
agttaactagagaatcggtgaaatcatcatttgtatatttggcttgaacgaaactaattcaagctacgaagctcgttgacccttttaattgtttaatt
tataattattatttgttttatattaaattgttttctgcactaggttgctcgtatttaatgacaaaatttgagagaatgccgaaccaaagtcttgtttaag
tcatgggtgttactggcttcattggagcccttgttactgtttctttactaaaaaatggataccgtgtgcgtggtacggttcgtttaatggagaaagct
gatgaatttgttcgtttgaacccagaactaaaagacaaaatcgaatttgtcatcgtcaaggacattagtgaatcatacgcccttaaggatgtcatc
aaggactgtgactatgtctgtcaatttgcttctcctgtctccgtttacgtcgctgactacaagactcaaatcttggaccctgctgttaacggtatcctt
agtttcttggaggcagccgccactgaaccaaaggtaaagcgtgtgatcatcatttctacctctgctgctaccgttaactttactgcagaccccaatg
caggttatacctataatgagaaggactggaatcccgttagttatgaggaggctgtaaatatcaaagacggtgacattgcttagcttgcttctaaag
atctgtctgaaaaggattctcgtgaatgggttaaggaaaaacaacctcatttcaatatttgcaccatcagccctccctatgtgtttggtcctcccatc
catccatgaacagtatggaacctaatgttctaactaagatgcaaccttctatgcaaagtaatcaagtcgatgttcgtgacgttgccaatgcctacgt
ttttgcacttaagaaccagcaaccgcgatcgtttgttcgtttctcaaggtcctttcttcaccgatcacataaatagagttcttcgtgagaaattccccg
aaaagaaggatgtcatttccgaaccttctaatgtccccttcaacgaaaaactgtacaagtgggacaactctctctccatctctcggtttcgaatact
acagtgaacagcaaaattacatcgacactgccgcaaagctttaggagcgtgctaagaaatttgcttcttaagaatatcttttcgacttgtatttcag
aaagacaatctatctcatttagccagaccagtcgaatcttccaatctggagaagtcgactgtggattcaaagcttataaggaagctaacgtaatct
gttcctttgtaaggaaaagttccttttatctggaagatttccgttgcgaaggctgttcgatttgtttaaaacgtatttgctttgttttgtttactaattgt
aataattatccttttagacttatgacgtttgttagtattcgaaaatatcttcttacaatggcgtattgttatatgtataattaacaaaacatctatatgg
acaatatcttaaaaggtattaatccgatgacgccaaaagcgatagtggaatgaactgatataaacatatagttgcttactaatacagccttttttgt
ccatataacgacgaaatgaccaactttttttacgatctaaaccaaacactctaaggaataacaatgaacagtcttccccttcaaaacaaagttgca
atcgttacaggagcctcacgaggcattggagcaggaatagctgagaccttggcgcgtcgcggtgccaaagtagccatcacctttacctccgacag
tagcattgacgcggtagaacaattggtgaagaaaattaagggttttggcacttcagcagatgcaatcaaaattcaggcagatctacgtgatatttc

aagtactaaacatattgttgataccacagttaaagcttttggttccaccatccatatccttgttaataatgccggaatcacaaatgaccgaatatcg
ttaggaagtcataccatggaagattacagaaaaatttttgatgtgaatgttcgtggtgtctttttttatgacagaagccgtagtgtctaatctttctca
cggcgataggggcagtattattaatatcggaagtattggtggaagaatgggagtccctcaaaactctatatatggtgcaagtaaggctgctattg
aaggttttactagatgttgggccgccgaattaggtcctcatagccataccgtaaatcaaattaatccaggagctgttgacaccgatttgctgaagg
aatatggaagtggaggcagacagttcgcagaaaaaatgactccatttgaaaatcgcattggattaccttcggacattgctgaggttgtcgcttttc
ttgctgaagatagaagtcgatggatcactgggcaggtgatctcagcttctggcggattggctatgtattagatggtttagagaaaattaaagttaa
agcagaccatcgtataagcagtattcaatctgatgaactaaatgccgttctttcacgtgttggaaacttttgaaagagtttttttataacgatcgattc
aagcccttacttcagtaaataaaggatgtcgtaaatggatttatttttggacatggagagatattcatttgcgattctgaggtcgtcgctgcagacg
cttaatacttatactcaactgggttatctaatattcatgtattcaagaagaatcaactacacgatagatcttgttaaggatattctcttcgatatcctt
ttcatacttaagtttggagtttttaagctcttttctaaagttcacagtagaataatggtaattcttccataatgcctctcaatgtttatcctattgtaatt
atatttaaaattaaaggttatttattgaaattgattcgtttaataaatttttattagtttaaagccaaaacaagatcggaagattacactgatatgtc
tataagatcgcgtttactgtgatccattgaattaaccagatccctgaacagtactatctcatccttttgttttatggtgtttgctaaatttcagcaatat
tctaatacacaatacattgtaatgattataatgttgacaaaccaactatcctgacactgcggcaccaaacattcatagagctttccgttgatatgat
atctttacgttcgcaaggatattgaaaaaatacaaagaggttttcatgattcgcattgtctatttcatcattgctctcaaaaggatgcacattcttac
cttactcacctctattggaaacctatagacatgttttctaattgtctttattaacattggatgtttctccttgcaatcctatgcaacttactcaggtcatt
agaaaagagttataaattaagaaaaatagggaaagtaagaaactgaaaagaaaagaatttttcttctttcatttgaattcttcttgtttattactgt
ggcattacgtctcgctaaactattcaggacactcattctatatcgttaatatgacgtaacctacacaacctgtaaagtgcttctactcagcggcaag
taatttcttcttttccctgcgactcgaatttcagctgaatttgggtttttacaacaaattccacatatttatgagatttttacgtgtaaatagtaaaaac
aattgatttttaggctctttgtgaagatttgagttgacgacaagtgttcttaccgtcattacacatcatcgtcttccctggtaattttcaggcttggatt
tttttatatcggttataagaacatcgtggatcggttgcattgagcgtgtatttgtgattggacaaatacatttcttacctggttaaaaaaatcatcca
aaaggattctgtttgcgttttgtaaacgtctacgtcttatcgtcatttcgtggtttaacatttctactcttcctctacttatagtatttgtaaaaggttaa
aaaactttacgctgttgtcattggcatagagttgattttgaaagttttagaagcttatggcaacacgagcattcataactataattttattggtttagt
gacttaccttttgatccctgaagtttaatactagtttcgtctggtcgttattcctttttgcaagcatgacagcaaaaaaaaagagaagttgacgaaga
aaagcgtcgaagaattttagaaagaaaccgcattgcagcttcgaaattccgacagaaaaagaaggaatggatcaaagaattagagcaaacag
cgaatgctgcttatgaacaatcgaaacggctacaaatgatgctttctcagctacaacaagaagctttccgattgaaaagtcaattgctcgcacatc
agggatgtccctgtagtgtaaaaatccgatcagtacttactgattttcaaacagcgcataacgctttgcaagcccagcatatggcttatcgccctat
gcagcctcctccatccgacaaccttttagatccggttgtatctacaagtcctaatcacacccattcatcgctccatagtatgcctccacagccttcca
acggtcaaatgcaacctccttcgcaacccactaatcccaatcaatcatccatgtttcccccgtctggcttacctggatcattaggtggctccatttaa
tgtaaaataatccatgggtttcaattatggaacatgaattcctggaatgataccgctcaccttattcaacacttgtttaagagtttgatacgctggag
accctgctaagtatcccaatcgtcgggggtcctatcctatatatgtattccccaattcatttgacatgatgagtgtaatctctaaaaaaaggttctcc
tcagagaaaacacaggatgcatagattgatggcttatgtgtgattatcggttctttttttttttttgtatattggcataaaagttccttctttataatgat
ggtttttttgtttattgttattgattgactagttccttttctgaagacaatggttcatggaaacttgtgaatctaataatacattgtattgaagacaaata
aaggtgaatgaatctaatgttagtgaaatgaaatcttattaaatttgtactactacactgcaaagtagaaaatactttcacaaacaatgagcagct
tgcattccgattatgactttgcattgaatttttattgacataaacaactaaccagcaactggacataacgcaattatgataccctttcttatagtata
atactactttaaggagcatatcttcgtaaacgtactacaatttgccgcactcggatacagtaaacaatttgaaatttacaccaaaatagcagtcac
agtcaacaccaagaaaatcacttggaaatgtatagggagggctgaattcaccaacaaccaaaacaccagaaatgggtcgtgttattagagcac
aaagaaagagcggaggaattttccaagctcacactcgccttcgtaagggagccgctcagctccgtactttggactttgctgagcgtcacggatac
atccgtggtgtcgtccaaaagatcattcatgaccctggtcgtggtgctcctttggctagagttgctttccgcaacccttaccactaccgtactgatgt
cgagaccttcgttgctactgaaggtatgtacactggtcaattcatctactgtggtaagaacgctgctttaaccgttggtaacgttttgcccgtcggtg
agatgcctgaaggtaccattgcctccaacgtcgaagaaaagtctggtgaccgtggtgctttgggtcgttcctctggtaactatgtcatcattgttgg
ccacgacgttgacactggcaagacccgtgtcaagttgccctctggttctaagaaggttgtcccttctgccgctcgtggtgtcgtcggtatcattgccg
gtggtggtcgtgtcgacaagcccttgcttaaggccggtcgtgcttatcacaagtacaaggtcaagcgcaactgctggcccaagactcgtggtgttg
ctatgaaccccgtcgatcaccctcatggtggtggtaaccatcaacacgttggtcactctaccactgttccccgtggatccgttnnnnnnnnnnn
nnnnnnnnnnnnnnnnnnnnnnnnnnnnnnnnnnnnnnnnnnnnnnnnnnnnnnnnnnnnnnnnnnnnnnnnnnnn
nnnnnnnnnnattcatctactgtggtaagaacgctgctttaaccgttggtaacgttttgcccgtcggtgagatgcctgaaggtaccattgcctcc
aacgtcgaagaaaagtctggtgaccgtggtgctttgggtcgttcctctggtaactatgtcatcattgttggccacgacgttgacactggcaagaccc
gtgtcaagttgccctctggttctaagaaggttgtcccttctgccgctcgtggtgtcgtcggtatcattgccggtggtggtcgtgtcgacaagcccttg
cttaaggccggtcgtgcttatcacaagtacaaggtcaagcgcaactgctggcccaagactcgtggtgttgctatgaaccccgtcgatcaccctcat

ggtggtggtaaccatcaacacgttggtcactctaccactgttccccgtggatccgttcctggtcaaaaggctggtcttattgctgcccgcagaactg
gtctcttgcgtggtgctgccgctgtcgaaaattaggtttctcaatgaaaagttttgttccggctatagaagaaagtttgtgtgtgataaatgtagtta
ttgttcgtaaatgatgaagtgaggtataattggttatagtagtacagtaaggtttcaaatgttgtagaccagatgaagattgcaaacagtgttatta
aaaattgtgttgaaaaaagaaacaatagctatcaaaatatgagaaagcatagatggaatacagcatctgtgattgctttttgaacatgaggaa
agaatcgatctgttatgaaagttcgttaaatcttgaaggagtagtaggagtagtagtatacaaggttcctttattcttcattgagacaaaggtggtg
tatccataaacatcttagcacctgggccaggaggatatctttgaccgacgacttcagcactgtcggaagcttcacggatttctttgatagtctccga
agagagttggatgtccttggcagcatagttttcttccaagtacttgatacgctttgtgccgggaatagggaggaaatcgtcgccttgagccaaaat
ccaagccaagctgagctggcccgaagtgacattgcttttagcagcaatagattccaatttctttacaagttcaaggtttttataaaagttttctttct
ggtagcgaggagcaattttgcgaaagtcgccctcgggaaagtcatcgggagatttgtaagcaccggtaaggaaaccacgaccaagaggagcgt
agcaaacgattgtgatgttgttttcacggcaagcttgcttaacaccaatttcaggacgctcgatttctaatgagaaaggagagtactcaatttgaa
cggcactaacggggtaaactttgcaggcacggcgaatggtttcggcactacattcagaaagaccaatgtagcggatcttgccggcttcaacaaa
acccttaagagtctccataataatttcgacaggggtttcaccactgaagcggtgaacatagtacaagtcgatggaatcgacattcaatcgtttcaa
agacatatcaagcgccttgtgaatgtattccggggtattattcaagataccgccttcttccgtcttttggtagccaaacttggtggcaaggaagattt
cctttctgcgaccggtttgcttgaaccaacggccaaggcagtcttcgttcgcaccaaagccgtacatgtcggagctatcccaaaaggtacagccca
tgtcggcggcacgggttaagacagcttgattcgcttcttcagaggaaggaccatacatcgcatgcaaacccatgcatccaaaaccaataggaga
gactacggtgtctccaatcttgcgagatgcgacgaccatattaatagcttttataaaccaacaacaaacaacaataaaaaataccacacaaaca
ctaaatgagttagtatctcgataaacgcatacatgggtggaaaacaaaaggaaataaattcgaataattattagacgttcttttcatttctttttac
atttgtttttcctttttcattttctttcgacaaattccttttcctcataattccttgctttttcttaccttgtatgaatgaacgaacgtactgtttggcaaac
gattaatacaaactgtaacaagattccgttggtataaaaagaaagaaacacgaccctgtatatcggtacttctatctcatccacgataccggaatc
tgaaataaagataatgacgttctatcaactcgttgttcctaaaatttacctaagaacataggaaaagaaagaaaagaaaagaaaagaaaagaa
aagaaaaagataaagaaaaaacaaatgtaactgttttcactttcgttgggttcacactgaattccttgcaacttttgtacaatcaagttgtttgttta
cgattcgtttcaagatcttatctgatcataggtagcacgttactcatatgcgggacaatctgataagcagaaaagtaaacaaaggacattgagaa
aaagaacaaaaacaacaagttggacttgatttcttttttcttcctttttcttttctctgtcgtttcttttgatttctctgtagtttgtatttacttttctttag
tttacgcgtctccccttagcaagaaccagtagataggcatagagatgcttccaggttatctccatacaacatggatcccgtttaaccaaagcacct
aaatgagcactcctggaaaaaggccaggtacgagaaggaatcgaaaaatcacttcatgtagagaatgccatcgtctgaaattaaaggtactttt
actttggaataactggaaaatggatgactaacgttctagtgtgatcgtgtttggccttgttccaattgtcgaaaacgaggaattgcccatttatgcc
cagatggaattgtcatttctttgaggtaggtcctttttttttatggtcggtggacggaggcgagttctgagctcgtgaaccaaaccggcttggtagaa
tctgttttcggtttccgttaaatctttcttccgtagttcctccactgaattttttttacattgctaacaactcccttttttgggatatagtgataaattgatc
aagctattgttagttagaatttccaagctacacaaagctgtcaagtcctacgatgaaaaacatgattccctgttcgtagctgaaaaggatcaagaa
gtaattcagtctttatttgataaatacttggatccggctgagcagcctccaccatcatcatcgtcatctgcgcagactcaatcatcagcatcccaga
accagtccatggtgacagaaaacaatgacaccattctagaatcttcaccgtctgaggaaaaaaagcttgcagacgtttcgcaaatgcttggccgt
ctcaaagtaagttctcacggccatttcaattattatggtgcctcttccagtcgatcttgcatccctcaagcggacccaaatttggaagaagacgaat
tttttacccttcaaaggtctttgttatacccttcttcctcttcttcttcccttctccattggagccatcgcgttcccgacgtattaaatccaaccaatttat
gcgacatgctcccagtcccccaatttgctgtccgtcttttagacctctattttgacaatgtaggatggagctgccatgtcatacatcgcccttcctttg
aaaaggctcgtctcattctatacgctcaggatccctctcggtctccgccccaacctcgtttccttgcccttacctatatgatttttttgtctcggtacgct
ttttgcttctccggcgctggttcgaaacaaaaaatcgctagcgcatgagttttaccttcgtgctcaactctgctttgatatctcgtactcttccttcgtc
ccttctgtcgatgctgtcgttgttctcatcctcatgtgccagtactcatacttctgtgattgtcttgaacgtacaaactttttcctggaactgtattggaa
cggccgcacgtattgctgttgccatgggaatgcaccgtgacggagaggtatttgaactttccgcgttccaattgcatatgcgtcgaatgatttgggc
agagctcttgttcttggaccgaatgctttcgatgagtttaggaagaccatatgcgattagtaacgatcaaaccaacgttttgcctccttccaatattg
cagatgctagcatcaaacctgatagtccagtcatcccagaaccgtctcctttacgaaccgaagctagttttactattgtcaaagctaaagtatctaa
gtttttggcgaatgtcttaaatcgggccttcaaattcactcctcctagttactcggaagtacagactttaactaaagaattactggatttggaaaatg
agctaccagaatattttcaagttaccgaagatacaaagaacgcttctccaatcgttatcatggaacagtattctatcaaatttatcatccttcagtcc
ctactgtacttgcatcgcccttggtttgtacgtgccattacgcgtaccgaagaaaggaatcagtacgccaagtctttgtctctttgcacttctgtctcc
catgatctcatccaaatcctttattcattgatgtgcttagttcccatcgagcctcttcgttggtgggtatttcgatttcattctttgaacgcaggcattat
ccaaggagcttatgctttaagctttccaagaacaaaatatgcaatgacagcctataatgatttacgttatatgtgtaagatatttgagcacctcaga
gacgggtcctctttttccaataaagatttttcgttcttaactagcttgaaagcgaaggtatctgaacgttttcaattgactagtcagggaattgacga
tgaatatcttgtagaagaaattccctttcttcagtttaatgttccgtcgtctagatcaaacagtcgatctccggacgacgctaccgaaaaagctgaa
caggcaaggcaattgaatggacttgggatttcactggaccaaactacgatgactcaatccgaatatttaccatactacgaacaagaaaaccccct

tttgtttgattctctgtcttggcatgctccaaagaatgaatctgagctatacggtcccccagaaaccggatttttggatgctagcaactggatgactg
cagaggatgaaaagcctctcattcccagccttgctatgagtttggagcagtttttctgggaaacggatacgaaagcaaacatggaaaacctctttt
atcaacatgttagttgaagaataatcttactttttctatcccttgctttctgatgtctatgaacaagattaccaagagaagttagtgtattatcgacgc
cttttacatttttgttttttttttatttggttatgattcaacgtttttcttttctctgttttatttctttttttttatggatttctgtgttactttaatttaaagt
cgttggattttaccaattatatcaagccccggcgcttttgttatttaagtgattgtatttttttacaaaacaaaaaagtcttttatgtatattcagaatat
aattattctctaaaagttcatggtttgagcctttctcattcatctgacaaaaatttccaaaactgatattgtttgtttggagtcctttttgtatttaattc
gttattcaaaattttgctttgagatagttagtgaaatatttcttaattttcagttttagttaacatttaggaaaaatctattttgtataatacaatatgct
aaagcgttttagttggtaagtatctatgaaatgctgcaaaaataaaactctaccaacttaaagcattacgtttgctggaattgttaactagaactga
atggcttgatatgcaccggctttagaagtaaataaattcatttgaacaagacaagtcaacatgccaacaccagcctttgctccttcaacgacggga
atatcagggcccactgcttgttgaacaacatctaacatatgagccattccagcacatccgagacagataacgtctgcacccatttcttgggttgctt
tgcgagaactctgctgtacaagagcatctacttcagtatgcggcttcgattcaagttccaagggagtcaaacccgtggacacaactccaccgaaa
acgctgtcagaaatacccatggcccgaacaccggccgttaataatggtccataacggtttgttgttgtaacaatcgctactttacgacccaccgac
aaagccatgaaaatcgaggcttgcataatgccaagacaaggttttctataagcttcacgcaaggcagaaaccagagggtgatccgaatagcaa
gcgattaggaaggcatcgcattgaggaggatgacacttgagatatttcataacaaccgatgtcgagataattccatcgtacatggattcaatagc
agcgggtccttctggaggacaagtaaggaaatgcaattcgacattcggcggtgtacaagaagctaagaccttttttacattgttggtcatttcacc
cgtagaattgggatttataataagaatgcgtaccatgtggaatttagcgaaaaagaagggcggtaatagaaaagctgaacctgtatagaaaact
taagtaaattggctggctttgtgaaaccccaaaagagcttaagcttttctagttgaaaacaaaccaaaaaaccaatctaaaattcaaagtgagat
gattctagagaaggaacgagaaatcaaacaaacgtcaaattctttaaaaatgactcaagattaagaatgaacttcgattacttcgtccttatttat
aactgtttccttcttttctcttttccttgggataaaacttcgaaacacaggtgattaggacggtatgcgaaactttttcaggttaaatgtgtcattact
gaagaaaacaaaatgaatatctccgttatagaatctcccgaaatgtcggttaccttttaatctaaactgaagagacatatatagagaatgctgcc
cagacagtattttggaaacaatctttctaccaaatcattacaagtttttttttcagttgaaaaacaccttttaattattgaaataagagcccaccgtt
tgtgccaactgaaattttgtctaatcgtactaagaggtatttgtttgttttatataaatctccagatacaaagggttaagatactgcgtctataagatt
ccaaaacattatcaagcgtttataaagggattttctttttcttctcagtttttaaattttcttttgacttttatttttgcttggtgttagtctttgagcgatt
cttataattgtaattgtctagtaagatctcctatagtgaacatttacaagatgtctactgtagctgttgaacaaatcgcacccaagatcggcgatgc
atttttctctcaaaactgcgtggatttgatttctcgtgatttaggtggtcaaattattgcccgaagtgatgactttttcgcatcagcagaaaatctaat
tacgcctactgctccgattcgcagggaaggtgtctttgtagacactggcgcttggtatgatggatgggaaactagaagacataactctgaaccctc
cgactgggtgattgccaaggtaggtgcttccagtggtcgtattgctgggattgaaatcgatacagcctactttattggtaaccatgcgccggctgtg
agtgttgaagctacattttctcccaataaagttcccgatactaatgcattttggaccccgattcttcccaaagtatcgtgcggaccttctcagcgaca
tcttttccggctgaaaacgccttcggatcaaagctatacacatgtacgtttacaaatgtatcccgacgggggaatcgctcgtttccgtgtgtacggg
aatgttgctcctgtgttccctgccgacttggacgttcgtctggatctggctcatatgtacctgggaggtctagtggttcagtgcagtgaccaacactt
tggacgaaaagataatcttatcttgcctggccggggcgttaacatgggcgatggctgggaaacagctaggagccgagagcctggtcatgttgatt
gggttatcgtgaaacttggtactaaaggctatattgatgatgtccttgtcgataccaatcatttcaagggcaattatcccaaggaagtcatcttgga
ggcaactgactctcctgaccatgtccctggtccccattgtatttgggagaccatcttaacagctcaaaaactcggtcctcatatggaacataccttct
ttgatcttgataacgtttctactcctaagacgcatgtccgtatgatcattgttcctgatggtggtgtaaagcgtctgcgcatttgtggccgacgtgctt
ccttttaagacaagtgtttttttttttatgaagacgtgaaaacaccgcaaaaatgaaatttttatgatcaagcatggtcaacatgtgaatttgcactt
cgtaatgaatgatgttgctctcggctcctctatgaatgacaaagaatgaaacagcagcatcgaaaggatcttttgtctattcatacctttcttgcatt
ccttcccaggaagccctagtattctagaaacgtttccctatgctgtaggattatttatatatgtttatttatttcttcatagtattggaagcatacgttg
cctttctttacggccttctttggaccgatatattagtctttacatatcaatgaaaacaatgattcagaagcagagatgcatttctttcttcgtaatttat
gaatttgagttttaacttaaggataatattccttcatactttaaaggactttcgtctacggccacacctaggcgaaaacaccagttcccgtccgatc
actgcagttaagcgtctgagggcctcgttagtactatggttggagacaacatgggaatccggggtgctgtaggcttttctttttttgcttttctttttta
atacgtctactactgttgcttgcaaccgttttctcgatagaatttttgcaagctgttcctttacaagtgacaaaagactaattacaagttcaatgaaa
agaaatggaacgatcgtattcaattgcaattaagaataagaataatagtaataataatcattaagaacctagtatgatgccaatgatctcagatt
acggttcctttcaatgagcggattgtatgcatgcctttctattgtccacttttctccgtcatttccatttatattaatacaaatcaaaagcatagaaat
aaaccttcatcggaataaaccgcttgtaatgttctcttttttcatttactgactttcaatcggaagagttaagactcgtgagaaaagaaaggcaaata
agtaggcattaactttgagtggtaggattgctctgaagctcgatttcttcattcgtgtttgtcggcggaggagctaatgcatcatctctctgttgatat
ctgggaagcgtaggcgaaccggtgttttcagctgagcgctcaaatcccattccaaggccaaaaacaagaacgttataaattccccaaaagatag
caccaacgaaccatgcgagaccttcgaagccaacactaacgaaccatgcaagaccgttaccaagaccaaccacgataaagattataactttag
cggcaatccgtattgtcaatatgaaaggccaaataagtgctaggctttattagtaacacacattctaataacgtttgctattaacaacaatgctcca

cttaccatagaaaagagcaaagaatattgcccatatcacaagccaaatgcctaaaggtccaagaagggccaaactgtatccattttccatatata
cttgtttcagaaaagaaaatagttcgaaacgataggagattgctaatgtacaaaggaatgataaagccaatatcaaacagaaaaacatcgattt
acttttaacgactacattttcagggaaataatgatctgtcttcttcctaaagtcggtaaagaaatcattgaagctatcaaattcagtaattatcgtttt
tttgctttgtttggctttgacagaagttgaattggaagttttgtttatgtcttttggtgtatacaaaggaagtcccttctcaaggtcaacattgtaaaca
ttagcatggttgtacgcaggagggtcgcttgaatcgaatttcagctccttttccgacatctaaaacaaataaagagaaatttcaacgaataattag
caaaagagcgtcaaataccaagctgtcgatccgtattcgatgcaaaaatttcgcaaacgcaaaactcatcagaagtagcagccaaaactaaag
aagctctacctggtcattccaaaagcacaaaatggaagtcaaaatcagcccaattggcaggaattgaagaaataaaaagttaactccagcaag
aaatttcgcatcacttacgcaacattttgctcaggatagtgatgggttcagttacgctatctcttatagcgattattgttaataaatcgttaaagata
cgtttgagtcacaaggaacaaataacaagtttcgctagtttcgcgtttgtttacatagcagatctcacttttctgtgaactgttaacaatttttactt
gaaaatgactaacttactttcataccgttaaagttactcactttccatggatttttcgtattcatacagcaacagcaaacgaacaaaacacgaaa
ctgcagacacattaactaactgctttttattagactattggataacagcctgcaacaaaaaagcagctttcacggattccttaggaacaaaataac
ctataaattatttttatgaatcttctttttgttgctcaaattaaattttaacttctgtttagattcttgtaattgaattagtatttcccatatttcagttgaa
tcgctttacaagaaaataattttgaattcgattgagacaaagaattgtatgcatagtttcttatactgtgtttccataccaaatattggtgtttattttt
atttatttaatgtccgtactggacaggattgtcgttcaataaactactcgagtaaaccatgttgacaattagtgtcttttctaatatcccgaggtttgt
ttctaccgtataatttatcatacaaaacctatcaacacagtttttttccttacatcctttcaagtgtttacgatttcgttattacatgacggctgcaggg
acaacgttgtattgaattagtaagatcagatacttaaaaatcgaaaataatgataacgaggcacattgatctcgaaattctatttaagagatataa
aaagtaaaataaaataacatttgaactgaaagggattaaatttttcgcaacagtcactacgaatatgtcgtcaccgaaaaataaatattgtttatg
gtttaataaacaactctgatcagataaaattgggttgtaaaagtggaaaagaatagttggtaggattgaaactacaatcgaaggatataataaa
aaaaaaaagaaatatgcagccattttcttttcatcatttttgttcctcagtgatgtatgtaattatgtatgtctgcgaaagaaaaacgatctgataag
agtcctttgttgttctttttccatagatccttgattgttttgattagatagtatagatacattgtgagactgtaaaattatcagatccaataaagcaaa
taaaagtcaatcatgaaaatttgcgaaaggaaacgataacaacggaggtgatataatttatgatatttttaagactcctctatgctcttgttttata
gtgatgcgttaccaaaaaaaaccagatttcattaaggattaaacatattgtggtgaaacaactagatattagtatgcaagtatataaaataagat
ataaggcaacaatttaatatgttaaaagaaaagggggaaacaaaaggaaagaaggaatcgcataaagagggaaaaattacatgaactaagc
agtttgacggacttctgcaatagtatccggttgcttgatttcttcttccactttattggtagttttcaattcttggatttcaagggaagatttttttagatt
gttcgctagaggtagaaccagttgcctctagatccttgccgcctttaataccagcagcagcggctttctgagcgaagcgacgctctctccaccacat
taacaccatgcaggcggcgacgaacaaccaataaaaaatatagctaagaatggtaccataagtaccagtgttcatccaacccaaaatggcgttg
aagatcatgtatccaccatttgagttcatttcgggatttccgtagttaacgtgccaaatggcttgttgaaagggataggaaccaggtccatcaccca
attcagcggagtcacctccggtcaggcgactatagtgatcaagctcaaaatagtagacgctcttagccatgagaccagcagaaattagatagag
tatacaggtagaggcaatcagaaaccattgcaagttcattacatttccacctctataaatgaaataaccaatcaggcagccagtgatgagaccac
aaacaacagggagaggaaaggcagtggccggtgtttctaaaccgacaccaccaacgaaaacgacaacttcaaggccttcacgaagcactgta
aagaagggcagcaaaaacatggcgtactttttaccccaactactaataccacgagcctttcgattcgcgatactacgcataagctttttctccatt
taccttgaagatgcgaaacacgaagcattgcaaatcccataacggtaatcaaaatgactgcaacaagtgaaaagacaccctcccatatttcttcg
gaattaccccaaaggtctttacctagtgcataaaaggcaccaataaatccaccaccgatagccaagcaaataaatagtgcgacaaatgatccaa
cccacacttgtatcataaatttacgttttagttttggatctgatactttgccctcgttgctcgtcagcgtttgtgtgataaatgacatgagcactgaca
caatgatagatgcttctagtgtttcacgcagcacgataaagtaaatcgggacagaaaacacgttgtttcccatatttcttcgatttctttttgagttt
actttgaaaagttcgttcctaaattgattgtttacacacgttggacgaaaaaagttcactccacctcgtttatactactttatcagaataacggagct
taatagttcttccttaaatctgatatgggtctcaacaatggaggcttttcaccccattttaaactgttttaggttgatgatttgggtttcaatgctggtt
ccatctggcatgatctgataactaaagcacgttaccatggatattccgattggctatgtgtgtgtccaagtttgcatttcttgatcatcaaaggagga
ggagtgcaaatttagacaccacacacaaagtggacaaaataatcagatgagaaaatctgcgtataaaataaagtccttagggcagcgatgccc
accctctatatcagatcaagccggcttaggtagaaaacccaaaagaccttaccttgttgaacccaaactttgttctttttctttttcttttatttttttgtt
attcaggagcattagcttaagctctttggtttcctgttccttaacatttattgtttatatattcgttcttttcttcttctagtttatttttggtttatcaaact
cattttaaagttatactgatatcctttctttggagactgtttgtttgtatttaatcgcataatgacctttgctaatccttttcctaccaaaaaaggccttt
tgtctttggtgttgatcttcagtgcactatggaaaagtgctctagccaaaacccgcttgtttgaatggaatgtcacggatattgataatttggacgtt
gaccaatccggccatcctcgttgggttatgggcgtcaataataaatggcccattgacccacttgtcgtcgattacggcgatcaagtcatcatcaag
atgaacaatcaacttcgtgacaaccgtacttgtagcttgcactcccatggtatgtttcagcgccataatggctatatggatggtgtcccacaaatga
cacagtgctctattcctgctggctctacctactactacaactttacagctagtcaaactggtacttactgggttcactctcacgatatgagccaatat
cccgatggtcttcgcacttcatttgttatcaataatcccaatgaaccatacgactacgacgaggattatatcatcagcttaacagactggtactacg
aacccttttacaaacttgttcccgaaaaatttacaacttggcacaacccaactggtgccgaacctgtgcccgacactggtctcataaatgatgctgt

caactctaccttcaaaatggaaccaggaaaaacttatcgattccgttttgtcaatttaggcgccttcaataattacgacgttatgcttgaggatcac
aacatgaccgtcatcgaagtcgatggtgtatacaccaagcctcaggaagtatcctccattcacataaccactgcccagcgttacagcgttcttgtg
accgccaaaaattcgactgatcgtaattatgctatgacggcctatatggacgaatctctctttgaccaaatccctgataactataatcctaacgtga
ccgcttggctttcatataactcggagaccacttacaattacgccgactcagtcaatgagattgacagctatgatgatgcaaagttggagcctttata
tgatgttaactggggtgaggccaatcataatgtctctctctggtttgacttctttactcttggcgatggagcaagctatgctgaaattaatgaaaaat
cattcaaatatccaaaagttccaggtcttatgattgccaattcaactgactatgacgactacaacatgaagcccgtcacatacggtccctatacca
atgcctttattttcgaatataacgaaactgttgatgttgttatcgacaatcatgataccggaaagcatcccttccatttgcacggccacgttttccaa
gttctggaacgaggtgaagaggaagctggtgaatacaatgatcaaaccgaacacaatgtatatgaaaaccccgtacgtcgtgataccatcgaaa
ttcttcccactagttttgttcgtcttcgcttccttgccgacaatccgggtgcttggcttttgcattgtcatattgaatggcatatggaaagtggtttagtt
gctacatttatcgaggctcctgatcgagttcccgaaatcactgctcccgaattcgtccgtcgccagtgtgtgatgcagaacattgatgtttctggtaa
cggtgcaggaaacgcattcaatatatccgacctgaccggtgctcctgctcctcctggtgaaatgcctgctggttggacatcaaaagctattgctact
gtggccatgtgtgttttagcagcctgtattggtatgggtagtattgtcttttacggcattcaaatccatccgttgccgactgaggaacttgacgatga
agaagatttaaaattagctgctgaagaaaatgcagcaaatcttatgaaatcttctgatcctgtaaaataggttttaggtccttttattttgattttttt
tgttggtgaatttccctaatagtaattactgggtttttctatttcattgtctctaggtactaatcttctcaattaatcgtcagtttagttaacaattataa
ctcaaaaatttgcattgttcatggaaattatttttcttgactttttatataatttatacaagtggataatttcactgaagagactaaacagcgcaaag
taatagtcattttataagatatcatttcctatatttttaagaaccagattcgtcttgtttattagttcagcgaaaaccagtacttacctgaatatcatc
atgtttttttgttagctactttgtaaataacttatagtaatcaaaccaaaatccacgatgctaaggattgattaaggaaagacaattaatgagaac
caagactattattatgacaaaatttttttaagaaattcacaaatgtttactacgtacactctgtcgcgtaaagttctctcttccttgttggcacgacggt
ttatttattcactgtatcatttaatatgcaacatatattatggagaataaagccaagtccagcaagttcttttcttgcatgatatcgtttggaaagtaa
cacacgattttacaaagcttgacctttgtagttattgagttggatcataattcgtgattgatgagagcaagaggccgtaatgggagtttacggtata
ttaagtaaatggaaattcatatacccttggaattcggactaagatatttaggaatttcagtatgatctttaagttacctagaatgcttgtttttttcaaa
gttttcaaaacttgtttagcaaccgcttcagcactatttgtattcgatccggtaataatatttccatcctcgataacaaagccacctgtatgatacac
cggcgaacttttaaagatagcaccgctttccttggctatatcctcagtagaaggaaaatgtaatttcttcataacatcatgcaccccgctttgaattg
aatctctccaagtacaaccggttatccttcgattccaacacaaggtatgaccatcgtctcttgtagtgcaggctaataaaagaacaccttcagcaa
ctgtagcaataaccttgtctttgttaaacatagctttcataaacccttgaccatctttacaatgagaataattaaagtagcatccatatcccccagag
acgataactgcatcgtagtcattgggattgagacgactcaagcgagatattgtcttcaatttgatcatcaaaatgctctccggatcgttcagaacat
ctagcgcttcctttgtggcatagggttcccgtactgatttacaatcaaacccatagtctccagtatcagaagcaaattcaacatcgaacccattattt
tcaaggatatgaaagagttcaaagatctctggataacaggctcctgtattatcaccatcatcataaaatgggccataataacttgtcattaatatta
aagctttccctaaaagcgaaggcttttcctggtgtacagttcgattcatctttgattccttaatcttttctccttttattcttagaattaaatattgctatt
ctattccttgcttttgcaatgaattaaccaacaccaaggaatgaaaccaaaaagacaaaattaatttagtaacaaacctcataactctttaacata
tttaaatcgtgcattacgaaaacttatatttaaagcatcatagtgatcataatgacattgaacagatctgtcgctagtctgcttaagcttcctttgta
ccgaatgtcgtatactaaaaaccattctttttgctgtacatagctatcgctttatctctgtgcttaagctgttatttgagtacaatacccatctacagg
atgcaataatatcgatagcaatgtttgatccttattactacacttctacccactaaaatgaaatttagtccattaaaaacaaatttttatttgctaaat
taaaactcaacctcaaaattcaaaatgattggagccagctcactaaaacagagaaaggaaagacagtagaaccattaaggtcaacctataata
tacatcaaacggaagtcattcataaacaaaatcaatcaaagcttttctttttttaagcagaacacatctcacacgactcggggttatttatagagca
agcaatgaccttttcattataaatgtcaacatcctcagaatccttatcctccttcttttcagcctttccgtttttaacaggagagcgattctcttcagaa
acttgcttgttacgctcacgaaggatcataggatcaacagtgaatttaatagcagaagcagcagccatagtacgcaaatagtacataccggtctt
taagcccttcttccagccatagaagtgcatacttgtaatcttaccgtaagagggatccttcaagtgaacgttcaaactctgagattgatcaatgaat
ggaccacgatcagcagcatagtcaataacagtcttttgactgatttcccatacagtcttatagaggtccttcaaatcttgaggaatctcagggatag
cttgaatagaaccatcgagcatgacaagtttgttcttcatctcttcattccataagttacgttcaacaagatccttcaacaaccaagggttgacaatt
tggaattcaccagaaagaacacgacgctgatacatgttggaagtgtaaggctcaaaacattcgttgaatccaagaatttgagaagtagatgcag
taggcatgggagcaaccagcaaagagttatacagaccgttcttggcaaccttagcttttaaaccagcccagtcccacaattcagtaggagtaaca
ttccacatgtcatactgcaaaataccctgagaagcaggagagccttcataactctcatatggtccatttttttcagccaattcagtagaagcctcga
cagcggcatgatatatagtttcgaagatttgaatgttcagcttcttcgcaccagcagattcaaaaggaagacgaagtgcaaagaaagcatcagc
gagaccttgaacacccaaaccaataggacgatgacggaagttgcttttacgggcttcgggtacaggataatagttaatatcaacaatcttgttca
aattacgggcaacgaccttcgtcacgtcgtgaagcttttggaagttatacttgccatccttaatgaaagtaggcaaagcaattgaagcaaggttac
agacagcaacttcatcgggagcagaatattcgataatctcagtacacaagttagagcagcgaatggtacccaaattcttttggtttgacttgcgat
tgcacgagtctttgtaaagcatgaaagggttgccggtttcagtttgagattgcaaaatagcataccaaaccttttgagcaggtaattgacgacgtc

cacgattatccttttcatacttttcgtacaaagcctcaaactcttcaccccaaacgtcagccaagccaggtgcttcgttaggacagaaaaaggtcc
atggctcattcttttcaacacggcgcatgaataagtctggaatccaaagagcataaaacatttcacgagcgcggaaatcttcctttccgtgtgttttt
ctaagatcaaggaattccataatatcagcatgccaaggctcaaggtaagcggcaaaagcaccaggacgtttgttaccaccttggtcgacatagc
gagcagtattattaaatacacggatcataggaacaagaccatttgaagtaccattagtaccggcaatataagaaccagtagctctgatgttatgc
atattaataccaataccgccagcagtcttggaaatcatagcacaagttttaagggtatcataaataccttcaattgaatcatccttcattgtaacaa
ggaaacaagaagacatttgaggacgaggagtgcccgcattaaaaagggttggagaagcatgggtaaaataacgcaaagacattaagttgtaa
gtttcaaggacagcttcaatatcttcaccgtggataccgacggccacacgcataatcaaatgttgaggacgctcggcaacctttccatcaatacgc
aataagtaggaacgctccaaagtcttaaaaccaaaaaagttatatgtaaagtcacggtcataaataatagcactgtctaattcatccttgtgtttc
ataacaatgttgtatacctcatcagaaatcataggagcatgctttccagtctttggattaacataatcgtgaagcttttgcatgacgacagaaaaa
gccttgtccgtttgcttatgcaagttggaaacagcaattctggcagcaaggatagcatagtcaggatgctttgtagtcatagtggcagcagtttcag
cggccaaattgtccaattccacggttgtaacaccgggatagacaccagaaatgactttctgagtgatggccacaggatccacatggtcggaatca
agaccataacaaagtcttgaaacgcgagcagtgatcttgtcaaatgcaaccttttcctgatgtccgtctataagaacaggttagctttgcttctaaa
cactcaaaaaggcaatgaaagacataacgctttccgccacggaacgttgtataaagaccatttgtagctagctcaagacaacagcgttacgcaa
gacacaaaggacgaatcaataagatttcaaactctctttttctttaactgaaaaagatttccgagttttccaggtctttcaagaagcttaaacaacc
ttaaaatcttcgtgaaaacaaacccatcagttcatttctttcaagcctttatttattctccttcattgacataaaaatatacttacctctcttgtagaca
aacattgtatttgtttaaaacatagaagtggaggaggtgtggattatatatagcaaatgaagaacgcgctggtacgcgtcgcgttcaaattaacta
atacacgcgacgcgtcatagtagcatgatcttcgtaacataccatgtaaataaacctgaaggaacaaaatggtaaatacgccgctggagatattc
aatactgttcgtggccttacgcttgcaactttaagcattcaaaaaatgaaaattctggtacactaagaaaaaaaaagaatcctttcatcgcgtctc
aaaagcgcggaatttgaaaagaaaagtcaaatctgttattatagtttagaaattcacgtttacaaatctaccagataacaatcagataatctcac
gatacgcgtgtttattagctacaaacaggtcagaatcatttgcaccgcgtgaatgaaaacacgcatcgcgtttgttgacgcgtcgtttatcaacaa
acaaccgtgtttcattcacgcgacggaagaaaaaaaatatacacaccatgaaccatgtactgaatattgctagtaaaaatagaaaagacaacct
taactttgtaatttggtctttgcaagttagtttgctctattcgttgatgaattcactacaatactaaaatttgcggttgctcttataggaatgattgaga
attttaaattttgtttaagatgtttttataacatatatgttctagatagccataattaactgttatactactgtcataacttctttcgagtttagaacaac
acttatttttttgaaactgaatatgaaaatctaggagattggcaagaagtttgtatgaaactgaaaccttttttatctgcaaccggagttcctaggtgt
tggttgaaattgttttcgttgtatatatgaaaggaaaactggattaataagaagtattgaaacaagctcatgaaactcggagtcccatgatcctga
agctcggacgaagataaaattacgaaccgaatatatgtatattgtcatataagataattggcagcattaggtaccaaatttttgatttacgctagtt
tgttgctaacttcctaagagactgatgacagaatgctttctcaagcttaaagtacggcatcaaccattcggcaatttgcttaaaaaacaagtataat
acgcacggaggtttgcaagaatgggcaaccttctgcttccgataggacgctaataagcaattgctattgatcaaactcccgtacgacaagtatgct
atacctaaatcttaaacttcgaaacataggtaatgccattggatggggaacttggcactctttatattgcttgatatctcactaaccgcgcatccact
atcagcatagtatgagttttctaaacagcattttcttaagttactttgtgtttttttctacaagggagaaatctaaaaatctaatattcactgtgcagat
ttttcaatttccgataaaataaaaatagtgaccaagctagacgactatttaaagaaagttaaacaataagggttctcaaaaagttcttcaaatgaa
gactgaggcatgcaatggggatattcatcatacaaagaaatgtaaaaaaagcaagtctctcttaacgagcaagaggacaccagaagcaaaga
aataagaaagtaaagatcactaatagcgcatgcaatttgattataatgtgttgctttgagacgattgcggagacagaaaacttaccaaacaaact
tggatcaaaacatctcataaaaggatgacccgagaaaaattcaataaaaagccgtgcccagacacaggaatgcatttcaccccgacagagaag
ccatacagaaaagcatccattcataaaacacagctagcaagaagggataaacggaaatgtacaggtaaattacagatatgataataaagatag
agtgaaaagaatcggaaacgatttatctagatggaaaagatatgatgatattccataggagagaggccactttacatcaaaatgcaacgcttctt
ctttttgctaccaccagaaggcttcgtctttggcttatgcttcaacatagcagcacgagtggcagattcaaaaacttcacgaactccttgatttgtct
tagcagagcattccaaatacttataagcaccgatcttttgagcaacggcttgtccctcttcactactaacgggatgctgttgagtcttagagagttct
tcaatgatctttggatcattacgcaagtccgccttgcaagcgacgagcaatatcggaagactggagcagaagtgaaggacttcagaaatccactt
ctcctggacgttgtcaagggaatcaggggaatcgacagcgaagcaaataagaatgacgtgagagtcaggataagacaagggtctcaaacgatc
ataatcttcttgaccagcagtatcccataaggccaattctacgtgacgtccatcaacatctacatcggcgacatagttttcaaatacagtgggaac
ataaacttctgggaaggtgcccttagagaagacactatggaaggttagtaaagagttgaagaccgaaaaaaaaagataaaactgtatggttta
agaaacgaaaggggcttaaaaggaatggaaaatttatagagcccttataagcaaaaaccacagcttataaggctaataacgataaaaaatcga
ttctcttacaattcatatcaaaccaatatctccaaggaacgcaaaggaagatgccccttatgtaaatctttgacacgttcaaggaaagaactcttt
gaatccgttcaacgacaacgttttcattcataactgaagccctgtgactagacctgctcaagtcaagaacaagccctttctgtgaaaaaggcatct
gaatatgaacgaacctaaaaccttttcttcttcaaagaaccaagccaaaaaaatcagacaaagcgaaagaagatcactcttgtcaattttcttcg
gcagaatgccgtccgtttccccaaaaccagatcatttacttacattaaaaggcatgttttaccacatgcaccgtctcccacaataaccagttttcta
cgaagttcagttgccattcctaaattcttttttttcctgctctactatgaaggcaaaaacttaaagtagattgttgtttatgactaaatttgagtcaccg

ctctttccttcgttatggcagtggttaacttatttactctattctttcggaccccgaatgctgagcatttaccgagactcaacaaatcgtcgtatagca
ccttgtagtgccttaaacgttatttagatagcgcagagatgataattaaacaagttgaaagaacgctagagattgcaaaactaattggctaagaa
aatttctaaatctcttactaagtcacttctcggcttctcgaaaccccgaactcgttgctcttcatttatgaagtacaacgatgtacaaggtaccaagg
cgcaaacaaagaagagaaagattcaacttcgtagcgaatcgttaaatatgtaaacaaaccctaaagaacaatcaagaagtttgggccgaatcc
tgcaaaacagcctagtgttgaataagccttgacaagctttatttttggttgcttgaaagaaacacgaagaccggctttatgtttcattttgctttttga
gccatcgaaattcgtgtgattttcgacgtctgggctaaaccccagagtacttgcttctttatttgctgtaatagcttacctcgttttctctttcttcctgt
atcgtcattaagaacgtgcttcccttgaataaatagaaaacttccgttatctagggcattttgcttcttagtcctgctcttctcgacttactcgtctatt
ctattctccttgaggtttcttctgtagccaattcactctaattctgtttaagttcttgccatagatccttttgtctttcgttgtttatattatgtctacttcac
aaccgcaatctcgcattaatcctaacgatccaaggttcgagcgctatgattgtctgccctgtcgcctcataggtgctacagcatttacagggcttgg
cgcttatgcctactggcagtcaaggattcccaaaagtaccccacattaccgtacaaaacggttaggcctattgtcattagccacaggatttatcag
tgttggactgtatcgcttggttaattaatgcaccccattcttgaaatagttctccaaacaattctttctcggtacaccaatatatacttatactgtcctt
gacagctttacaaaaccattctatcgtttattaaatacggtattcagatttgtatttacatttaaaaataaagataaagataagataaaaacaatta
aaacaggaactggacaaaaagagagtatcttaaaacatagattcatataaaacacgaaaagttttcttaaatctcttttgaaatgacaacgtaaa
aataaaacttataagcaaaagatatgcaatatgacgaaaagaatgattgtccttcgaataaataagtaaggtaaactcatgctcgtatcaactat
ttaggggttttcttctccgagatctgacagtatgccgtctttttcataatgcatagagcggttccatcttggcttagtgaacttgcacattcaagttca
acgaaatatctcggcattggatttatattaatatataaatccactttgtattgctacatacgcttcttataatgaaattaaaaaagtgggaagaatg
agactaatagaaagatggctcaggcaatggtcttcgtcgatgtcctccgttgtaacctaaagattcgatggcactaggatccctgtaaaattcgttc
gtgttcgttccgtgtaatttagcatatgaatcttcttcgatagcttgccatggggagttgtatttataattagaatatgtgttcgtggaatcgtcatga
ctcgaaaatggattatcggtcgtacgcaagtctgtcgagccccgcaatgttgttcctgtaaatcctggatcatggaaaacattcatctcagagcttg
gatgcttgggaaaatcttttagagaagaactcttttcattagattcatccaagatacgataataagtaccattctttgttcttcgtaacaatactacg
atgttacgaatgaagataccgacgattaataccaacatggtaatagcgttgatagcgaagaacacaatggcaataacctgacgagcaagttcgt
tcaccgtgaacgctttgcacatgattagaagaaatacaccactaatcatatttacgatagaaataccaatgtgcatgctatttaaatgctttgttgc
atatggtcgagcaacgaccataaagaaaaataccacaacactaatggcaaacatagcacaaccttgtaccaccggagaatcttgaccgaaacc
gataaacatcgatcttatcaaaattacaataagcaaaaaaaagctcaggaagaaaaagcgatgtctgtagctcacgtacatgaagccccaccg
catcaaatgctgagtgtccgaatacaagttgaacaatgcatccggatctgaaatagcaccagtcttgcgaataatttgcatagtacgcgcaaaag
cgtatagcaataaaatagccatatctacaattacatacatagataagaatgccaatgctgaagagtcacgtgtatatatttcccacatacataag
gcactcatttgccagaaaccaagaaagatggcccggtaaaaaacaccttttgcaatagctttccaatattcacgagcacgttgtctttttagtgccc
tatctttagcaataaggtagtacaactcaagtacgattcgagacaatatagcgatacataatgtaaacccaaggagaattataaagaacgaaaa
gccggtaataaaaaagtttgtcgtttcaattcccatcatataggagagacgtctgatgccacgaagggttatggtaccagaattagaacttgaag
aggaacctgaactagaatcccttttggaaaagagggatgcagaatctaacccacgttttgcgagacccacgttgccatgtgaacccatttcaacg
aggacagaagggtcaccaccagtagactttacataccaggtaaacacgtcctgcataaaccctattttaataatacccattgaccagatgaagtt
acgtccccaactaaagacaatacctggaagaggactcgccatcatggcatacataccttgcagctgtgcaaactggaatagcgcaaccatgttct
cgaaaacttcccacattgctggagtttgaaatatgggagataaaagcagaaacaagagcaaaggaacaccgatcaccatgcacagaatccaat
acacagcatgatagtacacagagcgaccattctcgacgttcgtcttcacacaggcaatcctttcattcgaacttgtatcgtacatgttgacggtaac
ttttgcatccaagtcaggcactgtataggcaatgctaggaagctcattgacccactttgttacttcacctgtcaaatcgtggtttccttgcaagattat
gtttccagcctgcatagggcaaaacccggcaatattcatgtcacagggatctatcgttttctctaatttaaagccataagcgctaacattcacgtgg
attgatacattactagacacaagagatcgtgctgaaacgtcaaagtatatagaggtattacctgggtaatacacagcatctagtgtcgaagcgga
aaactgggaatcggtcatacatgttgtcaacgatttgacatataattgtttcggcttcgctcgcaccgattgaatagcaaaaaaaataagaaaaa
gggagagaattgggctttgcaggagcctcatattggagccaagtttccggttgaaaacttttctcgttcaccgatatctgaaggctctttggctaat
gttaggtttcttacccgtttgccgtaacctattttacgactgagttacaagatacccacacgtctatactatgtataagaagggaaaatgattctga
aagaaaagaaggaccaaactgaagagtatgttaagcagagactgctgaaatcccttcaccagtcaaaaggtggcttaaaaggtggcttaaacg
gatcttgaaaatgattgcgttaaaagaggtaacaagccggtggttactccaaaacttcttaataaagagtatgcattatcgtaatatggttaattgt
caatagcatatgcttctaacaagtattccaagtacgaacaaattgcgctaaacgagaagctgcactgcaaggagctcagtaaatgatactgcga
gtgaaaacatagaacagaccgggtttttttgctacaaagaaaactcaccaggaacctatactgccaagcggtgtttcaaagatgggcaaaacgga
attgaagcgtaaaaaggctcagtccgaagcgaaacctgcagaggaagtacaaaaggatactgccgtagtggaacaggatggaaatgctacgg
aatcagcccccgagaaaaaaaaggtgaaaaaagagaaaaccgccaaagaggaagagaagagtgaaggagaggatgcttcggtagaagcaa
acttcaaggagtttgagaaccaagaaaatgaagaagagggagaagaagaagtccgtggaacagcggatttgactgaaggtatccagggagag
gttgaaaacttttcacaactcaatctttcggagaacacgcaaagagcaattcaagatatggctttcgaaaaaatgacggaagtacaagctcgtac

gatccctcctttgcttgctggtcgtgatgtcttgggtgccgctaaaaccggtagtggtaaaacacttgctttcttgattccagctattgaaatgctata
tgctttaaaatttaagccgcgtaatggtacaggtgtcattattgtgtctccaacccgtgaattagcccttcaaatttttggtgttgccaaggaagtctt
gaaataccatcatcagacttatggtattgtcattggtggtgcgaatcgccgtgctgaagctgagaagcttgtgaagggtgtcaacctattagtggc
cactcccggtcgattgctggatcatttgcaaaacaccaagggattcgtttatcgtaaccttcgttcgcttgtcattgacgaagccgatcgtatcttag
aaattggtttcgaagacgaaatgcgtcaaatcgtcaaagtgcttccttctgaggaccgtcaaacgtcgctgttttctgccacacaaactacaaaag
tagaggacttggctagaatctctttgagacctggacctttgtacatcaacgttgattcctcgaaagacacttcaacggtagaaggtttggagcagg
gttacgtagtcgtagattctgacaaacgattccttctcttgttttctttcttgaaaagaaacttgaagaaaaaggttatcgttttcatgtcctcttgtgc
gtctgtgaagtacctggccgaacttctgaactacatcgacttgcctgtgctagatcttcacggtaagcagaagcaacaacgccgaacgaacacgt
ttttcgaattttgcaacgctgaaagaggtatcctattatgtacagatgttgctgctcgtggtttagatattcctgcagtggactggattgttcaatatg
atccaccagatgatcctcgtgattacatccaccgtgtcggtcgtacggctagaggtaatcagaagaggggtaagtgtctaatgttccttactccttc
cgaacttggcttcttgcgctatttgaagaatgccaaggttcctctgaatgaatacgagttccctgctaacaaaattgccaacgtacaatctcagttg
gaaaagcttgtttccaagaactactacctgcagcaatctgctaaagatggataccgtgcataccttcaagcatatgcctcttattctctaaaatcga
tttttgacattaataaactagacttagcaaaggtagccaaatcctttggttttgctcatcctcctaatgttaatatcaccatcggtgccagtggacgc
actgataagaaagaacgccgcggaggctacaaaaacaaaggaaatgtttattcaaaacagcggtcttctactatgtctcaagatgcaggtcgga
actggagtcgttaagacaattaactaactaattatcattacgttcaatgagttttatactatacatttttttggaatacaaaaggttatttaataggc
gtttcacttaatgtgaaatgatttgtgaaatcaacttatttcatgtttggtaaatacggggttcaatatacaatactttttctacgagtacggtttga
aaattcacagaatgcgtatcacttctaagtaactactagagtatggcaagtaaattgaaagagcaacaaaatgtacaaccatgtggtatccaaa
attgttgtcaaaaaaatttgaaccttctgctaaatgattcctttagcgagatatagcaaggtgcgttatatcaacaattgccattattaaggcaagt
gctcactgccaagaaggtcattttcacaaatccaagcaggctcaaccacaacagaccgtcaggtttcaggaatttgttcttctgttgtgatattaga
agaaatgagtgaacttggggtttctggttcaccaaatgaaaatccaaatgttgaaaaaaataatgatggccctgcagaagacggacgcgatgta
aacacaaatggaatgaatgaagaagaagaaggtgaaggtgaccgtttggataataatgaacgccatgactcttctgaagaatctgagacagac
gaggaagctgaacgagcaatccgtgaagggttcatagtggaagatgaagaagatgaagctcctcatgaaattatgaagaagaaaaagcgtaa
gaagcgcgtcgaggaagaaaaaggacctcaaggtatgttggatgaggatgatttagaattggtcatggaaaatactggtcaggataatcgaac
aaaattaaaacgactgaagagagggcgcgaccaggaggaagaccttgaaaaaattttctcagaagatgaagatcaagaagaaaaagaagaa
ggtgggagagaagaggagattgatggacctgaccgtggatatggaagacgcaacggggttatggatgaatttgcagatttcattgaacaggac
gaatttgaagacgaagagcctcaagaagaaaagtatgatgtgacacctcaagttgaatccgttaggccggaggcattgggcatatccgaagatg
actacttccaaatttatgaaatttttggtgatggtaccgactatgcttttgccttggaagatgaagacgttgaagatgaactacaggagcctgttag
tctaaaaacgatctttgagcccagcgaacttcaagaaaaaatgcttacggaagaggatgaaatcgtccgtatcaccgacgaacctgaacgcatg
cagttatacatgaaaagaaactccgattgcactgatcaagaattgctcgatgaaagtcattggataaccaatcaacttttggaaaagcggcttga
tataaatagcgaattacttgagcctttccgatttgcagtacgacatgccgttcatttcttcgttcgtgaaagcctggaagttccctttatatggcaac
accgtcgagattatattgttcattatgaccgtcaacgtgattctatcaccccgctactaaaccaagatgatctctggcgtattttcaacttgtgtacc
agatattggtctcttcattccaagaagaatgatctgatgaagttatatactgatttaggtatcagtgatgaaatggtaattacattatgtcaagaag
catcttctttggaaacagtggatgacataaatgattatcttcacttcacttattccgaacaaattcgggatcgtgctttgttggtgggtactggtctgc
gccgtcctcaagtatcgaaatattccttttacgaaaaggctaagaattctcctctttatcatcttgtgaaagaatttggtattagtgcaagagatttct
catttaatgctatccaaggtgcacgtcttcgctttgttgaagatgtatcagtaccacctcaagagcttgcaacatcgtatgtcaccgatgagcttacc
gatcccgaactggtcctgcaaagagctcgcaaggtgtttgctgaagagattgttcatgatccctactttagaaaagcatttagagataaactatttc
aaacaggcgttgtatccacgttagcaacgcaaaagggagttcgtaagattgacaatgagcatccatattacgaattcaaatacttgaaaggcaa
gccattgacatcttttgaacaatctcctattttattccttaaaatgttaaaagcagaagaagaaggacttgtacaaatatcggtcgacttcaatgat
aagaatgatgtctttaatggctttttgaattgattttatccgataactattcagagaacgccactaaatggaattctcagcgggagatggtattaa
aagatgtctttaggcagttatcaactttagcaccaggcgcaattcgcgaaacgctccgatctaaatactcggatgaactaggaatgctttgtcgtg
cgaagttatattccagattagatcaagcaccttacgagccttcaacgaaaaattttgatcgcggtactattccttcagtcttggctgtttcaaatggc
aaaggcgaaccatctgatgcgatcatttgtcttttcgttaatgatatgggcgaacccgctgattcattgaagttgcctgattttaaggaccctgcaa
atcaagctatgtttactgactttgttgataaagtcaacccagatgtgattggtgtttctgggatgagtgtttctgcccataaagtacgtctgaatgttc
aggtttctttgagctctcatgatcccatcgacattatcatggtaaacgatgagatcgctcgtctttaccaaaactcttctcgcgcaaccgatgaattt
ccaagtttttccactacatctttatactgcattgcgctggctcgctatgtgcagaatcccctattcgaatatgcggctttgggtcatgatcttttgtcttt
gtcatttgatccttggcagcatcttttgtcttcagagaccttgtggagatatcttgaaactgcattggtggacgtatcctctcttgttggaatagatatt
aatgaggctgttaataacaaatacgaggccaacatccttccttacattgcagggttaggtccccgtaaagcagactacattttaaagaaaattgc
aaccctaggcggccgtattgataacagaagtgacttaatttctaagcaaattgtcaccaggaaaatttttatcaattgctcctcattcctcattatac

ccaacgatgaatacccaaatatggatatccttgatacaactcgtatccataatgaagattacgaactcgctcgtaaaatggccagtgatgctttgg
aattagacgaagaggacattgaagaattagaagctagtaagggagttgtatatcacttactagaggaaaatgaagcggccaagcttgatgaact
tgtccttgaagaatatgctgatcaattagagcgtgagtttaatcaacgtaaaagaagtacattagaaaaagttcgtcgtgaattaaaggatcctta
cggggaacaaagaaatatgtttcatagacttactccgtcggaaatattccttatgctaacaggtgaaaatccaagaaacttgcgccctgatacgtt
agttcctgttaatgtgaagcgtgttactaatcggtttgttgctgtcaagctcgactgtgatgttgatggcgacattaaagctgatgaggttagtgatg
actatattcctcctcctcaactccttcaagcaggtcagactgttgaggctatcattatctcactaaatgaacaaaattttacagcagagctttcctta
agaaacagcatcctacagactgtgctggctaaacaaaaggatcgttcccatttgacagcttattgggatatagaagccgaagatcgtgacaatga
gaaaatgcaagctgaaactcaggctgaacagcgagtcgctcgtgttatcaagcatccgttgttcaaagacttaaatgcttctcaggcagaagcat
atttatctaagttacaagttggtgacttgataattcgtccgtcatctaaaggttctgatcatattgtagtaacttggaaagtcggtgaatctgcgtatc
aacatattgatgttttggaactggataaagaaaacgaatttagcattggacaacgacttatcgtaaaaggtcgttttgaaaacatgacatatgagt
atagtgatttggatgaattgattgtcatgcatattaaagcaattgctaaaaagatcgatgaaatgtgcatgcatgataaatatagacgaggaagt
cgtagtgatactgagcattggctagaaagctattctgaagctaatcccaagcgttcttgttatactttctgctctgatcaggaacacccaggatattt
tgtcctttgttttaaagctggggtaacttctcctgtgttggcatggcctgtgaaagtaattccgaacgcttactttttgcaagggaatgtttatgctga
catgaccgctctttgcaatggatttaaacttttgtatgctgcgcgtgcaaaaagttctagacgctaaaagttttacctgttctatagatatcataatta
tcacttgatgtcttacaatacagcaaaaattcaagtatccgacacgataactaccattatacttcttacgtctttctaattttatataattcattttga
taacttaataaaattatttgttcaggaaagtatgctataactattttatatgtaaatagatactacgtaatatagattcatttctttactacgcgtcgc
gacattagtcgcgacgcgtcgcgttgccgagtcatattaagtatgtcgtgtacaaacgtaaacaaaacctataccagaataaactagttttggata
tgaatacagaacatcagagtccattgatggattctggagaagcagtgaataatgttgaattttcagagaacgacaactctccaaatgacctccta
gagacaccacgaactcgtgtcaagagaatgctggcgaagtttgatgatgaatatgagcctggtgctaccataattgaaaaagatattccttctaa
agacgttggagatgaccgtaatactcagatggataaagacctgcagtccaaatttggatcagcttacgagcgtgtaaagcgacaactacagaat
gagaagaaaataaataatacatctgaaaaagaaaatcatatggatgaaagtttacgaaacgatagtgacaattcccaacggaccccaatatta
agttctcctgtagaacaagattcagctcaagctttaccgcttgaccgtgaatctcggttaaagcaattagttgaaaagaaaaagaaagaattgaa
atcacaacaagaggaccttttcaaccttgaaggagataccgacggcaatgacggttcatcaggtgccgatagtaatcaggaggatctgggatat
gagagtaaatcgtatggaatggaacgtggatatcgaaaagcctctagaaaagcacttcttgaacttcatcaaaatactgcgagactgacgcgtg
aagtagcgagggaacccgaaatcaagattggcaaaaaagtaactataaacgatttctttgataaaattggatttaagcctaaaacacaaagtgg
catagacatgtcaaatgagtcaccatcagcacagaaaacacctaatgagatgtcaacagagaaaaaggttgtcgccaatcatgaagaggaggg
aaagaataatgcagacccattgactcctaatgattcttcagaaaatattaccgataagggaatggaaccaggtgaactaaaattgcccgatttag
gtcagtcaagtctgttcaataatatgtttccaaaagaaacacctccaaagttatctattaaggaaaaaattcaaagactttctaaaaacatatata
ctggaacttcagattcggattctgatttggaaattgagactcgtcccaaaacagtgacgctggatacgatacagtcagtcaaatcatcagaaaat
attgatactgttgctgataggttaaaaagacttgctggtattcggtcaccctcgaaacgaaacgccaagcttgaagaaaaaggattcaatttcaa
agaattcaatcgagatttgatgaaaagagcagcaatgatgtcaaaagctgaaagagaagaaacagaaaaagagttggagagtcagggacttt
tggcttctggtagtgaaaaagatgaagaagagcaactggggtttgttgaacgtgctagaaaggaagcggaaaggattcggcagaaagagcag
gagcttgatggaggtaaatcttcggatgaggatgaagaaattagttctgatggcgttccttcagataaagcccctaagacattaatttccgacgta
attattcaggcaacgcaaagcgagtcaactgatacaagtctgaagaaaaggaaaaacaatcgcgttgtattagatgatgatgaaatggacgaa
caggactattcttcatcggacatgagggtggtggattccgataatgaggatgatttacggattcatgacttttcccaatcatcaaaagaatgtaag
gaagagaaagaaactaaagaagattcctctgctttctatttacaaggtatgcttccttcagattcacagctctctattatcgatgcaaatttcagtca
gcctcctcctaggtgggcattatcgaatagtacccagctagaagatgagactcaaccaacccagatagatgccgtggttcctacccaagtggatt
cctcagtaccgactcaggtagatgcgactaaaaaagatgagccaagccatttggtaaaaactcaagttgataatgataatcatgaagctactgct
cttcctaaaaggaatttactacagaggcgtccaaacgatgctgactcaaatgtcccttttacagaattcgtagaagatcaggcagaagaatctga
agatgaatatgctggattgggaggagcttccgaggacgaagacggaaatgaggaattggaaccagaaatcaagaatatgattgatgatgaaac
aaagcttaagaaagaagaaatagcatctatggctcagtatgctcgggatcaagaaattgatagggacagtaaattagtcgaacagctgatgaa
agatgtaactaccggtggacttcgaaaaagacgtagaaataatgtaggcttattagatgatagcgaagatgatgatgatgagcatagcgaaatt
cgacgagaaaagcttaaggagcttcgaagaaggaagttgatggagcatgaaaacttggagatacttggtggagaaaaaaggaaagcattcct
ggctacagttgaggataatctactaaacactactgataaccttaagtggttggacaataacgatgaagactctggtataggatcttctgagcttgg
agaagagcgtccaggatctgatcctgccgaaattgatgctgacgaggatgcaattcgtgaagaagacgaacttttggagaaagcatcacgaaat
tatgttgatcgctctgaactaagtaagacgatctcaaagacgtctttgaatggaaaggatgacattgttctaccagcaacacttaaggcaatgatg
caaaaatcgaagaggaataaagtagatcaaggtgtcgacgatatttcttcttcaaactcaaaaacctccactctcgtttcctccacgcaacgaact
agtggtcgtaaatttaaaggtctcatgcaggtttcgaattccaataacaagcaaaagtctcagacttttggaacgcaagctacttcgactagctca

gctattcctaataaaccgaatctactagcttctctcaataactttactgattttgattgacatgaatgattgatgatatgataaattttatcaagctgg
tcttgattgaatttttggttttgctttaatacacgtaaaactgctttataataaaaatatattttaaaaacccattatacgccattagtactttcaagtc
tattagtatttgaagttaactgactttattgacaacacaatgtttaaaactcgtaatgatgaaatacaagccagattcttattttcatacctttcataa
caccttgccaagaaagccctaccctaaataaagtcaagtagggtagggcgcaaataacagtcacgatttgctattcgtaaatggaacatctaagt
caaacaaaccatctacaaaaatggtaagtgaatatgagaacaaaaggaggataaaaacgttaacattattaggcccctaagaagaaatggtct
aaaggaaaagtgaaggacaaggctcaacacgccactgtctttgacaagaacctcattgaccgtatcaacaaggaggttcccgccttcaagtttgt
ttctgtctctgtccttgtcgaccgtatgaagatcaacggtgctcttgctcgtatcgccatcaaggacttggccgagcgtggtgttattaataaggtgg
accactcctctaagcagtacatttacagtatgtggcgacgttcttttttaaaaatttttactaaccagacattagctcgtgttcctgctacctctgcc
taaacatggaagtgttgatatgaataaatttttcaacttttgagttcattgtgagagacaagtagttgtttttgtaattccctttacttagtagtaagt
ggtttgtgtgggatttttgtgcttccaagtagaagagcaaagcagatcggcttttgttctgtaattcattgtaggttgaaccggtgaattaatccagt
gcggaatatgtttctgataaaactgagatggataggttcttcacctttgttgagtagattttaattcttctaaatcagcaattcttgttgcatttgaat
gaccgaataggacgcagtatgcatgcaaaaaaagaaatactgcatgtttcaacgagaacagagagctactatggattgcgatctctgttcatatg
atgtactaaaagccgatgtatatagaatcgaattctgtattttcaaagtccaccattcttcatttcttgtttgatccttcgttataaaacttttgtttacg
gtttaggaacctgcgttccaatacactccgtcatcctttgtaggtacattcaaacagtttgttctacctcggtaaaatggttaccgaggtatctgatc
gtgtacaaggctctgttattagtaattacgtttggtctttatttactcttacggaatgtgttgaatatcgcaatcctttaggcctataaggaatttgag
aatttgaccaaagaaaactcccttggaaagaggaaatatccaaagtgaatttgcatcgttttatgatgagttccttgaagacaatcgctacgcata
atggtgcatttcatgcggacgaaggtaagggaaattaaaggacttgaagccaggaatgaagttggacagttattgacatctggtgtacattgaaa
acgctttaacaagaggagatatgcttgaaggttgacatttttatgttatggtccagaaaacgaagttctcatttgacggattaggatgttgaaattg
atactgaggttgtagcctgaatagaggtttgttgatgttcatgatgaatgaaataaaaaaaaaaaaaaaaaaaaaaagacttaataacttaacttc
ctgcttccgcgtgcttggtttacttgatttgggatttgaaaagctaacttttacttatagctttagctgtttacatgcttcgtagtatcgaaacatatcg
taacgccaaagtggttcgctctagggatcctcagcttttagacagttgcgatatcgttgtagatgtcggcgggaaatatgacggtgtaaagtatttt
gatcaccatcaacgtgaatttagcgatacgttttcttccaactacaagactcgtctttccagtgcaggtttggtttataaacactttggtaaggaggt
gatctcttcggtcttagcgggcaagcaactttctcaagaagacttggacatcttgtatgaaaaaatctatacttcgtttattgaaggtttggatgcaa
atgataatggtatttcaccattccccgctgatttgactcccacattcaaagccgccatgagtcttccagaaatggtgtctacttatatcccctcttgg
aactctgaaaaacaagacgaagagtccttcttggaatgtttccataaggcctctgagtttattggcaactggtttgttcgcaccgtcaaccattacg
catcttcttggttacctgcgaagtctttggctcgtgaagccgtcctcaaagcaaaggattcttctattttgattatggacaagttctttccttggaaga
gtcacctttatgacattgaaaaagaacttaacatagagggtcaattcaagtatgctatttattcggacggaaagggctggcgtgttcaagctgtcg
ctatcgatcctacttctttcacttctcgtcttcctctacccgaaccatggagaggtgttcgtgatgaaaagctttctgaacttactggaatatctaact
gtatttttgttcatgcatcgggtttcattggaggaaatgccacttttgagggtgctctggagatggctaagcgtgctttggctttttagaattttcgtca
actaggtatcctcaagaaattttgctaaaaacattggtctaaattttgggaaggatcgttattctatggattgttattataaatagtagtttagtcta
attatgtttaaaattgttctaatactatggtaatgctcttcgttcacctataaaaaatcatgccttctgcaagatctttacacccagaagagtcacttt
tcaaaaaaatcggtaaaaataaagagaacgtaggatgctcagcgatagggcttagtatgcttgatgatgttcaaaacttccgctggtatcattcg
caattgaactttttcattgcccgaagaaatggcttttcgtattactgtactgctaatacctttcccatttcactgtccattttaacaaagctaatccta
gatgaccaggcggtagccacgttttcaagttcccctttctcaagcttagcagagtacgcttcctgcatctcagtcgttaaatcatttgtttctcgataa
aagcagagaatttgagagtgcgtaaaaaagggagtaagagctttttgaatggatttttctggataatatttgcaatccaaaattcgcgtcaatgtg
tcaaaaccaaccaaataaatttgttctttaggagaacagtatttcgacagacatacacatttgtcggcaaagagcgcatacttacaaagggaaat
cgagacactacagttcttaatacgctgttttagagcttccagcattgctaatctaatgtccaaggatgctggagcctcggctttgtcagcgtttgcaa
tgctcaaaagcaaaattagataacttccttcaggaacactctgagacatccccaaatgagcattgtgaggaggattaaaggaactatcgagtact
aaatccccattcgggtactctattagtattacttcttgtcaaggttattgtagcatttaccgtatagattagcttcttttggaggttgctggtctaaaaa
ttgaatctcgtcttgagatgctctaaaattgctaacaatcgtcttgtaattatctagattcaacatatatgcaaatcctttgggaaaaggaagatata
gaatactgacaaatttctatcgttaaggtagtcaaaccgttcttctcaacaaatcttttgttaaaacattattatatgtaaataccttaagcagttat
atatatgtacgtatataaaaaggtaaaaggcttgagaaacctcacaatcttgattctcaaatttcctacactacgcgactgaaacttaggatctct
acttttctacatcaagaccatatttcaaaacattaaattaattaagtaccaaatagcatggaaatcaactgttctcgtaccaaaatatccaatttga
ttgcttttcatatatcctaaaatccctatgatatttttcaaaatataaaccacaagcgtctttttttttataaaaaagaaaacactcaataaaaaatg
gtgaacaagaaaaaaacaaaaaagaaatgaaaaatctaagcccacatctttctgaacttggcatcaaattggttacgaacttcaaggaacatac
agtagaccttgaaaaggttcatgtccatcttgtcttgcaattcaggagaagcaagagaagtggagtaatttccgttcatgttttgcatccagtcagt
gctaccactttcaccttcagtagacgcctggacagcgttagaatcttcgagagcactcatggcattttcaatatcgtcatcggtaaccaaattgagt
aaattaccaatacagctgcaaatggtagctagaaccaaactgaagtcgttcaacacaaaccagatttcagattgcttcaaaccattctgctcaact

aaaagatcgacacttccatgagtgaagaagtcaagtagataagcattgagggatcccttattcacttcgaaaacaggtaagcgagaagttttga
ggaatacaccatcgggtgtttgttccataaaatcggaaacactcttggcagtggcatcgctgaaaccagctaatccacaaaagtcggaaaccac
accagtgggtaccgtcttggaaagtttagctgttccatcaaactttatattggataaaggcaacttattctcctcaacaccaacattaccatgttcgc
gatagtaataagcatacgtcttatacagattcaaaacccgttcgtcgtgtgcatttaatacctcaacaatatcctgaggaagagaaggcaaaatg
atttctgaaggagcgaattcatcacgattcttcaatgaaagagcaaactgctcgttaatgcgaatgcgtccgaataagtggcagaggatagtaat
ggtatcgtttaaagcacccaacttgtctgaagaatacttgtcggcaatcttatggaatataccggcgcgaagtaagttcatgaaagcatagttgct
aggctcggtataatacagatgcatagtaagattggacaaattaataggcttaccaaagtcatccaacaatccttcacgaagtagatactcaataa
caaagcgaaggtgatgattgacctggttgctcaaaacagcaccgttttgcatgaacttgggttcatccaaaatcgagcgaatagtattacgtgca
aaaggagcagcttgtgaacctgagatgagttgggataattgtaagaccaaggaggtagtaagagggaattgaccttgaatatcccataattttgt
ggtcaacaaacggtaaaccttatgcaaagggacaccaaggaatcctacatgacccaataaatcaaaaccacgacgaccagcacgaccagcag
cttggtggaaattcagggcgtttaattgtaagttatcacccaagaacacaacagtacggcaaggcatgttaatacccaaagacaaagtacgagt
agcaatgacaacactgagttgaccacaacggaatagcacttcaaccatttgacgatagcgtcgattgagaccagaatggtgtacaccgatacct
ctataaagagcgtcgacaaaccattcaggaacgacttgacgtctacgtaaagattccacatcacgatataattcttccttgccgtaagtggatttg
gcgccagcaaaagaaaactctggggaaggttcttccggatcgaagaactcaatccagctagtatcaccggattcctttaaggcttgttctttctgct
tttcagtggctgtcttttcgaatttttccatttgtttagcacgttgaccagcagcagcttgccatttcttatactcacgcatttttctttccattcttcag
agttttcacgccaatcacgttccttttcgactagacccttgaaaacagtttcggcaagatattcacattcttggcgatcgtaattgaagcacaatgc
gggtaataactcttgagtatgcaaatctttaagcataggcaataaattggacgcaatagcgtccaaagagtaagcagaattatacaattgaagct
gactattgacaacacgttgtggatcattacagaagtgtttaataagggcttgcaagggagagttcggagcaaatgcattaggcaaagaagccca
tgtgtcaatagtggctttaatcttcttctcataacccacaacgtcaaccttcttaatgaaagggatgtcctcaaaatatctgtcagggtccagtctttt
gctgaccttgaaatcacccttggaaactagcttcatagcatagtacaattgaaggcaatcacgaggttcaaaagccaaatttgtggtggtaccttc
agagaaagacatagctgcggcaggatggattagggcaatttggccattgtcaccctctacatgtaatccattaaactctttagcaccgccgtaaac
aaactttctcaagtctgagtaacgatgttcgtgaacaataagatgaataggattagcatgagcacgctgaagagcagaaagccatgtatggaatt
cctcagggttacctacagtggccgaaagtgcaacaatcgggcaaggagcaagtaaaagtaattgttcttcaaccattccgtcttccatttgtccaa
tacagtgaatttcatcaaaaataatacgtctaatcttgggacaccatgcattggccaaagcaggttgcaaaagcatagtttgtaaaacatgggg
acagtaattaacacttggcagttagttggattgttaatacgatagtcacgagtgtagacggaccaaacagtttgaccagcatgagggtagtgtttg
ttaaaacgagcataaacctcagcggatagttggttcaccaaagccttagtaggagcaacatagatgactacaccctcatcattatcacgtaaaac
cttctccatagcataaaaggagatgaaggtcttacctgaagaggtaggtgcaacaacaaagacagactcatctttgtcaagaatatccaaagtct
gacgctgccattcatcaggatcaaaagcaacccggtcatcaggtgcgcttcccatagatctttccatatactcaccgaaatggagcaactggaag
gtaggagaagtgaaagggacttccaaatcaaattcttcttcctctaaatcagggagcacaaagggaaaagataggggacgggtttcgagtttctt
ggctttgcggataacattttcaaaacccaaagcagtaagtgtatcgtcaagagcagtcttaacctgcttagtaatgggcctggggtagacaatact
aatttcacggaaaatctccaccactatgcgaatattcttttccttagattcgtccgtaacacaggccgctgtccagagggaaattaagacacgaat
gcggtataagcgcatttcgttctcaatgtaaggatcataggttctagtgttcaacaattgatcaatacgattagagcggagattcagatctttaata
cgaccaagttcgttcctagtttcccgccacatatagtcgttcttattttcttccttctcagacttcttggcagcaatttcggcacggatttgttcggcttt
ggagagtttaacaggtttacccttttcgcggcattttccttatccttagccttggatttgtttttgggagcttcttcactttgactcttggcttccttaag
ggtttgggtgaaaatggtctggcgcttcaattgaccacctttagcaccggtaagtgagctggcatacttttgaatatggctgataaagaattggtcc
tgacgacgcattctatcaacctgccttttatctttgttactgtcaccggtctttttagttgcactgatatcgtcgtaacgcttagaggtggcagacttat
cattgatacgcaattttgagaaatccaaaactgtctcaacaggttgaagttccttgggctgaacggcccattcctcagtgttgtaaaactgggaaa
taacgggatcaccgtatggaaccaaggaagtatcgatttcgctagcgggagcctcaatgggagtacaaagttccttagatatggaagaaaaaga
agataaatcaaaaagatcaccggcatcgggaattgcatcaataactaacttccaattggagagagcatactcacggcagatatcaggaatgtaa
aattcgccaagagcaacatcagatactagacgaacaaataagcgagcatcgaaaaggtcatggaaaccgtctaccttgctatctaacagattga
ggatggaagaagcacagcctaagtatgacgaaataaatttttgaatagctgggtcctcgtataaggggaatgaaggttgaggacgggaattcaa
attcagatgttcaaggacagcagctgaaatgacgatcattttagctagatcacggcttaatggggaagatgattcgtttttttatgagccaagcagc
agcaagagtctgaacacgcattgcattcgtaggacaggcatcaatattgacttcaggcaaatctatcgattgacggtttttgccgaatttttccaaa
taatcagaaattaatttggaagcttcctctacagcttcggtagagcgagtaaaaaaggaaggcaatatacacatcgaaatcttattatcgacaaa
ctcgtaagatcctaaaagagcaacaggtacgccatcggataatgcaaagaaaataaaagacttaaataccatcaattgttcatcagtaatagca
aggcctccttccttggtatttttattgttgtcaaggaccataaaaccgtaaggacggtgtttggcaataaatacactccattgagagtctgttaagg
atttgaattgaaatacagggacatattttcgaagatgacggatggctactctacggaataaaaggagggaagagcgcaaaatgtcatcataatt
agagtaaagcgcttcccagtttttgaaaaacacaatacaaaaattcagtctggcacttttaaacttggccagttctttttctaaaagataaatagca

tacaatatttgaacaccatttttgcctaaaaattggcaatcttgtgcaaggatatggaagattaaagaatcaccatcgataagaaagagttcttca
ccagcaaaatcaccgattaaatctactgaacgataattttgtaccgttgttagtctggcaatctcctcgctcatatttatgaaagtgaatgggagaa
ggaagaagggttgcatctaacatgctctttagccattggtgtcacccttgttacttcagccgccaatacccattaattctaaatataaacaaatgaa
agaataggtatataaaataaaaaaaataaaaaaaataaaaaaaaaagagagaaagtacataaaagtatagattaggagcatataaattaga
agtttaaccaaagtaatattaagtatgctgaacaattcatcgaatgatagtaaatgccgttagcatgataaacaattttccttaacatgctgaccat
ggatggagaaagcaatgagagcaacatctagctctttagttttagacttgtaacaataagaataatggtcccactactcaaacccattgtctgtga
cagagcgactatatgttacttctgatgaatgaagcagctttgaaatttttctggctaaaagcaaaataccacttgcatcagagggaaacgatatac
aagaaaaaaaagcaaaggagcggaaaatatattagaaaaaagggttcattcaattgatttagcttaaaagcattggaaaccgaactcacaaaa
gtgaattctgtaagaattagcaggatctttggattacctttgggctttcttctagcggttataattttgcttatattattatttcttacatattcttttcttt
aaaaaatggcgcgaatcactaacatgggaaagagaaaaaagttcttggaggctacgccctaccaagcaaaaccagttgaagctgcgagagat
gactctccaaacaacaacggtgatggctctgctgaggaccctaagaacaatatgaacaatcgagcagactcgaagtcggatcagcgacagcga
aagagccgttcagaacatcgaaggataaggagacagaatatgagaatgaaggataagttttgttttgcatgcagaaaaccaggtcatatagtac
aacattgccctgaagcgaaatcagaggtctccatttgcttccgctgcggaagcaaagaacatactttaagtgcctgtcccaaaaagggaccattg
cagtttgcagtttgctttatttgtcacgaaaagggtcacttggctggtcaatgtgagaagaatccccatggtctatatcctaaaggtggtggttgtaa
gttttgtggatctgttcaccattatgccaaggactgtgaccaagtacgcaaggaggatgttagttacgggcatgttttggcaactgcaggtacgac
aggtgccgatgaagatgtctttcatgaatatgcaagggaggttaataggccttctgcgccaaaaccatcttctaaaccagcaaagaaaacggtta
cgttttgagcgctgaaatttagttgaaaaaaaagaaatacggggttttagtaggtacggttatgtttgggacgtgagtcccgatctatggtaataa
atcaaagtcaagtcaagaatgtttaggttcacgtgtataaaaaaaaggctttctttccaagatgatagactcattaaagatatatccgatttatgat
ttaaaaaatagtgtctaaaattagtttatcattgatgtttcaattttttttttctgtgaaatggctcgctttaatagatcttatgtgtgtagagagcttca
caacagtaaagattaagtgtacatcagcaagctttttttgatttttattttatgaatcgacctagcatataatattggtgggcattaaaagcagaaatt
atcgtacaagagtccatagtatggcagatcagcgaacttccaaacgtgcaagagcagaaacaggagtattcagccagtaagaataaaaaatga
attgcgcggccaggtttcgatcctgggacctacgggttatgagcccgtcggagttccactctcctaccgcgcacttttcggtacaccttgcaactcg
aacttacgaccaggattgagtcataagtccgctctaccataggaccttttcttgtactattgcctatgtagtactgtaattaacccgaattctatgat
aaagtctgtaagttaataaattagaaagaacaaaagcacagtacaaactaagtagttcattattaagaaagaagggaaaaaattcttgtctgaa
agttgcaaatacctccgttttatgttcttccctacgaatgcaagaaaaatcgaatttttttacatgtcactttccaaggccttttccctctaaaatatttt
ttagattttatagatttgattacataacagctgtaaaaagagttcaaccattttctatagaggtatcaatttttgtgaatttattttctttttataaaca
aagaaaatcatgatctgcgccgatgaaattaccgatgatcagcgacaccctacacttcatcaaaggtacaacggaagtaacaaccgccgatcat
atgaaggtcggcagtttaagtattaacagcaatgtacttgactacttcttcatatcgcgaatttgcatttacattacgaagatctcgtttagtgggaa
ttagacgatttttttgaaaactatcactcaattcaagtcaaatcatagttaaatctgtaagaaaacgtttgtggtttcgcaaattgcctttgtctagag
aagtcgactacttttgttgcctcaccttgtcaatttatcgattgcatcgacaggtctactcttttatcatcttgttgattgacttgttcgatttcaagtttc
gtggagaaggtttcaagggtttgagagtcgttgttaaggaggatatctgaatcctaattactcattagtggctttagcggtggattttttttggactg
ggagataaacaagtaatacctttatttattttattatatattttttttttatttcgctactttgcttcattaatattgtcgagaagaaagcgagattgcat
ccgtccattatagcaagttaatctgtatctcgacttttgtagggaaaagacaaggagaagaactagcccttaatttttttctcgagaaaaacagtga
ttttctcttttcaagttggctttttctatttgtttggggaatagatcactgtgctttcttagctagtgggttctcttccaatttttttatctgcttattgtgcgg
attctattgaatattgaccatcctttgtttttggacgagttaagaagcttataatactttcttttgctggttttagcctgaaatttgtttattattttttttac
tgtataacctacagtgtgcttattctatatcatgtcaacggaaactcctgccgtcgaaggaacctgtcgggcgtcgtacactgcgtcttctgctccca
cgaatccttctaacgtaaactctatgaatgaccaacattctgcttctgttcccccggcctgtccgtatcctcatgtcgttggcccttcttttgtacctcc
aaccacacattcgaagctcgatgatttgccaaggcaacctcctttgaacaatcataaaaataaccttgaaaatattctccctaccatgccaggctc
ctccattcctttggctcaaggtcattcagatcattcactgaacagtagtctttgttgttgtactactgttcatggtcatcctcacatatcccctacaatg
attaatccttcctctgctcattatagacttcctcctatctccaccatattaggtggatctatggcagaccctgcaataatggcggctgctgctgcttca
tctcctcattatcatcctcccaaccctaattcaccggatgtgcctctacctccaggcttcctttctccttacaaccctttttcttatgcatatttagtcgc
aaaagcaaatgaagctgctgctgttgcagctgtacatcaacatcagccttccaaatccgctgatcaacatgctcagcccgctcaacacgttgatcc
ggttgccccatcgtcaattccgccatctgcgaatccttgctcggcggtaacgccgcaccatccgaacagcagctttctaacatttccttttcaaccac
cctatctccctttcatttgtcctggttgtgcacaaaattcaacttcacaaaatcgtttaaaccctcaaccaaatagcgatggattcatacgctgtccc
gctccttcggagcatgagtcgttcgtttcccatgctggaacagaagcacaagaaccaacgaatcgttccagttcaaacgaagcggagcctcaaca
ttccgagagcgcttctagtagagtggtagacatgaatcgttttggtttgactcctgacgttcatccctcatctcaaagtggttttattcccgaaaata
cgtctgcagcttctcaaccaggatatccgcactcgaatggcttccacgatgtttttctttctgagctttctgctgcagctacccttgcacaaaataatt
ctaatactactgcagcttcggagggaaaaagtcaagctaataaagattcttcttccatagagaagagagcacattttgccaggaggcattcaaat

gtccgaaaatcgaatcagcaaagcaatacaatctcaagttccaccattcgatatcgttgcaccgagtgtcttcaaggcttttcgaggccatcgagc
ttaaagattcatacttattcccatactggtgaacgtccatttgtttgcgattatgtgggttgcggaaaagcgtttaacgttcgcagtaatatgcgacg
ccatcaacgtattcacggtgcataactatccactgacttgaggtcttttctgtacaataggtcacgagttcctatgtttgtttttgtttttcgttttggtt
tttattttattttattttcttatgatgttatggtactgttttagaaaaggagatttgtcatttaataagtgatgaatcaaacctatcgaacctgtctcgtt
tacctaagtactaagtactgaaggtcacgtgtaatttactgtcttttctgaaaaaagagctagtgcacaaatgctctttttgaaaagataaaatcgc
aaatcacctattgtaattggacgctttataggtaatgacatttttctataagtttcagtaaatatttatttatacacgatttctggctgttgaatgattt
ttatttgcatgaacagcaatttattggtttttttttttaattagtttaatgaatagaaacattacagatcttttgctaacctgaaatgtgcaatcagcat
gtgatcttactgggtacaatgtatggccatattcctcaaaaatatttcatttttaagtcaattaaacaaagaattggtgaattaattacaataattgt
ttgaaatcccgagtctgcaaaagtaaagagtaaggacttacttttcccttgtcccaatcgacaaggcaaggacgacgaaaagtacggaaatcatt
ttggtatttatttaatgcgtcctggacagaagtagctcccgaaatcagtagatgactttgatccttctgtatattggagagcatgatactaatttgat
cttgagtaggaggatccaattcgacctgcaactgatatcgaaattgttcgttattagttctttcaccaagaatcatatattctgtaagtttttcttcaa
ttactgtattacgttttcgagcgaagagggtcaatattcttttcgtcgttaaaccaaacatgttgagaattaacagttatttctcttcaacaaaaaca
atgaatataaaatatacttagagagaacaaaggattcaatccccaagtcaacgtaagcaaggccttcaaaactgcaggaaacgctaggaagca
ccttcaacgagaaaaattttatacagcagagaaataagtaaatctttttaaaaagctctcctttaagttgaaaggtaaaaggttacttgtttatgag
acagattcctagacttggtttcacgtcatgtaaggaagagatttgcgagtcttattacggactgtgctaaaggaggaaaaagaagtataatgagt
ggaagggagattcatcaagagaaagaatcttaggaaaaggtagggcataaatagaagtagaaaggaaaacgaaagacaaaaaaagagtcct
aacacgtcagttagtttgtcttactttcttgggtagagtcattctttttgtcgtcggaagcactttgttcttcactacgaaaggcaaataaagagggt
aactctttgcggcgccaagcagtgaaaagaggtgcaaggatagagacaggactgaggtagaggagtgctggttgtgcaacacgaaaataatac
aaggcaaagttggtgatagcaagtcctaggatgtaagcaagaagggtccatttgaagtatggggtagatgtattcgtattcttggacgtctgcgc
attgctaacatcgaaacgacgcatcaaactgaccataagtccaggcataacaatatcgcccaaaccaagcatagagaagcgaccaggctttga
aaaattgggaatcacgtatttggctggaatgtcgatcccagttgctacagtgaccatgacatcggtgccaaaaacaaagtaaatgtcgtaaacaa
aaagacaagctaaaagaacagctccagtagcaaaggaatcaattcgcatcatactaatggaattggctgccaaagtccacgcaagcaagttcg
aagtgaaccaatgctttgtttttacatagtggacagtaattacagctgtcaaaagtaaacaaagagactttccaagagcagatttgggattcaag
gtccgtaaaaagcacatagctgctgcaaaactgccatagccctgtaaaatcatctggatatactctttcccataataacggagaatcaggtacat
gcctacaagcgtaagcccaccaaacacaggaaataatgctgcaagtttcttattaattgtatgcttttcttctggatttgtttcgcgtgttgaccattc
tgagccaatgtaaacagtcgcgattgctgcagatgctaaaatgattccttccattctctcaaaaatgggtaaatgcaagaccaacaggggaact
tgcagcaaaatacgagcctttggagtattggcaaacgataaaattgttttcctgtttttaaagaaaccttaaataaggaagcatcctcattgccgtt
aacaaaaatttgcatactgcacggtattagatattttcttaagcttactatgataccgtaaaagaaaaaagtaagagacacgaaaaaccttggag
gctatgaacaagtccttaggaaatgattttctctcttttctttttttttcctgcatccgactaacggttgctgctcccatacatgtcttcgtagtttgtattg
agccattgactatggaatctgtgaaatttgaacccattattatttatttatttatttagtagttttcctacatccaagaatgattactagcatgatccat
ctaagcgttacgctttctatatttggaagattggatggtagcaaggaatccatggaaaggatactttagagtggaaaggcatttcaaagaaaagc
aaatgtttatgaaaactttaaagcaataacttccattgagccaaccgaggaatagagcagcccacattacgaacaagacaataaccttggggttt
tccacccagcgtcacggattttatactagcaatgcctgaaggccttccagcagtcagattaaaattagcagttgtcacaaatccagataaggcatc
agccttcggacaaggaggatattcagcagcttctgctgtaatttttttgtcataaatcagtttcttccaaccatcaatttgatcctggtcgatactata
gatgcgagcccgatcgtgtaaagcacctctttgtaccattcgaatttcaactcttgaaaacgtctgagcagttcggtccatgacatcggaatgtgcg
tcatcagagaagtattcccaataacaacaaaatgggtcaccaatttcagagaaattcttgccaaaattttcatagttaactcgtttcgccggtggtc
gtcttctccagtgttcctcgtttcgctgctttcgttgaatttcatatgcctgtccgctctttgtatcaggaaaatcagcaggaaaaaaaggcatcttttt
ttcgaaagcaatttgatgcaaatttttttagccctccaaatcgtacacctttccgatacatcaacttctgccagaaaaactttgctgctccccaaggca
atatgacagttaacccattccatttcggtcgatgagtaatgtaaataggcactaattcgttttgaacattcaacgtcaagtgccttttctgttgaaca
taattaaaaatatcaaaagatggtatttcatgtttatcccataaaccaattttgctatctaatacttcatcgtttctgatttctttaccactttcattcat
tctttttttcttcttgtctcaatttagattcattatttaccaatgatgcatcaaacccaatggatgcgccaaatggaagagaagtagtaggtaacgtgg
aaattgacttccatgttcctttttctgaagactgaatgtctgggaaaatgtcatgaagaactaagttagcgtcaggacccaagacgtcgaatgaag
ccaattccaatctccaatcatgcatagcaatttgatcatctaaaacggcaatgcttgataaatgattccacgcatctaaaaaagcagaagcatgc
agtcggataactaattgtactacattttcagaattacgaaccggaatttttgttttgcatgcctcgttaggattgtgccattgtaataaacaaggaca
taagagctctttcgtttgagaatttctgacataactggtgaatgaacgataagaccgggtgtatgcagcggggagtcctgtggtaaaacattctcc
gaacgtttctaggagaacctttaaaggcccactaatggaaactaatggttgataagacatatcaaatgcaatggcattcaaacagaaagcagct
ctatgagtaggccggtaagacttttcagttggactttctggaattgcataaccccatgcattaatcatgtgagcacgttttgaagtccaaaggtgag
ttggtaaccaaacttttcgtaattgccgtccggcaaacttcccgcttactaactttactgcagctaaggatggaatcctagaatcctcagttgagtgt

tctgtttgtaattgcccagagtttgtaatttgagaacgtagaccttgacgtgctaattttctgtggagtcgttttatgaatctttttaggcgttgcttgg
aaggaataattggtgttgtagcagaagaactcagctgcatttcataatacgcacggtcccgtagagccttgggaatccgtcgaatattatgagaa
gccgcacgtcttctaagacagcgtggaagcgcttgaaatgcacgttgagcactcgagtgcttcgaccgttgcatagcatcttgtaatgcagaaatt
tcaaaagatctggattcgacaaagcgtttaatgtccacagcaccgttctttaatgctggagattctacctgaatatgtctagcatcggcaaatttcg
atctcttcacatttgtatctttcgattgtatttttttgacccgaccgtttcattttcgtatagatgctctaaaaataaaaacgaaggaaaatgtacagtc
gtaaatgaagtagggaatgcaaacaaagatctaacctcagaccataaggtttcttactgatagatgggttttaccaagaaaataaaactaaaga
atacctgaatctctgtttatttttgttttaattccaaaataatagcaaaaatcctgaactttgaaaataaatattcaatcctggaacaaaacgtattg
tacactaagggaaagccaaggccaaaaatttcaccacaagcaaaaccaatttcatgttggttttgcaatacttttaacgttaatgaattacaatag
tgaattagaaacatttgatattcaaaaagaagaaattttgaaaggactggcttcttctttgccggatgcttctcctaaaggaaatccagatagccct
atttttcctttactagatgtaataaactcacacccagactgggtaacctcaagttcatgttctggaagaatttctatctatgtacaaggattaacctct
aggaagggaggtggttactggctttttgtatctcacgaaccatgcacagatttacctgctccccttaatacggaatctgtagagtatggaaaattgc
ctgagtcaccatcagaaggatccagagagattcaattttcttttgagccgatgatattacatgtccaaactcgatccctggagacagctcagcactt
acaacgagtagcaatgagctgtggatttcgagaaacagggattcagggaagcaccaacaaatatatcgtagcaatcaggacgagcctaaagat
tgacgttccaattggagttgtagatgcttcagaaaggattcagctatttgtaacaaaggagtatatgacatggcttataaaaaggacattagagta
tttcgctgaaaatcaaaggcgaatggataggatcaaggaccaattggaaacacaggttgcaaagcgactgaaaccaagacgaaaactgaagt
gagacaaaaatgtagaaaaaactcttaatttgtgaaagcgcagcttcagtgttcagtgagacaacaggttggctgtgcacagaaaaaccatatg
tttaaggggagcagcggctggaaaatagtaatactatcaataaaaggagtatttactggtactagatgatttttataattttcctttgtaaaatgtct
aaccatccatcctcagaaatgagacttgcttccataatgtaatctcgatgccatgtatgattgtattcatagatttggcaccgctgaatacgagtag
agtccataaagcaaaaaaatctagctttccatttttctttgatgtcgatttccaagtgggtgtaacttaagtcggcagagatttgtatggaagaattc
tcttgaaaagtgttattgagttgagaaagtaagtttttttcgacaagcatgagcagttaatagtttgtgaactctccatagaaataaacgcagtctat
ctggctgattcgaggccaagtactcgtctgcccaaatatctagacggaaaaagttggggagggtgtatttgtaaattgaaagtgcatcttcgaaa
acacgaaaaataatgtaatggggagcttcaaatgcatttccattgagggatgcatattggaaggaagcatctgggttctcagaaggaaacatgg
tttgcatgggatgcatcatgaaatcgaaacgagcacccaagagaggaatcaaaggagcaggacaaatttgaagagtctttggattctttacaag
catccttttcctagattgttcttctggatcgtgtggaatgaagaaggaaactcctgagagacgttcaaagttgagcgaaggaacttgaaagggtgg
ttgtgtaaataacttgattggatctggcaaatcatgcttctgtttcaaacgtttttcttccaattcttttagcgttgctaataagttttgacgtttcaact
ctagcgaggagatttcctccatcaaagtatcttggtcatcatgcccttctgaggacataatatcaatgaaaaatgaaaatgaaagcagttcaataa
agaatagatatagttatgggattcttaaagcacctataagagtaacctattcaaatggaagttataacaaacgtgcataaaagtcgttgaaaaca
aaccaaatttagacaaaatcaaaggcctaaaaaaaactatcaaatgctcagcaatcgcgaatttctattatcgaatacaaaaaccaattctttgt
ggtgtcgactctaaaaccatacaaatataatcttaaccaggcaaattcaggaaatgtcaactcaactctgagacaatataagaatgtgtggaaa
gggtattctcttatacagagcaaacattagaagaaaaaacataatgaagacatcaacattgtaaacaaatgctgttaatgattgaaatggaaat
aaattaagtccgcgagtttggtagtataatatggattctatttctttcatccattgttgctggtttttttcttgggattttctatggaagaagatttaaca
gaataaacatttgtattgttttccttttcgaattggcaatttgcttagagttccaactaccaaaagaacttacttgatattttgaatacttctgtgaaat
tcgggtttctacattaccttttttttatttattattttacttgagtatccttcctttgtttatatttacatactcgattttcaagatttttgcatctccttgcatt
ccgtttgtacacgtaaacaatcaggtggtggtttccgcgaagtacaagtaaagcagcagcatcctgacgtttcgattcattatgctatccgaaggg
aatattctctttaagagaccttttgtttaccaacaaaaacgtaaacatgcgaaacacccaaagaataaacaaatcctttaattaactaggaaaac
caacatgtcattctaatactatcttcgttaaaaagaacaaaaacaactcgtcaaatcgtgcctctccttttctgtccgttcagcatagacatgccata
attcactccaccacctacaagcacatttgcactccctttacgctgccgatcaccagggtacaaacatcataagatgaaaattttcgcgtaccttatt
aagtaacaaatttgcagagcgagttttggagaaaatgaatcttttgacaaatcagacagagaaggaaaaaaaggaaaaatgtttggatgaaac
attcagatcaaagaaatagagacattgaacaactaaactctgtggattgtattttacaatgaaaactagagagtctaaataagcggcgacgcga
ctcgaatgtttttcaacaaacgttccattccaagtctactcacgcccgaacccacttttctgaacataccacttttatcgaacggaaattttcatccat
tagaggacaaagtatgggtgagaaaaaactcacccattgggtccagtaaaatgcgtctagctttcctattcgtttgatacctttttttttgtttatttc
cgattgtatcgtttgtttatctttaagtttgtttttttttactttttgggtttttgttgttttatctgacaaaacctgacatctatcagatcgattttcattggc
tgtttgcctctggctagaagagataagcatctatggtcagaaaagaaagataagggaataaatgtaatataaatggactcttccccgttctattta
agaataatcctttcgattcttcaacgtccattttgacacaaaatcgttttgtcttttgaaacgtagaacgggggtttttttattttaatttttcatttgttt
attttattttccttttgttataagtgtttcctggttaccgttcactttctagactttgaggaaccaatttttttgacgaaagtttggtttttttacgattttga
agcctctgaagaccatagattgtttttatcgaggggttttctttttatttgttttttttcctttagttattcctttttttaggggaaagatattcgctcgtaa
ttgagctcttggttctttaaagactttattgtttctaataggtagccatccgtctatctttatatcgggatttgtggtaattcatagtctcggtagaaaa
aaaaaagatttttttttattgatatctttagaagttattccgaagaacgtttcccatttttcattgatttgtctttactttttttcctccacacaaaagcaaa

gcaaaacagaacagaacagaacagaacaggacaaaaaagtttagaaacatattgtcctttcctttgtgattagttagtaatcatgttgtctcaatt
aatacaaaatacttctgattctgtaaggacggccactcctgaagaggaagaacgtctttgtccagtcattgactttggccccttgctagccaacga
gccgggtgcgcgagagaagattgtcgagcagctccgccttgcttgtgaatccaccggctttttccaaattttaaaccatcctatcggcaaagatgtc
atcaaaaatgcttttcgcgcttccaagcatttttttgaactccctttttgatgagaaactgatgttgagcaaggatatgtttagcaaccgcggttatga
gttgctcaaggactttgttttagaaggtgaagaagattcttctctagacggagtggttctcgacgatgatctatctttccctggtagctcttcctttac
tccttcatctcttggttgcccgattccttctgctcagtccctttccacgtacgctgaaccccctgactcatcaccgactacttggaacaccggcctcgg
tgactctaccccatctaccagcaacaatgccgctcttcatgattctgaatgtatgtctaatgaattccgcgaaagcttttattttggtaatgacaatct
ctccaaggaacgtctcctccgcccttttcaaggtcctaataaatggccttccaatacaggtgctaattttcgtacttccctcgtccagtaccacgatg
aaatggctgcttttgccaatcaagtcatgtgtcttttggccgaatctcttggtttagcgcccaacgcatttgatgaattttgctccgatcccactactg
ccattcgtcttttgcggtacccctcatcctataaccgcaccggagtgaatgaccatactgatgctgatgctttgacacttatggcgcaagatgaagt
tcgtggcttgcaaatcgtagatcctgtttcacagtccttcgtcgctgtttctgcacgtcccagtgctttggttgccaatttaggtgatatcatggctatc
ttaaccaacaaccgctacaagtcttcagtgcaccgtgtttgtaataccagtggacacgaccgctacaccattcccttctttttgcagggtaacattg
actatgtagtggcccctttgcctgggctaggtccctcgaccactgagcctattgccgtggaagacttgttgagggatcatttccataacagttatact
tcacacactacaaccacttctttggtttgctaattcgaatcccttttgttccgttgtattctatacttttcctttgaatccgttagaagtttcccaatgac
tttacatcccgttaattaagctgttttaataattttctatagattgctaattgtttttccctaagggctctgactgccaccgatatctcgtttcttcagg
acggttttgagctttcaaatttacgccaacgtttcgttctttttcaacgttttatttccatacggcttgcttttaagcttctgttatattctgctcattttac
tttttcttttccgttatagaaaagaaagaataattggatgtcaacgtttgcgtgcaatcgtcttttccctgtgtaattagatcgttcgttcttttgtttat
gtttttcattttgttattattataaatactaattttgcttgctatgaatgaatgtattgttgagctacaagtaggagtagtttttaataaatagatatcat
tagatttcattcttgaattatttgctacagaagactaatatcgattccatgaatggttaaacatactaatactgccttagttaacgtttaattctacat
atgtaaagctatcgattttcaaataacctaggatatgtgtataaatttactaacattggttgacgaccctctcataattactacaagcagcaaccct
ttcaagtcgcactccgaaattatatgcacaggagaacgtacgatatccacaataacgagaacagacgagctatctaccttcgacgtaaacatacc
aaagaaattaccacaaggattgacatagcaaatcgttgattcggtcattcagcgaatgtaaaagtaaagagccgaactctacgaaggtttctaca
aattggtggaagcaagttcattggagtgcgttcaaccaagcttcctttgttgacagggttacgtcacaaaagaaccttaacggaaaaattttccaa
tggtcgcttccacccactagccggaagaaagaaatgacgcaatttccggaaaattaagggttgtaggcttgttctggttatcttgacagacatata
tagagcaagggctccccgtgagttgtttccttctctgaacacactacacaccacaacatctacgaatatgagtgctactgataagtccgacaacat
tccttccgctgacactgttcagatggttatttctgacgttgatggtaccctttttggatgcccatcaccgtttccacttccgtacctaccgtgccatgagt
tacattcgtcaaacccgccctgacttccctattgtcttggctactggtaagcaacgctctgctgttgacttgatccgtatccccttgggtttggatgcc
ttccctgctgcccacgttaacggctgtatcctttacaacgaaggtaaggttgtctctgccgaatacttggaaccctcggttgtcatggaggttgttga
agccaccaagcacaaccccaacatcgctaacgtcgtttacgatgatcaaaatgtttacatcttaactcctggtcgtgaagacttgaagaacgttaa
gcgcttggaggaaatcggtgaaaaggttgtctggtccatgccctttgaagaggccaaggctaaggttttgtctggtgaaatcaaggtcatcaagat
ggctgtctgtgaagaccccgacaagctcgatgttgtccgtgatatcttgaagcccttccctaaggacaaattcactactacccaagctttggcttac
tgtattgaattgattccctctagctgtaacaagggtactgccctccaaactatcaccagcaagatccttcctcacatcaagaacgagaatgttatcg
cctttggtgatggtcaaaacgatttgaccatgttcggcgttgctggctggtctgtcgccgtcaagaacggtatgcaaatcgccattgataatgcccg
tgccgtctcccgtgttggtaacgatgaaggtgccgtcggtgaagtcttggagcgtgtcttcaacatccctgaagactacactcctcctgagtacaag
ttttaaattatgtgatataccatgactaacgttgccctccgctttcaataaccatttgttattttgaggtgttgtttatttacgttgcgttcgtttccttttt
tgtttaatgcaatctttttctttgtacctttgtcgatatttccgattggaagcaaaggttcggagcattttctaaagatttttgtcttatctttttagttc
cttgcgcgcgactttacgctcatagatacgcatagccaagcttcgcttcaatgtattattcttctgcatctgtcctttcttgtgtcttgtttcgtagctct
ggtatgtgtagttagttattgacttcaactttagacagtgagtattatgttttaactacaaatcaacatttattcatttatttattaaaacaatttcact
gcgagaatctgtaattcataacgtatcatcattatgttgcgggattcctgcacataagatgatcaataaatagagtaatttaggtttctatgcaata
aaaataaatagctaatcatggccgggaaaatgaccatgcttctggattccatggatcaaacatttgttcttgagtcaattgaaaaatatcggatcc
aagtggatcatccaactgaaaaatatcactttggagagctgataagtgagaatattgtttgcccagctcttttaataaagaagacgaacggccaa
tagccatttcaacaatatcaaaaccagctgcatgaagtgttacacagtgcctcaaaaaagcatctaatagggaaaaggcccttgctccataagta
ttactttcatgaaaatctaaatgaaggcgaatcagaagggaaacaagggttgaaaaatgaaaaccagcgtaaggccagaaaaagtcagttatt
gaatcaggagaaatttccataaaattggaaatggcttgttcaacacaagtaaataaatcttccgactggaattctggcagatgaaatcgaagcgc
ggttttccataataaaatctccacagcgtcaaactggatcctaagtgatgcagctccaaggccagattcttcaagtttagaaggacgagcggaat
gtagacgactaaaaaatccgctaatctggtaagaaatggagcgattgttcttgtaatgcttgcttagggctcgaatggtaaacaaatcttgtaata
tatcttgcagtaccatactaagttctatcattttcaagaacgttttgaaagacgtagaagaagtaatttcgagaggtaaagtctcgttagaatttat
ctttgggacgacataatcatcctcatgaatattcgttgctaaaccaaaatacatggagtaccacttgtctgcaataaaaatggcccaccatatcctt

tttctgatagtttttcttcatttgggatttcccagtttgtacaatcgtgattaaaacctagcaactgagacaaaacagtcgttctcgacaggatactt
gtgagactatacatattagaaagcggacgtccaattaaatacaacaaagctgcttgtattgtacttagtcttggttgagtaaaatgaaagtttaaa
ccatcttcaactaaattccacatctttcgctgatccaaagggggaacattagaaagcctggaatctttattccagtaaattagcgataatgaatata
gagcagctaaaagaatagacggaacgttcctcgcaccgtttttgtaagagagaaggaagcgcggcccatggagaataggatatgcaggatgaa
tatgagtaaaatacaaatcaactagtatacggcctgttgtttcatcaacatgggtagctaaaaggtcgtgcaatgcgcgctcatagcgagcgaaa
tcactatggcggtcgttctcatatgcaggatcaagaatatacataacaggggatttcgagtttgaagaaacatactttaaagtcatattatcaaaa
gagcatacaccgttttcaaaggagaaatattcacgaatcaagtctatgtcagaatcacaggtaggacccaacacatagggataattaagcgaaa
tatctaaacccagcttgtcgaatttatcgtcagaggtatgatacatttcggtaattcctgccaggttcagaggaactctctgagagctttggttgcttt
gacttttcagatttgctttggctaatgcagctgtgctttcaggaatatccaatgaaggctggctacggagaacagacggttggttgttaataaagta
atcatcgccgtatgatgggttgtcaaaagtcggatcatcagaaatagagtcggatcttttcttgggtttgctgcgttttatgggcggtaagtggtatg
tacaaggtatcttgagcgttaggcataaagcacaagaattacctacaacttcaatgacacatctcgctttccgaagtcggcaattgtcacaaggtc
tggtttttcttgaacgatatggtctttctaagcttccttcagctggaggtgcgttcatggaaagaattgtttgcaagaaagaaaattggataaaatg
aaacttgaacaggtaaaaagataatattctaggatgtctttgattttttgtaatgagtgtaaccgccaattcttcgaatttaaaacagtttagctga
cccgctgggaatcacaattttgaattctatattttatcaattattctatgctcggcgatgatgtttcctttaaaaaccaaacaagtttaccagcagtca
aggacggaggagaatgtgatttcacgcctactctttttacatatccaatcaaaatatatgtgaatattttgattttaatagttattttttaattgttcga
gcagttgaaatggaggattccttaagaattgggaatttggatatcaaggtggttgtcagcagaaactgtctgggaaggaagattgattcattctag
ataatgagataaagtactcaaacggcaagaatgaaaaacattaattgcatataaactaagagtcaaagaaacagcggttcaaagaagtcttta
catttggcatttcataagacagtgtttgggtctttcaaattggtaatttatagtttcaggtcacattttataaagtaggtaagcgtttcaagatgaacc
aaaaaaaaacatgtggttattttcctcacagtttacaaaagccaaaaagaacatgtccaatgaatagttgatctgaagaaatcgttgcatcatcct
gggtacagaaatcaatagttgtttccaacagtgtactaatgagaagagagcattgaatagggaggaacaggaccagtctgtacgaggcatctct
tgccaaaggttatgcttgaagatgtatacaggataataaatcccagcatcactcattgttaaatttataattcttttatgctttttgggacactccttt
tctatgctgaaggccatcgtctggtaagggaatttgatttgatttgtgttttgcactctaggcaccgtttctgtgattttgtcgagctcagctttgactg
tttctttttcctcttcagaaagggtgcacatgtaagataaagaaaaaaccattttatagatggcattctctggtacagagcaatgagcaaaaggaa
aagcacagtagcgagccttttgaaagccgtgtccaggaaataattgggcccattcccaagaattgaatgatggtgttcccgcacgatacttgtcca
atagtttatttatttttctacccaaggtacagattcgtctcggacattccaaacaagacctagggctccccttggcttcaaaacacgatgcatctcc
tgcagtgcttttggattggcaaaccaatggaaggagttaccacataaaatcaagtctatgctttcgtctggtaaaggaatctgcgttgccgagcct
actaatgtttctaccgttggatatttcttttgcagctcttttagcatactctcttcagggtccacagagataatcttcttaggatgagcagcttttgttc
gaggaatcaacttccccgtacctgctcccagatctaaaatagtagaattgtcatcaacccaaaactcgtcaacaagccaatctgttataccaaatg
gaaattctggcctacctagttcataattttcaatatcagtttggtacttcatagtcttttgctttttccggacattttcgtgaaaattgacttttgatag
gaactgcaataattcttccaagttccatataggtttcgtagaaaatttatatacagtggtcaaactacttcaagaaagaaatacattctgctgagta
gttataaagataaatagaaaataaatttattaatgcggtgaaattaggagatatatgaaacacaatccttgttagacgaaatctagcaaactgat
aaaaatgaatgtctctgaatgagaactgtattgcattaggaattaataagggtgtacacgcattaaacaaacaagaaaccagaagatcaaaac
gaaggtaaagaaaaaaatacaacatttaaaaaaccatacaagcatattcattaagtcatcaaaggttctcctacgcacccatcaactcttctactt
cctgatctggtcgccattctcgttttgataattcgtcttcaaaaacgctagttggaagctcggcccgatccatagaaataccattttcttctagtcgat
caatgatgtcgttggagacatgcggttgaaagaggactttacgatcgttgttgaatgcatttgcagaagcaacggttgaatatgcatttgctagttg
agctttcgtgggcaaagtcaaagattgcaattggtcctcatctttcgttaactcaaattcatttacaaccccatcgtcttgagcagcttctaaggtttt
tcgtaattcttctctatggtagacctcaaggcgctttaaaaatgaccgtgatactttcgtctcagaactatctggtcgataagattttgcaacaacaa
gaaattggcgtggatttaaggcatctaaagatccaagagtctcttgtaaggcttctggctcgtcctcttcacttaatctataaagacactgaagcca
ctgcagcagttgggttaacggcgtaagatgaggagtaagggagacttggaagtcttctccaggatagtcaataggctttttgagccttgcagagtt
tgtctgagaccattcttccaaactggaaatattgaagcgaatttccatagcggcagctcgggagtggtatttttttagtggaaaggacaatgttgaa
gagacgcgcagctagccaacgatacagtcgttctatacactgaatacgtaagagaggatgaacataaaatacttctaatagatgaagggtggaa
gcaatgactttagtaatattacgtggtgaagctgaaggggtgctgcgaggagacgaaggattatccgtgacatgacgacgccttaagaaattcca
gcttcttgaatgaagaatagagtccaaaccctgtattcctgtatacttgagcatgccttcttctagattttcctccatacggcgaatggcatctttgca
caccaatagataaatttcggatactagttggcagcattcacgttgtatttctccagttagttcttccaatctcgcatccctctgaagatagaatgaaa
gcagacaaacgtttgctagccacatgacacacaaaaccatatcctggcaattcgcttgtatgacttgatgaaccgaatcgagaaagcctgccaaa
agatcatgtcctatgttcttacttttcggcatatacgagtaagcaaatcttgaagccagataaaacagtttcgccggagctaatcgcaaagacggt
ctctcaggccgaaactcttgcacaaataattgaagcatagagcctatttctctttcatcaaaaacaaaatattgccaatcatagttattatcattaat
accaacaccttcctgatcatattttccagaattccaatttgcagaatccaggttgtgtgtttccaacataatgtttccgagatctaactccaaaccca

agccttgattcgctggtattagatcatttccttcttgtaatgattcctgcgttcctttgggagcgtcattgttccaaatacctacggtattgaagatgtc
attttccagattgtaattactggatttcaaccgattctgggatgaattgggcattactttttcgttcattccatcaaaagaagaagtttttctccattg
aatgagccattgtcatccaatcgttccatttcatgtggatcttttctttctctacgttttgctttcgacactggatacacttactctacaataatgaacg
gtgaactttcggtgatttatcgctaaatatcgaaagcctagcatcggtattcggacaaggatctccggtattcaccgaaaatcatactaaagagga
aggacaaaaaatatgtgaaaaacaaagagaaccggaacgcttggactggagtccttttcaacgaataattcttttctatttcatttgaaatatttg
cttccaatacgccataaaaaaaaaagctcctgtaaagaaccatcctggcgatcattgtacaagaacttttggaggatcttaatgaccttttgctttt
atcgttactttactctgtttttacttttgaaattgtttgtattcaattactgagtactttgcaataattgaaatttggcttgttgagtctctcttacgtttgg
tagaccttttccttcttctcgcattctacttttcctctttcttcttgaacgaagttaattgatctattttgtgaatttcatctgtgcggaaaatacaataa
aaatgagctcgaatcctgtagaacaaaagcgagaacaagcgcaaatggactcaataacgaaaccttccaatccagagactacgatgtctcgttc
agaaaaacttacatatgtactcattcacaatcctctaattcctattgggtgcgtcatgactgtaggatcgttcctgatttcagggtatcacttgagaa
gaggcaacgaactcaaggcaaatcgatttatgaagtaccgtgttttatctcaaggatttactcttgcagctgttgcgtttggagttttgtttatgaat
cctaaacccaaacctgctcctcctaaaccatattaaaatttctcatcccaagtcttttctattcgcttgagcttctttatttccttattctattggaacat
ggattttcagagaaaatgattttttatgtgtttcttttcaattcgatattctttctcgaggattggagaatcaaagattcccttctcaatttatgatacac
tgaagagtttagatcataaaaacgattttactcaaaaaatacaataaatgagcaaatcgagctcaattcttcaagaccctttgagcattatttcta
atgcaactttgacgcatggaaatgttcatcaatgaattacttcaaagcagaaagtaaatcattaaaggttcagagaaagcttttcatttaagtacg
tgctacttcctttcagtgaatctttttttgtacatctcttgtttccttgctgtcttttggacccttaaaaattaactacatcgattcattgttgctctaattc
ttgttactaatattcattgaaacgatttcaaaagaataagtaaaagagagcatttcggtcatgaagaatgttcatagtaagcaatagctgttgaag
atcccttcgagcgaaatcccaagcaagttattcaattactggtatgaatataaattaacctataatgaatctgattattcttgctaattgtttaataa
aagcgacttctttgaatattatcatcttaacgttccaaatacgatacctcaagtaacttgaaaagtgaccatggaaagcctgtttttttggtttccact
cttcgtagccgtatttaatctaaaaacgaagaaagaaaatcagaaatttccttttctatttattataggctgataactttgtagaatccattcgttttc
caaccagggcagttcaacaattcctattttgcatgaagacagtacaatccattcttaggcaaccgatctttttttctcagaaattatggcttcgtagt
tactcaacaaaatatcaggaagctccttcttcctggaaggcaatgctgaatttaaaacatatttacgagcattcgggcgaaatccatcagaattgt
gttaatcgaaatatgcgtgcagtggcagaaacgctaccaacaatcaaatctttatacgaagagcatactgccgttcgaaatcaattaaccccata
cgtcagtgaaaagaacaagattactggtttaatcaagtcttctaaagatggggagcaccgaagtttttttggtctctgaagcaaaaaatataaaag
taaaaatcgactctattgaaaaactatcttctgatatccaaaagaaactatatcattattgcctaagtcttccaaattccactttgcctagcgtacct
ttagggtccgaagacaatgctgtttgcgttgcttccataggagaacaaaaaaatttgaatgaaaagaatttttatattaaggatcatttggaacttg
ctggtgatgctattgatctgctcagtgcttcggagactagtggtcatgcattctgttacataaaaggggaagtagctgaattagaaatgtctcttatt
aactatgctattgactttgcgaaatctagaggatggtctctcgtgattcctccatctatagtccgctctgatgttgcacttgcttgtggttttcagcctc
gggatgaacagggtgaacagatatatcaactagaaaaccaatacgaaagcaactcgcctaagcaatgtcttgttggtacagctgagatcagtct
agcctctcttggattcagacagacttttcatgatctaaaggagaaacgagtcgttggggtttctcgatcctatcgacgagaagcaggtgctcgtggt
cgagataccaaagggctgtacagacttcatgaatttacaaaggttgaactttttttcatggactcatccgtctcaatcttcgagggtatttgacgata
taataaaattgcaaatcgactttatacatagtttaggtctttcggcaaggtaagctcacgttgctctttttttataaattttcgaaacaagaacttacta
ctaactcttgatttattctagggtattaaatatgccaactcaagaacttggtgccccagctgctcaaaagtatgatatcgaagcatggatgccttcg
cgggcgaagttcggtgaaatttcgtctacatcaaattgtctagattatcaagcacgacgcttacttacacggtataaaaattctaaggattctggat
ttgttcacacccttaatggaacagctgctgccatccctagacttgtcatcgctttgcttgaaagtcatcagcagcctgatggaagtgtggttttacca
caatgcctcattccctatttaaagaaagaaaagctttttgttccgaatgaaaaagaagtatccgaatgaggccttaataatgtaggctccaaatga
aaggctagcttattatcatttcacccttccaaacaaggaatacggaaaggtgattttgtcaatctttacaaaataaaatcaaagcttcttctgatca
tgataatatatcatatctatacttgaactttcatttatgaatttctcgttacttggtaaagaacagtgttggttttgtccgctacatgtaaattaatttg
cagagttttgttaactttggtagcatcagcattttacaaacaatatcattatgatgtttttttttctttgtcttcgtctgtaaaacgccgctaactaag
agggtagtttgttgcacaacaacgacagatgagcatgccaccaatgtaaactacaacaatgcatccaaaattagttgttgaaaacatatgtcata
tgattcctttataaataatcaagctagaactttgcaaagaaagtaagctttaaagaacatggcatggttttcatatataggatcttgcatcgtaaat
aaatgctttagcttcagtcctttggcaaagcaaagcaaagcacaaaaagaatccaacaggctttccagcttatcgaagtttatgtaaaagattatt
aaagtctagatcattcaaaaaaaatctacaattgttcaatcccaataccaagagctgtaggctttaagtaaatcagttattgtataaccccgcaag
aaattatttcctaatacccatattttatagaatacttttatgtatgcagatacttacgtatcgtaaaagcgcgcgtttaatattcaagagaaattttta
tcaacaaacaattaatcttcgttcattgatgatacttcttcagaaacagtaggtttaccaagtcccttcttacgcttataacgttcattaacgacctca
acagactcttgtcgcgctctctgtagaattggtacataccttttagatgttttaagttctccttccccgatattcacgctgcttccggtgttttgtaaaa
ttccatattgagaaatcctctgattagctatatccagcatgaaggatgctatctgctcacgaatacgaagagtatgccattgatggtaggtttttca
tatagatgtttcgttatacggggggctgttgtctcgacatctgttggcaaaccccattcgatattatcaaacatacaatcgtatagaattaatggaa

actcactggccatatcgtatattggtttactttggactttctggatatccaataaatcactgatgattgaaacaggctctaaatgctgaccaactaaa
aataaaacggccaccatgcatcttacttgatgccaaaggaaagccgttccttgcagatcaaatgcatatagctcggttgtaggatcaacacaaat
gatttttgagcttaaaattcttctatgataattggtaatttgttttgaagcatcaattttacaaaaatttcgaaaatcgtgttctcccaagtagagggc
ggctgcctcgttcatccggtcaatgtccaatgttccaccgggataagactttgtaaacaagtatttgtaatgtctttgtttacaggagaagcgagca
ttgaattcaggaggaggaacagcatatcctgtgattcgaatatcagatggtaatattctgtttaatacatcgcaatagacaataggtcttcgttcgg
aatgtcgcacgtttagcgaaatgacctggcccatagcagaaacgcccttatctgtacggccgcatctactgaaattgcatgttgcaggactctcaa
tgagatgagctttcaataaagcttcgaatatttttccttctacagtaggtaaaggtgtaggttcagtctgatatgctaatccattataatcccagccg
caataagcaaatctgaaagccaaaaagatcgtttttctagttggaaagggcgtggaggtttcactttcttggcaggcaaagctgcttgctgagta
gaagcattcgagtcctcatttttatgaatacctgatgtttctagttctttttggacggtctgctgagttccagttgaggaagcttgctgttgtgtgtttc
ctgcagagcttctctgtaactgttgatccaactctaagatttttgttatgagctggtctttcgaccattcagcatacttggatgctgacattcgaagac
ttcttgcgcacttgagaatcttgaagtctctaaatttgcacctggcccgactatactttccggtagggtaggtgttggatgaatccaagtataatact
tggacgaatactgataaaaacaatttatacgacagctcaaaagacaaaggagttcatgggcattttagtgttgcttatttatggtaatcatggaat
tctcttgaagagatgaaaaccctcaaatagacattaaacgtatagccgcaaactttcgaatcatccattcagtcgagtttttttttcttaaccatatt
tatggttagttggtgtttttttttttttgtttatttagatgaaaacgccaacgtgtctttcctcaaagatgacttctcaagtcattcgtaaaatccatag
ctaccattgaagaaaaagaatctttttttgcattgcaaaataactttcaggtagaaaggaagcgtttccattagccttataccgtgtttttgtctttcct
cgatcgcccaaatatttagaatgcctggttcatttagaaggaaagagaccctctgttagaataaaaaagaaaaagaatcaatagaggtttcgtac
ttcatttcattttgttcttggttttccttatttgtttacagatatgtaaacaaaccgtttgttactcgaccgctactcatttttgaggttgctgaaatccaa
gttgcacagagttagcgtagtaccaccagcagaaacgattgataattgcgcgttttgggtagattcatgtttcaaaatattttaaaaaaccttagg
atcacgaatttatttcgaaagaaagctacagaacctgaattgtttttttcctctttcgaaatctcctgtaaaagcactccgattacgagcagagcata
tccgacaggtttacaagtgtccgatatcaggaaaaccagtttcttatgaatgtccagagtcaggattcccttctcactgcagtaaagctcattggga
acaagacaaagtccatcatgccttaatccctaaattacgccaaatcaatgaggactatcatgatttaactaggccaaactctatccctcagcttgc
caatcttcctggcttagccgagaaagacgttgccattgctctttggtcctgggattcttacttttatactagaggctttcctagcattaattctgaaag
gacatcacgacatttatcaagtttgctgacttatcctatgactattggcggcctcttacacaaaaactccccatacaaccttcaaaatggattgaca
cctcaaggacttcaatccttagccgttcaaagatacatgctgaatcgccctacatcagcccaatccactgatccccgtcctacaaggatatttgttc
ttggtgcccgacttgaaagtgccttgcctcctgcactatggcttcagggccttaacttcttatttccaggccgtctgtttcaacttcattttgtcggacc
agaagttgtgaaacctgcaaatatgagtgatcttccctctcctctcacaattcattttcaccaagactattatcataatctacacaagacaggcacc
tttgaacctttcgatccctactatgatactttctttatacccatgcctgattttgctaatcctgaatattatagctcttgggtcccgacgttgcatgattt
aattgctacaaagtgcgcagtatgggttactagtccttcatctgctcaaacgaagcgggacactagtatcttacaagatgtacttaaggattcttat
gaaaaacttcttttgcctcaaaagaacgtttttgctagtcgcgcttggaatgtcaatgatgaaaatcttcacaacgtatatcatgtaaaccatgaag
tttacggctttcgttctcttcatcacaatgttcaaaatgtttaaaaagctgcatgcgccaggtctcttagaatacagttcccatcattcctcatgtttct
acggttcttaaacaaaacatctacttctttttgctaataaatctgcttaatttagcatttcaaaataagttatacaaagtatttctccaatggatttctt
gtatttctcgcccaagtacatcttgtatacatacagcaataatcaaaatcgtatataagggaataaaagaatattatcaatattattaaacgtaaa
catgtttacaagaaacaaaagttctatacttcactttaaccaataaatcatcaaaaaccaagaatcttataaataattcgtcatcattattaagtttt
tccaacgaggaagttagaatattcaaagaccctccccgacacggacgtattgaccttggtgggtagaaaatttgaaatcggcaggtgcttcaaag
acaagcacaacggtgcttcctagttgaaatctaccaagctgttcgcctcgatttatcggcactccattcaaaataggtgacgcatcactataggct
gcttcctgaaaagagcctagtgacttcttgcgtagtaccattctgtttgttgacaatgtaggatcacaatcaattactatactgccaacatttgtagc
acctacaggaatcatgctcataaatccatgcttatagcgaccaagtaaagcaactcgctcattcaaaacgaataaattatgcagtcttcttgccaa
aaacggagagacgctgaacaactcaccggaaaagtgccttcttgactcaatcacccaatcagcaggcgaatggaaccgatgataatcgccagg
tgctaaataaataacagaataaaataaaagattattaggttgaatatttctccaaggtaaagagtcaatgtcgacgacatctgcttcctgaaccca
gacatcccaattcggctttccgttatctaaaattggatgagttgtaccgttcgagtcatctaaaggtacatcatcgtcatcctccataattgaggtgt
aaaaactatcagtacttatatctgatgcatccgacagaaccgaacttgtgtcacttgtcacgtcctgaaaagcattaaatggacagcaattgtcat
agtaagagtcacgtgtggaattgttattattccaagtctcactttggctttgagtatctgagccagtcaaaggatctttggtcgataacccattatca
tttcgaaagtgactacatctgtcatgatatggagggatagaataatttgaagcaaattctttatgctttttcaacaaacttgaagttatatcttgtcta
aaatcaacaatagcgggatcaactgtagaaacacctcccaaaagcgcttccaaggaataagatagtccttttacgtgttcaatgtagttattatca
acaacgccttggcaaacaattctaccatccacaggtgaaacaaccaaagaatccggatcgactggacgaacttcgggacgaagctcacgacaa
aagaaatcctgaaaattgcgataatgagttaaatctgggtctttcaattcatctaaattacagcgaaagacccaagaatacaacttaaatcctgg
atatcttaaaaatgtaggtaaattggcacgattgaactgaccccaaatgcgcgacaaacttcgtaagggaagggagctcaaaaagtaaacttgc
caggagctgtttggacggacattgatagaaagacgacccagtcgacttcttctacgaattcgatgaaggcgattggaaagacgcttccattgtag

tgaaacacgaacattgactccgttcaatgcctttactccgttcaaggcccgaacaccaccaccgatgccacggagactgacttttgtccagcggtc
tggatgaaaaccacgaaatttttgtcgagagcgcatgaaattaaaagaaagtttgtcacaaacgacaggaaaaaaaaggagaaaccgtataa
aggagaaagaagacaattaaaaatacttctggtaaagggataaaaataaaatttgcccaagattggtagaggatgggaaatctgaactctgat
cagatagtttgttaaatgattaaagactcaaaaagactgtaggaaagttataaaatgaaagccaatggtaagaaacgaaggaatgaacaacac
ggtgggaaacaaagcgttccttggtaccagcaatggtaaagtagatacaattccagtcactctaaaggcatgtacaacgcaagaaattaattaa
aggaagaggaggaggagagaatcaaaactacagtgtcagataattctttttactccagctaaagcacgcaaaattacaaagggtacaaaaatc
aatatcttttcagtcaaacaacagatcaaagtttgaaaaagaacatgaagagtatccaaagaaaatgaaaatcaagtgtaagagtgaaggcttg
taaagaagagataggcagaaattggagacgaaacaacgtcctctaatccttcgcaacgcaaatctgtatactgatgacagaaacttctttttaag
taagttcttgtggtctattcttcacgaagagtatgtaaacaaagcctttgaaagggaattcttgttgtatactgttaggctctggcatcaacaaaag
cacgatagtagatcaagtggtggaaaagggaacagaactctgtgggggagtctgatacagaaacatgagaggataacgtaacatagcaaaat
gccttaatctaccgagattgagcattgccgagagagaagacagaagtaaaatttcacggatagacagtggagggaatcaagaattgaatgaaa
tgaaataaataagatccatttattttcaatctctgaaacaattaagcatcttgaagaggttcgaatggagccgaatagatgatagctagacagag
agctataaacgtaggatggactgtaaataaacataggaagagtagacgaaaaacgaagcattcaccgtttttaaggatctcctttttaatagct
gtaaatcatgtttgacatcttcttcagaagcacctgttagacctttatcgacagctttttcacgatcacgctccgctcgctccatagctcgaaaatctc
gatctcgggcagcagagatattcacattggccattgtcaagagaggagaaaaaagagatagctcgacattggatggtcttgcagagttgactga
atttcgaacagtctttttatggactcgggaatctctgctgtctcttctgtagggaaaagtaaatagtcgtcaaagaagttgagagttggcaattgat
ttgaagtggaattattggatgatgcatttcgacgatgtcttaattttcgacgatctataaattgggaagacgcatcatgtagaagaagcgaggaag
actcattggcaatatcaaacatatccttggcttccatgatgtgtcgtttttctcagccaagtgtgataaaaccatagcttgaagagagttacagcg
ctgctcgtattcttctcgggcatccttcagttctgcttcatattcttcttgagctctttccattaaatggcgtctccactgttcttcaatttccaattcttct
tcttttttttcttgcaagccatgaaacaaatccaatagcgttctacaggtgccatttcgtatagctgtcaactcttcctgtaactcttgttttttgcttc
gaactcactggcagtcagtggcatatccaaaaattcatcatcatcttcatttttcatatccaaaaccctggatagaacgtccatcttggcttggcttt
tttttctgttgttttgcttttgaaaagcaactgttgaccttttttcgcaggaaaagaagtgacgctggcatcagggttcgggttgtggggtttacgaa
acaaagttatcttaacgaaaaggtgagtttttatcaaactaaaggcagagctggtcaaattgtgaaaggaagaaaacgagagcaagggattatt
attttattttattttgatttataaaatagagaatagagctttaaagaaaagaagcattcaaatatatgtggccattgtgattttaattaggggccgac
cattacggactctatgaattgtacagatacaaacttttgatttttctttttcactttttttacaagagggacatcttataaaggagtgaaaaaaatat
cataaaatatcattactgaacagcaaagaaactaaagaaagacaggtgggaaaattcagtagagctaagcgttttcctttttaatttttctcggga
gaattgttcatctttgtacgagcttgagaggcttctttattgctaatcaaacgaccaacggcagggtacttttgatcgaaaaaagccttccagtcat
caagtgtttgcttttcttcgtcgtttagatccgaccaatcatccaagtcatcagcttctgaatctggaataaattcgtcatcaaaactactttttgcga
gtccacgagttgcatcgtgtccaccgaatgcactgtaaggacctttaggtccataaaacttggatcccagcgttacattgtacacggtacctttgat
ggcaagaaaaactaaggaacggtggaccccgttaaactctcgaagctccaaagggctatagtctctccattcaggttgttttggttcaggctcggg
gatgatcgaatcttcttttctacgtttccatcttgtgatgactaaatacaagaccaatgtaacaataaagacgatttctgtcgaagccatttgaaga
ccaaggaataaccagagaatggaagcaaacaaatgttttcagggttcacttaggttttgtacaaatatgttcaaaaacaacgaaaaatctacga
accgctcaaacaggtttcgttttctttcttcctttcttcctttttttccttttatgctttataaggaaacaaccaaattttgatttgtagagggagaaaaa
ggaatcgttcttactcgaagcgaaccaacagtttgcgaaaagtaaacgtagcgatcgttccagcgagaagaaatatcatacaaaaaacgttaaa
acgattaaaaaacattcgtttataggaaaacgaaggaaaatcaaaccatttcccaagggaaaaaaaaggaataaaccaatggaaatagactg
attataattaagaaaactacgaaaacaacaaaagtcttcccaattgctaaagacacccaaatcgatacgcttggaaaactattcgtacagcctat
tgcctattttattgcccaagctaagttaggccgtacaagaccaaagcagaaccatcctaccttagagccatatatacctttacgacaaagacgtgt
aaagtggatgcaagaccaaagttgaaaaaccatctgaatagtcatttcaatatcctccttcagaaagaatacttatggctgtcattcagaacgtgt
ttttcgtttcctagctgcttctttttggcagctcaaagagctgggtttattgattgagccttctggctcgtgttcatcatgaaaacagcgcgagtcgaa
ctcttgtgctttgagtaaacaaagcttacaagaatccgctcgtacaaaagttaaatcgttataatttctatttacgagattatttataagattacgat
ggtcatgaaaagagaaccggatatggatctctgactctcttgaaaatcgaacttgttttgctactacagaggaatacacatgtttaagttgcccaa
attcatctgtatgttcgactttataacaatggactatacttccgatgattgcctggtttgtttgtctttggaatagctttcaaattatatttattacattt
atatttaatactataattaggatatgatattagtgaaatattttcatgtttttctttcactctactatgaaaacatgtagtagcctgccgactcactac
aatccaccgttaaagggttgtagtatagagtactaagatgactacctgcgagatgcaatggtctccgcacaactttggcacttgcaaagggttata
tctttacaagcaatttgacatgtgtgcgctggagaacttatgcgctgccctcggtcgtcatgtctctctttcgttaaaaacagtcacatgcacaattt
ggaagatcacattcgtgggttgtaaatctttggtgtcctttctttttctttcggtttttcgtagagttaaatacataaattatatagaaaaccatgttt
cccaccaacacattgcctaatacaagtctttttgaaggtgataatggtcgcttaaatataagcttaaataaatagcgaattgctccaattaccaatg
ggatcgacaaactcgtcgcttttcctagaactttagtaaaagagaattgtaccgtttataacgaaattaaaagaaagagtttcttcttttctgctttg

tgaattaatttccctataacttccctcgtaacaattctttaaccttaacgatagcgtttcattgcggtaaatgagaattcggtacactctctatttctct
atttgaaaaacgaattctagtgtttagccatgttcattccaagctacaacgaagcatacgccaaactccattggaaccagcatactttgaagaaat
cttttgaaaagaagcgtatcgatcgggagttggccctatcttgaacgcattttgtatactataacgaaacactgtggaattgttagggtattaaggc
acgaaagtacgactggtcgtcctggaaaaatatactttggattctcctttatattcttggtgatagtaatttgactcttttaatctatcaaactggatg
tggcgttgtttccggttgtccctttgtattaacctccggttttgtcggtctatttcatcattattcgtaacatctggaaaattggtcgttgtttcttggat
ataaaaccttgtaaaattcttcaatatggtcagctttcgtcgcgtccgctggtcgaacttactagagcaattttttctcccaagggtacgttcttgaac
ttttgggcattgtggcactctaaaattggctctgaaatgctctactcaaagcaaagttttcatacaatgtattctggtcctgtttattaactcttgcagt
ctctttttggcttatgtagtcttcgtgttgggtgcgaggtcatcgtttggtttgctatcattaacaaattgtctggtctttacggaattgtatccctatttc
agagtaagtgctccttccttcattacttgtctaatctaattcaaactaggttccgctgtctctgcttggcagattttcatgtatatgacttctattttcac
cctagttctcttttcttggttagcagtttacattccaagggtatgttttaactctattctcttttcagggattttctgtgtgataatcattatgcattcgttt
gctaataaaaaaaaacaggatagtgtgccgcatgcactcatattgttttattcttatgcaatcgattttattttaaatatggtctttaccgtcttgtttg
cattgagctggtttgccaaaatgctcacttctactcaatctgctcaaggtgtgccggactcccagcagcctactagcctttgggccgtgtttttcgaa
gcagaatctattccttctttacttttgctaaccttttttacctctttaaagctatactttactttaataaccctttcttactctaataaattaatcgtcgac
tctggcatccgaccccaaactctcccaagctcgtttatgggtcgcttaactcgcattttgatgaagccctacattatcgcagctaatcgtacctattt
gagaaatcacgccaaacgcttcctagaaaatattgagcttcaacaacgtcttatggatgaaattgtttagaaccaaagtaatagttatttctacctt
caatacacggttatttaggtttttttgtttcattattgtgtacgttcgtctgtctgttaattattatatgcaaccctttcaccattattctaattccttttt
caaccatcaacctgtctataatcgcaggatatagaagctttccactacttttcaccttttaataaatgctttggttcaaaactatcaatacagagtta
agaaacgctgatgccacatgtcatagattgtatataatatacaaaaatgatatattaatctattgtattcaccgttaattaacttcactctattaaaa
attaagaaggaaagagtattttttttcgtacaaattgcagtctacttgttaaaaactgcaatcatgcatacttatacatagcggagaagctttcaaa
ctttatacgcttctgaaaccaaaattttagatattttcatgaaaagaagtatttgctttccaaaaccttcgttaaatcaaatgaaccttgtgaaggta
gagttgggatcctcagcggtacccattttttatcagcatattcagttgatatccacaaatcataccttcttgcaacatccttctgaatcatatagttct
tatatcgctttcgaaattcatttctcaatttttccattattttctgtgaatgtaactcaacaaaagcttctcgcaacagactacctaacttctcgacatc
gcatgcatgagtccagtaggagtcgtggacggaagcaaatgttatgttggctgccttcgctttcaggcatgtcataaacaaatgtgttgcatccaa
agaatgcacaaaattaggaggaaaagcagccgcttgttttctaggttcaacggaagctactctatccctgtcctcaatacaaactgactggaggtt
tgtctgcacttgtcgctttttaaagtttctataaggctgaataatggggaaaccgagaggagttgtccaaatagtgggcgtcaacttatccttatga
ccttcctcaatgtattctgccggtaaagaatttgaaataagatacgagcaagtagataaccaatcttgaatctcatgagctctttcaaataaggac
cgaagggctcgaaataccatcttggttaaataactagagtaattgttaagcatatctttcgggatatctcctcgcttttctaattgacttataatctga
tttttggcacctacataagtaacgccataaacgttagtcatcacggtgggcttaacaacgcttctgtcgattttatctttcaaagcagaagccatctc
atcgcctttcatagactcatgttctaatttgcttataacaattttagctacagcatcatagacatctttaggacgatcattaggagatagattaacttc
atgagcaccatcggggtctcttcccaaagccgcataatgctgtagcccattacaagtcccatcttgctgtacaggtacgtgacaaacataagtctc
ggggttttcggagcgcgttgcttttgcaatttctgcaattgcggcaagcgcttggaaaggatcttcagcagatgtccaccatttacgtccatccaag
ggacgatcatacgagtcaaagacttcttcaatgttgtcatccacaaacttttgacgagtagcataatcaactttgctattaccaaacaaattagcta
ggtgaactttcagccaattgagtccattaggacctaacggctttccttcagaaaactctagtaaaccacgacaaaaatcgttgttcacatggtgca
aatgcgcggacattgggtatactcttcctcggaaatccatgttatgaggaaagtagaatctttcgtttagatatgaattagcaatctcaagtttata
attaaagtcacagcgttgagagtgtgcggttgccttatctctagttagcttatcccttagactgtaccacgttgcacgatcatttggatttaaagcttg
cttagggtaaggtggaagattcacctctacgtttttgggaggaatactgagcataccttcacctgaattccatattttgacgataacatcaaaagta
aagcgatttattctccagctcacatcaccaagtgcgcttaaagcgccatatacttttttttaaatgtcctttacgagaagcttctttcacataatccaa
ctgttcagtacaacctttcaaacggataattgactctcgagagtaataataacctcctttgtcccattgcacccagggtttcggacgtaccaacatt
gggagcatctgtggatgaataagagggttctgaaattcacttgctattaattttaagagctcaactctaggacgaagtactcccgactttcttccat
ggatatattgatacgtatgagcgaacgcggacacagactcttttacagattccccggtatccgaacgcttaaaattaatatccatctttgcagcctg
taaaaacaatgatatcgcaattgcaccaatcttggtagtgacataccttggccagctctccaaagcgtgtttccatttcccatcgttctcatcggcat
tacgtctcaactctttaacggcagttctaaatgcacgaggattattaaaaacatactttagattatcccttaaacggatatgtccctctctttcttctg
cttgtatacgttcagataagaattcgcgttcaaagctccgcccaacagcatcagtcaaccgagaaatcaataccccatgcgtttcctcgatttcttc
ttgactagaaaacgatgaaatagagcgaattacttcaatgattgttagagcagcaagtttgtccgctttcaaaagcttcagaaaaggaccacaaa
agccctcagctatatatggatttcctgcatttgtttcaattctatggtaaaaagcatcaagacgtttattttcttgattaaataactccttcaactctac
aaaccactggtaaaacaatgcagaagcttctttatttttgggtaaagctcctccgcgttggaaaatttcctcatactcagccttccataaatcaata
gcagaagcaatcgaagatttttctaacaacttttgtctcgccaaattgacgagttctgagctaggcaccttttccaatgatacttcatcaactggta
actcttcaggactagtagatatattatttagtgactttagtaaggcatccagacgcattcctttgctcttaattttatcaatataaacatgttcagtcg

ccggtacaggtttcaatcgagactctccttttactgctgtatttcgtataagatcgttctcagtcaaattagaagattgaaaaccaaaaagagctag
ttggctattggagagagtttctaaaggaatgttgaggattctaatgatcgcttgtatttgttcgctacttaaaatggacgtctgggataaaatatcaa
caacttttcctttcctgtatctccattccacagccattcgatgaagaactggataaccagaatcctttccttcttttaataaggctgcctggaaataat
aagcataaatcgtggcattacccggaagctttgactttttaaggatcgctctcgttgttcgaatcatggaatccagagaaaaatcctcagaattgat
tagatgtcgaagataaaattcaatgtatgggtacatattttcatcagttgcattaaaattggctatgcgacccaaaagctgagcagaacggacaa
aatcattcgtgaatacacatgcctcgtatagactcaccatttcagacttttgatccgtgttaattttgaattcatacttcttggaaggaagaattggct
tatacacgttgacggtttcctcagcattttgcaatggctccgtttgtattagccgccgaccacgcacgttgactctacaaaatcgcgtccgtaaaag
acatcgctgaaattgcctttgagagaacatccttcatagcataagtatatagttgtcgaagtggatacctacttctttaaggctcttagggaatgga
cgtgcttggcagacttgtgctaaaagactttactgctaaaggacccttggaattctcagcggacaagtaacaatgttacctttagaaatgtaaaca
gaagtaggaaagttgcagcaaaaagccaattctgttgtctttttttgatcattataagacttctcatacaatagaaaaagtaaacaattaagtgga
aatatattttgctggaagcatggaattcattttcatatgcttattccacaaaatgacactaaaagtctgatgaacacaaaaacaaatctaaaaata
aatacaacgagcatatgaagtttttcttttataacattaaagtaccattatttgtttctcatacattcaaagggttgccagctaaaggataaagata
aaaatacaaaatcataataggtaaatagccttaagcttggtcaatcggaattcgttcaatttctctagagatcctttttacccatacatcccgttcct
gtctagttctagcaagaaacataaacctgtaatagtcagtaaagatcctgagcaataatacttacttgtttcctttttgattgggggttttcgatgaa
atattgtaaacatctggaaaagcattcgttatggaataatccaatccattttgcgagtaaataacaaaagacagcatttcgtctttatcaaaagac
ttccagccatcttcatgacatttgggtaatcgattgcttgtcccttcatttttcgccggtccctcgcaaactcagtgattgtcgttaaaccggtataa
atataagcgtttttgaaataaattgaagtgaatttctgaaaatgcgtctttctatggagtttaccaaatttagttctagtagcatcccgaaaaaatat
gattcttcctggaattaaaatagcatagcatttctcataaagatttcctgttcgcatatatagcaccccttgagcctatattgtgattagcatttataa
aatcattttttttttcttacccgaattatcctgcatcctaggatgttgcaatagttatggatgtgtgtacttgctatacattctgagacttcccaatgatt
actgtgagatgcaacaacaggctcaatagatgagtcaatatgcaatatgctaatgttagtgtccctgacataatagaattcttgtaaatcaaggtg
caaacgctgtttccaataacgtgcagtctccccaagcttttcaatccatatgtcccttgttttctgattatagctttgaaacagtaaagacataccatt
caccatacgtacttcaaaaacgttgttgtcagttccactttcttccggcttaacaagctcgactttcgacaaatccaaaaacccatttgctgatgag
agcatatccaattcacgccttctctctgtttgaagcaaagcaagtaattgctcttttgtagaactgtctatgtcattgtcaaaccagggagttgtctc
gctaacatcttttaatgggtcaaacatggactgatgttcattcgtttcggagtcgtgaagaaatggaaaatccatttgcttcttaaaattttccatta
aaatggtaagaggcaggacataactgggctcaatggaaagaaaaaggtgattaaaaaccgtgaagtacagctttttgtgaaacagtctaccaa
aacgccctcttcgtccagcgctcgtcgtcaagcgaatcaaatatccttcgaacggcacgggctccttcatttttgagccgtttctgaacgtgacaaa
cgaagggtaatgatttttaggtcgtaactcaagctggctgatatctttagcagctagtaatcctacatacttaaggctctctacgtttgtaatccact
caatacggtcatacgttctccagcacaagcctacgctttgcgttttgaaccattggtttacgatttgagaccattcagggtttaactttaaaacattt
aagcaagaattaacaaaatcggtctccgaaatctcccaatttttcaaaactgtatcatgaatcgtttgtggagaaaccttatccgttgaatcagcat
tttttatatgcaaagctgtttcattcctttgccgtgagttagcatgataatttacgacggaaggagagttgggacaagtaaaactgagacgcaaat
gaatatgaaatgctgggacacctatcagaaaagaaggtccgggagtgaagtccaatgcacggctgacgatagcaatccattcatacgcagaag
tagtacactgaaaatttaaaaggaaaatgaaagttttgtgttttatgtagggggaccaaattgccagcgagttatcgaacgaattatatatgttcc
aaaaggtataagagggagtcaagaacaaagaaaatagacagtttttcgataatgtttatgattaggttcattttctgaaatatagtcaactactg
ttttcatatattcttcaccaataaaatctaaccgaacagtccctttctcagaagtttttacggcgataactgtacaaggggcccaggaaagtatggt
ttttcttggaatccgagaagcaatcatttcgttgaattcagtagggattaagttggaagaagtttcaactcgcaaaaggacttcttcagtccgaaaa
acaatgtccgaagatggagcgcttagttttgcatgttcattcgtacctaggacacgaggactactttgctttaaagaaaatcgaagacgacgtgaa
gttagagatcgttggtcagacgaccgacgagactgaaacgtttggttacttacagcggtgaagggacgctgtacatcgaaagaagaacgaactc
gagcagagtcagaagagtagggtatgagagtagaatcacggttgttagaatgcatggagttggcaaaggagtctcgtttggatacatgtgaaga
gcgagtacgatggtttggacgatgactagttaatggaggcctagcctggtccattgatggtcgagtagtagaggcataggagtggatcctgctcat
agagacttcatcgactgatctgttaacatgagtatgcaaccgttggaagagttcatgatttatgggaggttcaacggaaatgtcataagaagcctg
gtgtcgagagtagttttgaaatcgtttttccaaaagccagtagatccacttagaaaatttgtcgggatcggaccaatgaatagacgacgagttaa
atcgcgtaaatgctcaacagatttttggttgagccaagcaaagtcaccttctcgttcattcattgcattgtcaagtcagacgatataagcaaacga
atacttaagaaatatatgtattactattgcttttataaataaatatatatatatttaaacaacaagcaaatcaagtagctttcgacccttgtattgttt
acacaagatacactgaggaggcgaaaagtaaacaaaaacaaaacgctgagtaaacgtcagctcaaaaaaaaaaaaaaaaaaaacaataga
ctaattaagttattcactgaacttgtaaaaacaaaaagggaatttagtaaacacttctttcataaggccagtagccagatggctggcgagaaaat
gttcaaacccaaacaaggcaggcagaggttacgaaatcagacgatagttgtatttatgtttgcttcagtgaaaggtatcgaatacttatttcctctc
ctctagagtgttctgaatcgttagccctaacgacttgcttttctgtgaatggtaggtacctgtgatgatagaaacggtaattagcgctagggtacttt
attcaatgtaacgtattaaaaggaatatgttatctatttacgctgcctaattattgtatcgttaaagctttatacgagctttgtaaagcgtcctattct

ggttagcaaattttagcgaaagcggattgtataaaaattttcttacgtttttgttttgaatcaacgaaaaaatagcgagaagttaagcaaccaatc
aaaatccattataacactaaaaatttagcttctcaatacgattggccaaagccttttcttaagcttaggtttacttatgattggaggttttgaagcat
cgtttgtatgtgacatatgtcattggatgcagcatagagcctataggccaattacacaaaagtgcagatgtggtcacttttctatatataaagttgg
aattccctcaattttaacatctcagaaacaagttaaaggttgctttttcaaaagagtttatttaacttgtttatttatcgttctcttttttttgaatacac
atttttgtaaagctaaataacaatggctgaagaagaacatcaaatcgactttgaaggtggtgaagccggcgcctccttgaccttccctactcaat
gctcttctttgcgtaagaacggtcacgttgtcatcaagggccgcccttgcaagattgtggaaatgtccacctccaaaactggtaagcacggtcacg
ctaaggtcaacattgtcgctgttgatatctttaccggccgtaagtacgaagatgtatctccttccacccacaacatggaagttcccgttgttaagcgt
gatgactaccaattggtccacattgacgatggcttcttgtctctcatgacttctgacggcagcaccaaggatgatgttcgtattcctgagggtgaaa
ttggccaacaaatcgaagaaggcttcgaggagggcaaggatttgattgtctctgtcgtctctgccatgggtgatgaaattcctctcagtgttcgtga
ggctccctccagctcttaaactggacatcaccgtgtatgcgtagtggatgcaaagctaccgtcaaattgtatatgttaccatgtaggtaatgccgat
agaagggtagacgattcctttgcatgtcatccaaggagtatgactttggaggtggatgtttccatctttttttagttgttgacgctcatacatgtttctt
gttattacgaatttcttaatggatggataggtcaaataaaaatggaatcacatacgttagatgatgagcataatgaatcattgtaatattaaagta
gaatgttggaatggtaaaataagagtaaaccagtcttggatcacttttgtctgtctatacagtgccgatataatatagagatgatggttaatttattt
tgttttcaaactgcgtgtaacgtaaaagtcatgttttctactactttttctgttctttcattcaatgccttgattaattggttcattcattcatttacgatg
gattgcgcatactcaatttaagcctccttgctggaaatttgtccgacatcgattgtaacttgcgcgagtaacagttaaatcatggaaataacgtaag
caagatggttctgtttcagggaattcgtacaggaccgtagacctgtgaaaacatccttaccacaatttttcttttacggtttcgttcgttgttggtttta
actaccccgataatctattagcaagtttattgaaatcgtaagtgaaagagcactgccggcaagggtttgtcatacggcaatagctttgaagcttaa
tatctaacttacacaataaaggtacatttataccatgactcccattaagactgcagtccttggtacaggcatgtctgccttcatttttcatttccctttc
ttggagtcgtccccagaaaagtatgagatttatgctgcttgggagcgtcgtgcaactcccgaacactccaaggcacgtaatgcctatcccaacgtc
aaggtgtatacccagcttgacgagcttttggctgattcaaatgtagaattggtggtcgtatctcttcctcctgatgctcacgtggcagcagttactgc
tgctttaaatgcaggaaagcatgtcatttgcgagaagccatttacacccacatatgaagaagcgaaagggttgtttgagttggctaagtccaaga
atctcgtattagccatctatcaaaatcgccgctgggatggtgactttttgaccgctaaaagcgttatcgacagtggccgtttgggtgaagtcgttga
attcgaatctcatattgaccgtttccgtctgttccgtaagggtaactggaaagacgtacccagtcctggttctggtttagtttatgatttgggtgctca
cttgattgatcaagcagtgcttctctttggcgttcctgaatctgttacaggaaaggtgttttgccaacgtcaaattcctcccttggaagtagaagaca
actttactattgtgttacattatcctgctaaggagaacaagcttcccgttgaagtgatcttgcgctccactagtatttcttgtggggtcgccttccgtt
attgcatcaagggtacccgaggcacgttcttgaaatttggtgcagatcctcaagaaggtcaacttaataagggcatgaaaccctcagaaggtggc
tatggtcaagaatcttctcaagactatgctaagctatggacggttcctgttgatgccgatgtgaaagcacttcctgcacctactttcagtaccgtgcc
tactatttccggaaattacctcgcattttatgaagaggtttataatgtcctctccggcaagtcctctgccatttcagttctacctgaacaagtcttgac
agtggaaaagattatcgaagctgcctacaagagttctgaaacttcggcttctgtgaagattgcctagattacatgcttatgtttattgataaatttta
tttagaaacctcttcatgcaatgaatgattcctctattcgatcaattaatgacagtagacgatatgcgtcgtaaggagttggattgatagaaatgca
attgctaatttcatctatctactgccatatgaaaatatgatcttgggagaaaataaatcaaactaaacccaattggtgaatttttaaacccgaatct
atattgatgtatgagtcgaatggaattccagactaataattaaaaccaatggcttccacgtttataagaaaaacctgataaagtgccaagttctca
atgtcgaacggattttgattagcaaagaaattcacatgtcactaaggatggtgaaatgatcaaatttttaattggctattagagaactagtctatgc
aaaaaatcgttattctcaagtaaggaactaaatctcttccgtacaaaaatttctggtggtgaagaattcttttatagaatagcctactacattacaa
ttctgcatttactagataagctaacttaataagcatgctattaaacaaaaaaactatcattttctgccttgacgttatttttgatttattaatttctaca
tgaattcaaagatattattatacacgtaatctttagtaggaatggccaccggagcgctgaggattgtataatgtcagtcatgattgccatcgaaat
aattgcccaaaaaaaattttaatcaaaagctctgtcggccatgagaagtccaatttaatacttaaatatagcaaaagccgcaactatgtgttaaa
gaaatacgtttgaattcagaagtgaaagcttttgtctgatcttattttctttttggaattatataaaaagaaaaaaaaagaattactttcatttactt
tcttcgttcttgtgttttgaattaaaaatagattagcattcatcgtaatgatgcttattccttctgtgttgacggctgcattagctctcttgccgagcac
ggcctttgcagtaaataagtcggcctctgaattaacttcatacaatcccgacacgttaccctataatgggaccatgaatttctgcattggtgctaag
aatgacaacggtgcttgcaaaagtactgacgattatttggccgatttgaatgcattagctaattacacagacagcgtacgtacatatgctacgagt
gattgcaacacactggagaacttgttgcctgctttatctcaagcatctcataatttcagtgcttacattggtgtttggcctactgacgatgcccacttc
ggtcaggagaaacaagcattatctcaatttttacccaagtatggtgttaacaacatcaaaggtataactgttggctccgaagttttatacagaaat
gaccttagtgcgagtgcccttgctgatcgtattaacgatgttcgtggtcttgtcaagagttttggcttcgatgtccctgtcggtacagctgatagttgg
aatctctgggctgctggtgccggtgatgcttcgatccaagctagtgatttcatcatgtccaacgactttccttattggcaaggtcagaatactagca
acatgaccaatactttcatctcagacactcttactgctctagatcgtgttcaacatgttaagggtaccaacaacgtacaatttttggttggtgaaact
ggctggcctactggaggtccttcttatggtgaagccgatacttccaacgatatcgccaagcaattttaccatgaagccttatgtcaaattctcaaga
aaggcattgacgtttactactttgaagcgtttgatgagtcttacaagggtaccgacagcaatgttgaacctcactttggtgctttctatagtaatcgc

gttcctaagttctctttgaactgcactcaagcatcttaagttccttccatcgttatttatgtaaatatgatacctttagcatagtttaatctcatacata
cgtctttgcattatttacccgtctttctggtcgcacgcagtaaattgaaaaatgttgtccgatcactgttcataagcttcaacctaactttatttatgtt
ttcttccttttcttcattccagcagctctaacccatgtgtttactagtgtgcaacttgcacttcgctatcaatgatagcgagcccagagaactgttgta
aacgtcagtacggctcatatcaacaagaagcgtctgcaacctgtaaagagccaaatacaaaaaaaaaatgccaattctcagattactggtggtt
gggtatctaatattcatgcaaagcatatttgcttttgcatctgataataagagtttgctggtaggtttcattctctgttgcatactgaggaaaattaga
ttgctaacgttgcatcaaatagacggaagatattatggcttcgcaaatctgcaatggaatgtacgccaaagatggaaagaaatcggaaattaaat
gtacgtttttgaagtggtggtttgcctggaatgcagtcgagaaggatttcaagtaaactgggttaattggcttgttttacagtaatagagatattgg
ataaatcataagctatcaaatcttgctattctggactttaaaatgtcaaatatgacttactttatgcataaccatcatgcaacatcagtcttcaatcg
ctttcaactaactttttttcttctagtaaatgtcgatgaacttaagtcaaactttgatggtgcgattcgcttgttaattttcaattggaaagaagttgatt
cagttggtttagttggtgaagaccagcaacgtcattatatctgtgattatcctgaaatagaagctgatttttgcagtgaagaacaatatgggctcta
tctggtggatgaaaataaacctcacaaatctgttttctcggatttcataaatgcgaaaacattggattcaccttttgtctaccctgtggatgagactg
gattatattgtgtcttcactgcaccattggagggtgccaaagaaatgtatactatcagtgttacttgggagaactattttggtgagcttgacgctact
gaatatccccatttgtttttgaatcccgccttgcttattttgaacgttattgtttccatttggtggtcattaattatgtttcgctacagacatgatcttctg
caagttcaaaagtacatttcaggcgtcgtcgttctttctttggtatgtggtttcatttcttctggatactttttctatgccaacgcaaatggttactcaa
cagcaagtaaagtatttgccttctttttgtctatctttcaggctgcccgtcaatcctatttcggttttctcgttctaatcgtctccttgggttattcaatcg
ttgtcccaacccttggcagcctccttcgtaagtgccaaatccttggagctctacagtttgttgctctgagctttttccttacttctttatttgtgagcccc
aataaccaagaatctctcgtcttaatgtttgctgctcctatctttttattcaccattttcgccatgtttttttggatcgttttggctttgaataatactatt
caagaccttcgtttgcgtaaacaaaccgtgaaagctcaaatgtatactcgtttatgggtcgttatttgcttcggtataattgcctatggagcaattgt
cgttactaatgctatcttaattggaattaatggtcaaatgaactactacttgaaatactggaagttactttggttcttagattacggatatgctgatat
tctttctttcattttgatggtcaccattttgtatatatggaggcctaccgagaataaccgtcgttttgctatgagcgaacaagttgctcaagatgttga
tgaatttgaaatgacatcctcagtttcccatgaaagtctgcccatttatcaagagcagccaggttcggaagcccagcatggtacacgaagtggaa
atgctgatgaacatcaagctctctttgctgtggacgatgaaagtgatgatgatgctccgtatcgtgtgaacgaaagcaagcaaaagcctacttaa
atgtgcactccgtttcgataccttgaacgtcggcgagtctgtatttaatttggaaattagatctttttttcgacttaatcatgaaattctttagatgtcttt
atattttttctttttttagttagttacaataaattaaagaatactattaatgatgtaaagaaacagagttatatgattcttgtcccaaaagccctccat
ctctttgacaaaaaagatgttgattttgagttagtgttaattaaaggcatatactccataacgaaaatatcaaaccatggagtttcatcaatttcttt
gggaatttgatcacttgaaacgcccttggacaaaagtttggttacccgttcttccaaatttccccaaactaaaaatagtctttcccgaagtctaagt
aaaacttgcttttcaacttttaaatcgcacggatatatagtagaggtattgaagagagcttgagcatcttcactataaaagataaccgggagtgtt
gtttttgatgcatctctcacaagaaagacaaacatccatttgtactctgaagacatagaatccgatgtagtaacgcaaaaactggttaaatctcta
ggccagaaatcgacaatttgtaccctgagtcgatgttttcgaggcgatgtaatagagttcagaggagaatgcagaatagtcgatatggtagtaat
tggaatttctattgaatcattaaaaacatgagcagttatattagaggtcttagggtagttaatttcatgagagatgaaaggattaacttgatcgtca
acagaattggcagcgacttcggggacttcttggtttttttacataatctcttttccttctctttaaactctccaattcaggaacattaggatcaacctttt
caatgctgcttttatatttttggccaggatcgccatgcataacacattccagttgaccaattcgatcaagcttagctcgagcatttttttatgaggaca
tagtctccttcctgaacatagttacgacaatagaaatcatgctcatcccaaagaatacaccgtacagaataacgaccctgaggacctttccatcgc
atatgaggagcagtttcgcgagttacgggatagaaaaattcattttcggaaaaatctgtgacataaacagtaaagtttttttctgtataccaagttt
taatgacttgggcatagaaagtgaaccgctgggaagggcgaattttggagagagtgacgaccgagttttcacggtctttcaaagccccccattca
ttatgatgatgcacttcaatggcaggagtttggctttctacagtatggtgctcgaattgtttttgccaacagatgcctagtcttttcgcaaactcaact
tcctcggcccttacatgaaataaacgaaaggtattactaattgagctttccaaatttctagtccaaagttgaaaggtaaagtcagtcttagatagcc
cttgcacgctgtctctataaggccgaattgttatatgatgcagcagaagaatctgcccttcttctgtaatttctggaagatcgttttctttttttactgaa
caaatgtatacgtagaccttcagtacgaggataacaactagggtcccaaagctctattgtcactacccagtctaaagacgattttgggttagctttt
taatagcaaaacaaaatgaaaagacttaaagtaagcgaccataatcccttccaccatatccttctagatccttatatgaacctttgagcctaaaaa
acttttgctttccttggatttacttgtcttttcctctgttcatactcaccacatgttcctctaagactttgtttcttaggggtaaatgttttaacaatgcca
aataaatttgtcacaaacctttgggacttaacctcgtgacatattctgacagatcggtattgaaagttctaacatttattagcaaatcccttcatcca
tgagaccttcaatgaaatgaacttacgttgatttgaaattcaccttggtttaaatacaagtcttcaacatcatgttgtatagaggatacctccatcaa
ctttgtcgattaagtatctacaggagtttcgaatggtgcttggatagttgaacaagatgcgttttctaaggacgtaaaaaagccttcaagtttacaa
taaaaacacagaaaaaatcatccagttcacaggtaagaagaagaaattattgaatagatttaagaacgaaagtgtaaaataattaaaaggaat
aaatggacggagctcttcctattgttttatgatggtctcatataatttgtgccatctgaggtacctaaaagagtttaattaaaaaaacaaaacacat
ttcttttgttttcagttaatcgtaaatgtcaagccgcttactgagaactgttatgtaaaggatcccagtgacgaaaggtgcaagagagaagtgaaa
gggactaattcattaatcagtcttattcgttgagaagacgaaatattcttacttgcttcagcagaactataattcgtcaactgccattcgaatttcatt

taaaatcatatgcaacaagctaacaaaggaaccatttatagggaagcgtttaaacaaacctacaaacttcttctttcaaaagaattaaattcaaa
ttactgcttaaatttattcaattgagctaccttattcgtctgagacattctttctatcgtgtatggtagcgctaaattttttggagcacgaccaaagct
tcaacttagttccttgaatttgagcaaatgctttgtgaaaacaaccttgtttttcaggttgtactatttgatctttgctgtcgaatggtcgtaactataa
caagaagttatctttagagtcctcatttgatacaaagttttctactctattttgagcttttattcttctactgtgctctcttgaaatgaagtaaggttggc
atcttttctaataggaaattagcgttgtcaacttaaacctatttacaagacttcagcttcacaatagtattcaaagttccctaaagggaatggatcct
gcaaaccagttctgaaattggatacaaaagcttcaaaaaggagtttttacttcatttttgctttgtactttgccgctattaacgttacagtaacaac
cagccaaaggaaattgacttgtctcaaataaatcgagcgaactggaattacttattttcataaattttaagcgtggagcatgtcatcaaaattcca
aataaatctattaaacaaaacagataatatatcttttagctgtataacatcactctacgattctttctttatgactgtgaaggaatgcacacataaa
agaacgggtcttaaaaaagaactttaagagttaaaagaacaaagaatgaaaaagaaatgattagagaaaataaataaataacacaagagttc
aggcaccaattatatactgatataaatatcctccaattaactgtgcataacttcgaagtccgaatatcgtcaataccaacagagcaggaagccaa
aaagaaaaaagcattaaatccagtccagctttctttagcgtttttgcttgctcttgaatggcttttgtttcttccggagtcaatgcgctcgtctttccac
gaccaacgaggcgggattgccatttgagtgaccaccaagcgcttttgttatgttcagcactttttgaggaatcatcagaaatcacttgtggtacatg
tccctcgtcggctacttcagaataaggaggaggtacatcatcaggaatagcagagatgcttcgcaaggtagaaaccatagctttctgcataagta
acggtacttcactcatatagttctggggtaattcctgtaaagaaggaaataaggttgaagaccgtgaccataatttaccttttatttcttcaacagtg
cttgattgtttcaatgttataccattttccgtatcagccttttcatcaagcaaagtaaagctttccttttctttctccgcaactaaactcttaaactttgt
actagctaaatcattaggacatttgtcgttggcaccggctaagtccggcaatgcacctttcgaaagtaaacgtaaatagatatccaaatgttgata
gagtgcagcataatgcaaaggtgtgtaacctaacgaatcccttttatttacatcagctccagcgttaataagaaatgttgtagcagaagcaagac
cagcggccacggaatagtgtaataatgtacggccagactcattcaccacgtcaaaatctgtaggaaacgttgtctttgtctcctctacaatagata
aagcggccaatacagagttttcaagactgttcgggaatggaacattatgttcttttgcaacttgagataccatgcttttcaaagactcaatcctttcg
ctcataaaatcatggctagtttcacagtcttggttagaggttggtttaatagagtttgtaatatactgagcaaaatcatctctccaagaacttagtag
tttttagaaagcgttaaaggattctgaatagctcccgtcagcttcaaaccaaggacttgtaccgtcaacttaaaaagttcattatcgagattatcct
cgtacgtaaatgtaaccgttgaactgcccgagtccataggatagttcacaaattttacgggaaccgtctcggccatcgttgcaggaggacatgttg
ctataattgttgaatcactccatgagaaagtgacagcggctggcgtctctccaaataagcatactaatccattaaagaaatttgctcctaaaatag
taacttcatagccacccataatggatcccctatttggtataacacgagaaatgagaggttcccttgactgaggagggcttggtgtggcagaggctg
aactaatcacgttttgcttattaaattcgggtgttctaagaattggctgacagcgttcttcatttgacatataaggacctattttagcgtcatcgttttc
caaaaatgcatttggtaattgagtgtatggagataattcatgaggaatgtgtctcgtatccattttcatggcttcgttgttgattgtatcaaggtgaa
cttgatggttattagtagaaggacttgccggataatcattggacacatgaagcgttgggatattaggaacaacattcgttataccatcctcctccag
aatattagaaacttcagagaccatatcggacttagcccattgctgaggttgaagataggaatttatctcagacaatgtctttcttcgcttttcggtag
gtctgtttactgaatcaactgaagccggtattaagttttgtgttaccttctgttttgtttgatgattggtagaattcatttttttcccttgtttgtccacttt
aggtgcatttcgagttttgtgatcatcaagtacggttatgggctcaggaaaggaaagatgagctactaaatttgaggaacaatacaaatcccatg
ttaaacgaaaatagggacaagactgatgcgatgagtagcaagtaattctagcattaatatgtgcaatttttccatattcatttggctcaagcggaa
caaactcaggaccagtaaaaaccataatcatttttcgttcatcgatatcttccaatttagggaattgattaagaagtttaatttgaaagttatgcttt
tcttctggtgtagcatctgccatggtacgctcgcaagcttttagtttagtgtagttaggcatacaagcatctttagttgcttgagaacgagcatttcgc
ttcctttcacggagtaagcagcgaatacagcatgtatgaacaacattgttagtatcatcaccagccatcacagtcggtattaactgcaagtactcg
cgagggtcagcgtaggttactgatttctttgaagatcttttcttctcttcacgatgggcccaattaggtaaacggatatatttaaaaggtacggcacc
atcattagcacttcgaatttgtatttgaaatagcatatttgtctccactcttgacctccatttttgttcgttaacagcaggtacgtgattaacagatata
atataatcttgaagccccgggaatggagcagatgatatagggaaagattcgccttcagaatccaaaagaggtaactcagcggacaaattttcatt
cgcaaaagttggattacttagatatgaagggtttattactgcagaaggaaaatcggtagctggcaaattagggaactgcttgatggcgtctccatt
tgagtataattcagtagtaccatcaagatggtcattcgcaacttgcaagttcgtcactggaggaaaaacggagggattggaaaactgagagttgc
tgataaggaaagggtaagccgtatctatgtgaggctgcatgtactctgccgaacgatcttgagattcttcactgtcatcttgagaatttgacaggtt
tggtgaagaatcaatcgagagagaaggcggaacttccaacaattttgttgaagattcttgtggtaactcgtcaaagttgaagaattctcgcatggc
ggcatctccgttttctgtctcatattcaggagttgaaaaagtaaattcatcaaaagaagatctacttccatcagaactcatcttcgtattcttctcag
gttcccatgtttcctcattcttctctatagacccttgcttcttcatgaataattcttcaattggaaggccgacattcatgttttaaaagccctataaagg
aagacttgttcaccttaaaggtaacttatcaaataatattatataaatctagtagcaattcgacctatatcgtacagttttagttttagtaaacttgtt
ggttaacgagtggttgggatgtcatgggtatccaacaatagcagtataaatgcaaattcccagactaagaattgcataaatatacacatgaatgt
ttttattgtattttttttcgtcgcctacttttattattgtcacaattggttgaaggatttcaccgttcttaatggacgcggtttgctgagctttcatctgaaa
agcagacaacattcatttatttacatctaaacttcattaaatcattatatcattagaaagtttaaaaaaaaaaggaaaatcgtagaactgtatcgtt
atcagtgtttgatttggtggagcatgcgagttcctaagagttgtaattcaagcatccttttctaagactaactgaaaacatggatgcgagccaaag

cgtcctttactctttccctgacatcaggagatggatggttctgtagcaaaaacaagcaatcttcaaatcctagttcgcgtaaaattttggctccctgg
atatcctcttcagtaggagttgatccttggtttgaccataataaacttatgcaaagccatattcctgctaaaaggattcccgaatttaattctaacgt
gtcttcttctaatacatcaaacgcaggacgcatctccaaatcaaactcatcatcctgaagttccatttcgctatcacctgccgagctggaattatctg
acacatgttcaattcgataacgttcagcttccttgagcataatatcttttacgagcaaaagcatgtttttatcttctaaaataatatctcgaaattcat
cgtcactagaagcaacatgcaccaaagtgaaaattccaggttccatcaaagtcgggttctgtgtcaagagtttctgataaaggatattcaataaa
actggtaacgatacaacttcaagaaaatcataaacaccttcacccttgggacacgcaaaatttcgaaagacttggagaatttgttcctgaacacc
gagatcttcatcatcacaaagtttcagaactctatcgcgtccaagtttttgaataataccctttttcagtttctcatcattctcataaacgctatttctc
aatgcccaaacagctttctttcgcaaggacttgtctttggcagtagtatttgctaccagaacgtcaataaattcaggcgttaagaatttatgacgaa
gtggagagaattttaatagtaaattacagactacggccatcgctgatgattgtactctcacgtccgaatcaatcaaaagtttcatcaatggctcatt
aagatccgcctctgccaatcccgtccttagaatatatacgctccttgttaggcttagtatgcattcgcaagcagcttttcttatatttttttgattcgtta
gacaaggcatcaactataataggcaaatagttggcatccacaaccatcttcctatattcgtctttggaagttgtcagggcagcaatgaataaaag
aagagactttaataacagttagaaacaggattagaaataaacaacatacctccattataagatcattttgttcttttctattagttggaagccttaa
agcatcagttaaacaactgacctccgttacgggaaattcctcattactatatttcacgactttattcagtagttccttagaaaatgttataacattcg
cattagctgcaaccttctgcattgcttcgttttcatttaccaacattgccaacagacgaggtgctatctgttgagtttcttgggattgagagaaaagt
cgaattagaatggggataactataagcttaacttcttcttccgaactctttccgagaatgccgcttttatagagagtcactatacaagagagggcc
aacaatcgaattggagaatgatattcccgagctaagctaaacagctgacaaacttgttcgctcatgttgtctccaaacaatacaagccttactttt
tcaagttttccagtttttcaaaatcatcttcctctccaaactcattaacttgaggtaggttcgtacgccaggagaacgctttgaagtatgaatttaat
atagcgagtgaattaacaatcaaagtatcacaacttttaaaaaatcggtacctgactgattctttcagttttgatgtatgagcgatagtagagaat
acggtattacagaaggaaggtaccgaatccacctgtgggtttttcgatgaaaccaaatagtccagaactgaagcagctaaagaagagattcgga
tgtcatcaaacataggatatttcttctgtgatggcaaacggtataagtcgaacaggcgattaaagagacttgagaaatgcatttgagtcacgaact
cggcagcttgatcaaagttaagcatgatcgtaaataccttcaaggttataataagcatgtttttggaacattatcttttactgaaagtaactcaata
acagaaagaaggtcctcagggcgcaaaaaatggacaaaagctttttatcgttcgccatgagcgaatttagtacaataaacacctcatatttttct
tgtaaagaagcctctttagaagaaaataaatttaacaaaagctttggggcctcagctttaattgctaactccttcctgtcattccttcctataaccca
gtttttgaatggaataatatttttcagtagttcagactggtttgagaagatcaaagaaagtttatcttcattaaaggattcgatgttctcttccatgtt
ggtaattccctttgatgtagagtaccgtaatgtttatagcttttaaagccagccgacaattgcaaattaagatcgatcgtaactatttttggtctaatt
aattacaaactcagagaagaaagtatctttagcgtcgttgagaagttggggttttggatttgagcaacactaatgaaaggactgatgaaaactag
ttgaaggatcatttctaaaatactgaatcaagtccaatcaattgaagaaattgtaatataaactttcatcaataataagataattaattttattaaa
tatcttgatgtcaaagcaggaactctttaaggaaatttatattacaaacttcaaaatcagatacaaaagttttaaattcagtaatttaaacgttatg
ttttattgaattaaaattctacaaatggaagaattcaaatttcattaagaattagttttggggatataagggagtaggcgcaattctcctttttttggg
gtcgagaaggttcattatctttaacattatagtctttagacaatttgagttccttattaggttcagctggagtaggaagagttacaaccggtgttgct
ctcttcgtcggaatagcaacctgctgctcgtaaagattgcccaactcttcaggtgtgaatttaatgctagaacaaaacccgtcaatggaagtcatc
aagaggatagtaccatcgtcgctccaggccaagtcagtcagtgtagagtaatgaaggtttgcagctctataaaacggtttgcaggattgggtatc
ataaatataaacggcatcctgacaagcaattgcaaaaatcatcctataagggatttcaatgcaagtcgttggtggactgtgctttgaagcttgtctt
agctcatacaatataggagaaaaacgcacagcaatcgctggttttgagaacccggttagagaagcaactggttgtctggaaatacttgatctggt
aaatatgtacgcggtatgcatcgattctgcatttggctgaccgtgtaatcgaagtttgccagctggagtaattaacagcaagccatctggagaaaa
atctggacgacggaaaaatgaaactagggtttcattacagtacagagaataagtaattttagacgcattttctgtgccaccctcagatacaacatc
agagtctaggtttatttgagaatcggaagtcaaaagattagcatcattgcaggaattattttcttgtaattttttttcttcaacgctgctgatttgttcc
gtcgggatagaagacggctttgaattttgagcattcgtgtactctatgcgatgaatacgtgatttgagatttaactgggggaatgaggagtctctgt
tttgtttttcaaatttgtaaacgcataatgagcgatcgctgctttgagtgactacatatttgttcaaaggatcccaagctacgccctgaacatagtga
aggtgatcaaatttttgaaaaagcaattgccctataaataattagtaaatcaaagctattttgaagtatttatattacctgtttgcgtgttgtatatcc
gaatactattgtccattgcaccagcaactatgtaactttcatcttgagaccagcaaatatcataaacctctgcacccatagaacgacatacggtttt
caccttccagtattctttcgcaagcgctagctcttcagcatcatctgccaatgtagctgctggggaggaagagggaacccaaagaaagatagtac
cttcgtctccggcggtagccaatacatcccctaagttattagtacaactaaacaatttcagtaatgtctgataaagttaccttgcgaactaaaccga
acaacatttacggcttgtgcatgtcgactaagcgtagaaagatattcaattttcggagtcaaattctcatcatcacatgtatatttccaaacctatca
tgttagtacatttaactttaaaagaatgttctgtcctgactaacttcctccttctcatcatccattcatacccttattttaccatcacctccacatgtagc
aaatctgtttttttccattttttgaaaatcgacggaataaattggcgtatggtcgtcgttagcatcgtaatgccatcttatttgtaaaacttctgttttc
attctttttcctaaataagggttcctcgattcaaataattacttgtgtttaattggaatctttcctttaaattttcacaaagttgtttgcgaatagctttc
gaagaaaccctgaaaatttacaatcctagttgtaaaaagtgctcttcatgaaacaggatttctattcaagtaaaaaggacaacagagccttgtag

ttattagaaccgtaactttatatattctaactcttaaaaaaggaaagcagcacgaagatggttagtctgcacttttaatttcacttgttgtaggtctc
aagcttagagcaatttttactatcaattaagacaatacgatatgtcacacgatcagtcgttcaacggaatatgaggatcgtagcgatttataacgt
tttgtgaagcatactaatcttgattaccctagtctgtaattgttctgttcttgggtctaatgcacattaacactcaacgacgttgtaagagaacgagtt
aagatgatatatactattttaaagggctaaaacaagccgtttggtaaatactgggattaataaagactgtacaaaatcactttgatcgttatcttgc
acctgccaagtatttcttgaattccatattcagcatcacacttcaaactctcacctgaatagttcttaggactaatacgctttttttcggagatttgttt
aagccgtaaaagtaatttttttgaatttctgaacttggaacatcgaaaattttttttaagaaacgcaatttccgtatttgacgcgtccaggattcatta
tcgcgtttatattgtttcaatatgctgaaaaaagaggaaggacaaaagtgtaactgtccaacatgcggtaaagaagttgatttagtagacataaa
tgctcatttagattcgcactttgaagtacctgcaacatcatccccttcaaaacaagtttctcccttttttcagaaagaaaaaaatagccctttaaaac
gaaacactccttcttcttttccttctcagggatctttcactccttcatctacaaaaaagccgagagcttctaaaagagacgtacgacctctcgctgaa
cgagcaagaccgactaccttagatgattatgttggccaggaggaattggttggtcctcgtggggttattcgaaatatgatagaacaggacgaatg
tccatctatgattttatggggaaacagtggaacaggaaagactaccttggccagaatcattgcggctactacaaaatcgaggtttttggaagtttc
agctacttcaatttcggtacaagagtgtcgaaagatatttgaagaatctcagaatcatcttcggttaactggaagcaagactatagtatttctagac
gaagtccatcgcttcaatagagctcagcaagatatattttgtatgaaatttactttatttacgaaaccttccttactaatatttacatagtgcctttgg
tagagaaaggttctatcacattgtaagttacaaactgttatgtacgtttctaactatttcaggatcggtgctactacggagtatgtattattatttttg
ctggaaaaactttattaatttgtaggaacccttcttttcgtcttaactccgccctcatttctcgttgtcctgtatttgtcttaaaaaaattggacaaaac
tcacgttttaaaaattttggaacatggttgccaattagaaaaagatcgactcggtgacataccgaatatcgactcttccatattggaatatgtttcg
gctatcactgatggagatgctagaatggctttaaattgtttggaaatggctataggattaatgcgtcaaggtcctattactttggaagaaattaagg
aaaagttggtacgctcttcagctgtgtatgatcgagtcggcgatgcacactacgataccatttccgcttttcacaaatctgtacggggttgtgacgtt
aatgcatctctgtactatctaggacgtatgttagaatctggtgaagatccattgtacgtcgctcgtagaatggtgcgcattgctagtgaagatatag
gagttgcagataattctatgcttcctttggcttcaagtgcatttacgtctgtgcagcaggtcggaatgccagaagcggatgtcatattagcccattgc
gcagttgctttaacgatggctccaaagtcggtggatgtttacaaagggtataataatgttaaaagatttctatctttgaatcccaacgcaggcagg
gctgaaattcctttgcatatacgaaacgcacccactaatttgatgaaacagcttggatatcataaaggatacaagtacaatccagactataaaga
tggtcttgttaaacaagaagtaagtaataattttgtagatagtcactaacgctcgtagtatatgcccgaatctattcgagatgccaaatttttcaga
ctcccagctgagctattggaagatgaagatttagaaaaatgataaattaatgaaaaatattattatttgataaatgaaatacataatgatttacaa
attgtttgcctcttctaacttgcaccgtttcttgaatttccctcttgaactaaactggctcattgccctagatatggtagatacgcccgttaagcagga
aaatgatgatttatgtttacatgaaataaaaaatgaatcgggtgtaagtttttcaaagcctcagaaaaccaggaaaccttcgaaaaaacgtagcc
atgaagttgaactggagtcgacgagactttctccagccttacagactttattcggtgtatttaagatcattaatacaacatatacttttctttattcaa
gaaattgtttaactacaacgttttccttgttaaactctagcgtacgccagtctttaaaaagagatctctcagtcttggagttatcccagcttaaaata
atcgtaccagacctaattgagttagattacaagtcattcgacagcctggctatggaaatgaacaaaaacgtttacgcagatatacctaaacaaat
atactctgatgaccaatcctttcaaggatcgagttacgtcctaacagtttgtttaaatcaaacggttgaatcttctattaaacgtcaaaaaagggag
ggtcctactatgaaatcacggattcagaaagggaagtttgatataaacgcattaaagaaagaaattgacagtcgcaatacaatttttttaaaaca
tttacaggaatttataaattcttgtggtgctaaaagcttagatccggataagcatttgattcaagaagctgaaaaaacgataccttctcctcctgat
gttatagttaatcctccttcgaagataggtatggaccatgagcaaaaggctccatcttctattgaagatgtcctagtgagcttgaccgaggaatcta
cttatgaaggtcaaatagttcaagaaagtctttatcaataccctgccgttgaaccacgatttgggactttgaagcgtaatctttctcaaagtttggta
aatgccttatttagctctagaaatattgaaaggatgtatactcaccaagctgatgccataaatgctctttgggagaatcttcatgtcatcgtttcaac
tggtacatccagtggaaagtctttgatttaccaaatacctattttggatgaactcgaaaaagatatagaatcttctgctttttttgtttttcctacaaa
ggctcttgcacaagaccagaaacgatctttaatggacgttttaagctttatgccttcgctggctcatatccggattgatacgtttgatggggataca
ccaattgaagcaaggaatgaaattatagaaaacgcgagtatcatattcacaaatcctgatatgttacatcagactattttaccgaatcatcatcga
tggaatcgctttttttaaaaatttaaagctttttgtattagatgaagcacatatttataatggtatttttggtgttcatgtcgccatgatcatgagaagg
ttacgaaggatagcagaatactatggcaatgatcggtatagatttgtttcttgttcagctacaattgaacaacctgcacggcatatgaacatgattt
ttggagttgaaaacgttaaactggtgaattacacaacttcgccttccggtgctaagcatttcttgatgtggaatcctcctttagtggataaaaagaa
catacaatcaggcagaaagccggcaatcgcagaggcagcaaatttacttatttacttggccgagaggaaagttcgtacaattgtttttttgtcgtat
tcgaaaagcttgcgaatcactcatgagattggtaagaggacagctcaaagcgtctcataaagagtatttattggagaaaattcaaagctataggg
ctgggtatacggttgaagagagaaggaaaatagaaaaggaaatgttttgtggtaatctgtatgggattgttgcaacgaacgccctcgaattgggt
attgacataggttccttagatgccgccattaccgttggtttcccgtgttctttgtctaatctacgacaacagtttggaagagctggacgacgaaata
aatcctcattagcgatctacgttgttgaaacattccctgtagaccaatattatttacaacaccctatgttgatacacacacaacctaacgcagagtt
gacattggaccttgacaatgaggttctgattgcaagtcatctacagtgtgcagccttcgagttgccaattaatgagatgagcgacagccgatttttt
gagggcaatttatccgaagcatgctctactcatctggtaaaaaaagaaaataatttgtttttcctcacccaaaatgcctaccgtttcctgcagtcc

aagtacaaatacgcaacatcaatgaagacatgtatactttggtggacgtaaccaataataggaatgttgttttagagcaattagagccattccga
gttatgcttagtgcctatgagggagctgtttatgtatatcaaggaaagacattcattgtgaaaactttgaatattcctagaagattaataacttgta
acagagtagatgtagaatggtctactctgcaaagagattatactgacattgatcctcaacgaaatctctcgagaaagaccctacaatccgctaac
ataaatgtgtttcacggaactgtctcggtggcattacatgtgtttggctactttaaagtaaataaacaacgggatatactagatgttgtcgatattgt
ggataatcctgttgaaatgattagccgcgggttttggattgatgttccatggcacattgtggaaatcttatcgtggaaaaaaatcaacggtgcggc
aagtatacatgctgctcagcatgctttgatgtctttaatgccgatattcgctgcaaacggcgggaatgacatacgaacggaatgcaagtcaggcg
aaaaagaatttaagcaagaagctagtgagcgaagaaggccatctcgattgatattctacgacaattgtggtgaatgcggtggggccgatttgtgc
aaaaaggcttacaaacatatcgacgaattgacgatcatggcaatagatcgcctagaacattgtggatgcaaaatacgtgaaggttgtccacaat
gtattacgagcatacaatttgaaggaggaatttgctctggggaagtattagacaaaggtaggttttataatacttcggtttactaatagttttctag
atggagcattaatacttttaaaaatgctagtcggtcagaatgtcaatcttgaccttttcccagatggccccgaaatagacgagtaccatgcactaa
ggacacttataccaacttaatactattactataattaatgtttcttacgtctattctatgaagtgattctaactatatgtcaaaaaattcgtgaagctt
ataacctaagttcacaaccctaccctataaccaaggatggcatccactccgtaccatacgttcctcttttaccaaatacgaagtaaagtatttcgta
taaagaagatcactctttgatctaatataaataaattaaaaaaaaaagcaatgagttttgctaatttgttacgaaattcgagagctgctactttgac
aaaatgttttcaggaatctagtccatctactacacatagtttgttccctttgcaacaatcattaacaacaaaaagagcagacggagctcgtggaga
ttggggttgtaaacgaaatttgccgcggataaggacaagatatatatccgtaaatgagttggatagtgtatacaaccaaatggaatttcagtcga
gtgctcgatttgttcgccttttacggaattggtttgatgctgggttcgtaagggatccggcagagcaggcaataacccaagagttggtaaaggaat
ccaaacccatccagtcaaattatcgcaatcgatttggtttacaaaatcagaggtcgaatgattccagtcctatagaaaattccaattttaggaggtt
taatgttgcggctggtatacagtacagtaatgtacctttaataacttctcacgttacacctaagtacgagtctaattgcggatatcatcgccgcaaa
ctaatcgcgcataccttaaattcagacgcttctaccgcacactacgggttaggaggtttgattcttaggttgcccaagactcgcgtcatgtctcgaa
atagaactttaccacttttctctaaggaataccgcatatcgtataccatgtacaaaaatgggcaacttcgtattgaaccttattcaggaccttttctt
ggaggacaaggagtctttgctatggaacctgatgagttgaactcctttttttcataaggatttgaaatcggtagccgaagaacctgttatgacaagt
agtattcaaaaagtaaggaaaaatccggcagtcaagttggacgttgaagacgaatttgatccatcacccaaaactccattgtaatgtcgctctctt
tcttctgtttgtcccaacttataaattatctgtcccctctttaatttcctttattttcgctgatctacatgttcggaacttttgttattacaaagtatctaat
gttttcctttctactttccagctttacttcctgaatttttgatcggttacggtgatgttccatttctctccggaaccttcatatgttatttattacttgtaaa
tggttttctgtttatgatatcaatatttttgattagcctttcactatccttcgtttctagtacaactaattttactgagtttactcatcacttcaatcatga
gccagaactcaaaatgatcttaatatttttaagtgcaaaattcaaaggaattatcaatactaatatctaagctattgtgtattaaataaaaatgtga
tagctaaaataatggctatatgaatacttaaaaagatatcattacaacagagctgtttatgtgaattagatacatacaacctgaagtttttctcaga
cttaacctaaatactgtcagtaatgtctatacatatatttgctaaaccaccattcttaaaatacaagtaaatagcatgtataatcagataccatata
taacatatactattataagtcggcgaacggaagaataaaaaatgcataaaatgaacgtaaatcatccaaaaataaacgcttaaaagtaattata
aagactgcttttggactagttccagcctaaccatgaacgccgcattgtacgccaaaccgttatttcaccactcttcaaatcaccaagtttgtaacta
actaaagaaaccttttggattcgcgtccttgttctaaagcgcgaattttcatctttgaaacttcccattctgcaacgataccgacgtggaatggatt
accaacgtcaaaagcatatttgtgacggagtgtgcctttttgaccactcaacctaacattatcaaaagtgattatatcacctggtctaatttcgccat
tttgttggatcgtaacatttgagtcattggcgtacacaacggcaccaaaagtacgcttattaattggctcaatgttttgtgtaagattaccaagtata
gaattaacaaaggtatatgcgctaccatctccaattgtaagcccttgagaagctttggcttgtctcaatatggtgttaccataacattcaaattcttg
ggcaagtcgctccttagacggctgaactggaggtgccagttgtaattgcgaaagctgagttacataatgagtagcaggattaaaagtagaagtc
aggactgtctgactataatcatgaaacaatatgtgtatagacttaaaatgcttgtttggacctgtcgtgccttccctaatcataaataaaacagagt
cattcaatttcaagacggaattaggcaccgtactagagtcaagccaccatttctgttcagcacaaggatcaacaatcttagctacataagcgtcat
taaatgggccattcgtggcgacgatagaaggtcgtttaacagaagcagtggatctaattgatttttgtgatggtctctgactgagaagaggagggt
tcatgtcaggggcttgccgtatagaaatagatgaaatagatttctgcgagacagaatcatttcgttgtggcatagtagtgggacttacttcctgttt
gatgggatcaggagattgtggcacactttcactcgaactactccttacatcttgagaaaaaggagaaacaggtataggaggtacatccgtcgtgg
gagggggcactggaggtacagctgcagatggggcaggtggaacctgatgacttaagggtggaggaactggaggacttgaagttggtgctggcg
ggggcactggttctttcgatatttccgatttgctcacagaaacaggaggtggaggtggaatagatggaggtgctactggtcgcggacctgatgctt
tcctcccactagaagatggacgaacaagtctagtaggtaaacgcatactgtttcctaactgtgtaatatcctgggtcagggaattattctgattagt
ttcagaagccggtaccgcagaagaattggtggaatcataatggaattgttcagaatgagtgttgggtatcggaggaggagaaattgaaggggct
tttggttcagtatttacaggggccaccggaattggaggtggagatgccacaggcacacttggagttggtgctataatacttgtcctacgctcgcttt
gagtaaaattcattgaatcattactctcctcgacaggttcatcagatactgggagcgcaggaagaggatgctcagtaagagaatcaggagtatca
ggttcatcaggttcatcagatagaacaggtcgaccacccgaagtagatattgaaacaggatttcgcagcgaagaaccggtagacggcttggatt
gatggcctggaataaatttcgttttcagctgtctcttcggttgaatgaactcactatcaccaaattctccagaaggagttttgggtaaagggggagg

actaaatatacgaggaacagcaggtaaatcatctaaatcctcgttaaaatccgcaatagttttggttttgttgaaccttatggaaggaagtgatgg
acgacgcgaagaacgctgagagggagctacttcagcatcagaatcataagcctgggattctgagacttctgtgatacggctaggtaacttccgct
ctgcaaaatcattgttattgtaagaatccttccaatcgggggattgttcacgactattcaaggatcgaggaggagtaacaggtgaattcgcggcag
actctacgtcagcagccgtaccggccacggtctcctttggaggatcaatggagtcaataactttacgaacagcatgagcaggatcaactatagaa
gtgggagactggcctatagaagaactattggcttgaggaggcttaaggcgtgtgaaaaggtgagtctgcgaaagaggcaaaacttcttctacct
gagaatccttttgattactaaagtcagtgataattggaggaggagacgtttgttccttttcctcaggccaaccagactcttcagttccaggaatatg
catcgctaaaccaggaagttccatacttggggaagctgaaagagaagtattagatgcctcatcgtcggcatttggttccactgtttcttctatgttc
gtagaaggttcaggggaagcttgcatagtcacggaagacattcccgagggagcagcagagtctcgtggtttgctgggattcgtctcgggttccgc
gtgatcattgataggttgatattcagattcgggttcagaacccttttttaaagactttaaacgaggaggaggagccattccgggtaggccaaaagc
atgcataccaaggccaccactcatctttgccatacgttcacgaagagcgatgcgacgagaaagttctggatcaatgtcttcatgcttttcctcttcg
gacttttcctgttcttctaattttcagaagcttcagtaaaagaagaatcagaagcagcatcctccaaatcttgaggagcggcttccttagatatagt
agattcaatcatgggacctgttgaaggagcttcggaatgctcttttagagaatgatcccttgataaggaaagctttgactgaatttcttgagcgaca
gcagtagcagtatcagtagggattttgcgagcctccgcagcaggttcggactttatgttgtcgaaggatgcgggctccgtagaaggaggagcagg
gggtgcagtagagggagtttctgctgctgccgatgaagtaggatcagcagaactatggactacaggagcctgttcatcggaagaaaagtgagaa
ggaacgtcagtagcagggaacgatgaattggacattgagaaaaatgaacttgaaataagaacttataatatatagtaagccttctttaaaattcg
ctgaatttaacagcggtcaaaaggaatatgtatccacaccggtcgaagaaaatccagtgctttgggagatgtttcagatggaatagatgacggct
atgaatcagatgacgataatttccaagaggaatcgagcaattttggatgcgtaagaaataccgatgatcttgtttggattaatgaatgaccgtgat
aaataaaaaaaactaagaaaaaaaaaaaaccttgtacagaaataagaaagaagaaggaaaatgtaaatattgtgaaaaaggaagaagggca
gttattccctgtttacggtggtgttgttcaaaatgatggtagtaggtaggtgtaggagcagtggttgttgagggtcaccactcttaaggttgtaactc
aaagggcacctgaataagagtggcaggatgtaataaggtaattaggaggaatgaggtcacaaggccaagattcttgataaggtgaaaaagaa
gaatgcgaaccgagtctgcacttgtaaacaaagaacagaagagagtcaactagaagaggaattgagctgccaaagagagcggcaatagcaat
gaaaccgagatttgaaaacgaaacgtaaaataaataaataaaaagggaaaacaaaaaaatacagtggagaaagagaatgaaagtgatgctt
ttcttggttctacagcctttaaaacaagagtgaaaacaattagtcaatcgagatagttgatatatacaggtgttgttaattatgaattttcatcaaga
caagatagagagttaaaggcattttgtttattgctgctaaaaaacaaattctcaaaagatacaatggatcagtaaaggaattaaaactggatttg
gtttttttttttttttttntttttttttcttttttttttgcaaagaaaaagcttctttctatgggtatatattgaaagaggaaatgattttttttgtaaactta
gggcaaggtctcccatgcttgaactgttataataggagttattttgaattaagttctttcttctctaattcctttcaaaccttttgttttttttttttttcttt
tctgaaaataactttccatgctactatgtctattgattctaccattgtcatccaagatatacaacccatgaatgaatgaaattgacgataatttcata
agaattcatcacttgcctctttaattcacagctactttctgcttagtatgaaatagtgtttcttagtatcgaaataatatgatattatcgtacagtccta
attaaaagaagaaaatatgaattagaaacggactcgaaaacagcaatgttcacctttggtacttgtcgtccgatcagctcttaggtcaatttcctct
tctgtaggctttctttagtttgattttatttactaacgaactttaatgaagcaaaaccggcattcttaataaacatggaaaaagattcgaatcgtccc
gtgttttgtttaattacttacaaaaaaagaaaattccatgttgcatgtataatgtatgatgtttcctatcaaaataaaaaaggtctggaaactccatt
gaagtattgacttagaggaataggctcccacgcattgtaccttactgaaggaaactattgaaagggcattattggatgaaaatctctctacctatc
gttggtatgtgtaaaagctgttagtgtaaaatagtggaagatcaaagttgtgatattttattttacgatgtataacgtataggagtccgtattgaatt
tgaagagtgtggtttagaaattcttctgtatgccaaacgactgaggtcgcattcgatgtctgctaataaatcaattttgaagaaaaaggagagcgc
acaagtaaaaagagaagcccaattaaacgaatggtctaaggatcttttactagaagcgaaaagtcggttggaatacgacaactttgtattggaa
tgggttgaaaaactggtggatcccgtcgatgaacacacgttgaatgaagctcgtgaatatctgaaaaaatcagattatgatcaaattgtcaagga
acgaaagctcgctcatatatgcggctatcccatttgtaacaacgcgccccgacaacaaacgcgttttcgccttgaaaatcaaggaatggttcgtta
tgctggactgaattattattgttccaaggattgctttttgaaaagttgcgattttatggtgaatctgagtgacgagcctctttggattcgcgaacgag
cgcgacgaatcctgcaacatcgctttgatccgaaaaaagaagcaaactttcaagcacgacttgaatccgaagacgtggagcatgtacgcttgttg
ctgcaagggcttcctgtcggttccaaaggcgttgtcggtgacatcgtcgaacatcctccaaattcagcagaaaaaaatcaaaaaccaagttgatt
catttgaaaagaatttggaaattggtttattaaatgaaaacatttcgtcctgaacaaagcgcaattcctctttgaagtactttatgattccctattcc
ctacttgatcctcctccttactgcacaagtaccacactgcatggtgtgagaaaagcaaacaggcaattttcttggaactttgaaagagagtattatt
tgataatttatctaatagaaaaagtaattactggtgggaattacccaacttctatttgtgtggaggcctcttgcgtttatagataatatcatggcaga
aatcatacatttgctggaactaaaggtttccgagctacaaagggcagaaacttcgcaagaattaaagcttcattttcgtgaatttcggcgattatg
ggaatcagcccgtgtggaattcgaatactctgctgtggacttggatttgattctacgctatgaaaaatcagttcaggagtatatacgattaaataa
acggttcaaagataagattgcagctggaattccttggatttcgattgcgtctgagcggaatgctcttcctgaggttcctcaggaatcatcagaaaat
atcgaggttttgggagcgaatgaacctgagcttttgacgacagacgatatgcatttgacaagtgcaagccaagttactgctgcaatgagagatctt
catgtacaaatggttcaagcggtggacatgagcgctgaaaactctaatgaactcgcttcaagcaccagtttattggaaagcttgcaagaaaagta

ttttagtgtcgaagacgtattgtatgcaagcaaaaagattatgagatcgcttaagctgaacgatcaaaaagattattatcttgttatctcagggttc
ggctttttttatgtttgttttgttttatcttttatggaaaagaattatatggccaattttatctatgtttttctggtttttaaggtgatcaattttttttttcct
ttgctgatgatataatctcggggtacagtgtgcggacatttatgagctgactttgtattctgtttgataatattttatgaaagtgtattttaattcgttt
acggtttgaatggtattatgatttctcatacatagtaaaagtcgtactttgatggattgttttattgagaaatcagatgaaaacggaaattctgtata
agaaaaaagaaaaatctagtttattgaggatcataattaatcaagccagaaatggacaaccaaggttagtcttgatcaaaagtataaaatcata
aacagattgaatatcctttttgttttactgttttgaggtcgttttcatgataatgattcaaaacattgtaatcagtaaatacgaggctgactaatgacc
catagaacgaaatgcttttgaaatggtttttttacggatttgtttatttacgttagtaagcaagtcgcttttcctacaatacggaaagatggaccaaac
gaaagttattttaaataagtaaaaccctttttgttttcattgttttagtaaaacagtttgctattttctgtcgtttatcaaaagcgaaagcgtgttttttct
aagaatgattcactttttttcgtttaaaaaaaaaaaaaaaaccttgcatttgagaaaaaaaaccttttcctttatttaagaaaaaaataattaaagaag
aaactgtagtgatttctttcccatttacatacttttctttgttctattgaccattgttgtcatagtccaaaattattggtaatccttgttacttatcatacc
atgggaccaaaattggcctatatttaaactgtggtcagagccataatttcaaatacgacttaaatagatcgccttgaagaatactactaagaaaa
aaaataggtagacatcatgcccgaacaagttgattatatggaggatgcatctttcgctgccaatatgagcacatctgttttacatgcagacgatcc
tcttgcaatggaaaacgatgtagcaccggcactccatctgtccactacatatacatatccaggaacacctgaaactttgcagccgttccaaaaatt
ggcagaggaagactttccttactacgttcgtatttgcgcgaaaaattgcgaccttacagaggcggctctcagttccattttgaaagcccctagtgtt
ctgtatggatctgggttggctgcggtctattcgttaattgcttatttgaaccccaaaaacattgccgtccataagcctggctttggtggatactctggt
acggttgccattattcaacgtatcaatcgcttgacaggattaaaaacctcgtacattgatggaaagtgtgatgaaattcatgaaggcgacgtcatt
tggttggaaacacctatgaatcctttaggtattgtctttgacattcgctactacaaggagcttgccaaaaaaaaagggtgccatcttggtcgttgattc
tacattcgcgcctcctccaattcaagatgccttagctttgggtgctgattatgtggtccattccgtgaccaaatacttgggtggtcatacagatgtgtt
ggctggtgccacagcctcgtataacacagctgctattaaggctctcaaggcagatcgcgcttatatcggcacaatcctgcatccccagcaatgcta
tttgctcttacgctcgctccgtacattccctctgcgcatcgccaagcacagcgaaaatggatatctaatagctacccacttgaacaaacttgctacg
gattccgcctttgcttccaaacttggtatagatagcagcttgattttagaggtgtatcatgattccttgcagaaagaagactttgtttctcaaaacttg
acaggtggccattcatcatgcttcagcattctcgtaaagtcagataaagttgccaaacatgtctgctgtgaactgagatatttccatcatgctacat
ctctcggtggtgttgaaagcttggttgaatggcgtagaatgaccgatcagcacattgacccaagactcattcgtatatctattggtattgaggatgc
caatgatttgattcaagatttcaaccgagtcttcgcttctctcagctcctaaatatggtgcgaaatgcttatacaacagtcctttatgatccttcatag
atctttttttttaaaaaaaatttaacatatacagctaacattgattcaatatatacaatatagataaattctataacgatttcccattttgtttcgaaatc
ttgtacttcaaatttctacatctttacgtgtttcataattatttatttcttacttgcatcgtaaaggatccaactttgaattttcttccctctcaatcaaag
aacgttgttacttgtttgaaaactgtagtacccttcccggaaactaggtatacctgagcaaaagtgactttggtttaccacggattccttggagattt
tggatttggagcaaaatgccaactatcgcctgtgataaggaagcattatataaagcattgggaagaaattacagtatgtgaaaaactttgtggaa
taaaagccatctggaatcggaagctaacatataaaaccagcaacccaggaattcgatgaattatgctttaagtttggtattgagctagatgagga
tacaacaaatgacccagaaagagatccagcagagcgtcctgctttgaagattgagattgctgctaatcgctatgatatgctttgcatggagggca
ttgctcaagcactgaatgtctttaatcgccgtatggatcctccacaatataagttactccctgctactacttctcttaaaatttctcccgaaactgctg
atgttcgtccttattgtgcctcggccattttgcgtggtgtgaaatttgatcccatcagataccaaagtttcattgctcttcaagataagttgcactcta
acttgtgccgccaaagaagtttggtagccattggcactcatgatttgtccaaagtcgagggtcctttcacctatgaggccttgaaacctgaagaaa
tcaactttgttcctcttaatcaaactcgcagcatcaacggaactgagctcttggatttctataaggatagcaagcaccttagtcgctacttgcatttg
gttgctgactctcccagataccccgtcattttggattccaagcgtcgtatctgctctcttcctcccattattaacagcgattttagcaagattagtgttg
acactcgtgatgtattaattgaatgcactgctactgacaaaaccaagcttgacatcgtcatgaatatgatggctgttatgtttacttgctattgcgat
gaacctttcactattgagcctgtcgaagttgtttccgagcacaataactgctcccgcatgactcctaacttgaagcctgttcgtttcgaagctgagac
tgactatttgaaccaatgctgtggcatttctttgcctcgtgaggaagtttgcaaccttctttctcgtatgctgcttcaagctcagcctgatccttctgac
gaaaagatcattttggtcgacgtcccttgcacaagagctgatatccttcaccagtgcgatatcatggaagatcttggtatttcttatggttatgacaa
tctcaaacatacctatcctgaccaaagtgttacctatggtaagcctttggaagttaaccgtttatctgatatcgttcgccatgaagctgctcttgccg
gctggtccgaagtcttgcccttcattctttgctctcatgatgagaactatgcttggttacgaaagaccgacgactctcgtgccatccagctagccaat
ccaaagactttagagttccaagttgtccgttccagtcttttgcccggtattctcaagactactcgtgaaaatcgtactcatgccttgcccataaagat
tttcgaagtcagcgacgttgctttcatcgatgaaactcaagaacgcatgacaaggaacgagcgtcacttctgtgctatccaagctggtttgacttcc
ggtttcgaacagattcacggtttgttggatcgtgtgatgcttatgctcaatgagaagcgcatttttaatcccaaggattctgacagttccggttattg
gattgtaccagacgacgatgcaactttcttccctggtcgttgtgctgctgtatactatcgtaaagacccaggcaccgctggtgtgcgtgtgggtaca
tttggcgttattcatccccttgtactcgagaaatttgaactgtccattgcagcatctgctatcgaactggacttgacactttgggtttaattttgattaa
caacttaaaaatagaagcaagaaaattattatagtccttactgtgtgaaacaatcgttgtacctatagaaaatacatttgggttttatactaagcat
tatttatttgacgatagccaaaaaaaatgcttcactgtatggtaataaaagaaattgtctacggaatgattatttgagcaagacttacaagagaaag

cagcttgtataagacatgctaaacaccagcaattaagtatgtacaatacaaacaataacaaaaaagggacttggtgctcatatttcagtttcctac
gtagctaatagggcaaaaataggacaagtaaagttgcttatttagtatccataggaagcaagtttttcgttgatagaagaagcagatttggcagc
agtaaccttcttttgcttggagaccttggcttggtagaaagcagcactcttgacctttctgcgttcctccaacttagagacaacgtcggcatacttcc
agccaacctcagaagaaaggcgaccgacagtgcagtacttacgaccgggcttaaggcgtaagacacgaagggcagcagggacgacaacacg
cttttgcttgtcaaaaggaggaggaacaccctcaacagcttgcaagtgttccaaagcagcttgaccacgggggctcttgtgaggaagcataccac
ggacagccttttggaagatgcgagagggagcacggaagtggaaggcaccacgagtagggttgtaacgacaagacttgcgcaagtaagcaaga
tacttaagtttgttacggaagaagctaccggagatgttcaactcttcacagcgaacaacgacaaccttttgaccgcccaaaagctgcttggcgact
acagatgccaaacgtccaaggagatgactaaatttcttgtcagtataatcctctcattttctataatctcaatcaattcttaccccttagcgtcaatc
acaacgaccttttggaattccgacattgtgtgctggatgaattgttgaactttgcagaattgtgcacagtattattgtgactgctttttaggcgaac
atctagaataatgcatggcaacgatgcacacagtaaacaatttacatagtttaaaaaaaagctcagtattgtaacaagactatcgaatctttgttt
ttgcacgtcatgttgtttattgctggaaaatacttcaccttgaagtttcaaacgatttcattctcatttaatcaatcccataaagttctacgttctcaat
accaaaccatctagcttttaaattacagcatagatatctatgtaacagtatcacttacctctgtcgttttctactttttctgaaactactacagaccaa
ctcaatataattcatcaaatcgcttttaattcacaaaacctatggaataagaatctacttcttaccaccagccttttgggcagccttggtaaccttag
cttgaccaggggcagtcttctcgacagccttgatgacaccgacagcgacggtttgacgcatgtcacggacggcgaaacgaccaagaggagcgta
gtcagtgaaagcttcaacgcacataggcttagtaggaaccatttgagcgatgcaagcatcaccagacttgacgaacttgggggattcttcaagct
tcttaccggtacgacggtcaatcttctcaatgagctcattgaagcggcaagcaatgtgagaagtgtggcagtccaaaacgggagagtaaccagc
agaaatttgaccggggtggttaagaatgatgacttgagcggtgaagttagcacagcccatagggggggtcattcttagagtcaccagcaacgttac
cacggcggatgtccttgacggaaacgttcttgacgttgaaaccaacgttgtcaccggggagaccagcttcaagagattcgtggtgcatttcgacg
gacttaacctcagtggtgacaccagaaggagcgaaggtaacaatcataccgggcttgatagtaccggtttcgacacgaccgacggggactgtgc
caataccaccgatcttgtaaacatcttgaagaggaagacggaggggcttctcgacgggacgtgcagggggctcaatggcgtcaatggcttccaa
aagagtcttacccttgacaacaccagccttagtctccttttgccagccttggtaccaggtcatgttggtggtaggctcaatcatgttatcaccttgga
aaccagagacgggaacgaaaggaacagtcttggggttgaaaccaaccttcttgacgaagttggaagtctccttgataatttcctcaaaacgagct
tgagagtagttgacggagtccatcttgttgacggcaacaatgagttgcttgacacccaaagtgtaggcaagcaaagcgtgctcacgagtttgacc
gtccttggagataccggcttcgaattcaccagtaccaccaccaataataagaatggcgcagtcagcttgagaagtaccagtaatcatgttcttgat
gaagtcacgatgaccgggagcatcaataacggtaacatagtacttaggagtttcgaacttccagagggcaatgtcgatggtgataccacgctca
cgttcagccttgagcttgtccaaaacccaggcgtacttgaaggaacccttacctaattcggtggcttccttctcgaacttttcaatggtacgcttgtc
aataccaccacacttgtaaatcaagtgaccggtagtggtagacttaccagaatcgacgtgaccgataacgacaatgttaatatgtcccttttcttta
cccatgttgtaaaataaaaaatcgcaagatgtagcgcttggcttacggtgttgcagacaagggacgtagcaggatccaatttcatatattctgcta
agaaggcagtcacattgttggcatggaaactctaatattggatggcaaattgccccacgttctttatagtgattcttttatattgaagacaatatca
gcttacaggggtattcagacattaatcccttcaccagccaaatacctctctatagcgatctttgaatttgtgaaaaaaatgtatacaatagttaaga
tgttgtgataggctgaatggaataactaaaggaggcgagaatcaggtaattagaaaaaaaaaatttcctgcaaaatttttagtgctctgtttggat
ttttgggagagatttacgcaaatacattttgtgtgcgaatcaaatggagggactgctagtcaaaacaaacaaaaaatagtatctaggaacaatta
cagtttagtctctataaagctgttcttccttatgtttgcttttgagcaaaatggttataaatactcaatatatagctaacagcttagtacttacggtaa
tatagcaaagtcaacacattaaaggggttcattctagttcaattataaacaaatattgattaccaacagcagaaatcttttatttggggatacttgt
tgggcctgttcctataattatcaccagacatattcatcaacagacattgattatatatttcgaagcttctgtacttattaaagcattcaaacaatcat
aaaaagatttctataaaaaaacaacaactgtaacagagaagaagaactttatccgtaaagccttcattattcaaaccttgcgtgtgggataaaaa
catacaccggaccaatccctcgtctatttcgtttattttttttccaatgatcaaacaaagaggtgaatttacttccttggaagttcggtaagcgttcgt
tttaagcatgttgtaaatcagactaatgacaacaaagtaccttatgaactttatgctttcttctgtgattgcaattattacaaaaatttccggttttca
acacttcgtcttcacccaaagtgagaacatttcaatcctttttgaacactagtactacttgtgtactcgtataagcatttttataaaacgaacttttg
ttgacatcattaccgaatgaatgggatccataaatcacaatagcaaaaccttttaaactgtttctttcgccatgcactacgattcttccatagtaata
tcacgatcctggcgactaggtcgaggtgaaggactacgactgcgagaatccaaagaggttctacgaggacgtactggaggttccaaaaaagca
aagtcaacttccaatttcctgtctaatagcattagagacttttgacgaacaacctcttcagcttgctctagttcagcatactcgatcaatgcatatcc
tttgacatagcccgttcgtcggtccaaattcaaatgcagatttttaacaggaccaaaatcagcaaacaaatcttcaagttgctcttcagaagtctct
ggatgcaagccagtgacgatgataatgtacccttcaacagatttggcaggacgcgtttgttgactcattgatggccgatattagacaacaacgcgt
ttctttttacagaaaaaagcaaatttttgcaaggaagtaaaaaacgaaagaggtaaagaaaaataaaataaaacagacaaacgaaaggttatt
cgtttaaaaaagacctttgagatgagattgttggaagaattcagtaaacgtgtattgttgtctcactcgcttctttccctagaagtatcgcggtcgaa
tacgttataaaagcattaaagacactacctgtctcattttcctatttcgttaacttggttttatgaaggagaacgattcatgttgcatacaaaaacag
tcacactcaacacaattgctcaggaggtacaaaaaagaacctgcgcagaacaaacgaaaaaaagatcaaaatggctcaacgagttacctacc

gccgtcgccttgcttacaacactcgtagcaacaagaccagaattatcaagactcctggtaacaaagtccgctatttgcatatcaagaagcttggca
ccatcccccgttgcggtgacacaggcgttcctttgcaaggtattcccgctctgcgtcctcgtgagtttgctcgtcttccacacaacaagaagaccgtt
caacgcgcttatggcggttgcttgagtgccaatgccgtcaaggatcgtattattcgtgcctttttgatcgaggaacaaaagattgttaagcaaaag
cttaagcaaatgtcttcccaaaagtaaaagggtaaaggttgagccggaaagctctctttgagctgctctatctaaataacttccttgattatcggaa
attaaataagactattttcggtgcgtataagctgaaatcaagtagctatctttaaaagtagtgtgggattctatagcagctgtaaaagtaaacaaa
tgcggagattggagtactttataggtaaaagcatttaggacacacacatggaaacgaaatcattattcaataactaaataaagagaggttggta
atgtggtttagccataatgagtgcttggagtacaacgatgaatcttggcatggcatatagggcatttcaaatcgactatcagttctttacgtgacat
aacacaagcatagcagaaagtatgaccgcaattagcactgtatggattcgtgaggactgagttggtctcgtcacgtaaaatacagaatgcacatt
cattcggctggcaggaaatttttttgttgtgtttcgaagttctttgtacttggaccaaagggttgccggcaatcgggtttcaaacaacccatcgatt
aatcccataacaatttgtcgcatgataattgtttggtcatttgcacctgtggagccttcttgatgagtgtcgaggtctggaagtatattttcagtactc
agtgaactttgaaacatattggtataagtgttttgtccattaaaagcaagaagagctttaagcacggcgataagagtgaacaacccattacctgc
gcgttcaatttttcgaattagactgtttgtgctttgagaaagcgaagctgagattactttccagagcattaataaatacaaccatgcatttcctttac
ggtttttaggaataatcccaaactaatcacgtatcgcaactgctctttccaaacaggccttgtagctattacaagtttgtcgtactcttccaccaat
gcttgtatggtctgtatttgagtatcagcgcttactactgtttcccagcgagtcgaagtcatcgttgcaatgaatccaatttataaaatcgttgtttat
ataattatttgtcacgctgaattaatcctctcagttaaaaatccttgtcagtcatccacgcatatcaataaaataagagtttcatagatttatctata
agagtttagcggtcctttctggttataattgaagcagtcgaaataagaccgatttaatatgggttgaagtccgctcttaccaacaacaaatatcaa
atgacagaatgtcaattttgactgtaagaaaataaatcataaacaattaactaactgcattgtattagggatttcgtaaattcataaaatccgtca
aatcgtctaaagaacgaaggaagggcagtaaaacttcaggttcggaaaagtccggtggtacagtcagccagcaaagtcataaaacaaatgaa
ccttcttctaattctacggagtttcctggacttgaaaggtttcaagtgaaggaaaagctgggagagtaagtggattattcggtgtcgtgtaaaaga
gccagggcattaacggtgacatatagtggagcctatagcaaggtatataaagcgtattccttcgatcttcaagaagacgttgccgtcaaagtcatt
cgcaagtacgaaatgaattcgaaacaggtatgaccattgaatatgtttttagatgatgctatacagttgatcgagaataatgtggagaatttctatt
atggactatgatttttatagcatcaggatcaacggtagaatcaaaatcagctaacttggactagaaaagcggtgtacacaaggaagtgaacata
atgcggagaattcatcacaaaaatattgtttctctattggattttattgaagtgaaagatttcttctatcttattctggaacttgctgaaggaggaga
aatatttcacaagattgttcatttcacttacttttcagaagaactagccaggcatgtcattatccaaatagcagaagcgatacaacatcttcatgat
gtatgcggtatcgttcatagggacatcaagcctgaaaatgtgttgtttaagccgactaattatcatccctcagagaattaccaggcgcctcctttgg
atccaaacaagcgtgatgaaggtgccttcatacccagagttggtggaggtggtattggaaaaattatgattggtgattttggttttagcaaagttgt
ttggaacacaaaaacatctactccttgcggaactgtgggttatgcggcgccggaaattgttaacgacgaactttactcaaaaaatgtagatatgtg
ggctttaggctgtgttttgcatacgatgttgtgtgggtttccacctttttttgatgataatgttagagtattagcgtccaaggttgtgagaggcgaattt
gagttcctatctccttggtgggataacatatcggaatctgcaaaggatttagttgctcatctacttaccgtggatccaagagaacggtatgatatcc
accaatttttcaacatccatggattcgcggtgaaccgaagatgctttcagttccatcccacaagccaaaattctatacgcgttctatagattctaca
tcttcttcacctggcagaatgacatctgacttaaaagagcgaccaaggagaacaatgatttcccgtttgtccagtgcttctatgcataaatcaccct
ccattactacattgagtgatgcaatgatggttgcttacgacgttcgtcgctcaaactttccaatgagtcatgcccataagaaatccaaaccttcaag
agctaactcagtagcggagctaattgttgaagaagattctggtgatgaacaagcaggaaacagttttccctttagcgatatttccaatagtgatga
tagagactcttttcagttggatcttgctcaatcatcccttatttctagaagatctgctcgacgacgtccaatgtaattttgagtttcaggccatttgaa
atggcgttcatttatgtatgaatcgggttggcttggcataacatatctcctggtaacattattttctttttcatttcttctctctctttcatgcggtgacgc
ctagcataacaatcgttgctcgtttttaagtctcatcattttattaactatgcgaaacggtcacgctttcaaaggaccctacactctttaatatttttct
tcctacttttctatattcttctggtatctagtaatgtataaaattttctaactcactttgctatttgtaatgtttagcaaaaaaaagtagtgcatttgtag
aaagaatgttattatagccagcaaatgatgtagaagttgaaatatgaagaatacataatctatgccaaacatcacggaaagaaattatcaccag
cagatttctttttcaagagaccatgattatgaaaccgcccatagttcatgaataaagctgctgagagtgtctaaagacaaaattgattcataagg
tatgcatcatttcaaaattaaatttaataagttatggatttagaaataggatgtctttaccttttatatagactgcccagtaatctgatcagggaagt
gccattgagtgcgagaaaaagggaaacgaaaccagggaataaggaaatgtaagtaaaaagcgaagaaagccattcaactcctcgagtatctg
tttttctatcagattgggtataattatgatcagatagagccattacatttttctctaagcattcttttggaaaaaatacgatgtagcactaggtatatg
caatcctaaattccacagaggaggtgccaatttgacaagaatagatttaacggcccaaccgattagaaaaagcaaaagtaaaacaatagtattg
aagacaaatgtttcatgagaagtaaagacgtatgggccaaaggtaacctggtatcgatagtattgcaatcgaagccaattcatacgctctggctc
atatacgccatcataatctttcttagccttgatgattactggttttcccatcctgtttgacttagttatcccgcgacagcttgaaagaaagatgatgct
gtgaactaatgtataaaaagagctttaaaaacaatccactttcctttgatttctaaggaaatttatacactctgggaagtgggttattggtaaataa
tgcttgctgtaagtctcagaaggaaaggaattatacctctttacggtaatattattttcatataatagaaacgaagaaaaataaagaagcgacga
aagtacacaaacaaaggattattattctgtaaaaaaatttgaaaaaataaaaataaataaataaatgcatacgatgggtaaggagattatcatg

gatgaaaaagaaaaaatttgttcatctggaggaaatgttttgtcgtggtaggcacaattctgtttgtaaacaacgaaagacctggaaaaggaaa
ctcaatgtaccatagctgttcagcgaacggtttctacgtcggaattttgtttctcttttctgatttaattggaattgaactttattgaaaaaatgagac
attcgtaaatatcaaccataaacacttacgtatggtaaacaatgcgaaggcaaccataataagctgcaagcttttcagataaattatttggtgtttt
ctttgttgagccgtgcttataatttcatagtatagatgctgcagaagtttaaggtcaacttctttatgttttcaaatcgtcaagatgaagtatatccaa
caacactttacccaaaacatcttacaaaaaccacaaggaaattgttgtcgattactaataacctattctaatattctgattgaaatatttttcaattt
tcgtggcctacacgtataggaaagtgtcagatatattgattccatattctatatggtttgctaaattcagttgatttccatttttttccagcaattcattg
aattaaggaaataaacctatgttctttttaagctgtactacatggtttattgcgtttttatggctatttgagggaattcttggatatggagtcaactcg
actgaattactcgagttgaggtatggttttatataaaagaaattaaataaataagttttggattttctatttactccttttttagagaacttactcgaga
gttgttttatcatgggtacgacaattatttgaatttggcatttccgcttgacgaactagctcccttgtcttgtaaaggtttaggaccagactttaaaaa
tccccttaatcttggcatcaatgatgttcgtggaaattaccttttaaccctgatagatactatagataccttgatcgttttgaatgatcgccaaggctt
tcatgaatctattcaaaggatcattcaacatttgtcttttgaaactgacacgaaagttcaaatatttgaagctactattaggattctgggaggactat
tgtctggtcatatctttgcttctaacccagagtacgggtttcaacttcccgggtataataacgagttattggatttcgcaaccgaacttggtgagcgt
cttttgcctgccttccggacacctacggaaatcccttttgcgaggataaatttgatgaaaggagtgtctcctgaagagaccaacgagtcttgttctg
ccggagcatccagtctcatgcttgaattctccatgctgagcattcttactggagaccagaaatttcgaaaggcagctgaaaatgcctttttttgccat
atggaatcggagaagcagtattggtcttttaggaaacagcattgatgtaatgagtggaaaatggatttatccagtttcaggaattggcgctgggat
tgacagtttttatgaatacgctctcaaatcgtacatcttacttgatgacgtacgatttcttgctgtctgggaagagtctttgagcagtattcgtcaatt
tatgtcttcgacagaaaatcattactatcagagcgtttatgcatatagtgggaccaccgcaagttactgggttgactcattgggggcatactttccc
gggttactagtcctcgctggcgaactagaatctgcaaagaaatcgcatctatactatttttcaatttactcaaagtatggacagattcctgaacgat
ttaacgtttataccaagaccatagaactacaagggtacccattgagaccggagtttgctgaatccacttattatctataccgtgccactcgagatcc
gttttatttgaacgtaggaaagacgattctaaacaatatagaaaaaaatcttcgtacgtcatgtggatttgcttccttggacaaccttgaaacaggt
gtacgttccgaccgaatggagagtttcttccttagcgagaccttgaaatatctgtatttactatttgatgaagagaatatatttcattcaaaatttca
agactttctttttactaccgaaggccatttattacccatcaatagacaaacacatgaagcccttgactttgaccaacatcgccatccgggaacatgt
acgttagattctgatgaccattctatacagcctttattttttttctaaagttgcgtcccgagaagatttcgactttatttttcaaatggtcgagtccaact
gtgatggaattgccgaggatttaattgatccttatggaatttgcgcaagacctatgattgcagatgtcgtagaggttctttacggaaacgttttatta
ccgccatttaagccaacaagacgtgtcatgaacgacatatacgttccaacattgatcgggaacagaattcgcttgataaaatatatcgataatga
aaactacagtatttttaaaattggagcggaaaaggtagaaaagaacgatacggtctacttgactgaccctaattacgcaatgtttatgagtagag
atgccctgtatgcgacaaccgcttttgtgcacatagtctcttctacgggagaagaagcttactccgaatttcttcatattcatgaaggatcttttctgc
aaccatttcaatttccgttgataactttgaactacagtttgtgcgacaaattgcctgtatctttaaagtcggatatggcttatatagctcctacaggta
actgcagttggctgcagcaagcaaaaaatgcacaaaaagctggaatgctcttactattggatcaaggttccaaggtcaacaaaactgaactgat
gcgagattcgccttcttcctactttttcggcttgattaagccttatgtgcctccaacggtacaccttgatatgaatcaaggttatgttctccaatggaa
caacatgactgcgcaaaggattcacaaagacgcgccttgttattttcaaaactctttaattgaaaacttgtatgtgtgtgaaacatgctcagaaga
acaaaaagtatactaatggaaaaatgggtaatactgagtactttactctgcttatttatttgtttcaggtatgactctataataaaaagactgataa
attatctacaacgagcatactataattttcagttaaaaaattacatggagaatctcttaaaaccaatagttctattgcgaaaagagtgctcttcaag
atctagtatacaagcaaaattactatccaaaaacacaaggagcacctggtcagtgttatgaaaatttgaaaaacccacaagaagccattaccac
cgaaaattccttaataaacaaatatgaaagcttagatggtttaagcgaattcgctgcccttaggagcctcagattgaccactgaaaagagtgaat
gtaggagctttaaggcgtttacgaccttccaaaaagcccaattccattaggaaaacgtgacccaacaaactaccattcaattgcaaaatgagttc
atcggcagccaaagcagtaccaccagtagccaagatgtcgtcaacgatcaagactcgttggccaggcttgatggaggatttttgcatagcaaaa
gcatcttcatcgtattccttcttgaaagtaatagtggctaaatcaccagggagtttgttgggtttacggacggggacgaatccagcattcaaacgc
aaagccaaagtaggaccgaaaaggaaaccacgagcttccaaaccgacaacaacatcaatttccttaaattctttttgaataagttcgactaaca
aatcaatgaggatgccaaaggactttgggtcagagaaaatgggcatgatatcttcgaaaaggattcccttcttagggaaatcgggatattgagcc
aatttagagcggagaaattgaatacgatcggtagacattatggttcaaaaagaaagaggaatgagggataagaattgaataatttgccaagttt
ggcgcttgtttatttttttaaaatttttttgtctttctacgacaggtagcaggactgcttttattcaagtaacatttcttgaaaacgaaagcgcgaagcat
cagtattcattattggaagctaaatctatgtatgttatttgttatttagaattgttttcatcaggaaaaaaaaaaaaaacaccatttctcaggtcatg
gatttatatgcacttgtactaggggaagaaataaataaaacaaggatgaaaaagtagagattacaaaaatccgttgaagcatcctaaatccact
cgtgtaactttgtaattacaaaaaaagtgaaagaacgtataaaccattaatgaagagtttgtttatgtgaaaaataatttgtttatcaatgcagtat
tttctctttcattgagggtggataacaaaccattaggaaggcgttaaatagtattcggtttttcattatagctcataataagactcaccacgaagat
gcgtatgtgaatcaacactcaaaacggtttaactgttcaatagtcatagagacgtacatactccaagttttgatgaaaaggtttgtttatgccaaat
ttttcttttgatttaacactcacttgttcatggtctttgatctcattcttctatatatgagttttgctttgatcttgtaaagagcagtttttttatttttttttgtc

attcgtttagtataatttggtgttttggtggaaaaacaataatattagaacttgaaaaatggcgtcttcaatttcaattcgacagtttttttgtttgggtt
agcaagactataggtatatttcaatatatttccttggtattattggcagcttactatgcttatcccgtttggtttgggatcttttcttcttttggaatcgg
accatccgactcgaatggttcattcacaattatgggggttgccgacccacaaattgaaggtaaccataaggtcgaggccaatggattttttaaagg
tacagttgatcttttagggaatgatctatatttgcgacacttggttcaaacgaacaagttttggggaaagcccgacgctatggttcttttgggtgatc
ttgtgagtttccagcatttagacgttacggagtttcaaaatcgtattgaaagattaaaacgtattacaggggccgcaacattcaatatcaacgaag
cagctgtatcctctcctcaagtcaatgaaattcccgtgtggacagtaccagggaatcatgatgtcggttatgggtatgagctatctgaagcgcaga
cccaaaagtggaattcttctttcgggccattgaattgggttacgaaagtctctgttgctggttccccagtacgcattcttggtattaatagtttagctc
tggataacgttcaattttatcctgagatggcctcaaagtttcgatcttgcaatagtctttcatgctctgggatccttcctctctcaagggaagccgttg
atgtatggacgtttctctatgagcaagccctacagccgcagatccccactatccttttaactcatattccattttataaaccagcaggtgcttgtgtc
gatagcccttttatttctcgttcagacaacaatcgagtccgtttgcagaatcatatctcttttgaggcaacgcaacatatctttcatttattccctagt
atcgccttgatcctttcagggcacgatcatatgggttgcgactataatcaccctaacggggtaattgaacatactttgccgagtgtcatggggttttt
tggagggaatatcggttttgtggagttgactttaaggggtatcccttcttttcgcagaaatctgcacgcaaaattctgtaacctcctttcgattctccc
cttctcggtaaaagatttgtgtacagtgaaacaaaagcattcgggcctttttggatatgacttttactcgcacctttgcaggcccttcctacatttg
gtgggctcttcatatttctacctgtgttcttacggttttacgtcttctactaatatccttaatttaaaggcgaattaaatatcctaaatttaatttcaaaa
taaatatttttatgcactctatttgaatttgtcggtattcaactgtttgtacatccagcctcatggactatgtctaagtgtaatcgaaaccgtttttcag
ccatccttcaagttttccttttgtttttggttcgtttcgcatgatattccactgtttatttactaaaacgtcataaatgtggtttgtctatactgttttattg
cttgtttactaccagcacactttggttaccatggtaacctaattttgcgaatttcgtaacacaatttacggaaactataaaagacttgttacagtccc
taatgtagctaggatttgtttactttttgggtctcttgttattgtttactttttttggaagactataaattacgtttccctctatttataagatacccttgtc
caaacccgtatacattgcttattagagaaagttgctctattcattcggcgttcctatttgtcgatattaagataactttataccctctatcccctacta
gcaacaagtttatttttaaaaatatgagacatattacgttaatcgctactgcgcttactttgctgcgtttatgttctggtatggctgttaacaagcctt
ccaatcattctatttggctttctggtaattcagaggctttgcaatggtctgtcgttgatacagacccattgcaagcgcatgtttacgttgtaaacgaa
aaagtttaccctgctactagtaaatatgcaatgactgttgacacgttcaacgagagtgccactcttcctcctcttgagcttacgcctggctcaggat
atcaagtaaaccttgtgtctattcttaacagtaccgttatttatgcccaatctccccattttactgtaatgagtaaggaagaatcaacgggttctctc
gatgataataccttgcgtgctcctttacttcagaaattaggtagtaatacctcaagcactggcaactggaccatgattcctaaccctggtcagtcat
ccactgtttccaaatccactagtatgttcactagtgtcaccagtattggtgctagcggtgcttctactagcagttccatgagtgacggtccttatttta
ccaatactagctctctaactgttcatcctacaagctcattcgtggcttctactacctcttttaaacatactgttacgtccgtcccttataactcaaattc
cacttcgctcgcatcagttacatccgttcctagttataccaatacctctagttcggtgccttctaaaaacaccactgtttcttccccaagtgtctcctc
acccgtggttgcttcttattccaatgttacatctactcctagctttaccagctctctgcccgctttcacgaacaccgctgttgctatcaattcttccgca
aatgtctcctcatccatgactacttcttattccaatgttacatctactcctatctataccaattctttctcttctatccccacatcttccgctaacaagac
ctctcttcctcactttactaattcttctgtcacttccgctccttcctctgtgagttctaccgctgtccctcgcttcacaaactcctccatcgtgacgaatt
cttccttgacttctactcccttgacttctgatagctcggtttctgtttccactgttactagtcaaaacaacggtactggttgttctgcctttactaccgta
accgtaactaccccctagttacatccctatctcttctactcctacagtggctttaccttctgctgctcctagaccttctgatgccacttatgccgatcctt
ctgacactacctcctctgcaggccaagatgccggtagtgcttcctctactcaatctttgcatacctcggtggtatcatcttctaaatcgatatcctcat
ctggaaacagtaattcaaggagcgttcctagtggtgcctctggtacagtaagcattagctatgcttcccttcttctcatcttaatgtcgatgattgtcc
ttttgtaagattaatttcataaggaatgagttgaagctttggctgaatttatttcttgtatgctggccatacaagatttgcttcaaaatattgagagtc
atattttattatcatttcaattgatacccaaggatacccatttatcatgattacatgattattttcagcatttttttgaaaaatgcaagcaggacttttg
tttgtatactttcttttcatgataattgaaatcttttatctagcattgggcatacatggtgacgggcatacatctttcatggttggatttattaccatttt
atatatttttttcttacaaagtgctagttgcattactggagaagtctgatgaatttattggatacttcatgttttatttttattgccatttcgtcaagtggt
gtgtttacttttaatctctataattttctagtgtacatagtttattggttacatctttcgttattcatagtttactttctttctagattcaagtgcatagaa
aataaaagtcatactaaagcccaaatatccaagagacttaagtaattgagaaagatatgataactgaaaaaactatatcagtaaacaatcaaa
caaatacctatacaataactatactgtgaaaagaatttttttttttacacgaaaaagaactttcattgaatacggcaagcgaaaaaaaaaccgaata
aaagaattacaaaggatgagaaaagaaaagattttcttttcttccaattctcgatccttcaatttttacgaactggtttagaggtgacgtgaagcgg
ggtatggtatttcggtgtaaagtgacatacggctatgcgattatttaatcctccctaaaccaacgacataaacaaccttcattgtagaaaaaaagg
gtatttttaagcctttgaagttaagaaaaacaatttgaaagtaacttttacaaatttgggacgtttcttacaaagcaaaaggagcgtagaaaacca
atgaaccctcacgtatcgaattcacccagtaatttcaaacgcgaaaatgtatacgaattatctggcgacttactggatcctcaagaattgaaaagt
ttaggagttagcggacgcatcatttatgtgttacggcatttaccgttcaaatcaactattcatggaaaaaaatcgtacatgggaattcaaaggccgc
cgaggagccacgacgatgtattccagcatgaattggcttgcaaactccacttattggaagacgaaacttgtgggatggacgggtgcgatcccgac
ggtgtctgatgactcgaatgaggagaatgcggagcctgtaacgaaacttactaaaaaagatttgaaaaattttgaaaatgcctataaagaatgg

aattctgacaagcgtactgaggatgtcccagtttggcttactaattctttgagtaaaggagaagatgacgaagaagttgtcctttgtgagagccgtt
cttctcaatctcgctggcttgcttatgctgaaaatgtgattcgtcccttaatccattataaatattggcccagcacagaagtggatgagaacgaaga
gcaatggtggaacgactatgtcaccatgaataaagcctttgcggagaaaatatatgagtcgtataccccaggtgatttaattttcatacaggatta
ttctctctttcttgttcccaagtttttgcgcgaaaaactctctgatgctgtcattgccttttatcatcatcaccccttcccaagctctgaaattgcaaggt
gcttccctagacgtaatgccatccttcatgctttattaaattctgattttgttggattcgaggattattcttacgctcgccattttatcagctgctgtagc
cgtgttataggtttagaaagtggacatgattgggttgcctatgattccaaacgagttataattcgtcctcttactgtaggaattgatgttccacgaat
tattcgatcaagtggcaatgctgctgtttcagcaaaagttgaagagctcacgtcgaaatttgcgggtatgaaagttattgtaggtcgcgatcgttta
gacgaaatctatggtgtccctcaaaaaattctttcatttcagcgctttttaaggcaatatccacagtggcgtggcaaagttgttttagttcaaatcac
gatttcttcggctttccgtcatccaaagctctcttctcatattcataggcttattgatcaaacaaactctctttatggctccgagaattatgctccaatt
atttattatgaagatcagatagaagctgttgattttttgctcttctcacgtttgccgactctctcttcattaatagcattcgtgagggcattagcaata
ttgcattggagtatgttgtctgccaaagagatcgctatggtgtagtaatgctgtcagaatttacagctacgtccgcgttgttgcacgacgttcctcta
attaacccgtgggatcataatgagactgcagataccataaatgaagtcttaaatatgtctgttgagcagagaaaaaatattcaacacgagtctta
taagcaggtcacaactcatacgatgcaggcatggacctcttcattaattcgtactttggctaataaattagctgcggcaaaatctggtcagctgac
accagtcttggtttctgataatgctatctctttatacaaagcggcttctcgtcgtttgttcatgttagattacgacggtaccttgactcctattgttcga
gacccaaatgcggctataccatccgagaagttattagatagtctcaaaaagcttgccgttgatgaacgaaatcaggtatggattatttctggtcgt
gatcaacagttcctgcgacagtatatgaacgacattaaaggccttggtctttctgctgagcatggcagttttgttcgtaagcctcacagcgatgaat
ggacgaacttggcagaattgcttgacttgtcttggaaggaagatgttaaacggatttttcaatattttactgatcgtacccaaggttcctttattgaa
gaaaagcgttgtggtatgacttggcattatcgcaaagctgatcctgaaaatggatcgttccaagcacttgaatgtgaagcattaattgaagagat
ggtctgctcgaagtatgatgttgaaatcatgagaggtaaagcgaacattgaagtacgaccctcaagtataaataaaggtggtattgttaaacaga
ttctgtcttctttctctgatgattctatcccatcttttattttttgcgctggcgatgatagaacagatgaggatatgtttcgctctttacataaaaatggc
aatcgtttccatgagaaatcctcgtttactgttacagttggttctccacataagcttacggtagccgattggtgtattcctgaagctcaaaatgttgta
gactttcttgctgatcttgctagcattactgactgaacaatagcaaatctggtattatgaattcagtaaagcaaatatttattcttattatatgaagg
gactagcaatgaattgagttttgctctgtgaaggtctctaatatcagctataaaaggaccttattaatagtactaagttattatcggtgtttatgatg
attcctactcagttcaaatttgtgaatttaagttcgatgttataaaaaagttctctttagtgatctgttagttgaataaaaaacattaacgtctatata
atatattaaaaacttgctctttttatcctagaaataacgaaatgatagattaatgattatatacaagaacaaagcataaacgtctcaatgcattaa
aatagttgatcgcacttcaaaattactattacttagagggtgataaactgttgccagttacattggttcgtataaaaggaaaccgtttaggggaag
ctgatgccatctgaatcggggctgaggatttttttggtaaaggactttgttcagaattgagattaccgtctccaatcttactttttggtttggtaggag
aatcatcggttttgttaatgtcgtttccattttggtgattgcttacaagaacaacgttatcgttctgatgtttgaacataggaataggttttaaagtta
gatgatctggcttgatgccagactcaataaacttgtaaaaccggtttgcatattcagccgggggaacggctgagattgctgactcagaatgattga
ttcctttccataaatgttcagctttttttacgtaggaatactagagatagttaataaacgaaaataaaggcttagatgaacgaacctttgtaagtaa
atcaataataccgatataaaaaatatgattggcaggttggttattttcattggtagcttgaaaccctccatcatcactatagaacataaagttactt
ctcctaacaaacagttaataaaaattttggcaagtatacatactccataaattggttgctgggaagacgattgcatgatctatccaatgaaaccgg
tccagtgctattcacgacttgcctaagaacgtgagcatttgattcaccgacgcctgcagacggaacttggtgttgtgatacattgggatcatatacg
gataatatgctatttcgaattttatcccgatttccgcgagtaagatcatgaatacctactaaaagactgtagtccataatacccaaagaagaaagc
aaatcgatgtcagctttaacctgagttaaaaatatctgacgttttaaaggtccaaactgtaaatgcatatctcttcttaaccaattcgaatccttcat
agtgcacatggggttctgagatggtaaatcttcatttagttcgcgtcccagcgtcgaacctttcaaatcaaatgtttgatgtatgtcacgatgggga
ggaaagagattattcatgaccacaaaatgaattttgcgtccaaaaggaagtttcactctgtgtaagccataaaactgtgagatcaacgtgttcgg
attatttttgacatgctcataataatcataaagaatttctcgtaaaaatttatgttcggaacggtggatagttttgataatgaagcgatagtcgcgtg
aaaagtagaagaaagaaccactctttccaggagaatcaagttcggagagaatgtatttactagtaagcgagacgagataatccgcggcatctaa
atggaagagctgtcgtaaatggcgaaatacccaaggagcatagtctttaaatttgaaatcgtaccttgcggaaggagtcaactcgttaccggttat
gtcaaaggtatatttgtgtcgagcagtaaagtcggccggggtcagagcacgatccattttggcttggcatcgagaaacccctacacgaatacctgt
gagcatgttatacgcagtaacgtaattttcgtggccttctgaaacacgtgttcctactaaaacgcaatcgtcgtccaattcctcgcgccggcgtctc
atgttcaatcgcttttgtttcacagcctctgcccaactagccgagccaatatcaggaggaatttcatccaaagccgagttcgacaaactggctttac
gatcatcactgcgtttggcacctgagtcattcctggaggagggggtttcggggtgcaaacgtcgattcggaagggtcgatctgtgatctgtggtttg
aggagctgggtttttaggagaagttgcttcagctgatgctgcgatgttgctttctagagctcctgcatcgaaagaagatgggctatcatacgaaaa
ggaactagtcgaagttgcagtttgaggaacggaaggctctgtagaatcttcaaatacttttgtttctgtaaacaattgcagcgaatgcatatcagc
gatcttaggagggcaatccaagctagaacgagaaaggatttttccttctgcttgatctccaaactgagaatgagaggagttggaagcggaatgct
tttcgccacgaattagcgtctccggtttttcttcatgagtagaaactgtttctttcggtatggatttggaagggataaagttgtttgagtcaattttcat

caggatttcaatgaaacataagtaaacaagatgattaatattttaaaagtacttatcccgatgttcccacaaacgaagctctagtttcttgcagttt
gtatcgctgcccccttttccaaaggttccaattcgtttcttcgtttgccagtcacgtaaaaaaccttttaacgtggtttttttaaataggaattttccaat
aaataaataaaaacaacaagtttctgctgtttccagcgctttctctaccttttctatgatgcttactgtgtggtgttctctaggtgagtatcaatgggt
tcagttaccgcaattctccgaagaacaccgagaagacgatttcttagtttattccatataaaaaaaccaatcttgaaaggttggaaaaagaggttt
tcatgtgaaagtatttacagagaaagaaacgaaaacaaacagcttgaaaatctgggtctacacaaaaatgttttgcaatcgtctgtttatgtaag
atgtaaacaaaattggatggaatggatgattgggttattcgcgtcaaaagaaaggcacatttgattaaaagcaaataaataaacatagaattaa
acatagctaatgatagaaacatctggttattgagagaaaaaaaggaaactaatcaaatgtagagcaaacatttcttaatgacaaaaatagatca
aataattatcaatgtcaaattgcaaattgcaatagaagaaaaaagttcaactaagagaacaaacttgaataccttcgaatggaaaattgtttcct
ctagaaagatgggtttttattgaaaagatatttctcatgaaatgacacttctttaccagattttcgattttcgattttcgatccctagaaaagagtca
aattcaaagaaatgaattcatttgtgtgtttgatcgttgtaaataacacgaaaaaatgaaaacgatcgttttcgtaaactgcataaatatctctaga
ttgtttactagtgcaatcgaccactgttctgtatacctacttataatcctttaaaatgtaaacaaagaaacaagattcatacgtatcgaatggaaaa
ggaaaacttgggggttttcatggagtaaagtgaacgacaaggagcttggaaaggaagtactgaaaacggaaactacatcccttctaccgtttgtt
ttcacattttaatacagagacaagaatctcataaaacagggaagttccagttactcattttctaaaaagaaatatatgaatgcctttcatttcatttt
tattaccattcttttgattttttaacagagtttatagattgttccattttctacttgtaatgaagcaaccccatggaaaactgttactatgaaggaaa
ccctatttgtaggaagaaatgctttttggttaacgagaaaactggattccatggttttcctcaattttgaaaagtaaaaggaagaaagaataaaat
tttgttctataaggattttatgaacgcaaagaaaaataataggaagcatgcaacatcatagtcaagtatcagctaccttaaagtcgctagaggctt
tttttgtttctgaaaatcattttcaagaaaccaaggaaaatgcaccgatcgtacaaggtaactctttttttcatgacgcttataaaattaacatgaaa
agcttgtctagagaatttggggacctgtgagtctttaaaccgtgtaccaattccattgttcatgaacatagcctttatcgatcattgttttgctctagg
agtgagtacaattccatccatgaacgatgattcgaacttgaccctctcgcagcttatactttgggatacgcaccttattagtagatctttacaaaggt
tatcatacattgaaaacgagagaacggaatgttttcatttgtctacttcatctagcaataaggatgatgagcgattggcccaagaaatcaacttgg
atgctgaggctaagaaactatatgctgtggctaagacagggattctacgttgggtatggaataagagagggaaaaaagttaacatcggtttaga
tgatatttcacctgttagaacagaggcacgttgatttgaaatcattttcagattttctcgatacttggtatgcggatagctcaaacgaaaagaaggt
attagaaaagataacaacactagacgagaaaaaaaggacccaaaaaattttgcactttcaatcggagatgccatgggtcagaatacatagcat
ccttggacgttatctattgtgtaccaaattagaattagagatatttcatggttataactttcagcaatcaaaaattttaaatttcttttaaatttaactc
gaatttttttttgtgcgaggcaaaaaaaccttgaatgaaaggaactctttttttaatgatcgttttcatcattcatgacaacatccaataccatacaaa
gtacattccatggtcatttgctaagcaattgatattctttcggcaatctctcttagtgcttgcttggcttccacatctgggaaaacagtcaatgcatcc
tttgcatttttttctatagccattcagtaattctttttttcttggttttatcctctatgctctccgagaggtcttcatgtgctacgtgcatagacaaacccag
gtacctgccgtaagtaaaacaagctttattcatataagcggaagcattgttcaagatgctagcagataagcaagccttcgcaggaaggaatgca
gattgaagcaaaagcatccgttcactttgggaattcgcgtgagattttggtttcatgaaatgtccctccaccaagtttgcaataacagaggccata
agctctgtaacggcactactttctagatgagaaagatcaacagaagcttttcctaataaataatcaccaaccaatgttgctagtttgagctcttcgg
tgctttggtcttctcggatattgatatgtataagattcgcggcatgaattagttctgtgacacgtgataactgtttgtatctttgaaacatctgttgact
tgtatctccttcgatcctggaagctcgtgacattaataaaactaaaagcggatggaaaaactgagtgtttgaacgtctgtagggttcagtaattcg
gtccaatagcggattattccccgtccaagcatttagttgcttatttagggattccatttcctcagtcaaaacatttaaaagatctgcatgccatctgg
aatctcgtgaaagtacaccgacggcctttaaaagaacggatgcccaagaactacttttgttcacagaaaagtaagaggatattagagaaggatt
tcgaagtatatgtagtggtaaacccatttggaaagaagcttaatgtcggatttgctgtagttgtatatatgtgcctgtatagacaattgccaatttct
ccaaaatgggttagagccgtatgatagagatgcaaactaaagagactgataaaatggtctacaaaatacaaattataaagaataaaatgaatt
atttactgcgtttaaaatagaatcggtttttttataaatccaattcattattgttgtactaaagtaactgttacctttatctgcactcgaaaattgcaaa
gggagtagtaggatgggaagtaagcttgcggtgaaatacaaaaatatacgactctggtaatcgtttaaataacttcaataccaaacagaagaac
taaaagctgaaatacctgaagctgaagagaagagctcgttgtgaatatatttcattaggatttacttcattcactagagaaataaaatcataaaca
caatatctactgacaataaaaaatttttttgtttcttatgactttcaaaagattgtaaaacttatccggcattgtattaaggccagaatcaaatacctc
gagaacgaaggattgatcgaatggcattcagttgaaaggaaccccattccaaaatatcttcccgaatattgttcaaggcctttgcatgttgaactc
tatgttgaacaaaatcataagtagcttggaaattgggtgaattatctttcgtcataaacagttcggaggcactgtagatggctgacacagcagcgc
gctttgtgtaccaatgaaagtcagcagacttgtcctgagcaaggtaaaggatctcatccgaaagatatgctaaggattttacagatgagggaaga
ttggaaggagttacgcaggttgcaatcatatgtggtaagtgctccaaaatatcacgattggcttgaagacgagaccagattaaatgtgttactttttt
ctgtagtactaggcaaaattgtaaggtgagatttctgttcccttaaagcgtaacgttgctgtaataagaaatacgagatgagatccattggacctg
agggaaagagagctttaggaagattgctatatccaagcgagcgaccacctattgtgattgctttttcggaaaagcctaattgtgggacatgttcca
aagcatgcttcaagatttgagcctttttaccagaaattggagggcgagtttcaaaggtcgcactttggtaagaacgcttagcgattctcaatggaa
gagcataagaaaaacaattgggcaaatgcgagactctatgagctcctttgaaaccaaacattaggacaggatgtaaaaaacccttaggatctga

caaaagacacaaaaaaggctagctttatagaaattatggtgttaaacactacctgaattataagagaaacagtttaaatatatttctctatttttcc
tagataaattgggatggtgcaaaggacaagggcaagtctggaggatcgcactatacaatttcctcagcgactctgcaaaacagttactgttagat
ttagacttgagacaccctccaaaaactgcatatcagcttttgtaatatcctttttctatttaaaaatatttaaaggtcagcaaaatttatcactcgca
gacatcgaatagcaactcttgataatcctttccattgctcctctgtcctttattaggttataacgcagcatccaaaatgactcctcctccatctgagat
gcatatgtatgttttattttccttctagaaccgaattagtgatggattgcttgtacaagatcactaagagtaagatacagacctgtattaaccccatt
atattagcattttattaattcaggaaggtgtaatgtgaatagtcgtttcctttttactaccgttttgcgcaactgttgctaacatttccattggtatagt
ctgatcccaaaactcgtatttggagtgaccattgcagtgtacgatcggccattgaatgtacgtaaacagaagatactgccagttctataattcttta
cttattgacttgtgctaacatatccgtagatataattggcgtctattctactggtcaaggtaaattctcctcagtcagaaattaatggaaacggtgtt
gtacagaactaacgttaaaattattttagaacatccaaacagagaaccaatggatatctctcgtttctacacatttttgacgatatttacgattgtg
tgcctctggtgtacgaccgtcatttccgtgcttatagtaagtgaaagcttcagcatctactgtttgcgaattttactgttctctcaaatattcttgctga
cactgtctagttcctcacgataagcagtggctactgttccaagcccaagaatacattaagacaaacaagccagttgtttagaaaatagaaatgga
tgttctcaatattggatcttctaatgtcgcctactgattatccttgtagttttccttattaagtgaactgctgaactgcaacttttagaataaaaacgca
taggaaatccatccatcgttgtttgttgattttgtattccaatgtttttattatttttgttactcttgctcacaaaaagcttagagtgcaagccggcatc
ggattctatttttcaccaattgtttttctatgtggatgagcgaaagttcatttgcttcttaattaatgaaggttcataaataaaatagatgtatttttgg
aacgcgtacaatcgcataatttttcttctttagggacatctcttgcaacaaccatattgcacaccaagggtgcagatgctctcatctttttctattgaa
tgaaaatctcttacgttcatcatgcgactagatactttaacttgcttatgttttctttttgtttacatctgataaaattttacgtcctttatttccttcgtta
actgttttcggtaactgaaagaagtttgctatcatactttgcatacagtaacgcaggtatcacctatcgcatgatgatggttccttgtatgctgttgg
cgtcattgcggcttatcaaacttaactgctcctactccggtctattgaacttaccttctaccattcaccaagtaagcaaacgtcttgtaaactaaatt
gtagttcaagtacagaaggaagtttttgagttccattaagaggttctctatctgctttctttacaagcttcaattggtggttctctgatttaaggaaca
ctacatttagatacattctttttattttttttatttccgctttactgattaatctttatcaattttgcgaccatgcaacttcaacaatctttcctctattttttt
acttacatgtgcagtttctgctcagtacatgggtagaattgattatgaacaagatggtggagacatagctgttgtcgatgaaaaatatggtgaagt
ccatgccgccgatatacctaaacctgaagatggaggatacttgaatcctgttcttgtcccgtctacagaaggaactaacaatccttcggtaaagga
taagataggaaatattttccgaccagccttggacttgattcaggacgtggcttatcgcgttgacgaaatgttcgaagacgccgaagatgagcttga
ccgagcggagtccgctatcaagcagaaggagatgaaaggcaagaagaacaaacactgcccctgcaaaggtcgtaaagcaaatgacaagggct
ctaaggtgggtgatgatgcttcttaccttaatgatcctgaagagaaacccaaagatcgtgaccataagaaagatttcaaacataagaagccttgt
cataagaagaagggccatcataaagggccgggttctgacgatgaggttttccctccccctctcccaaagaaaggccatcatgaagggccaggtt
ccgatgatgagtttttcccttcccctcctccaaagaagggccatcatgaagggccaggttccgacgatgagtttttcccttcccctcctccaaagaa
gagcaaggatgccaaaatgaaacatcacaaagctaaagaggagaaaaagggtccaaaaacagaggataatgaatttgatggcacccaacgc
gatatcctcgttctagaagcttttggcgagcttatgcagaacaatgctgaaaatattgctgatgagtttgccagcttcttagacgcccttgacattga
atattttggtaacatgcccgttcatattgcgaaagatgtccgtgaagaaaacaggaagccaaaatatgaggaagagagtaattgggatttgaac
gatcttactcctgaacaatttgcttatctcgaaatgcttaatgctgctggcattgaccctaaggctgctatccaacaaccttcttcctctcctgagttc
gattacacaaagggaagtaaggaaaaagaaggtgctgtctttgtcgaacaagaaaacggcgaacatgtcaacatgaaagctttcccggatcac
acattgcgtattaagaaatcaaatccggagtcccttggtattgatactgttaagcaatacactggttaccttgatgttgatgatgatagacatctttt
cttctggttctttgaatctcgtaatgatcctgtgaatgatcccatagttttatggttgaacggtggtccaggttgctcttccatgactggtttgtttatg
gaattaggaccttctcgtatcaatttgactactttgaaacccgaatataatcctcacagttggaactccaatgcttccgttattttcctcgatcaacct
attggtgctggcttcagtaatggtgatggctctgttcttgacaccattactgccggtaaggatgtttatgctttcttgtctttgttctttgccaagttccc
tcaatactcccaccttcctttccacattactggtgaatcatatgctggtcattacattcctcaatttgcaaaggaaattcttgaacacaaccaaggtg
ctaacgattttgttgctaccggttatgaacaccagaaatcttacattaacttgaagggtgttgcaattggcaatggtcttacggatcctcttattcaa
tactattattatggaaagatggcctgtgaaaactcctacggtcccattatgccacaagagcaatgcgaccgtattactaacgcatacggtacttgc
tcaaagttgatcaccgcttgctatcaaaccggtttcactcctatttgcatcggtgcttctctgtactgcaacaatgccatgatgggtcctttccaaca
aactggtttgaatgtctatgacattcgtgagaaatgccatgaccctgagaacatgtgctattctgaaaccggtgctattgaagagtacttgaacca
ggaagatgttgtcgaagctcttggtgttgaacaaggttttaagggttgcaatatggaagttaacctaggattttttgtttaagggcgattggatgcgc
aagaccttccgggaagacgttactgccattttagaagagggtttgcctgttctaatttatgctggtgatgctgattacatttgcaaccacatgggca
atgaagcttgggctgatgctttggaatggagtggacacagagaattttacgaaaccgacttagctccctggagccctgccggttctgaagctggtc
gtggtaagacctataagaacttttcttatctccgcatttatgaagctggtcatatggtgcctttcaaccaacctgaagctagtcttgagatgttgaac
caatggttgaatggaaaccttcaattttcagcttaattatgatatgaagttatgattaacaattcaataataatttaaatttttttcctgttttggtttgt
gattattatattcgtatttttctgatttcctttagtgaatgatacttaactaatacatattagtaagttcgcaaataaagatttgaaaggattggttata
atataatgaggtattttattacggtaagtagaccttattatacgacatttacaaaagacacgggtcataacattaatatcattcatatatatagtgg

ataaaggtaaagcatatacaagcatcgtgatcaatcagaaaatcataaagcaatcaaaatagtatttccgaaaggaaaacaattaaccatctag
aagttctgtaaaggtctggtagattcttctccagctgcagtgatagcatcgatgtctgcctgctcgagcttcacacttttgtaagataagaattcctt
catacgctccactttgcttgtagtagtgacagggaaaacgccctgggcatttgaccaagctaacaaaatcaaagaatcggaaacattgtatttact
agcaagagacttgacaagtttgctaacggttccatcaggatccttttttgaggggtagaagagaactataaccttcgatgatgatatttttgctttta
cagtattctagcagagacttcatatgcgtatagacttgaggatggaattcaatttggttaacacgaggagggacggtagctttctcttttatttcttc
caaatcctttacataaaagttagagacgccgatagaatgaactcggccttgaccaagttctttttccaaagcttcccaaccttttgagagaggaat
atttttctctttaaaaggtttaggggaatgtaacaagaaaaggtcaagataggtaagccccagtttcttaagagaagtagttaatgcagccgaaa
tgtcttcaatactagttgtaactttcgaggtgataaagagtttctcacgaggtacatctaagtctttgagggcaataccgacttcttcttcatttccat
atgcttcggcacagtcgatatgagtaaatccgttttggactgcccattttaccgattctacaacttccgtctgaaatcgggatttatcgtttttgtata
aggctgtacccacaccataagcaggaaacggaatatggggacccaaagcagcaatcagcatatttcgtgtgtatagtttatgaaagttaagatc
aacagaatacaaagaaggatagacttgcaacaaatttcaatcgtctttcagtgaaatatactgaatatcgcgtttataagttaggcgaatgaaat
acaaagtaggagtatagagtttttagcaaagtatagcaatcttatagtataatgaaactaagctttccctgagtgagtgtgtgtagtaagggactg
tagcattttatatagtcactgcaccaaagcttgatgtcatcattcatataagccgataaaaggtagatttgacgtgaatactgttaacttgcggtgct
tgcggtgtaccagattaaaaagtgctatcaacaaacgaaattgtgagaccacttatatgcgtaaacaaatttctctgtattttgacattaaaatcg
gacatttcgttttccataccttattgaaattaaccataattaatcaggaacgcatacttatgtgaagaagcttaaaattggctctcatgtttgtgtag
gcatgaaaaaggaagggtgtccgagtggttatggagctagtttaaggcactagtgcgaaagctcgtgggttcgagtcccacccctttcagtaattt
ttatcatttttactatgaaaactagttatcgatgtttcgcaggaaccggagggaatcagaaggatagttgtaagtagcttatcaaagcgtcctatta
tttattgaagtcttctacattacttaatacgagacagtttgtctcctaacaaatttcttatcaacaaatattattctttttatatactctgtgaaaaacc
aaatgctcttcataggaagatacaatcaatacataataaacaaaaaacagaatacaaaaggtcttcgggaacgttgctcagaaagaattagtaa
aaaggaattccacacccacgaaaacccttgaacacaacagataggaataattaattaggccactttgggataataaaatgtacaaaaagaaag
actcagctaattcgaaaagcattaccacgaggaaagtaacgactgaacggatctgatgcaaagcaaggaagcaaagcagatatcagtaagact
taatataacgtactgcacaagaatcaactgtgagacgaaggttttaacgatagcagcactttcctcaatagcgaaggttccgcttacttggtcttc
acagtttccgatgtgttgttcttactcaacataggtccaacagttccaaaaacacggtcccaaaacggactggtgacaccaaatgcagatttataa
tccttataatggtgatccaagtgccaagtttttaactgagtgaaatatccattgggcaaccgtctgtggtgcaaaaagtaatgagtcaagtcatag
cagacataaccaaagacaccgccagagaaaccagcaatcgccatatgataggggaaaattaaacgaaccaaacggtaccagggagtagcaa
agaaaacgaacagggctggaggcattacaaggcgatacctgtcagcaggcaagaaatggtgaacaccgtggaacaaaaagtgaagagtaag
gaacacgggatgatccggggtatactcatccaaatgaaatagacatcggtgcataaagtattccacaatcgtccagacacctagaccaataaaa
aagaagctgacggcgacaggaacaggaataccggtacaagcctgcaagaaaaagtatgtgatgcagggaatccaaatcataggaatcatgta
ccaaggggtcttactaaagggctccaagaagttaccaaacaaaggagcagagccacttcctttataatgtcgaggacgatggacttgatcgaga
taaacctctttggagatattgctaaatagaatctgcggcaataatggcttcttcaaatctaagaaatgatttttttcataatctttgtcgatattggtc
ggcttaacaacaaaatcattaccatagccatcaccaatattttgatccactagatcactaaattctttatttttaacaggagccgaggtgttctgca
agactttacgaatcgtatcggcatctttctgtttggcagtgctgctagcgctatttaaaagaccatcaacatctttcttgtgataccggagggcaact
tcgctaacttcagggttggcgtgaatttgttcagttactatatattaaattagcatgtgatccatcactgaaataaaaatcggtccgaggaaaacgc
atgaacgccggtttcagttagaaatgacttaccatcatactcgacaccatctgataaaatgatacaacggttggagagactagccattattgcaa
gaaagccaagtacaaagcttctatagaaacttaactacagagattactaaaagaaagtattccttgctaataggcgtcctgtaaaattcgaattg
cagcgatgacacaaaaagcaagcttacaacacaaaccaataagtatgaggataaatcaattcttctttttgaaaaaaagaagtaacaatgcaa
caacttctgaatcgacaaatccgtcaattgtcaaaatcgcaatattttgtagacggcaggttcttgctcgccataaatataggtatactccagatgc
cccgcggtcaaatgcaaggtaagggtaaggctcacatgaaaaaatcaggttacttttcatcaaactccctcggaaatattaataccgaggtttac
gaaaacgcggtaaacgatcctcctctgcctccggttcggttcggttcccttgtgatactgccaggacgacgaaaacgaaaagaatcgccggattt
ctgtatcaactagagcaaattttgtctaattaacgtacatttttaagtatctatagttgtttacctcgtttggtttacttgtgctatttcgttttctcatag
tgcgatttagggattgctgtgtcgaagtttcctcttgtcctcacaatttgtaatcaaattgcttttgtttctataagatttttttcagtgatttccttcaa
ttggtctgtttcatctcaaagttgttttagaggattgcttccaacatgtatctagatgcagaagccttggcagaggctccctaccgtaagcaagagg
tatgtagtatcaatgatgcccatgcggtgcaaaattgcaattatgttcataagatgcgcatcgatcttccgatcagcatagcataactaacattcta
tcagaactcttctgaaaacactaattcacaacagcaacctgcctcctcttccgcctctcaccaacccggtgctggttcggcttcttccatccccagcc
ctcctaggtatgggtctaatggagtaccgtctagttccgtttccacctctcataaaacctataaagatcatggcaatgatggtcgattgacccctcct
cctgcttctatggggtatcgctacagtgaaagcaggtctcgccccactcgcttatctgatgaacgcgagcgaccccgccgttcttctcacaattcttt
aagcccaatttctaggagaactcgctctccctcgcggtctccttcccctggtgcttcccgtcgctaccgctctcctagaagcttacgtgacaacagtc
ctcgatcacgtagacggtactctcgcagtgaccgttcctatcgtcgtcacgaggaaagtcctcaaagccctcctcgctattctagcagacgtagtcg

ctatcgagatgatcatcgttctcgccgacgaggtgttgaggatgattttgagcgccgaagccgacgtcgcgatcacagtagaggcaggtctcgcg
gccgtgatccctctcctatcaacgaagacgatcttgacagacgtaccgtgtttgtttctcagctagcaaatagattaacaactagagaattatctga
ctttttgaacaggctggtcctgttcgtgatgctcaaattgttcgagacaaggttagcggtcgctccaaagggtatgaaacgaataatcttcgttat
aattactgacaacttgcgtttagtgttgcgtacgtggaatttcgagatgaagattctgttcaacatgcactttctctttctggaaaacgtcttttaggt
attcccgtcattgttcagctcacagaagcggagaaaaaccgtaaagctcgtgaagcagcagagtatgtgttttcatattataattatatggaataa
atggaataagccttcgtaactaacttttcatcagaacgactcgagcagccaatgcggaaatcccctttcatagattatgtattgctaatattcacttt
aaccttagtgatgaggacgttaaagccatttttgatccgtttggtgaaattgaacttgttcgtttgcagcgagatgaacaaaatagaagcaaaggg
tttggctatattcagtatgttgcttattgtctaattaccaatatactgaccaaaggtttagatatcgagatccgatgtgtgctcgaaatgcgcttgaa
aaaatgaacggatttgatttagctggaagaagcatgcgggtttgtttaggaaatgataaatttactacggagacaactagttccatgctaaagcg
atttgatgacatgctgaatcgaactgaacgttctcattatcgacaagggcggacacgtcaaacctccgtttctgcatctagggatttgtctccagaa
gcagaacctttatctccaagtgagcaagaaggccgtcctatttcacgtgatgagcttatgaaaaaacttgctcgaattgacgatgctagtcctgttc
cgtatgtctagtttaaattaactagtttcagcaatactaacgaaactatagaactatggacgtgcaccctgatccaactcgatatcggtctgttattt
tgaaaaatatgtttgatcctaaagagtaagtctgactcaactgccatcattcttagtactaacggtctgttctagggaaacgtctgctacttgggtcg
aagaattggaacaagacgttcgtgaagaatgtgaaaataaatatggaagagtaatgcatattacggtagtgccaagtgaagctggtgaaatttt
tgtcaagtttaagacgtttgaagaatgcgaaaaagctgttcttggacttcatcagcgatggtttggtggccgaaaaattatagcttctaaaatttct
gaagctgattataatatcagatttcctgatgctaagcatgtttaggtggttaatctcctgtttgggcaataaacaaaggaggctatacaatggatgg
aagagcatgaaagatacaagatggtctaaaactcgatgaaaagaaaataatttattaaaggctaatcaaacgctactttatcttttgatgcaagt
gaagaatgaatttacatagtcaaaatcgcgattgttccgcgctttaataataggactgaatataataaccttgaaatacgcttaaaggactttatat
tgtttaatataaaaagacgcctgtcttcaacaaactattagaagcaaggaaataacctaagtatagcgtagcgtaagcgacccctttcacttcgtt
tccttagtcttaaattattagtacggtatcttttccttctgctatatcagcttttgggagtcgcgctacgcgtcttaatcacagggttcgtgacgcgccg
cacatttcatcaaaaccctaaccccgctaagtcaaatatatatattgagtcgcgcgacatggcttttgtaaatatccctaaaagagtatatctcagc
taggataccatttaatttataacaagacgaatcaaaatgtctggaggaaaatctggtggtaaggccgcagttgccaagtctgctcaatctcgttcc
gctaaggctggtttggccttccctgttggtcgtgttcaccgtttgttgcgtaagggtaattatgctcaacgtgttggtgctggtgctcctgtctatttgg
ccgccgttttggaatatttggctgctgaaattctcgaattggctggtaacgccgcccgtgacaacaagaaaactcgtattattccccgtcatcttca
acttgccattcgtaacgatgaagaattgaacaagcttttgggccatgttacgattgctcaaggtggtgttgttcctaacatcaatgctcaccttttgc
ccaagcaatccggcaaggctaaccctagccaagaactttaaataatttatccacaaggtttattggtttgttttgtttgccttcttggatgtcttatttt
gatccaatttccctcatggctattccatagaggtgtcgttttgcagggttttgctacgaagtattgagaataccgaattcagacgtatgtcataaacg
aaagaggtttatcttgtaaagttttcccgcaaggtctgatatcgcttcataatagccgcctgtacattaatgcgtttacaaatgtaagctctctttga
acctctacctcagagagaagcaatccctggagcatttgcattgtaatctcaacatttgtgtagaatttcttctaatggtttttcctgtttattttatagc
ctatgacctggtgaatgtctgtcgtctaggtgtaatttgttttcttgtttattcattaatgaaaatattcatgcctaattgtttggtttctggaaaatgga
agtatggaaattgtttatgtagattggaaagcagtaaatattagtttattgaaaagtaatggattcttttctttatatgaagttagttaaatagattg
atttaaattagacaacgggtaatacagtgaacataatgagagaacaaagagtaaataaattaaaagagttgcagaacacccatgactttttcgt
atacagtaaagacgataccgccgctaagaattaatcgagctagacgaggggttgcaccggaccaaaatcgcaagacgccatcctgtgtgaaga
ccttatatgcacagtggaagctattcttgtattcttgcttagcggttaaagattgcatgcgactcttgatcgtatcaataggttgcgtgcaataaact
gtaataataccagctatagaacctatgaggaaggtggatacagtaccaagagactcatcaggaggaagtcggctttgaagtgattgtttgatgct
gttataagctgtgaagcgtacaccagagttggtagcttgacgagctactgtgggagccaaaccacgatacaaagctgaaatacctccctctatga
ggatagatttggaagcctgaaggaatccactaaaacgaggattgggcttcttcccttcatcaatgatagcagttttgatactttcaaagggagtca
aaacaagtatagattctgcaacgccggcccccaagccagcaagtactgttcgaggcccgcttagatgaccattttcatcggcaagtgtctttttgat
actgtcgaaagcaaaaaaccgtacagccgcttttaaggagtttccgatgatgactgttgagcaacctttgtaccattctaaaccaaaacgaggaa
gctttccttttacaccctcgacattgcgatacaactgaaggcgagttttggcaaattcagctgggtaagtaatggaaatttcaactgcaccagcaat
accgccagctaaaatactcttcccagggccagggtgatttgcgttcatgatctttttgtcattttgtacagttgtagccatttttgttttaaggaattgc
taaagataaaacaactgtgtttgtcttctgagaaaaaagactcactggaaacaagaacccgttcaaagaagtcttttacgggaattgcaagtctg
cagacgagttggtccctatgaaaactaccaaattttcttttttttgttaattacccaaaagcccgcaatggagataaaaaatatatgcgtttaaaa
gctaatattttaggaaaactatttaagaaggcggagaaaccagtgtttaatatttctaaatgatgaagggtgttttggctggttcactgaagcattc
agaatcgaagtagacgttattatgaaacatttatgaagaaactagatacttaaattagaagaaatacaaacttattagaaatataaaaggaagt
aatcattttatttctaattttcttcaaatgacttcatggtcatggatacatacggttgaagcaagatgctatcgtcaaaatagtcgctttattttctata
attcattactactctgataattcaaaagaggattcttctatgagagctgccaaaccttctggacgttgctttttttgggattgtagcgtcgatacctat
gaatgattgtcaggcctcattcattcgtatcggattttgaagaaatgttgctatctcatccgaacttgtgaatacatttgaatatcaaggtttcgctct

tatttctgcgtgtataacagaacttgaattgaagatgttcgtatgtaagcagtcgtacagtctccagcagaatacagcataaaactactagtagcc
agaaaagcgttgacaaaaaggtggctgccttttagcagaattatgtcctttctccgttaagctatggaatgaagagaactcccaagttgaatagta
gcttccttggtaagggtgtatataaaaaaggcaaatctgcttaattgcgcttcacgtatgcattcaaaggaaaaggagctgattgaacttgacagt
catcatcacctactccgttttcatagttaaaagcagctaacatagtttcctttccaatagtgaccataggagcatgccaaaccccagcggcataagt
gacaccttgatagcctttagcaacaaagatttcaacgtgatcccaatcaggcaacccatcaggagcatctggagcgacagcaactaggtagctgt
cctcctcagcgtctctgcaaaggggaataaacgtctgcgttgtataagggtgcctttctaaaacttttatttgaaaggctggtgaatcttcatcggc
aggaacaacagacggtttggtggaaaagaaattccaagttgctttaaaggaaggagtggaagatttactatagttatttacggattcagataccc
caagatatttcgtggctgtaccgccgttcgctactaccatagggccattatcttttggctgaactaccgttccatatggagaaaagttttcagacgta
aatggcttgggaaaaaggccttttggtaccattgtaagtgtgcgttgccgagttcaatggaaatggttagcttctaacttacagcatatctagttag
tttgtagtcccagcaaaggaataagattaacatacctaaaatggccggtgttttgagatagtaatctttacatgtgaataatagaaagctttgttca
tcttcgaggccgaaatgccggacaggaatagcattagtgaaagaaatagtttattatgttttatagttttcttcagtgagctgaaaatctattttcaa
tgacttctattcaatcaataataatattgaagtacttcaagttattccaagttcagtctcattaagggcatcataactatttaaaaggaattttacta
gtatctaaatcacatgtaatctaatcatcttaataaaagattgtctgccttcctaggaaagtctttgctactaccatatagtttagtttggttacaacc
ttagcattgggctgcttttgtgccaggcgtaaggatcaattaaagagcattccttttaataagggtgcttaacagcccatgcattggcaatctgttct
cgagtagcaacccaaacaccttctttggaagtaatgtgctccattaacttttgcaaaccgcgaaaacgacctggacgacccatcattcggcaatg
gagggcaatagtcatcatctttggagaaccgttctgtccctcttcatacagaacatcaaaggcgtcacggcagtaggtaaaaaagtcttccgcttt
acaaaacccaggggaaatagcaaatttcatatcattaacatcaagggtataaggtacgattaaaaggcccttgtcatccttactgcccttatcttc
agccaagggatcagggacccagtaaggaaggtcatcattgtactcgtccgaatcgaaaggttgaggtaaaccaaactcgcgatagacttggca
aacaattttacgggtacgaagactagcacgacctgtgtaccaactacgaggaggagtgttagtaggggaagcgttctggatagccaagattgttt
tcttgatatgttcgtattctacttcctctgaaacattttcataattaatccaacggtaagaatgagagcctacttcacatccagcttcttccatagcac
taacgaccgcgggatttttctcaacagcctggccaatagcccagcatgtgaagggaacctgatgcttcttgaacaagttcagtattctccaaaagc
cacatcgtgatccgtactcaaaacaagactcaatggaacgctcgcgaacgggacgattagggaaaggcatcaaaccaccctccgttaaaaacg
tttcaaatccctcgtcgccgtacaacaatgaacgctccccaccctcttcgtagtttaccacaaaggaaaccgcaattttgctattgtttggccaagg
gtttgggtccacaattcgtccagcagctcccgttaaatcgcgactttcatacatagtgcctcaaacttatactataaatgttttaacagcagtcaatt
ccaatttgcccgacgattaactttttagtgaacgtatgagcctgtttatttgtcaataaaattttctacataataaccgcctttctctcttacctactct
agcaaatccgttcagtataaatattgggaaacggcgagttagtgaatgacttacggtctttcttcaagtcataagttctttatgaccattaagtgaa
actgataatgtttttttgctagatatcggtgattatcatctaaagcagtttcaagattttgaatctttcacttttgtttaatgactttattttcgttaagtca
taccttacttatggttaagagaagcgaacttatgtatgtacaacggtatatatatatatcgagcagaataattacaacccggctgttgtatcattct
cgttttcctattttcttattttttcttttttttctctttatttttgtaaagtgacgggaaacactttcattgattgaacttttagacgagttcatcggtttctt
tcctcatgtcaattatacggcctatcacgaatgagactgtggtatgtggttttcatgaatatgctttaacttttgtagcataggatttgctcgggtcaa
gtaatcacggatttagcatcagcagtgaaagaactggttgaaaattctcttgattccggcgctacaacaatcggtaagaccttttttcttcatttcct
attcttactttgtcgcctagaggttcgctttaaaaattatggtcttgatagtatagaggttttggacaacggttccgggatttcaatcgaagaccaag
attccatcggtatgaattacaatcaaggggaatgttttatagtctaattattttcctccaggaaagaaacactatacgtcaaaaattgaagcattca
gggatttagatactctgcaaacattcggatttcgtggagaagcactaagcagtctctgttctgtttctaatgtgacaattacaactgcaacttcctca
gaagcaccaaaagggttccaactaaaacttgatcatgaagggactgtgatcagtaaatctacagttgcttcacaagtatgaggcttttctccaatg
aaaattcttattaacttaaaatagaaaggaacaatggtttctattaatgatctttttttccactttacctgtgaggaggaaagtgtttgagcggaattg
tcggcgcgatttttccaagtcaatttctttacttcaagcgtatgctgttatctccacagaaaagaaatttataatattccatcaaacaaagggctctg
gaaaagttgttcaactttcaacaaatatgaactcggatatgaaaatgaatataatgaatatattcggtacgaaaatatcaacgaatttaattagat
ggaaagatggtttcatagatggatatatctcgagaccacatgtcggttcagttcgtttgtcttccgataaacaaatgctatttatcaatgggcgacc
catccatttgccaaaactagtgaaaacaatccaagaaacttttaaaatttacagtttgtctcaagcccctttttttgctattaacctaacgttgactg
aaggtacgcacaagtctcaatatgagtgtgtttttaatatgtgtttaggaactgtagacgtaaatgtgtctccggataaacgatctgtttttctttctg
aagaaagttatatcatagatttcgtaagggaatccttacaacggttatgtgaaggcactgcacactcgctgacagaatctaaagtacaaccctct
agtacaacatcatcttcgagcccttatgaattacactctgcttctttttctcagcccaatgattcttctcaacagcaatcgctagctacaattgacttc
accaacgacgatgacttgattgatccccatacgtctgacaattcttatccaagaaacaagcgaaaactttctatttcaggaaaaacggccgaaag
tgatagtggaagtgccccttcacttaataaagctttcatgcataaacataaatcaaggcctggtgcggtagatgacaatttagaaagtcggttttc
agcttctcatgagaaaatgcgacaacgcttatcagcatttgtttcaaattctacttcaaaccaaccacaaacggctgaaaatttaccattatccccc
tcgtctgcaaaaagtacggcttctgattctttaaaaagtactccatcaaacgagtttgaatcatcattatctcctttccagagaagttttcctaaatct
tcctcggtagtagatgaacgtttattttcttcttcttcccctattgaagcgaacgaaaaaccattctctagtccaattccatcgaaaacagtcgcatt

acaaaaacacaaatatttcctcggaaaacctaatgataatctaaaaaaaataaaagaaaactttgaggtttcttttgatgaactggagaaaaac
ctgacttataatgacgatatttcgaaagaacctgccaaacttggtttaattcatgatatttctgatgccaatcaagaagagcacctaaatctcatca
ttcacaagaccgatttcttgaaaatgagaatagtcggacaatttaatcggggattcatagtagtcacccatgagaataaccttttcattattgatca
gcatgccagcgatgaaaagttcaattatgaaaaactaaaggatcgaatcaagttgaaaccacaagaattggtggcacctaagcaactcaacttg
gcagccactgaggaaattgtcctatttgagcatatgggaatgatccgtgagaagggatttgaacttctggtggacatggatcaaccggtaggaaa
acgctgtaaattagtagccgttccttctggtaaaaacgttgtatttgatgtgggaggtatgtattctgttaaaattatttccagcccagtactttacta
acctcacgatagatttattggagatgttaaatcttctttcggagcatccacgtatcaatcctatcagtagcaagttagaaaggatgcttgcgtctaa
agcctgtagaagtagtataatgattggtcgcgctttgacaatatcagaaatgactacggttgtgagacaccttgctgaactaagtaagccgtgga
attgccctcacgggagaccgaccatgcgacaccttttcgtatgaaaaagtctaattcttgaaccgatttctttaatcacctttactcgtggctgttta
attgttgttgtttacattaatttattatttcgtttttgaataatttaactctcattttgtaaaagtaaataaacatccactcaaacaaactgctccggga
atagtttctactgcgaccaaccttgattcgttaacaactaacatagagaaataatagtgagatattctaagttaagacagtttcttcttatatacgat
tttgaagaggttaattaagtaaacttttgcttgaaatctgtcaggaaaagttaatcttaggtattgtttggctggaagccatatcctgaactataaac
aaacctataacaacataatttcagtacagtgagtatgaaaaaacaacttttccattctaatagacaacgtaatatcaaatatcacgaagaaaata
gtcggcttgagacaattcgcgatggtttaagaaaggtttcactatgtccaagcatggaactattagctgtcataacgaatggaaatcgtctaattg
cgtttaggaataatggacagcgaatttgggatgttggatttgaaggattgaatgctactgctctttgttggcaccaagatggtattattttacatgtc
ctatgcaaatgggaaagctatcgtattataagctaacgttgaaaaggtcatcttttggtagtgggatttgaaaatggagaacttaagtttatagatt
cgtcttcgggtcatattgtcgaacagcgtccggcttcaagaaatattcctataacaatgattacctggatggttgaggattcttctaatttttcaatc
aaaacgatttttctttcgaccctatatcttatatgcccttgctgggaactcttcctagctcttctaaagggtaagtctttgaatgcattaacttattatt
gacaacggctgcagagaacgagtattttcttccaaagcaattgctgaattctttaagccatctagtcctgaacgctctaaagctcagaaaattgaa
cttttaagcgtactcgatgaggaaggaattcgccatataaagtaagtcactttttctacggccaatactaatgaaattaagcatgttttcgtcttata
aaattggggaaggtgactcgctatactcagcacttaatatggtatgtttataatttattttagagcattttctaatctgaaagaaattaccaaagtcc
catcatatttgcaggcagatggcatatcatgttttaaactgtgctgacaaggaacatagcagtctccagacactatatatgccattgctggagaaa
gggctgcaaaacattgtagatatagccacaatgtcgacgaaaatgcagttgtttgtgaggtatatcgaagaaacagtagatgcaatgtatgaag
agttcgataatattgccaaagctgaaaatagcctacgaaagtcttttacccaaatttttgacaaatatgaaaataatcctttctctcccgagcttga
attgtatcaatttataatgaatgggttaccaactcccatattgagggaatggttgacagagagagtcggcgatcgagtcttaaaaaattgggaaa
aattgatagtaaatgctaaccggtctttatcgacattctgtcaggaatttatactacctgcttccgaaagactggggatattgcttaacttgatacga
ggaaaggcaatttggggagtcatgaaaggagatagtttacttgatccaaagttaattgatgaatgtttatcttcgctttcatcacttcaaacaattt
cctttacatttttatcttcgcttcagaaggaaaatactcaaatgaaacgttatgtttgttggctaaattacgctatgcatgaatttattacgactgaac
cagcaacaattcctcctcaggaaatcgttgatcatatacaggattgtgtaaagtatattcggaattccctaatgcattctcagttgacaccatatttt
atgggtgtcgagaagcttcaagaagcaagagactcaaaatctaacagtcctacgtttcaggatgttataaactctttaagactttcttttgatgtca
tttttagttatccatccttggcttgccgcagccagtggatgaaaacagcgaatattcagttattacgagggaacgattgggatgtatctgcttgtctt
tttcaaccagaacctgatatttcaattcaaaaatccatatattataaaagaaagcttccaggaatttatatatgggaatgcagtctgcaaaatgga
cagatgttaccctatctgtgctgtcattttgaactggatgcctcttatattctccaaattgaacaacttgataatgcttcagttaatgttttggatgcga
aaatctattccagcaacatactgataattctagctgaatatcaaaacaaaacctttatgtgtgaagtttccatagaaaaaatacccttcacatctat
tgataagtcaatttgtgatttcggaaagttaccagaacatatgaatactattccccttattccagtccgtgatacaatatgggttcaagtgttcgacg
atagctttgcaccttctaaattcgactatatagagcaaggcacgagctcctatggtattgtttactctgaagaaacccaaaaatatcgatggtttaa
aatatgagcatgcgaccaactttcattttagaattttatgataaaaaggaattctcgcactcttacgccttattaactaattatttgaatctatatata
tacaaataataatgaacatgctaatcattcatatactttgttgaccactggactacgttgtatctcgttaatttaagaaaagttcacatttttcactta
gatacgttcaattcatcgagagttgtgtttcattctaagagacacaagatggatacttttccgtctctgtttctgtattcattgacaatacaaaatagc
aattatgtgcagagctcgtgcgtggcatctttatctggaaaaaaggtgcaagagattgttatagctacggaatctcggcttttggtttataaagtgg
atccaaatgatggtcaaattagctgtattttgaatcataattgctttggaatcattagaaatgtcgctccccttcgtttaacgggcttcaaacgcgat
tacttggttgtaacttcggattctggacggattactattttgcagtatgaccttagtgctaataaactggttcctgtttatcaggaaacatttggaaaa
tcggggattcgaagagttgttccaggagaatacttggctgttgatgccaagggtcgtgcagcaatggtagcttctgttgaaaaaaataaattggtt
tatgtgttaaatcgagatgcggaagctgatcttacaatatcttctcctcttgaagctcacaaagcaggaaatatctgctttagtcttgtgggtttgga
tacagggtatgcgaacccaatatttgcagctatagaagttgactacagtgacatggatcacgacccctcaggtcaagcttatgagaatgcagaaa
aggtcttgacgtattacgaactagaccttggacttaaccatgtcgtcaggcgttggtcaaaggtggttgatcgtagctcttatatgcttctttctgtgc
caggaggcaacgatggcccatctggtactcttgtaatatccgaaggatggatttcttataggcatttacaaagaaggtttcatcaggtacctataa
tgcgtcgaaaaggcaatgaattggatgagacagttactccgtggacaggaacaaaatccaagaacaaaagcctacgatatcctttaattgtttcc

gctgtaatgcacagaatgaagggatcgtttttctacttgcttcaaactcatgaaggtgatttattaaaacttaccatagagcatgatggacaaggc
aatgtaagcgagttacgcctgaagtactttgatacgatacctctttccgtacaactcaatatcttaaagactggtttcttattcatttcatgtgaaggt
ggaaatcatcaattatatcaatttgagaaccttggattggatgatggagagttagagatttcttctctggaatatcctgatgattctcttatcaccac
tggtaagaatgtattttaccaagttcgtggtttacagaatttgtcgttagtcgatgaggttccttgtctctatcccgtaacagattcatcagtagttaa
gagtccttctttagatgacccaactcagatatacacaatttgcggacgtggtaatgcttcttctcttcgtcgtttaaagcgtggtttggaaacaactg
aagtagttgcctcagagatccctggtgctcctgtcgctatttggactttgaagctgtcccgcaatgatatgtatgatgcttatataattctgtcattta
ccaatggtacgttagttttatccatcggagaaaccgtagaagaagtgtctgacagtggattgttatcctccgtttccacacttaatgtgcatcaaat
gggtcaagattccctccttcaagtttatcctaagggtattcgacatattcgttctaataaacaagtaagtgaatggaaactgccggctgatttattta
tcacccagtcggctattaatgacactcaaatagtaatagctttgaataatggtgagcttgtatatttcgaaatgagtgatgacattgaaggtggtca
gttaaatgaatatcaggaaaggaaaacgcttacagcgaatgtcactgcactcgctttgggacctgtacctgaaggttcgaaacgcagtaattttct
ttgtctcgcttgtgatgacgctacagtacgagttctatctctagatctttacacgaccttagaaagtttgagtgttcaagcattaagctcacccgcta
gttcattatgcatcattccaatggtggttggtgggtattctactttgtatctccatattggtttgatgaatggtgtctaccttcgaactgtcgtggatgt
aacctctggacaactattagatacgcgtactcgatttttgggtccgaagcctgttaaactatatccagtttctataaaggaccaaaaaacagtactc
gctgtttccacgagagcttatttagcttattcttttctacaaaatctgcagctttcgcccatctcttattcggcaattgaacatatttcctcgttcgctag
tgagcaatgcccgcaaggtattgttgctgtacatcacaatgttttgaaaatttttacaattgacagtctgcaagatgatttgaaatcagaaatatat
ccgcttatgtgcacaccacggaaattagtgaaacacccagaacttcctattttatgtattttgcaaagtgaaaggaatttcactagttccaaaaatt
atcaggacttaggaagtcattctttgtcgtctcgcgcgaataaggacttgagtccactacaggaaatggaatggtcatcatatatatctatatttga
tttaagatctaagcaaatgattcacacttgtaaactgggtgataatgaagcagctttttgtattgaaatggtgcatttcaaatcgaaagatgaatta
ttcatagtcactggctcggcaacggatgttaatctggaaacacggggttgttcacatggtaatatacgtgtgtatcgacttaaagacgagggaaat
agccttgagttaattagtcataccgaaacagacgacatccccatgtgccttcgctcctttcaaggaaggatgctcgctggtgtaggcaaatactta
agaatatatgaattgggtaacaagaaagttctaaggaaatgtgagacggttcctgttccgctttttataacccgcttagacgttcaagggagcaga
attatagttggtgattctcagtgttctattagatatgtagtgtacaagcatgatgaaaaccgcttcgtcatctttgcagacgatcctgttcaaaggtg
gtgcacgacgtcgactatggttgattatgatacggtggcaggaggtgacaaatttggtaatttgtggcttttaagatgtcctgaggaagtgtcaaa
gctttctgatgaagaaaattctgcatctaagcttctgcacgagaaagcttttctgaattcaacccctcacaagttggaacttttggctcatttttatgc
aaatgatatacctacatctatggagaaggtacaattggttgaaggagcaagagaagttttgttatggactggtcttttgggaacggtaggtgtctt
caccccctttattaatcaagaagacgttagattctttcagcaaatagaatttttctgcgaaaggaagttccattgctatctggtcgggatcatttag
cgtatcgcagctactatgcacctgttaaaggtgtaatcgacggagatttatgtgaaatgtactatcatcttcctcgcgaaaggcaggaattgattgc
gaatgaactggatcgcacggtcgctgagatttcaaaaaaaatagaagattttcgtgtacgtagtttttagggaatggcttatgaatgaagaaaaa
gattttacgatttgaatcaaaaatttttgatgctatgaacgaataaagatctgataatcatctctcgtatgcagttttcgacacacctatcgttattta
agaagataagttatatttataagctttaaagtaataatttccacggtacttccgatatagttcactctttaacacgaaattgatcatagtgaaacta
agcttgactcttgtttacaaaaacactgacatcgtttcgatgctttagtatgcttatactagcgtataacagccagggatgatacctaactctaatag
tagcgtactcttgtgtagtttcttcgtcctcttatttctacgttatatactataagagtttaagctacggcgctaaatttgttttggggtgaaaagcagt
cacagaccgacaacaccaagaccaagaggaatgcgaaatttcctccaaccaccaagaggtgcactaagcaaaatggcagaatccgtagcccgt
ccatcagttttaaatgctgctgatgatgatatcaagaagcttttagctgccgactgccacatcggctccaagaacttggaagtccgtatggaaccct
atgtttggaagcgtcgttctgatggaatccatatccttaacttggccaagacctgggagaagcttgttctcgctgctcgtatcattgctaccattgag
aaccctgctgatgtttgtgttgtttctactcgtgcttacggacaccgtgcagttttgaagtttgctgcccacactggtgctaccgctattgctggccgtt
tcactcctggtaacttcactaactacatcactcgcacctaccgtgagccccgcttgatcattgttactgatcctcgtgctgatgctcaagccatcaga
gaggcttcttttgtcaacatccctgtcattgctcttactgacactgactcctgcctgaaccatgttgatgtagctatccccactaacaacaagggcta
caaggccatcggtttgtcctggtaccttttggctcgtgaagtccttcgtctccgtggtagtatctcccgcaacactccttgggagatcatgcctgacct
ctacttctaccgtgatcccgaggagattgagcgtgaggaggaagctaaggctgctgctaccgctgctgctgaagaggaagctcaattggctgctc
aaactgctgctgccgaattcgaggtcactgacagcgctgctggtaccgttgatcctaccattttggataatgctaccgccggtcaagttggtcaaac
tacttgggaaggtgacgctgagtggaataccaccggtgctgctccctccgaatgggcttaatttctgtgtcttattatcatacagttaaaaaacatt
cactgttgaaagttctttcaatccaggttatgctaataattaaaagccttttggtgtatatgtggtagtgagcaagccaataatgttgatgtatatgt
actagcaatgtgaccttgtagtagtcgtactggtaatacttgtgtactatagtaattagaaaaacaaaaaagtgaattcagtataaattgtactga
attggattcgctttccaatgcatcatggtgagataaattttccattaaagtctaccttaaaatagtttcttctagggtaaacaacgtgaggaagcaa
tattcatttataaagttaatcagattcatattactagtaaaagtttgctagtaccttactttattagtttctcacctcaacccttctgtaatcctacgac
aaacatctggctgtagtctgacaacctgcaatcttccataatgacaatggacaagggaaagggtcgtgctatggaaatagataatcccatacagg
atggatcaactcttccctggtatgtattgtgaatgaatcgtgttcggtaatgagaatgttctgttgtgcatggattgcatttcttaatgtccccaaatg

tattttatgctgaatccagaagggaaactctgtctaagatgagctactttatcgaagcgaatgggaatacatggacattgggtttagtcgagcgtg
aaatcagtttcatactaacagttgactagggtggaaaaatatcgtccttctggattagaggatgtagttgcgcataaagatatcatttctacatgtg
agttattaatctagctgttcatgttgaaactcatgtgcgctaacctttagtggatcgatttatcagcacaaataaagtcccccatatgttgttttatg
gtcccccaggaacaggaaagacatctactattcttgcttgtgctcgcaaggtttatggccctcaatttcgcaaccaagtaatggaactgaacgcta
gtgatgaccgtggtattgatgctgtcagagaacaaataaaaaactttgcaagcacacgtcaaattttctcgtaagctaatatttttgtttctatatag
actaaatttttactctcaaactaacacccgattcagatcttctttcaaaatgattattttggatgaagctgatgctatgactctggctgcccaaaatg
cacttcgaagaggtaagtcaatgggttgttatacgattatgttaatggtttgcctttagtcattgaaaaatataccaaaaatgttcggttttgtatca
tttgcaactatatcaacaaaattgctcctgctattcaatcacggtgtacacgctttcgttttcaaccacttccttctttagaaattgaaaagtacgttg
accacgttatcaataatgagcgctgtaatatagattcagaggctaaaggtgctcttttgaaattatcaagaggtgacatgcgtaaagcacttaata
ttttgcaagcttgccatgctgcttacgaacatattgatgagtcctcgatttataactgcgttggtcatcctcatccgtcggatatagattattttatca
agtcaattatgaatgaagaattcgttactgcttacaatggtaaggttttatgtttaagaataaacagctaacattctttagctatcaattccattaag
cagcaaaagggtttggctttacaggatataattacttgcatttttgaagcattggacgaacttcaagtaccggcaaatacacgcatattcattttag
atcaattggcttccattgagcatcgtatgtcttttggttgctcagagaaaattcaactgagcgctatgattgcaagtattaagtctggtgtggatcta
gcagctaaaagctaatgaagtcctcacatttaagaaaatagtgattgacaagttatcaccaattcagatagaaagtaataaaagaaactattgta
aatgcatactaattaccgctgagtatccttaatcttaaattcctttcaaaatatattttccccaaaaccctaatattaaacaatcattaacctagtag
agatgctttaaataaataaaccactttttccaagatatttttaagaagactgttcaatcagaaagaattttctgattatatcctgaagctgctgctgc
gtaaagggctttgtcagatagtcgttcatacctacgtctaaacatctctgcttagcggacttttggatatccgctgtaagagctataactgggacaa
acttatccacagtttgtgaactctcatagtcgcggattttgctaaccgcttcggctccatcacagattggcatgtgatagtcagcaaatataatacc
gtaatagccatatggcttggaaataaatacttcaacaagttctcgtccgtccttcacagtttcaacgtgaagccctaaattcattaattgcttgctta
ggagtttccgagcgatcaaattatcttctgctattaaagcaactttcgtttttaaaatctgcagttcttcagaattgaatatttctttcccgtgtgcttt
ggtggtagagacttggagctttgaagtatcggaaaacttaccataattagctctctttgttaatagaatactcatgttttgaattaacttcgataacc
gaacaggttccgctaagtataaaacgcgattcaggtttttatcaagagtagcggtgaaaggatcaacgtttattgtagactttattttggttcgtcg
caaagaaggcattagaactatcagccatgtgttttgaagcaacgggttcgctaaaacctccataatcaagctctctgattgcttgttacaagcttcg
atacaaagaaagtcatatggtttttcgttgttctttgcttctagtaaaactttctcaacttctttcgttgaaattaccgtattagtatgagcaagagaa
aatagtgacttaaatagagatcttttagtaataaacgattcaaccactaacactcgggtagactttagtgtctctattatgctggcaaatttctgatg
agtttccttaaaatgttcacgagataattcaaaagtggccttgtttaatcgtacgtaaaaccaaaatgtagacccttcgtcgtatacagatttaaca
ccaatattaccatccatgatcttgcaaatttctaagcaaattgaaagtccaagacctgaaccaccataaattcgagtagtactgccatccacttga
gtaaaaggcctaaataaaagcttcatctgttccggttttaacccaattccagtgtcttcaatttcaaaatataaataacaatcagattctggtaattc
aaggtcgtctttaatccagcaacgcaagagaatatgaccctcattcgtgaatttgacggaatttcctagaagatttgtaataacctgtcttagttttg
caatatcaccgcccaaaagtgcaggaacatttggataaatatcgtaagaaatttgaacaggtttatcggctattgaaggatacaccaactcaata
caatccgcaatattttcttccacgtcaaaaattttgctcggctcaagttttattttcccagactctagctcgctgaaattgagtaaatcatcaataatt
tgaactaatgacgtgcagctttgcttagcagttgagacaatatcatactgttcatcgctaagcttagtatcactaagcaacgaaagcataccataa
aaacttgagaaaggtgttcgtaattcatgggacatatttgccaagaagttagctttcagattcacagcacgtatggcagcttcccgggcttctttttc
atcattaatatctatgcagactataatccattttctattgttagactccttttgcagaggcaacccacgacatagatgccagtgaaaattaccatttt
ttcttaaaagaagttcaatttctccgaagtcttcgcttttcaaaatctcaagtttcttctggaaaagtgagtgttgttcagggtgtataaacttcttcc
aactaagcgactcgtccgcgtcaaccattccgaaataatcatgcttggaagcatttataaattctatgtctccaatttcggaatctaaagtccaaac
gatgcaagggagggaatccaagagcgttatacattgttcttcaatatttttaaatgtttgatacgaaagccgttgcgattcaatcaacttgacattg
tcagttattgtccgacgaagattttgaaaaaggagagcatttgatatgcttatagcaatttgctgagacaaaagctttaaaaacattacgtttccaa
ggtgcattaagttacgaggaaatcgaagatacaaggcaccaattacactttccttaaattgcaaaggtataataatgaaagaattcaagctatgt
tcttcctgaagccaattcataacgttttcgtcctttagcatagaaacactttctagagaaaccgtatgcgcattgttgaacacgtctgacaaaatatt
tcccgggattttaatatcggattcatttaatggtacagaaggttctaaagcttcactatactctgaattaccaaaggcagataatacatactcagag
tcactttgcaaaataaccgcagcaaaaccgacctgggaatattcaataatcagctttatgactgtagtaagtaaagatacaagcctcaatttgctt
gacaaaagctgcccgcatttaatgactgatataagagaaacaatatccaaaagtccatcttcacattcattgttacagcacgtttccttaccgcctt
cattgtgctcactactaatatcgcaattcgcaaggaaattaagttcctttttttggaataaaaagtcatgcgatgttagactaaaatcgttaatgtcc
tttttcttaaataactccggtatttcttcgatgacattatccatcatagtcgatttcgatgaataagttggttgattaaacgataaagaaaatctttcg
gaaagcaactcctttaatttattttccactccgtagcatcctatgtctccataatgattaagagcgctcagataataagattttgccagaaaagtgta
agaagccgtgaaataaaaatcaccgataacttcaaataccagcgccaattcgatcttattgaatccctgacgctcggcatctacagcgttctcaaa
atgtatcgctgcattcatgaaatccccgtcgtttcggcaaatcataccttgcagaaagtaaacccatagtaagtaactttaatttatagttatagg

caaaatcctttagtgctttcaaaacattgattaaaagtaatgtcaatttttgcctctcctcggtagaaatatcctttttcttatatagaacttgtgcag
cagaaaggccaataaaacctctcacaagcttataatattgctctagtattttaccatccgaagcctctaaaaactctggagcgacttgcaccacat
aatcataatgcttaaataatgtacacagataaatcaaagccgaataataccaagtaaagtaggtagatggcttcttgaacaaaaattcttttgat
aacgactcttgagatttttgatttgtcgaaagaatattattcgggtctttattatcagtcagaccaaataatgccaacaatacgtttcgataaaaata
caagtaatggttaaaaggtttttcaacttcccatagtagtataagcgtttcggcgttttcttgatcaagcaaaagttgatgtatatgatctccatgga
aaagtcgatttgccgtcaagcgagctagtacataagaaccatatattttgtcaccaaaagttacgcaatggagtaaaattccctctaatgttatttt
cggtttcacctgatacatatcaagctcattgctaatcatccatttaagaagttcagcgtgaacactggcatttctacctcttaacgtatgattcgaca
actgcagttcatttaccttcaaaaaaacctccggcctccttcttttaacaaaactatacgaagccaaaaagaccagtgaatatctaagagaactgt
cgccatattttaaatatagctttgcgactaaaaaagcaaaatagtaatagtacgtttgtgagctattgaataagataaagcaaacgtcagaaagt
aatgtggatattatgtcaactttttctggatcaataggcctgcgccgctcgacttcgaaattgcaagatagaaattcagaagataattcttcgaata
gctcgttcaatacttcttcgttcggatttttttccaaaggaaagttaaggcgatctaaaatctgtatgactatttcaataatattttccgatttgtctcg
atagcattcgattttagctaacagtcttaatgctggaagatagtcagatatagaaactgagtctaagtttattttactcaggacagcgtaagaattt
ttattatctttgttccaccaataacacatggctagaccaatacgtagccttaccatttcttggttgcttaaataaccataagacgctttctttggcaaa
agaaaaaaacaggctttcagtagttcgatagcatatttgtacgctccatattgaactaagtggtcagatacagtttgcatcgccataatgtagggtt
ttgtatttgagaatttttttacaacacccaagccttttaaaacgtgatgaatgtcattgtattcaaaaaattttcttgctttcccttcgatcaacgcatg
ggctatgtgagcgtgcatttttttctgcttgccctgcgctgatacttgacagaaagccttcccgtaatttacaagaagtaaatttacatctagttccatc
ttcgtaaaaagtaaaaatgttaacagaactatcaagtatttcactaagcgggattaataagcctagtggttccgttatcgaacacaataactcgta
tggaaaaggttcaataagtaatgcagcccaactcaaatagtttctagtttttttcggaaaatgcatctcccaaataattcgacatgactttggttaat
tcaaaagatggtagattttctaatctttgttctattaaccccagatcatatgaccaagaattagtcttctcattaagtttgaagcacccattcgtataa
gcggtattcataacaacctttagaatcaaagggttacctttgctaacattgtgtaaaagttgaaccaaagggtctaaggcttgagtcgggtaccgc
aaatagtaatccaaaaattcagtgatactttttctactaaaaggcttcaccttgatttgagtgaccatagcatacggcacttcgtttatatacaaag
gaagattgtcaaaagcaagaggatcaccccaagccattatcattaagattggtaatcggtgagctataattccacgaataagtgaaaaagaaca
atcatcagccagatgaacttcatctaaagcaataatagttgatcttatccgacatgtaaaacttaagatttcgagaagacagacaatgaaaccta
aacgaccgctagcacttcttatttcttgagtatctctttcttttattataatatgattttcatgtttacaatggtgattatatttaggggacagaaggtg
attaagttcgggaacacatttgaatagtgatcccatgtaaacaagttcgtctcctaaaaattcccaaagggaagagaagtaattttcaaccgtctc
tttctcttcacgaagcacctgacgtaatatctgtgagacgcaatcaaacattgtagagaaaggagaagcgtgaataccttgacaagagcacattg
cgaaatagccatattttctcgcttctaccgaaacagattgcaataaagtggttttgccgcagcctttttcaccagtaatggcgagaatatgacaata
tgtcgcctcattttccacaaaggaattattgaataggtgctttttcttgatccgcacactattttttccacttgtgaaaatctccctttttgaagaaaaa
aaagtcaactcttggaatatttgaacgtaatcagacgggttattgaggatgagttttgggaaattcggttggcttttactgtcagaatttaacggag
agtcatcgaacttgagacttattggtttaaaagaagatgaatactgtaacagcagctcgtagaaaagaatgcaaagatcatcagcaacactaaa
acgttcgtttatagacttgttggtcatttttttttattacattatcaagtgcaatgggcacgtctggtcggcgggataagaccgaagggaactgttgtg
tatgtatagattgaactatacgcatcggttttcctccccatggatagcattttgttaataatacgtaaaatgcgacacctaaagaataaatgtcgga
acggctatccaatggtcgacctacactgccaagttgttctggactgaagaattggagcttatggtgaatgtcatctagttgatatagcttctgccaa
tttaatgatgatagcgtaaaacgaattggtgaaacactggttccaatggttaatagtttagcatggataggattgtcaatgttaaaatgaaaagag
tctggacgaatttctccatgaaaaaactgtttatcatgcatataagagagcgtgtgacaggcagctaaagcgaatttataaaatagtcgcagatc
aacaggacgggggtcagacaaccagttcttcacgagatcagggccagggtcatcatacacaagcattcctagagatttatttaacagcaatctgt
actttgggcatatagtatagctattgcccccgagaaagacgataactgacgcaacaaaaagcactcattttcgagcttgacgttttgcaaggaa
aacttaagaattacttcctttttcgtctccttgtgcaatgcttttacaaaaattgctgttaataaagaagaaggatttgacctttccctttttgaaatca
gttcaaaatcgtgcaactctggaagtgcagcatcaatcgctttattcaaaagcttaaaaccactcatccttactttatttcacattaaaaagagata
aacaaatgataatcttccagtttacatcctttcgttctgaagtagtgattgataaatttcacgtaaatatatgtatacctcgtaccaccttgtgctttt
agcgattatcgtcgagaacagtaagttcaataaagatatctaacgaagcttgcaaagctaagaaatgtagtagagacaaaaacagtaacgaaa
gaagataactgtaaacgtttttagtaatactgtcgactagaaaacggtatgattacattaaggaaaaaggtcgaatcagcttacagtaacaaatg
tattgataaatgcaaagaaatgaaaccctaagtcagggattaagataaagttaatatatgaaagtaaataaaattccgaacaaaaacaaaaat
ataggaatcgaaccctataagagagaagaaaaagcacatgtaagaggcaaataaaattaaaaaagaaaaaaattgataaagaatgttgaaat
taaaaatgacaataaataaataaaagctttcacaagaaagactatttacattcctaaaagatcgtaaactggtaaccagagaatgccccattcat
ttccctttaaatttaaagcattatccacacaagactgaggatcaaacataacgctgtcaaggaagcaaatatctgacgattctgcatctgaatgat
gagaccaactggtagcaaaagcataatcaggcagactgatgatatgtgggttcaacattacatgaaccttcttttctttaagataaaactctttaat
ggtaaaggtctcattatacattttagtggcgttcaatgtcataaagtctgaaaactctgaatatccgcgaagaggacgtctattcattagtgattct

gggacatttgattcatagagctctttaattagcgataaaacaaatggatgcctcggagaagccataaaaaagtcatattttaaaggtttagaagc
caagaataaagggtggtactccatcatgtatggattaaagtagtgtttagaattaagcgaagcagacctttcagcaacttgaagcgcatgatcaa
aatttttcaagatctggaaggaacgagaaatccaaagacctccatactcgtaaaggcctattaatgttgcccattgtgctcttgccggacgattga
acattttccatgaatgtacaaaagaagggaagttcttactgattaatctttccagatcttcttcagaccacttaacgtagtcccagtcaggatgtctg
tagcggatgtcgacttcaaattccgtaaactgggacgagtacgtattggattccgtagtaaacgtatggatgattctagggattttacgattccact
tttctacgaccttatccatcggcgcatggccgtgagtattgagaaaatcagacccagcaatagcatgatgaaaatgaagctgctcgcattttccctt
tttgtctacctcatttacgcaaataacttttcggtaaggatctacatcgcggtcggtagcgtacgaaaatgcattttctccccattcttcataaataa
atggcttttcctggagacgagattgaataatatgcaaagtatttagtccataatttttaaagagaccaaatattactataaaagcaaggatgaaag
ggataagaaactttagcagtttcgatattacaagtccataatggggtttttcagcgtccatactcgtgctacaagaattatcaccacgtcttaatga
accataagggagcagaatcttaaaaaaatgaaatggatcttcgttgaaagtgttccttttcctcttacaattcgaattccctttcaacaaaaaaa
aagaaagaaaaaaaaaaaaaggaggggggaagaaagttgttgttggttaaatgaatgaactgtgtaacacagatcacatctaagcttagcg
aatttaaatgcttattgaagatagcatatgtgtgtttactatgcgttagtaaacactgacaatcaaagaaacgtaaagattggtttttttttttttgta
aaatttccttccatattgtaaaatcaattaataatagctttatctcccaaaccttttttgaatttttttcattgaacaagaacgcgtaaaagcctttgttt
atgtagccaagcatataatgggttgtgtttttgaaaattgcaacgcctgtttttaaggaaatgaaatataaaataagtgcgaaaaaaagtaaaga
aaataagaacaaagatgcctattatggtgtttccaagcattagaagacaactattatgcataccaatgacctacttatataatgggaaactaaat
gaatcacaataatatgttgagtaatctttctcgtatgaatgatatttttattcttttataaacaaagtactcgctttcatattgtacgcataacagaat
ctttcaaaaacctcatttttttattattaaataccgtacatcatttgatgtctcttataaataaagattttgtttgggtctgttttgcaaagcaattattc
caaacattcagcatctaaatacccaagtgcatttcgtatgcgaaatacccaaacagaatttgctttgttgctataatcttcaaaaactcattcttgtc
tctttttctattgattgtatgggttttaaatcaaacactaacaccaagaattttgttcatccgttcatctcgaagctactacaacattttccttcatgttt
ctcaagggtccttttaaggtgacagttcccaatttgcagtctttaacgaaaccttttttcataacaactcctattttctatgtaaatgctgcaccccatt
taggtcatctatatagtttggtcttgtgtgactcaattgctcggtttcagaagctttatagaacagatcgaccggtgtttagcagcactggcacggac
gagcatggtttaaaagtgcaaaccgttgcccaagaactaaataagcatccattggacctttgtaatacaaactcacaacgattcagggatttggc
tgtccttgcgaatactccatatagccattttatccggacgacagaccaccaacacgcaacagctgtcaagaacttttggaatcaactaaaagaaa
agaattatattgagtttcagcaccatgaaggttggtactctacgagcgatgagacgttttatcccgaatcagctcttcagtggattgtggacgaagc
gacagggaagaaaacgctagtatcggtagaaacagggaaacaggtgcaatggtccagtgaaatgaattatcattttaagcttacttcccttcaat
ctcgtcttattgaattatacgaacaagaacccagctttgttcagccttccatataccatagccaaatattgacagaacttaaatctgggcttcaaga
ccttagcatctccagaccacgctctcgcttatcttggggaatccccgttccgaaagatgactcccaaactatttatgtttggttagatgctttagttaa
ctatcttactgttactggatatccctggatttcatcagatcatgtgaatggaggatggccagctgatgttcatataataggtaaagacatcattcgct
ttcattgtatttattggcctgcttttcttatggcagctgggcttcctttaccgaaacaaatccttgttcattcacattggacaatgaataaaatgaaaa
tgtcaaaatcacttggaaacgtcgtgaatccttttacgcttatacagaattatggggaagagattgtcaaatactatctgttgaagcaaggaagac
tgacttcggattctaattttgacatacaaggactagaaaaagactacgagcacgatttaagaagaaattttggtgttctcacctccagaattcaat
ctaaaaaattatatgtaactgaagaaactcaaaaagaatggccatccttatttcaaagagaaaaatatcaagagtttgtgaatcacctatctcaa
cttccagacgcttgtgctgcttgtgtgcaccaaggacaagtgtttaatttgattgaaaacatacaaaatactttgcgttccatcactaagttacttca
agaaagtgagccttggaatttggacagcaactcgtccacccgaacagacattctgatgctcacagctcatacgctacgtatatgtgctattcttttt
caaagcattatgccaaaaaaatccgaagaacttttggaccaattgggtgttgctaaagaaagtcgcagcttccatgatgcgaaggataccttcat
cgctactcaatttaatctcggaaaaaaacaaaagaatcatttatttcaaaaaagacactagcattcattataaatttatcattatccgaaactttaa
tcaaaaggctaagaagatatcccaaatttcccatacgtctacctgattattgttattatcaaacatttggcgtagtcagttcagaactgtctaatttt
ctggggaatactaacgcctgtacgtccaagttcctcaagttaatcaatgttataatgccagttttacggaactctggaacagacacgagtcggatg
gattttgtctcactattaataattttcgttccaaattttggttgatttccacataaataaagatttggcaattcgtccataacgaaggtatctcgatca
gtaaagggatagcaccctaaattttaaaagttagtaatataagaaaataaaaaagtaagagagaaaaaaaacttacatagggtatcgggacta
gtaggggttatgtgattccataagacggtgttttccatcaatcgcaaagatgatttatgagggtgatattttcgaagatcattgatattctggccact
acttgcaagagtcagattatcactaatagaaagccaggtaggattggttgttgtttgcaaggaatttccaatccatgccttcgcgttctgcagcagt
gctggatgtagaggttgctgaggaagaatatttgtactataatcatagggaccaggaatcaaggttacatctattgtagcacaaagttgatctaga
agagtatctaattgtattgttggattgggattatagcttgaagtatcgtatccgtagcgctttactttctttttcccaactgggacagaaggagtcgat
gagtcagaaatttctatcgcaggagcaagacaattgccaaggatcatcaaccgtttgatggagctaggatcaaaggtagaactcatggatggaa
gagcaccacgcaagtactcgatgatttggtgcagaccaattccctcaataccatcgttagaaaggcctaagccactgacgactgccataaactct
ggagattcctcctgtttagagtctattgtcataggaatggtgtcgttattggagagagattcgggaaagcatacatcaataacgacgaagcgaccc
gcctcatcctcatgaccaagagcagccataacaacaccagtgacgacaccaacatcatacataagcgatccagtacaatcaacacgaccatagt

tgtcctcaagggaatactggatttcgtctgagttaccgttttcgagagagacatagggtgaatcctcggaagcagtaatgatggtgtgaagttgag
tgacatcgtccataacattaggctttaatttagaggtcatatacgccgtaccgacgacccagcaattctggtcatttcttacatccaaaacgcggtc
gagttcaagatggtttggccatttcttccgagcggcactgacgacttttggacgaagcgcagttaatcgcgcgaagtatatactcgaatattggtg
attaaaggtctgctgagttaagccgagctggagttgatttaattcttcgcaggatacagaagttcgaacagcagacatgcttcacagaagcgact
ccacaaaagcgaaacaatattaggaggttcagaaaaaatataagtcacaaaatttccttccacaacttgatgggggaaaaacaagccagacag
atatatttagcaaagatacgaagaaagtgatgaatacaaaaagaatggaagaaatttcaaaacgaaaccgaagaatcaatctgccaactcttg
taataaaaaagaaaagaaaaaaaaaggcctcgttctaattatgaatgcaacaacggtgtgcgttgaaaaatgtaaacaatacctgtagtgtact
aatttgtgtggtaaactgaaaggcagaagaataaacctctttgttgagagacacatccaacaccaagtccattaagaacaaatccaacaatatc
aaacaaagaaaatcaataaaaaatgaaaatcaatatatattaacaacggaacactagaagactagcattcctccaagcgatcgctttaagagct
ctacgaatacgatatcatatatttgacaatgtctatagtgatcaagccaagggaatgcaacaaacgttaacacacaaatacagatacaagtgga
aagagcatacagcaatgcgtaataaaatgcatcgcactaagtagaaatgtaatggtaatgccgaaaagtgtttagtggacagaaaagaagagc
tcgctaactggaatgtagtggagtccgataattggctgatcagctgaagacacgtttgattcgtggagaaggggacaagtttcgccactgcgacc
aacaaaccagctttatgcaatccaagtgggtaaaaggtggggtaggttcatcggtgttaccgagaagcaaacaagaagtcggcacctgttatga
gctagtaattttcgagatttctcaagcgaacaagtaaaggtatggaatggtgatgctatgagagatacctccagttatgtttcctctttccttcttgc
atcatcgattcgcattgcactgcaagcaaacaaacccttttgtgagagtcatgctgacggaactagtaatgatatgtcttgctgtgactttgaattgt
tgtaattctgattctctgttacggaaagaataagaatcatggatgaatttcgtcttgtaatgtgatattgattacctacttatgatgggttatttacaa
gatcgaattggagtaacgtgtatgtgagatgattggcatgtttaggtttggtacattgacgaaacaagacgaacaaatcacgagtaaaggagaa
cggacgttccatacaacagacaacccttttggagtgggtcccttcattcctgttctgtgcccctctctctatgctacgcatgcggcttggcaatatca
ctactcctgtttgtctgcctgtctaccttaaggagaggagaaatggtatgcccaaatacgtactaaggaacaaacataatagattagggaatgaa
atgaagctatcgataaaaagaaaaaaagagatctgtttaaacaaagtcgatgaattgttttattcaccctatgctctctaaatactgtgagagaat
ggaacaaagatcgttatcttgttgcaccataaattatcttactggaagtgaaagtttagaagtgctgcaaacgcaataacaacaaaccttttgatt
gctagtgattatcttgtatgttaatgaatgataagggtagatcaacaaacagaacgaaacagcataaacaaataaataatcacaagcaaaaaa
aaaaaaaggacaactaagtcccgctccgctacaaagtttctataggacgaaaccattgataggataatagacctgtattcaaagtttaccaagat
ctcaaaacaaacataaatggtccaatgatggcttaaaaagaagatgactggattgttgtaaggcttgttttgattttgcgaaatttttttagcaatgt
aaacaatcagccaattgagagatacaaacaaaagaaaattttttaagatgaagtagaacacttgtttaagaaatcatttttcttttatttctttca
tatcacaaaaaaaaatttatttgtattttttaactctctaagcaaacatttttactccaccgtctaggaaaaccgatttgttgcttggctggcttatagt
ttgatattgctgtttgtttgttatttcattttgaaaactttgtttgtcttggttctttcctctagaacgcaatcgaaaccaaaacattagtcttcgggaat
aaaataatgattgtttaattcgcaagttaggtattcttaattcgtggcttatcacacctgttgtcagaagggggggtggctatcaaatcacagaggtt
ctacaaaaatgcaaaaacgtcatggctttcaccagggtaagggttaaatgataatttttatataaagagtctacctccggatcgtttctataagca
ccactttcggacttttatattcctatacacgctaggtctatacttaatcatttgactttgtccaattatatttattcatcctcttttataatgtactctcgt
gttcttgctatcgccgccattgttaccatggccttggccgtccaagctgccaacagcactgccaacagcactgctccttttggtaactctacctctta
ccaatctggtaatacctcaaacaccaccaacagctcctcctcttctgagtctggttccagctccagcgctgcctcttctctcacgaacttcagctctg
gtgccttcgtcattgccatgattgctgttgcctgctccgttatgtctctttaaatacgtacaatatgcccttagcatctcttctgttgaatgtaaataat
cgctgcgagtatcttactcactgaccataaatacctctagtagaggagtcaataatattgcctacaaattaccgacgttttctagtttatttgttgtat
gttgcattgtcgagtatactacagtgggataatgctaagtgcgattgtatttacatagtggacacgtatgggacattgtggtataaatactgaaata
agaaggattgaattttaaatccgctacgatcttttttattgcttttgttcattttttttcttaattctttgctggattcttgatggttgttcttgattgagag
tggaaataaaagtagctaaatgtagcctaggatgataatgtgagggtttacctttgcaattactaggtttctttatctgcttctgattcttgaattctt
aaagtttgtttcattccctcttccatcttgatgtcaagggacttcaacatgtgatgacgttgccaatgtcgaccatcgctttcatagtgttaacagttg
gtccctgatctttgtgaatgatactctctacctgtgtaacctgattaataggcttgttttgccttgttgtcgagtcgaattctacacgggtcagatgaa
ttcatgagggctatcgtcatagtttctttttttaattcgcgtttgcaaatgtgtattgtttatttttttctcatagtttttaaaattgcgataatgtatgctat
aaagatgttgtaagaatgttttaagaaatacgacatagggtgtatttgaagaataagtatgatgttaaggaatttagttgatttgatgagaaggat
aagtgataaatgttgagagggtataataatctactttgttctctatatgttcattgtatgtggaggatagatggttgtagatttattgggaatattctc
atacatgggagagatttgtcatgggcgttgtgatgattatatacttaatatggtgagctatacgacctaaccgatgaatacgtgtgtattataagtt
agcaaggttattttgacacccaaataagagtcaagtttgttcgttcgtaaaaaaaggggaacaaggaaaaagggtgagaaagtgagaaagaac
atggtgctattcggtattgagtgcttgttcgcactgcactacagcttgttaaggtgtgttatgacaaaatgaaaaatttgaaaagaaagcaacgaa
tgtcaacgatccaagagccaaatactgctgcaagagcaagtataaaaagtacccggcatataaagagaaaggtacgcagagaaaaaaaaaa
gttagattgtctggaacttgaagtcgtcatgcagccttaactttttaaggatggttgctacagaaccataaagtcgataggtgttttgtgctatcaca
taagtatgctatatatttatattaaatatgcttactgttacatttcttgcccaaaatgacaaaggaaataatgggatctgtagacaaaacgtattat

ggaaaaacaatagatttgtaaaggttgttgacgatgagagttccaaaccatttcttagaactttaacaacttgatgacatgaatggagttgagtgc
taactggtttgtttttttagtgtactttgtttccactatctcttatattgttacgactgactagtgttatctttcttcaatagctgatactttttggatctgtt
tataaaacacactatccagtatgcctcctaaaaagcaacaacagaccgaagtgcgtgtccaatggggtcgtcccggtaacaatttgaagactggt
attgttggtatgcccaacgtcggtaagtctacattcttccgtgccattaccaagtctgttttgggtaaccctgcaaattacccttatgcaactatcga
acctgaagaagccaaggtcgctgttccggatgatcgttttaattggctctgcgaaacttataagcccaagagcaagattccagctttcttgactgtc
tttgatattgccggtcttaccaagggtgctagtactggtgctggtttgggtaacgcattcttgtcccacgtccgtgcagtagacgccatttatcaagtt
gttcgtgctttcgatgatgccgaaattgttcacgttgaaggtgatgttgatcctattcgtgacttgaccatcattcgtgacgagttgttgattaaggat
gctgaattcgtcgagaaacatcttgagggtcttcgcaagattacttcccgtggtgctaataccttggagatgaaggccaagaaggaggagcaagc
tattgttgaacgtgtttatgaatttttgactgtcaccaagcaacccatccgtaagggagagtggtccaaccgtgaggtggaattgatcaatacttta
taccttttgaccgctaagcccgtcatttacctcgtcaacatgagcgagcgtgacttcgttcgtcaaaagaacaagtacctccccaagattaaggctt
ggatcgacgaatacagtcctggtgacgctttcattcccatgtctgtttcttttgaagagcgcttgactgctatgaacgaagacgaaattgctgaaga
atgcaaacgtcttcaaaccaagagcatgcttcccaagatcattatcactggctataccgctttgaacctcatcaactatttcacttgtggtgaagat
gaagttcgttcttggaccatccgtaagggtactaaggctcctcaagcagctggtgtcattcataccgattttgaaaaggctttcatttgtggtgagat
tatgcactatcaagatttggtggaccacaagactgaaaacgcttgccgtgcagctggtaagtatatgaccaagggtaaagagtacttaatggaa
agtggtgatattgctcactggaaggccggaagacgctaaagtaattaaatattgtgctttcctagaaatataatttttgagggaaaatgatgcta
aagaaaagtttgaagtggtttttgttcagtattcaagtgattagttgagcttgagatgtagtatgagaaaaaaataagagtaagtaacgtatcaat
taacattctattaaatattgtacatccaagaatattggtatatgtgaaagcaattagtgaagattattatttccaaaatttttcggcaaggtaagtt
atcgcggattctctatcgatgtttctttttctttttatcttcattacccttttctgttcaggcgttgctccggttaaaaggacagcttcacgggccgtag
aattaatatgtttgaagcggtcaggtccaatatgagtacctccgaaccaacgtgaaacacatacaaagacattttctgcatcctgtaagaagttag
ttaagttaagtattttagagattcagactcctacgaaccatcattgtgagtagatgacccatgcgcgatcctgctgcagattcaccatcgtcatcatt
gtcttgaatgacatttccacgctcagaaattacacgataagcgaccatattgtgatttgcctgggaaacattagcaatctgtattaatagtaaaag
aggaagtgatagtttgtcaaacaacatgcagaagtgttccaaagtatttccccaaaaagtgtgatgaagcataccttggcaaccttttttgttcatat
acagttcttccaaagcctttcgaacctgctctgggctataaactctagtggcatgagcagcaaaggtactctttctgtcagtgatcgcttgtgattct
ttccagtcaaatgagggtttccattcttcttgatgagtttctggagtcttagcaacgtattgatttttatcaagcattactggtgtatctttgtctgattc
ttcttgcttcttgtgctcgttctgcatgacttcttgttcggcttcgatatccactagttctttcagcaaatctatatatgaaaagatacatacatcaccg
gtagcaaccgacagcagaacgtcttctgcaagggctttatcaactccataagcatctagcacgatgggtggagactctggatagtcggtgggaaa
ttgaaggttaagacgcactctgctgtcgggtatactcagcgtgtaagtgtaactctgctcggatataggtaaaaggcaagaaggataaatactttc
tagggcaagtaactcatcttgaaatgcttcgttgtcttccatttagattttcttgaatgtgatgtgaagtttcaaaaaaaaaattaaaagaaggatga
attaatattttctaaaagaaaatatatcgtcaaaaattattgatttatcaacaattgtttgctattttgaaatttttgtttttagtaggtggaattggtg
ctcttacgaaaattcatatgttttgagtgagacacttcaagaacagaagtgaacgtttttcgcttcattctgtattattctttacaagattctagaagt
aagggcttaaaattgtctccttgtgtatcatttcaatttatgaatttgtttcaacacgaatacattttcacgaaaagagatacagattggtcatgact
cattaaaattaccgtttataaatttgttttacatttttttttacttttatactatgttttaggagggttgacaccatcccaagacaatgatgttttttgta
tttacggacatgtaaacaaacttgtatgatttgataaaaagaaggacgaaaaggaggggaatcagaatgtcatttattcgaaaagaaacggtag
aaggaagagaaagacaaaccaaaacaagaatgatcgaagataaacataagggtaggaaaataaaataaattggtcgaaccaaaatggttcg
aatgaataaatacataagccaataaatatggatgtactcgtaaaaaaataatgagtggtttacaaaattggaaaagtagaaatcaaggatactg
gtttacctaagtatggatgactgaacgagaacctaaaagacttgttagtagtgatccaaaggaacggctcttaccttgggaagtcctgcatcgact
atgagattcacagtggatggagatgatggggatgcttcaaagatttcctctgtccatatatccattaaccgagcaactgaaccctgctctaccgaa
acagatacgggatactgaaagttggaattcgagcctccattgcataagcaaacaatgcagccgggacgttcgaaggcaaagttgtaatcgtcta
aatagacgcaacgagataaagtggtgcaaaagtcggggaacttttcgataagagattgtcgaacatggatcatctttgagagaacattgaacat
gaaatgagtggtatcgtattgactgctccaaagagcatttctgttttcaggatcatcccctccaaagaacatttgttcttgaccttgaaaaacaata
gggattccgtctatatacaaaagggtgactaaggtattcagtagtaactgcagagaatggttagttatgttaaaatatgaaagtaaaaatgttgat
tcgtaccgaagaatccgatgtcatgttgagaatccgggggacatcatgattctctataaagtttccgaggatagtagtatcaggaatattggctcg
ggcctccaagacagctttttcataaaattccttcatggacattcgaggaagttgagcaaaggctttgcgaatttggtagtacaggggataattgat
ggaagaaggcatgtaccgaaggtaatctccgacgaacttgggatccccatgaaagacttcaccaaaagtaaaaacgtttgcggcttcgataaag
ggaggataaaattctctatgaacatgcttcacagtatcaatacgaaggccatcgaaatgatacatttgcactaggtcgcgaatccaattttgaaag
aacttttgaacacgaggttcttcagtgcgaatgtccataagaccgacggtatcgtctccgagccaaccagtctcgcaatcgacggtgtcatcgtag
ttttggacatactttctaggatggtagtctgcttcagagttgaagggacagtattttgaatagtcaatttcaccagagccagcatgggccatgtggtt
aacaacgatgtcaaccatgcatagcatgttgcgatcatgaacttgtttaacaaggtggattaagtcttgctttgtaccgaaatgtggatttacatca

agcaaatcctgcgcccaatacccatgataccccttgaccgtagtcggtaacatcttcaatgttttttactatgggactaatccaaatggccgtgcatc
ctaaagcctgaatgtaatccagtttgcgagcgatcccacgccatgttcctccgagatacatgcgaccagtggagggagcattcgaatgatcctcat
cagagaaagcaaaacgatccgttaggatttggtagatgcattgggatttccagcgctgggtactttgctgcaatgaagagcacatttacaatgctg
ctataagaggaaaagatgtaagaaaataggaaaagaaaaaaagcacgaatgaagagagaaaaccccaaaaggaaagagaaaaagaccga
aagaaaaacttggagaatggaagaaagacaagaaaacgaaaagcaaaattggtattcagacgtcagacatcaacaactgtatgcggttgagg
ggaaaacgatagaaaagggtaaaatgaaaccaaggcctactgacaggaaaacaaagaaaaaaaaaaaagaagaaatattttgaaaaaccg
atctgaaagaatgaaaatttcagaatacgctactgaaaacattgatatcgtataaaggagattttaaagacgatatgacgaaggaataaaaaaa
aattaaatgaatcagactctaccgaaataagaatttctttattagctataagtacaggtccattttgtaataaaaactcgttttgtttgtggattaaa
tatttatttatttatccatctatctttatttatttactaacttggacatacgtagattaataacacagttttaataagccactggtaggcttcggtatgg
cagctaacgaattacactaagcctcagtaaaaattgattccattcaagactgctattcggtatatatgtctatgatggattctttctatcgtaatcttt
aaacatggaaatatcaaactagcctctcgtaggagaagtttctattaattttcaggtacgcttataattaaaaatacttatatttgaaacacttata
atgaaaccatgttaccgaaaaaaattattgtaagaacaatcatccttcatcaagaagtatacaaatcccctattgctagcttccatcacttatccta
atcaatatatatcggttgtttaatatccagtaagaaaaagaaacatagccgtattgcaaaaccaatttgcgttacgtaggttccatatcagtgtag
acaataatgagtttgtatggatgcaactaatgcaactaatatgagctgtttccttcttatggtaacaaaagtgcgtgcatttctctccttggcattcta
taaaagagagacctaaaatattttgaggatatgcttaattatgatcaaatcttaatgaatactgccctatctttcattcccagttgagatttctgttat
gatcaacatatcctttatgccagatttttatgctctcacttgggtgatagaagcatagacttgaataagagcatagatgatgggtagtaacatatca
tcatcaagaaatcgagaataaggatatgagttgggaaatgagttttcatgaaaggtaacagaaccaaatcgaaggattcgtatcattaaagtcct
catttattacttattatttattatttattatctataaatttagtgtttttgcaaactgaaaattcaagtagagtcacatttgattcgcgttctcatatgac
ttttctcgaaacgtaactagaataatgtcgaccaaggaggacaaccataaagttggataaactacagtatgagctttcaatactaaaccgtagac
tacaaaatgcaaacactatctcttgcactcctctttcttctgtccaattccttcctgccttttcgactgaattcctgaattcttaaatggaaatatcaca
ccggaaagtaacaaaacatattccgagacacaacccttacaccttaatgacacccctttctatgcccacgatatcatagaaaccctcagactagtt
gtaaattaaaaaagtagtattatgtcattgctaattcctattgtaaccctgaaaactacacaaagcctcctgctctcttttgattcatggtgaaatctt
cgtcagagaaatgtcccctttttttttttcaacttggctgttccatatacgaactggatccaccaatcagagacgtttacgtttgatcgtcatgcttct
aagaacagattagaaatatctagatagctctcctgatccaaattagccaattaataaaatgcatgccctgcgattttcgttgattgaacgacagca
cacctcgataaatcggtagcttcattttattggctgctaaaaagcccggactttaaagaccagacgtcattcgcccagactaaacttttgttacct
ctaaaagccccttgaagctttaacgtatccacaattgatgtccatcgctaaaccacgtaccatatatgtcgctttcaacgtaattagatgcaaaact
ggtctttttaaacatgagctctgcacatttcaactcctcatcatcatgccaataaccgaaactccttccacttatatatctccgtccagccatcccttt
gacccgctactgtacactcccaaactttgaactttttttttacctctcccttattcccgtgaaagcctacctgtcttttgtttacagatactctttgaagt
ctttctttggactgaggtttggttgtttttaatttgtgaagttttttttttcacttcgcactgcgacgctacttcgctgatccatcttaagcttttccatag
ctgtatagaaaagagaaattaacgtttctctaaataatatatcttgcgatagcgcctgttcgttataccccgtttttttactgtagtttgggtttcccac
tagtattaatttttggatttttgatacgataccatttgagttgattaatactccctgagataattttttcggctctataaaggaatacctaaaagtt
ttgttgtgctctatctaatacctattttttgtcctaattcttatattcttgcacaatgaagatatcttctttcgcggttgctcttttgattgcaactccatt
tctctctatttcatctgccgtttccaagggtcctgccgatggctacacccttgctaaaagggcaggtggtttctataccaatagctcgacgccactat
cgtctagtactgttgcttcttctactcctttgagcagttccgcactttctagcactcctgcttcttctactcctgtaagcagtactacaccttctagcact
cctgcttcttctactcctgtaagcagttccgcgccttctagctctcctgcttcttctactcctttaagcagttccactcctgctgccgctgccactagca
gcaacaccacaagcagcgctccttccagcacttctgcttcttctactcctttgagcagttccgcgccttctagctctcctgcttcttctactcctttgag
cagttccgcgccttctagctctcctgcttcttctactcctttgagcagctctgctagcaactctaccactagcgctccttcaagtacttctgcttcttcca
ctcctctgagcagctctgctagcaactctaccactagcgctccttcaagtacttctgcttcttccaactcctctagagcagctnnnnnnnnnnn
nnnnnnnnnnnnnnnnnnnnnnnnnnnnnnnnnnnnnnnnnnnnnnnnnnnnnnnnnnnnnnnnnnnnnnnnnnnnnnnnn
nnnnnnnnnnnnnnnnnnnnnnnnnnnnnnnnnnnnnnnnnnnnnnnnnnnnnnnnnnnnnnnnnnnnnnnnnnnnnnnnn
nnnnnnnnnnnnnnnnnnnnnnnnnnnnnnnnnnnnnnnnnnnnnnnnnnnnnnnnnnnnnnnnnnnnnnnnnnnnnnnn
nnnnnnnnnnnnnnnnnnnnnnnnnnnnnnnnnnnnnnnnnnnnnnnnnnnnnnnnnnnnnnnnnnnnnnnnnnnnnnnn
nnnnnnnnnnnnnnnnnnnnnnnnnnnnnnnnnnnnnnnnnnnnnnnnnnnnnnnnnnnnnnnnnnnnnnnnnnnnnnnn
nnnnnnnnnnnnnnnnnnnnnnnnnnnnnnnnnnnnnnnnnnnnnnnnnnnnnnnnnnnnnnnnnnnnnnnnnnnnnnnn
nnnnnnnnnnnnnnnnnnnnnnnnnnnnnnnnnnnnnnnnnnnnnnnnnnnnnnnnnnnnnnnnnnnnnnnnnnnnnnnnn
nnnnnnnnnnnnnnnnnnnnnnnnnnnnnnnnnnnnnnnnnnnnnnnnnnnnnnnnnnnnnnnnnnnnnnnnnnnnnnnn
nnnnnnnnnnnnnnnnnnnnnnnnnnnnnnnnnnnnnnnnnnnnnnnnnnnnnnnnnnnnnnnnnnnnnnnnnnnnnnnnn
nnnnnnnnnnnnnnnnnnnnnnnnnnnnnnnnnnnnnnnnnnnnnnnnnnnnnnnnnnnnnnnnnnnnnnnnnnnnnnnn

nnnnnnnnnnnnnnnnnnnnnnnnnnnnnnnnnnnnnnnnnnnnnnnnnnnnnnnnnnnnncctgcttagaa
cgtttcttccactcctttgaccagctctgctagcaactctactactagtgttccttcatctactcctttgaacactactagcagtgctttgcctacttctt
cccctgctaagaacgtttcttccaccccatttgttaacagcactagcagtgtcttgccaacttccgctcctgctaacaacaccgcctctactcctttga
acactactagcagtgtcctacccacctcttctcctgttagaaacgtttcttccaccccatttgttaacagcactagcagcgtcttgccaacttccgctc
ctgctaacaacaccgcctctactcctttgaacactactagcagtgccttacccacctccggtcctgctagtgctgtgagcaattctagtgctactagc
cccgtcacagttagcactaccagttacgccaacagtactctgcctgttaccagcactgttactggcaccgaaaccgtgactacttctaataatatta
caactactgctactgtaccaacaactgaattgtcgactgttactgatcctagcagtgcttctgcccctagtacaagttactacagcactactgtgac
gacgactggtcatagctctacgcctgttactgagactcgtacttacactagtattgttaccgtggacggtacaagaactgttgttggtacatctgttg
ttcctacttcggttacttccactgatcttacgactaatactgtcactggcactgaaacaactaccgtctctcccagtgttactgatactgcgaaccct
agtggtactgatactacaaacgcttcccctgctaacactcaggccgtcactaacactaacccctcaactgagacccatatcttcactactactagc
gttggtacagctgctgaaaccaagaccttacctcgtacagtctctttgactagtactgtggttgcgggaactactacttatgtggctgccacttcatc
tactgtcccaggttcttccgctaccgttgcccctggcggtggctcctccagcttcaccaacggtacttatcccgtctccaatatcactagtactcttcc
agtgagcagcaccgcgagctacagtaactacactacagttgtaactggtaacgcaacttctacattcccttcctcttacttgtctactggtgacagt
actttcgtaacctccagcactcaagtcagctaccctggaaatgtcagtagtagtctagctagcggttccaatcagagcggtaacattacttccggc
atcaagaccgcatctcaaacctcctccaatagcggaagcagcagtttctccgctcaaccttcggcagcctccacaggatccaaggcctctatcttc
ttgactgccctttcagtcgttgcagttttcttcttcctctcttgaagcggtgaagctttaagttgaagagatatttatcaaacgtgttttaaataatttg
cttgtttatattatatatatatgtatttagtattggaagggatttccaaggattcctttttttttacttcaatattttgctttaacgggattgcctcgattgg
ttttttttctgatatttttttgaaattctttttaaataatattttttggttgaaactcatttgagaacaagcgtcgtatgtactttttttttcagagcagtaga
acaattgtgagttccgatcatcttagcaatggactttttttagtcttagctagtatacctaaaagtgtttaagttcaagttcttttattgtactttgtgat
tgtttacaagaattacgtttagtttggttcgttactttttaaacaacactaatagtcaatctatgaatcgccattgaatcattaaaagttgtggttttgc
ctagtcttttataccttttttcttatcaagatacttttctttgatgaatgggaaagaggggtaaaccctggaagaaaaggggatgagacggaatttta
gatagaacaaagcaaacaaagaggggagagcagcattgaggtgaggggaaaagattatgtgaaaatagggtcttagaaggttttatgtacgg
gtttttagtctttgatttctctctttatttcgttctttcgttctttctctctctcttattttttttactactattattattattattaatattatgtaacagttttc
tattacaatatttcaaaagtatcatcctataattgccatcgctgtctgtatggtaatttgaaattgcgaaaggaaccaaagtaacaggtaaaggttg
gagaccatattgggcaaaatagatgagaatgatgcatgcaacctgctaagaaaatcaccatctgagagccatacactacagacattggttaaaa
accattttgttggtgacgaaagattttaaaaagcagctctatctccgtggactggaatggagttgtatgactgaacgattaacaattaaagtgaatt
gacagctacagctggatacctacctacgtgtcgaaatctacaaagtaccatatgaagcgtctcggtcgtggcgatgcaagaggcggatcaaaag
cgagcggccgaatcaacgtatggaattgaaaacgtttatgtactagaattgtctttggatggcaatatcttatatggtacagaaaactttactcagt
tggttgggtatgttgaattgagatggagaggttcgcacctgcttgtggtgttagacggtcggacagacgaggtggtataggtatagaaggatgga
tgctaacaaaacttgccgtatagtattacattctcgacattaattggaaaatgcgttcgaagcttactactagacgacgaaaagtttgaatgggct
acacagcaactactcgaagatgactcgaagagcgttcgagtctcttttacacttactccatcatccgagccggaagaacagattttttcgaagat
gaggaaatggaagtaccgctagaacaaccccttgagcttgattgtattggaatcctcatccgggactcgcaaacaaatgatccttctcatactttg
tgggttatgaggcctctcaagctgttcaccgaaattaaacaggaaatcgggcaacagctcactcaattcctcggtttcggtgcccatttgctcgccg
aacatcttcaaaaaataaaagaaatcccgtttccgaatcccttaccacccttcgagacgattctctgtcgcgtatgcgatcgtgaaatccaaaatt
ggttctttgaacagcactctgagtactgcttgcttacccatcatgcagaagcgcgtgtgcaaagagccaacgacatgctaaatgagcaaaggaac
cttctacaaatcctcttagatacattggattctacttactcccaagactatcctcacgtgtattatacaaatatggaattagggaccgtacttcctcct
tctcttcatgatttcttaaaatccagttcttctcaaaccaagaaatccgagaaaatccgccatttcctctacaacatgcttctagaaacccttcgcttt
attgactgttccctcgccatccacttaccttccttaccctctgttgaagataatgagcaagatatgccgtttagctctgttcgtctcctttctcctgcta
gcgaggtcttgaatgcaaaatcattatcatggtgtttgccccaagttgacgaccctggtctcagaaagctttttgacgacagtaatgctcttgtaca
aaagaagcttgatgcaaatgctcgtctaatcaacatcatgtattacgcggaacgtttacgttgtgaagtcgaagaccaagtccaaaccatagttg
aacaatacgttcaagatcacgatacccaaccttcttctttaaataaagatgtcctcgttccctcttcgttagaccaacttgaatctccctctccctctc
cctctccttcttttcatcccttatataccgacaccccaacctcagcctatataccaagtgccaatccctctaatcggcttccggtgctttctcgggcaa
attttgaatcggttcccccggttggcgaaaaagatagcccttcatcaatcaagaatattcgccagttttcctccactcttggtccttgtaattccccct
ccagtactgcgaacaccggtgttcctattacctcccgtagaaaatctgtcagcactatgaatgcgatttacggtgtcgggtcgtacacctcggatgc
atcgccaaagtttagaccttctttttctctagagcgaagttctttatcaaaacatgccgattcccccaatacacctttaagtaaacaggtaggtatct
ctaccattgcccccaactccacttccaagggacatccttctatacaagattatgaaattattaagcccatcagccgaggaacatttggtactgttta
cctctcaaaaaagaaatctgttggcgaaatctacgccattaaggtcttacgcaaggtggacatgattcttaaaaatcaggttgccaatgttaaggc
tgaaaaggctattttaatggcccaggaggaatctccctttattgctaagctctattacgcctttcaaagtaaggattacctttaccttgtgatggagt

tcatgaatggtggtgattgtgcatctttgctgaaatcattgtacgctattcctgaatcttgggcaaagatttatatagcagaggtggtgttaggttta
gaacacttgcacaatctcggaattattcatagagacataaagcctgaaaacatattgatgagtagttctggtcatctgaaacttgctgacttcgga
ctcagtcaaatgggtcttagaacaaggcaattccggttacaaaaagcaaagtccgttttatcacctcctagttttcagtcaccaacctgttttccag
aatctaatgaaccactgacaaattcccctttaattttacctacgagtgttggccttcacaacctttcgtcagatttaccccagaagaaaaaaaacga
gtcttttctttttcttatcaatcagaaaatgaagggtttggaagctcaagtagtgaattcactaataggaatagtgaatcagagtcagactttggat
atcagaaaggctctcgtaatttatttgaccaatctgttcctttttcaaaattgcagatgcacctaggaaatttgttggaactccagattatctggctc
ccgaaacgattcaaggaagtactcaagatgatatggtcgattggtgggctttaggttgtgtatttttgaatgtttgtttgggtatcctccttttcattc
tgacacacctgaaaaggtattcaataatattgtagctaaccgaattaactggcctgatttggacctttatccttgttctgaagaagcacttgatttga
ttaaatcctttttaaaactgaatcctgaagatcggcttggagccaaaggaattcaagagattaagcgacatccatttttgaaggaattaaatgga
atgatctactaagtcatgaagctccctttgtaccaaaccttgagacacctttagatactgtttatttcgatgatcgtggtgcagttaacacggaaag
gaacgttactccgaatgatacggttgatgaagagtctgtccactcatctggatctggccgtcgcgtacaatcatctttgagtcggcaattgcatagt
cgtgaccgaagtatttctggcagacgccatcgtcggtttagcgaaaacatgaatgaatttgatgagtttggatcattttcttacaaaaacttgagtg
ttttggatcaggcgaataagaatgctattgaaaaaattaggtccgagttgaagtccaagctccatataatgcttcctgctgcatcttccgctccaaa
tagtgaagtttcttctgcaaaggctcctgatctacaatcatacagcccaggtgttccaagcttcccctctgtttttcctctcaggaatctacgcctcg
aaaagcatccggcgttagcgctgaacacgattttaaaggtaaagttgaatctactaagcctaatacgactgatttcgtcaaacagttgcatctacg
caagcatagcatgacaaatgtatcgattgcttcttcagaaagtgatgatttttttgtcccttgaccttttgcaagctaaaaatatttctaccgaccaac
tgacaatggtaaaatctgatgaagttcattcatccccactttcttctccttcagttaattctccgacgatcctaacctcccttgctcccttaaaagtag
ttatatgtgtggacaaagtcgtgtctttctctgaacttataaagctcttaaagtcttacaggtttcaagtaacggttgtagatgacgaggataaaat
gctgagaatactgatgtctgatgaaaagttttctctcatatttttacaactcgacctgactcgaatttcgggaatttcaatcttgaaaatcgtaaggt
cgagtagttgtccgaatagaaacacaccagcaatagccttactgccttccaggattgatattaatgcggcgattccacgcttgttcgatggccgttt
atatcttcctataaacggttatcttcttcgaggttatatagctcggctgtgcaaccgataaatatatagtccactttttgttttaggattcctcgagtg
acgattttgcaaataattaaactattgggatgttacgatataaccttactactatggtgaatatttagaggtcttgatttatgttacctttgtttagaa
atgttttgttttagttaatttatttcgtatgaacaaaaacactaacgaaatatataactgtatatatcaatatatttattcatttttctgctggaaaacc
gcatctttatttttttccaaaaatcacagattttggcaaaatgaaagtacaacagtacatatcctttcgaatgttagacttttaagctgccattatca
gaattaaacttaccttttttggagctacttttcgaacatagtgcaaacttaattcttcaggttgcaaacgatatcccatggatgcctcaattcgctgta
ttgttagtaatcgttgatatgcaaaccttacttacgtttagtatatcttcatccggaggatcgtatcttgagaaacaatgaacatggacctttggaag
tgaacgattttcaaataagtgctcttttccttcgtaacatccattaaaggcatctaaaaattccaaagcagagccaggaaggttcatcacatagtg
ggaaaatactggcgggataccaatgacacgtttggaagaagaagcagtatcttttgagtttccttttgcaagctttttttggaggagggagctctat
gatttcctgcttcgaaaacctcaacagctcttcagcagatgaacggatgaaatggcgaccatcttgattataagctttgacaaaatctccgacctg
ataatcgttagtaaaacactaagaaaactttactcaccttgttgatttgaacgttgtcaactaggctttcgtagcttgaaggattcaaatcattcgca
aaaacgattacacgtttttttacctgctggacatgcaaaaggaccaactccggccataacatcgcaaaccgcttctccttcttggaattggcttacca
agcgatcatgttcagtgctgagacgagaattccaatacaccttggaaaaatcaaatcgaaatttgcagttagattcggactttaatagttagagga
atcttaaatgaaaatgacatacctgaactaccatgaaatttgggtctccggctaagacttccatctgaaatgttctatatttggtgtcgatactatct
gtcttgttcacaactgttttaatagaaggattcttatcgagaataacttgacctataaggtacttatatggaagccattcctctcgtaaattcatatg
agcaatatgacctactgcagtaaatccagaaggatgctcttctttttgacctggaggaagaatggcatctaagatgtcctctttatttattgagttag
tttatactttaaaacgatcaaacgcggacacaagcttttcacgctcacataccagctctccagtagtcataatctacggtaagcttaaatggaacc
aattttacattattttccttaataaatgactttgttgtttccgaaagtccctcagtccctatccgctcattagcacacttttgcatattgattctactaca
tggtgcataccatctaatgtagctttttgatttaagagtaccaatttacggtcttcatcttggtatacatgttttactcgcggtaactccaacaaatcc
cttgagtcgtcagttgtttaccttaataagctatccgacgtacttgcgacaaattttgttcaaaatacccactttctggggaagaactcgagcagcta
cgagatcaaacgactttttaaaaaggttcttatcaatagctttagagaaaggactcattttaatcggatctaagcgtaactctccttccataatgttt
tagtaatgttgaagagtccttggtagaaagctactttcaatatctcaacagaactatattaaatgtctgctaaaaaaaacttctaagagtattgcta
ttatatcctaagaaaaaaaggactttcatataactatactttcgagtcctgaaatctctgctagaatgctccatgctctaatcgtcagaagtttacta
tttccaccgttaagcgaaatattcctgctattgccttttcgatgatgcaaggaccataaagacgcgatgttctcaatgactcttccgtctgctaagaa
cgagttcaagttactttaagtactcgtactccctgtctttgagcattatgaattttagacgtgaaatggctccggttttgaagtgctgaaaaactttg
gttccagaaggaggaaccgatttaactcttattgaatgacatgagtgctagctatagcttataatcatcgtacttttcaactagagattattatttat
tgcctattcttttataggtcttccgtcgttggtcttattgtttgtttacgttgtgacattttgacaacaatggcagtcaattgtttttgttttgatttataca
tatatactacttttcttctaaggttcgctcatcctttcccattcaccattacttataatttaaaggaattccgttttgtgcactataaatttctacatcaa
agaacttgttaacatcaaggtgtttgtagatctttatttgttttgatttttgattatcaatcttcacacagtaagtacttaaacaattgaagatttacct

ttgtgaaagtcttgaaagtggtttactttatcactattgtgtcaatattctaaaaagtgtgattgaattataattaatacgattcccttcagaattgca
ctttcatttctatctttcttactttttgagtttgtcttttgctcctggaaggaaagtttttatttttactaccagcctgatttttgtcccttaaagaaagtt
gttttgaatcgttcataatgttgggtgacatttatggaaacagactttttctcagaacctttgtcttattcagacattgcttcatctactcattcatct
ctgactagttcacccattaaacagtttcttcatcaactgtggaaggttgttactgttaaaggttcaaaaacggtcgaggaaccagaatttcctgatc
agccggttcaaaacattaacgttgaaattcataatgtcgaccggttctatccattgagatatatatcttccattacagaaccgatgaggaagagat
atgaggaacctacagacattacaagttctgatcttctattcaatccttttagtcatggtttatcgtctattgtgttcagctataaaaaatccacttattt
aaagttaggtgcttccgataattacaatgatatgtttgaagatgcacagatttacaagtatggtctctgcatgaaacgcgcacgcagtaatatttta
ctggcaatgaattactcttccaaaatatatctgaaactatctgattatttcaagcatggaaaagctcctttatcggtccttgtgcaccatgtgatgct
atatgaatactatcctgaggaatttcaagatgtactatggtcggctacgcagtttcatgtcatgcatcaagtacgactgaatcgtgcaagtttactt
caggcgagggcctgttaccaaaagttaggggacattcgtatgtactttatagatcctgcggatatgtatgttccgccagattctttgaattggttaat
tatctgcaccaaagatttccagggtctaattcaattaaaacgtactatggatgcaaaaaaactatttcaaagaaggtccaagcacttgactttagc
gcagtttgcaacttcgagctctgacctgagttggctatgtatgatgttgagcataggacgaaatgctcgtgcattgccaatacatggatatctgagc
gcaaaaaaagttttatatccaagtatcatacctgataatgtttttgcatgaggcaagtggattctgttttattcaaagagattcatagcattcatca
atttttagaatttcaagggcgcttgtttatggacctccaagattgtcaaacattccacagagaatacgagatttatcattgttatcgaacaggaaaa
ccgttatatgagagttacgaagaaaaagccagaggcgatgacccttttgttatgagaagttatccaaagatgaattttgaagtatcgatcaactct
aacaaggacataattgaaaaatataagcaagtttttgagtataaaagaaactctctcctcaggcctgtccttgttggaaatggacagaaagcatc
ataaatttactaatgcattttggttatcatacataatataaaaatagtttccaggcagcaacgttgatgaatgcgtttaagcctaattacgatgttta
tgaattaattaataaaattatttgttgtttatttagattgtttctgtattgaaagttgaaaggactataaagtacgtatataattatcggtttgctctgt
ttgtttatttacaattttatgacgctccaacaaggggcagaatgctactcttgtatctgtcgcttattgttcgttaatatcatttagtaacgatttaact
gaattataagcccttaaaagctttttaacgggactacctaacgttgcgttaataaacaatactcttttaccaaagtacattgaacagcattttacca
cgtacatataggaaggaaataacaaatactcaactctctaaatacaattaccatttactaaagcatatttgcattcataattgtcaacttagaactt
tcataagctggtggtttctttgaatttttttgtattctgtctctagcgaaatctctgctttttagaaagagtcattattgtttaatgcaaaattggagct
atttattctaaaaaaacaaattgacaggctgctcggcgtgtcccattggaatgatcagataaacagcaactgtctgttacacaaacataatgaag
gcagttctgtttccgggtcagggtgttgaatggcgaaggtggatgcaaccgcacctttctaacaaaattgttgaaaagagtttgatcgaagccgaa
gaagttactgaaatccctttgagaaagtatattctggacgctaagcctgatcatggtttaccaaaattgagtacatttatagctcagcctgccatcc
ttgcttgcaccataggactttttcgtgcatttttccaacgaaagtcaaagcctgtcctttttgtaggacattctttgggagaatattcggcactcgtag
ctagtcaagcgctaccttttcaatcggcactaaaacttgttatggttagggcaagagcaatggatgaagcttgccgacttactcaagataagactt
ctatgctcgctttaactctgtataatcgatcgacatgtgcaattaaatttcaggaagacgtaatcaaagcaaaaatgccttttcccttgatagacatt
gccaatgttaattcttccagacagatcgtgctttccggaaactacgaacagcttgaaacgctttcaacttccatacaatccaagtcagtaaaccttg
gtagagtgcgaacaaattggttggatgttgcaggtgcgtttcactcccattatatgcttcctgcgatggaaccattggaggaagctctccaaaaaa
ctgaattcctcacacaggaaacggagacgagctttatctcccctagcaatgagcttcgactgcctgttgtttccaacgttacggccgacttgtatcc
aattgaagcttcaaaaatacgaaagctacttcttcttcagtgcgtaaagcccgtcctttttggcaaagtttatctcggctgaagtctgaacatggg
attactcaatgctatccatgcgggcctggcagtacaatgcaatcattggcgaaacaaaatgagatgcacgtcgaggagcagcattattgaatgca
gtttaacatgttggtactcatcttgttatttgtcgaatccaaaagcatcgtataagagcgttagtgtataaatcttttgcatactagaaaaatcatttc
tacatatctatatattgtgaaaatagaatgttgagctcaagcattcaaagtaattacatggttgaacatacatgtttacaaatcaattatcattactc
ccttactatatactaataataccggaaggaacaattttgtcaatacgtccactcaataagctaatatatttagagctggtacactaccatggatttt
gaatatgctttcacaaccgtgccatttaaatatatttttgacttgctattcaaatgacaagacaagacgaaggcaagaacacaaaccccaaatgt
atgcatttacttaagcttcttcttagggcggagttggttggtgtgaccacactttttcttacggcagttggtggcacgaggaggaaggcgagcatag
cacttacggcagatctgcttttcgcagttgtgcttgctggccaaagccttcaaagagggctcaatgataccaccacgaagacggagaaccaaatg
aagggtagattccttttgaatgttgtaatcggaaagggtacggccatcctccaattgcttaccagcgaaaatcaaacgttgttggtcaggagggat
accttccttgtcctggattttgctcttgacgttgtcaatagtgtcagaagactccacctcaagggtgatagtcttaccagtcaaagtcttgacgaaaa
tctgcatgttggtggtggacaaaggatctctgttgtcgaattattcctctagttttgggcccaaaaaagcagtcacgaccagaacccttgaaacctg
atacttcatttggtaaactgaaataacgaattacagtgacattcaaactctcgttaaatgatggaaattgtcgttttcataaggcatgggaatccga
attttttatattgaatcattatttaaatttctaatgaattgtatgtaagtcggaaaacctgcttcaacttttccagtttcatgggtgacctactcggag
acattttcgagttttcattgagaaagacgagtaaagaattgtgaaaatgtagttataaagctagaaagaatttaattttgtgctttttcttttgtaaa
gtaagagaaggaatttcaaaaaatcatttggtgatcattttcgagaccgatttacgatattgtgagtaaacaaagtctgagatgaacgaaagcgg
tagaaccattctattaaaatctttagatatcaacgacgagcataaaagtttgcataagaggtaccataagctcagcagtttttggtcaagagtgat
tttgatatgaaaggccggccaattgacattctcaaacgaaactagttaaccgaaaatcaaaagctctttaaataagcgttgaaagatatatatttc

aatcgattcacaatttgacttgttttggttttcacgacaattgattcatattccttttccatatcaattcaatatagtcatactcaattgcttcataccttt
tccccatataaagagtattctaacagccttcatcatcctacgaatccttcaagccatctctcggatgtctaaggaattagtaagtcaagaatgtttta
cccataaatgatttgaaacaaacaatttccatcgtatatatctagttcctcaaaacgttggtaagagagcataaatctcttaatgggaagtagtatt
gatgcaaaatagtattttcttctttatgaccattcagtattttcaattggaagagaaagagtcgcaatcatactactctttttcttagttaatcggtga
tcacttgcatatggcctaagtatagaatggtcaaaagatcagaaacaggttcaagttagtgtaacatctcaagcatgatccctttcgaaaaacaa
cattcgtcttcctgccaatccaaccttttttttaaaaaaaaaaaaaagcttcaaaaaaatcaatttaacacacacactttgagacgctcgccctgaca
tcttcgtcgaaatcgggcagtgactttcttaaattccctattgaaaaatatctgttatgcagctattaaaaggatgatagcttcataatggtcaccgt
atatcgagtatgatcagcagcattctaaaattaaatccacagatactcatgaagactttaggctttaaagaatcttaaatgattttattaaaaaaa
aaaacagctattttgaaactaccaagagaatgagcgagtgatcacaactaaccgtgtagaaagcccgcaattcattgtgtgaatgtagcagagg
aaacaaacgtaaatacaaaaaaaaagttctttatttacgaattcatacgaattgagtatctcataaagtcttaatcaaacaaacaaactataagg
aaatggcgacattttctataagaatacaaaagctttcgcgatgccttggtatctcaattgtctctttaatagagacatacgacaagaaattacaaa
atataaatgtcaaccttgtcagaagccacaagtaccatcctcattcactaaatattctacgacggatgagttgtcgatttgtttacgacctatttgga
atgatagctttgtaatagaagacgaaaatttcaggtaaaactcaagctgcttgaaacaattgtttgtggttttcttgatgtaaattaccataggaaa
gaaaaagaaaactcgacaatcatacaagaagcaaagctatatataaaagattctacattttattttaaagtcaaccgtatcgggcattcagggca
ttgaaaatagtgaattcatagaaaaagacaagaaaatttacgagagttatcagtccaatattactttttcatgttaaacaaagtaactaggtaata
gtggacataatggaaaagtaggggacaataaatcaaagatttaaacctagctttcttgactagaaacgcttgatgagataaagaagagagggg
aaatccatttacataaatgagcaagttttgcaactactgaatgcattgcaaacaaatgagagatatacataggcataaagaacaggaaaagaac
tcaaagtctctccattctctccaaaatagcacgagtgaattcctcagtgttagcagttccgttcaagtctttggtgacaacctttccgtcctcgataa
cagaataagtggcagcattaatcaaatcggcatactccttcaaaccaagatgacgaagcatcatgcaagcactaagtatcatggcggtaggatt
ggcttcattacgtcccgagatgttcaattgagcatgacgacagccaggttcaaaaagggcatgctcatgtccaaaattagcaccggggataacac
cggggccaccaacgagggcactgccaatgttactcaagatgctgccgtacaagttgggagtgacaagcacatcgaattgttgagggcgagaga
cggcctgcatagaagcattgtcaacgatcaagtcattagcggtaatggactcgtagccagcggcggtgttgtagaaggttttacggaaaagacca
tcagccagtttcataatattggctttgtgaatacaagtaacggtctttctgttatttttaagggcatagtcgaaagcaaattgggcaatacgcttcga
ctttggttcagtgataattttaagagactctaccactcctggaacggattgatgttctaaaccggagtactcaccttcagtattttcacggataagag
agaagtcaacaccgctatgacgagtctttactcctgggatgttctttatgtgcgacaaagacgcaaaaaggtccaattctttgcgcaaagcaacat
taaaagatgtgtgtccgtcgcgttctgaaggagtataaaagattcccttcaaaccaaccttattccggttcaaggattgaattgcttcatgcaaagc
tacttctgtcggcttgtctgttttgtccattcccgtcacatcgatctcttcaaattctaccgggacattagcagccttgaagatagatgcaaccgattt
ggtagtttcttttccggtaccgtcgcctggaatcagagttaccgtgtactttccaccatattttaaaggttgaaatactgatgacctcctaactgctgt
tttgaacatatcctacttgctgttttctaatcaacttgggtcctttcaacgtattttgttgttggtagaggaaaagcgggtaactcgctcgatatccctt
atttgaatccgacgatgatgcctatatcctatagtgcaatcaaaacccgcgtaggcttacaaagttctgttagctaccttgttacctatttgcgaatc
ttgctaagacaaagtcaaacgaacaagtcaaagagacagaaaaaagaaagagaaggtaagaacttttctttctcctcgatttcttcgttcaaaat
tacttttatagaatccgaagtttgcgtattctcgcacactatgaagcatatattactctttacgaaaatgattgtatataaacattacaaagcgtcgc
tacgaaagctttatgttacgaaaacgaaaagaatcccgctctacaactcacattatgaacgaaaaaggggcgacttaattcataaataaaattat
ccctaatgttttacataactttcataagaaaattaacgacgataacgtctcaagcaaagatggtaagataaaacggttcggttcaaataaaacaa
agacaaatgaaaataagtttgtacctatgcatgcatacatttgaccatagcatagcatttcaaagggcgcatgagtaccaacggaagcgtacatg
aggttagatatgaatgggaatgtagaaataaagaaaagtaaaaacaaagatctggactttttttctttattaaagaaaatttattcttgaccgaaa
ccaatggtttcttcaacagcaatagacaatttatggtccaaatcatgcttagatatgtatggcggaagatccaaccggttaaagcaagtatgtgct
tttggtagttttgatggttcgccagacttctcaattgtaaaccttcgagggccatcgcttccctgtaaatccttgaatccattcacaggaatacggct
cgtgcccgtagtaaactgcaaaaggcgagacttcttttcattgctccattcattcataagctcccagaaccatttgataatttggtcattgtcactgt
atgaacgataatctgtatgtttttttccaatcatccatatctacctctgagataccaccaatcaaaagctccaactctcgttcatcaaatacatttatc
aaatcttggggaataagttcactaaatccatcatgaaatgcattaaattggtcttctatgcgtttctgcacacgccaaaccgtcaccaaatccacat
attcttttttgttttcttcagtcacttcaatattacgtccatccggctttaaatcaatcgtgaccacttcgccaaaacagttatcttccacgctgaacgt
caaatccagaattccagtaatatcatttttcaaaatccaaactaagcttcgatagtactctgcatccatgctttccatgtcctgtagagttattttcttt
tgcaaaatcattttgtaaaaagagacgacaaagaaagcatcaacgaaacgtcgatgaaagacagctagtccgatcacacgtccaataaactta
aagtaatttaagtgttcgggattgattccagaatgcgggttgatttgcaatgtatagttatccatagaagaatattcaaataaacagtaaaaaggg
ttaaacatttcatgtgacaacagataaaagtattcacgcgatagaccaccataatccagtccgtcttctccttcaaatttaatcatcaaacgtttttt
tagatctgacgctgactggcgcatgatttctgcatatgaatcctcaaagatgctgtttcgtcgaactttaatatgacattgaccaggtaaaggatgc
aaagctggttgggaaaggaagtaaataagttttcgtctaaaatcacgtttgtactgtggtacattttgatccaaagacgaaggaagacgtggatc

atcccaagtggttgttttcgtgttgtgatcaacaaaatacacccgagcagtattggtaagacgcatttcccacccacttggtagtggtcccaattgt
gatacaggttgctgttgaattgtcgcattgttcggaccaccatatgtccgaatatactgctgtcgacgtggatcaacccaagtggtagttctcgtatt
gtggtctacaaagtatggacgaccttcaggagtgtatctctgttcccatccaggaggaagttcaccagaaccagcagttgtagcattcgacgtcaa
catactggcttcagtacgtctagcaggaacactagaagaaggctgaaggccttctgtaactgcattggacccaccgtttaccatttgatgttgatc
attacctatgatagcgcccaaattagggcgtatccatgttgtgctccgagtattatgatcaacataataagtgcggcccaaattatcagttcttcgct
cccatccaggtggaagtcttccatactgatcttcaaatgaactgtaagtccgtggttcaacaggaacagcggcagaactgtcagccgtacttcgag
aaggtgttgtgctgaccgaactagtaacgccgccattaggcaatactctttcatttccagcattagaggtacgggagctagtaccggaagcagca
actgaaggagcctgtaatccggactgggcagtagtcgaaagatttaatatgatttttccgtgaaccactgtattctcgttggatttcttcagttcacg
agtcaacatttcgtctccgccgactgccagatctatcacgtcgctaattcgaacatttactacaccaagaaatccttgtcccttctttttaaacttcttt
tggtcaaagatctgaacagcaatcgtgctactatcgaccacgttcctgataaatgttagttaaaacagacaaagtaaaagaacgataactaggc
atcaaatgataaacacagtcatctagcatttccaaggtgtttgataaataaaacgacaaccgcttcaatgtctaccgtacagaggtttacatacac
ttcaaatgtctcattccaatacggatttaaagttttctttatggcagtagtagtatgtgtttgttctccatctacagttaacactgcaaagggatcagg
aaatcgaaaaacatcacgtttataaagaccatcagctgcaacgactaattctgttagcgataaccaatttacaagtcatatacctgttactcgaag
ctttcgggccctgatacaaatagttagttcaatgtttataaagccggtatatgaaacgtactgattagaacttgacatatgtacggaaaaaatattt
tgttacacaaatcttttttctatttctcgtttaaaggaattttgtccagaaaaagtccaatcctctgtctgcgtttacgccgtaaaagagaaaatcga
acacaccaattgtccttgaaaagtactttatggaatcgaataataaatattacttataagaatcaaataccaatccaaattcgtgctttttaggcac
aatttgaaatccgacggatttcgcgtggtaagtaagaaaaaccaacttaataccacgcattttcatgagcttttctccagtagataagccagagtg
tttaatttcgttattgtgaatagcgaattataagatagcgcgaaatcaaacaataggaataaaatattaaaattataacgtacaatgcgattgatg
aatggttacaaaacaatgacttaggaatagtgtaaatagggaaacatctatttgttttaatatattatcgaagaggattatgaggtgtatagcgtt
gaatagattgcagtttcataaatcaagtatatgaagcaatttgggaaatagaatttggaaatagatctgaagcttgtgtattctaagattcaaata
accattagctttgatgcaagacaaatgaaaacgaatacacaactccgtaaacttattgtacaaatactttatgggccttttcaaccaaacgtgaat
gacaaagaaggaatccaggtgacaagaaaaaccatttgaatctttataaatccaaatcgtgatctccatacatcaaatatttttgacatagaaaa
catgaaggttgcatgcacaccttgaattcccccgtcgttgtatccttttacacaaaagggatgctctagacgaaaacaaaccaatgaaaacgaga
aaataagtaaataaaataaagaaaaataaacaaatgcagaaatatcgtggaaagaaggttatgcgggaataaaggaaagatttagagatgtc
aaacgatcggacaaaaacacctgcaaggctagacgctagacaagcgtctagcttatattttcggttttttaaaattttttgtgaaaataaacgaa
ataaagaaagtttcgttctataacaacaactggtaatatcaaacaagcaaatacccaaataaaagtaagtttcttttgtttgtgtcgagcgctcaa
tcataggaaaacatgtctgttcactaaacgatcgtcttctttttcaagacacataatcttaagaatgaggacacatggtaagcttaatttatacctc
acagttcacccaccataaagacttataataatcatggctgctataaaccagtagcaatgatattttgtaaagaatattatagcattctataatattc
atgagatgattgaagttaaatcatatttttacggcgagcctcaccggaagtgcgaggtggcatctgactactgtcggcgaatggagagtaattag
gttggcttgcaacactttccgttggagaaggacggcttgaatggcgggataacaatctggaggattcagaagaattaggaggagcctcactggtt
tgactttcagtatcgctttcgggaatggtagctaacgggccttcaaaataagattcagatccactttgttcagctgtttctgggttatgctcatcaaa
aggagaagcacctaaaccctcagaaaaattacttccattgtcggcagcttgaggaccagctgcattctctggaggatcaggttgagtaggattgc
caataggtctaaccatacgattaccaggaggaggaccatttggtgcattacgattagcagtagctgctccgcccgccaaacctgcgccagcaccc
gcggcagaggaggcggcagcagctgggttaccagctgcagcgtttgtgatgttaggagaagcagtggaatttggagctgtaggtgcagcgttag
gtgcggcaacagctggtctggtagcaataggacgaccagaacccgtgctggcgacagctggtgcagggcttccactccagccacggtatctgtca
ggggaagaggcgcggttagggccacggaaattgttgttttgattgggattgaaaccatattcctcaacctcttgcatacgttcagctttaatacgtt
tagcccgttggagtctcttccaaaagaggacacctaatacaattaaaatgagtaccacaccgataggaatggcgacggcacacccaatgacgac
gcttttatggttgctatcactactctttttagagttagaaggaagactaccagaggaagaaagcggggttgatttcgaactagacgcggaagagat
tggggaagaagaagtattccaattcatagaaccagtatgggagacatacgaggtcgaaatttcggtaacgaacgagggtccgtcgctgtaaacg
ctactaacgatggtaccagaagaactgactaaatccgagggtgaaggagaagcggaggaactagatccagagctgctgttgttatcatcactgc
cagtgttgcttgttgcttgactcgatgaagaagtggtaggagaagtgttttcgctagaaatgctggaactaccagcagcagaagagggggagctt
gacacaggcgtggacgatgactcactactggataaaggggtggaggagaaagaagcaggtgctgaagtagagctgaatgtattactcacgctt
gtagaattcatggcaacagcaattaaatccccttctccgtaaaaagatagttttctctttgattctgagtatataaaagattgaaggaatttttctttt
atctaaataaacggaagcgataaaaaaagttgcgtggaattgcttcgctatgccttttttttcgttccttattcaggttgattgtagttgctgaaggaa
agtaaagttgctattgtggataggtttattagggaagggtagtgcactttactgctcagaaaattgtcctgggatattccaagactggaaaaggga
aatacagttctttattttttttatttgtttttttattttttggaaaggttcatttagtgaaagaaactttattttggggaacatgttaccaacaatacataa
aggaagtcggtttcaatcgaattctcagaggtaaacactgtaaatttgaaaaagaaaactgaaaagaaatatgtttgaaaggtgagaatcttga
aaattcaaaaccatgtaggccttcggtttggaataccatgaactaagaccaattcaggcaccaccaaagtaatattcgatatggagcgtaggatc

tcaagttttactcctcagtacgaatattaccaactgtacaaaagttttaatttaccataagtcatttgtagatctgctgtgtgctatcaaaagaaagt
acaatttaccagaaaggaaggaaataggattaagcaatcattcattatttcaattaggataataactctatcaagtccaaactagattcttggata
tgaaaagcttgtgatcaataaatatctcatgtaattaagtaaacctcgtttaagaaaaattaaagtcatataatcatgaatatgcgaaaattcaac
aaatcaatgatatacaaagtagtgtagtgcaaaagaaagaaattccttggttaaccaaaaaaaatgcctgcatattccatttatatctgaaaaga
tccgttaaaatctaaacatgccaaattcgtcgtcactagaatcccatgagtgtcccattaaccttcgcataccagcgtcctctgcagttgcaagtcg
cggaatagacacttgaggatgatcaagaggtttgtagatagatgatatacaagtgttgtaggaatgacaagatttgcatttgtgtccaaggaaat
gatagttggtatcagatcgagagttgcaatcattacagtgtattgtagttatccatgtattgtaaggatagggcatggctgtcgctcgatttccac
gtccaggatgcgaaacatggaattgacgttattaattgtctttgagcatgtcggacagcgatagtttgtcttaatatattcaccataacaacgctgg
tgcaatggatgagaacatgcgagaaaaatcactctttccgtcgagttgaacatgtattctccacaaatagggcagttgcaatctgtacttcgttca
atgcaccgatgagtatcatagacagcgataggaaggcaaataccacaagtctaggaaatattagtaagcttgaattaacagactctgctgtctta
ccttgcaatggaaataatcatcgcccagaccacgacctattcgacagatgccacagtcatcacagtgatagctggatttcgatggatcatcatccc
atagtttgcacttttattatctgttagtcaaattttaactcatcctctatggtccataccttgttgcaataataacgagccatggactcgtgacaaaac
ttgcagttctgagctgccggctgcgctttaaagcaaaacatacaaagcatattttcggtagcagttcgattgagagtatgatcactcacttcattat
ggcaatgtctgcatggaaaccattgtttacaatcagcgcactgcttcttacagcaacgcatataatgcttacacccgtaaataccgtactcctgatc
atgaaacgttggttttaaatccttagaacttaaatcgtctttatttgtcgtcgagcgtttcttatccttgcgatactttgcatatccagacattaaaagt
ttctgaatcgatttgttagaaatcaaacctttttgactaagtttctttccatacctgcataacagacgcctttcgtttttcactaaattgtgattgagcc
tgtatttgctgaatcttcttgcgaaccgaatcttggtcattcaggtcaactccacgcagaattttctcatcttcttcatcttcctctagtattcgtgctttt
tcagacaaaacggaactcactcttggaaggttttcgccatcttcttctgttatatcattcagtaagtctggaggcaattcaaatacggacgttaattc
ttcagctgcctcctcaaatatctgcgcaatttctcgcagatattcagaccaagacataagtactagctagccttcaaaacaccttggtgattattaa
tgtttttgtaaacggtctgtttgtggaagaatcgttattcctcgttacatctcattaaaggttttgtacatgaagttaattacgttaaagaacaacaga
aaaaccatttaatacgcgattgaaacaatcttaaagacatagagatatactgatttgaaggactgaagagatgatgagttccaagcttcagaga
aggatgcttaaggtcacattattatttgttaaatagtcgctgcaagtgaaatagtatatacacgagtcaaaagggaattatagtattaattgtttga
tttttaacgtctatcaaagtatacaagacggcgacttttatcagagacgtacattatgtataagaaaggatatatataagtataaacaaaaacgt
aatagatacccttaaaagtaatattgtcataacgatttgacctttttgaatgaatttaagaaaaaaagatggttcatctttttccacgtcgttttttc
cttcccattcatgccaactaccttgcatatcttttaaactgtcacatgttagtcaaatagtaaatcaaataattgagtgggaacgtacattacagctg
gttcaggagggggcgattccgaaacaaaatattcatgcatcaaagcctcatgagctgatggacgatgagcaggattaagggataacaatttctg
acatagatcaattgccgcaggtgtaagtttttctcggaaggtttctagaaagcgacaagacttttcatgaggtggttttaaaaggttataccatggc
atatttttaacctctggccaggattctatattgggtgttcccatcaaatcgtaaataatttctaactggtgtatttcatctttcccttggaaaaggggct
ttccaatgaaaagctctatgacaatacatccggcgctccagatatccacagcactatcatactttgtttctcctaaaagtagctccggaggtcgaa
accacaaagtaataactcgattggtgtaagttgcgccagccttaggataagtcttaaaccttgcaagaccgaaatctgcaaactttaaatctccat
tattgtttagtagtatgttagatccctttatgtcccgatgaatcacaccgagatgatgaagataatccaaggcttcaaagagttgtttcgacagatg
cttgatatttcctggtgtaaaatgcagttgagaattaagtaacacacccgtcaaatcatgatccatgtactcaaaaaccatattcacagaatctaa
aaatgatgttagtacgtaccaacctatatgatgcgtactttttcaaccaaaatttctaataatttaacaatgtttttatgccgcaggcgctgaagaa
tttttacctcacgaactgtagtgattggaaactgaaatgatgttagcaaggtaataaacaacagatagttgtacacatgaaattacgcaccccatc
cttttcttgttctaagcgaattcgcttcaaagccactaattctcctgttacattattaattgctttgtatactttcccgtatgtgccttcaccaatctgat
ctactttttcgtacgcagactttccatacgtataggtatagatcggttcttttggtggcacacacgtaatgcttgggggaggtgaattcaaaaccatc
cttttagaaggaggctgatcaaatcgtgaacgtctagaaggcgctggctcatgcggaatattaaatcgaaaacggctaggttgattcccacggat
attaggatcgcgatgagagataccagagctggtaggacttgatttctttgtagaatattgatttgatctaaaatcttctggggtttccttactaggaa
gattgcttgatcgagactgaattgagggcatcttagaagatgaattggctgatggaaaacgaggtaacccagaagggtgaggaggaggaggag
gaggaggaggaagagactgtttagattgaggaacctggtaagtagaagacgggggaggaggtggaggggaccgaggaggagaggtaggact
tgaatgggaacgtgaaaagaacggccgcttgcgcgaacgatcaaattcaggggaaatcggggatcgttgaggagaaaacggaggttttctata
cttatttttctttttaggacctcgggatttgggaggtctaccgttgaatctggagcgggaaggacctgtactatggtgaaatccacccccttcggcgat
caaactcgtcttgattggaagtcgtatccattaaaactctatgtccgcctaaagcttcctcgtatgcatcagagcgtctatgtttttcttcgttgttata
tgaattaaggccatttggctcggatccccgccgacgagaagataattcttttgaataagacatggttataatataggattaacaatacaaagaca
agcttgacaacagtaaaaaataaaatgaaaaccgacggtatcacaaaagcaaataatttttttcctttacaagtatttttggtatagaaaagggaa
aactcaaaaaccgattattggaatcaatcaatcactcatttaaagaaggatagggatagaatccttcaattcacaagaaaatcaaagagtaagc
tgaatcacggctcaaattttttacaattaatgaaaaaagaagttcgcaaagtaaaaagtgtataatccttgtttgttaatgattattaggagaagc
aaagaaatcgaaaatttttaaatagataattcggtagatactttttgaatatataaatagcaagaaaaacgatggcagacctcaatagagtattt

aagacaagaaaaagcggatgtattgagttacacttagatgaacatgattaacttcttctcttaagaggaggaataaatgaaggtctttcaggatg
ataatttaaagggaacaaaatatcttcaaggctataacttgatttccaggtttttttttgtatctttacaatttttgttgtcggtgctttatgtgtaggg
aaggaggatcgtgtcgtatagcgtctgcactgtacctacttcccgacttagttaaccaccagcaatggtgattctatagaaaattggttgcttattat
ggtcatagaaaaggtaagtattctgtttatttgagattcttattttgaagcataaattactttttgctcacatattacagaaaattgatggtaggatg
gtatactcgaaaagcagctctagacacttagaagaaaaggaaataaagctttttttaaaaaaatgtgtaaggatagaacaaacttttgataaacc
gagtgagcatattctttgagtcgatctgatgaagcaatgaagatacatctggtaatccgcatggatccttaacctttcatatatgaaataatctattt
aaggaatcaaatgtggattggaaaggcaagtctgagcttcttattttagtacattcctatgagtatgatgaccaactagacagatctcgtaagcaa
ctttgttttactgtcagacacagccagcgtttgcaggctcctgttgaagggagtattcgtctgctggttcctttgtcttgattgtttccttctaattttaa
tgaaacagctaatattttagctttcagaaacacagtacgtagtaccactgacgaagcaaaaatttggttagaataattgttgattatgtattgaga
attcctcttggatagaaatgaatgagcatcaacatcatatatcctattcttatgttgtaagcgctctttgtttgaatcctttttttatgtttacagccaag
ctaagagagtatgaacaagttatttcaaagtaataaagaagggatccatgagaaaaaaaaaaaaaaaaaatgtataaagaattattacgatggt
cattcattctgttttacttgtaaactcctataaactctacactgaaacgaaagtgattcattcatctcaaatctcctttttatttttttttttaaattattaac
ttgatagctttttttatattgtataaagataatttgtttgaattcgatttctttttttcttatttgtttactaatacaactataaagacatcgttatcgtactt
aggaccgtcgttgcgaaatcgttataccaccgtggatttttagaaaaaacaacttccttttctgtgctgaggttcaattatttgaattcatttcgtttg
tatttttactttttgctcctttgtattctgaagtttattcttatttttcggaccagataccacaaagtttcacctgaagaagtcttgtagagtcagtaagt
ccaagtccatctggaacaactaggctaagcacttaaaaatggaagatgatttgaaaagaaattcaaactcctctgaattcacgagagatgaaga
aaaaggccatactacgtcggtagtgaggaatggtttcaaggataccacgtttctaagctcggcggaaaaagaagaaagcaatattttaaaagag
aatgacaataattcacatacgcccatgaactctaccatgaactctaatcctgatcgaaccatgtctttgcccaaggagaatactcctaatcatgctt
cagaacgaagtcccaagcacaataaggaaggatccgaaagtcagcctcctctcccagacgaaccagttcctgaagaatatcaacgtcgatcac
aaacagaatccgaaagtcaacctcctcttccagacgaaccagttcctgaagaatatcaacatcgatcacaaacaaaatccgaaagtcaacctcn
nnnnnnnnnnnnnnnnnnnnnnnnnnnnnnnnnnnnnnnnnnnnnnnnnnnnnnnnnnnnnnnnnnnnnnnnnnnnnn
nnnnnnnnnnnnnnnnnnnngatcacaaacaaaatccgaaagtcaacctcctcttccagacgaaccagttcctgaagaatatcaacatc
gatcacaaacaaaatccgaaagtcaacctcctctcccagacgaaccaattcctgaagaatataaagaatctgttgtcaatgattctggtcttcctg
ctacttccagtacagaggcatgtacgcctcagccctcgatagcttggtcgaacgatcaacaagtggcagcgatttgggatacaaacgctcaagca
tactacttttgggaccgtcgtaccaataccaccagctgggaaaatcctctagaagaaaatgactcagaagacgaatcatcaagcaagtatgctgc
tagagtacaatttaatcgtctttcgggtaaatacatgcccgagtgggcgacccccgagataaaaagtgaggaatacaaggcctacaaacacatg
gagcagttctttgatgtggatgctgctatgtcaagtacacaaggaaaaagtcttttggaggagcgtaaaaacaaaagatacacgagaaaacaaa
tggaagaaatgaagaaaaaaaaaaagaaaagaaggagcaaaaacgtcgagcaatgtttgacattgcttccgatgataaagatttccggcgaa
gaaaaatcattcgctactaacactttttgtacattattgctaagtcctgttctcatgatcctggtagaactgttatagaggacttttactattttgctag
tagttcattccacgtagaatttcgttttcaacttcctgttgtaatttatttaatagttaatttatgttttctcaattgaaacaacttctcattatttgatttc
agtcgggttttgtttgtttattaccgaaatacagtgatttttaaaacgtcctgcaccaacatacgaatccagcgaaatccttcaacatagcaaatac
caaacgttttcaatggcagcatatccttccagtgctaggatatcatctgtttctttaaaaaataaagtccttcagactccctgttttatcttccctactt
ctaagggaacaattcctcatttaacacctgacaacataaatcacaatgaggttccggttgtctactttgggctagaagattcgtaagttttttcttaa
ttttaataataaacctatagtgctaattttactgtgagtgatcagattagatttattagaaaagtcccctattttaaacgcggacggtgagattgaca
agtgggtagcaggaactccttctcaatctatcttaatagctcctagaagaagcagtccgcttccttctgtgtctgctggccctcatcatattcaagtc
cttacctcaagtggattgagaaaactgacaaacgaagtctatgtaaaggcgattaccaagcttcgccctgaaatagcaattcctctgaatgataa
tccctcaaatgctcctggtgtaaagagaaaacctaaaatgatagagcggtcagttacctggaccgatgaattactgaccgttttgcaaaacaagc
agcttacagagaaagtgaaggttttctttcccattccgaacgttgaagctcctttttttgaatcctttaatagaacgttttcgagaaaatgattaccag
caaaatatttcaggattagcctgctatgggaatttacaatccataccttctgaactatccatttatccaaaattgtttcttcgtcctttgttaactccttt
ggcaatcttacaaagtattcatcaaggcgctgatatcgtaatggctgatttagtaaatcaagcttccgatgctggtatagttttcacattttctttccc
tcctcctgtagaggacctacagaatagaaagttagagctggcttgggatatgtggcataaccgttttgctacggatctttcctctctacaacctggg
tgtaattgcaatatatgtcagtcatatagccgggcttatgttcatcatttacttcagacgcgagagcttgttggttggattatggtgcaacagtatgtt
tttttatcataaaccacctcataaaatcactactaactttcactttagacacaacatacatgttttccacaacttcttttgtggaataagagattctat
agcagctggaagctttcttgaagatactgaacgatttgaaagaatttacgctcctaactttcctcgaactactggtttaggaccacgaaagcgagg
ttatcaaatggatttgaccgatgttcagcctatcgaaaataaacccaactgggttcaactaaaaaccgagacagatgatgacaagacctcccata
tgattcagaaaaaggaaaatgctcaagaatttcaagagcctgaattacctgagaggtaatgtcttttgccttttcaattctaacttatgttttggcag
agatactaccgttggtaattttgctgatacctacgcagctcaaagtgaaaataaaagtggatctgaattggaggaagatttgcttgctgagcttga
cgaaatcgacgatagtgcttatcgagaaaaccgaatgcaatcattgaaagaagagtatgtaattacgctaaatcaagctaatattcgatagattt

gaaagagtttccgctgccaaggaaaaaggtcatatgcaatatttgacagttgaaagtgaaagagaagtaatggatttaacaacgtacgtcagat
tctatattagtttattctaaccttcttttatagctcttcaaagcgtgtagtcatccatttctttcatcctgattttagacgctgcaaaataatggattcgc
accttgaggtaaagaataaagttaaccaggaaaaatgactaatatgtttagaaaatatccaaaagtcattgggaaactaagtttatacgtatcga
agcagcaaatgctccatttcttgctttaaaattgggcttaaaagtgctaccggtagtactttgttacgttgacgcaagtctcgttagtaaactggttg
gattcgctgatttagggaataatgacgattttgatacacccgtgttggagttttggttgttaaaatgggctgcaatagaaaagataaaggagaata
ttcctcaagggaaaaagtcaattatgggatttaaggatacgaaaaatgaatcagaagacagcgatttagattaatttatagaaaagatttagata
aagtaccaaaaagaaatttaaatacaatccatgatcataggtaccacctttttacggctagcagcggagttcctttggttaaatacaactatagtg
ttcccttcaatgtttgctaagagttctaaacctaagtctttttgatgtttctccaaaaatttttcagcaaatgatcctccctgcttagttcttctatataa
tatcatttgacgacgaaattcttccttttttgcttagggaaagtccaataattacaagatctgtttgcttctcttcggcaaattccttcaattcttgttgc
caattattccgcttctcgacaatccaatcaattccttttcctatgctgggataggatatagacacatttcctggagcgtattgttttaaatctcgatgt
aaaagatccataaaagaaaatcctttaaagcttccttttctgtcttttaaagtattgaaaaactcatcacgatcccatccagatggaacgaactgg
aataactgttctgctacctcaacatcagtatctgtagtcttttcatttttgagatttccagtatcgataagtaaaggaccaagcgctaaaacggctat
atcttctgcttccttttgactttccgttgattcatgaagagacttcagtaaatccatgaaatattgactcacaagagttgaacaacttccgcattcttt
gataattctagggttggcatgtaaataattaccttcgtctttgtggtgatcaattacaccctccacgcttcctttgaatttagctacttctttacggtct
agggcattgtgatcaaccaaatgaataggagacgagtagaacctttcgggtactgtcgcaacttcattaagaaatattaaattatctgtgtgtaat
tttagcatgtcaagaagacataacaactctgggcgaagacgaagctcatttttaggaatattaaaaaaagggacaacgcacctttcacgaagctt
atgttgcaagaaataggcgtaaactatagaagtagcacagctatccaagtctaatatatatgttaacgactggtagatcattatgttacatacctg
cggactcattaccactaacaaaagtcaaatttttcgtttgcagattagctgaagtttcagaaacaatttggaaatattgtttatagttactttctaac
aatttggccagttttgaaggaaatgacatggccttcgtatgaaaaagttgcagatagcaaataaatagggtagacgacagggaagaggcatctg
atattctccttcactatactatcgttgaatgtaatttttttatgttacttttacatcgagaataactatttagtcatttataacattctcataggaatatag
tgcattgcttatgcccttgaattctcatattatcaataaaataaaaagcatagcagggtttgtaaggactgtatagaaatcattattaacactatcat
attatactaaatctaaacattttcattatggtctttacagtccctttgattctaaggaaacgtattagtgagtaattatagcataccataaaacatca
aaacgaacaaaaattgataaaatttcaagccaattgaacgacttccccccttattttagaattctagtttttacttgatcttctgtcttcatgcggtcac
attgtcagaggtattttgccctatggcacgctcaaacttttcgagtacttccggacttccaacataaccacatttgctagatattgaatcataaaagt
aagacaagtctttctttataccgttccattttgcaggatcctgagcgaggtctgtgagttccaattgcaatctgtcttcaaacatctttatgacatttg
gaataacacgaatgcaatctggctcagagtaacggtgtaaaactttatagagtttgaccaagttcttaatgagatccttcatataatcatgaggct
cttcattatcccatgtaactatgataatttgaccacagcaaaagtttgatctttctttcatgagagcaataaacttctcagttatctggtcatgatgta
aatcaaaatcgttctcgagactgagcaattcttgagtttgtctaatcccagaatattttgatagaaacttgaccattgaagttgatagtgcttgaaat
aaagtaaccgtttgtgaagctaacgcaatatgtttacctaaaactctactgagtcctgatgaccgtacagttccagctccaagaattaactgatat
actcgggaatttaaagttcgcaaaaggtctatcatgccatagtacaaataatgagagagtcttgggaaatgatatactaactgtccatattggttc
atagtctcaagtaaacgtacagaggcattgaccatatgaattgattgattttgaagaagacagtgttccgacaatgctgactctccttcctcttctg
aacgtttcatgtcatctgcactcggttcttgaaaaaagatccaatcatctggaacttttgttgcgcattggtcgatatgagatgccaatctctgtactt
tctggctgactgaaatagcctcccaatgttccttttcataatctaaagcaagcagttgaatattctttatttgatgatttcgaaaccatttctgtagttc
atgcataacaatggacatgaaatcgttttgtgtgatgccacataaattttcaagttcattataaaacagggaatgcaacgtaaacaattttaaaca
atcctgagaagaatagttgtctaaagcagcgaactgtgcgctatttatgttggttactttttgtaaaattaattcggagctagctgtcattaaatcgt
tcaaaacaatagaataatgatactgttcttcatcttttctggacaaaatatcaattagaagtttaagctgttgtcccagtcggcgtaaaaaattccaa
agacacgaaatagattttgcgagcatgttatagaattctgaaatagacggactttcattggatgcagccggtaaggattgaaaagaaggattttt
tatcgtggagaggttagtagtactgatactttgacgactgacctttccattaagagatttcacttcagcagaagtttgtgttatcaacgactctaatt
ccttaaacacagatgttttgtatatagttaatccagactgcagaccatctgtaaggagaagacaatcaatgaaacgttctaatttttttcataaaatc
tggagaaaagtccgtaacgagaggtaaaggctcagtgttgacccgtagctttttatttttcttcaaataaatagtcaataactccaacggagatct
ctgggatgaaaaatcgcgtatgtcctgaataaggatttcgggaaatttctggattacagtttgagatatggtattataaagtgagcgttggtcttct
aaaagttcaacaataggaggtagagaatggaaatcaattctttcttgcttagactttaactctaactgggcagtgatggaatggatcccgtctatc
gcatttaagaagtgaccgtcacgggcggattggtgagtttgattgctcaaatcaaaaagcgattccaaaaacgacagtgactggattaattgatg
aagctgtgaacaagagcgttcctgcatggaaatagcttctcgttgttcttcacgggcttttgttatttgattgagattctctaaaaagccatgagtat
tttgtaaacaaatctttccatcctttttagattttttaaattttccataataatgggaaaactatcactagcattctcaatctcttgcaacaaatgaag
ttctacaatatccagataccaagagattttctcttgcaacatggagttattcaacaaaagttttctactgccatgttgagcatctcctgcctgagtaa
tgtcgacatgttcactcgcaatgtcaaaagtctgggggtcatcgaggttgaaatcatcctggaaatatatggatgggatggtagatagagggaaa
ttatcaaccacgctaggagtaggaggcggaagctcctcacgatcaaatgtaatggtctctgagtttgatgatggatatgaagactcacttacgag

acctcctcgagtcgtagacacggaattgcgatgcgcaagtgactcatgcacactagaaccagagttataattgatgcgcttagcatgacgccctacatagcgatgaaagctagtttgcttctgcttggagtaacgagtgtattcatgagagatctgatgcaaataagcctggaaatcgtgaaccggaacaatcgggatgggagaaagagagacaggagtgatttccgttctagtaggtgtataggatggaaaagaaggatttatagggtcctctagaagagatttaaacgataatgccgatgctgttggatcagcaaatggtgacaaagggacttgaaaggaagcctctgtagaaggataggttaaagaaggggatggagcaattgattgttcgtccatcaaggccttgtagaaggcagtaattcgccaatttctagtaagtggaatgcaattgtctaaatattctggttgctcagtgtaagcccaacgtatagtgtggttcgttcatctgcgtaaaacgccaaacatgaaacaaacgatctaggaaggataatagctttggtgcatgtcgcatgcacaaaatcaaaaggtgaaaaggcactcatgtcctataataagccatccaaacgattttgttttgatttgtttacaatattctacagatcaatgatctctctttatagttatatttgcatataagatctgataaaatctcgacgtaaacaaagagaaaaacaatgtgatcgcgttgggcagagcaccctccaagcacacgtttcacttttattttataatactttcttttcttttttttctatttttacttcaaggcttgtaaaagctctcctaaagcctagcagaggaatacggttgtatccaacaattccttcgagattgctatactactttaaacgtttcttaccgtgaatgaaaggcttatgaatagtaaataaggataccttccgatgcaatgttctagctgcatagtgagtacagacgatacttcaactctgtcctataccttttaacaatggtttcaatattttgttaaactggctagtgaacaacgcatgccatcctattcgctttggccgactttcgtcaagttgcttcttactctgctcttgtttcttgtctttttctttctttaatttttgaactgcagggattcattatgaaacctctggtgagcgctggtttaggtacgtgcattttgtggcgatttcagccattcgttttggtttcgaccgctctctgtaattgctcagttgttaacaaatccgtatagcccaaggttgcacgggcttatcgttgattggaatcatatttctctctgtgctttcctatttattcgctcatgaagctgaagctttgatgcatgatttggctggtagtggcttaacaggatctcaagtcgctaaaacttgcattggcgccgtcgccatatacacagtaagtagtgttgctatttcctgagcaagtgcctaactttactaccaggttttctttttgttttgcgggtcgcaagtattggttgctcggtaccagaatcgcgttcagctttcgtagaatccttcaggctgttttctttcttttccttcccttgtttctatatatgtataaacaaacttccgaaaatggtggaaagatagtttgattcaatttctacaattgcatggcccctttagattttaggcttgctttccttttcgtttgattcagtgtcattcgtaatattttaattctttctcggttacgtgagggagagagagagagaaataaaaagagaaaaaaaaaaagaaggtgaattcgtatagcatgttacgactaagccgacaaagtttatggatacatgtatatgcactatgattcattattatatgaattttttttcaaacttcctactcttttaatacaaacttttctaattacctatctacgaaatgaattgccttaggagaaattaatatatacggttcccttctctttgatttaatactacgattactactactctatttgcttacttttactttcagtcataattgtgtatagaaagaataaagcaacactttttttattcacagcgtacttatctggcagtattatgaaaggctattttaaaataaaataacaaatttttagataccagaagcagtaaatggaaggacagggaatttgcagaaacgtcctacatctatgccacgattagcccatccatccaggcggctaatgttatatctacgccatacttggtccatagccccagtcaggctttggacgtacttttcggtgttgtgagctggtgtaggagtaattcgaagacgttcagtcccagcagatactgtagggaaattaatactctgcacatatatatgatgattttgcaataaggcatcactagcaagcttggctaattgagcatcaccaaccatagcagggacgatatgagtatcgttagaaagaacgggaattccgatttcagacaaggattgtttcactcgtcgaacagcagaacgctgttgttcgcgctcgacgtttgaggtttttaaatgctcaatagctgtttgagcaccaaccataacatgaggaggtaaggaagtagtaaagataaaaccaggagctagagaacggatcatgtcgacaaagggagaagagccagcaatgtaaccaccgacggcaccataagacttggccaaagtacccgttataatgtcaattttgtctagcaaaccaggagtttcttcagcaacaccgccgccacgaggtccatacatacctacagcgtgaacctcatctaaaaaggtaatagcaccatatttcttggcaagatcgcaaatttcggaaataggagcgacatttccactcatgctgtagatggattcaaaagcaattacctttggacggttcagaggaatggaagccaatttggcttccagatcaacaagatcattatgtttaaaaatcatttttttcacagcgagaattacgaataccattgatcatggaggcgtgattcatttcatcacttaggaaaacacaatttggcaatttcttaccaagagtggagagagtagcatcatttgcaacatagcaactaccaaaaacgagagcggcaggtttttgatgcaagtcggcaagtgtctgttctaaacgtatagcatgctgattgtgcccagcaatattacgagtaccaccagctccacccccatacgtgtctatgcaacgatgcatggcgtcgctaaccttcttatggccgcccatgttgagataatcattgctgcaccaaacctgaacacgggtatttggctctgccaaatgggccaaaggatattcctgagctagacgattaatgttgttgaaataacgatacgatttatcacgctttttctttcgaagctcttcttgataaaaggtttcgtagtcaaattttgtagcacccattggagagattccagaagcatttcgggctgcacttggtagtgtttcttgtttgatggaagcaacctcgttaggtttgccagaggcttctttgactgcttgcgcatgacgacacataccagagcttcggtcggtcacaccagcttttaaatggatctcttccagagaaggctccttggccatatgtttggattgagacttaaatccacgatgacctcggaaaggaaccttgtcacctgtaggaagcgcgcctcctataacggggcaatgagatccagcgcgaaccagaccagtacccatcattctacggagggttgtagcgtctgtcttgctgacaaagggcaacgcttggtagcagcttttacaacgtgttccatggttttctttttaataatcctataaaaccaacagaaaaataaaagtcaatgagaaaaaaaaaggaaaaaagaaaaaaaattccttcgtttttttttaataatataaaaggataatattccaatccgagcagttttcgaaagtactgcgacgacaaaaagaaaaagattgtgtgtgtgtgttgttttttttttggaatagtctaacctttggtattggtcaaggtcgtcttcaacacgcttgttttaaactcgttttcgagaagcaatcaaaaaagaggatccagaaattgaaactcgatcttgttttaataaccctaaaaagttttcgttagaacaactcgatttcgttcgtaaatacgagaatattccgatttatacctttgtgggtggtggtgaaggttaaatgggcatggtagtcgaaaccgccttagtaatactttgcccgattcttgcgggaatcactggcgaacaagcgacaaatgagcgaccgtacaatagagcgagacccaacactagaagaaaacaaagaaagaaagaaaaggtaaacatgaggaaattgaatgagattgaaatgaaacttttataaaaataaaccaagtaggaaaagaaaaaggaaaagaaaaggaaaaaaagaaagaaagaaaagaaaagggaaattggttttatgttgatgaatccaaccttctatagattactggtatcgatttcgcaatttatgttttcatgttttatagaaaatcgaatctcaacgactttgttgatttttataaaagaattcaaagagtttgttaatttgttttaatgactagtgaaaaaatgaaattt

gattttgtatccatgttaaaaaacacgacaaatagaacctttcttgctttataccgagtagagaatgcttatagaaagtacaaaaaagataaaaa
ggtaaaataactaaaaaacaagttttataacccttttcttttttcatatggatgcttcgacctcttacacttttctcattcaatcatgtttataaacag
aaactgcattcctaacaatatcaagtaacccaagtagaatagtcattgtaaacttcttatgtagtagaaagatgtacccatttttgtttatcgttgac
tcatttgtagacattaattgcaactgtcatataaaattaaatcatgatgacaatgataaaagatactgactcattatataagtttatgcgacgccaa
aacataaaccaaagaaatacacttgtacgttgttaccctgctcagaaaagcttgcaaaccaaaatccttgacactttcagatgtaaaaaaaaa
aggcctctatttacattataagaaagactgtaattgcatacattccaagacaaaatagatcaacggtggaaaataagggtttaattaattttattat
tttttttaaaaaaatataacatataaggaaaatatttaaagcgtaaacaatccacatagaccaaatggaattaagattggaagaagaaatcttg
agccaaattggcaaagatacagacgtcccagacaaatcggcaaagtatcaagtccatgaatccattcaagaaaaagatgatacattagatgac
actgattctattgatagaaaacccccattcatgagtgctgccctcatcctattgaataatttggtactctcgatttcattttctattgtcattccaacct
cgaatagctatgtcaaagtccttggaggaagcgaagttctttctggtctcattattggtcttcctatgattacggcgctcttctctttataccctatgtt
actggtgtcgaatcctaaatctgctaatggctttactttttataaacgtccctacgtctgctcatgtgtcactgcgctcgttggccatatcctttattcg
cttgcttttaaagccaaaagcattgccctcatactagtcagtcgaatcattctaggggttgcatgtgtcatgtatctgtaccataaaaagttcatgtc
cgacagaatcctcgtaggtgccaagtacagaacttttatgagtactctcaacacaataatgctaactgctgggattgcaattggtccttttcttggc
ggtctgatggctaaaacttcttcaaattctcacaatgccgtatggaatcaatatacttccggaaattggttgctcgctatcttttggtttgttttatttg
tcatttcttctttattttataatgatgttgatagtaccattcctcctatccctgccagaaaccaaaacgacgattccaattctgaagcccgaacaacg
catccccgtccagattttatctctcttttaaggatttttgttgctggttatattgctttctcttgcttctttacaatggaggcatatcaggcttccatccca
atctatactgcctacctatacaagtattctccttttcaatcgggaaatttccttgcacttgcatgcctaattgatatcccgtttctccttttattctctttt
ctttctcggtggatcgaagatcgaacaattacattgataggtattttaatttcggttatgacagtcgctgttcatctcatagtcaacagacttcaaaa
agagtatctacagctttatttcactttattttgttttgtctttttggaacttccattgctttttcacctctcgtatctcttcttacaaagcagcttcatcca
gctcatcatttgctagcaagcgttgtgattcaaataagcatagctattgcagacaccaccggccctgttttcggtggtgcaatccgtacggttacttc
tcttggattcctgataactttactcgcattacatgtgttggcaaccatctttatgctttgtatttggaaaaccgctaaggttaagactggttaattcttt
ttttttgtttatataatatatttacaatgttaacgaattagcttaatttttgttttgatattcaatgtagatgaatgcgaccattaataaaataaaaatt
atctttctaaaggcgcttatcgacctttaagatatgttaattctttttccatttcttttttttttgggtaatagccgctttcctataatgcttttagcgatta
tggcctttcatatcttttgtactccaaagtaacaatatcattttatataccttatataaatgatgtaccttttttatgtctgtagccaagtgtcataatta
agtaaaaagtataaataaatttagatgatttcgttcaaaaacttgttccaacgaacataagaaagaacaatttatagcgtcaatcgcttagtgtta
tcatatcttggatgtaaacaaacacgtaccccatttctattcaactattttgcaaacagcacgagtagagaggccaagcaaattattattagaaac
aaaattcgaataatcttttttttattttgttttaaaagtcaaacgacaacagtttacgacacagaaaggcagctacaaacgctgccaaccctccaat
tctcaacacccaagcgacactgttatcgtttatgtcacgatggcttccttctgaatgaataaatactatcgtagcgataatggtactcaaaacttga
ggagctgaaataaacacattatgcacaccaatcatttttccgctgctctcacgtaaacccagcttagcaacttcggaagaaaaaagtgaatatgg
aatccaaagagtacaagcccaagataacccacaaattgctaccatcacttgtgcctcccaagatgttcccagaaaaaaagtacttagcatacag
atgccaaataacacatgagaaaaaagccataaattcggtaaagtaagacctttaatttgtacttttgttgaaaatgtgctaataatagaatgattt
gaaggcatcattctctgcaaacgggatgtagatgcagaaggagcaggagaaggagcttctgtaggctcggatcttagagaagacctttcactag
gttcagctgaagatcgtggtatatattcagcaggtcgtaaatgcctttcataggcccttgagctttcttcagttgcagtctcgtcatcgtcatcatcat
catcatcatcatcatcaccctcatgttcaagaagaagaggtaaggctgtatttgaagccaaagaaacaattgcaaacaagagcaatgcaaatga
tccttgacgtgttgccatgtcccagtcctcctcatggcctttaggtgcgtgtctcagaaacagagtaccaacataagttgttatatagaataaaaat
ggaaaccatccaaagtaagcaaaaaactgtacgtagcatatttttcgcaaagagtcaggaaggcttgatatggtttgtctcattgttccaaaaaat
tccagaatctcggacaaaactgactgatcacttccagaaacaggaaatgatctttctttaacaattaaagacgtaatgccaactgtcattacaaga
aaaattgaggccattgtgcaaaatgcttgaagctgggtaatatttaagaaagcaaatatccggtataacggaagaaatcccaacaagtatccgc
aaacattgccaataccaatcattcttcctgcccaagagtttgcatcatgttgttgatctgaacgaacagaatcgacaattagggaacgagcacttg
ccatgactgaaaatacattagagaaatatccaactaataataaaaaaacataccaatattcacagcaatgtctaaaaggtaaatactcaaggtt
gctaacacaatggttacacggtttgcagttgtacccttgaatagtagctcactcaaatctggggcccaccccatcaagaataaactaaaagtaccc
aataagctagctcctagcataaaaggtcttcttcttccaagtttcgactgaaagcgatccgaaagtatcccagatattggttgaataatgatccccg
tcaatggaccagcaatccaaataatggatgtatattctttgcgaagaccgagagaaaatagatatggagaccctgatgtaaataaattagagttt
ataaaagaaataaaagaaaagcctcacttaccataaccaagttcaacagaccctattattatagcatgttagtattatcaagacaacattgcgaa
atgttcgataacttacaggttagctggactcccaaaagagacttgaaataagttagcatagtttccatagtcgaaaacatcaattgatacatacca
ctgttaaagcaattaaataccatatggatctgaggggaataggatccttgtatggattttctaagtccacattatttgaatcatcattgtttctcaac
agatgagagcgctctggataaagctcgctagacattgtgggcaaagacaggaaaagaagggaaccttatgtgatgacagtttaaataaacaag
gcacaagaaagattcaacaatagcaggtggagcaatatttgcaaaaggcaggttctaataagagaaagagacccaagatggtcctgcaagaa

agcaatcctttgtttatacttctgatgttatctttctttttattgctacccaatcgtgtacttttatggtattgtataaaagtaatcagaaaatcgtttat
acgaataccatatagaaaattgaacaatgccgaaagaaaaaaaccaacacagtcgatgtgaatataatccaaggtttcagcttccaagtaaaa
cgaatgaaaatgagtattcaattctctagacatagcaattgttaatatttcattcgcaatcagcaaccaaattggctctgtaaacattatgtttatga
gattcaatgaaaagaaacccaattgaacagcggaaaaaaaaaaatgtcgttctttatctaatgaaatagcaatttactcaccttaaactacgaaaa
ataaaggattctatactccttggattttatacgaagaaattggtttctagttggcttatgttctccccagtttgtttctgtaacactgacgtacagaca
tacagatccggaaggatattagcagcggcgtcttttgcattacggtatcgtttttttacgacaccaagcaatatctaacgcactagatttgtaatgta
aacaaatcagaaaacacgcagtagccttgcgttagttcccttgttttagttattttttagtttgatttcatcctctataggtgtaagtgaacatagcaa
tggtagtatggtgaaagaatatttccttctgactcttcctctagtaaacaagcaatcgagttttctaggttgaggaaatactcgttcgagtgttaatt
aagttgcttatctatttgtttttcttttcagtagaaaagaaaggaaatggctcccttgttcaatagagacgtagaaaaaattggtctgcacagaaac
gggcatgaaggatatttaaatacaaagacacattttactcagtctgtagtgggtttttggacctccttttctttttttaattcatgctttcgttcattgctt
ttctttcctttttttctttgtttatagaagcttgttgtctttaactagtaaatttttcaaaaacctgtaggtcgagcatcctttggaatcgctagtctttca
ttatattttttttataaatcagtacaaatatttgaagaaaactcttctgtaagtatgtgtttacgagtccacaattggttttctcctccaccctccaaagc
aaacttctaccaacaactagaacgctcgacaagaaaagatgggggacaatccttgggttctccaggaacaggtcctgttagaactttcagaagt
gataaaaaaattcactttctgaagattctcaaactcgagcagctgctttaagtgtatgttattggatatcataaaaagctctgtagacttgcttttaaa
gaagtttcattttctttttttaacatactctaacatgtttgaatagttacttgaaaaaacaaaggacattcctgatgtaaataattatctcgcctgtata
ttgatcaacggtgctgaactaagcgtaccaattcgttctgctgctggtttacttttaaaaaataacttgcgtgtttcaaacattgaacttgcacacgg
attgcaaagcctaaattatacgaaatcgacggttgtacgcggtttatgtgatcctcagcctttaattcgcggtatttctggcaacgttataactacgc
tgattagtcgatgggggggttagtgcatggccagaagttctaccacaattgatggatatgctttctggctcaacatctagtactactcaagaaggtgc
tttcagcgcacttacaaaaatttgcgaagattcggcccgagaattagatagagactttgatggaacgcgtcctttggattttatgattcctcgcttca
ttgaattggcgcgtgatggaaaccccaaaatccgtacagatgctttgttttgccttaaccagtttcttttaatccaaagtcagtctttgtacgctcaca
tcgatacgtttctggaaacttgttacagcttagctactgatccttccccgaatgttcgaaagaatgtttgtcaggcccttgtctatttgcttgatgtac
gtcccgataagattgctccctcattgggtagcattgtagaatatatgctctactcaactcaagatatggaccagactgtcgctttggaagcatgcga
attttggttagccatctctgaacaacctgacctctgttcttctttgggtccctatctcgacaagattatcccaatgcttcttcgtggcatggtttactct
gatatggatcttttgcttctaggaaatgatgccgatgattacaatgttgaagatcgacttgaagacattcgtcctcaacatgccaagggtaagtctc
gggtaaccatggatacggagggtcctattggccgccaaggagccgcaaatgatgagtttgatgaacttgatgaggaagacgaagatgatgagg
atgaatttgacgatgaggatgaagatgctgatgctttcatggactggaatttgaggaaatgctcagctgctgccctcgatgctttatcttctttttgg
aagcagaagctacttgatgttattctccctcatttgaaggaaagtttgatgagcactgactggaaagttcaagaggctggcgtattagcattgggt
gctattgcagaaggctgcatggatggaatgatacaatacctgcccgaactttatccatatttactttccctgttggaaagtaataagccgctagttc
gcacaattacatgctggacccttggccgttattcaagttgggcaacttgcttggattccgaagtggatcgtcaaaagtatttcattcctcttattgaa
aagttgctgagaatggtggttgataacaataaaaaggttcaagaagccggttgctctgcctttgcgatcttggaagagcaagccggccctgctctg
gtgcccttcttagaacctattttaactaactttgcttttgccttccaaaagtatcaaagaaaaaaatttgctcatcttgtatgatgctgttcaaacgctt
gccgattacgttggtcctgcacttaatgaaaagcgttatgttgaacttttgatgccgcctttgcttcataagtggtctacacttgttgacgatgaccc
aaatattttccctttgctagaatgcctttcatcggttgctgtgtcccttaaagatggatttgctccctttgcagcagaaacatatgctcgtacttttcgg
atgctccgcaacaccttgtacctgattgacgcttctcagggcgatcctacgattgatgttccagatcgggactttttagttaccactttagatttaatc
agtggtattattcaagcactcggaagtcaaatagccccccttaatcgctcaggcagatcccccgatgggacaaatcattggtgtatgcgccaaaga
cgaagttgccgaagtccgtcaatcggcctatgctctactgggtgatatgtgtatgttttgctttgaccaagttcgtccttattgtgatgcgttgttatta
gacatgttaccacaaatgcagatgcctttgttgcatgtcagtgcctccaacaatgccatctggtccgctggagaaatggcattacagttgggtaaa
gatatgcaaccatgggtgaaacctttgctggagcgtcttatttacattatgcagtctggcaaagcgaacagtacagtactggaaaacgttgccatc
accacaggcagacttggtatgtttaatccagagttagttgctcctcatttggaattttttttaccaaccatggtttgaaattatcaagaccatcggtga
aaatgaagaaaaggactctgctttcagaggattttgcaatatcctggcatgcaatcctcagtccttatcatatcttctccctatgtttgttgtatgtgt
cgcagagtacgaaaaaccttcgatggagttgcaagacatgttccaaaagattttgcaaggatccattgaaatgtttaatggaaaggcagcatgg
caagcttcaccggagatgttggcacaaattcaactacgttacggggtttaagaaaaaggcatgcatatacaccgatggatggtcgaatacacac
gtaaacgtttgggaaaataataattcatttttttttaatgataggaatacggtatattgaatctttacataatcaatttgttctatgtgaaaaaaaag
aggacccgctatggtaatatggatggataccctgaaaagaaacctcttcttattcttattttctataggttgataggtagagaataaaaataaaaa
acctttttttttttttgtctcgaaggataaaggtattcaacaattaagaagaaaaataaattgaatgtaagtaaagcttgtgcttacacttgctttca
atttctagtgctgcttatggctactaatactatacatgcctagctttgtcacgagttgtgaaacataaaagctgtagtatgatgaataacttaacacc
tggaaaagattaaattacaaagaaaaacagcaattcgatttgtagatttaattcttcaagcgtctttatgctatgtggattttcgtgaaagtcgtac
gaggtgtaacatacaacaaggcgtgtatttattcaaggtttttcaaccaaaacatgtcaatactttcataagccaaactcattctaccatgagaaa

cttcgttattttctcttatgctgaacagccccatacataattcctttcaccataatacgatttttgttaccgtaataaaataaaatagtatgatttgaac
cgctaattacatatcatcgaaattccatgaaaatttgattaacgagaaagaaaaaacatcatttcttccgagagactcctacgtaacagccagatt
tcattatcatctttatcgctaattttccatgtcaacttgttaacgaatttgaccagatatatatatagaggatgaccgctgtaatctatcagtaaccac
tgtcaaggaagaagttaaaatagatcttttgaaacattcgaacttgacttgatcatagtaaaagaacgaaattttttgaattaaaagaaactggta
cattttctttttttagaacaagaaacattatatcggcaatgttaggacccttttttgaagctgaacaacggcctcaagtgccctcaattcgcttatggct
catatatgatcaaccgtagcaaatgctttgactccgtgtatgcagctttacaatgtggttaccgtcatatcgactctgcgcaaatgtactttaatgaa
ggagattgtggtagagccatccttcaattcatgaaggacactggcacgaaacgtgaagacatctggtttaccagcaagctaaacaacttggctgg
ttatagcgcgacactcaaaagcattgacagcagcgtgaaggcttgtggtttaggatacattgacttgtttttgcttcattctccttatggagaccgcc
tcggctcttggaaagctttggaagaagctgttgaaacaggaaaaattcgtgctattggtgtatccaactttggacctcatcacattcagcagttgtt
ggaccatcatcctaagattgttccctgcgtcaaccaaattgagctccatcccttctgctctcaaagcaaggtggttgaatactgccaagcaaaggg
aattgctttggctgcctatgctcctttggcccatggtgaaaagtttggcaataagcaattgattggcattgccaacaaatataacaagacagaggc
acaaatcatgatccgctttttactccaacgtggttatattgttcttcccaagagctctactccttctcgcatcaagggtaatggtgaagtgtttgatttt
gaactttccaaggatgagatggataccttgtataatttggatgaagactatcattccgattggaacccatgtgtttcttctctctaagttgatttctct
cttggttcgttttccttgaggggagaaggtaaagaagcttgaaaacaaacaactttatagatttgtgatgctgagatgctttaatgatattgatttat
gatttagcaaatgtgaaagaaaaaaaaaaaaaaaaaaaaaaaaaaaaaataatgtagtaaaatgggaattcatggatcctttaatggtatgtaatt
ctacaaatctatgaagaaacatcaatcctgttgcttttgcaatacaacacgtctaagtttcgtccgttctacctaaataaatgtgctcttttcaaagt
aatatgcatcgtgtataaaataatcttgcatcttctttactagctacattcgttgactctcgtcttagttcatagtaccattcacgctaccttgcttaca
tacactacacagcgccagagaaatcctcgaaatccttgaaaatcttgcataaattgtacttatttgtactatgattactccttcttttcgtcaatattg
ctttaaacgctcattctctttttctcctgctcgtcttcgtgatgtccaacgtttaaccttcacagggaatttagtccgtgatgctgaacgtttgcaatcg
caacgaggtaatccctacatgaaatacacagttgcttccaataatggaaaggatgtgcctgctacattccgcacggtattcttttttgatgaccgcg
aatttgatcgtatggaagctatcctcaaaaagggaaaccatatctttgtagaagccaatactgtttatcgaaaggctccttccggcgattcctccaa
ccccactccgttatcgttgagtctccaacatcgtaagtcattccttgctcatttccctctgaggatagattgctaatatgcttaatttcccatactcag
aatacctttttgctaacaaaccatagtcaatgttcatatactaaagcgagctcgctcccttgccgaagaagagcctgctgaaaaagacactgcgtt
tgaaactggacccctccccaacagcttttagagcggttttggctatatttagctaactaattgagtgtgtacagcgattctaactcattccttgttcgc
tttgatgttctttgagtactacactctcaagttcactcttgtgtctttacctgtatgcttattcctactttccttcatcgtttctagtataattaaattctgc
tattactgtcttcactttctaagaccttgttcttctagttttcgttgctcatagttatttgaagaaatattggcagcgaatttttctttatgaaattccttg
ttggattgtttacacaaaaagttgtgtatttattcacataagaaccgcataaacatcgtttattttatacaacaaacgatgcatccggtgcttctaaa
agactttttgactctgttccttttccttcgtactgtttacatttgctggttttctttaaacccgtttaacgtaaatatcgttatttcgctgcatttttgtgga
tcgcacttcccctctgtcgcaaaatggtttcgttaccttgttacttattcgtgactgtattctgctctaggcgaaattagcaaattggatttttacgaga
gctatcaacatggcggtttcaggaggcgagttgaagggtaaaaaacgtaaatctggagactcccataaggagtccaagaagagccttcaggttc
tctctttgaaggaatcttccttgcccccgtttgttacgacaatatccggttcttaccctcccgaaggaactcgatttcagcccattcacgcagacgga
aagaaaacaaacgatgtctcgcttattggaagaaccgagcgtgtccaatttgaagccaaaagcaagcctactagcaacgttgagtccaattact
gcttggccattgctgacaagcatggtcgtgtaaaaaagttggttcctacccgctaccttaataccctttgagcgaaaaattttagctttgaaagaaaa
ggatgagcatatgaaaaagcaacgtggtgctgttagtggcacagtgatggaacaacgcgccaaccttggtttagcctttggtactaagaagaac
caaaaggcgattatggaggaatctgctaatcgtgtcaaggctgaggcgttaggcgatgttaaggaccaacttgttagtaatgttcaaaaggccac
tgaagctttacctacccaagaagagcttaatgcctatcaagcacaagatcgacccattcctcctgttcacatggatgcagaaactgttgaagaag
cttacaaattggatgacataattccaaaagaagaaatgtctgctatttatatcaagccattcttggattctgccgaggacaagggatggtccaaaa
tgttgccataccgtcattctgtctttgtcaatgagcgcttctctcgattattgtcgattgaggaagttgatcacaagcgtgctaaaattctctactatat
ttcactattgcaagcatttttgttttctcgtcgctctgtcacggatcgcgacactttgcgtagaaagcttgctgaccctccggagattttaattgatgg
ccttttcaagcgtttcacacaaacaaccggaactggtgcggtgcaagtttctatgcgtcaggttgatcgtataatgtgctatctcattgtcttatacct
tatcgttgacaattactcaacggacattctcactttagctaatgacttgaacattaaggtaatgaaagcaaatgaactttttaagactgtgggatgc
cgaattatggcttactcagaaactcaacgtatggctcttggtctgaacaagacagaagcgaagaatcacaagagggcagttttgaagatacccct
tgaattccctagaccccgtcgtggtcgtgctagaaactaatccaatgatgggctttgggattctgtatcttgaaacaagctcgccgcatataaattg
tttacatcgaaagtgaattattgtcaaaaggttatccatcgtacgggtttagcttcgatgcgttgcattgctttgctacgggtgatgaaatgaaagat
tggtttatatatatggtcttaatgggtaaaaaagtaatataaatatatacatatggattggaaagcaggaaggaaattttcaaattattgtttttat
ataattttcaataaaatcaattttaagcaaattcatgtggtcaaaaaggaggatatagttttgatgatattttttcatgtacgagataactgatgatt
gaatagaatgtacaccatcgaccttttattgaaaagaaagaggataagagatctaactataagggtagtattagtaaaccatcattggaagaat
aataagagaaccataagaaagggaagggaaggaaaggaaagaaatggggttacaggatgcaaaggaaaggacattaattttctttagtataa

gtagcccataaagaaacgagctcatttctagtaataggttcagctaaaaccaacccatctcgaacaacttgctttgcttcgtccattcgatgcatcg
cctctaaagcctttgctttccgataatagcattttccgtaattattacggatcttcaaggaagcatttgcatccgccaatgcctcaggaaactgtgag
agcgatagttcagttgctgcacggttacagagcatagtgcttacttcttcacgagtcagcactgcaggttcccaagagcaacgattgagtgctaat
tgtaaagcaagagtgtataactttttagcttcctcaaaccttttccgcttgaaggcttcattggctgtctgcttcattttattcacttgagtagagaga
gccaaatttggttttgctgggggaatggtggcggaattgcaaacgggtatttgaaagtagagatggatcttgcataactgtttaatttctcctgaa
tgaactgctcttcttccgtcaaattctccaacttcacttgcggtgcttccaattgttcggtccttggagccattctgaacacgatctgaaaagtaactc
tatgttagtatggacgaaccagtgtatgttttctttcttctttttccaggaccctacaattataggctctggatacagtgagcatcgtcgcttttgcatg
cacagctttcaggtcttggtattacgcatcgcagtaaatcaggcttgggcatacgctaaagtatggtttcttaaataacgacccaaaaggacatgc
ggtattgttacaacaattataattgtaaagaggtggttgctgttctggagaaaaaagcatgccttaccttctctaacgaacagctgctgctgaaact
tgaaaaaagcggactttgcgaaaaccattcaaatcttggaaaatccggaaaccatggactcctcaccaaatgattttctaaacaaggtcattgga
aaacctgtagtcattcgattaagttctggtgtggattatcgtggtatgtgaggtggatgttggatgttggacattgtcatgatagctggaggacgat
gtactctagatgttgttcgttgcttcacacttctaaactgatggattgtttggaacgctgtacgctaatgttgcgattggtatataggaactttgagtt
gtcttgatggttatatgaatcttgctttggaacaaacagaagaatatgtcaatggtaaaaagacaaacgaatacggtgacgcttttattcgtggta
acaatggtaagtcatagctgatcggatgaatctttggtgtgttcaagagatgaatgatctaacctggttttcagttctgtatgtttctgcattaaatg
agcaatagtttttttttctcaattcatgttatgacaattacgcctctttgattgattaagagtaaataaaaagaaaagccttttactgggttgtgttcc
ggacaattgcaacgggattgtgcaaatggtttagaattagtacttgtgaacattttatcttatacatcatattatactctgtctccgttatttatatcg
ataatcaacatgtcctttttttatcattatctgtaaaatatgctgttcagagtagtcaaataactaagggtaaagttcttgttgcatttctcgtcggtgg
ttggatctgttccttctttgagcagcaaagaatttgacagatagaaaaagcagcagcaatatacatacaaaaatacttatgaaaacatataatat
atggctttgcatccaaaaatatgcacttgcgtcggacccatgccagctactaccgtgaacgtgggagaaaaaggaaacgggcccatttccataca
gagccgggggggagcgttcgcactttgctgtgtgactctgacaaatactgacaaaagataaatgaaaagaacaaaggacccgtgctcagcaaaa
cggttgggtatttggtaaaataccggtgattaaacttggacagagaagtaatcactttgtagagaaaagcatgtcttggagtagcggcaaaccag
tcattgctgtaaccgattgggtcagtaataggaagcagaaaggtggcctcatcaagtaaaggatccatagactttttacagcctacatccaaatcc
atgtatacgcctccgtaatggtagacaataaaatagcgaacagcatcaaatcgttgaatgtcataaggataactatgatagataggcataaacc
attgataatttgtttccataaaagatagggccatttcatcggtccatagttttactgtccacccatcttcttcgggatgttgagttcggcatgaattta
cagaaacagaccacttatcaggaatattctcgtctttccaaatttgatggataattttaggtatcttcgatttcgtttgagaagatgtagaagaccct
gagctagcttccatctcttgtaaatttaaatgcaaagtactggcctttccattaggtaatagatacacatacttttgacaatacgcagaaagcttta
gaaaaataaatacaaaagtaagtaaagaaattgtcacaataaacagcttgcgagttcggcgtaccatggccggtgactttgaaaagaaaaaaa
ggaaatgagaaaacgaaagaggatgagacgataccaaaaaaatgaagaactaaatatgatgatttctggaaaagaaaaagaaaatatttgta
actatagaggaagtcgaaatgaagtgattaagagagggaggaaatgcggattggaaatcaaggaaatagcatgaattcaacaaaataagcaa
agaaaagtatgcaattttctctaacaaatagaaacaggaaataattgagggataagaaggaaaatgccaaggacggtcaaaaggaagcaaaa
tagtaataggtgttttctcggtttcaaaataaaaacctcgctatcttttagaaatatatatataattaatgagaactttgaaagaggaatcctaatcc
tgtttatagagaacttgggttttcgctgaggcgaacagtagcaaggcgttgttctatacctacctcctttggtggtagtagtatgtatgtaattacga
taatggttgtaaggtaaagttaaagagtaaacgagacaaatcataagaaaacatatttacaaaccaaatatatcgtcaatttcatcctttttctttt
ttgatttttcttctttttcttgggtgctgcagaaggagattctaaagatcgtaacgggggaatatcggttgcttgaacacctcctcctatggattgtg
cttcggcagaaggtaattgaggggttcgtatggatttcatgtctggagatggagagctagtaaggtctgttctttgagtaaaggatgattgaggag
tagaggaagatagttgttcacgagatataacaaccccgaggtctgatgcagctttttctgtgtctttaatagcttgctcttgcagggaaagattcgg
tgaaaggattggagacgggaaagataaactagcttcatattccattcctcccaaaatataccaaacacgagataaaataccaagaaggagaca
accaagccctgggtactggccatgggcaattacgttggtaaaaagagcgtaagattttgggcattgttgcaataacaaagatttcgcagtctcgtt
tccagagagccattgttttaacctacgatgaagcatcataacgtgcctccaccaaatcgtgagacggtgttggtttttattcctgtacacgattttct
gtaacaatacagcatctggctgcaagacatccatagttgggaatgtcaaaacaaagcgcgtgcgttcatattcaataaccatctctcggtaatac
ggtgaagtctcggagagttcggtgactattgtccaaaggctttagtaggatgctattcacaatagagtctatatcatcgtgaaataaaatctatgat
ttaaaaaagacaataacaaaaaaatatatacgagattacatactaggatgatgtttaccttggtggaacccaagaatacagaaagtatgttttct
ttccctatgggttctacctagctatctggatcatagctttgtcaacgtaactcgattcttggtttttttataaaaagagaccactagcgaacaagtag
taatcgaaatgtgtactcgaacaaagggaatatctcaatttcctcttgtttactacatttggcataaacgtatatacaatgcaatgaacaaactatg
aagctgcgaatgtgatgaaaagtaaggcaaaaataaataataaaataaatggttccattcctttattattcgctttctctctatcttcttgttcctttg
ttgtattccgaaacaatagttactttttcatgtttacaaactctgcttatttccaatgatgttcgtttagataactttaaatacgtactatctaagctca
aaactagcgagttaaaaatttaagcttatcacctaaatgagaggtgctttccctttttttatgatatcagtattggatatttctcggcaacaattctaa
aagtattcctctgcaagcatcgttgaaattggaggtttttacatgatttcattaagttcaaaatttttcttggttgtcctttacaatatttaaatctaga

gaagaaacaatgggtttgaagaacttcccctcgtgggcattaacgaaaatctttggtcttgaaagtgaacctaggcgagtgctctcttctcatagc
aattgctctgatgaagaacaaaggttattaacagaagaagaccattcatattttgatggtcaaagttacgtggaaccaaatattccttctttatatg
gttccatggtgcctgttcctcaacaaatccaacaacaccatgttcacccttggggacaaatgttggatgatcatgctcatggattggctaccgtgcg
acatgaatttgcggaattgttgcgattgggttcgccaactgtcatagcctatctacttcaatcctcagaacaattttccacagtttttagcttaggcca
tttaggaaaagaatacttggcggcatcttctctttcgacaatgacagctgccatcacagccttttccatcttccaaggaatcatttcttctctagaca
ctcttgctacacaagcctttggagcaaacaaaccgtacaacgttgctacttactttcagcgctgttgctttatccttcttcttttacatattcccattgc
tctcatctggttaaacctcgaacacatccttgtttttcttcgtcaagatcctaaggtcgcgtatctatgtggccgttatatgaaagtgttcatctttgcc
gcgtaagttctaattatcgtttgtctcaaagattctttatactgacttttctagtcccgcatatgcatcatttgaagctatgaagcgctatcttcaatcg
caaggcatttttaagcctattacttgtgtcctctgttttgctgcccctttgaatgttttacttaactacctattggtatggcatccaaaaattggctttgg
gtttctaggtgctcctaccgccgttgcaatcactttctggtttcaaagtatttgtcttttgtcttacgctcgattctcgtcttccgtcataccttggaccc
acttttccatgcgtgctgtacaaaatttaagacctatgttaacactttctttttatggcatgttgatgattgtgaccgaatgggctgcttatgaaatga
ccagtttaggtgccggttatttgggaacagcttcattggcctcgcaatccgtccttctcaccaccacctctttggcgtttcaaatcccttttgcttttgc
cgttgcctcttctactcgtgtcggtcatcttgtcggttccggcagaccgaatttggctcgtatatgtagtcgagtagctacttgccttgctgtcataatt
tctatgactgacggtcttttactattttgcttccgtaattattggggccgtttcttcacttctgatcaacaggtgctcaatatagttcgagatatattcg
caatcttgtgtatctttatcgttttcgatgctctcaacgctgtcgggggcggtttacttcggggtattggcaagcaaagtattggtggtgccatcagc
atcattgtcaactacttctttgcccttcccatcactgttatttttgtcgttgtcttacagttaggtttaaaaggtatttggatgggtatgattgttgccgtt
acaactggtatcacttgtcaatataccgttcttggacatattaattggcaaaagattcttcaagaggcccattatagaatggctcacgtataaatgc
ttttccaaaattggtctacagtcctttatcttacttccacagctgaactttctttataaattcccaatgtatcttggatttctgctttcacccttctctttat
tcgataattagaatactttttctccgattacctttctctccgttaattagttcctggcccgcaccatctgtatacagttatattcgcctgtaagtttgga
gccttgaatatcagtatttggtgcgacaaattcatttgctccgtttctcttaatattctgactagacgggcgatcatgctgtactttctgtaaggcattc
tattttacaaatagatttgtaacttattagagtactatatagagcgctgttcttttagttcggcataataaatatcaataaaaaaagaaaaaagaa
aaataaaattatcttgaaatcgctttgattttgattttaaataatattcttaagtttgtctggaaacgctgacgctttttcttcatgatcattactatga
actcatagtttgtaagtataaaatttcggtgaagtatggataatccttgcaaaatgcagaaatacgataggtatgaaaataaaacgaaattagttt
tactaatagtaggcaagtacggatttggaaagcagaaggccaaaatgccattgagcaatcacatatttgcttgctaaatgccaatacagtgggtt
gtgaagcactcaaaaatacgattcttccaggtgtggatttctcgtatggtttgtttttctaacaaattacctaggaattcgtgagtttactgtaattga
tgcatctgtggtagatttgaaacatgatggatccaacttctttgtgcagtacgatcaagaaggacagagtcgagctttatgcactggtaccttatta
caacagttgaatccctcggtagaaatgaactatcaacaaatggtacggaccttttgggttgtatttgagaataaaagactgatcatgtccttagga
gcctgagaaactaattgaagaagatattgagtttttttcaaggtttacaatcgtttttgcttcgaagctaaaattgaatgctatgcttcgacttgaag
ctttcttgagatccaaaggaatccctctcatttactataattctatcgggttcgtaggtataattcgaatctctgtccccgaatatacaagtatgtcta
attttaattcgttttctaacgattttaaaagccgttcagtctcaatcggaggtccctttagatttacgacttcgaaatccttggcctgaactaatagat
catgtacgctccttgaacgttagtcagatggatggaacaaaaagaaatgagctcacgtatattgaacttctaattcattatatgctgcagtttgaag
cattgcatcaccggaaaccagaaaattcagatgacaggaaagttttacagggcatgattcgaaaggatataaatggacaagcttctgaaaatta
cgaagaagcgctatcaagctattggagagtttttcaagagtataaggtaaagcgatgtgtaagccaacattctaacaaaacagataccttctaat
gtgatggaagtattgcaggatgagaaaagtcaattcattaacgaaaagtctaccgatttttgggtaatgtgtcattctttaaaatgcttttatgaag
aaaaaggcactttaccattgttaggtacacttccagatatgcactcgaacactcagcgttacttgagtataaagcatttgtaagtttaagactattt
aactgtttactgacaaatgaaggtacaacgaaaaatatcttgttgattttgccgctttcaaaatatttgttcaacggacattacagtctcaaaacag
acatgtggatgatataccagaagttatcataaaaagattctgcaagaatgttttaaacataaaattccttcggtacagatcaatacaagaggaac
ttgagtctcccaaacatctcggtatgtaacagcattgtggttatggaaatctaacaaatcctacagattcatttctttctgacgaaaactctcttattc
cttggtattttgcatttcgaatctcagagcaactcttaagttctcccacacaatcatcactagaaaaagaatctccagtcgttaagtccttagtggac
actctctttgaaaagtattcattaaaacaagaatataaaaagaaagttctaaaatgcattgatgaactgtatgctaagctattcgaatgaagcact
ttaactgactgtaaattaggtctcgagcgtctgattatgaagttcatccagtttcctctctagtaggtggaattgctgctcaagaagcgattaaggta
tgtaaagattgctctcaagtatatattttgaacctaattctcacatccacagattcttacaaagcactatctacctctcaatcacacttgcatttttga
cggagtccttagcaaaactgaaacattcaatatttagaaaccaagaaataagtagcagagaattctatttcatgattctagcgagaagtaattctt
gtacagcataagtgaaccgcctaaagtaaagttcaccttagcttagctttatagaaatttaaatgcacaaccaaatgattcccactcctgatacttc
ttgcttatcagaagacatattccaggatgtatatgaacctgcaggtattcatagtttctgttaatattttactaattttgaataaagaggatacatttg
ctttgttggacgctctagaggaggataaagacaaattgtgtaattttgcagatgaaagactactaattactgcagaaatagggtaagaaaatatg
ttgacactttgcgttaaccgttcgtcttgaaagttgtggatctggatgtgtatcgtcgtttttcaaaagtggaatattacaaaacagaccaacaagt
aagaaacttgactataaccgttttgactaatgaacagtacattttatgacagatatttcaagtgccgcttgtaatgcagcagcttatgtggtaaata

aaaatcgagaagtatacaatgacagaagtggcgcattcttaactgcacaaacttgcttcttagaaggattaaggattaaaaaaagcattgatgtc
ttactttttaatcctccttatgttcctaccgaatttgaggaaatccctaaagaagctaacatagcttccacttgggcaggaggagctgatggaatga
atgtaacaagccaattgttggaaattttggatgtaagcatatcacttttctctgtctgccatttctgacttattcatctttaggacatactttctgcaga
cggaatcttttatatggtcgccgttgctcgaaataatccagcaaaaatccaaaattatatgtctcagtttggttataattcaaaagaagtgttgcga
agaaaggcaggaagagaaacactatgtgtattgtgtttttcaaggagcaatgctttattttcttaatattattatatcggttcttttaggtcatattta
atttcattcaagaaagctaagattacaactaattgtatgacttgacttaagtatcgattcgtaattttccttgatgaacatgtgcacgagcgttaaca
caagctggaatctcttcctcatagtgagaaatgataaccattgcttgggaaggtaaaagatttttatttaaccacttgtgagcttggtgcatatatct
gatatctacaccttggaaaggctcatccaaaaccaaaaaacgaggccttttaattatggctctacaaaaaagaataaatctttgcatgcctgtag
gtaattgggacaaagggatattctcaaagctgcggagactcagttcatctaaaagttttctaatggcctctgaacggctctcagtttctcttgggag
tgtgaaggtagtactccaagcacttaaaagagtctctctgcaggtaaattctttaggaaaatgtgtgtgaacttcaggggactagtataatgttagt
aaaaagaccatttagggataacagtaacatacacaatgaccgacgttttcttgaatatcgaagactgaaattcctgttcctggtccaataaacttt
ccaaaaaactttatgtctgtagcaaagagctttggatgatcaccaagggcgaaagctaagagagtagtttttccgctaccattgctaccgcttaaa
gaccatcgttctccttctttaatagtccaattaacgttctctaaaacaggtttgccccaatacgtacaattcagattgttcatttcaatgatcggctta
ccaatcttaggaggtttagtatcttcgagctgaggttttgtcaaaactgcggaatttaaggatggatgatagtgatccttgactccttggtaggttat
ctcgtggttatttagctcaacaatatgagtcacgaaatcaggtatttcgtcctgttgtcggagggaaagaacaattttaggagagcaaacgctgga
catatttcccaaaagattagaaacaattgaacgagaagcaacatctaacccagctaacctttgttagtactgaagcaaaggctaagtatgtaaac
gtaccatatggctcatctagtaatagcaattgggactctaataataattttacagcgagcatcgcccttctcgattgtccattgcttaaattaatcaa
actagattcgagtaaatgctccaacctagtaattgaggctgcatattgaactttttccaatgctttttcttcgggacctctgtatgcatcaagtaacc
atttttgcaaactaatatcttctcgtttatcgcggtgcgaatgatacctttctgaatagtaagcagcacgctgaagacccgagctttgaaaatccaa
aaactgaatcgcttgccagggacttttgttagtatctgatagaaatgggtatttaaaatttgtagcaggagaaggtgtatacgatccttttaagcac
taaaatcagtcagtaaataatacattatcaggaatagtcgggcataagtaaccagcactctagaagaaagttatgcttttcaatgcatctgttcca
gtgaatggaactaagtacaatagtgatcgcgcacccttccaagaccattcttttgagcctcaacttactttaagaaaagtagttcttccactcccaa
cagcgccgaccacggcccaattttgatttttggttatttcgaaggaaatatttttaaaaactggaaaccggccatccaaaaaggttgttttggcgat
tcgaacgaaagaagccatactttcgaacgtgtacacaacttatgattacggtttttattgagtgtcaattataaaattttgttaaatttaaagaaga
taaaaataattaaggtgtaaaacgctatacagtttgatctctgattttgagatataagaggtagtttcgcaaaaaaataatggagacagcagtaa
attgcatcgtacggcaattacaacgtgatgatctttcagtgaatctgtaaggattaattaatacttagagattactaacgatctagcctcattaaat
ccataaccgttacgctacctagacttcgaaatcagcgaaatgtttacctagtatttggaagattactgcattgcgagcccttttggagactgaaggg
aaatgttcctaataacttactatcattgattcatggactcctagaaaaccaaacagcttcccatgatcaacttggaaacactaaggtgctgcagct
aatgatggaaaccctagaaattatgccaaaagataaagatatcattgcttcaagaaagttactattgatgggtgctttgtatagagccttaaggtc
aaatccacattttacttgatgtatgctttggagcaaaaagtaaaaaaggcacttaggaaaaggattcaaactacacgtttaaatgaaatagaag
ctttagagattatccagatcttatttcaaagctttcattcagacactggagtgaactggaattgtttatcgctttttcacatcctctagtatgttctttc
ctgaagtaaaaggattaaatcactgactgcttataggaatggtattcattaacttggaatgctctatcttttgctttttcggacaatgctgaaaatgc
agttttgccggcaattgtgctatctcgtttaattagtcacttttcgcatagaacgttagaagacttgttggaacggtaagcatcatttatcctgtcgta
ttcattaactttttttctcagactgatccatttagctggtcaattaaggaagatcccaaatgcgctgtcttcggtttctcaaacaagatccaacttttat
atctttgttatttttctagatgcaattagccaaaaacagtacgcaaaagttggctttaccaacagagatcagaaaacttgcctacagacgcttcgtg
tgttaagtgaattagaatggggatataaatctatatcttcgtcctggtccactttggattttatttttgtagttgttttgaccaggggtaaaaatatga
ttgctccatatatagaatccgtgggccatgcaattcatcgttttaagtcagaaaggaaagagttgaagaatactttgctttcaggtatcccactttc
atacgctttgcttctgatcaagcagatgctaaaattagaaatccaacagcaagtttcatccaactatgactctcaagttattccttttacgagctcaa
ctatggaacagtttgcaagcatttcaaattcttgcttgtttttgttccacttcttaagacaggattctcaaaaatccccttggaagcagtacatgctac
aactgagtctatacaatttcttattcgcagatcttttctctaaatattcttcagaatctccagctattactgaaaaagaaacgatcatgatacatcttg
attgcattttcaatttcgcccttctaccggtttttgaaaatgatttacgggacattttatgctcaagattgcaattgtatgaagaatctgccaaagaac
tttttctggcgagtctttttctattatcaagaataatattgttgctaatttggataactcatgtctccttttcaaggcatccataggcctgctaccattc
ctgactgaattacaacaacgtgtatatctcgagcaatcgcaacttacagtcgataccttcccaaaggattaccaaaataccataattcttcttgtta
ccaatcagctctctacccttccatattcatctgcctcatctttacttgcttactgggcttctttattgattcctgctgctcgataataagatatcgttaca
gctcgttatttgatttaagggtatacaccgtagtaccaaaaatcaataggtacaggaattcagacgaatactcatctttagcacatttctatagcaa
aatgtttatgactagatcaggtatgaaatccaactaaactaaggctaatatcttagaatacgatagaggtgtgaatacgttttcacctgaaggacg
attgttccaagtagaatatgcgattgaagccattaagttgggatcaactgcgattggggtgaaaacaaaagagggagttgtgttgggcgttgaaa
agagacttagtagtcctctaatggagtcatactctgtggaaaagttgtttgagatcgacacgaacattggttgcgctatttctggtcttacagcaga

cgcacgcacgattatagatcatgctagggtgcaagcacaaaatcatcgcttcacttatgatgaacctcaaggagtggaaagcattacacagtctg
tttgtgatttggctcttcgttttggtgaaggagaagaagatgaggaacgcattatgtctcgtccttttggtgtggctcttttgttggctggtattgatga
gcatggtcctcaactttatcactcagaaccttctggtacctatttccgttatgaagccaaagcgattggctcgggcagtgaaccagcaaagaccga
attggtcaaagaataccacaaagacatgactcttcaagaagcagaagtattgattcttaaagtccttcgccaagtcatggaagagaagttaaact
cgaaaaacgtacaacttgcgagtgttacttccgacaagggattccacatttataccgatgatgaaatggctgacgctgttcgtcgtgaagagcac
agatcagattaaggtatctatattttttactatactgtctacgagccttatatacgactagagttgcattccttttcctttttcatgctccaatgaacgg
ttttacgaaaaatagctatctttctataggtgatcatccgcgagaagaagccggtgattataaagaagaatataagagatgacagatgcaattag
aatgtaaatgatatctataaagatagtgatttgtaaatactttggctgtaggattgtcattcataacaaggtacaccagattcaatagaaatgaaa
tttatattaagtgattatttctagtgagaacaatatcaggtactttcgttcctataaatatatgagattggctatatactgtaaaatgcgatccctcta
ataatttttccatttacctcatcatatccggcaattctacaaagattgcatcaggcaaagttggtatttaagtatttgctgtatcaaactgaaaacag
aagaaaagttaagaaaaagatctgcttatgcttttacgcagacaatttcaaacggcgtttgctcctgtccagttaagtcatctttacctagtgacta
gacagcaatttcgaagcttaagaaatgttcaggtttgtttcaagtctacctttacgggagctagtataaagtatttacctccaagagtcacaccagt
ataccaaggtacgaatgccaatgacatataaactagcttacagtatgactaacattgcggttctcattagactgcagatctcctcaatctatagga
gggaaagcaaatttaaaacaattacaatggccgcaaccacccagaaatgttttgatcttgaagaaaacaatggacctaaaagtcaatgagtgct
ttacgagattggtcgagcatctacagaaagtttatcctgagatttctttaattgttgataaagaagctgcgaaagcttttccacagtatggattgtat
acctgggaagatacggaggatttggagaagaaggtagatatcgtgcttacagtaggcggagatggtactattctacatgcagcctctttgtttgca
aaatcaggcatgccacccgttttatcattttccctagggactttgggatttcttctgccgtttgatatttcggattttcagacggcatttgcagatatgt
atgagtcacgctcttatgtactaatgcgaatgcgcttatctttgactatgaaaagaaaaacccatgatgagactacttttatcatgaatgagatgca
tatccatagaggtctctctcctcatatggctgtcctgaaagtattcatcaacgatcgttttctaactgaagcaattgctgatggaataatcatatcta
ctcctactggatcaactgcatattctctatcttctggtggacctattgtgcatccatccataaatgctctcttgcttacacccatttgtcccagctctctt
tcctttcgacctgttttattccctgattcttttactattagtgtggaaacttctgaaaaatctcgtgtccggcctcagctttcggttgatggtcgcttgct
tgggttgacggatatcggacagcgtattcatatttacagtaaaaaagataatggaatcccttgtgttattcgtagtcataaagaagacgattggat
gactgacatcgttggactccttcgctggaaccacccttttgatcgtaaaggatggtaaggaatttttcggctaaaagatgaaaacagcggtttttttg
gttttacataaaaaaaaggagacagaaaagaaatttattcaatttttctttataatatcatgctccttcctttcttgggtttatttatcatgatgtttta
gaattatcgttgaccttgtgtttttatttataaatggaatcataataataaagaatactataacaattggaatctgatttgtttttcattctgtttgaag
tcattcacaaatccgtatttcttattatacttgtatatatcgcaaagtaaacacatgaataatttcaggtgagcattaagttagatagagctgtgtat
atctttttctgttataattgttatgatttttgtcgcatctttaagcagcttattatatttagatttttctgaaggctttttgtcacgtatcttcgtaaacaaa
ctaaatgaaaacttactttcagagatgaaaaaactacacattatatctccaaccgaattttcaattgaagcaaaaagcgcatctttgaacggttgt
ttctacttttacttttacttttaccttttgtttatttttattaaattgacccgacttttgtttaatttgtcacccacaggaagtagcagcactgaaaatcac
ctcgaatcttcttttatagaaggccccacaatagcgagaagagcgatggcaattaaggagggacagaaagatacatggaaatcatcaaaaaatt
actcagagaatataatttacatatttcatatttttaaatagaaaggtccttttgctttattcggttcctagtgattgggtagcatgtgacctatccgtgt
ggaaatagctttgaacgcgtccgcctcgacttatccattcccaacaacaagatttggtctgggaaactgttccagaaaaaccttatcttttacctgt
attatatatatataaacagtttcttaactggtataaattcgtgcaatctttgacatctttcccgtttttgacgcgtattaacggtgtaaagactgtact
gaagaagaaatcaattcaaaaggacgagttttgatcaagaaacgagttctcgttgatttatctttagaacaaagagccatactaggaaaactttc
ctttttctaggactgttgcatttatcttttagagataccgcaattcgttcagggtataattcatacatcgtcttttctgactaagcttttgcggaattacc
ctgcattataattactcctcattttcaaatcacgatctttacgttcatctagttttattgtataatcatggttgagactggttatgcatctcctcacgttc
atcaatcggtgagatcgttggctttgacttatgcccatctttgtccatctattttaattccttcttcacaataccttttggctgctcaaatacgtgatga
gtttcaagctaaatatgtagctccatctgctgatgctgtcgaaaaagaaggagctgaattagaatttgaacatgagcttcatttagtcgcccgtttt
ctgggtttggtcgcatcaaaagaagaaaaaagctcgggtcagtataaccaacttttgaataccattacccttgagtttgaacgtacgttcttagca
ggaaacgaagtccatgctgttgtccataatgtgggcttgagtgttccagcgcaaaaagatgtcgtttgccattactatcgttctagagctttaactg
gaagaggcatgaaattccacggttccgctatgttgaatgacccttcagtcaagttggctgccatttttggaggccaaggatatgaagactactttg
atgaattagccgagctttatgaagtctatgctccatttgcttcggaacttgtgcaaactctctctaaattcctttttctcttgtacaaaatgaacaag
catctaaagtgtactctaaaggactcaatgtacttgattggctcgctggcgagcgaccagagcgtgattatttagttactgctcctgtctcccttcctt
tggtaggtcttactcagcttgtccaattctatgtcactgctcaaattttgggtcttaatcccggtgaactcgcttctcgttttaccgctgctactggtca
ttcccaaggtgtcatcgttgcggctgctgtttccgcttctactgatgacgagtctttcattgaaaatgcaaaggtagctttaactactcttttctggatt
ggcgttcgctcccaacaagtctttcctaccaccactcttcctccttccgtagttgcagattctgtcgccaactccgagggtaatccttctcccatgttg
gctgttcgtgatcttcctttagaaattttgaataagcacattaaaattacgaatacccatcttccagaagatagaagagttttcctcagccttgttaa
tggtcctaaaagctttgttgtcagcggtcccgctcgttctctatatggtttaaatctttcccttcgtaaagaaaaggcagatggtcaaaaccaagctc

gtataccccactctaaaagaaaactgcgctttgttaatcgtttccttagcatctctgttcctttccataatgcataccttgcccctactcgctctttactt
gaaaaagatttgaagggtatggaattatctcctcttagatttcctgtatattctaccgatgatgctagtgacctccgttctgaacatccatctaactt
gcttatggccctttctgtaatgattaccgagaaagttgttcattgggagcaagcttgtagtttcccagaggctacccatatcgttgacttcggtcctg
gtggcgtttctggtgtcggctcttttacccgtgctaacaaagatggtcagggtatccgtgtcattgttgctgatgctttcgatgccttggatcttggct
ccaagactgagttatttgaccgtgagatcaagtctgtaaagtttgcccctaactgggtagatctctacaagcctaaacttataaagaataagcttg
gtcgtgtatatgttgaaactagactttcaaaaatgcttggcttgcctcctctatgggttgctggtatgactcccactagtgttccctgggatttttgctc
tgccatcgctaaagcaggttacgcttgtgaactcgctggtggtggttactttgatcctagaatgatgcgtgatgctattcataaactctccttgaaca
tccctcccggtgcaggaatttgtatcaatgttatttacatcaaccctcgtagttttgcatggcaaattcccttgattcgcgaaatggttgctgagggtt
accccattcgcggtgttactattgctgcaggtatcccttctttggaagtagctaatgaattgattgaaaccttaggtgttgaatatatcggtttgaag
cctggatccgtcgaatctattaatgctgttataaacatcgcaaaggctaatcctaccttccctattgttatgcaatggactggtggtcgtgccggtgg
acaccactctttcgaggatttccatgctcctatcttacaaacttattctgctgtccgccgctgtgacaacatcgtccttgttgccggtagtggtttcggt
ggagctgatgatactgaaccttacttgaccggtgaatggagcaccgcattcaagcttcctgctatgccatttgacggtatagtattcggttcgcgtc
tcatggttgctaaggaagctcatacttctttcagtgccaaagaagcaatcgttgctgcacccggtgttactgatgctgaatgggaaaaaacatatg
atggccctgctggtggcgttgttacagttatgtctgagttaggtgagccaattcacaagttggctactcgtggtataatgttatggaaagagcttga
cgataccatttttgctctacctcgtcccaagcgtgttcctgctttgcttgcaaagaagcagtacattattaagcgtttgaacgatgattttcaaaagg
tttatttccctgctcgaattgttagacaattgtcccctgagaagcttcaatttgaagcagttgatggtgttgaagaaatgacttatgctgaagttttat
accgtaccatcgacttgttatacgttacaaaagagaagagatggattgatgtttcgttgcgtacctttaccggtaacttgatgcgtcgtattgaaga
gcgtttcaccaaggaactcggcaagcatacccttatcgaaaacttcgaagatttgaattgcccataccccgtggctgctcgtttccttgaagcatat
cctgaagctaatcaacaaatcttgaatacccaagatgtccagttcttcttctctttatgttctaaccctttccagaagcctgttcctttcattcctgcca
ttgacgatactttcgagtactacttcaagaaggattctctttggcaaagtgaagaccttggggctgttgttggtgaggacgttggacgtgttgctatt
cttcaaggacctatggccgttaggcattctacgaaggttaatgaacctgctaaagagctccttgatggcatccatgaagctcacatcaagcatttt
gttgataattattataatggagatgttaacaaggttcccactgttgaatatttcggtggcgtgaaacctgttaacttacctccttccgccgtggattcc
gtttctgtcacaaagaacgctgatgaggaaatttatgttataccggaagccggatcccttccttctgcaaaggcttggtttgaactcttagcaggca
aagaagtttcttggcgtagagctatcttcactactcagcgagttgcccaaggttggaaacttgaacagaatcctgtacgcagaatccttgcacctc
gttatggccagcgtgttgttgttaagggtcatggaaacgatgcggtcctcgaaatgtttgagctccaatgtggtgagcatgtgcttgctgttcgtctt
agttatagcaatgacaccatcacggtgtctatgtttgaaaatcgcaatgcccttaaaaagaatgtaaatcttgacttcttgtttaagtatgatccttc
tgtcggctatgcgcctgttgcagaaatcattgaaggcagaaatgaccgtatcaagcatttctactggcaattatggttcggcgatgagccataccc
tgaaagagcttcaatcactgacacatttactggtcccgaaaccgtaatcacggctaatatgattgaagatttctgtagaactgttggaaatcataa
tgaagcttatactaacagagctaacagtaagcgtatggctcctatggatttcgctattgttgttggctggcaagccattaccaaggctattttcccta
aggcaattgacggtgatcttctacgtcttgttcatttatccaactctttccgtatggttggaactcctttggtcgagggtgataaggtcactaccgctg
caacaatcaccggtattgttaacaatgattccggtaaaactgtcactgttaagggcgttgtataccgtgacaacaaggaagttattgaggtcatta
gtcgcttcttatatcgtggtaacttcaccgactttgagaataccttcgaacatactcaagagacacctatgcaactcactttggctactcctaaggat
gttgctgtactacaatctaagagctggttccaattattggatcccagccaagatttgtctggttctgtactcactttccgtcttaactcctacgttcgtt
ttaagaatcaatctgttaagagctctgtacaaactaaaggtatcgtattatctgaattgccttctaaggctattatccagattgcttccgttgatttcc
aatctgttgattgccatggcaatccagttatagatttccttaaccgaaatgcaaaacccattgagcaacccctggaatttgaaagtggtggctaca
gcgtcatacaaacaactgatgaaggttactcacctgtttttgttacccctcctactaactctccctatgctgaagtttccggtgattataatcctatcc
acgtttctcctattttcgctgcctttgtcgagctccctggtacttatggaattacacatggtatgtacagctccgcagctactcgtaagtttgttgaaa
ctttcgccgcagagaacgttcctgagagagttaagcattatgaggttactttcgttaatatggtccttcccaactctgagcttattactaagctttctc
ataccggtatgattaatggtcgtaaaatcattaaggttgaagttttgaacaaggaaacagaagaagctgttcttgttggtactgctgaggtggagc
aaccagtgtctgcctatatattcacaggccagggctctcaagaacaaggaatgggtatggacttgtatgctacaagcccagttgccaagaaaatc
tgggatagcgctgacaagcatttcttgtctcactacggattctctatcattgacattgttaagaacaaccctaagggtattacaatccactttggcgg
tactaagggtaaacaaatccgtagtaattacatggctatggcttacgaaaaaattaatgatgatggctctacttccgttgttcctgtatttaacacc
attactgaggagtctacttcctttacattcacacatccttccggattgttgtcggctactcaattcactcaacctgctttgaccttaatggagaaggcc
gccttcgaggacatgcgtgctaagggattagctcaacaggattgtgcttttgccggtcactctcttggtgagtattctgcgttgtccgcaatggctga
tgttctttctattgaagccttggttgacttggtgttccttcgtggcttgactatgcaaaacgcagtccgtcgtgatgaattaggacgctctgaatatgg
tatggttgctgccaatccttctcgtgtttccccaacatttactgatgctgcattgcgtttcatcgttgatcatattggtcagcaaactaacttattgctt
gaaattgttaactacaatgttgagaaccaacaatacgttgttagtggaaaccttatgagcttgtgtaccttaggccatgtcttaaacttcctaaagg
ttcagaagattgacttccaaaaattgattgaaactcttacaatggatcaattgaaagagcaattatcagagattgttgagggttgtcatgagaaga

ctttgaagcagaaggaagaacaaggccatctagagttagaacgtggatttgctactattcctcttaagattgatgttcctttccactcttccttctta
cgtggaggtgtacgtatgttccgtgaatacttgcttaggaagatttcacctcatcaaattaatgttgccaagttacgcggaaagtacattcccaact
taactgctaagccatttgaaatcagcaaagagtacttccagaatgtttatgaattaacgggatcccaacgcattaagaagattcttcaaaactggg
atcaatacaattcatagagatgttttgtaatcttttgctacttatggcaggagtggtttaaaaagtacttaatttactcacttattttgatatatagtct
tcgtttaataaggagtgtattgttatctctaacaatgcgcttagatttactttttcatttatggtacttaagtaaaattggttatctccagtctctacgt
gaagcggtggtttatgatttacttgaataagataattttaatagaagaattgaagttgtgtggtattgcagcttaatttcaatcatatatttgtccttc
attaaattactactgcaagcatttttttattttcaaattctcatggatatgtgtaaaatgttcagagaatcatagtgcaatgtagcgtgtctcggtatgt
aagttactgtggcacaattattttcgacattcctaaagtgaaaacaagtacaacttgaagacctgtcctttaccaatgcttcgtcaagtgtaagtta
gataaaatttcagtaaattggtatactaacgagaatagaaagcctaaaaatgcacgcactaagcgtgtaatggataaacgcgagccgaaagtc
gtagaaggagcgaaaattgctcttttcttaagaggaaacgcgacttcgcagcttgttgttgatgctttacatgacttgtacgctttaaaaaagccttt
tgctgttaattttcaaaagaaaaatcccattcttccttttgaggatagctcttcactagagttcttttctgagaaaaacgatgccggtttggctgtttg
tgctactcataacaaaaagagaccccataatttggcttggattcgctttttttgattatcatgtgctggatatgattgaactgggcatagtagactata
aaccaatttcttcttttcatactgtgcctattgtgcccggtacaaagccgatgatgcttttccaagggccttcttttgattctcatcccgtttttcgtcat
gttaagtctttatttttggactttttccgaggcgaacccatccaaaagttggattctgcaggtttgtcttatgtaattgttgtttctgccggtgaagcca
gcgacgacgagacgcaagctttaccgaaagtgaatttccgggtgtattccacaaaattgttaaagtcaaacacgaatcttcctcgtgttgaattgg
aggaaatgggacctcgactcgactttagtattcgccgtgttcaagctgctcaagcagatgtcttaggtgaagcgttgaagaaacccaagacccaa
gaaccaaagactaaaaagaatgtggatgttgacttggtcggcaacaaggttggccgcgttcacgtggatcagcaagatcttagcaacttgcaaa
ctcgcaagatgaagggtttgaagcgttctgctgccgaagaagaagaggaggaagaagaatcggaagtcgaagacgaagatgttgtttccgacg
ttgagtaagcaaaaaagccaatgtttgcttcagccttggaaaatccatacggatacaatattttgtttttggaaaggcattttggaaattcatggttt
tctttttgaatttggttcctggtttatataaaggtaatttaacggtcagtttctttgattttgggaagcagtctaaaatttagatataaatatttgtctgt
ctctatggtttctataggaagttttaataaattttaatttacatttagaaatactcgagttctaatagaaagtactggagtctctagtaaccagatctt
ttagtcgttttaagcaagttgcactattccaaacggaatacgttcagcgtgttgcttagggcatttggatgtatttctttaatattttaaggtgttgaa
cagacctcagaatggtagtaaggagttactgacttcttacgaatttcttttctggcctagcaaaagagaggaaatcgaaggaaattgaatgaaaa
taaaagaatctagtaaagatattaagtcagaaaccccaacttggtatgacgggtctcttgtgaatatgctaacgaacatctactaccttaaacata
cttcaggtcaaaaaacaaaaatgcaattgaatacttcgagagacgttaacccagcatcagtagagcgtctcaaaagtgacgctaaattaatggt
agtcattaaagctgtggacatggtaaacaaagaatttgattagatttgtaaagcaaaggtgctaatgccaagtgattaggaggaagaaatgcaa
caagatgcagtacatacaggtaaaggagagaagcttaagaaagaaactaatggacatttaaatagctgtgcaagcgatggagaaatttactgtt
gaaaaggtaacacgatatttagaaagaaaaaccagctggcggcggaaagaaactaatagagtttgtttaggacgttgctgcgtttgtaaaaaga
gagttcgacaaaaagtactctcctacttggcattgtatcgttggtcgaaactttggttcctatgtgacacatggtaagaagatctcttgttgctgaat
tccttgatggtagcgtccaagctaacaacattcagaatcccatagatttatatatttttatttgggaacgattgcgtttctgctatttaagtctggttg
aacaaaaagagtttcgtcgtgggtcggcattatgggtccttatttattttaatttggatagtttctactgtattctatgcaatctgaaattcctagaaa
aaaaagtatttctctactatgagtgttttcacttatttaatttctagaaagaaaccttatccatatgtttctagtgctacttatgccgccgaatttacca
attgtttgggaattgaagggtttcgtaatggaataggtatctgctgctgaatgtcaggaccaagttgtctatttggcaaaatctctgcatttgtctta
atcttattcaggctgttgatactggttataagattcctaagcaacaggagattgcaaaattcttccattggtttgctcgctggatatcttgcaggaag
tgaaacttttgtgagagaatcgttaaaaacttccaaatctattactttgatgtctgcctatcagtggattcgttttgtaaataaaaaaaaaagaattt
gaaaaggctcttattttattcgtttgtgaaaacctcacataattgtaactcatgttttaaaactgcaatagaaaggtccgatactgtccaagaaaga
ggagaccacagctgaatcaaaaccataaacccaattgaatgctaaaactgcaaaattcgattctatcgtctaacccactaataggctcgattcca
ttccagtctttgtcgacgccattcttgaacatcatccactcgactcgtaaaaccttttgaaataatttcgtgttcttattcaatattcaagcttttagac
atgttcttttcattgattcaaatctcattttacttgcacacactcatacctagtagcaagcctctttcacagctatccgctccttacctcttcatctcttc
actgccatttcgaaccgccaaaaacccttgcattcgtagcattattaatattgtatacctatatgtttataacattttatatttgtttcgccgaagttag
ccagaagtgaattgaaccaacgcgactacgatcttatctcattcctggtttatggtacagaagccacccgtactgtctttgcacagtaattaaccta
aactactgccatttactaaaaaagacgttttctttttttatatagagcaattgtttgctcaatttctctcttttcaccaacacaaagccacattgcacc
acttcttacaggctttacaaagtacgaaatcaatttgaggcagatttcgttccctgaacatttcttttgtatgttttcacatcgtctgtcattaatttctg
taacctagctcacagatatcctggcttcaaatttgttcgttacttacttcatacgcaaagagtcatttttctgcttaagatgggaattagtattgacgg
agaaaaatatatccgtaatctagcttcttatatctatactcacgaaaaacgtttagcaaatgcttccagccaatttaccagtacttcaattttcagca
gccagccagttcgattgcatctcaccctccaccatttatacttcttattgactcaatttgaggaaatcaatgtgaatacgggccctcttaatatccgt
attgagaatctgcattccgaggcatttccaagcgactacacttctttcctcagaaatcgagcatctagaaaagtagactttgatgataatatgagc
atgaagagcgtcgccaccattggtagtatgctttctcaggtgtcttccatgtggagccggttctcaactgcttcaaacgagttcacggagcatagaa

acaggctaaagttactttctttgctgcgctatatttattcttcttttaccaaactccccttttttaagccttgtccataacccgaaagcaattctaatctct
cagtttgaagagtttcctctagatactgcagtgcccatcacggtatttaagaacctatcttctttggaagttcgcggttacgatgtaagatccatcttt
ggatggaattgtctttcaataggtttgaaaagcttaattctgcatcattgtgatctggtggatttgtccgaggtgtttatcaagttggtccttgatgat
tctgatctataccggtttcgttcatctagacaacagcaattcggtcaatcctcctctactgtcaatcaaactcgttctccaacaatgtcgatgaatctc
cggcgctctgtctcgcttgggtcaaaagatcaatcaagtccacagcattcaaataatttccaccgcaccctgtctcagtctgtattagttttgtcaaa
agaaggcgttgattcttctaatcttagtgtcaaaagtaatgagcagttatccgacgcccaaagtgtattgtcatcccatgcttggagccaactccta
tattttcgctgctctaattgcaaccttaagtatatatccgaaaatacatttctacctcttgtctcccttttatctttggacttaagcggtaatcagcttac
cgaaattccctctgctttggctgaactgccgcagttacgctctttaaacttggcttccaataaactcgataattgtcgtactttctatcatatagaaat
acctaatctacaaaatcttgtccttgcgtgtaatcaattgaaggatctgtcgggtttggaagctgttccttctctggaaagattcgatgttcgggata
atttgattgttgatccagttgaattccgtcgcctcattggaaatacaaattttgaagaagcatatatagcttcaaatccatttataaagactcattcc
tcacatcgaattatgattttcaattattttcgagagtttccggggtctaaagatattttgcttgatggacgaggacctagcatgcttgagagaatata
tttatctgagaaggcaaccccaagtacgagccaaggtggccagccctcaggagggagcgatacaaatgccgtcattcatcctacaaagtctactt
tgaagaaaacaagcaaaacaaggattgttgatttgtcgttacccccctgccaacgtttctcttgcacgtccagaaagtgatattatatctactcaatc
gggatcctccattactaaacctggaacctcagaatctagccatgatgctggagaattatttcgaaaaaagattgaagctttgcgtcaggaggcag
gtaatgagtggatagaagctttagtacaagaaagcttgacgagacacaaaaatcatacagagcaaagcttgaaataaatcgtatccttcatgtt
acttatgctcatggataatgccacttaatctacgattctaatttctcttatggacatgatgtattggtttttttttatttatgataatttttacttttattta
tagtatccttctgaaatgtacataaggtttcttctttttttttcgagtttatttaagtttcattatattgctggatatactaatcccggacgttacaagtta
tttttaggtgtggttcaatcggttcctgacattgttaattgaaactgaggttgactatgcggcttcctatcaccaattttgtttatagaaacgacgtcc
ccgtaatacttggcaaggaaatttatcacacttctgtatactcgaaaaaagactcgtctgttctctttagaaacaatacgtggttttcatcgcataat
gtgttgcaagcacaatttgctctgcttttgaaaccattgttatggcatctctgtgaaagaatgctgttaaatttttccttgctcttttccttagtccccgc
tgatctcctttatttttgttgagtgtatttgagtttaatccagtttgttgtttgaagttatggacatgaagaaggcaagatctgccttcctgttaatcta
aaccattgaagaaatacgttttatttgaaagtattccctcaatagttacgagcttgtgcatgataagatgaaaaacaggcttgtatagatggaattt
ctgtaaatatatttcttccattcgttagcctatcaactattttttttaaaattgattttgtttcagatcttcatatttgggttaagctccttacgtttgcaa
gcctccggtgtattatgtttgtccaaacttattaatgagatattaaaactatcaaacggtccttccagctttttttaatgccctataaccatggataca
acaaaaacactataatatatttaaaaaatattaattatagaaaaccatttaggtggttaaactaaaacaaaaattgttactaaaagagaccaaat
ttactcttttggaatgaaaaatgaaaaaataaaaataaaagtaaattagaaaaatagtgaagtgtacttgcatctcctgttacatgaatttggtttc
tgtgctttcaagtcttatgcaaggaaaattcttcttgaaccgtaatgaacccctatgcggttctaaacctggaggaagatgaattatacactgatga
acaagtaaaagaagcttatcgaaaagctttaatacaatctcatccggataaacgaaaagatattctgcctggtcgatatagtgttgaccaaattc
gtgaagcttttcaggccattggaagtacggaagatcgcgagaattttctcaaaaccaaggaatctggtaagtaaagaaacggtatgccttaatta
taagaaagatgttaacttgtgtttccagagcgcgcacaatattattctgtggttgatttgagtgaatttgatgagttggaagatggctcctactacta
tccctgtcgttgtggtgagttaggtggttatgtcgtaacggaagagaatttggaaaacgaagaaagcattattccttgtttgggatgcagtttgact
atccaagtagcttatgaggtggtagaaacggacggaagtgaacagtaaaagtggtcatttttcatctaataattcaaattctatttaggaacaaa
agacccgaacgagtgcttgtctttgtcgttacgtagcaaaactgctgtcaacaaaaactaaactacacatggtgctcgctttttgtttccttagcggt
cttttccgagaagcatattactattgaaaccatcgtttggaattttgctgttcttagcaatcatatatctatcagcaagcgtatataaaagggcgtga
cattgcaacagttgtcaaaaaaataaaacttcaatttctgttgggcatcgttgattccaaagatatatacgaatatagatttgtctatgaaaggacg
aattttgactctgtcttttctatgtctaacttggaattggaaagaaagcagcatatttgagactagataaaaacacttttcatagcatgaagaaata
ttcctatttccaatagaaattataaatgtgaaaaaccttttgattaattcaacatctatcctataggattattctcttcttgattttgtttcatagcatta
tttaccaacgaatttggcagtttacaacgttatctatattaccgtacctttgtgtagttgcttctgttctttccagcgatgatagtcactatagggatgt
ctgccgtagtttgttataatagataattcgcactcacagcagcttctctaatgtcctgggcgattctggcagtgtctggagcctgtctactgtacttcc
acacactgccacactattcctccttcttccatgggcttccaccaacctacagggttagggtttggaatcttcgaggttctttgtttacagaatataaa
caggacgaacctaccgtcttcacgtatcgtcacaaaaggtagaaggcaaagcagctaacaaacctcttacgaaggaacgaattttttttaattaaa
atttaaaatctgtgattttcctttttttggagcttactgagggaatcgctttacgagcacatcatttttgcatattcaagctcactttaggatttaacgaa
atttctattcatttgttctatcatttatatttttttatagaaactcaaaatgtcaaacactgagactttcaagttcgaggctgaaatcagccagttgatg
tctttgattatcaacactgtctactctaacaaggagattttccttcgtgaaatcatctccaacgcctccgacgctttagacaagattcgctaccaaag
tctttctgatccccatgccttggacagtgaaaaggaactttacattcgcattactccctataaggaaagcaaggtcctttgcattcgcgatactggt
attggtatgaccaagaatgacttgatcaacaaccttggtgttattgccaagtccggtaccaagcaattcatggaagctgctgcttctggtgctgaca
tctctatgattggacaattcggtgtcggtttctactctgcttacttggttgctgacaaggtccaagttgtcagtaagcataatgatgatgagcaatac
atctgggaatcctctgctggtggtagcttcaacgttaccttggatactgagggacctcgcttgggtcgtggtactgaaattcgccttcacatgaagg

aagaccaactcgcataccttgaagaaaagactattaaggataccgttaagaagcacagtgaattcatttcctaccccattcagctcgttgttactc
gtgaagtcgagaaggatgtccctgaggaggaagacactactgaagaagtcaaggaaggtgatgataaggctcctaagatcgaggaagttgac
gatgaatccgaaaagaaggagaagaagtccaagaaggtgaaggagactactaccgagaacgaagagcttaacaagaccaagcccatctgga
ctcgtaaccctaatgaggtctccaaggaagagtatgcttctttctacaagtctttgaccaacgactgggaagaccatcttgccgtcaagcacttcag
cgttgaaggtcaattagaattccgtgctgttctttttgcccctcgtcgtgcccctatggacttgtttgaggctaagcgtaagaagaacaacatcaag
ctttacgttcgtcgtgtcttcatcaccgatgactgtgaagaattgattcctgaatggttgggtttcgtcaagggtgttgtggactctgaggatttgcct
ttgaacttgtctcgtgaaatgctccaacaaaacaagattatgaaggttatccgcaagaacctcgtccgtcgctgcttggacatgttcaatgagattg
ctgaagacaaggagaacttcaagactttctatgaagctttcagcaagaacttgaagcttggtatccatgaggatgctgccaaccgtcctgccttgg
ctaagctcttacgattcaactctctccactctactgatgacatgatcagctttgaagactacatcaccaagatgcctgaacatcaaaagaacatct
acttcattactggtgaatctaagcaagctgtcgagaactctcctttcttggaagttttccgtgctaagaagtttgatgtcttgttcatggttgacccca
ttgacgaatatgctgtcacccaattgaaggaatttgaaggaaagaagttggttaacattaccaaggacggtctcgaattggaagagactgacga
ggagaaggctaaccgtgagaagctcgagaaggattacgaagagttcgctaagcaactcaagaccatcttgggtgacaaggttgagaaggttac
cgtttccaacaagattgttggttctccttgcttgttgactactggtcaatatggctggtctgctaacatggaacgtatcatgaaggctcaagctctcc
gtgatacctctatgagcgcttacatgtctagcaagaagacctttgaaatcaaccccaagtctgctatcattgctgaacttaagaagaaggttgatg
agaatggcactgatgaccgttctgttaaggacttggcaactatcttgtttgagactgctctcttgtcctctggtttctctttggatgatcctagcgccta
tgcctctcgtatcaaccgtctcatttccctcggacttagcattgatgaggaagaagaagcccctgctgccgagcaagctgctgagccctctggtga
gcaagctgttgaaagcaagatggaagaggttgactaaattaattgaagtaatcaggaaatttgtcaagttgtctatgcatcatgccttttggaagt
aaacgactttgcagtatagcaagtattgtatggtttaataatttcgtcaaaaaagtttcttttcatgagttattttaattagagactagataatagaa
aagagtctttttatattatttaatggtttattatctatattgcatgaatagtaggtacttatagtgttgaagtagcaaggatttcttagaaatctgtgat
agtaaagacgagaaatgtgaatggataactttgcaaagtagtgttattatagttctgttatgaatgctgggtgtgatttaaagaaaatgtaagttgt
caataagcgttaaagatagaaggaataaatggaatatgcttatttggtcataataaaactgtaagatacctaatacatatttgagcacagtctttg
gtgctattagacgaattcagacgtcgtctttgtaaggacgcttttgtcaactggaaagtcaaatttcacgaatttcttgaaaacaatgtgagtttttt
ctttgttccccattgatgaatggctgacttgcagaatttttgccttgacatgtattgtttatgcttgaaacgtgacttttcatcagcatacaacagaga
agtgtagcaagcagttccacataaagctgtgtatgagtttaaaggatgagagagagaaaagcccggaagaaggattattagctctggaaatgg
aagaaagctttagtgtaatagttttaccaagccagtttcgtctgtttataaagtaatcctcgggaacttggcctaagaagaacatgaaaatagaaa
ggaaaacagtaaacttgtgttgctaaaaatttgtcaacaccctaagtgatatatctgtacaaattcacaacacagtaggcaaaggtaaagcataa
agaataggaatgaaaacgggtttggagtaacataaaatagcaaaaaaataataataatttaaaatcctaggatataaattcatgagagtca
atagcgctcgtgcaaggcaaaaaggtaaatatgaaggaaaaaatacgtttagagaaaaaaaaagaaaagaaggatagtaaagaggtttagtt
gttgtctttagagccagctaccgcgtccacaaatttttcataataaaacattccatggctcgtgtgtgaacggagaatggactcaacgtctttggca
tcaatacgatctcccatggtggacaaggcttcttttaaaagaccaattgggatttttccagactgagtgtcatcaaaagttgcaaaagcttctaaaa
ggtccattctaggagagatacgacacaacattgaacccataaaagtaaggaaagaagctaaattaataggaggacgaacagaatcgaacattt
cctgaactacttgactagaagattcttggtttagagaggtaagcatagttttcaaatcatcttcgccgatatttccatcgccatccttgtcaagcagg
gcgaaagcttctttgagttcttgaatttgagaagaggtaagttgagcaaaagcaccagaagaagcccttacagcagcctgcgctgctactttttca
gaagtagtagaagaaagaggaattcttttctcgcccaaagagttggatttatctgtagccatcgctgaatttttgtatgtctaacctatgtaaatac
gagataaaaataaaaaagaaaagaatagatctttgaaaagagatgttctggaaaccttcgaactggatatttggttatgggtgaaaaggaagat
ttatcaaggaaaaaaaaaataaaaaacaaaaaaaagtgaaaagatttaagaaaataaataaatgaaaatactgcccttaatccttcacacag
aagaaaagaaagaatgggtaaagtgtgcttttggacagacgaactttttttcttggtgaagggtattctttggtttttgttccaaccaatgtgtaaac
aaacatttcagatcctcgggatggaatgaaattagagaatccaataacgatcaaacaaatcatttttccttatgcaatccaatggtttcgttttgcc
atgtttatgttgtataattacatggcagaaacttacaaaattaaatgtttggtcatttgaggcatgccatccaacatcgttttctaaaagaagagca
aataatttttttctaattgataaacaggttcctattgttccagcgcatcgctcataatctgtttttttgtgactttgttcattcgcagtgtttcactgtagta
gtagtaacttccagtggaataaaggtgaaggtttactgaagactcgcacaacaaaaaaaatgtacaaaatatttaaatagtttagattcctacgag
aacttttaacgcccagattttaacgtattgtaactgacttatttgtttatatcgttctaattaatatgataactacaaagatcattcattttctttattat
gatagtgatgaagaagtaaaatttagtaatagttcagttaattcaattctttagagtgaaaagaagacaggcttctcattttacacaagtcagtctt
cctctttcccttctcccaaagtttaataattgatatttgttgcataagtaatttttaaaatggatttcaactggaagccaacagactatataatgctgt
atatatattttatacttactatttcaacgtctacggccacacctaggcgaaaacaccagttcccgtccgatcactgaagttaagcgtctgagggcct
cgttagtactatggttggagacaacatgggaatccggggtgctgtaggcttttcttttgttgctctttacttctttttacgtagactgttgcttgcaacc
gttttctcgatagaatttttgcaagctgttcctttacaagtgacaaaagactaattacaagttcaatgaaaagaaatgaaacgaacgtattcaattg
caaataagaataagaataatagtaataataatcattaagaacgtaggatgatgccaatggtctcagattacggttcctttcaatgcgcggattgta

tgcatgcctttctattgtccatttttcttcgttgcttccaattcatattgatacaaagcaaaggcatagaaataaaccttcatcggaataaaccacttg
taatgttctctttttccacttactgactttcgatcggaagagttaaggctcatgagaaaagaaaggtaaacaaattagcattaactttgagtggtag
gattgctctgcagctctatttcttcgttcgtagttgtcggcggaggagccagtgcatcatccctttgttgatatccgggcagcgtaggcgaaccggtg
gtttcagttgtatttgcatagtctaatccaaggaaacgcatgaaaactatataaaatccttgggcaacagcataaataatatcaccaagcaatact
ccaattttatatatgacaccaccaagcaatactccaatattaccaatggcaatacacacaaagattatggagtagggcaaggctcttattgccccg
atggcaattggatgaaagactgccttttgttagcaaacaattgcattttttttaaaaacaataacaaaaatgattcacttacaatagaaaaaagca
tagaatattgcccatatcacaatcaaaagaactaaaaaaccaagaagggtccaatcgtatttaatttcattaaaggcatttttttagccaagaaaa
gggttcgaaaggataggaaattgctagcgtacacaacaacgaaaagattgctgttgatagtaaacgatagtttctgtgtttgaggaccacattttc
gggaaaaccatagtttactatttctgatgttttgcagtgaagctcctttatggactcaggaaagctgctggattcagtcattatcgtttttttgctttgt
ttgactttgacagacgttgaattggaagttttgtttatgtcttttggtttatacaaaggaagtcccttttcgagatcaacattgtaaacattagcatgg
ttgtatgcaggagggtcgcttgaatcgaattttagctccttttccgacatcctaaaaataaataaagagaaatttcaatgaataattagcaaaaga
gcgtcaaataaaagctgttgatccctattcgatgcaaaaatttcgcaaatgcaaaactcatcagaaatagcagccaaaaactaaagcagctcta
cctggtcattccaaacattcacatgaaagtcaaaatcagcccaattggcaggaattgaagaaataaaaagttggcttcagcaagaaatttcgcat
ctcttacgcaacattttagctcaggatagtgatatgttcagttgcgctatctcctatactgatatatagcataaatcgttgaagatacgtttgaacca
caatgaacaagtaacaaatttcgctaatttcgcgtttgtttacacctctgtaaacaaagcagatcccatttctctatgaacttatgaacaatttttta
ccttgaaagtgagttactaatttttcgttgcactaaagttattcaaacagaagaagtaacaaacaaaacacggaaactgcagacgcattagctaa
ctgcttttcattagacaattgagtaacagactacaaccaaaaagcagctttcacgggttcttcaggattaaaaaaacctataaattacttctttgaa
ctgaaggaactgtgcacggccatacctaggcgaaaccaccagttcccgtccgatcactgcgattaagagtctgagggcttagtctcgtttgtgcta
tggttggagaggtgctgtaggctacgtctttttcataatttatccatttttttcttttaatttatacactttctcgattaatatggaaaaaacgtacaaac
tggtttatacttgctgcaggatgttaccctaaaggtaacgattaacaaagatctcaataaatcaaatggaattacaagttcaagaaaaaaggaaa
tcaacgatagtattcaatttgaaaataaggatattaattgatggatgtaatgaccaacgacccaaaaacgttaggctccagtaatgttctagcaa
gtttaccaactccatgcctctaaaggcgacgaaaaaaagcttcaattattcatattaaataattggtttttcattgcaagactttactatcatactaa
tacaggggcaatttgaaaactaaaataaacttggaaattcgatgtaaaccctcaaagtaagacagtaatgagtaataacaaaaccaagagcttc
aagtatctctcctcattcatctcataatcagatatattgaaataattaccataggctaaaggttcagaagaagacaaaaacaaacgaatggaact
aacgtcaaaaagtaggattggtttgcagctctattgctttattcgtcaacgttagctgaggaggtgtagaattcttttgttgatatctaggaagcgta
ggcgaactatcgttctaaattgagctatcaacttcaaatccaagaagctccatgaaatagagataatatacactacaaagaagtaaaaaaaaaa
cattccgccaatagcaactaacaatccaacgaacacttctatcatgtcaactgctatacaaatggacaaagtaatcctaagtatacatagggtga
tgacctttacaaatcgacaacaataaaactaaaaccagatgctaccgtttgtaagcaagccagcaatcataaaaatttccgcttacgcgtgatac
caaaaattgctgcccatagttcagcataaatgtctaaagtggcaatttcccaatggtgcttgtcttgtcccagaactatgataacataagcatagat
tttgtatacttagatatcagacatacatacccttcctataagactacggatataaagcgaacatttttttaatctttaatgattactttttcccaggaag
aaagcggtttattggctctataaattcctttagaaggaatttaaaccgctaaattgagtcattttcaactattctctctcattgattttggtagaagtc
ggattgcaaggtatatttctatacctttgggttgtccaaaggaagtcctttctcgagatcaacattggaaacttttattaagattcgactgttccgac
attttgacagaaatttaaagaatatgcagcgatttaacagcaacggagtcgaaaaagcaaagtttctgttctcattgttaaaactcgcaaatcgct
acagaatggaaaagttcaagagaaatggcagcagaacctaaagcggtttccttgctcatccaaagaagctcaaattccaaacaaaattattcca
attatcggaaatagaaggaaaaaagctggtcctagcaggaaaattcgctgcccttacgcaacagcaatggtcaccttaaagtcataaaggcaag
taacatgtttcataatgttcgtgttgtttacatctgaatcaacaaagatgattcccttttttatgaggtttttcttaagaaatttaagtttttttcataatc
taaaaaagtaaaattaatcatcagaatatattctactcgcctttgattaattgcttctttatttccatgtcaagtaagatacgctttggaaactaaaa
accagggatatttcatcttttgcttttgcgtagagggaaacaacggtcatattttctttcgatcattgcttttaagctaatatgctcgttcttgtcaact
gtcttaatttcattccttaatgatcataaagcaagggaaacaaccataaaattaagtccaaaaccaggcttcaattataatttcactgatttggtat
agtttaaaaggcgcttttgctcgctgaaatcctagcttttcttatttgattcttattcctaggtgactttccagctactaaacttctttactgacatttcc
atttatgagagtgaagaaccattctacaagatatccaactatctttaattttaaagtttattttatatatctatatctatatacacttttcttttacatat
ttactcatttctttcgatttatctcaaaaaaaatttccctcttgtctatttattaacgaaagcaaaagagtgtcactaaaatgaacaagaaagctccct
ataggactgtaaataaataaatagcctggttcatatctgggaaaaacgctttgataaaaaaaaaaagcatttttagttggttctaattcttctgtatc
aagtttaagagcatttttttgggtctctctttgagtaataaaaaagcgacagcaaaacttgatcgtacaaaacctttctaaacgagcttacaagaga
aagaaagcaaaagcaacattgtatatcaccatgtttcgatggtcttcaacctatacctttggtttgttatggttaatcgtcggcggtgaagcgtatc
aattgactactataaaacaggatcataaagaaacgatgaaatactacgaggcgaaaatacatcatctgaaaagtttacaaagagaaaagtgat
gttttaagattcctttttccttcaagtctattcttaattttataggctctccagtatgtcttcccgtgcttcattgggaattacagccagagaagtaata
ctatgtattcccattcttctttgaagctgctcatgcttgacaacaatctcgcaaaacgttcctgtggtttttctattttgaatttctttgtacgctctttca

tacataacctttgcattctttaataaaacagattttgaattttctgcaatagcaaggggaatatctgtcctttgaagctccaaatctgctctgctaatc
catattctcgtacggctagcatcataagtacttgccagtttgagtgcttttgtagcttggccaaatatattccaagcccttgtcaagatttccacgga
cgggactgggttttgttgtaactgtgcttgaattaacgtgtcggcaaattctgcgtaggcgtctgccttgtcacacaataattgaatatggatgggct
tcgtcggagaatttgacgcctgtaacggattttgcacgttgatttcaggcagttgtgacagacggtcatcatagacttgtagcaacttcacccatac
ttgttctgattgttgccccgataatacgaggcgcttttcccttgccaaatgccaatccataaattttaaaatatcatacttttccgaaataggctgaa
cgatttgaattagattcttaaagtcggactctgtgaaaagacttgtgctcgcagctaaagcagcgatttctcccaaaagaacagcagagtcttcgt
cgctttgggatacatgtagtcgaaatttctcaaaatcaatgtttcctaatcccgaggaagcaatttgttccagctctagctttttctgatgaaattcgt
cttgctgaagcattgaacatttcataattgcatctaaagcagcttggatatggggtaatgcgagttgctcattaccagcttctgtcctactttcagca
aggtatttatgcgtttgcgcaagattaaaatatgcttcagaatgtgtttccaaacttatggctttttggtgcaggtcaatgctaatttgcagatattgg
gccgactgactcgacagcatatcgcaattggaaaccatattcaaataaagtctagctttgttaaaccaagcatcatagtcatttggcctggctgtc
aaatactcatcgtaagctttggaagctttttcatagaatcgatagcactagaaaactattagttttgacctttattaaactgcgaaatgaaaataac
cactttcaattattgtttatgaaaatatgtgaatctgctatttttgcaacttaccttttcaggatctagccgccagcggtctccactctcttctagttca
atggcttcttcaaagacgtcgtctattgactgaggcttttgttgctttgtcttcttcgctttagctgcttcacgtagaatctcttttcttgaattactcatg
acaaaaacaaaaatgattaatcagtacatataatttcgttaaatacaaataaaaggctagaaaataaaataagtcaggttttgtcttcctataca
cagaggtattttagaattatgataatattacattacacgtcctacttgcgaaattttgcacctactaaccaaaacctatcgtttgaaaaagaaaggt
tgagatttacaagttaaaattggtttactgaaacaaatcaattaaaaaaaatttggaattcaatctttacaccctacgaaaaaaggtttctctataa
aacactcctttaaaaaaaactttttttgcttgtcttttggttctactatccaagtaaacgtgatcgtcatagaaagattaccaagtgttgattataatgt
ctcaaaaaatttccgttctttttgtatgcttgggcaatatctgtagaagcccaatggcagaggccgtctttcgagacgaagtaaataaggcgggcg
tgagcgatcgttttgaaacaattgacagctgtggtacaggagcatggcacgttggcaactggcctgatcctcgtaccatcgatgtgctgctagcaa
atggaatacaaacagatcatttggctcgtaaattgaagttgacagatttcaaaaagtttgactacattttcgcaatggatcgatccaacttgcaaa
atatcagacgagtgtgcccaagtgatgccaagccggaaattgcattgtttggagattatggcagcgcaggcgtatccaaaattgtggaggaccct
tactatggaggagatgatggattccaactgtgctatgaacaactggtagacttttcaagaaactttattcggaaagtaattggatagaaaggtttt
attgatagaaaagaaattataaaattcataagttatatatcgattacaacatgttagagagcaaatattcatacccatgacatgagacgaatata
atgaatgataagaaaaaaaaaacaacgatatcgtaacaaaaaagagagcgagaggggagaaagagttatgctttgcagtatcaaagtcggtc
atcttgattagattgtttagagtaaaaataatttaaatagaagagaccgaacaatattgtcgattgtagtagacgagaccactagagtgtgccag
gggtatggaaccatgctcaacacgatcaacgtttgcgacaaacaagagatggtcttgtacaggtatagagtgaaggatggaacaatgtaagag
accaagactatccaaaagatgcggaacaccttctttactaaaggaaacttcagatggagaaagggtatctgcggtagaagaagagggagaagg
agggctggatatgtcctcttgaacagaagaaagagagggagaaggaaaaacggttggatgattcggcttggaaaggatggaggcccattggga
gtgacggaggatggagctggaaagaaggtggacgacaacacgtcgggattggacaatggcagaagcagttcgggaaggaattttaagattga
aagagacaacaggattcggtgaaagagaaacgggggtgaaagaagaagcggtcatggcagcttgatgaccgttaggaaaaccgctggaaag
gatgacgacgggttgggcaaagcgtcgcatcaaagcccgaaattctgcatgatgagaagaagaggagggaagagaggaagaggaagagctg
aaagaagcacgaaacggaagaacggtagggtacgaagaaaaaacggggaaaagatgatgcggatgaggatgaagggatagccaaggacgc
attcgatcaacaacaaatcaggtccaatggcaatggaaagctgctaataaacaaaaaatgagtaaaagaaaaggatattgcgaagagaaaga
caaggaatatacgttcacttttattagctagttttcaatgaactatttttatatttcgtcccttttatttttttccatgaatggtgttgtaactagaaagtt
tgttgaatgactgatgattacgatcaaggtctgaccagatcaaaggtatgaacatgaatgaccaggtcaggcaggtatggtaagagaaagcata
tgtcggtttcccttcgcttcccacaagtttaaactcttcaacctttaagggagaaatctaagaacgaagtgactttagggaaaattcgccaataaa
gggggtttcatccccaaatagagaataaggagagttaacgaattaaaaaattactcacttaaaaaaaagaaaggaagggagacgagacaaac
ctagagacggccgaaaaaaataatcacacgagaaagaaaattgcactacgacatgttgtttttctatagccgacagacgtaaataaacaaaca
gataagtttcattctacgtttcagtttgcggttctattcgcccgtatttgtgtcattgacaaccagcatttttaagtttgtcttcattctgtctgcttatac
tggatgggcgttgctctgtctcattctcctcattctcctcacattcccgccttctcttccactagcatctttcttcctgatatctttgcaagctcgttactt
atccttatcccatactccagtttatgccgttctgtgttatccaacccaataccgatgatcctataagacgtgatttcgtccggaattttcttgaagaaa
cccgataagacaagacccatccaggccagaatcgtaaaacgaaggatagcgaaagacgacgagaaatcgacggtaaagagatcatgtcactc
tcgcatacaaatgtagacaactagaatgaaaacaaaaaaaaaaaaattaagacggaaactgaagacagtaagaagagacaagactaattgttt
catcgattcttgtgaatcattacctaaagtttaagtttcccgttacaagattcgggtcacatgatgatatggaaggcagatgtcatttaacgtaata
aagcgtaactctatagtatttttcaacgaaccagataaaaccaagtttaaagcttgtaggcaaatcttagacttgcctgccctgcctctcagctaac
taccagcaaccgcttcaaaaaagtcggttttgattaccaggcctaatttttgccgaaataaaattttcatccatgaatttgtagttcaatgagttag
tttttttagaaagtaagtttttgtatgtcttgcgtatttcatctccttcttcaaccttgaattctttattttttcagttacgataccccttcttctcgtgctttt
tgcttctccagcccactctcagtcagtgtaaggtgtaagcttttactcaaccctcttcgcattacgttccccctccttccactacgaatctttttccgtct

ctggattcgtacgacccatgctacccttatctaccggtaacatgacataaattaagcatttgattcgctgtcggttcgaaaccagcacagtatggtc
caatcaagtatattctctacctatctaccttgtctagacgtttcttagcgacgaaatcatcctattgccgacctccgttgcttctcgttacacgtgacc
acttttgcaccgttcctgctttttcgtcctgccaatttttggtataagtaggctccacctcccctacgctcccaagttcctttcaagcccgaaagttttg
gaacactatacttaacatagcttagtgaattcgctaactactgttagaagaaaaagaaacacaacaacctttgtgcttgagaacttctgtgtttca
actgttagaaaaagataattctttctctgtcaagttgacattgtcgttttcttcccttttttcttaactttcatctacctattgttttgattctctccagatt
ccaaaactttacctattctccgttgttcgtttgtgcgtacttcagaacgggttttacaaggcttagaatcaactaatttttcttttaatccttcccttata
ttttctcacggatttgcttcaagcattttagtgtgaaactatctttttagggctttctttctgattttgcttctgtgttgtcactgttgcgatttctgaatcc
tatatatttggataccaatcatttgattcccaaaatacccgttcgtttttcgagataatttttgagcttacgctcttgttctaattcgaaaacgttcgtct
ttctgtttttcgtttttcgtttgtttcaattcccctaaatatatcccttaaactctaattgttgaaaccttctttgtatagaagtttcttaaatttggatttct
aataactttctttttggatctttgtgctgcaatactttgcatctgtatcctgtaacattcgttcataatggctgaccacgtctctaacgttggtgaatac
caagatgctgaaaagcaaatgcctcaagagcaagaacaacctacccaccaacaggctgctccttctgaaccttcggaaggagatgaagagccc
gatgagaacatcgatgaattgatcgaagaactcttttctgaggatgttcacgatgaagaagaagaaacggacgaaactgcagctggtgaagcca
agcctgtacctgaggaacttcttcaaactgatatcaagactggtttgtccaccagtgaagttgaagagcgccgtaagaagtatggtcttaaccaaa
tgaaggaagagtctgagaaccctgttcttaagttcatcatgttcttcgtcggtccaattcaattcgtcatggaagctgccgccgctcttgcagctggt
cttcgtgactgggtcgattttggtgtcatttgtgcccttcttatgcttaacgctgtcgttggtttcgttcaagaatatcaagctggttccattgttgatga
gttgaagaagtctcttgctttgaaggccgtcgttgtccgtgatggccacgtccaagaagttgaagctaaccaagttgtccccggtgatatcctccat
atggatgaaggtactattctttgtgctgatggccgtattgtctctgaggatgtccatcttcaagttgaccaatccgctattactggtgaatctcttgct
gtcgaaaagggtcacggtgaccccacctacgcttcatctggtgttaagcgtggtgaaggtttcatggttgttactcacactggtgactctactttcgt
cggtcgtgctgctgctttggtcaacgccgcttctggtggtactggtcactttactgaagttcttaacggtattggtactgttcttttggtcctcgtcttgt
tcactctattctgtatctacactgctgctttctatcgctctgttcgtatcgctcgtcttttggaatacacacttgccattaccattattggtgtccccgttg
gtcttcccgccgtcgttaccaccactatggctgtcggtgctgcttatttggctcagaagagagctatcgtccagaagctttctgctatcgaatccctt
gctggtgtcgaagttctctgctctgataagactggtacccttacgaagaacaagctctctctcggtgaacccttcactgtctccggtgttagtggtga
tgaattagttttgactgcttgtttggctgcctctcgtaagcgcaagggtttggatcccattgacaagtccttcttgaaggctttgaagaactatcccg
gtcccaagtctgctctctctaagtacaaggtcattcaattccaaccatttgatcccgtctctaaaaaggttactgcttatgtcgaatctcctgaaggc
agacgctgcgtttgtgccaagggtgctcctctctggattttgaagatggttgaggaagctcatcccatccaagaagacatcttatctgcttacaagg
agaaggttggtgatttggcctctcgtggttatcgttctttgggtgttgctcgtaagtttgatgacgaagaatggcagatcatgggtatcatgccctgc
tccgatcctcctcgtgatgacactggccgtactatttctgaagcaaagcgtttgggcctccgcgtcaagatgcttactggtgacgctgtcgatattgg
taaggaaaccgctcgccaattgggtatgggcactaacatctacaacgctgaacgtcttggtattactggtggtggtaacatgcctggttctgaagt
ctacgactttgttgaggctgctgacggtttcggtgaagtcttccctcaacacaagttcgctgtcgtcgatatccttcaacaacgtggttacttggttgc
tatgactggtgacggtgtcaacgatgctccttccttgaagaaggccgacactggtattgccgtcgaaggtgctaccgatgctgctcgctctgctgct
gatattgtctttttggctcctggtctttctgctatcattgatgccctcaagacttctcgtcaaattttccaccgtatgtactcttacgttgtctatcgtatt
gctttgtctcttcacttggaaattttcttgggtctctggattatcatccaaaaccgtttgcttaacttggaattgattgtcttcattgctattttcgctgat
gttgctactttggctattgcttatgacaatgctcctttctctatgagacccgttaagtggaacttacctcgtctgtggggtctttctactgtcgtcggta
ttgttttggctattggtacctggattaccaacactaccatgattgctcaaggtcaaaaccgtggtattgtccaaaactttggtgttcaagacgaagtt
ttgttccttgagatctcccttactgagaactggttgattttcattactcgttgcaatggtcctttctggtcttctattccttcttggcaattgtctggtgct
gtgttgattgtcgacattctctctaccatcttctgtatcttcggttggttcaagggtggtcaccaaacctccatcgttgctgttctccgtatctggattta
ctccttcggtgtcttctgtatcatggctggattctactacatcttgtccgagtctagtactttcgaccgctggatgaacggtaagcctcgtgaaagca
gagacaagcgctctatcgaagaccttgttgttgccttgcaacgcacttctactcaccatgagaagggcgatgtataagccaacgttctaacataat
tattgtttatattaaacgttctgttctttcatcccctaatatatatatagagagtgttttattaatttcaatatgccttggttttgttaacggaacatgaa
ttgtccgttcgattagtgaaaatatatctgatttaattcgatatttgatatctaaatgaatatacgaagaataattgtttataagctagtgaatataa
aaacaatttaattcattttggtgtctgcaagtatatttttcggtcggtgtattataatgcccaacttgtttaatcagaaagtgtgtattatacttaagtc
ctaaagccaagtggattgttttcgcttaaactattcaactgtggcatgaagaagcctaatgaatgtgaattaatttctacagtgatatctagtcgac
gtcccttttaattgacaatgaagataatattgtaagtgttgtagtttgttggtagagcatcgtatttgttaatagcaagttatcctgatttgttgttaaa
tttttcagtaacagcgacattggcttttttttttcacttgttttagttgtgacatataaaaatatcgattggagcaaacatgtatattgacaaatgtggt
ttcgccgtatatttgtaagtggaatgcagtttgggatcaattgtagatcgcgcaaattttgactagaaccatcttattttattgaccatggatatgga
cccctatgaggttttgggagttcaaaaaaatgcgaagaacgaagagattcgacgagcatatcgtaaaaaggcactacagtttcacccagaccga
atccgggatgaagagaagaagaaatctgcgagaacagaattcgataaagtagcacttgcttatggcgtgttaaatgatgaaaagaaacgaaaa
cactttgatactacaggctctataaatttgagtgaggacggtttggaatttgattggaaggaatggcttgatgaaatgtatagcggcgtcgtatctg

gagatgcattgaccgagttcaaagcatcctatcaaaactcagaagaagaaaagaaagacgttttggaggcttataaacgccggaaaggatcga
tggacggcattttggaggaggttatgtgtagcgaaataagcgatgaagaacgctttagaagcatgattgacgaagcaatttcccaaggaatcgtt
cctaaatataaacgctacaagccgaatgaaaaagctcgcaagaagcgcgttcgtgcatctgagaaggaggctcaagaagctgaagagttggca
aaagaactgggacttgatgagaaactcaataagcgtaaaaagaccgacgaaagcggcgaagaggctttaggagctctcattcgttctcgacag
aagaatcggatggacgacatgataaccgcattggaaaacaaatatggagaaccgaaatcacagtccaagtcgaaatccaaatccaaaaaggt
cgagaaggaaaagtaaatcttaggaatgcctttctgaagcaaaactaaactgtgttcctcaaaatatttttgcgcatgaaaatattagaaccattc
tttaatgtttgcagaaataccaaaaatgtctcattcctttaaaacgaacatggagagaaagcattcttcctatgtaatcaatcaaatcacctaaag
ccttctgtttttacgatattaaatcattttctaataataatgttgtttattgatctactgcatgctgtagccggccacagtgaagtgtacctaacttact
tatgagaaaaagtaattatacactaactggtactaatgtttacaggattgaatcttacactcaaaaggattctttttatcaacaatacaataaagat
agaattttattgacattcttagtcgaacaaaccaaagcccatctaaacatactgttagcatatgtgttctctttgatatcattgttctttgcatggcat
ccatcattagtggaaactctaaaggcaaaaaaatattgtgtaaacttacgtcttcatcagattcttcctcttccttggcttcttccttcttggcttcttc
agtagcaggagcagcgccggcggcagcagcagcaggagcagctgcagcaccaccagaaggaacagttgcgagcttctcgttaccggcagcaa
tcaactcttccaagttcttgccttcaagttccttgagcaaagaatcaacacgttgggattcaacctcaacaccaacggtagtgaggacattttcgat
atcagaggcagagggagcgttctttccgccaacggtcaagaggaggtaagcagcaagatacttcattttccttttgtattttgtatgcggttgacgg
cgcgttgttgtagcgagtcttgcagctaactacttttccatgtttgactctcgtgactgtttttttgtttacgaaaaaggcagtcacaaaagttttggtt
gtgaggaaaataacgagtaccagttaccattcacctggatcaaacaccatggcagaggaaaattacgaggaagccgtacgtgctgctgaattaa
ggaagagaagaaccttccgcacctttgcctatcgtggcgttgaactcgagaagctcttggacctttccgctgaggaattggtggaattgttccctgc
tcgtcttcgccgccgcatgctccgtggtcttggagccggtgcttctcgcttcatcaagaagcttcgtaaggctaaggctgaggctcctgtcaacgag
aagcctgccgttgtcaagacccacttgcgtagcatgatcatccttcctgagatggtaggctctcttgttggtatctacaacggtaagcttttcaacca
agttgaaatccgtcctgaaatgatcggtcattatttgggtgaattctccatctcctacaagcctaccagacacggtcgccctggtattggtgctactc
acagctctcgcttcatccccttgaagtagatttgtttgaaatatatatttctctaatggcaatatttgatttcatgttaagcattgtaacttaagttcga
tatatatttacgcggtcacgatgtactcgtaacagtccgaaaaacggccgtagaatgctactcctttgtatgtaaattttggatgccaactcttctaa
aaatccatattcatctcaattctataactaatgaatcattgcttactatgtatttcagtaggtccattctgaacatattcatcaaacgtaacataatgt
gaggcaacagactcattaaaagacgtcttcgcgacaatactacctcccaagaatgccaattcttcaaacatagcattcgtgctggcatctggtttc
caggaaggaaaataatccggtaatgttttgcagggattaatgagcggcggatagggtgtaggaggattcgtagtcataccgataatagagtctgt
accttgggctacataatatggctcgagctttcctgttaattgaaatctaaattgtaattgctgaacgagggcttcccgaaacccgcgaatgcgagat
ccacaacccattacgataatattctcccataaatcatttcgacgattataatcgacgctgttgtgtttaattgtcagataaattgcttccggcagact
gacgctgtggtttgaaggacggtggaagagttcctgacatgctttgaaacgttccctaccgattatgcaatctccaatcggtgagaggtttatagtt
tgaaactcgacttcactattctcgggaatttttctttgagaagaatttcccttccgctcttcttctttccgagcaagatatgctcttgtttgaccggtag
ccacaattttcccaatgtcttcgacgccttcttcatctttgcttgtattgaattcctcctctcgctttgtttttttctttagatgcatcttgctcctcccgcaa
aacttgagtgatctcgctacacttaacttgttccgcaagcaactcggtaatgtgctcttgatcaacatgctttcctgttgcttggagtgtaggcagag
attctaacattagcttttgcaggtgtaaagtcatgtttcttccacctaacggaatttgctgtgtcgcagaaaaaagaatctggccgtccaaaatagg
agtgatatctgtcttttcataaccaacgtcaattacaagcccatgcaaaacaccaatggcgtataatcccatcaatgactcataaccaattgtaaa
tgctggcacatgtagctcttcaaaaaagaattgagtagcaagctcccgatcgtaaaaggaccaataagttggtattaataagcatacaggatatt
gcgaaaaagactccactgacttactgagaatagtagaatagaggtgcttccagaatgctctcaatgcgtcccaatcaacgacatatcctttttgta
tgggttgaatctcatttggaagaggttcccaatcttcaacgttacagtcagattgaaaagaggcactaaattcttccttctgagtatctgcttctgcc
gcatcgttagcagtcgacgagtcttgatcctgtaattctgctggcttttccagaatttcgttttgctttgaatcctcttcttttggagtcttctcttgtttt
aatccgttctctttttttattgctgaacatatattctcctttttcattctttccaactctggtcctcacgcgaattgttggtggtcctaatgattcagcaag
cccaagaacagcgcgatgatataaagaacctacttgtattacaatgatatggtcatcgcgaaaggaaggcatgactttatgagtgcagattcccc
cgtagctgtgttttggactacttcctagtgttagtagtagtacagtggaacttaggaaagaaaattatgtagtttgagtagcaatagacgaatttgt
aggtgaaaatgaatttgtatcaaatattactaataaaaacaaaaacaaacatggaaaaataaataggaaataggcgtcttgacaagcttttcga
ctttgcttccataaaacgcttcaacttacacaagtcatggcatttattaaccaaaaatggagcactaatcaaataaaagacgattcaatattattat
cccttttttttctttcatattggaaaagtttccagtcacgaagcctcgtgatctcagtaggaaaaagaccaatctaatttacaaaggcagaaacatg
ccagacacaaacaagcagagaccagcttgaattaaaagagaagagaataactttaaaatcacacaccctagacatcaggatgaacgaataat
ggcatgtcagtgcaaacttgtaacaaggtaatttacaaagcaaaattgttctaagaggaatagtaaaagaagtttgtcaagccgcgtcttctctct
ttcttagaaacataatagcgttctctacttgcatataatatatatatatatacataggcagaaaagagagaaggaaaacgcaagaaacaaca
actaatgataatcaaataaaactttataggagggttgcaacggagatcgggtaactaaggattctaaagaaaagaagtctgttcaatttctcgat
gaagatggttccgtagaagattcagcagcggaagctcgttcccaatatactatagatcttccaacattattgacggacgactttactgtttttaaag

aatccgctgaagagacaacattctgtaatcgatgataaaactccaaaacactgcctccaacaccaaagtataaatctttgccagcgataaggga
gatattcctagtgttttctagtagcatgtaaataaaaggttccaaggaagaaatagagtatatggtttcagaagccaagatcagagaaaagtact
gatcaccgtaggactgttgaatgatactttgcatttccgagccccaaggccccgacaaaaactcgcaagtaatgctgttacgggcaaaatcatcg
ctgaattcttggagtaaactctcagacacgttgattgtgccgtgttcttgagatgaatcatgctcctgtgtattgaagtaccaatttaacaacaagtt
tggcaatgttatataacgcaagacatcaatgttgtagtcttggaaaattaaagtgctcggttcacgtcgttggtaaagctcttgaaaacaagtcaa
gattggcaaggcacttccacaaccaagttccaaaaccctagtcaagtttcgacccaagatattgagctttttatttcatggcataaatcaatactg
cactcccatgtcttgaaacctccttcatacaccttgggcaccaagtcgtttgcactgtccagcaatttcagttctataggcgtcgattccagcgagtc
attctccattaattgcattttcacgtcccataattctctttgaactagcttataaggtagcccatggacacacgcatacgacagcttggatggaatat
atttcagcatacgaccaagtgaaacagattcaatagggacaggtgcaggatctttgttttgttctccctgcagttcctgtggctttggttcatcataa
aagccaaatgtgaacccttctgcctgcattttcactgttttctcataaattccttggattccccagtccttggaacacgccaaagacattggtaagga
ttgcttgagcgaattgcgcagcgcgtttcgtctcagtaacaaacaaaatgaaaaaagaaatttaaacttgaaaggacaagaagacggctagtct
aaaaagggactggctgttccaatgatcttttgtctcttgcagatggatggaagcgagcaattgttctctttgaaaggggggttttcaacgaagagta
aaatttgtattttgaaaggaagaacactagattccttaggataaagtaagagtacactaccttgaatctatttcttttgataaaccgcatttttgaat
gtcctgcttaaaaacgttccacttgttggctttgcatgcaaatatgtagaaaaaccatttatgtagacacattctaaaaagcatagcctttcgtgtat
ccctttatggaacagaaaacaagaaaaacacgatgaaaagtgaacaagtccgaaactactctgcattcacgttctttttcttttcagcttgaagga
caacatttgagtcgtcggagaaggggacctgaattgtcttcctagattgatccacgttccaaaaaacaatccttaagataactgaagaagccaaa
tcatagacttcatgtaaggatggtcatgtcccagtttttatagagccaatgctcagaactccttaacggaaatcatatctcaatttgtaaagagaga
attcttatttttctttctaatacaaagaccaaaatagatcccaataggtgaccatgtctaggatcctgcaacctgtttcgcttcttttccctgcctagta
gctcttaagaaaatatgtctaaatacaacattcaggaatttgaatccaattttgacactgcttttgttgatttctttaatcaaaaaggatataagcag
tcttactttgttgataatattcgatttctctttggtttcggctgtgtcattcccgctgcgattgcatttggcttggagcaactccatgatttctcaaagtt
aaaggagcatcttttgagtcttttactagtctactttgccttttccgggcttttaacttggtggatatcctttgtgaagggaaaccgaatttatacggg
tacaagtcctgaccaaaaaatatcaatcgacgcagagactacccttcccttgaaaccaaatcctcatttagatttgacaataagggtaaaaagaa
acaaaaaaactgttccaatagctcataagctttctatatcagttgccgacatatttgacgaagaaggacaaatatcccaagacgctttttcaaaac
aaatttcgagcttctacaaatcttatattgaaaacgagtaatatattcgtaccggagagctttttttatttgaaataaaatttgagccgctagctcta
ctttcgtgcttgctttaatagctagcttcccctgagtttcatttcttttactctacttgtattctttgcaaattcattctctctttaacacttgcgagatttc
ttctattacatatccagaaatttgcattcattatttctacctttttctacctttatcgtttttttaacacagctttgatcatgttggttccaacggcattcac
tcatgtgctctttttcttgtttcttttctcttcttcgtttcagcttagtacttgttacaacaacaaagggactgtctcggatggtcagttatttcagcagtg
tgttgcctctctttctgagacagaaaataaccaagacataaattcatctcttgagacattacgagatctttctcatgactattctcttggccaggaag
tgttaaaaccgggtttaagagatcatctactaaactatatgaataactttacaagtccagaatcaactcgatctttggcttctagtgtctttgcttctg
cctttggcaataatcttgctgttcatgaattgtccgccgaagctaatctcatgcctttgttcatacagtccttaaaatatgagccatctttatcggttgt
actaaaaaagctattcttgcttagtaaagctgtagcttccgcatggtcagcacaagcctttgtccagaactctggcatagataccatttctgacttat
attccacttggcttcaaacgtcaaacaccgatgtacctaaaagtattcgtgagctacttcttggtagaattgctattctcttggaatcattacatcctt
acctttcgaagtattcaaaagttactgagagcttggttccgaattggtgctctgtactacaatcacaagttcagtcaaactggtcatcctactctcca
tcaactttggagcttctactacgtacaattgtgtctatgcaagctgaatgggaagcatgccctagtacagtgttttacgaatggcttaatgatgatcc
aattgtaaaacatcatttgattcgacaagattcgggctttagggaagatttccttgatattataaacgaagctttatcccttccttggccaaaaaag
tattttatttaaaggatattttcaaatatagatatataaaaatatgtttcctatgtataccatttatgtctggttcgacttgcttccgttctcacaacact
agtatagtagttagtaacttcaagtacattcatatataacaggaaaacttaccgtttaatgtcatcaaggcatggtaccaattcacgaattactttc
gctttgaatggttgtatatgaataggagaaaccagttctggtagcaaacctaaacatttcaaagcaagtaatcgaacggcggctggattgttgag
gtctttagggatagctagcaaaggtgggattaaagagttcatatagtcagaaattaaggaaggtatttccttaatggcagtcataataacattaag
ggcagaatactgtacgccactatctgcaagtactaaacattgtaggagtagaggtaatacagtaggcatatcaggaataacaatatctttaggta
cattacctatgacaatcgataacaccattaaatagaaaggcttttccttggatgtaactttaggtatggcttctaagagtttaggcatcagttccgc
gtaaaatttttgcttataaagaattctctcaacgtaataatttgttttgttcaagattggatcatctttgatgataacagcaaacgcatttacaactga
tgcagcatcttccaaatgcaagcattccagtaagtaattgactatattactagcagattgaagctttctagaaaccaaagccttcgcaagccagga
tagaatttgcaacgctgcgtgagattgccctttgtctttggattgtgcttttccgtacagagatgtaatagccggtgtttgcagataggtattgatatc
ttcatccttactgcatttgttcaaaagtgaagataataagcgcaaagccgataacatgacagctggaatagtggcttgttctaccatctcaataca
cgaattcagtaacgtttcatgcaagtctttaacgacaggaactgatctttgaagaccattcatacctccctgaaggagcaatactaatcctgcata
cttctgatcagacatgggctttgcaaggcagaacgtagctgaaagctgtctcacttgcacatcgtcttgtattagactagattcctcactagtaaaa
taaagtttataaagttcctcggagaagcttatctgatttttcaagggacaaacccgtacaatttcgttgataatttgtgaaacactagtcagatcttt

gatgctgaaaactgaaataggagcatacttaaaactagtagtatacaaccaaggtaccaaatgatctaagtaagaagggacgtcttctctctttt
caatatttcgatttcgaaaagctagacaaagagtttgaagtacgatgctggcatattgagagtcatgtttagccacgttaagttggtctttcaaggc
ttcaaccaatctaataactaaagttttgaataaactcttttcttcagatagggttatcaatatgtgaagcgactgtttgaatgtagcatcttcggcgg
agctgattttagatgatatctcaggaaataaagaaaaagcatgaggaaaaaccaccaacaaaataatgtcaggcttatataaaccaatgtctttc
agtttatcagtagcgctttttcttaaaggatcatctaagtcaaaggccaactcaaccaaaaattgaacaatcatcgtgagttcgttcatgtccaaat
atccctttacggtagaaagagtatacaacacatcgactgcggaagttcgcaaacttacttcatcctttgcaacacccattaaagatccggacatga
aactcaaccacttgtttttatgtggtaacaaagaattggggcagtctacagaattttgacttctccatttcccgtacaacaaagcgtcacatttaaa
cacataattcagaaaaactaacattccgtgtctcttttcgagagattcggactctataggcaattctaagatgtacggtaaatatttctcacaacag
taattgaaactctccaaggaaacagaagcaattgcagcgaaaacagcaccaatagaagaggcaacttttacagtgtaatctttagaaaaatatt
caaaggactgagttaaaagaacaccaacaaagttggtgaagtttgaggataacccgttttccttatacaaatttttcgtcagaagattcaacgcac
tcaacgcggtattctgcaatgttgtatcagtggattccaagatctcttgctttatggattcccaatactcagtgcagctccataaataagaagttgga
tgccaattctggcagacctgaacaaaaatgttaagcgcatcaattttaacgcgtacagcagaagcttttagtttcttgaataactcaggaagaagt
aactttgaaaaagcatcggttgcaactaaggtgttcctaaaaaatgtcagcttctatataaaaaacaggtttccgtacttcaactgttctttcaaat
catcggtggttattgttaaaccttgtgcgtcaggaggagctcggaatgtaatggggaagtagcaataagtaatgtcaaagaattgctaattgatat
tagtaaacatcatcatatcagtagaaaaaatgcaatttaccttctcaaagccacctatagggaatcgtttcaatatttttttgcagcatagagaaaa
ctaaaaatagatttcttggatccttttcccctgcaaaagttgtacatatgcctgcaaagaaagcatctttagtatttggtgaaatatctgaattggtt
aaataacaaactttgaaaataatttttcacttaccaaaaaggtattcgtcgataattgtatcaaagattttcaagatactatatcgagtttttttgcga
atatttaggcatatcagttgcatcaaaataactaaaagcgttagttaattttattgataatctcaatccataccagtagcaaacatccacagctttg
ggggatggaaagttgtccatgtgaagtaaagcaacaattcctagtgaattttccattaaggtaacttcatcgtccaaacgattaagataaaattga
agcaatacagatactagagggttaagttaacataactctccttcactatcattacttaccatgctttacaggcaatcggtcttttggcaatccttcca
agacctgagaaagtaaaagcaaagctaaacaaggttagtataaattcaagcttaactgaatttggctaaacttaccttttcctcgcatcgaaaca
ttcgtattagtcaaatagtcaccaattgaagtaacgacatcaacaattccaatttttttcatcaataatatctataaattaaggaattagtaacagtc
gttttctaactttatattcgcaataccttttatgatatggtttacaatgtcctttgcttcactctcatttttatccactgtaaacaggtatgaagcaacta
agctactattcattatgataatttttaagaaatcttagctagaatccaaatccagattccaaatgtgtaggcaaaatgctagacctataaatattttt
cacttcgtaattccaaagtccacaaaacctagtgattaagcttttctttttttaaacaaaatatatttatgaaagaaaattgcgaaagcaactttgta
aatgaaatgaaccaaaattggcctacaaagttgttggtaagaatgtatgaggaatttggatgaatccatagattgagcaaacgagctaaataac
ctttttatctaagcgagcacagctcatatatgaatgatctctaaactaaaaaagttgctttttatcattcttttgctttttgctttttttatttttttattttttt
aaatgaatgaagaaaaaatgatccatttatttaaactttcatattccatccttgactcccgttaagttggttagagctgcatagcgcatcgctatttg
gaaaactaggtgaatttggactgaaagagttaaaccagcacaaataaaggaggatgcaaagattggcagtattgttacatttctgaaccataaa
agcgatatttaattaaataagatatcaaggtctctctcttaaattggtatggaacagccagaggcagatgagcatatagcgcgtcttaatcaagct
cgcgacattgtcaaggaggatgaaagcctttttcccgaaattgtaaaaaacattctttctgttgcaaactattctgattacaggtataaaaaaatgg
atgtcgagtttttttatggtttgggttttctagtaaaaaggtcgactttgatcaaaaagttgaattggctgtcgcttgcttggagacaataaatactctt
ctttcttttgataataaagatgtgaaaaaggacataatcttatgctcttgtacactgtatccactcgttttctttcactgttgcatattcccggacgac
actcattcttgggaagcaatatcaaaagtaaagcaggaaatattttccctattcgattcttctaccaatcatctaaaaattgcatgtgttaaatttat
ctcttccgtcattctagctcagtcatccggcattcgagatcctcgtttagttacaaagtctgatgtatcactttccaaggtccctacaaatcacccatt
tttgaatcctacttctttgaagcaggaatctttcgaccttcttcaaaaactgtttaccgtgttttcgtctaaggcaccaagccctctttttattacgagt
gtgctcaacgtgcttccagtaatctcaaaaagaagaaaggagctcggtcctttcattgttgaaagcctgttggagtttcatttacctagtcccaaag
aaactagtgaagagcccggtacattggacaagcttgcccgtcgttgtattgaaaaaaacttgagaataactttacttcatttagttaaaaatgctg
gaatctcttcttcgcttatagaaaaaatacatgcatatctttctggtcaaatctatcaaagtaaaacagaagagcatgcaaagaagcgacaagct
ccggtaccagatttcatatcttcaaaacgtgctaagtctactgcagtgcagagtttaatggacaggatacagcctcgtgctcttgaagaagataat
gccgctttaaacaaccctttagtcaatatctttgcttcacaagcagccacgaatccattggctagttttgatgtgacggcaattccagtagaagtcg
ttacagagatcgtactggcttgtcttcgtcaactagataatgattactttcatcaacaaattaatatgcttcgtgagcgagttcgttctttatctgagc
ctgagacgttggggttggatcaagaagttgacgaagaagaagatgatgactatgagcctcctgaggttgatgttcagacaattaatgcaagcgtc
gagcgggaagcggctagatttgaaggtgtcactccttcggctgtcaatatagatacctttgaactaccaactccggaaaccctctcacctatggca
attttagagtacttccacagttccttgtcacgtctttttgactattcatctcagtttgaaagaactattgtttcttcttcaaatttgcaaaatcttacatt
agagaatgtagataatactgtttgggataaacatcattggtctcttctacttcccaggttgtgtactcgtggcttgagtaattatcaacctgttaactc
tgacgaacagcaaggacagagctcatttactttgtcttccttcgtgcgaggacaattattcacatatatagcttcgaactggagagcttgcaccaat
ttaacactgaattggctttcggaggaatggtataatgaccgtcttatgatggagaatgatacgaatgaagacagctttgtcaaatgggaagggcc

acaatatgaaagatgggctttacgtgtaatcgatagcgtacttccttatcttgaaatgaaagacaaagtgtttatgatttttctgtcagaattgcctg
agcttacagatgctatgattgaaaagataaagtttatatgtctcgaccctgacaagtcgaaacttggttttatgactttacagtaagtgcttgtctgc
ttattctagttattctaatcatgctaatatgttagatatctgattaaatttcgattaccggcgagaaaccaatgcttggatctgttacaaaaaatgtg
gtgggaaaatggtaagcaaattattccgatgttttcaactaataatgatcagatgatgttcgcaaatctgcgcaaactttgttggaacgatggcga
cctggttttgtcaaatcagcatccgaagaattgtctcagtctaattcgcaacctgcagacggggaatcgaaaacctcagaggaagttggcgctta
ataaaaattagcaatcgttcggataatgcgttggaaggttaatgccgtatatataagttatatcatttttgtggattgatatccttttaagtttttttc
ttagtatcatgctatttatgttgttgttgtacataatacgtcgagagctcttacaatgaatagtgttgaaataactttttatgcagctttttagattgta
acagtcggggtttccatacgtgctaattactctattgcgaacctgcgtcaacgggttcctgaaaccatcagttcatatgagagcacacacttccata
cttttacacgttgtaactagctttagttaataatgaacagtctagctataagtcttatttttgaatcttgtacatctgatatggtttcataagttaacg
cagtatgagcctgaacgaatcgttaaacaagttagaatgtgaggtctaagaaaagcaacttcagttgaaaccaagaaatcactggatttgttatt
aagtataatcagcttctatgagtcttcaaagtgcaggaaaggttgaaatcgaaattcaggaaacacaagatgaaataggtaagggcgcgtttgg
cacatatggaatgaacagaagaattaacgcgaaagaacatggaaaagagaacgtcaaagagttgcaagatatggcaaatacattggaaaatc
tttctgatagtataggaaaaagaggaattcctagaaacagaccttctgacatgaagggaagtactacgagaaaagcatcatctggacagatgat
agcggctatgcagaatgaaattgatcacttaaaggtacagacggatgaagtgaaatcacaactgaatgttcaaaaacgaagaaagaaggaact
ggagtccaagaagctgaatttattgaacttgctagaatcactaaagatgcaagaaacatcggtcgccagtatggtgaatcgaaagcagcgtacg
atgaaagaagatcaaaagatgcacagagattttgaactacagatctcgaaactgcacgaggagaaagaagttttgaaagaggagattcaaac
acttcaaaaccagatgaaggcagctcaggggaaaccgaaacttataaaagcttacagaaagatttggaggataattatgacaaaatcgcttca
gagaatttgaagaaggatccgctggttggtgaaaaagtgaatgagctaaatgaaacgttacaaggagtggaagcaactgttttaaggcaattgg
aagaaaccaagcaagtagaaaagccattgagagattccatgacaaatagcagtggtgaaaaggaaaagatagcacattcgttggaaaaagca
aaaaatgacgcccatgtttattcaggctttattgaaaactatcttggccagttatgttccaacttggacgttcagttggatcgcatagaaagcaccc
ataaccagttgagcgaaatgcaacagagccttgagatggacgatgaagagtttgcaaatttgaactcaagtcttgtaacagacacgaccggtat
ggattctatctctgaatcaaactatgatcagtctttctctatcacggagtcataaaagaaatgtacatgtttctgggaaagaattgtgaaaggtgtg
tgcttaaacgatctttttatgactctatactttatttctacaaaaaatttccttctttttctctgtatattaattgattataatgaatacccactaaacgc
ttttgattatacaattattttacctgaaatattgcatcgtaactcgaagacaacatcaaagttaaatttatataaaattttcttgacaatttcagcagc
gtattttatcctttttgtttcgtcattacaaagaacatagttcattcatagaatcaacaaccaagtgcaaattttcgaaaaacgtgtttagggtgact
ctggtcattctagctgaagagcagaaatatttggccactagataatttccttctagataaagtgttctagcaacttgatcttctttaagctcacgatc
aggtgctagcacctgcatacacaacttagcatcttgctctgagggaaaaggaactcgtacttcactagacaatggtttgttagaagcaagttgtttt
cttaatagaaaatagtttcttaaatagctctgaagtcttacactttatgaggaagagaattcatattccaatagaagattttcaatgtattttcgact
agtccaaagaatactatttttcagtcgttttccagaaaagaaaaaataaaaacaaacaaatggtaaaccacaaaagtaatccaagtgtctctttt
ccaataatatgatcgtttttcgtccaaggtcggcccttggtatactcgactaagtagattttaacgctgaaaagcacagaaagaaaatgaacaga
atttaataaaaactaccagacgaatgaaggaaattgcacaaccagcttaaaatgataaatctctatccgatttcaagagtctctttttctcttctgt
caaaaatgatgaatttttccaatacgaaaactaggtaacattgtacccggaggcatcgtatatatactaccgtcgcgcctacctatacgttaccaa
agcataaatttaggtggagatgagaaaagacatcagtctcttttggtaagttttgtaacgttaaatgtagtacaaaatgatgtttgaaaaattctta
tctacattgcctgaacaatattgctgaaaattcttttcctgattgtactatttcatctttttgcttccttgtacaaatactaacatatactagtttgtagg
tagaatttggtttgaaatatattcttttgaaagaaataaggctgaaatcaataaataaaaatcgataaaatgaaaaaaccagtcgtcgctatacg
ttacacaaacttttcgtaagtgttttatgtcggttgaaacaagtgaaaaatgtagaaacaagaaattgtcattaccaactcagaatccattcatag
atgatcaatacacaagaattgagagaaaattggaatgctaaacaaaagaaatacggggttaaattagacccaagcaagaccatcaatcatacc
atagaatatttactttaaatctacgcgtgtgtttgtttacagtcgcgtaaacaaacagtttaaagaaactgtaactacgcgacacgaatttcctacc
cttgctttctactctttactttcatgcatgcatttcgttgctttcaagaattccattactaaatttcaacgattatactataaagcgaaagaacgcaag
aaactacactcactcattttccacatacaagacaaaagatcagattttcaaagatgccagaaaaacgtaacaaggtcgtgcccggcatcgcaat
caggtatccttccatgcagttttctcatagagaaaactgggatggcttttcaggttatacacgaaatatcatcatacttcacttgaatgtggaagga
tgggaaacaaaaagtttttccgtctatttatgttcaaataaaccctaaccctgtagttaacgcactcgaagcaggttcctgtatgtaatagggatg
ttgttgcgttgcgtttgtaaacaatagatcacccaatatttatgttactaaaataaagactgagggaactgttcaatgtatcccgaaaatacaagc
atattgttttgaaattagccaagttgtatatacgaatatgcttccataagatacattgaatagctgtccgtctgatatagagaattttaccgttcaca
aagcattcctgtagaacccaagcatcaaacataaaaaactgttacttggttgttactacaggaatgctttgtgaacggtaaaattatattttctttta
tttttatttaaactggtttattagaaacttctgaaatcctcatttttattgactgtatttgtaacgaccgagaaaaaagatttgtttacaacccccattt
actgtactaccctgtatcgctctattcatttagcgtcaagtcccaagttgctaacaacaaacaacgaacagtgtaaacaaagcagtattcattcga
ttcccctttttgctgctgaaagaggcgaaattcttctttatataatattattcttgataaatgaaaatctgaatgcctgatcaatttgatgaaaactt

cgttggtgtggacgataacataaaaaacatggcccgagtgggaatggcccttgatcgtgagctaaacgggaatacgtattcatatgatttaaaag
aaaacagaaacccttcgtatgcacagaataaagactccttcgaaccttttttcaaggaaacgcccaaatcgaaaagtatcaataagcatttccaa
gattttacgatgaatggatctttaagtacacagccaagtgtagaaaagccaagaggaagaaacatgaattcctttgaagaaaatagcttcaacc
ggttaagaaacagcagtaatctgtttcacattgggtctcccgctttgtctgatcctggaagcctcaatcgagcaacggaggatacgtcgagctatc
gaaataagtttttgtctgcttttcagagaaagagggatccaagcaaaggcgaagaagtcatgttggatgaatctccgtctttactgaggaaagga
ttggaccagacacctctaacaaagcgtcaaaacaggaatttattattcgattccccaagtgaacgtctcccaaagaaacccgaaactccatggcg
gaaacctggtttgcgatttcaacctactccgcagccttcgaaacaggcactaaaggaaacgccgtcatcattaaagacagcagccaaactgatg
gagcagttggacctggactcgaacgatgttccttttgattctcaaaaccaaacgtcctatcgtcttccaaacatgacaaacctttctcaactggtcc
aagatccagcaactgaaaatacgactcgtgcctcccaaagcggtcgcttgccaaacctagatactgtaccaatagcagaaacagatgaaaagct
gttttatgctcacaaagctctagaacgaaagctcgaggcaatgaaacaagaacattctgactatgaggaagagattctacatcttcgagagcgg
attgatcatcttacggatgcgtataatcgagaaaagaagagagcacgaagcttagaagatcgtatgtcgagagaattaatgatgaaatatgaca
ataaagaatccgaaagtgtcgatccctccataacctcaaggttgctagcgactacgaaagagaaggaagcgcttatagaacaagtcaaatcgtt
acaagagcaatacgatcatatacagcaagtatataagaatgttttattagaccgcgaaagctacatcatgaggttaagcacgaaggtatctgag
aaccatgaactaaataatgaaaatcaaaagttgaaggagaggcttcttgttttggaaggcaaagaaaataatacagatgaactaagggatata
aaagaaagaaaccttcctgattgggaaaacaaccaaattttccgtagcgaaacttctggaggaagaaacttgaacgaaccaaaggaggtggat
agtggcttgaaaaacaaggaaagtatactacctagtcacaagcaacactctccacccgaaaatcaaaaccttgtgaaggaactgacgaaagaa
atcgaaaagaggaaggctctggagctaaaactgctttcattacaaaataacggaaaacaaaagaaccatcgatcaaaaagagttcatgtatca
ccaactaccaacaagaagaaatggaagaagggtgaggaatcggattttgctttggatggcgagtccgaatggagcgatgaaagcgatttcttgg
atacagaagacgaagaagtagaagaagaagcaagaccaagtattcgaaggaaagaacctgttaaccagaaaaggaaacacttggatgattat
tctggattagtggaagacagttatttaggagaagaaggtttagattggagccctatgttacaaccagctttaaataacaccaaagagagagctca
ttcacctgagctaccaaagcatatccttgatcaagtagatcatatcataaactgcaatgctgtgcataagcctgaacagtgtacagtgtgccacgg
acgccagcaagagggtccggcagttagtgatctctgggggaaaaatgaagagcctttgcatccggatgtgacaatgcgaccgagtcagcctccg
cagaaagcgttgaaggatgttatggagcaactgacgaaggagttgatgggtttgaagaagcaatatgagaccttctcgaagcgctacaattcgtt
gactccagaatatcacagacgcagacgccaaaccctaaaggtcaagctgatcaagattttagagttgatggaaaagaagtcagaccagatttac
gctctacatgatatacatattgctgaaacgttttgtgaagagtaattataagccggctggtacggttgcgactactaaaacaatatggttttcctaa
ttgttttatatgaatttttaatcttaaataattttttgtaataatgaagctagaatagcatcgatatcaacgactattatttcttaataagtagcgttttt
tttttttttgtatactctataatttcatcttttaaatcttgagacaaagaagaaaacaaaggactcggccagtggttcaacatatttcgttttggtac
ttgctttgtttacgcgtaaacaaagcaaattcgggtagcatgcaccgctacttgtggtcttttgatagtgtagttcgcaactatatttcattggatggt
ttgtgatgtggacggccatcatcttcttcgcaaatattttaatgttacctaagagccgcctccgacccaatagaatgacgcatggaaaaactttgag
ctagcgttttcggaaaatggtcgaagctcttagaaaatttttgaagtcataatatcatgacctcccctgaagtacgtaataccacaagtggacaaa
ccgcgcacaaccgcttgtttacatgcctgaatgtgcccaaaaagaggacttcaacgagtgatttaccaagaaaattttcattaattatcctaaata
aaaatccactgaatatttccttttcccccatcaaaatatgtttctacgaacataaacaagagtgtactaaaatccctaaaaaaggtgttgtcttat
acacaattctagacatgaagtttctatcaaatgataatgtatattcaaatttgcgggtcaatttcaggcacccgaggtaactagatcgttgatgccg
gggccctccgagagaacattttccgaaagctttatatataggagttccccgtgcaaccttttgcttctaccaaccatcaacacagctcatcaaagta
agtaaaaaaatgcaacgagcattgttaagtacttttacgaaatacagaaaagcaatgcttggcgacattcagggatgaaagaacattataggtt
actaaccgaatttatttagatgtactccgcttccaagtctcctaagcaatggattgcttctctcaactctgagttggatgttcctgccgtcgaccgtcg
tacttctattatctgtaccatcggtcccaagtccaacaatgttgagaccatgtgcaagctccgtgatgctggtatgaacatcgttcgtatgaacttct
cccacggttcttacgaataccaccaatccgtcatcgacaacgcccgtaaggcctctgccaccaaccccttttccccttggccattgccttggacac
taagggtcctgaaatccgtactggtttcaccattgacggccaagacttccctgtcagcactggtcacgaaatgatcttcaccactgatgatgcttat
gctgagaagtctgacaacaaggttatgtacgttgactacaagaacatcaccaaggtcattgaagtcggccgtaccatctacgttgatgatggtatt
ctctccttcactgtcattgaaaagattgatgacaacaacttgaaggtccgcaccaacaacaacggtaagatctcttccaagaagggtgtcaacttg
cccaagaccgatgtcgatttgcctgctctttccgagaaggacaagtctgacttgcgcttcggtgtcaagaacggtgttgacatgatctttgcctcttt
catccgtcgtgctcaagatgttcacaacatccgtgaagttttgggtgaagagggtaagaacatcaagatcctttgcaagattgagaaccaacaag
gtgtcaacaactacgacgagattctcgaagccaccgatggtatcatggttgctcgtggtgatttgggtattgagattcctgcctctcaagtcttcgtc
gctcaaaagatgatgattgccaaggccaacattgctggtaagcctattgcttgtgccactcaaatgctcgagtccatgacctacaaccctcgccct
actcgtgccgaagtttctgacgttggtaacgctgttctcgacggtgctgacttggtcatgctttccggtgagaccgccaagggttcttatcccgttga
ggccgttacctatatggctgagactgcccgtgcttccgaggcttccattccctacggtaacctctaccaagaaatgttctctctcgcccgtaagccc
gtcagttcccccactgagactactgccgttgctgccatcggtgcttccatcgagagcgatgccaaggccatcatcgtcttgtctgtcaccggtacca

ccgctcgtctctgctccaagtaccgcccctctatccccatcatcatggttactcgtgatgctcaacgtgctcgtcaatctcaccttaaccgtggtgtct
accccgccatctacgagcagcctgctctctccgactggcaacaagatgttgacgctcgtgttgccttcggctgtgagcaaggttacaagatgggta
tcctcaagaagaatgacaacgttattgtcctccaaggtgccgtcggtggtaagggtcacacctctatcttccgtctcactaaggctgagtaaatgta
tcactagtctttgaactattggaaacggtaggaaagtcggtaaacctaagctgacatttattttattcatatttttggattcttaatggtatcgatagt
tttatataccttaaatgattaacttttattggttaagtcattgttttatatatggaaaaaattagtatttaacgttgaaaggtttactgaatttcgtact
gctacgtattcaatgttgtgtaaagtgtagtaataaagaaaagtacttagattatgtttttttntttttttttttttttcaagtttcaatgtgatcaacct
gtagagagtagtaagtagaagtttgtatgccaacgactaagaaaaggtaaccctttcctccctaatagggaaagttgtgattaattttgtttggaa
taacgactttttttactgatattgtaaggtgaagtgaatgaaatctactggaaagcgcaatttttcatgggaagaagtggctacaagacaaaagaa
gagctacaagaaagcttcatagggaaaagaagtgagcaactttacaatagatgaataaaattaactttcaagataatcgtttcttaaccaaggc
aaaaaatcaccaagacaaaagcagtagttgaatcctagatttttttttcttctcctaaacatgcacgttgagaggaacaaggaggcgtagaagaa
gaggattcttctgtttacttggctaaacaaagataaagttagaaaagagactgattgtttatgagatcggttcaacgtaccatcggcagcatttgc
aatgtagttgacagcatcagaaacagatgtaatttcatcagcattcttgtctggaatttggatgctgaattcctcttcaattgccattacaacttcaa
cagcatccaaagaatccaatcccaaatcattggaaaaagaagcagtaggagtcacctggaacaatggtcaataactaaaaaagtacaaaaaa
agaaaagcaaaaaagagaattttgactcttcttctaaacaaaaacctctatcgaggaagaactcaagcacacaaaacaggcattcataagaga
aatctaatcacacataccttcttaggatcttgaattttgtcaaaagaagaaaccacctttagaattcgttgctcagcgtcaggacgagcaacactgt
aaaaccgtaagggacgaaagttggtcataggacgaccagcgacggccaaaggtcgaaaagatgcagccaaacgagagaacattgtgcttaat
ttttgaattgaagatatatcactcgaataatttggcgttttggtgtcggtaaacgatcttggtggtacaagaagggatctttacacgactggattga
atttagcagttccgtcaacgaccagataacgaactcgaacgaaagctaacgcatttaaaaggaagctttcgtcctcgtttctgcaattcatatcta
agggattgttttagttcacttgttttctgttacgtatacaacaacaaaaaaggctatataaggcagcatctcggctttccttttaaccagagtcaacc
tcctcctttctcttcagctttcattaccttcttactgaactcctttcaacaaatcagtctatttcagaacatttcaattcaccttcacaatcagaaacttt
ggttgtctggtgattgttaaacgaattagacatttgttaaaggaattcgtctatctttttcagccagttctgcccaaactcggctgtaacttttgtcatt
accccatatcatattgattccaatcctccggaaccaatacaagaagcattcatatacccttttactttcattattaggaaaaactatttttttgagaatt
tcatttagcacctttaaaaaactaacacaatgaacgtcttgactcctattgctgatggtattcccaatggattcgctaaaagagaccatcctctaca
acccaaggcaatttccgtctctgatcaacctatccaaactaacaagttctatggtaaccttttttttggaagaccaaaagtttccaagtttcccccaa
ccatattcagtgacatggaggaacaaggatgatggcttctctggtctaggcatcagtcatacagaagccagccagtatgcatatggacctgatcc
tagtaaaactccgtgtgagttctatatcaatcccttaggattattctcgttgattatttctgcgaatgagttttctaacggaaacaaactttccgcgag
taatcctcgtcatttttccgttctagttactctggaaccttcctttttatctgatggaaacatcacgattcctatcgttactggaagtgcattcatcagc
gccatttacaagaatttgaccccagtcttcaattcgtccattatggtaaagtcgcttgataagagtgactacagtggtggctctaagtacaaggtca
cgttggaggataacaaggtttggctcatttatgccttcccttccgactctagctcttttgatctaaaaacagacagtaaccgtaagttagttgcatcc
tccaaatttaacggacttgtccaaatttgcaagattcccagttctagtgtcgatgacggcacaggtgacaaaacttttgacaagtatgctggcgtct
atgcaaccggcatcactttaagtgctaatgtttctgacacaactggtgagtactggtttacttttgataaagctggtgacaaatctaagagtccact
aatgtatgctctccctcaccatcaaagtacttttggctctgatacccagggatcaaagacgggtatggctttacagagcactgttaagggcactat
gattgcttatggagcagataaatggcatttagtggaagaaaatctccccaaggatgtatggtttgtcccttacccttggaatgggggaaatcaaaa
caattctcgagaagtattagatattatccgttctgcttgtgggaaagacgtggattttgatgtcgtgactgcaacagatacaaatagtatgtatgcg
tctggtaaaatcctcgctaaatatgctcaaattgctttggtggcagccaaagtcttgcatatagatgagtacatgaagactagtattgaaaaattg
aagcaagccctccacagatttattgataacaagcaacagttcgctcttaattacgataacacctacaaaggtatcattagtaccgcaggtcttgag
gatccccttgccgactacggtaatacttattacaatgaccatcatttccattatggttatcatatctatgcaatggctattgctggcttattagatcca
gattgggttgcggatccccaaaacgagtttgttaatgctttgatgcgtgatgtagcaaatcctgtagaagatgataacaactttccttttttccgatc
ctttgactggtttagtggtcattcttggtctaaaggtttgttcgcaagtggtgatggtaaagatgaagaatcaacatctgaagattacaacttttact
acgctatgaaactttggggagcggttagaaaggatgaaaacatgattaaccgtgcaaatttgatgctgggagtaatcagaacgagcatgaatga
ttatatctatataactcccgagaacaatattcagccagaaaaaatacgtggcaattactgtgcaggaatcacttttatgaacaaggttgactatac
cacctatttcggtactgatgagtacttgaagcaaggaatccatatgatcccaatcactcctatcagtggttacgttcgtactccttcttatgtaaagt
caagctgggatgctaaacttgctccaattatcaacagtttttcgaacaattggactggtatcctttattcaaatttagctattaccgaccccaccgct
gcatggaacgagtttgtttctcccaattttgaggactctaatgttgacgaaggtgcttcaagaacttggtacttgacattagcagcctggatgggtg
ccttgccttaaatgctatatgtgataatatgtttggcatatatgaaagaaagtaatgaaagaaacgacagttatgaacaggcaggcttagcaatct
ctttacagagagtgcttttacgacaaacctttagaagttagtctaaaataaatttacaggctacttgctacggcaaacgtagtgcttttacgagtgc
attgaggaagtaaaattgattacaactttaattgcattcctctacattttttttgtgaaatttaaaatatatatatatatattaacaacattattaccag
gtacttcagagaaagtttagaaggacattgctagttggaataggcgaggttaggaagatactgccctacacataaacgtaaatcaaaagtggttt

taaagtgagaaaagagtaccagagacttgtttaaaagaagagagaaaaaaagaaatattttacagcttttcaaatatacgaatgagtagaaat
cttgctaatcttggtaagaaggaggtcgaattaacattcttttccctctttttagttctggagacgttactctgtgtttaaaaagcatcaaggttcata
gagcaagtaaaaagggcttgttcacaacagtgttctttgggaaattcctagctagaaatagcatagcccttccatactgtcaagatggcttcaatt
gtttattggaaatatctaatgatgttctaataagtctctcttcagatatatagcgagttatgtcgcctttaggagggcatctatccattttgtagacat
gttattcataatgagttatcaaagatcatcattcaatgtatatgttattcattgagcgttcctattcaacctcaagacccattttaccctttttctctga
catcaaagaagcaaaatgtagagtgtcaaacatacatatataggtatacatagaagaacaaagtttcctatggcggagattaccattctcgtaat
cctttcgtataaaaaaagagaaaaaattctgcatcatcatagaaccaaaagtcaattcacatctgacctgtcgatttaattcttgattttggcagac
cagtcaagccaagatccagtataatttccaacactaaaacatgttaataataactctaaaaatggaaaaccaatttgcacatactgatcataacc
tagaccggacaatatcttagaagcagtcgtactacgtgcaccagatcggcagtaaacaaccacactgtccttaatggaaggtttatgaaagccat
aagtgtccataaactccttctctccaagctttaacgcatcttctaatttactaacaggaaggttgactgccgtgttgatggatcctgcttcatactcac
ttggctcacgaacatctgtgatacaacatgtcaatacaagagtcttagaagaaatactattttgtgttttcttaccaattaaaatgggacctttgctt
tcagccttggctcgtttactcaagtccctaacctaccaatgtattagcaaaccccactccaatgcaaattcatgtccatttttttttttttatcgtaaa
gagcaaacaattctcaattaaatcacgaattgacccttgaagacagcaaataattattgttacacttactttttcgaaatcatagattgctactttac
ttcggaaagacgctgtttgaaaaaagcgcgagcttaatccagtaggcattctctgaattactctcacaaacatactcaacaactatacagtacctg
agccaatccaagaatggaagggtattggttaaaggtaggtgatatggaaacctgaaatcatttttgcttaggaatatcaaataactcatgatgct
tatttcttcataataataatgataatcatgtcattcaagattatatttcaaaattggatttcctaaagcgaaatctcaatattcaattcctacaaaag
cgtgaacagggccagatggtcttcttatttctattaactagcttccgcaaaaccattaaaccttttaaccagagatataccccattgacatctgttct
tcatcaggtcatgcttggtaaccaatggcaatttactgaattttgtcctgcaagaattatggctttcaaagaagaataaaatctataatctccttatt
ttctaacttgcataaatatggaatgaacactccaattcattctttagtgaatacacgtcctcgtaaccaaaaatacctttataaaccatttcctgga
atattgcaaagtcccattaaggcttacaattaatgtgagaaaagaatgctaaaatttcgtttcggccttttcagaatacttcttttattaatgccata
cttgtagatttgtttgagtagaatataccgaaccaaataaaatctttttctttttctttggttttatttttgaatcttattgagacagtagacagaaga
ttctttaattcgcatttttggctagttgaatatgcccaggaaagccaaatcatattaactcagagttcgtcctttatacactggattgtatcgttgatc
ttcgaaattatagcatttatccttttataaagtcagtaagcgaatgaccgataagtcttcagaaaacgtactctgctttcaactgttcaatttctgaa
tcatgatctctggtttccgaagatagggataactgttcgatttttattgtctaagcatttgaagaatgagcaatctgtacttgactggccatatgtttc
ttactttctgtagcttggatcgctccaatccttgcagttgatccacataaccctgtaattgagggctactcaaattcgcttgaagctttctgatatcca
aagaaacctcatttaaaccttctgttaccagttggacgacgatccccatctgctctaatccaattgtttttcatggttcgccttcaatgaccttttcta
ggttaagccagaatctttgtgctgtacacttactcctcaaattctttatatagttgtgtccttgtatcttgcgcttttaaaatcttctgcatcgtttcctcc
attttggaagtatggtcaaaagcaaaaacctctacattcgtaaacatcgaccactgcagaattcgatcgcattctttttcttatattcctatcaataa
caagaacataaaagggttcgaagtttttaatagttcaattcactgtaaaaatattccaaagaattaaaactacatatcctatgctcattgaacata
aaaaccattgataaaaaaaaaggaaattaagaatcaataaaactacttcgtataaatgttccttgcttcctattgttcccattatttacactcttcat
tcctcttatgcaaccttccacatttgttcgaaacttgtgaaaactctcaaacgactggtttttaggaaattgatgatcaaattagattagaacaaaa
agaaaatatgaaacgatgtcgagaggaatctgcgtgtcttgagatgaacacaaagaaaaataaaaatcaaatggactgtacgtagaccatatt
aaattgttctggatttgttcatttaacattacagtaaaaacattcgttggtttatttttatgcatgatgatcgcagcaaagatactactggtttgatgt
tttcagaaaaggtaaactttgtaacaaacggagatgaaacttcttgtgaaggcccttgcgacttttgcgaaaaatcaactcttctacacgattgcg
atcgctgtactcgacccgtctgcagaaactgttcgacgcttgtgtaagtcccataccaaaggtctttggattaaaatgctgatacggtatagttata
tgaaagaacagacaaaagcaagatgtttagactgcttttaattatgttattatagatctaatttacaacatccgggaagcacgaagtcttaccccc
agtcgctttttttctcggaccttgcgatgattgtaacgggtttttggaggtacttccatttggagattccatttcgctcgtttctatgtttctcgattgttcc
gttccctcattgtgaagagaacacatgggtcgttcagatacgtttggactagataaagagctggcttttgtgaacattgaagatttctcattggaga
cggtccttcttggtatttttctgttccctatatcttttaccgaaatcccatgcagtttattgtttgaaccgtcagtcagtaacgtctgctttccttgttgat
gaagaaacctagttttacctacgtctgatgtcaactgagggtcttccttctttgaaaagaattctctaatgtcgaaatttcccttccttttgagagtat
tttctttatttaatggttcatctggaacttccatgaactcttgatccctagctcttttctgtagttccttctccgccagtgaacgagaacatacaaaagc
gggcttttcggtagaactttttttcatcctgatatgtcttattcggtcgatcacttatttcttgtatgctaacgttttgaacggtgccgtactcctttcgatt
taatattatactgatttcagaagctaactgattattaaatataatatccctttctgataaatatttccttgtttcatagcttctcagaaaactactaata
tcctgctttgagctgaaattctcattatcgttgtaggacccgactgaatttttgaatgacgatatatacaataaagcttgagctcgtgttacgccaac
atatagcaatcttctctcttcatctacgtcctctgaacgtgaatgagggataatattttcacagacacaaggaaggaaaacgactggccattccaa
gccttttgcagcatgcaaagtagatatagtgacctttgaagatttttcttcttctttgtcgttgctcgctaaagtgacatgtccgagaaacttttgaag
agaagtttcttgtggtccttcctctaatagacccacctcttcctgttcagcaagaacatcactttgctgaaccaattccatcacattgtcccatttttca
tcatatgtctctggatttttttgcgtaaaaagtcatagtatttaatttcgcgcagaaccgaagatattaagtcactaactgacttctgtttgtcatga

gattctctgtcgagccgttctattaaatcaataaaatttttcacagattttagaaatgatttgtcttgtctttgagataggaggatattgttttcgctta
ctttgactaggatttgccagagtggaagatttcgccgttcactttcttcaagaaggcgatcaacttttgtcttcccaatattcctagtaggcgtattta
taattcgaattaacgatgtcggatctcgattagcaatcactttcgcgtaagctacaacatcacgtacttcctctctgtcaaagaacttgtgaactccg
accatccgatatgatactccaagctctgtcaaagcatgttctaagctccttgtcaaacttgacgaccgcactagaatggcaaaatcatcatatgta
aataaacggggacagcattccacgatgcgttttatttcacatgcagtccaatatgattccttctgattcgtctcgaattctcgatatgatggcattaa
agacataaaaaaatcactcttaagtcctttttgcggtcttgccttgtcttgggatatgatggagaatgctagttccaaaatggaatacgcggaccg
atagttcatttccaaattcagtacttgtgttcctttaaaatcctttgtcattctatttaaattttctatttcagcacttcgaaaaccataaatagattgat
cgggatcaccaacaatagtaatagctgtatccttttgagctaaaagctttaataaataatattgaattctgctagtgtcctggaattcgtcgatcag
aatatgtttaatgttcgcaatacatgacggatatgtttgaagcaaaagaataaaatgtaacaacaggtcgtcaaaatcctgatgttgttagtgaaa
tgctactatcctccaaacataccattaaattgttctttctcagtgttgtctgatatagcttatagagctcggtggattgataactttgaagttccggtg
gaggagcatccattcctcgtagtaaaattagttcagggctattagcttttgcaagtaagccagaagatttcattttgatattctggttaagacatttt
cgggtgttaattcttgtcctcgaataccactagctaaggaattttctgcctttttaagtgtagccaacagtcgcttaactatagctttcctatttcaata
aaaattaatatattcaatcctcaagtctcaaacttacgcgtcgcttctgtcagccaccaaccacttcggagctagcccaatgtgctttccatataca
gacaggtacttatacgcaatggaatgaaacgtaccagaaatgattttggacgatttttcgtttccaagaagtgtatggatcctttgtttgatttcgtt
tgctgctttattggtaaacgttgcgactataagatctcgaggattgcagtttgcaacttgtaataaataagcgactcgggaagtcaaaacgcgcgt
ttttcctgaaccaggacctgctaaaatttgcgtatatttatgagcagactcgacgctgagtcgttgttgagtgtttaaagactgtaaataagacggt
gcggacgccattcagtattttactaacattagaaatcttacttaaatagaatatctggaaaaaagagtgcttgacctgtaatttgggacagagtag
gaaaggtttggttgttttcagtacaaacatatatatgtctaataataaatgaaacgcgtcgtgaaaagtaaacaaaattccgttaaaaaccgacct
gaaaagagaatggggtacttcatatcaataaaaaacgcgtaaataaataatttataatcctctcaggacgaaatatcatgtcgaaaccattacca
gatccatttttacgtttattgatgagctacctactcggatactaccaatactagaaataatccctatgcggaagataaaatgaattactaacctattt
ttctttatttaacgcagaaaacaatgttgagaaaaacgttccagcttttcagctgacaatgactgtcacccacgtaatgtataaaacctcaccgagt
tcaaagtatcttacctcactctccttcaaacatttgcctatttcaaacgaataaattacaaaagaaaagctattaaattttggaattttgaaaaaaa
aggacaatggataaaatgcgtaatgctactcacgctggctcatggtatatcgataatcccacttcacttgagaagcaacttggaggctttacatat
gcttctgatcctccaattgaaggaaaacgattcataatatcaccgtaagtgcttgggattgacgaatggcaataagtcgtcactggataattggct
gacagtatgctttagacatgcaggatacatgtacagtggtagtacagcgactcaggcgttccaaagaattaattttgaaaacagaaaacgagttt
ttatcctgggaccttcacaccatgtatttactcgaaagtgtttagtgagtcaagccgaagcttgcataacaccctttggaaatttaacagtagatcg
agaaggtaagaaaaaattactttaaatggaagaggaagaatcaattcaaagtttgctaactgtcaaaagtatgtcaggatattattgagcaaaa
tgacaactgtttcaatgaaatgagcctaacggccgatgaagaagaacatagcatcgaaatgcaatttccgctactagcttaccaccttcaaaaac
aaggctgcctcgatcgagtaaagattgttcccatcatggttggtgctttgagttcagaagctattcaaacagcagcaaacatcctttgcaactacat
tgtagatgaaaaaaacgtctttattgtttcttctgatttctgtcactggggacgtaggtttggttacactatgtacttgaacgaaacgaatgatttgg
aagatgcgattatcagctttcgaagaagaggttcaattgaaaatccaaaaatttatgaatctatttccgctcttgaccacatgggtatgaaaattat
atcaacgaaatcaagccgagagtttggcaagtatattcgattgacccacaatacgatatgcggacgttatcctattcagttaattttaaagtccatg
gagctagctcaagtgtccaatcaatttgagtttattgcttatgctcaaagtaatcgtgctagaaatgtgtccgacagcagtgtgagttatgctgctg
caattgcatagctaatacagctatggtcgctaagtattaggatcgtgttttatgagtcctacaaatttttttattaatgattcgtttttatgactattatt
ccaattatgttccttaattgcctactgcattttgttttattttcatcgagtttcgatttatggttaatgttgcttgacttttgagattagaattgagcagat
gtatcatgctaaatgtgatcatgcgagcaaatataatatttttcctacgacgacgaaagcagcagattataaccttaataaatccgcagtcatgga
tcttactttattagcttcgctgtgttaatatttatggagtaaaatgcaattgtgcataagaagtattttaaaaggataaacaaaatgggtttcctagc
taaaaaaaataaaaaaaaattcttttattttttggaaaaccgtgcttgcttcctatttactcttatatgctgaagatttgcagttttcggaaacgtaa
cagctttcaatcaattcgattaaggaatttggtgtaatttcgttgcgaaaatttgattacaagagaatgaattcatcattagcaaaacatgcttgcct
tagaattcacttacgagtgatatgtcatacggacgttaccttacgaaagtcgcatatgtaacaaattgaatttattcagagccattgagaatttaaa
gtatcttgattcttctaacatttattctaacagtcgtcgaattgaaatcaattttctggaataaatcgttcgcgtacatcgattgaaggtcttagttcgt
gaaggaaacacttgtcactttatagaaatacttgaaaatatccataggaaaaaaggtttggattcctgtgaacgagtattcgctcctttcacaccc
gttttatcgaattttgatgatagtagtatacgtattctggtttcttccttaaactagtctcattatttttttggataaaagttgagaagagcagtaagtt
ttgagaaaagattctacttaatcgatatttgtaagtttatttgctctcttttttttttctctttacgcaaatctttttgggagttttgttttgcttcaggaa
tctattttgattttgtttttcgaggaataagtccttttgggcctgattgtttattttacaatgcctcctcattccaggttatcgtatggtgcggataacta
ttctccaagagaaattgacaaccacggttttgtaggtaatatgcatacttcagccatgatatccgtggatgggtctgtggaaatgatgtgttggcca
caattcgattctccttcggtatttgctcgtatattggatgctagagctggacacttttccattacccctgtagaagaatcaagctgtaagcaaatgta
cgaaccgtcaacaaatgtgctacatacaaagttctattctgagcgcggtgttgttcgactgcttgactttttttcatcgacaatgggaagattatgag

ccactatatccttggcttattagaagagtttgttgtatcagaggtagcactcgcattaagatggaatgctttccagcattcaattatgcaatggaga
accatgaaacaaaaattatgagagcccctaattatcttcaggccgagttttatcctgcttctggatcccctcattatattttggatgctatcccagca
gatggcgatatttcaattcctttggaaattactcgaccatcggaaagactcgttgatggtggaggaattacttgttatttggagttagaagaaggtc
aagagataacgtttgtatttcgacaaaataagttaggtcctagtgttgattatatagcaacgaatttgcttgataaacttgaagattcgactaaaag
gtattggagagcatggattcagcagtgtacttatactggccgctatcgggaatttgtccagagaaacgccctcaccttgaaacttttaatttacgaa
cctacaggagcagttgttgccagtccttcgttttcattaccagaagacttaggtggtgtgaggaattgggattaccgatttacttggattcgtgactc
tgcttttacaatctatgccttagcacagcttggattccgagcggaggctgtcgaatatatgtcatttatataccacatattaaaaaaaaagaacacg
gatggaggtataaatatcgtttactctatccggggagatacagagggtcttgaggaaaaagaactaaatcatctgcgaggatactatgattctcg
gcctgttcgtatagggaatggtgccgttaatcaccttcaacttgatatttacggcgaactaatggatgctatatatatttatgataaaaaatgtcaa
ccaatagcttatgacttatggttagaagttcgacatatatgtgattacgtatgtgccaattggaagcgacctgacaaaagtatatgggaagtacga
gggcaaaataggaactttttgtattcaaaaattatgctttgggtggccttggatcgggcacttcgcattgcctttttgaggtctcttccctgtcccaaa
cgaatagaatggatggatatcagggatgaaatttatgaaacaattatgcaatcaggatacaatgtagacaatggctattttgctcagtcttttgaa
aacatagatattctggatgctggtgttcttgttatgcctatggtttcttttatttctcccacagatcctcggttccttagcactatggataacatcatga
aaccattggaaaaaggtggattgatgagtaatgggttaatattccgctataataactttgcatatgaagatggtgtgggaggcgatgaaggtgctt
ttacaatggtatgtttttggctggtggaagcactagctatggccggctgtcgaggatatccaaagcttatatccactgctgtaagtatgttcgagga
tttggctcgttattcaagtcatctatctatatattcagaagaatgttccctgtccagtgagtctttgggcaattgtcctcaagccttcagttcaattgct
gcgattgctgccgctcatattctggataaggctctttctgtaattaacgatgtttaatgaaagtccagtcttttaatttaaaagttataatgtattaag
tactcaccatagtttctctcttattctgtcatttttcctcaatcttttcttaatgatttgactacctatatcaatcccttacccctttttttataaggaaag
caaagaataaattaagtaagaaaagttggattctttactaatttttacgtaaacaaatttttatcttccttcatttgggggggaggtattcgaagaacc
atctagtttgctcaaaatcattatattactattcacgactcaaatactaccctaattacactctcttttctttctactttatcatgtacattacatataat
tgcaaatttttacagtaaatcagtgcctttttctcctccaccgtttgtttactagtgtactccttatcgtttaatgcacttctttaattcgcgctattcacc
atttgaaaggtaggcaaatttatcaaagtaaaacatgtccattttactagcattttgatcatagttatataattatttatggaaaaaaatgagtatg
gacgagagcatttactctgcaaatgatgaattggtgaaagaacgcgaagagtcagagtctttgaagcttgaagcaattgtgagctcaaactttttt
actactcccaagagctagtgatgcttttcacagttctttggaaaagggctatcatcctaggtaaaatcagtgtatggtgttttgtttgtataaactaa
ccaggcgtcagagttatcttcagtgccctcaagaaaagtcgggagtcgattataaatggaagcctttttgaaaagtattcggctaaggaaaatca
ttacctttcgggtggtattctccatcctcattaccgttctttgggaactgctaccttatgtgtcggccctcttcgatttgaaaatactagttttacattcg
tacattattctaattcagttaaagaaccattcaatgctccaaacatatcttatgtgaatccccaatataatggtttagattacagaccaaacctgaa
aagaccagctccaatgatgagcgcaggaggagagactatcgatgacaaagaaaaagattacgaaaaagatttactaccacctcctcaatatag
acccccgtcccctaaaaaacccgaagactatgagagtacacctggagaacccgtcgagcctctaataacagatgaatcaatacagcacttgaga
atacgagctcaacaagatcccacaataatgaatatactgaagaaaatagcgaagggattgggaactcctgaacagtgcattcttcttcataatga
gctaataggacctagtacttttccatcaccacgtcgtgccaaaaagccgcctcgtaaacgtataactttaacgttaaatcaacatgacaaagatga
gtttgtaaacagtcttcttagtgataggaagactgtccgtcagcatttcgatatcatttttcagttcaatgaagcacccgaacaacaatggatatta
ccacgtggctctgtactttcgcatgtttttaatacttccacaaaaagtctgaacgctttaaagctgcttttccacgtataccaagctactcaggaacg
gtcagtgactgatattaaaactgaaatacaaataaaagatttcaatcctatacttcgagcagcaattgaatctctagtgagcggaagtcattcaga
acgcctattaatggaaaaacaccggcgactctcaactccagaagctaggtattatgtacaatactctgagcgaaattaacctttaacgtatatgat
tttaaaataacactgtaccatataaaaagaattcagcgtttctatagtctaagattgacatgtagccaacatggaataattacatagaaaacaag
gctttacgggcttaaaggattatgaccgtctctaagactttggtggatttttggtagagttttccttatcctttttttcttttcttcttatcttgcaagat
taggttctcaaatactctatgaggatcatcagggtcagcttctacctgactaaaaatggtaccaatttcaagaagagcattcgcacgcttcattctt
agatcaataggtaggctctttcgtagaagattgttgcaagcatcccgtaatacaccttgaatgtcgtatcgagcgccacaccagctggcccgcaaa
acttttcctgtaatatccatttctaattgtgcgcgttcttcaggtgtagtactttcatccattttaccctccaatttttgcatttgatcaatacttaattta
gcatccattgctgcagaaagagtatcccaagtgtcacgagcaatctttccttttttctttcatccgactccaaaaaccgccaattccaaatttgcttga
tttgattgtgtttcgtgctttttgagtatagacatcacccatagcttgtaagatttcgacaccgaatgattcaattcgaagtgtttctgcctccttttcat
attgttcacgaagtgcctgaagtccttcttcagtagagctcttttcaacccaactgtctagacgtttttgaagatttttcgtaatttcagaaattcgttt
cttcctgtcttcgagcatttgcttttcccgtttagcagcagcttctcgttctcgaatttgcgcatttttttttcgctcattaaattgttgctttatagtcggg
gttgcttcccctgaatcaagaagaagctgttgcttattcatttgattcatctcttcctgcatggcttcctcctctcggaacatttctctgacaaaagaa
agctctcctacccattcacgaaaaggggcaccaccgaacagattggcaaagaaatcatacgcgtcagtaaacccttgctctggaactgccttttct
tttccatgctgatcgtatcgagatcgaagctgttcgtctcctagcacttgatatgactcgctgatttttgaaacctctcgcttgcaccttgagggtcct
ctgggttcttatcagggtgatacttaaccgctagtttcctgtacgcctttttaatctctaaacttgaagcatccggagacactcccaatatttcgtaat

attctgtgtcgacaggcataattaaactaaccttatagaatcaaagttgcaaaaactcttgctgaaacgatctagtttaccaaatcagtagtacttg
ttttgaggcgtcgcgcgtcttgaaatttgaaacagcttgaagggctttgcttataagaattttttttattttacttttctcttttttcctttttctttaatca
atgaccgcttgcaattgaaaagcaagttttgacgagagatgatcctgtaagtctttataaattacaacgacgaaaagaaaaagattctttctttaa
aaaaagagaataaaaaatacaaagaagtagaatgtttatttcaaagtcagatcctacagttcgtatatggttaaaggtaagtatcttactaatga
gaaatatgagtacataacataattgaagtgagccttcgtcgactgtgacatttgaagttacatccgattaaaagtaatggagcgaaaaagatgat
tacagttctcatatttgttatcaatttacttggcacaaactcttgattactcagatgaaagtgaatgatgcttcatcagcaagtttgttgcctaaccga
tttatatggaaacgatattctttgatctaattgtttttaagataattcacatcaaagtgagcattctttgacacacataaagtccacgacttgcgtttc
ttcaccttaacagcttcataaaagaagcgataggaagttacaaataccctaaacaaaatccgttcatttattcatttctatcttgtttatttctactga
tatagtgtttatttacaaaaatacctcaacgtcacttttctgactataagctcaaacaattagcgaagcttaatgctcgcttacatcacttttaaaatt
acaggtaaatgtatgagaattccaaatggtaacaaaatttgccttttgcctatactgaggtcccatattgagtagtctttgttttttttagagtttaat
tataaaaaagtcgttaatatcaggataaatggttgcacttttcaagaatctaggtataggaggcaatgctggaaaagctgctgatgacaccaaga
agccattaccaagaagaggtaaatactccgttgccccgggatactctcaactagattgggccagaaaagtagccagtggtgaaaacttatctgta
tgtaacaattatttcttgtaatgattccttagttgtccgcaacgttggtttcctaagagtatagggggatggaaatctcatacctcactctatttggaa
agttttgaagttcactatgtattttttttatttcaatttcatttagctctttatcgatctaacatatctttagggagtggataaaccgacgagagttact
aaagatgagctaaaaagacattgttcggaggaggattgctggatatcaatcaaagggaaagtttacaatgtaactccttaccttaattatcatcct
gccggtgttaagaagattgtaaataattctggcatagatgctactcaagtctttagtaagaaaaataattctactagcttgcatagactgacgcttt
ttttttagtgatgaatcacgcttgggtaaatgaggaagcaatactaagaaattgttttatcggttacttgacttgaagatatccaaaagtcctttact
agtcgtctatggaatactaatatactttttctctttcaggttctattaatttctcaatatgcccttatatattattttctgtgtttttttctttcttagacaat
accaattctgcctgtaccataatctgtttgtccttattacttaaagtccgaattataattttcttaacaattgccatttttcctagaaatggtgaattat
aaatcattagtttcattagttgatgcctttcgtcttcgaaaagaaccaagcattgattttagtgctccacgaatttctttaactaacgacagtatcag
gggaatcataataggtgcaaataagggtgcatatattccatactttgattcatcaggaaaataaacagtggttaccatacttgaatgaaataagg
ccttttgtgccttttcaaacgcattcgtactatgggaaagagttagatttaatttcctctctgaaagggtattgtatgcaagatccaacgacttcaaa
gcctccttcacatatgactgtatttctttaggtacagccatattctcaatggacttgataagcttagcaaggtttaggagtgtatcagtcgttccaac
caggctttccgctatcctttgccttagcattctctccattactactgggcttagactgaatgacaaaaaatcatccacacccaatagtaaaagaaga
tctcttgatatagtacgaaactgagacttcatgtcagaatcttgcaagtaattagaagcagaattattagtatttgtaatgataatcgaaccccatt
gaggtaaaagcattgaattcgttgcaacttctttaccatattgatcttccaactttaatggctgtatctctttgggaggtacatataacacaaaatgt
aaagtaggctccctcacatttgggtttactgagagatatttttcaaaattattaacaactttagggaaatcagcaaatgctgttcggtactcaccttc
tttaaagtaaaccggtggatcgtcgacaaagtattgtacctgagactctacgcttaaattcattatttgagaaagttgctggattaaaggctgtaaa
tacttggcgattgcgctctcaatttcccaacctctaagatcatgggatccttcacccaaaaataaacttaatagaatacggtattgaggagaaaat
tgaattgcccgcttattgtaaaactcaaaaggaaggtttttttgatgtcaaccgtgaatactttgcggatatatccatgatttctggactaaaaacctc
atataaacgattaactaacataatggcaatctcattttcagaaaaattctccttgtaatttaagtgtagatctcttcctttccaataccaagagttttc
agaagattttgctaaacaaatggcgtagtcggccgcttctgatgttgcaaactggatattataaaacgaatactgaggttcttcatttatgagcttg
tcagaaagatgggttaactttcccgttggatcacccgtaattctgaatgttggggtaaatcgtaggttggattctagttgatatctggtattttccatc
tcataaaaaggcagagaagcccgatagtacgtagttgttttccaccagattggaattcccagtaaaataatcaactgtatctggttagccctggct
gtttcagtaatttttatataaaacgaacataataggtaagcattatatatcttcttagtttggagtcatcattactaaaatcctttttttcttttccttttt
cattctttgaagctcgactatatttatgagaatccatgaatcatattttatggtgaagtaagtacttttggtgtaagcaattagtgtgtgtatattcaa
aattcaacaaagtaaaaacaaattaaaaacaaattggaaaattgacggttatattattcttagaagttattcttagctaactcttttcttatagctgt
tgaactaagttttataggatcactattttcaggcattgtagtacgtgcaaaattaagaatatcgatgcaataaatatctagttcaggcaatcctcttt
ttcttcgttccatctgtacagcatccgcacctttgacagtttcttcagaaacgagcaaactgtcaatctcgcgagttgttatagtagggccgaaagg
atcgtttatagttacaatttcggattgaatatttttttttattgaatgaaggaaatcataaacggaatttttcctcacttcaatactttctaaatacgct
ctctctattttgttcaagagtaattcatttcctagaagagtaagctcgtttgcactgttacagcagtattgggcataccagaaacaccaacataaac
tttaactatcccaatccatgcagataatgtaagtagaactttatgaccgacatgtaaatggtcaaacgtccctccaactgaaataatcttgattagt
attcgtgactaacgaaaaccagcgtaccagctgcaatacgattgcttattttcttttcctcattttcaatctccacgaagctctgttcaagctcacga
ccttcaagcacttcggcctcgatatcgagtacttcagtatttgatttcgagattaagcctacaacagcagttagatttgacagaatatgaattaaag
tactaccatcataatttttagtgcagtaaatggtatcacatagattcgagatttcttgaacagaatatccagaccaatcttcgaataaaacgacagt
aggaacgacattgaaaatagaagggtctttggtagtaattgagcagagatatagttcacttaaagctttcgaaagcttgaaaaaaatacgttcat
tcctttcttcttttaattcaggactgcatacccaaatatatagtctattattgcgattctcactcagccgttccgctacctttgatacggtagacaaata
ctttgtaatagtttccaaataaggtacacgaatcgcgattaacgtatttttacccattcggcagcttttcttttcgtgttcaagaggaagtgaatagt

acgataccagcattcagtacaatgaagaagtgtagatgaaaacatagcaaacattcttacaaaatttttgtgttctaagtccaattatgtttcaat
tgctcaagcaatttatatttctaccgaatttgtcactttaacatatggaactctttctttctacttataacttgaattgggagaatcctacacaatgag
ataaatgatataaagcttttctttaattaattgtcatagctattgacgattaacgtatgacagtgaacactcgaaaaaccatttcaataattggaaa
tttatgtggaatgctcagcaccgaatttttcaaggtattggactaaacgttcagtttgttcgacggtaatagcattatacaaagaaacacgaatacc
gccaatactacgatggcctttcaattgcatcatatgaagtttctcagctcctacgaggaactcattttcgagatcttgatttatgattctgaacgtaa
cgttcatacgacttcgagcggaagaatcaacgaagttttcatagatagggtacttgtctaacacggtgtaaaccatatgagatttcttcgaagaac
actgctccaagaatggtaatcctcccttttttccatatattgaagccccaagttaatagcgtgaagagtagggatcggtagagtgttgtaaagact
gttgctatcggccataagtttgtagtcacccatagtagtagaaacagggataccgagcttgtgaagatctggaaccaaagcacgttccaaaatga
agtccctgacaaagaccatcgtgataccggcagggcctgcgttcttttgagcacctgcgaaaatgatatcatgcttggaaaagtcaattctacgag
aaataaagttcgaagaagcatcacaaacacgaatagcgcctttggggatgtttggaggaggctcattaaattctacaccgtggatggtctcatta
tcacaccagtaaaccaaagaggtttgaccagcgggagtataattgagatcagattctttaggcaaacaaccatactttccagtcaaatctctcgtg
tcgcatgccacatgacaacgaaatccaagacgctgtgcttcgttcaatgctttttgctccatgaaccggtaacgatatagttggctaccaattcctt
ggcattggggttgtaaagtgcatgatgcgcatatacattatacaggacggcagcaaactgttcagtaccacctccttgcataaaaagaatacgga
agttatcaggaagtccatacattttacgaagagtttgttcagtacgattcacgataccagttccttccttactacgatgggatatttctcctacaccc
atgccaaggttttgaaagtttacaaaatccttggcatattcttccacgacatccgtagccatacctgctggtccagcagcaaagtttataacatctt
gtctagtaggcattttgtagaaaagaaacgtccaaagaatataaaaataaaatttgatctgttaggcgttgtaattgctaagatgtagcaaccatg
gtggagtaccatataagatgatagatgctgatgtcgtaaactgatgaagaatatcgtataactatcgtcattcttttttttttttttttttttttttttt
ttttttttttatatatatatatatatactactacgtacttacttactttactttatttatttagttacgttacgtactactacgtacgttaccgttaccgttt
accgtttaccgttaccgnnnnnnnnnnnnnnnnnnnnnnnnnnnnnnnnnnnnnnnnnnnnnnnnnnnnnnnnnnnnnnnn
nnnnnnnnnnnnnnnnnnnnnnnnnnnnnnnnnnnnnnntaacgtaactaataagtaagtaagtagtaagtaagtagtagtcgtagtagta
gttacgttaacgtagtattagatatatatatattcttttnttttttttttttttttttttttttttttttttggaatcattacataaaaacattttgcaactgc
taaagctttccattatttatgttgcactatgacactcgttagttcctccatgaaaggctttttcatgaacgcataatttttggttgaatcttctcttagg
atgcaatttttaagaattgaagttgctaataacaggtttcatctttcgtgttcagtgcttagagaaacaatctaaggacgtgacaatgaattctcat
ctagaagaaaaaatcaataactggagaagtaatcagttattgctaaaggagagaatagtattcacttcgaaattcaaggatgttgattctataag
atacatagccgggttggatatatctttcgacaagacaagcccgaaggctgtttcggctattgtcatttacgatcttcaaaatgctcaggttatatac
aaagattacttagttattgataacctcagagaagattatgtccctggctttctgtcgtttcgtgagattgaatattatttacctttactaaaaaggatt
ccccctagttattgctttgacctcataatggttgatggaaacggtattttgcacccaatacgtacgagaaaattaaattgagacatagctaatttat
caaccagaattcggcttggcatgtcatttaggtgttttaaccaactcacctactatgggtgtagcgaaaaattatttccattgttttggactaaatga
atctttagacgaccatagacaacatctttcaaactacatctcgactaacaccagcaaacccccttttattcttgtattctatcggtgaaccatccattg
tcctaggagctgcagtttggacaagaaaggaatccaaacgacctatatatgtaagcataggaaatcagataagtttggatatcgcaattcaattg
gtgtttcgaaccagcggcgtcgtcagcaaaattccagaaccgatccggcaagctgatatatttgcaaaatttgtgattcatgagcaagcttgtagc
aaaaaggagtaaacgttcatacacactataatggcgtactattgttagtctatgttgcaataatgaaatgatttgaactatataaaggtacttaa
attagcattaattaattcacccctaaaccaaatctaataaatcatacaaacataagggaacctaacgaagagaaaaaccatcctctgggtatcat
aaaaggtacaaggaagacatgaataaaatgaaataaaaaccaaaacaaaaagcatgaataaaacataaaagaaagagacagaaatacatg
ggatcaaacttgtaaaatacaggcaaggaaaacagcaaggcaattatacaggaggcatctgtatagcgtgctcatagggggggaggactcggcc
caacactactaaacataggagggcattcttcatcccaactcacgccaagaccacctctttctgtaagaggcaagttaaccttcattctcaaaatgc
gagcatttgaattgactgggcgtgcgttaatttcctccacaacaatcgtctcaaatatcaactggtgagctatcgaaaggctatattgaccatccaa
cgtaacatcgcatataggtttcgataaaagtgaagtactaacaggtatttcaaacaatatacgatcgccatcgaccttccacccagttctgtgttcg
tcgtttcccaacaatctagtttcacgatatacatgtggttttcgacttccttcatgttccgtgcatgggttaatctgagctttgatttgctcctctatccg
ccaggatacttttgacaaatgccatgaatattttgtattttgaggcctacatccagtaagctgaaattgtatagggatgcagcttagagcgtggatc
accggaggcatctccaacaacgcgactagagaagtaggaggaaagaggcgttgagcgactttgtcagtgtttaaaataatcgatcgctttatgtt
tagcgtgtgctcaagttttacatttgaattagcagaaacatcactggttactgtagccaaaagaaagtatcggatattaacaaatggggattctgt
cgtttgagggaaatgccccgggataataaaactaaatggccaggtatgcgtaccatgagaaaaggagaatgtacttgaagaaaaattccaactt
tcaaggacatcaacaattgcacgacaattgggacagctctcacggacaggatttttttgtatgtaatacacgcatgaatttcaaattgacagaattg
gtttgaatagcatgttcaaataccgtcatctttagaagacctgatgctagggcgccagaggatacatccggagctccgtacataatcaaaggagg
tgattccatctgaacgtctagttcaagaggtcggtcagctgaacttgaagatacattagaacgacgaataaaagaaggcatcaataataaaaac
gtcgatattgtaggtgtttaccaccgaaataattgaatctcgagagaaagaacaggaaatcgtaagatatatagagtctttatgcaaatacaaat
caatattatttttctcgtttttttgatataaatcctacaatgaaggttagatctatgttatgaaaagagaaaaaataaaggataagtaaatcgagcga

tagtacccctcataacagagaagaaaaaaatgaaaatattctcataccttacgataaataacaaacgtagatgaataagaagattaatctaata
agatagaaacaaaattccaattctcttattctcttgaaaactccaattctgtttactttctgaagcaaatgaacgacaagtgtgaaatcctaccaaa
atcaagtgatgattttagaagcgaatgcaaggtaaaaatttcctttaagaagttatttttcgacttcttatacagtgaataacagccaattgccaag
gaaatttcacttgctacgcggactaggtcccagcagggtatttgctaaaagatacttattgcaggcgtggaaaatacgaccacatttaagctctcc
gcactgtaggcctgggtataccacggtactcccgctgcatttccgatacttcgaatgtattgtaatattatataataaagtattatcagttgaaaaa
aatgtaaacaaagggaaagcaaagcaagggttatgtaaaatacattctgtgaggtattcaaagtaggttcctctagtcgaataaagaagaaaa
gaaccttataagattgtaaaggacgattgtaaacttattcttgtacagacccatatctggaaatatagaaaacaaaaatggtatctttattaagaa
gaactataacaagttgttaaagtcttcaacaagtggtgttgatgatagcttgaatacttaaacatctggtttctacgttgacggatacacctctgctg
cttttccctcgacaccttagtagtgtatcgttaaactatcaaggcaagagtaaatgattttcacctatgttaattttttttgcttacaaacaattctagc
attcttacggttcataaattagttgataccaaataatctcctatcacgaacaaacttcctgcataaagagagtgtgtgttatttcctttttgtttttcc
gtcacttgaaaaatgtcttcttacaccgataccgtgaactgtatgttacagcatcctgccggcatagataaaattgctgcattaatcgtaaatgttg
ctcgtttaaatcccgttgctagtaaggcaaacagcgaactggtttccatgttgaacgagttccgatgtattctacgcctacctggattgtacaagctt
cttgtaaactttgaccgtagggattccttagaatcccacttgagcactgcaataaatgtctgctactatctcacagaaggccttgcatttttgggaaa
caagcaaattgttccaatgtccaagactagagaagaccagctatcgcttatttcttgccgtttttggttattggataccctgttaactgtttatcaatt
atataaaaaagttcgcaacactcacgatagacaagatgaattggacctggctgccaatgtcgctagcctcccccctgtgcatccactggtctatagg
cagtggacttggacttagcaagcaacaaataggtatcttaggactcttcagtactgttgttcaagtacgaaaattacttagggctttaaatgataa
gaaacaataaagtgctgaaattatacggtttttttacttaaaaatacatgacttacaaaagcagtaaagacattctttctcaatcctggtatcactcg
aatttccgatttcattgaagaataatacttggtgaaactgggttaattatacatctattatttactttacaaattacgtcctacctccaataaacagtt
cctcatccttcctcacagatgcaaaaacttatgctactctcattctgtgcctgtttatcacatttagctttccatactaaataaccatgaacttgcatat
tcgatatattttctcttgtgaccagaaaccctaacctactgtgaccggttttccctcaacgaggaaggaagagactggctgtaacaatttttcatcc
acaatcaccaccatgcttttgttcaaggacgctatttctggggatgagctcgtttctgatgcctatgacttgaaggaagtcgatgacatcgtctatg
aagccgattgccaaatgctcaccatcaagcaaggtggtgatatcgacattggtgccaacccttctgctgaggatggtgccgacgctactgaagag
ggtaccgaaaccgtcaacaaccttgtctactctttccgtttgaaccccactagctttgataagaagtcttacatgagctacatcaagggttacatga
agactatcaagggtcgtctccaagaaaagaaccctgagcgtgttcccatctttgagaagaaggccgtcgaatatgtcaagaagatcttggcaaa
cttcaacgactacgacttcttcattggcgaaagcatggatcccgatgctatggttgttttgatgaactaccgcgaagatggtatgactccttacatg
accttcttcaaggacggtcttgatgctgagaagttttaagcttcaaaacaaatcttttggggttatattacaaggtgataagtagtctgaaaacatg
ttatttacgacataaataatactacttccataatatgcattttctgcacaacgaaaatgaagggttataaatacaagaagtcgctcttcagtagtac
tttaaaattggtttcattaatatactatcgtttactccttgacatcttttgtagtcataagcttttcgaatgtagtaactagaagagtaggcattgaat
acgtagttgcagcagggtatgagtgttcatatactggtatatctttgaaaaatgttacagcatgaatatgatcgcttctgactttcttaatatcagta
acaactatactgttcattctaaccattgtattagaagcactttgagtaggtcgtttcaagcatttcaatttgttttcttttagctcaaatatttgaaaag
gacttttatctttaaagaagaatccttcttacaaacgatccccagatgaagtaagctctccagtagtatttaccaactaccacttcctttgacaaggt
catgacagacattcacgagactcaaaagggagctcttgtatgataataacgaattgatatttttttcataagtttctttactaatcctactgtccatag
aaagaaattcaggctttcattcgttctcgaacaagttacgatgttttgcctacttcatttcgtctgattgttttcgatgttacgctgtttgtaaaaacat
cattgtcgctccttgctttaaacagtgagtattcaaacattcttatctgatgttaagcctacggttgctgaagctaactctttcagacattgtcagtgc
tccgttgtgggatagcgaagcaaatagatttgctggtctgttaactatggcagactttgttaatgttattaaatactactatcaaagtagctctgttc
cggaagccttggctgaaatcgataaattccgtcttttgggacttcgcgaagttgaacggaaaattggtgctattcctcctgaaaccctttacgttcat
ccgatgcatagtttaatggaagcatgtctagctatgtccaagagccgtgcaagacgtgtgccattgattgatgttgatggcgagacaggaagtga
aatgattgtttctgtcttgactcaatacagaattttgaaattcatctccatgaatgtaagtatgcatttttttctatcagtagtaactgtcttctagtgc
aaagaaacatccatgctccgggtgccactcaacgaattacgaatcgggacttggtcaaatcttgctacaagtacgatggatacgaaggtttacgc
agtaattaaaactcttgcagagcaagctatctctgctgtcccaatagttaatgatgagggtatgcttaaacgctttcctttaagtaattactaatcat
ttctattagggatcctaataaatgtttatgaatctgttgatgttatgcatttaattcaagacggcgactataataatttagatctaactgtgggagag
gcacttttaagaagaccatcgaattttgcaggtgtacatacttgtcgcgatactgatcaccttgatggtatttttgatgctataaaacattctagagt
gcatcgactttttgttgtcagtgaggattttcaccttaaaggtattttatccctttctgatatacttaattatattgtgaatgatcaactttaaagagta
taccatcgaatgttatttctattaatttgttgaaattgcctcatctatttgaacaaaaaaataaatatatactatgtatttattttccaatcctttgtact
tcatatttcacttgatgctaccgttttacatgccgtttatcttttaataactagggcgctattttcgagatcattttagcctgttctctctcctttaagagt
cgaaatatttaattttctaactttattctcaaataagtatggaaatttaatgatcatatcttcacagaaactattaatgcttatgaatgactttccaac
agaggctgtaaagagatcatgaaactgcgaaattagttattattttaaacatatcttacgaaaatggatgatttggtggcataatggatattatatt
atgaggtattcatttttattcgaattctacttttaaccgtaatcacatggcgtttcgcctatcgctgtaactgacatagacatctagagcttcaacac

ccgtgttatctatttgtcaaaggaatggttgggtactatagaaatacaaggtaagattattagatatgaaataattctcgaagttggctcatagtgt
cttcagggcaagtaacggtatggccaatagtacgaggatcctcaaaaatttcatagtcattgcctccggtaaatgttttatcaccaaagaaatgga
tggtttcgaatccctccttctgaacgtgttgaaggcagtaagtcttgtcccagccagtagggaagacatcaaaagaaatttgaccaccaatagag
aaggttaaaccataatcaccaaacttctcgcgcaaggcattaaccatattcaaacgaatattgtgaactttatcaaattgctcaaactcccggcgc
tcttcggtgctagcattacgaccgatgggagaaatgttaaccataccattacggaattcaacaaaggtaccacgcttaacagggatgttcaagtc
agcaatgtagtgtaaacagaaattcacaagcttttggtatctttcttcaccaatgtattggataaaagattgactggcaagctgttgaccataacgg
taggcggttaatccattttcggcaaaagaataatcgaaggaattaaccacattctctccgctaacgctgagctgctcttgttgtttgctcaagtcact
acctccgacaaatccaacagcaacaacttgacgaagcttctgcaagcagtcaagcatttcaggagagatgttctaatattgaattgttagtacata
tggcagcaaaatacgttttagaaaccggcagcatcacagagaaagctactttcattatatttttcgcaatgaaaatttaacaagcaataacaaga
aagagaatgccagttttcatatttttgcatttttagatttttaagtatcgacaggtccaacagcccgcccctcttgacgcagcaaacgaaaaatgt
gttgacttaccaaacgtgaaggagtcaaagttccatcaacatcaaataaaactaaggtttttggaaatctgcgttcttctataggaatgacggcca
ttttttttacttcaaagatgtttgttgtgggaaagtttgttttcaagttgacgcagtcaacataccctactgcgtctgaaaatgatatcaagttttgagt
caaggtacaatactttaaacagaataaaacagaattttatttaagtttctgattaatattatttgtaaacaaattggaattcggtactccagattctc
ttcattcgttgttttcatgttttatgtccaacacaaaattaaagaaaaccagcgtttaaagtagctaaagtagagtaataatttatgtttgcccgtag
caatatgattatatggctttactctcgctaacagttaaggtataatcatttgtttagtataaatatatttttgaagaataatgcctatacataatatac
gcatatgctcttatatcaaatcatattgttaccagtgctaacagaacatttggatacgcaacatattgtttacgaaatgaaataatagaaactgagt
tatgaacactatgaaggttatattcatttattgaaatagttttaggaaataaaatagaattaaaagcgaatagagtagtagtattgaagttttctac
acagagaagtaaaaaaaataaaaaataaaaaaaagcaatcgatatcggatacttctaataggcgcatatcctcatatcagtaaagatagaatc
aaaatattataagagcatatgttatgctacaattacgtccacttatattactcaaaaatgttataatatgataattcatttctcttaaataaaagcat
acttagcagttcttaattaggcaattgaaattcagctgcaagtttctcactcaatttggatacaagtgtcagctttaatagtgaatgatggtcattat
cgtttactgaaagtatcattcaaacagcatacatttccttgttctaaaactacaattggttcacacaaaataattaattcttattgaaagcttagtat
aacgcggtttagtaataacagtttcatcgtttgaagatattgtaatcattgagaacgaaagatttgatgagccaatttctttttgatgcttctttcttt
ttgtttgatcttttgcccgatacatcttggcagcatggaaagagaatgcagcttgcaggtttagcatccacaataaacccaggaagttggctttttc
ctcttcagagtagaacagggataatttccttgctgactcctcctcgtttatatttactgtaggttctttctcgtttataaacaatacatattcatcatca
accaattcttttggtatttcatttaaatgtatcatgcttgttttatcatccttttgacttgcattttcaattgtcgttttgtttatatattgaagaatatttgt
ttcatcaagggtttccatactttgtacaccagcccattgaacgttttcaggaatcaaagaacgctctgctttatttgttatttgaggagaattttcctt
gtcctcttccgagcgtagcttatcgcgggaggtacgtagttcaaagctagacatgtagacaattgtttggttattcgtaagtttgttttgatcgtcga
actgacctttatttgatctcgagctcgttaagcctcgttgaatctctttgttttgtgcatccaaagtaaacatatgaatctggttctgaagtataagta
tttcgttttcgtaaaaatgtaatttttcatttgaaaggctctgtatggaatcaaggtagtagttgtatttttgcatctgcacttcgcgaagcttctgtag
cttttgattttccctttccaaaagtgaaacgtgtttttgtcgaaccgagagtgttttttcagtatttttcagggtcactgtatcttgtctttcttgaagttt
cttcagtaagtcgacatttgagttttgcaaggtctcgagttggacagtttgatcatgaaactttatatttagcgcttttatttcttgcagacgaagaga
agaagacgtgttccattgcctttctttctcatttaattcgtgttcatatttctgacatttatcagtaagagactcggccttcacagtttgcctagcatgt
tcaaacgttaaatccttgaatcttttgacgctttctttaagtttctcttgaagagcatcaaattttgcacatttttctttcatgctgtcattctcctgagtt
acagcatttatttcggaatttaatttctcgctctctttttttggcctgttcaattccagtttccaattctttcattaaaccctttagttttaaattttcaccaa
ttatagcctccttttcgtcctttaatgttgcgcattcccgctgagatttatccaaagcgctagcaatttcctcgtacttggtttctaggtttatgtgttgtt
cgggtaattcatattcacggatttttttcattttcttcttcaatcttctttaataatttcctctgtctttcgctctcttcattttcttcgctttcaatgacagtg
tgtgcttcttcaggaatcggctccaactgtttacggcttttcaacttctttttttaaatccgtgttttctgctgcaaaatcttctatctcttctttgtaattct
gacaagttatctctaactcctccaagtcgttttccaaagccataatcttttgggccaatagacctttttcgacgttgagaattcggttctcttcctcga
ctttttgacaatcatttcttattatatttattgttttagagagattctcgatgctagattttgttagtacgtcttcttcagagtgtttctccagttgatact
gcaattgttcggcttcaaaatttgctcgatcgacacttgctataagtgctttcctttccttatataaatgtctattctcttcttctttggcttccaaatcat
cctttaaaatattcgcattgtgctgatgtctatagttatcttcggtgacagaattcaaatgccgtttgagtctgtcacattgagaagataggactgat
ttttctttatcctgttgccttattttaagttgatgcatcttcttttctttcgttaattgaaatacgtccatttccaattccttttgtttatctgttataggcttt
atgctctgttcttgttgaaaaatctgtactcttagacaatgttccatgtcttttaggctttcattttcctgcttaagagctttgttctttgaatctgatacc
tgaaacgcgtattttgagcaaattagtttgcattccacgttaaacatggcctgattcttttcttggatgaactgaaggacattcttgatgaggttttca
attttcaaaacggtttgtacatcggttgtctcaatactcatcaagctaatggcttcgtcactagtaataagttgggtaatgtcctttacacgagtactt
tcgtattcttctgtcttcttttctattagactttgacattgtttatcttttatgactttaattgtggaagtcattatgaattttgctgtattaaccggtgcat
tgacttcctcttaggaatctggttactttaacttgacgacgactatcgatagagtgtttctttttttcgaacaagtatttgataagtttggcttttgttaa
atggaatgtttacgctctgttaacatttcagatggaaggataagctcatgtaaacataaatgtgtaaacaataacaaaccttgagtactgtttctcc

cttccctgttcacattatatatcacgtggattgacttttaaaaagagaaacttcgtttcaattgtaatacgcttaccgaccgacttgtatatatgatcc
ataaaattctcttacaatggtacaccaagaagattaacttggcagcaaagactgctatcaaaatttatctataaaaaattatctctttcgaaaata
aagcattcaattctataggaacatgtcacacacactttgtttcaatatgattgtactgcaatccaaattgtgaacaaatagatatatacatacaaa
atagcatttaacgaattctagtgaggttgaagctatattgaataataaatctttcaacacctaaccataaccaaatacagaatggcaggaaaaag
tattgtttcaggtataaagaatgagttttttttgggaattgagcgcgtctaacaaacagggaaaccttggaaagcggagaaatcggcatatcgtag
gtcaggattatctggtacacaaaaatcttcatatgagaaacgaatggaagagaaaagaaagatagacgaaataaaagagcgcgaaaggaaat
taaaagaagaaaaagacgaggagagatctgtaagtaaaatgatctataaaattttcgacttatattaactaagtttttaggcacatgctcagagg
attcgtgcacgccgcgaagctaaagctgaaaaagaacgaatggagcttttgcaagctaagcttcatcaaaaggtcattgatcgtcggagacgca
gagaaaagcgcaacaaaacgcttaaagagcggtgattttaattttgtcttttatatatataatattttaataaaaggtttttggtgggtttttaagcc
ttttttatgcgtttcttttaaggaattaatatttttgttcttgtttttgtgtaatttcaactaaaggagtagagtccggtaaagccgctggtacaccgcg
gatattgatttcatctacccattttttttatcaaagcttatagtataaatgttgataatgtgagaaatgaatgaatactctgcacaattttttttttttc
ttttattaaataaaaaggggccgaaattttgtgaataatgaatcaggaacttatagaacgtttgtttgagcatgtaaagctcagcttagatttttcct
actgtccatattcaaagtttgcagtaggagcttgtgtattatgtgaagatggaaaaactttcgtgtacggtgcaaacggtaagtaatctcctcaaa
agaatttaacttacgttatagttgagaacgcatcttatggcgctggcgtatgtgctgagcgagttgctataacgaaagcagtggtaagtataacta
gacaaaaatttcaattgacaaaaaatatatatagagtatgggaaggaaacgttttatggcgttaggagttatgagcgacaaaggaagagttact
ccatgcggtatttgtcgtcaagtgtaagtaaattcaagcaattttacaaatgtatttacagctaacagaattcgcgaattttccaaggatattgatat
ctttctgtttaatcctgatggtacttttcagacgaaatcaattgcagaggttcgtgatacgagttcagttgagatgaaactaacttctgttcaagctg
ctcccagattcctttggacccgaagatttggaataaagtttagcaaaagccgatttattacgtctgcaaacagattcaactttgaacctctttcacg
agcatgcatgttgtcgtagtgtcatcgacttttctatataacgtgttaggaatgcttttacaaaatgaattatccttacccgccacgatactgaaatc
cataactccttgctgctttagaaaacggtgaatcccatttggtatttttggaatttgtgacagcagtcttgcaaatggaatcagccaaaattcttgta
gagttttcatttttagtattatttgataaaaccttctcagcaacttgccgaagtgactctgaatctagattgtcgaacactccgtctgtagctagtata
atcaagtctccattctgtagttgatgttgagttaagtcactcatattgggagtaatttcatccctcgatgagtaagaagacgggtaaatagcaagtt
gatagggcatattaaattgaagaacttgaggagttgattcatacaatattgatccgtcacgaaggattaagaaaccagaatcacccaaactatta
aaccccggttagtaaatgaatcaaaaatcactaaacttactttagagaatgaagtttacctgattgcttgtcaaaaagtgtggcgcatgctgtcga
ggagccagctcttatactcttgcttttttttaaagaatcaaaggcgttagtcataacagaaagggggtcaggaaacttattgccaaactttttgaat
tccttctcaagttctcggattaatccccaagagaactttgatgggtctataccaatatttgtccaaccaccgaccccatctaatacaacgttgaaag
catattttgagttttctagattactaaaggcatcttctcctgcatctggatgatcaagatttccggaacttacgaacgaggacggcttttttttggaaa
ctagttgctgagctaatcgtatatataaaattcttctttataaataataccgacttcaaacgattaccatgaaaaattggttgtattgacctcgccaa
aatcatagtgatttttgtgtattatatacctttgttgcttcgtatgtagctttgacggtgaattacacacaaacaacttgtatatatatataaaaaaaa
agcgatctttattttgcaagtacgtaaaaaagttttggtttgcgagaaatattttaagaaaatatttatcacactttgaaggctgcggtaaattttgg
gacaagggccacctctttgttttctttttttgtttacttgtgggtaccatacaccaaggcaattaaaactgtcgtcaaaacattctcctttttttcttctgtt
tattaaatgtatacgagactttcattccaagattagctatagttatataaatttgacccggatgaaagaaaagcatctcactgtaaaaagttaattc
aatttcatttatcaaaaaataacgaaggagtttttgcactaaccatagagtgctttagctgatcagaattgacctaaattttcgttgatttttttgtatc
tcacatgttataagagatttttgtgtgtactcattaaaaaacatgaaattaaatagctacataaacaaatttcatatacaattagtaaattttggtct
aaaatagaaatggatttttgttttataaacgacgatttattcatctcattctaccaccaaaagctgatgtgtacaaagatatatataatcttttaccg
atacctaagggagatcggttgcagacaaggttcatcgaagtatttcagccaatagatccttgactttagttatctacagccaattatttaccgagaa
atagaaatgacggatattataaagaaaagtatttgctctcgaaagaacttggttacctttacgtcctataccaaatttaacgagatttagttcacat
taacaccaatgatggcggcattcactcttccaccatctccccctctaaagaatgcatcgatttcaaacgataaaaacggcgataaaaaaaaggtgg
aaatacaagattattttcaacaaatgtttgtttaagaaagatccagtctactcaggtatgtcagtataaacttttggctcttctgatgtatcaacaac
tcatccggtactgtccgttcctggtttaagccctatttaaagatatatgttgaagtcttctatttcaaaatgcaggtttaactaacattaacagcctct
acgtgctaaacaatcctctacccccgaacaagtcaagtatccgattgtcgaccacacttatgatgcaatcgttgtaggtgcaggaggtgcgggtct
tcgtgctacttttggattagctgaagcaggattcagtacagcttgtataaccaaattgtttcctaccagatcccacaccgtcgcagctcaaggtggt
attaatgccgctttaggtaatatgaccaaggatgattggcgttggcatttttacgacactgttaaaggctcagattggcttggtgatcaggatgcta
ttcattacatgactaaagaagcaccagaagctgttttggagttagaacattttggtgttcctttttctagaactaaagacggaaaaatctatcaacg
tgctttcggtggtcaatctttggaatacggaaaaggtggtcaagcttatcgttgcgctgctgttgctgaccgtaccgggcactcacttcttcacactc
tttacggacaatctcttcgtcataacaccaacttctttattgagtactttgccatggatttactcatggaaggtgatgaatgccgtggtgtcattgcta
tgaaccttgaagatggtagcattcaccgttttagagctcacaaaaccgttctagctactggtggttatggtcgttcctatttctcttgtacctctgccc
acacctgcactggtgatggcaatgctatggtttctcgtgccggacttcctttacaagatcttgaattcgtccaattccatcccacaggtatttacggt

gctggatgcttgatcactgaaggtagccgtggtgaaggtggtttttttgcttaacagcaaaggtgaacgattcatggaacgatatgctcctaccgct
aaagaccttgcttctcgtgatgttgtttgtcgttcaatgactcttgaaattcgtgaaggtcgcggtgttggccctgaaaaggaccatatctacttaca
actttcacatctcccagctgagatcttgaaggaacgtcttcctggtatttccgagactgccgctatttttgctggtgttgatgttactaaagagcctat
tcctgtcttaccaaccgttcactataacatgggaggaattcccacccgctatactggtgaggtccttacaattgacgagaatggaaatgataaagt
tgtacctggtttgtacgctgctggtgaagctgcttgcgtttcagtacatggtggaaaccgattaggcgcaaactctttgcttgacattgttgtttttgg
aagagcttgtgctttacacatcaaggatactttggagccaaatactccacacaagcccctagctgccgatgctggccttgaatccttggatttcctt
gataaggtccgtacttcacaaggtcccatacatactagtgaaattcgcctcaatatgcaaaaatcaatgcagagagacgtttctgtcttccgtact
gaacaaactcttgaagaaggtgttagaaacatcgaaaaggttgacaatacttatagtgaactgggtattaaagatcgtggtttaatttggaatact
gatttaatcgaagcattggaattgcgtaaccttttgacatgtgctgttcaaactgcccatgcagctttgaatcgtaaagaatctcgtggtgcccatg
ctcgtgaagattatcccgaaagagatgatggaaaatggattaagcatacattaacctggcaaaaaaatacgactgaccctgttaccttgaaatac
cgtggtgtcgtcaatactacaatggatgaaaacgaagttaaagctattcctccatttaagcgtgtctattaagcggctttttttccaggagaatcaat
tggatttatctagttattgatagtataattttaagtgttcacctgctatgaaatcgaaaatgtgatttatgattcttatttgatttgctggcgataatct
gtatatcttcattagggcattgagtagcaagtatatatatgtatctaaaagaataagaaacataaatgattttgtaaacaattcaccctttttacctta
ttttttatagtatcacgtagtgaaaactcgatgtaattcgttaaatacgaatgttatacacacattcagaaatacgcgttattttgttctgttaataaa
gttcgaattgtaagtaatccaccattgcttttgaggataatcactttttttatttatgtttgcatttctacgacaataaaacatataaattcacaagatg
tcttgcattgacaagatgtccattatgggcattagatctttcgatagtcgatctagagaaagtattcagttttttttctcctttgacattgatcgttggtc
aaaatgggtcagggaaaacagtaagtgattacctttatttcgtgatttctaattttaagacaatcatagaatgtttgaaatatgctacaacaggatt
gcttcctccaaacacgaaaggtggagccttcattcatgatccgaaggtaagttcatatacacggaacattattgcttataactatagctgtgcggtg
aaaaagaggtactcgcacaagtgaaactcgcgtttaaaaacacgaatcaaataaaaatgatatgtacgcgatcattacaattgttagtaaaaaa
ggccacaaggcagcagaaaacccttgatgggcagctattgattgtcaatgacggtgagcgcagtactatcagtaatcgatgtgctgagttagatt
tacaagtacctctaagtctcggagtcagtagagcactattagattatgttatattctgtcaccaggaagaaagcttctggcctctgagtgaaccctc
taacttaaaaaagcggtttgatgagatttttgaatctatgaaatatgcgaaagcccttgaacagctaaagaatgtaagacgtgagcaagaaaatc
agattaaagttgatcaagccaacttggtgcatttcaggtcggataaagaacgggctgaaaagattaggatgcaacttcacaacacgcaaaaacg
catctcatttataaaccataggattcaagatttggatcaaatgatatccgagacagcaaaattacaagatgagttgttcagatccactgaagaata
tgaacaaaaagaagtcgccatatctcagttggaaaatcactgtgcttttctgaaatcaagtgtagaagacttaaagatccatatggctgaaatga
atgaccctaccgaagaattggaagagtcatctaggaattttaatggacaagtagaaaccgaacagaagcttcttttggaacttgaaaataacaa
ggcttcaaccgaaattcagataacagaaaacaggagaaggttggaaaccctctccgcaagtttgggacgtgctgaaggagagctagtttcgctg
gaacgaacgaagcgtagaaagcaggaaactttagagaatatcgcttcctcacatgagttctcggaacaagatgaagacaaaatcataacaaaa
taccttgaatatctggactcaaatatatccgaaagtgatagaactatacagtatacacgagataaaagaaaagaaagaggtgctcgaattgagg
gccttaaagctcatgaattgtatatgcagaaaaccattacaggtttggaaagttctataaggaataatgaaagcaatatggaaagtattcagtca
cgtgttcaagaccttgaacatatggctcaaacgagggatgctttattagaagagataaaacaaggggaaagagaattccaagatgtgagaaag
gagtacgaaagaaatgaatggcatataaagatcaaggatgaaaaaacgaaaatacatgacaacgaaaagaaacttgaaaactttgcttcatgt
attgataagtttcatgacaatgccaacgtccactcaaaattacagctgttagttgagacgaaaggaaaattaatggctagtgcagaagaagccca
tgaatttttcaagcaccggttctttgaattaacctctcttgatgttcatgccgtcagtgaatttgatgaaaaactaaattcttctttacaaaactcaca
ggtatctgtagatgctataaagaagaaacagaattccgcgaatgctactgttgttgaacttgaatataaagttaatacttgtcgaaaagaattatc
agatctggagtctcgaaataatgaaaaggctcagttaactcaatacttttcgatcaacttggatgaacaccatgaaaatattcaaaagttagactt
agacatcgaggatagcaaacgtacagctcattctctacaatttggtataagcttctacgaaaaggcaattcagatgtctaattcacaccatgcttg
tcaactgtgtcaaagaatgttcgagcatgatgaagaagctgcctttgttgaacattgccgctcaatgattgaaaatattccggggaaattgtcaga
ggtaaccgagcaattgaatagcttgaataagaaaagaaaagagcttggtgatataattcccctcattgaggaagcaaggtcaataaaaaattct
ataaaagagaatttggatactttaactacaacagaacaacagcttggaaatgcaaaagagcaagctgtcgctttagatcttgaagtaagaaata
ttgacgcaaagcatgaggaattggaaaatctaaaaatgaagttgtccgatattaagcgtctaaatgaagaaattgaaaagtgcaataaacacat
ttcagaactgcaggctgagctatcaccctcctacaaagaattagatattaaccaaattactcttgagcgaaaaaaggtggctgagatgaacaaa
accgcaaacacgaatttagaaaaaattacttatgaagaggccaagcaaaaaagggagcttgacaaaataaatctcgctaataaagacaggga
ggtcaggttttcagatctgaaactcaaaatcaatgagttacatcaactagaagttgaaattaaaaactgttgtgaaaaaaagataaagatggaa
agggatctcgaagaaagtaaacagtcctgcgaggaacagcgtccattattcaacaaactcaatgaagaaatgcgtagtttggagaacgagcttg
aaagaaatgaagaaaaaagccgatcactgctgcaggataagaacatttttcttcaacaagttcaagattgggctagagagttgcaaattgatat
aaagtctgaactagaacgtttagtaaaagagaaggacagtcttcatcgaacaattgaatatgatttatccaaattggatactttcacccaaaaga
taaaaaatattcaggaaaaattggctgatttgcgttctagagaacggaacatttctgacaatttacgccttcgtcgattgcatagtcagtttgtgga

agctttagaaagaaaaaataatctgaagtctcaacaaacacagagtaataggcaggctttccgtaatgaaatggagtctttgaaaatgcaatat
ggagcgttaaacgctgagcgtgctggtttactaggcgagtgtcgacaacttgattctagtgttaagcaattaactcaagatttaaatctcgaatata
aagattcagatgaacgttatcgccgtcaattaattaaaacgaggacgactgataaagccaatgatgatattgggaaatatgcgaaagctctagat
attgccatcatgcaactccattcattaaagatgaatgaaataaacagaatcatagatgagttatggaaacaaacttattgtggtacagatattgat
acaatcttaattcgctcagacgctgaaggaaagggaaaccgctcttataattatagggtaagtattttaaaattcggtttttttatgctaacgtata
ggtttgtatggtaaagggggatgccgagttagatatgaggggacgatgcagtgctggtcaaaaagttctagcctgtattataattcgacttgcttta
gcagaatgtctaggagttaattgtggaattctagctctggatgagcctacaacgaatttagacgaggaaaacattaccagtttagcgaaaagtct
gtcgaaaattgtcgattttagaaggagacaagcaaactttcaactaattattattactcacgacgagcaatttatccgtttggttaatacagacgcg
tattgtggttattattataaagttaaaagagacgctaatcaaaagagcattgtaagtatctgaattatttgatagaaaacaagaactaaccgaata
tcagattgtacgagaaacgatgtaagcattcgagctgctttagattaattggagtactggagttatgatatatcagaagtgcacgattttatcaga
gaaaagtagataacaaataaaaatcatctgaaataggatcttataaaagttattattgttattcgtctaattttaattttcggtccaaactatttgca
aacagtatgtttattttgggctatagcgatagacaaaagcaatctcaagagcttttaataccatggggaacgtaagtatctctcaattctttgattct
tagaaaattaatcctataagaaaggtaagaattatccacggtctttgtcgtccacttgcacaatactatacagcgcaaaggagctgcaaagaaaa
tagttaacaaacagaatgctagggtaattgtcaaacattcaacttttatctattgcataagtcagccctgatgtctttaaaccaagtatttaataact
tacagcaatagggagtgattcacatgcatcggaagtgctaataaacctatgcgcagatttggctgtgtgccctaagagagaagcaagaagtaaa
aagacggtaatgaattttgttctcgacgacatgctttcatttaggataataggcaaataatattaaataaaaacgcaaattgaaagtattctgttgt
tgaaatcaaaaggtttaccaataaattaatacctgaaaagtaagagggaggttgtgctaattcttcagatacgtctaagtctagtacaagcagaa
cgaatacggagatactcctcattatttgtgttattcttaaattaattcagaagttattatttttttgttattattttttttcttttttttttaatactggaatag
acttttccatcagttgtaatgttgattaaaagggccaaaaattaaaagtgctgtaaatgttagacatgggtgtttaattgtgcatttctttcggtgttt
ttcatgccttagtatcttcaactacctgtatattttttttattcaacatttaaatgatttatactatggatacagcgacttaaaactaactagatagatat
gttttacagtttcttatattcatacaattaaatccgttgtggtccaacggctaggattcgtcgctttcaccgacgcggccggggttcgactccccgca
acggaaatgtttttgattatagttttgagccatcaatctgtttcaacccatgaggagggtaacatacatgggattcctggacattataagctaccatt
ccgcgtctctaatgctttcgctttaaacaagtcgtttactactaagcttcttttcataggtgcattacgaaagttatactacgaggaactctgtagatt
catttaattcattagatctttgcttattataagctattataggcaattagaagctattagaggttctattataacgctttaataatttcccattacaaa
atgtttcgtcataaattcgtacgcatcataaatttccttcctagattcagttgatggtaattttcttcttcgaatgagattgttccatcttgggcaagat
gatgcttaaaactccattggtgaattgggcttcaacgcggtcagaatcaatgttggtcgggatgctgattgttcgagagaaggaaccaaagcgac
gttcggaccatcgttggtttcctttatcgccttcgttgttgcgttcattcaacgattcaccagaaacggtaagttttccttcgtcgtaatggacttgaac
atcttctttttttgacaccaggaagctctacatcaacggcaacagtatctttgccttcgtgaacatcaatggcaggtgataaatcgaaattgcgatttt
gacgttgaaggcgggggaataactcaaaaagtcagaaaacgcgtcttcgaatggatgcaattcaaagaagggttgaagagacatttttataa
ataaccttaagaaatcggattcgcttgcaagggtttaaataataagcaataagcaataagcaataagaaaaaagcaataagaaaaaagaaaa
aagaaaaaaagcctctcaattctttaacttttgttgtcgttgtccaaattacatttgctgctctcatatttatacttgagcaggacggattttgaacctt
ccacaagaatcagctagtactttnnnnnnnnnnnnnnnnnnnnnnnnnnnnnnnnnnnnnnnnnnnnnnnnnnnnnnnnnnnnnnnnn
nnnnnnnnnnnnnnnnnnnnnnnnnnnnnnnnnnnnnnttgtttattatgtgtgaataaacaaataaaaaatactactaacttta
aaaaaaacgaaagcttctcattaacatacttgtaataaacgaaatataaacgaaaagaatgaaacgataaaccaacatttttttgcaactgtaga
acatattgttacatgtatttgtcaatttcatttcctacttcgtagcatcatctatgaacgcacaatctatttacatgacaacaagaagcagcggcgtt
atcgataaaaattcaaggtttatattgttattgcgcttgccgtgaaattttacatgtcattcaagtatgagaagtatatgaattttacaaaatttcga
ataatgagtgatatgtatttatgtggtatctcataaaaatcttatattctcatatctcttatttcaagtaccttgtgtaattaatattatgtatgtatatt
ttataacataattacagttgtctacggccatacctaggcgaaaacaccagttcccgtccgatcactgaagttaagcgtctgagggcctcgttagta
ctatggttggagacaacatgggaatccggggtgctgtaggctactctttttgttgttctctttgtctttgttccggttttcttttgctttttttatatcgttt
cataaattgaattgggatgtcttcagaatcctggaatttcctcatattttccaacgatggaaatgacaacactgtattggagggttacaataatgtt
caatttcttatttattgctctcttttctttaatgcttatagaagtgaactaccaaagattgattgtttgatttacgagaagagagtagagcgaaattgt
gaagttgatttctttcgttcaagcttttcttttcttccataatgcgcgtgttttagaggataaacaaagctttctaggtcctttgagtatcagtacaaaa
aggaagatctactctttcttacactatttaagagcgtatatctatctatatacccatttagacactattcctagcttattaattcactcggaaaattag
aaaagtaagtagaacagggccaatatggtgattttcggttctattatcaatacatcaactaactcgtacttggacccatccaaagttgaggagtcg
agcggatttctgactttaacccatttgcgggaagaaacaagtacgaatggataacggatttattgcctcttctgaatccagggttccctagtattgg
aagcatttgtcaatatgacgaataaaacagagttaaaggaaccttatatttattataagcgagcacccttattgaaaccttacagactttcatggtc
tgaaaaatacaccttgctcattggtttagcttactattgaaaatttgagctacatcttttacctctatttatttttgattcctaatgcttctttttattata
tatacatacaagcttaattgcaatagttatcaaaactgaaaataaaggaaatcgaatctcacagtaaatgatgttaacttactattaatttcgata

aataaatataagtatcttttatatatatatatagttttagtggacttttgaattatttaagtcaaccgtttcaagttgtataagcatattcagaaacag
gcaatgtttatttttaaaataagtagcaaaccctaccaaacaatgaaacagaaaacagaaaacgccatgaaaaaaataagtgtatactaatgat
atatatagaaagaataactgcaaaatcaaaccaccattttgctatttaactcacatctgtcagcaccttttgactctactcatattcttaggaaatac
tatgataaattcgtcttatagctgcatacattgaccttgagatgccctatatacttcggtcttgacgatagtatacatcattaatacctttaagagat
aaacggtacatgaaatctttccggctgactgatattacaaacgagtacttttctaagattttcaatgttatcataatactttataattttttcgttttc
gcagaaaatgaaagagaaagaaaaaatatttccgttgcggggagtcgaaccccggccgcgtcggtgaaagcgacgaatcctagccgttggacc
acaacggatagctgttagacagcacatttagagctatttatagctaaaattaatactacttgtggctatcatagtccacgcaaattaaaatcagttt
atgtaaaaaatagaatttcgcaattaagcataaaatcatcaataaaaacccattttctaggattttgtgactcaaaagtaagaaaccccataatat
catatactcgaaaggaaaaaataagaaaaaatcaagttaatagttaacttcaggatcgaaattgtagagaaaaagggaaaaaacctcgtttctt
tttcgattttattgttttaaagggtaattcaacattttatccataaacatggattcagatattaaccaagtcatagcagtagttatagcctctggcgg
attagtttttttttacttttttttttacacgtttcacacgttagctattgaaatatcgccagatcttacaagatatctgtgtatcaatctgcattgacgct
tatcatttatttttaaattctgtagagactctataatatatctttctttttccctcttctgttttccgccttcgtaattattcgttttaactatcttgactccg
gatagtcttcaaaatgatgctgggatgtactaagacatcgttctcattagggttcttctaaccgcaaatagaaagagtccatcgaaaattacgcaa
tttactttactacatgatagataagacgacaaataaaactttacaaggatggtcgcatgatgtagttggttatcatgtcgatctaataagtcgaatg
tcgccagttcgagcctggctgtgaccatgattttttttcacttttgttttcttttttttccctcatcaaaaatttgacgttttcttacgtttattaatagtttt
attttggtttttctttcttttctttaactatatttatttttattcattggggatctcatccatactgcagcttttacgtagattcctatttcctgttataatg
gtgtttttttttttttatactttctaatttatgctattctaatctattctattctttttcgttttctttaattattagtcaaataaaaaaaaatatgaaaaaa
aaaagtgaaaaaaagaaaatgaaagagtgaagaggaaaagaaaaaaattggacaagccagaactcgaatctgggacctcttccatgctaag
gaagcgtgataaccatctacaccacatgccctttctgatgtcttctttcccaaannnnnnnnnnnnnnnnnnnnnnnnnnnnnnnnnnn
nnnnnnnnnnnnnnnnnnnnnnnnnnnnnnnnnnnnnnnnnnnnnnnnnnnnnnnnnnnnnnnnnnnnnnnnnnnnnnn
nnnnnnnnnnnnnnnnnnnnnnnnnnnnnnnnnnnnnnnnnnnnnnnnnnnnnnnnnnnnnnnnnnnnnnnnnnnnnnn
nnnnnnnnnnnnnnnnnnnnnnnnnnnnnnnnnnnnnnnnnnnnnnnnnnnnnnnnnnnnnnnnnnnnnnnnnnnnnnn
nnnnnnnnnnnnnnnnnnnnnnnnnnnnnnnnnnnnnnnnnnnnnnnnnnnnnnnnnnnnnnnnnnnnnnnnnnnnnnn
nnnnnnnnnnnnnnnnnnnnnnnnnnnnnnnnnnnnnnnnnnnnnnnnnnnnnnnnnnnnnatactttctaatttatgctattcta
atctattctattctttttcgttttctttaattattagtcaaataaaaaaaaatatgaaaaaaaaaagtgaaaaaaagaaaatgaaagagtgaagagga
aaagaaaaaaattggacaagccagaactcgaatctgggacctcttccatgctaaggaagcgtgataaccatctacaccacatgccctttctgatg
tcttctttcccaaaatgactatagaaataattattatgatgctgtactcagatttcctgaggatactactatatgggtgtgtttgaggccattatgtag
cctgtattagtcgttaacgtagagtactgatgtttcaaattcttattataaagcaaagttcacttttgtgatgaaggtagatctttttttgcttgtctaga
atctaggatctgttatttttctcttaaaattcgttattgtacattctgttacgttctgctctcacctgcatattgctctgacattgacaaaaagtattcaa
tccagatgtgcagaaacaattccgcttctataggttgctatcatataattaagtagctctcactctaacagcgaaggccgtgtggtttagatggttat
aatttctgcttaacatttgatgttcgcagacggtcccaagttcgagtcttggcacggtcatccatacctttttattttttcattttcagttcgttttaccagg
atgctgcctttctctcttttcttttcttttcttttctctttttcctttttcgagcttgcttaacaatgctttctttcgtttgtatcacccaccacctacttgcgta
caaaggtcatacaaccatatgagtttaaaatcaagatcttcaaccacttcgacaacgtaaacatgaaatctaaaacctaaaacccaagatagga
aaaaataacaataataatacaaaannnnnnnnnnnnnnnnnnnnnnnnnnnnnnnnnnnnnnnnnnnnnnnnnnnnnn
nnnnnnnnnnnnnnnnnnnnnnnnnnnnnnnnnnnnnnnnnnnngtgtatctcttgtcaattcgttacataaatacgtactagacagc
atattgcatactgaaacgaaaggtcttaaccttctaagactaattttgcttattttgaagccagtattatattttacaagcttaaaggacaagaaga
atggattgaaaacccagtctatggattgctttcacacaacacagttttgtttcatttcagtatcatctcttatggtggtatatcaaatcttttgctcatc
tttaagaaacgaatagtaagtataatataaattgagtataacgcaactttgacgtagtattgtgtactactacgcagtacagtacatatattacttt
atacagtaaattcaatataataaactatgctttattttgatactcaacctttacaagacgatttattataatgcacaatataatatatgagatttcga
cgtactattagagcacgctgtatgttttgactaaaaattgaaaagtgagatttacagaacttttgttgattcttctaatctgattacctgttgaatgtt
cttttagtaaacgagaattatagaacacggtttctttttttgttttctttttttgttttttttaccacagtttgaggttttttaatgcgtagaattataaaggtt
ttttatttgcatctctagagtagtagtaataattctttgagtcctcaaaagctgagcatttcggctggctttttttactcgtatacgacaatggaaagaa
catcaaataagtgccagctcctataaaaacttaaatctcttataaacttacatttggtttgctcaccttaaattcgacatctttcgttagtatcgtccg
tttctatttttctatatgagagagagaaagagagaacaatgatgaatctcctgttaaatacgatgtatgatcaaatatagttaacattttgaggata
tactcttattataaagtggtgatttttcgaacatttggctaaacagttataccattttaactttggcatcaggctttcttctttcaaaacaagtcattgc
attcgtgttaaacaatctcaacctccctcttataactataatgtacattttggtaaacagcaataaacgacacatcttttatttatagctatagaagg
ggttgttaggctacaagaccggacaaaataaaggatccatttggagagaagcttttatttagctagaagttttgcttcttagttcatgcaatccaaa
tgactttcatttatgatacatctctcctgaatgcaaaagatcgaatcgttgttcgtatttccattatagggcggaggagggaatgatcgttgcataac

aattctgcttattgaagaggcatgatcaacaacggaaaccaaatgctcttcgtaaggattcttacatgatataactaaatttaagatgctctctgga
aattcaaaagtggattcgttactattaaagcaatcatcatatccgcatcctcaagtcaactgtagtcattcttcacgaatctgaaacagtaaaaccc
acagagttttgcaatttggaatactttctatactttaatacgtaacaaacactttgagccagttggaataacaagagggtagctttcgaagctttttt
catataattagtcactttgatgatgtctactactttttttatagtcgttgtggttgttaaatttggacttgtacaagagtagaacagtatattttatgaaa
gacttgaaagactagctacttgtcaatttttcagctcgatttttcagaaaaaatgccggattgagcgtttctcattatatttgggtgaacagtaccag
aagataagagctgctaagctaaggtatcaaaagtctccattgacagttctatttaagaaaaatgtgccacctttcttggaacccaatgtagggtaa
gggacaaatgtaagactaagctgagcaactagagaacccgttaattgctgcaatcattcatcataactttttttaaacgtgaaagaatcaagaatt
atgcgaaattacttttcctttcatggaagtattgtcaataatgaaacgaagtacttaccttttacttttgaatttttttgattcgaatccatatcaaaaa
ccgcttctgtttaccaatgatgctcgtttttcttcaaaacggcattgtgcacgcgtaatatgtgatataacagataagggggttattggtatattacg
tcagctcttatgtcccaaaaagttctcgtataagtctgtgaatcccaaactgtaagttctcaataaacgaacgagacagaaagctgacatagcact
gtcatgaaagtaagggatctctaaaattagtgttccttctcaattaaagaaagggaaaagggatgcatgaaaaagtgcaatctagaacaaacat
aaattcatacaaagtatagcctaaaagctagttacgatattgcctgttgcaaatatctcatatcgtaccaacactccatccatccatccaactttcat
ggggctaccctaaaattataatattagaacagattcacttttgcaattccttaaaccccacgtcagttttctgcaaatcctccctccgttcctgtgaa
gtgccccttaatagcgcttacaatcatgttactttttgttatactgttttatgctgtactaatgagaagtacaccagagtcaactagtcaaaacagag
tgcaaacaaaaaatccatatttgattgaatgatttacgcgcgaacattctccatcttctaatttgttatgtctggctttgttatgaataccttaatgtta
agtatagcacgctgtttttggggctatcgggtggcttaagtgaatacgagaaccgacggtcaatatcggaaacagtcacttaaagcgatggcttgt
atagtttgactgatccgggtagcttgaaacttttgcaaaatgaaaattaatataaagtaccgttcaattagattgattggttaaaggattgagaaa
gtgttgtgcattcgacacgactgaaaatgcatttcaaaagggttagtgtattgctattgctattgcgtcttggcaagaaaaggtaaagataatagt
gcctctttcgacatgtcatattatacaatattataaagaagtttaggtagtaaaatgctaaatgctaaatgctaaatgctagaggcttcgataagtt
gttgctttaagaagannnnnnnnnnnnnnnnnnnnnnnnnnnnnnnnnnnnnnnnnnnnnnnnnnnnnnnnnnnnnnnnnnnnnnn
nnnnnnnnnnnnnnnnnnnnnnnnnnnnnnnnncctgaatgcaaaagatcaaatcgttgttcgtatttccattataggacggaggag
ggaatgatcgttgcataacaattctgcttattaaagaggcatgatcaacaacggaaaccaaatgctcttcgtaaggattcttacatgatataacta
aatttaagatgctctctggaaattcaaaagtggattcgttactgtgaaagcaatcatcatatccgcatcctcaagtcaattgtagtcattcttcacga
atctgaaacagtaaaacccacagagttttgcaatttggaatactttctatactttaatacgtaacaaacactttgagccagttggaataacaagag
ggtagctttcgaagctttttttcatataataagtcactttgatgatgtctactactttttttatagtcgttgtggttgttaaatttggacttgtacaagagta
gaacagtatattttatgaaagacttgaaagactcgctacttgtcaatttttttcagcttgatttttcagaaaatatgccggattaagcgtttctcattat
atttgggtgaacagtaccagaagataagagctgctaagctaaggtatcaaaagtctccattgacagttctatttaagaaaaatgtgccacctttct
tgaaacccaatgtagggtaagggacaaatgtaagactaagctgagcaactggagaacccgttaattgctgcaatcattcatcataacttttctaa
acgtgaaagaatcaagaatcatgcgaaattacttcaataatgaaacgaagtacttaccttttacttttgaatttttttgattcgaatccatatcaaaa
accgcttctgtttaccaatgatgctcgtttttcttcaaaacggcattgtgcacgcgtaatatgtgatataacagataaggggttattggtaaattacg
tcagctcttatgtcccaaaaagttctcgtataagtctgtgaatcccaaactgtaagttctcaataaacgaacgagacagaaagctgacatagcact
gtcatgaaagtaagggatctctaaaattagtgatccttctcaattaaagaaagggaaaagggatgcatgaaaaagtgcaatctagaacaaacat
aaattcatacaaagtctagcctaaaagctagttacgatattgcctgttgcaaatatctcatatcgtaccaacactccatccatccatccaactttcat
ggggctaccctaaaattataatattagaacagattcacttttgcaattccttaatccccacgtcagttttctgcaaatcctccctccgttcctgtgaag
tgcctcttaatagcgcttacaatcatgttactttttgttatactgttttatgctgtactaatgagaagtacaccagagtcaactagtcaaaacagagt
gcaaacaaaaaatccatatttgattggatgatttacgcgcgaacattctccatcttctaatttgttatgtctggctttgttatgnnnnnnnnnnnnn
nnnnnnnnnnnnnnnnnnnnnnnnnnnnnnnnnnnnnnnnnnnnnnnnnnnnnnnnnnnnnnnnnnnnnnnnnnnnnnn
nnnnnnnnnntaaaattagtgatccttctcaattaaagaaagggaaaagggatgcatgaaaaagtgcaatctagaacaaacataaattcata
caaagtctagcctaaaagctagttacgatattgcctgttgcaaatatctcatatcgtaccaacactccatccatccatccaactttcatggggctac
cctaaaattataatattagaacagattcacttttgcaattccttaatccccacgtcagttttctgcaaatcctccctccgttcctgtgaagtgcctctta
atagcgcttacaatcatgttactttttgttatactgttttatgctgtactaatgagaagtacaccagagtcaactagtcaaaacagagtgcaaacaa
aaaatccatatttgattggatgatttacgcgcgaacattctccatcttctaatttgttatgtctggctttgttatgaataccttaatgttaagtatagca
cgctgtttttggggctatcgggtggcttaagtgaatacgagaaccgacggtcaatatcggaaacagtcacttaaagcgatggcttgtatagtttga
ctgatccgggtagcttgaaacttttgcaaaatgaaaattaatataaagtaccgttcaattagattgattggttaaaggattgagaaagtgttgtgc
attcgacacgactgaaaatgcatttcaaaagggttagtgtattgctattgctattgcgtcttggcaagaaaaggtaaagataatagtgcctctttcg
acatgtcatattatacaatattataaagaagtttaggtagtaaaatgctaaatgctaaatgctagaggcttcgataagttgttgctttaagaagaa
cggatcactaatttttctcggcttatcgaattggaaagctgcatgcaggttatgatttgccagatcatttataatacgatcgaatactatacttgaaa
caatagatgcttattatattccactgatccagcagtggatacgtccactttttttttctgtttggatgccgcgtgtttgaaaatgaaggagggatacgg

tgacattaagcaacaaaagggcaaaacaaacattgctctaggaaagtagttgattcatggtctatgattcatatgtttatgtagttacctcaagca
aaaatgcttaccttttgcaaggtgtaaacaccttcgccgaaagtaagacttgactgactgcttgtaaaaaaagagtataaagattttaatgcttgg
gtacagtaatttgaatcttatccttcgtttggacaacgactcatagctttagtgcatgcttttggtactaacgaaaagccttggtttgtgtttattggtt
ttcgtttatgagtttaaaacggaactaatgcgacgaatcatgatgcccacagatgacttttagattcaatttgtaaggtgtcaaacaattagcttag
aatatgtcatttttgttttgtgttactgattgcgatgaattgtatcgaattgctgtgaaagaaatccattaaccatcaacttataagaaactttaacc
ttttagttacaattgtatatgtcatttttagcgaagtttataaaaaaatgaccttcgtttgcactacaatctctaatatagtgttagaaacaaccaaa
gttaaagcagacactgtagactaggcaaaacgaccaacaatgcatacctcaatcaaagttcgtgataaggctcttgacccacaaaaaatccaat
aaattaagatagatgtcacatacatttgatcacagtacgatgcgatggacattccaaggggattatccttttcaatagcgaatacgagaacgtac
gtagatgtaatgaatttttcatagaatacttcagaatttcaaccagaggatatgccaatcgtacaagaatatgtttcatgaatcaatatatgatcg
gtttatttgactttttgaaagcaagagaatgtaaaaaatttataaatgacattgagacgatcgttgtctcacagcagactataaacggaaaccctta
agcaaaatatactactactgtaaatgatttgctcatataaaactcctaaggcagcagtaaacatttcatcactcaagtaaaaatatgcccaaatta
ttgggataaaggattgaaaaagaagtatgaaaagagatgtctaagtgccgtatagagctctcgagcatccaaatgtaataatttttacactcgta
aaatagtggatacaaccaattctagaatattgtactatagcgattaaggttaaagttaaattaattccttatggtacgacattacaaatgctttgttt
caaaatttaataatcaaaagacatcatattccttatctgtgtcatagcatataaggtggccataaccgcgtcacttcggaaaaactgttaattactt
tattcttataaatttgctcttccgagttatctattacgtatttagaagtaacttgaacgtaaaaccaaactaagtaaatgaacgctcttgttatgcca
ctcaattcaactttgttttggtttacacgtctataatagaaaaagcatactagactattggaacatcgttttctttccattatcaaattccttttatatta
catttctcaaactttatcaacgatgaagtattgaatgattgcaaccaaagcgagcgaacttacgttggatgtttctatataattgcgattgcttcgat
gtatctatccttcnnnnnnnnnnnnnnnnnnnnnnnnnnnnnnnnnnnnnnnnnnnnnnnnnnnnnnnnnnnnnnnnnnnn
nnnnnnnnnnnnnnnnnnnnnnnnnnnnnnnncatagaatacttcagaatttcaaccagaggatatgccaatcgtacaagaatatgttt
catgaatcaatatatgatcggtttatttgactttttgaaagcaagagaatgtaaaaaatttataaatgacattgagacgatcgttgtctcacagcag
actataaacggaaaccctaagcaaaatatactactactgtaaatgatttgctcatataaaactcctaaggcagcagtaaacatttcatcactcaa
gtaaaaatatgcccaaattattgggataaaggattgaaaaagaagtatgaaaagagatgtctaagtgccatatagagctctcgagcatccaaat
gtaataatttttacactcgtaaaatagtggatacaaccaattctagaatattgtactatagcgattaaggttaaagttaaattaatttcttatggtac
gacattacaaatgctttgtttcaaaatataataatcaaaagacatcatattccttatctgtgtcatagcatataaggtggccataaccgcgtcactt
cggaaaaactgttaattactttattcttataaatttgctcttccgagttatctattacgtatttagaagtaacatgaacgtaaaaccaaactaagtaa
atgaacgctcttgttatgccactcaattcaactttgttttggtttacacgtctataacagaaaaagcatactagactattggaacattgttttctttcc
attatcaaatttcttttatattacatttcccaaactttatcaacgatgaagtattgaatgattgcaaccaaagcgagcgaacttacgttggatgtttct
atataattgcgattgcttcgatgtatctatccttctactatttatctcatttggtaaatcgcttgcgaaaacaattgggatggcgatagattcttatgtt
gataattgtggtaattcaatgacgtttgccctgctgttgtcatattaaaagtattcatagaaaggttggggagaatcatataacaggaaatgtatta
tacaacgattgttttccctgatttgtgaaatggaaaactttggtgaatgattcatgaattttacagagctcaacacacctgtagcatagtcaccata
gaatcgtataaatacgaggcttttgctgcgtataaaagaaaagtactttcacaaagatttcattcggaagaagagcagagcggatcaacttgttg
ataacaaggcacatcttttccattcaaacatgacgtttgcacggaaaccattcaacttccccaaaagaaactaagatatccgtttagtaataaaca
gtcagtacgtatattgtcacgggaaaatactccgtatagtaataagagtgtaagtggaatacagttataatcgctttaagtgtcaagctgaatact
ctaaaataagatactaattatccggagtgacatatctttagtaacgtccctggtgatactaatgaatatgttattgaacaagtttattagctctgcca
gcaaaatcttcagttaggaaggttttgtctgataaggacgggcgtttataaagtaatctaaaactaggaattaaaaacaaacaataaggtttaatt
aatttttgatgattgtttaatctttcagtctgttagtttattaacattgatatacagtaatatactatggtttatattaatattagggattactctgacat
atacacattcggttatttctcagagaatatacattattattactaatataataatacaccaatgttacccgacaatcatgcgaatattatataatagc
aactgacttagtattataatctgaaccggtgaggaataaagatatacagaagagaaggatgaaggttacagttttcaataaatctaaacaatagt
agcttaatgtcgcatggagcggagctccagaagtaaaaaatcatcataggatacttgtcgtggtcgaatcagacaaattgttgccaacttaattgc
cattagtcattcaaatctactttcgaaaatatgatgacaccgaatcaccgattgaacccttaagctgatgatataaaataaaaaacggcccacttc
ttattaacatcgctaaagaaattataagtttactagaccacagtaataggatttaataattcacattcaaagttttacaattagcttttagttctaat
gtaacaatgcatgcgattggactggatacccaggaaaagaaacgacgctcggtaataaaaaatgttaaaattaattaaatccgttgtaaatttat
tttataagcttagattaaaggactagagaaaaacgcaagcagatcttggcaacataattatttctgtatcggcttatttcttccagatacacccgaa
gggatggatggaaagaataatgtcatttggaatttaggatatcaaaattcacttaccttttctagttatcttcatttaagtgaatctttttggttctta
tattatagacggtaacacagaaatactcagcagatgcacttggaggttttctatattatattggacagtttatacatggaacttatgccttttttttttt
tgtccgtttagaaaagaacattcttttacctacgttccagaataaatgagacgacctaaaagtgtatatacgtataatttggaatagataatgggg
caacctttttttattttgactttttttcattttgttgctcgtggtataatgacttaaattccttgatgaggtcaagctttggcattcattttggtaaatttaa
tttttaggaatatgatatggataatgaaaaatttgataaagccgcttttcttccttatattttgcaaagaaaaaaatcataccgactggataggaac

acgagtagttaataattactgaaattaaaggctgttcaagtttgtaatataaaaaggagtatctatatagcttatatacatcattattattcagttta
ttttatttgacaaaaagcttgcctaaaatacgacttcaaatttaatgccttgctggaattgacatatgaccgttgtattttttgtgcatcaaaatcta
tctacctagcaaaagaatgctagacgagagttcttattagatttcatcatttctaatttgcgtaaattacctcagtataaaggcaagttctactatta
ttcctttaaagcacaaatgcaactagtaaatgaaagtcaatatattaagtcttcgttataaacaatcgctacctttgtatttactagggctcttttta
atgtaaatatcgtaagttatttttagcaaggaaaatctgaaaagttgtgaggttattcttcggatttatccacaaattttaggtagccttaagtcagg
attatttggtttagagcgatatgtaattttagacagcaagtatgagcgaaggtagatgatacttcattattttactatcaagtaagcagtgtgctatt
aacggaagcagcaagactttcagtttaattcgtttcgagccaaagatttgtaaaaatgtaaaaatgctaaccaaagcattccgtagccattgagg
tacaaattaacgcagtactactatttagtttagcagaaatgtattaataagggcaaatttttctatacgatatattcagaaatatcctttctgtcag
cttcacttactgtaggtttattcataactagcttactgccatcgtacaatagactacctaatgtttagctcagtcttagataactaaaatgttgataca
aaattcatcagttatgcttccgcaatatatatatcaaatggtaaatttttgatttaaaaaacactactttcatctaaattggaaagtggctgcaacta
acattcacaacaaatagctgttataacggctaagcttagaaataacaatattttctgagcgaattaacaaaagattaaatgcaactaaaggagg
gttttcataactttactatcaaaagcctggatagaaactactaaaatttattatatgcaagaaaacttgatgtatctgatgtagtttgatctaattta
ggcttaatattggatattagattgatagaaattgaagtttgaatgggttcctcttaaataccgtaagctatttactttcttgtcacttgtcctagctact
tcagtcttgagtttaaacgaatatgaaaaaagtatattctacttttgtaatactttgaataagtaattacacatgacgaatagcgtctactatatatt
aaaaggtgaaaagcttaatcacttgagtgtgcttataatcacttacgtggacttccagaatacctagataatataaaaagggtacatttaaacaa
agatatctgtgtattctttaatttgctcagacttaactgatatagtttttactggtttttccatggcagagattaccaaataaattttcatttgattatt
ttttttttaggtacacttgataaatcagaatagcatataaagctataaaatttaggaaacatagtagaagctttcaacacttttttagaataaaaacc
ttaccttaaaaatttccgcaaactttaaaacaaaatttttgacgagggtagtaagagcataagtattctcatcgcttataacgttgacttgtagaca
taggcagaaagttacataacttttagaaatttagtatctattattttagtgccttgcaatacctacagaaatgaaaggaataacctaaggactaaa
tgatagactaatcacaatgtttacattatttgaaagccgaacacatccaggtttatgtaatgaattttttccttgcgataattataaattgcacatga
gctcttggatacttagtatatataaataatatgttagagcaatttgttcatttgtttttttatttttaaactaatcataccaaaacgtaaagaagccaa
aaaaagttaccggcaactctttcaatgtaaagaacgaattactatgctagctaaccaagccacctattattatttaaagctttcagtttttagaaaa
ataacacaacgaaaagacattatcgtgaagaaccaactggatgaagcaaggcacgttcgattattgaattcccaataaaaaataattgataaga
agtttaaatatttggtctacagcataacgctgaaaaaaggtttttttataaccgttggtaaaagcagaggcctctagtaaatgaatatttctgagt
cactttgcttataacaaataataacacataagagtagttaacatgattttgcaacgttagactaatagtgccaaggaaattcttgactattataag
ataaggaaagggttttattattcgcatacatttaggcgaaacgaaagtacgtgctgctgattaaaagaaatctttggaacatattttggtttattaa
attcttgaaagaagccttcgcaacagaaagaagattactgaaatttttaatccagcgagcaatgaatttatcatgtatttgacatatgcaaattata
agtagaagttctaatgatctgtaaggctgataaaagtaattaggatgaatagcactactatgatattatgacaagtaagctcttgtagctcagtgg
tagagcgcttgcctagtaagcaagaggcccagtgttcgagtcactgctggagcattcctttttaaacattatatctttctcatgtcttaagttcagct
cgaagagcagtgctgtttgttcaaagttttcttgtctctctaatccaccgtatactaattttataaacatactgggtttattttattgcttaacagtaac
tgaatttttgatacttactacctttggaatgttagatttttttataatgattttaaaaaatatgaaatgatatttacagaaatggttaacattttccc
atagagctgagctagctaccttgtttttatcaaagtaatcttacagcacgaaatagtgaaagaaccatacaagctatcagtcgattgctacgaaa
tgcgctgtggagtgcatgatttcttttggagttattaagctgtttgtctatgcttcgtaaggaatgtgaaggaatcggcgggtgcatactgttttcaa
ataggtaaagaatctactgaagttgaaatccaaagtaaatacataaatacatgttcattaggctttagcgaatgtttgggtctttttctaggctaag
atatgcacaggagccaaaaatttgtgaaaactcatacaacttgaaggttgctatattggtatttctggaagataaactctttcgctgatcagctcca
attactgatttttttgttacgatgaagtcttcgtttgggtaggcggcatcataaagttcaatcaggtttcattcactgattgaaactgccattgtatatc
tgtactgccaaagctaaaatagaagaatcatcaagtaaatgtctttatctcaaaggaaaaagagctctttcttataaattattnnnnnnnnnnnnnnnnnnnn
nnnnnnnnnnnnnnnnnnnnnnnnnnnnnnnnnnnnnnnnnnnnnnnnnnnnnnnnnnnnnnnnnnnnnnnnnnnnnnnnnnnn
nnnnnnnnnnnggtaattgttttaatttttaagggttgttcgaatgtaacagtgttgtaagtcgttgagaagatgaattgaggtcagtaaaattg
ttgtattaatccatatatttcttcctgaaatgtttagtttgagcgttacgttagtttctgggaaaaaaatcagtgaatacatattgaattcgttaccca
aacaaagttgtttgagcagctccgcctaccttttgagatgtccaactctgcttgctcaaattcatcgaagctgttcctgtttcatttaaccttttcccta
tagtagcaaaggatgaaacaagctagtagataacttttgaccaaattgcctcgtttgtttttctttatcaagtccttcaaagctaataaacctcatga
atgaaaactttatttaaactcctaaattagatccattaattcattgtgctcatcaatactctgcttaaggaccaaattcgttatcctattaccatttttt
ttctcatcaaccggctatttagaaatttaagccattccaacacaaattttaagtaaattgggcatagaatgactacgtcaatgaagaagcaatgca
agataacctgactacctgttgttgtaagcctttctatttgttggccgtgtcttaaacgtagataatgccttccaattatttaaataagcaatggtgaat
atataccaaaaatgcggctagtgtattaaagagttacaacaaactgctctagttggattcggtgagaggatgaacgataaagagccttgttttcac
ggtagtaatggggtaaggatcgttcaaaagtatgtttataaccatgttatgaaattattcccaaatattatttctgttcattaaccatttatagtgcgt
tacgtcgcgtattaggatcaaagttgtcggcaaaagattatacgctttgtgttgcttggtttggacttgaattaaattacaatctcataaaggata

gccaacgaagcttccaagtgcttctgaaattccttgactctaatcattgatatttaaatgtggtaggtttgaccagatcaagaacttcttatcacctc
cttgtaaattttcatacttatactaaagcaattataagcgtaacgtccgtgtatagtgaatgaaacaacctaacccttattcaaagtttctacaaat
acgagggtaccttgctctctggagcaaaagtattacaagcgctggtctctttatttgctatgtcaaagtcttcagcgtcgtttaaaccagcaaacag
ttcataaaaagcttcaccgttgccagttaccaacagccctaaatatactacacaatatgtcaagagagccttggagatgctgtcacttactcactg
cataacaagtttatacattcgaaagccaagaaaactaattttcacaacacataaaagtaagcatgcatactttaatatatactaattggcgaaga
tgtatgagattatgcaggtatcccgcaaaaatgaattgaattaacttctatcatgcttggcctaaaaaggataattaaaggttatgtagggtgcag
tgattttgttttattctccgtttaaaattatttcagattatttgacccagtacgtttgaatgtttttcatgtccactcatgcacattcttgttgtgaatag
tgcgtttcaaatagaagcttaactcatcgaatcattgctgttaggtatcagatattctttcgacaagcaataccgtttctgttaaactaaattcttatg
gcacttgagagtagccagcgagaacgcaaaggaagacaacgaagctatgataagtgaagcgtgttcaatgactactcatataaattgagataa
tttgtttcacaaggtttttctcgcatatacgaagaatgtgcttaaatataaggagttaattagcaacgttacgcatttgccggctttaaggttcaaaa
atgatgactttagtctttttgaaatagttagactctaaattcgctacattggcaatgatttttacgttcctcacgataacaaagttttccagggattta
ttaaccaataaaatgtacgactcttactttatgtaaaaccgtataaaatataaatactttatacaggatatttacgttttacaaaatttcagttaatg
aggttcattagtaaaatccaaatcctgttattcggtttcataaaagctgaaggaagattttatattgctcacaaagatgcagaaacgatagtacat
gctcttaaaatcacacaaaagctagattgaattgtactagaggttatttcatttatcctactattgaatgtaaaatgtgagaaaccttactttacagt
tgttagtactaaaaggctaatactttatttataattgactttttaaaaacacgatcaaggaatgaagtctttaggatattgtatttggccagaatctt
tctcagaaactaaacaaggtcatttaactttattaaaagctggtgatatattgcgtaaaatttgccaaatgcttagctttaagatacagaaaagcta
gtaaagggtttaatgaaggtataattcaatcctaagagactgtttgaatttatttatttttagttcctttttatttccttgaaatggagtataatcctc
aaaaaaggtttttggtagtaaaacctaaaaaataaagtgataaaggaaaaatttgtaatcctccgtttctagcattacttctcattcaggtcctttt
aattaaattctgcgttattaagcttaattaactggtatctcatctactttaggttcattaagcctaattgttacaatgcttaacgtgagtacatcagga
ttaagtttactgaatgccttttgcacggagtatgatgattgcgactattttaccgctattaactctatctatattagtaggtgggttaatcactttgttt
actgatagaaatctaaacacatcgtcctacgctggagaagatggtggtaactcagtgctataccaacatctatcttggttctttggtcgatggccct
taagttcattaattgaacttactcatcgtgccataagctggaaccttatgcagttttgctatactctatacatgacggtaaaaaatttaaattgaattt
gaatgcaactttttactaatggagaaacgcgtctcaaagtagtcggatttaccgtgaggtgcttgctaaataaaccgaaactaattgcagaagct
cacgttgctctattaaaaggcccttttatcttattacttgaagtcgactgctttcagttgtgtctaagaaatactcgatatcgaatactatgttaatat
agattcattctctatctcttcaacgaaccgataaagaagtagttgactaaaaactccatgttagcctagggaccccgacgttcaagaaatactggt
cggatggtatatttatttttgacacgaaaaatttaaaatagcccttttttaatcacggaaccacgaggaaatcaggaataatttataagaaaga
gctctttttcctttgagataaagacatttacttgatgattcttctattttagctttggcagtacagatatacaatggcagtttcaatcagtgaatgaaa
cctgattgaactttatgatgccgcctacccaaacgaagacttcatcgtaacaaaaaaatcagtaattggagctgatcagcgaaagagtttatcttc
cagaaataccaatatagcaaccttcaagttgtatgagttttcacaaatttttggctcctgtgcatatcttagcctagaaaaagacccaaacattcgc
taaagcctaatgaacatgtatttatgtatttactttggatttcaacttcagtagattctttacctatttgaaaacagtatgcacccgccgattccttca
cattccttacgaagcatagacaaacagcttaatannnnnnnnnnnnnnnnnnnnnnnnnnnnnnnnnnnnnnnnnnnnnnnn
nnnnnnnnnnnnnnnnnnnnnnnnnnnnnnnnnnnnnnnnnnnnnnnnnnnnnnnnnnnnnnnnnnnnnnnnnnnnnnnn
nnnnnnnnnnnnnnnnnnnnnnnnnnnnnnnnnnnnnnnnnnnnnnnnnnnnnnnnnnnnnnnnnnnnnnnnnnnnnnnn
nnnnnnnnnnnnnnnnnnnnnnnnnnnnnnnnnnnnnnnnnnnnnnnnnnnnnnnnnnnnnnnnnnnnnnnnnnnnnnnn
nnnnnnnnnnnnnnnnnnnnnnnnnnnnnnnnnnnnnnnnnnnnnnnnnnnnnnnnnnnnnnnnnnnnnnnnnnnnnnnn
nnnnnnnnnnnnnnnnnnnnnnnnnnnnnnnnnnnnnnnnnnnnnnnnnnnnnnnnnnnnnnnnnnnnnnnnnnnnnnnn
nnnnnnnnnnnnnnnnnnnnnnnnnnnnnnnnnnnnnnnnnnnnnnnnnnnnnnnnnnnnnnnnnnnnnnnnnnnnnnnn
nnnnnnnnnnnnnnnnnnnnnnnnnnnnnnnnnnnnnnnnnnnnnnnnnnnnnnnnnnnnnnnnnnnnnnnnnnnnnnnn
nnnnnnnnnnnnnnnnnnnnnnnnnnnnnnnnnnnnnnnnnnnnnnnnnnnnnnnnnnnnnnnnnnnnnnnnnnnnnnnn
nnnnnnnnnnnnnnnnnnnnnnnnnnnnnnnnnnnnnnnnnnnnnnnnnnnnnnnnnnnnnnnnnnnnnnnnnnnnnnnn
nnnnnnnnnnnnnnnnnnnnnnnnnnnnnnnnnnnnnnnnnnnnnnnnnnnnnnnnnnnnnnnnnnnnnnnnnnnnnnnn
nnnnnnnnnnnnnnnnnnnnnnnnnnnnnnnnnnnnnnnnnnnnnnnnnnnnnnnnnnnnnnnnnnnnnnnnnnnnnnnn
nnnnnnnnnnnnnnnnnnnnnnnnnnnnnnnnnnnnnnnnnnnnnnnnnnnnnnnnnnnnnnnnnnnnnnnnnnnnnnnn
nnnnnnnnnnnnnnnnnnnnnnnnnnnnnnnnnnnnnnnnnnnnnnnnnnnnnnnnnnnnnnnnnnnnnnnnnnnnnnnn
nnnnnnnnnnnnnnnnnnnnnnnnnnnnnnnnnnnnnnnnnnnnnnnnnnnnnnnnnnnnnnnnnnnnnnnnnnnnnnnn
nnnnnnnnnnnnnnnnnnnnnnnnnnnnnnnnnnnnnnnnnnnnnnnnnnnnnnnnnnnnnnnnnnnnnnnnnnnnnnnn
nnnnnnnnnnnnnnnnnnnnnnnnnnnnnnnnnnnnnnnnnnnnnnnnnnnnnnnnnnnnnnnnnnnnnnnnnnnnnnnn
nnnnnnnnnnnnnnnnnnnnnnnnnnnnnnnnnnnnnnnnnnnnnnnnnnnnnnnnnnnnnnnnnnnnnnnnnnnnnnnn

nnnnnnnnnnnnnnnnnnnnnnnnnnnnnnnnnnnnnnnnnnnnnnnnnnnnnnnnnnnnnnnnnnnnnnnnnnnnnn
nnnnnnnnnnnnnnnnnnnnnnnnnnnnnnnnnnnnnnnnnnnnnnnnnnnnnnnnnnnnnnnnnnnnnnnnnnnnnn
nnnnnnnnnnnnnnnnnnnnnnnnnnnnnnnnnnnnnnnnnnnnnnnnnnnnnnnnnnnnnnnnnnnnnnnnnnnnnn
nnnnnnnnnnnnnnnnnnnnnnnnnnnnnnnnnnnnnnnnnnnnnnnnnnnnnnnnnnnnnnnnnnnnnnnnnnnnn
nnnnnnnnnnnnnnnnnnnnnnnnnnnnnnnnnnnnnnnnnnnnnnnnnnnnnnnnnnnnnnnnnnnnnnnnnnnnnn
nnnnnnnnnnnnnnnnnnnnnnnnnnnnnnnnnnnnnnnnnnnnnnnnnnnnnnnnnnnnnnnnnnnnnnnnnnnnnn
nnnnnnnnnnnnnnnnnnnnnnnnnnnnnnnnnnnnnnnnnnnnnnnnnnnnnnnnnnnnnnnnnnnnnnnnnnnnn
nnnnnnnnnnnnnnnnnnnnnnnnnnnnnnnnnnnnnnnnnnnnnnnnnnnnnnnnnnnnnnnnnnnnnnnnnnnnnn
nnnnnnnnnnnnnnnnnnnnnnnnnnnnnnnnnnnnnnnnnnnnnnnnnnnnnnnnnnnnnnnnnnnnnnnnnnnnnn
nnnnnnnnnnnnnnnnnnnnnnnnnnnnnnnnnnnnnnnnnnnnnnnnnnnnnnnnnnnnnnnnnnnnnnnnnnnnnn
nnnnnnnnnnnnnnnnnnnnnnnnnnnnnnnnnnnnnnnnnnnnnnnnnnnnnnnnnnnnnnnnnnnnnnnnnnnnnn
nnnnnnnnnnnnnnnnnnnnnnnnnnnnnnnnnnnnnnnnnnnnnnnnnnnnnnnnnnnnnnnnnnnnnnnnnnnnnn
nnnnnnnnnnnnnnnnnnnnnnnnnnnnnnnnnnnnnnnnnnnnnnnnnnnnnnnnnnnnnnnnnnnnnnnnnnnnnn
nnnnnnnnnnnnnnnnnnnnnnnnnnnnnnnnnnnnnnnnnnnnnnnnnnnnnnnnnnnnnnnnnnnnnnnnnnnnnn
nnnnnnnnnnnnnnnnnnnnnnnnnnnnnnnnnnnnnnnnnnnnnnnnnnnnnnnnnnnnnnnnnnnnnnnnnnnnnn
nnnnnnnnnnnnnnnnnnnnnnnnnnnnnnnnnnnnnnnnnnnnnnnnnnnnnnnnnnnnnnnnnnnnnnnnnnnnnn
nnnnnnnnnnnnnnnnnnnnnnnnnnnnnnnnnnnnnnnnnnnnnnnnnnnnnnnnnnnnnnnnnnnnnnnnnnnnnn
nnnnnnnnnnnnnnnnnnnnnnnnnnnnnnnnnnnnnnnnnnnnnnnnnnnnnnnnnnnnnnnnnnnnnnnnnnnnnn
nnnnnnnnnnnnnnnnnnnnnnnnnnnnnnnnnnnnnnnnnnnnnnnnnnnnnnnnnnnnnnnnnnnnnnnnnnnnnn
nnnnnnnnnnnnnnnnnnnnnnnnnnnnnnnnnnnnnnnnnnnnnnnnnnnnnnnnnnnnnnnnnnnnnnnnnnnnnn
nnnnnnnnnnnnnnnnnnnnnnnnnnnnnnnnnnnnnnnnnnnnnnnnnnnnnnnnnnnnnnnnnnnnnnnnnnnnnn
nnnnnnnnnnnnnnnnnnnnnnnnnnnnnnnnnnnnnnnnnnnnnnnnnnnnnnnnnnnnnnnnnnnnnnnnnnnnnn
nnnnnnnnnnnnnnnnnnnnnnnnnnnnnnnnnnnnnnnnnnnnnnnnnnnnnnnnnnnnnnnnnnnnnnnnnnnnnn
nnnnnnnnnnnnnnnnnnnnnnnnnnnnnnnnnnnnnnnnnnnnnnnnnnnnnnnnnnnnnnnnnnnnnnnnnnnnnn
nnnnnnnnnnnnnnnnnnnnnnnnnnnnnnnnnnnnnnnnnnnnnnnnnnnnnnnnnnnnnnnnnnnnnnnnnnnnnn
nnnnnnnnnnnnnnnnnnnnnnnnnnnnnnnnnnnnnnnnnnnnnnnnnnnnnnnnnnnnnnnnnnnnnnnnnnnnnn
nnnnnnnnnnnnnnnnnnnnnnnnnnnnnnnnnnnnnnnnnnnnnnnnnnnnnnnnnnnnnnnnnnnnnnnnnnnnnn
nnnnnnnnnnnnnnnnnnnnnnnnnnnnnnnnnnnnnnnnnnnnnnnnnnnnnnnnnnnnnnnnnnnnnnnnnnnnnn
nnnnnnnnnnnnnnnnnnnnnnnnnnnnnnnnnnnnnnnnnnnnnnnnnnnnnnnnnnnnnnnnnnnnnnnnnnnnnn
nnnnnnnnnnnnnnnnnnnnnnnnnnnnnnnnnnnnnnnnnnnnnnnnnnnnnnnnnnnnnnnnnnnnnnnnnnnnnn
nnnnnnnnnnnnnnnnnnnnnnnnnnnnnnnnnnnnnnnnnnnnnnnnnnnnnnnnnnnnnnnnnnnnnnnnnnnnnn
nnnnnnnnnnnnnnnnnnnnnnnnnnnnnnnnnnnnnnnnnnnnnnnnnnnnnnnnnnnnnnnnnnnnnnnnnnnnnn
nnnnnnnnnnnnnnnnnnnnnnnnnnnnnnnnnnnnnnnnnnnnnnnnnnnnnnnnnnnnnnnnnnnnnnnnnnnnnn
nnnnnnnnnnnnnnnnnnnnnnnnnnnnnnnnnnnnnnnnnnnnnnnnnnnnnnnnnnnnnnnnnnnnnnnnnnnnnn
nnnnnnnnnnnnnnnnnnnnnnnnnnnnnnnnnnnnnnnnnnnnnnnnnnnnnnnnnnnnnnnnnnnnnnnnnnnnnn
nnnnnnnnnnnnnnnnnnnnnnnnnnnnnnnggcgtatcggttttctattgttttcatttttttgtttcttgtataattttgttgctgaactctc
gaacttactctaaacaacatacgttttgcctaacaaagaaacagattacgctattttaagaaaagccatataatctgtatatctacagtcgaaaat
cttcagactggaagattcaagaatgctggatacaacctaattaacctttaaaataacatacaaaaaaaaatcgatatttcaaaatcaaattgcaa
tatccttggaaaacaagaaaataacaactgtcttgtttccacactgtttgtaactgacgaatggaatagaaggtaacatacaaattaagtgggaa
tatgcgagaacgttcgttccttctatcgtacaaaagaggaatatttaaatcatatatcagtttctagatatatctagaacagcgagtttaatcagat
aaacaagcgaagcattcatgaaagcaaaagataaaaaaacaagatttaataaataaataattaattaattaataaggaagagtaagagaaat
cagcaatctgaacgaactacctcgaaatctctggaagaaccccaagtatcccaagtcatcatatttggaaaatactagaactcatataaaatgttt
gttattttctgtatgctcaggtataaataggagcgaagtagagacgattcgacaacaattaccgaaaaagaataagtgtgattttgattcttgcgt
gaaattagcgagtgttgtttacaatgtctcttcaaccattcttcgatttatacccattccaagacggactttccgactttttgagttattcccctcgcgt
acagcgccagaatcgtgttggtgacttgtcacctgccattgatgtccatgaagacaaagacacggttgctgttgatgtagaacttcctggtgtcaa
aaaggaagacgttcaagttcattacgacgaaggaaaactgaccgtttctggagaatcattcaatgaacgtaaaagtgagggagagaaaggaaa
ccaacgttggtccgaacgtcgctttggatccttctctcgaacaatcggcatccccagcaggatcgattccgaccgtattaaagcccaatattccaat
ggcattttgagtattgttttgcccaagattgaacaatctcgctccaagaagcaaatcgccatcaattaagctgaagggctagcggtactgagtttat

gatgatatgattgtattctaatgagaatttataatggcataatggattttaatatagcatagcgttttaatgagagtttatcacactttaaagatact
catttcaatattgtttataataagtagcactgtggctaaaaataatgtgagctatgaagtctaaaatctaaattgtttagtcgttgtgactctttattc
tttgcataattttgcaatgtagtctttaataatatgctcattgatgattttattggtgggtggtattttcggattactggttggtgattacagactgttca
ttcttagcttgcttagcgtgtagcatttcaattcatttagggtaggtcgcaaagagctatacctatatctatttctttctttctctctcttttactttttca
cacaattgtttttaaacctacctgtatagctaagctacccgactaatatattgtctattgtctcctttcgatgttgtcaaaagtgtgtgacttgtttttac
gatttcagtcgcactgttgggtataaaacaaatgtaagtaatagtgagatttgtttcaatacaaatttatgttgctaaaagagaaaaaagaacaa
aatgaggagcctacagcaccccggattcccatgttgtctccaaccatagtactaacgaggccctcagacgcttaactgcagtgatcggacgggaa
ctggtgttttcgcctaggtatggccgtagacaactacatgtacataataagctatatatataacataaattacacaaggtacttgaaataagagat
atgagaatataagatttttatgagataccacataaatacatatcactcattaatcgaaattttgtaaaattcatatacttctcatacctgaatgacat
gtaaaatttcaggcaagcgcaataacaatataaaccttgaattttagtcgataacaccgctacttgttatggtcatgtaaatagattgtgcgttcat
agatgatgctacgaagtaggaaatgaaattgaaaaatacatgtaacaatatgttctacagttgcaaaaaatgttggtttatcgtttcattcttttcg
tttatatttcgtttatacaagtatgttaatgagaagctttcctttattttagtannnnnnnnnnnnnnnnnnnnnnnnnnnnnnnnnnnnnnn
nnnnnnnnnnnnnnnnnnnnnnnnnnnnnnnnnnnnnnnnnnnnnnnnnnnnnnnnnnnnnnnnnnnnnnnnnnnnnnnn
nnnnnnnnnnnnnnnnnnnnnnnnnnnnnnnnnnnnnnnnnnnnnnnnnnnnnnnnnnnnnnnnnnnnnnnnnnnnnnnn
nnnnnnnnnnnnnnnnnnnnnnnnnnnnnnnnnnnnnnnnnnnnnnnnnnnnnnnnnnnnnnnnnnnnnnnnnnnnnnnn
nnnnnnnnnnnnnnnnnnnnnnnnnnnnnnnnnnnnnnnnnnnnnnnnnnnnnnnnnnnnnnnnnnnnnnnnnnnnnnnn
nnnnnnnnnnnnnnnnnnnnnnnnnnnnnnnnnnnnnnnnnnnnnnnnnnnnnnnnnnnnnnnnnnnnnnnnnnnnnnnn
nnnnnnnnnnnnnnnnnnnnnnnnnnnnnnnnnnnnnnnnnnnnnnnnnnnnnnnnnnnnnnnnnnnnnnnnnnnnnnnn
nnnnnnnnnnnnnnnnnnnnnnnnnnnnnnnnnnnnnnnnnnnnnnnnnnnnnnttattctgtatttaacctttaagatttgt
agtttcgctttcctaccatagacactaactgttccaacacctttgtatccgccgttcgttttaaaaagcaaggatgtgctaaaacaaagtaatctac
aagttgtgaaagaatgtttaatgttacttattgataaaatcctaatattcctaagtattacccgaaattcgtagcgctgaattattattaggtattac
atgaattggagacaaaagaaacgagaagtgtaaacacaaattcaaacaccaattatattcacatgattaattagtaattgtattcgaacagagg
aggaataattaccgagaatgtttccaaacaaaatcatcaacgagggtgatttttccgagtaagtccgtatatttgcgttaactagccaatcaatttt
tttctatactaagattagcaataaccatatattctaattcagtttcaacttaacagatacaattaccaattacgaaatccacactcaactatattaat
actcatcaaacattacttcgatgaatgatagcaataaaaattttctgaaaggttacttcgtcgctgacacagtcaacattatacctttataaatagg
tatctgaagggaaatatagtttcgctcttgatgctagtttaaatagggctgttgtacatttacttatatattttttacttccttttattcagtccatgtgg
cagaaagggtttataatgcgctagagactctcggcgtattctcgatgtaacagagttgtaaacttttaaaaaataaacggttctctgaatatctaa
acgatatagccaaaaatattagtttaaatgctgtactaacataagcgggcatgtggtgtagatggttatcacgcttccttagcatggaagaggtcc
cagattcgagttctggcttgtccactttttactactattttttaagtggatattttttgcataaacgaatgtctcacgttattgacaacgcagtctattg
ttgtgtacatagcagatgtcactaatcgtgcgactctctcagcaccctcgatataacataagattgttattttttatcgttacatttttttagttaccttg
aacaacatcaattgatcatcgacttaatcgaactctctactaataccttactatgctcagtaggtattatgcaagtagatacaagtggtttttcatag
gataagtacagaagtttaaggaggtttcaatgtaatcttaaaaggtgtggacgattagtacatactttttagattcaaagctattttttttaattcttta
cgtaaatgtaaaaataaaattactcattatttttgaacaacggggtcactcactagagaagcagaagaaaatacctcgaatagatactttttcacc
atagctaaaaatagcttaacttctgcatttaattgctagtatccttttaagcaagggaataagttaattgtcataaggacgcaagatgttccattat
acgaagagagtatttgtttcgagatgttagaaagctagcttaaaaaaaagtcatcagccttcaacaaacattcatcccataaatgtattggtgtct
tctttacaactatggttagctaaggtatccatttctgcattaaattagccaaagaagcggaataaaagaagcaatctaattcgcttgaatacatgtt
attctacatcattattggcaagaaaaatgtaaaggaatgtactaacgagaaaacattgaaaatcgaagacgaggaacctacaatctatgtatgt
atgttgtttatgcaagatttaagtttttataggagctggcacttatttgatattctttccattgtcgtatacgagtaaaaaagctagccgaagtgctca
gcttttgaggactcaaagaaatattactactactctagaaatgcaaataaaaaacctttataagtctactcatctaaaaatctcaaactgtggaaa
aaaaaaacaaaaaagaaactgtgttctataaatctcgtttactaaaagaacattcaacagataatcagattagaagaatcaacaaaagttctgt
aaatctcacttttcaatttttagtcaaaacatacagcgtgctctaatagtacgtcgaaatctcatatattatattgtgcattataataaatcgtcttgt
aaaggttgagtatcaaaataaagcatagtttattatattgaatttactgtataaagtaatatatgtactgtactgcgtagtagtacacaatactacg
tcaaagttgcgttatactcaatttatattatacttactattcgtttcttaaagatgagcaaaagatttgatataccaccataagagatgatactgaaa
tgaaacaaaactgtgttgtgtgaaagcaatccatagactgggttttcaatccattcttcttgtcctttaagcttgtaaaatataatactggcttcaaa
aataagcaaaattgtcttagaaggttaagacctttcgtttcagtatgcaatatgctgtctagtacgtatttatgnnnnnnnnnnnnnnnnnnnn
nnnnnnnnnnnnnnnnnnnnnnnnnnnnnnnnnnnnnnnnnnnnnnnnnnnnnnnnnnnnnnnnnnnnnnnnnnnnnnnn
nnnaaccataagagatgatactgaaatagaaacaaaactgtgttgtgtgaaagcaatccatagactggtttcaatccattcttcttgtcctttaag
cttgtaaaatataatactggcttcaaaaataagcaaaattgtcttagaaggttaagacctttcgtttcagtatgcaatatgctgtctagtacgtattt

atgtaacgaattgacaagagatacactaccatagaaatgctgcatgcaagaccgttgagaaattattcgacttactttttgtaaaatagtgctttc
tttgtgaaataagaattcaagacaagctaatgatagagttacaatgcttgctacagtgtgtgttgtgcaaattaacattagtattaaaacagtatat
aaagaatataactgtaaacaacacaagcaattcttccttagtataggggtagtacaccagcctgtcacgctggcagcccgggttcgaatcccgga
gggagaggattttgtttttgctttgttattttttcccatcttgggttttatgatttacgttgtattgtttttattaatttttgtttcaatatcgtaatttctg
ttcttcctgctttcctacttttgttcgagctggttgaagatcttgatttttatctcatatagttgtatgacctgtgtacgcaagtaggtagtgggtgatac
aaacgaaagaaagcattattaagcaagctcgaaaagaaaaggaaagaaaaggaaagaaaagaaaagcaaagagagaaaggcagcatcct
ggtaaaacaaaacaacaatgaaaaaagaaggtatggatgaccgtgccaagactcgaacttgggaccgtctgcgaacattaaatgttaagcaga
aattataaccatctaaaccacacggccttcgcttattaagctggtgcttataaatttgacaaaggtataagttatgtttcattcgtggatgtttgaca
cttttaataaatccagaggaaatcgataatccatttagaaatggtagaaataagatgatttattacttctttttgaaaaaatacacgtactgactta
tgacttgtggtcgaatgtaagcaaagtttatatagcttctgataaaataatagcgaataagcatttctgacatctcttctaaggcaaccatctcgat
cgtaaacagcattaatcattgagtagactacttacattcctgttaaagatgaagcaatttctttaacttcccttgtgtattagcttcacttgcgacga
aaatcagataatgcgacttaataaggaacaccgaaaagaggtgaaaatagccaagaaacgcatattccatcatgaccaaatttcctaacactta
agagctatcctctatgactaattaatttttcgcttcaatctggcgatattattgacatttaaataggttaaatataattactcctactctattgttatat
aatcatcacatgcttaaaataaaatatattaattaactatgcaacttacttaacacacactagtcaaaacttgttttatacgtcctttggcatcttta
ggcttttctttatctgtaagatcctcataagtatgcagatttgtatccaaatcaatgtgagctaaagatggtatgcgtgatctgtcatagatcttatat
ccaataaaaaagaggacgaccacaatagcggctaagtaaccttggaaaaagtcacccgcattaggtttaccgccgacaggaaaaagtgataca
taaaactgagctattaaacataaaatgttgaaacccaaaccaatacatgagccccagatgcccataggtgacacaaatccgagagtatctaaag
agcgaccttgctttttcatggcaagtctaaagacaatatggcacaggtttatactaccccaagaaaagaagtttgcaagaccagatatagcaagt
aaccaattgaaaatcgttgtacttttacctttagcttcgttaatataagcgaagaatccaaacgaaagaactacaatcatggccattaaagggcgg
cccagacgatctgtataaccaaaaaaggaaggtgcatgcttattcagggcaagagcgtgcaatgtacggcttgctgtaaaagtacaagcattcg
cgactgaaataacagaaattataatgacggcattaaagacggaaggcagcccttttattccagcttctttgatagcaaggacaaatggggaaac
actattgccactactgttattcatcaaattaggatcatcaggactaaccagtaaaccaaccataagtaagccaacaacataaaaaatggcaatac
gccaaaaaacctgacgtgtagcgtgaggtaaggacttctgggggttttcagcctcagcagccgccaaaccgacaacctcggtacctgaaaagga
aaaagctgctgtggtaaacacagagcagaaacctttgaagccatgtctgaagggttgattcttaataatactaccaccgatataacctctatgatc
ggttgggacaccgccacaattgataatgattgctaaaataataaatccggtagtagctataaccttgattgtcgacaagagaaactcgacttcac
cataacctttgacgccaaacaagttaataataataattaacaatagaaatatggtaatccaggcagcactgttaatttccgtccagtacttaaaag
tgatagcacaagtggttaactctagagggaaggtgacgaaataattaaaaaagtagttccaaccacaagcaaaaccccaagcagggtcaataa
aacgcatgacatacgtataaaagccaccagccagcggataagcaatagccaattcacctaaagagtatactgtaaaaaacatcatgataccgat
caatgtataatcaatgatcacagaagcaggaccgcctttagcaagcgagctaccggaacccacgaaaagacctgtaccaatggcaccaccaat
ggcaatcatctgcatatgccttcctttgagttttctttttaaggcaacaccatcgtcacgaacagaagttgctggtttaaaatcgttaaatatactttc
gaaaaagttttcctttttggttctaaatctaaaagttcaccatgggcaacgatattttcgttacttggacccaacttcttttcatctttattgtcctgat
aatgcgttttgtctgaagggtatttttcgagaccagtttcatttatagattgcgacatttttttataattcttccaaagggtaatagaacgtatgctttcc
cgattgacacggtgcaaaaaaattaaaatgatgatataacaaaaaaatcaatgaatgtaatcaaggtctatcttccttttggcatcaattgttaat
ggacagatattaaaattgcagaattaatgggtaaacaaatctcgcaggtcaagtcaaaaccgaatgtttaaagaatcctactcacaaaaacaaa
ccttcaaggaaacgaagacacgaaaagcttaataaaaattatgaaaatgtaatttttccttttcttgaactaaagataaagaacaattgatatga
ctagaagacaagggcttttctttctttagtagctcaccttttttgcaaataacaacccttgtatgtatatacggaattagaaactatctatgcacatttt
ttttaaatgaaacgatataaatactcaaaatatgtgcaaagataagtgacgtttttatggagcagataagattatgcttgcgtaaaaacgtcaaa
gttttaagaggcgaaatattactttttgtttcattaacttaataagtatacgtacaaatatatatatgttaagatataaacaaaagaacaccagaaa
caattagtgttatttttcacttcttgtgaagagcactattgttgttaccctgataagcaaatttccttcaacccctcgtttacagtatctaccccaatat
ccacatatgtctgttctctgatagttgaagatctccatatgaacccctgacaatcaatttaggaatgtgccgaataacccttctggttgatataacca
tcagtgctccatacaaaaaaacaaaaaaaaaaaaaaatactgaagacttacaaatactatcaaactgtctttatgaaatcgaatttagtctatgaag
ctttattcaaaattgcaatagttttacagcatccagtaacaaaaatttttattccccttttcctacttttgtttaagcaagattaaagctagcatcagg
gaagtgttcaaacttctcatcaaggttaccacgcttgttcaaaaacttggcgtagcagccatcagggatgacttcaccgcgactacgcttctcaatg
aattcagaacggttgtattgagagaagccccacttcttggaaacaataatttttttgttgtccagggaacttgtattggcaacgacggagggcttca
atagcagtattacggttggagtccttggtacggatagagatcaagatttggccaatcctgacacgggcaaccaaagcgttgggtttaccaaaagc
gtgacgcataccttgttgcaatctatcggcaccggcacaagaaagcatcttgttaatacgaacaacgtggtaagggtgggcacggacacgaagg
tggaaggcgtccttaccagccatcttcactaagtacttgttggaactaatacgagcggcttcaagagcttcggaagtaatttgttccaactcgttag
atactaagtgaatacagagggggaactcgtcgacaccggcacgcttacgacccaagtcaaagattctgatcttgggatcaggaacggcacggtt

ataacgagatttggggtaaggcttgtttttaatgtaacgatagcaacgtgcgggtctacgggccattttcgattcaaaggctttatgggtttttcttgt
ttttacaattcctttccaatacccatctagtctttcctctcgatgaacaaaaaacagtcacaccttttgttacctcagcgggtaacgtacagtttgtttt
tgtaataattttttgacgattctgtttagccgttatagggatattgtttgtatttacacataaagaaacaaacacggactgttgatttttgactttaat
atttatagaaagtcactaatttctgggaactatgaaattatctaataagtaactttataagtctccattatacaatctgttttcttttcatgtagtactt
gataccttgcgtagacaaccaatattagtagatcttgtaatacctagcgaatgaacgccgtatccgttatggttcgggagtaattttacataatgaa
atctatacaataaagaatataaagcttaagtattgtcattatttgagttttcatcttatctttttttgatctagtgctagactttcgtccacaaattggc
aagtcggttctgtcagtaattttccacaacacaccacagcataatttaaatatgagccaggaggctctcacgacggaagaatacacccagcgcct
tgagcgctggtcggaaagactaaaacgccaaaccatttctcaccttcctgcggattatagccgccccgtaccttcaagattagtggaagccgtttc
tgaaaagacgctcaatgatgagacgaaggctgcattgatcaatatttatgttactgcccaaacaaaaggaattaatgctactcctttcaatatatt
gctgacattgtttatcgtcctagttagtcgtctgacaggtgacgaagacattagcattggaaccagctctgaggatgctgttccttttgttttgcgga
cttttattcaacaaaatgagtccttctttggacttttggaaaaagtgacagaccttgaaaggtcctattcggttgacgctgtggattttactgatttga
tcaatcatttaaacagtaaactagcgggacatgatgaaactcgtaaaactcttgctcatattcgtttttacaatgcaccagatacgccatccgaag
gctttttatcttcaaccagcatggatgtcgatctgactgtcttagtatccctcaagaagccggcggaagagctttcttctttacgttctaaatttacttt
tcctgatctacaactgaaaattatttacaatcaacttcttttctctcaaaatagagtcaatattcttgctgaccagcttttccgacttgttatatctgct
tcaaaaaacattgaagactccattggctctttagatttaatgactgagtctcaaagctgcgttttaccggatcccacagttgatcttgactggtctgg
ttttcgtggtgctatccaagatatttttgctcaaaacgctgccaagtttccagaaagagaatgcatcgtagtgactccttctgttacggaaaatgttc
cggttacttcttttacctatcgccagattgatgagtcttcaaatatactggctcaccatcttatccaaagtggtatagaaagaggcgatgttgttatg
gtttatgcctaccgtagtgttgatttggtagtagctgtcataggtgttctcaaagctggtgctactttctccgttattgacccagcctatcccccagct
aggcaaattatttatttgggcgttgccaagccaagaggtttggtctttttggctgatgccggcgttgtttctcccactgttgttgactatgtggaaaag
aatctagatcttaaaactttcgtaccctctctgaagttgtcgaatgacggtactttgactggtggtgttcttaaagatagtaacattgatattttatca
agcgttaagcacttaaaatcccaacaaactggagtcgtcgttggaccagatagcactcctacacttagttttactagtggtagtgaaggcgttcct
aagggtgtaaaaggacgccattttagtttagcttattattttgattggatgtcgcaagagtttaatctttcggagactgacagatttacgatgcttag
tggcattgctcacgatcctattcagagagatatctttacccctttattccttggtgcatctcttattgtaccaactgctgaagacattggaacccctgg
acaacttgctgaatgggccaacaaatataaagtgaccgttacccatctgactcctgcaatgggacaattactttccgctcaagctgaagagcaaa
ttccatctctccatcatgcatttttcgtgggtgatattctcactaaacgtgattgccttcgtttacaagtgcttgctacaaatgtcaatgttgttaacat
gtatggtaccaccgaaacacagcgttctgtttcttattttgtggttcctgctagatcaaaagactctacattcttggaatgtcaaaaagaagtcatac
ctgctggtaaaggtatgaaaaacgtccaattgcttgttatcaaccgccatgacagtagcaagatttgtggtattggagaagttggtgaaatttatct
acgtgctggtggtttggcagaaggttaccttggcaatgaggaactgacatccaagaggttcttgcagagttggttcgctgatccctccaaatttgat
gatcgcactcctgaagatgccttatggaaacaattttggtttggaattagagaccgcatgtatcgctctggtgacctgggacgttaccttccatctg
gtgatgtagaatgcagtggtcgtgctgatgaccaagttaaaattcgtggttttcgaattgagctaggtgaaatcaatacccacctttctcgtcatcct
aacgtacgtgagaatattacacttattcgtcgtaacaaagatgaagagcctactttggtttcttatgtcgttcctcaaaatatggagaaggatgatt
acggtagcggtactgatacggacgatgtagtggttcaaggtcttcgaaagtatagaaaggttattcaaaacattcgagagtatttgaagaccaaa
cttcctaattatgctgttcctacagttatcgttcctttaaatcgtatgccactgaatcctaatggaaaaattgataagccggctttaccttttcctgata
cttctcaattagcagctgcctctatttctcgttctaaaaacgcggttactgaggcactaaccgttgctgaacaagaaatgaaagatatctggcatag
cattattcctcatgctactgatttaaataagaaagcaaacttcttcgacgttggaggacattcaattttggctacccgccttatttttgagttaagga
agaaatatgctgttaatgttcctttaggtttaattttccgcgaacctacaattgaacggctggctagagaaattggcaaacttaaatccggagaaat
ggttgatttatatgatgtacctaaggaagaacctaaagataaagaagttgcatatggcaaggatgccatccaactttcaaatatacttcctcagtc
attccctaccttctctggtttaaaggctgaaagccccgggacggtgttactgactggcgctaatggatatttgggtgtttttatacttcgcgatctaat
gactcgtaactcaaatattaaagttatagccttgattcgtgcatcttccgaagagcacggcttgcagcgtctcagggatagcggtattgcttacggt
gtctggaacgagaactgggttaagtcgatttcagtgattaatggagatcttgctttagaaaactggggagttgccaacgaaaagtggaacagctt
atccgaggaagttgacgttattatacataacggtgctttagtacactgggtttatccttattctaaacttaaaggacctaatgttatgggtacgatca
ctgctctcaagttatgttcatttggcaagccgaagtcattaagttttgtttcatcaacatctactattgatactgaatattatgtcaatctttctaatga
aattacttctaaaggtggtgatggtatccctgagtcagattctctttcaggttcagctgatagtttacaaaccggttatggccaaagcaaatgggtt
gctgaatacttagttagacaagctggcttgcgcggtttacgaggagttattgtccgtccaggttacattttaggtgattcaaaaacaggagctatca
atactgatgactttttggttcgaatgatgaagggttgcattgagttagggctttatcccaacatcaataatactgtgaacatggttcctacagatcat
gttgcacgtgtaacgacagcttctgcatttcacccaggtgaaggagttactgttgctcacgttactagccatcctcgtcttcgattcaatcaatttttg
gcaacgttgagtaaatttggttttggtacaaagcaatccgaatatttgaattggcgaattgctttagaaaaatatgtcgttcatgaatctcatgaca
gtgcattatatcctttgctacattttgttcttgataatctcccatcgaatactaaggctcctgacttggatgatgccaatgctcgtgagatcttaaaac

gtgatacagagtggaccaaggttgatgtatcggctggagctgctgtacttgaacgagaaatgggactttatctgtcttatcttgtttccattggattc
ttaaagaagcctactcaagaagctgagaaggaattaccaaagataaaattggatactttaactatggaaaagcttgccagcgtcggaggacgtg
gtggcgctcctgtttctaagtaaaacatattttgggcattagtgatatattaaaagttcttcaaaaatttaatctgttagttagcgatgtttttcatcat
attctatgcaagaattattaaaataaatatcagaattaaatgctggagatataggtacttccaacgccaagtctgctggcgacaatgggggtttaa
aattattgtcgttaaattgcttagtgtcgtaagttgtattttgaaaatcgtaaacatcagaagaccttaaaggtacaggaggcggcgtgcggttttc
cataaccggaaaatctagtgagtcgaaaggtgacgtgattatagcttttttttaaatagatttgaaaatgtaaaagtaaaagttttcaagaccat
gagttttgcagcatcagccagtagagcaagtaaacctaaggcacaaacagctaaccataataaaataaacaataatgattgtttagtctcagat
attactgacttttcaagggtattaaggactttagtaagaccgcttgaagtagtttgtgaaagtgtcgaatttgacatgagagcattctttaaagtttg
attactgatgggttcacttaatacgtccccaggaagtagtggtaagtttatatgagacttattatgaacccaagtaagaacttaatctgttagcaaa
tgtgacattgtatggaacgaacctttttcaaaattttcaatcttatgatagagtaaacagtttaaaacattttgcacggctttattcaatattgtatctt
caaagatagaggatacggtagaattcacttcattcatgaatacacttaaagtgtgatttaaagaaagtgtagtatgcgatatcgacccaaacata
ccgtcgttaatgcgcctttggttagctaaaatagcctggtttgtattattggaccaatcatatgatatattttgtaaagtatattgcatgtcttgataa
gatatattagagaacgtgctgtgagacgtctctaaattgttcaacttccgcaaagccaataattggcacaagcaagaacaaagggaagcaattc
ctacaaatagtaagatggtggcaggcggataaaaaatgtatgaaataaaccatcgcaggcgaaactttaaatgcgcgggaataggcagaaaa
gaaatactttgcttcacattccaaagcaaaggagattccaaataggtcactagatctcgagtatcttcgaacatgttcttttctatatattcgtcaat
gagagaagctctccgcttcagtctgtaccatttccaaccttcatagaaaaagtttatcaagattaaaccaatgatagaaatcagtaagcttataaa
aattgtatattttaaagtagttaatacggtgtaaactgagcttgatgagtcgtccgcattattatcggaacaaacggtgaattgtgtctgtttaggtt
gagatattgacgaattgtcgaatgtaaaatttccaatatctttcaaaatagagtacctagccagttttaatgggccactgattgcattttctgtgacg
tttatagcatgatcaaaatttatagaattgcgaagactttcaagcttggtatcataataagagggaaggcgtaaatttttttaaggattggatctgtg
gtattgaaacattaggcaaattgacgtcttcgcctaaccaggaagcgatttttcgaaacgaggattcagctttatataattcactgttaaagccgct
aacagtgtcttctattccttcagaaactgctttaagagtactatttacaacatgttctatttcttctcctacgtcaaccattgcatctagggcaccgttt
aaagccaattgcaacaaacataaataggtaccgtaagtgaattcaataaagaaaacaatggttttctcagctgcttctattgtatcaattagtcca
gataaaagcaagcctacagtagaattgatgcttttctccgtaagcttaacaacaacgctcgcaatcatgccaggagaattcagaattgaattcact
ttttcttgcattatattacaagaataacttttagctgccgcctcggatttggatatatcatctatctttgattgtaagtttcggttgaatagaagaaatt
gtaacaaaatatataagcaacatattgtccacggagtaatccaggcttgggagagccttgcctttagtcccaagtaagagttcttcatctatgagc
tctatacagggtatgaaaaaagcaatttcctagttttggtcgacatatcgtattgtggagaacaaagaaactttttcttatggtatgggacttaagt
ctctgacgtaggaaattatacataataaggaaaaaagtaatgcaaagaagaacccgattaattacgcacaagactcacatgggaaatgtcacg
ggagaaatctcacacgttatgagaatgtttattgagtggaagtaataagtacaattgcaaaattgtctattggaattggacgtgttagtactacgt
agaagaatagtaatatgtccagggatctgattgatttactaaatgaaaacgactaagagtcaatcttatttagcctgatcagtcaagttttctgatc
taaaagatttcgactgtagacacacaaatatatattgttgtatatatttgtaatcgtaattggcttccttgattaaaaacaatgattatgtaaattaa
atataaatcagtatttctgtattcaagtcggaaatcgcatattcaatgttttgtttacttattataaagcacaattctttaggcttaatcaggcgcctt
aaacattgggccacaagacctgttataactcatttaggtgcttaaacagatttattgtaataattttttttttttttttccacagttataagtaaatag
aaagagggaagaaaatcattaagtaatcattgttgaatgcgtttattagaaaacaatacagtgattcttcctaaatgcaattatgtgaatttttgac
aaataaaccaacaatcgacgaacagataaatatgtttgattatcaaaagacattgactaaaataaaaccagtaaaaccggtttcatcattccggt
aaataatagctacaataatatataataataaatattattttttggtttttttggttgcagaacaagaaaacaaaagaaaagttgaacctatacgcta
catacttctttgagaatttttgactctagccaacaatcaatattccggatctgtgaatccttaaatcataatggatgaaactattaagccaaggttta
tcgataaaggtgacatgaaattgcaagtaataaaagtatttcagacaatgcaatctttgaaagagcaattgagaattttcattcaagatagcatcc
cgaatgagtttataattatcaacatttttttagagtccaactttaatgaaaaaaaagaaaaaatagtaattgaaaatgaaaaggcttattgtttttt
aactaggtactgtctgtttatatcacgaacagtcgcttaatcaaatgtacaagataaggtttattgtcgttagcgtgaatatgttgaatttcgtttga
gagtgttatcgtatttaataaatagtgattttaacgtttccaatatcagttttacgtagataatatttctttgctaatatagataggagtgcaaactttt
cgcttgaacttggcatcttacctatactttccctgggcaattttttaaacactgaattaagcccaaatttggactctactttggaaaacaccctcttt
cgatactttttgtaattctttttcaccatgaattctgctacagttcctgaagaacttcctacttctgtaagtaatatgcttcgctttaaatcaagttggc
ttgtccatgctaacgaatacgataggtttcttcttatttcgataccatcctgatccttgattttgggtatgtaactaaatatgttcgtgcttgatatcat
gcttattgagcaagtacattgaattacttcgcgcagatgccgattaacccatgatttttgattgcgacagttattaacatatcctagatctcaataca
gccatttgattgctagacgccttcgtgaactccatgtttacgctgaaatgttaccatgcactcagaagattgctgagcttccttttaaacccaagggt
gtaatcttctctggtggtccctactctgtatatgatgaaggtgctccccatgtcgatcctgctgtgtttgaacttgaagttcctatttttggtatttgcta
cggtatgcaagagcttgcttggttgaatggtcgtggtgttagtgcaggtattgaaagagaatatggtcctgctatggttaccattactggcgaaagt
aaatctgaggttttccagaagtttttgagtctatgcccaaggagtttgaagtatggatgtcccatggtgataagttgaatgctttacccactggtttt

gaatcaattggtcagacgaagaactccccatacgctgtcattgctcacagcactaaggctatttttggtattcaattccatcccgaggttacccata
ctcctttaggcttgcaattaattaagaactttgctcttggaatctgtgaagctaagccaagctggtcaatggaaaactttgttgacaaggagattctt
cgtattcgtaagatgatcggtcccgatgaacatgttattggtgctgtttccggtggtgttgactccactgtcgctgccaaagttttgaaagaagctat
tggcgatcgtttccatgctattatggttgacaacggcttgttacgtttaaacgaagctgaaattgttcgtgaaaaccttagagaacacttgggtattc
aattaaccattgttgatgcttctaacgaattcattggaaaattgagtggtgttactgatcccgagagaaagcgcaaaatcattggaaacacctttat
ccacgtttttgaacgtgaagccgagcgtattgtgaagactaccaacggcaaggtagaatatcttctccaaggaaccctgtaccctgatgtaattga
aagtatttcttttaaggggccttctcaaaccatcaagacccaccacaatgttggtggccttttggaagacatgaaactcaagcttattgaaccattg
cgtgaacttttcaaggatgaggtccgttctcttggcgagcttctcggaattgagcgcagccttgtttggcgtcatcctttccctggtcccggtttgggt
atcagaattttgggtgaagtaaaccgttctcaacttgaaattgcccgtaaagctgaccatatcttcatcactgaaatttgcaatcatggatactatg
acaaaatctctcaagcctttgctgctcttttgcctgttaaggctgtaggtgttatgggtgacaaacgtactcatgaacaagttattgctttgcgtgcc
attactacttctgatttcatgactgctgattggtttgacggatttagcgtcaagtttttgaagttggttagctctcgtatttgcaacgaggttgctggtg
tcaatcgtgttgtctatgacattagcagtaagccccctgccacggttgagatggaatagacaccaggatttgatttcatttgttttcgttaccctcact
actgtaattatagcttttctttggttgaagaaaaaggttgggagaattaaggagtaataatcaggtttagatttattatattgctcaagcggttttac
acatataattaaacagatggaagatgaagaaaagataaatttgacttaagcattcatttatcaaaacttgtagtataatagaaattcaattaatat
ttaaacaatcgttatacattaatcattaacctctttgtttttattttatcctttttttgataacttgcagagggcgtagactagtagtcaaacgttattg
ttttttttctcgactacatgaacatgtcactagaaccgggaggcataaactcgtcattatcgggctcaccaggaaacttaaatccctcaccgggt
cttgtttttctttgtgaaaaccgatttccaaagggatcttgaaaagctcgcaaatcattgggcgatgttggatcatacctagcaccagaaggtacat
aaggaatgtttggaggtccttcttcaccgacagtacttgttccttcctcaggatgaaatataggatgattaaacgtaggatgcattcccccactgttc
ccggaagcgccaacaccagctggataaagatcatcagcaccaattgatggaaatagaggcttctggttaggcaaattcccaaaaggagctaaa
attccaaaaggagcatcgctgggctgtacatctacgaatcgaggatgggtagtagattgtatcgtgttcttcggttcctcagatgacggttcatcct
gattttgagcctgaatatcttcgtttgatgggagcacatcatcaactaaagacttaagattccaatcatctgccagttgttcgttgtgaaaagcccg
agtagctgttttatttgaagtcgtattgagacacaaaacatagaaccaatctttccagaaaacaatctttaccgaaatatcaaaaacattattcac
attgtaaataaattctgcaccatctgtataatcttttggtatttgtgttaatacttttccatctggcaaaacacatttttggaatgtagttccggctatc
aagagtgctctatgaaactttccttgaactctctcaaaatattggttcttatccatagcaaaagtaataaacccaactctaaggtacttgatatgtc
atatcttcaagtgctaatatatatatatagcagacatttaacgccttcatacttggataatctttataatatatataattatagaaaggttttctacat
gcactgtatatatatatatatatatatatatatttgtatagattagttaaaggcgaaacttggcttactatttcatgtgtataaagttttataaagt
aagaagttaagattggtaactagaccataaataaagctgacaagacacaaagactgatctccagtagggctcacattgcctaaaacatggatta
aattcgtggaatagggatataatgatgatacatgatgaaacaaataaggaatcttctaaataaaggcacaacttctctgtattcttcatgattaag
gctacacaggattaatcagaggcttgttgtaatttagcaagcatcgtcgataactttccattattacgaagccttgtaaggtctgagtttcctgatttt
agttagcatccacaataagtacactagattgaagtagcacaaaaattctagacataccccccaataaattcattcttgaagaagatgcttgggaca
gttccttggctcgtcttttccataagatatgctaatgaaaggtaagactaggaaaatataaaagtcactcatgttagaataccttgaacttcggatc
cagaatctaaaccgtattaatatacaacgaaccgttttgcaagatacatttgaagatgttcattctactcactaaaggaaattcctttaatgatcat
acatacctaataagtctaattcataagatttgaaaggagcagtatagtcagttaaggttttttttgccgctgaacagtagggacagtaagatttgct
aaagacggtaatcggattgcttgcaatcgctttttctacaaataattttgctatagagctcataattatagattctacttttatgagtaagggttggg
cgaatagatataggtttccgtgcagaatacagaggatgatgaagagtttcgtgtcgttaagcagatacaatgcattctgataatgaacgagaatt
aacttactatatttacaattctgcagcgataaataagaagatgcttacaagttactaggttaatgagaaaaaaaaaagcaacaagaaggagga
aaagataatgatcaagaaaaggaaaacttaacgttgtcacctctttatgttatgaaagatgtcaagacacggtagtatatattttattttcttttcta
atggacagctaaagactggagcttgttagaaagctcaatattttgatcattttccacagaaggaaggcccaagtctttgctacaaaagtgttagca
tatgttagttttgacaaagacaagagttttcttttaaattagaagcggccaattcatccactctttttttaaaactgtacttactgcattgcgtaggcg
tcttctccatccgcatagtatttactctcgatcccttgaacactaaagaaaatgttaataggatacccagaatttttttagcatactcaaatttcaaac
tgtcacggtacaaatgaagagcagcacgattgctctttctcacatgaagactcatgtacctagcaccatatacctcaaccatagctctttctgaaa
acgttagtggggtcaagatagtaaaaaaatgcaaccatataaagaaggtatcagaggctgtagctttgatgtaaattgcccaatttgccttggtat
tggagaaggacctctctctaccgaaagaaaatctcgtctaatcaaagcgaaagaggctcaaatagattttttccaactaccatttcttttattttg
cttatgaaatataagcttaaagcgctctgatttcttcaacatatagaaaactgaacttacgactctgaaccatcaatcgtttcgcaagacccaactg
tctataagagcgcataacggataagctagtgacatgtccatgaggaattccatctttgggttcttcctccatttggctaatacatagccaacgattc
tgccttttggatttgtagcaacataagaaagcataggccaagatatagcatgataaagatctataatggaagttaatgtttagattatcaacgtaa
ctggagttattttccaggaaatagtcaaacttgagattgttccttacaatatttcaattgatagttttccggcaaattattaagattgcaatgttgcat
tcccaccaaatcttcaacgcgagcaggacgaatatccatttttatcaaaaaacttggtgctagtatccacaaacgtgttttcttatattttggtggtt

gacttttggtcgtctggtgtatctcagaatgggtaataccacataacttatggaagagcttttatccgcacttctatctttctttctttttattcaagtat
ttactacatgctttgaaatcaaagggtaaaaaagaatttagtaaaatctaacactctaggagttgacaagtatcgcttataagccattcaattttag
gtaacctcctaggaaacaaactcctgggaactagcttcaaagaacgaccttaacgatattgaattccaaactctccagaaatgttcattgagaca
actcaaagtttgtttatcaatgaaatgcagtgccatccagtaaacggatagcgtaatattcgtaaactccttattaaacaaaagagtactttctcaa
ctatagatgtatgtacgtttagaatccaacaccgaacacgaagatacataaatggactggatgaataaaaacaaccagctttacatttctctgga
tgcttttaaaagggtaaaggtaaacaataaatatggaaaacaacgatctaagtatccacaagaagtgaaaaaaagacagccacttcaagataa
aacgaatcaaacagcaccaatacatcaaaacaaaggttatccaaacattctaccggatgaattttcatatatcaaaactccaaaacgtaaagga
tcagcaaggtcattccaggacaagagttttgtagatttagcaccgctggatccagatttgttaagaagctttcgacctctagcaacgtcgacgccc
aagaaaaaggttaaaacgaagccagagcaagcaaggaatttaaaagatcaggaagaaagtgaatttgacgagttagcagagtggtttcaatc
agcttctaattttgaactcgacctttctgaaccttctagccagaaacaatcaaacttaaattgtcaagaagtcatcttaaacaaatttgctccttttg
aaacagaaacagatgaaattattttgttttaaactttacatctatgcacattgcagcttatgggcaagaaaacttaatgtattctgcaggaggaaa
acattgtgttggtgtgttttcctaaagaaaatatcgagttgctttgatgcaagcaactccaaatgaaaattacaaggacaaggatatttaaggtaa
tcaaattaggtttttattataacattcctttagtgtcatgataaatcttgagcgaagattctcgactcgcagtaggcccatacattatttaaattcaaa
ttacacaagctagttgtaggcgtatatcggtcattgcaagcaaacgaaagaagaaaagtaatcattcgtattcctatgaaatgaaataagtccgc
gaaattacgttgaaagcctagttataactaatgatgaattaaacttattctggctttctgtactgcatttttttccactcgaacttctccatcgaaaatt
cggtaaacgtataaaataagaacagccccctgaacatccattaatacgaaactgggtgttattttggtatcatcgttagcagccgatacggtcggt
gctcctgttgcggaaccaggattcacaaagaagcaattatccaattcaaaggcagaaaacttatgcgtatttccataaagaacaatatcagcatc
catttctctagctagtatacttaaagcttcaggactgtcctgagggacaattaggtgcccgcttgtgtatgctactttgaaagagcccaatatcaact
ttccagctatcggcgatttgttttctatgtcataagctcctcttactagttttaaatctgcgcatacatgctttaaatagtcatatatagatggactcgt
caggtttcccaaacatataatttgattgatttttccaggaaccaaaagctgccgaaactgtcaaagttagtcaattatgcttcaacctagaagagct
gaattgattcgttcaattcacctaatatcatgcataccttttcagataattttggtgatcgattaggaatatgaaaatcaccaattactagcacaagc
attctatactattattcctttatcaaactttcaattcttcgcgaattaaaaaactcctcccttttgtggtggttgtgtactagtcacgtttaactataata
gctatcaacgaaactccattcggtcttttgatctttttcaaatccaatcttttcagaggatttagtaaatgctaaattttgtctttaaaagaatacaag
ttaatccataaccttcctgtgtgggttgtttccaaacaaataaataaacaacagtcattgttactgagcataccattttcctaatatctttggaaaac
tgaaccttacaagtctgtatatcaattgcactttttatgacaggaacatttcataatagcagattatggctagatggctgtttagatttcttccattat
ggtaatactattttcatatgctttgacaaaaggaaaatgtactgatatgtgaatgataggacacagtaatgctattttacaagctaagcagttagg
cgaccaactagttgtaggtgttcactctgatgaagagattacacttcataaagggcctcctgtgcaaaacttaagtgaaagatgtctagcagcaat
tacttgcaaatgggtaaatgaagtcgttccttacgccccgtatgtatttgaccttgcgtggatgaggaaatacgactgccaattcgtggtacacggt
gatgatatctctactgacgctgaaggaaatgattgttatcgtcatgcaaaggctgctaatcaataccttgaagttaagcgtacccagggtgtcagt
actacagaactcttagacagattattaaactcgctgccattatcaacttacaccatttctccatcgaatataaggacttatgaaccgttattaaaaa
ggtttgcttctgctcctaacggaacagatccctacactaatatatttatacaatcctccaatggtcctgaagaattagtctctggaatattttccgat
aaggtaaagttgagaaagtccgcaatctacctagacggcgactggagtttgttcactgggacacatatgacggctttaaaggcttgtgcagaggc
atttcctgatttaccgatagtcgttggagtattttccgatgataagtgctctgaaaagccagtattgaatcttttagaaaggacgcttaacctttttgc
agtgtaaatatgtttcggctgtaattattgatcctccttctgcaagaagctttgatacattgaaatacttgaaagcgaaatttgatggtctcattgac
aacttttcgtatcctaaattctctgtaaaggaacaagctacatttcaagtgactcttaacaggaaaccaactgatagctttcaaatactggacaag
caaggatctgacacaattaaacagcgaattgtgtcaagaaaacatgagtatgaagaacgtcaacgcaggaaacttaacaaaaatggaacaga
aaataccactattcgcgcatgcgtcgctagttgattattaactataattattctaccttctacaaattgtcaactaaatgttgcattaagcatcatcac
ttcctcaacatttatggaagcatcattaaaataacccaaattagacaagtgtgataaacatcaaaagaaagcataggtattatacactcattttca
tctttatggggccccaaggttcgtatccttcaataataaaatcttctatactaaattgatcaatttctccaatgtcagttgcaccacgtctaaatgata
acgtagggaaagactttggagtgcgagccaattgggtctggagtgcctcaaaatgatcattgtaaatatggcaatctcccatgacatgaataaat
tcagcagcttcatagccacacatataagcaaccatatgggtaaggatagaataagatgcaatattgaagggaactccaagacccatatcagctg
agcgttgatacatcatgcaagataactccagcttcccaccaggcttacttggttcattcacataaaactggcaaaagatatggcatgggggtaaa
gccatctcaggaattgctagaggattccaggctgataagattatacgacgatcataggggtttgttttcaatgtatgaattacttgtgcaagctgat
cgacacccttacccctgtaatcggcatcgcaaccgacataatcagcaccaaagtgcctccattgaaacccataaactggtcctaagtcaccggttc
taaggtgattcaagcctcgctggtcaaggaattccctacttccattaccatcccagatatgaataccttttcggtcaagtggtttgcatcagtatca
ccagatatgaaccacaagagctcttctaaaacaccacgtaaaaagacacgtttagtagtcagtaaaggaagagtctgattgcgcaatgaaaagc
gcaactgaggaggggcaaatacagaatgagtaccagtgccggtgcgatcagggcgagaccttccgtactgcataatataacgaatcaaatcta
aatattgattttcttcatgactgggtttcgaaggaactcgaatggaggacaagccagattgaagagggggctgagattgagcaagctgacccga

atgtatgcttggagcaacaaatttcgcagacgctttcttggctgaagaaggctgagaagtgggaggttctataatggacgaagagttactagaag
gcaaaggagaagctttcggagtaacgtcaacgtaagaaggcggattgttgttctcttcattgctttcactatcttgttctttagagtcttgttcatcat
cttcgttggattcttcattttctgtaccgctggactcatcttcatcctgattctcaatatttttcacggcattcggcaacactttcttatgttcaagatgc
aagtgttccgctactttagctacaatgtttcgccattcagccatgccacccattccaatgtctccacaagcaaggactttttcaataggcaaaatga
tttctgtatttttgtaaatacggccaattgtttccatatgttcttgggttatagggttggtccacatcatcgtgttcatagcgggggcaagcagcattg
gtttatgaagcgcccaagctcgtaccaaagaagttagaagattatcacaaataccattagcaattttagccatggaatttgcgctcaaaggagca
atcaacaacaaatgagcccagcggcgtaattctatatgcgtgataggactatccaaaccgttccagcaagtccagtcatcaacgttcgtatacac
cttgactccaagagacgtgagggcttcggtttccacaaaattgcgagctgggtctgtcaaaataacttgcacatcgacgctatcgtaagttaagag
cgccttaacgatcataatcaattttatagctgccaccgatcctgtagctgcaactaggacatgatgcttcgaatccgtaagttgatgctccttttcca
taatcataggattcttgacagctcgcgttgcaaaccttgcatgtaagggttgagacatgctgaaaatgaacccaatcttagtaaaaagaaagaac
ctttatattataaatgacctaaaactgtcttggtgaagaaaaggatgtttccctaaacaagttgttttagcgacttctttaacactcgcgtgaacgat
tgcgcagcatacactagccttcggtatatataaatagaatttgaatgtggcaaactacaaagtacaaaaaaacatttagtcagactataaagcat
agaattttgaaaaacatagtattatcgactgtttgatattctaaacatattcatcttaatatatctgtttggactgtacagatcactacaacgaacag
ctgaaaaaaaaattacatagtggaaacatacataagtagagcaataaccccttctaatcgctacaaaacaaatcaacagcatacaaatcttaac
cgcaaaagataacaaatccatttattgctaataagcttagaattggatagcagcaccctaaagtatgttagtacaagtttaccagacatttccatt
gaggccttgagcagtatttcatatgttcgcaatatcaaaaatggtgaaataaattgtgttgctcgcacagctctcgcttttgtattacataccttggt
cttcttctcaccaccaagctcaaagtgcttgcaacgcttcaagcagatttggttcttgtatttgcaagcaacacattctaaacgaaggacaaccttc
ttggtaaccttggcctttctgtggaagacgggctttgtttgaccaccgaaaccactttgcttgcggtcgtaacgacgcttaccttgagcaaccttaga
gtcgggacccttcttgtattgagtaactctgtgcaaggtatgcttgtggcagttcttaccaggacagtaggtcttacgagtcttgggaatgttcacca
ttttttcttgtttagggttgctggttggcggtgattaagagcttgacactcaaaagtttcgactcaaattgagtgttatgcctatgtgactgtttttata
cactttagggtaggttcatttctggacttcattgaacttctcattaagatatttaccgtacacaatcgtactgcgttctacttgacaaaatcatagtaa
agtaaaatgatactatgaatcagttttactgaagttagttacaccaagtataaaagatttcattcctgtattatggatgcactgcttctatatatcct
ggtaaagcacttatttatcaatgttgaaagtcaaatattcagtaatcccaacatactaggtttgtccatgaggtaaggaagtgggtccttctgttaa
ctgtgaacacggttgatataatttaccttactaaagcctcttcatatgcaatgtttgaaatgatgtaagtatagaaaggcaatccaatgaatcattg
accacttccgtatacatttcttttgccagattttacgtcaaggtgacgactttagctgtttttataagttgttcataaaaagcagaaaagattactctt
cttcataccgattgtccttgaaatattagttattcaatttgaaagctaccattgacttcaaccaccgttaacgaaaataacatactccttgtttttca
ttatgtctttagtatatagccttgtgccttatgttcgtcctagtgcaattgtcttaggtgttgcctttgaccatgctgcttcctttatggtttatggacctt
taatgggcgacctttggaagaaagcgtaagttaagacctttgcttttcgggcaatagacgctcttataggcatgcaatactaactttgtttttctaat
tagaatgtcccctactcctcaacatgtccattctgctccttgcagtcccatcaaaaaggcaactataacctattcgagtaatctcataagcgcatgc
gttcagacttattccattgctgcactcttgcaattaactggaaccgtaagtatgaaaggtgctttttatgtaggcctttatgtctttggagcatcagga
ttgcctgatatggttgactacatatttacggaaaagcgaggatgttcctacacattggtaaaaacaatttcttcgtttgtgaaaactgtcggcatga
gcgttgtcttgttaggctatggtatccgccaaaaccgagcttaacgaaattttagtactttgttttccatcttttctaaaatttaactttaaggaacgt
acaaatcagttagtaatgactaaatgaatttatgaacctcgtatattataagtattgtattttgttcctaatttatccatctctttgatatttgcaaaaa
taaattatagaggccaaaacttcttgccaacttgtctttaaagaacatcccttttaacattaaatttatgttaacgaattattctcatcttcagcatcc
tgaaacgcttcgtagtcgtctgtcgaaagggtatcgtttctgcgatgatgtccaaccctggcaatatcttccttctcgttcaacgtacctttctcttca
gcgttattgggattagaaagagttgtagtcgctggtaaaacaacatcacttctattttctacgttttccggtaattcacttgagactccatcgtgctca
ttatctatttgagaaggatcattttccttctcgtttaatacatctacctcttcagcgttattgggaccagaaagagttgtggtcggcggtaaaacaac
accagcactgttttctgtattatcttgtaacccatttgagacataattatacgccgtgtttatttgagaaggatctagaatacccaagtgaacatcag
taggtgagttttcatcctttcctcttcgcttctcttcttctgtggaggtaatagaaatcttctccgtattgttaaaggcgtccttccataaaattttccga
ttttgctcatcttgtactgagcgacggcgttcaacttcttcaacaccaccaatatttcgtaaattatcatctttaataaactcttttagttcttttgcgaa
ctcatcttcatgaaatagaactttctttcgttggaaggcttcaaaacgttcaatagcctgaagtctgacatcgctatcaggcctcaactttaaaacat
ccatgtgaatcttttcacgaatttctttcatgtctcctgcagagggcttcacggctaaaggcttgctttttatagaagactttgcttgaagctcggcga
tggcagaaacatttgataaaacagtcgtaatcgagtctaaatctttgttcaaatcggttataaggttcgtatgattatccaaaaactcttgctcttgc
acaatggcattcaactctttttcccattgctttttccatgtcggtgttacttcttctattgctttttgcaattcatgaatactactttcaatggattcctttt
ccttaaccaaataagaaatttgatccaaaccagggcggacttttcgaagcaaaacgtcagacctcaagagctcaatttgatcattaatgtcatcg
aacctttcaactaatgatttagcaacagcttcaatggtatctttgcttttcaagatatatccacggtcaaatccaaatccagaggccttctcattaat
tatacccatctgttttctaaattcattcagttgtaacggaattttcgtgaaagatgcagcaaacacttgctttaaagaagctaaatctaattgcatgt
tttttacttgttgataattcgtagcatttgtcactactttatcacttttagcggttttggaagcagaggaagactggattgttgcatttgtaatatgctc

actgcctaacttcctttctataacattcagccgttgaagtatttcctcaaatatagaagaattatttattccatcgtcttttgaaaagatttcataagg
ccgagtggcttcgcctacagcttcaacattgtcaaccggtgattcttctgggacttttaattgtttctgagcaataattaatgatttaaacttcatgtc
tgagtagtcttctaataaataattcactttagtatcgtagtcttcaatgtaaaattcaaaggggacatcttcgaattcattctcaaatttagactgaa
aaagggcttgtaatttctcctgggtatcaactgcaaaaggagaaatcgctttacgggtcctgttgtttagttttaagaaataaacgaaattcctgtg
agcttgttcaaactctcccttaacttcccctttcttatcccaaggttcatccttgacgttagcaaaattctgaacctcctttggcgtttgcgagttatcg
tctgcaagttcaacaatgccagatggggacggccgttcatcaccagaagtgtaatcactatcgtgcttttcttcattattggatgttgtttcgtcctta
tcctttggcaatagtgtactattgtttctaaccaaacgatccagttcttcaagtgaatcgttttttttgctttgatacatttcattgattgatatttcagc
gtttaacccagaagaaatggattcttttgtctcagcgtctcctattttaagctgatcctcagtttctttattttctcgaacccgagttgattctctattcg
gttcaaaattactcttctcagatactcgtttactttgttgtaaatcgtgtaaatctctgtcgctctctttctcgactttgttgtattcctcaaaagattcc
acttcttgtgaaggccttcttatttcctgcgaattttcaaatctcgatcctaaacttgtagatgcttcattcggaattggggaagtgaatgaacggcct
ctcggtaataatacaggagtaccagaaggtaaaaagttgggagaagcagttttaccggaagaaaaagacctcgttgcagaagaggttgtcata
gatctttgaggggatcgttgatcctttttcggcgaaatgggggtatttagattattcatttcataagagttataattacgttgactagggtaagaagg
agaaaaagattgcctatctaatttcattgaattagaccgggaagaccctctgttaaggtttcgacctttattgagatcgttccaaccagtttcttcaa
aaaaagggcctaaggttacagaacgaggttctattgttgggttactgactgaagaattcgggggcttgtagtctgctacttgcgagtcgttcaata
aatcacgcacagattcaaccgttacctcgtttttctgaatagtatcatttaatccttgttgagtatttttattcatcttagaaaagcgtttactagctcg
tcgttcaagagaggatgattgcttaagagcgtctaaagcatgaggcgtatcagaaaaagctagagggtttagagagctttttcagaatttaagct
tttattaatgaaattatactcattaatttctctcctttgagtattttcttccgtcgactctcgacttgcctgggactcctttcgaggtctcaaatatcgtg
atttatatagagactgcttatattttaagcttttgagaagatgtatgataatctctcggacatgtggaagatgagcttgaagggaatccgttgaagg
attttcagctagtgtttcttcaagaatagaacgtaattcaaggggaaaatcatccaggtctaaactttctattcccacttctttaaaaatttttatagt
aatgttgaattctgcaacaagacggacataaatagatgagacatcgtcggctcctgcaagccctttgctccaccttgttaagctttctaacaactgt
tttgtaatcaccagtagttttgtaacggaggcttcaattgagttagctattggcatgaacttatcgccagaatagtttctagcagagattattgatgg
tgtcctttgtgaactggtaactgaaggatgacgttgcggcgctgagcttctggaattcgctctctgtcttgaagaagataattcttggtcattgttttg
aaaagacatcttagaattacatgaaaagtcaagccttaagccaaaacgatgaataaatatttatacgcataagataaaaacaaaatattaagaa
aataaaatggatgcactgaagaatatagattttgctataatctggagatggttgcggtcccactctgataagctgtcgtgaaagagaacttaagg
gtataaacgaaaatcaagcttcaaattcgtcaatacttatcaaaatgaagagctaatatagcaaaattctatcttaaacttttaaaagtatacagg
cgttaatgagaattaataaaagtacattggcaaaactaacttcattatgaacgaaaggtagctgaagcaaatccagaatcaggtacaaataagt
acaaaagcataaaagtatgtacatatatatatatatatttaaaaacccaggaaaccgtgtacaattacacattaccgttcgctaatcaatatagac
aaatcataaagttgggtattaaagggtatgaatataactttttgaaataaataagagaaaagacagctgaccttagctatctgaattctcagctac
gggttctgctgttctggcacgtctttgctttcgtggtttggcatggtcagtaggcaaattaaaatctcttctcatttttgtgcgaagccctggaagggc
ataattgacacgctttttctctcgtctactccttccatcatttaattcctcagaagcggagcgacttggctgctcagggctatcgggagacaccgtttt
atttgtacttcctggaggcctgcctctcttcgatttttttaccggtggttcttctttttttccttctctataatgttgtcatctgcggcgtgcgtttcgaaa
aatctcaaattggtaacggtggacagtaattggaggttattatacgcagattcttcattttgcgttagatttaatgtttcctttgtggaacctctatca
ggtggtgcacggttgttcagaatgccctgaagccaaagcgggttagaagctgttgagtcgttcctactatttccaacttcaccgtgtcgaggaggc
tcacttgaatatctatcattcggtacccttaaggcacttaacggctcatcgctatctgaagtatctgaagcagcagatatccttgataatgagttttt
cctcttttccggtaagatgtgtgaatccagatgagtacttaatctatgagattcttgtacattcatgtcgatagtacctggattctgccccgatgtagg
aattggttgaaaagctaaagtcccagggttttgcatgatctcagaatcagcaggatagcctgatgccgtatcaggttcttttgaaggcaattgttga
ggctctatagtaaggctaaacggatttgacaaaggaatatttggattttcatgaccggtgtgatatgaatcattacttactccaacttcctgggaac
tcttggattttgaattttgaggcaattgcgtctcaacagaagcttcattagctgtattgcgggaagaagaagctgagaggtgtcgttcattattaac
agggacagtattggaatttaacacattaaaattatccgaacttttggaatttaaaacgggtggctggttgtgcaattcattacggacattgcttcca
tttgtttcaaaagattccggggcgtcgggagttatttcggctgaatatttgacattcgaggaaacgttattttcttctctggactcgttagatgatcta
atgatttcggaaggatgactcctagtttttttcttcagaattttcttgcttttcgtttccttgatgcttatcagtgagtgaacttaatgatttttttggagcg
ttggtcgaaggctcgctttgatgcccatcaccatttaatagacagcttaagtttttttattgttgcatcctcatttgcctcttttaatatatcctcagtttc
tttaacaaaggtttcctagtaaaatggttaaaatgggtaaacaaaagttataaccttacctcatccagatcttcttggtccgtttcatactctcttga
catttgagattccgaagttcgaaacaagtctatttgacattaacaactgaaaaatggataacagctatcttaccttgcgcatcttggcgatattgac
ttaatgattttagaaatgactcagctccaccttggcattctgacaaaatagaattaagcccctcttgtaatctttcgtttttaatttgttgggactctaa
ttgttcttctaaatttattgcgactgatcgaaggtctatgttttctgacaataacctttcattttccaattgaagttcttttatacgagccgattgggctt
tgcttattctagattatatgttaattaaaaattgattagtataatgaacaaaacttaccgtattatttccttgtattgacgaagctgtttcttcttagct
agcaatgttagcaggcgttatttcaattcacgtacattcttccaaagtcaaagagggagatgctgttgacatgttcttttgtttttctcatttaattcc

atctactattaaattttagtccaaagtataaatgcgaagtgctgtgtgcttgtcagttggtgttgaggtacaagcagttgacgctaccattcagacc
gtccaagttgaaagttcgcgtgttcttaagaaaaacggaaatatatgataaataaaagattgaaaaagttacagttaatattgaaatagcatatt
ccttattatagaagaaagtaataagttctcgacttcgaggtaaactcatcggttattctactttacagatagattgcattctttatttctcaacatagt
gtaccatactttaattttcaaatgagctctttgtaaaacataatgaaaacagaagaaagtgatcagcattataatccccaattttcaacctcattg
cttaattcttcttcgttatcatcccattcttgcatccactcctcatcaagttgatcttcggtttcaacatcaaatgcagcagcatcagatctttcatggg
ctaaaatattatttgctaactttcctttggtctcttcatcgttgtttgaaccttgaacattagaagatcctatagaacggtttgcgctgctttcttttggt
cggggtgtggatgacggacggaagaagatgttaagaaaagaattgctagatacttcaattggtttgtcttcttcacctttgttaaagtctttgattc
gattaagaaatttttgagtaacctcctcggatgaaatgcgtaaagatctaaagcaaggttagtaaacaagaagtttttaaagatttttgggtctcat
actcattatcgtcaattaatagaggcaccacgcacggaaggaatttagtagccagttcttttgttgaaaaatagccagccgtggctagcataacat
ccaaagcggcttttcgggcatcagcaataggatccctaagtgatttagacaatgcagctactagaacacgtattcggacctaattgagttagaaat
gtcacgcattaaacgcttacatgttggtccaagtattctgaaatcttcccaagacaaataatcgagttaattctaatggagggttgaggatcattct
gtagtaaagcaagcgtccgcagtaggtcgttatttaacgctttttttagaaatctaccataatggttagaaaaatacatcatataatttcgtggctta
ctttcggtgcgattgcggaataaagcaagattgccgaaaattttagttttgagtcattaaatttgacaaaattgcaatataacagaaaagcgtcat
cacttgttaaggttggaggcagaatatcattatgcttatacaactcgttcaccagtaagcaccgtactctgttattcgtagtctttagacaagataa
gagagggattccaaaatcctttttcaaagtccttattatcatttgtaacagatgctatagaaaataaaatttccgaagcatcagtagatgtatctgct
gttttagctacctgtaaaagtaaagttaacatacctgacttaagggctaaaaatatgacaggcaatattctttttttgaacataattggaagacaag
aaagacaaataggttttaagcaatctgtaaaaatgataagcaaacaatacttagacaagcaaaacacatactcaaaaagcttttgacaatcacc
cggattttggatttgcaacttgttaatcatatcgaccatgacgataaaattggatcgaaaaaatccaccaggtctttcaccaagccgtaaaaattc
agatataccgatctgttgttttggctctggcgagatcaatttttttaatgagttgtgcagattctatattagttagataagactctttatacgtaaaga
ataaaccgaatgcttacagcaggtatgttttctctttggctaaagtcgacggctttagtataagtcggattaaaaacttggtagaccaaataagta
aactcgatacggtctacttgtgaaaagtccctatgcacaagttaatgccgaattaatatatagccttaccgatgcaaaacaagatccctcacaatg
gaagaggctaatgaggtaaagggatttagtgcttgaaagtttttctacatttagttagtatttccaataaaagtttcaaacatactttgaaggtgtc
aggattggagctagtcaaaaagaagtcacccaaaacccattcaaaagtctcactcaaataaatagaagacaattggacatttccgtgaatgcaa
ttctgttcctcaaggaaagacagtgcagactgtgaatgaaggttaacatgattaaatccagcgatgtattcttcatcaacgtacagccattctccat
aagccacatattttctgataattattaagcgagctcaactgatttgacaatggttggacaacctcggtagctatgtaaagtgtagtagactaagca
aaaaaagtagattagcaaccaagttctcccatcagtaaaacatacatctttgtgggttcccaatattttaattatacaaggatgtcgtatacctttg
agttgatcagcagattgttcagcaagccttgtcaactcgggacgagctgctattggaattgaaaagatactgcacgtctgacctgtttgctatatat
cgtttgtaagcatagatatcatacttattatctttcagcaagcttacctttatttttccgttatgcagagtccacaccgattcagacgccaatggtacc
ttttgatccaaatcataaggaaacagaaaattcgcctttgaaatgagtgatgcagcagacttaaagaattccattgattaaaagtaaagccttac
aaagattgaagaataaaaagcctaagaagtctatgactatgtgaaatttaggttttcaaagaaaagcaatttgaacaaaggtaaacgaagtag
gctcacaaatatatgattttctatacaaatcctcaaatagaaaattaggcagcttccaagagctgaagaggtagtgctctaaatacctcactcgca
aagtattgtgaaggtgttcgccagtgtgaatgcacaaaaaatcaactcatagaaagctagagagcctactggttttcactcatacccttttttagttt
aaaagtattttacttttaggaaatatagagcaattcttgtgagatcgcttgtcaaaaattttgtttacatcaattagtgattgctctctttatgatatat
atataaactaatgcaatatacgtataacaggccgtgtggtttagatggttataatttctgcttaacatttgatgttcgcagacggtcccaagttcga
gtcttggcacggtcatctatttttatatcccatgctctcattgtaagcatgaaggctgcaaagaacaagtttttttagatcgtttttgttttgtaaattta
tactgttcgagatattaaaaagaacagtatgtttaataatgactctaatcatattgtctttttacataaagtaatatgcttctaaaaacgcgtttagt
attgcaaaaagtatctagtaatatgtttattatgaaatgctatatcatatatatatgcttttagctgccttcattgtcaacaattcaacaaaagcaat
aatgaatgggtattatttttttactttttttatgaatctctcacggttttacgtactattatttacttagtaatttttcattctgtacaagtacatccttccat
gccttaatcaaacatcaatactggatggttagactactcaatttttagaaaaagtaaaaatacgaatttgatttttttataaaatatatagaacaaac
cagttttcgtgttcattaaattgtttgcttgacgtacctaaccattttgtacacttctgtcctttcacaaatggttaagccaaggctagttttcttggcta
tcgcaggaccaaaaaatgaacaattgtacctagaagtttttgattctgttgaagattcttcattatcaaagtatcaatatttgagcgaactaagttt
ggatgttattcaagatttaattcatgattcagaaaggacttctaatgattgcttcttaggactattgggcgtggaagaggatataagtacctacgca
ttttgtagtaacacaaatgtaaaattcatcttggcagtccgttcatctgatttggtagttcgtgaatcagaaatccgacaggtaagaataagccaac
gtacaatgtttctctataaaatctgtgtattaatgcgattgacttagctattgcgaagaatttatactatccatactcatgctgtgtgtaatccattctc
gctagagctaactcctgacactctgaagcattccctttattttaatgagaccctacggcagctaaattctgattggagtaatcaataagtgtttacgt
gaaacatcgtgctgaagctattcatatattcaattgtgtttattccagttcaggacgcagtttagattaacaagaatcacttttctttcataaactata
tgtacatctatccatttcgtgtttatagcctaacttaaatatcgcataactttacctttttatgtattaagtgatatgatactaatattaaccgacaaa
aagaaccaagcaacatacaaaccctttttttccttaagccatgtcataaacctgcaatgagcctgaatctccagaacgggtgattgatgacaagac

catctgtcgcaaccaagcgttacggaccgcatggtcataacgcttttgacttaggaattgaggacatcctcgaattaatcgaaaatcggtttcccc
atgtatgtctaagtatggagcgggcacgaccataccggcaccgctaattccatctaaaaatagaattccacacattttagttaaaaagtaaatacc
tataccatcagagcattctctagagtgaatattacattcaccaggctgtctttgtgctgcggtagtagtttgccgagcactaaagcaaaccacactt
ccacattgcaaacaaaccgctggtaaaattggagtcttatgacacatgcagcaaatagattcttgcattatatccatcaagctctctagcctagat
ggaagcttcaaaagctcgtatataccaggatattccagtcttggtttattgacttttgtgtcgttttcagcataatgctggcaccaaccattaattaat
tgcagttgttctgagcactgttgacttcccaaccttgtatatacttcatccaccatgggaagctccattagctcgcaaagttgggtaagttcagtctt
ctcataaaacgtttcatcaatagacttttcaaaatagatattgaaaagactgtaatgcaccaaagaaacccgccttaggaatgggagcataaact
tctcaaccaatgtcaataatttcagaagagtagcttcgctttcaagaatgtggcacttttcttcaatgccgcatgcacggaatacatgtccacagaa
ttgacagaatgcatttttagctgcatcgctatatttaatggaaggttcggcaaaagtctccgtttgttcgacgtagcaagcagtctcaaccacaattt
tgcagatattagcaatatagtaaagttcagaataataagaaatatgtccctgcttctcataaataatcattagagatacagttctaacaaactcatt
gaatacatctgaagaaagcaaaggtttaaagcttcctgcttccaaagtattctggtttttgagcaaaaattcctgaatatctgattttccataaaac
aacttgccaagttgacgattttgagtcttaattaaatcggaagtcgcatcatcagagctgttaaccttatggcataaacttttcatgactgcttcagc
aaaagataaaagaaatgccgaattaatagaaccgatgttgctaatccagactggagcttcatggctctgactgctaggtgtaccacgatagatta
cttctaatgaagcaaggctataacataacgaatctaacagttcatctggtaagcttccttttgacaaattgtcatcactaagctttgatgctgaaaa
ttccataacagaaaaatagcgctggatagcatcttggttatcgtccatacggttttcctctttctggataacagcactttgattagtgttaatccaag
gacggcttaaacaattttcagcaacctttttagcaacgtccttctctgtgttagccatacaaaattcgaaactctcattattttcaatagtaaaactg
cttaaaaatgcttccagatttttatggatgccaaaatcattaagacgaagacaaagtctaggtgtatgagtcgtggggattattacatttgataaat
aaccgcaaagagggcacataaaataccgagtagcaggtgtagttttatgcttgtcatttatatatgttgaagggtcacttcttctgaggagccttga
actggtgtatgtgttaaaacactgagtgtgcataagatgaccacaactagaaacgtaaagaccttcaatattatggttaacgggatatgctccca
aaatgcggctcttcgtttcatctaaagcaaccttttcagtttttcgccaaaaggacgatcaagtacttgcgcattgaagttagaaggtataacaaa
tagctctcgaagaacctgctttgtatccgctgtatccctcaaaaatttgctctttttatggtacccagcatcccatatgctgcttcttcattacaacttt
cttggcacaataagcaatttccacgcaggtgctcatgttgcttaaacggtggtctgtacgtatattcaccatactcatcctcatcatccatgttaaaa
tcactattttgtgccataaaggaggcttgctgggctcggaattgctgcataatatttttctgtttttccaatgcaatccgctttttctcttctacacgttt
cgcctcatcaatagctggctcttcactgcctaaatctgccgagtttatttgagtatgttgtagaatgctagcgcaagcagtagagtcggatttttca
aggaacttaagaaatgattagctttagcatgtaatgattttaactttgaagaatttatgactttaaaacataattctgctaaacagcagctggaagg
accggtatcttcaccatctggagaaactgactgcttctcatgacaacacttcgtgtagaatgctgtagaaccagtaaagtcattgatcctagagga
aattaaacaaagctggaggacctcattaataattgtctcactaacgtccacattttcctgtttagcggaaataagaagagcatactcaagagagc
gaagaagggttagataaaaaacatcagaatttaccagcgcaaagaatatatcttggccgttcttctctacaagcttgaagttgggttcaataaca
gcagcttctaaggggatgttctttttatcagacaagcgtttcttcaaaacagcctcggcctcttctctttcattcttagaatagtaagaactataagg
gtttattaaatcaaagtactcatccttcagagtatataaaccaaaatcgtgaagcccatcaggaggtttatagttagtcatctcttctctaatggaat
caaatgatgaatcttcaactaaattgcctgaaattttatataaaagcgccgaataagctaaaggtccaaaacatagttcgtgggccaatctaacc
cgtattgtgtcttgaatactcattctcaataagctttcacgttccgttgttaaagttatcataaacttcaacaatgaaccaagaagtctagggattttt
tcggaatcgtaatgctcatgttcattaaatttcctatcaaatacccaatccgtaaactcaattctttgtaatatagccttcatccctacatcaggattt
acttgcgaaagcactctctgaatcaatagtatatctttatcaaaagtatactccctgtttcctgggaagggctcacggaattcttgaaggtacacat
aaagttgacgatagaaacgagtagtaagcaaaaggttatgtccatttcgaacccaaagattagcatctacctgcatcaggaaagcaattactctc
aatggatgatcaatcaatgcaaaaaaggtttcctgatcaaaagatcctaaaacatccgaatcacagtaggaaagcactgtcgctaaagcccaat
gtagaggatgattaaggcttatagggtcaactgatacagaaaaaggtttaagaggaaaccgggaattgaatgaatcagacggatcatcaggaa
cacattccaacggttcgcgtggatcggaaacatcatctttaggctgagtaatggcaattaccaagacctgaacagcctgaagtaagtcattgcttg
atgcagtctcataacacgaagcgacttgctcactcaattccgataatctatgcatcaagaaaatatttttaaacgcgtaacatcccactggacat
gagagtgcgtagcgcgtttttgaggagagttaccttggaaaacgcgcaacaagtctatccactgatggatatagcgagaatctttagtaaccaaa
tttttacttgaggaacttgaagcaagtatttcaagtcgtggaatagattaaatgttcggcggctctttaaaacttggtcatttaactttatcttttgta
ccccttcggtttcaccagcacttggttgcacaagaagagaaaccgcggctgaattaatatccgttaacaaatcataatccttaacgacagcctcgg
caatactaggagtagtaaagaactgaaccgaaaggaacatgactgaacgatcaggttcccgatcagctagaacgaacgcactggcaagatgct
tgtaatgaatagcaaatctaatacccataagccttttaaaagccaagtttctgttaaacggaacaacatagagtcttgatagcagagtccgcaatt
ccttccaaaacttcaagtcaaaaactaataaataatctaaacgaagactaagatatggagattcagctacatccttaaaatttgaaggtttgtaag
taggaggtttttgccaatatgagttttgaagaggaacttgaaaagccatattgggacgaggccttcggcgtgcggtaatgatctgacgcaagttgt
ttagtagctggggattgaattccggtcttatagttactacagtctgagcagtatttggtggaggaggcaaaggcgttgtagcttgttctggcgttgct
tgttcagtattaccatgcgtaacattgttttcctgaacagtagtattttcttcttcttcttcttcttcttcttcttctccttcttcatcttgttcgtcatta

acgtcattcattggggaatctatagcattttcgtcgtctaagtcatgtagcagctcgcgctctgcttctgaatccatagtaatggcattttcatcatcg
tcattttcagcaccagtagctcggttaaaatctattccggctgtttctgcctcaccatcattatcttcagtttccatgtcggtaatgtcagtgggtagat
tattattctcaaggttggcaatgactcccaagagttcttctgcagcatagaactcatcttcactgtcatcgtcattagcaatttccatcggaagattg
ctaaagttcaaagacgggtcagtgggtttctctaaaccgggagaccactgcttcagcatttcattacaaaacacagtctggatataatttgggaag
tagcagacatgagaatctaacaaatcctcaagccagtgtaataaagtactgcagtcatcctcgcgaaaaaaatctctagcagaacgaacattaa
cggcgagattattactggaaataattctggcaattctcaatagctcttggatagaggtggaagttttaatagaaaatcgaccgatagaatctaccc
gttgagcgacttgtaaaccaaagctttcgctggctgtattcacagcacgttgaacgcaatgaacgacaccttcaaaggtgttaaattcatcgttcc
acagcattacacggaaaacatcacaagatttatcaacggaaccatatttagcagtgcttaggcgagacgcctcctcatcagccaagacactttcg
acagactgttgggctttaagattgatagatgagcaagaaaaaacatcaagtacaaagtcaagtacaattcgaactgtctcagaaatcgatttttg
tagctcagcaggaatgcatttctcaggagaactatcatcttttgtttctgagtggctgtgaattttgcaagggagatcgactttccatgcctcaggat
ctccgcaatcacatacgccactgaaagaagttgatatagaaacatgcgtttcatggccttcatgatttgtagaacgaaagcagggtgcacacaat
acgcaggtactgtcactcccgcaagttttacagcgatatataacttcgccatctcggaaaacatggccacaaacttcactgccggttcccttgtttt
gtagttcatcaaaatattcaggttcagctcgttgcgagctagaaagagaaaaacttgatgtaatactttgtggctgcccttttaagaagtacttcca
attgttttcgtcataattcagcaaagatagatatacttcttgtaaaagatgagctttcgattcatcgttggaatcataagcatgtctttccgcactctc
caaaagatacctttgtagtcgggtcggagtactctgcgcagactgctgtgaaacgttgtgttctagcatgaacgccaagtggtaagctgtaaaacg
ggaaaatttacaaaatccaaaaagtcaggacaagaaaatgaaaaagtgacttaagtaataatccttaagtgcctttctctaatataaaataaag
aataaaaaggtactattcaatagatgtaaagtcttttgcaaaagtaaatttgaagtatatttaccacttccttagtaactaactaaaggaataata
gaagcacatatactgaggttgctgatctcaatccaatcgactaaggatcctatcagtgctttttcgcaaaaaaaatcaatataatctctcacgaaa
taccgtcaaacaaaactttttttgcttcctatttctccttaccctttatggctaaatgacaatcgccttaatcaagacaggaaagacgtattatgaag
ggtaacattgtaactcgggagtaaagttaaaatttttaaagtgctgctatcaatgctggatttatcaaattgatataaatgctttacgaaacatctgt
gacctagctacgggtcttcaataaacaaacctgataacttttggaacttggagtatgtttcagcgagtaagaatgatatagcggaaataggtacat
gaattttgaatttattttgagtagaaaaagaaacgtatcaaccatttcaatattgatttcattaactcttgagttagaattagaacctaagaaaaaa
agtgtcgcgaaccgggatcgaaccggtgaccaacagatcttcagtctgtcatgctcccaactacaccaccgcgactttcgttgtaatcccttttagc
atataagtatttatataagacagtaatgatgtatgtttttttgaaagtggtggctgttaacgtggctgttaacataaacaatccagatgcagaccagg
aggacggagtgatccctgttgagaatgaccgcaataaattggacactttacgtttactttgcgttgaatatcatttcgtttaaaagtatgaacaaga
tgaagactgtgtgtgtatgataaatttttttatttttggattctcaattagaataaataacaacaggtatcatttctaataaagaaggaaaatacg
aacatattgactgcttgtaaaaaaaagaaaacagaaaaagagaatttcaaatgaaatttactataggttgctctagacatttatttcctttctactt
ttttatatttacttactatacaaaaagtagcaataaaaaattgatgaaaaaaaagggcccaaccggatttgaaccagtgacctggggagctgcaa
tccctcactctaccactgagctatgaacccttagttatgatatgataaatggataaattatataacttaagggaaaatgaaggcacgtagccaaca
tgaataaaaaagtattggtttaaactaacaatctatctttgttcttctacagcgtcaattgcccttatgagatctgcattattttacttttaggatgctt
cgttttctaatttcgatcaaaaggaaagaagcagtagatcattaaaaagcgaacatctagcgtaaatactttgtttgcaaacgactacggtacatc
cctggttactatagaatatacgtcttgtgaaagaatcattgcctttttcaactaaccaatttcgtaaacagttcatatgctaatgatatcaattgcaa
ttcattccagcccgcctcctaatttatactaatctgattattcgggcatttctaaacaatattggttgttacaacaaaatttgaagaaccaccaattc
atgagtttcaaagttgacctccgagtgatatgtcttttgaagctaaattaggtttaaaaaagctcaaccatattcaataaggtgtcgcttggtattat
ttcatcttcacaatggggaagaagatcgcaaatcttttcaaaggtttcaatccgaagtctccttaaatcttcgaatcccaatttctctttgaaaatac
ctgtaatcatttgaacccaaaacgtttgatgtttttttaaactttggctgtaattcagtttgtaagtaataaatggccttaaccggatccttaaatttta
gctcatagaacctgagaaaagttagctttggttctacaatcctcatacctttggatatgcaattctaaaagaacttgttcatatactgtatcttgacc
ccattcttcattaatttggagtttccaaaagtctttcagtttcattggttttaaaggatgagagctagcatttccaccagaaatgtagaatgtttcagt
ctcccaattaactgccaaagcatgaccaaacctgtcgtaaattagccccttcctgaaatttgaaaaaaatagatcttaccttgcacaaggatgcgg
attaccgattttctcatttatatctataacctttttccactgcttctgttcgatataataagcccatatacttggctgcaggattttatgatactctcgtt
gttcatatccatagaaggcgaaaatcctcttttttttttcatcaagtgaactccgttgacaaaacgccggatatggacaatctttgccacattgggac
caatcatggctttgcgcttcccaactggaagtgtcaataatatgcatgtctgcaaggaattcctggtcatgggtttgcccgccaaagacataaata
agatcttcatctgctgcgtattccatacaatggcccattctttcggtaagcaaattcatgttggcattcaaagatttaaaagcaatcaactttctttcc
caaatgccataatctacataatataaataaaaggtctcaaagaccaagtcccctggatcccatttgcatcctccacagacatatagtatattctcct
tttcgttcaaggtcatagcctggtcaaagatagccattggaccttgctctatagatgtgtctttagacaagcaagtccagtatcctttttgatattgt
atttccaaaaatctggtttgacatctgggcttacaagacttcctcctgcggtgccgaaataattaccaagtaaataaatacattgccgcgaagaat
ctaataccattcgatgacatacacgttttcctggaggtcttaatctgttagaaatataaaggtgactaaaatcatactgattaggtcccaggaggct
ccaactttgagtttcgacggagaatttccaaaggtcagaaaacgtctcaattccgtcccatccaccgtaatggtaaatactattgtcctaatttgttt

agctttgtgatattggactataaatggaagcttacttgaggattataaaccatctggtgtccacttcgaggaccgattccacgactaagtaaagag
atttccttagtgagtatttttggctgggcttccttcttccatctttgacttaattctttccaaaccttctcttcaaataagcgaatggcatcacacaaat
cgcctgactcgcagattgcactgtaaagcagatttaatggcgaaagcatataaggtatttggtagtggtgagcgatagctgaacatacctgactat
ttcctttgtacaagaaataagaaagcaaatattttatcgcttttgtttctagttccttgactcgttgtttttcagtggccgacaaacaatgaggagcat
tatatccatgcagttcaacataccatatactaaggttaaattcaggggcatgtgcctgtagaggaacgattttaatatatttgcaagcaaatggaa
aaacatttgagttctctttttgtgactgaagagtgaaagcttctgtgtttgagtcattggtaagaccggctttaagaagtagtaaaagatgccactg
atctctgccgccatagactgcaaatctaacttgttaaaaacaaactccaaatatcgattgtttaggctttacatactctttcaaattacaaagatgc
ggtgcacaatattttccgaacaaaatgtctgtgaccacgcagggttcttttaattttaataagataaactgcgtatctagttttgagaagaatttgg
agtttgcgcaactccaacggctttcgacttttcgaggttcatgtttggcaatattgtacggatgatatttcccagaataacttgaccagtcatgaata
gtataaggaagaattttcttttccattaactcaaatactaaattccagcctgtttggaagacttacctaacaagtggttttgtgtaagttctgctacg
aatgtaaacaaacatgggaaagtgcatttacgaaataaaaccgagaaaatcacacgaaaacgaagtgaatacttttggaatagggtttcatttc
taaaggaagaaggtctactaatttaaacgacacatttttgattcttttcagtttgtttacacgttaagtaaacaaagccgaaacaggatgagagga
ccattcacagacgaataaaaagattaatctgacccctttaaaagaataaaaagagcaacaaaaaaagcctacagcacctcggattcccatgttg
tctccaaccatagtactaacgaggccctcagacgcttaactgcagtgatcggacgggaattggtgttttcgcctaggtatggccgtagacaattat
acatttcactaaaaacctttatctcttgacgggtgattaccgtgtagacagtcttatcattgctttcaatataagtattatgctcaattctgcgatcag
tgcgaacgttacaacactttcaatccatgttttttctgaaagcccaaagttatgtctgcacataaagtgcagtctacttcttgaatcggttagaaaa
tttttatacgaacaacaaccaagccacgtagaaaatttacgcgtcatagaggacctgttgatgccaactctcattctgttttctaggaagggttgt
gatgttggttattgaagttggtcttttgtctagtaaacaatacttattgaatgaatagcaaggtacattcagacttgcggactcagtaatgagtatgt
ttactttttcttgtttgtttcaaagtaatcgaagtcaagcttaatgcatctcttcttctgctttttattgggtttgtatttagctttgtttgtaaataggaa
tggattgtttacattttgttaccccagcaacaccagctccttaaagtaaaaggacgcacacgtctttatttataaacataacaatcattcctcctaaa
gatttttactgccattattaggcgccgctataccgttaagctttacatttctaaaaaatttcacagtgttttagttaacggtattttctaattaacaaca
attcacagcttaacgacgagagtttcataagtgtaacttaatttaggcgtccctcttttgatattgttttgtgcaatcggtttcattccattcctttcttt
tcgtaacgttgcatatcttacaattaaacaagtcttctttgtaaacattggattcagttttcgtttatcaagatttccttcaaaaaggtaaaccttaca
gaaaagatatatcttggctgtcttctattttgatttctggtagaaagtatggggcttaaactacttaaatttgtgtttatgggatcggccgtcttgtgt
accttagtaggcggtcttttatccattctctgtattatcaaccaaacacaacataacgttgcttttcaaaatatatacttcgttcagctcaacaccacc
tcgatgttttctgttaacaatgctaccgagcttacaaaatcgataggagatcaaattcaaggtgatctggcaactcaattgtcttctgaacttcaaa
atgataacgagcagattcaagctataatcacggagctggaaaagcaaatgaaacaaatttcaggcattcccgattggtattctattggtctctgg
aattactgtaaaggcaacgcaacagactacacaaatcctacttggtgtagtaagccttcgccaaaatactactttaatcctatcaacatgctcgaa
aacgatctttcgaatgctgcgaaaacccagatcaatctccgtttatctgacgaagccgaatacggtctgaaaatttccaaagggatttgttatgctc
tgcgagctatgtatatcttaggttttatgtttttcattttcacacttgcttcagtattaatctcttggtgccctttttttggtcctcttttagtctctgtaagc
tgcttcttcgctgcagcctttacatttgtagctgcagtaatggccattgctttttatcgggttgctagttctaaactcgaggagaatctaactatgttg
aacattccaattatcttagggaaaaaaaatttacgcatattcgtttatttccgcagctgctggtttggcagctctagttttgttctttcttgccaatttaa
gtacttattccccctgtttaattctacttatttattaggttcatccatttttttattatatggttccttcactcaaaaaccactttctgttactttcgtacaatg
atacaatcgtgtatctattccagcttatttcgctctttcgctatttttcttcatattgcttaattttcatagattataataaattattcagcacaaattcct
atcaagtagtctatagtgcaaaaagatattgtatcaaaagagtgtatctgaaattaaattttcatagttgtcaaggaaattctttgtcctccgcgatt
aattctctgatccatgttaaataatccattccctcagtgcgtttcttcatgtttgtttccaaaaaaattcacaaaatttggacggacttggcttttcatta
agtcttccactgtaacataggattttccatcagcaacgtcacaaaaggcgtacaaaacttgacgggcactgtcacgatcttctagttcctccatcac
gacttctgtaaaacgttcgcatgtaacccccttcttcgccattcgatgctctctcaaataaggccataccttcttccgagtcataaactaaacccaatg
aagccaatgcagcataaaattccagctcgttcaacatatttgacttcttcttctcgaaatgacgaaagacggtcgctataccatctaattcttgcttt
gagagtgtacgtttctctctttccaaaatttcattttgcaagtactttattttatgagcaataatttcagctgtaacactgtattcgtgttccaaatcat
cataggaaaacaacgtgtagtcgttttcttctatacctgcttccttacaattgtcagaatgtaccttcacgctagctagttcgatttccaaagggccc
agatgctcttgtaaaaactcaacgtgctctaactgatccctccattctccttgaaggttagaaatctcgtttgaaatggttgagagcatttttgagaa
actgtttgcacgttctgcaaatgaaatacgcatgctttctttaatttctctcatacgggtattaattaacttggagcgatctgcttcctcagtcagaaa
atctttccattcgcgttccagatccacgatcttcaagccagcaggaggctcatatattcgaagttgataagttttcaaactcgtctgtattgttgctag
tagtgactcaagatcaattttctcttttacccattctcgtttttcagctgctttaaaaccaacgaaatcccgagaatgagccttaacgtcagaatatg
tgttctcaaaagtagaggctttccagtactcacgaactcgagaaatactctctagaagtcgtttcatacgagtctcatagtcttcctgcatcgcacgt
gttaacattaaaacttcagtaaatctctcaactctcctcgctgcagtctcaactttatctaaagtagaaaaagcatgaaagtattcggcaatgtaag
tcataatactgcgttcatcgggacgatcgacatcacaaacgtcctcgacatcaatcaaacgagggataccaatatgctcttgggcaatatcaaat

gctagttgcatattttttcgattctctttttttgtctagttcatggtagtttaacagatctggtcggtgcttgtgaattaaagcgcagaacgccaagccgt
ttgtccaacttctagagaaatcatgaatctccacctctgggcgatattttgctgtttttcgttgacaccataacagcaaaccttctttagcagtcaaa
ccttcttcattaatatcagcaattgtgaatcttaaaatcaaagtccagataagaccgagaatcaattttagatttccatcaacaatgtctaaaataa
attagttttcaatcttatgaaaagagacgaaatatttccctaatttgataatgtcaaccccattcagttacggtttgtccgtgtaaacaaagtactgg
aatcgcaaaactttataggaaaggagttgtattaccatattatttctttattattcataccagctggaccaatgtttgttaaaggcattcctttactctt
aatatattccaaggccttattgacattttcaagtttgtgtaaacggagacgaggctttcgattgtattttcctaattgttcatcacctattatttctaac
agctgaatcaacagtattccgtctgataaatctttcccaaggtcaaaaaccgcaggtaaatcgtgtaatgaaagttttgtgttaaacctgtatttat
ttcgcgtgttaattcacatctttttgattatttacttaccattttgtaaaagttctattctgcacgttctgccattgatttgcttgcatcaaaggtaacttt
tcgtggtaactatgttttggtgagagtaaatattacaagaaggtagggactataagttaggggaagttataagttgtactgattgattcgtatcaat
tttatatcttctaaaataaatgtatttaataattatatttttaagctttattaacttgcgtaaaacaagcagcgtccatatccatgtcgaattcgaaaa
cttgtcagtaataaagtcctatattcgacatactcttcctttgttattcttagattcttataatatattagattaaattagctgctagaacttgattctct
gaaaatgaacactttacatttctcaggatttgcctaggatggctctgtaaaaagacaaaaccacattattattaaggcaaaaatcaattttaagtc
acggataataaaaacttggttcgtaaaattagttttgcatcaaatgaatgcaattcattgaaaaaaatacaaggtacgcccttgatataatgccttt
ggaaaagtcgtctcctttattttattgaactctgttaaaacaatataaaaccttctttttcccttcaggtaaataccaaatcgtgaccaaaactcgt
gaagtcaccaatgtattttacgcttgtccatagagttcaaacatattctttctgtgaaaaatagaattaaagaaagaagagttgcttgtaacctttc
aaaataaagcagtatacgcttcacagattgaaaagtcgtcccgaactagaaaatccagtttctaacgcaaaaattaaggaataattaatttttga
ccctccgaaagttcaactttagtctctggagtagtcccttgaaagtcatcatcggcgtttgtggagttaatgccaaattttataaataaaccagtcca
tttagcttcatcatcttgttcttcattcacctgggatttagttaaagtcttaatcctttgtcttaaaggtactttctcccaagataaaaagcaatgttcg
cctgcagaaataggtattgactgaaaagcttccaagggaatggacacattttcctctaaaatacgaatttctactccctcaggaacatcaaccggg
agtaaaagcacgtttttctgaaaagtaaatacttgagatgttccttcttctgcaaaaagatcagtttccgccaaatcgcaaacaaatgtattagaat
caaactcggcggatatagtcgatgtaaattgtttgcagatgtcctttaattcctgttgttctacagccctaaagttcaaagacatatttctggtagttg
aaggattacgctactgagtgtagcatatggatatagtgttctaaatatggcataggatttagtagagcaaatgaaattagctacagtccaatactt
ttaattgattagaacagaaaaaaaaatatttattttaaacgcttagaatcattatggcattactaaaatattgtatgacctttctaatttataggcaa
cgaaactttcacttcttcggactacttcttgagacatctcgttccaacaaaacgggtttaataacttcttcgtcaaagcgtgaaaaccattgggcttg
gtcatctctagatgcgtggaaaaaatgattgaatgctccagcagctttttggtactgttcgaaagtattagaaattttctttgaaggactagcctca
ggtaatactcctgcatgctcataatcctctggcaccactggccgatcagggacagaagaatcaagagacgcattggaaagctccatagctccatt
ttctaattcataatcagctgtggcctgccatggaagcatggaaaattcggtatcgctatccacatcggcagctacgccggtctcaatgtttaaaatt
tcaagcatgcgagaagtagttccaccgaaaatgataagagacagcacgactacaacaagcgttgtagccttcaacgtctgggcattctcaccttc
aaagccagcggccaaagcgacgcctacagctccgcgaagaccagcccagaaaagcatcatttggtagctatatgggatatggtctatgagagtt
ccgtccctttgacgatgaaatttgttcaataacttacttaaagggaacacgttcatatatctagaagctgttaccgcaatctatacaccgttagtata
ttttcgtggtagcccatatcaacatatacataccgtcgtgatcaaaataaatataggcttatagaccaaatcaacctgtgtaaataaggacattcc
caaataaataaatataaagttctcagataattgagcaaggacacggaaaacgtatttagtgctcaattttgctttgtaagacatattataaaaagc
ataatgtttcaaggtaattccgcaaaacaaaagcgaaacaacaccagacatgtgacatccatttgagaaaaaatatgaagcatacgccataag
caagatgatacaagattctatatgagggtaacgtcgcagataagaatatttaagaaccagagccgtcataagtccaacggaaacaccgattaac
agactgatgaaaaaagtgataataaaaattgttataccgttaaatagggtgaacaaatgaagttgttttccttgaaactgttgcaaagtttcaaac
ataacaatagcaaccgcgtcgtttaaaatactttcgccaaaaataatggtataaagcttttgatccactttataagaattgaaaatagcaagaacg
gttacgggatccgtggctgataaagtagctcccatggataatgcttcgacaaaggacatatggaagttctcaaagttcaaaaacgagaatatgta
aaccaatattcccaaagtgaccgcagatataaaggtaccggcaaaagcaaatgtaaggatagtccctatatttcgaaagaaatttgactgttcca
aattagcattttgaaaaccaatagccccaggaagctgagtgggctatgagaaaaaaacatacctgatgcaactcgtagcccgagttaaggataa
ttggcggaagtagaacattaaaaaaatatgtagagtgaaaagaaaccatattttgaataattaatccaggagaaacccgaataattaggccgac
aaccataccaacaaatacggaaatgaccgtttcgtgaatggcgcgaatcttctttgattgaacatagtaactagtaagaagagcaccaatcagg
agaacaagaagaatgaacagagcccagcttgaatacagttcttgtaaagctggatccttatcatcctcttcaccatcagctcgtcggataactagg
caatgttagatttaattcaaaagagttgtaaatgatacacgcaaagcaaagctatcggctgccaaggccaaggaatctgatgaatatttacattc
agagagttaaagcttgacctgccaaaatcgtcagtcaaactcaccatttactatggaagcagcatccatgggaacaaagaggataaatagtttta
atgtgatagcacttattcgtaaaggattcaacggctgcagaaacacggtatttggtgtacagagtttcttcgagttttaagagaaagaaaatttcta
aaatatatatactttttatactatgaatttatagatatcaaatcacacgaatttattcaaaagagtaattggatttctgctttaattcttcattaaaat
ctacagttgagggaaaatgtttgttactttcaatatgttaccttgctacttgtcgcttgaaggattatttgtttattatcatatttcagaatgtgaagttt
tttgagggatttcctgagtttttgtataaataatatacttgtttagctttctttttgaaaagtagagacggatgggtttactgagagactatctagatc

atctttcattaactagtcttgtaagcatgctagacagacatatattacgttctaaagaaaggctattcattacctcttagcaagaacagagttgttta
taagacaatcgaagctgctttggttttaagtgcgtgcttatgcgagttgattcaagaggaagtaaaaagcaaatcgcttgaatctgaccgattcct
gttcacaaagaaaacatgacagagaaacaagaagtaaaaataactaatcaagtaaaattccttttccgtctccaatacaacatcacgagggaa
gcataattcgttaagggaaagaaagtatgcaagtatccagaaagaatatacttaaactgtttaagggaattcttcctatgataaatagctttaaga
cggttcgaaaaagttatttgccatttgcgattcgtaaacgagaatcaataacaaaacaagatgatagcattaccattggataattgctataactca
ataaaggtaatcatagttgtatctctacatatccaagaagaatatcattctgcgtgaatcaaggttcatttaatttgaaaacattcaccacacacat
agaaccctagattccgatgtttcagtctcaagaacaaggcaagactgtagaaagaaaaggacgggtgggatctttcatataaaaaccaaaaaat
acgtctttctcattatatattcaacgaacagccacaagattcagcagccatttatggtagtctaacaaagcaataaaacgaaagcaaaatagaaa
atagccaaagaattgcatgtgtaaaagaagtgagaggggagtttcaaaccaaaggaagataacgtacaaggttgtcgggagcaactttgacag
aatcctgtgaaaatgaactaattggaagaagagggggatgcatttcgaccttgctgttgtcgtttatcgtcaataacaacaattgaagagggccat
tcagacttagcagtatcatcgtcttgagagttgggctgtgatcctacataaggattctgcatagcaggacaagggagaaaagaaggaagtacga
cagtcgcagtggcaaagagagcagttgttccaaatagcagatttaccaacgaccgacgctgggatgctctgagtgtacgaaacatagagaaaa
gtatacaataataaggaatctgcaaggtattacataagacaattcatgtcaaaagatttgggtaatacccaaaaaaaaaagagaaatcgggag
aaggcaggcaaatgcaagggttctgacttgtctttcataggaagaagaagaaccaataagagaggtaggaatgaagaagtaggaaaacccgg
aagaaaagcatgtaccaagttataaggaaaataatagaaagacttatttattcagaaaattctttttcttccgttcattcttcctcatttcttcgcaa
accagatttcctcgttacgacaatccttactgccaagctcaactcttttaaataatgctcccggccaaaaaatctattgtaacactcccatagtaga
atatacttattttaacggttaatattccgtatagtaatactattaccatattcaaaagcatgcgtttaaactttaagtagatatctgctaacctacgat
aattatgcttttattttaataagcatattgctatggtaactgaaataggccgaatatatatgtaagaatagaaagaaaacaaaagaatctcttcgtt
agtttttccttttatttttttttagtcgtttttttcttttgtaaaaattaggtgttatcctcttgagttgcttttattcaccatgagatcattgacgaatatt
tcatttttttcggtatgactaaaaattccattgacttcatttgagtttctgttgaagtttcttttataaaaaagccccagtcttctctctctgactgccaa
cgttaggtctgttcattcaatactcttttattcaaataaaatgacaggaaaaccggctatttcagtaagtaaaacgtaaaaagttaattaatgctttt
tctttcaagaaaatgaatcatggtaaaaaaagaaagagcaaaaatatgttagaagcagaagaaaacaataccattcctataaaagctatatact
aatgaagtctttagttactcagtgcatctgttgcgaaagccctcgacggagagcttatgtcgactggagccttttccgtagatcagtaagtcctaga
taatatgcaggaacactagttgtagactaaccattgcttttcttttaagactcatggaattggctggtttgtcagtttctcaagttgtataccgtgaat
atccctcttctagtcatgccaaagtacttgtctgctgtggtcctggaaataatggaggcgatggtttagtagctgctcgacatctatggcaatacgg
ctatcaaccaaccgtatattatccgaagccatcgtcaccagatctgtacaagcgactttgcaaacaacttgaagaccttgacatacccatccatac
tacccacaattccaagatacttcatcaacttcttggtcagtcttctctggtagtggactctttattcgggttcagctttaaaggacctgttcgtgagcc
ttttggtgagattatctctgctattgttgattcaaagcttcatgtagtctctgtcgatgctccgtcctcctggggtaagttttcagtgctttcaaggact
acctgttctaacctatgcagaaattgacgaaggtcctcaaaaagaaggccctatgaaaaatttcaatccaaatgttttgatttcccttactgcaccc
aagccttgttgcaatttctacaagggtaagcattacctgggcggtagattcgttagcaaagccatcaaggacaagtatcacttgcaattgccctcc
tatcctggaatggaccaaattgtggatattacggatcagcccttagctacagtataaagctcttactgcccttggcaaccttttttgtctcaagccca
gtcatgttcctcattttatgattataattacatacaatagctttttttaaaatttctaatgttgatatgttgtttttactgaaggttgcttctttatagcga
aaagaacatgtgctcaaactaaggtacaaatttcacaatttagttgcattccaacactacagaataaaataatcaacatttttccaaaatttcgttt
tcaaaatggaccactgacgactttctccaattcactttcctagcttcactgctgtagccggaacgtgcaggcaaccactacgtggccatttcactga
gagttttgaaaatacctttttggagtccctttttttgaagaatatcctaattttcttattttccttatttggattcgaaagatttctgacgaattctatatt
tctagaggaaaatttttttttcttctgctgacccattgtcttggtacatcttagtaagcaataaactaccaggattcttttttaggttttcttatttgaat
cttttcattccaccatttccaccatttcgcagaaacgtaaaattgagaacatgactgatcctgccaaccgcgaggagttattccctgaaattggtaa
agctgcacagaatgtttctggcgccgatgaagaaggctctggagttcaagaagtcgaatctttgtgtatgcaatgccataataatggtactacaaa
actgttcttgacagtcatcccttactttcgagaaattattttaacgtcttttgaatgccctcactgcggatttaagaatgctcagatccaacatgctga
aaccgttcaaccagagggctctagttttaccctccttgtcaatgagaaagatgatttgaaccgtaccattgtcaagagcaacgaggccgtcgttcg
tattcccgagctccatttggagattcctggacgtcttggtcaattgaccacaattgaaggtgttttaagcaacattcacgatgatttatctaacgatc
aagatgctcgtaaagaagccgctcctgaattgtatcagcagatttcacaatttttggagcgcgttctccaataccgtaatggagaggctcctttcac
tttcatcctcgatgatgttaccggcaatagctgggttgaaatgagacctggcaaagacggaaatcgctggactcaggtcaaatacaagcgtactc
tcgaacaaaaccacaagttgggtcttgtcaacacggatgaagatcaaaaccatgaaacttccagaaagactgatgcccccgccactttggctcat
gatgctactgccgtcgaggttgatcccaacgaagttcacactttccattctacttgtccatcttgctcgcatccttgtgatactcacatgaagcttttg
gatattcctcacttcaaggaggtgattattatgtctaccgtctgtgatcattgtggttatcgttccaacgaagtcaagaccggtggttcaattcctgat
aagggtcgcaagatcactttgaaggtcatggatgccgaggacctttctcgtgacatccttaagtctgaaactgcttccttgcaaattgctgaacttg
gtttggacttgtaccctggtaccttgggtggccgattcaccaccattgagggaatcctcgctcaaatttatgatgagctctatggccgtgtattttctc

aagagagcgattctatgactcctgaacaagttgcaaactggcagcaattcctttgcaatctgactaatgctagagagggcggtactcaattcacc
gtcgttttggatgatcctttggctcaaagctatcttcaaaacttttatgcccctgaccctgatccaaacatggtaattgaagagtatgagcgaagctt
ccaaacgaatgaagatcttggtcttaatgatatgaaaactgaaaattaccaacagccttctaccgatacaaaaaaggagtaaacggttagctag
gcaagtttcttaggctttttgagttgttattattatgggtttcggaattccatgcatttttttgatacatatacagggttaataaaatagtgctttttactt
tgcctgctttaggtattgtagctttcttgatttacaagttaacttgctcgtcattttagtaaataatcaaacaattttaaattaagactgtcaaagtag
accataaacattttgtctattcgttagtattattttttttctttatctattttacgctattatcatttttctttttggcgtagtttcttttatttcgaaaaccgg
gattaacagtatttcgctgcatttcataattccaagtttaaacgcgacacgatttaattacactttctctctgccatcacacattcaagtcgaaaata
taaacaaacactcttttcactctttggtgaactgtctctctttttcattttattaattaattataaaacaaaactttttacgattgatgattcaagaccct
acgaattctccccgccttgatgctctatcgtctagaaacaaggaaaatgttcctggaaagcttttcaaaaagttcaaatgtccatccttaataatat
ctgaaaaacgaaaggaattgccattaagaaaacgacctaggattaattacaatgaatatggttctagcgacaatggaagtgattctgtctatgtg
acggaggagagagccggtctagcggccattaaagaagcaaatcgctgtgtactttcccatgaaagaaaagatcctgacacagtaataaaaaaa
aaattctctgtaccgaaaccaataaaaggacatgaagacctgtccaaaattagtttcagccggccacctcctacgttaggaatgaaacgaagag
cagactttattgctaggccactatatgatcctgctgatgaatttgcaattgttttatatgatcccactactgacactcacgaagaggttcccgatatc
aaagaatttttggcagaaaagcgaaaggttgaccaagaagcaaaatccaaaaaaggaaaaaaacagatactcgagaacgagtctgacaccg
aagaagaagctaatcaaagtaatgattccactcaacctcttaatgaagagacaccaaccgagtcagcagagtcggcaacaacggccaaaggg
aaatttaaaagacagaataaaagcttaaaagacttacttggaataaaaaaagagaagccaccaccaccacccgtacctgttgtaatcgaccca
aagctaactcgcattttacgtccacatcaaattgagggtgtgaagtttttgtacaaatgtgttacgggacgtgtggatcgtggtgcgagcggctgca
ttatggcagatgagatgggtctcggtaaaaccttgcagtgtattgctttgctatggactttgttgaagcaatctcctcaagctggaaagccgaccat
tgaaaaagcaatcattacctgtccgtcatcgttagtaaagaattgggctaacgaattggtaaagtggttgggaaaagatgcaataactccatttat
cgttgatggaaaatgctcaaaacaggagttaattacagctttgcagcaatgggctcaagtgcgaggtcgccaagttacccggccagtcttaatttc
tagttatgaaacccttagaggttatgttgattgtttaaagaacacagagattggcgttttattatgtgatgaaggacatagattaaaaaatagtgac
tctttaacatttaccgctctagacaaactaaatgtccaaagaagaattatcctttctggtactcccattcagaacgacttaagtgaatatttttcgtt
gttgaattttgcaaatccaggccttttaggttcacgacaagagtttcgaaaaaactatgaaattccgattttacgtgggcgtgatgcagacggctca
gagaaggacaaaacgatcggggatacaaaattggctgaattggcaaaaattgtaaatcgatttattattcgccgtaccaacgatattctatctaa
atacctacccgttaaatatgaacacgtagttttctgtggtttgtctgatttccaaatgaagctctataaccattttgttacatccccagaaataaaca
aaattttaaaagggactggcagtcagccgttgaaggcgataggcatcttgaaaaaattgtgcaatcaccctgatttgctgaatatatcggaagatt
tggaagggtgtgaatcgttatatcctgctggattcattccgcgtgaacatcgtgggcgtgaccgtaacattgattcttctctctctggaaaaatgttg
gttttagagagaatgcttgctcaaattaagcgagaaacagatgataaaatagtattaattagtaactatacgtcaacactagatctatttgagcaa
ctttgtcgtgtacgtggctataaagctttgcgtctggatggcacaatgaatgtcaataaacgtcagaagctggttgatacttttaatgacccagaga
aagacgcatttatttttcttttgtcttccaaggccggtggttgtggtatcaaccttattggagccaatagattaattcttttcgatcccgattggaatcc
agctgctgatcaacaagctcttgcacgggtttggcgtgatggacagaagaaggactgttttgtctatcgctttatcgcaactggcacaatcgaaga
aaagattttccaacgtcagtctcataagcaatctctatcgtcttgcgttgtggatgaagctcaggacgttgaacgtcacttttctttggacaatcttc
gccatctatttcatcttaatgaaaacactatatgtgaaactcatgaaaccttcaagtgtaagcgctgtcgaaatggcaaacaatttattcgggctcc
tgcaatgctatatggtgacaccagtacttggaatcatttcaccaatcctactctagagaagattgaagatcatttattgaagcgagaatgcggtaa
ggaacagattactactgtatttcagtacaaatctcattagctctgtttgtggatatattcgtttttttctcctaccatgtttgaggaatgattgtttcatac
actctgcatatgtacagtctatttaataacttgttttctatttttatcgatttttatggtacttatggaattaagggtttacttatgctttttttaagtgcag
caacgctcaaaagtaattgctttatttataacgacgaaatctataatataatttttgtttaatctaatatatttctcaataaatgtcactatccgtgat
aacaggagtgcttatggaaactatgctgcaacaacaaaggtatatatgaaattttgcaaatcattgtcttaggcagtattacaaattaaagagtat
aactaccactgaatgcataatattcgaaccttggataaaacaatttatccaaggtatataagggcgcttcttaaccttacttaagatatagcttact
cataggaaaactgcactttataaatgtgtattttgtgatttaaagattgcaaacataaacaaatatataatgtatatgataactaacgacataatat
atatcaattattgaataataatgtttatttttagaaattgtacatatttgctctagcatagcttaattcatcgacaaaaaagaagaaagtcaagtttt
gagatataatacagtaatttacaagggctcaagcccagtccgtaagtccttcacaaaaaccctgtgtattcagttttcatcatatagttaagcaac
gaacaacgcttatggatttgggaagcagatgttcgataatgagacaaagatcactgtgtgaaaagccatacggtccaatctgtttaaaatagaat
ttttatgacgataatagagatgttagaaaggaatacataaatcgtcaatgcttagtgaaatctgaagtttcacgttcatattaatctttacttaaag
cttttgcagtaccaacggcaccttgcttttgctggtaagaaggcgtaggaccagatttccgaacacttttcacgacatcatcactagcaaagccag
gtaaacggatattccagtttctttccaagtgaagctggacgtccttcatgtccaatgtgtcagacttgcgatgctttgcgagacgacaggaaaagtt
tgttaccgactcaacaaactcgtcggcaatttcaagcaataattcttcaacttcaggttctagacgttgctcagaatcaatttgttgtaataagtcgt
ttaattttcttttagacaaaaggcgattgccattgtcaagttcgtaatgaggagcacgcgcaagaccaggtgtgctaagacctacatttccagaag

cataaccaccagtcaaagttgcacgtccaggaggcatagcaaagggaacaggagatttcgcatcaatttttcctaagtgattattcgtcataaaa
gaagagttttgcattttgctatccatactcccagcctgctgaggagtagacgagccggcatctgcaccttctacattcaaagtgtttgccacgtttga
gtcagtaacagcagtacttcctaaaggattatcaaaaggtgctctttgctgatttgcattaggttgcgactgagaacgttgcaattgaggagtggtt
gctgcacttctagcatcgttacccaaagtgttgtttggcgtaacagaaccgctattgtttacggttccggataattgtcgattttgtaattgttgatttt
gtagctgctgaatagaacgaattccattcgaaatagcagccgtatattgagcaatttttacagtcagttcagactcttgtttcgccaacgtatctcg
agtagaagcatcaagattctcatttgttgttagctctttcttgattctttcatgcgtctctttcatctctgttaaataattctgagcagtctgaagctgcc
tatgtcgaacaactaacattgtttgttcttgaggactcaactggtgtcgaggacgaggcggaacagcatttggttctggagtacgctgttgaaatg
atccgttagcttgcaattgttgtaattgttgctgatttggtaactgtcccgattgcgcgtgtgcttgaagctggcggcgaatagcttgtgcctgtaaca
aatatacctgaccttgaggagtatttaatcccaaattttgagcattttgttgatatatagacatagtatgtaacagatttgtaagctgatttgcccgtt
gttgattcaaagctgcctggtttacaggaccactagctgggcgctgaacaggcgttcctggcccggaatgttgtccattcattttatttatttaacca
ctatacaagataagatgttgccgaacttcttttcgtgataagagtagctttcaccggcaattccaaatttggcagtcgacaggtcgaatatagtatg
cgtattacaacgaaagtacaatcttaacaaaacgtaaccaaaagtagaataaaaaaaagacattaattttactagaattaagagaaaaagattt
tattataaaaggtcaaattctggtttttaaagtcaacctcatacatagatattggtacaaattacaaattgacaatgaaacatagtggagaaatcc
agtacttaagaaagaaaatcgtacattatgtgaaagtggttctaatgaacaagagacagtctattccttcttaatatctttgccatcatcctctatgc
tctcctgtgaatgccacatagaagcataaatcccatttaatcttattagttcttcatgggttccttgttcaaccacttgtccgtctttaagtacatatat
gatatcgcaatttcgtatagtcctcaatcgatgagctataaagatacttgttttctttgacttctgaataagggaattcacattatgaaggagttctct
ctcagttgaggtatcaagagcagaggtagcttcatcaaaaaatagaatgtctgggtttttaataataatcgagagacagctaaccgctgcttttct
cctccagatatcattagtcctcgctcacctacttttgtgtcgtacccgtctggaaaggtttctataatgtgatgaatctgagcttgcttcgctgcttga
ataatttcttcttttgaagcttttggatttccgtaacctatattgtacaaaatagtatcgttaaataaaggagtatcttgtggaactaccccaattgcg
tctcttaaggactttaatgtcattaaagttaatggttgcccatcaatcataattttgccggaattggcatcataaaaccgaaatagtagtcttaatag
tgtagactttccgcatccactgggtcctacgaacgcaactttattgccagctggaatagtgaaagaacaatttttaagattgggcggttgggatta
tatgcaaatgaaacattatcgaactgtatagaacctcccttcattaccaagtttctggcatctggggcgtctttaacctgtatatttatctgttttaaa
gaaaacaaatgctccatgtctgtgaatgcttgacgcatttcacggtaaaccgatcctaaaaagttcagggggattgaaagctgaaaaactaactg
attaatcataactaaatcaccaacgcttaagtttgatgaaacgattcctcgacatcccatccacatcataagagtaagagcagtcgagaatatgat
tgcctgtccactattcaaaaaggctaaggatgaagcaacacgaatatttgccttttcaaatgcggaaagatgtttggaatatcggttcaattcgta
cggctcattgttgaatgtcttgacagcttcataatttatgagggactcaatggcaacagcagaagccttgttatcagctgcatttgcttgacgccga
aaagttgttctccaagatgtcgtcttcaccgtaaacaaagaatacaatgcaactgtagctgctgcaataccagcaaaagaaacaccatatttgta
agttaaaatagaactgaccatgccaatttccagagtaatggggataatatgtagcacgagtgaagataagacgaagctaattcccttagtccctc
gatccatagcacgagttagacttccagtttgcttcgataaatgaaaattcatatccaaatttagcaagtgttgataaacattactggaaacgttttt
aatggcactttgagatacagaagcaaagacgctattccgtaattcttgaaatacggtagaaaacaccctagctaggccatagcctacaatcaca
gcacctacggtggaccaaagggcacccacttcttgaacgactgctgtattcatggtgtcgataatagtcttgaagtaaaacgggacttggacatta
aatatttttgcagcaacgagaagtaccaaagctgagactactcgcacttttagattgagcttacctttgggccaaacatattgaatcatatctttaa
ctatcatccagctcgttacgtaggtgtctttcttcttaacttctgaattagcagaatttgcagttgacacaccagtatgaactttctcatttttttggct
gcatcaaaaggttgcttttctgcttttaaagaccccgtagggttgggtgtctttctaaggtaagaaacagtggaaaacttcaaagatgtagaattg
cccaaaatagagcatttattcggagctaatatttggtttacagagaggggccctttgtatacataacgtatgggaagttcattaatagatcgtgata
tttgccttgagatagctgcgcacagcatctttgaaaggccagagtgatttttctaaagggtaataaataagaacaatttaattccgtttcgcaaact
ttatatcctttcctggacaattcttacaatcaaaaacaatcaaatgaacttgaaaggtatgaacaatgactcaaaaatagactagctttcctttttg
tctttcttaatgttttgaaaaattcaattcagtctgaccaattttatgtgaattcggtgtaacgaaagtattgtcgcaaagtgaaaaataaaaggca
agtccgtgaacagatattatattcaaaaaattcaagatgtaagctgtttggtctacttccagaagcttgcttctggatatagtaatcaaggaagca
atcgacaagtggtagtagtcctggttcatagaccacttcgtgcttacgttaggagacctattcagtaagaaacctcgattagtaagccgttatattg
atcagaaaggaagttcggataattttaagtacgatctagatttttggtcaacagaatcgtcgaaatccgaagacggtttacgatccatgcatttact
gacattctaagctctctttgaggtaaaaaaaaattatcatatattattcttttatctttagtagaagaattaaatctattgttcgtattagaggaaaa
ggaatggcagaatttttaaagcatttgaattctataagtgtttcttctgaaagacttattgaacctgagcagaaaccggctacgagatttactgatg
ctctgcttcatgtgaattcaattacagatttaattcgagacgctgagaaagaagaattaatcaccgctgaagcgacatctttaccaaaaggaatcg
aggaaaaattcaactcggagagtcctgcagatcacgttgcttgcattgaagaattgttggatatttaccctatgcaaggcggacgagaatatttgg
aagccttagttgagaagtacaatacacatatgacctctttggaaaatctggaaagagtactaatagaacaaaaggaaagattacatttgtttgaa
caaagacagaaggatcaagtttctgcgagagaaaacatattacagcgtgaaaattctgaaatacaaagacttgaaaacgaaattgaaagggta
aaactagagctggaaagatattcttaggttcctataaccgtcgctcttgcacaaaaaagctttatagtcatatttgagaaatggaaggagccgagt

ttcctaatggaagtcaggaaaattgcccagaattttctgagttctttcaagaggatagattgcttccatttaatatgcatcccgattttcaatcgaga
gaggtatatgaggcttcccagatacattcgaagccagagctgacttttgatgaaagtcaaaggcatcgaagcgacatccgaaaaagtgagacaa
gatattcctactttgatgtcgatttatttaagcttcaattaagccgcacgctttcacaatctacgcgacacaaagaaagtagctgtatgaaacgagc
aattaccgagctggaggagtttgaaaaaagggatgccattattagcaaggaaaatagacgtctttgtttagaagaactccttcaaaactataaaa
acgaaacgcccacggattgtaaccaagaagagaacattgattataccgattctaatgatatctaaagagtaccttacaaacagaaatttcccgct
ctatatcagtaattgacttttgattcaatatctctttcatcagtatacgattatactatcctttcaaatgacactaatagcaagacactaatagcaag
acactaatagcaagaccaaacaggtagtatcagttaatgagtgcatagccttttgctttctgagattctcgtaaaccaacgcaaatccataaatcg
tttgtttgtttacttttcttaagcaaagttggtatcattggaaaggctcgggaaagttatcactattcttcggataatcaccccttttaagaacagaat
tactccttgaaggagtaaacaaatagcctttcattggaaaccctattcttgatttgtttagcttgtgagaattcatatatgtcaaacatctgttacttg
ggtttgtttacatttgggcgtcataactgatataaaagggaaaggtattccagtcgattttctccagtcatattcatcataatttaaagaaaaacca
aaggatacatacttttatatataatgtcacagcctactcgtgtattaaccgccgatgatggtttaattgacgataagcttgcccagcgcatcacaga
gcacttgcctcctaggtgtgctgctgccgaagtatggaaaagggtgtattctcttgagcacgacggagcatcacttcacactatgtatcagaactgt
atgaaacagaaggaagaatccggaagccctaagggtgcttgcattttggtcgcaaaggatgtgcatgataatgtttttggagcctatgtagatga
gtatttgaatccacgtcctcattatgttggctccgatgaatcgtttttgtggaaatgctattctgacaataatagattccgtgcatttccctttgttgga
gactcaaactttgttatctattgcacaagaagtttcatcgctttaggaggtggaaatggtcgctacagtctttggctagatggaactttggaatatgc
ttactcggacactacgcctgctttcaaaaactctcctttaagtcaccgtagttgccaaaatcagagaatctttattgtgaatgttgaactctggactt
tagaataagtctattctttcttccagatttcacccttgatgtttttgttcatatgctttttttagaatcaagtacggaacgatgaattggttttatgtatt
ggagaaaaatcctataaagcatgcgtcttgtgatattgagaaatttttacttacataaaagtgttttctactgtattttgttctcttatgggtactctgt
tgatcaacacgaatagcttcccttttttttttcacaaccgagcttttcaaggttccaaaaagggaatctgtaattacggagttgtctggattttatgaa
gttgatgattttttttatttacgttgtctgcggctgaatttttctttggtttttcttttttttcttgggaattcgtaacaaggcttccataaatgatgatga
tatgctgtttcactctatttttcttgattttattctgtataaatatattttatgactgtagcaaaggaacatttgtaggtgtgttcattactcattagtcac
ccaagtattggtaaatgagagacgaaaattaaagctctgcgtttcatctataaaccggttctcttattttatcggtatgtataccgttttatttttgga
ttctttttgtttactatatagtgacacagaatgcagtttgttaaagaaaggaatctagtaaatgaagcctagcaaaaaccaaaatttagttaaaat
ggctccgtcttcgaaaggactcaatggcaagttagagaagccaaaaagtgctttacaaggtgtgtttatatcggttttattagtacaatcactgac
cgttagcaattgtattgtcggactcttacaactatcgttttcgtcctttaacattggataagccaagggtaagcaaaacagcaataggagtcaata
gctaatgtgtttagtgcctccttcctttggcaaataccccattgattgaatacacatttgagtttttggccctagcaggtgtacaagaggtttatgtttt
ttgctgcgctcacgccgatcaaattcgtagttatatcgagtatgtacttgcctattttgtatattcttgctaacaagtctcagaaattctaagtggaac
ttggcatcctctccatttagcgtccataccattgtttctcgtgaatccctctcagttggtgatgctcttcgtgaattggataccaaacaattaattactt
cagattttattttggtttccggtgatgtggtttcaaatgtgcctttaaaagaagttttagaagagcatcgagcaagaagggaggtagacaaaaatg
caattatgacaatggtcgtccgtgaagctagtccctttcatcgcacaagagcaagtacagaaagctctgtttttgttattgacaaaaagacaagtc
aatgtgtgcattaccaagctaatgagcgtggaaaacattatgtatctatggacccggaaatttttaacgagcacgaggagcttgagattaggaac
gatttaattgactgtcaaattgacatttgctccaacgatgttcctgctcttttcacggaaaattttgactatcaagacattcgaaaagattttgtttat
ggtgtcttgacgtctgatttactcggcaaaagtattcattgtcatgttaccaaagacaattatgctgctcgtgttcgtagtttgcaaacttatgatgct
ataagtaaggatgttatttctcgatgggtctatccgtttgttcctgacagcaatttattaaaccaaagctttacctatcaacgataccaaatttataa
ggaagatgaggttgttttggctcgttcttgtgaaataaaagctcgtaccctcattggtgcttttaccaaggttggagatgcttccatcgttgctaata
cagttatcggcagaaactgcataattggcaataattgcaacattgatggtgctttcctctgggaaaatgttaggattggtgataattgcagaattgg
aaaggctattatcgcaaatgatatacagattggacacaattgtaccatagaagatggtgccattctcgctgctggagttatcattggcgacaatgt
taatgttactgaaaacttgcgtttgacaccacatgaaagccactcgcaaggaatacccaatgaccctgctttggtaggagaacgcggtttgggac
aagaatatcacgctgaagccgattccgaagacgaggaagagcttatggaagcttctggtcttatacatcgtgttgaagatttaaatctctctgact
cagaaatctctgagctttcctcttcctctgaagaagacatggattttatccctttttctgccagacgtgattcggccaacactatgaactctgatgatt
ttgatgagggagattttaataaagaagcccagcaaagtattgagcgcgcctttgaagaaaaccattctattgaaattgccgcattggaactgaat
acacttagaatggcaatgaatgccaattaccatgaagttcgttcagctatcattcttgcgattttaaagcgtatttcatatcttgattttcctgccaaa
gagtctatttctaaagttatgactcgctggggtcctttacttctgaaacttacgtttaatcatgaagaacaagtcgataatcttttgactcttcagaaa
tattgcatccgtttgagtatgcaaaagcaatttttgcaattgttgggttatctctatcaactagaaattgcagaagacgacgccgtcctggactggt
acaacgatccacggtccgccgaaggtgaattagcagttttgagagacaattatggcaagcaatttgtcgattggcttaataccgctgaaagtgaa
agcgaagaggaggaagatgatgaagatgacgagtaaccgatacgtattttggagaaaaaaaaaggaattttgactttacgtaagtaatgggttt
ctggttttattgtttataggaatcaagttgaagtatatggacctttggaagtttgagtttgagatatcaatttagagtagtggcatgaatacacaaac
attagcaaaagttactttgtatatttaattttacattcatactagtgctataaaatgctaaagcacattgaatcttaaaagtaagcatataacaattg

aacaatagtattaaacccattataattttggggtttcctttatataaaagagaaaaaaacacataccttaactatattgcagcaaaatatatttata
atacaagtcataaccagttaaagcatctagaataaaaaattaatgaagcaaaagacctgctttgtaaataccatgcaaaaggttgtattgtcatc
ggtattattgtggaggatgaataccagactgaacagggtcctgcaacacacgagccggaaacatcggtgtgtcggtttctaaataaaaagaaac
aggctgtttgctgtcttttaaatcctctcctggtgagcctgatgataagctaacagtagaattttgttgtaacaaattcattaattgctttttcaatgta
tgtagtaatgacgatttggtaaaaggttttactagaagatcagtaacgccatgattaaagtaattgacagcatcgtccatggatagattagacgta
atagccactattggagtattttgatcatattggcggataaggcacgtaactgagagaccatctagttgaggaagtatgaattccatcaagatcaag
tcgtaaccaccactatttgctttatttacagcgcttataccatcaacagcaacctctacttggcaatcaaaaagagttagaaaatcgatagtcatcc
ttcttgaaacttcatcatcctcaactaggagaatacgaggctgccttcgccaaatacctccagtaacagacatattagaagccattaaagaatag
gaatccgttacgggatttgattttgctaaagactctaccgataattgggaatacgatttagatatggatggattttgctctagatgagggctgtatgt
cggttcagtaggttgaggaataggagggtatttttcataaatagagaaggcagggtggtgtggtaaatttggtggtccattcgcagacaaagatg
tatttagcgaggtagcagaagccgttccattagtctgcttctccttgccagctagatcataatctgaagaaggtgtcgcatataatgatctgtgagt
gccttgactggcaagggttgaatccatagcaatcttggacgcagaagacggtgttgccaatggttgagacgcaggaatatgaggcatagacgag
acaggagtgagattttgtccctttgtgatggattgatagccatcgattaatttttgaagtggatgagaaggaacaaacatggagttggaagtagtt
tgttgctgattattgctttccatatcacataaataagatatgaggctaccaatgagtaagtctcttgactcaattcctcgtttgagttcgaacagcttt
aaaagaatagtttggtaactgtttgcaagaccagcaaggttcgtatccaaagatttttgaaagtcatggataccatcaactcgttgccgaaggtttt
caatggcgggagtagcagcgtcactcgacatgttacgttttgaaggagccttacgctgaagaaatgaaattagcaatcttcaggaagggtgatct
atataagcatacctttatattatctagcagatctttatgatgcagttgaaaatcatcatggcgaaattcccaagcctaaaaccgtgtaagttggaca
tgaaaagtaaaacgctagattacttacaccttcaccataaatggaaggagccccgtcctcattacgcactttgtgaaagtcatatctaatttacgg
gttagtaacattcatagtaagcctcgaataaagacctacttatttaactgtcgaacaaaactagcaaagttgctgtgcttaaaatgtcgaggtaaa
atcgtctttgtaaactcgttcgtctaaaacattcagcattagtattttattaatacatatgggaaaagaaaggacaagcctactattaggcagaaa
gaagagaagttgaacggaaaaagcttgtagtaaaataaaagaaagaaaaaggagaagaaaaaggcttcatcaacaattcgtgatgcaagta
aaaaggagcaaaaaaaattcagaatggaaattctttttttgtttaaaaaaaaaaaacaacagatcatttgccattgccttgcaagactgctaagt
cgaatgatcaaagtgtaaaaaggactttgaaagtctgcttgcttttgaagttgataaaaaaagtgagattgcttttctagaaatatatatataatg
atgataacagtccgaatgaacgtccaaatgagaattcgacagaatcgtaatctgcaaattgaatagaattgttttatttcaaaacgaaaagcgat
ttttgtaataaaacaacccgattcatagtgtttcactagaccaattttcttttcccctcttaaacgcttttcgaggatttcagaattctctttttctttctt
tatttcaaccggcaaaatccgtcaaaaaagcttacatccaacacaataaaagaatctccagtatcgctccagcgcaaaatatggcgatactcgg
gttcttccaacatgttgaagagttttcgcacctagagactctgttagcagctaaactcgaattcctgatgcctgaaccgcgtcatattcgtacaaag
tcggaggacccgctcgaagacggcatcttctctgtgttttagtgaccaaaaggtcgattctcagagacgtttgatattaattattcgcaacaatggt
gttggttcctggtaagaatagttgacaaggggacatggctatgttacttgtttgttctttttctacttgccgcctccagcgtttgaaattattgtgaag
cgttttataccttggtacactttgttaggtgtcgttatatacgatgattactaacgaagggaaaaaggatgaagcaaagatatctacgtgggtcagt
agatacaaagggattaaaagaacaaacggagcatccaataccggattaaaaaaaaaaggaacaaacaaaccgaagaagcagcgattctatt
cggccttttaaggatttgaatacaccgactcatttcatatagaatgctgtacacacgttatgttttatgctccccagaagccatttgttgcctacactc
tttccattataaactatcatgaatcaggactgcttcctgtttggctgaatctcttttgtttacatattttatctgatgcttgtgactacttactttacccaa
cgagaaccgagcgaccgctaagcagaccaataaccgattccttcggcatcagataaaatttttaatatgatgatgtcaggggggattttcttgcttc
atctgataatgtttggagttaagctgtgatggagcaagaatttttttccaaaatttcaattgttattcatgtttttcaacattttttttcgttaatctcttgt
tttaaatcttaaatttagttgttcgagcttttttttatataatgttttcgcctctaccctatttccgagatgatcttcctttttgacttttcgttttcattcgtc
gataggattggatcattgacatgcagatccactcaggtcaagaccacgactgacaaccttttttcttcttccattctctcctttctaacttagatttact
tcaatagacttattcccaagttttgagcctttgttttggagaatctaatcgactggatggaagtatcagctttataggcgaaatttatatagacgaat
ttccaaacgaaaaactttccttgagacttgggttctcttcatcatagttgatttattcaagttatccgtccgtcgaagctttcaccattcactcggtgtt
gcttgattcaaacgtactatcaaacgatagacgagtatccattttccgtgatttgcatatctgattacaaaggcttttttttttgattgtttccttttttgg
ttctaacttggttcaacagatttcttttttgtatcggattcaatttgattcatttttggaggatcaaacagttcatttcagttcgtcttttcttaatcggct
tatagatccttggtttatttctttgggcttcatttttaattctttatttttttatttttcttaataactgcttttctgtctttttattattctctcttcgatctatc
atcatcataatcattcatcatgacttatcccaccgaagactatgattacgaaggccttcccgaaaactcacccatgtatgctcacttacttgccggt
gccttttccggtatccttgagcactccgtcatgtatcctgtagacgccattaaaacacgcatgcaaatactcaacggtgtttctcgcagcgcaactg
gtaatgtcgtggcttctgcttacaaaattggctcaaccgagggtgtcttttctctttggagaggtattagcagtgtcattatgggtgctggaccttccc
atgccatttatttctctgttctggaaatgttcaagtccaaattcaacgcttccgctgatcggccgctcatcacatccttggccggtgcttgtgctacta
ccattagtgatgcctttatgacccccctttgatgttatcaagcaacgaatgcaacttcccactaccaactacagatctcctcttcaatgcgctcttaca
cttttccgtcgtgaaggcgtgtctgccttttacatttcctatcctacctcgatcgccatgtccataccttataccgctattcaggttggttcctacgatgt

tggaatgaactttttaatcctgaaggcgtttacaatcccgtttctcatatcatttctggtggtcttagtggtgcagtcgctgcaagcctgactactcc
tctggatgtcgtaaagacgcttttacagacccgtggctcttccacgatccaagaggtgaaagaatgcaaaggttgccttaatgccatccgaatcat
atataattttggtggtgtccctgctttcttcaaaggtatcaagcctcgtgttatcgtttccatgccggccactgccgtttcctgggctgcttatgaagcc
ggaaaagagattctgtctcaagtatccagtcccccggaagtctagtctatattgtttttacctactttttcttctatccttgttcttaacatccgataat
gtggctctttttatttttccgctcttcgaaaggtttgaactgatagctttttgtttacaacactatttgtatatgtatatatatatatcttttgagctaata
ttttttttttattcctatctgtcccccaaaaaaggcttaaatccgctactacatgccttgtgctttcttttatattttttttttatggttataaaaaaaaag
ggtttctttttgttaggttattagaagtttcaataaaaaaatgcatttttatttatccctaaggccaaacctttttatattgtcgtgtagcatcatgtagc
ataaaaactcaaatccatattaacaaaggagatataaataaaaaacggctgctttctgtagacgttactgatcccttttagactattcttgtgtagca
tgtcaaggattcatattcatgatgacaagtcataaattccctagacagaatcgtaaaacactaggtttttctttttttatctttaattcactacgaaatt
ttgggaattcttttcgagttacagggagtactatgagggagcaatggataaagtaggtgatttcctcctgactaacaaactaaaaatgacattgca
aaaaatataaccaattagatggaaaaactaaaaaaaaaaattagtatagcacaatcacacacagtctatcgagcgtacagctgttctggagtacg
agcgttcctccttttctgttcgaaaagaagcaaaaattcttcgaagattctatacctcattagcagaaagaaccatactatttcacatactggaagc
attattcctataatgtcagctattatgcctaaaaccaaatacttgttcgaagaaagaatattctgaagcccttacggctataaaaaatgtagctcgaa
atagataatattatatgtacttccttaatgagctccattctttataattatatttatggaaacaaaagaaggacatattcctactcaagattgggaag
aataaacgggtttttatacttattcgtctctataattatatgaatacagaacttgagctatgactgctaacagatgaatattgaacattcggcaaatc
caagacgaaagacaaaaattgtaatctaacagctctgcagctaccaaaattagtttatgacagagtgagaatgcccaaactattatgtatataaa
tcatatcttaacaacccttatcgagtttggtcttttattcccacaaaaggtatcttccaatccgattacctttcttgaacaggacgcctaaggataag
cgttgtcttcaatccatatatgaaatcaatcttatcaaaaggagtgtgtatatctttcccacaagaccgaactaaatctgcttttcggtagaacgctg
gttaataccagtaatgacaaaaaaaaaaaatacgctagcggaaaggtaaccacgcatacacagcaaagggaggaaaaaaggtgtaaaggtta
agaaaagtgtttgagcgttataattcacaagcatttacaaacgttctatagttatgtacagttgtaggaaaaaaccatgcataatagacagcctat
caaagtgtcctataagtcgagatattgcttccatatgtttttctttagttgttcttcatcgattcccattgtgctggggtgtagcatttctagtgacgat
tagtatgacaggtcctcattaacaaagaataactctcggtcgataacaaagagaaagagacaaaaagaaaccccaactttcgtatctatgggaa
aagcatctaacatacctgcgtaaacgcatgaacctgtttcacaatttcctcaagctcgtggttgacaaaccgatggcgagcgagagttgtttttcct
tcaaataaaggcgaaacaatgactactacaaagttttgaccgcaccctccagataaatcttggatttcctgaaatcagagttaaatcatgctaacc
cataagagaaagacttacaacatgagtaggttgtagcttagattgaattaaaagttctaattgctgtgcgtttaccattctcaaggtcttggaatat
ttgggatactcaagtggtgaattgcaggtttcaagtttgccagctaccaactgaaagtttgcgattaaattttcagtttaagcataatgacggaaat
agaggtaagaaatcttgtaaatgaaaaaactaatcattctataggatcgtattcagggttggaatgatggatttgaagaattgttaagttatatac
ctgcaaaactgtattatcgagaacaaacgacggtaggtgcttttgaacaggaattgacattgaatgaccgttaacatgctgttagaaccaatgga
agcaaaagaagtcgtcttctgaagaaaaaaaggaacgtcgcaaaatgaagttctctttggatggtttaaacgacgaagaggaatctcaaaaag
aaggaaagagacctccaaattggagtgctgaaaaagataacggggaagagaatgaagattctgatcgccgtgctgaaagtatgtcacagatac
gtggaaaactagcttccaagattcaggacatgcgagaaaagcgcaaagctagcaatctggaccagaaaaagcagaataaaaaagtatccacc
gaagagaagaagactgcttccaagaagaagtcccccaaaaatcaagcttcgtctcgtgctggtttttagacaaagttagacatactagcaaagc
gtcttacaaagggtaatataggatattaggagttgtttacattgttttttttttttggatttttacgttgcgctcggtttccgttttggaaaaagctgaca
ttgctatctgaatgagcagatatttaattttacgtatatttgttcattaggaaaggaacaataaaatatggttgaacggttcttgttaataatagtctt
tcagtacgacagcatggtttgtgatacatatccaaaactcccatgtcttgttcgaggtcatgggatattgtcatatttatgctaagttcattctttgta
tcatgatgttgatatttcttattatttaaattcatctacagtacagttaaaatccctattttctctgtcgtgaaactaatcaatcgctctttgtgtttgta
gaaatccgttacgttgaattgaatcaaaagcaaatgtaaatagaagtgcttactgttgcaagctttgagcccttccttcatttgtagcaaacaaac
gcaaattttgaagtaaaaaactgtttggcctcagattttcttatgggtttttatttcatctgtggtttttctctctattctaagttaatttaattgtctact
atctaagttggtatttcgtccatggccatatcgcttttattttcgcaatattatgtgtcaccctcaacataccatatacttaaaaatcagatttttaggg
taaacgatagtgaaaagtggttctaaactatttctcatggtttattcttatataagactcaatagttctttctgagattgtattttgttccatttctcga
agtttataaacaaacaacttcaactcagaaagacccttaacttgcagctatctgtgagctcggatttgtattttattgacccgtttactgaataattg
gctttgttctgtttgatacaacattcacagtactttgaggagtgtctctgaaagagttggaatgctatgggggttgttgtactgctgcaaccgttcaa
ggcgtagagctcacgaagaaaaattggttcttaaagaagaggaaaataatttttcttgttcggctactcataaagagcgatccagtaaaaattcc
cgttccagtttaagcatagaatggacaacggaagctcttcaacagtttatttaccaatattcagctcggcgaaatctttccaatgagcaaagcatg
tctttttcatatttttctgagtcttatgcacagctatttaaacacatatattacagcgacagccggtccatgacagaagcagatgaggctcaggctat
tgttacgtttaccatcggttcctatttatacaaatacagcaatcccaagtccgtattttggaaacgcttgaccgaacatatggaaaacattgaggac
gatggtacatgtacagcgcatcagctagaatttcgaaaaaaggttgcacttacacacgtcggtcatccggcttttcatttcctccccttccaggata
aacgaacttttcattacatatggtctttatttgtttcttcatattttatgaatgctgcttctatccctccttccccccaacggccagctgcagacaatac

cgcgcaacgaaaaaataaaagatccacaccacaggaaattaaagaacgagaagacgtcgatgttttaagagagaaattcacgcagcttctttt
agaatgtcctgatgaatactgtgaaaactggctacaacggttatatattacatgtattcttccaaaaaatcattatatttatgttttagatgaaagtc
ttgactttttagaatccattgaatccatgaagcagcagatcgttaatcgaaaagctgcagctcttgactagagacccgttttagattcgagctttctg
tgattcgcttcatgtatatattctttttgtctttactttatgtttgtttgtttgtttgtttctttttccttcgaaaaatgggtatttttatgagaaactataat
cgtttagaatataatgacgattctttccttgcttgaattaattcattttgccttttctagtcatttgacatgaacaatttaacaatcgacaataaatgtc
atacacgtaccaaagtgaaataccacacttattacacttggcaattttcttctacaacactactctgcagtcgtgtatacatactcatctgaaaatg
gttgcctcccatattaccagctacagaacaacagttacaagagcaaatatatccattaatacaaaaggtagatttttaacggttgctcttggcaac
acgctcgagttcatccttcttcttaatggcataagagttagaagaacccttggcagcgttaatgatttcttcagccaagcactcagaaatagacttg
acattacggaaagcagcctcacgagcaccgacggtgataagagcgatggcttggttaacacgacgaagaggagaaacatcgacagcttgacg
acgaacggtaccagcgctaccgatacgggtcgagtcttcacgaggaccacaagcggcaacggcgtcaaccaaaatttggagagggttttggtcg
gtcaagagattgatgatctcgaaagcgtgcttgacaatacgagtagccaaaagcttcttaccgttgttacggccgttcatcattaagctgttggtca
aacgctcaacgatgaaacaacgagccttacggaaacgcttggtttggaaacggccagcggtgtgaggaaggggttgaccattgccaatggtgat
ataatcaaccaaagagatgtccttcacttcaacaccctcaaaagggaacttgttaaaaagtttgatgctgccattctcatcaagagagacgcccg
gggtgagactcgacgtagccatcttgcgttgtggtttttttcttagtttccaactacgactaagagtatggaaagaaactgtgactgttttcataccct
accctaaacaaaccttttcgaatgactttttttaaggagtataataattcaggtgaatagcgatatcgtaataatgtataatatcccacgctagattg
cggtaatcaacaaagcgtgcttcctgtgtaatcaaagaattaggaaatgttcagttaagtttagaaatgaaatacgagaaccttgtatgccatgat
aacatatttcgtagaaaaaattacaggattgcagacctctggacgaaaaataccaagtcaaacctcaaagtaccagaagggtactatcaaagta
atttcaagaagccatttccttcttgactttacccaacaaaacactgacttgatttttcataacaggctgaccatgcttcgtccgttttgacaattgttt
gcgagtctctatgcgttcatgaatttttcgatcatgttcttgctgtttctcctgtttcttctttatggcttcttcttttcttcgccttttatcttcggatacttc
gtatagctgttccaaatgattcttccttggtgtaggcttaggcgcttcttcctctgtcctctttttaccatactcctcaggtttgactgttttgtaataatg
ctttttggcttttgcttttaaaatcagatcttgcttgatttttttggccttcccttgataggcatgctcaggaagcactcgaaatttgaatccttctttgc
gtttgctcgagtcgtttttttgccattttccaacacggcttttagtttttttctaatgggatccctatttacttttatttctccaaaattgacgccctaataat
ttgtattctcgtgaataatatttacaatggctcctttcctttgtcgcttttggcaacttcccagaaccactttttcttctcagtttctcctatcgttacgctt
cgttgactttggcaatagtcttttagcgagctcgaaaacataccgtatttcgagttgacaagaactcaagctcatcagtggcatacttggcataaac
aaagcacgaagcgaattttagctttaataaatatcgagaagctgtctgttgaactcgtctttctttacttaagaagaatttgttctcaagtcttatgc
cgattggagcagtgagatacacatcttaacgcatttgaagaatttgtttgtatcattatttttttcttcatacaaaacgaacctgtcctcttttttttttt
ttctctcggttatattttgacaaagcacggtttatgacttcgtaattggctgctttctaaatccaaggtaattcttctatttattcttttctggacttgtaa
ttctcttccatatcgatagtcctattcatgatttggattctgtttggttttacataaactgcatccgctcgccctctttgaaaaataagttgctctggtat
ccattccttttgtatatacgattaaatgttatgtaaacgaattgtctctccgtacacgcgacgttcactcttaatcaaccgtgctttaacgctgagtgt
tttatataggtgcttgcactttaagcaggaatcgaaaagcttacctttgtctgtagccgaacgagagttaaaatcgattcccacgagtgcacagag
aaaatcgtttgtaacctccttgccaagctttcagtatcctatttctgtgagcagtgaatctgaaaaggttccttcaaacgatgaactcatttaccgtt
atctgaagactcttcccgaattagtgttttcggatcctcatattcaagataatttgaatcagctatctaattctaactcaccttggttttcagaatcgg
ctatttttctgcagaaattagccccatatttttaccttccttcaaatgcatttcttgaaaaagcaaacttgtactttcaatatcgcatgctaaaaacttc
ttcgattcatcatcttgcctttgccatttatgatgtgttttatttcttcttcttaaagaacgataatcgaggattccgcatgcaagcacagaaaattgtt
ccctttatgtcctttttttatgcatataaacatgcccgattatgtctttcatttgtacatgaacttacctaaaaaatttcgggataaaattgacattacca
tattaaataatccgtttcgcaaaatttttgctcatgctttttctgctatagaaaatcctctcgttgtcttacgttcgcttaataaggctgatgccaacaa
acatcgtataaggattctttattttcattatttacatttttttattcgttcatggagaacatcaaaaagttgttgctctctctcatgaaatattcaacaatt
ttaattgggttgctagaggtccttgtcaattattgatgaactcttattttcagcaaaataagttgaatgcggcggtacaagtatttaatgaagtctcg
gccaagaagcacacgctccaatcaaacaaggtacttttaagggacctattaagaatcattacttcagctggttattataatcgagactcatttcat
atggttcttaagattattttaaaaactggatatataccttctcttcaaattttctcaaggctccttacggccatatcaaagtataatatgccagagga
tcttttgccattgatcaatcagtatagaacacagttcaacaataggttaactccgagcgtttttgttgctgccgttaaagcgttggtttattgcggtga
tataaacaatgtaaaacagttttatcaagaagctcaagctaccggagaacttaaaaaccttagacatttgttcaatttgttcctcacatcaacgact
gtaagccttgacgcctccatggcaatgaatttgctaagaacattcaaaaacgataggtcttttattgatgaatcaactttggtgagctgcatcacct
tgttttcgcgaagaaaagatttagatgccatggaaaaagtttataagtatatatcggatttgggcgttccgactcccgctgaaggatatgcagcac
ttctagatgcttatattgaagcagctaatgacaaaaagattcaattatgccttgaaaaaattgaagagcttggcataggtaataatcctagtgtga
atcgcatgctcatgaggtatgcactttataaatatgactatgagttattacaacgttgcactgctgttgcacataaatatgataaaggggacgtg
attatctatatacaataatgatgctctatcacactaacgttggtgaagtcaagcaagctcttaatgtcttttctacaattcaaagaccaaatgttatt
cattttcaattgtggttaccatacttggtaatttaaagcagcttgatcaaataagatcattagaggagcgtatgtcttcattaggaatacagacga

ctccattgtctttagctgcttttattacgagttactgtaaacaaggcgccgaaggtttggcacaggccgaacgatatatgaataccatttttcaaacc
aaagaacgcagagctgctattttcaatccacgaacggcaaatgatcttcgatacccagtatctttattctccccattgattcgggaatatacctcttt
gggagatatggcggaagcaaagaatgttcttgcattgtatctagattaccattcaaagagtatcacttcgcaacctgacatcccctttgctatggct
agtatgcaactgtattgctctttgcacgataacttacttgctcgtaaattctgggatttaattttacgggtcgcaaggcagaactttatgtcaacgaa
agttgaaaattttatatcaggtaaggatgacagtgcggatgaagaaagtgaaattgttgctgctcataaaggaagcctgaacacttcagcagaa
acgtacttttctttcttagccagtgtcagcgcctttcacgaattagataaagaatggcttcgattaagaaaatggggtgttgattttgacgaatcctt
gttgaataaacgtatcgtatggactttatacagtggtcgaatagacatggcattatctgaattttgcgagtatttcctcacaagggaaaaactgtcg
gaatacattggagaagaggaaaatacttcgcgtattgctaggtttttgttatcacctaactctccggtttatgcaagcacctgggacgtacttcgag
aaaagattgctatggctaatgagaacggatatgttcgtactcagaatgccgaattgatggctgcaacccagtattttcaattttggaaacaaaaaa
atagattgttgtttacaattttgtcgggactttagatactttttgattgtataataatgttaatattaataatacttccatttataaatccttggtacaca
ataatgcagtaagaaaggtaaaaaaaaaaaaaaaaaaaaaaaaaaaaaaaagaatattattagtactaagtaaacgtttaactttaccgtttg
gttatactaataaaagtaaagttaggttaacgaacggaacctatttggtttaggnnnnnnnnnnnnnnnnnnnnnnnnnnnnnnnnnn
nnnnnnnnnnnnnnnnnnnnnnnnnnnnnnnnnnnnnnnnnnnnnnnnnnnnnnnnnnnnnnnnntaataataatatata
tatntgtatattatattatatatatatatatatatactatataatacttacgttacgtacaataataaaagtaagaaaaaaagaaaaaaaaaaaaa
aaaaaaaaaaaaagaaatattatttagctagaaacgtttattccttggtatcataaagaatggttacaggaccattgttggtaagttgaacgttca
tcatggcaccgaaaacactgaaaggttagttatgataggagtcaaaatagaaggtgaaacgcgtgtaattacccatctgcaaactgttagcaat
atgtaaaatctaaaagacttacttttaactttgtcgttgcccaaactggtcttcaaaccttccagcacagattcataaagctgaacagcctcggggc
ctttcatacttctgtggaagtcgggttttgcacctttatttactcgtgcatgcaaagtaaactgagaaactatcaatggctgttagaaagaatctggt
ccaattgagcagtatttcaagcataccacataagatttctccttgaacctcaagaatcgtttgcttccagggttggtcttgggcattttcgaaaagcc
ttaatttcagaactttgttgcatagcttatctacatcctccatagtatcatcttttaatatgtcaataccaattcacaaatcattttttttcaaagaagt
catagaagataggaatactctttctgaatagcccttacctgaaccaacaccgacaaggatgcaaagtctataaacaatgtacgatggttaacaat
cgataattccagtgtttggagcactattacttaccctttttggacggaagagacgacactattgtcaactataatttacttgttagcttatataacca
caagaaaagtagagaactcgcattgggaactcatacctgtcacagaagcattcaaaactttttgaataacggctttcatggttctagtgaaatttc
aacagtttatgatttcttaaatacaacaaaaagcggaaaagaaagaagttcctcaaattctatacagaatatgtatttggcgaacttgtttgtagg
aatgaaaaggcatcctttaaacacgcttgcatgacttcaaagtgcttcctcgaatttctgcaacatagttggttttatttgataaaaagaaaattga
aaaatgaatacgacaatgaagagactatgaaaaaaaagaattacctaaagaaattcattttatttttgcctttgacttattaacctggatggcata
acacggtctattaaaacaaagcacaaagcaaaagaagcagctgaatgttttcaatcacccgttgaaaagcgaatgaaatcaaaaggcaacatt
gaaataaaaataataaagacaaagttcaagatacatagaaaagcgagggccaaattcacttcttaattcacttgagaagtgatcctatcagtgc
aacataaaacagcacgagaattgctgagaattgaggtatagaagagtaagatagagaaaacaattttacgaaaagttgacaccgcatccatta
tgcagtttacctaacttaaagttgagtgtttcatcaagtcaaacatgaaaccaagtaagtattttcactaaaagcgcaatcttgtatacagttctctt
tagtcagaaccgtacacactgattaaaaatgacatctatataggaattgcaattaatggaatacattacgaatagccttcttgatacgcttaaaca
aagacttcttagcaggagcttccgtagcatttttagttttgttctcagcatcagcagcctcagtaccagcttggccagtggtgggcttggcttcagag
gatgccccggctccagcgggggcaccagcaccagcaccagcaccagcatcagcatcagcatcagcatcagcaccagcttccttagggccctttg
acttgggagccttggcagattttgtggacttggaagggctgagggaattgtctccttccttggaaggagacttttgggcatccttagctggtgatttt
ggagcagcatcacctttagcaccctcagtaacaggagccttatcagaggcctcagcctcagctttgtcagcggcagttgacggggcttgagcttta
ccagcagcagattctggagcacccttggcggcagcaccagcaccggcagtaggttcaacagcctccttggtagcggtagtatcaccagcaccgg
cggtaggttcagtagccttcttggtagcggcagcatcagcagcatcggaggcaggcttggcagcttcggtagaggtaccagcaccagcagcggc
agtgtcggcaggtttagaagaagtttcaggctgcttgacggaagcatattgtccagaggaagcaggttgttcagcatcagcgggagatgtcaagc
taccaggcatggttataccttcagtagagtcttctgcgtgtttgctggtggtggtactttgtgcggcagtagaaggagcggtagaagtgccctcagt
agtgacagtagtggtatgagggacggctgagtcattgtaagaaaagtcttgtgcatcctcgaaagcgatctcctgttgtacgtcagtgccgatttg
ctcagtcatggccaaactcgtggagaagggacctacggggctagcggatgcatcagcattttcggaagcggatgtagccttgctagcaaaggctt
ttgcatcaacatcgggcttaacgttggaaggagcgttctcgtcaccgcgcttctggttgtactctttcgaaatcatattggcttcaggctcaatgggc
ttgttttcagcgacaggtgcgtcttcagtcgtattttcaagattctttgcaacaccagtggttggtggagatgaaacttcagtgaatgtagtagtctct
ggtttgtaagcaccggatcgcatttcgttcgaaccctgtgtttcttcggtagcgttgttcttggaagtttctaaaacgtttctagttagtaagtggtttc
ctgggcaggagaattctttggacttgtatctaaagggatacaagtgactggcatggctgtgaggtatttctttcgtaagtcagccacgtttaccgct
gcgcttccttcgccttcgttgaatatatttaaagagaggaggaacgcgcacattatagattctagttttcacttaccaatttcttcagcagcgctggg
agcttctgtaaatgcagacttttggtcttcgctgtgaatgctgctattgtcgctgctgcttatttctacggcagcagggctagcactagtagcttggtg
agggaagtctttagcagcagcatctgttccgtgctctttttcctcttcagcttcagttatggtctctgtacgcatggctgaagaagggttttgctcaga

agtagagtcaccagcagctgcagctgctgcactcttcttcttcttagaagccttcttcttggctctcttcacggcctttttcttgtctttcgaagagggt
gctgaagaaagggaaacaggttcactgttcaaggtcaaatcgccctgtgcgctcgtggacgcgttttcagcaacgttttccttgtttgctagcgtttt
ttctaccatttgatctgttatggtctttgcttcttgcgttctactaagtctgaaaatggtagtagtggatggcagaaggtgactttcgcttttgctgaat
tccaaaaatttacaaggacgcgcacgcgcgctgagcttcgtgtttagtcataaagaccgttctacaaaattctagccaaagattagttattcctttt
actttagttagaagacaaattattcataaaataaaataaacgaagttcgataataaccaccaccatttggtaattcctacaaaactggcgttacat
tggttcatgcctgcttttctttacttttctacatagtggagtagaattatttacttcacttcacttagttaagttgaatttagtctagtttagttctttcttc
catcagtagcaacaaatcattggcttcttctataaacctttttcccccatagtttcttctcccatagttcattttatttacatttcatttacttaggtcca
gtgaaaaatttttgccaaccttttcatctctcttcttcctgcttgcgtagcatcagtatgtgaaacacatataacatcattacactttcaatttatactt
gaaaacgctttagaagaaggattcgctaagactccgttcttcgttgctaagtagaaacttttattggttcaaatcgaatcttcatatattgtttctaa
aaccttgctttgtaacgctctctctcgtcttacaatcacatcgcaagcaaaatttatatgaaaatgaaataaggacaaaactggaaatgcaataac
ttaacattcgatccaaaactcacataccaactatcgaaaatctaccttttctttcactgaaaagaaccaaacttctatccgtacagtttacaccacct
tttcaatctttcttgtaaatagcatcattgtctgcttccattttacgtgctaagaattaaaaaatatcaatttcctatattggagaatgtacgtcaaatt
aacaggagaaaaggaaaaaatgctaaattagaaacggaaacgcaaatggaaatagaaatagaattagaaataaaagtaaaaaaaaataaa
aacgtaaaggaaaaccgaaccttggaaaggaatagagacaagtgaaattgaaatgcttcaaaaacaagcatctcctagcttttccacccttcgc
catgagaaataaagttaaaaacaaaaaaaaaccttaaattgaccatccaagaaatgaatcaaaacaaacttccaataccacatggaaagaaa
tgaggtagggtttaatatgattaaatgttccatccaaaagttaatcttgtaaaatataaattgttggcatttttagtcttcatgtattaaatccttaaa
gatagttgcggaatctcgtcaaaattcataccattgaagatactcattacgaatcatcaataaaattcgtcctttcttggacatacatgcagcgattt
gcattgtctaattaaattattacgcgtcgtatcgttgttgcttatttatagaaaataacatttgtgaaaagtcaaattcttcatatgcaatttatgcttc
ggttgttccatgaggagattcaaggcgcctctgttcttcctctttcttgaagtccagctcggcccgaggctgttggcgcatcaagtcaatggcattgc
ccaaaagtcgattgaaggtaggcttgtcaaaagtggccagctttaggcgcgttttcgcacgcaccgttgctgttctctctgtctcgtgaatcagagc
taattctccaaagtaatcgcctttacccagtgtgaccacgacacctttgccttctttgcacacctctgcttcaccatcttcaattaaataaaactgatt
tccaatgtctccttgacggattacaatgcctccggctggataaacaacggtttgaagggcatctgcaatttttttgacgctggtatttgtccaaacccg
acaaaatgggaacttcctccagtaaactctcgtacatacggcgctggcgaaaagcattttcaaatacgatacgatggaagcttgctcgatccaga
gcatacaaaatacagtcgggagtcttggatacaacggtcgctgcccgaggtgcgttgtacatcaaagcgagttcgccaaagtattcccctggaga
tattgtaccgacataatcaccataacctgtggccagtacttcttccgtcgaaatatttggttcgttacgtttgtacacatcaaattcgcccttttctac
aatgtaaaagtagtcaccctctgcgccctgtacgattaatgttacaccagcttcgccaatacgcttttccgtcatagcactcaacacttccaagtat
cgctcttcatccaaatttttaaacaaaaagttgtggcccactgctttcttcaaatgttccagctcttcaggtgatttcaagggaacttgtggtcgagg
tagcctcataaatgagttggggttcatagactcggcagaaacggattttcttcgcaagaagttgtagttctgaggatatgaaacgttcatttctttc
agtccaccggcttcaggagtgtgcagcttttcaggacccaccgcttcaggctcttctggcatttcattcagatctgacaaaaaatcattttctaaacc
atcttcgctaatgccttcactcattacttcatccatgcgaagcatctctttacgctcatttctcagtttactagcaaagaaatcgaagcaaagttgga
ggacatcagatggacactgctcgttcacaagggtttttaactcctctatgagcttgtcaaaagaagcatttgcaaccatagcacctaatcttgaag
attattcgtttttttcactttttttcagctttggttcgtgattctcgccttgattctttctttaatatcaatacttgtgtttgtctagtttccttaatcaaaaa
cacttcctcgtttacttgttcttcttgatatatgctaagatcaagttggtcagttgagtgcttcttttagttgaccgacggtgacccaatatctacgtca
ttatatacgctgtactccacagcagcatttctgcactccacaccgggatcaggtatccatgagcgggactatcgtttcttctaaggtaaattgataa
caaagctcgttaaaccatttacgaactcagaacgaggttataaaagaaaacaagaaaagagaaatgcgacaaacgatgcaagtcagaaatca
tttaaacatggacgagaacgagctatgcttgagtcaaaaattggaatgagaaatggatctttatttacttatttactttttatgagtcaaaagttgt
aacgtaaatgatacgtaaggtgaaattgcttttcatgagtagtaactgacttttattatatacaaacgcatgaaataactaacggtccaagagatt
ttgaagaacacacaagaaagcaagattgtgggccattaaaatacccttttagaaaacactgctaatcttaagtgcgttcaaaaggagggaatatg
ggtgggtaaaatgttaggttagtggtatagtgaattaagatagaaatattaaaatcggaaacattaagataaagacaacgtatattcacatatca
acaatcgacaatcctggatgccaagtaataggaagagccactgctggtagaatcgttatcattttcatcattcattacaactttggatgaatcccat
tcggaaaaagaaaaaaaacaaaggtccaatgcatgatcaagaattcatagtcaggtaaatgaagacggtgcttcatcaaattgtcgtttcactcc
tcggagaacatcccctgttgaatgcattgcatcttggtgactttccgatcctccccttcgctctctcctccatgctccgaatgcagactaggtgttgt
atgaaaggaactttcttgtgctcctctactgggtgaataggcagtatatgaggacatcgtaaaacttggccgcttctctccactcgattcatgagat
gctacatacagaggccgatccgacgtcaagacagaggtgtttggtacaatgcctgtttgtcctgaagagcctggcaaggtagtagtcaagtttgc
agttgcagttgatgcagtaactgctccggggggtaatgggtcttctatagttcggccataaggagagtctttggattgagaatgtacaggcggtcg
tacatacgggggaggaacagacgtcacagtggaaggcggtggtgtgggaatatgaaaaaaggaagtgtttggttgaatggatgaggaagatgc
tgtcgagctagcattttgattcacagtagcggaaactgacgctttccttctcttagtttgctgactcatatcctgttgtggagcagtcggggttggtaa
ctgttgcttttgcagctttttggctagggcgacggctaatgcatttgctattgcagtggcctgtgctgggttgttcacagcagccggtcccaattgtcg

attaatgtgatcttgtaaaagtgaaattgcatgcacggcctgctcgttcggtgcttgctgttcaccatttgagttccggaaaaattcagggttcaag
gctaattttccatttagcataataattggttggatgccagactgtaataatgatggtttttcttctttggttgctgatccattgtcctttgcgatagatg
ctcgttgagaagagtcagaagtctttgaagaactgattttaccgcctgcagtaccatcacttcgatagggttcaatattctcatgctcaaaagatgc
agctcctgtaggagtagatgcaggtttcttagttttccgaaacatattcccttcgaactgatcaatataagagggatccaaaatccaaaatgatcct
tttccggactcattttgcctccttggaattcgtataaatgctttattcagagataaattatgcctaatggaattgtgccacgctggtggttgatatcga
taatagggccatgtattggaaatccactcgcaaatatcgcctaatgtcaacttcttgttagaattggcaataagggtacgtgcaatcaaatttgcgt
aagaaaggttaggtttttggtagatatcaccataattttgtggttgacttgattccaaacctggatgcaaaggcaatgatgtcgaaggatgattcg
attcttgatcaacagaaccatgttttgtgggaatggaactagtaggaggagtctcagagagatttcgtaaagtttcagggttagcagatggtaccg
acgaaaaagcgtttgatgactgacgctgaataaattgaggagacacagtctgtggtttgtattcggaagagaaggagagagattctaatggatg
aggatcaattgcaccaggatgctcaaataaatttgctggtgcaggctcggtggtcataagagggggaggagacaatttagtagaatccagttca
acttcttcctttacagaagtatataaggaatcttgattttggtcaatggacggttttggttcgatcaccggagctacggtcgtgttcatcctatgtgag
ccaggggcggcagcttcagattttctttgctcctgtttttgtagcccttctggaagtaaaaacgaaaaagaaatttgtccaatttgaacgcgagttc
cactatgcagtgggacggtggacccgcgttcgacgaattcgccatcaacaaaagcaccgttcttcccgatgacagataattcaaacctctggttg
ggaaaactatatgcaattttagcatgctgacgagagatggctttcgtgtctcccagatgtacatcgcaatcactagaattactggcttttcgaccca
ttgtaacttgcagggtctgaacaaagaaagaaaacttatcaaattctagtttagcataggcttgaaccctgttggtacctgatttctgctccgctgc
atcttgtcgaactgcgtcaggcatgccgacacttataccaaaccttcctttcctcaatcttgagaatatcaaagccaattgccaccgcaacaaag
cgcgcaagttattttctatcaaaaaaatagattactggcgaaatgaaaaggggatttcgcccttttaaccgaaacctatacttttccaccgaatgtg
tagttgatggagtggtagtgatttggtgttagtaaaaacactttcccacttcacaacatctggcgtaaacccaaacaggtgtattttggctcaaattt
gtttttcttttaaattttcttttataatttcaagatctgagggaaatttttcttttctcaagttgcaattggtgtatcttgagatttacgtactcaacaatc
tttactacaagaattcttgaaacagacagacttaaaggtgattaatattatagaggaagatgtcaaatttcttgaagtggtttggaggagccggtc
gtagcaaagatgtgacaaaggatacgatcactagatttcaggatactttgtctatgtttgaaaagaaagaacaaagactagagcgtcaaattgct
gaacaaaatgagattgctaaaaagaatgcgactacaaacaaaagaggtatgaacgaacaaggcagaagaaatgctttaatgaatattgacga
gattactaactgctttcagcggcattggcggctttaaagaggaaaaaaatgtacgaaaatgaactagagaagatagagggaagtcgtatgaata
ttgagcagcaactctataccattcagaatgcgaatctcaactttgaaactttacaagctatgcgacaaggtgctgaggctatgaagcaaatccaa
cgaggtgtagatgctgacaaggttgataacattatggacaaaattcgtgatcaacaagctgtttcagaggaaatttctgctatgattagtacaccc
ataggcttgaacgcagacgtggatgaagacgaactggcaaacgaattggacgaattacaacagatggaactcgataacaagatgcttggagct
gagaagccacccattcatgctcctggggtccctacaagcacccaagttgacaaacttccttctgtaagtaaacctcaagtagatgaggaagagga
attaagaaaattgcaagctgaattctccctataattgtaacgttatatatatataatgaaaagccctatatgcattaatgcttaacgaaggacaga
tgcaaatgaatttagatgggtgcattatgctgcttgttgcgatgtatcaataaacaatataaattatgtttaatttacgatgaattgataagtaaagt
gagaaaagattgaagatggtttatagtttatactctcttaaaactaaaggctactttcctacgaaatgtttttaatcagtttcgataataaattgctc
aggcaaagaaattaccattgacattatgaggatggatgaccttaacatagaataaaatatagtcaaaacctttgggaagatacgtctaattgcca
gaaggaacggtcacaatccaaagtgtaaatatgagccaaaaacaaaaaactcattctatttcatttacttttgagatggatgaacataaggcttg
accaacatttcggaaaaggatttcaaagcctcagggccacgaacttctgcagggatttgaggtactttaacaatgtgaaagtcttcgtataagtct
tcaatttggtctagatacttttgctgcatctttctacgagcaaggcattgaggacaagttgtagtaggatccaataaaagttggttaactacaatgtt
ctttacgtcaatctcgtatccaaccaattctatacagttagtaaaatagaaaaacgactttatgtgcatatagcataacacaaagcgtaaaagga
atgccgtaaatacgtaccttgaatcattctttcagtttcgtaaagcgagaggaattcagaaatgcaaacacaaacaaaggtagtgagatctgcat
tcttaaactgttggttcacttgagaaatgttttcacgcatgccttcaagtttgccaaaaatatcttcttggtttgcgttgacgcccataacactactca
tttggttgatcataggaccaaagcgactagagaggccaccaagtttgccaagagccttttcaaggacagttgggaaattcaaaaatctcaatgta
tgaccggtgggggcggtatcgaaaatgacacaatcaaattccatagatttaacttgtttcaaaatttcagcaaaagcgagggcctcatcgatacc
gggaattgtaaaagctaaatcttgcatcataccagacatgggattgtttggattttgctggtcagcttgctctgtcatttcttgaatggattgattag
ggtcaatctccatggcactaaggttctcaaatccaggaaccttcgtggcgtcttttccaaactttgttccaaatgcatcagacaagttatgagcagg
gtcggtagatatcagcaaaacagaagaacgaaccttggacatttgaatagccaaagagcaagaagtggtagttttaccaacaccacctttacca
ccaacgaaaacccatttcaaggtcttttggtgcaacaaattctccaaagttccgggtaaaggttctaacgacatgatggattaaagatactgtaca
aaaagtaagaaatttgactttgctatttctaataaacgatgaatactgcgaaaatgaacagagacctgttttatggtggaagtatgattttctttgc
tagaggatgtgttgcagtatgtatggagctggaagaaatataaagaaagtaaacgaaatcccaaaaatgaataagaaatttaacaaagaaaa
agaaatttaacctgaaaattgcatatgtgcattttttttacttgattgagaaagtatcttagtacgccagaaggcgccatataatgttggaaacatac
atgagggaaaatctaacaatttaaaaggtaaaacctgcatgtataagtatatcaaaagattattttatttacacaatccttggaaacccttgaaga
aaaaaaaaaaaaggaacgaatactttttacatagttgaccataagactggttggtattaatttcgagacttcatcaacagtagaacgtttgcaata

gaagcaaattaaataagtcaaataccagtgaaagcattcctgtttaaacggaggaagaaagaaaagataggagatgcaagacttggtttggaa
acgagtgaaaccaattttttcatcagattataaaggataaaaaagaaaaaagaagagaaataacaacggtaataaatatgtcatagaagtca
aaaagttgctgctaggccaaaagggagagtaacggatcattcaatgaaaactctcaatcttggagaaggccctatgtgtataaccctatgtctca
ttttgtaagcaatttattgaacggataggaacctagcaacaacctataccggatgaatgttcaccataactgtccttgtcccaagaaaatagtaag
tatcctatccaggcgccaagaaaggtgaataaaattacgtatccattgaacgacatgacaataagcatgagaatagtagctgctccgtaatagc
acatttgaagtaaagcacgaatgaactgttggaacacattcacatgaggagtaccactaggaaaaaacaatcgcatgggcccttgaagcgtttt
accattcgaatgctgaacaacgtaagcgtcaaacaatctttggattctcttgaaggcctcaatggcaatagcaaagaagatgatgcctataagag
agcctgcaaatttgtttccggtataagcgtgccaagactctgatagaaaacatgagtcgatggtatgccaattccagagcatgctcattttgcaag
agtgtgaagaagagtgtcccatatccataccacccataccactcatttttgagctgaaggatccgaagcactgtataactccatttgtttttttgttc
aagagcagaaataaacaaaatgaatgatcaaaaaagatagaaatttgatgaaatgctttgtttactaagtataacgaatacgtttatcgttaaaa
ttacaatagcgtatcgcgcttcaatgcttaatcaaaggaatcctatgctggtttcatcgtatatataagtgcaggtatgagagcgttgtgctttcaat
cgagcgaaatttgccttggcgaaaataccgctcttatagtgagcacgaacgtggtggatgtacctctcagtaagcttaaagagactcatgtatgtt
tctatgtttcaatttgggatagcgatatttccgtagattaccttacattggtttacgggatgtgaagcaacatggtagaaaagtgtgttatgtaaaat
aaggataaatttagcactttcttgtagtgctgtagtaaacaaagaggtgaaatgaggttttataaaggaagaaaacggttgcttagttttcacata
tgatatcaatgagaggaaatcatttgttgtccataaaatcgattaaatatagagacattttggagttcatttccacttaaacatactaagtggaaag
aaggataaacaaaacttggattaaattataaaacagatgattcaaataggaatttttagctctggtttttatttatttttctatatttcaatcatcttct
ctttcactccctaggtttacctattagagaagctttcaaaatctggtttcattaccttttttttttttttttttttactataaaccttggattataacttaa
taaatgtaaatatacatataaatataaatatatatgtccatatctgacttgcaattcttttgaactcccgaaaatataataaataaagaatcaatgg
attaaccaactaggctattcgcctttgtaaaattcaatatcacttttgcttattatacagaccgtgcagtttgaaaacgaaaattatttgaaaatgca
gtatttgttagatcaaatacagtaaacaagcaacctatcagttatgtcagtgtactaattttcaccagggcctgctagaaaaataatactcgattga
atctacgaaccaactataaagatcttgataattttcagtaaaagaaattttcatttttaatttatattttttatatttttaatacaattttatacaatt
ctcgatcatctgaatttcataattgagcaacacaaactttttcgcaaaagtaacatagcagaggtgacgagctttttataattgtcttttctaacagt
tcacttgtttctattataatttatctcttttattgtcttcttttcttgctacaattaacacaacgaaacgtacttccctattttgtaaatactttcaaacatt
tggcaattagcttcttcgcccttaaccaattttatttaattataggattccgtaaacaatgggccgcttaaggaagagatttaatgagaaaggccgt
cagagcggcatccaaaagatgcttaacctgaaaaaggcacgattgcatcgctctgtacgagaacaagacgatgcctctccagaacacccaaaa
cccatccttgaggagcctcaaactactgattccaacgtggacgtaataacggatgaagataaaaaggaaaaagaggagaggagacgccaatta
caagaccaattgatgaaagaaaatgaaggcaccactatgtctagtaaaaagaaaaagcggttggacaaatatatcgtaagttaaatttagtgta
tctatcaattttgggtgagaaatgctaacaagcttaggaaaataaattaaagaaagagacctctgcagctttaatggcgaaaattgccgaggaac
gcgttgacacatctctactctatagtaccaaggacctaggaaagccaaggactgctaaggaacgattacagagatccttgttggaagagaagaa
gggattaccaatctctgatccttctagccgtctatatacgtccgtcgaagtgaacgagtctcagccatcaacgtccgatacgagcatagctagcac
ccctacaaacaaaagtgcccccggttctggtttaagattcggattctccagcacttctgctccctctggggctgtcactgctgctcctttgaaagttct
gcctcccaaacggaagagatcatttcgatgggggaagaacgaagattttgatgatgctgatcatgactctgatgaacaggattatttaagcacag
atagtgaagaagaagacgaagctgatacaggatctgatgatcaatcactctcagaagacagccttattgaaaacgacaaagaggaagctgcgt
ctgacaagaacgttaacggtctttcaaaagaagtcattgaggcaaaaccagtgactgaggaaaaacctgaagctgtgcagcatcccgatcttca
tagtgaagaatttgacctaaattcgtcagaagaagaagctagtgctgatgaaagtgatgaagctaacgagaacgatgtttctgaaagagctaac
cgtttcaagtcttgggcaacacaacaacttcttcctggtgactcattcgagcatgaagagcaagaatctgttatttcagctcaaaacgaccagatc
cgtgagcgcataaggaatccgagaaaactcaaaagaactgaagaagatcctgatgttttacctgttggtgttgacgaagtgaaaaatcgaaaga
caacgtacaaaatcgtaaatcgtcctcccgagattcaagaatccagacttgcacttcctatagttgcagaagaacaaagtattatggaacatgtgt
ttgctgatgatgtggttatagtctgcggtgctaccggaagtggtaagacgactcaagttcctcaatttttatttgaagctggattcggttctcctgaat
cagataccccctggtatgattgcagtaacacagcctcgtcgtgtagctgccgttagtattgctaagagggttgccgaagaactcacaggcttttcctc
taaagttagtcatcaaattcggtttgacagtactgttaatcccgaaacagctattaaatttatgactgatggtgttttgattcgtgaattatcctctga
tttttttgctgagttcgtattcggccatcatcgttgacgaggctcatgaacgtagtgtaaataccgatatcctgcttggtcttctaagccgtatcgttcg
tctgcgaagagaaatgagtgagaatgatccaaagataaaacctcttaaattgttgatcatgtctgccacacttcgtgtttctgattttgcagaaaac
aaacagctttttgctcaatctcccccaattttgaagattgatgctagacaatatcctgtctccatccatttcaacagaacaacgaagccaaattata
tagaagatgcatatgacaaagtgtgtaaaatccataatcgattaccggctggtgcaattttggtatttttaacaggtcagcaagaagtggagcaat
tatgttcaatgctccgaaaacggtttattcgatcttataagcctcaacgtaaacagaatggacgtgttgctgtttctaggaaagaaatttcgttaga
aaatgaagatattcaatctgacaatgaagacgaagagaaaaatatctcgccaaaattagacgctattgaacttgacaaagaatctgagccgatg
catgttttgcccctttactctctattgactactgagaatcaattaaaggttttcgaaaaaccaccggagggtcatagaatgtgtattgttgctaccaa

catcgcagaaacctctatcactattccgaatgtacgatatgtagtggactgtggaaaagcaaaagaacgcgtttataatgaacatacctcagttc
aaaagttcgaagttagctggatcagcaaagcaaacgcggatcaacgtgctggtcgtgcaggccgtactggaccaggtcactgctatcgactctac
tcttctgccgtatacgatagagaatttagtcaacattctttgccagagattttacgcgtaccagttgaaagtattgtgttgcaaatgaaaagtatga
acattgataatgttgcgaattttccttatcctactgcacctagtcgtgcaagccttcaaaaagcgttgcaattactatcagtactaggcgctattgac
cgtgaaggccagctcacaaaattaggagaacaaatgtcgttgtttccgctttctccaagattttccaaaatgcttataattggtcagcaacatgggt
gtcttccttacgttattgcattggttgcagcgctttctattaatcagttgtttgtgtccaagcaatcacttttatatgacgctaatagaaaagaatcag
tgtcggaagaaactgaaattattgaaaacgaagacttgaagcaacgtgaggaacataaaaataggttacgtggattttataacgctctttttaaa
tttcaagctatagatacagattctcctgccctgaggtacctaagtgcagtatgtgcttatgactatgcagccgataaaattaggttttgcaaagaaa
attatcttctttttaaggctttagaagaagtatttaacctcagaaaacagttaattaaccttttaaagaggtatctttcgagtaccaataatgatttca
ctaagattcaactgaagcctccgtctgatatacaaatcaaggcattacgacagttcattgcttcttcctacattgaccagattgcgtcctacgacca
agaaaagggagggtatgtcacattatttccatgtggagctgaaacggcacgatttgttccagacacctctcctaacttccagcctcagtatgctgtg
tatcaacgtcttcacgaaagctctactggacgtgtgtatatggaacctttgacatcggttactggacaacaattggcccgactggcaaaaaattct
agcttaatatcttactcaaaacctttgtcttatcctcctatccgctacattgacgatggtgttaaacgggagtgttatgttataccaattttgagttca
catacgggttcaggtggagcttcatggaatcttcctgctgaaaaagttttacaaaagcgtactaatggaagatgggaaacagttcaataaatcaa
caactctgttatattaatcttttgaagaacgagtgaaaagttgactagtcccgtgagtgcttaattatacttacggaaacaaaaactagtagaattt
tggaatggttaggtaggtattttgggataattaataatacggatttttatatatttttcagaattaaataaattataaacgagaaaacaaacagcga
aatttaaactaagggaagcttggtgatgcaggattcgtactcattcgatgtccattgcatcttggtcgtggggggaagaagaaaatgttgtaggac
tactggttgtcttactagggatgttaatatcgttaaattcatcataatcatcatctagttcttcaatcactacagaactggatggttcatgcttcgcaa
agtctttggaatcagaaggcttgggttcttcaaccgtcgaatacggtgtaatttcagttgggtagtactgaattaatcgaggttcatcattatccttg
aaatgagaatcgtaagcccaactatttgatggtcgaaactgtagcatggaggaaggaaaataagaaggagagggagatttgagagagagtac
ctttaaacgatttgggtgacgaactaaagattttgatgcatcgggggcaaaatctaattcttgttcaggtgcttgttctaatggacgacccgtagac
agatcgactacttgagttgtgaaatgtggatggttggattctaatgagaccttcttccttggcaaaccatccgtttgtgggacttgctgcaactgag
gggtactcgtcggaatcgttgaagagctgttgtctggagaaacgagcgactgttgtgtcaaatcttcatccatgagagtctcatctgaacgagatc
gttttcgagaaccagcaggtgaagctgagacaggaggaaacgtttgatggaaggaaggcggcacatctggttgaaccgagtttgaactgaacc
gtggtcttttagcaggaaaatgtgaatcaagggacgagaagggagagactaaagagatctgaccctcttcagggaataaattattatgatcgga
aggtctgtctcgcttcatataaaataaccaaatgacgccaaaagaaaaataaatagatataataaatagatataaataaagacaatgaattcaa
aaacctttgaatcaatcagacttttcgtttacctatagaaacgaaccaaaaacgaaacgtaatttaccaatcgattgtttcacaatttttaccttcgt
atccagcaaaaggagtgagtttttttattgtaatttacttaaattcccgaacttttaataaaaagatgcttgcattgagtcaattccctcgaggaacg
aaaaaaaggactgtaatccagatactttttctttaatcatctagaaaaatccatatagaaatttacgccagagtccattgttcgtctacaatgagtt
aaaattgtacttaaaaaaaagagaaaaaaaaaaaataagtataattaaatataatacatttaaatgaattcataaacattcggtagtgtagcaag
tcagctatagctatagaaactatggcagtatatgtagagcagtttcaaagtaaaatagcagatcaaaagaaataaaaagaaacgtaaaacata
agtgaaatagacctctttattacgacgattccataaaggcggatgcgatcgtcatgttaggaatcccgtgaatgtcctatttggatgattataggaa
atgaaagaaaaaaggaagggcgctatccatcataggagtggagggttgtttatataaaaaagtctcaaagatagctgttgagactgatcgtaat
agctttgtttttactgcagcgtcagcgatatttgacaggtcctcgggaagccttgggaaagagtatagcgttcataactttggtattgatttggtatg
ctccgccaatctcctttgtaccccggtgttgcaagtccctttccttttatgttacaatgcaaaatcatcagaaaggacatggcatgaaacaaaagtt
ccattactcattcatcctttccaatcctaagagtttttacatgctcgaagtgtagattgctcagcagtttgtgaaggaatttgttttatttacccacagt
attgaccattgcatgaaagggaattttgacaccagaacaagagctgactccatacacattcatatagcaaaccattcgttttctagaccttttgctg
ctgcagtacagggtagaatcgcatcgtttacaatccaataccagaaattgtttctctgaaagacgctgtttctagcaaaactctggatggacttag
aagcaatctgctttggtactattgtttccttattttttttcctttttctataaactgtcattgaaatgagataactatacagagacatcctttggaacaaa
cctatgaaactcgacaagctgtgggttgcttttgtgaacaaaacttttcctatataattttccttcttttcaaaaaaaattcacgttcttttgcatcgag
taattcccattttagtctcttcctctgcctccctcagaagagaacaatagcaagaaagcaacaaagaaacagttggtctacattctgtattcataca
atttatttactttctttctacttatttaagaaaaaaaaggaaaactaaaacctaattttgatatatcgtattttattttgcaaatatagattccatggag
ccattagaagaagaattggtaaaaattttagattttgaaaagtatggggccttgacacctataccacaggacgatggagtaaatccattagcaaa
aataaactattccaaagactacgaacagggaatggcttattttcgcgccttagctgccaagcaagaatattccatacgggcattcaacttgacctg
ctttcttattatgcataacccagcccattacacggtatgggcctaccggttccaaactctgaaacatattccagaactcattccgcatgagttggcgt
ggcttgactcagtcatcgaagactttcagaaaaactatcaggtttggcaccatagacagaaattattaggaattacaaaagactatttacatgag
cttgaatttacaaagcgcatgtttgacctagatagtaaaaactaccacgtttggagttaccgattgtggattttgcagaattttcatgactatacaa
aagagttaagcttgattgacaatctgctagaagaagatgtgtataacaattctgcatggaaccatcggtttttttgttcttttcgaatcaaatgctaat

cacgagcgaagcttgcaacaagaactggagtttctttatgtcaagctctctcaagcatcagacaatcaaagtgcatggaactacttgagtgggtat
gtcaacgttcatttggaagttttctactaacttctacagaattttgaaaaaagcaggaccaaatcaagtggcttggattgcaaatcaattagaaaa
attacttccgtcttcgaacagatttcttttagagtttctggcggaatatgatcctacacgttcaaaagccatttatgaaaacttagcaaacgaagtg
gacagagataatcaagcgctatggaattggatgattcagcaagtgcctcagacttgaaacaaaccactagaacttgtctaggattgctggaaatc
tccgttagaaatgaaacactaagaaaccagataataacaaaaacgtaatgaatatattccttacaagtgacggaaagataagcacaagaaagt
aaaataacaaagagacaagactttttgaacgaccagattaaacacacgttgcattgtatgctatgaagtaaccgagttgaatacacgtaaaaag
aatgacatcttaaatattacttatcatcaagcttaattcgtttcatatactctaggagcctttaatatttttcgttagtaagatacttatcgtttcgaaa
aggaattcctttccatctttgcaacatactcaggatcattttgaataccagacctttcgaagacttcacgaattgcctgtgataattcaatgaattca
ggatctcttgccaacttaagcatcaccagaggtgaaggtttcacacccggttttaaaccatgcttttcacagaatacacggagacgatcgatttttt
cgacaagctctgggtcattaaggacaacagagctcatttttttcttctatcttttgcatcttatcgtccaatcccaagttttcgccaaacatattatcgt
ctggctttgtcgttgcatagcttctccaaacaggacggacgatggcaggtcgttgcataaaataaaccaaagacttcgaggcagttctttgagaaa
gcatacgaggaaccaatttcaagaaatgcatgatggttgatattagaagggtggtaaaggccaggtacggtacagtaggtagggtatagggga
acaaaatgatttcactttttgtactatcagcaacttattcaacagctttaaagactcttattcattcataatccgaaaattttggctggctatgaagta
gtgaccagactcatcttgttgcaacacaaggttatcactataagaaaagcgttagaaggaacgaagggtttttaatacatacgaaaaaatatgc
gctttttatctgcaaaacggatactgcctgaaaccattactagtagattggcaaccgaccccaagatgctttgacagtcatagtcttccactttagc
ttttgtaggaggaaagcttgcaatcatggataagagatctgtagattgcatggggcgaccattccaactgatgagactcgaaggtcgataaaact
ctggaagcttgtctcgtccattttcaagacaaacgtagtaacgatgtacacaattttgggcggctataaggatgaaatttagtattccgtacgatttt
ttagcttttaagaaattaaaaacttaccctgtgttgcaaactccattgattgcaagaaaacgtttgtagaggaacttggtagcttgccaaaaacga
gaccaacttcttttgttattgacatttctcaacgaagaaagaaagaattgggtttcgttgaggaaaaggtaacttctaacagtttttgatggattact
gttttgatgcattgctgcaagcaatagacaaggaacgtttctactaaggttattacgaccagtctgaaatgaagaaaggaaagaacgaagaata
atacagtttcatttctaactttaatatacaaaaccattacgaaattcaatggttatagtgttatacgataaataatatgcatagaggaaagtacgta
gagagaacagtcgtgaagatagacttactgagtatttttacttttgtttggggcccgaagatttggttaggcattcaaaacaaaatggttttccaca
gtgctctttggaatgatgctttaccgaaggaacagaagtaggaacagaaatctgagatgcagaaacgggtttggcgacgaaactttgagaatcc
cgatatacaaatgaaggacgaggagaaaccatggacaagagggaaacaaaattcctttttttcgtagaagtagacaaagactttagccgcttag
agaatgtagagcctcttttcggcggtctttctggagtttggaaaggatttttcagaccttcctgaagagtgatattgtttgtgtgcggttctgcctgag
aatctttcggcatttcattcattttcgcattcactaaaggagttgagtcgagattctccttgacttcaaggtttgactttgtattaggagatagcgagt
acgaaatatcgtttgagtcttttgataatgactttcgggtcgcaccttctgaggatgaggatgaaacagtgtgtgaagaaacagaaagatgggaa
gagacttgactagcgacatcctgttctttcattgttgtatcattgcaagtgtcttgagtttcttccttagagtgcgacgtcctcgagaccgttgaatga
tttgatggaagcgaactgccaaccttgttctgaaaaaaggatatcagaggaatatcatcggctatttccaaatcacattgctccagtcgtgcagct
gttgaatgatattcgtccttagccagtttgctggagggaaaagtaggcgccgcgcgcatatgttcgtgagcactaccaagctcatcttcaggtgcc
cagacatcattttatttttaatccaagaagatgacctcgatttacgacgatttttccagtggaaaggtcaatttcgtcagctatgtcagaagtgtc
gcgtccgtatttttcaaacaaagatgccaaaaactgctcgtaacgtttctgagaagcacgtcgcctttcaagtaagtcatctgtcgccgaaagctg
cataggatacgcgactgcagacgtaggaccattttcctggaaaggatgagattctgagttaccaaaggactcgtggccttcgttttggaacaaatt
catcaaaacgagtctttagaaatcatgggttgataaaaaagtggaaagaaatcaccaattcaaggctcattcgcacaagggaaaaactgggat
gagcatgtaagtaggtaaagagtaaataagaccaagaagcatcatctatccttatggaaatctgagaataagtgtagcaatttgaagagaacag
aatcaaatgaaaacgagttcattttgcgttgacattttctaaacaagcgacttttgttcttacgatccaggataggcaaacatccttgtaaaagtca
ttcaaaaagccttacatccacgtcgctaattctaccgtcgattttcttaataaagaaaaactcttgatgtgtgaacacttcaaaaatgattatcttcc
gttggaagaacatcaagaatataatgaatggacgtaaggggatttaagccatccaaacaaaaagtttattcttcattaatatttccataattgtac
ttctttgtcctaaaagctatagctttcgatacaacaattcgctttcatttcatgcacaaaagatgaaggcctccaaagcgatgctattgtttttcagc
atataaactttatttctctattgtttctattgttttgaaactaaaaactaaaaagcaccaaactttctttgcctacccaagcaatctttcagacttgaa
cccattgtgtttcaattgtctctaagcaccgttacttcacctaagcgagtttcattcctgaaagaaagctaccttagaaagcactaagctgatccag
agccattctgtttctcgctgttcttctcaatgccctttgtttacaaggtgttacctatcttcctctacttatatctcattttcaagaaaaggaaactcgg
atctttttttacaaaacgaactttattacttgaatctgtttttaataaaagtttataaacttatgtctttaaagtccctctttttttcttcgctttgtttacat
ctctttgttccaaaagagacaatgctacctcaagaagtaacataggttctctgcttcattgtgaatcatcgcccattgtgaaagctaccaacaatca
tggttttgctttgggtattgtgcctattgtttgggcatattgtttgaaaatacaatcatgtcctttgtgtattgtcttcctaaaacgaaaaaaactatat
aagctcttcatagaatgcctaatatgaccagcttcggccaacgtgcgtctacaacaacgtctttcgacgcgatctattacatccgtgttcttggctac
gatttattttgaaatatcttttaaagcataacttttgattattctctcctttggatatcttatagattttgacgttctttgcataattgtttgtttgaaaac
aatctatacattctttttatttttttagacatagacgatttttttttttatttttttcctctttacgaattttctttgttttaacgaatttcatgatgactttgatg

acattttttctccttcctctctttgcgtttgctttgatatcacctacgtttgcattgtaccctaaaaaggtgtcttatgtttctcaagatggtcccttcgat
aatgccaccatcggcgaaattttaaatggttcgaaaaacgaagcgacatcttgctcttgcccttctcatgtattgtcttctgacgttcctctctattcg
gcaaaagaaaccatcaccgcttctagttattggacccttgtcacaacacaagttactaccactgcttatgttccatttacttctcaacttgcctgtgtt
cccacaactgattcatcttcctcttcttctctcactagcagcacgactggcagttcatcttcctcttccggaggttcttctccagccccttcttcaacttc
tgcgtcttcttcgactgtcgttcctactccctatcaggcgccgagccccagtcccaatccaagtcccagtcccagtcccagtcccagtcccagtccta
gccctagcccaagtccctcctccacagccaccccatctaccacttctcaggtgagctccactggataccсttcggcttcttcatctgtacagactgct
actacgagctcagctagtaccccaactccatcaactagctccactttggtttccagctcccagccggtatccactacaagcctaaccagctcttctg
cttcttcctctaccccctccacaattcaaactgggaggtttcctcacacctctgtcctctctttcttaaccgagagcttctctactatatacactacgac
tgtttcgagtgctagttcctttacggacgtcactactataacttctaacgtcgcttcctctacacctattattagtaccatttcttcttcaccatctgttg
caacaacttcatctgtttcaccttcttcttccctgtctggaagcggaagcattcctaacaatggaaagcatggtgttgtctctactgccactcaactg
actactagtggctctcatgtatacactacaacagatctgactaccatttatgataccgtctataccacaagcataggcactaacacttttgctacta
ccgttacttcttatgatactgggagcgccttcacgactacaactggctctgcgagtcaatcatcatcttcaatccctatttcatcgatccctatcacat
ctacgagttcttcttcaagtgtaggtagttcgactcctttgacttcttcgaccattctttcatcaacagtcgtcgagaattctagtacggttgttatacc
cacaacaactaccgcattcttcgcatcttcttcaactgaatactttactactacaggctcccccacccctgagcattccgtagtaattacctcttcttc
tgcgaagactacctctgcccccagtgcttcaacttctattgatacatcgagcagggttattacaagcgccactaccagctcatcctcaacgccaag
cttggtgactacttctccaaacgttacttgccatacagagactgatactttgactactacttccggttctgttggtttcacaagcactattacttcggc
atctggatcagtccctggaactgttatcgttgacgtccctactccctcttgggttactgaaacagtgacttctggttctaaaggatttacctctaccat
tgctactcctgtaggcacatccgctggtactgttgtgattgatactcctagtgctactccctcttgggtgactgaaacaatcacttccggctnnnnn
nnnnnnnnnnnnnnnnnnnnnnnnnnnnnnnnnnnnnnnnnnnnnnnnnnnnnnnnnnnnnnnnnnnnnnnnnnnnnnnnnnnnnnnnnnnnnn
nnnnnnnnnnnnnnnnnnnnnnnnnnnnnnnnnnnnnnnnnnnnnnnnnnnnnnnnnnnnnnnnnnnnnnnnnnnnnnnnnnnnnnnnnnnn
nnnnnnnnnnnnnnnnnnnnnnnnnnnnnnnnnnnnnnnnnnnnnnnnnnnnnnnnnnnnnnnnnnnnnnnnnnnnnnnnnnnnnnnnnnnnn
nnnnnnnnnnnnnnnnnnnnnnnnnnnnnnnnnnnnnnnnnnnnnnnnnnnnnnnnnnnnnnnnnnnnnnnnnntgagacagtcacttccgaatcta
aaggattcacctctactattgctactcctgtaggcacgtccgctggtactgttgtgattgatactcctagtgctactccctcttgggtgactgaaaca
atcacttccggctctaaaggattcacctctactattgctacccctaccggcacatctgctggtactgttttgattgatattcctagtgctactccctctt
gggttactgaaacagtcacttctggttttaaaggatttacctctaccattgctacccctaccggcacatccgctggcactgttttgattgataaacca
gtacagacgacaactactacaactactacatttgatggattaaatggtggttataccagttcatacggtggaagcgccactgagacaatcctgatt
ggtacccccaatcactcagttgtggatatcttttaaaatatacaaataaatggtcactttaatataataatttttttagatttttagatttaaaataaaa
cagtcttttgaaattttatgataatataagttcaaagtattagtgcgatctataagttcctattcatgtgtgtgttataagctaaagaagctgcagca
aggtcttccctggagtctcctacagacttgaacagggttatagcactccgagggtcggtcaagtgagcacgtcccttgtgatgcttgttgcataagt
cgaataaatcagcttgcacatcatcgatggaaaatacaccctctttcacgggtgttaacagatcacctgcttcagatagtgcaccggctcttgtatc
cacaaagacactcgctttagaaacacaagcagagtcagcttcatgcatatttggagtaaaaccacctactaggtcaacatgagtaccgggttgga
tccatgagcctttcacaataggagatgtactcaacgttgcacaagaaatgatatcgcagactttcacgacgtcttccaaatcttcattacgaatag
cgtgcaactggatagaaggaaagtcatgacaaattgattcaatgagagcaacagcattctcatttcgatgattccaaatagaaatggtttgaatg
gggcgaacagagcaatgtgcgtaaataaggcttctagcaacttttccagatcccaaaatgactagcgaagccgcatcattcctagataagtacgt
agatgcaagcgccgaggcagcagccgtccttcgagacgtgagttcccctccgtcaagttgagctaaatgagtaccagtatttgcatcacttaaaa
ggtaatgactgaaaaggctgggaagattctttttggagttgtttggaaaaacatttacctgtttaacgccaatgtactcaccaggagaccaacaag
gcataatcaacagagtattatggacttcattttcatccacggagcttttaatcggatagtggagtctggggggacattcgactggttttgtgaagat
gtccctaagcgcatcgattaaggcgtcccatggtaaataactaattaatgcttctctagaaagaaagatggtaggtgagcttaatttattattcatg
atggtactgtattataggaaattagagaggatcaaatcagttcaaattatgatatatctggcgagaaattgataaaaaaaggattgtttcttatat
attaaattactgggtcacttttgtccatttgtaagattcacaacgaagaatgtacgtcggtttgaagtggaaacaactttcaaaatataagttccat
aaattatacttccaatagtttcagtaaggcaaagtcgaaaggtaaaaagagtttcctggacagcaaaatagtcgtaaagtattgatgatggtaca
gtcaacaagtactgccttgtaagtttacagatactgccggatattacttggacattgaaacaatgtactgaataaccagctatatctctttagtctg
aatgaatatatggatgtcaagattctttgattaagtttggattgtgaaatttacaattctgaaacaaatttaataaagaaggaaatataaatgaac
attaattaagaaaagcaaactataaacgacaaaaaaaactccatcaatattgaatgtagaaggaatcagttccaaacatattttgcaaagcaca
aaaagggagaaagtaacgtgtaaaaacccaattacattgatgcttcagtaattccattgttctcacttttcttcttttccgagtcttgcttattatcta
cggtggattcaacgggctcaacggcagtgagttcaactatggcgttggtagcacctgaagatataccagggctgtcgttatgcatgtcggaatcc
aattcttcaggaagaatgagattaaggaatatagaaatgaaagcaccaattgcaaatccgttttccatgacaagggtgatggcatccaagaaac
cggtaaggcctttgttgtttccagaatatgtaaaaaagtagctgaaccaatttgggaccaatatagaacccatacctaatgttagggaagcggtg

aggataaagcgattacgacgattaaaagggatttgggcgatgatggctataccagaggtcgtgacggaggaaaacaaaaaggtagtcattcct
cccagcacggaactaggaatagccacaaacacggcagcgaacttaccaaagagacccatgcaaaacaagatgaaagcacagaaaaacccgg
ctcttctattggcacacttagttaggctgattacaccattattttgggcgaaggtagtgaggggagtgatagtcataagggcggctatcaaggagg
atgtaccgtcacagagaataccaccctgaattcgagagtcaaaatctggaccatcgacgtccagcatagagacgtctgaagtagcagtaatgtc
cccaatggcctccatcatgttgacaatgtacatagcaagaatgggaagaacggcagggccgtaaaggcgaagtcggaaagtgtggacccagtt
aaacgtgatgacgggagcagcatcgataatagaacgatcccagtaatgagtggcagcagaaatgatcatgccgacaacaagaccaatgacga
cagaagtggtcttaactagaggaggaccaaaacgctcgatgatgatgatggtagcaaagacactgaatcccaaaccgatgaactgggcgctac
cccaatacagcggatggggagcagagtttgaaggacacatggcggtgcggctcgtgcagccactaccgccagcccaatcctcaagaccagtcat
gatgagagaagcaccaattaacaaaacgacggggccagttactatgggagggaagaggcgtttgaggaagcgggaagggatgaaggacatg
gcgatttcaagcaaagagcaaatgcagcatgtagcaataaaagcaccatagccttgaggacagggaagcttggtgccgtcagaggcggttgga
caatagccattttgatacatttgactgagtattttaggggcgacactgacactagtaaaggatataccaagaacggagagcatgccggtgccaat
gtaataattggttttggggattctgatgcgagcgatattgatgagggtcaacagagcagtactgatgagaccggcagagacgaggtaattggttt
gttcaatagtaagattggcactagcagcaatgatacgaggggggggaagtgacgccgccaatcatggccagggcatgttggaagcccaagagaa
gggctaagaggacgggtgttttggcgttcaaggaaaagaaaggagggtttttggtgtttggctggtaaaggggatggcaggaagcaagaaact
atagtcataatccccgatccaaaagtctttggtgaagacagagtggagggcattggaacacttttgaccaaaggagggaggagaagggtatgca
tcgtcataagcaaatgagttggacatgatgaacgtaaaaagaagtcagtaagaaaagtacaggaaagaaaagcgaaaacgaggtaatcaaa
gagaaagaaaaagagcaaattagaaaagaaaacaactttgattcaaccgaaaaatagaaataaataagatagaaaaacgaaaacaaaaac
aaaacaaaatcaaaatcaaattttaacaacggagatgaaagacctgataaaaatagctttgataaataagattgagaaacctatgatgacttgc
tcttttgatgatttcttttgttgtaagcgttttgactatataaagatggtttggctacgaagggaccaacgagaaagagacgaaagatggaaattta
atgatgtttccaaatcaactcccagttagaaagacagacagtatagaccaagctcttcaattcgaaagttattccaattcagttgttttcgatttca
agagttatccaagatgtacgatgagctaccaaagagtcacaactttttcaatcaacgaaacaatgcgttaaaggatctttttgagccccaagttg
gacacaaagcaagggaaaggcaggaagacaggaagaaccctcgaaagtcagcgataagcctcgtaatttatacttaaacggcagaaagaac
aaaagccccaaacgagtctcttgttggactcatctctccattttaacggcgatagaaggacatcttgtttgcttctccagataaaggttttgtggaat
acagagggttgcaacgtcaagagtaagtaatctacatttccttatctattacccccctagattagatcgccttcatgggtatcagaagaagcgcaag
gagaaaggatttatctaatcaaatacggcgtaaaacaatgcaatgagcgagggtaagagaatataaggagaaaaggaggaaaacggtaaaa
aaataaggcacatttgaaattcgtttcggaaaaggagaatagcgtttgatcgttctcctacataaaatggtcaacaatgcgaagcgcgcacaca
gcagttgattgtaagagcgagagatacaggagtcaggaaaaggagagacagttggaaagaagcccttctatcaaacctggtgtagcgtaacgt
cctactagttggctggtcggttgaacctcttatcattgtcctgcagcaaacaagtgaaactgtttcttaaacaaaaagcaagagtcataattataag
aataacaagaatatcataaacaatttataacttatcaagactaaataagacactacttactccataaaaaaataaccacaagcgttcgccaagtt
tgttcctacaaaagtgtagggtgtgctaaaaaaaaaagtctccaccaagtattctggtctttcaggttttcctttacgtcctgccttagctccctcca
acaactgtataccagaccatcgctggcgtttgttgtcattcataaccctccaccattccttcccttctggcctgtcaaatcttccaaacgttaacgctt
ctcatttccctgtttctgtatcctcttcgtttttataattttcttttctattccactttatttttggttttgttcgtttttggggggtttttttggttctctcttta
ctgttttgagtgattctttttctattgcttttttttctgtaaatttgccattgtttgtgttttgcatttatttttccttctttttttttttggaaaaaaaaccaaa
agattttttttctaaacgttttcttttcaccgcgtaaattctcgacttttcttactttacttagttacttactaaaacttacttaacgatgtcgccttcttctc
attccctcgaaaaccccccctccacgaaaaacacgcctctatagttccagcagggattccccttacccttcttcttcgtggaatcaatccaattcaaa
actcgatgatgtgaattctctaaaatccatttctctaaatgatcgtcagggtctaaaatctcgttttaccgtacaatcgcatgatgagtccgaggag
acacaagacccagacttcactctagtacttccctccaatcgtctatacatcgttattcccggtctcatgctatgcatctttctcgctgcccttgatcaa
accatcattaccactgccatcccaaccatcgtcaacagttttcatgatagctcttcgtcttcatggattggtactgcttattcgctcgcagaaaccgct
gtccttcctttcgtaggcatcatgagtgaagtcgttggtcgaaaggctgttctctacacctccattgtcatctttctctttggaagcgccctttgtggtg
ctgccaagagcatgatatggcttatcttgtgccgtgctgttcaaggtatcggtggtggtggaatcatgtctttggtaactatcgtcatcgccgacata
acacctcttcaaacacgctcttactatacaggttgttttggtgccacctggggtatcgcttctgtcatgggtcctttgataggtggtgccatctcccaa
aaaacgacttggcgctggattttcttcattaacctgcccaccggcggtattgctttgactacgcttgtcttatttcttaatttgattccaaaaccaaga
acttcttttacccagttcctgaaaacctttgacttcgttggcatcatcacgattactgttggtgttgttctctttctgcttggtcttaatcttggttctact
actggtgtttggaaacacgcaaatatcttatgctacctaataatcggcattctttgcctggtcgtgtttgctgttaatgaaacgtataccaagcgtat
aaagattatgcctccttccgcatttaaaactctctcactcacatccgttatggtaacctccttttttacactactacattatgtctaccatcacatattac
attcccgtatactttcaaaacataaaaggcgatggtccacttatgtccggtgtccacaccctctcctacgcagtcgtctcctctgttacctctgctatt
agtggttttagcattggcaagttaagaaactataagtactttatgattggcggttggatcattctcttggccggttctggcgccatgattgctatttac
tatgacactgaattctccagggttatggggttcattgcgctgacggctgtcggagtgggcaatttgtttcagcctaatctaattgcactgcaagcatc

cgtccctcctgcaaccatggcaacttcttgctccgcattcatgttgcttcgaaatatgggttcctctattggcatttccattggaggagttatttatgat
caacagttatctactttgctcaagggcacgtcttacagtaaagacatgacgttttcgcaaatccaggctattacagatgtttcgaagcgtaacttcg
ttttgaaagcctttgcagatgccattagaatgatttggatcgtcaatgtgccttttgctgcattaggagcaatcttgtcatttttgttaggcagaaca
aactaagccaaagcgtgttacaatacgatgagaagaaggaaaagatttcacaagatggtcgtgcggtagaaaagccataaatatgaaggagat
ggcatggatacttattggagtgtgtatggacgtttgaaattgggtttgttttggaatgggtaattgtctatttatgaaacattgtttgttgttatattag
taatagtttatttttagtccctccttgttaatgttaaagggtaataccttctggaatttttttcgctacgaactaataaatgttgttttcatggaaatcc
acaaggatattaacccataaaggctaacggtgaatttcctatgcctctttcaaatatggcttcgtcttgctaagtaccaaaaagaatctgattcgta
tacctaagcccgtggtttgagtgattcatatgtgcttgacaaggaaactatctctggtttatcatagcttcccaaatatgacttgtaaactcctaattt
caccaagagaactactcaaaaagaaaattgtagtttcatcgaaagactatagaataaatagaaactcttcgaagcttaagactgtcaaattacta
aacctatcatatgcagctttttaccactagaaatcgagttttcgaaataattctcatgaagtattaaaagtagaaaaatgtttctgagttagataatt
ggtttttcctgttattgaaggaacttggcaacagcattttccataatgtaataatcttaaattggactacctttatttatttagaagagaataaactt
tttgttttgattaatagcgagtttctgtttacatgatcattttatggaggttatttacaataccatgagtagagaatgttcaggtttcacaccatccgtt
aaccacatttttattaaacaactatatctttaatacatggaagcatcgggttgaaaagcatagaaaaacggtatttctatattaatatctatatata
ataccaaaaaaaattggagatgggttattgtagtgggctaacaaatcttttcaaaatggtgctgctaaagttctttcagaatggattacaaattttt
gcgcttctattgatgactcaagctcagtggcttaactgggccatttacaagctgtctccatccaagttgggttatgttcagtttgcattatatggacta
atggtatttacttatttatttgcgaatgtaacccctccaggatccccagtggtatgttagaatttaacagattgaagtattaacatgcagactgcttt
gtttgagcctaaaatcaatagccaatatatgaaaagaaaagacgggatgcctcgttattgcggtaaatgtttacagtacaaggctgatcgaaccc
atcactgttcgcgctgcaattcatgcgtattacgcatggatcaccattgtctttggtttcgcaattgtatcgggtttcgaaatcataagcttttctacct
tgtttgcttgtatctgacgctgtatgctcttacaactgtttacagtacgtttatggctataacgaaacatttcaccgcagcaaatgggaccataagcg
ctttgtatttaatattttggggctcattgttcgcatttgcgtttgggctcagtatcgtaatggttgcatttacaatctatcatacttaccttcttttacaaa
atctcaccactttggaatctatgaattcaagttggtccaaatatgccaagtcaactcagccctttaatgttggtctgtataataattggtgtcaagtg
atgggatcggctccttggttctggtttattccaattagaaactcaatgggaaatggcactgtttatcctatcaaccctaatgcacttccatacatttcc
acttcagaagaaaaatcagagacatcccataagaagataccattgcctataagctatagagaagatgaatgggctagtgatgaggaagagtat
gcaatgaaaagtcgtcgctggaatcctcatcttggtcagttcgagtggcttgatgatatagtttaggtactgatgggtttcttatatcccgtttccttc
ctttcataactttttacgccgaaacaggcaatttttcgtataatttttttccgcccttgtttgttcttgttttaattcgttcgatatacattgtcaattcttgg
atatatttattcttagtgattcatttactcgtttcatgattcgcttacgtcttgttcattctttgaattcaaaagccaactagaatgaaaagaaagaaa
aaaggaaatgatggaagacttttgaaaagagcataatttattagtaacaaaagttcttccaagttgggcaaatactaaaaagtttccgccatatt
ggaataaaatattttaattccgtaagtatgttttaggatagccttccattcattcccatttcgtttaggtagtataatgattttttggggttatttagtt
taatttatttagtttattttagtttagttcagttcagtttagttacttgggtttaattaggatagtttgtttagtttcaagttcataatgcatatttatttat
ctgttcagttataaattatgttttgtttttatatttacctttgcaaccttttttttttttttttttttgccatagtaaaacgtatatcatgtaggcctttcaa
aaaggatactgtagtatgcttatctagagaatataatttttttactatagtaattcaatttatgatttcaaaagctattgatgcttgttctatactttgca
ttgtttttcacttgtaacgaattgtagcaaagttctcatattggaatggcgagttgtgtcatttattgcttttcattgctttgtttttttttatttttttcctctt
aaattagcaaatcatttgacctttatatcttgatctgctaaaataaaccatctatttactgctaattgaatgcaaacttatggtccgtaactgttacac
atagattgtaatgcgccgcaaatccatagagcttcagaaagcttcttatctataggttaatttacattgacgaagaacagccactaaaagtgctac
tacttctggattacgatttgttgtcatcttcagaggtaaacaagcataccattgcctcgctgtaattcatctatcatagataaaatatcgctttcatac
ctcaaccttatagctaatgttctatcttcctttaccatccgtattcgagtttgtttaccttaaaaccgacgatagattagaagatacttgaacgtatat
ccttcataacatgtgagatattagtaagctaaatacgaaatttcaaaagttttcttttcttatacaaaacaaaggattcctattcttccaccgacata
tctttctcatcgtaccattcatgtttttgatttttcgtaactcgttagtatgatatttcactagagctcgcaacacatcgttaatattcgtatataaaggc
tcaacccgcaaaatttttaattggtaacaatacccgggacaactttggaagcaatcgaaatgtctgctgctaagccttacaccgaagctttagatc
ttcttaagaagtatgaaaagaaagatggtttaagtattgacgatttaattcgtcacaacttccaaggtggtcttactttcaacgacttcttgattttg
cctggttttattgatttcgtccccaacagtgtttctttggaaacaaagatttctcgcaatatcactttaaagactccgttcgtcagttcccctatggac
actgtcactgaagaccaaatggctatttacatggcacttttgggtggtattggtgttatccaccataactgccctgctgaagagcaggctgatatgg
ttcgcaaggtgaagaaatacgaaaacggttttattactgatcccgttgtcttctcccccgagcacactgttggtgatgttttgaagatcaaggaaac
caaaggctttagcggtattcccgttactggtatgttattatggtaatggattgaaggagagtttttaggtttgctgctaatgatgtctcaaatcatatt
aactttcgttcttccgcaatgacttaaatgtcatggggacaacatttcgattcctgaagtttagcaaacgtttactcacttaaccttgtgctcttctttt
attcataatctatccattgtttttggctttttttactaatatttttagatactggtaagcttcgtggcaaattgattggtatcgttacttctcgtgatgtcca
attccacaacgacgttaaatccaaggtaactgaggtcatgactaaggaaaacttgattactacccgtgaaggcattagcttggagcgtgccaacg
agatcttgcgtcgatccaagaaaggcaagcttcctgtagttgatggtgacttaagattggttgctttgctttctttgactgacttgatgaagaacttg

catttccccaatgctagcaagtcccctgaaactaagcaactcatggttgctgccgctattggtactcgtgacgatgaccgctaccgtttgaaactttt
ggccgaagctggtgttgatcttgttgtaatcgactcttctcaaggtaactcttgcttccaaattgaaatgatcaagttcatcaagtccaacttcccta
agctggatgttattgctggtaacgttgttacacgtgaacaagcagctaacttgatttctgctggtgctgatggcttacgtattggtatgggtagtggc
gctgcctgtattactcaagaagttatggcatgtggtcgtcctcaggctactgctattgcacaagtttccgagtttgcctcccaattcggcgtcagtgt
aattgctgatggtggtattcaaaacattggtcacatggttaagagtcttacccttggcgccaccggtgtcatgatgggtagtttacttgctggtacta
ctgagtctcctggtgagtattatgtccgtgatggacaacgttataaatcctaccgtggtatgggttctattgctgctatggagggtaccggtgcaaat
aagaatgcttccactggtcgttacttctctgaaaatgatgttgtgcgtgttgctcaaggtgtgtctggccttgttgttgataagggttctcttctccgtt
tcttgccttacctttatactggtttgcaacacgcttgtcaagacattggtgtttgctcccttgagaaacttcatgagtctgttaatgctcatgatgtccg
tttcgaattacgttctagcgcagccattcgtgagggtgatattcaaggttttgctacttatgaaaagcgtttgtattaaatgggtatataattttttct
ttcggtttgttttagtgatcttttgtgttcgttaaattgaatgcgtgtgttttgggaatatttgtgaataatagaaatgtatttaatatgtctgatggtgt
tgtaagttcgtactcttatgtcgtattagtttcatgaatatatgggaaacatttaagtaataaattattcatttactcgtttctaaatattgtatatgca
atatatccttatgttatcataacctttttaagtgcgcagtatgttgctcgtaaactgtcattcatttgtttttgtgaatcctttgtaactaaaaatgatc
ttcgaatattggatggattggaaagacgcaaggatgtcaaagaactaaggttttggtgattgtttccagttggtagattaacatcgtttgaattcaa
agtagctggagtttgggaaggatctttttgcaacgtttgatgttgatcgaaactaggcttttcaccttccactttattagtactagagataggacttg
cgtccagatctattgtatccaagtgaggtaaagaaacttcttcattcgaagattccgctccgaaaatgtttgccatttccacatccagatttgatgtg
gataagggctccaaattatttggtagtgattcatccagctcaactgtcttatcctcttcatcttctggtttatcttccagaagagaagattgtggaga
atctaaagcctcagtaagatccaacgtattgcctttcgaagaaatggttaaaggaaaagaagggggtggaggcatgttctctatggaccaagga
atattctttgagtgcgactgttctggagagttataagcagaaacgcttgaatctgaagtagacattgaatcatcaaaacgcactcgtttttttgggtg
atcttttaggaaaaacaaccggctttttaggtgatttaaccgtaaaccttctaggcgaatttgatatctttggcaacttagaaggagatgaagatga
ataggtagggatagacgtttcctttgcgagacgttgcctagccttatcgtagtcttccatgtggtaaagttgatacatgctatgtttatccttagaac
caagatctgataaccaagttttcagcaggctactcagtacagcttgatttttttgcttggattgggtaagtgacttaaaggaaatctccatcaacttt
tgcaatacaggagtgtaatcatcgactttgctttgtgcatccaaaatttggttttcaattatggatttcaatagatcaatttcatcctgtaagatatcc
gcatacatcatagcattattttctttaaaatagcttacaacaggatcaacacttttctgaaaggcatcataaaccttttcaggattaacgtcagcaa
tgatagcagacctttgtattaacaatgattctaactttgattttgctaaaagtagcctttcactggatgagctgtcagcgatccgttcgcaagaagat
atccaatattttagaatattgatagaatcatcatagtgcttgactttctcaacttttccaattgtgtctttttgcaaaggtaatgtttccaaaaaggta
gcacgcagtaaatctttagcttccattaactttgattcgcaagctttagcttgcgcagctgcttggttctttgcggttgctgctacggaggcagacga
ggcaggattcgctaatctgctgtcgagttcagctatttgttggcgaagatccactatagcttttacatattgactaacatgcctgtcaacgctaatca
tgttcctcaaaacctctgtttttatgtttttagcacgattagcatattttaaggtattatgggtttcttcatagtggaatgatgagggagaaatgcata
ctatcatcacagttttgcaattaccaccaaggctaaatttgagtagtctcgttaattttgaatcacggtaaggaatgtgtgcccttctgtgtggatcg
cacaaagcatttatgcagtttcccaaagccaaaagcgatttgttaatgttggcaccctccaataaacggcctcctcggtttttagtagcatttgcac
gttctgagccagcaaggtcaataacggacaaggtagccaaagcgtgatcttcattaatacctgcagtacgcggcttttgtaccaaagaaacctgc
aagacagcatgagatcgtgaagaggcaacatttgctgctgtgggtgacatggttctatttccatttccacgagcaacaatttccattaattcttcaa
gagaggtaggagataaagaggttagtcctggaacggtaattcgacggtcagcgtcctcgcgcagcgttagaggtttggactgagcaaacggttg
ctcagtcagcaaatcccgaatggtttcattataaatttctaagtaagatacgtagatactaaatatttttttcatccctcaagtcgtccattcgttcaa
aaagctcctttaaagtcagatacacaatgccgggatcctctttcgttccggtgattgtatgagttttaccacaaccagtagcaccatacgcaaaaa
ccgtggaattgaacccatctagaatattgtccaagagagggcgagcagtttgctcatacacggcttgctgcgaggcagtttcatcgaatactctgt
cgaaagcataacgaagatcgcgaccaaatccagtaaagtttcctttctaccggaaagtcgagagatgttacgctgaggagcagacattcgtcta
ccgccagcgtgcatggaagaagcatcgtcgggaggatcaaagaccagtacacggtcatccaaaacctttaccactcttcgaatccctcggcatac
tcctggatttttaaaagaaagtgaacttgctagagactgcccatctgaagacgcaagtaagttggcggattcaatggtactaaatggacgaactc
gaactaatcgtcgttaatattcatcttacaatacgcaaaaaggaattttccaaacatacctgcgacagagatggacgattgtcttgacatcgaaac
tcgtcgccgcacaagcaatcatccaagtaaaaagtagaattttatggaagccagatttgcaacccaaggaaatcgaactttgaatatgaaagaa
tcaagtagaagaaaatggtgaaaatgagcgttctaactggttttcgtcctgctgtttcaactttgttgtttaccaggtaaacacttgttgacgatgtc
agtataccaagtggttgttatggcaatggtggtttgtaaacaaatgaacgcgtaaacaaccagcaaaaaaaatttcagaaaagactgtataata
cgtagataaacattaaatacaaaagatacagatctttttcttaaaagagcgaaacggaggcttgttaagtggaattctttctacgaatgccacttg
ttgagtttcatagcaaaagtgtcaccagaagagaagagaagagaagagaagcgaagcgaagctaacaattaacactttagaagcaacaaaac
ttgatgaaaaagctttgccttttcctttaatcatcttctgcatacacattttagtgcgtacttccaagctaatacgataatctgcatggaagggatttc
attagggcgaatgaaaaagaagcaacaaagctattttatgctggatcgattttctttttaatttcacaagttgaaaaacccgcattaggattctgg
taaggtttctgccatataaaaaattgtaacaaacataaacaaacgaacaaatgtgcaagtgtatatctatcattatatactcgttttaaaataaa

aataaaagccattgtattatatataacagcgtgaaaaagtcaacaggaaaaagaaaaacgaaaagtatattttgtgcaaatatcgactttctgaga
tatttatttatttatttatttctcattatataaagtttctaaatcgtagcaaaaatagatctgttacaaagaaaagtacaggagcaaagacatttgca
cgctgttatcgaagaaaagagtagacctatttccattaagaactggcccgtcctcatcactaagcagtctttgtatcctttcatctatgtcattttcta
aaaatcgatacgatcgatcaagaaaggaacgatccgtttcacctccaaggcataggaattacgcacatagagtccgtaagacaaggcagtcggt
aagtatcttgttttcaaatttccgcttctcatatcattaacttggaatcttaggaggagaatgacgaaaatggcgacgaatacgatacacctagaat
tcgtgaatccagagaaaaagcatttctgaaacagcaaaaacttctgaaagccattataagagtgaaagaagatcgagctggtccctttgaaaag
cttttggtaagcttctctttgctttcacagcaagacaatgaatcttggattcataacggaagcatagatttcactggtttggatatgtttgatccaata
tttgtgatcaaggtgagttttgactttattcaatctatattgacgcatatcagcttgcgccagttgaagaattaaatcttcttttaaaaaacataccgg
aaattttgtcattaacggatgatgctacaggcgtaaacttttggaaggtaaggttcttaaaatttatattaacttactgaatgattagtatgtatccc
aattggtcaattcgttcttagaacatgatcaatacggaaatgcttcaagaagcttgaaaacggttgccaaagacattcaaaaaatcctggccccg
aaaacttttgagcagttggtaaatttggaaaagcaagtacatgcgaaactccagaatagagaaccaatagacacggattattgggaggatttac
tcaagtcgatacactcttacaaagcagcagcatatttacgcaagttattttcccagccgctacaattacggaagcaggaaatggctcaacaagaa
atggaaaaggcgatttctgatgtaaatttcttatacaaacgccaacaacttacgcctacttcgaaaccttataaaatacccattgatcccgatcca
atttttcgaattgatcccgttcccaaaacgacgcaacctattgagcaaaatgactatgaagaaaatttggtacgtttcatgcttccacaaatatatt
aacatatgttttagtccgaaagatccgagattattagaaacagcatctatattcctacatcgtttctcattaagaagcaaagcagacagcctcaca
agtacttgatacctgacaccgaagaagagttttataatactgtaagtggaaggctcgaacgtgaataccttaaagctggaggcgaagaagaaca
agaaatgtctggtgactttgttgagggaccagtaagagccattagtgaagatggaattccgttaaaaaagcccaagtattttaatagggtcatgtt
aggtttcgagtggaattcgtataaccaagcacattataatgaagcgcatccaccaccaaaagccgtacagggctaccggttcaacatttttttatcc
tgacttgattggcagtgaccggtcacctacttataagattgaacgtgggccaagagttgacaaagagtcagcaatgacacaggataatgtttgca
taatacgatttattgcagggataccttaccaagatatcgctttttgcattgtggataaggaatgggactatagcgccaagcgtgaacgagggttta
agagcagttttgataatggtgttttatcactacattttcagtttaaaaaaacacatcataagcgttgatgttcggttggttcatatgtcgttacacga
gttgaaccaacattaatgtttttacagtgcggtggatatgactaaaaggtgaaaaccgtattgattgctagaaattaaacaaaatgtagctaataa
aaaagaacatttgcttaattagaaagaggagaagagcatcaaaagaaagaaacaacgaaaatgatcgtgcaaagttgtatcgtaagtggaaa
aggttcaatccttttttagccttaatagttccggttaatgttttgtttaaatataatttgcgagcttaccataagaagctttcctgtaaaataaacagaa
agcaaagtaattccacccatggcagcgtagtaaggataaaccatgagagcgctaccaggtctctaaaattaaatttattagatatgtttttcatgc
ctgcagtcggcggtatcttttgaaaaatgacatacttggagataagtaggcttatgaatgctttgcaagaagcgttgacgttgaacaacaagttt
ttcatctattgatgagttagatttcccttttttttactattttcacatacgctgacgaatatacaatgaatttccagtacctgtcaaaagtgatggttga
aggtagtatcgggtacaacccgcatgttgaagaaatgcagtaccctaacagacctagagaatcctcatcttcagtaataagaagttattcgagttt
tttgaatatgaaaactacttattagcactatagcagaagctcattttaatgattttcatatttccaatgagagtatcgaaaaaaagccagacttttatc
caaaacacaaacaattatcgatactcatactcacataaccgatagaaataatagcagtttttatagacccttcttgcctgatgtgctgtttacctttg
tatatgagaaacgcaaagaaagttgtttgtcctcacaacaataatactagctgcaaatacattccatgatgatgtttttatcattcagatggataaa
atactgtcagaagagtagtttttttcaaagagttacctgaaaggcgcttgaaatagtccgacatataaaatttaggatgcttcttataactgaaaag
aaaaaccagatatataaaactgatttacgaatgaaattttttattagttctcttttaggaatatggacaggagtctcatagcacatagtttctatgg
aaattccggcgccttagtttgtttaccaaatggaaataaagttcgcttaatgcttttatgaaatcactaatgctcagcaacattgagggaactagca
atctctaaaaggagaagctttgcactttcttttttcctttttgttatgatcgcctattttttttatagataaattgcggaagtagattctcattacattcctt
ctacgtggagttatacggcattgtctattgatatcagaatgtaaacaaaagacaaccgagtctattgacatgtactaatgaaatcgaaaactgact
tttctctggaagaactatgggaacaataggaagcttccgaccttttttttttcttttttatatgaaacaagcttttattttttatcgtaaaagtttgactgct
cgggttgcaaggactcgttggtaacccgccttgttacctttggtttcctataatttttttacttttggagagctcctaccttcttaatcttccctcgaccat
actatttttagacaaggccagtaaaagtaccttctcagctttcgatcctttcatctaataagatactcttctccttgtcgtagttgtcgattcctttcaa
ccaacattccgggcttggcttgatcgtatttgctgctgcttttacttgtttagactatttcataggaagggtgtgcctatcctatgctttcttggttactt
tcttttaaaactgtggtttctttttgggtacgcttttgttgaaaagatttggtttcaacatcttttcggggttttagtcataaatctattcagttatattta
tttcctctttttcgttctttatttattctttttttttggtcattctttgtttgaaatttccttcttttaactttcaattttgctgctcttctttgcattgattttaaac
cctataattcaaaacaataaatgcgctgcatggctcttgacgagtttttttttgtctgaacaacctatgtataatcatgaatgaatcggtcttaccttggttat
cacccatctctgaaggaataagcagccctcccttccaagatttttgacttacctccttcctcttcatgctctttttcattatcttctattacttctggctct
gatgatacgtccgtattgaatatggactcgaccacagatgctaatccttatgctttgccaaagttgctctcaaacgtcaagcaagaaactcccctg
caaacccaagacgttccttccaaagggtctgacgtcttaacagcttctctcgtgattggaaaacttggttatgcaaagttgattcaccagcatcgta
aactgtctcagtccacacgacgtaagcaggatcgtggccttatggcaatggtcttatcaaggtctgaaaaacaagaggaacgagaaaatcactc
ttcggacgcaagagatgctatcaagagtgctcttcgaaagagactccgtcgccgagaaggacgggttcaaaaagctttaaagcctgctcctattt

tgatttgttctagatgttctgctactttcgatcatcaattcgctctttccttgcatgagaatgattgtttcactcaaacaaacttcaaattatctgacttt
ttcgtaaacgacttgatgggaaattcgctttttgaataaaaataatactatcgttatcaatagtgcacatgaagctttaaccttcgtaaaaggcata
atttactctgaaatatgccgtacaaaggttctcctggtttttggaactaaaatgtttgctccctaatgatttacacgtcaaatttagtctacaacaatg
cttactaagtttatgaaattattcttcgttctttttgcaaaaaatctcaaacaaaaatgttactcatattcgtactataaattttggtgaaaaataata
atcttttcggtcacaataacaaagtgttgcatacaagaatagtcaaagaagatgtagtaatttggttgtacatgaaacatattacaataagctgtt
attaaatgcaacaagtagacggaagtatgtaataaggaccttggtccaactaggaaatgtttttatggaaaaatacgctttacctcaaaaggagt
ataattttccacatttggatgctccgaaagtaacttttcagtgcaatcatcatattggcttctgaacaagtgagaaccaacgggttgtaaagaatat
tgagtgcagatatcacgtatattcacgttctccttttggatcttacttaaatccagctcaactgatggatctcgttgttgcatgactaaagtcacgata
cggtgcttatcttctcctttatgcattgtgggaggggtatatgggattaaacagtttgaggggcgtattgctaaacggtcagtagccttgacgggtat
gttagaagctagccataagcaggatgtttgaaattgattggtttcgtaatttggcacatccagatctagcgtggctatggtatagttatgttcttcgg
tgctaaatggtagaatctggagcaatggctcagagcatgtctgctcggttggtaaaatcgaaccagggacgacattggttgattcatctttggaaa
aactcaaacgaacatctactatgggattaaacgatccaagtcctaaatctttgatgaccttcatttgctctaatctttgaaggagtagcaatttatc
atgactttcccacttttgttttaacaaatggagaaatgccggacgtgaaaagtcgtattcaccatcataaaacttggatcgaacgcttggaagatt
aatctccgaaagaactcttagcatgctaatacgagcagtttctttgccagcggagctagccaatgcttgctctaattctttacgctttttctcagagt
attcttcaacgactaccgttgcgagccgatacgcgtcatcttgagctttcttataaatattccttataggaagaacccttggcaccgcatttttaaaa
gtccaaacccgttgcattctatatggcttagtccattcaaagtgtagaagtttgttcttgtcttaaacctcgccgggttacaatagtaagttgttacct
tgtaagtgaaatctctgtaaattatagaagaatttattaaatccatcttgtctatgaagaagctatggatttattagttctcttgtgttccattgctaat
gactctagaaagatcagcttattcgaaaaggttttaactgagcagcactataaccttaacgaatgggcattgaatcagctttcaagtcccaagaa
aaataaccttataaagccactttatctagacttatttgtggccgtttggacccagctttttataattacaagtaattcttgattatgtgcaaggatatt
atataactgtttccataattactatgattttcgttaagtagatataattaaaagcctagtattattgagctgaaagatctgtaaatcataaacaatcc
ctcaaaaagggatgaatcaatacaatacaatctgataatcattacttctttaatagctgattctttttgtaaaaccgattatatctgcatggaacaa
cggattctatgacgctttaaataatattcttttgacagtataaaaatcaccgtcatgcctaggcttgtagctaaagtagatttattttcaaatttaaa
gtacgcatgtatttcatgaagaaaaaaaagaataaaacaccccttggaggaaacaataattaacgaaaaacatatattaaactaccacagcag
aatctgatagaaatcctgtaagaccatggaaacttcaaaatgaatgagaaaagaaggaagcgaatgccatttggtttaaaagatttttgttttttt
gcttgctgtcccaataaaactctcatttaaataattgctcatattgcagtttccgcagttgatatgctaaacaaataatgattgtgatttctatgaaa
atcaatattcacattgtttacaaattggcacattgccttttaagttgacgataccaatctggggtagggagtaaatcggcctattaaataactaaga
ataaaaaggcgatcacgtggcttagtgaaatatagcgaacatagtaaaaagtgcgacaatgatacagaagaaaaccattatttttctcttccat
taaaaatttccatcataaatagttaatattttctacaaaaaccacagcttgtttgcgttgaacagatcctacttctgttgttttgtttttggcaccgac
aaacatttcgagactgtttgtttacaaatacatgacgcaaaccactagtcattgtattcactatataaatatctgtttattagttttgtgttttaccaa
aaacaacatcattctctgtttaatctacgtactcattttgtaattatcgcagtctaatacgggggccattgatcgagcttcttaccaattgaagaaag
ttaaaaataagtcgagacaatcaccaattactgtgactgtattatttatacttcgttcggtactttgggctttgctgtcgccttggtactctcaatcctt
caaactgtctgaacctaggactaatacttatactgggtgcgtgtactaacatacacaggccctaccaagtatttgtactttcctattatataattatc
ctttttcgtcaataattcttttttttcttctttccgccattctttatctgttttcctttcttgtttttttgtttacgttttgacgaaagaaagtccaagtcagtc
agctagcctttgctgcttatgaaagcatcattcattcttgctagatttcgcttctttcgcttccttccgtgtatatttttctgcgatcgtttgcacttttcct
aagttattaacctgtctggtggatttgcattcaatattcaattaccgcttccttaaaaaaaaaagagtctctgcttactttccttgtttatattttttgat
ctttttgtaccgggatttttttttccacgtttcccatggattttgcaaatatgtatcttggctttgacaatttgggcagtgaaagttttcatccaaacaa
ttcatctgtccatcaagatagcaatcaatttccttttcatccttcccttcaagaaccaggaccacctgacatgtcagcaaaggcctccgttgtttcga
gtgacttggggatgtacgatatgaatggcatatctcttcaaggaatgaaagcgaaagatcaatttcaaatgagagcaccatctaagtacattcctt
tagttggcgatgcaagcttatcttcatatcaacgcaatgttatgttatcccctgatgccaaaatccctgagacagtcagtctttcggaagtatcaaa
agctcaaaattatggagaggcagtaccattaagatccgagaaagttgcccctacactgatgaatgtgcaaccctctcaattacctttggatccttct
ctagggttgagaaattttcagcgaaacccttccaaagtttttttcattcgacaaaggaaaaagtactccttatcccttaatccccgaaaattcttctca
tttagaaccttactctatacattctcctagctatcgtgagtctccatcaatacagtcgtctttttcaaacccaacaacccctcaaaagcaaaagacta
tcactcctaatagttcaaattatagttcgcctaagctggatcaaggaagcatatctgaaacgaaacgaacgcctcgtattcgccgcagtgtaactt
ctttagatgacgactgctctgacaagagaaaacgtttcttagaaaggaacagggttgctgcatctaaatgcagacagaaaaaaaaaactttggac
ccaagatctggaaaactctgccagagttgcttgcgagcaatcgaaagctctgagatccctagtcgctcagttgcgggaagaagttttatgtttgaa
gaatcaattgttggcccatcaagactgtggatgcgaaggcataagacaatatctttcttcagaggcaatgggcatcatgtccgctctccaaaagc
actgaatttcaattgcctctagtaacgttcatttgagaacaggataggttgttattggtttcgaaatattcctcgtaaatataatggagtccgattttc
atgttcttctaattatccatatttcatcttttaatgttcttacatacgttgatgctgcctatggagaatctttctaattgtatgttcttaatacttacttgtc

cgagtttatctttgcatttgttcttcttttgcttgcttctaatgtaaaagcataaaaattttctgcatgtgtaataagggatcgttaatacttaaattcta
atatagtctcaattaatttcttcgtcttttctattcatggaaatttctgctacgccataaatatgcttagtctaagttcatagtattgtcaattacttgta
cgcaatgattcttctgtgtttactaagaaatcttttcgcatattattgcacaatgcgttaagcatgaaacaccatttccataccaatttcgataattgt
aagtaaatcattagctatttggtctctcttttttttgttctttttttatttattatttagaggatgccgccatcaaatacttcggaccatgaaacgcctcc
catcccaggaactttggtctctagagaacgttcacaggttataagaactcgattggctaaacttttaggtaccaataaagcatttccaggatccca
gcctgtttctttcgctcataaacatatacaaaccttgaaagagaaagactattatgtatgcgaaaagagcgatggtatccgatttttgatgtatata
accaaggacccttcacgacctgataaacccactgtttacttaatagacagaaaacctagttactatcacatatcgaatttgatatatcctatacctа
cagatgtttctggtaaagctatgcatacggaaactgtgttggacggagagctgatattagaccgattcccagatggaaaaaagcaactcaaattt
cttgtatttgattgtttagcttgtgatgggaaactatatatgaatcgtcctttagataagcgaataggggtatttatggtaaacatcattattccactt
cagcaatttatgaggaaattcccaaagtcagtaagcacgtttccttttctaacagaagggaagaaaatggaaagatcatatggcatagaaatgct
gtttcgggatattattccgagtcttcatcatggaaatgatggtctcattttcacttgtgttgaaacaccctatattacagggacggatgtcaacctttt
aaaatggaaaccaaagacaattaacactcttgattttatgttaaggctggaatttatcgaagttgatgaacaagggaatgtcgattttgatgcgaa
gcctcaattccaactaggcgttttccatggccggaattcatactcttattttgcagatatgtatgtggatgaggaagagtgggaaaagctaaaaag
ctacaatactccacttcaagatcggattatcgagtgctccttggatgaagaaaatcgatggaggtttttgagatttcgagatgataaaagcgaagc
taaccatatctctaccgtggaaagcgttctgaaaagtattgaagatgctgtttctgaagaagatctacttcgtgaggcatatgcgattaaacaggc
atataatcaacggcatccgaaaccaccaaacaatgataagaaacgaaaagcagacgaggatgctgtacaggatgagaaagtcaaatcggagc
gaaaagaaactgcttccggccttaaacaaaactttcatcaggactgatgcgtgtaaataatttttttgaggatgattcttttatttcttcctaacaga
tcatctactaatttctaccatcttttatttcttccttaaacagttgctttttccattgcaatttctcaagaatcattctttgagaagattatttattaacgc
atattttcctcctcttactgaacctcgttatagaaataacgagtttgagcagcagaggctaaactattgacttgtaaaaacagtcacgttagcactg
gaaaagtagtccggtcgacatttgggcttagtacacgtaccatacagagcatcaggagcttggacggttaaaatgggtcgcgttattagagcaca
aagaaagagcggaggaattttccaagctcacactcgccttcgtaagggagccgctcagctccgtactttggactttgctgagcgtcacggatacat
tcgtggtgtcgtccaaaagatcattcatgaccctggtcgtggtgctcctttggctagagttgctttccgcaacccttaccactaccgtactgatgtcg
agaccttcgttgctactgaaggtatgtacactggtcaattcatctactgtggtaagaacgctgctttaaccgttggtaacgtcttgcccgttggtgag
atgcctgaaggtactattgcctccaacgtcgaagaaaagtctggtgaccgtggtgctttgggtcgttcctctggtaactatgtaatcattgttggtca
cgacgttgacactggcaagactcgtgtcaagttgccctctggttccaagaaggtcgttccttctgccgctcgtggtgtcgttggtattatcgctggtg
gtggtcgtgtcgacaagcccttgcttaaggctggtcgtgcttaccacaagtacaaggtcaagcgcaactgctggcccaagactcgtggtgttgcta
tgaaccccgtcgatcaccctcatggtggtggtaaccatcaacacgttggtcactctaccactgtcccccgtggatccgttcctggtcaaaaggctgg
tcttattgctgcccgcagaactggtctcttgcgtggtgctgcagctgtcgaaaactaagcttcgttatctaattgtttcaaaatgctcgctatccaagc
tacctttgttttgggttatgtaatgtttataaaaagtaatacaatgatggctgtctgttcatgtgttatgagggcaagaaaaatatttagacatagta
gctggaattgacgttgaatgagagggaacaaaaagcgtcatacgtagtcttagtaaatgtccttatatccttgattgtatttttagtctcgtcccaa
atacctttactttaagacatagccgatgaacaaacaacattgttacccattatgtattaaaattcgtcgcgtcttgatagccttcttccctccagtata
atgctttttgcttagtcttattaaggaattgtttattagttgataaattctgttccattgatttttaatttgtatgttcttttgtaggtattcctcaactggc
tatggaagtctgtgtctagtaagaaaaaatattgacaatatcaatgacagcgactgacttgaatacagaaactcaataagtagaaagattcgttt
caaattaatttcttaaggacaattgccattgaaacgtctgctttgcaaggcagaatctgaaaagcttattgttgtttgggaattcaaacagaaaatt
gttgtatacactaaatcaaagtcaaaatctatgtaatagagacaacatttggattttcttcactatgaagcataaaaactttagaaagcccagtgt
gaagtcagatcaaatttaaccgtgattctataatctgtatggcaaatacagaaaatatcgttttgagaagtagctctttggcactcttgacacgaag
gtgtgtataggccatacacaagatgtgatgtccccttttgactttcggttccctttcaattcgtctcatttacatacatattccttacaaaatatgata
cgaccactccattttgcctgcttataacaaaggataatatgaaaagtcaccggatacactccagaatccagctgttgtaccaactttggatattctg
ataccacttctctcactgttaaaataaaaaacaagctgcttaaggcgaagttcggtatatcttgaaactcatccgtttgggttgaaaggcattgaa
gaatatatgcctcataggattaaatatttccggcgaagtacttcttacaaaaaaagtgatttaacttgtctttttgttcttaatttcctctaaacacc
aagctataaaattggtgtatgatcacttaattcacccaacaccgcctatcgaagagtgacatcacgcaacaatgaaataagtgagatcagacgct
aactagttcatacaacttggcaattacaaaacggtataactagtgcttttataggatttgctttttgttggtaaccatgggtgaagaagctgtggga
gctcaacactctagaaagggttctacttctagttcgtcatcaataagtaaaaaaggtttgtcaatgctgctgggactttaactttgtagtttgagatt
ttctacttggagctagatactaacaaaaaaataataatagatatacaaacaagtttaaagcagcttcagctactccaaggtacttaaaaaccattt
tacagtgggaggacgaatcagtcctcatccattagcctgttttctattcaaaagatacatatactaactcttggcttaatagcttttcaaaatggag
atattgctgaagtagacgctctacttcagaatcatagcaaggaagattgcacttatgctttgtttctcagtattcaatgtgcaaatgcttctatggtt
aagcatattttatccgtttttgatgtagatttgaatgcttatgataaaagtcaaaatacggtatgtacttcttcattgctttatgggaatcttcctaatg
gaaaactttttacatacatttagtattcctagtcgaagagacatgtattctactcaatagttaaaacatttgctaactatatctagccccttcatttag

ctgctatggcaggtcgccaggacgtagtggaaattttgttattacaccccgacattaactataacctcctcaatggaaacaatttaaagccttggc
aggttgcatcttctaatgctttgatggatttcatgaaaggtaagggtgtcagcttttcaaattctcataactaacgtaaaaaggcttctgtgccatgt
atacaaaggaaacagcccgcgaatttaaacaaggctttcaggagcgaaattttgatacattggactatttgatgagacataatgaatataattct
gcaattgatttgaatgagattgatattgaatctggacaaacctatttgcactccgcgacgaaggcgaaggacatagaacttgtcaggtggctgctc
aatcataatgctgatccttatcgccgagataagttcggaaagcttcccgttgattatacaaaggatgaaaacttgcgtagtcttcttaggaatcagc
cttctgaaacaaactctgatctatcttcttcatccgaccaaaacatttccggttacttgaaaaaatggaccaactataaatcaggatataaattaag
atggtttaatttgcataacggcgtgttatcttactacaagaaccaagatgatgcttcttctgcctgtcgtggatctataaatttaaacttagctcgtct
agtttatgatcctaaacaacctactgtatttcaggtcattgggaggggttctgttcgctacagtgtgagggctaactcacctgtagaagcacgccga
tggatcaatgccatctcgcatgctattgaacatggtcaggataccgaaagaaaccaaaattcagatgctgtatccattggtacttacgatataccg
atacccgcaaagcgcggtatgagctttagctccgaacatttaccctggaacgaaactgattcttatgctggttcagaggacgaacatgaagaaat
gccaaatcgggagagttatgaatttaatatcaacattgctaaatacaaagtggacatattacagagtttgattaattcagcagtttcccatgagaa
gtacaaacaagacccatctcttatgcaggtgtttgaaggaattgaaaatgcttttgccactttacagaagactgtaatggaggttctacatcttcaa
catgaagcagaaatttactataaacgaaaactagaaaacgcaaagtcaatcaatgctttatgggctgaaaatttgaagacagtcgttgaagaac
aagaccaaattgaagaacgctatcacaggagtgaaactcatcgcaggcggactaaaagagcacttcgtaaattggcttcctctgtggaaaataa
tagaaataatggccaaatcaacgaaactgcatctttatcatctacctctttgtcgagtgattttactgaggaggatcaagaagaagatagagaga
catttttcgatgccaatgctaatcaaactcccgctcgtatccaatctccctctaaagagactcctcctaccgttggccggtatcctacagaagacag
tcctgttcctaccgccggaaaatatccttctacagaggcacctgctcctaccggtgatcaatatccttctgcggaggcacctgctcctacgggtgac
cactatcctccctcaaaggcacctgctcctaccggtgaccactatccttctgcggaggcacctgctcctaccggtgaccactatcctcctgcagagg
aacttgctcctaccggtnnnnnnnnnnnnnnnnnnnnnnnnnnnnnnnnnnnnnnnnnnnnnnnnnnnnnnnnnnnnnnnnnnnnnn
nnnnnnnnnnnnnnnnnnnnnnnnnnnnnnnnnctcctaccggtgaccaatatcctccctcaaaggcacctgctcctaccggtgac
cactatccttctgcggaggcacctgctcctaccggtgaccactatcctcctgcggaggcacctgctcctaccggtgatcaaagttcttctaaagtttc
atcccaaagaaatgaacaaccagccttacaaggagagttacctgaaggtgctgaggatcaacaaaatatccccccttcttctgctcctggtgttgc
aggtggcacagagaaacaagaaaagaaagcgccatcaaatgaagctgaagcagatcctctttttttcggagcttcaaaaatcattccatggttat
gaagcacctcttcgattaaatttaggtgttaatgacgatcgcccaaaaatcagtctttggggcattttgaagggaatgattggtaaagatatgaca
aagatggctttacccgttagttttaatgaacctactagtcttttgcaacgtgttgcagaagatatggaatatacagatttgctcaatttagcgtctca
acaggacgatccagcttggcgtgttttatacgttgctgcttttgcagcttcagaatatgcaagtaccattaatcgtgtagcaaagccttttaatccat
tgctcggagaaacttacgaactatgccgtcctgataaaggataccggttcgtgattgaacaggtcagtcaccatccacctgttggagcagcatact
cagaatctgtgaactggaaatattatggggaatcaagtgtaaaatcgaagttttatggtaaaagctttgacatatctcctcttggcacttggtacct
ggaacttaaaactccctcaggcaagacggagttgtttacatggaaaaagattacctcttcggtagtgggaatcattgctggaaatcctaccatag
ataattatggtcagatgttggttcaaaatcattctgatgatgtaaactgtatcttggattttaagcctcgcggctggcgtggtacccatgcctatgaa
gtaaaaggaagtgttcaatccaccagtgatgttcccgagtacacagtgaatggccattggaatgataaaatatttgcttataattccggtagcaat
aagacaatgctatggcaaaaccatgagaggcctccccgtccgttcaatctgacacctttcgccatctccttgaatgctttaacggacgaactaaag
ccatggttaccacccacagatactcgattacggcctgatcagagggcaatggaagtaggacaatatgatcttgccgctgaagagaagaacaggc
ttgaggagaaacaaagaggaaaaagaaagatgcgtgatcaaggagaagtgcaagaatggagaccaagatggttctccatgaagaagcatcc
cattaccggtgaagactactgggagttcagtggtgaatactggaatgtacgagaggaagctggtaaggcaaaattgtctggtcaatcatttgaat
ggcctggtgttgagaatatttttgaaataaacagtaaaagtattgtctcagtatttttcaagatagtcccaagtcccgttaattagcaatacgttac
aaaacctaaaaaatacgtgtaataattaagccataactacgaattacaactagaatatttacgaagactataactatattaagaggacagtatga
tgaaaatttcattttaagcagcagtaatagccttgcgagcagccaagttgcgagccttagcaccttgtttcaaagaaacacgggcaaccttttcttg
agccttaggaacggcctcctcttcctcagcaacaatgatttgaatgtggcaaggatgagacaagtaagcagtgacacgaccataagcacggtag
gtacggcggtgttgcttaggagcaccgttgacttgaatgtgcttgatggccaacttgtccatatccaaacccttagcctcagcgttagcttcggcatt
cttcaaaaggtcaaggataaccttgacggacttaacgggccaacgagcttgggtgacaccgaattccttgccttgagcagtacgaccaacaccgc
cgttgaagcgacggaagggaacagcttgcttgtgttcgaggacattgtccatgaaagcaaaggccttcttcaagttcatgccattaagggtgaag
gcaacttcacgagagttcttaaagtgagttctcaagtaagcaccgctggctttggcgcacttggtctcaacagcaggtgaagcggaatagcgggc
cattgctacttttgttgtggtaaaaggctagtgctatctgtatgggttttgtcttcgtggtttctgttgtttcgttttgtgactggttttctcaacgcaggg
tagggctatttattgcgaactacgcgtaaaaataaataaggagtaacgtttattgtttcgataagataggtttagcacatacgttggggtaacgta
caagtgcggtattcagaatgattttaccgtacgttgtaaggttgaaacttttctttaccaaaatcgcacagttggaaaacttccattcaacctccctc
gttgcattcattttgccttagcatttgagctacggcaatttacatcaacgaaggacgactattgtcaagggcaaggttcctacatatgagcgcccga
gattcatgtgatcccaaaacacttaggaagttgtgtgaacaaaaatatgacaagagtacagcacttctttccatcttaaaagagaatttgacggc

aattggaaatgagttggataccttaatagagaagaatcaaatacgttttcacgctgaaaatgggaatgtattggaaggatcgaaaggtttaaccg
aagccaagaagaagcgaaagaattcgaaacgattaacaacggacgcgaattctgggaaactggaaaatcgcaaggaatggaagcagcttttg
tctgttaattctacgattgctgcccttataggcgaaattttgctgacagatgtttcttttgctttagaaaaggatgctttaacttttatatggacgaag
gttcattatcgagttatttcctttttgaaacataaaattgttcagacgtctaggaagaataatatagaggtacttgggtctttagtgactctatatttt
cattaccttgacacttgcgttcatttttacgtttctttcgccttcattttgattaaagtttaccgcttgcaatgccttgagcctttagcatcttatcttgct
actcaacttgccttcaaaactacgaaaaagtctcgaaaaataaaggttgaatcgggaagtgctttgcagcaggatactcttttaaagtctttgtttt
cttctatttttggtttaaaaaatctgttttccggtactttgagttccattattcaaaatacatcaacatctgccgatcctgagacagctactagacgct
ctgcatacgtttttagtcctttatttgattcttctttgcctgacaaacaagctcattacctgattagtactgtatatggttctcttatttatgtaggtgata
tctatcgttatttggctcaggctcgttctccaaaattgccggattattctattgctagaaagtattactatatggctgcttatgctgttccaaattgcgg
cattcattatcatcagttaggtttaatcgaagtacaaaatgctgattcatccgccacaaactctcctcgaagtcgttttcaaggactatacaaaagc
tcgtcttctagctcgcttcatacaaatgaagcttcttctaaaccgatggttgaagatgctaatttgttaggaatttcctatttctttttggcttctatttc
tcccaacggtgttgctgcaaaaggtgcttgtaacagccttcttattgcacttaagcgtattttaggaaaacccagttcttttgacccatctttagtaat
gtatcaaaaggacaattctcttgtatccttatttcttcgactttatgctctggcattttccgactttgactgtatgtcttatacaagctcacttgtaaag
cgagttcgatttttattaagtgattctttaaaaaacgccaagttgtcttacgaaacattattgagaatggcttactgtgcgcttgctgcaaggtgtct
cgcatcgaagttagggtatagtgaaacacaagaagcaaataagagggttccttctgcgaaactagctacacagacgaccataatgtttttgaag
tacttctggatgcagtgaatggtcagcttcctttgcatgcactaaatgtaagttctttgcacaaagatcacccacaaccttatggagtgctcctgcat
aataaaatcctttccccaatggttttacctatatgcacgttggtttctttctgcctaaagcatggagatacttggctgacaaaagatattcgagcagg
tctatcacagatctacaatcgttttaacttctttttgaaaacacaaagcaggctttacctactgaggaatccttaagttggagtactcaatgggatg
ttggtttaccgtttttctttcccatgcaacaagtgtatgatgtgaattacgacttgctgaaattacttatgacaaataaaatttaccctctaggagag
aaagaggaaacggaacgtcaaaagataaacaaggatcacgcatcgttgaaactatctattgactctttgtatatattgttaggtcgacatgcaaa
tgctcaagcttcatctgtgtccgtatcatctcctgcaacccctcacggattctatcggcctttggagtcaacaggtattcctgcttaccctcagttcag
tgcatcgtctttaccatctgaccaatcttcacctacatcaccgttttattctggcgcttcttctgttttacaaccgtcatcgagtatcgcaagcttacaa
tttggtagaatggtcgaccctagaccatcgcagccaatttcaccttctctatctgaaaacctgactcagatgacacattcgaagcttatggcttcttt
tgatgccacacgagaaaaggatgtaagtaggccagggacacctttctatgatccacatttacgttcacatcgaacatcgatatcaaccgataatg
aagattttcatcaaaataccccgttgaatggtgaaaggattttatttcgacctattggaagtcctaagccacagccaaattcttctagcgatacttct
gatgtgcagcgtcaactgtttttatcagaattgttaaagaaaatgattaacatcaatgatacttgaagaggcttgtcgtacaattttcttttcttttctt
ttttttatttttttttaaaaaaaaagcggccttgaaatgaggtaggagcgtatgaccgcttgtaacgcttgagaattaaaaaatgaagttaaggaa
aggcatattagtatgaatcacccgacgtttgtctgtcaagatgatggaaatgattttttttaatggtaataaaaaggacacgtagaagtgttcgcgc
taaattataatgacaagggaatcgaatccttgtcattgtagattctttcaatcactcctcgttcattctgtttctgataacctaaagaaatgttttgtta
tccttatagtactcagcaagtgcatagatactattattcctaggaataagggacatgcaaaaggttgcttactcttaaagtagtatatgagagtaat
gaaattggaacatatacaaagtaaaattttgaatctaacaagtaatggtacatatgatatacgttgtctgaaccttgcaatccagtatttaattttc
actacaattgcttttctactcgttagaatatttttttaaaaatattcttggcaatgtcaccggaataatactgaaagtatggatagaactgagaatag
aatcaggaaggggtaattttactccataatgttgcaaatgtaaagtattatctgatgatatcaaaacttgtctggcaagcaacttttaagatattct
actttaaggatacacaaggcatgttgtaaagtgcttaaaaaccaacggtaccattcgtaatagttcataaggcacctgaacaccatgcttatttat
ggaagattataccgcctgtcttcagctttactaccctttgtttacggtgcgtcgtctgctttttgcatttttatactcaaaaatatttatatccaagttaa
aggaaacgttggatgggtaggaactgatcgtaattgaagtttacttaccgaatatctcaatctctgaaacaaggatacttaatagttgatttttggt
gtgttcgagccaattttcctaatcagctctctttctacttttataatatagaacgcgtaaaaatctgatttcatttgaaagaaaggggaggcttctgtt
gtaatcatgagtatgattcatttcaaaagccgtcaaagtctttactcgtttgtacagaatcgagcatttactacaaagcaaaaacttggaaaaaat
agtcctttaccaggatgggatccccactcacaatactttcgtccaggacctacgatggccaatcgtttagaagaacatatgaatgatacgattctgt
ccgacgtcttagtgggaacttataaacacaatgttgattttgcggattcttctccccaccataatgagccagcagatcgactggttcgatggactgg
tgacaatccatattacaaaaatcgaccccctcgccattttcgtgggtataaacccctcttgcctattaagcgaccagtccggccagaaaatcttccc
agcatctccaaggtttctataagtactatggtcaaggaagcgcttcaagaaaagtctcaactattgagcactattatggctttccgttccatcaccg
gtgtggagccagaaataataaaagctagaaaagatgtttctccgtggagattacgatccggttttcctgtaggagcaagcgtgactcttcagggc
gaaccaatgtataatttttttgtctgtcctttccgagttggtccttcctcagcttcacgactttaaagggatttcttcaagcgttggagatcaaacagga
aatatctcttttggtcttcctggaaatgctttgcctctatttcctcaaattgaagctgtatatgaaatgtaccctcattctcttcccggatttcatatcaa
tatcgtaacaaatgcccaagatactcgattggctagattcgctataagcagcataattccatttaaaacaagcaatcccgaagatgatgttcgtta
atacatgaagaatcctattagcataaagaattagctactgatttcaatacttaatactgctttctttcctcaattacaaaccatatattattttctgttt
ccaatttctacaaaaaagttaataaaagaagaaagaattaaccattaaataatttttaaagaataaaagaaagaaaatcaaaaattaaagagc

ggcaatgcacaagacgttaatacaaaagaaataaaaaaacatcaacattatgtatcgtaaaaatcgtaaagtcatttactttcgaaacattagc
aaaacaaatttttactgtcacttgattatccgattttaacaataaaaggatgctggagcaattccgaggctgtaggtctttcatggcaatcaatggc
aaaagttttattcaaaaagtcatggacggagggagtggcattgggaggatattcaggagggatattttgtcctattttaaaaattgcttgcatttga
tcacaagtgggatacggatggttgctagtaagcatttctattactaaacagccaagtgaccatatgtcagttttctcagtatgttgtgtttgcttgac
cacttctggagccatccaaaacgaggatccttgcaaagacggccgagaggatctaattttatcagtgggattttccaattccaatttttttagagatg
ccaaaatccgagattttgatttttcctttgttatcaaccaatatattagcacctttaatatctctgtgcacaatgcctttggaatgaagatactgcaag
ccttctaatgtttgctttatgaagtttctgaccagaccggcctcaaaacttccatacatttcaagcagtccggtgacagaaccgccaggaacatatt
ccaagaaaatgttcaaaaactccgaagttaaatttgatccgaggtattggacaatgtgttcatgagccagctcctgcagtaaagctatttcactga
caagggacttaaaaagactagaatgtcgttctttagatttttaaactcactaatgcaaatttgtttgacggccattaactcaccactagatgcattc
ataccgagataaacttgcccaaaggaaccagaaccaatcaatgctccgcgaatccacttgactggctgttcttcaatttcttcttctgttacatttgt
atcttcacttgtggtggtcggagtaggcgaggcaggtgatacttgttccacgaaagacggagacggtatcggcgacattttggacgtgaccgatg
gaagttgaatgtttcgtaagctagctgtttgtaagtctgtaaccctatgtctttcagaaagtttctttgaaaatgaagaattttgtacaggtggggttt
ttataggaacggttggtttgcttttcttatcaatatccgattcgttcaaaggcgtcgctctatttatgccttttgaatcctctaacgtatttatttttaatg
cggacattgatctcagaagctctaaatgttggcgccgcaaacttctcgattttggtagaatctcctttccaaatgtattttcacgagaatgatgaag
gttttgattcccaacaaatgaattatatagcgtttttttctaataactttggttgatgttcagggaaaaagtgagaaatccgtgaactgataagttcac
tcgggggtctttgtatggatagacaagaagcatttgtcctatccgaaggaaagagggtttttttgtatgttcttagacaataaagatgaaaagttttg
tgggctattagaattgcttggcaatggctgagcggattctatttcccaggatttacgaagatcttcaaaagaagggcaaggcttggattccgaaac
tataataagtctttctctttctggcgacgacacgttgtggcatatttcctgaaagtcttcttcaaagatgggtcgaatttgactacttttcgtgacgca
gatattatactttatgccatccggcaagcaaaattttcgtaaagccagattcaatgtttcccaataattcccaaaagactgaattgctcggctctgc
ccattgcaagcaataaaccttagaatgcatggtttcggggtatctcgtaagctatttctttcttttaagaattgacgacgtctttcagcatcagtaat
cccaagctcttctagcaatggatgtgacaaccggattaaatgtcgtccctctattttatgttgtgcaaacgcgtcaatgttttcttcaaatccaacttt
tttcagccatccgcagactttctctactgtatagttttccatgaagttttgccagttgtagagattaaaacaccgtcgctttaccgcgaaccttttatg
gaagttataatagctggctcttcgttaacagtgagtgaggactcgacttaattgtccaaatagacctctaccgcatcagatcgaaataagcgacgt
tgttgcactttgcaatacttattcagcactctactgtgctttaacgatacgaagcctatttattcctccacttcgttcaaaaaatcctttcatttgggttt
cttatacattataaaatcaatggcaatttctttttcaatttttattttgtttacaatgaacaaaaaatatttaaatgctcagtaacctctaaattttagt
aatcagcataactaccacctgttcgcaatatattcaatatcttattccaataagacaacaacaaaactccaatcgacttttcatgcgttcgtttcaa
gtaagcactaatgcaacatagtactttaggtataaccttgttctttaaaatggattaaactcaaagagtttcaaaacttttaattaaacaaaatttat
agggtttcaacagcatggatttttcattggtttcaaatatttgttttatctcgaatgtttcacttcaccccaactcaacctgatgatcgcactagacta
caaaaacatataatcatccccatacggaataaaaacaataatgctgaacttgaatagctccaggaatataagccaaataaaccatcgtcatcgt
aagctccaaagtccataagtaagcgttctcccaaccttcatcccctcagcctttcttttgttgaaaaaaaaaagactaaagagaacgagatataac
ctctaaaggaagcacaagccattccgcaaatacgaaagcagctacacgcaagagccatatacaatcctttcatacgtgtaaggtataacggcta
atacgatccaagttcgagtatttcgaaattttccagatgaaagtacacccgataatcataaaatatcgaaatcagaacaaattagaatcgccgtc
ataacctgaacaagcatcagactaatgcccaacaacatgcatgttaataataaaaatcgattattaaaaactcatcatcgcatgcgaaatcaggt
agtccattagtcgactttataattcactgaattttttttttattcagagttctactaaagacaagcattcattattgcttcaccgaacatgggtttgttg
cagaacaggaggttaagtgagaagtaggattaagcatctctcaaaactactcaagtgagaagattttcctttcacaaaaatcctcattcaccgtc
ataaataaatagaatatagtgttttatcgcgccaaaatttccaattacaacatcatccgttttcctcgatcatgagagagccaaatgtaacatagct
ttatattccaatactactaaagttcgaaaaaaaataaaatcttgaaagaatccaagccattaattaggtggcccttatatcataagtccttgatcat
ttctaaatacctgattcgctttatatcaattacttattctcagtgttcgatccaaagtaatcatccatttccttatcaagctcctcagccgatttcttgg
gacggctattacccttaccacgacgaatgtttcttcttcttgtggacttgggcttgctagatgtattggctttagcaggagcaacgcgagcggctaat
gaattcaattgacgactggggtcaaggataatctcaaccttcatttttctggtgccatcaaccacacgtccttcgtactgttcataagcacgagtgg
catcgccaggacgagaaaagataacagtagcaatgccctttgagcgaccatttgggccataagccaaagatacacgcttgcaaggtccaatag
actttacaaaaagttctctaacttgaggctctgtaacatcagcaggcaagttagatacgatgattttagattcctcagagattaccgattttatggta
gaagcagtatcgttagagggtttgtttgctttaggggctttaggtttgctaacacgagaacggcgctttctaattcctcctttgggctttgaagcaat
gatagcatccaatgattgatctaattccatagacattttcaaaaagttgtggtatggtggttggttgctgctacaattttcgtggcgcgtccttaaac
aatcttctgcctgggtaagggtaagggcaatatataacgatgctttgctcaaggacgttaaacgggcgaacaaagggaagcagatccttcaatg
gaataaacggtttcatataacatagcttttacaataagaattattcgacattagcttcaacatacttacaatatagtttcaacgacgcaagctattg
atgcggttttagcctgtaactattcaaaacagaaaggaaaaacattttatttacttttcggtttgacattcatagcaaagatttcttagaaaaagaa
ctatactcgaaatacgagttagagtctcaagctcatatcaatcatatgataacgaggagcgattttcccattgctttagagatgctagaaattgga

catcacattagtagacgaaaatgattcaaaggcctgaaccaccattcatatacacaagtgatatagcaagagcaaagccgaacttgaattcgac
ttatataggcctctcacgattgatcagagttacgacaataaaaagaaatcaatgttatcaagtcaaccgtattttatcgtaaatacaatccttagga
aacggaaagcaagttgagctactcccaagcaggaacaaaatagataataataatcaaaacaatttgcgaaaagtagttagctcatcagctata
acaaacacagttgcttcacgatactaaataatataagaatttcgggatgaactttcacccaagcacctggacattgacagataatgtatataaag
tatcagtatattgcaaaaatctatatcgaatggaaacggttaagccagtgtttgcattttaattaaaaggtgctcataacaaaccaataagaacaa
aacaacagcaaacaaaagtccaataagtaactaatactgataagaagattttttaccaattttagttttattttttttggtttttttctttcagat
acagaactagctcgaaagaacaaggaaaaactgaatgtccttagatcactcaaccagaataaaacgaataaaatcccatcaaggggcaacag
ggtaaatagtttaaaaagagatttgtgaagcattatatactcaaatcagtaatcgatttatgtcatagcatagcgggtgtacattggaaagtatcc
cacctgcgaatacggataatagtaattgtaatccgagcaggtacgattcactgatggcgagttagtagcaaattgaccagcgccttgagattgat
catgattattgttaatactaccattattagaggaagtaaataatgcgtaagaagcactccaccattcccaagccttctgaccacgaggacccgggc
ttaagttccaagacatgcttactagccagtcttgttcgaacttgtttaacagttgggttctaaatccagttacttgagaccatgattggttggtaaaa
gtgttgtcatccaaatatttgttagcaaggattaaagcacttgtaagtaacgcgcaaatttggaaaaagggagtctcctttgtccagtggtttaaaa
attcagaagactgggacgcagcatccaagcgatcagagatataggacaatgccaacaagcaagaggcacgaggaagacgggttgaagtcaa
gacttggagaacgaatttacggaaggaaaagacaaggctcggcaaagagttcgctagagaatgcatttgagcattgttaagaaatcgagcgta
gagtttgcaagtcatgatagaaagatactcaaccatttgatccatcttgtaatccaagttggcagatacacctcctgtataactaagctcatcttga
tccatagcactgtcttcggcctggtattgaggagacatacgatgtcccacaggaggagcaacgttgcagacatttgagtcataacggttgcctaat
gctgcaggggcattagcgcaagcagcgggattaccggcttgagcagcagcaccagcagcataacagttaacgggaccggcatttgctaaagaa
tcgtaataagtcccacgggatccaggaattaaagaagaataaggaacttgaccagaagggttcaacactaaagggtttccgagagcaggatttt
gggcagaaggatgatatgccaaaggatacttctcgggacctaaatagcgttgaggttggccggccgcagggatggactgctggtaaggaggcc
aataataatttgaaggtgcaggagcaggtaggcgttgttgaagcctatgctgtgaaagataaggaccagcagcagaaccaggaacaccactgg
aagagttggatgaagtacggagatggtgagaggtagaagaagaagggatagaaatgcccgacttacctggatacgaatcgggcacggaaaca
ccaggcaaaaaggacggacgacgctggccaagcccgtacatgggggtatggctcaacagagcagcaaccgatgcagcagagtttgcattatta
ctagacaaaacgggggcaaacataccatagccgggggcagcccctgaaggctgggcttgcgagcgattagtgtgctggtaagagaaggccatt
gtcgaatcaagaatgtttccaaagaaaaaaaatgaaggaaatagatgaaaaaaaaaacagaaagagtcctctcgggacaaaagaaaacaaa
ggcttcttgttattcgaaaacaaatggatgatacacgcttacacacactatcttagcacaaaacccgactaagaaatgcttgttccgcttgcaaat
aagacgaggcttaataaaaagctcgtttgatgcaaaaatctgctaaatagaaggataaaagcttgaaccttccaaaaagtggtttttgagttgtt
ggttgtgcgcaggaaaaatctgcgcgcaccagcaatttaatgggacttaattttctagaatataaaaatctagaaaactaaggtctgtgaaataa
cgcgacaacaacgagcctgagcgaattcaggatttgcacgaaatgcggtaaagcaagaaaaggtcgcttaacaaatagagttgggtagaagcg
aactgatttggtgtttgctaatagaagattaacaatccctgaaagaaattcgagcaaaacaaaggagagttgaagattgaaactcaccgatata
agagatgcaagtgtagaaaagaagaagttgagggtccttaagagtgtttggagaagaaggacaccaaaaagggtaacgaaaacgtaaaaca
atcagaaaatgaatgcactgttacaagaggaatttacgagcaagtagatgagatgatgagacttgcgagaaaaaaggtaggtagtaaaataaa
tgaatctttgaccacgtaggttgaagagaaggatacaaaacaaagatggcaaagaaaacaagtagatgaagtagaggtagagcgagaaggg
gtacgctcagtctgcttttaaagagttcagttgactaaaaaactgcataaagtcctcaatggacgtgagcaggaaaaaaaaaggaacaagccc
aagaaaccacaaaaacaacctaatgaaaggctggttaaaaagttgtgacaaaggtggaagaaggctattgctgcaatgctttaccatgttttga
atatgtaaacaaacaattcatcatcgaagggtctggccagttcgacagcaaatagcaaatcttaagtcgtctacgcaaacatatttaccaaagca
cactcgagtacgcaaaatatttactggatcgtctattttacgctatttgtccaccacaagaaagagggaaaagaaaaggggagacacattcagat
ccttccttcattgaggaggattgttggttccttgtgacgaaccagtattcgataggattacaggtcacagagtacagagtacagagtgcaagttttt
ttttacttttgttttgcttgttttgtttcctattattgtctgtctttttggatctgtacataatcaattgaaacgagatttacagcaacatcgagtatagt
cagctttgttttgtcgtctattgatcgtcaaatcaagctcgttacaagaaaaccccattcctctatctgcggtagataatttatcttgattccttttggg
ttatctgtcagtgaagccgatcaaatacatgacacgacctcctttcatcatccctccaagcatttggactgcgtaagaatgatttagtccatgttcgg
caaacgcggtaatggtaaggctctctcgtggtctttatggaaaataacgaaactatatcatctcgcgttacaaatttgaactatgcaaggagcctc
cgtaaaccagatttcgtgaatccttcgattttgaaaaaaaaagtcgacctacatcaagctaaatcttacgtcgttgtgtttcggtccggtcttcgacc
tgagaggtcttaaacgtatcgtgtcggtagtgcttcggttaggttgattttccggctcaaaatgaaaataaacgcttccattcgcagtcttgacatac
atcttacaaggaacaaggaacaaacgtttttttcgtttgtttatggattcgaaaaattctgtttatttccacaaaatcactctttttatccttttattttcg
ttgtcgactaccaacttatccatgacctaacgttacctaaatagactgggcccatcggcagctagccaaactaaagctgccttttcctatggtaaga
agaataagctcaaatggcgtatgccattactgtcttattcttctttattcccatttaccgaaccctattttattttcccctaaaatattatacgtaatgt
atcacatggtaccttattattcttgtcctcttttgctctatgggatgctcgtatggaatcgccacttcaagagccatcgttctgcttaaagcctatttgc
ttctccgcttttacccatttgtctcttaaccattagggtgtctgcagaaagctacgtagcctaattctctcatatatattaggatcgactccccgacta

ctccctttttccaagaattagcaagcattgggtacattagaagtttacggatgagaatccctagaagaaagaattttctttactaacaacattctta
aaggttaaatttctctttttccttaaccttctctgtttttcatcgtgccattcggatttttgacacggtagtattgtcgatactgccagtgtaaaattttttg
tgaactagacaagacaagggcgtattgacatactctttgtcgtgatttcatctttttttatatatttatatacatatatagagaagttctcggttcttgct
gcgtttatactcattttgacgcctttcattttgattcattgggtttcatccgttcatcaaggatcaccaagttaagttctctttcttttcttaccgtttattt
aaattttgtatccatatttacttgttaaagcgaaattttcttgagtttattttcttgtcgtgtttctcttcccgggttttaagatttttgtttttgaattgagt
tttatttctcctcggtgcccttctgttggtatttaattggtttatttaatttttctttttttatcttttatatcttttattttggttttccttctccttctccccttttt
ttcttttttgattatttccctttttccatagacgttttcttagatttatccatccgactatccatccctcttgtctctttgtaccaactctcctgtttgcgtt
gcgtatttttgtttctaatttgcaatctttccgttttacctttggttcgttggtatctttctcttcaattcgctttctctttttttattttatcctcttctacccat
tttgctttctcctattcgttaatttattaactggttccttgtcctttttgttttaatttcttttctatattgaccataaaatgtccaataaccccctcaaatac
cgctgacgaatcggccagcgttgcggcttcgtcttcttcgcccaactataatgcacactctactacaaacaaatatgcgcgcccgaatgtgaaaaa
tcctgatgcaccgcgcccctacaagtgtccactttgttcgaaagcattttatcggttggaacatcagactcgacatattcggacccatactggtgag
aagccccatgcctgtacttttcctggttgttttaaacgcttttctcgaagtgacgaactaacacgccatgctcgcattcacaccaatggcaactctcg
cagaaataatgctatggccaattccataaatgtaaaaccatttgagccaaccccggtgaatcagtctgatccttcctccattgcctcctttaatcatt
ccaataacaattcaactagtgtacccatgccaaacacacaaccctataatcctgctattcctcaaattggtgtaacttatgcccccgtctccaatcc
ggtatccagtctccccgtcgccttcccttaccctttcccggttgcctcttcagcatctactcagccccagcacgctttcgttcctacatataacactac
acaccagcctgtaggcgttcctgtttataatcgtcctgcggcctctgcttttgattccctccgagccaattgtgcagatgttcattcctcttctgtccct
caaaaatcagaatctgacacgaacttgtcctccatgaatgaaatgtatatattggcttcagctgccgctaaccaacttgattcagctgctggcgca
cctaatgccatttataattcgtctcccaaacaagcaatacccgatccttcctcctcatctttgccgtcattatccaatcttacctcccctagcaagtttt
caaatcctctgggaaacctgcctccgtcttcttcagactcatctccaagtcacggtgctccggcttcaaaccgtgctttcgtgaacggtccaccgtta
aaacaacctaatagctccttctattccggagcatcctcgcatacaccgtatctgccaccaagatcgagtagtagcacatcactacattcgatgtat
agtcttgtgcctagctcgcatgctcctccttccttgcgatacgctcattataattatcttccttattctcgtcccagtgtttctggaaatggtgaagaag
agccctttagtggttctgatttcaatcattatcgatatcaacgtcgttcaaggcctggctctccatgtagcactgctccaagtagtcctacattctcaa
tcagaagctttagtcctacacccgatgttacacctttaatgactcccgcacattctccaaagatgagagcggccgattctgatacgtcttttgtgcag
ttaccttctatccgatctctttctcttcgtccttcccagcctcccgtaattcctcctctggagtgtgatccaaactcgctgtcctcctcaacaccttcttc
agcagcggtatccaggaccccgtcgtctgcttctttaagtgcattgtcgaatgttagtacaggagcgaaaccatctaacgcttccaacgttggacc
cgttcgtatttcttctaaccgtcgaatacgcaagatatcatcatcctctcgtttgagcgtgaataatttgttatctgcctctccgccttcacctgcttct
gcttcttccactaaatcatcacatactatgccacctgcattttctatagggccgttaacacctcttacaaaaccgtagatgttttaatatacttacttg
atgtgaaattgaaagattacaggaaatcgttatatacaatgcggtggttttatctttttactttatatttgtgattttatttttatttgtatgtacatgttt
gtataatgaatacaggatgcttgtcgatatcaatctggctttcagtaggatgttttatttgtctaaacggagtagtaaatttacacaagattgcaaa
attcagcattaaaatgtctttcacggcttatcagcgctttcaatatgatattttctttcaaactcatttcattgcaatgtgtaagtacattcagttacaa
aatattaaccaatctatttacttctaaatagtgttagcatttgaaaaaaagggaaaaagaaaagcaaaagaagttgatccaaactctcagactg
acgtgcaaatcaatgaataaggatcagcataataagctcagcgtgaactatgcattttcaaatcatcaaagaatttagtttttttttctttcttttttat
gtaaaataaaaaaaatccaacttctatctgcaatttttttaaacaacttacctgttccaaaaattcttagagctagtagaactaatttgaacgtttttg
tattgatgaaagaaacagtcacaaagtttgtttcggcatgacgcgacacgacgcgacgcgaaagtaatttattaagtcgaagagaaatcgcaaa
gcacaaacacaaggaacaacatagaaaatattaacggacagattcttttatttttttgtaaatgagaattggcattcactgcagtagtttcgtgata
gtctttcttctttcgtaaagcgtcaagttcttgttgtaaactgctaatttctgcatctatggttttacgaaccgaatccagatccgtacgaataaacct
atggtaggaaagttagtacaagaagaagaaacaaaggaaaaggagccgtagaatcctgagataatctgtccccaaattgccattaccccccag
aagaatacaaacaacatacatttttccaacaccgccgtagacggcagcatcttttccaacgacatctaattctttctttgccaattccttgattctac
aagagcgttctttagccgttacttgcaataaaatcgatcgcaattgttgttgcgaatctatggccttctcttgaatctgagttgccagctaaaacatt
tttgttagaacaacaattctgcttttcataaaaagagttttcccacctgttccatagttgaaagcagtaaaattcagagttttattatagaaggtgaa
attggaaaattggttttttcggaacagaaaagaaaatcagaaataaacaagaatctaagaaaaccggttaaaacgatctcaaatgctccctctg
ggtttgtttatggtagaaggaactagcagacaagcaccgtacttgttgttagtagcactatgtttagggaggctttgctatatttgcttattcttggct
aaggacaaactgcaacgcaaagacaggttcgtacataatgggaggaaagtgaacctggtttcttccctttgcagacgaaaagtaaaaataatcc
tatatatatatatatttaattcaaagaggaaagcaagttttgtaattgaattgaattcttggataaaactttggcccggtctttaaattttttgctcgta
atatcaattgtgattgagttctctgaatttggttattctttttctgtttcattatactttcagagcagcttgtcttgtaccgttttccttgtaaacaaactc
tagtctttctctctacatccaagaagggcgggcattgtttgtttctacggtttttcttttgattccctagttttcttacaattttcgaatttaaaccaattc
cctaattagactaagtagtatgaatttgccagaacctactgctattcagaagcctattcctgttgaggaatttctgcaaatcaaagagcaatatga
gtcagaacatcctttgataacgttacaaaccaagttcaatttggcatgggctctcgtttgctctgaagatcttcagcaagtacaacaaggtctcact

ctgttttgtaccatatacagagactttgctgaacgtcgtttggagtctttgtactacatagcgctaacgcagtacaaaatgaagcaatatgaagaat
cacgtcggtatttgaacatgctgcttgcgaaggatccctctaaccctcaagcattgaagttaaagtctcttttgtataacgctgtcacaacggaagg
atacattggaattgcagttgtagcaggtgcagttgtttctgccgccgcattggttggctgggcttccaagcgtttgttttccagacgacgatagtgct
tacaggactgcttaatgtcgcttatgattcctttttctttgttgtaatgaatttttttctttctttatttataatagtaatttccgtatggtcaagtttgttttt
tatacaaattttttttctcttacatacggctttttccccctttgattgattagacaaaaaagggtttttataaagcttctgagctaataaagcatgaaa
tcgaactaatcaacgaaggtcacattctatcgtacacataaaattttcataaattaaagatgtaagcaatagtctaattgcttacaagaaaactgt
aaatcatccgttaaagtactaacgccctggtaggttttcttctaaagtcagtatatctttcttctttccctctaagtgttgctgtaaaagagcagtttgt
tgctctaatctttccaagttacgctcaattaaagaataatcgggacgtaagcctccggcctttaggtcggccaatacgccgtacttgtaatctaaaa
gtttcaataaatctcttcgtacaagctgagagcgactttggagaatgctgctctcatcaacttctctctgtaactttgtgatcttttgttctaaagtag
ctagctcatttcgtagtccaacaatttcccagtcagagtcatttacgtcttttggagtctctaaatcgcttctaggacgctgaattgtcaacgtggaa
cctacgctctgcgactctcgcatagtatgcgatgggtcgttagcttgaagattgtagtcgatatttcctttcttaaaacttgcgcgaagaccgaaag
gaacgtccttggggattcggaacccttgggaacgttgagtgaggatatgtaaaaatacgagacattgatctttgtcaatggtttctgctttttgagg
attgactagattccatgctttcatgatttgtgagcgagggatccctagatgttcgtaaacgggagcaaaagagttgtacgaaacagcgccatactt
atcggaatgtgagctatatacatcggtatagcgcattctatcagaaggtggcatataccagtcaaacccatcttttagaatatcgtcttctggatca
tctatctcttgagaagcaacgttcacctcatcgcttccttggagggcttcacgggcagctattaagtgttgtttggaaggagggactagctcatccg
gaatttctggaggaacatcatggtatatctaaagactgttagcaacacgtctcattttgttttacgaattgtaccccgttaatgatgttaaaagtaat
cttcatagcaatgctaaattcatcaaaatccagttgtccatcttcatcaatatcttaaataaaataaaatgttagcccttcgaattgcagacgttcgt
gaatgagttatcatccatccattcactgtagcagtacctaccgaccaaatcccaaagcttggctagcttgtcattgccgagtcgactactttgaagg
atactgcaagctttttctcctgaaacatagccgccttctggatgtaggctacgaaaaatttcccagaatttgtttttttcctgaggatccatctgtatt
caagtaagaaatcctaatttagcaaaaaatggtagatgagtcacaatatttcctaataaaatataaccgtttttctttataaaaaataaaaaaact
tcaaattcaaaaattcaaggttttgtttactttcgggctctccgggtaataccgggatgtgctcggtccttccgagggtaaaggtaagggtcaggca
gatagtcatcaagaaaaagaaggaagtgtaaatttcataggtgtgaaaagtttcacctctgccaataccagcttatttagacaaatactaaaaaa
tgcccagatctaggaggtcaaaggtcgttaccctttcacagaccgataaaaaaggtcatgaaggtaaagcaatgctgtatactggcattcaagaa
tctttagacaagtttgactatgtaagtaaacaaggaggatgaaaggcaaaacaaacctttgatgatatttcaatccgtttttatggaacaatagtg
ttttaaattggagaaatgaagtgtgtggaagaaggaaaaaattggttgctgcacattgaatggtagcaagcccgaaaggaaatggattttttttttg
tagaaatatcatagaaaaatttctgagtctttgaaacaagaaaaggatgctaactctatgtagatgtgggtattcgatgttgccaatatgcgtaac
acttacttgaagcgtgttcgtgatgactggaaggacagccgtatatatatgggtaaaaccaaggtcatgggaaaggcattgggatataccgctga
agaggaacatgccgagaatgttagtaaactttctaaactccttcatggaacggtgggcttgctcttcaccaataacaaacccgaagacgtaactg
gctatttcgagtctttcgtccaaaatgattttgctcgtgccggtacagttgctcctttcactcatatcattcctgctggcccagtatactccaggggtg
gacagattccagttgaagacgatgttcccttggctcatactttggagcctcaagtgcgccaattgggtatgcctactatgttaaaaaatggtgtcgt
tactttactcgccgactttaccctttgcacagaaggacaagagcttgatagtcgtcagacccgtcttttgaagttatttggtgtcacagctgccgagt
tcaaggtcaaattattgggtgtttatactaaaaagaatgcttctgtgcagttatttgaaactgagcaagatgctggtaatagcaaagacgacccca
tgcaataaagcgtatggcttgcttttctttttcacctatcagaaaataaaggtcatcctcatgaatcttgtttttcaccaaaagcttttgtgcttatgga
ttgttttggcatattacttactcttttggaattcaaattcaaaaaattttttttttggaaattcaattttggaaaactggatgtatgataaagaaagaa
aaaatatggatgggaaaacagtctactataaaatacaatggctctaatttttttaagggctgttctgtggctttttaatttcttgggatattaaggtat
ttgattgaatggaatttttttgaatatatgtatagaattaagtttttttttcccaggtgtaatcatgggctcgtaaagctgaaaaacgcttgataaaatc
gtatatgataggatattccacgtaatttggcgattaataaaagcaatcaacaaaagttcttttgttctgtctcaacttttgaagagcatgaataacg
gactggtcattccaagcttcagaacctcataaaaccatgcgttttccattaccaacgataacatgaatatggcagcctatgtagccgttattggaac
taaggacaatccagtgtatgaattggaaatgggaggaggaactgaaaagcaagacaaggtaatattgattttataaaatggtccttcatgactg
acgctttcatattagggcttgggcgcacacctgaatcaatttattgtacatgcaagtttagaccttgtagaacaagtacaatggactagtaatgcat
tgtatgtttatttttatggaagttatcagtctctaatggagctccactagttatgtccgttctattgaccaatttcacgagatgtacgtttctgcctatat
taccccgtccagtaagtatccagtttcttcaaaactgtcaaatcatgcaagcaatttgttagaaaaagttaaagaaggaaaaggcgtttctgacaa
ttaactctcattctagatatgaagtttatgctccttcatcaggcacaaagctctgatagcattaaaactttttttccaggaacttcatgagctatatatc
aagacgattatgagtcctttttatcaacctaatgaacccatcaagtcacaagcctttgacttaaaagttcggtcgcttgctcgaaagcttctgtgaa
tagtcctcttttcttgtgtttaaaaactgtttaatgtatgtttaccctgttggcaaacaggtccattcaaaatgacattcgttcgtagtgtatgttccag
agattcattgtatggaatttcaaacgaataaacagatcaagtttaatatttttttgagatatagtagtcgaattcggttatcgtcgcaagtttacctct
atttgatgatacagtaaacacttacagaaattgtttttgagtatccattactgatacaattgatcaattgataggcgactatttttatttaccgtgaac
tagctatttattctttcattcttaatatcttttttttccagctcagcaaaaaaacattcatatgtgtttactttctgttacctttgaatcgttactttgagcg

tgacctgacaatacttcacaaaaccacaaattcaagttctacaaatttcactagagtcaccaccactcgactggacatggcgaaactaagtgaat
tggcgagcgaccacagctctgaggaggaatcagaagtgtccagtgcagaggaagcgtcttctcattctcccgaggtttcgagtggaaaaaatgg
caaagaagtcgatcaacccaagtctaccgaaaaccatgattcggattctttaaacgagtatgttcgtgctgaaggtgaagctgtagtatcaaaag
agggattagatcaggaatccggtgtgtttttggttcgtttacctcctaaaatggcattagaagatataactgaaatgaacttgggacaaaagcctt
cgatagccgcaagctcgtctacagcagaatcttttcagctgcgtcaagaatctttaggatctgtacgtttgtttgtacctgatgaaaagggcacata
ctctaccagcgaaataagaacgggcttcgtcgtatcagagacacctagattacttttgaatggaaaacccaaggaaaaggaacaaacaactgat
gatgctgatggaaagatccctcaaatcaagaatttaaaacaacatttcagacctattggagctgtagatactacaccgtctacgaaagaagcagt
accttctacaagcgttacggaacctacaagcactgaacagcaatctacagaagaaaagaaggaagagcgatccgaaggtaaaaagagatcac
gagagaaaaaggaaaaaggaaaagaggataaggaaaaagaaaaggataagaagaaaaaacacaaaaagtcaagtaaaaaggaaaaaga
ttagattgctcgaataagcgtaatattgtcacctttgagtaaaattcgaccttgacgtttagtgttagttaattgcattcattaatcaatcaataaatt
ttgggtttctgcgggattttgaatatggaaagcgttggaggccgtagattagtaatcattcataaagacactcgtttagtcgtaaagtgtctagaat
acacacaaatcaaaaacagagtgccgcaagagaaattttggggggactagactaccatatggctttctttgattcattaattctcttttccttttcgg
ttctttgtcaacaagccttactttccttgaaatcccagaaaccactctgcataacttaccaaggtcacgtcgattctgtttcgcatcaatctgaacgg
cgtcatctaagataatgttcataaattcatcgaatccctaattatatatcagtaaagagtacatctatgttttcggcagataattgcgcgaaacgac
gtgtcaaatatcgctcaacgttacagttggaagcgcaacttctccaatatcagaatcatacatacccgaatctgtccttcaattcgtaaatctactt
gttcaaaaagccatatctgaactggggtatgttgttgtaacaacttgaatacaaaattctggaaaccgccgttagacaacaaaatttgctatttctt
tcaacgtacaataggaggaatcataacctttttgaacacgtcccgacatcttgaatgtttttttccctgtaaacccgaaaatatgaaattacctgcg
tccttcaaggtattggtagtgtccaagtagcgtatctacgaaacaggggttggtacgaaatacttttaggattattatctctaaaaggtgaagaga
atgaatcttatattttatgatgacaatggttttaccgtgtgctgcttgttttggtgttcctctatataatgcttcatgagatggagacagaatgctgaa
ggttggaatagtcgttgaaataatatataaagagagttcaatgcttcaaagctactttattataatctcagttcatccaggatattgtcctaggattt
gctacctttcttcacaagttcgtcgactgatgagtgcatatattagcgtaaataaaggaagttaaactaacaaacaaaagatcaattttctcgcca
gcattgctacggttcgccttacttaaagaataagtgaaattttataaaatccgaaaaccaagcaaggagtgctgaccactgtctggaggtgaaga
atgaataagtatatcagtccctgatttctttctcaatgccaataataaatgtgaaatggaacatcctttaagatgtttattaaatggaccaaaagtc
aaaattgactaccaaagtatccaaaatcgcataaactaagaactacataagatgaagattttatttctcttctttaagggaagaataaagttgtag
aagcctttgagcgaacgagctgtttcttcacatctaatctgcaccgatattttcttacacagattggaagcgtcgaagttttaacgggggacgaag
aagatgttgaaagtatttagctgttcattttcaacaaaatattgttttgagtatgaatcgtatggtgaatcaagtacgagtgagcatgcgaactcac
ttatccagaaaaaaaaaagaaaagccgttctacagctcttcattcgtaaagagagctgattaaaaagttgagaatcatttaaaatatatgatcctt
attgcatacgaataaaaacctttaaattttttttgctttcccaagtttgtttacaggttaacgatttctgtctgattcgtaatagcttcttgtgccccct
aacacactcgttatccatagacgtagtatggtaaataaccagaacgcgtttttgtaaatggaatcaaccaaattaccttaacaaaagcgtttcagc
tttcctcatggaagagttacataatgctttaacaaccctctatgcgagtgcggatcgcaatcaaaagttacaagcgaatacctacttggaagagtt
tcaaaaaagcgtaagagtcagataagttatttacgtaacaatttttgctaacttgaattttaattataggctgcagcttggcaagtatgttttcaaat
tttgaataccgctgaaagtacgattgaatcaaaactctttgctgcacaaacactacggcagaagattgtgtatgactttcatcaactaccaaaaac
ggttcatgccgaattgcgggactctatgttgcaacttttcgtttcctctaaagatagcccaaggccactcttgatatcattcgctgtctgtatggccgc
aattgctttgcatatgacagagtggcataatgtaatcaatgatgtcttccaggcttgcagcagtcatgatccctcaggtcgctgcatcctgcaattct
tgtctgttttgccggaagaagcagctgatccacgaaaaacatctttgacttgggaagagctttgcactcgtatagacgaactgttgcgggataatg
gacccgctgttttagaattacttttacaatatgtgaattgcaatacacatgctgatggaacctctaatgttgatttgaacctagtattgaccagtttg
acatcctggcttcgcgaaatacctctcgacaaagttcttgcatctccgttgatagaattggcttttcggtctgtagatgacgatatgctcattgacga
tgcagtcgaatttctctgcgctaactttaacgaaacaagggaagtagatgaatcgatggaaatgattatgatgctttatcctcgtttactacagctt
cgtccaagcattttcgctcataaagatgacccggatgcctttcgtgcaattggtcgccttttcacggaggcaggggaatcttgggtcgtcttaatgg
cccgaatgcccaatgaatttctcccattggtagaatccgttgtacaaatatgtgcgaacgatgaagaattggaggctatcaaattcacgttcgcttt
ctggtgggatttgaaacagatgttggaactggaagcgtatgctcaagcacgtgctatctatgcaccactctttttggagttggttggtattgtggtta
gccatttgcattatccacgagcggataatcatcttctcaatgaacaaatggctcaagctgaagttttctttgatgatcgtgagtctgaggaccggttt
cgtgctttccgtcatgaaatgggtgatgttttgaaagattgctgtgctgtcgcaggagtcactccttgtctacttaaagtttctacaaacttttagaa
actcttgatgcaaaggaatcaggacttccttacttttggcaagatattgaagctcctttattcgctttacgtgctatgggccgtatggtcccaaatga
tgaagacaaagttattgttcacttgctgaggtttcttcccaagcttcctcaaaacaataaagtacgttatgctgcaattttgttcgtgggcagatata
ctgaatggaccgcacagcatccggaatatttggaaatgcaattgaattatattgctgagggattccaaattccatgtaaagaagttcaaagtgctg
ccgctcaagcgatgaagcatttttgttctgactgccgggaacatcttgtaaatcatcttcctcaattacattctttttatctcagcatgagagagtcgc
ttgatgctcagtctgttctcgagattgcagagggcttagcgcgcattgctgctatccagcctattacgaatacctatcaatcaatacacagttttatt

gctccttctttgcagaaaattgcgttgagccaaaccaaaacaggacacacagaaccagagattgaagcgctagctgatgaagttgatatacttat
tatctttttttcaattgtctatccccttagttcctcacaaggtattcatcctactattcaactttgcttggacatatggccagtcgtggctagaacgatg
gacatagttcctagtgcgttagtctgtgagcgaatctgcaagcttttgaagaatgttatcatgacttttcgagaaaaagctatagtcctgctgccttc
tattgcagaaaccctggtgaaaggtttcgaaagtacacagtttggctgttttctgtgggtaagtggagcatgcgttcgggagtttggcatcattgaa
atgaatgagcagacccaaacgtctgtttggggttttgtagaaagacaatgcacaaatatgttttattatatgactaagaaatcaccaaaagaaat
acccgatgtaattgatgatttcttccgatttatgatggatgcacttttggcaaatcctcaacgggtatttatgagtgagatgttggagcctttaattca
tgcatcattagtttctttacaagtgaatcaagacgaagctctttgcagtgttttggtttatttacaggatctcgtatcgtttgccttaagcacacctcc
atatactctttctgagccggtttcagaatcacttataaaaaaattacgtttgctagtgctgaataattctcaaccaatgttcagcattttatttaatgg
aatggcgtttatttttcctcgtaacaatgtgtccgatgcttctgcggtcttaattcctttaattcgtttaatatgcatggagaatgctagtctttgcattc
agtacttgacgagtgtattggatcaacttccacaaggtacaattggccaagaagagaagatgaaatttttggaaactttttcccagcattgtgcttc
ttctgaaatgcctcgcttgcgtgctcatttacaggattggacgtctatgtatcgcagaagggtactttcaccgcgatcaaagggcatcgcattgtca
tcatagctcctttcaagagacggcgaaattataagaaatagaaaagaacaactcgttaactgatgatatggtatgaatggatagatggattgatc
ttaataaatctcgtttcctgctgtacttgccaaatagattcggatatcataagtacctgtttataatgttggctattcttggtataatttaaaactaaa
atctaaaaaatggttcaagatgtttaaaaaaagaaagaaattccttttattgcatgtagaaaggaatgcaattcaattttccgtcataacaaatga
ctcacttatttatttatttatttattgtttattaacgcagaaagatactttcgttaattagctttttcgttcattacataatgatctacatactccaatgct
tttgttggtttgctttctaagataccaacatatatgcaccgtgggaccaaaaaggggacttcttaagtcactgtatgaagtaatttaggaatttacc
aacaacaaacagctaaaactatcaaaaacgaacctttggtttttgatttgagtgtttcgaaaacaacttttattaaaaatgaaagttcaaggaaa
acgtaagtaaccctggaagtcataaacaagcacttgaatccatcaacatgttacgtcaattgcaagtttatggagaaacagtgtttactgaataa
gtaattaacttgaaaacatagccatcactgaagcgaaaccccatgaaacaggaattgttcggaatgtgaattcagtaaaggaattgactacgac
attagacgacaagacaaagactttgtatgatgttttagcaaatgctgttaaacgatatggagataacgacgctgttggatatcgtgatttgattaa
ggagcatgttgaagagaagatgattacgaaggttgtaaataatgaaaagaaagagatccccaaaaagtggtcgtactttgagctttctgactac
aagtaccttagctttaaccaattctatgaaaagaccatgcgacttgctggtggcttgcgcaagtttggtttgaaagcaggtgacaaattagaactg
tatgcttctaccaccagttgctggctcatgatggctcatgctgctatgtctcagtcaatgtccattgttacagcctatgagactttgggtgaagaagg
tcttttgcattctttacgggaaactgacgttcatgctatcttcacggaagccaatctcttgggaagccttgtcaatcctttgaaggaagtcaacttgc
attacattatttatcgtggtaaagctaaatctgaagacattgaagccctgaagcaatctcgtgcagacattaagctaatttcctataatgagctcga
gtctttttcggaacccgctactcctgaaccccctttctcctgatgatctttgttgcactatgtacacttctggtagtactggccttccgaaaggtgtattg
ttgacccacagaaatatggtagctttaataagtgctgtttgcaagatcattcctgaagttacctctaaagattatctgttggcatatcttccattggc
gcacattctagaatttgcttttgaaaacttatgtttggcttggggtggtacaattggttacgccggtgttcgtaccttgactgatacgaactgcagaa
actgccatggtgatatcctttctttccgacctactattatggttggtgtccctgccgtttgggaaatggtccgtaagggtatagccagcaagttggat
gctgcttcctctgtcaaaaaatctgttttctgggctgcttaccatactaagcttagcttgatgcgacatggtatacccggtagttcattcttagatgct
gctgtctttaataaaattcgcactgccggtaccggaggtcgcttgcgatatgtattgtcaggtggttcagccttgaatcctgatacgaaaaagttttt
gagtgtcgttctttgtcctgttttgatcggttatggattgactgaggtcagtgccgctgccatgattgaggttccttctaccttcaacgttgatgactcc
gccggtgtcattttgccttgtacagaactgaagctgattgactgtgaagagggttcttactacgtaaactccaaccctcctcgtggtgagattttgct
tcgtggaccttctttgacaaagggttatttgaaccgtgaccaagaaaataaagagagctttagcaaggatggttggttcatgacgggtgatgttgg
agaattgacgcctcatggattactaaaaattattgaccgtaaaaagaaccttgtgaagacccaaaacggtgaatacatcgccttagagaagctg
gaatctcgatatcgttctagttctcttgttgccaacatttgcgtttatgctgatcaatcgaaggttaagcctattgccgttatcgtccccaacgaaacc
actgtccataaattagccgtggagaaagctggtgttgacgaaaacacctcttgggaggatatttgtaagaacaagaaggtgcgtcatttggttttg
gaagatcttttgaagattggtcgttctcaacattttgcgaatattgaacttgttcaaaatgttgtccttgtccctattgaatttaccccagaaaatggt
tttgtgacagctgctcaaaagcttcagcgtcgtaaaattatagaaagctttagcaaagatgttgacgaagcttatgctgagtaaacattgattctct
atgaacgaatgctatgattttaggtttaaatgattattttcaggttgtaataatgcgtgttaatgtttatagtggactgtttgtttgtgcagggatgaa
tttctgaagactttggattcataaatataaatcggtttttttttattttgttttgaagctacgaagcttgtacgaaaccgttctttttctagtgaaatat
gaagctagttcatattgctttactaaatataagatatttttcgtgagattctagaaaagagttgtaagtgtgaacttagctacgagtaatgattcaa
ggtaatttttgtaatttgtatactctggaaatcgctttcctgaacagcttgtttttagcatcgctcatctaacgcgtttcaaacttctatctacactgaa
agtatagagaggacagcttttgtctgtatctcatataccgatactgtgcggtgcgttatatgaagcttcgctttactgtacgcctctttatttcttgac
aaaatttcgaagcaacggactttgtattttacccatacccctttattctctattcaaaatgaatacatcgtaagttactagagaatgagcgcatttttt
actaacatagcaacagatcattggattgggcagatgaagaggaatacggtagttctttgccatccattcaaacttttgacaacccagatggcacc
aagaccatgattgagttccgcctggacgacaacggcaaaaaggttaaggttacgagagtaattaagaaaaccgttgtcaccgaacgcgtacag
catgctgtcgctgagagaaagaaatgggaaaagtttggacaagaagcaggcaaggccggtggtgtagacaccagaacaaccagtgtcggaga

aaacgttcaattacgccttcaacttggatggactacttcgaaggaagaagaacaggaagaggctgctttggcaactgccaaggtcaaggccaag
agtggatctgttgtacgttgccgtgcttgtaaaggcagccattttactgctcagtgcccttataagagcattattgggcccgttgatgaacctcctttg
gagactactcccgctaccaacagaatcactggtgcattgggtgagaagcgctaccttcctccacatatgcgtggtacttctacccgtgaaggaggt
gactctatgttcaagcgtgagcgtgatgatagtgcaactctccgtgttaccaatctttcggatgatacccgtgaagaagaacttcgtgacttgttcc
gccggtttggtggtatccaacgtgtatatttggcaaaggacaaggaaactggacgtgccaagggctttgcttttgtcagctactatgatagagaat
gtgccattcgtgctcgtgaccgtttggacggatttggttggaacaacttgattcttcgttgtgaattttctaagcctcgtgattaagcatcttttacgc
gtttcgatctacgataaaaaacattctcttctaaggatctgcgctttctaattggatttacaagttcttattgttttcttatgcgcacctgatctttgtaa
atcttttggaaactctttttagctgttttattaatttctcctggtttatgcttgtactgtacaagaaattcgttccatattttcaatgagcatgtggaag
attcgtttatacattttgggttcttgatgtaggtgaccaacttctttacttaaaaattttatctatgtaatcaatggaatcacgtttcgctcctttctgta
tgtatacgaataaagaataagatatgttggctcggctctttggagagtaatgctgctaacaatttccaagtaaaatgttttagtagatgtaagactt
taatcattgttcgcatgctacttattctttggaaagctgttccgtttggatgctttagctaaaacaacaaattaactgaactcatacgtattactaaat
tttcagtaaaactcgctttactctcatctttttcttaatttggtaaaaagatttctgtcttttaatgatttgcaaatttattattgaactccgagtacttga
atatatagctgctatttctattacctacagagaaggccgcttaactcagttggtagagtgtgagattccaaatcttgaagtcaagtgttcaagtcac
ttagtggtcatttttgttgaataatcctccaaggactggaagctaaaaatgaataacagataaagcaaattaaagaactaaggagttggcgaata
aatgagcatactgttattctagattcgtgtccctcatcttctattccatataagcgtacaatcttatggaaaaagcgagagccaatatacaaataat
tcgatttttaatgattccgaatttagatcattaatttcattttatgaaatcacaaatatatacttactttagatctagaagctgctcttgtgttgtatat
agtagtagtagtagccgattcattagtacattcaagtaatagaattgatctgtaagagcattttatctttcatgctgttatggaaatgttaaatgtcc
ttttgtataaaatttgacagttcaaaatggtttctctatctgctgtcgttacgtagattgaaaatgaagtaaattcaacaaaaactcgaaatgcatc
atacagtcaggtatatattacgccggtgaatgtcaaattgatcaacgaaacaattgtattttaattcagaaaccagaaacaagttatgatcattac
ttccaatattatatcaaaaaggaaccaaaaaaatggcttgtctaaaacttacgaccctttcctatttttgtccaaccttcatcttctccttcggcaac
aggggcagcttccacagtatttggtgcttcagcttcagtattcacaggtggctccttttgatcagttactttaacctctgatagttgttcagcaacagt
ttctacggaatcgccttctttcttctcgctaggagcatcgttagtagaagttgcactgtcacgacggacggtctgagtgcgcttagcaagcttttcttc
aacgcgcttgagcactggagtattgtcaactggcttagcaccaccgaaaggatcggacttaggcttcgtagggggatttgaggttgtcgaagaaa
cctccgattgttcattcgcagcagaacgaggcttcaagttaagacgaggacgcgtgctagggtcacgagagggcataggaccgcgacgaaccca
atcacgctcctcacggggactctcacgagggctttcaagtgcaggatcacgtacagcgcggcgtttcatgaagccatcacgggaaccactgtctct
gcgttcgggaggaggaagaggaccacgacgaacccagtctccggaagtacgatcatcacgaggagcagcagcacgacgaggctctgcgacag
taatacgaatagcacgtcccataaggttttcaccagataaagccatagcggagctcaaaaggtcggcagtctcaaattcgacgtagccaaagcc
acgagagcgctcagtcatggggtcagtgacaaggcggacgctggtgacaccttcaccaaagaagtcggcaatatcggcttccgtcaagtcaaag
ctcaagttgccgacgtaagcggtgaaaggaggctctgtgggaatgggcacggcttcgcgttgaaagcccatgtcgcggcgaccctcgcctgccat
aggcctgccggctggttcgtaagtagaagaaggagcttctgcaacagaagggctattacggaagccaagacgttcttgagctagaataaaaagt
ggttaacttgatgctatcctttagaaaaggaaacgttctctttccttttgacccctcctgcattcgaaggaaggatccacccaatgtttcttcaaaac
caataatacttacgcaaagaaggcaaatcatcaacgtcgtcagcccagctagtcgagccaaattgctcatcacccaagaaggcggtgaggctca
ttttcgtattctgaatagcgttaaaacaaacgtttcaggcgctaacaaacccttttttggaccttttctttgttaacaatccatcataacaattactcac
taacccattataaattcaaatcttttggtcttgctcttttcgtcaaaaccgttctgtcttgttttttttacaattcagacgtttcctgtaggtcaggcttaa
gtttgttacgatatgcctcgaaacactgtgactgctcaacgtaaatttgaatcaaatttttttttgtgtcgttccgtcaccatgagatgaatttaacgaa
agtttaaagtatgaaaatacacgtggtatacttaaaatgagttagtttagttgcttctaatcctcctgctaacagtgcaccttgttagtgccattaga
aacagtagcattgtaaaggcagaagaattgcttttcctttttaaattgagcaatcgtaagatacattcataattttgttcttaatatagacgtaaaca
gtggaaaagtttctttgtttctactccatttaggattttgggtagcaaatgccgacgaaagatcctggcaaaccagatctacacaggttatataacg
aaatgaaacgttctattcaccagaatctgcttcactgtgtgtcatcaaacaataaatttaagtgttcgttatagctttgttaacttcaatgtagagaa
ctcgaaacatagaggtccgtccctgtactgaaccgtatggttgaatgattgaaaaaccgcatatatgcacacaaaaaaggagtatagaaggtgtt
gcccctagcctaatatgaggaaattaaatcacaaattgaatttgggtaaagtaaacaaaccaatatcgtcacgtttctctaaaaaaaataatcct
gttgggaattgatgaaggtaaattcatagagttttcaagtacttatggttgtcggagtgaaataatcaaacaaaaagttcaagaaatctaaagac
cgattaaaccaatgatccagctagcagattcccaaaaacaaatcattgctttttaccgatttcaatagttctctttcacacatgagctttttgacaaa
ttttctacaagcaaaggtattgcaattgaatggtaaaggacaacaggcgactacgcgtcgaacagtgtaaacaaacgcttaccgaccaacagtg
atctatggtaaagtggctaggcaaccaaactatttgacgcaattgagcttaacgaaagacaggcatcgttattcttaaaaaacaacaccaaggcc
taaaaagcaagcattattatttagattcgagattctacatcaaatgtaatcaatttgagcatatatttttttagattttagctttaactaactaaatata
tgttgatgatttcttatgagcaagtttctttctttcatccggcaaattttgtatcaaaaatttaccttcactaaagtcataaagaaatgaagaaagtat
tttgtttgtttgatataaaaagggaattctcattgtattatttttaatatattcatctttttgtcttcacgtttttttcgcttttctctcatcatgtactgtaaa

taaacaagtagaagtgatcgtctacaaagaaaatgcaatgtagaagcccaattctcttcataaactaaaaacttaagttgaaatgtagctaatat
ttaaagattacatcttattcatggttctaaaagagacattttctatttctagcttagctatagaaaaacagaaagaaaccttttgcacgctcattctt
agttaacagtagaacaacgcaaaagaacataaattttacctgatactgtgaccatccctaaagtctgcaatcgtctctattcacaatacagccttc
aaactaaatgttcacatcatagtgacaaattgagtagcaacaacaaatctgcgatcattctcagcaaaaggaataattatcgataaagtctatgg
caattaatttactttctaccaatactataaaggaataaagaaaataaaaggctcggaacgtatcatattttagacatattgctggatagaaaatta
ttggttttctctactccttttccactcttactcttactaaaatcactcgaggtaattgtattataattaagttcattggtgtctaaagatcatcatttttct
ttttttttttttttaaatcatcaccttggtgtactaacattgtccgaatcagcaatcatcaacagagtcctatgcaaaacagattttcattcatgtgg
aatccataatggaacttcttttgcaagatgggcacatcaaggaaaatatgttagaagacccgcagcggagcaagacaccatcaagccacgaag
gaattcctaaaagaagaagccctgttaatgcgaagagactaccaaaacgacctactcgaccaaatctgttgactgtggctcctcgaaatactgaa
gagaaaaatacggattactcaattgataatgattccgatgatgattccccgaatacctcatcctcactaaacggccagtctgaaaatgaatcagat
gaggcggatgatatcagtttggccaacacgtctgtaaattcttcctctactgctgctgaggttgctgatgaagaggagtcgccaacgaagcttgat
gaaagaaaaattcaatccctataccctaaacataaaaatcatcccgtatcaagtcgagaacatctacgttatgttcctgttcataagaggcgaatg
ccaaaaaaagaaatgacacttagccaaaagatttttgtcgcacaagtgataaatactggttccactgaaataccaaatcatagaaagagattag
ggaaacgctttaataagtatgatccatccaattacaagagttctctctgggcttctgaagttagtgcaagtgggaaatacctagcgaccgcagga
aaggactccaatattagagtatggaaagtcattgagtctaaaaaagatcgtagttcttttttaaagaagagactaaaaggaaagaatcttatttt
agcggtttcagtatctttgagccagaaccagttttaagctgtactggccatcgagctgaggttctttgcgtatcttggagcaaaaacgactttttgct
aacagcatccagcgaccgtaccgttcgcctttggcatcctagactaaacagatgcttggccatctttcaacataatgaaatagtcacatgcgtttct
tttcacccaacagatgatcgtttctttgtatcaggtacattggaccatcggatccagctatggtctgtcttagagcacaaaaccttgcaatggaatc
aattagaatattcaatttcagccatttgttttcaacctaatggtcagcggataatcgtcgggatgttttacggaatgtgtgcattgtttgaaacagaa
caacttcgttacgtttcttcatggctcattcatcggtcatcttcacgaaatagaaaaagtagaataactggattacaatgtacgaatgccacccctg
gagaacttgattccgaagcgtatgttttggtttccacaaacgacagttctattcgactttttgatgttaaaaatagatctttgattttgaaagtggatc
aggatcaccgcataaataggcaagtactttctcgtctcaacgaagacaataattatattattagcagaagcgactccaacgaagttacattatgg
catctcccatacaaatttctgtcttcaacaagtaaacgaagaggggttgtgcgtcatttgccaatggaaacatttcgaatttgcacagaaagactt
acctctgcgatcattgccccctcgagaacagctgtagaggttgcccgctcggaaggatattcctttcctggaaagaattggaaaagtgcgttttcc
aaagtttcagagaaaaccgctgatctacgccccattgatattatcatcgttagtaatatggctggaaaaataattgttttgaggcgtgattgcctac
aagaaagaaaaaagtcttcccttaaaggctctgtaaaatttcgaaaattcgttcatgactggttgcatatttaatttttcttctctttattatttttaat
ctgatgtctaaaaaagatgtatgattaaccttatacccttcatcgtaaaaatattttgactgatggagtaaaccatatacctgatcaagcccagaat
actacgcctgaatgagcggtcaccagttgggcatgccgtggattaaaacggactatagaaggcacttcaggacattctactttttgagtcacagg
aatacagctttgcggaactgaaggtattgtatccttgacctgtgccattagttcatgcatattccaagtgtcggaatgttgatttgatgatttcatctc
aggcaattcgtagatggccaggtgcttctcatcatttgcagtaaataaatatttactgtctggagacatacaagcagaagagtaaaaagaaaggc
ttttactagcacttttagcatttgtcctagctggtacacgtaacaattcctgtccactataagtatctaccacgtaatggtaatcatttccagtagtca
acaaaatatatcttccatcagtactgaactctatattcgaaatgcttacatggggagggaggaatgtcaagggaatctcctgaaatggccgtgaa
tcgaaagtctttatgttgtaaagagagatcttatatttcctttcactcacagatgcaaaaattaagccggtagcatcaaaggcagccacaacaggc
gagctcacatttagtaatccctaaacaaagtcagtacaaggatcatcaattttgctaccgacacatatcatacctgacaattaggagaacgaaag
tcccatagtcgtatcgtattatccatcgaggcactaagaaatatttcatcagaaggtgaaacatcaagagaagtgacagcttgtttatgcccggta
aagtaccgaagatatcgattcgtaactacatcaaggtagcggattgtgtcatcttctttagtagaagcatgaattagagaattagaatgatgaga
aaaccgacccaaatgtgcaccatatttcttactggcgagctctttgatgaatctactgaagttaataaacataaatccgttagaagcaaatattttt
tttataaaaagtttcagctatttgacttactttcctgttacacaattgtatagttttaatgaatcatctgtggaagacgccaaacaaagttctccactg
tcgtcgaaacttacagaagtaatttcaccgcctgattctgtatttcttaaccattttctgggctctattgaagataataagtcatatgttattggaatt
gttgtcattctgtaatttataaactcaaaccgttttgttaaagcattggtggacagacgaaactgaggattggttttcaacataaaagccatggccg
aatactggaaaagcattcccaaatatttttgtaaatactgtcaaacagtaagttaagcaatgctagctacattccaagtatactaatgctatactta
gtttgttacagatactcagctcattcgacgggaacatgaacagagttttaagcacaggaatgcattaaagcggtttatggatgatattcattcatct
aatctgaagcggttaagtcaagctaaagagagctcgccttcgattcctgcttataccccagagccgttctatgcatcttatgaaaaaccaaagaca
gagaggcataaactacatcctgcaaaaaagaaaaatacactagacgattgggaaatttctgatagtaatcaaactgtctcttcttcaactagcta
cattcctacacagactcaagccattcaatatgacgaatcgactaattcagaaaaagagaagaaggaaggatatacttcaaaatctagagaaaa
agaggatttctcttcattaaaaagaaatcgagaatctatcgaaaatgaagagaaggcttcctttcattttaaagtaaggcaaaaaagtttagactc
agaggaaagctctaaagatagtacagtcaaagaacaaaaagaggaagaggaagagccgaaagtaattttcaaaaagaaaaagtcttctgccc
gtcaaaatctaaaatccgctaaaagggctaaagattaagttcccgttcccattcatgtagaactaagaatctaaaaagaaattatttattttattat

ttttataaatagaaagtgctctttttctataagttaacatgatatccataaatgttaaggacgaacttgcaaaagacccatagggaatcagaaaaa
aagaaaaaaaaagttaaatcattataaaagatcgtatgacctatatttcgtccatagtagtagctttcagctttatttttccaattcactaatgccttct
ttctttttgtaaaacaatatgtacgaagaagaaaaatcatattgaagtgctgcaaagccatccactctttgaacgatggaatcatcaattcgatac
cattgattctcgctaaaaacatttgatgtatagtgaccattttcaagttgaccataatgactgacaaaagcaaataaatcgtaactacattgatcat
aatttatccctgattggaaacaaggaggaatgagcatattactaggaatccgaaagttcaattgtagaggagtatcaatttttttccagcctaaca
tcgtgttgcgaaacctttgtatctgtattatcaagtaatcagataaatgagatatgttcataattttctttgctggcctttgcttttggcaaacagggc
aacaccaagcgttatgtccttccaaaacttcaaaagccataaatcttcggagacattcatgtaagctaacaaaaccaggtacatctggaatggga
atagcaagagaagtaaaaggagcgaacgtagtagacgttcgttgacaaactgtacactgtattcgactacaaagttgaccagagaaaatctttt
caactatactcagattatgtcgaacatgcaaattccattcaacatgactaaaataagagagaggcagtttttctcgagcacttaattgcacatcgg
aaagaggaggtatggggatgcgagaggcattcgaatttaagtcttcatgaattttatcgagtacaaagtttagaaactcttgagcatcgcactga
ccatctacactgaaatctctgtttagagattgaacaatatctaggaatcgttttggagagatgattctttttcctttcctttgaaaaacttctttaacc
aaagagtgaaaagcaaaagtcattttcccaccagatcccaatggattaccagtgttaatggctccaagggaaggagatttctgatatataggggc
aataagctctcggcatgcaaaaatacattgcaaaacacagttcatatagcaagtgttcccaagattagtaagaccaataggcagatattcggga
atgtttttattttcatcaatctggccatttgacatggaaataagtaactgaggcttccattttagggatattagaagagaataaatatgatatgaaa
catctatgtcttccaagtagttttctatgctataacatataacgatatttggaagaaacggggttcttttaaggtaatcatgaacttcagcgacggat
tcagaagaaaatagcctaacaggaatgcagatggttgaatccgaaaaactatactccttagaactttcctcagagtaaatattgaggaataaga
ctttactcgatttatccttagccaatttcatggctgattggattgacgtacaaggcaggaaatgttttactgcaagtctactttttaaggcaagtattt
tctgaatgaaaaccatcatttgctacatttgtattagtttaatgagagacaattaacgtatagagaaggaagcagaacttgctgatactgcagtag
gaggacgtataaaacacctattttaaaagaatcgttccatcccatttcctctcttgaaaacgtaccttttggaagtgcttaaattctggataagcag
cagatcgaactgagatttcaaaccggcacggtatctcatatgccagcttgatagctttcagataataaatataagactttacaagtaagccattttt
ctctaggtttatagcctgcgctgtaagctttgaaaatcgttgtctgaagaaacctacctcttcgagccattgaagtcctcgaaatgtgcagctttgca
ctgtttcatcgtcatgaatataaacgaaagctttgtcaaataatatatcaaaataatcatcggacatctgatatccgaagtgctagtttactccatat
aaattatacaactggcggtgcgagggcttacacaaagctaaactacagaaacaccaagtatttcgcagattaacgacaaactaagtttgaatga
cactgaattgggatttacaaataagaatattctgtcaaaggctacgtccccaaatagtgtaagttgaattcagaagaggcataggccttacgagt
actataactaacggattgcctttatacatagtgactcagatacagaagtttacttaacagtgcaaacaaataaaggccatattttgctgattgtgg
ataaacttgtaccataccatgtacaatttgttcttgaagaaggccaggagaatgtgcaaagcgcagtcaatgaatatgtgaattcttctttcaaaa
atccaaatacaactataaccagcttattcaattcatgcttacaaaaatgggatacttttgcgaaagcaaggttgaattcatctaaatctacttcaga
gcacgaacaaggtgaaaaggatgcttccaccattgaaaaaggacatgaaaaatccattgaccaagtttcagataagttttcagacttgaacatc
aaacagaaagatatctccaacggggttttccgttctggctccaaaaatttgaggatacaatctttagattatgaaagagaggaaaaggacttggg
aagggaattattaaaaccaaatagtccaatttctaagcaggtgttatctgaaagggaaagtcatgatcaggatgatgttcatcaagtcacttcttc
tcaaaatcttcagaccccgaaacctgctagaaccgtacaacttgaaggaaacgactacactcttgaattaacagacctcaatttgctgaacgttg
gtgtactaaaagcaccaaggttgcgattggtggtaaaatgtgttcgctgtcactacggcaatgaggctactgtccaggaaaaatgttcattatcct
gttcaagatgcaataatggtcttcaaatcatatggttaccaggaactgtttatgtgaattcagctagattgggtgtacttgatttgatcggatgtgca
cccgttgacgttctacccattgattgttttgtttcttgtttggaatgcactgatgagcaaataatggcttttaaaggtctttcctccatgcagcctatgg
tccaattttgcaggtcatgtaagacgaggacattagttcagtatcaggatacaaaattccagttacttaaacaacgagaacaagccctcggttcca
agaagccttccaaaggaaacaagaaacaaaatcttgggattgtaaagggtacgcctcttcctaatcgtggagcttgtgagcactacaagaaatc
atatcgatgtatgtatcactatttttctcaaagaatgttcaactaataataatagggtttcgttttccatgctgtaatcgaatttatccttgcgataagt
gtcatgacatcgatcaggatcatttatatgagtatgcaaataggattatttgcggttattgctcctttgaatctatgtataccaaagaaggaacatg
ctcacattgtggaaacttcacaacacgtcgccaaaaaagtgctttttgggaaggtggacaaggaatgagagatccaattcgaatgagtagaaaa
gaccccagaaaatacaagagagttatttgatacgaatgatggaatacaaccctgtttctatagctcaagaaaagtataatgaaatttttatgatta
tagcatatagaacaaaatacaatcgaataaggctagacattgtatcggtttattaggtagattcatcactttctccatattcaaccaaagctctcgt
agaattgttcccagaatgctttgtctgctcctttgttttaggttcgtttacatctcccacaattgatattttcgaaaaatgagggtcatttcgaagaaa
acctcttggagtgaatacatcatcgaaactcgcaaaccttttttgccttcttatttttcattaaccttttttggtaatctggatttgttaatgcttgagagc
tcgtacgctcatgacgagctaactgctgagcgattctttcatcagaaggagtaggaggtaaaagactcagtccaagaccagcctgtttcttaaact
cactttctttttcttcttgttcctttatcatctttttttttatctgtataaaatgttagtagtctatacgaaacagtaagtcgcaataccctaaaatctctt
cgaagcttttgggaaacggtaaaaggatctgcccattgcctctcatttctttcataaatcaactccaattctgtatttcgtgtctctttttttcttctgtgt
agcgatactttttttccagtgcctctaatggatcttgaatttcagcagtagcaacgttggatgatgttacggtatcggcagcctcagctttcctttttcc
cccagtgaaaacaatgtactctgtgttctagcaacatgttgcgttagcaacttgtaacttatgctatggaacttacctttgggtctgtgtgtatctcaa

gagaattagagcaaagatgacaatgaacagtaaaagaccacacttttgaagagtaataagcaccaatttgttttttctctgcattaaacctagtgc
cttggtggattaaattttcgcattgattgcaccatactgcaaaaggcatctcaaatcgaactacattcttagtttttgcccgctttctaggtttattttc
ttcatcaggagggtaatacctccccatgttgaatccttgcattatatatttgtatattattgcagcatcctgtagatttatgcgttggtagaaggattc
ttggaatccagtacgtttcttaagaattcgagtaagacctaatgaatagatacgatacaatatagtgatatcgcgtaacttacaataaataagaag
tgtcgtgctctccatttttgacatacagcgtataatttggcatcaaaacttgattacatggactattcagtagacaatcgatatgaaatgtaactata
ttatgctttagaaggaactaaatctttaacccattttactttttcagtattaggaggagctaactcccaaatgtcttgagcgaatttggaatacttgtg
tcgaagacataaggacatagcaattgcaattgtttgagccgtaggtaaaacattaacttctttcatttcacgtaataaagacataatttttttccattt
gattcaactcaacataaagtcgaagacgttcatggaaggcctgaatcgtagggggccagggaatctttgatctgtagaacgcccagtagaagtc
agcggatgctaaatcgcgcagttgcctgagaatgaaaagtacggtgattaatgatcgtgaagtgaccgcacaacattggccgggtagcgtagga
tgaggctcttgaacaggatactgtactggagcatccacttgctctagtaggattctcagagccaaaaacttgtggctttcgtgcttagaagtgctca
aaattcttgcataagagcaaaggaggtcttcatgaattgcaaattcattttccttgtacttagaaattgtaaattcccacaaatcacgagaaaactt
gtgcatctcttcaagacgatcaggctgtatttgagcaatatccagataaagatacataaattttgaagcaattgatgagtccaattttgttttggga
tgtaaaaggatatcgttcgcaagttcgacatctttaagtttcaaacagcattgagccaagacgttgatatggatcgcatttggagctatcacggaa
ttgggtcgatataaattttcgtagactttttttaattaattcagtcaacttctccttactgacagctaaatcactgaccaacagagagtataaaccctg
taacaaaattgtgtaagtgaattcattcggtgcttgcatcgatttttttcattagattgtatagagaccaagcagtgttatattcgcccttccgcaaat
gatattctaacaaatgattgtatgcgacagttttttttagccaacgatttttttaaaattaaatctagcgcggcttttggctgttctccttttgttaacttg
gcaacttttttgggccaatttatagctttcatttcttaaatttaagtcatcttttggcttctttgatcgacgtatgggttgtttgtcggaaccttgctctga
aatcagcttttctttgcgttttcgagctaactttctcgcattactgacttgtgtaactaatgcagtatgaaatgtctgaactctcccaagttttgacaca
atccccgccaaatttctagaataccggagaaaaaatgggtagttgcatctgagggattgctaatgggacgtaccagtctagtatgtcttgaacaatt
tactcctataataacagaagcctgtaagcaaaaaacgtagctgatggtcgctatctataaagtaatgaatgaaacgctacaataaaaagcatac
gaatatgtaaaaatgcttgctacaaaactatgcgacatgatgaaatagaagtaagaaaagatatagttattttgaacatctacaccaaaattaaa
gcatctttctaacaagagtgttaatgtgctcatcacgcttaccagtatcaccaccctcgatgaagtgagtgaacttgcgggctctgaagccaccca
aaggagaagagagttggaagggccagatgaagttggaggcttgcttgaagttggggccaacagtgtaaatttcatggatcaagtcctcaacaga
catgatggagtacttgcccaagtttgcttcaatgatggagttgtcagtaagagaaatacgttgcttgttgatcttgccgaaaccgcgcttgtaaatc
aaatcacggacggtcttcaagttggggacaccgtaggtaacataaggctcagcaacttgaagcatttccttggtggccttgttgaacttgacgaaa
acaccattgttgatttgaatgagacgcaagagttgcataatcttacgggccttgggagggatgttgttaacaccacgaagacggataacgaagac
caacttgggttcgtcaggaacatagaagttgccctcggcacgagccttacgaccgagttcaatttgctcacgctcagccttgcggtattcgacatc
ataagcctcagcacgcttagcaatgagctcacgcttcttttggcgaagctagaatgatgttagttaagggttcaaataaaatttttatgaatgaaa
ataaagtagaaccaaagcaagtctttcttgataagacatagtatataaatgaagagaaaaacaaactcagtcccgaaagatggtattattggaa
gcacatcgcaaagctagctatccaaaatttttgtcatcatgttgtttttccgcataaaacgaaatttttgcttgctaagcataaacaagcaaaaaga
ttttacttcatactatgctttgattcatccctttcagataaaatttttgtctttgtcgcatttacttacacctttcttttcagcagcagcagcaacacgttg
ttcacgagattgctcctgggtcttcttcttcttcaaaagagactcgggagcaaagacttgctccttggaagggaccgttgacgatgctacagccatg
atggtggatgtctagtggtgaagtttggtttccaaatcaaagaccacaactttccccaagagcagtcacaaagtttggtcccaatatcgtgactgtt
ttttgggtagggtaacacgaaatttacagagaaaagaaaacacgttgttgtaaagtatccggaaccaaccacagaaatggctaccaacgttggc
cctcaagtccgctctggagagctcgtctttggcgttgctcacatcttcgcctcttttaacgataccttcgtccatatcactgatttgaccggtaaggaa
actatcacccgtgttactggtggtatgaaggtcaagaccgaccgtgatgagtcttctccttatgctgccatgttggccgctcaggatgctgctgtca
agtgcaaggaagttggtgttactgctcttcacatcaagatccgtgctactggtggtactgctactaagaccccctggtcctggtgctcaagccgcttt
gcgtgctttggctcgtgctggtatgcgtattggccgtatcgaagacgttactcccatccctaccgactctactcgcagaaagggtggtcgccgtggt
cgcagactttaaatatgttttgggttctgggacttttggattattactatcaaatctcttgtcgtatatgtgcaataccttatgtattgctgttacaaaa
gggtaccaactgatttgatggtaggtattcacacaataaaataaagtatcgaaatcctttcttatgatgttgctgaaatatgcaaagtgcagatat
aatacaaaagtagatgaaagattgtaaagagtgagtattgtaaagtctcttttatttagtaggtaaggaccggttcaaaggtacgtaggattgtat
aagattgaaaaatgcaagttgaatagatcaataagtatgaagtgagaaaattaaaaaacctttcctgcagtggctgctgcaggaaggaagatat
tgtggaactgtttcttaacttaaggaaacttgggttgtaattttcatctgaagaaaatatatgtatttgactacataaagtcacgtttatttaggctaa
cctttactaaaaatgtttcctcggattttggtttctattattagtgttgtattaaattagcctactttatcaaaaaagaaataaaggaaaatttcatgt
cataaaatccttctcaaatacttggcttccatatagggtccaccgtttttgtttacttattaatctttttagaagaaaaaaccacctggcatcttcaga
ggttcgctagaacgaaagagaaaataagaatcaatgaataaaaataaaaacaaagtttttttttctactatcttcgtatttggattcttgaacttg
taggtagcatttcataggagtaaacaaaaaaatataagtgttatgaccttgagatttgcacgcctgacgagctatgacaaattaagaattattttc
gttatttttgaaaatatgaatatttctttactgtgtcttttgggaggaaaaatgatgccttattgatcatattaatagattgagctatatcaagaaaat

ggaggaccccaaaaggcatagaaacaggcaattcaagccctttattagtgagaaatagtcaagttcaaaaacttccatcctacaactttttttttg
ttcctgttaggattttctaatgtaaacaaaccaaacaaactcatcactacatagaaggcattctttcatattaatcttttaactctcatatgttacact
acactattgctattagtgtgccctagccaagtattctattcgtaaaaataaactgtgactgcttttacgagtggatcccaaagtcgaaacttttctca
ttcagcagttcaccacagtagaaccaccatccacgagccaatatgcagtcagttcagtgctttggaaagaaaggcaatgccacggctgtcgctca
ctgcaaggccggaaagggtttgatcaaagtcaacggagcccctctctctctcgttcaacccgaaatcttgcgtatgaaggtttatgagcccattttg
gttgctggtgctgataagttcgccggcgttgacattcgtgtccgcgtttctggtggtggtcatgtttctcaaatctatgccatccgtcaatccatctcc
aaggctatcgttgcttactaccaaaagttcattgacgagcactccaaggctgaactcaagaaggccctcgtcacttacgaccgtactttgttggttg
ccgaccctcgtcgcatggagcccaagaaggtaagtgatttgctatggagaattcaaaacgacagttttggtactcactattttcttctttgtttacta
actctatatttagttcggtggtcacggtgctcgtgctcgtcaacaaaagtcttaccgttaaaaagtggttggctttgttcattatgtatctatacaatt
gattctccttccttaccacttatggactttcatttttgtaagaacttatagagctatcattgtaagaaatgtgaacagagagtaatttgtagaatgttt
aaatatagccaagcttcgttcgtaactcactaaaacaaattacctttgaatggatgtatacaagtgaatgagattacaatggtccatggcgaggg
aagagtaatatctttcgatgcaaactctatgttgttgtatttttttccttatattcgcttatagaagctaaaataagaatgctttaagagatagtaaac
taaagtacactacggacctgcatttatacacgtttcagctgtgagcgaatctttgtctgatccctctaccgagaggaatgttaatttatgtttaggaa
atatagaaggtagaagatggcaaaatcaaaatgggaaactttagaaaatgaccaatttgcctctgaaaatcaaaagtcagagcatgaatggaa
aaagaaacgcaagcttgaaagaaaacggaaacgtgaattactagaagaggaagcaactaaaaaagacaaaaatatattctcgtacaggtcgt
atttatcagcaactgtcccgcgattggggtcttgtgacaatattcaggattatgaaattcttaacaaaattgaagaaggatcctacggaattgtcta
ttgtgcaaaggagaagtcgacagaaaaattagttgcattgaagaaaatcaaatttaatcctaatgggtttggttttcctatcacgagtctaaggga
aattcattcattatccactgttcgacatgaccatgttatggaactgaagaaagttgtggttggtgatgacttacaagacatatatttagttatggagt
ttatggaacatgatttaaagtttcttctggatgacatgccggaggattttcttatatctgaagtaaaaacgttaatgcttcagttgttgtcatctaccg
caaccatccattatcattggtatcttcatcgagacctaaagccgtcaaatttattaatgaataacacaggagagatcaaggttgcggattttggttt
agctcgtcctacgtccgaaccaaaaccaaagttgactcgcgttgtcgttaccctttggtatcgagctccagagcttttattagggacaccaagttat
ggaagggaaatcgacatgtggagcataggatgtattttcgcagaaatgctaaatcgtactccgctttttcctggaaagaatgagctggatcagttg
cataaagtatgtgcttctcaatatactttcgtttttagtttgctaaccactagatttttaatgttttagggtatcctgatgaagagcaatggcctcaat
atcgactattacctcatgcaaataaaataaagctcccaacggtaaaaacaaagcctaggttacgcgatgttgttagaaacttaacagctaacggt
tatgatttgatggagcgccttctttctctggatccagcgaagcgaatttctgcgaaagaagcactccaacatccctttttctacgaatcccctcgacc
aaaagatccaaggtttttccctacctttccaagtaaagccaaaggtgaaatacgcggtggaaactttttgtctgccaataccacgcaagaccctgt
aaagtaattgagtttatttttgtcagtttctacttagaacatctagcgaaagaaaaaactatatatatattgcttggaatgataccaatgtttgatta
gttgaaaagataaacagtgattgtctttgttcagtacaggcctaatatttatttcaagaagctttataaaaatgtttgactaaatgctgagtaaaca
attgacggtttgcctactctcttgttcaaaattttcattactttgacaacgtaaatgtctttcgaaacgaagtgcattgcttaagcaactaattatgaa
tcattaaaattgaattaaacttcctatttaagtgttactaaatattaattgagtagaattatgaatgtttacattatgtacataaatttcgttcaatga
accaacaaggcattgttgggtcttctacattcattctacactactagtttctctttcctttttgtttgttatatatatcaacaacttgggtgcttgatatt
aaaacttgtgaaaccgccgcacattaggatcactgttcaactatcctaccacagtttaccaactatcaaaataccaacttagaggactagtagcc
gagacggcaaattaaaatcatactttcctctttgattttggtgtttaacgcctatttaattcaaaaagatggcaaggctgttaacaaaatccaatca
agtgattgattttctatccaaccagctttctagaagaaatgctcgacacttttgggaaactcaagagaatttagaatcgtctaatcctttactacaa
gaaccacctgagaactataacagtcttaatatttcagatttacctccattatcaagaagtgttaccgttgctgaaaatgaacagtctggagaggca
ataaaattgggaacgtttgaaggttgctttattcctactactttaaatgtcttatctattttgctctacttgcggtttccatggatcgtaggagaagca
ggggttttaaagtcattattaatgctagttattagctacaccataggattcctcacaagcttatcgatatcagcaatatgcacaaatggtatggtac
gaggtggaggtgcgtattatgccgtttctaggtccattggccctgaaatgggtggttctattggctttatttttatatcggtcagattctaaatactg
gtatgaacatttcgggatttgttgagcccgttattggcttaatcggaaagaattctggaacaattattaaactttttcctgaaggattttggtggaac
tttgcttatactaccgtcgtattaatcctttgctgcagtttgtgctgcttaggttctgcgacatttgcaagagcatcgaacgcactttttttttattatcat
cctttctaccatatccatccctatttcagctatattggttcgtcctttcaaaaatcctagcttagatctctttttttacaggagtgaaattgtcaacctta
actgataaccttttctcttcatatacaaaggatttaaatacgaacattcaagagtcttttaaaagtacatttggtgtttttttttcctgcaagcgctggtt
tattagctggtgcgagtatgtccggtgatctgaaggctccttctcgtgcaattcctaaaggaactctttcttctttatttacaacgtttctcttatacttg
actgttatcctttgtatcggtgcatccgttactcgatcaggccttttgttcgacatggaagtccttgaacatatcagtttgcatcctttgcttatcatttc
tggaatattagccagtggtgccttcagttcttttatgggaatctttggtgctgcaaaactgcttcaagcaattgcccgggacgatttaatccctggttt
atttctgtttgccaaaggctctggacagcaagatataccatttcgagccattttgctaacttatttcattactcaaattagtctttattgggacataaa
catgctttcttcgatgataacaatgacctttctcttaacttttggctttataaacttggcttgccttttattaagattaagcagcactcctaattttcgtc
ctaccttttcgttcttcaatcggttcactaccggattcggttgtgttttgtcatttggcataatgttttatattgaccgaataaatgcgtttgcctcgtttc

taattgcgggattactaattttcgttatatacttcacgagtcccccaaaaaattggggagatgtttcgcaaggaatcatttatcaccaacttcgaaa
gtatcttctgcacaccaataaagcaaaagagaatatcaaatattggcgtcctcaaattttattgctcgttaacaacccaaaccgctcggagaatat
aattcgcttttgcaattctttgaaaaaaggtaaatctaaatgtagttctaagcatatgctaaccatgtagggtctctttacattttggggcatgttatt
gtgtctaaggattttcagggaagtatggaagaattaagaaaacagcagcaactatggcacgaattcatccttgagagagatattaaggggtttgt
tgatcttactgttgcaccagacgaagtgtggggaattcgtggtcttatttcgtcggctggtttgggaggtatacgaccaaatatcgccgtactcacg
tttattaatacaagatataaaaaaattgatgcagctaaacgtaaaagtctcaaggatgatgatgattcctcaagtacggaagatatgacagaatc
tcagatagccgaacatgcaattttacctgtcaaatgggttcaaattttggaagatattttagctggtctcgtggacgtgatggtgacttatggatttg
atcgactgcaatggcctcagacaaaaggaacaaagcgatacattgatatgtttccggtacacatggcgtcgaatctagaatcaaataggaacaa
aacaaggcccatgtatgctaccaatttagagacttatacaatggttttccaattaagttggatattgcatacgtcacctgaatggaagcaaggttg
cacattacggctacttactattgttgaacatgaagatgaaattgaggctgaaaaacaaagtttgcagcaattactagagacattccggatgaaag
ctgagataaaagtcttatgtctgtgcttgagcaatttagatgcttataggtatattctaaaaaatgagcccatttcagctgagaaatccgctaatat
agagttcctgctgaaagatgatctttggtggaatgaactttctagacaccgtaataattcaacgacctgttcaaaaagtactataattcaacgaaa
ggtttctaattacagttttggatcatctagtgttgggcaactaactgctccgccattgagcattcctttgagtttccgacttggtcaatccctaaaacc
agatcacccttttactcgatcttcgatggaaaatgaatactatactcctcctaaacttcatcgatattactctgatagtactgttgaattacctaatga
cactccaccgcaatcacctgaggactatgatgatcaaagcatagaaagcaaagaagagaattcagataaagacgctgagttaacctttaataat
ctttcttcaagagcccagtacatcgttatgaatgaaatgattcgggaacacacagagaatacagcagtattatttactgttttaccggcacctcacg
ccaaaacacatgaaaactttaccaagtctgaacaatatgtggatgatttgcttatctttatgaaggggttacctccatgtggattaatccactccaa
aagcctgaccattacgacagcattatagatttttggcagaactataccgaataactgtactttatttattcatgaaattaattatttatttttgattt
tcctacataaacatgatgttgaaatgtatgtagaaaaacagaagggcctgaaagagtcaaaaccaaaatgaaagaggtaaaaaggggtaaaa
tgaaaggtatataagatagaatacaacaagagagaaaagggggagagtcagtattggcaagtgcatacggagcacgtaaaggcattaggcat
tcaccactggttcactgctttcaatagtaggagaagaggaaggataagagggccggaatccgcgagtagtagtgatcctcttggagtccattttg
acgcattcgggccagtcggcgtattcactgtccgtttggtctttccattgaatagtgacggattcattcgcaagatttttcttctcaaaatatcggct
aggtcggcaattgtgttaggaacctgttgttgcatcacaaagcggtttctttgtgatatgggcatagccaacagttccttacttaatggttgaacag
accgatatcgcctataatttagtaattgagataccacggatgtaacaactcgttcattttcgaaatctgccttcaaaattggaacccagtgatccttt
cgaagccttgcagggcgcattccgttaggagcagttccttgttctttctttgcgaatggcaattgaaacaattgtgttggtttcaaagcttgtctcata
gaaaacaaaacttggtttgtttgtatatgtcgaaataaatacagcgatttaggcacaaaattcattttcgagggctccttacagctttccaggtata
cactgtcttagcaagacatgaaatgagtctattgcattcggtgaatacttcggtaatatatcccggtgtacaaggaaagtcaccgaagtctccggc
atccaaggtcgtaagtaaaaataaaacaaccggtctcgaaagagcatctctcaattatcaagaaaaaagctggagtgtttgtagccatgttaga
atgtctcatgattggcaaggaagtttccgagtttcggtgcttaaatcggttcaaataagaaacattcaggatcagcgaaacttacatgcgttaact
atagacactgcacaggatttatggaagcgtcaagagcagcttaagtatgctatttcacgagggacactccacgtctttttgtcaattcatattcccc
gtatgagttttccggtttatattactgaatgtagcgagaacgcagtaggtggtgtttcttatgtaaaagaaaggtttattaactatctctttttctaga
accacattttctacatagacgagaagatacgacgcaaggttttccagaaatatcggaatccttcttcgtttattctgcgaagctggtgctcgtcgca
tgctgaaggaccctttcgtttgcatcgagaatggaatattcaaaaaggaggacagcattttgttcatatcggcaatgatgtaaggaaaatagaaa
agttacagcgatatctaattaagcttttggctatggctatcgttcgttgctttttctaaaggttttaataactgtttactaacttacagcctatatctgc
cgtatgcgaaatccgcaatggcctggtatgtgactttgcggacggcgtatatctctatacggaccatcgcaccgttgataccgaagatagtcttgat
aaaagtatgtctacgatgtcgttggatcgacggaaagacgcatactcttcacaggccttactacgtatcctcaatctctctcaatgtattcatgaac
ttgagcttacgcagaagaaagttcgtaaagacctagtaacgagcgagactcagatttcaaaaaaaagccattgcttaaagtttacctacagtaaa
caccttttcttctctctcaattgcattaccgtctgtcgaagtatttagcagcatttcagaaattaagcgatgatcttcaaaagaaaaaagatcaagt
tgcgcatcagcgctctcaattggaaacgcatcgagcacttttaaagttgcgtgaaactcaataccaggaagcttttgctcggttaaaagcgttatg
ggaaacaagttcagaaatccgaattctcatagctcatgcaaccagatctcacattaagcaactttgcactgtttatccagttcacgtaattgatgat
cagccccgtttatttacaatacgtaaccttcggttttgtctgcttccaaatacagtccctccagcggaactggctgctgcgataggctttttggcacat
ttggcgtttaacatatctcagtacttagagtacgattttccttatcctgtctctccagctggttctaggtccacaatattggatcctctttctcccaactt
agtgaaccgttcctttcctctatatcccgctgcacgagctccccacgcatttgaacatgctctttatctacttaaccataatattatatttcttctggct
cattatggcttgcctaacgatcaaccatcagatataatgactaattgtgaaagacttttacaatttattctttctggacaacatctttcttttgttcaa
gtcacatctgttttatgatatttctgtgttgtaatggtgttattttcttgtctcttgggaacagctgtttcattttaattagcctactatgtgcgcctttaat
ttaatttattaccctcgtgttcattatttccccattttcaactgcacataaacgttagtacagcttagttatttcatttactagaattgaagtaagtcttt
tatcatgaaaatagacagaaagagacagtattatttattaatgagtttagtaaaatacccactcaaccgtatcgtaaaaaaaattaaatcgaaca
attaaacaaaaaatttaaatactttttttgaaaattattgaaacaaaaaccgtaataattgtttcaagaataagctgggggagctggtcctacact

ctcaaataccggaggacattcttcatcccagctaatgcccaaaccagcagggcttgtaagcggttgaggaattgacactcgtaagatacgagcat
ttcccatattactcgttgctccctttcgtcgattaacaatagcttcgatgattaaaaaatgagagatttttagattaaatcgaccagaaagttctgca
tcacaagtaggttcccggaaacctgcagcgttaatttggcaagataaaaaaaactgatgatcaactaatttccatccagattgcaagtcttgagc
acttagtattttttctcttggactgtacgaaagcggacattttctttatgacggtcacaaggcttgggagtataatttaaatgttcctccaagcgcca
agtaacccgagtaactccccattcgctcccatcgctctgaacaaaaccagtcatagtgatttccattagggcgacagtgtgagggtgtaggatact
agacatggtgatgttagcaacaagatcggttggaggaaaaatccgtttgtgaatcgtatctgggccagggacgaaagctctttttagctctaagg
gatacttcatggtttcgggtttataaggggaaagacctccatcaggtgtagcgtagcaaaacatagcctccaagtaatactcgacagtaatatgcc
gtgtagataatgtagcaggtagagtaccaggaaaaagccaactgaacggccaatggtgtgttccggtgggaagtttggaatttgtaataaacatc
cactcttgtaacacttccgtagatcccccacaatcaacacaatgagaaataggggggatgatgaacagagattttctgttttaattgaagtattaat
gagtgaacatgaacaaacggttgatgaaggattgtgagtttaaaaataccagatgccaaggcgccggtggaagtttccggagggcccaagagg
acaagaggaggagactccatccgtacgtccatttggcaatgggaatcacgggaatggctagacgcacggggaagagacagtttcaaaggcatg
aagaaggatgaaccaaggaaatcgtaatcaaaaaaaaatttaggttattctaaagaagttgctgcaagagcgattgcaactataaaaaagtcg
atcaaataatgagcaatgggaatcggtaaaaaaaaaaataaaaattaacaggtatggcgcatctacactcaaagacaaaagaatactaatga
gtgtcaatatagaaagaaagtgcgtatatcactaattgttgacataaattctaaattagacaattttaaagagatatgtaaaattgcgtccaacga
taacgcaaaggaaaattgataatttttaagacacaaaaaaaagcaacaataacaaaacaagaagaaaaaagcaatgacttttaaaaataacg
gaaccgacgatgtttgtacgggacctgtcggtctaggaatgaatacagagaaacatgcaccgtcgagtcagagagtatacaaccaacaccttttt
ttttttcttaaaaagcgccaggagggcggtaaaacgtaccgacgagttgatacaacaagaaaaaaatatgatgaatgatgagtttcgcagaccg
tttcgtttcaaatcccgtcagaaacgatcgcgtaaaacgtcaagagtttggaagatcacaaatgatccacaacctttaaagaaacggagtccttat
gagcggatgaaatttccacaaaaagttcaaccaattccccaatctttctgatgtttgttgtctgcaggaaaaaattattgaacactacaattgcata
aaatcatatacggaggttccctgcatttcttaagttatcgtttgtcttctgtatcttttttaatgagcaccttatttattgttttcagagttaacgaagt
atatttgtttcgctaacgtgttcgctaactttattggatggaaccaccgtgatggacttgaaccaaccgcattcgctctaattcgtacttatcctcttct
aaacacatcgccactagcattgattcaaagtccttcctttaatccgtcgaaattcactaaaattacgctttacgggctctgaaacaattttgtgattg
ttcaattctctggatagccgtttttaaaaaggttcgttggatccgattctttccattcgctgctggtactgcagttacgaaagttacttttgctgagtt
gatatttttccctttctacgctgtaacagtggcgaacaaaccattgggggttttacatttacgcttccttcaaaagacgttgaatagttttgtcgaag
gcgtcgaaggctgtaatctgtatcatcaatttatagactatatagatttctttttgtttttttttttatcctccccttcccccccctaatttaataaccta
ataattgtcctttaatttcttgaaagacgagttgagcgttatttgcattggtttaagtttccgtttgttgtcttttaatattctttgctagctttatcttttttt
tttgcgatctttattgatcggtgaaagctatggctactgtccaaccgtcgaacatgcattctgcttctgctgttcgtttccaaatttgctcgggttcatc
cccctacacaaatcgagtccgtccgtcatacgaattaattgaagctcctactcgtcaagcaacatctactgttaactccaactttgatgttaatccc
atctcatcatctgaacgacccactctccccctgaaacaccataataatatccctcccccctatatctcctgcgttgagtgcacgaacttcgactccag
catcgcgccagtcaaaacaggaacaaatcgaagctgcaaatgctgcaaccaatctttctcgcaatcgacctgcaaacttgacgaaatcagcatt
gcagaacagtaatttgcatacattaccagcttctcctgtccctccatattgttctaatacttcttcccttaccaatgatccttctaaagaagctgctat
atcgaaagatcactggaaaccggattctgaagcaaccgtctgtgcgttcccttcttgcactgctcgttttggtttattcgaaaggaggcatcattgtc
gttgttgtggcgatgtgttttgttctactcactgtaatcgctcaattccattgacatttgatgttcgtttctcgttatcggccgcgaagctttttagagcc
tgtgtatcttgtttttacgattacataaaatggagacaatcttttgagttgggttctgcttccaatgtcattgtgggtactgaaccccatgctccatctg
aatctacttataaaaacaagcaagtctctacttctgtacctatccccaaggttgatgctggaatgtcgaagactgagcttccttctgaaagcttggtt
ttaggaactgttcccgataattgggtttggagtaccttttaaatctatcgtttaaccttgtcgccttctatattcgttttagcttctgcattcatagaatt
ttggatttaatgtatcgtgcatattacaaacgatgctaatatttgttatatctggttgttggtaccagtacttgatgactatgtgattctcttatcttta
aactttcgaaccgctggctaacttgctatatatggtatgctatgctcattccttatcgttacatgaaactcgctttagattcggtatcttttatttctatc
tgtttaatcattttcttttccgataccgattttgttatatactcttatttgcttaatttatgtggcttagaaggaaatgtcgctataattatctttaaatct
cgtctaatccttgagagatttcgctctggttgttatgttttgtagattaccctttttttgttttctttttttttgattttttttagatgatttaatgaataaac
aatgcttactttaaaacgagcgttaaagaacagaattctggtataataaggaattttatttatgtatgcacaaacaaaatgaagaaaaggcttcat
gtgagtaggaattctgggaattaagtaaaaggattgtagaggtttttgttaattaagactaattgaatacatcaagtattacagaacattttacttc
aaaaactcagtccaaaatttcggttataagagcagtaccaacggtgtttttgccttcacgaacggtaaatcgttgacctttctctatgacaatagga
tgaacgagattgcaaaccaattcaatgttatcaccgggcataatcattttgtcagaatcgttggggtcaggatgtgtgagttcaacagtaacatct
gaggtgcgagtgaaaagttgaggacggtagttgtcaacgaaaccagttcggcgaccaccttcttccttagttaaaatgtaaaaggaagctttgaa
ctttcggtggggagcaacagtaccaggaacagcgacaatcataccacgcttcaattcgtcacgcttaacggaacggagaagaagaccacagtta
tcaccagcaacggcagcgtcaagttgcttcttgaacatttcgataccagtgacaatggtcttaatgtggcttccgtaacccacaagttcaatttcag
cacccttcttcaaggtaccgcgttcagcacgaccggtgacgacggtaccacggccggaaatggaaaaaacgtcttcaatggccatgaggaaagg

catgtcgacgtttctttcaggggtagaaacatgatggtcaacagcatccataagcttggtgatactttggagaccaatttcgggattgcgaccctcc
aaagcacaaagggcactaccactaataattggagtgttatcaccgtcatagccgtattcagacaaaagttcacgcatttccatttcgacaagttca
atcatatcaggttccaccatgtcagccttgttgatgtagacaacaatgtcattgacaccaacctgacgagcaagaagcaaatgctcacgagtttga
ggcatttgaccatcagtagcagaaacgacaataatagcaccatccatagtagcagcaccagtgatcatattcttgatataatcagcatgaccggg
gcaatcaacatgggcataatgcctgttggcagtttcatattctacgtgtgcggaagaaatggtaataccacgagctttctcttcgggggccttgtca
atttgactgtaatccataaagctggcttggccaagctcagccaaacccttggtgatagctgcggtcaaagttgtctttccgtgatcaacgtgaccaa
tggtaccaatattaacgtggggcttcttacggacgaacatctagaaaaaaaaaagttagtgacatgataaatgaagaaaaacttaaggagtgaa
ggcgtattccttattcctttgagattgttcgcgagcctaccttgtcatccgcataagaacgcgacggaattgtaaacttgttaaaacatcctgtagcc
aaagggaatttggttaagcgagaacaattctggcgtaagccctggaagagcaacgaagaggttgcttttactgctttcatggtgagaggaggtat
tggtaaagagaaagatgaatgcagggaaggtttataaaagaactccgtggaggacgttaaaaaccgtttattggaatggaaaaagttattctttt
tttttgtaataagtaataagacaaaaaaaaaaactaaatagaagaaataaatagataaaatagaaaaaaagagagtgggttgaaaaaagggaa
tggaaattttacatttatggtgcgagggtgtgattgttttggttcacttgtgagttttaaaaaataaggaaacggacaatgtatgaggaggaaaac
gaaaaaatttagaaagttgttggtttgaattttgttggcgacctacgttgctagaaaaaaaaagtctacttgaaaggttgcatgcagatttataag
ggctggtagagaaagatagaggaaatatacaaattgaattctaagagaacgcaaaccaatcaaaagttgctagtttcgtttgagaaaggtaaat
aaataacgacagttatcaattatttcccaaaaactagacctatcccaggctagcaaaacataacagaatctattaaggggactcaaatgcggaa
accatgggaaataacatatcacattagtaacagatcaaaaagattgcaaggaagagtgttatggtgtcggaaactcgagttttaggtacgaggg
ggtgcaataaggataacggaatcacctagaagaaatgttagacaagagaggaactagaaaggagaactatgaaaagtgaagcatggtcttttg
ctctccaattggaaatctccggtaaaggaaggaaaagaatgatggaaccttggaaagaacaccccaagcttctagaaacaagaccaatgataa
gggattacttttcaaaatcttttatgtataatccactgagtaaggtggaaaacttaccacgaacaaaaagcatctcataatgttttcgaatgtttttt
aatgcttttcccttagcaggttcttcttcatcaataatcgtaattacttcttcggcatctcctagaaccatattctacaaattcaacagtaagttattga
cccattcagcctcttctcctgtggaagcgcaataatgcataccagatgctcgtcataagcctggaaaactagttagtacaatgcttcttctcctttcg
ctgctttcttaaatacatacatgaagacgtccattcaattcacgatcaccgcgtaatttcacataaacattttcatcaagtgaaaggcgaaccaaat
caagtggttcagcaacctagattcgttagatgctttaacagacatttcgcttacagactgagtagattccatggtaataattaaaaagtttttcctac
tttttcaacaatcaccggttttggcttttggttagcaaggcatacaggaaggatgtgataggtaacaacgatgtcttgttgaattctagtgatatcga
aaatatctttttaatcatcgttctagctcgctattcatagcttcatcacagtaagagaggaagggtaccaagctgaaagcaacgcaacccaatttc
atttaagaaatgctgatttcttttacaaaagctctgccagtatggagagcaagctgctctcctctaggaagcaaacttttgggaatacgctggctga
gtttactgagtgatctcaataatcgtcctaatgaaaaaaacagcaaacgcgtaggacgcggacctgcaagtggaaaaggtaagacaagtgccc
gcggacataaaggatccggacagagacggggtagaagaattaagcctggctttgagggtggtcaaaccccgattacaaaactgtttcctaaaat
agggagaagtactagcgaaatcaaacattatgtcccattaggtcttggccgggttcgcgcatggattatgaatggcaggttagactcaacgaagc
caattacgatgaaagagcttttagatagtcgatgctgtcggggcatcaaggatggcgttgaattggtatcagacagtaaatctcaagatttggatg
tcccaattcatattgaagtctccaaggccaccaaagaggctatatcctttgtagaaaaggctggaggtacggttaaaacggtatacttcaattctc
ttggacttcgtgcccatttgcatccagagcgatttcgtaccgttcctcgcgcgtcgcttccttcatcgaaaaaggatatcttgtactataccaatccc
gaaaaccgtgggtacttgtcaaatgtcaatttacgggaacactattacggcgatcagcggtttccctatgaagctttaccagatgaaaaggcata
atagtaactgttggaaagacacgcttattatgttacaaagcattttgctatattttccccgctctcatcctttctttttcgatgttatcttcctttaatga
acgcattgtgagttgttagatggcttttaattaaagagttttaaatgtgaattccgatgcaccttgttacaataataaagggtacaattttggattgc
gttatgatttgcattttgccataatagtagctctggaagttgacgatttttactttctgtattctaaataataatctatcagaacattacgatttcatat
tgaagtagcaaattaagaagctcaatttgctctggatataagcagttcctaaaaaacactttacaatggaacgaaaagtatggaagtagaaaga
aaatcgtttcctatttcaataaaagctctaatcttgaatatccattgatttttgacttcttttgtttactaaaataaggtacccttgcgcgagttcatgtt
ggatacttttctggtagggaagcatggaacaagaacaagcaagttcaaaatgtcagaatcctcaccgtttattaagaaattagcagcaaacggt
atgaaaggaagtggaaagaaatgaagtaactaatagggaagatcgaaaaactagagatgaggccttgatttctctacaaaagtttttgagtcga
aagaaaaaaatttgagcgacttgacttttttaaaactatggaagggtctcttttactgtacgttgacatgttgaaaagttggtggaaagaaaaaatttg
gtcgactaacgcgaaaaactaggcatgtggatgtcggacaaacctttgtatcaacagaaattagccgataatttagccggattggttgcaatagt
gcatgaggaaaacaggatgttatttcaggctacgttttgggaaacaatgggtcgagaatggccaggaattgatattttgcgcacgaataaattct
atatgttgatgcgaagattctgcgctgctgctttcttggaaataaaagcaaaaagtgattcggaggattcattgaagcaaatggttgctaaataca
atgaaatgtggatgtcggttcccttcaagtatgttgctgtttaggagcaagtaagcgccatgtcgttgtctcgctgcttcttttcatttgatgaattaa
tttcttcaaaattaatttgctaacctgtgtagtcctaacaatttttcgtatcccaacggcatcctctttcacctagcagatatttggacagaagaactg
acgaaggcttttgacgaaaaggttcctaaagctgattggtacttgccctttgattcgttaaggaaaacaacaaaaaatactgctttgaagaagac
gctacctaagcgtttggagagggttgcagagtacactaccgctgacgagtcttcataaacgtctagggaaaagggctatgtgaagtagaaaagg

gttattttgatgattgggatactttttgtgtaatataataaatttagacttttgggttcaatttggtttggttatcgagaagaatgttctgtaggaaata
tttacaatccagatccgcaaggcgtataaaaaaggatgttaggaggaccaaagagagacctaccaaaaaacaagggctgttctgggaatcgaa
cccaggaccaccatcaccctaagatggtatcataccactagaccaaacggccgctgttagaatgtagatgcttttgtacaatctgtttgccttcctc
tttttctgacatggatacaaaattaaaagaatctagcgagtgccgcttttgacaaataaatttcaaatatatataaaagcaaattattgaacaaaa
gaaatcttgattcctgtcccttcgacagtttcaatatggacccttcagtcatgcaaaacagcagaaagaattggcaaatttcgctggcttttgaacc
attcacgtaaatttttatttctatttctatttaaaggttcagttcattttattgttttcttctcaaaatatctttcgtccttattttgcttttatatttattttta
tcttgtacttattgtttactaagtaaacacaaggtaaatcttacgctataccttatacctccctattgcaagctcaagggtaacgcgagtagactac
ggcgacaagatgaaatcatttaccctctaccaaacaccatgtctcaatttccagcattagaagattttgatgtaggattaggatctgctcctgaag
gaaaaagtgagcaaactgacttccttgaacgggagaaactggcccttggtgaagatgcgaatcaattcgaaacattagaagacaaagatgcgc
ttttaggctttgaaaatgatccagaagcagaaaagaacaattttgagaaaaactttcctcctatcgatactgaaatgcagacctctggcactttttc
ggctcccaaaactccttacatggcccaagcggaggtgcaccctcctgaacatgaatcagaggattcggagcccattcacaagtggaaagaagag
aacgaacgaagaattcgtgaaaaggacgaagcaagtcgaagactttatgaatccaacgtagaaaaggcaagaaaagccattgatgacttttat
gagaactataacgacaaaaaggacaaaatcgttgcaaagtccagagaggaacaaaagaaccttttagaaaagaatgaagctgcttcctcagg
cactactagctggcaacgtattttgaagcttgtcgatttgtctgataaagccgaagctagcggccgctcgactcaacgtttccatgaattgcttact
agtcttgctaaagatgaatctgcccctggagctggcggaaacgctttgtctgcttaaattgtacgcaagggttcgaaccctgtttggcagaatattg
caagtatctcttttctaattaagcgcatttttgatatttttctatgttcaatgaaacatttctgaatctttctttttttgtatcacgagaacctctttcatttg
tgagtaacctacttttataccgctatatatctcttatctgtcaattcagtaatggataatgaaccggtatgtcattatgttctagtgtactgttacatga
tatacaacttgctgattttaccttttctcgttcgttctggaattgatatggtctgagaaagttcgcaatgaataaaattcaataatgttgattttccctt
cgacttctctccttactcgtaaaagaaaagctatagtctctcgaaaccttgatactttttgaagataaatctaggatactcctggtgtggattcaatt
tttttaatgaagtctacccgttcaattaacgattgaacatatctcatgctgcttctctactagctaataattcaatgaaaatatctattaaatataaaa
acaaaacaaggataactgaacgcgttagccgtaacttcacgtctgggcattcgtcttctagttcgtctattattattgcttgcacggtttgagcaagt
caatccaataaaccaaaaaagtaattgtctacctaccgaggagctacatcatcagtcctccccaactgccattttatcaatcgataaaagtaagtt
cctggttgagagagttcatcaaactttcctttttcgagaatcttaccctcagcaacgactaaaattaggtcagcgcttcgtatggtagctagtttgtg
agcaattgttattgtagtcatatttcgattctgcatcaatgattggatagtacgattaaccatgacctccgcttctccatccagagcagaagtggctt
catctaaaatcaagaatgcaggagtccgaaggagtgctctggctattgcgatacgttgcttttgtcctccgctgagttgcaaaccacgcattccga
cttgggtatcccaagtatctggaaaagaatatataaacccacaatcagcacgaagagcagctgcctctatttcctcttgagttgcattttgattacc
ataagaaatgttatctctaattgttcctgtaaaaagaactggttcttgaccaacaacaccaaagtgacggcgccactgttgtacattaaaggtaga
aatgtcagttccattagcaagaattcttccagaaactggattatagaatcgcaataaaagctgagaaagggttgattttccacctccgctagctgc
aacaatagcaatattcataccgggttgtatttcaaaggagagatctttgaaaattgtagattttggtctcgtaggatatgcaaaaccaacgttctca
aaagtcaatactgaatttcgaatggaattaggaactggtgttccagtggttggagtaatttgaggaacactatccaacaagtcaaacaaccgact
agctgcaccaagacctttcattatgtctgtaaaacacccagaaagacctatgatacttcctccagcgtaaacagtataaagcaagaaggagctta
gctggccaacagtaatgtcaccagtagcaaccattttaccacccaacgcaagtatagcaataacggttgcattcccgaaaaaaccagtggtgcc
aaaaaacataccacttgccaaggcttctttgcgagcaaggttgaagagattgcgaatataattattataacgatggacctcctggcgttctcctaa
gaatgcttgtgtggtacggacgtttgctagtttttcctctgagactcgagtaagatcacctaaagcatcttgggttctacgtgacaatttacgaacgt
attctccataaaagaaagcacctaacgctattggagggacaatcaaggacatataaccagtaagcgttaaagatgtatacaacatcatagcaat
cccagcgacagcactaacagtacttcttaatccatcactaagatatgtagagagtgactttccaactatactactgtcagtggttaagcgagaaat
gatgtctccatgtaagtgagaatcataaaaacgcccatctaaagtaaggcatttagcaaagagacgagaacggagtcgagatatgatattttca
cttagaagtcgcagtgtcaagattcgaccgaaattgcaagcagaaccaatcaaaaacagaccaagtagacccatataaaggtgttggaagga
atccccatgatttctgtaaatccaatttgagtagcgccagatcctgcatccagaatcttacctacaacatacggaatcgacatggaaattgaagag
gaaattaacagaagtattgcagcaacggtaaacttctttccttgacctctagcaaggacaagaatattaaaaaagcttactttttttaggttcggga
ggacttttgggaacagactccccggaagactgactaatgctttgcttctgtccttctggacgcgatgggttactaggattagaaaagctattttgag
atcgatgaatttttggatttggaggaatattcggattgcgtaatcctttggaaccagaagaaagtgtcgagttgtatcgaatacaagttgtgggaa
cgaatggagaatagatgccatatgagggcagggaaggatacgggaagtgcttaattggttgaaaggaagactgaagtgggtacctgggtaaga
aaggcaaagatgttcgcaaggtcggattcatgaatgcatggtgatggacagagaacatacgatacaagtttttaggagaaataatactatccttc
gattgaaaagactccttcgagtgagaggaagcaggaaaagattgtgttacaaacgatgcatgactcaaaagagaatactttgtaggaaccgata
tcttggagcaaaagttcacttgaaacgaaccaacattattcaaaagaaattggcattggaaccgacggggatggactgcatttttacgaataagg
tcgacaaccatttgctttatacatgaagacaggaagagtataaacacttacaagaatgaataaaaacaaattcagctcacctttttcaaatggatt
cttgtgacgacaatggaaggtttcactttttaccacttgacaaaagttgtggtaaagatgtaattcgacaatcaagcttgattccaacaaaagac

tgttcagtttccttaagtggaatttttataaggtctaagaataaaatgaacgctagtccgtttgatctttacttttaacggttaggcatctcgttcattt
ctggaggaaaaatttaaatactacctttgtacacagtatttatcttctaactagtcataatgttttgaaatcctcttttctattcacgtattttcaacgtt
ttgtcgcaaaaacaaaacgttgaaactcactttttcgtgaattcttggatccattaatcaacaattctgaaaagttcatgttcatttccaattgaaaa
aataaaagtttttatgaaattcaatctctattcacataaatatcatctagtatgtctgctttttcgacgattttctttcagtcttgccttcaccatttcc
agagaaatctagtatatataagccaataccgaaacttgaaggcaaccttcttaattcaacttcaaaccgagaattttattaaaacggtgaaatcct
tgataactatttccttgagtaatactttgtactcttggtttcgttgatatacaggcttgctttgaataattgcttcttttacgttttgaaagctcgccaaa
cggtaaaatcgtcgaatttttctttttcataagaatttgaaaagagctcatttgttatttgttgtttttgtgttacaatttgcattcattactttagtgcat
catggcaatagattatagtaaatggtatgcagctaggaaagaagaaagataaacgaagcacataatattcaattgtgctagactaacatgtttgc
agggacaaacttgagttgagcgatgataggtaagtgtaatttctccttttcttgatagcagtgtggccgttcatggaacggagtttaaccagcttga
ctttccaataagtttttttcaaggttgacgctggtatgctgcagtctacttttatgaatgatgcataaggactttttttcaaaccttactaacaaatgttg
tagtgatattgaaggttcgtttatatatgacggatttagttttctaattttctctagtacatcccaatgttgacaaaaaatcgtttattcgctggcgcca
acgtgacattcacgagaagcgtgcaatgagaaaacaaaagatggaagacatcaaaagcgctgtatcaatgaaccaaagattaaaggatcgca
ttgctcagatgtatacttatcttcaggagtattccccatccgaaccactgacttacctttcttccttcttgcaatcgaaaagtcaagaagattctgaac
aagccgtagaaggaggaatgtcttatcatcagatgctagttgctcttttagataccattaagaaggctattgaagaggagaacaattcttcgctag
gcattaacgagcaatatatcaagcatcttcagagccatgagaaacgccttgacgatttgatgatgaaagccaaggaggagtacgatgaacttga
gtccgagagcaagaagtatattacttcagaagatttgcacattggatttgatagcacacacgttcaaaagaaagaggaagagtccaaaaataaa
aagaaagagaaagaatctgttcaagtcatcgaatcccttaacgatcctagtccatctactcatcaacaagaggtcggttcagcacaatcttcatcc
gccattactaaggctgaacaagaggaatctgattcggatagcgtttcattgtctgttgatggaactaagttctcccaaattgcttttggtgactatgc
tgcttccgaatctttcttaaaggaacatcttcatctgctagctgaggaatcagaatctgattcaattttacttgaggcttttaacgctgaactggaag
gtaaaccttggttagctaagcaatttgtccatcaagccttattactcaactattgccgacaacttggccctggtggaattaccatcttctttcaacgg
tatgctttttcacaacacttcaaaacttcagagctaacagaaattgtagtgttcaagatcccaatcaccaagctcggcgcttattcttggaagatgt
tcaaagtacatataaccgcattcacgagcgtagtcacgctatggcaaatgaacaagctcaaaatggagaaggtgttgaacagattcaactctgt
gccgtggatgccaataccaagctgtccatcataattcccgagaaagacgctacagagcctgaggtgaaagctgcacgcgtgacttttgagtcatt
ccccccctaaccttcaaaaagctttggaatctaataaccttgataaaataaatgctgtccttggtaaaatgcccattgaggatgccgaggaggttgt
cgaaaaactcagcagcactggcatgcttagtattgaagaaggaatcatagataccacgaaaggtgaaactattcctgaaccttctgctcaacaat
aactttttttcagttagaaactgctctgattattatgatcaatgaaatttaatttcaattatcgaattgatgtaatgatatttcagtccaacttaacaat
aaacggatttactattattattattatatatatatatatatattttttttttacattagaattatacttaaactcatattaactgaatggaggccattaaa
gcaacatggtcgctgggatatctaccctctagaggttctgcctctttcatttcgtttgaatgaggaattcgaagtaaacttttcagctttataccgtca
acaacttcatttacagttttctctgttccagaattccggtccataacaaaaatatagtctaaatggccttggtaagtattagcccaatttgtgaaagc
aggatgctcaaatgtattctttgcattttcaggatcgacgtgtttgtaaccataagaatataagctaacaagtctaggattttgtttgtgttgctcata
aaaagaactgtaatgcggtacaaattcattctgcacatgtaggattcgctttttagatggggtagatgctgaagtgatggaagtaggagtagaaa
catcactttcggtatcattttctgattgcttagctggcgattgatattcagcttcttcttttccttctaattcagactctccaaacacgtaattcatactg
cgttcaataatatcggtagcacgctgacaaatggaaaaaggcttcgtagtcatcgcaggatagtttgtatcaaaaggttctgtattgaaatcgcca
gcaataattacaggccaatcttgatgggcctcagctatagccttcacctcctttactaaaatagcaccctggcgaagtctttcgtaagagccataa
ggatgccaaaacaaatgagtagtagcaagaaagatacctctggaaggattatcagcacgttctaatgtaacacaactgccaatatttttttgtattc
attcttcctggtaactcatggtgatcatcatagtgaataacgtctatttttaggtttttaaacagagagtttctccaaaaaattaaaattccatgtgtt
tttccttcagcagcttgaaaaaccaagtcaaaacccatgctttccatcagaggtttataaaaagaaggaatgaattcggcatcaacctcttgcata
caaccgatatcaggagcataaaacttgaattcattttcaagaacttttgagcggtttttccatttcagggcttctccgctatgagggaacatggagc
gacgaatatttgtctgagctaacacattataggtcatgactttaatatttacaggtgatttattttcatcaaaaggagcgaaatttggtaaggtaag
catgtctctttttataaaatcggtattgaacgcacggggttgatcttgctgaggcatgaattgtttaagagcagcctttttttgctataataatgttaac
aaacgagaaagaattataaaaaagtcgcttcaactagtaggtcaagctcaaaaagatttgtaacatacccatcttctctgcctttttttggcgctgc
aattcaatatactctggtgttacaaaagaaggagcacctttttttggagccattctttgtttctggtttagaatcggccatgtggatacctctgaaaaa
agaaaatttcaaagtggtgtgtggtaaaggtgcaacggttgtagctattaaattcgcagatggataggattcgatgaatttcctcaagcggaagg
cacgaataagcatgaaagaagaaaacaacagtctgccattctaacgtaaagaatgattaccagaatcctttcacggttttggagaaaagtaaat
ggttcaccgaatataacgtatacttgcgtgttcaccttggtgctttcggtttgcttgtaaaaagctagccatagggataggatgaaacagattcttct
tttgatttccttctgacacttcttccgtttgagattgtgatccttgcacaaaagcaaactgatcgaccaaatcatccaaagccatatccttccaagcc
ggttcatgtcgtagctgcttacttctcatgagaattaaattcgcacttgaatgctgaattgtaagttcatcttctggcatgggaggcaggtattcgag
acgaacttctacatcatcaatgggcagcttgtcttttcgccattccgaagcaaaaagaaccactcttggttggttcatatgtatttgacaataattgg

aatccattgaattgacttttgctacggttcctttcaattacgaaaatctgcggttctgtcacaagttggttttctccacagctgtaaacaaacgctacg
aagtccctcctccgacaatcagaatttccaaaactattgaaattctactgtctctgtactgttaaaaaagctctgattacgattacaactatcgattt
tctaagaatatgagctgagctacaggcgttgaaattcctgcttcacttctgcaacgaaaataccgaaactttgaaatttgagaagtatttaagaga
gatatatattcttaaactactttataggtttaacctatgtttatatttatccttgtccgctcttttataaacaatagtagcctatgtgagaacggccttc
cttaaggacacataaaaacatccattggcactggtttctttacggctttagtgaatacagttaaaaagagacagtcctgtctagaactgtaaactg
taaacctataatcagaagcagtcaagtctgcatcactgaccagtcatcgagagtatggatgtatatatattccccaatcctcctcttttctctatctc
tctttataaacacaaaaatcgaaagctttcatagcctagtcttcactgctttgggcacagcggattgtttatttacgttaggcttttgaaatccccatt
tgtttacaatatcgccaatctccatttataacaaaaccattcttacgtcgacgaaataagaaaagccaatactctttcaaagaaatcccttgaaaa
atcccactttgacagtcatctcaatcattacaagttgctctcttcagtaagcctagcgtcattccataagctaaacctataactttttttgatactccat
cgattttgcagtcttcctgaatatggattcttacgttttgtcattttcggttccagatccatcttttccgccttcagaagaaacagccctttatgcaagc
aagtgcaatttttatggaaaaaatccttacgaaagcattagaaaagctgctgaactacgctgtaccgaagttcagtctctttatccggtaactgtta
atctacatgcgaagcataaaacgccagacataagtgtttcccgcaatgagatagtaattgtaatcgcaggagcttatgaaagtgtatattcagcc
aaaatatacatccttcagtcccttcccaagcttataactagtcgcattttgcttgatgaacctctggaaaccctcttatttgatgaagaagggttcat
gtacgaagacgcgatgaagcatttcgatacaatctcagaattgacgggagctaaactataccttattcatcgttcactaagtagtggtatagaagc
tgaagtaagcacagggcgtgggttcagttatcgtctgaatgaacaagaaagctttcgacaaaagcatttagagtttacacgtttagaatttggac
gagaagaagaagctgatagctcatattttgtggtttttttacggtgattactgtagcgtggagcatgcacgtatcaagtttctgactcttcttgatgaa
cttcgtggtctttcagttttaagtattcccatatccgcttcttttcagccaattcttatgggaaaggcgtattacagctctgcagggctaaaagctact
gaaacgatgaatatgtatacacttccgttcttttctgatatgctacctcgattgcaaaatatgccggaactcaattcgtcgagattaatcgttactgg
agaatccgaaagcctaaagcgtttagaagaagcgttttattatgctgaagaacatttaaaagtattcacgcacattatggaactatctttcaccaa
gcttatcgaatttttaacgaacgaaattgacgaactacgacttattatggaagaaaattcttcttttataagatttcccgattacttcaaagaggga
aaaggctcatgcacagaaaactcacacatcaagatttattcctcaacgcttgttaatgccgaaagaaccgctcttcgtttatccaaattatcgagt
aaatatgttgaaggcagaacaaagtttgatgttcatgacagcgaagagtttttgcgaaagagaggttcttggaaagatttagcatttttagaaaa
gaacacatatagttctaaatcttcctctgccacttcactcgtgagttcctctactgaaggtaacagtgcatcattgcaatcaccaggaaaattacta
gtgccacctacggaatacatgcttcagctagcaattatcacaatggcttctggagtagagatgcttctaaaaagtaatgaaatcacgtatttgggc
caggaaaacaccacacctattgccatggaaaaagcaagcaaggtttttcacaagttcggatcttcccaatggcatcaaattcttcttgacgcccca
gccaaagaccttgacttcatatctggaaagaagaatggcaagttggacaaagtgaaacaacaatgcaagtttaattatcataacggagacatta
ttttctgtccaatgtcttcgactatattcacagtcgacatctatggtgatgaattagaaagagtaataaagggaatgaatactatgcttcttgaattt
ccttcagaaatgcactttttttattccagaagagatacacaagaaacttattgggttccgtggagagcaaattcaaagagtgacgaagctatacaa
cagctatatagagttttccacgactccctttgactgttacggacacaatgttcttataagaactccgtcaaagttttccgagaatttatgggcagtac
ggtctttattcgtcaaaaccgcagaaggactaggttacggtgtccctaagtatttatatgctgtctaagtatagatgacaatttcgcgcagatactg
ttcttgagcgttgtttttaccgtcttatgtcatgcgaatgttggttttgaaccgataagagaacacacctcaaatgccttttgtgatgttcttgtacacg
atgttgtaactttcctttccttgattagactttatttcatttgaaatcgtaaattctaaggtatacatgccaattcccagatttgattacttcctttttttcc
tagtaagtcatgaattaaataaccggaaatccttggaaaaattcataaacgcaaaaatattcgatggttagattcttacaataaaatacaaaagt
aaaaaaaaatctcaacattttagagctcctcttgggtaaatcacattgcagataaccatataagagaacaacctaacctgtataggtaaaagatc
tgctccttttgaaccatttgggatttatagtaaagaattgaatcaaacagagttgtgtcttagccaaagtactaaaggcttgaaaaattgtcaaagt
tggaaatgatcaattcaacgagaccagacagaccgttttcattatataataaagctatagtacaagtgattttagtgcaatgtaagtgagttgcag
ctacgcgtaatttacagggtagataactttacagatctcaatagcccccatgaatgatatagccttctcgaaatttacttttgtttctttcgaactttg
gaaaactgatgagacattaccgaatttggaaagttcactgtctttcaaattgtcgcatttgatgcaggattggacaaatgctgttactttacgtcatt
acaaacaatatttcgagtgtaaagaagttcgtttgtttattattatttatttaactgaattagcaggtatcatctcaaaaatggattcatactatgaa
gtatatcaatgaaactttggaaaggagaaaagcacactccttcttcgtttccctacctgacttgaacgaatgttatgagaagagcataagctttga
tatcactcaactgttacgaaagtttatactttgaatagatattaatagaatagtcgctgagaatttctcagaattagcaaagttcaatgtaccctcgt
tcattcgattaatcgacagacaaactccctcgtaacgagaaagctaaattgagttgcaaaatgcttgagccataattttagggtagcagcaccatc
ctaatagagcgccataaccaaacttcaagctcagttaccctttcccaaccatcttacctagtgtctcttgactaggcaaatgcttatcgagtactga
gaaactaaaaaggaaaaaaaataagaaaaggaaaatatatttaaagttttttttttggtaggaaatttttgtcatatttatttgttttcagttgctttt
ctctttttggtgaataggaatccttctaccagattttgaaggttcttttcgttttcattttggttctgtttttggttattttgcttgttgtcactctttaccctt
atccttccttactgctattcattcattctctttttgtacagctttttttctatctaaaataaaactaggatggctacacagccattgaacttttgctctaaa
tgctggaatgtaagttctcattttgaatgccaccacatttctgaaaaagttccatttggagggattagactaactccgctgcttacaggaagtaaa
aaacgattccacagttttcgtttttggaaaatactggcatgtaaactggtaagttcaaatcatttggccaggatatgctaataatttttagcttttcat

gtagctgttgtgataaaattctggattcatcatctgataaatatgtttttgaacaagggagtttgtattgcaacgattgtgtaaagctctgcaattac
tgtgaatctcctatccttgagattaccgtaatggcaggaggtcaaccttaccatccatcttgtttttcgtgctcaaattgtcataataatctggatagt
agcacgtatgcaagatccaaagggcatatgtattgtctaccctgtcttcgctatgttcagatcaatccccagtcatccactgaagacgttattcata
aattaacacgggttccttcgaatccttacgaaggtacgttcttagcagttcgcttttcttcattgtctggatattttactaactaattcacctcatcagg
tatctgtcatttggagaagactcctcaaaaaatttcaaatactatagagtcatcctcttcaccttctgaacaagtatactctcccagtggttttcgtcc
taagctttcaatccagacgtcatcgcctgaaaatataaatgcttccgttcctttacaaccaattcaagaggttttagataatcaggagtctaagcca
agaccctgtcaaccacaagtcttcgtttctcctgtcaatcctatcactccaggtgcccaaacgaataatatcctttctggatcttttgagtctgccaa
gtctggcttttccaatactatcgactctaaccttctagcttccgaatctctccatcctaatactcaaaactctattgagagagatatgcaaatccattc
tccaagaagcgagcagtttgtacatccgaagtttcaaaaagctgctcctttttattttcaatctcaatctgctaccaattctccttccaagcatggtgc
tcttaacagtcgtacagaaacgtcacctagcaaattaaggaccgcttttcgagacgtaaccaataatataaatggccagacctctaatgttcgga
atagcatttcgaacaatagagatctcagacaagagacttttggagaacaagattattcaccgacaagaggtcaatttcctcgatatccatccaga
ttgtcaaaaaagaacccttgcctttcaaatgacttaggcactcaaccttcacgtaatgcgataacaccttcctctttgactttttctccccgtgctaaa
agctttactttatctcaaggcaacgcaaaccatatgtttgagcgtagtcgaagacgcagttctttggttcgtgccagtgatgtattttcatccaacgt
ttttgagtttacagaagaacatgtcaaggatctagttgcggaagtatcatatcttcagacagaacggggcgctttattacaggaaatttcaacctta
gcatcgtttactgataccgtcccgtgtatgccagctgaattattggagcaagagttagtatcaaatttagtggaaagatttgataacctgtcgaaat
cattccagtcggaaataacatccttgctgttacgacgggatgccttgtcagctaccgttacaaaattgcaaaatgcatataacacagcaacagaa
gaaactgcctatctgaacgttaagaacactgaattagcgagcctaaataatcaattggagagagaactttcgactttaagagaatctgcccagag
taaaagaaaaacatcgtttgggttgtttggtagaaaatctggcaattcaagctattcaccttcgccttctcaatctccgcgtgaatcatcgtctaggt
tacagatggttgcctcgagccttgggttcagacctaaagataaggaaagtaataaagataatgaaggctttaaacgtaactcgaaacttgatctt
agaaaaagtctatcgaaaaaattccaatggaagagagaatcaagagttcctacaccttcaagtatcgttgaggagactggagtaggagaacag
gaagataacgttcctgaagctggcttttgtaaactgtgtggaaagtactcgacccaactgggaggtcattatcaggattgtctcagctccacaatt
gatcagcaatatcaaatacaaagaaacaatggtacgctcatttttgactacttcatattgttattaacctaactagcttcttctgcacctttagactct
gagcagtttgacagtgtaaatcaaacattgattaaagttccttctcttattccagcatgcataaatttcgttgagacttatggcttggattatgaagg
tctttatcggaaaagtggtgcaacgtcccaaatgaaaagaatatggacattgctgcaagaagaagacgttgctctccacccatcagatgacattt
ctgctgtgacttctgtactaaaacagtaccttcgaaatctcccaaatcctattataacctttgaagagtacttttcttttattttagcaggaagttgta
cctcgtttcaagaaaagcttgaagggtttagaggcgttattgaaagactaccttccgttcactcgacaatcctgcgcttgattataaaacatttgtcc
aaggttgctaagtatagctctgaaaatttgatgaattctaagaatttagctgtcgtattctctcccaccttagtgcgagatcccgcaaatgaacgtg
atgtcgttgacatgacaataaagaattatgctctttcctttttaatagattacgctgacgaagtctttccttgattacctcaagccggcaagacgatg
ttttattatgtcttccatgtcattcttttcctttttttttttttctggtctttaaaaacgtttacaaattacgatctttgccctgcttaactaatggcattgct
tctattcatttctgcccagatgcacgtcgttcgttatataatttgctttttcatacatataaggtttgaaagcttcactttttcaaatgaaaacagaata
tctttttggaaataacactactaaacaagtgtttacttatatgggagttccaaccaaataatatctcctactgataataccttctttaattaatttaaa
atgagcacaaaatctaatgatttcatttcttatttgtattctgttgactttttcctttagtttactccgtctttaattgtcttttttttatatggtttttatt
tatttttacaaatatattcagtagctaccaaagacaaagtataaatggaaacatggtttattttaataaactagttctgtgagtcttgactctttacc
gtctgcaggcagagaaaccacttctcggactcacaacattcaggacatacaggaaaacttattagttcgagctgaaagaagtattttaatgtttta
gtatttctaaaaaattcatatataactgtatgattccttaaaggtgcaagtagaatggcaaaagaattcagaaagtgtattttggggcccgtgaat
gcaggatataccctggcttattcctctttattaaaagaccaagttgaccgaccttttctgtttaacccgaaaccattccggagctacagagctcgta
actttttaaatatttacagagaaggttccaatgtcatcgatcccccagaaaaggtaggccgattagattataaagagacacataaaatgcctgca
attgattgcttactagtggattcttgtattacaaagacgcggaaaacaataaagatgatgaagtctttattacataaagagagctctgtcatattgc
taaatcaagatatctctttatggatggaatacagttctatatttgaagatccgctgaaacgacccaaactttatttagggaagttccagaatttttca
acgaacagaccgaaaaatattattcaaactagctctcttccttacctaagactgtgtgaaatgccaaccagcccaaacttcgactgggacatatc
aaaacttcaagaagtcagttgcttaccgccagaattggatcactttctaaatatggaacctcttggaaagacaaaacttcaaagtacaaagcaat
ttttgactgaacaggtctgtgacttaatggtattaatgacgaagagccttcccaatcctcaaatgaagaggaatgccatctatttatggctagacgt
aatatcgaagttaccattcttcccttacttagaaacgtctcttctaagctttgatgcattgtatcagtctacttgttttactcctacaacatatttgaat
gttcccccttagaagccaatatcatctcaaagtaccattgcatgaaaacactcttgaaattcgcaacctttcatgttaacgatccttcactccatac
ttttttgaaccatttgttaaatttctctgtccgtaactataattccctattccctttctcgacctctgaaccaccttctaatttgttttctatgatcgaaaa
aattccaatcccaaatgtttctgattgaggttatgttagtattcgttcgttttgcatgtctacgaaatctgtttttttcaacctctttcctccttgccaact
ttctgatatttcttccttgagaggagtactgatgattctactttctttattttaatcgaattaggacctgtctgaagctcattcagcaaattggggcgtc
ctttcaccaaatgccttttacattcttactcctcgagtaaatgacaagttgaacagaagccagcggaacccatctcctttctcgaatcttttaaaatg

aatgcattctttttatttttttctttttcctattgaataaacaaataattcgattcatacttggtatttattctttttacccaggtaacacagttacctggg
aagcataatgtcctttgcatgcaagggtgcagccaaactcattaaaaaaccaaacaagaaggatttttggggtaagaggctgtattagtacgttc
tttcgtctttttttattttgtttctctcttcttaaaactcaaaaaggtttcttgaaaagtatatatcgttcgttttcacgctgagaaaatgtctgaagcagc
aaagtttttttggtgccgtagaagaagaggacgatcgttttcaacatttttcaagtgaagaggaagacaatgatataatctctagagctacaattcg
ttcctccagaaatgacaaaagcagcggtgggagcgcttatagaggttttacagaaaatgtgtctgaagaggacgataatgaaagtgacgaaga
gaaaaatgaaagatacaatgctgaagacaatagcgatgtcgaactttacactggaggcgattttgatgaagagaatgataaagagaacgcaaa
agctatcaaatcagaaaagccactgaataagatttctttagaagaagtcgaaaagaaaaagaaagctactaaacgatcgggagttatttactta
tcgaaagtccctccttatatggcacccaataagcttcgccagcttttatcccagtatggtaaaattggtagaatataccttgctccggagtctgctca
aaaacgtgccaaacgtctgaaaggcggcggaaataagagaaccatgtacgaagagggctgggtggaatttgaaagcaagcgtgttgcgaaaa
ccgtcgctgaaatgttaaacaccgaaaaaaattggcggtaaaaaaactagttggtattatgatgacatttggaatatgaaataccttcctaaattta
aatggcatcacttaaccgaacagatcgccgcagaaaatgctgccagggccagccgattgaaggttgagattgaacagggccgtaagcaagtaa
aggagtacatgaagaacgttgaacgtgccaagatgattgagggtattcagaaaaagagaaaagagagaactgcttcttctgaagtcccttcctc
taataatgatactcccgttgagactggcccttccaacaaagaaatgcgccgtttcttcgatcaaaaaagtgttaccaataaacgcgttatgcctaa
aaacgaaaatgagcaaagaaaagttgaaaacgttctcaataaggttctttaatacttttacttttagaaaatctctaagcacagtttgaaagtttg
gttatcatatggacgtcagggtaataatagtttatttaatttttgcatttataaacgagtaaaaaaaaaaaaaatactattgtatcctttaaaggtctt
ataaacatctaatattatatttataccatttcaagtttgaatggatgttcatcattagtcataagcttgcatcatttctaacgttttcatcccttatagc
atgaaggtaattgtctgtggcattcatagcaagtcttaatattagtatttttcaagatagaaatgcaatcgctgcatacattggaatttccacatgca
cgacattggcttctccattccatcagagaaaatcgagtatcgcataaagaacaaagatcagttgtcgtatcacgttcaacatgtacgttggaggtg
gtatccggtaacatccaagcatgaacttgaccaagattatctccaatgaatataccacgagcttgaccgctacatgtaatgcacgtaatagtctgg
cgggtaggctttttgttacggccaaaaccacttttgtgttgaagttgacgaataagatgcaaacgccatttcggcttatcaaaaaagttattggatt
ccaacttcactttggactcaagcctttttttcccaaatgcgtacaataccatcctcgtggccagttaaaaataaatctttattcaaccactctgaatttg
caccagtataaaagcaagaagagcatatgtcctcatttaaattcgaattgtaaattcgactaagagtatcctttacaaggactttgccgtttatatc
tataatacagcagcaatgaccagctgaaaaagcaatttccccattccttgcattgacggatatggtctgtatcgattgcctgaagacacgtactga
tcggacaaattctgcacggttcaagtcccaaaccattaaatgaccagtttcatcaccgctaacaatgatactgaacgctttacaaacagctacgga
agtaatatttgccctatggccatataaaactttttttgaagataatcgtacaggtttcgagtccgataattgccatagacgaagggttaaatcagaa
gatgcagtcaaaaaagtcctttcatcacatactaacagatgtgtgattttatctgaatgcaactcttcaaagacaactaatttagaatcggtctgct
ccattaccaattctacatcactacttacaccattccatattaaattggaataagacgacaaagggacacaatcagcagaaaaggcacgaagagt
atctttcgtagggtcatacaggatatgagcaatttttttcgatattattattataggtgaaacgctttgaaataataatcccatgttattttcaaaacg
gccaaataagtaagggcccaaaggagtgtctgtaaaatccggcccgcgaggaggatgtggctttttgaaaacctgcttgggagtttgcccaaagt
tgtgaataattcccactgcagccgccaattcaaactcattttcgatattttcaaggtcgattgatccttgatatgaaagattatggaaaacgtttgtc
gattcaactgcttcatcaccccgctgcttataaccgaacacaagatcaatccattcatgaaggtgtgaactcacgtacttgctttcaagagcttcac
gattttttggacaaaaatagctggatcaccttttgcccatggtggcagaatgacatcgcttattggttttgtttcattctgccttgctccaaagtcaa
agccgttactgttgacaaacatttcgctaagataaaagaactcaggaattaactctctaacatctgccatattttccttagacgaagaattccaagt
ttgctcaattgagtagaataacctatctgcatggtcaaattgtcctccttgtagagcgaggtatgaatcaacaaatggtcttaagcgtatcaaatag
gagcagacaatcatagcagacgaataatgagtaccatagtgaaatggcttttgttgcaggtcacctaagcttaataataattcgtaacgctcttta
aactgagcttctctgggagggtattgtgcacccattggtttcgataagtctctaaaagtcctaggattgcttaaatcgagttcttcactagtataatc
agcaatcacccaaggaaaaaccggatactgggttaagtcattatacgtgcgacccgcaagcgtgttcactatctgcaaataattaaagtttgaga
tttcgtgtctttcccatcttttggtagccggatgtccactgttaaaagatagaacatttgctagccttgacccaataaagctcgttttgttagtagcttt
tccaagatcacgactcatcgatgtagccaaatgactagttgaaagaacatccgcaccatgggcctttgctattagcttttgataaagtgcatctcg
gctcttagctgtagaaagtatagtcaaaaaacttctcccatccttaaaaaaaagctcaattcctacatctcgtaacaagtaaaagcgtttgagcac
taaagataagtcagaaaaattccaataccagctattttctctagaaacagtaggaagcgtattcctcgaagagtttattgattgcatattaagaag
ctgtaggtacggatctttttcatcagtcacgctgctatcgtttatatcgacgatttcattatctgatcgcaagaaaaaattatccatcaaataaagat
attgttttcctaatagcaaaataccttccatagcttctaaaccaaaaatcctagaaacattatatgcgtcttgtatagtatctcccggaacaatgga
gcgcataacggttctatttttatcttctttatcctcttcatcctcattgtcttcatcatccttaccaaaggttggttcattagtagtatcagcctccgaat
catgtaaagtatcttcattaaatggaaccattagcgtaatatcctctttcacttgtgtgcgaaggggaatttcagagctcacagaagaatcaaagc
tccgatttcctgcagttgacgacgtttgtcgttctaattcctgttttggattcatttcttttggaggaaggatacatggtctcaaacgattcctcatcctt
tctgatccttcagtagaatccagttcccataaatcgggacttgaagaaagaatcgagttttcacccgccaattcactctgactataaaatacagct
gacattacaaagttctcttgatctgattgatcctggaatagcttccgaaatcgggtgcattccaacgaatacagacttgaaatccaagcctcataa

gaatgcgacatttcattaatgagattttgttcgctggaagctattgtcaaacttttcttcaaaaattctaatcgtgttaattttaaagatgacagttcg
tcttcagcagccttatgttcttgctttataaaatcttcccaatgaaaagagaagcacgtttgcataaatttatcggcttcagccgtattttcttgtagc
caagaaagaaactggtcagactgtaatcctgtaatggtttgcacattttgactaaacgagaattcggttctgaaacttttgttcacttgatctaaaa
agtatggagattggatatatagcaaacgccaaacgtcgacagcgcttaaacgtgcccttgcgttgccgaattttagtactatatacatactgtacc
aaagaaatgggaaaagttctggttgataacttttagatacaagtaaaggtgtttgccagtagagtattttcttcataacatgctccatggcagctcc
atcattcctttctttcgttcgagcaagctctaatagaatcaaccttaaaaagcacccgtataaattactaatagtatactttgcgtttttagggaaaa
actccaaatggttctctatagaatcaagtttttcaagcaaatacccaatttcatcaatagtgcttaaacaggattcttcaataaaaccaacaagtct
taaccttaagtactttgtaaacatgtagctgaaattaattaacacatggggattcgttaaaaacatcttatcgtcctttactgactcaatgagctgtt
gaaggatcaaatgaaaaacaacccgaacgaatgaagatttttcgcttttgtactagagggcaaagaaactattaacttggagaggcttaacccc
tgacccgcgacgattcgatcataagcgttagtcaaaaaggaaataaaaagaccattaatcagtttctttgagttagatccatctataacagtatttt
cgaagaacgcagtagtatcgaattttggtgacaaactaggctgtagattactttttgtagctgttcatttaacagttcctggcggacacgattggtt
aaaggggttactacccgtctgttagatccacttactgacattttcctaactgatcttcttatgccctgactgaacgctttcgaagatgagctacgagg
cagtgggtgacgaacatgctccatcaattcacgaatttcttgttctggaactatatcttctttaggagagaaaaaaggaaacaccattcgaattat
gtgttcattcaaatcaaagtttagacttatattaagagaataagaaatctgagcatcagtcaagagaagtaatgtttgctgagcgtaatacaaag
attcgttcatttgcacatccgcttcagaaagacttaacttctttagcaccatataaacagaatccagtaatatcactatcatctctgaaataggaaa
aattcgatctttgtaattcaagagcatgaaattaagcttttcttcaaatgtgcaatcacttttgaattcaattccccgaacaccaagacctaatgcta
aaaggattaagttcaaccacacctcttcgatatgaatttggttaggcaagctacttttcaaaattaaaaatccgtgaaatttctgtgagaattttga
aataaagctggagcccaaactacacacggactgatttagcaatcttaaaccaaataaaaaataatgactatcaggttgccttataagaaatagt
aaccatgctggagtaatcatagaagagaaacgccgaagaaaagatggttcttgatcactctcaattaaaagatttacaaaaacctcacaaaccg
catggcccgattcctttatatccaaatactcacctacctcctcaggtcttttaaagtttggagaagcgtttgaaagtcggtttgttcgagaacttcttt
tcgtagcaaaagctgcatatatgatgtacgttacaatcgttcgaacattatctgaaacaaaagaagcagttaatagaattttcagaacaactttta
gaaggggaagaacatccaaacgaaataatcgacctctcattgcagcaagcaactttttttaaaacttgcatcctcattaaccgttttatattcaaag
cggaatactcattgttctcaatgagtgaaacaatatagccaagttgctgaatcactaaatgattatttttgctttagaccaaaggtcaaaatcaag
catgagataacgaaagagaaggggattaataatgaagggatgtttaaaacgttcatgatgaataccggctttggacgaaattgatcggagtgttt
cctcggaaattaatagatgtgactttgaacgaaggagtttagctaaaatttcgaaagaatgaccttgttcaacttgttccgaaaagcgccaattgt
cttcgatcgctgaaagaaaaaacaacaacccaaaattgagctcgtcctctgtttcagctatggaaactagcttcaatagtatactagaaccacca
acgttatatagagcatcgctgaaagatggcataacgacaggagtaacagaacctgatgaaactccaaaactaggctgatctatcgaaaagattt
ggttatatgatggaactgctctgtttatatacacataggagtaaaactgatcttgcaagttcttcagtgtatccaaaacgctaaatgaactgaagtc
agataaccgtgggtcacttatattgatatagtttttagcacaagcggtgaaagctatggaagaagacgagatacttttaatatttctcagaacatct
tctgcattcaaattcgatgcttggacttctaaacttaattcggcagatgctttataggttaaaaactcatttatggaaccttgaaaagaaccagtat
aattggggcccaaaccgtatataatagcaatctgagaagaaagtaatggttccgtgaaaaatctagtcgttccgatcattaatggtaataaatta
gcagacgaatcattcgtctccctgttcttttcagatatatcactttttagagaaccacctttccgctgattgttatctgcagacgtttgaaataagcctt
tggaacccatagaaggacctagagaaatataaagtggttgaagtaaagattgaatgaaaggatatgaacagggcgcacgttcaacaaatgttc
catcaatgtacaaatcaagcatagaagacaacttttcgtttctatgaacgagagttatcaaacgccaagtaggtgttttaacactgttaaaaatttt
tgaaaaagatctcgtcaaaggagtacagtaattgataatacaagcgtctttgcttgtggcatatgaaccgaaatggacgctgattgaatttgtaga
tggatcaaccattacacgaagcaaaggagatttcaaaaaatcggtgaccaaaaatattgtcgttggaacaaacttcttatttatattaagagtttc
catcaagcatccagagtgtaaccataaagaaatcgaatatccattgtcaggagttacgggggtgcttaaagtgaaatcaatggaggagtaacct
ttgacaagagggttgaaataaagagccggagatactggctttgacaaagcttcggcgagccagtatcgtgctaaaggagactttccattgatata
aatatttaggatctttgcagtattattgtttgacagcccataactcgcacaattcaatgtaatatcataaagcaagtccttcaaaggttcagtaata
gtataattgtttggaggatcaaataaataaatcaagagataatccattacttttgaggaagatattttaatagcattttccggagaggatttgacca
atttctgcagatgatgtaaaagcaagtttagaatggatatcgaggggcataagagaattacttggggtaaaacatccgcaaaagccagatacttt
tctgcggcattttggaggcacaattctactgaatttataagttcggacgacgaatagtttgatttgcaagtaaaaaacgaagtacctttctctgatg
aaaggttactgtcattcacagaaaatgctaatattgtcagcataagtctgcactttaggtaagtagtggaagggtttttacactggaatctgggga
attaaagtcatcatctcttgaactagctgtcaaactgcttttgtccgacccattagaaatatctaatcgttttagctcatttgtggaagaatcggaac
tcaaagcagatatagcaggcccttcaatactttcttcgtaagataaaagtagtctttggacagctaactcaatccaagtatctatatgttttcttgtc
aacagccatgaacgtaaaatcgtatatgaggtagcgatgctcaaaatttgtaaacagcattcaaaaacttctaagctttggaatgaatgaaacgt
caattttgtcaaagcctttttattgcggtaaaaccatgatctcgtatgaacagttgcggaaaaaaatgaactagagatctccgatttcattaaataa
tgaaactctttcaactgttttaacaatgtagcctctgaatcgcatcctataataattgaaatcaattcaacacacttcgaatatgtaagggtcttttc

agtcatgacagttaaggtcttacaagacaaaaaagcaaattaataatttaagaaataataattgcaaaggtcaaaggttcaacatgcaacctct
attgaaagcgagtcaatgcttgtcttatatattgagatcatatgacccgatattcatgaaattatttgaaaattgtaaaagttatttccttaaatggtt
tattgtaacgttactgctttctcaaaccgtccttttcgcttttaatcgatatgtttagttaagactgtgttgtaggagagtaagagagctatatatgag
ttcctattatccttcaacgaaaaagaccttcaatactaaacttttaataatatacaattaatctgtattatagaatactatgagttatagcttacttat
gaacactattagtgtatagaagaaaaaggtatttagcaatgacaaaatcattttacctgaatggtgaatacttgctttgactttatgcgataagaat
tgacttcacaggacaaaataaactgaaacataaattgaaaaacgataagcgcatctaccttggaaagtacaaacttgaattatttaataaataa
catagaactggaagccgctacagtaatcaaagcattaaaatagagcaacctattcatgggtagctttcaattaggaaactattaggcaaaccact
catatcgtatatattctcaatgtaaggacaataaaaaatgtcaaaacgaaacgtacacaatgggaacctaccctttaccagaagaaaaatttaa
gttacaaaatcaaaggctaggttatccgttcagattatccttcatcaattcataataccctagattcgtgaaaaaaatagaaccgcatttcgttact
caccggctcaatactaaacggagaattcataaagaactatgaagtatccaaggtgcatccattcagcccaggttaagtaagaaataagagaaac
ccaattctaagcaaaaattgatggcagtaaaatcaatgagtcattcgaatagaaaggaaaaaagcatatgagaacacatgataactaaccaca
gaattgctggatgaaaataagtcattgcttctaattaaaaacaaacagcacacataaacggtgatcaaaggagacattggatgggataagatag
aaatgaggtaaaagaataagtaattcggctaaaattattaaatttagttgggtctttgaatatactgagcggtgggtgtaggcatatcactgatta
caggagcggtagttttcacttcgcctttgtcatcaaatgactgtttttgatagtttttgaaggatgaacgttcaggcggcgacgtaacctgcgctgat
gcggccaattgatgttgcagctgctgttgattttgctgatcctgctgagattttctcttcaaagtccaatcaaacatatagtcaaactcgtaattctgt
ctgcaaaacaggtctcggaagagtttgcgaagataagcataatctggcttgtcgtcgaaacgaagagagcgagtataatttaaataaatggcaa
attcctgaggaaaaccacggcataaagcctcagtaggagtagagattttcttttccatgatcttttcgtacttttgtttctttgttgcagccttcaacc
cttgccaaggaagacttccgcggcataagtaaaccaatacgtagcccaatgactccaaatcatcacggcgcgattgttcaatacccaagtgagtg
ttaatacttgcataacgagcagtaccggtaaggttcttgttttcacggtaaggaatatgaagatgggttttgtggtcccgatacttctttgccaaacc
aaaatcaattacattaacctgattaccgcgttttccaatacccatcaaaaagttatcaggcttaatatcacgatgcaagaacgacttggaatgaat
aaattcgattcgtgatatcaactggtcagcaagtagaagaactgtcttcaaagagaactttctactacaaaaattaaacaaatcttccatcgatgg
tcctagaagatccataaccatagcattatagtcacattctattccaaaccaacggacaaaggggattcctactccacctgacaaaatgcggtaaa
ctcgatattcgtattccagttgaggatgtttggcccttgtagactctaacttgattgctacttcctcaccagaaacaatgtttgttcctagataaatat
caccaaaggagccacttccaattttacgtccaatgcgatatttgtttccaattcggaggtccaacgccatcttggaaacgatatcgtatgagtttttt
tttttatatcagagagattgaacctccactactttcagtgtattcgatccagctaaaaaaaaatgttggtgaaggaaggagtcagatttgcgttgttt
gcacagactagtaaattccaccgtaattacacaaactgtagtttaacgtagtcaagtgagtcaaggtccttatcgtagtaaacagtttcataggta
aataaataaaaacaatattcacttattttaaacaatacattttacatttattaatgatgagaatgaaataaacattatggaatttttttttctttttttct
ttttagtatagaagaggggagtaacagaacctcatttttaacaattttctaagaaaacgaacgtaataaaaaatacagaaccatgcaggaaaga
tagtccaagagaaaacagagtaaattaatggcatttattagaatatggagtccattgacactgtatttgggaaaatcaagtttcaaccataacaat
aaactcatcaaagtgaaagacacgtagaagtaaatgtataaggtccatcgtctgagcaaaaaaaaaaaaaaaaaattaacaaataacagcttg
aaaagtaaagagtgagataggaaaagaatgtgtcactgaaacgaagaaaacaaagagaaatgaacgaaagaagattaaatacaagataaat
atatggataaaaagagcgaaaaactggccaaacgattgaggcggccgctatagccaaaagttgtgtacaatcaaagatcgaagaaaggaattt
tttgttttaatggtgatccaacgatgggttcgtaagccaaatatttcttcttggcagattccagttgttttcgttgacggggagataatttggataattt
ggatggactaaaaggagaatccattatccaagtaccggtttcctgaaagcgaaaaacaccatcccttacacttaatctttttctcgtggaagccac
attatgagaagttgcatttaagtgagacatggacttttccttgaccaagcgaacattttcagcaaacgatggattcaaaacatattcaattcctccc
ggtttgttggagctagttttcggattgtgacgagacagtacatctagattctgtaaagaagggtccgattccttttcatcctagatatttgggatctt
gcaggcatagctttggaagacgtcaaaatatttcttggtatcgaatgcttgaaattcatgcggaaggaagactcgtcaacccatttttcgttgaat
aacaggccaggacccattctggcgagacgaaatttattgtgctttccgaggatacggtatcgagcactaaatttagcgtactgtttttggcagga
ccatgtgagtcgttggtttttgtttactaaattgtggtttctgatgaatggtggccccaagttcgaccaattgccgaaccagacaattatgatccaac
aaacttttctcaattctcacacagacagaagcagaagacaatggcccttggtccttaatctttgagactacctgcgtggatactgacatatcatgta
ttgatttcgacgacaacgagagagacacagtcggcttccaaaaatcagcataagcgtcttccagttgttcttcagtaatcgcagaaaaatagctat
cttttggtggagaatcacgacctataggaaaaagatacttggaagatgcaaagggaattgaaggatgagtaactttgttagacgcacgagatgc
atcaccgaatggtttcgctggaaaactagcaccgtcaagactgtttacgcgctcatgtcttctatgagaggtatttacgtctggcacagtaagtagt
tgtttggaaatgggctccgtgttgaatttcagggattgtaaagaatgcaagctatgacgacgataatgtccctttctatgcattcgcggattcggca
aaacatcttgttttgattccagcgatagcattaagccccctagtaaaacacaagacccatgtaggagttagtgttgggattgctaaagaaaccttttt
ttttcccgggtactacaagcaaaaaccatgagatgtgatataacgtgccttcctaaatcccgttatttataatatggcgaaacttagaattagctta
aaataatataagattaataaaggaaacagcactattacgggctattctaaatatggctgttatcgccgtctcgttaaatacttgtcaaacttcacaa
gagctcattaaaatgatttaaggaaaaagtattcgttgcagacggaatatggaaagccaatgcgaatgcaaaaggagaggaatttggtaagag

aagagggaaaaaacaaaggtataagataatttttaattacggaagaatcaagaatggacagaggaactaaggaaaaggatcggtagagaac
gcctacctccagcaccctaggtatacgataactcctgcaacagcgatgaagaagggggaaaggaagaaagctcgattattgtaccagaagcctc
gtttcgtagaaagctttgcttcagctgtaaaagcacgcgctttccaagactctaaattcgctttggccaattgagcttccctgatggcagcatctgta
gtatgcttatgggacaatggaagaggagaagaagtactagtggacttgtttggatcttcaaaagaagaaggagaaagtaaatcttgatctggtg
acgatgtggataaggatccacgagtgacagatgaagactcgccagacttgatgctatcagaggttcgaagagaagactttctgtctaaataaat
agaattattgatgaaaccagcagtcgttgacatacctcggacaccatgctctaaactttctaaattttcattcaattctacacatctctggtactgaa
ttgccatttgacgcatgatcgagtccaacatgacttctgtctctagaggtcgaccagggcccattgaagcaggaactccacgtgttagtattcgaat
ttgtgcagatactttttatgaatgatttcttcttcattgtagatatcgacaccaaaatccaaaacgtctcctgagttcaatttgcaaggtgcagaag
ctttgttttcagaagacagtcgatttccattaacaaaagttccattggaagactttacatctcgaacgtaaaccacaagggtgtctttttccagccat
atttctgcgtgttgtcggctaagcaccttggaattaaagtataggttggatgatgctggagcggtcgatttattcgtatgtcttcctactttgtaagta
acagatggtgagagcactagtttttttcgtttgaaaagaatcgttcaatggagtcagtgtaattacggcagacatctcctataccataaaaaccctttt
aaaacgatgcagttgctctgtaattttgcaaaaaaacaaatgaaattagaatgtaaatataaaaatgggaagcaagaaaacgaacgaccaaac
tttgaagattcccaggattgtatgttgtcaattccttttctgtattgaaagtgtaaagaggaaggaaatgttttcgttccataaaacgttcacaacaa
tatctatgtattatttagaagaattcttgttacatttggaatcaatagtaaaaggacttttcatcagaaaatactgtcctgatcgttcaccccagatg
gattcagtagaatcggcttgctgttacagatgtagtttggagttggtttttattatcttcatatagtattcgcaacataaaagttataaactcataata
ttgaactcttctgttcgtatatacataatcagattctatgaaaccccattcaacaacatggaaacatcaaagttatttaaaaaggatgaagtcgtcg
caaaaaaaaatagttaataaaaaagaacaatatattacagaaacaaggaagcgagcgttacaaccaaagtttcgacttcgattgtgcaagatt
gcttgcatctcgattggcaatttatgccgatccattaagatcaaccgaagactcaacaacttttagtctgtaggaaatatcatttactttcttttgcaa
agcagaaagcttcgcagttacggcaagtaaaataggatcttgcgaaaacgcatcgattcgttgagaagtatatggcaatatcttacttgtatcaa
gactgctagatttcaaagagtcaaggattgaatttgagccaaacaatcggaacaggtgaagacatacagaagatgttgaaacgctgaaactca
gcgcaaggtttggtggtaatggaggagtaaataggttattagcggctgttttatgcacatgtgatggtggatgagaataggaagaggatgggttg
ctaaaagtcgacggtaatgactgggaagaggaaggctgcatggagatgttgctcgcttgtgacactaagtctggcacagatgacttagactctgc
ttcttccgtattcaataagaaaacaatggctcgtaaaatttcctctagttgttgtattgcgcggccggtgctttctttgattcgcagtaaaacgtacag
caagtatcgcaggtagtttacaagatttagtatttggtctaatcgtaaagtatttggttcttcaagcttgatcatatgagtcgaccttcccacttttatt
gtaagattgatacggtcaattaaagttccttttctagagacaattcccttcacggcatctgtctttggcgatgagaatacaaggactgtagggctttc
tccttcaaccaattccaatgcctctttcacttcattaatcacagagtcccaaaatcccggtaatacattgtgttcaaaccactctttttctttgtgaact
acaaaaacgtttacatctgtttccatcgctttacttacagcaattgagaaatttagtatcccactgtatcagctaaagggtttaaaaatatcgaaga
tgtgtttttttgggatgctcacgaaagttgctgtgttagaatgacgataactgagctcaacagatcgaaggatccacaattttcataacttactgttta
ttttagaaatgcagaaatcattttgacagttactcatttggaaaacttattttgtaggttactttatcataaacgtaatatgaaaatgaagtttgaag
attacttacaacgagagaagtatcagaattaaccttaataaaaattaatttagagagtcttttgtctacatttttttcccaaaccccttgaagaaatgt
tcgattcatactaagatttcgtttacgattgtcgaccgttcggttaactcgatccagcgagattaaacgaagaccagcattctccatccattgattaa
gttcttgttcaacgaaaaaatatgatattgtcccatcctgacgaacaaaggttcgctcatctatgagagaaggatcggagctttgttgaaacttctc
ctgagcaaagtctccatcacagtaatcgcgaaataccagatgtcctttgggattcaatactgatgacagattatgaatagcattctgttgcttttca
cgaggaagcgctgaaagtacaaaaatggctgtcacagtatccacaaaaccaggtgagacccattgttccagccgatcttctgttatatcgcatac
gaaaggaacaactttggattcattaaattgctcatgctttcttacaaactcaatggcacgggggctaaaatcacaacaataaacctttaaatttgg
ttgtacctccagcaatggataaaccaaatttccaactccacagcctacttccaatagtctcaatggatccttgttaggtaatccaaagtatttatca
aattctctatccagccaatgtcgatctcgaaaaaatcgagtctcatttcgtttataaaatttatcccatgatttttttggattcccgtttgtatttttcagc
ccaaaaaggactcaatgtatttttcgattccataaaattcaaaaactatggattctcaaatgtagctcgacaaagaagttcaatatcagcaaaag
cgtctaactcgattattccttactttccagataatatatacttttcaaatacttctttctaaaagtaaaaaagacaaaaatgactcaaaagctgcaa
aacttttcgaataagagaagtttgctcattactcgcagtacttagttcaccgactaccaccaaaaataaaggtcaagctcaaatagatgaatcga
acctcagaaagcgtggaacctactccttcagataaaaatagtacccattggagtcgtgaatgggtgcctgtagtcgtagaaacctcctctgctgaa
aacgatgtggacaatgaagatgacgataattcaaagttacaacgggtctttatggtaaaacgatgggttcccaaaaatcaacaaaataatgaga
aaaaagacagtagtccttcaaaaggtcaatgagcaatctttcatatatcttacagcaaaaagctctagagttgcaaggtcacttgacctttaggat
gaacaatggcatgtgtttcagtaacctcatatagaacctctttgtcatcatcctcaggtagttcattcgcgcttcgtttcaaaaagtggttaggtcctt
ttgtaaccctcatgaatccagtaattcgacgagcatttccacttgttaatggtcgacacgaaatgcatctggtagctagatgacctatacttttagca
aaggatatttggcgaggtaggggtgctccaaccgaaacgttgatcacagcccgacctgatgtcatttgcaactcaaagacagcttcttgtaattga
gttgcctgtagagaattcgttgataaaagtacatcaatatcttcaattataagaatagaattgtttgtattaaactcattttgctgtaagcaggctat
aataggcgcaaacaactgtttgggattgtttttcacctcgttggcctgaactttacttggaataacagcaggagcaaacaacatgctatatccatc

aacatagaaaaaattcggcttcgtccttacgtccgttccctatgcctaaggttagtatgacaaattttttgctactcaaaattacttgcccattttcga
agagcattggtatgttcatctattgttttactaaatgaaacaaagcaaactttcagatgttcacgaatgcaatgatgaagatagtagtgaatcaaa
aaggttactggtgtttctcttgttcctaccagtaacgtcaaggaaaaaggctcaggaattggtggaatgtgctgcgcaatcgaagtcatatgagct
cgtctttgtttttcttagctattcgtatcaagtactaaatttccactagccgagaggaaatatacagtgagtggtagatggcagtaatgtaggtagta
agtagtacatgactaggattcagtcaaaagtccagggcaagaaaatgtataagaatttaagtagagaagaaataaacatttgaataaaaaaaa
aaagaaagagaagtcgtcgttcttagatgatgacgactgggtagaaataagtcttgctaatactccaggctcgggaaggacaattttgaaaaag
tttatttgaagtgttctaagatacagacgaaaaagcactggaatgcagtttcttgagcttcaaaaaatagcagtgtcggaaattctatataaaata
aaggaatgagagaaagaaaaagaagagatctaaataggaacctttggactcaagtcagacacaatcctttctttttgtttaaaaggtagcttaa
acttataccatttcttttcgacgagggcgtacataactaacgtttttgggtctacacaaaactgtcaaaaagttgatccatacgctgttatgataag
gccgaaagtcttctgtttcagtatttttagaacgcaaaaattcgtgaacattttggccgcagccgatcaagtagcactggtaacccaaaagaattc
ctggaaagatggatcccaatgcagcatatattccaagaaaaaaagacacgccggcccacggtctttgtaaatgtctggcaaaatttacatcaga
gccataccgaaagctggcaaatgcaatataaaaccccaagaccgtcaggtacaaagcttcgaaaaacaaagaaagcaaaaagataaagtagt
atcgatagtttcttcttccaatgcagttattaagccaggtacaatggtggtcgaggtattcaacacaattgtcacacaaatggcagtgactagcac
gaggcgggcgatagagacggcatgtatggcaataaactgttgtaatcaaaaaagctgaatcagaacgggtagatccgactagaattcttttatct
tctggaatcaccgaccaaggatgcgtaggatcataaattaaaatgttggagtttcgaggaagaattccagggtcagaagtagagcacttcagcat
agaaaccaatactaatgcatagatgtaggaaaatgttataggaatagctggtgacacatgatgccataaccaaaaggcactaaaaacgaaaaa
cagcacacctggaaaaagcaaggcaaacagtgtaagtgcaaatgctttaaattgcgtagacatttggaatctccctcctaagaaaaagacatta
ttgcctggaaagtgcttgtaacgtctctcaatttgatgcacatctgatccatgcggcgtctgtttagtaacattttgttgattttcagtcttttcatcgta
tacaatgggattagtaggatctgaaagacctgaaaaccccggattccagggttgtttccatgggtatctcaaggcagtctcttttgggagatgatg
aggactcatacttggaatttagtccatacaaatcattgtaaaacagagtttgtttaaaattgtgagaagagcggtactctttgtgtgagtagtgttct
attgccgcgagtatggcgactcgagttggtagtaacttcaaaacactaaaatgcaaacaggcaagtatgtttgtttacactgaatgtggaataaa
ataaacgaaaaactaagaaatggggatgcttacatttaaaaatgaacagcggcaaacgctgaatgaattataggaaatgatgatacgctaaga
atgtttcgaggatgcatagtgtaaatacaagggaaaaacgacgctcataattgtgagactctggacgctgtttttttaattccattttgagcgcatga
aagcgccagcgtctctctacatacatgattcttgcaaaatagggtttcttgtttgctgcaatggatatagggatgaaatgggacgaagtaagtcgt
gcaactatgaccgacaagaatcaaaggtttttttatctacagataggaaattgaggctatattcgttggctgaggtatactagagtatcttctgttca
tatcatgtttggtctcagagaaattcacattctctcaattattaccatacaagacctccaagttagatctgcatcgtttctctattgatttttctattga
aaacacactcgctcaactgcctgtttatatttactatttacatctcttatgagtatacggaaaatggcaaaagtcgttgttaccattaactttatattt
ctataaaagaaagcactagttctttaagcgaaagaacttggttcgttaattatgaattgcattagaaatccattcgtctatgcgaaaagtacttgtt
gctgtccataaggattgcatcagagaataaagacgccattgcatatcataaagacatatacctggattggacgaatgtattcaaaaagatttgttt
ttattttcggtactacctatacgtcttgaatgcgtaagtggcaatcatagtgacacttgcaatacttgatgtctctttcatcattcttgattcaaaagc
aagtcaacagctcaatttgtgttgttgaattcagaaaagatatagtgtgagtgtgttgaattttgaacttgaacttgaaattggtttccagttatatc
caaagtttacatttaccaattgttacattcccgtcgacaacttttgaagttaattaccaagaaaatccagtacaatggctactatattaagaagcat
tatgtcgaatcgatacgtccgatggacaaaagaataccctgaattatttgtatgtactgtgtggtttgcaagtaataatcttaataagagaataatt
aaattagattgtgtcaaaaggattatgaaactaaatcatgatttctagattacctggtgtgtcatgacatatacctttggagttgctggctacatgct
tggtatgtctcaaaaggtgaaacctttgttaattgtttctcatgaaatgtgatttgttaataactttacaaaggacaacgaggattgttgactgaaca
tgataaacaagttcgcattccgcgcgataacgcacatccttgggaggacccaaatcaagatcaaggaagtctcctttacagttacaagtattttcc
tagaggagacagatccaaggaaccaaagaagtcaccagctccaatccaatatagtacggtgactaccaaggggattcctcaagaacttattgaa
cggttttccaagtagaatgggacggttatgcatgctcgtggtttatgactttttcataagtgatcttaggttatatttttatttatgagtttctttatttc
acatcgaaatctcttttatgaattaaaatgtaataaattgttttgaagcgcgattgaacttgcttcttttttaatgcaaatctttttttgttttatgttg
taaccgtaaatgggatatacgactttttttctatattgtgaaactgtttcatacctagtaacgtgcatcaagcttctttgcaatagtaatatatctgct
taagaaacattgtaaactaatcatttaatttccaaattacttcgaaggaaactactctttattacatgaacaatccttgagtgcatcgtattgcgtct
gtcgtctttttattttcacttcctattaccactttaaccagaatttagggacaacatatataggcatgtcgcaagcggtcgtagtcctttcatcttctga
accaaaagctgtagaagctgttttccgtctttttgaatctccacaattacatgaacttggtgatggatggaagcaaatctctggaaaacttttaagc
tcgtttgaagacgcgaaaacagaatgcgaccatatctctagtgaagaaaaagtagactgcaattgcttaaaatatgaagcgttttctaccaagaa
gaagttggtagtttttgatatggactcaactttgattcaacaagagtgcattgatgagattgcagccgaagctggcattcaaaaagaggtctccac
cattacttctcttgcaatgaatggtgaaattgattttcaggaatctcttcgtcgtcgtgtatccctactggagggattgagtgtcgatgttattgagaa
gatcattgcaaagattacatttacaccaggagcaaaggaattgtgtcgcagcttgaaaaaattgggtgctacacttgttgttgctagcggtggattt
atccctatggcaaattatgtcaaggagcagctcggtttggattatgcttacgctaatatggtttgttattttccatactatatctcttttactaacttga

cttagttggaattcagtcatgacggaaaatttttgactggtaaagtacaaggtgccattgtggatggacagcgcaaggcggaaattctgcgtgaa
aagaagattgaacttggattggatgtatttgagacaatggctgttggagatggcgccaatgacttgatcatgatgggagaatccggcttaggggtt
gcatacaatgccaagcccaaagtacaacttatggtacgttccattcttcagtttcatatatttttaagtactaatgtgtttaaaggcttcttccaaaat
taatcagacttctttacaaagtgttttgtatttattggggataaacaaggctgaacaagagcgctttttagcatgagcattgaaaacgttaattgat
gtagataacttgctcatttaggcaacaatttcggatgaaggacgatttttttgaaattgggttcatatgcgcttgatctatctatagaagttttctttc
aagtgtttttctagatatcccaactttttttctttttcattcttttatagagtttttctgttagttttggaatccttttcattatacatttgtgatgaagcgtc
gatagtattttgttttcagcattcatcaatgaaaaaatcatcctaatttcacaaacctttccaagatcatattcgatcgatagaaaattcattcatttt
attaaaattgattgcatgttgattgcatagaaacacctctttaccactgtccatcactttgaaacgaaggctgttatgttgagcactccattgctacg
attatcaccagatattttaattgaaattggtagctatcttgatgctgaagaccttattcatctaattaagacatgtaaacggcttcataatgtgtttga
gaacaaaacagtttggctaagattatgtaaacgaattgaaggattagtgaacgttgacatgactgaagatgcaaattataaaagactatatctaa
gatggaagaagaaaggatatgtttatacttggggagagaaaagctggaatcgtttgggacgattgataaataatatacattttcctcatcacaag
cctattgctttaacaggttcatttgcggtgaaagctattcaagtagcagtcgggtatgtctctacttttatgtgaatcataactgtggtttgtatgtaa
tgctaacaaacaatagtggctacggaatatttttgctggacgagaacggaaagttgcgcatcactggaaaacggacgaattcattttcacaagtc
gtaccggagttggatcgcgtttttttggattcatgctggtcgggaatactgtttagcgatgactgatgggggtaatttatggcaggtaggcgtgaact
ctacattgtgtttgacagaggaaaacgttttgtcggagtatagaggaaagattacgatttttcgaagtggttgggactctctttcagcattcgtcccc
caagtaggcttcttactatggaaaactggaaaagaatttgagcctatgagatatgaaatttttcggggacgagaactgtttgatgtttcagattatg
ttctttgtgctggatttattatctttacgacaaaagaagaatcgacggttttccggtttgatctcgatgggaattatgaacagcaagcaaaagaact
gacgaagtttcgaattcctattcaggatggtccatggaaacttttcgggagcttttatacctttagttgtatctctaatgatggtaacacggtataca
tggggaatgaatctacgaaagaatctgacgccaagccaattgttcacgactttttacaaaatcaggggttaaagcagctggctcatggagattat
catcatctcattttaaaaaatgatggaacggtttggtcctggggaatggaattgtcgaattctggttgtctgggattaggaacattgcataaccaaa
taggggacgacgtcttaatagacgtacatggtcgtcgagtgacggtgctgaagcctcgaaggatatttctcaatgcaacttgtacttcaatcgctg
cgggcggctggcaaagtgcgatgatggcgatttccgataaggtgttaccggatgcaatggagcctgcaaggatgaccagcttatccttgactcac
ccaagcgttcctgtgttgcattttctccgtagcagacgaaatgactagacgcatgtacaatcttttataaacgcgtatgtcttttattacataatgatt
tacgagcgtatgagaatgaaaagtttgacacgtttatttgatggatggatacgattatgaatgaaacagccaaatgatctaccgctaaggcgaag
ccccgtggattaaactatgaaattataagattcattatcattataacttgtaatttttttgttttacgaccgaacaattttatgaaaagtcgataaaaa
taaaaataaaataaaatatatgaaaatttaactattccatttcaaacctttttgaaaaaaaaaaaaataatctgttatcaaagaatccgtaacaaag
ctgtggagtgttgattaggcgaaaaacaaaaaccgacatagaaataaactatacattgagcgaaaatatgactacatgaatagaacaatccaa
gagtttatttccaagaggccttacctatactctacacaaagcagacagctataactgccaagggaaagacgctttaaagccctagaatagaacg
aattcaaaagagatttgtgcgaatttattataatacctaacgcaaaggatttttttattatgtaaagtaaaaatttataaataaacgaaaccggcc
aaagttatttactgctaaaaaaaaaaatttcggccttggatgaaacaaagctccgaaatacaccattctatccaaaaccatacttataataaggaa
gtacgcttattgctctggaaatataaagctttttgttaagaaggtgctgttagttgatttttacttcccattccaattatatgtattaagtgataacatt
tgttgattggttttaccttctttcaaccctttctcttacaaattctttttttttctttttttcaaggcctaatatttgccagggattcttaaagtatccatcctt
ccctgaataactttatatgaaacgaaacttgtgatttgaacagtagcttgaaatcataaatattccaatgtatccaattagttttgataccaacgac
aagctatacttagagccagatttaagaggcatcttgcgtatttgctcaccatttttcttctttttgctgcaaacgcatcaactttttcgagcaaacaga
ttggtatgtgctgctttgtgtgtgttagtcgaaacccaatgctatcgtctatgtaaggtgttccatcattatcgtgatacggactatctggccctttcct
tggtgtatgggtttctcatgatgcagagtttgcggacctgtattatttgcttgcaaaacacaactccccgtcgcgtcaatagcatgatcaacaagaa
gggtaccaaaggatcttattacgacgctgtacaatatgtatgtagtgttcgcaaatacggatggaccgacaatgctgtgcctgaattcgatcctcc
atactttccttaccgaacttgggtgactcgaactattatatatggggcatgttattggctttgcgtggctttcttgatctggtataattcgcagtcgaa
gacaccttatgaagtgaatacttggcccttacgtttacttctcccttttattagatcactttacgtttatttggttttgaataactgttattatacgatga
ctgtacttagtattccacttagaatttgggacatacgcgactttccaccattgatgaaaaatctaatcaagacgaaatcggttcacgatttctggtc
gagagactggcatgtatggaccaagccatacttccgaacactaggatgggaacctgtttacaagctcaccaagagcaaatttctaggaagtttag
ccgcttttcttgtttcaggtctgtaccatgattttgctttctggagtgtcataggtcattcgaacccaggaatactttttcagtttgcgataaatgccgt
ttttattaagtttgagaagcgctttcctatattgcaaaaaattccattcttgccaattttattacagattatacttcacactgaactaggaatgggaaa
ggtaatggtgcagcaaggttggggtcctttcgtgagaattgagcattaatttattccaactcttgcttatgctaatctcttgccaaaaataaaatttg
tatcaaagaaactcaaagtggttccatacggctggcttggaatcgattattcacaactgaccaattaatcaagttccaccaattggagatgtatttt
tccttgttattaatgccataatcttgtaagtaagcatactgccataggttaacgcataataatggaactatttcttttgtcctggttttaggaaattct
gttccccataaaaaagcgttggacgcattgtaagtacaaagtagagaaaagagagagcgctcgggactatacactagccatagccatccatcgc
caaaaatagagttgccaacttcaattagctgagtacgaagttcattgaaagatccaaaggatgcatcgattcccggctttaaatttactgctccaa

gatcagcatcctcagaaaatcgatcctgagaaattataccagaaaagaagaagtcatggttgactaactgactagagtagttaaaaaccttggtt
tttcttggatcatttacgttctgaaccattaagtcggaaggaactttgtaaggaatctggtgtaacttgcattctttgtgtagctcttccctcaaacga
ttatcgtaagaaaagatggttttaacagcatctcttgatagaaaattcttgtaaccaacatctaatgctttcgtgaacctggtttgtaagttcatcgtt
ctatatttagctcttgaatgaagggaaaaacactacccaaaaatatatcacggttatatcggtggtagctgcacaaaattataaagataattcaat
tttacgttgtagaaaaagattctacaaaagaaatggtgtcaaaatgtatccgcgacgcgtttcaaattccttagacgatgtttgctatatccaggtt
tgctagctcgccaagtaaataagagcaagtagcaatgtcactcgcaggtttacagaagaatttcgttttctggttttcatgggctttttctcttaatat
atataggctttgctatgagtgatttgactctggagatataataaatattcaaaatttgtgtttcctctaagtgcataaaagattccgttgagtagggc
tcatttactacattctcagtctttgcatgtcataaatgataattacaccaaataaatatcatactactttcttctttttaattctaataaagccaagaat
cctcatagaaacctactttctacatatactttatttacacataaaaccgagtagattttgggctgcattagtaaacaaaacttgaaacaaacaaaa
atttccctttgcacttgaaactctttttgctggctttgtttttacactttaagcaagttataatatcgttttataggctaaaatataagatgagtccaag
acaaaaaatcgaataagcatcgataaaaaaaagtgtaatgtaaaggtacaaaaaagaagcctacagcaccccggattcccatgttgtctccaa
ccatagtacaaacgaggccaggccctcagatgcttagctgcaatgatccgacaggaactggtgttttcgcctaggtacgcccatagacagtatctt
cagttcagaaaggaaattcataggtttcttttgatcctgaagaacccatgagggctactttttggctgtaggctgttactcactttcaagtgcaaaa
agttgttcaacagttcacagaaaagtgagatctgctttgtttacagaggtgtaaacaaacgcgaagctacctcttacttgtttttgtgactcaaatg
tatcttcaacgatttatgctagatgtcattataaaagatagcgcaactgaacatatcactatcctgagcaaaaatgttgcgtaagtgatgcgaaat
ttcttgctggagccaactttttatttcttcaattcctgccaattgggctgattttgacttccattttgtgcttttggaatgaccaggtagagcttctttagt
tttggctgctacttctgatgagttttgcgtttgcgaaatttttgcatcgaatagggatcaacagcttttatttgacgctctttttctaattattcgttgaa
atttctctatttatttttaggatgtcggaaaaggagctaaaattcgattcaagcgatcctcctgcatacagccatgctgatgtttacaatgttgacctt
gagaagggacttcctctgtatacaccaaaagacataaacaaaacttccaattcaacttctgtcaaagccaaacaaagcaaaaaaaacgataatta
ctgaatttgatagcttcaatgatttctttaccgactttaggaagaagacagatcattatttccctgaaaatgtagtcgttaaacgtaaacggatgttt
taccttttgatatcggttttatcattgctttgtgcattagcaatctcctatcgtttcgaactttttttcttttctgaaacaagtatatatggaaaatggata
cagttgggcccttcttggacctttaggcatttggcttgtgatatgggcaatattctttgctcttttctatggtaagtggagcattgttgttaatagcaaa
cgctattagaatgtgtgtgtgttactaacaaaacctagaatttatttggattatcgtattgatagtgcggattgtcgctaaagttataatctttatcgt
ggttggtcttgcatggttcgttggtgctatcttttggggaatttataacattcttgtttttttgccttggaatgaaatttgagagctcagctgaaaacacc
ggttcgcctacgcttcccagatatcaacaaagggatgatgcactagctcctccgccgacaactacgaacgaagaaatagagctgcaaagcaatc
ctacaactcaaagttaatgctaatttgtttacctttcttttctcatgagtcttaactcttccgatcgaaagtcactaagtggaaaaagagaacattac
aagcaatttgttcggataagtatttatttatatgcctttgttaagcatttttgtgtttaattttgactacaaagtcatccttgtaccaacatgaattgga
agcgacgaagaaaagtggacaatagaaaggcatgcatacaatccgcgcattgaaaggaaccgtaatctgagatcattgggatactaggttctta
attattattattcctattattcttattcttaattgcaattgaatacgatcgtttcatttcttttcattgaacttgtaattagtcttttgtcacttgtaaagga
acagcttgcaaaaattctttcgagaaaacgattgcaagcaatagtcgtagacgtataaaaaagaaaaacaaaataaaaaagtaaagagcaac
aaaagaaaagcctacagcaccccggattcccatgttgtctccaaccatagtactaacgaggcctcagacgcttaactgcagtgatcggacggga
actagtgttttcgcctagctgtggccgtagacattcgtagcattattcccgatggtactatagccagattatttttgttgcaagatttttttctttgtcatt
cattgcattttctcttattaaagaccaatttcttcatttttcgcagagtaaaggactggaataacagctgttttgccaacgatgaatttagctacttta
tctagtttatccaatagttgtaacgtttcgtataggttgcacttataaatcaacaatgataaacataattctcaatttcgagagaactgggtttgacc
ttaaaaaagaaataataatatggcgattatgcaaataaaaaatataaataaaacagctgaaatctctgagccttcacgtcgaccgcggtactctg
atttatcgtaaagcacagctcaaggatctcaaatagcttcatagtaaaaataaaaagaaaaagctttcagtttcggtcaatgcatccatcaatgat
ggcgagatctaagtcttcgatctggtatactactttgatttcctatttcctggagtgttgaaaactcgtaaaaattatcatgagaacattcgataaat
ccattggcatccgcaacctttcttttggaagacgtttaaggctcattttcatcatagttacaaccatatccctccaaaaatgttttcaattgctgtgga
tttaagttgtcgatgtcggagatagttctcaactcgggtaggtctttataatgttttcagatctctatgctgaggtccttgttttgttagttttggcttca
atgtttctgatgcgtgataattgttcaccgttatgggaaaaaaaactgatagaaaaagcgtcgaatcgtccttccatcccagataatcgtccttcca
ccccagataatcgtccttccaccccagataatcgtccttccaccccagataatcgtccttccaccccagataaacgtccttccaccccagataaacg
ttcagtgatgctattttgtccttctctaagctcttgcaacatcccacgaatttcagacaagttatcttgtgtaagagtctctcctcgaagtttggatata
actgcttcattaaggctcaccaatttgcttctaaacgaatcaaactgatcctggggaatcgcctgtgagaattcagctatgaaacggcttaaggaa
gcataagaatcatcagatggtggaggaattccaaattggttcgtaaataaatcttgactcattcttgaaaaagggtcagcgaaagcgaagtgtgt
agcagctattggtcacaatgtttgtttattggagttgcgaggcgaaaagataagtggaatgagggattccttcaactttgttaaatgtttaacgatt
gttaacgcattgatggtcgtcggacagtatgctgctgtcggtattacgaaagataatttgttaagattatggttcttagaatacgccgctggtcacg
ataataatctcacccgatatgagaatgtttatcgagtataataaacaacttgagttgtatcgtttttttgaattgcatttggttgtattacatatttagg
agctataatgtccaggatctggttaactaaatgaaagtttgaagacgatggaatttattatccacactagtctaatattccaatctgaagattctga

ctgtagacaaacagtttatatagatcttttgatacagttaacgcaatctgttttatttgtcaaagtcctggtggtcatcataattcctatatttcatatc
aatattctttattgatgatcgaaatacaatgtttttgtaaatgataatcatcttttgttctctcatcgcaaactattttcttttccttaatttcttaattctg
atgggtttgtttctcgtaagctcttaatctcataggttgcatgtcatttggcagattcaattttgactattccaatcgaaactttttttttagcgtaaag
agcagaggaaaaagcagcatttatagagtcataaactttttaatcaagtcttcaaatgaaaagtaacagattcatgattttgcaatttcaatagaa
tccttggtcattgtattactttttgacaaactcgccatcttaaactgccatttcatctttcggtcaaagcctatagaaaataatatgttgtattgattga
tactagccattgatactttaaacaacttcaatcaaaaaggaggaagcagagatgaccgtaaataagcaccaaaaacaacaggaaagtgagtag
ctaataagggccaggctaactaacaatcgcgactacggaaacaaaatacgcatgctttaaaatataaacaaaagctcctcgttagacatctcaa
tctcaactggacgctgacaataacaaccattcaataaaatgggcatagcataaaaacattactttgtatggtcgttcaaagtcggagaacctgtat
ctccaaaagtttgcatatctatttcttgctctcttgcagaatgcccaacaaactttttttgaaaggtttgctttggtagggcttttcccaagaggagaac
ttggcatttcaatattgttaggtgcagttgttggagtctcaacgcaagcttctagctcgtcaagttgaacgggatttgttgagtctggatcaatcccg
aagctttgattagttctataaccgaaaatgattgtcaaaggcagtataaattttctccataagtcacgccctagaaatacagccaaatctggaacc
atagatatacaccaaacgatgatgtccaacgcaactaaacactcaagttagcaagaaatccaacgtacttcaatttgcttttccgatgaatgcaa
atcacttacgagaaattattacagcggaaataaaaaataaaactaaaatcaatccaccaagcaaaggtaaaataattttcttagacaaaaatga
atcctttataaactctgacaatttatcagaatatttgaaaagtaaaacgaaaataaaagcagccgtataaacataacacagaaaacatcgaaaa
tatcctgcttctctcaatgtgcccgctggagctagtcggggacgtaccggcatgcagatgaattcgaggaaagaatttctccataatctggctggc
actgtttctgtcatgcgttcatgcgcttcattcgcatgaactcgattatcaccggattcgtctccattactcatttgaagttgttcgtaggaaggagga
ataggtgtaaacaaacccttctccaaatcatctccactgttataacaaacttttgagtaacttggcaaatccaattgaactgatcttttcctcgactt
gtcgtattcagtgaaattgatgtcgttggggtccattttcctacgttattgtcatttttccaagcttcgttcgtccattctcgctcttcacttataaatga
acctgaagtctcataaatccgagtttagcaatttttcccttattttaccattaggtagctctctttttttgctccaattgaggtttgttttgtgcaaaaag
agaatgcgacgatgagatgggaaaagtcatttctttctttttgcgagacgtgacgagactttgtaaaagggtagttagtacatatttgccgtactgc
agtatcgacgattcgatttaacgctaatcaaaaccatccttatacaacttttgctgcttgtgtttctattcgttaaatgaaaaacattaacaatggat
tggagaaaatgtctacagaaaagtaaatgccgtgattttcatataagcagcgagcaaaacgtaaaaaaagtgataaaggaaaagaagcctag
agcacctcggattcccatgttgtctccaaacattagtacgaacgagaccttcagacatttttacttaacaagagtgatcggacgggaactggtgttt
tcgcctaggtgtggtcgtagacatcctattacttatattttttagaattatttttataattcaaaaaaaccctcattctgtcggattcggcaaaatctc
tcatggcaaaacattcttccttctcaggaatctcaccgatactcaaaagtcgagcaagacaagtaaatcttgtggtatgatattggccatgataaa
catgagattaagagcggccgtttggtctagtggtatgataccatcttagggtgatggtggtcctgggttcgattcccagaacagcccttgtttttggt
aggtctctcttcggtcctccttcgttttaaaaactgggttattttcgcatagttttaaggacacatacgcgagctggtttctatagctcaagcatttgc
aatgtttttcatgcaagtgctttctacatctaaggaaaaggtcaaatttcactacgacgttgtggttgtattgctcgctacgcagaaatcatgctctt
gaaacggtatgaaattacttggcaactgcgaatgcgttcaataacaagaaattacagatagctttatgcaatgccaaaagattcattctatatcca
aaaaaagaagaactcgggatatttgatgagcgactgctttctctttttgttcaatgacattatgtttatcaggtccaacctctcatacaattttaatgt
tgataaatagatttcatctgcctcataacttgcttcttatttttcttttacatttaaaatccaccacgaagagccaaaactagatgcaaaacactacc
gccttctaaatgatagctttcagcatacttgtcgtccgccctaaattaccattagaatcatcctcaaatcctccttcgtacatttgttttcctgcataga
ttaaacgctgttgggatggaggaataccttccttttcttcgactctttctttgatacgagaaactttgtcatttggatcaatatccaattcaatctgtca
acaattcgtgttagtccttatccttgacgactctgctttacgagtataaaccaacctctttgcctgttaacgtctatcatagtttttgttaataattgtct
gcatctttaatttttacaagtttaataattttcctttacctttactttaataagcattatataaataatattcctgtcggattgacaattttgtttgttgg
ataatagggtgggtggtagtaggaaagtagcacgagaaattcacacttgtctttaacaccacatagtcctttagcttttacaaagatctttcacag
gtctggaatcaaataagcaaggaatgagttttcaagacttgcaaattgatcaatggctgtaagtcttatatgttgttcagctggttttttttctcatca
gaaattaagtcagaaactttgaggaaatcatccagctttcgttttaaatttcattgttgactaatttttatgtagaaaagatgcggttcacgctcgcg
ggtttactaaaatgacgcctgttcagacaaacaccattcctctttttttgagaaacaaagacgtcgtcgtggaagcagtaactggttcaggaaaaa
cgttggcttatttattacctgctattgacaaaattttgaagcgagaatctgatgagcacggtttgggtgcacttatagttgctcctactcgtgagctg
gcaaatcaaatctttcatgtcactgaagagctattagtatatcagccagaagagcttgcggatcagaaaaagcttgtacctgatatgtatattggc
ggaaaaggaacactgtcaaatgatttgacctcttttcgaaaaaaaaatcctacgattgttattgggacaccaggtcgtttaaacgaaatgttggga
caaatttccagtaaaaatctagctattttaattttagatgaagcagatactcttattgacatgggttttcaaaaagctttgcagtccattatcgcaca
attaccaaagcaaaggcgtaccggtcttttcagtgctactatgaatgacacggtttcatcatttttaaaagtagctggccttcggaatagtgttcgtg
tttcggttacagttacattgaagaaagaggacaccagagcaccatcctctttgtcaatccagtcatatgttttacctcccatgtttaagacccaagg
aatgcttcatatgctccaaaatcaacagtatgataaggcgattgttttctttctttcttgtgcaactgtcgaatatttctactctttgttgtctcagatg
aaacttccatttgaagtgattgccttgcatggcaaacaagctcagtcgaatcgaagtaaaaattacgaaaaatatgtaaaaagcaacaaaaaat
gtgttttacttacaacagatgtcgcatcccgtggtttagatatcccaaatgttgaccttgtgcttcagctggatccaccattggaccctaaatcctttt

cacatcgctgtggtcgagcaggacgtgcaggccgttctggattagctcttgtttttctaaatgaaggaagggaagaagattatgaagagttgctga
gggtacgtaaagtacctattaaacgaatggatactcctgaagcactgttggatcgaaagcatcttgacgaattaactaaaaacctaaggagtatt
gttacaaaagaccgagatatctacgagaaagggttaaggggatttgtctctcatgtgcgtgcatataccaagcaccaagcatcctttatatttcgt
ataaaagacatggatttacatcagctagcaacagcttattctttgttacacttacctaagatgccagagttgaaagatgctccgactgccaatgat
gtttttcagccagttgatattgatacccgagcaatagcttttcttgactcatctcgtgaacaagctcgccaacggaaacttgaggaacaaaagagt
caaccggagaaaccaaaattagaaaggaagccaaaagctgaagcctggtcaaaacaaaaggagatgaaagagaagcgtcttgtccgtcgcg
aaaagcggaaatcaagacgggaatttgataaaacacaaaagagagtaaaaatggaagaagaacagcaaggaacccgagagaatagttcttc
tgataaagtaaagcatggtcaagaagatgacgtttccgacgaagaaaatctttccgagttagaagatgattatcgtgctttgcaaaaagaaaag
aaaaaacgaggttccacttcgattgcatttgatggcttataatctttttttggggaataataggttttagtgtatggttataatgggatttaaaaggc
taataatatgcacgattcgtgataagtttaggagggagtaagcaatttattgtatactaagagtacatgcgatagggtggagttttgtttgatcgttt
caatatacttaccgcatggttctatttcagttgtatgctgtaaaataataggaagattggtgagaattttggtgaaatcactgactttgtacaaaga
atttgatactacgaggggatacttttattttcgtttatttggatggattttgtttatttatactcaattgcagattacaattattagcaatattatatgcc
tttccatacaaatattcaatgatactaagggtcttctgagaacagtttaagcggatatccctttttgttagcatacagtagcaatatgtgataaatag
aaatgttctacttacagaagaaggcagcaacgatagttcctgtacactgtatggaagcctacaacatcaacaaacttctattgttgcccatctgct
gaagattattgatagctattgtatattattgggattaccatgatattgcaaatacgcacagcagcgacgagactttagaaaaaataaaatattttg
aatgtgaacagttctagtttagaaaataagaatgtattagttctgaagctttgcagtaattgtttacgagggcaataaagtcatcggattcagttac
gatcggggtttctttttttagaaaatccttagcaaaaataaatgtcaaataaatctcaattggcaggaactaaaggaaaaaagctttccccaaca
ggaaatttcccatcacttatgcagggttgagttacaaagacaagtaacaattttatgatttcgcgtttgtttacacttacataaacaaacccactag
ggttctttcatgaagtttttctttttattcatcatctttttcaacctcaaagtagaaaaactcggctttataactaagctttacacatggcttttgaatt
ttcatgccatgtaataaagatttcaatatgcaatacatggaaactgcaaaatgcattgtctatatcttctttgataccctgatataaaaaaactttta
aacctaaataattttgaagtcagtttataataaaatagcaaccttcattttccaaagctctcaagagttaaaaacaccaacaagtaccttctttcta
gcttacaattgtctacggccatacctaggcgaaaacaccagttcccgtccgatcactgcagttaagcgtctgagggcctcgttagtactatggttgg
agacaacatgggaatccggggtgctgtaggctctccttttacggtttatatttttatctagcctttgctattcttccaacataattttacaaattttata
gctgttggttatcaaacgagtttttagaggaagaaacaaaacgcagtctgcaagattggtttttataattgaaattttataaaaaaagactaaatt
cttattggaacgaatttatgtacagattagcatgaaatttgaatgggatgctacaatttattagaaatcgagcttccgtagagctcttaagtcggtc
atttcgtaaatacaatgtgccagagtcttcaggacaactgctgcaagacctagggtttgtccatatgtccatgtcaggtgtgtttcacctcttacccc
ttgggctgagggtacaggaaaagatatgcaggctcatccaacagtcgatgaaatccgttggagctgctcaagttagtttggctcacttatcgagta
aagaagtatgggatcgaagccatcgatgggaaacgagtggcagggagttgttccgattgagtgatcgaacggagcgagaattatgccttgctcc
tacacatgaggaagatattacccaatgcatagccaatcttgtagaatctcaaaagcagttgccgatacgggtataccaaattggacgcaagtata
gagacgaggcaagacccagaggtggattgcttcgtggaaaggagtttgtcatgaaagatctgtacacgtttgacatagatgccgaccaagcaaa
agaaacctacaggcaggttttgagtgcatatcaccaatttttcaaggcagtagggttaccgttttcgatggtgaatgcagatacagggaatatcgg
tggaaagctttctcacgagtttcattatcgcagtcaggcaggagaggatacgatatgtgtttgtccttcgtgtgagttctcaacgaactcggaactg
ttgaaggagatacaaccagaaacagcgcacccttgtcctaagtgtggagacgagttggctttgtcctcttccattgaagtgggccatgctttttttgc
tgggacaggtttattcatccaagttaggtgcaaacgtagaagtgaaccagcggctccaaccattgtatatgggatgttatggtatcggcgtttcaa
gaatgattgcggcaattgcaaatgttactaaggattccaagggacttgtgtggccgacgaatgttgctccttggaaggtgttggtggtcccgacgt
cttttaaaaatatgcaggcagcagaatccgtgtatgatactgttgcgaatgtagtcggcaccgacaacatactgttggaagatcgcagcaaccac
tcttttggatataagatgaaggacgcagagctaatcggttatccttttgtggttattgtaggctcccaatttcaggaacaaaaactgtgtgaggtcc
atgttcgagctacgggaggacgacaccttttacctgtggaatctctgcaccagattctcttggggaatttttataagcccatctttaacctcgatga
atgagaatcaacagaaatgaaggagtatgatacagaatttacgactaggggagagtctaaatgtccattctataataccttaccttttctttactt
ttactttttaaaataagcataaatgtaaacaaatatcatattcctgaccctcgttaatacaatttttaaacagggacactatcaagctacttgata
ctatggctaatcatcatcacaatagaaaaaaaaacaaaaacaaaaccaaacttttaaataaggaaaatagaaaaataaatactctattgtcttt
gtttttgtttcaatactgtccatacacaacagattttccctgaaattcttttatttgtttatctcaagttacaagccccaagtcacaaattccattgtttt
gtatcgactcaaaagaaactgataacaaacgaggcggagaccactacaagtggaccatgtaaattcaacaacgttacgttccgtacatttgtttt
atcatggttttatctcattgattatctcgtttgtttatctaaatcacccgtcctatcgcttcttatatatagaggagaaagtatgacaaagcatcgaac
agaaagagaatttcgtaatagctactaagggaattctgttgaatctccttgcaatcgccacggactttactttttctttgtgttcttttggatttccgg
agtttcggacttttattctccagttctcatatatacggtccttcttgccctcgcaattatagcatctggcgatcattcatcagcagcacattaaactat
aggtaagtaagaaacggaacggtggtgctttacgtgatagaagcgagattgaaactatctgttaacaatcgctcatctttgtggcattcaagattt
actgaccaagattcgtagaaaacgatcgttgacatcatgtcgaaattcgcagagtttcaactccccgtcattaaaaatgaaccacctaagcattat

ggacctaacagtgctgagcgagaacaactcatcgccgcctacaaagaattagagtcacaattgcctttgtccatccccccttggtgtaaacggaaa
ggatgtacagactagtgacattggcgaacaacgctgcccctttaaccacaaaaagattgtagctcgctatcatcgtgccaacgcctctgatgtcca
aaaagctatcgagtcagctcttgaggccaagaaggaatgggaaaacttgtcctttgccgaccgggcagcagtcttcttgaaggcggctcacttaa
tcagcaccaaataccgctataagctcatggctgcaaccatgatcggccaaggtaaaaacatttggcaagctgaaattgatgctgccatggaaatc
atcgatttttttgcgtttcgattgcaagtatgctagcgaattatactccgtccaacctcctgaaaactctttaggtgtctggaaccgcaccgagtatcg
tcctttggaaggtttcgtatatgccgttactcccttcaacttcactgctatcggaggtaacttggctgctgcccctcttctcatgggtaacgttgttctc
atgaagccttccgattatgctgtcttgtccaaccgcatcgtctatgatattttccttgaagctggtcttcccgccggtgctttgcaatttgtccccggtg
atgccattgaagtctccaaagcttgcttcaaccactatgactttgccgctcttcatttcactggtagcacaaacgtcttccgttctttatggggaatca
ttggtgaaaatgttgctcaaggtaaataccgcacttatcctaagattgttggcgaaaccggtggtaagaatttccaccttgttcaccactccgctca
aattcgttcggcagttgtcaacactgttcgtgctgcttatgaatatcaaggacaaaaatgctctgcattgtctcggctttatgtttctcgcacttcttg
ggaagctggtttccgcgaccaactcgtgcaggaagtgaaagctttgaaggttggcgctcccttggaggacctttccaattttgtcggccctgtcatt
caccgtggcagcttcgacaagttaaagggtgtcttggagcgcgttgctactgacagcgaagtggaagttttggctggtggtaaggctgatgactct
actggttactttgtcgagcccactgttctgctttccaagaaccccaaccatgacattttcaaaactgaatttttggcccgatcctcgctgtttatgtct
ttgaagatgattctttggagcaggtctgtgatttggttgattctaccaactcatatggtctaactggttctatctttgcccaagatcgtattgtcgttcg
taagctcgctgagcgtctccgtaatgctgccggaaacttttacattaacgataaatgtaccggtgctgttgttggtgaacaacctttcggcggtgct
cgtgccagtggtaccaacgacaaagctggtagcggtatgatcttgtcacgtttcgtttctcctcgctccgtcaaggatacctttgcttatgccgaatc
tgtgttataccccctctaacatttaaaatattagacgaaaaagaaattttcatgaaaaccatcaaaagttttgagtttattaaaatattttgtgcatac
taattccgaataattttggtgcatagatcgacgcttgtagttatgatacagaacatagtaatgcaataaagtttctactgtacggtattgtctcccat
ttggtatcctgtttgtttcaacgaaaggaatcgtaccgttgaagagagcgcgttttagcagacaatgtacaataattgatagactttaaactaaata
aaatgctctgtgtttcttttagattatcctcatgtttgtcttacagctctctgtagtgtgtattagaatctgtttgcatgtcaatacatactgtctcatag
ccagctactttctttctacacctaccaaagcccttgacccacaaaatgttctcatgggaggcaagtttatcaaatggtaagaaactgtcgtttatga
atgttttattaacataaatagaactgtattttgtccgacagtacttttatagtggcagctatgacaagttgttcgaaatcgatacttcgtctttatccg
aaaagggacttgaattaacagaattgtatatggctagagcaaagttggccttgggagaaagtattgaatctattcaaggcacgcttactcaaaag
tccgctggaggagccgctcttttggcgttggcaggtgaaggaaacatggaccttttaattgagcagtatggggattctgatgctgctgtacaaaca
ctaggagccatctatttaatccacagaggagcttatgatgaggcgttaagtttgctacaaaagtctgtcgaaaacttagaagctgtagctttggaa
acttatatccatctttaccaacacaagttagaagcagcagaagcagcgattacaagatgtttggattgggcagatgaagaaattgttttacaaatt
gctcaatcatgggcaaagcttgctagaggtggtttagactcgtataatgacgccttttatgtttacgaagaattgaacggtagtgaaagcaatcct
cgtgttttaacttgtatggcttgctctgatttatgtttattacgaacacaagaagctctcagttctttacgatctgcccttgaattgcaatccaactatg
cacctgcggcagccaatttaaagacagccgttcttgctcttggtcccttagctccattggatacaaagcaactctatgaaaattcttcttgcggtgca
tttcttacaaagcaattagatcaaaaatcccaagaatttgatgcattggctaaacaattcctagctaattcttctgcttaactgttgtgcattctttttc
gaagccccatgtttttatgatcttcgctatgtagacgaattcttctttcattccttgccaatcctcatgtaccacagacctgagtcaaaacatgtagt
tgtccttggtattcctttttatatttataattcaatcctttctttttctttcctttttttaaccaagtataacattgtatcttggcttgcaacatatggtag
cattttgtaaaatcatgtaggattcttatacagcctataaaagttttcatttgtatttctactctataataaattgactgcaatcatggtttgcaaatgg
aaccttttcgagttcaacgagttcattagtctttgcaagttgtcatctgcatctacttccatgtctcagcaaatacctacaacgcatagtatattagtc
tgaaaatcaatgtttttccaaataaaaaaaaagactttttaggtaatcttatttcttaaaatcacgctttcatgaaagtttatcaatcgcgtgtcac
gcacactgtacaattgaaaagctgtatatttctatgacaagaacgctcctttttaatttaaaccaaaaaccgaagagtcattaatcgctcaagga
tgttcagtcgcgacatatcataatgaaaacagtcgaagaagctctttaaatatgatgtactaaaactaaagcttgcttaactgctagtttgcgcttt
aagaatcaaagacgcagaatcaaacatctatataggatgtattgtctgggattttttttacaagcggattgaaatattcaaggattaggactcttct
atcttgctaccgacctctataattcaacagttcttgtgaagaaaagaaagatgcttgtcttaaacgtgaagtattccatccattcgcaaggctgtgc
cgttggcagattcaattaggacacgcacagtgtctttttaaggaagctatcacaactttgaacaatgagttttcttagtcagcctgtctgtttttttgtt
caaaggttcctatcgccgatcttgtctcgtcatcgacgtattatacatcacaatgctttacaattcgttgtcgggaatcaggtcggtttgttatcaaca
agatatcgggtattttctatctataaaagctttggttcttcctttttttggtcgcttctaactgatattgacgttctatacactccctctggaagtattat
gacctgaactgctaataaagaagataagtatcaaagagttccttcttctttttcataatactttgtgttcatactgctctctcgaagtcgtcaatttcc
accaatctttctattgatttctaaacatcgattacaatatccgccgccaatcggactagcttgttttagtatcaataactttagaattctaaatcctat
atcaacggactttctctcaagcgcttcactgagctacggagtctgaccctcaagtatcttcgtcaacgggatataggctggataatttccaaatcct
caaagaaacctgctctgatgttttggaaattgttatcttttcttcttttgatttactaaaaattatttgttttcccttctcttttcatgccaattatccctat
aattgcatattcagtaacaaatcgagcctccgaatacattcaccctctttttgagaatccataccttgtgcgtttaaagctgactatagcttttataa
agcaattctttaataatctcttttatttctgctgtctttactacgctacaacatgggatgtatgtccagtaagtatacagattcctcaggcggagaagt

tagtaaaaaaaagctagttaaattagaaaacccaggaagagtctcttctcctgaaagcgaaagaaatacagtaaaggaacattggttgaatatc
gtcttgcgcggtaaaccacagaattcgaatgcggctgtgtcaaacgccaatggaaatggcaatgaaatcaaagttttattgcttggtgctggtgat
agcgggaagacaacgattatgaagcagatgcgtcttctctatagtccaggatttaaccaggctgctagaaggcaatatcgaatcatgatttttgag
aacataattacatctgtgtgcttacttttagaagccatggaaaatagtggcatgagtttattgccagaaaacgagaaatatcgaccgatcatattg
agaaaacacaccattcaattgaatgaaccttttcccgcggatctttacgaggcattgagggctttaacgctcgatccaaaaatacgggctgcaca
aaattgcggctctaatctttctcttttggataacttttattattaccaggaccatatcgatcgaatctttgacccacagtatttgccctctgatcaggat
attctgcattgccgaattaagaccacaggaatttctgaagaaacattcttactaaaccgtcatagataccgtttctttgatgttggtggtcaacgctc
ggaacgcagaaaatggatccattgcttcgagtccgtcacagcattacttttccttgtttcacttgctggatacgatcagtgcttagttgaagataata
gtggtaatcagatgcaagaagcgttgttattgtgggactcgatttgtaattcaagttggtttgcggaatccgcaatgattttgttccttaataaattg
gatttattcaaacgaaaagttcatatatctccgattcagcagtatttccctgactaccaggaaacaccaaccactccaacatttgtccaaacccagt
gtccgttttctgataatgctgtaaggagtggtatgtattatttctacttgaagtttgaaagtttaaatcgcattgcttctcgtagctgttattgtcatttt
actactgctacagatacgagtttgctacagcgagtcatggtttccgttcaagatacaattatgtcaaataatttacaatctttaatgttttgaggaag
acaatgactttttgatgaaaatactatttaatcatactagtgatccctcaagtaactgcgctgtttacttaaaacctcattgaatctctatgtcgcat
atattttgcctgtaattctttacactcttcttgcatctacctccttttttgaacgcttaagttttgttgataccgacagttgggcattccttttgttcttgga
ctaatttcgtccactgtttcattcatattattaattattctatagtgaagactttttttgagtacgagcacaagtccgattcacgtgctagcgttttacga
gaatgctgatacactatatgttaggatgttatgtgacgattccgtcaaaagtaaagcattctctaaatacaaggtataatgtctgaagcccttgaat
tccttctaatcaccaaccttgtttgtgtataatttctcaagataacatttggacgtttacaatttccaatcaattgataccacgttgcattcgataacg
ctattttgtaaacaaacaaaccagtgtatgagaattcgtgtaaaacattcgaccaaagtaaaaacaatcaccgtcgagaggagaaggcatctgc
tgcaaaaatcatatgaagtagcacgtaaatatgcataagcgagaatgaccagaattcagtctcaaaaatttgttttactttagaactgaagaatg
cgaatcaagttggcttcttgtaaatcaatgaatgtcctttcttttatatgttcctttttttaaaccttcattcgttttctaattatgtcctactgtttaatag
cgttcctaagtcgagtttgcttcaatatgtgttagtctagagagaaacctgaaaggcttttagcctttcaggtcaaaacaagccagttttataacag
gtataaacattttctccaagcaactttgtaaatgagtatctttcgtgaagctctgaatggtaatcctttattctattcacagatgtcaaggttttagct
gctgattgtgctcgtttttttgatggtacgattaaaagaatatgaagacacataaattatttctatctgctaaaatatactaaagatactcaattacaa
aattgcttggaaacagtctcctccttttccacacccactatcctcgagcgtcaaaaccgtaagacattgagagtaaagagggtttcttttaaatagc
atacttaatgtaacttttttttacatacagttttttttattatacgctctttaaatagctatgagtcgggatggaggatttattaattaatgctcgacacca
tctatgtatgcggaatcccaagttgattcgaattggtcttcgtcaaatagaatctattttacatcatattgcacgacctagtaacattaatgataaaa
tccccaaagagatttttctaaagttgcaagactctcatctgtacaactctgttgttccttgtatctatgcattggacagccttttggattaccagcaga
atgaagaagcctatttcgaaaattacgtctttattcaacgccttatggatgatttgttacatgtgattgagggcctggtactaatccatcccctttccc
aaacatttttcgaggataaaactgctttacatctatttatacaggttttagaaccaaaccagattacaagccttcaagtggcagctttgaaggctct
tgtatgtgtcatggtggaccgccctcttgttgtccgactatttgagtccattggtggtctgcatcaaatttgcatgttatttaagcatagacaaacga
gccaggatacgcgtttgcaaatccttgagttcttttacttttatttatctcctgaaccttacaatattgaggatttaccatatcgcaaaacacggaag
gataaacaaatttatctttcaaaatatctttctaatgttcaagacctacggaaagacttggatacttttaagccatttggtaaactggacgagacttt
tgactgaaattttggaaacaactattctttctttcttgcgttactacaccaaatagctctcatacagttttcacaatatatccataaataaataaatta
attatatttagcttgttaagtcaattcgatcccaaacgtccagtatccaactaaacgaactgtggagaccgatgaagactccatcccagcatgtga
tatcagccaaatttcgtccttctcctactccatcacgcctccgtaaaaaactaaaagatggcaatctttgagacaacaaaaatgtagatgccgttgt
gccactgtttggtgacataccataaggtgtctgactaggtaaattcgaggaattagataaggaagaggcattagaagttggagaagcgaatgtcc
caccggctgaggaggatttattgtgcgacatgggatagtttgtggtagttttcgtagacgacagtcgaaggaactgaaccatctgagaaaattttt
caacaatgaagctatatctgtttattaactcgaattcattcttgctcagtgcaacgaatgcataccgtcgcaaacctccacgagcatgctgatcatg
caaggaaaaggactgagcaatcacgtatccattctcgtcgtctccaaagaatagcgggtttgacatggattctgaaatactcgacgacactgaat
actcacaactaaggcatcggaccatcagcctcctcaaaatggagtatttgttctgatttcgcgggtattggctggatatgtatacaatgtcagggtt
tttggtagaaagggaaaaaaagtcgtggaggaacatctggttgtataaattcacatggaagaactgtgctgcaagtaacacaagcggattttcgg
gataaaggactttggcgagctgagaaaactgagccggctgaatgaatgcgaggagattcagttgcttttgactgcctctgattactgtcttcaggg
tcacttttgaacaaagactctgtagaacttaacataggatcttccgaaacttcagattcagcattatcttccgatggtgctgaagatttagtagcaa
tgggtgtatcaaaatgatcacccaactcagaacggcgcaatttctgagtacagaaaattattgacgggccttccaattcacagaagtgtccaaga
gaaaagatgacatccatattacaaagtggaaagaacactaaggcaacgaatctaaaagaaattgaccaaaaaacaaactttgtattgatccgt
aaaccattcagcggtataataagaaactcaatccttcttagaaagctatttacattcaaaaatggcaaaattgttgcaacgggcagcgacaaatg
actgttaggcctgcaagatcaactaatagtaagcaatataatgtttgatggcgcatagcagaagtaaggaacttctaaagaattcaaatataaat
atattaatgatcacatccaataaatcttaaaagtagctattattatacgtgcgatcatatgtataggaatgaaaaactttcgattctattttccattta

ttttttgagcctggaatcttgaattgtcttaataagcgctagaattaattaaaaatcactaaattatacaaatttgtattattctccaacgaaacgaa
tgaccccgtccgtcagaaagaattccttttctttttaacgaaagcttatacggcaaagaagcctttaaattaagaaaaaataattgagtttttaaac
aaaatcctgcagctcacaggaattttagacattacctaaggagaggtggtgtaggcggaaagaagaacgtccaaatggctaactccatcgtcct
aacattcgaatacgataaattaatgaaaaacttcatgagcgtataagcagtatattacaaaacactttatcatggcttcttatatatttgattgaac
caatatatatatactactcttaataatattacacattaccaacttagtatcaacaaggtctagtgtaaaaccaatgttattagcactatattatctatt
ttcaaaaatgatgacttcttgactttgcatatcgaaagagctcctttctttttctgtcaacaacagttattcagtctaaatggtgcgcttatccttaaa
gtcaggaacatcataagtctccggaggatcgtccttgtacttgaatatatagtccttgatttcatccatcttatgagagtaattggaactcaaattat
ggcagtttacggcgacaacagggttgtgaagagagtgttgcaatgcttgtctagcaacatcatagcgtgaagtgttgttaaccatcatcatggtga
aaggagtggttgtagtaccttcttcctggtatccattcatggtaacacgagagggttcaatgcggtcaaaaagcaagctcttcaagtccatgacat
aaccgtggtagttaaagtgaacatggcaattcttcgtgaacaaggcgtcgaattcaagttgagtcattccgtgaggatgcaaactggaaagcacc
ataaggtcagtgacgttaacaacacgcacccgcaagctagggatatcgttcgtcaacatttcagcagccttaaccacctcaaacataatttcatta
ccaataccgactaaaacgacgtcaggacttacgccattgtcagtactgcagaatttccaaacactagcaccagcagtgcagtgtgcttcagcttct
tcaatagaaagataacttggttgaggattcttactagaaaccatcaagttaatggtgttctcggaacgcatgcatctggccatcgtagccaagaaa
cagtttgcatcagctgggaagtaaacacgactaatgccgggtttgaaggagagcatggtagtgataaatcttggtgattgatgagagtatccatt
gtgttcttgacgtgcccaagtactggaagtcacatagttcaaacttccaaattttccatgccatggtaattcaaggcccatcttgataaacttgctgt
attgaattaacatactaacgacaattgtcatgaaggcctcgtatgaggggaagatcgcagtacgaccagtaagggtgtaaccttgcatgagacct
tggcaaatgtgctcggaaaggacttctgtgacacggccacccttggtcatcaatgttgggtcagtttgcatagtgcgatcagtatattgcaagactc
catcaagtttgttactctctagttcatcgggagagaagatacgtaaggtggtattgttcgcgcgtacaagttcgcccatatatctaccaacaacctg
agtagctgatgtcttttcacctttgggtacaccgaatttcttccagtcggttacatttaatggtgtgtatgcattataagtttcatggcgttgacccata
cgcttattaacacgaggaatatactcgcgaattcccctagttggaagtccctcctcgtccaagaattcatcaaagcgatagctcgatagccaatctt
taagcatcttacgctgagtaggatcggttcgagcggccttcaaaggaacttgatgagcatggtaggttccttccaaaaatgtgcccttcaaagtttt
agggcaacctttgcccttgggagagcgaaggataatcataggccatctaggcttaattagttcacgtttcacgcgagcatggtgttgaatatcacg
gatagtttcaacggcccagtccatggctgcagccatttcacgattgaattgatcagtatagttgacaatagcaacttggtaaccgaaaccactgaa
aagagccaatagttcaaaatcatccatacaaccatagatagtacgctcagaaatcttgtaaccgttcaaactcaaaatgggtatgacagcacca
gactcggcaggatccaaaaacttgtgagtttgccatgagccggccataggaccactttcagcttcgccatcgcctataacacaagttacgatcaa
gtcagggtgatcctgaacagcaccataactaacagataaagcataacctaattcaccaccctcgtggatagagccaggacattcggcatttacgt
gtgaagggaaaccaccaggtaaggaaaaggtacttatcaagtttttaagaccttcgcgattaaattggtactgaggatagaagggaccaagtga
gtcttccaaaaacaaagctgccaagatggctggggcaccatggccaggaccaacgacgaagatcatatccaagtcatatttattaatcacacgg
ttgcaatgagagtaggcgatacttaagccgggacaagtaccccagtggcccaggagtcgagtcttgacgtcatcctcggtaaggtcaccaccagt
ataaatgtccttggagaggaaaattaaagagcaagccaagtagtcgcatgctcgctggtagcgatcgacggcagcgacttcatcgtccgaaata
tttgaggtatcgaggggaacataaagacaagataactcatgaggaagacgagaaggcatgctaggaggaaaaggttgtttttcagcaatttcaa
cttgctttgacaaagcttcaggggatgcattggggatatcgtcagcactcatattggacacaaaaatgggataataataataagtttaataaaag
aaaatcgttaaccgttggaaatcgtaaataaatagaaatcaatcagtaaacccaaaatataagtaatagcaggtctaaagaagtcgtaacaaa
aaattcgttatgtgaaaagtcgtgaatcgcacggaaacgttttgttgcgatctaaatgtataacaacaaaagacgagaacaagaactgtacaaa
cgtttgttaaaattgatcaaaaaggaaaacaaacaatgataaacccttgacaaacgttttaagataataagctggcaataaaaacaataactaa
caaggaccagtgtatgaataaatcaaatcgacaacgaaagaatgcaaaatctgatgcaattgtcttttgcagagccccccggctatgggagcgg
ggcctatatatacttaaaggccatcggataatcgaaaacattcttgtttctaactagtatacttcaccacaacatcatcatacgttgcttgcttttatt
tttcttcatggtctactacgtatactacttacgtagatcgcatttgattttacattccatgatataagagaaacaccgatcgcaaaggaagagaata
ggtctttaggtcttgatgtgttcaaggtgacgtataccggccttgtgtaagctgggaaatccccaaaacgttcactttatattaagcagtacaatta
agaaccgagtaagatgtattgtggaattttcttagcgaggttatagtatttctgaattcgatatttggtcggccgggcatgaggaaagaaagggta
tggaaaaatagtatgtaaggcgcttctaacaaaaagaaggaaaaacaaaaaagaaaaaaaaagaagagaggcgagtcaattgaaaggaaa
ttgaattagataaaagatcattcgaagaatccgcgtctgaaatttgagaaatcatgcttatgaacaagaatccaattactgaaacaaagtgttctc
atgattaaattgtttttcatagttgttttgggagagaatatggcaacgagacatacccaaggatgacatgaacaagttttacgcgtcgattctttaa
cgtgcgcgtttttcgttccaaaagatcaaagaataaacaaaacaaatagaattttacaaattagtgataaaagggaactctactttgagaagtat
gtaccatttagaataaattgtttgcaattcagaaaagagaaaaccaagtaggttgagtttacaagttcgtctactgtttacttgaacatcgtactgg
agtgcatcagtataatagttaaagtgtagcgtaattgagctttttaaagtttgtgttacgcagtttggtatttggaatgtaaacaaaggatcatgaa
aagaaaaaaagaggtagagaaatgctagtattgatgacatggtctctatggaaattgtccagaaaagaaaaaaaagttattctatacatgagat
cgttttccaatttttttttctttcaatcattataagtaaagactcttacagcagtgagttactaggtgaaaaaaaaagcattgcaattctattatgtca

atttatatcatatcctccccttaaaattcgtttgcagcatatccttatttattttaatcacatatgctttaaactgcacttccaccacagaattctatatg
gtacatcaaaagatccagcttccaatatcttttcctagaaatatcattgcatttcatttctcttttataacatattttgatcaaaaaattttggtttgttt
gtgacttcaactttgcacttctgctattcttttccctttttcctcatgtctggtaatacatcttcattcaagcgaagtgctagagaaaaatgctgggaag
caagagacttttattttcaatgcctagataaaaacgacattttggatcctttgaaagacagtgaacgggcgtctcaggtatgcgactctgagaaga
acaattttgagtccaattgcgtccagtcttgggtaaattatttctgtaagtttcgtgttcaacaacataaacaacaagaagcaattcgaaaactcga
ggaaagtggtgcccgtcgtttataatgaaagtttccttccttccttcattttcaaatttcaaccaaaactctgcttgaatgaaataatttctccttttttg
gaattatttgctacatacaatctcttctcttccctgtcttctgatgaagtaccacagactaccccttctctatatgcaactaggaataatatagattta
cctatttcatgaatataaatctttaatcgttacttaaagaaataataagtttccatcctgttttgtgacatacaataaataaatcaatgcccacgatc
atttttgaggacgaaaacaaagcaaacaggaaaacgaaagaacaaacaagtaacttacacaggagccactgtgagcttgtttgcttcctcacgt
aaccatggaatctgtaagatctgattcaacacttcaacggtagaagcatccacacagttgattccttgactatgagatatcaaaagattgtggatg
gcgcgaatgatttgtttagttttgtagtgaggggggagtctgacctgagtattttgtaataaagggagcaatacttgtaaataagcatatctcagct
ttgctagttcattaggaatatcattaatttcccggattaatacatcaattagtacgttgaggtcgttagtgtaaaagatttcatatgttttcggagtag
taaataagagatacagctgctttaaaattagcatcctagttcgaggatctcctgttcctagttagcttccatcttgaaatccccgttcctgttgatcat
cacagtcttcttcgtcccaaatactctcattacatactttctctgtttaacataaagacaagtccttcattgtaaacacctgtatccactttggaagct
gatagtaaattcatgaccccattttggatttcgacgacaccggtagaggcaagcttgacgagcataaattgctcgttcaaactcaagagtagatgc
atacacatagaaaaattttccacgtcttcttccgcccaagtaaattcggctgtagcaagcagcccttttaaaaacgattcatctgccgtcaccaaat
cagtaagaggaagtcgctgagaacgacacaaatgatacatcaaacataatccatagcgaaaggatatatacatccctttttctgagggatctcta
aaacgaatgattgcttgcacaagagcaccaaaaaagccacttgcaattagtagcttcacacacttgtcatctaaccgaatatctaaaagaaaga
atacaacgataaatttaataatatggatgtcttccatgaaaaagaagcaatctaacatgatcgtttgcactgtattcttgtccaacttgaacaatgg
actacgaatcagtttctcagcggccgtttgtaattggatttccgtaattaaatacggctctattccagttaaaactccattccacccgctctctcccga
caacttttgtgctcacacaacgtacttccatattttaaaaagaaagaaataagcctagaggtagcaacttctacctctaatttgttcgatacagttg
aaacgagttcgtctacttctaattctctttaatcagctttcaattccctataattttgcctactgaaccagttccttgaaactgctctgtaaatgaatcc
cttagatccacaccaaacacttaccaatccaaaattgtttttcgttttctaatgaataaaagacatctgtaaactccatttacaagtttaggcaaga
aacgcttaagagagccttccttttgggaatattggtacacgttcgtattatccaacgttaatagaataagcatttttaccgaaacctaattgttcgct
aagataactcaattatactttataatattcttactgataaccagcacgaatatagaagtgtcgtacctaaaatcctcttcgttgtactgatttttttcat
tataaacctcttcatttctctctttgtttttttttttatatatataattttgataattatagggacatctaaaactaattattcctggattcattagaagca
gaagaaatttttgcaatgccacgtataagaacatcacggacaaaacggtaagttttcacggttatacacatttcaaaacatggtcctaaagtgcta
atactggtcttatttagtcctccagaaggattcgaagagattgaaccaacgctactagaattccaagagaggatgagagaagctgaaaatacaa
ccgggaaaggctcaaaatctgagtctttggctcctatttttcaactccaccatcaacgcagtcgctatgtttacgatctttattacaaacgagaggc
catttctacggagctttacaactggctactgaaacaaaattatgctgatgctaacttgattgccaaatggaagaaggtacgattacaaatacttttg
acttcatttactaattgtttcaggctggttatgaaaagctctgttgtcttcgttgtatacaaacaacagaatctaaatttggttctacttgcatatgta
gagttcctaaagccaagcttggcaaggacgctcctagtacttgtgtacattgtggtatgtcttgttttaaatccgcacttttactaataatatacttag
gttgtcgaggttgtgctagtggtgattaaccatgtcgggaaactcaatcaaaaatgaaaacgttgttatgatgacactctcaggttcataaaatta
ccatttaaaatgctatctagtgtttatagagaaatttcgataaaagaacgtcagtgtacatagaaattatgcctcgtgaaggtttcgtagaagaaa
gcccctatatagttatttcacattccctttttttgcttgcaaccatgagtttgcttcaatatcagaagcatgactcgttagtatttgctgaaacttttgca
gcatttcgtttttttggcatattctttcctacctcattttgccattctagcataatttttcgaattatggcaataccctctttctttgcctgtccttcaccatt
ttccattggttgagcttcatcttctgcttccaactccatcgctttcgcttgtgccatttctatgattttggctgggaaatgcgccagttccgctacatgta
taccaaagcttctatcactcgctcctccgcgaacattatataacagtgcaacatcgcgagcattagtatctcctacatgggctgtcacatgtaagtt
tttcactgttggaatttcttccgacattcgagtcatttcgtgataatgcgtagcaaaaagacaaaagcaatttatctttctcacaatgtgctcagtga
tagcccatgccaggccaaaaccatccgtagtgctagtaccccttcctaattcatcaattataataagagaacgcggagttgcggacctcagaatc
gtggcagtttccagcatttctgccataaaagtggagacgcctttcaattgagaatctccagctccaacacgggcgagaatagcatctacaacgtc
aatagtagctgagtcgcaaggaacgaaacaaccaatttgagccataactgtgataacaccagcttgtcgaatgtaagttgactttcctcccatgtt
tggtccagtaattataagcatctctgattctttctgtttaagtaaaatgtcattgggaataaaattaacatcatcctgtgcttccaaacaaggatgtc
ttgcttgcttcagctcgatacaagcgcaatgggaagagcgcaaatgggatcgcaattcattaatatcagggttttcttccaagcctacgacatcaa
tcatttttttcttggttatcagtagcagtatccacttccaaccttggatccagtattgttggacacacatatgcggtaaccgcaagcgtacttgcaaga
gcaaaactcgttataacatccaaatgtgatacaatctgaccaactgcctccaaaggaggactgtatgttgcagcaattttgatgacttctcgagct
aggccattctgatgatatctataactttttttgatagtccatatatgtactattgaggttatgaagacgcttggtagtaaagtatactccattttttttga
gttgataattctgaataacgggaactacgtccccgtaaacaaccagcttctgctcttgtgagacgcaaacaccatccatataaatgatgctgctca

agatgcaatttttctcggtatcctgttgaagatcaacacccgcttgcttatgttcgtcaaagattgaattttcaagttcctcgagcctttgccgtaaa
tccaataattcttcatcaaattcggcacgaataatatatttatgagcatcaagtgcctctaaatcaatagtggtttccaccagctcaataagttttgt
tagatttttacagtgggtagatagtggcgtcgtgtagacttgatcgattaactgtttgtgtacactctttaaggaatctaaagcagtgacaatattcg
gaagagctttggccatttgataaatacgaacaacatcttccaaacttgaagacccacggataaggcgtcggcaaagtctaggaacatcagggat
ggatcggagaaggtgatcatcgtctaacaaaagctgacgaacttctgggtcctcaataaaagcagatactaacttgtgtctcctcgtaatcgcctt
tgggtctaagagaggctgaacgatccatcgtcggagttgtctcgcacccatgggagttctacaatggttcaacaaaccatacaagctcatcgtac
gatgcgcatttccatttgctgggggtaacaagtttaatgcacgtacagccgctacatcgagacgcatatactgttccaaattatgttgatatagatg
aaactttcgtgaaagatccttctgtaactcaacatcatccgaagggattgggtcgaccaggtttggtcctaaataccgaagtactgcattacatga
gcccatagcaagctgaagacttaactctggaataagtgcatgtgtgacgggggtatccaaaacctttgcaagctctaattcaacgtctttgggggga
gaagtatgaacttcgtattccggtaaccaacgcaccacaaccttcgataatatttctgaggcgatttaattcagcactagtaatcgatgtacccgtt
gcggtattttctccgtcttgctgagagataagacattctttggcaccggtttggacgagaagcgcttcaaaatttgtgtaagaatccgagtcaacga
actcagagacacctattttccttgtaaaagggtctataaaagttacgccaacgatacgttgatcttgttttacacgcgttgttaccgctagcaatata
gagcctgcagagttatctacattaacggtaggatcttttgttgtatcactgttcattaatagatcctcaatcatttgcatgttaccaggacttgcttga
tttagcaaatcaaaccccgtcttgcttttgttggagccccaaatttcgactcgtttcgcatagtttgataaaacttcttcagcgaactttatgaacaac
gaaggaaacatagtacagaaagagagatttgattgattgtgatatttcaggactgaagtcgtatggtatgcattttgagcaacaaatgaagcgtc
ttctccaatggccacatatgtttcctaaaaataggtttagtataagtacaatgtattttaaagcaatgaaaagaaaaacgagcataagtaaatttc
cagagacaatagcctagttcttcatttcctgccaggaatttcaaaaatcagaaaattaaaccacgtcaacctcgaattctgtttcttttgtctttctat
agtgttaaaatacttactccacgatcaaagacgcgtagcgtgtttgagtctttgggcatcttctcgtaaaatgaaaacatacgagactcatcggtcc
gttctgaagagaatattagttacatatgcttcattgcatcaaaaaatcaaagcgtaccattcaaagacgcgctatttgagcttttggaagacatctt
gaaaaattgggatagaagaagaccttagtatttttctaccgcaatcgtttaacctttgcaaatatgtaattatctattgaaaaaataaaatagaac
gaaaccgaaatgaaatgaagtattgtactgccaatccagctggcgagtttggtaaagacagcctgaaattacctttcaacgaaacaaccttgtttt
ttatgcttgctacttgaaccttcacttgatatatttttatttatatttcctaacattttacaaaagtttctattctaaaaatattctcacagcgaaacata
cgttatactagtatgtatatgtttgtacggactttgaaaacgattcaaattaagtctcttgattcatttacggtgttcttaacttcaaaataattcatcc
attttagcattcatttttggacttaaaatccatttaccgacatttcttagcttattctctattagacaggattaaaacttcggttactgttccacgattg
aaaaacagttttgaaacaatatcaacggctaaaaagtggcagacatccagttattacatattttctttcactggaatcaacactaattttaatagat
taaagccgttacaaaaggtgttccaatagttatctagtcctgcttacagttaaagaaccgtttatttcgtttttctaaatcctttaaccaaaaagcttc
caacgattgattattgtatatcgcttgtctctgtcacttacctctacagacaaaatcatgaacttgtttgagactactctttatgcaaacggagaaat
ctttctactctctgagcatcttggtcgcatgaaacattctgcttccactttgggttattattggcctggggaagaagcagtgaaagaaaaactcttac
atgctactgaaacacttgaccaagccagggtatgccgcgatggttcttagattacgtgaaccacagacttgctaattgctcaatagttacgatggg
agctctcgaaagacggtaaaattcatgttaaggctgttcctgtcgctgtatcgaacgaacagcctttgtatactgttttcttggatacccagccaagt
gatactcagaacaatgttacatgcattaataaaactacctcgagagatatctacagttatgcggtacaaagaacaggcatcgaatacgccaaac
accaggatgtcttgttacacaattcaagcgggtttgtaaccgagggcaccatcttcaacgttgcgtttgaacgacacaaccaatgggttactccaa
acctcaaacacggtacgtcgaacatattgctccttgagcgaatattttggacttaatatgcaaaaattgcaatggcttactaaccttttaaataggt
ctacttcctggtacaatgaggcagcatctcctatctactggttacattgtacaagacaaacaaaatgaactacgcaaagaaagtttaaaaaatgg
cgagcagattctcttattcaactcatttcgtaaaatatgcaaaggagtcttaattataaagccctaagctttagttatctgcttaagaagaagggtt
catctatagatgttaataaactagtcttccgttttgtacaactgaatcccatcaaggcgctagtcataaacaaattaccgttacggcattaagctaa
cgcttatttcacttgacggtagatccatttttcaacttactcttctcttgccgccggccatgttggtaacgttaacgaagcggcgaacatatacaaga
cgcttgtgagcacggcctttaggttgcttaggcttttcttgcttctcaaccttggggcattgagattttacctaacaacggtgttagtctctctacatgt
aacaattctcacgcaattcttacctttcctgcacgagcgagagatccgtgaactttacccatgttgttggtagtggctggatgagtgcgactaccgc
ataacacctttctcgcgcaacccaaaaagacagtcacaaatatgaaggttacttgatattgggtaacattctttgtttacaaatgtgcttttgtttac
gtctcttcattaccattatccaagtattcagtgaaatgagttttgggtacgagtatagagtcgacgaagctcatgaagcacaagagtttttgaggta
gtaagttgttccttaattaattcgcggcattcgctaacatagtttgtaaagtatcccaactttggcaaaaccttcagaaatccccgaatctttggaag
cagataaaacaataattcatactgggtctgaagtagacagtaattccgatagctgttcagtagtttcctgtgatgacacatacccgtcgcgaaacc
acgttgatgaaaattccggttcggaattcgaagacgattcccctcttgcaacgttatcaatgtattcagcgaatccttcatatcctaactctccagta
aaaagagacttcttttcaactgaatacgaatccattgtagaccgtgttaataagagcaatataactgataaagtgacgagctcttcaaaaagact
gttcaaacgaaatagggatctcttagcaaggcaattccttcatgctttagacaaggaagtctttcatgataaaatttctgagtcggcccttgataat
gaagttaaagtatcgtggtccaagtccttcaatactacggcaggaagagctactcttaaacaaccacggtcaaggaccttccttggtcaacgtgtt
caagcgtacattgagctttcagaaaaagtcgttgactgtgaatataagctttataataccttagcacatgagtcgtgccatttagcttgctggttaat

tgacaaagaaatgcaatctcctcatggagcatgttttaaggcttggtaagttgtttcgtgtaaatatatgatcgcaaactaaccagatgatgtctta
gggcaaaacgtttgatgaacaagttcccatcaattgaagtaacctcaaagcacaattatgatattgattataaatacaaatggctttgcatgaatg
aggcttgtaacaaactgtaccaaaggcattctaagtctattgatccgaagaggcatgtttgtgcaatttgtagaactggacttcttcaaattgctcc
cgtagctaggcttcttaatcccttccagctttttatgaaggagaatatgggaaatataaaacgacaatatcctacattgtcacacaaagaaataat
gcacaaagtcgcttctgaatatcgactcgctatgtcaatcgattcccagaaatcagactctcctggtttccttaatgtgatgcagaaatttgatgatc
tatcattaaaaaataaaaagaatgtgatgattcatgctgaaatataatttggttaatgatttattattaatggctttttttcttatttacggcagttaatt
aacatccccaacacctcttgctcattcataatatgaactaactttatattattaaattgtaaaattaatagaataaatctgtacagatctttttataac
cattgtatccagatacaacatcccaatgaggaagagaagaaaataaaagagaaaatgtttaaactgaagaattcttggtaagagatcggtgctt
atgaatggctggctgaatcatattaaacttatcaaacgaatttcctgcgtcagacttgccaaacacgaaaggtaaaattaggagaaggaaaaat
atagcaaataaagtaaagtacagagtggcatagcggagcacgatctgtcggcgtaacttgctttgtctgatggtgtaaacaggaggacgaatttg
cctgctgggacgaagccagaacaaaatgattgaatggagtttatcaatatatggaattaaaatgatgggacactgaagaaagagtaacagatg
gctgagaagaaagtctccagcaaacatgttaatttcagtagtcttacatatgaattctctccaaggctgagtcagcaccatgtatccgaaaccttgt
ccattccataaacctgaccaccacgctaaattagcaccatcatgacggaattctctagataagaagcaaattattaggattttgtgggcaaagcg
atgaatggaggtaatagcaatgaacccaagcaatgtcgtactaaaagtccatccttctaagtaaccaagaacctcaaaaacaacaatatctata
aatacaaacaggatatgcgcagctccagccatgtaaattccaacctttctcgctttaccaccaaccactgggcgtaagataataccaagacagaa
gaagagaagagcgataccagcactaaggacaagaggaacacaagacataataatcaaccgaatgaaagcattagtcctttcagattcattatc
attgcccggctgagaattggcaaacaaaaatggaatgcaagtgaaaaacacggtacctaaaggacctagaatttcaccataaaacattgtcgta
atgcgagcacgaggagagtccatagaaattttgtcggctttctttccatatattttacgcttatatccagtaacgcgagttcgtgtaagctggcaatt
tccaatccaagaattggaatggttgcgagaattctcacggaaaatccatctaataaattctctataatcaacaaagaaatcagtccaggcaaatt
gatgagggttatacaagaatggactaacacaaagagcaaccaaagtaatccagaaatagatataatgaggcaaccaagctgtgatagatccgt
aaagtagcataaacataagtctagaaccaaagtacagtgaaggtccactaaaacgagaatataacaaggcgaatggtacacgaacagtagca
aatcctctactggtaccaatgtaccgagcaccaccaaatgacaagttagcaatcagactttgagcataaatctgacaagtaaagatttcaaagat
gggtgataaagagaaaatttgtttagatacacggattaccattctgatggcaccacgttcattcaactcgcaaacagctaaggggacgaacgcaa
taccaaagacaatgaaaattgacaaaatacaacgtttaagccattctaaaactggttgaagttggtaacaaccctgaggactaagaggagcagt
aaccggattaaattggcgatatttacaaacaggtaccacactaaacatcgcaccgaggttaataatgaccagcataagcaattgcaacgaaaac
ataatgaccatgttattaacatggaaaccagcatgggcatagaagaacgataaaaatctgtcgaaaggtaattgagtacctagatgaaaatatt
cacgggaaagcatttgttcagccataccagtaccaattttcgtagtaaagttgagaatcgagccaaaaccaaggtcacgacctttaccacactga
taataatcacagtgcttgattctaccacccctctgtaaagcggtcataccagcataaatatcttcgttgacatgcaaacctttttgagctttagatac
accaccacgggtcgtcataaacaaaacattgataaaatcaggatggccataatgcaacttgccaccaattacagataaaatacgagcaaacata
gtaccaaaagtctgttctttggctgcggcgacatcaccgagcataccagtattttcggaaaaaatatattctcgagcaccaagaatagcaactgg
atgattagacgtggactttgcattcacagaataaggattatgcataggaggggtaaattgctcaaattccgcgagaatatttctaattttaagaca
ttcttccaaataattatcctggtttgcatcaaccatttgaacatattcaccgcgaatgtaaggaattgacatattttggttatcagatttaccatcacc
gagaattggattaccagatagacgtatacgatatttagggcgtcgcttaccattctccattataggacagtggccgtcaatcagtacagagtaaat
tcctctttcttcagctggacgagtaggtggatcttcatccatgtaagcaatttgaagatcagggtacgctctgagaagaaattctgcattttcagctt
cttctttagtgaacttagcataacgttgcatggaaacacaaaaacggaattttctgttagccatcacgtccaattcttcgtctaatcgagctggatc
accgtttgtccattcaacgagttcagggcattcagtacggtataagagcttaattgctctactgtagttactaaatccattgattgtacgataaagc
gtttgagtacgcaaagatgcccaaattcttgttcgcaaagtgtactccggtgtagcagatttaaaaccaatgcagtaaaatggaagatcataagc
cttagcctgcaggtcattcttttcaccatctatggttgagtccactgaatcattttcatcggctagcagcttcgtgtcatcaacaaagtttttccattca
attggatataattgctttaagtactccaaaagagttacacgagaaagctggtcttcttcacgaattatttcacgtaaagacagcaaaatttttttctg
aataatgaggtactaagactgtaaacgtaggcatagcatccacagaacaatgctttggaattgactcagccagtgattgagcaaaaaatgaaat
tctcctagcagcctcagaattagcaggaaaaagatcctgtctatagtgcttcacttgctgggagtaaaagaaatttggcgcacgaagcgtatgat
atccagccttctcagaagggacttgatgatacagcaactcctgaatttggtccctagaaataaggtgttctctatacatggagatgattatagcatt
ccaaacctgagaaatcaataccttgggtttaaaggcaatttccatatcattagtatagagaagcttggtataaattcttctaggaacacggtaaaa
aagctcacgccaaggagtccacacactaattcccaagtagaaagcgtaacctaaggagtaaatggtactaattaaaatataccataggtaagta
tccagaaagaacaatgacagatcagtaagatacagcaaactaagaagaatttttggttgagccttgcataggccagaacctaaaatataatcac
cgcaatcgtaaggacgcatagcacctaagaagcgaatggaatccgaaaggttgagtgcaagaaaaaagtaagactcaagatacttagcaaca
agaaccgtaatccataagcaccatgaaatgaaagcaccacccttagaggttgaggtaaaattagaagtaaaatatcgattagctaaatcgtgac
cagagctttttgtggtaaatcctacaagatttcttaatggcgtgacagcaaagtaaaccacacataccaatgaaaaaaagaaatgtacgatacca

accactaaacgagaaacataagtctgttgatggtccgttgaagcaaaaataaaaatagtaggtgctatattgagtaacagagcagttagaagca
tcagaaaactaggccataaaggtctagcacaatgatagcgtcttgggacatgaagatactcaaagctaacggcgagaagagtaataaaagtag
caattgcacctgctaagccacatgcagtccagcgacaagctgccggaggcttattattcattcgtatatgaaaatgtgaagtgtacaagttggcgg
aattataagcagaataataccaatacgcagaaatgtgcataacccagattcgagaaaagttggtgatattatgaaaccatgatcgatattcgtag
tatgtcttgtaaaaacaagatttccattcaacattacctaaatggcggtaacgcataaaaggaggtagatctaacaaaggagttttatcagtacat
tttatttcttcaaggcctttataagaccaaaagaattggttaatgtcatcgtaaccaatattgttagcgtgatctcgttcgcggcgaacatatttacc
atccaggatttcaaagagctgagcatggaagaaccgatatataggagagattacagaatccaaaaaaaattctttcggtaaagccgaatccaag
tctcgagcttcagatgaaatataatagtcgtaggcgcatttaaagatgaagcaaagacattcaggcataaaacgaacgttattagcctcacccca
gcacaaaaagtaaaggcacagttggaccatcattcgatgaggagaaaagctagccatttgatctctccaggcttgttctgcctcttttaatgtcatt
ttttctacgatcggtgatgacatatcatggaacccaatggcatggccatcattaaaatgtgaagcaaaataccactttgcaaaattggcgtaagta
ccaccaataacatctgcgtgaagagtaaggagtgcttgggaaggagacatacgagaggcacgagaatcgagtagaaccatgatataatcaaat
atgttacgcatattatccttttgaaatccaagtttatttgtaaggtcaagtagtatttcctgaacaccctccatagttaccgggggttcttcagcagct
tggttccatgaagggtaagcgacaggactagcgtcgacattaaaggagctactatcataatcagactgaaggtcatcatgaacagatggatcat
cataaatcaagcgttgcaatgagttcatggaatcattttcagacgtgtaactcgtagcatcgtcttcaaaaaggccgcggccttcttgctctcgcca
atactgatccattttatgaaaatcaagtaaaatggatataccagaaaaagcaaaagtttataaaatgaaaaatgataaaagatgcacaaaaga
aggaaaggaatgataacgaaagaccgttaatcgaataaaaaagagagaaaactgtagagaaacgtaagtatgggcgtacttgttcacctaga
aattagctctgaacatacaccgcacgaaatgtcagaagccaaaaagatcaggaaacgtcctcaaatggccaagaacaattatacgattgaaaa
aaagaaaaaaagtggataagctagaatatgataaataaattacaaaagtatcgtagacgatgccgaaatgcaagatttatctttaactttcgatg
ctgatttagatgcaaaataaaaagatagatgcttttatgcaataaaaacctttgcaagtgttgagcagttcttcttaaaaaaggaaggttttcagc
atatgccgtactcgaagtgtacaaaggaaagaaaaatagaaaaaagatagtgtctgggataaatagaaaagaacgaactcagaaaagaaag
aaatccgtaaatcacttcaagccgtaacaataatgaataagctatacgaagggaatgaacgcgggtccgtcaaatttttcttctctatgccggag
aaagagaaaccgtgatacctcacctaaggaaaagataaaaactatctgtataaatacaagatagatataccgaatgctatctattccctaaata
gacgatacacggaccaaaatcactttctaaaatacaaaaagtcgaaaaagaagcaaggaccggatgttgagcaataacaattctatagatgac
ttgaagtcctttttaaatggtcggactaacaagaaatcaacaaatcaatgtttcttgccaacagataccaaattcctagtttgttgtttgcaggttc
ctgggtctgggtttttccttacctagcttaagcctagaaggaagggtagtgcaggtttctttgtttgaacttgcagctgcataatgcaggcttagaac
aaccaggggtcgttacctgcttaagcatccgagcgaattagaggaaggaatccgttaacagcgtcatgtaaacaagcaacgctgtaaataaata
aacagagagcagaaacagtacctacttttaaaccaaaagatcaaccgaattatttaagaggctgcagggttgcatttttataggaaagattcagc
ataaaatgcccaattaactttttttcatcgcatctctttctgatacattcaaacattattggaaatgtagcgataaaacgtccgctttcatataaaca
atatacctttcacagttaacattcaattacggacttacctaataagaataaatttcgctttctttatacacgacaattgtgtttacagatacaaagcg
ctttccaaaacagaagaatatgcataaatccacgatctaattttaaaattattattagaaacaacttatacactttatgtaccctttgccaagttctg
ctcacattcgagtgtactttatttttaactaaactacttcttatagtaaccaaaacagtagtaaacagtgaataaagtcgttgaacgatattgaca
gttttgttcggcaatttcataataggtgatagtgaagcttcaacaatttttaacagtaccattatattgtttagtgaaaccaaacaactttgcttatttt
ttatttcagcgcatgtacttcagtaccgatttgtgtatagtgtgtagtaaggaaataccttattctatttttggtacacgtcagtcgatgcttaccaa
ctactgatatttgatctaatttttctttttggaaacgatcgcgttcgttttataactgggctgattgtataataactattaacttcatcgaaagctgttt
gataagtttcccaaaactcggttcttgttgcgaaaagctactaataactttgaaatttgagagaaactcgagaataataagaccataataaaattg
tactgtatgtttaatggatttataatcttctttttttacccttttcctcttaagcattctatttccgtgtttcattttctgagtatatagacttggttcttttc
ttatcgattttgcgtttttgtgcttttctttcttttaatttgttaaatttataatactcacttctacatttgtcactcttttggttggttttccttttagaagga
attacttgtttttgatttccttttgatatttgcacactctttgatttcgtgttctgtttactttttgtttctatcaattcttctttttatttgaaaccttcgtttt
cagtatccttacaatttatttttgggggttttttttttctcaatttgctcattgaccttatctcgtttgtctctctttctttgattatttaaatattttattat
tatgtcaacgcctaaagtacctcccaccccttcgtctcctcgtcgctcaatccagcgtattgctgatgcgaaaaataagtctcttaccttcaatcgcc
gtagattagggttattcaaaaaagcttatgaactttctgtattatgtgacgccaaagtcgtggtcttggtttatgacaccaaaaatgcctgccatgt
atactcgtcagaagagcccgaagaaaagcgtgataatatgttgcgtacatttttggataaagacttcattacaaacgacccttacgtaataatag
tccccagaatcttcagacaggtgaggcgcttccttcttggcaaaatcaggatgattacatcgcttctgtgactacatatcgagctgaaccgtcccgc
aaggactttatagactctcctgtggatcttgatagtcaaccctatgctatgaagtcctccacctcctatcattctgttcccaggaaagagaaaccga
aagacaattcttttccgcccttaaatacaaactccgcacaatcgtttgatctaccattttctccttcttctagaggatcaaatgtcgatgctttaccta
acgactggatgaatacgttttcggatcaatcttggacttctccattaccgaatacagatgacatcttatcaccttctcctgctcttcaacataataatt
cccctatggatgttgacaaatttccacaatatccttctgttcactctagacgtaccctttatggttcccgaagtcgccctgagtattcttctataaagc
gtacacaatcgttaaaaactaggcgcacgacgaaaccaaaagttacccgtatacataccgcacatccttccatcgatggtcacttcgactatatcc

ataatagtcctttatcttctggttctaccatagaaagtttatctgctaacccatttattccccctgagcctcgttcccattctattgaaattgaaaacta
taactgtttggacgataattttagccctaatacttgttctgatacaatacctgaaatttcattgtcggcagataatgagaatcttaatatttttaatga
tactacaggagcctctaattctcataatacgttctttgattcccaatctctgaatgattctaataatgtcgatatatctgatgctgctttaatgcaagc
aaatgctgattggcacgcgctcaccacttctcacatagatttggatttagaacaccaagatacatcttaatcgttaccatctcctaactgagctaca
cgctgtttacaatcttttgaggagtttatggtaatcgtagctacttttgctgaatggtgctgtaaagaacttactcactagtacattcgttaccaagtt
agaataaatatatatatatatatatatattaatatgttaatatattaataaatttcttgtctaagttaaaatttgtctcccggtaataccgtcaatt
aattgtactgttgcatgatccttttttaagagtagcaagccttcttagatgtcccaatgaatgaggtgtccattcccatccttgattaagactagctact
aataacttccttgctaattctaagaacccatgcttagaaaacagagaaatcataagcgtagtaatttttttgttcgtatccctttaaatcagcaggtat
aatacccgtatccttataccggtaataatcttgccaagcaccatatccaaaaataaagtcattgcattgtatagaaacgtaaattgcgaaataata
gactaatatgttcctttttgtgataaattgcttttccgtatccagtttttcgatgaaatcaggttgacaaaattctgtagaatagacatgtttgaaaag
ttctaaattcttttgtgttgaaaatacatttaagtaagataaacttgttttggtaacgacttcggggtgatacttcttcgtgtactcgcgtgtatacac
agcagcgtaaaataaatccgatttcgtacttatattccgtaaaccaaatggcaacctatagctttcatcttctcctaaatttttttttcgcaatttcccg
gatcctatcgaaacttggaatcacagaagattcctgtacattggcatttgcgtaagctctgtaagcaaaattgaataatcgttcaatactgcaaatc
ttaggtaaaactggactttgaacccagtattcagaatacaggtttttttaaagcattgacgttaccgctttgagcagcaaatcttaaaagctcaaaa
gatgtttgtgcgttcggggttaaaccgaactttgagaactctgataggtaagcaaaagcaaaaggatgatacttttttgtccgccgataaagcatga
attaagttattgattgtaagctcggttggcttcaatggatttataggtcgcttaatcatatcgttgtacagctcgagagctttctctggtctttttttgaa
ttgcacaaagatatattaaggaatcataatcgcgattatctggagctgtgttaattgagacgaacttcatatagtcaaatagagacattgcttgttg
agtcatttgagctttgcaaaaggagcccaagcaaacgcttaaaaacgaatgctcaggtaatttcatttcattttgataacgctggacaagctccat
taattcgtcgtagcgttcaagttgggttaacgcaattgcttgtgcacagcacaaatctgaatgtaaggtagaaccaatgttgttatatcgtttcaaa
acttccaaagacataagaggtaacttctttctattacacgcaaccatgaactgatagcataattcgtgccaaatgcctaaatttgtatttccaaggt
tctcaaggattcgctgcatattatttaccttcaacgtagtaggtaatgctgcgacaaagtattctacttgcgatttagagactccttgaaagcaattt
gggtcatttagtaaagcaacgactttctgtttccaagcgtcaaacttttctttacttttctcattaaacttcaaccatttgcgatcattacctacagctg
ttagggaactatatgtgtcggtatcattatggtcaagttgtagcctttctgaaagaataggtcccaaatagaaactagcgtcaggcaaagctttttg
ttcgtcctgagttgcaactttggcattggttttgtctgcttgttcaatagaataaagaacttgctccaagtccttttctgataagtcaatagagggcta
tatttgttaataaatatccttgtaaacaaaaagtcatagcataaataagctgcagaagcaacctacgctaaaagcataccgagtcgttaattcgat
tatttcttatttgttctaagtttttttctaggtccttaatcttttgatcaagttctcgctgagcggcctctaaggatgctaagttggaagcaaagcatcga
aggccatgcttttcatcgaatttacgtcgccattggaatatatgacagtaattcctatttattttaagcactttcggtaatgagtaatgtactttagca
attttttggatatttcgcgatgttaaccatttgaacgccatgtgcgactgtattctattgttttgcacttttctttattgcattcctagaatgtttgttaac
tagttaaattataaattcagcctaaacgtttctattagccttgcaaatttcagttgctgtgaacaaattggtaactgctgtggttcaaaattgggagt
atgctgctgtgaagcgaaactccatttttttaatcaaaaatacttgcttcaagttcatgaacaatagaaacatttattacttattccttctgaactaac
aactaaaacgaatcaaatcgaattaccttcaaaatagtacgcaaagtttgatatcaattttcgaaaagaatcaaaagaaacagtaaacaaagca
atatgctctacaacatttatttctacttcattaagatccttcattagcccaagaatagaacataatctttcatggtaactgattcgtagttgtaactaa
agatatttgctataggtttttttattttgactatgaaacttgtacaactatttcattcggagaaattcattttgttgacgatttcgaaatagagctccaca
aacatgagatgcgtccttaaggttgatgatattcatgagtacttgtaaaagaaaaacgttattacggaaatgcaggaaaaacatgtatgaggatt
agttagagaaatgtgagtgtcggtaacaaacatagcaatttctattgatgacaaggaaatatcaaaccattaacaacccagaaacataaataaa
tatgaacatagaaaacaaaaaatcatcatttttaatccaaaagtaccatacgatcctctgaataactatacatgttattttttgtagaatcattggc
acggacaatattgcaagtagtatctagtacagagccggttagggactgccgggaatctaaaaccctggtttgaatatcacctaggatagaagaa
cttgcggaacgagcgacaggagaaacggtgatgggataagaaatttcgacatttctagcacgataaagcctcacctttagtaaaagcatgtaag
tgacattatagagatcagaagaagtagtgaacccttgtatagaggaatcatccaagtcaggatcaggaaacacttttgacagcgtttgcgaaag
accatcctttcccaagcgcacatcgtcaagatcttgttcagctttaagtattttctttgtacgaacaataggcgaggaggtagaagacggatgacg
aacagtgacacattccaccaaagacagtgacaattgctgcactcggatcttggatccattagaaggcttaatggatatgaacaccgtaagcaaat
ccataggcccaatacattgttttggaatggatgcctccacgatgactctattgtctatgcttgcttcttttatatagattggtgaattatacatacccc
aagaggagagcgtgtcgtaacgatgaatgacaatcggaacgcaagtacaaacatcttctaaaccagaaagagtaagaatagcccgcatctcgt
atgttgttgagcaagaaataccggaacgaacaaggtcgaatgtcggagtgagcacttctccctcagaatacttcatttcaaacggtaaatccatc
gaccatattctataagctgaagaggactttgtaggtgacctaagtgagtctgttaagagcgatgaggcgggcaatgacgaattgtctgaagtttg
gctaagaacacgagaaggcagtgaactacttcggaacacattttgagtgtgtggagggggtatgggcggctggtttgaggaacgatgtgagaca
ggttgatcgggtatataagactcgtaaaaatgttcattaaaaaaggaagtattagacgaaggagacaaaaggtcgtcactggcttcggaattttc
ggaaaggcgaaggggtgtagtatgtttttttttagaaggaggaggaaacaagacttgttgtactaaccgctttggttcagacaaatgattgctcac

gcctaaagaaggaacggcaacagattcgattttccaaagctccaaagcaacttgtataatagacaatggttgatgaatctctttgcttcgtacttg
cacaaggcctacgattctaggagctgatgcagggattccattatagccagcttgaaaatccaaatgcggcaacggacgaatttcaattggagaa
aagtttttaatatatgtacagggagctgtcatgctctagggaaagtcacatagactgtggtttggatgattttcttgagtttcagaaagtacaggttt
cagtagacaagcaactacctgggttacttgggttacgacagaagtggggagtagaaagaagacgagatgagaagagaagaatttgaaaagaa
tttttctcttccgtattttgcttttcttttttttatggaacccaattcatcaagcggtatattcctgttgtgccaatcataaacttggggtacaacaattt
tagcgcttgccaaggtgaatgatagtattaaattgtttcttgcagttcaattgagaagaaatttcaggagctttcacttctttgcaaccgttcagtcg
caagtgattttcattgcttacactcacagaaaggaggaaattcgtctgtttgtaggagttctgtcggagtcttttcaatctaccgttttgttcctatatt
tcttacttgtttttatttttcagtctctctatccttacaagatgatcaccaacatgtttatagctgaaagctgagcactggaattcatatttgacgaat
tcattggtaagctttgaagagaaaaagaaaataaagaagaaaaatgaatattgaagtatattgaaacgactttgggttcttcagaattggtttgtt
taattacatacactactacaactaccactaatgctacaagggtagacgatgaaggacactatcatatgaacttaacatgatattgatttcgctttcg
ccaaagtcttcaaaacacagaaaagatagataattgttcattctctttttccttttctttaattttcttcaaattagctgaattcaccggcgcccttcaa
gtttggaaatttcccttcaaggtagcattgatacctacatcagtgcttgttcatttcaaactcttcacattcactacacattgggaattctctttcaggt
ggtatttttagtcaaagttttgtcgttttgggattagcgaaaggtatctaagcgattttcgcgtctattcttcacttctgctcccccagtatcagcaatc
ctatttcctactccatttgcttttgtgaatgacacacaaacgtaatgcgacaaagcggtcgcatccagatgatgctgcatctttagaagaacaaag
aaagcgaaagaagacgttcttcaacaagtttgtgggcatttgttcccgtactgtaacatatgcgaaaatcttcctcacgcgctcttcaatgacggat
ttggacagtactgctggcaatactagttcatctttatctgagaatttggataatcatccttcagaccctgtcgaagtccaagaaaatacgcacggcc
agtcgcttgcttccctcgcaacaagttcagggaaaaatgaacaaatcaaatcaaagaaccatagccgttttcagagtagggatgaggaacttga
tcctatcgccctacagcgtctcttcgatccaagccttcatagctccaactctgatggacttaagagattgtctcctcaaagttttcgtgcgtcccgtct
acaatatatccctcgtcctcatcgatctgttagacgcttatcaataagtgatcgcttaaatttagccgtatcacaagaaacagctgagtcctcatttc
cttccgtcctttccccgtttcaacgaaacgagactgaaggtttggtcagctctcacgatccatcctctatttcttctaatttctcatcccaaatccctac
tccttacatatccctatttcctgccacttctaaacaagaaaacactccaaaatctcatgaatccaaatttgtcacccccttcttcacaaattcatgattt
aaaattacaacctagctcttctcacttaccttttactgctacaatgagaagaactatcgataaaaattcaacaccttggcattttcctccacgtcttg
tatctacagagcagcatccccgtccttcgcgctccccttttatcatcagacttagataatgaaaattctcttttgcgattgattcgtgaacttaaggaa
aagcagaccgaagcgtttcatgactggaatgaaattgatttcttgcagctgaaaggtcttcacattactccccctcctgttcgtcccaaatttgtatc
tctactagaattccctgaggatgctaagaaacgggctttacggcttttaaatgatgccgaccttcctcgttcggcttacacagagcctataataact
aaatataatattccaataatgatacgagaccttcggaccttgaaaaataagcattggctaaatgatgaggttatcaatttctatatgaatttgcttt
cggatcgctctcgctctgatccttccttacctcaggttcacgtatttaacacatttttttacacgacactacaaaaaaagggttatactggcgtccgt
cggtgggccagaaaggcccatatgagaattgcggattttgactgtgtattagttcctgttcatctagacgtccattggtgcatggctgcaattaata
aaaagaaaaaaagattcgagtattgggactcgctggccggaagtcctggtaaagtctttgatctactaagagagtactacattgctgaaactaaa
ggatctgttgatattacaaattgggaaaactacgtcgatcatagttcgcccagacagcagaatgggtatgattgcggtgtctttgcctgcaagaca
gcggaatgtctggctaggaatgttcctttacagttttcccaaagagatatgccagagttacgattaaggatggcagctagtataattgacgctcgg
atatattaattattgatacagtgtgtttttttatctttttccttcattgtttgttacaatctctcacttttatttacatacatctatggtaatttctaggggct
ttaccaaaaaataattcaaaccaatattcatataattttggtagcatagatttgctcaaagaaagcgatgataacacgtagcataatgataaaaa
caccttccattcataattcacaacgattcatatccataaattaattgtttaggcactcaattgacgagcatccagacccatggcgttcaaaaataga
ttaacattcgcaatacgtacgacggcaatctcttcaggagctcgcttgcttgtaataatattatcgaggactctatctctcgtttcagaaaccaaaa
cgaaatgttgatcagaagaagcagcagtagcacaagcagccaaaaattcccaaggatacgattcatcagcataagcacgagcattaggcggag
gaaaaacagtcgcaaaatggtctttaagacggtcaaataaaccattaaatatcatctccctaaaatcagttagtacatacaactaatacagtcaa
gtcaaaatacataccagttttctttgtcttcttcagtacacgatccggtttgcttaagaatttcagcacggcttattagcatagttaaaaacgacagt
ccaattttggtttgaattaggtataatgcattggatcttccaaggagctgagaaaataaggtattaacgtttgacatacccagctcattcacaatcg
taagtaatgaagggataacaaattgagtaaatgcattcatttctgtcgcaataccggcaggtaactcgccatttacatcaaaagttccctttaaaa
cgatattaagagaatcaacgttgcaaacgattacagtgagaatagcaaattgttgatctttcgaaaggtgaggtaacaatcgtgggagaagacg
cattcctttaggatgagaaataacagacaaaaatggtggacgagccttggaagcgtcgtctaatgactcattgatatgaaggacattccaaatac
tttccaattttaggttaagattacttttccattcattaaatgtctcttcatcctcaatattagaaacagataattttcggagagattgttcaaaatcaa
gcaaccaatcataaatcctctcaattgcttgtaaaacatcctttttttgtagcaaaccctttgaaagtggtttctttggcgttgattgtttgttcagaag
ggcgtttcacatttaaaagttgtcgaggggtacggacagtgtttatagctattttacctaaagcgccctcgagggatagctgagtagttttagggcg
ctccttggcggaataaaccaatttttgcaattgctgctgcaatctttgcatggggttttctgaacgtcgtccgcgacgacggttgctactgggaccca
agtacgtttggataaagtgactagaatcctcttcagagggattacgaccacgaattatagaataaactcggtagtaaaaatcatccgattcggga
tcttcagacatcagttgggatatctgaatacgagcaatgaaagacttatcactgcgagtcatcaaaccggcacattgtgccatgagcatatgatct

ctttccaatttctggcgcttttcatccaagcttctagcttgagcaaattcctcttgttgaaaaaggttcaaaagtggaacaggaggtactgattcggc
accatacaccatattagtgggatatggaacgggggtacttgaggtggaggaacgttgggatttgcgaataattgcgaaggctgcatcatagga
ggtaagggcaagccaggatttgcaacattcgattgttgaatggggaacgatacatttcctatactggcaacagcaggatcgagagctgcaaaag
tatcatgggtcaaagtagaaggagggggaacctgaacggtttgaggagccacaggggtaacagcaggcatcgctgaagcaacaggttgttgcg
gtttaggacccggcgcattaagggaattgaataactgttcctcaagctcttgcaagctgaaaactttttcgttggtttgcgcacccagtcgattttga
aacccagaaacctccggaggggctggttgaacaataggcttctcaggtacaatgttgtcccatatagatgccatgggtctcaaatccgaaagcat
attagtattgactgaagtatcaacagtttcctcgacattcctttctccagaagcagcaactggattgacttgaggaagggaagaagcttgcgtggtt
tcactaatgatcttgtccatgggacttttgtaatcgtcctcctgttcatcttccagtttgctagaagcctgcgcagtttgattgaaaaaatcaaagtcc
cgacctttatattgttagatatttgcgttaaaacaataaaggttaacttgttaacaagcgacgccatgatgttgcattatcacatttacataccaata
tttccggcgctaacaccaaaggtttcgtcattcaaatcgtctccctcctctactaatcggtctgctaagtcgtcgtacgtttcttcaaaatcaattcta
aacgtccacagtcagctttttgccaaaacttgtctagttatcgcataagctgaaaacagcattcatacccttcttcatcgtgtgctccatctttagga
aacaacgcctcctttggaagggttgtatcgaacccgaaaaacgacatttttcgagttttctgtctcgttacgacgttggtggttaagagcaactaag
agccatactactactatacggtggagatcgaaggagcagcgtagaatgaagcgtaaggatattttccttttccatttataaaaagcaaaatgatta
tttatttattaaacttactgaatattttggtttaaaatttgtagcatctgaataacttgcatattagatatctttgcagcatttcattcataaacaagta
taagtgactaaataaaatcaataaatttttgtcttcattttgtcaatggaaacaaaggaagagctcatagtttcgaatgtcgaaaataacatcagt
ccagttttgcttattggaattaactaagtaataaatataaattcaaccgacgattgtggggccaaataaattataggctttctcgaaataactgggg
agtttaaggatcgtttagtaaggctctttccaatgtcaagtcgtgagatccttgttcgaagaaccgccggagccccataccatcttatcaaactcag
tttgctgcttttgaaattctctttcttcttcgctttgaacctttgagtcagtagcatctctcggccaatcccaggacgttttctcttgattttcatcctcga
cagggaaagagaaatccttttgttcttttccatttgtacgattcaaagcgttcttttctcgaatacggtcccatgcagatttgggagactcttcctggt
tggaagaatcctggggcatggatatgcctaacgtgtttgaatcattagaagaagcttcctgttttccatttcccaatggattctgtatctgaccaggt
gaaggaatattcgatgaaggaggaaagtttgttctttcttgcatcatagcacgctgtcgaataagtgcttgttggattttccgtaagatgtccggac
catcagggcgctttttcacttgagatacattgtatcccatggtgacgatctgggcgatctatattaaaatttccgttagaaagaagacgagacgaa
aagctgacgaaacaaagcaaaccagtgaagtgaacatggccgccgattacaaaaaagacataccccatttccagctatacctgatatgacaga
cgtagcgtataatcgaaggggagagattttgcgctttcgtgcttaaacaacgctcaaagttagcaacagcactatacaaatgtcgaaaacagaa
ccttacccagaacgattgggataaaaaatcctccaatgattccaataaacttatttcggcgaaccctcgatcgtgattcgtacaataagtgtttttg
ctcttctgagaattcattctttgggtcattgagcattctctcccaaaacacatccattttgtcttagtttatgaaaagaaaacaattgaggaaaatca
agaaacagaatagacagagtgaagacactgaatctgactgaactaatcaatcaatggcaaacgatcagtgaattattgcttctacgaccttaaa
agctggtaaatacggaccaaaattgaacgaaaaaacaaagtctccttttgttttcgaaataaatttaagaaacacttttccctagtggaaactttc
caatgaagtaagtaccttcctaggaataaagagactaaagcataatcccagaagaaaataatcctaagcgcatatgcttaaattcagatacaaa
tatatgcttttgtgcgatacaaggaagaagaaaacggatattcaaccgaacccaagaaaaaaaaaaatacaaaagaaaatcttttatgagtcat
aaaagaatcatactacatattttccatttttatttggcctctcgtttgtataactgttaaaatgcgttggagtttttaattagatttacaggaacctgct
agtaatacgttaaatgaaaaaaagcaagctaaactattcatattttctatattccaacagtttcgcaaattaccagaattgaaggaattcgtgtaa
tttatcaaatattagaagaaaaaaattttgcaatcctaaagggaagtgattgatttgatgcgctttttctttttggagacaaaatcccaacctttacg
agcaaggtgatcttcgcactcaaatttgctgttctgtagccattggatgcgtgttgctttgcttcagaaaatatagattactttgaaaataattgaatt
caaagagaaatgtcgaagcaatcgattgatacttctaatgaattcaagacccaaaaggaattagaaattgatcctgagtgtgcgcttgagcacg
atcgccaaatgttgcaacagctgtctgaacatgcttctgatttggattcccctaatgtggcttccaatggcgatattcctcttgggtttgacctttctg
gcattccattaaatatgaccccagatttctatctgcgaatgaaccaaaatatggaatacgcttttaatcagccaaatcctgttgcctctcctcaatat
ctacttcgcagttcactagtccctactttggcgaatctcagcaatatcattttgtctatccttggaaagcctgttcaggaggcttcggccatcgtcacg
aatcccgcttcagaaatgggaatggcttttacaaagttaatgagcatgtttcgtatgatcaaggacatatacaccgatgattcatttatctaccccg
cggttatcaacatgcgtactccttctcagcgaagtgccgttcgtcgcgcaaacttggcgattttcttgtccgccgtttatggtgctcttcagattggttt
ttttcatttaaacgaaaattttcttgaagtgttcgctcctgatgaagccaacatccttaccaatcaaggtactctgtacatggaattaaaaacacag
gcttacatttctgccatggcccaagcagaacgaccaaaggaagatattttaaacgatcttttcccttccgatatggctcagcgatttttactgcgtcg
taatgccaagatagacgacaagcttacttatgttgagaaacaaattttggaaaagtgtgctgctcggaaagaacgattatcgagttttactccgca
ggaagcattaaacgaagtttatccttggggaaaatttttaaacgagatagcctgctatatccacaacaatcactcatctattgcagctattcctgtt
cccaatggtcatgtaaaacggaaagcaaaaagtcaaggtggaagatataaatcatgtgacgaaaattcctctccatcagagtctagttctgactt
gacagatgggttagcttttggccttccaaccgccactttggatgcaacttctgaagctcataatgtttccagcgttgttttatacgatcaagttcgaa
acatgactagcgccggtaatggcagtaagcgagctaaaaaagttgcaaatagaaggacatggactaaggaggaggaagaagctcttttagatg
gactcgatttagtaaagggtcctcgctggagtcaaattttggagctttacggacctggtggtaaaaaaaatgaagttttaaagcaccgtaatcaag

ttcaattgaaggataaagctcgaaacatgaaactttttttcttgaaaagcggacaaattgtgccagctcctttacaatgcgttactggtgatcttcg
gagagagtaaaattgtgaagataagcgtccgcctttttcaggagttttatttttttatttttttattgagtttgaatggtttgggttatttatataattttat
tttaatgtctaaggaatttttgggaataatactttaagactgtttatcacaatagaacactcacgggtatcttttgtaacaatctcggaagatgatttt
gcaatagcttgttcaactatatgggttactctttggtaatcgttacgaaaaacttggagcaaggtgggatctcgacgatcaaggttctgcattttcgt
gctaattgaaatagccagttccttaaaatagaacatgctaattgtagcaccgtcaagaatgaaattgggttgtgcttgaaagtattctaataagtt
agtataatatggttgacaaacggtagtttgtttaagtgtcttctcccaaaaaacacattgagatccaatttttgagatttgataccatccatcttcaa
atttagaccagattgtagaattggcagcggtggaatcagcagatgaatgtaggagcctacgagttgtaggagacaattggaatatagactcaaa
acaagcgtttgctttttggcttctagagctcaacatctgagacagatttctctctaatagatatttgtctagtaaaaacagagtagcgcaagcattg
gaaaaagttgaggaatttgctgttaatacattcgagtcttcttgtgctaacaaaaaactcgacgtttcttttagcatcctagagtgaattccaatatt
tgatttatgcaacgactgtacggaagactggcgcttctaaaactggttaattgattgcaaagcactaacaaaagacatacaataaggggatagc
gactaaccagttggtccatttcttgtaataatgtagctgctcgaaaataattttcaaatctgattcaattagccatacgaaacaaaattggattgat
gaaaagatttttttcgaaagggcataccgttcatgttcgttagaaacactatctgcaaaagctactaataatttctgcaggtcaaaatagtgatgaa
tccagtataagctggtataagtcgaatgaagattcacggttgagtcatgtaatcgagaagcagaattgtagggcacggtaaccagagaacgcat
tctttcataaacatctgtgagttgcttcatttttttctcgaacttccgttagtttcgattgattttgaaaaatggattccacattccctgcgaggttctgtt
catttgaactgataaaagcctttctctgattttcaatttcctttatagcatatcctaaccgtttttttagcgactgccttgtcctctgttgcgtctgatgct
gcatggaccgaagctaacaaacgtctagcctgttgctttgggtcaaagtcatgagctttgtagtcagaaaaatcatattgaaattgcatgaaaac
gaaatataaacgatcaaataaaggtatccgcactaacacagggtttcctttgggagattgccaattgtacaggtacaaccaactataaataaata
agtggaacgaaagatataattttagtatcaaacaagattacacgattcacataaagatgtcaaatcgctttcaattatgttacaatttggtgtcgtt
cttgggtttcaatcgcataacgacccattccaactcggtaagaaatattgcaagctaaaacctatacaggtaaaaaggttatgttttcgaacgctg
ttaaattgatttcatattttttttcgttcctttaggaaatgaaataaaatgaaatacatttcgaaactccagcatattatcaaatagtaagcatatttg
gatattattaaatagagaagcatatattgacaatatgatgcatttaatctaatttataagcatatgactatatgcgctacttatgaaattagcgtata
tatgcttatataagcatataataagtatacacttaagcatatgctagcaagcttatcttgttaggatatttgtaggaaccacgttatttactcgctcct
ttgtaatgaaagaaattctatttgcatacttttattagctccattatattgaatcttattaaaattattttataatttttgtaaatagtgttcctttcctct
gttagaaattcttgagtttcggcagtgcagttcagtaacatacatcgtgcattgtgcattacagtgactagcgccaaactacgaagcagtttccccc
ttcgcactttacttctacctgcacctcttacctgaggagcgcctacgagaatcatattcgttgatcaagataggcattcccatcatataacaatgtcg
ttctcttttggtaagtacttgaaactctaaagctacattgcactaacacgcttcaccaggccaacctgcatcgtcgtcaaatacaccttcttttggta
caacagggttgtttggtcaacaaaaacaaaataccccctacatcgaacttaggttctggatctctgtttggtggagccaatacagcacctaatccttc
cagtgctcctcctgcaggaggattaggttcttcaatgtttggccaacaacagcaacaacccgccagtggtggtatcaccggctcttccacatttggc
tcacaacctttaagcggacttagtgctactacgactgcggtcccccccttcttctaacactacgacgggcacaggtgcaacctcgtctcaacctctttt
ctcctggagtacgaatgccgcgacagcaaagccttctgaaatgggcatgtcatctgcaaatccaaatggatttttgttatcttctaccatgtctcca
aacgcagcagttagtaatgctcaatatggccctgcacaacccccttccattgaagaacaaacacaaaaaatattgaacgcttggaatttgaaaca
tcctgactgtgctttccaaaggtacttctataataaagttcccgttgagcaggcagctctttacgtcaaacctcctactcataatcagcaaaaatgg
gatgaagctgttgcgaacaggcctagtaacagtgttgtgccagtgctcgctgtgggatttcctgacgttcaaaagagaatgaacatgcagattaa
ccaggtaaacgcttatcgaattcgtatgcgagagattgttgaaacacttgaaagattgggcaataagcatgaccttagctccaatataaaacttgc
ggaagcaaagaatcgccacatacaattgtctgagcgtatccttcgtttagccattaaagtgcatgtcttaagacatcgtggttatgctctgaaatcc
aatgaagaagacctaaggaaaaagctcgatgatcttgctaaatctctaaacaatcctgaagtatttggtcgtttgaatgagatttgggcacgaatt
actttaatctttgagggagagaagatttcagatgaacaacggaattcccttgcgaaaggcgtaattgattggactaaaaacagtgatcaactaca
aaccatcacggatgttttacgtgatcatcaaaatggattatcgtacgttgccaagcttatccaggaggatttgggtaccttgagtaagcaactcgat
gaccaaacaagtatgtcaaagtaaatctttttcaatagtggtattgttgggtttttcttttcttgatcatgtgctgccagtagtgatctatgaaatgag
caaaggtactgtggttttgtaattttattttgggtggcttgctttcccatttttcccttgtcaagtcggtggtcatattatttccttactattcccatgaaa
tgaattgatagtttttatgtttctgcttgttgtttggtacatcctgtcatctaatacttatgatttagactttagcagttcagctcttttaattttattatgc
aagcttatttatcacaaaaatccatatatcattaaaggccctgaatttaaaaatcttttctttacaccattaaagaaagtgaaccgcactgttatcat
gaaagctgtttactttaaaggcatcatcaccttgaatcccctatgtgaactcccaacaaaatatgaaaagtgttaaaaattgaattttattgattgt
ttgccatcatcatccgagcaattctttacacacataagcagcgttcaagcaatcttaagtgttgtttgttacagaaacgtgcctgtgaatagcttcag
ctttaagagcgtaagttcttgacaaaggagataacaaaaggagcttaaaagaaaagttagcatgtatatttgcagagagaatatagctttctcat
tcaaacttactcaaagcgagaacggcagcaatagaagcacctttctttatttcctagataaaaccggttaattcaactgtccgatgcccttccaaa
aggtatccttttcgcaagacatgatcacatacggaaagagcaaggttatcatattgcttgatttgttctttagaaggacgaccagcaccagagaac
atttcgcaatttcaaaaatgtcagtttagggaattttggtggaagaaactaatggagcatgaaatatatcctgtggtattaggtagattcgataacc

tcagtagatatcgttttgatgcggtaaatggaggataacagattgtagcctatggaaggttataatgaatagtgaggattcccgatatatagattc
aacaatggacgcctgtttcaagtcggcaaacgatacttttgccaacgactaactggagggaaaggaaacaatcaatttggactattcgttggattt
taattcatcttctcgttaatttactaagaagtaagttcattaagggtttggaagatgtctgaattagatggccacgatgctgaagtacggagtgctg
aaggagaggcgaatcaggatgctgcgcagcaagagccttcaacagaaaagggtgaaagtgctcgtgaaaatgtagacattgatttttctgaag
acgaactttcagacttggatgaagatcaattcgaaaattttgatgaaacgaaaattggtcgtgaagccgatgatgaacctaccatttacaatttac
cttcatttagaaaaaagcaacactctgaggatatcaaaccaagtaaagtggaaagcagcaaaaaagaacgacgtgctcggaggggtaggtca
cgtcgtcctgctcaacgaggagctttggacgagcttgaagggttggaagaaggagaccgccgtcctgccgaagaagagcttcctatggatcctac
tgtggccgctaagcgacaactggatatgcagatagatgcagttttgaaaccaactcgtcagaaaaagcgctccaacgaagataatattgaacaa
ttagcagatgatgaagtgcttcgtttgcgcgaacaaatgcgtttggctgcgttacgggatgcagaactgaattcagaacgccttcctgctaccgaa
aaactgaaaatgcttccgcttgtagattccgtgcttcgaaaaactcatttatatgataccattctcgacaacaatttgttggattccgtccgaatgtg
gttagaaccattgcctgatcgatcccttcctgccctaaatattcaacaatcattgctcgatattctaacgaagctacctattgaaacagagcatttgc
gtgaaagcaaaattggccgcgtaatcttattttacacaatttccaagaagcctgagccattcatcaagcgcattgcagatcgccttgttagtgaatg
gagtcgtccaatcataaaaaggagtgcgaactaccgcgatcgcgctgtgggtattgctaccttgaaccctgagcttataaacatgcgacgtcgta
gagatcttgccattgctgaggatgatgtcaataatcccaccggtaggattggacaaacagttattcctcgatccatcgattctagataccaagttgc
acccagagtgcgtctcaatcaatccgggttgtctgtcgctggccggttgaagcctgctgatacggaacaagtccgtaaattgaaggcgaagatga
aagctgttcgcagtaaaaatgcaaagaagagtggagttagtatcgaaggtcgaggcctataaaatttttaaaaaaaccattcgaatattctgta
ttgctttaggatcactccctaaatccaatactaacagggaatggtcggtaacaagcaatattatttaatttatggactcgtgctaatactttgcatca
tcgttgaaaggaacgtattgtgagaagtctgggaagtgttttattttctaaattcttttgtaatgttatacatgaaatgtgtatatataatgattaata
aatatggtcatagcaggttctaaatgttttgtctgcattcagcaactgcattgtataataaaattaccctttttgaatgcctttttcatcctactatgct
agctacatcaatgtccctcactgcccaagaaatagaaactaagtccttgtaacttggtatattctttctgagtcgagcttttcctctctacttggtatt
cggtgatttcggagtttaggagaattccatcggtcattttcaaaagatagatcggtaacctatagagcttagttcaccgagttcagatcagttagga
agaatatggtagttcaaggttgataagcgcatatcaaagacgttaaaaatgagcaggtgtaaggagtcaaaatcacttagagtttgctttataac
tgtgactaatcaaaatcagctcgcttattaaacactatgaagccgctaagcgctttattaccagaagttgaaggaccacagacccgcgtatgtaac
tttgattgaggccctaaccgtcctatggaaaaacaacaaaacagtagtttgctaacttttgaagtatcaactcattgaaaaattgggttggatggc
cggttgtgttgtggtttaccaggtcttgctaaacatgcctgtttacggtgccgcaaaacgcgatttcgtggaccctattaagtatgatacatgaaaat
tatttcattttatgtaatgctaattcttctatagcgtatggcgtgtgctggatggttcttctgcatcgtaagtaaacaaagtcttgaatgttggatggg
aagagactagggaaaaaggggtttttgaattgctttcgcttttatggattcttaaaagatgtcgattaccctcctacagttagaagaggaagcaga
gaatcttcgtaacgtttataatttccagccgaaccagattcttctttctatttgacaacattctttttggggggtttttatcagcattctaacaatagca
gtggtgccttggtcactggtttggcccctatttttctttctggttttctaatgcaacttttggctgccggtcgtaaaattgctgttaattttaatttaatca
gcgaccgagtcatgtttcaaaatacccaaaaaggtttgtaattagaattgtggttcaatccgttggctaatacctagttttctctggtttttgtacct
ccttttatccatcgcatatgtcgtatcaggttactacggccacgtttctgaacttggtattactttatctctcattttcgtagtccagttgttcataccag
gaatcatatgtatttatttatgcgaactcgttgaaaagggatacggcttaggctccggacctgttcttctcttgtcgtctcatatcgtttcttctttgttc
tggcagatactgagtttcaaccgttatcaattgaccgaagacattagtcaatatgaaggtactcttgttggttttgtattttccctgttttcttataagg
aaaaattttatgcccttcgatcaattttcttccgcactgagcgtttaaatttctttagtctgttggcttgcgtaggtgcatttgttggcttcatttaccttt
tgtgcatgagaatcgatatctcgattcgttcttcccgtgttcgtggtttccgtcaaaactttcctttgagattgctctatacttccgccagtcccttcttg
tgtatctattctgttgtttctcatcttttagttttggccttcttcattcacagtatgttccctaactctgtctttactcgctttttggttcaatacacggaat
acgaaactgatcctattcgacaactgcgtcttacgggtggactaatttactactttgttcctcctttgagtttatgggaaaccatcacttgcccaatcc
acactattgtctatgctgtatttttgtactctacatctgtgtatctttctctcgcttggatgaatgccactggtagcggacctcgtgatattcttgctttct
tcaaggaaaaccaacttataatagctggttttcgtgaagtgagcatgctaaaggaattggaaaaaatcgttcctagtgctgctcgcatctctgcttt
ggtaatgagcagtttgagcttacttgccggtatcactgcttctacgttcggtttaggagtagtcattgcttccgctattgtatttgcttctttcgaagtc
attatgggtgcaaacccttcacttgctaatggactttaacgttgaactaactatttagtagtatcaataatagacagatttcatgaagaaaagctttt
gcgtctttgagctaatagcgttttagtattttcattatgtctacaaaaattctcatttcatgtctattcactgtgaaaactgttgttgtaagtcgttctat
gattaagaaactaagtaggtaaggtagtccacaaaatttaactttctaaattaaatataagtaaactgtttttttctttattttcttttttttttgcagta
gaatgaaaattctaagtcgaatgatatactgttagatgatctcaaccgcaaagatcagacgagcattgcatggtcctaggaaacacaccaccag
aaggttcagtctcaggctataggaaaggtatacaaaggaatctttttatttaatttttttctgtactttttggagttgaaatttcatatcatattcgttttc
acgcctgctctggatgttttattaaagctgaactttgaagacttacaatccttgtatctctaataaagttgatcatatcagccatttcatcgtagtctt
cactgatacaattattttgcgttatcgaagttgcagattttctcatcggattgaatattggtcttgtcttttccagtgaacacttgctttcttatctctttc
tttattttgaatatgcctacgagtagttatagcagtaatgcaaataagtttgagctaatccattgtaaagaattggcctggaatctttcacgatctca

tttaacgactcagtacaagtcattttacatgtgctttgacgatggatcatttgggtatattcaatttgcgtacggaaatcttggtctgttcgtaaaggt
tgctcccttaggatggacttactatccttcaaaaaatgaaacggtccaggtgcccatagttgctagctctacttatctatataggaatatgaaggttt
ctaaggatcgctatagcgttgatgttcaaaaacactctatgcattttgatgtgaaaatgcgagcgtggcatgtgatgattgaaggaatgtatgatct
tacaatacatatggaagatacggggttttctttaatggattttggccaaaacgatataaagggctacatggagcacaaaacatttccagtaaata
ccctggaaggaacggttactgcacatggaagtacaaaaaaggttaccggcaccgccgtattcattgatgcaatgttctttaaagaaaagtttact
aatctgggacatacgtggcaaaatagtttgttgtttgacgcgtccagaaagaatgttttaacctgtttacattattttcctcgtgattcagctgaaac
ttttcgatctcaagctagtttgactttagatgggaagctggtggccgttcttttaaataacgaatataccacagaaggtgttttacaggtggatgga
atacgataccagttacctacccatattcatcgcattctctcaggttttacgttcgatgggaaaccagtcagcgtgcacttggcagcagatctagaaa
aaattgaaagcattaccgatgttttaaacatttttccaaaatgggcaaaagacattgtatctatttgggctggtcttccttacgtcttcgtgtggttgc
aacatttagtagctgaggtttacatagacgaccagttcgtttctactttatctgggtgccattatctggagcagacttgtgtccatatgccctattctt
aaagagaaaaaaaaaagggagaagtttttcccaccgatactgcgtcacggttttttccggttcctatttttgcaaaaccattcctcctaaagattta
cacaagattcatttgtttcattttctctttttcatacacggggatatctcatttgacttcatctaataaataaattttttggtctttcttcttacatatcat
attcatgatttgttgggttgctttcaccttatcccttccttttagatcattttatacccagcaatttcaggtttttggttggattttcttgcatgtttccttg
ataaataatttgtggttttcctatatttggttttgtcttttacgtttttcgtccgcttattcaataaggatttatgattgttcaggttcattagactagcac
cttttagacccaattattattaataaatttttatttatcaaactcacaaaatgaatctttcaactctctcttcctgcatttattttcatttcccattcacat
atagtgcacttgagcgtacatccctagttactttcctactatagaggatgtggtacttggagctgtcttttaccacactctctgttcgcaatgtcgtct
gagtacaagtatgatgaacagggacaattctttccggtgtttttgctggtaggtgtgtcttgcttcatccttcctatgactttttctacctttttggtcg
aattagtaagtgacactatgagggaatcagctttattaacatacacggatgtgtagcgtctaaaaaaaatgaatctgtttcggatccatttaccgc
atatcgtccgaaagatgttttgaaaaagaaggaacccaaaattcgattacggtacgtaaaattaggaaaataacgtgttgatgaaaaactaatt
ggtccctttggtttatagacattttattattttaattggatgggtcgttattgggtatttgtcttatcgcattgcttataccaagttgagcttcaacgtct
gggatccatacgaaattttgggtataccgaaaagcatggcagtagacgacattcgccgtcattacaagcgactttctatcaagtttcatccggata
aaattagaaacatggtcaatacgactagagaagaggttgaagctaagtacatagaacttactagtgcgtatagagctttgacggacgaggaaac
tcgtgaaaattatgttctttatggaactcctgacgtacctcaacacattgatattgggattgcacttccaaattggattgccgacaagaacaatagc
atttacgtgcttggtttctatggcctcatttttggtattttcttgccctatagtgtcggtcgttggtggtatggtagcaggacttatacccgtgaccaag
tgcatgttggaagcgtagatgagtggtttcctaagattcacgagaatttggaaatggaagacattttgcagctgttagcttctagcaaggaacttc
aaatctttcattcttccccggaagcttcaaaaaagactgtcgtagaactcatgaatagtcatctaaatcggaaggataagtcaaatcccgatatgc
aacgattgctttcggaaacagatttggtaatgaatgcattagtatcggttatcgctgcatacggattttctaaaccaaccgataatgttttcaaactt
tggcagcatattgttcaagcagttccgttagataccccacttcctttgttgcaattgcctcatcttcattttgaagatgctaaaaagcttgcttcacgg
ggtattactcgcatccctcaattcttacaatcacaaaacgagaaaagtatgtcttgtttatcaggctactctaaaaaacaagttcaagaaatggta
gaagttgccagcaacattccccgtatttcaatattggatgctaaggtactagtggacggagatgatgttgtcactactggagcgattgcgaatttcg
ttttgcgtttaaagtgctcttatggggaagaaattatgccagaagttagtgttgatggtacagaagttgccactgagcaagacaaaatcgatgcgg
aacgtattcatatggaaaaggatgaagtcttgggagatttggaggttcttcctcatgcttgggcgcctcacttcacgcaatatcacaagactgcat
ggtgggtgtatgtggtagatccccgtcaaaacagagtgattattccaccattcagcattacggatattccacgcactacgcgaaccattcgtatac
ctttccaggtacctccggtagaaggtactttcaaatttcaaacgcatgttgtatccaactcataccttggagaagacattgtaagcaatttgacaat
tatagtaaaagacgctgcggtgatacaagacgttaccgttgaagaggatgaattttctgaagctgaggatgattcattcgaaaaccctaagcatg
gcgctgaaagcatggaggatgatgacgaagaatctgacgaagaggatgatcctaatgctaatggtttcaacacagatacttctgaagatgatga
gtaattatgcgccaaacactaacgatgcaaaaaaaaataccgatttccgccaagaagtgtcctttagtccattcttctttttttttgtttgtttccagttg
tagctttctaatcttattttacgttcttatataatttgcaagtctatgcataggaataaagatttgtatatggccacggaatctttttacttaacggcga
acaattacttggtttgcgatactttcatttttttcatagcatatttaattgtggaatactttcgaattggagttttccaattgaggcactcttttgataata
gaaacatataaattatggattgaacttttacagctcattagtattatggtctttatagtctataaatgtttctaattgtaatatgtgaatgttatgctgg
atccaattatacatatatatataggtctatggaagccaatccatgaaatgtgcataagactcaaataaaatcatagtaaatcatgtaatatgtatgttt
atgttcattgtacatcttttgaacggaaggccataaattgccaacacaggatttcttttgactggagcaaagcacttggagtcagtgtcaaattggg
gcaaaagtttatttacagcgaatacaaacataacaaggaatttcttttattaaagtaatattttctgaatgatggcagcagcaaaaaaaacttcatg
gaagggaattttataaaaaaacttgggtgtcccaagcggatattggctccaatggtagatcaaagtgaattgccctggagaatattggcaagaag
atcaggcgccgacttgtgttatagtcctatgtttcattctaggctgttcagtgaaagtgaggattaccgtaagaaaatgttttctactcgagacattc
ctgaagaaaaacccctaataattcaattttgcggtaatgatccagaaattatgctgaaagcagccaagttagcagctccttattgtgatgctgtag
atttgaatttgggttgtccccagggtattgcaaaaaagggtaaatatgggtccttcttgcaagacaactggcccgtaattgaatctataattaaaa
ggcttcatgaagaactaccaattcctgtaaccgccaaaattcgcatttttcctgatcccaaaaagacgttggaatatgcaaaaatgattctcaatg

ccggtgcttcattcctagcagttcatggacgtctccgtgagcaaaaaggacattttactggagtggctgattggcagcaaattagtatgctccgtga
aaacctccctgctgataccgtgttgtttgccaatggcaatattctccatgcaagtgatatcgaccgatgcattgaagctactggtgtagacggtgtt
atgtcggcggagggaagcttatataatccaaaaattttcctccctttaaatacccctttgaatatactatatcctcgtgtcgatgacatctgtgatga
atatttgaacattatacgtgaattcgatcttcatacagattatagcaccctttctggcatcaaaggtcatatgttcaagttaatgagacccttattga
gcattcacacggatatccgaagcatgttggcgactgaatgtacgcctcgtgattttgagagctttcctcgtgcggtagctgctctccgaaagcgttt
agatgaatgcaaagagaagggtgaaatttgtgatgaagaagaaccgaatgaggagaagaaggatgaactaggatatcccaagattccttggtg
gcgcgtccaaccttactttcggcctcttacagaaccgtctgtgactaaacgaaagctcgaagaagcgccagtttcagaagctcctgctaagaaatt
gaatgtagcagtttgagaatgtgggttaaaatagcattcttttgtagaaagcagaaaatgagaaaagaaaaatagtgggaagttttggatattga
ttttgttagaataaataggctgtataatattgttattgaattaacttctaggagctaaacataataagtaacaaaagtaaaataaaagtgaaacaa
gtgtaaaaccaatagcaagtaaccagtgtttgataaacgaaagcaatgaagaaggatgagaatccgatatcatcaaacttcctacggaaaagtt
attgaatgtaagcagattacactctaagtaatcatttcgattttctaatcgaattaaggaggatcataaacgtttcaaacgaggattataaagtagt
aatcataaacataggatacatattaagcgaagaaatgaaggaaaaagtgtaaaacaacattataacgtaaacaatatctaatgaaaggtagca
gcatgaacagggcatccggcagcacctgtgcttagccagtcgcgataacattttaagtggaacatacataagcagtattcaattcgcgcaactttt
tctcctggggagaattcttcaaaacagactatacattcttctccaatacaaggtgaggagtccgttaattggacactaatataccgccgagcgtgc
tttagaaactcctttatatttggtggagaagatccgttatggctgaagcaagaagcaacatgcgcttctctttcctcttgtgacttgaattttgatag
agaaacatagcaaactgggcattctaccaagtcatcttggttattcgcgtcatttgctgaaggattgccttcaccatgaggcagaggtaaccgggt
ataggctagaacgggaggagttgattcaccatccatacaagtaggtatcggaaaaggaatatcgagagttttcagttctgataattgacgatgac
aatcgccgcagacgcggacagtgcaccacacaggatcttcttgagaattatctgtatcatcatcttcggccccatcaagcatctcatcgaaaaggt
ttgattgatcaggtggctccgttggatcaacagagatacttactgaaggaagttcggcagtatagttagaacagttggcgcaatatacctttccac
accatctacaatgatgtcttcgtcgaaaccagttgaaagcagattcacaacgaggacaagaaacagcatcttcatctgcttgccattctgcagcg
ggagtatttataagaagccgacacaaggtaggaggatatgctgactcactttcactcatattgaatagacggcaacatgtaattttttaaacgtc
ggaaaaatatatatctagatatatatatgtaagtaagtagttgatcttctcggtttattgtaacaaaactgccaattagcttgattgagctaaactat
gagtcgagaattaaaattggattgaatcaggagtcaatcgcttaaacagttaaatcaaatttgtatataagttcaaataataacaatttctttgaa
aaaaaaagccgaaatagtacaattggcagaatgggcaaggtttatatataagtggcaggaagaagacagaattggttcacgaagaagcaaac
ataaagttggtcttcaatgataagcggctgttctatcaaaagattaagaaaaaaacataaaaaaagataagaacaatatttcttaggaaaggta
ataaacaaagcgaattggaatgttatcaaaggttttccaatagggctttctgagcagagctttcagatgatttgagtttctacaaagaatgaatca
gttgattctttctttcaagtgaatcttacctccatagcacggttaagcttctctcgagaggcttgacagtggtctcttacagtgacttgaaagcaatct
acgcgttgctgaaaatcattaacggtacagttttcttgtaagagtttttcttcttcagtttcaatttcttctattttagaagaaatctgcttgaactcgg
atagtaacagggaaagtgtctttgacgagtcttgatacaatttctgaataccctgaagtttagactgatttttctggtaatgctgaagaattgtttct
ctaatttattagttctgaagcaattgatgtttcatacttggcttggcattcaatgatatacgattgaaggacgcgatcgtgatgtttgctgaaagaaa
taacagcttgtgaaacaaaattcgcgtgtcgattgctcaattctgtttgtaaatgatttactaaatcagacattttaatttacggtaaattgcttttttt
gtgatggggaaagagcaagtttgttctggcgttgagtttatgtttacatggtcctattcgcgacgcgtcttcttcttggaatcagtgtattaaccaga
gtttaaatgatagaaaaagctgtggttttgcatataagaagaaattatatcctttaaagatgattgaacgcgtcttgaatgatttgttttttttcaag
accaatttcagtcctttcacgcatttttttgaagaataaggctacatactttttttgttggttattgtaaagtaatattttccaccgtatcttgttcaacttt
catgaatacaaaggtaattacatctcgttgataaagcagactttatgaaagcgttaacaaaaaagaatattaatttttgatgtctacattagaaga
tgaatagagaagaatgtattgaaagagacagaaacagagaaagaaatctgctttaccaaccagagttttgtttgtttatatttcgaagtgttactt
atgtataacaatgttacttacttgcttacttggtagttacaaactgacttatttaaacaaacactaagggcaaaaacctccccggctctatgggattt
tccttttcgtaatatttacaagcaatactacaattattatatctttatcaagagtaccgtttgttcataaaaggtttggctttacttgcatctgggcaga
atggaagaccctttctatgaagtgaaagcgtaagtttgagctcccttccttgttatatgaaactttttgttttccgaaatagaactaacgatgagata
gcgatgctttggctcaaatggaaaatgtccgtaaactatttgaatctttcatgaagagtcgaaatgcagggattccagcacttaacacggagcttt
cgtttgcaattgaagagctgaatgaaactttagaagacttgaaaggagctattgagattgcaatgagtaattctgaacgatacggtttggatgaa
caagaattgaaagcgagaagaaagtttattacagacctcgacgtcgaattaaacaacttaactctacaaatgaatgccgctccaagcactgatat
tgagcctggaccaaatagcacagatatggcgaaaacagcattgactgaagacgaccgccactctcgaagacaggtgcagcaacaattatatca
acaacaggatgttatgctggacggtgtttatgatactgttggtaatatacggagtctagctcaattgatgggtcaagaattaggagaacaatcaaa
cctgatagacacattggatcacactatagaaaatactaatcataaactgcgtcgaggaatgaaaaggttaaagaattttacagttgccagtgccg
gtaagaatctccattttggaaagttgtactaacggaaatacgcttttagattcaaagtcaggttgttgcattactgttttgatctttatattaatagttt
tacttgtactcattgttcttctgtaaacggttttacgtgcaatgacccttatgataatcaagcatacatggattcacgctacatgtattttatccaaca
gtgtcgcttaaacggaatttgaaacccatgagattaatctatccccattgtttgtaactctatttttggatttttagagggtggctcttgatctgtaa

aggttagtaaaaataccaaaactacttgatatgatatgacatgactagacttactatctccttgtgcggcctcatcttctccctctccttcatcatcat
catcatcttcttcttctaaatccactgaaccttcttcatcgctagcttcctcggtcatggcttcagtgaagtacttgacagcattgggaaacaaatctt
cagccaaaaagatggtgagagaagcaccgtcaaaatcatcgtcatgttcttcaccagtccaacggaaatagtcaaagaaatttggagggctatc
ttggttcttgcggaaaaggctctttccagacttccacttgatgtcgacaggctttgaaacaagagaggtgttggtttttccaaaccctcttcgtcctt
cgtggtgctttctttcactcgaacttccttaaccaaactaaggttatcatcctccaaataatcgttaggttggaaagtcaaggtgatgcggacatca
cgaggatcttccttattgtatctttcgacatagatgttttccaacttttccaagaggatgctgtcttcaggagtgatgtattggcctaagcaagggtta
gcaacggtttacaaaacaaaccgactccaacgaacatactgatttcatcacctgcagcttcaagaacaaccacccagaagttgcgaatgtttttc
aagatatcacgacgtttctcgaaaagaggcttgaacatttcattctgcttctttactgtaaaaccggttagaaatatataattgagctttatgtattg
ttatgacaagcaacctacagatttcgagttcagcctttccgaaatcttgttcaagattagcaaggctctcaaaggcttctgcagcatccatgttttat
tgaatagttaatttggcacaaagttggaattttctaaatgtcctgctggcattctttcgatatttcgtgtattacccttactatgaagtgaaaattggtt
tgtttacgctgagtatattttacatttgctgtttattatgagcattggaactcttatttagatgtaggtctatttgagcttactccgcttctttttgaaat
ccgttagtccgtttcttctgctgcgccggcgtagtaacacgaaacgtattataatggttagtatcatcattctgacatgcatatgtttgtttccactcct
acatgactactagtttcagtggataacatatcaagaatcctgtgccaaaagttgtatagctagattcagggaaaatgaaataaatgcagcctgcc
agccctcaccagtcaggtgttgctcataatttcggttgatgataattacgtttttttttatgatgggatgaattcaagatatgaaattaattgagatca
gcatgctaaaacaacttacgaacattttcaactagtctattttcgcctaaaccccgactttgcttgagagctttttttctttggattggtctatttaaatt
ggtatttattacatattcaaaatgctttagtattctaggtcattagttacgagttttgatccttcattaaaaatgtcaatctgtctttaaggggaggaa
gagttactgaaattttatagcatatacatattttttttagaaatatctatgaaaactactaggaagtcaaagaatttgtgtagctagtaagtttctaa
tacattgggattatatctcttccacctaccgtactactaagtgtcaattgaacattagagctaaacagcagttaaaggacaattgattatttccaatt
gacattcttcttaaagaattgttcatggacactgcgaaacgcttggaattatgtcaggaaatacagcaaaatgaaatcgcggctttgaaggcaatt
ttcatggacgactttgaggagttgaaatccaaaaatgcttggaatgtaagtgattaatatttttgctttagctgaacttttgtaattaggtatcaaat
ggccgggtcttcgctattcatttatgttctcgtagtgcaagtgcaaaaagcattgcaaaagtcgatctctatatagagttgggaagaagttatccct
atgtcaaaccaacagtgaagcttcaaaatggagaaaatgtactgaagtctcaggtgcgcttcctgttaaaaaagttggatcaaaagacgaaaga
gttactgggtgaggaaatgattttcgaacttgctagtattgtacaggaatacttgaatgattggcagtcggacttatcctctcagttttctagtttgg
aagaagagcgtgctgttcaacttcaacaggatagagagcgtgctgaaatagacgaacagctaaaattagagagagagaaggaagctcactac
gaagaggaaaaaatcttacaaaataaaattcgtgacgagcttcagcgtcgttcttatgattctccgcaatcgcccaaggagaagaggtcggatg
acaccattaaattgtcttttaatcgactgccttcttctgtttatttcgactgttccatctctgtaaaaaactgccaagattctctcgtcgaatttcataa
agtaattccattatacacgatatcccgttcccctctttctaaattagcactggcaaaagctgatgtcgatgaagtcctattaccagattgcgtattca
tgcttagaatgatacggtttacgaacccttattatagtactgaagcgggcaaaaaattaattcaagatctggaaagtgaactagaaaacttgaaa
gtcattcgacatgatttgctggcttctatctatgagtatcaattgcagcgtgaattagaaagccatggttggcgtctgtatatattacaagaatactc
taataaactcactttggcaggcttgttacaaactattgtgacactggatgtagagactgtccgaactttttccaataatattttagaagggcttgctg
aacttcatcgactaggaatatctcatcgcaaccttcatttagataatgtcattttatttcaatcgggtcacaaaacctttgctaaacttatggattttt
gttttacaaggactttacgagatatgaatgcgttatttccttttgatacaggatcagagagtatccctggaattttgccagaagatttgtatcctcca
gaagtcagtgaaacctcttttatgtcggcttctcgcaaaacagacatatggtgctttggtctcttagtcttacaaatgctctgtggtatgcatgtcttg
caaaaattctcttcgataaagcttttgttcagtcatgtcattcctttactacctggaccatatcaacaacttattcggcgttgcttaatgcgtgatccc
aaaaagaggccgacagctacggatttactttcaagtcaagtcataagactcgggacatctattttgtctacagttgagcagggatcatcaggaag
aaatccccgtatgtcatatggtgggcaagacggaataatcgatatgttatacagaaaaagtgtctcccgctatgagactgattttgaggagattga
atttcttggcaaaggtggatttggtgaagtggttaaagtaaaaaatcgtattgatggtcgtttttatgctataaaaaaactcttgctccacaacaat
gacaaagaaaacagtcgaattttacgagaggtaatgaccctttctcgtctccatcacgagaacgttgttcgatactataccgcttgggtggaaact
gatacaagtgagacttttaaggaatctgattccgaggaagactcatttgcaccatcatcaagaataactagcgatattcaacgctcaagtaagta
caccacagatgatttttcagatcttgatatacattttgagggtgagacttctagtccagaaaatcctgaattgtcttctgaaacttcttattctgcttc
caatatttccgatgatatcaatttgggtgaaagtgaaagcgataaagcgacaattttaacaagtaaaactcaagagccaattcttaacgctacttt
atatgttcaaatggaatactgtgaaaagctatcgttacaggatatcatcagagaaaaacttccagttgacgaaatatggcgcctttttagacaaat
tttggaagcattggcatatattcacagtcgaggaatgatgcatagggatctcaaacctggaaacgtgtttttagatgaaaacagaaatgttaagtt
aggagactttggcttagccacagaaaatgagccaagtcagatcaccaatgataaactaaaggataagaatacggttgatggggaacttacttcg
ggagtgggaacggctctttacgtcgcaccagagcttcttaaaaatactagtggattacactatgatgcgaaggtggatatgtatagtttaggtatt
atactgttcgaaatgtgtatgtcattttcaacatctatggagcgaattcggttgattgatgccatcagatctccttccatcttgttccctaaaaatttcc
ccttctctctaacgagtcatgaatataaagtaattcattccctcttgcaacatgatcctactaaaagaccttccagtcaagaattgctggaaagtga
ggctatacctcctaaagttggcgaagaatatatacaagaaggcttgaagttgttatcaaatcgaaatagtccgtattattcaaaacttttaaaagtt

cttttggtcaagtccccgatagacataaagattacacatatgacttcaatctttcagaagacaacaatgccatatcaagggttttagaaagagga
tgggatagtcttcttgctggcttggttcgggatcatatagttcaagtctttcgaagacacggcgcaaaggagcgagaatcacaaacgttatttcca
aagtctacacagtatggtgaaaataaggctgtggtttcattattagacagagctggagcacttttacaactcccctacgatactgcgttgccatatg
caaggaatgtagcccgtaacgctgtcgaagaagaaaaaacttttctaatagcggatgtctttagagaatcttacggtggtggtaaaccaaaagct
ataaaaaagatatcatttgatttaactacaaattcagacaacttagactggtacgacgccgaaacaatcaaagttttggatgaagtgctgcttga
gattccatcactagttaacagttgtatctgggtaaatcatgctgatattttttctggaatccttgactatttacaaatttctagagaaaaaagaaacc
gcactagtcaaattcttggtcaaaagaaccaaggtttttctttttcgcagattcgaaaccaactcagaaatgaatctttagtgccttcgacgacttta
gatgacttgaaacagtttgactttttcatgacttttgaggaaggtttaaataaaataaggagaatttttgggaaatcgctgacgattaaaatgcgc
aatgcattagtatatttagagaaagttatgtttttttgaaagggcttaaactttcacatccggtttattttgttcctctttgcgtccacaaccatgaat
tttatgaagggggagtaatgtttcaagcaatacacactgccgaaaaagccgagcttctatgtgtaggaggaagatacgataagcttgttaggtta
tttgatcctccattaatgagaacgacccgaagaaaacatgttattggtatgagtttgggattagagagtattgtgtcctcagtatcgcggtatatcc
atttccactctaggaagcaatccaaagtgcaaacaccgtcacccataatgaaaacattaggctcctggttacctcggcgagtagatgttcttgtaa
ccagtatcggtaaagactctttaatggaaaaatgtgccttacttcaggaattatggtccctgaacattcgcgctgatattgttttacgtggagctact
agtttggaagaaatagttacgcgtttcaaaagtgaaggtgtaaattgggttctagtagtaaggcaaaaaaacacgcaaatggaacattctgtga
aagctcggaatataatgaaaaatgaagatgatgaaattaaaatagacgagattggtgtttggctcctaggagaaattaatgagcggaagcgaa
atgagaatttatcccaaacaaggcagataattgattccgatatccaagactatgggaaatacaatgattcatcatctgatattgatttggatgtgc
agcttgttcccttgaaaggtgctagtgacagaaagtataaatggaagcataagcaaaatgcaatgaacaaagtcaatgaccttgtacaatcagc
aatacacgatagtaccaaagacgccttggcattagcagtagactgtgatgctggagccatgcaaggactgaaaagtacaagtcttaaagatgat
aattcttggaaaaagttgattgacagctgtcctgcttctcaaagggagtatatgcaaagactccaaacgaaactcacaaatttggctgagaaagg
aagaaatcgagtttggatttgttcttttcgaaccaatgaaatatatctttacggtttaaattaattaattttgatagaataatagaatttcctcttgac
tcatcttgtcatctctctagctataaacaaatatgcaacaacgtacacaactagtaaacacggtttttacaatttcatatatttttttgtttaaataaaa
ggatatttgaaattgtttcaaaacttgaatcaaaattcgttaacagtagctccgcttgaagtgaaaaagctcatttgtctaaaacaattaatgttca
aggattcagacgaaccttttctgttacagctttagtcttcaatgatctctttaccagaaagacaattgttcaccaaattggaaacaaaaatagatgc
aatttgtccaaaattctatattgatgccgggaatattcaggtccttcatgaaccgagtgagttttatcagactcttaaggacttaataaaaaatgca
gagaacaggattttcctttctacattatacattggaaaagaagaaaatgaattcgtaaacgtaattcgacaagcaatgattgcaaagccaaaatt
acaagtgtttattttggcagatcaattgcggtcaacgagagaatttcctttcagctcttccgcttctctgcttaccaaattaagggaagaatttcctg
aacgatgtgaaataaaactctatcacactccaaacttacgaggtctcaagaaaagattggttccaaagaggtttaatgaaggttggggacttcaa
catatgaaaatttatggagcagatgattccttaattatcagtgggtaagtattcgttttgagtgcttcttctgatgagttatattctagtgcaaacttg
tcaagagactactttaccaatcgtaaagatcgttactatctttttcatgataaaaatctatctgaattctttttttcacgttcactctacatttgcaggat
tatcttttgactgcctacccagtaaacatggatttctgccgacaatagaggacagccttgcatctcagtttagactagagtggaataatgatgttcc
taatcctctaactaattctagggaattcattaaagaagcttcgaatagacttcaacatctattatgcagtagtcaagatcatacttaccaaagagat
tacaaaagacctcttacttcatcatacgggtcgagttttacggaagatactcaacatgaaaataaagcaatggaccaaaactcgtatgaacgaac
cgccattgtatatcctctttttcagtgcatgcctgtgctgacaggacaagtacaatcaaccgaagataaagtgctggacttacttggaaacctcata
atgaatgaaaagattgaatggacactgacagctggctattttaatgtttatccggaattaagaaggcaattactaaaaagtaaggggacaggag
aagcaattgtggcgtcgcaacaagctaatggcttttacaagtcgaagggtccttcgcatatgattccacctgcttatcagtacattgctgagaagtt
tttgattgacgcgactcgaatgagaaaagatatcgatgtcctgcaatggcaaagaccagggcatacgtatcatgctaaaggtaaataagaacta
aaagcttttaactaacattctaggattttggtttacctatcctgggaggtcaactcccttttttaactacgataggctcgtcgaattatacaagtcggtc
tcgacaactggatttagagtcaacgttggtaatcatgacacaaaaccaacaacttcaatcttcattccaaaaagaggtacagttgattcggcaat
acgccaagcctatgggatcctgcaatctggaaaaagtaccagtgtatgtaaaagcgttggcagcacttttgcgaaagaaactttaaaagtttatt
aaagaaatattcaatgtaaaatattagctaggaatctctttctttttctataatttcgatttctggtattctaatttttcatttacgagaatatcatacta
gtcattcccaccgcctacatattgtgatcgtgaattgggcattatatttctgtaattgttaatcatgcctcttcaattctcatttggagatggtttaagc
tgtaactgtttaaatctaaggatcctaggaatcccgaatgacacgaaacagaaatgggttcctgttcctagtgaccttattcgtattgtaagtttac
accagtacttggcaaaaattaacattttagaaattatatagcctatctcaggttcatcatatagaagggaaatcgatagttgcctgtaagggatgc
tgtatttcacttttttgtcgtgaaggaagtagtacacgatctgaagctgtatctgaaagaaaaagaagatgttaatgtagaaatttacgtttacgaa
aaagcaaagcttctctctaccttcaaagatacaaaaactgtatctcagtttggaattcgtatggacatgcttagttcgtttcaagacattacaagta
atgaccaagaaaatttaagaactcaactggacccggaaatagatacatgtcttaacaaccttgtttccgaaagaaagaaggaaaaccgacaag
cattgctggcattccttcgttcccagcagaattcttatgaagagtatacgaaaaaactcagacgggaggcatttaatttaaatcaaaataccttac
acgaacccaaaattcggtatgaaggcgagatgaaggcttttgtgtctccatttcgtgaaagtttacctaatggcaagtctgaagtaaaacactctc

atcgcactcgaagaagcaaatctaagaagcatgttgcatttaccgataaattcgaagtaatctcttctgataaagagcaccctactgaaccggct
ggatcccataaacaaagcttaaatgcaccgcaatttctagaagccttgtcaaacaaagataatgatggtttagaagtttcctctccaacaaaaga
cgattacaattctagtgaagaacttgcatttgatataagtttcagtgattccaacggtgatgaacagccgcttgaaactactcttcccagcggcaca
ggtgaatcagataccctgacctccgaaaaacattccaaagaaaaagatgtgttcatggaattagactcttcttattctgaacgcctgcccatgacg
atacaatcgtccagctatacggaacgtacatcttcgtcaagtctgagttcggactcttcatttgaagcaccaacggcatcttttgttgaccgtaaag
aaagatggtcacgtatgttgcaaaaggcagacgaacatagtcgtagtgtacgtgctaagagtatgggctacaatttagcaaatgatgatccaaat
cttattacctcttataagcactctttttttatcgcatggttggaagagtctaaattaacgaggatccttagagcgcttggaatatcttactttaattttta
ccttttatttttgctataatttttggatgaatatgcacctgtcacgaatcagtttaatgcaaatagtcatgatttttattaatgataatactctcaacat
ttgtgaacgatttcagtagacaatgaatactttagctttttagtcgaatttttacccatagcaatttcctcctcgatcgacaaggctaatactcatgta
ttacgtttagaatcttcaagcagcttaacatatcatcgacaattatgcttgaaccattgcagaatggttgagtacaatgtacgagtttcatcaaaca
taggtttcaaacagcatatatacctctacgtgacttggcagaagttaccaccccagatacaaaagtacaaaagtacaatagtattttttttttgaaa
aaaatatcagatcatcagcgaatacttatgatagtcatttaaaagtatatcatgttaatcggaaaaaaaatatggttgtaaagaaaaaaaaaaagct
aagtcattttaccaagcgcaacatgaattttatttagatgttagaagggtaaagcatgacacgaagagatgcaccgaaggtcttaggaggaagg
ttgtggacaaaacgagcacggacaacaccagagtttccgtgaggacgagaaacggtactaatcatagttagtgatttgtgatttttttatgtattatt
ttcttcttcgttcacgaagagaagataaaagtcagcatagcaacaaaagagacaagccctgaaagaccagaccagtataagaaagcaaacgtc
gcattcagtgtaacgacgggctgccatgcttgactttgtgtaaacgagtatacttacccccagataacacggatcttggatccacggataggtttgt
tgcacttgtaaacaaagcaaatgcgcttacccaagtaaaattgagcatcttccttggtatcgcagccttcaaccttaaccaaagaggtgtttggttg
aatgtggtgcttactgcgttggtaagacaagtgcttggctttaacgtataatctgtgtccttgggcaggcatatttgcttggtaaaagggagttggtt
aaagataggtagcgtacggtttggtttggttgtgactgttctgcagtacatggctagtaaggctcgttgttctttttacggagtacggtacgttggcc
tagtaaacaatataaacagacgtacgatgaggagttttctctttgctagaaaggaatcgtgagagaaatttactagaacagtatacatacgtgtg
ctgagtaggagtgtaacttcaggaaaggacaaaataaaagaaatccatcaccatccattcttgtcagtcctttttctaagtacgttaaacgtgcac
cgtataagaagcgacaaagaataacatgaattcgtttcattacagaaggactcgaaagcctcaatgacgagaggaggagcatcggtttccttga
ctgatttcatttcattttcactgtttacctacgtatccaccttgcaccttcgtataaaaagtaacacatctcactgcagccctttaattgtcaaaaggtt
gctactgtcgtatggagtatttttgttcaattctttgagatgcaggaggaagaggatgatttagcatttttttacgaaaccaataaaaaagcctccttt
aaacttagcgaaacggttaagtcatccattgtcagactctggtcagttcctttttgaacgttgcaactcaaaagtgtcgtttactaacagtctctaga
tgaaaactccgaccaagagccttcggtcgaaaagcctcctctaaagcacattcgttcggaaagtaaaaattcctctaggacgtctactctagcca
atacgaaggcagagccttctgtttcgtctaccttaccttctgctgtttctccaaggaaagaagaggatgatcctttgagcaacgtacgttcatcagg
gtcttcgggaaacacacctcatccttatgctacaagaactgcaatttctgctttccgaagcaacagtcttaacaccattgatactccaccccagacg
cctttcttagatgatgactttacgaaacgacttcaagagctggatcgtcaagtggaagaatttcagaacgtatcgaaggaacaaacggatatcga
tgtaaatcttgtcttacgtgataccagcccttccaaagatgctgctgctgccggtctgagatcccatagcttaccgcaaacggactcctccaacttg
gcagcagaagaccccccttgcttcgcctcaaatagaccatgtacctgtagtattgcagttggctgtgataggacaaaaggttcctggaagcagcac
gttcctgcctgctgattgggagtctccgcttctcttcaaggtaaaatccaatcagcagttccgtcgcgtccgaaatgcttacacagtaagaaaacaa
gttgaagatgttgtttttgtcttccaaaatcgacggctctgggactttggtacccctaaaggcgctggaatgctaaaaattgacacgcgcttggtga
ttcatgccttccacaaagcagattatgaatttctgcagcagcaaaagtcgcaagaagaagcaaaacttctttcacgggaagagccaaaaacaat
tcccgtaaaacgacacatatctattttagttcgaccgaaaaaaggacaggatgttcacctttcgatacctgtagacactttgattgaagatgttatt
cgaaagtattgtaagcaaacagaggttccatttcatgaaggcatacggctagaatttgaaggagactggttagacccaaaggacagcttggaaa
atacagaaattgaggacgaagatcaaattaatattctactttaaaaaaaaaaaaaaaattcacagggttccacgttatttatattttctcacattatt
gcataattttgttttattttagattacgaccattgttgtaaatagatattcataaatatatcatcactttttcccaaataaaggatataatatttttggt
accaaaaaaaattaagcttaagcaaatgcttctgttggaatgactccttggtttttttaattctaagaatgaactcatggcttgtgaatagttccagtt
attctgctcacaacacattctagcaaactgatcatttaatcccgtttccaattttaactttaaaaccatttgttcttgcatatcttggttgggcaatgca
gcaggaccagcctgctgatcgttggttgtcgcggggaccacattcggagtagaagctgaatttgcatgagcaatccaagaaagagaaccaacat
acgagcggacagtcaacatatcgttgataactaacacgcctgttggtccaccaggtaatataatcataacacgatcgaagcttcttgtgaggttttt
attttggggctcttcaaattttccgtgtataataatttttatggaactgccgatagaaggaagcatcaagttccatccctcaattaaccattcggaag
aatctgacatatcgtgccgagtttggggtaacgcgtttccagcagcaacaatagcttcatgcccagcgtacaaccgtgatgcctgttcatcacttga
gcggacatgtaacaagtttctgcttttctttttgtaaattccccattttagcgaatcggatttggaaggaacaaccttggatcgaatattgcttgaatt
tagagaaacagagaaggtggcttgagggctatatatttgttggataagtgctgtacgatcggtatcccatcctttgaaaaaagcctccagaaaag
gataaatgagctgctgggtttcgggtttgtcaaagaagggagggaaggccgggaaaggcagagaagtatgagcagccaaaacttctgggtgaa
ttggatttccatcaagtaatcggagcttaggaaatcttgcaaccatctccatctgatactgtagagactggtttgcaaatgtggtttgagagattgg

attgcctactagtaccagctcttgcaaatccggtaattttgttttgggggcccagggttccagatctttgagtgaagaaattcgattatttgcgaggc
ttaagtttaacaaatgaggccatgtttgggagagagtggtgacagaggctacagattcgatgttgttatctgataaattaacagatacgacatcag
aaaaatgaatagaagcgaccttcattaaagccggaaacattttgcttttcgtagaagcttctgccaaaattcccatttgttttaaaagagggtcctg
ttgcaagtttcctaagtccaagaatttggtttcggaatggtaacgagaaacaagaaattgctttaatttttcaatagtttcatttgtggatgaaggat
tggacgtggcgttgtcttttgatggagtcaaaggctgagaagatgaagaagagagagagcgcaaggcagacggtgatcctgaagtagctaagc
gacgagttcgctttgactttgatttttttcaatgcagaagcagctggagaagaaccagtgatgcttcgaatggctaatcgatcaccggcaaaggctg
taccgttcaagccgtttagcttggagaaatcctgacttttcacgactgctgtagcagaagcgggagtaaaaacgaggttcataagacgcacttttg
cttttctccaaacgaaactgatcaaatcgtcttctgtagctttggaatgacccgtaatgaccacggaaattggtggatcatgatgtgaccgattctt
ggtaggggttctcttggcttcaagtggcttgtcgattgccatctcattttccttagcagcatttttacgatcacgtttcctacgtagcatcttcacaatt
ccacagaatgctgagagattggtaaagtgtgaaaacgttttaggcttaaggcaagcaaccgacatctccttcctcatccgtaaccgaaaatagct
tatcctttttaagctcaaacattaagcgaatgctatgaaacaaacgtataaacaaatcatataaggtgaaaacgtggaatggtttgccggctgatt
tcccgtgttttaaatacaaaaggaggtgagaataaaatttctttcaacattacgatgttgaattgactactcttttatctaatttaactctagatagg
aatttctctgtcatggttgtattaaaattgtttctttttcaagattccatcaactaaagcataaacaaccagccttcttcccatgtacctaatcttcctt
cgtgtggttgtttttcaagtgattttagggtagggttataacaacagtcacgtacactgattataaaagccatggcagagcttcctttttcactatcg
tgcggttatataccgacgcccacattcgtgttgctcgcttttcgttttatttatgtggaatggtttgagcgtcaaacgtgggccagagtcaattggac
gtctaagtacccatacagtgatcttaaaaaatctactgttcactgtgggtcggtggttcagaaatgtctaatagatagatttttttaatagttaacaa
tattatcaacaagaaatttttttatttcaaaatcaatatatcaacgagtagtgctaagagaatgaagtataggagagtaattggtttctgtttgatttt
ttccttgtaccttctactagcattgtatttttttatatactgattaaacttggtggcattagcgagtgttcatttagggtgggtctatcgtatatggaggt
ggtacgtaggacagctgaaaaggaaaccgacgaaagaggagagaatagtactaggtgcatgtctcaattcagtcttggtgtcggctcaatccta
ctagcaatgcttacttacgttgacgacgttttcctttctttcctcgtcctgattttcatgatatccatgactgttgtctattccaaccattcacccaggaa
gaagaaagtcaacaattgtagtatttaggagtcgcttttgatcgctcattctcagtttaattttacaatttactatcaaagactatcaatgaattgatt
tcaagttgtctagtgaatagagaaaagtcactttatttatttatttttttttctttttaagcaaagatatgaaaatagacaagcaaaaaaatatttaa
aaattctataactttcctcaaagcggattgtctgttcttttattaactgaattatattttaatccttcgtttgtttgtttacatattacgaattgaattaaa
cactttaatcaaagttatgggatccattcctgacactaagggtgtttttcatggagggcttatagtaatatgccattaagtcccttttagtaatttca
tgaccttatgctagcattattcttgtaagattctcttatcagacattcctgataccctccctcgtacctttgcgagcagcatcacagtactgtgttcccc
taatctacttacataaacacaaacctgacttcaacaactctcctcgtccaagtaatcagtatcccaaatattcataaaaaaagcattttgcacagtt
agaatcttggcttgtgatttcaagtataaaattcacagtagaagccttttgaagaggtgatagtgacatatttgtacagaaataataggtttcgtac
tatactaaaggggttttaattgcttagtaacccttgttgagagcccgttagtttggatttttattttcttttcaatttttaatcaacgacttactcagttg
ctttctttttgctttccactatgcaagtaaccgtaccagcacctggagctgcctctgcttctgcttcaaaggacgaaaatcgtaacgttttgccaaa
aaacgtcaagccaatccattatgacctcagtctctacccagacttggagacgtttgcttacggcggtaacgtcgcagtctccttggatgtaatcgag
gattctgacacgattaccttgcacggtattaatctacgcattctgagcgctgctttggaatggggaaatcaaaccatttggacgaataacatcacct
ataaagatgaacgcattatccttcaattctcatctattgtgccagcaaatgctgttgcggtcttgaagcttgctttcactggtgtcatctccaagggt
atggaaggtttctatcgttctagttacgtagacgccgatggaaacaccaaatatcttgctaccacccaaatggaacctacaagtgctcgtcgtgctt
tcccatgctgggatgagcctgctctaaaggccacttttagcattgacatcaccgcgaaatccaactatactattctaagcaatatgaatattactga
agaagtcgtcaaggatgacatgaaaactgctcgttttgctaagacttgtcgcatgtctacttatttattggcgtggatcgttgctgaacttgaatatg
tcgagtgctttactcctggtaaacactgccctcgtttgcctgttcgtgtctacactactcccggtttcgccaaccagggcaagtttgccgctgatttag
gtgctaagacgttagactttttttctggtgtcttcgacgaaccataccccttacccaagtgcgacatggtagctattcccgatttcgaggctggtgcc
atggaaaactggggtctcgttacttaccgtctctctgctgtcttggtttctgaagattctgctgccaccgtcattgagcgcgttgccgaagttgttcaa
cacgagttggctcaccaatggtttggcaatttggtgaccatgcagttttgggacggcttatggctcaacgaaggcttcgctacttggatgtcttggtt
tagctgccacaatttctatcctgaatggaaggtctgggaatcatatgtcaccgacaatcttcaatccgctctcgctttggatgctcttcgctctagtc
atcccattgaagttcccatcatgcacgaatatgaaattaaccaaattttcgatgccatttcttactccaagggttcttgcgtcattcgtatgattagta
agtatgttggagaggatgttttcatcaaaggcatccaaagatacatctccaaacatcgctacaataacaccgtcaccgaagatttgtggggtgcct
tatctgaagaatctggtcaagatatttctaccatgatgaattgctggaccaaatataccggttatcctgtacttagcgtctcagaaaccaaagaag
gaaaactccgcattgagcaacatcgattcttatctaccggtgatgttagccctgaagaggacaagacactttactgggttcccttgaagttaaaga
ctctcaaggacggaaagcccactgtggatgagaaggttgtcttgacagaaagatctgttgagcttcccatttctcaagaatctcatgacgcttaca
agctcaattcagaacaatctggtatctatcgtgtacaatacaccgctgatcacttgaagaaacttgctaaggtcgccgttgaaaagccagaatttc
ttagtgtcgaagaccgtgctggtttgatcgctgatgttgcctccttgtccagagctggctatggtcatgttagcagcactctcgatttggccaagact
tggaaagaagaaccaagctttgttgtatttactgaaattttagctcgtctcaacggtataaagtctactttgcgctttgaaaagcctgaaattgttga

tgctttgaaaaaattcattttggaaatctcttccgccaaggcacacaaacttggatggacattcgacagctcagatgatcatattagtcgccagttt
aagagctcagtctttggctttgccggtgtcaacggtgacgagaaggttgtctctgacgctctcaaaatgtttgaagcatacgctgcaggagatcaa
aaagctatcaacgacaatctccgctcttctgttttcattattgcagtccgccatggtgagcctaagtgttgggaccaattgttggacatatacaaga
agtcaaatgacccgtatgttcgcactaattgccttagagcctttggttacactgaaaacgatgcatacattgagaagactctcaaacttgctttgga
tcctattgttaaagatcaagatttgtacttggttttgtcaagcttgtctggacacaagcgaggtgtcctcgctatgtggaaattcgcaacgagccatt
gggatcaactattgaagcgacttcccattgccggtaccatgcgtggtcatatcgttcgaatcattaccgcaggatttactcataagtcagatatcga
caccattaaaaagttctttgctgaccaagatatcaagctgtatgaacgtgctcttcaacagtctttggacagcatctcatccagctctagcttcattg
agaagtccttgaacgacattattgaatggcttaagacgaacaagtacctttagttcgaagtctgataagcaagcgataattaaatacaatttttac
gaaatatgaaaaattgatagacagaaatgacttatttcactataaataaacgttttcttacggcacttttaggtaaatcatgctttcacctcctactt
ctaaaccttaccgttcacacaaactttcagcacaaaatatatatatatatatcaatttcattctaatattcttgtaaggtataaactaagaagtact
actataaaagaatttctcgcatcgtcggcccctacgccaatcgcctaacttaataggtcataagagcagggttctttgtcaacgagtttttatctcg
aggtcgcaaagtcaaaaatatgactctggcagtaaaatacgtatcgaatcacatatttaccgtggctgacctcttacatttttgattcaatgagaca
aggtgcgatccacgcccttttatagctttgtcggcttctcgttagggtagggttattctgtgactatttttttctttgtagaaaatttacgtaatgcgctg
tttacgatgtgtttaaacagtaaaattggaaagaaaacaacaattattaggagaataaaatttaataaaacaaaagtaaaacaaaatataaaac
gaaatagtagattaagacttaataataccaaatgagaatatgataatcagaaaagacataatatatacataagaaagaaaatgcagtacggttc
attaaagaaagaaaaagaaaaacagagaagaaaagagagacaagtaaaggtttactaatctgcaagaaatgaaaatgcaaatagaagaaa
atgagcaatatacagaaaaaaagttaagagaaacaagtataagacaaagctttatatgattaagtgttaccctcggtagcaaccaaaggataa
gacgccttcatacaaaaccgcttttcattgccggacaaagtttgacagtaagcacgatgctcgtacatttttcggtaccattcaattaccgaagccg
aaagttgatttgttttgagcttgtgcacaacaagtgcgtcaaaacgaaggaaagcctggcgaggattatagccttcgctatgcaaaactagatcgt
acagattgtatctggtaaacttaatattttgaacatcgccagacttataaatattataaccggtttgaacatcctcccatggataaacgatttcacg
accactagcgatgtgatgggcgatgtctggtgaaacaacataagcaaaaccgcacatataagggaactcacggacggatgtcggcaactcttta
cgagtacaatctcgtccaaagtaaaattcatcctcaccaatgtgaggacgaagttctgagaatagcaatggtaagttgacatatgcatcatcgtc
aacttttgctataaaatcatattcatctcgcggagcacctaaatcctgaaattccttcatcccaggaagttcatgggtctcaattgtttcattataga
caaacgttccccctgatactgtatgtttttttaaaagatgaccatcagcaacatctgaaagcaaaggatatgatcgatatcccttcaaaaatgtttg
aaagtatacaatactttttccacgatccacgttttccccaatagggagaacttcaaggtcaccgtactttcgctgctcttcacgaatagcagccaat
ccttcatttgtatctggaacacctaagacaaacttgacgtcaatcttgtccttgctagcaaattccctgatgtagtggtggtagtcctcacgcaacag
acgtcttcgttccacattgtttgcatgtgagaaaacgcccaaacacattcgtaagggacgaggagccgtgctctggtttgtatataaaggactaag
gaacatctcctccaatagtaaagccgtctttgtttcagtgcaccatgaccggtacatgatgagaaaaaagaaaacagttccaaaagcagacaac
accaaatacgagaaaaaaaggtttttgtttgcgaccatattgaaaagaataagaaaacgaaagaattacttcaattggaagaagcgaattaaa
atacactatgcgaacctaagcttcagaaaagaacataccaacagaaaaaataaaatgggaaggcacaccaccttcagccgactttctattcttct
tgtagcaactgaggtaggtatacacaaagggtaagtgctttctacctgcttcaaaacaacgtaacttgtcgacatatggaataacgtaggaatgc
aaaaggtcgttaaatagtaaataaatacattcttgggcaattttcaataatcctagttgtttttttatttaaagtatcttcttattaatttgttgtcctct
ctgttcacttaagttaaaaaatacattcatattaggctgcttcacttttgcaagcaataaaaaaaaggcgagaagatatcccaaaagaaagggaa
ctgtcatgtttaatttaagcgttgtttttcaataatattcgtaattataataattaaaggcatgaaatatctatggaagtatttgggcagttaaatatc
ccataaaaaaatagtagaattatccattatattccaatacgagatgccgttccacagttgtttccttaccagcttcctcttctccttcaaaacgaagt
ttacaggaaccattcggtaatgttcgtattacctctgccttgtaaaatgtggttgtttctggatagcgtgccagcacggccgttccaggactaagagt
gggcaatgaagctgttttcggtgggatataaataagattacttgcaacggttttatatacttggcctgcattgccgtcatcatctggttcgggatcct
gaacttcatacctaatgtcaagaaaggttagaactggatccatgtatgcattacaatcattgaaatttgcttctaccagaaaaaggagctacatgg
agtctaccacataacaatttgtaccaacttacctctgcttagaaccctcccctgtcaccttgacaataatacattgaatccattcaccccccttcagtt
cgtggcaagcgaaaagcgacagacattcccgaggttagtgcaggaggaataaatatttgtgaagcgcttcggtttcgccgacttctcgttaacgg
gctattcataatagaggctgcggacttctcatttgctttttctaagatgtggataattacttctaaagattgtatagcaggatctaattgggttttctg
gtcttttgttgcttgtaaaccacgccgacaagcttcaatccttgcttccaagtctgctttttcatcctcagccgtttcatggcattctttttgactcccca
ataacgtctttataggttctaaatcggcacaaaatttattccacatagaatcaacatcttctgaagcattcgggcgacccatgaagcttttaatcat
ataactaacacctaggatcttttgttaccaaattttttttgtggtgcaacatcttgctagactacttcacagcaattcttcgacacacactatccttca
acaaaaatagaatctaaagtttattctattaccatataaattgtttgaaaacaatttgaggtttcatttttcttgttctttacaaaacagcgttttagat
acttaaaaattatatactaattcgatttgtttgttggttgcctaacaaaagataaagaccatttattgtatgttaattattcaatatacagtcctttca
tgtagaaagtaaagcagctaaaaaagcatgcaaacttcttccatcagccaattacttcactattagaccaaaagacggtggttgtgcttgcaaac
taggaaaatgaacgtatagagaaaatttatagacgcaatttataattctcggagggtaagtattgtctcgacaacatcgtaaacaaacggcgcat

aaataggggcatattcaagtacatcattttcaaacacttttaaaagataaatcattatccattccaaatgagcatttaggcaaaaaaaaaatacg
aaacaagcatatctttcttagtgctttattcgaatgatgcaaatcatgcaaaagaaagattgtaacaaaaatacataaaggaaagtattctaattg
cgcattattgtttcgcccagtttcgtatgtagcagaactacgtgacaaaaatcagaagaaataaatacaaaaagaatgtatcgtgaaaaatcatc
taggatatttcatctaattgaacataattgcccggaaattggccttcagaattgtgcaaacgaccaatccaccattcctatatcttttgttagtaaat
ttgaaaccacagaaggaagtcaagaagaacagaaaagggttactcacattttgattgtcagatcgtgaaactacctcgatgcgatcaccggcat
ggaaggacaaatcaccggcagcctgtgctgtataatcatacaaagcagtaacgtactctacaggtggaacagccggtttgaacttgggctttatt
ggtggagcaggaggagccgatgcggtagaagctgtgaaactagcaggcttttcgggggcagtagcagcagccatggcggacggttgagcacca
attgaggtgtaaggagggggaacttctgaggttgccgccgcactactagtaggggaagaagcagaaacacttgtagcatcaccagcgacatctt
cacgtttgttagaaaaggaagaagaagaagatggcgaagaatgaccagtgtagctgccagagtgcttgcgagcaggcctgcctgtcttgaatcg
agtaattgtcagagcctcagtacggtctcttacatcactgcgtttttcttcgtatgctgtgataatgtcggaattcatgtcgaaatagggaatgttga
agttactcattttctgataaagaatccaaaaaatattgagctgcatgtaatagagtccttggaaaagggggcaatgaaatcttgctcaagttcaa
aaaggcgtggaagctcttctttgaccatatcattataatattcatactcctgggatgcttgatcaaaggaggtttcagcctcgtaaaggtgcttttca
tccttaagcgagcgatcctttttttcttgtagtctctttagggtagcacgatgacgatcgtaatcaatttgtttatgatcacgcttcgtgagaaactttt
cgactgacctgataatatggagcaattccgccgttggttgaacaatgcgggtttcaataacctccaattcagacgctaaactggactccagctcgt
ggacgatgtctttataagcttctgctgcctgaacaccttcctcacttccctccagacggtacgactctgggtcagaggctcgaccgctaataggatt
gtatatttctatgcaagcatccgcaaacccaacttgatgattcaataatccatgaattgcttctgtgtattttgttgtgtcttccgatagcttcttggct
tctgtttcaagtgatttgaatcgtcgtcccgcatcttcgtaaattgggtctttcgtatgttctccaatgttgaatttactacgaaacgtctgtggtgcac
ggaccaccgactttttaaatcctttccagctcattcagatctgtctagtttatgtctcaattgggtgttgttgtcgatgggaagaggaacatgtacgtc
cctccctccaatcttgtccttctcatcatatcaggtactacgtttcctatcgttatctacattaacaaagtatttgacaatatcggtaaacgcacttca
acaaactttgcttcatctcctaaatgtttataacgcagtgtaagatacatgtgacatacaaaataacaaaccatcgcaaaaattccaatgcgttat
aatgattattgccgtagtggctaaaaaactcttcttccatactttcagttccacagtatcaactatgcataggtggatatcagcagcgaatgccaag
aaagctgtataaaagctaacccaccaagtctgtttattaaaatatttttccttaattcagaagattcgagtttttttctttctgtcttttagccacccaa
ctttagccccaaggtttgtttttgctacctcacagagctcataatgttacttcaaaccacaatctctgcctttcatttgactctggagtgccgctgagc
gtttggattggctccaataaatttggctgttttgtgtgtctcactatatataggcataacgctcttggaattatttcgctgctaattccttttacttctac
tctttctcgacggacaacgcttcgttgctgtttccaaatttcgtgcttataaaaaagcgaggtttttcgtcggattgttttctttgattatactttcatca
tttttatcgctggtttcattgttttattgaaataggcttcgttttgcactttctgtgctaaaagtcctttttactcttgagaaagagtgaaccactattac
taagcaagtattctttaatactgctacaaacgtcgtctgtgggattgacggatcctgtattttaaaggacttgtcgcctcctcccagagaccataatt
ctctgatagttagacattgctgaagctttttctagtgctttcttgtctatttctttactttgtgatttttttgttggtttttccttactttttgtgataattctc
tttgaagcatttctttattctgcttcatcgatatttgctttttgtgtttcctatcggttggtccatttcgaattctcagtccatcatttccattacctaagtt
atgtcgagattcagtagatttatgactctttctcaaaaagctcttccaaggacgtcttcgtgtttgaaccgcccttggactcgagctggcggcgtcgc
ctcctcacgcttgttctcttcgagctgctctcgtttttctacttctccttcttcccaagacccttctatccttaaatcctcggtttctcccacggcagctgc
ttctacagcagcagctcccgctaccgctacaaccactatggagacacccactgttgctaaagcgaaagctgctttagaggccatttctggcacaca
ccccatttacatggactttcaagccacctctccccttgactatcgcgtgttggatagcatgcttccttatcttacaggcgtgtacggaaaccctcata
gtcgtacccacgagtatggctgggaggccgaaaaggccgtagaaaaagctcgtgaagaggttgctacggtcatcaacgccgatcctcgtgagat
catcttcacttctggtgctacagagtccaataacactatactcaaaggtgttgctcgtttctatcaaaatagaaaaaagcacattatttctgttcaaa
ccgaacacaagtgtgtacttgattctttgcgtactttgcaggaagaaggctttgatgtcacctatcttccagttcaaacaaacggcttaattagtctc
gacgatttgaaaaaagcgattcgtcccgacactgttctcgtaagtgtcatggcagtcaataatgagattggcgttatccagcctcttgaagaaata
ggtaagatttgccgcgcaaacaaagtttttttccacacagacgctgctcagggctttggcaaaattccccttgacgttaaccgtatgaatatcgact
tgatgtcaatttccggccataagatttatggacccaagggtgtcggcgctgctttcattcgccgtcgcccccgtgtccgtcttgcacccttattgagc
ggtggtggtcaggaacgtggtcttcgcagtggtaccttagcacctcctttaattgtgggttttggcactgctgcccgcctttgcagggaagaaatgg
cttatgatcacgaacatatttctagactttcgaagcgtttgattaatggtcttttggctattccttttacttctctaaatggtgatcctaaatcaagatt
ccctggctgtgttaacatttctttctcttatgtcgagggtgagtctttgttgatgggtcttaagaacattgctctttcttctggctctgcttgtacttctgc
ttctttagagccttcatatgtccttcgggctatcggccaaaatgatgaagatgctcatagttctattcgatttggtattggcagatacactacagagg
ctgaaattgattacgctcttgataacgttcataaacagatcaacttccttcgaaatttgagtcctttgtgggatctagtgcaagaaggcgttgatctc
agtaccattgaatggagttcacattaatggatatgttttccgctgccaaagaaaaatgtcttttcagctttctttaagttaccaaaggttaatgagct
tatctacgactaaaaattatataattgattgatgttatttgtaatccctttttatattcacatttagccgaacattgttgtatgtttttataaaattgattt
attatgctttatttaaaatgaatcatataggtagtattatggtatcctaacataatgtgctgaacacgtctaagaaataaattatgtatatgtatatg
tacatggttttatacagtgttagaatgaaaactctcatgtaaccaaaacccaaaggctttttttctttttcatcgcttgatacttgcttgcttaaacatc

tcaaataattctcctgagaatcgaaaacactcatctccccaaatagacattgcactgcttggatcccccccgttgtacattttacagtttgcaaata
cgcgcaaaacttctcggtaaacttcttctatagtagtaagattgccttcacgaaccatatttttaatagtcctcaaatcctgagggtggaaaattag
cgaatcatagtcttgagcctctttccggttgacgggatggctaaaaggcgccccataccgatgattggagatattatccaaaacgggaaggatca
cattttggaactttttgagattggtcttttcttcgtcctgcgaagtttgtcgtctactggcggcccgagttgttgctcctgtagctacaggaacagaag
cagcagcagttatatccggagcattagataacgttggttcttcaggtaacggttccgatgattttcttttcgaccgtaagtttttggaaactgaatct
gtgacagttgttgtttcttgtacggctttttcttccttttctttgattttcgatttctcttccgcctgttgctcaagaattgcatcataagatttcacacaat
tatttagctcattgaatagatcattcataacgtcctgtcgtagttgcgatcgcttgtccaactttgtttcatcatctgtaacagagctgctcttaccagt
cttccctccggtttccgaatctgtaaaagtctgatgataaatcctttttaattcttctgtgttcagaggctctagcaaaaacggactctctgatacttg
tttcagaatcttttcttcattatccccaagttgccgttttaaatcgagacactgtaaaagagcaacgttctcggaaagattcatactagggatgtctc
ttcatatcctttatgaaaataatatttcctgtaaagagtaaatgctcgataacacagctctcttcctctctttttcttcactttgagcctgttccaccgtc
tgcagtttcccacctataagaatacggaacgcaagttaagtacttatcttatgaatgtgttgctataccctagagcgtattaggagaatgggttcaa
gcgtcttaattgattaacgtaatgctacacgaacattttaaattacgtgtctaaaggcatgtcagaagccgacaattcaagaaaaaacaaaattta
tcctggtggatagtgagagaaaataaaggttcattgcatactttgttagaataatgaagaaatctggatttcttcacgggtatgtgagttcataga
actgtttggatttaaaatatagtacgtttatttacgggacatggttgcccagaatgcttctcgtttgtttcgtaaatcatagtcaactataagaaaac
atgattccaggacgttttatgctgtgaaagactcgtaggctaactattatagacaaacttcgtctccgatgcaatctctcactgggtccttctcatctt
tgcagatggatactgggaccaatacttcaatggttagcacaatctttgaacccccggatttgactaccattgacgcggatcttttagtagtcattaa
aagtctccaaaaaagagatatcacaaccaagtgtcgcgcactacaagatttaattcaatggagcaaaattgctcagtttgaaaatgaacaatttt
tggacgcctgggccgtttactttcctcgtttggctattgaaacagatcgacgagtacgacaatatgcttttacatttactggtctgttatcttcaaact
ttcaaaagaaacttgctccctggctaaaagcctacatcacgccctggatgatgggttttttgatgttgataaaacagtctcctttgctgcccgtaat
tcactcagcaaattactctcagaggataagtggccgcatgtttggatgaaatacgggaatgttcttgttgataatattgttgatatccttatgtatga
aactgttgattctatgtctgatttacggtttatttcaaacgaagacgcagaatctaaatatgaacgcacctgtgcttgctgtttctctggtctctctttc
ttgatcaatcaaaacttaatgaacggcgtccatcctgccgatgggaatactaagtcacaaagctttcacgaacattacttagaggcaatctccgta
ttgcttgaatctaaacaattatgggagctcccttcttcttctcatggaatcattaaactccgtttgtttcaatttttgcaaaatcttatgaatttcgaca
aagactttttctcctcttacaaaggtaaaattttaaatctcgtgccgcgtatattttcccgttccgacagtatatcgtgcgtgtctttgttaaagatgat
gtcctcattgttaaccgtttatccttcttcttctgccactttagcaaatcacccaaaacgtcctatgcgaaaatgtttgcagaatcttcttcgtaaacg
attagctctcccaaattctggcttttacacaacttttatgacttttatgactcgtttttcggctgaacagcttgtcccttccacagatgatttaatcgaa
ttttccaatgcctttatggagactgctcaacaagaacagcgtgtctttgcaactgaagtttatagctgtcattttgagttcctgtcttttgtctttaaca
cttctttggattctcgaattaaagatacagtattagaaattctcaagtctatgtttgttggctattttgaaggaaccatgattccccaatgtcctgtttt
tgagttcgataagtgtttacagagcatatttcgtaaaagtgaaattttttccaacttgtggaacgaggtactttacaatttctatgaatcccatccgg
tacctgactcttcgtcgtttgaagttttctcaaggtctgttaatcttgccgtgcaaactgctttgagttacaaaaataacaacaggtcttacgatata
gaataccaacgtaatgtccgaccttgtttacaatttttgatgcagctgtctgctagtgaaaatattcaatttgctagtcttgctgtttcgcatcttacta
agttttgtcacatttttgagaaaacaaagtttactcaatatgtgcagtcggatttaagcgagtatttggtaaactttttgccatctattatattgactc
aacagacgaagccgtcatatttggatttgcttcacgaaattgtctatttacttagagaatctgataatatcgataatctttggtcctccgtcattgatc
agttgatgaccgaaccattgaacaccgaagtcctcaattcacttcctcttagcttttcagatccccgcttacgtgataaagttcctttggtggattcc
cttgtctcttattacgatgagaccgtgtctaatttgattagggagaagcattacgattggaacttaataaaacttaccatcgacacaaaagacattc
tcattcctgctaaattggttgagaaatttttaaccttgacctctaataattttcaagtggaattaccattgagtgttttggaaaatcacttaaatgtttt
atgttgcgcgttcaaatcattacgccatgacttggctgaaatatttgcacaaaatgaaagcctttatttattatacccttttgctattgatagacccca
aattctctagcattgatggtcatttgcgtgatagtttaattgggcttttcaatgcggaatgtcaaatgaatgacggttttacgaactctctttgcaaag
cagtctccgactggagccgtaagaccgttttagaaggcaaatttgatacacataaagcagcgcaaatcgtcgctgaactcgcaaagtcgcataa
aagcaaaattcccctatctttgtttctctatactgaaaacttttggatttccacattgactccatctgtaaattattttgctaagacttattttattgatc
aagcaccttgttttggattctcagagttgattgagaaaaatcagtctgatgaagttgtcgatagtaaagcaattgcacaactttttcatgcttcgctc
tactcgttggtgttttgtgaatacgacgaaatgagctctgatcttttgctgtatatatctttgtcgcatgcaattttcttaatttatgtccaaaatggaa
ttgaaattttctctgacatacctattgaaagtgctaatgacttttgtcaaagatgtgagaaattgttcaggagactgtttagcctagcacctttggac
aattcacttaccttttattcaaatttccttgttcagcatcaaagcagtgttggacttgctatccgtgagctgataaagataatcacttcttcttctgatg
tgatgagtgtctgtgccggtcaagctatcacaaatctactgaagatagcttcttcaatatctgttttaagctctgatgaattcgaaacgttatgtagc
agcttggatattttcgcttcaaaatccgacatcctagtcgtagccctacttgaaggatttaaaccacttgaattggattcaggaatcgttgaaaaga
taaggtttagaagtgccagtcaattatctggtaaattggtttcaagtgcttctgtaaccaaagctttgatgacactgaatgcatctacaccgactca
ggatgattcaaaaccacttttgcccattacccgttgtccactactcttaacaagtcttactgaatgggcaattgataacgctacaaaatctttagatt

tgttatcattgtctctgttatttcaatttctgcaccggtttgttccctccgtccagaagtttgccggttcttactggaaccgaatttttgaattaataaaa
tttacacttaaactttctccggtggatattcctatggtaaagtctatggagctttacgcactacgtctatatcttacacttacaaagatagcggaaat
gaacgacgatatatatgatggactcgttgaacatgatgatgatataaaatatcttggcctgaagagcttcctttcttaccatgaatccaacctaaa
atcttcagtgaccgctggactttgcagcacctaccttgtaagattattagacaattgttcgcataaggctatagcaaaaattccttaccaggaactt
tatcctaatttgcttggctttctggatttatcgaatgaggccatgtgcatgaaattattgcaggataaaatctcgagcgaaattaaagacttgacgg
tatactatatggttgaaagtacttctgaaccagacgtttctttcgctccagagttactctctatacttattaattacgctgaaaatcctttaaatatttt
aggtgaaatagacactatagagcctgctctacgatcgtatcttctagcatgggatcttgttttccatcattttgaagaaacgacttatcaaattaag
ctggcactcaccgaacagttgttatcgatggatctagtgaggccattgcttgcgatcatagtagaaattctccagttgtcctatgacagacctgtcg
atgtaagtaattatccaaagacatcctataatcttttggactatgcttctcctttagatcgaactcgatgcctgtgtacgtatatatattatcaatgttt
gaggcatttgtcttcagcagttcgaggatactggtcagaaattaagaatagagcttttacatacacagttgagtcattcactgagtctaatgtatct
ccacttttgatttctgcctcacttgatgatgtagagaaggcgatttcttcttcaagttttgaaacgtctggagataccgttgtaaaactgaataggaa
tacgaaagaaatttcctttatatatcacgttgacgagcataaacttgagatggcgattagaatacctttggcttaccctcttcatcaagtacaggtg
gagggaattgaaagagttggcgtgaatgaacgacaatggcgcgcatggattctcgcttctcagagtattcttacttcccaaaatggctcgataatt
gatgccttgcaacttttaaagagaaacatttcgatgcactttgaaggtgttgaagaatgtgctatttgctactctgtactgtcggttgaaagaacac
taccaaacaagcgttgtagtacatgcaggcataaattccatgcttcttgtttgtataaatggttcaaatcgtctaatgctagccggtgtccattgtgc
cgtagtagctttacatttatttgaaagcaaaaagtttcctttgtttttgtttaatgacgatttatatagagtagtttaatatctaaactttagattttttt
ggctagatttttattgaaatttaaaaattctatgtcttagtggtttatgggagtcatctgtttacattgcgtacaacgagtataatgaaaaagtattca
tatagtaactctctaaagcttcaattaatttttgtttaaacaaaaagaaggaagttggtaaagaatgttgaattgtttttgttttgaaagaacaggg
aggggtgacggggcactgactttacaaacaaactacagcctttgtttatccgaaaagttcatgtaatcaaaacaaagataaataattcatttcagc
gcaaccagagacatcctagagcaaatatgtccagcattcagagttcagaattcagttcaaacaaagaaggtcaaaatgagactcttaactatttg
gacatagaaaacatgtttacgtatgcaaaacttttaggcagagaataatggaaaactaaccggcaggttacttaatcatttgcaagaacagtatt
gcaagagtatacctgaagaaaatgcagatgatagccgttacaagttaattgaaaatcaatatggatggtgtaaagaaaccagtgttttgatatcc
gcagttttcgttatgattttggattccatacatgaattcctagaaaaccaaggaaaagttccaattggctcagctaagggagaaggatttaatcac
caagatacaataagacttttcaaagaattagttgaagaaagtgaaaatgtaaagcttcgtcatttcttttctgcgttatcgtgtgcaattgaaggac
tacagatcacgaactatcaaattcaagacgcactagagatgaaagacttggaagatcaaggagacttggatggatatgagattgaagcaagca
attttgtagagaggttccaaaattgcttgaaagagttcacggaagttggaatttatgtacaagcattagggaaaagcaaaaacaaacgaccgtg
cttaggaatgtatcaatcatttggaaaatctagtgtagagcaattacaaaggcagctaaatagctacactagtcgagttatagaaccgttactcgc
agggactgaacaagatccaaattcatcggaaactccatcctcctcttccgacggcagactgggtgcgaggatgaaaacagagagtaagagttcc
tttacaaagctcttcaaaaagaaacgctttaatcgatcccagaaagaactgaaggaacaggaacctgttcgcgagtaatgtcgtcagcaaggca
atgcaagaagcagccagcacaatccatatccaagtgttattatttattaagctcaagattagatcagcataaaccgttccagtcagtaattattttt
aatttttaattctggttctttgtgtctgtaatagaatgaacataagtgtcaatcggaagaaggtttatttcgacttttctgcaataactcacgaatttt
ttctgcctcctcttcttccatctttcgtcgatactctgcagaagagctttttgttggagagctttttctcgattgacctttgctttctcgttcctctagaa
aatatacgttagtttccttcatataattgttataatgtgcgttagtgtgtaagtacatctttatttaagcaagattttaggttatattttatagagttgc
attctcctaaaaactttccgtaatgtttatggcattcgtctaaagccaaaataagttctgcgcattgcttctgcttgttgatatcaagctgaggatgc
attggtaactgtttgttaaagagcgtatgaagatacgtgaattcgtgctctgcgtggaaagggaaaaacgctaagtacgttgaatttcttttccgtt
ttctgtaaagatactatcaaaaaaagttattgaataataaaggaatattaaaatgaaaataactaaagcaatttcgcgtatgacagtagacgct
aaactcaatcgcttgtctatagagagtaatagtagagaccttttgacatggtaatttcaaaaaggaagttactgttatttagagatcgtccagagt
gcaagaaaatcctgcactagggtcaactggaaggcctttgggatatccatttacgacgctgtaaaatttacgaacaaaaatgccactgaatggat
tttcgtgcgttatgaatgctttttgaaaagcgaatttcctaatgattcattgatgactcattgacctgagcatttgtatgctacgaaatccttgagtag
aaactgctaattttcggccattcgaaacaataactaatcttttcaaagtttcctttgttagaaaagataaggattgaagacaacctcatctttccga
aggatccttttgtttagcatagaatcgccttttcgaggactaatgtttacaatgtctaataaactctttattcggcatcttaaactttttgatttgcgta
aattgactcggtctatccctggtttcttttattgttatgaaactttaattgcttttgttattatctctttttggatcctcattcctttgttttctcttttgttta
cgttgtttgttccttgatatgtaatatactgttacttatcgcgtcgcgtccttggtagatactttcgtttgggaaatcaatgctttgtcctttaccataac
aggcccaaaatagtaccatgtaaaaaatgcatttttatgagcttcaaaggattctttgtttcttttgaaacattgaaacaatacgaaacaatatgga
aaaatcaatggtatatataatggagacaatggcacacaatagaccattgttctacagccgctgaagattcagtaagaatagaaagcctacgaac
acttctaagcgaaatagacatttgtgaatcattgtcgagtctgttctgattctctaaagttgtttcgttcattgggttttggttttcctatcccattcatc
tttcttgccattaattttgcatccacttttgatgaaatatctctaaatttcttgttacgcccttttctgcattttaattatagacacataaccttcgtttg
gttacgttttgaaagaccacgtttttgttcatttcttgtagagtttttgctctttttctttttttttttaagaatctttcttgctttttttactcgttctctgct

tgagtatttatcaattagacaattttactgccgtttcgtttataagcatttccccttacattcgctttttaactttactttcttttatgatgtttttagttac
gtacttcttccttcccctctttcttttctctttagtttcttcctccttggcaaaattcgaagaccaacaggaagccgctctttccaagaaaacgctttact
cgaaatccactaacaatgatgttgtcagtattaaccttgatatccccacttgtacctatgaccatgtcttgtcttctgatgtcactctttattcagcaa
aacaaaccgttactgcttcgagttattggtctcttgtaacaactagtattacaactactgcttatgaaccattcacttcgcatcttccttgcgttgctg
ctacaagcgttatatcttctttagcttctagtggtccgcctgcaagccctgcttcatccacttctgtcgctagtgtcactccttcatcctcttcttcgaat
gttatctctacgaccgcttctggctcttccggtagtccttcgacgagcgtttcatctacgaatagcgcaactgcttccaccaccgtccacagagcgg
aagttccctcttcatcagcttcgacatacttgtcttcgagcttaaactctcctagtcccgctccttctacgtcgtcagttgactcttcaagctccagca
aaactactccttctgctacagtgccgtcgagcaatgtttcaagccccagttcggttacttctccacctttaacttcgactcctaatggaaacaccgttt
ctgcttccactcccaggtccaccgcaactgaatcatccaagtcagttggttctaccactgcaactaccgccacttcttcagacgtaacttccggtac
atcctctgttgcttcaacaactggtacttcaagttttcctacctctcagacgactactgcttcctctaacactgaaaattccttgtcatccaataccgtt
accgcttcttctacttacccttcaagtatatcaactgattacctaaccagtgcctttacaactactgcaacttctgtctcggcaactacaactgggga
aactgatacgagaactatttatcgtactttaacatccggaacattaagtacttcatcagtgatttttaataatagcactcctatttctggatcattacc
atctggatataaacctgctataccgacttcaggttcttcaccggtgaacaactctacgcctcgttctagtaatagcgttcccgtgacatcaacctcg
aagtctgtacctccctctactagtgcaacgagtaatgcaagtgtttcaactcccactcctgctgtaccgacttcaactgtcgccacaagcagttctc
agttgacgtcttctgccccttcttctactttaatcacgtccgcatcttcaagcactacacctgctccttcaagctctgttccgttgactagtggtaatag
tgcatggacatctgtaatttcatctgcctctagcacggaaattgtttctagtcaagcgtctacaactacctgggcatctacgtacgccacgacaact
actatctctggatctgtcgtttctacaacgatgacttctagcgttgttccccaaacctctcctccctcttgggtgactgaaacagtaacttccggttct
aaaggattcacaactactattgctactcctgtaggcacatctgctggtactgttgtgattgatactcctggtcctactccctcttgggtgactgaaac
agtaacttccggttctaaaggatttacctctactattgctactcctgtaggcacgtctgctggtactgttgtgattgatactcctggtcctactccctct
tgggtgactgaaacagtcacttctggctctaaaggatttacctctactattgccacccctaccggcacatccgctggtactgtactagttgacattcc
ctctgcttggataacaacaaccactacttctggatctacaggatttacttcaacgtattgcgacaccaagtggtaccaaaaccggaacagtattag
tagatgttccaaactccaactccctcagtcttcccttcatgcaacagccgttgtaactctgacaactccttccgcattcaagttttaaatgacgatat
ctctccttcctacgtacatttggatgaaacaattacgcgatagcatcttccaactacacaatagcgagcacacttcgtgtatgattnnnnnnnnn
nnnnnnnnnnnnnnnnnnnnnnnnnnnnnnnnnnnnnnnnnnnnnnnnnnnnnnnnnnnnnnnnnnnnnnnnnnnnnn
nnnnnnnnnnnnnnnnnnnnnnnnnnnnnnnnnnnnnnnnnnnnnnnnnnnnnnnnnnnnnnnnnnnnnnnnnnnnn
nnnnnnnnnnnnnnnnnnnnnnnnnnnnnnnnnnnnnnnnnnnnnnnnnnnnnnnnnnnnnnnnnnnnnnnnnnnnn
nnnnnnnnnnnnnnnnnnnnnnnnnnnnnnnnnnnnnnnnnnnnnnnnnnnnnnnnnnnnnnnnnnnnnnnnnnnna
ggatttacctctactattgctactcctgtaggcacgtctgctggtactgttgtgattgatactcctggtcctactccctcttgggtgactgaaacagtc
acttctggctctaaaggatttacctctactattgccacccctaccggcacatccgctggtactgtactagttgacattccctctgcttggataacaac
aaccactacttctggatctacaggatttacttcaacgattgcgacaccaagtggtaccaaaaccggaacagtattagtagatgttccaactccaac
tccctcagtcttcccttcatgcaacagccgttgtaactctgacaactccttccgcattcaagttttaaatgacgatatctctccttcctacgtacatttg
gatgaaaacaattacgcgatagcatcttccaactacacaaatagcgagcacactttcgtgtatgattctgatagcagaagaatagtaacgtgctg
tgatgtgaaacccgtctacagaatggaccgagacgacaagaaagcgtactctatgcaaatttacaaggatgatgatggtcaattccaatttaaat
actcagataatggagtattccattcgttgggactcttaaccttgacagatggcagagttggtgtaaccaccgacttgaaaaagtttaagccctttta
ctcaaatggtactgaaatggaacgttctgagaatgtcgttttaagagcggtcaattattaaaaaataatggtataaaacgctacattaatccctta
atgactaattgtcgccctgttaatattgaaagcctttcaaactttaattagacacgctttctagacttatgtgaaacacgacgataacttcttgaata
aaagtactcattacttgaatagaatatacggaatttcttaatgaaatattaaaattttagagacatgttctatgatttcttgtaactatgaataattc
acacctcgtaacctttttctatagtacaaacaaaatacaaatgcactttttgacgtttatgtgaaattgattccttactgtaacatcttttccattgac
cttataaagatgagattttcctttagtatccttaatagaaatctcacatacagaataacaagaatatacgagttattacttacaaaacaaaagctc
ccttcggtataatgtagaaattcattcattgtaaatgattacaactgcattgttttacattttaccgaagggcatttttctcgttatgtaatataaggtc
gctgaaagcattagcttaaaggaagctgtagtccctaaagtatagtactcaaggtcaatagtatccgttttcaaatacatcaaaaagacggactt
aaaaacgaagcctttctctttgtttatttatataacattaatttttgcttgctgtctttcgagaggcttcgggaatgtctggacggcgaaatgaaacc
acaatccgacatctcattcagtctgtcccaggaaacaacatttgtgcagattgccatacgcgtggtgttcaatgggccagctggaacctaggtagg
tggatatcagcattccttggctaacttggataggtatatttttgtgtctgcgatgtgcaacaattcatcgaaagcttggaactcatgtttcaaaaata
aagtcggtttcgcttgatcaatggacagatgagcaagtaaatgtaagctgaaacgtatgaaagattttagtatcgttttctaacttgctcctagaat
atgcgcgactgggggaatattaacgccaatcgttattggaatcccagtcctctggatcatcctttgcctatgaacgctcatagtgatgataagtaag
tttgagaatgagcggataccattggaaagaagcttttgaaaacatcaataactaacctgctttcttcagcgtaatggaaaaatacattcgcgataa
atatgaaaaaaagctattcttagaggaaactacgaataaaaaaggtccttctttgcccccctagatcaaacgcgtctgctaattctgctggcgcctc

aagttctcgatcgaattacaaatcatctcttaaagctattcatgacatgggttttacaaatgacacaattaatttgaaagcgctggaagaggcaca
cggtgatatcaatgttgctgttgaaaaaattgttcaggagttgaaaaaaaacacatcaaattctgcctcttcctcttccatgaaaccaaattcaag
atcctcttacaaggctccaaagatatcatcagcacgtctttctagaagaaataaaaatttacacgtgcgcttcgaagatggttcgaaaccaggcg
atgaggacggtagtgtcggtgatacgcgtgcgacatctcctactttaaatccatttgaacagatgatggcaatgacaaatcagggtatgtctgttg
ctcctggtgttgaaaccacttctagtccgtttttagggctccggtagagcctaatcaacccttgcaaccgtcgatgacaggtcccgcagcaacttc
ctcctatgaaaacatgagcatgccttcttatagtatagactcctccagcatgtatccacaaaccggttcatcatctgccgcaccaccacctgctcaa
cctttacaacgctctcatactggggttgcttcctatgattacaattaccctttcaattctaattattcacttccttcaaacacaactggttctaatccctt
ttatgactattcctcagcagcgccttcgacgcctggcatgtctgcgagcacaaattatcccaacaatggcaacagtttatattatggtaactcttatc
caaatatgacagataatatctcattgccggatatgagcaagttatctttaggagagtcaaacaatgcaccttcaaatccttcatcgcaatatctaa
gttctacgctttcacctcaatacggtgcatccggcacaaatttctctcaatcatctacagatccttataacttgagaacagatgtttataatccttca
gtttcctcttacagtgcccaaccctcaactttgcaacttcagccgactgggttggctccccttcttccggcgaaattccacttcaaggcacaggaca
accgtttatgcaaagtcaaatggggatgcaatcaaataattatcaaatgccattgggaaatacttggatggaatacggtgacgtgtcccagcaag
gtaccgcaatgccaatgaatgaaatgaactatcctggggtaatgaattacgaccccaatatgcctatgaatactggttactatatgcaaggatata
acaacacaatgatgccttctgaaggtatgtatcaatctgctgatgtctatggagatccaatgaaccctggttcatcgggaggtatgggaggcccag
cgagcagcacgtcgaatgcagataactacttgcaaaggattatgcatggtaagcgataagagtttgaggaaagaaagaaaaaaaggcgtgaa
gccgtttttgaccttaattgttagggtactgaaagaatttaacatacaatcattatgtttactttttttcgttctttgataacgtttgtttacttgcttttc
ggtttctaatgttcatacttatatccatggcacatattcgtgtgataagcatgatggatggtctaatgggccatgtcttttggtccaaatttatttacg
ctcatttattacattctttggccatactctgaaggcaattactgcatttgtgagatcagtacagcgaaatgaaactggatccgcataagtgctatat
gaatttaaattatgatgatcatgcatggaattatgagcctagcatctgttttgcataactgttattgaagacatacctatatatatacatatatatat
atatgtatatgttgtttttgaatgttcaggtacattaatgaaaaattgtttttggtatttaaagatgtgtcgtttcaagtcgtacaattatagctagctg
ataaatccagaaagggcatcaatcctcatttcctgaaatcaaatttaaagcttcagataaacgagcttttaacgtcgtacacagtcagggtgaatc
tgccaacattgagaaatacaatcttcaaggtgatttatcattaaaatcaagatcattaaaccggtcttttacttgcaagagatcttgagcgcacaa
gtaaacacacatgtggttacaaatctgtttgacttcttccaaattcaatttctttggcaattttgagagaaaaatcggtttaaacttgcagaaaacg
aacaatcggaagctgtaaaaaaaaaaaaaaaaaaaaaaaaaaaaaaaaatattatatatatatatattactactacgtttacgttttcgtttagtt
ttaagttttttaannnnnnnnnnnnnnnnnnnnnnnnnnnnnnnnnnnnnnnnnnnnnnnnnnnnnnnnnnnnnnnnnnnnnnnnnnnnnn
nnnnnnnnnnnnnnnnnnnnnnnnnnnnnnnnnnnnnnntttacgtttacgttacgttagttagtaagtaaagtaaagttaaacgtttaaacgttaa
cgtaacgaaacgaaaacgaaaacgaacgaagagagtaaaaaaaaaaaaaaaaaaaaaaaaaaaaaaaaaacaagttcttttgaacagtt
tactaatattgttcatccagttttcttgttgaatttttttcaattgtaaggtccctgcttaggcatttgaaaccaacacaacaaacaattcaagatac
tctcaacgaggatcaaataaccgatttctcgctgataagtcagcaaggtctttaatgatgaatatttgatcgacatatataaaatcctgttttgtttg
cttaaattttcgaattcgccactacttaattgtgtctttgaggtatttttacttactttcattaggtcaagtcagggactactcataccgtatcactttca
tatactcttagttgatttggagaacagtgaaaaagatgagtgttcctaattcgccaactgaagaaaccgctttactaggttcagacatagtgagca
aggatgatacaaaaaaccattataacaagtggacgattttaccggcattgtggatcggcgggttcttgtcggctttggatatgaccattgttgcca
gtttgtatccagtgatcggcagcgaattcaaattaatgaataatgcttcttacatagctacttcctatttaattacgaataccgcctttcagcctttgt
acggacgtttgagtgatatattcggccgccgtgctgcagttgtctttgcaaacgttacttttaccatcggaacctttttatgtggtacgtctcgtagcc
ttgttgggctttgcattgctcgtgcaattgctggaataggcggaggtggtctcggaactatgtcttcaattatttctagcgatatcgtttctcttcgtga
gcgaggcacttggcagggaattactaacattgtttggggaatcggcggtagcttaggtggtcccctcggtggccttattgctcaaagatggggttg
gagaattgcatttttttccaaattccacttggtattgtctcgactatcgtggttgcttggcgggttcgcatcaaacccgttatccggggttccaatgc
cccattattgtcgcgaattgattacctgggcagctttttgcttgtcagcgggattagtttactgatcatcaccttcagcttggctggagatgcgtattt
ctggttgtctccgattatcattggattgttaacctcatctctgttactcctttatgctttctattgggtagagaaaaacgttgcgttggaacctattgct
cctgtcttcatcttgaaacaaatcactcctttgaatgtctgtctttgtaatttctttaatgcattttgtggtttcgttatcctttatgaactccctttgttttt
tgaagctgtgctgtttatgccatcctctgaggcaggtgtccgcatcttccctcatgttatctttacttcgttgggttctgtcttaggggtctttacatg
aagaaaactggtagatatcgtcctttaattttatttggatattttggcatgtggacgagcattgtcgcctttagcgtacttttatcatttggtcaacgc
attccttcaatttacataactttgtttttgagtctgtccggaacctcttatggtatgaacttgacttctaccttgattgctatcattagctccttggctcc
cgaagaacaagcagtggctactggtctttcctacttattccgtgccactggcagtgttatcggtattagtctttctcaaacggctacgctgactcttgt
actgagtaatttatcaaagcaacttgctgatgtcccaggaaaagacgatctcatccgacgtttgcgtgaaagcatcagcattattcctgaccttcca
gaaaatgttcgtcgtcttgtcatccaatcttatgttaaagcattctcggtttcctttgtaatcgtgtctggcttcgctcttgtcggaatcgtttgctctct
aaaaatccgtcaattctacttgcacacatctgtagaccgcaactaacctttgcttgtttctataatttagatttagactcgatcagggaacgaggaa
ccgattatgatttttgaataattggtttgttagtggtagagaaagttgattttcctaagattgaaagcttgttatatcctgtaattaattaatcactct

tttttatggaaaagcttacacgtttaaattccctgttcttctatgaaaatgcacttcttggactttttgctttttagttttgttattgttctataggtcatc
ggtgttttaatatccatcgtttatgctttttgaaagtagaatgtgaaatgaataaaatttgttatccgaatacaaacaggttccaatcttcaacggct
caatcgggtcttgcaatctaggaatatgaatttacaagcaaattttctcgatgccacggaatctaaatgctatttttattactttaatacagcatcaa
aacaagaaaaaagtaaaaatcaatgaatctacacaaacagcaaagagctttattttgcatctcttagtattgaaagatcgtaataaaaattccat
acaagacgatatatcatacaagggaagaaactaccgatttcagttcaattcctctctgtatgaatatcttcatttcgaggtttttttccatcaatacac
tgtaagagttttctagcaaagatcgtatatttacattacatcactcggaaaaatttagcagaaagcgtacatataaaataattcaacaccaaacca
acgaaaaaccaaagttcaaaaaatggctgctacaacggatgctaagacttacaagttgaaccacacgatggttcgtgtgaaggatttggaaaaa
agtctcaagttttacacagaagtttttggtatgaaattgattgaccaatgggtttttgaagaaaaccagttttcgctttcatttttggcttttgacggg
ccctctgctcttcatcacggtgttgaacgttccaagcgtgaaggtatcctagaactcacccataactttggcacagagaaggaagagggacctgtg
tatgtcaatggaaacaatgaccctcaccgtggatttggtcacatttgcttctccgttgataacatcgaatccgcttgtgcatacctcgagtccaagg
gcgttgcttttaagaagaagctcagtgatggcaaaatgaagaatattgcttttgctcttgatcccgataactattggattgagttgatttctcaagac
gaaaaacgatctgaggctcatactgaaaacttccgcttcaatcatacgatggtccgcgtgaaggatcctcaagccagtattgctttctatgaaaag
cttggtatgaaggtcattgacaagtccgaccatcctaatggagagttcactctttacttcttagcttatcctgcagttcaaacccgtcataaccgtga
aggcatcctcgagttgactcacaactggggcactgagaagcaagaaggtgctgtttatcataacggtaatgacggtgaaactaagggatatggt
cacatttgtatctctgtagacaatatcgaagctgcgtgtattcagtttgaagaaaaaggattgcctttcaagaagaagttgaccgacggcagaatg
aaggatattgccttccttctcgatcctgacaactactgggttgaggtgattcagcaaaagtaaagacacgatatcatgattacatttttttaatggta
aataaaattacgctgaaaagaagactacataactaaagccactaaactcttccttaaggtattgaataatagtaggagaatttaatttaagttgc
aatttattttgactgtacactcattctataggaaaaaaccatctaaaagattccctccctcaaactagagttttcattctagcagctaattcttcaatg
tgaagtgacaaagaaaactctcataatttttcaagtccctttgccaaagctgcaccactttgatcatgttttatcctttaaagtttcatacacccgcta
cggaataagcttacacaacaatagtgagagaaatgcatcatcaaatcgattaaaaggtcttagtaaacgtcgagccaaaggagtgagacctatt
actaaaaggtttcggaggtattaattaaaaagaaaaaaaggcaacagttgctaaaaaaagcatcggatcgtgagtggatagtaatagaatatc
gttcaacatggctaaatgcatggacgttaaaacacttagatcgtattattcccgctcgaaagtttcaatgtcaaacaagcatgccatatacaaaaa
ataaattaaaaccattacttgatcatagacataaatttttttgaaatagaatagagataccgcaggatacacctggcttgtctattattccacggt
cacagactttgcaagatttcgaggttgatcaacattatgtccacgcaaaaccgctaaccaataactcaataactgaaacggaatgacgtttagtat
accctgcaaacagtcgactgtcttgggaactacaaaagtgggtaaacctttgaacatatctgaatttttttgtcgtaatcacaaccggctttccacct
cgagctctaacctgctcaaaagcatttaccgctttcgggtattgaaaatcatcaggaacaagcataataatgggaaggccttcttcgactaaagct
aagacaccgtgcttgagttcacccgcaagaataccttcagcatgagtgtaggaaatttctttcaccttgagagcaccctccatcgcagtcgcatatt
ggtagcctctgccaattattagcatgttcccttttttccaatagatgttgggaagcatattcttttagttgggaattaagttgaagagtttcttgtacttt
ttctccaatttttgctaagccgtcgatgatttcgatacggcgatttgcctttgaaacagaatctcgagacaagtataaagccatcagcgttaatgca
atgtattgggaggtataagcctttgttgaagcaacgcatatctcaggtcctgcatttatatggacaccacaatgtgtttttctcgatatgctagaacc
gacacaattaacaataccaatcgttaaagcaccacgttccatacaaaaactgagtgccatcagggaatcagctgtttcgccggattgtgagacga
aaacaaccgtgtcgtcacgaaatatgtttgggcatcgatccatgaaatcagatgccagctctattactactggaatgccagtgagctcttcgaaaa
caggacgaacagcaatacaggaatggtaagaagtaccgcaagcaagaaaaaccaaacgtcgactttggcgaataatgtcgtaatagctttcta
gtccacctaaagtaacgactctatcagcgaaattaactcgaccacgcatggtgttgagtaaactatcgggctgctcacaaatttccttaatcatat
agtgatcgtaattgcctttcatggtgctcataatctccatctcaagcgtctcgattgtacgcgtagtactcattccctcctcacggcgaagtcgatga
acatgtaactcgccgtcgcttacatgagctatatcgtcgtcttcaagaaacatgacacgcttagtgtattctatgatgggagtagcatcagatgcaa
aaaagtattctatagggcccggtatcccttcttcagtgacaaaggcgcgactttgggcacggtgtagcaaatctggtttatcaccacggagcatac
cgcttgtgcccggaggcttcgtgaaccggggatgtaaagtggatggtgctgccccggaaaggccatcggaagattcaggaaactctacatcgac
aaaatcaaccttcaggttttgagaacttttgacacctacaataagaggagaaccacgtcgagttgcaactatttcaccgggatagtgactgctctt
tataagaagagcgtaggccccttctaattcgcgaaaaagaagtttagcgagagaagtaaaatcgatgccaggtgtagtgtcgtagatgagttttg
cgagcttggcgacgcactccgtatcggtctcgctttcgaagaccatgcctctggactcaaggacggttttcagttctcggtaattcgtaagaatgcc
attgtgaacaacaataaattcgctatgaggatctgagcgctgagggtgacaattgatgggggaaggttgaccatgagttgcccatcgagtatggc
taatagcacaatgattgatgaatttggtgtccttgttaacgttggatcggttgatagtgttttctaattgactgacgttaccaacctctttgaacatca
atgcatcttgaccctcatctccatcgacggcacaacctgcagaatcataaccacgatattccagtcgcttcaagcctagagtgaagtgttaattatg
cggtttcaatcctacaaaaaaagctcataacggaagcagaactgagtcctgccggggaagaagagttctaaacagacatgtaacgaggacagt
catccattaaatgtacgcagatggaaccagctcatactttttttagattttcttttcctcttccaagacaattgtttcttacctttgacaagcgtatcaa
ggatgtaaccgcggtctctttcgactaaatagttgatgtaaccaaaaataccgctatatagcatttagcaacttcattcgtagtaaaaattttcata
aaccataccacatccagaaaagaaagaacgaaagcaaacgcttatacgatattggagaagtacaggttaatttggggataatggtgtgtctattt

ggttaaggagtctaaacaatatcttaccttgatattataatactgagtacaacgtacgcaaggaaagtggaaggaataatcgctggttttgagac
gtttttagcgatgtgcaatcataattaaactgtttacaatttatattaatttggatttaggtcatttttacaaaaactgactgtataagaccctgtgta
gtactgtttgttgattttagtgatacagtaatattatactgtagtggtttcttaagtaataagtaaagcaaaaacaaggtacgttttacaatttttata
atcaactgcagcgtatttaaagaaatgcgtttataataaagctgaatagataagaagccttgttgtgaaaactccttttcctgaatatgactttcct
ctttaatttgtatgcatcgttcgaaatttgacctattagtaaataagtaaataaacaaattgtacatcaaaaataacacacatagcccatacactg
gatggcttactcttttattttttatatattatattatatgatttatttttctttctccgtcttttgtttgaattgtcagttccattttccatcgactcgttcc
atcatcattcctcttcgctcaaatatggacaatatttcagtttatgcttttgctggattttcaattcgacaaaatccgaccctgtggatgattttcgccg
acggtttttccttattcataccaagtaacccaaatacctcaagtaaaaaaaaaaaaaccaataaccaaagttgtgccttgtatacaaggtaaaa
ctatttcaaagtaacaaaaattcttgaaacggcctttttcttgtatttgagcctccttctttctttttcttttcttttttttttcagcaaatttgctttcgg
cattgtttattgtttgctaactgctaccttgggatcaaaatttaaattaccgtgaagtcaactatagcaaatcactacttgagctgtggaaaatgttc
cttcgtttatttcgcttacttcggttatgaacaaccaccaagttacgtggtgttcatgaacatgtacaataccaacaacactgggatggcaaaagac
tggcaagatgcgcatttgggtcaatgtcagaggatcgaagaatatccaaatgtagctattaattatccaggaccttatgtaactcattctggaacc
atggcatatcatcctacaaatagtacgtaaatcctagtagtccctgttatgcatcaccaaaatgagaataaaattcttttatttggttgaccttaata
ttttttattgaaataaaacttattaacacgttcagcttccttttatgctcaagtacctacccatactagtcctcaaggtaccctttacccagtatataa
ttctgtaagcccagtatatgatcctactactggacgtgttctttatcgaaatttaggacctcctccaacgacttcgatacctgcaactcgacctggac
tagctgtttatggtccgtcgcagcatttgcaagctccccctgctccttcgacccatccaggcgagcgtgcttcttctgcagtagcagctggaagctca
attgcaacaaacggagattatccatcacaatctgtgatggctccttcaggagactctaatatctcccggcccacaaatatacggtcatctacttctc
cctttgttgctaatcaaaacattcctctcccagccggcccctcgagtcaaccatcgccggctaataatgagtttagacaattaaatgtaactgatgc
tctgtcctatctggatttggtaaagttacagtttcaccacgaacctgaagtctataatgaatttcttgatatcatgaaagagtttaaatcgcaggcgt
atgtgtctttgttttgctttagctacaaatattccaactccaactacaagcaaaagttttttttttttttaattttttttttctttattttttactaacttcaa
taggattgaaactcccgaggtcattacccgagtatctactttatttgctggttatcccgcactgattcaaggatttaatacgttcctaccacctggct
acggtattgaactaggtcctgctgaacctggtaaacctcccgctgtgcatatcacaactccacaaggaccactagtaattgcagactggaaccgtc
ctcatgcttctgcccaaccaacttcaggttttccaaatgtagctccttatccaacagccaactcgcagcctgacctcccatcccctggtgtaatcccc
ccttctgctcattcttccacttctgttccatacaaatcatctccttctccgatgactccttcatttccctcggcagatgcatctgtaaagcaagccgctg
acttagatcatgctattaattttgtaaataacgtgaaaaataggttttctcacaaacctgaagcttacaatgctttttggacatattaaaatcatat
caacatgatcaacgtcctattcaactcgtttattatcaagtttctcaactgtttgcggaggcacctgacttattggaagagtttaaacgatttttacca
gatgtatctcttaaaactacttcagaaaaccaagagaagcctattgctgccatttcagagcctgtgatacaagcttctaaagtgtctcctgccgcaa
caacttcttctcttcctcccattggcaaatttgctcctccagctccaacgaaaccttcccaaccagtagagaaacgccgtgtagagccagccgtata
taccagaagtcacgcaaaacgcacgagaactgcaactacagccgccgaagaagctactccaagagccttcaatgttccaattgcccaaaacaa
aaacccgacagaacttgcttttcttgatgctgtacgtcagtatttggtcaatgagtctaaatataacgagttcattaagctccttgaattatactctc
aggaagtatttgacaaaaatgcactggtcgaacgttgccagacattccttcatgataatgagcaattaatgaattggctcaaagatttagtaaagt
gggatcctaatgattgtattcccgttccaagaccaagggttgacttaacccagtgtaaaagttatggtccaagctatcgtcttctccctaaaattga
actcttattgccttgttcaggaagagatgatgtatgttggacaattttaaatgatacttgggtaagccatccaactttggcttccgaagactcgggtt
ttattgctcatcgaaaaaatcagtttgaggaatctttacacaaattagaagaagaaagatacaaatatgatcgtcacatcggagcaaatgtgaga
gccattgaacttcttgaaatgaactaccgaaaaagtcaaaaaatgactgaagtagagaaggaaaactggactcttcccgttggccttggtggtaa
gtctcccagtatttatcagaaagtcataaagaaagtttatggaaaagaggcagcgcaagaaataacggaaaatttacagaaacatcctgccatc
actatcccaattgtgctgaatcgattgaagcaaaaagatcgtgaatggagggctttgcaaaagcattggaatgagctttggcatgatgttgaaga
aaggaacttttaccgtagtcttgatcatcaaggcatatcattcaaatccactgacaagaaaaacaccactcctaagtttttgattggagaacttcg
gaatttatctaagcagcaaaaaaccgaagtagaggaaggaaaaacgacaccatcctatcaatatttacttgaatacaaggataagtcgattatt
attgatatggctcggctatttggtgtttttttagtccatgtttcaacgcattcgatagaagaccgagaaaagctttctagctttttgcgttccttcttat
cacttttctttgatatcccttatgagtcatttgaagagtacttacctagtcattttaatgacgaggattcagaaactgaatctcagcttagcagtcatg
ataagacgaagcgttcaaataccccccagttctcatggagtcaacaatggcttggggttgctaaaggatatttttaaaaagtcacaaaaaggcccc
cgggaaagtagaagcgtgaaatctgaatctgttcccgaatccgccgaagaaacccccgatcccaatgatgtggatgagtaccctactgatgctga
agaacctaatgatttggcagccacgtctatattttcaagtggtgaagtttgggtcaactgtaaatttaccgattcggacggaaccctgttggatgat
ggaactaagttatctgatcgtaatgtatacaatttgtttgggaatatgggactttattgtttttttagattatttcatattttatatgttcgtttagaaca
actgaaaggactcgaggaaaaagcatatcagaaacagcatggtatcaagcctaaccctgttgcgcttgaattagggcttatacgaaatccttccg
agcgtttgggatttccacttccttctgctgataccgtatatggtcaagctttagaattatgcgagcgtataattgaaggagaaatagatcaaaatgg
atttgaagatgccttgcggtgtttatatggaacacatgcttttaggatgtacaccgttgagaaaatcattggctctttgatgaaacaattgcataca

ataactaccaaccgccggatagcacaagtatttatgtttttgagaaagatcgagtgcaacgaaaaactagtcctcgacagcaaattatgtatcgt
gtacaaacagaaaccattattggacctgatgaagacttatgttgtatagattggaattcggaaactcaacagtcagctattcgtttaatgggaaga
gaagatttaactatggcaaatctaaagaccgatgcagagaaatggtgttactacatcggcagctatataatgtctagtccaaccgagggcatttt
gccagaacaagttcggattcctttttgcatcgaaatttaccaattgatgatgttaatgaagaagacgagaacaattatgtagtgcgcaatgctcat
gcaatactcacgaatttcctaaaatgtgatttggcgctcacaatacctataaatacggtgaaaatagtttatgaaccaaacacggaagatgttttt
gctcgtaactctaagcttgtttatcctggaccatttgaaaaaataagaagctatagactgtcccgatggcatgaatggttagaaagtgaagatgga
tggtctagagggttgaccaaagagaaattgaaagagattaaagcagctactcttgataccttatttacagaggataatttgcgtgcctcgaaacc
ggaaaaaatgacagtctcagaaggcaaaaggaaaaacggattgaacggtggaactggtaatggtaggaaatccgaaaagaatagtttacaaa
agtctggatacagggctagagggcttaaatctgatggtattgccgaagcagaacggataaagccaagaattgatataaacgacgataatacgtc
tgaagaaacaagtcaaattgacggagaagatgatgccaatactattcaagatgaggatgaagcagagttgtctaagggtcagcaaccagttaa
agaatatgatgaagagactagtgaagttgctgacaatataagtccgaaaaagaagccgttaacgaaaaacgtggaaggtgaacaagaaaagc
cagaatctgataaccaattcggtgaagatacgtctgccactagtttaaataagttagatgctgatatggaggacgacaaaacagatatggaaga
gcaagctgatgagtcttttgatggcgacaatacgatggaagatgatcagttgaaagaataatatattactccacaagccatttacttacaacaatg
gcttataaaaagttggttcacttttgtttttcatttatgcacgttatatcatgtatattctgtacgtttagttcttaccttttgattattataatttgcatta
tttaatagaaatctttttttgtacacatgtttcttcattttgttacataagttactttactgttcagtctagtctgcgtttacaaatccttttcagttttgttt
tcggattgtttattcttttctttttattcgtcaaagtaaagtataacgtggcacatacacgaagaaaagaaagatcgtctcaaataacaaggaaat
cattgatccaccctacttcatttgaaggtgcatatttgttttagaacttaccgatcaaaatatcagtacactataaaatcgtttcattttagtattgctt
ttttttttttcttattttttttttcaattctaacaaatcactttttacttgtatttaataaaggatacctaaatagctttcctttttttaggtgatctgaacatt
acacatttggtaagtaaaactgtaaaagaaaacaaaacaaaggaaatatcattttcaaaaacaccaaaaaaatcattgtttggtatcttttagcaa
aattaaagctcaagatggatgatttactaatgtctaatactgttcagttgctcaattatttccatgggtctgcttttatatacgacagtctttcaaggt
tgaagtgatttattctatttttcgaaggattccaaagtttgattatctacgttgcgaagttactatattatgtgtaactcgaaggttcgaagaagcaa
cagtgcaatttcacatttgaaagaaaagtgtgaactggatgatccaaacgttgttcaaacgaacgaagtgaggattgagaagtctgcgattccttt
tcaaaactcaaaactcctcttttgggatgaaatagagccctggcaacaggataaccattacattatttcagcataccggccagctactttctctttt
acaaaaagcttcaagactattctctatttacacaatgaatctataaacatttggtcgcacctcttcggagcaatcatgtttcttttcttcattacacgt
tcagaaaggatacttgtaagagaaaccacaacgtctcaggatgtctacgtttttatggtatttttttataagcgctctcacaatgcttctgtgttctagt
ttttaccataccatatcgaaccattcaagtcacgtttcgaaatatggaaataagcttgattatttgggtattgttattatgatcgttggttctttcgtgc
cttgccttcattacggtttcgcttgccatgccagctttcgaacactttatattgggacaatcttttctataggtgtagttgtcgcatttacttgtatgctg
gatcgctttagacaaccagaatggagaagcttccgagctattctatttgtaatcatgggtttatttggtatatttccagtattgcatgctcttttcctat
acacaattaacgatctactcatgcgaatgggtctaggatggctacttcttcaaggggtattttacattgtaggtgccacgatttacgccaaaaggat
accagaaaagtggtacccaggtaagtacgacatatttggaagtagccatcaatggtttcacgtgtgtgtgatcattgctgctctttgccattttcga
ggcatttttgtagcatatgacttcttccatgaagcggctaaatgttaaaaaggttttatacttacatatttgatttatcgttcatttatttttttaagaag
ctcaacaaagactgaatttcccgaataaaaggtaccttgtttaaaaacaggatctttgggaaaatgattttgaagttacctatttgaaggctagca
cgagaggaggagtgtaaaaaatatatcatcattcatttatatagtaatcaaccttcgttacatacagcttcaaacgagggtaaagaattcatcaa
aagacattagatatcattttgttagcctgcgtataaacagaaagcaagcgttcattcttttcacgttcacttttaatattttcatcctcaatcaaaaga
gaatcaaaaagctcttcacgataaaggctgcttacaagccgattctgtacagcatctttcgatgcattaacaagcaggtgcatgataaccttagga
acctgatcgacgattatcttcctagttaatataaaataagacgttattaattcttgaatcaactcaacttcaagttcttcacgctctgtcatttcttcta
atgccatagattcaacctcgctagaaagatcagtcattttaacaggaaagttatcagcgaaagttgatcgtggttgtgtgttggtttgatcactgag
caaattgacaggctcattcatagaaaagctgttcgacaatgctctgttatgcatgggtgctcgcttcaccgaattgttggcaccaaagacgtaact
aaggaatgtctttctttcaaggttgtccgaacccatattttgtagcttcctatgaggcgtaatagctgcaacgtcatctgagtttgcaatgtctgtgat
agaaccatcagggttctcattctttgtagcatctattgcttgatttgaagaagtagccgaacgacgacgtgtagtaagctgctgatttttttctatcaa
gcacatttgacatggcccccctgaacacctaggaaatcagggtggttagtattgatgtaagccatttgtattgaaatcaaactttccacgaacgaaa
acgtaggacttaggttttcacgaagtaaatcgctcacagtctcaattaaggcagtttgaagctttggaaatctagacatatcagagtcaccagagt
aatgacatattttcatcaattcttcgtaaacaagctcaacgcattcatggcaaggtggagcaagaagagctaattgaggctttacaagtatatcaa
aagccatttctgaaagaaacaaagtagctctagaaccagtgctgttgagaatagctgtccgaatatccgttatagacaagttttgtaaaggatca
atggagtttaaagttgtggaaaacacattgttaaaaatggaatataaacgagcacctccagaaagctcctttgtaggtatgctggaagaattacc
atctatactggcaataaatgaattggcaaacctgttcatagcctgtaaaagtataattccacgctgacttttatcagaaattttaaagacaccataa
ttttccaactgggactgggtttgagaaattaatgtactcaaacgtgctttgatgtcgggtaaacgatctcttatatgggcaataagtaaattgcttaa
tgttttagcaagatagaaggtcccgcatcgatttacgatagctcgataggcaggatgatttgcaaagaagctattttcggattgcaaagcatcact

caaagacttgtgggaaatgatatcagattgagaacgattaactgttggtataaatcctaatttcagaggatatacgcgcccacttaaaatatccat
tgcattggttccttggtccatcaagtcaagtttagttaaaacaccaattgtccttttgcctttcggatcaacgagtcgggcaagttttaatccttcact
attgacgatatcaaaatttgccggagaaacagctaatacgattgaattaggtttactgatatactccataatcaacgaacgagtttgagcttcaat
gtctgtaggttgatcgccaacagggattttcgtgagaccagggagatcaattagagtgagatttagaacgtgttttgagtaaattttgaggttaatc
ggtaatttattaattcctttattagcaccagcgactcgcaaagtttcattttcgatttcagcacgaacttttccgaaatctgtgaaacggacattggg
taaatggagaaactcagcatatccttcccggtcgctttctttttccaaggaagcagaggaaatgttggcattagagatagaagaatcttgctgggc
atcttcaccaggggcattcatatttgaatcttctttcaagttgataagttgtaagattaacggtcttctggtaacgattcctgttccacggggtagaa
aatctttgccgactagatatttgttagtttaaagtgaagggttcatccgaacaagcagagttacttacctatattttccaagacggacgattttccgc
acgattgagaaccgaccacgacaatggagggtaaatccaaaaagtcagaaccgatggtattatacaccagatcctgaagttgattgactaaggg
aatcaatttttccatattagtaacccgtagtaatttctcagagaaacgcgctcaacaataaataacaaataaaaactgctagaatgagattctaaa
gaataattcgaaatgaaaatggaaaaaccaagacgtccacagagattggatcgattttcgtttcgacgtttaaaggatcaaaaaagatcaacga
aattagatacgtagaagagaaaaagacttaaatcaaaggtaacagtctttttaggatccttcaagtcaaagaaaaagaagtttcgacgcaccaa
cgagggaaagagtagcgagagatacagaggagtactaggtagagaaagcaaggcgtcagttggtatagtatatcaacaccactaaggaagta
tacaaagtcttagaaagcagttattttataatccaaagtagaaagaaaaaggaagttgcatttaataattagaattcacgattcctttttgagcca
aaatcttttgcttcatgtagcaacaattcgtatcatgcattctttcgtgcaagaaggaaaataataagcaacagcagcaagtagaaaggatttga
atggaggtagctttctccttatttcttcctctctacgatatctgttttctttcagtcatgaagcaaccattcgttcatataattatctctccatccaagag
gttgtatcaagcacaataaattgcaatttcttaaagggtaccaaagctccctgttttagaagctaccgttcttctcatcctttgtttacataggctttt
gtttacatcgtagtaaacactttgaataacatgggaaccgtttgactgttccttagcttcagaggaaaagcaaatggctttttcactcgcaagatct
tttttcattctatatctggttcctttttctcctttctatgccttttgaatttgttccctacgaagtgacaaactactgttcatactgatttatagtttacttta
agatttcatcaggcttttcggtaccaactttctttttatatttttctactcttattctttgttaaatacatccgaatcgcgggcccttttcatcattcctatt
ctactttcgttcctgctctgaaaagtaaacaaaacaaacaacgaaccacgctacatattcctattcaaaaaaaagacaattaggtcgattccgaa
agagctcccatactggaaattccacttttattcttctacgctttctatgaaatagtcattcttcaaattctaaatcctcgacatatcctttgtaatgaat
cacctaccgtcgatttactgacaagcttaggttagggcaaacaacagtcacaatttttcaccttgcaatagaaacttcgtacatctttttttgcaaca
cctttaaccagcatcattcaaaatggcatctgtggttaccaagaagggaaaaaagagcggagatactatcaactccaagcttgccctcactatga
agagtggcaagtacgtcttgggctacaagtctaccttgaaggttctccgctctggtaaggccaagctcatcttgattgccggcaactgccctcctct
ccgcaagtctgagcttgaatactatgctatgctttccaaggccaatgttcaccactactctggttccaagtaagtttttcctattcaagtattcagtta
tagaagcaatactaacctgtacagcattgatctcggtaccgcttgcggtagactctttagagtcggtgttttggctattactgacgctggtgactccg
acattttggaagcttagacaaaatgaataagccaataaatttccataatttatgttgctgtggtttcttatatattttatgtaatattgtaactgatta
gtttttcatcttgaagcagacctcgtaagctaaatagtacccagaaacaagaactcttggcttattgtttaatatcagctaaaagtatggtaaaaac
ccgtagtagtatttatggctgaatgtcggcatttacaaagcgggtttttatggaaatatccagacttataccaaacgaatcttatattcatatttctg
acctaaagtgtcgtatcgtacagctaacattatcacatgtgcaaatagtatcctatttaatacctataaacagaacaataatctgaaaataaatta
aatatataatttaccaacattgtattaagtccaattagtttcgttggtttacgcatacacataaacatttagtcactcattaccgaattcataaaaaa
ctcgtaaaccgaattcgaatagtttatagatttcaaatataatcccaaggttacaatcaaaaaagtgattctgaatcagacaaaggtgatctttga
cattcatcttccattttgaccacattcaatctgtccacgacatcatccagaaaataggaaggtattggcgggatatcattttcaacaatcttcatgtt
ctcatcgtcattcgttgctattaagccatccagtttttttttacgttcattttccaatatcagttcattatgcttttctataagctcctgcatctcagtaga
aggtccaactcctaccttctcacgcaatttggttacccaaggatgtaggtacagttcaatagcagtgggacgtctctctggatcacaatcaaaaca
gcgttctaaaaagtccctagcaagacttgtaatttgataactcgaagggattgtgggggtatgcataaccgcaatatgatacataattgcccattc
attgtccatctgattccaaggagtgcatccagccagcatttctaagattacacatccaagtccccatatatccatagaaccaaaccttcctttttag
ttcctatgatgatctctggggatgtatacataggtgttccagctaagttgtgcatctcaagctggatatcctcaacgtgagacgcctgatcttccgga
ggaggtacataaagggcggacccaaaatcgctatacttaatattcccatttctatccaacaatagatttgctggctttatatcacgatgtaaaacgt
gtcgtgagtgcatataacacaaaccttctaataactgcaaaacgtacatgccaacgaccatctcatcttctatacggccataggaaagcaaatcg
gacaaagatccaccttggcataattccataaatatatataccttttctctatgaacctcgacaccataataagaaactatgtttggatgattaagttt
ctccaacaccgtcatctcgttatgaatctgatcaacagcacttttaagagagcgactgtcttgaattcttatttccttaacggccaaaagatcacctg
tttcaagattcacaccgctatacacatctccaaacattccacttttaacaaattgtccttgctgccaacgaatcgtaatattggatagagaagaagc
aagtttaataaagtctctatttcgatatactgaaacatcgagcacatgtccaattgctaaccgctcatgcaacattgcatttctttcagcttcaatctc
ttcaattcgttgcatcatttccttttgcaaaactgtaattgtttcatcctcctcgatagtatcataattcaggtaatttctcgaagcctcatccatgttta
ctacttccaattcatcaattgttttagatttagcaccaagaatgtcaaaatgatttaggagcaaagacattgttttgcccaccttatctcgtaatttct
caaaattggcgtcgtcaagcagcaatatatttgttccatggagtacaaccattaaaaactctaaagcacttaccgcccaacgaaacgtttttctgt

caagaggatcacattcttcacaaaggaaagcaacccaatcaatcgctaaaaccataattttttttctgaagatgactatccaagtaggaatcaaaa
ttcaaaaatcgcataattcgttgactaaattctgctgcgaaagtaaacgcactatatattaaatctgagcaattaaaccctttacatgcctctcgga
cgtaagtggtgctatcaacaacagtagaagcgatacgaatgcaaagtttgttaaatcgaatatagtccctatagacgcgcgcgatattagaccga
cagtttacgtgagcgctcaccaaacccgtgtttaaatcttgaaatacagcctttgcatcttgtaaatgagcagcgtcactagaagtaacaatccgt
atatgattgtcttttaagtcggagtgaaaatctggaatatcaactttaattatccttcctttccaaagcaatggttgtttggtacaaataattaggac
ataagaattattctccttagtgtattctatatcattattcgcagtgcttgcgattaaagatttgattgcatccggtctttctcccaaagatgcatccgc
aataacgaaaacaccatcacgttctaaattggcagtatacgttaaaaaatggcccgttgaagacaggcggtcgaccacttcatttaaacaattat
tgtggacgacgtattcagcagcattttggaatcgagagttatacacatcggaaaagcgtaagatcttacggaatcgaatttgggtgttcttaagaa
gcgttgtgaaccagtttgttagagacattattgaacgctcttcaggccctttcaaatgagattcaaggtgacgatgtacattaattaaaagacgatt
caataagtaaccgaaatggctagccatttggtattctcctccatctatccaacccacatacttgtcaaggaaagcccattcgttctctagaaaatct
atttctttaaaataaaggtttctgttactagaactcaattttatagcgagcaacttgaaataaaattttaaagattctaataagatgtcattgtaata
ggcatcaacatcttgaggtaaatcccaacctggataaggccttgctatgtcaatgtattctaatttaacacgcacagcaaccgataaagttattttg
aaatcatctagtaaagaatcaaccatgaatgagtttgaccctttttactttttttggcatacattaaccgaagacgcaatgcttcatccactaatttgat
aggaaaatcgaccaacgcatttagttcatcaccatatgtcggaagcttcatatcctcaaaagctttagcattctctataattgtttgttttgcctggtg
aataagacgatttagagtagtcaatgttctctgctcaaatgttttctgtaaaccaaactgccggaatatacgttccacaaaggacggcgaattagt
gatttggcttgtcaaatcaaatgtgtcatttccaatccaccttcgaaggattacagtttccgtatgaacattctccttcacattactccatgataccag
ggcattcaacttgtcattaaattctttggaagcatataaaggagcgtcttctctcatttcgcgactagaaatataatagctttcacattcttcgacttt
atcaaggagttcagcaacctgctcaaacgcggatttttgacaacgttctttaggttgaacttcaaaattgataacctcttgtaagacgaattcaacc
ttctcccgattacttcggatgacattggcttggtctgctgcaagacggccgtgaagccaagcctgaagtcccaaccagatatctgtgatggtttgttt
actatagccactttgtttcgtgtcacgcatcctcctcttttccgactgcatgacatcgccctttaacaccgaagaaagcattaagtgccattcaagtc
gctctcgcttttcgggattctggagatcaacttcgctaaataaaatgcttgggacgttgtaaaaagcgtcaatatcaaattctggataagcagactc
agaaaaatcagtatcgttatcactttcttcttcagaatgatgaatggatgccatagaatgcgacgaattgtcatttactgcagctaagtcaagatcc
acattattgtaatacgaattttcctgcacaccgtaaatgtctgaaggtttcttatggtggttacggacgaattcggtactttcgcggggtcgagtgat
atgaggataaatgtttatattggaatcggaccgatggcttctatttccgacattatgcggagagcggagcatggatgaatagtgttggtaggtatt
atatgaagtgcgaggaagagttgaagggctcgtagtaagggaatgagctgtgatcaaagaagggtgatatgaattattgcagctcttagaatgg
ccaaatcgctccccgttcttcctgaagaagaggaaggaaaggaagaactattcataattcgttttgaagactcataatcgtctgcagattttgaa
ccagagatgttgacggtagatgaaatttgagcattatcaagatacaattgctgtaatcctaaatcgaccggatcattagaagtatactgagtgctc
tcaaaccccattagtatcaatcatataccggaaagattggcaaaggcgactcagtctttgagttaccgtgtgtatccaactttaatgatgtataggt
acaaaataatttgcaaagttattaactgattccttggcttccaacgaaacaaaaagcaattctaacttacaaagctcaaatccgcttcgtataaat
caaatctggagttcaatgcaatcttcaattttgatataaataaaaatattaagaaaattccttaaataaggatttgtaaaaacaaagtttttggagg
cgttcacaaaggttttggctgttgaaacaggatactgcagaggaagaataatggcacactaccaaggaacaacgtcgtaaacgcgtggaggaa
cataggaacatcagaaagttcgcttacaatgagcttatatgttgtaagttggtttctattatattgcagtatgccaaaagagaatatttttatctcctt
attatgaggcagagcctatgctcctgaaacgtatatttagtttatttttttaaagttatttcctaaagcaatacacgttttaacttttcatagaaataa
cacaatcttccaaacagttttcattttgctactattataatgaacatctttaagagaagcatagatcattggaattgggtttttttactcagaatgaca
attgtttacaccaacgaagtaagcggttgtctaagtgcgtattgcgtggtccaataacttacaaagctttttaaatataagtacaaacaattactct
aggtctgttgcataataataaaatcggttagtttggtcacatagcagtaagtatcttttgatttattccatatgatcgtcttccatttaaatcatttaaa
aaatagtgaaatcttcattcatttttttcaaatgcaaaaagaactgttcacagctcattaaagataagtcataatgcatcctgtatcatacagagta
tcgtaaattaaagaagtcatgagtggaagtatttgtaggatgggaaacgaatagcaatagactgaaaaggaaaaagaaaagataatcgtactc
gtattcgtattcgtactcgtcttacttattggaaacgaaaggatgaagcggactcaaaaccagcatttgtttgttcacaaattcccagaagcctctg
tgtgtttttcagaaagacaccaaagatcgggacaagtcactactaattacaaacgtctgatggtccagagtttgtgcaaggaaaggaaaagaga
gaaaatgagaaactcgacgactataaaaggaaaaggtataggttatgagataaaaatatcaaatgaatagattttcttcatcaatcttaaatag
aaagcagaaggtcaatgcagaaatgaaaggaaaaatgagacatatttcaaaaagttttatgtttctgctcgtatgcacaagttgtcgtaaaatca
ttgaaattctaaaataggaaatgtcccatcaaccacaggatccaattccagatactgaaaatgatcaaatttgtaatgataatagtatccggtgat
tcgtccagtccgttgggaaaatgcaatataatagaatccttcaaaactgacgtcggacgaagagtttctgcttcgaaattgaaaatcattcactttg
tccagcatcgccaattccttccaccgcatatagacagtgcctttcctccaagggttgaatggcttaatcttgcgcagaggtactttccgctttgcata
ttgaaaagctcctagccgtttccaatgtttatagtctgttgattccgaagcaccccattccggccaccgtgtcaggaacccatacttgtctccgatga
tttccgcctcaaaatatgtcgtgagtgaagaattgtttccactcaggccactaatcttaaaaaacccacagagcattgattgctcaggattcacatg
taatattgtcacggctacatcaaatgtcttgttgccatcggatttttgaattccatgaaattgagcaccgtttctcatgaacgacgatataaacatcc

tgttctactttatcttctttgcaattcctaaaagaacctcaatttgtcttccagttggcaatgctagacttcaaacttttcttaaaaccaagctaaatat
ccaaactatcagatttaaaacgcggagacacgaaataacaagaaaaatgatgataactataagcaattattctgctaataaatgaaaaacata
ggcactttcaacaactgtcctttgtctaatgtttacttgtaggatggttaatgctgctactactattactactactacacctttctgtccctttgtagtgt
acacccacgagctccattacccgcaaacctagtttttcgagtttttgggaaaaagatttagatattcttaataaatacttttgcttttattttcatgg
ataactggcagattcaaatctgactttaaccccatttctatcgagtgttttcaagctgaacacataggcaatgtaaattcgtcagtgatgaatgagt
ttttgtcattcttgttccaacaaggtcgtattaacgtcgcttgaatgctgaaaaagtttttggccaaacatgaatttctgaggcgaatttacaaagtt
catccatgtatatacaatcaattcatagaaggtattttataaatattacgattccttagctattattgtatgaaatcaaagaaaacatgaattttcta
atatctatccaaatagattccgtaaccgccaaacgatactttgtatattaaccgttcattattcctttaatggcatggattaaatcatctgcatatgg
acgttgttcaggattcaactgtaggcatgcacagactaaattagacagcctcggttcgtagggattttcggatggaaaagagtattgtgcgttaca
aacagctaaagccaaagaacctccctgactgacttgcttttcaaaaggagtgtagccaaacatgattgtatacaaagtgcaaccaaaactctata
tttgttagcttacgttccttccttgcgttgctttttttttctttctttgattacccatatatctgccttctctgtaattatagaacctggtcttacatggaaaa
gctcgggagctcgataaggcatagtacagttttctgaggctttatcctcaaaacttatagcctctgagttatttgaaataatagaaggaatcctaca
gatgcttccaaaatctgtaagaacggcatttctcatatcgtcgctcaaaagtaagttttctaattttaattctgttagttttctcctctgcttgcgacgt
accaggtttcacatctccatgaagatagctattggttctctttttttttcatgggcttgttttctaattgtaaatcaattaggtttgttgtatccactgcat
cttcagaagcagagtcctcaccccgttatggagatatttcaaagcctgtaaaagacatagggaccagctcaagataattcgtttgggaataaaa
gtattctttttatgttgactctgaacaatctgcgcaacagtgccttttttgaaaaaagggaaacataatataaacatattgaacaccatcgacctcct
tttgcaattgaacatgtatgctttttagtaaattggcatggttgaattcttcatgaaactttgcttctattaatgctttttgaacaccttctcgacctga
atggcatttaatctttttcaacgcaaacttttcaccactgttcacattctcaacaagatacacaaacgcatatcctctatacatatattaatttcctttt
tcttctttttctgtattattggcatacccttcaccgagtagttgaagcactttatactgctcatcttttaaaacgatcagttcattgttgagtagcgaag
aaattaacggattgatagaattccatgtattctttaaaaaggattgcattttcttgtattccaagggtgttccaaatttaccctcgtttaagtggcatc
tagttaagtactcaatgaaggccactttacaaagtgccatctaccgccatgcatgcacaagaaaaaaagccgtgaacagagtatgaaaacggatg
agaaactgcagaaacatggcattagaagattggttattaaacgattaaaaaatagcattaccatagcttacgtgacgccggaaaatcagcagaa
aagaacaaataaaagatttgtccttccagagcaggtaaggacaaaaattcaacacagtccgggagtctttgagttggaacctttacctagaaatg
gaattgtgtactgtacactattgaatggcgaacgaattcaacccaatgactttgtgtttgtgtcctcgccgtttgctggcgaaccatttcaaatagct
cgaattatgtcttttgaaaaatccgaatcttgtcttgggactaacttgtatgactctgtccgtttgaattggtttttttcggccacgtgatattcaacga
aattcttctgatacgcgtcttttgtacgctagtatgcacagcgatatatacaatattggattcatccaagaactcgtaagcgtgaaacacaaaaca
gaaattcacaattttgatgaatatacaagacagccgaaatcatttcattatgaccgcctgtttgatcaaaatatcaataaggtcttcgacttactac
ctgtccataatataacaaatctacctaaacacactcttttccaactacaaaataactatgatttcgctattgtagagttagggaaaggacgagcctt
gatggaagcaccccgactttgcaacgtctgcgacgaatggtgttctttagatttttcggtacaatgtgcagactgcaagagacattatcatatgaa
atgtgtgcatccgccgttggtaaaaaagccgcctcatggttttggctggacttgtgcattatgttctgtttcacatcgacaaaaaaaatcttccctac
gaaactccgctacattgatgacgcctagtgaatcccaaccaatagaaacatcagtctcttcggtaaaagaagatactgatactaatgatgaaact
aataactctgacatgcatacaccctcaaacttttcttccatggcgactcctgataatcatatagaaaaagatgatactttgagctcttctactgaagt
ggtcgtatcacccttagaatcagtaccaggcagttccttttcaaagcttcgccgcctcccgtggaacatgcgttacatagatttacgaagtgacttt
gatgatgaaagtgaatctgacttgtatccttcccgcgtccgctctgccacatctccagggttttcttctacggttgaacttcattcaaaagctcctag
attactgactactgcggatgaagagattgatcaaaacgtgcgttctttagaaaatgccaaatcagacctgccctcttttttttgccaaatggccattct
taaaaaacatacatttaaagggatttctattcccttttttttgaaccatccctacgcggaggaatgcttttagttcctctagagcttgatgattcggatt
tagatgactatttacgaaaaagttggaatcaatggaagaacttaaagattgcgatttctccattcgtgcttttagatgtctccatggcattcttatac
caaaatgagctgaatatgacgaatgctcttgacaatttgcagagactcataacaggaagtgattttccaaacgtggagtccctacaaattgatga
aaagagattcggggagcttgtgaaagcctatggagggtctttgcacgttatacatcgttctttggaggcccgctgctctttaaaagaacttttacaa
ttttatttggcgtggagtatatctgcgaaggggactgcaattctgaaggattttgctgcaacgaaggaaagagaaggaagttgggaagaaaagtt
cacactgttttctaactatgagctttatgatccttctaaattactaagatataaaaagcctatggtctgccgcaattgccaaagtcgcaaaagctct
gaatggtttgtcggtccagggacggataacactaataaagataagaataaacttgttatattctgtgaaaggtgtggtattatatggaagtactat
gcaaccacctcgcaaaaagcattaggtgttgagtttggtcgttcgactgagaaagttgtcaaaagaagaatattagaatgggagcaattgtttgc
taatatccataatataggcaacagtcaccatactcgtgacgatagcccaggtgtatcgccatcactgactattgatagtcataaggctaacaatgt
taaatcagcaagcgaaagttctccgcgtcttcaaagcccacttaatagaaatccgagaaatggtacttcaaagatcaaagtcgaagcttcattac
cggcattgataacaaagaaagaatgcaatttatgcggaggaaacaactacgacgatttaattggatgtcaccgatgtggattaattgttcacaca
tcgtgccagggcttaaaaaagggggcagacgatcaaccgaaattgatgtccatgatatctactagtcgatcaactagaagtgcggcgaacaata
acaccaaatctcatcattctatagaaagtactcagtggttatgtgaaagttgtacctttgttacgaagtttggacttgatagtacccctgagtgttta

ttatgctgtcaaacggatccgcaattatcaatgaaaaggactgtagaaggaaattgggttcatatgttgtgtgctgcttggacacctggtgttataa
tttcaaacgatgaatctgagcccgttctaggaattggtagtttaccatcggatgtttgggctaatacttgtgaaatatgtcaacttggccgtggcgtg
gttgtgtcttgccataataataacgcatcgcttgctcatataagctgtgcgttaaaatctggatggaacctctgttttgatttaattcgaagagatac
gcctaaatacaatattgaaattgggatgtcgacattgtcgttttttaacagctactcggtcaccagcaatgaagcggatttatctccttacctttcttt
gaagccagtcgtgtttcgcggccatgctaatatccctcgtaatcttatacctcgcacaagcacaataccgtcgctgggtaaaaattcctggaaggc
ttatttagaaagtatgaacagcaaacacgcattctatttcttacaagcagctcaaacttatacacccttggaagagcaacaattatttgaagaaag
ttgtagcaaatgttcgacgacaatgagtccatattggtggccgggaggcctttgtcatatgtgttatttccaccgttccccggaaagtgagttaccc
attacgaatgtttaatttttttggtattttacgggatatatttatttaatttattttgggatgggatatgattatttctaatgtgcataaatgtatgagtg
atgtttcgcattctctttcactttttcgttttcgttttctttatttagtactgtgattgagttaataaaagtttttggtatatggcattgtttttttaaagact
aatatgtgttggctactgtccactcatagagaatcaaaaggtataggaatgaaatactgtaaaaacatttatcatatttaaagtaatagacactttt
aaaattaaaattaaaataatacaagttggaacaattaattacgtcctggtaaaatagttatcaagctttaataaataaccacgttctctagtataa
taataatccaccctatggtacacatccacaacaaagggtatatgacatggttttgttctatttttataaatatttttacatagtactgaaattatagtc
atcattcataccaactggtaaattattaggagcagtaataggaagttgcacgattggcatagtgctgttattagcaccctgatacgccacgggcgt
tttaggatcagcagaagaatacattgggaacaaggagaccttagggagtaggacaggatgatgctcgcttaaaagagacttgtttaacatcgga
tccttcacagacattggcacggaggttggggcaatggaggatgcagggggtaaaataggagacaaattactattctttaaggaccgagtaaaat
aagagccagaattggcatgcattctctcatgttgccgtaaattatctcttcgtgaaaagcgtttcccgcaaaaacaaacaaagggcttatcggtag
tgtgaagggaatgaatatgcctacgtaaatgctcggatcgcttaaattttttctgacagatatggcaaacaaaagaagtgccgcctggtgaacga
ggggcttgtattttggaacgcgacgatcttcttgttttgtttgggctctgtttttttacttaacactgacttttgtgcatcgtctagcagtacggggtccc
gcagagacggtaaggaaggaacattttctacgtctggtttttcagaggcagcccttggtgaaggagtgccgaagaagaacgtggaggaagtacc
tggcagtttctctttcctctcccgggtatccacagaagaatcaggagagacgatgggcaaactgatattaacaggggtcagattgggtgaaggag
gtaagtgcgaaaccagcgtgctataagaaggtacacttgcagcaacagagtcactggaaggttgtgccataacgagagagggctccgaagaag
gacaataatctctgggaaaagaaggaagggtaaaagaatccgaggaagtcaagtaagaacgctgcctacgcaggtcgttggtatgaggaaga
tggtaagcttcggaatcaacgtgaggtgacttataattggggtttaaagaaccagatgaataacgtttatatagaggtctgtcagtataggcaggt
gctggatgagagagagggagttgatgaccctccatgtcgggacaccaatcattagcagggtctttgccaaatgcatagtatgaagaagttccggg
atatgtattggagtagtagggagatgaggcataggattgtttgtgtgcatcaaaagtatttgagtctatgtgagtgctgataggggctgaggtaga
aggctgtggtggagagatgtttgatttgggagatccctgagagaatgcagggagaaaagaggtaagcggttcggataagggaatatcgttgga
gggctgggcaggattgacggcagacgagagggatgaaagagaaaaggaagaagtaatggcaggaagggaatggggatggttgggagcgga
aaaagaagagtgtggaggagggacattgatggaagtagcagaggagtttgaagaagaaggatatgtaacaggagcagaaggatatgaatac
aaaggattcgaagaagaaggaagagcgacttgttgggaaggtgcggtcgaagagggattggagggataggtgtaggggaaagagtagtttgg
gttaggtggggcagacaaaaacgatacgttgttagacataaaagggaatggacgactggtagtagagaaggaaaacaataaaagaataacaa
ggattttaatgaataagagacgaaatgaaaaacacagatgttagaccctggtcatgcaaacgagctaagaagagagagagagggagaaaga
gagctcaagtagataagtaaaaaaattaacaataaagtgaacaaaggcggcgacagaaccagtgacagaattcagcccgtatgaaacccaag
aaggaagaatgaaacaaggttaccgataacgaatcttttagaaaaccagagatcgacaggattgaccaaacgaaaagacagaaaacgaaca
ccaaaaccaaggcagagacaagagacaagagaaaaacaattcaacccagaagaagaaggaaatacacaaaggatacagtaaatacacaac
caaaggatgtgctaggagttctttctaaagcaaatagacagatgcaaagtctgaatataaacaaaaataaaaagacgagaagaatgatagaaa
cttgacaagtcaaaaaagtagaaagaacaatgcaaatgaaaaagaagaaaacgagcctagaccccaagaaaaaaagtaaacaaactaactc
tagggaaacaagagagagctaccacagtaagagattgagaaagtggacacaggaaaggaaagacaaaaaataatagcgggtaaaggctcg
gcgggcaaaggaagggtatacaaaaaggaaataataataaacccaattattatatatgagacgaaacacccaataaaaaaaagaaagaaac
aaacgaaataagaggacctaccaataaagtgtgtaaataaatgaaatataaacacaaacgaaacgataataggtaggtaagaatcctaatga
aacgagataatagtttggctctgcaacaaagagagaccaacaccagtatataaacgatggtttcacgatcattcaactaacaaccccctgaaatgt
gcggaattcgctaattggctcggaagaaacaagaaacgaaacatcaataggataggctaaacagtatatctcctcccttttcgtcatgtatggtat
aaactgggcgggacctttataaagtttccccagataaagctaagaaaacgatgacgtaggcgacttataataagaggggaaaaagagggggtt
catattacgaaacaaggtccagaaaccccttacggaaaccatcgtagtgtatccgaagaaaagaagggcaaacccaaggacacaaggtaaaa
gaaagttgaaaagtttttttttctttcatgcaagtaaaaagaaggctttttacttgccactggatacgtgtcaaatttaaccatgtcgatttcattctg
tacgggttcaaaactttggatcttctcgctaaatttgagtgtctgtcatggctcggagtacacgcatctagacaacttaaattgactgcgtgcttgca
ggaagaattgcaacatacaaccaagtcatgaattaacaagacattctagctttttttggagatgcataaacaatatgtttaaatgttctttatgcaat
tgtcaatacacggctggattgattcatctctttcttcaacattccttttcaaaatttttcttttatttctcgtcatcgttatccgaaagcagcggggttct
gctcggttgtttttcaagcttattgggcaagaagtcagatagtaacctcctccctgacgtattcctatgctgattatggggaataatctccaataata

gggttagtatgtaccccagttgaaaggtactgaaacaaaaaataaaaaaggataaaaattacctggggcttttgggatttctttgttatattctgg
ggcaggagaaaaggcaaactttttgcatacccttagggaagcgttggtttcaataaggagaattaaattttatcttcttccattttagagtttgata
gccaacctccttatccttagatttggtctatttcgggaaaacaacttgctaaagaagggatggggatgatgacttcgcaatgagcaattaaatttc
ggccgttatagttcaataggaattaaagaccgaatagaatatgccctttcaccttttgtcattgctattatattcttagttcgcatccttactcccaat
ctaagcgggcctggtctagtctagtctctcgtccgtcacatactcgctacctcatacgttcaccttgctcgttatcaaacattaatcgctttgttttcgt
ccctaagtatctcgacgcatccagacaataggaaaacaaagaaataaagaaaatcgagagaaaacacttctgttgatcggatgattattggttt
gttgattgttgattcgttaaaatagtacgctatccagtcgttcccccttttgtctccttctttatccgggttgcttatgcttacaaaggatcattactcctc
tttttaacggaaagttgttgatcacgtatgttgctgtgctctcgcttccttcttctgggttagcaacagacgacgggatcttcgatgtttacggggtga
attctttgttgctatttggaaactctggtttattcacttcccaaaatcaggagtatccgttaagcttcttctcgtttatgttgctgctcgtgttccaatga
actttgtcattgtgtatctataagaaaaagtacctcagaaaaaatcaacaattatatgttaaaaaaaaagcttctggttaacaaatgattctttgca
ccagttgcataaactagcgtattcattttctctccaacgtgtatgttttttttttcgctaaaattcccaaaaggttagatcctcggctttcggtcaggagt
atgctcgtttcatttaaccttttttgtcattagttcctcctccgaattcttaatcgaaccgtttccctaattgctatgaaccttaccccagggaaccgtcc
actcgtagtccccttcaattcactcgctttccactctaaggatcctactccttcgtctttcctatgcgggttcaagtgatatcgtataaacacagtaca
atatgtcaaaccaatatttcagcaatgaccctatgttcaaaagtctactgtcgtacagtttcttgattgcttgtcacagggtagagagtgcttcttgtt
tgtactcatccatatacacactttggttaacatcctttaatgatgataaattgtcttcattacgtggtccgtcttgcgcgttgtcggtttgattaaccct
aaaaaccgtaattcttcgagcccaaacagaaatcgagactgtaattcaacgctttccttgtttgaattctcatccacttccattggtacgtattgcct
gtctggtttggcgttcatttcatctttgggtaagtcggcttcgctttcctctgtgaaccttcgttttgtgggcagccgctcctccgtagccaacacttgt
aagttttctgaaaatgccatgtaccaatcatacaaattcaaaaatgctcctgactcactatacagcttgtatagaatagataggtccggcagccat
ggtttattctgttttaaattcgcttcatttttctcttctgtgtcacttatcctctgcataccaatgtaatactctggattcatgcacgagctatgaattac
gcttcgttggtgtccagacaaaaacccttgttctagcggtcgccgatagtcaaagaagaaaacctcataaaacggatatgcatccataccttgcgt
tcccaacatcccatgctcaatctcttcatccaacaattgcactaattcttttaaccaccttgagtaccgaagaatgctcttgtcaaatcctgtttccat
aacttttcgagtatgttccgttccgctataattcttcattcgcatctccaccgtttctagatacttatttttggaagaatccacaagatgttcaagctca
tccgcaaggttgagtcctttacccactctcggtccagaaaacatatgcttattcaggtgttccatacaagccagtgcttgatgagaatttaatcgca
atatatctttccgaaaagtttcatattgttcgctttcaaaccatagcccttcaagaaaaacctccatatactctccaaatgctagctttgttggcctac
tcatatgtgtttccagctcctgccagcatttcacatgcttacgcagaaagctttctcgccttgccagttcctggaatgtgcttttaatgtactccaaaa
acacatcgtcattttccaataaatcttgaataattttttttcccagatgttcccatttcccgtaacatctgttctatccgatgctgaaagcttggaacag
tgcgtaatatagatacgtaaaaggtagagacagcacctgtttggttattctgaattaaataaggtaaaggactaagagcatttccatagaaatgt
gtaagtataccgtagcgtaaacaaccaacaactttttcaacgctccaggatctttcgtaaaacatcgaccgaaaaagcttcctagttctccatcca
aactttaaacttagattttcctcgacgagagtaaatatactttcaaggatatctgtcgtggctttcatgttaaatacttcgggctggacatggctaaa
aaagacagagtcaagtgatgcatcaaaaagttccagaggtacttttaaattgaatatcagataaacatgatgatccttggacttttgcgaaatcat
attgagggcttccatcaaagcatttagtaatcgcctatcacagtcctctacattctgtaaaaccaaagtaatactttcccctaactcttccatatcat
aagatagaattccaaggcccaaagacccgcctttgatttgtcgaaagcaggattttagatctctgtggttcttgagatccagcacaacgaatctctt
taatccaagctgataaagctttaatttaagctgttcaaaaaaccgaagatgaaatgtgtttctgcttccatcacacaatatcgcggcctttagttttt
gtccttctggattactcttcagtgcatgctctgttagttgttgaatcactttggcattgctctctgaagttgcttgatgaacaacactgcttaattcttgc
atgacgctcttgcaaagtttctttcgtaaattgacccattccagaggttcttgattccctatcaaaggaacaaataggttgtctagctgaagaggct
gtttttcttctttcctacgcggttttacataataacatccctatggttcaaccattagtttcagaaacacttctcttactactcatcctaaaataccttgc
taacggtgtcgtgttttaaaacagacgccatttttttaaagatttttgttttaggttaaatactaacaagcgacacttaataattttccatttaaaaact
gaagtcaataggtttttcgtttagagccgcgactgaaacagcagctcgcatcgctactataattatcttataagaataagctacgcttaaccccac
aaacatctgtaaaaagaactcaatatcctaaatttattctgtaaaatatattcgataaatgaataaataaacatttttttatttttgattacttcagag
aaacatagccatcaagacagaatatacatatttatttctcttcatcagaagatgcagacatagtctcagcatcatcttcagattcttcatcttcaga
actagggtctaaaccttgaacttcatgaatacttttcaccgtaacatcaaatccttcagattggaactcagaaacggtctggtctagtcttgagagt
gaagaggtgataacagcgaaatcgttgtttggagaaaagaaaggcaaacagtactttttaaggttttccataaattcatcaatcgtaacagcggc
aatcctttccaagaataggcgatcccattcttttcctaaatctttaatcgaaagcaaagtaaatgagtttttagcggagtaaaaggcattattttca
agttctgaaactgtactgtatgccatacactttgccgactccaagtcaaagctgttaactgatacctttccagaaatgattgaatttaatagatcttt
gcttgatgaccaagctctgtaaacatcagaagaagtcaaaatagagtaaaataataaccctccgtcaatgtctattcccatattgaatccgtatgc
taaaccagaaccacggattctgttccaaaaaggaccgtccattaaacccaagtaatttgcaatcaaaactatgatggggagatccggatcattcc
agctttttataccaggaatggcaacaactaaatcactgctttcattagaaggcataggtataatagtgagagagcttggagatttaaattcattctc
ttgtaaatagagtctagaaaaggaaggagaagaagacaactgagagtcgaagggtttgtttcgaagaatctgagatacaaagttggtccatga

agaggcagcatcgttcgttcctagtatatcacctatcacatgaacgcgagggcacatagattgcaacaaatggtttctcaaatcatcaaaagcag
aaaaaacttcggaagcattatcgctgagcttttgacggagttcacggagaactttttcttggcttaatgtattcaaagaatacttcaaagagttctc
attgtacaacctaatgtcaacataggaaggcagaacatactcagcatccctcttttgatacggaatatcggccaacaattgatttataatagaaag
taacctattcttgtcccatacagtagcagtcaataatgttccaatccaataaataccctttttggtaattctctctagttacccgacattctaaataaa
ataactctcgcttcgtctcatagtaagaatatgaacaagaagctttatcatagagcaaagatgcatcaaaaagcacgacatcccgttcaagtcgc
tttacaacttcttcgtgagaaatgaaacctacattttctagttctgcaggagctgcttgtatgtatttagcaaacacggaaagatattgctttaaagc
aggtgggatatcagcggtgtcgaaatatgtaactatgctaataaacgatgaatctatatgatcaaactgtacatatagaggaagctcttttccgtc
ggaatcaatatatttttgaacgtcattttcaaaagattgtcctgaccatttcgttttagcagttgtggaattgtaaaagcatatttgactaggatctgt
tattttgaaagaggagaccaaattatagggaagtttttgctcattcttcgcctttgcacaggcaagattacgccctaaagcctccaaaccttctttac
caagagagttcaccctgtcggtcaaggttttgtttgtttcctgttttactctttcagccatttcatatgacggcaacgccaagaccgaaattgaatgg
ttttcgataaaagtagaccggatgagatctacccaatcctttttcagaccatttcttcaattcttcattataggataaaccttccataaattttgaaata
tctgatccatccctgctaccataaacatgatcaagggtaaggatcttggtaaataaaatgcgaggagacacctctagggcggttaggtattgattt
ttttgagtatctaggtagtcacgaagtctatccatgtcaatggtttcgatgcttgataaaagggatagaaccttctcaaccaaaccatcgatcttctc
aacaggaacgctgtcaagaaataactgaatcgagcaagggtctttagggaaaacgtaaaagtatataaaagagcagaatggatcctctatttcg
acaaaagcctgtttaaggggagacaccgctgattcgctgagaaaatcactaattgtctgtatagcgaagattttgaattgatccattaaaggagg
accgttccacgcaattgagagggaacctgtagattcatcttctgaagaaaagttcacgcttttgactatggaattggtgattcgagtattatcgtctt
tttgagaccaaggtcgcgtccagtcctcaggcgtctttaagtcattatcaatgatatctctaacaattgatgaagaaatctcaagaaggcgattttc
ctcgatggagccagcaactacgagacagatattgctaggaacatacattgctttgtggtaatcacgaatttgttgaatagatagcttacgaagttc
atcggggtgtcctcctgtctcgtaaaaatagccgctggagggaggatactgattcgttcggatacagtcaaacataacatcaacttcagaagtttg
cgaatcctgcatttcggaatacacaactcctgattcctctccaaactgattcgtatgatacacttctgtgcagaatgcctcctcgctaagcaaagga
aataacacatgatcagcaaaaactggaagtagtcttaaaaacccgtcttgctcagctgatgataattcataaacggtacagtcaacatcggtata
tgcatttatatcaccacaggctcttccagcaaaatttgttaggatgccattcataggatactttttactacccatgaaacacagatgctctagagtgt
gaggacatccagaattgtcgtgagcttcggtagctacaacaaacgaaccattgactcttgtagtgggagtattgacacacgcgactgtaaagcca
gtggcatcgttgacccatttttttcacttcatagtttaagagccgctcagaagcaatttgtcgaaatttcaaatcaaatttctctgaaaccatttgtacg
ataattagccctgttcaacggatattataaacgaactttctccagttaccaaaccaaaacgatagcagttgttgatcctctggaagaaatacctta
gaaacaaaaacaaaaaacaattagaagaagcgtctataagatctatcctacttatattgttttaaacaaatctgtttaaaacaagtaggcgtcct
aaaggtggctcttggatttttccatacttttgttggttaggcctgaaaaaccaaaagtaacgatgttactttaatggtcggtttgtttatttacatgaa
tgaaaataaattcaaaaaaatgtattacagtctctatatatttaattgggatattttggatattggaatactgttttttttggcacattactttcatgtc
ggtagcttcaatagcaaaagctttggttgaagttgtttcacatgattatcgacattagggacagtcatgaaacgctatttgttgtgtcttaaatatag
atcaatgataaaaaggaaaagaaaagtaagaatatttgggtaggaattcgtattcattgaattgttaaagtaatgtgctaacgagacaaagttg
aataaaatctcgacgaaacagatttgatagatggaattgaagtcctattcataaattttatatacttatatatatatgcaaatatatttattcgttta
cactacctaagagaattgacgattttatgatggaggtactttggttcatgaaaatcgataaagcggttggcattaacttgataatttcaaattacgt
ttcaagcgattaatagctttgatatacttggttcaaaagaaaccgagtttgtattttggctaagtttttttagactcgataaaaaactccaccaaaag
gagagcatgctatgcaacttggtggaaaacaagtaaaagaattcatatatttatctttgcagtaaaaagtaagtatgtcactagtaaattacgaat
cagacgaggagaaagaagcacaagatttcgagaaagaggaatctttatgggtcccatctaaaacgaataattgggaaataccatcatcgcctg
aaggaaatgccgatagccttttggagaagaagattgaacaatttttagcattaaaaaggaataatgtccactttcatgctcgtcttgtagacaatg
aaaatttcttaaatccccaattattagatactttgcaagaatatgcaggtatatctgaaccgacggcatctatgatgccttcagcatcttggactcct
catgctcttcccagcgaagcttatgcaaagtctttacgagcggctcaggatgacttaatcaaaaaacgagagcaaagtcagagaaatcggacag
aaattgcgttccaaaaatcgtcatcataaaaattaccaaaacgcctgtgctacaacgatttattcattacatgattgatgaacgtctctcttttagac
aacccactttactactcttactttctatattagaggactcaatgaataccggactacccatttcttttcttttttttttcttataatcatccaccaaggagc
gtacaaaagtgatcgtaatccggtagaatataatctccacatacagagcaaaggttatcatggatatgaataataaataagattgatcaaaaaa
ctagaaaacaaaattattatataacatacagaatgaaaacctcaagaaggagattcgtatttcctctaaaaactcttcttgaggtttctgttaggc
aatctttttcatcattaaatgcccctacattcgtaatgtcatacagctaagaggtatttgataaaggggctggactaggagctaattcctgcaaaga
taataggatgagatgtaggatacgttgtaaactccagtatccaacttttcgaattcttttcctgcgaagttcctggtttaccacgtcttctgcaaacg
aatcatatacctagcaaagagttagtatattcgtactttaactggccaataccaacctccaatgtcctatgaactccttgggaacaattttgtcttaa
cagacacaaatagggtcaaacttacctgcgaagatatttcttgcagtacaccactatcataaatggtccagcttccatcaaagtctgacaacggtt
ttgataaatgacacatgataggaataaaagtacgggtagaattaatgtaagcgacaattggtcgaagtaaagcattatttacataacccatttga
ttcgtgaaaatgggtatagccgctttcggattatgcaattgaacatcaacatcaaaatgtacatctggaactggatcttctttcttctctgttaatttc

gcattcattaaaacctgatggaaataacgcttaattatgcttttcgcaggctcttgaggacaagaaatatttactgaaaagtcaactgatccatcat
taatccacccgaaagctcctgaaacccctcgattcaaatgatcaatagctagattatcaattcgaagcctgctatgtcgcatgtctggcgattgttc
gtacggcttcatataaggtgaatacggcttgagctgcaatctgtgtaccgtaaacatgctgttgtcaaagcttcccgttaaattcgtagcattcatta
catcataaaacaaccattgcttcctgagcttgggaagctcgcaaaagaaaacgctgacaggaaactgcctaaaattgtcaggttgcataagcgt
aactcgtaaatcttccaacttgaagcgttcaatttcaaaatctccccaattatgttgtcttcggtatgatctggggtctatagaaacatctgattgca
caaaccttcgatcaacgacgccgcggactccacttaggacgagctccttgacgattccttttccattcaggaaccttgcaaaactaaaagaaacat
ctgccttatctatggataaatcaaattgggtatagtttccttgcttcaataaagcaatgtcttcagtgaccgaggacgtggaatcgtagctctggcg
caaggccataaattctttttcgtagtttggcttatctgatggattgtgtaaattcttggatgaagcctctggcctccttcgtacttgaattttgttaaat
gtgataagaccctttctccaattaggaacaatagcgttctcaaagacaacctcaaaacccgtgtttttttgtcattaattgtccaatccatttcccaaa
atactcttgagccgacactgtattgagtgtgtataacaataaagaaaagaaagtagtcgttccaaggataatccaaacaatgtggctgacgagcc
accagctgaagaaagctgtaacattatcaattgttagaaatttgttttgcctaaaaagaacattctttatcttaagaaaagcccgtgaaagcaatg
gtttctttggtggtttcaatgaggcagcaggtgacggaggaggtaaacgctgttctgaagggatttttgtggagtacaaacgaaagcatccttgta
atttttgacagcccttctgcaaacgtatggtaccgagagcctggtcggtaggaagaaactttcggtcattctttacatatcgaacggaatgagttgc
gttagacgtagacagctggcgacaatttctgttgactcctaatccatataaagttgacgaataagtaataacaggagctaaccaatgtccgtgag
attgcacaagtctcgcagatgactttacaataggtataagcatcaactagaatttatcagataaaattagctatctggatatgtttcgatcataaga
agactgttttcctcggttaataacagaaaaactcaagaatagagtagatgagatgactcttggagaaaaacgtatacggagtggtaaaggatgt
caattacagatttgcaagttaacatgcaaatgaatgatcaattcaattgtaaaaagaatgaaaaatgtaatttttcactttttccagcagaaaaac
gcgcgaggtaaacgataactttagtagcaagtttatgtagaggtttcaaaaaggagcttgattctatactactagttttgtctttgaaaccgggtttc
agcaagttcgttcaatgacaagttttggagagccctgattcacggtaaatatattctggatgtgttgtagaaataggaaaccaatttttcaatccttt
caaaaaatataaaaaaggaatatatttaaaaaaaagttaagaaaaaagaattttgtagcaattactttgggttattatctaaaaaccccactgca
agtacttgtactttaccaacaaccacgctttgctagcatcccaaattatttctctatcttttacagaaagaatgggacttgttcaggtatgcttttaca
ggttgaagatggaaagatcaattcagtttcgagtctttttgttcgttttgcagaagtaggatgaattgtaaaaacggtagtcataccagacatgaa
agtcattgatgatttcgcttttgtaaagattttgttcttttactaactgacggctgtagcaaataatgcatgtcctggactttccagggtttccttggaa
aacagtgattgccagtttctctattggaaaatatgtgtgggatctgtacctgcaacgcagacaggctccttacttattgaaagagagtcctcctacg
gttttagcggaacatgttgacgaagagaaatatcagaaagctttagcttacgctagagacaaaacttttatgagcacgattgtttctacttttgcat
tagtgactgacctattgttgattaaattcaacggcctttcttacttgtggaatgctacaaagttttcttggatggatcgtttggcggcctctgcttctaa
gtttagtttgtcagttcctatttcccactcatgtgtgtttttattcgcacttactgtctactcttgtatcatccagcttcccttttctttgtactctacctttg
gaattgaggaaaagtacggatttaacaaatccaccccaaaaattttctttatggacatgttcaaagagctcaccattggcggtgttttaatgagttt
gctggtttctacctttgtaaagattactatgaattttggtgacaactttgttctctacgcttggggtgcctacattctctttggtttggttttacagtcca
ttgcgccttccctaattatgccccttttcttcaagttcaccccattagaacagggttctttgcgtacaaaaatcgaaaaacttgctgcttccatcaaat
ttcccttgaagaagctttatgtggttgatgctagtcgccgttcggctcactccaacgcatttttctatggtcttccctggaataagggtattgttttatt
cgataccttagtaaagaaccacactgaagaggaattgcttgctattttgggtcatgaactcggccactggtacttgtcacacaacttgatccatact
ttggtagattacggtatgagccttgcacagttatttatgtttgcagcattcttgcgcaacaacagtctttatgaagcatttgactttacgaaagagaa
gcctgttattattggccttttactgttttccgatgcccttggacccgtgtcctcgcttttgactttcggttccaataaaatttcccgcttctgtgagtatc
aagcagatgcctatgccaagaagcttggtcttgctaaggacttgggagatggtttgattcgtattcacaacgataacctttcgcccttggagtacg
attccctttatacctcttatcatcacagtcatccaattttgaccgaccgcttggatgctattgactatatgaacttgaaaaagaacaattgaacatta
aagaatttctacatgcttctaaaagtgaaatgagtatagttatgatttccaatgcaatagatttttttatccataaagcgtttgaactttttgattcgc
cttatttccaagcgaaggagcatagttataacgagtagacaccaggaaaaggaactatactaaactgtagattcaaataaattcaattcaacca
attaccacatcaaattcctatagcagcttgtttctcttatttttttcgaacaatcatcgcttcatttattaatatactcttgcttccaatgaatgctcacag
acttatcagattagcatctttatttcctcgttggatactagaaacccaggaagttccgcagccagcatttgattgattcgtaagcgaatctctaaagt
aagctcgtaactcaataatgattcgtcgctaccatcagataaaatttcaaagctccgtaaaaccccgaattataagtgcgaaaaaaaataaaatt
gaatgaacggatgtaaagaaccaatagaaaaaacagtcttcaaccttcataaaaggcattgtcaaccctcctttatactttgattctgactgacct
ccttaacctttgtactcgagaatcgtctcatttccatcaaccagctatagaacgtcgtaggtcctgtaaataaacaaaggaaaaagaacccacgat
caattttgaaaaaataaaaaatttgaattttctttcagggtaaagtttattctattggaaaaagaaacatacacaatggagcctcatcgtccagca
atgcttaacaagttcattcaaaccaaccaggtttctatccttaacttgcccttttccggtggtcaacccaagaaaggcactgaggtcgctcctgaat
tgattgaacaggccggtgtgcaagaggatttggaaaatttgggctactctgttcgttacatccaaaacccttccttcaagacccctcctaagattga
gggtcccagcaaggacttgatcaagaaccctcagtatgttagcgatgtgtgccgtcaagttcgtgataccgttaagaaggaattgagcgaggaac
gtcttgtcgtcaacgttggcggtgaccactctttgggcatgggtaccattgagggtgttcaagctgtttatgatgatgcttgtgtcctttggatcgatg

cgcacgctgacattaacaccccagaatcctctccctccaagaatcttcatggctgtcccttggccttctctcttggctatgccaaccctgttcctcccg
agttcgcttggaccaaacgtgtcatcgacgaaaagcgccttgcctttattggtctgcgtgatttggatccccaggaacgtgccttccttcgtgacca
caacattgctgcctttaccatgcatcatgttgataagtacggcatttccaaggttgtcgaaatggctttggaacatatcaatcccggcaaccgtcac
cctgttcacttgtcttttgacgttgattcttgcgatcccatggttgctccttctaccggcactcgtgtcccaggtggtttgactttccgcgaagccatgt
atatctgtgaagccactgctgaaaccagtaccttggttgccgtcgacgttatggaagtcaacccttgcttaggcaatgaggaggataccaaaaac
actgtcaacttggctcgatccatcatccgtacaagcttgggtcagaccttgctttaactttctgattctttctatggctattatgatgtttcaaaaatat
aatatatgataaattatttttattctagcaataagactaggcttttgggggtttcgtttcataaaaaaatagagtatatcaataaatcttcttgcgtgc
taaagtacaggtacttcaattaaaagtaaaataaaattgcaagtagaaccgatgacagaattcccaaaaatcccataaaccactgattagtttga
accttcatgctcttgatttcatcagctaatagctctacttccttatatatcttttgctccgtgtcataaaccgaattgtttctcttaatccctgtatctttc
attcgtcctttttccaagttaagagttaatcgtacttctgctagtgatgtcttgacatcgtcccggacagacgatttcatttttttcaacttcccctacga
gcgattgcgagttcctcctaactttgtcaaactcttgctcttcaatggcttctaaatactttctgatttgaataaacgtctttttctgctgaaaagacac
agactcttgcttcgtcttaaatccaatgtttttttccaactcagtcaatctaaaccgtcgtgttagtaatttcattccactagatttccattgataaaca
tacgcttctgcagtaattctttgcattaattttgtgaatgctctcgctttttcaggcgcatatccagcttgcatcaatcgagctttggattcctgtacaa
cagattgacttggctcgtaagaagcagaaaatctttttagagacattatatctgatttgtatacatttggacgaagtccaccaaatccacaaaaga
tttcctttcttagtatccttgacatgatttgtacgaccgagaattggaccaaaacaatgctttatatttattcaacttcttttaaagcaatatatcctta
attaaaacatacttggagttttgaatacaaattcgtcaaaagtcagtcagtaatcagaatggtgtaaggagaaagtcaacataatattcttcatat
tttgaacatgaggaatatacatactctttataatagaactcgagaataaagctttaagtactaaactttttttattttctgcttttcctttgctttttgctt
tgcatagtgaatgaatgtaaattatatacgtcttacattatgagacgttatgcatgattcttaaagaccaagatgagtttcattaattattataatta
aaatagaatgcttataaccaagaataatcctttgtcatcatcattcggatgaatagcaaaaaagaccaatctaatagaaaacgaaatagaaaat
ctcaaaaaacttagcaaattttggtctatcttctaatctcataaattaataaacaaggtaatatttccaatgaactaaagtaaagtcatatatgccg
taaagatcatagaaacaaagccaataatccacatggcaacgtcgagggtacgaagcttccaagtcgtcgcacaagctttataatgtagcatagg
aggatacatataaatcaatggtatacaacatacactaccaaccatactgacaaattggtctaaacgagaggagccactatttaattagttagtaa
tatccgaaataaaaaattgatcaaaacttacgcccaagaaacaagtatagcgaaaatgacaatgaatacacgcaaagtgtttttcctccacttca
ctttacgattgcgtttaccgcttcgcgtaaatattccttgttcaataatagctattgcagggaataattgtaacggcgtactaagcaagatggctac
agcataaagaaactgaacaacaactgtgaaggaactttgaggcatatttaaaatgacaactgtttgtactttggagccaaaagcagcatacgat
agaaaaccaattgaaataaataaaagggaaatagccagcataactcctgtcaataatttaggaagatttttaggacttgccatttgttcttgaata
ggaagaattaaacaaataccttcatatgtaaaaatagcaacgccaataaaaaggctgaagtccgttttgttgaacaaaacaacatcagcaactc
cttttgttgctaaagtgaaaacatcccaaaagtagaggtataagatgccaagaagaataaaagcatcggcgatcaatgcagtagctgagagttt
cgatatttttcgaattaatgacaaaggaacaaagactagaaattgcagaaatatgaagatggcaacatggtactctttatgtgatgcgataacag
cccgaacacatgcttgtaaggtactggccacaaatgatatatatgcactagcaaatccaatctagagtggttagtataaattacgttgtcaaaggg
acttacttgagaaacgacaatggaggttaaaattgctaatcgcattttggggccatataaaccccaccaatgtcaccaaaacttccaggaatttt
ggaccgcgtttcaatgagcaaaagaaaacagatatgggataggagtcctactaccagcaaacatacactggaaaagacaagaccacccagca
aaaacctatgtaaatattagcaaagttctcggtttttttttggaagaaacgatattaaaacgtacgctttaggaagaaaaagcacaccagttccgac
gaatgacttcagtaaaagaaggacagccttcccattggaagcactcccttttggcattgctttatggcgagcttgttggccatcaagcaggggttct
tgctcatacaaatcagaatattgaatcatcgatggagtaacgcgtctgggcatcgcaaaatcttcttcgtcttcctcatcttcttcactcaattcttca
cctgcaaagtgtccataaacagacagaaactcaagaaaatttcgatttaagaaattgggtttaacggaaaagttactaggacttcccaaggaaa
tgctactaggtcgattacgaaggaaatctcttcgcataccgcctgcttgtttcaggtcttggatattcctactcgcatcgctggaattgggattcaaa
gctgaaaaagaccgagagcgtccacggcgtataacttgattttgatctatttcttcttgccaacgatacacttgtcggtggacatcaccgccctgca
aatataaggacgaaaactcatcgtcttttggacccttttttgtttaagtggcgacgaacgatatttgggtcttctgtggttaggatatcgtcagaagcc
agtcctaaacggtgtggcattctttcctcacgcgcaagcgagccataattcgggtggatgggcctgtcatactcaggtgggcgactcgacagcca
attcgattgagaagaaggtgtatcaacagttatataagaatctttctgtggaggaacaaattcttcctgtagaggcgaatgggataaccgatgaat
ctgctgagatcgtctacttgaatatgagccaacgatattgaaattattgctttcgctgcttcgtcttttgatatttattgacctgggagcactcatatc
agtacgtttttttgaaagagaacgtcgattgttatgattaagttgccagattgagacagatttgcgattcctttcaagttgtattccttggtaaaggtt
aaaagcgctaaatcattggggtttcaataaataatacaacaccacttctaagggaacatatatagacgtatctaagtaaaatgcacacaaactat
gtgagctcaatgtgcaagggcacattgtcacctaagtttagtcaaaccaatttctatgtcagccaacttgccaaatgcataattctttttttaaaatta
gacatatttattcttccttaattttctaaaaaatagaaaggtacccatattaatttgtacttacaattttgccgaacgcaatttagaggaattcaaaa
gagtagaccaacaggaacgttttctcaaggcagataaagattgttttggtgagtttataaaaggtgaatataataaggccaaagtatttcttaaaa
aagcatctaaaaatagcctctcagattcaattgattccctattcttcataattgctctactggaggtacaagaaacatttcttttttttctctatcttag

actctttggtttccactttgaagtttgcatgagtttttttacggcaaacaatacgaagctttgtaacgtcctcaaaagtcaccactactatgccaaata
tagtaaaggaaagattgactacacctgttcggccacctgtgttcaataataaaactttttataaaagaacacttccgtctcagtgcttggcgtttga
ctcacatgagggtaaacaggttttcttgagagcacttcaagaaggtcatatggaaaattacttttctttggcccagcaaatggttactcagaatga
accagccttttgcggactaggtactttatgtatgatccttaactcactaaaggtagacccgggtcgcttatggaaggggtcttggcgttggtatgat
caaaacatgctagattgttgtcgttcactaacggacattgaacgagacggagttactcttgaagagttttcatgtttagcaaactgcaatggcttgc
aaactgtgacgaaatgtgtgaaagacgtttcctttgaagagttcaccagagaccttatcacctgttctacacaagaggacaagatcatggcaattt
ctttctgccgtaaagttctaggacaaactggagatggccatttcagtcctgttggagggtttagtgaaaaagacagcaaaattctgattttagacgt
tgccagattcaaatatcccagctattgggtagatttggaaatgatgtacaaaagtatgtttcctctagatgctacgaaccatcagccaaggggata
tgtgatattggaacctatgcagcatccagtaggcgtgtttacggttggtctaaataagtacagctggaaaaacgtttcgaaccacctttatcatgga
attgatgctgcaaaatccgtacaggatctaatttctcgcttagattctttaaatcaatcatccataccgctcatacagcatcgatccaacagttcctt
gtctgctgagtttcagcatttctgtgatgtccttgaaaatacagaaacctataaattagtctccaagattacacattcagatgcaaagtatttaactc
tggctttttggtctatttttctctttccgtctttcaaaagcactgcaatccttccctcttaaatcaactcattccatacttgaaggaaatcgacatcaa
cgagattcaaacacagatatatgtattaaaagaacagctagaaagtcttacgcattgttgtaaagccgacaagccttgttcggcttccgactgttg
taagagtttgtaatacccttttttctctttttttttttgttctagagtttagcatttttttagatttggtaattatatctatttattcattcttctattctattattc
atctaaaacttggttccttcgtgttatgaatcattatcaattcttaataaatcaagtgaaataaaaataaagaggcaagacataatcaagttgcaa
aggaaaaaacttcgccgtaatgacctgcaaaaccaagtaacgcacatgggctaaataagtaacaagcgacatggcttgcatcatggaagcaaa
gctactcttaacgatattccttgtcaaaagcatttgttaaatcttccacggtttttaagaatcatgtctcctgatcagattggtttagttatggctggcc
tcttgtcccttggtggagctaagggttacttcagcaagggaagcaaagcttctctcatcgcaggtgttggtcttggcggtctttatatgtattcctca
agactcatgaacaccaattctcctaatggtgttcctcttgccttggctgcatcagccttattgcttgcgtctagtgttcctcgtttagcgaagactaga
aagcctcttcctttaatgctcactgttctcggtgctgcctctacagcctactactatagacaatgggtataaatgttattgttcacatttcttatatcaa
tatatgtatgttgatgcaataaaaggcttgtttgatatttctattttctacggttcatttcatcagttcaagttagtaatatcattcagcgcataaaaa
gtacagtgtaaaaatataaaagtatcataacaatttcgtaatttcgagcatatacttgctgctctgtctcagcattggctaatgagtaaactcgaac
ggtgtctaaagtgatcaatatgaatccgaaacactacagcctaaatgctgattagaaaacctagcacttgtattgtagtaaagaagtcctttttctt
ccatcaccagtatgtttctttaaaagcttgaaattggggccatgtttgatgaaagcaatcgctggtttccaactttcctttcgcttccggaagccattc
ttggattttacgaatccgcttttcgcttgaaggatgagtgctagcaaaagcaagtgcaccgcccatacttgattctgcagcagacattcgctcccac
atgccccttgcagcttcaggatcgaaacatgcctgactcatcagcatcaatccgatgtaatcagcttcgccctccatctttcgggaaaacggtaaa
agtagtccaaagtttaataagagatgagaaagctgtcccgacaaatccaacgaagctgctattaagtaaaaaatacagctaatagctctagtaa
aagcgatttttctgaagagtgccttgcaacttgatgtgccgtttcgtgtcccaatacagcggctagtccatcttcgttttggcatatgggtaatattc
cttcaaagacaaatactttaccaccaggaagcacaaaagcattggctgaaggatctcggatcacatgcaactcccacttcaaatctgaaatccct
gaaacagcgatgatcctctttaaaacacgagaaacgtacaacgttgtaggatgataacttggcagcatacgatctccatactgggacatgatttc
tttgtaagcactttgagccatctttcgctcaaagtctaacgatacatcattaaaacgtctacggttgcttatgggaacatattctagatgcgtaaagt
agtagactccgcctgttgcaataagagcaccgacgattaattggaaacgcttgtctcttggatttgggctaaattcccgatatttcctggtgccggt
cccgttggaagaccaattgggaaaggaagcataatgtctggctttcgtcacttgttgaaaagtaggagtaggtctcttcaataagaagggagatt
gttgtctgagcgactgagtaagagtagaaaagagggtttttcctttactccggatcatgcttgaaattgaagggaaattctactttttcgcgatctta
cagtatatacaatacaatggtggtgtaaataaatacgaggtttgtaatattataatatcgaagttctgagggctacgctaacaattattaaaattg
aagactatatacacgaaaaaggaaggaaatagaaatatagaaaatatgctgcccaaaatctgattcatcctcgcttttactattcatttttccagg
aagaagtcgttgaagaccgatcgtttgaacagatatcatgagaaatacgtttgtcctgacgagcagttaagacagaagaacactgtaagcagaa
gagtagttcattatgacaggccatgctttgaaaaaggatagagatggtgaaaaatccgttatggtcgaagaacgagtgttctcaataaaggtttg
aaagaaaactccaaggaggatttcaaatttgcggtagcacatgattcgttgtatgtttagtaaaaaagttgtcatacccatagagaagttttcttta
ggggttcagcgtaaccatcgaacaattaaataaaaacacagaaagacaaaagctgcacccaaataaaatatgactttgtcttgccttgaacgcc
tgttgatagctgagagcgtcgtccgtgttatgcccaacgtatttgctacatcaagtatcttcttctttgtcgactttaaaattgtttttttgttcaaccaa
gtcaccaagtagcaaacggccgcgttctaaatactcgtcaatctgggtttctgcccttcctaagaaatcattttcacgtaaaagcccttcctgcctg
gtgatgttgggcaagccattctcaacgacttcagcatccgtcattgcattttcgccgtaagggttgtcgggagctgcattcgcatatcctcttcttcc
aaacaaatcccttctattttttgcatgggcagcctcgcgaacttggacctttagttgctcaaaatgctccgataattgggaatgtttttgacgaaact
cttgaacacgcacagtggctttatttttcttcgcaggtatcaattctcgcttcaccatgctattgtaatcctcgatagagcgagttagagaaagaaa
agatgccgatatttggcctagtttcgttagtcataatgaattgaccgttcttcaaggaaagtttcgcttcttgaacagaagaaaagaaagaaacat
cttttccatatgaatttctttttaccttggataccagccagtgatatagcgttgctttcatcttcaacaccacgctcaaattcgtccagatccttttgtat
cgactgaattaacttggtacactaacgatcacagttaatttttctttcagtaatatatcatacatggttatagagggtattactcattatttaccagga

aaggttccaagacaaatattcattacgtcctctctacatacatggtgtagtagatgatttatcggcaagtaacgatgttactcgttataaaacgaca
taatgggaatgtaaacaaaagatatattcatatagagacaaaatgggcgttgattcataaaaagacataatgggtaatggaccataataagaca
taatgggatggtaaaccaaaggacaaatactataaatatggctaagcaagctgatcaggggaatttacttttaaccttgtacttaattgatttatg
aagagtatcagtatcgttgcaacaattcattaatcattattactttctatgaagaaaaagaaatatatcaaaaagggaatgaaaaattaaaaaga
aacaaaaaacctacatttatccaattatcgtaagtacctaacacatttgaaaaataattaatgacaaaaatgtttgccagcaaacatttggcttat
gccaaacggcgttttctagaggagttattagacgagcgtacgcgcgaacgaggctttttccaaaaatattgcttgtgaactcgacgagaagaacg
cacaggacgcataatcggtgcataacgctggcgacgttcatcatcatcatcttcttcagggttgtaaaaccactcactccagagaggatcaccatt
ggcatctgttccgtatcgtcgaacctccttcaggtatgggtattgcttagcaagctcgcgtaaatggtcctggacggcagccaaaggcataccactt
ggaaacaagtgagcaagcactgtcaatgccgttgtagaagtagtagaaaaaaccaactcatccaaaatgagtcccattaataagctttcgggag
cagaattttcctttgaggactcaacataattatcagaagactcagtagaatcgccgttttcgtgcacacaagtagaaaaattagcattttgttcgtc
attagtgttaggagtcatggtctttgcattttcatcattaacgtatggtgaaacatgtaaaatggcagtaggaggttcttgattgtcctccatttccgc
gtttgcaataaggttctttacgttttctctgttcgccaaacattcaccgctttcatccatgctatcatggcgaacaacaataggttcttctggtgttggc
actatgctattgtagaactcattgtcatgctcttcttcgtcttgatatacctggatgtcttggtgtgcatggtttgattcagagctaaagaattcggtgt
tggggttagcgtcgagatgaggcacttcattttccgtctcggctattgattcattcatcatagacatgtcaacttgttcgtgagtggcatgtggattg
acttttccgtcttccattccaacctgaataagtctttccagcatatttcttttccgtgtagactcactttcttcgataggagtgcttggaaaatttttgga
atttgctttggtcaatgcctgagcagcaaaaagttcaggatcagcaacgaactcttgaggaggagaagaaggcaacagaggaaggtgtccgcg
agtcgatggggacatcggtgcttcattttcatttgcgtagtcataatccgagttttctccttcggtatcgaaagggtcttcgggccagcccagtagca
ttcgatacccaaacacatccagtatgggacaactatcaagtcctggacgaaacaaaacgacgcggtcattctttttcatatgatatacggctttac
gaagagagtcggtaaggatcataccgttccagcccacacactcaacgatcatacggtcgagcgtgcatataacgattgcatgaacacgtgaaat
agccttgtcagctgtggaaaggacatggtcgcattgttgggaagagcgtccgatcttgataacgcgagacacgctcattttcacataagacatgg
gcgtcaaaggttcccttttcacggcggagatgggtgacgatggttgatagaatgtattactggaaggctccggagtcaaaacgaattgacctaaa
atagattagtaagatgttttctgaaaactgttccagctacgctgatagacaagcttgaaggttgtagatctaatcaattacagacattgtgtagaat
gaagttgaaaattactacccttcttgagagacaaggcacctacctataggaggattccctacgttaagagagccattgggatgcttcgacatcca
gcttttcttatgaaaagtactaggagacgaggcgagtggagaaggaagtgagaactcattctctgtaactgaagccattggagaaaatgaaaga
tattaaaattatagaaacgattggaaagaggaagagagtaaaaacaaaacagagtccttcttggaaacgcaccaaaatttagataaggaaatg
taatgttaggacgaactaacaaagaaagatgcgggggagtgaaacaaaggaatgaaatttaacttaaaacacttcaatgaattaccttaaatac
ctaatgtttaaagaaatcgctcaaatacgtttcatacgtttggcataatccgggcggaaggatgtcaacaaacaaagggaacagcgtacacttga
cgaagtttctttagtggatttgcaagtgctgttgaagcaactgacgcaaagtctgctgaagaaagaccctgaaacgccccttgcaatgtttatatat
atgaagtaccgcgttcgtgtcgcgacgcgtcgcggctgaaattgaaggagcaataagcgtgaacgcgttttctttttcgtgtccagtggattccaa
cgatttcattaaaacgcgacgcgttgctaaatgatatagaattatgttatttgtaattgttgtttatcatgcgcaagcgtagaaaaacatgcgttgta
aaaaaaaaaagaaaaaggagtgtctgtaacaaggcgaaaggtagtttatatagagggtgaaatcatacctggaaacaagggataggagttttc
ttttacgaagggttgtcattgtcatagatctgttttactttccaaccctgaaaaaacaacgtgtcgcaactgcatcacaaagaagaagtgggatact
taatcatgttggatatttttaaagatataaacaatatgaaatgatcacaaccgattcatatttgagtttgaaaatctgctgcggtgtatgtacgctg
tgaaattgttatgaaagcaccactttctggaggtttcttctttcctcgtaggaagcttgatgtagaggataggaatgggttctatttgagtttgattct
agttttagtcattcgattttctttctttggaggaaggacgcgttgtttattaaggtaatttagcgtatccatacaattgtcatacttttctttcaactcaa
tggaaagtaaattctgtatgataagcctaagaaatatatatcgtagtactgaatcgtttcacaaaacctttagtaaaaatcacaccaattatatttt
acttcctactgtgatttgtgtgcgaattcgttgcatccaattgctgttttccgctttctttcttaaagaatcaggctgacattaaaagcgttccaaccac
tttcttccttcggaaagaacttttgttacgaaacacagatctacgttttcaattttgttttgtttgtaaacattcgaattacgagcttttcgacttctata
cgcgttaatttttttaccaatttttaaaaatctgtatactctattacacataccgctccctgtaatactgcggtaaaaaggctccaatacatggaaga
aagaagtttcgagttcaatcaagaaacagttagattccttatgtagaatcaagcaaataaatatataaattgactttattattttttttggatgtact
tactgtatttataagcattaaatccaaaaacaccagaaatcgctctctcatttattaccgcactaacagtctgtcttcaaccaattcgcaccaggac
gtgaagaagtgtttaattcttctttttgtgaaatacttgttgtaaaggctcttgcgttttttttccagagcttaattaaattacacatctaggtatttcta
tatcctataataggtatgtgaattgtgaaaaattttgcatcaggagatgtgctggcatgtctgctgctagacttgagaaaaaatataggcttttcca
aagcttcctgaaacatatgttcttaaattgttgatgtgtggatttccagtaggtttttcctaaaaatcgagtcttgtttcagtttgcagaagcacagca
cagtataacgttcattgaatactaatcaatctttgtagaatcatgtcttccaggacgaacgttgttggtatcgatttcggtaacagcaaaactgttat
tgctgttgctcgtaatcgtgctatcgatgtcattgttaacgaagtttccaaccgtgctactccttccttggtctcttatggcgagcgttgccgtttcctt
ggtgaaggtgccaagtctcaagaggcttctaactacaataacactgttggaagcttaaagcgcttggccggtcgtactctcgatgaccccgagatt
caacaaattgagtctgctttcatttccgctcctctcaccgaggttgatggtttggtcggtgccaaagtgcgttatctcaatgaagaaaatgctttgtc

taatatccaactcattgctgcctacttcaccaaaattaaggctattgctgaggctgagttgattggctccgtcagtgacgtcgtcatttctattcctgc
ttggttcaccgacattcaacgtcgtgccattttggaagctgccaacgttgccggcctcaatcctttgcgtttgatgaacgacaacgctgctgctgcct
tgacttatggtattaccaagactgacttgcctgagcctgaggaccctcgtcgtgttgctatcgttgaccttggtcactccaactactccgtttctatca
ttgaattcagccgtggccaattccacatcaagtccactgtttgcgatcgcaaccttggtagccgtaacatggacaaggctcttattgaccatttcgct
gccgaattcaagcaaaaatacaacattgatgtcctatctaactctaaggccaccttccgtttggccgctgctgttgagcgtctcaagaaggttcttt
ccgctaatgcatccgctcacattaatgtcgaaatgatcatgaacgacattgatgccagctctttcttgaagagagacgaatttgaagaactcattc
gccctcttttggagcgtcttacaggacctcttcaagaagccctttcccaagccggcctcaagaaggaggatcttttcagcattgaaatggttggtgg
ttgcactcgtgttcccagcgtcaaggagactgtttctaaattctttggtaagccccttttccttcaccttaaatcaagatgaagccgttgctcgtggttg
tgccctctcttgtgctatcttgtctcctgttttccgtgttcgtgacttcagcgttcatgatattactccttatcccatcactttcagctgggaacctattcc
tgaaaaccctgaagaagataccaacttggaggttttctctcgtaacaaccctatcccctctactaagattcttactttttaccgtaaggctcccttta
cccttgatgctttctactctcaaaaggaattgttgcctggcaatatccgccccaacatttctcgttacttgatcaatgacgttaagcctaacgctcag
ggcgatttgagcattgttaagattaaggctcgtcttgatttgcacggaattttgattgttgagcaagcttacattgttgaggagcaagaagttgaag
agcctgtcgagactcctgctaaggaaggtgaagaagaaaaggatgaacctgttaagatgcgcaaggttaagaagttggtgaagactgctgatct
tagtgtttctgttgaggaagaacgtcttcctgaaaaggttttgcagaagtattctgaagtggagcatcaaatgattgctgccgataagcttgttgcg
gagactgttgatcgcaagaatgctttagaagaatatatctatgatactcgctccaaacttgaggacatttatgccccttttcactaatgaagaggaa
agctctaagtttaaagaaatgcttactgcttacgaagactggttgtacgaagaaggcgaagacactaccaaggctgtctatactgccaagcttga
agatcttatgcgtatcggaggtcccatccgtcagcgttatttagactctgaagaggagaaacgtcaaaaggctcaagctgagcgggacgccgcc
gcttctgcttccaaagctgaagctgagaagaataaaaatcaacaggaagtaccctctggtaaatttgaagctggtactggtggtcgtgctcctcct
cctcctcctgctgagccagttgctgaggaaaacggcgatcagcaaaatcaaaatgaacaaatggaaattgatgaacagaaacaataaagaaat
ttaggtaccgtttgcttatgatgttttaatatagttttgttcatcttcgggaggaagatttttaaaagctttattttatcttcggagataattaggattta
ttaaaagtagttattgttctataaagttttacatttatgaaagcatgtatgcagcatagaagtaagcgttttcaacataattatctgtggatactaca
tggtcaccgtaattcgataatatatgtaaagagtgcataattccaattatagaattaaattaagaagtttatactctaagattatcactgttcacca
cgctgttaatcaatataaacaacgtgcaggtgtaattgcagcaaaagagtatacaacagacaaaggtcaaactaaagataacaggtaaaacgg
aaaagaaaaaatgcccagttaaacatatacagggaaaccaaagaagttataagataggaagacggagaaaaaggatgtagatgtcagaaatg
gctagcatagaatgctatataagagggatagttaattaaagcttactttaaagtaccagcaaattttgtagatgtcgacataaatgagggccaag
atactaaattacataataaggcatttcgtttttttcttgtgaggaacaggaggatcgagtgctgccacgatagcttcatcgaacacgttctttaaac
ctttttgtgttaaagcagaacattcgacgtatttaacagcccctaattcacgagctaaacgttcgccttgctcgtgtgtgagtgtgtgtttatgttggc
gagctaacttttgctgaacagaaggatcgtcacgtaaatcaatttgagtaccaacaatcaagcagggaacaccagggcaatgatgatgaacctc
gggaaaccacttttctttgacgttttcaaagctggcaggagatgtgacgctgaagcaaaccaagaacacatcagtttgaggatacgataaagga
cgcaagcgatcataatcctcttgaccagctgtatcaaataacccaagagtataaggctcatcgccaatcatgacggtaacagcatactataacag
ggttaacttcttgcacgctacactctgcaacattatgaatcgaaatcaattttaacgcgaaaataaacaaactgaaagatatcctctttcatatagt
ttatttccaagtcaattatcatatacattgagggctcaacaggctcaatacaattgagcctgtatcccgtcaagtgcactttttcgtgaatccataac
attttatctggtaaagcttccacatacattgtcaaagactgtaggaacatagtcacttgggaatttatttgttgtgtaagaaatcagcaagcaggtc
tatcatgtgtgtcagtaaataaaagccccattttctctcacaagagcctattgcaaaggtatttattggtttattctttaacttcagtgatttaaattat
gttatactcatcttaaccattcacaacctttgaggattctttcaagaataggctcagtgcatatgacatgaactacgtaccttaccaacagcaccgt
caccgacaacaacgcacttaatagttggcatgttgcttaatattgactcctaaacaccttcgtaaaagcaaattctaacgaaactggtaaccttaa
gtccgtctgcagacacggtgttggttgtaggtaagaagcaaagccagtatgaccgtcatatccagttattaacgatacaggctgatattgcaattt
acaataaagagatttaacgcgagtttatgaaaatttgtgaagcttgttgaattaaaaaatcctttcgtgactactgtacggtcgttcagattcgtta
acgtgtagtttaaaaataagcgaaaggcttgaagtcatttgattatttacaatatgaaaattataaacattgcggaaatagtcctggtattaagctc
aatgaaatgaatgttttcctagagtaaaagtagaatagtatagatggacgaaactataaattcaatgagagtttaagattcctttcctggtttaca
atatcagatactaaaaaaaaaaaaaggaacataaaattaagtgatcttctacaggaaactgatgatggatattccaaaccagatacattctaaa
tagatctaaaacatctaaatgacgcaaaaaatcgtagtttataatgaaaggctttattcctttcaaaaacgatggattcctctaaagcataatcaac
ataaaataaaccgttgcaggaaaacaaaccattttgtaaacatgctaataaatatgaatcaaaggattaagcaatataatatatcttgcttataa
aattcatgaagcacagacccttcataacacatattaaataaatactctcaaatacaagttgctaaaatcaattgtaacatatcctgttactcgtcgt
cctcaaaatcctcagaggcttcagaactttctgaagctccgctagactctgcttccgcataattcacctgctttgtagaaactctagtactcgctcta
cgaggtagatgaacctgatttttatcttttatgttttgctttttcgcttttggttgtcttgttccggtgcgcttcctttttattttttcacggttcccgttcct
cgtcgagtttgtttggctaagtagttttgcagtactaatcgtggaagttcggaccatgtgttctatggcggattctatctcaggtgtaaggaaattctt
tgcaaagaatttttgcttctcttcagaagttggtagaggcttgaaaatagtagatggtagtttcacttgcttcgggtaagtagttaaactccatccca

tgctttgcgccttagtttgaacaattttctgtgcaaaatcactcagcatatagacgtactttctttccggttcaatagcatcataattctgctttacccg
ttgcaccagatggaaaataatcggaatattttcactggaagccacattttcaacatagaattgtatatacttaacaaagtcaaagacattttcatta
tcagacaatgagatgtctggatgatgagagagtaaatgaagcaaataagtgataacatactccatagtataattgcgcactttacgaaagtatat
cacttgcgagcatatccacaaagacattttcgatattatctcttcttcggggtcatgaacaacaaggaatacaattgcatagaactgcataggaag
ctttcgatactgcaatctttttttgtaagttttgtatataggcgtttcggacatcaaaaagaggatcttggcataataatgaagttcgtaaataggtat
taaaatccaaatattcagcgtaaattgggatactggctaattttaacaaagattttcctgtaatcaatcgtagatgagctttacccaatcgcgaggt
atttttatgaggactaagctcaccttccgttaacaatattacatttaataatttcagaacgggcgcggctatctccaatgcttctggagagcgatatc
cagctcgtaaccgattaactaaaacttttattgcaagtactttgcaacgatataggtgatccaaggattgaaactcacaccattcatcgttttcgac
atcatcttcattcgtttgtggaaccatttgaattatttccttaaccaagcattcagtgataaaatcagcatgcttttcaacagcatctaaacgatata
aaaacagttgcgttaaggccgacagacgggttgacagagaattatcgtaatgtaatccgtcaacgatttttgatacgaatgaatccatatcaaag
aaattatcagcaagcgctaacacagtagctgcttgctttgcttgaccatctgttccttgaaaacaaagattcataagggtattaattacttcagagt
ttacggaaaagttcttcttttttcttagcaaaatgtgaaagagtctttaagactttgacgtccgctttcagaccaaaagatgaaatcatattttccata
ttagtaaacaactcttcgtaaatctcaggatgaatcaatggtaattgctgaagtaaaagctcagcagaatttgcatattgaaagttactttgtgaac
ttccaatcttattgaccagcttcattacagttgttttattaaaaacaaataatgcacaacggtaaaccagtaatcgaaaacattcaaaatgttggtt
actcatgctggataccattcttttaaataagtgcttaatatttttttaaaacagtttgatagtcattccttggtgaaaatgtttcgattaacattttatat
gcctgccggtcatttgtatctgcaaacttttgcagcgatgattcatttaaagaaggattgacagtcttcgaagagatagctcctattacctttttttaa
cctggaagtcatttcttcttcgttttcatcaataacaccaccattatagtttttgcaacattcaataaaatacttacagtatccagaaaacctgagttg
tctttgctgtaacataaaaaaaaacgcgcaaagctttctctgagaaatgattcatggcaactaaaagtctgtttacacgagtattcgtatcagggttc
aattcctgaagtacaagctcattcatcgcaaggtcaacagtagtattagtagcttcatcatttatataaaatacttctagcaaagaagaaggtatc
caactgaacaggtgtagactaacctcttcaccagcttcaagcccaggaaacgcaacatgataaattttacttaacgtttgaatggcttgaattcga
actgaaagctttttatcacgaagtctatcccccattaattttaatatttctactggaacgtgaatttttataatgtccagcgacaaatgaccgatagc
ttgcaaagcagcaatacgaaccttttcatcagtatccgcaagctttccctgataaagttgaataacatagttagttatgtctgaagatgccaaagg
gttccttaatacatccataagcttttctacgcagtccacgcgacaagctgttactttatcattcaggcgaccacaaaaggcagaccagaccttagg
atagtccgaccaaagagagtgattaggaagcatcagacggactgtttgaattgcgacacgtcttacactggtttgctcagcttgaagctcgttttc
aaactgagggacaatattgagtaatgttaagggtgcatattcccaaagttgaataacaagattatgtgcgaatatgaattctggagttgtttttttca
tccgaaacggagtctttagtatcaaagacgatatcagaaaaatattgacaaacataacgttgtagacgatcagcagaatcacggaagatattcct
tgctaactggaaagcaggaccgtggttctgggagtctggggaacctatgtattctcttacaccatgacccgaaattaattgagcaaaaagaatgtt
catggcgatctgaggtacgctattaatttcattaattagttggtgaatgatatcaagcatatagaactcaacgtttttgttagtaccctttcttgagat
atcgaaaaagagacgaaatatagaaactagaaaggactctgcatttggcaaatcaaccatgagtactgcagacttaacgtttgataaactttca
agtattcgataaacttgaagatagtacgtagattcgggattttcgagtccgcttaaaagcttcagtattacttggaatatttcttccagttgggataa
tgtaaatggagcatctggggcacacaatcgaagaagctccacgatacagcaagcaacaaaagagcgaattcctttatctctatggtgtagaagg
ttctgattgactaatgatgaagcaaccggcagcaaagaattgcgctctgtagtttcctgttccatatctgacaattcatccagtaaactccttaacct
tttaaccaattcagtagtagcaataggtttatcatgacttggaataataaccttattaaatttcagtttcgacattgtgatcataaaacctctattaa
ctaatacaaaagaacgtccgtaaagctttaacaagtaaaagcatttgtcaatgtaataaatacgatgaaactatccgctggatattaggtattcca
gaaaagataagaaattagaaagtgttgtgtttgtgcgacgcgtcgcgttttcatcagtttttaattaattgtcacctgcagtccgttattagctaac
aggtaacgaagtaaacagacagtctaatactaacaggaaaagcaatttaaagtacgatttcaaatttttgcactaacaaaaacttatgtaaaaa
gcaatgtgcctactatcactttcttgtcctttcagaaagcccaagagaaattcaatttaggtgtagtgcaagtcgggttacagtaaagtacaaactt
acatgaacttttcgtcgtataataaagttttgattgaattaaattaatgatttaatatccaagatcgaagcctggtcgtcgattcataaaaaaaaaa
agtgtgcagaaggagaataaaagtaaagaaagaaaaagaaagcagcaattaaactgatcgaataatcaaaggaacagaaacgtactttgctc
gaaataaataaagctttttgtttgtgttaggaattaataaaggacagaaattcataaataattacctagtatatacaggataaacaaaggagtatc
ggttcatacgggacaaagtattatgcaaaagtataatgccttttatgtcccttctccaagttatgaagactcttcaaagttaaagggtcagacgtac
ttacaattggggttactgtttccaaaattaagccggactgtttgtagcgtgaaacctcattgcctccttttctatgtcgaaggaaacccttgaataag
aagaaaaagccctttccagataattctttcttaggcttcgataataagatctttaatgagttagcagcttcacttctgcgccttgtttcataactcgta
gttactaatgatgtaactttcatttcaacgggtgctagcggtagtttccgaaggttgttgtccactgtgatcgtactaaagtgcctctggtgccttctt
acttcatcgaatttctttggaagttgattgggtttgcgaagcaaggaaaccaaccggtttttgcagttgcctttagggtataagattacagaagcct
gaacaccattgctaactagtgcagacgtctttattggctgctttaattcaatactaccatgcacagcagctttttgttgtacgttatgatcacgtgaa
ggtatatctagagaagagaaggtgctaaccgttgaacttgaggacacgctgatttcagatttttgcgaaagaatcccatccacagggtactttaa
aggctcaaaattgttataaagttcaaatggctgcctatgagctcgcaaataaggatgactcattattccaaacatatgaagtctttttgaaggatc

aggcactaaaatcttacgcagcaaatcacgcggttcttccttgatgtactcgggaaaaataagagatgtggtgcatatgtaccggtatagccgtac
aacattctcaccattcggattatggggatcatcgtcaaaaggcaaataaccggcaagcattgcgtataatataacaccgcaactccagatatccg
cctgggtacccgcatatgatccaactttgcagtttaccaactcgggtgctgcatagcaaggtgatccgcaagaggttgtaaataaatctggtttag
cattagttatttgattcttacgagaaccaaaatgaccaaatgttgttgcaaatccaaaatcggcaattatgatattttttttgaatccagcaaaatg
ttctccaatttcaaatctcggtgaacaacccctttggaatgcaaatatccaacaccactaatgagttgtgcaaataatttacaagccgtctcatcct
cgagtcgtcttgaagctaaaatatagtcaaacaattcgcctccgctcacataatccaagacaatgccaatgtgatgtttggttttaatgaggtcaa
cgtacttcacgatattaggatgatcatcaacttctctcaggatcgtcgcttcacggataacccttcgcagcaaaggacctcctttgattacacgaat
tattttgataacaacttgaggtgttgggtgcttctgagaaggagaggaacccggtaaaggccatcctagttttacttttccaaattcacctctccca
atagtccgcccaagaatgaaaggaccgaaacaattatttttgatgatttttcaaagttcgagctagaatctcggaactatccatatgcaattctg
aaagagaaaaccgatcattactagctagtgcacaattagcaaaagcaggactagctttatgctgccacaaaaaatcaagacgttccttatgttca
ggaaaatgtgcaatgtattgctcattctttttctttatcacctgaatattggtggtatttttcacgtatgcttttacaccagagattcgagagcgattag
gtgaatgcagtgaagatatttcagtcactgtttctttcggactaccatacaactcaccgacagcaaacgagttattacagaataatggggggcgtt
cttttgtctctgatcctttgtggagttcggtgtttttttccttctgatagttgacaagcttgtttgacaacttcgtctgttacagtcaattgattagatgatt
tgaagtacttggattgttttaactgtgcagaaagtaaggacttattagagaaattcaaaccagcggaactgtctagcacagcagacgtgtttaaat
tagacgagttttcaactttcaatgaggaagaacaattaacaatagcattttcatgcgccttgttacccatttccaaagattggtcctttgagatagg
atttacaaagtcatcctgagagaaaactttgttagcgatagaagaaggttgaaggactttatcctttgaggaagctttccgaggagaatggatatt
atttgaattcaccttaaaattaaaaacaggtgagtccaactcaaattgattattattaaaaggctctcctaaagtttggtcaccataatgctttataa
gcacctttgccgaattattctcagttggaaaagaggatcctcgaggattgtctttaaaaaaaggaaaagaacaagtagagtgtgcaggtagagt
attgaaggctaagtagtccggcggagaggtaagtcttagtaaatcatatagggaatcatcttttgttccccatttctgaaaagaggtatcattttct
ggagaacaggcagtctgtggcagtacagaagtaggggtgtttaaggaaaattgccttttcgatggttccggagtgtgaggggaatgtataaaag
ctttatgatcatatgaatgaatttgcgaaggtgagtcattgtgaactctgtgttgtatattatagtccagcgatgcattattaggtagagatggagg
atgctgaaaagaagatcttgacaaactggaaagtgaagagtcaggaagagcacggatggtactttccaccacgctgcaatgtttcagatgagag
tttaacgaagggtacccttgagaaatagggattgaatgaccaacagatgaagtgtgcatttttttgttaaggtatgtacaaatcatatataaagaac
aaagaaaaacaaaggaaagattaagaaaagaaagaagagagcctgcaaagactgaaagaccaaacaggagaagagataaataattatcaa
tatgccgaagagcgtttaaaatattagtatgggacgaatttcctcgattaactcggtattaaaatgaaaatccagtccagattagaaataaaagg
gaaaagaattagtttggcaggattcaggaatgaaaaaacaataaattagtagggaaggataaagtcgcaaatgtcaacgcaaatattgaattc
aaaatctccagtattctgaccaatagtgacttataaatgccaaggaaaggtcaacaaattaaaacgataagttcggaagtgcgaagggtgagta
tgcgacaggcataggcattgacaaaaaaaaataaagtgataaccagaggctggtctgaaaacggaaccttttttgcgatagcaatatacagagaa
attaagaatctgaatgatatgacaaaataaaaagatctaaccgtctttttagatgagattaatgatcaccgattgccaaaatatggaaacaaagg
attcgttaaaatttcggcgtttaattgcaaaggaatatgaaaagagatgaagattgaagtcaagaaatagatcttgctttgtgaaaaaaaagaac
ggaaaaatttaaaaaataatcaatataaatatcgtttggttgggttcaaataggacgaagtaggactgaatacaacagatagaagttacaacac
aatgaaaaattgaaacagagaagtggaaaggtgctcgatcggaaaacaatgttctgccaaaaacggacaattaagcgactgatgacacaggc
gagtccggtttaagatttaataaagctaaccgaaaaagcaatccgacagattttactctcggttacgctattttatagatttcctgccttgaatcag
aactggtttagcaaagaaaagtgacacaattcgcaatgctttggggaagagattattttaagcagacactctaagtatggtacaaaaacaaactt
ttttagataattgcacaaaatgagaaaaagatttgtaaaaatcgacaacttaaaccaaatagaagtttgattgacaaaatattaaatctatcaag
aaaacacgaataatttggcaaactttttttttttactcttctataattatgttttcaagctgcttttatttacttattttataaaattccaaaattaatata
ctctcaccatctgtgaagagtggtgtttgggaaacgagacgtatgtaatgtccgaaaactgatatataactctggcgacgcagtgcatatatcatt
atattgaagctatccttgatctttctgggtaaataaatatatcaaatcaatttgtttttttattttctatatatctgtaactttttgaggaagaattttgtg
aagatcataaaaatgctcaacttgctcaatttccggggtttttgtgggcatcaatcgttttcgccgattggcaactcaaacaatccattggtaagag
acagacaagaagctgttcccagttcttcgttgtagctagccattggtgaacatttaagcatcttgtaggaaatcgtttaatccaaccctattatttac
atttgcaaacatgaatctaagaaattcgtcaaagaaatgaagaaaaagccttgatatttctgttaaacactagtatggctgctttctacctttcata
ggttatccatttttatattctttcgcttttagtaacatgtttgtttgtatgattcaacttcttatgtgctttagatttcagagttcaagtaaccgaaacatt
aactcttaatgtgtgaatcatcaaaataaacattcattcggttcccttcacagcatgaccactgcgtaggtaaaagaattcataggctttggttcta
gtattgtaacattcctttgtaacgatatgacatcttttgatatttacaagttgaatttttttaatttgtatgtgcataggactcacggtcttttgcttcttc
agtcactgtccagtactatccgttctccaaacacttcctgacattcatattaccttttcaccaatgagtgtgattcttgcagatttttttacagatgattt
caaatattgtttttgcattagtaatattctttgcatttttcctagctttgcccacacttagtgcaatcgttttggatgtttctgtttgcggctaccttgttgt
tcgaagaatattgcaattgggatctaaagcgctcccaattaccaataaacaacatcaaaaaacctgttgatgtcttcatcaagtgttttagcgcctc
ctggctgtactgactagtgtgtctgacaaaagtgaatttaacgtaaattgcttgtttttcttctttttcacgaatttttaggttcaactcaattgtcctgt

tctcttccctaattcttccgctaatctatagtgacatggatgcatcaagttgtacaattacggtacctttgtagtatatggcaagcgaaaaatcatca
ttatctttcgatttttcattcttgttaatgagatgaatcaagtattattgaatgttatgtaactgcataaattcatactacgatgtatacacctcccgttt
tatcatgacttgcttccgtttattctcaccaagatacatattaaaatgaacagaatctaatcctcatgaaactgctgcctaatccattcttctccaatc
tttctttcataatgcttttgaagggcatttcttcggaatgctagtttatctgctcgttcattcagtatatctaaccccgttttccgcgcttgatgctgtag
tgtttcttcgtgtatcttatccaaagattcaaattgctgttgtctttcactctcatctaacgaaaattggaaaaatcctatccctttcgtacgaatttctt
tggaggagtcgtaatgtacagcttgttgctcgtcttgtcggtgcagattctcgatacgctgctcatcaaccttatattccggcatatattctccctgaa
taacattttccggtctgtagtcctcttttgaagggagaaggacgtctcccgcttcatagatggatactttcctagttcgtccaaattcatccgttactt
ctataaactcattctcccgatcttcttcagcccacttcttcgtaaaatcaactagcatctcatcaccatcagcaacttggccatgatcttgtaatagct
gattatatttttgagctttatgctctaaacctagcctacttttcctcaaatcctcttcattgtcatccatattctttaggtgtattcctcgtttctgttcatt
gtttgacctctttcggggtttttcttcctcgttatgcctcttgggtctttgacgatttgaacttgcttcatcctttttttattcttgcaacccttttgtttgttgt
tccatattctgctccatcatttactcttcgcttcttttcctcttcaaatcgagccttttcctcggctaattcaccgaggaaagttaagttacttgttgtgt
ctgttcggatttctcgctttttctgttggtccattgtaatcagttttctaacacaagttctttcaaatagaaacttcaatcccaatgaatggtaacttcg
atatcataaaaatactatgacgattcagcgtaagcgtagggcaaggtaaccgttgaatttttttcatatggtactaatgaaactaattgttttgtaag
attttcattccaaatacagtaatcttccacaatttacggagtttgttctgtctctaactagatggtggtgacatgaggactaggtttgatttgttatttt
tattaagtctctgaaatcagtagtttgttaattcgagttttctaactagtatgaaagaaaaaaatttaaatatagttttcttgactgtgcgtgaattga
gactttatgagacattgaacttgtacaagggcatttgaattttcctttttttacgaaatagccatataagcttattttaccttggtgcattctaaagaga
tgattgaattagctttgaatgattagggctacagatgacaattgatagttctatgctggaatgttccctaaacatcatgtgtggatgtagcaatgaa
agctctagacggccaccataggacatctggaacagaatattatcccacatcattcttcaacttcctcataaaatgtaggcaagaccgctaaaacc
gtatccatgatcgttatgtcattacttaaaaaggtaaacgatgcctccaggtgtgtttttaaagccattgattcaataagctctggttcaaagagaa
tttctacctctttttttttcagtttgctgaggataacttgttccaggaggattccagactgcaaacttggcattgcacaaagtgctaacttgctctaaat
ggatcaattctttggacattagctctaccgttaagtattcttgatcaacaactttcgcttcaagcgcatcggaacctaaaactttggttatgtgtgcct
ttgcttgctccgtagaaaatctaacgtgcccatcaggctgaaacccttctctgtttgaaaaatcactattaaaaggaagcgtatttaaggaaccttt
ttctttgcttccacctgctgtgcttgatggctgagtataacgaacataaacatcagcattgtcccataatcctttataattatcaacaaaatagtaag
ataaggccctttgtaaaggacctggaagagaaacagaaaacttcttatcatcaataagctctttttcggcttgaattgccaaattcaaagcactttg
aacattgtcttcatgttccggtactactttgttgtgcaggacactctttaaaaaattttgcactagttgagaagccattctcagctcattttcttctgta
acaccggctctgtagagaagatgggaagatctaacgttccagttaataatgcatcatttgacgcagagggaacacttacagaaagacagctacc
acccaagcaaaatcaacatcatattcgtctaaaagatcatactgaatgtagtctacggctgtttggttttcagtaacttgagcatcgtcattttcatt
caaatccagacctcccgtgaatcccttttggccagttgaaacacctcctaaagcaaggtacattgtgaaaagtcgatacctacttgcatcattccat
ttacgctttcttctgtacttctgaatgaagcgttccattcgtctaaacatagttagtcaatgaaccaattggatcagtatcacgaacacaattatagg
aatatctctgtaacaaattagcgcgttaatctccagtttagacatatcatacgtacaggctataaagcttttggttttcagctgtctcctccatagtga
tttctggctcctggaaatcttcctcccagccagaaaggtcctaacggttcggttactattgattcgtttccataatcttcatactacatttcttttcttgt
ttctttctccctgtttctttgatggcccattaacatcgctactttttgggacattcattggagctttcacgtatttttcttgttccagtaagtcatccatta
ctttgactctcttaagctggtggttatgaataattagccatatctactactttttttatcaggctttttgttttcaccgataacgtcatgatattttaggct
ctctggtaactcatgaactaaaagtattttaaggaaggaagcatggacaactgtagtctacataatgctctgtttataggtgaattggtaatatact
agacgttagtgttctaaatgcgctacaccacatgaatgctacgatatttcttgacagtaagcagtaatgaatgtcagtcttcgtttaagcgcttagc
tgatcttggaatctttgattatttttaagatatgttaggtttcagcattattgattaaaagtaccctccttcattctgtaattcatattatattaccctag
ctccaaaaaaacctgttctaagtagtatcaccaaatcggcatctgaataagctttagaagcataacagtttaagtaaattatactatgtatgtattta
tacgatacacattcccaactctttattaaaaatgagatacgcttctcatatatgccattttatcattagcagatatgaaagattagccttcaaaacat
ttcctcgcatttcccataaatagttgtagatacaaaattataaaatgtatttttattcaaaaaccatgaacccaaatatatgtctcaccaaaaccga
cataaacccaaaaaaatcccacaattaataaaacccgtactaacaaggttctatatttgaaactttggagctaaatatgctgaatctggaataaa
ataattttcgtattcatttccacaaacccatactttagtcgaatgtaaccttgttaccactgaattcggtaacagatccgcggttaggattactagaa
cgagcacctccacggccaccaccgccgcgaccacgaccaccgccgaagccaccacggcctccgcctccacctccagtacgaggagtagagaaa
tcgagacgagaaggacgtccagcaatgaaatgaccattcatttcaacgcagcgcttagcagagtcaatgtcggaaaaggtaacgtagccgaag
cccttgagacgaccagactgagggtcggtgggtaaacgaatggattgaatatcaccgcatgtagcaaaagcagtacgtacatcttcctcggcaa
cgttaaaagatagatttcctacgaaaacagtgtcactaggagaagagagctgatcaccaaattttccggcacgttgttgagaataaggttgagga
ttggaaggacgaggagcagaaacatccaagttaacagcgcggccatcaatttctttctgaccgctgacagcggtagcggcctttacagactcggg
agattcaaaatcaacgtaaccataacccttagagcggccactttgaccatccatgataacacgagctccaacaacagtaccgaattcctcaaatt
cagtacccaaccattggtcatctacattccaagagagacgaccaacgaaaagcgtgcaagattcgttagaatcagcgggcttagcaacctttgca

ggcttctcttcttcaacagtttcggccttacgtttcttttcactggaatcagaagatgcgctatcagaagaggattcggattcagaagaagagtcag
aatcggaagaggactcttcactgctgctctccttcttctccttcttatcttccttcttttcttcctcagactcggagtcggaatcagaatcagaatcgga
agaagattcttcactgctgctttccttcttctccttcttatcgtccttcttttcttcttcagactcggagtcagaatcagaatcggaagacgactctttct
tttcttccttcttctccttcttgggagcaggcttttcttcttcagactcggaagaggattcagaagaagactcctcactgctgctttccttcttctccttc
tttactgccttctttttatctttcttgggagcaggcttttcttcttcagactcagaggagctgctttcggactcagaagaactggattcttcagaagag
gacttctttgaagactttgcggccttcttgggagcaggcttttcctcctcggattcagactcagaagaagattcttcagaagaagatttttgcttcttg
gactttttagaaccagatttggatttctttttttcgacaactttcttagcagccttcttggatacatctttatttttagatggcttttggattgctcccttct
taggagcaacctcctttttgctggaggacttcttctcttcagatctaatttttttcccatcttttaaaatttattcagaaagatatacgctttggttaag
tgaggttctgcccgatcttcgaaatataaattttcataactaagataatataataataccaaggatttttaccgcacccttgcagtgcgataacacg
gaataagctttttataacgcagaacggtatttggaagaaaagaactctatcgcttctaaaaggactgaactttctaaggactttatttggattttatt
gatcgtatgtatatttttgtagaattatttatgtgaatacaatgtcgtacaatctgaaatagtatttaagagggggatttgacatattgaataaaaa
aaaaacattcaaactaggattcattagtaaagtggtgacgaattagccaaacgaagcaatgaatttcctttaaacctaattatgcaagttaaaac
tgcataaaatagcgatggtaatgaggaaaagaaaggaaaagtgttaactgaacagtaaaatgggggatgacgatttgccgaagctaacatcgg
tgaaatttgtaaagatttgcttccaacattcgaaagttgtacattcaaaaaacaaaaacatgtctaacttaagaagtagccataagggccttcagt
ttggagatggctaaaccagggttcagacccttagggcaagtacgtgcacagttcatgatggtatggcatctgtaaacagacatagaattttgcatg
gcacccaaacgacgagccgtaccttggtctcgagaatcaataatccatctgtaagcttgcatcaagacagctggacctaagtactcttcggaattc
caccagtaggaaggacatgacgtagaacagcaagcacacagaatacactcatataaaccatcgagtttttcacgatcctcacgagattgtaaca
gttcttttcctttaggaatattattattttgcaaccaaggctcaatactcttgtattgcttgtagaagtaggtaagatcaggaactaaatcctttataa
cgaaacaatggggcaaaggatatatttttgctggcttctcatcattcttaatgtgacatagacaagctaatgtattgctaccatttatgttcatggca
caagatccgcaaatgccttcacggcaagagcgacggaacgtcaaagttgggtcctgttcgctcttaatcttatttaatgcatccaaaaccattggt
ccacagttctttaaatttactacatacttttgtagcttgggctttacttccgggcgttcaggattccaacggtaaatctcaaacgtcttcagtttttcct
ctgctgcttcaggtttcgaggcagtagtcgaaacatttgactcagtagcaaatcttctggcactaaagagcaaagaatctctgaaaaagggtcgt
acagcttgaattcgacggcacaacatggtacaaaacacggacctagcgtttggcagttggtaagaggtggaaaaggcaggctttgtggttggctc
ttaaccccaaaatcatagcgccttcgattggccaacaaggtatactttgaaaaccaaagttccagtattaaaaaattgtataatacatttttttaatt
ataaatttctgtatacactgtcatctatttcattgaatttttgttcggccaccctctggctataaattatagtgaaggtttgcgagacaaaggttcatt
agtcgaagtccattgttttgccatgaaatgaacaaaaaaaaaatacataaatataaaaaaaatagaaagaagagaaaaaggtatttaatgtta
gtatatatttatatatgtacgtatatttttttatctattttttttttttatgggttttattggctcccaagacaagcatgcaatgagcaactatatgttagc
gttccaatgtcctgaggaacaactacgcatcaaaacgttcattaaatttatcaataggtacatagttaaataaatatatatagatatataaatgttt
gtttatcattattatttttaaaaaattggtcactcgcttagataactttagtgataacgtttgtatgtaccgagattatagtggttttcgccctgtttgtt
atatgttatagacactttctggttgaatctttaagattagaatataatttacttcattctatctgtgtgttttataaaaaaattatttaacgatggttcta
tctgaaagtcaatgttattcagttaatcagatccttggacgttattgctattcttccacgtagtaccaaatcgtctaattgaaatacatccttataaat
tgtacttaatatttccacttgttagacaattgcaataacgtgtgagacttctacagtgacaaaaatatcttccaaactgtaaacgatcataggaatg
accttaatgttcatatacgaaaaagggccaaataaaatatgttatggtttcaaaaaaaaaaaaaaataaaaaaaatagaattcttttacctaatta
tgattacttcttaaaagttcattgtactaaaaactaatacattcgcaacccaccagaagctgaaatcacttgcccagtgatccatcgactactctcg
tcaacaagaaaagcaactacatctgctatatcttggggcaaaccaaaacgattctcgaagggcgtattttctgcaaaagccttggctccttctcca
agtccctttaacatatccgtgtcaacggctcccggattgacttgattgacggtatggccttgagggcctaactcagcagcccaacatcttgtgaaac
cttcgattgctgctttgcttgcacaatataaggggatctgcggaactgcatttcttccagcaatgcttccaataataataattctacctccatttttgg
gaagataaggcactacggcttctgccatgaaaaataccgcacggacattcacatcgaaaattgtgttgtaatcctcaatggtatgacttcctaatg
gaattcggttgaggacgagtccggcattattgacaagaatatggatggtggaaccaaaagcttgaactgtggtatcaacaatatgtttagtacttg
aaatatcacgtagatctgcctgaattttgattgcatctgctgaagtgccaaaacccttaattttcttcaccaattgttctaccgcgtcaatgctactgt
cggaggtaaaggtgatggctactttggcaccgcgacgcgccaaggtctcagctattcctgctccaatgcctcgtgaggctcctgtaacgattgcaa
ctttgttttgaaggggaagactgttcattgttattccttagagtgtttggtttaggtcgtaaaaaagttggtcatttcgtcgttatatggacaaaaaag
gctgtattagtaagcaactatatgtttatatcagttcattccactatcgcttttggcgtcatcggattaatacctttaagatattgtccatatagatgt
tttgttaattatacatataacaatacgccattgtaagaagatattttcgaatactaacaaacgtcataagtctaaaaggataattattacaaatagt
aaacaaaacaaagaaatacgttttatgaattaaagaacctatttcacaaaggaaagccaataaacaaagaaatctttgagatatacagatatag
atacgttagctgtctcagcgaggatatatattcgactggtgtggataaatcaagatcatcgaagcatttaaccggatccctggacattactagcgt
ctttatacgtagtaaaacagagtccaatcaacgtgtacaattctacaactgtagttattatttccacttgataaaacttctcataacccgtgagagtt
actattataacaagtggaatccaactgggttagttgggtgtttgcatcaaggagttttaacgagaaccaggagtaaccggcattaaatcgctgttt

gagcagttaaagcgatttttgaaaacattgaaacaccaagtgtttgtaaaatgtaaagtgttcgtaattatctggttgttgaattttcgttaaagcg
gtatggtgaagaaaaaagtaaacagcaaaagaacgtaggatactggaaagaagaacaattaaaccaatttttgtaaaacgagattttcccgtta
aaatcgaaagcgcttccatattactaaaaagaacaacccactcgacaatcatggcggctgccggaatatcatagtcccaaattaccataaatacc
ggcagcattttggtactggaagataaaattacagatccacacgccgacgtccaatgtttccatcctatgagaacattcatgagcgcaatgataga
gatttggtaggtagcagtttcgaccaagcagagaagtaataaataaagatattgtctcactattggcaatgatataaacgagttttctgtaatcat
gctgggaaaagcagctgcacgcttctccagccgtgaccaaatcaaaaaaacgtcaaacaacacaatcaatatggagaaattaatcacctatac
gcgtaagcttcttatgtttctaagaattctccacaaacacttatgtatcgtattatagtcttacattgcgaaagttccttgcagacaaagaattaaat
agtaaatgccgatacacctgaggcttcattaaaatcacatcaatacttattaatacaaagtccagctcaatgtatttatcagcaaattgcttgcaat
ttttctagaccagcgttagcgcgtctgtgaattactccattgtctcaagatctcgcacgcttcattcatgacgacgtttttttctgatctatttctgttgta
ggaaatagaatcaaaagtaaaaatttagatccctttcatacgtgtatgcagctatctataccgcatggccattgacgattcacttacacattgtgct
aaccgaatattactcgttccccgtccatagtccgtaaacaaagaatcaactgtagtattacactcgacacatttcatctctagatggaagattccat
gttgtttctttccaagtcttgttttggtacgtacttgagtattgttactcgggtaacaataacattcaatagatgtttgtcaatatataaacctttattc
atggaagctgtgctttttcggtttttattcatttacagctactgttgttcaaaaggagtacaacgtcttcattactttgaaagatactcttatgacatat
acaaaaaaaaaaccaaaataggattgtatcaatagtcagtaaaattgaattgaaggatgttctctcgtaataaataaaatatgcaaaaataaac
ataaaaggaaaaaacaaggatgtatcaaaaaagctttcaaggagaggcatgggaacagacaagggcctatgacttcaatggtggtcttttgcat
tcgtgtttgaaaggataaattggcgaccaagtttttgttaaacattacgcatacgtttttcaacgctgggattagaggagtcataattagaaataaa
acaaggttcgcaagaacaaatcctgaaagctaaaggaaggacgaacgaaagaaaaatagcacacattgacattataagttgtagacgattaa
cggaataagaagaaagaatccagtagagtttctgtattctaaagtgtcccttgatagactcagaagatgaaaatgttttgaaataaaaacgagta
ttatagtagaacaaggcgtttctcctctcaatgaagtcgttgttccaatgatgatagcaaatattcggttttgataattactgaggagaccaacaca
tcgtacttgcccggagcattgtcaatacgagaaatgattaagccaattacgtgttgcgtattttggtccaaccatagtgtgtcaggttggcctgcctt
gagagattctaaccaagaacggcgaatacggataggcttttgaaagcaagaaaataaatcagcacgagaattgtcaattgaggaacgggaaa
gatcaactgcaacacgaacattaaggaaaggaggaagcgattctccggggtatcgttcaaattgtacaaggtctgtctcctcctcaaaagtaacc
tgaaagatgttcgacggggaagcctcatctagctggaatacagaatacccatcgtattgtgaggcataattggtacgatgagcagaaaggttaca
aacgatttggtcagaagagacatttttaccaaacacacggagagcatattggtccaaaaaagacagacgggcccgaatgatcttcttagccacg
attgtcaatggatagctagcaaagctggattggatgacatcgtgagccggcaaatactgcataacagagttgacgacctcgataggaagcaagg
gttcattcgcggggattaagttaaaattaggtgtagaatggggaacgtctacgacagcttgagccatgacagaaattgtttggtgtccaattaatt
acgttaacgaagaatgcttaaagtgaaaacaaaatagtttgtctatacaaagacaactgatcaacaagcgaagatggagaaaaaagaaaact
gtgtaaaattttactctataaatcaagaccttgtcaaaacgttatcgaaatcccttaaatttgaataagaagacaggcacaaataataagaagctc
agaaaaatcccaaaactcaagcgacaaaaaaaaaaagtgaaaacggaatctaaacaagccctgaaaatctttacagatgcaaaataggagcg
actgttcacttctttatataaacaagaatctattgccccccattgcaaatgccggaaaaatgataatagagttaaaataaaggtactctgcttgtta
cttttcggtaataaagataccaagcctttacgtcatgattcttcaccgagatttttgtttatacggatctttttcaagcccggttttcagcctggtttatt
tgaaaataaaaaaaaaacaacttccatccttcattcaatggacccaagtttttgtcgtcgacaaggaagcaagaaaacaaagaaaaagagact
aattaacatcctcatacgtctcctgaacgaaaatggggaagcctatacaactaccgtgaatagacccggatcaattcctttgttaatctttgccgat
atctaatcgaatttcttccgtttcggcagtcatactcacagccaccatctcatttgccgatttcagcctcggaacgcaacgaataatgtaacactcg
gcagttataggtgttttcggcaaatatttgtcgatctgtttccccatgatgctatcgtgtcgaccatgatgatgggttcatgatagggcaataggcac
gaaaagattgcttatatgagttaaggtcatctgaaagctgttatgtgagatatagcggaatatatctaagttaacgcaaaccatcggaaaaataa
agtagtatgcttcgctctcaattggttgactttttttctcctctatgctcgataaatgctttgcactagaagaatcggttgcttggtaatgccgtttctc
ttgtttccatctggccaacagtacttgactacaagccaggctgggcccagagaattgatttaactctggtacataacttgttaatggcgtagtcgaa
gctttttcttgaaatttcctagtttcttttgcttctggtgttttagattctttcggggacagcgacttgtggctaatgagctccttgggactagccgatgc
gaaagaacccgaacctccagactttgtggggaaaccgtgtccgaccgagtgctcgttacgatccggaattacctttataccgtcagaacgagatt
gtggggtaacacttcgcaatgcactgtcttgagttttggttgctattaatgatgagactgaagcagagcgaatcttgctaagagatgcatgcgagg
atgcactgtcttgttgcagacttgcgatcctctgtcttatttgtataatttttggattcttgctgagatctttactgtggttctttgaagaagaggtgga
atcgacgggggactcacgagtcggtgtgtttgttgtagcccgttgtttgggaagtgataaagcgccgggcaatttccttgccaaagcggctgctttt
tttccgtcggaatcaatatgaatgaaagaatgcgaattctccccagattccttgttatgtacaactggctctttaggagttacagagttggcttctct
agggattgaagtatcaatttcctcaaaatggcttttgtttctgagaacgtttcctttcgtggcttccataaccttctcttcttctgcttgtgttgaatcct
tttgttctacctccttggatgcgtctagtaaatcatcgttagagggaccacttattgtggaagggatatccaagcttcttctttcttgaatagaatagg
gcttgtttttcatagaaaccgtagattcaatggaaattttgggtggagtattggattgctggtttgacttaaccacagcttcctctgaatgattgtcga
atttttcatctttctcctttccattgaattcctttgtaagaggcatttggctttgcttttgcactacataatcagtaccatgtgagacacttctagtgagt

cttgtatctgtcgtttgcggaacttgtttgggacgtctacgggatcgtggatgcgctcttccttttgtcatctaatcatttatgttagcagctttctttaa
atagattgcttagtagctagcaagagaataaaagcagagtttcctccgtttgtcatcaccgtacatgcacaagactcgaggctttttgatctttttct
acttcatcctgcttttccaattgtaaatctgattttgccattcctggtggctgtccccattttaatgttaatagaccctgtcgtatctgttccatctttga
agaatccatctcacaatttaaataaggctgaaacttataaatataaaatgaaataaaaagaaagaacaaaataaaaaaccgttatctacgttttt
ggaagaaagaatcctcttgaggtggtaaactcctttacgccttgacggtagtagtgtcaagggactcctacaatagggcacatgcaagaattgct
cttagtaagtttacgttcaggatcctaaaaaatctaaaattcatcttcatataaaatagtaatttataaagaactgagtccatataacttcatacac
ccaaggaaacttcgctgttaagtttttaatctgtacatccattcaaactggctaattattgagcttccaatcacgaaataaatcattagcttattcta
attctactcaaaattatattaaaccgtattttatttttattccactacattttgctgagtttttgtttatacgctttgtgatctaggaatttgttacctccgt
tcctttcgcaaaatgtcagagtttattaataaaaatgttccgtcttcaacaaactcatccttccaatacccagtacgaatgcaaaattgaggaaaa
cgaaaatccagaattttcataaatttatagttggattactctttttgataaaatttagctcattttctgggttgcttttgaattgctttcttggcgaatc
tgtatttaatataatctgatataaaagtgctagcagaaaacttgccttttctaattcttagttcttctgctttcacacttcgtttgtttgtctagttaata
gttgcctttttgtcttcttcggatgtctacattttaaaaacagccttctaccgctaatttttccaaatatacagaaatatcgaaaacctttcttccttctg
ttctttttatattcaaatatcggatttcttttcttagggtgaaaaaaatcattatttaattgaccaaaatactaaccggttgtatttttgtaaaattac
acgaaatgtctttgccaaatgtgaataagctttctcatgatggtcgtgttcgttattacaattgggcaaaaacgttcaccaatgtctcttccggatttt
atactcccaaaaacgaagaccaactacgagagattatagtggacgcaaatgcaaagcgtaaacatgttcgtgttgttggcgcaggccattcccc
gtcagacatcgtttgtagtaccggacatctcgtttctttggacaaaatgaacaaaatggtttctattgaccatgaagctcagacggttacggtccaa
gcaggtattcgctataatcagctgcaagtcattcttgaaaaattaggctactctctccctattgtcggtagcatttccaacacttccgttgccggtatt
atgtctacatgcactcatggttctagtcttcaacatcaggtccttcctggttatattgagtcattacgtgtgatgctcgcagatggctcaattgtcact
tgctcgcgtacccgtcaaaaggatgtttttcagctgctcaagtcagtcttggtgctttgggtgttattgttgacttgaccttacgtgtggtgcctgctt
ttgatttaatagctacccaagccgctataccagtttctacattatttgagaaatggaaggcaaaccagttatgggaggctgcagaattcgttcgag
ttcatgtttttccatacgcgaaccgtgctgtcgtctggagtgcaaacaaagttaaccctaatagcgttccccatactcccaagcaatccaatttccg
aatcaaatttaactcttttatttacgaaaatttgctttacattggtaatctatttcctcggtttatgcctactttgcagcgtcttgcttatcactttttctat
ggatggaggaacggtcatctctctaccactgttgggcccggatttgatgttatgcagatgttttgcttcttttctcagcacgtcactgaatattctgtt
cctttggacacagcttcggatgctttagaacaagttatcaactatactattggtcctgcccttgagcagggaatttacactcatcagcctttggaggt
tcgagtagctgctcctactcctgaagatgagtgctggctttctactgcttctaaggttcctacttgctatattgagagtatcatgtatcgtccttatcac
agcaatgtcaaatacgaatcctattttaatgccttcgaagagattatgtgtggtttcaatggtaaacctcattgggccaaagaattccatttgaata
agaatcaacttttagaactgcatcctaatctctcaaagtggctagaactccgtaatttattggatcccaaccgcatttttttggaataattacatgcgt
cgtcatttctcttagatgtgatgactgtacatgaatactttttaaggctatgccttgaaggttgtactcacttattgaagctgctccgtttgtcttgaat
cttgagtctcttttctagcgaaatgtttgcgtgtgtttgtatagacgaacaccaatttacctgtaaagtagtcgtataggcaaatagatattaaaaac
aagtaggagattgatataatttatgttattttacttttccttctttatcttgtaaactcatagtctttcatgtgtcttcaacatttcgaaaatactcatttc
aacgacgaaatgtcctggtaatacggtaaatggtggactttttagaaatattgcaagacttatccaaattctgtagtaaacgaatccatagcacat
tcttagactaacggatttgttatattttataaaagctggttttatgtatatatataccttaaattgaattaagttgaattgaattgaagctcatcacct
gcattacaaaggacgagaatcgaatgctttctttttgtcttaaatcgcgctatgcttgcaaagttattcaaaaaccctataaaaatctaaaaacttt
acgttggaacagtacgactgtaagaactagatttgctccgtcaccgactgggtttctacaccttggatctttaaggactgctttgtttaattatttgtg
ggctaaaaaatgtaatgggaaattcattgtacgtctagaagataccgatcaaaagagaaaagtcaatggctccgaacagtccatttatgatttat
tacaaacttttggacttcaatgggatgaaggaccgattacggggggccctttcggtccttatgagcaatccaaacgattgcacatttactcggaat
acatttccaaacttctagcttctggaaaagcgtataaatgctacgacgaatgttcaacgcagtctcctaaaacggacaagaatccctatgtcgtcc
gatttcgctcggaagaaggaccaataacctttgttgatactgtctatggaaaaattacgattaagagaaatacacccaaagtacttagtgatgatt
ttgtgcttttgaaatcggatggatatccaacttaccactttgctaacgtagttgatgatcatttaatggggatcactaatgtaatacgaggtgaaga
atggattccatcgacaccaaagcatattcaactttacaaggcgtttcattggcgccctccgacatattctcatattcctttattgacgaatcccgatg
ggtccaagttaagtaaacgacaaaatgatgctcatgtgactagcttgcttgaaaaaggatttgagcccatggccattcttaactttctcgcactaat
gggttggtcttccagacaaaaaaacgacgttatccctttaccacaacttttggagctttttttccattgatagactaacaagaggttcttgtatagtcg
cattggagaaattgcaattcctaaacaaacattatttacgcgaaaggatgaccgatccgaagcaactcgaacaaattgttgctcaaatgcaaaa
aagcttgaaagaaaaatatcccaattctgtatctagaatttccgactcagagtacgtttgccgatgtctgctattgttacacaatcgtgtacgaact
ctgccagaatttgtagacctttgtggctattttttgaagaaccttcacaaaatactatacaacatagaatgcctttggcgacgaatgtttccgttctt
ttatcgcgtcttcttcatgagtttcaactttgttcctggcaaccccaagttcttcaaacgatggtttcggatgttgccagctctttcgggatcccattag
gaaagctacaatctttgattcgccaaattctttgcggatccgttcctggcgctaatttagcagatacaattcatttactcggtcgagatactaccctt
cataggctagaggcttataatagaaccatatcataattacctcctacctaataaagctcgaattcaacgtttaataccattttcacgcttttatttatt

acccctctgcaaggcactgcgttatacattgaattacgttctccaagaaacttaccactgggaagacctcctttacaacagctgtacatgcagctg
atttctttcttcaatattttccatcaatgtcggctcaaaaacgattatttgttaaagttgttatactgggggatagcggtgttggtaagacatgtctca
tgaatcaggtatgtcccgttcatcagggattgatgctagaggaatcaaaatttatggaaccgcatgtttataacgcggagtagaaaccttttttggc
tgcttataaatgttgtttctctaaaagtgcattcaaagacataaaaatacagggttctgaggagattgctctactagaaaaattgtggtttcgatttg
aataccttacacgacgtgtattgaatcctggtcggttttgaaatggagaatatcagaagaacgaaggattatccgtttcagtgttcaagaactctc
agcatacgaagtgattccccctcaagctcaagtcctatcaagttgcaaaatgctaaccaggaaaactagtttgtgaatcaaaagttcagccgtga
atataaggcgacaatcggagctgactttttgacaaaagacgtgattatagatgggaaatccgtcaccctccaagtatgtgtaatcttaacatattc
ctcttattgaattgacactaatatattcagttatgggataccgcaggtcaggagcggtttcaaagtttaggcatggctttttatcgaggagcagatt
gttgtgtgattgtgtataatgtaaataacgccaaaagttttgaatcagtggagaattggaaacaagaattcctcttacagacatctcaagttgaat
cctcctttccaattattattgtcggtaaccaaattgacaaagattcttctaaacgggccgtgtccctgaaccgggctttagcttattgcaaatctaga
cgagaaaatccgctaccacattttgaaaccagtgccaaggataatagcaacgttgctgcactttttgaacaagtcagccgatacgctctagaaaa
cgaaacaattcatgatgattttgttaatgatttttctgaaccccttatcatttctaaaccccctaataattctaattgtaactgttaattccttaccaatt
ctggcacttttactgttcatttcttttcgtttgctgtctgttccaagttgaaacataattaaagttgtatcgttcacgaatagctgccatctacgctgat
gcgctaaatacactctttctttcgttgtctattgttattactaacatctcggtacttgctctatattttataaatgttaaataatttagatttaatgaattt
catcaaaaagaatgcgtataatacaaatgtcagtataataataaactctctaatatccttcctcgccccattcgtcctccataggcgcgtagttagt
gagattctccattggcatgagaggtcctaacttgtttgtggtttgtggcgatgctgggatggattttataggagatattttcattggagaatagggaa
ggttctcaatgtcttcttcgatggtctcatcgatatcattctcttctagtcgtgctttcctaaatttttctgacacatctttaaccatatgatcattgttaa
ttattggctttgacgggctgttggatagaggctttgaaattgttctcggtgaagctcgaggagttacttgaggacgagcttgaaataaaaaaggag
gatggtgatcaagtgcagtggtttgggatgacggtgtctgttttgtattagttcttccaaaaaggggcgtatttccaaaatctggtgatggtgttgttt
tagtgttttcatgtttagcgctctgcgatcgagctccagaaaatttattgttcaaatctaatcgtcctgcggcaggtagccttcggttactgaatgctg
tagtgcgtggttcatcagcactaaccgatcgagcagcaccaaattcattagctttttgtctagtcagcggtgtcttagggcttgccattggacgatg
attgtttctggatgtaggcgttgacaaagcagcaatcctacccttctgaggagatttattcgtagtttgactaggggttgtaggagctttggaactat
aagtgttgttcgttcggacattcgatctggtttgaggtggttccttaagcttctgtaaaagatgctcaccttcaaacataaatggtctctggttttttc
ctcccaagacgagatttctggaataagtaatgcttgtactttgggtaaatcccttgatagacgtttccgaatttttcctcgcgtaaaagcaatccgg
gttccctgcgacctctttgcataagacgagaagagtcatttgcgcttgcctccagttcacgtttcccttctaataaagaagagtatttagctataagg
ttcaagaaatgcttattttctcttgcttcgttttccaagaacttcagatattgttcatgcgctgatagcgaatcttcagttagattttcttcaaaagcag
gagaaaaagaacgacgttgctcatcagtgtaaaacaaaacatcccaagctttctttattttcccccgagcatcttcaataaacacaggaatatgct
gtttctttaattcttgcaaattctgtaattgtgtttcccatttggccaagtcttctcgatacactccagacgattgttctaaatgagcataatcagcagc
gttaatattgagtttctcccaaagatcatgaacttcatcacgaaaagcatttatctgcataacacacttttctttcatgtttactaacatatccttttgc
tcgtagagtttggttataagtaattgtgggacgcaaaattggggaggtcggttcatatgggtgtttttaagttcttgaaacgaaggttcgtctattga
ttcgacgcctatctccatccataattgaatgatttcagactctaaattatttacgagctcaagtcgagaatctagttcctttcgtgcttcttcattacg
gagtcttaaagaatccagaaatacatctgaaacatcagacaaaaatgcattctcgaaatcgggaatggcaaatggcgttcctaattgtttacaag
tttgctctagctgttggtataagactataatgttaattacgggccgttagaatgttaaccttacctttaatagtagacactttcttgtcgtattgagcc
atatactgactttctaaatgagtaagttcatctaggcattgaattagaggaggatttatttgaagatccacatcagggtcatctagcttgagtgaag
aagctaaaaattgaattgtatcaatcttctgacgacaatcgttttcaagattctttcgacattgctccatttcatgaagctgagcgttcatagctgtc
gtaaacaattcaaatagggaagaattgtctccatctgttccaagaacctgatgcaatatttcaatcctttcaaaatggttttctacttgttcacgaaa
atttgtccaagtataattttgtcattttcgtttaagtatggctcctctgcaacgttatcttgactattttctatatccatactgtttcactcaattctgta
caattccaaaaattcctatgcaattttcttttcacgaagacgaaaagtacacttgaaagaagaaatttaccataccaagactaaagagaaaccct
tagcattagaaaatacgaatgttgtactacagtcgtagaggacagcccccgagtactgtggtttaaagtagttgaagtaggattcattaacaaga
gacaactggataaatttataaaaaaggccatttatttaaaaaaacattgaataaacaaattaaaatgtaagttagctgctcttaacgtctaaggc
aaacattgctaaaatatatctacaactcaagccacatatagatgaacattcagctcttggtttgagagagtacaagtaattagtttgaagtttgaa
caactatatggtagacgtttgttaaaaaatatggatagctggtttgccttttaaaagtggttattgacagacctttctatttgtagtttgtgaattata
acatacaataaactcaatttttgccagcatttatttattaaaagcgggagaggtctgataattaaaagaaatcaaatactaggaaattcatgaaa
catgacacgttccaaaattaaggaataaaagccgtaaatagacaaatcaatcttgaaagtgtcaaacaaaaacttgataaacaaaagctctcta
gaattgaaacgtgaatcatctcaaagtgagagctcggatagaggaccgggaaaacaaaacgtcaaaacaaaaacttagatcattgaaacaaa
agaatggaataaaaataaatcaaactatccaacatggtaaaatgaagaaccaaattatcaacatagtactgtcgagatttctggtatgacccag
caatatggagatataatagagcaggttaggaaagtcatagctgaatttgcagaggactcgaaaacatattcatcatactgggcttgggatgagat
ataaattgagctcgtccagcggaatgtacagacagtttaccagctctacgcatttgtcgttccaatacatagacccggaaaaagtccgagtttaat

cgcggacgacgcatctctttgcgtacaaggacttcattccatggaagcaaaggtgtatctgatggagaagaggtatgtaagataccgtcctccat
aaaccagttatctctgttcagcttttgcaaggtcatcctgttactcggacgagtaagagtcggttctataggttcttgtatagctggatttttacgata
cataacgccagaaatactagcagaaaaagaacggagccttttcttgaatgtagagcctaaatccgacaagctgaaagtcgaagaccttttacgt
aaggattttcgtccttgtaacgtagaagaaaagctttgggaggattcaggtgaaggaagagaagaagcttgacgaacttgctgaatgaactccc
aggagacagcaagagggtgatcactgtcggcagcttcgaacactatagatgagaattagtaaagctactggcgtagtataagaatggtacctgg
atagtttggaacgtctcttttaggttcaaaagagacagaatctaaataataaaccaatgactgactggatgcctgactaactttagagtccgattt
acgatttgtcaaaacgcgaccacaacaagggttggcacagtgatgatgcagcaaaaaattgttgaaaaacaatgtctttctggagttgtttgttaa
agtaggacctgaaattcttgtttgaggttgagtagagaagaggaatgcgaaatcatcatcattgtgtaatggttgagaagaaggtgataacatat
gatttaagttggcatgaagaagtcttttattcgatagcctgctaagacgatctcgacgatgaattttgaactttaaagggactgatatcgtagtgtct
tgcaacagttgaagggtcaagtctccagcttgaggataacgatgatgtttggaacttttcctactaccaacagacgagtcctctttttgtgagctttt
ttctgacagagaattgcaattgggaaaatcggccagaagttctccctggctcgaattgtatacacagctatcgagttctacccggttaaaaagtct
gatattcataatccaacaagaccaaagacccaagaaaaggaagatatcttcattaagagatgaaaacaagagaaaaaggaagggaaaatag
agatccaattgtcggaaccttccgagagaaaagaaaatcaaagaaacgaaaccgaatacgaaccgaaattaataaagagataagaaaaata
gtataatgaaatccaaaacttcaaaatgaatcaagaaaagatcgtaacccaagtacctaaacaacaatgaaaccgtaaaccaacaaaagaaa
gaaaataaattcgtctattcgacgatagaaaaagcaataaacaagggctttcaaagaaaaaaaaaattaaagtaaagaaataagctgaaacg
tctgaaattggttaacaaacaaacaaccttatgcttgcttctaatgcatgtacggagaaaaaatagaggttatttgtaacaggatttttttccgcc
aagtaagagtataaagaaacgacgttcgattacaggagcaaaataacataccatcgtttttccaaatcgactgcttttagctttaggaaccgaaa
gaacccgggtggcataaagttaccgacatctggagaacagaaacggttacggcgaatgcgttcggcttgatctaaagaaacgaatatggaatct
atgacttcttcccgggcactcttgcattgctttagcgttaatgagaggaatcaaaaagcctatgcgacatgatgaggaaaaaaacgagcatttcctt
gcaacaaacggtaaagtaagactggcaacgtaatacgaatttgggttgtttatgagccaggtcttcttccataactataagaaacgatgctcaatt
cgtcttctatgaaactttactgcgatatcatacaaaaaatttcgtcgataacatgatatttggtggcaggaaacctgcaagtctccagtaaaatttt
atttctctttgtcagttttaaagaaatggaagaatttccatattttttctttttcgtttgaacagcctagtgaaagtccagtttaattgtttatatcatac
tatgtatattatatcatgaggaatttcagtcaaaatttacggaagatttggaagatttggaagcatttacatttattttgaattatttttgttttcgtttt
gaaggaatccatcatcatctgtgaaaggaaacgaggacgacgcatgaaacctgtgtatgagccatgtacgaatcatcatcgtctttggtctttggt
ctgttgtagggtatttctgtagtagcctttttgagttaactatgaaaaatatatcttatagctttctcaagcgtttacaaaccgtcattttaccgttttcgt
ataatttcaaccaacaaacctctttaatacgatgcttctctttgatcacattcgatggaaatcggactgtatcaaaagtgaatgttgcgtgtttgtaa
tatagacagtgaattgaacctgatttattaatcaattgtctctcagaaaagtaataagaagagttcttgtgaatttatcgacagatccgaggaaag
aaattgagatattgagaatcaagacttcattagcgtatcatatcgccttatccaatttggttgtgattgtttacctttattttatcacctttcagttcctt
atgggtctcgtgctttccattccttctattataggaagctccttgacgggcatcgttggcattggtagcagttttatcgtcacttcgctgttaagtctaa
caaactatattcagagtactgtaggtgcgattattagctatgcagttttatatttgctgaattcgctgctgtcttggtgcatgctgagctcctggttta
ataataaactgtcgaagctatcagctgggtacttgcaatttgattgccaaaatgatggaaaatgttacagtgtttttgctgttcatcgcttgtcttttg
cccttgtgatgttccattgcctttttgcctttattctaagtctttgtaattctcaatctacagtttctgcgaagattcaaaatagtttgtggccattcaaa
attgttttgtggattattctagtcacaacttcatttttgatccccacttcattcatttctttttggggtaatgttttatctgtaattgggtctgccttgttca
tcgtatacggcttactgcttctcatcgatttcgcacatacttgggccgaaaaatgtgttgaacgagtattggttactgattctacctcttccaaatttt
ctttgatcggctctactgtaggcatgtatctagttgctttggtgcttacgattcttgcatatgtctatttctgcgcttcttcttgttcgtttaatcaagcca
ttaacaccattaaccttctattatgtattgctgttagctgtatttcagtgcatcctattgttcaagaatataatcctcgatcgggccttgcacaggcttc
tatggttgcgtgttacacttgctacctaattctgtctgccttagctaatagaccagatgaggaaaaatgtaatccatggagtggatccgctactgga
actcgagagtttagcaaagtaattggagcagctttcacctttttaactatcgtatacagtgctttgagggcagtttccggctcggataaagatgaag
gttatgaatatgtttacggtgatgctcacaatttatctcgcgaaagaagtttagagcccagtttagataatgacgacgtcgtatcaaattcgtattct
gattacaattatatttggtttcatgttatttttattttagcagcttgttatactggttcgctccttaccaactggaacacaatgaatatctatgaaaaca
aagaaaacgatgtttttgtacgtataggattttcttatgctgccgtgtgggttaaaataataacttcatggatctgccatttactatatggtatgttttt
cttatgatggtgtttgttgtttactaatcttttttagcctggacttgtttggctcctatattctttccatatcgttttatgatttgatgatgtatcctagca
taaattataatgatcctcttttaatttcagtacattaagtatttggaaaatgtattcatgatgttccgtccttgtaataaaaagctggaggttattttat
tttttttttattcagaaaaacggaaaacctagaaacgagaagataaaaaaaaatcctataatgttccggactttcatgttgtctctagacattgt
acgagcttaagctataatgagtctcagcttaattgcaagatctgacgaaaacaggtatggatgttgtcgcagtaggcaagctgtagacaattggc
aattaggtgttatttatatattggtcatgttagtctctgaaggattgtgaaaataaaagtcattttttagttacataccggtctcaaaagattaaaga
ttgaatattaatcaatgcattttgcatatcgaatctttacttcattgcatgaaattcaaaagcaatgtgcaaagagcgagtaatcttaagcttggta
acaaagtcgaggtttctactttgaaggtaagaaaagaatatatgatttttataagaaagcttcatgaaaggacactattggttttgtttatgaaag

tgtaaacaaacgcgaaactgaaattcgttactttcctttgtgactcaaggctgcgtaagagatgcgaaattcttgctggagccaactttttatttctt
caattcctgcccactgggctgattttgattaaatcttgagctattttagatgaaagattgagatttgaatttacattgagctaaatccctttcaatttc
gcctttcttagcggcttctgaacatttgcggttttgcatcgaacaccgatcaacagcttttatgtgacgctcttttgctaattattcattgaaatttctct
ttatttatttatttttaggatgtcggaaaatgagttaaaattcgatttaagcgatcctcctgcatacagccatgctaatgtttacaatgttgatctcga
aaagggacttcctttgtataaaccaaaagacataaaaaaattgtcctatacaacttccttcaaaaccaaggagagaaagaaaaagataatgact
gaatttgacagcttcaatggtttctttaccgactttaggaagaatatagatcattatttccctgaaaatgtagtcgttaaaagtaaatggaggttctt
cagtttgatattcgttttatcattgctttgtacattagcaatctcctatcgtttcaattttcttccttggttattaactgtatcaattgaaaaaggatacg
gtcaggctttaccttcaggcatttttcttgtgatatgggcaatattctttgctcttttctgtggtaagtggagcgttattgttaatcgtaaacgttattag
aatgtgtgttactaagaaagcctagcacttatttggcctttcatattgacagtacggattgccgctaaagttataatctttatcgtggttggtcttggc
aacggtcttgcatggttcgttggtgctatcttttggggattttataacattcttgttttttttccttggaatggaatttgagcgctaagctgaaaacaccg
gttcggctacgcttcccagatatcaacagagaaatgatgcactagctcctccgccgaaacttaatgctaacttgttaacctttcttttctcatgagcc
ttaactcttctgatcgaaagtcaacaagtagaaaaagagaatattccaagcggtttattccggtgaatatttatatgtctttgttaagcgttcttgcg
tttaattttgactacgaaatcatccttgtatcaatataaatggaaatgacgaagaaaagtggacaatagaaaggcatgcatacaatccgctcatt
gaaaggaaccgtaatctgagatcattgggatcatactaggttcttaatgattattattactattattcttaattgcaattgaatacgatcgttttattt
gttttcattgaacttgtaattagtcttttgtcacttgtaaaggaacaacttgcaaagattctatcgagaaaacaaattacgggaaatagtcaacgta
taaaaaaataaaaagagcagcaaaaaagtagaggagcctacggcacccgggattcccatgttcgtctccaaccatagtatgaacgagaccttc
agacgttttcacttaactgcagtgatcggacgggacctggtgttccgcctagttgtggccgtagataattgtttcaatctgaaaagctaaatatttat
cagctataactactaaaaaggcttaggttcttctgcatgtcatatctataaggaagaataataattcctatctaactaaacataatttatcacaatc
tgtaaacctaattacccaattttattgcgttgaacgctatttcaaaaaacccagattccatacctataatactttttatcaagttctctatcgaattctc
ttattcgagctcttcatcgctcagagaattaagctcgtcatcatcattctttaaagcaccttttttcttctgacgcatgtcttccttatgattttggtcttc
caaaattgcttcacgtagaagtctacgctgctcttcacgaatttcacgttgaacgacaacgtaggcacccaaaggtgttttatcccattcctcattc
gctaagaaggtctcaactgaatcgaggttagcaggactgaatttttgtttagcacgatgagttaagacaaactcagtttgctgttcaagcttttgga
ccaatatcagaagagagcgatttagcttgacatttttacttttctttgcaaaccgcttagcacgaacaattactggtatgacaaattcggggaaagc
aatgcttgtagcatataaaacataatattcccctaaaagttccaacatctgattagttatgccatcttgataaacgcgtgtcctcaaatacgcagtt
ggcgcgcgaatttcaacttcaaaatccaaaggttttaatgtgctaggcttgggcttccttgaaagttccggggaatccaagacatcgaacagtact
ggcgccataggaatgaaaagaccggtatgtcttgagaggtaaatgagacttcgtaaaacatgaaatctcatgggatatagttgagatgaggggg
aaatcatcattgcacctagagcaatttgaactaaagggtataccaaagattgcatttccactgctattccctttttcgcggctcaaccaagtagcccg
acttaacaagcgagaccaaaagtcaattgcatgcacatacgaccagctttgtacaagtttccttgtctctggctttggctgatggattgtgtttctga
gagtaatagcaagttgtcgaatatatcgaaaagtaactaaataacagctttcgggatttaacaaaaataaatctgcggaactatttttcataaaat
taatagccgctaaagtgtgaacagttgtataagaacaaatgcgaatcatggttaaatacatatgcttgaaaacaaattccagcaaagtcaagtct
gccgttaagcaaatactacgtaaaaccaaaacgcatgaaactctaacagaatcatcacggttggtactccagacctcaacaacagcctccacaa
attgtttcaaaaactttcgataagtgatcatgtaaggaaggacattttgagtttcacgaagtagtaaagatatatttttagcatcagtcattccttcc
aaaaatcgcaagatggaaaatccataggacttaagcataggactgaggcgagacaaaatcttattgtcagtatttacaattttcctaccgcttttttt
ctgtttgcacgggaacatgatgattcagcacacgtggaacagcttgaatggctaacaataaaaggtcgttaaatactctagaatctgttattgagt
atttcaggttcatcccttcgtcctcattcaaataagcggctgccttaaatgcttgaatcaccttctgcaaagttgtcaacgacttttgattatttaaat
gatcccgccatttactcagcatatccatagtaatctcagtttttcctacactagaccgcttgcctccttcagtctcatcctcctgcaatactgcatctgc
attgaaatccaacaggtcgctatcatttttctttaaataattgtaaaagtctgggtccttctccttcaatgaatcaagttgttcttgatattgctcagca
tccgcctcttcttcgtcagaagagctgctttcctcttgttcctgagtgactttttcttttctgttcttcttcgacttggagacccctttttcagtttcctgat
ctttcggagaagggtttttgtttttctgaagtgtagttggttcggtcacagcagagtcttcaaaatcaccgtttaaaaaatcatccatagtcatatctt
cattctccgactcaggggtttcattttgggatttgtccaagaactgtttcacctcattctgtaaaccctttttagcgcctgaatttttatggactccagt
ggaattggaagtctcggaagatttttttatttattttaccatctttttttgtttttttaattgactctttcttgcctttaaaatggctgatatcttcgacatata
gaatttggaggtggttggtttttttccagtggtggaaatcggtatgcctagtatcctacactaactcaatctcttgtaattttggttttttggtgctatg
agatctttgttttaaaagtaagtattattagtgaagaattatacatttctttaattttgaccttcttttgatgaccattggtttccaagaggaattcgttc
ttcatgaagattcatgaaattcccaggtctgaagcaaatactgtaaaaggaaaaaagcatatatcaacaggatttggaattgaatctgattcttcc
ttgctgaatgattcttccatccaaaatagtagtgtgcagccatcaagtcgcaaaccactatccgatgattttaatgagccattattgacaaatatac
cttctgagtcattttgcgaaccggaaaagcgtgggaggagctttgcaaggaatccttgttcctcagggaagggaccaaatggggataatgatcaa
ggtgatgcttatgcgcttgaaaatgaaaacctttcaattcaaccttcttgcctagaaaaccaattagataagtttgatgtctctgaaggtcataagc
agggactgcccaagacgaaaatccaaaaagagaagcgaaaagcaggaagaccgaaaaagttgtcttacgtagcagaggatatactctcagaa

gggctttctcataaacggaggcgtggtcggccaagaggttggagaaagcatcctgaaagagatgtctgtctggaaatgattgatgctcctgttga
aaaacataacccaaagagtgtttttgaagcagttgtcgtacctttaaattccgttaaaaattctttaaaacaggaaattgcaaatgagccaaatga
tagaacctgggattttgaacaccattcaggcatgaacaccgacgtgaacatgcaagatggcgaactggaagattccgttcccggattgacatttg
attctagcataggcccttcttcgtgtgacttacctaacaataatgaatcattgaatgttactaagtccgatccagactctgataccaacttcagttttt
ccgatgatgatttcgctgtattagataaaattgagcatgagtttgaaatacatgatacggatcagcccaagagtacttctccttctagttcaccctcc
cataggattactttgtgtgaaaataatgaagctctacctcagaagagtgtcacagaaataaaggaaatacagaaaaataatactcttaatagtcg
gaaagagaacaataacatttttcaatctagcagcaaagcaaaagcacaccatgccgggatgcgttttcatgtacggccgacatttaatggtcgaa
gcaaccttgaggctaaatccattggaaagccttctaattttaaatatccttcttttctgaaaagttcaaggaacaacgaaaatacatcaagtgcga
gaaataagccccttgttcgcagcccttttgctcaaaacaattctggtgatcgaatcaaacaaaccctttcaaaaccatttcgacctcctctgaaaaa
agatgcactacataatattattgaggaaggaacagaaccagaatctggtcctaggagttcaagaatgatgcatcttgatgacgctaatttaacta
atgatggtcattcttctgtagtcagtagattgcaatcagaagtctccaatttacaggaccaagtatccatagttgagttagcctatgatcttgaaaat
acccaggaagacgaagtgaagttgtacgaaaatatacaaagatggagaaggtcggctcagttagccgttgaagtccttttccctgtattttcgtta
aagtttacaacaatgctgcaggaagtgccagaatctgttttgccaagtgttgttgatgatcttcgttcaaaaccatgtaacattgggacgtatcttg
aacaactgaatatccctttttctttattaaactataatccggaaaacgattcttggggagatgaaacttaaatacctttttttggaatgatttttatgt
ataatgtgttctaggtaatgaaatgattaatcaattcggtactatctgaagtcaaatatatttcatatatttttattaattatcttttaagagaaattta
gagttttacgaatacgcccatattgttctgatccgccttcattatttacaccttcaccaactactcacattaggtagcccaaattcatcgtcccgaaa
acgtaaggtagaaagtaaaagtatgagttccaagaaagtcgcaacagctgttattgatttttttaaatcaagcaaccgctactagtgctgtttctga
cgaggaaaaagaaagtttagaagttgctggtacgagactatagattatgcgaagtatcaatgactaaacagtgtagctcaatgtatcaaagattc
tttcaaagttgagcccaatgaaattactcctggttcaggaaagcgattagtggatgcatacgaagcctacgaacagcagcatcctgttgaggcat
cctctactgcttcaaataatacagatgaagccgaaaagttgaaagcacaaggaaacgaagcaatcgctaagaaagagtaccagtctgctgttga
tctatatactaaggctattgaattggatcctacttctcctgtctattattcaaaccgtgctgcagcttatagtcaattaggacaatacgagtctgctgt
tgaggatgcattgacttctttgagtattgatcccaaacattctcgtgcttatggtcgtcttggtcgtgccaagctttctcttggagatgctggtgctgc
ggctgatgcttatgaaaagggattggagctggaccccagtaacgatgttttgaagcgtggtttggaagccgctaagtctcagatgaactcctcttc
tgatgttgatgctcaagcctcgaccactgaacagtctcgtgatggaggtgctggtggaactggaggcatgcctgatttgggttcaatgttctctggt
ggtatgcctgatttgggctcactgatgaataatcctggtatgtaatatatttatgtctgaaagataactaactcgtatcagctatcatgaatatggct
caaaacatgatgcaatctggcgctttaaataacatgatgaacgatcctaatattgctaacatggctcgcaatttcggaagtggtggcggtatgcca
gatttgagtgctttggctagtaatccccaattgcaaaacttggcccgcggcttcatgaacaaccaaaataacgggaattccgaaaacaactctca
ataagatactctatgacaataaggatacttaatgtttaggtttcttggattaagatatattcatatgcttgctattgaatgagatcaaatttttaaaa
aaaattattcactgaaaactagtcttatacacattactgaggaaatcttaaacccaaaaaaatatttgtaaatttgtaacaaaaagttgtctttaat
tgtcctgtctttcatcgccagcatcgtggtaatccgactcttctaatatgtcgctagcatcagaaccttcatcattttgttcagtagcgtcctcatcggt
tgcaacaaacaaattcgcaatgaaagaacgtaaattagacataaacgttcgatgttggggagaacttgaatcgcccaaatcttcttccaatctac
gcattagttcttgatgaactggatggttagggtcctggttaacttcttgggcattatggttagcgtcagtttcgggctgattcctaaacaaggtattg
ctaagataattagctagacgatgagaagtatcacggccaaatacttgaggttcatccgtataactatctggaggcaaaggatcgaaagacacaa
tggtacgctgtaaaatcctttgcggcaagaacctttgtaatgttctttcattcagcaaaataagatgtcttgctaaattttccgttatatcagagtatt
gtccctgaattggttttaaggtaaactcttcaaccttcaaaactgattgtaggaaagtcaaaacatcatgagatgaccagttgtcgcgagagcgca
ttgcgtatagttctgagtttagttcctgtacaggatcttgaggttcgggaatactaagttgatcaaaagtcaatcctattgattctagcaatttaccc
agcataaagggagcacgttcgattgctgcaagcatagtcgagcgggtttcttcaatatcaccatacatcttgtacatagctaatgcagaagaata
aaacatgttaggcattttagttatttgattagtattttcaaggtaattagcaaaattaactacccaaccatattctccacgtcggatagcgtaagtat
ctatacaagaaccaattgcatatggatcagaaggatctaattgtaaaagaaccttgcaaaattcgaaaacggttcgccaacatccacgactttgt
aagctttgcatgtaccgccaaatacaaagatacagccttctgtttgtggggaaaataaaaggaagacgaacagtacctgtagccagattaaattt
tggatgcaatgcactatcaaaagcaaataaagcgcgtgcaatcaggtcaactggcaattcatgatctccttgttggtcgataatttcggcgacttg
taataaagtgtcaatatgaaatgggtgtgaacgcaaaagcataaataaattattcggatcgtaaatctgaacataaaactcaaaagtttcttgga
cttcttgataggcacgactctgggtaaattcgaaaaagcacacaccatctgaggattgctcgattaaccgcatactaagaccagatctagctaga
acgggccatccttcttgaggctgaactaacacgtatcgctttctccttacattactacgtctagcattcactggtctttttcaacgattcgtccaaaa
attttccggatctccaagtcaggatttaggaaagttatgttgacacttactaactttgctagctcactatttagttttgtttcactaggaacgttatctg
ttgctggagctccttcttccttggtttttttggattcgtcgccatacttaagcttcagttcagcaacagcttcatcaatctcctttaattcatcatcaacc
gtatcctcttttggtttatctacaatttccgtatctttgcctgcagcttcaggcttgttcttcttcttttgttgtttctttctgttcggttttctttgggaatcc
gaaagcttattcatattctttttatttccttcattatttgaatgtatatcatcttccaattcactcgtgtccgattcttcaagagtctcagttttgttgcta

acctgtgcattcaatatgtcaaacggattcaagaccttctttttttggtttgaatgagaaacatcctcatctgaatcagtttcagattctgacgactct
gaagactcaaaaattagctcttgctgtcgtctttgtaattttcttaatgctcgtgtcgacatagagaaagattggttttagtctgcttcagccaaaac
gatacaacaaagtatcggatttaggcacaattttcgattaaattgtcattctgtaaaattttgagaattttagaagttaaaatagaatacatgtaag
tcaaaaatattatggatcgaaaatgttactataccatacgttcaactagaagtcatcatgataaacgaattcatcgtgctgtatactgtatgatagt
ccataactaaaaaacaaaaaaccccttgagaacattatctcaactttcttaaagaaaagcagtgatggcttctccaagtccgagttagtgtgtact
aggtccatgaacacagggagtttcaatgttgaaatcaattaatgtacaaatgtctggtacgatcaccagcaacttagtagggaaaatacatgaat
agtcagatacttcattcccttgcatcaaaaagataataaatcttgaagcgataatttcttatatgacaaggatttaaacagagaaatatgaaacaa
agtcatagctttatatttttactttaccatagatcagattccaatacgactcctcttcagatccttctactaattccgcagagatgaatggaccggttt
tattgtgctttcgatgggcacgaatatcattatgggaatcttgcttccttttctgattaaaaaagcaacttacacacaaaatagcctgttcaggagat
ttccatctcactatggcctctgtttcgtcctttctatagtcaatgtacattggttcgcaggtagctgtggggttttgacgggagaaaacatatcggagt
aagctttgaatggtgcttttatttgtcaatggatgtaggttaatcaacctcgtcaaaagtccttttgggaatgatcttttagattccgaatgtttcgttt
gcgactttttacagtcatcctctaatggttcaaattgacgttcagagctttcggggtatttgactgaagatgatgatgctcgatcaggatttgttcgg
aacgactgatttgtaattataggttcagtgaagaaagcacttgaagaagatttcatcgtatcagcctgatattccatatacattttctctcttttggtc
cattcttccctcgatagaaggttccaaccttgaggaatgacaagagatgtgagagtctcctgcgatattgaatgagccagtaccacaaaggcata
tccttcaggagtagggataatatcaaggtagggaagaaatccagaaaagcattctcgtagtagcccagccagcataaatggcttgttagataga
gttgctggaaacggttccacatatactattcgctcgaataaatccaggttgggatcaagtttatattcattcctgattatggaatgcgattcttcatc
gaaagtaaatagttcatttttaaatttttaacacattgttttaagtttgcttgagtaggggacggttttaacccctgccaaaattcttgtttgttgatt
aaataggtcaaatgaaatttttgtgtctctctttccctatagaatagtacttcaatcatacattctactgctcgaataaactctggctttaagtcgttt
atgttcttaaacgcgcttcttgccaaacgagttgaagaagtcgaagtagacccatttatacttttatgttcctgatccacgggtgtcttattcgataa
actaggtcttcttgttagtctattgcgaacattgacttgtcttggtagaaacatcagacaatcgataaaggtaagtatctagtcaatttaaaacttta
atattctcaaaaatcaaacaaatgtacaatattctttataatgacttgatttatctaactggattcaaacaagttctgaagtggaagacaaacagc
aaaccttgcctgcgcaaacaccaccagaaatttggaattggtgtttttctgaagtgaatttttaaagaaatgtccaaaagaaaagatatcgatga
agaagtttctgctgatcctacggtatgcgcgaaaaatcagaaatacatatgagcgtgctttagctaacaattgctttaggatttgctgcagtttatt
aatctaggagccggcaatgaagttggaagaagctgtcatattcttcaatacaaagggaaaacagtgatggtatggatttcaggattaagtttcca
catatttccgactgtctctaacgtttataatagctggatgctggtgttcatcctgcttacaatggtctcgctgctttaccgttttacgatgaatttgatct
gagtactgttgatgttctattaatcagccagtaagtcgtattaagctttcccaaggctttagtaagccgtggaagaacttcctgcaagcgtctctaat
ctagtcatttttactaatggttataatagctttcatttagatcatgttgcatctttaccctatgtgatgacgaaaacaaattttagaggccgtgtattc
atgactcatcctacgaaagccgtttgcaaatggcttttatcagactatgttaaagtcaggtacctaatatttttgttaaaaacagttagttctaacat
acagtaatgttggcgtggaggatcaattgtatgacgagaaggatctgacggctgcgtttgagagaatggaagcagttgattatcattctacgattg
aagttgaaggagttaaatttactccatatcatgcaggtcacgttttaggtgcttgtatgtttttttattgaaatggcaggcgtcaaagtaagttagtag
gaaagaagcaagatatattaacttaatagattttgtatactggagactactctagagaggaagatcgacatttgcatatagcagaagttccccct
acgaagccagatatcttaattacggaatctacgtatggtactgcttctcatcaacctcgtcttgaaaaggaagctcgtcttctaaatattgtgcattc
tacgatccgtaatggtgggcgtgtgttaatgcccgtctttgcccttggaagggctcaggaattactccttattttggacgaatattggaacaatcact
atgatttacgtcctgtacccatattgtatgcatcttccttagctagaaagtgtatggccgtttttcaaacttatgtaaacatgatgaacgataatatcc
gtaaggcatttgctgaacgaaatccctttatattcagatacattaaaaatttaaggaatttagatagattcgatgacatcggcccttctgttattgta
gctagtcctggtatgcttcagaatggtgtatctcgaactttgttagagcgttgggctcctgatcctcgaaatacattgttattgactggttactctgtg
gagggaaccatggcaaaacagataacaaatgagccggttgagataacatcaatgtcaggtcaaaagattccaagaagaatggctgttgaaga
gctttcatttgctgctcatgtcgattatcttcagaacagtgagttcattgatcaagtgaaagcagaccatgttgtacgttgcaactgaaatattctta
gatgtatatttactaacgagtctagatactggtacatggtgagcagacaaatatgggtcgattgaaaagtgcactgttaagcaaatttcataatca
aaagcttgatagcaaggtttatactcctagaaattgtgtacctttatttctttacttcaagggagaacgccatgttagagtaagtttaagtcttgaaa
aaccgagttggctttattaatggaaattaactaatatttttataggctcttggaaaaattgcagtccgcaaaccaactgatggggatattatgagtg
gaatattgatacaaaaagacgctgtatacaaacttatgtcttcggatgaccttcgagatttcagtgatcttacgactaccgttttaacccagaagca
agtaatcccattcttctctagtatggagctggctaagtaccacttaaatcaaatgtttggacatgttaaagaagagcggacgaaatctggggaatt
gtgctatactgtcatggatgctattcatttgagcatgccaactgagcacaaactgactttagaatggactggaaacatcatgaatgataccatcgc
agattcagtgattaccgttttgttaggaattgaaagtagtccagcttctgtaaaatgtacgtgtctaaatatgtcctctatctttattgttaaattaca
aaagctaatttttagtgacaaatcacaagtgcattcatgggcacattgacgcaaagaaatcgaaagcagaggaccgcataaataagttaatga
tgtttttagagagtcaatttggcgaaagcattacaaagtcgaaaaacggagtctacataaaaattgatcaatatgaagcttctatcgacttttcca
ctatgaaagtggaatgcaataatgaaatccttcgaacaaggattgttcacgttctttctcgagccgtaaataccattcttcctttttcagaaccactg

aaagaaagtcttaataatgtggaagaacaaaaaagtaaagatgactcaagtgaagaaactgaagacaataaagtaacgcctgttcatgaaga
aaaaaaagaatgagctcatctaatcattatataggattttggatactttgaaaattaattttattgatgtttttaagtgaacaaatgaatacttagg
tataacacatcagatatcaatttgtaatcgtttatagaatatacagagacaggagagctcaatgcaaacatataaataataatctttgcttttcttc
tctttttttattttatttttttaaggagcattgcacaagggcgaaagaccgtgtcaaatggtttctgctaaaaataaaaaaatccgaaaaatgtct
taatgtttttgaaaatgcagaaaataagatcctataaatcaactgaaaactgtctacttaaattgatttattaagatcaaaatctgccatcatgtg
cgagaaaaatttcgtatcctccaacaaacagcaaaatgtcaaacaataaaattcctaattcgagattcgatctttctacctattggggacgagttc
gcgtacgtaattatgtctaaaaaagaaaaaaaaatcagctgtggtcctttatttcctgattattaacagttatagcatgcaatggacattactgatc
caaggtaactactaagaatatagattgaatgagttgaatagtttactaaacgctatttgcatcttagaactctactatcgacaaaatcagatattga
taatgctgtcaagactttacaagagtacgaaaagggagacggcgatttaaatgaaaaggtttggaatgctaaaaagggtatgcttatgttacgtt
aaataaagcataatttgattttaacgtgctcacgaatatagttgttgattccaccttacatcctgatacaaaagagcctgtgtttctacctttccgcat
gtcctgctatgctttggtatgtctctactttttttccactattaatagtaatattagaccaacttgtttgttacagctggaatgcttcaacccaaccta
ggcactgctggaaccatcttctggcagtggacgaaccagtccgttaacgtagcattcaattctgcgaatgctaacaagtccactcaattaacatta
ccacaaatggcgaaatcctacatgtacgctgtcactgccagttgtggtgttgctatcggccttaacaaggttgttcctcgtattaaatttttgtcttct
tcatcgaaagcggttttgggacgcttgaccccttttgctgctgttgcttttgctggtgttttgaacgtgtttttgatgcgcggtgaggaattgcgtcag
gtaagtattcttctcataacgactcttcttgcgaatgaatttatgattttttttctaacgattatagggtatcgacgtttttgataaggatggaaactct
ttaggtaagagtaatcgtgctgccttatatgcggttggagaaacagctctttctcgtattatcaatgcctcacctattatggtcattccccctcttattt
tgatgagacttcagaaaacaaactggcttcgcaagaaccctaaatttactattcctgttaatttaggattggttggtatcacttccttaattgcggtg
cctttggctgttggtgtttttcctggtagagagactatttctcccatgaaactagaatccaagtttcacaacttgaaggatgaaaacggtaacagttt
aaaaaaggtggaattcaatcgcggactgtaaatatctaggttatgcggaataactctccgacattataccccttgttgattatcaattttttttttgg
gtagacaaacttgctttgaacaactattcagggtaagcttaaatcgatctttttgattctatttaaaacgatagcaaacaaatttagaaactgagat
attcaaatccattgacatacagaaaccacaaaagcaaatttgtttacaatatagtgcaatccaacaactctttttcttggtttaacgcaaagcaac
gtatcgttatggttatgccagtcatgggcatgcgtgcacgtcgaccaaagtttctatcttttaccaaccaccaagtaaagagaaattggtaaaatg
gcatctgctcaggaggatttaattgactacgaagaagaggaagatttggttcaggaccaatctgctcaaggaactactactacagcagaaactac
tgaaaatggtgaaaaatccgataagaaaggttcctacgttggtatccactccactggtttccgtgactttttgttaaagcctgaacttcttcgtgcaa
ttactgatagcggatttgagcatccttcggaagtgcagcaggtctgcattcctcaatccattttgggaactgacgttctttgtcaggcgaagtccggt
atgggtaaaaccgccgttttcgtgttatctacccttcagcaaattgaacctgttgacggggaggtttctgttttagtcttgtgccacactcgtgagctt
gcattccaaatcaagaatgaatatgctcgtttcagtaaatacttgcctgatgttcgtacttctgttttctatggtggtataaacatcaagcaagatat
ggaggctttcaaagacaaagctaaggctcctcatatcgtcgtcgctactcctggtcgtttaaatgctcttgttcgtgaaaagattatgcgtgtgaac
aacgttaagcatttcgttctcgatgaatgtgacaagctcttagatagtgtagacatgcgtcgtgatattcaagaggttttccgtgcaactcctcctca
aaagcaagttatgatgtttagtgccaccctttctagcgaagtacgtcctatctgcaagaagtttatgcaaaatcctttagaaatttacgttgacgat
gagactaagcttactcttcacggtcttcaacaacactttgttaaacttcaagaaaaggagaagaacagaaaaattaacgacttgcttgactctttg
gagtttaaccaggtggttatctttgtaaaatccgttagccgtgctaacgagttggatcgtctcttacgagaatgcaatttcccttctatttgtattcat
ggtggtcttcctcaggaggaacgtattaagcgttacaaggctttcaaggactttgataagcgtctttgcgtagctactgatgtttttggtcgtggtat
cgacattgaacgtgtcaacattgtaattaattatgacatgcccgattctcctgatacttatcttcatcgtgtcggccgtgctggtcgtttcggtaccaa
gggtttggccattaccttcgctagctccgatgaagactctcaaattttggacaagatccaggaacgctttgaggttaatatcactgagctccctgat
gaaattgatgtcagctcttacatgaatgcttagttttattcatagaaacaaaaaaattttcttatattcccttatgcttttcaataccttcctgtgcgat
cttcttagtgcgacattgcgttacaactgtttatatattattaccttcttgtacacttattctgttttaccctcgttcttttctgtcctactactagtggtga
ctttatactttctttttggtgacattgaggcctctgttattcattcttttgtatttctagctagtgagaatgatgggttgaaaattctagcttatgcgta
gtagcgtgcatacctttatcagctttaaacatttatgtatgatttacagatcctgtacgtatgagatacattcattaaaagacataaatccattgata
ttaatgtcaggttcatatacttcatgaatttgtaatcaatctccaaatcataactaaaaccataaaaaacaattcactccggttattaaattatgagt
taacataatatacaataatatttttgatgaatacaatttgtataaaaaggtaacaagcaacaaataatttcctcgtctagattcaaaaatttatgcg
tcttcatcagggacttcttcaacagtaacattatggcgaacagaactttgagaaacgggacttgtggaagttgtattattcttctcatcactaggctg
attgcttgagagggcggcttctttaacattcgttgctgatttagtggaaatatcattgggaataccttgaacattatctgttgaagttgtactggtagt
gttttcagggtgaattttgttagggtcttgtgcctctgaagtagcagtttgctcagctttagaaccagcattctctttatcaattggaacagctgaggt
aggttcatattcaggaggtcccgattcatcatcgctgtttactatagaatagtgtccgctagacaataaagtttccaaaggatttcttgtttgggttg
aagtttcttcaggctcaataacttcaggatcaggatttaaagcagcacgagtcatcttctcaaacgcagaattagcaggagctgcttgagaagaa
tcataatgttttctagcattaccgcgtttgtttaaacgacgttgagcacccaataagtattcatttagttctgaaagctcggtacgagtcaaatcttc
gtcggaaaggctctgctgtgaacgctttggagctttttcatgtgataaaggaatttccgctacacgaggaccttcatctttgataggctgttgaattt

gaatataaatttcctctggctttccgctaggagtgtttgatgctctagtaatgggtgaatcattttgcacaggctcaggaaatgacctttgaggatac
tcggtattttctggtgttgttgtgcttgggtcttcttcggaagaataaagacgttccacaacgggatgattaatgaaaaattgatggaaatcattag
gaactattaattgcgtattggagggatctgacaccaaaatccttcgtaatagcctcctggcttctttttcgcgtcggatatttctttgagtttcgtcatc
ctcaaatcttccttgtacgatagtacgtacaacttgttcaaaggctcttcgttcccgtaattgttctagatagtattcctgtaaacgggcctcctgttct
cgctcgtgtaaatattcacggaacttccggtgttgaatttgctgtagcacctcctgtcgtgcgtcttcgatagcttgtttccgaaatgctagttgtcgtt
cttgctggttaatgacctctatttgttgatcgagttctttaagagcgagttgtagactgtgatcttgtacgtgttggtattcattcccgtgagggtcaat
taagcgaaaaaaggctgccatttctagacctgtcttttcgtagcaatgaaaacaaaggaatacaaagtttgtttatagctttttgatattggcggaa
gtatagtttatgttactcagtcatggctcctttatgtcactaactctcatgttaccagaatgtaccggaaacatctacaacgttttaccagaaaactg
atcttcatcaaaaattaagtttcgttcgcttaagtttaccttaaatatttgttcttgcatgcaaatatatatttaattctacatctaggtatcaagaagg
gtctttttttttttacagtactaatgagaatctgatattacagtattaacaaaagaaaggaatgaaataggataaattattaacaccataatattct
aataaacaacaattaaataaaatcaagcaagataaacatattcatcacgtccctgtcgctctagatattctttttcaatcaacatatcaatacatct
tttaatatccgagacttgaggggcaaatctgcttttgatttgatcaattgtctccttgaccaaaacgacatgcttaagcgtccttctagctttcataat
acgcacgatggcagattgcaatagcaactttcgatcttcctcaatagttttctgggtttctacaacttcctgtttttgctccgatttgatgggcaagtt
gagttgaacacgaatctttttcatcttaaagtttttgttaatagtatagacactctccggctctccaagcttctttccttcttcaagtaacaaaaccttc
gcgcgtaagaaaatgtttagaattccaactaatatgtcatgattcaacccgctcaactgtattaattcttcatacttatacgaatcacttttattgtaa
agtagcagcacagccatttgaaaggtagatacttgaaacacataagtggtggtggaattgggattgattcgtgccttgatttctcctttagacaaa
tggaaaagccagcttaatttccgaccactatggcaggtattataataactttcaaaccgttcgtaaagtgattttaattcgggtgggatatgaaaac
tagaggaactagctgttaacggccagaacgacgttcccaaaaccatagcatagaaatcaatgggcgacttttgcatttctggaagttgacgaaaa
gaatcggtcagctcctgagacaaagaaatatcttgaaacattctctgcaacttgctggtgtactcgaatccacagacttccttcaattttgaaagca
ttccggattctgcatcctgagagtttgacattccattgaccaaccgtttagccaatagcttcgtatagaagttctgaaacacatccttatcctctacgt
agcgaaaaattatgataatggaagataagatattttcaatatcatcaacctcggccgccttgctgttctttcgtaagacagaatcggaatattttgc
cagtaattcgggagagcgcgacgaagaaggctgacagactacattacgattgataatttctctgcacgatgtatccaaagatttcgtgaaatcag
catcgccttgaaaagcgtcctcaaccagccttttagacgaaagatatgttgacaaaaggctctcaaggtagtctttaggatctacttcagatacctt
gtcgccttcaggcgcgattctagcaacagcaaggaatccggatcgctttacgaaatcttcaaaggactttcttaacggttgtaagccgtttggtgtt
ctagacattaatcgatacatcctaacaatatcgtcatgacaattttcatcaagcatacgcgaaaagtcattatgcaaaacttcggaatgcttagta
attagttcatcctcgacagcttccaacagtggttttaacgtggactcatgcaaatacaaatgtacaagctcttcttcctctgaaagacggctctccg
cttttccaaggtaatcggttatagaatgcgttgagatatactgagaagattcagtagcatagtattccctagaagatgaaatgaaccttgattcaa
agaatgctttgtaagaattgaggttggcctttgacatatctgctttgtcaaaacttaacgacgttatagaatctacacagactttcacgtccttcata
tcgacaggttcgtgcgaccgttttctgttaaagacgtataataagcttgtaagtatatcatcgcagatatacgcaaacacatgatggtaccaagat
actaagcaaagcgtgtagatatcgtaaaccacagtatcagaaaaccggtttttcaattttacccagtatcggttaaggtatccaaaaagatgatg
aaggaacctagaggacgttgtaaaacgagtccaatacttagcgtaagccgccaaacgatcttcatgtgttgcttcggaaagtgacgtctgttata
aaagttaatagaactgtggtgaaatagaaaaaaaaaaaaaaaaaggaagtttgttgaaactacaatcaccagaaaacacttaccaatcgtaaa
ttcgtcaaatattctttgagatataacaccaaattattgtataaagcttcacctaaaacgtttgcggctgtttctaggttagtccataaatcacaagg
agttaaaataaaaaagattgcccgtttatcacagtaaccaagcatacatactctgatcgctgaaactctctactgacattgtttttgaagcatcggc
acaataattatggatagccctattccaaagttagccaaagtcttgaatttctcagtcccttttatgaaatgcataacatacgtatagagctccatat
agcgagtaattgacattccttcatccaatcgatccaaaatatgcaacgcgccatctttaagaaaatcccacctattttcgattgttagtttcagtcac
tcttgttaatcatatgacatacgttccgtttaaagagtcatatttttcaacatgaggaagatcattattgtttttctcggcaccgtcatgaactctatt
atcacaatagaacgattaaaattagattcttcaaatctacactacagcaaataagcaatagtggttggcgaaaagctagtttcgtccgcaagcttg
agtttggtttggtgtataagagaggcctaggatcgtatagtgaggctgaatgtgatgtgtaatctagtatacgggagtatagcgatttttatatatg
aagattcaatttcctagcaatgtttaggataaatcatagagaaaatacgaaattattcggtctgataatacgaattgagtccatggattgccttcgc
attcgaaggacagcaaatcaagacttcttgttgctcgaatataatattatcatactatccgaagtagttttcataatataatctttaaaaatacaaa
ggaaacgaacacagcgctggtatcataattgaaaacattgaagaaattcaaatgaaaattctatattcttatgtttgaaacgaaggtggagctgt
tgtatagctgatgcaaacagaactcgatgaaaacgcccaagatatagacgaaataaaatcgaaaataacggtggatgaacagtaccattcaat
caatataataatacaaagtcatttctttataacagcgctcttacagtttggagcattcatggctacaggatttttgttacccaacttgtttctaggtca
caagtaacaaaaggcgcaagacacctaacgtctatggctgttcgcttcggctcgattctgacttctccctctactactactaccactacagaaaag
ggtgatctctattgagacagtataaaagaaaggggaacggagtttgactaggaatccttttgcattttacttttgagaattgagcccttttcattgac
aaagagtttgctacttttccgtataagcagttagtctttaggaaagttcatttgcgtatattgaaacgtttttgatattgattttaccgattaaaaggtt
aaattgaaagaaagacgcgactttcgattgatataggcggttctatcttttggaaatttctattcacgacatttatatttccgcgatatacaataaac

tttgtatctaatatttatttataaccatttaccttattctttgcttgatttgccgaattcagagttcgcgcaaacgataaaaataccttacaatcactg
tttagtgttgtgtcactcgttatattgatttcattttcctcatctttttctacattctttggtagtattgaggccttctgtattaagcctcattttgttcttttt
gttgctatctgtcgatttttgacgcttttcctattctttttcgattttataccattgcaagtggaaatcgtgaaagaacaattttgatgcttaattagagt
actggaagagctatctttttttctttcctcctcttacaagttttgcggctttcacaaagttcgtgttaaaaatttcccccaagttcaagtttctactagaa
tttggttctttttctaagtcattatatatcctaaaacttcttcttctataagaaactataatgtacgacgacacacccagaggcaatcagcctcctgcg
aatgttaatgatcttggttcaatgtataattcgcgagataccagtgcttcttcgtttacaaacttaaacgattcttctgcccgtctcctgcataatactt
ctaatcctaatttcagcagtggtgcactagctgccggagtaggtggtagaagagctagtggctattctcgcccgagtccccaagcatacaataggt
atcctaatggcgatgcaagtggctctgacatgtattcaacgtctcctcctcaaaatagcagtcttgtagatgatattgaaaacattaaccgcttgga
agcagttgccttcgccggtcctacagccggtgatcatgattttgcattaagcaaagaagacaaagaaattgatgactttttgcactatcctttacca
acaaaggatgctaagaagttaagctatttcgtgggtggggaaggttttatgcaattgctttttcttatcttccttatttcaggtaccggtatgcttttc
attggcttgccaattcttacctataccggacataatgctcttgcaagtacatctgataatggtattaccaaccataaatttagaagcttaagtcttctt
cgccaccgttcccttgtggatcctgatactcctcaagatgcttacaagaaagacactaaaagctctggtactttggacttggtttttttccgatgaatt
caatactcctggtcgcacattttacgaaggtgatgatcagttttgggaagctagtgacttgcattatgccgctactagtgattatgaatattacgatg
ctgacgtaccgacgacagcaaatggtacccttagacttcgaatggatgcattcttctctcacaatcttaatctcaggagtggtatgatcacaagttg
gaacaagatgtgctttaaaggcggaagaatagaggtttccgcttctcttggtggcaaaccaggaactccaggtttatggcctggtatttggacaat
aggtaatttagttcgtcctggttatttggctacttctgacggagtttggccttatgcttataatagttgcgatgctggaatcactcctaatcaatcgga
tcccactggtttatcgttccttggtggacaacgtctacctaattgtgcatgcgaaggccaagaagataaacacccgagtattggaacaggtcgagg
cgcccctgaaatcgattgtttggaaggaacttttggcagtggaatcccttataatggatctattaatatggaaagtatgcccgtcgcttctcaatctc
ttcaaactgcaccttatgatgtgtattactatcccaattatgattatgtggttgtttataaccaaagtgtttcatccgtaaacggctggactggaggt
gtataccagcaagctttgtctgttgccacacaattgaataatacttggttgagcaataatgcgtatcaaacatacggatttgattatgaacctggct
atggaccagagtcttatatttcttggttcgttggtgatgcatatacttggactatgtatcaacctgctattagtcgtaatggaaatgtgggaccacgt
ggtgtaagtgaagaacctttgattatagttttgaatatgggtatttctcccacatgggtttacttctactggaaccaattaactttcccacaaaccca
gtacatcgactatgtacgtatttaccagaatacttcagattcttcaaaacatgagattgggtgcgatcctgaaggatatccaactacagagtatatt
gaacggcatcctaatgcctatcgaaactataacgctactagctgggagatggctggatataaacgacccaagaacaaacttatggatggttgtta
agctttcaaatacttatgattttcttgaattgggtttctaattgattttacctctcttcactctttttttcttttcaattggcattggtattcattttactcagt
tcctctctttttctttctttttatacattttcttttatttgatgcatattatgaagcttatgtaattttattacatcctaaaatttaaaagtgttttgttctttc
tagtgtgtttaattttagttcaatgtaaatgccaagaatataaaaaatcctaaactattaattaacacgtagacaattcccattcataaagtagata
aaagtaaaaaaggaatagatgctcttcacaatctatttcaataaaattatgtacaaaatgattcgtacttttacattgcaagcactccacatcattt
aaatgttcatacccgaagaggactgcataggaaggatcgttggggtggcagtagaagactgaaagggtgaggcattactagaggaatcctttga
atgtaagcgttgctcaattttcttcttggccgcaatgcattctcttttcataaacagtttttctttataagtttcttgagcaaatgaatccaattcctgag
acttaactcgtacatgttgatctaaatagtcaaaatgggtaagtttggaatcaattaacttcagttgcgtatcgatggcttctttcatagtcgatgac
atccgtttttcctcatcagaaaagtaaacttcaggaatatcatagcaggcactgtctatatccatgccttcagttttaatacttgagagagtctgatc
tttagtttctttgtttaaatcattgccctcttcaattggctgtgaaaattttaaggaagaaggagttaccatttttgctaataatgtcacgacagacaa
aatgggatttttatcttgtaaggaccgtaccccaggatatgaagcagaggaggattgaaaaagctccttatctgttgaaggaagattcagaaact
ttaatatacactgctctatggatttggtgccaacgtgactagaaattttggaccagtcatctggaaacatatcgaggccttcaaataaaagaagca
tttcctgtgaactccaatgtgcatcgtcctttttcgaagaagattcgattttcttataattctgaggtgtcgtcggcggaggaaatctttcttggtcata
gcaagttgaacaaagcgaataggagagatcggaatcactagtatagtaagtgccgccaaggatgccaccacaagaataacaatgagtgggag
atgtttcttctttttttttcaacattttgactttctattgcagccggagacggtcgtggagcattgcccttcagggcctcctcgacctcagagtcatagat
atttttgcggagtatagaaggttgtaaattatatggttgggtacgaggaattgaaggaggaggaggaggggcaagcggcgctagccctcttggg
gtatcagccaaaacttgaaaatggcttgtggagggagggccaatcttcgaaggtcgtgtagaaggatccacattataatttataagtccccactgt
tccaagaatcgatgaacacgcaaaacagcgcaaacatcaccagctaaattacgtctgcaggcagtgaatgtcaggtagacttttggatttagtcg
aaaagcgcatatcataaagtcgcgatattctttgtaaacttgcggaattttagaggaatttttattgtcaaaaaactcggggttaccgttcctctcaa
tttcatgtatattattgaaatcaaaccactgggcccattcagggacttctacgggtggtaattgactcaataaaaactctttagcttcatccttcggtt
cattgtccgccaattctattttttcttcatcaatatccatagggacattagaatctgtagagggttgtactggctcagtcattttgtttcacgtttgtctt
aaaagtgttcagaaaaaatgttcaaacacctgtaactaccgccaaatgcacgtctttaaatcctcaaatattaagaatgtttaaaatttagaaaa
aagagacaataatattgcatgaaagagcgctgtgtagtgtttgccaagaattgatggaatatacgaggttgaaagaccgaaagggtgtacagtg
gtaccagcttcattacagtaatttctatgatcgatgaaaattggaaacagaaaagttcaactctttcctaacatgctgaatggaaattttgtttacg
agataatacagcttgtagacatacagccaatgttgttcataatatcacaaaagcattcataaatggaagcaggacagaagcgagagacatgaa

ggctctcctaatgatttaaatgttactttgaataaaataaaatgatatatttcttgcaggagaagaaacactaaacaaaaagcatcaaagaccta
gccggcaaccatgaatgaatttacttgctcagattaaaagagtattagtgtcgagatataataaagaaaacgattgaagctcatgaaaagtaac
acaattacggagaaggattgagagtgctagtcaatgtgctaacaaaaaccggattcaattgggagccagcaagcattcttcaaaatctaacttct
cgtgaaatctagcacatgatagtttttactcaagcacttcttctatagtagggtttctgggactaacttttgaagaagtatttttggaacgaggttcg
gcagcagtagttgtagcagtagagggctctttttccatagagctaacggaagtggaagattttttaggactcttctttcctttcttggacttcgtatct
ggtttagcgttatgccaagcccagactggttgaaaactatcacaaaaaccgacgtctgcgagtagattagggaacaaccagagacccgggcttg
caacaatggcagtgatacaataaagtataaagcgaaaaactaccaaagcaaaaaacaaaacaataacacccaaaccggccatactaagatac
caagctcccctacgcatagacataggccacaaagggaatagaacaagtgctaaaacaagtaaagcaaacaaaactgccaaagcgataactcg
cttttgtaaaggctcatacaaccagacgtaatgcatgtcgtcctggaaatcctgtgttcgattcacttgcagttcgacaatctcctgcctttttttgtttc
ggtggaagcttgtcgacccgaaccatcatagagttcataatcaagagttttaacacatcaatggcgtcttcgcgcgttgaaatagggggaaatcct
tttacctttttttggaggatacgcatctgaggttaaaaatcgcataactcttttgattctgaaatagtaaacacgttttccattgagaatcgcaggtttc
gttttcaactctgggcgagatttcagataatttgtgaattgcatggcaaatgcacgcgtttcttgatcattctgggcaggtattgtaggagtactcat
gctaaatctcctttctatgaggtttcttttggtttttgaagaactggtagatgttgtggatacattcgtatccacatatataggtaacaaagttcgttct
tctagtgtaaatagaacatgttagactctacgctaatccaaaagaaatactgattcgaatagtaaaataaaaagactaatatattattttctacga
aaagaaaccctccttgagacggagaagttttcaatatatgagcagtatatcgtcatattaaaatattttctagtctttagtatgatatacattcaaa
attagatggtccgtatttccaacatttgagctagagtttgtatgatcgagtacatgagttcagaaaaaaagagcattatgggttaaatggcatgaa
atcgttcggatcaaattagacatgtttggctctgaaacatgacgaggacgggaacagtcgttaacaaaaagaaggttgtatcatttctaataagtt
aattcatagaagattttgaaattcataagtcaaatatcagccaaagcagccttaacaaagtaaaaacaaatgggaaggaaaatgacattacaa
accattcagttcttggatacttctttcaagtcgctggatttcttcttgtaagggttttaggctttgcactaaagtatccacttcggaaaactttcgtttta
atttggcttctcgtatcatattttcaaccaggaacacctgttcctcgatcacaatcttcatttgcgtatattcattttgctttttttctgcttcttctttcctt
gctgatgcgtcctgttccatttgcgaagaccaagtttgtccaacttggagtcgaggaattgcttgtaaatgaagagctgtttcttgcagaaagctctt
tgcttcagcacatatagagtgttttatttgttcttctgcttccgtatttactggataagacgaaattttttttaaccgtagattcatacttcaaacacaag
tctgaaaatctttgacgaacgtcttttgtttttttgtagcaactcttgggtaactattgttccactcaataggtctgcaaggttttctttgtataatgttat
gtagttttgaagcgcatggcgaaacacttgtaagtggttaatgtgtaaaatataacccggaatatggtccttgtctttctgaaaggcgagagttttg
aaataattttggttgcaagaattgcaaattaaaagattctggaattggttcgaatggagaatttcaaggacacaaaaccgacaaacaactcttcc
gcataatcgacaatgtcgccgacggcgagttaaagtaaaaacattcccacattcaggacatgtttccacttctgaatcttcttgccaattcacaatt
tcctgttccaatttctttgctttattcttaagtaaagcatcaaaaacggataccttttcagaggaccataaagactcaagtcgcttcatgcgcttatct
aaacgaagaaactcgattcttctcttgtcagcaattgacttacgaaaagcttcaaaggtgtgatagcgggaacgagtttggccatgcacatcattg
tagcctggccggttttcgtaacattgtctgcatatgcgggaccaaaatccaaattccggatcgtagtctgctgaaactgagagcttcgcttggtata
aagtatgaaagttgcagaatacataaccacacttgcgacaatgaatacgaccattcatgaaattaagaattttgtcgcaagtgggatcatggcat
ttcatatcgacaacttctgcttgccagtgttctctcgtaacaacagaatcggtaagctttgtagcttcgataccgattgcaccagtccgatctccattt
tgttcgtaaggttccagttttagtaatcgttgggccgcttttgcgtgcaaggtggtcgctccattgattgttctcgagaaccaggagttgacgaagtc
ttgatgctttggctgtgtttcttcttccgtggaaatatgagttatgtctatgtgatcatttaaagactgtaaattgggcaattctattccgcaaattggg
cattcaacagatttttggatactggtatattcgctgaaactctcgctgcgattcgtacttcttgaattggccgatggaactcgagcgccaattcgtctt
ctgccattttgcattctagcttcttttgcgagttctctctgagtattatcaattatcaataaggataagaatattcttctcttcctttcaacttcctttttct
tttcctcctgtcaatcataaaaaagagcgcttcaatcagtccgttcttctatattatacgttattgaaacctggtgaactgttcatatatgaatcaaa
atacagtttcaataggaaaaatatctaattataaaaattaggacaataaaattaaaaaaggaaacttcgagatatcttgaaagtatatgtacaac
atgagttgttaagaagccttgttcattgctttcttgtattcttgatgcaagccgtcagcaataaagaaatacaagcgaataataatgtaagtaaga
aaaacaaaattgaataaataatagaagtaagtgataaaactgggtctcctttaaaagaacagtgctcttcatgctgcatgtgaacgaattcaacg
tagagttcttgaccaaaagaaaagagcagaagaatacaatcaacaaaggtaatctttatacattttaaagaatcattcttcctttaaaagcatttt
gatgcaatttctgtctatttactcgctccattacactaataaacaaacagaaccaaatcgaaattagcaaagaatgtttcatattatatctgtagaa
agattatatctaataacgaaaagcattcagtaaagttttaaagcggttacgcccgtggaaaacgtcgaatatatccatagaaggagaatcgaatt
cataaatacatatttcagtaatcctctgtgtattccaatatataataaaaaatgtttcatagaaaaggaaagtttgaatgatgataaagacttctct
cttgatatacaggattacaagtttcttataacttgggttttaccacagtagttcgcagaagcaaaaacattgagaaagccggaaaagtggaatat
aattattgaaagaaaagaatataaaagtgatttctaggctaaaaaaaaggaaattagacgattatgagcgaggatttggtatatacattggaac
gacgatttcgacaagaacaatcctcttttctagacccattagaactttgcaaagttgaaaagaatctcgattatgccaggaaaaaactgtgggaa
gaaggcaaatgccaattggaaaaagttggagaaccagatggattatcgagtcgaatcatgaacctgcaaggacgtatcaacgatttgagagtg
gcacttgaagctaagatagagcatgaagaaaagaaaatcaagatggaacaagaaaaggaagctgcatcgacgttggaggaaattcgtaaaag

ggaaacccacgaacgacaacaagagcagcatttacaatatggaaactcgcagctgttggaccatcaacggtccgtccaaacagagatttccga
gtccttacttcatatggcatccgttttaaaagaaaatgcaatctcttttacaaatgctttggtaaatgataatcaagtcgtagaacgggcaggatca
ctgctagacaaaaatgcaaaaagtcttgacagtactcatcaaaatgttcaacacttcacaaaatctaagaagctctctttctggttacaaatagga
atggttctcgccgtgatcgtttcgttcctagttatgatgtttatcttacagttcacgaaaaaccaaaattaatggaaatttacatttactaacaacgg
acccgtatttattaattctctacttgtgaaaagaaataaaattgactttgtttaactttctctatgttaagtttagtttaccagtcaacattacaattcc
cactactgtaaataaaaaaaggtaagccgataggcagcaatagtaggtgcatactatttgaaccttcccgctttctttatttttctttttgcaaata
ctaaccccaattaagagtaaacaaccattaacgaaaattgaatgtaccaaccacttgtaaatctcatcattgcgcaaaaacgctaaaactaacta
ttgatttttctttttatacacccaaaaaatttcattaatcttggctcttggctcttctcttggcaagacgcttaccggtaccaaagaccttcttgcctct
gttcttacgttgcttgcgttgttgacgagcaaccttttcaaggggctgggcttggccaacacgaaccaaacggtagtgaggctcgaatttcttcaag
gcctcgttgctatcatagatcaaagcaaatccagtcgaacgaccgccaccgaattgggtacgaaggccgaaagcagttatattctcggcatcggt
cttatacatttgagccaacttttcgcgaagctcattcttggaaaggttggccttgccggggtgcaaaatgtcaacgacctattcgtcgcagttagtc
atcattgaaaacatagcagaatgtcttatttgcttaccatttgcttgcgttgaagcaagcggttagtcataaacttgcgagttctaatggtcaccgcc
tcactcatattggtcttgctgtaacttcgtagcttgcaagtttcgaaatttactaccagaaagaacagtcacgtccaaagtgttttgacaataccgc
actgcaccagggttctataattggatctgtaacgttgtgagcattacattgatatgtggtaaagttgcataacgtactttagcgtttttaacgactg
ggaatcatgaacgagaaagagagtatcatacttaccaagtaaacacaagagacatagccataggaaagaagtaagtagcaatggaaaggtta
ttgactccttttctcttcttgaagaagaaggagacagaaagaattgactgggtagagccatttacgacatttgtttcaagaatctatggcaatagca
ttgatatagacgatgagatcaagaattttaactctctacgcgataatatgttgggagcagatgatacaaactctggaaagaatgtgatttactcgt
actatggacaattggactatttatcgtttagatttcccactggaggaaatggaattgacattgcattcgaatggaaggatagcttatccaaaggtg
attcttggtataagcagtcgtcattagcctttgagcgtgcttgcgtcttatttaatttagctggtttgctttcaaggttagctgctactcacactactcg
atataccgttcaggattataagcaagcagcaaattacctgcaatgcgcttctggaatctttaagatgattcgcgaatctttttacatgctcctggaa
atgattttgactcgcaatttctgctagggattcatcacctactcctcgcacaggcccaagagtgtgtacttggtcacatgtcgatgtccaagatgag
ctcgtctcttgcagcaaagatagctaacagtgctgcaagtttatacgaatcatctgttcgtgcttttgaatcaatggaaccatctacaaatacctttt
ttgtctctttagcatctgcgaagaatcagactttagaaggaatagcatgttatctgatggctcagagttgtaaacaagaatctaaatatggattggc
tattggcttttataataaagccaaatcaaagcttcaggctggccaaaaatcattttcatggttaaaaccagaccccgattttatacataagcaaag
cactactgaatatacgaattatctatcaacttttataaaggataatctaaaaaactccctgagcagtacggaaaaagataacgattttgtttttcat
gaacctgtcgttcgggaagctgaactcccacaaattcaatctttgcaagcactcccttatcttactttagacagattatatagtggaaaggagtctg
ccaaatcggtagttggagaagacttatttaaagcatttgttcctagtgccattactactgctatttccttgtattctgaagaaactgcaaagctttatc
ggtcagaacaggaagaagtggaacgacaaaatacccaattagcaaccgtgtttgcttcactggatattgaacatctacaagaactcacgcgttc
agattctagctcatctgctttaccgaaggaattggcagatgccagagcaaaattattgaatgaaaatttccaagaagctttccagatgctgaaag
acaaaccaactcagttaaaacgaattttgattcagtcagaatccgaactggacaaagagagtttggagaattcgaaaatgagctcccgttttggt
gcagcttggacgttgtcgcctagtcaagtctttgcgtctccttttcgtcaacagattcattcctttttcaagatctggagaaagctgctgtaatagat
aaagctattctacagcaatataactcgatcaaggaagacattgagacgctttgtgacataaataaaatggatactctttttggatcagcggcggtt
tctttgatggaaagctctaacgttccaaacttgttagacttaccggatgacaatactgagttagaaagagagatgaagatccagctagatttacta
gaggagcttcaagttcgtgtacaaaaacttgttccaagtcgtgaagctactcttaaaacgctccaagaaaaatgcttgcatgatgatatatctgaa
ccgttaatgcaaaacagcaacaaaaatgagagctctatagccattgaacagcttttcgcccaacaactaaaaaagttcgaccctatgcgcaatc
gtttgcatggatcttatcgtcaacaacaacttcttctcggagaaatgagaaatgccgttcaaaaattaaagcagaatccgaatttcattaggaagc
tggaatcttatactcaatcttttaccaaaaataaagatttgtctgttagactcctttcgtcagcaatcactgcaaatgcaattgctgaaggtacctca
aacggattgaattactatcgttctcttgaagaaagaataggagattttgattccaagctgaaaaataggttactaagtagaagaaaggaaggag
ctcaatgtttagctaacatgacttccaataacccttctcatttgtcttccaacatgcgaaatcttcatctttagcagagatgatttcaggtaacaatcc
cttgttacttcttttctttgttcccataatggaattgacaccaatggcaggttttgacaaaaatctgaagtgttttgcatacccagaatgatggttgaa
cttactcagacgcaagctttgcaagaagaaactgctttagtcgttgctcctttgttgcaaaatagaccgaagctttttaacttcaacaagcattacg
attatttaaagaaaatgttagaatcgcttcctagctatttttcagttttggatgcatctaaggcttggatgatttattgggagctaaattccttagctg
tcatgaataagttggatggtgccgttaaaacgagagcaattagttctcttcgtcaattacgaggagtaaatggtggtttttgtggtggaaatggtca
acaagaacatgtgttaagcacttatgcagcagtattaagtatttgcttgtgcggtgatgatgaagcttttgctcttatcgaaaaagagaaactacta
cggtggttttattctttaaaaaattatgatggatctttccgcgtaaaccatgaaggcgagtctgatgctcgttcggcttatgcagttgtttgtgttgca
tctcttttggggcttcctggtgatcatgatttattcaaggatactttagaatggctttgtaaatgtcaaacatatgagggaggattagcaggcgttcc
atacgcagaagcgcatggtgggtacacatattgcgctttagctgcaattgctttgttacaaggagtccataacttggatattccaagacttgtaaag
tggctattgcagagacaagattccgctcttttcggtttttccggaagaaccaacaaacttgtggatggctgttatagctggtgggttggcggaagcc

atcttatattagcttctggatttggtaatagatctgaaaacgtaacgcatggcttattttacaattcagaaaaacttctaaaatacattcttcaatgct
gccaaggggcctctggtggtttacgtgataagcctcccaaacgacctgatcaataccatacttgctactgtttactaggattgtcctctatagcttat
gactacaggatacaagataaccattggttaattgagccttcttgcttatattcgtctttggatttcttgttgccgactcatcctatttattgtatacctttt
gggtttggaaaaaaagatgttatcttactttaaatctatttaactttcgaacgcattttaagctatgtggatgttttaatatttgtctatgattcatgaa
atacaggaatataatgtttcgatctaatgaactattcgttttcataaataaatagatttcattccttgttatcattcatataatcaaataatcataaaa
tactcataagatcgtaatatcttctgttgtagaaaaaaaaaagggggggagaagaattagctatgccatcggctgtgtctaagttgaggaaaagca
ttctttatcccaatatttatcggcatataaagcaagaaggtcagtcatagaatgtctaccgtcttcacgccagaagtaaagttcccaatcttctttgg
tatacaaggcctttccaaccaatttgttaagaatataatttagtctcgggatcacaaactctgtggtcacgagatcaactcgttgaggataatgttc
tatatctagatgaatattcactttggagcacaaatagtttccggaatcataatgtggaactggaagatgaattgtatcatcgccatcatctcccata
tcgtcatgtgtccagtagccataggacacctttttaggttctgattttgtataagttaaagtctcagtaacggtattggaatcaccaggtaaagtag
aagatttatcaaaaatactaagatatccactatgctcaagccagtaaggaaggtccttcacaatgaaccaggggatgcttttcaaaacataaaaa
acatttcctttaaaaggggaaacttgaaataattcatccaaaccaaaggagccttgctgcagaccaccacgaattccgcctgtgtttaaaatgata
tagtgaggcatatggcttcttgacttgttgacaattatctttggaaatatttcgctttcaaaaagattatatatagagttggatgcatttcgaggaac
ttctgaaaatccgtaatctttcggagagcaacccaaaggatcaagtaagtttagtcgccttctggcatccgcaatttggtgtgaaagttctaaccct
tcacttgtatcaaacttggactcgtggactttggagtggaacacaaatccttttctattccagtcaatgtaagaacgggaaaatttggaaggattgg
taaaaatataagaggcttccaatattttcctgggataattgattaatggagaaaataacgaaaaaacagaataagaagagattgggttaactttc
gaagatgtaaaggaaggaacacctcgaatagaagcccaaccagctgtttcgcagtaacgtcctgattgaagcccagtagctttttcatcgtatac
ggcatagtcgcggatatgagaatgacccccaggacttgaattatagtatctggaaattcctttcgaagttttagaagtagtagatctagttcaga
agagtcatgtacaggaaaatgaccagcaattaaaattaaatcgatgtctttcaattcttgtaattcattccaccaaggctgccgaagagaagcctc
cattgaaaggacccgggctttgtcggatccacctgaaaatgaacccacgaaaccaagactaaggatccgaacattgtggggagtttcaaagaca
gcatatttttccccaaaagatgcatacataccagcttcttcatcgtatatatccacattactggccaagtatcgcccattccaaaaaggtataaaat
tatggtatgtatcattcatgacaattggcaagtatagttcatgatttcccgccacaagcagatcataaggtacatttgttaataaatcgtttgtccaa
gagccaggaggatctgtgaggtctgataaacctgtcccatcgtgaagatcaccagtgtcaatgacaagcaggtcgacgtctttttcttgagccaat
tgtttcatacgatggatgaaagaaacaaattcccctaaatctgcagagtatcgtttgtcatgtgcatgtccagcaagccaaccatgcatatctgttg
tatgaagaatgtttacctgtccccatttcaattcccgacgaagtgtttctggtaagacagcagctaggcaattatctattaatatcagtaatgaaac
gagaaactttagaagctggtttctcattttaagtttggttctgaatggtagatacagaatgaaatcagagcaagatacagctgcattaggaatgct
aacgaggcaactagtgattatcgatcgtaattgtcttgtggtatagaaatacatttttattttctttggaatattttcgctaattttcttttacttctgta
aaaatgaaattagcctttagcaatttgtttacacctgagagaaatcgtattcagaaggtggtcatcattgctggaaaggaactaagagaataagc
aactctttacttaggttgaggatgctagtgatttttttacttagaggaagtcgttttataaataacaagttcaaacatttaagacaattggaaacgag
ttttcctatttttatttacttatcatactattctaaataaaatagtatgatacttgttctgggactcattagaaaatactgtactggtaagaaaatgctg
tggaagtgtggaatggttattataactggcagttcgattcttattcgaacattacttcggggaggaagtactttgggtaataagtttgcttgctgaat
atttcatcttaccgaaacagtattcagcaaccttctccatttcagatggagtcttgttagtcgatgtttttagttttctttacaagtctctagtatatcat
cgattgatttttttatctattacagatggaaaaagttgcaaaaaaatcgtattgaatgggacgggactcgaacccgcgagctttcgcactggcgcc
taaagccagctccataaccactcggacacccatccgattatggtagaacaagactgtataatatatatattcagtttccatgtttagattattaaaa
aaccgtgttacgtttattgtttattagaagactaaaatgttttatgagaggtagtgaattttcattgttacaaaaatgaaaaagttgggattctacga
aagaattgattttacgaaggcatgaaacagaatgtattacatttttgtatcttcattccgatgtaatccttaccatggaacctttacgggtttaagta
aaagaaacgaaaaaaaaaaaaaaaaaaaaataaaaaactaaactacataacaagcatatctcatcaaacaattaggaatctggattatttattt
tggcgtaatttttttcatttatttatcgcacaagtcgcttaataatgaaaacactatgacatcaaggcagtaatagaacgcccccgttatgtcccctta
acaaacaaagattgtacgtatttttctgaatagcttgcttaaatgccacacttgaaaagtatttaaaattatcaatactgaaggctgtgcactaaaa
gttttcacataaacttacacaagttggacgcaaacaaaaaatagctcttttaatatcaaacccgctaactatagaaatagttgcctaagcacacttt
atatccaaaacactaaataaataacaataaataaagaatagagttagatcacttgataccatttgcaaagaagtacggaaaaggctgcaaaca
gtaaaagactcccaagaataccaaaaaaccaattaaggccaggaacatcttgacctggaacctgaacgttcataccccaaagacctgttaccag
gttcaaaggaattagaattgtacccaaaatagttaatcgcgacaggacttcgtttgtttcgtttgacacaagtgtcatatttatcgagatttgagcc
aagtagttagaatgacttcgagatagaatttttttcgtaatgattcaagttttgaaccatcgttactatatgatcttgaacatcaccaagataaagag
caatctctcctctaggtgccacttgccaagattcattgcatcgtttggataaaccacgcacaacatcagctttattcgcaagcaatctcagtaagct
cataataagttttctgcattcaccaacacgtttcagcatactctcctgaggcttcacctctaaaacgtgctcatgatgaattcccaaaataatctcat
caatagtatctacctcatcctcaattttctcaataaagggagcaaatgcatctgtagtatcatccaatagggcataggcaatccaatcagcgtttat
agttaaatacccattcagttggcgaattctccgacgtacattagccggatgcggtgttgggttcatatggaaggtaatgattccatcgcgaaaaac

aacaagataaaagttcagcggttttaaatactcaggtccagaaggaagctgattgaaagaccggaatgtgacaaaatagtaagatcgaaaaag
ctccaccttttcacgatcttcttccatagtaatatcttccacagtcaaaggatgaataccaaatgttttagcaaggatgcgtacttcttcttctttggg
ggacgagacatctaaccaccaagttccgccggaacggaaaagatcttcgaacgtttgattatcatgagtaagttcccacatctcatgagaatgga
ttgttgagggtagctcctctgtaaagaacgtaaacctgtaagggcggtcaaactcagactttcgtaattcagcccagcgatcgcgaatgcgatatc
taccattgacaagtaagggttcgctcattttcctattgcgctcctttcgctggtcagtttcttcatccgtaaattcatcaaaagtttccttcatctttctg
gcccactcatccagaaaggagaaattgggatcacggagataatgcgtggaaataggtaagaacaattcgtcacgatcttcgacaccctcttgag
tctcttcggtaacctggttatttgcaaattgttcataatgacttgaagaagatttaggggatttgccaaatgaccgtggagaagagataccaaagt
cacttacgtcgccccttggctagcgggacgagattcctcttttgatggggttgagatggtttcaggatgactgacaggcataggaatgttttcggg
aaagtcgtcatcattatcaggaagctttttgcttgaaatttcggagaggagcaaacagagcttggcattgaaggagggttattaacgtctttggc
aattttttatcatttgtttgatatgaattactttttgatgatcaagcaaacgagattcctccgttaattcatcttctgagctttgcacatcttgcagac
tgggatctaggggatggaaaggacgtgagcgattcgagaaacgtccatgaggattccttgacatactcaaatatctcagagtatcaaatgcggg
attaagatcgtttggcacagatcgagaaacttgatcgtctcccacaacagcattatacggtacacgaggttttctattcgaattaatatccatcaca
cgtgctgaagaatggctaacgttaatgccaggacgattggatggacgtttgggcgatgaggatgggccaggcattgtgggattgtttacaatatc
atctgtaggcattcaccctttatttccagttatacattaagatttttctttttctaaattcctattaccctttaaataataaatcaatcaacaaaacca
agagaaaaacgtagtttctctgatggtatatttggaggaggatagacagtggatgggttacaccaacgtggtcaagaataagtgacaattataca
gaaggaagtttacagttatttcaggatttcgcatacaattcatttcttcctgtttacatttatagcttataaaatttattcaaattcaaaaattttttttt
tcatttctatgtctatgtatccacctgtcgaaacaaacgaaaagtgcaaaatatgcgatgcaaggcagacacggtatctctttttacacataggaa
gtctaggaaatgttcagttttaaaagtttccaatttcttctttttttttgaaataccgtccttgtctaagtttcttctcgagttatcataatggacaacca
aatgcagatatttacaggattgcgatttcatttcagcttagtaacaaaaaagtacaattgaagcctatacttctgatacctaactatatagatgagt
aatttaataaaaaaaatcaaaagcagcaaatagtgaacgatttttatttttttcagcgaaacttctattacctttttctttgtttatttgtttacgtcta
atatgacaaaacgaaatcaccaactatcagtgcactacacaaacgtagcgcagttttataaagtgcgttaatttgctttgtggctcattatttgcgt
catatatacagcgtactatctagccgacactacaccatcctacactcaccttttaccaactcctaccaacagacaaatttccaaagttgtgcaaata
tgagcgattctcgcgaaaattctgtgtaccttgctaagttagctgagcaagctgaacgctatgaaggtaagaagctctaatcatccttgaatggat
aaggtttatcacttgagaattccgcatgacacgctaggatttattgaaaaaatatagtaaacgtgatttaagaattagtttgtgtagtttttttatgg
attgtttgtgttttggcttgtattgaaacgacgcttcgatgggagagccacacagttaaggacgaaggtttgttctttatacttcatcttccgagctct
gaagacaacatcaatatcccatttcatcttgaagtgattttgaaaatggtgggaacagtttatattgaattgtcagctacagaactgtgaagactc
gttttcgagtttttttgaatggaagcagcgagggtttggtgaaattctctacttttccttcgtcacacgtactatttgttccttttaactgttgcccgtttc
agaacgctttttttgtttcgctttatcaagtcatttttttctgcctttttaacttttgggcggctgcgggattctctattgattccatttcctctatgatctcg
tttgaataattttactaactttctgtagaaatggttgagaatatgaaaaaggttgccagcttagatgaaaagctcacagttgaagaacgcaacttg
ctgtctgtcgcttacaaaaacattattggtgctcgtagagcctcttggcgtattatttcatctatagagcaaaaggaggagagcaggggtaactcc
aagcaagtcagcttgattaaggaataccgtaataagattgaagaagagctcgctggaatctgccaggatgttcttcaagtgcttgaaaagcatct
aatccctgctgccggtactggtgaatctaaggttttctattacaaaatgaagggtgattactatcgttaccttgccgaatttgctgttggtgatatttg
caaacaagccgctgatagttctttggatgcatataaggctgcctctgacattgccgttgctgaactccctcccaccgaccctatgcgtttaggtcttg
ctcttaacttttctgttttctactatgaaatccttgactctcctgatagagcatgccatttggccaagcaagccttcgatgaagctatctctgaacttg
atagcctttcggaagaatcttacaaggattccactttgattatgcaacttctccgtgacaatttgactttatggacctcagatgcagaatacaataa
gtcttccaaagaagaaaaccctgctcctgtatctcccgaaaacgagtcctctgaaccaaagcagcatgccgctgactcttcaaatgcttaagatat
gaactgtttctttaatgtcatgagtgcgcaactttttctattaaatgtttaaaaaaagttcaagcgctgttcttactatgcatatatgagttgataccg
atattttttactagtctcgtagacatttgtgtaggtctttattgacatggcaacgtgctatttctttcatttattcctttcttatgtctatcattgtaacag
cagagcctattgctttccaaacatatgggaacatagtctcctcatttgccatatagattaaggataaattatattattattattcactatgttgacccg
catctaaagcccaaataagtacattaattctttcttgtctagactggaaagtgactgtatcttttcacttcccattgtcttctggattaaatcacgcttt
ttttcttgcactgcaacaatcctttcttcaaccgtattctctgtgatcaagcgatatatcgtaaccggtttgcgttgacctattctataaactcgatcaa
tcgcttgatcctctagactgggattgtaaaagggatcttgcaaaatcacatgattagcagctgttagatttaatccaatactaccacattttaaaga
tattaacattacagtaatgttcagatcatgagtaaatttttgaatagaatgatccctttgtatggatgacatacttccatcatatcgaacatggctaa
catgctccgtccgcagcatgtgagaaacgatgtctaaatattgagaaaactgagagtatactagaatttttttcttgagtccccttggcaataacact
tttaatgatctctagtaactttaacattttcgatgaaggagtacattcactcaacctcttcccaaatttcttgagaaagtcttgttcatatcgattacg
ccatacgagaacttgatctatatcatttttttgcttcaaattcctttgctgttgcttcattgtgttttcgaagagagcgaagctgacaaattgatttcaa
gcttccgaaaactgccagaatcgtatcatccgaaattaaatgatcctttcgaatagaccctctacacatgggacacttacaatcaggttgactttgg
tttgtcgggtgttccaaaagcatttccatacattcctgacaggtataatgcccacaaggaataataaacacaggacacaaggtagaatcaagaca

aacactgcaaataaaatcttcaattccattaatccgttcgatcacagaaggttcaaattttttacataactctcgcgagaagtcagaatcacaaac
tcttaaaacagaggcaggcgccttaagttttaaaagccaaggatgacagcataattgtcgcaaccgaagcaaggagactaaaacgtttccatac
gtagaagagcgatccaagacgtagttggctaatagagattgcgcttgattcatttgctccgcataagtactacgttcttgagaagtaaatgatatg
ggaacaacttgaacgtgtttgttaggaagctctataattcgtttatcattaatttgagtatttttttgttcttcggaggacgacggcacccaacagggc
gcgcaagcgttgcatagcagcatgcacaatatcttccctataggatttgaggggacgagaaaaatctttgacaaacatactccaaacgcaataa
ggtttaactctcaagaatttaatcaaactaaataactcatcgacgttattctgtaaaggagtaccggttaagcaccaccgataaatagattctagtt
gaaagcaagcattggcagcctgggtattccgattgcgaattgtgtgtgcttcatctaagataactcgaaaccatggggtatctagaaaaggcgta
gaagcaggaggggcaagtcgaccactacgagagtggtggatgaaaagctctttttgtcgcatttcttgagataaggtgctataagtggtgagaac
aacgtcgaaatgagcaagttgttcactagtgtaacttcttttggaacctccatggtgaatgtagatggataaatgcttggaatgatggactttgagt
gagagttcattcgcccactggcttaaaagagccaatgggacgacaattaatgtggttttacggctcatatcgacagaacggcgagtaaggagca
aggcaatggtttgaacggttttgccaagccccatgtcatcagctaataagccgccatgttttggactttgttccatggactccatccaattgtgtcctt
caacctgatgagtaagaagggatacaagaaggccagctttcgaaaggtgttcttgggcttggttggcatcaagattctcaagattggatgtaaaa
caatcttctaagaggttgtgaagttcatcagagacatgctttgggactgcaactctttcataagaaggagaaagaggcggaggtggaggaggag
atggaatggagtttgactggtgactaagagcctttgcagtaatgaaagggcccaaaggaaattttttagatggaggagatgaaggcggcgactc
tccatcgaggtcgattggatttccttgagaaccagaagaggaagatgctggaagggtagaaagaggaacggtagaagaagacgcagaagggt
tggaaacccgagaagggggggaaaacggaagattcctaaaagaccaaagacgttgttggcgccagcgccggagggaagattcgtgaggaga
acgcctttttagggaagctgaagaagaggagccagctgaacgagaaggagaaacgaaagcagatgaaacagaagagggagaggtaaaacg
aggagggtttggtgagtgcctcagagattgttcatttctgaaaccaactagttagttaaaataacttgtaaaatacctcatacgtttgaattggttg
agaggaggtagaatatgaaatatttgggagagggggagaagaaggtcggttcaattcgtcttgcagagaacgagcaaaggcttcatcagcctc
gagttgatcgggcaaagtggaggaaaaggcagcagggtctaaagcaagcggtagagacgacatctcacaggcgaaaatgaggataacatgct
ttcaattaaaaaataaaccataaaagtttaaaagcataaatgaaatcgagcgtaaacaagcgataatacacaaagtagataaacctctaaaatt
agtcgtaaagaaggtggtgaaagaaagatgtcacgttaaattgcgctatttcgaaaaatttcttaattgtgtaatactttttatgcgatgggggtta
aaattacatctagggaatcaatggcgttcttaatcctcattcgtggctgttactttttttgtttaccttttattgcaggactttgactgaaatcctccaac
agccttctctacgtatatggggagaatttgaacttcttattagaggccaggtccttcaagtcctaacgttacctcgaggaaaagtttgttttagtgac
ctggtcgagaccagagaaaaaggactatgaggggttacgcctcgcgctaccatgaggagtagtatgacctcaccggtcgtgattgactcgtcaa
acccctccttcttcaaaccaatcaaaccctattgatgccacatttccaagtctctgataaccgaagcgacaagcactcgtttactattgctataaga
acatgctttcgtcagaaatttccacaaggatacatttttttctaaattgcatgcttctttattttatattcaaatgataaaaattctcggactcaaaac
ccatacatctcgttttctcatttgtggtcaaccgtaacaaatgacataattccattagctttctttcgcaagaaataagattggctcggtctccacatt
tctagataccccttcggctgacttatatgttaagggaaatctttcttcaacaggaggcacggactcatagtaaacaatgacaacttcccatgggaa
agaggaacaaaaacaaacaaacaaacaactaacacaagataatcagcagctaagaaacaaatataagtaaaatttataaacacttctaaaat
acaatcaaaaacacgtatttcgagtataaactgttgtttgaagaaaaatttaggaaccgaaactttttttcaattttcaattttcattctcctcattctc
ctcttctagtgatgtcatcctttcttctctgagaagagcgtgcacgcatattcgcaagtttcccttacgcgttgtttttctctatttaacgctgtccgaa
gcccgtccttgctctttacctttggttttctactgttttactgttataccctccagcgggtttcttctttctttcatcgttttcatccaatttttttttggttttt
ctttttgatttagctgttcacaatatacccgtattaaagatatcttgatcttggttatttacagctaacttggtttggtgttcatcaatagtagcgacgc
gtcgtagctttattcacgaagttactttttgatacatctcacttcctcttatttttttataatctacttcggtttatttacttgtctttctaattcctgttttatt
tgctattcgtatacttttcttactgtttcttctgtactattttggtgccatttttttcctccgaattccttatttacgaaataactcggactttatatatcgga
ggcgtgttattttctcttgacacgttcattctcattcgctattgttgttgacaactttcggttgctttctgggttgtcttaaatttttgtttgtgttacaattt
gccacgccactccttcttcctctatcgtaacgtatcgtttgttctcgttcaaagtgtcctcttttgcactgtttacatattgtattacgctgtctttactct
tcgttatttcagcaagtatataaatacactcagcacgcaacgtctctccttcttgctttgtcctcccttttacacgttttcccccgtttctcccgcttttta
cttggtttttctttagatacactcagctttggaggtttgtcattccgttgcttccttttgtggctgttgttatttactgacaaactctttgtgtgattctctc
aaagtcgttttaaatcacatatcttctttactctttctcaaactacattatcttgttattggtgaagggtccgtattgtttttttcttttttttgtttctataa
cattttactggtcctcgttcgctacctatttctcacattcgcatagtatttcgtctatcgctaacaaccttcaaatatacacatatatcaatttatatcct
tctttcttttgaagtttcccctactttttgaggttatcttcaatcccagattatttttcggattgtcatttttatcttgtcttattctttctctgtggattttca
gttttcctttgggtaattctttctagtctgctttcatctaggatcttgaggtttttatttgcatctccttttgcatttgttcatatttctttttttttcctttggtt
taaaagataccgtgggttttcctttttttgtattacacttaatttatcgcggtcttttctcattccttctgtgctaaagctttgcatttttgtgtttgttttcct
atagttttcttgtgataatattaaaaactaacaaatctttgacttgtttgctttttcatctttttcgtttgaaaaggtttctgtttagctttttttttccgttt
gacccgttttgcatcaactttttgattgacacggttatttcgctttagtttcataaaaaatttcctttgtgtttttctttttttgcgttacacagtctcgcaa
atattcgtttgagttccggatatcattttatccctcttattgcctttatctatatttgctcttttcctttctctttttgtcgcttgtaaatgtcgattgattgct

tatatcaacgctctttattccatccctctttcctgcctgctaacctgccttcttcctctgaagctgtttcaaaaatgcccgcttcatcaggtcctaccttt
tcagattaccagatagccccacctttgctgaaacgcccttctatggcctctcactactcatccgccacctctcctgtctcgcctatcagctttgaacca
aagaataatgacaccctttggatgggcgatctcgagagctggatggatgctacatacttacagcaactctgggcctctcttggtgaacctgttaac
gtgaaggttatgcgttcaaaaactgcatcttccgacgccttgattagctattgctttgttcaatttgcttcttctgctgccgccgaacacgctctgaag
aaatatagtgatactgtcattcctggcgcgcattccctttttaagctcaattgggctactggtgggggcatccatcacgctaactatgtcaatcgag
atcccgaatatagcgttttcgttggtgacttacttcccactacccaggattccgatctctttttaacttttcattccatctatccttcttgtacgtctgcg
aagataattgttgaccctgttacgggcttatcacggaaatatggctttgttcgcttctccaacgaaaaggagcaacagcatgctcttgtccacatgc
aaggttacctatgtcatggtcgccctttgcgtatctctgttgctgcaccgaagtctcgtgcttctatagccgccgattctgcgttaggtattgtttctaa
tcccacctcgaacagacaacccaatcaggatttgtgcagcgttgatcctttgaacaccactgtttttgtcggtggtctttcgcatgatttgacagaga
aggacttgcaactttgttttcaaacttttggcaaaattttgaacataaaaattccttatggtaaaggatgtggctttgttcaatacaatgataaaact
tccgctgagcatgccattgctgctttacaaggagctttacttggatcatcacacatgcgccttgcttggggacataatacactcccggcctctgcttt
ggagcgatccaacgacggtgatattgaagaaacaggtctttcagctgttggccctaccaatgtcccttctggtaatatggttggaaccgctgcaag
cggtctgaagggtgctccagtctcacctcgaagtgcagttgctgcccagtctttgcttcccaatgcaattgtccattccttgaatggaatggaacctt
tgaatgtaactcctttatcgcctcctcctttgtctcgttctgcttccatttctccggcattgagtggtagtgcttccggtttgacgcctctttcttcaaact
tttcgcaccatggtggtcaacaattaccctcctcagttttgcatggcggtagtttgaacaccgctcccaaagctcaggctaacgtaaagctccctga
atggttacagtcgtatgctggggagaatcaaccttcggctggaggtcaagatcttttggcttctcgcatgtcatctttaaagctaatggacgatgat
gcttttgcttactctgctacttccgttcctggacgatccagctcttggtatcctttcaataaggaacgtcagtcttctatggttgatgtttctggtgaaa
gggaaaagtttgagtctttaatggacaaacctatggttggtgcaggtatgaagaatcgtttagcccgcccctattcctttggtccgaatggttcaca
atacttacaaccctcttatcgcattcctcgtgatgtatgagtttattgaacatttctgttgattcatttttagtttttctttacttctaatggtgtttgtaa
tttccatatttataataatcgctcttttgaaggaatgatcaatttccactgttgctatcctctgcattgttaaattatgatttctgtttttaatttcttttg
atcgattgagggtagtttacttttggctttgttggtttacgatgactattttcctattatattattccctttttgatgtttccctgttttgtcttatatttatt
ttgtcttctgtgctgtttctcgttgtaaagtttcattgtcgatattttatcattacccttgttttaagttattatcattactagcggaatagggttttacctt
acttcgtcattttgtttatttttttctattcatttgacgtttatttcgagcttttctctttcttattgttcatagaataaattacagtgtttatcatcatgag
ttatataatatcagattgtatgtaggtcataagtttctttttttcaagtttcataaatggatgtcaattgagaaataatggctgtctaaaatcaaaagt
aaattacgttcttacattaaaaatgtcgttttgccaagttccaatttatggagagtacagtttcgctttatcataattctaaatttttgtaacgctatac
gtatatagatcaaaataggtcaaaggactagcagactacaaattgaaaatttcaagtgaggatagcgaacagaaattggagagaaatgaggtc
ttccctgaaaaaagcaaagccaaaaatcatccaaacaagtgaaaatgaagaagaagaaacaggaagcgatgcatccgcgagtctttcgaatc
gtacgtacagaaaagtcaataacgccaaaaagtgaacaactttgttaactgattatagaaaaaacaacattgaggaaatctaaaaaaaggccg
ctagtgaaagggaccaagcctgtcaatttacaagatgaggcaagtaaatatttgatgaaatgtttgaactaacatgtaacagcagcttaacgaa
agcattccagataacgataaagaaacaccattacttagagtaaagaagccgaatcctattgtgacaacacaagatttgtaagactaactctttgg
aaactatgaacgagacaaggctaatcagtaaatgcatttaggatatctagaagaagagaggaactgaattcatccagcggatatactgaaaact
ttgtcaatgaactaaaatcacggagtcggcaaactccagttcagtatactgaacaagagcactttaggaatggagaacatttaaacaaacctact
gagagcattagctcacgtatgcttaatcaaggcatctctgaggcttccatgatacaagaattaaaagatcgtaaagaacgacagagaaatattg
aattgatggaagataattatattgctctagacactggtcagcaggtataccttcctggaaaagctactgatggagagcggctactgcaacatgaa
gatgaggtacaagatgaaggttacaatggatacaacgcgtacgtagaagacgaaaagagtctccgagaattacatcggtttagtcccgaaacgt
tacgaaaaagatctattcaaagtgctcttgaggaaaacgaagtattcattgatgaaggtgaggacggagctatggctgatcccatttatgcatgg
gagcaagctcagattcagaaaggagcatttacggatgcttctagattaacaaagctacctcctaagtttccgaatatacttaccattgaagagca
aaaacagcgtttaaaagaagctgttgcatctgaaaaacttcaacaagaagagcgcgcacaaatcatgaaaggcttattagatcaagagaatga
gattcaacaatcagaagaacgaattaaacagtcctttatggagctgaataatcgcatgctcgaaaacctacaatcttcttccacttaattctcctgt
gtgtatattatgaatgaatcgattttgattgtttacgctcgtatataacaagagtaaaatttcgatgtcttgtctctgaataaatgactgttatataca
aaagatgttttgtccgcttatgtttctaatatttttcttatttactgagaattcattgctctcaaaaactgaatattcagcatttgaaaccaagtaacat
gttagctatcattaaaaacgatgaacttggctactaatacaaatcgccaagaactttacaaaaacataacttcaatgggttttaaaagaagttact
cctctttaaactacgatgtttcgagaaaaaaggtcttaaatgtcctagagattcctctgaagaaactcacaattgaagacccatccgatggtatgc
gagatttgaagtctgagaaccattctatattatgtgattgttagaatatatattaacaggtatctgtagatcttgatgttgaaatggttcctcgtaac
cgtatcgcctattacaaaaacaaatacacagttgtcgtagaggatttggatgctgaacttgaggaagaagaggacaataaaaagaaaaatagtt
cttctaatgcagaagatcaatcgagagtaaagctttcggttatatctcctttagagaaaaagttgaaacgagatttattttgccttctctttgagcgt
ccaacagataaaaaggaattgccttgttctggctactcttcaagctgcaaatctgttccttctgtaactttgcccgaatcaaaaaagctttttgaag
aaggaaagttgtccttcaccacgaaccgtacgaaagggtctagaagatcactcgaatcctcatacgatctcgatttgggctgatttggagtacag

cctttatcgtttacgattgctcaaattctttgccgttctgagtgcgataaactgtcgacaagaccgaagtcgaaacttcaagtgcattggtatactat
ggaaggaactacacaatcttattatttattagtttttatttagttctatgtccaaatattgacatttttctacttcatctctggactccttacttaccgtat
tacaatgcttaccctattctgaacttataagactttgccttacgcatgaactcgatttcttcacttcaatttgggatttctggcttatactttttcaatta
tttatgtcttctttaatggtgttttaaaataaaagtttcatcactgattgctctctgttatgggaaattgcaagtagagacatgccttcaaattcatac
gaattaaagttaaataattacagtgatagtaatagaaaactatccccaagtatttcaagatcaagatcttcgactgttagctcgtcagtgaacact
gaatcgacgggtgctcaagttgaatcctccagtcatgcaattgtgaactcttctttgtcaaaaagtgaagactttcaatggaataagttttggataa
gatgggggcgtttacgaagaatttcgctgcaattggaaacctcggaatcgggacagaattcacctttaggaactggtacttgtataacccattctg
gaaatttattagcggtggggacttccagcggatgtgttcttttgaataatctaagaaataatacatcaaatacgcttgtacccgaactttctagttct
gagaggtttttgatgcctgtaacttgtcttgcaatctcacatactcatagagtactttgtcaagggcacggggatgggaccctgtttgtatgggatct
gactcagaatcctcccgtccaacgacatgctattcgtcaaccttttcaggaatctgttttaacgcatttggtgttcaacggactttctgacaatgttct
cttatcggcaagtgcttctggaaaagtttttgttcatgaattttataattttctaataaataagcatcatacagcttgggaatacgactttccagacgt
tcaacctacttttattgatcgtttggtcgtcgatgtttcaagcatttcttttgaagaattatatctaacatcagtgaaacatacttcgtttatctttctcc
aatcgctttcctgtcttcgaatcatttccctttttcccaaattgtcgttagtttataaacttaggtactccgatgttcaggctaattctgcctgccacgct
gtttcttctttcgttgtacctacctctggatcgagatccaggacgcgagctttcttctgccttggctacgattgtcatgtacgcgtatatgcattaacct
tacaccaaggagtgttatcggtagagctactaaacaaaacagattttagtactcccgtatggaaggtttggtatcttcctaataataatgttatcttt
gctctgttgaaggataagagaatactatttatgaacggcattactttaaacatcttgggtgtatcttctatttccgagtatgaacttcttacctgggat
tggtatcagtctgcgttgacgatgcaaggaatttctctaggcgtctttccgtatattagttcgagcttttctttatctcctagatacttatttttttcttacc
cctgaatcggtcactgtaggtacatttatttcactcaatgatcaactacagtttgcatctgaaaaaataaatataatggatacgcttaagttaggac
tctgttatcatgcgaattcaaatctatttagcaaatcttacaaccaaccggatttgagccataagttaatggatttgctgaagaattacattcgaat
attggtacaaagcttcccttcctcacggtccgtagaatcatctagcactgatcctcttgtaccacaagatgaattagaacaattattgcagcgtgct
gaaaatatgaatccaggttgttataatctcgtcctttattgtttgagagctgccaacttcttaaatggtttacagtcattattagactatattctacctt
gcttagctgaaacagaagtgtcttatatttttatggaagcattactggatatggtacttcaacagaactttaaagcaccttctcctgatatacagaa
attattgttattatatctagaagaaaacaggcggctagcttatgctgacaaattaataccggcaattgaacatcgatatttagacctcgatttctca
accagattttctacttataacggattatttaattcactgtgttatgtttcgatttatgcattcaatgattactcaacgcctcttgttaaatttcttaaatt
attagttgcagatctaaatgattcgtctgaatggattgagtattgtgtgaataatggctttcaatatttattaaactctctcactggctttaagtatcct
ctatgtgagcctaacagtgtgaatgaggcgtacactgtcacgcatactcttttaaagataatcttctccgtcaacattttaccattcaaaatatcggt
tcccgaatctgggtcttttccttatcttcgtctcttttttgaaaaagagagcaagctcatttttttgagtgcctagagaatgcctttgactctccttattttt
ctttagacaaggtaaccattgctaaacagcccgttgatactgtcagtcgtcaatggatgatcgagtgtctatcgcaaatatttaatacaaccagttt
tcaatccgacagctattacaccttccttgcgaatacagttgctaaatactccaagcagattttactcccacaatctttttacttaaaaatcatggaag
ggttatgtaatgttgaaagatatcatattgacaaccagggaattcagaaagctgtagaaaacttactttcggtttacgatccaccaaacattgagg
cactgttaaactgcttctgggataaccatttttatcatgctatcatgaccgtgagttttaatcatagcttttatgattcctacttccaagcaatattggc
tctgtgggatcaaaaagattcttcttcgttaattttcaagtcctccgaagctttaattactatgttatttgacaacttgagaaatctaaattccacacc
tgtctgctataataaatacatcaagtgtctaataagttttacaatggacttcttaaatgtcgataaagttacatttttaagactagcctcatctcggtt
aaaaaaatcacttcctaagatgatcgaaaaatgcatgtctgctaaatccgttcaattgcaagtattaggaattttatataatctttctacctatgag
gaattacttaaatggtttggtcgtcgtcgtttgctatcttttttcgaaatagtatgtgctacttctggggactatgctgtccttaaattattgcaagtctc
tccggaatttaaaaacttggaaggattgataaatattttgatatattacaattgcttagaggcaagcatccaaatgtatcgggaactaaaacatta
caatcaagctttatctcaagttctaaaatatgttcggaccagagttttctttgaatcaataaatggcgtgaagaaagttgtacctaaaaacttggaa
cgtgtaacttactatcttcagattgtcgtgcctttataccaagcgtgcctaagttgtcttccgccagaagaagcagctaatctctgggtacaccttgt
tttcagcttgatagatgtttatgcaaatttatctcgtaactgctctcaaagtactcctgacgtcttgatgtcctttgaagattctgtattgggctgcatt
gagggtttgttaaattcttttcttgaactggtacagtcggacaaaaatttattaaacattgtggttttgaagatttgtacaaaatcctctgaaagtcg
cgaatgtaatacttatatgcgagatctgttgctgaaatttatgactgacgtccatgtgagctgcgatatatatcgagagtgcgcttcactttgggata
gaaagcaattttatgatacaaaaagttctttaagcgaaagtacagtgggagtactaatagatgagccggataaaagcaaagaaattgctatgaa
aaaagaaaggataaaagttttactctcagggcaagttcttccaactagtaacgagtaagcatgcagcaatgatcgggacttttgatacaatacct
gataatgtatcaataatgaatacatgaccttgcttctttttctttttttatggtttttttactagtagaataatattttcctgtagtgttcaacatgtgactt
atattaaatgcaaaatgtagggttcgttatttatgagcaattaaataaggataatatgaatggagggtagcagctttctttctttttttatttactttac
tttattttatttttttagccgcagtaaaatatccccaagtcaaaaacaaaacattaaaaagtgaaaataagttaaaagagaacaactactcaactct
cggtaaagttacttagaagtagaggcctctttagcagcagagatcagacgtgtgagcatttcacttgaagctccttgaccagccaatcgaccaaa
tacaacacaagcaagcaaactgcttcctcccaatcgattctctccatggaggccaccaacgatttctcccgctgcgaataatccttcaattgggtg

accttctttagataagactctagcttgcgaatctgcttgcacacctcccatagtgtaatgcagaacaggctcaacctgaccaacaaaaaacttgtc
cgaaagctcgaagggttgtttaccaaactgttctcttccgaataagtcttttatttcgcctcgagaggctttgttaatcgtagaaaatgtattttcgag
atccgtggtcgtgcagttgaggattttggagagttcttcggcatctacttctcgcataagtcccttaaaactgtaaaacttgacaaagttggcaacc
tcaggaacgcacttagcaggtaaaactagttgcacaggagaattcagtttcatcatttcagctgtgacatagtcacgatagccaagttcgttgcaa
aagcgtcttcccttatctgtcagtaaaactgcgccagagccacggagagcctcggcagctaaaaacttagtcatggaagaaggatctttgggatc
aacccaaccagtaggatgaatttgaatgaggtctaggtctacggtgtttccaccgattgagctaaccatcttatgaccatcaccggtgcaataagg
cccgtttgtggtacttagtttgagggcctcagggtgatgcaacttcaaaagggagtcgtcggagaaatcagcagcatatcctcctgtagcaagga
ccacgggtccatagacggaacggtgggttttatcattcaaatcctcatactctacaccaattacggatgagttggaggagtcggttagaaggcga
gtaacacgagtatttttctttatttgcaggcgatcgggatgagcagcggagtatttttctgttttgtctacaagaacaaatgctagaggtttgaaag
gataatccgggtgacttccacgatgagtgcgaggttggctatggccagccaaaagagaaagttcattcatctgaagaccgaagcgttctgatagc
caattaacagcgctattggatttgcttgtcaatgtgtgaagcaattttgggttggctttcgacttggccgaaagaatggagtcatggtaaaatgtttt
tacgctgtcggacacgttttgatcaaattgaagttgagtagaagcagcattgatcccactagcagcttttatgctatttcctccaaatgcagctattt
ttcaaagttagtacactgttatatataagtaagcaagaaacagccaattcattaacagagaaaaagagcaacccaacccaacgtaaggactata
tacgtgcatgcttgcaaggatgtatatatttacgtcaaaatgtattccatgaaaactgataaaaagcaccatgtcatgtcatgccatgccatgccat
ttcgtagccgacttcgacttcgactttcctgaaagatgatgggcacgcttattgctcaatcctctgcaaaacatacaagaccgttccatattcaata
catacaattcttatctaagagcaaaacatttcccctaattccaaaatcgtatttgtagctgaaagaccagctaaaccaccgccaataacaattgc
ttgtgtgttattgcttactgttgacatacttgctttgttcttgaaggctgtttttgcaaatagtaggccaaggaatgcaaacagggtcgttgttctctta
aataacatagaagaaaagttcaaatcggatgaatgacatagaaaatttcaagaagaaaagtctgatgattctttttctcttaatatgatcttacga
taaaattgcaatgtgaagtataccgaacctcaaggagacgaagccaagaaaatatgaagaaaatataaggaaaagaaaaagaaaaaaaaaa
aaaaaggtcattttgtgatttttcgtacttggattgctacatgttgttcttttcgtgaagctttctatcccaagtacagttttcaaacagctgggcaaa
gggcagaatggacggtatgcaatcctcgaaagctttcagctgaagctagaaaaggttctcaacggttccaaagtgggttctcattcctctttcattt
agttgacataggccgtttcaagataaattcaatcgcacaagaaacatttaccatgaagcctttcttttccttctcttttttttgttaagcaacaaaga
aggacttctgttacgaagttacttgatcttttttttcgatactattttccataaattcacagctacgtaaaaaaggccgcttaactcagttggtagagtg
tgagattccaaatcttgaagtcaagtgttcaagtcacttagtggtcatctttttttctcattgttgaaaagagccttggtaaatttgttttatcgtttttac
tttttaaggtctttgaatttttttgtttttttgtttttttatttttttgttttatgcatcaaaatttctcctgctatcaagaggcttcaagtttacgttccttcctgct
gcccattacagccttgtacctttactgacaaagaagaggattgtttttgaacctacagcaggaaatacgaaaggaactgaaactttatagacaaa
caggaggattttttttttttttttttttttcaatatgccagaatcaaggaattcagagtttgacccaaatacaaagaataagaatgcagatgaaatgta
taatgagctcaacaaaattcccttcttcatgcagtctttagatgatgttgaagaagagggacaggggaatgctcaactggatgccttaaaggctct
ggcttacgaaggtccgccgcacgagattgcgcagaatttccgtgaacatggaaacgagtgttttgctacaaagcaatatcgtgaagccgccgagt
attatacgaaagctttagctgaaaaatgcggaaaaccagaaattgaaattccctgcttttcgaatcgtgcggcgtgtaatttaatttttgaaaatta
tcgacaagtgctgaacgattgtacccaagtccttcagcgtgacccacaacacaccaaagctctgttccgttctgcaaaagctcttgttgctttgaaa
cgatatgacgaagccgagctttgcctgcggttgttttctacttcgcaacaaggagatcctgctgttcaagctctgggcaaagatttaaagcgaaag
cgagaaacatttgaaaagtttgaatccgaaaagagaaggattgcagaagaaaaagtaacagcagcaagagtcgttatcgaggccatgaaaga
acgtcgcattcacaccaaaacttccgaatatcctcctgatttgggagatgccagaatcactatgagcacattcaacgacccgaaaagcgaaatgt
atttgcctactctattattatatcctttgaccttgcagtctgattttcttccctctgtctctgaaaataccactcctcaagagttattagaaacggtattt
tcagaacagccctcctgggattctgaaaacttgtaccgacctgattctttagacgtctttgccgcaacttctactggtggtctcgttaaggtcggaaa
acgcgttcccatattacaggcacttattcatccaaagactgtattgacggatggtcttgttcaatttcatgttgttcctaattctcagacttctgcgttt
attacttcctggaaacaaaataagagctcttgaggttaaggcttaaaactcaattgtggacttggtctgctgtcgttctttttggtgttctcgaaaac
tagcattagttgaaaaatggatttgggttattttggattttgaaattctattttatgtacaattaaagctctatttcaattcggtatttttcataaaatg
acctagaacaaattcaaaccataccagtaaagccataagcattcaaaaatctttattcatataattcattccaaataatactttattattttaaaca
accaaaagcttttctgtctaaatacaaaagcctatttgatttttaccaacttcctcgtcttaacctactacacctgactagtatacgtcgacttgatcc
tacataatttgctaatttttcttgattcataatcattagtcgaacttaaattaattttcctttatttgaattttttttctatttttcggctttgtcttgcctcttc
gtgttgtctttcgattgaacctataaatcgaaaaggctttaggaatttcatctaagatggatgctctcggtttcaagtatgtattctctatgcacaaa
agtcactgattcattcattagtacaaacggtggatatattgaagccttaattcgaggattcaagtctgctttacttacagaacatatctatgggaatt
tatcacaatgcgaatcattagacgatttacgtttgcagctttcctcgacggagtacggtagctttcttgctaaccagcaaaagttaactagcagtac
gatagctgccaaagctacagagaaattaatcgacgagtttgatatgattcgacgtcaggccgatgagaaattatcaaaatttatggattttataac
ttatgcatatatgatagacaatgtaatgcttctagtgactggtaccgcgaatggccaagacacccatgatcttttagaacgctgccacccgttagg
gtggtttgagactcttcccgccttgtgcgtagctacaaatgtggaggatttatacagtgttgttttggtagaaagcccacttgcaccttactttaagg

attgcctctctgcagatgatttggatgagaagcacatagaaatcattcgaaacacgttatataaagcttatttagaagatttctatgaatattgcca
agaaataggatccggaacttttgagacaatgtctcgtatcttagaattcgaagctgatcgacgtgcaattacggtaagttccatacccataaaacc
agagcactgctatctaacatactttaagattactattaactcgtttggaactgatttgagtaaggaggaacgaaacaatatgtatccttccttcgga
cgcttgtttccatttggtacttcgatgttgtctaaagcagataatgttggcgatgtagagaacgcttgcagcttagtgaaagagtacagtgctttctt
tgaccaagcatcacagaaatctctcgatgattactttttcgaaatggaggtcgagtataacaagcttgcatttttgcaacaggtaagaagttttcaa
tcaaaatttagcgccgcatacttactttgaaattttagttccactatggcatcgtctacagctttttaaagttaagggagcaagagattcgggtatgt
gattcttcatgcaagcacaagtagacatgttttactaatgcaagcagaatataacgtggattgctgaatgcataagtcaaaatcaaagagatcgg
gctttaaatatcattccaacaatttaatttttgctaaataataagacgaaaccagatgaacgaaacatgctcaggctatataaatatctccttttctt
tcatgcatattcctagaacaattgaaagagtttacacttgtgtagcttcaaaacttcgatcagatactactccttatgcacattcggattcttataatt
atgaagttgacaaaaactaaaacgaactgattgcaatggacttcatttataatatgatccaaagtgaactgttgtaccctagtatagtactgctca
attcttgttaatttgttaagaattcgcttcaatttttgaatgatttttcctttcatctgtaataaaaaatagaagaaattctcacgattagcaaatcatt
cgacttgaataagattgttggtttacgaaaactcgagatccaagaagttcgaaaatgatacaattatcttatttttttctatttctttttctttttccag
tttattcatctatgcaaaagtgtttctaatttgaaaacaaagacgattgccatccacaggctgttacagcactcaatttctaccatcacccttaaaca
gcaagaagtagcgaacattggtttggacgacagtcttttgagaaaaagcaattgtaagaaaagaaattattctttctccaaaaacttacaattgg
gattatccattttgaaacaaaagtaaataaacgttggtgacttgagaccacatacgacgatgagttcatctgatgagggattgttccctcgtggac
ccctatatttagaaaatttagtcactcctttttgcgaaaaacttggactttataaattacccagacatttccatgtaatccttctaagtgcgttgtttta
tcaggctataaatcttctttctccatggatttccaataagttgagtcgccattataaacacttgtccaaacgtactaaactaaactgggattctcacg
ttgttagctctgtacaaagtgtgtttctcatttcgttgggctatcttatcctgaaggaagtcaataactttggtgacaagctctttgggtattctgttta
tgctggtgatatttacgccttggctgatggctactttctctgggatttgtatgttactcttcgttatgttcatatcactggcataggctttgtcgttcatg
cagttgcagctttgttcgttgtgacattcagttatgtaagtaacttaattactaattgtaagttttgttaacagttaacagtccccttacctcatgtact
atggaccaacttatttgtcatgggaattaagtaccccccttcttaaacattcattactttctagacaagaccaacaggacgggatccactctccagtt
ggtcaacggtattttgctcgttcttacctttatttgcgttcgtattatttggggttggtactctgcttatagcactgcaagtatgttattttaaacttccat
acagtcaggatactaacataaatatagtggaaataattagccgtgttactgaaattcctcgtggcttaagtttgttttacttagctgctaagtaagtt
ttctactctatttttagtatactaactcaatcagcatgtctttaaattgcttaaatcttttctgggttttcaagatgatggacgctattcgccgtcgtgct
caaggtgaaaaagtaagccctgttcaaactgccaactctgaatttgcaaagaagaacatttaaagtactggacgtactttttttcgtgtgggtgagt
ataccttttcagctttcatttatttccttcttttcaagcgaacattatatttattttcattgtctctatatggaaattttcaaattctttatgttgttcgctt
gcaattctgcatatatataactaaaaataaacgtacaactagagtttctaaatgaaagactttgcttgttttttttatattgttaaagactttgcattat
acttaataagctatcttatatgaacactgtttttttatatccagtcctatttagtttcctataacggtacgcatgtattagatatattgtaagattactaa
acttcattcaacgttcagatcaataaaaaccttaaaattttacgatgttacctgaatcatgacaagtgcaaatccgaaaaataaagcagatacaa
tgattaatgagttagaattccttgttaatttactggaatgcagatatacatatatatacaacgagactgtctagcaaaatatcctgataggatattgt
gctggtcgagctaagcaacttgtttatcgatacatcttagtcaatcaggcatgttgaaatttattgagtacctataacacctactgcatctcaaataa
ttagtgtagatacccgtacttaattcaatgcgaatgttcaagattcatcatttatcattattcccatgtcaatttcactttcgtctgacatggtgatatc
accattataaagggtattaatttacttaaaagaaataaaagagacaaaggaaataataaggtaagtcataagaaaagattaaacgtaccacgg
cttttactcagtccgaaattggcaaatatttaatggcttttgttcccgtcagggcactgcgagcacgaattttgtctccgaagaaatagaaagagta
gattataaagggaatagtgatgctgataaatgccatagtagcaacggcccatcttgtagtcattctatcgaacattatgatactaatgtgagcgaa
aacggcagcaccaacgaaagaagggagagtaaaagcagctacagaggatgctgagattataggatagcaatcagttaaataatttagaatac
aaagccaattatgtgaattggctacactagacatggcaagacccacaatgggaaccatccaatgaaacggaggagagcaggtaaaagcaaac
aaaaacatacctgctggaaaaagtaatgtaaaaaataacgaactagtaaagcgaacctctggaaccgcgactccatgattgttttttttgcttctt
cgaaaaatccagtcttgaataggctgatagcaaaacaaaacggaataggctactaaaccagcaagatatgtacaactagcacccatgtaggac
attctgtaagtcccattaaacacaggccagatcgccgccaaaaagaagtaactgataccgtaagcataaaagttataaaggcccatagtaattat
gataggttctgtgaaaatcatgcgaaatcccatagtactgatgaactttatattctgaaatacctgtttagttgtaaccttaggaagagtttcaatg
gttcgcccttttgctatttcatacctggatatgacctttataggtagagtttcgggacaaagaaaaattacgaataaaaagaatccaccgacgatg
atattgatataataaacccagcgccaaccaaatttgggattcgaatcaactcctgtagccatgggagcaccaatactaccacctgcacaggccca
aacaaacaaagacataggagttccagacttatcatcgggaaagatgtctggaattcctccagcaacatttgtcaaagctgtacttccggcgagac
ctatgatgaagtgggaaataaccatttgaacaatatttctgggtaatgcacaagatatgttaaatatgacatagagtaatattgagaaaaaatag
acaggctttctaccattgatatcggacaaagggcccagaaacataggacccaatccactgcccaggatgttcatgctaagatttaaagtggaaac
ctgtggggaaacgtgaaagtggtctcctacagattcagaaatgcttccataggcattactcgtccaaatgacatatatttgtagagcgcaatatac
aatcataatcaaccatcgcttccaccaaggccaattttgagggtgagcaggatgatccggggatataagatcaacaatgctactaatgacatgct

cctcttgctccttcttttcgttgatactagatctatgatcgatattggaggaagagtgtgaacccaattcaagatccgcaacttctttagacgttttag
aacttgtcttgtttaatgaagaaaacatattgagaagattcatctttggcgatttgaaaacactttcaacataccatccaaacagaaggtactttcg
taaactcaaaaaagaaataaacaagaaaagaaagaaagtctaatagttgagctaatttatttgcaatcagaattacaattcaaataagggtcca
ggatgattcacgtgtttaccaaaaaaagcgcaaagaacttcaaagaatattaagagaaatatatagacctatatttcgttttcttaaaaggcgcc
atttccttcacatataaataaatcttgagtaattatactacgcagactaccggtgcagcagagtgccgatgggaaccatcatccatataccttcca
agttgacttgtctaacacccgtgaaaagaactgttcacgataaaactttccatatagagatccttgaagtagaaaaaacttgcaaggcattggga
aggtatagttgctaaggaaattttcttagtgcatgtatccgtgaaagcatacagacaagacgtaaaatagctagacagaaattaaattagacata
ataaaaatgaataatataattcctaaaaaattagcatctatctgccgacgatcgaatttatctttaaaagtaatgggtaaagcaccctaaattatt
attgtccttgaggcataaagcaaagattcacataacgtttccgagcttaattgtttccaagtgcaacgtattgtagaatgctttcttataaggaaga
aaaatgatccataaggatgatatccctttgcccgagtttaccaaattatacgaattggatcgctgaggaataaattaagtaatttccaaaattaat
ggacaacaataaacaggttcccaatgagcattacgtttccaagaggccaaggaccatatgagaatgaaacgtaatgaactaaacactgaacta
aaaagaatgatcttttttggattgaaagaaaagacgcagaatcatttttacgctcaaatcatcttcaaggttgaattcgacgaaaatattgctgtt
atttgagcagtactgtaaacgtcctccatctttcagttcaaccccatttgaaagagaaggtagtcgcaaagggagatgaaaagagctttaaatac
attccttcttgttcaaatcatgtttctgagacaacgtttttgggttacttacctgtctacaacacctaagaggaagaaatataattataaaaaaggc
accttacatatatatatatatatgtatatattcattaaaaattccatttctttgctttttgagtcttaaaagccagcaaatacttgcacagcttcgttt
catcattatatgtattgggaatgtccgacctttaaagcaccaccacagtaaataaaactatgtaaggaaaaatggaggaaaaacgtacgaaaat
ctgcttactagaaaatagacacttatttttggaatttaaaaaatcattttgtgtctttctagtacatactcattgaaaacctttcacgctaacatgtttt
ccagattcgtccattttctccaccaatattccttgcaggaatgaaatgattcatggaaggtgtccttacgttgataaaggatgcatctttcaacaca
aatcacaggtatggcgttcgttcactgatagtcaagttgcaagagccatgagcctagtgtttgcttgtctgtttggatgttgaagattttatagtttgc
taattagcttattctagaacaattttccaagtggggattggaagtctttgaataaacctgaacaagcacctctttcctcagttgcttcttccagtcga
ctcagcgtcaattccccatcttttactccgtctttggtcatcaaacctacagatgttccttcaatatccagtagtcaaagagaatttactccattgacg
aaagcaaaatgtatgtttacctaaatacccttctgcaaagcttacagttttcagcttattcttctgctttgtcgtccggaaaaaacggagtagctaca
aataggatgaatagtcctcctaaaactatttcattgacgccttcaagctctagagctgctgtaggttcccccgagaaatcatttttagctcgtaacg
ctgcaccttttttcaccgaaggccacctctttgtctcttaaaaatcgttccgctatgtcatcgtactcttctgtgaatgcaccttcgttctcgtcggctaa
aggggctgtccctgcaatttcctctccatctcctggtttagtatcctttttcttcttcagtgcagcccagaaattatttgagttcatctattagtgggtca
gaaccggttcaaggcgttgaaatggatcgtcatatcgtagctagatttccaccttttcttcataccatggatgattcacagaagaaaaaactccag
actttccttgcagatgatttgcaaggttggcttttgcatctttccagagaatattttcatttttccagtccgtcgagattgccagcccatattttctctta
tcacagcttgacccctcggcgcaccataactacttcgcttcctgtattgggttattcagtcgctacttacaaagtaattgatggtgataatgggaaa
ccttatttatttgttcaattacaaaagtttcatttattacgtgataaaaatatcacgaacgtagtaccatggacgaaaatacattctcctcatatcat
ggatataagagaggcatttacaactcatgcttttaggcaaccatgtaagtttttttcatgtcctcctacatattgttaacctttactagcaattgtgttc
gtatacgaatactctccttcatgtccctcgctttatgacttgtttttctcttctccagtatttcgtaaaaaagctgcatctttttatttctcgaagcctctg
ctaaatactcaaaaagtgctttggataattgctagtcaattggttagcgctttacacaccattcactctgccggtatggcagccaaaatgattacag
ctaaaaacgttttaatggtgggaaaactacgagttgcattatttggtctagggatttatgatgtgattttggattattcaaacaattccttgggagag
ttacagttggaagactgtcgaaatcttggccttttgcttttgtctttggcttgtggcatagaaaatatatctattgcgacagcaagagatcatatcgc
aaatcttgactttcggggtgattttcagtcctcctttgttgaactgattaagagcttaatattggatgacgggattcgaattgaccatgtattaccatc
tgttataccttatatcattgaaaattatgagaatagttctctgtatgaaatcaacctttgctttactttcaattcaaagcgctaacacaatttagaatg
caagatgtttatcactctcgcttagctgagcaggtagaaaacgatcgattgttaagaatacttttaaaattcgaattccttgatgatcggcccgagt
ttcttgaagatccagattggtcaccatgtggacgtttttggattattcgcttgtttaggaaatacatgtttcgtatcaagcaatataccaaaggcgat
gagcatccaataagatctcaattacttgatacatccaaattaacggaaaggaatattgatttgtctcaccttgtacaatgtttaaacaaggtaagt
attatacttcatagccgatcttacctaggatggagtaactaacgaactagctggatgccggaacggaggaacggatacttttagaagactcttgc
aaccgcataatagtcagttataaagatgtatgtaatgatttgtctgtatctctacagtgttattgaccagaaaaataggtgaaaacagcgattaat
actgcgtttatggaactggaatatcgtgtctctaataatgttagtataaaaaaataaaaatttttttatggaaatcattcattcaaaagagctttcaa
acttttgcaagttaactaaccagctactaagataattgtatataaaatagtgaggtgtccaacttgttatttttaagaattttgtgttagacatatga
atttaagaagcctttttacttaatcaaaataaaaataaatgacgaactactttatatcaaaattttatggcttgtaaatgaataaagtccttggctac
ccctaattaactagattgttccaagaaataaacaacacaagaagtaaaagtacctattattttgttttcttattttcgaaagtgtggttttagtggaa
cattgtcgaggacgtagcggtatagtaaacacttctcctctaccatccacattagtaggttttcacaaaatacctttttcttgtagaaagaaaggagt
ttaattctgttattcagaaatattttcaagacagaataaatcagattgttttacggttgcgaaccataaaaagggactggaaagttgaaggttata
atttggaaagcgtaaactccatactttagagaaataaggccttttgaggatttggaaaatgaatgcgggagagtttttggctcaaacactctcgcc

agatgcaaatctgcgtctgaacgcagaaaagcagctggaaaatgctgcccgtactgactttgctcagtacatgcttttgctcactcaggaactagc
caatgaaagctctgcttcatacattcgtatggctgctggtttggcactgaagaattcgataactgcacgggagactgctcgtaaagtagaatatga
acaattatggcaatcactacccattgaagtgaaacaccaagtaaaaattgctgctttacaaacattggcctcaccagagcatcaggctggtcaat
ctgctgctcagctaattgcagcgatcgccgcttatgaattagcactcaaccaatggcctgatttaatggtcactttggttgccaacgttggagatgg
tcaaccatcttctctcaaacaacactctttacaaactattggctacatttgcgaagctgttcctcctgaggttttgtcaattcagtcgaacgctatttt
aactgcagtggttactggtgctcgcaaggaagagcctgacgttgtggttcgtctagctgccttaggtgctttatatgattcccttgaatttgttcgtga
taacttcaacaatgaagctgaacgtaactatatcatgcaagttgtttgtgaggccacgcaaagtccggaagcccctgttcaaacggctgcttttgg
atgtttggttaaaattatgcacctttactacgatttgatgcccttttacatggaaaaggcgctttttgcccttacaactcaagggatgtataatcctaa
tgaacaggtttctttgcaagctgttgagttttggagcaccgtttgtgaagaagaaatcgaagttaatcttgaagttcaagaggcgcaagaactcaa
tgaagagccttctagagaaaaccatgaatttgctagagcagcagctgctgatattttacctgttttacttgagcttctttgtaagcaggatgaggat
gctgacgaagacgactggaatatttctatggcggctgctacttgtttgcaactttttgctcaggtcgtcggtgatcttcttgtgagtcctgtattggttt
tcgtcgaacaaaatattcagtctcctgattggcaccaacgagaagcagctgttatggcttttggttctgtccttgaaggccctaacgttgttatgctt
actcctttggttaatcaggccttgcctgttctaattcacatgatgaaagatcccgtaatttttgtaaaagatacaacagcctgggctcttggacaaat
ttcgaattttgttgccgaagctattaaccctgaagtccatcttccagaacttgtttccgctcttttgactggtcttactgacaatcctcgtattgtcgct
aattgttgctgggcttttatgaaccttgtatgccattttgctcctgaggatagtcatcacttgtctttaatgactccttattatgaagctattattgcag
ctttgcttcacgttaccgatcaaaaaggaaacgaaagtaattctcgaacttccggttacgaaactttaagcactttcattatttttagtgctgatact
gttttgccaatcatagccaatgttttgaccgtaatcatgtcacgactggaaatatccatacaaatgcaaaatcaaatcttggacattgaagaacgc
gctaatcacgatgagcttcagtctaatctttgtaacgttcttactagcataactcgtcgctttggacctgacattagatcaacgtctgaccaaattat
gactttattgctacaaactatgcaaaccgcacctaagcaatcagtagtgcatgaggatgtactattggccgttggtgctatgatgaattctttagaa
gagcaatttgaagtatacgtttcttctttcgctccctatctatccgttgctttgagtaatgagcaagaatatcagttgtgctcagttgctgttggtttgg
ttggcgacttggctcgtgctcttaacgcaaaaatcctaccgtattgtaatgaatttatgactatcttggttcaggatctacaaagctctgtattagac
cgtaatgttaagcctgctattctttcgtgttttagtgacattgcgctagcaattggtgctgcttttgaaacctatttggaagccgtaatggtcctttac
agcaagcgtcgtccgttcaagcccccctggagctagtttcagcatgatcgattatgtagatgctttacgtttgggaattgtagaggcatttgttggt
ataactcaagctgttcgtactgataatcgtttagacttaattcagccttatatacatagcatgtttgccttgctgcatatgatcaccaaggatcgtga
gcgcacagaatccttaactcgtgcagcattgggcttattgggagacttggcagagtcattttccggtggtcagttgaaggaatattttgcagcaga
ctggataacggaattgttaaattctggaaaaacaaaattctgcactcaacagacaaaggatattgctcgttgggctacagagcaagtcaaacgtc
aagctagattttagttatggaaaatgaatgcaactgttattaggtttagagggaaaaataaagaaacatggacacacaaagtgggagtaagag
aaatgaatgttgaaaagtgtaccataactttacatgaaacgaatcagttttcattgcaatcttcaatataccaagcttaatcttataatccatctata
aaagaattcgcttaagaaaacaaaagcagatgctgattactaatcgatgttgttgatttgatagaatgcatctattgatgtattttatacagaccta
taattatcattttgcttttgaaagtaataaactgttgtttgaattgaccttgtagtcacccagtcaccattcgcttgacttgccaaaagaccaattttg
acgtacttctttaacgatgttccgtttgcaaaaacattactaatcacagcggatatttgtaatagaaaagtaactgaagtcaaatatttccattaag
ttaattttgagagaagattacctgtcgacgacggattaattttttttttgagttccatgtactgtattacgttgatttttcgaaatttattcaatttacctt
aagttacaaaagatttgtaaattatctttcaattgctatatcccttttcgatctttcgattttccttgtttaaacgttgaattgtttccatataaaagta
tacccgggcaagggtcacgaaggatcatacttttactttttactatttgaatattttatttaagccaatcgattaaaatggaccccaataatcgacat
tcaatatccgacttacccaatgctgctgttcagaaaaaccctgatagctcaggcacttttcccggttttgaaataccagataaccgtacatcaatgt
caaaaagcttcagcaatttgtctatgacaagtcatcacggtagtcatgatttatctatggagtatacgagaagtccttctacagtccgttcttcaaa
gatacgctgggcgcgaagaaaccctaggtttgacccagataagaggaagcgtcagtatgatcgtgtgaagcactctgcttccaataactgcaaa
cttcatctttcattgcagaaccacgcagctgctcaactacgatccgactctttattattgcaagcgaatgacaaaagagatttgacgagcctggtta
caagagttaaagagctagcagcaaaactcagtgctgccagattaacttttcgttttcgtcgagttttactcatttcaaacgatgattcgaattctgta
aaagcagctgttcaactggcacaatggcttttggacaattttgtagaatcatcagctcaacaagattttgattccgaatctaagggatcacaagct
caaatgtcatcatcagattccaatgatcgttcagaaagtcctccgtttttggagatggactgcagttccgcaaaaaacattaaaagccaattttga
tttatttagaagacaagttatactctcttccagaaggtgaaaatcttgtccctaaggaaaatgtccgattttggtctgctgaactttgtactcaacaa
cccaatgtttttgactgtgtgattactataggtgatgacacaacggctttacgtgccagttggctatttcaagatgttgtccctcctgttatttcatata
gcgttgcaaatccaggctttcttagtgttctccctataaatgattatcagcagactttagatgtgatttttcggaaaggatttactgtcaaccttagga
tgaggtttcagtgctcaataatgcgttatgttggcgaaaagaagacacacatatgcgaaggtcagttctcggtcctaaatgagcttcttatcgata
gaggtccgaatccttttatgattatgctagatttatacgttgataatgagtatataacatgccttcagtcggatggcgtgtgcgtgtcgactcccact
ggttcaactgcatactctgtagcagcaggaggctccctatgccatccaggaatcccagccattttaattagtgcaatttgtccacatagtctttcgtt
tcgccctattattttaccggacagtatgaccttgagaattgtcgtccctttggatgcccgaagtaatgcttggtgtgcatttgatggtcatcatcgtgt

tgaacttggtttgggtgactacatttctgtttctgcatctccatttcctttcccttcttttaaccgttataaaaattcaaccgattggtttgatgtgttacg
ccatacactcaactggaatgaccgcaaaaatcgccaaagagctcatcactcgaaacaatttggcctgggtctacaagggtaacaactaatcact
ctttttggaaagttttgtgcgatatttgttatttgatcgttctcattttgattcatgtttttatgtatatttaaattagttttatgtcaaaataatgtctaa
ggagcgtttactttatgctttttgaggcctaatttagacggtgtcgtatataaaataatccaagcaatttgtaggatttgtaattcttcgttagggtaa
gttacaagactattaaatggtatggctcaaaactttgtcagcaattattcgtaattcatctcattgaatttacctagcataaacttcatttataatga
agtggtctatgagtatattagattaagcatattcagagatcaaaaagtaaaggaaacgaaggtaaccagagagattaaagattttaagaatatt
attacaacgcaggccatgaaaaattataataaaaaaaaaataaacaagacacgaaacaagtaaatatgttttagctgaaaaaaaataaatgtc
gtaattcgagcggccatcagcttaacaattttgataatctaggaagtcactgtacacatgtacaagatcaggcgtaatactttgtgaatgctgcga
tcgacggtgatccagtgctgcagacaatgatatgaattctctacagtcctcgcagtatgcgactctcaagttggccaaccgtaaacgcagtttttcg
ctgacaattgcagcaggggtatctcgatataaagggcaaagagtagctgcagagggaggagcaccgtagcctaaagcggtagcaacaagggc
atcggtaccttttagagctatatgttagtaacaaggggccaatagaacagtttttatgaaaaaaacgtacctttagaaaatcgtgaatcatacga
gggatgaataggttgttgaacgagggaggaagcgatagaaacgtcagaaggcatagaactttgtaataacgaggaattaggattgtttggagc
aaacatgttcatggagtgactataggcgttaggggtacgagcaaggggatgagtggcatagtctggttgacgggcaaaaggatgatgattgtgtt
gctgttgagagacgttcgcgagtgactctgcagcgtctactaaagagctatttatatttcgtgaaacatcaggagtatggacaccgttcgttaaatt
gttcgcagcagcaacggcagccgcagcgacagcagcgttggtagatgcatttggatcgccaacttgagacggatgtggaccaggcgttaagcta
tcttgtgtattaccccgacgaaatcggcgtctggaatcagttaaagaatggatatcttcgtggggaggaactttattaatctctgggggaatattgt
ttgggtccatagccaagaagctcggacattgcttagcgtgggaagcaaaatttactggacgacgccagttctgacggccgcaaacacattgaaa
acggccattttcatttctcctccaaacgtacctacgtcgaccaataatgacgactgtttcgggttgatgatatgtccgtttatgatttgttaaaccaga
ctgtttggcaaaaaccatgtcacatttgtcacatctatacatagtggtagcagtaggagaaggagggataccggttgaagggtgtttggggggat
tcggggttgctgttgcagggctaattgttgacggtggaggagcactatgagtaggaccagggttggttgcggaagatcctgtagatattggacctt
gacctgtagcggtggtggtcgcagcaacgggtacggaggatgtagtaggagaattggtaggatcttctttttttaagttcgggtcgtccataatttg
cctaggagcaatggacatggcacggaaaataaaaagagagtgaaaaataggagattccaggtgtgtttgtttatggatgtaaccgcaattctaa
attctaactccagcactaggacggacgaaagaatgtgaatcgaactatgcaaacggttcaagaagacaagaattattacccacgtaagtgtctc
gaaaaacgaaaatctatatacaagaaaacagaaattcccgaacaactatgtgggaaagacagaagaattggaaaaaaataaaacaaaataa
aaaaataaaaattaattctaaaagttcaggcaagcttgaaaaaaagccttcgacgatgtaagagaacttgatggttttaacgtttactttctagag
aaaggccgaaattccaaagtggcgaggtgctaacggatgtaccaatgctgaattcaatcttgcaccaaacaagcacctactggatttactggacg
cttcaacaaccttttctcttcctattccttgggagttagtctttctctgttgttcgttttgttactagtggttcttttctttcttgtgacctgagcaaacatt
gacatggaatcaccaactccaaaagaaagaccaccatttcttaaatgtatcaaaatgatgcaaatatataataagacatacctttcttacgccgt
agaaacacgttttactcgctttttttcagcaaaacaaatttttttgcgtttggagtagtgttgttggtaagtagcaggaaagtagcaaatgagtagca
aagtgataatacggtaacgaaacgagtcccgctaaaaaaacaacgagtaacaccaaagagtaacattacatttcgtctattaaaggtattaaag
gaagagaattcatgcaggtggaaatgaatgagattgcataaaaaaaaaaataaaaaagaaaagaggaatagttaaaaattgaacattaccgtg
actcagattggtggttccgctctctgttttgtttttacgtataaaaatccatcaacaagcatcatagttctaaaagtcgttgaaagaaacaaaggaa
ggaagaaaaaacgacatttcgttttagttgagtgtgtaggccacattcattatttcgttgccggtgaattctctttattgtatgagaattgattcagct
tcaaggtccatttatgaaataggaaagaaacttttcaactcggaatcgagtttttgggtaagaatctctttgctcctttcttttccctttgcgcttaaa
tatatcgggtcccttctttctttcttgtgttattgacagtagttgcaaacatttttctgaatttttactgttgttcctgttgttgaaaatggactcgaatg
actgtgtcttgtctcttatcgatgagctagaacaacgacaacaaactctctctcgtcttcaagctcaattgcaacaggtatgtgatttccttcccatg
gatgctaacagttatgtcaaggcgtttctccaactttcgaaaaagaaagctcaacatccgtcgtatgctgctcaattacctgctgccgtgatttcca
agtatcctcgatgctttgctgtcttcgatgccatggaagaatcttcatcgaaaaacaatccctctacatcttcttcttttcagattcgtctgcaaagcc
atccatccatgtccaaacccttttctctgccgatgagctttgatattgtgggccatgactttttatcctgtttgcaagatatcatgcttctatgcaaatc
taacaaaaccattcagtctttattggaatccatgcaaaaagaatcctcaaattgaataggtttctcttgcattcatgaaaaatcaagctagttgctg
ttgtcaaaaacgactagagaagagacagcgggacctcctgccttttgccaattgagcttttgagtaaaatacgttcccatacaagtgacctttcgtt
cttttgttttctatattcaatgagcatgctccttcatcatgatccgctaaactcgcccacctacaaccgatcctttggcaccggtaccataaggaaaa
ttgaataaaaaaaagaaacgtttttttacaaaccgtctatgatcatggttcttaaccatgcatacccaagtgtccgacgatgcctatcaaaagcgtc
gcccaaggtttaatcacacacacgcctggcttattgaaagtttctattcaatcaaattgtagagtatcttttcgtttattttttttaccttcaaaacacg
caatgtagaatgttccttgatgagaattaaaaattccttaatctggattttaaaacagaatgtataatattaatttttttatttttttaatgttttcttttttt
ttattctttttataaacttggtattctttcataatagcacttgctttgttcacccactgaatatccgctaccacaatacattccttccttactttaccacc
aactgttgctgtccttttggaaagtgctatatctttgatagtttaaggcagcagacatctcttacgatacgaactttttaaaataaattaaaagctttt
caagatgctacaaattcaaaagacaaatcttttgcgaaacttgcgcacgttctctaccactgctggtttgaaaaatgctgcttctttgtacgagcaa

acaatacctaatttaatgattaattcagacaccaaagttattttccaaggttttaccggaaaacaaggtaccttccatgctcagcaagccttacaat
atggcaccaatattgtcggtggtaccaatcccaagaaggctggtagcacccaccttgacaagcctgttttcggtaatattgctgaagctatgagag
aaaccaaagcaaatgcttctggcatctttgtccctcccccattggccgctggtgcgattgaagaggctattgaagccgaagttcctcttgtagttgc
cattacggagggcattcctcaacacgacatgcttcgtgtttcggatatcttgaagactcaaaataaaactcgtttggtaggaccaaattgtcccggt
atcattcgtcctggtcaatgtaagatcggtattatgccttctcatatccataagcccggccgtatcggtatcgtttcccgtagtggaacattgactta
cgaagccgtcaaccaaacaactcaagctggtttgggtcaaagcctcgtcattggtattggcggtgatcctttccctggaaccaacttcattgatgcc
ttgaaactgtttttgaatgaccctaatactgatggtatcattttgatcggtgaaatcggtggtagcgctgaagaagatgctgctgaattcataaagc
aagccaataagagcaagagcaatcctaagcccgtagtttctttcatcgccggtgccactgctcctaagggccgtagaatgggtcacgctggtgct
atcgttgctggtggtaagggtaccgctgccgccaagtttcaaagcttggaagacgctggtgtccgtatttctcgctctcccgctacccttggcagtct
tcttgtggaggaattgaacaaattgaaccagtaaatcaagtcaatacatcgaatgtctgctattacgatgtcctcgctttcttttactcttcactcag
atgaatgagtcagtacatatctcccccctctctcttatgttatgtatgaaagtagaataaaatcattgtgtaccgttctactttcgagttaatatagtta
acatacatatgattaataatgcagcaaatgcaacatcattgtgaacatggtcatttcatttttttatgagtaaatagctaatcatgtttgaactagatt
cttctagccgaactatcatttaaatcattgattttccatctatgtatcaggtaatcaccgataatacttgaagctttttataatctcacgtttgtaactt
gatatcataataatttttatataaacgttcatatgaaatcaatcgacaaagaaaacaattaagattattataacattcgttctttttttctagtcattt
atatcataagtgtgcatcatatagctttgttttcaacatcgtacacaaaccaaaatcccttattttaccgccattccaaattaaggctttggcaacat
cgcatccattgtgctatttttttgcaacccatttggaatcgacaagcttatgacgctctgcatgcaatagcttgtaagcgatacggatggtagaatca
ctcacacccgtaacctccgaaatttctttcggagtcttgggaaatcccattaacgcagaaatcatgtaaatcccactcgcagcgatcgaaataggt
gagcggcctgcaagtgtaccctcttgacccgcacgacgtgccaactcggcagcggccgattgcacggtcataggaagcagcaagcggttacaaa
atcgaaccatgagatcctcagccgaagtagagctactttggatgtattcttgacctttcaaggcatcgacggaattatggagagcaccaccctcag
taagcatacgttgtaatgtcttatagacacgtcctatctctttttttgggaacattggtcaaagtacaaatttccataaaggttctaggaaccttcccct
gacgacaagcaatataaatacaggcagcaatgatcgactgcgatgatttacccttcaacgccttgtgatcatctactcgtttatacaactgtttcgc
agtatctgcaattaccttgggcaaactaatcgcatcgcacattgcaccaatttctttataagccgtcaaaagattcttttcaccacgtacttgaaccg
accgtccctgtgccttggctaacattgctccagttccgtcgtgcgaagaaatggccgtatccaactgagatccattcaaaagagggtttgcaacttt
accaacacgactgggatccccacctgcttcatccgaatttgaaaatgtgcgccactcacttcgagtatcgataattcgatcgcccaaaaccaacct
gtatattcctgttagtaactactattctcataattagctaaataaggagaattcgacaatcatgtaaatgacgaaattcatcgatttctctcaatcaa
agcacatttcatccaactcacatgattgccaaataccccgagaatgaacacacccgcagcttccacaaacggtatcaccactcgaaaattcctcc
acaagattaggaggatcttctcgacattcagagcaaatcattttcactgaaagcatggtcgtaggcatacctgcaaatgttggtgcgttcatggta
gagggcaactttcaaatcaccgtttctgggaaatttctatcattgccctcagcatattcaataagctttggtttcccctttaaattgccttgttatttta
tttagcgtattacttcttacatatcgtaaaatattgttcaatagcattagtgcactcactattcatcgtttcgtgtaaacatccaatgtatccgtctgaa
aaacaccgatacttggaaaaatgagttcgtcaaaacgatgaatgtctttttggatatatatatataaatttaaaatcaagaaatgaattgcttcatc
gtaagttaaacgcgtacttcatcatcttgttttcacctttcgtagtatttggaaaggacaactttgcgacttgtagctctttaaatcaaacacaggta
acaatgttctccgattttcgaaagtaattttgcaaagttccctatgaaaagcggaaaatccatgggcttgaatctatggctggacttccagatattt
gtacctaacttttatcaatttatttatcattgtatttgccaactcagtgccttagctatagacccattcatgatgacaataagaaaggatcataattg
atgtgaacaaaataagcaagatttatgggatcattaattctcgcattgcattgcattgtacaaactcatttagagcccatgggctgttgtctattgtt
tctatagcaattttattacatctgcgtagcaaatagaaaactacatatgatactatacaacgaacggtatagcagtatcaatttaaaagaaccaac
cgattgctttttcacccgaaatatatgtttatgtatatgaatatgtatatacatatatatatccacataaatagagcgttctagtttaaacactcata
cgagtctttgcacgagtgctaccggatttcgaagagtcttcatgagcgctaaactcaatggtctcagtacgaggcaggtttaatatttgcttaaagc
gattgccaatatccaaaaggatctcacggttcgtacaagtctttgtccaaacagccaaacgatagaaaccctttctcatgttgacaacaactccca
tcacttcttcgccagtaggatctaaagtttctccaatagcagaaagtacctattttattttcgttagcatagacatcctgaacaaacaacgggcaac
gaagtaaaaaaaggacgacagctccaaccaaacatggagatttttactcctcctttaagaggagacatcgaaatctatcttcatagaatcccgtt
accataattcttctttacataccgtagtaagccacatttcatctaaagcgtttcctccacgacccttgttttggaaagcccatttaccaccgtttttatt
gtgaatatcttcccattcaggacgtacaccctcacggaaaaaggagtagtcagattttatgggtaagctggaagcagcattgatgttgttgtgaat
tctatataaaacacgcatgttagcattcttaaagatcttgctattattctttctttttctcttacttacccccaaaactcttccacgctgttaaaggttat
tatgttttttttgaagttcattccattcaagacctggagtaggaggcatcaaaaaccataatgtccagggtcttgccaatggatgttttaaattaaag
cttaatttatcatcaaaaatagtttttgcggtctcttcctgggtttcgaccgcatttgatgcggtctgtggctctttttgttgttcagtctgcatggttttg
gctagaaagaatgaaccccaccaaattttatagcgatgctattgagtatggaaaacccttgaaatttgtactaatattttagatattggaatggaa
ttgagccttctgtaaatggaatgttaggaggtttaggaactgggcgtgtatgtattacaacaagacaatccttgtttatggaagctgctactagtta
ttttggattctattcgactattgctccataaaattacaagctatatgaatgaagctaacgccacaagattcctcaagttgatgcgattgcctcccgta

cttttttgcgtcgtttggattaccgcctcaaaaagatcgagagtttaattgctgcagattgcggagcaggaataggaagagtaacggaatctgtgtt
gcttaaagtcgcctcgcatgtagacattgttgagccggtggaaaagtttgtgtcacaagcaagaaccaatttgcaggataagccttgctctttcatc
tgtaccggattacaaaactgggctcccgagaaaaaccgctatggtaagccaacttgatagcgactgtctacagaggaagcagatgatttcagct
ataacttgtttttcccccaatgctaataatgacttaggcttaatatggaatcaatggtgcctatcccaccttaccgatgatgatttggtagcttatttg
ctctcttgttcagaatcgttatgcgaacatggagtgatttgcataaaggtagttgctagtttcattcagtagaagattctaacctcttataaccttag
gaaaatgtttcatccttcacggaaagttttgacgaacaagatagcagcgtcactcgcaccgatgactcgttccggaaaatatttcaaagagcgaa
tttagtcataatagcggagtctctacagcacggatttcctgacgaactctaccctgttaaaatgtttgcacttgttccaaaaccctcaaagtaagac
aaaaattacgttcaatcagaaagaatagaatgaggagtaattggaagagtaaatttcattaatattatgaattggtattgtacataaacatataa
atcataaacagaaaagacacagcaattgagattctatgaactgcttgatgcagcaggcacgagtttttttcttttcttttttttagcctttgttgacga
cgagcttgctattgctgcgttcggtacgacaaccgtagcattcttaacgtcttctgaatcattaaatttttgaagctcttgagaagcagatattgtaa
acattaaatcccttaactgatctgataaatcagaaatttgctgctgcatagagacaatttcctcaactttcttgttatgcctagcctctaaggattga
attcgcatcaacaaattctcgttcatgctcttttcttcgtcgtaatcatgcctccattggattatttccgaagaaagcttcttgattttgttatccttcttc
ttcaaattaccttcagcaacatccacccgcgaacgaagttcattattagcatttgaagaggctgtggtaatcttggactttagagttatttcttcattc
gaacgagttagcttttccgccatagaagatagttgtgattcataaaatatacgctgagattccaactgcgaagttaagatttgagtgtattcaagcc
ccattttctcacggatctttaagtttgactggtcgaaagcagaactttctccggccggctttgaatcattcgatagttcaaccacttttccatctagct
cggactgcagcaagcgatggacgtagctatcgcctgcataatcccaaacccgttgactttctaaattcatagcatagcaatgattggtttcaataa
aatgctgctttgcatgagcatcatgatatctgccacagccaatattaccgcagataagacacatccacagatcctcttcacatgagcaggcaaag
catttcccagaatgacggagggcttgatctttttgggtatatctacaaacggggcaagaagagttcccccatttttgtaagcattgacaatggaag
gtatgctgacaagcaatagtgataagtcctgttaccgaagagtccattctctcaagacataccacacatgtaggaagctctacaagggagggcgt
aggcggagccaaggggcgtgagaaagtagatgctcgtgaagtaatgttcatcaaaggtgtaggggaatctgaagggctttcaacttgaatctca
tcaacgtgaacgacatggcatgtctcgggctctatttgactgaaagcttttccattatattcagaaaagaaacgaattacactgacttgatcaacg
aatttcaaaagagcgagaatccgattctggactttcgtcctcaacaggcgaatatgacttatagacctacaactcttttctcccaaaaatccgagt
atatctgaaggcgacatatatagagggatagccagaatagcaagaaggagtccgggtgcacggaattcactggtcatatctggaatttctttgta
caaatgaataatcccatgacccagactaacttcttctacccttgggacatgagtttgctggtggtccatagaaataattgagatttcttccaagcga
aaccctgcgtgttcttttggaggccacctggtgtttttttggatttattttcgtttttaatgaaaatatatcttgaacgggattttcacaaacaatttt
tatgtaataatgcatgcattgacagtttctacaaaggtggatggcaaaatggaagtcagtataagcaataaaatagtaaatgaagaaagctgat
gaaggactaatccataaggatagaaatcatataaaaattaaaagaagaaaaagcatacgcacaagttcatctcctatcaatcgaaagaagatg
aaggaaatagccagaagagttatgaatggagtaggctgaacagcataaggagttttcttttgctgtaaaacagtctataagaatttcaaaaaaa
ccgaaaagtaggaacaagtagcgaaaagactttcactcaatgtttaagcactcataagaagttccgtagaaattggtttagtatgcaagaagta
atttaaacatagacaatttcatctttatataacaaccattgggctccttcgtagtccaaaagctacctgttgaagactgtaagccctgaaggcttta
actcataaaagctaggataaacaacttatagagctatatttatttataaaatacttgtttaaaagaagatgccatattcgaaatggagaatatcag
atttaaaaaacgagtgcacttccatacgaacctctcttttacaacgctcgtttcactagcggtgaaagggtagtctagtttgccattgtatactaaa
gtgaagtcgattacattataaaaaaagtggataaagtagcaggctggaacgattcagaacgatttcttaaagagaaaaaggaaaggaacgtcc
agggctctgtagaagtggttttgggatatttatgtgaatgaaataagtagaaagaaggagactacatctgatgattttacatgtctaaatttggca
attcaaacaatagccatgccagaggaaaacacacaagaagttggcatgaatctgctactagaattagccgatggtataactttttattctgttgaa
agctttgttttctcactcctatttagtgctggatgcaacaccagacgatttgtcttcggattttactaaaatccattgtttagaagaagaaatcaagg
gtatgtgatctccgtaaatgatgaatagtacgccaattcaaatatgtgtactaatcaccaatttagaaatgcaaatggctttggatgcttccgttca
caaaatcatgagctcaaatgcaacaccagaccaaagatcttccataatacaaaacatcgttaatcttgtagagaagaactcgccccttcttcata
aaaaagctcatatatccatgcatacagaggctgtgtccaagcggaatttttcgaggctgcagacttgctttcaggcaatggaaatgatgtatcctc
attttttattccatatgctgctgccccagagcctctagtaccccgcagtgggaaacgattgcctactagaacgtcgaagcggcgacaaggaagag
aacccgactctagggacacaagtgagaaaaaacagaaacatgatggtattgaatcctttatcccctcttctaactttacagatcaacaatcagac
gcttccacagtagcaggttcaagtgatattccataaaacaaaattcattatgtgacaagcgggtccttgcacattggtctactttctttttataaaaa
atttattttcctaattctctctctctctctcttgtttttctccttcctcttctcccatgacagtattcttttacatgattttggatagtcaatagtcaata
tgtagtaccgctttggtccactttttactgtgctactttattggcttctcattcattttgctaagaataattgcattgtgtctacgttcgctggtccaaaa
caattccccatgttcgtctttattgtttaagtaatatatgaatttattcatgacttcatgtcatcctcataatccctcaatgtcttaaatggactataac
atgtataatttgacagtataaccaaaccaagaagcctgcatttgacaggaaaatcaatatagacaaaatcatcctgagaagtaaattacagatg
aatttatatttccttgaaactatagtaaatattagaagaactctataaacctctattctttgcttgatgaattgaacttccttggaagatgaaacatg
ccaaattttagaatcgccatttcccactaccacttttgaaaatgggggatcgaatagagacatatgcttactttaaaaagaaattgggtaagattg

aaattgacttggttttgctaatttataggtgttatatcagtggacagcaggttgaaatggaccgctgaaggaaagacgaatccagcgattgatatc
tcttttgatgcaatagcaagtaaggatagctatactgtttcattaaagcttacagaaaagatttgcaaatgacaccagcatcgtctcccaagatcat
ggttcgtgtttttatcattatgaacaaaggcgaggagcctactcccttggtgtttagctttacgaatacaaataatgctcgcggaaattgtgagatg
gtcaccggagagctccaaaaagcaatcactaggcaacgggataatacagtttccgatgagaaatcatttggagtggaatccctgaaccagaag
aatctcttggaggatattgacttacaagaatcacttttgacgagcaacagtgagatgtttcaaacatttaaagaagcagtcatgaaaggccacttg
agtaatgagcaattttggtctacgcgattacatttgctacgcgcccatgctgtcgaaagatcacaacaacgcggaccatataatgttctatcttcta
taaagccaaagactgtggataaccaaatgaaggtgtctttgacccgccaacaaattcatgatatgtttgaacaacacccgcttcttcgaaaagttt
ataacaagcatgtccctccattagctgaaggagagttttggagtaggtttttctgtcaaagctgtgtaaaaagtttcgcggagatcggattactcc
tatggatccttatgatgacattatggaccaatatttagaagccaatgagggtattatttttaccaatttcaactttgctttttgaatttattgctaacac
tattgtagaagattcggctaggaaggatgaacctcctccatctcatgtactcgatattgagggaaatttacaaaacgcaagtgtacttgctgaatt
acgcccggacattacaatgcgtatagataaagaggctgttccttttatgaaaaatatcaaccagttatcagaacgacttttggagaaaagtctgg
ggtaaggaaacagtagaaagtctatgctgactcgactataggaattcaaaacgactaaagactgaaactgaaaatcaatatttgcaagagtctg
gattccatgatttagaagaagatcgcacagattccaaagtcatattgaatatacaagaacaggatcgattcttagaaacaaattttgccccttcgt
caaagcagtctatggaagaacccctgccgcctctggaaactttgcaaagtctttaccaagaggaaaatgtgcaactagatagtatggatcgcgac
ataaatgcgttggcagaggctgctcaacagcttacccatgctatgagagaaaaatatgagttcgagactcatggttctgatatgaatattccaaa
agaattaaaggaagaggtaactatgtgtcatactgtaagtgtcgagttcttacaccagttttggagtgccttgcttagtccaaactatgctgataaa
aaaatcatggaacctctacagcatgcattattgaattcaaagactcgggtggatgccatagtttctcaagcgaaaaagcaaaacgtcaatccgtc
ttatatctatcaggtaaggtttatttcgtctcgtgtatacgttgctaatgagaatgttcatagttggtttcctcaatcctttcatccattgatgttgcatt
aggggaatatcaaagaagaatatccatcactccttcaacttaaaaggagttttcatcttcgttttaatcatgatgcttattttacgactgactattta
atgaaccatcgataatctgactttaaaagaatcgataccgaacgaatgtgttggggaattgggttttcagacattctttagtactgtaaattagaa
aaaacatgcaacttcgtcagaatacgaataatacccatttgtaaaaatggttcataaaacaaatttatacttcagctatgaatttcaatattctttct
attaaatgaaataaaactataaatatataaatacagtttcattctttcatattgcacttattagctgtataatatatacctggtgtaagtgcactaat
gctagaattgttttgtaacaccaactcacatggaaagagacctgaatctttctttaataaagaaaagacagttgctatcctaattctatataaaata
ggtctttaaaaaggtagttttataatgtttaatgttggagccagtgtgttgagggctgttaagttccctatatttcaaggcatacgaacgacatatttt
acaaatacaaaatctatctcctgcgaccgtctgcgctcatttcaaagtttaagttatcaacggaataagcaaccatcgtccgcagcaaatggagg
aaatgaatggagcagtcctggcttagaaaatctacttgctcaactacaggaagcggttggtgaaacaacaaagacgaatgatgatatggtagca
ggtcctcgagaagaaaaattggaggataactttttggatactgtttggcctgaacctgcgaacccagtggaagagatcatcaatcggcctttccaa
gtagacccagctgtgcaacatttagtcaacatgattatgagggacggaaaaaaaacaaaggcagagaaaattgtggctactgcattgaccattg
tacaaaaagaaaaaggaaaaaacccagtggtattgctgaggcaggctttagctgatatttcacccatcatgaaattggtaagttacactgacgg
gttctttttataatacatttacatactaacatgcgataagataactgctaaacgattcaataagggcgtcgagttccctattcccttgaaagagaa
gcaaaggaggaggatagccattaaatgggttttggattcatctaaatcgcatagttcaaaacgactttcggaaagacttgctaaagaaattgtcg
caattcttgataataccagttcttgtttcaaaaagagggatcatttgcataagatgtgtctggtcaatcgtggtaatgcccctattcgtgtatagtttt
ccttatctaccgtttaatgtatgtaaacgtgtacctgctgggaatcttttcattttcatcgatacgctcactccatatactttgaaaattttattgtaatg
tatgctaaattcctctatataaataagatcaataatatgcaatctagtcattagcaaagcttaataataaacaacgtgtaaggttgtagtaatcatg
cccttgttttgttttggatctaagatttcataagtacatgtctcaaatgccattaaaacatcctcatcagaagtaatcgtaataaaatcaccatcttc
atctatatactttagccggaccctaaatgaaccatcatccttcataataccacttaactttatcttgtgctccactttgcctagcaattcaccataact
aatatttcgcggaacaacaagtacaaaactcgcttcatggagttgtaaacggaccataactggatcgcttcttaccaaagaagctgcatctaaag
tatccgattcacccctatgaggaacagatccggagtcatatttgtacgtatcattgtaagataatcgattactggaatgatcactttgctgtattttg
gaaaaatctggcatatcctcataaatcccagattctgaatcatcaacttgcgagtgctgtctagaaatatttgagcgactatagttatagtcgctta
aaagcgaattcacctgacttagatgagaagtttggctcgctcccgagggattggatacagtcgatgaggaatcgttaccgacaaactctgaagcc
gtttgcgagtttctggaaacggacctgcctaagtcctcgccattgaggcgtaaatctgggtatccaagcttatcaggactagcacgttgatttcctct
agaatagccgtttttagaaaacggactaggattggataacggggctgaaaagtttaatgtctgagtcatagtatcatcaagtgaatgtgtgcgcat
taaatatggttttgaagggctaccaatcttgatagatccggtattatccaaaccatggtgacctaaggaagtagatgatgtgttacggacgtcttta
gaagagctatctgtttttttatgagcctgcttaaagttgaaagccacaacctttggctttcttcattccttaacttcaatacaaaagattcattatgtg
ggtcacctctccaaaagatgtgaagggcataaggatcggcatttgtattcggaacaacggcaataacattagaaaccaaaattcttcctttaagtt
gaagagtatctagtctctttggctttttatttatgcttatgctgcgtgcctgctttttaaaggggaaagttctttgcaacataaaagtatttttcgaat
agatagacgtaatactcccgttctatatcggctttagagacgctaacgacgtcccataataaaagctgtccaaaaaaattcaatgagtagcctttc
caatcaataacgcgctgctcaagctcatcaatcgcagtcttattttcatgaatacgcctggtttcgttcactttattagctatacgtacgacacaagc

cattccttgtcttaattcttcttcatgtggatagttaagaggagtgtttttaattaactgagacaaaagaaggggatatttacaaattctttggatgg
gcttgattagcaacgcaggaaactcgtaagaaggttccaagagatagctcaattcgctgagttgtgcttgattatcgatgattaattgctgagcag
tggggtaattcaaacaaaatacttgataaacagaaaagcgctcttctaaagccaaaaacaatgcacctattcgttgctcttggggtggtaatgac
agattcatttccaacccaactagaaaccttctttgaaaatcaagaatttcgttaagattggtaaagattttcagtatcgtttcaagggataagacttt
gcgctgctgcaatgcagtcatgaagactgacagaaattccaaatcttgaatatactttaattcagtttcgtaaatttctgcaagaaccctttgacca
ctttctataagataatgaagcatacctggtgatttttctttaggagtttccaaaagacgaccaggtggattagtgggtaaattttcaaatttatctag
caaagtctcaacggtccacagtgcttttattaatgaagaagtagaatcgctaaagatctcagatatggaaaagagaacattatccccaaaccta
aatttttttgcacatcaacagaaacctatagagctccgctttacaagcatttgtgttttccaaagtaactgaataattcacatttacgggttgttctg
gggaaactgcattgtataaagcgcaaagagggtagccgaaacggcagaacaaccacaattcagttactggatcatcgtacggaattccattaat
gtgcaaaatcttaaagggagtaacatcagcaacggaatgtataaactccagaatattttgaagacctgggatttgtagtaaacgcttccgtatatt
ttcgcaacgcctgtataaagatacagtattttctaaagtatttatgactttagaaggaggttgaagtaagcgaagcttcatatactcagaaatacta
gtatcatctttgttaactaagcttgtggtactagaattcacataagttgggagtgatttggaagaaactatttcctgttagctctagaagttgaaaga
aaaacataaaacacgttcatagaaacatgctttcttgcatcctcagcttatcctgcagatgacttatctatatatacctttccttgactgacgagttg
gcattcttctaagctatcattcaaaacctttttttctattttaaacaaaatagatatcaattttatccttctccgaactgaaggatagagtcaaccgct
ctcatatagtacaacgcttggtttaagcgacaaactcaaaagctgagtaaaacacgattaacctaaaaaaatcatacacaaaaatgatattttca
atagatttttaagcagaaaattcctttatttccacgaattctagcgttttaaagcaattgtagggaaaactaggatccttggtgtattgtttaagtgg
caggacaagtggtttagtggtgtcgctacttagcataaaataattttacgtactctaccgcattatctgaaaagggcgagagaaagaaaaataaa
caaacttgaagagcatggtaggaatcgttaggatgaaagaaaaggaattcttgtaatttgatataatcataataaaaaacaatagatttacgaa
aagacagtgaagcttgaatgcgacatcgtaattatcaaataaaaatcgttagaaaagatattcattgggaattgaggaaccttttaacgccaaaa
accaaaaacaaaatataaaagaaaccaggtatataggatataaaaagaacggaacgttgaacgctcaagagtaacgaaaagattgagctcat
tacagctcaaaacaaagccggagaataaggatatattaaaaagcttatatctatagaaggaatacaagattcagctcaccttcattacaaaaga
ttatcgtctgccccatcattgacagaggaatagatattggatagtttctcagacagcagttcgcttcgaaaaaagtattgtcgtaattgaagcaag
caatcgggagggatgtttccagcattgcttatttctcgtagatatgtgttttgttctgcacttagtctttgccaatctagaagaacattcgccaatgtc
tctttattcaaagaagagtcagagactcgtggaggtggttgatgcagttcaacggatttcatatccgatggttgttttgcgagggatttgctaattcg
ctgaagatcagcaagaatggagtaaatagacatgtcagaggaagtactcggatcctccaatagatcaagtctttccaactgcgattgcacttccc
tcaaagatgtatatagggttgtgagtacgtctttggaaatggagaatttttttgctttccttttctatatgtaacgtcccttgcataagtgtacccacta
gtttcgacaaacgactgacttcagcaacccgccatttcccttccatatcctctaacaactttttggaagcttcgaaatctgcaagcggcgtcttgaa
atcatcggacttgttcctctcattgcctattgcttctaggactttggcgtacgctgcttgatacttaactttcttttccaaagtctctttttttgctcttaa
aaataaatatttctatgcaacctgttagctatgattctcaagcgtcacttttgtgcaatcgtacattttgcgaatcagtgaaacctgcatcatcatgc
agcttttctttgctactttccaacagagattgatttaaaacatacgaataatctggcgataaaaccataactagtcaaacgacactcaaaggcttct
accgagtttcattcttactgaaaaactattgtttgattgttacttgtcaatgtcgtttcaaggtaacatctagtaaacagagcgaacatgaaaacaa
atataagtaaaaagcagataattctaaacaaaaaaaaaatcaatgaagtggtctattgtgtggtttttctgtaaaaattggatttctaatggaaag
gctgaatgagaaagatcctttttcggattcttcaatgatcatcgttcatatatttgggtcataatgcatatcgcattccacattccttcttctggaggg
gggagatcatcgattacaaagaagaagatttaaattttaaagaaagtatgcaagctaaatactctatagttggtttggtcgccttcgtgctttgtct
gctttggtgaatggaatgaaaaaaaaggaaacatctcacaaacaaaagacttttcattaaacatgtagtaaaagccaatgagctcttattttgta
caactattagcttttttttttcactagctagcttcttttgctttacaaacttatacataaattcctggtagcacacacagggccaagtatatttgaagatt
ggagtatttttttccaagcttttattttcgttcatttatttattctatcatcctttagttatactgaattcaatatattgcatatgtcatcgcaatgactcat
gttgaaaactttttgaattttctttagtatttaatttcaaagtaaaaaaaaaaacaagaaatttataattgattgtattacagaaattaaaaaaagct
gctgtcttccttaagagttatcttcgtctttgatataatcattaggcattcccagccttagcacacttatcctcgacatgtcacgctcactgtatagta
agtaaaatatgacttgtgtagcaagtaatacagactaacaatggcttttaggatctgaggacgtctctttggtgcagttatacctgccgacggagt
ccgtcaggcccataatgttggcattaggggaattgtcaactattcatttcaaagatgtaagcattcatctgacaatatattggctaacattgaatta
gctaaacaaagatgtggtagcgtttcataggtcttttgttcgagaaattcgacgtttagatgaagctgaacgtcaactacgttaccttcattcagag
attgccttaaacagcatcaatattcccgagcatcatcttcctaaaaattatgaaattaccttagaaacgaccacaatcgaggatataattgagaag
atctctcgcttggaggtgcgcgtcagacaactagtggagtcaagccgagcactgaacataaagtatcttcaacaattagaatattcaaacgtcttg
tcaaaagttgattcctttttcgcaaaaagtaccaatgctcataatcctcttaatgtcaatcccgatagtggttccattatggctggagaagacgatg
caactgctccgcttatcgagaacgctttagaattaggaactactggttcttttaatagtgaagatactggagctcgaacttctatttctcttgattttg
ttacaggtattatcccgtctgtgaagtttcaatttttggaacgaatcctttggcgtactctccgtggtaacctctatatttaccaaacccgcgctgacg
attctgtcatccatggtaccgaagatgctgaagaaaaaacagtgtttttagttttcgcccatggtactcaaattctttccagaatccgtcgaatctca

gaaagcttaggtgccaccctcttttccgttgacgaagattcctccattcgcgctgggcaagttcagcaggtgaatggaaatatcagtgacatttttg
ccgtattagaaaacactcgctctgctctttacacagaacttgctttcattgccgaacacatttcttcctgggaatctattttacataaggacaaggtt
gtttatcaagctatgaatttgtttagttatgatcaaaaccacaagtgccttatcgctgaaggatggtgcccaaccactaaccttccgctggtacaaa
aaacgcttcgtgacatttctgatttagttgattctcaagcgccgtctattcttaatatcattcgtacttcagaaactcccccctacctatttccgagtaa
ataagtttactgaagcttttcaatcgcttatcgattcttatggggtggctacttatcgcgaggtgaaccatggaatcgttgctattgtcacattccctt
ttttattcgctattatgtttggtgacttgggccatggcgcaataatggcttgtgtagctctaatgtttataatgcacgagaagaccttggctgccaag
aaggatttggatgaaattctaggcatgatcttttacggccgttatatggtacttttgatgggtttattctctatgtacgttggtttcatttataacgattt
attcagtaaaccaatgtctatctttacttctcgttgggcatggcctgtaaaaagcgaagatactgctgccaaagctattcaaacgggcacatatcc
aataggtattgatcctgcttggcatagcgcggacaataatcttttgttcatgaattcttataaaatgaaagtttctgttgtcatcggtgttatccatat
gacttattgtctcttcctttctttgtcaaattatcgatttttaatcgtaaattggatatatatgccgttttccttccatcccttatattttggaagcaatt
tttggatatctcgttgttacaattatatataaatggagtgtggattggaaagctaacaactggcagcctcctagcttactgaatatgctcattctcat
gtttttatcacctggaaaaattgaagagcctttgtttagaggtcaaaaacagttacaaattatccttgtccttgctgccttaatttgtattccatggct
cttatgtgctaaaccttatgctttatggcgagaccatcgccgacaagatgccaagtatcaatctgttgacgattcacaaaatgttgatgaagctga
agtcttgcttgacgttgaaaatgaggtgccccaagaagaaggttttgattttggtgaaatcattattcatcaagtcattcacactattgaattctgct
taggttgtatctcccatacggctagttaccttcgtttatgggctttgtcgctggctcacaaccaactttcgaccgttttgtggaacatgactttagcta
acgcatttcgaatgacaggtgtagttggttccatcttcatagtaatccttttcgcgttttggtttatagttacgtgccttgttttggtggctatggaagg
tactagtgccatgcttcacagtcttcgtctacactgggtggaaggcatgtcaaaacactttgaaggtgaaggttatgcttttaccccttttacctttct
gcaatcggcggctgactgaatagaatgaagtttcctttgattaatattccaaaaattgagccaaactaataagttgcttttaattcaataaataacg
ttttatctattcatacatggtttctcagaagtgcgtcagagaactcatctatattattttgtaattattttcattcaatagtttctatttaatttaggaat
gtcaaaaaaaaatgtttcaaaacaattgccatgttgtcctaccatagcaaccaccttcctctaacacacctaaattttctaactgctattttcgaata
tttttcaggaagcataatcattactgattattgaacgaaacaacttgtttattgagtttaaagcacattttaaactaaactttctccgaaaatgggtg
aagactcatcaaatggcgcatctgttaattttgcagaaaagatcagtagtacagcgaagatagattcctctttggacaacttatttaagcaatctgt
aaggtaactttatattttattctagctaactaacttcaaagaaaccaatttcgcgtcctgccataaaagagcctactaaaataacattaccaagtac
gaatgaaaatgcggatgaaaacattgaagagggagtcaacattgaagccgaaaaaaaggtttcaaaagaaaagaaaaaacaatctaaaaag
aaaaagtctggtttggacggtgacaaccttgaggcggattactttgataaattaagagaagaggaagcagtcgaaaaggacactggtagtgag
gaagaaaagaaaagtcctaaagatgaaaaggaagaggaaaaatccgaaaatgataaacaaaagtcagaacaggagaaactggaacagag
agctttaaaacatcaacaagatgtaaaaattcaacaagagcttcaaaagtctgaaaaaactatttttgtaaataatttaccttccaaggttgtttta
gacaaggtatggaacatattttaaatatacttgcttctaaccaaaaatagcatgaatacaaagccttctctaagcactttcgtcgttttggggctat
caattcgatcaggtttcgtagtttggccttttctgaatttttgccccgaaaagttgctttccttgaaaagaaacttcatgctgaacgtgatacagttaa
tgcttacatcgtttatgatgaagcttcatctgctagaaatgcgttgtcattgaatgccaccgtttttatggaccgtcatcttcgcgtggatagcgttag
tcatcctatggctagggatgtgaaacgctgtatcttcgtcggtaatttggcttttgaagctgaggaagagccattatggcgttacttcggacagagt
ggtgaaattgaatatgtgcgcattgttcgagatcccaagacgaatttgggtaaaggatttgcctatattcaattcaaggaacccctttcagtcgaa
aaggctcttttattgaatgaaaaacagatgccagagggaagaaccttacgtgtttcaagggcgaaagttattaaagccaatgctcctgcttcaact
gcaaaacccaataaccaaaagggtcgtactctacaaggtagagcccgtaaattgatgggcaaagcaggaaatgctttggtcaaagaaaatttgt
cgttagaaggtcatcatgctaaaccaggtgaaaatcctctagtaaagaagaagaagcctagcaaaaagcgtaaggaacgtgctgatcaatgga
aacaaaataagtccaaaaaggtcaagaattaaaatcggacatctcagtgaaaatcaacttcgctaatatgggttaataaaggtttttgtgattta
ggttctttatatgcaacttgatatatatataataataattaaaatctctttgggagtcattcttgtgattaatgaattataaacgtgcttggcatggag
tatatttgtttacattgtaaaggactggcgtttgttgatgttagctcctttgcgaattctcaatggttattgcttgttaaacacgctaacataattgaca
aagtgtgtagagaaaatgcaaatcttggggtttattattgcattactgaccttacaaatacaagaaaaaccgtttgtttcctgctcgataataaaac
gcacagtcaacgctcctgagtcaattgtcttacagacactccacaccagatattggtcatttaaggctcatgttcctgtcaactatgatcccgattct
aagggtgagagacgataaacattctaggccgcaaactaacataccatataggcttcatggtgcattgtgatggtaacaatctatacatgggaga
gtctccaggaacacgtttactgcgggtcgagtataaagacgatctaacattgcatacgaaggatcgtaaagctttggtgtatcacccagaaaatc
ctcttgctttaaagcacgaagctggttccccatttattattgaaatggtagaagaaagaagggaaccagaaagtccttatttgctattctattctaa
atctcaccattttacaacctgtaaagaaaacggaagatggattatttactccggagacttggacatcccttcactcgattgtcttcccatagagctt
gtcggaatccgctataaaggatggcgaacacatgataaaaagccttttgctcttaaagaccctggggaggacggcttggatactccttggaatga
ccaattgattcctatgtgagcctcaactttcgataatactctcaatatgaaataccatgaaagtaaaacagaaagaagattgacaataaccttcgt
agtacttgtacttcaacgatgtgggcttgattttcaagaactgtgagagagcatggatgtaagatatcccacttggagttgagaacagagtattttt
cagaaaaaaaaattagtaaatcaaatatattgccacggtaaaagcctgtaagcttcattgaaatctgtatcaattttgcttttgaacaggcacatt

gatttttcaaaagttgaaagtaaaatataacttcagtcgtaatttatatcacatcaactaatgtaagcattagcattctgtcattttgatattcgtag
ttttcttggcaacatcttgacatcttagaatcacaggttataaacttaaggaagttctatcgttacaagtaagttcttaaaaaacatataattcgata
ctgatctttaaatgattgaagtagtccttgtttctttcctttagactggttagattttactacttgctacagaacaaagacatctgtgtgaaatgagttt
tgtgaatgaaaaactgaagcttgaaaatattcgttcagcgttaattcgtcaggaggatactatagtctttgatttttggaacgtgctcagtttcctg
taaatgagaaagtttacaagaaaggaagtgaaggctgcttaaacttgcagcataccgataagagcttcttagaatatcttttacacgaggaaga
gaagctttactcgctcgtaagacgttatgctagtcctgaagaatacccttttacatcggatttgccagaacctattctacctgatttcaagaaacctt
ttccattacataaaaacgaggtgaatgttaataatgaaatctttaactattatgtgaaggaaattttgcccaaaatctgcaccaaaggcaatgact
ctgacaattttggcagcacagcagtttgtgatattcgatgccttcagtctctatccagaagaattcactatggcaaatttgttgcagaagcaaaatt
tttgacaaacccagaaaaatacaagaaattgattgaagctcgtgactcagaaggaattgtgaacgagatcgtagatcccgtacaggaaggacg
ggtgttgaaacgccttcactataaggcactcacttatggtcgtgattctgcggaccctagcaagccaactgataaaattaatgctgatacggttgtt
tccatctataaggtatgcatgctttcaacagtctcttatgttttaacttgtcttactaactactatgcaggagtacgtgattcctatgactaaaaaggt
agaagtagattacttacttaatcgtctaaaggactagacggtgttcttgtaatgatagatatcttacacaataaatcctatatgaggcctttaagtt
atagagaaatgaatataaatatccaattttattaaaaacggatgcaaaataaatcctttttgggggatttttaaataatcattacttgttttatttta
ggaagtcattatatatcttcatctccgcgcctgcgcgatgcaggccacacttttcgtaaaattttacattagtgtcggaacaatcaagaattaccttg
tagcaattcaccttgaaagccagtttcgtaagggcttgaatcagaagcttgccaatagagcgtttctgaaactcaggatggacgacaacgtcttcg
atatgtccgcaatttccattaccacgtataaactttctttccacaaaaagtgtagctgttccaacctagaaaagtcagtagatattcatcaaatgcc
aaacgctctgcttactactttatggctttctttgtcttcgacaactatagtataataagtgtctgggaaattttgttttaaatccataaaacgcttttga
taagaagccaaagttgggtgaacggtacttgctaccaagacatcgagtagatccagatactctacaaagaaaaggtaagtttatgaatataaac
aaaacaagttgctcacctgtattaatatcactaagatatagagggcggaccgtaatatctgtgcgcccattcaaaacttctgttgcgggatctttca
gcagaaactcatcaaacaaacactccgatttaactgtctttctagatccttcaatctcttctttctgttgaatcctagaagcaacacgaatgttctta
gtaacggcaggatgatacccaaaaacaggactcgtttccagaaatgcgaacaagggtgcaaagaaaatagactggaataaattatcaagaag
agcaggcctctttttctcaaaaacaccatggccgataaattgtaaaatccaacttacaatatgtaataggatagcagttgtattgactgttgattga
gaccaatttcgaaataacaattttggcaacgatattgagaatatatagagaactggagcataaagcaagccatctacaaaatcaacggacagat
agaagccttggtatatgataccaactaaaacagccaagttcacatggaaaggagaattgaccaacgttacattgaaattggagagtaaagttaa
agctgagcacaaaagtaatggaatacacactttagcgttagaactgatgtttcaacaaaaccatgatataccttggtggattctaacattgacga
gattggaatgatatgctgcataaaacctatagcttctactaaactttggacattattagctatttccatagattgaaatgcttaccatcgacattatt
attgagaggtttgaacaccttgcttggtaagtagtaggtaaagatgattcacatagtggattagagcatctgaatttcaaaagatgtaacacttag
atcataaacaataggttgatccttacttcctatatttgttatgaacgttaaataccattgacgattggcggttgaaacactagaaacaggattgaga
ctatagacagaacaaaactactaaaaattaataaaggcagcaaaaaaacatctcatattttatttgacaaagttttagctttcaaaggacgtattt
ataaggaactaagactaactacataaatatactaggtaaaatcttttcttgcaaaaacaaaaagaaaaaactgaaaagaataagtcattatgta
cgataactgaggttaatgataatattttcatcaaaaaagagcatcaaaatccaaatggattatctggcgtagcttgagttatactcgcctttctggc
attcggatttccattgtggtctattccagaaaaagaaggttgatagtaatcagaaggctgctgcatattttcatagcctgcaggcatgtataaacca
ggatttgatactggatcattggacgtctcattaggcatacccccataaggttgaccatatccatatccagaaccatatgcatccataccgttgttatc
gtatccataagccatttgttgtgaaggatcatagtagttttgcatataaggttgttgcattcccatttggccttgcatatctgctccataaaaaccttg
ttgatccataggtgcatacatactttgttggtcatatgcaaacgaagtgggcgcaacattataatacatcgactggtcattaaaattaggttgcata
ttatacccgtaacttggttgcataggagcaggagtgaaaggttgctgatatccagtgaactgattctgtaaaccggttggttgagatgctatatctg
tcctctgaccttgcataccagacataaagtcatgttgctgaccagtattttgaggcattacagatctatactgctgaggtgtaaccccggttgtttgg
tattgcattcccgttggctgcatcgcaacacctgtcgtctgactattcataccggttggttgatacgacatcccagtgggctgagcaggaaagccag
ttagttgagccttaatggcagtaggctgagaagctatgaaatccattggttgttgatgtccagttggctgcatagataatggtgacatgtgagcttg
ataaccagtaggctgactgggtaaagcactgacaggtggttgaaaacctgttggttgtacgtacagaggtgattgaaaagggtctgagtaggca
gggggagcagaagtgaaataggcattttggatagtaatgccagtaggctgaggtgctatagctccttgagatgatttttcattctcttcagccttctt
gtttgaaacatctgtagcggacaccgatgaagcacgtggagcaggctcaggtgcctttactttttcggacaagtctaattttgcatatcagattca
agttcggtcttgtgttggggagctgaagacgatcgaatagtaggttcttcaataggtttatttgaccaagcatcatcctcaaattgttgattcacagg
agcagcaggcttgctagtcggaaccttggagggtacaggctgtggtatttgcacttcaggagtagctggagtagaaggagcttccgtttttacttc
agcaggttttaatacagcaggactagaaggcactccattcatttcatttacatgcttcatgacacgaatcacatcgccttctttgagacctaaagtt
cgtaacgtttcaggcgttaaagagggaatgcacgtttcatccaaatgttcattgttgaaattacgggtatagcggttacagacagtgaaatcaaca
ccgcaacgtagaaagaagtcaaaccaatcatatgtaattttaggtctctcttgaataacatcaattttatctttagcttcggtggtagtaggaggag
ctagagctggtctgctttggggacgagcgggctgagatggagggatatcctcatcattttcattcactggtttactacggggctccttatgtgtttctt

tgcggcgttcagagcgagaataaactttcttgccagtcataagctccacaaaatcaaggtctttgtatgacattttcgtacttggtacagagatctt
aacgccgttggacttgtgtaagtgaattttatcatcactaaaacctaaaaattcagcatcaaccttgaaagcgccggttctatctgtccaagtacga
agctttgtatcatcgggcttaatgacagagtgactacgagatcttctgcgagcttctttcttttcatgctttggttcctctttgggttcatgtcgagaag
atttcctcgacaaaccaccattggacggtttggaagcagatctttcatgcgatgtagaatcaggcttgatgaaactggctggaaccacaccctcttt
tccattcagacgacatttccaccaatcactactagcagtatcatttacgatgacaactcgatcatttgcactaacagtcaactcgtcaggggcttcg
gcctcaaatccatagacaacagtacccatcttatcggtagttgaaggagcagccgatgatccttcatcagcaggtgatctaccactgttgtataca
gtggaagcttcatagaaaccggttcttccaccacgggtaccagatgtttcggaagcctcgttcaacttgtacaaggcggaatcagaaggcaagtt
aggcataggcttgccactagcttcggcgatttcacgaaggccggctgctctagcagatccagctacatcacctaaagcacgaatgatgctatcag
cattaccattgctattagcatgcaaatgtaaggaggaaattccgtcctctcgtttatattcgataaagacatgctttcgttcagcagagtaatttata
aggtcagtgacggaaaaaacttccgccgactccatgctctttgtgaaagtaagtacaacagatttgttattgatagcgaggatcccgacgcgcttc
tttttcttgccaacaacttcacgaacttcccatttcctaaaagctggatcatcaaaggaaacgttgtcgtccatgttgctttcctcctgagatccgcct
gcacgaagtttaatgtgtcgaccaggatctccataagcgtctgaaggagaagtaggagaggaaggcatgttggtgtaatccttcgatctgcttgg
agtagcagtggcagtagtagtgcttacgctaggtctgggaggttgtacgggaggtaaagggggtggaggaggggaagaaatagactttgcagc
agtaggctcggcgggagcagggggaggagggggcggcggtaaagtttcggtccgcggaagagtatatgtaggaggaacctcttgctctctgcta
gaagaataatagtcatcaggttcatcggcgtccgataaatcttcttcttcaacaggaggcgggtgaggtggtggagatgtgtaatcttgaggtaaa
ggaggaacaggaggaggaggaggagaagaaacggatacagaaggttgaggatgaggaattggtggtggtgatgatggttgcatttgctgttga
ggaaggggagattgctgagaacgagcaggttgtggttcatgctccacagaaggaggaagcggtggtggagcaggggcacttggcattgctgta
gaagctgatgcatccaaaggttcaagatagtttcttggggcaagtccatattgttgtttatatcttgtcaaaacccaatcttcatcagtgcttccata
agcatccaacgcctcgtcagctggaaatgagacctcatctattgattgcttttcataaccatacagacttttcatccttgctgaaggctcatcctgaa
agctggttagcattttgtttcatttttgaagactttgtatgcttttccattaatgaactagtttgctaaacacaaatcacatcacaaaagaaaagaca
aatagaataacataaaaagagtcgcattcgaatcaaatacatacttcagcaatatagttagaaggaacgatgccaatttcttcatccttgtcgga
actattaacatttcgcttgactagataccaatcatcctctcctttctgcaaaagacaaagtatttcgtcttccacaattgataattctcgttctgtttct
ggaatatgttcatttggatcgattggttccggttcataactgtacaaaaccttgtagatacctatgattggtaaatttgccattatgacaaatcgaga
ggttgtacagctttaattgaaagaacgactaaatcttggtggaaaatatacgtcggcgttgactttggttgatgacctttagccaagtgatcaatta
cctaagggctcgttaactctctgaaaaaggttttacgtaatagataactccacgggtatgttttcttaaacaaaattaaacagaataaacgcactg
cgtcaataaataaaggaaaataaaaaaacatataaataaacattcgcagcaatttttattcgtaagagaattcaagaagcatcgataaaccca
tggttttcatgctttttctttgttcttttttttttttttttttttttagtgttgcttggatagttttaagctcaggaaaaaaaattgaatcaattactaaga
gaaagtgaagtgtatcacggagtattcctcaatccagactgtaagcagatggctttctgaacattatcgcaaaaaagaaagattacagtgacgg
aaatgaacacgtttacgattatctacttgccataaacaaaaaatcatactgagtgatataaaaaaaattaattggacacactggggattgaaccc
aggccaactcgcatgcaaagcgagcatgctaccactgcatcatatgcccgcagttaactgctgaggttcaataaattatatattgcctgtattacg
gtttttgttatttgtccaatccataaagcttcgctgttttatggttacagttgatgtttacaaacagaatgtaaagttgttcattggaaagcttcaacg
atgctgtagagtgctagtgtaaatgatcccagatagtacgcattttcgaagtacagatacaaatggccgacctaagtagagaagagaatcgaaa
atctgattctgtatggactcccatatcttttcatctactttactttagttaaatttttcctttttctttcctgtaagctcaataagactctacctatctata
atgaatgtacatttggtcctgtctagaacgcgcaacttcttcgagtttttcgaatggttctttcaataaacagtcttaagaataatcaatttaaggaa
ttgttactcggcaatgaatacaacagacaataccgtttgcttcaataaatccacagcatatgtttactaacaaagtagaacgcgtcgttatcttcaa
ttcgggctgccagtccactcccctcaaccctaccctaaaggttaaactgaaccgtcacacacaacaggctggaactttaccactgttcttttgcttg
aaacaatacagcaatggcggcttccatcattcccaaagatgtttccttagacgaaacaggacacatcaagttgttcaacaagttccctttcgaggg
tgttgaagtcaaggatatttccttggtgtaagtgatttttttttcgagggagagaatggctttgtgaaaaatggttccttggactatttcgcctttttccg
ttgattcctattcaataataacttaatttcccttgctgtgttaataaaagaaatcctaaactaatacttaatttagtgattatattactgttggtaatgg
acaaccccttcctcacactgctggccgtttccaaagtaagcgcttccgtaaggctcgttgcttcatcgtcgagcgtttggccaacagcttgatgatg
aacggccgtaacaacggtaagaagcttttgtctactcgcattgtcaagcatgcttttgagatcatcgctcttttgaccgaccaaaatcctcttcaaat
tttggttgatgctgttgccgcttgtggccctcgtgaagactctacccgtattggtagtgctggtactgttcgccgtcaagccgttgatgtttctcctctc
cgtcgtgtcaaccaagccatcgctttgatcaccgttggcgctcgtgaggctgctttccgtaacgtcaagtctatttctgagtgcttggctgaagaaat
catcaatgctgccaagggttcttccaactcttatgctatcaagaagaaggatgaactcgagcgtgttgccaagagcaaccgttaaaaaacacata
cgttgtggtatggaggatcattgtttagtcataatacagtttttgcttggtaatttatggttgagtgttcgtagcgcaatggaaactatccactggttt
aaataagcctctaccctctaagggaacagtagcatgtaagaagtagaaaacgcaaaatcagttttcatattcatttctgtagttgtctataaacaa
gattgtagaatgttttcataactaagtcgtttttttcattcaatattatttgctcaagaaaggaaagcattgcttttgagagaacacccatcgctatga
gacgaaggtaaagcctcaaatgaatgaaacaaaactgtatgcttcgtgaatctaatctataagtcatgagaattcgcattggctttaaaaacgag

cttcaggtaagtattacgaaaactatgctccctaaattaaaaaaacgaagtatacagagggcccaaaaattaaattttgagagatacatgaggg
gatgaagagagagatgaactaaaagaaaaatactactagaagtagaaacagattgcttcaaaaccaccagtaataaaatagatgcaaggact
aggacaataacccccttcccagcattttaagccatatacttttggtaaacaccgacgaccgagttaaaaacgaccaaaaggataccaccgccagg
gcctaaacggagtactttggggacaaagcccttgtaaagagcagtaaagccttcctccttagcaatagtaaagagagcaggataagcccaattg
tacttggggatttgaccggcgatacgaggagcagtctgtacacgggatttgacgacatcaaaaggggtacttagcaaagtaccgaagatgccac
caatagtaccggcgataagattattgcacatttcaccagctctattcgtagcaggttgaagagaattgcggactttttggataaggccaaagtagc
cagcgttccaaacaacatgacgccacatggttgcctcaaagccgttgtaaagggccaaaacaccttcgtgcttgatcaccttcgataagcaatca
aaagttccattgtatttggaagcattgttgacgtcctgaagacggattttcatcaactcaaaggggacgacgacaaaggattccgtaaatccagc
acatgagccagtaagaagggaaaggctaggtgtggacttcttttgtccaaataccttgcgccaaaattttccatacgcctcattggaagcaaattt
tagagcacgtttgggggcctccatcataacaggaggaaggatacctcggtaaagacgactagctccttccttctgaacgatcttctttaagcagtc
gaaggttccggtgtattcagattttccgacagccaactgcatacgtgtctttaccacatccaaaggatacaaagagagcacctagcaagttagag
caaactccatgaatacaacagaaacacagtagagagtaaatacagcccaaaagcactttaatagtgtactaacaaatacatgatagtcgaaaa
gatttgtaacccaaagttttcgtgcaaagatttcttttttttttttaattctgttgacctctataaaccacccatccataaaaattcagatcctgtttgc
aattccactaatgttgctattgtctttaagcagtattacactacctgcttgtctttctgttttcaacacaactacatacctcggaaataccagcaacag
cgccagcagtaaaagtcacatgaaatggaatatcgtcagctggcattttgaatgttggatgtatgcacctgtcgagatcagatgtccaagtgtttg
gaagaagctaagttgtagaactcgagagatctgatcaaggattcaaattgataaagccccgtatttacccgtaatttgccttaagaagactgata
agcgccgatgacctttgtctctttaaaaaagccaaaaaaattttctgcctcgtagagcaacatgttactttaagggtgtaaacaaacctatccgaa
tgtacgtgattgaagaatacttgggaattcttgcaagtgcgaaactggtagaggtaagttatattgacgaataaatttagaaatactttaaaaaat
gacagattgaaaacaagaactcattttcatttacaaacaatacgaaacataatctccgtacgaaaatgaattaagagttctagtgaaactgatgc
aagctatcgaacagctatgttatttcataaacaaaagatcgaaaaataaaggattataagaaaaaggctaaggtatagaagcatagcggtcca
aaaaaaagaagaaagaaaaaagagttccattgtcctttgattgaaaaaagaaaagaacagagtattggaatcttcaagttgtttttctttgatc
gaattccaaagacacaataactcaataaacacaagcacggcaagacctaaattcattgcaataaaaccgattgtggttatattgtaaatggcac
ctccagtgatagcgcctactgtatcagccaaagagacaccaacctggacaacgatgctcaccaaaatttgaaacttgggatgaagctgcttcgaa
gaaagagagactaacggagcagagccgacactaaaaccaaaggacatcacagagtaaaggagaaagtatggctgaacaggaattttatgca
cggcatccaaaatcaaatgcaagacaagggctataattccgatcaaaacaccaagtaacatgatttgacggtcttcgagccatttggacaagaa
ggtactcaacagaataaaaggtgctataacgagggaggaaagcgctaagaaattgcctgtttgaaaagcattataattgtataacctacccgcg
taaataggaatacttgtctggtatcccatgatggtaaagtaacaaacgaaagcggtcaaaaacaggaatgaaacaaaaatttttgaattaacg
acagacgaaccgtttgttctccacttgttccttctctgtgtgttacagatctgttattctctgggaggatctcaataaagaaacttttcaaaaacacc
atgtacactccccatgcgattaacataaaccaggagccagcagtgtattcattccagtaggggctgtgaatgtttcgagacgctttggccaaggta
cctcctataaacgcacccgacatgaaacccaaagtttgagcaaggatattcaaggttgagagaaaggtagatttatttcttccgacaaattccttg
tcgctcaagtactttttgtggtataaaaacatcgtaaacccgataccattaaacatacgtcctatcaaaattaaatatatccaatgagcccgatatg
ctaagctgtatagcaaatgtccaataatgtgcgcaaagcaagatgtaaacatgggtcgaaaatagagcgtgtacccgtttgcggattttggatttg
cgaaacgaagcattgggtacattaatactaaagagacaagagttggaataccaatgacaagaccggaaaatgcatccgttccacccaagtattg
cgtataggcggctgaaatcggcagggctgttgttgcagacatttctgacataaggttgttgatcaatatgagagcaatggagcgctttggaggata
cttatctgggtcttcttcaggataatatggtccagtttgtgttgttgttatctcatcttcatcttcaatttgcgtttgcttttgcttacttggtagggtatgc
aattctatgttttcagtaaaaggttcctcattcccagctcggtttaacatagagtcccttctcaatggccgcgattcaggaacatgccgacgatggct
cagcatgacccccttcattgaattccgaatcttccattccttcctgttttcagaattaccagttacttgctcaaatattcccttaaataagataaaaatc
aaaaaagtctactttaagtttaccctactcttatgtctccagtgccctgctttaagtgaaaaaaaaaaaaaaaaaaaaaaaaatgacagagtaa
gaagtaggagttttacattcgaaagattgatttttcacattgatcagatacttcgaagcgacactttaatcaggataagcattagggtgtgtcgctc
aaataagggctgaaagtaaaataaaaaagaatgtaaacaaacgatttaatagaaggaacaatttgctaatttactacattttaattctttcattac
tatattcctgtttgcaaatactttgcaaacacttttacgccgctatcaagaggttcaatcataacgaatctcttaatagaagaaacgatcagcgcga
ggtatgaaggtgaacaggaaaacctatcactccctcttacgaagttaagcgatctctttctgctacaagcttactcttggttctataaggtcaatag
ctttagcccaaggcgctagtaaatcaagaatagaaaatgcacaccatgcatacttacgtaccatacatttttttatatctaagtatatatactagaa
aagtagaatagaaagaactttctttaaggattcttgaaaacaaataagcgatcattcaataattcgtacatcgtccgtctcgtatataacgatcgt
gaatcccactaattataaacagcaccagttaacatagcccttttgacgaaactattgaaacccttttgtatatcatttattcatttattattattattttt
cttttttattccttcctgattccctctgggaatgattacaattaagccgccaattgtcgtaaacgaatttcatcttttgtttgtggaattatattgaata
ttttgctgtttttactatatagaatggaagaacaacttatccttttgcggaaaggtaaagcgtataccaaaaaaacaacaaacttcgtcgaatact
aactatggaatgaactagagtttttgcaaagactctataagaataagccagcaaaggatgctagtcgggaagaaatagctagatggctcgagaa

tcgatcccaagaggtctcaaaaatgactgacatcgtcaatgaaggcaaagagcagttagcaccctacgatagaatgaagtatatggaacaactt
tcggacttgatcgttcgattacatgaaacacggtctgaaatttttcctcgtcctccttttcgttttcatcgatcctaccaactgcagcagcaagcaaa
accagtagccaacaggcaaaaacgcgaagatgtcaaggtcaaacggtttgagaatgaaactctttttttgtcaaatttgtcaaacgaaactctag
ataatagtctcttcgatcaccaagaagaacggccgatcaaattaattataagagatttacaagggtgcaatgtgtatattcctaactgttctgcggt
tcatattgatagctcaaaagactgttcctttcactttggcaatattcgaggatccctccatatttcccagttaaaaagctctcaaatatgtgcctcttg
ccatcaggtaaggtctctttcttctatgatttttcaaatttccctttgttcccttctgttgtatttactgacgaaatcagtttcgcttacacgaatccaat
cacctctccattcaactgcattgccaaacttcacctgttttagaaggatgcagcaacattatttttgagaaccctagtgtgcatccaattttggatttt
tcttgggcgaggccggatgcctcgcccaattttaaagtaaaagcatagcttttactacaagcagctatgagcttccttaagaattgaattctagtac
tcgctgcactctcctctccttatttaggaagctaatgttgctaccaagctatcgaaaagaacaaaatctcatacgctttataattccatccgttacttc
cactcatattctactcgattaaatatttactgtcgtttatcgaagactttcttttgtggacttcgaagtactgtagaaactgaatcgaaaagacgatc
attcaacaaaaaccaatcttttctgagccatttctttcttttactacctttatatataagtaaaaaacgaaatctcatgtcttggccttttaatgttttc
gttatttataataagagtaaataaacaaaaatggctttcaagtaacaagtaaccttctcgtatttttttacgcatataaccttttttactattatatagc
atcagaaagaacatacgttacctaagcaattaccaaattatttcaaaagttaattccaacatttccttaaccatttcagaagctttcagttaatacc
agaaagaattgctgtatacctatacatatattgaagattgcatcgtaattgtgtgccaaaattgatatttttaggcttctatgacaaacgaatcaca
ggtatttgaattgtagcttttgttgaagtattcattactaattgactgtttccatatagccagaccaagaaactcttgaaataaattcatctcaacatg
cttctgccgatagaaatgttctttctgcggcttttcacccgccagaggatgatgatgttgaaaccgatgaaagagctcctttataccaaaatacacg
gcagtctatggatacggaaccagataccattgattcagtgaaccatccgtcagctgaaaatgcaccttcagctgatcaatcccgccattcaccaca
agctgcttcaagctcaacatctcgtcaacccagaacccactatgtagacagtgtcttttccaacctttctgcaaaaccaagtgcacaacctgagaa
agagaatgaagaacctcctgtaagtcaacaaaccctagctttccgcagacagttttcttttcatgttcatgatatatattatcagtataaaatttgtc
atgaaactagatgaatgattactaactttttttctagacatatgaagaagttgcagctgatatcgcgcctccgtattgggataccacaatgataattc
ccaactttggtactaacgaaatttacattgatggaatgacggtcggttcaccaattgcttttttttggagtgctctagtatctttttttgtttcccttcgtg
ggctttctcgtcacttacgttttgtctgcaacccattcgggtcgctatggatcgcaggttggtttgagcatgaccctcttacaacgaggatataatat
ggttgttgaaagtggaatgcttcaaaatgatgataacgataattcctatgattttgatgaattaccgcaccaaaaatttataggttgtattcttttaa
tcacaggttggtgtttatttttaattgatactattaattttattcgagtccgccggacgaagaatgctattcaacaaacaaatccagagaatctggct
gctgaagccattgtttaatgtttaccgcatttattcgcaaatcctttacgcatttatcgttttgcatcatcttttttttttttcttcctattccatcctatatc
ctatttaactttaaattgtttctctgttcttgaggccattttcaatggtcttttatttacgactttaccaatgccaatatttgaaatgggtaactatagaa
ttgtttaatttgttatgtactatataatgatttctttttttttttttttttttttttttcattcaaactgttacgctcataatggcttaactggggacccagat
tgaaggataagaaacgcttttttattatttttaattgaaatgtctaaagtaggattcaatatcatcagtaaacataacatatgaaatatcatgcata
aactctataatccaaagttttgagtccaaaattttgaaatgactgctcactttacttctaattttcacaggacagatctgggttgcgagacagacact
caataagatgcctgcagtctttccttacgtactaaataaatgttaatacacgctcttagtttaaaccagattttaacttactgttagcatataaggat
tagcggtatttcccgtagtgtctgcaaaaacctcgttttcatcaacctctgtaatatattcgtcattatattttcgaaaaacgttagtgttgaaatcac
gcatatatgtccaataatgtccaaaagttgcctgtcctcgatgaataaaaacggctaaaaggtcataactgtgtttcatatggccatcaaacaaat
gcattctctcttcctgtaatcgtatttcttcctgtttcatagccttgagctctacttttatttttttcagtctcttcctgaagaagttgaggcaactttttgct
tataggcaatcgtgagcgtttttgatcatcagcccaagctgagagtgtagttaatgtatctactgaagataaaagttgtgaattcgtagtcattaga
aatttttgacgctgttgcagttcaccaattctttgtttcatgttccaatattcatccaacaatggagccatttgcccattttcatcttccatatagcggt
ccatatgtaactctctcccaaattcgacgtaggcattggatttaaagggttgaccggtacttcgatcaaattgtactcgttggatttgtagctgcaaa
atcatcggaagttctttaatacacaaagatcttttagtagtttcattgcctaaatcaagtgttaccgtctcaaatacaccatccaaaacatcatataa
ggtttgtctttctgtatacaaatcgacaatcagatgcgaatacaattcttcacttgatctggttccttgactatcatcatgtaatgtttgcttggtttta
ccataaaataatcttcgaatcaaatcttcatctgaatttttttgatccttcgttatctttcgtcgggcctaaagaaacctcaatttgaaataaaacgtg
atcaatgcattcggcaacatcctgctggcgtcccaaatccagagatgaatttatctcctcttctgatatatcaaaataagctatttgtgtatcttgttc
attgttagaaggcaaattgactaaatccgaagcggaagtcgagcgactgctgtttgaaggagcagcggtttgttgagtgtgttgatggctctccaa
agatgcattgcttaattccattgggtgaattacagatgaggatgttgaattgacttgttccatagttaacgggattaaagccaaatacgccaacttt
gatgatggaaggatagatgaagatttagtggtaataagctgctgaaagagagaacgaagctcataggtaaattgtaaagcgcgtaaaaattca
atgcggtttacgctacgaccaccaaccttttttgacagctcctctgtcttttaccatagaaatgtctttattatggtcaatccccaaaactgcatcacg
aagaggcttaatgataaaataatattggagcaaggaattcaaataacaaaaatttcctgtgttttccaatcctacaggaacatttaaattgggtgg
ctcctcaggaacgtccaggttaccatgttccaaaaagtgcattatcagttttgaattcctagatttgccaatgcaattcaatgcagacttcaagcgg
tctgatagctctggttgatcctcgaccgcaaagttataaacattaattaacatatcatccgaaacgtcacggtcctggactgcaagtgtttcataag
cgtcgtcagtggtcatgaaagagtcagggatttggggaatataagaagggtcctgagaagctaagaattcatacaaaacggaactatttctttcc

tcggcaatgcattttaccgcctctattgcttttaaagaatcgtcctcaaatttgaccaatgcaacagaagcgaccataccgtcctcagtatctggag
taattccaagccaagtacaagcctcgtcgatactataaaatattcggtgtgtagaacgaagatattccaaaagtcctttactgtttctagcataagc
aattgtttccagattctcgcgctgaactcgtatcgattcaggatgcttttctatgaacttcagaaacgtgtttaatatatcctcatccgtatatttgaa
attatgggctaaagcagaaacttctgtgactaaagatagagaagaataagctgcttgtatagtgtcactagaaacaagaccaatgtttctatccg
attgaagttgatccttcagcgctgcagtttttctgagattataaatatgtgcgagagcatctacataaatccttgcattatccgggtcattctctttttg
tttgttataaaaaaaacaaactaaatcatcagggtaatcattcgtaacaccaagcgcaataaaagcagggtcatcgggcaaagaagcactgttt
tgatggaagattgaagtgtacatgggagcaacactgttataagcgggaacatgtaaagcagaaattaaataaggacgggctaattttgcggatg
catttggactagggacgagaggatcctttaatccatctcgatagttatttgccaatataatgagctcatttcgagctaacgttaaatttatttttgct
cattagttaaaggttcgtttgcaatgcttggaacgaaatttacgttttcagaattaaagtgaaatgataaaaggttcataagtttttaatatgaggt
ccttttccaacgtgcttctcaaaagtaccttccggagcagcactgatagtacgatgatcattattgaataacacattgcgaagaaacagctccaga
gaagcaaatgttgtatagagggaaggatgcgaagatcgaggtgactgttgttgagaagccgatttttttgcaattaaagaagcaaaggaaggac
gtcgagagtctatgatgtctttaatacatgaagcatccaaataactgtctctccattcaactttcacgcttatttttccatcttcagacttccactgca
cagcagagcatactgatgaattaggagtgttctcaacatgttcattaagcagttctgtttcctccatcatacagaatgcgattattttctctggtggg
gtaaaatccaccccaggttcctttattaatggaaaaccgttttctttttgtatttgtacgtgcatatgctttcgggttttctggcaaacgcaccagaag
tcataaggattttgctcaacggaatcaacagaagcgaagtgcaattgccaatgatatttagcattatgagagctccaagggttattccaatcttta
gaagatccgtcaaagacgtctaaatcttgtattaatcggttagggctcttcctaattttaagaccctgcatatcagccattcgatgaatatcaaatat
tcaggtatttaccgatacaaaatgagatagtttatgcaagaaataagggagatattgaagagaaaagaaagaactaaatattgtggaattggaa
gatacacagagggtcaactgcatcatctgcaatagggcatgataagattatattaaaaagaaacataatattaaagaagcttggagtatattata
taaataacgttaaatctaatatagcaaaaagcgttgaaagcataaattaacatcatatatatattagtaaacaaaaaagcaagaggaaacgaa
gaacgcaagaagaggatgaaagattcggtctgtactttgagacaagagaattcgttacgggagaaagcataacttattctttacaaaaactcca
agaagaaaggaagaaacaagaagattatcatatgcaagtcccaaaaaggcgtttgaggaaactgcaatgagaagcttaaagcctttttttacta
agaacaaataaatttttaagcgacagaacaaaaagaagttataatgaaaattcttcgaataaggttattcaacacgctaactttgatagcaggta
ttatcgtagaagaccaatagttcggacgctgctctagacaagatatattaattctaaagaaaccgtcaaataacgaatcattaaaacacagcgct
aaaattcctgccgaacatgattacgattcaccgtttgttcgaagggaagaaagcaattcaaaagctctcgctgcttcagtggaagcattaggtgat
gaatcttgctgagagttatctaccatatcattaacaactggcgttgtgggtacacttggtgacaggctaggagtagccatgggaagcggtacatag
ctgagcataggactggcaaacgattgtgaagccataggagaattagatgttgaaatagggatgatgggagaagggtaagccatcggagccgtc
gtaagggtaggaatcatacttggtaatagagggtttttcggctgaccgtaacgaacataagaaacttgtaaatgtcttccaccaaattgataaccg
tttaaatattcaatggcatcaccagcttgctgctctttctcatattgtacaacaccgtagcccctggaacgaccattcgattcataagcaattttcgct
tgcactacattgccaacgtttccaaaaaggttaaacaaatcttgattaacggcggcccacggtatctaaatcagttagcattcgcgccatagagca
aaatataaaaacatacattaccaacgtggatgcgatttgatggggcaccgtgtgagaaggcattatctacaaactcatcaaatgcagctggtaca
cctaccattgctggcatatacatcggactactcatcatagaggaagcatcatgcgaataactagggaatccaacgccaggaacagtgtatgaatt
gtttctatgcttggcaaagcgatccaatcgcacttcgagtgtgcgacccatgtaatcagtgttgtttagcgattttatagcatcgtgtgcatctttcga
ggagctcattaaaacaattcctataccacgggaacgaccgtcccggttcatttgcacgtcggcacgaacaacagtgccaacttgtcgaaacaagt
ctttcagatcttgccaacggacagaatagggaagctgtattcgtttagcataagcaaacaaatcctttcaaacatacatttcctacaaataactgtc
gttctgtatcattttctcgatcaaacatgtgtccatgaggcaaaatacgagaaccaaatttgggttcctgttcacgatcctgatgcgagttagcaat
caaaaatgatagctacatattccatcaacaaaaaaatgtcaatctgtgcttgctgataataataagtacgtacttctcggacataaatgaggcga
cccataaacttttggttgctaagtgtttccactgctctttgtgcttcttcactagtcgaatattctatgataccacaacccttaattcgtcagctaccta
tgtactcccattaacgatgttccaaagcaatgacaataaacactggatatcctagacagcaatactcatcgatagtataccgtcattgctcacaac
gttgtatgaaacgtaccttggaaagaccattcggtagattgaggatctcggcatacaatacgtgacctgcctgtttcataaaatccttcaattcata
ccatcggacttgaaaagaaagatttcccacgtatacacggcgttcctgccgactgttttgcttgacagtcaccatatagggatctttcaaaggaaa
tccttcttcgtgatagctccctcgttttcgcatcccgctgacatcttcggagcttcttggagacatggatctttcatggtaagttgccggcaatgccgt
atcctggagatatgatggatctgatgggcccaaattacccggagttacatcggtctaactttgttagtatttgtctatatagctcttaacatcactta
cccattccatttgtccaggcatcatttatttgttggtgaaaggcataaatattcagtccatatgaggaatcatttccaagcttatcacaatgctaaaa
aataccttattgggattaatggagtaagctactcttctaccgtgttaataatagtaatagtattgtataaactcattattataatattgcttcattttcg
tagtatagaagaaaacacatccttcttatgatatacacaattgcatggaaagccatttgcaacccaaagaagaatccatatactagaataaatta
aaaagtctaaataaataatttcgttattcctggtctgttatacgaaaaaattaacgagtcaatggcaggcattcatactgtcatcgtcatcgatgaa
gacacatgaaggaacaatataccatttcaatagtagcaaaaggaaatataagccatagagaaatgtacataatcagtgtaagtccttaaactca
acaacagacctaccgaataaaaagaataagatagttcttttgcactagttatcgcattcataaatataggctcataattttatctttgctttagtgat

atagtagaaaataattaaggcataggaagcattagtttcaatattaatagaaacaccagcattattagattcttgaaactctgttcatatatataca
gactatgcttgttgtccaaattctgtattccaccgatcatatcgctctaatccctccaaattcacactaggcctaattacttttagactctgcaggaag
tcttgtaggttcattggacgaatcaattccatcttcgtaaatagcagtgattctcctaaagcacgcaatggacccatagctgcgtctttcgccagag
ctgtcaaatcagacccagagtaatatttggtaagttgaacaatactttctatgtcatcatccgttaaggaatgcttttgatgttgtaaaagattcttt
atatgaactcggcgtcccgtatcatcaggtaaaggaatgtaagtccgacgaacgaatcgacgacgagcagcctcgtcaattgaccatggtaagtt
cgtggcagcgagtactagaactctgggctggtcggccgactctctggaagcagcagctcttgcaagtgaagaccattgaattaagaattccgtttt
gatccgtcttgagctctcatgttcgtttccatcagcgcttctggctgacaataatgaatcaatttcatctacaaagatgatactaggcgacagtttttt
cgctaatgtgaataacgctcgaaccaatttttctgattctcccagaaatttggatgtcagacttgatgccgagattgagaagaaggtagattttgat
tctgtcgctaccgcacgagctaacattgtttttccagttcctggaggaccgaaaagaagcataccacgagctggctctcgaagaccctgaaataa
atctggtcgcaaaaaagggtatacaaccgcctccttcaaggattccttcgccgaacctaatccagctatgtcatcccaaagtacttcatctcccttg
acaacgatttctctcagaatattccttcctaactcctcgtcaatttctggacaatcttgaagtacttgctcttcccgactcttcagagtacttatttctgt
aaatggattaaaacttgcattgccaacctttctgtaccagaagaaagctcccaatcgtcattcacactacgggaagacgcagtatcatttacgga
tgacagcgaaagctcagatggagataataaatgtcttagaaagggattattggagttggttggacgcaaaaaagaagaagatgatgatgatac
acgaaattcagggatttcaagggaagcaggttccgtttttgttagcgtatttgagatggaacttgaagaggaaaattgggaaggcattcctgtaat
agaacctggaggagatggagatttacgaaaattctttggaatcacatttgttaaagtctctgagggatttagctttggaacactggttctacgcttc
atatcgattgacggagaagtaaaacgagaagactgctgcggacgcatactttcaaaagagcgtgcttgaggataagcatggtaagcttcagttt
gaggattatgtgatttataagcagcactcgaagcgatgtaggcaggtggtaatggatgatctttctgcttggaaggagaaaatagcccccaagca
agtaaggcggctttattcgcattcgtccgtcgcgattcttcagatggagggtttataggattggtagttttgggtgacgtaaggaatttgcttccaaa
taaagcgtttttaatcttcgaggattttgaagctttcggtgctttattaccctctgtttctgaaggaattggacgtaatgatgtacggtgcgctgaaac
tgaaggagcgattgacgtttgactagaggaactagcactgctggactgttcggtccctgcagacgaatctcgtctctcctgactagccgtagcccg
atgcttcgccggcttttcttcttccttgtatggtgaataagtactgaattgaggctttttaagttgttggagtgcagcttcttgcagcacaacctcgttt
cttaattgatcaacttgcgtcgttagctgttccaacatgcatataagtgcttcggcgtactgtcgttcttctggattgaggacttccaaattctctttag
aaaatagaaagctaggattatcaagtatatttttttggctttttcaaacatcacaattgcttccacataatctgaagattgttgcttgtgcaatgcta
cctttgcataggattgagttgcattgtaacattgttctaagctcatttttagcattctcttctcctacccttacaacaaaccagatgaatcaaccgca
aggaatgattactatacacagtaattcttaaccagaacaagaaatcttaatgatcaaagcaaacaatataaaagcgcagattcagacgacttgt
acaaaattcagtatatgtgaaaatactttctcaaagtgaaattcaggaattctaaaacccagtcgtattattcaggaaaaatacaaaaattaaaa
aggtggttatgactaaattagtggaatatactccaagggacgtggtcttttgaagtttctactcaaccttcgcatcaaaactcagacctaccttatg
atggtactatgacgcagaaaaaacagtttttcctcatggctttgtgaacacattgtaaaggaagagatacgcaaatttccaaatacaaaaaaa
aaaaattaatataaaataaaaaacgattaagttaaaaaaataacaggaatacggtgaatgaataatatgaaaccgatttacctaaaccgaactt
gtctatgccaaaactagatgtagtaaatttagtgtattcatttttttgcgactacaagagtgtccatgttcataatatcttcataaacttttgcaaata
ataagttaaacgtaaataattttactcgaaaagatttatcaattggcaaaaatcatatgtatatataatcaacccaaccataaacacaagaaaag
aaaaggggaatgtcgaaaaagttctgaagtctggcttagtgaaagtaataaaaaaatccaaagataacgttaacatcaaccataaaccttcata
cacaaacacaacgtacatgtatgtaaaggaatgcgcgcctttcgtaacaaaaaatttagaaagtaaacaagttagcagatagcgaaccaaacg
ctactgaacttgcctacattccaactctaccaacttaatcgttggacatagaaaggatttgcgcagatttcgtctttaggacaggggttggtctcttg
gggtggctgtaatgttgaatgcgttgtaattcaccaacaaagctttgaccccagaattgactagtgtatttgctgacgtagcgatacagtttttgttg
gtttgcatctctttgattatctggcaaggttaatgcttcgtgaatggcattcgctagctcctccgtattccaagggttaattacaatacttccatttagg
gattgagctgcaccagcaaactcactgagaattaaagcgccatgacgttgttgttgagtacaaatatattcgtatgaaactaagttcataccgtca
cgagtagacgtgatcaaacatacatccgaaatgttgtacagagcgaccaattcgtcaaaccgaacgctcttatgcaaaaagtggattggggtata
ttctatggttccaaaacgaccattaatgcgtccaacgagttcgttgacaactgctcgtaaattttgatattcttcaacatcttgacgagaaggtactg
caacttggaccaaaacgactttgccaacccattcaggatattgttctaagaagacttcaaaagcatggaatttttgaggaacacctttaatataat
caagacggtctactccaacaatgactttgactccttgtagacgacgttctatggtagagatccgctcctgaacggtatcagatcgtaaggcatctcc
aaacttttcgggatcaatgccaatggggaaggttccgacgcttaccatttgaccattgtactcaacaccgtttggcaaagtgttcagtccaaggatt
cgagagcaagcggaaaggaaatgacgagcataatcataggtatggaaaccaaccaaatcgcaattcaggacaccctcaagaatctcattgcga
acgggtaaaacacggtaaatttcactacttgggaatggagtatgtagaaaccaaccaattttgatatctttcagcttatttccaattaattcacgta
ataattgaggtaacatcataaggtgatagtcttggacccagatcagatcaccatcgcgcaagtttttaacgatggcttcagcaaaagccatattgg
ctttttataagcctcccagttttcctcgtcaaaactaatttcaccagggtggtagtgaaacaaaggccacaaaatgctgttactaaaaccattgta
gtgacggtcagcaagttcatcatccaagaaaacgggaattgcactgcactcgtctctcagacgatcgacgatcatgggcttttcgtcctcagggat
ttcctgtcctatacacattgtgttagttattgcttaagaagatccataatgaaaacacaagaaaaggaaaacaagtttggactcgagtaaaacgt

ataggcctttcaaacctgattaactaaacagcaacgttttcctatccattttctccataattgccttttcttggaatactgcttcatgacataccacac
catcctaaccattgaaatgtcattagtttttcagaccacttagggcgctgaccagaccacctgaagacatgctaaagtcataagttccattttcttt
acgcttaatggtaatcggtaaacggttggagacgacaattaaacgtcgagtttcggtattcatgtcttccgcagaagtttttgctaatgtttccaaat
tcgacataattgaagtccttgctcttttataaataaagtatcgtaaaactgaaatagataacgttaatttcccttggaaaataaaagttttgtggttt
gggagtttgtaaacggtaagtgttgttagtagttattatatgcaattcgttttccagaaaatgcgtaagaggggtatgatgctatatgcattagagg
ggaacaaatatcaattaccaaaaatctttaagttaattttattcaatttttcttttatttttttcttttcatttctaatttttattttgaacgcctgataa
aaaaattatacccgactaattttgtacgatatggaaattagaagaagcactgaatagtatgatatacggaagagggaaagacatacaatgtaa
aatattcatgacaagaggaggacaaataataaacgtatagagccattcgctactgcttcactttttctagatgtacatttatgcagtataattcgg
tttctatggaaccaaacaagataacaagacatggaatgtataattttggaatccatagtaaagtatattagttcatcacgaaaattgtagaatata
gggtagcaattcataattcatgcagctacagagacgaacatctaggttaatggatctatgtcaaagcaaagaaaaaaaagacttacactcaaca
tcttcttcactacacagagcacatctatcgatacgacattcaataatattttctcaaaagttgacaatatattttgttaaaaataccaaagttacaat
gttcggtaacgatctttagtatgacgtagatgtaaacaaaccgaagtcgtaaacagcgaaatacaagaaaagattccagaaaagtaagtagctt
tcttcatagtggatgccaacctatgccgtttatgcagaaaaaaaaggaaatcataaacaaaaagacattatattatgaacataaataacgaaaa
agaaggatatttttacaacttaatgacagatacgctaaaaatcaacgacatattcccatcgtccttctgccagtcctcttgctgtgtcgtctaataaa
acatcgcgtcgacttcgcacatacactgttcgatccagaaccttttgcttataggaagaaagcatcttgtctaaagtcggactttcacataactcac
ttaaattctctacagcaagctctagatcacgctgattatcttcaaaaatctcagtttggttgtttctcacaagaaaataagcaaaagcgtacgtcca
ttttagcgtctgccgacaatcgaacaaggtatctacagcgtttttcagaaattgtacctccacccacgataaattagaatcaacctgcatctgggtc
atacgttgataagttttgtcatataactgatgatcgagtttagcagactgctcatgatttgcaaagcgattgtaataatgcaggtagcgctctagag
atgctcgcgaccgagtttgcgcgtctctggcattcgacccactttttcctcatagcggttacatgaataccaattcgtgccatgttcgctccatgga
cccaaacaaacccaacagtattcatatttgcatttttacacgtcatatgattgcagcctccatttttctcaattgtggtagagcatttgggacattct
tttgtgttggcgtggatccaatttgccgtctctgagtcatcttgacatttttgtaaccagaccttcacgagtgggcatatggcaggctgatgattatca
cgaccacagccaaagcaaaattgttttccacaaagacatgttaccgtcgggacaaccgagtttaagctactttgggaaacatgacattcaatggc
ataaggacaatcgggagcagggcaccaacgaaacatatcgctatcgtcaacaaaagatcgatccagcaaccgagaaaattgatagtgaaattt
ttcatctaaaaggtttaaaatcgactgctgcgaaattatctgactacatccttcttcaggacattgaatcatagtttctccttcttgaattctcgacac
aagataatgttgataacaagaaagacagaaatcatggttgcaatccgcactgaaaaagggaagcaagccctcatcgaaacaaatagaacatgt
accttccttttctgcaacttcatgctttttttcatcactttgttgaacaccagccttatgaagcgttcgctcagggtcatcgacatacatttctaacaac
ttttctcgattccatttgaaatagcgatacaaacccaatgcttgctctcttgtcaaagtgataatggacgtcaattgctctatttttttcattttgccaga
ccctaagatctttaggactaagcactttgtagtctacatcgtatgctttccgtcgtttcttttcaaccgtaaatccatagtcatcgataacaacattat
cagcatcaaattcttcctctaaatccgagttttcgaacacctcgtcatcaaagtcttcttcttcttgaaaaaatccttcgtcttcttcatatgcatcaga
catgatattctacgacaatgcctttatctgtcacaaagttactttctcttcgttaatatcctcgtagacagtcaaaacaatccaattccagtttaaaa
cttccaatttaggatgaaaatcgaattctctctcttaagcttttataagaatacagtactgaatgggttcaatgtgaatgaaaccaaaagttaacct
gggtaacctacggacttttggcgtgttcaaggcgaaatcccaagcttttcacaagtcaaattcgtttgagcaaaagaagacacgaaaataatctg
tagaaataaagaaatcgacgtatttactggtagccacaatacgctataagagaccaaagtgttatttgcagaggaagagtttggtggttatcata
aatgtaagcgagtattttcttaaacctattttacgtaataataattaacttacgctaaatgtttttgggggcttggtagactacactatttgattaagt
accagtaggtgagcaggcaagctacgcttacttggaaacctccagacacggtaaaggaaaggattagcacctattgactcaaaaatagaagta
aaacatataatttattgttaatattgataaagaaagaagctttaacttttaacatactggctaaaagaagctgcttttacaattaaggcatagtaaa
caaacgtggctgaacggatcattggtttcgtaacatactattgatggacgaaagcaatttatacagcgttttagaagcattaaatgttgttcacgcg
cctgaatctagtcgagaagctcgatttgaggctcaaggggtaagtggaagcttcttttcatcaattgtagctattggacttactaacatgacaatag
ttactagataatctgaaagagtctttcgataacagtggctcaggtttccaattactcaatctaaatggtgaagttataaaagctcgagggtacaaa
attgatattcatgtgatccgacattttgcattgagccttttcgaagcaagcattaatagtaactggaagaattatgatgatcaaacgaagcattctc
taatttcattcctttgtgccttttctttgcaaaactcagatgctctctccgtttattatgttcgtaacaagcttgcttctgtctttatagaaatagtgaaa
cgagattggtatagtcatgtttggagagaagattttgatttatttttgcaatccctctggggtatgaacgtggagcatcgccaactttcttctttcata
ttacgtggcatcatggaggatttatatcagtatgatgaccctgtcgcatcccttcgctctcatgttctgtttaatgcgttaatcagtgttttatcctcttc
tcgcactcttcacaagttatatccatccggtctcccttattctgtatctattcccgaaaataacgaaggatggttgaaccgttggtcatcgacacttg
aaagtgaaaccgatgcactagaaagtctgaaatgcttcaaaagttgtctttcttgggttgccacggattccatcattgaagccaatattgtgccccg
gatttgcaacattttagttaatggacctcttcaattgaaaacgcatgctattgattgtatatatatatgtgttactagaagcatggaaattgacgatc
ctctttgggctacagtagaagaattgctttcgtctgagggcctatatactcttcatcaagtatattcaaacatatctcagtcgatacaaattgatgac
ttatcttcttcttctggtgaatacatcttgcttaagaaacttgccgaaaccatcgttgctcttggacaatacaactatttggattctagtcgtcgaaaa

gctattaacctggacttcttagacacttactctgctttgatccttgaaattatgaggcatccgagcttattaattagtgccatttctcaacacttttggg
tcttagctttaagggatcctttaatatcgaaacacgataaattccaacaagtttaccctcagctactcagtatcgcttcagaacgattacttcgtttc
gaagatgctgtggttgatatgattcctaagtcacctatagctatatttctagctgaagacgttgagggagttgctgctgtgcattctttttgtggtaat
tatcgaagatttatgtttgatattgttcgtctcacagtttccataaagccattagaatctctgagctgggttcaaaataattttcaaaatatattgatg
aacaatattaaccagattcaatccgaaatggctttttcatcgaaaacaactcctctttatttgattatggatgttggattttcaaccatagaagcttc
acttcatggaatcacacgctggaatgaaaatgtaactgatagttcaacctatgaacttattttacaaagcttgttcttatggtgcaaacaattagtt
gagatcgagtttaaggatcctatgcttataacccgacttattagtgttttagttttgttcacatcaattttggctcgtgaaaatacaaatctgcttgga
gttgttttggagaaaatcatttctgcagttacttacaataactcaccctccttgtacggattttctgatcttcagaaagtcaatgaaatgcgaagtag
gtgttgctttgaattagtcagacttggagaattaatgccaaatccattgatgaatatatttgatcagctacagagcactattgaccaacttggcacg
tttacgactcttagcggttcagaaattgtgatgttgaaaacatttttatttgtaatttctcaattctccgatatcaatcatactctcaaaaatgagtttt
tcgaaaagttggtaggtcctgttgtctccacctggctcgatgcgcaacctcctgtaagtactccggtggaatttttaaaccacgtgaaactgcctca
aatgtccgaatatctgtttgcaaaatatccctataatgcggattatacgaagtttgagctggatgcagatgcatcttcttttcaaacggatttggaa
gacagccggaaatggttatggcccattaaatgtttaggtaggttctgtgaagctacgtacagcaatagacggatacatccttctgaatttgatgaa
caaaaaactttatggttggtgatccttccgaacatcgttcctggattattaaaattaattgagcaattgcactgctgctacgatccctcttttatctcc
tcgttaggaacacgtaattctacgatactccaaaaaagtatagttgaacgattttggcttcatggtgttagccaaatttccaagcatcaattcctgg
aagaatcatataaaatggatgtttctgcaaacaagctaatacattcctttggacatttcttgcgccgactgagagagtattgttatggtgcaatcgc
ttccttcatgagacttggggaaccattcttcgcaatccctggtttatcaaccgctttccttacttcatttttttaaccatgcttctggtttgagtcttcacc
aatggacatctgttgtcaatgttattatcaaaccatactgtctgcattgtccccctgagctccgtgatgattgtttattaccgcttcttcctccgttgctt
tctgaattggatcggaaattggtgaatgaatggcgaagtataaccgagagaggacttcttgttgaagaagatactgaggaaacagaagatctttc
ggaagaaatgattgaagaatctttacttcgtcatcttacatatgctacagcgaagctgattacggaaacgtttttacaaattacacctacacaatct
cgttcaacgatcacttcagacttcgtggagaaagagtcatcgaccagtggatcttccaagctatctgattatgtattggataatgcgatcatctgtg
aacccccttctaagcacactttgccatttgctagttatccatgattcgagaacagttacgcttgttgtgaatgctttcatagctattgttcctttactggt
ctcagagcgtacccattcgatagtccgagaattcgtttgtcaaaaagtgtttcaaactattataatggctatcaatgattcttactttgagcaattgc
agtctgactttatccgtctggcttgtataattctttcttactctcaaggaattacagactcatcctttagagtcttggcaagtattccagctttggcatc
acaggaaaatttgttaagtacattcgcgaatagattccgcgaagcatctacgttgaaaatccagaaagcattacttatgagattacttaattccgg
acgaattgtaccgagaactgatagaagagctatgaactcagctgtgcttgatgtgagcgctaaagaaatgttgaaacgattcgaaaaaagtgtt
agtctacaagatgatacaaacaagggtgatattctttcgaaagatgaggatactgggttggctaatctctttggttaataaaagaaaggaaatta
aataataattgcctttaacatggttatctaaatgtacattgttaaagcaattggatatgatgaacacccgaagcttctttggatgtaatctaatgtga
tatgttgcgatggttaaaaactcatatttttataatcattttatttagtttatttttagttgctatacctatggaaatactagtaacgaataaaaatact
acgattatccaatattgaacgaattaaaatgtcttcatttgagtaagcactccagatcgaatactagtaaacaaacgcagacattttgtatatatat
gcataactgatttctttcattctatgaagataggaaagtatagaatacttgcatctttgtatgtttttttccaatgaaacttgtttagaaaaaaaagt
aaagaaaaataaacaacaattgaaattaccaccaaatgacaaaattgatcctttcttttcctaatttcataaagttggaggtatctttcaccatctt
atatcaagtgtttaccctaaaggccggttcgacctggcccaagctaccataacaagctacaacctccgacagccataaaattcagttttcgcgga
aaacaagctatttttggagtaatccaagaccatttggatgcagtttaagctgcgtctttaaaaacggttgatttgcagtggattgtgaaccaatcgc
tcagaaacgaagatgacgaaaaaacacgcttttaaaaatgcagaagatatgaaaaaatatcacttgagtgaattcaattccgaagctactcccc
tcactagacccgcaatgaaaagtgagtttctgcggtattatatctgtacggctgctaatcaatatactaggcttacaacatgttccatgtaagttctt
tcgccaaggaacctgtacaagcgggaaaaactgtatttttagtcacgacttggaatctgcgttcgaaaaaactgtctgtaaatattttcaaaaagg
gaattgcaaattcggctccaaatgtgcattagaacatatccttccggatggaagacgtgtaaaaactcgcgccctaacttcatcaatgcttgccgg
ccctatgaatcctacagatcaagttgcctctacaatggtaaatccaaggttttcgaaagaaatagctgactcggaactaagaatggaaacaccaa
tagagaaaaaaccggaacctgtaattgaatcatacaatgcgatgcatatggcttctactggcgaaaacgctttgcctaccgtgtcagtggaatcta
ctaaaaaagcaaatgctccaccagaaagtaataatttggaccaagaaaatatttcctttgtcccttcaaaaactaatgataatcaagatcaaactt
catcacctaatgcttataatacatctatgaatgcctccatattgccatcaacaaaaagagttgtatctaatccctctatgaagcctcccttagttgaa
ggatcttcatctttttcttcgggtcctggaatgttatcctctcctcctttaaccccagagaatcatcaaatgttttatttaccaatgaattcgttgagca
cgggtgtgcgtaatctcaaattactccctcaagatgctttttccttttgtttaaggtcgccttctttggatcgttcccaaagtgcctctattcttcctaga
atatcatccggtacttatgccggaactaacaatattggacgctttgcaaaaagtccacctccagtgtccggacatggatcaaacggtttgaaaaat
ttagggaatagtggcttactttattcttcgtctgcatctttcgataatgtgcatgcattccctagtcgtcgaagtgtccctaacctgatgtctaccctcg
gaacaagtccatcaaattttcatgcttataattcaaaaaacacagataagactcattttagtaaattgtctgcgtcccaaagtttattcccttcaaca
tcgctatatggctcgtctgtaggaacatctcaagatgaattgttaagcactgatggagctgacgaatttgctaatgaagaagactttatccctaata

gtttgcaagaactcctaactccggctgagctagaacgaaaaatgagtcacggtgatgattttcccagttcttcgtctacaaatcgatttatatcgaa
agcgtcatctggtttaagtagcaataatgctacacccttcggttcggttaatggaacaccaacctcgagtaggtttttttgctttctttgaacgaaata
aggaaggaaacaatacgtctactagagtgtcaccagctgcaatctcaaaaatatcccaagattcgttgaatcttaaccatacaaaagcaatgcat
tccaatctgtctgctatatctgaggctttcgaagcaaagcttaatgtaaatggaaaaacacgtgaagaggatttagccttaaaaactccattccaa
gcaaacgaaaaacagcttccatatgattcagcgttggacgaagaaacacaatttcaaatggatgaagaatgatttaaaaatttgcgcaaaaagt
tactttatcgtcatgttttactaaaaaaaataaagtaataagctacgctctaatatttgctcagtacaaaacagtctactgtcaagaatgtcgtttaa
aaactataatgaaatgtatagaactcagaagatctttttttatcttcaaatactattacagcaattctatattaagaaactgtttttcatccttgtaagg
ctactccactctgataccattaaagatgttttttataaaaataacaaacccatttttaccgggaaatctcatagaaaataacaggaggactattgta
ataaaggagctttgtgtaatttttgtttgttaaaactcgtacattaaagttggggatcaactgtaagaagttatcaattgtttcctgtcttcgaagtgc
agcggctaaaagaatcttacaacgaggattgacttgagtaaggtcattcatcgtagtcgctaaacatcgtaaatcaccttcgtatgtctaaacatc
aggatgccctgaatgaaaatatttgccatgtgtccaaggatgctcatatgacaaatattttgtatttcagggcaacgcaaacagttagtacgtcttt
taaatgaacgtaatcaacttacaacgtctgatgcaaggacaagctcagcgtaaacaggcggatcaacaccccaaagcaagcgttgaacagaaa
gcgcacacttgttcatatgagcgttttcttttacaccctgacaaactttattatctccgtcagtggccaatacagaagctcccatttttgagcataata
tactgactaagccagtaccagcaccaagttccacaactcgggtccctggaggtatagggttctgatatagcagttccgcaagagccattccagctt
cccaagtacgtgtgccagtggtcccttctcgtaacaaaatattctgagactcgtacacttgaactgaataatcacccaaggtatattgcacaggag
gtatttgcttgctcgagagtgactctatgtatatgtacaaaagcgtctcgttgggctccgcatggttttgacagtgtatatcaagtagtcgaaaatag
gatttcaaaaataacttagtatagatagatggatacaaaaattcacttaaataagcctgtgcaaagggcttagccaacgtcagccattcgttgtca
gatgttacccactgaaactgatccaagctgacttgctgcaagtattgctgtttaactttttgtaaaaattcaggaattgtatccatcagaaaaaaga
atcctaataaaacccaaaaaataaatatattctatcccctaagtacaaataaataatggttaatatcttcctttaactcttaaagatcaaggcgaa
cttaagcaatgcataagaggaagaagaccaaaagtttataacgcctacgattacgagtttgttgtttgacaataaagcttattgttgccgttaatct
tgaattataatacatacaagagaaccgcttgattctcagtctatttcctattgacacaaagttttatcttctgtaaggattatgaagtggtcaaatgg
atttttacgtcctttttgattttattacaataatttgcctccttctggtggtgacttttgccgcggtacggacgcgcggaagagccgaatccatgcttct
cgtattttattgtcgcctctaactatatggaatttgtatcgaaacgaagagaatgaagctttggttacgttcatttataggtggatttaagttatttgtt
gggatccttattattgttctattttctattcctagtctcgttagttatataatattttatgacacggttattcctcatgcagtggtacaatatcctgtatat
tttaattacacgtaagtttctaggttgatgctttctttgaggatgctgacacattgatgtatacagaactggaagcgattacccctatgccaaagtga
atttggatcacttcagcatagatcctaggcttcctggaacatctatccttcaattgagtatcccccgttctcgacgtaaccgagaggtaggaaatttc
atgatttcagtcgactttcatggaagaaccgagcagacaacgataaaatctgtttccagaacggtcctttttccacattattcaagtatacacgaac
agctgaagctcgtactttgctttcctctttatctagcggggatcttagaagagcgggatacagtaagcgtaaagcttttggagtctgaaatgtttgg
gaaacatcgcagtgcaattaacacactatcttcacgtttctatgttggtgatagtcctaagagtgcaagtattcatatccactctaaggatattgag
ttttatgatgcaactctagcttttgcatccaaactacacggcatgcggtatgttgttaaatcttcagataagattttgctaatataatacttctagatg
gttcatgtatacacataaggtatccgctttttttcgtttttaccgccctgttttggtttactgggatatgtacaaccttcattacttacttgatcatttcatc
tacatcaaaatctcggaaaagagttgcttaatgattcaaaatgttgtaaaagaaaacaagaatatttatgtcttataaactagctatgatacgttg
tcacgacactaataccttgaatgagtcctatgaatttatgatttgattgatgatcctcgacactttcgaaaattaattttgtagcactgattataatta
tgtttattaagcgcacattagtagttgtttcctaggaaaagattggcagatacgccctcattaatataattgcgttcattaggcatacattcatcatg
aaggttgagccaggttgttttcattatatgatatttgaattaaatcttcgaaaaatgacagtagaagtcgtattatttgctgacaaaaagctgctaa
tacaatatagcaaaaatcctgaagtgtaagaaataaggacatgaaaaactgaacaatcaccgaccgccatttgacttttgtaaaacctgctaact
ttattttaactttttcggaaagaatgacattcgatggagaagctggatgttcgtcaatgaatgaaataacctggtttgtaaatgaagagcctggggt
aagttcatcttcgtctataacactaccttctctactagactgaatgctcgaatataactcgggtgtatttgagtcgtctagtccgggcatcgagatttc
atatcgtttttttaaattcatgcaaaagaggggacatgtttgataagctctgtatgatccacttttgtattccaatttcttctgaaagtatggtgaatcc
ggaaatcaattctttgttccgtaaacgttctgtctcaagagagcttgtcagatttaccaaatccctgtaaaggttttcttttggttccacttgatatttc
ttagatggagattcgttagtttcctcttcaacgaatgatttcacaggagaaaagggtattgacaaaaaaggggtaggagaagaaacagtgagttt
ttcaaacttgctaggcgaaggagagtctaaagacttttttttctaaactgctaaattttccctttcgaaacacttccaactcgccctctagctgttggc
aatggatttgtaatttatggttctcaaactttagtcctgataaagcatcttcgtttaacaacgactcagaagcagctttgtataattcgttgaagctgt
ttgctttttttctctaattcttccaaagcactcgcatatttgctttctataatggaaagttgcttctttccatggataagttcatttgatttcgctataagct
ccgattgaaagttctcattttctgctcctagtgcttcgtttttactattgagttttctaatttgcaaattttgagcctcttgtgttaaactcatttctctatg
ttgagtttcaagggaacctaggtagtgagtcaattcgtcaatttttccatttaacagatcgacaattctagatttatttctggcttgatctcttgcttga
tccagagaatcttggagttccttcaatcgcaagttttgacttttgactgtattattttcgataccatagttgctgaattggttggctaccctgtcagcg
gcagaaggtagaggattcctgtaattattagtaacaaataccattttcttttttttttgcataccatgtgagcggagtcgagtgggcaaaaggggtac

tgatgatattgggggatttcatctctagttgttttgaagtagtcggtagaagaagcttttgagcttgaatcttagacggactttcgatggagtcaaaa
gaggaatcctccattgcatgttcctgagcaggtgatgatgcttgtgagtgctgtactaaaggaactgattttaagacaatttgctccacggggtctt
tctcagtgtcagcatttccacttttactcgactgtactagctttaagtatacttctcgtagattttccttggtaatggagagatccgggttcttttggac
aaactctgatgtagcacgcttttggaattcgtttaaaagaggaggagcagaagttgaacgtagaagctcaatgtcatctataaccgaaaggaatt
tgtttaagctaatctacaatttgttagcaatgttccaatgatgttgtttcaacaaggattcaattggaagccagaatgctcgaatccacatacttttc
ctttctttccaaagtcgtaggaatcaaaaattaaatcaaagtcttgcgaacgctgcatgacgcgcgaagatcgcgcgttttcgaagaatactgcaa
atgcaacccaaactctttggccacgtactaagcgatcctcaattcactttctagtaactaaatgatagtatttttcaatagaagaaagaattccaaa
aaaaattgacaatgcctttatattaaataacacactgacattgtgttgctgctatacagccagaagaaaggaaagcagcagagcgagagacaag
cctatttattatgaaaccacctaagctctgaaatatatgtattgtaagtattaggctatttcattcattattaattagattttaagtaacacgctgcac
gtagcttgtaaagaagaaacgaaatttcaacaagcactaaagctgagaggtgctacaaaatacatacgaaaggccattatctcatgaagatga
agatgaagctgaagctgatttcatcaaggaacttgaaagctacccagtagtgttttggtatgctgacgttttctgtttaggctttccataattaacag
cagtaaggaatgcttaaagaaactacacataattcatgttaaatgaattaagaatatgtatccattgacagcgaatgcttccaagtgtaatgccat
ttgattattcttatggactgtatgtctatactttccattcaaaagagggtagggaaggtttcttaaagtaactgagcttttacctttctaatgagaaaa
cataaaaagtatttctcttccgatatgactgaacgaacgcagaaaagattgattacttcaattcgagagcgtactcatctttgttcatattttcagaa
ggagcaattcagtcaactaaagggatggatgcaaattattaagcttacttgcatgaatcagaatgcttcgttgatcctcaaaattggaacgagaat
gaaaatagaaaacatcaactagtgaacaagttcattgtattttcacgaaatacgaataattgttctattgacttacatactttgaatttctctggaa
caagagctgaatgagcttcctagtgtataaaacgattctctattaattaatatttttgtttacatacatagctgcgttacttttcatcatgttgttactt
accaccaccaccaaatgcttctttgaaggcctctttaaaaccgacttcaattccctttattgtttgagttttcaaggataataagaggattattgtac
aatttattagaaatatttatttgccgtaattttccaggaaaaaattgaggaattctgtcggtttgtaacgtaacatttgtactattctatggtattcgc
agaataagacattaacttaataaaaagaagaaatttcgtttatgctgatatatattaattgctatgagtgataatgtctttatggaaccagatccat
gggcttcaaatagcacttggggatctcccatcaaacccctgacttttgaaaccgctttaggtatgcatagaagccagttagtaggaaactttttcta
atagtgctaggaaactcgtcaattccatctcagtataaagaatgctgggaagaaattcaaaaccttgatcaatttctttacccaaacccgtctctat
actccaaaatatttggaatacctgaccaagttatttcagaatttttcgacttgatccagtgcacgagcgaacccttgcaacaacgacagtttttttgc
ccttttagcacttgccgccttttatcaactcggagttcccactactttggagcaacttcaaaagcaaagaaatggtaaactttctatattcaggtttc
gatcgatgaattactaactgttcatttcagttttacctatccttcaaaggtgtaacctagacccgtctcttcttcataatgaaactgaagcacccctct
tctctcatccttccacgattagctctcgtagaacatcttcaaacccatttcttgtctcctctattccgcaattacaaaatatttcccctttccctaatttt
ccttctactccttctcctacagaaagaaaggggtttttatacaattatgataatccatacaggccttcctcttctttttcttcagaatcagcccctgttg
ttcccgatacgtctccttctccaagggagggagctgccgaaaatacatcttatcctccgagaaagactacacatgataactttggtaaaaccaatt
ctcctatttatttgtctcaacctttcacagagccttcgttagattctttgcctcagtctttaatagattcatcaaagaacaaccaacgtaactttagtcg
gcgatatacacatacggatcctttctttaacaaaaacgagctaaacgttgatattgatcttgaacctagtggctctctcttttaccgccataacaatt
acataatttcagattcacgcaatactcgagaggtgttaaggcgatatagtgacttttattggtatgtactatcgtccgtgtattctacttcaagtactc
aagaatactaacggatctttaggcttcactcgtaccttgtggaaaaatacccttggagaagagttcctttactacgtaagtaaaaatggtttgcttc
cgctcaaattttaacaatatttatagcttcaaaaaagttccattgtaagtaattccatgctttggcgatgctttctcctaacagatgtagttgccaaa
cgaacagcatccagcagaaacacatttttagaacatagaagacaggggctatatgattttgtaaatgacgttgcttatcatccaatcttttcggaa
gacgaaattgttcaagtatttttcaaagagcctaatgtacgagcttacattgctaaaaaggaggaatctatacgaacttaatactaactgtcttag
gtgttcaaaaactggagaaaagaaaattctaccctcataaaaggaagcatcgcttccattcttcaagagccaatggactctgtacctgatgtatcg
gaatcatgtaaagagcggcttttaaaagctggtactgcttgtacaatagcaattaataatcaggtcaatattattaaaatttttgaaaaagctattt
gtacagtggatcaccttcatgacgatatcaatcgtttacaaaactcctttaagtgagtataaaagttgaatttcattttactaatttatttagctgctt
acttgacagtggtatctatcatcgagttttcaattctacttttgcccaaagagaaagcaataccatgtctttggtcagtaggcatgcctacaatattg
atacattactgcatcaacgaagttttacattgaagaattcatttataattaatcttactcgggaaaccaagatgcttatatcattaagggtatgtcaa
agtgtatgtatatgggttttaacaattagcaattgattgagagaatgaccatcccatttggttctgatttaaccaaagttaaacatttaatatctaat
gatgagaatttattaagagaagctacgataaacgattcgactaatggaagaagctctgctattcttgacaggagccccgaaaaccgaattgaaa
acagtttgcataaggtaaatattattactagaagtttatttaacacagggtttagaaaaaacaattttatgccgatacccttcggcaaaggttccga
attgatcaatccctacaagaagaaattcagtatctacagaactacataacaatactcgggaacccttacattgactattgcaaacagcacatcaa
gtatgaagaggaaggtttgaacatatggcaatctctaaagcgtgacttcgaaattttatacgaataatctaatgtattgaaaagttaatagctata
aaagaatatacaaacaaaaaaagaaacctgtatgtaagtaataaaacgagacttaaaatcagactgttgctagctcttttgaaatcaacttcgc
actttagtgtaaagatgactttaaaataagcccaaagtaagatctacatatcttgagtggggagatgtttaaagggaagaaatatcgcggccaaa
tagggaagttctgatccaatctaacaaaacgttggtacggttacgaagggaatataattctgaaagataggcagagcgccagaaaaagaaagc

aagataaccgctggcatggatttgcttatcgaggaaaggaacattcaaatcagcaattgctttttcatgaccaacataggcaagagaaccttggt
gagaatacttgaaaggacggaataccttcgtagatgcatgcttttcattagcgagagagataagagcagcagaatctaattcctcgccaatggca
atatgtctgtcgacacgaggcttctcgaagtcaagggagccaagtttgttgaaaagctgaccaaggaatgcaccttgttgagaagccacctgagc
agtaggggcgtaagcagtagaagtgcagtcaccgagagcaaagacgtctggcataccgttgacttgcatgtaatcgtcgacaatcaaaccacgg
cgattggtttgttcgttcaaagactcgatgagattcttgatgagaggacgaggtctattgcctccagcccatactaagagtccataaggaattgatt
cttcaactttattaccttctggatcggtaacttcgacaatgatgctatctgaggtcaccttcttcaaggcagtctgggttctgatattaatgttggtttt
agcgaaaagagacttggtatattcacgcaacttgctactgaacatggggagaacggagggtaaagcttcgatgagagtaacagaaaaatcgtt
agcaagttcaggataccaagatttaagatcagcgttgatgaaatcagccatttcaccggcgaattccataccagtaggaccaccaccgacgacta
caaagtgaacataacggcgacgggtttcaggatcaaggtccttgaactgagcctgttcaagacacttcatgatgcgggtgcgaatcttttgggcat
cccacatctctttcataaagcaaccatgctcttcgattccggggatgccgaatgtttgggtctcagcaccaacagagcaaaccaagtaatcatagt
taatttgctgaacaaactcctgaccatcagaagtttttctcctaatctcgactgttttgccatccttgtcgatatcggagcactcggcctcataaaac
ctgacaaaatgagacttgtgacgaagaagatagcgaattggttggacaatgctgcggctgtggacagaaccggtagcagtagagggcaagaga
gaggtaaaaaggaagtaattccttggggaaacaacgactacgttgtagagagagctatcgacggttcgaaggatggaagtagcaccccaacca
gcacccaaaatgacaagagtctttttagaaggatcagggaaaggttgtttaggatcgggatgacgaaagttatagatggaaacaccatagaaa
aacacactagcagctacaatacctagaccaactcgcttagcgagcttccagcggttcttaaaaggtgaagaaggggaaggagatgcatctttgg
ctgatgatgcatttcttttaggactggaagtgttgaaaaagcgtgccttaggagagatatatgtcttggaagcaaacacagaagaatgcgaccttc
tggcaaacggcgaaccgaaaccggaacctgcgacgcgtctggtcaaagattgagaaagaagctttgccaaggccattttatattaaatttcggtt
tcaaaaggaaggactgtttatatactgttagatagagagagaggagggggaagtatctaaaattgaataagaagggaggggaaagaaaaaac
aaacgaggagttagttgttctcttttggatccaacaatgaacactgtaagtaacctttttaatgataacttaaatgaatttcttccaaaaactaacg
aaagaaaaaagaaaacaaaatgaaaacgaccaaacccacttcacctgggtactaagatttaaataaaatttgaataaagggggagggaaaag
ttaaatcacactataaaccaaatctgataaccgtaaagtagacagtaataaaaaagccccacagctaagctaatttattacgcaaatatgattttt
gcaaacaggaatccttccttggactcttttgctttcaagaaaatgtgtatacagaagaaaggttatggtttgaagtggaatataaaaaaattgtcg
agtctagatactcttatacctctagaccactggaaagccagaacaggatgactgctaaaaaaaggatttttgtaccctcaattgctcaccacatta
acctttaaagcatttttgttggggtatgcagaacaggtgacaaccaattagcgaaaggcttgcaagagggacaaactccgcatttcagacagcga
cgcggtttatgtaaacaaataaagatccggattatctttaagatttttggggtatcagttcaagttggctgttgatgcataaaggtttagtttttgtt
tttactttatttttaacgatttttacatagttatttactttattgatatatctatttttgttcacttcgtctatctgtagaattgaatatttgagccttttcct
ttttttccggatttagcatttatcgcgttttgcaaaaattattccgtgggctactcggtcaatggtaatgtgccgagtagcaaatagtatcacgttgcg
taaagcgaaggttgtgatgtaacttgttttcatttaagcaatatcttgtcctgaaggacacgtcatgttgaacactaaatgaacgttattgcatctaa
gcgaatggattacattcttatgtgtcgtacgaaattcgcctggccgacgagatgaaattgaatggtcgaagcgttcgcaagggtaaaatatacag
cgtaccttgcattggtattggaaggatgcagtgagatacagaaactgaagccttatgatttgctgttctctggattcttaatcactatataactatta
tagtaataataataattataatggttattatatactaatgatcattaattttgttatcatcagaagattatttacatatcgattagcttttgtttcg
tccgaacgagggaaaccttattagatgctacccaccttaccaaattgcctatcggccggttgctcaaggagaagtttgccaaaaggttacggttac
ggttacggtcacggttatagttatgaaggatggacgatgtgaaatctccatacagtcaaatagcctgtgactttggagggtaaacaaagcaaagc
aaaggaaaggaaagaataaacattggtaaattgttagtttcctttcatgctcattttcatcattaataaaaagaaaacaccttcttaatctttctctc
tttctctctcaagatacatatacatattcgtcattttctgctcgttccaggtattctttgttaatgagcttttcaatagtcgatttcacatcagaaacgg
gtggaacaccacgatcctttgaactctcaatagtcatgtgcacgagctgatcatgacgaagtctttctttctgtttcatgatccgcactaccgtggct
tgcagctcaaactgcctgtcttgaacgacttgttcttgaacttcttcgctttcttgtcgttcgtctttcaaatatatttgattgattttaatacgatacag
cttatctgtaaattcctcattcacatagaaagtggtgtccggctttggaacttttccctttgggtccatcaaaagcggtcgaacacgagtgcagctta
aggattgcaaagttctcgtcaaatccgcgtccgacagttctgtcgatttttgtagttgccgatacgtaataccctccccagacgcgcatcgattaaa
ttctaacaaaacacatgcttggaataacgaaatgctaagctctttattgccaagaggaaatcgcgctttcacgagacaatgccctagagaaggaa
gccatcgaatttgtttgctagtttgcttcgttaaataaaatttctcaaattgctgtaaatcttcttgcatgtcccccggcaaacgtacatgtccttccgg
gtacgatggccaataggcttgggacagaacgttcacatagaggtctcgatgaaggtttttggacaattttgactgtttaaacgaacttgaaaactc
cttcgacaggttgatatccttaaacattccttccaaagcgtgcgtaaactgacttccacacgtcctttttagcatttccaatagcatacgctcgttttg
tgagcttgctgatttgttgagtaaaaggcgttttgcaatatcacgcttgtaaaacgcttcaaagacgtctttacttgaaacatatcgaaatatatcta
gtagagtttcaaaaatttgctgtagaggagtgccattggttacctgctcaccataccgcagtaagtagtcaatatactttgcaattgacttagctgg
agcttctctacgttttcctgatgcaccgtttataaaagtttcgaatgatttacgcatggcgtatgataaagcttcatcgttcgaaaaagcgttttctac
aacggactgcaaaaaagtatgcaaagacagtaaattaggaactaaatggtcttcgttttttttcatcatttacaagctgaaacccaatttcggtgag
gcattcagaccacggttggactaactgagaagtttctgaagtcaattggagtagagaatataaaagtttgcacgaatttaaatcgcgttcttgcac

aaatttttgaatgccattaaccaaggtagataaatggctttgaatcaaacgcttctgtaatgtttgtagaatatcattttgatggcgaaccaaatca
aattcgttaatcagcacagattcgtaatctaggcattgttggacatacatcaagtattcattcattggtaacgatttcatccgatcctcggattggga
ttcatagaactggttcaaaaaaacaaggtacattggtacaaacacgggactataggatttgacacgatgaagcattgacattgcttcttgtaggc
aactacgatcaataggctttgcgcaatgaatatcttcataaagtttcagaacgtcattcaaaaaaggaacttgaatggccttggaagccaagagt
ttatcacgaaacaaagaaagagcgaaatcttctaccgtaggaaagtctgaagattttgctagaaacgttttatccatgtaatagaatacgttctga
acagtttctacacgctccatccattgcttccaatgtcggacaacgatggagcaagcattgatatcgtccgtcttgtgggaaatggaggccaagcaa
gcatctgttagaagctgaataccagatgtaaagtgattttgcaataaagtgaagcaacgagaagcctggctggccttggttagattttccacacct
gaatataactcttgataacctgtctttagacctaatcctgccataatgacttgcagagcaatatcgagttgttggatcataacattataatacttttct
tggcgctgtttagacttcagagggtcaaaccctttgatgacaattttttggcctccatgggcatcctgaatgtattactgacttctattgtagcctga
taaattagcaaagaggaacaaataaaagtactgtgccaaggatccggcaagaatcgtcaacaaaaccttgatattcacgaaattcaaaagcct
gttgattgaaatctatttgacgcaaaaaatccaccttttttctgttttggcaataaaaaccaaagtggagcaaaccaaaatcaacccaaagagaa
ttaaacaaattatatttaagcacaagagcaatatctcgactttaaggaaaaggacactgtactgccaatctccttagcaaaactgtacaaacaaa
aaagttacttttaagaaacgaacgaaatagtaattttgcaaatgcttgtgaacgcctatacaaggtaagaaaatatagcgctcttcaatatgtcaa
ttacgttaaatgaagaaatggatccttcaatataaataagcgatcttttttatgaaataaccaactttttctttcccttccttttggtggatcgctgct
aagaattgctaggaaagcctacttcgtcttagcgaacattggtattacacgaattttctatatacttatacatcaaagaaatattatacaaaaaatt
taagggaaactgtcagaattgtcaactgtatttttttagaacaacataattcccaaaaactttccttgatccattaatttgctttttttcctgtaaaaatt
tctaatagaattgttaattgtccattggagtgataaacgttgttgtgtttgtgtttaaatgcagttgttgttttaactttggcatagtttacacataaat
aattccaaacatcttgcatacgtatcaaaacaaaaccgacgtcggagttctatacctttccttgttcttatatcccttttgtgattcctctcgttccgtt
attgcaggttaaatttggagaatggcatggagtcagctagaaatcagtaaaccgcacttagcgtatgctatcttgggagggttcacctctctcttta
tgctttgctcgttgattataaaagaaaaactatatatgggtgaagctagtaagttttcggctattaggcgagttcatctctaccggtatttattgttct
ggtaactgtgtgtctcatactcatttctataacatgctgttctagtagttactaacctctatagcaattgcgacgatttgcgggttgatttttggtcctc
acgttgcgaaaatattcgtacccacgagttggggtaattcggattatattactttagagcttgctcgtatcctgttaattgtacaggtgtttgctgcag
gtgttgagcttccacgggcttatatgctgagacactggcgtagcatgttaattatgctgcttcctgttatggtatttggttggctggtctgttgtgggtt
tatgtatgctctgattccccgtctcagttttctcgaagcccttactatatctgcgtgtgtaaccgcgacagatcccgttttagcatcgtctatcgtcggc
agaggaaaattcgctcgtcgggttcctggtcatttacgaaatatgcttctggccgaatctggatgtaatgatggcatggcaatcccttttctatacct
cggtctctacttaattatcgacaagccagctagacatgctggacgcgattgggtactaattattattctctatgagtgcgtttttggctgctttcttgg
agctgttattggcgtaatagctcgcaaggctataaaattttcagaacgtaggggtttgatggatcgtgaaagttttcttgtattttactttgttttggc
cgtgttttgtagtggtataggtacgattattggcgtcgatgatctacttgttgctttctgcgccggtgccgctttttcttgggacaaatggttttcaaaa
aaaacagaagagtcccatgtctccaacgttattgatttgcttctcaacctttcattctttgtttatattggtgctattatgccttgggaacagttccata
tgcctgaattggatcttagtgtttggcgccttgtagttttggccatctgtgttctggtcgcaaggagaattcctgccgtccttgcttttaaaagattaat
cccagacgtcatcaattggagagaagctttgtttgccggtcactttggtcctattggtgttggtgcgatatatacttgtctcttggctagagctgagct
tgaacatgacgaaaccatacctaccgctgagcttccgcgtgagggtgatgctaactattatgttgttaaagtaatgtggcctgtcgtttgctttttgg
ttcttagttcaattatcgttcatggaagcagtattgcgtttttcatgcttggcaagcgcataaacacaatcgcccttactttaaccaaaactagagat
tcccactttgcttttaatcttccaagagttaatcaaggtcaaactttacctttaaagcgaggaatcagttttcgttctggcgctgcaccctctatagttt
cttcaataagacgtcgccatcctgttaatatccaagaagatgacgaggcgtatgacgcgtcatctgttatttctgctcccagccctgcacatactcg
gcttcagcatgttgagtctcatcccgaagatcaggatgaagatagagaagaacctgttccttctgtcaaagattatgatggtgagcgtcgtcaatc
taatgaagaagatagagaggaagagatgaatgaaggcaacgaaagttactttattgggaatgatcttgtgtgtgaagattcacacggtaatatt
atatcacgttcacgaccaggagatcaagaggctgaaagacgacccagtgtagaaagcaagggtgcgaaggagcaggatatgcttaatgatggt
aagccacaatccagattccgtcggtttttcaaaaatcttcacgacgcatttgagcgtaattaccacgtaccagatagtgacgatcgagatcttgga
aaagaagaagctgatattgaaaataacgttcctagaagaaatagagggatatctgatgctttttcgcgtgaaaatagtattgaaagacagcgcc
gtgaggaagttctaggaaatatgagagacagtgaagatgaagatgaggaccgtccgggtataaaccctagagagtttgtaaaccaaaacaatg
aagcagttgatttacttgatgataaccagtctgatcactttcctgagggacatagccatccagcatccgttccttcgcaacctgaacattctccaga
taatgaaggggggagaagaagaagaagaagaagaagaatcccaacaaaataaaccaaatattcgatttttggatttacctactcctcgtccatcc
ggtcgtagaaagtcatattcttcttatcgtcgcggttcaattggaagtggacatcaaagaagttagttttatgtaacagtttttcaaaaaaaaaaaa
aaaggctctttatttatttattcatggtccttatttattcattgcaaagtgttaagaacagttttctgtttaatcgtctttgtttcgtatagcgctcgcttt
tttcttttgaccctaaaattcagcaaaaagagctagagggatttgaggatcagacgtttcctcggtgtaaaacatcatagcttagtattgttggaaa
tcactaatactaatttataactaatacattattgtctacatatttctattcgtattaagtggataaaagtattaggtatttaacaaaagcattgtgaca
cagtgtactaggaatcattacttggcaacgagtatggatattaaacagcataagtaataatcaacaacgaattatttaccaagatcatcttcccat

tcgaaaagtggatcatgtacttcacgggatgctttttcagtggctctccagacgcgcaagacattgcgacccgaaatgtctataagttcatctaca
gagagtcccagatctgctaatctgctaaataaagctgggtatttcgagacgtcttcgagtcctcttggaaccatttcgataccatcataatctgcgc
ccaggccaatatggcgataagaatcagtaattttagcaatatgctgaatatgttctactactgtctctaatgtcgtattttcaggagtagcgctgata
aacgcagggtagaagttgaccataacaacgccgtctgtttctttaacccttaaaaggacatcatcaggaacatttcggggatctaaacaattagc
aactatttacatcacgtcagatgaaagaataaacttacgattatggatggctttggcagagctatgggagaaaaaggctggtgctacagaaacat
ctaaagcctggtgcataacttcgggcgttacatgggacaaatcaaccatcatgccgagacgattcatttcacggaccagagctcgtccagcttctg
acaatccaccattagtttgcttaccaccgacagcagcatcagcgaaaacattatcacaattatgagcgagcgtcgcataccgcactcccaattcgt
agtagcgtcgtagtatagatgggcttccagcaatttggtgcagaccctcaataccaatcatacttgagatgcgtttttgtaagaagttatgttttaca
tctttacttttagtagccaaagagaagtcgctagggtacttgtcaatcatacgcttaatcagatcaagttgctgcaaggtttgctgaacagcttcatg
ttcgccagatctattccatgcaagaaagctatctggagcgagtggaggacattcaacaaaaccagaccaaaattgcccacctatatggccacttc
taagacgctttatatcggtatgaccagtagtatggtgaaggtcgctttgagccaatttgttatcgtaattttcacgaataaaagtgggaaagtcatt
atgaccatcaattaaaggaaaacgagtcagtacaaagtgagctttttgctctgcagtcgaaaatgaagaaaatatgaagaattgcggtcctttat
aaaggatgacaaagaaacatgctgaaaaaagacataagcttattctgatatacttagggagccattgtttctgcggatttgaagactgagtaact
ggaaaaggaattgcagacttttcttcagacggcatgatgaaaaagaaattatagccgtaggtcaataacgtaaatttgaaaagatgaaaaccttt
tcaatattcctaacttacttatatctactggaaccgtaataaggagattcaacttggtcttgaaaattaggtgttgtaataggcaactttcttgcaca
atctataataaacgcaattgcctccttcgaaatttggacttcctgtttactttttattctttttttacaattactagaaatgaacaaatgaacgcaaaag
aacgacaaaatacatgttgagaatttgaaacagaacagagatacgggatcaatgtggcctcaagctcgcttaatcaattaagttcattcaacaat
aatgaattctgatgtatccgttctcacttgctaaaagatgaatacagaagatgttgttttcggtccttttgtttactacggtactacgtacgtttacatt
aataaacatgtttgcatacaaagtctcagtggtaattaagaaaggttgcacattgaaatagtaaagtataattattatgaacttgatggatctattc
ttccattggaaaacgaatggaatttgtattgacaatgattgctaagtcatataagccgcatgaaaagcggaagatattttcggtgtttacaattca
atcaaacttcatgtcctttatatttatgtgtagtagttaattagcttttattcttcataaaccaacaaaaaacatgaaacataataaaggtgaaggc
gaactcattgaaatacctaagtatggaaccaattggctgataaaaatattaaatcatttccctgttttacagaaagttcgaaaaaagtatttcctta
ttgctgaacaatatcatatagttcgtcaagctgttcaaaggataagactttcgaaaatgcaccaagattttctcccacataatccagagtatcaga
gcaaggaattggaataattccggcttttcctgtaccaaggataaaacttaaggcgacttccgttaaagtcatatcatttcgtattgcgaaagaatc
aagagatgatactttggaaagaatcatcctaaccgcttctcgatcttcattattagataatatatgtttctctttattctttaattcttccataattttgg
aatctcccgttagtataccatgtaataatggagcagtagcaataatgggaattccaaggttatggcaaagcgcttttataccgttcacttcaatatc
tctagaaagtaaggaatattccacccgaacagctgatatattagcaatgtcacgcactctttggatgacttcaagactaggttcccataaacctat
acaacgaacgactcctttgttaacaaagtcattcataatcttaatgaattcttctgtcattggtgcgtcctctaccacttggcaattaagcaagtcta
tctttttcagacctcgcaaagcggatgtaatctcttttagagaattgaggaaggcttctgtatccgaatcttctatctttccaactgttacagttaaaa
agactcgctcaatgttttctggatatttgcgaaaatacctactcaagagccccaatgtccccgatgataatgaggaggaaaaaacttcgccagcat
cccagagattggaaccatgtaatagggcattattcatgcaaccaaaggcttcttggtcggaaattggtatctctcgttgtactagttttcttaatcct
agcccgataggtcccacgagaaaataatttatgtacggcatattcaattaaaagagacttaacccaagaaattaaaataagtagattaataaaa
attactatatgatcatgaatatatgtgtaattcaatagatattctttgactttcaatagagataaatggtgaagtaaggtcaaactcgtttgctgaat
cgtattgataattaggattcaattaattaaatatatatatatatatatatattcatgagttgttagaatgtcagaacggaaagtgcttgaaggaatg
cgattctctaaaaatgaggtaatgtagacatttaaagctccatacctaaataaagttaatataaacgaactgaaaagctattgcttacgtacgctt
acattgcagctcttagctaaacaagcgaaaatagcaaccatgtttaggaaaggtttgatttgtcggtaatcaagttcaataccgaaataaaaaac
cgtgatgactacaagaatgtgcttttggggaaaaaaacaaggctatgtacaagaaactttcagaatgttcggctacttaaacccatgaaaagcta
tagcctaatttcatctttaattgttaacaagaaaagataataatggacttcgaaaagcaaagctcaccatctatttataacccgcatgtatcaaaat
ctgttttatagaccaaatataacagtattttactattcattataagtcagactaccgtgaagaaagggaagacatcagaagatgtttaggaattttt
ggggcatagaaatcatagagaaaaaggttgacaaagattacaaggagctcataaaacgaaaatggaatatttatgcaaagtattgtaaagtctt
tgacttacgcatctgcacttccaaaatgccgtccataaaatgttcatgagtaattttcgtatctccctgtcgaagagcaatcataccagcttctacac
aaacactcttcaacatagcaccgttatattcatcagtacttctagccaattcttcccaattaatatcatcagcaattgccatcttacgagaatggata
cgcaagataccaacacgggcttcttcatttggaagtggaaattcgagtttacgatccagacgacctgaacgcaacaatgcaggatccagagtatc
aacacgattggttgcagcgattacttttacgcggtcatcgctactgaaaccatcaagttggttcaaaagttccaacatggttctttgaacttcacgat
cgcctgctttttcagaatcgaaacgcttcgtaccaatggcatctaattcatcaataaatataatagctggactttttttcttttgccaaagcaaacgca
tcacgaactaattttgcaccatcaccgataaacatttgaacgagctgcggagcagccaattttaagaaagttgcacttgattgagcagcacaagc
acgggcaagtaaggtttttcctgtacctggaggaccgtacatcaaacatcccttgggggggtttgatgcccaaattctgaaacttatcgaattgctgc
atgggcaaaacaattgcttcaaacaattcttcgatttgttttgataaaccgccaatgtcagagtaacgttcggtaggtttttcgtcaacttccatggc

cttaacacggctatcgtattccgaaggcaacttgtcaataattaggtaggagtccttattaacaccgatcaagtcgccaggatgaagttcttcggg
ctcaactagaccgatcaagggaagaaagatggtttgacgcgtcgacgttttgatgacagcagacttgccaacacgggtggcctctaagttttgatt
tgctgattcttggacgtctacttcttcaggcgccatgtccaaaatttctactacgttgccaaccaaatacggaagcatcttgttatttacgaccttctc
ttggttttctttgatttttcgagcatcgtgctttttcgtgtgtcaatctctgaaattcagatttcatgacctaccatcaggttagctttcttctccaaca
taatacataatatttcaaaaaggaacgatcctactacagaaaaaaaggacgattcagttcaacgttttcagcgagcagtccatcgactcaggaca
aatctgaaagctttgtttaattcaaattattaccgtctttctttttagagcattttctcccttcatgtcacttactttaatgtcattttctaaaagccttgt
ccgactactcagttcgtcagggttggcgttcagaatttcttgctcaattccatccatgaaatcatcatggctggaatctgcttgctgtgagcgattca
acgcgtctaattcctcaagtgttgacatttgtgttccggttaagttcacgtatgttctttagagaagtcttagtaaccagaggtacgagcattgttaa
atcaaacttggaattttatacatattttcttttaaaaaattgtatatgttttatttgtgtaattaaaatgtggccttttgctttaaatacagattgtaca
atcattcatactctacaatgtcaaatagaccagtatagaagaaaagattgttttcaaaggcatatctcaaatatcaagaaaaggagtcaatgtaa
tttactatttttacgaaaacgaacatagaagcgctcatcaatgctcttcgctttgttttgtttttttccgcatcatctttttttgtttacacctgaataa
acaaaaaaaaaaattcatcaacacaaaacacaaggaggccaccaaccgctttcgattctttagaaacaacttaaccgttcctttttttttttttctat
tcttttgagcgatacatgaacaagagacaatctgaaaagcagcaattttgcgatggctccgtattcgacagaaaatgattacgattatggattatt
atcaacattgacctctcataaaactaaaataattatttcttcatgattgtgtaattcttttgaataaaaatatttataaaaatgagtaaaaaaaaaa
gaacgaccacggcgagatttgcactcgcgatctttggcgaatccttaaccggaaaccaacgccttacttctaggccacgaggccgctctgccttca
aggtaaatttaatttattatattccgttaagagaatattttacaggagtaagctttctattaaattatgtaaaatgtatatggcatgaaaacatgtttt
accaattgaaatatggatacgtactgttttattgtaactccccttcatggaaaaatatctatatttcttattctttaaaaagtttgagcaaaacatctt
tgatatcagatttatgttgctttcagaaaacgaggatagtagaaaattcctcctacgaccgttagattcttcacaaattttcaacgtaagtcaattta
ttaaatagtagcttcaaaattcgttgcaatcatctcttcattgctttagctttgaaatgaaaaccggtaataggaatatagactggagtagctatcc
aatgatttgttttctttagaagattcattaattgatttagtaatttatttgttaaattattcggaatttttattaatgccaaattccaacctgatggattc
ttcccatgcacttgatttccacttaatataacgcgaaaaaacccgttacaattatatttccaaaagtactgagctctacaagtagataccccaaacc
aattcttgtctttaatctttagaggaggaagcggttcaagtaaaagaaaaaatggaagatttagctagaaagtaccaagaacaacagagcggta
aggattttagcttgagaatactgactcactatttatgattagaattgaccggatatgttgattcgataaaaaagcttgaagcacaattacaggaaa
acactaccgtttataatgtatgttagattcgattgcatgtattggatgcgattgcactgtgaaggatcaaattttcaaatgagctaacatcggtagg
aattagacaagatggatcctgaatccaatatatataaacaaattgggccaactcttgttaagcaaagccaagaagaggcaaagacgaacgttca
aacgcgacttgatttcatcaagaaagaaatgtacgtttcatctgctccatatcctaaagcaattacaaaggaaacaggttcacttaggctaatgta
tccagtgctcgcgtggaaaaacaaatcgagagttcaaaggaggaatttgtcaagattaagtctgctatcgttcaagcgcaaaatgaagcggcttc
caaagcaaatgcttcttcttaaaacaaaaagtaattatcataaatattatacacattatgtaccaaatgacatcgactcacggttcttaatcatggg
tcctatccttgtatcattcaccttctttgcgaatcgtaaactattaagggtatccattatctgagactttagcggtgaaacattcacaaacataagtgt
ttttgcattttctcccaaagcgtatctcaacaaataagtaagctataatgtacttagttagtatcttttcataatatgcggattcattacacatacctt
ggaattcctataaggtatatgatttttttcctttaccagcaacgtaagttgctaccccctagtgcatggattacatctcccaaacaactcaaacttttgtt
gatagcttgcgtttctcgtaaacgttctccttctgctttgctgaaacttagacgttcggatccggccaaatcaaccaaatttaacgttcctctacaaa
cttgattcatgctttcattccatccatcaatgtacagcgtaaatacagtgtgagaacgagaggagcgttcattagctttcgtagctgcgataaaacg
attttctgctgctttccccaacattttgtagaccatatcttcatcctcaaaatcaatcacccttacattatccactgtagtgcgtcgctctctttcatcat
ggtgaatgtcatgcttttgttgttgcatcaaaccatttttgctcaataaatcataaatcttttcattataaatctctaaaaattgcccttgtaacttata
tctccatcctttttccttaagtaactcgacatactcaaagatttgcttaactgctcgagttaccataccatcattcgacaacatggtatacgactttcc
ggatccagtttgtccataagcaaaaacacttacattataaccgtcaatggcactttgaactagatgagtgatttcttgaaatacagactcattcgta
gcttcaggaggaaatacccgatcaaacgaaaaaaaaatagcttttatcgtgtacatgaccaagagatgattcaatattaggtcctttgagtaacaa
ttcccgaggctctaaagaatgactatctgggtattggaaaatagattcaagctcatctgatttttgacactcgcgaggtaaaaaaggtcgtaccct
acaaaaaactcgaatgttgccttttagctcttggatagtgttgtgtaattgccgccgaagagactcctcagcaaagagctttttcgataatgattca
tttgacctagttagttcgtgcactttacaatccaattcggaaaaagtatgacctttgtcatttattatttgctggttttctttcaacaggatgttaagtt
ggtcatttttctctaaagccattgtatattctttcttttttctcgtacaattcatcacaagttcggtttaattcttttcgaacctgatcaagctctttctgca
tagaagaaatatctgcttctatcttttgttggtaccgcttgaattccatatccttctcggaaagcagcaattcgtagtgcgctttgctcgcatcctcttg
ctttcttgattcttctttcctcctctcaaatgaattcacgttctcttgaagctgcgcattatatgctttttttagatcgcggatcctcttggacaaattctg
ctgcgcttcttcaaacgaatgcttatactcagtagtcatgttaacaatttcttcgcgaacaataacaatctgttgctgtaactcgcgttgcattttttt
cgcactttctcttcatgatactttatattgtcaagccgttcttgaagttttgctaattgggcatccttgtcgtttaccaattcccgaagggtttcaattcg
tgaaccataaacagcgtctttttctgaaacttgagatataagggtgtttactaaaaaactttgtcagcaaacttttctttagcttgttatacaacgta
cacaagtctttatctctttccacgcattcgagaagtgattgtatttctgattgatgactcctatccttctcagctagggaagtcgctgtgttagaatat

ctctcttttaggtcccagtaagcagactctaaatcatgaacttgatccttgtcaatcaaagatgaagtattcatcaaagaccctctagctgcctctac
tacacttccggcatttttctttatcagattgatttttcgaattgtcatggagctttccaagggactatggctgttattaccacttgatgccttgtgggatt
ctagcatcgcctggactttttctagccattcctctttatctttagatacaccttttaagcttctttcatctttggaagctgaattggtccttttgagttgac
tacaaccttgaaacttcttgtttctcgcgtctgcatgcgatgttgagttagcgacttccaccatagatgaagattttagatatttccctttataaggat
ttgcattttgctctatcacatcaatggcttcctcttcagcctgctttcgtttccttagagaatcactagataaagcggctgcattcttagcaaagttag
ctatgagttataaacaaagaattattcaatacctcaattaccatttcatggattcaagtagaagaaaagagaacagcagacttgtcaatttcttttg
cttccttccatcctttctttactgttgtttttctccagtcctcgccgggtttaacgagctacagcactgaagctttgaatgggcttaagctatttccgttc
agttcgttgagaattaccaccaaggaagggtgggacacatgtcagatcctcttgttttgatcctatgagtattgaatttgtgaggtggccggattca
tattatatcggcattgatggatgctcttacccttcgaaaagtttaactggtgcgaaaaacttccctgttggtcttcatcttataacttggacaccttcc
aatggtgtaggcttagtatctggagtattctttttcatttggaaaccttctgcttacacacttacttacgatgcatcatccgagaatgcattaattgag
gaagtagattgtgtatatgatgacaggttttataactatacttgcgcaggaaactgggatcagctggtgaaattcatacagcaaaagcatttacag
aagatatttcagagcgaaggaccaatgtttttatggatacttctacttacgcaaatatagatttaaatgcagctgagtctgagctctttcagccttc
agagaaggacactgtattacgattaacaaaattcgatgttagaaaaagctggtctccctcttccttaggtcctgaaagatctttgcaagctattgac
aaaacttttatgtttcaacgactcgtacaatccgtatggaataatgagcatgcttcagcactagctgaattttcgttcgcctaccttgcatacgctttt
ttttcccattatgcagcattattgcattggaaaaactttttggagatgctattgcaatcttataagttagctgagaccgagccagattattattctact
tttctcggtacgtttttttattcccttatagaaccctactaatcatggctgcataggcttgttccaatcacaattcgagacattacatgatcgtgacatt
gaaacttctgctctttttgagggtgactttatgcagtccttgctggaaaacctgttggaacgcaagtcggacaacagccttcctctccaagttaata
atgctttagataacttagtgaatgctgtttctgaagtagtcaacatacaggaaaatccagaagacgctgccccttattatgcggatgatgccaatg
ctgctattgattacgaccaagaagttcatgaaattggacattatgtgatagatgatttcaacaatgagagtaattgaggttaaaagaatgccatgt
tctgctactttttttctacttatcactttacttaatccttacagtcttgtaattaaaaatcaactgaaatttcttattatgtttctataaaaagaaaccaa
atagacgtcgaggactttgtccaaaccagccatacaaacagtacgacaaagaagagacgaaagtgctcacagtctgcgaccacgacgaccacc
ctttctgcgggtagagtcggtagggatgggagtaacatcctcaatacgaccaatgcgcataccagcacgggctaaagcacgtaaagcagcttga
gcaccgggaccgggagtcttagtagcggtaccaccagtagcacggatcttgatgtgaagagcggtgacgccaacttccttgcacttgacagcagc
atcttgcgcggctaacatcgcagcgtaaggagaagactcatcacgatcggtcttgaccttcataccaccagtgacacgggtgatggtttccttacc
ggtcaaatcagtgatatggacgaaggtatcgttgaaagaagcaaaaatatgagcaacaccaaagacgagctcaccagagcgaacttgaggcc
caacgttagtagccattttctgttgttgccgttccaagatctgttaggcactccgcaggtttcttcctctgattcttctctacatgtatactagggagca
gtcacactttgctaggttagggttacccataaccgatgtaagtaagtataagcgaaattgttaaataacataataaacgataatacgaatttacaa
acgataaatttcattcaaaattgacctattaatgaattaattagatatgtttatttctctactacattctaggataactgtatgtaaaaaagtaggcc
accatacgaactgaaacgaaagtagtcaaaaataaatgatggcagaaaaacaaaaaaaggggtattgtgaagcttcattagttatcatttgttc
aagaatgaaaaatgctagccatatctacaaattcacgagcattcttgttcattaatttagacagactgttactcagcgcattgcatcgcaaaatgc
cgttacgtgttgcgccagttgtattttgcacggctttcagcaaaaatttgatctgattgtaaagcatcttgtcatcatattcaaaccgcatattacccc
ggcaactgccattgcaacaccgtttgccgtagacgttcatttgcctagttcgatttccacatgaagagtcatcacatactaaaacaccctcatagta
aagatttataaagtatcgaatttgagtagtgaactgcaaaacgattgtcaatgtggggtactcaaagccgcaatcacattcaactgtattcacaaa
taacgtttcttgcagtgatttaatactaggcatcgtaaatgagaccgcaaaacagctgggacatcttaaaaccatcggtgaaacattgataaacct
ctggtcatccgataaagtgctttcgtatcgttcaaaagctgatgactctgatgcttcatatgtataatatcttttaggatccagtcctaaacattcagc
taaacgagcgcggttggtgccttcgataggtgcaataactctttcaataggaggaagaatttgatgtgtcagataataattataatcgatagttat
gttgttatttgggttcataacatcctgtggactacgagcacgatcagcaggatgtccttctgcgtcggagcccgtgatgataaaggtatcacatcc
ccagctcgtacattctctccttttgctttctttttaatgctacctgtacaaaaggcattgttttaccattcggatagtcttcgggatttttgcctaggcg
gttaaagataataaatttatttgctgtaaagtttccattcttcatttgatcagcgaaatcagaaagatagatgtgtatattactgacgaccgtctctg
taagctccccagataggatctgatccaagcaatatttgctagcttcttttgcaagcatacagtattctcgacgcttcatatcaagaccttttacatct
aatactgaatttcctttatcatcaacctgtaaagcagcgtactttttttttagcatgtagtagcattctttgataaacattatcgatgtcgatttctagct
tcctgtatctttcattcaccttttctttaaattcgttaccaattataatagcttgttgcttatcagcaacgttcgtattgagcataacagaatccgtatc
accgtaaataacctgcagccccatagcatcagcaagttcttttgtgttcattaatgcttctcttcctttataagtaattaaaacagcgaggggtcgag
catagaaacgagatttggtatagcctagacaaccatacattgagttagccgtcagcttaagtgcttgctgctgaatatcccattgtaatagctgagt
tgggttagccgttttatctttcaacaggcctttaattagtctacggcgatcaaccaaatttgcgattagacgagggaaaattccctgctcacttgaa
gcggtagggatattaggaatttgatcttcgccatctttcgtaggagcagagcgttgtaccgtagtgaagcagatgttgtactcctggataatactag
ggtacaaactattgaaatccataactaatatacatgtatcatatagacctttctgaggttcaaaaactaaaccacctttatacttatctttttctttc
ccggtgtttcttcctgaaactctgaatcttcaagatcgagaatatcgacgttccttctgttgttatactgtttatctggaacaacataaccatttttctt

aaactcatgcaaaagaatgaattcgtttctttcagctcgggtaccagccaaagttctagcccatgagttaccggcgatattagttagattttttgat
agttgcaacatttgtatttgaaatcctacagctgcgataaaataagtgtcaatctcacaatgggtaacataatcaagcaaaccacgagctgtatcg
gtccatgactgtaaagccttttcttgattgatatcctgccttttaatgtttaattctttctggacaatttcggacaagctccaagattgtactttaacca
tggatcttccaaagtcattagaaaggtcacttattaatcttccacatgcaacttgtctttccacaaattgttgactactcctattataagagcgcggc
cactcacctcgacgaagacgcccaatgctatgccaattatgaattttacggtcttttaaccgagaaagtagaaccgaatacatagtttcaaaatca
tgacccacataaacatctgggtcgtaattctgtattttacttatgaatgaattcaataaggaaatctcagatctttcacagtagataggggggtttatg
attgcgagccatattttcaaaaccgttaggaaaaatctgtttaagcggacgaataatggtaaatgtaaacgatggcatatcgttggctgaaagtcc
tcgatcaatctgaacattctcgtaaactctagcgctaatcataaccatttcttgtttgtttgatgcgggatttataagagtacggaaagccaaactca
tcactgtaagaggaggtgtctggggtaattttgagtcaattgctactgatattccttgaggagaagaacagccgatttccacacggcaccaactag
cattccgtacggaatcaaaatttgggttttgtatctttagccaacagggtcccataactcttcgcgtgaggacaaactgttcaaacaaagaagtgtt
tgtaccaaatacatgagagaatgtgttacctttgagatcagaaggaagttgagggtaagaataagaatataaaacttctaagtaatcggtctcct
ttggaacgtcctctaactcaaatgcatacttatacctgcttaatttggacttccattcttttacgcctaacttggaaagaaatcccgagacttcatcat
acactgccttctgttgatccaaggaagatgatagttgactattagaagaagctggtaaaaagaataaagatcgcataataccatcaatttttacg
aatgaagagacaaagctcttggatttttatcataaacttttccaaacaggcagaggctgccaaatacttctgtataatccaaccagaaaaaattt
aaagatccatcttgttctactatatcgctgggagagacacgatgacctttgttgatagtcgcaggatcggggacttctaaattgcgtaggtgagag
ttaagctccgtaaaggcagaggtatctaactcggatgacaaaggtggtgcaaaagaagatgggagaatgggtgtagctggaagaacaggttcc
acggctggtctatccagggtggttgccgcgcttaagtctcggactgaagcatcattttcttcaagttgattggaatcttcaggtttttctggaagcac
tggctccatttcgatatcgtctacaagggcttcttcatcatcggcaagtatcggagagcttggaactgctacatcttccgtactttgaggttgacgag
ttgctaaagaagcagtaggttcattttctattgctgatgatgactgcgtgatatttggatttgaaaaaaggtttgtggatggtttcttatcaactgtag
gcgtcgtctgggctttacctttcgagagcttacttttggtagacggggcatccattagatcttgatcaattgaacccaatatttgagccatgaagtca
tcatcattgttggactgatgcttcgtactatgattactaggttgaatgggcttggctttgaaaaaggagctaataggctgactgttattgtccgctaa
agcatgcttagtttcttgacataggttagtatattcaattgatgaaaaaggggttactacggcgagagtcttttatgtcaagtgagacattcaagttt
agcatatggcaacttacttttctttttgtttttgacgaccctgtttcttcagcttcctcttcatcatcgtcataaccgcggtcccattcgtcgtaaccatt
gtcaacgtatcctgcaccattgtcatctaccacaaagtcatcttgatctaaacgctgtcgtacaaccttcaagtattcttcctctgagactttgtcgta
aagattatcttcttctttattttcataaacggtagctctagtttttccagcttcacgtaatgcacgaagttcagcaaattttgagttcgagtttactgtc
gcatttctttttcgcatgatcctcccttcacgacctaagttataacgatttcttaacagtaaaaaattttttcagtggaaaagcctttccttgtttccct
atttctgaatggttgaacgcgctgagatatgctcctggatgtaaacaataaaggtcgcgtctatattgtacctatttatcgttctctcgcgttagagc
tccttcaaacgagctctaactccatttctcgtatttagggtagggttgctaaacagtcacggttggtatgtagtacgaaaacaaacatgtgccaaat
tcgatttttgccgacaatggttgcctcctcgaccgtcccttctgtagcctctatttttgcacctgagtctcttcgcaagaagactaagactcaggagc
aagctcgtgaacagcgcgttgctgctgctgctgaaaagaaatctgtaagtatacccaaaaatagttagagcagatggttggaaacataatgatta
gtgcaagaagtagtagatgttgcacaattggagtaatacaatttggttgacaattgatcgtaccttacaagagtccaactattcattaccttgctca
gataatttctatcattacagattcagtatgctgaccaggaatagggtctccaaaagaagcgtgaacttattgctaagcgtgctgaagcttatgagg
ctgaataccgtgctgctgagcgtgagcaaattgagctcgctcgtaaggctcgtgccgagggcaacttctatgttcctgaagagcccaagttggtct
tcgttgtccgtatccgtggtatcaacaatatccctcccaaggcccgtaagattatgcaactcttgcgtctcttgcaaattaacagcggtgtcttcatc
aaattcaacaaggctaccaaggaaatgctccaagttgttgagccttatgtaacttatggcgttcccaaccgtaaggctgtccgtgaattggtttata
agcgtggttttggcaaggttaacaagcaacgcattcatctctctgaaaatgccatcatcgaggctgctttgggcaagtactccatttactccatcga
agacttgattcatgaaatttacactgttggtcccaacttcaaacaagcttccaacttcatctggcctttcaagctttcctctcctttgggtggtttcag
agaacgcaagttcacccacttcattgagggcggtgatgccggtaaacgtgatgaacacattaacagcttgttgaagcaaatgatttaaaatgttg
tttttaagttgtgaatgggcgctgcttctatagggttatataaagattgtaatttataataaataggaataaccgctaggtttaagtggtcagtagtt
tgttcaatttttgtgtgtttttggtttaggaattcattttcatttagataatttctaaagtcttgctattcaccttaaaacgatatactactgatgggttca
agctcacgtcaatactattgagcgatccggaactgctttcgtattaggaaattcaactccattcattataaaagcattaaccactatgaatcgaata
actctatatagcaatggctcgaccccgttactgagatatcctcataaacgagaattatggatcaatgtcaatgattattgcaaaaaggtgctactta
gaagcaaaccgtcatacggtgaaatttcgagagaaacataatgttcagttttactatccgagctcagacaaaagttttgtatattaatttcatgtat
ttacaccgtttgaatctatcttgcaattcataaattatggggaatttatgaaggtgcaaatccgctacgacctttccataatgttaaatccctaaaat
tgagatgaagtattaccagtattactcattttgtttataagattttggcaaagaacaagtaacatgtcacgcagaaggtgacatgctcagtcgaag
taacaattacaatcattgtcgacaatacattcgtttaggactcagtttcatttaaagattgcagtaaagggtccttgtttggcagctttttttctactttt
gttaaaagagactcaataatgccaacaagaacagccataataaatcaatgttgccccgttttattagccgagaacgaacagcttaacatttgaag
gcatttattttaataacaatgtaaatcaatcatcgtaattgcctacattcttacttatttataagatacgatacgaaatggttgatagtatcaagattt

ttacgaaccttccacttagggagctccactaactgctcctccgttatctagcatcatgaaacaacatcaaagacgctccaccgttatctgtatgcta
caccatctataaatactggcgtatttgcgcacgtaaatagcgtgcttggaaaaatttctgtttatattccttagatttgttcttattatatattttttgtt
tgtccttttcattgcctttgttgttgcatagaattaaaaatgagtctaaagatccatattgaccgcggtggcacattcaccgatgtaatcgctactgtg
acagatgattcgcgagagccaattgtaataaaattactctcggaagacccaaataattatgaagatgcttccattgaagctgtacgacgtgttttg
gagatttaccatggtaaacatattcctaggaatgaaaaactagacaccagttgcattagtcatctccgttgcggtactacggttgctacgaatgcct
tactggaacgtaaaggagaacgatgtgccttcgttactacaaaaggcttcaaagatggtcttattattggcaatcagtcccgtcccaacatttttga
gttggctattcgtagacctgatgtcttgtattcaaaggttatagaagtcgatgaacgagttactttagaagattttgttgaggaccctaccagctata
aaacaaatattgatggctctgactcatctttggtgctcggccgttctggagaggctgtacgcattttaaagaaaatcgacgaagatgcggtccgaa
aggatttacagggtttaagggatgaaggatttaccagcgttgccatttgtttggcacacagttttacttttcctgatcacgagatcgctttaggcaag
ctcgccaaagagcttggtttctccaatatcagtttgtctacagaattgatgcctatgattaagtatgttcctcgagccacatctgccactgccgatgc
atacctgtctcccgtggtccgtcgttacttgtctggatttcaatcaggtttcttacacggacttaaaacccagggacatcctaaaggtgttcgctgtg
aattcatgcagagtgatggaggccttgccgatgttaacaaattttctggtcttcgagccatattatccggtcctgctggtggtgtcgtcggatttgct
cttacttcttatgatgagaacactaacatcccggtcatcggatttgacatgggtggtacttcaacggatgtatctcgttacggtggtgcatatgaac
atgtctttgagactactactgcgggcgttactattcaatctcctcagctggatatcaatactgttgctgccggtggtggctctagattgttttggaaac
acgggttatttgtagttggcccagagagtgctggcgcacatcctggtcctgtttgttatagaaagggcggctatttgactgttacggatgctaatcta
cttttaggtaggcttcttcctgaatctttccccaaaatttttggtcctactgaagatcaacctcttgatgtggaggctactcacaaaaaaatttcaaga
attaaccgacgaaattaattctagtatttcagaatctcaaaaaatgagcgtggatgaagttgcttatggttttatcaaaatagctaacgaaaccat
ggctcgccccatccgttcattaactgaggctaaaggacatgatatttctgttcatcgcttgacctcctttggaggtgctggtggtcagcattgtgttg
ctattgctcagtccctaggtatttctcaagttctcgtgcacaaatacagctctattctttcagcatatggaatgtctttagccgatgttgtgtctgagg
ctcaagaaccggcttccttcttacttacttccgatataattccagaaattcaggagcgtttcaaaagaattattgagaatgcacggaagagcctga
acgagcaaggatttgctgatgaaaaaatctcctatgaactttttttgaatcttcgttaccaaggaacagacagcccgttaatgattaccaaacctg
ccgaatcttggaatttcaagcaagcttttctagaaaggcacaagcaagagtttggatttcttttcgataaagagatcatcatcgacgatattcgcgt
tcgtgcttctggtaaaagttttgaacacaaggaacccagcgttgatgctcaattaaaagagttaaaattcacaccagtttcaaaatcactatcatct
acttctaaaaaggttttctttgattcaaagcgtatggattgccctatctacttattggaaaacttgcctactggggttgaaattgccggacctgcgat
tatcgtagacaaaacgcaaaccatagtaattcctccagggtccgtagctcgaatccttcattctcatgtactaattgacattttatcgaaggaagga
gtttctgatgattctcttgctaaggcggaagctatcgatcctgtttatctttccgttttaggttcacgattcatggctgtcgctgagcaaatgggtcgtg
ctcttcaaaaaacaagtgtttcgactaacgtgaaagaaagacttgattactcttgtgctttattcgatgccaacggagatttggtcgcaaatgcacc
acatatgcctgttcatttaggttctatgtccacttgcgtgcgtaagcaggcaatgttgaacaaaggaaacttgaatccaggtgatgttttggtaact
aatcatcccagctttggtggaactcatttgccagatattactaccattaccccttatttcgaagatggagaaattatcttctacgttgcatctcgagct
catcatgctgacattggtggtatattgcctggtagtatgccttccaccagtaaagaactttcagaagaaggagctagtattaagagcgaaaaatta
cttattgatggtgttttccaggaagaaaaaatgattgatctgctttatagtgagccaggaaaggtagaaggcggatctggtacacgatgcctacgt
gataatttaaatgatttgaaagcccaagtagcttcaaatcaaaaaggtattaatctcatcactactcttattaaagagtatggtaaaaattctgtct
tacgttacatgaaggctattcaggacaatgctgaaagttctgtgcgtaagctcttacttaaagtgcgggagacatttgaagagcaagatctttacg
ctgaagactttatggatgatggttctcgaatttctttacggattacgatagacggtaaaacaggtgatgctatctttgattttgatggcactacagaa
gaggtttatggcaataccaatgctcctgaagcagtaacttacagtgctataatctattgtttaagagttttagtatctgagaatattcccttaaacca
aggttgcttgttacctattaaggtcaaaattcctgacaattgtttccttaagccttctgaaaccgcagccgttgttggtggtaatgttcttacttctcaa
aggatcacagatacaattctgaaagctttccaagcctgtgctgctagtcaaggagatacaaataacttgacattcggtatgggggggaagaaacc
atgagactggagaagttgttccgggatttggatattatgaaaccatatgtggtggctcaggtgcatgtgatggtatggatggtacttctggtgttca
cacccatatgaccaatactcgaattactgatttagaagtctttgagcgccgttatcctgtcattcttaaagaatttagtatccgtaaaaattctggag
gtgcagggaaatacaaaggaggagatggtgttgtccgagatattgagttccgcattccagtcacgttgtcaattttgtctgagcgacgagcatatc
atccttatggcatgaaaggcggtgaagatgcgaaatgcgggaagaatatctgggtaaagtactggatagatgaagaaggcaaacgaaaagtcc
ggagaattaacttgggtggaaggaacacatgccacatagctgctggtgatcatattgaaatccatacacctggcggtggaggatatggattggcg
agcgaaaggatcgagaacaaggaatcgaaatttgtggataattctgcttttcgggcgaacggaagtttgtcccaacgacactatatacagcacac
aaattaacgcttaatagcagactcagcatgaaaacaaaaagaggatatgagagatggatttttctgtcgatgtactacaattgaatttaatgatat
taaaaataaggctaataatgactatcatgattttacgagagaataaaatgttttggaaaactaatagcacattgtattgtaaattatggatgattc
atgtttaaatgataaagactcgaatcatcattatcataaagcagaactcaaaacacaggaaagaacaccggaaaggagaggcgaagaatcag
gcatgtaaattaagtgattaatgaagaaaatatcggttggggaatatatacataaacaagcattatgaaggacaaacgagatgacaaagtaat
acgaagcgagctattcaagcagactgttcgagaaagcaggagcaaaaaaattagaggaccatgaatggccaggagcggattcaagggatgag

caaaaaggcacttgaatcttcgttttgcataaaaggagacgtgggaaagactgcttcagtaggaaaggttagatcatgatcattaaaaaagaaa
aattggtccgtgtaattagagttatatgaggcactgcagtcctcgccttcgagataagactcactaggcggctccttgtgaatagattcggtattgg
aagggatagggaaaaacgaagaacgggtttctatggagggatttacgaagctagaatatggcgagggatcttgaaaagaggaagaaaaaga
gagaggctgagggaaagggaggcgttgtttgaagtgtgtgtcttggaaagctgaagctgattgaaagtcaatactacttggggtggaagaagac
gaagaaggcgaaggggtggagaaagttatattggtagcaaaagggcctgctataggagatttgacgtgttgggatgatgaatttgttaagaatg
aagggtgcatataagggagagccatatgtgggagagtagcattatagctttcgaatttttgttgataatgttgaatgtatacctgcagggtggagg
atttgatttgagcaaatcggtcaaagctgaaggccaaaacgtagagttgcgttccaggaatatccttgcagcgctgatgcagaatatcgatcaag
tgctccatgagctctcttcggaggcctttatcgataggaaaagaaggatgaatcatagaaaatggattaaggaaaggaatcaacgggtgaaaat
aaaaaacaaaacaaaaaaaaagaaacacaagagaacaaacagggattgtatgagaatggagaatgaaaagaaaaaagaatcaaaaaaaa
taaattgcgcagaaaagtcctacaggttgaggtttttaccttaccttgtgaaatactgcagtggagcaaaaagagaagctgaccttttgtagtgt
cttgaaaaactaagaatagtgtcagtcaagatcatgcggttctcgtcaggaatttcgttctcttttcccaactcgagagagaccttttttaccaaca
ttatcatcgtcatcgacactccagagatattaaggatatcgggaagattgatagaccattgacgtccaagaggtcagtacgacgcgatgcaatgt
ctcctccatagtgtgtttttcttactgataagattggtcttttggtggttggttgaaccatggcaaaagcaagggaaatcaaagatgaataaataaa
aagaaataagaaataagaaataagaataagaaataaaagaacccatagagatttttgccgttggaaaaaacgaccctccttcctcacctccata
aggaatgcaaccaactactacttttagtttcacctaggcaattccgaggagtagaaacctcgtttggattgttcagatatgaaaaggatgtgtgttc
tttgttacaaggatgataaggaggatgaatgaataaatgcctatatcgaaagtttccgatttatgagaaccaaaatgtagacgagtttcgcctctt
ttctgcttaaaattcgtatacgtacgttataaaattacagccatactggaccaaggaggcccgtaattgaagctttccatcatttcggcctaggcca
gagctcctagaataatgatgtttcatcagcttatctcttattctcattctcatcttatcgagctttgtagtatcaattcagtgatgcatatcgacgatcg
cgcacgtacgaccattcgagtctttcccaatcaaatttggtttgatccagcgtagcaattggtttatcttgctgttatttttcgtaccaaacaattgaa
cttgatacctcccaaacctcttgtgtcccaagtactcctgcttttctgggaagcaagcgacttttgtgaacattgtaggaatacagcgtttgtgtcttt
atccatcaacaatctccatcgtatgtctgggttctttgtttgttttctctctccccatttctttctctgtagttttctttaaatattgttgagacgtttggtct
tcctactttcggttatttctttcctaatttctattttatactctctttcatactcccgtttgggggacctcgtggcatgtcctcttcctccactaacaatag
tgacaatcccaacgatgggacaggcaccaagcgaaaaaacagtcaagatctagatggctttcacaaacgaccctttatttgcgatctttgtgaaa
agcgtttcacccgccttgaaaacctcacccgacataaactttcacatgatgatacaaaaagtatccattgtgagtattgtcaacaatcatgcaaga
gaacggatcttctcaaacgacacatacagcgacaccactctggtgtcctcccaaactcttcttctcatcttgcttacgaacaacttccccccaaaac
taaaaccaatggacaacatcccgataatatgaaaattgttctcccgtcttttgatgccgtcgatcttccgaaaacgtatcatcccaatctacggccc
tacttcaccgtccttccgttcctcccttctgactttcttggccattatatcgagagttactttgaatggtttcatcccattctccccattttgcaccatccc
tcttttcacatcgaaggtactgccgcctcatttatacgttcagttagcttgatcggttgtcattccactggtattgaaagcgatgctactttgggccttt
tgttttgggacagcggctttcacttgttgcagctctatactcaaagcgacattcaaaagagcacaagcctttgggtgattcaggctcgcttcttgttt
tgcatctcctcccttttttgaaaaaacttccacttttgctagtattggtcaagccttacttcgcgatctggtttatgattttcgagtactgggctggacgg
cccttccttctattcccgagaattcttccctttccgcttttattgattatgaatctatgaaacgtatcatcttttgtacatatattttagaatatcttctgg
ctgccttttttcaaccgaaacccttcccttttcttttcaagaacttcaactgccccttcccattgatgaaattttttgggaatctgctaattccttgcaaag
agaacagtggaagcagacatctccttccttccacgattgcttaggcaagctctcttatggacctattgccttaaaggaaacaagaatcactggtct
agctctcctttgtgctatctatgagctaatccgtaacgatgtaaagaaagaatatgttattggctctttgaaatcctctaagcaggtatatgaggcta
tgcttcaacatttacgcatgagtattgaatgctccattaaaaacaagaaagttttgtcgctatttgttagtttatggaatctagtacgaacgtattta
catcttcatgatcctttgttgactatgtcaaagtccttttatgatttaaagagtgctgttcatttctttcgaaattctgtagaaagtgacgacgctcatt
ttcataccttttcctgagttttacaacataagtctggctctggaagggattttatgggcacttgatcatgcgtcagtcgtcttgcaaaattccaattttg
acataccgatcattcactttgctgtctttcggttggttcttcaaggcttatacctcttgaaagattgtcgatccaatacgctgttcactcatacaaata
cagccttcttgcggaaacaaataacagtctgctttagcggtcgccagattcttatggaagcttgggatacttctgatggtattgtgaagttggcttca
gtcctaaaaaaaactttttagatattgaaggaggttggggaacaggtgctttactttccgaaatctttaattgtttgtaagagcgatagtccttttatct
cttgtacactacttgttcttttgtctttatttcggttttttttcatactccttctcaatttatttgatacacttgttctattttttgatataatgatacttaatgt
ttgatagacctaaatttaattaaattatctgtaaaaccaaagtacacgagaatacagaacaaaatcatcactagaccacgacgggataggaata
tttagaagtacatgaaagagaagaacaaggtcgttatcctaataagaatgcaaaagggtattcaaataaggaatctaatagaaatagttcgtga
taggtaaaaatatgtaataattgtttacaaatagtagcgaaaatcatcagcataatctccagtcaaagtaggatcatactcaccacgggcacatc
gttcgtcacgttttttattagcatttcttaaggaaaagaataggacaatataaccgattaaacctactaaaacacaacccagggtaaaagaatga
cccagaatataccggggagcatttgtggaccggtatatttgaccggcaacaataccagaactattggcaatggtttgttgaattccaattgcagtc
gctcgtttgtagtgtcctgcaacattattgttgagccaacccaaattcatgcctgggcctatatagcaaccaatggcgacaataaaacaagctgtg
aacttggctgcatttgaattgctagctataagaattatgtagccgacggctgcaatgatagaagcacttattaaatagggcgaacgaatctttgta

cgatccgaggcatttgcagctatgagaaaagcaataactccggcgatatatacagggattgtcatgtaatttgattgaagacctgtaaatcctatt
cctttgacaatagttggtaagaaggaggaaaagccatacaagaccatatctgcgcaaaactgactagtagcagatacatataccttgagatcttt
gaaagctttgcgaacttctttccattcaaatttctgttgacccaagtatcgtagattagcttccttgcgaagactagcaacttgtctttcttgaggatt
caagaaccaagacttttccatgtcgtcgggtagtaaaaagagtgaaagaggaacccctataaaagaaattaagccttctacaatgtaaatccatt
gccaaccagctttattaccgacatggacttgttcaagagcgtaggcaaagagaccaccaaaggcgccagctaagccagaagcgcaaaacaagt
aagatagacgctggcactgttcttctctcgtgtaaacagtagtcaaataaagattgagggcaggaaacaagcagccctccagcagaccaagaat
aaggcgggtaacgatgagaccgccatagctagaaataaaaccagaaaataggacaactatactccatgcaaatgaaatgaaggccagcatgc
gggagggtcggaagtgttttacaagaatgacgctgggcgtttcgacgagtatgtacaggacataaaagatactaatggcaacattgagtcgttca
ttatagagattcaaatcctctgtcatgccagcgacagaagcattgccaatgtttgagcgatccaaaaaggccatcaaatacaaaaaggcgatgat
gggcgaaatgacgaggtccatttttttgagtgcacctttttcaacgcttttgttaatgacattgactacttctcccgtctcgactacgccttctgactcg
agactatcaatgttctgttgtaaactatgctttttctccgtgttactaatgccatcatgagttgagctcgctatgctcttagaataggaagtttccata
gactctaactcgtccattctttcaagagtcataatttatgctatttcttttctctttacaataaacaaaataaacgattctaatataggccagaaaaa
aaaggccacaaaaagcctaaacaagatacaaaaaaactcctatgaaaatcctttttttacttataatgtcatgaaatcttcgtttcttcaggtttgg
agtggtagtatccttaagagttgacaatgtcaatagaggctccaaggagctttatctcttccttatgaaggattgtaagctccttatgaaatatgat
ggctcacattagatacttgctacttgacgcttttctttcttcccttttgtttcatcgatagggagctctcaactcccgacaatggactcgatactatcag
ttacttctgaggtaacaccgataaatagtatgagttttatcttcatcaggcaaattacgttcctaaagagaatcattgaaattaagtgatgaaaatg
taaacaagaaaaagtatgtcatcaacgaaaagtgtgtttccgtatccaaggaacagttgtttgtttttgtggaattgagtaaagctaaactgaatg
atgctgtgctgtgctatactttgctgcttgaaaagtcaaagcaagaaggttccataaggaagcgtaaaaaagtgattgatccaaggtagaagagt
ggatctttgaaagttcgttgttaacggtcgaaaatcccttggataaaaaacaccttcgttttggatttctgtaccggtcttactgacggctggtaaga
attaagaagaattaagattggtttgctttcaagctattaaatatagttgaaatttctgtttaaactcgcttgtttgtgaacgttcttgacattgcttctg
tttcagcgccgttcgagcattgtgcaggaggaaaaggaggttttcgacatcagcaatgtcccaaattacatgaaacgatttcaactttctcccaaa
caacattcgacaacttattcgcaatcggcaatttactggttctacaacgggctgggcagaaggctatatccaagcaaaatttttagttctacgagc
aaaatatgctgatgattttggcaatttgtgtctttgtaacgcactcctgtgttctttgcttggagagacagttgctggtgatcccaggaagctttcccc
ttcaacttaagctgcgaatttcaattttgcaacggacatatcattttactgcgagtacatcaacggcaaattcttcaaggagttaacctgcatttcaa
aacagtggagcaactcttatgttgggtttttgatcggctgttctttttttctttgaagcggctcttgttgttgccaatttgattcctcgtcatctccccag
tggaaatcctcttcctatgtctaagacaaatctccgtctttgcccgtccggagtcatttcaggtatatttgtctccatgcatccctaccgtaaaaaag
acattatgaaataaaatattagccgtccaaatccgtaagatcacaatttatctgcaaatatttgaattatggaaagattcccccctaaaaccatgga
atttcgtttgacgtcttgttcaaaacatggcagacagatgattgcttgaaattgaattttgatagaacagctatatgacgatttacctactttatacg
catgaaaacttcattcaaaacatatctcactttttcactgtagtcttctttgtgctcctatcctatgattgtttacgagcgtgaaataacgaagtttgat
gctcacattaatgaaaaaactacgaaagctacaagtgatatttgtaccttaaaatctaccaacatggactcaaaacctcttaattctttgtttgtttc
ggttcaactttttgtatacacttaattagctgttattctctttaagttgaggacgatatctcagcagctcgagcatcgtaaataacccaaccactgtg
ttctatttagtgagaacactcacaattaattttcctctagtacctaatctaggaggatatgtgcgttataatccaaggataacgggattgtctaaaa
atggactgagcgagttagcagtagtaaactccctaaatatcgtctaaactgcctttagttttgcgatggcgtttgcccttgcaatccactagttctat
gagatagtaatgtattacttaccacctatacctcttgccatgtagagccaattgcctggggctgggagggcgcaaagcaaataagcaaatcggta
tcgacaacgtaatgcaacctgaatatcgtttcccgataaaatttcaaatcgatgaaattcctgtcttttgaggatgtagagttacggttcaaaacgt
tgttatgatgtcgaaattaccagcacctgtttactctcataaacccggttatatgtttactactgatgaacagcaccggcattgatatttgtttctaag
actgtctagctcgtgaatgttttcatttttgtgagatattgcttttattattgatatcgcatgattgcaccttttccatctgtctcctgaaatagaatttt
tcctctccttcgttcccttttcattagacaatagaatagaaacaagtaggtaggtcatgtgctttcatataaaaaaaaaataaaaagttatgatcaa
attccttaagatatatgtcctttgagcaaaaacaaatgcattcacgacatgccgaatccagctgcaatttttttagttccattcctttctattttacagc
tttctttctttttattctattcttttctcgagtctttacttttttttttactttcttttttttacgtcttcagtgtttcaaattgggtcatcattacatttgtagatt
cgcttactttgtaattattttagcgaaactttatttcacaaatgaaaaagtgccgtaagtggttggtaataatttcacatatccagctttcttgaactt
ttccattcttcaaaatacctcattgaacatgactaccacgaactcaccaacagtgtcgaagaagactgtgtatttaatccgacatggacaagcgct
tcacaatgtaggccctaatgaagaccattcgatccgtgatcctgttttaactgacttgggaattgaacaatccaaagaattatcaaagcaactggc
tgacaaaaaaataaagtttgatgccattgtttgctctccaatgcgtcgtacattacaaactatggagcttggcttgaaggattatctggcaaaaga
gggtgttgagaaaattcctgtttatgtcaaccctttgtttcaagaagtaggcaacttgccgtgtgatgttggcttggaagtcaaagaactctcctcct
tgtatcctcgttatgatctcaagtactgtgtagacggtatttatccagagaaaaaagatatctatgatgtcgacgttgacattagcggtataagatc
caagtatgctttggaatacttatctgctcttcctcacgagaatattgctgtgattagtcatagcgcctttattcgcttcttgttgaagaaaatcactcg
agatgttgacatcaactttttacctcctgaattgacttttaacaattgcgaaattcgtgaatactttttgaagcctgatggaaatgaatttaaactttg

cgacgttgagtaaattacagaagcccagtaacttatcatgtttcgcccggtcgttctttacatcatttacagaagcgattatggaccataagagac
agaacatttatgaaatattcatcttacgaattttgtaaatatgaattttttttctcgatttctcgataatgaatgtggaaattgatttgatactaatgaat
atactggataagtctagtttgcgctttgtttctactcacgcctaccaatccgtgcaaagaatataggatactctaatttgaatatttatcaaaactac
ttgtgtgttgtctttcagaggaaaaaggcgtttggctaatcagacgcttgcagaaacagtcaagagtcatcaaagtaaaataaaaaatgaaaga
aaatttgttaaatcccaaagtatgctcacgcgccagaaataacttatccgcacagaactgtcatgaagtatattcctgcatactgaatttcagcaa
gtttaaaatatatcctgcttatgaaatatgttgaaagtgaccgcattgaagccaatcttcattgcatgtttcattatataacatctttgttggtttactt
tagggtaagatgcgaatgacaatgcaataacagcgaaagaaacccttcatacagacttgcggtcagcattcgtactaaagctaaataataaagt
atgagtaggggattgacagaaaggattcacacaaaatgcctttcttctctttttttcatcgtttaaaaagcgaagtgacaaaattataatatacgaa
caaggtattggtatggtttgtgtacaaagaatcaggaaaagaatatgttatgagttactgaagtattgttcgctgtttatacatttggattagggtg
acaactaaattccaatattttgaatactaagtcaatgaatagcaaatctgagaggtgatgtacaaaatcgatattcaagccaatttagaaaacgc
ttcctctttaatgctttaggcatatttataaaaaaagaatgaaagcaacgttctcaacctgaaggaattttttcaatagagcactgaaaaaatatat
cgagaaatgaaacttatagcttaccaaaaggacttaagcagatgacttcaaatagacaattatgaaatatgatttcttttttttttgatgttatgtttt
cattagattccttgttttaatttccaaattcgatgatcttttacatttatctattcaccttgcaacactcaaaaaggggtacaaaggagtagtttgaaa
tatcatatcttccatcgttttatagcgtctctggaaacatagacatagacataaacataaatgcttatagttttaccctaagacaaaaaaacattga
gatatgttgctcgttgtcatttttgtttacggtttacgtaatttcaatgaaaacaaaaacagagaaatcaaaatcatttgtacctgaggagtaacct
atgaatgagaatttgactatggagaactcaaaataaaaatatatccttttattattacgtcttggcttttcaaattcaattcttgacccttgtatcaaa
aagagacaatggagcgtaacaactttcgctaacagatgaaactaacttagtaattgaaattaataatgaaccgggatgcctctagcaaatatca
attgaacaagaagtaaccgccacaaactatagtcaagtttcgccatcaatgttattctagatataaacaattgaagtcttactttttttaaaaaaaa
aataaggtagtatactttcaaatggatttcatttttatctatcttaaatacttatatatttattcgcaactattttgagacttgtagttttcagattgtta
cattgaagatatgtcgaggagcaaactgttcaagccaattcaagtgggaaatatgtttttaaaccatcgtatcgtacacgctcctatgactagact
acgagctactgatgatggattcgttaccgagttgatgaccaaatattatactcaaaggtcatgtatcccaggatcgctattaatttcagatgcaaca
tttgcaggagaaaagtctggtggatacccaaataatcctagatgtttcacgaaagaacacgctaaggcttggattcctgtgacgaacgctatccat
cgaaataaatcatatatgtttatgcaattctggcctcttccgggtgaaattaaggatgagtatagaaatgatatccagagattaaaagaaattgca
tataacgactgccctatggatccagttggcttgcctgctggtgtcgagtccttcgataccgttcaggggattaacatatacaagaaaaaatatatg
agcaaacaagatatacaagagcatatacgggattttgtcaatgcagcttctttagccatccatgtggcgaaggcagacggagtggagcttcatca
agtgaatggatttttgctagatcgattcgtcctcggcggttttggcaatcaatgtgatccaaattatcgtggatcagttgaaaatcggtgtcgatttg
ctctagaagtcatggaagctgttgttcaagaaattggacaagacaaggttggatatagaatctcaccgtattcagcttggatggaacaaatcgac
agtgtagagacccatatatacctaatgaaagagatttccaggcgttttccgaaattggcttatgtccatgcaattgagccccgacaatattggaat
ggtcatatgatgttgaagtccgagttgaatacgtcttacttacagaattattggaaaggaccttttattactgctggtggctatgatcctggatcagc
aaaggaagctgcagacgagcgaggtacactggtagcctttggcagacaattcattgccaatccagatttggtatatcgtattcagtataatttgcc
tctaaacccttacaaccgctctttattttacgcacccaaaaatcctttaggctacattgattacccatttagccaagagtacctatatgcaacgaaa
ggaaaaaacaataaacacacacaggtgtagtcaaaacattttttttatccttttatttttttcttatcgcctatgaagctacattgttccatgattcatttt
ggcctttgtttaaaatggtatcgatatccatgatccaatgccgacacatagcaagtagcgaacgtaattattgtgaaaggaaaaagcatacaaaa
gaacgacaccctatcactccaaaaattatcgggtataatacaaaagtgagacaaagtccctctaattcgaagcaaaaacccttttgtagatggagc
ttataagaggaaagataagactctacctgagggtttttgatgacatcgttcaccatgaaggaaggtaacccggtcattgctaaaaattcgtgagc
cttatgaacagggtacaaaacattatcggcaccgcggccatcaaagaagttcccgaattcgttgaatgcctcaaaaggagcattccaagttgtgg
aaaacatgtagtgtttgccatggagaagaccacctttaccatagttcttggttggatttacacgagaacggccgtcgcttgcatagagctttccata
tccaatagtaaagacttcgtcgatgtatttctttaatggccaaggaacacccatccaccatccagggaattgatagatgacaacatcagacgaaa
caaacttttcaacctcctcttcaacattatagccttttgtgacggtagtctcttgaatggaatggcctaaacctgtcaaggcctctttggcaacacttt
gaagtgtctcgtttaatttgccctggctatggccaaaagggtgagatccattaacaagcaaaattttcaaacccatttcttcgtctaaaaattaaaa
ataagagcgtaaccgttaagtgtatagacaaaatggccgacctatttaagtgtgcggaaaatgactacttcgttactttgcttattcagcagccgtc
tgctcaatttaccatcataagtaatttcattctctaaattagcctttaataaaagccaattttggaaatttatcttcgctgttaagaagcttgcggattt
cgcaagaggaaaaatagtcgtatagagaaactaagtgataagtttgcatgtagtacttactatgagagaatttaaatgacagagataaaaaaa
aaaaaaattaaaaaatgaaaattccattgctatcgtggggaatgtagaaatacagtatatacaatatagctcgagaaggagctaaaaaaacat
aaaggaactactagtcaacatatctacaaagaaaaaagaaagattgacagaagaagcgatgtagccatctggctatgaatatggatgaacaaa
ggaatgaaaaatgaagggtatgaagaatggagtaataggcggtttaataggcagcagcaaaaatagacttgacttcgtccagggtaggttgaa
tagggttggtagcaccacaagcgtccttcatagcattctgagagaggttgtcaaagtccttttcaaaaacgcccaagtctttgagataagggttga
cgcccacttgctcgttcaacttttgaatcttctcaatagcagcctgggcagtgtgttcttcgcagccaagttgagcagcaacgtcgcccaaacgagc

agcggctccagcatcctttgagttgaacaattggacgtgaggaagaagaatggcattgcaaataccatggggaatattgtaaaacccaccaagt
tggtgagccatagcatgaacataaccaagagaagcattgttgaaagccataccaccaaggaattgtgcataggccatcttttctcgagcttcttcg
tctttgccattcttaacagcgcgattcagatattgaccaacgagttccatacacttaagggcgcaggcatcggtaattgggttggcagcggtggaa
acataggcctcgacagcgtgtgtcaaggcatccatgccagttgcagcggtcaaagagggaggaagtccatacatagtttcgggatcgttgacag
aaaggcaaggcatagtgtgcttgtcaataatcgacatttttatatgacgagtttcttcagtgataatagcgaaccgggtcatttcgctggcagttcc
agcggtagtgttgatggaaaccaaaggaagttgaggctttagcgacttatcaacgccttcatagtcagcaattttaccaccgttggtagcaagca
aagcaatacccttggcgcaatcatgagccgaaccaccaccgatggacactatggagtcacagccgaattccttaatgagttttaatccatccttaa
cattggtgacagtggggttgggctgtacaccattgtaaatataaacagtagaacccctttcttccaaaagccccttaactttatcgcaaagaccga
ttttaatgatgccaggatcagtaacaattaatattttttgtaaccactcatctttatttgattagcggcttcgttaagactacctttgccaaacaagtt
aaatgaaggaatgtaaaacgcgctgacagaagaagatctggtaggagtactagtggaaaataaattgtaattcgaagctgcgaaaaatcttctg
ctaaattcagagcgacatgatttccaaggcgaggagcgtctacaacaaacagaagacgctgccgtggaactaaagtgaacagagcgaacgttc
tggctcaacaaaggttgggcacgagaaatccaacgattaatgagcataatgggaacgaaggacaatgtttcaaatgtctttttttttcaataata
aatgatttaatctatagacctgggtctttttgagcaatgattctagctaataaataacaatcaaaggagctaacaaaaatgcaacaacataaaaa
tactttttttgcttacttttgcgatttcttttgattgatcagatttgcttttaaatggaaaccaaagtttaaaaagcaccaatcagatatataaggattc
tcaaatatgaaaggtatatggctaaagatatcgatgctatcgtggagagtaaatttaaatcttttttttcctatgatttgcctactcagaggcggag
agtgaaatgcgtggataaaatttttcctcggttcgctaaacttgatccaatcatacggcaggaaacacatcagcacatacgtagtgcaagattata
taaaatgatttgcgagccttgcagatcattttccgttgaagaaggaagctgtagtcttaaaacaaaattcttggaaggaggaccattcgtaagaa
aattaatgaaaaattactttttaatttatacgaaaaattcccaatttagctctcaaccagaataaggtcgagcttttttttttttctaagatcgacgc
acattctttgcttacagtgcgggacaattactttgctattacacaagacacgacgatcatcaaatgtccgagagtagataatcttgatatgaaaaa
cgaattctcttttccctcaagattatatcggtccttgaacaagtcgattgcaccgtaaaacctttgtagtataatatattactatatcaaaatattttg
aaatgatccgaagaaaaacaaatagatggaagtttcccatacaaaatggtagtcggattgtatttacaagaaattagttcaatttttcttacacag
accttactgagtaataagatttattaatggacgtaaaagatttccaacaatgatgccatttttttttttatcgttcaaaactttatctaagaatacgtt
cagtaaaatttcctttgcttgaatttgaaaaagtcaaagaaaattcaacatttttcccttttgcctttttgttttcatacacatcaattgtaatcaaatt
tcacgttcgttctcattttatttcgttatcataagggcagttaatctggatgttgtcaattaatcttgccttgcctaaccaagctgccgcaagtaagac
catgcgctttgtttcatgattgcaagattctagggtatcagcgtctcgaatttcaatttgatctggaagaaatccaagcttgcgcatttcggtattgg
actcttgaagcaaggtagcaaagtctcgctgaccttgagtcactttcctcaccattgcagataatatcttatataaatcaggagcgacccttcgttg
ttcctccgttaaataaccatttctagagctcaacgctaaaccattgtcacaacgaacagtcggaacgccaacaatctcgataccaaaatccaaat
ccgtcaccatttttcgtataatcgccaattgctggaaatctttctcgccaaagcatgccacatccggttgtattatatggaataacttgcttacaattg
ttgtaacaccacgaaaatgacctggtcgactagcaccttccagcatttccgataactttggagtttccacgaatgtaatacctgcggaaccagttg
gatataattcttgtgccgttggcgtaaaaactaaatcaacagaaaattcctttagcttttcgtaatcatcctctaaagtatgaggataagcatccaa
gtcttgttggttgttgaattgcataggattgacaaaaatgcttacggcgacttttgttcccagttcttttgcccttttcaccaacgttaaatgtccctca
tgcaagtttcccatcgtcgggacaaatgcaatccgttctccttgttgtcgccaaaattgaacttgtttccgaaccaaatctttttttccttcaataatc
atttttgaatttttatttaagtattaataggtatgctcttttgctggataaagcccttctttcacttcttgtatgtatagtcggacggcagctcgtatgtc
cccggttccgctcagaaagtttttcacaaaccgtggagcttttccactggaaatgcccagtgcatcatgcataacaaggatttgactatctgtgtgt
ttaccagctccaattccaatcgtaggaatcgaaacagattctgtgatttgctgtcctaacgtttcaggaatgcattccaacactaatatttgagctcc
agctttttctatttgctttgctgattccaaaatctgattagcagcagtttcatttcttccctgaatcttgaaacctccataaatattcacagcttgaggc
gtcattccaatgtgtccacaaacaggaacggatctttcagccagttttcgaatggtgtcataaatccaacttccacttccttctagtttgacaacatt
tgctccagaacgcataagagatgctgcgtttttgcatgcgtcgtccgcattgctataactcatgaatggcatgtcagccatcaacaggcaattgggt
gcaccgcgtcgaacgcttctagtatggtaagcaacatcggaaacgctcaccggtaaagtcgaggtgtggccttgggctgccattcctagcgaatc
tccaacaagcataaccggcattccctgttcttcaaatagcctcgaaaagctggcatcataggcagtaatacacgagaatttttctttagcaagcttc
cactgacgaagtacagaagtggtaattggtttcgtcaacattatcactagaaaggcgatccgtacgttttaaccttcaaaatcaatggattaaaat
tcctttttttgtattcaaagacttttacgcaaaagtataaactgaacgattaccgaaagaattgtttctcagagaaaactcaaactacgatggaaag
acgattctcttgggtatggtagtgaagacggcacaaaaccgaaaaaagcggcattcataaaattagctgcaggtactcttaatttttctaaaaga
ctaagactttaaagaaagtcatattttttttggggatcatagtggaagtttatgaaaagtgaaaatttgacagttgcttcaggtattcaggacacatg
aaagcaatgtatataaaacgaagaaattcctaaaaaatttaggataggcctcattatacgcgttcttgcaacttgctcctctgacaaacgaattca
cttcagctacagttgaagtaccctaggtttgggattcacttacattgcatggctatttctctacattaaacaaaatgaaaaggtgtcattacaggttt
acggtccagattttcaaacaaaaaatgacacccatgatgatgatcaaagctgctctataaacggatgtaaagacctttaacaagacacgtctcttt
catctgcaaatatgattcctgtacttgaaaggacatatataatacattcctatttgttgaactttgacggaagacgaacttgagtgactaaataaat

ccaaaaatcacaacaagagagatttaaccggaaaatggaattcaatgtaatcttagaccaatctacaatttgaacaaaaggcgcagaaagaaa
aaaacacaaataaattaaagacagaaccaaacgaaaacccttaccatacgaggaaagatgcgccaagaaaagaataattagttaaacattcc
tcaccaagaaattaactgaggttcatacaaatccaatgtgccaatctttaggaaactttggggtcgctttttgtttcgctatcggaaaactttggaa
ccttgcatccatcttaaaaagttatttcgtatgctctgatttgtttcaaaaacttgatgctttatggttagttgtaagtgaattgacaagtttgattggc
atgacatcgatggagatgaaaaagacttgcagcatccgatgtttttggtgaagaactctcatggaggaattgtaccatcatatttggaattgatat
acaatgggcccgataaattgaaaacgaattcgcaagtaatggataggtagagtcattcatactcgcagaagtaatatttaggatggctgacaatc
gccaacaccttggctcgtttcccttggaataaaagccagtatttgaaacgcaaggaccacctgtatcatcgctaaggagcatcataccagattgta
tccagaatagaaggatgttctctattttgttactgaagcattgtaaaggaatgaaacatcctcaacaatttggaatcaagggaattgccaaacaa
tatgattctatttgatttttagttttaattttgggaattctccaaatccaatgatgctctacattagatgtttataaaggataagaatgtatatctttca
acttgattatttcgcaagttaactgattaggaacgtcaacatgcatcttgtatttactctactataattcaaagaaagatcggaaaacataagttcc
atctgtaatactagaggcatttagaattgcgctttgcttatttactaataattgtccctagcgatgtttaattatgttccttcgtggatcagccattttta
tatcgcttatagtccagctaaaaaaggttggattatgcgaagggggtaatgtttacaaatgtatagaaaaacattcgccaaaaaaaattaataa
tttaactgtgtatgcaaatgaaaacgttaaaagcgattcaaagctttcatatcggtacagcataccatttattaagacttcttttgaatagcaagaa
aaggtttccaacgttggggtcgcgcctttttttttataaagcaatcagcaatcagcaattagcaaatgaaaggatttttaaaacattctattataga
acatataacaaaattaactaatgatctagatatttgataaaccgcaaaggagaaaatattataaggggagattaataataagtctaatttgtttga
tattgatgaaaggcgagcatttctccatatgcacggaaccattctgcaagatacacaggagcaaattcagagctaccaatattccaaaattttcca
tgtgcagttttcaaatgatggtattcatcaggagagcaccaaacaccattgtaataaggatcaacgcgagtccacggaaaccatccatgctcctc
gtaagcaagattgagcaaactttgctccatcatatgagcattttcatatttatatggataagcgccaatttttaatagacgcttaaattgctcaatgc
tggggcgaatgagcatcattccagcgttgaaggatccaataggttcaggtgggatcatatggtgctctcctctgtcagaaacggctgctaaaaggt
aaggataaaaatcatcgatgctcgcaccgtattcttcaaaattttcttcaaagtagtgttctgagtcatcttttggatccttgtagcgacgttgctca
gttttaacaggagtgcgtggttttccttgataaggggtatcaaatatatcgtcgatatttttcagaatcagaatgtcactatcaacaacacagagttt
gtcaaactgggtttgctcaaaaatacgaagctttgtaaacatgtgttcataccgcttggaaacgtcatggtccatggattcaacgtcgacaaccat
gtttgatggagaaattggatcaaccatgatgatagaagcaccatcagcctccagacgacgaattttccattcgtcaaccccgcggacaaccataa
tgtgaatgggatatttactttttgtaacaggatgatgcttcaagcgatgaactaggaccctagtcgcattgaaataagggtcattttcagaggaag
gcggggtaagtaatgccataaacgtatacttgttagggtcaccctgaagatccacaaatgcttgttcagttgacatttgtccctcaaaggcatgca
aaacgcttggggataaaagattaaaatggaatgtaaacgtacataatccgatgagcagagctgtagctaaaaggatgaactttgagcgacgca
gaaaatgagtaatttggccaagtcgaagttgtgtcatattaatcaaaaacaaggaatcgactgaagaaagtgagattgaaatagaaacgaaaa
caaaaaacgaggaaatgaacgactaaaaaattaaatatctgggtattattcgcacaaagccaaaaaaaagaaggaaaaacaaacgaaaaag
aacacgagtgaaaaacaagtctataggaaaggaaaaatgttattcacaagaaaaacattcaattactaaattcagatatccaaagtaagttatt
atttagttgtacatgctttttctttaattaaagaaaaaaaaaaaagaatgtgtattttggaaattctcggaaatccttgaccctaaatactgttttgaa
ggtgtcaaaccacgtgcgcgaaatatggacagtagggattcgagccatttatattggtgatattgtagtaaattttctctttaaaacgattaaaaat
aaataaataaaaaaaaaacattcagaattttgtatttctttatcaatagttggtttttagatttttaaaaaaaaagaacaaagtcttttgtaagaat
gaaatcctttcattgaaaaaaaaacgaaacaaatattcgaatgaactttgctatttgtgcaaatgaaaaagaaagaaggtgaaaagaatggac
aagatggataacaaattttatttctacttcgcattcgtttagagacctgacaaggtagtaataagtatgcaatattttgcgagatttttattttccaaa
taaataaagctaccttaacggttcctctattccacaacaacaacagagcttgaagaaatgcgttgaacctcttgttgggaaaaccaggcagatct
agtactatgaacatgattttggatcatgtcattgagcgcttcttttcattatctctattcaattgttgacgaacttgacgaactcaagaggaccttttt
accaagtccaattttggatggctttacgaaaggagccgctataaatgtgtattgtaacaatgagtaatgttggctagcaataagacaaattcatga
ctttcgactttcatggtagccttctaccgaggcctattactagctttatgaaagcgatagcttcgctattgaaaaggtagaaggaacttaattgggt
gccgcactttcgcttgcgaagacgagaaatgcaattagcaaccaacgagtcttgatagcaaagttacctaccaaggtcagattaaagtgtctaaa
gtgcgaaaccccggacgatacaattcgttgacggcatgataaaaacaactgcagaatggactaaaagtgaaggatcaatgaatatcctgataa
aaaaaaaagagaaacaaaaaaatgtttttcattactcttttcatcttcgctgagcgtgtgatatttaacttcatttgatgggagaaggggtaaagg
acaagggaaacaagcacgcgtcttctttcagaccaagattcgtagcatatagaaaaaaggccgaatggttaaccacaaaaaccagagcgaggc
gataatcgcgtgtcaatttatttcgctattttcaaagacaacaaagcgaccagcaattaaattaaacttttagctgccgaaactggcaaaagacgg
gaggtttctttgaaaagagagacagacctaaatatacaagagaaacaggacccagtttcttttggatcagatctatatgtttaagcttcttaccaa
cagattttaggaggagcgaggatggacacgaaaacattcctgtagtttcagaacattcgagtcctacatcatgactatgctaatcaattcgcggat
acttccaactaatgagaatggttttcgacgcgacatttgtaataagatacattggtttatcaaattttcaaattttagttttaagataaaagacaaa
ggtttcaaaaggaagaagacataaatattataaacaaaagcgcactgttaaaacaagaggtgaaaggtttatttttttttgcaaaatttttttat
tttttaatttttttgtaaaattttataggtactctaagagatctttacgaaattacaatcggcagattagattactcccaatcttctagaaacaacaa

cttctttggccttttaattatccaaatggcctggccttttctaaaaccgaagcgatttgtcaacgatagtaataggaagaattgttgtcatgattatta
ctaacgacataaccaaagtatagaaaacccgaaaataaaccccgctgctgcaatcatccgatccgttaaaccaactacgtgccgcagatggaga
gagaaggaaaaaataatatatcgtaggtaaccgattcgtttgctggaaggggtagaatgtttcccctctttttttttttttcttctttggaataggaag
acaaattccgtaaagcggggaatcatcttttgaccgataattggctaatcgattatcggttgcatcgtgtttgacagtgaaaaaaattatcatatgt
catctggtcaaagactcaaaaccttataagtaaaggttggtaagctccaccaatcttctcaagtaaggttcgcatagttacgttcccgtaaaccta
gggttttatattatgaagttctctgcttggtttctttctttggcctctgcttcgctttgtgtctcttcggtagctcacgctcaaaccttaacttactccaat
cccaatgtgaatctgagcaaagagaatcccatggtcatgaacatgacccttggcgattgtactccttacggtctccctgtaaacagtgccatatat
gacattcagtacaagcttacgggtgtcttttctcgtgattgggactttgtgaaaaaagctattgaagaaggcaaatatctggatgctgttgctgctg
ctaacttggtaaacccggatggaggtgttgccaacttgacttatgatagaatcattgatatgatagccaacgaaaccgatcataaacaagttgaa
tccctacaatgtttgaggaatttaattgacaagcaacaaaataatgtcatgactcttatggctgttcctgactctacaccccatagccgttttagtga
tactttagatgcctctattgaccggctaaatactgcaggcaaatccaagcgtggttacatttggggtgacctacgtaacgttggcgattgctatggt
agtcatgcagctgttcgcagtgattgtggtcatgccattgccaatgcacatacaggtaaaatatatataagcaatagggaatcttcgataaacgtt
tacggcacctgtttcttttcaagtgatactcaaggtgtagggacacatgagattgaaacagagattctcaagcaagatgcatggatggtttttgga
actgtttcaacagaaatgatgatagctctaggaacagtggtgaagttgatgactataacaatctagccagggtttgtttgagtgatcgcggtagtg
gttgttaagggggaaaaattcacgttcctttctttttttttttcaagaccgtctttctttttggaataacttataagcaatctttttccctttgtaaggg
atttctacttcttcccttgccctatagcaaaggtgtcgacattgctaatgatcgtttatccccgaaggaacgtatcgattcgtttttcgggcgttttttt
ttaattcccatctataattccgtttagaccctggaccatttcgtttaggattccctatagaatagtatgtacacctacacaaaaaataaaatctatat
gtatagtgtttatcgtcagttctactgtagttgtttgtatatttgtttacttgtattatatttaatttttattatataaataaatcgaggtattatgtcattg
tccttttcattgatacccgtaaggttcgtttccccaccccatgttatcatcactcgtggagctatatatataagcgtaggtacttccaagtacaggctt
ttacagacaaaagaaagcaaacgcataaaatctaagacttcacatggttttttctcctttcattaataattaatttatttattccgtaatttcattttt
ttgcactctttttctacttaacacttcatttctcagtttttgtttttgtccttttgtaaattttttttcccactggaacagctttcggctattatatcttgatt
cctattgtcaaccatgttgctttcatttgggataagtgctctgctgtttgcaggtacaactcttgcttctgtgccgtacaatcatacagatgttagttct
ggacaaatatatcgaatagatgatgccaataaacccatttttagaaaagaggaaggcttgatttttatacctgttgaatctaatgttcaagcgttcg
cttcttgtaatatattgaatcctacttacgatgaatctgatccggcttatgcgaaaatagttcaaaagccaaatacgagtggaacggttcatttgcg
gatggttcaacacccagacttatttcaagcttataaacatgccattgatggtcgtgaggattataggtttttaaaaaatttgaacccaaattctgat
gaagatttttacaatgttcagttaaattttacgtccggtacggattcaggatactatatgacgaaagtcacagaaccgggcatggtttgttttcttgg
ataccaggaagttgacgcaactgaagagcctgaccctataatatatatgcaatacaaatctggtgaatacagagatagtgaaccagaacatcgt
atttttgtaggtttgtcagtgatttccggcatcataggtttgttttggttcgtccgctgcctttgtaaccctagaaaattcacatttactcaatctctttt
gtttctttggtactttgcatttctggcaaatcatcctatgaaacgagccttgtttacagacaagaatgtttttaaccatccttatggcttcttaattccg
ttatgctcttactattacggtgacggagtcgaacgttctttatttaattcgtttgctctggctcttgcactcaatatgggttatctccgacgtagttcaa
aaaagctggttttcttggtcatattactaggttgggttcaattgctcttcatcgttatagcacctttgattttccctattcttttcttatttggttctcctaa
agcaacgcctttgcaactaacatggattctatttaattatggctatcttttctcacttttaggaatcaggatctgccaggtttaccgaatgcgcctcaa
actggggggttgcacttggtgacaaatacatgaaatctaagaaatttgttgcgcttctattattctcaatattggtgagcatgatggttcacaagcct
attgcaccaacttttgatgtatcttctgagatcgcgaaaactgttaatggtgttctgtccttctgttttcttgcctggatcttgaatgcatctggcaaag
tgtcggaagacaacgaaccagagatcttgcaaaatccttacaaagacgatcctgaaacgggcttgcttgctgatgagaagttttgaggggtttca
ctcgcttacatcgtgttctttcatattgtttgagaatcttaatagtatacttagcttgcatcatttacaattttcataaaaataaatctttataccaattt
aactttctatttctatcaaaacgtcgtattaaccattcccaaaatttttaataaataatgtacactttttcagacttcacctattaagtaaacagagtt
tgtatgtcttccaaagactgaacctgttgttcttccttggacattccaagtgaccctgcaataaacttcttgcgttcttgaactttcagcattcgttcct
ctacactgtctcgaacgataaaacgagttactacaacctgtttttcttggcctagacgataaacacgatcaatagcctgagattccactgaccagc
tccaccaaggatccatcattagaacgtgattagcacaggttaagttcaagccaagtccaccagctttgagggatatgttaagaatattatttgttg
gatcactccgaaattgttctaatgaaatagatctttgttcctgagacattctgccatcgaagcgagaatgcttgattcctagagttttaaatatttctc
ctatgtaatccagaaaaccagtaaactgagaaaagataactattttatcgggggaagatttgtttaaaactttctgaatatactctaaaagtacta
atactttgatagactgatatcggttccaatggttccagtgtaggcttctatctttcaaaattaaacctgctgacctaatttctcgaggctcataaacat
cgtccgcttcaaagggctgacgacaagtatgacaaagtggaggagaaatatcccttttcttttggtaacttatgtggtctttcaagcattcccgaca
acagacatgtttgcattttaatacaagaggatcttgaactggttccgaacagcaaataggacattctggagcaagagcatctagtgtatccttgtc
tgatatttctgtaatatagccaggaatcggtcttttctgtgatgaaaacatccaaattaagtcatgcagttcattagagttagtatccgtttccatttc
tttttcatttttttccactgttttcgacagaagtaaaggatgacaacacgcttgtcgaagccgtaaaagtaagcctaaaattgcagtataattcttaa
ataaagttcctgcagcgatactttcatgcaccctgcttcttgctttaacgtatagtgaatcatatattcgtcgttctgcttccgaaaaatctagatata

tcgtttcaattcttcggtcgggtaagcaaactataggatttccagtgacatcttttgtttcctttgttcttcgtaaaacgagtggttcgagaatagatt
gcacaacgtttaaagccttcaaaatgtcctttgcttcatagggcacactaacgaatgtacgccagtaattgtagttgcaccaaggttctatttgtaa
aaattttatcaaactgtacaaatcatccaatttattaacgattggtgttccggtaattacccatcgattctgagctgatattgcataacaggccttag
cggttttgctgcttggattctttatggaatgaccttcgtccagaatcactcgataccagtgaacagaaaacaatccacaagtatcggcactattagc
agactctgaaagtaaaacgccgtagctagttataaccaaatcaggaacattgttagtatcagtaagagatcgcaaatttacagcggtttcggaac
cgtagtaaaccagtgttttgaagtgtttagaatcagaaactttaactgactcggattgccattgatccaataaagacataggtgctaccaccaaag
ttgtctttgcagctatgggaaggctgttagagaagggggatttttcaattgaattttcgagaatatcggaacccccttctagcatgaactaatgaaag
catttcaatagtttttcccaatcccatttcatctgctaagattcctccacggtgataaggagtcattgaagggaaaactaacgttatatcaccggtgt
ataaatttacgaaaaaccgatgaggttcattatccttagaaaagtgataatcttcgggagacagtccaggatccataggaaacaaaaaattgattc
cataatgggtgcaaatttgaagatgtttctgacttggcatcaccttcttctctttgacacatccaataaagtgcctgtttttgatactcccgtaaatct
aaatgaaatccttcaggaattgaataagtagggagatttgcctcatttgaacgcgttcgatcatacaatgtagttaactgttcacttacgacatcgt
catcttcagcgatatctgaagtagaggactcagacgttgtaggatttattgaagaagatgaggataatagaagatcattaatttgtatgtttgatcc
tgcattagagtcctcggaatagggctctagagcaatccagctgaataaacgaagcaagtgatctctcttgttctgggtatctataaaagcagcgg
gatcatcttcaataaaagcagttttggctgaaaatggggattgatttaatgatggatgggaaatatcaagaaaacaataaatttggagtgtaaca
ttgcttcctaaatggagaaattcatctgcatatatgcaactcgcttcaaacgaccaaagtttttgagacattaaagtacataagacatgagcagtct
cagtaggcagctttcctatttctttgtgatcactattgaaaaagcgaattgagtttgtatacattggaccagatgaagtacccgaattcgtctttgaa
cgctttcgagaacctcgatattgggtttttgtaggggctttttgacgttcaaggcacacgcgttctccagcttgcagaacattagatccagatacaat
tgaatacgcttctacgccgaaacagcctacatacttgcggactcggtttgatttggttaaaaaagagaaggaagtgagggaagcattcctagaag
atgaccttgctacattaggttgtttagcccatgagtttatatttgaagagttgttcgtttctggtgtcagagcttcctcgtctttcgacttcgtagtggt
gactgtctgctgagagttttctagtgaaggagacgtttcagcagtttcgtctttagattcgactgaagtcccagataggggattatcgtaaaagatg
ttaatagcagtctctacatctccgttcgaggcattgtatagatctcttattttgcgtttcggtataattgcttcaccaagaattgatgttatatgggact
caaagagattaggagactctacattcacattatttttatccaccgattccagagtaccttcatgactattctcttcagatcttaatgcctctatactag
agatagcagattccggttcatcatcatggaaaaagcgaggaggagattctgattcatcgttagtttcagtttctaagtcaaggtttctgctaggact
tcgtttcatagtttccacagcctaaaaacaaaggaaaccctcagcaaaggtaaatttaatcaagttgctcttcagtaaaaaaagagaagcttaaa
atgaacaatcacgagtagaaaacaaaaaaaaacaattagaagtataagtgttcacgctagtaattgtatagctttttatggtcgtagtgtaagaa
acattggataaaatgaccagcaacgatagaaaaataattgtttttaactcatcgacttccaatggataaaaaaaaatggaataaattaactatc
tagataccacagactaaaacgtctgtgaatcctactgttgtgttaaaggcagattttggtcgcttcacaagtttagtaagtttgacgaggcttcact
aatccagataagacattttaaagtgtttgtatgttctaatgaattgggaagctaatgcatatttgaaagtgagatttgaccttgctagttgaataca
aagttatccagtcaacacctgtataaaaaagcattctgctgttcgacctcggtcctaatccaagaaacacaatgcaaaattcgataaggattaca
gggcatcgttagaaacattatagaaacacttcgcctaaagaactaactatactaaaaggatgctttaccaatgaagaaaaaagcccaaagccag
gttacttctacataagtcaactttcagagctacttactacaattgccttagtttagatccattcaaaatcaatcaagctttctttttttttacgctagaa
acattatagcacgcactacacccttacgcgggttacacgatcaacacgtatacaaccaaaagatcacacgtttttattaaagactttaaacagact
ccaaaggagcaacatcactgacaaccttggtaccaacgtcttgagactcaccgtgttgttcaagaagtctttgagcgtcgtgtctgggagatttca
agatcttaaccttgcgaacgagaacgttttgcaaaggatagattccgtgagtcgccttctcaatggcgcggccaatgacttcgggaatcaactttt
gaacaagctctctcaaagagcaagaagcagtttggttttggatgacctcgaacatcttttggcggacagcacggatttgagaagattgagcataa
gtggtctttttgacttggttgggacggcggctggtaaagccaatgacaaagacgcggcagaggtaaccatcagtggtcttgacagtttggtcagcc
tcgatggtggtttgccatttacggacaagagaacgcaacttgtcagaagtcaagctaagaccgttgaagctagtaaggcagttcttaccttgcaca
tcttcaacacgaagtctaaccttacggaaagcgtgctcctcatccttttggagatcggcaagagaaacctccatacaacgacccttcaaggcatcg
ttggcgttctttaaaccggcagtacggttgacgagggtcttaccaacattcttaacttcaaagaaggaaggagctttgatttcgtaccagtccttac
gggcaaaaggatcaacagcacgctttttaattcccttctttcctttggaaagtcttttattctttccaactgccattgcggtttggtgttagaaggaac
tggtctcctctgtccagacaagaagcacttctgtgactgctttttttagtgaaacaaaaaaaatgtcacacccaaacatttcgtatgtaaaataagtaa
aaagtaaaaataaataaaataaataatcaatcaagtaaataaataaaatactttctttactcgtatacagcaattgatacttttcgtttataatgct
aaaaagtagttttcatgcagcatataatattatacagaaatacgtgtaaaaacagtgatgaagcaacccaagtttgaatttcgtttatcgacaatt
cggccttttaaacgttattaacgcaattaagtccattataaacatattcctgacaagttattcgtttttgctagtgtccacctaagcaattctgtgtgc
aatgggtttgttcatagtgtaaacaaataaaggaaatgtatggagatatcactctgttctattgtgcttacgggccttcaaggtacattctaaaaga
tattattctgaagacttaagaacggtagacatggtagtttaccaaatgttatatcagtcgaaatttccagtagaatggggttgcctacgttaaaagg
tgaaaaatatggaaatgcgtttttttttttttattttcttagcaaatttatgaataaatgaaaaagtaagtcgtacaggaacacatttgtctgattcg
actttgctatggtttcgattttattttcatttgtttacactgctctatttcaggactacctcaacaactctgtctacaccatacaggtacacaggatattt

tagcggtcttttcttacattgcccttggtgcattcaaacaacaccaaacactacactatcgaatccagctcttacagtcgatgaactaggaatttaa
tcaaatgtgagttgtgtatacaaaacgaccaattgttcctttattcatgacaaataataaaagtactcttataaaaagccatagagagttctttaaa
aatgatgctgttgtttcttgccaattgctttttacaattactaggcgtcgttttcttcctttaggatttttccctcataaaaatgattccactggagcgg
ccgagaaaaacccatatgcacctcctgctgtaattgatagagtcgttttcattgtcatcgatgcgtttcgctccgatttccccttttctgattccttcca
tatgccatttcttcaatccattgtcttcaataccactcatggccttggtttctcttcgtacgctagatccccaaccgttacgatgcctagattgaaagc
tcttacaacgggaacaattcctgggtttttggatgtcttacttaacatagcagagtcggaatctacctcaagcttgactggacaagatagctggctt
caccaactaaacgttcatggaaaaagtattgcattttatggagatgatacatggcttaagctttttccaaatgcctttactgcctatgaaggaacta
ctagcttttatgtagctgactatacagaggtagataataatgtcactaggcattttgaacatttgcttccttcttcattggaccaacttccctgggata
ctttgattcttcactatcttggtgtagatcatattggtcattttcaaggaccttccagtcctttgatgaatataaagctaaaagagatggatcaaatc
gttgcttccctttaccgttacttggagaaatatgatcagattaccaatagtcactctatgatcttcttggtcggtgatcatggtatgaatgaaatagg
caatcatggaggctcttcagtaggcgaaaccactgcagccctttctatgctatttccttccctcgatgtctttcaaagggactcacttcacgaaccac
ctccagaaaatccctatcaacttatgagtatggttgaacaggtcgacattgttcctacattatgtctcctttcaggaattccaataccaaaaggaag
tgttggccgtatttttcacctgctattcagctttgggagtcaaaggcggaagccaagaatgctttattgagtaacttatatcaattgctatcactaa
agcattctccagaaatcacttccaatgttatagggaatttcgaaagtgcaagcaacaatgaaatattggaagttttgcaacaacttcagcaagaa
ctcgttgtaaagtcgtccgattactcgttgacgaaaatggctttagggattaccatactgggtttatgcaccattcttaatctgtattttacccttgcg
cagttttcctttgttgatattctgtgcttaattcccttcgtcattctgatagttgtgtatatgttctcaagtagctttattgaagaagaacaggtgctttg
gtatttctcttgtctttcgttaattttgttccaagttctcaagcaaacttctcttttcactacgggttatcaacttcttctgctcggcattatcattaggtg
gaatcaaaccggccaaaagcatgcagacgttcatgatttggtggacgattatatagctccaagttcttttctcaagtttatgctctgtatttgctcta
gctttatgccctcttttcgctcaccttctccgctgtcgtttctttcttcttttttgattgcatcgtacaaacttttctctgctgattctattcatatccctctg
ctctcttcggtaaaatctattaattatgtatcatttgcacaattgatttggtgtacactaattgctggtgcattagctactccaaatttcaagaatattt
gttgccagctgtcattatttctcttgttattgacaagattagaaaatatgggccctttatgtgttatatgatttactacaaaggacattaccgaaggaa
ggtatctattcatatatattattttctataatggagccgttggcatttttctctttgggcaattcaaactctttggcttcaatcgatttgtcgcaagccta
tacggggattaaatcttacagtctttattcagtagggtttctattgttttgctctgtcttttcaggaccattctggtggagcattcatcagcctaagatg
tctatgcgaaaatggctaagcccagctttttttcttttcaagtttcgctttgtttattttgactctttcttgctacttctttagacatcacttatttatatgga
gtgtattttctccgaaactcttatatcaagccacttggacaattctatacgggtttacaaaatttttattacagagtctactaacatcactaaaataat
aaatgggttttgagcatgagaaaagctttctgtgtgaatcaactaccgttattgaagtcagatctaatgatactctttatgcttcattcgttatgtaa
ataggaaaaaaactaataattaaacagacttacaaattaatgtagatgtagtagttgccaattccgttgtttggcttcagcgtatttgtttttttttcg
atccttcaaaactaaattcactagttgcaagatattttttgaattccccttgctcaaacagttaatttgtcaatcaagcgagtatataatgccctacag
tttgaactgctgttgtagaaaaatagaaagcttgtaagacatacaaagaattcgtccatccctataaagtttttgctgtaagaagacttcaatgtaa
aaactacaagttattattggtattcttatttctttttagcttagtacctactctcaatgaagtagtttttgattacttttcctacaaagtgtaaacaaag
aaaccattgacaagaaacgatgtgcgaaaatccttctaaacaattaaattgatacttagtttattgagtttatactttaatctgaattttatgcgaaa
aaaaagcttgaatgttcgtttttctatttctttattttattgttaccttctccaagtaacacaggtaatgtcgcacaagagacaagtgtagaacctga
gaaaacccatcatcctccggaaatctcttccttcttaaatttttaagaaaaccatggctgatccttctaaaaaccctattgctgatttgagcgaaact
caacagctggaactctcaaaattcattgaaaccgaacagcaaaaggtgaagttgcaacaaggtacgaaatcaagactttaatagtttttagggg
gatgctgaccgttacaaatttagcaattcatcaatttacatctacctgctggcctaaatgtgttagcaacatcggaaaccagcttgacaaaagcga
ggagcaatgcttgcaaaactgtgtggaaagatttttggattgtaactttcacattattaagaggtaagttggtaacattaaggattcatcaacgtga
gtcctgtggctttcttaattcatgataccaaagaaaccgttcagcataaatagagtatgaagaaggttttacattttaccagagtactatggcgata
tggacggagatggttattcactgatgattgtttcgtttgccgagctaaccagtcagtcttgaaactaccagaaaatagacaaatttttgaatgctaa
tgaataaaaatgtcattgtcataaatcgcgaagttaatgataaaacgaatgcatctcatcatcatcgtaaaaaaataggtaatcagatcaaatgg
gaagaggtccttgactatagttttggagtactttgtaaagtaaagaaaagtggaacgctttctagatactttatatcttctgaggcagcttcctaaa
aggtgtcgatttcattcagtcctgtgatcgacatctgttggtgtatagtttaactggattcaggatgaggggaattcacaggatttaatggtgaggg
tgtgttattaggacccagagtggttgacgatagagatcgaccacttgtccttgaaaggccagtattttgctgggaaacactcggcgttgattccgat
ggcgggttggatggtgtactttcggcagtagaaacatttgatggtgcagggaaacgaacctggaatatgcatgcagggagattcgggcattgag
caataacgtgcactggatacccaaaagtatgcatgactcctcgaatgaaaccactagggaaatacaagtactgcacggtgaattagcttttgtttt
ttctgataatgaatatgtaggaaaatgaaaaaacacttaccggagttgtatactctttcatttctgtcccggtggcagctgtcatcttagaaaacca
aaaaaaatagttgtccgtaatcacaaaagtcccctaggttcacggttagatatggaagtacaatgaattacttgtatttcatacttttcgattcgttt
tcaagttgtccaattgtttacgaaaaactatgggccataattctcgacataaaaaccgcataacatctatactctccacaattcgatttctagtcaa
taaaagcctacatataaatcaggattgtcgagatttttgctcctcattcattatgcagtcaaacggacaatcgttctcttgctgttgaaaagggcaa

aatcaagcaaccccaacaccgttagtttgttttctgaaaaaagataatgctgctcatctcatcttctatattcttaagatgaagcgaagcgtacgcc
atgcatcgattacattcaaacagcttccaccattttagactataatagcagtaaattttacctttctgtaagctttctccctacttggaaaccaatgtt
ctctaaaaaagtataacctctttccatctgctcatcattcgaaagggccgtttcttgcttttcagacaaacgtctggccatatgcaccatttcaatca
ataaaaaatcaaaagaagccaagtttacagacattggcaattgctgatggacaatcatcttggattctcaacacttctattcaagtcttaggagta
aggtgttttcgcatgctcactagttgccgctactattgacagaaagtgtgttttaggcctaaataccaaagttgaagagactctttttctttgcacttg
cttattatttcaacgtttttgttatggtttttacccttcttttataatttatgcccggacgcaattaaagctccactttttttgtctttgctattgctagttg
aaagggtgaatcctctattcatctcatgaatagctttgaattcaccaggaagcaagacttagtatccattttattttggttcaattccactttcaatca
ttgattccaatataaaacttttgaagtaaaccaacgttaaaattattattattattattgttgctattaattatgtgaagaaaaattaatgatgat
gattctggttgttgaaaaccgattctttttgattaggccgttcccagttgaaagtatgaaacattgaaatcaagaaaatcaagaaccgctgaaag
tgcaacgacatattaaacacaacacgttgcactttgcttctttattcattttgattagggggaagagaaaggagaaagtctataaccggggggg
gggaagatttttttttttttactgcataggagcgcaagtacaaaacaaatttttatcaccataagcatcgtctaaacgtgccacagttggccaaaa
ttttctttcactgagtataggtacgggatatactgcacgttcacgtgaataagggcggttccattcctctgaaactagatccttttgaggatgagga
gcattggtaagcaaattattatccttaggttgtttaccctcctcaatttcacgaatttcttgacgaatggaaatcaaggcgtcacagaaacgatcca
tctcgtacatactttccgactccgtgggttcaaccattagagtattggtaataggccaggacaaagttggtgcatggaaactgtagtcttgcaaac
gtttagcaatgtcagtagcctcaatactggcagtagccttgaattcacgagcgtccaagataaattcatgagcgcagcggttgttttcatttgaata
gacaagcttatagtagggagccaaacgagcggccatgtagttggcattcaataaagcagccttagaagcatcacgtagtccatcaaggcccatc
attctcatataagcccatgaaatgggcaagatactagcagaaccatatggagaagaactaacggatccaatagactttgtaccaccacatttaac
aacaggactagaaggcaaaaagtcagctaaatgacttttaacgcaaatagggccggcaccgggaccaccaccaccatgaggaatgcagaagg
tcttgtgaagattgaagtgacatacatcagcacctaactcgccagctttacacaacccgaccatcgcattcatattagcgccgtcgaagtaaactt
ggcctccattttcatggataatgcttaaggtctcctttactccatgttcaaaaataccaaaggtgctaggataagtaaccataaaagcagccaagt
tatcagagtatttctcagccttaacctttaaatcatccagatccaagttaccattgttcaagcatttgacagaaacaacctttaaacccgccatattt
gcagaagcagggttagtaccgtgagcggaaacaggaatcaggcaaatgttacgataatcttcacctatagatttctgataagcacggattacact
tagtccggtatactcaccagaagccccagaattcggttgaaagctagcagcatcgaatcctgtaaggttagtgagcatcaattccaaatcattaat
taagaactcataaccttgcacttgatccttaggagcatatggatggatattggcaaacttgggattcgacacggggttcatttccgaaactgcgttt
agtttcatcgtacaagaaccaaggggaatcatggcatgagctagagacaaatctttggactgaagataacgaatataacgcatcaattcagtctc
ggagtgatacctattaaatacaatgtgttgcaaatagggagtttgacgttgtaattctttaggaaaagcagaaacaactaattcatccacggtatt
aacagatttaccgctgtttagcttttcagtgaaagcttcaggacttgaagaaatctgaaaaacgttaaataattggctcaaatcctcgtttgtgata
ctctcatccatagatatagaaatagacgtatcgctgactttgcgcaaattataaccgtgagctaatgcccttttttattaaagcatcagcagactgta
cattgattgtcaatgtgtcgaagaaatgattcttattcaagattttgaaaccagcactttccaaaccacatttcaaaacattcgtaatagcatagatt
cgagcggcaatctgtctcaaaccttcaggaccatggtagatggcatagaatgcggacatatttgccaaaagggcttgagcagtacatatgttact
ggtagccttttcacgtcgaatatgttgttctctagtttgcaaagccaatctgtaagcaggatttccaagacggtccttactgagacccacgaggcga
ccaggcatttttctcttgaattcgtcattacaagcaaaaaagccggcatgaggaccaccaaaacccattggcagaccaaaacgctgcgttgagcc
gacagcaacgtcagcaccccactcaccgggcgtcttcatgagagtaagagcaagcaaatctgtggcagcgaccacacgcatgtttgaggcgtga
gcggttgatgcaagatgactatagtcaaaaatagagccatcagcaccaggatactgaacaagaataccaaaagcatccttttcgttggccttgat
aacttcagtggagatatcgactacttttacatcaatctcaaaaccgcgtgcgcgagtgcgtagcacagacaatgtattagggtaaattttagagtc
gaccaaaaaagttttacgctttttgcggtcgctgcccataaccataaccatagcttcaccagccgcagtggcttcatctagaagactagcattactg
atgctgaggccggtcaagtcggtaatcatggtttgataattcatcatggattctagacgtccttgagagatttctgcttggtaaggggtatactgag
tgtaccattctggattttcaaatacattccttataatggctggaggaattttgacattgtaatagcccataccgataagagtttttaccagctgattct
tgttggccacattttccactaattcgccaaactcgctttctgagtaatttggtgctgtatgagatttatcagtattgattaaaggaaaatgcttgttgtt
attgccattagcacgaacgttttctggaataacgtcgttcaagaatgattcaaaatctttgtatccaagcgtagaaagctgatactcttgctctttct
tagagggaccaatgtgacgacggccaaaggtatccaaaggaggtacgatattctttcgtgaagtgttattgagactacatgttaaatgaaagctc
ctccttcttaacgaaagagaacgattgctctgaatacttttgactaatccttgcaaggaaatagtccttctggacgaagcaaaaaccatatcggtc
aagaggcctgtttccaaactgaaaagacacagagaattatggaattaaatgaaaattaatgtatataccaaatcctgcgtatagataattaaga
attgacgatattagtagtgatgtgaattatcactaattcaatatgcaatttcgagcatttaccaatcgtttttcctacttaatactgaagcagattatg
gcagttggcaaactatataaatatagaaaaaaaagatatcatgattgtcctgttgcagatagcaaaataatccccgtgtgtatttacacttgttac
ctttggcactcgagtaactcgaaattttttttgtttacgccagtaaaaaaagcaacttttgaaaacgatcgggttataattaccgttttacagatcgt
ctctcttctacttccatgtttgtcttagtatatagagcctatccagttagcagttttgtttgttgaacagtccttacttaacttaatcagataaagtagg
cggttttctttttttactttatgagccttttctttctttaataagttttagtttatttactgctatttagactaggagcttgcacagtaaaaatcccgttcat

gctattcaaatcaatctttgaaacatctatagttttacgactcctacttgctagttatatcgtagcatttattccattaatatggcccttatgggcaaat
atgtaaaaagaggcattatttcttctatccaaatttgcccaacctctaagacttattctacgtttgctttccaagtttttcaatcagtgatattttaata
ttttaaagatacattggtttttgctgagttacaattgcacttttaaaaaactaaagtttgaggaaaaattcagacagttccaggtcccaatgaaaat
ccaaatgaaaacattattatatagatgacctagttactttgtggatatgtcagccttaatttttaaggaagaggcacgacgagccttgacaacttc
cttcttttgcttttgttcagcaacgcgcttggccatcaactgagcaaattcaccagcagcctcacggttcttttcagcttgtctgcgcttgagagcgaa
gcggtgacgtttgtgttgaagggttctgggagtaacaaggcgttggatcttaggagccttagtgtaaggcttcttgccttccttcttgggctgaacct
cacggcggatgacgaattggcgaacatcgtcttccttgctcaaattgaagaaacgacgaatcttggaggcacgcttaggtccaagacgcttgggg
acagtagtatcggtcaaaccgggaatgtcttgctcgccttgcttaacaatggcgacagcaagaacagacaagtcttggttgacgatgcaaccacg
aacggacttacgcttgcgctcaccatcacgacgagggcgatagcaagggtgaccagcacgaagcaaaagacgaacacggtggggaagcaag
acaccttggaacatggggaaaccttgcttatcattaccaccggtgatcttgaaaacgtagcccgcaaattcggggcctacagagtctgcgggaac
ttcttggcccattctcttgtccatgaagacgcggagacgacgatcatcgtcgatttcaatcagcttttgagtaccatttgctggataagaaatgttca
acttcattttgcgttgaggttcccggcaggttttctcactgtttgcccagtttcctgtacgggaaaaaagcagtcacgtgttaacgaggaacaaatg
actgcgttaatttaggtttaccctttcgcttatttctacacttattttgtttttcgaaagtaaataaaaatcaacttcacattcgatgatctctgtttaca
ttaaatagatcgttcattcaatgttataatcccgatttattattatctccattatagtagtactttaatgaagttttcgttcgtctctttgtagtcgtcgtg
caaaaagtctttctccttcaacgctgacttgtttagctaaagtctttctaaagaatatgctacgaatccttattgaaagcttaaagtgataattcata
tgcaatttgcggcatgtttgcttttctttacggtgattagacaagaccttggattaccagtaacgtagtaatactctcattttctgtgcaatttatgag
acatgcatatcttgcatttaattccatggccgtagtcagttgttcgtctttaatcttttcaataccagtcagagaaagtcctaaaatacaagttgatat
aaaaaatgaagtatcacatgcgaatagtctttttaaaaatatcctttttgatttgagttatcatttcctgcttattatggttggccggtaaggatgat
ttgtttacttttgtctatagttgttagactcattaaatcaatcactccgtccacaaacgaggcaatttgcgaggacagtacactcgctgcagtttcaa
ttggatttattatcgtcttttaggaatgcattctcctttatcaactctatatggaaatgcagctccggagcgatcgattgtatctgcggcttcctcgccc
ccaggacctatattaaactcttctaatcatttttgagatataatcggtttcctagccgtcgtcctggtggaagaagaaacgtttcaacggcaagaa
cagacttggatcgttttctccctttgtcctcgagctctgatgctctattgctatgtcgacaggatcccctggaaaatccatatttacaaaaaaaagat
accagggatcactccgacagtcgtttggagaaagattctaattgcccttacaatcgtttgctggaacatgttttagagattgataagacaagtcat
gtatttcgtttttctccagaaacatcttctccttccagcattattccaataaccttagagtcaaaaaccaatacatgcaaaaagaagcatactccgg
accaaattcttccttttcgaatccttgacgctcccgagttacgagacgatttttataccacattacttgcatggtctccgcgcggtgacctagctattg
gtcttgccgaaaatgtgtgtttatggacagaaggattaggacctgttggtgttttggaaaccagcatgtacgatgtaagtagtcttgcctactcgta
tgacggtactattcttgctgcaggaagaattgatggaaccatccagtttttggaagaggggaagttcggcccagaatttccatttatcatccaggc
gatatcggctgtatggcgtggaaacctgtactggacacgaattgtattataatcggaaaaggaaatggagaggttgtaatctatgatatactgtg
gtccgattccacgattaaagccacgaaggttggaacaattactactgctcatgaagagcaggtttgtggattggcctggaatcatgacggcactc
aatttgctacgggaggaaacgataatcgagtttgcctttttaattcttctgaatggcagaagccactttttgtttggcagcacgatgctgcagtcaa
ggcaatatctttttgtccctggcaaaaatctttgctcgctacaggagccggctcacacgacaaacatgtccgattttataattgcaacagcggaaa
aaaaatatctgagctctattgtggtgcacagattacttcaatccattggtccccaaagtataaagagttttgcgtaacttttggttattcggtggaaa
gtgttcaacatcgaattgctatttatagctggcctcatttaaaatgcctcgtttctgttctaccgagaatctctgatactcgatgtgtgcattctattat
gacttgtacgtatgatgatgtacgaaagaagcaggttcctggtaacagtattgtcgttgcaagcagtgatgatactgtaaagttctttgatctttcg
gaaaactgttcctggcatcacgatcgagttttatcctggaacggcatattcgactgtcaaattttggaaatgctggaaggcatggatggaacttcta
gttatcaaatacgttaaagttccttttcgtatatttcaggtatttttttttttttttttaaacatgcctttttaaaaagtccatctccggatttctggtgttt
tgccttttgtaagtgtctattatttttgtgtaaggaacagatatttaataaaatttatcaaactcctttttcttcttctccattagagcacgtacttaaag
cagtgtttattttattttctgttcattccatcgaatttctcatatacagcctctaactgtcatttgcacctaaccaaataccaaggccaacgcctttcttt
gctcgttcatcactgttaatcttgtaattcaactctcgaatatggaagacatagatctcggctttttagcggaaggtagccttaagcaaagcgatttg
ctggacgtggaatgtagtcgagttggaggtcctcctctacttcttcggtcgaaagatgtagcttctataaaggagaagtgtggctccaataagaac
ttcttattgcaaatgtatgcacctcgaaataatgagtcctcttttcatcgtattatctacgtctttgtcgacaaagatgggaaagctcaagaggaag
gatggacaaaggggtaagtactacaacaatacaaatgaaaagaatacgaagtttatggaaagctccttcttctgtcagtgtggtccatgatcaag
tcaaaagatatgcacttttatggatcacttcaaggcacttgctactgcttctactatttattctaatattttgctttattatcaattattgtaattaacat
aatttttttaggattcgtgtattcagaagccaagcttcgaaaacagaattcaaaaatctggaccttacactagaagagaatttcaattcaaagctg
tgcagtccacgagaaatagaagtggattctgatccatctgatgaaccatcagaagttcagcaaaaagatcttcaagacgtaaaagtggaaaccg
aaaatccaactaatgagccttatacaaaagccacaggtgatacttcctttctaaagttccaaaagtgtatatctagagctccagatcaaatactgc
gctattatcattctccttcttcccagcctttatggtgcaataaggacggaatccctaccagtgttcctgattgctcttgcggtgccaagcggatattag
aagtacaaattgtctctacattattagctgagttagaaattgaccactccgcagaagatgcccttgactggggtgttcttaccatttacacttgttct

gaatcctgtgatttagctaataaaggtattgcagaagaagtatgttggcttcaaaagttttctgatcaaggtattgaagcaccagagtaagggaat
tatggtgtttctctgttctattagggaaattgatttctggtttggttattactatttcttttgtttacatgtcagtagcatcattatgtatatcacttattttt
caacaccacttaacacactgaacgatcagatgtttataaagtagaatagattgaggcaacattgaatagtaacagcatctttttacctttcttttttt
tgccaagtcaaatttaacatactaattgcctcctttagataaccatcccataatagctcatgcacaaatttctcctagcaatacaaactactcacat
cttacttttacaatgaaaacgcaaattaaaaagatttataagtattgaagttgttcaaattaatttagctgtactgtaagactgtttaacgataactt
tgtatagaacaaaacacgagtcaaacagaaaacacattcatagttggtataaagagaaagagaatgaataagaatgaatgcatttctgttcaac
cacgtttttttgtttatacgatttttttgtctttgtttcatatcataattgatagcgggagacaaaatcaaacgtactatttccagaatcataaatatattt
tcacgtcctttttatatttagaacaaaagaaaggaaagcaaacgaaagaagggaaaataagaagtcaaaaaaaataagatcgataacgaaagt
cagaaaagcgtttgaaaagtttaaaaagcgagcgaaaaaataaaacaaagaaagtcaaaacaacatttagcaaatacgtatcaaataagaaa
ctttgacttcgtaaatcaaaggataaatgtcatgtacatattgttcagcagtgcaattcgtacaaatgaaattgggctctcgaccttaaagagaaa
cgtacttggatccaagctcactaagttcaccagcggcattgaccaaagcgttgttctcaccaacagagcttccaaggttactagcaggagcgaac
caactgtaacgctcaacagcaccgttgttttccaaaaaattcaatgctatcctggaagacactgctgacatcactcatactaggcaacttactaagg
tcccagtttgtgcaagcaaactcagtgacccagatgggcttactgtaagtagaggacaaactgttcaccaagtccttgagttgattagcagaagta
ccgtaccaatgagtagcaatgaaatcaatacggcaatcagagcatgattgcatgaactttgaaagccagtcctcaccaacgttgctaatagcagg
agaaccaaggtacttaatgccgcctgattgacgaaggggagttaagtattgcttatataaggaagcggcataaccagcatctaaagaagaaag
atcaggctcgttaaagccaatcacagtagcaccgttggagaaagtagaagaagcactctcgataccatcagcagaatgttggctttgaataaact
caaaggaggaatctaagttagaaggactggagccccagttataataccagttagcaacacccttaaatttatcagcaactgagggactggtacc
ggggatccatgccaagccacgcttagatgacttatttcctgaaggtgtggcagaagcagagctagaggtactgctagaagcagagctagaagca
ggggagctagtgctataagtattgagattagctgccttagcattaggagcttgaatgtcaccggaagggagtggggcactgctggaagtaatggg
agtgcttgcatcggtggtgctagatgcggaagcaagggcagcagaggtaggaatagcactagaactgctggcaacagaatccgtcgaagaagc
aggctgggcagcagtagaagaagtagaagtagcttcagcagatatagaagcagcagcaataggatagctctcgttcaaagccatattgggagc
aacggatgttgaagtagcagcaatagtgttgttattccatggagcagaagtagcgttcattttgaagaaaggagtagttgcaaaactgcttggag
cggttgaagatgagaaagatgtttgataagaagtaacaggggatgtaccgtcagcgtttgtactcatgaagactgttttgtatatagtttcataagt
agtcgtaacatcgttaccgccgcgtctatgatgacggtggttaagagggacgtaaggaagggcagaagaaatgcccaacagagccataaaaag
aaagttgataagaaatacgcgcatcttgctacaaatgaaagattaaaagaaagtaggatagcaaacgcgtcaatttagtttttgtttttataaatc
taaaagaatgaaaagagtcgttttaaaagaaagacttataaggagggtataataaagagggtattgggcttgagtgtttttgaccgaggtagaa
tcagccgctccaaactcttatataaaacgaaagtagtcacaaataaaaagtacattataaaccttaaaaataagtttgtttcaaattattgagtaa
attgtatttttttactttcgagagtttcgacaaactctgcgaaatatggggctgaatctgacacgacagctacaccggggcctcctagttgaatatcg
tagacagagtttttttaacgaatctaattcagtaataacatcctcaaccatctcatttgaacagtcagtgttcctcaatgtaactgtgcatcctccacc
tcctgcgccagttaactttgtcggacctatggatttggtagcatttataacttgctcaattgtaacatgagagacgtttaatgcctcaagcattttttg
gttgatgaaaataagctcgccaaggacctctcgaagtcgaaatacatccgtcacggtttcaagggcttcgattgcggatcgtgatatcccatcaat
ggtttgaaaaatggaatcaattacaagaggtgaacgctctcgaagtcgaaatactctctgaatcaattctttagtactcttgggctgctttgtatcg
gtaaccagaacggacagaggattctcaagttttaagaattgcttcatggcagtcgtcttagccgtggactttcggaaagcaattaggccaccatga
gtagcagcggcattgtcaattccgctaggactgccctggatacaacattcaccaataaaagaccaagcttctataagagtaagggtttcagctcg
gcccaagctttctccagttattggtggctgaatgaagccaaaaaataataacaaacatgttgcgacatcgactgaaacagtcgcgctgcttccca
aaccagcacccaagggcacttctgaatttattgttataacacagccttgtgtaggggaaccaatctgtaaatacaagtacaagatgcacagcatt
gatagaaaaactagaggactctttttctcgtttgtcaatagcttttccaacgctgccacgatctcgttattgagagtgtttggaggatgaaatacgtc
ctttgcgggtacagctttccaaggcaaagattcaagaggccatgagtgtttcgtccctatatctcgcatctttatgacaatctcgggtttttcgctag
gttctattctacaataggttcttagagaaatagaagccgctacagctgtctaagatactgttagtattcttgagtatacagaaggcttcgaaagacc
atttagcaaactgaaagcaagaaatttggatgaacttctcctaagcttgggataaatcaacttgtaactgttaaattggagcacagttgaatttgtt
ttttttcttataaaaggatacaagagaattatttataaagcgattcttcataaatgacaattccatgtaatgagaatcgttcaacaccattaacata
aataaaatgtacatcatgatttcgtattaccaatgtttttttaagtacaaagaataataagcttaaaataattgcccagtagcatgatccttaatag
agcctgagacgtcaacttacagcaccgtaaacaacagcatgttcaccaaataggattgtcttccctggagaggatacaatgaaacttctttccatt
gacttataagtgatttcttagagtaagctcaactaggtttatggcgaaggacaagtgcaaattcttatgttgaaccccataaaagaaagatggctg
gtaataagatggctggtaatggtaaatttaagcttggtaaggtcctcgcttatttacatgcttatgctaaaataagtgttgcttatacagtatcaaat
ctgtttagaaaaagtaattgattttaagagtaaataaaaccacttccagttattgctatgctataaagagagtagctaaaaaaacgaacaaaaga
tagtagaaattaagaattaaagtatattagaggatagggaacatataattgtacaaagttttaggttaaatcattcaaaccattttatggttgtgta
acattaggaagaaataagcaacttcttcttttacagaagctatactcctgtacaattcgtagaaataaatagactatgcatcctacaaaacggac

tgcagaaaatgcaacaacactccagaaactagtaaattgttaattaccattggcataccaatactttcacttacagaaatgctaagtaatagcttg
tttgattgttagaaatagaaatcccttttgtctggttcaagtttatgactaccgcaaccagagccaggttggaaaaaatccaagccaatacaatga
atgttctgaagtttttataaaaatcctgcttctttaatgatatatccggagacaagagagttttgtcaaccggacttgctagcaagcgcacagatttt
tcgtacatatcatcgatgtcagattcattcgtgggaatcgcaatttcaacgaccgtcccctttggatttctttttgcaatacccaaatcatttgagacc
gcatgttctcctttggtaccccaggacacatcatgcgtgttacaaaatgcgtaaacacttaatatgtttgtgtacattggcgacagaatcaggtatt
gaacaaaagatgtcagcatatgccagggatcgcaagaaattatagaaattagtgcatacattccgtatgtcgtagttaaagacactactatgttc
acgaaaatagtattgttggctaaagcatatccaaccgatgagttttcgtattttgcaacatcaatcgcatgaataattcctcgatatgcaatccaag
cagagcaaaataatagatagcacattaacaaaccatacagcaccattgtcgttgtataaaaaattgtcgagccatgtggacgatttcccaatgcc
aggataaaacaaggaatcaataaagccccataaatccagttcactacttttgcaagtacagctccagggggggaattttgcttcttttggggctacg
ctagacgttaggacataaaagatgataaagaaattaccgacattaaaccatgaatacaacatgtgtagaaaaagataaaaaaaactcgacaatt
agcaaaacttttcgaccaaatgtatgagtactctgaaaaatccgataataatgaaacacagaatgaatagcagcaaaaaagcttccatttaacc
aacgtctcctttgtaaaatgaactcaggtacacgatcggggacatcggtatccgcatacgcagacttgacatagtgcaacgtccaagcttcatggc
gtttcgatactaattcaaagcataaaacacgatcttctgccaaatacatattcgcttcaaaaatccctgaacattcaccattttgtaactctccgcga
aaatagctttccagcgggccattaccattttcgtcgttttgaagggcttcaaaccggtaagcgctaaaggcacccggaagtacagagataaaccc
gagtacgctttccaaaggcttgtccaataagttactcattttatattcaaaattttgggtagcaactaagggattcaacatgctagatccatatttcc
ccttcatagtgacaacttcaccgcatgcaccggctactcttggatttacatcgaatgatttccaaagatggtatatggattgatcccctggtcgtgta
ccgacatccaataaaacgcaaacagttggctgcagaactgggcaaagcgcttgaaaaaaccatcggtgagagttaattttctgtttatttttctcc
tttaaacaaaaaaccacctgcacaggaactataccttttttcggagcctttaaattttaaattaggatcgatggataattgggttgtatactcatatat
atgagcattaacttccttttcatttactctgtttttggctatcccgtcttgataaactccaatggatgccaagtaagccaaagtctttggatgtatattg
gttctgccatcggaaattatacaaacgaccaccttttccaagcatctactccccatactggagagctccttttcttacaaaggtaagaaatgttttt
cataatgctatgcattgtacgtgcaaataaaatttcgtcttcattaaacatagtaatggcaatgcaaatctctgtcttccggggctgagcaaacaat
ggctgtcgaagcgtaaataaattcttaacaaaatcttgaggctcacaagtggcggccgtgtaacgaacgtaggaaaattctcgatcttgctgctta
ggcagactagcaagaagcattttggggacagggcaatccaataccaagtttcctctgaagagttctactctctgaatagtctttttcgctttagcc
gaggttcatgctttgactgttgtcgctcaggttgttgctgatttgatatattcggggtatacgaatggatgggattgggttgtgccgtgtatccttcaa
aaggaggactagattcttctccttctttaaccatatagtcatctatactaaagtcatctatagagggagcttctgaagaagcaatcagatgtctgta
ctctttgtcatcttcgtctgcttcagataactgagggctagatccatctgaattttggtacattagagttggaaccttcaataggctagcacgatttgg
cggttcagtggtgagacctgctgcttcttcttcatctggcggacgtggaagagaacgtctcatccatcggctcattttgctttcaagaaatcaatgac
tgagaaactttcaattgaaaaaaggataatgctgtaaaaagattatagatgctttggaagaataaggaagcaaccaaagattgattttctcaaa
gaacgcaccctctttctatataagtaagtaatacaaataaagatctcttgaaattctttaacttttgtatagtgaacagtacagcagtgatattctct
caggaagtatgtcgaaaagagcgtttattacaagccaacagaattgcttaccatgaaacgaaagaaatgaaagctttaccgttgagtaaacaat
gcaatggagaaaagctggctttaaataacacgaaatgttttaaaaaaaaaaggtaaatatttacgtggaccatacttactgtttcacactaagttca
aatagtaaacacagaaagttattacatagttaagaaagaagggtgttgagtaacagaaaaatacgaagaaaagtaaaaatagtcgtgaataa
attagagaaggctttcgaaatttctttttggtgaacaataaatacccgttttcggtcgatcttgttatgctgttcgttgaagccttttgctaaaatcttc
cagaaacaccatttttttgagatttctataaaaacaatggagtgtttttttagacacctttttttcttgggattgatcgtggaagcatccaataaaggaac
atgaggacattgaattgactgttgaccatattcacaggaataaaaataagaaaatatcctttccaactatgctttggaagaggtttcttttccctca
tacctgctgaatctgtgtccaagtgggacaatttgattaacatgaacgcaacttgggacgtaatttggcttagactggtcgaaacagatagtaaga
atcccttacatcaattaaattgtttgtgtatggcttcaggggtatccggaatagtctgtctcaaattttaaaagtccatccaacatgaagactagaa
atgtgctttggaataatttcatagatgtttcattgatagaaacatcaaataagcttgccttgaaaaaagaataatgcttactcgctgtaggatcttgt
aatcgtcaagtgtctgatcttttttcttggtacatttcctcaagaaacaaggagcaaagcaaaatcccgttaaccttttaaaaagaaatcttaaact
ggccagcatcagccgatatgcttcgagcctttctttagtttctccttatggacgaatatcataaaggtcgttggctttttcgcagggaccatccaatt
ggagtcacttttagtttttattttcttaaatatgttctataaatctctcttattttgcaaaatggcccatcgagagctgtttcgacactggtctccaaag
taccactttcaacaacttcgtttacgcgtctgagaccgaaatgggaaactttcagataactcttattcatctgcctgctacccttactatataataga
aaaaccacttctttataacaaactttctgtcaaatgacatatattcgttagaacaatattactatccctttcatattatgagaatcaacactggctttc
cctcgctagcactcggtatcccaaaatgcttgtccacacgggtgttggaatttgtcgagtgccacaaatcgcgtttcgtagtctttttgacatttaga
ggaaatttacctctaactttgttgaataacaaagttatttgttctgttgagaacattgggatttagctctgcatccctagggtattcttggttttttttct
cgaatttttttttgacaaaaaaaaaattgattcgtctccagatttcacgaaaatttgtatggaattgatcatccataggaatctggattggatagaaga
tgttaatggagcccgatcatttacgtacggcagtcaaccgttcgatgacttcgcgtgaaggtagaagcaaaaaccggtttaatcccgtgacggga
gccaggctcgctgctggagtgatagcgctttcggcggataagcgatatgttctgatggtttcatcagcaaaaaagcttccatgctgggttgtgcca

aaaggtggttgggaggcagatgaatcgatacaacaagcagcgttgcgtgagggctgggaggaaggaggattaattgggagaatcacacagtc
actaggttgctttaaagacaatcgtccaacggatacggtcgatcgaaggaagaagtacttggagcaacaaatcatgaatgcccaccagcagcac
agcgatgatggaacctccgatccaactagtacattgaccgctgagacagaacaaaaactgacttccatcgagaagataatgattcctccccgtgc
ggaatgtgagttcttcgaagttcttgtggagcggctggaagactgttatcccgaaatgggaaagcggcacagaatgtggatgtcctatgcagaag
ctaaaagggctcttgcaagccgaaaagatattttggctgctttggaagaaagttccatcatcaaggaaatggatgaaagaaatcatatttaaagt
gtctttttaatcatgattgtatcgtttttctctttagtgtttttctcttcatgaactatcatccatggaaaagactgtgtccttttaatggaatcaatta
atgagggtaattttgatgtatggctaatctatgttgtggataacggtttgtcccaattagttttgccgctgttatttgttttatgggatatatatactcct
ttttatattcttatttctctctttcaacgttcctatcaatatttcctacacttctttttagactgaacggaagaagcttctttcttcgtttacgtctatgtg
cattagcttcgtaaataaaacaatggaagtcacgggagtttttgtcatttttatgattgcacccatctcgcataagtttctgaaatccttcttttacttt
tctttccacgtatgattctttctactcttttccgaacagttttcaattttttccttatttttacgttttcattctcattataattaaagaaactttagttgtc
cgttttactattcactgtttcttgatgttctcttctaccactcgttctttaaccacaataaggttgtaaaaaaggagcatgatcttctttttgctttttct
tttgctaatgaatattaaaccactgtatcgtttttttgttctacgcgtgcactcataatagacacgcagcttcgaacccaatcctctatggaaaccgc
tagaacagcatgtatacagtagcgaaggtaaaagatacattgtaatttatagatcttgcgctaataattctcttgcatgggtatgaatggctttgaa
tgaaagaaatggaatggataggagatggattttgtcaaagttttgtataggcaactaatctaccgtttttaatgtaaaagtatttttttttttatatt
gtgttttctttctttttcattgtattgtattttctcgagagttaaatctcgtgcgtcgctgaaaagaaatcaacagaaaaaagactgttcaaacaaaa
gtagcaaaatgagttcataggaaaaaacaaacgaatctgtgtaacatgaaatggaggaagaggacaccatgagcaggtgataataaataagc
atgctttttgagatttaaaaaacgagatctaactggcatgaggggatgcgcgagaagaatccacgtcggaaattacgatacgaggccccgtatct
gaattatccaaataagtatttccaagttgatcgagtcgaagttgaaggtcatgggcaagatgtaaagaattaggagactcttccggaataggtga
ccagcctaacggcggagatcctggaggactgataagccagtttttttcggttttaggaactttaagattgtagttttgcatgagtgagggatccttc
gtcgacacatctggaacgtaatggcagtcaattggggcgccggttggcaaagaggtatgttgtagagtatgaagagcctggcgttcggcccctga
atcctcataaaaggccatcactttgcccagacccttgatgacttttgttgaacgagggttggaaaccgagtgtatgaattcttgcaattctgcaaca
gaactagaggtcgttgtgaagactatcattgcaaaggaagagaacgaaaagattcaagcagaaatgtcttgaaagagacccacagtaatatat
aaaaaagcaggccaaacgatcctcagttccaagaaatagccgacttgcggtgcaatgacaatttcaacaatgaaaaagaaaactatttagaag
aaaacactggaagctttaaaagggctatatatgggttaagacagaagaaatactaataagtatagggaccttacgaacacaccagcaatgaag
ggttgagaagaacaaaacctcagttcagccatgaatatggacactaaagaatgaaaaacatatgatttcctcttttggtataaataagaaaaag
aaagggaagggaaaaggaaatccttcttttaccaattgccagtgaatcacctagaaaacttgaaacaactacgagctgatttcctttcctaacag
aacgttgtgatatgtacgatggaaaaagcttgtggaaagagatgttgtttatgttttggcccctgtgagaaaaggttattgttagactaagtggaat
ggattccgatagatcaacagactctatttctctattttcctctctcctcaatatccgtttattttattttacactttttttataggccctagttcgtttggaa
ggatgtttgctacgaatctcctacctgatattataaaagacaaaccaaggcagcaaggacgcgtttgtctttctgtgtattttccttatcttccttcta
acgtggcttctcaattacgacccccttctccctgtttacttcatggaagctaccctaccgacttttcgtatcaaaccaaggatgatcgtctaaagactc
ttagctcggtaaaaatcgctcaacataattctagtcttcatcacttcaaatgactctaatggaaggtattttcaaggactcttcttcctttaagctgtg
tgtgaaattacggatcccgaaaccatcctcgtgataacttgacatccaatatactgtaggctgcagaccctgggaaattcgtgtccatctcttcttg
attctaagaatgcgaggctttgtatcgtaaggcttccactgctctgctcgtctctctaccttcgtttcttcttttccacccaactttgccttgagcgaatc
cccggaagatagcaagctcgtttgggttcgctatttcgagatagattcacaagcatatatctcggagaatgaacaagtataaatagagacgcaag
tatgacacttgaattctaccaacaagaaaactagaatttggcttacgattgttgtacaggtttgttctgaaaaataaagaaattcgactgtatttttt
gcagcattatatttggcagatcccgttacccttcattcttgtttttttgtgatttttttagaaaagggaaccccccgtttttattttattcagattgttttactt
cgtccttgctgatatcgtcgaaaacacggtttgtttatatcttggtatcgacgtgcatttttcttcatatctctaattcgtgtcataagaatcttcttttg
ctttattgaagtttgaaacccttttatttttcttgattttgattaatccttttctttttgcgtgctatcatgacgttcgagaaaaaagtcatctttttttgatt
tggataattgcttataccctaaatcgtatgccattcataacatgatggcttcgcggatcacagcgtattttgcagacaagttaaatattcctgagga
tgaggcggagcgtttacgcgaaatatattatcgtcactacggtatcgccatccgtggtcttgtccttcatcatcaaatcgacgccgttgactacgac
aaacgtgtggaccaatcacttcctttggaaaaggtgatcaagcgagatgatcgtttgcgagagatgctacttgaacttcgtcaaaagtataaactc
tgggtattcaccaatgcctacattgtgcattgttcccgtgttttagctctcatggggattgctgattgctttcacggggttacttattgcgactatgacg
ccaaagacttaattgccaaacccatgccggaaatgtatgcgcgtgttatgaaagatgctggcgtcaccaacaaggaaaactgtatttttttcgatg
attcttacggaaatatcattggggcaaaagactttggttggaagtacaccattcagttggtcgaacaccaagagcctttgcccgaacctcaggcg
ggttctcatgtaattcgtgatatccatgaagcaaaggatttttttagattctatattcaaagatgatgattaaaaaatataatcttcctatgcattccct
tctccctttattttctctcaatgctttatccttcgaagaaccctccactgtcaatagactgataaacgtgtttatatgttcttttattcggatactaacga
ttacgagctttccctgcgtttagcaactctttctttttacttccagttcctgaatacctttttaggaggatttcatacgtgtctatgaattcgttatgccg
ctttatcatcttatgcaaactttttatgcctttcacgtgtagagctttttacgtttccttttattttatgagccattcgttttagtgtaatgaattgtttattt

ttttttgtaccatgttactagcctatatcattgcggcatagaccatcgcttttcatgaaggaagtagattcgtagctatctatctttcaagcttgtatct
ccatttggatctttgcaatgtgtataattgaaatactttgaacgaactgctatatccttagcgcaattattatgtccttttaaatcaagcttataacct
acgccgagcatttgcttctttataaatgatttatatcaaggtacgctgatcgagatattagagttttatatgggtaattcaaaatgcagacagtgga
gaatatatatattactatctaagattgtcaattttactagtgcaaacccaaactttacactttaccaaagtaggtgttcacagaaccatcaaagaaa
gatctataaaaaccttatatctatagaaataatacctaatacaatcccgttgggaaaggacctagcaagaaagaaattatcatataaatgagacc
ttgcaaacgacggcattgccataataactcataagagaacttaattaaaagtccaaatacatacatatcaagggataaataagaacctagctttt
taaaaaaaaagagaaaaaccatttacgcatagtctaataacagcaagaacattattaacacatcatttcgggaaacttctgaaacgctttcaag
aagtgaaaagatcagttaaaccatatcagatccaataaagtcgagccattttaaatactaatgggtcatccaagcagtcatggatggcttcattct
tgttgatcattattataattgtgaaaagatagcacttcggaatacatcattaccttccaagtttccacaggcagatcactgtcctggaaagaccaat
cgaatatttcgtcagcgactggttcatcagtaggatcatggtaaggagcgagatattcatgggccaaagcgtcagcagcactaatacgcttacga
gggtcaaagacaagcatcttctctagtaaatcaacggctgcagggtcagtgtttttaaatttttctgagaaggggaccttttccttcttgggaagcg
attggacaaaacgtaaagtgtttttgctacaaatggtttcaatgacttccataggtggcgtccccaacagttcagtgattatagagaattggttaac
gtgatcgcggccaggaaaaagaggttttccttcaatcatttcagcgaaaatgcaccccgcactccaaatgtccacttctacattatatttttgccat
gtaagcatgatttctggagctctgtagtaccgagtcgaaacatatccagtcatctgaggatcttgaatacgggcaaggccaaagtcacagatcttc
agatcacagttctcattaatcaagatattactaggcttcaagtctctatgaattacgcccgccgagtgcacaaatttcaatccccgcagaatttggt
atagaaaatattgaataaattgggtttctaatggcctcgacgtcaacaatctatgaagatcggtacccaacaattccgtgacaaaataaatatctt
caaaaggagaaatgaatatatcgcttagactgataacattttcatggcgcagatgtttcaacagcttcagctcccggtatgtcctttttgctaaaac
tggtgtactaaacggtttcatgatctttttgacagcaacattcattccagtaagctgatcctttgctgagcaaaccaatccgaaggcacccatgcca
atcggctgtaggtcgctgtaacgcgttgtaatttcaaaacaagtaccaaaaatctgtgtacgaataaactccgccatatttagccttctgtcgttgg
ttttcaccagggtaactctcgctgttcttccaatttctaaaccaacgggttcaaagtgctagtgctatatggacccttttttctgtaaaaggaagctct
ataattattttttttaattgaaaatcgatcaattaaatctcgttaggtcctagtgaatgaacgttgttaaaggtagatgctaagcagagttccctttta
cacgtaaggaagtatcctataatattggagtgcagagcgttattgtagattagggtcggatataacgagtagaattaaatcgttaacttatttaaa
gcattggaagaaacgagaatttgctgcttgaatgttgtaagcaaaacatggtaggaaaaattgtaggttgagagcaaaacaataggtttgtcata
tagacgaataaaaaaacgaaagaatatgtaagaacaagtggagcataccaaaattcagaatgagttgatctttgcaacatgcaggcataaata
aaaagtaggatacacacgtccatgcaatttacaggtttttatagagaacgctacaaaacatgaaggataaagaagtcgtatattatcattaaaca
atacagaaaaccatacaggcaaggaaacgatcgtttcacgagttataaagaaaactaagtaagaacgataacataagggaagaaataaaaat
gcgcctttttttgatttttggtttaccctagataagtagtaagtctattgcaatgcatatatcttaaagaaagaatgagaaaatagaaataatcgtta
acgtcccaaatggtaaaaatcaagatcaagatgtcaatatcaagtagtgaaacatgcaatataggccaagcagcatatcctgcgctcgacttcat
ttttaatgattgccgcaatttgcttgtgggcatacaaaggtgagtcctaaagacctaaagaatcatcaaacgaaagaaacaaggaaatggaaag
agaaagggaaagaaaagaatgacaagagatattggctaactgcttattattcgttaaattcattaggattttatacgcctaggctttcgagataat
ggaaaacgacggactgccgtaggtatcaccgtcatcatggaggtttccgattgcatagatgattttcgggaagcagcatctcctttagaatgattc
ataatggtaaagtaatcggattctcgatcttcattttgcgagtcagtagaattggaattgggtcgagacatgtctgttccgtacgccagtgacggtg
ttgacgactcgacatcactcagatgactcgctttgggagatcccgatgaggaaggagtcttagatactgcttcccttccttcggccattatgcgcgt
tttatgatttcctggactctggtttggtagtacttttcgagaaagttgatgatcgtaagaatttcctaatccatcaaacgattttttttaaagattgctga
atatgtacgagtgatgaagagtgaagagaacgacgagagcgacgattcgttaatgaaagcatatccatattgtcagaagaaatcgcacgggctt
ttaaagcattcgcaggtgattgcgttactccaccgacatggtttttttgaggttccgtaaacatagccgttgaaggacgcgaaggaggggtcgaat
ctagctggtctgtggacatcgtaaatgactgaggatgcttcaagatactagtacccttattttttacgggtacagttatgggacgagctggcaaatc
tgaaggcccccctatgaattgacagcggtgtaggacactgaatatgtgaaagcaaaatgcttatagtgtgcatgtgaggagctttaacatcatgac
taacagccaattgaactaggtatcgcaaatacgacacatactccatgggcttgtgctgaatataatccttaaacatggggttacctgaagcatga
gccaggaaggaatcccgctgtttcgaaatccttttttccgtcaatagagccatgaaaaacggatgcaatggagtttatttccgataggactcctttgt
gtagttcaaaaatttccggttgttcaagaagcttagcggcgttaggattctgcgtgaggacagtcaagggaaaaagggaaataggaaaagctag
atcctctatttgaaacagtataagtgaatcaacaatctcacaagggattcctccgttgcatagaatgttcgctaaaagctctaatctatcaaaatgt
gcagaggatccgaattcgctcaagtataaaggcgtgttttctccatgatgaaactcattgacgtccctttggacaattcccgagtgaaatgaaaag
gaaaaaatgggattttcagggaaatgcttttgaaagctctcttcgattccaattgatccagtagtattgtagacaatacaagtatgtcccttggtaa
caactgactcaaggacagacggtaaatcaaacgaaaaagggcatggtttaatggaaataagaatataatcaaacacttttccttcattcgcaag
atcaataattttgttagcacgatatgtaggccgatattttcgggagccaaagggcttcgaatgaatcgtgagtgtagatattggggcatcaggaac
attgaagaataaaattgtttcttgacaagcaactgatgtactttgttgaagcctccatcctagtaaggtagtgatagcagcgtttccgatacagaga
acactgatgcatggctttttttcccgagtccattataagttcacaaatgagagcaatagaaacctatagataaattaaatgattataaagagagtttt

ttaaagaaagaataaagttgaagtaataaattatgatagtcgtttgacagaaacaaagggattatagattccaatgattaacagaaatgatccat
ccggaaaagtctgaaaatagtaagattcaaataagattgcttagatgtcaaatttaatttctttttccaaacaaagctttcgtagtatatgtttaaa
tgaagtcgttacgtggaacgagaatataggtaaaaatacaggtgaatagtatgattctaaggaagatcgatgttgtcaacggccacttctttgtct
caggaatactttctattaaaacaattatctttttcagtggtttgttatggtcgtttttaacgctgagcaaaaagtcagtgtattcggaaggagaattc
tttcaaggcgtcaatagacgttctcgagaaggttatagtatgatattattcaaagtgccggagaattcctaatattatcgaatattacatatattta
taacactatggagtatattgtttaaggacgctacctgtaccaagacaaaagaagaacaggtgaaacacaaatccaagcaataagaaaaatcca
ataattcatgagttagaaatagcttttactttattaggatagagctattattgagatagctatctccagaaaaaacatccagtaagatagtttttgc
aggtcgagagaatgacttgtttatcgatccatctttgtcaatcaggctcatgggaatatgtagagcaagagaattctattccattccaaattcacac
gcctatttaccattccttagtccattcacaattctttcatacttctgatatacatattcactgtctcctatgttatacatatataatttctacatttttactt
acaatgtaccacaaggcataagaaaagttcgtgaataaaaaataagttatttttagagcaagaaaaaaaaatgataatccgaaaacgagaga
atagaacaatggataatatgctaaggagagagtttttcttgctcatgtaaaaaagaaaaaaaaaaaaaaaaaaaaagaactcgtgatttc
gtttctgacttcttcacatcttttatagtagccttctccgactaagtatttggatcctgagagttattaatcatataaaaaacttcttttgtaactcgtcc
gactctgagttcaagttcatgttttcttgagctttttgacttcatacaaactacgatcaataaaggccgctaaagtaacacatccgcctaaaagaga
taggacgtttgtcaagaagcctccaagagtatttccttggatttggcgatgaatgacgcgcataggactaatatcaatgtgaaaccataggccag
gaacaccgcctctgtgattaatatgagagggaagtttctcttcaggaccaccatgaattggtcgttcatgttgtgtcacagcatagcgattagtatc
aataattcctgtaccagcttttaatgggataaactgatgagacacgcatttgataaagtattcatatctgtagtcgggaatgttggttttcttcacca
tattgtctaaagggttttctttagcaatagaaggatcaagaggaggaccaaagctcaaatgatgaatggtatggctcatatcatgtagctcctttttc
tttcgaaaagtcgcgagtatcgtgtatgtgatttataccattttgggacgaccggccgggggcaatgtgaaagttacctgccatgcggtttacgaat
aattgaccggccagattgcaaccctctactttttgagcctcatagttttctttaaaatgttcttctttacattgtttgaacgcatccacatcgccgatac
cccaattagctcgaccatatgcttcacgtaccgcattgcatgtgttgcaacagccaggagtatcaacaggagcaaagtcggcagcaccgtaacaa
tcaccacatgtcttttcctcaggagtggtatttaaaggatcttggtcgttctccggatcattcagcagtaaattgtctacggaaataacttttccttca
ggggaaagcctagttttagaaacagaatgctggacgtcttgttgaaattcaccgctaatatccaaaacgtccacacttaaaagctggcatggaat
ctttgcaaaggtaacgttgaaattaatttccatacgctcaccatgagatgggtttacgacgatttctggaacaacaaccaccttacggtattgaatc
cattctaaaaggattacaaagagaacaatagcggaagagattagtgtaataagaccaccgctggcggtttttatacgagcgtcttcaacggtctt
ttgaaaaaagtcaaacttgcgaagaggagatcgaaaagccatttccagaaattaccttgtatgagaatctatcagaatataaaaagaagggaa
aaaggtatcctgaggttgtcacggaaaaaattattaagaaagttacaaagacggtaaaaaagcgtatccaagcttcaaaaatgtcgatctttaat
agaaaaataagaaaatattatgaaaaaacgagtacatcagtttagcaaactacaattaaacaatagatgccaatagcgaaaggatactatgaa
ggtagcaggatatcactaggcacacatgtgggagagaacagtctgaacaaaacgctgaaattcgtttaagaattctcaaaagaatgatttttgta
tagaaaatattaagaaaataatgattaatagaactttataccctaaaaatttcagctcgtttttggtgtgaaatcattgtaaatttaaggacaccat
gatatgtacaaaaatattcgtaacttttttttttcaatttattttctagtatttacggtttttttatatattaatatgaaataaaaaagaagaaaagacaa
ttcatttcaatatatttcgagactttgaaatcaaataaatatactccccccccccaaataaaggactctccggtcatcatctagaaaataacctgttt
tgatatatttttttttctttaaagataatgattcaactctaaaaacaatatcttctcctatccgttctcaatttcgactttttttctattcgtactgtttgttt
acaaaagtaacatttcttccttgatacttagtatcatattaaataccttgataaaatgcatttattgaatttcgcctgttttctcttcatggtttttcaag
atctatttggaatttttttcttcatttctattttcaaactctctgctcgagtttttttccttttgagatctatacgtcgttaaatgcactttatgtaaacaaac
ccctcctgctccttcttcctgccatttacctaccaccagtctggagaagactcttccaacaaaagaagaagcattgaagagaaaacacaggtatta
aaaattcactaggaactttttgtaaaaaacaaaattaaaggcgtgcaattttctccaattgacgcgttctttctacttgcttatagcgattcaagaca
caggagggatctcctatcttcttatttcattttaactttttaaaccacgcgttttatagcctagacaacaagtttttttacgacttcatatttcaccttcta
cttccctaaaatcggaattcttcgtacgacgttttcgttagaattccttatataatctcctttttttagaaggcgctatattaaaagagcatcgataaa
aatcggggtctttttactttttatttttttcttttaataacaaagcagttacgtggtcattctcgtgaacagctgaagcttgcagtttttcaccttttgac
gttttcaattgcacggagcttacggacttaaccagcctgacacacaacgtaattttgcaactcgcgtaaaaaaaaaagggttacattgaaacgat
catacagacgtatcagagacgacattaaaacagtgtaaattgtttgattggccgattattttcctacacttcaattatccaagccttttctaacctcg
ttgaccaatcctccgtctttattataacagagtgttccttatattgtggtcgatcagttgtgcgttgtcatcatcgtcttcatctaaattggaaatttca
cacgacttgatatattttaaccacaaaatttcttcttggtttttttccaatttctttagttttactaaaaactttttctttttcttttttttttttctttctgttct
tgccaatttaagtatttgcttgaaaatttttcagcatttctttttttagttgtcacgttttgtcctgtcatttcatccgcggatttgaagcttgacaaatac
caaagaattttccctgctgttaattcctatcaaccgtttgaacaaaaaagttaggttatttaaaaacactgaagcttactttctttcgcaaagtatttt
ttttcctcgtattctctaagctgagaatttttttatcccatatataatgtttcgtgcaagattttcaaggttttggaatcaccaagctggtcctaaaacag
tccatttttgggctcctgctatgaaatggaccttggtgttatcaggtattagtgactatacacgtccccctcagtacctttctgctcgccaatacggtg
ctttgtgcgctaccggtgctatctggactcgatggtctttgattgtacgcccaaaaaactactttaatgcaactgtcaacttctttttggccatcgttg

gtgccattcaactttcaagaattttcgcgtatcaacaacaacaaaaacgcatcaagccaaatgctgaagtaaaagaaccaaatcaaaatcttgct
tagcgcttttccagctaagtaaagatatgtattaagacaagcattaacaggaagctcattttcctcgtttccttcatcatgactttttcttatcatttg
tgttgtgacataattgattattaagcctgactaactcttctgttggaggcttttttgtttttttcttactcttgaaactttcttgaaagtttgagcctttgc
tattttctctcactaatttagggtacgggaagtgtatttgctcattgaaaaaaatctgtcacttgttatttgaaggctctactaggatgaaccaaaaa
gaaaaagaaaccaaataaatctccaattgctatttctggtttcacttatgttattcctttggagtttattttatctattttctcgttctgatttcattgtgc
atctatttatgcatcaatctccctacgagtacgctttcgtttttctttccttgttttcaaaatatatcctatcattttaatctctgccattgcttgacgtac
cataatgctcaaattgaacaattggttcgtataagatttgttgcatggcaaagtttacgttatccctctctcctcttttgatgtctaaacgatacatttc
tttttagtttttaaaacgagacaatgatggtatatatataaatatatatacgtatgcatgtatgctatgtttcctctatgcttatgcttataaaccaaa
cggtcaaggatacaatgattataaacaggttttagtatgggtacaattcgatttaactaccagtaatgtgcattcgcaataacgaataacttctca
gttatttatttctctgaagttgcaaatatcacttgattaatttttagtcttgctatgaaatttaattaagacatatatttccgtaagctatagtaatctat
actaaataacaaagaaaaaaacacttcatcaaatgctgtcaaaacaaaaataaaaacaaaatttcacatgacagaaaatcattcagaaatgac
gggtaaaagcaaaaagcaaaaacgaggaaagtaacctcaaaaacgaaagtgaagtataaaaggcatcaaaaatgaatataacaataattta
cataaacagaaataggtaagtaggagcaagaagtctttcatatgcaatcaatcaaatttcaagcaagcaagcaaagatattttggcgggcccttt
cacattattgagcactgttcatcacgcttcttgctatttttctttggttgcgatttcttagtagataaagattgttcttccaagcgcttttgagccttcac
aaaaccttgcttggcggctctgatgtaccaactatgggcggcagcagggtcagcagaaataccaataccttgttccatcataaaacctaccgcaa
actgagctttagcaagtcctttaacagcagccttatgggcccagaggagggcttcttcattatttttgagaagaatgtcttccgctcctgttagatac
catccagataagcctaattctgcttctccataatcttgctcagcggctctagtataccaaaaaatactacgccttggttctggaacacaattcaata
acccatgttcaaagcactctcccaacttgcactgagctgcaggcaagccgtaggaagccgataacttataaagctcaaactttctttcagaagata
cagaaataccaaaacgcttggagcgttcgtaaatcttagctaattcgtacatcgcacgtaccgcttcaggcctctgcgtctcacaagcacgttccat
atattctataccaagaggtacgtcaaccttttggctcaacaaaccattcaaataaatgagtcccactcgaaacatagcctcgacaatgtccatctc
ggctgcttttcgatataacagcacactcttgcgagcatcaggcttcactccgataccagtttgaaggcatacggcggttcggtaagttccaagcgg
atgccctttctttgaagataggctgtaaagctcaaatgccttttgcacatcacgctgaaatcccaacactccttggctataaaattgaccaattaaa
aagattgcttcagccacagaatgttgtgttttgttaggaaaagcgtgtttttttagtaattcaattgctctagttcgatagaccgaaattctagtatta
taagtagcttgatcaggagccaaaatgggtttgttttccaaagtttcaagagcatccaaatataacttagccaaatcaacctcatctaaaggactt
atacctctactttttctcaaactctgctctgcagtcatcaaatcagcatgtgtaacattctttggtggaggaattaatagcttatcatcgggatggca
aggaatagcatcactgtctggttcagaagtattcatttctaaaacagcgggagatgacgagttatcaattgacattccaccaatgttttcttctattc
cttttacatcaccagttaaatttgaatcgatgtcgttcataccatttctagaagaaagatttggtgatcctcgagaagccattatggagttacccaca
ggagtgctttccgctcgagcaatgggagcgggagaaggtggaatattgcgaattattggaacaggtggaatgggtgcatcagtttgcaaagagg
aaggaatttttcttaaacgtgcagattctcctcgcaagtcatctcgatctgcagctttaaagttagcattatgttgggggacgtgttgctcttcggttg
aaacgtttattggtatagggccaatactttcagcggaagtaggtaccaaagaagtttggctattaggataacggggttgagggaatggagatttt
ctgttcttgcgatttaaattctgtgagctaatgtaatcgaaatctgaacgtaaattcaggtgatttggacttgtgtcaaatgctttcaaaccttcatct
gatggacccagtgagcgagcggaatcagtaaaagatcggacattgggctctgcatccgaagaagcattggatggagtatacgactcgacgtcta
aatttgcatcttcatccgactcggactgaccttccaagttttcattttcttccaaaatattgtttctggagacggtttgatcgtcatcataataattatc
taagaaactatcgaaaccaccgtaaggaatataactgattcgacgtttgggatgggagatagaattgactcgaggagggggcataaatttctctt
cttgattaggaatttgggggttaaatctcggcgataccacacggggagtaggccgaataatttttataggcatgtcttgtcttgcagtctctatttttt
ccttcccagggtttctattattaggaggaaactctgaacttcgggataatgcattgtcatccatttttactcgtggatttgcagcggcttcagggtctg
gcattctcacttttccttgagacaaagccacaggttcatcttcgttcgttttctgtgattgttcttcttccattggttttattgcggttcgtttgactgcctt
gtcgtttggtgaaatccctcttgttacatcaaatcgcttggacacgtcgattcctgccgttggcgttggatccttgctcaaagtaggatatcttgacgt
cgggcggaacaatagcgatgccccagcagtagtggcatttcctctgccacgaaattgagcaggcagtccctctgctcttgcaccgggtcgaggac
tttcagccagcttttcatgttttggttctttccttgaattcagggagccgtgcatgaacggtataatacaaattaccaacaagcaagaagcaatcga
agtggaaagaagatcaatggacttgctcgttgaaaaccaaatccaccaagtccagatcctgaggttagtaaataacgatacactgcactgcatta
aacaagaagctgtaagtaaatgttttatcttctaaggattgtcatgaaattggtagctttagggtaattttcactttgcttttgtcttctcgtttcccgt
gggattcaagtcatatattaaaaaaaatcttcaaaagtaaaagaaatgcacaaaatagtaagttgacaatttgattcatatacagcagaaagatt
tagtgttcaaaacaaaaccctattggctttctataacaaaataatatgcaattcatacatccagagcatgacatttcgacgtgggattccaaaactt
ccaagacaaatcgtcatctatttcacatcatttcaaatacgggcattacattcataaaatctggctccaaaagacaattcaaagcaataagatcgc
ttgctctattcaaaattcccagactatgccttttacaaaataatgacttctcaaaggctgtttacaaaagagttccaaatgcagaagaacctgaca
aagaaggaacaccagcagaaggaatgggatgattgtttttctggtgcttaaaggtaaagccaataaaggcatttcgtcttggtttattaaatgtat
ttgacatgcgagcaatcgccgcttgcttttcatgcacttctttatacttagacatgtcattttcgttcgtaaagtcatcaaaataccctgcatcaatct

ctgaattcaaatcaggaataaatggaggtcgatacgctgcacgtacattatcccattgaatttgatcaaaatatggatgttgtttaacctgattcaa
agaacggtagcgataagtagggctagttatacagttggtaacaaaatcccatgcatcgtctttccagttaaactccaaatctcgaggatcgtcata
atgtggacgttgaaagcatttcttccaattttttagattggaccatgtttcattgacattggaaccggagaacggaggaaatccagaaagacattc
atataaaatgcagcccagagaccagtaatcgactgaataatcataattttctcctcgcaaaacttctggcgccatataatcaggagatccaacca
ctgaatgggcatagacaggatcctgagccaacaatgttcggaatacttgacgacgttcacgcatacttctctcgggaacggctacattgttcactt
cttgaagtcgtaccttcatactttcaatcttttgtttactgattataccagagctaagtccgaaatcagtcagcttgatatgcccagaagcaccgaca
aggaaattctcaggtttcaaatcccggtgaatatatccaagtttatgcagtgcgtcaattgccaaaaacatttctgtagcatagaacttggcatgat
gatctcttaaaacaccgctgttgctcaataacgttctaaaatctcctcctggtacgaattccatggccagatagatgtttgaaaaatcttggaatgc
atacaacagcctaaccagccaatccgagtttgcacttgtgagaatatctcgttccgtaagtacatgacgaatttcgtccatcttgtgcaaaacaga
cttgttcattacttttaaagcgacaatttccttggtatctctcttctttgctagccaaactgatccataaccaccttgaccgacttgagtaatcgtttga
aaatcaccatgacttatacgagtacgacgctttcgaagatatacgcgctcgcgaccgttgtagcgcttttctaattcacctcgtatagtggtagact
cttttgaaagctgttgctcaaaaagatgcagtctttgctttctagaattcagataatgcaactgctcaaagtaattatcaagaaaaaacatctggca
tatggtcgccattcttttaacctttggccgtgtgattttctctaattcctccaacgaaagattcatagtccccacattccaacttggagcttgaccaac
ggagtcaaataatcttgaactcccatgctgtcggtcccttgatcgctcaactgccacaggatttggagaagcaatcgggatcttgctcggtgatttc
tgagggctcctatcggcatggttttcttttgcaacaggactgtttgtaagcttcagctctcccattcggtcttccaaagcatcagtaagccgacgagg
atgagattgattaggttctataagtccatgtcgcttcagataggcgtcaaatttttcattagcagacgaactaagttttgaacttaaatcaaggtca
aaagtatctttttgtcccatcttatagttaactaactaaaaaaataattgtaatagaaatttataaatcaaaagatgagctgttaactatttacaaa
gagcgatcggaaaaggcatgtagtttgtttatggtaaatagtaacttccccttggttgctagcaaaaactgcatgtcgacaacctaaggaaagga
gttggaacaaaaggaaatttcaattaatcaattttagaactataattatgaattaatattttcaacaaaaaccgaaaagattatcgtcaaaactgc
ttacttatgtcaacatcgttgattggtcttctgtagtaaaatcacacatacgataaattaaatcctttagcttaaacaaatgtttatttaaatgactag
aacataacttttatctatgaacaacatactcataatactctcatcactatatttactcaatgtcataatagtcagctgtacttgacagctgaccgtca
atctttcacatcaaattgtttaccgtcatcttcttgatgatacattccacgtttgtagtaaatgtaattcatctaggttgaagtggttgttctgatgaag
aaacagatcctcgctttggaagaaactcaggtggtcggttcagtatttcatcagaaaagatgggtgacccatccatgaaacttggagaataagag
cgaccaaaagtattggggatgcttggagtgagcgacaaggaatgcgtatccaaacgtacaggatccttgtcctttttctgcatagtcgcaaagctt
gacgttgacatgttaaatggaacttgtaatgtagacgatgcccaatctgtagaaacttcggggctctcaatggcagcttcttcatcgaattcatgca
aacgcttggtacgatagctttcgcgaatgatgtcagtatcatacggccagaaaagcttttgcacagtcatgaaaaggaacttcggaaacaagcaa
gcagccatcgtaccaaatagtactgcccagaagtttggagttctgaatatacgagaagcagacttgtagaattcaatggagtaccagctctgaga
ataaacgcctgtccagaaccaaaaagttatacaacttaaagcccaaataccgactgagaaaacatcccaattagcttgattcaaaagaacatat
gaatcaactaccataattgtaggtgctgcaacatacacacccagatcctgcattcccatcgtgtcacgcccattttgagctgctactgtcacattatt
tataacgagaaaagaaaaaaagaaacatattactgactgatagaaaccatcaatcatgtatccaatgaatatcttacgcgccgaattcaattgc
aaaatacctctcttgtacagctgaggtatcttaagtgatacttcagcattcacatcttgatcgtaaacacccattacgatgacaggcaaagatgag
aaaatcaagttgaagagcataatgtacgtgtagtcaaagagataatttgcatcaaagttattgtagatttggaaccaaaagagcgtaaaagtcc
aaacgacacttttgtaaaagaaattattgaccatttctgctatacgattataatcccaacgtccatgaacaagaaccagcttgctcaaaaatctaa
attgtgcaatcgcataatcagcagacatcgcagctgctcttccttcctcaccaacgataccgataccaatatcagctttttggatcatagccacatc
gttggctccgtcaccaatagctaatgtcattacttccaaactttcccttacaagatgtacgacatcagctttttgcgctggactaacacgacagcag
agtacagacttacattgcttgcataacaacaaaaacttcgttctcattgatccctcgaggacacgcttcaaaacaccaccatcgataattaaggca
tgagatccagcaggaacatcgtgatccttctttgccgcttcaagctcttcgttggagcctgataagccaaagtacctataaagataatctgctaaa
atgacctccagttctggagttgagacttcttgatcaacatcaaactttatcaactccatactagcgtctagaagattgcaagaaaaaccgatgtta
attgcggtttccattttatctccagttaacacccaaagcttaataccagcattccctaaaagtgcaattgagtcaggaacaccttcctgtaaacgat
cttcaatagcggtaccaccaagcaaggtaagtcgtgattcaatcaagtcggcaacttcagcaatctgttcctctctattttcaatggcagaagagg
caacactatatttttctttccattcatagtattcctcctcagtaagttcacgttttgcaatacataaagtacgaagaccttcaagggcaaaagttttca
ggtgttccgcagtaactttttggagatttggtcgctcaccttgttccagcctttcataaataacgctatctgcacccttgcagatgaggacataacga
ttatctggtcctctgacaataacactcattctttttctggcagatgtaaaatcaacagtatccatcaatttaaacctatgagtctcaccaagtgcccg
agttaccatgacatccctacgttgatcaaggaaaacaaatccaacgtcacgagctgtaccaacgagtgcagcttcatccggggattgagctttgt
aaacgatacgatcgccaactctatcggccacaacactatgacacaaagcaagtgcaagaaaaaactcataacaagccaacgactgttcttcacc
agctttgccagtcaaatcatgaacgaactgagaagaaataaatgtaagattttcatccaccaaatacttattgtcataaatatttctcatttgagca
atcatctgcattcggtcacgatcaatgaagctacgcttctccaacgtaagctgctctgaatcctttccttctcggcgagctatacccgccattgcttct
gtgaaggcttctccgtaggcaatgccattgatagtgcacttttaaattccataacattttgagtaagtgttccagtcttatcagagaaaatatattc

aacttgccccaaatcatctgatatattccaagatttaggagtacaaggatacttgagtttttttataatacatgtccttgtcaaaatagatgaaaattg
cttgaatagttttgacaatttcaatagaaatgtatagggagatcggaaccaaattttggaacaagacaacacttgtaaaaaaggtaactatacca
tttttggcggcagatccaccaatggagccccattcaaaaaagtaagatgatcgcgtttgtccacgccaagcaataccctcgactataccacaaac
gagacacattgcaaataaaataatgaaattcaagtaaacattccagttcaagtttctggtaatacgacttcgtttcaaaggaggagcaccggaat
tcagcatgatttttgtatcatcaccagtgaaaacgactacgccaataacccaccttgtatttcgaagaacacatcctcgtaaaagcagattgtttaa
agatgcgggttccgactgaacatgtgtttcatcaagcatattggtgtcggtatgcccataacatttgcaagctccgttgtattcataaaggtttgcgt
tggggggctcgctttcaatccagaaacgagatcgttcacacgaaatttcgtcaaccacatttttacccgcgctcaaagcatgtcgaattttgagatt
cgtttctccgtccaagtttttggtttcaatgtagcaaataccttctggatcggatgtattgagaatgataatatcggcagggatttcttcattgtctga
aattttaacaaagtcaccaactcgaacatccttccagaatactttttttaaaatggcattcaccagtaccttcagcttgataatccactacatccattg
cccgctgttgatgcatagcggcgattccgcctgatgcaccaaattcctttcccttgggatcctcaaaaggatccttcatatcaagcgattctcggaa
tacggagtcatagcttggtctaccatcagttggaaggtcttggagaggaaccggatcggatacagtattttcttctttctttcggttcttcctcatgat
aaacagtcggaaaagagcagacatatactttttgaattttcgaaagtaactgacatactcggtcctaatattcggattatggtagttgcttaaacg
gagcgtcggggtgttattaagattaatgtcgagcattgtacgacgaaaatcttcgatagcatctttaaccgcagtgatagcaacaacaacaatca
aaggcacagctgccaaaccaggattcacttcattgccgaaaagtttgatgcactagtttacaagttagttattgctcaatgatcatcttgaaacgta
cctgaagaatcacaagaaacaagaaaaagaggtttgcgacattttgaaattgtaagaaaatattcttgggcaaaaagtttagaggagtatactt
ggcagtacgaattttgttccttggaaaataacgttttgcctcaccagtatcatctagttcggattccggtaaaggcaaattaaaatacagggatctg
ggaagacgttgtagaccactccaagactccaaactactagtatcgtgttgttccaactcaacctttgcggcatctgccaaaggtatatttagacgat
cagccagtctataaagcccaaggcgaaagccctttaaactggggatccagcgcttggacttaatgcttctagtagaaggctctgacatacaagca
attggaacagatgaggtgtagtatatgaaagagaatgaatcaagtgtcaatagaaaaaccaaagaggatgtgaaggtacggacaggaaggag
gaaaataaaaagatagtttcagcaacgacaaacttctcacaaagttaatttatatacaacgaactaggctgttttcaaataaatactttcaattac
gatatttgtacttgctctcaatacagcgacttccgatggaataaaacgacgggtcggaaaatattgtcgcccgaattcaattgtcgtcaaactctta
gacgcctcaattcgatttgtgttaaaattgccgattaaaaaattaacgacgaaaataaaccaaaaacccaatttctatttgaaatctctcaaattct
gcaaaaagagagaaacaaactgaagaaagtgtgtctggctgtggtcagattaacgacgaacagtaagtaacaaggatgagacaaggtagcga
aaacccgtgttgaaatggtagtgacgagccgagccttccacgtcactttggcccggatggtagtatcggccaaatttttccctagtacgatatctgg
tgtacgattataggataatgatgaaaggacgcagtgaatttacgaaacgacgctcgtattacaggactggaagcaatggtaatgagcccttgtct
[truncated: 1,030,712 more chars]
