# Supplementary material for: Assembly of Schizosaccharomyces cryophilus chromosomes and their comparative genomic analyses revealed principles of genome evolution of the haploid fission yeasts
Source: Sci Rep. 2018 Oct 2;8:14629. doi: 10.1038/s41598-018-32525-9 (PMC6168568; doi:10.1038/s41598-018-32525-9)
Supplement: Supplementary file 5 — Supplementary figures [file 41598_2018_32525_MOESM5_ESM.pdf]

Supplementary Figures

Assembly of *Schizosaccharomyces cryophilus* chromosomes and their comparative genomic analyses revealed principles of genome evolution of the haploid fission yeasts

Lajos Ács-Szabó, László Attila Papp, Zsuzsa Antunovics, Matthias Sipiczki and Ida Miklós

Department of Genetics and Applied Microbiology, Faculty of Science and Technology, University of Debrecen, Hungary

Correspondence: Ida Miklós, Department of Genetics and Applied Microbiology, Faculty of Science and Technology, University of Debrecen, 4032 Debrecen, Egyetem tér 1., Hungary

E-mail: miklos.ida@science.unideb.hu

Tel: +36-52-518-600/62056

Supplementary Figure S1.

a

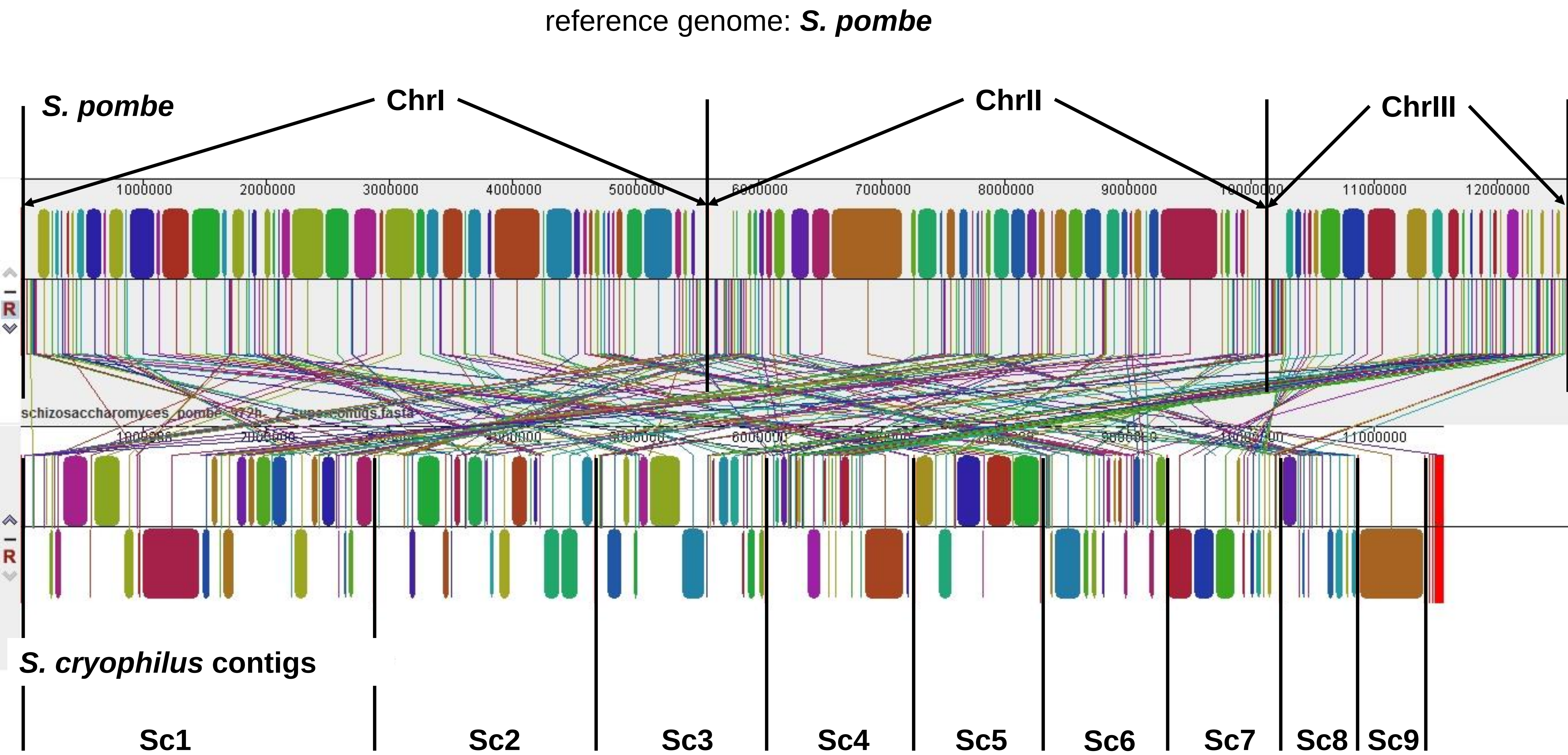

Supplementary Figure S1.

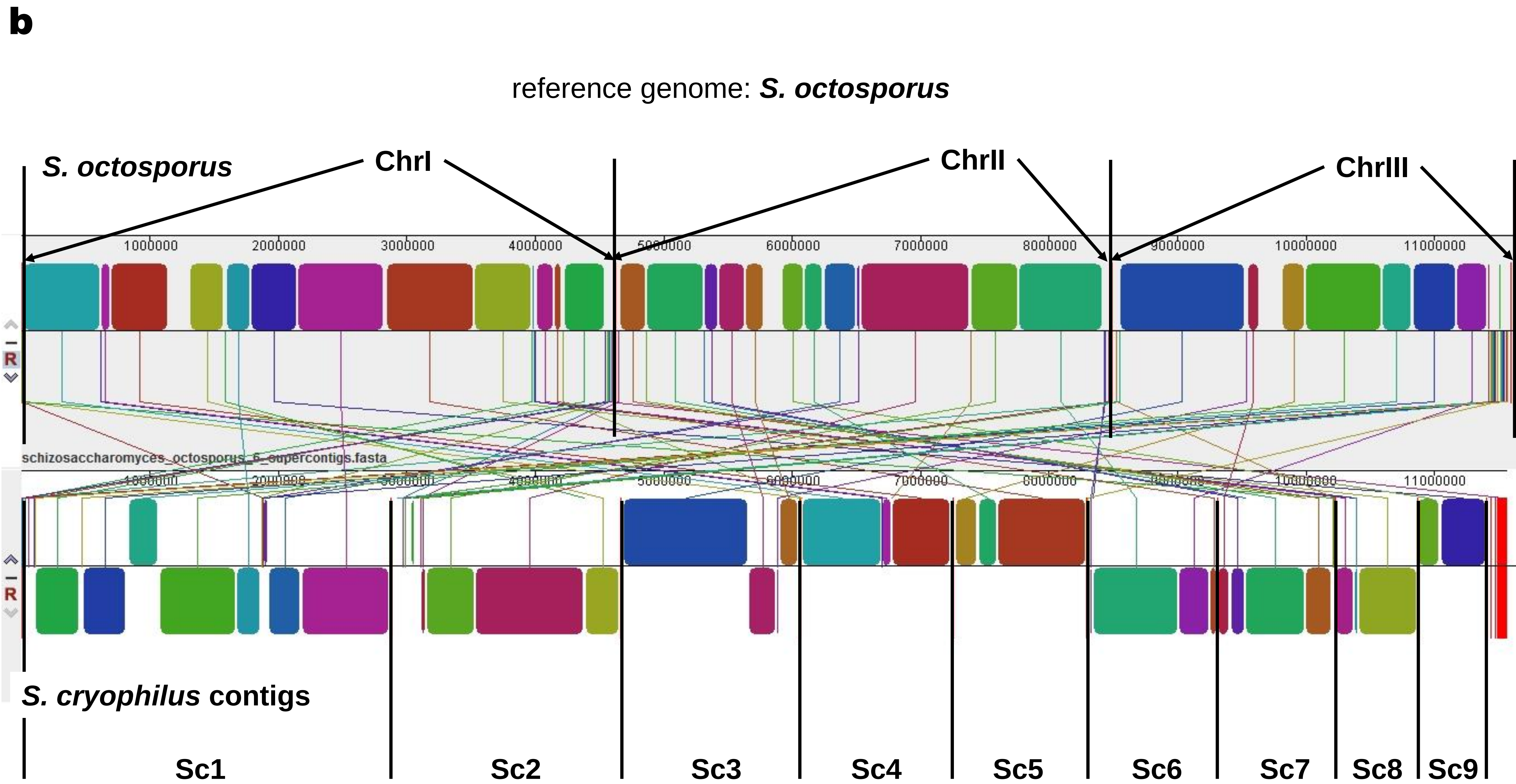

Supplementary Figure S1.

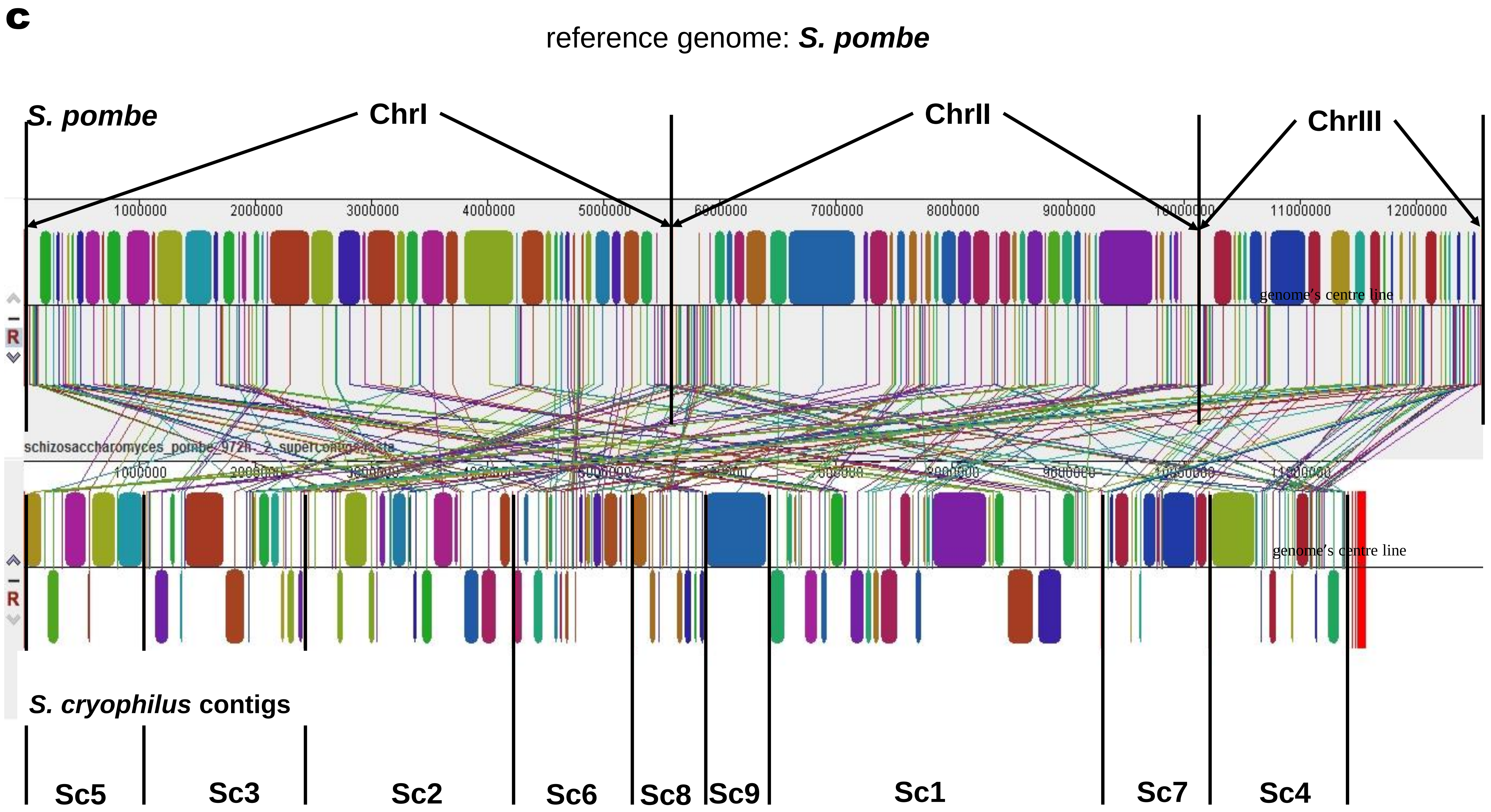

Supplementary Figure S1.

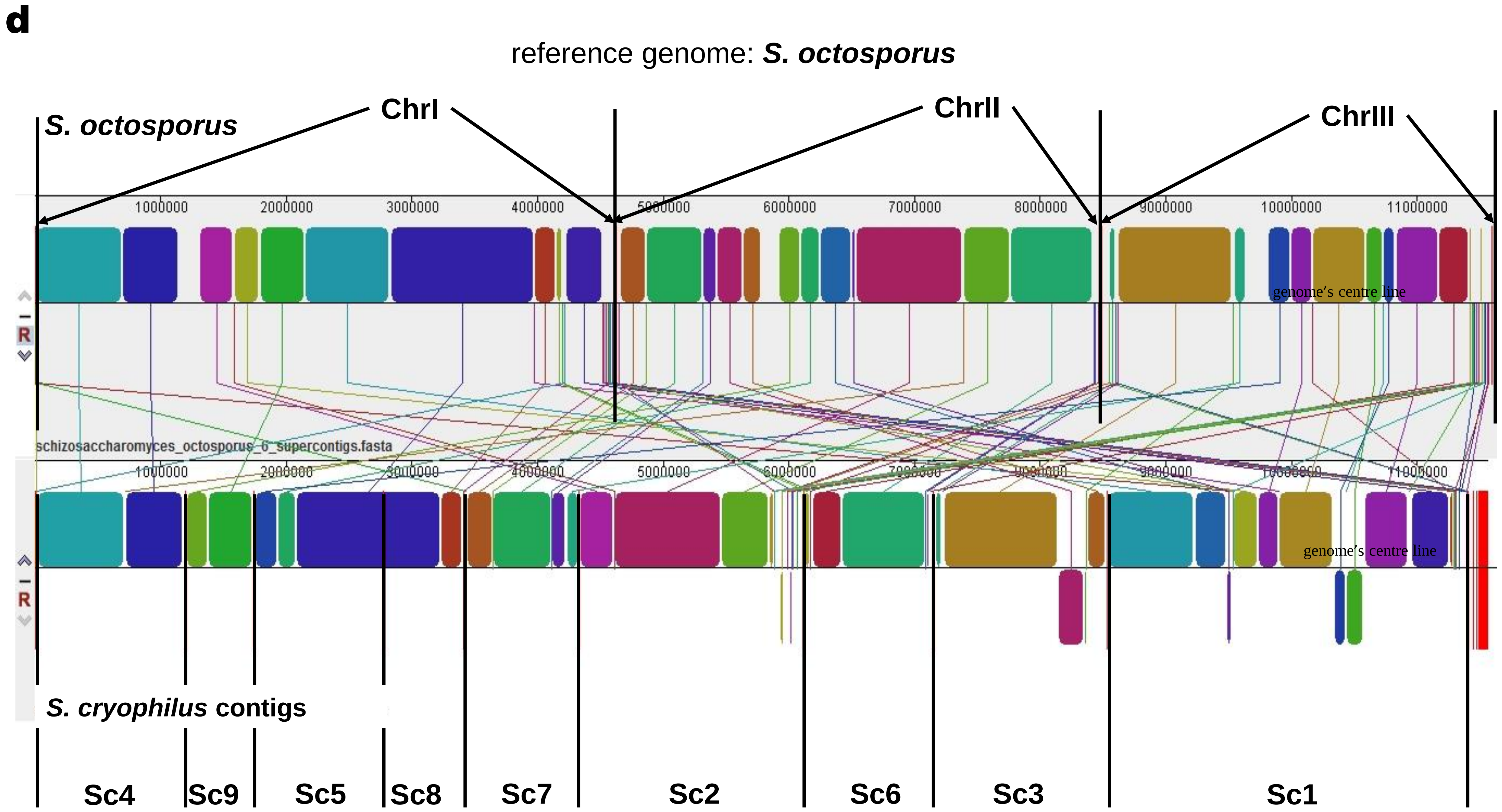

Supplementary Figure S1. Pairwise whole genome alignments with Mauve. Colourful rectangles represent locally collinear blocks (LCBs). Sizes of the rectangles refer to the genomic extensions of LCBs. LCBs below a genome's centre line are in inverted orientation relative to the reference genome. Vertical black lines indicate the chromosome/supercontig boundaries. (**a)** Alignment of *S. pombe* – *S. cryophilus* using *S. pombe* as reference. (**b)** Alignment of *S. octosporus* – *S. cryophilus* using *S. octosporus* as reference. (**c, d)** Reordering the contigs of *S. cryophilus* along the reference genomes using the synteny relationships.

Supplementary Figure S2.

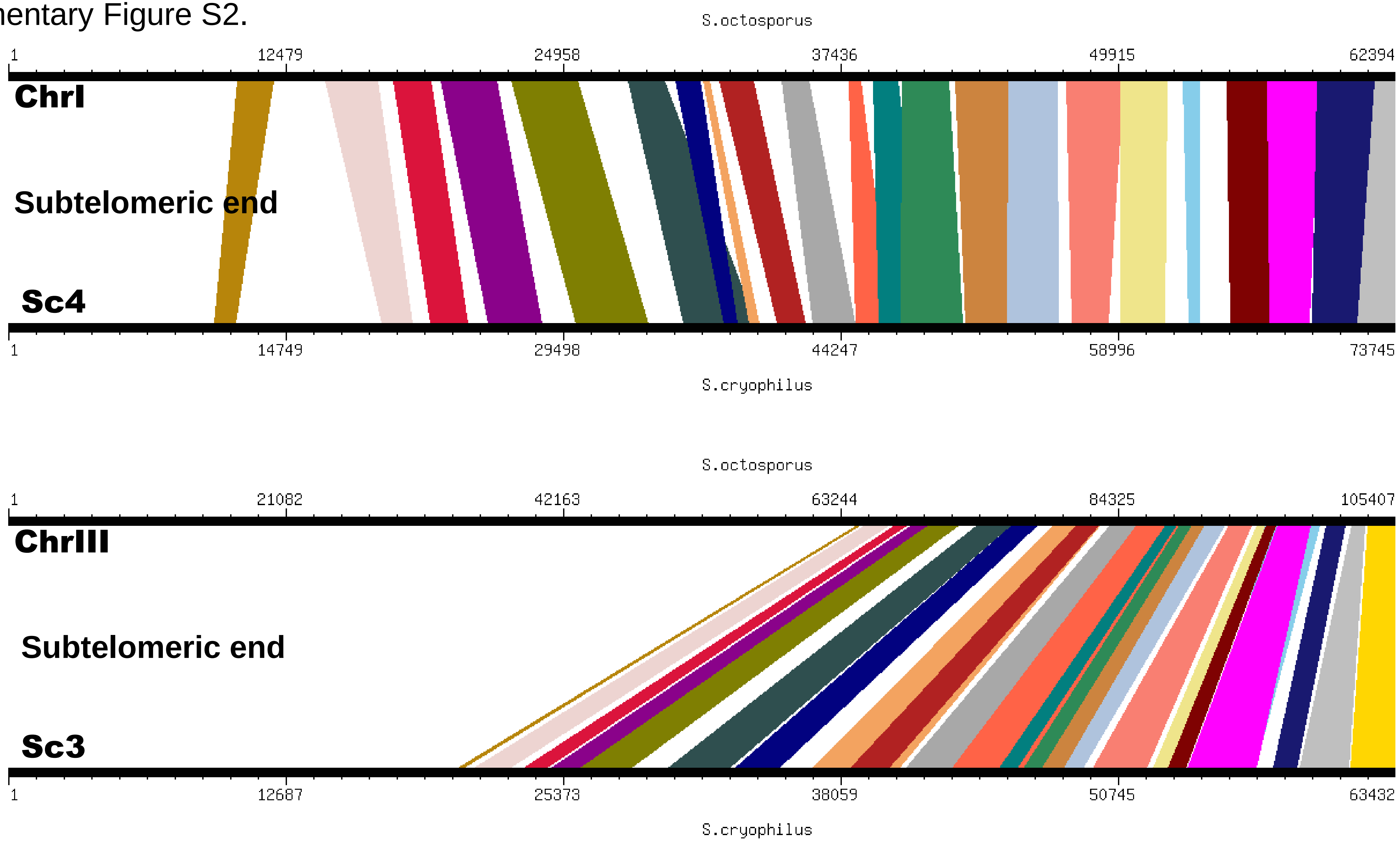

Supplementary Figure S2. Syntenic relationships of the subtelomeric regions of *S. octosporus* and *S. cryophilus* ChrI/Sc4 and ChrIII/Sc3.

Supplementary Figure S3.

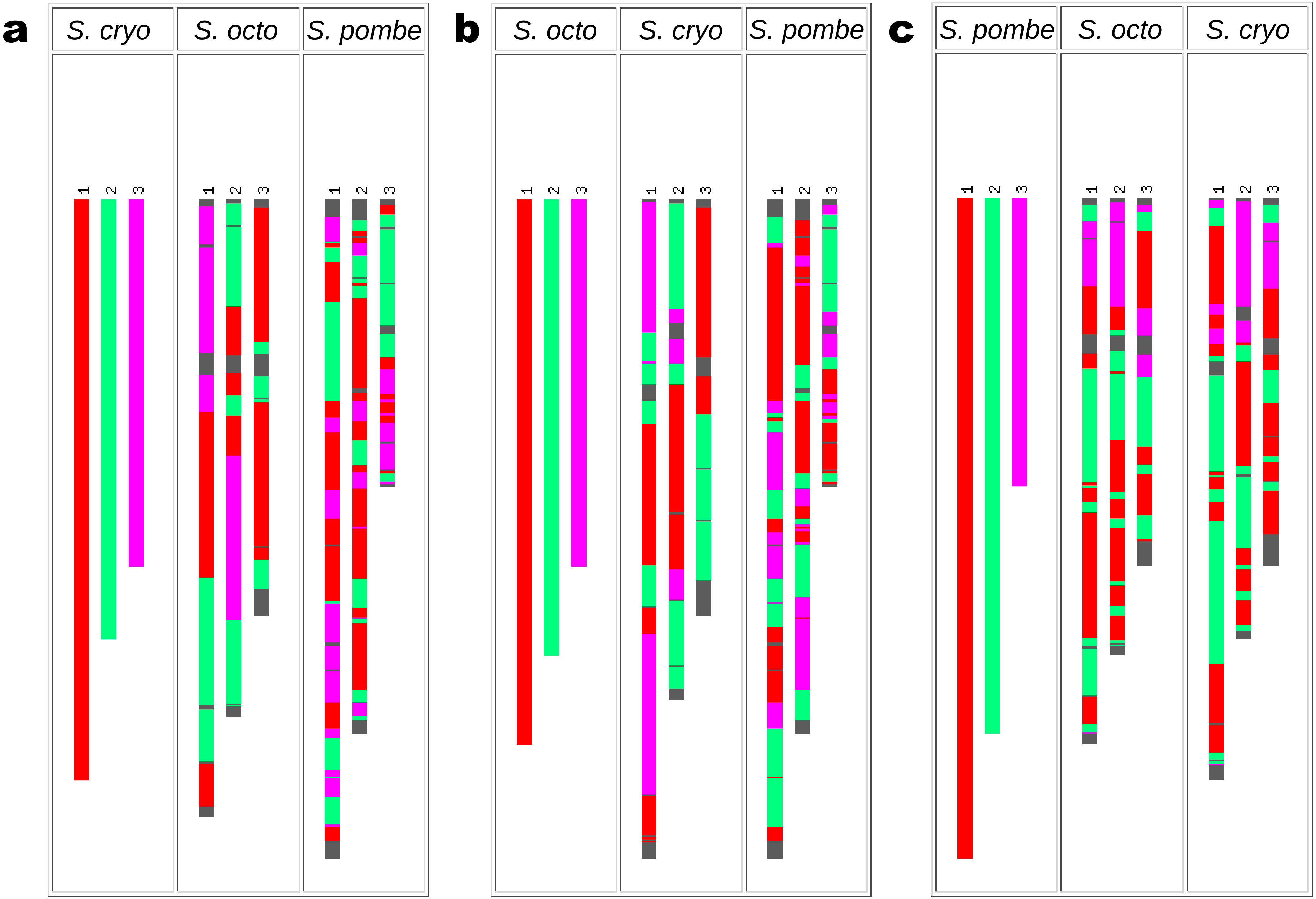

Supplementary Figure S3. Syntenic relationships of the fission yeast species depending 4580 1:1:1 orthologous proteins displayed by OrthoClusterDB. (**a)** Using *S. cryophilus* as reference. (**b)** Using *S. octosporus* as reference. (**c)** Using *S. pombe* as reference. The sizes of the individual chromosomes are not to scale except of image **c**. We observed that the chromosome lengths of the chosen reference genome can influence the representation of the lengths of other chromosomes during the image creation of OrthoClusterDB online platform.

Supplementary Figure S4.

a

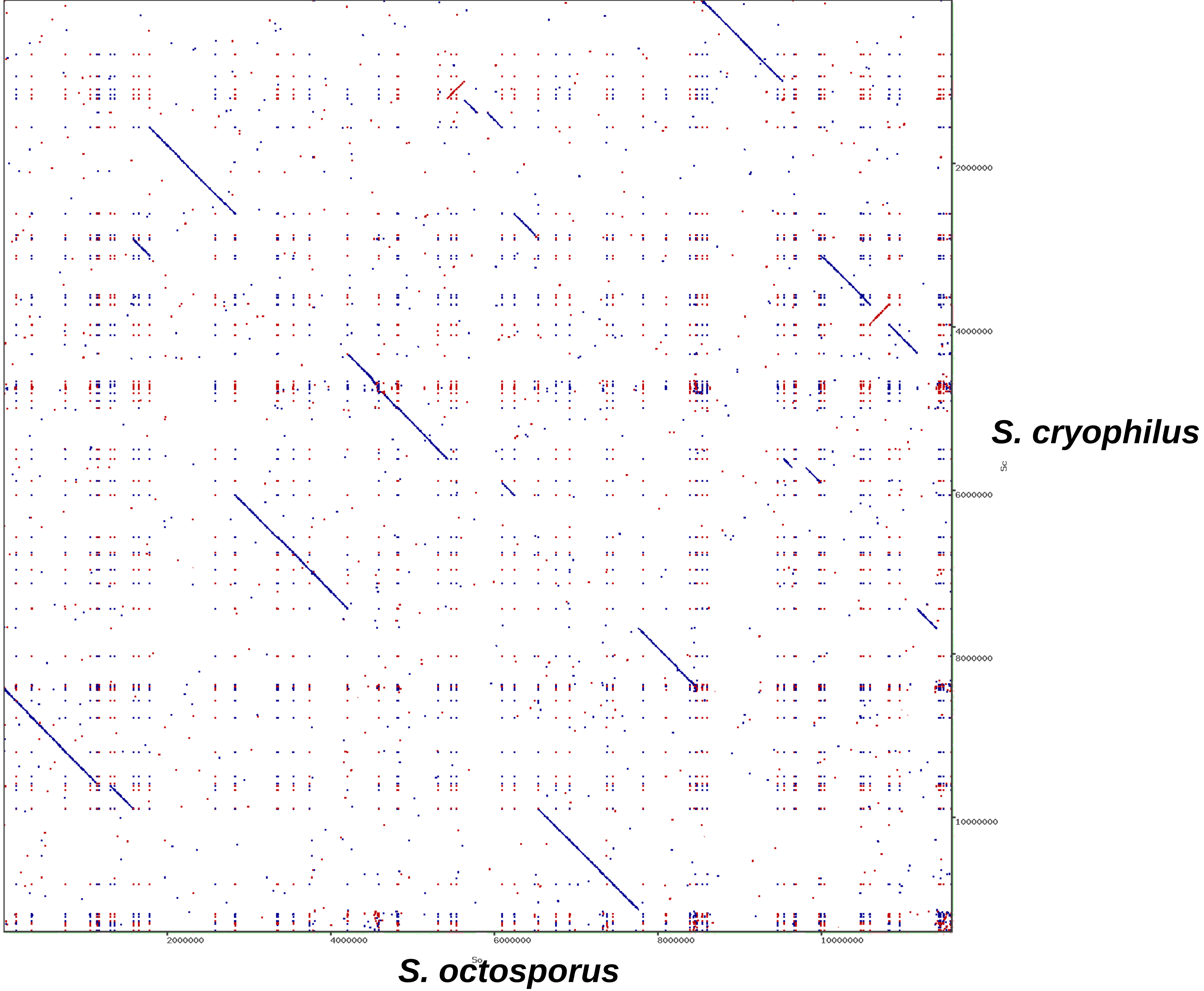

Supplementary Figure S4.

b

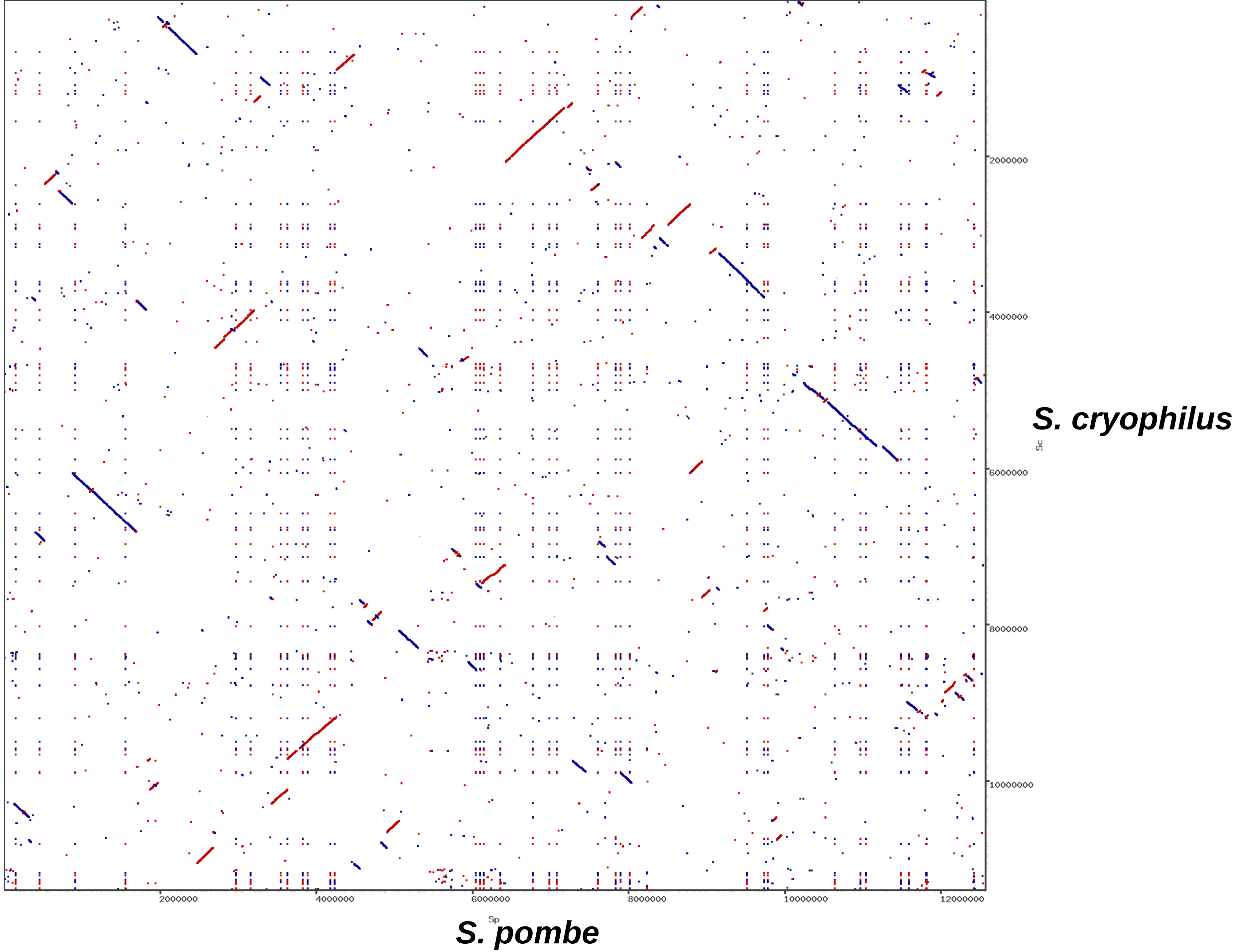

Supplementary Figure S4.

c

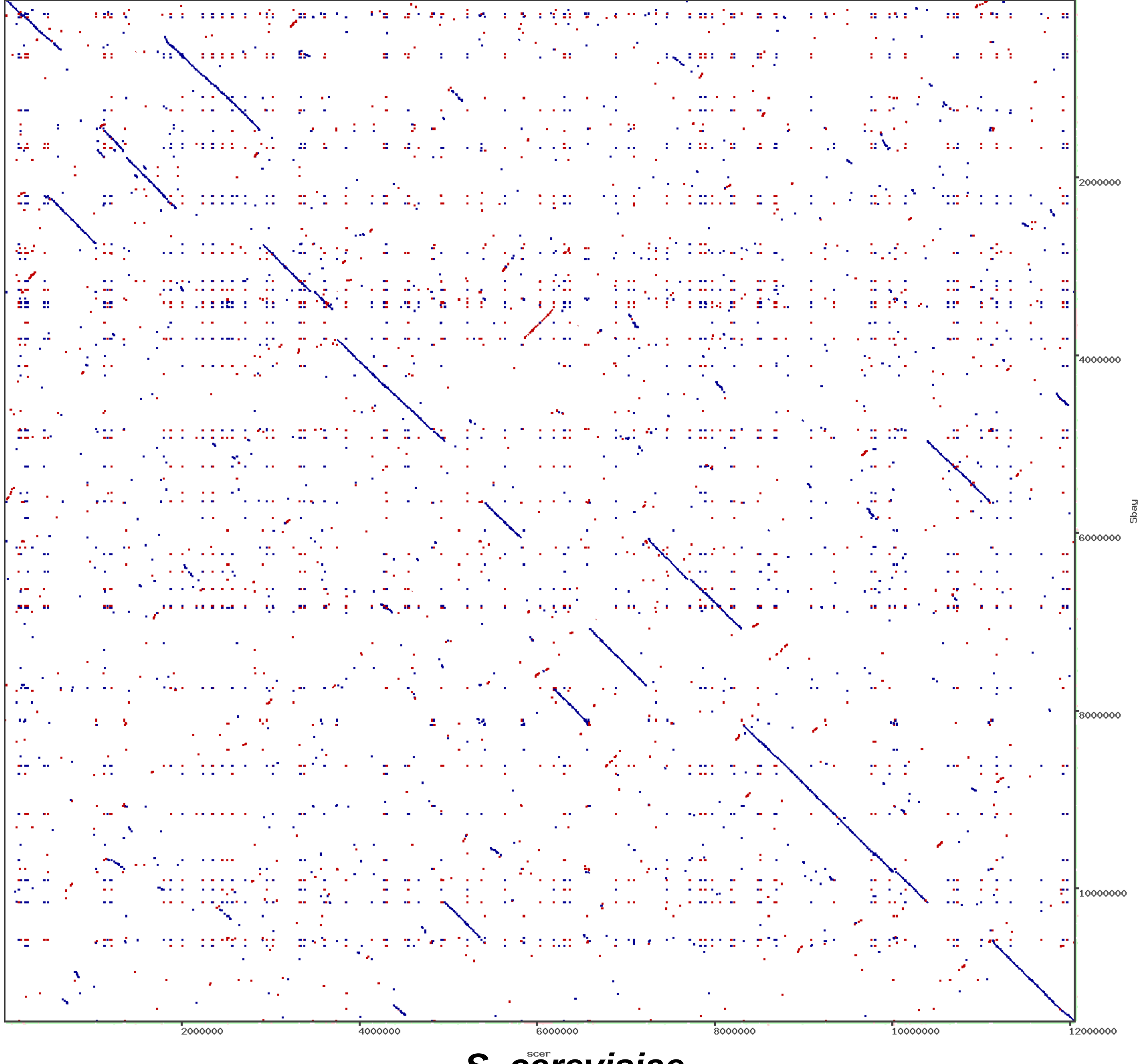

S. uvarum

S. cerevisiae

Supplementary Figure S4.

d

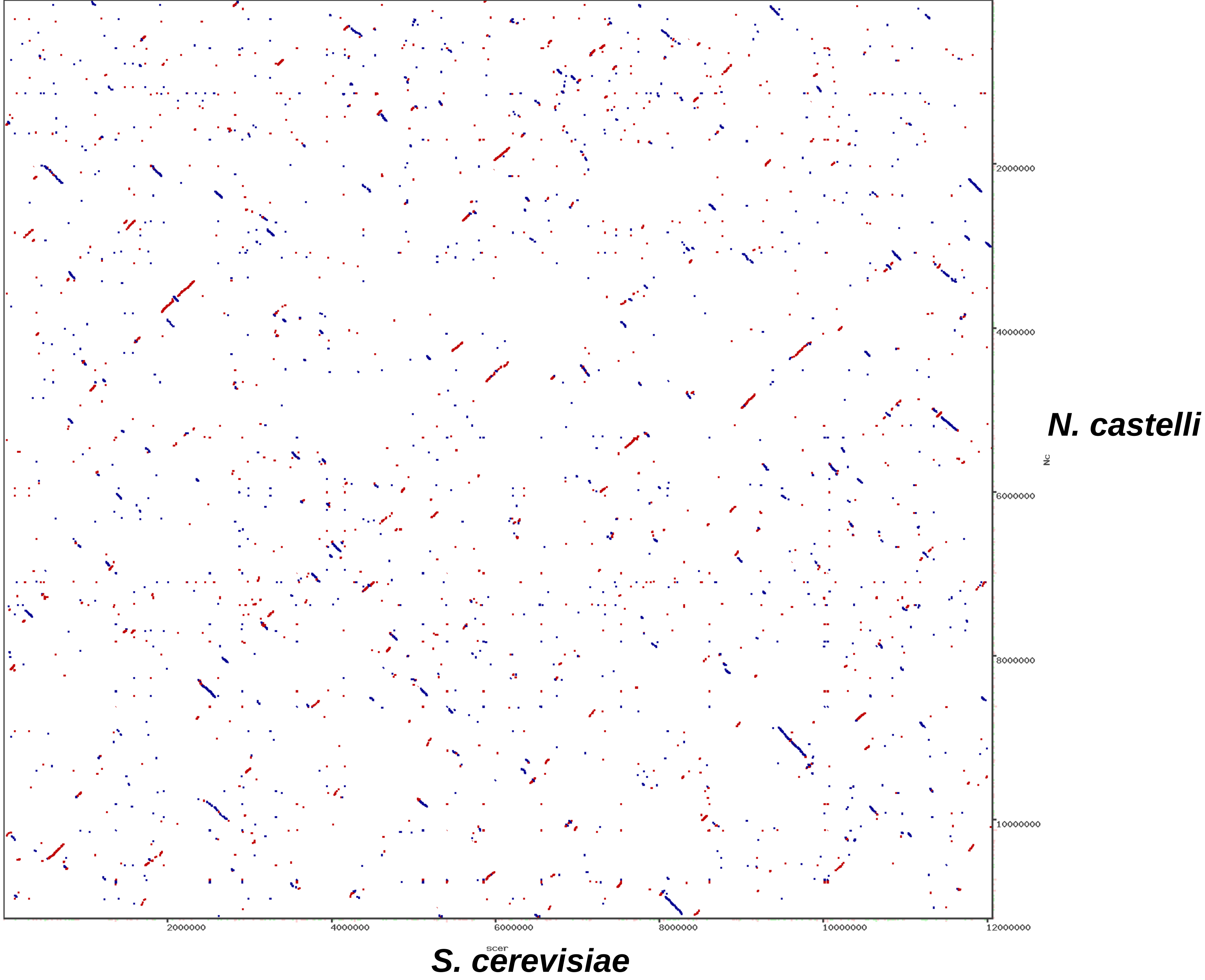

Supplementary Figure S4.

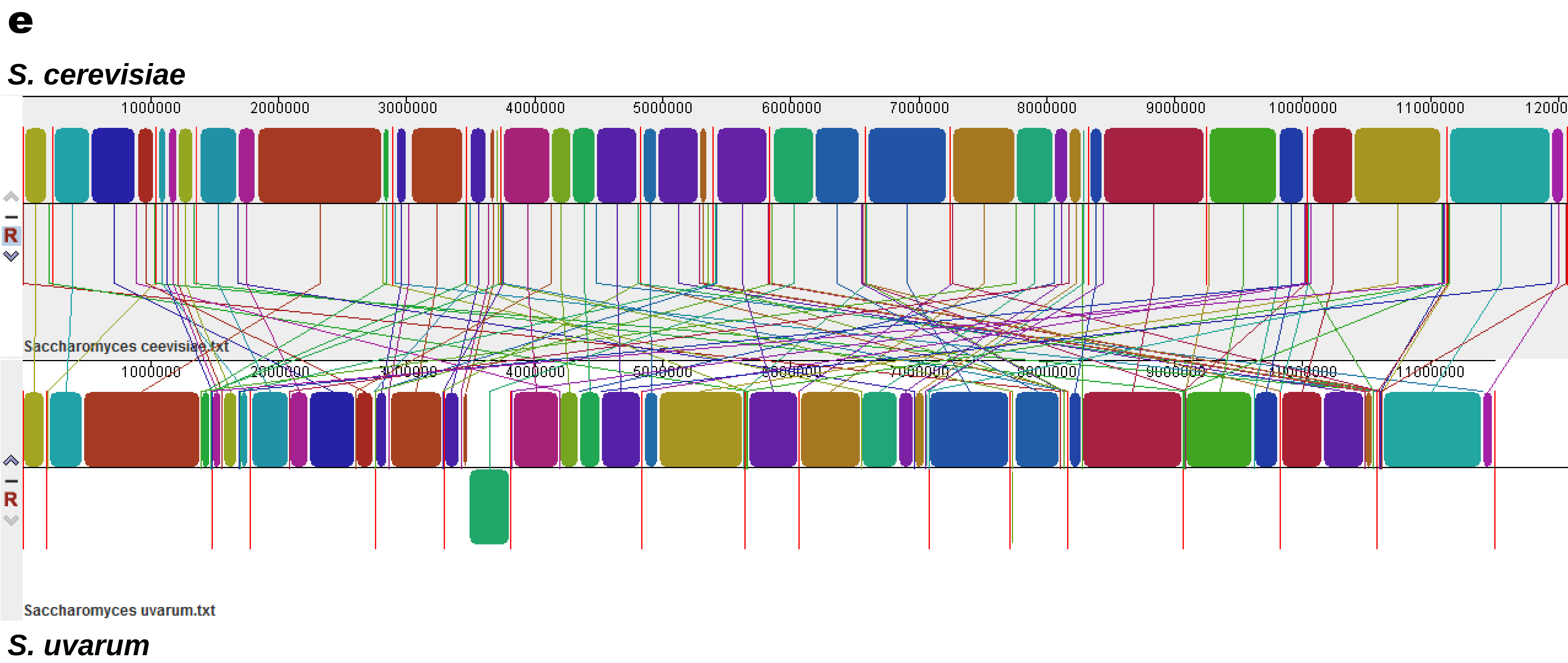

Supplementary Figure S4.

f

S. cerevisiae

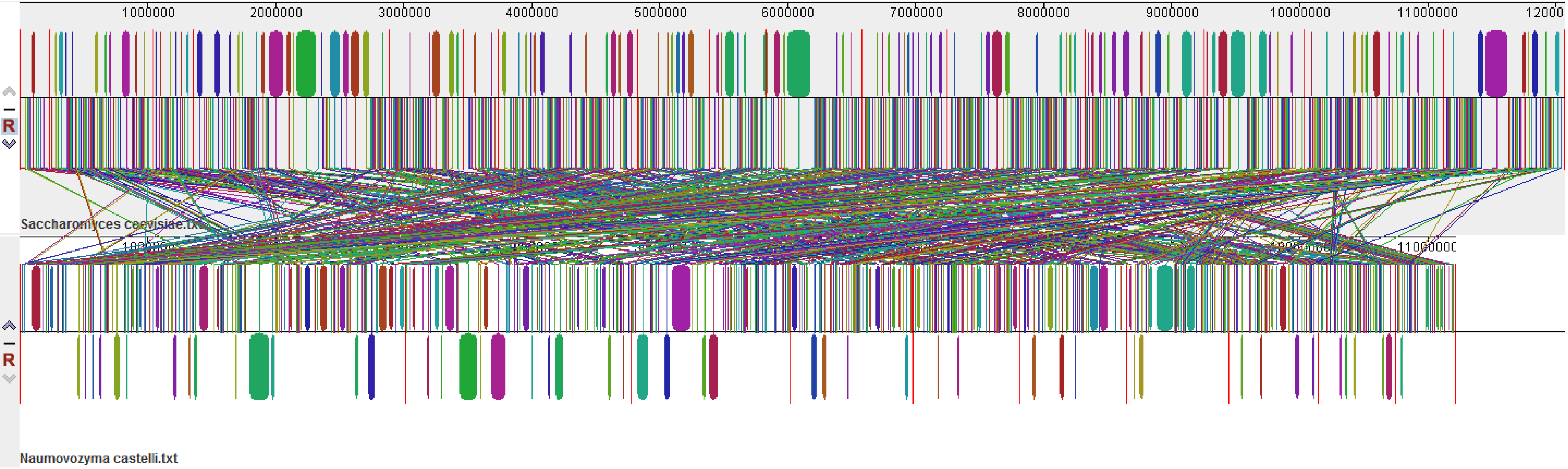

N. castelli

Supplementary Figure S4. Pairwise whole genome alignments with YASS and Mauve between *S. octosporus* – *S. cryophilus* (**a**); *S. pombe* – *S. cryophilus* (**b**); *S. cerevisiae* - *S. uvarum* (**c**, **e**) and *S. cerevisiae* - *N. castellii* (**d**, **f**).

Supplementary Figure S5.

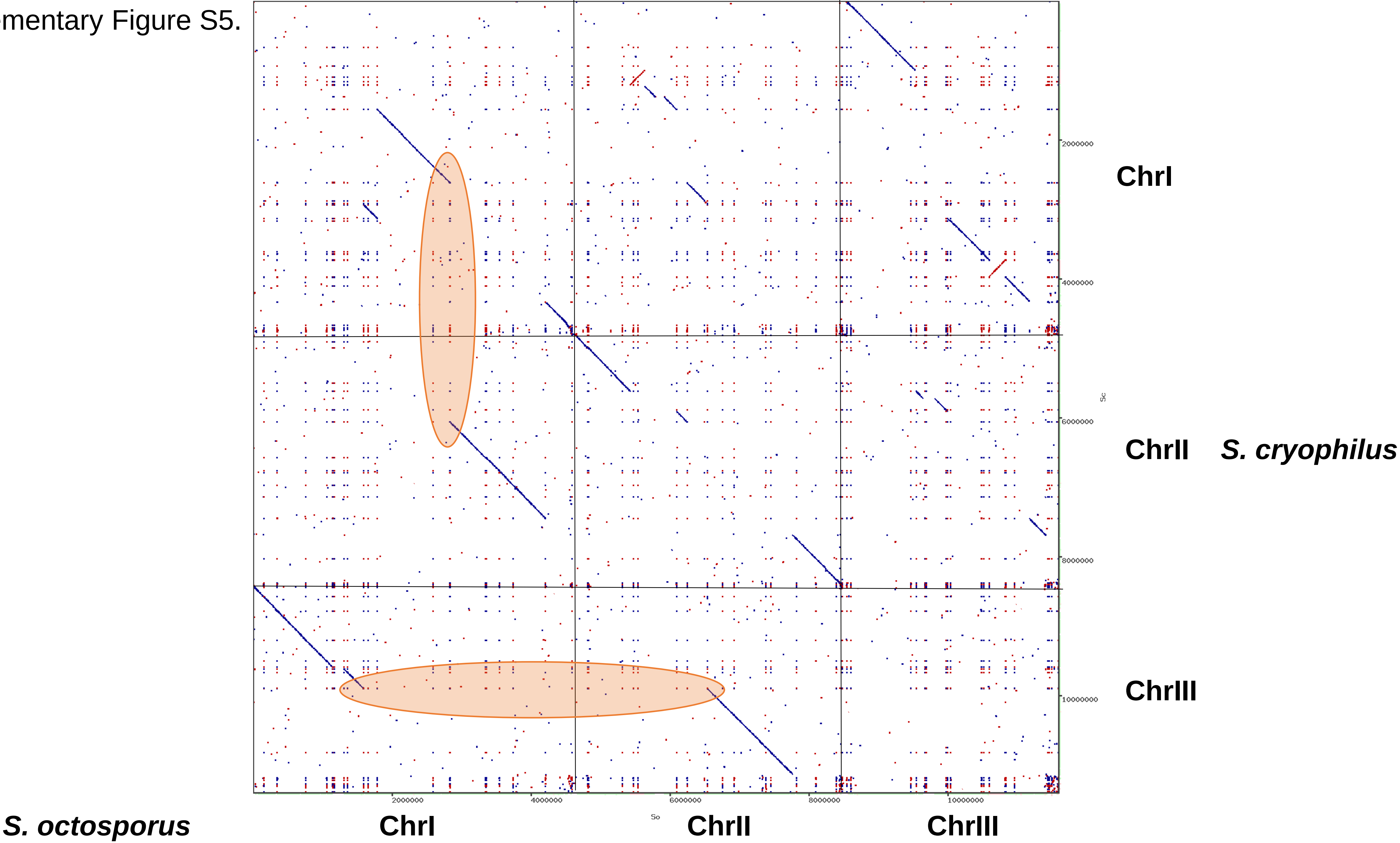

Supplementary Figure S5. Pairwise whole genome alignment of *S. cryophilus* and *S. octosporus* with YASS. Highlighted regions are examples of the disperse localisations of 5S rDNAs across the genomes.

Supplementary Figure S6.

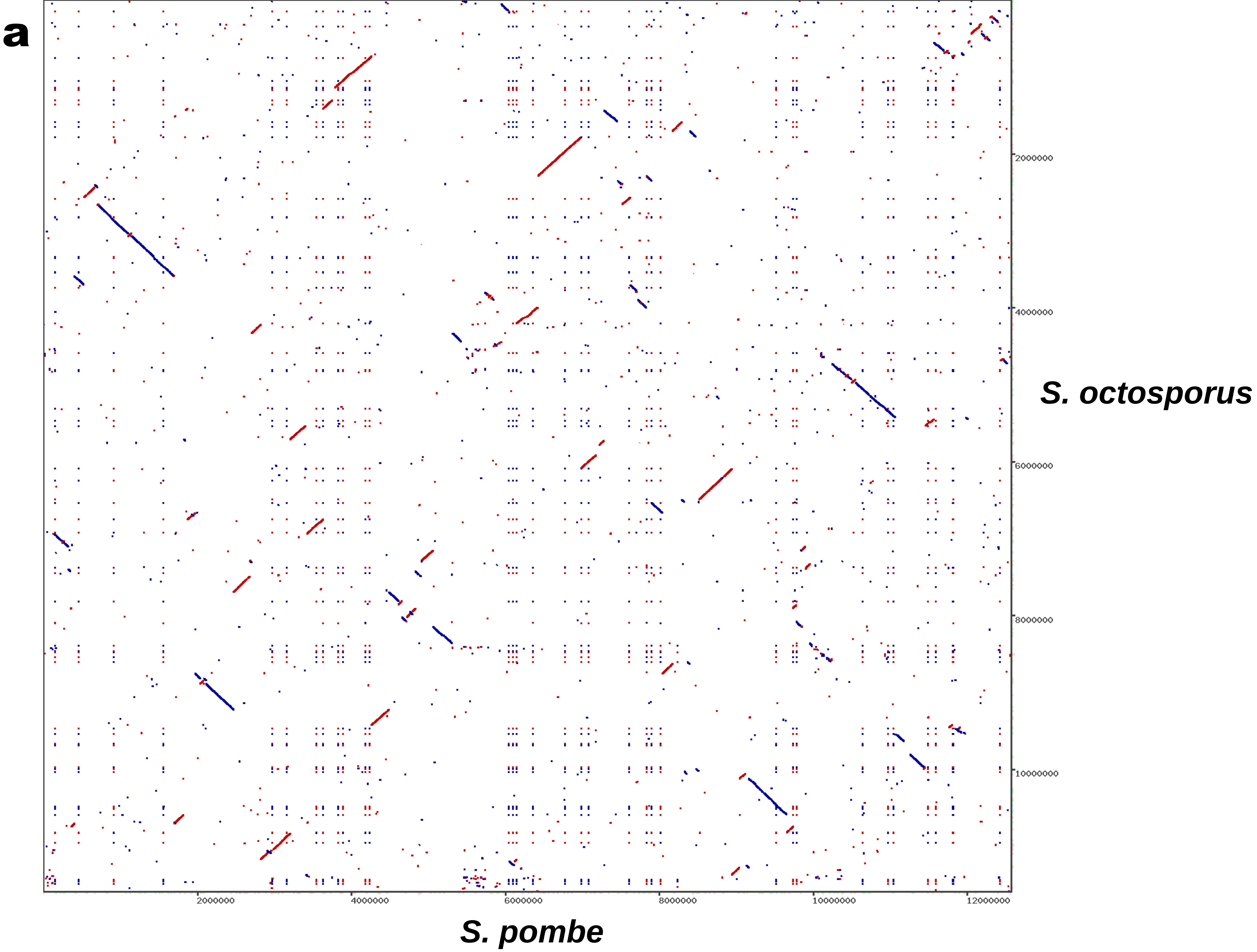

Supplementary Figure S6.

b

S. pombe

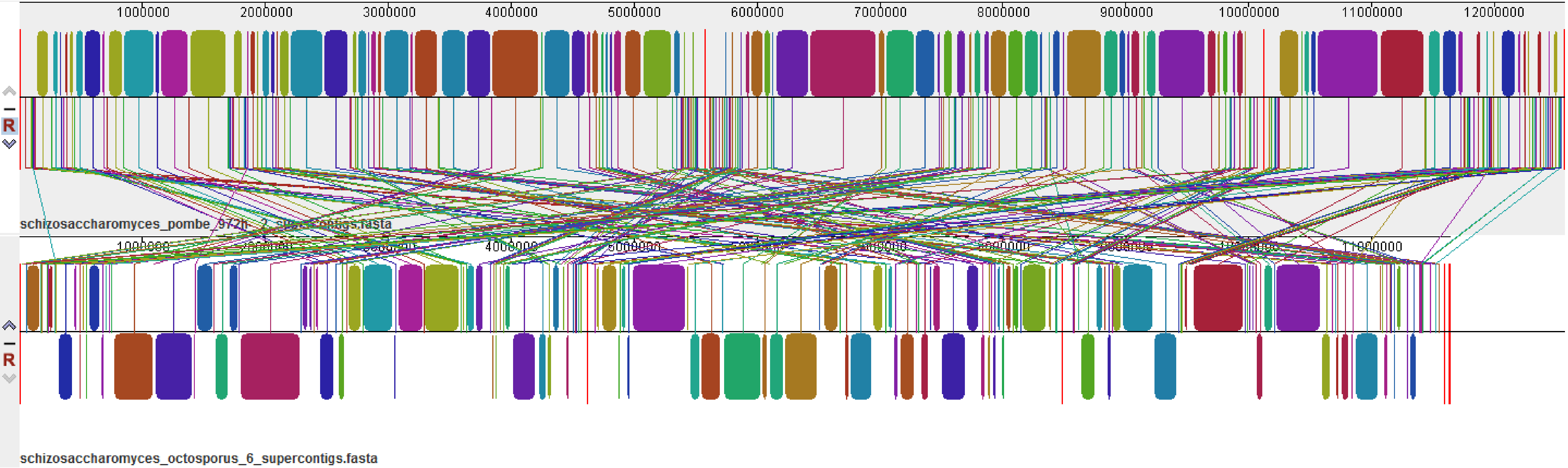

S. octosporus

Supplementary Figure S6. Pairwise whole genome alignment of *S. pombe* and *S. octosporus* with YASS (**a**) and with Mauve (**b**).

Supplementary Figure S7.

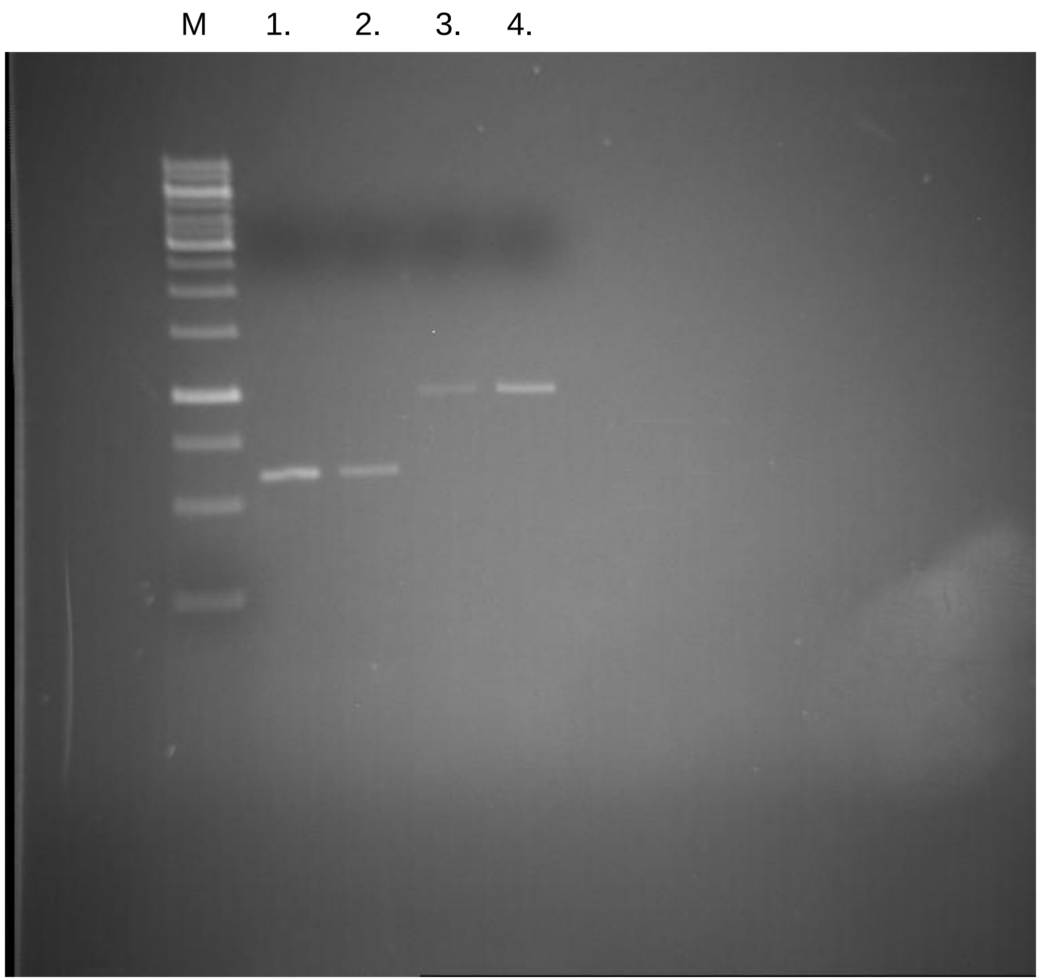

Supplementary Figure S7. PCR validation of togetherness of Sc9-Sc1 (lane: 1., 2.) and Sc5-Sc8 (lane: 3., 4.) supercontig pairs. M: 1kb DNA ladder. Gel electrophoresis was carried out in 1% agarose gel.

Supplementary Figure S8.

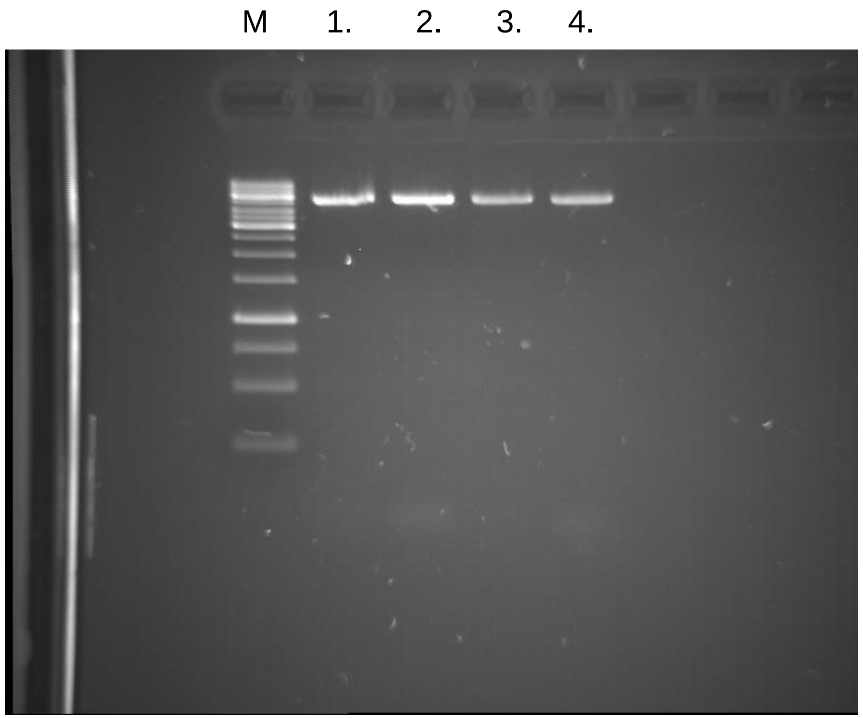

Supplementary Figure S8. PCR validation of togetherness of Sc8-Sc6 (lane: 1., 2., 3., 4.) supercontig pairs. M: 1kb DNA ladder. Gel electrophoresis was carried out in 1% agarose gel.

Supplementary Figure S9.

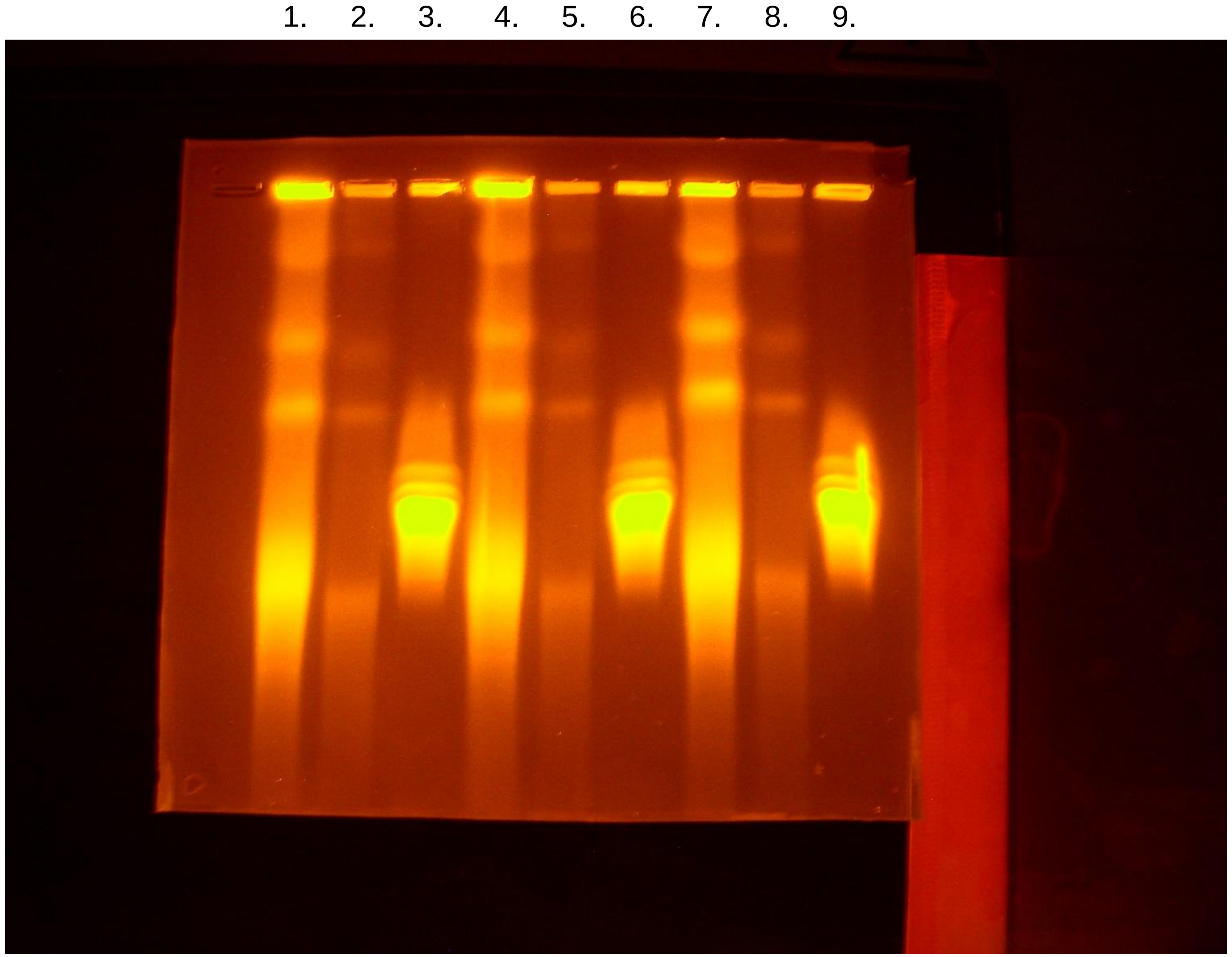

Supplementary Figure S9. Pulsed-field gel electrophoresis of the chromosomal DNA of *S. pombe* (L972) (lane: 1., 4., 7.) *S. cryophilus* (OY26) (lane: 2., 5., 8.) and *S. cerevisiae* (S288C) (lane: 3., 6., 9.).

Supplementary Figure S10.

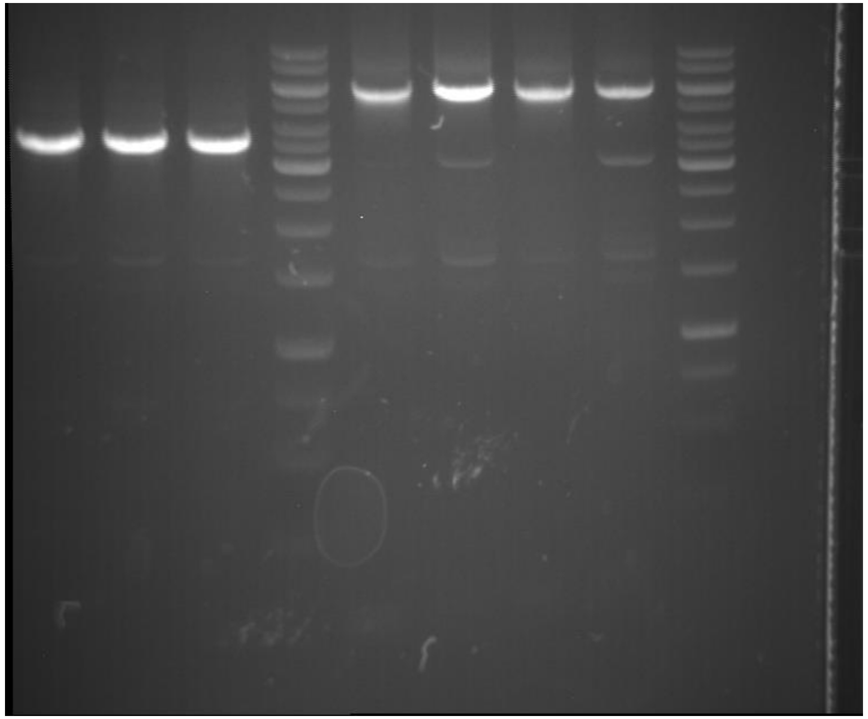

Supplementary Figure S10. PCR validation of the localization of rDNAs on *S. cryophilus* Sc7 (ChrII). M: 1kb DNA ladder. Primers 926-928 (lane 1., 2., 3.) and primers 926-50 (lane: 4., 5., 6., 7.) Gel electrophoresis was carried out in 1% agarose gel.
