# Supplementary material for: Assembly of Schizosaccharomyces cryophilus chromosomes and their comparative genomic analyses revealed principles of genome evolution of the haploid fission yeasts
Source: Sci Rep. 2018 Oct 2;8:14629. doi: 10.1038/s41598-018-32525-9 (PMC6168568; doi:10.1038/s41598-018-32525-9)
Supplement: Supplementary file 6 — Supplementary file 1 [file 41598_2018_32525_MOESM6_ESM.pdf]

Supplementary information

Assembly of *Schizosaccharomyces cryophilus* chromosomes and their comparative genomic analyses revealed principles of genome evolution of the haploid fission yeasts

Lajos Ács-Szabó, László Attila Papp, Zsuzsa Antunovics, Matthias Sipiczki and Ida Miklós
Department of Genetics and Applied Microbiology, Faculty of Science and Technology, University of Debrecen, Hungary

Correspondence: Ida Miklós, Department of Genetics and Applied Microbiology, Faculty of Science and Technology, University of Debrecen, 4032 Debrecen, Egyetem tér 1., Hungary
E-mail: miklos.ida@science.unideb.hu
Tel: +36-52-518-600/62056

General description: Here we provided the sequences of the concerning supercontig (Sc) ends and the confirmation of their overlaps and adjacency by Sanger sequencing. We highlighted the sequences as follows: light blue and grey represent sequences of the Scs. Green highlight show the positions of the primers used for the PCR and for the sequencing. Primers were designed to hybridize to the corresponding contig ends of the concerning Scs (see methods in the main text). Yellow highlight indicate results of the sequencing, which were in good agreement with the lengths of the PCR products in the case of Sc9-Sc1 and Sc5-Sc8. In the case of Sc8-Sc6 we did not sequence the entirety of the amplified product, we just validated the togetherness of these Scs. "+" and "-" symbols in the brackets correspond to the original orientation of the Scs available in the corresponding databases. Results of the sequencing are available in Genbank under the accession numbers: MH605091- MH605096.

Sc9 and Sc1 overlapping contig ends and sequencing

>Sc9_end (-) & Sc1_end (-)

atatgtgaattaagaagaaagttatgggaagatgataagttgctgcataactggcaacctagtcattctcattgatagaagtaaaaagacgtaac
atatttgtaccaaaaaaatgaatcgaaattatccggacctgtctggttcttgttcctgtatgctatacgctattgatcgttcatcatcgttctgattttc
ggcttgcttgatttcctgggatcctacgacgtatgtagttgcaatatcatcacaagttggcaccgtcaggttaaactcttcgttgaattttctataaaa
caatcagtaacgtgtccaaaagttaattgcaataggggaatgcatacccgtgcagtttacatccccaagctgttgctgctttagaagaaccagttt
gaggagggtactttcggattaatttttgcgagacaactgagcattcaccgcaagggaacaaggaagcagatgaaacaatataatgaaacaatat
atttctttcatatagtgtaggcaagcttggataattggctaaagtagtatggaaagactaaacaaataattagcatgcgagacatcaagactcatt
tgggcttacagtccaataatttttcatcatttcgtttctatttagattttataattagtaaagtaatgacagcaagaattgtcgtaatgaaaccattgg
gtatcacgacatagtttttgatcatttcattccttaccttgtctcttccttgtggtttaaaggagtgataacaacaacctcttcacttttctctttattttc
gccaagttgcctgagttcaggcatgtataggcgccattctttgtcaatcattacgctttgaaatacccatattacaaaacccacaattagaactgtt
gataaaagtatctgtatcctcttgttaaacaacatctttataaaaattgatacatcttcagcgtttaataaatggaagttgcggttgaagtttaagtc
agagggtactgatatttagtttgtttactttaccatcaatactgaagtttgccctacagttcattatatgtggcttcgtcaggcctctcattccacaaa
ggcaattagatgtggatttatgtgaagtcacaatattaccaagtgatttccaaggtatattactataaagcgtagatagtttatggccgccacagtt
gtgatgtcataattgggtccctcgttagcattcataatataacaattcgttataagaatggaatattagctaatatcagttaatattcataagcttaa
agaattgtgcttgataataagtaaacaaaaatttgaaaatgcaaatttctctttgattgcagaaactccgattaaaatttacaattcgtgattattg
ctattaattagagaagccagtcacgatggcaaatatacagaataatatatatatgtgtgtcgacagtcgaaatctttagatcggaagatttgactg
atcaggctaaataagattgacacttagtcgttttcatttagttaatcagatccctggacatattactattactctacgtagtactaacacgtccaatt
ccaatagacaattttgtaattgtacttattacttccactcgatttacattctcataacgtgtgagatttctcccgtgacatgtgaattgtcagagcaac
cactaatattattacaaattattttgtaataattatcctatggagtatattactgtataccaatgtttataaacaaacaaactgagaaagcagacgg
aaagtttaaccaatcatcaagaattagctcaaccttattgtttgtttacttactagttttagattactatataaacgccgttctcctcagccaaaatct
tcagttaggaagattttgctggcagagctaataaacttgttcgataacatattcattagtatcaccagagacgttactaaagatatatcactccaga
tatttagtaacttattttagagtatccagcttgacaattcaagcaattataactgtattccactcacacacttattactatacggagtattttcccgtg
acatgaatgtcgtaggactgtataattattttatccattttgtcacgggagaaatctcatacgttatgagaatgtaaatcgagtggaagtaataagt
acaattacaaagttgtctattggaattggacgtgttagtactacgtagaagaatagtaatatgtccagggatctggataactaaatgaaacgact
aagagtcaatcttatttatcctggtcagtcaagtcttctgatctgaagatttcgactgtcaacacacatatatatattgttctgtatatttgccatcgtg
actggcttctctaattaatagcaatgatcacgaattgtaaattttaatcggagtttctgcattcaaagagaaatttgcattttcaatttttttgtttactt
attatcaagcacaattctttaagcttatgaatattaacttatattagctaatattccattcttataacggattgttatattatgaatgctaacgaggga
tccaattatgacacctccctgcctaagagtctttcacttctttgaattggaagactcttattttcattgcggttcttctgttggagaagcgcgacgatt
aaattcaatgagctttgagtatgcctttttttcgaacgatgtctctaggaacccatgtgactgagcgatagtcataatctgagagttgcgatctatata
aacaattgtagtgagaaggcttttctaggttatcagcttctaaaatttcgtcaataacgatgttttgcataaaattgtttgagcttaagtccgcgttgt
aaggttcgagttcggatacatgaaaactagcaggatagcgtcttcgtatcgtttcaggaaaatttaactcgtagttatgttctcctatttttcttttta
ctgaaaatggaccaacaaatgttggttcaagtttatttgattgatggaaaaatcccactttgccatttttgactaatacctgatcgttaggttggaaa
atcggtggtggaattgtttttttggttgtaatactttgcgtatttatcatttgcttttctgatacgttcacgtatttcatcgatggattctgttctgtccggt
aaataaggaatatctagttcatccaggatttgcgtatcccaagcggtaactatagcttgaataccaaaagcagcattaattggagctatttttgtag
cagaatgctgtgaagagttataacataattgagctttgagaagataattgacccaatttgttaaatgtttgttcacgtagcaacgaagatatgact
ccaagatttgattgacgcg

Sc5 and Sc8 overlapping contig ends and sequencing

>Sc5_end (-) & Sc8_end (-)

gaaaatcctgtctccatgtctaaatgcatgaactcttggtcatgcaaaaaccttgtttttttcatgcaagaaaaccaagcaatagctacttccgatg
tgcatgggaggagggaatagattaaaaggaacaaaactcgaagtaattgcagttgtaggtggaaatagaagaaataccaatgagctagagtcg
cttctcctagtaaagtgtactgaattggagcagcctgatcggagagttcattaaagtaggcgattaagtcaatacaataggcggacggttctcgag
gatttgatttcttggattgttccaaagttgcaacaaccatgtattttgaagctttataccagaaatgatttggatctgcgcaagcacgaagcaaaaa
gacaaataccttccaacaagaaaatagctcagcataatatcgcttttggaattcctgaacaaggaatgcatcatcatttgagtatgaagagtattt
aagcataaattcatcgttgtaggagagaatcacatacaggacttcggcttcatcaaatcctgttaagtccgataacctccttgcaaattccttttga
gactttttcactgggtatgaatgtttatatacagacaaaacgcaatcttcttcttctttttcagtagcttttgaagcttgcacgcttccccgcaaagga
tatcgaatgttttggaatgcagaaaccctttcagctaccaaactctcaaacgtttcctttgaaatgctttcattttgagcttgaaaactgcttaagatc
caagcccaagagctacacatttagtcagtaaattgaacacaaaaacaaatcttgctaaattcaagacaaccacatacattagggagtcacccat
ggaaaaaagactgacgaaccgacaaactacaaatcaaaaaagaatgatttcaggacgaaagttgattccgttctaactcttataacgtctttcaa
ttgtttcttttgtatgggttttgcaacgagtactactactaagtcgcttaccataccacggaatagtgtcccacaatgtaagccaacgccgcgttgtt
aagattcactaataaacaaactatctctaaaaattcgataattgaaaaaaacgaatttacttgcaaacatgggcttcaagctgccacattttaatg
gacttcttattgaaaatgagagattgcttcttactaggttactttctgtttccttttagttgtatatttacaggtaaagctccaagttttacaaaacata
tttgaagagtatgaaagacaagctttatagttctttaacaaactaaatattggtacaacaagtgaattgacttgaatgaaaaaacgaaatcactc
gttttaatgaaggatcattgtttagtagcaagaaaaacgccgtctagttattatttaggaataacgaataatataattattatggcaatataaacta
tattaataaggtaataaacgtgtcacgggagaaatctcacacgttatgagaatgtaaatcgagtggaagtaataagtacaattacaaaattgtct
attggaattggacgtgttagtactacgtagagtaatagtaatatgtccagggatctgattaactaaatgaaaacgactaagtgtcaatcttatttag
cctgatcagtcaaatcttccgatctaaagatttcgactgtcgacacacatatatatattattctgtatatttgccatcgtgactggcttctctaattaat
agcaataatcacgaattgtaaattttaatcggagtttctgcaatcaaagagaaatttgcattttcaaatttttgtttacttattatcaagcacaattct
ttaagcttatgaatattaactgatattagctaatattccattcttataacgaattgttatattatgaatgctaacgagggacccaattatgacaaaac
gttcgttttgtattagaaattgtattagaaattgaatgaatacgttcatcttattacaacaaatagccccagtatactaaaaaaatcttaaccacaa
ccgttaactgaattgacgactcgtctttgagatattcgcaaagtaattttaaaagggtatattgtttatatgaataaatggaaatgctaaagagatc
gcgttctaaccaatatacttagagcttaaggtttggcaaacagaaaaggctctaaaacaaacgagcctttcgtcacgtattcattctgactataaa
aatcatccattaggagttttcttctactgctcaagaataggagctcaattgctttttttctttttctttaagggtgattgattttcctcatagggattctaa
tctgataatcacttcctatgcaataacccttcgtctcatggccagttagaaggtaaaaaaaaaagagattttgttattattgttcttattttcgccag
acagaagaaggttctggtctgaagagaatccaactcaaatccctttcctaaaatttctcttgtttcactgttataatataccttacttttgacattcca
gttatccccaatccattcgttacgctttttacgactacctttttttttttttttttttaagcttttggtctttgtcttttcaactgatcatatcttcgtccttc
accattcccatagtccatgggcagtcagcaatcactactccaattaaagaatgcttccttacgattaactgaggataagaacatccccaggacgg
ataatatatgggaaagcttttggtccttaccggaaagcttggaagaggtgctagaacaaatttctgttgttgatgttcgaaatgcgctaagtaaag
caccggaaaacatacatatgctggttgatatactattcaagaagctggtttctttgcagacggatagtgatttttctatacattctaatgaccttgcg
acaaaacatgtcctgaatagcatgcgtgtattgaccagattaataccccttttgtttgagtctccagaatgcgcttcttggtttaacaagtacatgtg
gttaccgcaggaaagcgggataccccgaggtgttaatattatggacactatcataaactatctatttctgactaattttacaatccctcccattgac
gaattcacaaaaggagttagatactgtatttgggaaactggtattgcatgccagtctcccttgaataaagaagtaaactttatggcgaattctgtc
gaggttcttcgtcttttgt

Sc8 and Sc6 contig ends and sequencing

>Sc8_end (+) & Sc6_end (-)

gtaaactacaattccgcgttagttacttcttgtccgtcaaatcccttcaaacaaagcttgtctatgatgtacctcattccaatatgtgttttccaataa
cttctccgcagcgttcaattgggttttcctttcttctggagaaagtttttcaagcttggtgcgaaatggggtatccaaagttttaggaaaagaaagcc
atgggcgcaactcgtcctttggccaaagagagacaatccgcgttaaacgactactcattcttacaaactgatttctttacggaaactgctttttaat
ccaacttactttgctcgcacatgtggaacctgtctgttggaccaatagttctttttgatttaagcattcttacgatagatcaccttaacctctttagcg
accgttaacacgtccgtcaaacttcactaaaaacgatgaatgtaaatagagaaaaaataatagggattctcgaatcactcaatttttgttcacgct
tttatatgcaacttgaatagatgttcttgtctatatgattccgataattcattttacgccaggaattgtgctactaaagagcgagactgcttgtgtcat
atcaaagccaattggtttgcatagaaaacataaacgcaagaaatctatttaccagtgcataatttccttactacctgttatatatttattgaattcag
atagtttcaacaagcggcgatgcccgagtggtctaagggggcagatttaagaggctttgcgccttgaaccctgctgttgtaaaacgcgtgagttcg
aacctcactcgccgcaaaatttcttttatcatttttatagcactaactcggtaaattacactataccaaggtaaggtaggaagtatataataggttg
agtctcctttctttaactactcggtaaagctactgtctattagtctcattaagaaattgtcataattgggtccctcgttagcattcataatataacaatt
cgttataagaatggaatattagctaatatcagttaatattcataagcttaaagaattgtgcttgataataagtaaacaaaaatttgaaaatgcaaa
tttctctttgattgcagaaactccgattaaaatttacaattcgtgattattgctattaattagagaagccagtcacgatggcaaatatacagaataat
atatatatgtgtgtcgacagtcgaaatctttagatcggaagatttgactgatcaggctaaataagattgacacttagtcgttttcatttagttaatca
gatccctggacatattactattactctacgtagtactaacacgtccaattccaatagacaattttgtaattgtacttattacttccactcgatttacatt
ctcataacgtgtgagatttctcccgtgacaaattatcttccgatctcgtagagtggaaattattgcaataaacttgcaaaagatatatataggatgg
aaatggactccgaaaacactgggggtggatacttcgctcaacatatggcaatacaagaacaagagataaatgctttaagccaacagataaactt
tttagttcaggctcaagaaaaaacggaagaaagaaatagccgaatggaaattgacgaagactatattccagaaaaacctctggacattatagct
tcggtacatgaaaaatatgggtctctagcagattcctatttaaatatgattcatccacttaatcctggaatatttgaaccatccgagcaatccgtag
cagtttttgggaaaaatctgcaaagWattttcggttacttgcctaaagaatttccannnnnnnnnnnnnnnnnnnnnnnnnnnnnnnnnn
nnnnnnnnnnnnnnnnnnnnnnnnnnnnnnnnnnnnnnnnnnnnnnnnnnnnnnnnnnnnnnnnnnnnnnnnnnnnnnnnnnnnnn
nnnnnnnnnnnnnnnnnnnnnnnnnnnnnnnnnnnnnnnnnnnnnnnnnnnnnnnnnnnnnnnnnnnnnnnnnnnnnnnnnnnnnn
nnnnnnnnnnnnnnnnnnnnnnnnnnnnnnnnnnnnnnnnnnnnnnnnnnnnnnnnnnnnnnnnnnnnnnnnnnnnnnnnnnnnnn
nnnnnnnnnnnnnnnnnnnnnnnnnnnnnnnnnnnnnnnnnnnnnnnnnnnnnnnnnnnnnnnnnnnnnnnnnnnnnnnnnnnnnn
nnnnnnnnnnnnnnnnnnnnnnnnnnnnnnnnnnnnnnnnnnnnnnnnnnnnnnnnnnnnnnnnnnnnnnnnnnnnnnnnnnnnnn
nnnnnnnnnnnnnnnnnnnnnnnnnnnnnnnnnnnnnnnnnnnnnnnnnnnnnnnnnnnnnnnnnnnnnnnnnnnnnnnnnnnnnn
nnnnnnnnnnnnnnnnnnnnnnnnnnnnnnnnnnnnnnnnnnnnnnnnnnnnnnnnnnnnnnnnnnnnnnnnnnnnnnnnnnnnnn
nnnnnnnnnnnnnnnnnnnnnnnnnnnnnnnnnnnnnnnnnnnnnnnnnnnnnnnnnnnnnnnnnnnnnnnnnnnnnnnnnnnnnn
nnnnnnnnnnnnnnnnnnnnnnnnnnnnnnnnnnnnnnnnnnnnnnnnnnnnnnnnnnnnnnnnnnnnnnnnnnnnnnnnnnnnnn
nnnnnnnnnnnnnnnnnnnnnnnnnnnnnnnnnnnnnnnnnnnnnnnnnnnnnnnnnnnnnnnnnnnnnnnnnnnnnnnnnnnnnn
nnnnnnnnnnnnnnnnnnnnnnnnnnnnnnnnnnnnnnnnnnnnnnnnnnnnnnnnnnnnnnnnnnnnnnnnnnnnnnnnnnnnnn
nnnnnnnnnnnnnnnnnnnnnnnnnnnnnnnnnnnnnnnnnnnnnnnnnnnnnnnnnnnnnnnnnnnnnnnnnnnnnnnnnnnnnn
nnnnnnnnnnnnnnnnnnnnnnnnnnnnnnnnnnnnnnnnnnnnnnnnnnnnnnnnnnnnnnnnnnnnnnnnnnnnnnnnnnnnnn
nnnnnnnnnnnnnnnnnnnnnnnnnnnnnnnnnnnnnnnnnnnnnnnnnnnnnnnnnnnnnnnnnnnnnnnnnnnnnnnnnnnnnn
nnnnnnnnnnnnnnnnnnnnnnnnnnnnnnnnnnnnnnnnnnnnnnnnnnnnnnnnnnnnnnnnnnnnnnnnnnnnnnnnnnnnnn
nnnnnnnnnnnnnnnnnnnnnnnnnnnnnnnnnnnnnnnnnnnnnnnnnnnnnnnnnnnnnnnnnnnnnnnnnnnnnnnnnnnnnn
nnnnnnnnnnnnnnnnnnnnnnnnnnnnnnnnnnnnnnnnnnnnnnnnnnnnnnnnnnnnnnnnnnnnnnnnnnnnnnnnnnnnnn
nnnnnnnnnnnnnnnnnnnnnnnnnnnnnnnnnnnnnnnnnnnnnnnnnnnnnnnnnnnnnnnnnnnnnnnnnnnnnnnnnnnnnn
nnnnnnnnnnnnnnnnnnnnnnnnnnnnnnnnnnnnnnnnnnnnnnnnnnnnnnnnnnnnnnnnnnnnnnnnnnnnnnnnnnnnnn
nnnnnnnnnnnnnnnnnnnnnnnnnnnnnnnnnnnnnnnnnnnnnnnnnnnnnnnnnnnnnnnnnnnnnnnnnnnnnnnnnnnnnn
nnnnnnnnnnnnnnnnnnnnnnnnnnnnnnnnnnnnnnnnnnnnnnnnnnnnnnnnnnnnnnnnnnnnnnnnnnnnnnnnnnnnnn
nnnnnnnnnnnnnnnnnnnnnnnnnnnnnnnnnnnnnnnnnnnnnnnnnnnnnnnnnnnnnnnnnnnnnnnnnnnnnnnnnnnnnn
nnnnnnnnnnnnnnnnnnnnnnnnnnnnnnnnnnnnnnnnnnnnnnnnnnnnnnnnnnnnnnnnnnnnnnnnnnnnnnnnnnnnnn
nnnnnnnnnnnnnnnnnnnnnnnnnnnnnnnnnnnnnnnnnnnnnnnnnnnnnnnnnnnnnnnnnnnnnnnnnnnnnnnnnnnnnn
nnnnnnnnnnnnnnnnnnnnnnnnnnnnnnnnnnnnnnnnnnnnnnnnnnnnnnnnnnnnnnnnnnnnnnnnnnnnnnnnnnnnnn
nnnnnnnnnnnnnnnnnnnnnnnnnnnnnnnnnnnnnnnnnnnnnnnnnnnnnnnnnnnnnnnnnnnnnnnnnnnnnnnnnnnnnn
nnnnnnnnnnnnnnnnnnnnnnnnnnnnnnnnnnnnnnnnnnnnnnnnnnnnnnnnnnnnnnnnnnnnnnnnnnnnnnnnnnnnnn

nnnnnnnnnnnnnnnnnnnnnnnnnnnnnnnnnnnnnnnnnnnnnnnnnnnnnnnnnnnnnnnnnnnnnnnnnnnnnn
nnnnnnnnnnnnnnnnnnnnnnnnnnnnnnnnnnnnnnnnnnnnnnnnnnnnnnnnnnnnnnnnnnnnnnnnnnnnnn
nnnnnnnnnnnnnnnnnnnnnnnnnnnnnnnnnnnnnnnnnnnnnnnnnnnnnnnnnnnnnnnnnnnnnnnnnnnnnn
nnnnnnnnnnnnnnnnnnnnnnnnnnnnnnnnnnnnnnnnnnnnnnnnnnnnnnnnnnnnnnnnnnnnnnnnnnnnnn
nnnnnnnnnnnnnnnnnnnnnnnnnnnnnnnnnnnnnnnnnnnnnnnnnnnnnnnnnnnnnnnnnnnnnnnnnnnnnn
nnnnnnnnnnnnnnnnnnnnnnnnnnnnnnnnnnnnnnnnnnnnnnnnnnnnnnnnnnnnnnnnnnnnnnnnnnnnnn
nnnnnnnnnnnnnnnnnnnnnnnnnnnnnnnnnnnnnnnnnnnnnnnnnnnnnnnnnnnnnnnnnnnnnnnnnnnnnn
nnnnnnnnnnnnnnnnnnnnnnnnnnnnnnnnnnnnnnnnnnnnnnnnnnnnnnnnnnnnnnnnnnnnnnnnnnnnnn
nnnnnnnnnnnnnnnnnnnnnnnnnnnnnnnnnnnnnnnnnnnnnnnnnnnnnnnnnnnnnnnnnnnnnnnnnnnnnn
nnnnnnnnnnnnnnnnnnnnnnnnnnnnnnnnnnnnnnnnnnnnnnnnnnnnnnnnnnnnnnnnnnnnnnnnnnnnnn
nnnnnnnnnnnnnnnnnnnnnnnnnnnnnnnnnnnnnnnnnnnnnnnnnnnnnnnnnnnnnnnnnnnnnnnnnnnnnn
nnnnnnnnnnnnnnnnnnnnnnnnnnnnnnnnnnnnnnnnnnnnnnnnnnnnnnnnnnnnnnnnnnnnnnnnnnnnnn
nnnnnnnnnnnnnnnnnnnnnnnnnnnnnnnnnnnnnnnnnnnnnnnnnnnnnnnnnnnnnnnnnnnnnnnnnnnnnn
nnnnnnnnnnnnnnnnnnnnnnnnnnnnnnnnnnnnnnnnnnnnnnnnnnnnnnnnnnnnnnnnnnnnnnnnnnnnnn
nnnnnnnnnnnnnnnnnnnnnnnnnnnnnnnnnnnnnnnnnnnnnnnnnnnnnnnnnnnnnnnnnnnnnnnnnnnnnn
nnnnnnnnnnnnnnnnnnnnnnnnnnnnnnnnnnnnnnnnnnnnnnnnnnnnnnnnnnnnnnnnnnnnnnnnnnnnnn
nnnnnnnnnnnnnnnnnnnnnnnnnnnnnnnnnnnnnnnnnnnnnnnnnnnnnnnnnnnnnnnnnnnnnnnnnnnnnn
nnnnnnnnnnnnnnnnnnnnnnnnnnnnnnnnnnnnnnnnnnnnnnnnnnnnnnnnnnnnnnnnnnnnnnnnnnnnnn
nnnnnnnnnnnnnnnnnnnnnnnnnnnnnnnnnnnnnnnnnnnnnnnnnnnnnnnnnnnnnnnnnnnnnnnnnnnnnn
nnnnnnnnnnnnnnnnnnnnnnnnnnnnnnnnnnnnnnnnnnnnnnnnnnnnnnnnnnnnnnnnnnnnnnnnnnnnnn
nnnnnnnnnnnnnnnnnnnnnnnnnnnnnnnnnnnnnnnnnnnnnnnnnnnnnnnnnnnnnnnnnnnnnnnnnnnnnn
nnnnnnnnnnnnnnnnnnnnnnnnnnnnnnnnnnnnnnnnnnnnnnnnnnnnnnnnnnnnnnnnnnnnnnnnnnnnnn
nnnnnnnnnnnnnnnnnnnnnnnnnnnnnnnnnnnnnnnnnnnnnnnnnnnnnnnnnnnnnnnnnnnnnnnnnnnnnn
nnnnnnnnnnnnnnnnnnnnnnnnnnnnnnnnnnnnnnnnnnnnnnnnnnnnnnnnnnnnnnnnnnnnnnnnnnnnnn
nnnnnnnnnnnnnnnnnnnnnnnnnnnnnnnnnnnnnnnnnnnnnnnnnnnnnnnncaaacaatttttatgcaaaacatca
ttattgatgaaattttagaaagctgataacctagaaaagccttcctcactacaattgtttatatagatcgcaactttcagattatgactatcgctcag
tcacctgggttcctagagacatcgtccgaaaaaaggcatattcaaagctcattgaatctactcgtcgcgcttctccaacagaagaaccgcaatga
aaataagagtcttccaattcaaggaagtgaaagactcttaggcagggaggtgtcataattgggtccctcgttagcattcataatataacaattcgt
tataagaatggaatattagctaatatcagttaatattcataagcttaaagaattgtgcttgataataagtaaacaaaaatttgaaaatgcaaattt
ctctttgattgcagaaactccgattaaaatttacaattcgtgattattgctattaattagagaagccagtcacgatggcaaatatacagaataatat
atatatgtgtgtcgacagtcgaaatctttagatcggaagatttgactgatcaggctaaataagattgacacttagtcgttttcatttagttaatcaga
tccctggacatattactattactctacgtagtactaacacgtccaattccaatagacaattttgtaattgtacttattacttccactcgatttacattct
cataacgtgtgagatttctcccgtgacagaaatataaagttacagacgaaaataatattgtaatactcatgaattgcagcttttcttcatgaaaata
cattgataaggatggcaacgcaaatagcctatccctttatgactatcaaggggggtatataggtgttggtctttcataaagtcttaaatctttgcatca
aaacgtccaaagagtcttgctttgagccagtcgaatagaaccaaaaattgagagcgaccggaaaccaattcgttcaaataagcaagtcgccaa
aaggtatttccccaagtaccatgcaaagaaagtactttattgagatatggaacgttgagatcagcaatggccttatcatgcccgacataagccaa
ggcaccctggtgtttgtattggaaaggcttcaattgagtgttagcaagctcttgcttcataacggagaattctttttcggccttcttctcgccaagctg
cttttccatgacctcaagtctatgcttcaaagcagtagcctttccggacctgttaagattagcagctaaccagtcaccctgctgatgagcgacttga
gcagtagcaggcaatcctgagaaagcacagtctccaatggcatacacatcaggcatgcccttaacacggaagaattcatcaatgatcaatcctct
acgagcaccactttgttcgggaatggaagacattaattgacgagtcaagtcacgggcagttataccagcagcccagaccaaaagtccataagga
atgttctcaatggtcttagaaccatctgcgttggtttttgaatgatcaattccttttcgttcacatccttaacaagagctt
